# Machine Learning Classifies Core and Outer Fucosylation of N-Glycoproteins Using Mass Spectrometry

**DOI:** 10.1038/s41598-019-57274-1

**Published:** 2020-01-15

**Authors:** Heeyoun Hwang, Hoi Keun Jeong, Hyun Kyoung Lee, Gun Wook Park, Ju Yeon Lee, Soo Youn Lee, Young-Mook Kang, Hyun Joo An, Jeong Gu Kang, Jeong-Heon Ko, Jin Young Kim, Jong Shin Yoo

**Affiliations:** 1 Research Center for Bioconvergence Analysis, Korea Basic Science Institute, Cheongju, 28119 Republic of Korea; 2 Graduate School of Analytical Science and Technology, Chungnam National University, Daejeon, 34134 Republic of Korea; 3 Drug Information Platform Center, Korea Research Institute of Chemical Technology, Daejeon, 34114 Korea; 4 Asia Glycomics Reference Site, Chungnam National University, Daejeon, 34134 Republic of Korea; 5 Genome Editing Research Center, Korea Research Institute of Bioscience and Biotechnology, Daejeon, 34141 Republic of Korea; 6 Department of Biomolecular Science, Korea University of Science and Technology (UST), Daejeon, 34113 Republic of Korea

**Keywords:** Glycobiology, Glycomics, Machine learning, Bioanalytical chemistry, Mass spectrometry

## Abstract

Protein glycosylation is known to be involved in biological progresses such as cell recognition, growth, differentiation, and apoptosis. Fucosylation of glycoproteins plays an important role for structural stability and function of N-linked glycoproteins. Although many of biological and clinical studies of protein fucosylation by fucosyltransferases has been reported, structural classification of fucosylated N-glycoproteins such as core or outer isoforms remains a challenge. Here, we report for the first time the classification of N-glycopeptides as core- and outer-fucosylated types using tandem mass spectrometry (MS/MS) and machine learning algorithms such as the deep neural network (DNN) and support vector machine (SVM). Training and test sets of more than 800 MS/MS spectra of N-glycopeptides from the immunoglobulin gamma and alpha 1-acid-glycoprotein standards were selected for classification of the fucosylation types using supervised learning models. The best-performing model had an accuracy of more than 99% against manual characterization and area under the curve values greater than 0.99, which were calculated by probability scores from target and decoy datasets. Finally, this model was applied to classify fucosylated N-glycoproteins from human plasma. A total of 82N-glycopeptides, with 54 core-, 24 outer-, and 4 dual-fucosylation types derived from 54 glycoproteins, were commonly classified as the same type in both the DNN and SVM. Specifically, outer fucosylation was dominant in tri- and tetra-antennary N-glycopeptides, while core fucosylation was dominant in the mono-, bi-antennary and hybrid types of N-glycoproteins in human plasma. Thus, the machine learning methods can be combined with MS/MS to distinguish between different isoforms of fucosylated N-glycopeptides.

## Introduction

Protein glycosylation is one of the most common post-translational modifications related to protein structure, stability, trafficking, and proteiN-protein interactions^1,2^ Protein glycosylation is divided into O- or N-glycosylation according to the amino acid binding groups, which include the hydroxyl side chains of serine (S) or threonine (T) and the carboxy-amido nitrogen of asparagine (N) residues, respectively. The heterogeneity and complexity of N-glycosylation are due to the various combinations of four kinds of carbohydrate blocks, including N-acetylhexosamine (HexNAc; e.g., N-acetylglucosamine, N-acetylgalactosamine), hexose (Hex; e.g., glucose, galactose, mannose), fucose (Fuc), and sialic acid (Sia; N-acetylneuraminic acid). These combinations are made by their corresponding glycosyltransferases in the endoplasmic reticulum and Golgi apparatus^1^. Various diseases, including cancer, involve the fucosylation of human N-glycosylation. This is due to two kinds of fucosyltransferase: alpha-1,3/4 fucosyltransferase (FUT 3, 4, 5, 6, 7, 9, 10, and 11) and alpha-1,6 fucosyltransferase (FUT 8)^3^. The former synthesizes a Lewis or sialyl Lewis structure on the “outer” arm of N-glycan, and the latter produces trimannosyl “core” fucosylation, catalyzing a fucose to the innermost GlcNAc. These fucosyltransferases are highly expressed in cancers, including liver, breast, prostate, noN-small cell lung, and melanoma cancers^4–7^.

Fucosylation of N-linked glycoproteins can lead to alterations in protein activity in inflammation, immune responses, and cancer metastasis. Core fucosylation has been known as an important key for structural stability and function of N-glycoproteins^8^. For example, core fucosylation deficient IgG has been reported to may lead to antibody-mediated cellular cytotoxicity^9,10^. In case of core-fucosylated alpha-fetoprotein, exhibiting an increased affinity for the fucose-specific lectin of lens culinaris agglutinin (LCA), is well-known a biomarker for HCC^11^. FUT8-mediated alpha-1,6 core fucosylation which interrupts the proteolytic cleavage of L1CAM protein by plasmin, plays a molecular driver of metastasis in melanoma^7^. On the other hand, detailed study of biological role of outer fucosylation in human N-glycoprotein remains uncertain. In mammals, alpha-1,3-outer fucosylated glycans of *Schistosoma mansoni* and *H. pylori* are involved in host cell adhesion^8,12,13^. For example, in human N-glycoproteins, alpha-1 antitrypsin significantly increases fucosylation in emphysematous lung disease, thereby now it is necessary to study outer fucosylation in detail^14^. Haptoglobin is also decorated with outer fucosylation in pancreatic and gastric cancer^15,16^. From these studies of N-glycoproteins with various diseases, it is important to identify the detailed structure of core and outer fucosylation.

Recently, liquid chromatography-tandem mass spectrometry (LC-MS/MS) has emerged as a powerful technique for glycoprotein identification. Using tryptic digestion of proteins and tandem MS, we could automatically predict N-glycosylation sites and their attached glycan composition^17–20^. In collisioN-induced dissociation (CID) spectra from LC-MS/MS analysis, features from several fragmentation ions from N-glycopeptides could be used to determine the type of fucosylation. Glycan fragment ions (B ions), such as Hex-HexNAc (m/z 366.1), Hex-HexNAc-Fuc (m/z 512.2), Sia-Hex-HexNAc (m/z 657.2), and Sia-Hex-HexNAc-Fuc (m/z 803.3), have been used to identify the fucosylation of N-glycopeptides from haptoglobin, hemopexin, complement factor H and kininogen^21,22^. In addition, N-glycopeptide fragment ions (Y ions) with Fuc and their neutral loss provide additional information regarding the glycan composition within immunoglobulin gamma (IgG)^23^. Using manual annotation with B and Y ions from the CID spectra of N-glycopeptides, we successfully identified 71 fucosylated N-glycopeptides from human plasma glycoproteins, e.g., vitronectin, alpha-1-acid glycoprotein (AGP), and IgG; however, the classification of fucosylation has not been performed^24,25^. Recently, a total of 973 fucosylated N-glycopeptides were identified from prostate cancer cell lines to indirectly determine the fucosylation type using multiple lectin enrichment and LC-MS/MS^26^. However, there is no software that automatically classifies one of the four fucosylation types as ‘none’, ‘core’, ‘outer’, or ‘dual’ from N-glycopeptides.

The deep neural network (DNN) and support vector machine (SVM), which has mainly been used for supervised machine learning, has advantages of simplicity in generating learning models without overfitting problems^27–29^. The DNN has recently been used in various fields, including the prediction of gene expression levels in epigenetic models, the sensitivity of molecules, the structure and activity of drugs, the sequence of peptides, and biological images from microscopy, magnetic resonance imaging, and mass spectrometry^27,28,30^. However, there are no reports of using DNN methods to predict or classify the molecular structure using peak m/z and intensity values from mass spectrometry, except for an *in silico* algorithm that predicts the charge and structure of 94 lipid metabolites using CID tandem mass spectrometry^31,32^. Using the SVM, plasma proteins have been predicted as biomarkers of inflammation with 77% accuracy^33^. Theodoratou and her colleagues showed that the SVM could be applied to classify different glycosylation types of plasma IgG in colorectal cancer prognosis^34^. These reports showed that SVM could be used as a classifier in the bioinformatics fields, such as proteomics and glycoproteomics.

Here, we used MS/MS combined with machine learning methods (such as the DNN and SVM) to classify the fucosylation of N-glycopeptides. The identified N-glycopeptides from IgG and AGP were used for training and testing the machine learning models. Models with the best performance from the machine learning methods were applied to classify unknown fucosylated N-glycoproteins in human plasma.

## Methods

### Materials and samples

N-glycoprotein standards of human IgG and AGP, 1, 4-dithiothreitol (DTT), iodoacetamide (IAA), and formic acid (FA) were purchased from Sigma-Aldrich (St. Louis, MO). Trypsin of Gold grade was purchased from Promega (Madison, WI), and HPLC grade acetonitrile was purchased from J.T. Baker (Phillipsburg, NJ). Water was deionized using a Milli-Q Advantage A 10 System (Millipore). The ZIC-HILIC kit was purchased from EMD Millipore (Billerica, MA). Samples of human plasma, with appropriate concentrations of K_2_EDTA, were obtained from the Korea Research Institute of Bioscience and Biotechnology (Daejeon, Korea), along with ethical guidelines for informed consent and approval. Human plasma collection from 10 men who provided written informed consent and its analyses were approved by the public IRB designated by the Ministry of Health and Welfare (Rep. of Korea, IRB No. P01-201604-31-001). The 10 men of averaged 34-years-old (24 ~ 44) were healthy volunteers without any other disease diagnosed by a medical doctor. In addition, no one of them has reported a big disease such as immune disease or cancer since the sampling time at 2016. All of the methods were performed in accordance with the relevant IRB guidelines and regulations. The human plasma samples were frozen and stored at −80 °C until usage.

### Tryptic digestion

Solutions of IgG and AGP standard proteins and 10-pooled human plasma were made with 1 μg/μL in 50 mM ammonium bicarbonate (ABC) buffer; this solution was denatured using 80 M urea at room temperature (RT) for 10 min^35^. The standard protein samples were reduced using 2 μL of 500 mM DTT at RT for 1 h and alkylated using 5 μL of 500 mM IAA in the dark at RT for 1 h. Aliquots (100 μg protein/100 μL of 50 mM ABC buffer) of the standard proteins were quantitatively analyzed using the Bradford protein assay and digested with trypsin at 37 °C overnight (16 h). The digested samples were dried by SpeedVac and rehydrated in mobile phase A (0.1% FA) for LC-MS/MS analysis.

### Glycopeptide enrichment

Prior to LC-MS/MS analysis of the human plasma sample, HILIC enrichment was performed using the ZIC-HILIC kit according to the manufacturer’s instructions, with minor modifications^35^. Rehydrated human plasma (30 μg) was diluted with 50 μL ZIC binding buffer. This solution was mixed well with a ZIC glycocapture resin, and 50 μL was transferred to a new microcentrifuge tube. Then, the tube was centrifuged for 1 to 2 min at 2,000–2,500 × *g*, and the supernatant was completely removed and discarded. The diluted sample was added to the ZIC glycocapture resin, mixed by pipetting 3–5 times, and incubated at 1,200 rpm for 10–20 min. Then, the tube was centrifuged, and the supernatant was completely removed. Next, 150 μL of ZIC wash buffer was added to the ZIC glycocapture resin; it was mixed, incubated, and centrifuged, and the supernatant was removed. These steps were repeated three times. Then, 75–100 μL ZIC elution buffer was added to elute the glycopeptides, and the tube was mixed, incubated, and centrifuged. The supernatant was transferred to a new microcentrifuge tube, centrifuged for 2 min at 10,000 × g and transferred to a new microcentrifuge tube (avoiding the transfer of any resin particles). The supernatant was dried in a SpeedVac and rehydrated in 0.1% FA for LC-MS/MS analysis.

### LC-MS/MS analysis

Prepared samples were resolved in mobile phase A and analyzed on the LC-MS/MS system of the LTQ-Orbitrap mass spectrometer (Fusion Lumos version, Thermo Fisher Scientific), equipped with an EASY-nLC system (Thermo Fisher Scientific), using high-energy collisional dissociation (HCD) and CID of MS/MS fragmentation^36^. Each sample (5 μL) was injected at a flow rate of 4.0 µL/min into the C18 trap column (75 µm I. D. × 20 mm, 4 µm, 100 Å) using an autosampler equipped with the EASY-nLC system and was analyzed at a flow rate of 0.3 µL/min with an analytical column (100 µm I. D. × 500 mm, 2 µm, 100 Å). The LC gradient started with 2% solution B (0.1% formic acid with 80% acetonitrile) for 1 min and was increased to 8% over 16 min, 35% over 74 min, and 95% over 9 min and then decreased to 2% over another 20 min. The LTQ-Orbitrap Fusion Lumos mass spectrometer was operated in positive ion mode, and the nano-ESI voltage was set to 2.3 kV. During chromatographic separation, the mass spectrometers were operated in the data-dependent acquisition mode. MS data were collected using the following parameters: full scans were acquired in the Orbitrap at a resolution of 120,000 for each sample; five CID and HCD scans per full scan were obtained; CID scans were acquired in a linear trap quadrupole with 30 ms of activation time used for each sample with 35% normalized collision energy and a ±1.6 Da isolation window; and HCD scans were acquired in the Orbitrap at a resolution of 30,000 with 20 ms of activation for each sample with 35% NCE and a ±1.6 Da isolation window. Previously fragmented ions were excluded for 30 s.

### IQ-GPA analysis

Tryptic N-glycopeptides of IgG, AGP, and human plasma proteins were identified by IQ-GPA as per the following procedures^18^. Raw MS and MS/MS files were converted using RawConverter (Ver. 1.1.0.18, 2014, The Scripps Research Institute) in the data-dependent mode and with the selection of monoisotopic m/z. The glycopeptide databases (GPA-DBs) of IgG and AGP were generated with their tryptic N-glycopeptide sequences with 351N-glycans, in which 2,106 and 4,212N-glycopeptide precursors were included, respectively. For the IQ-GPA search of N-glycopeptides from human plasma, we used 282 human plasma GPA-DBs, including 253,422N-glycopeptides^18^. We used a noise threshold of 50.0 for MS and 2.0 for MS/MS and a precursor mass tolerance of ±0.05 Da. We also used MS2 tolerances of ±0.02 for HCD, ±1.5 for CID, and ±0.8 Da for ETD and M-, S-, and Y-score thresholds of 1.2, 98.0, and 40.0, respectively, where less than 1.0% of the estimated FDR was used for true positive filtering of the N-glycopeptide spectra. We also used an IQ-GPA retention time window of 5.0 min.

### Data set construction

Following the IQ-GPA search for N-glycopeptide classification, the relative intensities of 14N-glycopeptide fragment ions (B_2_, B_2_F, B_3_, B_3_F, B_3_S, B_3_SF, Y_1_, Y_1_F, Y_2_, Y_2_F, Y_3_, Y_3_F, Y_4_, and Y_4_F ions) were calculated from.mgf files by our iN-house program (coded by Python 2.7). We used a monoisotopic ion peak when the S/N was exceeded by three or more times, along with ±0.02 Da of tolerance. Identified GSMs from standard IgG and AGP proteins were manually classified as none, core, outer, and dual fucosylation from their CID MS/MS spectra. Representative core- and outer-manual-classified N-glycopeptides of IgG and AGP are shown in Fig. S1. The training and test sets for the machine learning methods consisted of 433 and 393 GSMs with their manual classifiers, respectively (Tables S1, S2, and 1, and Figs. S2 and S3). Because dual fucosylated N-glycopeptides were rarely identified in the standard proteins, 65 (training set) and 64 (test set) GSMs from 41 additional experiments of AGP standard proteins were added. In addition, 671 GSMs were identified for the unknown data set to classify the fucosylation of N-glycopeptides from human plasma (Table S3, Fig. S4).

**Table 1:** Construction of training and test sets of glycopeptide spectra matches (GSMs) of N-glycopeptides identified from IgG and AGP standards and their classification of fucosylation types both manually and by machine learning methods such as the support vector machine (SVM) and deep neural network (DNN).

N-glycoproteins (IgG & AGP Standards)		Training Set (433 GSMs)					Test Set (393 GSMs)		
		None (%)	Core (%)	Outer (%)	Dual (%)	None (%)	Core (%)	Outer (%)	Dual (%)
Classification Methods	Manual Classification	170 (39.2%)	106 (24.5%)	89 (20.5%)	68* (15.7%)	162 (41.2%)	70 (17.8%)	96 (24.4%)	65* (16.5%)
	SVM Classification	170 (39.2%)	106 (24.5%)	89 (20.5%)	68 (15.7%)	163 (41.5%)	70 (17.8%)	97 (24.9%)	62 (15.8%)
	DNN Classification	170 (39.2%)	106 (24.5%)	89 (20.5%)	68 (15.7%)	163 (41.5%)	70 (17.8%)	98 (24.9%)	62 (15.8%)

^*^GSMs with dual fucosylation from AGP standard proteins identified and added to the training and test sets from additional experiments.

### Deep neural network

We designed a DNN architecture using Python (version 3.5.2 from Anaconda 4.2.0, 64-bit version) and TensorFlow for Windows (https://www.tensorflow.org/install/install_windows) based on the opeN-source TensorFlow DNN (https://github.com/hunkim/TensorFlow-ML-Exercises). We also used GPU support, including the CUDA Toolkit from NVIDIA drivers (https://developer.nvidia.com/how-to-cuda-python). Fully connected DNNs, which consisted of various combinations of nodes (8, 16, 32, 64, and 128) and layers (3, 4, and 5), were used for supervised learning with a manually classified training set from IgG and AGP. We used 14 ions as the first input (the number of features from the relative intensities of B and Y series ions) and 4 types as the final output node (which is the number of fucosylation classified as none, core, outer, and dual). Xavier initialization, which assigns the weights from a Gaussian distribution of random values with the node numbers of the input and output from each layer, was used for the initialization of weight values in each node from the DNN^37^. The Xavier initialization also performs better than the restricted Boltzmann machine method, as it uses a simple code for initialization^38^. We used the rectified linear units for each activation function and finally used a sigmoid function^39^. To prevent overfitting the DNN, we used the dropout regularization (value = 0.75), which is a technique that ignores randomly selected nodes during the training step^40^. For the generation of various performance models, each model was trained using various epoch times of 10, 100, 500, 1,000, 2,000, 5,000, 10,000, and 20,000; a learning rate of 0.02 was used with a gradient descent algorithm from TensorFlow. We used 10 experimental replicates for each DNN architecture, with different numbers of nodes, layers and epochs. After training and testing the models, accuracy was calculated with a manually classified test set from IgG and AGP. In addition, decoy data sets were created from randomly rearranged values for each glycopeptide spectra, which were tested five times. We found the categorical distribution probability using the Softmax function from the final classified fucosylation types; then, this was used to calculate the Pscore to select a model that can distinguish between the target and a decoy, according to Eq. (1):

$$Pscore=-\,\mathrm{ln}(1-({\rm{P}}1-12))$$

where P1 is the highest probability and P2 is the second probability.

### Support vector machine

We performed the SVM method using the R package e1071, which was used to classify the fucosylation of identified N-glycopeptides using the C-classification type for the linear function and probability modes. The input and output data were applied as in the DNN method. To determine the best-performing SVM model, we optimized the cost value with 20 numeric random seeds^41,42^. First, the cost parameters were attempted using a base of 2, and the 12 cost values 2^−5^, 2^−3^, 2^−1^, 2^0^, 2^1^, 2^3^, 2^5^, 2^7^, 2^9^, 2^11^, 2^13^, and 2^15^ were tested. Second, seven cost values 2^0^, 2^1^, 2^2^, 2^3^, 2^4^, 2^5^, and 2^6^ were tested as a narrower condition. Third, 13 cost values 4, 5, 6, 7, 8, 9, 10, 11, 12, 13, 14, 15, and 16 were used. Finally, 31 kinds of cost values between 8.0 and 11.0 were applied as units of 0.1. We calculated the accuracy using a manually classified test set from IgG and AGP. The decoy data sets were also tested five times, as in the DNN method. We also determined the categorical distribution probability from the final classified fucosylation types, where the Pscore was calculated using the same method as that in the DNN method.

## Results and Discussion

**Figure 1:**
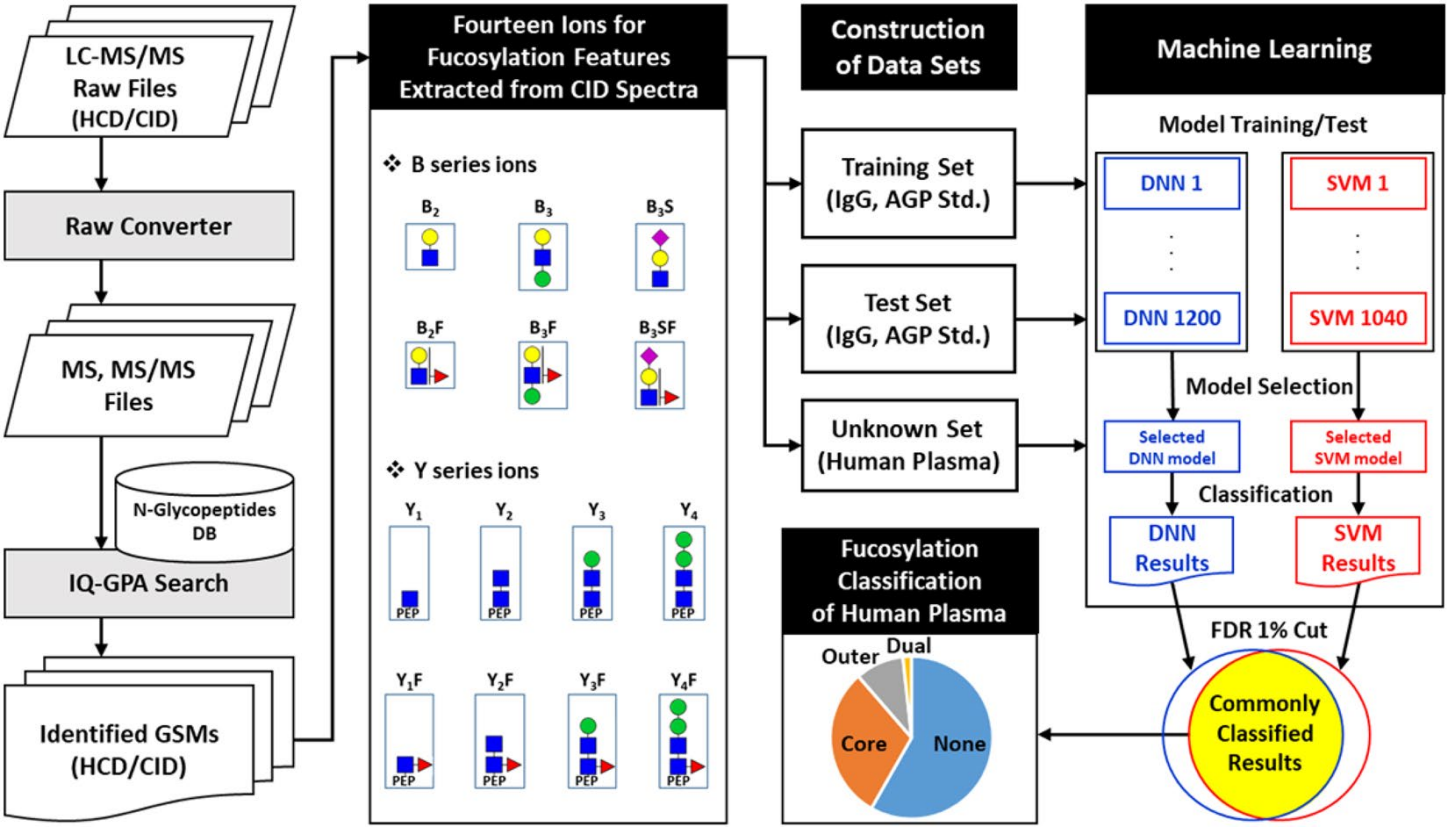
The computational workflow for classifying the fucosylation of N-glycopeptides using machine learning. The relative intensities of 14 fucosylation features extracted from CID tandem MS spectra of identified N-glycopeptides were calculated and used to classify fucosylation using the DNN and SVM. Training and testing data sets were constructed with N-glycopeptides identified from standard IgG and AGP glycoproteins using IQ-GPA. The DNN and SVM models were constructed with TensorFlow (ver. 0.12.0) and the R package e1071 (ver. 3.4.3), respectively. The best-performing model was selected from each machine learning method, and classified N-glycopeptides were filtered with <1% FDR using a random decoy. Finally, the DNN and SVM were used to classify an unknown data set from human plasma according to four types of fucosylation: none, core, outer, and dual. Green circles = nomannose; yellow circles = angalactose; blue squares = N-acetylglucosamine; red triangles = fucose; and pink diamonds = N-acetylneuraminic acid.

We present a workflow for classifying the fucosylation of N-glycopeptides from LC-MS/MS data using the identification and quantification of a GlycoProteome Analyzer (IQ-GPA) pipeline and the DNN and SVM machine learning methods (Fig. 1)^18^. We used IgG and AGP in human plasma as standard proteins to prove our concept, as IgG in human plasma is exclusively core-fucosylated, while AGP is mostly outer-fucosylated^43–45^. N-glycopeptide spectra identified from IgG and AGP using IQ-GPA software with <1% false discovery rate (FDR) were used to train and test the machine learning models. In order to estimate the number of false positive identifications, IQ-GPA calculate the false discovery rate (FDR) using a decoy database, which consists of a decoy N-glycopeptide by reverse reading of peptide sequence and N-glycan from the target N-glycopeptide^18^. Based on the B/Y fragment ions in the CID MS/MS spectra of each N-glycopeptide, the relative intensities from 14 ions, namely, Hex-HexNAc (B_2_), Hex-HexNAc-Fuc (B_2_F), 2Hex-HexNAc (B_3_), 2Hex-HexNAc-Fuc (B_3_F), Hex-HexNAc-Sia (B_3_S), Hex-HexNAc-Fuc-Sia (B_3_SF), peptide (Pep)-HexNAc (Y_1_), Pep-HexNAc-Fuc (Y_1_F), Pep-2HexNAc (Y_2_), Pep-2HexNAc-Fuc (Y_2_F), Pep-Hex-2HexNAc (Y_3_), Pep-Hex-2HexNAc-Fuc (Y_3_F), Pep-2Hex-2HexNAc (Y_4_), and Pep-2Hex-2HexNAc-Fuc (Y_4_F), were calculated and used as input data in the DNN and SVM. Four types of fucosylation (‘none’, ‘core’, ‘outer’, and ‘dual’) were used as output classifiers of the DNN and SVM, where the accuracy was calculated from manually assigned results for a supervised learning method. The relative intensity values of the test data sets were randomly shuffled and used as decoy data sets. Then, the area under the curve (AUC) value and FDR were calculated using the random decoy result to select the model with the best performance from the trained models. We compared the results from each best-performing model between the DNN and SVM methods using the test sets of the standard proteins of IgG and AGP (Table 1). Then, each best model from the machine learning methods was applied for classification of the fucosylation types of the unknown N-glycopeptides identified from human plasma.

To select the best-performing machine learning model, we used a test set to calculate the accuracy between the predicted and manually obtained classification results. In addition, we used values for the AUC calculated with Pscore from a single target and averaged decoy data sets using receiver operating characteristic (ROC) curves, where the decoy Pscore was averaged over five decoy data sets. A total of 1,200 DNN models (5 nodes × 3 kinds of layers × 8 kinds of epochs × 10 experimental replicates) were generated and tested, and the Euclidean length (EL) was calculated with the accuracy and AUC for selection of the DNN model with the best performance in the test set (Table S4). The longest EL value (1.40629) was from the eighth model; it consisted of 64 nodes and 4 layers with 10,000 epochs (Fig. S5A, Table 1). The Pscore of the target N-glycopeptide spectra was well distinguished within the decoy data, having an AUC value of 0.999 within both the training and test sets (Table 2). In the case of SVM analysis, a total of 1,040 SVM models (52 cost values × 20 random seeds) were generated and tested. A model (random seed = 435, cost = 8.7) showing the best performance with the longest EL value (1.40394) was selected for an optimized SVM model (Tables S5, 1, and 2 and Fig. S5B). Using an FDR filtering value of <1% with the random decoy method, the numbers of classified GSMs were compared between the two machine learning methods, and the DNN showed slightly more GSMs (Tables S6, S7, and 2). Next, we compared the union of 393 GSMs (Table S7) with the same classification results over the two machine learning methods with the manually obtained classification results. For example, NEEYNK_5_4_1_1 (no. 146 in Table S7) was classified as none fucosylated glycopeptide in both machine learning method, but it was classified as outer-fucosylated glycopeptide in manual. We calculated a sensitivity rate of 100% (true positives/(true positives + false negatives)) and an accuracy of 99.78%. The results suggest that this strategy could be applied to unknown data sets such as those related to human plasma.

**Table 2:** Comparison of Pscore histograms from the classification of fucosylation types between selected machine learning models of the deep neural network (DNN) and support vector machine (SVM).

	Training set (433 GSMs)		Test set (393 GSMs)		Unknown set (671 GSMs)	
	DNN	SVM	DNN	SVM	DNN	SVM
AUC*	0.999	0.994	0.999	0.998	0.998	0.986
Pscore cut <1% FDR**	4.623	0.982	5.559	0.303	3.415	0.692
Filtered GSMs***	433	417	391	387	657	626
Union of Filtered GSMs**** (TP / FP)	433 (433/0)		392 (388/4)		638 (626/12)	
Sensitivity (TP /(TP TPFN))	100% (433/433)		100% (388/388)		99.21% (626/631)	
Accuracy	100%		99.75%		97.47%	

*Area under the curve (AUC) values were calculated from receiver operating characteristic curves between the target and decoy.

**Pscores were less than 1% FDR between the target and decoy, where Pscores were calculated as the natural logarithm of the difference between the first and second ranked probabilities for classification of the fucosylation types.

***Number of glycopeptide spectra matches (GSMs) was filtered with less than 1% FDR between the target and decoy.

****Union number of GSMs were classified using the DNN and SVM filtered with less than 1% FDR between the target and decoy.

**Figure 2:**
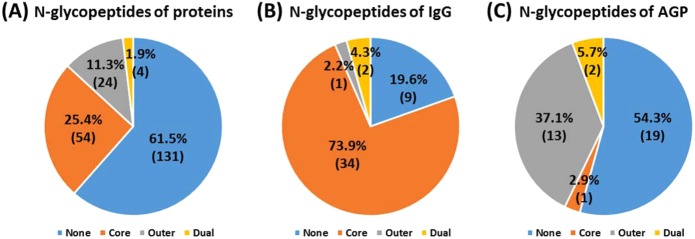
Classification of fucosylated N-glycopeptides of (**A**) total proteins, (**B**) IgG, and (**C**) AGP in human plasma.

From the DNN results obtained using human plasma samples, 218 distinct N-glycopeptides of 657 GSMs were classified with an FDR <1.0% using the decoy method (Table S8). From the SVM results, 211 distinct N-glycopeptides of 626 GSMs were classified using the same filtering conditions (Table S8). A union number of 213 distinct N-glycopeptides of 638 GSMs (Table S9) were classified as the same results from the two methods, where the sensitivity was 99.21% and accuracy was 97.47% (Table 2). This indicates that our strategy classifies well the fucosylation of N-glycopeptides from glycoproteins, including IgG and AGP. Approximately 40% of N-glycopeptides were classified with fucosylation, including 25.4% based on core fucosylation, 11.3% based on outer fucosylation and 1.9% based on dual fucosylation (Fig. 2A). Core and outer fucosylation were dominantly classified in IgG and AGP, respectively (Fig. 2B,C). Similar results showed that IgG is mostly core fucosylated, whereas AGP is highly outer fucosylated in human plasma^46,47^. We also manually confirmed the N-glycopeptide spectra of IgG (98.95% accuracy) and AGP (96.76% accuracy) in human plasma. We demonstrated that fucosylation classification using our DNN and SVM models with 1% FDR filtering was highly reproducible and could be applied to other N-glycopeptides.

We classified a total of 82N-glycopeptides with fucose from human plasma; this is the first report of 36 of these N-glycopeptides (to the best of our knowledge) (Table S10). Eight N-glycopeptides form alpha-2-HS-glycoprotein, ceruloplasmin, haptoglobin, kininogeN-1, and vitronectin, were confirmed with the study of liver-secreted N-glycoproteins, where bi- and tri- antennary glycopeptides were most common ones from HCC plasma^22,25^. Fucosylation of AGP and haptoglobin inhibits the biding with drug and hemoglobin, respectively^48^. We also classified 20 fucosylated N-glycopeptides from complement components C7, IgA2, IgJ, IgM, alpha-1-antichymotrypsin, alpha-2-HS-glycoprotein, AGP, apolipoprotein D, ceruloplasmin, hemopexin, and phospholipid transfer protein as core fucosylated. Core-fucosylation is a kind of N-linked glycosylation in which an alpha-1,6 linked fucose is added to the innermost N-acetylglucosamine (GlcNAc) residue. For example, the core fucosylated alpha-fetoprotein isoform (AFP-L3) was approved as a biomarker of hepatocellular carcinoma^11^. The N-glycopeptides of SWPAVGNCSSALR with core fucosylation, which were previously reported using the Endo F3 glycosidase and an LCA lectin approach, were also identified from hemopexin^49^. The core fucosylation of IgA, IgJ, and IgM from the Endo H treatment study was confirmed in our study^50^. In addition core-fucosylated N-glycoproteins are involved in a series of immune and inflammatory responses. However, 11 fucosylated N-glycopeptides from IgG1, clusterin, kininogeN-1, vitronectin, prothrombin, beta-2-glycoprotein 1, alpha-2-HS-glycoprotein, haptoglobin and hemopexin were classified as outer fucosylated. The outer-fucosylated N-glycopeptides of haptoglobin, hemopexin, and kininogeN-1 from human plasma were also reported in previous studies^16,23,25,50^. The dual fucosylation of four N-glycopeptides was classified in IgG and AGP, including EEQYNSTYR_5_4_2_1 and EEQFNSTFR_5_4_2_1 from IgG and NEEYNK_6_5_2_3 and ENGTVSR_6_5_2_3 from AGP. However, the spectra of EEQYNSTYR_5_4_2_0 from IgG and ENGTISR_7_6_2_4 and ENGTVSR_7_6_2_4 from AGP were classified as outer fucosylation. The core fucosylation of human plasma by FUT 8 occurs with high substrate specificity at the bi-antennary glycans^51,52^.

**Figure 3:**
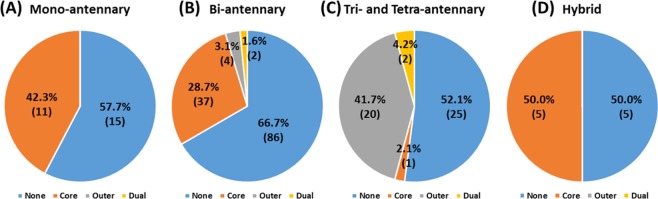
Classification of fucosylated N-glycopeptides of total proteins in human plasma by their (**A**) mono-antennary, (**B**) bi-antennary, (**C**) tri- and tetra-antennary, and (**D**) hybrid types.

**Figure 4:**
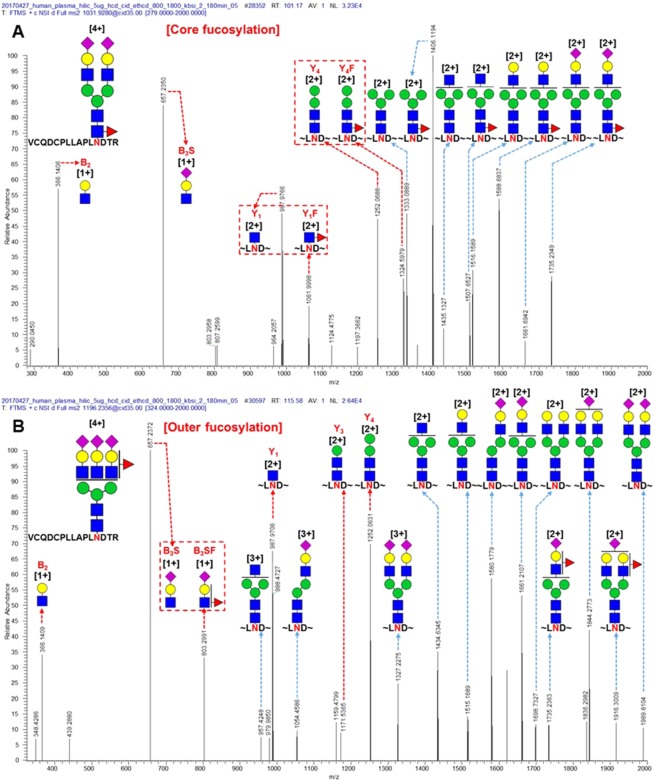
Representative CID MS/MS spectra of N-glycopeptides classified as (**A**) core fucosylation with bi-antennary type (VCQDCPLLAPLNDTR_5_4_1_2) and (**B**) outer fucosylation with tri-antennary type (VCQDCPLLAPLNDTR_6_5_1_3) of alpha-2-HS glycoprotein in human plasma (green circle, mannose; yellow circle, galactose; blue square, N-acetylglucosamine; red triangle, fucose; pink diamond, N-acetylneuraminic acid; red arrow, fucosylation diagnostic ions; and red box, pair of fragmented ions with or without fucose).

Most fucosylated N-glycopeptides are of mono- and bi-antennary and hybrid types and were classified as core fucosylation (Fig. 3A,B,D). Tri- and tetra-antennary N-glycopeptides from human plasma were dominantly classified as outer or dual fucosylated (Fig. 3C). Representatively, CID MS/MS spectra of N-glycopeptides of alpha-2-HS glycoprotein, which were not used in the training or test set, are shown in Fig. 4. Core fucosylation with the bi-antennary type (VCQDCPLLAPLNDTR_5_4_1_2), which contains Y_1_/Y_1_F and Y_4_/Y_4_F ion pairs, occurred, but B_2_ and B_3_S ions were not paired with their fucosylation ions (B_2_F and B_3_SF) (Fig. 4A). Otherwise, outer fucosylation with the tri-antennary type (VCQDCPLLAPLNDTR_6_5_1_3), which contains a B_3_S/B_3_SF ion pair, occurred, but Y_1_, Y_3_, and Y_4_ ions were not paired with their fucosylation ions (Y_1_F, Y_3_F, and Y_4_F) (Fig. 4B). Bi- and tri-antennary N-glycopeptides (N-glycopeptides of alpha-2-HS glycoprotein) were successfully classified in the training and test sets using our approach (Fig. 4A,B). Alpha-1,3/4-fucosyltransferases of FUT3-7 and FUT9 are reacted in synthesis of Lewis antigens. The enzymes might catalyze the fucose transfer to the acceptor substrate N-acetyl lactosamine (LacNAc), forming the tri-saccharide Lewis structure^8^. In here, according to our result, the enzymes also seems to have substrate specificity of N-acetylgalactosamine that linked as beta-1,6 or beta-1,4 with mannose to generate the outer arm branch of N-glycopeptides. Eventually, we demonstrated that the glycopeptide data sets of glycoproteins in plasma other than AGP and IgG for the training and test step were also well classified using this approach. Therefore, machine learning methods could be used to classify fucosylated N-glycopeptides from human plasma.

## Conclusions

Several algorithms are currently available to identify N-glycoproteins; however, they cannot distinguish between structural core- and outer-fucosylated isoforms. We demonstrated that the DNN and SVM machine learning approaches could predict the core and outer fucosylation of N-glycoproteins from complex samples such as human plasma. For the training of the machine learning models, a supervised learning method was used with manually identified N-glycopeptides from standard IgG and AGP, which are representative of core- and outer-fucosylated glycoproteins, respectively. In this study, we showed that the machine learning method can be used to classify fucosylated N-glycopeptides of IgG and AGP and other glycoproteins. Our method was applied to classify fucosylated N-glycoproteins from human plasma, in which 213N-glycopeptides from 54 glycoproteins were classified with an accuracy greater than 97% compared with manual classification. In human plasma, we characterized 82 fucosylated N-glycopeptides (54 core, 24 outer and 4 dual) from 22 glycoproteins: IgG1, IgG2, IgG3, IgG4, AGP1, AGP2, complement component C7, IgA2, IgJ, IgM, alpha-1-antichymotrypsin, alpha-2-HS-glycoprotein, apolipoprotein D, ceruloplasmin, hemopexin, phospholipid transfer protein, clusterin, kininogeN-1, vitronectin, prothrombin, beta-2-glycoprotein 1, and haptoglobin. In addition, we reported 36 unique fucosylated N-glycopeptides in human plasma that have never been reported (to the best of our knowledge). We found that most of the fucosylated N-glycopeptides of mono-, bi-antennary and hybrid types were classified as core fucosylation. Tri- and tetra-antennary types of N-glycopeptides were predominantly classified as outer fucosylation. We found that the machine learning of the DNN and SVM may be useful in distinguishing fucosylation types in N-glycopeptides. Combining mass spectrometry with machine learning approaches could be a viable solution for distinguishing structural isomers of biomolecules such as peptides, lipids, and glycans with diagnostic peaks from their MS/MS spectra.

## Supplementary information

Supplementary figures and tables.
