# Author Correction: Machine Learning Classifies Core and Outer Fucosylation of N-Glycoproteins Using Mass Spectrometry

**DOI:** 10.1038/s41598-020-60009-2

**Published:** 2020-02-13

**Authors:** Heeyoun Hwang, Hoi Keun Jeong, Hyun Kyoung Lee, Gun Wook Park, Ju Yeon Lee, Soo Youn Lee, Young-Mook Kang, Hyun Joo An, Jeong Gu Kang, Jeong-Heon Ko, Jin Young Kim, Jong Shin Yoo

**Affiliations:** 1 Research Center for Bioconvergence Analysis, Korea Basic Science Institute, Cheongju, 28119 Republic of Korea; 2 Graduate School of Analytical Science and Technology, Chungnam National University, Daejeon, 34134 Republic of Korea; 3 Drug Information Platform Center, Korea Research Institute of Chemical Technology, Daejeon, 34114 Korea; 4 Asia Glycomics Reference Site, Chungnam National University, Daejeon, 34134 Republic of Korea; 5 Genome Editing Research Center, Korea Research Institute of Bioscience and Biotechnology, Daejeon, 34141 Republic of Korea; 6 Department of Biomolecular Science, Korea University of Science and Technology (UST), Daejeon, 34113 Republic of Korea

Correction to: *Scientific Reports* 10.1038/s41598-019-57274-1, published online 15 January 2020

This Article contains typographical errors in the Acknowledgements section.

“(Research grant T37413)”

should read:

“(Research grant T39710)”

