# Supplementary material for: Machine Learning Classifies Core and Outer Fucosylation of N-Glycoproteins Using Mass Spectrometry
Source: Sci Rep. 2020 Jan 15;10:318. doi: 10.1038/s41598-019-57274-1 (PMC6962204; doi:10.1038/s41598-019-57274-1)

Supplementary information for:

Machine Learning Classifies Core and Outer Fucosylation of N−Glycoproteins Using Mass Spectrometry

Heeyoun Hwang[1,*], Hoi Keun Jeong[1,2,*], Hyun Kyoung Lee[1,2], Gun Wook Park[1], Ju Yeon Lee[1], Soo Youn Lee[1], Young−Mook Kang[3], Hyun Joo An[2,4], Jeong Gu Kang[5], Jeong−Heon Ko[5,6], Jin Young Kim[1,**], and Jong Shin Yoo[1,2,**]

[1]*Research Center for Bioconvergence Analysis, Korea Basic Science Institute, Cheongju, Republic of Korea, 28119*

[2]*Graduate School of Analytical Science and Technology, Chungnam National University, Daejeon, Republic of Korea, 34134*

[3]*Drug Information Platform Center, Korea Research Institute of Chemical Technology, Daejeon, Korea, 34114*

[4]*Asia Glycomics Reference Site, Chungnam National University, Daejeon, Republic of Korea, 34134*

[5]*Genome Editing Research Center, Korea Research Institute of Bioscience and Biotechnology, Daejeon, Republic of Korea, 34141*

[6]*Department of Biomolecular Science, Korea University of Science and Technology (UST), Daejeon, Republic of Korea, 34113*

[*] These authors contributed equally.

[**]Corresponding Authors. Email: jongshin@kbsi.re.kr; Tel. +82−43−240−5150
Email: jingyoung@kbsi.re.kr; Tel. +82−43−240−5142

Contents

(1) List of supplementary tables:

Supplementary Table 1 Glycopeptide spectrum matches of alpha-1-acid glycoprotein and immunoglobulin gamma, and their relative intensities as training set for machine learning….4

Supplementary Table 2 Glycopeptide spectrum matches of alpha-1-acid glycoprotein and immunoglobulin gamma, and their relative intensities as test set for machine learning………21

Supplementary Table 3 Glycopeptide spectrum matches of human plasma proteins and their relative intensities as unknown set for machine learning……………………………………..37

Supplementary Table 4 Models generated by deep neural network for fucosylation classification…………………………………………………………………………………63

Supplementary Table 5 Models generated by support vector machine for fucosylation classification…………………………………………………………………………………87

Supplementary Table 6 Glycopeptide spectrum matches of alpha-1-acid glycoprotein and immunoglobulin gamma, and their Classification as training set for machine learning…….107

Supplementary Table 7 Glycopeptide spectrum matches of alpha-1-acid glycoprotein and immunoglobulin gamma, and their Classification as test set for machine learning…………122

Supplementary Table 8 Glycopeptide spectrum matches of human plasma proteins and their Classification as unknown set for machine learning………………………………………...136

Supplementary Table 9 N-glycopeptide having both core and outer type fucosylation in human plasma……………………………………………………………………………159

Supplementary Table 10 Total fucosylated N−glycopeptides of proteins in human plasma commonly classified by SVM and DNN. A total of 46 fucosylated N−glycopeptides were validated previously (with references), while 36 were newly found………………………..160

(2) List of supplementary figures:

Supplementary Figure 1 Representative CID MS/MS spectra of N−glycopeptides of (A) EEQYNSTYR_4_4_1_0 from an IgG standard and (B) NEEYNK_6_5_1_3 from an AGP standard, which were classified as core and outer fucosylated N−glycopeptides, respectively. Green circles = mannose; yellow circles = galactose; blue squares = N−acetylglucosamine; red triangles = fucose; pink diamonds = N−acetylneuraminic acid; red arrow = fucosylation diagnostic ions; red box = fragmented ions pairs with or without fucose………………………163

Supplementary Figure 2 MS/MS spectra of N-glycopeptides identified from alpha-1-acid glycoprotein and immunoglobulin gamma for training set……………………………………..164

Supplementary Figure 3 MS/MS spectra of N-glycopeptides identified from alpha-1-acid glycoprotein and immunoglobulin gamma for test set………………………………………597

Supplementary Figure 4 MS/MS spectra of N-glycopeptides identified from human plasma for unknown set……………………………………………………………………………..990

Supplementary Figure 5 Model selection of (a) deep neural network and (b) support vector machine……………………………………………………………………………1661

Supplementary Tables

Supplementary Table 1. Glycopeptide spectrum matches of alpha-1-acid glycoprotein and immunoglobulin gamma, and their relative intensities as training set for machine learning.

No.	Experiments	CID Scan No.	N-glycoproteins	N-glycopeptides	Relative intensities of B/Y series ions														Manual Classification
					B_2	B_2F	B_3	B_3F	B_2S	B_2SF	Y_1	Y_1F	Y_2	Y_2F	Y_3	Y_3F	Y_4	Y_4F	
1	AGP exp_2	2144	Alpha-1-acid glycoprotein 1	ENGTISR_4_4_0_1	100.0	0.0	0.0	0.0	47.8	0.0	27.3	0.0	0.0	0.0	14.8	0.0	15.4	0.0	None
2	AGP exp_1	2066	Alpha-1-acid glycoprotein 1	ENGTISR_4_4_0_1	26.2	0.0	0.0	0.0	44.2	0.0	26.8	0.0	23.7	0.0	100.0	0.0	0.0	0.0	None
3	AGP exp_2	3657	Alpha-1-acid glycoprotein 1	ENGTISR_5_4_0_2	33.6	0.0	6.8	0.0	100.0	0.0	46.4	0.0	4.0	0.0	0.0	0.0	38.4	0.0	None
4	AGP exp_2	3691	Alpha-1-acid glycoprotein 1	ENGTISR_5_4_0_2	0.0	0.0	0.0	0.0	87.8	0.0	100.0	0.0	7.8	0.0	18.9	0.0	38.6	0.0	None
5	AGP exp_1	3850	Alpha-1-acid glycoprotein 1	ENGTISR_5_4_0_2	71.2	0.0	10.2	0.0	100.0	0.0	24.9	0.0	0.0	0.0	0.0	0.0	21.2	0.0	None
6	AGP exp_1	2127	Alpha-1-acid glycoprotein 1	ENGTISR_6_5_0_1	100.0	0.0	6.7	0.0	46.5	0.0	36.6	0.0	3.6	0.0	0.0	0.0	30.5	0.0	None
7	AGP exp_1	2266	Alpha-1-acid glycoprotein 1	ENGTISR_6_5_0_1	100.0	0.0	4.0	0.0	33.6	0.0	65.3	0.0	0.0	0.0	0.0	0.0	26.6	0.0	None
8	AGP exp_1	2368	Alpha-1-acid glycoprotein 1	ENGTISR_6_5_0_1	100.0	0.0	12.5	0.0	47.8	0.0	65.4	0.0	0.0	0.0	0.0	0.0	36.5	0.0	None
9	AGP exp_2	2404	Alpha-1-acid glycoprotein 1	ENGTISR_6_5_0_1	100.0	0.0	6.9	0.0	53.1	0.0	79.9	0.0	0.0	0.0	0.0	0.0	16.3	0.0	None
10	AGP exp_2	2273	Alpha-1-acid glycoprotein 1	ENGTISR_6_5_0_1	100.0	0.0	8.8	0.0	51.5	0.0	30.5	0.0	0.0	0.0	0.0	0.0	8.7	0.0	None
11	AGP exp_2	2122	Alpha-1-acid glycoprotein 1	ENGTISR_6_5_0_1	100.0	0.0	10.6	0.0	49.5	0.0	25.3	0.0	0.0	0.0	0.0	0.0	8.7	0.0	None
12	AGP exp_2	3604	Alpha-1-acid glycoprotein 1	ENGTISR_6_5_0_2	60.7	0.0	5.5	0.0	100.0	0.0	26.9	0.0	5.2	0.0	0.0	0.0	7.7	0.0	None
13	AGP exp_1	4033	Alpha-1-acid glycoprotein 1	ENGTISR_6_5_0_2	57.3	0.0	6.0	0.0	95.8	0.0	100.0	0.0	0.0	0.0	0.0	0.0	34.3	0.0	None
14	AGP exp_2	3938	Alpha-1-acid glycoprotein 1	ENGTISR_6_5_0_2	71.8	0.0	5.2	0.0	100.0	0.0	74.7	0.0	5.8	0.0	0.0	0.0	39.9	0.0	None
15	AGP exp_1	3756	Alpha-1-acid glycoprotein 1	ENGTISR_6_5_0_2	64.8	0.0	4.8	0.0	88.1	0.0	100.0	0.0	7.2	0.0	0.0	0.0	42.2	0.0	None
16	AGP exp_2	3776	Alpha-1-acid glycoprotein 1	ENGTISR_6_5_0_2	0.0	0.0	0.0	0.0	15.6	0.0	100.0	0.0	17.9	0.0	13.6	0.0	14.1	0.0	None
17	AGP exp_1	4838	Alpha-1-acid glycoprotein 1	ENGTISR_6_5_0_3	0.0	0.0	0.0	0.0	48.1	0.0	100.0	0.0	15.0	0.0	9.6	0.0	14.2	0.0	None
18	AGP exp_1	4749	Alpha-1-acid glycoprotein 1	ENGTISR_6_5_0_3	10.2	0.0	0.0	0.0	100.0	0.0	36.1	0.0	4.0	0.0	0.0	0.0	15.6	0.0	None
19	AGP exp_2	6210	Alpha-1-acid glycoprotein 1	ENGTISR_6_5_0_3	9.2	0.0	0.0	0.0	100.0	0.0	31.6	0.0	4.7	0.0	0.0	0.0	29.0	0.0	None
20	AGP exp_1	5085	Alpha-1-acid glycoprotein 1	ENGTISR_6_5_0_3	28.3	0.0	0.0	0.0	100.0	0.0	45.3	0.0	3.6	0.0	0.0	0.0	31.4	0.0	None
21	AGP exp_2	2248	Alpha-1-acid glycoprotein 1	ENGTISR_6_5_1_1	100.0	30.5	0.0	0.0	46.7	0.0	57.4	0.0	0.0	0.0	0.0	0.0	24.3	0.0	Outer
22	AGP exp_1	2178	Alpha-1-acid glycoprotein 1	ENGTISR_6_5_1_1	100.0	25.0	0.0	0.0	53.7	0.0	48.5	0.0	0.0	0.0	0.0	0.0	12.4	0.0	Outer
23	AGP exp_1	2305	Alpha-1-acid glycoprotein 1	ENGTISR_6_5_1_1	100.0	0.0	0.0	0.0	56.1	27.7	87.4	0.0	0.0	0.0	0.0	0.0	26.6	0.0	Outer
24	AGP exp_1	2013	Alpha-1-acid glycoprotein 1	ENGTISR_6_5_1_1	100.0	36.7	0.0	0.0	61.0	36.9	23.3	0.0	0.0	0.0	0.0	0.0	10.5	0.0	Outer

Supplementary Table 1. Continued.

No.	Experiments	CID Scan No.	N-glycoproteins	N-glycopeptides	Relative intensities of B/Y series ions														Manual Classification
					B_2	B_2F	B_3	B_3F	B_2S	B_2SF	Y_1	Y_1F	Y_2	Y_2F	Y_3	Y_3F	Y_4	Y_4F	
25	AGP exp_1	3643	Alpha-1-acid glycoprotein 1	ENGTISR_6_5_1_2	71.9	13.4	6.1	0.0	100.0	37.0	98.5	0.0	3.2	0.0	0.0	0.0	46.3	3.0	Outer
26	AGP exp_1	3953	Alpha-1-acid glycoprotein 1	ENGTISR_6_5_1_2	58.4	8.2	0.0	0.0	100.0	42.3	73.8	6.4	0.0	0.0	0.0	0.0	16.1	0.0	Outer
27	AGP exp_2	3502	Alpha-1-acid glycoprotein 1	ENGTISR_6_5_1_2	90.5	17.3	0.0	0.0	100.0	20.6	36.7	0.0	0.0	0.0	0.0	0.0	0.0	0.0	Outer
28	AGP exp_2	3805	Alpha-1-acid glycoprotein 1	ENGTISR_6_5_1_2	67.0	16.0	0.0	0.0	100.0	23.6	43.4	0.0	0.0	0.0	0.0	0.0	21.8	0.0	Outer
29	AGP exp_2	4775	Alpha-1-acid glycoprotein 1	ENGTISR_6_5_1_3	18.5	5.9	3.7	0.0	100.0	35.3	89.2	0.0	7.7	0.0	0.0	0.0	56.9	2.8	Outer
30	AGP exp_1	4665	Alpha-1-acid glycoprotein 1	ENGTISR_6_5_1_3	22.1	5.1	0.0	0.0	100.0	27.9	29.9	0.0	3.2	0.0	0.0	0.0	11.6	0.0	Outer
31	AGP exp_45	9466	Alpha-1-acid glycoprotein 1	ENGTISR_6_5_2_3	0.0	4.9	0.0	0.0	100.0	60.7	73.8	0.0	0.0	0.0	0.0	0.0	54.2	11.4	Dual
32	AGP exp_231	10181	Alpha-1-acid glycoprotein 1	ENGTISR_6_5_2_3	5.0	6.5	0.0	0.0	100.0	59.3	63.3	0.0	0.0	0.0	0.0	0.0	39.5	8.8	Dual
33	AGP exp_29	7877	Alpha-1-acid glycoprotein 1	ENGTISR_6_5_2_3	0.0	9.7	0.0	0.0	99.4	66.4	100.0	0.0	0.0	0.0	0.0	0.0	34.2	8.8	Dual
34	AGP exp_21	9470	Alpha-1-acid glycoprotein 1	ENGTISR_6_5_2_3	12.6	9.9	0.0	0.0	100.0	70.7	94.8	0.0	0.0	0.0	0.0	0.0	54.7	16.3	Dual
35	AGP exp_8	8661	Alpha-1-acid glycoprotein 1	ENGTISR_6_5_2_3	0.0	9.1	0.0	0.0	100.0	58.3	90.8	0.0	8.3	0.0	0.0	0.0	43.8	12.5	Dual
36	AGP exp_25	10476	Alpha-1-acid glycoprotein 1	ENGTISR_6_5_2_3	7.0	12.5	0.0	0.0	100.0	64.5	78.5	0.0	0.0	0.0	13.5	0.0	45.3	14.7	Dual
37	AGP exp_33	13182	Alpha-1-acid glycoprotein 1	ENGTISR_6_5_2_3	5.9	12.0	0.0	0.0	100.0	76.3	75.5	2.5	5.4	0.0	0.0	0.0	38.9	7.6	Dual
38	AGP exp_42	9926	Alpha-1-acid glycoprotein 1	ENGTISR_6_5_2_3	5.5	10.0	0.0	0.0	100.0	67.1	77.4	0.0	0.0	0.0	0.0	0.0	38.8	8.5	Dual
39	AGP exp_19	9707	Alpha-1-acid glycoprotein 1	ENGTISR_6_5_2_3	5.4	9.6	0.0	0.0	100.0	67.1	87.0	0.0	0.0	0.0	0.0	0.0	48.9	9.1	Dual
40	AGP exp_17	9678	Alpha-1-acid glycoprotein 1	ENGTISR_6_5_2_3	9.0	5.7	0.0	0.0	100.0	63.7	79.4	0.0	5.7	0.0	0.0	0.0	35.3	11.6	Dual
41	AGP exp_18	9689	Alpha-1-acid glycoprotein 1	ENGTISR_6_5_2_3	7.8	11.7	0.0	0.0	100.0	67.4	91.9	0.0	0.0	0.0	0.0	0.0	40.8	15.8	Dual
42	AGP exp_38	9793	Alpha-1-acid glycoprotein 1	ENGTISR_6_5_2_3	5.8	9.2	0.0	0.0	100.0	63.2	78.2	0.0	0.0	0.0	0.0	0.0	38.6	14.7	Dual
43	AGP exp_26	10331	Alpha-1-acid glycoprotein 1	ENGTISR_6_5_2_3	9.0	11.5	0.0	0.0	100.0	48.7	79.4	0.0	9.4	0.0	0.0	0.0	38.7	8.4	Dual
44	AGP exp_31	6869	Alpha-1-acid glycoprotein 1	ENGTISR_6_5_2_3	6.1	11.7	0.0	0.0	100.0	58.2	79.3	0.0	4.3	0.0	0.0	0.0	38.6	8.6	Dual
45	AGP exp_37	10109	Alpha-1-acid glycoprotein 1	ENGTISR_6_5_2_3	0.0	0.0	0.0	0.0	100.0	47.3	30.2	21.7	0.0	0.0	0.0	0.0	5.4	12.0	Dual
46	AGP exp_13	11998	Alpha-1-acid glycoprotein 1	ENGTISR_6_5_2_3	0.0	0.0	0.0	0.0	100.0	34.5	52.5	0.0	0.0	0.0	0.0	0.0	17.4	12.2	Dual
47	AGP exp_1	4751	Alpha-1-acid glycoprotein 1	ENGTISR_6_5_2_3	0.0	8.1	0.0	0.0	100.0	48.5	30.7	0.0	0.0	0.0	0.0	0.0	14.8	0.0	Outer
48	AGP exp_9	8629	Alpha-1-acid glycoprotein 1	ENGTISR_6_5_2_3	0.0	8.2	0.0	0.0	100.0	59.1	75.1	2.2	2.0	0.0	0.0	0.0	33.9	7.9	Dual
49	AGP exp_33	8034	Alpha-1-acid glycoprotein 1	ENGTISR_6_5_2_3	0.0	0.0	0.0	0.0	100.0	35.9	26.7	17.3	0.0	0.0	0.0	0.0	11.5	4.8	Dual
50	AGP exp_12	10883	Alpha-1-acid glycoprotein 1	ENGTISR_6_5_2_3	6.2	6.0	0.0	0.0	100.0	62.4	60.2	0.0	0.0	0.0	0.0	0.0	31.1	10.0	Dual

Supplementary Table 1. Continued.

No.	Experiments	CID Scan No.	N-glycoproteins	N-glycopeptides	Relative intensities of B/Y series ions														Manual Classification
					B_2	B_2F	B_3	B_3F	B_2S	B_2SF	Y_1	Y_1F	Y_2	Y_2F	Y_3	Y_3F	Y_4	Y_4F	
51	AGP exp_26	10629	Alpha-1-acid glycoprotein 1	ENGTISR_6_5_2_3	10.0	0.0	0.0	0.0	100.0	30.9	32.6	18.1	0.0	0.0	0.0	0.0	3.1	6.4	Dual
52	AGP exp_42	10185	Alpha-1-acid glycoprotein 1	ENGTISR_6_5_2_3	6.4	6.2	0.0	0.0	100.0	42.4	22.2	9.1	0.0	0.0	0.0	0.0	5.8	9.5	Dual
53	AGP exp_16	12458	Alpha-1-acid glycoprotein 1	ENGTISR_6_5_2_3	8.8	6.5	0.0	0.0	100.0	29.8	23.2	13.6	0.0	0.0	0.0	0.0	0.0	0.0	Dual
54	AGP exp_43	9940	Alpha-1-acid glycoprotein 1	ENGTISR_6_5_2_3	0.0	0.0	0.0	0.0	100.0	50.0	37.5	22.8	0.0	0.0	0.0	0.0	15.1	17.8	Dual
55	AGP exp_20	10302	Alpha-1-acid glycoprotein 1	ENGTISR_6_5_2_3	11.4	13.3	10.2	0.0	100.0	62.1	55.2	18.8	5.6	0.0	0.0	0.0	16.3	11.1	Dual
56	AGP exp_15	12345	Alpha-1-acid glycoprotein 1	ENGTISR_6_5_2_3	0.0	6.1	0.0	0.0	100.0	36.9	21.8	15.0	0.0	0.0	0.0	0.0	3.1	8.0	Dual
57	AGP exp_2	4761	Alpha-1-acid glycoprotein 1	ENGTISR_6_5_2_3	9.8	9.7	0.0	0.0	100.0	54.0	71.3	0.0	0.0	0.0	0.0	0.0	36.3	6.7	Dual
58	AGP exp_2	4640	Alpha-1-acid glycoprotein 1	ENGTISR_7_6_0_3	60.8	0.0	2.9	0.0	100.0	0.0	14.0	0.0	0.0	0.0	0.0	0.0	3.7	0.0	None
59	AGP exp_2	2168	Alpha-1-acid glycoprotein 1	ENGTISR_7_6_1_1	100.0	35.8	0.0	0.0	62.5	39.2	31.0	0.0	0.0	0.0	0.0	0.0	0.0	0.0	Outer
60	AGP exp_1	3674	Alpha-1-acid glycoprotein 1	ENGTISR_7_6_1_2	20.4	11.8	0.0	0.0	100.0	38.6	71.2	0.0	0.0	0.0	0.0	0.0	0.0	0.0	Outer
61	AGP exp_2	3280	Alpha-1-acid glycoprotein 1	ENGTISR_7_6_1_2	9.9	14.5	0.0	0.0	100.0	13.5	41.5	0.0	0.0	0.0	0.0	0.0	0.0	0.0	Outer
62	AGP exp_2	3574	Alpha-1-acid glycoprotein 1	ENGTISR_7_6_1_2	30.5	21.4	0.0	0.0	100.0	40.9	97.6	0.0	0.0	0.0	0.0	0.0	0.0	0.0	Outer
63	AGP exp_2	2931	Alpha-1-acid glycoprotein 1	ENGTISR_7_6_1_2	32.0	19.6	0.0	0.0	100.0	32.3	85.8	0.0	0.0	0.0	0.0	0.0	0.0	0.0	Outer
64	AGP exp_1	3385	Alpha-1-acid glycoprotein 1	ENGTISR_7_6_1_2	30.7	20.7	0.0	0.0	100.0	39.1	79.7	0.0	0.0	0.0	0.0	0.0	0.0	0.0	Outer
65	AGP exp_1	2790	Alpha-1-acid glycoprotein 1	ENGTISR_7_6_1_2	24.0	16.5	0.0	0.0	100.0	22.5	59.4	0.0	0.0	0.0	0.0	0.0	0.0	0.0	Outer
66	AGP exp_2	3895	Alpha-1-acid glycoprotein 1	ENGTISR_7_6_1_2	34.5	0.0	0.0	0.0	100.0	14.2	22.4	6.1	0.0	0.0	0.0	0.0	0.0	0.0	Outer
67	AGP exp_1	4688	Alpha-1-acid glycoprotein 1	ENGTISR_7_6_1_3	100.0	5.4	0.0	0.0	91.0	31.1	21.4	0.0	0.0	0.0	0.0	0.0	5.7	0.0	Outer
68	AGP exp_2	4696	Alpha-1-acid glycoprotein 1	ENGTISR_7_6_1_3	65.4	7.1	4.6	0.0	100.0	26.6	20.0	0.0	0.0	0.0	0.0	0.0	6.0	0.0	Outer
69	AGP exp_1	6102	Alpha-1-acid glycoprotein 1	ENGTISR_7_6_1_4	26.9	3.3	0.0	0.0	100.0	27.4	9.3	0.0	0.0	0.0	0.0	0.0	4.3	0.0	Outer
70	AGP exp_2	6114	Alpha-1-acid glycoprotein 1	ENGTISR_7_6_1_4	30.4	4.0	0.0	0.0	100.0	27.4	10.4	0.0	0.0	0.0	0.0	0.0	3.5	0.0	Outer
71	AGP exp_1	5897	Alpha-1-acid glycoprotein 1	ENGTISR_7_6_2_4	39.8	0.0	0.0	0.0	100.0	65.2	9.1	0.0	0.0	0.0	0.0	0.0	4.3	0.0	Outer
72	AGP exp_2	18684	Alpha-1-acid glycoprotein 1	LVPVPITNATLDQITGK_4_4_0_1	75.6	0.0	0.0	0.0	100.0	0.0	62.2	15.9	0.0	0.0	0.0	0.0	74.8	0.0	None
73	AGP exp_2	18849	Alpha-1-acid glycoprotein 1	LVPVPITNATLDQITGK_5_4_0_1	92.0	0.0	0.0	0.0	100.0	0.0	67.1	0.0	32.1	0.0	26.9	0.0	60.4	0.0	None
74	AGP exp_2	18402	Alpha-1-acid glycoprotein 1	LVPVPITNATLDQITGK_5_4_0_1	71.0	0.0	0.0	0.0	46.8	0.0	54.3	0.0	0.0	0.0	9.3	0.0	100.0	0.0	None
75	AGP exp_1	18035	Alpha-1-acid glycoprotein 1	LVPVPITNATLDQITGK_5_4_0_1	63.5	0.0	0.0	0.0	55.8	0.0	61.1	0.0	4.1	0.0	13.0	0.0	100.0	0.0	None
76	AGP exp_1	21226	Alpha-1-acid glycoprotein 1	LVPVPITNATLDQITGK_5_4_0_2	0.0	0.0	0.0	0.0	100.0	0.0	74.6	0.0	8.6	0.0	10.3	0.0	39.5	0.0	None

Supplementary Table 1. Continued.

No.	Experiments	CID Scan No.	N-glycoproteins	N-glycopeptides	Relative intensities of B/Y series ions														Manual Classification
					B_2	B_2F	B_3	B_3F	B_2S	B_2SF	Y_1	Y_1F	Y_2	Y_2F	Y_3	Y_3F	Y_4	Y_4F	
77	AGP exp_2	21895	Alpha-1-acid glycoprotein 1	LVPVPITNATLDQITGK_5_4_0_2	0.0	0.0	0.0	0.0	100.0	0.0	62.3	0.0	11.6	0.0	9.5	0.0	27.1	0.0	None
78	AGP exp_1	20953	Alpha-1-acid glycoprotein 1	LVPVPITNATLDQITGK_5_4_0_2	0.0	0.0	0.0	0.0	100.0	0.0	80.8	0.0	12.2	0.0	12.0	0.0	33.5	0.0	None
79	AGP exp_2	22177	Alpha-1-acid glycoprotein 1	LVPVPITNATLDQITGK_5_4_0_2	0.0	0.0	0.0	0.0	100.0	0.0	74.0	0.0	10.2	0.0	13.8	0.0	34.9	0.0	None
80	AGP exp_2	22181	Alpha-1-acid glycoprotein 1	LVPVPITNATLDQITGK_5_4_0_2	56.3	0.0	7.0	0.0	100.0	0.0	3.3	0.0	10.4	0.0	15.4	0.0	88.1	0.0	None
81	AGP exp_1	21228	Alpha-1-acid glycoprotein 1	LVPVPITNATLDQITGK_5_4_0_2	64.9	0.0	5.1	0.0	100.0	0.0	2.7	0.0	8.6	0.0	13.6	0.0	65.5	0.0	None
82	AGP exp_1	17744	Alpha-1-acid glycoprotein 1	LVPVPITNATLDQITGK_6_5_0_1	100.0	0.0	6.6	0.0	47.4	0.0	38.9	0.0	9.6	0.0	21.4	0.0	58.0	0.0	None
83	AGP exp_2	18417	Alpha-1-acid glycoprotein 1	LVPVPITNATLDQITGK_6_5_0_1	100.0	0.0	9.6	0.0	47.3	0.0	51.5	0.0	0.0	0.0	12.7	0.0	49.2	0.0	None
84	AGP exp_2	21663	Alpha-1-acid glycoprotein 1	LVPVPITNATLDQITGK_6_5_0_2	0.0	0.0	0.0	0.0	97.9	0.0	100.0	0.0	10.0	0.0	19.9	0.0	72.0	0.0	None
85	AGP exp_2	21947	Alpha-1-acid glycoprotein 1	LVPVPITNATLDQITGK_6_5_0_2	0.0	0.0	0.0	0.0	100.0	0.0	98.1	0.0	12.7	0.0	24.1	0.0	81.9	0.0	None
86	AGP exp_2	24574	Alpha-1-acid glycoprotein 1	LVPVPITNATLDQITGK_6_5_0_2	40.4	0.0	5.8	0.0	100.0	0.0	46.4	0.0	1.9	0.0	8.1	0.0	46.4	0.0	None
87	AGP exp_1	21160	Alpha-1-acid glycoprotein 1	LVPVPITNATLDQITGK_6_5_0_2	65.7	0.0	7.9	0.0	100.0	0.0	37.4	0.0	3.1	0.0	14.9	0.0	49.4	0.0	None
88	AGP exp_1	20785	Alpha-1-acid glycoprotein 1	LVPVPITNATLDQITGK_6_5_0_2	72.1	0.0	7.3	0.0	100.0	0.0	54.1	0.0	4.9	0.0	16.5	0.0	50.5	0.0	None
89	AGP exp_1	23576	Alpha-1-acid glycoprotein 1	LVPVPITNATLDQITGK_6_5_0_2	80.0	0.0	8.1	0.0	100.0	0.0	49.6	0.0	3.7	0.0	22.2	0.0	66.3	0.0	None
90	AGP exp_2	22166	Alpha-1-acid glycoprotein 1	LVPVPITNATLDQITGK_6_5_0_2	77.5	0.0	8.2	0.0	100.0	0.0	56.9	0.0	0.0	0.0	8.8	0.0	56.1	0.0	None
91	AGP exp_2	24680	Alpha-1-acid glycoprotein 1	LVPVPITNATLDQITGK_6_5_0_3	0.0	0.0	0.0	0.0	100.0	0.0	62.3	0.0	7.4	0.0	8.4	0.0	24.7	0.0	None
92	AGP exp_1	23647	Alpha-1-acid glycoprotein 1	LVPVPITNATLDQITGK_6_5_0_3	30.6	0.0	3.6	0.0	100.0	0.0	40.0	0.0	5.6	0.0	8.0	0.0	37.9	0.0	None
93	AGP exp_1	23454	Alpha-1-acid glycoprotein 1	LVPVPITNATLDQITGK_6_5_0_3	0.0	0.0	0.0	0.0	100.0	0.0	69.4	0.0	7.6	0.0	10.6	0.0	22.3	0.0	None
94	AGP exp_2	24316	Alpha-1-acid glycoprotein 1	LVPVPITNATLDQITGK_6_5_0_3	32.2	0.0	3.5	0.0	100.0	0.0	37.8	0.0	4.9	0.0	7.7	0.0	28.9	0.0	None
95	AGP exp_1	23791	Alpha-1-acid glycoprotein 1	LVPVPITNATLDQITGK_6_5_0_3	33.7	0.0	3.2	0.0	100.0	0.0	39.9	0.0	8.5	0.0	9.0	0.0	40.8	0.0	None
96	AGP exp_1	23496	Alpha-1-acid glycoprotein 1	LVPVPITNATLDQITGK_6_5_0_3	32.5	0.0	3.2	0.0	100.0	0.0	40.1	0.0	5.8	0.0	7.9	0.0	35.0	0.0	None
97	AGP exp_2	24786	Alpha-1-acid glycoprotein 1	LVPVPITNATLDQITGK_6_5_0_3	36.5	0.0	2.4	0.0	100.0	0.0	28.2	0.0	4.7	0.0	4.8	0.0	38.6	0.0	None
98	AGP exp_2	18341	Alpha-1-acid glycoprotein 1	LVPVPITNATLDQITGK_6_5_1_1	0.0	14.5	0.0	0.0	58.6	40.2	100.0	0.0	13.2	0.0	13.4	0.0	58.3	0.0	Outer
99	AGP exp_1	17678	Alpha-1-acid glycoprotein 1	LVPVPITNATLDQITGK_6_5_1_1	0.0	19.3	0.0	0.0	59.8	43.0	100.0	0.0	0.0	0.0	11.1	0.0	34.6	0.0	Outer
100	AGP exp_2	18352	Alpha-1-acid glycoprotein 1	LVPVPITNATLDQITGK_6_5_1_1	100.0	18.9	0.0	0.0	55.5	25.7	44.2	0.0	0.0	0.0	12.8	0.0	43.1	0.0	Outer
101	AGP exp_1	20837	Alpha-1-acid glycoprotein 1	LVPVPITNATLDQITGK_6_5_1_2	0.0	10.7	0.0	0.0	100.0	58.0	89.2	0.0	0.0	0.0	10.2	0.0	32.5	0.0	Outer
102	AGP exp_2	21492	Alpha-1-acid glycoprotein 1	LVPVPITNATLDQITGK_6_5_1_2	0.0	20.5	0.0	0.0	100.0	12.2	64.8	0.0	0.0	0.0	0.0	0.0	10.4	0.0	Outer

Supplementary Table 1. Continued.

No.	Experiments	CID Scan No.	N-glycoproteins	N-glycopeptides	Relative intensities of B/Y series ions														Manual Classification
					B_2	B_2F	B_3	B_3F	B_2S	B_2SF	Y_1	Y_1F	Y_2	Y_2F	Y_3	Y_3F	Y_4	Y_4F	
103	AGP exp_2	21811	Alpha-1-acid glycoprotein 1	LVPVPITNATLDQITGK_6_5_1_2	0.0	11.3	0.0	0.0	100.0	53.5	84.0	0.0	8.6	0.0	0.0	0.0	29.1	0.0	Outer
104	AGP exp_2	21715	Alpha-1-acid glycoprotein 1	LVPVPITNATLDQITGK_6_5_1_2	71.5	13.5	5.7	0.0	100.0	35.3	54.1	0.0	8.9	0.0	16.1	0.0	56.1	1.4	Outer
105	AGP exp_1	20848	Alpha-1-acid glycoprotein 1	LVPVPITNATLDQITGK_6_5_1_2	75.0	15.1	7.8	0.0	100.0	36.2	58.1	0.0	6.2	0.0	17.7	0.0	66.9	0.0	Outer
106	AGP exp_1	20524	Alpha-1-acid glycoprotein 1	LVPVPITNATLDQITGK_6_5_1_2	46.7	11.4	0.0	0.0	100.0	10.9	36.2	0.0	5.1	0.0	0.0	0.0	26.8	0.0	Outer
107	AGP exp_1	23819	Alpha-1-acid glycoprotein 1	LVPVPITNATLDQITGK_6_5_1_3	0.0	7.0	0.0	0.0	100.0	27.3	34.8	0.0	0.0	0.0	0.0	0.0	16.2	0.0	Outer
108	AGP exp_2	24491	Alpha-1-acid glycoprotein 1	LVPVPITNATLDQITGK_6_5_1_3	0.0	0.0	0.0	0.0	100.0	33.8	72.5	0.0	7.2	0.0	12.7	0.0	28.2	0.0	Outer
109	AGP exp_2	24741	Alpha-1-acid glycoprotein 1	LVPVPITNATLDQITGK_6_5_1_3	0.0	0.0	0.0	0.0	100.0	34.9	43.7	0.0	5.8	0.0	9.3	0.0	30.1	0.0	Outer
110	AGP exp_1	23564	Alpha-1-acid glycoprotein 1	LVPVPITNATLDQITGK_6_5_1_3	0.0	0.0	0.0	0.0	100.0	39.0	62.9	0.0	0.0	0.0	7.7	0.0	30.2	0.0	Outer
111	AGP exp_1	23449	Alpha-1-acid glycoprotein 1	LVPVPITNATLDQITGK_6_5_1_3	30.5	6.2	3.4	0.0	100.0	30.2	39.7	0.0	6.5	0.0	10.8	0.0	43.4	0.0	Outer
112	AGP exp_2	24453	Alpha-1-acid glycoprotein 1	LVPVPITNATLDQITGK_6_5_1_3	52.4	10.2	7.4	0.0	100.0	49.7	82.0	0.0	9.8	0.0	22.9	0.0	68.9	0.0	Outer
113	AGP exp_2	24527	Alpha-1-acid glycoprotein 1	LVPVPITNATLDQITGK_6_5_1_3	30.9	1.8	3.5	0.0	100.0	26.2	43.9	0.0	5.9	0.0	5.7	0.0	36.0	0.0	Outer
114	AGP exp_1	23649	Alpha-1-acid glycoprotein 1	LVPVPITNATLDQITGK_6_5_1_3	26.7	6.0	3.7	0.0	100.0	18.4	36.7	0.0	2.5	0.0	10.0	3.6	30.2	0.0	Outer
115	AGP exp_2	24762	Alpha-1-acid glycoprotein 1	LVPVPITNATLDQITGK_6_5_1_3	22.0	0.0	0.0	0.0	100.0	29.0	21.6	0.0	0.0	0.0	7.5	0.0	47.7	0.0	Outer
116	AGP exp_19	35459	Alpha-1-acid glycoprotein 1	LVPVPITNATLDQITGK_6_5_2_2	0.0	25.5	0.0	0.0	100.0	44.8	54.6	0.0	0.0	0.0	0.0	0.0	16.0	9.8	Dual
117	AGP exp_11	29920	Alpha-1-acid glycoprotein 1	LVPVPITNATLDQITGK_6_5_2_3	21.7	5.9	4.8	0.0	100.0	21.3	11.0	13.4	0.0	0.0	1.5	3.0	6.1	22.4	Dual
118	AGP exp_1	1604	Alpha-1-acid glycoprotein 1,2	NEEYNK_4_3_0_0	33.4	0.0	12.2	0.0	3.8	0.0	100.0	0.0	4.8	0.0	10.4	0.0	74.9	0.0	None
119	AGP exp_2	1620	Alpha-1-acid glycoprotein 1,2	NEEYNK_4_3_0_0	38.5	0.0	6.2	0.0	0.0	0.0	100.0	0.0	3.9	0.0	4.3	0.0	50.8	0.0	None
120	AGP exp_2	1459	Alpha-1-acid glycoprotein 1,2	NEEYNK_4_3_0_1	29.9	0.0	9.7	0.0	23.2	0.0	37.5	0.0	11.3	0.0	14.9	0.0	100.0	0.0	None
121	AGP exp_2	1456	Alpha-1-acid glycoprotein 1,2	NEEYNK_4_3_0_1	27.6	0.0	0.0	0.0	19.9	0.0	41.5	0.0	18.3	0.0	18.4	0.0	100.0	0.0	None
122	AGP exp_2	1955	Alpha-1-acid glycoprotein 1,2	NEEYNK_4_3_0_1	15.7	0.0	4.4	0.0	33.9	0.0	100.0	0.0	5.2	0.0	6.1	0.0	69.0	0.0	None
123	AGP exp_1	1707	Alpha-1-acid glycoprotein 1,2	NEEYNK_4_3_0_1	27.0	0.0	0.0	0.0	41.8	0.0	100.0	0.0	7.3	0.0	2.9	0.0	58.1	0.0	None
124	AGP exp_1	1461	Alpha-1-acid glycoprotein 1,2	NEEYNK_4_3_0_1	28.1	0.0	8.9	0.0	19.1	0.0	36.8	0.0	12.4	0.0	11.4	0.0	100.0	0.0	None
125	AGP exp_2	1582	Alpha-1-acid glycoprotein 1,2	NEEYNK_4_4_0_1	29.1	0.0	0.0	0.0	39.4	0.0	100.0	0.0	23.0	0.0	42.0	0.0	72.7	0.0	None
126	AGP exp_2	1774	Alpha-1-acid glycoprotein 1,2	NEEYNK_4_4_0_1	15.2	0.0	5.0	0.0	36.1	0.0	100.0	0.0	13.3	0.0	8.0	0.0	49.7	0.0	None
127	AGP exp_1	1730	Alpha-1-acid glycoprotein 1,2	NEEYNK_4_4_0_1	14.8	0.0	5.8	0.0	28.7	0.0	100.0	0.0	20.2	0.0	16.4	0.0	46.0	0.0	None
128	AGP exp_1	1571	Alpha-1-acid glycoprotein 1,2	NEEYNK_4_4_0_1	23.7	0.0	0.0	0.0	73.2	0.0	100.0	0.0	57.9	0.0	72.5	0.0	64.2	0.0	None

Supplementary Table 1. Continued.

No.	Experiments	CID Scan No.	N-glycoproteins	N-glycopeptides	Relative intensities of B/Y series ions														Manual Classification
					B_2	B_2F	B_3	B_3F	B_2S	B_2SF	Y_1	Y_1F	Y_2	Y_2F	Y_3	Y_3F	Y_4	Y_4F	
129	AGP exp_1	1598	Alpha-1-acid glycoprotein 1,2	NEEYNK_4_4_0_1	100.0	0.0	29.5	0.0	97.2	0.0	57.6	0.0	0.0	0.0	0.0	0.0	42.4	0.0	None
130	AGP exp_1	1432	Alpha-1-acid glycoprotein 1,2	NEEYNK_5_4_0_0	80.1	0.0	8.3	0.0	0.0	0.0	100.0	0.0	0.0	0.0	9.1	0.0	34.8	0.0	None
131	AGP exp_2	1404	Alpha-1-acid glycoprotein 1,2	NEEYNK_5_4_0_0	71.5	0.0	14.4	0.0	0.0	0.0	100.0	0.0	0.0	0.0	13.2	0.0	60.2	0.0	None
132	AGP exp_2	1622	Alpha-1-acid glycoprotein 1,2	NEEYNK_5_4_0_0	57.6	0.0	14.0	0.0	0.0	0.0	100.0	0.0	0.0	0.0	8.2	0.0	28.4	0.0	None
133	AGP exp_2	1871	Alpha-1-acid glycoprotein 1,2	NEEYNK_5_4_0_0	61.7	0.0	25.7	0.0	0.0	0.0	100.0	0.0	0.0	0.0	10.7	0.0	37.9	0.0	None
134	AGP exp_2	1963	Alpha-1-acid glycoprotein 1,2	NEEYNK_5_4_0_1	100.0	0.0	8.0	0.0	57.0	0.0	44.3	0.0	0.0	0.0	0.0	0.0	28.3	0.0	None
135	AGP exp_2	1960	Alpha-1-acid glycoprotein 1,2	NEEYNK_5_4_0_1	0.0	0.0	3.3	0.0	19.8	0.0	100.0	0.0	14.8	0.0	5.4	0.0	31.0	0.0	None
136	AGP exp_2	1776	Alpha-1-acid glycoprotein 1,2	NEEYNK_5_4_0_1	100.0	0.0	7.0	0.0	57.0	0.0	46.6	0.0	3.5	0.0	4.4	0.0	31.8	0.0	None
137	AGP exp_2	1772	Alpha-1-acid glycoprotein 1,2	NEEYNK_5_4_0_1	0.0	0.0	6.0	0.0	29.2	0.0	100.0	0.0	21.5	0.0	11.9	0.0	42.9	0.0	None
138	AGP exp_1	1942	Alpha-1-acid glycoprotein 1,2	NEEYNK_5_4_0_1	100.0	0.0	7.4	0.0	53.5	0.0	41.4	0.0	0.0	0.0	0.0	0.0	28.8	0.0	None
139	AGP exp_1	1717	Alpha-1-acid glycoprotein 1,2	NEEYNK_5_4_0_1	0.0	0.0	4.0	0.0	20.3	0.0	100.0	0.0	23.1	0.0	9.9	0.0	31.8	0.0	None
140	AGP exp_2	2848	Alpha-1-acid glycoprotein 1,2	NEEYNK_5_4_0_1	0.0	0.0	7.1	0.0	37.8	0.0	100.0	0.0	14.8	0.0	10.7	0.0	40.0	0.0	None
141	AGP exp_1	2822	Alpha-1-acid glycoprotein 1,2	NEEYNK_5_4_0_1	0.0	0.0	4.4	0.0	31.9	0.0	100.0	0.0	14.9	0.0	9.1	0.0	23.7	0.0	None
142	AGP exp_1	1937	Alpha-1-acid glycoprotein 1,2	NEEYNK_5_4_0_1	0.0	0.0	4.7	0.0	27.1	0.0	100.0	0.0	13.1	0.0	8.6	0.0	38.3	0.0	None
143	AGP exp_1	2122	Alpha-1-acid glycoprotein 1,2	NEEYNK_5_4_0_1	0.0	0.0	9.7	0.0	36.5	0.0	100.0	0.0	12.6	0.0	4.6	0.0	22.9	0.0	None
144	AGP exp_2	2130	Alpha-1-acid glycoprotein 1,2	NEEYNK_5_4_0_1	0.0	0.0	10.2	0.0	59.3	0.0	100.0	0.0	20.8	0.0	7.4	0.0	40.6	0.0	None
145	AGP exp_2	2280	Alpha-1-acid glycoprotein 1,2	NEEYNK_5_4_0_1	0.0	0.0	0.0	0.0	59.8	0.0	100.0	0.0	13.3	0.0	5.9	0.0	17.7	0.0	None
146	AGP exp_1	2709	Alpha-1-acid glycoprotein 1,2	NEEYNK_5_4_0_2	59.5	0.0	6.0	0.0	100.0	0.0	0.0	0.0	7.1	0.0	8.3	0.0	44.4	0.0	None
147	AGP exp_2	3443	Alpha-1-acid glycoprotein 1,2	NEEYNK_5_4_0_2	49.9	0.0	4.6	0.0	100.0	0.0	0.0	0.0	0.0	0.0	6.2	0.0	34.9	0.0	None
148	AGP exp_2	2710	Alpha-1-acid glycoprotein 1,2	NEEYNK_5_4_0_2	61.0	0.0	6.3	0.0	100.0	0.0	0.0	0.0	5.1	0.0	8.6	0.0	42.5	0.0	None
149	AGP exp_1	3310	Alpha-1-acid glycoprotein 1,2	NEEYNK_5_4_0_2	51.9	0.0	4.9	0.0	100.0	0.0	0.0	0.0	0.0	0.0	4.9	0.0	32.0	0.0	None
150	AGP exp_2	2806	Alpha-1-acid glycoprotein 1,2	NEEYNK_5_4_0_2	61.2	0.0	6.1	0.0	100.0	0.0	0.0	0.0	5.1	0.0	8.2	0.0	42.8	0.0	None
151	AGP exp_1	3042	Alpha-1-acid glycoprotein 1,2	NEEYNK_5_4_0_2	59.7	0.0	5.0	0.0	100.0	0.0	0.0	0.0	2.7	0.0	6.6	0.0	42.4	0.0	None
152	AGP exp_2	3146	Alpha-1-acid glycoprotein 1,2	NEEYNK_5_4_0_2	57.3	0.0	6.3	0.0	100.0	0.0	0.0	0.0	2.8	0.0	6.0	0.0	41.2	0.0	None
153	AGP exp_2	2545	Alpha-1-acid glycoprotein 1,2	NEEYNK_5_4_0_2	0.0	0.0	0.0	0.0	50.8	0.0	100.0	0.0	11.3	0.0	8.3	0.0	29.3	0.0	None
154	AGP exp_2	3159	Alpha-1-acid glycoprotein 1,2	NEEYNK_5_4_0_2	0.0	0.0	5.7	0.0	49.8	0.0	100.0	0.0	23.3	0.0	9.6	0.0	30.4	0.0	None

Supplementary Table 1. Continued.

No.	Experiments	CID Scan No.	N-glycoproteins	N-glycopeptides	Relative intensities of B/Y series ions														Manual Classification
					B_2	B_2F	B_3	B_3F	B_2S	B_2SF	Y_1	Y_1F	Y_2	Y_2F	Y_3	Y_3F	Y_4	Y_4F	
155	AGP exp_2	2710	Alpha-1-acid glycoprotein 1,2	NEEYNK_5_4_0_2	61.0	0.0	6.3	0.0	100.0	0.0	0.0	0.0	5.1	0.0	8.6	0.0	42.5	0.0	None
156	AGP exp_1	3115	Alpha-1-acid glycoprotein 1,2	NEEYNK_5_4_0_2	0.0	0.0	4.2	0.0	45.8	0.0	100.0	0.0	19.6	0.0	11.6	0.0	38.3	0.0	None
157	AGP exp_2	4501	Alpha-1-acid glycoprotein 1,2	NEEYNK_5_4_0_2	0.0	0.0	8.5	0.0	91.5	0.0	100.0	0.0	11.9	0.0	7.8	0.0	37.7	0.0	None
158	AGP exp_2	3738	Alpha-1-acid glycoprotein 1,2	NEEYNK_5_4_0_2	63.6	0.0	3.3	0.0	100.0	0.0	0.0	0.0	0.0	0.0	2.1	0.0	26.8	0.0	None
159	AGP exp_2	3470	Alpha-1-acid glycoprotein 1,2	NEEYNK_5_4_0_2	0.0	0.0	4.0	0.0	54.0	0.0	100.0	0.0	16.6	0.0	10.0	0.0	37.8	0.0	None
160	AGP exp_1	3387	Alpha-1-acid glycoprotein 1,2	NEEYNK_5_4_0_2	0.0	0.0	0.0	0.0	96.8	0.0	100.0	0.0	25.3	0.0	8.1	0.0	38.7	0.0	None
161	AGP exp_2	3794	Alpha-1-acid glycoprotein 1,2	NEEYNK_5_4_0_2	0.0	0.0	0.0	0.0	99.2	8.9	100.0	0.0	14.1	0.0	7.1	0.0	34.2	0.0	None
162	AGP exp_1	2977	Alpha-1-acid glycoprotein 1,2	NEEYNK_5_4_1_2	58.6	0.0	5.3	0.0	100.0	5.1	9.0	19.1	0.0	0.0	0.0	0.0	8.7	18.1	Core
163	AGP exp_2	3119	Alpha-1-acid glycoprotein 1,2	NEEYNK_5_4_1_2	33.3	0.0	0.0	0.0	100.0	0.0	12.5	25.4	0.0	0.0	0.0	0.0	4.2	22.4	Core
164	AGP exp_1	2420	Alpha-1-acid glycoprotein 1,2	NEEYNK_5_4_1_2	87.4	0.0	0.0	0.0	100.0	41.8	57.7	0.0	0.0	0.0	0.0	0.0	14.3	0.0	Outer
165	AGP exp_2	2514	Alpha-1-acid glycoprotein 1,2	NEEYNK_5_4_1_2	61.9	0.0	0.0	0.0	100.0	27.1	47.4	0.0	0.0	0.0	0.0	0.0	14.0	0.0	Outer
166	AGP exp_1	2651	Alpha-1-acid glycoprotein 1,2	NEEYNK_5_4_1_2	57.2	4.2	5.7	0.0	100.0	25.4	44.4	0.0	0.0	0.0	1.9	0.0	18.4	4.3	Outer
167	AGP exp_2	2787	Alpha-1-acid glycoprotein 1,2	NEEYNK_5_4_1_2	100.0	7.8	8.3	0.0	71.6	35.4	46.2	0.0	0.0	0.0	3.3	0.0	23.5	0.0	Outer
168	AGP exp_19	4351	Alpha-1-acid glycoprotein 1,2	NEEYNK_5_4_2_2	42.8	14.2	14.3	0.0	100.0	43.8	47.4	12.6	0.0	0.0	0.0	0.0	9.3	17.1	Dual
169	AGP exp_9	4179	Alpha-1-acid glycoprotein 1,2	NEEYNK_5_4_2_2	57.4	0.0	10.1	0.0	100.0	32.7	49.4	10.6	0.0	0.0	0.0	0.0	0.0	0.0	Dual
170	AGP exp_36	4733	Alpha-1-acid glycoprotein 1,2	NEEYNK_5_4_2_2	67.9	0.0	10.8	0.0	100.0	29.5	9.0	20.2	0.0	0.0	0.0	0.0	0.0	0.0	Dual
171	AGP exp_22	4457	Alpha-1-acid glycoprotein 1,2	NEEYNK_5_4_2_2	54.5	16.6	0.0	0.0	100.0	33.1	26.4	30.4	0.0	0.0	0.0	0.0	6.3	10.5	Dual
172	AGP exp_13	4971	Alpha-1-acid glycoprotein 1,2	NEEYNK_5_4_2_2	67.5	0.0	0.0	0.0	100.0	17.9	12.8	13.1	0.0	0.0	0.0	0.0	0.0	0.0	Dual
173	AGP exp_15	4698	Alpha-1-acid glycoprotein 1,2	NEEYNK_5_4_2_2	50.8	13.5	0.0	0.0	100.0	27.6	11.9	18.3	0.0	0.0	0.0	0.0	5.7	16.7	Dual
174	AGP exp_20	4497	Alpha-1-acid glycoprotein 1,2	NEEYNK_5_4_2_2	46.0	0.0	0.0	0.0	100.0	16.2	25.2	18.2	0.0	0.0	0.0	0.0	3.9	0.0	Dual
175	AGP exp_23	4556	Alpha-1-acid glycoprotein 1,2	NEEYNK_5_4_2_2	59.9	15.5	0.0	0.0	100.0	30.4	36.7	18.5	0.0	0.0	3.6	0.0	0.0	0.0	Dual
176	AGP exp_1	3630	Alpha-1-acid glycoprotein 1,2	NEEYNK_5_5_0_2	71.0	0.0	7.0	0.0	100.0	0.0	63.8	0.0	0.0	0.0	10.8	0.0	40.0	0.0	None
177	AGP exp_2	2140	Alpha-1-acid glycoprotein 1,2	NEEYNK_6_5_0_1	84.1	0.0	8.0	0.0	42.7	0.0	100.0	0.0	0.0	0.0	9.1	0.0	31.5	0.0	None
178	AGP exp_2	1970	Alpha-1-acid glycoprotein 1,2	NEEYNK_6_5_0_1	100.0	0.0	7.2	0.0	40.7	0.0	78.7	0.0	3.3	0.0	8.4	0.0	27.3	0.0	None
179	AGP exp_1	1919	Alpha-1-acid glycoprotein 1,2	NEEYNK_6_5_0_1	100.0	0.0	9.2	0.0	45.9	0.0	42.0	0.0	0.0	0.0	4.0	0.0	17.5	0.0	None
180	AGP exp_2	2289	Alpha-1-acid glycoprotein 1,2	NEEYNK_6_5_0_1	100.0	0.0	8.0	0.0	49.2	0.0	65.1	0.0	0.0	0.0	3.0	0.0	22.3	0.0	None

Supplementary Table 1. Continued.

No.	Experiments	CID Scan No.	N-glycoproteins	N-glycopeptides	Relative intensities of B/Y series ions														Manual Classification
					B_2	B_2F	B_3	B_3F	B_2S	B_2SF	Y_1	Y_1F	Y_2	Y_2F	Y_3	Y_3F	Y_4	Y_4F	
181	AGP exp_1	2100	Alpha-1-acid glycoprotein 1,2	NEEYNK_6_5_0_1	89.7	0.0	4.8	0.0	44.4	0.0	100.0	0.0	2.5	0.0	3.8	0.0	27.6	0.0	None
182	AGP exp_1	2242	Alpha-1-acid glycoprotein 1,2	NEEYNK_6_5_0_1	100.0	0.0	7.2	0.0	32.6	0.0	53.4	0.0	0.0	0.0	0.0	0.0	12.8	0.0	None
183	AGP exp_2	1967	Alpha-1-acid glycoprotein 1,2	NEEYNK_6_5_0_1	100.0	0.0	5.8	0.0	33.9	0.0	33.1	0.0	2.5	0.0	1.9	0.0	20.3	0.0	None
184	AGP exp_2	2204	Alpha-1-acid glycoprotein 1,2	NEEYNK_6_5_0_1	0.0	0.0	18.8	0.0	23.2	0.0	100.0	0.0	27.0	0.0	13.6	0.0	10.1	0.0	None
185	AGP exp_1	2159	Alpha-1-acid glycoprotein 1,2	NEEYNK_6_5_0_1	0.0	0.0	0.0	0.0	21.0	0.0	100.0	0.0	17.9	0.0	16.2	0.0	14.1	0.0	None
186	AGP exp_1	1703	Alpha-1-acid glycoprotein 1,2	NEEYNK_6_5_0_1	100.0	0.0	0.0	0.0	56.6	0.0	44.1	0.0	0.0	0.0	3.6	0.0	4.5	0.0	None
187	AGP exp_2	3251	Alpha-1-acid glycoprotein 1,2	NEEYNK_6_5_0_2	69.5	0.0	0.0	0.0	89.2	0.0	100.0	0.0	8.7	0.0	13.5	0.0	41.9	0.0	None
188	AGP exp_1	3403	Alpha-1-acid glycoprotein 1,2	NEEYNK_6_5_0_2	73.0	0.0	6.0	0.0	86.9	0.0	100.0	0.0	8.0	0.0	10.7	0.0	37.4	0.0	None
189	AGP exp_2	3713	Alpha-1-acid glycoprotein 1,2	NEEYNK_6_5_0_2	0.0	0.0	0.0	0.0	38.7	0.0	100.0	0.0	21.2	0.0	10.8	0.0	20.9	0.0	None
190	AGP exp_2	3572	Alpha-1-acid glycoprotein 1,2	NEEYNK_6_5_0_2	71.1	0.0	6.5	0.0	100.0	0.0	87.8	0.0	5.8	0.0	7.2	0.0	42.3	0.0	None
191	AGP exp_1	3672	Alpha-1-acid glycoprotein 1,2	NEEYNK_6_5_0_2	48.6	0.0	4.4	0.0	70.9	0.0	100.0	0.0	7.1	0.0	8.7	0.0	31.1	0.0	None
192	AGP exp_1	3157	Alpha-1-acid glycoprotein 1,2	NEEYNK_6_5_0_2	54.6	0.0	4.6	0.0	65.0	0.0	100.0	0.0	6.4	0.0	10.2	0.0	31.6	0.0	None
193	AGP exp_1	2840	Alpha-1-acid glycoprotein 1,2	NEEYNK_6_5_0_2	68.5	0.0	6.2	0.0	100.0	0.0	84.3	0.0	5.9	0.0	5.1	0.0	29.7	0.0	None
194	AGP exp_2	3880	Alpha-1-acid glycoprotein 1,2	NEEYNK_6_5_0_2	56.8	0.0	4.9	0.0	100.0	0.0	80.2	0.0	4.3	0.0	7.1	0.0	31.9	0.0	None
195	AGP exp_2	2944	Alpha-1-acid glycoprotein 1,2	NEEYNK_6_5_0_2	69.4	0.0	0.0	0.0	96.5	0.0	100.0	0.0	0.0	0.0	6.0	0.0	22.9	0.0	None
196	AGP exp_2	3063	Alpha-1-acid glycoprotein 1,2	NEEYNK_6_5_0_2	0.0	0.0	0.0	0.0	33.1	0.0	100.0	0.0	19.8	0.0	11.4	0.0	23.4	0.0	None
197	AGP exp_1	3976	Alpha-1-acid glycoprotein 1,2	NEEYNK_6_5_0_2	69.8	0.0	0.0	0.0	100.0	0.0	52.9	0.0	2.3	0.0	0.0	0.0	12.8	0.0	None
198	AGP exp_2	4584	Alpha-1-acid glycoprotein 1,2	NEEYNK_6_5_0_3	22.9	0.0	3.3	0.0	78.8	0.0	100.0	0.0	7.1	0.0	11.1	0.0	40.7	0.0	None
199	AGP exp_1	4491	Alpha-1-acid glycoprotein 1,2	NEEYNK_6_5_0_3	29.0	0.0	4.2	0.0	100.0	0.0	89.0	0.0	7.6	0.0	12.5	0.0	42.7	0.0	None
200	AGP exp_2	2086	Alpha-1-acid glycoprotein 1,2	NEEYNK_6_5_1_1	100.0	26.6	9.1	0.0	38.4	14.3	90.4	0.0	0.0	0.0	6.4	0.0	26.6	0.0	Outer
201	AGP exp_1	2075	Alpha-1-acid glycoprotein 1,2	NEEYNK_6_5_1_1	88.2	16.0	6.5	0.0	33.3	12.9	100.0	0.0	0.0	0.0	9.2	0.0	28.6	0.0	Outer
202	AGP exp_1	1898	Alpha-1-acid glycoprotein 1,2	NEEYNK_6_5_1_1	100.0	19.2	0.0	0.0	39.8	0.0	60.2	0.0	0.0	0.0	9.5	0.0	24.9	0.0	Outer
203	AGP exp_1	1677	Alpha-1-acid glycoprotein 1,2	NEEYNK_6_5_1_1	100.0	18.8	0.0	0.0	35.8	0.0	48.9	0.0	0.0	0.0	0.0	0.0	8.6	0.0	Outer
204	AGP exp_2	1705	Alpha-1-acid glycoprotein 1,2	NEEYNK_6_5_1_1	100.0	27.1	0.0	0.0	64.8	0.0	73.4	0.0	0.0	0.0	0.0	0.0	12.8	0.0	Outer
205	AGP exp_1	2780	Alpha-1-acid glycoprotein 1,2	NEEYNK_6_5_1_2	50.0	14.1	0.0	0.0	68.3	22.1	100.0	0.0	0.0	0.0	11.8	0.0	39.3	0.0	Outer
206	AGP exp_1	2462	Alpha-1-acid glycoprotein 1,2	NEEYNK_6_5_1_2	70.2	35.5	0.0	0.0	100.0	0.0	84.7	0.0	0.0	0.0	0.0	0.0	26.3	0.0	Outer

Supplementary Table 1. Continued.

No.	Experiments	CID Scan No.	N-glycoproteins	N-glycopeptides	Relative intensities of B/Y series ions														Manual Classification
					B_2	B_2F	B_3	B_3F	B_2S	B_2SF	Y_1	Y_1F	Y_2	Y_2F	Y_3	Y_3F	Y_4	Y_4F	
207	AGP exp_1	3578	Alpha-1-acid glycoprotein 1,2	NEEYNK_6_5_1_2	52.7	12.8	4.3	0.0	80.0	31.6	100.0	4.0	0.0	0.0	8.9	0.0	35.3	5.0	Outer
208	AGP exp_2	2852	Alpha-1-acid glycoprotein 1,2	NEEYNK_6_5_1_2	47.5	14.2	0.0	0.0	81.8	21.9	100.0	0.0	0.0	0.0	7.9	0.0	37.8	0.0	Outer
209	AGP exp_2	3164	Alpha-1-acid glycoprotein 1,2	NEEYNK_6_5_1_2	54.1	10.4	5.6	0.0	76.8	20.0	100.0	0.0	0.0	0.0	2.8	0.0	26.2	0.0	Outer
210	AGP exp_1	3070	Alpha-1-acid glycoprotein 1,2	NEEYNK_6_5_1_2	34.6	9.2	0.0	0.0	100.0	26.0	95.8	0.0	0.0	0.0	0.0	0.0	22.3	0.0	Outer
211	AGP exp_2	3788	Alpha-1-acid glycoprotein 1,2	NEEYNK_6_5_1_2	66.4	0.0	0.0	0.0	100.0	29.5	83.7	0.0	0.0	0.0	3.4	0.0	18.8	0.0	Outer
212	AGP exp_1	4387	Alpha-1-acid glycoprotein 1,2	NEEYNK_6_5_1_3	15.5	5.7	3.2	0.0	91.1	30.6	100.0	0.0	0.0	0.0	12.7	0.0	41.6	2.4	Outer
213	AGP exp_2	4474	Alpha-1-acid glycoprotein 1,2	NEEYNK_6_5_1_3	13.8	4.5	0.0	0.0	72.2	24.6	100.0	0.0	0.0	0.0	10.1	0.0	34.6	0.0	Outer
214	AGP exp_25	3991	Alpha-1-acid glycoprotein 1,2	NEEYNK_6_5_2_2	42.6	31.4	0.0	0.0	74.7	48.0	100.0	0.0	6.3	0.0	0.0	0.0	29.2	9.9	Dual
215	AGP exp_34	3654	Alpha-1-acid glycoprotein 1,2	NEEYNK_6_5_2_2	65.2	14.9	0.0	0.0	100.0	19.9	43.5	12.9	0.0	0.0	0.0	0.0	11.1	11.1	Dual
216	AGP exp_27	4318	Alpha-1-acid glycoprotein 1,2	NEEYNK_6_5_2_2	64.8	23.0	0.0	0.0	100.0	47.3	44.7	13.9	0.0	0.0	0.0	0.0	12.1	12.0	Dual
217	AGP exp_45	5124	Alpha-1-acid glycoprotein 1,2	NEEYNK_6_5_2_2	72.9	0.0	0.0	0.0	100.0	21.4	16.1	12.0	0.0	0.0	0.0	0.0	0.0	0.0	Dual
218	AGP exp_9	3879	Alpha-1-acid glycoprotein 1,2	NEEYNK_6_5_2_2	54.5	28.1	0.0	0.0	100.0	21.7	30.6	17.8	3.6	0.0	0.0	0.0	0.0	0.0	Dual
219	AGP exp_28	4570	Alpha-1-acid glycoprotein 1,2	NEEYNK_6_5_2_2	56.5	0.0	0.0	0.0	100.0	28.1	26.4	28.4	0.0	0.0	0.0	0.0	0.0	0.0	Dual
220	AGP exp_37	5184	Alpha-1-acid glycoprotein 1,2	NEEYNK_6_5_2_2	38.8	0.0	8.7	0.0	100.0	30.8	42.5	24.9	0.0	0.0	0.0	0.0	0.0	0.0	Dual
221	AGP exp_37	8937	Alpha-1-acid glycoprotein 1,2	NEEYNK_6_5_2_3	0.0	8.6	0.0	0.0	100.0	59.7	86.9	11.6	8.2	0.0	7.4	0.0	33.3	16.3	Dual
222	AGP exp_8	7429	Alpha-1-acid glycoprotein 1,2	NEEYNK_6_5_2_3	0.0	8.2	0.0	0.0	100.0	48.7	70.0	18.6	0.0	0.0	8.6	0.0	27.9	19.2	Dual
223	AGP exp_44	7860	Alpha-1-acid glycoprotein 1,2	NEEYNK_6_5_2_3	0.0	9.1	0.0	0.0	100.0	55.4	80.0	16.7	1.9	0.0	7.7	0.0	38.6	23.3	Dual
224	AGP exp_7	5921	Alpha-1-acid glycoprotein 1,2	NEEYNK_6_5_2_3	2.3	8.3	4.5	0.0	100.0	40.8	61.2	25.5	0.0	0.0	7.1	0.0	27.9	21.3	Dual
225	AGP exp_20	8454	Alpha-1-acid glycoprotein 1,2	NEEYNK_6_5_2_3	0.0	8.5	0.0	0.0	100.0	48.1	65.8	19.3	0.0	0.0	10.9	0.0	34.8	19.0	Dual
226	AGP exp_1	4426	Alpha-1-acid glycoprotein 1,2	NEEYNK_6_5_2_3	0.0	9.5	0.0	0.0	100.0	48.9	91.1	2.7	6.9	0.0	2.7	0.0	39.4	8.5	Dual
227	AGP exp_34	6967	Alpha-1-acid glycoprotein 1,2	NEEYNK_6_5_2_3	0.0	9.8	5.0	0.0	100.0	57.1	90.2	7.9	0.0	0.0	9.6	0.0	35.7	13.0	Dual
228	AGP exp_36	8720	Alpha-1-acid glycoprotein 1,2	NEEYNK_6_5_2_3	0.0	11.7	0.0	0.0	100.0	52.7	76.0	22.3	0.0	0.0	9.8	0.0	37.6	18.0	Dual
229	AGP exp_33	6963	Alpha-1-acid glycoprotein 1,2	NEEYNK_6_5_2_3	0.0	11.9	0.0	0.0	100.0	53.3	80.3	4.4	0.0	0.0	11.1	0.0	35.2	11.4	Dual
230	AGP exp_17	8293	Alpha-1-acid glycoprotein 1,2	NEEYNK_6_5_2_3	0.0	8.0	0.0	0.0	100.0	46.6	73.5	11.8	0.0	0.0	6.0	0.0	31.5	13.3	Dual
231	AGP exp_9	7529	Alpha-1-acid glycoprotein 1,2	NEEYNK_6_5_2_3	0.0	10.8	0.0	0.0	100.0	42.8	73.2	11.8	8.5	0.0	8.4	0.0	27.2	15.3	Dual
232	AGP exp_42	8943	Alpha-1-acid glycoprotein 1,2	NEEYNK_6_5_2_3	0.0	7.2	0.0	0.0	100.0	44.5	98.2	11.1	0.0	0.0	3.6	0.0	29.1	13.4	Dual

Supplementary Table 1. Continued.

No.	Experiments	CID Scan No.	N-glycoproteins	N-glycopeptides	Relative intensities of B/Y series ions														Manual Classification
					B_2	B_2F	B_3	B_3F	B_2S	B_2SF	Y_1	Y_1F	Y_2	Y_2F	Y_3	Y_3F	Y_4	Y_4F	
233	AGP exp_28	8807	Alpha-1-acid glycoprotein 1,2	NEEYNK_6_5_2_3	0.0	8.1	0.0	0.0	100.0	43.7	81.6	8.3	0.0	0.0	5.8	0.0	27.5	7.1	Dual
234	AGP exp_2	4447	Alpha-1-acid glycoprotein 1,2	NEEYNK_6_5_2_3	0.0	6.1	6.5	0.0	100.0	51.9	83.0	0.0	0.0	0.0	4.8	0.0	20.3	4.8	Dual
235	AGP exp_28	8651	Alpha-1-acid glycoprotein 1,2	NEEYNK_6_5_2_3	0.0	4.5	0.0	0.0	100.0	35.0	38.9	12.2	0.0	0.0	0.0	0.0	24.4	4.9	Dual
236	AGP exp_15	9766	Alpha-1-acid glycoprotein 1,2	NEEYNK_6_5_2_3	0.0	0.0	0.0	0.0	100.0	22.3	39.5	15.8	3.5	0.0	2.0	0.0	7.9	9.2	Dual
237	AGP exp_14	9919	Alpha-1-acid glycoprotein 1,2	NEEYNK_6_5_2_3	0.0	0.0	3.4	0.0	100.0	20.9	41.1	14.7	1.6	0.0	2.5	2.2	8.5	12.6	Dual
238	AGP exp_16	9944	Alpha-1-acid glycoprotein 1,2	NEEYNK_6_5_2_3	5.0	6.7	0.0	0.0	100.0	15.7	41.1	14.1	4.3	0.0	2.6	0.0	11.3	0.0	Dual
239	AGP exp_1	4393	Alpha-1-acid glycoprotein 1,2	NEEYNK_6_5_3_1	66.4	20.2	5.7	0.0	95.8	25.5	100.0	0.0	14.9	0.0	2.2	0.0	27.8	0.0	Outer
240	AGP exp_1	25092	Alpha-1-acid glycoprotein 1,2	SVQEIQATFFYFTPNK_6_5_0_3	0.0	0.0	5.9	0.0	100.0	0.0	15.7	0.0	0.0	0.0	4.6	0.0	10.7	0.0	None
241	AGP exp_1	2081	Alpha-1-acid glycoprotein 2	ENGTVSR_5_4_0_2	84.5	0.0	7.1	0.0	100.0	0.0	21.4	0.0	0.0	0.0	0.0	0.0	13.9	0.0	None
242	AGP exp_2	1631	Alpha-1-acid glycoprotein 2	ENGTVSR_6_5_0_1	100.0	0.0	7.5	0.0	35.0	0.0	43.7	0.0	0.0	0.0	0.0	0.0	14.5	0.0	None
243	AGP exp_1	2325	Alpha-1-acid glycoprotein 2	ENGTVSR_6_5_0_2	79.0	0.0	0.0	0.0	100.0	0.0	52.1	0.0	4.7	0.0	0.0	0.0	44.9	0.0	None
244	AGP exp_2	2363	Alpha-1-acid glycoprotein 2	ENGTVSR_6_5_0_2	90.0	0.0	0.0	0.0	100.0	0.0	93.0	0.0	8.8	0.0	8.9	0.0	32.0	0.0	None
245	AGP exp_1	2200	Alpha-1-acid glycoprotein 2	ENGTVSR_6_5_0_2	72.3	0.0	0.0	0.0	89.8	0.0	100.0	0.0	3.8	0.0	6.1	0.0	39.2	0.0	None
246	AGP exp_1	2417	Alpha-1-acid glycoprotein 2	ENGTVSR_6_5_0_2	65.1	0.0	0.0	0.0	100.0	0.0	95.7	0.0	3.1	0.0	4.5	0.0	23.7	0.0	None
247	AGP exp_2	4012	Alpha-1-acid glycoprotein 2	ENGTVSR_6_5_0_3	26.7	0.0	0.0	0.0	100.0	0.0	93.5	0.0	5.0	0.0	11.8	0.0	47.1	0.0	None
248	AGP exp_1	3871	Alpha-1-acid glycoprotein 2	ENGTVSR_6_5_0_3	26.7	0.0	0.0	0.0	100.0	0.0	69.1	0.0	4.4	0.0	11.8	0.0	62.7	0.0	None
249	AGP exp_1	4149	Alpha-1-acid glycoprotein 2	ENGTVSR_6_5_0_3	28.8	0.0	4.9	0.0	100.0	0.0	54.7	0.0	1.8	0.0	7.7	0.0	41.7	0.0	None
250	AGP exp_2	1588	Alpha-1-acid glycoprotein 2	ENGTVSR_6_5_1_1	100.0	34.6	0.0	0.0	58.8	0.0	63.7	0.0	0.0	0.0	0.0	0.0	17.1	0.0	Outer
251	AGP exp_2	2426	Alpha-1-acid glycoprotein 2	ENGTVSR_6_5_1_2	60.3	15.1	0.0	0.0	100.0	45.7	70.3	0.0	0.0	0.0	0.0	0.0	26.2	0.0	Outer
252	AGP exp_1	2302	Alpha-1-acid glycoprotein 2	ENGTVSR_6_5_1_2	89.0	27.1	0.0	0.0	100.0	35.7	73.9	0.0	0.0	0.0	0.0	0.0	0.0	0.0	Outer
253	AGP exp_2	3847	Alpha-1-acid glycoprotein 2	ENGTVSR_6_5_1_3	20.3	4.5	4.3	0.0	98.2	33.0	100.0	0.0	7.9	0.0	9.4	0.0	45.1	2.0	Outer
254	AGP exp_1	3758	Alpha-1-acid glycoprotein 2	ENGTVSR_6_5_1_3	15.9	2.9	3.2	0.0	72.4	23.2	100.0	0.0	3.5	0.0	0.0	0.0	36.2	2.1	Outer
255	AGP exp_1	4042	Alpha-1-acid glycoprotein 2	ENGTVSR_6_5_1_3	24.0	13.0	0.0	0.0	100.0	24.4	44.0	0.0	5.4	0.0	0.0	0.0	44.3	0.0	Outer
256	AGP exp_28	6954	Alpha-1-acid glycoprotein 2	ENGTVSR_6_5_2_3	6.2	8.6	0.0	0.0	100.0	61.6	67.7	0.0	0.0	0.0	6.4	0.0	35.2	9.1	Dual
257	AGP exp_26	7051	Alpha-1-acid glycoprotein 2	ENGTVSR_6_5_2_3	8.8	12.5	0.0	0.0	100.0	74.9	81.9	0.0	0.0	0.0	0.0	0.0	52.4	14.0	Dual
258	AGP exp_36	7102	Alpha-1-acid glycoprotein 2	ENGTVSR_6_5_2_3	7.6	11.3	0.0	0.0	100.0	64.8	90.4	0.0	0.0	0.0	8.7	0.0	34.0	12.1	Dual

Supplementary Table 1. Continued.

No.	Experiments	CID Scan No.	N-glycoproteins	N-glycopeptides	Relative intensities of B/Y series ions														Manual Classification
					B_2	B_2F	B_3	B_3F	B_2S	B_2SF	Y_1	Y_1F	Y_2	Y_2F	Y_3	Y_3F	Y_4	Y_4F	
259	AGP exp_27	6629	Alpha-1-acid glycoprotein 2	ENGTVSR_6_5_2_3	6.9	10.7	0.0	0.0	100.0	64.6	72.2	0.0	3.7	0.0	8.1	0.0	44.3	13.1	Dual
260	AGP exp_22	6653	Alpha-1-acid glycoprotein 2	ENGTVSR_6_5_2_3	8.9	8.2	0.0	0.0	92.9	56.5	100.0	0.0	3.3	0.0	6.8	0.0	43.2	9.3	Dual
261	AGP exp_14	7628	Alpha-1-acid glycoprotein 2	ENGTVSR_6_5_2_3	0.0	0.0	0.0	0.0	100.0	30.8	41.7	0.0	0.0	0.0	0.0	0.0	13.8	10.0	Dual
262	AGP exp_5	6180	Alpha-1-acid glycoprotein 2	ENGTVSR_6_5_2_3	8.6	10.6	0.0	0.0	100.0	55.4	70.9	0.0	0.0	0.0	0.0	0.0	44.4	15.0	Dual
263	AGP exp_2	1493	Alpha-1-acid glycoprotein 2	ENGTVSR_7_6_1_1	93.1	30.1	0.0	0.0	43.5	31.1	100.0	0.0	0.0	0.0	0.0	0.0	0.0	0.0	Outer
264	AGP exp_1	2034	Alpha-1-acid glycoprotein 2	ENGTVSR_7_6_1_2	25.5	16.1	0.0	0.0	100.0	10.3	65.3	0.0	0.0	0.0	0.0	0.0	0.0	0.0	Outer
265	AGP exp_2	2068	Alpha-1-acid glycoprotein 2	ENGTVSR_7_6_1_2	25.8	21.3	0.0	0.0	100.0	22.8	60.4	0.0	0.0	0.0	0.0	0.0	0.0	0.0	Outer
266	AGP exp_1	2173	Alpha-1-acid glycoprotein 2	ENGTVSR_7_6_1_2	23.2	13.3	0.0	0.0	100.0	34.6	58.0	0.0	0.0	0.0	0.0	0.0	0.0	0.0	Outer
267	AGP exp_2	5011	Alpha-1-acid glycoprotein 2	ENGTVSR_7_6_1_4	33.0	0.0	0.0	0.0	100.0	22.7	11.3	0.0	0.0	0.0	0.0	0.0	2.6	0.0	Outer
268	AGP exp_1	5112	Alpha-1-acid glycoprotein 2	ENGTVSR_7_6_1_4	32.6	0.0	4.3	0.0	100.0	25.2	11.7	0.0	0.0	0.0	0.0	0.0	3.3	0.0	Outer
269	AGP exp_1	14653	Alpha-1-acid glycoprotein 2	LVPVPITNATLDR_3_3_0_0	6.6	0.0	3.9	0.0	0.0	0.0	100.0	0.0	1.8	0.0	24.1	0.0	26.6	0.0	None
270	AGP exp_2	15353	Alpha-1-acid glycoprotein 2	LVPVPITNATLDR_3_3_0_0	8.8	0.0	4.0	0.0	0.0	0.0	100.0	0.0	6.1	0.0	33.2	0.0	37.8	0.0	None
271	AGP exp_2	15433	Alpha-1-acid glycoprotein 2	LVPVPITNATLDR_3_3_0_0	8.0	0.0	4.5	0.0	0.0	0.0	100.0	0.0	4.0	0.0	34.6	0.0	39.4	0.0	None
272	AGP exp_1	14732	Alpha-1-acid glycoprotein 2	LVPVPITNATLDR_3_3_0_0	6.8	0.0	4.1	0.0	0.0	0.0	100.0	0.0	3.3	0.0	30.4	0.0	34.9	0.0	None
273	AGP exp_2	12253	Alpha-1-acid glycoprotein 2	LVPVPITNATLDR_4_4_0_1	63.4	0.0	0.0	0.0	100.0	0.0	44.7	0.0	12.7	0.0	0.0	0.0	29.7	0.0	None
274	AGP exp_1	14693	Alpha-1-acid glycoprotein 2	LVPVPITNATLDR_5_4_0_1	0.0	0.0	0.0	0.0	4.9	0.0	100.0	0.0	10.5	0.0	8.6	0.0	13.7	0.0	None
275	AGP exp_2	15498	Alpha-1-acid glycoprotein 2	LVPVPITNATLDR_5_4_0_1	0.0	0.0	4.5	0.0	5.1	0.0	100.0	0.0	15.2	0.0	7.3	0.0	17.3	0.0	None
276	AGP exp_2	15349	Alpha-1-acid glycoprotein 2	LVPVPITNATLDR_5_4_0_1	51.9	0.0	0.0	0.0	100.0	0.0	23.1	0.0	12.4	0.0	0.0	0.0	60.8	0.0	None
277	AGP exp_2	12062	Alpha-1-acid glycoprotein 2	LVPVPITNATLDR_5_4_0_1	83.6	0.0	9.3	0.0	48.1	0.0	26.8	0.0	0.0	0.0	9.3	0.0	100.0	0.0	None
278	AGP exp_1	15375	Alpha-1-acid glycoprotein 2	LVPVPITNATLDR_5_4_0_2	29.2	0.0	0.0	0.0	100.0	0.0	56.6	0.0	0.0	0.0	11.7	0.0	52.6	0.0	None
279	AGP exp_2	15598	Alpha-1-acid glycoprotein 2	LVPVPITNATLDR_5_4_0_2	0.0	0.0	0.0	0.0	9.0	0.0	100.0	0.0	8.2	0.0	6.5	0.0	8.8	0.0	None
280	AGP exp_1	15027	Alpha-1-acid glycoprotein 2	LVPVPITNATLDR_5_4_0_2	72.9	0.0	5.9	0.0	100.0	0.0	33.2	0.0	0.0	0.0	8.5	0.0	78.3	0.0	None
281	AGP exp_1	15062	Alpha-1-acid glycoprotein 2	LVPVPITNATLDR_5_4_1_2	40.9	0.0	9.4	0.0	100.0	29.1	96.4	0.0	0.0	0.0	0.0	0.0	58.5	8.2	Outer
282	AGP exp_2	15539	Alpha-1-acid glycoprotein 2	LVPVPITNATLDR_5_4_1_2	35.2	0.0	0.0	0.0	90.8	33.6	100.0	0.0	0.0	0.0	16.1	0.0	32.5	5.0	Outer
283	AGP exp_2	12153	Alpha-1-acid glycoprotein 2	LVPVPITNATLDR_5_5_0_1	36.9	0.0	0.0	0.0	51.9	0.0	100.0	0.0	11.4	0.0	8.8	0.0	53.6	0.0	None
284	AGP exp_1	11548	Alpha-1-acid glycoprotein 2	LVPVPITNATLDR_5_5_0_1	58.2	0.0	0.0	0.0	65.8	0.0	100.0	0.0	11.8	0.0	0.0	0.0	22.7	0.0	None

Supplementary Table 1. Continued.

No.	Experiments	CID Scan No.	N-glycoproteins	N-glycopeptides	Relative intensities of B/Y series ions														Manual Classification
					B_2	B_2F	B_3	B_3F	B_2S	B_2SF	Y_1	Y_1F	Y_2	Y_2F	Y_3	Y_3F	Y_4	Y_4F	
285	AGP exp_1	11847	Alpha-1-acid glycoprotein 2	LVPVPITNATLDR_5_5_0_1	75.1	0.0	0.0	0.0	84.6	0.0	86.5	0.0	13.7	0.0	0.0	0.0	100.0	0.0	None
286	AGP exp_2	15705	Alpha-1-acid glycoprotein 2	LVPVPITNATLDR_5_5_0_2	11.2	0.0	0.0	0.0	100.0	0.0	60.4	0.0	3.2	0.0	0.0	0.0	30.4	0.0	None
287	AGP exp_2	12110	Alpha-1-acid glycoprotein 2	LVPVPITNATLDR_6_5_0_1	44.9	0.0	0.0	0.0	68.5	0.0	100.0	0.0	0.0	0.0	11.0	0.0	25.0	0.0	None
288	AGP exp_2	11937	Alpha-1-acid glycoprotein 2	LVPVPITNATLDR_6_5_0_1	100.0	0.0	6.0	0.0	41.9	0.0	20.9	0.0	3.3	0.0	8.1	0.0	42.8	0.0	None
289	AGP exp_1	11608	Alpha-1-acid glycoprotein 2	LVPVPITNATLDR_6_5_0_1	100.0	0.0	6.8	0.0	43.4	0.0	17.8	0.0	0.0	0.0	9.5	0.0	41.7	0.0	None
290	AGP exp_1	14686	Alpha-1-acid glycoprotein 2	LVPVPITNATLDR_6_5_0_2	0.0	0.0	0.0	0.0	9.6	0.0	100.0	0.0	14.7	0.0	9.1	0.0	5.5	0.0	None
291	AGP exp_1	14947	Alpha-1-acid glycoprotein 2	LVPVPITNATLDR_6_5_0_2	94.3	0.0	5.4	0.0	100.0	0.0	38.2	0.0	6.7	0.0	0.0	0.0	42.2	0.0	None
292	AGP exp_2	15460	Alpha-1-acid glycoprotein 2	LVPVPITNATLDR_6_5_0_2	88.0	0.0	2.6	0.0	100.0	0.0	28.0	0.0	7.1	0.0	0.0	0.0	34.0	0.0	None
293	AGP exp_1	14571	Alpha-1-acid glycoprotein 2	LVPVPITNATLDR_6_5_0_2	85.6	0.0	6.0	0.0	100.0	0.0	25.9	0.0	6.8	0.0	0.0	0.0	28.9	0.0	None
294	AGP exp_2	15123	Alpha-1-acid glycoprotein 2	LVPVPITNATLDR_6_5_0_2	78.4	0.0	5.5	0.0	100.0	0.0	33.6	0.0	9.1	0.0	0.0	0.0	33.9	0.0	None
295	AGP exp_1	18288	Alpha-1-acid glycoprotein 2	LVPVPITNATLDR_6_5_0_3	43.0	0.0	0.0	0.0	100.0	0.0	25.4	0.0	5.0	0.0	6.8	0.0	0.0	0.0	None
296	AGP exp_1	18623	Alpha-1-acid glycoprotein 2	LVPVPITNATLDR_6_5_0_3	44.5	0.0	0.0	0.0	100.0	0.0	26.1	0.0	4.9	0.0	5.2	0.0	0.0	0.0	None
297	AGP exp_2	19281	Alpha-1-acid glycoprotein 2	LVPVPITNATLDR_6_5_0_3	39.3	0.0	0.0	0.0	100.0	0.0	23.2	0.0	4.8	0.0	4.6	0.0	0.0	0.0	None
298	AGP exp_2	19603	Alpha-1-acid glycoprotein 2	LVPVPITNATLDR_6_5_0_3	33.7	0.0	3.5	0.0	100.0	0.0	20.4	0.0	4.8	0.0	0.0	0.0	0.0	0.0	None
299	AGP exp_2	18920	Alpha-1-acid glycoprotein 2	LVPVPITNATLDR_6_5_0_3	44.0	0.0	3.9	0.0	100.0	0.0	26.2	0.0	4.5	0.0	5.1	0.0	0.0	0.0	None
300	AGP exp_2	11797	Alpha-1-acid glycoprotein 2	LVPVPITNATLDR_6_5_1_1	39.0	24.2	0.0	0.0	79.1	48.0	100.0	0.0	7.2	0.0	6.4	0.0	17.4	0.0	Outer
301	AGP exp_1	11413	Alpha-1-acid glycoprotein 2	LVPVPITNATLDR_6_5_1_1	36.7	34.6	0.0	0.0	77.2	14.4	100.0	0.0	0.0	0.0	0.0	0.0	25.8	0.0	Outer
302	AGP exp_1	14674	Alpha-1-acid glycoprotein 2	LVPVPITNATLDR_6_5_1_2	0.0	10.7	0.0	0.0	100.0	52.0	75.5	0.0	0.0	0.0	0.0	0.0	19.0	0.0	Outer
303	AGP exp_2	15241	Alpha-1-acid glycoprotein 2	LVPVPITNATLDR_6_5_1_2	0.0	10.0	0.0	0.0	100.0	56.1	75.1	0.0	8.0	0.0	0.0	0.0	17.5	0.0	Outer
304	AGP exp_1	15014	Alpha-1-acid glycoprotein 2	LVPVPITNATLDR_6_5_1_2	0.0	12.5	0.0	0.0	100.0	54.6	93.9	0.0	0.0	0.0	9.6	0.0	19.9	0.0	Outer
305	AGP exp_2	15562	Alpha-1-acid glycoprotein 2	LVPVPITNATLDR_6_5_1_2	0.0	9.6	0.0	0.0	100.0	28.6	75.1	0.0	0.0	0.0	8.4	0.0	16.0	0.0	Outer
306	AGP exp_2	14877	Alpha-1-acid glycoprotein 2	LVPVPITNATLDR_6_5_1_2	0.0	20.3	0.0	0.0	100.0	15.1	74.0	0.0	0.0	0.0	0.0	0.0	7.1	0.0	Outer
307	AGP exp_1	14782	Alpha-1-acid glycoprotein 2	LVPVPITNATLDR_6_5_1_2	75.4	11.0	6.4	0.0	100.0	26.3	29.5	0.0	4.1	0.0	11.4	0.0	32.7	0.0	Outer
308	AGP exp_2	15289	Alpha-1-acid glycoprotein 2	LVPVPITNATLDR_6_5_1_2	97.0	18.9	5.2	0.0	100.0	28.6	42.4	0.0	8.0	0.0	9.4	0.0	38.3	0.0	Outer
309	AGP exp_1	14434	Alpha-1-acid glycoprotein 2	LVPVPITNATLDR_6_5_1_2	81.8	14.0	0.0	0.0	100.0	9.7	22.9	0.0	0.0	0.0	0.0	0.0	12.7	0.0	Outer
310	AGP exp_1	14441	Alpha-1-acid glycoprotein 2	LVPVPITNATLDR_6_5_1_2	68.7	17.0	0.0	0.0	100.0	0.0	25.2	0.0	0.0	0.0	8.9	0.0	33.6	0.0	Outer

Supplementary Table 1. Continued.

No.	Experiments	CID Scan No.	N-glycoproteins	N-glycopeptides	Relative intensities of B/Y series ions														Manual Classification
					B_2	B_2F	B_3	B_3F	B_2S	B_2SF	Y_1	Y_1F	Y_2	Y_2F	Y_3	Y_3F	Y_4	Y_4F	
311	AGP exp_1	18433	Alpha-1-acid glycoprotein 2	LVPVPITNATLDR_6_5_1_3	0.0	6.1	0.0	0.0	100.0	43.6	57.1	0.0	0.0	0.0	0.0	0.0	9.5	0.0	Outer
312	AGP exp_1	18550	Alpha-1-acid glycoprotein 2	LVPVPITNATLDR_6_5_1_3	33.2	6.0	0.0	0.0	100.0	13.1	19.4	0.0	2.3	0.0	2.4	0.0	0.0	0.0	Outer
313	AGP exp_2	19176	Alpha-1-acid glycoprotein 2	LVPVPITNATLDR_6_5_1_3	31.1	5.0	0.0	0.0	100.0	20.9	20.2	0.0	4.1	0.0	4.6	0.0	0.0	0.0	Outer
314	IgG exp_4	5355	Ig gamma-1 chain C region	EEQYNSTYR_3_3_0_0	12.3	0.0	10.1	0.0	0.0	0.0	100.0	0.0	11.3	0.0	26.3	0.0	50.8	0.0	None
315	IgG exp_3	4184	Ig gamma-1 chain C region	EEQYNSTYR_3_3_1_0	8.9	0.0	9.1	0.0	0.0	0.0	100.0	51.9	13.6	2.6	14.3	7.2	25.2	5.4	Core
316	IgG exp_1	3022	Ig gamma-1 chain C region	EEQYNSTYR_3_3_1_0	9.0	0.0	9.2	0.0	0.0	0.0	100.0	49.4	14.9	2.6	14.8	4.5	26.4	5.4	Core
317	IgG exp_4	5322	Ig gamma-1 chain C region	EEQYNSTYR_3_3_1_0	7.3	0.0	10.1	0.0	0.0	0.0	100.0	53.6	14.5	2.4	14.4	3.0	27.1	6.7	Core
318	IgG exp_2	3021	Ig gamma-1 chain C region	EEQYNSTYR_3_3_1_0	14.8	0.0	15.9	0.0	0.0	0.0	100.0	64.2	18.4	2.9	20.6	8.9	45.7	15.7	Core
319	IgG exp_4	5328	Ig gamma-1 chain C region	EEQYNSTYR_3_4_0_0	2.7	0.0	6.1	0.0	0.0	0.0	100.0	0.0	16.0	0.0	12.9	0.0	20.8	0.0	None
320	IgG exp_3	4222	Ig gamma-1 chain C region	EEQYNSTYR_3_4_0_0	3.0	0.0	6.7	0.0	0.0	0.0	100.0	0.0	18.1	0.0	14.4	0.0	21.4	0.0	None
321	IgG exp_1	3126	Ig gamma-1 chain C region	EEQYNSTYR_3_4_0_0	3.8	0.0	6.9	0.0	0.0	0.0	100.0	0.0	19.5	0.0	15.6	0.0	29.1	0.0	None
322	IgG exp_3	4091	Ig gamma-1 chain C region	EEQYNSTYR_3_4_1_0	0.0	0.0	16.2	0.0	0.0	0.0	100.0	72.6	18.7	11.5	21.6	8.6	38.8	21.6	Core
323	IgG exp_3	4405	Ig gamma-1 chain C region	EEQYNSTYR_3_4_1_0	0.0	0.0	11.8	0.0	0.0	0.0	100.0	54.7	20.9	6.8	18.3	0.0	32.2	9.5	Core
324	IgG exp_4	5187	Ig gamma-1 chain C region	EEQYNSTYR_3_4_1_0	0.0	0.0	9.6	0.0	0.0	0.0	100.0	60.9	19.2	6.9	18.2	4.4	31.0	8.5	Core
325	IgG exp_2	2997	Ig gamma-1 chain C region	EEQYNSTYR_3_4_1_0	0.0	0.0	9.1	0.0	0.0	0.0	100.0	54.0	18.4	5.6	15.9	0.0	23.0	7.0	Core
326	IgG exp_1	2937	Ig gamma-1 chain C region	EEQYNSTYR_3_4_1_0	0.0	0.0	14.4	0.0	0.0	0.0	100.0	72.2	21.9	9.3	17.5	6.2	37.3	17.5	Core
327	IgG exp_4	5471	Ig gamma-1 chain C region	EEQYNSTYR_3_5_0_0	0.0	0.0	3.3	0.0	0.0	0.0	100.0	0.0	36.6	0.0	12.3	0.0	11.9	0.0	None
328	IgG exp_3	4720	Ig gamma-1 chain C region	EEQYNSTYR_3_5_1_0	0.0	0.0	3.4	0.0	0.0	0.0	100.0	52.9	36.4	17.7	13.5	0.0	11.8	0.0	Core
329	IgG exp_4	5432	Ig gamma-1 chain C region	EEQYNSTYR_3_5_1_0	0.0	0.0	3.7	0.0	0.0	0.0	100.0	56.4	36.7	17.2	14.0	5.0	10.4	2.7	Core
330	IgG exp_1	3153	Ig gamma-1 chain C region	EEQYNSTYR_3_5_1_0	0.0	0.0	3.8	0.0	0.0	0.0	100.0	52.8	33.8	16.2	16.3	2.5	9.8	2.6	Core
331	IgG exp_1	3010	Ig gamma-1 chain C region	EEQYNSTYR_4_3_1_0	8.7	0.0	8.4	0.0	0.0	0.0	100.0	73.8	11.8	3.7	10.6	0.0	24.4	3.1	Core
332	IgG exp_4	5305	Ig gamma-1 chain C region	EEQYNSTYR_4_3_1_0	9.1	2.6	9.0	0.0	0.0	0.0	100.0	76.4	10.7	4.2	9.5	0.0	24.9	3.8	Core
333	IgG exp_3	4168	Ig gamma-1 chain C region	EEQYNSTYR_4_3_1_0	9.6	1.7	9.1	0.0	0.0	0.0	100.0	80.1	13.1	4.4	10.6	0.0	27.6	3.0	Core
334	IgG exp_3	4197	Ig gamma-1 chain C region	EEQYNSTYR_4_4_0_0	0.0	0.0	5.2	0.0	0.0	0.0	100.0	0.0	14.6	0.0	12.5	0.0	22.4	0.0	None
335	IgG exp_1	3062	Ig gamma-1 chain C region	EEQYNSTYR_4_4_0_0	0.0	0.0	5.3	0.0	0.0	0.0	100.0	0.0	14.9	0.0	12.2	0.0	22.4	0.0	None
336	IgG exp_4	5318	Ig gamma-1 chain C region	EEQYNSTYR_4_4_0_0	0.0	0.0	5.3	0.0	0.0	0.0	100.0	0.0	15.9	0.0	13.0	0.0	24.0	0.0	None

Supplementary Table 1. Continued.

No.	Experiments	CID Scan No.	N-glycoproteins	N-glycopeptides	Relative intensities of B/Y series ions														Manual Classification
					B_2	B_2F	B_3	B_3F	B_2S	B_2SF	Y_1	Y_1F	Y_2	Y_2F	Y_3	Y_3F	Y_4	Y_4F	
337	IgG exp_4	5181	Ig gamma-1 chain C region	EEQYNSTYR_4_4_1_0	0.0	0.0	18.2	0.0	0.0	0.0	22.6	100.0	18.6	11.3	20.2	0.0	28.4	5.8	Core
338	IgG exp_2	3046	Ig gamma-1 chain C region	EEQYNSTYR_4_4_1_0	0.0	3.0	11.7	0.0	0.0	0.0	16.6	100.0	24.1	10.9	23.4	4.0	40.3	8.6	Core
339	IgG exp_3	4407	Ig gamma-1 chain C region	EEQYNSTYR_4_4_1_0	0.0	3.2	14.9	0.0	0.0	0.0	19.6	100.0	27.6	11.7	26.1	9.4	46.5	10.0	Core
340	IgG exp_1	2894	Ig gamma-1 chain C region	EEQYNSTYR_4_4_1_0	0.0	3.1	11.2	0.0	0.0	0.0	9.8	100.0	17.4	11.5	22.6	4.4	36.9	9.9	Core
341	IgG exp_3	4075	Ig gamma-1 chain C region	EEQYNSTYR_4_4_1_0	0.0	4.2	11.8	0.0	0.0	0.0	7.8	100.0	21.5	14.4	16.0	8.0	44.8	9.6	Core
342	IgG exp_1	3281	Ig gamma-1 chain C region	EEQYNSTYR_4_4_1_0	0.0	0.0	12.6	0.0	0.0	0.0	16.4	100.0	23.2	11.7	20.7	2.5	32.4	8.8	Core
343	IgG exp_2	6163	Ig gamma-1 chain C region	EEQYNSTYR_4_4_1_0	0.0	0.0	6.8	0.0	0.0	0.0	9.8	100.0	17.9	12.8	10.0	4.1	43.9	7.2	Core
344	IgG exp_3	5170	Ig gamma-1 chain C region	EEQYNSTYR_4_4_1_1	70.8	0.0	0.0	0.0	100.0	0.0	63.2	36.7	0.0	0.0	0.0	0.0	30.2	22.8	Core
345	IgG exp_4	6142	Ig gamma-1 chain C region	EEQYNSTYR_4_4_1_1	69.8	0.0	15.0	0.0	100.0	0.0	59.3	47.1	0.0	0.0	0.0	0.0	27.7	25.2	Core
346	IgG exp_1	3787	Ig gamma-1 chain C region	EEQYNSTYR_4_4_1_1	64.8	0.0	0.0	0.0	100.0	0.0	41.5	38.5	0.0	0.0	0.0	0.0	18.4	0.0	Core
347	IgG exp_3	5195	Ig gamma-1 chain C region	EEQYNSTYR_4_4_1_1	0.0	0.0	6.1	0.0	14.8	0.0	100.0	0.0	16.2	9.1	12.6	0.0	23.5	3.0	Core
348	IgG exp_1	3774	Ig gamma-1 chain C region	EEQYNSTYR_4_4_1_1	0.0	0.0	6.5	0.0	18.5	0.0	100.0	0.0	14.1	11.2	15.5	0.0	32.1	5.4	Core
349	IgG exp_4	6150	Ig gamma-1 chain C region	EEQYNSTYR_4_4_1_1	0.0	0.0	7.2	0.0	18.1	0.0	100.0	0.0	11.2	9.1	14.4	0.0	23.2	4.0	Core
350	IgG exp_2	3220	Ig gamma-1 chain C region	EEQYNSTYR_4_4_1_1	79.9	0.0	22.4	0.0	100.0	0.0	27.9	28.0	0.0	0.0	0.0	0.0	13.2	20.2	Core
351	IgG exp_2	3226	Ig gamma-1 chain C region	EEQYNSTYR_4_4_1_1	0.0	0.0	17.3	0.0	27.3	0.0	100.0	0.0	18.1	0.0	5.7	7.3	29.3	0.0	Core
352	IgG exp_1	3099	Ig gamma-1 chain C region	EEQYNSTYR_4_5_1_0	0.0	0.0	4.1	0.0	0.0	0.0	100.0	75.5	31.5	20.9	11.4	2.9	9.0	0.0	Core
353	IgG exp_4	5428	Ig gamma-1 chain C region	EEQYNSTYR_4_5_1_0	0.0	0.0	4.0	0.0	0.0	0.0	100.0	78.1	32.8	20.1	11.1	3.1	8.5	0.0	Core
354	IgG exp_3	4716	Ig gamma-1 chain C region	EEQYNSTYR_4_5_1_0	0.0	0.0	4.8	0.0	0.0	0.0	100.0	82.9	33.9	21.5	10.3	2.8	10.1	0.0	Core
355	IgG exp_2	3050	Ig gamma-1 chain C region	EEQYNSTYR_4_5_1_0	0.0	0.0	4.9	0.0	0.0	0.0	100.0	73.3	22.6	12.7	5.6	0.0	8.1	2.1	Core
356	IgG exp_4	5299	Ig gamma-1 chain C region	EEQYNSTYR_5_4_0_0	0.0	0.0	4.5	0.0	0.0	0.0	100.0	0.0	16.6	0.0	14.6	0.0	25.6	0.0	None
357	IgG exp_3	4199	Ig gamma-1 chain C region	EEQYNSTYR_5_4_0_0	0.0	0.0	5.2	0.0	0.0	0.0	100.0	0.0	14.5	0.0	14.9	0.0	28.2	0.0	None
358	IgG exp_3	5257	Ig gamma-1 chain C region	EEQYNSTYR_5_4_0_1	0.0	0.0	5.4	0.0	13.0	0.0	100.0	0.0	11.5	0.0	9.8	0.0	24.5	0.0	None
359	IgG exp_1	2875	Ig gamma-1 chain C region	EEQYNSTYR_5_4_1_0	0.0	4.9	9.2	0.0	0.0	0.0	94.8	100.0	22.6	15.6	13.9	12.4	36.4	3.8	Core
360	IgG exp_3	4085	Ig gamma-1 chain C region	EEQYNSTYR_5_4_1_0	0.0	0.0	9.6	0.0	0.0	0.0	100.0	80.2	13.7	12.1	15.2	6.7	30.5	3.5	Core
361	IgG exp_2	3003	Ig gamma-1 chain C region	EEQYNSTYR_5_4_1_0	0.0	3.4	13.5	0.0	0.0	0.0	100.0	93.4	12.3	11.8	19.3	0.0	27.4	3.5	Core
362	IgG exp_4	5179	Ig gamma-1 chain C region	EEQYNSTYR_5_4_1_0	0.0	2.9	7.3	0.0	0.0	0.0	75.7	100.0	12.5	12.1	11.7	8.2	23.5	4.5	Core

Supplementary Table 1. Continued.

No.	Experiments	CID Scan No.	N-glycoproteins	N-glycopeptides	Relative intensities of B/Y series ions														Manual Classification
					B_2	B_2F	B_3	B_3F	B_2S	B_2SF	Y_1	Y_1F	Y_2	Y_2F	Y_3	Y_3F	Y_4	Y_4F	
363	IgG exp_3	4413	Ig gamma-1 chain C region	EEQYNSTYR_5_4_1_0	0.0	0.0	0.0	0.0	0.0	0.0	100.0	99.6	9.5	9.9	10.4	0.0	28.5	0.0	Core
364	IgG exp_3	5108	Ig gamma-1 chain C region	EEQYNSTYR_5_4_1_1	100.0	0.0	11.8	0.0	77.6	0.0	33.5	47.3	0.0	0.0	0.0	0.0	0.0	0.0	Core
365	IgG exp_1	3671	Ig gamma-1 chain C region	EEQYNSTYR_5_4_1_1	100.0	0.0	11.2	0.0	79.9	0.0	32.5	43.9	0.0	0.0	0.0	0.0	0.0	0.0	Core
366	IgG exp_4	6105	Ig gamma-1 chain C region	EEQYNSTYR_5_4_1_1	100.0	0.0	10.7	0.0	73.8	0.0	37.2	46.6	0.0	0.0	0.0	0.0	11.5	0.0	Core
367	IgG exp_3	5110	Ig gamma-1 chain C region	EEQYNSTYR_5_4_1_1	0.0	2.4	5.6	0.0	18.0	0.0	100.0	95.9	15.7	15.9	16.0	10.2	30.1	0.0	Core
368	IgG exp_4	6109	Ig gamma-1 chain C region	EEQYNSTYR_5_4_1_1	0.0	2.0	5.4	0.0	18.3	0.0	97.8	100.0	19.3	17.0	16.4	10.4	29.1	2.2	Core
369	IgG exp_1	3693	Ig gamma-1 chain C region	EEQYNSTYR_5_4_1_1	0.0	2.2	5.7	0.0	19.5	0.0	100.0	98.4	15.5	16.8	17.1	11.3	32.9	0.0	Core
370	IgG exp_2	3216	Ig gamma-1 chain C region	EEQYNSTYR_5_4_1_1	0.0	0.0	6.7	0.0	27.1	0.0	98.6	100.0	15.4	17.9	14.0	11.5	34.8	0.0	Core
371	IgG exp_4	5450	Ig gamma-1 chain C region	EEQYNSTYR_5_5_1_0	0.0	0.0	6.5	0.0	0.0	0.0	100.0	96.3	32.8	36.1	10.7	3.5	9.5	0.0	Core
372	IgG exp_4	7185	Ig gamma-2 chain C region	EEQFNSTFR_3_3_1_0	12.8	0.0	10.5	0.0	0.0	0.0	100.0	55.8	17.3	3.0	18.1	6.4	36.6	7.5	Core
373	IgG exp_1	4437	Ig gamma-2 chain C region	EEQFNSTFR_3_3_1_0	11.1	0.0	10.5	0.0	0.0	0.0	100.0	54.3	15.9	3.1	16.9	5.5	32.1	7.1	Core
374	IgG exp_3	5741	Ig gamma-2 chain C region	EEQFNSTFR_3_3_1_0	6.6	0.0	10.1	0.0	0.0	0.0	100.0	52.1	17.0	0.0	13.3	0.0	26.8	6.2	Core
375	IgG exp_2	3508	Ig gamma-2 chain C region	EEQFNSTFR_3_3_1_0	6.3	0.0	7.7	0.0	0.0	0.0	100.0	43.8	13.4	3.2	12.3	4.1	25.3	6.4	Core
376	IgG exp_3	5816	Ig gamma-2 chain C region	EEQFNSTFR_3_4_0_0	2.9	0.0	5.7	0.0	0.0	0.0	100.0	0.0	15.8	0.0	12.6	0.0	24.8	0.0	None
377	IgG exp_4	7313	Ig gamma-2 chain C region	EEQFNSTFR_3_4_0_0	4.5	0.0	9.1	0.0	0.0	0.0	100.0	0.0	18.2	0.0	19.2	0.0	35.1	0.0	None
378	IgG exp_1	4557	Ig gamma-2 chain C region	EEQFNSTFR_3_4_0_0	4.2	0.0	8.7	0.0	0.0	0.0	100.0	0.0	19.7	0.0	16.8	0.0	32.4	0.0	None
379	IgG exp_1	5662	Ig gamma-2 chain C region	EEQFNSTFR_3_4_1_0	0.0	0.0	14.4	0.0	0.0	0.0	100.0	66.1	14.2	6.7	24.7	2.6	36.6	22.9	Core
380	IgG exp_2	3469	Ig gamma-2 chain C region	EEQFNSTFR_3_4_1_0	0.0	0.0	7.0	0.0	0.0	0.0	100.0	48.4	16.1	4.9	12.6	0.0	19.3	5.0	Core
381	IgG exp_3	5721	Ig gamma-2 chain C region	EEQFNSTFR_3_4_1_0	0.0	0.0	7.5	0.0	0.0	0.0	100.0	48.9	15.7	4.9	13.0	0.0	19.1	4.7	Core
382	IgG exp_4	7480	Ig gamma-2 chain C region	EEQFNSTFR_3_4_1_0	2.1	0.0	9.2	0.0	0.0	0.0	100.0	54.1	18.0	5.4	16.3	2.3	24.4	6.1	Core
383	IgG exp_1	4661	Ig gamma-2 chain C region	EEQFNSTFR_3_4_1_0	2.3	0.0	12.4	0.0	0.0	0.0	100.0	57.7	20.0	6.8	17.1	3.7	31.2	10.5	Core
384	IgG exp_3	6073	Ig gamma-2 chain C region	EEQFNSTFR_3_4_1_0	1.7	0.0	12.0	1.8	0.0	0.0	100.0	45.9	15.1	5.4	18.1	0.0	30.2	12.3	Core
385	IgG exp_4	6975	Ig gamma-2 chain C region	EEQFNSTFR_3_4_1_0	2.4	0.0	10.4	0.0	0.0	0.0	100.0	61.4	23.3	8.3	19.1	5.6	31.2	14.6	Core
386	IgG exp_1	4325	Ig gamma-2 chain C region	EEQFNSTFR_3_4_1_0	3.1	0.0	16.0	0.0	0.0	0.0	100.0	62.5	23.4	8.6	21.1	7.6	39.4	15.7	Core
387	IgG exp_2	4229	Ig gamma-2 chain C region	EEQFNSTFR_3_4_1_0	0.0	0.0	15.5	0.0	0.0	0.0	100.0	73.5	22.1	3.6	24.7	0.0	55.9	8.7	Core
388	IgG exp_2	3496	Ig gamma-2 chain C region	EEQFNSTFR_3_5_1_0	24.6	0.0	0.0	0.0	0.0	0.0	100.0	29.7	31.5	0.0	47.5	0.0	0.0	0.0	Core

Supplementary Table 1. Continued.

No.	Experiments	CID Scan No.	N-glycoproteins	N-glycopeptides	Relative intensities of B/Y series ions														Manual Classification
					B_2	B_2F	B_3	B_3F	B_2S	B_2SF	Y_1	Y_1F	Y_2	Y_2F	Y_3	Y_3F	Y_4	Y_4F	
389	IgG exp_2	3498	Ig gamma-2 chain C region	EEQFNSTFR_3_5_1_0	0.0	0.0	3.9	0.0	0.0	0.0	100.0	53.3	35.6	18.3	14.2	2.7	10.6	2.6	Core
390	IgG exp_4	7309	Ig gamma-2 chain C region	EEQFNSTFR_3_5_1_0	0.0	0.0	4.1	0.0	0.0	0.0	100.0	52.6	36.7	16.7	13.9	2.5	9.5	2.3	Core
391	IgG exp_3	5766	Ig gamma-2 chain C region	EEQFNSTFR_3_5_1_0	0.0	0.0	3.7	0.0	0.0	0.0	100.0	52.0	37.1	16.2	14.5	0.0	9.9	2.2	Core
392	IgG exp_1	4520	Ig gamma-2 chain C region	EEQFNSTFR_3_5_1_0	0.0	0.0	4.0	0.0	0.0	0.0	100.0	56.4	37.9	17.9	13.4	0.0	9.7	2.3	Core
393	IgG exp_4	7220	Ig gamma-2 chain C region	EEQFNSTFR_4_3_1_0	14.3	3.0	10.1	0.0	0.0	0.0	100.0	75.5	14.8	5.3	10.2	0.0	33.5	5.5	Core
394	IgG exp_4	7274	Ig gamma-2 chain C region	EEQFNSTFR_4_4_0_0	3.6	0.0	7.9	0.0	0.0	0.0	100.0	0.0	19.5	0.0	17.0	0.0	33.6	0.0	None
395	IgG exp_4	7270	Ig gamma-2 chain C region	EEQFNSTFR_4_4_1_0	100.0	0.0	0.0	0.0	0.0	0.0	41.7	41.6	0.0	0.0	0.0	0.0	24.8	39.1	Core
396	IgG exp_1	4427	Ig gamma-2 chain C region	EEQFNSTFR_4_4_1_0	100.0	0.0	14.9	0.0	0.0	0.0	37.7	40.1	0.0	0.0	0.0	0.0	23.7	37.8	Core
397	IgG exp_3	5694	Ig gamma-2 chain C region	EEQFNSTFR_4_4_1_0	100.0	0.0	15.8	0.0	0.0	0.0	42.6	42.1	0.0	0.0	0.0	0.0	28.9	41.2	Core
398	IgG exp_2	3467	Ig gamma-2 chain C region	EEQFNSTFR_4_4_1_0	100.0	0.0	16.3	0.0	0.0	0.0	62.6	51.7	0.0	0.0	0.0	0.0	33.6	51.2	Core
399	IgG exp_4	6926	Ig gamma-2 chain C region	EEQFNSTFR_4_4_1_0	0.0	0.0	8.2	0.0	0.0	0.0	100.0	59.5	8.9	11.2	14.9	0.0	25.2	7.3	Core
400	IgG exp_3	5692	Ig gamma-2 chain C region	EEQFNSTFR_4_4_1_0	0.0	2.1	6.8	0.0	0.0	0.0	100.0	67.8	17.8	9.4	15.3	5.3	25.7	3.9	Core
401	IgG exp_2	6486	Ig gamma-2 chain C region	EEQFNSTFR_4_4_1_0	0.0	0.0	7.4	0.0	0.0	0.0	100.0	66.6	15.9	7.0	17.4	4.6	36.2	4.0	Core
402	IgG exp_4	7268	Ig gamma-2 chain C region	EEQFNSTFR_4_4_1_0	0.0	2.3	8.9	0.0	0.0	0.0	100.0	68.4	19.3	11.7	17.2	5.8	29.5	4.6	Core
403	IgG exp_2	3400	Ig gamma-2 chain C region	EEQFNSTFR_4_4_1_0	0.0	2.5	8.4	0.0	0.0	0.0	99.2	100.0	15.0	9.9	16.6	4.4	28.2	4.3	Core
404	IgG exp_1	4717	Ig gamma-2 chain C region	EEQFNSTFR_4_4_1_0	0.0	9.0	30.8	0.0	0.0	0.0	100.0	79.8	11.1	0.0	0.0	0.0	21.0	0.0	Core
405	IgG exp_1	5111	Ig gamma-2 chain C region	EEQFNSTFR_4_4_1_0	0.0	0.0	11.1	0.0	0.0	0.0	100.0	77.2	20.0	0.0	14.1	5.2	29.7	4.6	Core
406	IgG exp_4	7620	Ig gamma-2 chain C region	EEQFNSTFR_4_4_1_0	0.0	0.0	14.5	0.0	0.0	0.0	100.0	83.0	19.6	12.9	19.8	0.0	31.7	6.9	Core
407	IgG exp_1	6085	Ig gamma-2 chain C region	EEQFNSTFR_4_4_1_1	72.9	0.0	17.1	0.0	100.0	0.0	52.9	46.4	0.0	0.0	0.0	0.0	28.8	0.0	Core
408	IgG exp_2	5075	Ig gamma-2 chain C region	EEQFNSTFR_4_4_1_1	67.5	0.0	14.3	0.0	100.0	0.0	44.1	45.6	0.0	0.0	0.0	0.0	30.0	0.0	Core
409	IgG exp_2	4772	Ig gamma-2 chain C region	EEQFNSTFR_4_4_1_1	0.0	6.7	10.2	0.0	29.7	0.0	100.0	80.0	13.3	12.6	13.4	6.5	33.9	4.6	Core
410	IgG exp_1	6083	Ig gamma-2 chain C region	EEQFNSTFR_4_4_1_1	0.0	2.1	8.0	0.0	18.6	0.0	100.0	72.4	21.0	15.0	18.0	9.3	39.3	5.4	Core
411	IgG exp_2	4745	Ig gamma-2 chain C region	EEQFNSTFR_4_4_1_1	85.9	0.0	0.0	0.0	100.0	0.0	34.6	49.5	0.0	0.0	0.0	0.0	11.6	0.0	Core
412	IgG exp_2	5110	Ig gamma-2 chain C region	EEQFNSTFR_4_4_1_1	0.0	3.7	9.9	0.0	21.2	0.0	100.0	83.4	19.4	15.4	22.9	9.4	41.5	6.4	Core
413	IgG exp_4	8721	Ig gamma-2 chain C region	EEQFNSTFR_4_4_1_1	0.0	3.1	8.2	0.0	23.0	0.0	100.0	87.1	16.7	14.8	17.0	0.0	44.7	8.0	Core
414	IgG exp_1	4464	Ig gamma-2 chain C region	EEQFNSTFR_4_5_1_0	0.0	0.0	4.0	0.0	0.0	0.0	100.0	75.4	32.7	19.8	11.1	3.0	8.6	0.0	Core

Supplementary Table 1. Continued.

No.	Experiments	CID Scan No.	N-glycoproteins	N-glycopeptides	Relative intensities of B/Y series ions														Manual Classification
					B_2	B_2F	B_3	B_3F	B_2S	B_2SF	Y_1	Y_1F	Y_2	Y_2F	Y_3	Y_3F	Y_4	Y_4F	
415	IgG exp_3	5743	Ig gamma-2 chain C region	EEQFNSTFR_4_5_1_0	0.0	0.0	4.8	0.0	0.0	0.0	100.0	72.7	33.0	22.0	11.3	3.2	10.2	0.0	Core
416	IgG exp_4	7257	Ig gamma-2 chain C region	EEQFNSTFR_4_5_1_0	0.0	0.0	4.9	0.0	0.0	0.0	100.0	74.5	34.6	21.6	13.0	3.1	9.9	0.0	Core
417	IgG exp_2	3512	Ig gamma-2 chain C region	EEQFNSTFR_4_5_1_0	0.0	0.0	10.5	0.0	0.0	0.0	100.0	91.9	34.3	19.7	18.5	2.7	10.0	3.0	Core
418	IgG exp_4	7236	Ig gamma-2 chain C region	EEQFNSTFR_5_4_0_0	0.0	0.0	4.8	0.0	0.0	0.0	100.0	6.6	11.3	0.0	11.4	0.0	18.0	0.0	None
419	IgG exp_4	7214	Ig gamma-2 chain C region	EEQFNSTFR_5_4_1_0	0.0	2.5	8.4	0.0	0.0	0.0	100.0	94.5	18.0	13.8	16.2	9.3	28.4	2.4	Core
420	IgG exp_3	5671	Ig gamma-2 chain C region	EEQFNSTFR_5_4_1_0	0.0	3.1	7.8	0.0	0.0	0.0	100.0	85.4	17.5	13.8	16.7	8.6	30.5	2.4	Core
421	IgG exp_1	4373	Ig gamma-2 chain C region	EEQFNSTFR_5_4_1_0	0.0	2.9	7.8	0.0	0.0	0.0	100.0	93.8	18.2	15.2	18.1	9.4	30.3	2.6	Core
422	IgG exp_2	3436	Ig gamma-2 chain C region	EEQFNSTFR_5_4_1_0	0.0	3.2	4.5	0.0	0.0	0.0	100.0	92.0	17.8	15.0	17.2	8.7	31.2	2.6	Core
423	IgG exp_4	8669	Ig gamma-2 chain C region	EEQFNSTFR_5_4_1_1	0.0	2.4	5.9	0.0	14.6	0.0	70.6	100.0	12.7	7.7	10.0	8.6	21.7	0.0	Core
424	IgG exp_1	5940	Ig gamma-2 chain C region	EEQFNSTFR_5_4_1_1	0.0	2.9	5.5	0.0	20.8	0.0	96.7	100.0	17.2	17.6	16.7	11.2	31.8	0.0	Core
425	IgG exp_2	4872	Ig gamma-2 chain C region	EEQFNSTFR_5_4_1_1	0.0	2.7	5.2	0.0	20.4	0.0	94.3	100.0	12.6	16.4	12.8	11.0	34.1	0.0	Core
426	IgG exp_2	4542	Ig gamma-2 chain C region	EEQFNSTFR_5_4_1_1	0.0	0.0	7.8	0.0	22.8	0.0	85.7	100.0	10.2	13.3	9.0	0.0	17.0	0.0	Core
427	IgG exp_1	4447	Ig gamma-2 chain C region	EEQFNSTFR_5_5_1_0	100.0	0.0	0.0	0.0	0.0	0.0	99.3	19.5	9.2	0.0	7.0	0.0	6.9	0.0	Core
428	IgG exp_1	4468	Ig gamma-2 chain C region	EEQFNSTFR_5_5_1_0	0.0	0.0	7.1	0.0	0.0	0.0	100.0	100.0	0.0	0.0	4.7	2.9	2.7	0.0	Core
429	IgG exp_3	5122	Ig gamma-3 chain C region	EEQYNSTFR_4_4_1_0	0.0	0.0	15.3	0.0	0.0	0.0	100.0	85.1	9.9	10.5	14.1	0.0	35.0	4.7	Core
430	IgG exp_3	5130	Ig gamma-4 chain C region	EEQFNSTYR_3_4_1_0	0.0	0.0	14.0	0.0	0.0	0.0	100.0	63.1	18.7	6.6	19.4	3.1	37.5	11.1	Core
431	IgG exp_1	3739	Ig gamma-4 chain C region	EEQFNSTYR_3_4_1_0	0.0	0.0	13.6	0.0	0.0	0.0	100.0	60.6	20.6	10.5	21.5	8.3	37.9	12.6	Core
432	IgG exp_4	6100	Ig gamma-4 chain C region	EEQFNSTYR_3_4_1_0	0.0	0.0	10.8	0.0	0.0	0.0	100.0	57.6	22.9	7.2	21.5	5.9	37.0	12.0	Core
433	IgG exp_1	3712	Ig gamma-4 chain C region	EEQFNSTYR_4_4_1_0	0.0	3.9	12.2	0.0	0.0	0.0	100.0	76.0	17.2	13.7	15.7	9.6	36.7	9.7	Core

Supplementary Table 2. Glycopeptide spectrum matches of alpha-1-acid glycoprotein and immunoglobulin gamma, and their relative intensities as test set for machine learning.

No.	Experiments	CID Scan No.	N-glycoproteins	N-glycopeptides	Relative intensities of B/Y series ions														Manual Classification
					B_2	B_2F	B_3	B_3F	B_2S	B_2SF	Y_1	Y_1F	Y_2	Y_2F	Y_3	Y_3F	Y_4	Y_4F	
1	AGP exp_3	2234	Alpha-1-acid glycoprotein 1	ENGTISR_4_4_0_1	44.2	0.0	0.0	0.0	50.0	0.0	0.0	0.0	42.2	0.0	100.0	0.0	18.9	0.0	None
2	AGP exp_4	2128	Alpha-1-acid glycoprotein 1	ENGTISR_4_4_0_1	32.8	0.0	0.0	0.0	58.7	0.0	27.2	0.0	43.9	0.0	100.0	0.0	0.0	0.0	None
3	AGP exp_3	3807	Alpha-1-acid glycoprotein 1	ENGTISR_5_4_0_2	67.2	0.0	4.0	0.0	100.0	0.0	36.0	0.0	0.0	0.0	0.0	0.0	31.9	0.0	None
4	AGP exp_4	3700	Alpha-1-acid glycoprotein 1	ENGTISR_5_4_0_2	0.0	0.0	0.0	0.0	97.1	0.0	100.0	0.0	13.7	0.0	20.5	0.0	24.8	0.0	None
5	AGP exp_3	3870	Alpha-1-acid glycoprotein 1	ENGTISR_5_4_0_2	0.0	0.0	0.0	0.0	73.4	0.0	100.0	0.0	19.3	0.0	16.7	0.0	31.3	0.0	None
6	AGP exp_4	2230	Alpha-1-acid glycoprotein 1	ENGTISR_6_5_0_1	100.0	0.0	6.8	0.0	49.2	0.0	53.7	0.0	0.0	0.0	0.0	0.0	35.6	0.0	None
7	AGP exp_3	2593	Alpha-1-acid glycoprotein 1	ENGTISR_6_5_0_1	100.0	0.0	9.5	0.0	56.0	0.0	91.5	0.0	0.0	0.0	0.0	0.0	33.3	0.0	None
8	AGP exp_4	2506	Alpha-1-acid glycoprotein 1	ENGTISR_6_5_0_1	100.0	0.0	7.3	0.0	61.3	0.0	56.0	0.0	0.0	0.0	0.0	0.0	22.1	0.0	None
9	AGP exp_3	3937	Alpha-1-acid glycoprotein 1	ENGTISR_6_5_0_2	74.7	0.0	0.0	0.0	100.0	0.0	82.3	0.0	8.5	0.0	0.0	0.0	41.8	0.0	None
10	AGP exp_3	4259	Alpha-1-acid glycoprotein 1	ENGTISR_6_5_0_2	64.2	0.0	5.4	0.0	100.0	0.0	97.9	0.0	0.0	0.0	0.0	0.0	43.5	0.0	None
11	AGP exp_4	3951	Alpha-1-acid glycoprotein 1	ENGTISR_6_5_0_2	66.1	0.0	5.6	0.0	94.2	0.0	100.0	0.0	6.8	0.0	0.0	0.0	37.5	0.0	None
12	AGP exp_3	4070	Alpha-1-acid glycoprotein 1	ENGTISR_6_5_0_2	0.0	0.0	0.0	0.0	51.3	0.0	100.0	0.0	21.6	0.0	14.4	0.0	15.7	0.0	None
13	AGP exp_4	4267	Alpha-1-acid glycoprotein 1	ENGTISR_6_5_0_2	69.5	0.0	7.6	0.0	100.0	0.0	48.4	0.0	0.0	0.0	0.0	0.0	35.3	0.0	None
14	AGP exp_4	4825	Alpha-1-acid glycoprotein 1	ENGTISR_6_5_0_2	0.0	0.0	0.0	0.0	40.3	0.0	100.0	0.0	14.5	0.0	7.5	0.0	13.3	0.0	None
15	AGP exp_3	5053	Alpha-1-acid glycoprotein 1	ENGTISR_6_5_0_2	86.7	0.0	6.0	0.0	100.0	0.0	48.5	0.0	0.0	0.0	0.0	0.0	17.5	0.0	None
16	AGP exp_4	4869	Alpha-1-acid glycoprotein 1	ENGTISR_6_5_0_3	22.2	0.0	0.0	0.0	100.0	0.0	42.3	0.0	5.1	0.0	0.0	0.0	17.1	0.0	None
17	AGP exp_4	4888	Alpha-1-acid glycoprotein 1	ENGTISR_6_5_0_3	0.0	0.0	0.0	0.0	80.2	0.0	100.0	0.0	25.8	0.0	13.8	0.0	29.9	0.0	None
18	AGP exp_3	5086	Alpha-1-acid glycoprotein 1	ENGTISR_6_5_0_3	0.0	0.0	0.0	0.0	48.0	0.0	100.0	0.0	12.0	0.0	15.3	0.0	13.2	0.0	None
19	AGP exp_4	6322	Alpha-1-acid glycoprotein 1	ENGTISR_6_5_0_3	35.4	0.0	0.0	0.0	100.0	9.3	35.4	0.0	0.0	0.0	0.0	0.0	33.8	0.0	None
20	AGP exp_4	2442	Alpha-1-acid glycoprotein 1	ENGTISR_6_5_1_1	87.1	20.6	0.0	0.0	57.8	32.7	100.0	0.0	0.0	0.0	0.0	0.0	20.5	0.0	Outer
21	AGP exp_3	2567	Alpha-1-acid glycoprotein 1	ENGTISR_6_5_1_1	100.0	25.5	0.0	0.0	67.9	26.3	53.4	0.0	0.0	0.0	0.0	0.0	16.4	0.0	Outer
22	AGP exp_4	2098	Alpha-1-acid glycoprotein 1	ENGTISR_6_5_1_1	100.0	41.7	0.0	0.0	67.0	0.0	37.8	0.0	0.0	0.0	0.0	0.0	13.8	0.0	Outer
23	AGP exp_3	2250	Alpha-1-acid glycoprotein 1	ENGTISR_6_5_1_1	100.0	24.7	0.0	0.0	85.0	0.0	54.5	0.0	0.0	0.0	0.0	0.0	33.3	0.0	Outer
24	AGP exp_3	3715	Alpha-1-acid glycoprotein 1	ENGTISR_6_5_1_2	77.1	21.7	0.0	0.0	100.0	20.9	38.9	0.0	0.0	0.0	0.0	0.0	12.4	0.0	Outer
25	AGP exp_4	3820	Alpha-1-acid glycoprotein 1	ENGTISR_6_5_1_2	32.2	16.0	6.4	0.0	100.0	21.8	89.7	0.0	3.2	0.0	0.0	0.0	33.2	0.0	Outer

Supplementary Table 2. Continued.

No.	Experiments	CID Scan No.	N-glycoproteins	N-glycopeptides	Relative intensities of B/Y series ions														Manual Classification
					B_2	B_2F	B_3	B_3F	B_2S	B_2SF	Y_1	Y_1F	Y_2	Y_2F	Y_3	Y_3F	Y_4	Y_4F	
26	AGP exp_3	4074	Alpha-1-acid glycoprotein 1	ENGTISR_6_5_1_2	70.8	17.8	0.0	0.0	100.0	44.3	41.4	0.0	4.4	0.0	0.0	0.0	20.9	0.0	Outer
27	AGP exp_4	4161	Alpha-1-acid glycoprotein 1	ENGTISR_6_5_1_2	68.6	14.3	0.0	0.0	100.0	13.7	22.1	7.3	0.0	0.0	0.0	0.0	20.5	5.2	Outer
28	AGP exp_3	5005	Alpha-1-acid glycoprotein 1	ENGTISR_6_5_1_3	20.2	5.5	0.0	0.0	100.0	33.7	76.5	0.0	5.3	0.0	0.0	0.0	49.7	3.2	Outer
29	AGP exp_4	4831	Alpha-1-acid glycoprotein 1	ENGTISR_6_5_1_3	19.9	7.2	3.2	0.0	100.0	34.9	83.5	0.0	7.5	0.0	0.0	0.0	51.0	0.0	Outer
30	AGP exp_40	5698	Alpha-1-acid glycoprotein 1	ENGTISR_6_5_2_2	66.4	24.2	0.0	0.0	100.0	15.5	38.5	5.2	0.0	0.0	0.0	0.0	15.3	3.4	Dual
31	AGP exp_30	7912	Alpha-1-acid glycoprotein 1	ENGTISR_6_5_2_3	6.3	8.3	0.0	0.0	100.0	63.9	75.3	0.0	7.6	0.0	0.0	0.0	44.7	8.5	Dual
32	AGP exp_36	10024	Alpha-1-acid glycoprotein 1	ENGTISR_6_5_2_3	9.1	7.4	0.0	0.0	100.0	34.3	31.8	18.5	0.0	0.0	0.0	0.0	10.7	26.6	Dual
33	AGP exp_37	9868	Alpha-1-acid glycoprotein 1	ENGTISR_6_5_2_3	0.0	0.0	0.0	0.0	100.0	57.7	98.9	0.0	0.0	0.0	0.0	0.0	31.3	13.4	Dual
34	AGP exp_35	9853	Alpha-1-acid glycoprotein 1	ENGTISR_6_5_2_3	0.0	9.4	0.0	0.0	100.0	65.8	70.0	2.1	8.3	0.0	0.0	0.0	39.0	10.8	Dual
35	AGP exp_34	7974	Alpha-1-acid glycoprotein 1	ENGTISR_6_5_2_3	0.0	11.8	0.0	0.0	100.0	52.0	50.0	6.1	6.9	0.0	0.0	0.0	24.0	10.5	Dual
36	AGP exp_22	9866	Alpha-1-acid glycoprotein 1	ENGTISR_6_5_2_3	7.4	10.4	0.0	0.0	85.2	56.1	100.0	0.0	6.2	0.0	0.0	0.0	38.6	10.8	Dual
37	AGP exp_28	10186	Alpha-1-acid glycoprotein 1	ENGTISR_6_5_2_3	5.0	8.7	0.0	0.0	100.0	50.4	76.9	0.0	3.3	0.0	0.0	0.0	35.5	8.6	Dual
38	AGP exp_20	9990	Alpha-1-acid glycoprotein 1	ENGTISR_6_5_2_3	8.5	9.3	0.0	0.0	100.0	56.7	75.8	0.0	5.8	0.0	0.0	0.0	40.2	12.3	Dual
39	AGP exp_32	8077	Alpha-1-acid glycoprotein 1	ENGTISR_6_5_2_3	6.6	6.7	0.0	0.0	100.0	66.9	78.2	0.0	4.6	0.0	0.0	0.0	47.9	8.7	Dual
40	AGP exp_18	9968	Alpha-1-acid glycoprotein 1	ENGTISR_6_5_2_3	7.3	6.4	0.0	0.0	100.0	36.5	19.9	9.2	0.0	0.0	0.0	0.0	5.8	5.9	Dual
41	AGP exp_19	10035	Alpha-1-acid glycoprotein 1	ENGTISR_6_5_2_3	8.0	10.5	0.0	0.0	100.0	41.5	27.8	15.6	0.0	0.0	0.0	0.0	13.4	13.1	Dual
42	AGP exp_25	10759	Alpha-1-acid glycoprotein 1	ENGTISR_6_5_2_3	15.9	0.0	0.0	0.0	100.0	30.4	57.5	33.1	0.0	0.0	0.0	0.0	24.2	15.7	Dual
43	AGP exp_3	5059	Alpha-1-acid glycoprotein 1	ENGTISR_6_5_2_3	0.0	7.8	0.0	0.0	100.0	44.7	27.0	0.0	0.0	0.0	0.0	0.0	9.7	0.0	Outer
44	AGP exp_3	2283	Alpha-1-acid glycoprotein 1	ENGTISR_7_6_1_1	100.0	24.1	0.0	0.0	33.1	69.3	58.0	0.0	0.0	0.0	0.0	0.0	0.0	0.0	Outer
45	AGP exp_4	3157	Alpha-1-acid glycoprotein 1	ENGTISR_7_6_1_2	22.8	18.5	0.0	0.0	77.8	18.3	100.0	0.0	0.0	0.0	0.0	0.0	0.0	0.0	Outer
46	AGP exp_4	3437	Alpha-1-acid glycoprotein 1	ENGTISR_7_6_1_2	22.6	18.9	0.0	0.0	100.0	38.2	69.6	0.0	0.0	0.0	0.0	0.0	0.0	0.0	Outer
47	AGP exp_3	3935	Alpha-1-acid glycoprotein 1	ENGTISR_7_6_1_2	28.0	17.9	0.0	0.0	100.0	44.6	79.8	0.0	0.0	0.0	0.0	0.0	0.0	0.0	Outer
48	AGP exp_3	3613	Alpha-1-acid glycoprotein 1	ENGTISR_7_6_1_2	21.9	26.7	0.0	0.0	100.0	29.0	67.4	0.0	0.0	0.0	0.0	0.0	0.0	0.0	Outer
49	AGP exp_4	3719	Alpha-1-acid glycoprotein 1	ENGTISR_7_6_1_2	18.5	12.4	0.0	0.0	100.0	38.3	41.3	0.0	0.0	0.0	0.0	0.0	0.0	0.0	Outer
50	AGP exp_3	3281	Alpha-1-acid glycoprotein 1	ENGTISR_7_6_1_2	32.7	18.5	0.0	0.0	71.6	25.0	100.0	0.0	0.0	0.0	0.0	0.0	0.0	0.0	Outer
51	AGP exp_3	4937	Alpha-1-acid glycoprotein 1	ENGTISR_7_6_1_3	59.3	8.5	0.0	0.0	100.0	27.0	23.0	0.0	0.0	0.0	0.0	0.0	2.4	0.0	Outer

Supplementary Table 2. Continued.

No.	Experiments	CID Scan No.	N-glycoproteins	N-glycopeptides	Relative intensities of B/Y series ions														Manual Classification
					B_2	B_2F	B_3	B_3F	B_2S	B_2SF	Y_1	Y_1F	Y_2	Y_2F	Y_3	Y_3F	Y_4	Y_4F	
52	AGP exp_3	6409	Alpha-1-acid glycoprotein 1	ENGTISR_7_6_1_4	18.4	2.9	0.0	0.0	100.0	27.8	14.3	0.0	0.0	0.0	0.0	0.0	2.6	0.0	Outer
53	AGP exp_4	6024	Alpha-1-acid glycoprotein 1	ENGTISR_7_6_1_4	26.6	2.6	0.0	0.0	100.0	24.9	9.1	0.0	0.0	0.0	0.0	0.0	5.7	0.0	Outer
54	AGP exp_3	6155	Alpha-1-acid glycoprotein 1	ENGTISR_7_6_2_4	30.5	7.0	0.0	0.0	100.0	74.0	15.0	0.0	0.0	0.0	0.0	0.0	0.0	0.0	Outer
55	AGP exp_4	21712	Alpha-1-acid glycoprotein 1	LVPVPITNATLDQITGK_5_4_0_2	0.0	0.0	0.0	0.0	100.0	0.0	74.3	0.0	9.5	0.0	9.2	0.0	27.5	0.0	None
56	AGP exp_3	22375	Alpha-1-acid glycoprotein 1	LVPVPITNATLDQITGK_5_4_0_2	0.0	0.0	0.0	0.0	100.0	0.0	66.7	0.0	11.7	0.0	12.8	0.0	38.4	0.0	None
57	AGP exp_4	22014	Alpha-1-acid glycoprotein 1	LVPVPITNATLDQITGK_5_4_0_2	0.0	0.0	0.0	0.0	100.0	0.0	76.9	0.0	9.8	0.0	12.9	0.0	55.0	0.0	None
58	AGP exp_4	21900	Alpha-1-acid glycoprotein 1	LVPVPITNATLDQITGK_5_4_0_2	62.1	0.0	3.4	0.0	100.0	0.0	4.8	0.0	6.4	0.0	18.5	0.0	96.1	0.0	None
59	AGP exp_3	21916	Alpha-1-acid glycoprotein 1	LVPVPITNATLDQITGK_5_4_0_2	66.5	0.0	5.9	0.0	100.0	0.0	0.0	0.0	11.0	0.0	17.9	0.0	88.7	0.0	None
60	AGP exp_3	22208	Alpha-1-acid glycoprotein 1	LVPVPITNATLDQITGK_5_4_0_2	71.9	0.0	7.0	0.0	100.0	0.0	0.0	0.0	0.0	0.0	15.1	0.0	77.6	0.0	None
61	AGP exp_4	17971	Alpha-1-acid glycoprotein 1	LVPVPITNATLDQITGK_6_5_0_1	0.0	0.0	0.0	0.0	48.8	0.0	100.0	0.0	6.7	0.0	9.6	0.0	33.3	0.0	None
62	AGP exp_4	18169	Alpha-1-acid glycoprotein 1	LVPVPITNATLDQITGK_6_5_0_1	100.0	0.0	8.6	0.0	48.1	0.0	43.3	0.0	8.3	0.0	16.0	0.0	62.9	0.0	None
63	AGP exp_3	18255	Alpha-1-acid glycoprotein 1	LVPVPITNATLDQITGK_6_5_0_1	100.0	0.0	6.7	0.0	60.5	0.0	37.2	0.0	0.0	0.0	17.3	0.0	55.2	0.0	None
64	AGP exp_3	18568	Alpha-1-acid glycoprotein 1	LVPVPITNATLDQITGK_6_5_0_1	100.0	0.0	4.8	0.0	30.7	0.0	46.0	0.0	8.0	0.0	10.2	0.0	66.1	0.0	None
65	AGP exp_3	21756	Alpha-1-acid glycoprotein 1	LVPVPITNATLDQITGK_6_5_0_2	0.0	0.0	0.0	0.0	89.1	0.0	100.0	0.0	8.7	0.0	18.8	0.0	75.6	0.0	None
66	AGP exp_3	21804	Alpha-1-acid glycoprotein 1	LVPVPITNATLDQITGK_6_5_0_2	71.4	0.0	6.2	0.0	100.0	0.0	53.2	0.0	5.3	0.0	16.5	0.0	51.9	0.0	None
67	AGP exp_3	21566	Alpha-1-acid glycoprotein 1	LVPVPITNATLDQITGK_6_5_0_2	72.9	0.0	7.9	0.0	100.0	0.0	54.4	0.0	0.0	0.0	16.7	0.0	57.0	0.0	None
68	AGP exp_4	21554	Alpha-1-acid glycoprotein 1	LVPVPITNATLDQITGK_6_5_0_2	71.8	0.0	6.6	0.0	100.0	0.0	49.6	0.0	0.0	0.0	18.5	0.0	47.9	0.0	None
69	AGP exp_3	22024	Alpha-1-acid glycoprotein 1	LVPVPITNATLDQITGK_6_5_0_2	39.4	0.0	6.3	0.0	100.0	0.0	61.3	0.0	3.5	0.0	17.6	0.0	64.8	0.0	None
70	AGP exp_4	21868	Alpha-1-acid glycoprotein 1	LVPVPITNATLDQITGK_6_5_0_2	62.6	0.0	0.0	0.0	100.0	0.0	39.3	0.0	0.0	0.0	15.4	0.0	44.0	0.0	None
71	AGP exp_4	24860	Alpha-1-acid glycoprotein 1	LVPVPITNATLDQITGK_6_5_0_3	0.0	0.0	0.0	0.0	100.0	0.0	62.8	0.0	6.9	0.0	7.2	0.0	20.3	0.0	None
72	AGP exp_3	25815	Alpha-1-acid glycoprotein 1	LVPVPITNATLDQITGK_6_5_0_3	0.0	0.0	0.0	0.0	100.0	0.0	51.4	0.0	7.5	0.0	9.2	0.0	24.5	0.0	None
73	AGP exp_3	25633	Alpha-1-acid glycoprotein 1	LVPVPITNATLDQITGK_6_5_0_3	28.4	0.0	0.0	0.0	100.0	0.0	36.1	0.0	4.8	0.0	4.3	0.0	31.0	0.0	None
74	AGP exp_3	25301	Alpha-1-acid glycoprotein 1	LVPVPITNATLDQITGK_6_5_0_3	30.8	0.0	2.8	0.0	100.0	0.0	41.3	0.0	7.1	0.0	8.2	0.0	31.1	0.0	None
75	AGP exp_3	25591	Alpha-1-acid glycoprotein 1	LVPVPITNATLDQITGK_6_5_0_3	31.1	0.0	3.1	0.0	100.0	0.0	35.5	0.0	4.7	0.0	8.4	0.0	38.6	0.0	None
76	AGP exp_4	24579	Alpha-1-acid glycoprotein 1	LVPVPITNATLDQITGK_6_5_0_3	0.0	0.0	0.0	0.0	100.0	0.0	50.1	0.0	5.4	0.0	7.9	0.0	11.0	0.0	None
77	AGP exp_4	24847	Alpha-1-acid glycoprotein 1	LVPVPITNATLDQITGK_6_5_0_3	30.4	0.0	2.5	0.0	100.0	0.0	34.3	0.0	6.0	0.0	8.3	0.0	29.9	0.0	None

Supplementary Table 2. Continued.

No.	Experiments	CID Scan No.	N-glycoproteins	N-glycopeptides	Relative intensities of B/Y series ions														Manual Classification
					B_2	B_2F	B_3	B_3F	B_2S	B_2SF	Y_1	Y_1F	Y_2	Y_2F	Y_3	Y_3F	Y_4	Y_4F	
78	AGP exp_4	25276	Alpha-1-acid glycoprotein 1	LVPVPITNATLDQITGK_6_5_0_3	32.1	0.0	2.6	0.0	100.0	0.0	32.7	0.0	7.7	0.0	6.7	0.0	37.2	0.0	None
79	AGP exp_4	25139	Alpha-1-acid glycoprotein 1	LVPVPITNATLDQITGK_6_5_0_3	17.2	0.0	3.9	0.0	100.0	0.0	35.8	0.0	5.7	0.0	7.2	0.0	36.9	0.0	None
80	AGP exp_3	25817	Alpha-1-acid glycoprotein 1	LVPVPITNATLDQITGK_6_5_0_3	27.4	0.0	0.0	0.0	100.0	0.0	32.8	0.0	7.2	0.0	6.0	0.0	32.1	0.0	None
81	AGP exp_4	18062	Alpha-1-acid glycoprotein 1	LVPVPITNATLDQITGK_6_5_1_1	0.0	14.2	0.0	0.0	44.6	30.8	100.0	0.0	9.9	0.0	12.8	0.0	35.5	0.0	Outer
82	AGP exp_3	18440	Alpha-1-acid glycoprotein 1	LVPVPITNATLDQITGK_6_5_1_1	0.0	14.1	0.0	0.0	43.2	44.0	100.0	0.0	12.4	0.0	19.7	0.0	41.7	0.0	Outer
83	AGP exp_4	18128	Alpha-1-acid glycoprotein 1	LVPVPITNATLDQITGK_6_5_1_1	100.0	12.3	8.7	0.0	30.8	19.1	24.5	0.0	0.0	0.0	9.7	0.0	38.2	0.0	Outer
84	AGP exp_4	21470	Alpha-1-acid glycoprotein 1	LVPVPITNATLDQITGK_6_5_1_2	0.0	11.6	0.0	0.0	100.0	53.2	96.8	0.0	10.3	0.0	0.0	0.0	31.4	0.0	Outer
85	AGP exp_3	21715	Alpha-1-acid glycoprotein 1	LVPVPITNATLDQITGK_6_5_1_2	0.0	9.8	0.0	0.0	100.0	50.3	97.5	0.0	0.0	0.0	0.0	0.0	36.8	0.0	Outer
86	AGP exp_3	22028	Alpha-1-acid glycoprotein 1	LVPVPITNATLDQITGK_6_5_1_2	0.0	0.0	0.0	0.0	100.0	49.9	62.6	0.0	6.6	0.0	0.0	0.0	30.2	0.0	Outer
87	AGP exp_4	21473	Alpha-1-acid glycoprotein 1	LVPVPITNATLDQITGK_6_5_1_2	62.9	14.1	7.1	0.0	100.0	34.9	45.7	0.0	8.9	0.0	11.5	0.0	56.6	5.0	Outer
88	AGP exp_3	21787	Alpha-1-acid glycoprotein 1	LVPVPITNATLDQITGK_6_5_1_2	67.9	17.2	7.1	0.0	100.0	35.1	57.6	0.0	10.3	0.0	26.1	0.0	64.9	4.7	Outer
89	AGP exp_4	24923	Alpha-1-acid glycoprotein 1	LVPVPITNATLDQITGK_6_5_1_3	0.0	0.0	0.0	0.0	100.0	35.4	62.2	0.0	0.0	0.0	10.1	0.0	26.4	0.0	Outer
90	AGP exp_4	25175	Alpha-1-acid glycoprotein 1	LVPVPITNATLDQITGK_6_5_1_3	0.0	0.0	0.0	0.0	100.0	33.5	47.2	0.0	0.0	0.0	11.8	0.0	35.2	0.0	Outer
91	AGP exp_3	25409	Alpha-1-acid glycoprotein 1	LVPVPITNATLDQITGK_6_5_1_3	0.0	0.0	0.0	0.0	100.0	37.8	66.5	0.0	0.0	0.0	7.9	0.0	30.7	0.0	Outer
92	AGP exp_3	25437	Alpha-1-acid glycoprotein 1	LVPVPITNATLDQITGK_6_5_1_3	30.7	6.4	3.2	0.0	100.0	28.1	44.7	0.0	5.6	0.0	10.5	0.0	30.3	0.0	Outer
93	AGP exp_3	25420	Alpha-1-acid glycoprotein 1	LVPVPITNATLDQITGK_6_5_1_3	0.0	0.0	0.0	0.0	100.0	35.7	63.0	0.0	6.8	0.0	8.9	0.0	29.0	0.0	Outer
94	AGP exp_3	25702	Alpha-1-acid glycoprotein 1	LVPVPITNATLDQITGK_6_5_1_3	0.0	8.9	0.0	0.0	100.0	34.4	51.9	0.0	0.0	0.0	12.7	0.0	28.9	0.0	Outer
95	AGP exp_3	25364	Alpha-1-acid glycoprotein 1	LVPVPITNATLDQITGK_6_5_1_3	31.5	5.8	3.3	0.0	100.0	30.1	43.8	0.0	6.4	0.0	10.9	0.0	39.2	0.0	Outer
96	AGP exp_3	25708	Alpha-1-acid glycoprotein 1	LVPVPITNATLDQITGK_6_5_1_3	27.6	0.0	0.0	0.0	100.0	36.5	14.4	0.0	0.0	0.0	7.5	0.0	35.8	0.0	Outer
97	AGP exp_4	24925	Alpha-1-acid glycoprotein 1	LVPVPITNATLDQITGK_6_5_1_3	29.1	5.7	3.6	0.0	100.0	25.9	37.7	0.0	6.2	0.0	11.7	0.0	35.9	0.0	Outer
98	AGP exp_4	25177	Alpha-1-acid glycoprotein 1	LVPVPITNATLDQITGK_6_5_1_3	29.6	0.0	0.0	0.0	100.0	22.4	24.7	0.0	3.4	0.0	13.4	0.0	36.5	0.0	Outer
99	AGP exp_18	35503	Alpha-1-acid glycoprotein 1	LVPVPITNATLDQITGK_6_5_2_2	0.0	20.9	0.0	0.0	100.0	57.5	72.5	0.0	0.0	0.0	0.0	0.0	20.5	16.7	Dual
100	AGP exp_7	29857	Alpha-1-acid glycoprotein 1	LVPVPITNATLDQITGK_6_5_2_3	25.5	13.9	4.1	0.0	100.0	46.5	26.4	0.0	0.0	0.0	5.2	0.0	28.1	8.8	Dual
101	AGP exp_38	32062	Alpha-1-acid glycoprotein 1	LVPVPITNATLDQITGK_6_5_2_3	24.4	0.0	0.0	0.0	100.0	23.7	10.6	10.0	2.5	0.0	0.0	0.0	0.0	0.0	Dual
102	AGP exp_32	29837	Alpha-1-acid glycoprotein 1	LVPVPITNATLDQITGK_6_5_2_3	30.4	10.0	0.0	0.0	100.0	22.7	0.0	0.0	0.0	0.0	0.0	0.0	5.6	27.6	Dual
103	AGP exp_30	27103	Alpha-1-acid glycoprotein 1	LVPVPITNATLDQITGK_6_5_2_3	29.9	0.0	0.0	0.0	100.0	39.0	0.0	0.0	0.0	0.0	0.0	0.0	9.8	37.5	Dual

Supplementary Table 2. Continued.

No.	Experiments	CID Scan No.	N-glycoproteins	N-glycopeptides	Relative intensities of B/Y series ions														Manual Classification
					B_2	B_2F	B_3	B_3F	B_2S	B_2SF	Y_1	Y_1F	Y_2	Y_2F	Y_3	Y_3F	Y_4	Y_4F	
104	AGP exp_4	1589	Alpha-1-acid glycoprotein 1,2	NEEYNK_4_3_0_0	48.1	0.0	11.2	0.0	0.0	0.0	100.0	0.0	3.4	0.0	9.0	0.0	71.9	0.0	None
105	AGP exp_3	1567	Alpha-1-acid glycoprotein 1,2	NEEYNK_4_3_0_0	65.3	0.0	8.3	0.0	0.0	0.0	100.0	0.0	9.7	0.0	5.4	0.0	63.7	0.0	None
106	AGP exp_4	1966	Alpha-1-acid glycoprotein 1,2	NEEYNK_4_3_0_1	27.7	0.0	5.8	0.0	32.0	0.0	100.0	0.0	5.4	0.0	4.1	0.0	73.2	0.0	None
107	AGP exp_3	1408	Alpha-1-acid glycoprotein 1,2	NEEYNK_4_3_0_1	16.7	0.0	0.0	0.0	12.2	0.0	33.1	0.0	13.5	0.0	9.2	0.0	100.0	0.0	None
108	AGP exp_3	1944	Alpha-1-acid glycoprotein 1,2	NEEYNK_4_3_0_1	15.2	0.0	6.7	0.0	38.2	0.0	100.0	0.0	4.6	0.0	6.0	0.0	69.5	0.0	None
109	AGP exp_4	1425	Alpha-1-acid glycoprotein 1,2	NEEYNK_4_3_0_1	12.5	0.0	0.0	0.0	31.7	0.0	20.1	0.0	5.0	0.0	4.5	0.0	100.0	0.0	None
110	AGP exp_4	1549	Alpha-1-acid glycoprotein 1,2	NEEYNK_4_4_0_1	36.0	0.0	0.0	0.0	54.5	0.0	100.0	0.0	14.1	0.0	58.0	0.0	46.2	0.0	None
111	AGP exp_4	1798	Alpha-1-acid glycoprotein 1,2	NEEYNK_4_4_0_1	17.0	0.0	4.0	0.0	31.5	0.0	100.0	0.0	12.8	0.0	18.5	0.0	46.4	0.0	None
112	AGP exp_3	1787	Alpha-1-acid glycoprotein 1,2	NEEYNK_4_4_0_1	11.5	0.0	7.7	0.0	24.8	0.0	100.0	0.0	12.0	0.0	15.9	0.0	46.4	0.0	None
113	AGP exp_3	1532	Alpha-1-acid glycoprotein 1,2	NEEYNK_4_4_0_1	37.3	0.0	0.0	0.0	61.7	0.0	100.0	0.0	34.6	0.0	71.6	0.0	79.1	0.0	None
114	AGP exp_3	2054	Alpha-1-acid glycoprotein 1,2	NEEYNK_4_4_0_1	53.2	0.0	0.0	0.0	48.5	0.0	100.0	0.0	0.0	0.0	34.8	0.0	51.4	0.0	None
115	AGP exp_3	1357	Alpha-1-acid glycoprotein 1,2	NEEYNK_5_4_0_0	76.2	0.0	12.4	0.0	0.0	0.0	100.0	0.0	0.0	0.0	4.6	0.0	46.3	0.0	None
116	AGP exp_4	1370	Alpha-1-acid glycoprotein 1,2	NEEYNK_5_4_0_0	52.4	0.0	14.2	0.0	0.0	0.0	100.0	0.0	0.0	0.0	21.5	0.0	29.9	0.0	None
117	AGP exp_3	1780	Alpha-1-acid glycoprotein 1,2	NEEYNK_5_4_0_1	100.0	0.0	7.1	0.0	68.9	0.0	44.6	0.0	0.0	0.0	0.0	0.0	32.2	0.0	None
118	AGP exp_3	2771	Alpha-1-acid glycoprotein 1,2	NEEYNK_5_4_0_1	100.0	0.0	12.0	0.0	59.5	0.0	41.4	0.0	0.0	0.0	0.0	0.0	19.9	0.0	None
119	AGP exp_4	1807	Alpha-1-acid glycoprotein 1,2	NEEYNK_5_4_0_1	100.0	0.0	6.7	0.0	49.5	0.0	50.4	0.0	0.0	0.0	4.4	0.0	39.3	0.0	None
120	AGP exp_3	2047	Alpha-1-acid glycoprotein 1,2	NEEYNK_5_4_0_1	0.0	0.0	7.4	0.0	26.4	0.0	100.0	0.0	18.8	0.0	11.3	0.0	36.3	0.0	None
121	AGP exp_4	1800	Alpha-1-acid glycoprotein 1,2	NEEYNK_5_4_0_1	0.0	0.0	5.9	0.0	25.6	0.0	100.0	0.0	29.0	0.0	15.1	0.0	42.6	0.0	None
122	AGP exp_4	1551	Alpha-1-acid glycoprotein 1,2	NEEYNK_5_4_0_1	100.0	0.0	13.1	0.0	72.8	0.0	27.9	0.0	0.0	0.0	0.0	0.0	5.4	0.0	None
123	AGP exp_3	1778	Alpha-1-acid glycoprotein 1,2	NEEYNK_5_4_0_1	0.0	0.0	7.2	0.0	27.5	0.0	100.0	0.0	20.2	0.0	13.2	0.0	36.9	0.0	None
124	AGP exp_4	1546	Alpha-1-acid glycoprotein 1,2	NEEYNK_5_4_0_1	0.0	0.0	0.0	0.0	54.2	0.0	100.0	0.0	10.7	0.0	10.6	0.0	21.3	0.0	None
125	AGP exp_3	2936	Alpha-1-acid glycoprotein 1,2	NEEYNK_5_4_0_1	0.0	0.0	5.6	0.0	30.8	0.0	100.0	0.0	12.0	0.0	9.5	0.0	35.5	0.0	None
126	AGP exp_4	2035	Alpha-1-acid glycoprotein 1,2	NEEYNK_5_4_0_1	0.0	0.0	5.0	0.0	32.5	0.0	100.0	0.0	19.4	0.0	8.8	0.0	41.3	0.0	None
127	AGP exp_3	3261	Alpha-1-acid glycoprotein 1,2	NEEYNK_5_4_0_1	0.0	0.0	17.2	0.0	61.0	0.0	100.0	0.0	8.9	0.0	16.1	0.0	59.4	0.0	None
128	AGP exp_3	2261	Alpha-1-acid glycoprotein 1,2	NEEYNK_5_4_0_1	0.0	0.0	8.2	0.0	58.0	0.0	100.0	0.0	18.1	0.0	12.2	0.0	51.1	0.0	None
129	AGP exp_4	2401	Alpha-1-acid glycoprotein 1,2	NEEYNK_5_4_0_1	0.0	0.0	21.4	0.0	53.9	0.0	100.0	0.0	21.0	0.0	0.0	0.0	30.0	0.0	None

Supplementary Table 2. Continued.

No.	Experiments	CID Scan No.	N-glycoproteins	N-glycopeptides	Relative intensities of B/Y series ions														Manual Classification
					B_2	B_2F	B_3	B_3F	B_2S	B_2SF	Y_1	Y_1F	Y_2	Y_2F	Y_3	Y_3F	Y_4	Y_4F	
130	AGP exp_4	2242	Alpha-1-acid glycoprotein 1,2	NEEYNK_5_4_0_1	0.0	0.0	5.1	0.0	39.4	0.0	100.0	0.0	12.0	0.0	4.2	0.0	28.1	0.0	None
131	AGP exp_3	2809	Alpha-1-acid glycoprotein 1,2	NEEYNK_5_4_0_2	55.0	0.0	5.8	0.0	100.0	0.0	0.0	0.0	4.1	0.0	7.1	0.0	37.3	0.0	None
132	AGP exp_3	3521	Alpha-1-acid glycoprotein 1,2	NEEYNK_5_4_0_2	51.9	0.0	4.9	0.0	100.0	0.0	0.0	0.0	4.0	0.0	5.3	0.0	41.3	0.0	None
133	AGP exp_3	3173	Alpha-1-acid glycoprotein 1,2	NEEYNK_5_4_0_2	54.8	0.0	5.1	0.0	100.0	0.0	0.0	0.0	4.2	0.0	7.2	0.0	37.3	0.0	None
134	AGP exp_4	2755	Alpha-1-acid glycoprotein 1,2	NEEYNK_5_4_0_2	57.4	0.0	5.5	0.0	100.0	0.0	0.0	0.0	4.9	0.0	8.3	0.0	41.9	0.0	None
135	AGP exp_4	3183	Alpha-1-acid glycoprotein 1,2	NEEYNK_5_4_0_2	0.0	0.0	2.9	0.0	36.5	0.0	100.0	0.0	12.0	0.0	7.9	0.0	22.6	0.0	None
136	AGP exp_4	3580	Alpha-1-acid glycoprotein 1,2	NEEYNK_5_4_0_2	56.8	0.0	6.0	0.0	100.0	0.0	0.0	0.0	0.0	0.0	5.2	0.0	31.8	0.0	None
137	AGP exp_3	2809	Alpha-1-acid glycoprotein 1,2	NEEYNK_5_4_0_2	55.0	0.0	5.8	0.0	100.0	0.0	0.0	0.0	4.1	0.0	7.1	0.0	37.3	0.0	None
138	AGP exp_4	3294	Alpha-1-acid glycoprotein 1,2	NEEYNK_5_4_0_2	55.3	0.0	4.4	0.0	100.0	0.0	0.0	0.0	0.0	0.0	7.7	0.0	43.0	0.0	None
139	AGP exp_3	3360	Alpha-1-acid glycoprotein 1,2	NEEYNK_5_4_0_2	0.0	0.0	4.1	0.0	50.0	0.0	100.0	0.0	18.7	0.0	9.2	0.0	35.1	0.0	None
140	AGP exp_3	3834	Alpha-1-acid glycoprotein 1,2	NEEYNK_5_4_0_2	54.1	0.0	4.5	0.0	100.0	0.0	0.0	0.0	0.0	0.0	3.5	0.0	28.2	0.0	None
141	AGP exp_4	2755	Alpha-1-acid glycoprotein 1,2	NEEYNK_5_4_0_2	57.4	0.0	5.5	0.0	100.0	0.0	0.0	0.0	4.9	0.0	8.3	0.0	41.9	0.0	None
142	AGP exp_3	2718	Alpha-1-acid glycoprotein 1,2	NEEYNK_5_4_0_2	0.0	0.0	0.0	0.0	80.7	0.0	100.0	0.0	9.4	0.0	9.0	0.0	43.4	0.0	None
143	AGP exp_3	4731	Alpha-1-acid glycoprotein 1,2	NEEYNK_5_4_0_2	0.0	0.0	0.0	0.0	62.5	0.0	100.0	0.0	9.3	0.0	3.7	0.0	28.8	0.0	None
144	AGP exp_4	3485	Alpha-1-acid glycoprotein 1,2	NEEYNK_5_4_0_2	0.0	0.0	6.0	0.0	50.9	0.0	100.0	0.0	19.3	0.0	9.5	0.0	38.1	0.0	None
145	AGP exp_3	3685	Alpha-1-acid glycoprotein 1,2	NEEYNK_5_4_0_2	0.0	0.0	4.2	0.0	62.5	3.6	100.0	0.0	14.1	0.0	11.0	0.0	35.3	0.0	None
146	AGP exp_4	1715	Alpha-1-acid glycoprotein 1,2	NEEYNK_5_4_1_1	0.0	0.0	0.0	0.0	18.8	17.7	100.0	0.0	21.1	0.0	0.0	0.0	26.1	0.0	Outer
147	AGP exp_4	3137	Alpha-1-acid glycoprotein 1,2	NEEYNK_5_4_1_2	100.0	0.0	0.0	0.0	69.9	7.0	25.6	42.3	0.0	0.0	0.0	0.0	10.3	28.9	Core
148	AGP exp_4	2595	Alpha-1-acid glycoprotein 1,2	NEEYNK_5_4_1_2	82.2	0.0	0.0	0.0	100.0	20.6	20.5	0.0	0.0	0.0	0.0	0.0	20.8	0.0	Outer
149	AGP exp_3	2931	Alpha-1-acid glycoprotein 1,2	NEEYNK_5_4_1_2	51.9	7.1	4.0	0.0	100.0	21.9	57.6	0.0	0.0	0.0	3.6	0.0	18.7	6.2	Outer
150	AGP exp_3	2675	Alpha-1-acid glycoprotein 1,2	NEEYNK_5_4_1_2	100.0	0.0	0.0	0.0	86.8	23.7	37.6	0.0	0.0	0.0	0.0	0.0	37.5	0.0	Outer
151	AGP exp_4	2845	Alpha-1-acid glycoprotein 1,2	NEEYNK_5_4_1_2	33.6	4.7	0.0	0.0	100.0	29.0	39.8	0.0	0.0	0.0	1.9	0.0	26.7	0.0	Outer
152	AGP exp_27	4342	Alpha-1-acid glycoprotein 1,2	NEEYNK_5_4_2_2	65.9	0.0	0.0	0.0	100.0	27.2	32.0	15.9	5.9	0.0	0.0	0.0	0.0	0.0	Dual
153	AGP exp_18	4264	Alpha-1-acid glycoprotein 1,2	NEEYNK_5_4_2_2	54.8	0.0	0.0	0.0	100.0	25.4	19.4	18.0	0.0	0.0	0.0	0.0	7.2	0.0	Dual
154	AGP exp_14	4857	Alpha-1-acid glycoprotein 1,2	NEEYNK_5_4_2_2	97.3	0.0	0.0	0.0	100.0	56.6	56.5	16.2	0.0	0.0	0.0	0.0	0.0	0.0	Dual
155	AGP exp_10	4131	Alpha-1-acid glycoprotein 1,2	NEEYNK_5_4_2_2	64.0	0.0	0.0	0.0	100.0	30.1	29.7	22.4	5.4	0.0	0.0	0.0	6.3	22.0	Dual

Supplementary Table 2. Continued.

No.	Experiments	CID Scan No.	N-glycoproteins	N-glycopeptides	Relative intensities of B/Y series ions														Manual Classification
					B_2	B_2F	B_3	B_3F	B_2S	B_2SF	Y_1	Y_1F	Y_2	Y_2F	Y_3	Y_3F	Y_4	Y_4F	
156	AGP exp_16	4783	Alpha-1-acid glycoprotein 1,2	NEEYNK_5_4_2_2	61.4	0.0	0.0	0.0	100.0	26.6	45.3	11.2	0.0	0.0	0.0	0.0	0.0	0.0	Dual
157	AGP exp_3	3997	Alpha-1-acid glycoprotein 1,2	NEEYNK_5_5_0_2	45.9	0.0	9.5	0.0	100.0	0.0	58.3	0.0	0.0	0.0	4.4	0.0	19.1	0.0	None
158	AGP exp_3	2276	Alpha-1-acid glycoprotein 1,2	NEEYNK_6_5_0_1	100.0	0.0	7.8	0.0	48.1	0.0	90.8	0.0	3.7	0.0	4.3	0.0	32.8	0.0	None
159	AGP exp_3	2471	Alpha-1-acid glycoprotein 1,2	NEEYNK_6_5_0_1	100.0	0.0	10.3	0.0	46.7	0.0	51.2	0.0	0.0	0.0	0.0	0.0	14.8	0.0	None
160	AGP exp_4	2266	Alpha-1-acid glycoprotein 1,2	NEEYNK_6_5_0_1	0.0	0.0	16.7	0.0	30.9	0.0	100.0	0.0	14.7	0.0	9.8	0.0	12.4	0.0	None
161	AGP exp_4	2383	Alpha-1-acid glycoprotein 1,2	NEEYNK_6_5_0_1	100.0	0.0	7.7	0.0	36.9	0.0	52.4	0.0	0.0	0.0	0.0	0.0	17.5	0.0	None
162	AGP exp_4	1770	Alpha-1-acid glycoprotein 1,2	NEEYNK_6_5_0_1	100.0	0.0	12.5	0.0	75.0	0.0	46.4	0.0	4.5	0.0	0.0	0.0	14.6	0.0	None
163	AGP exp_4	2226	Alpha-1-acid glycoprotein 1,2	NEEYNK_6_5_0_1	98.1	0.0	7.8	0.0	47.0	0.0	100.0	0.0	0.0	0.0	4.8	0.0	29.0	0.0	None
164	AGP exp_3	2071	Alpha-1-acid glycoprotein 1,2	NEEYNK_6_5_0_1	100.0	0.0	7.7	0.0	44.5	0.0	57.1	0.0	0.0	0.0	5.1	0.0	26.4	0.0	None
165	AGP exp_4	1845	Alpha-1-acid glycoprotein 1,2	NEEYNK_6_5_0_1	0.0	0.0	0.0	0.0	38.2	0.0	100.0	0.0	24.4	0.0	9.5	0.0	20.3	0.0	None
166	AGP exp_3	3763	Alpha-1-acid glycoprotein 1,2	NEEYNK_6_5_0_2	70.6	0.0	6.2	0.0	100.0	0.0	92.0	0.0	6.6	0.0	11.0	0.0	44.8	0.0	None
167	AGP exp_4	3530	Alpha-1-acid glycoprotein 1,2	NEEYNK_6_5_0_2	69.6	0.0	0.0	0.0	95.6	0.0	100.0	0.0	5.9	0.0	15.2	0.0	44.4	0.0	None
168	AGP exp_4	3235	Alpha-1-acid glycoprotein 1,2	NEEYNK_6_5_0_2	73.3	0.0	5.1	0.0	88.5	0.0	100.0	0.0	6.4	0.0	11.8	0.0	41.5	0.0	None
169	AGP exp_4	3849	Alpha-1-acid glycoprotein 1,2	NEEYNK_6_5_0_2	61.7	0.0	7.2	0.0	96.4	0.0	100.0	0.0	7.8	0.0	13.8	0.0	39.1	0.0	None
170	AGP exp_3	3214	Alpha-1-acid glycoprotein 1,2	NEEYNK_6_5_0_2	0.0	0.0	0.0	0.0	33.2	0.0	100.0	0.0	19.3	0.0	11.9	0.0	15.8	0.0	None
171	AGP exp_4	3732	Alpha-1-acid glycoprotein 1,2	NEEYNK_6_5_0_2	0.0	0.0	0.0	0.0	65.7	0.0	100.0	0.0	43.9	0.0	17.3	0.0	27.3	0.0	None
172	AGP exp_3	4095	Alpha-1-acid glycoprotein 1,2	NEEYNK_6_5_0_2	68.4	0.0	5.3	0.0	100.0	0.0	95.1	0.0	8.5	0.0	9.4	0.0	45.3	0.0	None
173	AGP exp_3	3129	Alpha-1-acid glycoprotein 1,2	NEEYNK_6_5_0_2	22.5	0.0	6.1	0.0	85.9	0.0	100.0	0.0	0.0	0.0	0.0	0.0	26.9	0.0	None
174	AGP exp_4	2979	Alpha-1-acid glycoprotein 1,2	NEEYNK_6_5_0_2	65.3	0.0	0.0	0.0	100.0	0.0	92.6	0.0	0.0	0.0	6.1	0.0	26.8	0.0	None
175	AGP exp_3	4779	Alpha-1-acid glycoprotein 1,2	NEEYNK_6_5_0_3	27.4	0.0	0.0	0.0	100.0	0.0	88.6	0.0	9.4	0.0	11.4	0.0	39.5	0.0	None
176	AGP exp_4	4617	Alpha-1-acid glycoprotein 1,2	NEEYNK_6_5_0_3	28.8	0.0	4.3	0.0	100.0	0.0	88.1	0.0	7.9	0.0	11.8	0.0	43.9	0.0	None
177	AGP exp_4	1713	Alpha-1-acid glycoprotein 1,2	NEEYNK_6_5_1_1	100.0	42.6	0.0	0.0	58.8	0.0	47.9	0.0	0.0	0.0	4.2	0.0	22.0	0.0	Outer
178	AGP exp_4	2002	Alpha-1-acid glycoprotein 1,2	NEEYNK_6_5_1_1	100.0	34.2	0.0	0.0	48.4	0.0	81.6	0.0	0.0	0.0	5.3	0.0	22.9	0.0	Outer
179	AGP exp_4	2181	Alpha-1-acid glycoprotein 1,2	NEEYNK_6_5_1_1	77.7	15.8	0.0	0.0	41.8	28.0	100.0	0.0	0.0	0.0	6.1	0.0	27.0	2.5	Outer
180	AGP exp_3	2008	Alpha-1-acid glycoprotein 1,2	NEEYNK_6_5_1_1	100.0	20.3	0.0	0.0	56.3	0.0	44.5	0.0	6.7	0.0	0.0	0.0	11.5	0.0	Outer
181	AGP exp_3	2237	Alpha-1-acid glycoprotein 1,2	NEEYNK_6_5_1_1	88.0	16.4	5.8	0.0	33.3	24.1	100.0	0.0	0.0	0.0	0.0	0.0	32.0	0.0	Outer

Supplementary Table 2. Continued.

No.	Experiments	CID Scan No.	N-glycoproteins	N-glycopeptides	Relative intensities of B/Y series ions														Manual Classification
					B_2	B_2F	B_3	B_3F	B_2S	B_2SF	Y_1	Y_1F	Y_2	Y_2F	Y_3	Y_3F	Y_4	Y_4F	
182	AGP exp_3	3683	Alpha-1-acid glycoprotein 1,2	NEEYNK_6_5_1_2	51.9	9.5	2.8	0.0	100.0	27.0	69.2	6.1	0.0	0.0	4.5	0.0	27.6	6.9	Outer
183	AGP exp_3	3041	Alpha-1-acid glycoprotein 1,2	NEEYNK_6_5_1_2	49.3	17.0	5.1	0.0	78.6	18.7	100.0	0.0	0.0	0.0	13.4	0.0	39.3	1.8	Outer
184	AGP exp_4	3217	Alpha-1-acid glycoprotein 1,2	NEEYNK_6_5_1_2	70.1	19.5	0.0	0.0	100.0	35.6	70.7	0.0	12.9	0.0	0.0	0.0	40.5	0.0	Outer
185	AGP exp_4	2939	Alpha-1-acid glycoprotein 1,2	NEEYNK_6_5_1_2	24.2	15.4	5.5	0.0	72.1	20.5	100.0	0.0	0.0	0.0	12.8	0.0	38.5	0.0	Outer
186	AGP exp_3	3362	Alpha-1-acid glycoprotein 1,2	NEEYNK_6_5_1_2	56.9	16.9	0.0	0.0	76.0	32.8	100.0	0.0	0.0	0.0	3.8	0.0	47.2	0.0	Outer
187	AGP exp_4	2632	Alpha-1-acid glycoprotein 1,2	NEEYNK_6_5_1_2	46.9	0.0	0.0	0.0	100.0	33.9	81.9	0.0	0.0	0.0	0.0	0.0	16.1	0.0	Outer
188	AGP exp_4	3797	Alpha-1-acid glycoprotein 1,2	NEEYNK_6_5_1_2	84.0	5.6	0.0	0.0	100.0	30.2	99.4	7.5	0.0	0.0	5.0	0.0	34.9	4.0	Outer
189	AGP exp_3	3991	Alpha-1-acid glycoprotein 1,2	NEEYNK_6_5_1_2	65.1	13.9	0.0	0.0	100.0	34.2	86.7	7.2	0.0	0.0	0.0	0.0	46.6	4.3	Outer
190	AGP exp_4	3217	Alpha-1-acid glycoprotein 1,2	NEEYNK_6_5_1_2	70.1	19.5	0.0	0.0	100.0	35.6	70.7	0.0	12.9	0.0	0.0	0.0	40.5	0.0	Outer
191	AGP exp_3	4752	Alpha-1-acid glycoprotein 1,2	NEEYNK_6_5_1_3	17.8	5.3	3.6	0.0	89.3	32.8	100.0	0.0	0.0	0.0	12.7	0.0	47.4	0.0	Outer
192	AGP exp_4	4522	Alpha-1-acid glycoprotein 1,2	NEEYNK_6_5_1_3	18.0	6.1	0.0	0.0	90.3	31.1	100.0	0.0	0.0	0.0	12.7	0.0	41.2	0.0	Outer
193	AGP exp_32	3737	Alpha-1-acid glycoprotein 1,2	NEEYNK_6_5_2_2	54.8	28.1	7.4	0.0	100.0	22.3	49.0	17.0	0.0	0.0	4.9	0.0	17.3	10.7	Dual
194	AGP exp_27	3977	Alpha-1-acid glycoprotein 1,2	NEEYNK_6_5_2_2	35.8	17.5	5.9	0.0	100.0	12.5	22.8	12.2	0.0	0.0	3.5	0.0	7.4	15.4	Dual
195	AGP exp_35	4713	Alpha-1-acid glycoprotein 1,2	NEEYNK_6_5_2_2	65.2	16.0	0.0	0.0	100.0	30.1	57.8	21.0	4.8	0.0	4.5	0.0	12.6	14.8	Dual
196	AGP exp_33	3653	Alpha-1-acid glycoprotein 1,2	NEEYNK_6_5_2_2	49.8	15.4	0.0	0.0	100.0	20.0	40.7	15.1	5.2	0.0	0.0	0.0	11.0	0.0	Dual
197	AGP exp_39	4604	Alpha-1-acid glycoprotein 1,2	NEEYNK_6_5_2_2	55.3	17.6	0.0	0.0	76.7	51.0	100.0	4.8	5.6	0.0	0.0	0.0	28.9	8.1	Dual
198	AGP exp_10	3850	Alpha-1-acid glycoprotein 1,2	NEEYNK_6_5_2_2	57.2	17.7	0.0	0.0	100.0	0.0	18.4	8.6	0.0	0.0	0.0	0.0	0.0	0.0	Dual
199	AGP exp_33	4027	Alpha-1-acid glycoprotein 1,2	NEEYNK_6_5_2_2	69.8	14.4	13.0	0.0	100.0	19.6	36.4	32.9	0.0	0.0	0.0	0.0	0.0	0.0	Dual
200	AGP exp_43	4537	Alpha-1-acid glycoprotein 1,2	NEEYNK_6_5_2_2	71.0	13.5	0.0	0.0	100.0	26.9	63.8	23.5	0.0	0.0	0.0	0.0	18.4	4.7	Dual
201	AGP exp_10	4070	Alpha-1-acid glycoprotein 1,2	NEEYNK_6_5_2_2	68.6	14.1	0.0	0.0	100.0	18.4	10.9	41.6	0.0	0.0	0.0	0.0	0.0	0.0	Dual
202	AGP exp_3	2752	Alpha-1-acid glycoprotein 1,2	NEEYNK_6_5_2_2	67.4	81.5	0.0	0.0	100.0	0.0	97.6	0.0	0.0	0.0	0.0	0.0	27.5	0.0	Outer
203	AGP exp_37	4770	Alpha-1-acid glycoprotein 1,2	NEEYNK_6_5_2_2	53.9	15.0	0.0	0.0	100.0	15.7	42.3	35.3	0.0	0.0	0.0	0.0	11.2	20.2	Dual
204	AGP exp_42	4787	Alpha-1-acid glycoprotein 1,2	NEEYNK_6_5_2_2	74.8	20.1	0.0	0.0	100.0	60.7	99.8	14.7	0.0	0.0	0.0	0.0	27.4	11.4	Dual
205	AGP exp_38	4696	Alpha-1-acid glycoprotein 1,2	NEEYNK_6_5_2_2	67.3	10.4	0.0	0.0	100.0	14.0	49.9	20.3	0.0	0.0	0.0	0.0	9.2	8.6	Dual
206	AGP exp_28	4926	Alpha-1-acid glycoprotein 1,2	NEEYNK_6_5_2_2	29.0	9.8	0.0	0.0	100.0	21.4	43.0	20.7	0.0	0.0	0.0	0.0	3.7	0.0	Dual
207	AGP exp_16	5415	Alpha-1-acid glycoprotein 1,2	NEEYNK_6_5_2_2	61.3	0.0	0.0	0.0	100.0	32.4	42.1	23.6	0.0	0.0	0.0	0.0	0.0	0.0	Dual

Supplementary Table 2. Continued.

No.	Experiments	CID Scan No.	N-glycoproteins	N-glycopeptides	Relative intensities of B/Y series ions														Manual Classification
					B_2	B_2F	B_3	B_3F	B_2S	B_2SF	Y_1	Y_1F	Y_2	Y_2F	Y_3	Y_3F	Y_4	Y_4F	
208	AGP exp_40	4658	Alpha-1-acid glycoprotein 1,2	NEEYNK_6_5_2_2	80.8	10.8	11.9	0.0	100.0	30.4	90.4	36.0	0.0	0.0	4.9	4.3	8.1	11.1	Dual
209	AGP exp_13	5550	Alpha-1-acid glycoprotein 1,2	NEEYNK_6_5_2_2	41.3	0.0	0.0	0.0	100.0	28.2	35.8	13.9	0.0	0.0	0.0	0.0	0.0	0.0	Dual
210	AGP exp_26	5048	Alpha-1-acid glycoprotein 1,2	NEEYNK_6_5_2_2	58.3	9.8	11.9	0.0	100.0	31.9	36.8	29.7	0.0	0.0	0.0	0.0	10.8	0.0	Dual
211	AGP exp_40	4328	Alpha-1-acid glycoprotein 1,2	NEEYNK_6_5_2_2	68.6	14.9	0.0	0.0	100.0	23.6	45.8	8.4	0.0	0.0	0.0	0.0	0.0	0.0	Dual
212	AGP exp_43	4877	Alpha-1-acid glycoprotein 1,2	NEEYNK_6_5_2_2	60.2	10.7	7.1	0.0	100.0	15.8	52.9	9.9	0.0	0.0	0.0	0.0	19.8	10.3	Dual
213	AGP exp_16	9686	Alpha-1-acid glycoprotein 1,2	NEEYNK_6_5_2_3	0.0	10.5	6.0	0.0	100.0	65.4	99.0	0.0	0.0	0.0	12.5	0.0	40.2	11.5	Dual
214	AGP exp_10	7457	Alpha-1-acid glycoprotein 1,2	NEEYNK_6_5_2_3	0.0	10.1	0.0	0.0	100.0	47.8	69.8	15.2	0.0	0.0	8.5	0.0	28.9	15.3	Dual
215	AGP exp_15	9515	Alpha-1-acid glycoprotein 1,2	NEEYNK_6_5_2_3	0.0	10.6	4.1	0.0	95.4	55.7	100.0	0.0	8.0	0.0	8.4	0.0	38.7	9.8	Dual
216	AGP exp_43	8738	Alpha-1-acid glycoprotein 1,2	NEEYNK_6_5_2_3	0.0	7.6	0.0	0.0	100.0	45.0	85.5	14.3	0.0	0.0	8.8	0.0	37.5	17.1	Dual
217	AGP exp_39	8633	Alpha-1-acid glycoprotein 1,2	NEEYNK_6_5_2_3	3.5	9.2	4.8	0.0	100.0	46.3	75.0	10.6	0.0	0.0	8.5	3.3	33.2	14.0	Dual
218	AGP exp_30	3882	Alpha-1-acid glycoprotein 1,2	NEEYNK_6_5_2_3	0.0	5.5	0.0	0.0	100.0	27.1	22.9	35.5	0.0	0.0	0.0	0.0	11.7	24.6	Dual
219	AGP exp_27	8385	Alpha-1-acid glycoprotein 1,2	NEEYNK_6_5_2_3	0.0	9.9	0.0	0.0	100.0	49.8	78.4	16.9	0.0	0.0	10.3	0.0	31.2	23.2	Dual
220	AGP exp_22	8445	Alpha-1-acid glycoprotein 1,2	NEEYNK_6_5_2_3	0.0	8.2	0.0	0.0	100.0	41.4	78.7	0.0	0.0	0.0	0.0	0.0	21.9	9.5	Dual
221	AGP exp_35	8687	Alpha-1-acid glycoprotein 1,2	NEEYNK_6_5_2_3	0.0	9.2	4.8	0.0	100.0	46.0	66.7	21.6	0.0	0.0	5.1	0.0	25.2	14.7	Dual
222	AGP exp_18	8217	Alpha-1-acid glycoprotein 1,2	NEEYNK_6_5_2_3	0.0	8.4	0.0	0.0	100.0	52.3	86.6	7.8	0.0	0.0	9.6	0.0	40.2	14.2	Dual
223	AGP exp_24	8858	Alpha-1-acid glycoprotein 1,2	NEEYNK_6_5_2_3	0.0	10.9	0.0	0.0	100.0	44.2	83.9	14.0	0.0	0.0	6.5	0.0	33.7	13.4	Dual
224	AGP exp_29	3918	Alpha-1-acid glycoprotein 1,2	NEEYNK_6_5_2_3	0.0	3.0	0.0	0.0	100.0	20.5	45.4	21.0	7.0	0.0	2.6	0.0	14.1	12.8	Dual
225	AGP exp_4	4619	Alpha-1-acid glycoprotein 1,2	NEEYNK_6_5_2_3	0.0	5.6	6.4	0.0	100.0	35.4	33.6	21.8	0.0	0.0	0.0	0.0	22.9	15.6	Dual
226	AGP exp_13	10122	Alpha-1-acid glycoprotein 1,2	NEEYNK_6_5_2_3	0.0	3.5	1.9	0.0	100.0	24.6	41.1	16.0	0.0	0.0	4.1	1.8	12.7	16.2	Dual
227	AGP exp_6	5905	Alpha-1-acid glycoprotein 1,2	NEEYNK_6_5_2_3	0.0	4.5	0.0	0.0	100.0	33.1	83.4	2.7	0.0	0.0	0.0	0.0	28.4	8.4	Dual
228	AGP exp_3	4679	Alpha-1-acid glycoprotein 1,2	NEEYNK_6_5_3_1	56.7	10.2	0.0	0.0	100.0	22.5	59.0	0.0	6.9	0.0	8.8	0.0	25.5	0.0	Outer
229	AGP exp_3	5953	Alpha-1-acid glycoprotein 1,2	NEEYNK_6_5_3_2	20.0	7.4	5.0	0.0	100.0	23.3	0.0	0.0	0.0	0.0	0.0	0.0	0.0	0.0	Outer
230	AGP exp_4	26479	Alpha-1-acid glycoprotein 1,2	SVQEIQATFFYFTPNK_6_5_0_3	21.2	0.0	6.6	0.0	100.0	0.0	57.3	0.0	2.7	0.0	12.9	0.0	27.7	0.0	None
231	AGP exp_4	26406	Alpha-1-acid glycoprotein 1,2	SVQEIQATFFYFTPNK_7_6_0_3	9.2	0.0	0.0	0.0	100.0	0.0	16.3	0.0	1.5	0.0	0.0	0.0	4.1	0.0	None
232	AGP exp_4	1622	Alpha-1-acid glycoprotein 2	ENGTVSR_6_5_0_1	100.0	0.0	7.4	0.0	40.4	0.0	55.8	0.0	0.0	0.0	0.0	0.0	18.6	0.0	None
233	AGP exp_4	2301	Alpha-1-acid glycoprotein 2	ENGTVSR_6_5_0_2	100.0	0.0	0.0	0.0	71.9	0.0	98.7	0.0	0.0	0.0	11.6	0.0	45.2	0.0	None

Supplementary Table 2. Continued.

No.	Experiments	CID Scan No.	N-glycoproteins	N-glycopeptides	Relative intensities of B/Y series ions														Manual Classification
					B_2	B_2F	B_3	B_3F	B_2S	B_2SF	Y_1	Y_1F	Y_2	Y_2F	Y_3	Y_3F	Y_4	Y_4F	
234	AGP exp_3	2540	Alpha-1-acid glycoprotein 2	ENGTVSR_6_5_0_2	42.1	0.0	0.0	0.0	100.0	0.0	50.7	0.0	0.0	0.0	4.2	0.0	26.7	0.0	None
235	AGP exp_4	2449	Alpha-1-acid glycoprotein 2	ENGTVSR_6_5_0_2	87.1	0.0	0.0	0.0	100.0	0.0	57.7	0.0	6.5	0.0	3.6	0.0	22.2	0.0	None
236	AGP exp_3	2385	Alpha-1-acid glycoprotein 2	ENGTVSR_6_5_0_2	86.2	0.0	0.0	0.0	100.0	0.0	45.2	0.0	4.6	0.0	7.8	0.0	33.6	0.0	None
237	AGP exp_4	2552	Alpha-1-acid glycoprotein 2	ENGTVSR_6_5_0_2	49.0	0.0	0.0	0.0	72.2	0.0	100.0	0.0	2.3	0.0	1.9	0.0	21.5	0.0	None
238	AGP exp_3	2645	Alpha-1-acid glycoprotein 2	ENGTVSR_6_5_0_2	78.8	0.0	5.5	0.0	95.0	0.0	100.0	0.0	2.6	0.0	4.8	0.0	30.9	0.0	None
239	AGP exp_4	4068	Alpha-1-acid glycoprotein 2	ENGTVSR_6_5_0_3	36.1	0.0	0.0	0.0	82.4	0.0	100.0	0.0	5.3	0.0	15.5	0.0	64.2	0.0	None
240	AGP exp_3	4183	Alpha-1-acid glycoprotein 2	ENGTVSR_6_5_0_3	25.8	0.0	0.0	0.0	100.0	0.0	81.5	0.0	4.4	0.0	10.0	0.0	42.3	0.0	None
241	AGP exp_3	1431	Alpha-1-acid glycoprotein 2	ENGTVSR_6_5_1_1	100.0	46.2	0.0	0.0	24.3	0.0	40.3	0.0	0.0	0.0	0.0	0.0	28.5	0.0	Outer
242	AGP exp_4	1462	Alpha-1-acid glycoprotein 2	ENGTVSR_6_5_1_1	100.0	38.5	0.0	0.0	38.8	0.0	48.8	0.0	0.0	0.0	0.0	0.0	18.9	0.0	Outer
243	AGP exp_4	1575	Alpha-1-acid glycoprotein 2	ENGTVSR_6_5_1_1	100.0	28.2	0.0	0.0	44.2	0.0	74.4	0.0	0.0	0.0	8.8	0.0	37.7	0.0	Outer
244	AGP exp_3	2599	Alpha-1-acid glycoprotein 2	ENGTVSR_6_5_1_2	86.3	12.6	0.0	0.0	96.9	37.3	100.0	0.0	0.0	0.0	0.0	0.0	35.7	0.0	Outer
245	AGP exp_4	2306	Alpha-1-acid glycoprotein 2	ENGTVSR_6_5_1_2	61.2	20.7	5.4	0.0	100.0	31.2	93.6	0.0	0.0	0.0	0.0	0.0	30.3	2.2	Outer
246	AGP exp_4	3953	Alpha-1-acid glycoprotein 2	ENGTVSR_6_5_1_3	19.9	5.1	3.9	0.0	96.9	30.6	100.0	0.0	4.1	0.0	0.0	0.0	49.2	0.0	Outer
247	AGP exp_3	4068	Alpha-1-acid glycoprotein 2	ENGTVSR_6_5_1_3	20.7	5.6	0.0	0.0	94.3	33.9	100.0	0.0	7.0	0.0	0.0	0.0	46.0	0.0	Outer
248	AGP exp_23	6765	Alpha-1-acid glycoprotein 2	ENGTVSR_6_5_2_3	11.7	10.8	0.0	0.0	100.0	59.9	55.2	0.0	0.0	0.0	0.0	0.0	45.6	9.1	Dual
249	AGP exp_9	5961	Alpha-1-acid glycoprotein 2	ENGTVSR_6_5_2_3	8.5	0.0	0.0	0.0	100.0	68.4	90.6	0.0	0.0	0.0	0.0	0.0	44.8	11.9	Dual
250	AGP exp_40	7029	Alpha-1-acid glycoprotein 2	ENGTVSR_6_5_2_3	12.1	14.7	0.0	0.0	100.0	53.3	52.9	0.0	0.0	0.0	0.0	0.0	37.6	10.1	Dual
251	AGP exp_41	7205	Alpha-1-acid glycoprotein 2	ENGTVSR_6_5_2_3	8.3	0.0	0.0	0.0	100.0	57.8	59.3	0.0	0.0	0.0	6.8	0.0	35.0	14.1	Dual
252	AGP exp_38	7085	Alpha-1-acid glycoprotein 2	ENGTVSR_6_5_2_3	8.0	12.9	0.0	0.0	100.0	66.4	74.6	0.0	0.0	0.0	0.0	0.0	26.0	8.3	Dual
253	AGP exp_34	5844	Alpha-1-acid glycoprotein 2	ENGTVSR_6_5_2_3	10.6	0.0	0.0	0.0	100.0	75.3	92.3	0.0	4.7	0.0	0.0	0.0	40.5	14.1	Dual
254	AGP exp_42	7163	Alpha-1-acid glycoprotein 2	ENGTVSR_6_5_2_3	8.0	6.2	0.0	0.0	100.0	57.5	43.0	0.0	0.0	0.0	3.0	0.0	29.7	10.9	Dual
255	AGP exp_32	6001	Alpha-1-acid glycoprotein 2	ENGTVSR_6_5_2_3	6.6	6.0	0.0	0.0	100.0	72.8	67.2	0.0	2.9	0.0	0.0	0.0	39.2	9.3	Dual
256	AGP exp_4	1946	Alpha-1-acid glycoprotein 2	ENGTVSR_7_6_1_2	31.0	15.5	0.0	0.0	100.0	21.3	74.1	0.0	0.0	0.0	0.0	0.0	0.0	0.0	Outer
257	AGP exp_3	2390	Alpha-1-acid glycoprotein 2	ENGTVSR_7_6_1_2	22.0	9.8	0.0	0.0	100.0	24.9	82.9	0.0	0.0	0.0	0.0	0.0	0.0	0.0	Outer
258	AGP exp_3	2203	Alpha-1-acid glycoprotein 2	ENGTVSR_7_6_1_2	12.3	19.2	0.0	0.0	100.0	24.2	58.9	0.0	0.0	0.0	0.0	0.0	0.0	0.0	Outer
259	AGP exp_3	1953	Alpha-1-acid glycoprotein 2	ENGTVSR_7_6_1_2	38.4	12.3	0.0	0.0	100.0	11.0	75.7	0.0	0.0	0.0	0.0	0.0	0.0	0.0	Outer

Supplementary Table 2. Continued.

No.	Experiments	CID Scan No.	N-glycoproteins	N-glycopeptides	Relative intensities of B/Y series ions														Manual Classification
					B_2	B_2F	B_3	B_3F	B_2S	B_2SF	Y_1	Y_1F	Y_2	Y_2F	Y_3	Y_3F	Y_4	Y_4F	
260	AGP exp_4	2136	Alpha-1-acid glycoprotein 2	ENGTVSR_7_6_1_2	26.8	20.4	0.0	0.0	100.0	18.7	58.5	0.0	0.0	0.0	0.0	0.0	0.0	0.0	Outer
261	AGP exp_4	2157	Alpha-1-acid glycoprotein 2	ENGTVSR_7_6_1_2	37.4	23.3	0.0	0.0	100.0	21.2	59.5	0.0	0.0	0.0	0.0	0.0	0.0	0.0	Outer
262	AGP exp_3	5210	Alpha-1-acid glycoprotein 2	ENGTVSR_7_6_1_4	41.3	2.7	0.0	0.0	100.0	22.7	16.9	0.0	0.0	0.0	0.0	0.0	3.7	0.0	Outer
263	AGP exp_4	5084	Alpha-1-acid glycoprotein 2	ENGTVSR_7_6_1_4	29.3	0.0	0.0	0.0	100.0	22.3	11.2	0.0	0.0	0.0	0.0	0.0	3.3	0.0	Outer
264	AGP exp_4	15084	Alpha-1-acid glycoprotein 2	LVPVPITNATLDR_3_3_0_0	4.6	0.0	4.9	0.0	0.0	0.0	100.0	0.0	7.9	0.0	32.7	0.0	35.8	0.0	None
265	AGP exp_3	12281	Alpha-1-acid glycoprotein 2	LVPVPITNATLDR_3_3_0_0	9.3	0.0	2.8	0.0	0.0	0.0	100.0	0.0	4.5	0.0	35.8	0.0	33.9	0.0	None
266	AGP exp_3	12722	Alpha-1-acid glycoprotein 2	LVPVPITNATLDR_4_4_0_1	32.2	0.0	0.0	0.0	24.0	0.0	9.3	0.0	15.8	0.0	100.0	0.0	5.0	0.0	None
267	AGP exp_3	12725	Alpha-1-acid glycoprotein 2	LVPVPITNATLDR_4_4_0_1	29.1	0.0	0.0	0.0	35.7	0.0	11.0	0.0	21.9	0.0	100.0	0.0	0.0	0.0	None
268	AGP exp_3	15340	Alpha-1-acid glycoprotein 2	LVPVPITNATLDR_5_4_0_1	0.0	0.0	3.7	0.0	4.9	0.0	100.0	0.0	6.7	0.0	7.2	0.0	5.3	0.0	None
269	AGP exp_3	12615	Alpha-1-acid glycoprotein 2	LVPVPITNATLDR_5_4_0_1	44.4	0.0	0.0	0.0	81.1	0.0	40.9	0.0	0.0	0.0	14.2	0.0	100.0	0.0	None
270	AGP exp_4	14992	Alpha-1-acid glycoprotein 2	LVPVPITNATLDR_5_4_0_1	0.0	0.0	0.0	0.0	7.8	0.0	100.0	0.0	2.7	0.0	2.4	0.0	9.0	0.0	None
271	AGP exp_3	12307	Alpha-1-acid glycoprotein 2	LVPVPITNATLDR_5_4_0_1	100.0	0.0	14.0	0.0	59.5	0.0	30.3	0.0	0.0	0.0	11.1	0.0	76.9	0.0	None
272	AGP exp_4	15488	Alpha-1-acid glycoprotein 2	LVPVPITNATLDR_5_4_0_2	0.0	0.0	0.0	0.0	12.5	0.0	100.0	0.0	8.8	0.0	9.9	0.0	11.1	0.0	None
273	AGP exp_3	16069	Alpha-1-acid glycoprotein 2	LVPVPITNATLDR_5_4_0_2	32.8	0.0	0.0	0.0	100.0	0.0	47.8	0.0	0.0	0.0	6.5	0.0	45.1	0.0	None
274	AGP exp_3	15799	Alpha-1-acid glycoprotein 2	LVPVPITNATLDR_5_4_0_2	70.5	0.0	6.6	0.0	100.0	0.0	29.8	0.0	1.1	0.0	7.3	0.0	66.7	0.0	None
275	AGP exp_3	19503	Alpha-1-acid glycoprotein 2	LVPVPITNATLDR_5_4_0_2	46.0	0.0	0.0	0.0	100.0	0.0	44.2	0.0	6.7	0.0	0.0	0.0	61.6	0.0	None
276	AGP exp_3	15678	Alpha-1-acid glycoprotein 2	LVPVPITNATLDR_5_4_0_2	36.6	0.0	8.1	0.0	100.0	0.0	44.9	0.0	0.0	0.0	16.1	0.0	88.4	0.0	None
277	AGP exp_4	15381	Alpha-1-acid glycoprotein 2	LVPVPITNATLDR_5_4_0_2	95.5	0.0	0.0	0.0	100.0	0.0	30.9	0.0	0.0	0.0	9.8	0.0	75.5	0.0	None
278	AGP exp_3	15761	Alpha-1-acid glycoprotein 2	LVPVPITNATLDR_5_4_1_2	40.0	0.0	0.0	0.0	100.0	51.5	96.8	0.0	5.4	0.0	0.0	0.0	65.1	0.0	Outer
279	AGP exp_4	15367	Alpha-1-acid glycoprotein 2	LVPVPITNATLDR_5_4_1_2	16.3	0.0	9.3	0.0	54.2	20.4	100.0	0.0	0.0	0.0	0.0	0.0	0.0	0.0	Outer
280	AGP exp_3	12326	Alpha-1-acid glycoprotein 2	LVPVPITNATLDR_5_5_0_1	53.0	0.0	0.0	0.0	72.1	0.0	100.0	0.0	0.0	0.0	12.0	0.0	55.6	0.0	None
281	AGP exp_4	12020	Alpha-1-acid glycoprotein 2	LVPVPITNATLDR_5_5_0_1	54.6	0.0	0.0	0.0	89.9	0.0	100.0	0.0	5.7	0.0	12.9	0.0	78.2	0.0	None
282	AGP exp_3	15537	Alpha-1-acid glycoprotein 2	LVPVPITNATLDR_5_5_1_2	0.0	0.0	0.0	0.0	100.0	30.2	50.4	0.0	5.4	0.0	0.0	0.0	11.0	0.0	Outer
283	AGP exp_4	11892	Alpha-1-acid glycoprotein 2	LVPVPITNATLDR_6_5_0_1	46.3	0.0	0.0	0.0	67.3	0.0	100.0	0.0	0.0	0.0	10.9	0.0	22.3	0.0	None
284	AGP exp_3	12405	Alpha-1-acid glycoprotein 2	LVPVPITNATLDR_6_5_0_1	47.8	0.0	0.0	0.0	62.0	0.0	100.0	0.0	0.0	0.0	7.0	0.0	18.4	0.0	None
285	AGP exp_4	11843	Alpha-1-acid glycoprotein 2	LVPVPITNATLDR_6_5_0_1	100.0	0.0	8.0	0.0	18.3	0.0	15.0	0.0	2.6	0.0	10.8	0.0	34.1	0.0	None

Supplementary Table 2. Continued.

No.	Experiments	CID Scan No.	N-glycoproteins	N-glycopeptides	Relative intensities of B/Y series ions														Manual Classification
					B_2	B_2F	B_3	B_3F	B_2S	B_2SF	Y_1	Y_1F	Y_2	Y_2F	Y_3	Y_3F	Y_4	Y_4F	
286	AGP exp_3	12246	Alpha-1-acid glycoprotein 2	LVPVPITNATLDR_6_5_0_1	100.0	0.0	5.7	0.0	55.0	0.0	44.0	0.0	5.3	0.0	15.3	0.0	77.6	0.0	None
287	AGP exp_3	11939	Alpha-1-acid glycoprotein 2	LVPVPITNATLDR_6_5_0_1	100.0	0.0	0.0	0.0	48.1	0.0	7.1	0.0	0.0	0.0	10.1	0.0	16.5	0.0	None
288	AGP exp_3	15635	Alpha-1-acid glycoprotein 2	LVPVPITNATLDR_6_5_0_2	90.1	0.0	5.7	0.0	100.0	0.0	42.1	0.0	9.1	0.0	0.0	0.0	42.7	0.0	None
289	AGP exp_3	15529	Alpha-1-acid glycoprotein 2	LVPVPITNATLDR_6_5_0_2	82.5	0.0	6.2	0.0	100.0	0.0	32.8	0.0	7.8	0.0	0.0	0.0	30.2	0.0	None
290	AGP exp_4	15283	Alpha-1-acid glycoprotein 2	LVPVPITNATLDR_6_5_0_2	86.9	0.0	5.7	0.0	100.0	0.0	34.4	0.0	7.2	0.0	0.0	0.0	39.0	0.0	None
291	AGP exp_4	14929	Alpha-1-acid glycoprotein 2	LVPVPITNATLDR_6_5_0_2	78.7	0.0	0.0	0.0	100.0	0.0	23.8	0.0	7.3	0.0	0.0	0.0	28.0	0.0	None
292	AGP exp_4	15591	Alpha-1-acid glycoprotein 2	LVPVPITNATLDR_6_5_0_2	90.3	0.0	0.0	0.0	100.0	0.0	17.0	0.0	4.4	0.0	0.0	0.0	41.9	0.0	None
293	AGP exp_4	19268	Alpha-1-acid glycoprotein 2	LVPVPITNATLDR_6_5_0_3	36.3	0.0	3.4	0.0	100.0	0.0	25.0	0.0	4.9	0.0	4.3	0.0	0.0	0.0	None
294	AGP exp_4	19155	Alpha-1-acid glycoprotein 2	LVPVPITNATLDR_6_5_0_3	43.0	0.0	0.0	0.0	100.0	0.0	28.9	0.0	4.7	0.0	5.8	0.0	0.0	0.0	None
295	AGP exp_3	19311	Alpha-1-acid glycoprotein 2	LVPVPITNATLDR_6_5_0_3	41.4	0.0	3.2	0.0	100.0	0.0	24.2	0.0	4.9	0.0	5.6	0.0	0.0	0.0	None
296	AGP exp_4	18825	Alpha-1-acid glycoprotein 2	LVPVPITNATLDR_6_5_0_3	45.1	0.0	0.0	0.0	100.0	0.0	24.2	0.0	4.9	0.0	5.6	0.0	0.0	0.0	None
297	AGP exp_3	19438	Alpha-1-acid glycoprotein 2	LVPVPITNATLDR_6_5_0_3	0.0	0.0	0.0	0.0	8.4	0.0	100.0	0.0	16.7	0.0	8.5	0.0	3.6	0.0	None
298	AGP exp_4	11669	Alpha-1-acid glycoprotein 2	LVPVPITNATLDR_6_5_1_1	40.7	28.8	0.0	0.0	71.9	29.4	100.0	0.0	0.0	0.0	0.0	0.0	21.0	0.0	Outer
299	AGP exp_4	15088	Alpha-1-acid glycoprotein 2	LVPVPITNATLDR_6_5_1_1	81.4	66.9	0.0	0.0	59.5	44.8	100.0	0.0	0.0	0.0	0.0	0.0	0.0	0.0	Outer
300	AGP exp_3	12104	Alpha-1-acid glycoprotein 2	LVPVPITNATLDR_6_5_1_1	49.5	24.6	0.0	0.0	86.5	68.6	100.0	0.0	0.0	0.0	0.0	0.0	0.0	0.0	Outer
301	AGP exp_4	11993	Alpha-1-acid glycoprotein 2	LVPVPITNATLDR_6_5_1_1	65.0	36.5	0.0	0.0	61.7	53.4	100.0	0.0	0.0	0.0	0.0	0.0	0.0	0.0	Outer
302	AGP exp_3	15640	Alpha-1-acid glycoprotein 2	LVPVPITNATLDR_6_5_1_2	0.0	14.7	0.0	0.0	100.0	55.1	92.1	0.0	0.0	0.0	7.1	0.0	17.2	0.0	Outer
303	AGP exp_4	15128	Alpha-1-acid glycoprotein 2	LVPVPITNATLDR_6_5_1_2	0.0	11.2	0.0	0.0	100.0	62.0	85.0	0.0	0.0	0.0	0.0	0.0	22.4	0.0	Outer
304	AGP exp_4	15447	Alpha-1-acid glycoprotein 2	LVPVPITNATLDR_6_5_1_2	0.0	10.0	0.0	0.0	100.0	44.9	80.8	0.0	0.0	0.0	6.7	0.0	26.0	0.0	Outer
305	AGP exp_4	14800	Alpha-1-acid glycoprotein 2	LVPVPITNATLDR_6_5_1_2	0.0	12.2	0.0	0.0	100.0	12.5	76.7	0.0	0.0	0.0	0.0	0.0	8.0	0.0	Outer
306	AGP exp_3	15285	Alpha-1-acid glycoprotein 2	LVPVPITNATLDR_6_5_1_2	64.0	13.3	0.0	0.0	100.0	16.4	34.1	0.0	5.4	0.0	0.0	0.0	24.6	0.0	Outer
307	AGP exp_4	15172	Alpha-1-acid glycoprotein 2	LVPVPITNATLDR_6_5_1_2	92.4	14.7	4.6	0.0	100.0	31.8	35.3	0.0	0.0	0.0	15.0	0.0	37.3	3.6	Outer
308	AGP exp_4	14833	Alpha-1-acid glycoprotein 2	LVPVPITNATLDR_6_5_1_2	72.3	15.2	0.0	0.0	100.0	0.0	24.4	0.0	3.1	0.0	8.5	0.0	19.2	0.0	Outer
309	AGP exp_4	19281	Alpha-1-acid glycoprotein 2	LVPVPITNATLDR_6_5_1_3	0.0	0.0	0.0	0.0	100.0	40.2	55.1	0.0	0.0	0.0	7.9	0.0	14.2	0.0	Outer
310	AGP exp_3	19654	Alpha-1-acid glycoprotein 2	LVPVPITNATLDR_6_5_1_3	0.0	5.3	7.3	0.0	100.0	43.0	50.4	0.0	2.1	0.0	6.7	0.0	14.2	0.0	Outer
311	AGP exp_3	19081	Alpha-1-acid glycoprotein 2	LVPVPITNATLDR_6_5_1_3	38.6	0.0	0.0	0.0	100.0	16.4	21.0	0.0	0.0	0.0	5.5	0.0	0.0	0.0	Outer

Supplementary Table 2. Continued.

No.	Experiments	CID Scan No.	N-glycoproteins	N-glycopeptides	Relative intensities of B/Y series ions														Manual Classification
					B_2	B_2F	B_3	B_3F	B_2S	B_2SF	Y_1	Y_1F	Y_2	Y_2F	Y_3	Y_3F	Y_4	Y_4F	
312	IgG exp_7	6000	Ig gamma-1 chain C region	EEQYNSTYR_3_3_0_0	11.3	0.0	7.2	0.0	0.0	0.0	100.0	0.0	11.4	0.0	20.1	0.0	53.0	0.0	None
313	IgG exp_7	6058	Ig gamma-1 chain C region	EEQYNSTYR_3_3_1_0	13.4	0.0	11.3	0.0	0.0	0.0	100.0	64.7	19.5	3.6	24.8	7.6	51.5	12.7	Core
314	IgG exp_7	6726	Ig gamma-1 chain C region	EEQYNSTYR_3_3_1_0	6.3	0.0	12.8	0.0	0.0	0.0	100.0	66.3	17.3	3.2	22.5	9.6	51.1	17.2	Core
315	IgG exp_7	6394	Ig gamma-1 chain C region	EEQYNSTYR_3_3_1_0	13.1	0.0	13.0	2.4	0.0	0.0	100.0	60.6	16.3	3.1	22.9	10.6	44.6	14.9	Core
316	IgG exp_7	7005	Ig gamma-1 chain C region	EEQYNSTYR_3_3_1_0	3.6	0.0	13.5	2.1	0.0	0.0	100.0	51.6	11.7	0.0	15.1	5.9	35.5	17.3	Core
317	IgG exp_6	4370	Ig gamma-1 chain C region	EEQYNSTYR_3_3_1_0	10.9	2.1	13.4	1.9	0.0	0.0	100.0	56.7	21.4	3.7	21.0	12.2	41.6	12.7	Core
318	IgG exp_7	6311	Ig gamma-1 chain C region	EEQYNSTYR_3_4_0_0	4.3	0.0	9.4	0.0	0.0	0.0	100.0	0.0	19.3	0.0	19.0	0.0	34.4	0.0	None
319	IgG exp_7	6631	Ig gamma-1 chain C region	EEQYNSTYR_3_4_0_0	0.0	0.0	9.3	0.0	0.0	0.0	100.0	0.0	21.8	0.0	19.2	0.0	36.8	0.0	None
320	IgG exp_7	5973	Ig gamma-1 chain C region	EEQYNSTYR_3_4_0_0	2.0	0.0	8.0	0.0	0.0	0.0	100.0	0.0	19.9	0.0	16.7	0.0	33.0	0.0	None
321	IgG exp_6	4399	Ig gamma-1 chain C region	EEQYNSTYR_3_4_0_0	5.2	0.0	7.2	0.0	0.0	0.0	100.0	1.5	25.4	0.0	18.1	0.0	32.3	0.0	None
322	IgG exp_7	7845	Ig gamma-1 chain C region	EEQYNSTYR_3_4_1_0	0.0	0.0	7.7	0.0	0.0	0.0	100.0	61.2	10.6	0.0	10.5	0.0	36.3	0.0	Core
323	IgG exp_7	5295	Ig gamma-1 chain C region	EEQYNSTYR_3_4_1_0	0.0	0.0	16.9	0.0	0.0	0.0	100.0	69.6	25.5	7.6	14.6	3.4	32.2	21.9	Core
324	IgG exp_7	6085	Ig gamma-1 chain C region	EEQYNSTYR_3_4_1_0	0.0	0.0	8.4	0.0	0.0	0.0	100.0	51.7	17.0	5.0	14.4	2.1	22.8	5.3	Core
325	IgG exp_7	6421	Ig gamma-1 chain C region	EEQYNSTYR_3_4_1_0	0.0	0.0	8.8	0.0	0.0	0.0	100.0	52.4	18.0	4.9	15.6	0.0	25.2	6.3	Core
326	IgG exp_7	6739	Ig gamma-1 chain C region	EEQYNSTYR_3_4_1_0	0.0	0.0	9.2	0.0	0.0	0.0	100.0	51.5	18.9	5.1	15.4	2.7	23.3	6.8	Core
327	IgG exp_7	7490	Ig gamma-1 chain C region	EEQYNSTYR_3_4_1_0	0.0	0.0	15.5	0.0	0.0	0.0	100.0	73.9	17.1	4.3	19.9	7.5	40.7	19.6	Core
328	IgG exp_7	7021	Ig gamma-1 chain C region	EEQYNSTYR_3_4_1_0	0.0	0.0	10.9	0.0	0.0	0.0	100.0	52.9	20.0	4.8	18.1	0.0	28.2	9.6	Core
329	IgG exp_5	3033	Ig gamma-1 chain C region	EEQYNSTYR_3_4_1_0	0.0	0.0	9.8	0.0	0.0	0.0	100.0	53.1	17.5	5.1	14.8	0.0	24.3	6.1	Core
330	IgG exp_7	5764	Ig gamma-1 chain C region	EEQYNSTYR_3_4_1_0	0.0	0.0	18.7	0.0	0.0	0.0	100.0	66.5	16.7	0.0	19.7	0.0	34.5	18.2	Core
331	IgG exp_7	7667	Ig gamma-1 chain C region	EEQYNSTYR_3_4_1_0	0.0	0.0	14.1	0.0	0.0	0.0	100.0	60.5	10.0	0.0	12.4	3.9	29.6	11.0	Core
332	IgG exp_7	7268	Ig gamma-1 chain C region	EEQYNSTYR_3_4_1_0	0.0	0.0	13.8	0.0	0.0	0.0	100.0	73.6	24.6	7.5	24.6	2.6	42.9	20.7	Core
333	IgG exp_7	8322	Ig gamma-1 chain C region	EEQYNSTYR_3_4_1_0	0.0	0.0	16.5	0.0	0.0	0.0	100.0	73.2	40.7	26.6	23.9	7.0	15.5	9.3	Core
334	IgG exp_7	7954	Ig gamma-1 chain C region	EEQYNSTYR_3_5_0_0	0.0	0.0	2.4	0.0	0.0	0.0	100.0	0.0	39.4	0.0	7.8	0.0	13.5	0.0	None
335	IgG exp_7	8666	Ig gamma-1 chain C region	EEQYNSTYR_3_5_1_0	0.0	0.0	5.8	0.0	0.0	0.0	100.0	61.9	36.4	20.8	15.1	6.0	14.5	5.5	Core
336	IgG exp_6	4420	Ig gamma-1 chain C region	EEQYNSTYR_3_5_1_0	0.0	0.0	3.4	0.0	0.0	0.0	100.0	41.8	32.0	11.0	12.6	0.0	11.5	2.4	Core
337	IgG exp_7	8314	Ig gamma-1 chain C region	EEQYNSTYR_3_5_1_0	0.0	0.0	4.1	0.0	0.0	0.0	100.0	58.0	37.5	18.4	18.5	5.5	11.2	4.3	Core

Supplementary Table 2. Continued.

No.	Experiments	CID Scan No.	N-glycoproteins	N-glycopeptides	Relative intensities of B/Y series ions														Manual Classification
					B_2	B_2F	B_3	B_3F	B_2S	B_2SF	Y_1	Y_1F	Y_2	Y_2F	Y_3	Y_3F	Y_4	Y_4F	
338	IgG exp_7	6199	Ig gamma-1 chain C region	EEQYNSTYR_4_3_1_0	11.5	4.5	12.1	0.0	0.0	0.0	100.0	88.7	11.8	5.3	9.2	3.9	33.7	7.6	Core
339	IgG exp_7	6537	Ig gamma-1 chain C region	EEQYNSTYR_4_3_1_0	10.6	3.5	11.1	0.0	0.0	0.0	100.0	92.3	8.9	0.0	10.5	2.3	34.5	10.0	Core
340	IgG exp_6	4360	Ig gamma-1 chain C region	EEQYNSTYR_4_4_0_0	0.0	0.0	7.0	0.0	0.0	0.0	100.0	0.0	15.1	0.0	14.4	0.0	31.2	0.0	None
341	IgG exp_7	5761	Ig gamma-1 chain C region	EEQYNSTYR_4_4_0_0	0.0	0.0	5.9	0.0	0.0	0.0	100.0	0.0	14.0	0.0	14.4	0.0	33.2	0.0	None
342	IgG exp_7	6089	Ig gamma-1 chain C region	EEQYNSTYR_4_4_0_0	0.0	0.0	6.4	0.0	0.0	0.0	100.0	0.0	15.3	0.0	12.6	0.0	30.0	0.0	None
343	IgG exp_7	6427	Ig gamma-1 chain C region	EEQYNSTYR_4_4_0_0	0.0	0.0	7.2	0.0	0.0	0.0	100.0	3.3	13.9	0.0	11.6	0.0	36.1	0.0	None
344	IgG exp_6	4341	Ig gamma-1 chain C region	EEQYNSTYR_4_4_1_0	100.0	0.0	0.0	0.0	0.0	0.0	44.6	40.6	0.0	0.0	0.0	0.0	29.8	44.6	Core
345	IgG exp_7	5171	Ig gamma-1 chain C region	EEQYNSTYR_4_4_1_0	0.0	0.0	9.1	0.0	0.0	0.0	5.0	100.0	14.6	12.6	8.6	14.2	15.2	10.2	Core
346	IgG exp_7	5650	Ig gamma-1 chain C region	EEQYNSTYR_4_4_1_0	0.0	0.0	15.4	0.0	0.0	0.0	13.4	100.0	4.9	0.0	5.0	0.0	26.9	9.1	Core
347	IgG exp_7	6917	Ig gamma-1 chain C region	EEQYNSTYR_4_4_1_0	0.0	3.3	10.5	0.0	0.0	0.0	15.2	100.0	21.9	12.6	19.9	0.0	36.2	5.3	Core
348	IgG exp_7	5940	Ig gamma-1 chain C region	EEQYNSTYR_4_4_1_0	0.0	2.7	10.3	0.0	0.0	0.0	14.2	100.0	21.1	12.0	19.4	6.4	32.5	4.5	Core
349	IgG exp_7	8525	Ig gamma-1 chain C region	EEQYNSTYR_4_4_1_0	0.0	13.2	14.7	0.0	0.0	0.0	22.9	100.0	16.7	0.0	0.0	0.0	0.0	0.0	Core
350	IgG exp_7	6616	Ig gamma-1 chain C region	EEQYNSTYR_4_4_1_0	0.0	2.9	10.6	0.0	0.0	0.0	9.3	100.0	23.1	12.6	20.3	0.0	34.5	5.4	Core
351	IgG exp_7	7164	Ig gamma-1 chain C region	EEQYNSTYR_4_4_1_0	0.0	0.0	15.5	0.0	0.0	0.0	16.2	100.0	22.6	13.4	24.2	6.8	41.0	9.9	Core
352	IgG exp_7	6278	Ig gamma-1 chain C region	EEQYNSTYR_4_4_1_0	0.0	2.8	10.9	0.0	0.0	0.0	8.1	100.0	22.3	12.4	20.6	2.7	33.5	5.4	Core
353	IgG exp_5	3064	Ig gamma-1 chain C region	EEQYNSTYR_4_4_1_0	0.0	3.4	10.8	0.0	0.0	0.0	14.5	100.0	22.0	11.2	19.1	8.2	35.4	3.9	Core
354	IgG exp_7	7396	Ig gamma-1 chain C region	EEQYNSTYR_4_4_1_0	0.0	0.0	10.9	0.0	0.0	0.0	7.7	100.0	19.5	10.3	17.8	0.0	41.2	7.2	Core
355	IgG exp_6	4339	Ig gamma-1 chain C region	EEQYNSTYR_4_4_1_0	0.0	2.3	7.4	0.0	0.0	0.0	11.2	100.0	26.7	12.0	17.8	0.0	29.4	5.6	Core
356	IgG exp_7	9802	Ig gamma-1 chain C region	EEQYNSTYR_4_4_1_1	65.3	0.0	16.0	0.0	100.0	0.0	43.3	42.8	0.0	0.0	0.0	0.0	27.0	18.8	Core
357	IgG exp_7	9812	Ig gamma-1 chain C region	EEQYNSTYR_4_4_1_1	0.0	0.0	6.7	0.0	16.5	0.0	100.0	0.0	17.8	13.8	14.3	0.0	27.8	3.9	Core
358	IgG exp_6	4424	Ig gamma-1 chain C region	EEQYNSTYR_4_5_1_0	0.0	0.0	6.0	0.0	0.0	0.0	100.0	85.9	31.5	20.8	11.5	0.0	12.2	0.0	Core
359	IgG exp_7	8461	Ig gamma-1 chain C region	EEQYNSTYR_4_5_1_0	0.0	0.0	4.1	0.0	0.0	0.0	100.0	77.1	32.3	18.0	12.0	2.8	10.0	0.0	Core
360	IgG exp_7	5975	Ig gamma-1 chain C region	EEQYNSTYR_5_4_0_0	0.0	0.0	6.8	0.0	0.0	0.0	100.0	3.8	13.4	0.0	10.7	0.0	33.5	0.0	None
361	IgG exp_5	3029	Ig gamma-1 chain C region	EEQYNSTYR_5_4_1_0	0.0	0.0	8.3	0.0	0.0	0.0	100.0	99.1	15.4	18.3	17.3	9.0	35.7	4.6	Core
362	IgG exp_7	6087	Ig gamma-1 chain C region	EEQYNSTYR_5_4_1_0	0.0	2.8	7.6	0.0	0.0	0.0	100.0	90.8	15.9	13.8	14.8	8.6	29.7	0.0	Core
363	IgG exp_7	6423	Ig gamma-1 chain C region	EEQYNSTYR_5_4_1_0	0.0	2.9	9.1	0.0	0.0	0.0	100.0	92.5	17.8	13.8	17.7	8.5	30.4	0.0	Core

Supplementary Table 2. Continued.

No.	Experiments	CID Scan No.	N-glycoproteins	N-glycopeptides	Relative intensities of B/Y series ions														Manual Classification
					B_2	B_2F	B_3	B_3F	B_2S	B_2SF	Y_1	Y_1F	Y_2	Y_2F	Y_3	Y_3F	Y_4	Y_4F	
364	IgG exp_7	6741	Ig gamma-1 chain C region	EEQYNSTYR_5_4_1_0	0.0	2.9	8.5	0.0	0.0	0.0	100.0	90.6	16.7	16.5	15.9	10.4	34.8	3.1	Core
365	IgG exp_7	9750	Ig gamma-1 chain C region	EEQYNSTYR_5_4_1_1	100.0	0.0	11.7	0.0	77.1	0.0	37.2	43.6	0.0	0.0	0.0	0.0	11.3	0.0	Core
366	IgG exp_6	4859	Ig gamma-1 chain C region	EEQYNSTYR_5_4_1_1	0.0	0.0	5.7	0.0	16.8	0.0	100.0	98.4	15.9	16.7	14.1	9.9	28.1	3.4	Core
367	IgG exp_7	9769	Ig gamma-1 chain C region	EEQYNSTYR_5_4_1_1	0.0	0.0	2.8	0.0	17.0	0.0	97.4	100.0	15.7	15.5	16.6	10.9	27.4	0.0	Core
368	IgG exp_7	10599	Ig gamma-2 chain C region	EEQFNSTFR_3_3_1_0	7.5	0.0	8.7	0.0	0.0	0.0	100.0	49.0	13.1	2.2	13.1	3.5	22.3	4.6	Core
369	IgG exp_7	10686	Ig gamma-2 chain C region	EEQFNSTFR_3_4_0_0	3.6	0.0	7.0	0.0	0.0	0.0	100.0	0.0	17.1	0.0	16.3	0.0	28.1	0.0	None
370	IgG exp_5	3427	Ig gamma-2 chain C region	EEQFNSTFR_3_4_1_0	2.8	0.0	12.1	2.0	0.0	0.0	100.0	58.4	20.2	8.7	17.9	5.4	36.4	12.9	Core
371	IgG exp_7	10461	Ig gamma-2 chain C region	EEQFNSTFR_3_4_1_0	1.6	0.0	7.2	0.0	0.0	0.0	100.0	47.4	17.3	5.2	16.5	0.0	25.8	8.8	Core
372	IgG exp_7	10811	Ig gamma-2 chain C region	EEQFNSTFR_3_4_1_0	0.0	0.0	8.6	0.0	0.0	0.0	100.0	55.1	21.6	6.1	18.1	3.4	27.9	7.2	Core
373	IgG exp_7	10689	Ig gamma-2 chain C region	EEQFNSTFR_3_5_1_0	16.8	0.0	0.0	0.0	0.0	0.0	100.0	22.7	22.8	0.0	36.2	0.0	0.0	0.0	Core
374	IgG exp_7	10691	Ig gamma-2 chain C region	EEQFNSTFR_3_5_1_0	0.0	0.0	4.0	0.0	0.0	0.0	100.0	55.4	35.5	17.1	13.9	0.0	10.4	2.8	Core
375	IgG exp_5	3453	Ig gamma-2 chain C region	EEQFNSTFR_3_5_1_0	0.0	0.0	5.6	0.0	0.0	0.0	100.0	68.8	39.8	14.2	14.9	2.2	14.9	7.2	Core
376	IgG exp_7	10581	Ig gamma-2 chain C region	EEQFNSTFR_4_3_1_0	14.0	2.7	10.6	0.0	0.0	0.0	100.0	76.0	12.9	4.4	9.2	0.0	29.5	3.3	Core
377	IgG exp_7	10655	Ig gamma-2 chain C region	EEQFNSTFR_4_4_0_0	4.0	0.0	7.3	0.0	0.0	0.0	100.0	0.0	19.1	0.0	17.4	0.0	33.8	0.0	None
378	IgG exp_5	3408	Ig gamma-2 chain C region	EEQFNSTFR_4_4_1_0	0.0	2.0	7.4	0.0	0.0	0.0	100.0	66.7	18.6	10.0	15.9	0.0	27.2	4.0	Core
379	IgG exp_7	10720	Ig gamma-2 chain C region	EEQFNSTFR_4_4_1_0	0.0	2.5	8.4	0.0	0.0	0.0	100.0	65.8	18.3	12.1	15.8	7.1	26.8	4.3	Core
380	IgG exp_6	6063	Ig gamma-2 chain C region	EEQFNSTFR_4_4_1_0	0.0	0.0	13.7	0.0	0.0	0.0	100.0	95.3	20.2	13.9	20.7	9.6	44.1	10.4	Core
381	IgG exp_7	10382	Ig gamma-2 chain C region	EEQFNSTFR_4_4_1_0	0.0	2.8	12.5	0.0	0.0	0.0	100.0	85.4	18.5	12.4	17.3	13.3	40.1	10.3	Core
382	IgG exp_6	5157	Ig gamma-2 chain C region	EEQFNSTFR_4_4_1_0	0.0	3.5	20.7	0.0	0.0	0.0	100.0	77.1	17.3	10.2	15.6	0.0	35.7	10.3	Core
383	IgG exp_7	10722	Ig gamma-2 chain C region	EEQFNSTFR_4_4_1_0	100.0	0.0	0.0	0.0	0.0	0.0	31.1	36.3	0.0	0.0	0.0	0.0	20.0	46.1	Core
384	IgG exp_7	12133	Ig gamma-2 chain C region	EEQFNSTFR_4_4_1_1	70.6	0.0	15.8	0.0	100.0	0.0	62.6	46.9	0.0	0.0	11.0	0.0	28.8	0.0	Core
385	IgG exp_5	3739	Ig gamma-2 chain C region	EEQFNSTFR_4_4_1_1	0.0	0.0	8.5	0.0	20.0	0.0	100.0	78.5	21.2	14.7	15.4	8.8	37.6	4.7	Core
386	IgG exp_7	12154	Ig gamma-2 chain C region	EEQFNSTFR_4_4_1_1	0.0	0.0	14.0	0.0	28.4	0.0	100.0	70.3	19.8	11.6	14.5	8.0	29.4	5.2	Core
387	IgG exp_5	3735	Ig gamma-2 chain C region	EEQFNSTFR_4_4_1_1	64.0	0.0	18.9	0.0	100.0	0.0	38.5	52.7	0.0	0.0	0.0	0.0	34.9	0.0	Core
388	IgG exp_7	10653	Ig gamma-2 chain C region	EEQFNSTFR_4_5_1_0	0.0	0.0	4.6	0.0	0.0	0.0	100.0	74.9	32.8	21.5	10.1	3.0	9.1	0.0	Core
389	IgG exp_5	3400	Ig gamma-2 chain C region	EEQFNSTFR_5_4_1_0	0.0	3.2	8.0	0.0	0.0	0.0	100.0	90.6	18.1	14.9	16.9	9.8	29.6	0.0	Core

Supplementary Table 2. Continued.

No.	Experiments	CID Scan No.	N-glycoproteins	N-glycopeptides	Relative intensities of B/Y series ions														Manual Classification
					B_2	B_2F	B_3	B_3F	B_2S	B_2SF	Y_1	Y_1F	Y_2	Y_2F	Y_3	Y_3F	Y_4	Y_4F	
390	IgG exp_7	10548	Ig gamma-2 chain C region	EEQFNSTFR_5_4_1_0	0.0	2.6	6.6	0.0	0.0	0.0	100.0	90.9	18.2	14.9	16.6	8.6	29.5	2.2	Core
391	IgG exp_7	12088	Ig gamma-2 chain C region	EEQFNSTFR_5_4_1_1	0.0	3.5	9.0	0.0	23.7	0.0	85.7	100.0	11.6	12.7	10.6	7.3	22.4	0.0	Core
392	IgG exp_7	9711	Ig gamma-4 chain C region	EEQFNSTYR_3_4_1_0	0.0	0.0	10.5	0.0	0.0	0.0	100.0	54.8	19.4	5.6	20.8	3.6	31.2	10.4	Core
393	IgG exp_7	9698	Ig gamma-4 chain C region	EEQFNSTYR_4_4_1_0	0.0	3.5	13.1	2.5	0.0	0.0	100.0	79.3	18.8	13.1	21.7	6.9	42.3	11.9	Core

Supplementary Table 3. Glycopeptide spectrum matches of human plasma proteins and their relative intensities as unknown set for machine learning.

No.	Experiments	CID Scan No.	N-glycoproteins	N-glycopeptides	Relative intensities of B/Y series ions													
					B_2	B_2F	B_3	B_3F	B_2S	B_2SF	Y_1	Y_1F	Y_2	Y_2F	Y_3	Y_3F	Y_4	Y_4F
1	Human Plasma exp_1	18962	Afamin	DIENFNSTQK_5_4_0_2	100.0	0.0	8.1	0.0	94.0	0.0	28.3	0.0	0.0	0.0	6.7	0.0	30.9	0.0
2	Human Plasma exp_1	18960	Afamin	DIENFNSTQK_5_4_0_2	21.7	0.0	5.4	0.0	100.0	0.0	89.0	0.0	4.4	0.0	7.7	0.0	44.1	0.0
3	Human Plasma exp_1	18914	Afamin	DIENFNSTQK_5_4_0_2	0.0	0.0	0.0	0.0	17.3	0.0	100.0	0.0	3.2	0.0	3.0	0.0	11.3	0.0
4	Human Plasma exp_2	19315	Afamin	DIENFNSTQK_5_4_0_2	87.5	0.0	6.2	0.0	100.0	0.0	30.0	0.0	0.0	0.0	11.9	0.0	41.6	0.0
5	Human Plasma exp_2	19360	Afamin	DIENFNSTQK_5_4_0_2	40.2	0.0	5.4	0.0	100.0	0.0	91.4	0.0	4.9	0.0	7.0	0.0	47.4	0.0
6	Human Plasma exp_2	19306	Afamin	DIENFNSTQK_5_4_0_2	0.0	0.0	2.1	0.0	16.8	0.0	100.0	0.0	9.4	0.0	10.5	0.0	11.3	0.0
7	Human Plasma exp_3	18842	Afamin	DIENFNSTQK_5_4_0_2	81.0	0.0	8.2	0.0	100.0	0.0	52.0	0.0	5.1	0.0	7.7	0.0	42.5	0.0
8	Human Plasma exp_4	19406	Afamin	DIENFNSTQK_5_4_0_2	84.4	0.0	14.0	0.0	100.0	0.0	42.0	0.0	4.4	0.0	11.5	0.0	45.1	0.0
9	Human Plasma exp_4	19448	Afamin	DIENFNSTQK_5_4_0_2	36.2	0.0	5.4	0.0	86.4	0.0	100.0	0.0	4.2	0.0	6.0	0.0	40.2	0.0
10	Human Plasma exp_5	19011	Afamin	DIENFNSTQK_5_4_0_2	99.0	0.0	13.9	0.0	100.0	0.0	45.4	0.0	0.0	0.0	4.2	0.0	43.1	0.0
11	Human Plasma exp_5	19135	Afamin	DIENFNSTQK_5_4_0_2	40.5	0.0	4.1	0.0	100.0	0.0	58.1	0.0	0.0	0.0	0.0	0.0	32.3	0.0
12	Human Plasma exp_5	18647	Afamin	DIENFNSTQK_5_4_1_2	34.4	0.0	0.0	0.0	100.0	5.3	23.1	25.9	0.0	0.0	0.0	0.0	4.7	0.0
13	Human Plasma exp_3	8213	Afamin	FNETTEK_5_4_0_2	58.6	0.0	5.8	0.0	100.0	0.0	43.6	0.0	0.0	0.0	11.1	0.0	32.7	0.0
14	Human Plasma exp_5	7958	Afamin	FNETTEK_5_4_0_2	44.5	0.0	7.1	0.0	100.0	0.0	12.8	0.0	0.0	0.0	0.0	0.0	26.2	0.0
15	Human Plasma exp_5	12730	Afamin	FNETTEK_6_5_0_3	11.4	0.0	0.0	0.0	100.0	0.0	35.1	0.0	2.7	0.0	0.0	0.0	43.1	0.0
16	Human Plasma exp_1	1509	Afamin	HNFSHCCSK_5_4_0_2	100.0	0.0	0.0	0.0	88.8	0.0	20.8	0.0	0.0	0.0	13.4	0.0	75.9	0.0
17	Human Plasma exp_2	1548	Afamin	HNFSHCCSK_5_4_0_2	100.0	0.0	0.0	0.0	87.0	0.0	19.5	0.0	0.0	0.0	11.9	0.0	73.1	0.0
18	Human Plasma exp_4	1590	Afamin	HNFSHCCSK_5_4_0_2	100.0	0.0	0.0	0.0	87.0	0.0	81.9	0.0	21.6	0.0	0.0	0.0	50.3	0.0
19	Human Plasma exp_5	1488	Afamin	HNFSHCCSK_5_4_0_2	100.0	0.0	0.0	0.0	83.1	0.0	19.7	0.0	0.0	0.0	12.9	0.0	76.4	0.0
20	Human Plasma exp_5	1492	Afamin	HNFSHCCSK_5_4_0_2	98.8	0.0	0.0	0.0	85.4	0.0	100.0	0.0	23.4	0.0	0.0	0.0	58.4	0.0
21	Human Plasma exp_1	13729	Afamin	NCCNTENPPGCYR_5_4_0_2	63.5	0.0	0.0	0.0	100.0	0.0	71.8	0.0	10.8	0.0	16.6	0.0	68.0	0.0
22	Human Plasma exp_1	13720	Afamin	NCCNTENPPGCYR_5_4_0_2	13.8	0.0	0.0	0.0	97.4	0.0	100.0	0.0	0.0	0.0	9.0	0.0	21.8	0.0
23	Human Plasma exp_4	13975	Afamin	NCCNTENPPGCYR_5_4_0_2	64.6	0.0	6.9	0.0	100.0	0.0	73.9	0.0	10.3	0.0	20.2	0.0	77.1	0.0
24	Human Plasma exp_4	13811	Afamin	NCCNTENPPGCYR_5_4_0_2	16.2	0.0	5.3	0.0	100.0	0.0	88.3	0.0	0.0	0.0	7.3	0.0	20.8	0.0
25	Human Plasma exp_1	5154	Alpha-1-acid glycoprotein 1	ENGTISR_5_4_0_2	50.2	0.0	4.1	0.0	100.0	0.0	32.2	0.0	0.0	0.0	0.0	0.0	28.6	0.0
26	Human Plasma exp_2	5244	Alpha-1-acid glycoprotein 1	ENGTISR_5_4_0_2	59.3	0.0	3.6	0.0	100.0	0.0	46.0	0.0	0.0	0.0	0.0	0.0	38.8	0.0

Supplementary Table 3. Continued.

No.	Experiments	CID Scan No.	N-glycoproteins	N-glycopeptides	Relative intensities of B/Y series ions													
					B_2	B_2F	B_3	B_3F	B_2S	B_2SF	Y_1	Y_1F	Y_2	Y_2F	Y_3	Y_3F	Y_4	Y_4F
27	Human Plasma exp_3	5199	Alpha-1-acid glycoprotein 1	ENGTISR_5_4_0_2	56.5	0.0	6.6	0.0	100.0	0.0	30.8	0.0	3.9	0.0	0.0	0.0	33.0	0.0
28	Human Plasma exp_4	5332	Alpha-1-acid glycoprotein 1	ENGTISR_5_4_0_2	54.7	0.0	7.7	0.0	100.0	0.0	38.8	0.0	0.0	0.0	0.0	0.0	34.8	0.0
29	Human Plasma exp_5	5195	Alpha-1-acid glycoprotein 1	ENGTISR_5_4_0_2	55.8	0.0	5.9	0.0	100.0	0.0	48.8	0.0	0.0	0.0	0.0	0.0	35.7	0.0
30	Human Plasma exp_1	5094	Alpha-1-acid glycoprotein 1	ENGTISR_5_4_1_2	79.5	0.0	4.9	0.0	100.0	23.5	35.8	0.0	0.0	0.0	0.0	0.0	29.9	0.0
31	Human Plasma exp_2	5202	Alpha-1-acid glycoprotein 1	ENGTISR_5_4_1_2	64.0	9.5	0.0	0.0	100.0	20.0	20.6	0.0	0.0	0.0	0.0	0.0	18.1	0.0
32	Human Plasma exp_5	5163	Alpha-1-acid glycoprotein 1	ENGTISR_5_4_1_2	54.6	0.0	0.0	0.0	100.0	11.3	12.1	0.0	0.0	0.0	0.0	0.0	11.4	0.0
33	Human Plasma exp_2	9286	Alpha-1-acid glycoprotein 1	ENGTISR_6_5_0_3	43.3	0.0	0.0	0.0	100.0	0.0	5.8	0.0	0.0	0.0	1.8	0.0	1.5	0.0
34	Human Plasma exp_3	9084	Alpha-1-acid glycoprotein 1	ENGTISR_6_5_0_3	26.2	0.0	0.0	0.0	100.0	0.0	52.1	0.0	4.6	0.0	0.0	0.0	24.8	0.0
35	Human Plasma exp_4	9253	Alpha-1-acid glycoprotein 1	ENGTISR_6_5_0_3	50.9	0.0	3.9	0.0	100.0	0.0	8.4	0.0	0.0	0.0	0.0	0.0	10.2	0.0
36	Human Plasma exp_1	9587	Alpha-1-acid glycoprotein 1	ENGTISR_6_5_1_3	17.7	0.0	0.0	0.0	100.0	54.8	49.5	0.0	0.0	0.0	0.0	0.0	8.7	0.0
37	Human Plasma exp_2	8919	Alpha-1-acid glycoprotein 1	ENGTISR_6_5_1_3	68.4	4.6	0.0	0.0	100.0	18.9	17.4	0.0	0.0	0.0	0.0	0.0	0.0	0.0
38	Human Plasma exp_3	8894	Alpha-1-acid glycoprotein 1	ENGTISR_6_5_1_3	64.4	7.8	0.0	0.0	100.0	23.1	22.1	0.0	0.0	0.0	0.0	0.0	3.5	0.0
39	Human Plasma exp_3	9871	Alpha-1-acid glycoprotein 1	ENGTISR_6_5_1_3	9.9	0.0	0.0	0.0	100.0	22.3	23.8	3.6	0.0	0.0	0.0	0.0	17.8	0.0
40	Human Plasma exp_4	8976	Alpha-1-acid glycoprotein 1	ENGTISR_6_5_1_3	74.9	8.0	0.0	0.0	100.0	23.5	20.4	0.0	0.0	0.0	0.0	0.0	0.0	0.0
41	Human Plasma exp_4	9141	Alpha-1-acid glycoprotein 1	ENGTISR_6_5_1_3	21.5	4.3	0.0	0.0	100.0	34.0	84.5	0.0	6.1	0.0	0.0	0.0	49.7	0.0
42	Human Plasma exp_5	8869	Alpha-1-acid glycoprotein 1	ENGTISR_6_5_1_3	59.2	4.0	0.0	0.0	100.0	22.2	13.3	0.0	0.0	0.0	0.0	0.0	0.0	0.0
43	Human Plasma exp_5	9219	Alpha-1-acid glycoprotein 1	ENGTISR_6_5_1_3	14.6	0.0	0.0	0.0	100.0	41.5	38.1	0.0	0.0	0.0	0.0	0.0	21.1	0.0
44	Human Plasma exp_3	14454	Alpha-1-acid glycoprotein 1	ENGTISR_7_6_1_4	24.0	0.0	0.0	0.0	100.0	30.3	7.2	1.9	0.0	0.0	0.0	0.0	1.1	0.0
45	Human Plasma exp_4	13885	Alpha-1-acid glycoprotein 1	ENGTISR_7_6_1_4	38.7	5.1	0.0	0.0	100.0	37.9	5.6	0.0	0.0	0.0	0.0	0.0	0.0	0.0
46	Human Plasma exp_5	14875	Alpha-1-acid glycoprotein 1	ENGTISR_7_6_1_4	23.9	4.8	0.0	0.0	100.0	32.8	8.7	0.0	0.0	0.0	0.0	0.0	4.1	0.0
47	Human Plasma exp_2	13822	Alpha-1-acid glycoprotein 1	ENGTISR_7_6_2_4	27.0	9.4	0.0	0.0	76.5	100.0	13.9	0.0	0.0	0.0	0.0	0.0	0.0	0.0
48	Human Plasma exp_3	14493	Alpha-1-acid glycoprotein 1	ENGTISR_7_6_2_4	17.9	3.6	0.0	0.0	100.0	90.6	10.9	0.0	0.0	0.0	0.0	0.0	0.0	0.0
49	Human Plasma exp_4	14741	Alpha-1-acid glycoprotein 1	ENGTISR_7_6_2_4	22.4	6.3	0.0	0.0	100.0	85.5	12.7	0.0	0.0	0.0	0.0	0.0	2.7	0.0
50	Human Plasma exp_5	13807	Alpha-1-acid glycoprotein 1	ENGTISR_7_6_2_4	19.3	7.2	0.0	0.0	83.0	100.0	12.5	0.0	0.0	0.0	0.0	0.0	2.2	0.0
51	Human Plasma exp_5	14153	Alpha-1-acid glycoprotein 1	ENGTISR_7_6_3_4	17.7	5.1	0.0	0.0	76.7	100.0	2.5	0.0	0.0	0.0	0.0	0.0	0.0	0.0
52	Human Plasma exp_2	30047	Alpha-1-acid glycoprotein 1	LVPVPITNATLDQITGK_5_4_0_1	100.0	0.0	0.0	0.0	43.9	0.0	31.4	0.0	0.0	0.0	7.4	0.0	62.2	0.0

Supplementary Table 3. Continued.

No.	Experiments	CID Scan No.	N-glycoproteins	N-glycopeptides	Relative intensities of B/Y series ions													
					B_2	B_2F	B_3	B_3F	B_2S	B_2SF	Y_1	Y_1F	Y_2	Y_2F	Y_3	Y_3F	Y_4	Y_4F
53	Human Plasma exp_3	29500	Alpha-1-acid glycoprotein 1	LVPVPITNATLDQITGK_5_4_0_1	100.0	0.0	0.0	0.0	46.4	0.0	53.3	0.0	0.0	0.0	35.8	0.0	79.3	0.0
54	Human Plasma exp_4	29784	Alpha-1-acid glycoprotein 1	LVPVPITNATLDQITGK_5_4_0_1	100.0	0.0	6.8	0.0	46.4	0.0	26.5	0.0	0.0	0.0	0.0	0.0	51.1	0.0
55	Human Plasma exp_4	31478	Alpha-1-acid glycoprotein 1	LVPVPITNATLDQITGK_5_4_0_2	67.1	0.0	4.6	0.0	100.0	0.0	0.0	0.0	2.2	0.0	7.9	0.0	85.0	0.0
56	Human Plasma exp_1	32456	Alpha-1-acid glycoprotein 1	LVPVPITNATLDQITGK_6_5_0_3	31.4	0.0	0.0	0.0	100.0	0.0	32.1	0.0	2.6	0.0	0.0	0.0	31.4	0.0
57	Human Plasma exp_4	33045	Alpha-1-acid glycoprotein 1	LVPVPITNATLDQITGK_6_5_0_3	30.6	0.0	0.0	0.0	100.0	0.0	34.6	0.0	6.1	0.0	5.7	0.0	33.1	0.0
58	Human Plasma exp_1	30786	Alpha-1-acid glycoprotein 1	LVPVPITNATLDQITGK_6_5_1_2	70.5	0.0	0.0	0.0	100.0	46.4	39.2	0.0	0.0	0.0	6.7	0.0	51.2	0.0
59	Human Plasma exp_4	31353	Alpha-1-acid glycoprotein 1	LVPVPITNATLDQITGK_6_5_1_2	80.1	8.3	0.0	0.0	100.0	37.5	60.1	0.0	0.0	0.0	10.2	0.0	0.0	0.0
60	Human Plasma exp_4	32977	Alpha-1-acid glycoprotein 1	LVPVPITNATLDQITGK_6_5_1_3	32.6	3.6	0.0	0.0	100.0	31.0	47.1	0.0	2.3	0.0	0.0	0.0	37.5	0.0
61	Human Plasma exp_2	27598	Alpha-1-acid glycoprotein 1	QDQCIYNTTYLNVQR_5_4_0_2	73.6	0.0	8.1	0.0	99.8	0.0	100.0	0.0	0.0	0.0	0.0	0.0	41.7	0.0
62	Human Plasma exp_3	27020	Alpha-1-acid glycoprotein 1	QDQCIYNTTYLNVQR_5_4_0_2	61.5	0.0	0.0	0.0	100.0	0.0	74.6	0.0	4.7	0.0	0.0	0.0	59.9	0.0
63	Human Plasma exp_1	29130	Alpha-1-acid glycoprotein 1	QDQCIYNTTYLNVQR_6_5_0_3	30.3	0.0	0.0	0.0	100.0	0.0	68.7	0.0	0.0	0.0	9.5	0.0	40.4	0.0
64	Human Plasma exp_2	29848	Alpha-1-acid glycoprotein 1	QDQCIYNTTYLNVQR_6_5_0_3	31.2	0.0	0.0	0.0	100.0	0.0	76.0	0.0	0.0	0.0	5.3	0.0	25.2	0.0
65	Human Plasma exp_4	29531	Alpha-1-acid glycoprotein 1	QDQCIYNTTYLNVQR_6_5_1_3	17.9	0.0	0.0	0.0	100.0	18.6	75.4	0.0	3.2	0.0	0.0	0.0	42.1	0.0
66	Human Plasma exp_1	4603	Alpha-1-acid glycoprotein 1,2	NEEYNK_5_4_0_2	52.2	0.0	5.7	0.0	100.0	0.0	0.0	0.0	4.0	0.0	7.4	0.0	38.2	0.0
67	Human Plasma exp_2	4672	Alpha-1-acid glycoprotein 1,2	NEEYNK_5_4_0_2	49.3	0.0	5.0	0.0	100.0	0.0	0.0	0.0	2.4	0.0	5.8	0.0	39.0	0.0
68	Human Plasma exp_3	4633	Alpha-1-acid glycoprotein 1,2	NEEYNK_5_4_0_2	27.4	0.0	5.1	0.0	100.0	0.0	0.0	0.0	3.9	0.0	7.3	0.0	40.4	0.0
69	Human Plasma exp_4	4752	Alpha-1-acid glycoprotein 1,2	NEEYNK_5_4_0_2	49.3	0.0	4.8	0.0	100.0	0.0	0.0	0.0	3.2	0.0	6.6	0.0	36.7	0.0
70	Human Plasma exp_5	4319	Alpha-1-acid glycoprotein 1,2	NEEYNK_5_4_0_2	0.0	0.0	3.7	0.0	43.1	0.0	100.0	0.0	20.9	0.0	13.2	0.0	30.9	0.0
71	Human Plasma exp_1	4396	Alpha-1-acid glycoprotein 1,2	NEEYNK_5_4_1_2	56.3	0.0	0.0	0.0	100.0	0.0	10.5	37.7	0.0	0.0	0.0	0.0	6.8	29.4
72	Human Plasma exp_2	4457	Alpha-1-acid glycoprotein 1,2	NEEYNK_5_4_1_2	51.3	0.0	0.0	0.0	100.0	0.0	9.5	38.5	0.0	0.0	0.0	0.0	5.6	27.0
73	Human Plasma exp_3	4410	Alpha-1-acid glycoprotein 1,2	NEEYNK_5_4_1_2	49.9	0.0	4.3	0.0	100.0	4.6	15.0	33.8	0.0	0.0	0.0	0.0	9.6	28.3
74	Human Plasma exp_4	4528	Alpha-1-acid glycoprotein 1,2	NEEYNK_5_4_1_2	28.6	0.0	6.0	0.0	100.0	0.0	15.5	36.9	0.0	0.0	0.0	0.0	9.3	27.6
75	Human Plasma exp_5	4414	Alpha-1-acid glycoprotein 1,2	NEEYNK_5_4_1_2	51.6	0.0	5.7	0.0	100.0	6.1	14.5	34.5	0.0	0.0	0.0	0.0	8.1	26.9
76	Human Plasma exp_2	4828	Alpha-1-acid glycoprotein 1,2	NEEYNK_6_5_0_2	68.4	0.0	0.0	0.0	100.0	0.0	81.9	0.0	0.0	0.0	2.7	0.0	13.1	0.0
77	Human Plasma exp_4	4472	Alpha-1-acid glycoprotein 1,2	NEEYNK_6_5_0_2	74.8	0.0	6.9	0.0	100.0	0.0	84.0	0.0	2.7	0.0	6.7	0.0	37.9	0.0
78	Human Plasma exp_1	8272	Alpha-1-acid glycoprotein 1,2	NEEYNK_6_5_0_3	21.5	0.0	0.0	0.0	100.0	0.0	55.7	0.0	2.4	0.0	3.9	0.0	20.7	0.0

Supplementary Table 3. Continued.

No.	Experiments	CID Scan No.	N-glycoproteins	N-glycopeptides	Relative intensities of B/Y series ions													
					B_2	B_2F	B_3	B_3F	B_2S	B_2SF	Y_1	Y_1F	Y_2	Y_2F	Y_3	Y_3F	Y_4	Y_4F
79	Human Plasma exp_3	7818	Alpha-1-acid glycoprotein 1,2	NEEYNK_6_5_0_3	18.7	0.0	0.0	0.0	100.0	0.0	30.9	0.0	0.0	0.0	2.8	0.0	10.4	0.0
80	Human Plasma exp_2	4012	Alpha-1-acid glycoprotein 1,2	NEEYNK_6_5_1_2	54.7	14.2	0.0	0.0	100.0	0.0	44.7	0.0	4.1	0.0	0.0	0.0	11.1	0.0
81	Human Plasma exp_4	4103	Alpha-1-acid glycoprotein 1,2	NEEYNK_6_5_1_2	44.0	10.6	0.0	0.0	100.0	10.0	43.2	0.0	0.0	0.0	0.0	0.0	21.8	0.0
82	Human Plasma exp_5	3982	Alpha-1-acid glycoprotein 1,2	NEEYNK_6_5_1_2	40.7	14.5	0.0	0.0	100.0	0.0	50.2	0.0	0.0	0.0	0.0	0.0	12.7	0.0
83	Human Plasma exp_1	7468	Alpha-1-acid glycoprotein 1,2	NEEYNK_6_5_1_3	7.9	6.6	0.0	0.0	100.0	25.5	81.1	0.0	0.0	0.0	0.0	0.0	19.8	0.0
84	Human Plasma exp_2	7865	Alpha-1-acid glycoprotein 1,2	NEEYNK_6_5_1_3	54.9	5.9	0.0	0.0	100.0	13.8	14.1	0.0	0.0	0.0	0.0	0.0	1.8	0.0
85	Human Plasma exp_2	7608	Alpha-1-acid glycoprotein 1,2	NEEYNK_6_5_1_3	12.1	0.0	0.0	0.0	100.0	45.9	71.4	0.0	0.0	0.0	0.0	0.0	25.8	0.0
86	Human Plasma exp_3	8517	Alpha-1-acid glycoprotein 1,2	NEEYNK_6_5_1_3	23.1	0.0	0.0	0.0	100.0	35.6	59.1	0.0	0.0	0.0	0.0	0.0	26.1	0.0
87	Human Plasma exp_4	7652	Alpha-1-acid glycoprotein 1,2	NEEYNK_6_5_1_3	22.2	0.0	0.0	0.0	100.0	32.9	83.5	0.0	0.0	0.0	0.0	0.0	27.4	0.0
88	Human Plasma exp_5	7660	Alpha-1-acid glycoprotein 1,2	NEEYNK_6_5_1_3	55.2	5.8	0.0	0.0	100.0	12.3	27.6	0.0	0.0	0.0	0.0	0.0	5.2	0.0
89	Human Plasma exp_5	7574	Alpha-1-acid glycoprotein 1,2	NEEYNK_6_5_1_3	10.8	10.2	0.0	0.0	100.0	46.4	91.1	0.0	0.0	0.0	0.0	0.0	26.4	0.0
90	Human Plasma exp_1	7814	Alpha-1-acid glycoprotein 1,2	NEEYNK_6_5_2_3	0.0	5.9	0.0	0.0	100.0	22.1	8.8	20.7	0.0	0.0	0.0	0.0	11.6	13.9
91	Human Plasma exp_2	7938	Alpha-1-acid glycoprotein 1,2	NEEYNK_6_5_2_3	0.0	0.0	6.0	0.0	100.0	39.3	27.8	31.1	0.0	0.0	0.0	0.0	11.8	18.5
92	Human Plasma exp_4	7944	Alpha-1-acid glycoprotein 1,2	NEEYNK_6_5_2_3	0.0	0.0	0.0	0.0	100.0	45.2	42.8	10.2	0.0	0.0	0.0	0.0	21.8	11.2
93	Human Plasma exp_2	3426	Alpha-1-acid glycoprotein 2	ENGTVSR_5_4_0_2	51.4	0.0	0.0	0.0	100.0	0.0	20.5	0.0	0.0	0.0	3.5	0.0	30.7	0.0
94	Human Plasma exp_3	3353	Alpha-1-acid glycoprotein 2	ENGTVSR_5_4_0_2	42.6	0.0	0.0	0.0	100.0	0.0	31.8	0.0	3.3	0.0	0.0	0.0	36.9	0.0
95	Human Plasma exp_4	3468	Alpha-1-acid glycoprotein 2	ENGTVSR_5_4_0_2	59.9	0.0	0.0	0.0	100.0	0.0	21.1	0.0	0.0	0.0	3.0	0.0	27.0	0.0
96	Human Plasma exp_1	3108	Alpha-1-acid glycoprotein 2	ENGTVSR_6_5_0_2	66.9	0.0	0.0	0.0	100.0	0.0	47.3	0.0	0.0	0.0	10.0	0.0	7.8	0.0
97	Human Plasma exp_4	3240	Alpha-1-acid glycoprotein 2	ENGTVSR_6_5_0_2	85.9	0.0	0.0	0.0	100.0	0.0	56.8	0.0	0.0	0.0	4.0	0.0	6.3	0.0
98	Human Plasma exp_1	6030	Alpha-1-acid glycoprotein 2	ENGTVSR_6_5_0_3	23.2	0.0	0.0	0.0	100.0	0.0	32.9	0.0	0.0	0.0	2.3	0.0	32.0	0.0
99	Human Plasma exp_4	6123	Alpha-1-acid glycoprotein 2	ENGTVSR_6_5_0_3	29.4	0.0	0.0	0.0	100.0	0.0	31.0	0.0	0.0	0.0	4.1	0.0	23.7	0.0
100	Human Plasma exp_1	6005	Alpha-1-acid glycoprotein 2	ENGTVSR_6_5_1_3	17.2	5.6	3.7	0.0	100.0	34.2	81.4	0.0	5.1	0.0	10.1	0.0	57.2	0.0
101	Human Plasma exp_2	6119	Alpha-1-acid glycoprotein 2	ENGTVSR_6_5_1_3	19.1	4.7	3.5	0.0	84.4	35.2	100.0	0.0	4.4	0.0	0.0	0.0	53.5	0.0
102	Human Plasma exp_3	6041	Alpha-1-acid glycoprotein 2	ENGTVSR_6_5_1_3	68.0	4.2	0.0	0.0	100.0	17.2	19.8	0.0	0.0	0.0	0.0	0.0	1.7	0.0
103	Human Plasma exp_5	6036	Alpha-1-acid glycoprotein 2	ENGTVSR_6_5_1_3	76.4	5.3	0.0	0.0	100.0	23.0	16.0	0.0	0.0	0.0	0.0	0.0	0.0	0.0
104	Human Plasma exp_5	6055	Alpha-1-acid glycoprotein 2	ENGTVSR_6_5_1_3	18.3	4.7	3.6	0.0	87.5	31.6	100.0	0.0	4.2	0.0	0.0	0.0	55.3	2.3

Supplementary Table 3. Continued.

No.	Experiments	CID Scan No.	N-glycoproteins	N-glycopeptides	Relative intensities of B/Y series ions													
					B_2	B_2F	B_3	B_3F	B_2S	B_2SF	Y_1	Y_1F	Y_2	Y_2F	Y_3	Y_3F	Y_4	Y_4F
105	Human Plasma exp_5	5964	Alpha-1-acid glycoprotein 2	ENGTVSR_6_5_2_3	0.0	0.0	0.0	0.0	100.0	40.9	29.0	0.0	0.0	0.0	0.0	0.0	9.4	6.9
106	Human Plasma exp_1	10095	Alpha-1-acid glycoprotein 2	ENGTVSR_7_6_1_4	21.0	0.0	0.0	0.0	100.0	21.4	10.0	0.0	0.0	0.0	0.0	0.0	3.8	0.0
107	Human Plasma exp_5	10345	Alpha-1-acid glycoprotein 2	ENGTVSR_7_6_1_4	25.5	0.0	0.0	0.0	100.0	31.6	4.1	0.0	0.0	0.0	0.0	0.0	1.4	0.0
108	Human Plasma exp_2	10867	Alpha-1-acid glycoprotein 2	ENGTVSR_7_6_2_4	25.9	3.8	0.0	0.0	100.0	92.7	7.0	0.0	0.0	0.0	0.0	0.0	1.5	0.0
109	Human Plasma exp_3	10390	Alpha-1-acid glycoprotein 2	ENGTVSR_7_6_2_4	26.5	9.5	0.0	0.0	100.0	59.7	5.1	0.0	0.0	0.0	0.0	0.0	0.0	0.0
110	Human Plasma exp_5	10745	Alpha-1-acid glycoprotein 2	ENGTVSR_7_6_2_4	25.6	7.8	0.0	0.0	100.0	99.0	7.6	0.0	0.0	0.0	0.0	0.0	2.0	0.0
111	Human Plasma exp_2	10369	Alpha-1-acid glycoprotein 2	ENGTVSR_7_6_3_4	27.3	7.3	0.0	0.0	71.0	100.0	6.3	0.0	0.0	0.0	0.0	0.0	0.0	0.0
112	Human Plasma exp_3	10349	Alpha-1-acid glycoprotein 2	ENGTVSR_7_6_3_4	23.2	4.2	0.0	0.0	93.4	100.0	7.3	0.0	0.0	0.0	0.0	0.0	0.0	0.0
113	Human Plasma exp_4	10452	Alpha-1-acid glycoprotein 2	ENGTVSR_7_6_3_4	32.2	5.5	0.0	0.0	91.4	100.0	7.4	0.0	0.0	0.0	0.0	0.0	0.0	0.0
114	Human Plasma exp_1	10219	Alpha-1-acid glycoprotein 2	ENGTVSR_8_7_0_4	31.7	0.0	3.6	0.0	100.0	0.0	12.2	0.0	0.0	0.0	0.0	0.0	3.0	0.0
115	Human Plasma exp_2	28637	Alpha-1-acid glycoprotein 2	LVPVPITNATLDR_5_4_0_2	81.0	0.0	4.8	0.0	100.0	0.0	46.7	0.0	0.0	0.0	4.2	0.0	52.9	0.0
116	Human Plasma exp_3	28111	Alpha-1-acid glycoprotein 2	LVPVPITNATLDR_5_4_0_2	68.6	0.0	5.1	0.0	100.0	0.0	36.2	0.0	0.0	0.0	6.9	0.0	68.5	0.0
117	Human Plasma exp_4	28654	Alpha-1-acid glycoprotein 2	LVPVPITNATLDR_5_4_0_2	71.9	0.0	6.0	0.0	100.0	0.0	15.7	0.0	0.0	0.0	10.8	0.0	57.0	0.0
118	Human Plasma exp_5	27615	Alpha-1-acid glycoprotein 2	LVPVPITNATLDR_5_4_0_2	65.2	0.0	0.0	0.0	100.0	0.0	26.7	0.0	0.0	0.0	2.4	0.0	54.2	0.0
119	Human Plasma exp_1	27586	Alpha-1-acid glycoprotein 2	LVPVPITNATLDR_6_5_0_2	79.6	0.0	8.5	0.0	100.0	0.0	4.7	0.0	0.0	0.0	0.0	0.0	24.0	0.0
120	Human Plasma exp_3	27708	Alpha-1-acid glycoprotein 2	LVPVPITNATLDR_6_5_0_2	80.6	0.0	0.0	0.0	100.0	0.0	18.3	0.0	2.8	0.0	0.0	0.0	21.1	0.0
121	Human Plasma exp_5	27392	Alpha-1-acid glycoprotein 2	LVPVPITNATLDR_6_5_0_2	81.0	0.0	0.0	0.0	100.0	0.0	19.7	0.0	5.3	0.0	0.0	0.0	24.1	0.0
122	Human Plasma exp_4	30813	Alpha-1-acid glycoprotein 2	LVPVPITNATLDR_6_5_0_3	59.8	0.0	0.0	0.0	100.0	0.0	14.3	0.0	0.0	0.0	2.4	0.0	12.0	0.0
123	Human Plasma exp_4	30545	Alpha-1-acid glycoprotein 2	LVPVPITNATLDR_6_5_0_3	41.1	0.0	0.0	0.0	100.0	0.0	20.0	0.0	7.2	0.0	3.8	0.0	0.0	0.0
124	Human Plasma exp_2	30889	Alpha-1-acid glycoprotein 2	LVPVPITNATLDR_6_5_1_3	43.1	0.0	0.0	0.0	100.0	17.1	24.0	0.0	0.0	0.0	0.0	0.0	9.2	0.0
125	Human Plasma exp_4	30616	Alpha-1-acid glycoprotein 2	LVPVPITNATLDR_6_5_1_3	39.6	0.0	0.0	0.0	100.0	24.5	26.8	0.0	0.0	0.0	0.0	0.0	28.9	0.0
126	Human Plasma exp_4	30328	Alpha-1-acid glycoprotein 2	LVPVPITNATLDR_7_6_0_3	31.3	0.0	3.1	0.0	100.0	0.0	8.9	0.0	0.0	0.0	0.0	0.0	5.7	0.0
127	Human Plasma exp_4	29256	Alpha-1-acid glycoprotein 2	QNQCFYNSSYLNVQR_6_5_0_3	25.0	0.0	0.0	0.0	100.0	0.0	82.7	0.0	0.0	0.0	9.8	0.0	34.7	0.0
128	Human Plasma exp_5	28394	Alpha-1-acid glycoprotein 2	QNQCFYNSSYLNVQR_6_5_0_3	22.3	0.0	0.0	0.0	100.0	0.0	78.7	0.0	0.0	0.0	10.1	0.0	45.6	0.0
129	Human Plasma exp_1	2023	Alpha-1-antichymotrypsin	NGTR_6_5_0_3	36.2	0.0	0.0	0.0	100.0	0.0	23.0	0.0	1.2	0.0	5.8	0.0	38.8	0.0
130	Human Plasma exp_2	2300	Alpha-1-antichymotrypsin	NGTR_6_5_0_3	32.0	0.0	0.0	0.0	100.0	0.0	13.8	0.0	0.0	0.0	3.2	0.0	32.6	0.0

Supplementary Table 3. Continued.

No.	Experiments	CID Scan No.	N-glycoproteins	N-glycopeptides	Relative intensities of B/Y series ions													
					B_2	B_2F	B_3	B_3F	B_2S	B_2SF	Y_1	Y_1F	Y_2	Y_2F	Y_3	Y_3F	Y_4	Y_4F
131	Human Plasma exp_3	2083	Alpha-1-antichymotrypsin	NGTR_6_5_0_3	39.5	0.0	3.1	0.0	100.0	0.0	33.2	0.0	3.4	0.0	10.1	0.0	39.5	0.0
132	Human Plasma exp_4	2176	Alpha-1-antichymotrypsin	NGTR_6_5_0_3	38.3	0.0	3.4	0.0	100.0	0.0	34.2	0.0	5.3	0.0	8.8	0.0	41.9	0.0
133	Human Plasma exp_5	2213	Alpha-1-antichymotrypsin	NGTR_6_5_0_3	41.9	0.0	0.0	0.0	100.0	0.0	19.7	0.0	0.0	0.0	3.0	0.0	37.2	0.0
134	Human Plasma exp_1	2172	Alpha-1-antichymotrypsin	NGTR_6_5_1_3	32.9	0.0	0.0	0.0	100.0	7.0	2.7	9.8	0.0	0.0	0.0	0.0	3.7	24.7
135	Human Plasma exp_2	2280	Alpha-1-antichymotrypsin	NGTR_6_5_1_3	34.4	0.0	0.0	0.0	100.0	0.0	3.0	19.0	0.0	0.0	0.0	0.0	4.2	29.2
136	Human Plasma exp_4	2297	Alpha-1-antichymotrypsin	NGTR_6_5_1_3	35.4	0.0	0.0	0.0	100.0	0.0	5.6	13.3	0.0	0.0	0.0	0.0	1.6	25.7
137	Human Plasma exp_1	32504	Alpha-1-antitrypsin	YLGNATAIFFLPDEGK_5_4_0_2	54.4	0.0	0.0	0.0	100.0	0.0	56.5	0.0	2.1	0.0	20.2	0.0	76.5	0.0
138	Human Plasma exp_2	33340	Alpha-1-antitrypsin	YLGNATAIFFLPDEGK_5_4_0_2	51.8	0.0	4.5	0.0	100.0	0.0	46.5	0.0	2.2	0.0	5.5	0.0	65.1	0.0
139	Human Plasma exp_3	32826	Alpha-1-antitrypsin	YLGNATAIFFLPDEGK_5_4_0_2	48.8	0.0	0.0	0.0	100.0	0.0	40.2	0.0	1.5	0.0	6.4	0.0	57.5	0.0
140	Human Plasma exp_4	33074	Alpha-1-antitrypsin	YLGNATAIFFLPDEGK_5_4_0_2	69.2	0.0	0.0	0.0	100.0	0.0	29.4	0.0	3.8	0.0	8.1	0.0	57.5	0.0
141	Human Plasma exp_5	32099	Alpha-1-antitrypsin	YLGNATAIFFLPDEGK_5_4_0_2	54.2	0.0	0.0	0.0	100.0	0.0	50.9	0.0	0.0	0.0	17.6	0.0	75.7	0.0
142	Human Plasma exp_1	28824	Alpha-2-HS-glycoprotein	VCQDCPLLAPLNDTR_5_4_0_2	55.3	0.0	4.6	0.0	100.0	0.0	0.0	0.0	0.0	0.0	14.6	0.0	63.1	0.0
143	Human Plasma exp_4	29566	Alpha-2-HS-glycoprotein	VCQDCPLLAPLNDTR_5_4_0_2	48.6	0.0	4.2	0.0	100.0	0.0	0.0	0.0	0.0	0.0	8.9	0.0	63.6	0.0
144	Human Plasma exp_5	28442	Alpha-2-HS-glycoprotein	VCQDCPLLAPLNDTR_5_4_0_2	55.9	0.0	4.7	0.0	100.0	0.0	0.0	0.0	5.0	0.0	18.5	0.0	75.3	0.0
145	Human Plasma exp_5	28352	Alpha-2-HS-glycoprotein	VCQDCPLLAPLNDTR_5_4_1_2	34.0	0.0	0.0	0.0	100.0	6.9	60.2	10.8	0.0	0.0	0.0	0.0	43.5	34.6
146	Human Plasma exp_4	31529	Alpha-2-HS-glycoprotein	VCQDCPLLAPLNDTR_6_5_1_3	26.4	0.0	0.0	0.0	100.0	23.0	58.7	0.0	0.0	0.0	23.3	0.0	58.9	0.0
147	Human Plasma exp_5	30597	Alpha-2-HS-glycoprotein	VCQDCPLLAPLNDTR_6_5_1_3	32.9	0.0	0.0	0.0	100.0	31.9	69.8	0.0	0.0	0.0	0.0	0.0	62.4	0.0
148	Human Plasma exp_5	28906	Aminopeptidase N	VPVTLALNNTLFLIEER_4_4_0_1	89.5	0.0	0.0	0.0	37.1	0.0	0.0	0.0	14.8	0.0	52.1	0.0	100.0	0.0
149	Human Plasma exp_1	1530	Angiotensinogen	NCTSR_5_4_0_2	59.9	0.0	4.0	0.0	100.0	0.0	30.2	0.0	0.0	0.0	2.3	0.0	38.9	0.0
150	Human Plasma exp_2	1557	Angiotensinogen	NCTSR_5_4_0_2	55.1	0.0	5.3	0.0	100.0	0.0	42.5	0.0	1.9	0.0	4.3	0.0	38.9	0.0
151	Human Plasma exp_3	1544	Angiotensinogen	NCTSR_5_4_0_2	53.8	0.0	5.5	0.0	100.0	0.0	41.0	0.0	1.7	0.0	5.1	0.0	40.0	0.0
152	Human Plasma exp_4	1592	Angiotensinogen	NCTSR_5_4_0_2	54.9	0.0	6.2	0.0	100.0	0.0	54.4	0.0	2.8	0.0	4.2	0.0	40.6	0.0
153	Human Plasma exp_5	1511	Angiotensinogen	NCTSR_5_4_0_2	51.0	0.0	4.0	0.0	100.0	0.0	40.1	0.0	2.1	0.0	4.2	0.0	36.4	0.0
154	Human Plasma exp_1	3219	Angiotensinogen	NCTSR_6_5_0_3	36.2	0.0	0.0	0.0	100.0	0.0	29.1	0.0	0.0	0.0	3.3	0.0	42.8	0.0
155	Human Plasma exp_2	3309	Angiotensinogen	NCTSR_6_5_0_3	24.7	0.0	0.0	0.0	100.0	0.0	34.3	0.0	0.0	0.0	3.4	0.0	22.1	0.0
156	Human Plasma exp_5	3239	Angiotensinogen	NCTSR_6_5_0_3	33.1	0.0	0.0	0.0	100.0	0.0	26.5	0.0	0.0	0.0	3.6	0.0	41.7	0.0

Supplementary Table 3. Continued.

No.	Experiments	CID Scan No.	N-glycoproteins	N-glycopeptides	Relative intensities of B/Y series ions													
					B_2	B_2F	B_3	B_3F	B_2S	B_2SF	Y_1	Y_1F	Y_2	Y_2F	Y_3	Y_3F	Y_4	Y_4F
157	Human Plasma exp_1	25028	Apolipoprotein C-IV	ELLETVVNR_5_4_0_2	23.1	0.0	0.0	0.0	100.0	0.0	59.0	0.0	0.0	0.0	1.8	0.0	36.7	0.0
158	Human Plasma exp_5	24778	Apolipoprotein C-IV	ELLETVVNR_5_4_0_2	45.0	0.0	0.0	0.0	100.0	0.0	42.7	0.0	0.0	0.0	2.4	0.0	31.3	0.0
159	Human Plasma exp_5	24718	Apolipoprotein D	ADGTVNQIEGEATPVNLTEPAK_5_4_0_1	72.5	0.0	10.2	0.0	69.3	0.0	100.0	0.0	0.0	0.0	0.0	0.0	44.7	0.0
160	Human Plasma exp_2	25293	Apolipoprotein D	ADGTVNQIEGEATPVNLTEPAK_5_4_1_1	86.8	7.3	8.6	0.0	87.2	0.0	100.0	43.0	0.0	0.0	7.9	0.0	58.3	53.5
161	Human Plasma exp_3	24804	Apolipoprotein D	ADGTVNQIEGEATPVNLTEPAK_5_4_1_1	44.8	8.7	11.8	0.0	63.9	7.6	100.0	55.1	4.1	2.6	12.2	0.0	43.3	31.9
162	Human Plasma exp_4	25277	Apolipoprotein D	ADGTVNQIEGEATPVNLTEPAK_5_4_1_1	100.0	0.0	8.3	0.0	74.5	0.0	56.9	49.4	0.0	0.0	0.0	0.0	42.5	45.8
163	Human Plasma exp_2	27855	Apolipoprotein D	ADGTVNQIEGEATPVNLTEPAK_5_4_1_2	37.8	0.0	0.0	0.0	100.0	10.3	58.5	39.0	0.0	0.0	0.0	0.0	26.9	30.4
164	Human Plasma exp_3	24430	Beta-2-glycoprotein 1	LGNWSAMPSCK_5_4_0_2	62.1	0.0	0.0	0.0	100.0	0.0	33.9	0.0	0.0	0.0	12.8	0.0	64.5	0.0
165	Human Plasma exp_4	24919	Beta-2-glycoprotein 1	LGNWSAMPSCK_5_4_0_2	55.1	0.0	7.2	0.0	100.0	0.0	15.0	0.0	0.0	0.0	2.8	0.0	74.8	0.0
166	Human Plasma exp_2	15673	Beta-2-glycoprotein 1	VYKPSAGNNSLYR_4_3_0_1	51.0	0.0	0.0	0.0	48.5	0.0	40.1	0.0	0.0	0.0	3.6	0.0	100.0	0.0
167	Human Plasma exp_4	18459	Beta-2-glycoprotein 1	VYKPSAGNNSLYR_6_5_1_3	63.2	0.0	0.0	0.0	100.0	28.4	12.8	0.0	0.0	0.0	0.0	0.0	11.7	0.0
168	Human Plasma exp_5	22408	Biotinidase	FNDTEVLQR_5_4_0_2	53.7	0.0	7.8	0.0	100.0	0.0	49.7	0.0	0.0	0.0	3.5	0.0	38.3	0.0
169	Human Plasma exp_1	24313	Biotinidase	NPVGLIGAENATGETDPSHSK_6_5_0_3	19.5	0.0	0.0	0.0	100.0	0.0	56.2	0.0	0.0	0.0	17.8	0.0	54.0	0.0
170	Human Plasma exp_3	30160	Ceruloplasmin	AGLQAFFQVQECNK_5_4_0_2	55.2	0.0	7.1	0.0	100.0	0.0	71.8	0.0	0.0	0.0	0.0	0.0	35.8	0.0
171	Human Plasma exp_4	30413	Ceruloplasmin	AGLQAFFQVQECNK_5_4_0_2	70.5	0.0	0.0	0.0	100.0	0.0	62.3	0.0	0.0	0.0	12.0	0.0	64.4	0.0
172	Human Plasma exp_3	20507	Ceruloplasmin	EHEGAIYPDNTTDFQR_4_3_0_1	45.5	0.0	0.0	0.0	51.2	0.0	76.7	0.0	0.0	0.0	11.4	0.0	100.0	0.0
173	Human Plasma exp_3	20296	Ceruloplasmin	EHEGAIYPDNTTDFQR_4_3_1_1	19.4	0.0	0.0	0.0	57.6	0.0	16.2	0.0	0.0	0.0	0.0	0.0	52.4	100.0
174	Human Plasma exp_2	21574	Ceruloplasmin	ELHHLQEQNVSNAFLDK_5_4_0_2	91.1	0.0	9.3	0.0	100.0	0.0	88.9	0.0	0.0	0.0	17.8	0.0	86.3	0.0
175	Human Plasma exp_2	21244	Ceruloplasmin	ELHHLQEQNVSNAFLDK_5_4_1_2	78.8	0.0	0.0	0.0	100.0	0.0	52.5	0.0	0.0	0.0	9.6	17.0	44.6	78.2
176	Human Plasma exp_1	29132	Ceruloplasmin	ENLTAPGSDSAVFFEQGTTR_5_4_0_2	47.7	0.0	4.2	0.0	100.0	0.0	56.9	0.0	10.7	0.0	15.6	0.0	74.9	0.0
177	Human Plasma exp_2	29871	Ceruloplasmin	ENLTAPGSDSAVFFEQGTTR_5_4_0_2	31.7	0.0	0.0	0.0	100.0	0.0	78.9	0.0	0.0	0.0	21.9	0.0	77.8	0.0
178	Human Plasma exp_3	29403	Ceruloplasmin	ENLTAPGSDSAVFFEQGTTR_5_4_0_2	56.7	0.0	11.6	0.0	100.0	0.0	88.0	0.0	0.0	0.0	12.6	0.0	69.3	0.0
179	Human Plasma exp_4	29625	Ceruloplasmin	ENLTAPGSDSAVFFEQGTTR_5_4_0_2	52.7	0.0	7.2	0.0	100.0	0.0	77.7	0.0	9.8	0.0	14.5	0.0	94.6	0.0
180	Human Plasma exp_5	28716	Ceruloplasmin	ENLTAPGSDSAVFFEQGTTR_5_4_0_2	57.4	0.0	0.0	0.0	94.8	0.0	66.2	0.0	0.0	0.0	10.7	0.0	100.0	0.0
181	Human Plasma exp_1	11457	Cholinesterase	DNNSIITR_5_4_0_1	100.0	0.0	7.4	0.0	80.1	0.0	36.0	0.0	0.0	0.0	0.0	0.0	40.9	0.0
182	Human Plasma exp_2	11650	Cholinesterase	DNNSIITR_5_4_0_1	100.0	0.0	5.1	0.0	64.0	0.0	37.8	0.0	0.0	0.0	0.0	0.0	46.1	0.0

Supplementary Table 3. Continued.

No.	Experiments	CID Scan No.	N-glycoproteins	N-glycopeptides	Relative intensities of B/Y series ions													
					B_2	B_2F	B_3	B_3F	B_2S	B_2SF	Y_1	Y_1F	Y_2	Y_2F	Y_3	Y_3F	Y_4	Y_4F
183	Human Plasma exp_5	11547	Cholinesterase	DNNSIITR_5_4_0_1	100.0	0.0	5.6	0.0	62.3	0.0	35.0	0.0	0.0	0.0	0.0	0.0	21.4	0.0
184	Human Plasma exp_5	15800	Cholinesterase	DNNSIITR_5_4_0_2	66.8	0.0	4.4	0.0	100.0	0.0	47.6	0.0	0.0	0.0	2.4	0.0	46.8	0.0
185	Human Plasma exp_1	4485	Cholinesterase	DNYTK_5_4_0_2	51.7	0.0	2.7	0.0	100.0	0.0	24.3	0.0	1.1	0.0	0.0	0.0	50.0	0.0
186	Human Plasma exp_2	4550	Cholinesterase	DNYTK_5_4_0_2	46.5	0.0	4.5	0.0	100.0	0.0	25.1	0.0	0.0	0.0	1.3	0.0	41.0	0.0
187	Human Plasma exp_3	4501	Cholinesterase	DNYTK_5_4_0_2	46.4	0.0	4.0	0.0	100.0	0.0	25.2	0.0	1.5	0.0	0.0	0.0	36.9	0.0
188	Human Plasma exp_4	4632	Cholinesterase	DNYTK_5_4_0_2	63.3	0.0	4.8	0.0	100.0	0.0	19.2	0.0	1.6	0.0	0.0	0.0	43.0	0.0
189	Human Plasma exp_5	4514	Cholinesterase	DNYTK_5_4_0_2	44.4	0.0	4.1	0.0	100.0	0.0	21.6	0.0	0.0	0.0	3.3	0.0	35.7	0.0
190	Human Plasma exp_2	17003	Cholinesterase	ENETEIIK_5_4_0_2	51.1	0.0	0.0	0.0	100.0	0.0	38.8	0.0	0.0	0.0	5.5	0.0	34.6	0.0
191	Human Plasma exp_5	16747	Cholinesterase	ENETEIIK_5_4_0_2	44.9	0.0	0.0	0.0	100.0	0.0	36.5	0.0	0.0	0.0	2.8	0.0	28.8	0.0
192	Human Plasma exp_1	12515	Clusterin	EDALNETR_5_4_0_2	34.1	0.0	7.2	0.0	100.0	0.0	29.1	0.0	0.0	0.0	0.0	0.0	13.4	0.0
193	Human Plasma exp_1	16327	Clusterin	EDALNETR_6_5_0_3	19.8	0.0	0.0	0.0	100.0	0.0	57.2	0.0	0.0	0.0	5.7	0.0	34.1	0.0
194	Human Plasma exp_3	16379	Clusterin	EDALNETR_6_5_1_3	47.0	3.8	0.0	0.0	100.0	13.2	9.9	0.0	0.0	0.0	0.0	0.0	0.0	0.0
195	Human Plasma exp_4	16705	Clusterin	EDALNETR_6_5_1_3	0.0	5.3	0.0	0.0	100.0	36.8	74.0	0.0	0.0	0.0	0.0	0.0	27.7	0.0
196	Human Plasma exp_5	16430	Clusterin	EDALNETR_6_5_1_3	0.0	0.0	6.1	0.0	100.0	34.1	41.1	0.0	0.0	0.0	0.0	0.0	28.0	0.0
197	Human Plasma exp_5	5173	Clusterin	HNSTGCLR_6_5_0_3	57.7	0.0	0.0	0.0	100.0	0.0	2.4	0.0	1.4	0.0	0.0	0.0	1.7	0.0
198	Human Plasma exp_5	5336	Clusterin	HNSTGCLR_6_5_0_3	14.0	0.0	0.0	0.0	100.0	0.0	29.0	0.0	3.1	0.0	0.0	0.0	8.7	0.0
199	Human Plasma exp_1	27042	Clusterin	LANLTQGEDQYYLR_5_4_0_2	62.7	0.0	6.8	0.0	100.0	0.0	60.8	0.0	7.6	0.0	14.3	1.9	71.1	0.0
200	Human Plasma exp_2	27735	Clusterin	LANLTQGEDQYYLR_5_4_0_2	66.8	0.0	8.7	0.0	100.0	0.0	56.1	0.0	12.9	0.0	17.9	3.0	76.7	0.0
201	Human Plasma exp_3	27148	Clusterin	LANLTQGEDQYYLR_5_4_0_2	46.5	0.0	2.8	0.0	100.0	0.0	38.0	0.0	0.0	0.0	18.8	6.6	49.5	0.0
202	Human Plasma exp_4	27518	Clusterin	LANLTQGEDQYYLR_5_4_0_2	57.2	0.0	5.1	0.0	100.0	0.0	58.8	0.0	7.4	0.0	13.7	0.0	68.0	0.0
203	Human Plasma exp_5	26765	Clusterin	LANLTQGEDQYYLR_5_4_0_2	68.4	0.0	3.2	0.0	100.0	0.0	62.5	0.0	8.9	0.0	23.2	1.7	77.6	0.0
204	Human Plasma exp_2	29998	Clusterin	LANLTQGEDQYYLR_6_5_1_3	38.3	0.0	0.0	0.0	100.0	17.0	40.7	0.0	0.0	0.0	5.2	0.0	40.6	0.0
205	Human Plasma exp_3	29473	Clusterin	LANLTQGEDQYYLR_6_5_1_3	40.2	0.0	0.0	0.0	100.0	11.9	25.8	0.0	0.0	0.0	0.0	0.0	12.7	0.0
206	Human Plasma exp_4	29768	Clusterin	LANLTQGEDQYYLR_6_5_1_3	26.3	0.0	0.0	0.0	100.0	15.2	14.5	0.0	3.2	0.0	0.0	0.0	26.4	0.0
207	Human Plasma exp_1	14322	Coagulation factor XII	NHSCEPCQTLAVR_5_4_0_2	69.4	0.0	4.8	0.0	100.0	0.0	55.5	0.0	6.8	0.0	14.8	0.0	71.6	0.0
208	Human Plasma exp_1	14340	Coagulation factor XII	NHSCEPCQTLAVR_5_4_0_2	27.5	0.0	0.0	0.0	100.0	0.0	90.1	0.0	0.0	0.0	18.8	0.0	28.7	0.0

Supplementary Table 3. Continued.

No.	Experiments	CID Scan No.	N-glycoproteins	N-glycopeptides	Relative intensities of B/Y series ions													
					B_2	B_2F	B_3	B_3F	B_2S	B_2SF	Y_1	Y_1F	Y_2	Y_2F	Y_3	Y_3F	Y_4	Y_4F
209	Human Plasma exp_2	14554	Coagulation factor XII	NHSCEPCQTLAVR_5_4_0_2	79.0	0.0	3.2	0.0	100.0	0.0	54.0	0.0	3.3	0.0	10.1	0.0	67.7	0.0
210	Human Plasma exp_2	14620	Coagulation factor XII	NHSCEPCQTLAVR_5_4_0_2	29.7	0.0	0.0	0.0	100.0	0.0	74.4	0.0	17.1	0.0	0.0	0.0	19.9	0.0
211	Human Plasma exp_3	14357	Coagulation factor XII	NHSCEPCQTLAVR_5_4_0_2	61.5	0.0	6.9	0.0	100.0	0.0	65.6	0.0	8.7	0.0	11.0	0.0	68.4	0.0
212	Human Plasma exp_3	14322	Coagulation factor XII	NHSCEPCQTLAVR_5_4_0_2	30.1	0.0	0.0	0.0	100.0	0.0	91.4	0.0	14.3	0.0	0.0	0.0	31.9	0.0
213	Human Plasma exp_4	14648	Coagulation factor XII	NHSCEPCQTLAVR_5_4_0_2	66.9	0.0	6.1	0.0	100.0	0.0	54.5	0.0	9.7	0.0	11.2	0.0	72.3	0.0
214	Human Plasma exp_4	14646	Coagulation factor XII	NHSCEPCQTLAVR_5_4_0_2	32.1	0.0	0.0	0.0	100.0	0.0	69.7	0.0	15.2	0.0	0.0	0.0	21.3	0.0
215	Human Plasma exp_5	14392	Coagulation factor XII	NHSCEPCQTLAVR_5_4_0_2	67.1	0.0	5.1	0.0	100.0	0.0	49.0	0.0	9.0	0.0	14.4	0.0	69.0	0.0
216	Human Plasma exp_2	3044	Coagulation factor XII	NVTAEQAR_5_4_0_1	100.0	0.0	0.0	0.0	55.7	5.9	23.6	0.0	0.0	0.0	0.0	0.0	20.6	0.0
217	Human Plasma exp_3	2946	Coagulation factor XII	NVTAEQAR_5_4_0_1	100.0	0.0	6.5	0.0	55.7	0.0	32.7	0.0	0.0	0.0	0.0	0.0	7.6	0.0
218	Human Plasma exp_1	5719	Coagulation factor XII	NVTAEQAR_5_4_0_2	51.3	0.0	4.7	0.0	100.0	0.0	42.9	0.0	0.0	0.0	3.8	0.0	38.4	0.0
219	Human Plasma exp_2	5855	Coagulation factor XII	NVTAEQAR_5_4_0_2	49.3	0.0	6.9	0.0	100.0	0.0	59.9	0.0	2.8	0.0	5.4	0.0	49.3	0.0
220	Human Plasma exp_3	5778	Coagulation factor XII	NVTAEQAR_5_4_0_2	53.3	0.0	3.5	0.0	100.0	0.0	46.6	0.0	0.0	0.0	5.7	0.0	36.0	0.0
221	Human Plasma exp_5	5807	Coagulation factor XII	NVTAEQAR_5_4_0_2	54.7	0.0	5.8	0.0	100.0	0.0	50.9	0.0	1.8	0.0	6.7	0.0	40.1	0.0
222	Human Plasma exp_5	10110	Coagulation factor XII	NVTAEQAR_6_5_0_3	14.6	0.0	0.0	0.0	100.0	0.0	63.2	0.0	6.3	0.0	0.0	0.0	48.2	0.0
223	Human Plasma exp_1	16554	Complement component C7	NYTLTGR_5_4_1_2	54.6	0.0	7.7	0.0	100.0	3.9	10.8	33.1	0.0	0.0	0.0	0.0	7.6	31.6
224	Human Plasma exp_2	16861	Complement component C7	NYTLTGR_5_4_1_2	59.6	0.0	4.3	0.0	100.0	0.0	2.2	14.5	0.0	0.0	0.0	0.0	0.0	0.0
225	Human Plasma exp_3	16589	Complement component C7	NYTLTGR_5_4_1_2	59.6	0.0	3.7	0.0	100.0	0.0	11.6	34.3	0.0	0.0	0.0	0.0	6.6	31.7
226	Human Plasma exp_4	16975	Complement component C7	NYTLTGR_5_4_1_2	58.3	0.0	6.4	0.0	100.0	0.0	14.9	33.1	0.0	0.0	0.0	0.0	5.6	26.0
227	Human Plasma exp_5	16666	Complement component C7	NYTLTGR_5_4_1_2	51.0	0.0	7.9	0.0	100.0	0.0	10.0	28.2	0.0	0.0	0.0	0.0	7.5	31.0
228	Human Plasma exp_1	19516	Complement component C8 alpha chain	GGSSGWSGGLAQNR_5_4_0_2	66.6	0.0	0.0	0.0	100.0	0.0	42.6	0.0	0.0	0.0	5.9	0.0	63.3	0.0
229	Human Plasma exp_3	19270	Complement component C8 alpha chain	GGSSGWSGGLAQNR_5_4_0_2	73.0	0.0	4.7	0.0	100.0	0.0	40.8	0.0	1.9	0.0	9.7	0.0	73.9	0.0
230	Human Plasma exp_5	19483	Complement component C8 alpha chain	GGSSGWSGGLAQNR_5_4_0_2	80.9	0.0	4.6	0.0	100.0	0.0	45.9	0.0	0.0	0.0	6.1	0.0	46.3	0.0
231	Human Plasma exp_1	2149	Complement component C8 beta chain	NVTEK_4_3_0_1	30.3	0.0	5.2	0.0	50.9	0.0	43.6	0.0	2.7	0.0	5.7	0.0	100.0	0.0
232	Human Plasma exp_4	2309	Complement component C8 beta chain	NVTEK_4_3_0_1	51.8	0.0	6.2	0.0	77.0	0.0	43.5	0.0	3.5	0.0	0.0	0.0	100.0	0.0
233	Human Plasma exp_5	2192	Complement component C8 beta chain	NVTEK_4_3_0_1	47.7	0.0	0.0	0.0	92.7	0.0	68.7	0.0	6.0	0.0	0.0	0.0	100.0	0.0
234	Human Plasma exp_1	2116	Complement component C8 beta chain	NVTEK_5_4_0_2	57.4	0.0	5.2	0.0	100.0	0.0	34.3	0.0	3.4	0.0	4.9	0.0	44.9	0.0

Supplementary Table 3. Continued.

No.	Experiments	CID Scan No.	N-glycoproteins	N-glycopeptides	Relative intensities of B/Y series ions													
					B_2	B_2F	B_3	B_3F	B_2S	B_2SF	Y_1	Y_1F	Y_2	Y_2F	Y_3	Y_3F	Y_4	Y_4F
235	Human Plasma exp_1	2128	Complement component C8 beta chain	NVTEK_5_4_0_2	0.0	0.0	0.0	0.0	100.0	0.0	18.9	0.0	6.0	0.0	4.0	0.0	13.5	0.0
236	Human Plasma exp_2	2272	Complement component C8 beta chain	NVTEK_5_4_0_2	52.6	0.0	5.7	0.0	100.0	0.0	32.5	0.0	2.6	0.0	3.9	0.0	37.4	0.0
237	Human Plasma exp_3	2162	Complement component C8 beta chain	NVTEK_5_4_0_2	56.2	0.0	4.7	0.0	100.0	0.0	30.6	0.0	3.0	0.0	4.0	0.0	43.9	0.0
238	Human Plasma exp_4	2273	Complement component C8 beta chain	NVTEK_5_4_0_2	32.0	0.0	5.8	0.0	100.0	0.0	22.4	0.0	0.0	0.0	1.6	0.0	45.9	0.0
239	Human Plasma exp_5	2287	Complement component C8 beta chain	NVTEK_5_4_0_2	54.4	0.0	5.2	0.0	100.0	0.0	25.1	0.0	0.0	0.0	0.0	0.0	35.6	0.0
240	Human Plasma exp_1	21593	Complement factor H-related protein 3	FVQGNSTEVACHPGYGLPK_5_4_0_2	71.3	0.0	0.0	0.0	100.0	0.0	40.1	0.0	0.0	0.0	2.2	0.0	23.5	0.0
241	Human Plasma exp_4	21901	Complement factor H-related protein 3	FVQGNSTEVACHPGYGLPK_5_4_0_2	74.6	0.0	7.3	0.0	100.0	0.0	49.4	0.0	0.0	0.0	9.1	0.0	53.2	0.0
242	Human Plasma exp_3	15012	Complement factor I	FLNNGTCTAEGK_5_4_0_2	74.5	0.0	6.0	0.0	100.0	0.0	25.2	0.0	0.0	0.0	8.8	0.0	49.9	0.0
243	Human Plasma exp_5	15048	Complement factor I	FLNNGTCTAEGK_5_4_0_2	69.7	0.0	5.1	0.0	100.0	0.0	36.2	0.0	0.0	0.0	4.5	0.0	55.9	0.0
244	Human Plasma exp_4	3395	Complement factor I	NGTAVCATNR_5_4_0_1	100.0	0.0	9.9	0.0	78.3	0.0	58.6	0.0	0.0	0.0	0.0	0.0	43.9	0.0
245	Human Plasma exp_1	6679	Complement factor I	NGTAVCATNR_5_4_0_2	53.7	0.0	4.5	0.0	100.0	0.0	65.8	0.0	0.0	0.0	7.2	0.0	60.8	0.0
246	Human Plasma exp_2	6669	Complement factor I	NGTAVCATNR_5_4_0_2	51.7	0.0	0.0	0.0	100.0	0.0	53.3	0.0	0.0	0.0	4.7	0.0	43.0	0.0
247	Human Plasma exp_3	6787	Complement factor I	NGTAVCATNR_5_4_0_2	46.4	0.0	6.4	0.0	100.0	0.0	62.5	0.0	1.4	0.0	6.6	0.0	52.2	0.0
248	Human Plasma exp_4	6836	Complement factor I	NGTAVCATNR_5_4_0_2	59.7	0.0	6.8	0.0	100.0	0.0	61.8	0.0	0.0	0.0	8.4	0.0	54.6	0.0
249	Human Plasma exp_5	6774	Complement factor I	NGTAVCATNR_5_4_0_2	55.4	0.0	5.6	0.0	100.0	0.0	74.1	0.0	3.2	0.0	6.6	0.0	60.8	0.0
250	Human Plasma exp_4	17592	Desmocollin-2	ANYTILK_5_4_0_1	100.0	0.0	8.6	0.0	67.6	0.0	20.4	0.0	0.0	0.0	0.0	0.0	24.9	0.0
251	Human Plasma exp_2	22615	Desmocollin-2	ANYTILK_5_4_0_2	59.5	0.0	4.4	0.0	100.0	0.0	37.3	0.0	0.0	0.0	4.4	0.0	39.3	0.0
252	Human Plasma exp_4	22567	Desmocollin-2	ANYTILK_5_4_0_2	57.7	0.0	4.1	0.0	100.0	0.0	50.9	0.0	2.2	0.0	4.5	0.0	44.0	0.0
253	Human Plasma exp_3	24871	Endoplasmic reticulum aminopeptidase 2	DLEITNATLQSEEDSR_4_3_0_1	58.4	0.0	0.0	0.0	84.9	0.0	79.7	0.0	0.0	0.0	19.6	0.0	100.0	0.0
254	Human Plasma exp_4	25603	Endoplasmic reticulum aminopeptidase 2	DLEITNATLQSEEDSR_5_4_0_2	64.5	0.0	5.9	0.0	100.0	0.0	19.1	0.0	3.2	0.0	19.6	0.0	59.6	0.0
255	Human Plasma exp_1	24701	Fetuin-B	VLYLAAYNCTLRPVSK_5_4_0_1	100.0	0.0	0.0	0.0	28.5	0.0	32.1	0.0	0.0	0.0	9.3	0.0	48.8	0.0
256	Human Plasma exp_1	22837	Ficolin-3	VELEDFNGNR_5_4_1_2	46.7	0.0	0.0	0.0	100.0	7.6	9.0	5.7	0.0	0.0	0.0	0.0	0.0	0.0
257	Human Plasma exp_4	23285	Ficolin-3	VELEDFNGNR_5_4_1_2	34.9	0.0	0.0	0.0	100.0	0.0	23.6	26.1	0.0	0.0	0.0	0.0	14.9	0.0
258	Human Plasma exp_2	29232	Haptoglobin	MVSHHNLTTGATLINEQWLLTTAK_5_4_0_2	51.5	0.0	0.0	0.0	100.0	0.0	83.9	0.0	0.0	0.0	17.7	0.0	69.1	0.0
259	Human Plasma exp_1	27749	Haptoglobin	VVLHPNYSQVDIGLIK_4_3_0_1	18.9	0.0	0.0	0.0	28.1	0.0	61.8	0.0	0.0	0.0	14.4	0.0	100.0	0.0
260	Human Plasma exp_2	28444	Haptoglobin	VVLHPNYSQVDIGLIK_4_3_0_1	27.8	0.0	0.0	0.0	39.8	0.0	45.2	0.0	0.0	0.0	12.6	0.0	100.0	0.0

Supplementary Table 3. Continued.

No.	Experiments	CID Scan No.	N-glycoproteins	N-glycopeptides	Relative intensities of B/Y series ions													
					B_2	B_2F	B_3	B_3F	B_2S	B_2SF	Y_1	Y_1F	Y_2	Y_2F	Y_3	Y_3F	Y_4	Y_4F
261	Human Plasma exp_2	26631	Haptoglobin	VVLHPNYSQVDIGLIK_5_4_0_1	100.0	0.0	5.9	0.0	31.8	0.0	36.2	0.0	5.8	0.0	5.8	0.0	49.3	0.0
262	Human Plasma exp_4	26593	Haptoglobin	VVLHPNYSQVDIGLIK_5_4_0_1	100.0	0.0	8.1	0.0	31.9	0.0	39.1	0.0	7.0	0.0	7.4	0.0	61.2	0.0
263	Human Plasma exp_1	27827	Haptoglobin	VVLHPNYSQVDIGLIK_5_4_0_2	100.0	0.0	0.0	0.0	96.8	0.0	40.7	0.0	0.0	0.0	10.2	0.0	66.1	0.0
264	Human Plasma exp_1	27945	Haptoglobin	VVLHPNYSQVDIGLIK_5_4_0_2	66.5	0.0	5.6	0.0	100.0	0.0	0.0	0.0	12.9	0.0	10.0	0.0	67.7	0.0
265	Human Plasma exp_2	28700	Haptoglobin	VVLHPNYSQVDIGLIK_5_4_0_2	59.8	0.0	5.1	0.0	100.0	0.0	0.0	0.0	5.9	0.0	10.8	0.0	67.4	0.0
266	Human Plasma exp_4	28201	Haptoglobin	VVLHPNYSQVDIGLIK_5_4_0_2	100.0	0.0	0.0	0.0	69.9	0.0	18.5	0.0	0.0	0.0	0.0	0.0	44.0	2.2
267	Human Plasma exp_4	28408	Haptoglobin	VVLHPNYSQVDIGLIK_5_4_0_2	67.6	0.0	5.3	0.0	100.0	0.0	0.0	0.0	9.6	0.0	5.9	0.0	57.4	0.0
268	Human Plasma exp_5	27613	Haptoglobin	VVLHPNYSQVDIGLIK_5_4_0_2	64.6	0.0	5.1	0.0	100.0	0.0	0.0	0.0	7.9	0.0	7.3	0.0	65.0	0.0
269	Human Plasma exp_4	26569	Haptoglobin	VVLHPNYSQVDIGLIK_6_4_0_1	100.0	0.0	10.7	0.0	37.3	0.0	39.6	0.0	0.0	0.0	10.7	0.0	38.7	0.0
270	Human Plasma exp_1	27654	Haptoglobin	VVLHPNYSQVDIGLIK_6_5_0_2	79.3	0.0	0.0	0.0	100.0	0.0	46.8	0.0	4.9	0.0	4.8	0.0	0.0	0.0
271	Human Plasma exp_4	30252	Haptoglobin	VVLHPNYSQVDIGLIK_6_5_0_3	32.4	0.0	0.0	0.0	100.0	0.0	43.5	0.0	0.0	0.0	4.1	0.0	43.1	0.0
272	Human Plasma exp_5	29222	Haptoglobin	VVLHPNYSQVDIGLIK_6_5_0_3	34.4	0.0	0.0	0.0	100.0	0.0	61.3	0.0	8.7	0.0	15.5	0.0	34.1	0.0
273	Human Plasma exp_4	30058	Haptoglobin	VVLHPNYSQVDIGLIK_6_5_1_3	11.6	0.0	0.0	0.0	100.0	13.9	75.8	0.0	0.0	0.0	0.0	0.0	16.2	0.0
274	Human Plasma exp_2	30831	Hemopexin	ALPQPQNVTSLLGCTH_4_3_0_1	25.6	0.0	0.0	0.0	57.9	0.0	75.1	0.0	0.0	0.0	19.5	0.0	100.0	0.0
275	Human Plasma exp_3	30305	Hemopexin	ALPQPQNVTSLLGCTH_4_3_0_1	29.9	0.0	0.0	0.0	65.1	0.0	66.4	0.0	0.0	0.0	10.0	0.0	100.0	0.0
276	Human Plasma exp_1	30204	Hemopexin	ALPQPQNVTSLLGCTH_5_4_0_2	36.8	0.0	0.0	0.0	100.0	0.0	49.9	0.0	5.6	0.0	9.4	0.0	66.2	0.0
277	Human Plasma exp_2	30901	Hemopexin	ALPQPQNVTSLLGCTH_5_4_0_2	75.4	0.0	8.6	0.0	68.4	0.0	64.3	0.0	9.7	0.0	22.6	0.0	100.0	0.0
278	Human Plasma exp_3	30491	Hemopexin	ALPQPQNVTSLLGCTH_5_4_0_2	54.8	0.0	4.8	0.0	100.0	0.0	36.7	0.0	3.3	0.0	12.5	0.0	55.7	0.0
279	Human Plasma exp_4	30501	Hemopexin	ALPQPQNVTSLLGCTH_5_4_0_2	55.5	0.0	0.0	0.0	100.0	0.0	54.1	0.0	0.0	0.0	9.8	0.0	65.0	0.0
280	Human Plasma exp_5	29783	Hemopexin	ALPQPQNVTSLLGCTH_5_4_0_2	48.3	0.0	0.0	0.0	100.0	0.0	36.2	0.0	0.0	0.0	11.6	0.0	93.4	0.0
281	Human Plasma exp_1	22530	Hemopexin	SWPAVGNCSSALR_5_4_0_1	100.0	0.0	12.5	0.0	72.7	0.0	17.3	0.0	6.1	0.0	0.0	0.0	5.5	0.0
282	Human Plasma exp_1	25393	Hemopexin	SWPAVGNCSSALR_5_4_0_2	74.6	0.0	9.0	0.0	100.0	0.0	50.5	0.0	0.0	0.0	0.0	0.0	74.6	0.0
283	Human Plasma exp_2	25885	Hemopexin	SWPAVGNCSSALR_5_4_0_2	71.0	0.0	5.5	0.0	100.0	0.0	51.6	0.0	0.0	0.0	14.9	0.0	59.6	0.0
284	Human Plasma exp_3	25563	Hemopexin	SWPAVGNCSSALR_5_4_0_2	77.7	0.0	6.8	0.0	100.0	0.0	52.7	0.0	0.0	0.0	0.0	0.0	72.2	0.0
285	Human Plasma exp_4	25835	Hemopexin	SWPAVGNCSSALR_5_4_0_2	74.9	0.0	5.0	0.0	100.0	0.0	47.5	0.0	0.0	0.0	6.7	0.0	87.9	0.0
286	Human Plasma exp_5	25332	Hemopexin	SWPAVGNCSSALR_5_4_0_2	51.3	0.0	0.0	0.0	100.0	0.0	46.8	0.0	0.0	0.0	4.0	0.0	48.8	0.0

Supplementary Table 3. Continued.

No.	Experiments	CID Scan No.	N-glycoproteins	N-glycopeptides	Relative intensities of B/Y series ions													
					B_2	B_2F	B_3	B_3F	B_2S	B_2SF	Y_1	Y_1F	Y_2	Y_2F	Y_3	Y_3F	Y_4	Y_4F
287	Human Plasma exp_2	25764	Hemopexin	SWPAVGNCSSALR_5_4_1_2	80.3	0.0	10.6	0.0	100.0	0.0	18.6	31.1	0.0	0.0	1.4	11.2	14.2	61.3
288	Human Plasma exp_4	25753	Hemopexin	SWPAVGNCSSALR_5_4_1_2	81.3	0.0	3.8	0.0	100.0	0.0	14.4	27.5	0.0	0.0	0.0	0.0	12.2	56.2
289	Human Plasma exp_5	24981	Hemopexin	SWPAVGNCSSALR_5_4_1_2	66.9	0.0	0.0	0.0	100.0	0.0	9.4	29.2	0.0	0.0	0.0	0.0	15.9	32.7
290	Human Plasma exp_3	25158	Hemopexin	SWPAVGNCSSALR_6_5_0_2	81.0	0.0	0.0	0.0	100.0	0.0	36.5	0.0	5.1	0.0	0.0	0.0	24.0	0.0
291	Human Plasma exp_4	28467	Hemopexin	SWPAVGNCSSALR_6_5_1_3	35.9	4.2	0.0	0.0	100.0	8.8	22.4	0.0	0.0	0.0	0.0	0.0	0.0	0.0
292	Human Plasma exp_1	15042	Heparin cofactor 2	DFVNASSK_5_4_0_2	52.7	0.0	5.0	0.0	100.0	0.0	53.4	0.0	5.3	0.0	7.6	0.0	44.6	0.0
293	Human Plasma exp_1	15007	Heparin cofactor 2	DFVNASSK_5_4_0_2	0.0	0.0	0.0	0.0	77.4	0.0	100.0	0.0	7.2	0.0	3.9	0.0	20.8	0.0
294	Human Plasma exp_2	15333	Heparin cofactor 2	DFVNASSK_5_4_0_2	52.1	0.0	5.8	0.0	100.0	0.0	54.7	0.0	5.4	0.0	8.2	0.0	44.8	0.0
295	Human Plasma exp_3	15064	Heparin cofactor 2	DFVNASSK_5_4_0_2	43.1	0.0	5.4	0.0	100.0	0.0	45.9	0.0	3.0	0.0	3.9	0.0	42.0	0.0
296	Human Plasma exp_4	15380	Heparin cofactor 2	DFVNASSK_5_4_0_2	50.7	0.0	5.0	0.0	100.0	0.0	50.0	0.0	5.2	0.0	8.2	0.0	43.5	0.0
297	Human Plasma exp_4	15384	Heparin cofactor 2	DFVNASSK_5_4_0_2	0.0	0.0	0.0	0.0	85.0	0.0	100.0	0.0	13.5	0.0	4.8	0.0	30.9	0.0
298	Human Plasma exp_5	15145	Heparin cofactor 2	DFVNASSK_5_4_0_2	0.0	0.0	7.3	0.0	98.1	0.0	100.0	0.0	16.8	0.0	6.3	0.0	33.1	0.0
299	Human Plasma exp_1	20220	Heparin cofactor 2	DFVNASSK_6_5_0_3	24.1	0.0	0.0	0.0	100.0	0.0	0.0	0.0	2.8	0.0	2.1	0.0	48.2	0.0
300	Human Plasma exp_2	20659	Heparin cofactor 2	DFVNASSK_6_5_0_3	21.7	0.0	0.0	0.0	100.0	0.0	52.2	0.0	0.0	0.0	8.5	0.0	41.4	0.0
301	Human Plasma exp_3	20040	Heparin cofactor 2	DFVNASSK_6_5_0_3	21.2	0.0	0.0	0.0	100.0	0.0	40.5	0.0	0.0	0.0	3.1	0.0	37.4	0.0
302	Human Plasma exp_4	20540	Heparin cofactor 2	DFVNASSK_6_5_0_3	17.7	0.0	0.0	0.0	100.0	0.0	48.7	0.0	2.2	0.0	2.4	0.0	48.2	0.0
303	Human Plasma exp_5	20269	Heparin cofactor 2	DFVNASSK_6_5_0_3	17.1	0.0	0.0	0.0	100.0	0.0	48.6	0.0	0.0	0.0	3.2	0.0	42.0	0.0
304	Human Plasma exp_1	29227	Heparin cofactor 2	NLSMPLLPADFHK_5_4_0_2	72.6	0.0	0.0	0.0	100.0	0.0	30.1	0.0	0.0	0.0	8.3	0.0	72.9	0.0
305	Human Plasma exp_2	29952	Heparin cofactor 2	NLSMPLLPADFHK_5_4_0_2	80.3	0.0	4.1	0.0	100.0	0.0	35.6	0.0	0.0	0.0	20.6	0.0	69.2	0.0
306	Human Plasma exp_4	29698	Heparin cofactor 2	NLSMPLLPADFHK_5_4_0_2	67.7	0.0	3.9	0.0	100.0	0.0	42.4	0.0	2.9	0.0	8.9	0.0	72.4	0.0
307	Human Plasma exp_5	28803	Heparin cofactor 2	NLSMPLLPADFHK_5_4_0_2	63.1	0.0	4.1	0.0	100.0	0.0	36.4	0.0	0.0	0.0	4.6	0.0	73.3	0.0
308	Human Plasma exp_3	2935	Heparin cofactor 2	SMTNR_4_3_0_1	68.2	0.0	0.0	0.0	88.3	0.0	100.0	0.0	0.0	0.0	5.9	0.0	75.5	0.0
309	Human Plasma exp_2	3065	Heparin cofactor 2	SMTNR_5_4_0_2	69.0	0.0	5.4	0.0	100.0	0.0	50.4	0.0	4.1	0.0	4.0	0.0	36.9	0.0
310	Human Plasma exp_3	2996	Heparin cofactor 2	SMTNR_5_4_0_2	71.5	0.0	6.5	0.0	100.0	0.0	40.5	0.0	0.0	0.0	0.0	0.0	29.3	0.0
311	Human Plasma exp_4	3050	Heparin cofactor 2	SMTNR_5_4_0_2	75.9	0.0	4.8	0.0	100.0	0.0	47.3	0.0	2.5	0.0	3.8	0.0	39.4	0.0
312	Human Plasma exp_5	3015	Heparin cofactor 2	SMTNR_5_4_0_2	80.6	0.0	5.7	0.0	100.0	0.0	35.6	0.0	0.0	0.0	0.0	0.0	15.8	0.0

Supplementary Table 3. Continued.

No.	Experiments	CID Scan No.	N-glycoproteins	N-glycopeptides	Relative intensities of B/Y series ions													
					B_2	B_2F	B_3	B_3F	B_2S	B_2SF	Y_1	Y_1F	Y_2	Y_2F	Y_3	Y_3F	Y_4	Y_4F
313	Human Plasma exp_4	24610	Hepatocyte growth factor activator	DSVSVVLGQHFFNR_5_4_0_2	68.1	0.0	0.0	0.0	98.3	0.0	100.0	0.0	0.0	0.0	11.3	0.0	57.6	0.0
314	Human Plasma exp_2	12959	Hepatocyte growth factor-like protein	GTANTTTAGVPCQR_5_4_0_2	57.3	0.0	5.6	0.0	100.0	0.0	58.1	0.0	0.0	0.0	4.3	0.0	51.9	0.0
315	Human Plasma exp_5	12805	Hepatocyte growth factor-like protein	GTANTTTAGVPCQR_5_4_0_2	68.5	0.0	0.0	0.0	100.0	0.0	28.5	0.0	0.0	0.0	5.4	0.0	53.7	0.0
316	Human Plasma exp_1	26936	Histidine-rich glycoprotein	VIDFNCTTSSVSSALANTK_5_4_0_1	100.0	0.0	5.6	0.0	46.7	0.0	36.7	0.0	4.6	2.1	19.3	0.0	78.4	0.0
317	Human Plasma exp_2	27613	Histidine-rich glycoprotein	VIDFNCTTSSVSSALANTK_5_4_0_1	100.0	0.0	0.0	0.0	51.2	0.0	49.9	0.0	0.0	0.0	12.2	0.0	85.0	0.0
318	Human Plasma exp_3	27037	Histidine-rich glycoprotein	VIDFNCTTSSVSSALANTK_5_4_0_1	100.0	0.0	9.7	0.0	43.9	0.0	39.7	0.0	4.9	0.0	0.0	0.0	67.1	0.0
319	Human Plasma exp_4	27387	Histidine-rich glycoprotein	VIDFNCTTSSVSSALANTK_5_4_0_1	51.0	0.0	12.7	0.0	52.8	0.0	37.6	0.0	0.0	0.0	13.7	0.0	100.0	0.0
320	Human Plasma exp_5	26632	Histidine-rich glycoprotein	VIDFNCTTSSVSSALANTK_5_4_0_1	89.1	0.0	0.0	0.0	68.3	0.0	38.7	0.0	0.0	0.0	18.3	0.0	100.0	0.0
321	Human Plasma exp_5	26637	Histidine-rich glycoprotein	VIDFNCTTSSVSSALANTK_5_4_0_1	17.3	0.0	0.0	0.0	100.0	0.0	78.3	0.0	10.4	0.0	0.0	0.0	54.2	0.0
322	Human Plasma exp_1	28732	Histidine-rich glycoprotein	VIDFNCTTSSVSSALANTK_5_4_0_2	57.2	0.0	0.0	0.0	100.0	0.0	67.5	0.0	8.9	0.0	17.0	0.0	77.1	0.0
323	Human Plasma exp_2	29468	Histidine-rich glycoprotein	VIDFNCTTSSVSSALANTK_5_4_0_2	69.3	0.0	0.0	0.0	100.0	0.0	86.0	0.0	0.0	0.0	12.9	0.0	54.1	0.0
324	Human Plasma exp_3	28937	Histidine-rich glycoprotein	VIDFNCTTSSVSSALANTK_5_4_0_2	49.7	0.0	0.0	0.0	100.0	0.0	69.3	0.0	3.5	0.0	0.0	0.0	67.1	0.0
325	Human Plasma exp_4	29242	Histidine-rich glycoprotein	VIDFNCTTSSVSSALANTK_5_4_0_2	59.4	0.0	4.9	0.0	100.0	0.0	62.4	0.0	7.8	0.0	23.8	0.0	82.9	0.0
326	Human Plasma exp_5	28374	Histidine-rich glycoprotein	VIDFNCTTSSVSSALANTK_5_4_0_2	55.3	0.0	0.0	0.0	100.0	0.0	74.1	0.0	6.2	0.0	8.9	0.0	45.1	0.0
327	Human Plasma exp_5	17358	ICOS ligand	LFNVTPQDEQK_5_5_1_1	100.0	0.0	12.5	0.0	60.2	0.0	0.0	0.0	25.8	25.6	0.0	0.0	0.0	0.0
328	Human Plasma exp_1	20768	ICOS ligand	LFNVTPQDEQK_5_5_1_2	14.7	0.0	0.0	0.0	100.0	0.0	8.4	0.0	54.3	30.8	0.0	0.0	0.0	0.0
329	Human Plasma exp_4	21096	ICOS ligand	LFNVTPQDEQK_5_5_1_2	59.8	0.0	3.2	0.0	100.0	2.8	0.0	0.0	37.1	14.2	0.0	0.0	0.0	0.0
330	Human Plasma exp_4	21041	ICOS ligand	LFNVTPQDEQK_5_5_1_2	9.8	0.0	8.2	0.0	100.0	0.0	0.0	0.0	46.3	9.1	0.0	0.0	0.0	0.0
331	Human Plasma exp_1	15025	Ig alpha-2 chain C region	TPLTANITK_5_4_1_1	100.0	0.0	10.1	0.0	81.5	0.0	11.3	32.1	0.0	0.0	0.0	0.0	0.0	0.0
332	Human Plasma exp_2	15335	Ig alpha-2 chain C region	TPLTANITK_5_4_1_1	100.0	0.0	5.0	0.0	87.5	0.0	8.1	29.1	0.0	0.0	0.0	0.0	0.0	0.0
333	Human Plasma exp_3	14878	Ig alpha-2 chain C region	TPLTANITK_5_4_1_1	0.0	0.0	4.6	0.0	22.3	0.0	54.2	100.0	0.0	0.0	3.7	15.7	18.3	58.0
334	Human Plasma exp_4	15175	Ig alpha-2 chain C region	TPLTANITK_5_4_1_1	0.0	0.0	0.0	0.0	29.8	0.0	40.5	100.0	0.0	0.0	0.0	0.0	17.3	77.5
335	Human Plasma exp_3	18898	Ig alpha-2 chain C region	TPLTANITK_5_4_1_2	0.0	0.0	0.0	0.0	83.2	0.0	71.4	100.0	0.0	0.0	0.0	0.0	7.7	68.4
336	Human Plasma exp_3	15007	Ig alpha-2 chain C region	TPLTANITK_5_5_1_1	100.0	0.0	5.5	0.0	55.0	0.0	3.7	5.1	0.0	0.0	0.0	0.0	0.0	0.0
337	Human Plasma exp_3	14995	Ig alpha-2 chain C region	TPLTANITK_5_5_1_1	0.0	0.0	0.0	0.0	48.2	0.0	56.3	100.0	26.2	53.7	0.0	0.0	0.0	0.0
338	Human Plasma exp_4	15325	Ig alpha-2 chain C region	TPLTANITK_5_5_1_1	100.0	0.0	5.9	0.0	47.3	0.0	2.8	5.3	0.0	0.0	0.0	0.0	0.0	0.0

Supplementary Table 3. Continued.

No.	Experiments	CID Scan No.	N-glycoproteins	N-glycopeptides	Relative intensities of B/Y series ions													
					B_2	B_2F	B_3	B_3F	B_2S	B_2SF	Y_1	Y_1F	Y_2	Y_2F	Y_3	Y_3F	Y_4	Y_4F
339	Human Plasma exp_4	15315	Ig alpha-2 chain C region	TPLTANITK_5_5_1_1	0.0	0.0	0.0	0.0	34.3	0.0	45.4	100.0	11.2	55.3	0.0	0.0	0.0	0.0
340	Human Plasma exp_3	18979	Ig alpha-2 chain C region	TPLTANITK_5_5_1_2	72.2	0.0	3.9	0.0	100.0	0.0	3.7	2.4	0.0	0.0	0.0	0.0	0.0	0.0
341	Human Plasma exp_1	5651	Ig gamma-1 chain C region	EEQYNSTYR_3_3_0_0	9.4	0.0	7.0	0.0	0.0	0.0	100.0	0.0	14.0	0.0	13.6	0.0	45.5	0.0
342	Human Plasma exp_2	5984	Ig gamma-1 chain C region	EEQYNSTYR_3_3_0_0	6.9	0.0	8.8	0.0	0.0	0.0	100.0	0.0	6.9	0.0	16.1	0.0	48.6	0.0
343	Human Plasma exp_3	5945	Ig gamma-1 chain C region	EEQYNSTYR_3_3_0_0	10.9	0.0	10.1	0.0	0.0	0.0	100.0	0.0	11.4	0.0	8.4	0.0	41.1	0.0
344	Human Plasma exp_4	5801	Ig gamma-1 chain C region	EEQYNSTYR_3_3_0_0	9.8	0.0	8.5	0.0	0.0	0.0	100.0	0.0	13.8	0.0	18.6	0.0	43.3	0.0
345	Human Plasma exp_5	5913	Ig gamma-1 chain C region	EEQYNSTYR_3_3_0_0	6.0	0.0	8.6	0.0	0.0	0.0	100.0	0.0	6.3	0.0	16.6	0.0	42.6	0.0
346	Human Plasma exp_1	4903	Ig gamma-1 chain C region	EEQYNSTYR_3_3_1_0	7.4	0.0	10.1	0.0	0.0	0.0	100.0	84.8	7.9	0.0	18.9	3.2	64.2	13.4
347	Human Plasma exp_2	5416	Ig gamma-1 chain C region	EEQYNSTYR_3_3_1_0	28.9	0.0	9.3	0.0	0.0	0.0	100.0	82.0	4.4	0.0	30.4	20.5	76.7	20.1
348	Human Plasma exp_3	5245	Ig gamma-1 chain C region	EEQYNSTYR_3_3_1_0	19.5	0.0	0.0	0.0	0.0	0.0	67.8	100.0	0.0	0.0	12.6	7.9	44.2	23.8
349	Human Plasma exp_4	5932	Ig gamma-1 chain C region	EEQYNSTYR_3_3_1_0	8.3	0.0	16.3	0.0	0.0	0.0	100.0	70.6	18.4	0.0	21.6	7.7	40.2	20.8
350	Human Plasma exp_1	5791	Ig gamma-1 chain C region	EEQYNSTYR_3_4_0_0	3.1	0.0	8.9	0.0	0.0	0.0	100.0	0.0	17.0	0.0	15.8	0.0	37.9	0.0
351	Human Plasma exp_2	5966	Ig gamma-1 chain C region	EEQYNSTYR_3_4_0_0	2.8	0.0	8.7	0.0	0.0	0.0	100.0	0.0	18.6	0.0	17.2	0.0	41.4	0.0
352	Human Plasma exp_4	5765	Ig gamma-1 chain C region	EEQYNSTYR_3_4_0_0	3.3	0.0	7.7	0.0	0.0	0.0	100.0	0.0	17.7	0.0	18.3	0.0	34.9	0.0
353	Human Plasma exp_1	5706	Ig gamma-1 chain C region	EEQYNSTYR_3_4_1_0	0.0	0.0	7.1	0.0	0.0	0.0	100.0	53.6	17.9	5.3	15.7	0.0	23.7	6.4
354	Human Plasma exp_2	5595	Ig gamma-1 chain C region	EEQYNSTYR_3_4_1_0	0.0	0.0	13.6	0.0	0.0	0.0	100.0	57.0	17.4	6.2	13.1	0.0	30.3	19.4
355	Human Plasma exp_3	5880	Ig gamma-1 chain C region	EEQYNSTYR_3_4_1_0	0.0	0.0	7.9	0.0	0.0	0.0	100.0	52.8	17.1	5.2	15.1	0.0	23.4	6.5
356	Human Plasma exp_4	6195	Ig gamma-1 chain C region	EEQYNSTYR_3_4_1_0	0.0	0.0	0.0	0.0	0.0	0.0	100.0	66.9	3.6	0.0	0.0	0.0	18.7	7.3
357	Human Plasma exp_5	5784	Ig gamma-1 chain C region	EEQYNSTYR_3_4_1_0	0.0	0.0	7.5	0.0	0.0	0.0	100.0	53.4	18.1	5.4	14.9	0.0	24.8	6.9
358	Human Plasma exp_1	5580	Ig gamma-1 chain C region	EEQYNSTYR_4_3_0_0	17.3	0.0	7.0	0.0	0.0	0.0	100.0	0.0	10.8	0.0	9.0	0.0	39.6	0.0
359	Human Plasma exp_2	5693	Ig gamma-1 chain C region	EEQYNSTYR_4_3_0_0	14.7	0.0	7.3	0.0	0.0	0.0	100.0	0.0	4.5	0.0	5.4	0.0	37.4	0.0
360	Human Plasma exp_3	5646	Ig gamma-1 chain C region	EEQYNSTYR_4_3_0_0	15.4	0.0	5.6	0.0	0.0	0.0	100.0	0.0	6.3	0.0	6.5	0.0	39.5	0.0
361	Human Plasma exp_4	5772	Ig gamma-1 chain C region	EEQYNSTYR_4_3_0_0	15.1	0.0	5.8	0.0	0.0	0.0	100.0	0.0	6.9	0.0	7.3	0.0	35.2	0.0
362	Human Plasma exp_5	5618	Ig gamma-1 chain C region	EEQYNSTYR_4_3_0_0	14.6	0.0	5.4	0.0	0.0	0.0	100.0	0.0	8.6	0.0	7.8	0.0	23.0	0.0
363	Human Plasma exp_1	5687	Ig gamma-1 chain C region	EEQYNSTYR_4_3_1_0	11.2	4.1	11.5	0.0	0.0	0.0	100.0	90.2	11.6	3.8	8.4	0.0	42.0	8.0
364	Human Plasma exp_2	5992	Ig gamma-1 chain C region	EEQYNSTYR_4_3_1_0	12.1	4.1	7.1	0.0	0.0	0.0	100.0	83.5	3.3	0.0	4.7	0.0	31.6	3.5

Supplementary Table 3. Continued.

No.	Experiments	CID Scan No.	N-glycoproteins	N-glycopeptides	Relative intensities of B/Y series ions													
					B_2	B_2F	B_3	B_3F	B_2S	B_2SF	Y_1	Y_1F	Y_2	Y_2F	Y_3	Y_3F	Y_4	Y_4F
365	Human Plasma exp_3	5972	Ig gamma-1 chain C region	EEQYNSTYR_4_3_1_0	6.9	0.0	7.2	0.0	0.0	0.0	100.0	96.2	1.8	0.0	2.3	0.0	32.3	5.0
366	Human Plasma exp_5	5753	Ig gamma-1 chain C region	EEQYNSTYR_4_3_1_0	8.7	4.1	11.1	0.0	0.0	0.0	100.0	88.4	11.4	6.2	11.6	5.3	38.2	7.9
367	Human Plasma exp_1	9068	Ig gamma-1 chain C region	EEQYNSTYR_4_3_1_1	0.0	0.0	9.7	0.0	33.9	0.0	100.0	61.2	0.0	0.0	5.5	0.0	39.5	0.0
368	Human Plasma exp_2	9215	Ig gamma-1 chain C region	EEQYNSTYR_4_3_1_1	0.0	0.0	0.0	0.0	28.4	0.0	100.0	99.4	7.9	7.0	0.0	0.0	28.7	0.0
369	Human Plasma exp_3	9230	Ig gamma-1 chain C region	EEQYNSTYR_4_3_1_1	83.0	0.0	14.7	0.0	100.0	0.0	30.8	21.1	0.0	0.0	0.0	0.0	0.0	0.0
370	Human Plasma exp_3	8948	Ig gamma-1 chain C region	EEQYNSTYR_4_3_1_1	0.0	0.0	0.0	0.0	21.0	0.0	100.0	96.9	0.0	0.0	12.5	0.0	54.9	0.0
371	Human Plasma exp_4	9278	Ig gamma-1 chain C region	EEQYNSTYR_4_3_1_1	0.0	0.0	4.5	0.0	34.5	0.0	69.5	100.0	0.0	0.0	0.0	0.0	48.2	0.0
372	Human Plasma exp_5	9233	Ig gamma-1 chain C region	EEQYNSTYR_4_3_1_1	83.5	0.0	0.0	0.0	100.0	0.0	26.9	7.5	0.0	0.0	9.5	0.0	0.0	0.0
373	Human Plasma exp_5	9174	Ig gamma-1 chain C region	EEQYNSTYR_4_3_1_1	0.0	0.0	8.2	0.0	28.1	0.0	100.0	91.6	5.8	4.1	0.0	0.0	62.1	0.0
374	Human Plasma exp_3	5448	Ig gamma-1 chain C region	EEQYNSTYR_4_4_0_0	0.0	0.0	7.4	0.0	0.0	0.0	100.0	0.0	10.4	0.0	5.1	0.0	21.2	0.0
375	Human Plasma exp_5	5420	Ig gamma-1 chain C region	EEQYNSTYR_4_4_0_0	0.0	0.0	4.1	0.0	0.0	0.0	100.0	0.0	8.5	0.0	4.3	0.0	15.5	0.0
376	Human Plasma exp_1	5611	Ig gamma-1 chain C region	EEQYNSTYR_4_4_1_0	100.0	0.0	0.0	0.0	0.0	0.0	32.8	41.5	0.0	0.0	0.0	0.0	19.6	40.1
377	Human Plasma exp_1	4872	Ig gamma-1 chain C region	EEQYNSTYR_4_4_1_0	0.0	0.0	5.9	0.0	0.0	0.0	1.6	100.0	11.8	8.1	12.2	0.0	27.4	5.7
378	Human Plasma exp_2	6129	Ig gamma-1 chain C region	EEQYNSTYR_4_4_1_0	0.0	0.0	4.5	0.0	0.0	0.0	6.9	100.0	13.4	11.6	4.7	0.0	29.3	10.0
379	Human Plasma exp_3	5899	Ig gamma-1 chain C region	EEQYNSTYR_4_4_1_0	100.0	0.0	0.0	0.0	0.0	0.0	30.1	36.9	0.0	0.0	0.0	0.0	19.4	40.9
380	Human Plasma exp_3	4946	Ig gamma-1 chain C region	EEQYNSTYR_4_4_1_0	0.0	0.0	15.0	0.0	0.0	0.0	8.5	100.0	10.5	5.4	13.5	3.2	29.8	3.5
381	Human Plasma exp_4	6207	Ig gamma-1 chain C region	EEQYNSTYR_4_4_1_0	0.0	0.0	6.3	0.0	7.4	0.0	4.7	100.0	5.6	0.0	3.4	0.0	11.4	0.0
382	Human Plasma exp_5	5715	Ig gamma-1 chain C region	EEQYNSTYR_4_4_1_0	100.0	0.0	0.0	0.0	0.0	0.0	31.5	38.9	0.0	0.0	0.0	0.0	18.7	38.2
383	Human Plasma exp_1	9178	Ig gamma-1 chain C region	EEQYNSTYR_4_4_1_1	55.0	0.0	10.4	0.0	100.0	0.0	51.8	43.5	0.0	0.0	0.0	0.0	23.0	21.8
384	Human Plasma exp_1	9252	Ig gamma-1 chain C region	EEQYNSTYR_4_4_1_1	0.0	0.0	6.7	0.0	15.5	0.0	100.0	0.0	14.0	12.6	10.6	8.1	37.0	3.2
385	Human Plasma exp_2	9369	Ig gamma-1 chain C region	EEQYNSTYR_4_4_1_1	58.5	0.0	13.1	0.0	100.0	0.0	55.0	43.8	0.0	0.0	0.0	0.0	26.2	26.4
386	Human Plasma exp_3	9342	Ig gamma-1 chain C region	EEQYNSTYR_4_4_1_1	63.4	0.0	14.0	0.0	100.0	0.0	61.2	47.5	0.0	0.0	0.0	0.0	29.5	30.4
387	Human Plasma exp_3	9412	Ig gamma-1 chain C region	EEQYNSTYR_4_4_1_1	0.0	0.0	0.0	0.0	21.6	0.0	100.0	0.0	9.0	5.0	0.0	0.0	30.2	0.0
388	Human Plasma exp_4	9262	Ig gamma-1 chain C region	EEQYNSTYR_4_4_1_1	0.0	0.0	0.0	0.0	25.1	0.0	100.0	0.0	3.2	0.0	9.4	0.0	22.4	0.0
389	Human Plasma exp_5	9379	Ig gamma-1 chain C region	EEQYNSTYR_4_4_1_1	0.0	0.0	6.2	0.0	18.2	0.0	100.0	0.0	7.6	13.3	14.5	0.0	33.1	4.7
390	Human Plasma exp_2	6300	Ig gamma-1 chain C region	EEQYNSTYR_4_5_1_0	0.0	0.0	0.0	0.0	0.0	0.0	100.0	99.7	18.8	12.4	3.6	0.0	0.0	0.0

Supplementary Table 3. Continued.

No.	Experiments	CID Scan No.	N-glycoproteins	N-glycopeptides	Relative intensities of B/Y series ions													
					B_2	B_2F	B_3	B_3F	B_2S	B_2SF	Y_1	Y_1F	Y_2	Y_2F	Y_3	Y_3F	Y_4	Y_4F
391	Human Plasma exp_4	6672	Ig gamma-1 chain C region	EEQYNSTYR_4_5_1_0	0.0	0.0	4.1	0.0	0.0	0.0	100.0	87.2	31.8	20.8	9.4	3.0	7.0	0.0
392	Human Plasma exp_5	6286	Ig gamma-1 chain C region	EEQYNSTYR_4_5_1_0	0.0	0.0	0.0	0.0	0.0	0.0	100.0	80.7	21.0	20.9	0.0	0.0	3.3	0.0
393	Human Plasma exp_1	8778	Ig gamma-1 chain C region	EEQYNSTYR_5_3_1_1	67.4	0.0	0.0	0.0	100.0	0.0	6.3	0.0	0.0	0.0	0.0	0.0	8.1	27.7
394	Human Plasma exp_2	8934	Ig gamma-1 chain C region	EEQYNSTYR_5_3_1_1	93.2	0.0	0.0	0.0	100.0	0.0	8.1	10.9	0.0	0.0	0.0	0.0	8.5	10.4
395	Human Plasma exp_3	8958	Ig gamma-1 chain C region	EEQYNSTYR_5_3_1_1	100.0	0.0	0.0	0.0	95.3	0.0	7.2	24.1	5.4	0.0	0.0	0.0	0.0	0.0
396	Human Plasma exp_2	5454	Ig gamma-1 chain C region	EEQYNSTYR_5_4_0_0	0.0	0.0	4.6	0.0	0.0	0.0	100.0	0.0	9.5	0.0	6.4	0.0	17.1	0.0
397	Human Plasma exp_1	9244	Ig gamma-1 chain C region	EEQYNSTYR_5_4_0_1	0.0	0.0	0.0	0.0	20.6	0.0	100.0	0.0	7.2	0.0	4.9	0.0	20.3	0.0
398	Human Plasma exp_3	9198	Ig gamma-1 chain C region	EEQYNSTYR_5_4_0_1	0.0	0.0	3.3	0.0	14.2	0.0	100.0	0.0	8.5	0.0	3.3	0.0	25.7	0.0
399	Human Plasma exp_1	5809	Ig gamma-1 chain C region	EEQYNSTYR_5_4_1_0	0.0	0.0	0.0	0.0	0.0	0.0	100.0	94.4	11.5	5.4	4.1	0.0	12.5	0.0
400	Human Plasma exp_2	5741	Ig gamma-1 chain C region	EEQYNSTYR_5_4_1_0	0.0	2.9	7.1	0.0	0.0	0.0	99.7	100.0	18.4	16.8	18.6	10.4	35.1	3.7
401	Human Plasma exp_4	5782	Ig gamma-1 chain C region	EEQYNSTYR_5_4_1_0	0.0	2.9	6.8	0.0	0.0	0.0	100.0	96.7	17.8	15.3	15.6	8.9	32.4	3.0
402	Human Plasma exp_1	8799	Ig gamma-1 chain C region	EEQYNSTYR_5_4_1_1	0.0	0.0	12.3	0.0	32.5	0.0	91.0	100.0	9.3	13.2	9.0	9.7	13.0	0.0
403	Human Plasma exp_2	9326	Ig gamma-1 chain C region	EEQYNSTYR_5_4_1_1	0.0	2.9	3.8	0.0	19.1	0.0	93.3	100.0	7.3	12.4	9.4	6.4	26.5	0.0
404	Human Plasma exp_3	9160	Ig gamma-1 chain C region	EEQYNSTYR_5_4_1_1	100.0	0.0	8.4	0.0	43.6	0.0	10.4	11.3	0.0	0.0	0.0	0.0	0.0	0.0
405	Human Plasma exp_3	9256	Ig gamma-1 chain C region	EEQYNSTYR_5_4_1_1	0.0	0.0	11.9	0.0	34.9	0.0	100.0	92.1	15.6	12.2	0.0	0.0	15.0	0.0
406	Human Plasma exp_4	9273	Ig gamma-1 chain C region	EEQYNSTYR_5_4_1_1	100.0	0.0	3.4	0.0	39.9	0.0	4.8	10.2	0.0	0.0	0.0	0.0	12.2	35.9
407	Human Plasma exp_4	8887	Ig gamma-1 chain C region	EEQYNSTYR_5_4_1_1	100.0	0.0	0.0	0.0	88.1	0.0	15.1	8.7	0.0	0.0	0.0	0.0	5.3	0.0
408	Human Plasma exp_4	9393	Ig gamma-1 chain C region	EEQYNSTYR_5_4_1_1	0.0	0.0	5.4	0.0	13.6	0.0	98.4	100.0	10.4	5.4	8.4	6.1	14.9	0.0
409	Human Plasma exp_5	9296	Ig gamma-1 chain C region	EEQYNSTYR_5_4_1_1	0.0	0.0	9.3	0.0	16.1	0.0	90.4	100.0	10.6	14.6	0.0	0.0	29.5	0.0
410	Human Plasma exp_1	12580	Ig gamma-1 chain C region	EEQYNSTYR_5_4_1_2	26.1	0.0	0.0	0.0	100.0	0.0	9.2	12.0	0.0	0.0	0.0	0.0	1.7	2.3
411	Human Plasma exp_2	13332	Ig gamma-1 chain C region	EEQYNSTYR_5_4_1_2	39.8	0.0	0.0	0.0	100.0	0.0	10.7	21.2	0.0	0.0	0.0	0.0	3.1	0.0
412	Human Plasma exp_1	5106	Ig gamma-1 chain C region	EEQYNSTYR_5_4_2_0	100.0	38.1	12.6	0.0	14.0	0.0	10.0	17.0	0.0	0.0	0.0	0.0	0.0	0.0
413	Human Plasma exp_5	5153	Ig gamma-1 chain C region	EEQYNSTYR_5_4_2_0	100.0	40.2	0.0	0.0	14.6	0.0	5.2	12.7	0.0	0.0	0.0	0.0	0.0	0.0
414	Human Plasma exp_2	8733	Ig gamma-1 chain C region	EEQYNSTYR_5_4_2_1	74.5	31.9	0.0	0.0	100.0	0.0	20.6	31.2	0.0	0.0	0.0	0.0	0.0	0.0
415	Human Plasma exp_3	8719	Ig gamma-1 chain C region	EEQYNSTYR_5_4_2_1	86.1	44.6	0.0	0.0	100.0	11.8	9.1	51.5	0.0	0.0	0.0	0.0	0.0	0.0
416	Human Plasma exp_5	8692	Ig gamma-1 chain C region	EEQYNSTYR_5_4_2_1	83.8	30.1	0.0	0.0	100.0	0.0	13.3	20.4	0.0	0.0	0.0	0.0	0.0	0.0

Supplementary Table 3. Continued.

No.	Experiments	CID Scan No.	N-glycoproteins	N-glycopeptides	Relative intensities of B/Y series ions													
					B_2	B_2F	B_3	B_3F	B_2S	B_2SF	Y_1	Y_1F	Y_2	Y_2F	Y_3	Y_3F	Y_4	Y_4F
417	Human Plasma exp_4	6478	Ig gamma-1 chain C region	EEQYNSTYR_5_5_1_0	0.0	0.0	4.8	0.0	0.0	0.0	100.0	90.9	21.5	19.0	0.0	0.0	2.4	0.0
418	Human Plasma exp_4	13165	Ig gamma-1 chain C region	EEQYNSTYR_5_5_1_2	24.4	0.0	0.0	0.0	100.0	5.1	12.6	9.1	0.0	0.0	0.0	0.0	0.0	0.0
419	Human Plasma exp_1	13538	Ig gamma-2 chain C region	EEQFNSTFR_3_3_0_0	8.9	0.0	10.5	0.0	0.0	0.0	100.0	0.0	18.9	0.0	12.0	0.0	27.5	0.0
420	Human Plasma exp_2	13787	Ig gamma-2 chain C region	EEQFNSTFR_3_3_0_0	9.1	0.0	8.6	0.0	0.0	0.0	100.0	0.0	16.5	0.0	9.3	0.0	36.3	0.0
421	Human Plasma exp_5	13409	Ig gamma-2 chain C region	EEQFNSTFR_3_3_0_0	14.8	0.0	4.6	0.0	0.0	0.0	100.0	0.0	0.0	0.0	21.4	0.0	63.4	0.0
422	Human Plasma exp_1	13210	Ig gamma-2 chain C region	EEQFNSTFR_3_3_1_0	32.2	4.5	11.4	0.0	3.1	0.0	100.0	78.9	10.8	4.9	36.2	23.9	75.5	13.0
423	Human Plasma exp_2	13462	Ig gamma-2 chain C region	EEQFNSTFR_3_3_1_0	31.9	5.3	12.5	0.0	0.0	0.0	100.0	85.7	15.7	6.4	33.0	27.3	78.1	15.8
424	Human Plasma exp_3	13359	Ig gamma-2 chain C region	EEQFNSTFR_3_3_1_0	17.4	3.0	3.1	0.0	1.8	0.0	100.0	58.5	4.6	2.2	20.9	17.4	51.6	7.2
425	Human Plasma exp_4	13471	Ig gamma-2 chain C region	EEQFNSTFR_3_3_1_0	23.5	3.9	9.9	0.0	0.0	0.0	100.0	72.3	6.9	4.3	25.5	25.1	72.0	10.4
426	Human Plasma exp_5	13258	Ig gamma-2 chain C region	EEQFNSTFR_3_3_1_0	34.0	4.6	13.0	0.0	2.5	0.0	100.0	72.7	7.0	0.0	35.7	23.4	65.8	13.8
427	Human Plasma exp_1	13593	Ig gamma-2 chain C region	EEQFNSTFR_3_4_0_0	5.2	0.0	9.1	0.0	0.0	0.0	100.0	0.0	19.1	0.0	16.6	0.0	34.0	0.0
428	Human Plasma exp_4	13856	Ig gamma-2 chain C region	EEQFNSTFR_3_4_0_0	4.8	0.0	9.5	0.0	0.0	0.0	100.0	0.0	18.6	0.0	16.8	0.0	33.6	0.0
429	Human Plasma exp_1	13683	Ig gamma-2 chain C region	EEQFNSTFR_3_4_1_0	0.0	0.0	7.4	0.0	0.0	0.0	100.0	53.7	19.3	5.7	13.5	0.0	23.4	6.6
430	Human Plasma exp_2	13906	Ig gamma-2 chain C region	EEQFNSTFR_3_4_1_0	0.0	0.0	8.0	0.0	0.0	0.0	100.0	53.8	17.5	6.0	15.3	0.0	24.8	6.7
431	Human Plasma exp_3	13833	Ig gamma-2 chain C region	EEQFNSTFR_3_4_1_0	0.0	0.0	8.1	0.0	0.0	0.0	100.0	53.2	19.0	6.1	15.6	0.0	23.8	6.1
432	Human Plasma exp_4	13958	Ig gamma-2 chain C region	EEQFNSTFR_3_4_1_0	0.0	0.0	7.6	0.0	0.0	0.0	100.0	55.3	20.4	6.3	15.4	3.1	22.9	6.0
433	Human Plasma exp_5	13749	Ig gamma-2 chain C region	EEQFNSTFR_3_4_1_0	0.0	0.0	8.1	0.0	0.0	0.0	100.0	54.8	18.4	5.7	14.7	2.7	25.0	7.0
434	Human Plasma exp_1	13385	Ig gamma-2 chain C region	EEQFNSTFR_4_3_1_0	13.6	3.4	10.4	0.0	0.0	0.0	100.0	95.8	10.6	3.2	8.4	2.4	33.6	6.9
435	Human Plasma exp_2	13626	Ig gamma-2 chain C region	EEQFNSTFR_4_3_1_0	12.5	2.7	8.9	0.0	0.0	0.0	100.0	83.7	11.0	4.8	9.6	0.0	28.6	6.2
436	Human Plasma exp_3	13544	Ig gamma-2 chain C region	EEQFNSTFR_4_3_1_0	11.5	2.7	8.9	0.0	0.0	0.0	100.0	85.5	11.2	4.6	10.4	0.0	34.0	4.8
437	Human Plasma exp_4	13469	Ig gamma-2 chain C region	EEQFNSTFR_4_3_1_0	12.7	2.8	9.5	0.0	0.0	0.0	100.0	83.1	11.7	4.1	10.1	0.0	31.6	4.4
438	Human Plasma exp_5	13638	Ig gamma-2 chain C region	EEQFNSTFR_4_3_1_0	81.2	0.0	0.0	0.0	0.0	0.0	43.7	16.0	36.0	0.0	0.0	0.0	14.2	100.0
439	Human Plasma exp_5	13457	Ig gamma-2 chain C region	EEQFNSTFR_4_3_1_0	12.1	3.2	9.4	0.0	0.0	0.0	100.0	90.2	11.3	4.0	11.0	0.0	32.8	8.8
440	Human Plasma exp_1	17150	Ig gamma-2 chain C region	EEQFNSTFR_4_3_1_1	69.9	0.0	19.1	0.0	100.0	0.0	28.5	14.9	0.0	0.0	8.6	0.0	0.0	0.0
441	Human Plasma exp_1	16769	Ig gamma-2 chain C region	EEQFNSTFR_4_3_1_1	0.0	0.0	12.8	0.0	48.5	0.0	100.0	66.9	0.0	0.0	0.0	0.0	36.4	0.0
442	Human Plasma exp_2	17497	Ig gamma-2 chain C region	EEQFNSTFR_4_3_1_1	68.1	0.0	17.2	0.0	100.0	0.0	21.6	29.9	6.9	0.0	6.4	9.1	0.0	0.0

Supplementary Table 3. Continued.

No.	Experiments	CID Scan No.	N-glycoproteins	N-glycopeptides	Relative intensities of B/Y series ions													
					B_2	B_2F	B_3	B_3F	B_2S	B_2SF	Y_1	Y_1F	Y_2	Y_2F	Y_3	Y_3F	Y_4	Y_4F
443	Human Plasma exp_3	17150	Ig gamma-2 chain C region	EEQFNSTFR_4_3_1_1	77.8	0.0	17.4	0.0	100.0	0.0	31.1	33.5	0.0	0.0	16.2	12.3	0.0	0.0
444	Human Plasma exp_3	17088	Ig gamma-2 chain C region	EEQFNSTFR_4_3_1_1	0.0	0.0	0.0	0.0	24.0	0.0	100.0	75.6	7.8	7.3	0.0	0.0	69.9	0.0
445	Human Plasma exp_4	17558	Ig gamma-2 chain C region	EEQFNSTFR_4_3_1_1	70.9	0.0	17.0	0.0	100.0	0.0	28.1	23.6	0.0	0.0	7.1	8.8	0.0	0.0
446	Human Plasma exp_5	17204	Ig gamma-2 chain C region	EEQFNSTFR_4_3_1_1	72.5	0.0	15.9	0.0	100.0	0.0	34.8	17.0	0.0	0.0	15.7	12.0	0.0	0.0
447	Human Plasma exp_5	16818	Ig gamma-2 chain C region	EEQFNSTFR_4_3_1_1	0.0	0.0	7.2	0.0	37.3	0.0	100.0	73.8	7.4	7.2	8.4	6.5	52.8	0.0
448	Human Plasma exp_1	13687	Ig gamma-2 chain C region	EEQFNSTFR_4_4_1_0	100.0	0.0	0.0	0.0	0.0	0.0	21.9	28.0	0.0	0.0	0.0	0.0	16.1	31.7
449	Human Plasma exp_1	12634	Ig gamma-2 chain C region	EEQFNSTFR_4_4_1_0	0.0	0.0	11.3	0.0	0.0	0.0	100.0	92.6	14.8	17.6	11.0	0.0	47.3	13.2
450	Human Plasma exp_2	13908	Ig gamma-2 chain C region	EEQFNSTFR_4_4_1_0	100.0	0.0	0.0	0.0	0.0	0.0	24.8	31.2	0.0	0.0	0.0	0.0	17.9	37.0
451	Human Plasma exp_2	12874	Ig gamma-2 chain C region	EEQFNSTFR_4_4_1_0	0.0	0.0	12.7	0.0	0.0	0.0	100.0	85.8	12.1	8.0	12.6	6.0	38.7	11.9
452	Human Plasma exp_3	13706	Ig gamma-2 chain C region	EEQFNSTFR_4_4_1_0	0.0	2.3	7.6	0.0	0.0	0.0	100.0	70.8	21.4	12.7	17.3	6.5	31.4	4.7
453	Human Plasma exp_4	13734	Ig gamma-2 chain C region	EEQFNSTFR_4_4_1_0	100.0	0.0	0.0	0.0	0.0	0.0	40.3	41.3	0.0	0.0	0.0	0.0	24.7	42.3
454	Human Plasma exp_4	13504	Ig gamma-2 chain C region	EEQFNSTFR_4_4_1_0	0.0	3.5	5.2	0.0	0.0	0.0	100.0	83.3	14.8	11.7	19.2	7.9	35.5	6.2
455	Human Plasma exp_5	13532	Ig gamma-2 chain C region	EEQFNSTFR_4_4_1_0	100.0	0.0	0.0	0.0	0.0	0.0	43.0	40.6	0.0	0.0	0.0	0.0	25.4	41.0
456	Human Plasma exp_5	12719	Ig gamma-2 chain C region	EEQFNSTFR_4_4_1_0	0.0	0.0	4.1	0.0	0.0	0.0	97.3	100.0	7.9	13.0	12.7	5.4	38.5	6.6
457	Human Plasma exp_1	16956	Ig gamma-2 chain C region	EEQFNSTFR_4_4_1_1	0.0	0.0	3.8	0.0	25.9	0.0	100.0	85.6	7.0	16.6	5.5	0.0	22.9	2.6
458	Human Plasma exp_2	17547	Ig gamma-2 chain C region	EEQFNSTFR_4_4_1_1	57.4	0.0	0.0	0.0	100.0	0.0	39.8	58.1	0.0	0.0	0.0	0.0	26.0	0.0
459	Human Plasma exp_2	17308	Ig gamma-2 chain C region	EEQFNSTFR_4_4_1_1	0.0	0.0	5.3	0.0	23.8	0.0	100.0	99.7	15.8	18.8	8.3	0.0	35.7	0.0
460	Human Plasma exp_3	16967	Ig gamma-2 chain C region	EEQFNSTFR_4_4_1_1	62.5	0.0	12.8	0.0	100.0	0.0	47.3	51.7	0.0	0.0	0.0	0.0	24.2	0.0
461	Human Plasma exp_3	16948	Ig gamma-2 chain C region	EEQFNSTFR_4_4_1_1	0.0	0.0	8.4	0.0	24.1	0.0	95.8	100.0	0.0	0.0	5.6	0.0	25.4	0.0
462	Human Plasma exp_4	17600	Ig gamma-2 chain C region	EEQFNSTFR_4_4_1_1	65.1	0.0	0.0	0.0	100.0	0.0	57.6	53.1	0.0	0.0	0.0	0.0	31.0	0.0
463	Human Plasma exp_4	17369	Ig gamma-2 chain C region	EEQFNSTFR_4_4_1_1	0.0	0.0	0.0	0.0	16.8	0.0	100.0	93.6	9.9	6.5	4.9	0.0	22.9	0.0
464	Human Plasma exp_5	17237	Ig gamma-2 chain C region	EEQFNSTFR_4_4_1_1	61.1	0.0	0.0	0.0	100.0	0.0	53.2	49.7	0.0	0.0	0.0	0.0	33.3	0.0
465	Human Plasma exp_5	17009	Ig gamma-2 chain C region	EEQFNSTFR_4_4_1_1	0.0	0.0	0.0	0.0	27.5	0.0	100.0	56.1	5.4	4.1	3.2	0.0	20.7	0.0
466	Human Plasma exp_1	13534	Ig gamma-2 chain C region	EEQFNSTFR_4_5_1_0	0.0	0.0	5.6	0.0	0.0	0.0	100.0	85.3	27.7	23.6	8.3	3.0	13.5	0.0
467	Human Plasma exp_2	13785	Ig gamma-2 chain C region	EEQFNSTFR_4_5_1_0	0.0	0.0	7.2	0.0	0.0	0.0	100.0	90.8	34.0	20.6	2.7	0.0	2.5	0.0
468	Human Plasma exp_3	13927	Ig gamma-2 chain C region	EEQFNSTFR_4_5_1_0	0.0	0.0	4.3	0.0	6.7	0.0	100.0	59.8	14.1	15.3	2.0	0.0	2.9	0.0

Supplementary Table 3. Continued.

No.	Experiments	CID Scan No.	N-glycoproteins	N-glycopeptides	Relative intensities of B/Y series ions													
					B_2	B_2F	B_3	B_3F	B_2S	B_2SF	Y_1	Y_1F	Y_2	Y_2F	Y_3	Y_3F	Y_4	Y_4F
469	Human Plasma exp_4	13820	Ig gamma-2 chain C region	EEQFNSTFR_4_5_1_0	0.0	0.0	6.1	0.0	0.0	0.0	100.0	90.0	28.9	21.4	5.6	0.0	4.9	0.0
470	Human Plasma exp_2	17259	Ig gamma-2 chain C region	EEQFNSTFR_5_3_1_1	73.7	0.0	10.2	0.0	100.0	0.0	3.7	0.0	0.0	0.0	0.0	0.0	7.8	23.6
471	Human Plasma exp_1	13328	Ig gamma-2 chain C region	EEQFNSTFR_5_4_1_0	0.0	2.9	7.3	0.0	0.0	0.0	100.0	90.9	13.9	14.9	17.4	8.7	31.5	2.9
472	Human Plasma exp_3	16763	Ig gamma-2 chain C region	EEQFNSTFR_5_4_1_0	0.0	4.4	8.7	0.0	0.0	0.0	100.0	80.5	14.3	12.6	9.8	0.0	21.3	2.3
473	Human Plasma exp_4	13629	Ig gamma-2 chain C region	EEQFNSTFR_5_4_1_0	0.0	2.9	9.2	0.0	0.0	0.0	100.0	98.1	14.0	16.7	16.3	0.0	32.6	4.1
474	Human Plasma exp_1	16783	Ig gamma-2 chain C region	EEQFNSTFR_5_4_1_1	100.0	0.0	0.0	0.0	44.1	0.0	3.8	0.0	0.0	0.0	0.0	0.0	8.9	30.8
475	Human Plasma exp_1	16698	Ig gamma-2 chain C region	EEQFNSTFR_5_4_1_1	0.0	2.4	4.9	0.0	19.0	0.0	90.9	100.0	8.3	19.1	14.9	6.9	31.5	1.0
476	Human Plasma exp_2	17088	Ig gamma-2 chain C region	EEQFNSTFR_5_4_1_1	100.0	0.0	8.9	0.0	41.2	0.0	8.3	13.2	0.0	0.0	0.0	0.0	8.8	37.1
477	Human Plasma exp_2	17254	Ig gamma-2 chain C region	EEQFNSTFR_5_4_1_1	0.0	0.0	4.7	0.0	18.2	0.0	80.9	100.0	10.1	15.0	6.4	2.9	17.4	0.0
478	Human Plasma exp_3	16737	Ig gamma-2 chain C region	EEQFNSTFR_5_4_1_1	100.0	0.0	9.2	0.0	38.3	0.0	3.4	10.2	0.0	0.0	0.0	0.0	13.7	43.4
479	Human Plasma exp_3	16686	Ig gamma-2 chain C region	EEQFNSTFR_5_4_1_1	0.0	0.0	9.3	0.0	23.4	0.0	100.0	83.9	0.0	0.0	0.0	0.0	15.1	0.0
480	Human Plasma exp_4	17316	Ig gamma-2 chain C region	EEQFNSTFR_5_4_1_1	0.0	0.0	0.0	0.0	19.5	0.0	100.0	98.9	13.0	11.2	3.9	9.5	15.2	0.0
481	Human Plasma exp_5	16770	Ig gamma-2 chain C region	EEQFNSTFR_5_4_1_1	100.0	0.0	6.6	0.0	42.5	0.0	2.8	2.9	0.0	0.0	0.0	0.0	10.9	57.2
482	Human Plasma exp_5	16729	Ig gamma-2 chain C region	EEQFNSTFR_5_4_1_1	0.0	0.0	0.0	0.0	19.2	0.0	91.8	100.0	0.0	0.0	7.3	0.0	18.5	0.0
483	Human Plasma exp_5	20908	Ig gamma-2 chain C region	EEQFNSTFR_5_4_1_2	40.6	0.0	0.0	0.0	100.0	0.0	13.5	25.1	0.0	0.0	0.0	0.0	6.4	0.0
484	Human Plasma exp_4	16931	Ig gamma-2 chain C region	EEQFNSTFR_5_4_2_1	100.0	44.1	0.0	0.0	96.0	0.0	13.3	38.4	0.0	0.0	0.0	0.0	0.0	0.0
485	Human Plasma exp_5	16592	Ig gamma-2 chain C region	EEQFNSTFR_5_4_2_1	77.4	31.8	0.0	0.0	100.0	0.0	16.6	32.7	0.0	0.0	0.0	0.0	0.0	0.0
486	Human Plasma exp_3	13658	Ig gamma-2 chain C region	EEQFNSTFR_5_5_1_0	0.0	0.0	4.0	0.0	0.0	0.0	93.3	100.0	1.7	0.0	1.9	0.0	6.4	0.0
487	Human Plasma exp_1	16809	Ig gamma-2 chain C region	EEQFNSTFR_6_3_1_1	59.3	0.0	0.0	0.0	100.0	0.0	21.9	22.1	0.0	0.0	0.0	0.0	6.9	26.2
488	Human Plasma exp_4	17195	Ig gamma-2 chain C region	EEQFNSTFR_6_3_1_1	59.1	0.0	11.2	0.0	100.0	0.0	20.8	22.6	0.0	0.0	0.0	0.0	0.0	0.0
489	Human Plasma exp_5	16894	Ig gamma-2 chain C region	EEQFNSTFR_6_3_1_1	60.2	0.0	0.0	0.0	100.0	0.0	34.0	35.7	0.0	0.0	0.0	0.0	10.8	23.3
490	Human Plasma exp_1	10127	Ig gamma-3 chain C region	EEQYNSTFR_3_4_1_0	0.0	0.0	9.3	0.0	0.0	0.0	100.0	76.6	8.4	4.8	18.2	0.0	43.5	24.1
491	Human Plasma exp_3	9906	Ig gamma-3 chain C region	EEQYNSTFR_3_4_1_0	0.0	0.0	10.8	0.0	0.0	0.0	100.0	67.6	20.3	7.4	18.9	6.0	46.6	19.0
492	Human Plasma exp_4	10030	Ig gamma-3 chain C region	EEQYNSTFR_3_4_1_0	0.0	0.0	11.2	0.0	0.0	0.0	100.0	66.3	21.9	7.2	20.8	4.5	39.0	17.3
493	Human Plasma exp_5	10292	Ig gamma-3 chain C region	EEQYNSTFR_3_4_1_0	0.0	0.0	0.0	0.0	4.6	0.0	100.0	86.2	5.2	0.0	15.7	0.0	27.0	6.0
494	Human Plasma exp_1	9543	Ig gamma-3 chain C region	EEQYNSTFR_4_3_1_0	12.9	0.0	7.7	0.0	0.0	0.0	100.0	84.2	2.5	0.0	7.9	0.0	39.3	5.5

Supplementary Table 3. Continued.

No.	Experiments	CID Scan No.	N-glycoproteins	N-glycopeptides	Relative intensities of B/Y series ions													
					B_2	B_2F	B_3	B_3F	B_2S	B_2SF	Y_1	Y_1F	Y_2	Y_2F	Y_3	Y_3F	Y_4	Y_4F
495	Human Plasma exp_3	9595	Ig gamma-3 chain C region	EEQYNSTFR_4_3_1_0	11.6	0.0	10.9	0.0	6.3	0.0	100.0	91.5	4.5	2.4	8.8	0.0	33.3	6.8
496	Human Plasma exp_5	9665	Ig gamma-3 chain C region	EEQYNSTFR_4_3_1_0	13.3	0.0	10.2	0.0	0.0	0.0	100.0	78.1	8.6	4.8	4.0	0.0	34.7	6.1
497	Human Plasma exp_1	9505	Ig gamma-3 chain C region	EEQYNSTFR_5_4_1_0	0.0	0.0	6.4	0.0	0.0	0.0	100.0	95.5	10.6	17.4	11.3	6.0	30.3	0.0
498	Human Plasma exp_2	9825	Ig gamma-3 chain C region	EEQYNSTFR_5_4_1_0	0.0	4.0	9.3	0.0	0.0	0.0	96.4	100.0	12.2	19.1	7.4	5.3	32.0	3.2
499	Human Plasma exp_3	9490	Ig gamma-3 chain C region	EEQYNSTFR_5_4_1_0	0.0	0.0	6.5	0.0	0.0	0.0	97.6	100.0	14.6	8.3	0.0	0.0	15.5	0.0
500	Human Plasma exp_5	9610	Ig gamma-3 chain C region	EEQYNSTFR_5_4_1_0	0.0	3.7	4.1	0.0	0.0	0.0	94.2	100.0	7.3	18.2	14.9	5.4	24.0	1.7
501	Human Plasma exp_1	12957	Ig gamma-3 chain C region	EEQYNSTFR_5_4_1_1	0.0	0.0	13.4	0.0	39.6	0.0	92.6	100.0	0.0	0.0	0.0	0.0	40.1	0.0
502	Human Plasma exp_2	13111	Ig gamma-3 chain C region	EEQYNSTFR_5_4_1_1	100.0	0.0	6.0	0.0	42.9	0.0	3.0	3.4	0.0	0.0	2.7	0.0	9.8	55.6
503	Human Plasma exp_2	12990	Ig gamma-3 chain C region	EEQYNSTFR_5_4_1_1	0.0	0.0	0.0	0.0	22.3	0.0	100.0	85.6	17.3	17.5	0.0	0.0	22.1	0.0
504	Human Plasma exp_3	12972	Ig gamma-3 chain C region	EEQYNSTFR_5_4_1_1	100.0	0.0	3.1	0.0	41.4	0.0	7.0	6.1	0.0	0.0	0.0	0.0	13.2	44.9
505	Human Plasma exp_4	13137	Ig gamma-3 chain C region	EEQYNSTFR_5_4_1_1	100.0	0.0	6.7	0.0	46.1	0.0	10.4	8.1	0.0	0.0	0.0	0.0	12.7	55.8
506	Human Plasma exp_5	12944	Ig gamma-3 chain C region	EEQYNSTFR_5_4_1_1	100.0	0.0	8.9	0.0	49.4	0.0	9.2	7.0	0.0	0.0	0.0	0.0	13.1	49.0
507	Human Plasma exp_4	16943	Ig gamma-3 chain C region	EEQYNSTFR_5_4_1_2	49.1	0.0	7.9	0.0	100.0	8.3	7.0	19.9	0.0	0.0	0.0	0.0	0.0	0.0
508	Human Plasma exp_5	16636	Ig gamma-3 chain C region	EEQYNSTFR_5_4_1_2	36.2	0.0	0.0	0.0	100.0	0.0	9.6	17.3	0.0	0.0	0.0	0.0	2.4	0.0
509	Human Plasma exp_1	9753	Ig gamma-4 chain C region	EEQFNSTYR_3_3_1_0	8.9	0.0	12.8	0.0	0.0	0.0	100.0	51.7	11.8	1.3	17.0	7.5	36.0	15.8
510	Human Plasma exp_2	9935	Ig gamma-4 chain C region	EEQFNSTYR_3_3_1_0	14.7	0.0	15.7	0.0	0.0	0.0	100.0	70.7	17.6	2.7	19.3	7.6	48.2	19.2
511	Human Plasma exp_3	9928	Ig gamma-4 chain C region	EEQFNSTYR_3_3_1_0	10.6	0.0	15.6	0.0	0.0	0.0	100.0	78.5	17.6	3.5	12.6	2.6	44.4	20.8
512	Human Plasma exp_4	9975	Ig gamma-4 chain C region	EEQFNSTYR_3_3_1_0	11.5	0.0	13.9	0.0	0.0	0.0	100.0	68.4	14.4	0.0	18.1	9.6	62.9	15.1
513	Human Plasma exp_5	9897	Ig gamma-4 chain C region	EEQFNSTYR_3_3_1_0	11.2	0.0	10.8	0.0	0.0	0.0	100.0	73.3	15.4	0.0	23.5	0.0	47.1	21.3
514	Human Plasma exp_1	9787	Ig gamma-4 chain C region	EEQFNSTYR_3_4_1_0	0.0	0.0	11.9	0.0	0.0	0.0	100.0	62.5	18.9	5.3	21.3	4.0	40.0	16.1
515	Human Plasma exp_2	9981	Ig gamma-4 chain C region	EEQFNSTYR_3_4_1_0	0.0	0.0	11.0	0.0	0.0	0.0	100.0	62.5	22.5	6.9	21.0	0.0	39.7	15.7
516	Human Plasma exp_3	10070	Ig gamma-4 chain C region	EEQFNSTYR_3_4_1_0	0.0	0.0	11.3	0.0	0.0	0.0	100.0	59.7	18.5	5.7	19.2	0.0	37.5	13.8
517	Human Plasma exp_4	10210	Ig gamma-4 chain C region	EEQFNSTYR_3_4_1_0	0.0	0.0	11.1	0.0	0.0	0.0	100.0	67.4	21.8	6.6	18.9	5.0	39.3	14.8
518	Human Plasma exp_5	10108	Ig gamma-4 chain C region	EEQFNSTYR_3_4_1_0	0.0	0.0	10.3	0.0	0.0	0.0	100.0	60.4	19.1	6.0	19.8	4.9	36.8	13.6
519	Human Plasma exp_1	10485	Ig gamma-4 chain C region	EEQFNSTYR_3_5_1_0	0.0	0.0	0.0	0.0	0.0	0.0	100.0	58.8	33.1	17.6	9.3	0.0	5.5	6.1
520	Human Plasma exp_2	10657	Ig gamma-4 chain C region	EEQFNSTYR_3_5_1_0	0.0	0.0	8.5	0.0	0.0	0.0	100.0	80.1	42.1	14.1	13.4	0.0	8.3	3.2

Supplementary Table 3. Continued.

No.	Experiments	CID Scan No.	N-glycoproteins	N-glycopeptides	Relative intensities of B/Y series ions													
					B_2	B_2F	B_3	B_3F	B_2S	B_2SF	Y_1	Y_1F	Y_2	Y_2F	Y_3	Y_3F	Y_4	Y_4F
521	Human Plasma exp_3	10465	Ig gamma-4 chain C region	EEQFNSTYR_3_5_1_0	0.0	0.0	0.0	0.0	0.0	0.0	100.0	80.6	6.4	7.6	0.0	0.0	6.4	0.0
522	Human Plasma exp_4	10746	Ig gamma-4 chain C region	EEQFNSTYR_3_5_1_0	0.0	0.0	0.0	0.0	0.0	0.0	100.0	65.0	37.3	15.9	11.8	0.0	8.6	0.0
523	Human Plasma exp_5	10601	Ig gamma-4 chain C region	EEQFNSTYR_3_5_1_0	0.0	0.0	6.0	0.0	0.0	0.0	100.0	69.1	28.6	21.3	14.0	0.0	4.5	3.1
524	Human Plasma exp_2	9717	Ig gamma-4 chain C region	EEQFNSTYR_4_3_1_0	14.9	0.0	10.6	0.0	3.1	0.0	100.0	94.0	9.9	4.4	4.8	0.0	42.9	8.1
525	Human Plasma exp_3	9741	Ig gamma-4 chain C region	EEQFNSTYR_4_3_1_0	15.9	0.0	8.5	0.0	0.0	0.0	100.0	91.3	6.6	3.9	10.5	1.7	36.4	6.0
526	Human Plasma exp_4	9922	Ig gamma-4 chain C region	EEQFNSTYR_4_3_1_0	13.0	5.4	11.3	0.0	0.0	0.0	96.1	100.0	16.1	0.0	10.3	0.0	37.4	8.3
527	Human Plasma exp_1	9840	Ig gamma-4 chain C region	EEQFNSTYR_4_4_1_0	100.0	0.0	0.0	0.0	0.0	0.0	26.8	33.4	0.0	0.0	0.0	0.0	19.8	37.6
528	Human Plasma exp_2	10037	Ig gamma-4 chain C region	EEQFNSTYR_4_4_1_0	100.0	0.0	0.0	0.0	0.0	0.0	32.0	35.1	0.0	0.0	0.0	0.0	20.8	39.5
529	Human Plasma exp_2	10245	Ig gamma-4 chain C region	EEQFNSTYR_4_4_1_0	0.0	0.0	0.0	0.0	0.0	0.0	100.0	78.2	0.0	0.0	5.1	0.0	29.1	9.3
530	Human Plasma exp_5	9987	Ig gamma-4 chain C region	EEQFNSTYR_4_4_1_0	100.0	0.0	0.0	0.0	0.0	0.0	26.9	32.4	0.0	0.0	0.0	0.0	20.0	41.8
531	Human Plasma exp_5	10006	Ig gamma-4 chain C region	EEQFNSTYR_4_4_1_0	0.0	3.0	12.6	0.0	0.0	0.0	100.0	74.5	17.9	12.1	17.7	7.1	41.1	11.7
532	Human Plasma exp_1	12976	Ig gamma-4 chain C region	EEQFNSTYR_4_4_1_1	100.0	22.4	13.2	0.0	82.7	0.0	36.1	29.8	0.0	0.0	0.0	0.0	14.2	0.0
533	Human Plasma exp_1	13030	Ig gamma-4 chain C region	EEQFNSTYR_4_4_1_1	0.0	3.3	7.7	0.0	28.0	0.0	100.0	91.5	14.0	18.1	12.6	12.7	37.9	6.3
534	Human Plasma exp_2	13233	Ig gamma-4 chain C region	EEQFNSTYR_4_4_1_1	100.0	23.6	13.8	0.0	71.7	0.0	47.0	35.5	0.0	0.0	0.0	0.0	21.5	0.0
535	Human Plasma exp_3	13138	Ig gamma-4 chain C region	EEQFNSTYR_4_4_1_1	100.0	17.6	13.7	0.0	81.6	0.0	39.2	37.6	0.0	0.0	0.0	0.0	10.7	0.0
536	Human Plasma exp_4	13288	Ig gamma-4 chain C region	EEQFNSTYR_4_4_1_1	100.0	20.0	13.2	0.0	90.3	0.0	60.2	44.1	0.0	0.0	0.0	0.0	23.9	0.0
537	Human Plasma exp_5	13088	Ig gamma-4 chain C region	EEQFNSTYR_4_4_1_1	96.6	17.8	18.3	0.0	100.0	0.0	52.6	46.1	0.0	0.0	0.0	0.0	30.1	0.0
538	Human Plasma exp_2	10542	Ig gamma-4 chain C region	EEQFNSTYR_4_5_1_0	0.0	0.0	0.0	0.0	6.4	0.0	90.7	100.0	30.9	18.3	0.0	0.0	0.0	0.0
539	Human Plasma exp_3	9633	Ig gamma-4 chain C region	EEQFNSTYR_5_4_1_0	0.0	2.7	7.2	0.0	0.0	0.0	93.5	100.0	8.8	14.2	11.7	8.2	32.8	0.0
540	Human Plasma exp_2	26482	Ig mu chain C region	GLTFQQNASSMCVPDQDTAIR_5_4_1_1	93.3	0.0	0.0	0.0	85.9	0.0	62.1	100.0	0.0	0.0	0.0	0.0	69.8	73.1
541	Human Plasma exp_1	2772	Ig mu chain C region	NNSDISSTR_4_3_0_1	74.4	0.0	0.0	0.0	100.0	0.0	84.3	0.0	4.7	0.0	8.1	0.0	80.4	0.0
542	Human Plasma exp_2	2942	Ig mu chain C region	NNSDISSTR_4_3_0_1	68.3	0.0	15.9	0.0	100.0	0.0	94.8	0.0	12.0	0.0	19.9	0.0	81.4	0.0
543	Human Plasma exp_3	2842	Ig mu chain C region	NNSDISSTR_4_3_0_1	79.7	0.0	27.0	0.0	90.2	0.0	100.0	0.0	5.9	0.0	25.6	0.0	74.5	0.0
544	Human Plasma exp_4	2928	Ig mu chain C region	NNSDISSTR_4_3_0_1	67.1	0.0	18.4	0.0	100.0	0.0	89.8	0.0	3.1	0.0	21.0	0.0	61.1	0.0
545	Human Plasma exp_5	2834	Ig mu chain C region	NNSDISSTR_4_3_0_1	76.1	0.0	22.7	0.0	91.5	0.0	100.0	0.0	4.4	0.0	26.4	0.0	55.4	0.0
546	Human Plasma exp_1	3001	Ig mu chain C region	NNSDISSTR_4_3_1_1	83.1	0.0	16.0	0.0	100.0	0.0	30.3	41.7	2.6	9.2	4.5	14.8	38.1	93.3

Supplementary Table 3. Continued.

No.	Experiments	CID Scan No.	N-glycoproteins	N-glycopeptides	Relative intensities of B/Y series ions													
					B_2	B_2F	B_3	B_3F	B_2S	B_2SF	Y_1	Y_1F	Y_2	Y_2F	Y_3	Y_3F	Y_4	Y_4F
547	Human Plasma exp_1	2681	Ig mu chain C region	NNSDISSTR_5_3_0_1	52.5	0.0	20.8	0.0	100.0	0.0	75.4	0.0	4.9	0.0	12.2	0.0	69.0	0.0
548	Human Plasma exp_2	2834	Ig mu chain C region	NNSDISSTR_5_3_0_1	77.9	0.0	13.5	0.0	100.0	0.0	94.9	0.0	0.0	0.0	6.3	0.0	98.2	0.0
549	Human Plasma exp_3	2746	Ig mu chain C region	NNSDISSTR_5_3_0_1	63.4	0.0	15.9	0.0	100.0	0.0	74.5	0.0	6.8	0.0	14.7	0.0	80.8	0.0
550	Human Plasma exp_4	2838	Ig mu chain C region	NNSDISSTR_5_3_0_1	80.1	0.0	24.4	0.0	96.4	0.0	100.0	0.0	10.5	0.0	11.2	0.0	77.2	0.0
551	Human Plasma exp_5	2750	Ig mu chain C region	NNSDISSTR_5_3_0_1	70.3	0.0	21.4	0.0	100.0	0.0	83.0	0.0	5.5	0.0	12.2	0.0	82.5	0.0
552	Human Plasma exp_4	3061	Ig mu chain C region	NNSDISSTR_5_3_1_1	98.5	0.0	23.1	0.0	100.0	0.0	33.0	35.0	0.0	0.0	0.0	0.0	22.9	35.3
553	Human Plasma exp_2	3007	Ig mu chain C region	NNSDISSTR_5_4_0_1	100.0	0.0	8.6	0.0	78.3	0.0	67.4	0.0	0.0	0.0	6.4	0.0	53.1	0.0
554	Human Plasma exp_2	3177	Ig mu chain C region	NNSDISSTR_5_4_1_1	100.0	0.0	12.0	0.0	65.0	0.0	0.0	0.0	0.0	0.0	0.0	0.0	15.5	26.0
555	Human Plasma exp_2	3207	Ig mu chain C region	NNSDISSTR_5_4_1_1	0.0	0.0	0.0	0.0	34.5	0.0	93.9	100.0	4.1	5.9	2.8	0.0	7.1	7.9
556	Human Plasma exp_5	5962	Ig mu chain C region	NNSDISSTR_5_4_1_2	51.9	0.0	0.0	0.0	100.0	0.0	18.5	0.0	0.0	0.0	0.0	0.0	4.8	18.4
557	Human Plasma exp_4	3362	Ig mu chain C region	NNSDISSTR_5_5_0_1	100.0	0.0	9.7	0.0	65.4	0.0	89.4	0.0	8.9	0.0	0.0	0.0	0.0	0.0
558	Human Plasma exp_5	3507	Ig mu chain C region	NNSDISSTR_5_5_1_1	100.0	0.0	8.5	0.0	81.6	0.0	44.0	65.9	4.6	0.0	0.0	0.0	0.0	0.0
559	Human Plasma exp_1	2571	Ig mu chain C region	NNSDISSTR_6_3_0_1	62.2	0.0	13.5	0.0	100.0	0.0	95.3	0.0	0.0	0.0	8.6	0.0	37.5	0.0
560	Human Plasma exp_2	2777	Ig mu chain C region	NNSDISSTR_6_3_0_1	100.0	0.0	14.2	0.0	92.8	0.0	87.3	0.0	0.0	0.0	5.5	0.0	14.9	0.0
561	Human Plasma exp_3	3033	Ig mu chain C region	NNSDISSTR_6_3_1_1	60.5	0.0	12.9	0.0	100.0	0.0	30.0	32.2	0.0	0.0	0.0	0.0	9.7	30.7
562	Human Plasma exp_4	3113	Ig mu chain C region	NNSDISSTR_6_3_1_1	65.2	0.0	0.0	0.0	100.0	0.0	32.1	27.3	0.0	0.0	0.0	0.0	5.0	38.5
563	Human Plasma exp_1	19134	Immunoglobulin J chain	ENISDPTSPLR_5_3_1_1	48.7	0.0	8.0	0.0	100.0	0.0	11.2	19.4	0.0	0.0	0.0	0.0	10.6	46.0
564	Human Plasma exp_3	18908	Immunoglobulin J chain	ENISDPTSPLR_5_3_1_1	62.0	0.0	11.0	0.0	100.0	0.0	27.4	27.2	0.0	0.0	0.0	0.0	16.1	64.0
565	Human Plasma exp_4	19585	Immunoglobulin J chain	ENISDPTSPLR_5_3_1_1	50.8	0.0	0.0	0.0	100.0	0.0	11.5	17.7	0.0	0.0	0.0	0.0	26.7	48.7
566	Human Plasma exp_5	19137	Immunoglobulin J chain	ENISDPTSPLR_5_3_1_1	64.2	0.0	0.0	0.0	100.0	0.0	11.1	18.3	0.0	0.0	0.0	0.0	28.7	42.1
567	Human Plasma exp_2	19127	Immunoglobulin J chain	ENISDPTSPLR_5_4_0_1	86.2	0.0	17.1	0.0	100.0	0.0	39.7	0.0	0.0	0.0	0.0	0.0	38.5	0.0
568	Human Plasma exp_1	22460	Immunoglobulin J chain	ENISDPTSPLR_5_4_0_2	84.3	0.0	6.2	0.0	100.0	0.0	37.6	0.0	0.0	0.0	5.4	0.0	78.4	0.0
569	Human Plasma exp_1	22442	Immunoglobulin J chain	ENISDPTSPLR_5_4_0_2	0.0	0.0	0.0	0.0	38.3	0.0	100.0	0.0	16.0	0.0	7.2	0.0	19.6	0.0
570	Human Plasma exp_2	22909	Immunoglobulin J chain	ENISDPTSPLR_5_4_0_2	88.4	0.0	0.0	0.0	100.0	0.0	34.7	0.0	0.0	0.0	13.2	0.0	78.3	0.0
571	Human Plasma exp_2	22784	Immunoglobulin J chain	ENISDPTSPLR_5_4_0_2	57.4	0.0	9.6	0.0	100.0	0.0	32.4	0.0	0.0	0.0	3.5	0.0	11.9	0.0
572	Human Plasma exp_3	22200	Immunoglobulin J chain	ENISDPTSPLR_5_4_0_2	68.1	0.0	5.0	0.0	100.0	0.0	28.5	0.0	3.5	0.0	6.0	0.0	66.6	0.0

Supplementary Table 3. Continued.

No.	Experiments	CID Scan No.	N-glycoproteins	N-glycopeptides	Relative intensities of B/Y series ions													
					B_2	B_2F	B_3	B_3F	B_2S	B_2SF	Y_1	Y_1F	Y_2	Y_2F	Y_3	Y_3F	Y_4	Y_4F
573	Human Plasma exp_4	22776	Immunoglobulin J chain	ENISDPTSPLR_5_4_0_2	62.5	0.0	5.0	0.0	100.0	0.0	29.6	0.0	0.0	0.0	0.0	0.0	67.8	0.0
574	Human Plasma exp_2	15804	Insulin-like growth factor-binding protein 3	GLCVNASAVSR_5_4_0_2	45.5	0.0	4.3	0.0	100.0	0.0	66.8	0.0	0.0	0.0	3.3	0.0	38.4	0.0
575	Human Plasma exp_3	15524	Insulin-like growth factor-binding protein 3	GLCVNASAVSR_5_4_0_2	47.7	0.0	0.0	0.0	100.0	0.0	71.4	0.0	0.0	0.0	7.0	0.0	42.8	0.0
576	Human Plasma exp_2	3512	Insulin-like growth factor-binding protein complex acid labile subunit	NNSLR_4_3_0_1	82.6	0.0	0.0	0.0	87.0	0.0	34.7	0.0	9.3	0.0	0.0	0.0	100.0	0.0
577	Human Plasma exp_1	3342	Insulin-like growth factor-binding protein complex acid labile subunit	NNSLR_5_4_0_2	52.0	0.0	2.7	0.0	100.0	0.0	35.9	0.0	0.0	0.0	2.1	0.0	37.4	0.0
578	Human Plasma exp_2	3432	Insulin-like growth factor-binding protein complex acid labile subunit	NNSLR_5_4_0_2	60.6	0.0	2.3	0.0	100.0	0.0	26.7	0.0	0.0	0.0	0.0	0.0	36.9	0.0
579	Human Plasma exp_3	3351	Insulin-like growth factor-binding protein complex acid labile subunit	NNSLR_5_4_0_2	48.7	0.0	5.1	0.0	100.0	0.0	22.0	0.0	0.0	0.0	2.2	0.0	35.7	0.0
580	Human Plasma exp_4	3663	Insulin-like growth factor-binding protein complex acid labile subunit	NNSLR_5_4_0_2	61.1	0.0	3.9	0.0	100.0	0.0	20.1	0.0	0.0	0.0	0.0	0.0	19.9	0.0
581	Human Plasma exp_5	3367	Insulin-like growth factor-binding protein complex acid labile subunit	NNSLR_5_4_0_2	61.0	0.0	5.8	0.0	100.0	0.0	38.7	0.0	2.2	0.0	5.9	0.0	35.8	0.0
582	Human Plasma exp_2	8788	Inter-alpha-trypsin inhibitor heavy chain H3	ENLTAR_5_4_0_2	56.6	0.0	0.0	0.0	100.0	0.0	11.4	0.0	0.0	0.0	1.9	0.0	18.7	0.0
583	Human Plasma exp_1	13583	Inter-alpha-trypsin inhibitor heavy chain H3	ENLTAR_6_5_0_3	36.4	0.0	0.0	0.0	100.0	0.0	41.7	0.0	0.0	0.0	3.5	0.0	28.3	0.0
584	Human Plasma exp_3	13750	Inter-alpha-trypsin inhibitor heavy chain H3	ENLTAR_6_5_0_3	28.6	0.0	4.0	0.0	100.0	0.0	32.2	0.0	0.0	0.0	2.6	0.0	25.4	0.0
585	Human Plasma exp_4	13946	Inter-alpha-trypsin inhibitor heavy chain H3	ENLTAR_6_5_0_3	31.1	0.0	0.0	0.0	100.0	0.0	37.1	0.0	1.9	0.0	0.0	0.0	49.5	0.0
586	Human Plasma exp_1	22574	Kininogen-1	ITYSIVQTNCSK_5_4_0_2	68.8	0.0	4.0	0.0	100.0	0.0	50.9	0.0	0.0	0.0	11.8	0.0	37.1	0.0
587	Human Plasma exp_2	22988	Kininogen-1	ITYSIVQTNCSK_5_4_0_2	79.0	0.0	10.1	0.0	100.0	0.0	61.5	0.0	0.0	0.0	7.8	0.0	56.1	0.0
588	Human Plasma exp_3	22407	Kininogen-1	ITYSIVQTNCSK_5_4_0_2	89.4	0.0	10.2	0.0	100.0	0.0	56.6	0.0	0.0	0.0	15.2	0.0	56.0	0.0
589	Human Plasma exp_4	22942	Kininogen-1	ITYSIVQTNCSK_5_4_0_2	85.3	0.0	13.7	0.0	100.0	0.0	56.1	0.0	0.0	0.0	11.1	0.0	49.4	0.0
590	Human Plasma exp_5	22388	Kininogen-1	ITYSIVQTNCSK_5_4_0_2	79.2	0.0	7.0	0.0	100.0	0.0	44.4	0.0	0.0	0.0	7.2	0.0	34.7	0.0
591	Human Plasma exp_1	22471	Kininogen-1	ITYSIVQTNCSK_5_4_1_2	10.0	0.0	6.7	0.0	100.0	0.0	36.1	6.3	0.0	0.0	0.0	0.0	0.0	0.0
592	Human Plasma exp_2	26235	Kininogen-1	ITYSIVQTNCSK_6_5_1_3	40.1	7.1	0.0	0.0	100.0	18.7	44.0	0.0	0.0	0.0	2.7	0.0	10.3	0.0
593	Human Plasma exp_4	26128	Kininogen-1	ITYSIVQTNCSK_6_5_1_3	38.8	8.4	3.5	0.0	100.0	14.8	38.2	0.0	0.0	0.0	0.0	0.0	12.4	0.0
594	Human Plasma exp_5	25355	Kininogen-1	ITYSIVQTNCSK_6_5_1_3	40.7	3.7	0.0	0.0	100.0	20.7	41.3	0.0	0.0	0.0	0.0	0.0	16.5	0.0
595	Human Plasma exp_3	26586	Kininogen-1	LNAENNATFYFK_5_4_0_2	82.5	0.0	10.2	0.0	100.0	0.0	27.0	0.0	0.0	0.0	0.0	0.0	60.5	0.0

Supplementary Table 3. Continued.

No.	Experiments	CID Scan No.	N-glycoproteins	N-glycopeptides	Relative intensities of B/Y series ions													
					B_2	B_2F	B_3	B_3F	B_2S	B_2SF	Y_1	Y_1F	Y_2	Y_2F	Y_3	Y_3F	Y_4	Y_4F
596	Human Plasma exp_3	29339	Kininogen-1	LNAENNATFYFK_6_5_1_3	45.4	0.0	0.0	0.0	100.0	10.5	20.0	2.4	0.0	0.0	0.0	0.0	0.0	0.0
597	Human Plasma exp_2	25878	Kininogen-1	YNSQNQSNNQFVLYR_5_4_0_2	55.0	0.0	0.0	0.0	100.0	0.0	77.6	0.0	0.0	0.0	18.8	0.0	73.8	0.0
598	Human Plasma exp_4	25851	Kininogen-1	YNSQNQSNNQFVLYR_5_4_0_2	62.2	0.0	0.0	0.0	100.0	0.0	57.0	0.0	7.8	0.0	17.0	0.0	63.5	0.0
599	Human Plasma exp_5	25165	Kininogen-1	YNSQNQSNNQFVLYR_5_4_0_2	54.5	0.0	0.0	0.0	100.0	0.0	81.9	0.0	1.9	0.0	0.0	0.0	62.7	0.0
600	Human Plasma exp_4	25837	L-selectin	DNYTDLVAIQNK_5_4_0_2	23.4	0.0	8.3	0.0	100.0	0.0	6.1	0.0	0.0	0.0	0.0	0.0	3.5	0.0
601	Human Plasma exp_1	12687	Phosphatidylcholine-sterol acyltransferase	VVYNR_6_5_0_3	31.1	0.0	3.7	0.0	100.0	0.0	50.0	0.0	0.0	0.0	7.0	0.0	29.4	0.0
602	Human Plasma exp_2	12915	Phosphatidylcholine-sterol acyltransferase	VVYNR_6_5_0_3	31.5	0.0	0.0	0.0	100.0	0.0	28.9	0.0	0.0	0.0	1.8	0.0	25.1	0.0
603	Human Plasma exp_4	12966	Phosphatidylcholine-sterol acyltransferase	VVYNR_6_5_0_3	40.1	0.0	0.0	0.0	100.0	0.0	27.5	0.0	0.0	0.0	1.8	0.0	23.8	0.0
604	Human Plasma exp_5	12785	Phosphatidylcholine-sterol acyltransferase	VVYNR_6_5_0_3	39.5	0.0	0.0	0.0	100.0	0.0	30.7	0.0	0.0	0.0	3.4	0.0	34.5	0.0
605	Human Plasma exp_1	23404	Phospholipid transfer protein	NWSLPNR_5_4_1_2	48.7	0.0	6.3	0.0	100.0	0.0	3.4	12.7	0.0	0.0	0.0	0.0	0.0	0.0
606	Human Plasma exp_5	23324	Phospholipid transfer protein	NWSLPNR_5_4_1_2	58.1	0.0	4.9	0.0	100.0	0.0	10.7	17.6	0.0	0.0	0.0	0.0	2.6	29.1
607	Human Plasma exp_2	20184	Plasma kallikrein	GVNFNVSK_5_4_0_2	0.0	0.0	0.0	0.0	60.5	0.0	100.0	0.0	21.1	0.0	8.7	0.0	41.1	0.0
608	Human Plasma exp_5	19816	Plasma kallikrein	GVNFNVSK_5_4_0_2	53.0	0.0	4.7	0.0	100.0	0.0	41.2	0.0	0.0	0.0	5.6	0.0	34.5	0.0
609	Human Plasma exp_1	4851	Plasma serine protease inhibitor	NLSCR_5_4_0_2	54.5	0.0	0.0	0.0	100.0	0.0	32.2	0.0	0.0	0.0	5.6	0.0	31.9	0.0
610	Human Plasma exp_2	4939	Plasma serine protease inhibitor	NLSCR_5_4_0_2	55.0	0.0	5.2	0.0	100.0	0.0	33.0	0.0	0.0	0.0	4.3	0.0	36.8	0.0
611	Human Plasma exp_4	5038	Plasma serine protease inhibitor	NLSCR_5_4_0_2	58.5	0.0	3.7	0.0	100.0	0.0	30.0	0.0	0.0	0.0	5.9	0.0	34.6	0.0
612	Human Plasma exp_5	4877	Plasma serine protease inhibitor	NLSCR_5_4_0_2	49.1	0.0	2.3	0.0	100.0	0.0	40.6	0.0	1.0	0.0	2.1	0.0	33.9	0.0
613	Human Plasma exp_3	5241	Prothrombin	GHVNITR_4_3_0_1	41.1	0.0	0.0	0.0	43.4	0.0	100.0	0.0	3.0	0.0	0.0	0.0	86.7	0.0
614	Human Plasma exp_4	5405	Prothrombin	GHVNITR_4_3_0_1	47.1	0.0	0.0	0.0	25.6	0.0	95.3	0.0	5.4	0.0	2.7	0.0	100.0	0.0
615	Human Plasma exp_5	5223	Prothrombin	GHVNITR_4_3_0_1	41.1	0.0	0.0	0.0	29.2	0.0	100.0	0.0	9.9	0.0	0.0	0.0	82.4	0.0
616	Human Plasma exp_1	5324	Prothrombin	GHVNITR_5_4_1_2	82.7	0.0	0.0	0.0	100.0	19.2	12.2	0.0	0.0	0.0	0.0	0.0	14.2	0.0
617	Human Plasma exp_4	5480	Prothrombin	GHVNITR_5_4_1_2	72.3	6.8	0.0	0.0	100.0	12.2	4.8	0.0	0.0	0.0	0.0	0.0	4.1	0.0
618	Human Plasma exp_2	27844	Prothrombin	NFTENDLLVR_5_4_0_2	74.9	0.0	7.7	0.0	100.0	0.0	67.7	0.0	0.0	0.0	5.2	0.0	44.0	0.0
619	Human Plasma exp_2	27820	Prothrombin	NFTENDLLVR_5_4_0_2	41.2	0.0	3.8	0.0	100.0	0.0	56.8	0.0	0.0	0.0	0.0	0.0	33.6	0.0
620	Human Plasma exp_3	27242	Prothrombin	NFTENDLLVR_5_4_0_2	80.1	0.0	10.0	0.0	100.0	0.0	53.5	0.0	0.0	0.0	10.7	0.0	47.9	0.0
621	Human Plasma exp_3	27228	Prothrombin	NFTENDLLVR_5_4_0_2	47.5	0.0	0.0	0.0	100.0	0.0	52.7	0.0	0.0	0.0	3.0	0.0	40.5	0.0

Supplementary Table 3. Continued.

No.	Experiments	CID Scan No.	N-glycoproteins	N-glycopeptides	Relative intensities of B/Y series ions													
					B_2	B_2F	B_3	B_3F	B_2S	B_2SF	Y_1	Y_1F	Y_2	Y_2F	Y_3	Y_3F	Y_4	Y_4F
623	Human Plasma exp_1	28401	Serotransferrin	CGLVPVLAENYNK_4_3_0_1	40.2	0.0	0.0	0.0	85.2	0.0	58.6	0.0	0.0	0.0	4.0	0.0	100.0	0.0
622	Human Plasma exp_4	27606	Prothrombin	NFTENDLLVR_5_4_0_2	77.6	0.0	0.0	0.0	100.0	0.0	78.9	0.0	6.2	0.0	17.6	0.0	39.7	0.0
624	Human Plasma exp_2	29120	Serotransferrin	CGLVPVLAENYNK_4_3_0_1	57.3	0.0	0.0	0.0	83.8	0.0	83.8	0.0	4.0	0.0	6.8	0.0	100.0	0.0
625	Human Plasma exp_3	28600	Serotransferrin	CGLVPVLAENYNK_4_3_0_1	42.8	0.0	7.0	0.0	85.5	0.0	89.4	0.0	7.0	0.0	0.0	0.0	100.0	0.0
626	Human Plasma exp_4	28899	Serotransferrin	CGLVPVLAENYNK_4_3_0_1	28.7	0.0	0.0	0.0	59.7	0.0	82.6	0.0	10.6	0.0	4.8	0.0	100.0	0.0
627	Human Plasma exp_5	28083	Serotransferrin	CGLVPVLAENYNK_4_3_0_1	28.5	0.0	4.5	0.0	56.9	0.0	76.5	0.0	0.0	0.0	15.5	0.0	100.0	0.0
628	Human Plasma exp_4	26451	Serotransferrin	CGLVPVLAENYNK_5_4_0_1	100.0	0.0	5.3	0.0	50.8	0.0	46.3	0.0	0.0	0.0	14.3	0.0	53.2	0.0
629	Human Plasma exp_1	28364	Serotransferrin	CGLVPVLAENYNK_5_4_0_2	55.3	0.0	4.2	0.0	100.0	0.0	70.2	0.0	5.9	0.0	11.6	0.0	52.5	0.0
630	Human Plasma exp_2	29354	Serotransferrin	CGLVPVLAENYNK_5_4_0_2	75.6	0.0	0.0	0.0	100.0	0.0	61.1	0.0	0.0	0.0	7.0	0.0	54.5	0.0
631	Human Plasma exp_3	28548	Serotransferrin	CGLVPVLAENYNK_5_4_0_2	66.8	0.0	0.0	0.0	100.0	0.0	66.1	0.0	0.0	0.0	3.8	0.0	62.1	0.0
632	Human Plasma exp_4	29121	Serotransferrin	CGLVPVLAENYNK_5_4_0_2	52.6	0.0	9.4	0.0	96.0	0.0	61.8	0.0	3.6	0.0	5.5	0.0	100.0	0.0
633	Human Plasma exp_5	28179	Serotransferrin	CGLVPVLAENYNK_5_4_0_2	58.7	0.0	7.8	0.0	100.0	0.0	81.6	0.0	6.4	0.0	17.1	0.0	66.3	0.0
634	Human Plasma exp_3	23286	Sex hormone-binding globulin	LDVDQALNR_5_4_0_2	48.4	0.0	12.7	0.0	100.0	0.0	36.5	0.0	0.0	0.0	0.0	0.0	11.3	0.0
635	Human Plasma exp_4	13511	Thyroxine-binding globulin	VTACHSSQPNATLYK_5_4_0_2	17.4	0.0	0.0	0.0	100.0	0.0	97.2	0.0	19.5	0.0	0.0	0.0	41.2	0.0
636	Human Plasma exp_1	17101	Vitamin K-dependent protein C	EVFVHPNYSK_5_4_0_2	100.0	0.0	0.0	0.0	76.2	0.0	5.1	0.0	0.0	0.0	3.8	0.0	48.1	0.0
637	Human Plasma exp_2	17448	Vitamin K-dependent protein C	EVFVHPNYSK_5_4_0_2	100.0	0.0	0.0	0.0	77.7	0.0	19.9	0.0	0.0	0.0	3.1	0.0	52.4	0.0
638	Human Plasma exp_1	24845	Vitronectin	NGSLFAFR_4_3_0_1	85.1	0.0	31.0	0.0	97.7	0.0	100.0	0.0	11.3	0.0	26.0	0.0	0.0	0.0
639	Human Plasma exp_2	25338	Vitronectin	NGSLFAFR_4_3_0_1	78.7	0.0	12.7	0.0	88.4	0.0	100.0	0.0	5.2	0.0	23.8	0.0	0.0	0.0
640	Human Plasma exp_3	24827	Vitronectin	NGSLFAFR_4_3_0_1	58.5	0.0	19.7	0.0	89.4	0.0	100.0	0.0	3.5	0.0	11.8	0.0	0.0	0.0
641	Human Plasma exp_4	25323	Vitronectin	NGSLFAFR_4_3_0_1	57.7	0.0	8.5	0.0	66.0	0.0	100.0	0.0	2.8	0.0	8.8	0.0	0.0	0.0
642	Human Plasma exp_5	24584	Vitronectin	NGSLFAFR_4_3_0_1	56.2	0.0	9.7	0.0	73.2	0.0	100.0	0.0	5.0	0.0	11.2	0.0	0.0	0.0
643	Human Plasma exp_1	24613	Vitronectin	NGSLFAFR_5_3_0_1	68.9	0.0	19.5	0.0	91.2	0.0	100.0	0.0	0.0	0.0	16.8	0.0	59.2	0.0
644	Human Plasma exp_2	25113	Vitronectin	NGSLFAFR_5_3_0_1	83.4	0.0	22.5	0.0	100.0	0.0	99.1	0.0	0.0	0.0	21.2	0.0	50.1	0.0
645	Human Plasma exp_3	24606	Vitronectin	NGSLFAFR_5_3_0_1	68.7	0.0	12.0	0.0	100.0	0.0	95.4	0.0	8.5	0.0	3.9	0.0	55.8	0.0
646	Human Plasma exp_4	25094	Vitronectin	NGSLFAFR_5_3_0_1	73.3	0.0	10.1	0.0	100.0	0.0	75.4	0.0	0.0	0.0	0.0	0.0	32.7	0.0
647	Human Plasma exp_5	24407	Vitronectin	NGSLFAFR_5_3_0_1	69.6	0.0	20.8	0.0	98.7	0.0	100.0	0.0	13.5	0.0	12.3	0.0	62.0	0.0

Supplementary Table 3. Continued.

No.	Experiments	CID Scan No.	N-glycoproteins	N-glycopeptides	Relative intensities of B/Y series ions													
					B_2	B_2F	B_3	B_3F	B_2S	B_2SF	Y_1	Y_1F	Y_2	Y_2F	Y_3	Y_3F	Y_4	Y_4F
648	Human Plasma exp_1	24402	Vitronectin	NGSLFAFR_5_4_0_1	100.0	0.0	6.8	0.0	62.1	0.0	23.4	0.0	0.0	0.0	0.0	0.0	29.3	0.0
649	Human Plasma exp_3	24438	Vitronectin	NGSLFAFR_5_4_0_1	100.0	0.0	7.7	0.0	64.7	0.0	48.9	0.0	0.0	0.0	3.3	0.0	35.3	0.0
650	Human Plasma exp_4	25041	Vitronectin	NGSLFAFR_5_4_0_1	100.0	0.0	3.8	0.0	70.4	0.0	43.1	0.0	0.0	0.0	0.0	0.0	33.8	0.0
651	Human Plasma exp_5	24199	Vitronectin	NGSLFAFR_5_4_0_1	100.0	0.0	6.8	0.0	72.7	0.0	53.2	0.0	0.0	0.0	3.5	0.0	40.4	0.0
652	Human Plasma exp_1	27909	Vitronectin	NGSLFAFR_5_4_0_2	47.4	0.0	4.9	0.0	100.0	0.0	56.3	0.0	3.6	0.0	8.3	0.0	44.3	0.0
653	Human Plasma exp_1	27933	Vitronectin	NGSLFAFR_5_4_0_2	0.0	0.0	0.0	0.0	49.5	0.0	100.0	0.0	9.8	0.0	2.2	0.0	13.7	0.0
654	Human Plasma exp_2	28613	Vitronectin	NGSLFAFR_5_4_0_2	49.4	0.0	5.2	0.0	100.0	0.0	54.3	0.0	3.7	0.0	7.0	0.0	41.5	0.0
655	Human Plasma exp_5	27590	Vitronectin	NGSLFAFR_5_4_0_2	50.4	0.0	5.3	0.0	100.0	0.0	54.8	0.0	4.4	0.0	7.7	0.0	45.7	0.0
656	Human Plasma exp_4	28463	Vitronectin	NGSLFAFR_5_4_1_2	22.0	0.0	0.0	0.0	100.0	27.2	39.7	0.0	0.0	0.0	0.0	0.0	13.3	0.0
657	Human Plasma exp_1	24437	Vitronectin	NGSLFAFR_6_3_0_1	65.8	0.0	8.1	0.0	100.0	0.0	90.4	0.0	3.2	0.0	0.0	0.0	28.9	0.0
658	Human Plasma exp_2	24915	Vitronectin	NGSLFAFR_6_3_0_1	64.8	0.0	9.9	0.0	100.0	0.0	51.4	0.0	0.0	0.0	3.7	0.0	34.0	0.0
659	Human Plasma exp_3	24459	Vitronectin	NGSLFAFR_6_3_0_1	61.6	0.0	12.5	0.0	100.0	0.0	74.6	0.0	0.0	0.0	6.0	0.0	23.5	0.0
660	Human Plasma exp_4	24929	Vitronectin	NGSLFAFR_6_3_0_1	79.1	0.0	11.6	0.0	100.0	0.0	85.6	0.0	0.0	0.0	0.0	0.0	24.4	0.0
661	Human Plasma exp_5	24229	Vitronectin	NGSLFAFR_6_3_0_1	69.3	0.0	14.5	0.0	98.4	0.0	100.0	0.0	0.0	0.0	4.3	0.0	26.7	0.0
662	Human Plasma exp_2	31304	Vitronectin	NGSLFAFR_6_5_1_3	0.0	0.0	0.0	0.0	100.0	29.7	55.8	0.0	0.0	0.0	3.5	0.0	48.5	0.0
663	Human Plasma exp_3	30798	Vitronectin	NGSLFAFR_6_5_1_3	0.0	6.8	0.0	0.0	100.0	30.9	64.1	0.0	0.0	0.0	0.0	0.0	50.6	0.0
664	Human Plasma exp_4	31068	Vitronectin	NGSLFAFR_6_5_1_3	5.4	0.0	0.0	0.0	100.0	33.4	67.2	0.0	0.0	0.0	0.0	0.0	40.3	0.0
665	Human Plasma exp_3	21145	Vitronectin	NNATVHEQVGGPSLTSDLQAQSK_5_4_0_2	67.1	0.0	4.5	0.0	100.0	0.0	78.5	0.0	12.1	0.0	0.0	0.0	71.4	0.0
666	Human Plasma exp_4	21736	Vitronectin	NNATVHEQVGGPSLTSDLQAQSK_5_4_0_2	84.6	0.0	7.3	0.0	100.0	0.0	74.6	0.0	9.2	0.0	31.6	0.0	67.7	0.0
667	Human Plasma exp_1	16030	Zinc-alpha-2-glycoprotein	FGCEIENNR_5_4_0_2	41.1	0.0	0.0	0.0	100.0	0.0	4.8	0.0	0.0	0.0	2.8	0.0	11.8	0.0
668	Human Plasma exp_2	16224	Zinc-alpha-2-glycoprotein	FGCEIENNR_5_4_0_2	74.6	0.0	6.7	0.0	100.0	0.0	22.6	0.0	0.0	0.0	0.0	0.0	63.6	0.0
669	Human Plasma exp_3	15877	Zinc-alpha-2-glycoprotein	FGCEIENNR_5_4_0_2	62.9	0.0	3.9	0.0	100.0	0.0	31.9	0.0	0.0	0.0	1.9	0.0	53.7	0.0
670	Human Plasma exp_4	16245	Zinc-alpha-2-glycoprotein	FGCEIENNR_5_4_0_2	68.2	0.0	4.2	0.0	100.0	0.0	37.9	0.0	2.5	0.0	6.8	0.0	48.2	0.0
671	Human Plasma exp_5	15970	Zinc-alpha-2-glycoprotein	FGCEIENNR_5_4_0_2	74.4	0.0	5.3	0.0	100.0	0.0	24.5	0.0	0.0	0.0	0.0	0.0	45.3	0.0

Supplementary Table 4. Models generated by deep neural network for fucosylation classification.

No.	node	layers	replicate	epoch	Test_Accuracy	Test_AUC	Euclidean length
1	64	4	8	10000	0.9898	0.998971	1.406288
2	64	4	5	5000	0.9898	0.998640	1.406054
3	64	3	9	10000	0.9898	0.997255	1.405070
4	64	3	9	20000	0.9898	0.997073	1.404941
5	32	3	1	10000	0.9898	0.996905	1.404822
6	32	3	7	10000	0.9898	0.996827	1.404767
7	128	5	9	20000	0.9873	0.999068	1.404599
8	64	4	9	5000	0.9873	0.998478	1.404180
9	128	4	1	5000	0.9898	0.995934	1.404133
10	128	5	7	2000	0.9898	0.995934	1.404133
11	128	5	5	10000	0.9873	0.998401	1.404124
12	128	4	6	5000	0.9898	0.995856	1.404078
13	64	3	9	5000	0.9873	0.998265	1.404028
14	128	4	7	10000	0.9873	0.998109	1.403917
15	32	3	9	20000	0.9873	0.998109	1.403917
16	128	5	9	5000	0.9873	0.998071	1.403890
17	64	3	3	5000	0.9873	0.998019	1.403853
18	128	5	6	10000	0.9873	0.997896	1.403765
19	64	3	1	5000	0.9898	0.995397	1.403752
20	128	4	9	10000	0.9898	0.995189	1.403605
21	128	3	7	10000	0.9898	0.995073	1.403522
22	32	3	8	20000	0.9873	0.997533	1.403508
23	128	3	9	2000	0.9898	0.995034	1.403494
24	64	4	3	10000	0.9898	0.994969	1.403449
25	32	5	5	5000	0.9873	0.997423	1.403429
26	128	5	8	2000	0.9898	0.994801	1.403329
27	128	4	9	2000	0.9898	0.994775	1.403311
28	128	5	7	5000	0.9873	0.997248	1.403305
29	64	3	5	5000	0.9924	0.991971	1.403163
30	32	3	10	20000	0.9847	0.999579	1.403137
31	128	3	8	10000	0.9898	0.994445	1.403077
32	128	5	10	20000	0.9873	0.996925	1.403075
33	128	3	8	2000	0.9898	0.994432	1.403068
34	128	3	8	5000	0.9873	0.996905	1.403061
35	64	4	5	20000	0.9873	0.996866	1.403034
36	64	4	10	5000	0.9873	0.996834	1.403011
37	128	3	9	20000	0.9873	0.996724	1.402933
38	16	3	1	10000	0.9847	0.999255	1.402906
39	64	3	5	10000	0.9898	0.994199	1.402902
40	128	5	10	5000	0.9873	0.996646	1.402877
41	16	3	10	10000	0.9873	0.996575	1.402827
42	128	4	8	10000	0.9847	0.999113	1.402805
43	128	3	2	10000	0.9873	0.996543	1.402804
44	64	4	4	10000	0.9873	0.996471	1.402753
45	128	3	2	2000	0.9898	0.993940	1.402719
46	64	4	4	5000	0.9898	0.993927	1.402710
47	32	3	2	5000	0.9898	0.993862	1.402664
48	16	4	8	10000	0.9847	0.998835	1.402606
49	32	3	2	10000	0.9847	0.998744	1.402542
50	64	3	4	20000	0.9873	0.996141	1.402519
51	32	3	1	20000	0.9847	0.998647	1.402473
52	128	3	8	20000	0.9873	0.996018	1.402431

Supplementary Table 4. Continued.

No.	node	layers	replicate	epoch	Test_Accuracy	Test_AUC	Euclidean length
53	32	4	2	5000	0.9873	0.995986	1.402408
54	128	5	2	10000	0.9873	0.995915	1.402358
55	128	3	5	10000	0.9873	0.995902	1.402348
56	128	3	6	10000	0.9873	0.995804	1.402279
57	16	3	5	20000	0.9847	0.998355	1.402265
58	64	3	6	20000	0.9898	0.993215	1.402205
59	128	4	5	10000	0.9873	0.995597	1.402132
60	32	4	8	20000	0.9873	0.995578	1.402119
61	128	3	7	20000	0.9873	0.995520	1.402077
62	128	5	5	20000	0.9873	0.995435	1.402017
63	64	3	7	20000	0.9873	0.995351	1.401958
64	128	4	1	2000	0.9898	0.992833	1.401935
65	128	3	1	5000	0.9873	0.995261	1.401893
66	64	3	3	20000	0.9847	0.997773	1.401850
67	128	3	2	5000	0.9873	0.995163	1.401824
68	32	3	3	5000	0.9873	0.995157	1.401820
69	32	3	6	10000	0.9873	0.995151	1.401815
70	32	3	5	5000	0.9873	0.995118	1.401792
71	128	3	5	20000	0.9873	0.995105	1.401783
72	64	4	2	2000	0.9898	0.992606	1.401774
73	64	3	4	10000	0.9873	0.994969	1.401686
74	128	4	5	5000	0.9873	0.994943	1.401668
75	64	5	2	10000	0.9847	0.997475	1.401638
76	64	5	5	5000	0.9873	0.994717	1.401507
77	64	3	2	2000	0.9898	0.992198	1.401485
78	64	3	7	10000	0.9847	0.997145	1.401403
79	128	4	6	10000	0.9847	0.997099	1.401371
80	128	4	5	20000	0.9873	0.994510	1.401360
81	128	3	5	2000	0.9873	0.994497	1.401351
82	32	3	5	20000	0.9847	0.997048	1.401334
83	64	4	8	5000	0.9873	0.994438	1.401310
84	128	5	5	5000	0.9873	0.994432	1.401305
85	128	3	7	2000	0.9873	0.994386	1.401273
86	128	3	2	20000	0.9847	0.996957	1.401270
87	64	4	4	20000	0.9873	0.994367	1.401259
88	64	3	8	20000	0.9822	0.999404	1.401259
89	128	4	3	10000	0.9873	0.994244	1.401172
90	64	4	2	10000	0.9847	0.996717	1.401099
91	64	5	7	20000	0.9873	0.994011	1.401006
92	64	4	5	10000	0.9847	0.996484	1.400934
93	32	4	4	5000	0.9873	0.993830	1.400878
94	128	5	6	20000	0.9847	0.996400	1.400874
95	128	5	6	2000	0.9898	0.991311	1.400857
96	64	3	2	5000	0.9847	0.996309	1.400809
97	64	4	10	20000	0.9847	0.996309	1.400809
98	64	4	1	5000	0.9873	0.993720	1.400800
99	128	5	1	10000	0.9873	0.993707	1.400791
100	128	3	4	20000	0.9847	0.996199	1.400731
101	128	3	4	10000	0.9898	0.991123	1.400725
102	128	4	1	20000	0.9847	0.996096	1.400657
103	64	3	1	20000	0.9873	0.993506	1.400648
104	32	4	5	5000	0.9898	0.990961	1.400610

Supplementary Table 4. Continued.

No.	node	layers	replicate	epoch	Test_Accuracy	Test_AUC	Euclidean length
105	128	4	7	5000	0.9873	0.993435	1.400598
106	16	3	10	20000	0.9847	0.995999	1.400588
107	128	4	8	2000	0.9873	0.993338	1.400529
108	64	3	10	20000	0.9873	0.993325	1.400520
109	16	4	7	20000	0.9873	0.993228	1.400451
110	64	5	7	10000	0.9847	0.995804	1.400450
111	32	3	4	20000	0.9847	0.995772	1.400427
112	128	5	1	5000	0.9847	0.995753	1.400413
113	128	4	2	2000	0.9873	0.993143	1.400391
114	32	3	6	20000	0.9847	0.995681	1.400363
115	128	5	8	10000	0.9873	0.993085	1.400350
116	128	4	1	10000	0.9847	0.995643	1.400335
117	64	3	6	10000	0.9847	0.995643	1.400335
118	32	4	10	20000	0.9847	0.995610	1.400312
119	64	4	9	10000	0.9847	0.995604	1.400307
120	64	4	1	10000	0.9847	0.995584	1.400294
121	128	3	3	2000	0.9873	0.992982	1.400276
122	64	4	3	20000	0.9898	0.990463	1.400257
123	16	3	3	10000	0.9847	0.995461	1.400206
124	64	4	7	10000	0.9873	0.992800	1.400148
125	128	5	10	10000	0.9847	0.995377	1.400146
126	128	5	4	20000	0.9873	0.992742	1.400106
127	128	3	1	20000	0.9873	0.992684	1.400065
128	128	5	8	20000	0.9822	0.997727	1.400063
129	128	5	2	5000	0.9873	0.992671	1.400056
130	128	4	2	10000	0.9847	0.995248	1.400054
131	128	4	2	5000	0.9873	0.992625	1.400024
132	64	4	7	20000	0.9847	0.995176	1.400004
133	32	5	6	20000	0.9873	0.992509	1.399941
134	128	3	10	5000	0.9873	0.992483	1.399923
135	64	4	3	5000	0.9822	0.997488	1.399892
136	64	4	8	20000	0.9847	0.994995	1.399875
137	128	5	3	20000	0.9847	0.994956	1.399847
138	64	3	1	10000	0.9847	0.994911	1.399815
139	32	5	9	10000	0.9873	0.992328	1.399813
140	128	4	4	5000	0.9847	0.994814	1.399746
141	16	4	1	20000	0.9822	0.997229	1.399708
142	128	5	8	5000	0.9873	0.992062	1.399624
143	64	3	2	20000	0.9873	0.992049	1.399615
144	128	5	6	5000	0.9847	0.994613	1.399603
145	128	3	1	10000	0.9847	0.994587	1.399585
146	128	5	3	2000	0.9873	0.991978	1.399565
147	128	4	3	20000	0.9847	0.994548	1.399557
148	128	4	10	10000	0.9847	0.994516	1.399534
149	64	4	9	20000	0.9898	0.989401	1.399506
150	32	4	10	10000	0.9898	0.989336	1.399461
151	128	4	3	5000	0.9847	0.994283	1.399369
152	64	3	8	10000	0.9873	0.991680	1.399354
153	128	3	9	10000	0.9847	0.994030	1.399189
154	128	4	6	20000	0.9822	0.996497	1.399187
155	64	5	8	5000	0.9898	0.988941	1.399182
156	64	4	1	20000	0.9847	0.994004	1.399171

Supplementary Table 4. Continued.

No.	node	layers	replicate	epoch	Test_Accuracy	Test_AUC	Euclidean length
157	64	5	10	20000	0.9847	0.993875	1.399079
158	64	4	6	10000	0.9873	0.991214	1.399023
159	128	3	3	5000	0.9847	0.993746	1.398987
160	128	3	3	10000	0.9847	0.993661	1.398927
161	32	3	1	5000	0.9847	0.993655	1.398922
162	32	4	2	10000	0.9873	0.991052	1.398909
163	64	5	1	20000	0.9873	0.990929	1.398822
164	64	3	5	20000	0.9873	0.990923	1.398817
165	64	4	10	10000	0.9847	0.993499	1.398812
166	64	5	1	5000	0.9898	0.988352	1.398765
167	128	3	5	5000	0.9873	0.990845	1.398762
168	128	5	7	10000	0.9873	0.990780	1.398716
169	32	3	7	20000	0.9847	0.993364	1.398716
170	64	5	9	20000	0.9873	0.990754	1.398698
171	128	4	9	5000	0.9847	0.993318	1.398683
172	32	4	6	5000	0.9822	0.995766	1.398666
173	32	4	6	10000	0.9873	0.990709	1.398666
174	64	5	4	20000	0.9847	0.993286	1.398660
175	32	3	4	5000	0.9847	0.993240	1.398628
176	64	5	7	5000	0.9847	0.992969	1.398435
177	64	5	4	5000	0.9847	0.992904	1.398389
178	128	5	4	10000	0.9873	0.990256	1.398345
179	128	4	8	20000	0.9847	0.992826	1.398334
180	64	5	5	10000	0.9873	0.990146	1.398267
181	32	5	10	5000	0.9822	0.995202	1.398265
182	128	4	4	20000	0.9822	0.995112	1.398200
183	32	3	9	10000	0.9847	0.992515	1.398113
184	16	3	6	20000	0.9822	0.994956	1.398090
185	128	5	1	20000	0.9873	0.989848	1.398056
186	128	4	3	2000	0.9847	0.992418	1.398044
187	64	3	8	5000	0.9873	0.989751	1.397987
188	64	4	6	20000	0.9822	0.994717	1.397919
189	128	5	4	2000	0.9873	0.989615	1.397891
190	128	3	7	5000	0.9873	0.989531	1.397831
191	64	4	2	20000	0.9822	0.994587	1.397827
192	64	3	3	10000	0.9847	0.992043	1.397778
193	128	4	8	5000	0.9873	0.989408	1.397744
194	64	4	4	2000	0.9847	0.991913	1.397686
195	64	5	2	5000	0.9822	0.994386	1.397684
196	32	5	1	20000	0.9822	0.994361	1.397666
197	128	3	6	5000	0.9847	0.991881	1.397663
198	128	4	4	10000	0.9873	0.989220	1.397611
199	128	3	1	2000	0.9873	0.989174	1.397579
200	128	4	10	2000	0.9898	0.986623	1.397544
201	128	5	3	10000	0.9847	0.991674	1.397516
202	128	3	3	20000	0.9822	0.994134	1.397505
203	64	4	6	5000	0.9847	0.991583	1.397452
204	128	4	2	20000	0.9873	0.988941	1.397414
205	128	4	10	20000	0.9847	0.991441	1.397351
206	128	4	4	2000	0.9898	0.986339	1.397343
207	32	3	2	20000	0.9873	0.988663	1.397217
208	16	3	3	20000	0.9822	0.993629	1.397145

Supplementary Table 4. Continued.

No.	node	layers	replicate	epoch	Test_Accuracy	Test_AUC	Euclidean length
209	128	3	9	5000	0.9847	0.991143	1.397139
210	128	4	5	2000	0.9873	0.988488	1.397093
211	32	4	2	20000	0.9873	0.988320	1.396974
212	128	5	9	2000	0.9822	0.993376	1.396966
213	64	5	1	10000	0.9847	0.990845	1.396928
214	128	5	7	20000	0.9847	0.990793	1.396891
215	64	3	7	5000	0.9847	0.990793	1.396891
216	128	3	6	20000	0.9822	0.993266	1.396888
217	128	3	4	2000	0.9873	0.988151	1.396855
218	32	4	9	20000	0.9847	0.990735	1.396850
219	128	3	4	5000	0.9847	0.990702	1.396827
220	16	3	7	20000	0.9796	0.995681	1.396781
221	32	4	8	5000	0.9847	0.990288	1.396533
222	16	5	10	10000	0.9847	0.990243	1.396501
223	32	4	1	20000	0.9847	0.990204	1.396473
224	64	3	6	5000	0.9847	0.990197	1.396469
225	64	4	2	5000	0.9873	0.987582	1.396452
226	128	4	10	5000	0.9822	0.992625	1.396432
227	128	4	7	20000	0.9847	0.990068	1.396377
228	64	5	3	5000	0.9847	0.989861	1.396230
229	128	4	9	20000	0.9822	0.992334	1.396225
230	128	5	9	10000	0.9822	0.992308	1.396206
231	16	3	5	5000	0.9847	0.989764	1.396161
232	32	4	1	10000	0.9873	0.987128	1.396132
233	32	3	8	5000	0.9847	0.989718	1.396129
234	8	3	8	10000	0.9822	0.992062	1.396032
235	64	3	7	2000	0.9847	0.989356	1.395872
236	32	3	3	10000	0.9822	0.991693	1.395769
237	32	3	10	5000	0.9796	0.994179	1.395711
238	128	5	3	5000	0.9898	0.983988	1.395685
239	128	3	10	20000	0.9822	0.991570	1.395682
240	128	5	2	20000	0.9847	0.988961	1.395592
241	128	5	10	2000	0.9822	0.991415	1.395572
242	128	5	4	5000	0.9822	0.991298	1.395489
243	128	5	2	2000	0.9822	0.991240	1.395447
244	64	5	3	10000	0.9873	0.986099	1.395404
245	16	3	5	10000	0.9796	0.993681	1.395356
246	64	4	8	2000	0.9873	0.985995	1.395331
247	128	3	10	2000	0.9847	0.988449	1.395230
248	32	5	9	5000	0.9822	0.990897	1.395204
249	16	3	7	10000	0.9771	0.995817	1.395126
250	64	3	10	5000	0.9847	0.988255	1.395092
251	128	5	1	2000	0.9822	0.990502	1.394923
252	32	5	10	20000	0.9847	0.987990	1.394904
253	32	3	6	5000	0.9822	0.990437	1.394877
254	16	4	9	20000	0.9796	0.992930	1.394821
255	64	5	2	20000	0.9847	0.987724	1.394716
256	64	5	4	10000	0.9822	0.990204	1.394712
257	64	3	4	5000	0.9796	0.992554	1.394554
258	32	3	8	10000	0.9847	0.987148	1.394308
259	64	3	2	10000	0.9847	0.987109	1.394281
260	128	3	6	2000	0.9822	0.989543	1.394243

Supplementary Table 4. Continued.

No.	node	layers	replicate	epoch	Test_Accuracy	Test_AUC	Euclidean length
261	16	4	7	10000	0.9822	0.989524	1.394229
262	32	4	4	20000	0.9847	0.986811	1.394070
263	64	4	7	2000	0.9796	0.991835	1.394042
264	32	3	3	20000	0.9796	0.991810	1.394024
265	64	5	8	10000	0.9847	0.986436	1.393804
266	64	5	9	5000	0.9873	0.983833	1.393804
267	64	5	5	20000	0.9822	0.988870	1.393765
268	128	4	7	2000	0.9796	0.991227	1.393609
269	64	5	3	20000	0.9847	0.986157	1.393607
270	128	5	5	2000	0.9796	0.990968	1.393425
271	64	3	10	2000	0.9847	0.985814	1.393364
272	64	5	9	10000	0.9796	0.990638	1.393190
273	64	5	6	2000	0.9847	0.984739	1.392604
274	64	5	10	5000	0.9822	0.987174	1.392562
275	64	4	1	2000	0.9847	0.984545	1.392467
276	32	3	9	5000	0.9873	0.981716	1.392310
277	32	4	3	5000	0.9822	0.986805	1.392300
278	32	4	9	10000	0.9746	0.994270	1.392271
279	16	4	5	20000	0.9873	0.981398	1.392086
280	16	5	8	10000	0.9771	0.991428	1.391996
281	64	3	1	2000	0.9847	0.983652	1.391835
282	64	5	6	20000	0.9822	0.986067	1.391777
283	64	3	10	10000	0.9847	0.983354	1.391624
284	8	3	3	10000	0.9796	0.988339	1.391557
285	32	5	4	5000	0.9822	0.985652	1.391484
286	16	3	2	10000	0.9822	0.985484	1.391365
287	32	5	5	20000	0.9822	0.985387	1.391296
288	64	5	2	2000	0.9796	0.987964	1.391290
289	128	4	6	2000	0.9822	0.985303	1.391236
290	16	3	2	20000	0.9822	0.985057	1.391062
291	64	4	5	2000	0.9796	0.987303	1.390821
292	32	5	7	5000	0.9822	0.984649	1.390773
293	64	5	6	10000	0.9847	0.981683	1.390445
294	64	4	10	2000	0.9796	0.986662	1.390366
295	64	3	8	2000	0.9796	0.986539	1.390279
296	64	3	6	2000	0.9796	0.986332	1.390132
297	64	5	8	20000	0.9847	0.981217	1.390115
298	64	5	4	2000	0.972	0.993784	1.390105
299	8	3	6	20000	0.9898	0.975973	1.390046
300	8	3	2	20000	0.9771	0.988540	1.389941
301	32	4	10	5000	0.9898	0.975604	1.389786
302	16	3	10	5000	0.972	0.993117	1.389628
303	32	4	8	10000	0.9873	0.977565	1.389387
304	32	4	10	2000	0.9746	0.990172	1.389347
305	64	5	1	2000	0.9771	0.987608	1.389278
306	128	3	7	1000	0.9796	0.984377	1.388745
307	16	3	4	20000	0.9847	0.979165	1.388668
308	64	3	5	2000	0.9796	0.984144	1.388580
309	8	3	8	20000	0.9847	0.978582	1.388257
310	32	5	4	10000	0.9822	0.980952	1.388158
311	32	5	6	10000	0.9847	0.978291	1.388051
312	32	3	4	10000	0.9822	0.980563	1.387884

Supplementary Table 4. Continued.

No.	node	layers	replicate	epoch	Test_Accuracy	Test_AUC	Euclidean length
313	32	3	10	10000	0.9822	0.980414	1.387778
314	16	3	9	10000	0.9746	0.987912	1.387737
315	64	4	7	5000	0.9847	0.977721	1.387650
316	32	5	10	10000	0.9796	0.982771	1.387608
317	32	4	7	10000	0.9822	0.979877	1.387399
318	32	5	6	5000	0.9746	0.987290	1.387295
319	32	4	5	10000	0.9822	0.979605	1.387207
320	64	3	4	2000	0.9746	0.986941	1.387046
321	32	4	9	5000	0.9847	0.976678	1.386916
322	32	5	8	10000	0.9771	0.983852	1.386611
323	32	5	9	20000	0.9746	0.986274	1.386572
324	16	4	4	10000	0.972	0.988831	1.386568
325	16	3	8	20000	0.9822	0.978653	1.386535
326	64	5	6	5000	0.9796	0.981049	1.386388
327	64	4	9	2000	0.9695	0.990838	1.386251
328	32	5	1	10000	0.9771	0.983075	1.386060
329	32	5	7	10000	0.9796	0.980168	1.385765
330	16	3	6	10000	0.9746	0.984493	1.385306
331	64	5	10	10000	0.9847	0.974186	1.385161
332	32	5	2	5000	0.9822	0.976497	1.385014
333	32	3	5	10000	0.9771	0.981457	1.384912
334	16	4	8	5000	0.9796	0.978873	1.384850
335	32	5	4	20000	0.9847	0.973454	1.384647
336	64	3	3	2000	0.9771	0.980362	1.384137
337	8	3	10	10000	0.9847	0.972722	1.384133
338	64	4	6	2000	0.9771	0.980356	1.384132
339	32	4	7	5000	0.9822	0.975112	1.384037
340	16	4	9	5000	0.9822	0.975027	1.383978
341	64	5	5	2000	0.972	0.984966	1.383814
342	32	3	3	2000	0.972	0.984811	1.383704
343	32	4	7	20000	0.9847	0.970929	1.382873
344	16	4	3	10000	0.9796	0.976070	1.382870
345	64	5	8	2000	0.9695	0.985309	1.382304
346	32	5	2	20000	0.9822	0.972541	1.382228
347	128	4	5	1000	0.972	0.982577	1.382115
348	64	3	9	2000	0.9695	0.984856	1.381981
349	16	5	2	20000	0.9873	0.966345	1.381515
350	64	5	7	2000	0.9746	0.978932	1.381359
351	16	3	8	10000	0.9873	0.965911	1.381212
352	16	3	1	20000	0.9771	0.976167	1.381168
353	32	5	5	10000	0.9822	0.970903	1.381075
354	32	4	4	10000	0.9796	0.973486	1.381047
355	16	3	3	5000	0.9796	0.973182	1.380833
356	32	3	2	2000	0.9669	0.985743	1.380791
357	32	5	8	5000	0.9822	0.970314	1.380661
358	64	5	3	2000	0.9669	0.985212	1.380412
359	32	3	9	2000	0.9669	0.983949	1.379512
360	32	5	3	10000	0.9796	0.970916	1.379237
361	16	3	9	20000	0.9771	0.973053	1.378969
362	64	5	9	2000	0.9695	0.980330	1.378759
363	32	4	4	2000	0.9695	0.980142	1.378626
364	32	3	1	2000	0.9669	0.981638	1.377864

Supplementary Table 4. Continued.

No.	node	layers	replicate	epoch	Test_Accuracy	Test_AUC	Euclidean length
365	128	4	8	1000	0.9669	0.980188	1.376831
366	16	4	2	5000	0.9746	0.972250	1.376632
367	32	5	1	5000	0.9822	0.964474	1.376563
368	16	4	6	10000	0.9796	0.966746	1.376305
369	128	4	1	1000	0.9669	0.979417	1.376283
370	128	3	1	1000	0.9644	0.981483	1.376000
371	128	4	3	1000	0.9695	0.976070	1.375733
372	16	4	10	20000	0.9847	0.960621	1.375655
373	128	5	10	1000	0.9644	0.980330	1.375178
374	8	3	4	10000	0.9746	0.970165	1.375160
375	128	3	2	1000	0.9644	0.979844	1.374832
376	16	4	3	20000	0.9771	0.966662	1.374467
377	128	3	6	1000	0.9593	0.984234	1.374399
378	32	5	3	5000	0.972	0.971149	1.374014
379	8	3	1	20000	0.9796	0.963392	1.373951
380	32	3	8	2000	0.9618	0.981023	1.373850
381	16	4	2	20000	0.9669	0.975947	1.373815
382	128	4	9	1000	0.9567	0.985898	1.373780
383	32	5	3	20000	0.9847	0.957908	1.373762
384	16	4	10	10000	0.9847	0.957669	1.373595
385	32	4	3	20000	0.9796	0.962395	1.373252
386	32	4	3	10000	0.9796	0.961722	1.372780
387	128	5	9	1000	0.9644	0.976167	1.372213
388	128	5	7	1000	0.9669	0.973616	1.372160
389	128	3	8	1000	0.9593	0.980181	1.371500
390	128	4	4	1000	0.9618	0.977384	1.371255
391	128	4	10	1000	0.9644	0.974678	1.371154
392	64	4	4	1000	0.9618	0.976937	1.370936
393	16	4	2	10000	0.972	0.966662	1.370846
394	128	3	3	1000	0.9644	0.974069	1.370722
395	128	3	5	1000	0.9669	0.971427	1.370608
396	16	4	4	20000	0.972	0.966066	1.370426
397	32	5	7	20000	0.9822	0.955558	1.370331
398	16	4	3	5000	0.9746	0.962868	1.370022
399	128	3	4	1000	0.9644	0.973001	1.369963
400	128	3	10	1000	0.9618	0.975532	1.369935
401	16	3	8	5000	0.9822	0.954820	1.369817
402	64	4	3	2000	0.9644	0.972198	1.369393
403	16	3	6	5000	0.9873	0.948313	1.368963
404	128	5	5	1000	0.9567	0.978504	1.368483
405	16	3	1	5000	0.9796	0.954438	1.367687
406	32	3	7	5000	0.9746	0.959501	1.367658
407	64	4	6	1000	0.9593	0.974788	1.367650
408	32	4	1	5000	0.9746	0.959074	1.367358
409	16	4	8	20000	0.9746	0.958575	1.367008
410	128	4	6	1000	0.9542	0.978809	1.366954
411	32	4	8	2000	0.9618	0.971020	1.366725
412	128	5	1	1000	0.9644	0.968074	1.366468
413	32	4	5	20000	0.9847	0.945380	1.365056
414	32	4	6	2000	0.9542	0.975856	1.364842
415	16	3	4	5000	0.9695	0.959495	1.364024
416	16	4	7	5000	0.9669	0.960550	1.362920

Supplementary Table 4. Continued.

No.	node	layers	replicate	epoch	Test_Accuracy	Test_AUC	Euclidean length
417	32	4	6	20000	0.9822	0.944551	1.362679
418	64	3	3	1000	0.9618	0.965160	1.362569
419	16	3	2	5000	0.9669	0.959449	1.362145
420	128	5	6	1000	0.9618	0.964292	1.361954
421	128	4	2	1000	0.9542	0.971576	1.361785
422	32	4	3	2000	0.9618	0.963768	1.361583
423	64	4	8	1000	0.9491	0.976076	1.361439
424	128	5	8	1000	0.9542	0.971045	1.361406
425	128	4	7	1000	0.9491	0.975973	1.361365
426	128	5	2	1000	0.9593	0.965898	1.361329
427	64	5	10	2000	0.9567	0.968171	1.361113
428	32	4	5	2000	0.9491	0.975325	1.360900
429	32	4	1	2000	0.9517	0.972599	1.360765
430	64	3	7	1000	0.9491	0.974192	1.360089
431	64	4	7	1000	0.9618	0.961605	1.360053
432	32	3	5	2000	0.9567	0.965801	1.359429
433	16	3	7	5000	0.9618	0.960647	1.359376
434	8	3	6	10000	0.9847	0.936439	1.358879
435	32	5	2	10000	0.9822	0.938886	1.358758
436	8	3	9	10000	0.972	0.949343	1.358689
437	8	5	9	20000	0.9644	0.956983	1.358633
438	8	3	3	20000	0.972	0.949207	1.358594
439	64	3	1	1000	0.9491	0.971958	1.358490
440	128	3	9	1000	0.9593	0.960052	1.357187
441	32	3	5	1000	0.9618	0.956970	1.356779
442	32	3	10	1000	0.9542	0.964448	1.356708
443	128	3	3	500	0.9466	0.971149	1.356164
444	16	5	3	10000	0.9618	0.955578	1.355798
445	32	3	9	1000	0.9618	0.955513	1.355752
446	32	5	8	20000	0.9822	0.934295	1.355590
447	128	5	3	1000	0.9644	0.951563	1.354821
448	8	3	4	20000	0.972	0.940116	1.352258
449	64	4	3	1000	0.9593	0.951809	1.351369
450	16	5	7	20000	0.9618	0.948313	1.350688
451	64	4	1	1000	0.9542	0.953771	1.349139
452	16	4	9	10000	0.9771	0.930165	1.349048
453	16	5	2	10000	0.9415	0.965749	1.348738
454	64	4	9	1000	0.9542	0.952975	1.348576
455	64	4	2	1000	0.9542	0.952515	1.348252
456	64	3	5	1000	0.9593	0.947361	1.348240
457	16	3	4	10000	0.9822	0.923133	1.347921
458	32	3	6	2000	0.9746	0.930870	1.347726
459	64	4	5	1000	0.9644	0.941424	1.347719
460	128	5	4	1000	0.9567	0.948151	1.346947
461	8	3	2	10000	0.9771	0.925878	1.346096
462	64	3	6	1000	0.9517	0.951699	1.345907
463	32	3	10	2000	0.944	0.958770	1.345502
464	32	5	9	2000	0.9567	0.945522	1.345098
465	32	5	1	2000	0.9644	0.937300	1.344841
466	16	4	5	2000	0.9567	0.944694	1.344515
467	16	5	6	20000	0.9669	0.932865	1.343552
468	64	3	8	1000	0.9389	0.959488	1.342442

Supplementary Table 4. Continued.

No.	node	layers	replicate	epoch	Test_Accuracy	Test_AUC	Euclidean length
469	32	3	4	2000	0.944	0.953370	1.341659
470	32	3	7	2000	0.9669	0.929873	1.341477
471	64	5	7	1000	0.9364	0.958737	1.340157
472	64	5	2	1000	0.9389	0.953907	1.338459
473	8	3	1	10000	0.9593	0.933266	1.338373
474	32	4	7	2000	0.9491	0.943282	1.338123
475	32	4	9	1000	0.944	0.944700	1.335513
476	16	3	1	2000	0.9262	0.961651	1.335147
477	64	3	10	1000	0.9338	0.953816	1.334821
478	128	3	2	500	0.9237	0.963457	1.334718
479	64	3	9	1000	0.9338	0.952554	1.333920
480	64	3	2	1000	0.9186	0.965620	1.332759
481	16	4	1	10000	0.9593	0.924020	1.331942
482	32	5	7	2000	0.9542	0.928714	1.331543
483	128	4	3	500	0.9364	0.944422	1.329954
484	128	3	7	500	0.9211	0.957760	1.328807
485	16	3	10	2000	0.9567	0.921994	1.328664
486	32	3	3	1000	0.9186	0.959754	1.328515
487	128	5	2	500	0.9288	0.949064	1.327928
488	128	4	2	500	0.9186	0.958834	1.327851
489	8	3	8	5000	0.9593	0.916581	1.326792
490	128	3	10	500	0.9237	0.952191	1.326608
491	128	3	1	500	0.9211	0.953927	1.326047
492	128	3	9	500	0.9364	0.938485	1.325744
493	128	3	4	500	0.916	0.958154	1.325563
494	32	4	9	2000	0.944	0.926973	1.323032
495	128	3	6	500	0.9059	0.959967	1.319921
496	8	3	5	5000	0.9491	0.915409	1.318622
497	128	4	4	500	0.9059	0.956277	1.317240
498	16	5	5	10000	0.9822	0.876490	1.316416
499	128	5	1	500	0.944	0.916445	1.315677
500	64	3	4	1000	0.9364	0.923690	1.315313
501	128	4	8	500	0.9084	0.950022	1.314433
502	16	3	9	5000	0.9695	0.885988	1.313357
503	16	4	5	5000	0.972	0.879753	1.311011
504	128	5	10	500	0.9313	0.920576	1.309496
505	128	4	5	500	0.883	0.966830	1.309370
506	32	4	2	2000	0.9389	0.912334	1.309155
507	128	4	9	500	0.8982	0.951958	1.308812
508	32	5	10	2000	0.9364	0.914192	1.308660
509	128	4	1	500	0.9109	0.939236	1.308397
510	32	5	4	2000	0.9491	0.899818	1.307847
511	8	3	2	5000	0.9517	0.894004	1.305747
512	128	4	6	500	0.8931	0.950255	1.304076
513	128	5	4	500	0.9033	0.940142	1.303771
514	16	4	10	2000	0.9618	0.880051	1.303668
515	128	4	10	500	0.9059	0.936231	1.302760
516	64	5	8	1000	0.9237	0.917947	1.302247
517	64	5	9	1000	0.9364	0.902732	1.300680
518	128	5	6	500	0.9186	0.920751	1.300618
519	8	3	3	5000	0.9593	0.878037	1.300464
520	64	3	9	500	0.883	0.953305	1.299415

Supplementary Table 4. Continued.

No.	node	layers	replicate	epoch	Test_Accuracy	Test_AUC	Euclidean length
521	8	3	3	2000	0.916	0.920025	1.298269
522	32	5	8	2000	0.9313	0.903606	1.297622
523	32	3	7	1000	0.9135	0.921158	1.297311
524	128	5	3	500	0.9084	0.925878	1.297090
525	128	3	5	500	0.916	0.917895	1.296760
526	64	3	4	500	0.8779	0.954043	1.296498
527	64	5	5	1000	0.9262	0.901942	1.292805
528	128	3	8	500	0.9033	0.922065	1.290796
529	16	4	3	2000	0.9491	0.874450	1.290525
530	8	3	7	10000	0.972	0.846396	1.288864
531	8	4	1	10000	0.9593	0.860491	1.288682
532	64	5	6	1000	0.9211	0.899274	1.287291
533	16	5	4	10000	0.9644	0.852139	1.286937
534	128	4	7	500	0.9211	0.897837	1.286288
535	128	5	9	500	0.9135	0.904363	1.285440
536	32	5	6	2000	0.9237	0.893143	1.284884
537	64	5	10	1000	0.9542	0.860265	1.284738
538	16	4	6	5000	0.9567	0.856574	1.284132
539	32	3	4	1000	0.8779	0.935260	1.282739
540	64	4	10	1000	0.9491	0.862440	1.282417
541	128	5	5	500	0.9008	0.912398	1.282151
542	64	4	4	500	0.9084	0.904370	1.281825
543	128	5	7	500	0.9211	0.890611	1.281255
544	64	4	6	500	0.8804	0.929770	1.280459
545	64	3	5	500	0.8906	0.916795	1.278155
546	32	4	8	1000	0.9313	0.871466	1.275450
547	64	5	4	1000	0.9008	0.902732	1.275290
548	64	5	1	1000	0.9084	0.894904	1.275164
549	8	4	8	20000	0.9822	0.812890	1.274954
550	8	3	10	5000	0.944	0.856354	1.274550
551	16	4	6	2000	0.9644	0.830844	1.272937
552	64	3	8	500	0.8906	0.908306	1.272080
553	64	3	7	500	0.8575	0.935707	1.269194
554	64	4	9	500	0.916	0.876069	1.267499
555	64	3	6	500	0.8804	0.910806	1.266756
556	128	5	8	500	0.9135	0.875985	1.265635
557	32	5	3	2000	0.9237	0.864123	1.264884
558	16	3	5	2000	0.9135	0.874094	1.264327
559	32	4	7	1000	0.916	0.865574	1.260267
560	32	5	2	2000	0.9262	0.852987	1.259140
561	64	4	5	500	0.9389	0.832929	1.255111
562	32	4	4	1000	0.9186	0.854444	1.254552
563	64	4	10	500	0.9135	0.858283	1.253448
564	64	4	7	500	0.9135	0.857500	1.252912
565	64	5	3	1000	0.9237	0.844855	1.251799
566	32	5	5	1000	0.8728	0.896613	1.251277
567	8	4	6	10000	0.9186	0.846357	1.249058
568	64	3	2	500	0.8753	0.888047	1.246907
569	64	4	2	500	0.9135	0.846674	1.245528
570	8	3	7	20000	0.9466	0.805127	1.242691
571	16	5	4	2000	0.9135	0.841028	1.241697
572	16	5	10	20000	0.8651	0.889679	1.240938

Supplementary Table 4. Continued.

No.	node	layers	replicate	epoch	Test_Accuracy	Test_AUC	Euclidean length
573	64	4	8	500	0.9135	0.838070	1.239695
574	64	3	1	500	0.9237	0.826493	1.239481
575	32	3	1	1000	0.8804	0.872016	1.239159
576	16	4	9	2000	0.9059	0.843819	1.238017
577	64	3	3	500	0.8753	0.875422	1.237947
578	16	3	2	2000	0.9135	0.834709	1.237425
579	64	3	10	500	0.8651	0.884512	1.237239
580	16	4	6	20000	0.9237	0.819313	1.234704
581	16	4	4	2000	0.944	0.793906	1.233460
582	16	5	8	20000	0.9644	0.767218	1.232352
583	64	4	1	500	0.8906	0.851433	1.232115
584	32	4	7	500	0.8524	0.888766	1.231459
585	8	4	7	5000	0.8906	0.846733	1.228871
586	16	3	7	2000	0.9135	0.821857	1.228793
587	16	3	3	2000	0.8906	0.846610	1.228786
588	8	4	4	2000	0.9033	0.830734	1.227220
589	16	5	6	10000	0.9618	0.762057	1.227107
590	32	4	3	1000	0.9364	0.791601	1.226164
591	32	3	6	1000	0.8601	0.872113	1.224889
592	16	3	4	2000	0.916	0.812171	1.224205
593	64	4	3	500	0.9033	0.824784	1.223200
594	32	5	8	1000	0.8957	0.831420	1.222104
595	64	5	9	500	0.888	0.839183	1.221791
596	8	3	5	10000	0.8906	0.834003	1.220135
597	16	5	8	2000	0.9237	0.796159	1.219464
598	64	5	4	500	0.8575	0.863133	1.216678
599	32	3	6	500	0.888	0.826376	1.213030
600	8	3	1	5000	0.944	0.759979	1.211901
601	16	3	6	2000	0.9567	0.743294	1.211512
602	32	4	1	1000	0.9186	0.789503	1.211256
603	16	5	2	5000	0.9288	0.770112	1.206541
604	32	4	10	500	0.8499	0.856134	1.206356
605	16	3	4	1000	0.8728	0.831219	1.205282
606	8	4	1	5000	0.8244	0.878763	1.204931
607	16	3	3	1000	0.9033	0.795518	1.203661
608	32	5	5	2000	0.8982	0.801255	1.203650
609	32	3	2	1000	0.8753	0.825619	1.203244
610	32	5	2	1000	0.8931	0.800115	1.199088
611	8	5	9	5000	0.8295	0.865800	1.199033
612	16	5	7	5000	0.9237	0.762549	1.197791
613	128	3	10	100	0.7913	0.896011	1.195404
614	16	3	8	2000	0.8422	0.847568	1.194852
615	16	5	3	2000	0.9059	0.779073	1.194826
616	16	5	1	20000	0.9033	0.781060	1.194155
617	8	4	6	20000	0.8321	0.853991	1.192347
618	16	5	9	5000	0.9415	0.726453	1.189183
619	64	5	1	500	0.8422	0.839436	1.189098
620	16	5	4	20000	0.8295	0.851368	1.188654
621	32	5	6	1000	0.9084	0.759985	1.184385
622	8	4	10	20000	0.8295	0.844738	1.183914
623	16	3	2	1000	0.799	0.870294	1.181445
624	32	4	3	500	0.8372	0.826040	1.176115

Supplementary Table 4. Continued.

No.	node	layers	replicate	epoch	Test_Accuracy	Test_AUC	Euclidean length
625	8	4	3	10000	0.8321	0.826525	1.172832
626	64	5	6	500	0.8779	0.777499	1.172695
627	32	3	3	500	0.799	0.857183	1.171820
628	32	5	10	500	0.8651	0.788383	1.170447
629	128	3	4	100	0.7608	0.887568	1.169014
630	16	4	1	2000	0.8906	0.749963	1.164308
631	32	5	8	500	0.8015	0.838685	1.160084
632	32	4	10	1000	0.8906	0.743274	1.160011
633	16	3	6	1000	0.8397	0.798710	1.158894
634	64	5	2	500	0.8422	0.795376	1.158414
635	8	3	4	500	0.715	0.910372	1.157584
636	8	4	9	10000	0.8321	0.802563	1.156070
637	8	5	6	10000	0.8295	0.804550	1.155583
638	32	3	9	500	0.827	0.805858	1.154702
639	128	3	9	100	0.7761	0.854029	1.153992
640	8	5	1	20000	0.8321	0.798969	1.153578
641	32	3	8	1000	0.8626	0.765709	1.153425
642	8	4	9	5000	0.827	0.802971	1.152688
643	32	5	5	500	0.7888	0.839895	1.152228
644	16	5	2	2000	0.9186	0.695395	1.152128
645	8	3	8	2000	0.916	0.697097	1.151087
646	32	4	6	1000	0.883	0.735835	1.149410
647	16	4	10	1000	0.827	0.796017	1.147855
648	128	3	1	100	0.8092	0.813421	1.147370
649	16	5	5	2000	0.855	0.762621	1.145694
650	8	3	9	20000	0.8321	0.785036	1.143972
651	16	5	5	20000	0.8295	0.782724	1.140495
652	64	5	7	500	0.7939	0.818225	1.140074
653	16	4	8	2000	0.9466	0.632526	1.138482
654	8	4	2	2000	0.8372	0.770578	1.137846
655	16	4	3	1000	0.8601	0.744129	1.137321
656	16	5	6	5000	0.8321	0.774871	1.137020
657	64	5	3	500	0.8168	0.790766	1.136870
658	16	5	9	10000	0.8346	0.771743	1.136726
659	16	5	1	2000	0.8499	0.752766	1.135336
660	16	5	1	5000	0.8295	0.772352	1.133401
661	8	3	5	500	0.7684	0.833129	1.133377
662	16	5	5	5000	0.8295	0.771989	1.133154
663	8	4	5	2000	0.8142	0.787788	1.132930
664	8	5	5	20000	0.8321	0.767943	1.132310
665	16	4	9	1000	0.7761	0.823178	1.131350
666	128	3	8	100	0.7481	0.847723	1.130614
667	64	4	5	100	0.7226	0.868656	1.129917
668	16	4	10	5000	0.8473	0.746732	1.129392
669	8	5	10	5000	0.8066	0.790073	1.129079
670	8	3	7	1000	0.5751	0.971136	1.128648
671	8	4	4	20000	0.8321	0.761973	1.128270
672	64	4	3	100	0.7455	0.845250	1.127039
673	32	4	8	500	0.7913	0.799209	1.124673
674	8	3	10	20000	0.8295	0.759163	1.124455
675	64	5	10	500	0.7964	0.793466	1.124207
676	8	5	3	20000	0.8295	0.758354	1.123909

Supplementary Table 4. Continued.

No.	node	layers	replicate	epoch	Test_Accuracy	Test_AUC	Euclidean length
677	128	5	6	100	0.7913	0.798017	1.123827
678	8	4	5	10000	0.827	0.760329	1.123400
679	128	4	8	100	0.7634	0.823935	1.123232
680	8	5	1	5000	0.8142	0.773634	1.123135
681	16	5	9	20000	0.8321	0.753543	1.122594
682	8	3	4	1000	0.7201	0.860996	1.122434
683	16	4	8	1000	0.855	0.726026	1.121668
684	64	3	3	100	0.6997	0.876335	1.121402
685	8	4	5	20000	0.8321	0.750986	1.120879
686	16	5	1	10000	0.8448	0.734650	1.119553
687	8	4	3	5000	0.8321	0.748415	1.119159
688	64	5	8	500	0.8219	0.758898	1.118680
689	128	3	6	100	0.7583	0.821948	1.118310
690	8	4	7	2000	0.7939	0.786991	1.117870
691	128	5	4	100	0.7023	0.869258	1.117513
692	32	3	7	500	0.8397	0.737221	1.117403
693	16	4	2	2000	0.8753	0.692487	1.116104
694	8	4	7	20000	0.8321	0.740341	1.113776
695	32	4	5	1000	0.8295	0.742517	1.113284
696	8	4	4	10000	0.8295	0.740031	1.111627
697	128	4	3	100	0.7608	0.808979	1.110524
698	8	3	8	1000	0.8422	0.723728	1.110442
699	64	4	9	100	0.7125	0.850844	1.109771
700	8	4	2	20000	0.8321	0.733174	1.109024
701	8	5	7	20000	0.8321	0.732015	1.108258
702	128	4	9	100	0.7888	0.777946	1.107883
703	16	4	5	10000	0.9109	0.630143	1.107619
704	8	5	8	20000	0.8321	0.730746	1.107421
705	128	4	5	100	0.743	0.819390	1.106096
706	8	4	1	20000	0.8321	0.728350	1.105841
707	8	5	5	100	0.5471	0.960693	1.105553
708	8	5	6	20000	0.8321	0.725838	1.104188
709	8	3	7	5000	0.8244	0.734508	1.104145
710	8	4	8	5000	0.827	0.731517	1.104104
711	16	5	7	10000	0.8321	0.725275	1.103818
712	32	5	9	1000	0.8626	0.687126	1.102824
713	64	3	2	100	0.7176	0.836147	1.101858
714	16	5	6	2000	0.9186	0.607586	1.101357
715	8	3	6	2000	0.9033	0.629567	1.101047
716	32	3	2	500	0.7964	0.760154	1.100948
717	16	5	4	5000	0.8906	0.644148	1.099134
718	16	5	2	1000	0.57	0.939585	1.098963
719	128	5	5	100	0.6667	0.873369	1.098755
720	16	5	8	5000	0.8397	0.708460	1.098641
721	8	4	4	5000	0.8295	0.719260	1.097910
722	8	4	7	10000	0.827	0.720911	1.097106
723	8	4	10	10000	0.8295	0.716003	1.095779
724	16	4	3	500	0.715	0.828973	1.094724
725	32	5	1	1000	0.8702	0.664090	1.094652
726	16	5	10	500	0.7328	0.813162	1.094636
727	32	4	4	500	0.8499	0.688188	1.093587
728	32	3	6	100	0.7583	0.787483	1.093229

Supplementary Table 4. Continued.

No.	node	layers	replicate	epoch	Test_Accuracy	Test_AUC	Euclidean length
729	128	4	10	100	0.7201	0.821896	1.092729
730	16	5	3	1000	0.7557	0.788558	1.092202
731	16	3	5	1000	0.7735	0.770831	1.092008
732	32	5	3	1000	0.8168	0.723682	1.091274
733	8	5	7	2000	0.5776	0.924357	1.089980
734	16	4	6	1000	0.8397	0.694605	1.089758
735	8	3	3	500	0.542	0.944998	1.089397
736	8	3	5	20000	0.8346	0.699694	1.089095
737	32	5	4	1000	0.7786	0.760834	1.088616
738	16	3	9	1000	0.7786	0.760678	1.088508
739	16	5	7	2000	0.827	0.706492	1.087686
740	16	4	2	500	0.7048	0.826745	1.086394
741	16	3	7	1000	0.8397	0.685255	1.083822
742	32	3	10	500	0.8142	0.714119	1.082999
743	32	4	7	100	0.6565	0.860252	1.082139
744	16	4	1	1000	0.8397	0.682381	1.082007
745	8	5	5	10000	0.7939	0.733459	1.080851
746	32	3	4	500	0.8117	0.712805	1.080254
747	128	4	1	100	0.7786	0.748733	1.080194
748	8	5	6	5000	0.8066	0.716042	1.078573
749	8	5	5	5000	0.8219	0.698256	1.078463
750	8	5	6	1000	0.5674	0.917002	1.078348
751	64	4	10	100	0.7176	0.800161	1.074805
752	8	3	9	5000	0.8066	0.708273	1.073431
753	8	5	10	1000	0.5827	0.901281	1.073242
754	64	5	5	500	0.799	0.716139	1.072966
755	128	3	7	100	0.7201	0.794683	1.072411
756	64	4	2	100	0.7506	0.765470	1.072075
757	32	3	1	500	0.7735	0.740963	1.071134
758	8	3	7	500	0.5827	0.898348	1.070780
759	128	4	4	100	0.7735	0.740180	1.070592
760	128	5	8	100	0.6489	0.851407	1.070498
761	8	5	3	2000	0.5751	0.902712	1.070341
762	8	5	7	1000	0.5598	0.909854	1.068274
763	8	4	3	20000	0.8321	0.669438	1.067959
764	32	5	10	1000	0.8244	0.678295	1.067577
765	16	5	10	5000	0.827	0.674715	1.067319
766	8	5	7	5000	0.8219	0.680574	1.067099
767	128	3	5	100	0.8066	0.695162	1.064825
768	32	4	2	1000	0.8321	0.663067	1.063977
769	32	3	5	500	0.7634	0.741021	1.063904
770	128	4	7	100	0.7379	0.766201	1.063748
771	8	5	1	10000	0.827	0.667664	1.062875
772	8	3	6	5000	0.8117	0.685812	1.062636
773	128	4	2	100	0.7455	0.756651	1.062210
774	16	5	6	100	0.5827	0.888021	1.062130
775	8	4	2	5000	0.8244	0.669483	1.062000
776	16	4	7	2000	0.8346	0.656022	1.061566
777	64	3	4	100	0.6947	0.802420	1.061361
778	16	3	1	100	0.5598	0.901385	1.061071
779	64	4	4	100	0.7379	0.760549	1.059684
780	32	4	5	500	0.8015	0.691821	1.058781

Supplementary Table 4. Continued.

No.	node	layers	replicate	epoch	Test_Accuracy	Test_AUC	Euclidean length
781	64	3	9	100	0.6997	0.791426	1.056379
782	8	5	8	5000	0.5878	0.877409	1.056104
783	8	4	2	10000	0.8219	0.662575	1.055711
784	32	3	8	500	0.7964	0.690267	1.053908
785	32	5	6	500	0.7659	0.723255	1.053423
786	8	5	9	1000	0.5929	0.870119	1.052919
787	8	5	3	10000	0.8295	0.648033	1.052624
788	128	5	2	100	0.7303	0.755032	1.050434
789	64	3	5	100	0.6896	0.791219	1.049560
790	16	4	4	500	0.7099	0.772255	1.048969
791	16	5	10	1000	0.8142	0.660898	1.048669
792	16	5	3	20000	0.8321	0.638211	1.048667
793	8	3	6	500	0.57	0.880109	1.048567
794	8	5	1	1000	0.5674	0.876542	1.044159
795	8	4	10	1000	0.5802	0.867315	1.043488
796	8	5	4	10000	0.8321	0.629379	1.043316
797	32	5	4	500	0.7099	0.764343	1.043158
798	16	4	1	5000	0.8295	0.632429	1.043090
799	32	3	4	100	0.7583	0.715738	1.042737
800	16	4	4	5000	0.8346	0.624698	1.042499
801	32	4	4	100	0.6514	0.813874	1.042455
802	8	4	9	20000	0.8295	0.628479	1.040700
803	16	4	2	1000	0.7863	0.681507	1.040538
804	32	4	6	500	0.7812	0.684834	1.038880
805	8	4	9	1000	0.5674	0.870112	1.038768
806	8	5	2	5000	0.8015	0.660276	1.038444
807	128	4	6	100	0.6947	0.771264	1.038006
808	128	5	1	100	0.7328	0.734624	1.037627
809	8	3	10	1000	0.7252	0.741863	1.037437
810	8	3	7	2000	0.8244	0.628259	1.036506
811	32	5	10	100	0.6234	0.826221	1.035021
812	128	3	3	100	0.715	0.748325	1.034995
813	8	4	9	2000	0.7939	0.663287	1.034518
814	32	5	2	500	0.7125	0.749930	1.034433
815	16	4	5	500	0.715	0.747049	1.034073
816	32	5	2	100	0.598	0.843262	1.033777
817	128	3	2	100	0.715	0.743197	1.031294
818	8	3	2	1000	0.5623	0.863547	1.030483
819	64	3	7	100	0.659	0.790170	1.028907
820	64	3	8	100	0.6107	0.827710	1.028620
821	64	5	8	100	0.6005	0.834774	1.028323
822	8	3	9	2000	0.7455	0.708091	1.028185
823	16	4	10	500	0.6921	0.760024	1.027930
824	8	5	2	2000	0.5776	0.847374	1.025507
825	64	4	7	100	0.7125	0.737208	1.025247
826	8	5	9	10000	0.8168	0.619596	1.025213
827	32	3	2	100	0.598	0.832521	1.025034
828	32	5	7	1000	0.7557	0.691659	1.024439
829	16	5	5	1000	0.7303	0.715453	1.022356
830	64	3	1	100	0.7048	0.739856	1.021827
831	8	3	9	500	0.5674	0.848688	1.020889
832	32	4	2	500	0.7786	0.659868	1.020610

Supplementary Table 4. Continued.

No.	node	layers	replicate	epoch	Test_Accuracy	Test_AUC	Euclidean length
833	16	3	9	2000	0.771	0.668363	1.020368
834	8	5	4	1000	0.5573	0.854437	1.020121
835	8	5	2	20000	0.8295	0.592849	1.019579
836	8	5	1	2000	0.5852	0.834878	1.019549
837	8	3	1	2000	0.8193	0.601674	1.016496
838	16	4	2	100	0.5598	0.847995	1.016106
839	128	5	7	100	0.6845	0.749076	1.014719
840	8	4	5	5000	0.8321	0.578838	1.013629
841	8	5	10	10	0.3842	0.937772	1.013423
842	32	5	7	500	0.7354	0.696618	1.012961
843	128	5	3	10	0.5623	0.842518	1.012925
844	16	5	9	2000	0.7837	0.641739	1.012924
845	128	3	7	10	0.5903	0.822090	1.012070
846	8	3	1	100	0.5013	0.878627	1.011576
847	64	5	3	100	0.5598	0.841508	1.010698
848	16	4	7	500	0.5725	0.832793	1.010594
849	8	3	10	2000	0.8448	0.551632	1.008952
850	32	3	5	100	0.6947	0.729632	1.007458
851	16	4	4	10	0.4148	0.917144	1.006585
852	8	5	4	20000	0.8295	0.569890	1.006402
853	8	5	5	2000	0.5852	0.816982	1.004947
854	128	5	10	100	0.6819	0.737693	1.004579
855	16	5	1	500	0.7277	0.691698	1.003988
856	8	4	8	2000	0.7786	0.633801	1.003953
857	16	4	9	500	0.6768	0.739273	1.002289
858	64	4	10	10	0.4377	0.901236	1.001902
859	16	3	8	500	0.7252	0.690403	1.001285
860	128	5	6	10	0.5064	0.862207	0.999921
861	16	5	3	5000	0.8321	0.554429	0.999891
862	16	3	5	500	0.7405	0.669114	0.998025
863	32	5	7	100	0.4122	0.908494	0.997632
864	128	5	9	100	0.6412	0.764032	0.997438
865	8	5	7	10000	0.8219	0.562296	0.995839
866	8	4	4	1000	0.6539	0.750766	0.995607
867	128	3	9	10	0.5471	0.829031	0.993283
868	16	3	6	500	0.7252	0.677952	0.992741
869	64	4	6	100	0.7074	0.695951	0.992352
870	128	5	3	100	0.6336	0.762582	0.991453
871	16	3	7	500	0.5674	0.808403	0.987653
872	16	5	9	500	0.5878	0.792922	0.987033
873	16	3	4	500	0.5852	0.792378	0.985049
874	16	3	2	500	0.6412	0.747211	0.984613
875	8	4	2	1000	0.6794	0.712196	0.984280
876	16	5	9	1000	0.8066	0.561952	0.983053
877	64	5	5	100	0.5802	0.793013	0.982599
878	64	5	6	100	0.6768	0.709613	0.980616
879	8	4	6	5000	0.7532	0.627120	0.980097
880	8	5	4	5000	0.8117	0.545222	0.977816
881	128	4	3	10	0.3969	0.893110	0.977331
882	16	3	2	10	0.4835	0.847361	0.975598
883	8	4	5	10	0.2163	0.950871	0.975162
884	8	3	1	1000	0.7405	0.634229	0.974980

Supplementary Table 4. Continued.

No.	node	layers	replicate	epoch	Test_Accuracy	Test_AUC	Euclidean length
885	8	5	8	500	0.5725	0.785923	0.972333
886	16	5	7	100	0.4198	0.872761	0.968475
887	64	5	9	10	0.4122	0.875642	0.967811
888	16	4	4	1000	0.5598	0.788416	0.966941
889	32	5	9	500	0.7023	0.661875	0.965041
890	32	3	1	100	0.5573	0.787386	0.964655
891	128	4	6	10	0.514	0.816172	0.964538
892	8	3	9	100	0.4478	0.853622	0.963947
893	8	3	6	1000	0.5751	0.772339	0.962937
894	8	3	2	100	0.5496	0.789659	0.962092
895	32	3	3	100	0.6107	0.743404	0.962083
896	16	5	7	500	0.5674	0.774469	0.960076
897	16	5	10	2000	0.7048	0.651671	0.959906
898	8	3	4	2000	0.7913	0.537019	0.956318
899	32	5	1	500	0.6794	0.672889	0.956224
900	32	3	1	10	0.4046	0.866299	0.956125
901	64	5	9	100	0.5776	0.760147	0.954697
902	8	5	3	5000	0.687	0.662400	0.954328
903	8	5	8	10	0.4122	0.856794	0.950792
904	128	5	8	10	0.5522	0.773880	0.950692
905	64	5	10	100	0.6285	0.712462	0.950060
906	64	3	6	100	0.6819	0.659901	0.948924
907	64	5	7	100	0.7201	0.617285	0.948464
908	16	4	3	100	0.5725	0.755958	0.948277
909	16	5	2	500	0.598	0.735822	0.948176
910	8	3	8	500	0.5623	0.761643	0.946721
911	16	3	1	1000	0.659	0.679558	0.946615
912	16	4	8	500	0.598	0.733556	0.946419
913	16	5	4	1000	0.5827	0.739629	0.941589
914	64	4	8	100	0.7226	0.599946	0.939194
915	64	5	4	100	0.6336	0.692067	0.938299
916	8	5	7	100	0.4148	0.841145	0.937861
917	16	3	4	100	0.5674	0.744285	0.935896
918	32	4	9	500	0.6921	0.629567	0.935605
919	64	3	10	100	0.7303	0.584271	0.935259
920	8	5	8	1000	0.4885	0.796580	0.934437
921	8	3	9	1000	0.6997	0.619253	0.934374
922	32	4	2	100	0.659	0.660360	0.932929
923	8	5	8	10000	0.8244	0.434655	0.931966
924	16	4	5	1000	0.7226	0.587968	0.931588
925	32	3	7	100	0.7048	0.609042	0.931491
926	32	5	8	100	0.5776	0.730532	0.931289
927	8	4	10	100	0.4122	0.833408	0.929773
928	16	5	4	500	0.5674	0.734255	0.927941
929	64	4	1	100	0.7023	0.606252	0.927775
930	32	3	10	100	0.57	0.728823	0.925248
931	16	3	10	100	0.6209	0.682076	0.922358
932	64	5	2	100	0.6412	0.660134	0.920279
933	16	3	10	1000	0.7226	0.569457	0.920017
934	128	3	2	10	0.5776	0.715932	0.919881
935	32	4	6	100	0.5318	0.746473	0.916533
936	32	4	1	500	0.7354	0.545721	0.915764

Supplementary Table 4. Continued.

No.	node	layers	replicate	epoch	Test_Accuracy	Test_AUC	Euclidean length
937	16	4	1	500	0.57	0.715693	0.914940
938	16	3	8	1000	0.6641	0.628835	0.914583
939	16	4	8	100	0.4911	0.771064	0.914176
940	16	4	7	100	0.4071	0.816755	0.912589
941	16	4	6	500	0.6565	0.632740	0.911785
942	16	5	2	100	0.5522	0.722083	0.909026
943	8	5	10	10000	0.5878	0.692358	0.908223
944	16	5	5	500	0.57	0.704925	0.906543
945	16	4	7	10	0.3613	0.830727	0.905895
946	8	4	8	10000	0.8219	0.380935	0.905887
947	8	3	5	1000	0.5751	0.698761	0.904990
948	64	4	5	10	0.3817	0.820426	0.904872
949	8	4	10	5000	0.5802	0.694281	0.904797
950	8	4	6	10	0.2443	0.871148	0.904755
951	64	4	9	10	0.5674	0.702892	0.903327
952	8	4	9	500	0.4427	0.787354	0.903277
953	8	4	1	1000	0.5852	0.687152	0.902573
954	32	5	3	500	0.687	0.584238	0.901833
955	128	3	6	10	0.4529	0.778432	0.900597
956	128	4	2	10	0.626	0.643759	0.897943
957	8	5	8	2000	0.7125	0.543526	0.896145
958	64	3	5	10	0.5318	0.720516	0.895519
959	16	4	4	100	0.4402	0.776832	0.892886
960	128	3	1	10	0.4835	0.749736	0.892119
961	8	4	4	500	0.4606	0.761462	0.889931
962	8	5	5	1000	0.5573	0.691102	0.887809
963	32	3	2	10	0.4351	0.773161	0.887181
964	8	5	7	500	0.7125	0.526854	0.886133
965	8	3	5	2000	0.5751	0.673355	0.885521
966	16	3	3	500	0.6438	0.607683	0.885300
967	8	5	10	20000	0.5878	0.657771	0.882140
968	16	5	6	500	0.6616	0.583241	0.881978
969	32	4	9	10	0.3613	0.801125	0.878828
970	64	4	4	10	0.4148	0.774605	0.878676
971	8	4	10	2000	0.5802	0.659033	0.878041
972	32	5	1	10	0.4377	0.760672	0.877612
973	32	3	9	100	0.631	0.609424	0.877245
974	8	3	2	10	0.2748	0.831647	0.875872
975	8	5	2	500	0.4122	0.771459	0.874675
976	16	5	6	1000	0.7455	0.456325	0.874073
977	32	4	3	10	0.3791	0.786758	0.873330
978	16	5	8	1000	0.6539	0.577323	0.872289
979	16	3	9	500	0.626	0.607081	0.872022
980	8	4	6	1000	0.6972	0.523014	0.871568
981	8	5	9	2000	0.6514	0.576566	0.869914
982	8	5	2	100	0.4198	0.760704	0.868851
983	8	5	2	10000	0.5751	0.648797	0.866993
984	32	4	8	100	0.6387	0.586032	0.866816
985	8	4	4	100	0.4453	0.743365	0.866536
986	16	5	2	10	0.3766	0.774223	0.860958
987	16	3	6	100	0.486	0.710014	0.860416
988	128	5	9	10	0.4402	0.736897	0.858367

Supplementary Table 4. Continued.

No.	node	layers	replicate	epoch	Test_Accuracy	Test_AUC	Euclidean length
989	8	4	1	2000	0.6921	0.507650	0.858319
990	128	4	9	10	0.6056	0.606737	0.857252
991	128	4	8	10	0.3969	0.759584	0.857028
992	32	5	2	10	0.173	0.838885	0.856538
993	8	3	6	10	0.257	0.816224	0.855728
994	16	5	3	500	0.6209	0.581920	0.850969
995	64	4	6	10	0.3868	0.757486	0.850529
996	8	5	10	100	0.4122	0.742472	0.849219
997	128	4	10	10	0.4148	0.736878	0.845605
998	16	3	5	100	0.5216	0.664763	0.844971
999	8	5	4	2000	0.5929	0.600483	0.843866
1000	16	3	3	100	0.514	0.669224	0.843835
1001	16	5	4	100	0.5623	0.628810	0.843554
1002	32	5	9	10	0.4402	0.718146	0.842324
1003	16	3	10	500	0.6132	0.575115	0.840697
1004	8	4	3	1000	0.5471	0.636547	0.839351
1005	16	3	7	10	0.5191	0.655964	0.836513
1006	8	5	3	10	0.1858	0.815402	0.836302
1007	16	5	7	1000	0.6081	0.573652	0.835980
1008	8	5	9	100	0.1781	0.815985	0.835195
1009	16	3	5	10	0.4427	0.703022	0.830796
1010	8	4	4	10	0.4122	0.719940	0.829592
1011	32	5	3	10	0.4377	0.702763	0.827923
1012	8	5	1	500	0.4122	0.716554	0.826655
1013	16	5	3	100	0.57	0.598133	0.826234
1014	16	5	8	100	0.514	0.646589	0.825998
1015	32	5	6	100	0.5394	0.625300	0.825804
1016	16	3	8	100	0.5852	0.582315	0.825560
1017	8	4	10	500	0.4122	0.710733	0.821614
1018	16	4	6	100	0.5242	0.631548	0.820755
1019	8	5	2	1000	0.4122	0.708402	0.819599
1020	64	5	6	10	0.4606	0.677810	0.819499
1021	32	5	1	100	0.4936	0.651788	0.817599
1022	8	4	6	500	0.4453	0.685314	0.817280
1023	64	5	1	100	0.5369	0.609560	0.812296
1024	16	5	1	100	0.3155	0.748519	0.812294
1025	16	3	9	100	0.3944	0.709606	0.811845
1026	64	4	1	10	0.3282	0.740834	0.810277
1027	32	5	3	100	0.5725	0.571775	0.809124
1028	128	3	8	10	0.5064	0.627476	0.806329
1029	128	5	7	10	0.3053	0.744744	0.804893
1030	8	3	5	100	0.5165	0.616365	0.804163
1031	8	4	10	10	0.2697	0.757428	0.804012
1032	16	4	2	10	0.4173	0.686039	0.802987
1033	16	5	10	100	0.4326	0.676152	0.802698
1034	8	3	4	5000	0.6489	0.470544	0.801550
1035	16	4	6	10	0.2036	0.774851	0.801154
1036	8	4	7	500	0.5852	0.543047	0.798347
1037	32	4	7	10	0.2468	0.758988	0.798106
1038	8	4	2	10	0.2799	0.747315	0.798012
1039	8	3	8	10	0.3461	0.717305	0.796437
1040	8	4	2	100	0.4122	0.679791	0.795000

Supplementary Table 4. Continued.

No.	node	layers	replicate	epoch	Test_Accuracy	Test_AUC	Euclidean length
1041	8	3	2	500	0.4122	0.676016	0.791774
1042	16	4	9	100	0.4148	0.672364	0.790021
1043	8	5	6	2000	0.7023	0.358817	0.788654
1044	16	4	7	1000	0.5802	0.533393	0.788124
1045	32	5	4	100	0.5369	0.576469	0.787768
1046	8	4	8	100	0.4046	0.674669	0.786689
1047	16	4	1	10	0.4148	0.668253	0.786525
1048	8	5	6	100	0.4835	0.620004	0.786242
1049	16	4	10	100	0.5115	0.595860	0.785291
1050	8	4	3	500	0.5471	0.559233	0.782343
1051	32	3	8	100	0.5725	0.532668	0.781979
1052	16	5	8	500	0.6056	0.494662	0.781947
1053	128	5	5	10	0.4097	0.664252	0.780439
1054	16	5	5	10	0.2087	0.751063	0.779520
1055	16	5	8	10	0.3817	0.675880	0.776214
1056	16	5	3	10	0.1679	0.757247	0.775637
1057	16	3	7	100	0.5445	0.546472	0.771435
1058	32	4	10	100	0.5954	0.489948	0.771071
1059	16	4	1	100	0.4122	0.651529	0.770973
1060	8	3	2	2000	0.5725	0.507598	0.765122
1061	8	4	7	10	0.4427	0.623649	0.764802
1062	8	5	9	10	0.2824	0.706278	0.760644
1063	16	3	1	500	0.5547	0.517414	0.758557
1064	64	3	7	10	0.3868	0.651361	0.757552
1065	64	3	8	10	0.4784	0.587035	0.757283
1066	8	4	1	500	0.5522	0.513827	0.754283
1067	128	3	3	10	0.4173	0.627269	0.753396
1068	16	4	9	10	0.2265	0.716314	0.751271
1069	8	5	4	500	0.5318	0.530473	0.751141
1070	8	4	5	1000	0.5344	0.526996	0.750539
1071	8	3	9	10	0.3155	0.680807	0.750359
1072	32	4	8	10	0.4198	0.617880	0.746999
1073	16	3	6	10	0.3511	0.656851	0.744798
1074	16	3	10	10	0.0204	0.744440	0.744719
1075	128	4	7	10	0.4631	0.575057	0.738344
1076	32	4	3	100	0.542	0.500767	0.737924
1077	8	3	10	100	0.3562	0.646129	0.737808
1078	16	5	1	1000	0.5827	0.451877	0.737382
1079	16	5	9	100	0.4148	0.608421	0.736366
1080	64	3	4	10	0.3232	0.659299	0.734257
1081	64	5	8	10	0.4707	0.562205	0.733235
1082	8	5	3	500	0.4122	0.606077	0.732965
1083	8	4	8	1000	0.4402	0.585371	0.732418
1084	16	5	1	10	0.1985	0.693718	0.721558
1085	32	5	9	100	0.4097	0.592966	0.720738
1086	64	5	4	10	0.5344	0.474810	0.714862
1087	8	5	10	2000	0.4122	0.580528	0.711984
1088	16	3	8	10	0.3511	0.616585	0.709541
1089	32	3	8	10	0.2137	0.671387	0.704576
1090	8	3	3	1000	0.5191	0.470854	0.700834
1091	128	5	4	10	0.369	0.587857	0.694073
1092	32	4	1	100	0.6336	0.278785	0.692221

Supplementary Table 4. Continued.

No.	node	layers	replicate	epoch	Test_Accuracy	Test_AUC	Euclidean length
1093	32	3	9	10	0.369	0.584640	0.691350
1094	128	3	10	10	0.5089	0.467028	0.690720
1095	32	5	10	10	0.3562	0.589910	0.689110
1096	8	5	6	10	0.2443	0.644310	0.689070
1097	16	3	9	10	0.4097	0.550363	0.686115
1098	16	3	4	10	0.1603	0.665909	0.684931
1099	128	3	5	10	0.4148	0.544445	0.684456
1100	128	5	10	10	0.4097	0.541454	0.678989
1101	32	3	4	10	0.2417	0.632901	0.677483
1102	32	4	9	100	0.486	0.470926	0.676733
1103	64	4	2	10	0.4071	0.539952	0.676224
1104	128	4	5	10	0.4071	0.539116	0.675557
1105	8	4	2	500	0.4122	0.535089	0.675448
1106	8	3	3	100	0.4377	0.506549	0.669457
1107	8	4	8	500	0.5547	0.374085	0.669053
1108	64	5	1	10	0.3511	0.566219	0.666240
1109	8	5	5	10	0.3613	0.558391	0.665085
1110	16	3	3	10	0.3791	0.537576	0.657803
1111	32	4	5	10	0.43	0.497556	0.657618
1112	32	5	4	10	0.2901	0.587087	0.654850
1113	8	4	5	500	0.5649	0.329099	0.653772
1114	16	3	2	100	0.4427	0.479705	0.652764
1115	8	4	6	2000	0.5725	0.311093	0.651564
1116	8	3	4	100	0.4198	0.497834	0.651207
1117	64	3	1	10	0.2545	0.597349	0.649305
1118	16	4	5	100	0.4453	0.469967	0.647427
1119	128	4	1	10	0.4249	0.477556	0.639218
1120	8	4	3	2000	0.5751	0.259646	0.630996
1121	8	3	6	100	0.3613	0.516384	0.630230
1122	32	5	6	10	0.2621	0.573141	0.630227
1123	8	5	5	500	0.4122	0.470285	0.625361
1124	8	3	7	100	0.514	0.337257	0.614767
1125	8	4	7	100	0.4809	0.382774	0.614638
1126	32	5	5	100	0.5318	0.303660	0.612389
1127	32	3	3	10	0.1908	0.579020	0.609646
1128	8	4	6	100	0.3969	0.454182	0.603168
1129	8	4	5	100	0.2977	0.516391	0.596057
1130	8	3	7	10	0.374	0.464017	0.595977
1131	16	4	3	10	0.3766	0.461822	0.595909
1132	16	4	8	10	0.4122	0.424936	0.592013
1133	64	4	8	10	0.3206	0.493056	0.588123
1134	128	5	2	10	0.3028	0.498572	0.583320
1135	8	3	1	500	0.5064	0.287551	0.582346
1136	8	5	10	500	0.4173	0.404748	0.581344
1137	8	4	9	100	0.1603	0.555355	0.578027
1138	8	3	8	100	0.4071	0.407138	0.575753
1139	32	4	1	10	0.1654	0.551295	0.575572
1140	64	4	3	10	0.3639	0.443441	0.573640
1141	8	4	3	10	0.2723	0.503318	0.572256
1142	8	3	5	10	0.3486	0.438430	0.560127
1143	32	4	10	10	0.4173	0.368763	0.556889
1144	32	4	4	10	0.0382	0.547864	0.549194

Supplementary Table 4. Continued.

No.	node	layers	replicate	epoch	Test_Accuracy	Test_AUC	Euclidean length
1145	128	5	1	10	0.4097	0.359711	0.545203
1146	16	5	10	10	0.201	0.506536	0.544959
1147	64	4	7	10	0.4885	0.220435	0.535933
1148	8	3	10	500	0.4148	0.335897	0.533747
1149	8	4	1	100	0.4173	0.328866	0.531312
1150	64	5	5	10	0.486	0.199496	0.525352
1151	64	5	3	10	0.4046	0.329818	0.521997
1152	32	4	5	100	0.4682	0.228405	0.520942
1153	64	3	6	10	0.2137	0.474454	0.520360
1154	16	5	5	100	0.3868	0.342022	0.516327
1155	8	4	7	1000	0.4097	0.310148	0.513854
1156	16	5	9	10	0.3282	0.391171	0.510617
1157	64	3	9	10	0.3537	0.368083	0.510479
1158	32	5	5	10	0.4122	0.300766	0.510264
1159	16	5	6	10	0.1654	0.480916	0.508564
1160	8	5	8	100	0.4631	0.189137	0.500234
1161	32	5	8	10	0.2265	0.444982	0.499311
1162	64	5	7	10	0.3588	0.340209	0.494449
1163	8	3	10	10	0.3104	0.376875	0.488245
1164	32	3	10	10	0.2901	0.387468	0.484034
1165	64	3	2	10	0.3333	0.347532	0.481526
1166	16	5	4	10	0.3206	0.357218	0.479989
1167	8	5	2	10	0.0611	0.475413	0.479323
1168	64	5	2	10	0.2901	0.379504	0.477683
1169	32	5	7	10	0.2392	0.412337	0.476695
1170	32	3	7	10	0.3333	0.335399	0.472844
1171	64	3	3	10	0.1679	0.441201	0.472068
1172	8	4	8	10	0.2799	0.380067	0.472012
1173	64	3	10	10	0.4046	0.241976	0.471438
1174	8	5	3	100	0.4097	0.216272	0.463279
1175	16	4	5	10	0.2748	0.368387	0.459591
1176	8	5	9	500	0.4122	0.200403	0.458334
1177	16	5	7	10	0.1628	0.428141	0.458049
1178	8	5	4	100	0.0916	0.443117	0.452486
1179	8	5	1	100	0.4122	0.122124	0.429911
1180	8	5	3	1000	0.4122	0.121302	0.429678
1181	8	5	4	10	0.3969	0.139023	0.420544
1182	8	5	7	10	0.1807	0.373236	0.414678
1183	8	5	6	500	0.4122	0.031467	0.413399
1184	8	5	1	10	0.1654	0.371411	0.406575
1185	32	4	2	10	0.2417	0.317749	0.399228
1186	32	3	6	10	0.3359	0.207337	0.394737
1187	8	4	3	100	0.3562	0.155443	0.388640
1188	8	4	9	10	0.2443	0.296486	0.384170
1189	8	3	4	10	0.1858	0.329125	0.377948
1190	8	3	1	10	0.1756	0.331255	0.374920
1191	32	3	5	10	0.3486	0.080026	0.357668
1192	32	4	6	10	0.2341	0.267991	0.355840
1193	8	3	3	10	0.2163	0.280759	0.354417
1194	16	3	1	10	0.2087	0.286315	0.354305
1195	128	3	4	10	0.3104	0.160661	0.349514
1196	128	4	4	10	0.3282	0.112969	0.347098

Supplementary Table 4. Continued.

No.	node	layers	replicate	epoch	Test_Accuracy	Test_AUC	Euclidean length
1197	64	5	10	10	0.2799	0.200312	0.344193
1198	8	4	1	10	0.1654	0.221076	0.276101
1199	16	4	10	10	0.2163	0.162060	0.270276

Supplementary Table 5. Models generated by support vector machine for fucosylation classification.

No.	Seed	Cost	Gamma	Training_Accuracy	Test_Accuracy	Test_AUC	Euclidean length
1	435	8.7	1	100.00	0.987277	0.998168	1.403943
2	435	9.2	1	100.00	0.987277	0.998168	1.403943
3	435	8.8	1	100.00	0.987277	0.998161	1.403938
4	435	9.5	1	100.00	0.987277	0.998161	1.403938
5	435	8.6	1	100.00	0.987277	0.998155	1.403934
6	435	8.5	1	100.00	0.987277	0.998142	1.403924
7	435	8.4	1	99.77	0.987277	0.998096	1.403892
8	435	8.2	1	99.77	0.987277	0.998090	1.403888
9	435	8.1	1	99.77	0.987277	0.998058	1.403865
10	435	8	1	100.00	0.987277	0.998019	1.403837
11	435	9.9	1	100.00	0.987277	0.997935	1.403777
12	649	8.4	1	100.00	0.987277	0.997889	1.403745
13	649	8.5	1	100.00	0.987277	0.997889	1.403745
14	649	8.6	1	100.00	0.987277	0.997837	1.403708
15	649	8.2	1	100.00	0.987277	0.997831	1.403703
16	649	8.1	1	100.00	0.987277	0.997818	1.403694
17	435	10.1	1	100.00	0.987277	0.997805	1.403685
18	649	8	1	100.00	0.987277	0.997779	1.403667
19	435	10.2	1	100.00	0.987277	0.997753	1.403648
20	649	9.3	1	100.00	0.987277	0.997727	1.403630
21	435	10.3	1	100.00	0.987277	0.997695	1.403607
22	649	9.4	1	100.00	0.987277	0.997676	1.403593
23	435	8.3	1	99.77	0.987277	0.997591	1.403533
24	649	9.6	1	100.00	0.987277	0.997494	1.403464
25	435	10.6	1	100.00	0.987277	0.997443	1.403427
26	649	9.7	1	100.00	0.987277	0.997410	1.403404
27	435	10.7	1	100.00	0.987277	0.997391	1.403390
28	435	9.8	1	100.00	0.987277	0.997384	1.403386
29	183	8.7	1	100.00	0.987277	0.997332	1.403349
30	649	9.8	1	100.00	0.987277	0.997332	1.403349
31	183	9.8	1	100.00	0.987277	0.997294	1.403321
32	649	9.9	1	100.00	0.987277	0.997287	1.403317
33	183	9	1	99.77	0.987277	0.997287	1.403317
34	183	8.6	1	99.77	0.987277	0.997287	1.403317
35	435	10.8	1	100.00	0.987277	0.997281	1.403312
36	183	9.1	1	99.77	0.987277	0.997268	1.403303
37	183	9.2	1	99.77	0.987277	0.997268	1.403303
38	183	10	1	100.00	0.987277	0.997261	1.403298
39	183	9.3	1	99.77	0.987277	0.997255	1.403294
40	183	9.6	1	100.00	0.987277	0.997248	1.403289
41	183	9.5	1	100.00	0.987277	0.997242	1.403285
42	435	9.4	1	100.00	0.987277	0.997235	1.403280
43	183	10.1	1	100.00	0.987277	0.997235	1.403280
44	649	10	1	100.00	0.987277	0.997196	1.403252
45	183	10.2	1	100.00	0.987277	0.997190	1.403248
46	183	8.5	1	99.77	0.987277	0.997190	1.403248
47	435	10.9	1	100.00	0.987277	0.997151	1.403220
48	183	10.3	1	100.00	0.987277	0.997145	1.403216
49	183	8.4	1	99.77	0.987277	0.997138	1.403211
50	649	10.1	1	100.00	0.987277	0.997073	1.403165
51	183	10.4	1	100.00	0.987277	0.997041	1.403142
52	183	9.9	1	100.00	0.987277	0.997022	1.403128

Supplementary Table 5. Continued.

No.	Seed	Cost	Gamma	Training_Accuracy	Test_Accuracy	Test_AUC	Euclidean length
53	183	10.5	1	100.00	0.987277	0.996996	1.403110
54	649	10.2	1	100.00	0.987277	0.996996	1.403110
55	435	11	1	100.00	0.987277	0.996976	1.403096
56	649	10.3	1	100.00	0.987277	0.996931	1.403064
57	183	10.6	1	100.00	0.987277	0.996905	1.403045
58	59	8.4	1	100.00	0.987277	0.996847	1.403004
59	183	8.2	1	99.77	0.987277	0.996827	1.402990
60	59	8.5	1	100.00	0.987277	0.996821	1.402986
61	649	10.5	1	100.00	0.987277	0.996776	1.402953
62	59	8.6	1	100.00	0.987277	0.996769	1.402949
63	59	8.2	1	100.00	0.987277	0.996763	1.402944
64	59	8.9	1	100.00	0.987277	0.996743	1.402930
65	59	9	1	100.00	0.987277	0.996737	1.402926
66	59	9.1	1	100.00	0.987277	0.996737	1.402926
67	183	10.8	1	100.00	0.987277	0.996730	1.402921
68	59	9.2	1	100.00	0.987277	0.996685	1.402889
69	649	10.6	1	100.00	0.987277	0.996679	1.402884
70	59	9.3	1	100.00	0.987277	0.996627	1.402848
71	183	10.9	1	100.00	0.987277	0.996627	1.402848
72	59	8	1	100.00	0.987277	0.996568	1.402806
73	183	8	1	99.77	0.987277	0.996568	1.402806
74	59	9.4	1	100.00	0.987277	0.996562	1.402802
75	183	8.3	1	99.77	0.987277	0.996543	1.402788
76	183	11	1	100.00	0.987277	0.996543	1.402788
77	183	10.7	1	100.00	0.987277	0.996510	1.402765
78	59	9.5	1	100.00	0.987277	0.996445	1.402719
79	59	9.6	1	100.00	0.987277	0.996420	1.402700
80	649	10.8	1	100.00	0.987277	0.996368	1.402664
81	59	9.7	1	100.00	0.987277	0.996342	1.402645
82	649	10.9	1	100.00	0.987277	0.996245	1.402576
83	59	9.8	1	100.00	0.987277	0.996238	1.402572
84	68	9	1	100.00	0.987277	0.996232	1.402567
85	68	8.4	1	100.00	0.987277	0.996232	1.402567
86	68	8.3	1	99.77	0.987277	0.996219	1.402558
87	68	8.5	1	100.00	0.987277	0.996212	1.402553
88	68	8.7	1	100.00	0.987277	0.996212	1.402553
89	68	8.9	1	100.00	0.987277	0.996212	1.402553
90	68	9.1	1	100.00	0.987277	0.996212	1.402553
91	68	8.8	1	100.00	0.987277	0.996199	1.402544
92	68	9.2	1	100.00	0.987277	0.996193	1.402539
93	352	9.4	1	100.00	0.987277	0.996173	1.402526
94	68	9.3	1	100.00	0.987277	0.996173	1.402526
95	158	8.3	1	100.00	0.987277	0.996154	1.402512
96	352	8.5	1	100.00	0.987277	0.996154	1.402512
97	352	9.3	1	100.00	0.987277	0.996148	1.402507
98	352	8.6	1	100.00	0.987277	0.996141	1.402503
99	59	9.9	1	100.00	0.987277	0.996141	1.402503
100	352	8.3	1	99.77	0.987277	0.996141	1.402503
101	158	8.5	1	100.00	0.987277	0.996135	1.402498
102	352	8.7	1	100.00	0.987277	0.996135	1.402498
103	352	8.8	1	100.00	0.987277	0.996135	1.402498
104	158	8.4	1	100.00	0.987277	0.996128	1.402493

Supplementary Table 5. Continued.

No.	Seed	Cost	Gamma	Training_Accuracy	Test_Accuracy	Test_AUC	Euclidean length
105	352	9	1	100.00	0.987277	0.996122	1.402489
106	435	9.1	1	100.00	0.987277	0.996122	1.402489
107	158	8.6	1	100.00	0.987277	0.996115	1.402484
108	649	11	1	100.00	0.987277	0.996115	1.402484
109	352	8.9	1	100.00	0.987277	0.996109	1.402480
110	352	9.1	1	100.00	0.987277	0.996109	1.402480
111	352	8.2	1	99.77	0.987277	0.996102	1.402475
112	158	8.2	1	100.00	0.987277	0.996089	1.402466
113	158	8.7	1	100.00	0.987277	0.996070	1.402452
114	158	9.3	1	100.00	0.987277	0.996070	1.402452
115	352	9.5	1	100.00	0.987277	0.996063	1.402447
116	68	9.4	1	100.00	0.987277	0.996057	1.402443
117	68	8.2	1	99.77	0.987277	0.996057	1.402443
118	158	9.2	1	100.00	0.987277	0.996044	1.402434
119	59	10	1	100.00	0.987277	0.996044	1.402434
120	158	8.8	1	100.00	0.987277	0.996038	1.402429
121	158	9	1	100.00	0.987277	0.996031	1.402424
122	158	8.9	1	100.00	0.987277	0.996031	1.402424
123	158	9.1	1	100.00	0.987277	0.996018	1.402415
124	352	8.1	1	99.77	0.987277	0.995992	1.402397
125	59	10.1	1	100.00	0.987277	0.995973	1.402383
126	158	8.1	1	100.00	0.987277	0.995966	1.402378
127	649	10.7	1	100.00	0.987277	0.995960	1.402374
128	68	9.5	1	100.00	0.987277	0.995947	1.402365
129	352	9.8	1	100.00	0.987277	0.995927	1.402351
130	158	8	1	100.00	0.987277	0.995915	1.402342
131	932	8.4	1	100.00	0.987277	0.995895	1.402328
132	158	9.4	1	100.00	0.987277	0.995889	1.402323
133	932	8.3	1	100.00	0.987277	0.995876	1.402314
134	932	8.5	1	100.00	0.987277	0.995850	1.402296
135	435	9.7	1	100.00	0.987277	0.995850	1.402296
136	649	9.1	1	100.00	0.987277	0.995850	1.402296
137	352	8	1	100.00	0.987277	0.995843	1.402291
138	68	9.6	1	100.00	0.987277	0.995837	1.402287
139	352	9.9	1	100.00	0.987277	0.995817	1.402273
140	352	10	1	100.00	0.987277	0.995779	1.402245
141	158	9.5	1	100.00	0.987277	0.995766	1.402236
142	68	9.7	1	100.00	0.987277	0.995753	1.402227
143	649	9.2	1	100.00	0.987277	0.995746	1.402222
144	352	10.1	1	100.00	0.987277	0.995733	1.402213
145	932	8.6	1	100.00	0.987277	0.995720	1.402204
146	932	8.2	1	100.00	0.987277	0.995707	1.402195
147	932	9.1	1	100.00	0.987277	0.995688	1.402181
148	352	10.2	1	100.00	0.987277	0.995681	1.402176
149	932	8.8	1	100.00	0.987277	0.995681	1.402176
150	961	9.1	1	100.00	0.987277	0.995675	1.402172
151	932	9	1	100.00	0.987277	0.995675	1.402172
152	932	9.2	1	100.00	0.987277	0.995675	1.402172
153	961	9	1	100.00	0.987277	0.995662	1.402162
154	961	8.8	1	100.00	0.987277	0.995662	1.402162
155	961	9.2	1	100.00	0.987277	0.995662	1.402162
156	435	9	1	100.00	0.987277	0.995662	1.402162

Supplementary Table 5. Continued.

No.	Seed	Cost	Gamma	Training_Accuracy	Test_Accuracy	Test_AUC	Euclidean length
157	932	8.7	1	100.00	0.987277	0.995656	1.402158
158	435	9.3	1	100.00	0.987277	0.995656	1.402158
159	961	8.6	1	100.00	0.987277	0.995649	1.402153
160	961	8.7	1	100.00	0.987277	0.995649	1.402153
161	961	8.5	1	100.00	0.987277	0.995630	1.402139
162	961	8.4	1	100.00	0.987277	0.995623	1.402135
163	158	9.6	1	100.00	0.987277	0.995623	1.402135
164	435	8.9	1	100.00	0.987277	0.995623	1.402135
165	686	8.4	1	100.00	0.987277	0.995617	1.402130
166	59	10.4	1	100.00	0.987277	0.995610	1.402126
167	352	10.3	1	100.00	0.987277	0.995584	1.402107
168	686	8.3	1	99.77	0.987277	0.995584	1.402107
169	961	9.3	1	100.00	0.987277	0.995578	1.402103
170	435	9.6	1	100.00	0.987277	0.995571	1.402098
171	961	8.3	1	100.00	0.987277	0.995565	1.402093
172	686	8.6	1	100.00	0.987277	0.995565	1.402093
173	435	10	1	100.00	0.987277	0.995558	1.402089
174	961	8.2	1	100.00	0.987277	0.995545	1.402080
175	158	9.7	1	100.00	0.987277	0.995539	1.402075
176	686	9	1	100.00	0.987277	0.995533	1.402070
177	686	8.8	1	100.00	0.987277	0.995533	1.402070
178	686	8.7	1	100.00	0.987277	0.995526	1.402066
179	686	8.9	1	100.00	0.987277	0.995526	1.402066
180	686	9.2	1	100.00	0.987277	0.995513	1.402057
181	961	8.1	1	100.00	0.987277	0.995507	1.402052
182	649	9	1	100.00	0.987277	0.995507	1.402052
183	649	8.3	1	100.00	0.987277	0.995507	1.402052
184	961	9.4	1	100.00	0.987277	0.995500	1.402047
185	68	9.9	1	100.00	0.987277	0.995500	1.402047
186	352	10.4	1	100.00	0.987277	0.995487	1.402038
187	932	8.1	1	100.00	0.987277	0.995474	1.402029
188	686	9.3	1	100.00	0.987277	0.995474	1.402029
189	158	9.8	1	100.00	0.987277	0.995468	1.402024
190	932	9.4	1	100.00	0.987277	0.995442	1.402006
191	158	9.9	1	100.00	0.987277	0.995422	1.401992
192	59	10.6	1	100.00	0.987277	0.995422	1.401992
193	158	10	1	100.00	0.987277	0.995416	1.401988
194	68	10	1	100.00	0.987277	0.995416	1.401988
195	686	9.4	1	100.00	0.987277	0.995397	1.401974
196	158	10.1	1	100.00	0.987277	0.995384	1.401965
197	961	8	1	100.00	0.987277	0.995364	1.401951
198	686	8.1	1	100.00	0.987277	0.995358	1.401946
199	686	9.5	1	100.00	0.987277	0.995338	1.401933
200	961	9.5	1	100.00	0.987277	0.995319	1.401919
201	932	9.5	1	100.00	0.987277	0.995293	1.401900
202	649	8.8	1	100.00	0.987277	0.995286	1.401896
203	686	9.6	1	100.00	0.987277	0.995280	1.401891
204	59	10.7	1	100.00	0.987277	0.995274	1.401887
205	932	9.6	1	100.00	0.987277	0.995254	1.401873
206	158	10.2	1	100.00	0.987277	0.995241	1.401864
207	686	9.7	1	100.00	0.987277	0.995235	1.401859
208	68	10.2	1	100.00	0.987277	0.995228	1.401854

Supplementary Table 5. Continued.

No.	Seed	Cost	Gamma	Training_Accuracy	Test_Accuracy	Test_AUC	Euclidean length
209	961	9.6	1	100.00	0.987277	0.995222	1.401850
210	352	10.6	1	100.00	0.987277	0.995196	1.401831
211	183	8.9	1	99.77	0.987277	0.995176	1.401818
212	932	9.7	1	100.00	0.987277	0.995163	1.401808
213	158	10.3	1	100.00	0.987277	0.995151	1.401799
214	932	8	1	100.00	0.987277	0.995131	1.401785
215	932	9.8	1	100.00	0.987277	0.995125	1.401781
216	686	9.8	1	100.00	0.987277	0.995125	1.401781
217	59	8.8	1	100.00	0.987277	0.995099	1.401762
218	961	9.8	1	100.00	0.987277	0.995092	1.401758
219	649	9.5	1	100.00	0.987277	0.995092	1.401758
220	68	10.3	1	100.00	0.987277	0.995073	1.401744
221	59	10.8	1	100.00	0.987277	0.995066	1.401739
222	352	10.7	1	100.00	0.987277	0.995060	1.401735
223	932	9.9	1	100.00	0.987277	0.995034	1.401717
224	158	10.4	1	100.00	0.987277	0.995034	1.401717
225	435	10.5	1	100.00	0.987277	0.995021	1.401707
226	183	8.1	1	99.77	0.987277	0.995008	1.401698
227	686	9.9	1	100.00	0.987277	0.994982	1.401680
228	961	9.9	1	100.00	0.987277	0.994963	1.401666
229	352	10.8	1	100.00	0.987277	0.994917	1.401634
230	158	10.5	1	100.00	0.987277	0.994917	1.401634
231	932	10	1	100.00	0.987277	0.994904	1.401625
232	183	8.8	1	99.77	0.987277	0.994898	1.401620
233	686	10	1	100.00	0.987277	0.994885	1.401611
234	68	10.4	1	100.00	0.987277	0.994853	1.401588
235	961	10	1	100.00	0.987277	0.994846	1.401583
236	59	10.9	1	100.00	0.987277	0.994846	1.401583
237	932	10.1	1	100.00	0.987277	0.994814	1.401560
238	352	10.9	1	100.00	0.987277	0.994801	1.401551
239	686	10.1	1	100.00	0.987277	0.994801	1.401551
240	352	10.5	1	100.00	0.987277	0.994788	1.401542
241	183	9.7	1	100.00	0.987277	0.994743	1.401510
242	686	10.3	1	100.00	0.987277	0.994717	1.401491
243	68	10.5	1	100.00	0.987277	0.994710	1.401487
244	961	10.1	1	100.00	0.987277	0.994704	1.401482
245	158	10.6	1	100.00	0.987277	0.994704	1.401482
246	932	10.2	1	100.00	0.987277	0.994697	1.401478
247	932	10.3	1	100.00	0.987277	0.994665	1.401455
248	59	11	1	100.00	0.987277	0.994652	1.401445
249	352	11	1	100.00	0.987277	0.994645	1.401441
250	686	10.4	1	100.00	0.987277	0.994620	1.401422
251	961	10.2	1	100.00	0.987277	0.994607	1.401413
252	932	10.4	1	100.00	0.987277	0.994594	1.401404
253	435	13	1	100.00	0.984733	0.997061	1.401367
254	68	10.6	1	100.00	0.987277	0.994490	1.401330
255	961	10.3	1	100.00	0.987277	0.994484	1.401326
256	158	10.7	1	100.00	0.987277	0.994471	1.401317
257	686	10.5	1	100.00	0.987277	0.994471	1.401317
258	59	8.3	1	100.00	0.987277	0.994464	1.401312
259	435	2	1	99.54	0.984733	0.996976	1.401307
260	932	10.5	1	100.00	0.987277	0.994438	1.401294

Supplementary Table 5. Continued.

No.	Seed	Cost	Gamma	Training_Accuracy	Test_Accuracy	Test_AUC	Euclidean length
261	435	15	1	100.00	0.984733	0.996912	1.401261
262	649	10.4	1	100.00	0.987277	0.994341	1.401225
263	183	13	1	100.00	0.984733	0.996860	1.401224
264	961	10.4	1	100.00	0.987277	0.994335	1.401220
265	68	10.7	1	100.00	0.987277	0.994335	1.401220
266	932	10.6	1	100.00	0.987277	0.994328	1.401216
267	435	12	1	100.00	0.984733	0.996827	1.401201
268	435	16	1	100.00	0.984733	0.996827	1.401201
269	158	10.8	1	100.00	0.987277	0.994302	1.401197
270	59	8.7	1	100.00	0.987277	0.994276	1.401179
271	183	9.4	1	100.00	0.987277	0.994192	1.401119
272	649	14	1	100.00	0.984733	0.996711	1.401118
273	961	10.5	1	100.00	0.987277	0.994186	1.401115
274	59	10.2	1	100.00	0.987277	0.994173	1.401105
275	59	8.1	1	100.00	0.987277	0.994160	1.401096
276	961	10.6	1	100.00	0.987277	0.994121	1.401069
277	68	10.8	1	100.00	0.987277	0.994121	1.401069
278	932	10.7	1	100.00	0.987277	0.994108	1.401059
279	435	4	1	100.00	0.984733	0.996614	1.401049
280	961	9.7	1	100.00	0.987277	0.994056	1.401023
281	961	10.7	1	100.00	0.987277	0.994011	1.400990
282	158	10.9	1	100.00	0.987277	0.993992	1.400977
283	649	15	1	100.00	0.984733	0.996497	1.400966
284	183	15	1	100.00	0.984733	0.996471	1.400947
285	686	8.2	1	99.77	0.987277	0.993933	1.400935
286	68	9.8	1	100.00	0.987277	0.993875	1.400894
287	158	11	1	100.00	0.987277	0.993862	1.400885
288	961	10.8	1	100.00	0.987277	0.993849	1.400876
289	183	4	1	100.00	0.984733	0.996368	1.400874
290	59	10.3	1	100.00	0.987277	0.993817	1.400853
291	649	16	1	100.00	0.984733	0.996335	1.400851
292	183	16	1	100.00	0.984733	0.996322	1.400842
293	649	13	1	100.00	0.984733	0.996309	1.400832
294	68	11	1	100.00	0.987277	0.993784	1.400830
295	68	8.6	1	100.00	0.987277	0.993707	1.400775
296	352	9.2	1	100.00	0.987277	0.993700	1.400770
297	686	9.1	1	100.00	0.987277	0.993700	1.400770
298	961	10.9	1	100.00	0.987277	0.993674	1.400752
299	649	12	1	100.00	0.984733	0.996186	1.400745
300	352	8.4	1	99.77	0.987277	0.993661	1.400742
301	183	12	1	100.00	0.984733	0.996180	1.400740
302	932	11	1	100.00	0.987277	0.993635	1.400724
303	686	10.9	1	100.00	0.987277	0.993597	1.400697
304	961	11	1	100.00	0.987277	0.993525	1.400646
305	686	10.2	1	100.00	0.987277	0.993499	1.400628
306	352	9.7	1	100.00	0.987277	0.993474	1.400609
307	68	8	1	100.00	0.987277	0.993467	1.400605
308	352	9.6	1	100.00	0.987277	0.993461	1.400600
309	183	32	1	100.00	0.984733	0.995947	1.400574
310	932	10.9	1	100.00	0.987277	0.993389	1.400550
311	932	8.9	1	100.00	0.987277	0.993299	1.400485
312	961	8.9	1	100.00	0.987277	0.993266	1.400462

Supplementary Table 5. Continued.

No.	Seed	Cost	Gamma	Training_Accuracy	Test_Accuracy	Test_AUC	Euclidean length
313	435	10.4	1	100.00	0.987277	0.993202	1.400416
314	68	8.1	1	100.00	0.987277	0.993169	1.400393
315	59	2	1	99.54	0.984733	0.995649	1.400363
316	686	8.5	1	100.00	0.987277	0.993111	1.400352
317	932	9.3	1	100.00	0.987277	0.993085	1.400334
318	59	14	1	100.00	0.984733	0.995584	1.400317
319	59	15	1	100.00	0.984733	0.995571	1.400307
320	686	11	1	100.00	0.987277	0.993033	1.400297
321	59	16	1	100.00	0.984733	0.995422	1.400202
322	649	8.9	1	100.00	0.987277	0.992833	1.400155
323	68	10.1	1	100.00	0.987277	0.992807	1.400136
324	649	32	1	100.00	0.984733	0.995306	1.400119
325	352	2	1	99.77	0.984733	0.995261	1.400087
326	59	13	1	100.00	0.984733	0.995254	1.400082
327	435	32	1	100.00	0.984733	0.995248	1.400077
328	649	8.7	1	100.00	0.987277	0.992723	1.400077
329	158	2	1	99.54	0.984733	0.995138	1.399999
330	59	12	1	100.00	0.984733	0.995118	1.399985
331	59	4	1	100.00	0.984733	0.995040	1.399930
332	686	10.6	1	100.00	0.987277	0.992444	1.399879
333	932	10.8	1	100.00	0.987277	0.992282	1.399764
334	686	10.7	1	100.00	0.987277	0.992230	1.399728
335	649	4	1	100.00	0.984733	0.994756	1.399728
336	352	14	1	100.00	0.984733	0.994743	1.399718
337	435	14	1	100.00	0.984733	0.994730	1.399709
338	158	15	1	100.00	0.984733	0.994723	1.399705
339	59	32	1	100.00	0.984733	0.994717	1.399700
340	158	14	1	100.00	0.984733	0.994665	1.399663
341	649	7	1	100.00	0.984733	0.994620	1.399631
342	158	12	1	100.00	0.984733	0.994568	1.399594
343	352	32	1	100.00	0.984733	0.994412	1.399484
344	932	13	1	100.00	0.984733	0.994393	1.399470
345	183	14	1	100.00	0.984733	0.994380	1.399461
346	686	15	1	100.00	0.984733	0.994309	1.399410
347	352	15	1	100.00	0.984733	0.994238	1.399360
348	352	12	1	100.00	0.984733	0.994231	1.399355
349	158	13	1	100.00	0.984733	0.994225	1.399350
350	68	14	1	100.00	0.984733	0.994192	1.399327
351	352	16	1	100.00	0.984733	0.994160	1.399304
352	932	16	1	100.00	0.987277	0.991609	1.399287
353	686	16	1	100.00	0.984733	0.994056	1.399231
354	68	10.9	1	100.00	0.987277	0.991525	1.399228
355	961	13	1	100.00	0.984733	0.993998	1.399189
356	686	8	1	100.00	0.987277	0.991466	1.399186
357	686	12	1	100.00	0.984733	0.993959	1.399162
358	932	14	1	100.00	0.984733	0.993953	1.399157
359	961	14	1	100.00	0.984733	0.993894	1.399116
360	183	7	1	100.00	0.984733	0.993869	1.399097
361	352	4	1	100.00	0.984733	0.993862	1.399093
362	158	4	1	100.00	0.984733	0.993843	1.399079
363	686	10.8	1	100.00	0.987277	0.991285	1.399058
364	158	16	1	100.00	0.984733	0.993739	1.399005

Supplementary Table 5. Continued.

No.	Seed	Cost	Gamma	Training_Accuracy	Test_Accuracy	Test_AUC	Euclidean length
365	183	2	1	99.54	0.984733	0.993733	1.399001
366	435	128	1	100.00	0.982188	0.996050	1.398860
367	435	2048	1	100.00	0.982188	0.996050	1.398860
368	435	32768	1	100.00	0.982188	0.996050	1.398860
369	435	512	1	100.00	0.982188	0.996050	1.398860
370	435	8192	1	100.00	0.982188	0.996050	1.398860
371	435	64	1	100.00	0.982188	0.996050	1.398860
372	68	15	1	100.00	0.984733	0.993519	1.398849
373	649	2	1	99.54	0.984733	0.993467	1.398812
374	68	16	1	100.00	0.984733	0.993409	1.398771
375	961	15	1	100.00	0.984733	0.993383	1.398752
376	961	12	1	100.00	0.984733	0.993292	1.398688
377	435	5	1	100.00	0.982188	0.995766	1.398658
378	183	6	1	100.00	0.984733	0.993228	1.398642
379	686	32	1	100.00	0.984733	0.993111	1.398559
380	932	2	1	99.77	0.984733	0.993046	1.398513
381	961	4	1	100.00	0.984733	0.992975	1.398463
382	59	10.5	1	100.00	0.987277	0.990430	1.398452
383	183	128	1	100.00	0.982188	0.995429	1.398418
384	183	2048	1	100.00	0.982188	0.995429	1.398418
385	183	32768	1	100.00	0.982188	0.995429	1.398418
386	183	512	1	100.00	0.982188	0.995429	1.398418
387	183	8192	1	100.00	0.982188	0.995429	1.398418
388	183	64	1	100.00	0.982188	0.995429	1.398418
389	961	32	1	100.00	0.984733	0.992839	1.398366
390	932	12	1	100.00	0.984733	0.992807	1.398343
391	686	13	1	100.00	0.984733	0.992787	1.398329
392	686	2	1	99.54	0.984733	0.992625	1.398215
393	932	4	1	100.00	0.984733	0.992606	1.398201
394	686	7	1	100.00	0.984733	0.992554	1.398164
395	68	4	1	100.00	0.984733	0.992515	1.398136
396	352	13	1	100.00	0.984733	0.992431	1.398077
397	686	14	1	100.00	0.984733	0.992379	1.398040
398	435	7	1	100.00	0.982188	0.994620	1.397842
399	68	12	1	100.00	0.984733	0.992043	1.397801
400	68	2	1	99.77	0.984733	0.991971	1.397750
401	649	5	1	100.00	0.982188	0.994458	1.397727
402	68	13	1	100.00	0.984733	0.991894	1.397695
403	649	6	1	100.00	0.984733	0.991887	1.397691
404	352	7	1	100.00	0.984733	0.991829	1.397649
405	686	4	1	100.00	0.984733	0.991823	1.397645
406	649	128	1	100.00	0.982188	0.994231	1.397566
407	649	2048	1	100.00	0.982188	0.994231	1.397566
408	649	32768	1	100.00	0.982188	0.994231	1.397566
409	649	512	1	100.00	0.982188	0.994231	1.397566
410	649	8192	1	100.00	0.982188	0.994231	1.397566
411	649	64	1	100.00	0.982188	0.994231	1.397566
412	59	5	1	100.00	0.982188	0.994212	1.397552
413	932	128	1	100.00	0.984733	0.991648	1.397521
414	932	2048	1	100.00	0.984733	0.991648	1.397521
415	932	32768	1	100.00	0.984733	0.991648	1.397521
416	932	512	1	100.00	0.984733	0.991648	1.397521

Supplementary Table 5. Continued.

No.	Seed	Cost	Gamma	Training_Accuracy	Test_Accuracy	Test_AUC	Euclidean length
417	932	8192	1	100.00	0.984733	0.991648	1.397521
418	932	64	1	100.00	0.984733	0.991648	1.397521
419	59	6	1	100.00	0.982188	0.994017	1.397414
420	932	32	1	100.00	0.984733	0.991356	1.397314
421	59	1	1	99.31	0.982188	0.993843	1.397289
422	961	16	1	100.00	0.984733	0.991220	1.397217
423	183	5	1	100.00	0.982188	0.993713	1.397197
424	686	5	1	100.00	0.982188	0.993687	1.397179
425	158	32	1	100.00	0.982188	0.993584	1.397105
426	435	1	1	99.31	0.982188	0.993525	1.397064
427	932	15	1	100.00	0.984733	0.990987	1.397052
428	435	6	1	100.00	0.982188	0.993435	1.396999
429	68	32	1	100.00	0.984733	0.990851	1.396956
430	158	5	1	100.00	0.982188	0.993318	1.396916
431	686	6	1	100.00	0.982188	0.993318	1.396916
432	158	6	1	100.00	0.982188	0.993228	1.396852
433	68	1	1	99.31	0.982188	0.993143	1.396792
434	59	7	1	100.00	0.982188	0.993066	1.396737
435	59	128	1	100.00	0.982188	0.992949	1.396654
436	59	2048	1	100.00	0.982188	0.992949	1.396654
437	59	32768	1	100.00	0.982188	0.992949	1.396654
438	59	512	1	100.00	0.982188	0.992949	1.396654
439	59	8192	1	100.00	0.982188	0.992949	1.396654
440	59	64	1	100.00	0.982188	0.992949	1.396654
441	158	128	1	100.00	0.982188	0.992949	1.396654
442	158	2048	1	100.00	0.982188	0.992949	1.396654
443	158	32768	1	100.00	0.982188	0.992949	1.396654
444	158	512	1	100.00	0.982188	0.992949	1.396654
445	158	8192	1	100.00	0.982188	0.992949	1.396654
446	158	64	1	100.00	0.982188	0.992949	1.396654
447	352	5	1	100.00	0.982188	0.992794	1.396543
448	961	5	1	100.00	0.982188	0.992742	1.396507
449	158	7	1	100.00	0.984733	0.990210	1.396501
450	961	6	1	100.00	0.982188	0.992703	1.396479
451	961	2	1	99.54	0.984733	0.990172	1.396474
452	68	5	1	100.00	0.982188	0.992658	1.396447
453	352	6	1	100.00	0.982188	0.992651	1.396442
454	932	6	1	100.00	0.982188	0.992541	1.396364
455	961	128	1	100.00	0.982188	0.992502	1.396336
456	961	2048	1	100.00	0.982188	0.992502	1.396336
457	961	32768	1	100.00	0.982188	0.992502	1.396336
458	961	512	1	100.00	0.982188	0.992502	1.396336
459	961	8192	1	100.00	0.982188	0.992502	1.396336
460	961	64	1	100.00	0.982188	0.992502	1.396336
461	932	5	1	100.00	0.982188	0.992470	1.396313
462	932	7	1	100.00	0.982188	0.992431	1.396286
463	183	1	1	99.08	0.982188	0.991920	1.395922
464	68	7	1	100.00	0.982188	0.991887	1.395899
465	686	128	1	100.00	0.982188	0.991842	1.395867
466	686	2048	1	100.00	0.982188	0.991842	1.395867
467	686	32768	1	100.00	0.982188	0.991842	1.395867
468	686	512	1	100.00	0.982188	0.991842	1.395867

Supplementary Table 5. Continued.

No.	Seed	Cost	Gamma	Training_Accuracy	Test_Accuracy	Test_AUC	Euclidean length
469	686	8192	1	100.00	0.982188	0.991842	1.395867
470	686	64	1	100.00	0.982188	0.991842	1.395867
471	352	128	1	100.00	0.982188	0.991700	1.395766
472	352	2048	1	100.00	0.982188	0.991700	1.395766
473	352	32768	1	100.00	0.982188	0.991700	1.395766
474	352	512	1	100.00	0.982188	0.991700	1.395766
475	352	8192	1	100.00	0.982188	0.991700	1.395766
476	352	64	1	100.00	0.982188	0.991700	1.395766
477	158	1	1	99.31	0.979644	0.994082	1.395672
478	10	8.1	1	100.00	0.987277	0.986274	1.395512
479	10	8	1	100.00	0.987277	0.986125	1.395406
480	2	9.6	1	100.00	0.984733	0.988048	1.394969
481	2	9.7	1	100.00	0.984733	0.988035	1.394959
482	2	9.8	1	100.00	0.984733	0.987977	1.394918
483	9	8.3	1	100.00	0.987277	0.985426	1.394912
484	2	8.6	1	99.77	0.984733	0.987938	1.394891
485	2	9.9	1	100.00	0.984733	0.987821	1.394808
486	2	8.5	1	99.77	0.984733	0.987802	1.394794
487	2	9.4	1	100.00	0.984733	0.987776	1.394776
488	9	8.4	1	100.00	0.987277	0.985231	1.394775
489	9	8.2	1	100.00	0.987277	0.985199	1.394752
490	68	128	1	100.00	0.982188	0.990139	1.394657
491	68	2048	1	100.00	0.982188	0.990139	1.394657
492	68	32768	1	100.00	0.982188	0.990139	1.394657
493	68	512	1	100.00	0.982188	0.990139	1.394657
494	68	8192	1	100.00	0.982188	0.990139	1.394657
495	68	64	1	100.00	0.982188	0.990139	1.394657
496	68	6	1	100.00	0.982188	0.990139	1.394657
497	10	9.4	1	100.00	0.987277	0.985063	1.394656
498	9	8.1	1	100.00	0.987277	0.984946	1.394574
499	649	1	1	99.31	0.979644	0.992483	1.394534
500	2	10	1	100.00	0.984733	0.987387	1.394501
501	2	8.8	1	99.77	0.984733	0.987342	1.394469
502	932	1	1	99.31	0.982188	0.989796	1.394414
503	9	8	1	100.00	0.987277	0.984707	1.394405
504	2	10.1	1	100.00	0.984733	0.987219	1.394382
505	9	8.6	1	100.00	0.987277	0.984616	1.394341
506	352	1	1	99.31	0.982188	0.989660	1.394317
507	961	7	1	100.00	0.982188	0.989647	1.394308
508	2	10.2	1	100.00	0.984733	0.987109	1.394304
509	2	10.3	1	100.00	0.984733	0.986954	1.394194
510	2	8.4	1	99.77	0.984733	0.986863	1.394130
511	2	10.4	1	100.00	0.984733	0.986837	1.394111
512	2	9	1	99.77	0.984733	0.986824	1.394102
513	9	8.8	1	100.00	0.987277	0.984176	1.394030
514	9	9.3	1	100.00	0.987277	0.984150	1.394011
515	2	10.5	1	100.00	0.984733	0.986682	1.394001
516	2	8.3	1	99.77	0.984733	0.986643	1.393974
517	2	8.1	1	99.77	0.984733	0.986585	1.393933
518	2	10.6	1	100.00	0.984733	0.986552	1.393910
519	2	8	1	99.77	0.984733	0.986513	1.393882
520	9	9.4	1	100.00	0.987277	0.983962	1.393879

Supplementary Table 5. Continued.

No.	Seed	Cost	Gamma	Training_Accuracy	Test_Accuracy	Test_AUC	Euclidean length
521	2	10.7	1	100.00	0.984733	0.986403	1.393804
522	9	9.5	1	100.00	0.987277	0.983813	1.393774
523	9	9.6	1	100.00	0.987277	0.983703	1.393696
524	2	9.3	1	99.77	0.984733	0.986209	1.393667
525	10	8.5	1	100.00	0.987277	0.983658	1.393664
526	9	9.7	1	100.00	0.987277	0.983645	1.393655
527	10	8.8	1	100.00	0.987277	0.983639	1.393650
528	9	9.8	1	100.00	0.987277	0.983613	1.393632
529	2	10.8	1	100.00	0.984733	0.986125	1.393607
530	6	8.4	1	100.00	0.984733	0.986125	1.393607
531	6	8.3	1	100.00	0.984733	0.986118	1.393603
532	6	8.5	1	100.00	0.984733	0.986118	1.393603
533	686	1	1	99.31	0.982188	0.988637	1.393591
534	10	9.6	1	100.00	0.987277	0.983535	1.393577
535	10	9.1	1	100.00	0.987277	0.983503	1.393554
536	6	8.6	1	100.00	0.984733	0.986041	1.393548
537	961	1	1	99.31	0.982188	0.988572	1.393545
538	2	8.9	1	99.77	0.984733	0.986034	1.393543
539	2	10.9	1	100.00	0.984733	0.985995	1.393516
540	10	8.7	1	100.00	0.987277	0.983431	1.393504
541	6	8.7	1	100.00	0.984733	0.985969	1.393497
542	9	10	1	100.00	0.987277	0.983406	1.393486
543	6	9	1	100.00	0.984733	0.985944	1.393479
544	6	8.9	1	100.00	0.984733	0.985937	1.393474
545	6	8.8	1	100.00	0.984733	0.985931	1.393470
546	6	8.2	1	100.00	0.984733	0.985905	1.393451
547	6	9.1	1	100.00	0.984733	0.985898	1.393447
548	9	10.1	1	100.00	0.987277	0.983347	1.393445
549	9	10.2	1	100.00	0.987277	0.983321	1.393427
550	6	9.2	1	100.00	0.984733	0.985866	1.393424
551	2	11	1	100.00	0.984733	0.985795	1.393374
552	6	9.3	1	100.00	0.984733	0.985782	1.393364
553	2	8.7	1	99.77	0.984733	0.985749	1.393341
554	4	8.5	1	99.77	0.987277	0.983159	1.393312
555	6	8.1	1	100.00	0.984733	0.985704	1.393309
556	9	8.9	1	100.00	0.987277	0.983114	1.393280
557	6	9.5	1	100.00	0.984733	0.985659	1.393277
558	4	8.4	1	99.77	0.987277	0.983108	1.393276
559	2	9.1	1	99.77	0.984733	0.985600	1.393236
560	6	9.6	1	100.00	0.984733	0.985581	1.393222
561	10	2	1	99.54	0.984733	0.985581	1.393222
562	2	9.5	1	100.00	0.984733	0.985555	1.393204
563	6	9.7	1	100.00	0.984733	0.985536	1.393190
564	6	9.8	1	100.00	0.984733	0.985536	1.393190
565	6	9.9	1	100.00	0.984733	0.985529	1.393186
566	2	9.2	1	99.77	0.984733	0.985516	1.393177
567	6	10	1	100.00	0.984733	0.985471	1.393145
568	6	8	1	100.00	0.984733	0.985452	1.393131
569	6	10.1	1	100.00	0.984733	0.985400	1.393094
570	4	8.1	1	99.77	0.987277	0.982836	1.393084
571	4	10.9	1	100.00	0.987277	0.982810	1.393066
572	4	9.4	1	100.00	0.987277	0.982777	1.393043

Supplementary Table 5. Continued.

No.	Seed	Cost	Gamma	Training_Accuracy	Test_Accuracy	Test_AUC	Euclidean length
573	4	11	1	100.00	0.987277	0.982771	1.393038
574	10	11	1	100.00	0.987277	0.982758	1.393029
575	10	10.4	1	100.00	0.987277	0.982758	1.393029
576	4	8.6	1	100.00	0.987277	0.982739	1.393015
577	10	10.9	1	100.00	0.987277	0.982739	1.393015
578	4	9.5	1	100.00	0.987277	0.982726	1.393006
579	4	10.3	1	100.00	0.987277	0.982719	1.393002
580	4	9.3	1	100.00	0.987277	0.982713	1.392997
581	10	10.8	1	100.00	0.987277	0.982706	1.392993
582	4	10.4	1	100.00	0.987277	0.982700	1.392988
583	4	10.1	1	100.00	0.987277	0.982674	1.392970
584	4	8	1	99.77	0.987277	0.982667	1.392965
585	4	10.2	1	100.00	0.987277	0.982661	1.392961
586	4	9.2	1	99.77	0.987277	0.982661	1.392961
587	4	9.9	1	100.00	0.987277	0.982654	1.392956
588	4	10.5	1	100.00	0.987277	0.982648	1.392951
589	4	8.7	1	100.00	0.987277	0.982648	1.392951
590	4	10.6	1	100.00	0.987277	0.982642	1.392947
591	4	10.7	1	100.00	0.987277	0.982629	1.392938
592	10	8.6	1	100.00	0.987277	0.982596	1.392915
593	4	10.8	1	100.00	0.987277	0.982596	1.392915
594	4	9.8	1	100.00	0.987277	0.982583	1.392906
595	4	9.6	1	100.00	0.987277	0.982583	1.392906
596	4	9.7	1	100.00	0.987277	0.982577	1.392901
597	4	8.8	1	100.00	0.987277	0.982538	1.392874
598	4	8.9	1	100.00	0.987277	0.982486	1.392837
599	4	9.1	1	99.77	0.987277	0.982486	1.392837
600	9	8.5	1	100.00	0.987277	0.982486	1.392837
601	4	9	1	99.77	0.987277	0.982467	1.392824
602	6	10.9	1	100.00	0.984733	0.984752	1.392636
603	6	10.5	1	100.00	0.984733	0.984733	1.392623
604	9	9.9	1	100.00	0.987277	0.982149	1.392600
605	9	9	1	100.00	0.987277	0.982130	1.392586
606	10	4	1	100.00	0.984733	0.984662	1.392572
607	10	8.3	1	100.00	0.987277	0.982104	1.392568
608	6	11	1	100.00	0.984733	0.984636	1.392554
609	9	9.2	1	100.00	0.987277	0.981955	1.392463
610	9	9.1	1	100.00	0.987277	0.981858	1.392394
611	2	13	1	100.00	0.982188	0.986908	1.392366
612	4	10	1	100.00	0.987277	0.981800	1.392353
613	9	2	1	99.77	0.984733	0.984215	1.392256
614	9	8.7	1	100.00	0.987277	0.981657	1.392253
615	10	8.2	1	100.00	0.987277	0.981651	1.392248
616	6	9.4	1	100.00	0.984733	0.984131	1.392197
617	2	8.2	1	99.77	0.984733	0.984092	1.392169
618	158	0.5	1	99.31	0.979644	0.989013	1.392066
619	9	12	1	100.00	0.984733	0.983885	1.392023
620	9	14	1	100.00	0.984733	0.983613	1.391831
621	10	10.5	1	100.00	0.987277	0.981003	1.391792
622	10	10.6	1	100.00	0.987277	0.980965	1.391764
623	10	9.5	1	100.00	0.987277	0.980965	1.391764
624	9	15	1	100.00	0.984733	0.983503	1.391753

Supplementary Table 5. Continued.

No.	Seed	Cost	Gamma	Training_Accuracy	Test_Accuracy	Test_AUC	Euclidean length
625	10	9	1	100.00	0.987277	0.980893	1.391714
626	9	11	1	100.00	0.984733	0.983386	1.391671
627	10	10.3	1	100.00	0.987277	0.980809	1.391655
628	9	10.9	1	100.00	0.984733	0.983354	1.391648
629	9	10.8	1	100.00	0.984733	0.983315	1.391620
630	9	10.6	1	100.00	0.984733	0.983308	1.391616
631	9	10.7	1	100.00	0.984733	0.983276	1.391593
632	9	10.4	1	100.00	0.984733	0.983244	1.391570
633	9	10.5	1	100.00	0.984733	0.983244	1.391570
634	9	10.3	1	100.00	0.984733	0.983218	1.391552
635	4	4	1	100.00	0.984733	0.983185	1.391529
636	2	14	1	100.00	0.982188	0.985665	1.391485
637	6	10.6	1	100.00	0.984733	0.983075	1.391451
638	10	10	1	100.00	0.987277	0.980518	1.391450
639	4	8.2	1	99.77	0.987277	0.980485	1.391427
640	4	8.3	1	99.77	0.987277	0.980485	1.391427
641	4	12	1	100.00	0.984733	0.983011	1.391405
642	10	13	1	100.00	0.984733	0.982959	1.391369
643	6	10.3	1	100.00	0.984733	0.982939	1.391355
644	10	12	1	100.00	0.984733	0.982926	1.391346
645	8	8.6	1	99.77	0.984733	0.982913	1.391337
646	2	12	1	100.00	0.982188	0.985439	1.391324
647	8	10.1	1	100.00	0.984733	0.982888	1.391318
648	8	10.4	1	100.00	0.984733	0.982855	1.391295
649	8	10.3	1	100.00	0.984733	0.982842	1.391286
650	8	9.8	1	99.77	0.984733	0.982842	1.391286
651	8	10.2	1	100.00	0.984733	0.982836	1.391282
652	8	10	1	100.00	0.984733	0.982829	1.391277
653	8	8.9	1	99.77	0.984733	0.982829	1.391277
654	8	9.9	1	100.00	0.984733	0.982823	1.391273
655	8	8.8	1	99.77	0.984733	0.982823	1.391273
656	8	10.5	1	100.00	0.984733	0.982816	1.391268
657	8	9.5	1	99.77	0.984733	0.982816	1.391268
658	8	9.6	1	99.77	0.984733	0.982816	1.391268
659	2	2	1	99.54	0.982188	0.985354	1.391265
660	10	14	1	100.00	0.984733	0.982797	1.391254
661	4	13	1	100.00	0.984733	0.982797	1.391254
662	8	8.7	1	99.77	0.984733	0.982797	1.391254
663	8	9.4	1	99.77	0.984733	0.982784	1.391245
664	6	10.2	1	100.00	0.984733	0.982777	1.391241
665	8	9.7	1	99.77	0.984733	0.982771	1.391236
666	6	12	1	100.00	0.982188	0.985296	1.391223
667	4	14	1	100.00	0.984733	0.982752	1.391222
668	10	10.7	1	100.00	0.987277	0.980194	1.391221
669	8	9	1	99.77	0.984733	0.982745	1.391218
670	8	9.3	1	99.77	0.984733	0.982719	1.391199
671	10	9.2	1	100.00	0.987277	0.980155	1.391194
672	8	9.2	1	99.77	0.984733	0.982700	1.391186
673	8	11	1	100.00	0.984733	0.982674	1.391167
674	8	10.9	1	100.00	0.984733	0.982661	1.391158
675	8	9.1	1	99.77	0.984733	0.982654	1.391154
676	6	10.7	1	100.00	0.984733	0.982480	1.391030

Supplementary Table 5. Continued.

No.	Seed	Cost	Gamma	Training_Accuracy	Test_Accuracy	Test_AUC	Euclidean length
677	10	5	1	100.00	0.982188	0.985011	1.391022
678	10	8.4	1	100.00	0.987277	0.979903	1.391016
679	10	15	1	100.00	0.984733	0.982383	1.390962
680	6	10.4	1	100.00	0.984733	0.982331	1.390925
681	6	10.8	1	100.00	0.984733	0.982324	1.390920
682	10	16	1	100.00	0.984733	0.982279	1.390888
683	4	15	1	100.00	0.984733	0.982240	1.390861
684	10	9.9	1	100.00	0.987277	0.979611	1.390811
685	4	16	1	100.00	0.984733	0.982078	1.390747
686	10	7	1	100.00	0.982188	0.984506	1.390664
687	6	2	1	99.54	0.982188	0.984448	1.390623
688	6	16	1	100.00	0.982188	0.984047	1.390339
689	6	14	1	100.00	0.982188	0.984027	1.390325
690	4	2	1	99.54	0.984733	0.981411	1.390276
691	2	16	1	100.00	0.982188	0.983936	1.390261
692	2	4	1	100.00	0.982188	0.983923	1.390252
693	10	8.9	1	100.00	0.987277	0.978737	1.390195
694	9	13	1	100.00	0.984733	0.981249	1.390162
695	10	9.3	1	100.00	0.987277	0.978588	1.390091
696	2	7	1	100.00	0.982188	0.983554	1.389990
697	8	8.3	1	99.77	0.984733	0.980945	1.389947
698	10	10.1	1	100.00	0.987277	0.978362	1.389931
699	10	10.2	1	100.00	0.987277	0.978310	1.389895
700	10	32	1	100.00	0.984733	0.980867	1.389892
701	8	10.8	1	100.00	0.984733	0.980855	1.389883
702	2	128	1	100.00	0.982188	0.983399	1.389880
703	2	2048	1	100.00	0.982188	0.983399	1.389880
704	2	32768	1	100.00	0.982188	0.983399	1.389880
705	2	512	1	100.00	0.982188	0.983399	1.389880
706	2	8192	1	100.00	0.982188	0.983399	1.389880
707	2	64	1	100.00	0.982188	0.983399	1.389880
708	8	8.4	1	99.77	0.984733	0.980809	1.389851
709	8	10.6	1	100.00	0.984733	0.980744	1.389805
710	9	16	1	100.00	0.984733	0.980712	1.389782
711	4	7	1	100.00	0.984733	0.980667	1.389750
712	2	6	1	100.00	0.982188	0.983147	1.389702
713	8	13	1	100.00	0.982188	0.983088	1.389661
714	8	10.7	1	100.00	0.984733	0.980524	1.389650
715	8	12	1	100.00	0.982188	0.983056	1.389638
716	435	0.5	1	98.85	0.977099	0.988028	1.389577
717	8	8.5	1	99.77	0.984733	0.980375	1.389545
718	2	32	1	100.00	0.982188	0.982693	1.389381
719	8	14	1	100.00	0.982188	0.982667	1.389363
720	4	32	1	100.00	0.984733	0.980078	1.389335
721	2	1	1	99.31	0.979644	0.985102	1.389290
722	1	8	1	100.00	0.982188	0.982460	1.389216
723	10	6	1	100.00	0.982188	0.982376	1.389157
724	1	8.1	1	99.77	0.982188	0.982311	1.389111
725	1	8.5	1	99.77	0.982188	0.982214	1.389042
726	1	8.4	1	99.77	0.982188	0.982182	1.389019
727	1	8.2	1	99.77	0.982188	0.982104	1.388964
728	9	32	1	100.00	0.982188	0.982052	1.388928

Supplementary Table 5. Continued.

No.	Seed	Cost	Gamma	Training_Accuracy	Test_Accuracy	Test_AUC	Euclidean length
729	1	8.6	1	100.00	0.982188	0.982039	1.388919
730	2	5	1	100.00	0.979644	0.984506	1.388868
731	1	8.7	1	100.00	0.982188	0.981955	1.388859
732	9	4	1	100.00	0.982188	0.981903	1.388823
733	8	8.1	1	99.77	0.984733	0.979327	1.388805
734	9	1	1	99.31	0.982188	0.981845	1.388781
735	649	0.5	1	99.31	0.979644	0.984338	1.388749
736	8	32	1	100.00	0.982188	0.981761	1.388722
737	8	15	1	100.00	0.982188	0.981709	1.388685
738	9	6	1	100.00	0.979644	0.984247	1.388684
739	352	0.5	1	98.85	0.979644	0.984234	1.388675
740	1	8.9	1	99.77	0.982188	0.981638	1.388635
741	8	8	1	99.77	0.984733	0.979061	1.388618
742	9	7	1	100.00	0.979644	0.984131	1.388602
743	2	15	1	100.00	0.982188	0.981496	1.388534
744	4	5	1	100.00	0.982188	0.981483	1.388525
745	1	9	1	99.77	0.982188	0.981418	1.388479
746	9	5	1	100.00	0.979644	0.983943	1.388469
747	8	2	1	99.54	0.982188	0.981392	1.388461
748	4	6	1	100.00	0.982188	0.981353	1.388434
749	1	9.1	1	99.77	0.982188	0.981347	1.388429
750	1	9.2	1	99.77	0.982188	0.981308	1.388402
751	1	9.4	1	99.77	0.982188	0.981301	1.388397
752	1	9.3	1	99.77	0.982188	0.981269	1.388374
753	1	9.5	1	99.77	0.982188	0.981217	1.388337
754	8	128	1	100.00	0.982188	0.981165	1.388301
755	8	2048	1	100.00	0.982188	0.981165	1.388301
756	8	32768	1	100.00	0.982188	0.981165	1.388301
757	8	512	1	100.00	0.982188	0.981165	1.388301
758	8	8192	1	100.00	0.982188	0.981165	1.388301
759	8	64	1	100.00	0.982188	0.981165	1.388301
760	1	9.6	1	100.00	0.982188	0.981055	1.388223
761	1	9.7	1	100.00	0.982188	0.980958	1.388154
762	1	9.8	1	100.00	0.982188	0.980783	1.388031
763	10	1	1	99.31	0.982188	0.980706	1.387976
764	1	9.9	1	100.00	0.982188	0.980634	1.387926
765	9	128	1	100.00	0.982188	0.980589	1.387894
766	9	2048	1	100.00	0.982188	0.980589	1.387894
767	9	32768	1	100.00	0.982188	0.980589	1.387894
768	9	512	1	100.00	0.982188	0.980589	1.387894
769	9	8192	1	100.00	0.982188	0.980589	1.387894
770	9	64	1	100.00	0.982188	0.980589	1.387894
771	1	10	1	100.00	0.982188	0.980570	1.387880
772	1	8.3	1	99.77	0.982188	0.980524	1.387848
773	1	10.1	1	100.00	0.982188	0.980388	1.387752
774	1	10.2	1	100.00	0.982188	0.980311	1.387697
775	10	9.7	1	100.00	0.987277	0.975112	1.387645
776	6	32	1	100.00	0.979644	0.982693	1.387584
777	10	9.8	1	100.00	0.987277	0.974989	1.387559
778	1	10.3	1	100.00	0.982188	0.980097	1.387546
779	1	10.4	1	100.00	0.982188	0.980019	1.387491
780	5	10.6	1	100.00	0.987277	0.974807	1.387431

Supplementary Table 5. Continued.

No.	Seed	Cost	Gamma	Training_Accuracy	Test_Accuracy	Test_AUC	Euclidean length
781	1	4	1	100.00	0.979644	0.982460	1.387418
782	1	10.5	1	100.00	0.982188	0.979916	1.387418
783	6	13	1	100.00	0.982188	0.979896	1.387404
784	5	10.7	1	100.00	0.987277	0.974749	1.387390
785	5	10.8	1	100.00	0.987277	0.974730	1.387377
786	8	16	1	100.00	0.982188	0.979851	1.387372
787	5	10.9	1	100.00	0.987277	0.974710	1.387363
788	5	11	1	100.00	0.987277	0.974691	1.387349
789	1	10.6	1	100.00	0.982188	0.979819	1.387349
790	1	10.7	1	100.00	0.982188	0.979747	1.387299
791	5	10.5	1	100.00	0.987277	0.974594	1.387281
792	1	10.8	1	100.00	0.982188	0.979676	1.387249
793	5	10.4	1	100.00	0.987277	0.974522	1.387231
794	1	10.9	1	100.00	0.982188	0.979637	1.387221
795	1	11	1	100.00	0.982188	0.979585	1.387185
796	5	10.3	1	100.00	0.987277	0.974445	1.387177
797	5	10.2	1	100.00	0.987277	0.974438	1.387172
798	5	8.4	1	100.00	0.987277	0.974406	1.387149
799	6	15	1	100.00	0.982188	0.979469	1.387102
800	5	8.5	1	100.00	0.987277	0.974322	1.387090
801	5	10.1	1	100.00	0.987277	0.974302	1.387077
802	5	8.3	1	99.77	0.987277	0.974276	1.387058
803	6	128	1	100.00	0.979644	0.981890	1.387015
804	6	2048	1	100.00	0.979644	0.981890	1.387015
805	6	32768	1	100.00	0.979644	0.981890	1.387015
806	6	512	1	100.00	0.979644	0.981890	1.387015
807	6	8192	1	100.00	0.979644	0.981890	1.387015
808	6	64	1	100.00	0.979644	0.981890	1.387015
809	1	8.8	1	100.00	0.982188	0.979288	1.386974
810	5	8.2	1	99.77	0.987277	0.974069	1.386913
811	1	6	1	100.00	0.977099	0.984195	1.386854
812	5	10	1	100.00	0.987277	0.973920	1.386808
813	5	8.7	1	100.00	0.987277	0.973875	1.386776
814	5	8.1	1	100.00	0.987277	0.973868	1.386772
815	5	9.9	1	100.00	0.987277	0.973862	1.386767
816	5	8.6	1	100.00	0.987277	0.973849	1.386758
817	5	9	1	100.00	0.987277	0.973836	1.386749
818	5	8.8	1	100.00	0.987277	0.973836	1.386749
819	5	8.9	1	100.00	0.987277	0.973817	1.386735
820	5	9.8	1	100.00	0.987277	0.973817	1.386735
821	5	9.5	1	100.00	0.987277	0.973791	1.386717
822	5	9.6	1	100.00	0.987277	0.973791	1.386717
823	5	9.7	1	100.00	0.987277	0.973791	1.386717
824	5	9.1	1	100.00	0.987277	0.973771	1.386704
825	5	9.2	1	100.00	0.987277	0.973771	1.386704
826	1	5	1	100.00	0.977099	0.983943	1.386675
827	183	0.5	1	98.85	0.977099	0.983930	1.386665
828	5	9.4	1	100.00	0.987277	0.973655	1.386622
829	5	9.3	1	100.00	0.987277	0.973635	1.386608
830	5	8	1	100.00	0.987277	0.973609	1.386590
831	8	4	1	100.00	0.982188	0.978601	1.386490
832	4	128	1	100.00	0.982188	0.978459	1.386389

Supplementary Table 5. Continued.

No.	Seed	Cost	Gamma	Training_Accuracy	Test_Accuracy	Test_AUC	Euclidean length
833	4	2048	1	100.00	0.982188	0.978459	1.386389
834	4	32768	1	100.00	0.982188	0.978459	1.386389
835	4	512	1	100.00	0.982188	0.978459	1.386389
836	4	8192	1	100.00	0.982188	0.978459	1.386389
837	4	64	1	100.00	0.982188	0.978459	1.386389
838	6	4	1	100.00	0.982188	0.978245	1.386239
839	7	10.9	1	100.00	0.984733	0.975629	1.386200
840	7	9.8	1	100.00	0.984733	0.975494	1.386105
841	7	10	1	100.00	0.984733	0.975481	1.386096
842	7	9.6	1	100.00	0.984733	0.975481	1.386096
843	8	8.2	1	99.77	0.984733	0.975481	1.386096
844	7	9.5	1	100.00	0.984733	0.975461	1.386082
845	68	0.5	1	98.85	0.977099	0.983101	1.386078
846	7	10.1	1	100.00	0.984733	0.975442	1.386068
847	7	9.9	1	100.00	0.984733	0.975442	1.386068
848	7	10.5	1	100.00	0.984733	0.975435	1.386064
849	7	9.7	1	100.00	0.984733	0.975435	1.386064
850	7	10.3	1	100.00	0.984733	0.975435	1.386064
851	1	1	1	99.31	0.974555	0.985594	1.386056
852	7	10.6	1	100.00	0.984733	0.975416	1.386050
853	7	10.2	1	100.00	0.984733	0.975403	1.386041
854	7	10.4	1	100.00	0.984733	0.975403	1.386041
855	7	10.7	1	100.00	0.984733	0.975383	1.386027
856	1	2	1	99.54	0.979644	0.980460	1.386002
857	7	9.4	1	100.00	0.984733	0.975345	1.386000
858	7	9.3	1	100.00	0.984733	0.975280	1.385954
859	1	7	1	100.00	0.977099	0.982875	1.385917
860	59	0.5	1	98.85	0.977099	0.982700	1.385793
861	5	12	1	100.00	0.984733	0.975027	1.385777
862	7	9.2	1	100.00	0.984733	0.975008	1.385763
863	10	128	1	100.00	0.982188	0.977540	1.385741
864	10	2048	1	100.00	0.982188	0.977540	1.385741
865	10	32768	1	100.00	0.982188	0.977540	1.385741
866	10	512	1	100.00	0.982188	0.977540	1.385741
867	10	8192	1	100.00	0.982188	0.977540	1.385741
868	10	64	1	100.00	0.982188	0.977540	1.385741
869	7	9	1	100.00	0.984733	0.974937	1.385713
870	7	8.7	1	100.00	0.984733	0.974937	1.385713
871	7	9.1	1	100.00	0.984733	0.974937	1.385713
872	7	8.9	1	100.00	0.984733	0.974930	1.385708
873	7	8.8	1	100.00	0.984733	0.974917	1.385699
874	7	8.6	1	100.00	0.984733	0.974898	1.385686
875	7	10.8	1	100.00	0.984733	0.974872	1.385667
876	7	8.5	1	100.00	0.984733	0.974794	1.385613
877	1	13	1	100.00	0.979644	0.979844	1.385567
878	7	8.4	1	99.77	0.984733	0.974710	1.385554
879	7	8.3	1	99.77	0.984733	0.974607	1.385481
880	7	8.2	1	99.77	0.984733	0.974483	1.385394
881	8	7	1	100.00	0.982188	0.977041	1.385389
882	961	0.5	1	98.85	0.977099	0.982104	1.385371
883	1	14	1	100.00	0.979644	0.979378	1.385238
884	7	8.1	1	99.77	0.984733	0.974205	1.385198

Supplementary Table 5. Continued.

No.	Seed	Cost	Gamma	Training_Accuracy	Test_Accuracy	Test_AUC	Euclidean length
885	7	8	1	100.00	0.984733	0.974043	1.385084
886	5	13	1	100.00	0.984733	0.973953	1.385021
887	1	32	1	100.00	0.979644	0.978899	1.384899
888	7	12	1	100.00	0.982188	0.976316	1.384878
889	7	14	1	100.00	0.982188	0.976290	1.384859
890	1	16	1	100.00	0.979644	0.978834	1.384853
891	1	15	1	100.00	0.979644	0.978834	1.384853
892	5	16	1	100.00	0.984733	0.973713	1.384852
893	7	13	1	100.00	0.982188	0.976219	1.384809
894	5	15	1	100.00	0.984733	0.973532	1.384725
895	7	15	1	100.00	0.982188	0.976057	1.384695
896	7	16	1	100.00	0.982188	0.975791	1.384508
897	5	14	1	100.00	0.984733	0.973137	1.384447
898	7	11	1	100.00	0.984733	0.973111	1.384429
899	6	6	1	100.00	0.979644	0.978077	1.384318
900	4	1	1	99.31	0.979644	0.977922	1.384208
901	7	32	1	100.00	0.982188	0.975312	1.384170
902	6	5	1	100.00	0.979644	0.977734	1.384076
903	5	4	1	100.00	0.984733	0.972295	1.383856
904	1	12	1	100.00	0.979644	0.977287	1.383760
905	6	7	1	100.00	0.979644	0.977242	1.383728
906	7	4	1	100.00	0.982188	0.974581	1.383655
907	7	2	1	99.54	0.982188	0.974574	1.383650
908	8	5	1	100.00	0.979644	0.977119	1.383641
909	1	0.5	1	99.31	0.979644	0.976996	1.383554
910	2	0.5	1	98.85	0.977099	0.979126	1.383261
911	8	6	1	100.00	0.979644	0.976322	1.383079
912	6	1	1	99.31	0.979644	0.976258	1.383033
913	686	0.5	1	98.85	0.977099	0.978757	1.383000
914	5	32	1	100.00	0.984733	0.971000	1.382946
915	5	128	1	100.00	0.984733	0.970838	1.382832
916	5	2048	1	100.00	0.984733	0.970838	1.382832
917	5	32768	1	100.00	0.984733	0.970838	1.382832
918	5	512	1	100.00	0.984733	0.970838	1.382832
919	5	8192	1	100.00	0.984733	0.970838	1.382832
920	5	64	1	100.00	0.984733	0.970838	1.382832
921	5	2	1	99.54	0.984733	0.970139	1.382342
922	5	7	1	100.00	0.984733	0.970055	1.382283
923	1	128	1	100.00	0.977099	0.977468	1.382088
924	1	2048	1	100.00	0.977099	0.977468	1.382088
925	1	32768	1	100.00	0.977099	0.977468	1.382088
926	1	512	1	100.00	0.977099	0.977468	1.382088
927	1	8192	1	100.00	0.977099	0.977468	1.382088
928	1	64	1	100.00	0.977099	0.977468	1.382088
929	932	0.5	1	98.85	0.974555	0.979967	1.382061
930	7	128	1	100.00	0.979644	0.974024	1.381457
931	7	2048	1	100.00	0.979644	0.974024	1.381457
932	7	32768	1	100.00	0.979644	0.974024	1.381457
933	7	512	1	100.00	0.979644	0.974024	1.381457
934	7	8192	1	100.00	0.979644	0.974024	1.381457
935	7	64	1	100.00	0.979644	0.974024	1.381457
936	10	0.5	1	98.85	0.977099	0.976348	1.381296

Supplementary Table 5. Continued.

No.	Seed	Cost	Gamma	Training_Accuracy	Test_Accuracy	Test_AUC	Euclidean length
937	5	5	1	100.00	0.982188	0.970884	1.381054
938	5	6	1	100.00	0.982188	0.970676	1.380908
939	7	7	1	100.00	0.982188	0.970657	1.380894
940	8	1	1	99.31	0.979644	0.972955	1.380704
941	7	5	1	100.00	0.979644	0.972800	1.380595
942	9	0.5	1	98.85	0.977099	0.973402	1.379215
943	7	6	1	100.00	0.979644	0.970832	1.379209
944	59	0.125	1	97.46	0.959288	0.990003	1.378528
945	5	1	1	99.31	0.982188	0.964791	1.376777
946	7	1	1	99.31	0.979644	0.966876	1.376427
947	4	0.5	1	98.85	0.974555	0.971932	1.376376
948	686	0.125	1	97.46	0.959288	0.986876	1.376284
949	932	0.125	1	97.46	0.959288	0.986442	1.375973
950	649	0.125	1	97.46	0.956743	0.988825	1.375911
951	7	0.5	1	98.85	0.977099	0.967497	1.375054
952	158	0.125	1	97.69	0.956743	0.985846	1.373772
953	649	0.0313	1	96.54	0.949109	0.992897	1.373555
954	435	0.125	1	97.69	0.956743	0.985497	1.373521
955	9	0.125	1	97.46	0.956743	0.984338	1.372690
956	961	0.125	1	97.46	0.956743	0.982674	1.371497
957	352	0.125	1	97.46	0.956743	0.982318	1.371242
958	59	0.0313	1	96.54	0.949109	0.989634	1.371198
959	68	0.125	1	97.69	0.951654	0.987135	1.371160
960	183	0.125	1	97.23	0.956743	0.982065	1.371061
961	3	11	1	100.00	0.984733	0.951142	1.369077
962	932	0.0313	1	96.54	0.949109	0.986649	1.369045
963	3	10.4	1	100.00	0.984733	0.950599	1.368699
964	5	0.5	1	98.85	0.979644	0.955688	1.368591
965	3	10.3	1	100.00	0.984733	0.950437	1.368586
966	961	0.0313	1	96.54	0.949109	0.985989	1.368570
967	3	10.2	1	100.00	0.984733	0.950359	1.368532
968	3	10.1	1	100.00	0.984733	0.950236	1.368447
969	3	10	1	100.00	0.984733	0.950145	1.368384
970	3	9.9	1	100.00	0.984733	0.950042	1.368312
971	3	9.8	1	100.00	0.984733	0.949886	1.368204
972	3	9.7	1	100.00	0.984733	0.949750	1.368110
973	3	9.6	1	100.00	0.984733	0.949601	1.368006
974	68	0.0313	1	96.54	0.949109	0.985134	1.367954
975	3	9.5	1	100.00	0.984733	0.949485	1.367926
976	435	0.0313	1	96.54	0.949109	0.985044	1.367889
977	3	9.4	1	100.00	0.984733	0.949343	1.367827
978	3	12	1	100.00	0.982188	0.951952	1.367811
979	3	13	1	100.00	0.982188	0.951848	1.367739
980	3	9.3	1	100.00	0.984733	0.949116	1.367669
981	3	8.7	1	100.00	0.984733	0.949025	1.367607
982	3	8.8	1	100.00	0.984733	0.949019	1.367602
983	8	0.5	1	98.85	0.977099	0.956872	1.367599
984	3	8.5	1	100.00	0.984733	0.948993	1.367584
985	3	8.6	1	100.00	0.984733	0.948986	1.367580
986	1	0.125	1	97.69	0.956743	0.977196	1.367578
987	3	8.9	1	100.00	0.984733	0.948967	1.367566
988	3	9.2	1	100.00	0.984733	0.948941	1.367548

Supplementary Table 5. Continued.

No.	Seed	Cost	Gamma	Training_Accuracy	Test_Accuracy	Test_AUC	Euclidean length
989	3	9	1	100.00	0.984733	0.948935	1.367544
990	3	9.1	1	100.00	0.984733	0.948863	1.367494
991	3	8.4	1	100.00	0.984733	0.948863	1.367494
992	3	8.3	1	100.00	0.984733	0.948799	1.367449
993	3	14	1	100.00	0.982188	0.951272	1.367338
994	3	10.9	1	100.00	0.984733	0.948514	1.367252
995	3	10.8	1	100.00	0.984733	0.948423	1.367189
996	3	8.1	1	100.00	0.984733	0.948365	1.367148
997	3	10.7	1	100.00	0.984733	0.948287	1.367094
998	3	8	1	100.00	0.984733	0.948274	1.367085
999	3	10.6	1	100.00	0.984733	0.948235	1.367059
1000	3	10.5	1	100.00	0.984733	0.948203	1.367036
1001	3	15	1	100.00	0.982188	0.950838	1.367036
1002	158	0.0313	1	96.77	0.949109	0.983593	1.366845
1003	3	16	1	100.00	0.982188	0.950378	1.366716
1004	6	0.125	1	97.69	0.956743	0.975429	1.366316
1005	3	8.2	1	100.00	0.984733	0.947089	1.366264
1006	9	0.0313	1	96.54	0.949109	0.982441	1.366016
1007	352	0.0313	1	96.54	0.949109	0.982085	1.365760
1008	3	32	1	100.00	0.982188	0.948067	1.365110
1009	3	4	1	100.00	0.982188	0.947918	1.365006
1010	3	2	1	99.77	0.982188	0.947193	1.364503
1011	1	0.0313	1	96.54	0.949109	0.979793	1.364112
1012	3	5	1	100.00	0.979644	0.948617	1.363663
1013	3	128	1	100.00	0.979644	0.947297	1.362745
1014	3	2048	1	100.00	0.979644	0.947297	1.362745
1015	3	32768	1	100.00	0.979644	0.947297	1.362745
1016	3	512	1	100.00	0.979644	0.947297	1.362745
1017	3	8192	1	100.00	0.979644	0.947297	1.362745
1018	3	64	1	100.00	0.979644	0.947297	1.362745
1019	3	6	1	100.00	0.979644	0.946597	1.362259
1020	6	0.0313	1	96.54	0.949109	0.976406	1.361682
1021	3	7	1	100.00	0.979644	0.945574	1.361548
1022	686	0.0313	1	96.54	0.949109	0.976115	1.361473
1023	6	0.5	1	98.85	0.974555	0.949750	1.360802
1024	183	0.0313	1	96.54	0.949109	0.972755	1.359066
1025	2	0.0313	1	96.54	0.949109	0.968902	1.356311
1026	10	0.125	1	97.69	0.954198	0.963263	1.355865
1027	2	0.125	1	97.69	0.954198	0.963133	1.355773
1028	4	0.0313	1	96.54	0.949109	0.966079	1.354296
1029	4	0.125	1	97.69	0.951654	0.963515	1.354255
1030	3	1	1	99.31	0.979644	0.932839	1.352734
1031	8	0.125	1	97.69	0.959288	0.951570	1.351191
1032	3	0.5	1	99.08	0.977099	0.922253	1.343604
1033	8	0.0313	1	96.54	0.949109	0.948222	1.341616
1034	5	0.125	1	97.69	0.959288	0.937326	1.341198
1035	10	0.0313	1	96.77	0.949109	0.942959	1.337901
1036	5	0.0313	1	96.77	0.946565	0.942751	1.335951
1037	7	0.125	1	97.46	0.959288	0.928857	1.335293
1038	7	0.0313	1	96.77	0.949109	0.938899	1.335043
1039	3	0.125	1	97.46	0.956743	0.846843	1.277693
1040	3	0.0313	1	96.77	0.949109	0.821359	1.255165

Supplementary Table 6. Glycopeptide spectrum matches of alpha-1-acid glycoprotein and immunoglobulin gamma, and their Classification as training set for machine learning.

No.	Experiment	CID Scan No.	N-glycoproteins	N-glycopeptides	Manual Classification	SVM			DNN		
						Classification	Pscore	FDR	Classification	Pscore	FDR
1	AGP exp_2	2144	Alpha-1-acid glycoprotein 1	ENGTISR_4_4_0_1	None	None	2.0470	0.0000	None	7.2812	0.0000
2	AGP exp_1	2066	Alpha-1-acid glycoprotein 1	ENGTISR_4_4_0_1	None	None	3.1075	0.0000	None	7.3278	0.0000
3	AGP exp_2	3657	Alpha-1-acid glycoprotein 1	ENGTISR_5_4_0_2	None	None	3.7596	0.0000	None	7.2751	0.0000
4	AGP exp_2	3691	Alpha-1-acid glycoprotein 1	ENGTISR_5_4_0_2	None	None	2.9977	0.0000	None	7.2835	0.0000
5	AGP exp_1	3850	Alpha-1-acid glycoprotein 1	ENGTISR_5_4_0_2	None	None	3.4573	0.0000	None	7.2803	0.0000
6	AGP exp_1	2127	Alpha-1-acid glycoprotein 1	ENGTISR_6_5_0_1	None	None	2.8861	0.0000	None	7.3128	0.0000
7	AGP exp_1	2266	Alpha-1-acid glycoprotein 1	ENGTISR_6_5_0_1	None	None	2.2163	0.0000	None	7.2881	0.0000
8	AGP exp_1	2368	Alpha-1-acid glycoprotein 1	ENGTISR_6_5_0_1	None	None	3.0071	0.0000	None	7.2944	0.0000
9	AGP exp_2	2404	Alpha-1-acid glycoprotein 1	ENGTISR_6_5_0_1	None	None	2.0328	0.0000	None	7.2386	0.0000
10	AGP exp_2	2273	Alpha-1-acid glycoprotein 1	ENGTISR_6_5_0_1	None	None	2.5824	0.0000	None	7.2568	0.0000
11	AGP exp_2	2122	Alpha-1-acid glycoprotein 1	ENGTISR_6_5_0_1	None	None	2.7635	0.0000	None	7.2543	0.0000
12	AGP exp_2	3604	Alpha-1-acid glycoprotein 1	ENGTISR_6_5_0_2	None	None	2.9080	0.0000	None	7.2693	0.0000
13	AGP exp_1	4033	Alpha-1-acid glycoprotein 1	ENGTISR_6_5_0_2	None	None	2.7842	0.0000	None	7.2731	0.0000
14	AGP exp_2	3938	Alpha-1-acid glycoprotein 1	ENGTISR_6_5_0_2	None	None	3.0706	0.0000	None	7.3049	0.0000
15	AGP exp_1	3756	Alpha-1-acid glycoprotein 1	ENGTISR_6_5_0_2	None	None	2.8261	0.0000	None	7.3042	0.0000
16	AGP exp_2	3776	Alpha-1-acid glycoprotein 1	ENGTISR_6_5_0_2	None	None	2.4268	0.0000	None	7.0517	0.0000
17	AGP exp_1	4838	Alpha-1-acid glycoprotein 1	ENGTISR_6_5_0_3	None	None	2.5843	0.0000	None	7.1635	0.0000
18	AGP exp_1	4749	Alpha-1-acid glycoprotein 1	ENGTISR_6_5_0_3	None	None	1.8795	0.0000	None	7.2669	0.0000
19	AGP exp_2	6210	Alpha-1-acid glycoprotein 1	ENGTISR_6_5_0_3	None	None	2.2721	0.0000	None	7.2579	0.0000
20	AGP exp_1	5085	Alpha-1-acid glycoprotein 1	ENGTISR_6_5_0_3	None	None	2.2833	0.0000	None	7.2721	0.0000
21	AGP exp_2	2248	Alpha-1-acid glycoprotein 1	ENGTISR_6_5_1_1	Outer	Outer	4.0481	0.0000	Outer	6.7230	0.0000
22	AGP exp_1	2178	Alpha-1-acid glycoprotein 1	ENGTISR_6_5_1_1	Outer	Outer	3.0794	0.0000	Outer	6.7378	0.0000
23	AGP exp_1	2305	Alpha-1-acid glycoprotein 1	ENGTISR_6_5_1_1	Outer	Outer	2.1624	0.0000	Outer	6.5095	0.0000
24	AGP exp_1	2013	Alpha-1-acid glycoprotein 1	ENGTISR_6_5_1_1	Outer	Outer	13.5106	0.0000	Outer	6.7122	0.0000
25	AGP exp_1	3643	Alpha-1-acid glycoprotein 1	ENGTISR_6_5_1_2	Outer	Outer	4.1116	0.0000	Outer	6.5520	0.0000
26	AGP exp_1	3953	Alpha-1-acid glycoprotein 1	ENGTISR_6_5_1_2	Outer	Outer	1.3418	0.0025	Outer	6.0368	0.0073
27	AGP exp_2	3502	Alpha-1-acid glycoprotein 1	ENGTISR_6_5_1_2	Outer	Outer	3.9507	0.0000	Outer	6.7428	0.0000
28	AGP exp_2	3805	Alpha-1-acid glycoprotein 1	ENGTISR_6_5_1_2	Outer	Outer	5.0611	0.0000	Outer	6.7192	0.0000
29	AGP exp_2	4775	Alpha-1-acid glycoprotein 1	ENGTISR_6_5_1_3	Outer	Outer	3.5884	0.0000	Outer	6.1466	0.0050

Supplementary Table 6. Continued.

No.	Experiment	CID Scan No.	N-glycoproteins	N-glycopeptides	Manual Classification	SVM			DNN		
						Classification	Pscore	FDR	Classification	Pscore	FDR
30	AGP exp_1	4665	Alpha-1-acid glycoprotein 1	ENGTISR_6_5_1_3	Outer	Outer	2.8247	0.0000	Outer	6.5837	0.0000
31	AGP exp_36	9466	Alpha-1-acid glycoprotein 1	ENGTISR_6_5_2_3	Dual	Dual	1.9269	0.0000	Dual	6.2293	0.0052
32	AGP exp_21	10181	Alpha-1-acid glycoprotein 1	ENGTISR_6_5_2_3	Dual	Dual	1.3228	0.0025	Dual	6.1682	0.0050
33	AGP exp_26	7877	Alpha-1-acid glycoprotein 1	ENGTISR_6_5_2_3	Dual	Dual	2.3879	0.0000	Dual	6.1675	0.0050
34	AGP exp_19	9470	Alpha-1-acid glycoprotein 1	ENGTISR_6_5_2_3	Dual	Dual	5.0930	0.0000	Dual	6.2594	0.0054
35	AGP exp_7	8661	Alpha-1-acid glycoprotein 1	ENGTISR_6_5_2_3	Dual	Dual	3.8206	0.0000	Dual	6.2088	0.0052
36	AGP exp_22	10476	Alpha-1-acid glycoprotein 1	ENGTISR_6_5_2_3	Dual	Dual	4.9179	0.0000	Dual	6.2208	0.0052
37	AGP exp_28	13182	Alpha-1-acid glycoprotein 1	ENGTISR_6_5_2_3	Dual	Dual	2.2269	0.0000	Dual	6.1942	0.0051
38	AGP exp_33	9926	Alpha-1-acid glycoprotein 1	ENGTISR_6_5_2_3	Dual	Dual	1.5123	0.0025	Dual	6.1821	0.0051
39	AGP exp_17	9707	Alpha-1-acid glycoprotein 1	ENGTISR_6_5_2_3	Dual	Dual	1.1031	0.0072	Dual	6.1809	0.0051
40	AGP exp_15	9678	Alpha-1-acid glycoprotein 1	ENGTISR_6_5_2_3	Dual	Dual	4.0010	0.0000	Dual	6.1835	0.0051
41	AGP exp_16	9689	Alpha-1-acid glycoprotein 1	ENGTISR_6_5_2_3	Dual	Dual	6.0129	0.0000	Dual	6.2612	0.0054
42	AGP exp_32	9793	Alpha-1-acid glycoprotein 1	ENGTISR_6_5_2_3	Dual	Dual	5.4069	0.0000	Dual	6.2588	0.0053
43	AGP exp_23	10331	Alpha-1-acid glycoprotein 1	ENGTISR_6_5_2_3	Dual	Dual	0.9816	0.0071	Dual	5.5412	0.0094
44	AGP exp_27	6869	Alpha-1-acid glycoprotein 1	ENGTISR_6_5_2_3	Dual	Dual	1.3186	0.0025	Dual	6.0767	0.0049
45	AGP exp_31	10109	Alpha-1-acid glycoprotein 1	ENGTISR_6_5_2_3	Dual	Dual	5.3473	0.0000	Dual	6.3721	0.0056
46	AGP exp_11	11998	Alpha-1-acid glycoprotein 1	ENGTISR_6_5_2_3	Dual	Dual	2.9560	0.0000	Dual	6.0967	0.0049
47	AGP exp_1	4751	Alpha-1-acid glycoprotein 1	ENGTISR_6_5_2_3	Outer	Outer	2.0323	0.0000	Outer	6.3802	0.0056
48	AGP exp_8	8629	Alpha-1-acid glycoprotein 1	ENGTISR_6_5_2_3	Dual	Dual	2.1533	0.0000	Dual	6.1632	0.0050
49	AGP exp_28	8034	Alpha-1-acid glycoprotein 1	ENGTISR_6_5_2_3	Dual	Dual	4.3588	0.0000	Dual	6.2081	0.0052
50	AGP exp_10	10883	Alpha-1-acid glycoprotein 1	ENGTISR_6_5_2_3	Dual	Dual	2.9806	0.0000	Dual	6.1987	0.0051
51	AGP exp_23	10629	Alpha-1-acid glycoprotein 1	ENGTISR_6_5_2_3	Dual	Dual	3.6935	0.0000	Dual	6.1959	0.0051
52	AGP exp_33	10185	Alpha-1-acid glycoprotein 1	ENGTISR_6_5_2_3	Dual	Dual	5.8750	0.0000	Dual	6.2033	0.0052
53	AGP exp_14	12458	Alpha-1-acid glycoprotein 1	ENGTISR_6_5_2_3	Dual	Dual	1.7488	0.0000	Dual	5.7592	0.0095
54	AGP exp_34	9940	Alpha-1-acid glycoprotein 1	ENGTISR_6_5_2_3	Dual	Dual	4.1570	0.0000	Dual	6.4062	0.0057
55	AGP exp_18	10302	Alpha-1-acid glycoprotein 1	ENGTISR_6_5_2_3	Dual	Dual	9.0683	0.0000	Dual	6.3362	0.0055
56	AGP exp_13	12345	Alpha-1-acid glycoprotein 1	ENGTISR_6_5_2_3	Dual	Dual	5.5343	0.0000	Dual	6.2574	0.0053
57	AGP exp_2	4761	Alpha-1-acid glycoprotein 1	ENGTISR_6_5_2_3	Dual	Dual	0.1396	0.3864	Dual	5.7051	0.0095
58	AGP exp_2	4640	Alpha-1-acid glycoprotein 1	ENGTISR_7_6_0_3	None	None	1.8454	0.0000	None	7.2318	0.0000
59	AGP exp_2	2168	Alpha-1-acid glycoprotein 1	ENGTISR_7_6_1_1	Outer	Outer	5.4799	0.0000	Outer	6.7090	0.0000

Supplementary Table 6. Continued.

No.	Experiment	CID Scan No.	N-glycoproteins	N-glycopeptides	Manual Classification	SVM			DNN		
						Classification	Pscore	FDR	Classification	Pscore	FDR
60	AGP exp_1	3674	Alpha-1-acid glycoprotein 1	ENGTISR_7_6_1_2	Outer	Outer	1.3460	0.0025	Outer	6.6105	0.0000
61	AGP exp_2	3280	Alpha-1-acid glycoprotein 1	ENGTISR_7_6_1_2	Outer	Outer	2.4372	0.0000	Outer	6.5841	0.0000
62	AGP exp_2	3574	Alpha-1-acid glycoprotein 1	ENGTISR_7_6_1_2	Outer	Outer	1.4963	0.0025	Outer	6.6847	0.0000
63	AGP exp_2	2931	Alpha-1-acid glycoprotein 1	ENGTISR_7_6_1_2	Outer	Outer	1.9377	0.0000	Outer	6.6938	0.0000
64	AGP exp_1	3385	Alpha-1-acid glycoprotein 1	ENGTISR_7_6_1_2	Outer	Outer	1.6900	0.0026	Outer	6.6799	0.0000
65	AGP exp_1	2790	Alpha-1-acid glycoprotein 1	ENGTISR_7_6_1_2	Outer	Outer	2.3127	0.0000	Outer	6.6600	0.0000
66	AGP exp_2	3895	Alpha-1-acid glycoprotein 1	ENGTISR_7_6_1_2	Outer	Outer	0.7086	0.0164	Outer	5.1068	0.0093
67	AGP exp_1	4688	Alpha-1-acid glycoprotein 1	ENGTISR_7_6_1_3	Outer	Outer	4.0170	0.0000	Outer	6.7324	0.0000
68	AGP exp_2	4696	Alpha-1-acid glycoprotein 1	ENGTISR_7_6_1_3	Outer	Outer	3.2365	0.0000	Outer	6.6803	0.0000
69	AGP exp_1	6102	Alpha-1-acid glycoprotein 1	ENGTISR_7_6_1_4	Outer	Outer	2.5417	0.0000	Outer	6.6418	0.0000
70	AGP exp_2	6114	Alpha-1-acid glycoprotein 1	ENGTISR_7_6_1_4	Outer	Outer	2.5641	0.0000	Outer	6.6417	0.0000
71	AGP exp_1	5897	Alpha-1-acid glycoprotein 1	ENGTISR_7_6_2_4	Outer	Outer	1.1749	0.0024	Outer	6.5885	0.0000
72	AGP exp_2	18684	Alpha-1-acid glycoprotein 1	LVPVPITNATLDQITGK_4_4_0_1	None	None	1.8483	0.0000	None	7.2146	0.0000
73	AGP exp_2	18849	Alpha-1-acid glycoprotein 1	LVPVPITNATLDQITGK_5_4_0_1	None	None	2.6591	0.0000	None	7.3596	0.0000
74	AGP exp_2	18402	Alpha-1-acid glycoprotein 1	LVPVPITNATLDQITGK_5_4_0_1	None	None	3.9177	0.0000	None	7.3569	0.0000
75	AGP exp_1	18035	Alpha-1-acid glycoprotein 1	LVPVPITNATLDQITGK_5_4_0_1	None	None	4.2533	0.0000	None	7.3603	0.0000
76	AGP exp_1	21226	Alpha-1-acid glycoprotein 1	LVPVPITNATLDQITGK_5_4_0_2	None	None	3.1145	0.0000	None	7.2940	0.0000
77	AGP exp_2	21895	Alpha-1-acid glycoprotein 1	LVPVPITNATLDQITGK_5_4_0_2	None	None	3.2008	0.0000	None	7.3046	0.0000
78	AGP exp_1	20953	Alpha-1-acid glycoprotein 1	LVPVPITNATLDQITGK_5_4_0_2	None	None	3.3266	0.0000	None	7.3103	0.0000
79	AGP exp_2	22177	Alpha-1-acid glycoprotein 1	LVPVPITNATLDQITGK_5_4_0_2	None	None	3.2260	0.0000	None	7.3008	0.0000
80	AGP exp_2	22181	Alpha-1-acid glycoprotein 1	LVPVPITNATLDQITGK_5_4_0_2	None	None	5.5229	0.0000	None	7.3553	0.0000
81	AGP exp_1	21228	Alpha-1-acid glycoprotein 1	LVPVPITNATLDQITGK_5_4_0_2	None	None	4.6708	0.0000	None	7.3462	0.0000
82	AGP exp_1	17744	Alpha-1-acid glycoprotein 1	LVPVPITNATLDQITGK_6_5_0_1	None	None	3.4811	0.0000	None	7.3720	0.0000
83	AGP exp_2	18417	Alpha-1-acid glycoprotein 1	LVPVPITNATLDQITGK_6_5_0_1	None	None	3.3644	0.0000	None	7.3362	0.0000
84	AGP exp_2	21663	Alpha-1-acid glycoprotein 1	LVPVPITNATLDQITGK_6_5_0_2	None	None	4.0110	0.0000	None	7.3160	0.0000
85	AGP exp_2	21947	Alpha-1-acid glycoprotein 1	LVPVPITNATLDQITGK_6_5_0_2	None	None	4.3897	0.0000	None	7.3289	0.0000
86	AGP exp_2	24574	Alpha-1-acid glycoprotein 1	LVPVPITNATLDQITGK_6_5_0_2	None	None	3.6076	0.0000	None	7.2904	0.0000
87	AGP exp_1	21160	Alpha-1-acid glycoprotein 1	LVPVPITNATLDQITGK_6_5_0_2	None	None	4.0035	0.0000	None	7.3200	0.0000
88	AGP exp_1	20785	Alpha-1-acid glycoprotein 1	LVPVPITNATLDQITGK_6_5_0_2	None	None	3.7407	0.0000	None	7.3255	0.0000
89	AGP exp_1	23576	Alpha-1-acid glycoprotein 1	LVPVPITNATLDQITGK_6_5_0_2	None	None	4.1506	0.0000	None	7.3409	0.0000

Supplementary Table 6. Continued.

No.	Experiment	CID Scan No.	N-glycoproteins	N-glycopeptides	Manual Classification	SVM			DNN		
						Classification	Pscore	FDR	Classification	Pscore	FDR
90	AGP exp_2	22166	Alpha-1-acid glycoprotein 1	LVPVPITNATLDQITGK_6_5_0_2	None	None	3.7752	0.0000	None	7.3175	0.0000
91	AGP exp_2	24680	Alpha-1-acid glycoprotein 1	LVPVPITNATLDQITGK_6_5_0_3	None	None	2.5918	0.0000	None	7.2926	0.0000
92	AGP exp_1	23647	Alpha-1-acid glycoprotein 1	LVPVPITNATLDQITGK_6_5_0_3	None	None	3.4706	0.0000	None	7.2899	0.0000
93	AGP exp_1	23454	Alpha-1-acid glycoprotein 1	LVPVPITNATLDQITGK_6_5_0_3	None	None	2.5721	0.0000	None	7.2978	0.0000
94	AGP exp_2	24316	Alpha-1-acid glycoprotein 1	LVPVPITNATLDQITGK_6_5_0_3	None	None	3.1380	0.0000	None	7.2778	0.0000
95	AGP exp_1	23791	Alpha-1-acid glycoprotein 1	LVPVPITNATLDQITGK_6_5_0_3	None	None	3.7673	0.0000	None	7.3056	0.0000
96	AGP exp_1	23496	Alpha-1-acid glycoprotein 1	LVPVPITNATLDQITGK_6_5_0_3	None	None	3.3549	0.0000	None	7.2883	0.0000
97	AGP exp_2	24786	Alpha-1-acid glycoprotein 1	LVPVPITNATLDQITGK_6_5_0_3	None	None	3.1663	0.0000	None	7.2927	0.0000
98	AGP exp_2	18341	Alpha-1-acid glycoprotein 1	LVPVPITNATLDQITGK_6_5_1_1	Outer	Outer	5.8363	0.0000	Outer	6.3910	0.0057
99	AGP exp_1	17678	Alpha-1-acid glycoprotein 1	LVPVPITNATLDQITGK_6_5_1_1	Outer	Outer	5.4367	0.0000	Outer	6.3813	0.0056
100	AGP exp_2	18352	Alpha-1-acid glycoprotein 1	LVPVPITNATLDQITGK_6_5_1_1	Outer	Outer	4.8966	0.0000	Outer	6.7355	0.0000
101	AGP exp_1	20837	Alpha-1-acid glycoprotein 1	LVPVPITNATLDQITGK_6_5_1_2	Outer	Outer	2.8410	0.0000	Outer	6.5235	0.0000
102	AGP exp_2	21492	Alpha-1-acid glycoprotein 1	LVPVPITNATLDQITGK_6_5_1_2	Outer	Outer	3.2634	0.0000	Outer	6.5292	0.0000
103	AGP exp_2	21811	Alpha-1-acid glycoprotein 1	LVPVPITNATLDQITGK_6_5_1_2	Outer	Outer	2.4676	0.0000	Outer	6.3861	0.0057
104	AGP exp_2	21715	Alpha-1-acid glycoprotein 1	LVPVPITNATLDQITGK_6_5_1_2	Outer	Outer	4.3874	0.0000	Outer	6.7393	0.0000
105	AGP exp_1	20848	Alpha-1-acid glycoprotein 1	LVPVPITNATLDQITGK_6_5_1_2	Outer	Outer	4.7882	0.0000	Outer	6.7437	0.0000
106	AGP exp_1	20524	Alpha-1-acid glycoprotein 1	LVPVPITNATLDQITGK_6_5_1_2	Outer	Outer	1.6454	0.0026	Outer	6.6811	0.0000
107	AGP exp_1	23819	Alpha-1-acid glycoprotein 1	LVPVPITNATLDQITGK_6_5_1_3	Outer	Outer	3.0126	0.0000	Outer	6.5005	0.0000
108	AGP exp_2	24491	Alpha-1-acid glycoprotein 1	LVPVPITNATLDQITGK_6_5_1_3	Outer	Outer	2.7684	0.0000	Outer	6.5397	0.0000
109	AGP exp_2	24741	Alpha-1-acid glycoprotein 1	LVPVPITNATLDQITGK_6_5_1_3	Outer	Outer	3.2342	0.0000	Outer	6.5530	0.0000
110	AGP exp_1	23564	Alpha-1-acid glycoprotein 1	LVPVPITNATLDQITGK_6_5_1_3	Outer	Outer	3.3524	0.0000	Outer	6.4351	0.0029
111	AGP exp_1	23449	Alpha-1-acid glycoprotein 1	LVPVPITNATLDQITGK_6_5_1_3	Outer	Outer	3.3853	0.0000	Outer	6.7019	0.0000
112	AGP exp_2	24453	Alpha-1-acid glycoprotein 1	LVPVPITNATLDQITGK_6_5_1_3	Outer	Outer	5.3948	0.0000	Outer	6.7122	0.0000
113	AGP exp_2	24527	Alpha-1-acid glycoprotein 1	LVPVPITNATLDQITGK_6_5_1_3	Outer	Outer	1.5981	0.0026	Outer	6.6040	0.0000
114	AGP exp_1	23649	Alpha-1-acid glycoprotein 1	LVPVPITNATLDQITGK_6_5_1_3	Outer	Outer	1.5529	0.0026	Outer	6.6571	0.0000
115	AGP exp_2	24762	Alpha-1-acid glycoprotein 1	LVPVPITNATLDQITGK_6_5_1_3	Outer	Outer	3.4135	0.0000	Outer	6.6667	0.0000
116	AGP exp_17	35459	Alpha-1-acid glycoprotein 1	LVPVPITNATLDQITGK_6_5_2_2	Dual	Dual	3.0017	0.0000	Dual	6.1046	0.0049
117	AGP exp_9	29920	Alpha-1-acid glycoprotein 1	LVPVPITNATLDQITGK_6_5_2_3	Dual	Dual	1.5618	0.0026	Dual	6.3977	0.0057
118	AGP exp_1	1604	Alpha-1-acid glycoprotein 1,2	NEEYNK_4_3_0_0	None	None	4.2351	0.0000	None	7.3523	0.0000
119	AGP exp_2	1620	Alpha-1-acid glycoprotein 1,2	NEEYNK_4_3_0_0	None	None	3.2300	0.0000	None	7.2806	0.0000

Supplementary Table 6. Continued.

No.	Experiment	CID Scan No.	N-glycoproteins	N-glycopeptides	Manual Classification	SVM			DNN		
						Classification	Pscore	FDR	Classification	Pscore	FDR
120	AGP exp_2	1459	Alpha-1-acid glycoprotein 1,2	NEEYNK_4_3_0_1	None	None	5.6724	0.0000	None	7.3759	0.0000
121	AGP exp_2	1456	Alpha-1-acid glycoprotein 1,2	NEEYNK_4_3_0_1	None	None	5.0169	0.0000	None	7.3970	0.0000
122	AGP exp_2	1955	Alpha-1-acid glycoprotein 1,2	NEEYNK_4_3_0_1	None	None	3.9518	0.0000	None	7.2801	0.0000
123	AGP exp_1	1707	Alpha-1-acid glycoprotein 1,2	NEEYNK_4_3_0_1	None	None	3.2298	0.0000	None	7.2738	0.0000
124	AGP exp_1	1461	Alpha-1-acid glycoprotein 1,2	NEEYNK_4_3_0_1	None	None	5.6291	0.0000	None	7.3745	0.0000
125	AGP exp_2	1582	Alpha-1-acid glycoprotein 1,2	NEEYNK_4_4_0_1	None	None	3.4439	0.0000	None	7.3800	0.0000
126	AGP exp_2	1774	Alpha-1-acid glycoprotein 1,2	NEEYNK_4_4_0_1	None	None	3.4937	0.0000	None	7.1492	0.0000
127	AGP exp_1	1730	Alpha-1-acid glycoprotein 1,2	NEEYNK_4_4_0_1	None	None	3.2545	0.0000	None	7.2449	0.0000
128	AGP exp_1	1571	Alpha-1-acid glycoprotein 1,2	NEEYNK_4_4_0_1	None	None	2.6851	0.0000	None	7.3220	0.0000
129	AGP exp_1	1598	Alpha-1-acid glycoprotein 1,2	NEEYNK_4_4_0_1	None	None	4.2406	0.0000	None	7.3174	0.0000
130	AGP exp_1	1432	Alpha-1-acid glycoprotein 1,2	NEEYNK_5_4_0_0	None	None	2.3909	0.0000	None	7.3094	0.0000
131	AGP exp_2	1404	Alpha-1-acid glycoprotein 1,2	NEEYNK_5_4_0_0	None	None	3.4515	0.0000	None	7.3720	0.0000
132	AGP exp_2	1622	Alpha-1-acid glycoprotein 1,2	NEEYNK_5_4_0_0	None	None	2.8494	0.0000	None	7.1948	0.0000
133	AGP exp_2	1871	Alpha-1-acid glycoprotein 1,2	NEEYNK_5_4_0_0	None	None	3.5384	0.0000	None	7.2341	0.0000
134	AGP exp_2	1963	Alpha-1-acid glycoprotein 1,2	NEEYNK_5_4_0_1	None	None	2.8572	0.0000	None	7.2915	0.0000
135	AGP exp_2	1960	Alpha-1-acid glycoprotein 1,2	NEEYNK_5_4_0_1	None	None	3.0855	0.0000	None	6.9043	0.0000
136	AGP exp_2	1776	Alpha-1-acid glycoprotein 1,2	NEEYNK_5_4_0_1	None	None	2.8441	0.0000	None	7.3158	0.0000
137	AGP exp_2	1772	Alpha-1-acid glycoprotein 1,2	NEEYNK_5_4_0_1	None	None	3.3945	0.0000	None	7.1079	0.0000
138	AGP exp_1	1942	Alpha-1-acid glycoprotein 1,2	NEEYNK_5_4_0_1	None	None	2.8477	0.0000	None	7.2931	0.0000
139	AGP exp_1	1717	Alpha-1-acid glycoprotein 1,2	NEEYNK_5_4_0_1	None	None	2.9489	0.0000	None	7.0823	0.0000
140	AGP exp_2	2848	Alpha-1-acid glycoprotein 1,2	NEEYNK_5_4_0_1	None	None	3.5696	0.0000	None	6.9572	0.0000
141	AGP exp_1	2822	Alpha-1-acid glycoprotein 1,2	NEEYNK_5_4_0_1	None	None	3.0057	0.0000	None	6.9574	0.0000
142	AGP exp_1	1937	Alpha-1-acid glycoprotein 1,2	NEEYNK_5_4_0_1	None	None	3.3968	0.0000	None	6.8089	0.0000
143	AGP exp_1	2122	Alpha-1-acid glycoprotein 1,2	NEEYNK_5_4_0_1	None	None	3.3132	0.0000	None	6.7731	0.0000
144	AGP exp_2	2130	Alpha-1-acid glycoprotein 1,2	NEEYNK_5_4_0_1	None	None	3.6642	0.0000	None	7.1684	0.0000
145	AGP exp_2	2280	Alpha-1-acid glycoprotein 1,2	NEEYNK_5_4_0_1	None	None	2.6934	0.0000	None	7.2098	0.0000
146	AGP exp_1	2709	Alpha-1-acid glycoprotein 1,2	NEEYNK_5_4_0_2	None	None	4.2379	0.0000	None	7.3236	0.0000
147	AGP exp_2	3443	Alpha-1-acid glycoprotein 1,2	NEEYNK_5_4_0_2	None	None	2.9149	0.0000	None	7.2817	0.0000
148	AGP exp_2	2710	Alpha-1-acid glycoprotein 1,2	NEEYNK_5_4_0_2	None	None	4.1254	0.0000	None	7.3173	0.0000
149	AGP exp_1	3310	Alpha-1-acid glycoprotein 1,2	NEEYNK_5_4_0_2	None	None	2.8892	0.0000	None	7.2775	0.0000

Supplementary Table 6. Continued.

No.	Experiment	CID Scan No.	N-glycoproteins	N-glycopeptides	Manual Classification	SVM			DNN		
						Classification	Pscore	FDR	Classification	Pscore	FDR
150	AGP exp_2	2806	Alpha-1-acid glycoprotein 1,2	NEEYNK_5_4_0_2	None	None	4.0969	0.0000	None	7.3172	0.0000
151	AGP exp_1	3042	Alpha-1-acid glycoprotein 1,2	NEEYNK_5_4_0_2	None	None	3.6173	0.0000	None	7.3096	0.0000
152	AGP exp_2	3146	Alpha-1-acid glycoprotein 1,2	NEEYNK_5_4_0_2	None	None	3.8253	0.0000	None	7.3070	0.0000
153	AGP exp_2	2545	Alpha-1-acid glycoprotein 1,2	NEEYNK_5_4_0_2	None	None	2.9264	0.0000	None	7.1620	0.0000
154	AGP exp_2	3159	Alpha-1-acid glycoprotein 1,2	NEEYNK_5_4_0_2	None	None	3.1025	0.0000	None	7.2279	0.0000
155	AGP exp_2	2710	Alpha-1-acid glycoprotein 1,2	NEEYNK_5_4_0_2	None	None	4.1254	0.0000	None	7.3173	0.0000
156	AGP exp_1	3115	Alpha-1-acid glycoprotein 1,2	NEEYNK_5_4_0_2	None	None	3.3099	0.0000	None	7.1882	0.0000
157	AGP exp_2	4501	Alpha-1-acid glycoprotein 1,2	NEEYNK_5_4_0_2	None	None	3.8572	0.0000	None	7.2877	0.0000
158	AGP exp_2	3738	Alpha-1-acid glycoprotein 1,2	NEEYNK_5_4_0_2	None	None	2.4811	0.0000	None	7.2780	0.0000
159	AGP exp_2	3470	Alpha-1-acid glycoprotein 1,2	NEEYNK_5_4_0_2	None	None	3.3912	0.0000	None	7.2123	0.0000
160	AGP exp_1	3387	Alpha-1-acid glycoprotein 1,2	NEEYNK_5_4_0_2	None	None	3.2015	0.0000	None	7.3429	0.0000
161	AGP exp_2	3794	Alpha-1-acid glycoprotein 1,2	NEEYNK_5_4_0_2	None	None	2.0508	0.0000	None	6.9221	0.0000
162	AGP exp_1	2977	Alpha-1-acid glycoprotein 1,2	NEEYNK_5_4_1_2	Core	Core	0.5235	0.0490	Core	6.6365	0.0000
163	AGP exp_2	3119	Alpha-1-acid glycoprotein 1,2	NEEYNK_5_4_1_2	Core	Core	1.4007	0.0025	Core	6.6247	0.0000
164	AGP exp_1	2420	Alpha-1-acid glycoprotein 1,2	NEEYNK_5_4_1_2	Outer	Outer	3.6095	0.0000	Outer	6.6868	0.0000
165	AGP exp_2	2514	Alpha-1-acid glycoprotein 1,2	NEEYNK_5_4_1_2	Outer	Outer	3.1255	0.0000	Outer	6.6266	0.0000
166	AGP exp_1	2651	Alpha-1-acid glycoprotein 1,2	NEEYNK_5_4_1_2	Outer	Outer	1.2365	0.0024	Outer	6.3561	0.0055
167	AGP exp_2	2787	Alpha-1-acid glycoprotein 1,2	NEEYNK_5_4_1_2	Outer	Outer	4.1798	0.0000	Outer	6.6467	0.0000
168	AGP exp_17	4351	Alpha-1-acid glycoprotein 1,2	NEEYNK_5_4_2_2	Dual	Dual	3.5701	0.0000	Dual	6.2850	0.0054
169	AGP exp_8	4179	Alpha-1-acid glycoprotein 1,2	NEEYNK_5_4_2_2	Dual	Dual	0.6683	0.0209	Dual	4.6692	0.0092
170	AGP exp_30	4733	Alpha-1-acid glycoprotein 1,2	NEEYNK_5_4_2_2	Dual	Dual	1.8525	0.0000	Dual	6.0131	0.0073
171	AGP exp_20	4457	Alpha-1-acid glycoprotein 1,2	NEEYNK_5_4_2_2	Dual	Dual	4.3552	0.0000	Dual	6.3825	0.0056
172	AGP exp_11	4971	Alpha-1-acid glycoprotein 1,2	NEEYNK_5_4_2_2	Dual	Dual	0.1564	0.3745	Dual	5.1266	0.0093
173	AGP exp_13	4698	Alpha-1-acid glycoprotein 1,2	NEEYNK_5_4_2_2	Dual	Dual	3.2402	0.0000	Dual	6.3492	0.0055
174	AGP exp_18	4497	Alpha-1-acid glycoprotein 1,2	NEEYNK_5_4_2_2	Dual	Dual	1.0323	0.0072	Dual	5.2996	0.0094
175	AGP exp_21	4556	Alpha-1-acid glycoprotein 1,2	NEEYNK_5_4_2_2	Dual	Dual	2.1135	0.0000	Dual	5.9782	0.0097
176	AGP exp_1	3630	Alpha-1-acid glycoprotein 1,2	NEEYNK_5_5_0_2	None	None	3.2616	0.0000	None	7.2910	0.0000
177	AGP exp_2	2140	Alpha-1-acid glycoprotein 1,2	NEEYNK_6_5_0_1	None	None	2.3822	0.0000	None	7.2510	0.0000
178	AGP exp_2	1970	Alpha-1-acid glycoprotein 1,2	NEEYNK_6_5_0_1	None	None	2.2476	0.0000	None	7.2888	0.0000
179	AGP exp_1	1919	Alpha-1-acid glycoprotein 1,2	NEEYNK_6_5_0_1	None	None	2.6671	0.0000	None	7.2675	0.0000

Supplementary Table 6. Continued.

No.	Experiment	CID Scan No.	N-glycoproteins	N-glycopeptides	Manual Classification	SVM			DNN		
						Classification	Pscore	FDR	Classification	Pscore	FDR
180	AGP exp_2	2289	Alpha-1-acid glycoprotein 1,2	NEEYNK_6_5_0_1	None	None	2.4219	0.0000	None	7.2676	0.0000
181	AGP exp_1	2100	Alpha-1-acid glycoprotein 1,2	NEEYNK_6_5_0_1	None	None	2.0142	0.0000	None	7.2580	0.0000
182	AGP exp_1	2242	Alpha-1-acid glycoprotein 1,2	NEEYNK_6_5_0_1	None	None	2.2420	0.0000	None	7.2394	0.0000
183	AGP exp_2	1967	Alpha-1-acid glycoprotein 1,2	NEEYNK_6_5_0_1	None	None	2.5911	0.0000	None	7.3018	0.0000
184	AGP exp_2	2204	Alpha-1-acid glycoprotein 1,2	NEEYNK_6_5_0_1	None	None	2.9485	0.0000	None	6.7648	0.0000
185	AGP exp_1	2159	Alpha-1-acid glycoprotein 1,2	NEEYNK_6_5_0_1	None	None	2.4474	0.0000	None	7.0754	0.0000
186	AGP exp_1	1703	Alpha-1-acid glycoprotein 1,2	NEEYNK_6_5_0_1	None	None	1.6353	0.0026	None	7.2540	0.0000
187	AGP exp_2	3251	Alpha-1-acid glycoprotein 1,2	NEEYNK_6_5_0_2	None	None	2.4609	0.0000	None	7.3114	0.0000
188	AGP exp_1	3403	Alpha-1-acid glycoprotein 1,2	NEEYNK_6_5_0_2	None	None	2.6248	0.0000	None	7.3026	0.0000
189	AGP exp_2	3713	Alpha-1-acid glycoprotein 1,2	NEEYNK_6_5_0_2	None	None	2.6156	0.0000	None	7.1986	0.0000
190	AGP exp_2	3572	Alpha-1-acid glycoprotein 1,2	NEEYNK_6_5_0_2	None	None	3.0547	0.0000	None	7.3050	0.0000
191	AGP exp_1	3672	Alpha-1-acid glycoprotein 1,2	NEEYNK_6_5_0_2	None	None	2.7065	0.0000	None	7.2660	0.0000
192	AGP exp_1	3157	Alpha-1-acid glycoprotein 1,2	NEEYNK_6_5_0_2	None	None	2.6331	0.0000	None	7.2571	0.0000
193	AGP exp_1	2840	Alpha-1-acid glycoprotein 1,2	NEEYNK_6_5_0_2	None	None	2.8122	0.0000	None	7.2990	0.0000
194	AGP exp_2	3880	Alpha-1-acid glycoprotein 1,2	NEEYNK_6_5_0_2	None	None	2.9622	0.0000	None	7.2881	0.0000
195	AGP exp_2	2944	Alpha-1-acid glycoprotein 1,2	NEEYNK_6_5_0_2	None	None	1.7460	0.0000	None	7.2835	0.0000
196	AGP exp_2	3063	Alpha-1-acid glycoprotein 1,2	NEEYNK_6_5_0_2	None	None	2.6836	0.0000	None	7.1618	0.0000
197	AGP exp_1	3976	Alpha-1-acid glycoprotein 1,2	NEEYNK_6_5_0_2	None	None	1.8375	0.0000	None	7.2805	0.0000
198	AGP exp_2	4584	Alpha-1-acid glycoprotein 1,2	NEEYNK_6_5_0_3	None	None	3.2134	0.0000	None	7.2638	0.0000
199	AGP exp_1	4491	Alpha-1-acid glycoprotein 1,2	NEEYNK_6_5_0_3	None	None	3.4492	0.0000	None	7.2909	0.0000
200	AGP exp_2	2086	Alpha-1-acid glycoprotein 1,2	NEEYNK_6_5_1_1	Outer	Outer	4.1093	0.0000	Outer	6.6479	0.0000
201	AGP exp_1	2075	Alpha-1-acid glycoprotein 1,2	NEEYNK_6_5_1_1	Outer	Outer	1.7502	0.0000	Outer	6.5879	0.0000
202	AGP exp_1	1898	Alpha-1-acid glycoprotein 1,2	NEEYNK_6_5_1_1	Outer	Outer	1.2386	0.0024	Outer	6.7095	0.0000
203	AGP exp_1	1677	Alpha-1-acid glycoprotein 1,2	NEEYNK_6_5_1_1	Outer	Outer	1.4947	0.0025	Outer	6.6653	0.0000
204	AGP exp_2	1705	Alpha-1-acid glycoprotein 1,2	NEEYNK_6_5_1_1	Outer	Outer	3.5599	0.0000	Outer	6.7359	0.0000
205	AGP exp_1	2780	Alpha-1-acid glycoprotein 1,2	NEEYNK_6_5_1_2	Outer	Outer	4.3569	0.0000	Outer	6.6430	0.0000
206	AGP exp_1	2462	Alpha-1-acid glycoprotein 1,2	NEEYNK_6_5_1_2	Outer	Outer	5.6047	0.0000	Outer	6.7593	0.0000
207	AGP exp_1	3578	Alpha-1-acid glycoprotein 1,2	NEEYNK_6_5_1_2	Outer	Outer	1.3193	0.0025	Outer	5.8283	0.0095
208	AGP exp_2	2852	Alpha-1-acid glycoprotein 1,2	NEEYNK_6_5_1_2	Outer	Outer	4.5574	0.0000	Outer	6.6507	0.0000
209	AGP exp_2	3164	Alpha-1-acid glycoprotein 1,2	NEEYNK_6_5_1_2	Outer	Outer	2.7809	0.0000	Outer	6.5040	0.0000

Supplementary Table 6. Continued.

No.	Experiment	CID Scan No.	N-glycoproteins	N-glycopeptides	Manual Classification	SVM			DNN		
						Classification	Pscore	FDR	Classification	Pscore	FDR
210	AGP exp_1	3070	Alpha-1-acid glycoprotein 1,2	NEEYNK_6_5_1_2	Outer	Outer	3.8469	0.0000	Outer	6.6099	0.0000
211	AGP exp_2	3788	Alpha-1-acid glycoprotein 1,2	NEEYNK_6_5_1_2	Outer	Outer	3.1346	0.0000	Outer	6.6529	0.0000
212	AGP exp_1	4387	Alpha-1-acid glycoprotein 1,2	NEEYNK_6_5_1_3	Outer	Outer	3.3251	0.0000	Outer	6.4006	0.0057
213	AGP exp_2	4474	Alpha-1-acid glycoprotein 1,2	NEEYNK_6_5_1_3	Outer	Outer	3.4935	0.0000	Outer	6.3556	0.0055
214	AGP exp_22	3991	Alpha-1-acid glycoprotein 1,2	NEEYNK_6_5_2_2	Dual	Dual	0.7552	0.0142	Dual	5.6358	0.0094
215	AGP exp_29	3654	Alpha-1-acid glycoprotein 1,2	NEEYNK_6_5_2_2	Dual	Dual	2.0181	0.0000	Dual	5.9476	0.0096
216	AGP exp_24	4318	Alpha-1-acid glycoprotein 1,2	NEEYNK_6_5_2_2	Dual	Dual	6.3717	0.0000	Dual	6.2480	0.0053
217	AGP exp_36	5124	Alpha-1-acid glycoprotein 1,2	NEEYNK_6_5_2_2	Dual	Dual	0.0211	0.4802	Dual	4.7695	0.0092
218	AGP exp_8	3879	Alpha-1-acid glycoprotein 1,2	NEEYNK_6_5_2_2	Dual	Dual	1.4193	0.0025	Dual	6.0455	0.0073
219	AGP exp_25	4570	Alpha-1-acid glycoprotein 1,2	NEEYNK_6_5_2_2	Dual	Dual	2.8460	0.0000	Dual	6.2216	0.0052
220	AGP exp_31	5184	Alpha-1-acid glycoprotein 1,2	NEEYNK_6_5_2_2	Dual	Dual	2.5829	0.0000	Dual	6.1243	0.0050
221	AGP exp_31	8937	Alpha-1-acid glycoprotein 1,2	NEEYNK_6_5_2_3	Dual	Dual	4.8289	0.0000	Dual	6.3255	0.0055
222	AGP exp_7	7429	Alpha-1-acid glycoprotein 1,2	NEEYNK_6_5_2_3	Dual	Dual	3.8068	0.0000	Dual	6.3737	0.0056
223	AGP exp_35	7860	Alpha-1-acid glycoprotein 1,2	NEEYNK_6_5_2_3	Dual	Dual	3.3430	0.0000	Dual	6.3824	0.0056
224	AGP exp_6	5921	Alpha-1-acid glycoprotein 1,2	NEEYNK_6_5_2_3	Dual	Dual	2.0065	0.0000	Dual	6.4092	0.0029
225	AGP exp_18	8454	Alpha-1-acid glycoprotein 1,2	NEEYNK_6_5_2_3	Dual	Dual	3.4738	0.0000	Dual	6.3692	0.0055
226	AGP exp_1	4426	Alpha-1-acid glycoprotein 1,2	NEEYNK_6_5_2_3	Dual	Dual	2.0307	0.0000	Dual	5.9214	0.0096
227	AGP exp_29	6967	Alpha-1-acid glycoprotein 1,2	NEEYNK_6_5_2_3	Dual	Dual	5.0605	0.0000	Dual	6.2548	0.0053
228	AGP exp_30	8720	Alpha-1-acid glycoprotein 1,2	NEEYNK_6_5_2_3	Dual	Dual	4.1497	0.0000	Dual	6.3737	0.0056
229	AGP exp_28	6963	Alpha-1-acid glycoprotein 1,2	NEEYNK_6_5_2_3	Dual	Dual	4.6777	0.0000	Dual	6.1839	0.0051
230	AGP exp_15	8293	Alpha-1-acid glycoprotein 1,2	NEEYNK_6_5_2_3	Dual	Dual	4.3158	0.0000	Dual	6.2889	0.0054
231	AGP exp_8	7529	Alpha-1-acid glycoprotein 1,2	NEEYNK_6_5_2_3	Dual	Dual	3.7841	0.0000	Dual	6.2623	0.0054
232	AGP exp_33	8943	Alpha-1-acid glycoprotein 1,2	NEEYNK_6_5_2_3	Dual	Dual	3.5011	0.0000	Dual	6.2562	0.0053
233	AGP exp_25	8807	Alpha-1-acid glycoprotein 1,2	NEEYNK_6_5_2_3	Dual	Dual	3.3800	0.0000	Dual	5.9661	0.0096
234	AGP exp_2	4447	Alpha-1-acid glycoprotein 1,2	NEEYNK_6_5_2_3	Dual	Dual	0.7530	0.0141	Dual	4.9629	0.0093
235	AGP exp_25	8651	Alpha-1-acid glycoprotein 1,2	NEEYNK_6_5_2_3	Dual	Dual	2.7699	0.0000	Dual	6.0421	0.0073
236	AGP exp_13	9766	Alpha-1-acid glycoprotein 1,2	NEEYNK_6_5_2_3	Dual	Dual	1.8164	0.0000	Dual	6.1662	0.0050
237	AGP exp_12	9919	Alpha-1-acid glycoprotein 1,2	NEEYNK_6_5_2_3	Dual	Dual	1.2907	0.0025	Dual	6.2717	0.0054
238	AGP exp_14	9944	Alpha-1-acid glycoprotein 1,2	NEEYNK_6_5_2_3	Dual	Dual	0.6076	0.0320	Dual	4.6344	0.0092
239	AGP exp_1	4393	Alpha-1-acid glycoprotein 1,2	NEEYNK_6_5_3_1	Outer	Outer	3.8706	0.0000	Outer	6.6333	0.0000

Supplementary Table 6. Continued.

No.	Experiment	CID Scan No.	N-glycoproteins	N-glycopeptides	Manual Classification	SVM			DNN		
						Classification	Pscore	FDR	Classification	Pscore	FDR
240	AGP exp_1	25092	Alpha-1-acid glycoprotein 1,2	SVQEIQATFFYFTPNK_6_5_0_3	None	None	2.2758	0.0000	None	7.2026	0.0000
241	AGP exp_1	2081	Alpha-1-acid glycoprotein 2	ENGTVSR_5_4_0_2	None	None	2.8546	0.0000	None	7.2778	0.0000
242	AGP exp_2	1631	Alpha-1-acid glycoprotein 2	ENGTVSR_6_5_0_1	None	None	2.4291	0.0000	None	7.2559	0.0000
243	AGP exp_1	2325	Alpha-1-acid glycoprotein 2	ENGTVSR_6_5_0_2	None	None	2.8573	0.0000	None	7.3181	0.0000
244	AGP exp_2	2363	Alpha-1-acid glycoprotein 2	ENGTVSR_6_5_0_2	None	None	2.0666	0.0000	None	7.3137	0.0000
245	AGP exp_1	2200	Alpha-1-acid glycoprotein 2	ENGTVSR_6_5_0_2	None	None	2.3083	0.0000	None	7.2911	0.0000
246	AGP exp_1	2417	Alpha-1-acid glycoprotein 2	ENGTVSR_6_5_0_2	None	None	2.0692	0.0000	None	7.2970	0.0000
247	AGP exp_2	4012	Alpha-1-acid glycoprotein 2	ENGTVSR_6_5_0_3	None	None	2.8613	0.0000	None	7.2828	0.0000
248	AGP exp_1	3871	Alpha-1-acid glycoprotein 2	ENGTVSR_6_5_0_3	None	None	3.2116	0.0000	None	7.3028	0.0000
249	AGP exp_1	4149	Alpha-1-acid glycoprotein 2	ENGTVSR_6_5_0_3	None	None	3.2606	0.0000	None	7.2736	0.0000
250	AGP exp_2	1588	Alpha-1-acid glycoprotein 2	ENGTVSR_6_5_1_1	Outer	Outer	4.9649	0.0000	Outer	6.7484	0.0000
251	AGP exp_2	2426	Alpha-1-acid glycoprotein 2	ENGTVSR_6_5_1_2	Outer	Outer	4.4813	0.0000	Outer	6.6727	0.0000
252	AGP exp_1	2302	Alpha-1-acid glycoprotein 2	ENGTVSR_6_5_1_2	Outer	Outer	3.3411	0.0000	Outer	6.7594	0.0000
253	AGP exp_2	3847	Alpha-1-acid glycoprotein 2	ENGTVSR_6_5_1_3	Outer	Outer	2.8171	0.0000	Outer	6.2946	0.0054
254	AGP exp_1	3758	Alpha-1-acid glycoprotein 2	ENGTVSR_6_5_1_3	Outer	Outer	1.6581	0.0026	Outer	5.1196	0.0093
255	AGP exp_1	4042	Alpha-1-acid glycoprotein 2	ENGTVSR_6_5_1_3	Outer	Outer	4.8734	0.0000	Outer	6.6466	0.0000
256	AGP exp_25	6954	Alpha-1-acid glycoprotein 2	ENGTVSR_6_5_2_3	Dual	Dual	1.9626	0.0000	Dual	6.1322	0.0050
257	AGP exp_23	7051	Alpha-1-acid glycoprotein 2	ENGTVSR_6_5_2_3	Dual	Dual	4.2177	0.0000	Dual	6.2530	0.0053
258	AGP exp_30	7102	Alpha-1-acid glycoprotein 2	ENGTVSR_6_5_2_3	Dual	Dual	4.4136	0.0000	Dual	6.1742	0.0050
259	AGP exp_24	6629	Alpha-1-acid glycoprotein 2	ENGTVSR_6_5_2_3	Dual	Dual	4.0885	0.0000	Dual	6.2147	0.0052
260	AGP exp_20	6653	Alpha-1-acid glycoprotein 2	ENGTVSR_6_5_2_3	Dual	Dual	1.2355	0.0024	Dual	5.8424	0.0095
261	AGP exp_12	7628	Alpha-1-acid glycoprotein 2	ENGTVSR_6_5_2_3	Dual	Dual	2.4547	0.0000	Dual	5.8802	0.0096
262	AGP exp_5	6180	Alpha-1-acid glycoprotein 2	ENGTVSR_6_5_2_3	Dual	Dual	4.4363	0.0000	Dual	6.2476	0.0053
263	AGP exp_2	1493	Alpha-1-acid glycoprotein 2	ENGTVSR_7_6_1_1	Outer	Outer	5.6467	0.0000	Outer	6.6722	0.0000
264	AGP exp_1	2034	Alpha-1-acid glycoprotein 2	ENGTVSR_7_6_1_2	Outer	Outer	2.7196	0.0000	Outer	6.6521	0.0000
265	AGP exp_2	2068	Alpha-1-acid glycoprotein 2	ENGTVSR_7_6_1_2	Outer	Outer	2.4661	0.0000	Outer	6.6813	0.0000
266	AGP exp_1	2173	Alpha-1-acid glycoprotein 2	ENGTVSR_7_6_1_2	Outer	Outer	1.7041	0.0026	Outer	6.6402	0.0000
267	AGP exp_2	5011	Alpha-1-acid glycoprotein 2	ENGTVSR_7_6_1_4	Outer	Outer	2.3367	0.0000	Outer	6.6089	0.0000
268	AGP exp_1	5112	Alpha-1-acid glycoprotein 2	ENGTVSR_7_6_1_4	Outer	Outer	2.0611	0.0000	Outer	6.5772	0.0000
269	AGP exp_1	14653	Alpha-1-acid glycoprotein 2	LVPVPITNATLDR_3_3_0_0	None	None	2.8141	0.0000	None	7.1632	0.0000

Supplementary Table 6. Continued.

No.	Experiment	CID Scan No.	N-glycoproteins	N-glycopeptides	Manual Classification	SVM			DNN		
						Classification	Pscore	FDR	Classification	Pscore	FDR
270	AGP exp_2	15353	Alpha-1-acid glycoprotein 2	LVPVPITNATLDR_3_3_0_0	None	None	3.1758	0.0000	None	7.2984	0.0000
271	AGP exp_2	15433	Alpha-1-acid glycoprotein 2	LVPVPITNATLDR_3_3_0_0	None	None	3.2431	0.0000	None	7.3042	0.0000
272	AGP exp_1	14732	Alpha-1-acid glycoprotein 2	LVPVPITNATLDR_3_3_0_0	None	None	3.0890	0.0000	None	7.2646	0.0000
273	AGP exp_2	12253	Alpha-1-acid glycoprotein 2	LVPVPITNATLDR_4_4_0_1	None	None	2.9274	0.0000	None	7.3197	0.0000
274	AGP exp_1	14693	Alpha-1-acid glycoprotein 2	LVPVPITNATLDR_5_4_0_1	None	None	2.4356	0.0000	None	7.0137	0.0000
275	AGP exp_2	15498	Alpha-1-acid glycoprotein 2	LVPVPITNATLDR_5_4_0_1	None	None	2.7406	0.0000	None	7.0088	0.0000
276	AGP exp_2	15349	Alpha-1-acid glycoprotein 2	LVPVPITNATLDR_5_4_0_1	None	None	4.0226	0.0000	None	7.3444	0.0000
277	AGP exp_2	12062	Alpha-1-acid glycoprotein 2	LVPVPITNATLDR_5_4_0_1	None	None	5.1439	0.0000	None	7.3488	0.0000
278	AGP exp_1	15375	Alpha-1-acid glycoprotein 2	LVPVPITNATLDR_5_4_0_2	None	None	2.3111	0.0000	None	7.2810	0.0000
279	AGP exp_2	15598	Alpha-1-acid glycoprotein 2	LVPVPITNATLDR_5_4_0_2	None	None	2.3080	0.0000	None	6.8756	0.0000
280	AGP exp_1	15027	Alpha-1-acid glycoprotein 2	LVPVPITNATLDR_5_4_0_2	None	None	4.2682	0.0000	None	7.3362	0.0000
281	AGP exp_1	15062	Alpha-1-acid glycoprotein 2	LVPVPITNATLDR_5_4_1_2	Outer	Outer	1.0300	0.0072	Outer	5.6798	0.0095
282	AGP exp_2	15539	Alpha-1-acid glycoprotein 2	LVPVPITNATLDR_5_4_1_2	Outer	Outer	1.2773	0.0025	Outer	6.2349	0.0052
283	AGP exp_2	12153	Alpha-1-acid glycoprotein 2	LVPVPITNATLDR_5_5_0_1	None	None	3.0663	0.0000	None	7.3126	0.0000
284	AGP exp_1	11548	Alpha-1-acid glycoprotein 2	LVPVPITNATLDR_5_5_0_1	None	None	2.0510	0.0000	None	7.3090	0.0000
285	AGP exp_1	11847	Alpha-1-acid glycoprotein 2	LVPVPITNATLDR_5_5_0_1	None	None	3.9229	0.0000	None	7.3714	0.0000
286	AGP exp_2	15705	Alpha-1-acid glycoprotein 2	LVPVPITNATLDR_5_5_0_2	None	None	2.0980	0.0000	None	7.2744	0.0000
287	AGP exp_2	12110	Alpha-1-acid glycoprotein 2	LVPVPITNATLDR_6_5_0_1	None	None	1.8582	0.0000	None	7.2348	0.0000
288	AGP exp_2	11937	Alpha-1-acid glycoprotein 2	LVPVPITNATLDR_6_5_0_1	None	None	3.3524	0.0000	None	7.3352	0.0000
289	AGP exp_1	11608	Alpha-1-acid glycoprotein 2	LVPVPITNATLDR_6_5_0_1	None	None	3.3947	0.0000	None	7.3270	0.0000
290	AGP exp_1	14686	Alpha-1-acid glycoprotein 2	LVPVPITNATLDR_6_5_0_2	None	None	2.2535	0.0000	None	6.9449	0.0000
291	AGP exp_1	14947	Alpha-1-acid glycoprotein 2	LVPVPITNATLDR_6_5_0_2	None	None	3.2958	0.0000	None	7.3256	0.0000
292	AGP exp_2	15460	Alpha-1-acid glycoprotein 2	LVPVPITNATLDR_6_5_0_2	None	None	3.0793	0.0000	None	7.3191	0.0000
293	AGP exp_1	14571	Alpha-1-acid glycoprotein 2	LVPVPITNATLDR_6_5_0_2	None	None	3.2652	0.0000	None	7.3131	0.0000
294	AGP exp_2	15123	Alpha-1-acid glycoprotein 2	LVPVPITNATLDR_6_5_0_2	None	None	3.3663	0.0000	None	7.3204	0.0000
295	AGP exp_1	18288	Alpha-1-acid glycoprotein 2	LVPVPITNATLDR_6_5_0_3	None	None	1.9117	0.0000	None	7.2676	0.0000
296	AGP exp_1	18623	Alpha-1-acid glycoprotein 2	LVPVPITNATLDR_6_5_0_3	None	None	1.8812	0.0000	None	7.2664	0.0000
297	AGP exp_2	19281	Alpha-1-acid glycoprotein 2	LVPVPITNATLDR_6_5_0_3	None	None	1.8445	0.0000	None	7.2595	0.0000
298	AGP exp_2	19603	Alpha-1-acid glycoprotein 2	LVPVPITNATLDR_6_5_0_3	None	None	2.4175	0.0000	None	7.2384	0.0000
299	AGP exp_2	18920	Alpha-1-acid glycoprotein 2	LVPVPITNATLDR_6_5_0_3	None	None	2.5186	0.0000	None	7.2647	0.0000

Supplementary Table 6. Continued.

No.	Experiment	CID Scan No.	N-glycoproteins	N-glycopeptides	Manual Classification	SVM			DNN		
						Classification	Pscore	FDR	Classification	Pscore	FDR
300	AGP exp_2	11797	Alpha-1-acid glycoprotein 2	LVPVPITNATLDR_6_5_1_1	Outer	Outer	3.5085	0.0000	Outer	6.6678	0.0000
301	AGP exp_1	11413	Alpha-1-acid glycoprotein 2	LVPVPITNATLDR_6_5_1_1	Outer	Outer	12.1950	0.0000	Outer	6.6418	0.0000
302	AGP exp_1	14674	Alpha-1-acid glycoprotein 2	LVPVPITNATLDR_6_5_1_2	Outer	Outer	1.9970	0.0000	Outer	6.3895	0.0057
303	AGP exp_2	15241	Alpha-1-acid glycoprotein 2	LVPVPITNATLDR_6_5_1_2	Outer	Outer	1.3472	0.0025	Outer	6.5181	0.0000
304	AGP exp_1	15014	Alpha-1-acid glycoprotein 2	LVPVPITNATLDR_6_5_1_2	Outer	Outer	1.8188	0.0000	Outer	6.5939	0.0000
305	AGP exp_2	15562	Alpha-1-acid glycoprotein 2	LVPVPITNATLDR_6_5_1_2	Outer	Outer	2.6626	0.0000	Outer	6.5781	0.0000
306	AGP exp_2	14877	Alpha-1-acid glycoprotein 2	LVPVPITNATLDR_6_5_1_2	Outer	Outer	2.7646	0.0000	Outer	6.5441	0.0000
307	AGP exp_1	14782	Alpha-1-acid glycoprotein 2	LVPVPITNATLDR_6_5_1_2	Outer	Outer	3.3641	0.0000	Outer	6.7559	0.0000
308	AGP exp_2	15289	Alpha-1-acid glycoprotein 2	LVPVPITNATLDR_6_5_1_2	Outer	Outer	4.7531	0.0000	Outer	6.7752	0.0000
309	AGP exp_1	14434	Alpha-1-acid glycoprotein 2	LVPVPITNATLDR_6_5_1_2	Outer	Outer	2.9615	0.0000	Outer	6.7741	0.0000
310	AGP exp_1	14441	Alpha-1-acid glycoprotein 2	LVPVPITNATLDR_6_5_1_2	Outer	Outer	1.2021	0.0024	Outer	6.6818	0.0000
311	AGP exp_1	18433	Alpha-1-acid glycoprotein 2	LVPVPITNATLDR_6_5_1_3	Outer	Outer	1.5179	0.0025	Outer	6.4650	0.0029
312	AGP exp_1	18550	Alpha-1-acid glycoprotein 2	LVPVPITNATLDR_6_5_1_3	Outer	Outer	1.8265	0.0000	Outer	6.5966	0.0000
313	AGP exp_2	19176	Alpha-1-acid glycoprotein 2	LVPVPITNATLDR_6_5_1_3	Outer	Outer	2.1231	0.0000	Outer	6.6538	0.0000
314	IgG exp_4	5355	Ig gamma-1 chain C region	EEQYNSTYR_3_3_0_0	None	None	3.7077	0.0000	None	7.3127	0.0000
315	IgG exp_3	4184	Ig gamma-1 chain C region	EEQYNSTYR_3_3_1_0	Core	Core	3.9487	0.0000	Core	6.6559	0.0000
316	IgG exp_1	3022	Ig gamma-1 chain C region	EEQYNSTYR_3_3_1_0	Core	Core	4.5255	0.0000	Core	6.6590	0.0000
317	IgG exp_4	5322	Ig gamma-1 chain C region	EEQYNSTYR_3_3_1_0	Core	Core	5.0640	0.0000	Core	6.6557	0.0000
318	IgG exp_2	3021	Ig gamma-1 chain C region	EEQYNSTYR_3_3_1_0	Core	Core	7.4522	0.0000	Core	6.6333	0.0000
319	IgG exp_4	5328	Ig gamma-1 chain C region	EEQYNSTYR_3_4_0_0	None	None	2.8121	0.0000	None	7.0449	0.0000
320	IgG exp_3	4222	Ig gamma-1 chain C region	EEQYNSTYR_3_4_0_0	None	None	2.8009	0.0000	None	7.0744	0.0000
321	IgG exp_1	3126	Ig gamma-1 chain C region	EEQYNSTYR_3_4_0_0	None	None	2.9567	0.0000	None	7.1448	0.0000
322	IgG exp_3	4091	Ig gamma-1 chain C region	EEQYNSTYR_3_4_1_0	Core	Core	7.7764	0.0000	Core	6.6151	0.0000
323	IgG exp_3	4405	Ig gamma-1 chain C region	EEQYNSTYR_3_4_1_0	Core	Core	6.1788	0.0000	Core	6.6493	0.0000
324	IgG exp_4	5187	Ig gamma-1 chain C region	EEQYNSTYR_3_4_1_0	Core	Core	5.6524	0.0000	Core	6.6412	0.0000
325	IgG exp_2	2997	Ig gamma-1 chain C region	EEQYNSTYR_3_4_1_0	Core	Core	5.2609	0.0000	Core	6.6554	0.0000
326	IgG exp_1	2937	Ig gamma-1 chain C region	EEQYNSTYR_3_4_1_0	Core	Core	7.7189	0.0000	Core	6.6168	0.0000
327	IgG exp_4	5471	Ig gamma-1 chain C region	EEQYNSTYR_3_5_0_0	None	None	2.0324	0.0000	None	7.1991	0.0000
328	IgG exp_3	4720	Ig gamma-1 chain C region	EEQYNSTYR_3_5_1_0	Core	Core	4.5235	0.0000	Core	6.6477	0.0000
329	IgG exp_4	5432	Ig gamma-1 chain C region	EEQYNSTYR_3_5_1_0	Core	Core	3.7578	0.0000	Core	6.6413	0.0000

Supplementary Table 6. Continued.

No.	Experiment	CID Scan No.	N-glycoproteins	N-glycopeptides	Manual Classification	SVM			DNN		
						Classification	Pscore	FDR	Classification	Pscore	FDR
330	IgG exp_1	3153	Ig gamma-1 chain C region	EEQYNSTYR_3_5_1_0	Core	Core	4.0484	0.0000	Core	6.6485	0.0000
331	IgG exp_1	3010	Ig gamma-1 chain C region	EEQYNSTYR_4_3_1_0	Core	Core	5.4014	0.0000	Core	6.6302	0.0000
332	IgG exp_4	5305	Ig gamma-1 chain C region	EEQYNSTYR_4_3_1_0	Core	Core	5.2028	0.0000	Core	6.6253	0.0000
333	IgG exp_3	4168	Ig gamma-1 chain C region	EEQYNSTYR_4_3_1_0	Core	Core	5.6900	0.0000	Core	6.6232	0.0000
334	IgG exp_3	4197	Ig gamma-1 chain C region	EEQYNSTYR_4_4_0_0	None	None	2.8871	0.0000	None	7.0474	0.0000
335	IgG exp_1	3062	Ig gamma-1 chain C region	EEQYNSTYR_4_4_0_0	None	None	2.8884	0.0000	None	7.0485	0.0000
336	IgG exp_4	5318	Ig gamma-1 chain C region	EEQYNSTYR_4_4_0_0	None	None	2.9020	0.0000	None	7.0795	0.0000
337	IgG exp_4	5181	Ig gamma-1 chain C region	EEQYNSTYR_4_4_1_0	Core	Core	6.4000	0.0000	Core	6.5806	0.0000
338	IgG exp_2	3046	Ig gamma-1 chain C region	EEQYNSTYR_4_4_1_0	Core	Core	5.4592	0.0000	Core	6.5810	0.0000
339	IgG exp_3	4407	Ig gamma-1 chain C region	EEQYNSTYR_4_4_1_0	Core	Core	5.3591	0.0000	Core	6.5805	0.0000
340	IgG exp_1	2894	Ig gamma-1 chain C region	EEQYNSTYR_4_4_1_0	Core	Core	4.6903	0.0000	Core	6.5777	0.0000
341	IgG exp_3	4075	Ig gamma-1 chain C region	EEQYNSTYR_4_4_1_0	Core	Core	4.3514	0.0000	Core	6.5770	0.0000
342	IgG exp_1	3281	Ig gamma-1 chain C region	EEQYNSTYR_4_4_1_0	Core	Core	5.9190	0.0000	Core	6.5792	0.0000
343	IgG exp_2	6163	Ig gamma-1 chain C region	EEQYNSTYR_4_4_1_0	Core	Core	4.9212	0.0000	Core	6.5763	0.0000
344	IgG exp_3	5170	Ig gamma-1 chain C region	EEQYNSTYR_4_4_1_1	Core	Core	3.5161	0.0000	Core	6.6463	0.0000
345	IgG exp_4	6142	Ig gamma-1 chain C region	EEQYNSTYR_4_4_1_1	Core	Core	4.7292	0.0000	Core	6.6240	0.0000
346	IgG exp_1	3787	Ig gamma-1 chain C region	EEQYNSTYR_4_4_1_1	Core	Core	0.5229	0.0489	Core	6.6619	0.0000
347	IgG exp_3	5195	Ig gamma-1 chain C region	EEQYNSTYR_4_4_1_1	Core	Core	0.5350	0.0470	Core	4.7157	0.0092
348	IgG exp_1	3774	Ig gamma-1 chain C region	EEQYNSTYR_4_4_1_1	Core	Core	1.2600	0.0025	Core	5.4845	0.0094
349	IgG exp_4	6150	Ig gamma-1 chain C region	EEQYNSTYR_4_4_1_1	Core	Core	0.4984	0.0551	Core	4.9766	0.0093
350	IgG exp_2	3220	Ig gamma-1 chain C region	EEQYNSTYR_4_4_1_1	Core	Core	1.5491	0.0026	Core	6.6452	0.0000
351	IgG exp_2	3226	Ig gamma-1 chain C region	EEQYNSTYR_4_4_1_1	Core	Core	0.4865	0.0611	Core	4.6234	0.0092
352	IgG exp_1	3099	Ig gamma-1 chain C region	EEQYNSTYR_4_5_1_0	Core	Core	4.0869	0.0000	Core	6.6167	0.0000
353	IgG exp_4	5428	Ig gamma-1 chain C region	EEQYNSTYR_4_5_1_0	Core	Core	4.1221	0.0000	Core	6.6121	0.0000
354	IgG exp_3	4716	Ig gamma-1 chain C region	EEQYNSTYR_4_5_1_0	Core	Core	4.5403	0.0000	Core	6.6071	0.0000
355	IgG exp_2	3050	Ig gamma-1 chain C region	EEQYNSTYR_4_5_1_0	Core	Core	4.4673	0.0000	Core	6.6155	0.0000
356	IgG exp_4	5299	Ig gamma-1 chain C region	EEQYNSTYR_5_4_0_0	None	None	2.8925	0.0000	None	7.1176	0.0000
357	IgG exp_3	4199	Ig gamma-1 chain C region	EEQYNSTYR_5_4_0_0	None	None	3.0345	0.0000	None	7.1074	0.0000
358	IgG exp_3	5257	Ig gamma-1 chain C region	EEQYNSTYR_5_4_0_1	None	None	3.0639	0.0000	None	6.7920	0.0000
359	IgG exp_1	2875	Ig gamma-1 chain C region	EEQYNSTYR_5_4_1_0	Core	Core	3.6575	0.0000	Core	6.6001	0.0000

Supplementary Table 6. Continued.

No.	Experiment	CID Scan No.	N-glycoproteins	N-glycopeptides	Manual Classification	SVM			DNN		
						Classification	Pscore	FDR	Classification	Pscore	FDR
360	IgG exp_3	4085	Ig gamma-1 chain C region	EEQYNSTYR_5_4_1_0	Core	Core	4.6563	0.0000	Core	6.6238	0.0000
361	IgG exp_2	3003	Ig gamma-1 chain C region	EEQYNSTYR_5_4_1_0	Core	Core	6.0711	0.0000	Core	6.6119	0.0000
362	IgG exp_4	5179	Ig gamma-1 chain C region	EEQYNSTYR_5_4_1_0	Core	Core	3.1548	0.0000	Core	6.5952	0.0000
363	IgG exp_3	4413	Ig gamma-1 chain C region	EEQYNSTYR_5_4_1_0	Core	Core	4.9735	0.0000	Core	6.5987	0.0000
364	IgG exp_3	5108	Ig gamma-1 chain C region	EEQYNSTYR_5_4_1_1	Core	Core	1.6957	0.0026	Core	6.6331	0.0000
365	IgG exp_1	3671	Ig gamma-1 chain C region	EEQYNSTYR_5_4_1_1	Core	Core	1.3581	0.0025	Core	6.6370	0.0000
366	IgG exp_4	6105	Ig gamma-1 chain C region	EEQYNSTYR_5_4_1_1	Core	Core	1.5359	0.0026	Core	6.6482	0.0000
367	IgG exp_3	5110	Ig gamma-1 chain C region	EEQYNSTYR_5_4_1_1	Core	Core	2.7950	0.0000	Core	6.6114	0.0000
368	IgG exp_4	6109	Ig gamma-1 chain C region	EEQYNSTYR_5_4_1_1	Core	Core	3.2414	0.0000	Core	6.6038	0.0000
369	IgG exp_1	3693	Ig gamma-1 chain C region	EEQYNSTYR_5_4_1_1	Core	Core	2.7924	0.0000	Core	6.6111	0.0000
370	IgG exp_2	3216	Ig gamma-1 chain C region	EEQYNSTYR_5_4_1_1	Core	Core	3.2340	0.0000	Core	6.6097	0.0000
371	IgG exp_4	5450	Ig gamma-1 chain C region	EEQYNSTYR_5_5_1_0	Core	Core	4.7366	0.0000	Core	6.5987	0.0000
372	IgG exp_4	7185	Ig gamma-2 chain C region	EEQFNSTFR_3_3_1_0	Core	Core	5.5548	0.0000	Core	6.6491	0.0000
373	IgG exp_1	4437	Ig gamma-2 chain C region	EEQFNSTFR_3_3_1_0	Core	Core	5.2706	0.0000	Core	6.6520	0.0000
374	IgG exp_3	5741	Ig gamma-2 chain C region	EEQFNSTFR_3_3_1_0	Core	Core	2.9842	0.0000	Core	6.6553	0.0000
375	IgG exp_2	3508	Ig gamma-2 chain C region	EEQFNSTFR_3_3_1_0	Core	Core	4.1857	0.0000	Core	6.6644	0.0000
376	IgG exp_3	5816	Ig gamma-2 chain C region	EEQFNSTFR_3_4_0_0	None	None	2.8990	0.0000	None	7.0613	0.0000
377	IgG exp_4	7313	Ig gamma-2 chain C region	EEQFNSTFR_3_4_0_0	None	None	3.2226	0.0000	None	7.1981	0.0000
378	IgG exp_1	4557	Ig gamma-2 chain C region	EEQFNSTFR_3_4_0_0	None	None	3.1040	0.0000	None	7.1548	0.0000
379	IgG exp_1	5662	Ig gamma-2 chain C region	EEQFNSTFR_3_4_1_0	Core	Core	8.3928	0.0000	Core	6.6252	0.0000
380	IgG exp_2	3469	Ig gamma-2 chain C region	EEQFNSTFR_3_4_1_0	Core	Core	4.1895	0.0000	Core	6.6601	0.0000
381	IgG exp_3	5721	Ig gamma-2 chain C region	EEQFNSTFR_3_4_1_0	Core	Core	4.2076	0.0000	Core	6.6614	0.0000
382	IgG exp_4	7480	Ig gamma-2 chain C region	EEQFNSTFR_3_4_1_0	Core	Core	5.0854	0.0000	Core	6.6548	0.0000
383	IgG exp_1	4661	Ig gamma-2 chain C region	EEQFNSTFR_3_4_1_0	Core	Core	6.2986	0.0000	Core	6.6430	0.0000
384	IgG exp_3	6073	Ig gamma-2 chain C region	EEQFNSTFR_3_4_1_0	Core	Core	5.1286	0.0000	Core	6.6588	0.0000
385	IgG exp_4	6975	Ig gamma-2 chain C region	EEQFNSTFR_3_4_1_0	Core	Core	6.5437	0.0000	Core	6.6334	0.0000
386	IgG exp_1	4325	Ig gamma-2 chain C region	EEQFNSTFR_3_4_1_0	Core	Core	7.2769	0.0000	Core	6.6297	0.0000
387	IgG exp_2	4229	Ig gamma-2 chain C region	EEQFNSTFR_3_4_1_0	Core	Core	6.5823	0.0000	Core	6.6247	0.0000
388	IgG exp_2	3496	Ig gamma-2 chain C region	EEQFNSTFR_3_5_1_0	Core	Core	1.0991	0.0072	Core	6.6928	0.0000
389	IgG exp_2	3498	Ig gamma-2 chain C region	EEQFNSTFR_3_5_1_0	Core	Core	4.1640	0.0000	Core	6.6467	0.0000

Supplementary Table 6. Continued.

No.	Experiment	CID Scan No.	N-glycoproteins	N-glycopeptides	Manual Classification	SVM			DNN		
						Classification	Pscore	FDR	Classification	Pscore	FDR
390	IgG exp_4	7309	Ig gamma-2 chain C region	EEQFNSTFR_3_5_1_0	Core	Core	4.1690	0.0000	Core	6.6473	0.0000
391	IgG exp_3	5766	Ig gamma-2 chain C region	EEQFNSTFR_3_5_1_0	Core	Core	4.7030	0.0000	Core	6.6473	0.0000
392	IgG exp_1	4520	Ig gamma-2 chain C region	EEQFNSTFR_3_5_1_0	Core	Core	4.9268	0.0000	Core	6.6401	0.0000
393	IgG exp_4	7220	Ig gamma-2 chain C region	EEQFNSTFR_4_3_1_0	Core	Core	6.2717	0.0000	Core	6.6299	0.0000
394	IgG exp_4	7274	Ig gamma-2 chain C region	EEQFNSTFR_4_4_0_0	None	None	3.1146	0.0000	None	7.1818	0.0000
395	IgG exp_4	7270	Ig gamma-2 chain C region	EEQFNSTFR_4_4_1_0	Core	Core	6.8546	0.0000	Core	6.6455	0.0000
396	IgG exp_1	4427	Ig gamma-2 chain C region	EEQFNSTFR_4_4_1_0	Core	Core	6.5740	0.0000	Core	6.6357	0.0000
397	IgG exp_3	5694	Ig gamma-2 chain C region	EEQFNSTFR_4_4_1_0	Core	Core	7.2496	0.0000	Core	6.6314	0.0000
398	IgG exp_2	3467	Ig gamma-2 chain C region	EEQFNSTFR_4_4_1_0	Core	Core	9.6498	0.0000	Core	6.6084	0.0000
399	IgG exp_4	6926	Ig gamma-2 chain C region	EEQFNSTFR_4_4_1_0	Core	Core	5.3678	0.0000	Core	6.6516	0.0000
400	IgG exp_3	5692	Ig gamma-2 chain C region	EEQFNSTFR_4_4_1_0	Core	Core	4.0269	0.0000	Core	6.6355	0.0000
401	IgG exp_2	6486	Ig gamma-2 chain C region	EEQFNSTFR_4_4_1_0	Core	Core	5.1259	0.0000	Core	6.6406	0.0000
402	IgG exp_4	7268	Ig gamma-2 chain C region	EEQFNSTFR_4_4_1_0	Core	Core	4.5408	0.0000	Core	6.6387	0.0000
403	IgG exp_2	3400	Ig gamma-2 chain C region	EEQFNSTFR_4_4_1_0	Core	Core	5.0786	0.0000	Core	6.6001	0.0000
404	IgG exp_1	4717	Ig gamma-2 chain C region	EEQFNSTFR_4_4_1_0	Core	Core	4.1692	0.0000	Core	6.6185	0.0000
405	IgG exp_1	5111	Ig gamma-2 chain C region	EEQFNSTFR_4_4_1_0	Core	Core	5.5301	0.0000	Core	6.6205	0.0000
406	IgG exp_4	7620	Ig gamma-2 chain C region	EEQFNSTFR_4_4_1_0	Core	Core	7.6380	0.0000	Core	6.6235	0.0000
407	IgG exp_1	6085	Ig gamma-2 chain C region	EEQFNSTFR_4_4_1_1	Core	Core	0.7474	0.0141	Core	6.6521	0.0000
408	IgG exp_2	5075	Ig gamma-2 chain C region	EEQFNSTFR_4_4_1_1	Core	Core	0.5730	0.0407	Core	6.6548	0.0000
409	IgG exp_2	4772	Ig gamma-2 chain C region	EEQFNSTFR_4_4_1_1	Core	Core	3.6419	0.0000	Core	6.6271	0.0000
410	IgG exp_1	6083	Ig gamma-2 chain C region	EEQFNSTFR_4_4_1_1	Core	Core	4.3657	0.0000	Core	6.6317	0.0000
411	IgG exp_2	4745	Ig gamma-2 chain C region	EEQFNSTFR_4_4_1_1	Core	Core	1.6088	0.0026	Core	6.6470	0.0000
412	IgG exp_2	5110	Ig gamma-2 chain C region	EEQFNSTFR_4_4_1_1	Core	Core	4.6818	0.0000	Core	6.6237	0.0000
413	IgG exp_4	8721	Ig gamma-2 chain C region	EEQFNSTFR_4_4_1_1	Core	Core	7.0346	0.0000	Core	6.6196	0.0000
414	IgG exp_1	4464	Ig gamma-2 chain C region	EEQFNSTFR_4_5_1_0	Core	Core	4.0754	0.0000	Core	6.6154	0.0000
415	IgG exp_3	5743	Ig gamma-2 chain C region	EEQFNSTFR_4_5_1_0	Core	Core	4.1857	0.0000	Core	6.6216	0.0000
416	IgG exp_4	7257	Ig gamma-2 chain C region	EEQFNSTFR_4_5_1_0	Core	Core	4.3272	0.0000	Core	6.6188	0.0000
417	IgG exp_2	3512	Ig gamma-2 chain C region	EEQFNSTFR_4_5_1_0	Core	Core	5.8263	0.0000	Core	6.6018	0.0000
418	IgG exp_4	7236	Ig gamma-2 chain C region	EEQFNSTFR_5_4_0_0	None	None	2.0259	0.0000	None	6.2031	0.0051
419	IgG exp_4	7214	Ig gamma-2 chain C region	EEQFNSTFR_5_4_1_0	Core	Core	3.7300	0.0000	Core	6.6069	0.0000

Supplementary Table 6. Continued.

No.	Experiment	CID Scan No.	N-glycoproteins	N-glycopeptides	Manual Classification	SVM			DNN		
						Classification	Pscore	FDR	Classification	Pscore	FDR
420	IgG exp_3	5671	Ig gamma-2 chain C region	EEQFNSTFR_5_4_1_0	Core	Core	3.6328	0.0000	Core	6.6183	0.0000
421	IgG exp_1	4373	Ig gamma-2 chain C region	EEQFNSTFR_5_4_1_0	Core	Core	3.7166	0.0000	Core	6.6099	0.0000
422	IgG exp_2	3436	Ig gamma-2 chain C region	EEQFNSTFR_5_4_1_0	Core	Core	3.4724	0.0000	Core	6.6099	0.0000
423	IgG exp_4	8669	Ig gamma-2 chain C region	EEQFNSTFR_5_4_1_1	Core	Core	2.1532	0.0000	Core	6.5920	0.0000
424	IgG exp_1	5940	Ig gamma-2 chain C region	EEQFNSTFR_5_4_1_1	Core	Core	2.6620	0.0000	Core	6.6081	0.0000
425	IgG exp_2	4872	Ig gamma-2 chain C region	EEQFNSTFR_5_4_1_1	Core	Core	2.6045	0.0000	Core	6.6063	0.0000
426	IgG exp_2	4542	Ig gamma-2 chain C region	EEQFNSTFR_5_4_1_1	Core	Core	4.5137	0.0000	Core	6.5936	0.0000
427	IgG exp_1	4447	Ig gamma-2 chain C region	EEQFNSTFR_5_5_1_0	Core	Core	0.5484	0.0428	Core	6.4698	0.0029
428	IgG exp_1	4468	Ig gamma-2 chain C region	EEQFNSTFR_5_5_1_0	Core	Core	3.0054	0.0000	Core	6.5932	0.0000
429	IgG exp_3	5122	Ig gamma-3 chain C region	EEQYNSTFR_4_4_1_0	Core	Core	7.1024	0.0000	Core	6.6233	0.0000
430	IgG exp_3	5130	Ig gamma-4 chain C region	EEQFNSTYR_3_4_1_0	Core	Core	6.9948	0.0000	Core	6.6366	0.0000
431	IgG exp_1	3739	Ig gamma-4 chain C region	EEQFNSTYR_3_4_1_0	Core	Core	6.1291	0.0000	Core	6.6352	0.0000
432	IgG exp_4	6100	Ig gamma-4 chain C region	EEQFNSTYR_3_4_1_0	Core	Core	6.2809	0.0000	Core	6.6387	0.0000
433	IgG exp_1	3712	Ig gamma-4 chain C region	EEQFNSTYR_4_4_1_0	Core	Core	4.8934	0.0000	Core	6.6247	0.0000

Supplementary Table 7. Glycopeptide spectrum matches of alpha-1-acid glycoprotein and immunoglobulin gamma, and their Classification as test set for machine learning.

No.	Experiment	CID Scan No.	N-glycoproteins	N-glycopeptides	Manual Classification	SVM			DNN		
						Classification	Pscore	FDR	Classification	Pscore	FDR
1	AGP file 3	2234	Alpha-1-acid glycoprotein 1	ENGTISR_4_4_0_1	None	None	3.0467	0.0000	None	7.3061	0.0000
2	AGP file 4	2128	Alpha-1-acid glycoprotein 1	ENGTISR_4_4_0_1	None	None	2.4178	0.0000	None	7.3251	0.0000
3	AGP file 3	3807	Alpha-1-acid glycoprotein 1	ENGTISR_5_4_0_2	None	None	2.7201	0.0000	None	7.2825	0.0000
4	AGP file 4	3700	Alpha-1-acid glycoprotein 1	ENGTISR_5_4_0_2	None	None	2.9733	0.0000	None	7.3085	0.0000
5	AGP file 3	3870	Alpha-1-acid glycoprotein 1	ENGTISR_5_4_0_2	None	None	3.0426	0.0000	None	7.2961	0.0000
6	AGP file 4	2230	Alpha-1-acid glycoprotein 1	ENGTISR_6_5_0_1	None	None	2.8115	0.0000	None	7.3019	0.0000
7	AGP file 3	2593	Alpha-1-acid glycoprotein 1	ENGTISR_6_5_0_1	None	None	2.4356	0.0000	None	7.2806	0.0000
8	AGP file 4	2506	Alpha-1-acid glycoprotein 1	ENGTISR_6_5_0_1	None	None	2.5268	0.0000	None	7.2704	0.0000
9	AGP file 3	3937	Alpha-1-acid glycoprotein 1	ENGTISR_6_5_0_2	None	None	2.6265	0.0000	None	7.3227	0.0000
10	AGP file 3	4259	Alpha-1-acid glycoprotein 1	ENGTISR_6_5_0_2	None	None	2.9017	0.0000	None	7.2774	0.0000
11	AGP file 4	3951	Alpha-1-acid glycoprotein 1	ENGTISR_6_5_0_2	None	None	2.7598	0.0000	None	7.3073	0.0000
12	AGP file 3	4070	Alpha-1-acid glycoprotein 1	ENGTISR_6_5_0_2	None	None	2.5286	0.0000	None	7.2450	0.0000
13	AGP file 4	4267	Alpha-1-acid glycoprotein 1	ENGTISR_6_5_0_2	None	None	3.3441	0.0000	None	7.2863	0.0000
14	AGP file 4	4825	Alpha-1-acid glycoprotein 1	ENGTISR_6_5_0_2	None	None	2.5367	0.0000	None	7.1166	0.0000
15	AGP file 3	5053	Alpha-1-acid glycoprotein 1	ENGTISR_6_5_0_2	None	None	2.5936	0.0000	None	7.2821	0.0000
16	AGP file 4	4869	Alpha-1-acid glycoprotein 1	ENGTISR_6_5_0_3	None	None	2.1295	0.0000	None	7.2775	0.0000
17	AGP file 4	4888	Alpha-1-acid glycoprotein 1	ENGTISR_6_5_0_3	None	None	2.8992	0.0000	None	7.3139	0.0000
18	AGP file 3	5086	Alpha-1-acid glycoprotein 1	ENGTISR_6_5_0_3	None	None	2.5595	0.0000	None	7.1643	0.0000
19	AGP file 4	6322	Alpha-1-acid glycoprotein 1	ENGTISR_6_5_0_3	None	None	0.1959	0.0884	None	3.0169	0.0051
20	AGP file 4	2442	Alpha-1-acid glycoprotein 1	ENGTISR_6_5_1_1	Outer	Outer	5.9903	0.0000	Outer	6.6445	0.0000
21	AGP file 3	2567	Alpha-1-acid glycoprotein 1	ENGTISR_6_5_1_1	Outer	Outer	6.2204	0.0000	Outer	6.7155	0.0000
22	AGP file 4	2098	Alpha-1-acid glycoprotein 1	ENGTISR_6_5_1_1	Outer	Outer	6.2685	0.0000	Outer	6.7712	0.0000
23	AGP file 3	2250	Alpha-1-acid glycoprotein 1	ENGTISR_6_5_1_1	Outer	Outer	2.7425	0.0000	Outer	6.7528	0.0000
24	AGP file 3	3715	Alpha-1-acid glycoprotein 1	ENGTISR_6_5_1_2	Outer	Outer	4.9264	0.0000	Outer	6.7546	0.0000
25	AGP file 4	3820	Alpha-1-acid glycoprotein 1	ENGTISR_6_5_1_2	Outer	Outer	4.1306	0.0000	Outer	6.5384	0.0000
26	AGP file 3	4074	Alpha-1-acid glycoprotein 1	ENGTISR_6_5_1_2	Outer	Outer	4.3785	0.0000	Outer	6.7160	0.0000
27	AGP file 4	4161	Alpha-1-acid glycoprotein 1	ENGTISR_6_5_1_2	Outer	Outer	0.3033	0.0051	Outer	5.1012	0.0052
28	AGP file 3	5005	Alpha-1-acid glycoprotein 1	ENGTISR_6_5_1_3	Outer	Outer	3.4244	0.0000	Outer	6.2040	0.0029
29	AGP file 4	4831	Alpha-1-acid glycoprotein 1	ENGTISR_6_5_1_3	Outer	Outer	4.4536	0.0000	Outer	6.4357	0.0000

Supplementary Table 7. Continued.

No.	Experiment	CID Scan No.	N-glycoproteins	N-glycopeptides	Manual Classification	SVM			DNN		
						Classification	Pscore	FDR	Classification	Pscore	FDR
30	AGP file 64	5698	Alpha-1-acid glycoprotein 1	ENGTISR_6_5_2_2	Dual	Outer	1.4080	0.0000	Outer	6.2084	0.0029
31	AGP file 55	7912	Alpha-1-acid glycoprotein 1	ENGTISR_6_5_2_3	Dual	Dual	1.1845	0.0000	Dual	6.1175	0.0028
32	AGP file 60	10024	Alpha-1-acid glycoprotein 1	ENGTISR_6_5_2_3	Dual	Dual	2.3703	0.0000	Dual	6.4193	0.0000
33	AGP file 61	9868	Alpha-1-acid glycoprotein 1	ENGTISR_6_5_2_3	Dual	Dual	4.3160	0.0000	Dual	6.2311	0.0030
34	AGP file 59	9853	Alpha-1-acid glycoprotein 1	ENGTISR_6_5_2_3	Dual	Dual	4.1207	0.0000	Dual	6.2378	0.0030
35	AGP file 58	7974	Alpha-1-acid glycoprotein 1	ENGTISR_6_5_2_3	Dual	Dual	5.6080	0.0000	Dual	6.2328	0.0030
36	AGP file 47	9866	Alpha-1-acid glycoprotein 1	ENGTISR_6_5_2_3	Dual	Dual	2.3730	0.0000	Dual	6.0583	0.0028
37	AGP file 53	10186	Alpha-1-acid glycoprotein 1	ENGTISR_6_5_2_3	Dual	Dual	1.3466	0.0000	Dual	5.9687	0.0027
38	AGP file 46	9990	Alpha-1-acid glycoprotein 1	ENGTISR_6_5_2_3	Dual	Dual	3.5775	0.0000	Dual	6.1747	0.0029
39	AGP file 56	8077	Alpha-1-acid glycoprotein 1	ENGTISR_6_5_2_3	Dual	Dual	1.0872	0.0000	Dual	6.1637	0.0028
40	AGP file 44	9968	Alpha-1-acid glycoprotein 1	ENGTISR_6_5_2_3	Dual	Dual	3.9733	0.0000	Dual	5.9764	0.0027
41	AGP file 45	10035	Alpha-1-acid glycoprotein 1	ENGTISR_6_5_2_3	Dual	Dual	5.6217	0.0000	Dual	6.3302	0.0031
42	AGP file 50	10759	Alpha-1-acid glycoprotein 1	ENGTISR_6_5_2_3	Dual	Dual	0.3745	0.0000	Dual	6.3270	0.0031
43	AGP file 3	5059	Alpha-1-acid glycoprotein 1	ENGTISR_6_5_2_3	Outer	Outer	1.7562	0.0000	Outer	6.4962	0.0000
44	AGP file 3	2283	Alpha-1-acid glycoprotein 1	ENGTISR_7_6_1_1	Outer	Outer	4.6853	0.0000	Outer	6.2870	0.0031
45	AGP file 4	3157	Alpha-1-acid glycoprotein 1	ENGTISR_7_6_1_2	Outer	Outer	3.0277	0.0000	Outer	6.5702	0.0000
46	AGP file 4	3437	Alpha-1-acid glycoprotein 1	ENGTISR_7_6_1_2	Outer	Outer	1.5913	0.0000	Outer	6.6547	0.0000
47	AGP file 3	3935	Alpha-1-acid glycoprotein 1	ENGTISR_7_6_1_2	Outer	Outer	1.3361	0.0000	Outer	6.6560	0.0000
48	AGP file 3	3613	Alpha-1-acid glycoprotein 1	ENGTISR_7_6_1_2	Outer	Outer	2.1938	0.0000	Outer	6.6650	0.0000
49	AGP file 4	3719	Alpha-1-acid glycoprotein 1	ENGTISR_7_6_1_2	Outer	Outer	1.5483	0.0000	Outer	6.6272	0.0000
50	AGP file 3	3281	Alpha-1-acid glycoprotein 1	ENGTISR_7_6_1_2	Outer	Outer	3.1976	0.0000	Outer	6.5742	0.0000
51	AGP file 3	4937	Alpha-1-acid glycoprotein 1	ENGTISR_7_6_1_3	Outer	Outer	3.1320	0.0000	Outer	6.6870	0.0000
52	AGP file 3	6409	Alpha-1-acid glycoprotein 1	ENGTISR_7_6_1_4	Outer	Outer	2.1598	0.0000	Outer	6.6092	0.0000
53	AGP file 4	6024	Alpha-1-acid glycoprotein 1	ENGTISR_7_6_1_4	Outer	Outer	2.6743	0.0000	Outer	6.6412	0.0000
54	AGP file 3	6155	Alpha-1-acid glycoprotein 1	ENGTISR_7_6_2_4	Outer	Outer	0.4656	0.0000	Outer	6.4080	0.0000
55	AGP file 4	21712	Alpha-1-acid glycoprotein 1	LVPVPITNATLDQITGK_5_4_0_2	None	None	2.9336	0.0000	None	7.3032	0.0000
56	AGP file 3	22375	Alpha-1-acid glycoprotein 1	LVPVPITNATLDQITGK_5_4_0_2	None	None	3.4870	0.0000	None	7.3069	0.0000
57	AGP file 4	22014	Alpha-1-acid glycoprotein 1	LVPVPITNATLDQITGK_5_4_0_2	None	None	3.6594	0.0000	None	7.3052	0.0000
58	AGP file 4	21900	Alpha-1-acid glycoprotein 1	LVPVPITNATLDQITGK_5_4_0_2	None	None	5.1908	0.0000	None	7.3476	0.0000
59	AGP file 3	21916	Alpha-1-acid glycoprotein 1	LVPVPITNATLDQITGK_5_4_0_2	None	None	5.3898	0.0000	None	7.3534	0.0000

Supplementary Table 7. Continued.

No.	Experiment	CID Scan No.	N-glycoproteins	N-glycopeptides	Manual Classification	SVM			DNN		
						Classification	Pscore	FDR	Classification	Pscore	FDR
60	AGP file 3	22208	Alpha-1-acid glycoprotein 1	LVPVPITNATLDQITGK_5_4_0_2	None	None	4.5843	0.0000	None	7.3348	0.0000
61	AGP file 4	17971	Alpha-1-acid glycoprotein 1	LVPVPITNATLDQITGK_6_5_0_1	None	None	2.8148	0.0000	None	7.1189	0.0000
62	AGP file 4	18169	Alpha-1-acid glycoprotein 1	LVPVPITNATLDQITGK_6_5_0_1	None	None	3.6583	0.0000	None	7.3692	0.0000
63	AGP file 3	18255	Alpha-1-acid glycoprotein 1	LVPVPITNATLDQITGK_6_5_0_1	None	None	3.5472	0.0000	None	7.3410	0.0000
64	AGP file 3	18568	Alpha-1-acid glycoprotein 1	LVPVPITNATLDQITGK_6_5_0_1	None	None	3.4470	0.0000	None	7.3730	0.0000
65	AGP file 3	21756	Alpha-1-acid glycoprotein 1	LVPVPITNATLDQITGK_6_5_0_2	None	None	4.0106	0.0000	None	7.3154	0.0000
66	AGP file 3	21804	Alpha-1-acid glycoprotein 1	LVPVPITNATLDQITGK_6_5_0_2	None	None	3.7254	0.0000	None	7.3276	0.0000
67	AGP file 3	21566	Alpha-1-acid glycoprotein 1	LVPVPITNATLDQITGK_6_5_0_2	None	None	3.8454	0.0000	None	7.3202	0.0000
68	AGP file 4	21554	Alpha-1-acid glycoprotein 1	LVPVPITNATLDQITGK_6_5_0_2	None	None	3.5502	0.0000	None	7.3127	0.0000
69	AGP file 3	22024	Alpha-1-acid glycoprotein 1	LVPVPITNATLDQITGK_6_5_0_2	None	None	4.2481	0.0000	None	7.3183	0.0000
70	AGP file 4	21868	Alpha-1-acid glycoprotein 1	LVPVPITNATLDQITGK_6_5_0_2	None	None	2.3372	0.0000	None	7.3019	0.0000
71	AGP file 4	24860	Alpha-1-acid glycoprotein 1	LVPVPITNATLDQITGK_6_5_0_3	None	None	2.4052	0.0000	None	7.2948	0.0000
72	AGP file 3	25815	Alpha-1-acid glycoprotein 1	LVPVPITNATLDQITGK_6_5_0_3	None	None	2.6202	0.0000	None	7.2883	0.0000
73	AGP file 3	25633	Alpha-1-acid glycoprotein 1	LVPVPITNATLDQITGK_6_5_0_3	None	None	2.4872	0.0000	None	7.2762	0.0000
74	AGP file 3	25301	Alpha-1-acid glycoprotein 1	LVPVPITNATLDQITGK_6_5_0_3	None	None	3.3390	0.0000	None	7.2864	0.0000
75	AGP file 3	25591	Alpha-1-acid glycoprotein 1	LVPVPITNATLDQITGK_6_5_0_3	None	None	3.2909	0.0000	None	7.2888	0.0000
76	AGP file 4	24579	Alpha-1-acid glycoprotein 1	LVPVPITNATLDQITGK_6_5_0_3	None	None	1.9914	0.0000	None	7.2870	0.0000
77	AGP file 4	24847	Alpha-1-acid glycoprotein 1	LVPVPITNATLDQITGK_6_5_0_3	None	None	3.1487	0.0000	None	7.2823	0.0000
78	AGP file 4	25276	Alpha-1-acid glycoprotein 1	LVPVPITNATLDQITGK_6_5_0_3	None	None	3.5334	0.0000	None	7.2993	0.0000
79	AGP file 4	25139	Alpha-1-acid glycoprotein 1	LVPVPITNATLDQITGK_6_5_0_3	None	None	3.4774	0.0000	None	7.2766	0.0000
80	AGP file 3	25817	Alpha-1-acid glycoprotein 1	LVPVPITNATLDQITGK_6_5_0_3	None	None	2.8529	0.0000	None	7.2858	0.0000
81	AGP file 4	18062	Alpha-1-acid glycoprotein 1	LVPVPITNATLDQITGK_6_5_1_1	Outer	Outer	5.0517	0.0000	Outer	5.9627	0.0027
82	AGP file 3	18440	Alpha-1-acid glycoprotein 1	LVPVPITNATLDQITGK_6_5_1_1	Outer	Outer	5.6793	0.0000	Outer	6.3831	0.0000
83	AGP file 4	18128	Alpha-1-acid glycoprotein 1	LVPVPITNATLDQITGK_6_5_1_1	Outer	Outer	1.5347	0.0000	Outer	6.5834	0.0000
84	AGP file 4	21470	Alpha-1-acid glycoprotein 1	LVPVPITNATLDQITGK_6_5_1_2	Outer	Outer	2.5356	0.0000	Outer	6.3935	0.0000
85	AGP file 3	21715	Alpha-1-acid glycoprotein 1	LVPVPITNATLDQITGK_6_5_1_2	Outer	Outer	3.5811	0.0000	Outer	6.1429	0.0028
86	AGP file 3	22028	Alpha-1-acid glycoprotein 1	LVPVPITNATLDQITGK_6_5_1_2	Outer	Outer	2.6749	0.0000	Outer	6.0400	0.0028
87	AGP file 4	21473	Alpha-1-acid glycoprotein 1	LVPVPITNATLDQITGK_6_5_1_2	Outer	Outer	3.5554	0.0000	Outer	6.6331	0.0000
88	AGP file 3	21787	Alpha-1-acid glycoprotein 1	LVPVPITNATLDQITGK_6_5_1_2	Outer	Outer	4.0335	0.0000	Outer	6.7353	0.0000
89	AGP file 4	24923	Alpha-1-acid glycoprotein 1	LVPVPITNATLDQITGK_6_5_1_3	Outer	Outer	3.1163	0.0000	Outer	6.5155	0.0000

Supplementary Table 7. Continued.

No.	Experiment	CID Scan No.	N-glycoproteins	N-glycopeptides	Manual Classification	SVM			DNN		
						Classification	Pscore	FDR	Classification	Pscore	FDR
90	AGP file 4	25175	Alpha-1-acid glycoprotein 1	LVPVPITNATLDQITGK_6_5_1_3	Outer	Outer	3.9031	0.0000	Outer	6.5903	0.0000
91	AGP file 3	25409	Alpha-1-acid glycoprotein 1	LVPVPITNATLDQITGK_6_5_1_3	Outer	Outer	3.4040	0.0000	Outer	6.4387	0.0000
92	AGP file 3	25437	Alpha-1-acid glycoprotein 1	LVPVPITNATLDQITGK_6_5_1_3	Outer	Outer	3.3240	0.0000	Outer	6.6816	0.0000
93	AGP file 3	25420	Alpha-1-acid glycoprotein 1	LVPVPITNATLDQITGK_6_5_1_3	Outer	Outer	2.9512	0.0000	Outer	6.4908	0.0000
94	AGP file 3	25702	Alpha-1-acid glycoprotein 1	LVPVPITNATLDQITGK_6_5_1_3	Outer	Outer	3.7735	0.0000	Outer	6.6600	0.0000
95	AGP file 3	25364	Alpha-1-acid glycoprotein 1	LVPVPITNATLDQITGK_6_5_1_3	Outer	Outer	3.3483	0.0000	Outer	6.6917	0.0000
96	AGP file 3	25708	Alpha-1-acid glycoprotein 1	LVPVPITNATLDQITGK_6_5_1_3	Outer	Outer	4.5064	0.0000	Outer	6.6642	0.0000
97	AGP file 4	24925	Alpha-1-acid glycoprotein 1	LVPVPITNATLDQITGK_6_5_1_3	Outer	Outer	2.4385	0.0000	Outer	6.7011	0.0000
98	AGP file 4	25177	Alpha-1-acid glycoprotein 1	LVPVPITNATLDQITGK_6_5_1_3	Outer	Outer	1.4135	0.0000	Outer	6.6906	0.0000
99	AGP file 44	35503	Alpha-1-acid glycoprotein 1	LVPVPITNATLDQITGK_6_5_2_2	Dual	Dual	8.3284	0.0000	Dual	6.2756	0.0030
100	AGP file 37	29857	Alpha-1-acid glycoprotein 1	LVPVPITNATLDQITGK_6_5_2_3	Dual	Dual	1.0825	0.0000	Dual	3.3308	0.0051
101	AGP file 62	32062	Alpha-1-acid glycoprotein 1	LVPVPITNATLDQITGK_6_5_2_3	Dual	Dual	0.3593	0.0000	Dual	3.6388	0.0052
102	AGP file 56	29837	Alpha-1-acid glycoprotein 1	LVPVPITNATLDQITGK_6_5_2_3	Dual	Dual	2.1372	0.0000	Dual	6.2624	0.0030
103	AGP file 55	27103	Alpha-1-acid glycoprotein 1	LVPVPITNATLDQITGK_6_5_2_3	Dual	Dual	1.2700	0.0000	Dual	6.3782	0.0000
104	AGP file 4	1589	Alpha-1-acid glycoprotein 1,2	NEEYNK_4_3_0_0	None	None	3.8889	0.0000	None	7.3601	0.0000
105	AGP file 3	1567	Alpha-1-acid glycoprotein 1,2	NEEYNK_4_3_0_0	None	None	3.1356	0.0000	None	7.3649	0.0000
106	AGP file 4	1966	Alpha-1-acid glycoprotein 1,2	NEEYNK_4_3_0_1	None	None	4.0073	0.0000	None	7.3133	0.0000
107	AGP file 3	1408	Alpha-1-acid glycoprotein 1,2	NEEYNK_4_3_0_1	None	None	5.1945	0.0000	None	7.3855	0.0000
108	AGP file 3	1944	Alpha-1-acid glycoprotein 1,2	NEEYNK_4_3_0_1	None	None	4.1547	0.0000	None	7.2720	0.0000
109	AGP file 4	1425	Alpha-1-acid glycoprotein 1,2	NEEYNK_4_3_0_1	None	None	4.4489	0.0000	None	7.3593	0.0000
110	AGP file 4	1549	Alpha-1-acid glycoprotein 1,2	NEEYNK_4_4_0_1	None	None	2.9046	0.0000	None	7.3338	0.0000
111	AGP file 4	1798	Alpha-1-acid glycoprotein 1,2	NEEYNK_4_4_0_1	None	None	3.3192	0.0000	None	7.2531	0.0000
112	AGP file 3	1787	Alpha-1-acid glycoprotein 1,2	NEEYNK_4_4_0_1	None	None	3.5788	0.0000	None	7.1761	0.0000
113	AGP file 3	1532	Alpha-1-acid glycoprotein 1,2	NEEYNK_4_4_0_1	None	None	3.3344	0.0000	None	7.3729	0.0000
114	AGP file 3	2054	Alpha-1-acid glycoprotein 1,2	NEEYNK_4_4_0_1	None	None	2.6593	0.0000	None	7.2982	0.0000
115	AGP file 3	1357	Alpha-1-acid glycoprotein 1,2	NEEYNK_5_4_0_0	None	None	2.9281	0.0000	None	7.3516	0.0000
116	AGP file 4	1370	Alpha-1-acid glycoprotein 1,2	NEEYNK_5_4_0_0	None	None	2.9743	0.0000	None	7.2163	0.0000
117	AGP file 3	1780	Alpha-1-acid glycoprotein 1,2	NEEYNK_5_4_0_1	None	None	2.9095	0.0000	None	7.3006	0.0000
118	AGP file 3	2771	Alpha-1-acid glycoprotein 1,2	NEEYNK_5_4_0_1	None	None	2.9453	0.0000	None	7.2723	0.0000
119	AGP file 4	1807	Alpha-1-acid glycoprotein 1,2	NEEYNK_5_4_0_1	None	None	2.9420	0.0000	None	7.3169	0.0000

Supplementary Table 7. Continued.

No.	Experiment	CID Scan No.	N-glycoproteins	N-glycopeptides	Manual Classification	SVM			DNN		
						Classification	Pscore	FDR	Classification	Pscore	FDR
120	AGP file 3	2047	Alpha-1-acid glycoprotein 1,2	NEEYNK_5_4_0_1	None	None	3.3450	0.0000	None	6.9150	0.0000
121	AGP file 4	1800	Alpha-1-acid glycoprotein 1,2	NEEYNK_5_4_0_1	None	None	3.1833	0.0000	None	7.2323	0.0000
122	AGP file 4	1551	Alpha-1-acid glycoprotein 1,2	NEEYNK_5_4_0_1	None	None	2.8781	0.0000	None	7.2625	0.0000
123	AGP file 3	1778	Alpha-1-acid glycoprotein 1,2	NEEYNK_5_4_0_1	None	None	3.3179	0.0000	None	7.0321	0.0000
124	AGP file 4	1546	Alpha-1-acid glycoprotein 1,2	NEEYNK_5_4_0_1	None	None	2.7323	0.0000	None	7.1779	0.0000
125	AGP file 3	2936	Alpha-1-acid glycoprotein 1,2	NEEYNK_5_4_0_1	None	None	3.4094	0.0000	None	6.7885	0.0000
126	AGP file 4	2035	Alpha-1-acid glycoprotein 1,2	NEEYNK_5_4_0_1	None	None	3.3737	0.0000	None	7.0132	0.0000
127	AGP file 3	3261	Alpha-1-acid glycoprotein 1,2	NEEYNK_5_4_0_1	None	None	4.7644	0.0000	None	7.2561	0.0000
128	AGP file 3	2261	Alpha-1-acid glycoprotein 1,2	NEEYNK_5_4_0_1	None	None	3.9032	0.0000	None	7.2478	0.0000
129	AGP file 4	2401	Alpha-1-acid glycoprotein 1,2	NEEYNK_5_4_0_1	None	None	3.8444	0.0000	None	6.2108	0.0029
130	AGP file 4	2242	Alpha-1-acid glycoprotein 1,2	NEEYNK_5_4_0_1	None	None	3.2281	0.0000	None	6.9503	0.0000
131	AGP file 3	2809	Alpha-1-acid glycoprotein 1,2	NEEYNK_5_4_0_2	None	None	3.7883	0.0000	None	7.3050	0.0000
132	AGP file 3	3521	Alpha-1-acid glycoprotein 1,2	NEEYNK_5_4_0_2	None	None	3.7091	0.0000	None	7.3047	0.0000
133	AGP file 3	3173	Alpha-1-acid glycoprotein 1,2	NEEYNK_5_4_0_2	None	None	3.6805	0.0000	None	7.3044	0.0000
134	AGP file 4	2755	Alpha-1-acid glycoprotein 1,2	NEEYNK_5_4_0_2	None	None	3.9612	0.0000	None	7.3143	0.0000
135	AGP file 4	3183	Alpha-1-acid glycoprotein 1,2	NEEYNK_5_4_0_2	None	None	2.9492	0.0000	None	7.0072	0.0000
136	AGP file 4	3580	Alpha-1-acid glycoprotein 1,2	NEEYNK_5_4_0_2	None	None	3.1298	0.0000	None	7.2836	0.0000
137	AGP file 3	2809	Alpha-1-acid glycoprotein 1,2	NEEYNK_5_4_0_2	None	None	3.7883	0.0000	None	7.3050	0.0000
138	AGP file 4	3294	Alpha-1-acid glycoprotein 1,2	NEEYNK_5_4_0_2	None	None	3.1089	0.0000	None	7.2996	0.0000
139	AGP file 3	3360	Alpha-1-acid glycoprotein 1,2	NEEYNK_5_4_0_2	None	None	3.2683	0.0000	None	7.1904	0.0000
140	AGP file 3	3834	Alpha-1-acid glycoprotein 1,2	NEEYNK_5_4_0_2	None	None	2.7164	0.0000	None	7.2705	0.0000
141	AGP file 4	2755	Alpha-1-acid glycoprotein 1,2	NEEYNK_5_4_0_2	None	None	3.9612	0.0000	None	7.3143	0.0000
142	AGP file 3	2718	Alpha-1-acid glycoprotein 1,2	NEEYNK_5_4_0_2	None	None	3.2516	0.0000	None	7.2745	0.0000
143	AGP file 3	4731	Alpha-1-acid glycoprotein 1,2	NEEYNK_5_4_0_2	None	None	2.8649	0.0000	None	7.2210	0.0000
144	AGP file 4	3485	Alpha-1-acid glycoprotein 1,2	NEEYNK_5_4_0_2	None	None	3.4185	0.0000	None	7.1781	0.0000
145	AGP file 3	3685	Alpha-1-acid glycoprotein 1,2	NEEYNK_5_4_0_2	None	None	3.3264	0.0000	None	7.0648	0.0000
146	AGP file 4	1715	Alpha-1-acid glycoprotein 1,2	NEEYNK_5_4_1_1	Outer	None	1.3966	0.0000	None	1.3406	0.0175
147	AGP file 4	3137	Alpha-1-acid glycoprotein 1,2	NEEYNK_5_4_1_2	Core	Core	4.4033	0.0000	Core	6.6263	0.0000
148	AGP file 4	2595	Alpha-1-acid glycoprotein 1,2	NEEYNK_5_4_1_2	Outer	Outer	1.6209	0.0000	Outer	6.6598	0.0000
149	AGP file 3	2931	Alpha-1-acid glycoprotein 1,2	NEEYNK_5_4_1_2	Outer	Outer	0.4039	0.0000	Outer	6.1848	0.0029

Supplementary Table 7. Continued.

No.	Experiment	CID Scan No.	N-glycoproteins	N-glycopeptides	Manual Classification	SVM			DNN		
						Classification	Pscore	FDR	Classification	Pscore	FDR
150	AGP file 3	2675	Alpha-1-acid glycoprotein 1,2	NEEYNK_5_4_1_2	Outer	Outer	1.6463	0.0000	Outer	6.4449	0.0000
151	AGP file 4	2845	Alpha-1-acid glycoprotein 1,2	NEEYNK_5_4_1_2	Outer	Outer	4.1967	0.0000	Outer	6.6196	0.0000
152	AGP file 52	4342	Alpha-1-acid glycoprotein 1,2	NEEYNK_5_4_2_2	Dual	Dual	1.3905	0.0000	Dual	5.4731	0.0026
153	AGP file 44	4264	Alpha-1-acid glycoprotein 1,2	NEEYNK_5_4_2_2	Dual	Dual	1.2887	0.0000	Dual	5.5900	0.0027
154	AGP file 41	4857	Alpha-1-acid glycoprotein 1,2	NEEYNK_5_4_2_2	Dual	Dual	2.2529	0.0000	Dual	5.7397	0.0027
155	AGP file 39	4131	Alpha-1-acid glycoprotein 1,2	NEEYNK_5_4_2_2	Dual	Dual	0.0547	0.3259	Dual	5.9998	0.0028
156	AGP file 43	4783	Alpha-1-acid glycoprotein 1,2	NEEYNK_5_4_2_2	Dual	Dual	0.2861	0.0102	Dual	3.5318	0.0051
157	AGP file 3	3997	Alpha-1-acid glycoprotein 1,2	NEEYNK_5_5_0_2	None	None	3.2606	0.0000	None	7.2693	0.0000
158	AGP file 3	2276	Alpha-1-acid glycoprotein 1,2	NEEYNK_6_5_0_1	None	None	2.2554	0.0000	None	7.2937	0.0000
159	AGP file 3	2471	Alpha-1-acid glycoprotein 1,2	NEEYNK_6_5_0_1	None	None	2.5510	0.0000	None	7.2387	0.0000
160	AGP file 4	2266	Alpha-1-acid glycoprotein 1,2	NEEYNK_6_5_0_1	None	None	3.2710	0.0000	None	6.3965	0.0000
161	AGP file 4	2383	Alpha-1-acid glycoprotein 1,2	NEEYNK_6_5_0_1	None	None	2.4129	0.0000	None	7.2537	0.0000
162	AGP file 4	1770	Alpha-1-acid glycoprotein 1,2	NEEYNK_6_5_0_1	None	None	2.7529	0.0000	None	7.2872	0.0000
163	AGP file 4	2226	Alpha-1-acid glycoprotein 1,2	NEEYNK_6_5_0_1	None	None	2.1225	0.0000	None	7.2615	0.0000
164	AGP file 3	2071	Alpha-1-acid glycoprotein 1,2	NEEYNK_6_5_0_1	None	None	2.5933	0.0000	None	7.2871	0.0000
165	AGP file 4	1845	Alpha-1-acid glycoprotein 1,2	NEEYNK_6_5_0_1	None	None	2.5258	0.0000	None	7.2210	0.0000
166	AGP file 3	3763	Alpha-1-acid glycoprotein 1,2	NEEYNK_6_5_0_2	None	None	3.0407	0.0000	None	7.3083	0.0000
167	AGP file 4	3530	Alpha-1-acid glycoprotein 1,2	NEEYNK_6_5_0_2	None	None	2.5250	0.0000	None	7.3051	0.0000
168	AGP file 4	3235	Alpha-1-acid glycoprotein 1,2	NEEYNK_6_5_0_2	None	None	2.7107	0.0000	None	7.3021	0.0000
169	AGP file 4	3849	Alpha-1-acid glycoprotein 1,2	NEEYNK_6_5_0_2	None	None	2.9380	0.0000	None	7.3021	0.0000
170	AGP file 3	3214	Alpha-1-acid glycoprotein 1,2	NEEYNK_6_5_0_2	None	None	2.5093	0.0000	None	7.1399	0.0000
171	AGP file 4	3732	Alpha-1-acid glycoprotein 1,2	NEEYNK_6_5_0_2	None	None	2.3501	0.0000	None	7.3153	0.0000
172	AGP file 3	4095	Alpha-1-acid glycoprotein 1,2	NEEYNK_6_5_0_2	None	None	2.9665	0.0000	None	7.3124	0.0000
173	AGP file 3	3129	Alpha-1-acid glycoprotein 1,2	NEEYNK_6_5_0_2	None	None	2.7745	0.0000	None	7.2555	0.0000
174	AGP file 4	2979	Alpha-1-acid glycoprotein 1,2	NEEYNK_6_5_0_2	None	None	1.8342	0.0000	None	7.2779	0.0000
175	AGP file 3	4779	Alpha-1-acid glycoprotein 1,2	NEEYNK_6_5_0_3	None	None	3.0547	0.0000	None	7.2993	0.0000
176	AGP file 4	4617	Alpha-1-acid glycoprotein 1,2	NEEYNK_6_5_0_3	None	None	3.5054	0.0000	None	7.2919	0.0000
177	AGP file 4	1713	Alpha-1-acid glycoprotein 1,2	NEEYNK_6_5_1_1	Outer	Outer	6.1907	0.0000	Outer	6.7612	0.0000
178	AGP file 4	2002	Alpha-1-acid glycoprotein 1,2	NEEYNK_6_5_1_1	Outer	Outer	4.5189	0.0000	Outer	6.7365	0.0000
179	AGP file 4	2181	Alpha-1-acid glycoprotein 1,2	NEEYNK_6_5_1_1	Outer	Outer	4.4960	0.0000	Outer	6.4344	0.0000

Supplementary Table 7. Continued.

No.	Experiment	CID Scan No.	N-glycoproteins	N-glycopeptides	Manual Classification	SVM			DNN		
						Classification	Pscore	FDR	Classification	Pscore	FDR
180	AGP file 3	2008	Alpha-1-acid glycoprotein 1,2	NEEYNK_6_5_1_1	Outer	Outer	0.9040	0.0000	Outer	6.6846	0.0000
181	AGP file 3	2237	Alpha-1-acid glycoprotein 1,2	NEEYNK_6_5_1_1	Outer	Outer	3.9857	0.0000	Outer	6.4647	0.0000
182	AGP file 3	3683	Alpha-1-acid glycoprotein 1,2	NEEYNK_6_5_1_2	Outer	Dual	0.6520	0.0000	Outer	2.3465	0.0076
183	AGP file 3	3041	Alpha-1-acid glycoprotein 1,2	NEEYNK_6_5_1_2	Outer	Outer	3.8267	0.0000	Outer	6.6291	0.0000
184	AGP file 4	3217	Alpha-1-acid glycoprotein 1,2	NEEYNK_6_5_1_2	Outer	Outer	5.6858	0.0000	Outer	6.7108	0.0000
185	AGP file 4	2939	Alpha-1-acid glycoprotein 1,2	NEEYNK_6_5_1_2	Outer	Outer	4.1689	0.0000	Outer	6.5592	0.0000
186	AGP file 3	3362	Alpha-1-acid glycoprotein 1,2	NEEYNK_6_5_1_2	Outer	Outer	5.9786	0.0000	Outer	6.6195	0.0000
187	AGP file 4	2632	Alpha-1-acid glycoprotein 1,2	NEEYNK_6_5_1_2	Outer	Outer	3.0519	0.0000	Outer	6.5383	0.0000
188	AGP file 4	3797	Alpha-1-acid glycoprotein 1,2	NEEYNK_6_5_1_2	Outer	Outer	0.9666	0.0000	Outer	5.9290	0.0027
189	AGP file 3	3991	Alpha-1-acid glycoprotein 1,2	NEEYNK_6_5_1_2	Outer	Outer	1.5652	0.0000	Outer	5.5592	0.0027
190	AGP file 4	3217	Alpha-1-acid glycoprotein 1,2	NEEYNK_6_5_1_2	Outer	Outer	5.6858	0.0000	Outer	6.7108	0.0000
191	AGP file 3	4752	Alpha-1-acid glycoprotein 1,2	NEEYNK_6_5_1_3	Outer	Outer	4.3485	0.0000	Outer	6.5729	0.0000
192	AGP file 4	4522	Alpha-1-acid glycoprotein 1,2	NEEYNK_6_5_1_3	Outer	Outer	4.5515	0.0000	Outer	6.5690	0.0000
193	AGP file 56	3737	Alpha-1-acid glycoprotein 1,2	NEEYNK_6_5_2_2	Dual	Dual	3.2943	0.0000	Dual	6.1443	0.0028
194	AGP file 52	3977	Alpha-1-acid glycoprotein 1,2	NEEYNK_6_5_2_2	Dual	Dual	1.5624	0.0000	Dual	6.1574	0.0028
195	AGP file 59	4713	Alpha-1-acid glycoprotein 1,2	NEEYNK_6_5_2_2	Dual	Dual	2.1139	0.0000	Dual	6.2334	0.0030
196	AGP file 57	3653	Alpha-1-acid glycoprotein 1,2	NEEYNK_6_5_2_2	Dual	Dual	0.2827	0.0102	Dual	5.0696	0.0052
197	AGP file 63	4604	Alpha-1-acid glycoprotein 1,2	NEEYNK_6_5_2_2	Dual	Dual	1.2872	0.0000	Dual	5.3452	0.0026
198	AGP file 39	3850	Alpha-1-acid glycoprotein 1,2	NEEYNK_6_5_2_2	Dual	Outer	1.4422	0.0000	Outer	5.5338	0.0026
199	AGP file 57	4027	Alpha-1-acid glycoprotein 1,2	NEEYNK_6_5_2_2	Dual	Dual	1.9759	0.0000	Dual	6.2951	0.0031
200	AGP file 67	4537	Alpha-1-acid glycoprotein 1,2	NEEYNK_6_5_2_2	Dual	Dual	2.1094	0.0000	Dual	5.9658	0.0027
201	AGP file 39	4070	Alpha-1-acid glycoprotein 1,2	NEEYNK_6_5_2_2	Dual	Dual	3.2718	0.0000	Dual	6.3547	0.0032
202	AGP file 3	2752	Alpha-1-acid glycoprotein 1,2	NEEYNK_6_5_2_2	Outer	Outer	17.1242	0.0000	Outer	6.6901	0.0000
203	AGP file 61	4770	Alpha-1-acid glycoprotein 1,2	NEEYNK_6_5_2_2	Dual	Dual	0.1961	0.0886	Dual	2.8183	0.0051
204	AGP file 66	4787	Alpha-1-acid glycoprotein 1,2	NEEYNK_6_5_2_2	Dual	Dual	5.2580	0.0000	Dual	6.1772	0.0029
205	AGP file 62	4696	Alpha-1-acid glycoprotein 1,2	NEEYNK_6_5_2_2	Dual	Dual	0.7411	0.0000	Dual	5.7755	0.0027
206	AGP file 53	4926	Alpha-1-acid glycoprotein 1,2	NEEYNK_6_5_2_2	Dual	Dual	2.6902	0.0000	Dual	5.9886	0.0027
207	AGP file 43	5415	Alpha-1-acid glycoprotein 1,2	NEEYNK_6_5_2_2	Dual	Dual	3.0408	0.0000	Dual	6.0660	0.0028
208	AGP file 64	4658	Alpha-1-acid glycoprotein 1,2	NEEYNK_6_5_2_2	Dual	Dual	0.5906	0.0000	Dual	6.2158	0.0029
209	AGP file 40	5550	Alpha-1-acid glycoprotein 1,2	NEEYNK_6_5_2_2	Dual	Dual	1.2843	0.0000	Dual	5.3341	0.0026

Supplementary Table 7. Continued.

No.	Experiment	CID Scan No.	N-glycoproteins	N-glycopeptides	Manual Classification	SVM			DNN		
						Classification	Pscore	FDR	Classification	Pscore	FDR
210	AGP file 51	5048	Alpha-1-acid glycoprotein 1,2	NEEYNK_6_5_2_2	Dual	Dual	2.8658	0.0000	Dual	6.2137	0.0029
211	AGP file 64	4328	Alpha-1-acid glycoprotein 1,2	NEEYNK_6_5_2_2	Dual	Outer	0.7245	0.0000	Outer	5.5072	0.0026
212	AGP file 67	4877	Alpha-1-acid glycoprotein 1,2	NEEYNK_6_5_2_2	Dual	Dual	0.4370	0.0000	Dual	4.8946	0.0052
213	AGP file 43	9686	Alpha-1-acid glycoprotein 1,2	NEEYNK_6_5_2_3	Dual	Dual	3.9169	0.0000	Dual	6.1789	0.0029
214	AGP file 39	7457	Alpha-1-acid glycoprotein 1,2	NEEYNK_6_5_2_3	Dual	Dual	4.5920	0.0000	Dual	6.3316	0.0031
215	AGP file 42	9515	Alpha-1-acid glycoprotein 1,2	NEEYNK_6_5_2_3	Dual	Dual	2.4794	0.0000	Dual	5.9232	0.0027
216	AGP file 67	8738	Alpha-1-acid glycoprotein 1,2	NEEYNK_6_5_2_3	Dual	Dual	2.8096	0.0000	Dual	6.3243	0.0031
217	AGP file 63	8633	Alpha-1-acid glycoprotein 1,2	NEEYNK_6_5_2_3	Dual	Dual	4.2796	0.0000	Dual	6.2741	0.0030
218	AGP file 55	3882	Alpha-1-acid glycoprotein 1,2	NEEYNK_6_5_2_3	Dual	Dual	1.1139	0.0000	Dual	6.4533	0.0000
219	AGP file 52	8385	Alpha-1-acid glycoprotein 1,2	NEEYNK_6_5_2_3	Dual	Dual	3.3425	0.0000	Dual	6.3779	0.0000
220	AGP file 47	8445	Alpha-1-acid glycoprotein 1,2	NEEYNK_6_5_2_3	Dual	Dual	2.7107	0.0000	Dual	5.9312	0.0027
221	AGP file 59	8687	Alpha-1-acid glycoprotein 1,2	NEEYNK_6_5_2_3	Dual	Dual	3.8803	0.0000	Dual	6.3678	0.0032
222	AGP file 44	8217	Alpha-1-acid glycoprotein 1,2	NEEYNK_6_5_2_3	Dual	Dual	4.3774	0.0000	Dual	6.2607	0.0030
223	AGP file 49	8858	Alpha-1-acid glycoprotein 1,2	NEEYNK_6_5_2_3	Dual	Dual	3.9697	0.0000	Dual	6.2862	0.0031
224	AGP file 54	3918	Alpha-1-acid glycoprotein 1,2	NEEYNK_6_5_2_3	Dual	Dual	1.0613	0.0000	Dual	6.2511	0.0030
225	AGP file 4	4619	Alpha-1-acid glycoprotein 1,2	NEEYNK_6_5_2_3	Dual	Dual	2.5267	0.0000	Dual	6.3950	0.0000
226	AGP file 40	10122	Alpha-1-acid glycoprotein 1,2	NEEYNK_6_5_2_3	Dual	Dual	2.0131	0.0000	Dual	6.3470	0.0031
227	AGP file 36	5905	Alpha-1-acid glycoprotein 1,2	NEEYNK_6_5_2_3	Dual	Dual	1.8280	0.0000	Dual	5.6140	0.0027
228	AGP file 3	4679	Alpha-1-acid glycoprotein 1,2	NEEYNK_6_5_3_1	Outer	Outer	3.3886	0.0000	Outer	6.7341	0.0000
229	AGP file 3	5953	Alpha-1-acid glycoprotein 1,2	NEEYNK_6_5_3_2	Outer	Outer	2.1363	0.0000	Outer	6.5969	0.0000
230	AGP file 4	26479	Alpha-1-acid glycoprotein 1,2	SVQEIQATFFYFTPNK_6_5_0_3	None	None	3.3360	0.0000	None	7.2719	0.0000
231	AGP file 4	26406	Alpha-1-acid glycoprotein 1,2	SVQEIQATFFYFTPNK_7_6_0_3	None	None	1.2425	0.0000	None	7.2109	0.0000
232	AGP file 4	1622	Alpha-1-acid glycoprotein 2	ENGTVSR_6_5_0_1	None	None	2.3894	0.0000	None	7.2547	0.0000
233	AGP file 4	2301	Alpha-1-acid glycoprotein 2	ENGTVSR_6_5_0_2	None	None	2.1038	0.0000	None	7.3095	0.0000
234	AGP file 3	2540	Alpha-1-acid glycoprotein 2	ENGTVSR_6_5_0_2	None	None	1.7445	0.0000	None	7.2618	0.0000
235	AGP file 4	2449	Alpha-1-acid glycoprotein 2	ENGTVSR_6_5_0_2	None	None	2.2651	0.0000	None	7.3060	0.0000
236	AGP file 3	2385	Alpha-1-acid glycoprotein 2	ENGTVSR_6_5_0_2	None	None	2.6120	0.0000	None	7.3099	0.0000
237	AGP file 4	2552	Alpha-1-acid glycoprotein 2	ENGTVSR_6_5_0_2	None	None	1.9858	0.0000	None	7.2659	0.0000
238	AGP file 3	2645	Alpha-1-acid glycoprotein 2	ENGTVSR_6_5_0_2	None	None	2.4494	0.0000	None	7.2877	0.0000
239	AGP file 4	4068	Alpha-1-acid glycoprotein 2	ENGTVSR_6_5_0_3	None	None	3.2961	0.0000	None	7.3107	0.0000

Supplementary Table 7. Continued.

No.	Experiment	CID Scan No.	N-glycoproteins	N-glycopeptides	Manual Classification	SVM			DNN		
						Classification	Pscore	FDR	Classification	Pscore	FDR
240	AGP file 3	4183	Alpha-1-acid glycoprotein 2	ENGTVSR_6_5_0_3	None	None	2.6842	0.0000	None	7.2793	0.0000
241	AGP file 3	1431	Alpha-1-acid glycoprotein 2	ENGTVSR_6_5_1_1	Outer	Outer	6.5564	0.0000	Outer	6.7363	0.0000
242	AGP file 4	1462	Alpha-1-acid glycoprotein 2	ENGTVSR_6_5_1_1	Outer	Outer	5.5166	0.0000	Outer	6.7343	0.0000
243	AGP file 4	1575	Alpha-1-acid glycoprotein 2	ENGTVSR_6_5_1_1	Outer	Outer	3.0600	0.0000	Outer	6.7162	0.0000
244	AGP file 3	2599	Alpha-1-acid glycoprotein 2	ENGTVSR_6_5_1_2	Outer	Outer	5.4778	0.0000	Outer	6.6836	0.0000
245	AGP file 4	2306	Alpha-1-acid glycoprotein 2	ENGTVSR_6_5_1_2	Outer	Outer	3.7565	0.0000	Outer	6.5951	0.0000
246	AGP file 4	3953	Alpha-1-acid glycoprotein 2	ENGTVSR_6_5_1_3	Outer	Outer	3.4446	0.0000	Outer	6.3575	0.0032
247	AGP file 3	4068	Alpha-1-acid glycoprotein 2	ENGTVSR_6_5_1_3	Outer	Outer	4.3726	0.0000	Outer	6.2901	0.0031
248	AGP file 48	6765	Alpha-1-acid glycoprotein 2	ENGTVSR_6_5_2_3	Dual	Dual	0.7436	0.0000	Dual	6.1510	0.0028
249	AGP file 38	5961	Alpha-1-acid glycoprotein 2	ENGTVSR_6_5_2_3	Dual	Dual	3.4739	0.0000	Dual	6.2120	0.0029
250	AGP file 64	7029	Alpha-1-acid glycoprotein 2	ENGTVSR_6_5_2_3	Dual	Dual	1.5873	0.0000	Dual	6.1374	0.0028
251	AGP file 65	7205	Alpha-1-acid glycoprotein 2	ENGTVSR_6_5_2_3	Dual	Dual	4.5793	0.0000	Dual	6.2339	0.0030
252	AGP file 62	7085	Alpha-1-acid glycoprotein 2	ENGTVSR_6_5_2_3	Dual	Dual	2.3704	0.0000	Dual	6.1440	0.0028
253	AGP file 58	5844	Alpha-1-acid glycoprotein 2	ENGTVSR_6_5_2_3	Dual	Dual	5.3608	0.0000	Dual	6.2566	0.0030
254	AGP file 66	7163	Alpha-1-acid glycoprotein 2	ENGTVSR_6_5_2_3	Dual	Dual	3.3098	0.0000	Dual	6.1781	0.0029
255	AGP file 56	6001	Alpha-1-acid glycoprotein 2	ENGTVSR_6_5_2_3	Dual	Dual	2.3885	0.0000	Dual	6.2141	0.0029
256	AGP file 4	1946	Alpha-1-acid glycoprotein 2	ENGTVSR_7_6_1_2	Outer	Outer	2.3693	0.0000	Outer	6.6734	0.0000
257	AGP file 3	2390	Alpha-1-acid glycoprotein 2	ENGTVSR_7_6_1_2	Outer	Outer	1.8039	0.0000	Outer	6.5793	0.0000
258	AGP file 3	2203	Alpha-1-acid glycoprotein 2	ENGTVSR_7_6_1_2	Outer	Outer	2.0649	0.0000	Outer	6.6283	0.0000
259	AGP file 3	1953	Alpha-1-acid glycoprotein 2	ENGTVSR_7_6_1_2	Outer	Outer	2.6110	0.0000	Outer	6.6300	0.0000
260	AGP file 4	2136	Alpha-1-acid glycoprotein 2	ENGTVSR_7_6_1_2	Outer	Outer	2.6532	0.0000	Outer	6.6867	0.0000
261	AGP file 4	2157	Alpha-1-acid glycoprotein 2	ENGTVSR_7_6_1_2	Outer	Outer	2.8483	0.0000	Outer	6.7205	0.0000
262	AGP file 3	5210	Alpha-1-acid glycoprotein 2	ENGTVSR_7_6_1_4	Outer	Outer	2.7106	0.0000	Outer	6.6235	0.0000
263	AGP file 4	5084	Alpha-1-acid glycoprotein 2	ENGTVSR_7_6_1_4	Outer	Outer	2.3235	0.0000	Outer	6.6066	0.0000
264	AGP file 4	15084	Alpha-1-acid glycoprotein 2	LVPVPITNATLDR_3_3_0_0	None	None	3.2397	0.0000	None	7.2872	0.0000
265	AGP file 3	12281	Alpha-1-acid glycoprotein 2	LVPVPITNATLDR_3_3_0_0	None	None	2.9565	0.0000	None	7.2969	0.0000
266	AGP file 3	12722	Alpha-1-acid glycoprotein 2	LVPVPITNATLDR_4_4_0_1	None	None	3.4652	0.0000	None	7.3167	0.0000
267	AGP file 3	12725	Alpha-1-acid glycoprotein 2	LVPVPITNATLDR_4_4_0_1	None	None	3.3591	0.0000	None	7.3215	0.0000
268	AGP file 3	15340	Alpha-1-acid glycoprotein 2	LVPVPITNATLDR_5_4_0_1	None	None	2.4824	0.0000	None	6.6993	0.0000
269	AGP file 3	12615	Alpha-1-acid glycoprotein 2	LVPVPITNATLDR_5_4_0_1	None	None	3.6730	0.0000	None	7.3405	0.0000

Supplementary Table 7. Continued.

No.	Experiment	CID Scan No.	N-glycoproteins	N-glycopeptides	Manual Classification	SVM			DNN		
						Classification	Pscore	FDR	Classification	Pscore	FDR
270	AGP file 4	14992	Alpha-1-acid glycoprotein 2	LVPVPITNATLDR_5_4_0_1	None	None	1.8815	0.0000	None	6.8777	0.0000
271	AGP file 3	12307	Alpha-1-acid glycoprotein 2	LVPVPITNATLDR_5_4_0_1	None	None	4.6543	0.0000	None	7.3496	0.0000
272	AGP file 4	15488	Alpha-1-acid glycoprotein 2	LVPVPITNATLDR_5_4_0_2	None	None	2.3718	0.0000	None	6.8485	0.0000
273	AGP file 3	16069	Alpha-1-acid glycoprotein 2	LVPVPITNATLDR_5_4_0_2	None	None	2.1285	0.0000	None	7.2758	0.0000
274	AGP file 3	15799	Alpha-1-acid glycoprotein 2	LVPVPITNATLDR_5_4_0_2	None	None	4.2162	0.0000	None	7.3316	0.0000
275	AGP file 3	19503	Alpha-1-acid glycoprotein 2	LVPVPITNATLDR_5_4_0_2	None	None	3.5128	0.0000	None	7.3288	0.0000
276	AGP file 3	15678	Alpha-1-acid glycoprotein 2	LVPVPITNATLDR_5_4_0_2	None	None	4.8412	0.0000	None	7.3308	0.0000
277	AGP file 4	15381	Alpha-1-acid glycoprotein 2	LVPVPITNATLDR_5_4_0_2	None	None	3.2342	0.0000	None	7.3377	0.0000
278	AGP file 3	15761	Alpha-1-acid glycoprotein 2	LVPVPITNATLDR_5_4_1_2	Outer	Outer	5.2943	0.0000	Outer	6.5132	0.0000
279	AGP file 4	15367	Alpha-1-acid glycoprotein 2	LVPVPITNATLDR_5_4_1_2	Outer	Outer	0.4139	0.0000	Outer	0.6254	0.1400
280	AGP file 3	12326	Alpha-1-acid glycoprotein 2	LVPVPITNATLDR_5_5_0_1	None	None	2.5684	0.0000	None	7.2962	0.0000
281	AGP file 4	12020	Alpha-1-acid glycoprotein 2	LVPVPITNATLDR_5_5_0_1	None	None	3.4912	0.0000	None	7.3346	0.0000
282	AGP file 3	15537	Alpha-1-acid glycoprotein 2	LVPVPITNATLDR_5_5_1_2	Outer	Outer	1.8246	0.0000	Outer	6.3530	0.0031
283	AGP file 4	11892	Alpha-1-acid glycoprotein 2	LVPVPITNATLDR_6_5_0_1	None	None	1.8049	0.0000	None	7.2333	0.0000
284	AGP file 3	12405	Alpha-1-acid glycoprotein 2	LVPVPITNATLDR_6_5_0_1	None	None	1.7203	0.0000	None	7.2270	0.0000
285	AGP file 4	11843	Alpha-1-acid glycoprotein 2	LVPVPITNATLDR_6_5_0_1	None	None	3.2816	0.0000	None	7.3450	0.0000
286	AGP file 3	12246	Alpha-1-acid glycoprotein 2	LVPVPITNATLDR_6_5_0_1	None	None	3.9422	0.0000	None	7.3634	0.0000
287	AGP file 3	11939	Alpha-1-acid glycoprotein 2	LVPVPITNATLDR_6_5_0_1	None	None	2.0940	0.0000	None	7.2859	0.0000
288	AGP file 3	15635	Alpha-1-acid glycoprotein 2	LVPVPITNATLDR_6_5_0_2	None	None	3.3185	0.0000	None	7.3311	0.0000
289	AGP file 3	15529	Alpha-1-acid glycoprotein 2	LVPVPITNATLDR_6_5_0_2	None	None	3.2763	0.0000	None	7.3157	0.0000
290	AGP file 4	15283	Alpha-1-acid glycoprotein 2	LVPVPITNATLDR_6_5_0_2	None	None	3.3841	0.0000	None	7.3210	0.0000
291	AGP file 4	14929	Alpha-1-acid glycoprotein 2	LVPVPITNATLDR_6_5_0_2	None	None	2.7933	0.0000	None	7.3116	0.0000
292	AGP file 4	15591	Alpha-1-acid glycoprotein 2	LVPVPITNATLDR_6_5_0_2	None	None	2.9066	0.0000	None	7.3189	0.0000
293	AGP file 4	19268	Alpha-1-acid glycoprotein 2	LVPVPITNATLDR_6_5_0_3	None	None	2.4622	0.0000	None	7.2574	0.0000
294	AGP file 4	19155	Alpha-1-acid glycoprotein 2	LVPVPITNATLDR_6_5_0_3	None	None	1.8549	0.0000	None	7.2686	0.0000
295	AGP file 3	19311	Alpha-1-acid glycoprotein 2	LVPVPITNATLDR_6_5_0_3	None	None	2.4471	0.0000	None	7.2639	0.0000
296	AGP file 4	18825	Alpha-1-acid glycoprotein 2	LVPVPITNATLDR_6_5_0_3	None	None	1.8912	0.0000	None	7.2658	0.0000
297	AGP file 3	19438	Alpha-1-acid glycoprotein 2	LVPVPITNATLDR_6_5_0_3	None	None	2.1768	0.0000	None	6.9320	0.0000
298	AGP file 4	11669	Alpha-1-acid glycoprotein 2	LVPVPITNATLDR_6_5_1_1	Outer	Outer	5.4979	0.0000	Outer	6.6080	0.0000
299	AGP file 4	15088	Alpha-1-acid glycoprotein 2	LVPVPITNATLDR_6_5_1_1	Outer	Outer	5.2104	0.0000	Outer	6.5993	0.0000

Supplementary Table 7. Continued.

No.	Experiment	CID Scan No.	N-glycoproteins	N-glycopeptides	Manual Classification	SVM			DNN		
						Classification	Pscore	FDR	Classification	Pscore	FDR
300	AGP file 3	12104	Alpha-1-acid glycoprotein 2	LVPVPITNATLDR_6_5_1_1	Outer	Outer	1.2624	0.0000	Outer	6.6393	0.0000
301	AGP file 4	11993	Alpha-1-acid glycoprotein 2	LVPVPITNATLDR_6_5_1_1	Outer	Outer	3.5070	0.0000	Outer	6.6379	0.0000
302	AGP file 3	15640	Alpha-1-acid glycoprotein 2	LVPVPITNATLDR_6_5_1_2	Outer	Outer	1.6334	0.0000	Outer	6.5857	0.0000
303	AGP file 4	15128	Alpha-1-acid glycoprotein 2	LVPVPITNATLDR_6_5_1_2	Outer	Outer	1.8161	0.0000	Outer	6.0759	0.0028
304	AGP file 4	15447	Alpha-1-acid glycoprotein 2	LVPVPITNATLDR_6_5_1_2	Outer	Outer	2.8797	0.0000	Outer	6.5377	0.0000
305	AGP file 4	14800	Alpha-1-acid glycoprotein 2	LVPVPITNATLDR_6_5_1_2	Outer	Outer	2.5847	0.0000	Outer	6.4523	0.0000
306	AGP file 3	15285	Alpha-1-acid glycoprotein 2	LVPVPITNATLDR_6_5_1_2	Outer	Outer	3.2798	0.0000	Outer	6.7226	0.0000
307	AGP file 4	15172	Alpha-1-acid glycoprotein 2	LVPVPITNATLDR_6_5_1_2	Outer	Outer	4.0865	0.0000	Outer	6.7255	0.0000
308	AGP file 4	14833	Alpha-1-acid glycoprotein 2	LVPVPITNATLDR_6_5_1_2	Outer	Outer	0.6018	0.0000	Outer	5.9044	0.0027
309	AGP file 4	19281	Alpha-1-acid glycoprotein 2	LVPVPITNATLDR_6_5_1_3	Outer	Outer	1.8958	0.0000	Outer	6.5224	0.0000
310	AGP file 3	19654	Alpha-1-acid glycoprotein 2	LVPVPITNATLDR_6_5_1_3	Outer	Outer	1.5765	0.0000	Outer	6.5194	0.0000
311	AGP file 3	19081	Alpha-1-acid glycoprotein 2	LVPVPITNATLDR_6_5_1_3	Outer	Outer	1.3989	0.0000	Outer	6.5649	0.0000
312	IgG file 7	6000	Ig gamma-1 chain C region	EEQYNSTYR_3_3_0_0	None	None	3.6412	0.0000	None	7.3113	0.0000
313	IgG file 7	6058	Ig gamma-1 chain C region	EEQYNSTYR_3_3_1_0	Core	Core	7.2860	0.0000	Core	6.6321	0.0000
314	IgG file 7	6726	Ig gamma-1 chain C region	EEQYNSTYR_3_3_1_0	Core	Core	7.2997	0.0000	Core	6.6249	0.0000
315	IgG file 7	6394	Ig gamma-1 chain C region	EEQYNSTYR_3_3_1_0	Core	Core	6.3952	0.0000	Core	6.6356	0.0000
316	IgG file 7	7005	Ig gamma-1 chain C region	EEQYNSTYR_3_3_1_0	Core	Core	6.2576	0.0000	Core	6.6426	0.0000
317	IgG file 6	4370	Ig gamma-1 chain C region	EEQYNSTYR_3_3_1_0	Core	Core	5.3599	0.0000	Core	6.6353	0.0000
318	IgG file 7	6311	Ig gamma-1 chain C region	EEQYNSTYR_3_4_0_0	None	None	3.1927	0.0000	None	7.1958	0.0000
319	IgG file 7	6631	Ig gamma-1 chain C region	EEQYNSTYR_3_4_0_0	None	None	3.2644	0.0000	None	7.2262	0.0000
320	IgG file 7	5973	Ig gamma-1 chain C region	EEQYNSTYR_3_4_0_0	None	None	3.1210	0.0000	None	7.1706	0.0000
321	IgG file 6	4399	Ig gamma-1 chain C region	EEQYNSTYR_3_4_0_0	None	None	2.6992	0.0000	None	7.1853	0.0000
322	IgG file 7	7845	Ig gamma-1 chain C region	EEQYNSTYR_3_4_1_0	Core	Core	2.6563	0.0000	Core	6.6483	0.0000
323	IgG file 7	5295	Ig gamma-1 chain C region	EEQYNSTYR_3_4_1_0	Core	Core	8.8647	0.0000	Core	6.6145	0.0000
324	IgG file 7	6085	Ig gamma-1 chain C region	EEQYNSTYR_3_4_1_0	Core	Core	4.6632	0.0000	Core	6.6572	0.0000
325	IgG file 7	6421	Ig gamma-1 chain C region	EEQYNSTYR_3_4_1_0	Core	Core	4.8603	0.0000	Core	6.6559	0.0000
326	IgG file 7	6739	Ig gamma-1 chain C region	EEQYNSTYR_3_4_1_0	Core	Core	4.9418	0.0000	Core	6.6550	0.0000
327	IgG file 7	7490	Ig gamma-1 chain C region	EEQYNSTYR_3_4_1_0	Core	Core	7.7733	0.0000	Core	6.6148	0.0000
328	IgG file 7	7021	Ig gamma-1 chain C region	EEQYNSTYR_3_4_1_0	Core	Core	5.3999	0.0000	Core	6.6528	0.0000
329	IgG file 5	3033	Ig gamma-1 chain C region	EEQYNSTYR_3_4_1_0	Core	Core	4.9462	0.0000	Core	6.6571	0.0000

Supplementary Table 7. Continued.

No.	Experiment	CID Scan No.	N-glycoproteins	N-glycopeptides	Manual Classification	SVM			DNN		
						Classification	Pscore	FDR	Classification	Pscore	FDR
330	IgG file 7	5764	Ig gamma-1 chain C region	EEQYNSTYR_3_4_1_0	Core	Core	5.9973	0.0000	Core	6.6264	0.0000
331	IgG file 7	7667	Ig gamma-1 chain C region	EEQYNSTYR_3_4_1_0	Core	Core	5.6636	0.0000	Core	6.6374	0.0000
332	IgG file 7	7268	Ig gamma-1 chain C region	EEQYNSTYR_3_4_1_0	Core	Core	9.2273	0.0000	Core	6.6165	0.0000
333	IgG file 7	8322	Ig gamma-1 chain C region	EEQYNSTYR_3_4_1_0	Core	Core	6.2623	0.0000	Core	6.6217	0.0000
334	IgG file 7	7954	Ig gamma-1 chain C region	EEQYNSTYR_3_5_0_0	None	None	1.9710	0.0000	None	7.2589	0.0000
335	IgG file 7	8666	Ig gamma-1 chain C region	EEQYNSTYR_3_5_1_0	Core	Core	4.4717	0.0000	Core	6.6355	0.0000
336	IgG file 6	4420	Ig gamma-1 chain C region	EEQYNSTYR_3_5_1_0	Core	Core	4.1869	0.0000	Core	6.6615	0.0000
337	IgG file 7	8314	Ig gamma-1 chain C region	EEQYNSTYR_3_5_1_0	Core	Core	3.9949	0.0000	Core	6.6395	0.0000
338	IgG file 7	6199	Ig gamma-1 chain C region	EEQYNSTYR_4_3_1_0	Core	Core	5.9024	0.0000	Core	6.6097	0.0000
339	IgG file 7	6537	Ig gamma-1 chain C region	EEQYNSTYR_4_3_1_0	Core	Core	6.4891	0.0000	Core	6.6036	0.0000
340	IgG file 6	4360	Ig gamma-1 chain C region	EEQYNSTYR_4_4_0_0	None	None	3.1797	0.0000	None	7.0988	0.0000
341	IgG file 7	5761	Ig gamma-1 chain C region	EEQYNSTYR_4_4_0_0	None	None	3.2073	0.0000	None	7.1274	0.0000
342	IgG file 7	6089	Ig gamma-1 chain C region	EEQYNSTYR_4_4_0_0	None	None	3.1204	0.0000	None	7.0755	0.0000
343	IgG file 7	6427	Ig gamma-1 chain C region	EEQYNSTYR_4_4_0_0	None	None	2.9278	0.0000	None	6.8487	0.0000
344	IgG file 6	4341	Ig gamma-1 chain C region	EEQYNSTYR_4_4_1_0	Core	Core	7.4169	0.0000	Core	6.6368	0.0000
345	IgG file 7	5171	Ig gamma-1 chain C region	EEQYNSTYR_4_4_1_0	Core	Core	1.2775	0.0000	Core	6.5712	0.0000
346	IgG file 7	5650	Ig gamma-1 chain C region	EEQYNSTYR_4_4_1_0	Core	Core	5.3142	0.0000	Core	6.5710	0.0000
347	IgG file 7	6917	Ig gamma-1 chain C region	EEQYNSTYR_4_4_1_0	Core	Core	5.3699	0.0000	Core	6.5822	0.0000
348	IgG file 7	5940	Ig gamma-1 chain C region	EEQYNSTYR_4_4_1_0	Core	Core	3.5219	0.0000	Core	6.5807	0.0000
349	IgG file 7	8525	Ig gamma-1 chain C region	EEQYNSTYR_4_4_1_0	Core	Core	1.5077	0.0000	Core	6.5787	0.0000
350	IgG file 7	6616	Ig gamma-1 chain C region	EEQYNSTYR_4_4_1_0	Core	Core	5.2990	0.0000	Core	6.5818	0.0000
351	IgG file 7	7164	Ig gamma-1 chain C region	EEQYNSTYR_4_4_1_0	Core	Core	5.8877	0.0000	Core	6.5805	0.0000
352	IgG file 7	6278	Ig gamma-1 chain C region	EEQYNSTYR_4_4_1_0	Core	Core	4.5810	0.0000	Core	6.5806	0.0000
353	IgG file 5	3064	Ig gamma-1 chain C region	EEQYNSTYR_4_4_1_0	Core	Core	3.1976	0.0000	Core	6.5808	0.0000
354	IgG file 7	7396	Ig gamma-1 chain C region	EEQYNSTYR_4_4_1_0	Core	Core	6.2345	0.0000	Core	6.5788	0.0000
355	IgG file 6	4339	Ig gamma-1 chain C region	EEQYNSTYR_4_4_1_0	Core	Core	4.9840	0.0000	Core	6.5810	0.0000
356	IgG file 7	9802	Ig gamma-1 chain C region	EEQYNSTYR_4_4_1_1	Core	Core	2.9034	0.0000	Core	6.6325	0.0000
357	IgG file 7	9812	Ig gamma-1 chain C region	EEQYNSTYR_4_4_1_1	Core	Core	2.0546	0.0000	Core	5.3545	0.0026
358	IgG file 6	4424	Ig gamma-1 chain C region	EEQYNSTYR_4_5_1_0	Core	Core	5.3954	0.0000	Core	6.6065	0.0000
359	IgG file 7	8461	Ig gamma-1 chain C region	EEQYNSTYR_4_5_1_0	Core	Core	4.2298	0.0000	Core	6.6149	0.0000

Supplementary Table 7. Continued.

No.	Experiment	CID Scan No.	N-glycoproteins	N-glycopeptides	Manual Classification	SVM			DNN		
						Classification	Pscore	FDR	Classification	Pscore	FDR
360	IgG file 7	5975	Ig gamma-1 chain C region	EEQYNSTYR_5_4_0_0	None	None	2.7983	0.0000	None	6.7457	0.0000
361	IgG file 5	3029	Ig gamma-1 chain C region	EEQYNSTYR_5_4_1_0	Core	Core	4.9060	0.0000	Core	6.6062	0.0000
362	IgG file 7	6087	Ig gamma-1 chain C region	EEQYNSTYR_5_4_1_0	Core	Core	3.3486	0.0000	Core	6.6131	0.0000
363	IgG file 7	6423	Ig gamma-1 chain C region	EEQYNSTYR_5_4_1_0	Core	Core	3.7240	0.0000	Core	6.6136	0.0000
364	IgG file 7	6741	Ig gamma-1 chain C region	EEQYNSTYR_5_4_1_0	Core	Core	3.7266	0.0000	Core	6.6135	0.0000
365	IgG file 7	9750	Ig gamma-1 chain C region	EEQYNSTYR_5_4_1_1	Core	Core	1.1998	0.0000	Core	6.6489	0.0000
366	IgG file 6	4859	Ig gamma-1 chain C region	EEQYNSTYR_5_4_1_1	Core	Core	3.6351	0.0000	Core	6.6036	0.0000
367	IgG file 7	9769	Ig gamma-1 chain C region	EEQYNSTYR_5_4_1_1	Core	Core	2.6271	0.0000	Core	6.6036	0.0000
368	IgG file 7	10599	Ig gamma-2 chain C region	EEQFNSTFR_3_3_1_0	Core	Core	4.1634	0.0000	Core	6.6630	0.0000
369	IgG file 7	10686	Ig gamma-2 chain C region	EEQFNSTFR_3_4_0_0	None	None	2.9952	0.0000	None	7.1198	0.0000
370	IgG file 5	3427	Ig gamma-2 chain C region	EEQFNSTFR_3_4_1_0	Core	Core	6.5825	0.0000	Core	6.6391	0.0000
371	IgG file 7	10461	Ig gamma-2 chain C region	EEQFNSTFR_3_4_1_0	Core	Core	4.8434	0.0000	Core	6.6577	0.0000
372	IgG file 7	10811	Ig gamma-2 chain C region	EEQFNSTFR_3_4_1_0	Core	Core	5.3358	0.0000	Core	6.6482	0.0000
373	IgG file 7	10689	Ig gamma-2 chain C region	EEQFNSTFR_3_5_1_0	Core	Core	0.2593	0.0176	Core	6.6569	0.0000
374	IgG file 7	10691	Ig gamma-2 chain C region	EEQFNSTFR_3_5_1_0	Core	Core	4.8757	0.0000	Core	6.6418	0.0000
375	IgG file 5	3453	Ig gamma-2 chain C region	EEQFNSTFR_3_5_1_0	Core	Core	5.9495	0.0000	Core	6.6219	0.0000
376	IgG file 7	10581	Ig gamma-2 chain C region	EEQFNSTFR_4_3_1_0	Core	Core	5.7542	0.0000	Core	6.6301	0.0000
377	IgG file 7	10655	Ig gamma-2 chain C region	EEQFNSTFR_4_4_0_0	None	None	3.0965	0.0000	None	7.1944	0.0000
378	IgG file 5	3408	Ig gamma-2 chain C region	EEQFNSTFR_4_4_1_0	Core	Core	5.4044	0.0000	Core	6.6409	0.0000
379	IgG file 7	10720	Ig gamma-2 chain C region	EEQFNSTFR_4_4_1_0	Core	Core	3.8331	0.0000	Core	6.6409	0.0000
380	IgG file 6	6063	Ig gamma-2 chain C region	EEQFNSTFR_4_4_1_0	Core	Core	6.8782	0.0000	Core	6.6053	0.0000
381	IgG file 7	10382	Ig gamma-2 chain C region	EEQFNSTFR_4_4_1_0	Core	Core	4.8075	0.0000	Core	6.6129	0.0000
382	IgG file 6	5157	Ig gamma-2 chain C region	EEQFNSTFR_4_4_1_0	Core	Core	7.8828	0.0000	Core	6.6250	0.0000
383	IgG file 7	10722	Ig gamma-2 chain C region	EEQFNSTFR_4_4_1_0	Core	Core	7.1532	0.0000	Core	6.6384	0.0000
384	IgG file 7	12133	Ig gamma-2 chain C region	EEQFNSTFR_4_4_1_1	Core	Core	0.9195	0.0000	Core	6.6716	0.0000
385	IgG file 5	3739	Ig gamma-2 chain C region	EEQFNSTFR_4_4_1_1	Core	Core	4.8480	0.0000	Core	6.6248	0.0000
386	IgG file 7	12154	Ig gamma-2 chain C region	EEQFNSTFR_4_4_1_1	Core	Core	4.8266	0.0000	Core	6.6319	0.0000
387	IgG file 5	3735	Ig gamma-2 chain C region	EEQFNSTFR_4_4_1_1	Core	Core	0.8882	0.0000	Core	6.6437	0.0000
388	IgG file 7	10653	Ig gamma-2 chain C region	EEQFNSTFR_4_5_1_0	Core	Core	4.1761	0.0000	Core	6.6167	0.0000
389	IgG file 5	3400	Ig gamma-2 chain C region	EEQFNSTFR_5_4_1_0	Core	Core	3.1527	0.0000	Core	6.6149	0.0000

Supplementary Table 7. Continued.

No.	Experiment	CID Scan No.	N-glycoproteins	N-glycopeptides	Manual Classification	SVM			DNN		
						Classification	Pscore	FDR	Classification	Pscore	FDR
390	IgG file 7	10548	Ig gamma-2 chain C region	EEQFNSTFR_5_4_1_0	Core	Core	3.6594	0.0000	Core	6.6115	0.0000
391	IgG file 7	12088	Ig gamma-2 chain C region	EEQFNSTFR_5_4_1_1	Core	Core	2.7501	0.0000	Core	6.5970	0.0000
392	IgG file 7	9711	Ig gamma-4 chain C region	EEQFNSTYR_3_4_1_0	Core	Core	5.9052	0.0000	Core	6.6478	0.0000
393	IgG file 7	9698	Ig gamma-4 chain C region	EEQFNSTYR_4_4_1_0	Core	Core	6.5022	0.0000	Core	6.6204	0.0000

Supplementary Table 8. Glycopeptide spectrum matches of human plasma proteins and their Classification as unknown set for machine learning.

No.	Experiment	CID Scan No.	N-glycoproteins	N-glycopeptides	SVM			DNN			Manual Classification
					Classification	Pscore	FDR	Classification	Pscore	FDR	
1	Human Plasma exp_1	18962	Afamin	DIENFNSTQK_5_4_0_2	None	3.2012	0.0000	None	7.3120	0.0000	None
2	Human Plasma exp_1	18960	Afamin	DIENFNSTQK_5_4_0_2	None	3.5642	0.0000	None	7.2777	0.0000	None
3	Human Plasma exp_1	18914	Afamin	DIENFNSTQK_5_4_0_2	None	1.9651	0.0000	None	6.8079	0.0000	None
4	Human Plasma exp_2	19315	Afamin	DIENFNSTQK_5_4_0_2	None	3.3896	0.0000	None	7.3153	0.0000	None
5	Human Plasma exp_2	19360	Afamin	DIENFNSTQK_5_4_0_2	None	3.4571	0.0000	None	7.2888	0.0000	None
6	Human Plasma exp_2	19306	Afamin	DIENFNSTQK_5_4_0_2	None	2.5595	0.0000	None	6.7730	0.0000	None
7	Human Plasma exp_3	18842	Afamin	DIENFNSTQK_5_4_0_2	None	3.5056	0.0000	None	7.3175	0.0000	None
8	Human Plasma exp_4	19406	Afamin	DIENFNSTQK_5_4_0_2	None	4.0013	0.0000	None	7.3251	0.0000	None
9	Human Plasma exp_4	19448	Afamin	DIENFNSTQK_5_4_0_2	None	3.1897	0.0000	None	7.2668	0.0000	None
10	Human Plasma exp_5	19011	Afamin	DIENFNSTQK_5_4_0_2	None	3.7351	0.0000	None	7.3198	0.0000	None
11	Human Plasma exp_5	19135	Afamin	DIENFNSTQK_5_4_0_2	None	2.6304	0.0000	None	7.2599	0.0000	None
12	Human Plasma exp_5	18647	Afamin	DIENFNSTQK_5_4_1_2	Dual	0.1881	0.1810	Core	6.3430	0.0032	Core
13	Human Plasma exp_3	8213	Afamin	FNETTEK_5_4_0_2	None	3.0869	0.0000	None	7.2829	0.0000	None
14	Human Plasma exp_5	7958	Afamin	FNETTEK_5_4_0_2	None	3.0948	0.0000	None	7.2578	0.0000	None
15	Human Plasma exp_5	12730	Afamin	FNETTEK_6_5_0_3	None	2.3051	0.0000	None	7.2603	0.0000	None
16	Human Plasma exp_1	1509	Afamin	HNFSHCCSK_5_4_0_2	None	3.3609	0.0000	None	7.3389	0.0000	None
17	Human Plasma exp_2	1548	Afamin	HNFSHCCSK_5_4_0_2	None	3.2901	0.0000	None	7.3373	0.0000	None
18	Human Plasma exp_4	1590	Afamin	HNFSHCCSK_5_4_0_2	None	2.2217	0.0000	None	7.3754	0.0000	None
19	Human Plasma exp_5	1488	Afamin	HNFSHCCSK_5_4_0_2	None	3.3968	0.0000	None	7.3404	0.0000	None
20	Human Plasma exp_5	1492	Afamin	HNFSHCCSK_5_4_0_2	None	2.1302	0.0000	None	7.3795	0.0000	None
21	Human Plasma exp_1	13729	Afamin	NCCNTENPPGCYR_5_4_0_2	None	3.5782	0.0000	None	7.3416	0.0000	None
22	Human Plasma exp_1	13720	Afamin	NCCNTENPPGCYR_5_4_0_2	None	1.5136	0.0000	None	7.2859	0.0000	None
23	Human Plasma exp_4	13975	Afamin	NCCNTENPPGCYR_5_4_0_2	None	4.1851	0.0000	None	7.3504	0.0000	None
24	Human Plasma exp_4	13811	Afamin	NCCNTENPPGCYR_5_4_0_2	None	2.4856	0.0000	None	7.2729	0.0000	None
25	Human Plasma exp_1	5154	Alpha-1-acid glycoprotein 1	ENGTISR_5_4_0_2	None	2.6100	0.0000	None	7.2676	0.0000	None
26	Human Plasma exp_2	5244	Alpha-1-acid glycoprotein 1	ENGTISR_5_4_0_2	None	2.7767	0.0000	None	7.2817	0.0000	None
27	Human Plasma exp_3	5199	Alpha-1-acid glycoprotein 1	ENGTISR_5_4_0_2	None	3.6014	0.0000	None	7.2918	0.0000	None
28	Human Plasma exp_4	5332	Alpha-1-acid glycoprotein 1	ENGTISR_5_4_0_2	None	3.4008	0.0000	None	7.2785	0.0000	None
29	Human Plasma exp_5	5195	Alpha-1-acid glycoprotein 1	ENGTISR_5_4_0_2	None	3.0890	0.0000	None	7.2765	0.0000	None
30	Human Plasma exp_1	5094	Alpha-1-acid glycoprotein 1	ENGTISR_5_4_1_2	Outer	1.0780	0.0000	Outer	6.6303	0.0000	Outer

Supplementary Table 8. Continued.

No.	Experiment	CID Scan No.	N-glycoproteins	N-glycopeptides	SVM			DNN			Manual Classification
					Classification	Pscore	FDR	Classification	Pscore	FDR	
31	Human Plasma exp_2	5202	Alpha-1-acid glycoprotein 1	ENGTISR_5_4_1_2	Outer	4.0159	0.0000	Outer	6.7188	0.0000	Outer
32	Human Plasma exp_5	5163	Alpha-1-acid glycoprotein 1	ENGTISR_5_4_1_2	Outer	0.2813	0.0858	Outer	4.2881	0.0061	Outer
33	Human Plasma exp_2	9286	Alpha-1-acid glycoprotein 1	ENGTISR_6_5_0_3	None	1.1873	0.0000	None	7.2128	0.0000	None
34	Human Plasma exp_3	9084	Alpha-1-acid glycoprotein 1	ENGTISR_6_5_0_3	None	2.2484	0.0000	None	7.2825	0.0000	None
35	Human Plasma exp_4	9253	Alpha-1-acid glycoprotein 1	ENGTISR_6_5_0_3	None	2.1273	0.0000	None	7.2272	0.0000	None
36	Human Plasma exp_1	9587	Alpha-1-acid glycoprotein 1	ENGTISR_6_5_1_3	Outer	1.2482	0.0000	Outer	6.5134	0.0017	Outer
37	Human Plasma exp_2	8919	Alpha-1-acid glycoprotein 1	ENGTISR_6_5_1_3	Outer	2.7763	0.0000	Outer	6.6696	0.0000	Outer
38	Human Plasma exp_3	8894	Alpha-1-acid glycoprotein 1	ENGTISR_6_5_1_3	Outer	3.3595	0.0000	Outer	6.6970	0.0000	Outer
39	Human Plasma exp_3	9871	Alpha-1-acid glycoprotein 1	ENGTISR_6_5_1_3	Outer	2.0662	0.0000	Outer	5.6884	0.0031	Outer
40	Human Plasma exp_4	8976	Alpha-1-acid glycoprotein 1	ENGTISR_6_5_1_3	Outer	3.2726	0.0000	Outer	6.6990	0.0000	Outer
41	Human Plasma exp_4	9141	Alpha-1-acid glycoprotein 1	ENGTISR_6_5_1_3	Outer	4.3364	0.0000	Outer	6.4326	0.0033	Outer
42	Human Plasma exp_5	8869	Alpha-1-acid glycoprotein 1	ENGTISR_6_5_1_3	Outer	2.8203	0.0000	Outer	6.6410	0.0000	Outer
43	Human Plasma exp_5	9219	Alpha-1-acid glycoprotein 1	ENGTISR_6_5_1_3	Outer	2.9746	0.0000	Outer	6.4676	0.0033	Outer
44	Human Plasma exp_3	14454	Alpha-1-acid glycoprotein 1	ENGTISR_7_6_1_4	Outer	1.4333	0.0000	Outer	6.4557	0.0033	Outer
45	Human Plasma exp_4	13885	Alpha-1-acid glycoprotein 1	ENGTISR_7_6_1_4	Outer	2.0714	0.0000	Outer	6.6534	0.0000	Outer
46	Human Plasma exp_5	14875	Alpha-1-acid glycoprotein 1	ENGTISR_7_6_1_4	Outer	2.3154	0.0000	Outer	6.6387	0.0000	Outer
47	Human Plasma exp_2	13822	Alpha-1-acid glycoprotein 1	ENGTISR_7_6_2_4	Outer	0.2657	0.0921	Outer	1.9847	0.0177	Outer
48	Human Plasma exp_3	14493	Alpha-1-acid glycoprotein 1	ENGTISR_7_6_2_4	Dual	0.1916	0.1771	Outer	5.2620	0.0031	Outer
49	Human Plasma exp_4	14741	Alpha-1-acid glycoprotein 1	ENGTISR_7_6_2_4	Outer	0.1713	0.2027	Outer	5.8597	0.0031	Outer
50	Human Plasma exp_5	13807	Alpha-1-acid glycoprotein 1	ENGTISR_7_6_2_4	Outer	0.1136	0.2801	Outer	1.4068	0.0249	Outer
51	Human Plasma exp_5	14153	Alpha-1-acid glycoprotein 1	ENGTISR_7_6_3_4	Outer	0.1204	0.2733	Outer	0.2167	0.3628	Outer
52	Human Plasma exp_2	30047	Alpha-1-acid glycoprotein 1	LVPVPITNATLDQITGK_5_4_0_1	None	3.0847	0.0000	None	7.3419	0.0000	None
53	Human Plasma exp_3	29500	Alpha-1-acid glycoprotein 1	LVPVPITNATLDQITGK_5_4_0_1	None	3.4294	0.0000	None	7.3580	0.0000	None
54	Human Plasma exp_4	29784	Alpha-1-acid glycoprotein 1	LVPVPITNATLDQITGK_5_4_0_1	None	3.5042	0.0000	None	7.3278	0.0000	None
55	Human Plasma exp_4	31478	Alpha-1-acid glycoprotein 1	LVPVPITNATLDQITGK_5_4_0_2	None	4.5559	0.0000	None	7.3381	0.0000	None
56	Human Plasma exp_1	32456	Alpha-1-acid glycoprotein 1	LVPVPITNATLDQITGK_6_5_0_3	None	2.1446	0.0000	None	7.2666	0.0000	None
57	Human Plasma exp_4	33045	Alpha-1-acid glycoprotein 1	LVPVPITNATLDQITGK_6_5_0_3	None	2.7519	0.0000	None	7.2864	0.0000	None
58	Human Plasma exp_1	30786	Alpha-1-acid glycoprotein 1	LVPVPITNATLDQITGK_6_5_1_2	Outer	4.9940	0.0000	Outer	6.6917	0.0000	Outer
59	Human Plasma exp_4	31353	Alpha-1-acid glycoprotein 1	LVPVPITNATLDQITGK_6_5_1_2	Outer	2.5814	0.0000	Outer	6.7524	0.0000	Outer
60	Human Plasma exp_4	32977	Alpha-1-acid glycoprotein 1	LVPVPITNATLDQITGK_6_5_1_3	Outer	4.2956	0.0000	Outer	6.5843	0.0017	Outer

Supplementary Table 8. Continued.

No.	Experiment	CID Scan No.	N-glycoproteins	N-glycopeptides	SVM			DNN			Manual Classification
					Classification	Pscore	FDR	Classification	Pscore	FDR	
61	Human Plasma exp_2	27598	Alpha-1-acid glycoprotein 1	QDQCIYNTTYLNVQR_5_4_0_2	None	2.9614	0.0000	None	7.2824	0.0000	None
62	Human Plasma exp_3	27020	Alpha-1-acid glycoprotein 1	QDQCIYNTTYLNVQR_5_4_0_2	None	3.1501	0.0000	None	7.3254	0.0000	None
63	Human Plasma exp_1	29130	Alpha-1-acid glycoprotein 1	QDQCIYNTTYLNVQR_6_5_0_3	None	2.0247	0.0000	None	7.2639	0.0000	None
64	Human Plasma exp_2	29848	Alpha-1-acid glycoprotein 1	QDQCIYNTTYLNVQR_6_5_0_3	None	1.6573	0.0000	None	7.2715	0.0000	None
65	Human Plasma exp_4	29531	Alpha-1-acid glycoprotein 1	QDQCIYNTTYLNVQR_6_5_1_3	Outer	0.6920	0.0032	Outer	5.7018	0.0031	Outer
66	Human Plasma exp_1	4603	Alpha-1-acid glycoprotein 1,2	NEEYNK_5_4_0_2	None	3.7823	0.0000	None	7.3031	0.0000	None
67	Human Plasma exp_2	4672	Alpha-1-acid glycoprotein 1,2	NEEYNK_5_4_0_2	None	3.4410	0.0000	None	7.2947	0.0000	None
68	Human Plasma exp_3	4633	Alpha-1-acid glycoprotein 1,2	NEEYNK_5_4_0_2	None	3.6109	0.0000	None	7.2805	0.0000	None
69	Human Plasma exp_4	4752	Alpha-1-acid glycoprotein 1,2	NEEYNK_5_4_0_2	None	3.4622	0.0000	None	7.2965	0.0000	None
70	Human Plasma exp_5	4319	Alpha-1-acid glycoprotein 1,2	NEEYNK_5_4_0_2	None	3.0577	0.0000	None	7.2091	0.0000	None
71	Human Plasma exp_1	4396	Alpha-1-acid glycoprotein 1,2	NEEYNK_5_4_1_2	Core	3.4686	0.0000	Core	6.6267	0.0000	Core
72	Human Plasma exp_2	4457	Alpha-1-acid glycoprotein 1,2	NEEYNK_5_4_1_2	Core	2.9747	0.0000	Core	6.6269	0.0000	Core
73	Human Plasma exp_3	4410	Alpha-1-acid glycoprotein 1,2	NEEYNK_5_4_1_2	Core	2.8859	0.0000	Core	6.6165	0.0000	Core
74	Human Plasma exp_4	4528	Alpha-1-acid glycoprotein 1,2	NEEYNK_5_4_1_2	Core	2.9694	0.0000	Core	6.6094	0.0019	Core
75	Human Plasma exp_5	4414	Alpha-1-acid glycoprotein 1,2	NEEYNK_5_4_1_2	Core	2.6675	0.0000	Core	6.6103	0.0019	Core
76	Human Plasma exp_2	4828	Alpha-1-acid glycoprotein 1,2	NEEYNK_6_5_0_2	None	1.5338	0.0000	None	7.2905	0.0000	None
77	Human Plasma exp_4	4472	Alpha-1-acid glycoprotein 1,2	NEEYNK_6_5_0_2	None	2.9817	0.0000	None	7.2938	0.0000	None
78	Human Plasma exp_1	8272	Alpha-1-acid glycoprotein 1,2	NEEYNK_6_5_0_3	None	1.8533	0.0000	None	7.2729	0.0000	None
79	Human Plasma exp_3	7818	Alpha-1-acid glycoprotein 1,2	NEEYNK_6_5_0_3	None	1.2373	0.0000	None	7.2477	0.0000	None
80	Human Plasma exp_2	4012	Alpha-1-acid glycoprotein 1,2	NEEYNK_6_5_1_2	Outer	0.5440	0.0292	Outer	5.5776	0.0031	Outer
81	Human Plasma exp_4	4103	Alpha-1-acid glycoprotein 1,2	NEEYNK_6_5_1_2	Outer	2.2726	0.0000	Outer	6.6604	0.0000	Outer
82	Human Plasma exp_5	3982	Alpha-1-acid glycoprotein 1,2	NEEYNK_6_5_1_2	Outer	1.2642	0.0000	Outer	6.4002	0.0033	Outer
83	Human Plasma exp_1	7468	Alpha-1-acid glycoprotein 1,2	NEEYNK_6_5_1_3	Outer	3.1711	0.0000	Outer	6.4142	0.0033	Outer
84	Human Plasma exp_2	7865	Alpha-1-acid glycoprotein 1,2	NEEYNK_6_5_1_3	Outer	2.2443	0.0000	Outer	6.6509	0.0000	Outer
85	Human Plasma exp_2	7608	Alpha-1-acid glycoprotein 1,2	NEEYNK_6_5_1_3	Outer	2.9128	0.0000	Outer	6.2643	0.0032	Outer
86	Human Plasma exp_3	8517	Alpha-1-acid glycoprotein 1,2	NEEYNK_6_5_1_3	Outer	3.5965	0.0000	Outer	6.4410	0.0033	Outer
87	Human Plasma exp_4	7652	Alpha-1-acid glycoprotein 1,2	NEEYNK_6_5_1_3	Outer	3.5117	0.0000	Outer	6.3945	0.0033	Outer
88	Human Plasma exp_5	7660	Alpha-1-acid glycoprotein 1,2	NEEYNK_6_5_1_3	Outer	1.8211	0.0000	Outer	6.6535	0.0000	Outer
89	Human Plasma exp_5	7574	Alpha-1-acid glycoprotein 1,2	NEEYNK_6_5_1_3	Outer	3.0580	0.0000	Outer	6.4663	0.0033	Outer
90	Human Plasma exp_1	7814	Alpha-1-acid glycoprotein 1,2	NEEYNK_6_5_2_3	Dual	2.7445	0.0000	Dual	6.3752	0.0033	Dual

Supplementary Table 8. Continued.

No.	Experiment	CID Scan No.	N-glycoproteins	N-glycopeptides	SVM			DNN			Manual Classification
					Classification	Pscore	FDR	Classification	Pscore	FDR	
91	Human Plasma exp_2	7938	Alpha-1-acid glycoprotein 1,2	NEEYNK_6_5_2_3	Dual	2.1661	0.0000	Dual	6.4334	0.0033	Dual
92	Human Plasma exp_4	7944	Alpha-1-acid glycoprotein 1,2	NEEYNK_6_5_2_3	Dual	4.4708	0.0000	Dual	6.2846	0.0032	Dual
93	Human Plasma exp_2	3426	Alpha-1-acid glycoprotein 2	ENGTVSR_5_4_0_2	None	1.8731	0.0000	None	7.2709	0.0000	None
94	Human Plasma exp_3	3353	Alpha-1-acid glycoprotein 2	ENGTVSR_5_4_0_2	None	2.4586	0.0000	None	7.2860	0.0000	None
95	Human Plasma exp_4	3468	Alpha-1-acid glycoprotein 2	ENGTVSR_5_4_0_2	None	1.8381	0.0000	None	7.2730	0.0000	None
96	Human Plasma exp_1	3108	Alpha-1-acid glycoprotein 2	ENGTVSR_6_5_0_2	None	1.4949	0.0000	None	7.2846	0.0000	None
97	Human Plasma exp_4	3240	Alpha-1-acid glycoprotein 2	ENGTVSR_6_5_0_2	None	1.4893	0.0000	None	7.2857	0.0000	None
98	Human Plasma exp_1	6030	Alpha-1-acid glycoprotein 2	ENGTVSR_6_5_0_3	None	1.7183	0.0000	None	7.2491	0.0000	None
99	Human Plasma exp_4	6123	Alpha-1-acid glycoprotein 2	ENGTVSR_6_5_0_3	None	1.5883	0.0000	None	7.2492	0.0000	None
100	Human Plasma exp_1	6005	Alpha-1-acid glycoprotein 2	ENGTVSR_6_5_1_3	Outer	4.0699	0.0000	Outer	6.6372	0.0000	Outer
101	Human Plasma exp_2	6119	Alpha-1-acid glycoprotein 2	ENGTVSR_6_5_1_3	Outer	4.2219	0.0000	Outer	6.2132	0.0032	Outer
102	Human Plasma exp_3	6041	Alpha-1-acid glycoprotein 2	ENGTVSR_6_5_1_3	Outer	2.3959	0.0000	Outer	6.6709	0.0000	Outer
103	Human Plasma exp_5	6036	Alpha-1-acid glycoprotein 2	ENGTVSR_6_5_1_3	Outer	3.1376	0.0000	Outer	6.6910	0.0000	Outer
104	Human Plasma exp_5	6055	Alpha-1-acid glycoprotein 2	ENGTVSR_6_5_1_3	Outer	3.4194	0.0000	Outer	6.0137	0.0032	Outer
105	Human Plasma exp_5	5964	Alpha-1-acid glycoprotein 2	ENGTVSR_6_5_2_3	Dual	2.0031	0.0000	Dual	5.6464	0.0031	Outer
106	Human Plasma exp_1	10095	Alpha-1-acid glycoprotein 2	ENGTVSR_7_6_1_4	Outer	2.2149	0.0000	Outer	6.5984	0.0018	Outer
107	Human Plasma exp_5	10345	Alpha-1-acid glycoprotein 2	ENGTVSR_7_6_1_4	Outer	2.0400	0.0000	Outer	6.6172	0.0000	Outer
108	Human Plasma exp_2	10867	Alpha-1-acid glycoprotein 2	ENGTVSR_7_6_2_4	Dual	0.0397	0.3927	Outer	5.4306	0.0031	Outer
109	Human Plasma exp_3	10390	Alpha-1-acid glycoprotein 2	ENGTVSR_7_6_2_4	Outer	1.0236	0.0000	Outer	6.5064	0.0017	Outer
110	Human Plasma exp_5	10745	Alpha-1-acid glycoprotein 2	ENGTVSR_7_6_2_4	Dual	0.1233	0.2704	Outer	4.9290	0.0031	Outer
111	Human Plasma exp_2	10369	Alpha-1-acid glycoprotein 2	ENGTVSR_7_6_3_4	Outer	0.4145	0.0505	Outer	0.6781	0.1379	Outer
112	Human Plasma exp_3	10349	Alpha-1-acid glycoprotein 2	ENGTVSR_7_6_3_4	Dual	0.2023	0.1637	Outer	3.5855	0.0091	Outer
113	Human Plasma exp_4	10452	Alpha-1-acid glycoprotein 2	ENGTVSR_7_6_3_4	Dual	0.0229	0.4259	Outer	4.2648	0.0061	Outer
114	Human Plasma exp_1	10219	Alpha-1-acid glycoprotein 2	ENGTVSR_8_7_0_4	None	1.8071	0.0000	None	7.2047	0.0000	None
115	Human Plasma exp_2	28637	Alpha-1-acid glycoprotein 2	LVPVPITNATLDR_5_4_0_2	None	3.3886	0.0000	None	7.3154	0.0000	None
116	Human Plasma exp_3	28111	Alpha-1-acid glycoprotein 2	LVPVPITNATLDR_5_4_0_2	None	3.8520	0.0000	None	7.3295	0.0000	None
117	Human Plasma exp_4	28654	Alpha-1-acid glycoprotein 2	LVPVPITNATLDR_5_4_0_2	None	3.8197	0.0000	None	7.3231	0.0000	None
118	Human Plasma exp_5	27615	Alpha-1-acid glycoprotein 2	LVPVPITNATLDR_5_4_0_2	None	2.5115	0.0000	None	7.3119	0.0000	None
119	Human Plasma exp_1	27586	Alpha-1-acid glycoprotein 2	LVPVPITNATLDR_6_5_0_2	None	3.3957	0.0000	None	7.2902	0.0000	None
120	Human Plasma exp_3	27708	Alpha-1-acid glycoprotein 2	LVPVPITNATLDR_6_5_0_2	None	2.1748	0.0000	None	7.2918	0.0000	None

Supplementary Table 8. Continued.

No.	Experiment	CID Scan No.	N-glycoproteins	N-glycopeptides	SVM			DNN			Manual Classification
					Classification	Pscore	FDR	Classification	Pscore	FDR	
121	Human Plasma exp_5	27392	Alpha-1-acid glycoprotein 2	LVPVPITNATLDR_6_5_0_2	None	2.5468	0.0000	None	7.3023	0.0000	None
122	Human Plasma exp_4	30813	Alpha-1-acid glycoprotein 2	LVPVPITNATLDR_6_5_0_3	None	1.5010	0.0000	None	7.2462	0.0000	None
123	Human Plasma exp_4	30545	Alpha-1-acid glycoprotein 2	LVPVPITNATLDR_6_5_0_3	None	2.1682	0.0000	None	7.2592	0.0000	None
124	Human Plasma exp_2	30889	Alpha-1-acid glycoprotein 2	LVPVPITNATLDR_6_5_1_3	Outer	1.4401	0.0000	Outer	6.5431	0.0017	Outer
125	Human Plasma exp_4	30616	Alpha-1-acid glycoprotein 2	LVPVPITNATLDR_6_5_1_3	Outer	2.7019	0.0000	Outer	6.6066	0.0019	Outer
126	Human Plasma exp_4	30328	Alpha-1-acid glycoprotein 2	LVPVPITNATLDR_7_6_0_3	None	1.7674	0.0000	None	7.1983	0.0000	None
127	Human Plasma exp_4	29256	Alpha-1-acid glycoprotein 2	QNQCFYNSSYLNVQR_6_5_0_3	None	1.8630	0.0000	None	7.2671	0.0000	None
128	Human Plasma exp_5	28394	Alpha-1-acid glycoprotein 2	QNQCFYNSSYLNVQR_6_5_0_3	None	2.0980	0.0000	None	7.2619	0.0000	None
129	Human Plasma exp_1	2023	Alpha-1-antichymotrypsin	NGTR_6_5_0_3	None	2.1701	0.0000	None	7.2800	0.0000	None
130	Human Plasma exp_2	2300	Alpha-1-antichymotrypsin	NGTR_6_5_0_3	None	1.7819	0.0000	None	7.2571	0.0000	None
131	Human Plasma exp_3	2083	Alpha-1-antichymotrypsin	NGTR_6_5_0_3	None	3.1810	0.0000	None	7.2910	0.0000	None
132	Human Plasma exp_4	2176	Alpha-1-antichymotrypsin	NGTR_6_5_0_3	None	3.5288	0.0000	None	7.2991	0.0000	None
133	Human Plasma exp_5	2213	Alpha-1-antichymotrypsin	NGTR_6_5_0_3	None	1.9565	0.0000	None	7.2732	0.0000	None
134	Human Plasma exp_1	2172	Alpha-1-antichymotrypsin	NGTR_6_5_1_3	Core	0.3715	0.0544	Core	5.2192	0.0031	Core
135	Human Plasma exp_2	2280	Alpha-1-antichymotrypsin	NGTR_6_5_1_3	Core	1.6833	0.0000	Core	6.5975	0.0018	Core
136	Human Plasma exp_4	2297	Alpha-1-antichymotrypsin	NGTR_6_5_1_3	Core	0.8467	0.0000	Core	6.6110	0.0019	Core
137	Human Plasma exp_1	32504	Alpha-1-antitrypsin	YLGNATAIFFLPDEGK_5_4_0_2	None	3.4142	0.0000	None	7.3311	0.0000	None
138	Human Plasma exp_2	33340	Alpha-1-antitrypsin	YLGNATAIFFLPDEGK_5_4_0_2	None	3.8759	0.0000	None	7.3234	0.0000	None
139	Human Plasma exp_3	32826	Alpha-1-antitrypsin	YLGNATAIFFLPDEGK_5_4_0_2	None	2.7554	0.0000	None	7.3117	0.0000	None
140	Human Plasma exp_4	33074	Alpha-1-antitrypsin	YLGNATAIFFLPDEGK_5_4_0_2	None	3.2063	0.0000	None	7.3284	0.0000	None
141	Human Plasma exp_5	32099	Alpha-1-antitrypsin	YLGNATAIFFLPDEGK_5_4_0_2	None	3.0559	0.0000	None	7.3269	0.0000	None
142	Human Plasma exp_1	28824	Alpha-2-HS-glycoprotein	VCQDCPLLAPLNDTR_5_4_0_2	None	3.6994	0.0000	None	7.3186	0.0000	None
143	Human Plasma exp_4	29566	Alpha-2-HS-glycoprotein	VCQDCPLLAPLNDTR_5_4_0_2	None	3.5413	0.0000	None	7.3124	0.0000	None
144	Human Plasma exp_5	28442	Alpha-2-HS-glycoprotein	VCQDCPLLAPLNDTR_5_4_0_2	None	4.7553	0.0000	None	7.3411	0.0000	None
145	Human Plasma exp_5	28352	Alpha-2-HS-glycoprotein	VCQDCPLLAPLNDTR_5_4_1_2	Core	1.7844	0.0000	Core	5.8803	0.0031	Core
146	Human Plasma exp_4	31529	Alpha-2-HS-glycoprotein	VCQDCPLLAPLNDTR_6_5_1_3	Outer	1.4924	0.0000	Outer	6.3196	0.0032	Outer
147	Human Plasma exp_5	30597	Alpha-2-HS-glycoprotein	VCQDCPLLAPLNDTR_6_5_1_3	Outer	3.5771	0.0000	Outer	6.6235	0.0000	Outer
148	Human Plasma exp_5	28906	Aminopeptidase N	VPVTLALNNTLFLIEER_4_4_0_1	None	4.8943	0.0000	None	7.3673	0.0000	None
149	Human Plasma exp_1	1530	Angiotensinogen	NCTSR_5_4_0_2	None	2.8845	0.0000	None	7.2868	0.0000	None
150	Human Plasma exp_2	1557	Angiotensinogen	NCTSR_5_4_0_2	None	3.3498	0.0000	None	7.2893	0.0000	None

Supplementary Table 8. Continued.

No.	Experiment	CID Scan No.	N-glycoproteins	N-glycopeptides	SVM			DNN			Manual Classification
					Classification	Pscore	FDR	Classification	Pscore	FDR	
151	Human Plasma exp_3	1544	Angiotensinogen	NCTSR_5_4_0_2	None	3.3851	0.0000	None	7.2904	0.0000	None
152	Human Plasma exp_4	1592	Angiotensinogen	NCTSR_5_4_0_2	None	3.5201	0.0000	None	7.2919	0.0000	None
153	Human Plasma exp_5	1511	Angiotensinogen	NCTSR_5_4_0_2	None	3.0779	0.0000	None	7.2852	0.0000	None
154	Human Plasma exp_1	3219	Angiotensinogen	NCTSR_6_5_0_3	None	2.0576	0.0000	None	7.2765	0.0000	None
155	Human Plasma exp_2	3309	Angiotensinogen	NCTSR_6_5_0_3	None	1.5216	0.0000	None	7.2497	0.0000	None
156	Human Plasma exp_5	3239	Angiotensinogen	NCTSR_6_5_0_3	None	2.0113	0.0000	None	7.2742	0.0000	None
157	Human Plasma exp_1	25028	Apolipoprotein C-IV	ELLETVVNR_5_4_0_2	None	1.8419	0.0000	None	7.2561	0.0000	None
158	Human Plasma exp_5	24778	Apolipoprotein C-IV	ELLETVVNR_5_4_0_2	None	1.8519	0.0000	None	7.2626	0.0000	None
159	Human Plasma exp_5	24718	Apolipoprotein D	ADGTVNQIEGEATPVNLTEPAK_5_4_0_1	None	3.0953	0.0000	None	7.2788	0.0000	None
160	Human Plasma exp_2	25293	Apolipoprotein D	ADGTVNQIEGEATPVNLTEPAK_5_4_1_1	Core	7.5475	0.0000	Core	6.6285	0.0000	Core
161	Human Plasma exp_3	24804	Apolipoprotein D	ADGTVNQIEGEATPVNLTEPAK_5_4_1_1	Core	5.5294	0.0000	Core	6.6056	0.0019	Core
162	Human Plasma exp_4	25277	Apolipoprotein D	ADGTVNQIEGEATPVNLTEPAK_5_4_1_1	Core	8.0918	0.0000	Core	6.6219	0.0000	Core
163	Human Plasma exp_2	27855	Apolipoprotein D	ADGTVNQIEGEATPVNLTEPAK_5_4_1_2	Core	3.2671	0.0000	Core	6.4372	0.0033	Core
164	Human Plasma exp_3	24430	Beta-2-glycoprotein 1	LGNWSAMPSCK_5_4_0_2	None	2.8114	0.0000	None	7.3218	0.0000	None
165	Human Plasma exp_4	24919	Beta-2-glycoprotein 1	LGNWSAMPSCK_5_4_0_2	None	4.3671	0.0000	None	7.3283	0.0000	None
166	Human Plasma exp_2	15673	Beta-2-glycoprotein 1	VYKPSAGNNSLYR_4_3_0_1	None	3.8002	0.0000	None	7.3542	0.0000	None
167	Human Plasma exp_4	18459	Beta-2-glycoprotein 1	VYKPSAGNNSLYR_6_5_1_3	Outer	3.3453	0.0000	Outer	6.6889	0.0000	Outer
168	Human Plasma exp_5	22408	Biotinidase	FNDTEVLQR_5_4_0_2	None	3.4949	0.0000	None	7.2809	0.0000	None
169	Human Plasma exp_1	24313	Biotinidase	NPVGLIGAENATGETDPSHSK_6_5_0_3	None	2.3039	0.0000	None	7.2770	0.0000	None
170	Human Plasma exp_3	30160	Ceruloplasmin	AGLQAFFQVQECNK_5_4_0_2	None	3.2117	0.0000	None	7.2717	0.0000	None
171	Human Plasma exp_4	30413	Ceruloplasmin	AGLQAFFQVQECNK_5_4_0_2	None	2.8177	0.0000	None	7.3220	0.0000	None
172	Human Plasma exp_3	20507	Ceruloplasmin	EHEGAIYPDNTTDFQR_4_3_0_1	None	3.7743	0.0000	None	7.3481	0.0000	None
173	Human Plasma exp_3	20296	Ceruloplasmin	EHEGAIYPDNTTDFQR_4_3_1_1	Core	8.7971	0.0000	Core	2.7220	0.0163	Core
174	Human Plasma exp_2	21574	Ceruloplasmin	ELHHLQEQNVSNAFLDK_5_4_0_2	None	4.0604	0.0000	None	7.3424	0.0000	None
175	Human Plasma exp_2	21244	Ceruloplasmin	ELHHLQEQNVSNAFLDK_5_4_1_2	Core	8.7677	0.0000	Core	6.2263	0.0032	Core
176	Human Plasma exp_1	29132	Ceruloplasmin	ENLTAPGSDSAVFFEQGTTR_5_4_0_2	None	4.4363	0.0000	None	7.3476	0.0000	None
177	Human Plasma exp_2	29871	Ceruloplasmin	ENLTAPGSDSAVFFEQGTTR_5_4_0_2	None	2.9752	0.0000	None	7.3049	0.0000	None
178	Human Plasma exp_3	29403	Ceruloplasmin	ENLTAPGSDSAVFFEQGTTR_5_4_0_2	None	4.2624	0.0000	None	7.3152	0.0000	None
179	Human Plasma exp_4	29625	Ceruloplasmin	ENLTAPGSDSAVFFEQGTTR_5_4_0_2	None	4.7734	0.0000	None	7.3550	0.0000	None
180	Human Plasma exp_5	28716	Ceruloplasmin	ENLTAPGSDSAVFFEQGTTR_5_4_0_2	None	3.6370	0.0000	None	7.3408	0.0000	None

Supplementary Table 8. Continued.

No.	Experiment	CID Scan No.	N-glycoproteins	N-glycopeptides	SVM			DNN			Manual Classification
					Classification	Pscore	FDR	Classification	Pscore	FDR	
181	Human Plasma exp_1	11457	Cholinesterase	DNNSIITR_5_4_0_1	None	3.2609	0.0000	None	7.3146	0.0000	None
182	Human Plasma exp_2	11650	Cholinesterase	DNNSIITR_5_4_0_1	None	3.1470	0.0000	None	7.3214	0.0000	None
183	Human Plasma exp_5	11547	Cholinesterase	DNNSIITR_5_4_0_1	None	2.6129	0.0000	None	7.2863	0.0000	None
184	Human Plasma exp_5	15800	Cholinesterase	DNNSIITR_5_4_0_2	None	3.1510	0.0000	None	7.2977	0.0000	None
185	Human Plasma exp_1	4485	Cholinesterase	DNYTK_5_4_0_2	None	3.0058	0.0000	None	7.2998	0.0000	None
186	Human Plasma exp_2	4550	Cholinesterase	DNYTK_5_4_0_2	None	2.9621	0.0000	None	7.2817	0.0000	None
187	Human Plasma exp_3	4501	Cholinesterase	DNYTK_5_4_0_2	None	2.9822	0.0000	None	7.2804	0.0000	None
188	Human Plasma exp_4	4632	Cholinesterase	DNYTK_5_4_0_2	None	3.3595	0.0000	None	7.2994	0.0000	None
189	Human Plasma exp_5	4514	Cholinesterase	DNYTK_5_4_0_2	None	2.7796	0.0000	None	7.2757	0.0000	None
190	Human Plasma exp_2	17003	Cholinesterase	ENETEIIK_5_4_0_2	None	1.9921	0.0000	None	7.2773	0.0000	None
191	Human Plasma exp_5	16747	Cholinesterase	ENETEIIK_5_4_0_2	None	1.7928	0.0000	None	7.2619	0.0000	None
192	Human Plasma exp_1	12515	Clusterin	EDALNETR_5_4_0_2	None	2.7477	0.0000	None	7.2419	0.0000	None
193	Human Plasma exp_1	16327	Clusterin	EDALNETR_6_5_0_3	None	1.7832	0.0000	None	7.2571	0.0000	None
194	Human Plasma exp_3	16379	Clusterin	EDALNETR_6_5_1_3	Outer	1.6930	0.0000	Outer	6.5883	0.0017	Outer
195	Human Plasma exp_4	16705	Clusterin	EDALNETR_6_5_1_3	Outer	3.3589	0.0000	Outer	6.2351	0.0032	Outer
196	Human Plasma exp_5	16430	Clusterin	EDALNETR_6_5_1_3	Outer	3.1122	0.0000	Outer	6.0156	0.0032	Outer
197	Human Plasma exp_5	5173	Clusterin	HNSTGCLR_6_5_0_3	None	1.4568	0.0000	None	7.2249	0.0000	None
198	Human Plasma exp_5	5336	Clusterin	HNSTGCLR_6_5_0_3	None	1.6106	0.0000	None	7.2516	0.0000	None
199	Human Plasma exp_1	27042	Clusterin	LANLTQGEDQYYLR_5_4_0_2	None	2.9981	0.0000	None	7.3454	0.0000	None
200	Human Plasma exp_2	27735	Clusterin	LANLTQGEDQYYLR_5_4_0_2	None	2.3422	0.0000	None	7.3571	0.0000	None
201	Human Plasma exp_3	27148	Clusterin	LANLTQGEDQYYLR_5_4_0_2	None	0.3022	0.0781	None	7.2948	0.0000	None
202	Human Plasma exp_4	27518	Clusterin	LANLTQGEDQYYLR_5_4_0_2	None	4.1626	0.0000	None	7.3395	0.0000	None
203	Human Plasma exp_5	26765	Clusterin	LANLTQGEDQYYLR_5_4_0_2	None	2.9670	0.0000	None	7.3495	0.0000	None
204	Human Plasma exp_2	29998	Clusterin	LANLTQGEDQYYLR_6_5_1_3	Outer	0.7453	0.0016	Outer	6.3233	0.0032	Outer
205	Human Plasma exp_3	29473	Clusterin	LANLTQGEDQYYLR_6_5_1_3	Outer	0.4081	0.0504	Outer	4.3986	0.0061	Outer
206	Human Plasma exp_4	29768	Clusterin	LANLTQGEDQYYLR_6_5_1_3	Outer	0.4068	0.0504	Outer	5.9993	0.0032	Outer
207	Human Plasma exp_1	14322	Coagulation factor XII	NHSCEPCQTLAVR_5_4_0_2	None	4.1165	0.0000	None	7.3454	0.0000	None
208	Human Plasma exp_1	14340	Coagulation factor XII	NHSCEPCQTLAVR_5_4_0_2	None	1.7752	0.0000	None	7.2780	0.0000	None
209	Human Plasma exp_2	14554	Coagulation factor XII	NHSCEPCQTLAVR_5_4_0_2	None	3.7035	0.0000	None	7.3380	0.0000	None
210	Human Plasma exp_2	14620	Coagulation factor XII	NHSCEPCQTLAVR_5_4_0_2	None	2.7719	0.0000	None	7.3079	0.0000	None

Supplementary Table 8. Continued.

No.	Experiment	CID Scan No.	N-glycoproteins	N-glycopeptides	SVM			DNN			Manual Classification
					Classification	Pscore	FDR	Classification	Pscore	FDR	
211	Human Plasma exp_3	14357	Coagulation factor XII	NHSCEPCQTLAVR_5_4_0_2	None	4.1423	0.0000	None	7.3422	0.0000	None
212	Human Plasma exp_3	14322	Coagulation factor XII	NHSCEPCQTLAVR_5_4_0_2	None	2.8655	0.0000	None	7.3255	0.0000	None
213	Human Plasma exp_4	14648	Coagulation factor XII	NHSCEPCQTLAVR_5_4_0_2	None	4.2596	0.0000	None	7.3497	0.0000	None
214	Human Plasma exp_4	14646	Coagulation factor XII	NHSCEPCQTLAVR_5_4_0_2	None	2.8339	0.0000	None	7.3062	0.0000	None
215	Human Plasma exp_5	14392	Coagulation factor XII	NHSCEPCQTLAVR_5_4_0_2	None	4.1920	0.0000	None	7.3476	0.0000	None
216	Human Plasma exp_2	3044	Coagulation factor XII	NVTAEQAR_5_4_0_1	None	1.0246	0.0000	None	6.9818	0.0000	None
217	Human Plasma exp_3	2946	Coagulation factor XII	NVTAEQAR_5_4_0_1	None	2.3616	0.0000	None	7.2610	0.0000	None
218	Human Plasma exp_1	5719	Coagulation factor XII	NVTAEQAR_5_4_0_2	None	2.9777	0.0000	None	7.2797	0.0000	None
219	Human Plasma exp_2	5855	Coagulation factor XII	NVTAEQAR_5_4_0_2	None	3.8079	0.0000	None	7.2976	0.0000	None
220	Human Plasma exp_3	5778	Coagulation factor XII	NVTAEQAR_5_4_0_2	None	2.6945	0.0000	None	7.2779	0.0000	None
221	Human Plasma exp_5	5807	Coagulation factor XII	NVTAEQAR_5_4_0_2	None	3.4142	0.0000	None	7.2902	0.0000	None
222	Human Plasma exp_5	10110	Coagulation factor XII	NVTAEQAR_6_5_0_3	None	3.0024	0.0000	None	7.2864	0.0000	None
223	Human Plasma exp_1	16554	Complement component C7	NYTLTGR_5_4_1_2	Core	3.4255	0.0000	Core	6.6167	0.0000	Core
224	Human Plasma exp_2	16861	Complement component C7	NYTLTGR_5_4_1_2	None	1.1283	0.0000	Core	4.3467	0.0061	Core
225	Human Plasma exp_3	16589	Complement component C7	NYTLTGR_5_4_1_2	Core	3.7914	0.0000	Core	6.6281	0.0000	Core
226	Human Plasma exp_4	16975	Complement component C7	NYTLTGR_5_4_1_2	Core	2.9364	0.0000	Core	6.6340	0.0000	Core
227	Human Plasma exp_5	16666	Complement component C7	NYTLTGR_5_4_1_2	Core	2.9851	0.0000	Core	6.6275	0.0000	Core
228	Human Plasma exp_1	19516	Complement component C8 alpha chain	GGSSGWSGGLAQNR_5_4_0_2	None	2.7582	0.0000	None	7.3216	0.0000	None
229	Human Plasma exp_3	19270	Complement component C8 alpha chain	GGSSGWSGGLAQNR_5_4_0_2	None	4.1223	0.0000	None	7.3397	0.0000	None
230	Human Plasma exp_5	19483	Complement component C8 alpha chain	GGSSGWSGGLAQNR_5_4_0_2	None	3.2089	0.0000	None	7.3071	0.0000	None
231	Human Plasma exp_1	2149	Complement component C8 beta chain	NVTEK_4_3_0_1	None	5.0553	0.0000	None	7.3556	0.0000	None
232	Human Plasma exp_4	2309	Complement component C8 beta chain	NVTEK_4_3_0_1	None	5.0829	0.0000	None	7.3541	0.0000	None
233	Human Plasma exp_5	2192	Complement component C8 beta chain	NVTEK_4_3_0_1	None	4.3068	0.0000	None	7.3506	0.0000	None
234	Human Plasma exp_1	2116	Complement component C8 beta chain	NVTEK_5_4_0_2	None	3.6839	0.0000	None	7.3055	0.0000	None
235	Human Plasma exp_1	2128	Complement component C8 beta chain	NVTEK_5_4_0_2	None	2.1066	0.0000	None	7.2684	0.0000	None
236	Human Plasma exp_2	2272	Complement component C8 beta chain	NVTEK_5_4_0_2	None	3.4882	0.0000	None	7.2908	0.0000	None
237	Human Plasma exp_3	2162	Complement component C8 beta chain	NVTEK_5_4_0_2	None	3.5181	0.0000	None	7.3007	0.0000	None
238	Human Plasma exp_4	2273	Complement component C8 beta chain	NVTEK_5_4_0_2	None	3.2711	0.0000	None	7.2797	0.0000	None
239	Human Plasma exp_5	2287	Complement component C8 beta chain	NVTEK_5_4_0_2	None	3.0059	0.0000	None	7.2811	0.0000	None
240	Human Plasma exp_1	21593	Complement factor H-related protein 3	FVQGNSTEVACHPGYGLPK_5_4_0_2	None	1.8257	0.0000	None	7.2742	0.0000	None

Supplementary Table 8. Continued.

No.	Experiment	CID Scan No.	N-glycoproteins	N-glycopeptides	SVM			DNN			Manual Classification
					Classification	Pscore	FDR	Classification	Pscore	FDR	
241	Human Plasma exp_4	21901	Complement factor H-related protein 3	FVQGNSTEVACHPGYGLPK_5_4_0_2	None	3.7161	0.0000	None	7.3144	0.0000	None
242	Human Plasma exp_3	15012	Complement factor I	FLNNGTCTAEGK_5_4_0_2	None	3.6104	0.0000	None	7.3159	0.0000	None
243	Human Plasma exp_5	15048	Complement factor I	FLNNGTCTAEGK_5_4_0_2	None	3.5364	0.0000	None	7.3163	0.0000	None
244	Human Plasma exp_4	3395	Complement factor I	NGTAVCATNR_5_4_0_1	None	3.2423	0.0000	None	7.3147	0.0000	None
245	Human Plasma exp_1	6679	Complement factor I	NGTAVCATNR_5_4_0_2	None	3.4563	0.0000	None	7.3072	0.0000	None
246	Human Plasma exp_2	6669	Complement factor I	NGTAVCATNR_5_4_0_2	None	2.1825	0.0000	None	7.2836	0.0000	None
247	Human Plasma exp_3	6787	Complement factor I	NGTAVCATNR_5_4_0_2	None	3.7318	0.0000	None	7.2941	0.0000	None
248	Human Plasma exp_4	6836	Complement factor I	NGTAVCATNR_5_4_0_2	None	3.6998	0.0000	None	7.3039	0.0000	None
249	Human Plasma exp_5	6774	Complement factor I	NGTAVCATNR_5_4_0_2	None	3.7777	0.0000	None	7.3150	0.0000	None
250	Human Plasma exp_4	17592	Desmocollin-2	ANYTILK_5_4_0_1	None	3.1139	0.0000	None	7.2956	0.0000	None
251	Human Plasma exp_2	22615	Desmocollin-2	ANYTILK_5_4_0_2	None	2.9822	0.0000	None	7.2869	0.0000	None
252	Human Plasma exp_4	22567	Desmocollin-2	ANYTILK_5_4_0_2	None	3.2712	0.0000	None	7.2967	0.0000	None
253	Human Plasma exp_3	24871	Endoplasmic reticulum aminopeptidase 2	DLEITNATLQSEEDSR_4_3_0_1	None	3.7219	0.0000	None	7.3417	0.0000	None
254	Human Plasma exp_4	25603	Endoplasmic reticulum aminopeptidase 2	DLEITNATLQSEEDSR_5_4_0_2	None	4.2527	0.0000	None	7.3329	0.0000	None
255	Human Plasma exp_1	24701	Fetuin-B	VLYLAAYNCTLRPVSK_5_4_0_1	None	2.7634	0.0000	None	7.3379	0.0000	None
256	Human Plasma exp_1	22837	Ficolin-3	VELEDFNGNR_5_4_1_2	Outer	0.1021	0.3018	None	5.0225	0.0031	Core
257	Human Plasma exp_4	23285	Ficolin-3	VELEDFNGNR_5_4_1_2	None	0.4181	0.0493	Core	6.6229	0.0000	Core
258	Human Plasma exp_2	29232	Haptoglobin	MVSHHNLTTGATLINEQWLLTTAK_5_4_0_2	None	2.8451	0.0000	None	7.3110	0.0000	None
259	Human Plasma exp_1	27749	Haptoglobin	VVLHPNYSQVDIGLIK_4_3_0_1	None	3.7935	0.0000	None	7.3491	0.0000	None
260	Human Plasma exp_2	28444	Haptoglobin	VVLHPNYSQVDIGLIK_4_3_0_1	None	3.7801	0.0000	None	7.3500	0.0000	None
261	Human Plasma exp_2	26631	Haptoglobin	VVLHPNYSQVDIGLIK_5_4_0_1	None	3.2542	0.0000	None	7.3545	0.0000	None
262	Human Plasma exp_4	26593	Haptoglobin	VVLHPNYSQVDIGLIK_5_4_0_1	None	3.6057	0.0000	None	7.3650	0.0000	None
263	Human Plasma exp_1	27827	Haptoglobin	VVLHPNYSQVDIGLIK_5_4_0_2	None	3.0086	0.0000	None	7.3337	0.0000	None
264	Human Plasma exp_1	27945	Haptoglobin	VVLHPNYSQVDIGLIK_5_4_0_2	None	4.8218	0.0000	None	7.3528	0.0000	None
265	Human Plasma exp_2	28700	Haptoglobin	VVLHPNYSQVDIGLIK_5_4_0_2	None	4.6641	0.0000	None	7.3392	0.0000	None
266	Human Plasma exp_4	28201	Haptoglobin	VVLHPNYSQVDIGLIK_5_4_0_2	None	2.4168	0.0000	None	7.3266	0.0000	None
267	Human Plasma exp_4	28408	Haptoglobin	VVLHPNYSQVDIGLIK_5_4_0_2	None	4.4743	0.0000	None	7.3416	0.0000	None
268	Human Plasma exp_5	27613	Haptoglobin	VVLHPNYSQVDIGLIK_5_4_0_2	None	4.6410	0.0000	None	7.3416	0.0000	None
269	Human Plasma exp_4	26569	Haptoglobin	VVLHPNYSQVDIGLIK_6_4_0_1	None	3.2955	0.0000	None	7.3210	0.0000	None
270	Human Plasma exp_1	27654	Haptoglobin	VVLHPNYSQVDIGLIK_6_5_0_2	None	1.8866	0.0000	None	7.2905	0.0000	None

Supplementary Table 8. Continued.

No.	Experiment	CID Scan No.	N-glycoproteins	N-glycopeptides	SVM			DNN			Manual Classification
					Classification	Pscore	FDR	Classification	Pscore	FDR	
271	Human Plasma exp_4	30252	Haptoglobin	VVLHPNYSQVDIGLIK_6_5_0_3	None	2.0617	0.0000	None	7.2724	0.0000	None
272	Human Plasma exp_5	29222	Haptoglobin	VVLHPNYSQVDIGLIK_6_5_0_3	None	3.0873	0.0000	None	7.2948	0.0000	None
273	Human Plasma exp_4	30058	Haptoglobin	VVLHPNYSQVDIGLIK_6_5_1_3	Outer	0.8116	0.0016	Outer	2.8606	0.0164	Outer
274	Human Plasma exp_2	30831	Hemopexin	ALPQPQNVTSLLGCTH_4_3_0_1	None	3.6990	0.0000	None	7.3329	0.0000	None
275	Human Plasma exp_3	30305	Hemopexin	ALPQPQNVTSLLGCTH_4_3_0_1	None	3.6300	0.0000	None	7.3374	0.0000	None
276	Human Plasma exp_1	30204	Hemopexin	ALPQPQNVTSLLGCTH_5_4_0_2	None	3.5207	0.0000	None	7.3224	0.0000	None
277	Human Plasma exp_2	30901	Hemopexin	ALPQPQNVTSLLGCTH_5_4_0_2	None	4.7268	0.0000	None	7.3673	0.0000	None
278	Human Plasma exp_3	30491	Hemopexin	ALPQPQNVTSLLGCTH_5_4_0_2	None	3.9039	0.0000	None	7.3212	0.0000	None
279	Human Plasma exp_4	30501	Hemopexin	ALPQPQNVTSLLGCTH_5_4_0_2	None	2.7571	0.0000	None	7.3178	0.0000	None
280	Human Plasma exp_5	29783	Hemopexin	ALPQPQNVTSLLGCTH_5_4_0_2	None	3.4125	0.0000	None	7.3345	0.0000	None
281	Human Plasma exp_1	22530	Hemopexin	SWPAVGNCSSALR_5_4_0_1	None	2.8997	0.0000	None	7.2872	0.0000	None
282	Human Plasma exp_1	25393	Hemopexin	SWPAVGNCSSALR_5_4_0_2	None	4.3824	0.0000	None	7.3332	0.0000	None
283	Human Plasma exp_2	25885	Hemopexin	SWPAVGNCSSALR_5_4_0_2	None	3.6904	0.0000	None	7.3214	0.0000	None
284	Human Plasma exp_3	25563	Hemopexin	SWPAVGNCSSALR_5_4_0_2	None	4.0522	0.0000	None	7.3332	0.0000	None
285	Human Plasma exp_4	25835	Hemopexin	SWPAVGNCSSALR_5_4_0_2	None	4.2874	0.0000	None	7.3423	0.0000	None
286	Human Plasma exp_5	25332	Hemopexin	SWPAVGNCSSALR_5_4_0_2	None	2.3141	0.0000	None	7.2926	0.0000	None
287	Human Plasma exp_2	25764	Hemopexin	SWPAVGNCSSALR_5_4_1_2	Core	7.4996	0.0000	Core	6.5995	0.0018	Core
288	Human Plasma exp_4	25753	Hemopexin	SWPAVGNCSSALR_5_4_1_2	Core	6.6415	0.0000	Core	6.6146	0.0020	Core
289	Human Plasma exp_5	24981	Hemopexin	SWPAVGNCSSALR_5_4_1_2	Core	3.6350	0.0000	Core	6.6365	0.0000	Core
290	Human Plasma exp_3	25158	Hemopexin	SWPAVGNCSSALR_6_5_0_2	None	2.4778	0.0000	None	7.2993	0.0000	None
291	Human Plasma exp_4	28467	Hemopexin	SWPAVGNCSSALR_6_5_1_3	Outer	0.9650	0.0000	Outer	6.1299	0.0032	Outer
292	Human Plasma exp_1	15042	Heparin cofactor 2	DFVNASSK_5_4_0_2	None	3.6296	0.0000	None	7.3065	0.0000	None
293	Human Plasma exp_1	15007	Heparin cofactor 2	DFVNASSK_5_4_0_2	None	2.4679	0.0000	None	7.2658	0.0000	None
294	Human Plasma exp_2	15333	Heparin cofactor 2	DFVNASSK_5_4_0_2	None	3.7051	0.0000	None	7.3070	0.0000	None
295	Human Plasma exp_3	15064	Heparin cofactor 2	DFVNASSK_5_4_0_2	None	3.5426	0.0000	None	7.2882	0.0000	None
296	Human Plasma exp_4	15380	Heparin cofactor 2	DFVNASSK_5_4_0_2	None	3.6534	0.0000	None	7.3045	0.0000	None
297	Human Plasma exp_4	15384	Heparin cofactor 2	DFVNASSK_5_4_0_2	None	3.0723	0.0000	None	7.2999	0.0000	None
298	Human Plasma exp_5	15145	Heparin cofactor 2	DFVNASSK_5_4_0_2	None	3.6155	0.0000	None	7.3107	0.0000	None
299	Human Plasma exp_1	20220	Heparin cofactor 2	DFVNASSK_6_5_0_3	None	2.5196	0.0000	None	7.2760	0.0000	None
300	Human Plasma exp_2	20659	Heparin cofactor 2	DFVNASSK_6_5_0_3	None	1.9824	0.0000	None	7.2614	0.0000	None

Supplementary Table 8. Continued.

No.	Experiment	CID Scan No.	N-glycoproteins	N-glycopeptides	SVM			DNN			Manual Classification
					Classification	Pscore	FDR	Classification	Pscore	FDR	
301	Human Plasma exp_3	20040	Heparin cofactor 2	DFVNASSK_6_5_0_3	None	1.8391	0.0000	None	7.2531	0.0000	None
302	Human Plasma exp_4	20540	Heparin cofactor 2	DFVNASSK_6_5_0_3	None	2.4249	0.0000	None	7.2727	0.0000	None
303	Human Plasma exp_5	20269	Heparin cofactor 2	DFVNASSK_6_5_0_3	None	1.9257	0.0000	None	7.2552	0.0000	None
304	Human Plasma exp_1	29227	Heparin cofactor 2	NLSMPLLPADFHK_5_4_0_2	None	3.0472	0.0000	None	7.3321	0.0000	None
305	Human Plasma exp_2	29952	Heparin cofactor 2	NLSMPLLPADFHK_5_4_0_2	None	3.8160	0.0000	None	7.3332	0.0000	None
306	Human Plasma exp_4	29698	Heparin cofactor 2	NLSMPLLPADFHK_5_4_0_2	None	4.0721	0.0000	None	7.3386	0.0000	None
307	Human Plasma exp_5	28803	Heparin cofactor 2	NLSMPLLPADFHK_5_4_0_2	None	3.7659	0.0000	None	7.3314	0.0000	None
308	Human Plasma exp_3	2935	Heparin cofactor 2	SMTNR_4_3_0_1	None	2.9830	0.0000	None	7.3285	0.0000	None
309	Human Plasma exp_2	3065	Heparin cofactor 2	SMTNR_5_4_0_2	None	3.3049	0.0000	None	7.3017	0.0000	None
310	Human Plasma exp_3	2996	Heparin cofactor 2	SMTNR_5_4_0_2	None	3.0791	0.0000	None	7.2837	0.0000	None
311	Human Plasma exp_4	3050	Heparin cofactor 2	SMTNR_5_4_0_2	None	3.2189	0.0000	None	7.3017	0.0000	None
312	Human Plasma exp_5	3015	Heparin cofactor 2	SMTNR_5_4_0_2	None	2.6526	0.0000	None	7.2781	0.0000	None
313	Human Plasma exp_4	24610	Hepatocyte growth factor activator	DSVSVVLGQHFFNR_5_4_0_2	None	2.5483	0.0000	None	7.3049	0.0000	None
314	Human Plasma exp_2	12959	Hepatocyte growth factor-like protein	GTANTTTAGVPCQR_5_4_0_2	None	3.4340	0.0000	None	7.2964	0.0000	None
315	Human Plasma exp_5	12805	Hepatocyte growth factor-like protein	GTANTTTAGVPCQR_5_4_0_2	None	2.5424	0.0000	None	7.3135	0.0000	None
316	Human Plasma exp_1	26936	Histidine-rich glycoprotein	VIDFNCTTSSVSSALANTK_5_4_0_1	None	3.1621	0.0000	None	7.3606	0.0000	None
317	Human Plasma exp_2	27613	Histidine-rich glycoprotein	VIDFNCTTSSVSSALANTK_5_4_0_1	None	3.5137	0.0000	None	7.3534	0.0000	None
318	Human Plasma exp_3	27037	Histidine-rich glycoprotein	VIDFNCTTSSVSSALANTK_5_4_0_1	None	3.8993	0.0000	None	7.3563	0.0000	None
319	Human Plasma exp_4	27387	Histidine-rich glycoprotein	VIDFNCTTSSVSSALANTK_5_4_0_1	None	5.6899	0.0000	None	7.3581	0.0000	None
320	Human Plasma exp_5	26632	Histidine-rich glycoprotein	VIDFNCTTSSVSSALANTK_5_4_0_1	None	4.0033	0.0000	None	7.3486	0.0000	None
321	Human Plasma exp_5	26637	Histidine-rich glycoprotein	VIDFNCTTSSVSSALANTK_5_4_0_1	None	3.5932	0.0000	None	7.3097	0.0000	None
322	Human Plasma exp_1	28732	Histidine-rich glycoprotein	VIDFNCTTSSVSSALANTK_5_4_0_2	None	3.9004	0.0000	None	7.3436	0.0000	None
323	Human Plasma exp_2	29468	Histidine-rich glycoprotein	VIDFNCTTSSVSSALANTK_5_4_0_2	None	2.5156	0.0000	None	7.3033	0.0000	None
324	Human Plasma exp_3	28937	Histidine-rich glycoprotein	VIDFNCTTSSVSSALANTK_5_4_0_2	None	3.1945	0.0000	None	7.3245	0.0000	None
325	Human Plasma exp_4	29242	Histidine-rich glycoprotein	VIDFNCTTSSVSSALANTK_5_4_0_2	None	4.4665	0.0000	None	7.3492	0.0000	None
326	Human Plasma exp_5	28374	Histidine-rich glycoprotein	VIDFNCTTSSVSSALANTK_5_4_0_2	None	2.9516	0.0000	None	7.3047	0.0000	None
327	Human Plasma exp_5	17358	ICOS ligand	LFNVTPQDEQK_5_5_1_1	Core	2.9715	0.0000	None	6.7795	0.0000	Core
328	Human Plasma exp_1	20768	ICOS ligand	LFNVTPQDEQK_5_5_1_2	Core	0.7874	0.0016	None	6.7247	0.0000	Core
329	Human Plasma exp_4	21096	ICOS ligand	LFNVTPQDEQK_5_5_1_2	Core	0.9385	0.0000	None	7.0710	0.0000	Core
330	Human Plasma exp_4	21041	ICOS ligand	LFNVTPQDEQK_5_5_1_2	None	0.1318	0.2609	None	7.1801	0.0000	Core

Supplementary Table 8. Continued.

No.	Experiment	CID Scan No.	N-glycoproteins	N-glycopeptides	SVM			DNN			Manual Classification
					Classification	Pscore	FDR	Classification	Pscore	FDR	
331	Human Plasma exp_1	15025	Ig alpha-2 chain C region	TPLTANITK_5_4_1_1	Core	0.1638	0.2117	Core	6.6684	0.0000	Core
332	Human Plasma exp_2	15335	Ig alpha-2 chain C region	TPLTANITK_5_4_1_1	Core	0.0527	0.3690	Core	6.6914	0.0000	Core
333	Human Plasma exp_3	14878	Ig alpha-2 chain C region	TPLTANITK_5_4_1_1	Core	6.8810	0.0000	Core	6.5681	0.0017	Core
334	Human Plasma exp_4	15175	Ig alpha-2 chain C region	TPLTANITK_5_4_1_1	Core	12.0797	0.0000	Core	6.5603	0.0017	Core
335	Human Plasma exp_3	18898	Ig alpha-2 chain C region	TPLTANITK_5_4_1_2	Core	10.5653	0.0000	Core	6.5557	0.0017	Core
336	Human Plasma exp_3	15007	Ig alpha-2 chain C region	TPLTANITK_5_5_1_1	None	1.8911	0.0000	None	7.1459	0.0000	Core
337	Human Plasma exp_3	14995	Ig alpha-2 chain C region	TPLTANITK_5_5_1_1	Core	2.7258	0.0000	Core	6.5886	0.0017	Core
338	Human Plasma exp_4	15325	Ig alpha-2 chain C region	TPLTANITK_5_5_1_1	None	1.9013	0.0000	None	7.1110	0.0000	Core
339	Human Plasma exp_4	15315	Ig alpha-2 chain C region	TPLTANITK_5_5_1_1	Core	2.0224	0.0000	Core	6.5885	0.0017	Core
340	Human Plasma exp_3	18979	Ig alpha-2 chain C region	TPLTANITK_5_5_1_2	None	1.8801	0.0000	None	7.2415	0.0000	Core
341	Human Plasma exp_1	5651	Ig gamma-1 chain C region	EEQYNSTYR_3_3_0_0	None	3.4156	0.0000	None	7.2130	0.0000	None
342	Human Plasma exp_2	5984	Ig gamma-1 chain C region	EEQYNSTYR_3_3_0_0	None	3.7591	0.0000	None	7.2332	0.0000	None
343	Human Plasma exp_3	5945	Ig gamma-1 chain C region	EEQYNSTYR_3_3_0_0	None	3.4839	0.0000	None	7.0972	0.0000	None
344	Human Plasma exp_4	5801	Ig gamma-1 chain C region	EEQYNSTYR_3_3_0_0	None	3.4264	0.0000	None	7.2468	0.0000	None
345	Human Plasma exp_5	5913	Ig gamma-1 chain C region	EEQYNSTYR_3_3_0_0	None	3.6218	0.0000	None	7.1855	0.0000	None
346	Human Plasma exp_1	4903	Ig gamma-1 chain C region	EEQYNSTYR_3_3_1_0	Core	8.0122	0.0000	Core	6.6149	0.0020	Core
347	Human Plasma exp_2	5416	Ig gamma-1 chain C region	EEQYNSTYR_3_3_1_0	Core	6.6961	0.0000	Core	6.6283	0.0000	Core
348	Human Plasma exp_3	5245	Ig gamma-1 chain C region	EEQYNSTYR_3_3_1_0	Core	6.5144	0.0000	Core	6.5816	0.0017	Core
349	Human Plasma exp_4	5932	Ig gamma-1 chain C region	EEQYNSTYR_3_3_1_0	Core	8.0587	0.0000	Core	6.6199	0.0000	Core
350	Human Plasma exp_1	5791	Ig gamma-1 chain C region	EEQYNSTYR_3_4_0_0	None	3.3375	0.0000	None	7.1780	0.0000	None
351	Human Plasma exp_2	5966	Ig gamma-1 chain C region	EEQYNSTYR_3_4_0_0	None	3.3858	0.0000	None	7.2389	0.0000	None
352	Human Plasma exp_4	5765	Ig gamma-1 chain C region	EEQYNSTYR_3_4_0_0	None	3.1882	0.0000	None	7.1970	0.0000	None
353	Human Plasma exp_1	5706	Ig gamma-1 chain C region	EEQYNSTYR_3_4_1_0	Core	5.0007	0.0000	Core	6.6548	0.0000	Core
354	Human Plasma exp_2	5595	Ig gamma-1 chain C region	EEQYNSTYR_3_4_1_0	Core	7.3394	0.0000	Core	6.6336	0.0000	Core
355	Human Plasma exp_3	5880	Ig gamma-1 chain C region	EEQYNSTYR_3_4_1_0	Core	4.9492	0.0000	Core	6.6568	0.0000	Core
356	Human Plasma exp_4	6195	Ig gamma-1 chain C region	EEQYNSTYR_3_4_1_0	Core	3.7498	0.0000	Core	6.6216	0.0000	Core
357	Human Plasma exp_5	5784	Ig gamma-1 chain C region	EEQYNSTYR_3_4_1_0	Core	5.1326	0.0000	Core	6.6539	0.0000	Core
358	Human Plasma exp_1	5580	Ig gamma-1 chain C region	EEQYNSTYR_4_3_0_0	None	3.2110	0.0000	None	7.1457	0.0000	None
359	Human Plasma exp_2	5693	Ig gamma-1 chain C region	EEQYNSTYR_4_3_0_0	None	3.3073	0.0000	None	7.0872	0.0000	None
360	Human Plasma exp_3	5646	Ig gamma-1 chain C region	EEQYNSTYR_4_3_0_0	None	3.2310	0.0000	None	7.1279	0.0000	None

Supplementary Table 8. Continued.

No.	Experiment	CID Scan No.	N-glycoproteins	N-glycopeptides	SVM			DNN			Manual Classification
					Classification	Pscore	FDR	Classification	Pscore	FDR	
361	Human Plasma exp_4	5772	Ig gamma-1 chain C region	EEQYNSTYR_4_3_0_0	None	3.1364	0.0000	None	7.0841	0.0000	None
362	Human Plasma exp_5	5618	Ig gamma-1 chain C region	EEQYNSTYR_4_3_0_0	None	2.7984	0.0000	None	7.0039	0.0000	None
363	Human Plasma exp_1	5687	Ig gamma-1 chain C region	EEQYNSTYR_4_3_1_0	Core	7.1028	0.0000	Core	6.6086	0.0019	Core
364	Human Plasma exp_2	5992	Ig gamma-1 chain C region	EEQYNSTYR_4_3_1_0	Core	4.8045	0.0000	Core	6.6135	0.0020	Core
365	Human Plasma exp_3	5972	Ig gamma-1 chain C region	EEQYNSTYR_4_3_1_0	Core	5.9932	0.0000	Core	6.5996	0.0018	Core
366	Human Plasma exp_5	5753	Ig gamma-1 chain C region	EEQYNSTYR_4_3_1_0	Core	5.7770	0.0000	Core	6.6114	0.0020	Core
367	Human Plasma exp_1	9068	Ig gamma-1 chain C region	EEQYNSTYR_4_3_1_1	Core	2.1047	0.0000	Core	6.6468	0.0000	Core
368	Human Plasma exp_2	9215	Ig gamma-1 chain C region	EEQYNSTYR_4_3_1_1	Core	4.7062	0.0000	Core	6.5955	0.0018	Core
369	Human Plasma exp_3	9230	Ig gamma-1 chain C region	EEQYNSTYR_4_3_1_1	None	0.7896	0.0016	Core	6.6504	0.0000	Core
370	Human Plasma exp_3	8948	Ig gamma-1 chain C region	EEQYNSTYR_4_3_1_1	Core	5.3945	0.0000	Core	6.6117	0.0020	Core
371	Human Plasma exp_4	9278	Ig gamma-1 chain C region	EEQYNSTYR_4_3_1_1	Core	4.9594	0.0000	Core	6.5996	0.0018	Core
372	Human Plasma exp_5	9233	Ig gamma-1 chain C region	EEQYNSTYR_4_3_1_1	None	1.0723	0.0000	None	7.2606	0.0000	Core
373	Human Plasma exp_5	9174	Ig gamma-1 chain C region	EEQYNSTYR_4_3_1_1	Core	6.2737	0.0000	Core	6.6148	0.0020	Core
374	Human Plasma exp_3	5448	Ig gamma-1 chain C region	EEQYNSTYR_4_4_0_0	None	3.0554	0.0000	None	6.9059	0.0000	None
375	Human Plasma exp_5	5420	Ig gamma-1 chain C region	EEQYNSTYR_4_4_0_0	None	2.7446	0.0000	None	6.9433	0.0000	None
376	Human Plasma exp_1	5611	Ig gamma-1 chain C region	EEQYNSTYR_4_4_1_0	Core	6.8716	0.0000	Core	6.6426	0.0000	Core
377	Human Plasma exp_1	4872	Ig gamma-1 chain C region	EEQYNSTYR_4_4_1_0	Core	4.1214	0.0000	Core	6.5761	0.0017	Core
378	Human Plasma exp_2	6129	Ig gamma-1 chain C region	EEQYNSTYR_4_4_1_0	Core	4.9041	0.0000	Core	6.5755	0.0017	Core
379	Human Plasma exp_3	5899	Ig gamma-1 chain C region	EEQYNSTYR_4_4_1_0	Core	6.6111	0.0000	Core	6.6480	0.0000	Core
380	Human Plasma exp_3	4946	Ig gamma-1 chain C region	EEQYNSTYR_4_4_1_0	Core	4.2492	0.0000	Core	6.5741	0.0017	Core
381	Human Plasma exp_4	6207	Ig gamma-1 chain C region	EEQYNSTYR_4_4_1_0	Core	2.3068	0.0000	Core	6.5738	0.0017	Core
382	Human Plasma exp_5	5715	Ig gamma-1 chain C region	EEQYNSTYR_4_4_1_0	Core	6.4614	0.0000	Core	6.6510	0.0000	Core
383	Human Plasma exp_1	9178	Ig gamma-1 chain C region	EEQYNSTYR_4_4_1_1	Core	3.6092	0.0000	Core	6.6278	0.0000	Core
384	Human Plasma exp_1	9252	Ig gamma-1 chain C region	EEQYNSTYR_4_4_1_1	Core	2.1850	0.0000	Core	3.9980	0.0091	Core
385	Human Plasma exp_2	9369	Ig gamma-1 chain C region	EEQYNSTYR_4_4_1_1	Core	4.3809	0.0000	Core	6.6225	0.0000	Core
386	Human Plasma exp_3	9342	Ig gamma-1 chain C region	EEQYNSTYR_4_4_1_1	Core	5.4695	0.0000	Core	6.6172	0.0000	Core
387	Human Plasma exp_3	9412	Ig gamma-1 chain C region	EEQYNSTYR_4_4_1_1	None	1.0254	0.0000	None	6.3530	0.0033	Core
388	Human Plasma exp_4	9262	Ig gamma-1 chain C region	EEQYNSTYR_4_4_1_1	None	2.2294	0.0000	None	6.7849	0.0000	Core
389	Human Plasma exp_5	9379	Ig gamma-1 chain C region	EEQYNSTYR_4_4_1_1	Core	1.6085	0.0000	Core	4.7335	0.0046	Core
390	Human Plasma exp_2	6300	Ig gamma-1 chain C region	EEQYNSTYR_4_5_1_0	Core	3.7609	0.0000	Core	6.5902	0.0017	Core

Supplementary Table 8. Continued.

No.	Experiment	CID Scan No.	N-glycoproteins	N-glycopeptides	SVM Classification	SVM Pscore	SVM FDR	DNN Classification	DNN Pscore	DNN FDR	Manual Classification
391	Human Plasma exp_4	6672	Ig gamma-1 chain C region	EEQYNSTYR_4_5_1_0	Core	4.1492	0.0000	Core	6.6017	0.0018	Core
392	Human Plasma exp_5	6286	Ig gamma-1 chain C region	EEQYNSTYR_4_5_1_0	Core	3.4656	0.0000	Core	6.6053	0.0019	Core
393	Human Plasma exp_1	8778	Ig gamma-1 chain C region	EEQYNSTYR_5_3_1_1	Core	0.3065	0.0769	Core	6.1964	0.0032	Core
394	Human Plasma exp_2	8934	Ig gamma-1 chain C region	EEQYNSTYR_5_3_1_1	None	0.2469	0.1081	Core	2.7110	0.0163	Core
395	Human Plasma exp_3	8958	Ig gamma-1 chain C region	EEQYNSTYR_5_3_1_1	None	0.0821	0.3262	Core	6.7505	0.0000	Core
396	Human Plasma exp_2	5454	Ig gamma-1 chain C region	EEQYNSTYR_5_4_0_0	None	2.8121	0.0000	None	6.9588	0.0000	None
397	Human Plasma exp_1	9244	Ig gamma-1 chain C region	EEQYNSTYR_5_4_0_1	None	2.5362	0.0000	None	6.8520	0.0000	None
398	Human Plasma exp_3	9198	Ig gamma-1 chain C region	EEQYNSTYR_5_4_0_1	None	2.9776	0.0000	None	6.8466	0.0000	None
399	Human Plasma exp_1	5809	Ig gamma-1 chain C region	EEQYNSTYR_5_4_1_0	Core	3.9837	0.0000	Core	6.5966	0.0018	Core
400	Human Plasma exp_2	5741	Ig gamma-1 chain C region	EEQYNSTYR_5_4_1_0	Core	4.0015	0.0000	Core	6.6046	0.0018	Core
401	Human Plasma exp_4	5782	Ig gamma-1 chain C region	EEQYNSTYR_5_4_1_0	Core	3.9563	0.0000	Core	6.6050	0.0019	Core
402	Human Plasma exp_1	8799	Ig gamma-1 chain C region	EEQYNSTYR_5_4_1_1	Core	2.3825	0.0000	Core	6.5951	0.0018	Core
403	Human Plasma exp_2	9326	Ig gamma-1 chain C region	EEQYNSTYR_5_4_1_1	Core	2.8790	0.0000	Core	6.5984	0.0018	Core
404	Human Plasma exp_3	9160	Ig gamma-1 chain C region	EEQYNSTYR_5_4_1_1	None	1.3434	0.0000	Core	0.4912	0.2429	Core
405	Human Plasma exp_3	9256	Ig gamma-1 chain C region	EEQYNSTYR_5_4_1_1	Core	5.0919	0.0000	Core	6.6004	0.0018	Core
406	Human Plasma exp_4	9273	Ig gamma-1 chain C region	EEQYNSTYR_5_4_1_1	Core	2.7898	0.0000	Core	6.7004	0.0000	Core
407	Human Plasma exp_4	8887	Ig gamma-1 chain C region	EEQYNSTYR_5_4_1_1	None	1.1256	0.0000	None	7.0443	0.0000	Core
408	Human Plasma exp_4	9393	Ig gamma-1 chain C region	EEQYNSTYR_5_4_1_1	Core	3.1149	0.0000	Core	6.5933	0.0018	Core
409	Human Plasma exp_5	9296	Ig gamma-1 chain C region	EEQYNSTYR_5_4_1_1	Core	5.6482	0.0000	Core	6.5956	0.0018	Core
410	Human Plasma exp_1	12580	Ig gamma-1 chain C region	EEQYNSTYR_5_4_1_2	None	0.6358	0.0126	Core	2.2296	0.0177	Core
411	Human Plasma exp_2	13332	Ig gamma-1 chain C region	EEQYNSTYR_5_4_1_2	None	0.3947	0.0517	Core	6.5049	0.0033	Core
412	Human Plasma exp_1	5106	Ig gamma-1 chain C region	EEQYNSTYR_5_4_2_0	Outer	2.8284	0.0000	Dual	3.0856	0.0135	Dual
413	Human Plasma exp_5	5153	Ig gamma-1 chain C region	EEQYNSTYR_5_4_2_0	Outer	4.6493	0.0000	Outer	2.9382	0.0149	Dual
414	Human Plasma exp_2	8733	Ig gamma-1 chain C region	EEQYNSTYR_5_4_2_1	Dual	3.1380	0.0000	Dual	6.1025	0.0032	Dual
415	Human Plasma exp_3	8719	Ig gamma-1 chain C region	EEQYNSTYR_5_4_2_1	Dual	6.2931	0.0000	Dual	6.4383	0.0033	Dual
416	Human Plasma exp_5	8692	Ig gamma-1 chain C region	EEQYNSTYR_5_4_2_1	Dual	0.4636	0.0423	Dual	5.4829	0.0031	Dual
417	Human Plasma exp_4	6478	Ig gamma-1 chain C region	EEQYNSTYR_5_5_1_0	Core	4.2032	0.0000	Core	6.5967	0.0018	Core
418	Human Plasma exp_4	13165	Ig gamma-1 chain C region	EEQYNSTYR_5_5_1_2	None	0.0650	0.3496	None	3.9769	0.0091	Core
419	Human Plasma exp_1	13538	Ig gamma-2 chain C region	EEQFNSTFR_3_3_0_0	None	3.0048	0.0000	None	6.9517	0.0000	None
420	Human Plasma exp_2	13787	Ig gamma-2 chain C region	EEQFNSTFR_3_3_0_0	None	3.1988	0.0000	None	7.0375	0.0000	None

Supplementary Table 8. Continued.

No.	Experiment	CID Scan No.	N-glycoproteins	N-glycopeptides	SVM Classification	SVM Pscore	SVM FDR	DNN Classification	DNN Pscore	DNN FDR	Manual Classification
421	Human Plasma exp_5	13409	Ig gamma-2 chain C region	EEQFNSTFR_3_3_0_0	None	3.6436	0.0000	None	7.3220	0.0000	None
422	Human Plasma exp_1	13210	Ig gamma-2 chain C region	EEQFNSTFR_3_3_1_0	Core	4.7546	0.0000	Core	6.6179	0.0000	Core
423	Human Plasma exp_2	13462	Ig gamma-2 chain C region	EEQFNSTFR_3_3_1_0	Core	4.9152	0.0000	Core	6.6095	0.0019	Core
424	Human Plasma exp_3	13359	Ig gamma-2 chain C region	EEQFNSTFR_3_3_1_0	Core	2.1707	0.0000	Core	6.6492	0.0000	Core
425	Human Plasma exp_4	13471	Ig gamma-2 chain C region	EEQFNSTFR_3_3_1_0	Core	3.2842	0.0000	Core	6.6313	0.0000	Core
426	Human Plasma exp_5	13258	Ig gamma-2 chain C region	EEQFNSTFR_3_3_1_0	Core	4.2329	0.0000	Core	6.6252	0.0000	Core
427	Human Plasma exp_1	13593	Ig gamma-2 chain C region	EEQFNSTFR_3_4_0_0	None	3.1602	0.0000	None	7.1567	0.0000	None
428	Human Plasma exp_4	13856	Ig gamma-2 chain C region	EEQFNSTFR_3_4_0_0	None	3.1877	0.0000	None	7.1454	0.0000	None
429	Human Plasma exp_1	13683	Ig gamma-2 chain C region	EEQFNSTFR_3_4_1_0	Core	5.1659	0.0000	Core	6.6519	0.0000	Core
430	Human Plasma exp_2	13906	Ig gamma-2 chain C region	EEQFNSTFR_3_4_1_0	Core	5.2147	0.0000	Core	6.6555	0.0000	Core
431	Human Plasma exp_3	13833	Ig gamma-2 chain C region	EEQFNSTFR_3_4_1_0	Core	5.1621	0.0000	Core	6.6555	0.0000	Core
432	Human Plasma exp_4	13958	Ig gamma-2 chain C region	EEQFNSTFR_3_4_1_0	Core	4.8508	0.0000	Core	6.6503	0.0000	Core
433	Human Plasma exp_5	13749	Ig gamma-2 chain C region	EEQFNSTFR_3_4_1_0	Core	5.0638	0.0000	Core	6.6515	0.0000	Core
434	Human Plasma exp_1	13385	Ig gamma-2 chain C region	EEQFNSTFR_4_3_1_0	Core	6.3169	0.0000	Core	6.6022	0.0018	Core
435	Human Plasma exp_2	13626	Ig gamma-2 chain C region	EEQFNSTFR_4_3_1_0	Core	6.0165	0.0000	Core	6.6163	0.0020	Core
436	Human Plasma exp_3	13544	Ig gamma-2 chain C region	EEQFNSTFR_4_3_1_0	Core	6.1760	0.0000	Core	6.6166	0.0000	Core
437	Human Plasma exp_4	13469	Ig gamma-2 chain C region	EEQFNSTFR_4_3_1_0	Core	6.0051	0.0000	Core	6.6198	0.0000	Core
438	Human Plasma exp_5	13638	Ig gamma-2 chain C region	EEQFNSTFR_4_3_1_0	Core	13.5979	0.0000	Core	6.6837	0.0000	Core
439	Human Plasma exp_5	13457	Ig gamma-2 chain C region	EEQFNSTFR_4_3_1_0	Core	6.7197	0.0000	Core	6.6081	0.0019	Core
440	Human Plasma exp_1	17150	Ig gamma-2 chain C region	EEQFNSTFR_4_3_1_1	None	1.8163	0.0000	Core	3.9414	0.0091	Core
441	Human Plasma exp_1	16769	Ig gamma-2 chain C region	EEQFNSTFR_4_3_1_1	Core	2.6239	0.0000	Core	6.6388	0.0000	Core
442	Human Plasma exp_2	17497	Ig gamma-2 chain C region	EEQFNSTFR_4_3_1_1	Core	0.6535	0.0110	Core	6.6215	0.0000	Core
443	Human Plasma exp_3	17150	Ig gamma-2 chain C region	EEQFNSTFR_4_3_1_1	Core	0.4164	0.0506	Core	6.6189	0.0000	Core
444	Human Plasma exp_3	17088	Ig gamma-2 chain C region	EEQFNSTFR_4_3_1_1	Core	5.5236	0.0000	Core	6.6335	0.0000	Core
445	Human Plasma exp_4	17558	Ig gamma-2 chain C region	EEQFNSTFR_4_3_1_1	Core	0.5167	0.0351	Core	6.6084	0.0019	Core
446	Human Plasma exp_5	17204	Ig gamma-2 chain C region	EEQFNSTFR_4_3_1_1	Core	0.2103	0.1541	Core	6.6358	0.0000	Core
447	Human Plasma exp_5	16818	Ig gamma-2 chain C region	EEQFNSTFR_4_3_1_1	Core	4.4763	0.0000	Core	6.6335	0.0000	Core
448	Human Plasma exp_1	13687	Ig gamma-2 chain C region	EEQFNSTFR_4_4_1_0	Core	4.6841	0.0000	Core	6.6739	0.0000	Core
449	Human Plasma exp_1	12634	Ig gamma-2 chain C region	EEQFNSTFR_4_4_1_0	Core	8.9651	0.0000	Core	6.6064	0.0019	Core
450	Human Plasma exp_2	13908	Ig gamma-2 chain C region	EEQFNSTFR_4_4_1_0	Core	5.6975	0.0000	Core	6.6624	0.0000	Core

Supplementary Table 8. Continued.

No.	Experiment	CID Scan No.	N-glycoproteins	N-glycopeptides	SVM			DNN			Manual Classification
					Classification	Pscore	FDR	Classification	Pscore	FDR	
451	Human Plasma exp_2	12874	Ig gamma-2 chain C region	EEQFNSTFR_4_4_1_0	Core	6.7537	0.0000	Core	6.6097	0.0019	Core
452	Human Plasma exp_3	13706	Ig gamma-2 chain C region	EEQFNSTFR_4_4_1_0	Core	4.5580	0.0000	Core	6.6352	0.0000	Core
453	Human Plasma exp_4	13734	Ig gamma-2 chain C region	EEQFNSTFR_4_4_1_0	Core	7.1685	0.0000	Core	6.6389	0.0000	Core
454	Human Plasma exp_4	13504	Ig gamma-2 chain C region	EEQFNSTFR_4_4_1_0	Core	4.0307	0.0000	Core	6.6186	0.0000	Core
455	Human Plasma exp_5	13532	Ig gamma-2 chain C region	EEQFNSTFR_4_4_1_0	Core	7.0027	0.0000	Core	6.6408	0.0000	Core
456	Human Plasma exp_5	12719	Ig gamma-2 chain C region	EEQFNSTFR_4_4_1_0	Core	5.3680	0.0000	Core	6.5998	0.0018	Core
457	Human Plasma exp_1	16956	Ig gamma-2 chain C region	EEQFNSTFR_4_4_1_1	Core	4.5200	0.0000	Core	6.6132	0.0020	Core
458	Human Plasma exp_2	17547	Ig gamma-2 chain C region	EEQFNSTFR_4_4_1_1	Core	1.9544	0.0000	Core	6.6423	0.0000	Core
459	Human Plasma exp_2	17308	Ig gamma-2 chain C region	EEQFNSTFR_4_4_1_1	Core	5.9855	0.0000	Core	6.6070	0.0019	Core
460	Human Plasma exp_3	16967	Ig gamma-2 chain C region	EEQFNSTFR_4_4_1_1	Core	1.2720	0.0000	Core	6.6421	0.0000	Core
461	Human Plasma exp_3	16948	Ig gamma-2 chain C region	EEQFNSTFR_4_4_1_1	Core	4.7128	0.0000	Core	6.5954	0.0018	Core
462	Human Plasma exp_4	17600	Ig gamma-2 chain C region	EEQFNSTFR_4_4_1_1	Core	1.8563	0.0000	Core	6.6586	0.0000	Core
463	Human Plasma exp_4	17369	Ig gamma-2 chain C region	EEQFNSTFR_4_4_1_1	Core	4.3264	0.0000	Core	6.5999	0.0018	Core
464	Human Plasma exp_5	17237	Ig gamma-2 chain C region	EEQFNSTFR_4_4_1_1	Core	1.3790	0.0000	Core	6.6643	0.0000	Core
465	Human Plasma exp_5	17009	Ig gamma-2 chain C region	EEQFNSTFR_4_4_1_1	Core	2.5205	0.0000	Core	6.6503	0.0000	Core
466	Human Plasma exp_1	13534	Ig gamma-2 chain C region	EEQFNSTFR_4_5_1_0	Core	4.4742	0.0000	Core	6.6070	0.0019	Core
467	Human Plasma exp_2	13785	Ig gamma-2 chain C region	EEQFNSTFR_4_5_1_0	Core	5.1598	0.0000	Core	6.5954	0.0018	Core
468	Human Plasma exp_3	13927	Ig gamma-2 chain C region	EEQFNSTFR_4_5_1_0	Core	2.9484	0.0000	Core	6.6329	0.0000	Core
469	Human Plasma exp_4	13820	Ig gamma-2 chain C region	EEQFNSTFR_4_5_1_0	Core	4.8675	0.0000	Core	6.5985	0.0018	Core
470	Human Plasma exp_2	17259	Ig gamma-2 chain C region	EEQFNSTFR_5_3_1_1	None	0.3094	0.0770	Core	5.0246	0.0031	Core
471	Human Plasma exp_1	13328	Ig gamma-2 chain C region	EEQFNSTFR_5_4_1_0	Core	3.6278	0.0000	Core	6.6133	0.0020	Core
472	Human Plasma exp_3	16763	Ig gamma-2 chain C region	EEQFNSTFR_5_4_1_0	Core	4.6708	0.0000	Core	6.6184	0.0000	Core
473	Human Plasma exp_4	13629	Ig gamma-2 chain C region	EEQFNSTFR_5_4_1_0	Core	6.3000	0.0000	Core	6.6066	0.0019	Core
474	Human Plasma exp_1	16783	Ig gamma-2 chain C region	EEQFNSTFR_5_4_1_1	Core	1.1225	0.0000	Core	6.2242	0.0032	Core
475	Human Plasma exp_1	16698	Ig gamma-2 chain C region	EEQFNSTFR_5_4_1_1	Core	3.3061	0.0000	Core	6.6065	0.0019	Core
476	Human Plasma exp_2	17088	Ig gamma-2 chain C region	EEQFNSTFR_5_4_1_1	Core	3.2694	0.0000	Core	6.6741	0.0000	Core
477	Human Plasma exp_2	17254	Ig gamma-2 chain C region	EEQFNSTFR_5_4_1_1	Core	3.5326	0.0000	Core	6.5920	0.0018	Core
478	Human Plasma exp_3	16737	Ig gamma-2 chain C region	EEQFNSTFR_5_4_1_1	Core	3.7273	0.0000	Core	6.6746	0.0000	Core
479	Human Plasma exp_3	16686	Ig gamma-2 chain C region	EEQFNSTFR_5_4_1_1	Core	3.7306	0.0000	Core	6.6066	0.0019	Core
480	Human Plasma exp_4	17316	Ig gamma-2 chain C region	EEQFNSTFR_5_4_1_1	Core	2.0361	0.0000	Core	6.5920	0.0018	Core

Supplementary Table 8. Continued.

No.	Experiment	CID Scan No.	N-glycoproteins	N-glycopeptides	SVM			DNN			Manual Classification
					Classification	Pscore	FDR	Classification	Pscore	FDR	
481	Human Plasma exp_5	16770	Ig gamma-2 chain C region	EEQFNSTFR_5_4_1_1	Core	5.1806	0.0000	Core	6.6784	0.0000	Core
482	Human Plasma exp_5	16729	Ig gamma-2 chain C region	EEQFNSTFR_5_4_1_1	Core	3.6012	0.0000	Core	6.5930	0.0018	Core
483	Human Plasma exp_5	20908	Ig gamma-2 chain C region	EEQFNSTFR_5_4_1_2	None	0.3124	0.0758	Core	6.5782	0.0017	Core
484	Human Plasma exp_4	16931	Ig gamma-2 chain C region	EEQFNSTFR_5_4_2_1	Dual	4.3606	0.0000	Dual	6.3007	0.0032	Dual
485	Human Plasma exp_5	16592	Ig gamma-2 chain C region	EEQFNSTFR_5_4_2_1	Dual	3.1942	0.0000	Dual	5.8693	0.0031	Dual
486	Human Plasma exp_3	13658	Ig gamma-2 chain C region	EEQFNSTFR_5_5_1_0	Core	3.5743	0.0000	Core	6.5922	0.0018	Core
487	Human Plasma exp_1	16809	Ig gamma-2 chain C region	EEQFNSTFR_6_3_1_1	Core	2.1606	0.0000	Core	6.6419	0.0000	Core
488	Human Plasma exp_4	17195	Ig gamma-2 chain C region	EEQFNSTFR_6_3_1_1	None	0.8976	0.0000	Core	6.6071	0.0019	Core
489	Human Plasma exp_5	16894	Ig gamma-2 chain C region	EEQFNSTFR_6_3_1_1	Core	3.0612	0.0000	Core	6.6362	0.0000	Core
490	Human Plasma exp_1	10127	Ig gamma-3 chain C region	EEQYNSTFR_3_4_1_0	Core	8.4747	0.0000	Core	6.6135	0.0020	Core
491	Human Plasma exp_3	9906	Ig gamma-3 chain C region	EEQYNSTFR_3_4_1_0	Core	7.8947	0.0000	Core	6.6200	0.0000	Core
492	Human Plasma exp_4	10030	Ig gamma-3 chain C region	EEQYNSTFR_3_4_1_0	Core	7.6343	0.0000	Core	6.6250	0.0000	Core
493	Human Plasma exp_5	10292	Ig gamma-3 chain C region	EEQYNSTFR_3_4_1_0	Core	4.7886	0.0000	Core	6.6087	0.0019	Core
494	Human Plasma exp_1	9543	Ig gamma-3 chain C region	EEQYNSTFR_4_3_1_0	Core	5.8376	0.0000	Core	6.6179	0.0000	Core
495	Human Plasma exp_3	9595	Ig gamma-3 chain C region	EEQYNSTFR_4_3_1_0	Core	6.6713	0.0000	Core	6.6113	0.0019	Core
496	Human Plasma exp_5	9665	Ig gamma-3 chain C region	EEQYNSTFR_4_3_1_0	Core	6.4945	0.0000	Core	6.6217	0.0000	Core
497	Human Plasma exp_1	9505	Ig gamma-3 chain C region	EEQYNSTFR_5_4_1_0	Core	4.1667	0.0000	Core	6.6082	0.0019	Core
498	Human Plasma exp_2	9825	Ig gamma-3 chain C region	EEQYNSTFR_5_4_1_0	Core	4.6673	0.0000	Core	6.6021	0.0018	Core
499	Human Plasma exp_3	9490	Ig gamma-3 chain C region	EEQYNSTFR_5_4_1_0	Core	5.0442	0.0000	Core	6.5920	0.0017	Core
500	Human Plasma exp_5	9610	Ig gamma-3 chain C region	EEQYNSTFR_5_4_1_0	Core	3.2460	0.0000	Core	6.6018	0.0018	Core
501	Human Plasma exp_1	12957	Ig gamma-3 chain C region	EEQYNSTFR_5_4_1_1	Core	5.5918	0.0000	Core	6.6081	0.0019	Core
502	Human Plasma exp_2	13111	Ig gamma-3 chain C region	EEQYNSTFR_5_4_1_1	Core	5.0474	0.0000	Core	6.6796	0.0000	Core
503	Human Plasma exp_2	12990	Ig gamma-3 chain C region	EEQYNSTFR_5_4_1_1	Core	4.3601	0.0000	Core	6.6071	0.0019	Core
504	Human Plasma exp_3	12972	Ig gamma-3 chain C region	EEQYNSTFR_5_4_1_1	Core	3.7825	0.0000	Core	6.6895	0.0000	Core
505	Human Plasma exp_4	13137	Ig gamma-3 chain C region	EEQYNSTFR_5_4_1_1	Core	5.6060	0.0000	Core	6.6780	0.0000	Core
506	Human Plasma exp_5	12944	Ig gamma-3 chain C region	EEQYNSTFR_5_4_1_1	Core	4.3100	0.0000	Core	6.6860	0.0000	Core
507	Human Plasma exp_4	16943	Ig gamma-3 chain C region	EEQYNSTFR_5_4_1_2	None	0.3417	0.0652	Core	5.6595	0.0031	Core
508	Human Plasma exp_5	16636	Ig gamma-3 chain C region	EEQYNSTFR_5_4_1_2	None	0.5090	0.0351	Core	6.2862	0.0032	Core
509	Human Plasma exp_1	9753	Ig gamma-4 chain C region	EEQFNSTYR_3_3_1_0	Core	6.0099	0.0000	Core	6.6458	0.0000	Core
510	Human Plasma exp_2	9935	Ig gamma-4 chain C region	EEQFNSTYR_3_3_1_0	Core	8.4231	0.0000	Core	6.6235	0.0000	Core

Supplementary Table 8. Continued.

No.	Experiment	CID Scan No.	N-glycoproteins	N-glycopeptides	SVM			DNN			Manual Classification
					Classification	Pscore	FDR	Classification	Pscore	FDR	
511	Human Plasma exp_3	9928	Ig gamma-4 chain C region	EEQFNSTYR_3_3_1_0	Core	9.2761	0.0000	Core	6.6130	0.0020	Core
512	Human Plasma exp_4	9975	Ig gamma-4 chain C region	EEQFNSTYR_3_3_1_0	Core	7.8092	0.0000	Core	6.6252	0.0000	Core
513	Human Plasma exp_5	9897	Ig gamma-4 chain C region	EEQFNSTYR_3_3_1_0	Core	7.4211	0.0000	Core	6.6218	0.0000	Core
514	Human Plasma exp_1	9787	Ig gamma-4 chain C region	EEQFNSTYR_3_4_1_0	Core	7.3600	0.0000	Core	6.6303	0.0000	Core
515	Human Plasma exp_2	9981	Ig gamma-4 chain C region	EEQFNSTYR_3_4_1_0	Core	7.7668	0.0000	Core	6.6318	0.0000	Core
516	Human Plasma exp_3	10070	Ig gamma-4 chain C region	EEQFNSTYR_3_4_1_0	Core	6.8669	0.0000	Core	6.6375	0.0000	Core
517	Human Plasma exp_4	10210	Ig gamma-4 chain C region	EEQFNSTYR_3_4_1_0	Core	7.2553	0.0000	Core	6.6249	0.0000	Core
518	Human Plasma exp_5	10108	Ig gamma-4 chain C region	EEQFNSTYR_3_4_1_0	Core	6.5452	0.0000	Core	6.6354	0.0000	Core
519	Human Plasma exp_1	10485	Ig gamma-4 chain C region	EEQFNSTYR_3_5_1_0	Core	4.5552	0.0000	Core	6.6306	0.0000	Core
520	Human Plasma exp_2	10657	Ig gamma-4 chain C region	EEQFNSTYR_3_5_1_0	Core	6.3047	0.0000	Core	6.6080	0.0019	Core
521	Human Plasma exp_3	10465	Ig gamma-4 chain C region	EEQFNSTYR_3_5_1_0	Core	2.9527	0.0000	Core	6.6069	0.0019	Core
522	Human Plasma exp_4	10746	Ig gamma-4 chain C region	EEQFNSTYR_3_5_1_0	Core	4.3696	0.0000	Core	6.6271	0.0000	Core
523	Human Plasma exp_5	10601	Ig gamma-4 chain C region	EEQFNSTYR_3_5_1_0	Core	4.7886	0.0000	Core	6.6239	0.0000	Core
524	Human Plasma exp_2	9717	Ig gamma-4 chain C region	EEQFNSTYR_4_3_1_0	Core	7.8329	0.0000	Core	6.6065	0.0019	Core
525	Human Plasma exp_3	9741	Ig gamma-4 chain C region	EEQFNSTYR_4_3_1_0	Core	6.5584	0.0000	Core	6.6131	0.0020	Core
526	Human Plasma exp_4	9922	Ig gamma-4 chain C region	EEQFNSTYR_4_3_1_0	Core	7.1598	0.0000	Core	6.5981	0.0018	Core
527	Human Plasma exp_1	9840	Ig gamma-4 chain C region	EEQFNSTYR_4_4_1_0	Core	5.9481	0.0000	Core	6.6603	0.0000	Core
528	Human Plasma exp_2	10037	Ig gamma-4 chain C region	EEQFNSTYR_4_4_1_0	Core	6.3414	0.0000	Core	6.6536	0.0000	Core
529	Human Plasma exp_2	10245	Ig gamma-4 chain C region	EEQFNSTYR_4_4_1_0	Core	4.7664	0.0000	Core	6.6129	0.0020	Core
530	Human Plasma exp_5	9987	Ig gamma-4 chain C region	EEQFNSTYR_4_4_1_0	Core	6.3592	0.0000	Core	6.6535	0.0000	Core
531	Human Plasma exp_5	10006	Ig gamma-4 chain C region	EEQFNSTYR_4_4_1_0	Core	6.2104	0.0000	Core	6.6237	0.0000	Core
532	Human Plasma exp_1	12976	Ig gamma-4 chain C region	EEQFNSTYR_4_4_1_1	Dual	0.0183	0.4394	Core	6.1199	0.0032	Core
533	Human Plasma exp_1	13030	Ig gamma-4 chain C region	EEQFNSTYR_4_4_1_1	Core	3.2510	0.0000	Core	6.6132	0.0020	Core
534	Human Plasma exp_2	13233	Ig gamma-4 chain C region	EEQFNSTYR_4_4_1_1	Core	0.1700	0.2044	Core	6.3913	0.0033	Core
535	Human Plasma exp_3	13138	Ig gamma-4 chain C region	EEQFNSTYR_4_4_1_1	Core	0.3498	0.0599	Core	6.5442	0.0017	Core
536	Human Plasma exp_4	13288	Ig gamma-4 chain C region	EEQFNSTYR_4_4_1_1	Core	0.5918	0.0202	Core	6.6036	0.0018	Core
537	Human Plasma exp_5	13088	Ig gamma-4 chain C region	EEQFNSTYR_4_4_1_1	Core	0.6181	0.0172	Core	6.6383	0.0000	Core
538	Human Plasma exp_2	10542	Ig gamma-4 chain C region	EEQFNSTYR_4_5_1_0	Core	4.0764	0.0000	Core	6.5866	0.0017	Core
539	Human Plasma exp_3	9633	Ig gamma-4 chain C region	EEQFNSTYR_5_4_1_0	Core	3.3659	0.0000	Core	6.6032	0.0018	Core
540	Human Plasma exp_2	26482	Ig mu chain C region	GLTFQQNASSMCVPDQDTAIR_5_4_1_1	Core	13.7754	0.0000	Core	6.5742	0.0017	Core

Supplementary Table 8. Continued.

No.	Experiment	CID Scan No.	N-glycoproteins	N-glycopeptides	SVM Classification	SVM Pscore	SVM FDR	DNN Classification	DNN Pscore	DNN FDR	Manual Classification
541	Human Plasma exp_1	2772	Ig mu chain C region	NNSDISSTR_4_3_0_1	None	3.4908	0.0000	None	7.3423	0.0000	None
542	Human Plasma exp_2	2942	Ig mu chain C region	NNSDISSTR_4_3_0_1	None	4.2925	0.0000	None	7.3530	0.0000	None
543	Human Plasma exp_3	2842	Ig mu chain C region	NNSDISSTR_4_3_0_1	None	4.4543	0.0000	None	7.3405	0.0000	None
544	Human Plasma exp_4	2928	Ig mu chain C region	NNSDISSTR_4_3_0_1	None	4.2013	0.0000	None	7.3185	0.0000	None
545	Human Plasma exp_5	2834	Ig mu chain C region	NNSDISSTR_4_3_0_1	None	3.8757	0.0000	None	7.3264	0.0000	None
546	Human Plasma exp_1	3001	Ig mu chain C region	NNSDISSTR_4_3_1_1	Core	13.4360	0.0000	Core	6.5714	0.0017	Core
547	Human Plasma exp_1	2681	Ig mu chain C region	NNSDISSTR_5_3_0_1	None	4.9312	0.0000	None	7.3220	0.0000	None
548	Human Plasma exp_2	2834	Ig mu chain C region	NNSDISSTR_5_3_0_1	None	4.7134	0.0000	None	7.3460	0.0000	None
549	Human Plasma exp_3	2746	Ig mu chain C region	NNSDISSTR_5_3_0_1	None	4.7942	0.0000	None	7.3476	0.0000	None
550	Human Plasma exp_4	2838	Ig mu chain C region	NNSDISSTR_5_3_0_1	None	4.3101	0.0000	None	7.3457	0.0000	None
551	Human Plasma exp_5	2750	Ig mu chain C region	NNSDISSTR_5_3_0_1	None	4.8750	0.0000	None	7.3441	0.0000	None
552	Human Plasma exp_4	3061	Ig mu chain C region	NNSDISSTR_5_3_1_1	Core	4.8327	0.0000	Core	6.6260	0.0000	Core
553	Human Plasma exp_2	3007	Ig mu chain C region	NNSDISSTR_5_4_0_1	None	3.2789	0.0000	None	7.3278	0.0000	None
554	Human Plasma exp_2	3177	Ig mu chain C region	NNSDISSTR_5_4_1_1	Core	0.0358	0.4011	Core	3.4152	0.0090	Core
555	Human Plasma exp_2	3207	Ig mu chain C region	NNSDISSTR_5_4_1_1	Core	4.0804	0.0000	Core	6.5884	0.0017	Core
556	Human Plasma exp_5	5962	Ig mu chain C region	NNSDISSTR_5_4_1_2	None	0.4298	0.0451	None	1.2319	0.0291	Core
557	Human Plasma exp_4	3362	Ig mu chain C region	NNSDISSTR_5_5_0_1	None	1.5317	0.0000	None	7.2869	0.0000	None
558	Human Plasma exp_5	3507	Ig mu chain C region	NNSDISSTR_5_5_1_1	Core	3.4116	0.0000	Core	6.6223	0.0000	Core
559	Human Plasma exp_1	2571	Ig mu chain C region	NNSDISSTR_6_3_0_1	None	3.4304	0.0000	None	7.2828	0.0000	None
560	Human Plasma exp_2	2777	Ig mu chain C region	NNSDISSTR_6_3_0_1	None	2.4112	0.0000	None	7.2906	0.0000	None
561	Human Plasma exp_3	3033	Ig mu chain C region	NNSDISSTR_6_3_1_1	Core	3.7292	0.0000	Core	6.6263	0.0000	Core
562	Human Plasma exp_4	3113	Ig mu chain C region	NNSDISSTR_6_3_1_1	Core	4.3286	0.0000	Core	6.6198	0.0000	Core
563	Human Plasma exp_1	19134	Immunoglobulin J chain	ENISDPTSPLR_5_3_1_1	Core	4.3286	0.0000	Core	6.5838	0.0017	Core
564	Human Plasma exp_3	18908	Immunoglobulin J chain	ENISDPTSPLR_5_3_1_1	Core	7.5450	0.0000	Core	6.5698	0.0017	Core
565	Human Plasma exp_4	19585	Immunoglobulin J chain	ENISDPTSPLR_5_3_1_1	Core	4.5194	0.0000	Core	6.6243	0.0000	Core
566	Human Plasma exp_5	19137	Immunoglobulin J chain	ENISDPTSPLR_5_3_1_1	Core	3.7508	0.0000	Core	6.6637	0.0000	Core
567	Human Plasma exp_2	19127	Immunoglobulin J chain	ENISDPTSPLR_5_4_0_1	None	4.0765	0.0000	None	7.3081	0.0000	None
568	Human Plasma exp_1	22460	Immunoglobulin J chain	ENISDPTSPLR_5_4_0_2	None	4.2345	0.0000	None	7.3386	0.0000	None
569	Human Plasma exp_1	22442	Immunoglobulin J chain	ENISDPTSPLR_5_4_0_2	None	2.6698	0.0000	None	7.1184	0.0000	None
570	Human Plasma exp_2	22909	Immunoglobulin J chain	ENISDPTSPLR_5_4_0_2	None	3.2914	0.0000	None	7.3375	0.0000	None

Supplementary Table 8. Continued.

No.	Experiment	CID Scan No.	N-glycoproteins	N-glycopeptides	SVM Classification	SVM Pscore	SVM FDR	DNN Classification	DNN Pscore	DNN FDR	Manual Classification
571	Human Plasma exp_2	22784	Immunoglobulin J chain	ENISDPTSPLR_5_4_0_2	None	3.1929	0.0000	None	7.2658	0.0000	None
572	Human Plasma exp_3	22200	Immunoglobulin J chain	ENISDPTSPLR_5_4_0_2	None	4.1926	0.0000	None	7.3363	0.0000	None
573	Human Plasma exp_4	22776	Immunoglobulin J chain	ENISDPTSPLR_5_4_0_2	None	3.7719	0.0000	None	7.3260	0.0000	None
574	Human Plasma exp_2	15804	Insulin-like growth factor-binding protein 3	GLCVNASAVSR_5_4_0_2	None	2.8293	0.0000	None	7.2672	0.0000	None
575	Human Plasma exp_3	15524	Insulin-like growth factor-binding protein 3	GLCVNASAVSR_5_4_0_2	None	2.1626	0.0000	None	7.2766	0.0000	None
576	Human Plasma exp_2	3512	Insulin-like growth factor-binding protein complex acid labile subunit	NNSLR_4_3_0_1	None	4.4783	0.0000	None	7.3596	0.0000	None
577	Human Plasma exp_1	3342	Insulin-like growth factor-binding protein complex acid labile subunit	NNSLR_5_4_0_2	None	2.5670	0.0000	None	7.2784	0.0000	None
578	Human Plasma exp_2	3432	Insulin-like growth factor-binding protein complex acid labile subunit	NNSLR_5_4_0_2	None	2.5059	0.0000	None	7.2838	0.0000	None
579	Human Plasma exp_3	3351	Insulin-like growth factor-binding protein complex acid labile subunit	NNSLR_5_4_0_2	None	2.9909	0.0000	None	7.2786	0.0000	None
580	Human Plasma exp_4	3663	Insulin-like growth factor-binding protein complex acid labile subunit	NNSLR_5_4_0_2	None	2.4086	0.0000	None	7.2654	0.0000	None
581	Human Plasma exp_5	3367	Insulin-like growth factor-binding protein complex acid labile subunit	NNSLR_5_4_0_2	None	3.4008	0.0000	None	7.2933	0.0000	None
582	Human Plasma exp_2	8788	Inter-alpha-trypsin inhibitor heavy chain H3	ENLTAR_5_4_0_2	None	1.6195	0.0000	None	7.2561	0.0000	None
583	Human Plasma exp_1	13583	Inter-alpha-trypsin inhibitor heavy chain H3	ENLTAR_6_5_0_3	None	1.7366	0.0000	None	7.2567	0.0000	None
584	Human Plasma exp_3	13750	Inter-alpha-trypsin inhibitor heavy chain H3	ENLTAR_6_5_0_3	None	2.3873	0.0000	None	7.2504	0.0000	None
585	Human Plasma exp_4	13946	Inter-alpha-trypsin inhibitor heavy chain H3	ENLTAR_6_5_0_3	None	2.4775	0.0000	None	7.2855	0.0000	None
586	Human Plasma exp_1	22574	Kininogen-1	ITYSIVQTNCSK_5_4_0_2	None	2.8799	0.0000	None	7.2908	0.0000	None
587	Human Plasma exp_2	22988	Kininogen-1	ITYSIVQTNCSK_5_4_0_2	None	3.8698	0.0000	None	7.3166	0.0000	None
588	Human Plasma exp_3	22407	Kininogen-1	ITYSIVQTNCSK_5_4_0_2	None	3.8098	0.0000	None	7.3247	0.0000	None
589	Human Plasma exp_4	22942	Kininogen-1	ITYSIVQTNCSK_5_4_0_2	None	3.9370	0.0000	None	7.3183	0.0000	None
590	Human Plasma exp_5	22388	Kininogen-1	ITYSIVQTNCSK_5_4_0_2	None	3.2379	0.0000	None	7.2974	0.0000	None
591	Human Plasma exp_1	22471	Kininogen-1	ITYSIVQTNCSK_5_4_1_2	None	1.8137	0.0000	None	6.9649	0.0000	Core
592	Human Plasma exp_2	26235	Kininogen-1	ITYSIVQTNCSK_6_5_1_3	Outer	3.1875	0.0000	Outer	6.6558	0.0000	Outer
593	Human Plasma exp_4	26128	Kininogen-1	ITYSIVQTNCSK_6_5_1_3	Outer	2.2362	0.0000	Outer	6.6095	0.0019	Outer
594	Human Plasma exp_5	25355	Kininogen-1	ITYSIVQTNCSK_6_5_1_3	Outer	2.9445	0.0000	Outer	6.6014	0.0018	Outer
595	Human Plasma exp_3	26586	Kininogen-1	LNAENNATFYFK_5_4_0_2	None	4.3082	0.0000	None	7.3276	0.0000	None
596	Human Plasma exp_3	29339	Kininogen-1	LNAENNATFYFK_6_5_1_3	Outer	0.4257	0.0479	Outer	3.0904	0.0135	Outer
597	Human Plasma exp_2	25878	Kininogen-1	YNSQNQSNNQFVLYR_5_4_0_2	None	2.9909	0.0000	None	7.3209	0.0000	None
598	Human Plasma exp_4	25851	Kininogen-1	YNSQNQSNNQFVLYR_5_4_0_2	None	3.5936	0.0000	None	7.3364	0.0000	None
599	Human Plasma exp_5	25165	Kininogen-1	YNSQNQSNNQFVLYR_5_4_0_2	None	2.8651	0.0000	None	7.3159	0.0000	None

Supplementary Table 8. Continued.

No.	Experiment	CID Scan No.	N-glycoproteins	N-glycopeptides	SVM			DNN			Manual Classification
					Classification	Pscore	FDR	Classification	Pscore	FDR	
600	Human Plasma exp_4	25837	L-selectin	DNYTDLVAIQNK_5_4_0_2	None	2.6932	0.0000	None	7.1606	0.0000	None
601	Human Plasma exp_1	12687	Phosphatidylcholine-sterol acyltransferase	VVYNR_6_5_0_3	None	2.4783	0.0000	None	7.2607	0.0000	None
602	Human Plasma exp_2	12915	Phosphatidylcholine-sterol acyltransferase	VVYNR_6_5_0_3	None	1.6152	0.0000	None	7.2464	0.0000	None
603	Human Plasma exp_4	12966	Phosphatidylcholine-sterol acyltransferase	VVYNR_6_5_0_3	None	1.6391	0.0000	None	7.2510	0.0000	None
604	Human Plasma exp_5	12785	Phosphatidylcholine-sterol acyltransferase	VVYNR_6_5_0_3	None	1.8889	0.0000	None	7.2682	0.0000	Core
605	Human Plasma exp_1	23404	Phospholipid transfer protein	NWSLPNR_5_4_1_2	None	1.4838	0.0000	Core	2.2533	0.0178	Core
606	Human Plasma exp_5	23324	Phospholipid transfer protein	NWSLPNR_5_4_1_2	Core	1.8892	0.0000	Core	6.6405	0.0000	None
607	Human Plasma exp_2	20184	Plasma kallikrein	GVNFNVSK_5_4_0_2	None	3.2067	0.0000	None	7.2899	0.0000	None
608	Human Plasma exp_5	19816	Plasma kallikrein	GVNFNVSK_5_4_0_2	None	2.8883	0.0000	None	7.2793	0.0000	None
609	Human Plasma exp_1	4851	Plasma serine protease inhibitor	NLSCR_5_4_0_2	None	1.9411	0.0000	None	7.2768	0.0000	None
610	Human Plasma exp_2	4939	Plasma serine protease inhibitor	NLSCR_5_4_0_2	None	3.0582	0.0000	None	7.2839	0.0000	None
611	Human Plasma exp_4	5038	Plasma serine protease inhibitor	NLSCR_5_4_0_2	None	2.7445	0.0000	None	7.2840	0.0000	None
612	Human Plasma exp_5	4877	Plasma serine protease inhibitor	NLSCR_5_4_0_2	None	2.5400	0.0000	None	7.2744	0.0000	None
613	Human Plasma exp_3	5241	Prothrombin	GHVNITR_4_3_0_1	None	3.6308	0.0000	None	7.3439	0.0000	None
614	Human Plasma exp_4	5405	Prothrombin	GHVNITR_4_3_0_1	None	4.0221	0.0000	None	7.3744	0.0000	None
615	Human Plasma exp_5	5223	Prothrombin	GHVNITR_4_3_0_1	None	3.6401	0.0000	None	7.3589	0.0000	Outer
616	Human Plasma exp_1	5324	Prothrombin	GHVNITR_5_4_1_2	Outer	1.4871	0.0000	Outer	6.6284	0.0000	Outer
617	Human Plasma exp_4	5480	Prothrombin	GHVNITR_5_4_1_2	Outer	1.9911	0.0000	Outer	6.7178	0.0000	None
618	Human Plasma exp_2	27844	Prothrombin	NFTENDLLVR_5_4_0_2	None	3.3435	0.0000	None	7.2945	0.0000	None
619	Human Plasma exp_2	27820	Prothrombin	NFTENDLLVR_5_4_0_2	None	2.5963	0.0000	None	7.2613	0.0000	None
620	Human Plasma exp_3	27242	Prothrombin	NFTENDLLVR_5_4_0_2	None	3.7428	0.0000	None	7.3136	0.0000	None
621	Human Plasma exp_3	27228	Prothrombin	NFTENDLLVR_5_4_0_2	None	2.0922	0.0000	None	7.2763	0.0000	None
622	Human Plasma exp_4	27606	Prothrombin	NFTENDLLVR_5_4_0_2	None	2.5762	0.0000	None	7.3128	0.0000	None
623	Human Plasma exp_1	28401	Serotransferrin	CGLVPVLAENYNK_4_3_0_1	None	3.5444	0.0000	None	7.3396	0.0000	None
624	Human Plasma exp_2	29120	Serotransferrin	CGLVPVLAENYNK_4_3_0_1	None	4.0713	0.0000	None	7.3507	0.0000	None
625	Human Plasma exp_3	28600	Serotransferrin	CGLVPVLAENYNK_4_3_0_1	None	4.8354	0.0000	None	7.3531	0.0000	None
626	Human Plasma exp_4	28899	Serotransferrin	CGLVPVLAENYNK_4_3_0_1	None	4.5369	0.0000	None	7.3598	0.0000	None
627	Human Plasma exp_5	28083	Serotransferrin	CGLVPVLAENYNK_4_3_0_1	None	4.5316	0.0000	None	7.3399	0.0000	None
628	Human Plasma exp_4	26451	Serotransferrin	CGLVPVLAENYNK_5_4_0_1	None	3.2585	0.0000	None	7.3400	0.0000	None
629	Human Plasma exp_1	28364	Serotransferrin	CGLVPVLAENYNK_5_4_0_2	None	3.5721	0.0000	None	7.3129	0.0000	None

Supplementary Table 8. Continued.

No.	Experiment	CID Scan No.	N-glycoproteins	N-glycopeptides	SVM			DNN			Manual Classification
					Classification	Pscore	FDR	Classification	Pscore	FDR	
630	Human Plasma exp_2	29354	Serotransferrin	CGLVPVLAENYNK_5_4_0_2	None	2.5703	0.0000	None	7.3122	0.0000	None
631	Human Plasma exp_3	28548	Serotransferrin	CGLVPVLAENYNK_5_4_0_2	None	2.6902	0.0000	None	7.3142	0.0000	None
632	Human Plasma exp_4	29121	Serotransferrin	CGLVPVLAENYNK_5_4_0_2	None	5.2484	0.0000	None	7.3515	0.0000	None
633	Human Plasma exp_5	28179	Serotransferrin	CGLVPVLAENYNK_5_4_0_2	None	3.9919	0.0000	None	7.3309	0.0000	None
634	Human Plasma exp_3	23286	Sex hormone-binding globulin	LDVDQALNR_5_4_0_2	None	3.5697	0.0000	None	7.2500	0.0000	None
635	Human Plasma exp_4	13511	Thyroxine-binding globulin	VTACHSSQPNATLYK_5_4_0_2	None	3.1572	0.0000	None	7.3381	0.0000	None
636	Human Plasma exp_1	17101	Vitamin K-dependent protein C	EVFVHPNYSK_5_4_0_2	None	2.6825	0.0000	None	7.3180	0.0000	None
637	Human Plasma exp_2	17448	Vitamin K-dependent protein C	EVFVHPNYSK_5_4_0_2	None	2.7484	0.0000	None	7.3264	0.0000	None
638	Human Plasma exp_1	24845	Vitronectin	NGSLFAFR_4_3_0_1	None	2.5421	0.0000	None	7.3370	0.0000	None
639	Human Plasma exp_2	25338	Vitronectin	NGSLFAFR_4_3_0_1	None	1.9818	0.0000	None	7.3264	0.0000	None
640	Human Plasma exp_3	24827	Vitronectin	NGSLFAFR_4_3_0_1	None	2.6588	0.0000	None	7.2981	0.0000	None
641	Human Plasma exp_4	25323	Vitronectin	NGSLFAFR_4_3_0_1	None	2.0830	0.0000	None	7.2396	0.0000	None
642	Human Plasma exp_5	24584	Vitronectin	NGSLFAFR_4_3_0_1	None	2.1565	0.0000	None	7.2654	0.0000	None
643	Human Plasma exp_1	24613	Vitronectin	NGSLFAFR_5_3_0_1	None	4.0558	0.0000	None	7.3083	0.0000	None
644	Human Plasma exp_2	25113	Vitronectin	NGSLFAFR_5_3_0_1	None	3.7665	0.0000	None	7.3156	0.0000	None
645	Human Plasma exp_3	24606	Vitronectin	NGSLFAFR_5_3_0_1	None	3.5422	0.0000	None	7.3236	0.0000	None
646	Human Plasma exp_4	25094	Vitronectin	NGSLFAFR_5_3_0_1	None	3.1928	0.0000	None	7.2811	0.0000	None
647	Human Plasma exp_5	24407	Vitronectin	NGSLFAFR_5_3_0_1	None	3.8690	0.0000	None	7.3346	0.0000	None
648	Human Plasma exp_1	24402	Vitronectin	NGSLFAFR_5_4_0_1	None	3.0336	0.0000	None	7.3021	0.0000	None
649	Human Plasma exp_3	24438	Vitronectin	NGSLFAFR_5_4_0_1	None	2.9695	0.0000	None	7.3077	0.0000	None
650	Human Plasma exp_4	25041	Vitronectin	NGSLFAFR_5_4_0_1	None	2.6744	0.0000	None	7.3030	0.0000	None
651	Human Plasma exp_5	24199	Vitronectin	NGSLFAFR_5_4_0_1	None	3.0028	0.0000	None	7.3141	0.0000	None
652	Human Plasma exp_1	27909	Vitronectin	NGSLFAFR_5_4_0_2	None	3.5620	0.0000	None	7.2972	0.0000	None
653	Human Plasma exp_1	27933	Vitronectin	NGSLFAFR_5_4_0_2	None	2.5089	0.0000	None	7.1448	0.0000	None
654	Human Plasma exp_2	28613	Vitronectin	NGSLFAFR_5_4_0_2	None	3.5303	0.0000	None	7.2960	0.0000	None
655	Human Plasma exp_5	27590	Vitronectin	NGSLFAFR_5_4_0_2	None	3.6605	0.0000	None	7.3029	0.0000	Outer
656	Human Plasma exp_4	28463	Vitronectin	NGSLFAFR_5_4_1_2	Outer	2.7883	0.0000	Outer	6.4685	0.0033	None
657	Human Plasma exp_1	24437	Vitronectin	NGSLFAFR_6_3_0_1	None	2.8867	0.0000	None	7.2914	0.0000	None
658	Human Plasma exp_2	24915	Vitronectin	NGSLFAFR_6_3_0_1	None	3.5730	0.0000	None	7.2854	0.0000	None
659	Human Plasma exp_3	24459	Vitronectin	NGSLFAFR_6_3_0_1	None	3.3064	0.0000	None	7.2795	0.0000	None

Supplementary Table 8. Continued.

No.	Experiment	CID Scan No.	N-glycoproteins	N-glycopeptides	SVM			DNN			Manual Classification
					Classification	Pscore	FDR	Classification	Pscore	FDR	
660	Human Plasma exp_4	24929	Vitronectin	NGSLFAFR_6_3_0_1	None	2.8869	0.0000	None	7.2845	0.0000	None
661	Human Plasma exp_5	24229	Vitronectin	NGSLFAFR_6_3_0_1	None	3.0447	0.0000	None	7.2841	0.0000	Outer
662	Human Plasma exp_2	31304	Vitronectin	NGSLFAFR_6_5_1_3	Outer	3.7531	0.0000	Outer	6.4679	0.0033	Outer
663	Human Plasma exp_3	30798	Vitronectin	NGSLFAFR_6_5_1_3	Outer	5.2272	0.0000	Outer	6.4406	0.0033	Outer
664	Human Plasma exp_4	31068	Vitronectin	NGSLFAFR_6_5_1_3	Outer	4.2202	0.0000	Outer	6.2742	0.0032	None
665	Human Plasma exp_3	21145	Vitronectin	NNATVHEQVGGPSLTSDLQAQSK_5_4_0_2	None	3.7693	0.0000	None	7.3536	0.0000	None
666	Human Plasma exp_4	21736	Vitronectin	NNATVHEQVGGPSLTSDLQAQSK_5_4_0_2	None	3.6739	0.0000	None	7.3496	0.0000	None
667	Human Plasma exp_1	16030	Zinc-alpha-2-glycoprotein	FGCEIENNR_5_4_0_2	None	1.3881	0.0000	None	7.2177	0.0000	None
668	Human Plasma exp_2	16224	Zinc-alpha-2-glycoprotein	FGCEIENNR_5_4_0_2	None	4.0204	0.0000	None	7.3263	0.0000	None
669	Human Plasma exp_3	15877	Zinc-alpha-2-glycoprotein	FGCEIENNR_5_4_0_2	None	3.2344	0.0000	None	7.3103	0.0000	None
670	Human Plasma exp_4	16245	Zinc-alpha-2-glycoprotein	FGCEIENNR_5_4_0_2	None	3.4918	0.0000	None	7.3125	0.0000	None
671	Human Plasma exp_5	15970	Zinc-alpha-2-glycoprotein	FGCEIENNR_5_4_0_2	None	3.3247	0.0000	None	7.3049	0.0000	None

Supplementary Table 9. N-glycopeptide having both core and outer type fucosylation in human plasma.

No.	N-glycoproteins	N-site	N-glycoproteins	Classification	Reference
1	Alpha-1-acid glycoprotein 1,2	56	NEEYNK_5_4_1_2	Core	New
2	Alpha-1-acid glycoprotein 1,2	56	NEEYNK_6_5_1_2	Outer	New
3	Alpha-1-acid glycoprotein 1,2	56	NEEYNK_6_5_1_3	Outer	New
4	Alpha-1-acid glycoprotein 1,2	56	NEEYNK_6_5_2_3	Dual	New
5	Alpha-2-HS-glycoprotein	156	VCQDCPLLAPLNDTR_5_4_1_2	Core	[1]*
6	Alpha-2-HS-glycoprotein	156	VCQDCPLLAPLNDTR_6_5_1_3	Outer	New
7	Hemopexin	187	SWPAVGNCSSALR_5_4_1_2	Core	[2]*
8	Hemopexin	187	SWPAVGNCSSALR_6_5_1_3	Outer	[2]*,[3]*

[1] Hägglund, P.; Matthiesen, R.; Elortza, F.; Højrup, P.; Roepstorff, P.; Jensen, O. N.; Bunkenborg, J. J. Proteome Res. 2007, 6, 3021-3031.

[2] Benicky, J.; Sanda, M.; Pompach, P.; Wu, J.; Goldman R. Anal Chem. 2014, 86, 10716-10723.

[3] Pompach, P.; Ashline, D. J.; Brnakova, Z.; Benicky, J.; Sanda, M.; Goldman, R. J Proteome Res. 2014, 13, 5561-5569.

Supplementary Table 10. Total fucosylated N−glycopeptides of proteins in human plasma commonly classified by SVM and DNN. A total of 46 fucosylated N−glycopeptides were validated previously (with references), while 36 were newly found.

No.	Glycoproteins	N−site	N−glycopeptides	Manual	Classification	References
1	Alpha−1−acid glycoprotein 1	103	ENGTISR_5_4_1_2	Outer	Outer	New
2	Alpha−1−acid glycoprotein 1	103	ENGTISR_6_5_1_3	Outer	Outer	New
3	Alpha−1−acid glycoprotein 1	103	ENGTISR_7_6_1_4	Outer	Outer	New
4	Alpha−1−acid glycoprotein 1	103	ENGTISR_7_6_2_4	Outer	Outer	New
5	Alpha−1−acid glycoprotein 1	33	LVPVPITNATLDQITGK_6_5_1_2	Outer	Outer	New
6	Alpha−1−acid glycoprotein 1	33	LVPVPITNATLDQITGK_6_5_1_3	Outer	Outer	New
7	Alpha−1−acid glycoprotein 1	93	QDQCIYNTTYLNVQR_6_5_1_3	Outer	Outer	New
8	Alpha−1−acid glycoprotein 1,2	56	NEEYNK_5_4_1_2	Core	Core	New
9	Alpha−1−acid glycoprotein 1,2	56	NEEYNK_6_5_1_2	Outer	Outer	New
10	Alpha−1−acid glycoprotein 1,2	56	NEEYNK_6_5_1_3	Outer	Outer	New
11	Alpha−1−acid glycoprotein 1,2	56	NEEYNK_6_5_2_3	Dual	Dual	New
12	Alpha−1−acid glycoprotein 2	103	ENGTVSR_6_5_1_3	Outer	Outer	New
13	Alpha−1−acid glycoprotein 2	103	ENGTVSR_6_5_2_3	Outer	Dual	New
14	Alpha−1−acid glycoprotein 2	103	ENGTVSR_7_6_1_4	Outer	Outer	New
15	Alpha−1−acid glycoprotein 2	103	ENGTVSR_7_6_2_4	Outer	Outer	New
16	Alpha−1−acid glycoprotein 2	33	LVPVPITNATLDR_6_5_1_3	Outer	Outer	New
17	Alpha−1−antichymotrypsin	186	NGTR_6_5_1_3	Core	Core	New
18	Alpha−2−HS−glycoprotein	156	VCQDCPLLAPLNDTR_5_4_1_2	Core	Core	[46]
19	Alpha−2−HS−glycoprotein	156	VCQDCPLLAPLNDTR_6_5_1_3	Outer	Outer	New
20	Apolipoprotein D	98	ADGTVNQIEGEATPVNLTEPAK_5_4_1_1	Core	Core	New
21	Apolipoprotein D	98	ADGTVNQIEGEATPVNLTEPAK_5_4_1_2	Core	Core	New
22	Beta−2−glycoprotein 1	162	VYKPSAGNNSLYR_6_5_1_3	Outer	Outer	New
23	Ceruloplasmin	138	EHEGAIYPDNTTDFQR_4_3_1_1	Core	Core	[46]
24	Ceruloplasmin	762	ELHHLQEQNVSNAFLDK_5_4_1_2	Core	Core	[46]
25	Clusterin	86	EDALNETR_6_5_1_3	Outer	Outer	New
26	Clusterin	374	LANLTQGEDQYYLR_6_5_1_3	Outer	Outer	New
27	Complement component C7	754	NYTLTGR_5_4_1_2	Core	Core	New
28	Haptoglobin	241	VVLHPNYSQVDIGLIK_6_5_1_3	Outer	Outer	[23],[49]
29	Hemopexin	187	SWPAVGNCSSALR_5_4_1_2	Core	Core	[47]
30	Hemopexin	187	SWPAVGNCSSALR_6_5_1_3	Outer	Outer	[23],[47]

Supplementary Table 10. Continued.

No.	Glycoproteins	N−site	N−glycopeptides	Manual	Classification	References
31	Ig alpha−2 chain C region	205	TPLTANITK_5_4_1_1	Core	Core	[46]
32	Ig alpha−2 chain C region	205	TPLTANITK_5_4_1_2	Core	Core	[46]
33	Ig alpha−2 chain C region	205	TPLTANITK_5_5_1_1	Core	Core	[46]
34	Ig gamma−1 chain C region	180	EEQYNSTYR_3_3_1_0	Core	Core	[46]
35	Ig gamma−1 chain C region	180	EEQYNSTYR_3_4_1_0	Core	Core	[46]
36	Ig gamma−1 chain C region	180	EEQYNSTYR_4_3_1_0	Core	Core	[46]
37	Ig gamma−1 chain C region	180	EEQYNSTYR_4_3_1_1	Core	Core	[46]
38	Ig gamma−1 chain C region	180	EEQYNSTYR_4_4_1_0	Core	Core	[46]
39	Ig gamma−1 chain C region	180	EEQYNSTYR_4_4_1_1	Core	Core	[46]
40	Ig gamma−1 chain C region	180	EEQYNSTYR_4_5_1_0	Core	Core	[46]
41	Ig gamma−1 chain C region	180	EEQYNSTYR_5_3_1_1	Core	Core	[46]
42	Ig gamma−1 chain C region	180	EEQYNSTYR_5_4_1_0	Core	Core	[46]
43	Ig gamma−1 chain C region	180	EEQYNSTYR_5_4_1_1	Core	Core	[46]
44	Ig gamma−1 chain C region	180	EEQYNSTYR_5_4_2_0	Dual	Outer	New
45	Ig gamma−1 chain C region	180	EEQYNSTYR_5_4_2_1	Dual	Dual	New
46	Ig gamma−1 chain C region	180	EEQYNSTYR_5_5_1_0	Core	Core	[46]
47	Ig gamma−2 chain C region	176	EEQFNSTFR_3_3_1_0	Core	Core	[46]
48	Ig gamma−2 chain C region	176	EEQFNSTFR_3_4_1_0	Core	Core	[46]
49	Ig gamma−2 chain C region	176	EEQFNSTFR_4_3_1_0	Core	Core	[46]
50	Ig gamma−2 chain C region	176	EEQFNSTFR_4_3_1_1	Core	Core	[46]
51	Ig gamma−2 chain C region	176	EEQFNSTFR_4_4_1_0	Core	Core	[46]
52	Ig gamma−2 chain C region	176	EEQFNSTFR_4_4_1_1	Core	Core	[46]
53	Ig gamma−2 chain C region	176	EEQFNSTFR_4_5_1_0	Core	Core	[46]
54	Ig gamma−2 chain C region	176	EEQFNSTFR_5_4_1_0	Core	Core	[46]
55	Ig gamma−2 chain C region	176	EEQFNSTFR_5_4_1_1	Core	Core	[46]
56	Ig gamma−2 chain C region	176	EEQFNSTFR_5_4_2_1	Dual	Dual	New
57	Ig gamma−2 chain C region	176	EEQFNSTFR_5_5_1_0	Core	Core	[46]
58	Ig gamma−2 chain C region	176	EEQFNSTFR_6_3_1_1	Core	Core	[46]
59	Ig gamma−3 chain C region	227	EEQYNSTFR_3_4_1_0	Core	Core	New
60	Ig gamma−3 chain C region	227	EEQYNSTFR_4_3_1_0	Core	Core	New

Supplementary Table 10. Continued.

No.	Glycoproteins	N−site	N−glycopeptides	Manual	Classification	References
61	Ig gamma−3 chain C region	227	EEQYNSTFR_5_4_1_0	Core	Core	New
62	Ig gamma−3 chain C region	227	EEQYNSTFR_5_4_1_1	Core	Core	New
63	Ig gamma−4 chain C region	177	EEQFNSTYR_3_3_1_0	Core	Core	[46]
64	Ig gamma−4 chain C region	177	EEQFNSTYR_3_4_1_0	Core	Core	[46]
65	Ig gamma−4 chain C region	177	EEQFNSTYR_3_5_1_0	Core	Core	[46]
66	Ig gamma−4 chain C region	177	EEQFNSTYR_4_3_1_0	Core	Core	[46]
67	Ig gamma−4 chain C region	177	EEQFNSTYR_4_4_1_0	Core	Core	[46]
68	Ig gamma−4 chain C region	177	EEQFNSTYR_4_4_1_1	Core	Core	[46]
69	Ig gamma−4 chain C region	177	EEQFNSTYR_4_5_1_0	Core	Core	[46]
70	Ig gamma−4 chain C region	177	EEQFNSTYR_5_4_1_0	Core	Core	[46]
71	Ig mu chain C region	209	GLTFQQNASSMCVPDQDTAIR_5_4_1_1	Core	Core	[46]
72	Ig mu chain C region	46	NNSDISSTR_4_3_1_1	Core	Core	[46]
73	Ig mu chain C region	46	NNSDISSTR_5_3_1_1	Core	Core	[46]
74	Ig mu chain C region	46	NNSDISSTR_5_4_1_1	Core	Core	[46]
75	Ig mu chain C region	46	NNSDISSTR_5_5_1_1	Core	Core	[46]
76	Ig mu chain C region	46	NNSDISSTR_6_3_1_1	Core	Core	[46]
77	Immunoglobulin J chain	71	ENISDPTSPLR_5_3_1_1	Core	Core	New
78	Kininogen−1	205	ITYSIVQTNCSK_6_5_1_3	Outer	Outer	[23]
79	Phospholipid transfer protein	245	NWSLPNR_5_4_1_2	Core	Core	New
80	Prothrombin	121	GHVNITR_5_4_1_2	Outer	Outer	New
81	Vitronectin	169	NGSLFAFR_5_4_1_2	Outer	Outer	New
82	Vitronectin	169	NGSLFAFR_6_5_1_3	Outer	Outer	New

Supplementary Figures

Supplementary Figure 1. Representative CID MS/MS spectra of N−glycopeptides of (A) EEQYNSTYR_4_4_1_0 from an IgG standard and (B) NEEYNK_6_5_1_3 from an AGP standard, which were classified as core and outer fucosylated N−glycopeptides, respectively. Green circles = mannose; yellow circles = galactose; blue squares = N−acetylglucosamine; red triangles = fucose; pink diamonds = N−acetylneuraminic acid; red arrow = fucosylation diagnostic ions; red box = fragmented ions pairs with or without fucose.

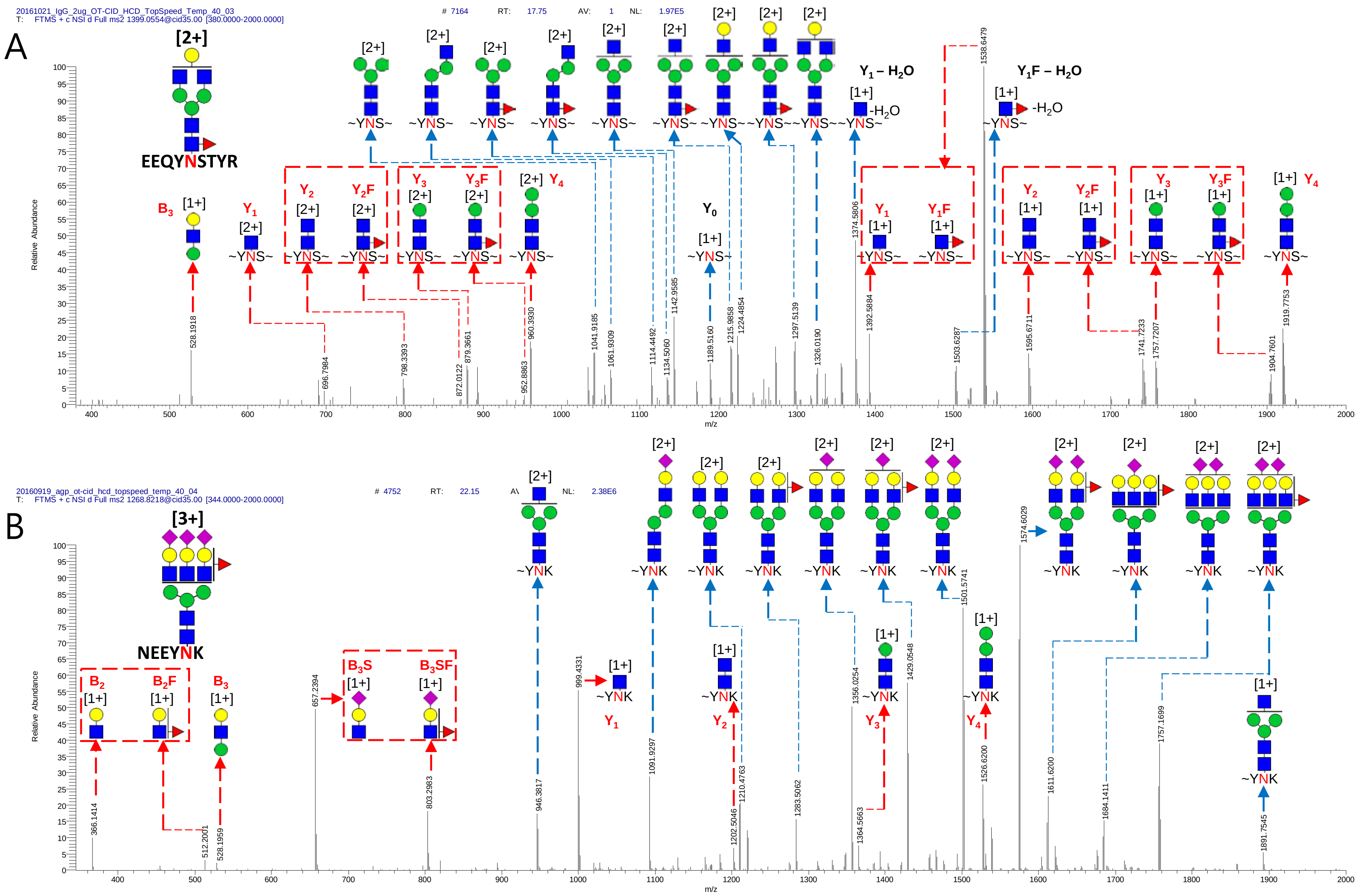

Supplementary Figure 2. MS/MS spectra of N-glycopeptides identified from alpha-1-acid glycoprotein and immunoglobulin gamma for training set.

Training set no. 1, Experiment: AGP exp_2

ENGTISR(=PEP)_4_4_0_1, m/z:843.34(3+), RT:16.94, Y-score:89.44

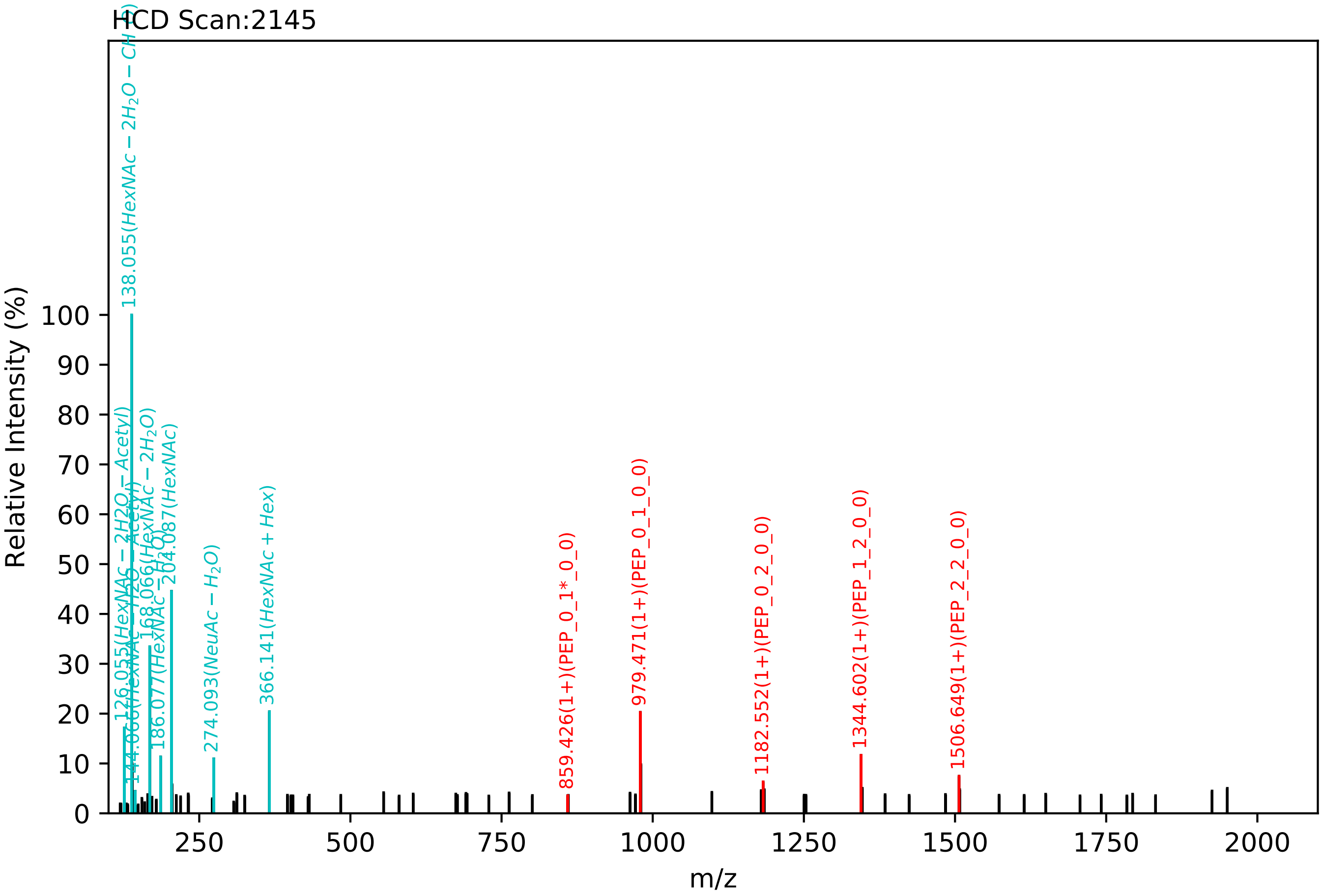

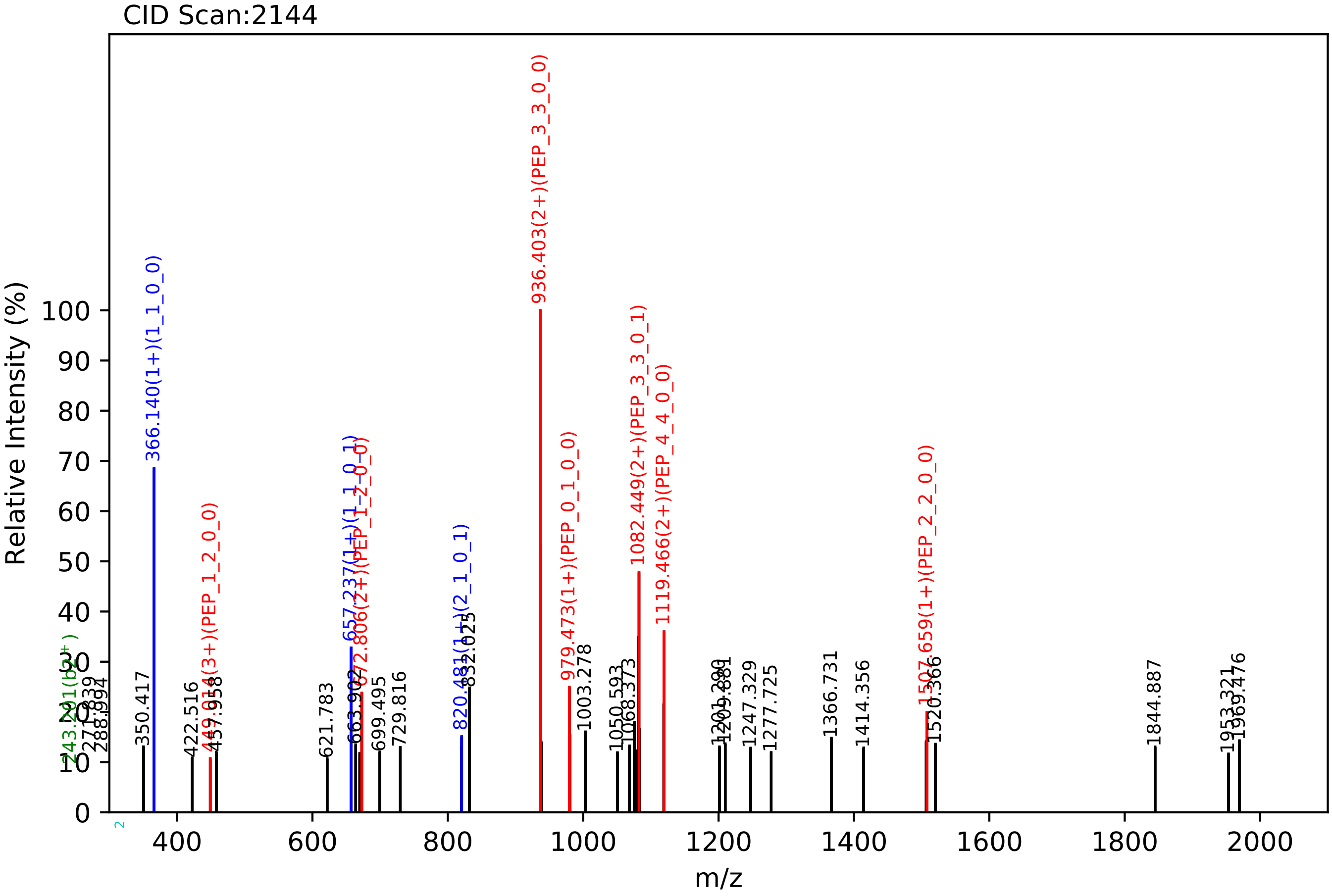

Training set no. 2, Experiment: AGP exp_1

ENGTISR(=PEP)_4_4_0_1, m/z:1264.51(2+), RT:16.60, Y-score:73.88

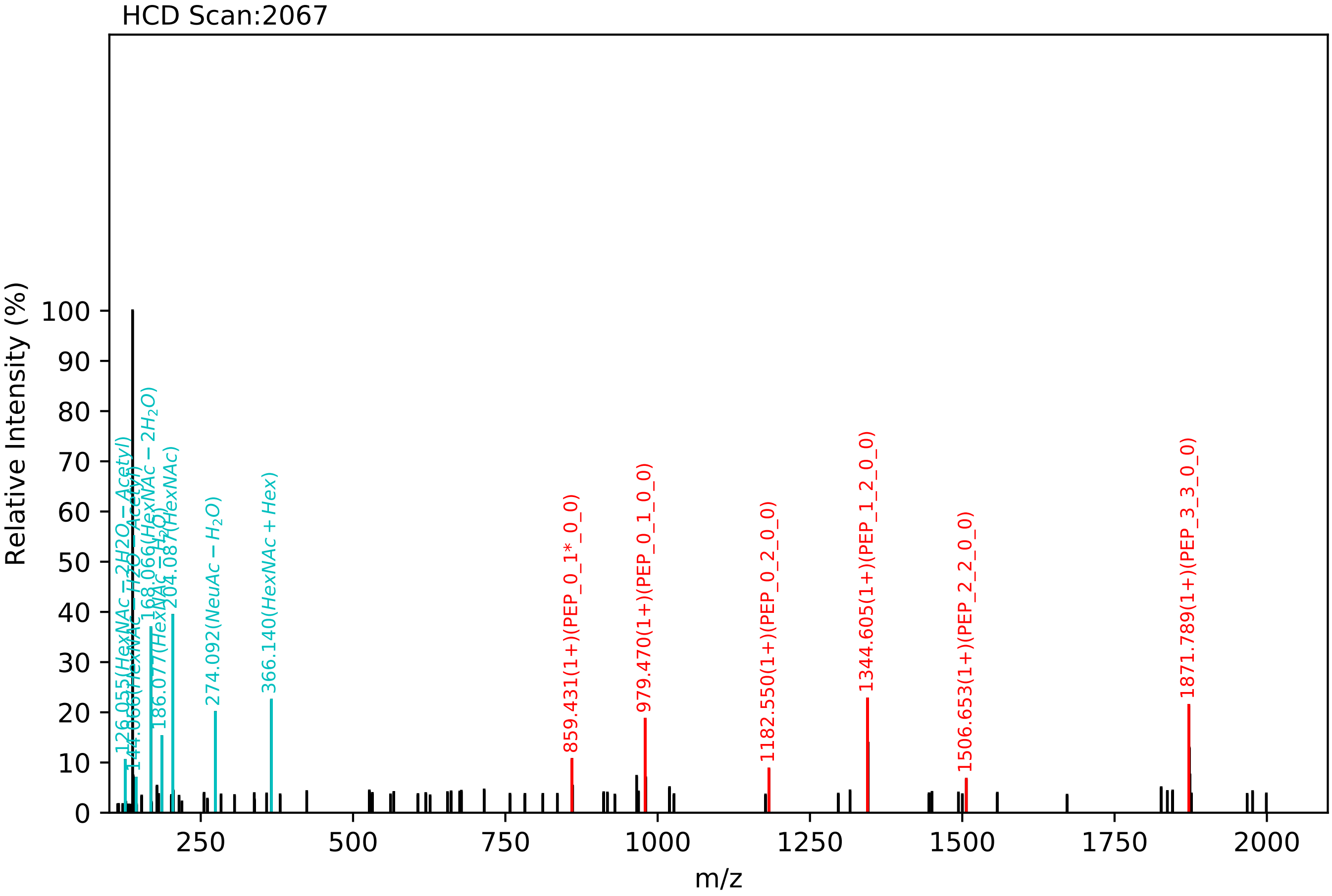

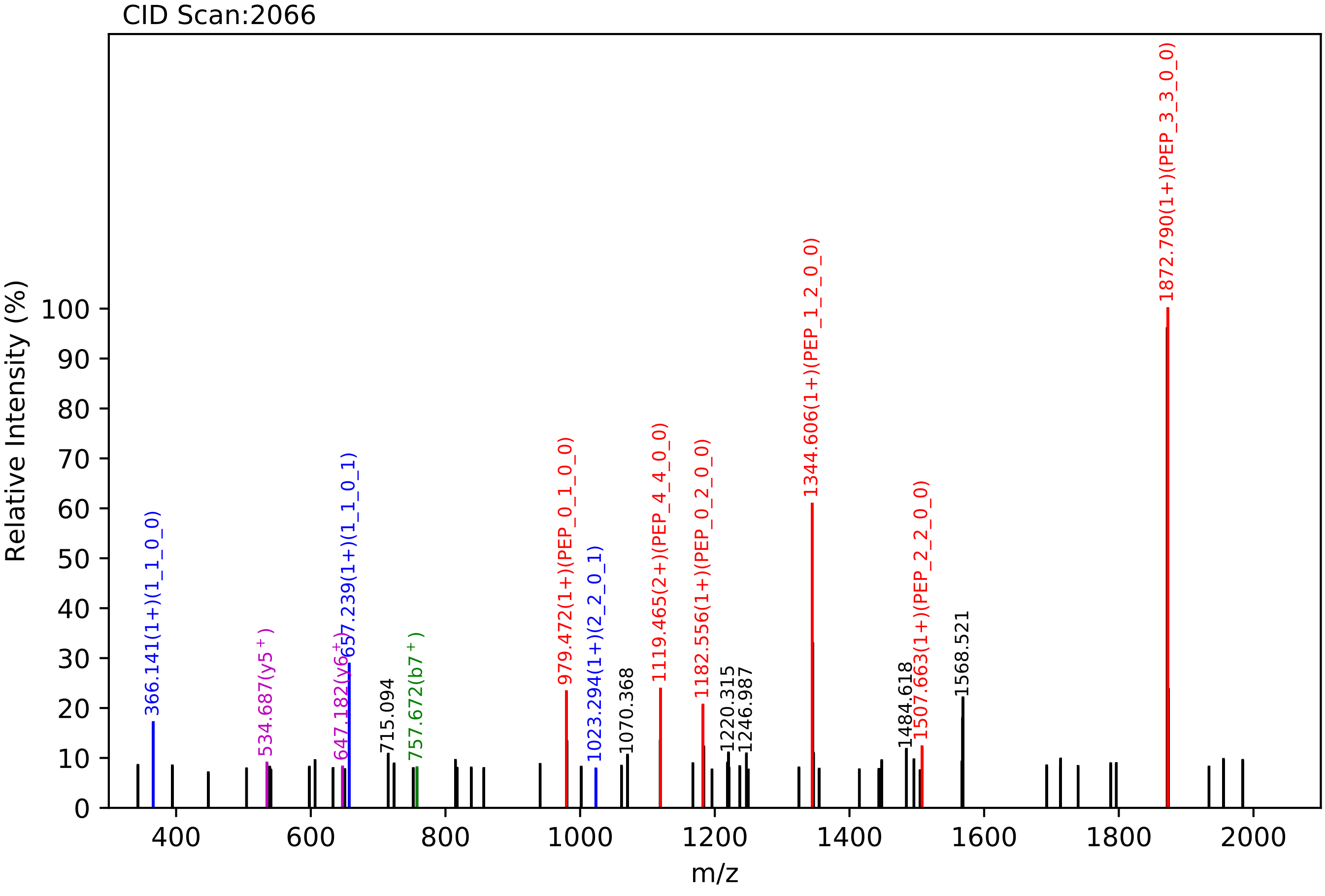

Training set no. 3, Experiment: AGP exp_2

ENGTISR(=PEP)_5_4_0_2, m/z:994.40(3+), RT:20.63, Y-score:93.52

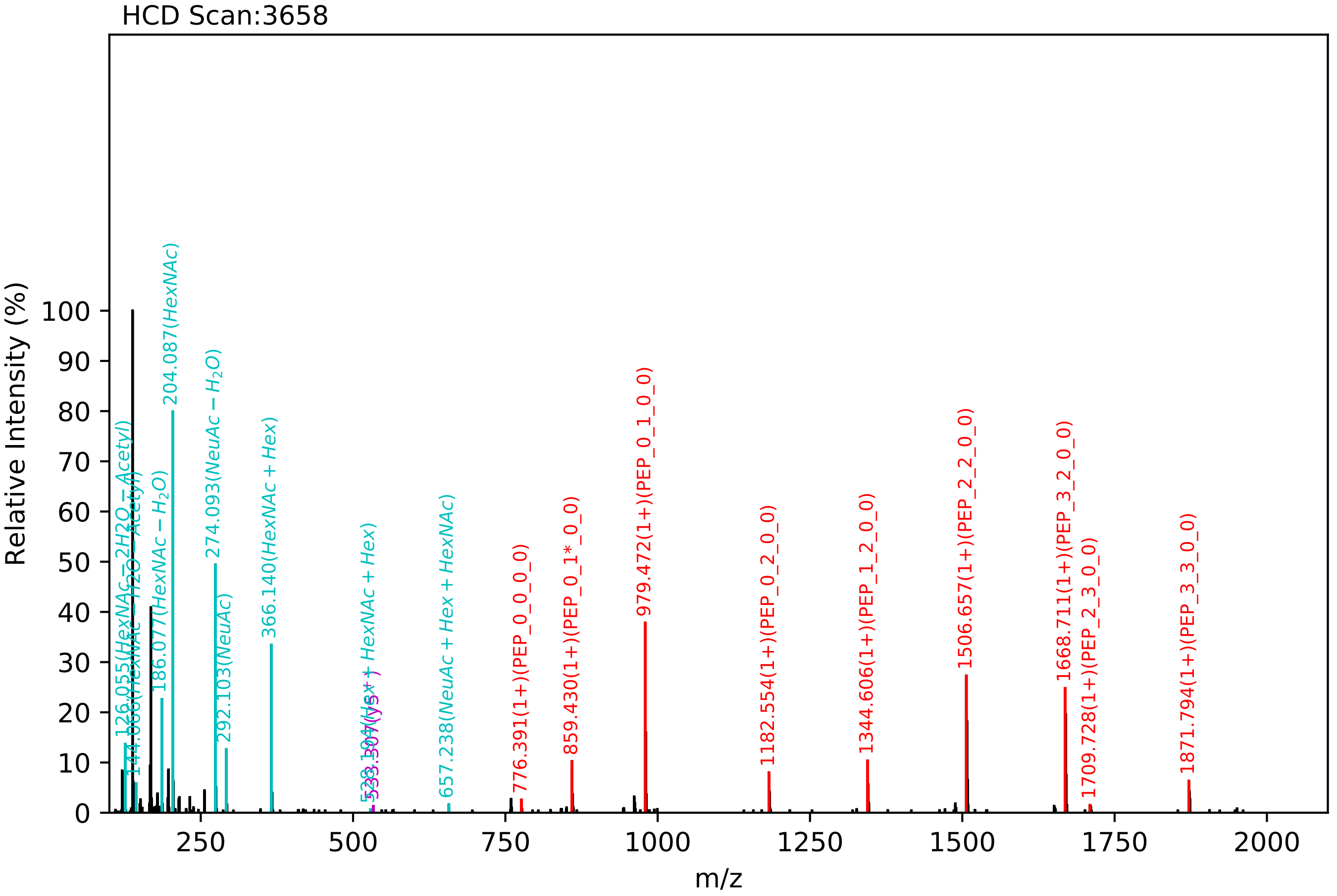

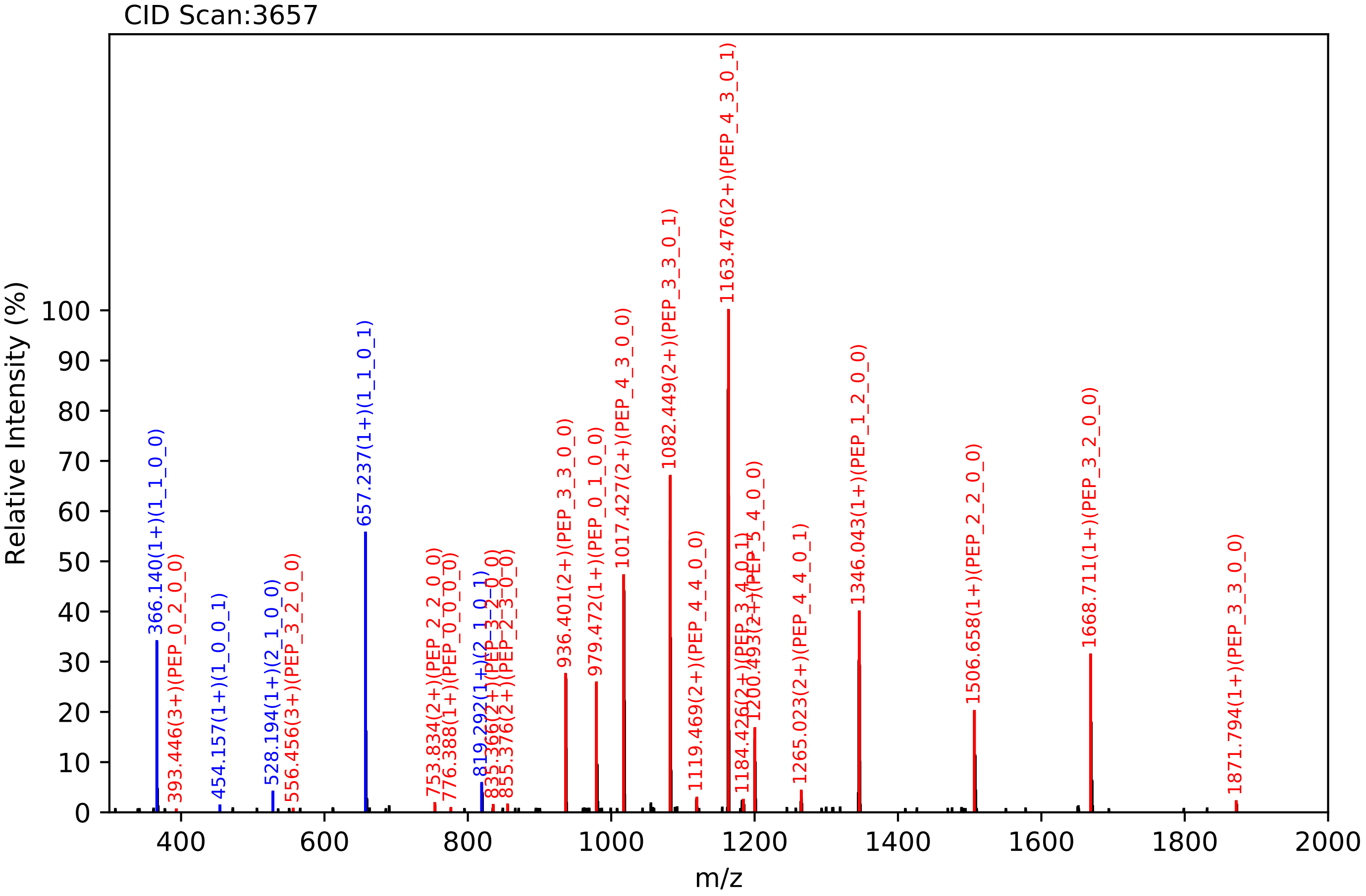

Training set no. 4, Experiment: AGP exp_2

ENGTISR(=PEP)_5_4_0_2, m/z:1491.09(2+), RT:20.70, Y-score:87.78

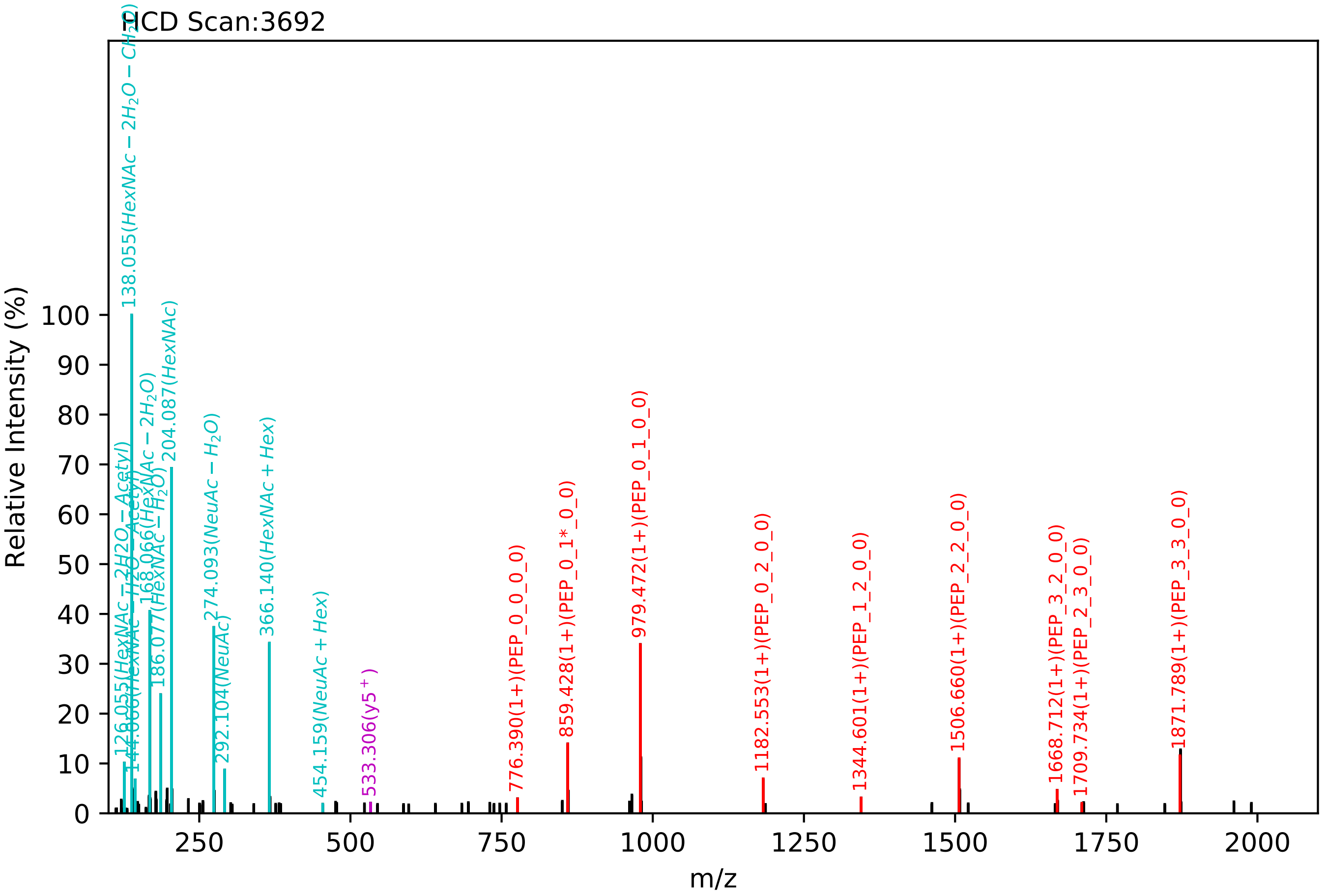

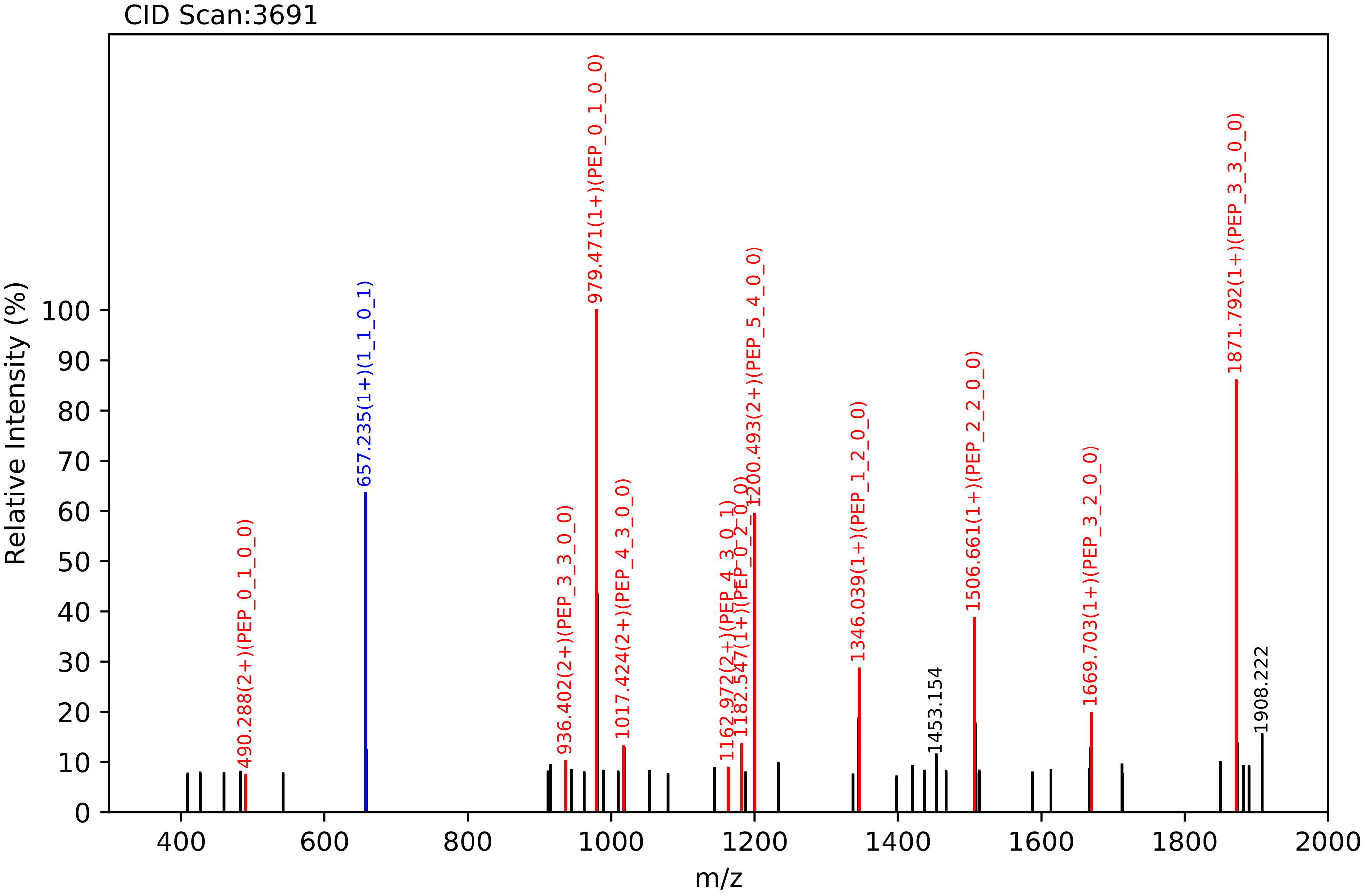

Training set no. 5, Experiment: AGP exp_1

ENGTISR(=PEP)_5_4_0_2, m/z:994.39(3+), RT:20.92, Y-score:85.60

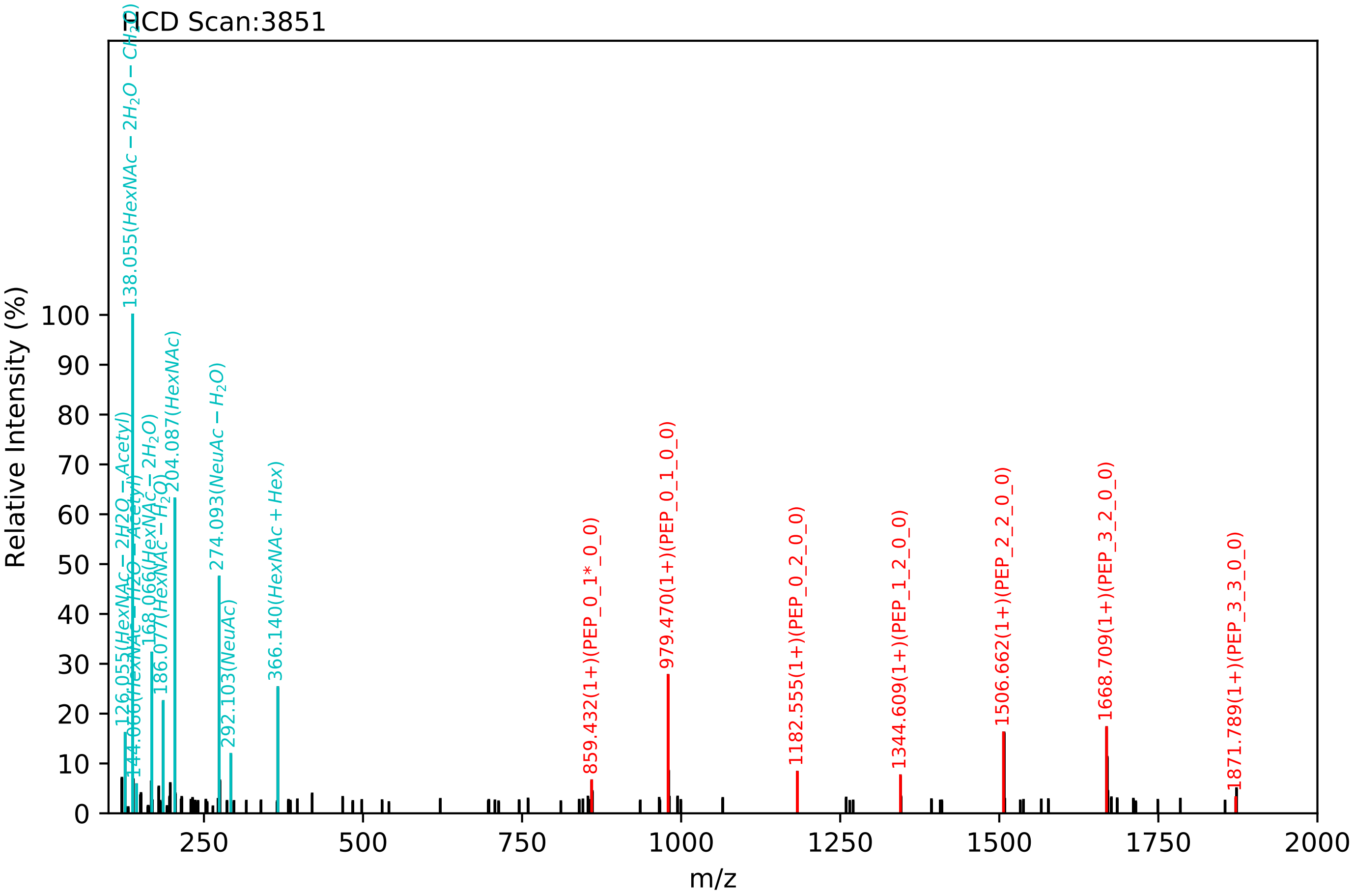

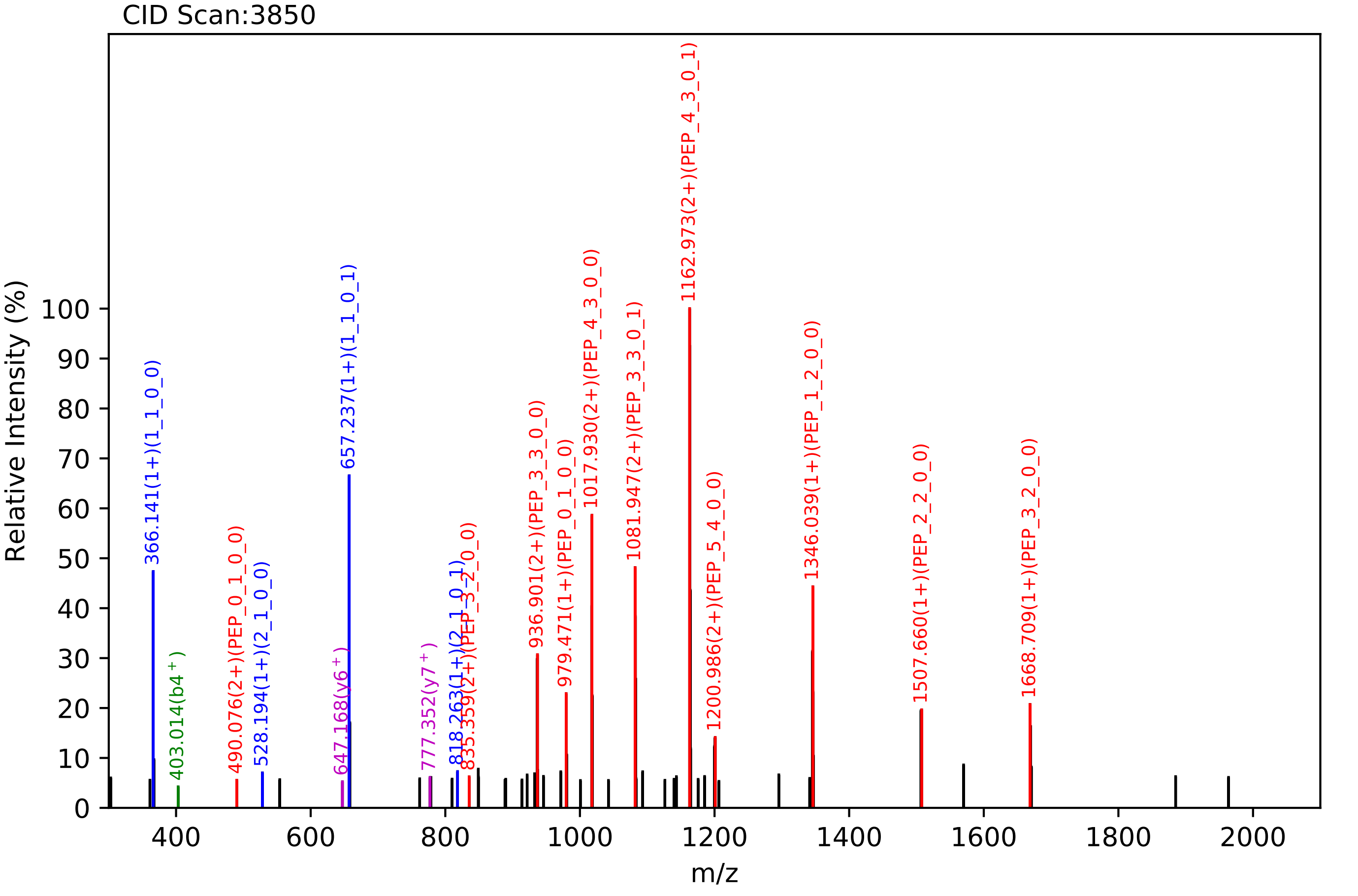

Training set no. 6, Experiment: AGP exp_1

ENGTISR(=PEP)_6_5_0_1, m/z:1019.07(3+), RT:16.83, Y-score:95.51

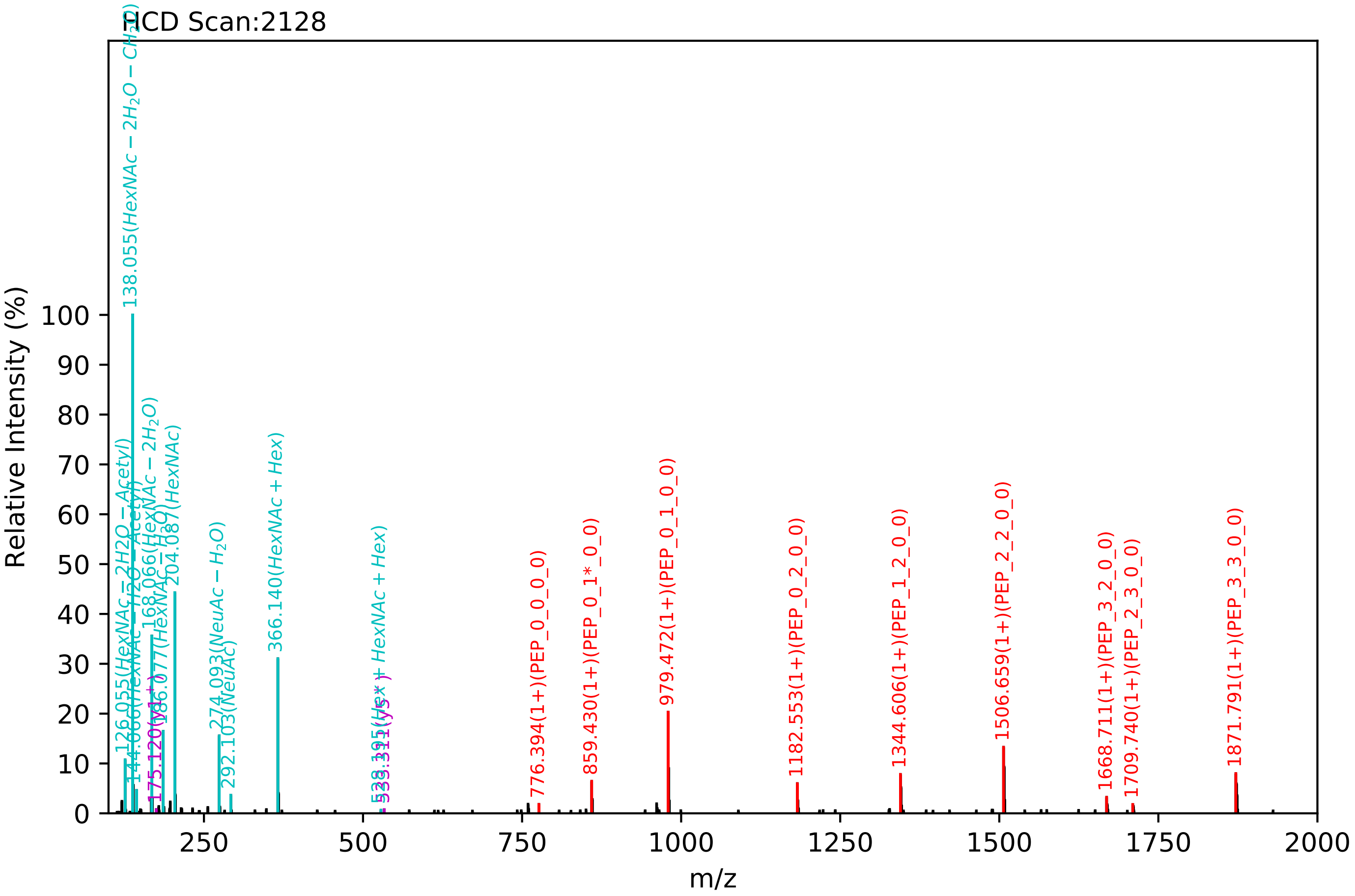

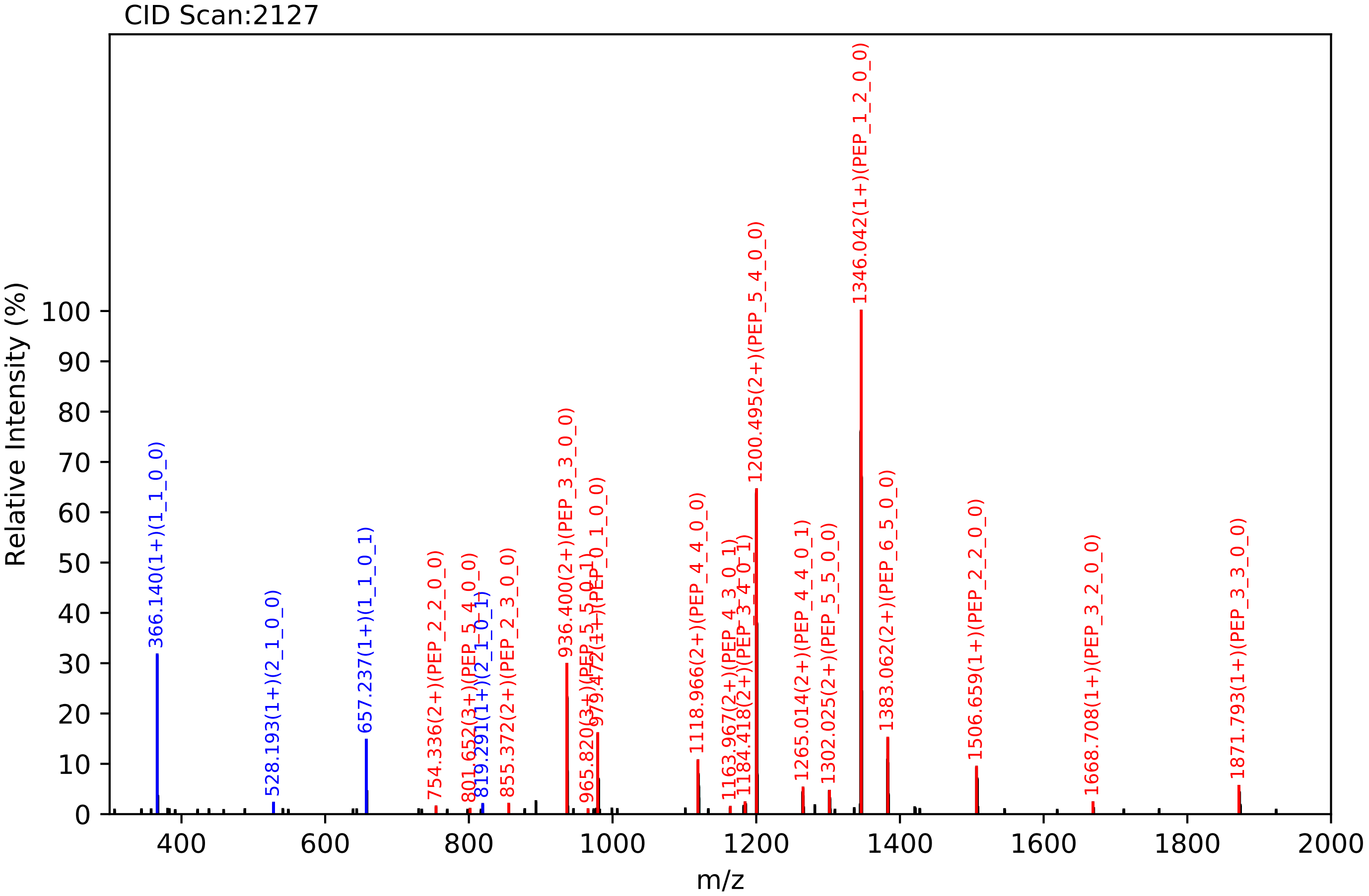

Training set no. 7, Experiment: AGP exp_1

ENGTISR(=PEP)_6_5_0_1, m/z:1019.07(3+), RT:17.35, Y-score:94.17

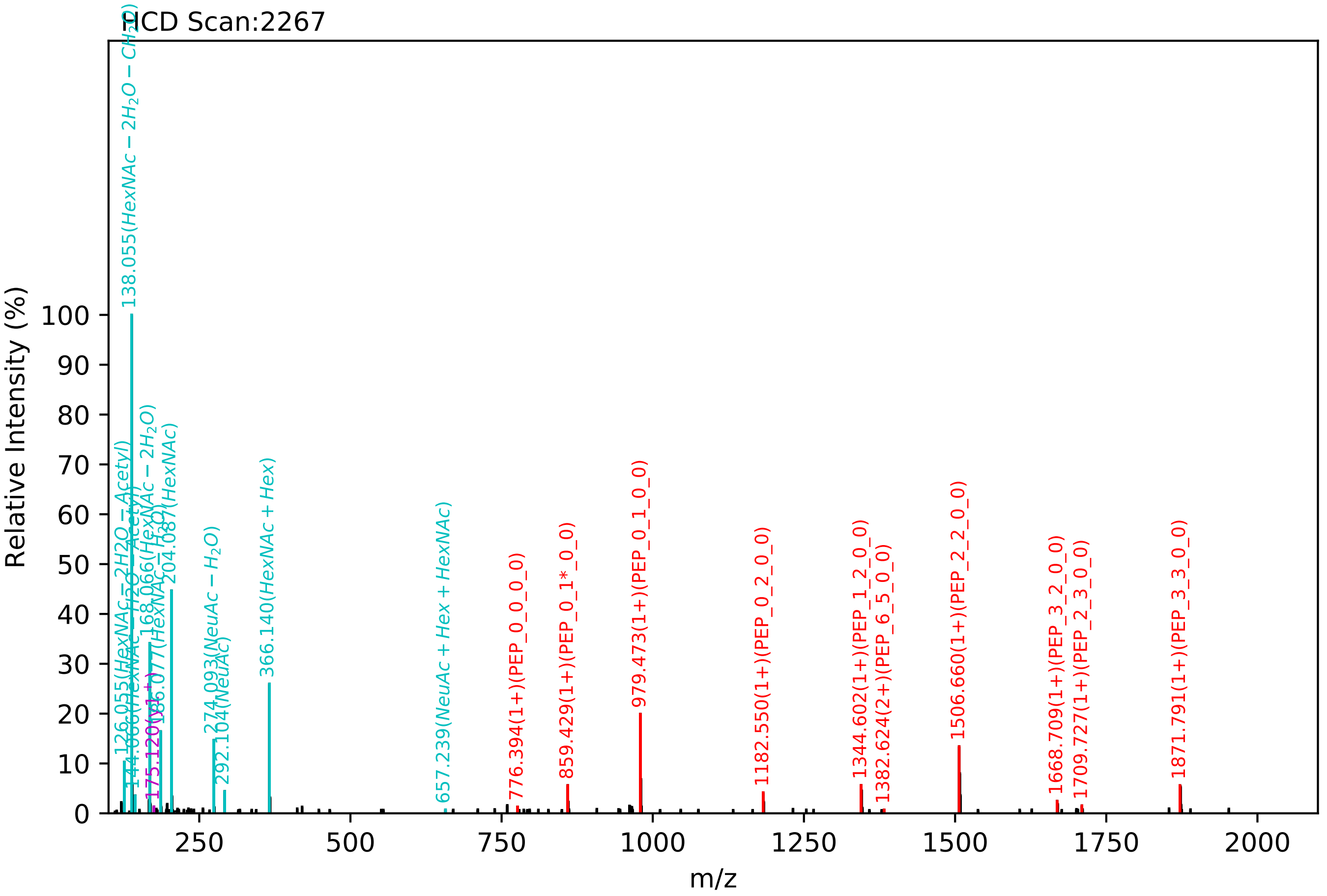

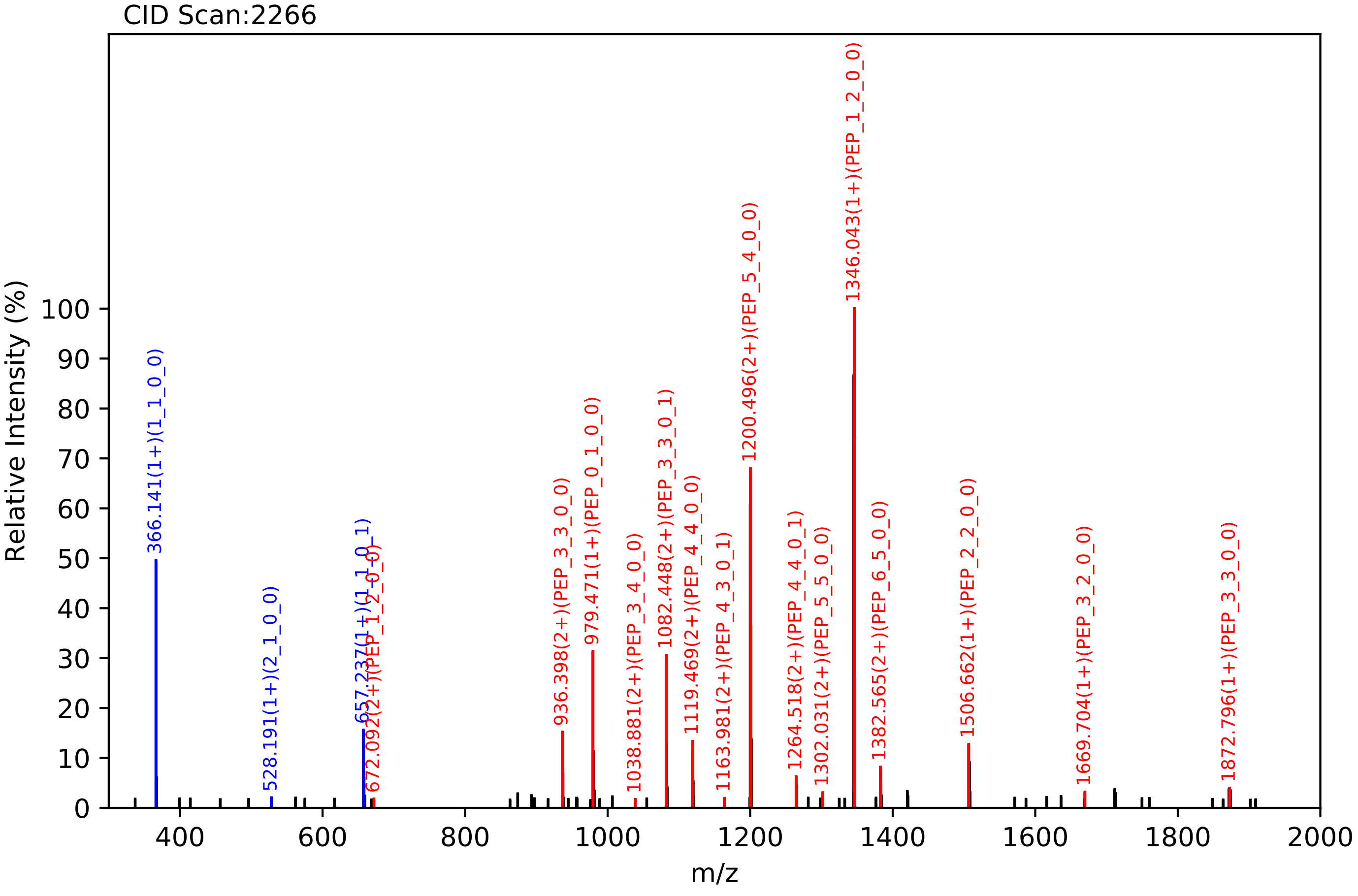

Training set no. 8, Experiment: AGP exp_1

ENGTISR(=PEP)_6_5_0_1, m/z:1019.07(3+), RT:17.86, Y-score:91.18

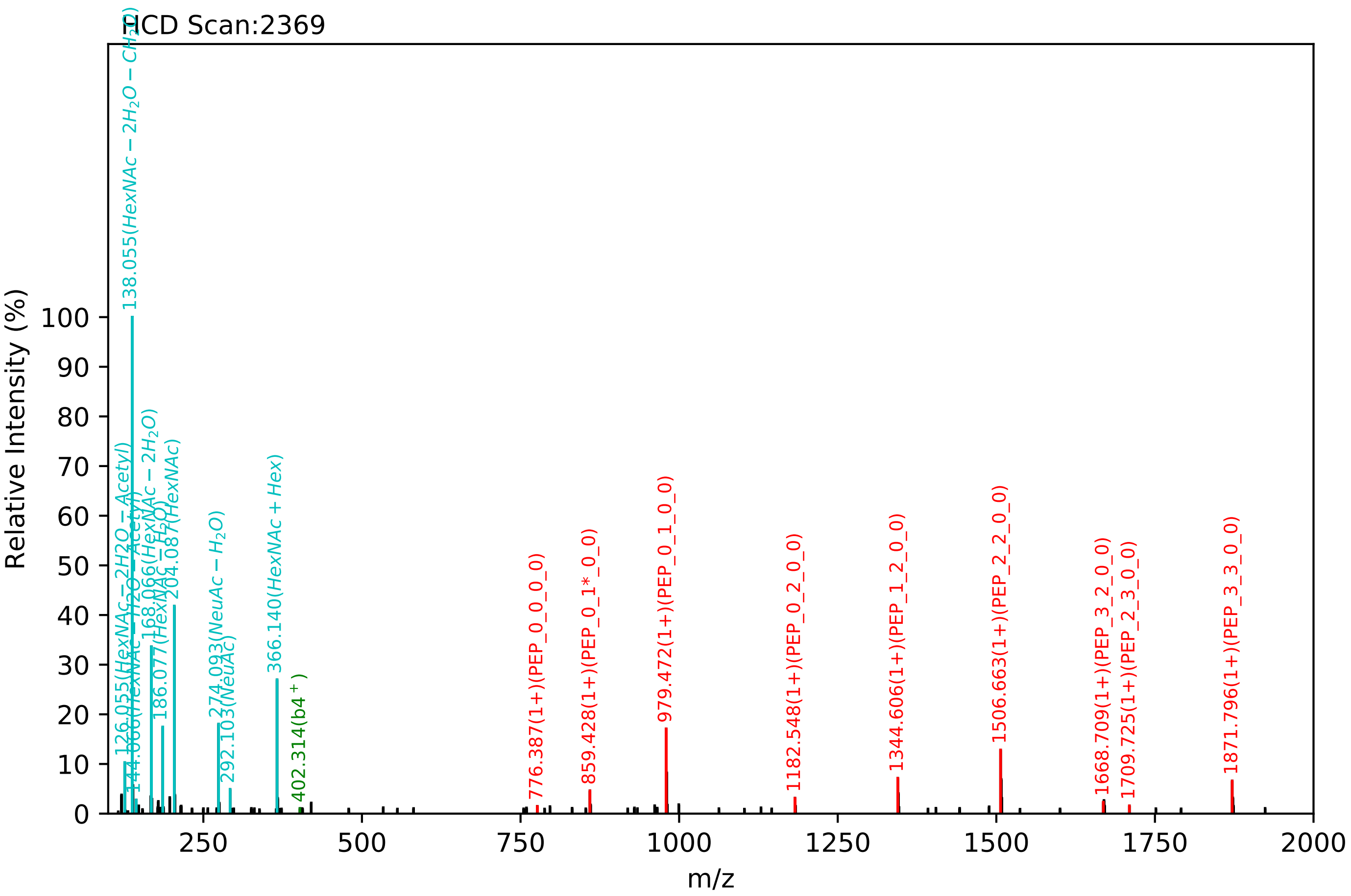

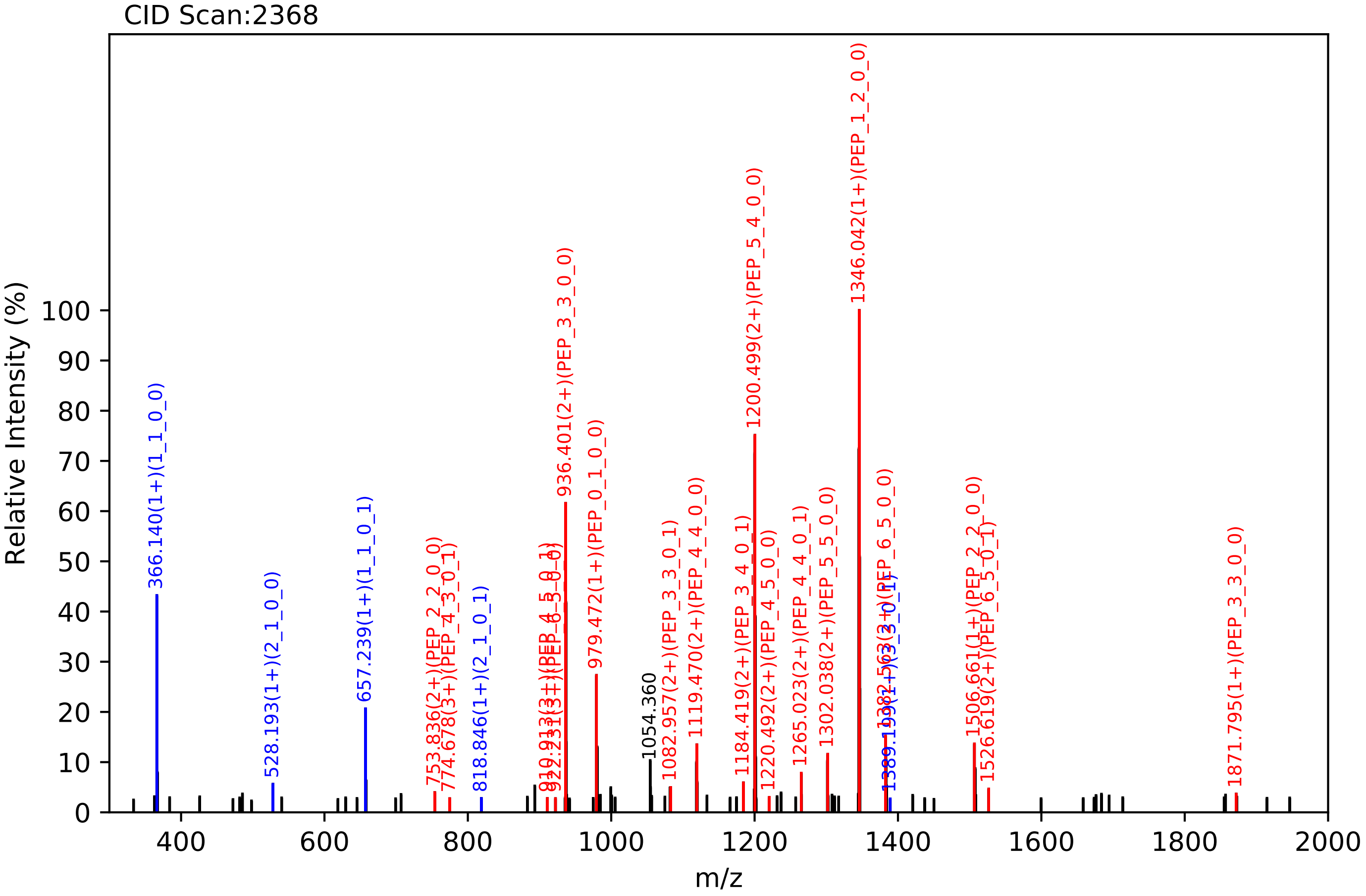

Training set no. 9, Experiment: AGP exp_2

ENGTISR(=PEP)_6_5_0_1, m/z:1019.07(3+), RT:17.88, Y-score:91.15

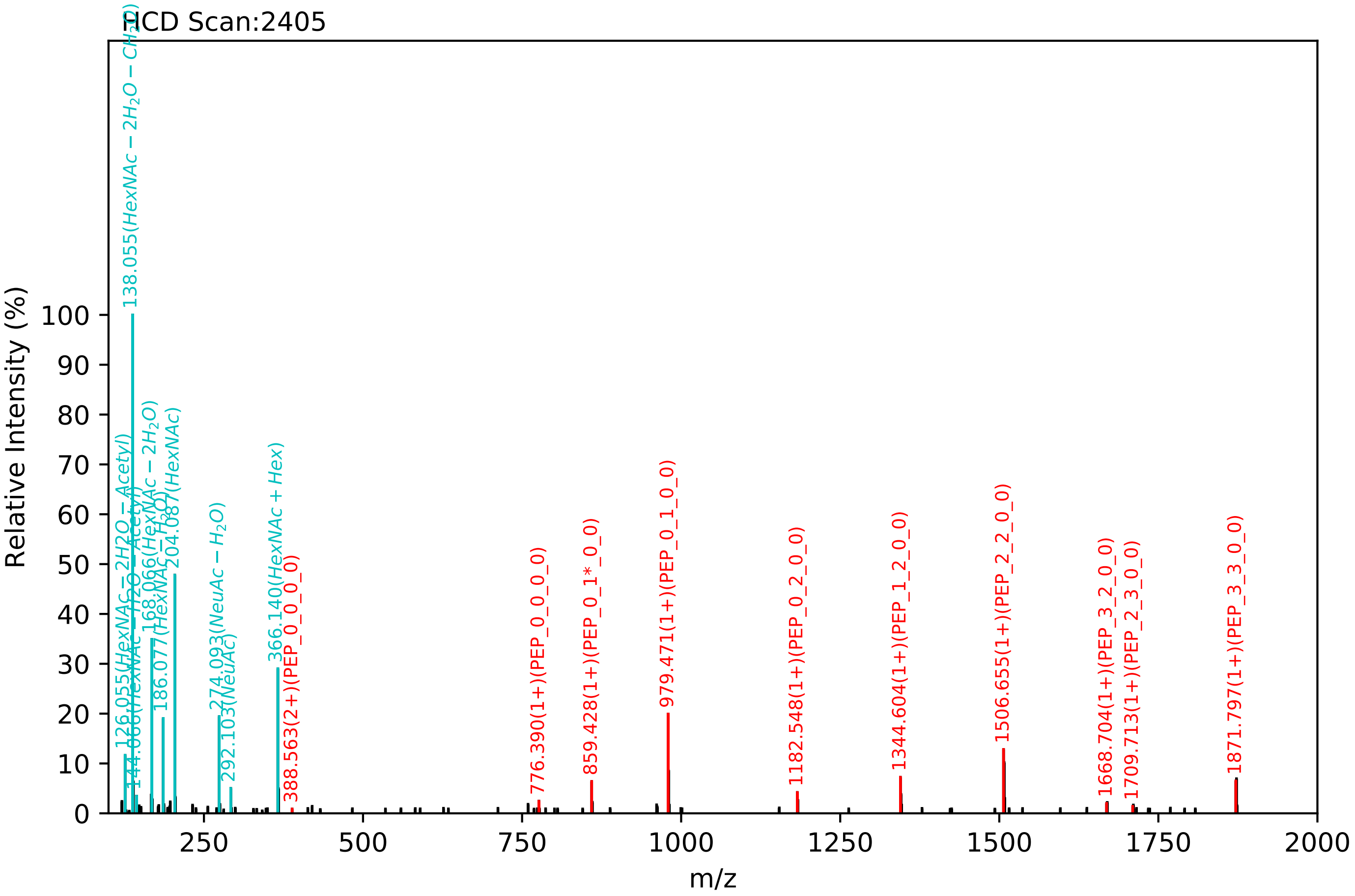

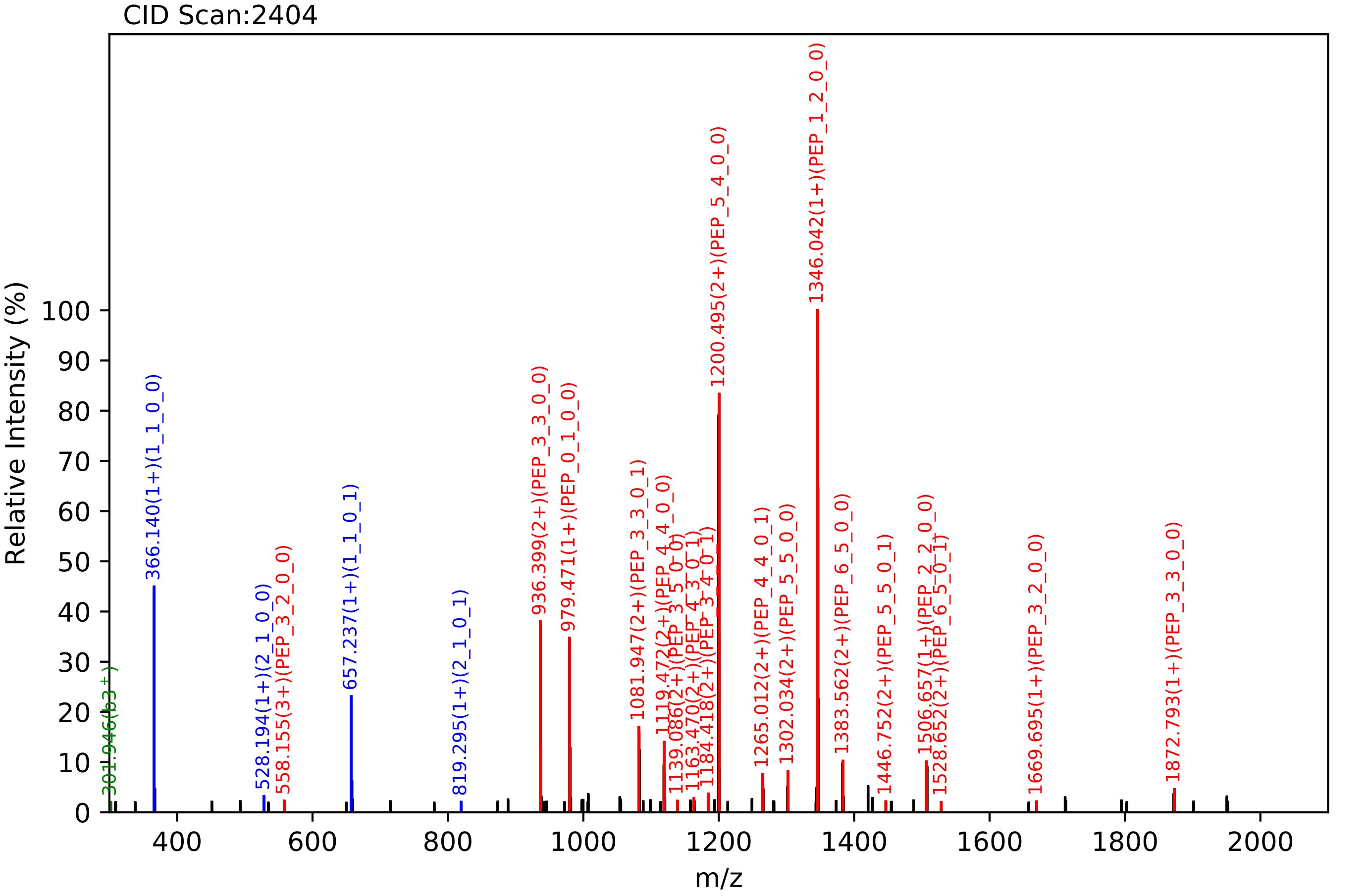

Training set no. 10, Experiment: AGP exp_2

ENGTISR(=PEP)_6_5_0_1, m/z:1019.07(3+), RT:17.37, Y-score:89.60

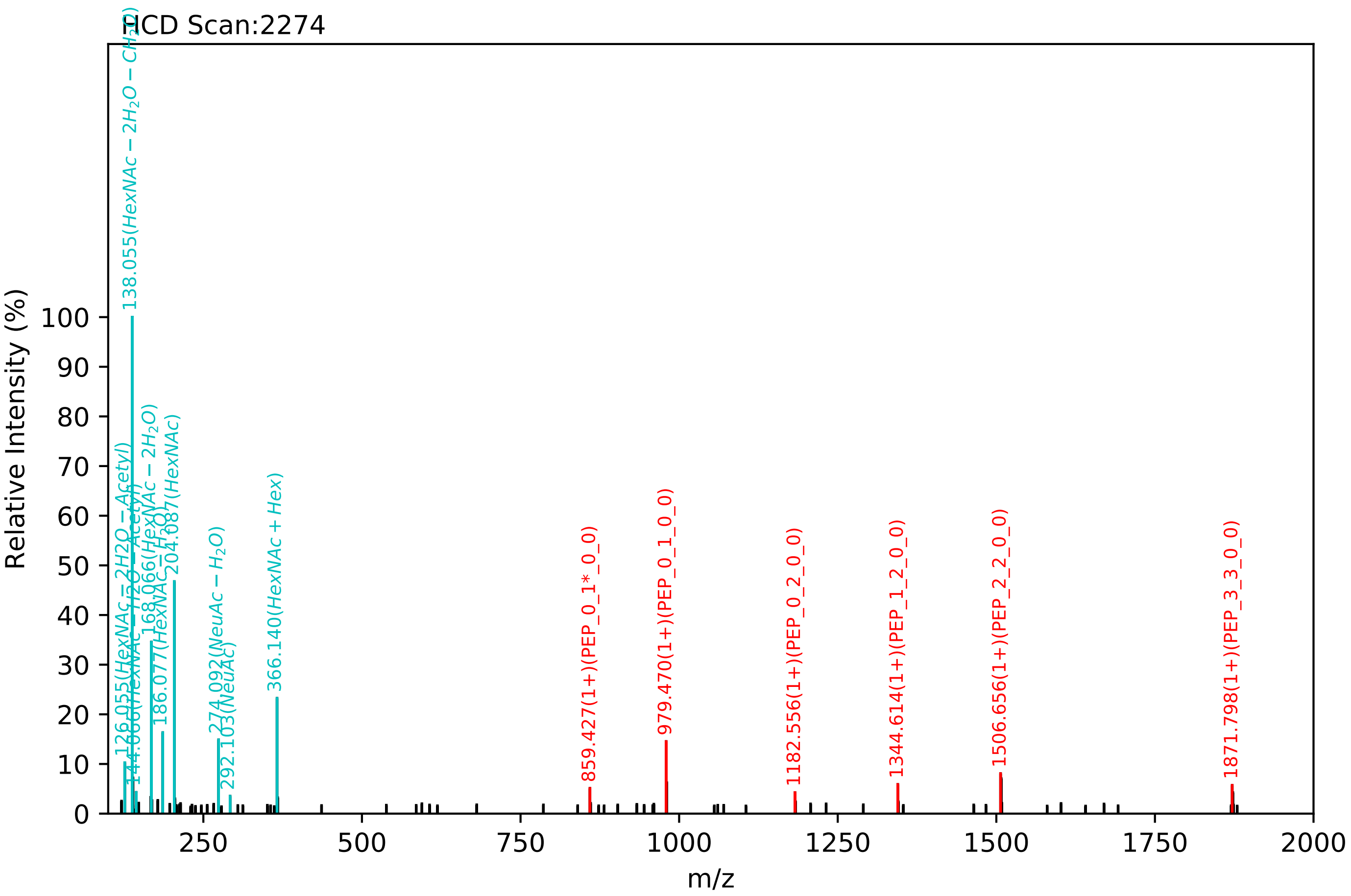

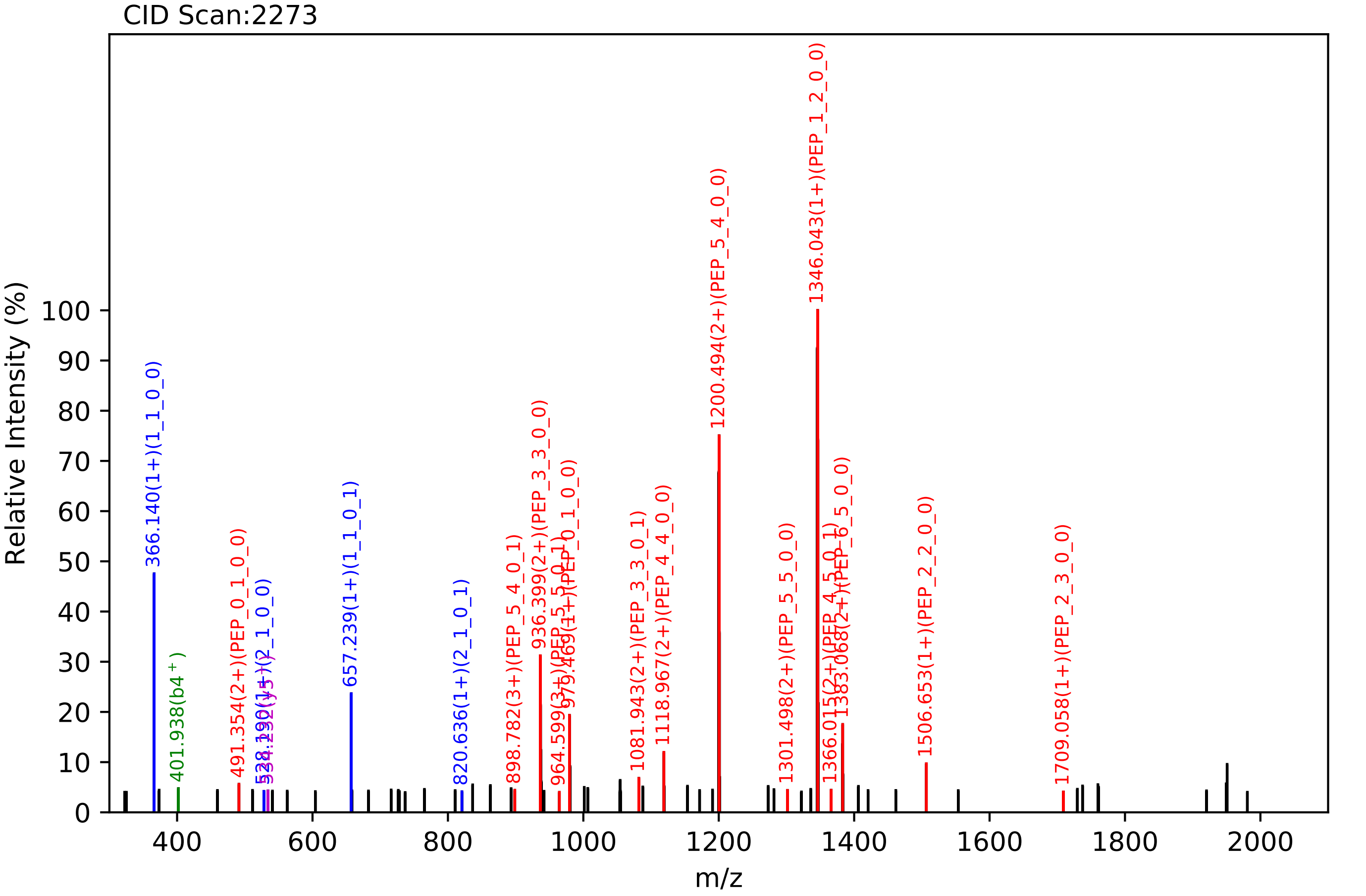

Training set no. 11, Experiment: AGP exp_2

ENGTISR(=PEP)_6_5_0_1, m/z:1019.07(3+), RT:16.85, Y-score:81.54

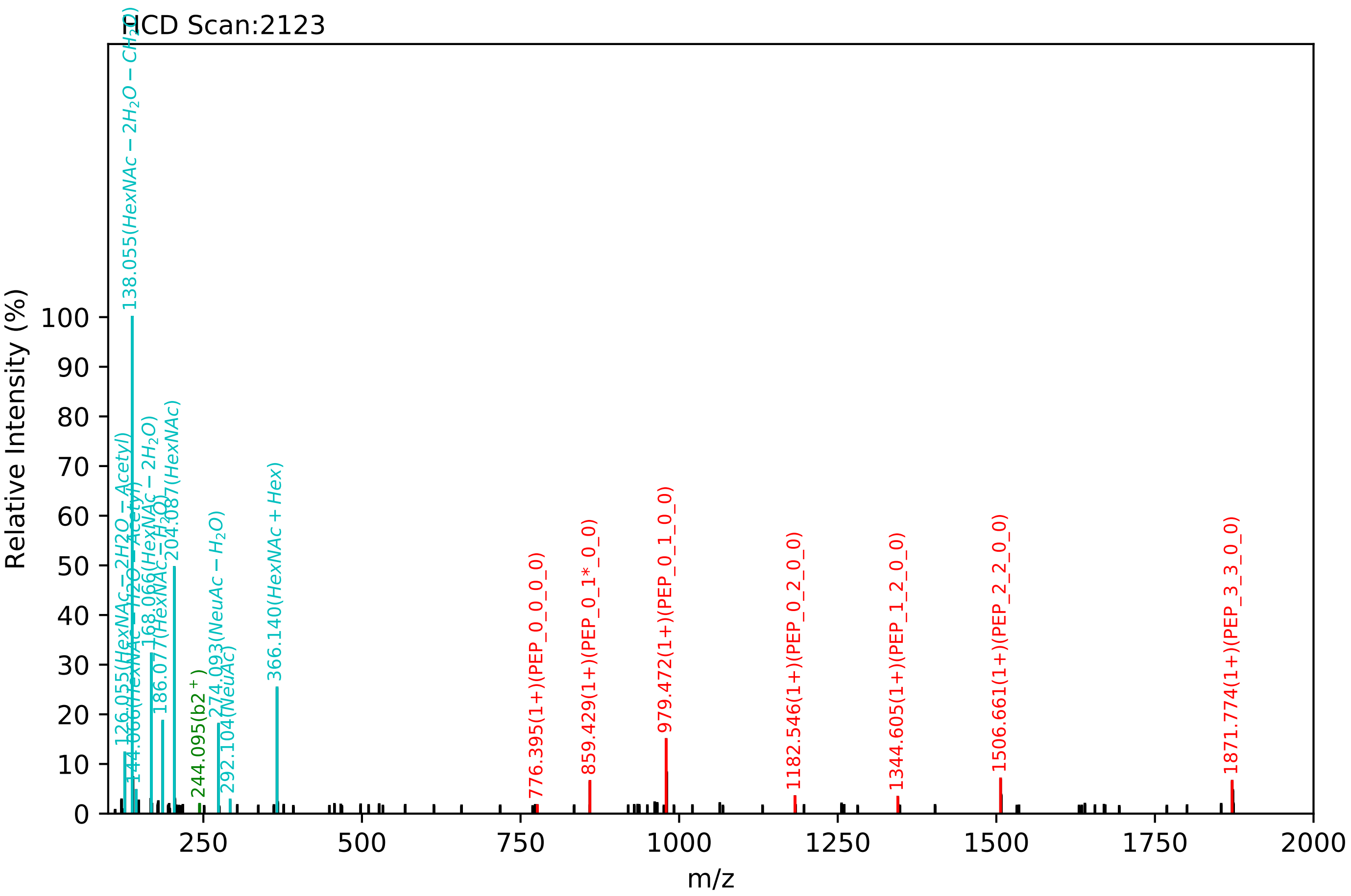

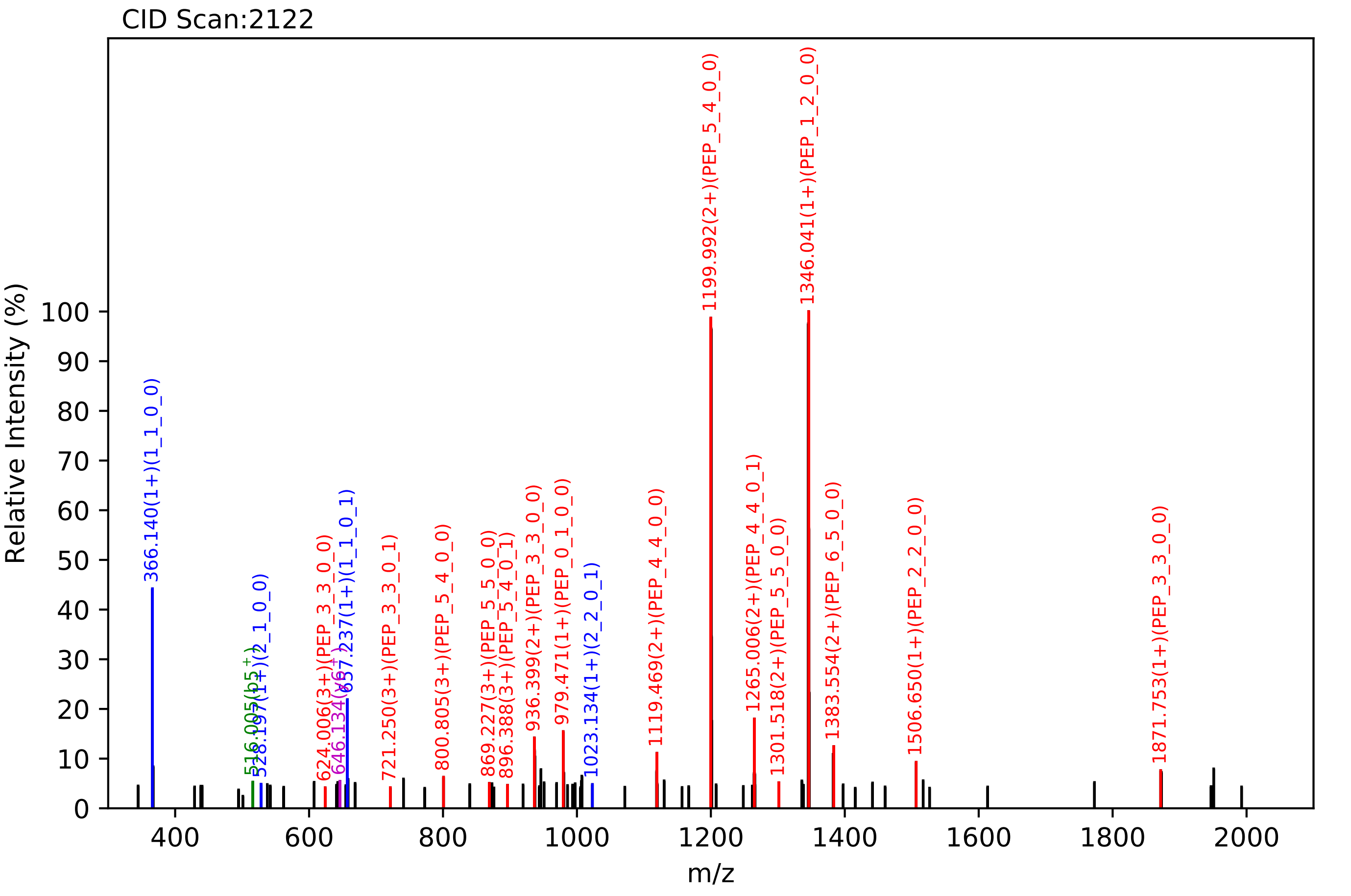

Training set no. 12, Experiment: AGP exp_2

ENGTISR(=PEP)_6_5_0_2, m/z:1116.10(3+), RT:20.54, Y-score:94.27

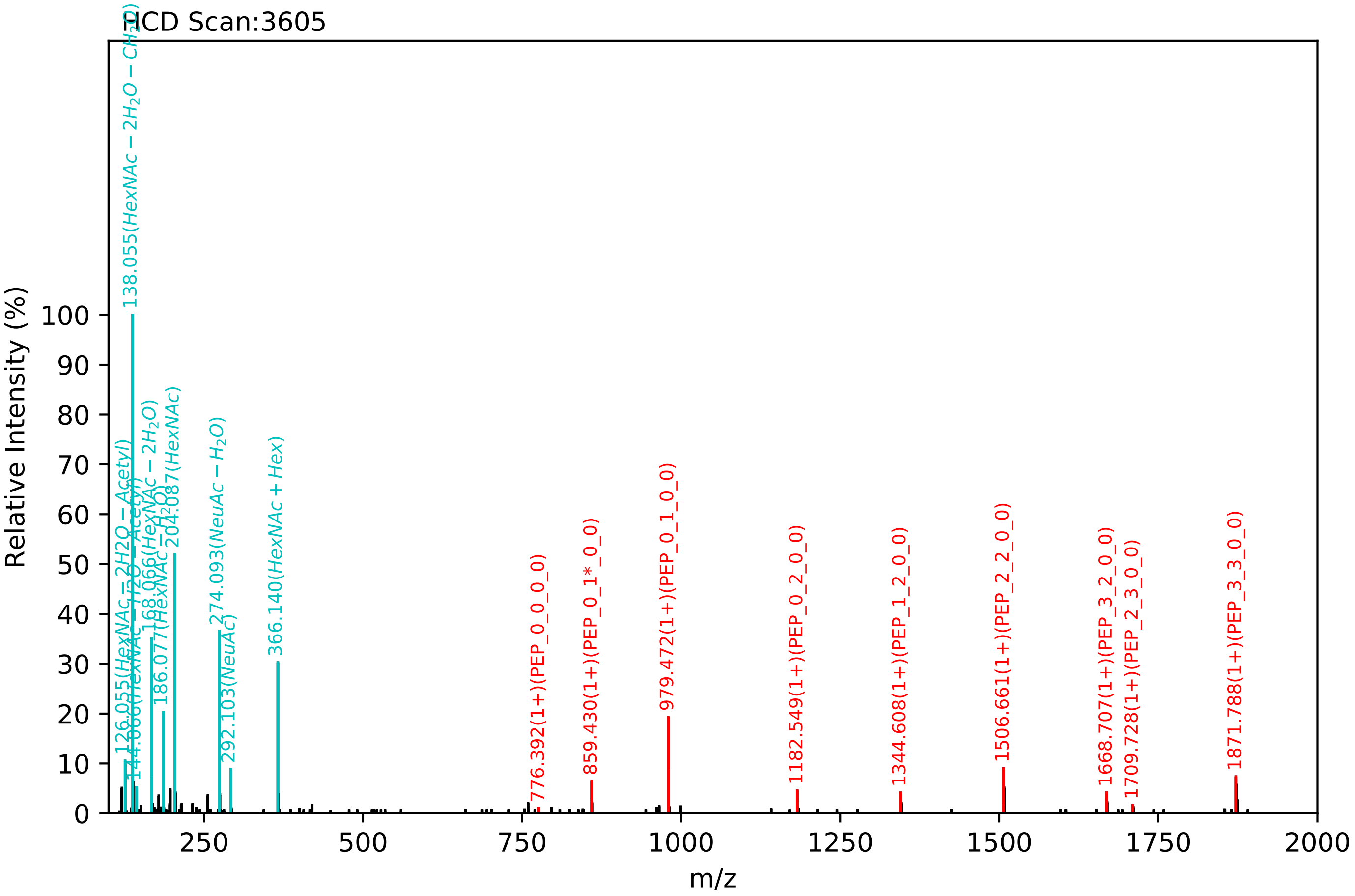

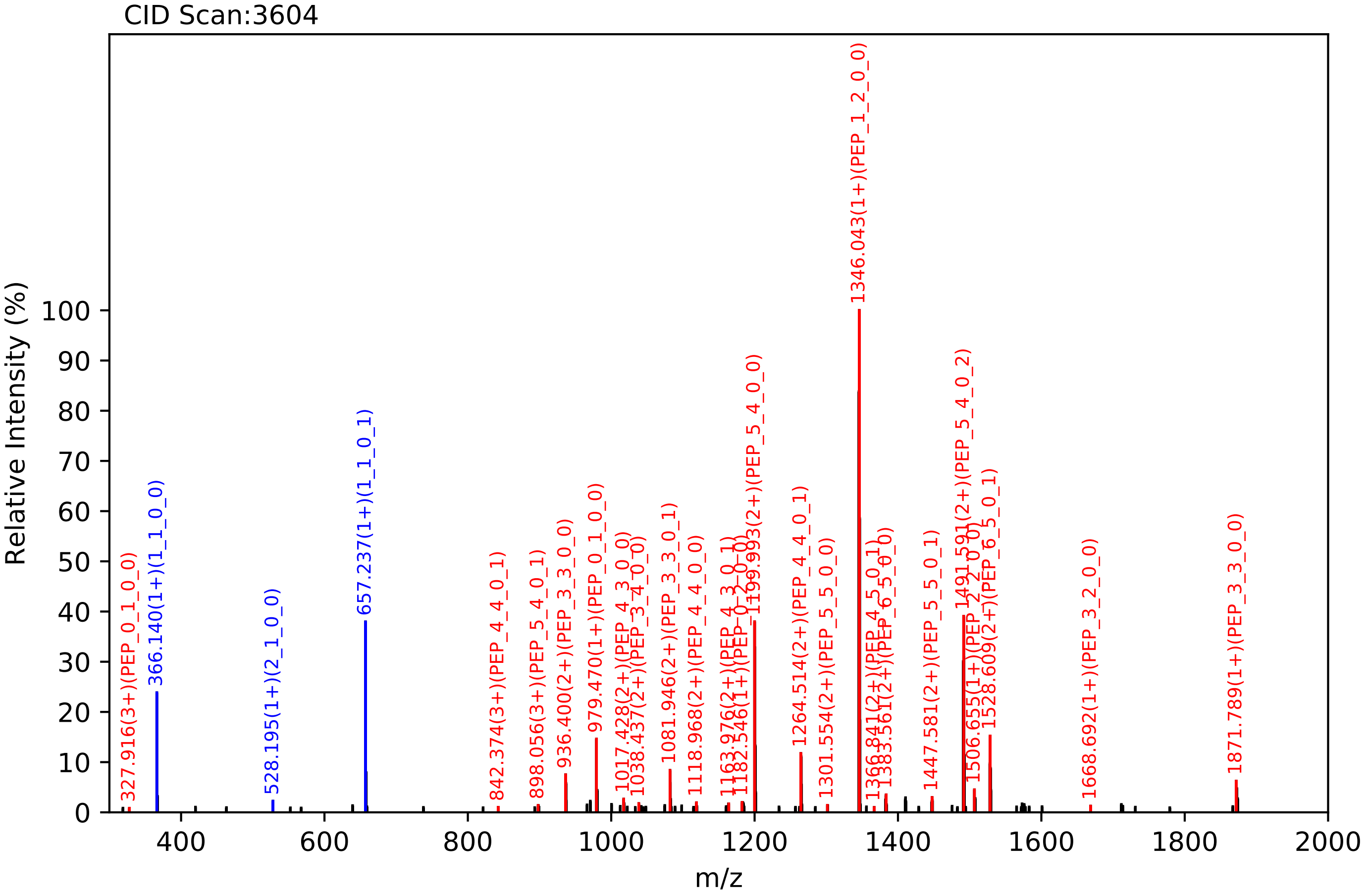

Training set no. 13, Experiment: AGP exp_1

ENGTISR(=PEP)_6_5_0_2, m/z:1116.11(3+), RT:21.26, Y-score:92.76

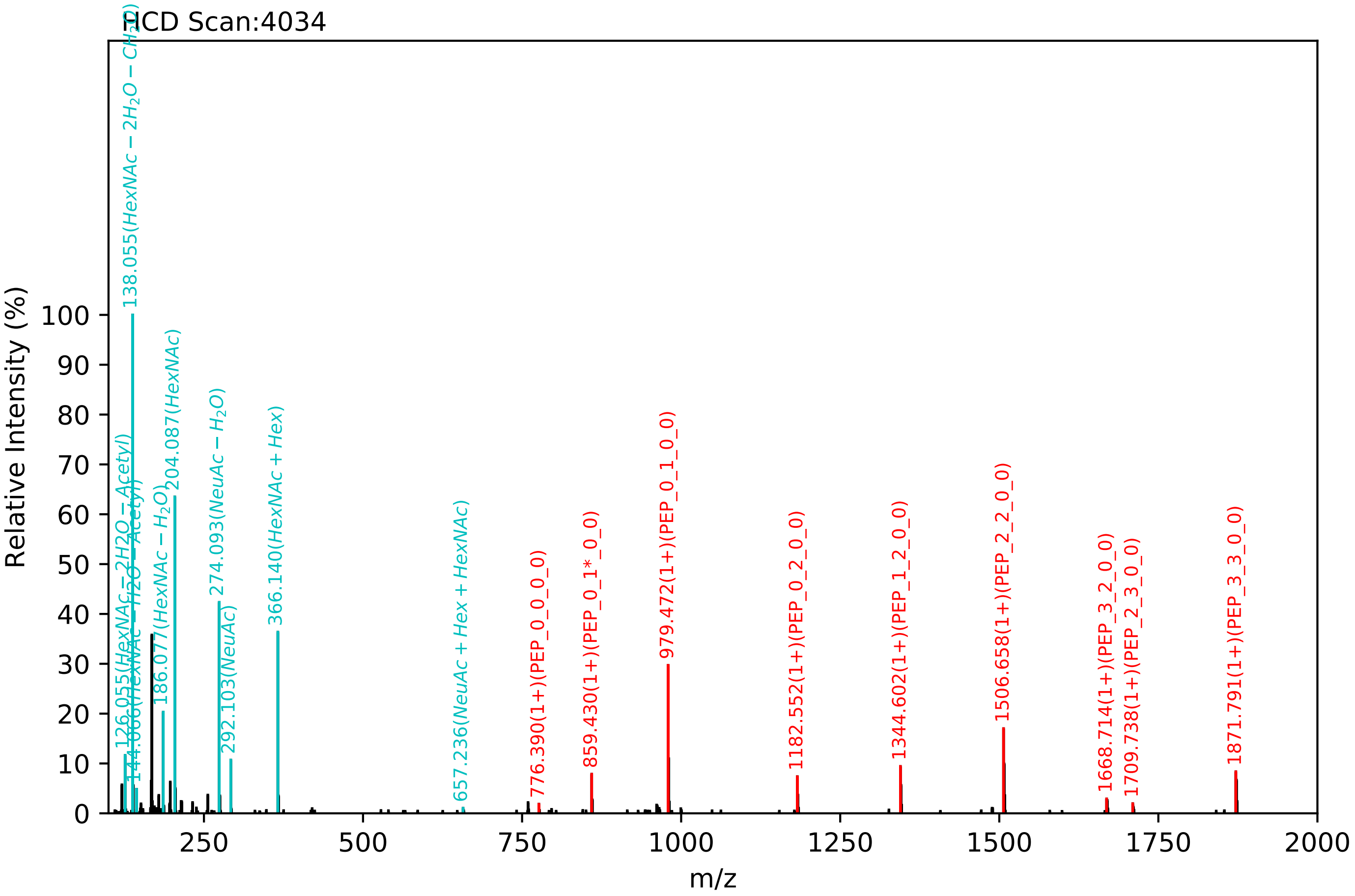

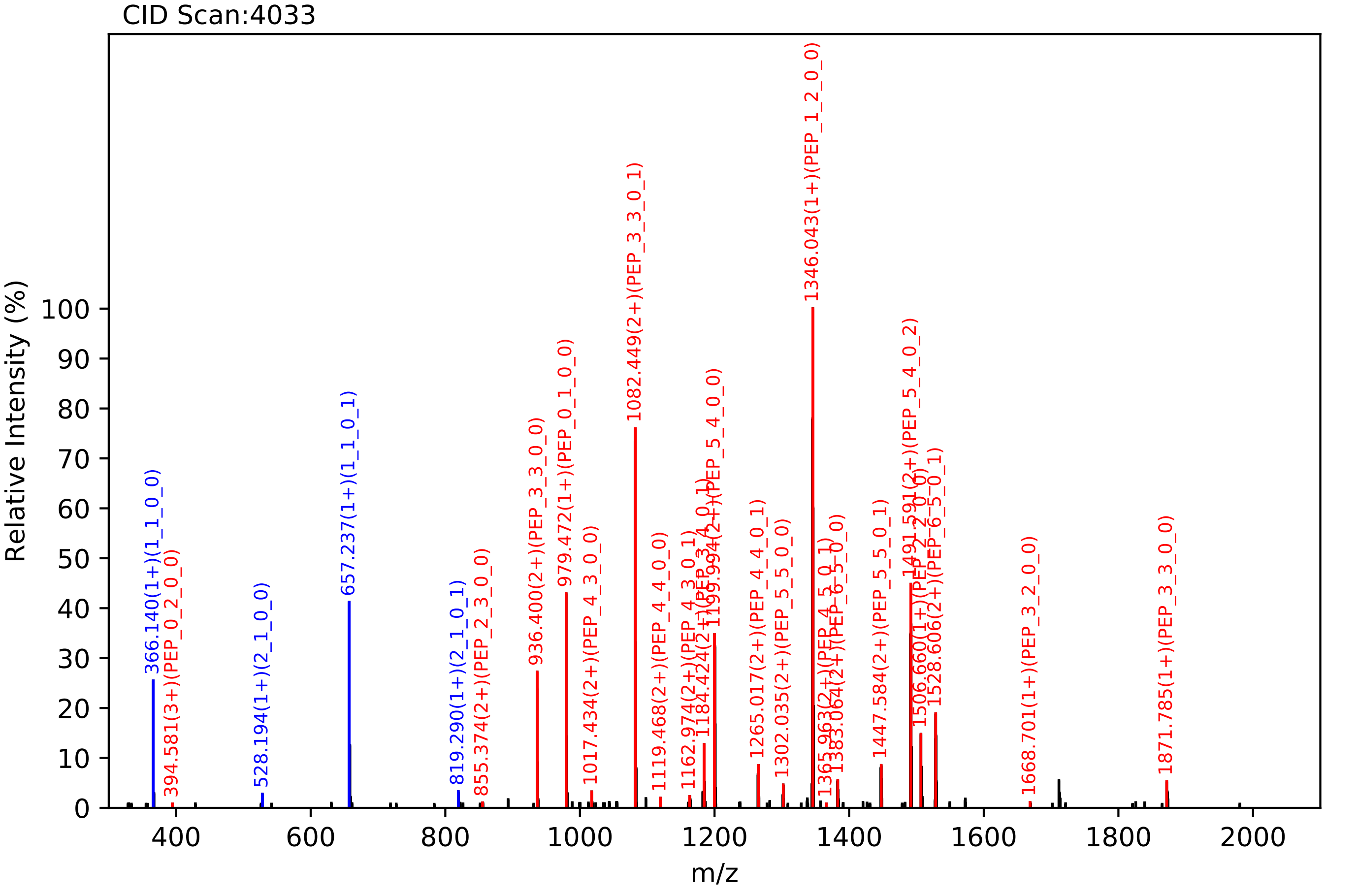

Training set no. 14, Experiment: AGP exp_2

ENGTISR(=PEP)_6_5_0_2, m/z:1116.11(3+), RT:21.15, Y-score:92.75

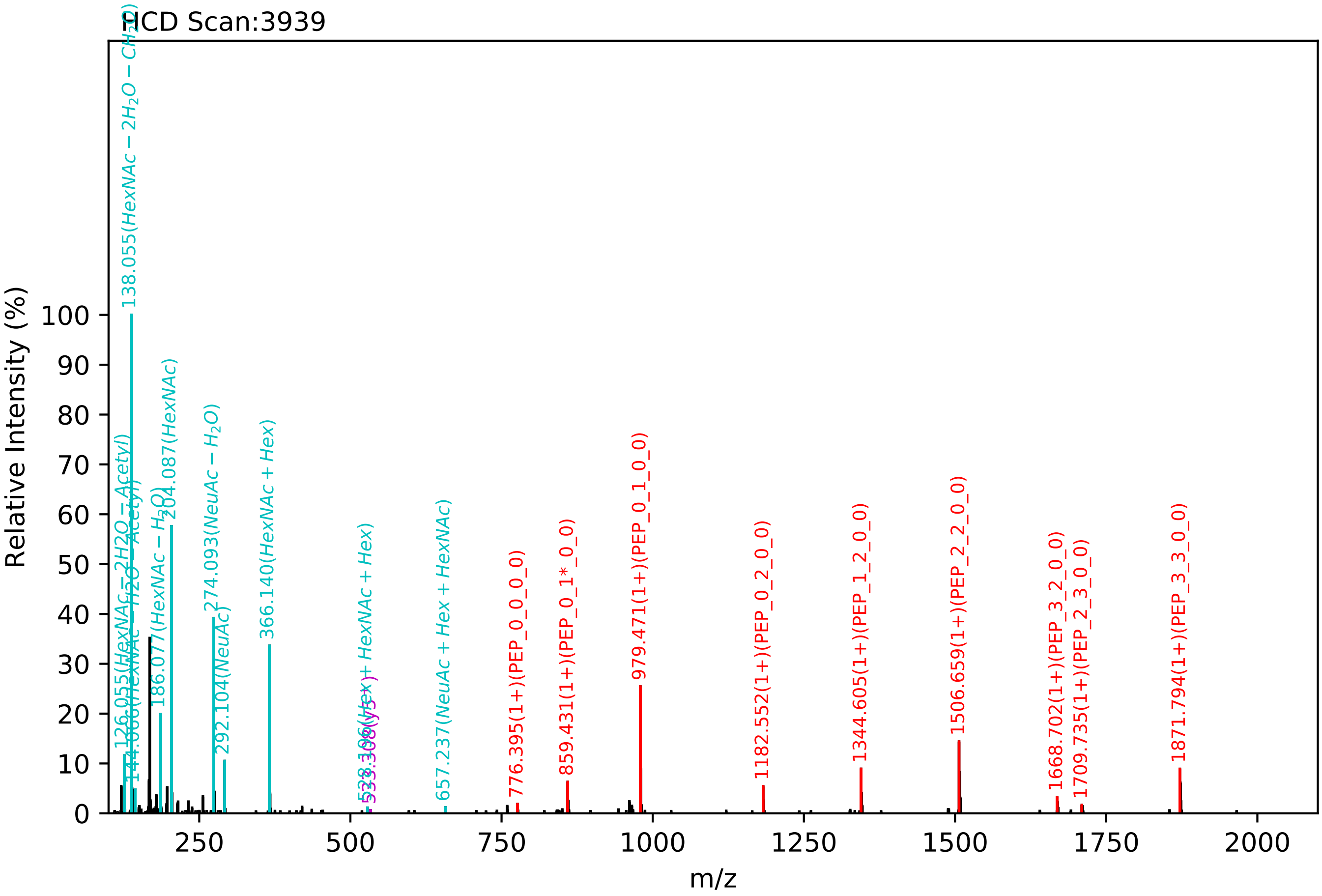

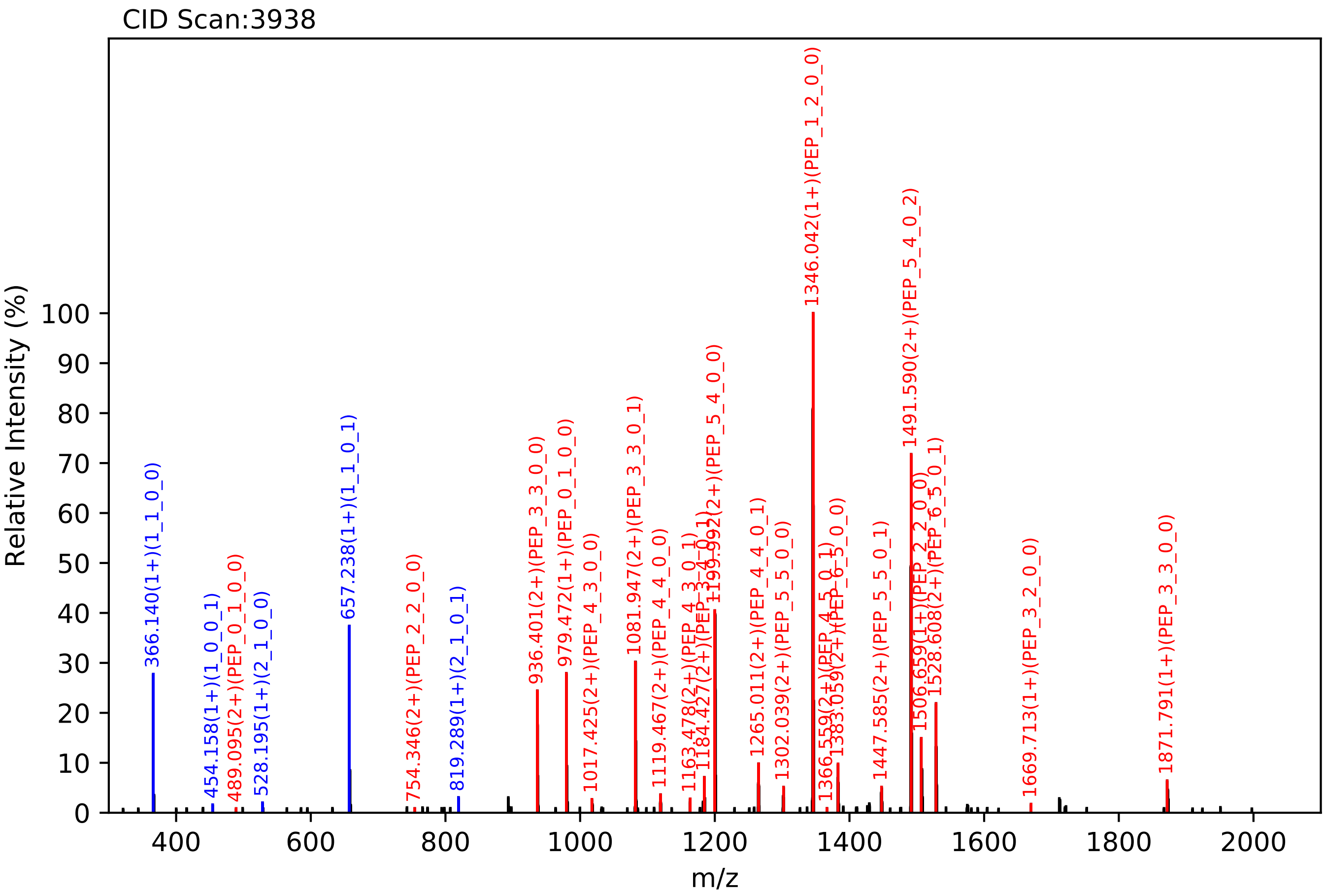

Training set no. 15, Experiment: AGP exp_1

ENGTISR(=PEP)_6_5_0_2, m/z:1115.77(3+), RT:20.75, Y-score:92.72

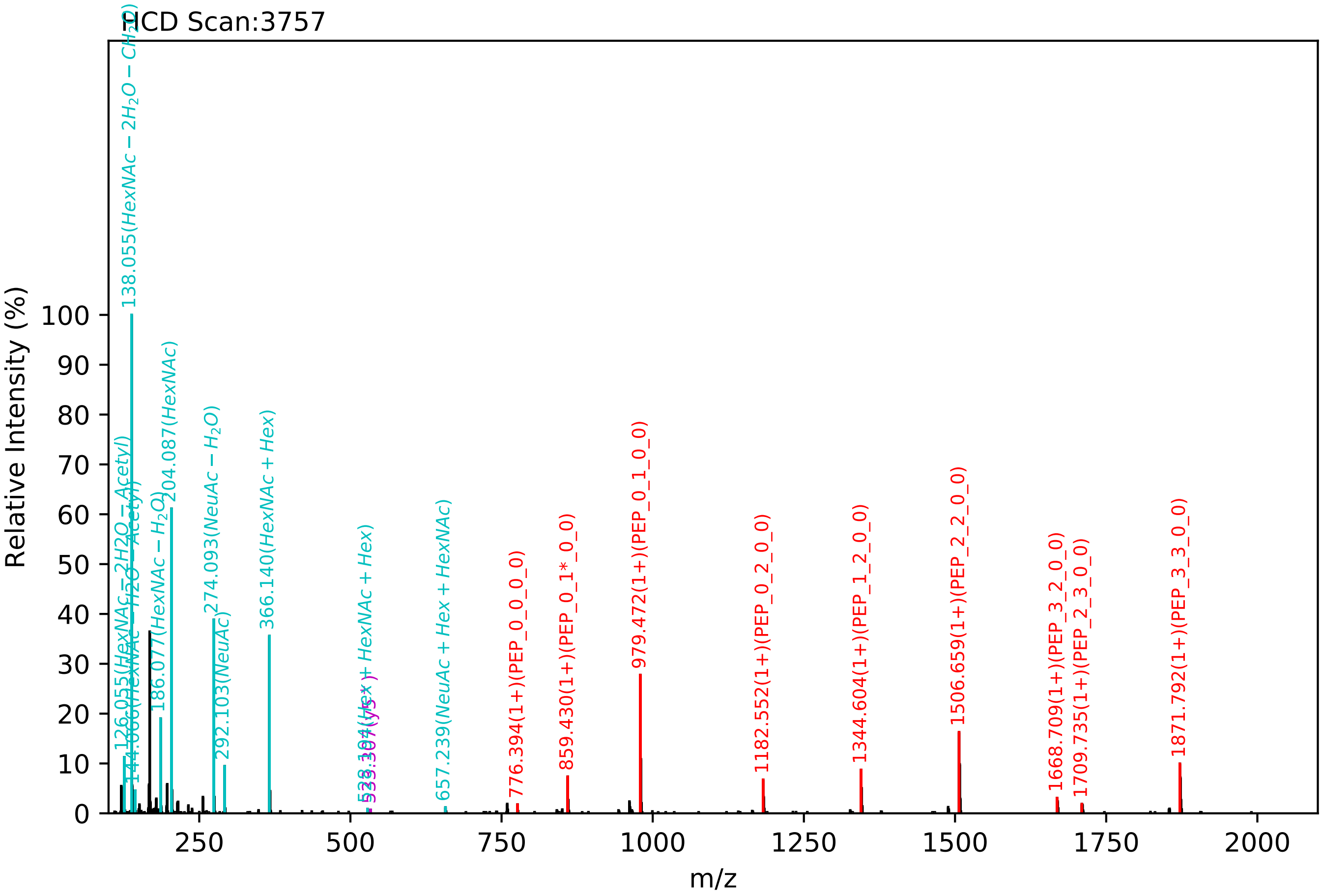

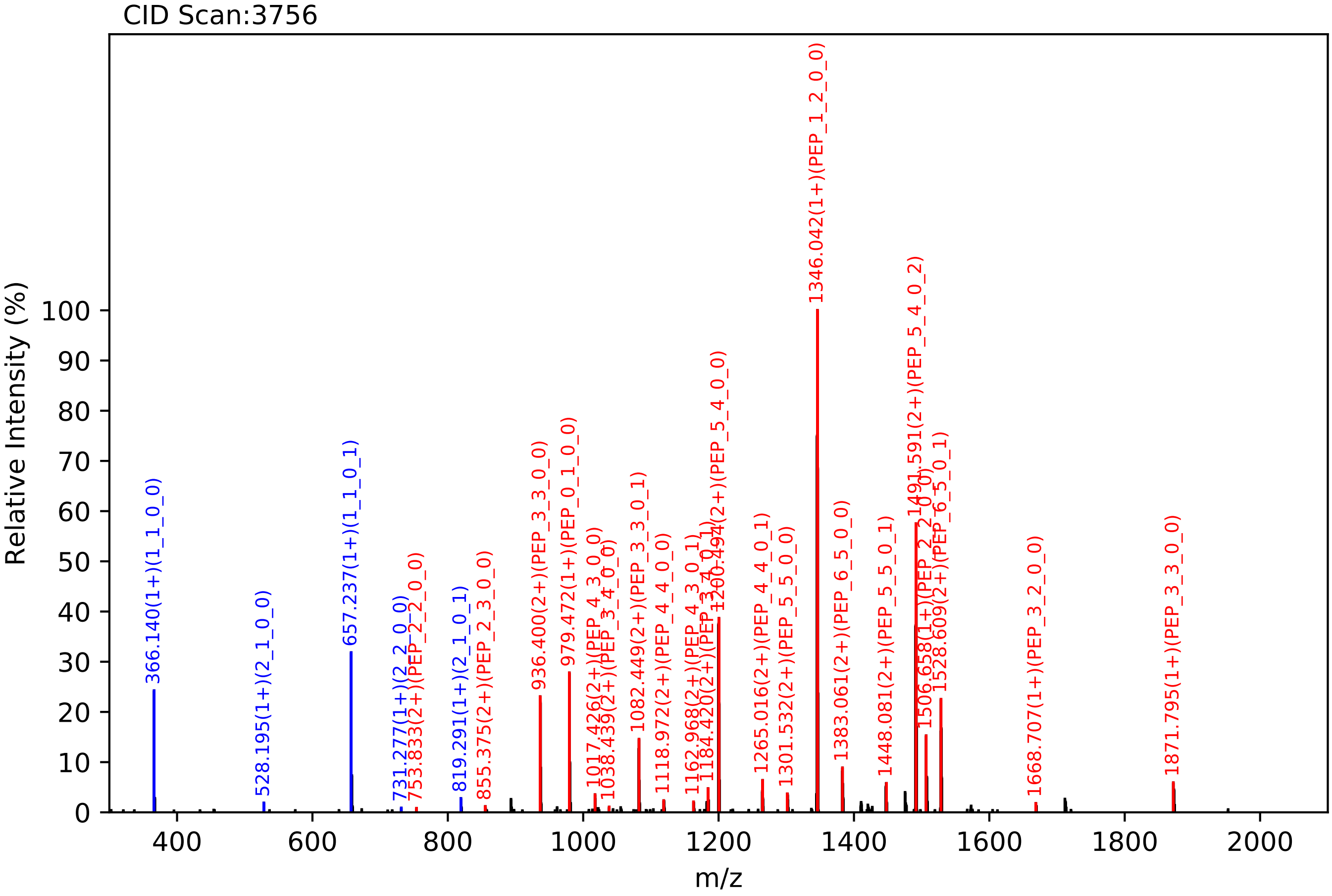

Training set no. 16, Experiment: AGP exp_2

ENGTISR(=PEP)_6_5_0_2, m/z:1673.65(2+), RT:20.86, Y-score:86.80

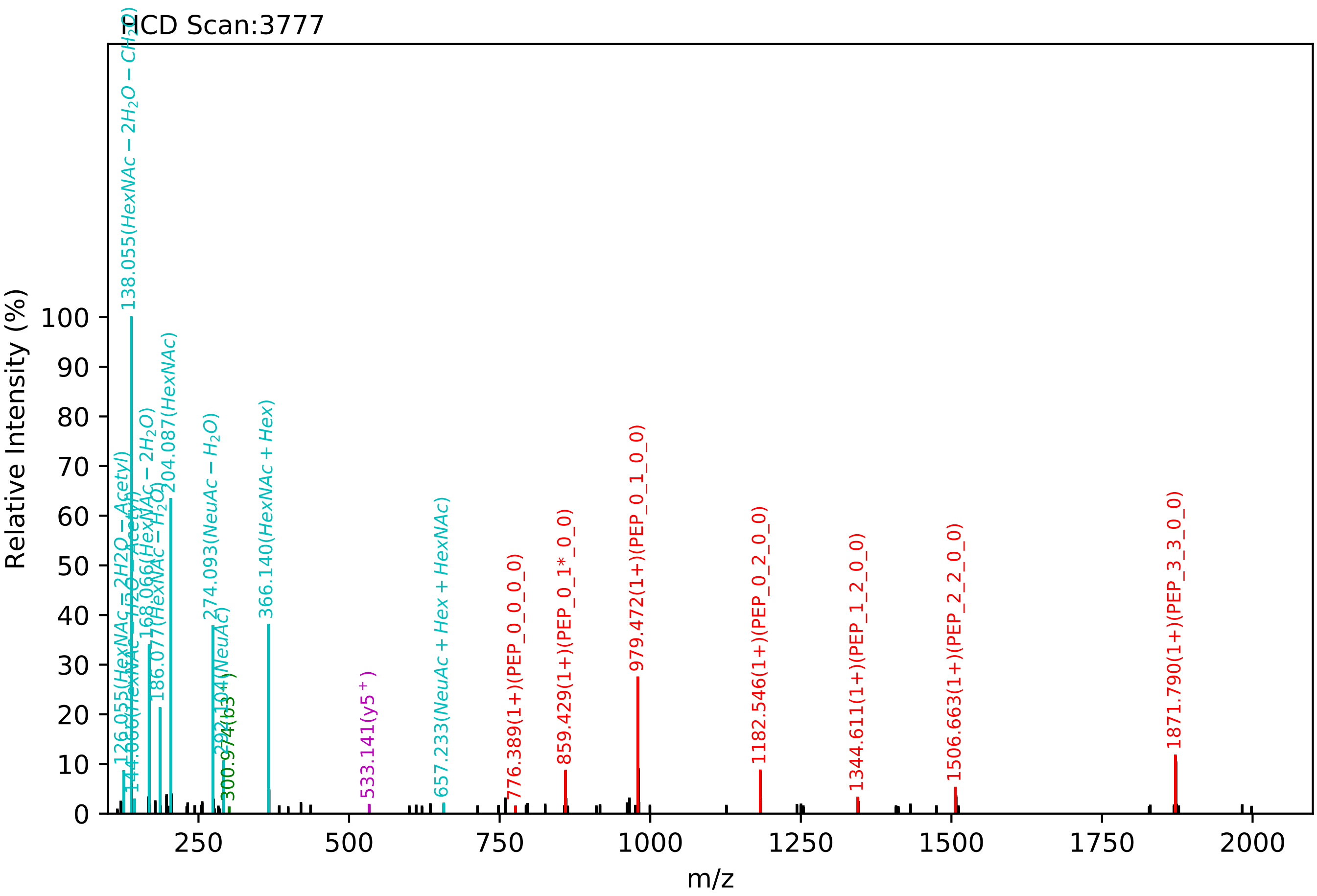

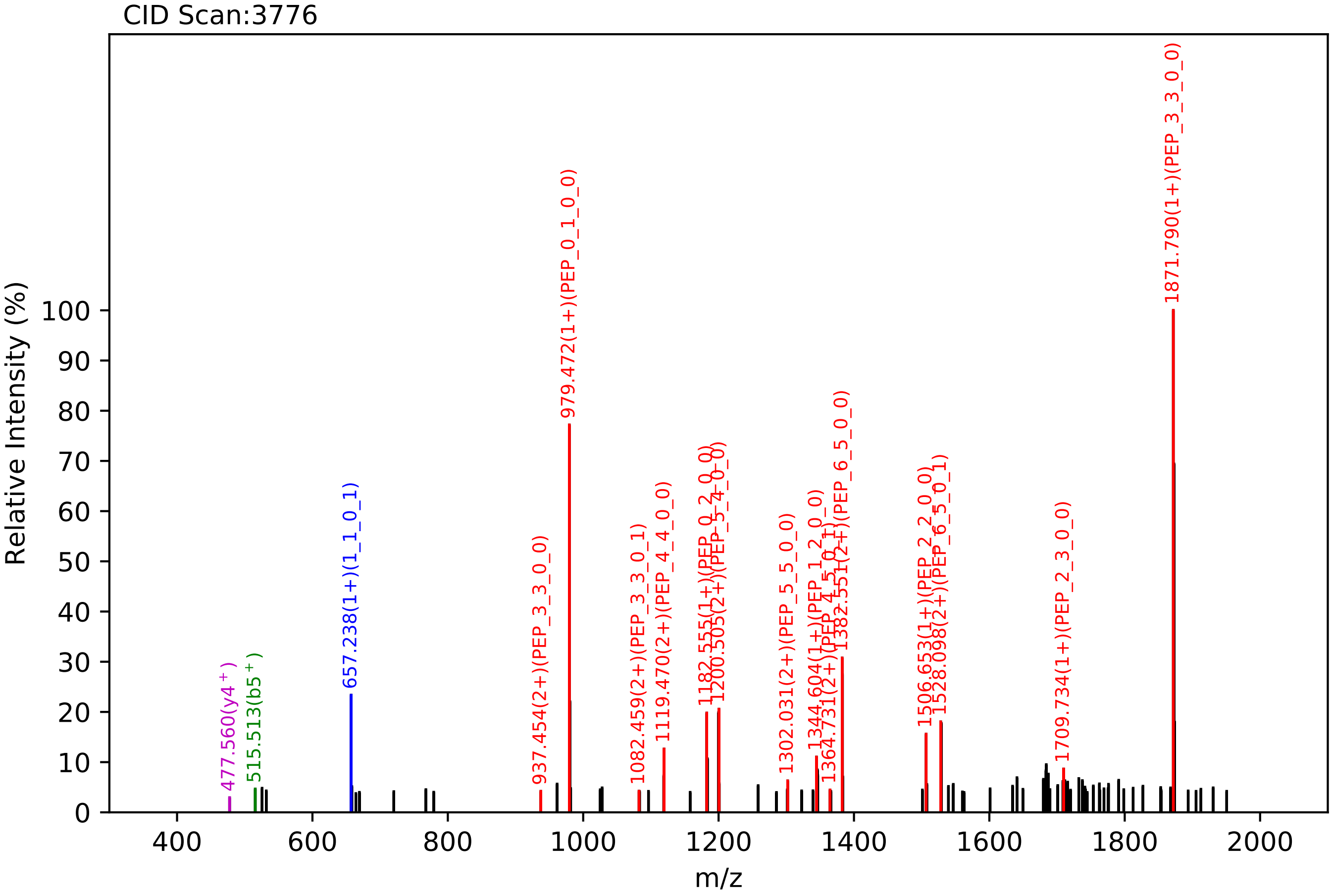

Training set no. 17, Experiment: AGP exp_1

ENGTISR(=PEP)_6_5_0_3, m/z:1819.20(2+), RT:22.74, Y-score:92.35

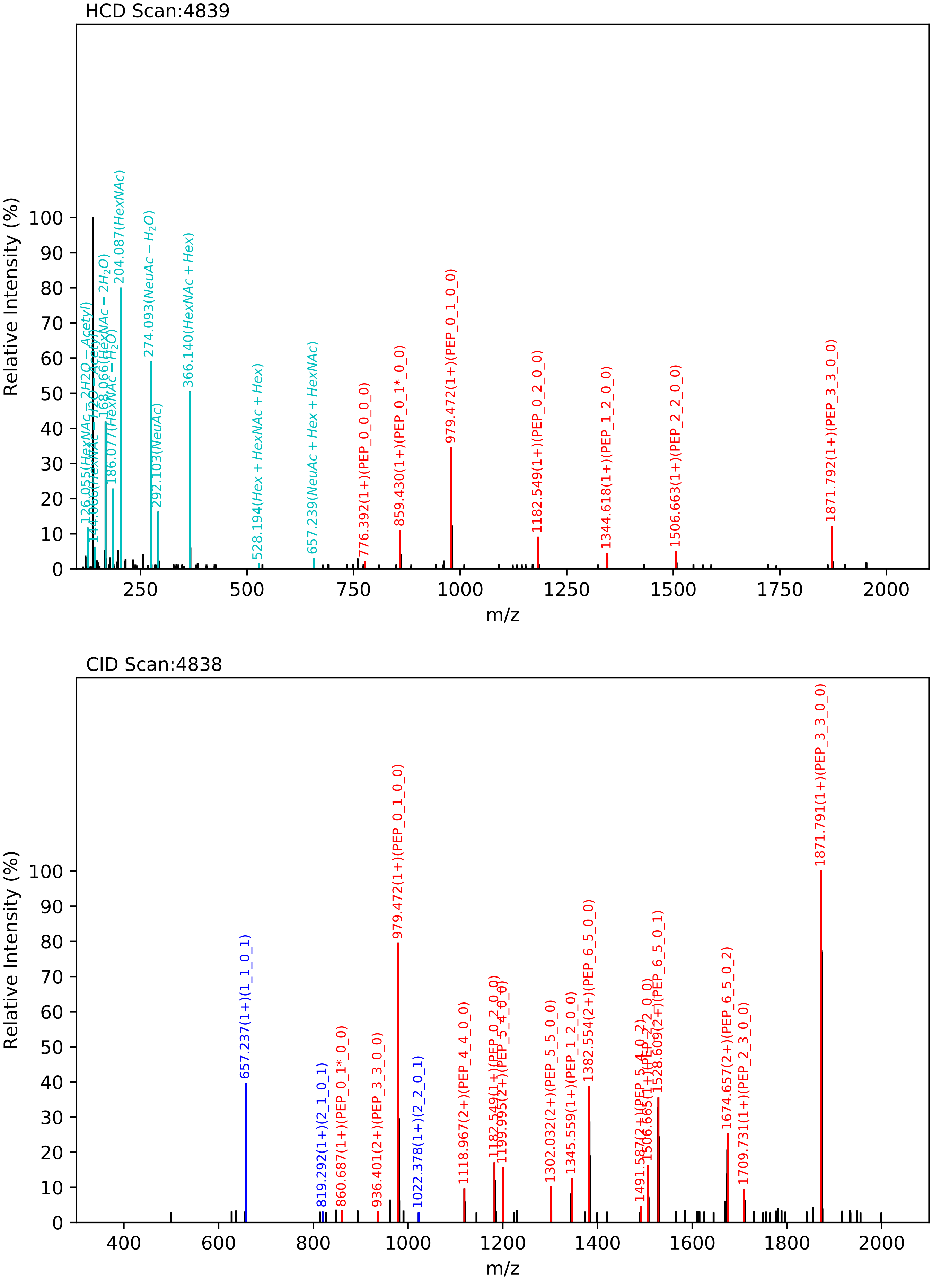

Training set no. 18, Experiment: AGP exp_1

ENGTISR(=PEP)_6_5_0_3, m/z:1213.14(3+), RT:22.59, Y-score:91.20

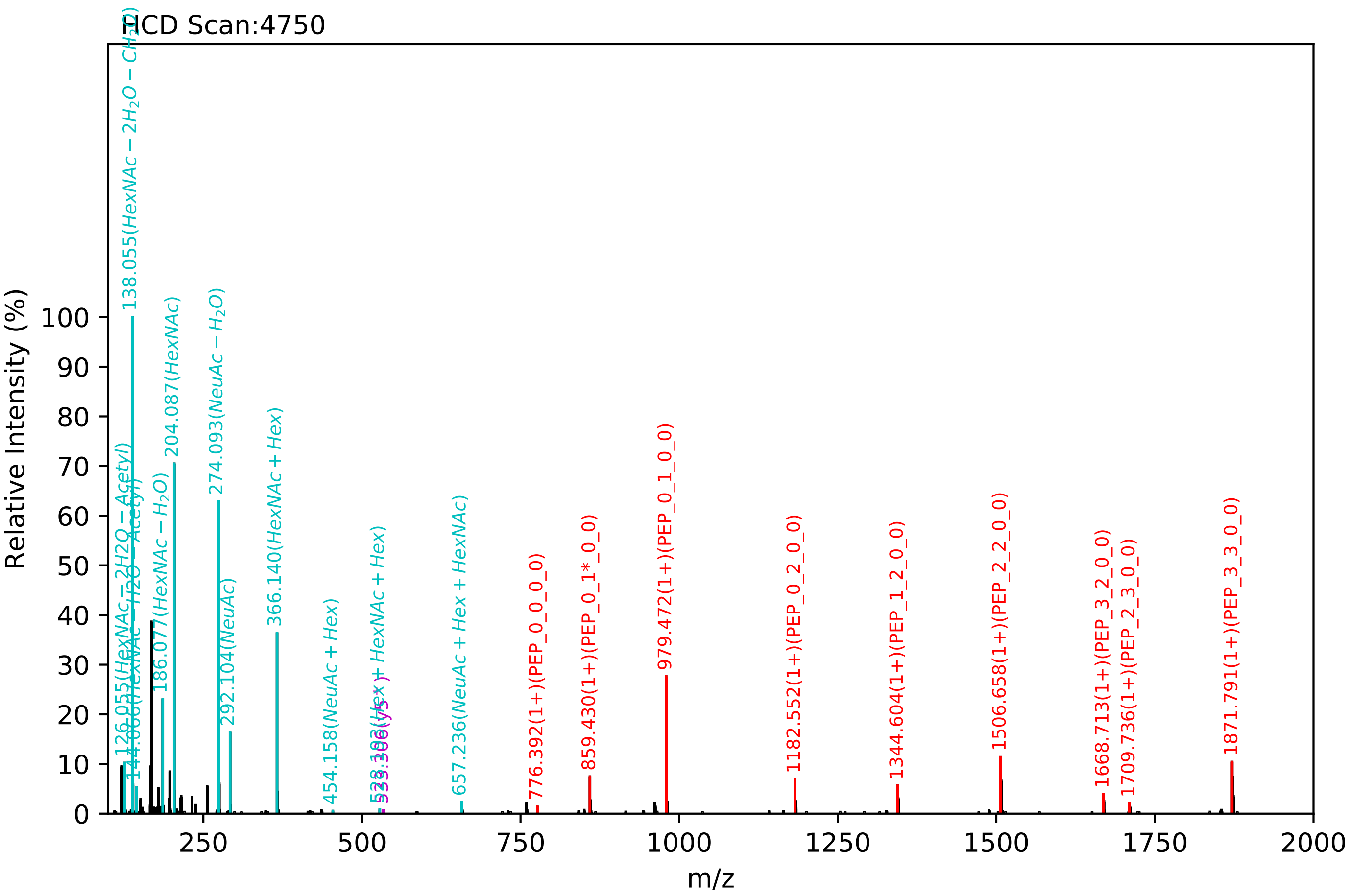

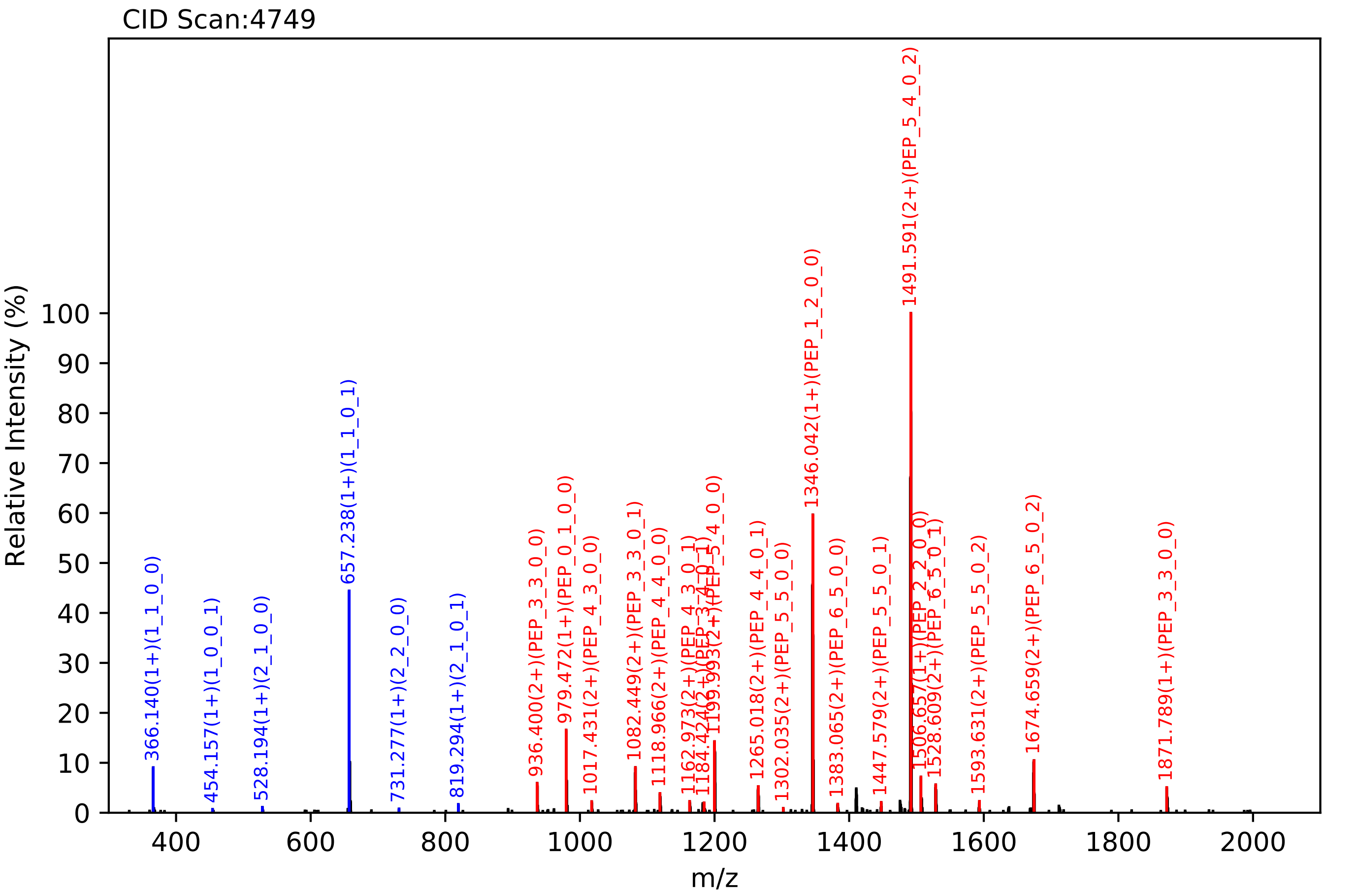

Training set no. 19, Experiment: AGP exp_2

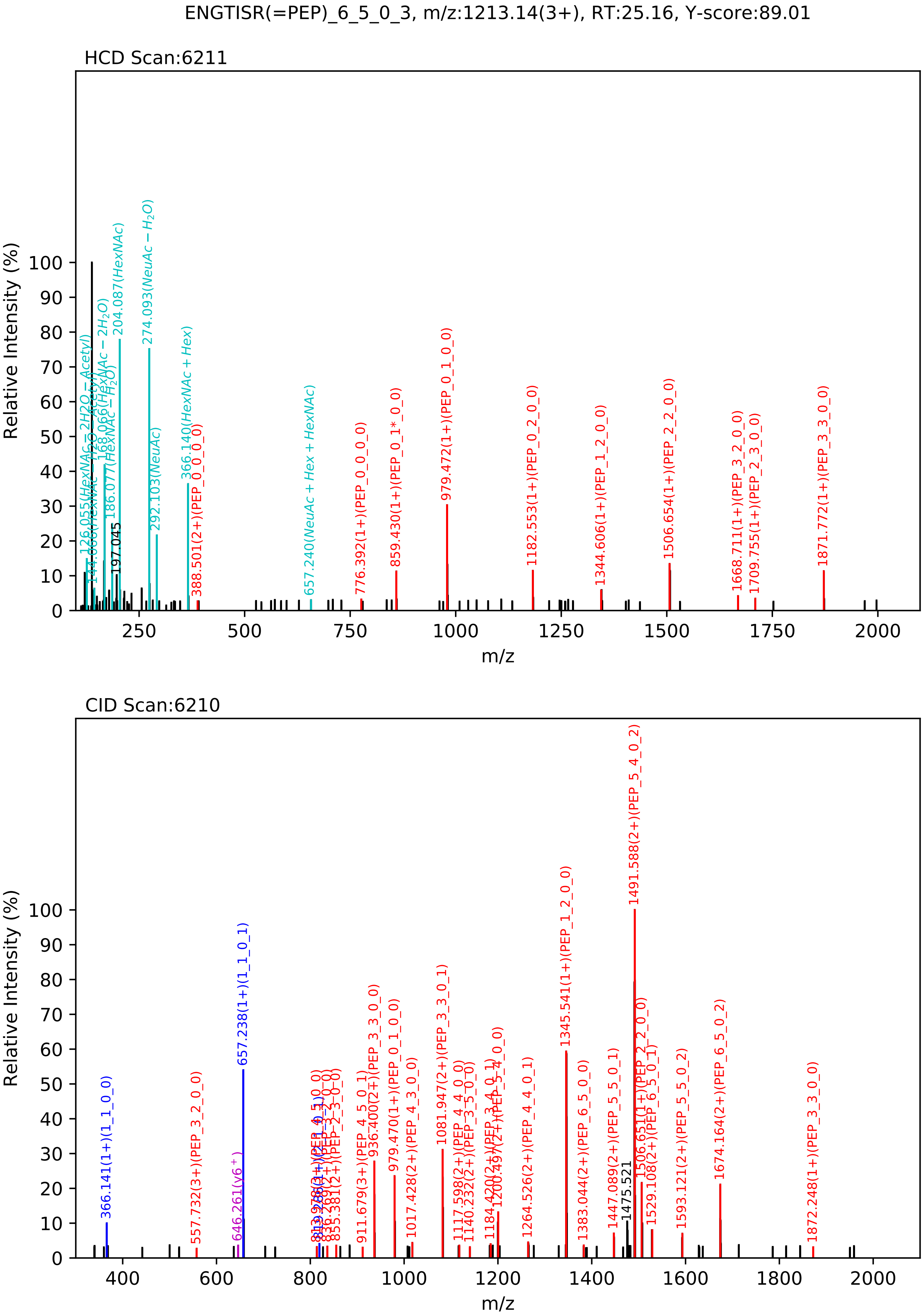

Training set no. 20, Experiment: AGP exp_1

ENGTISR(=PEP)_6_5_0_3, m/z:1213.14(3+), RT:23.17, Y-score:86.99

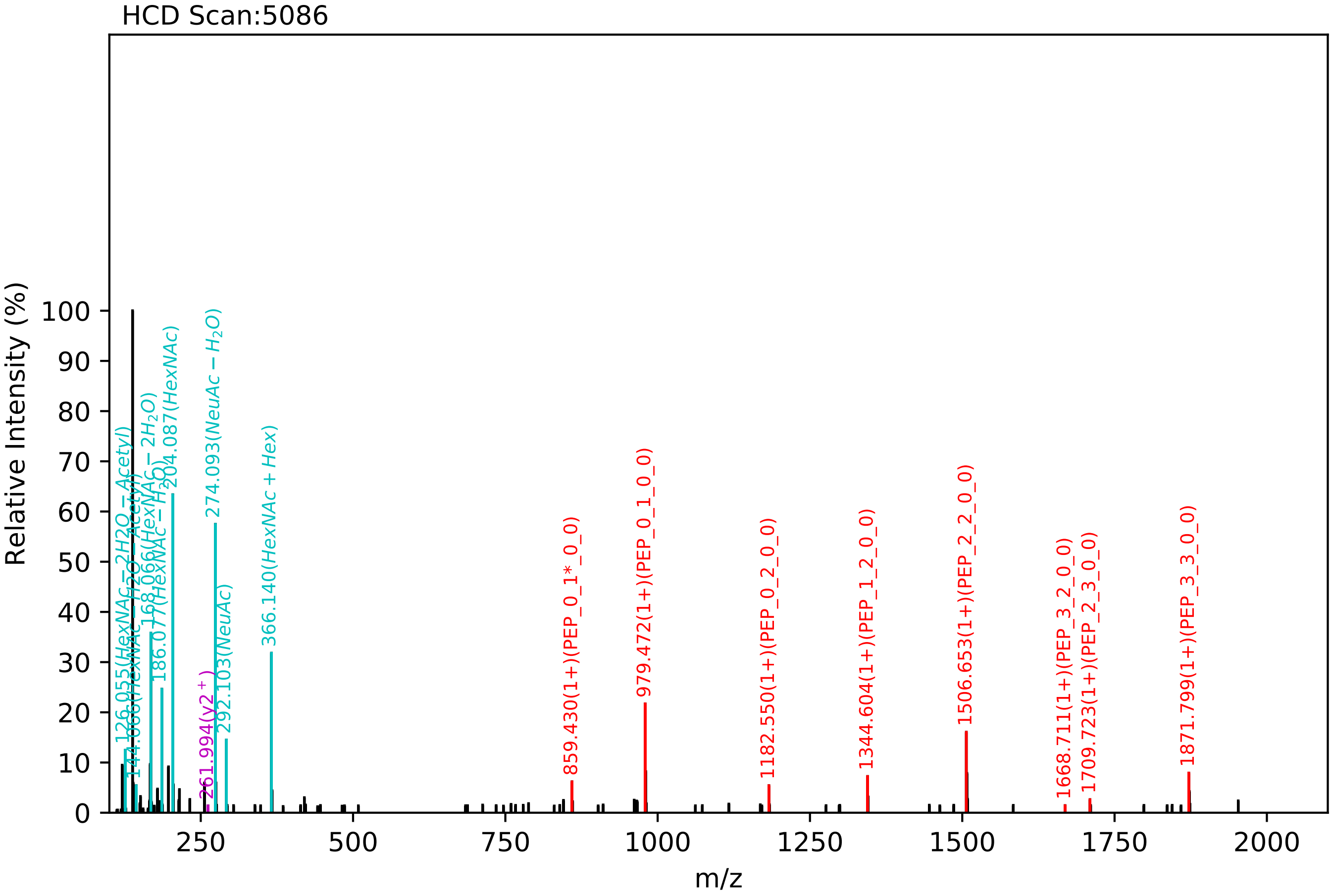

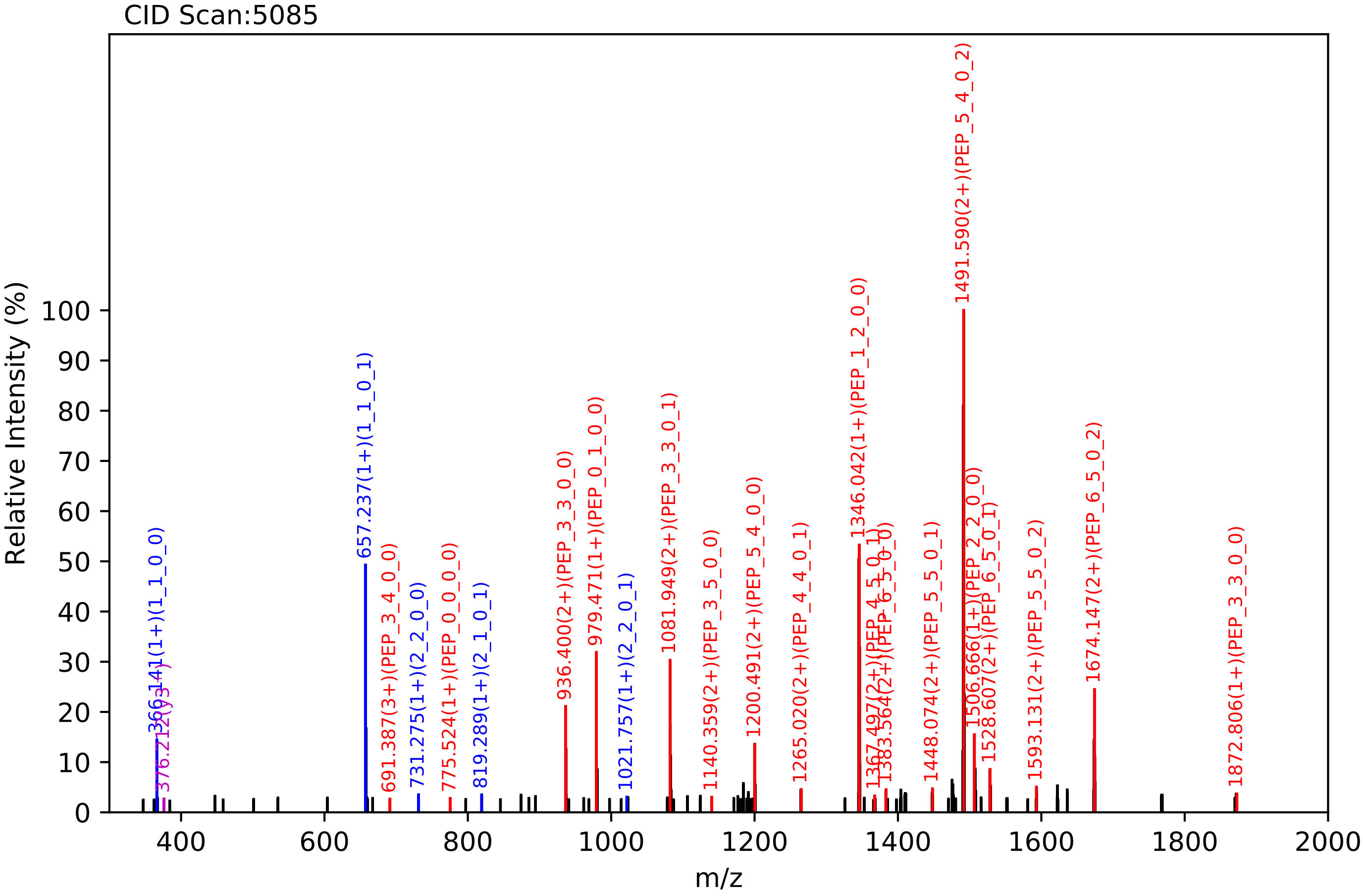

Training set no. 21, Experiment: AGP exp_2

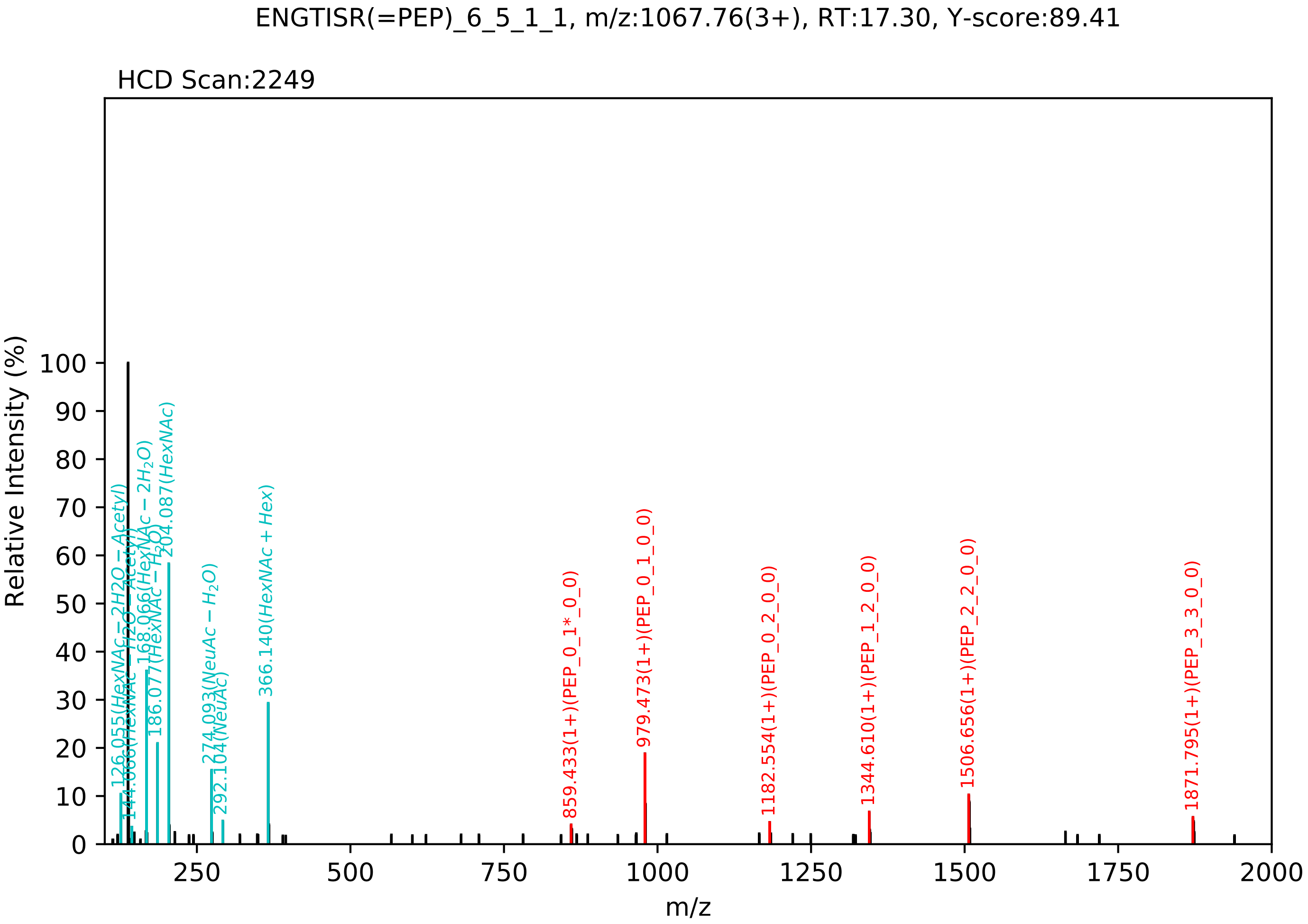

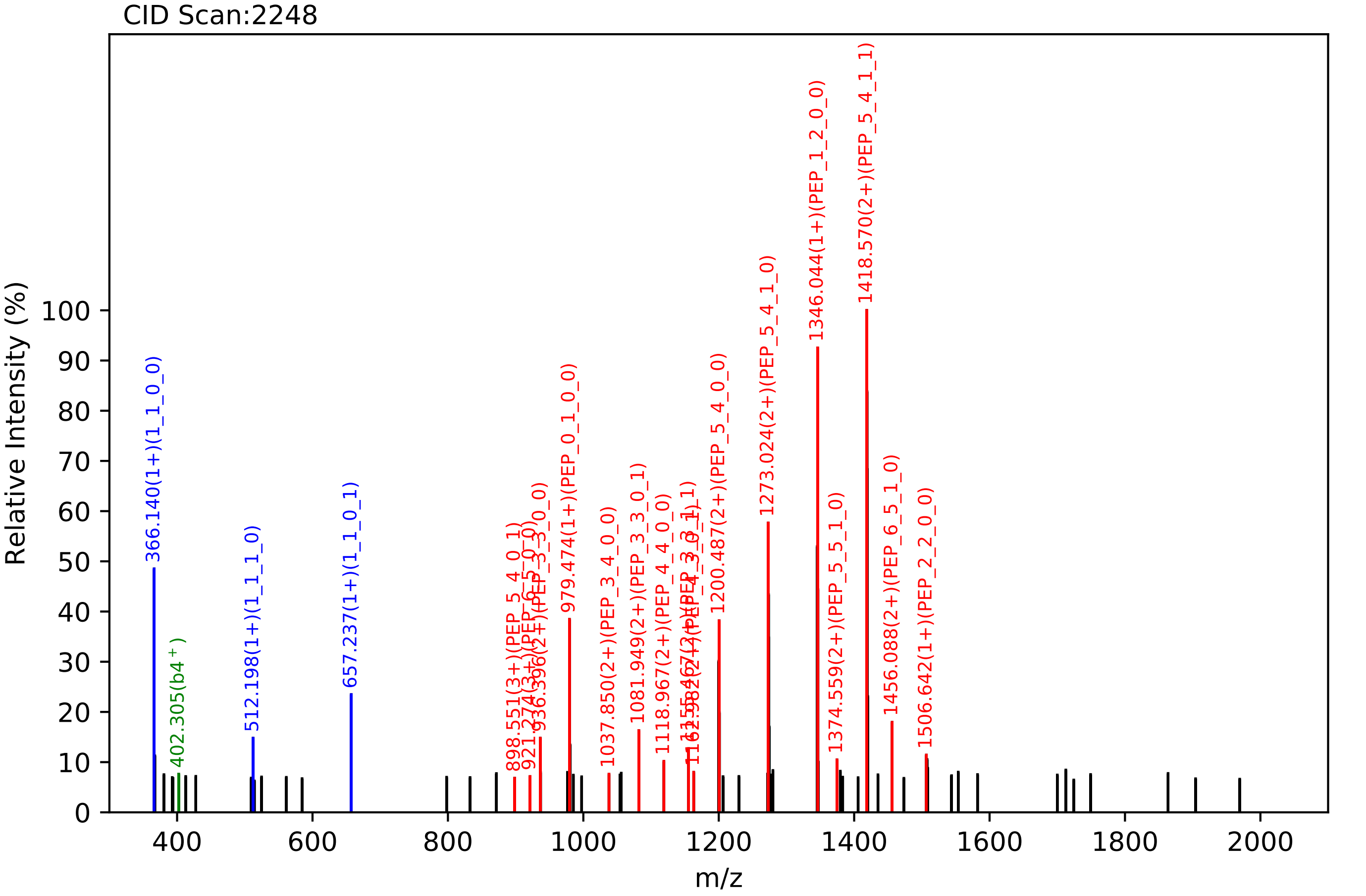

Training set no. 22, Experiment: AGP exp_1

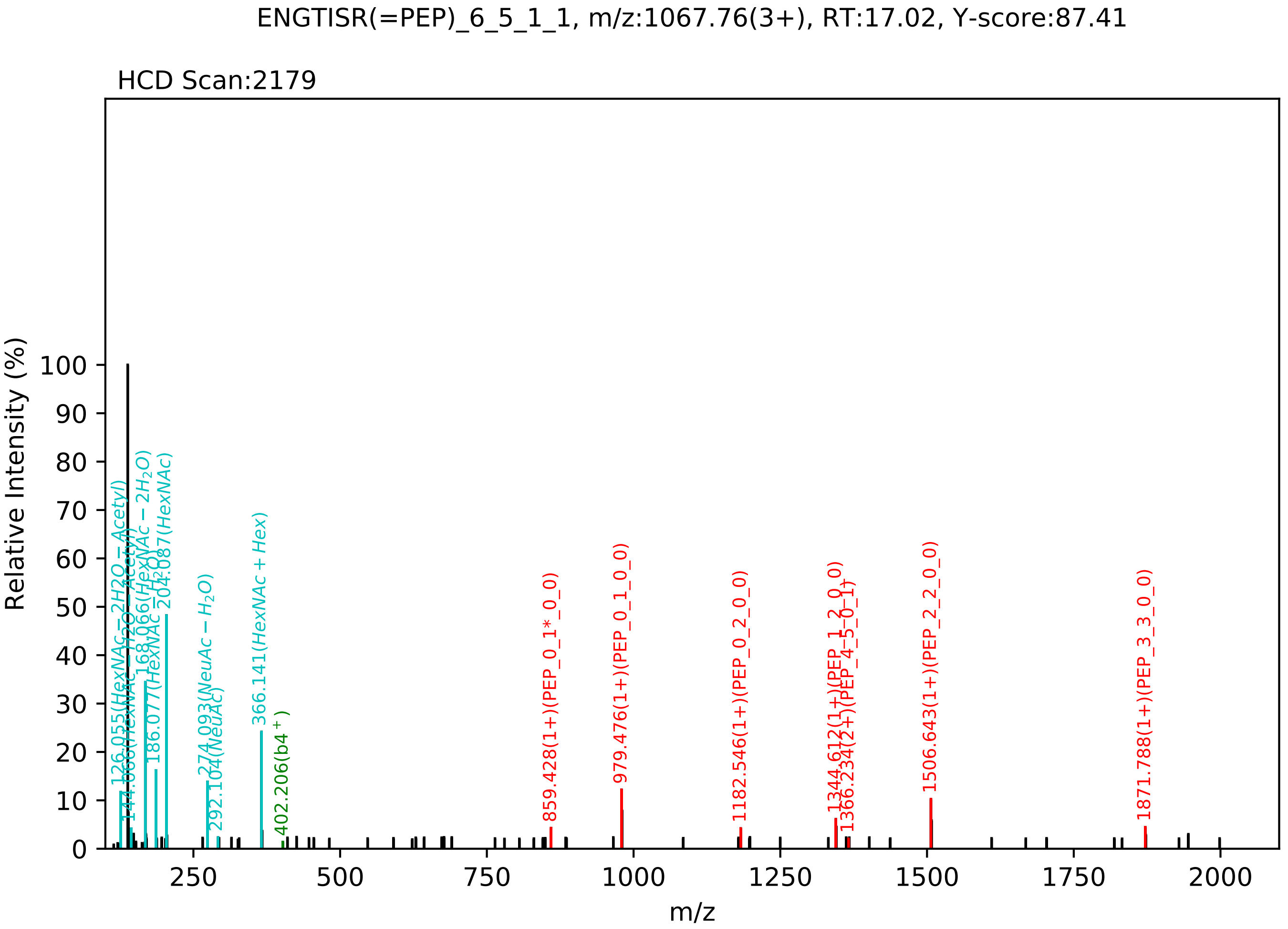

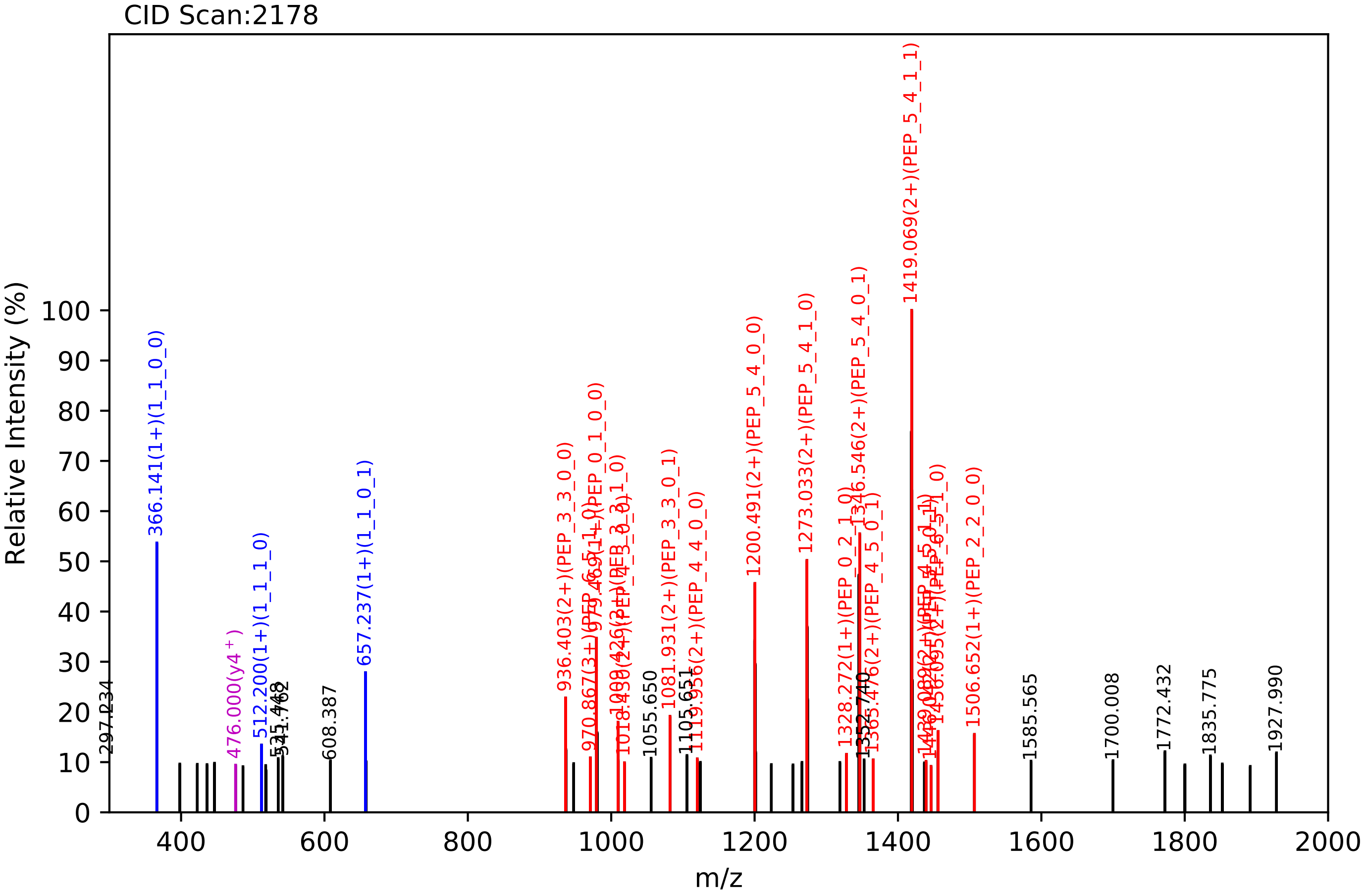

Training set no. 23, Experiment: AGP exp_1

ENGTISR(=PEP)_6_5_1_1, m/z:1067.76(3+), RT:17.53, Y-score:85.23

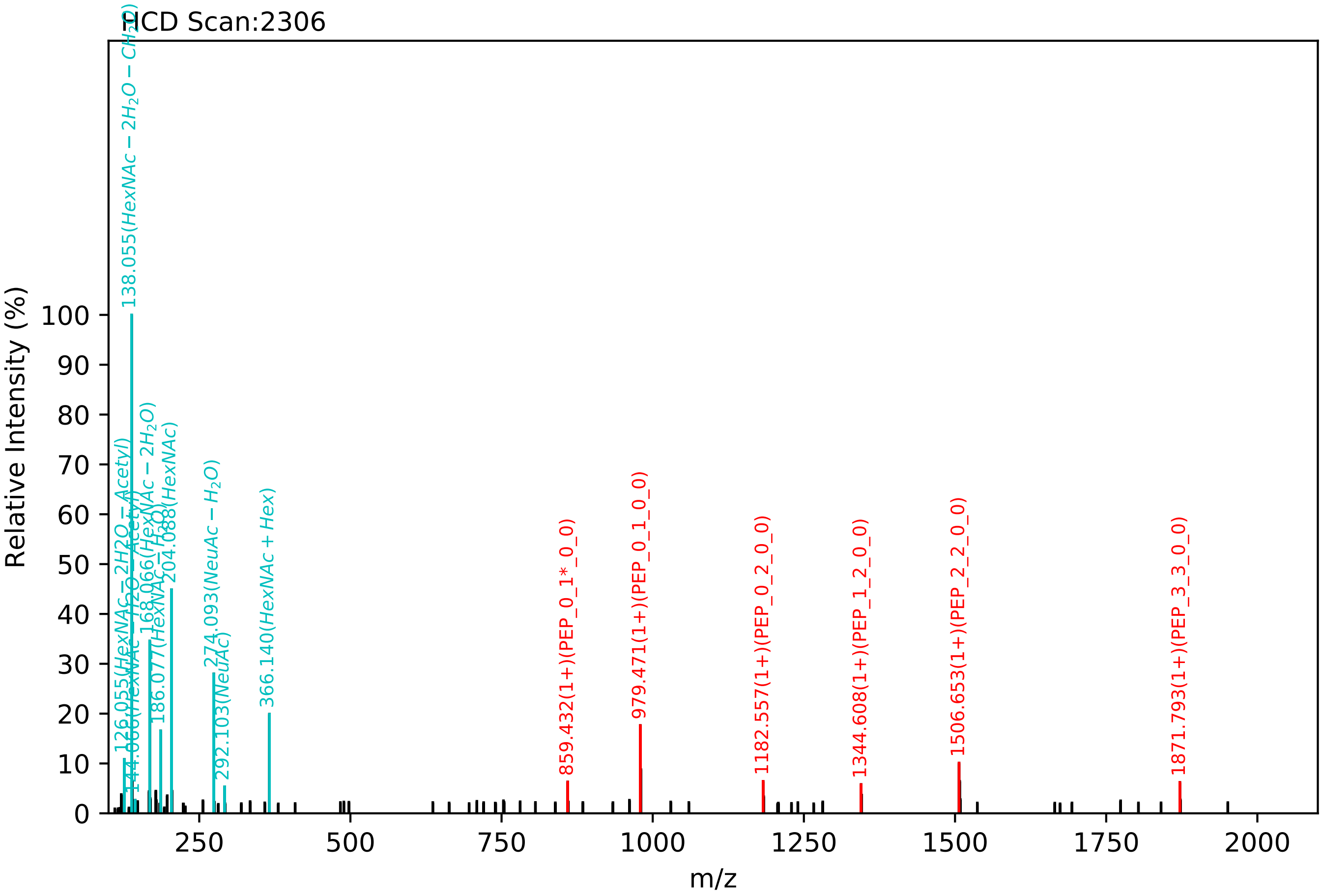

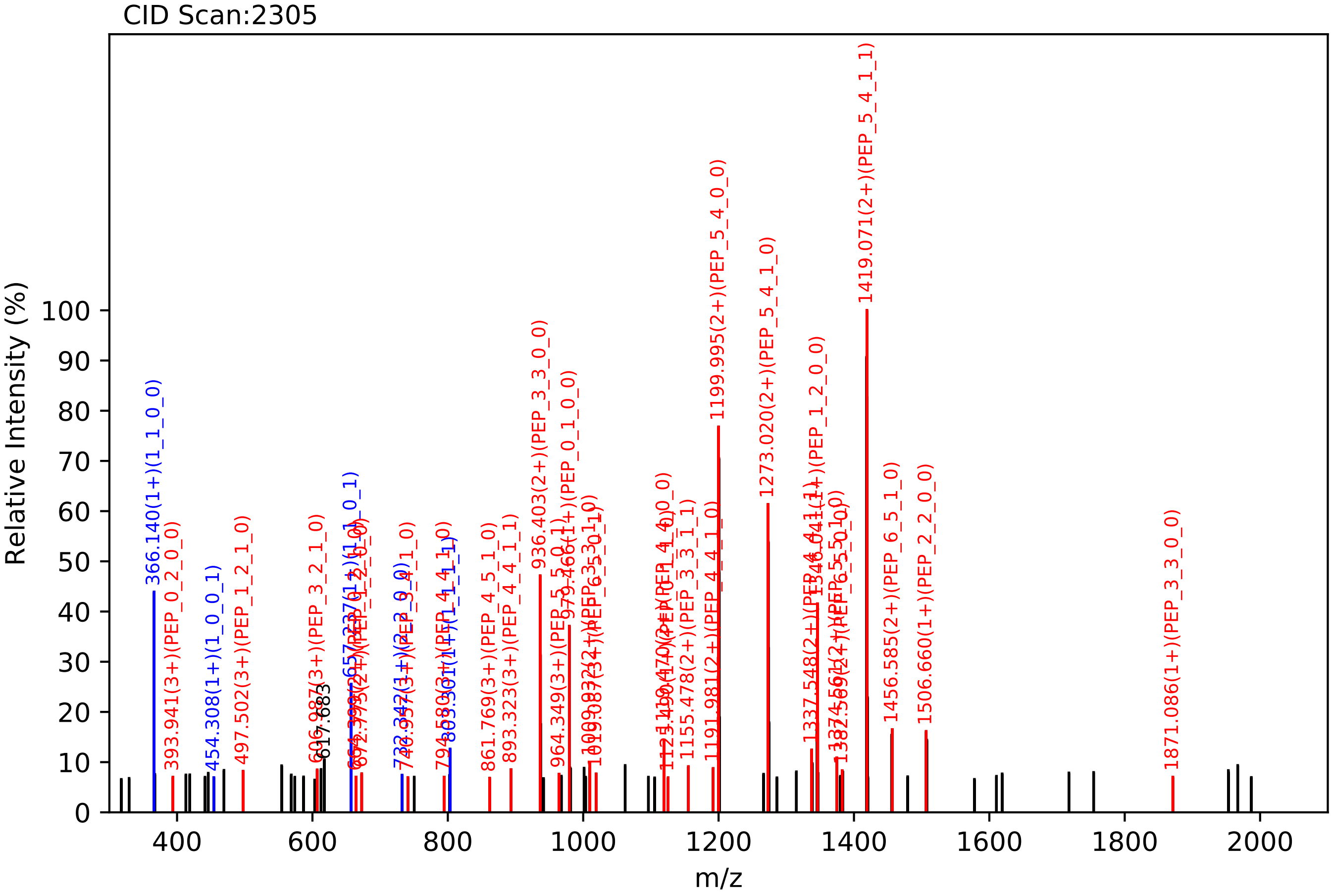

Training set no. 24, Experiment: AGP exp_1

ENGTISR(=PEP)_6_5_1_1, m/z:1067.76(3+), RT:16.47, Y-score:80.85

HCD Scan:2014

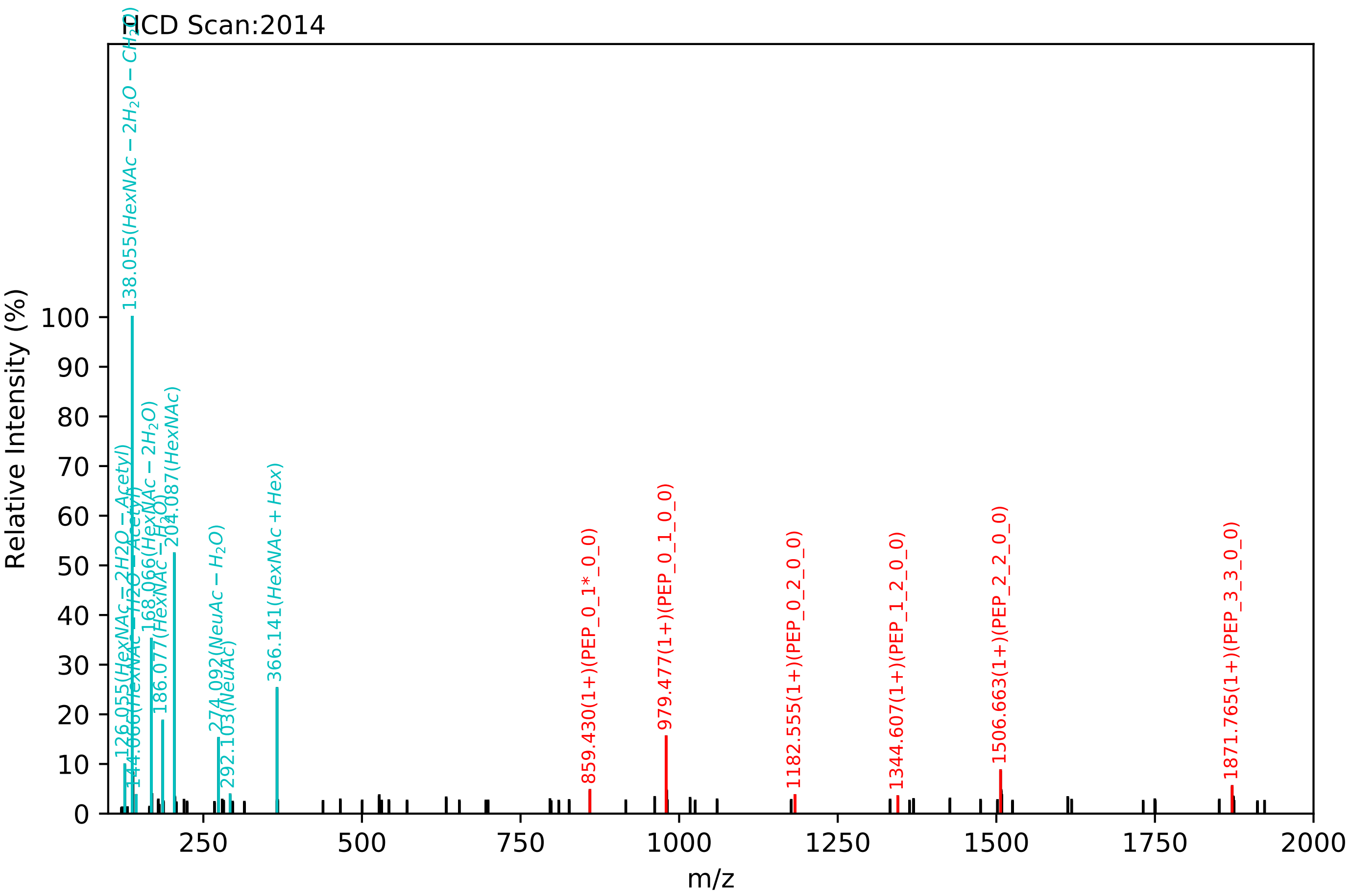

CID Scan:2013

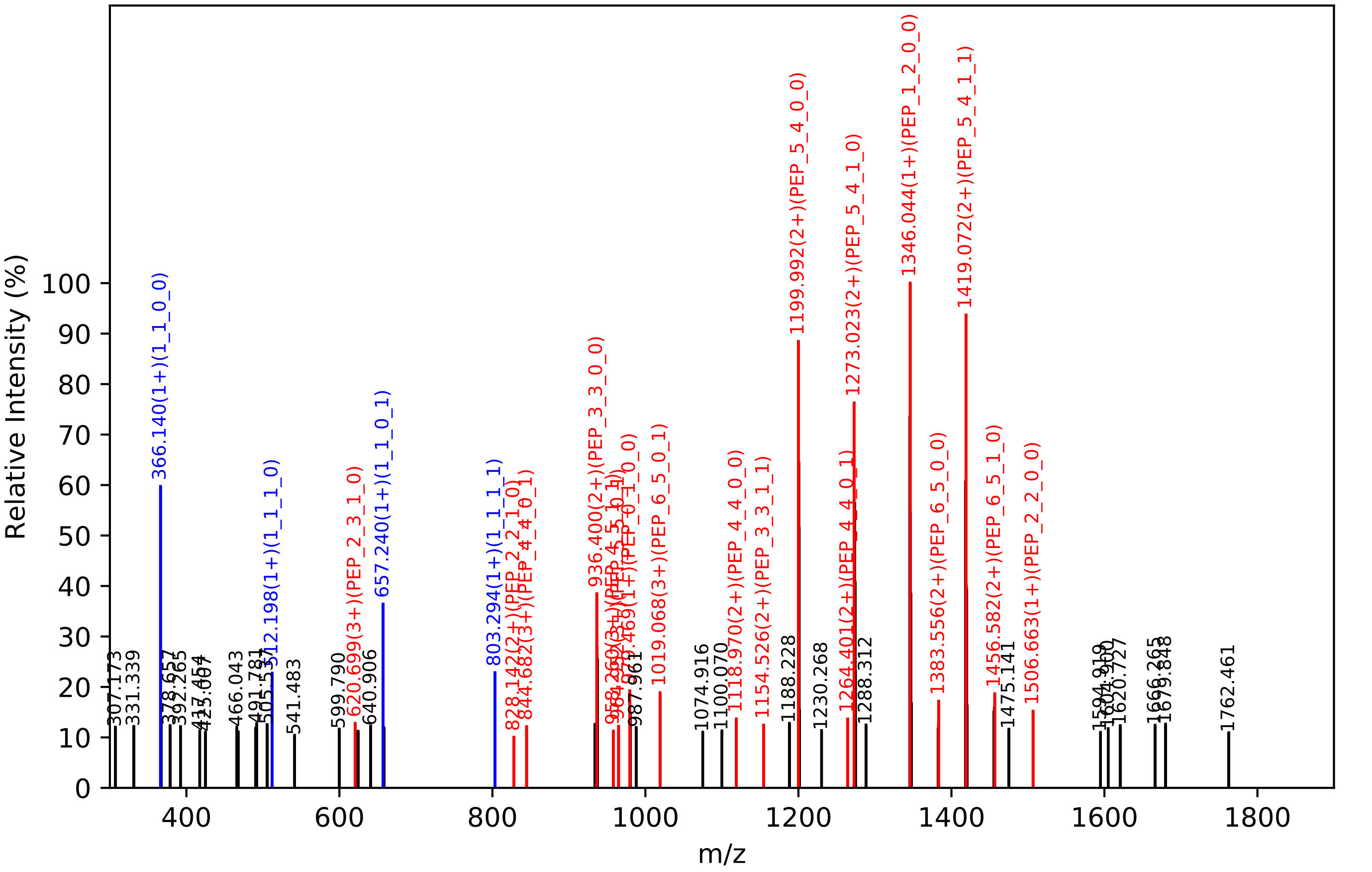

Training set no. 25, Experiment: AGP exp_1

ENGTISR(=PEP)_6_5_1_2, m/z:1164.79(3+), RT:20.55, Y-score:87.82

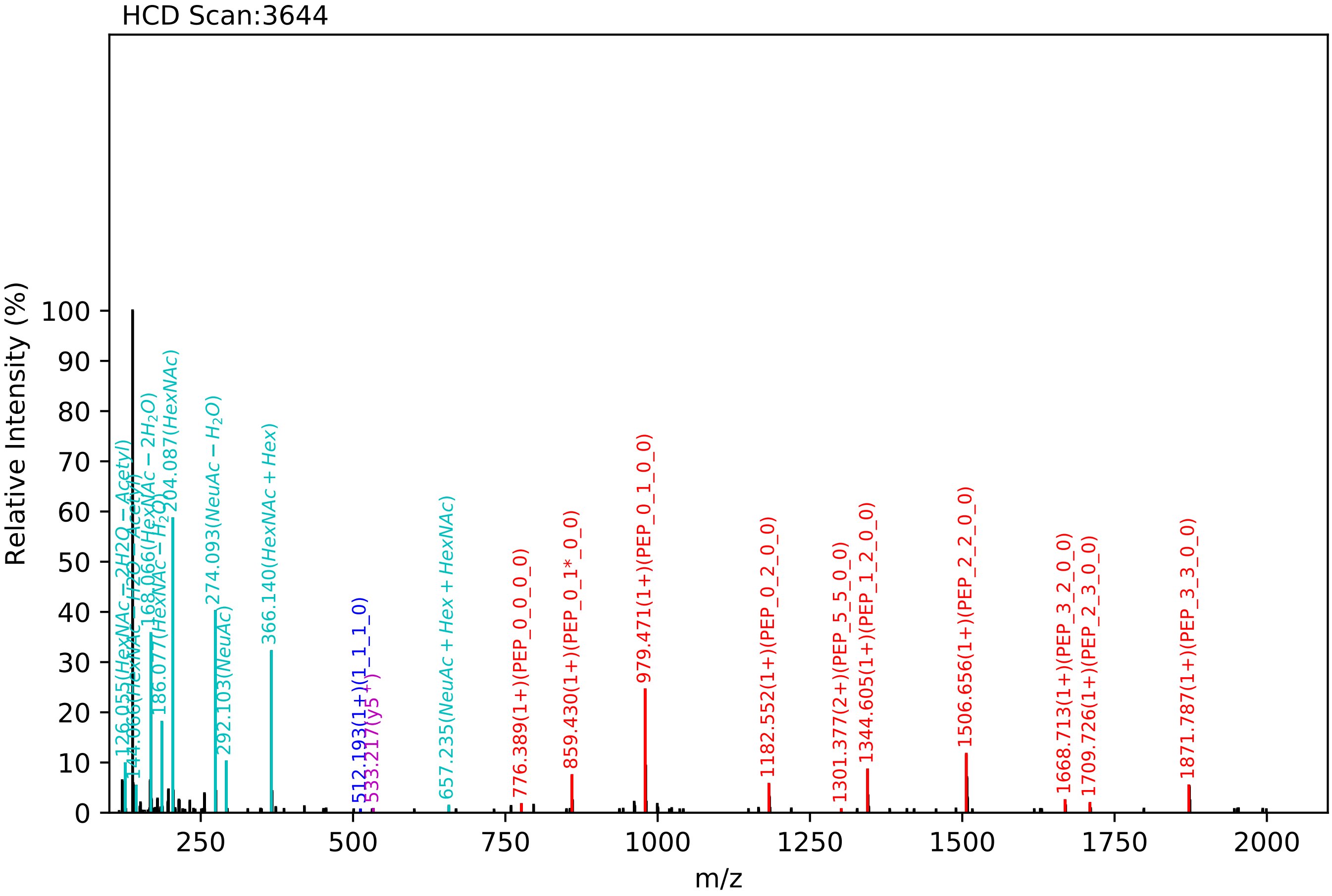

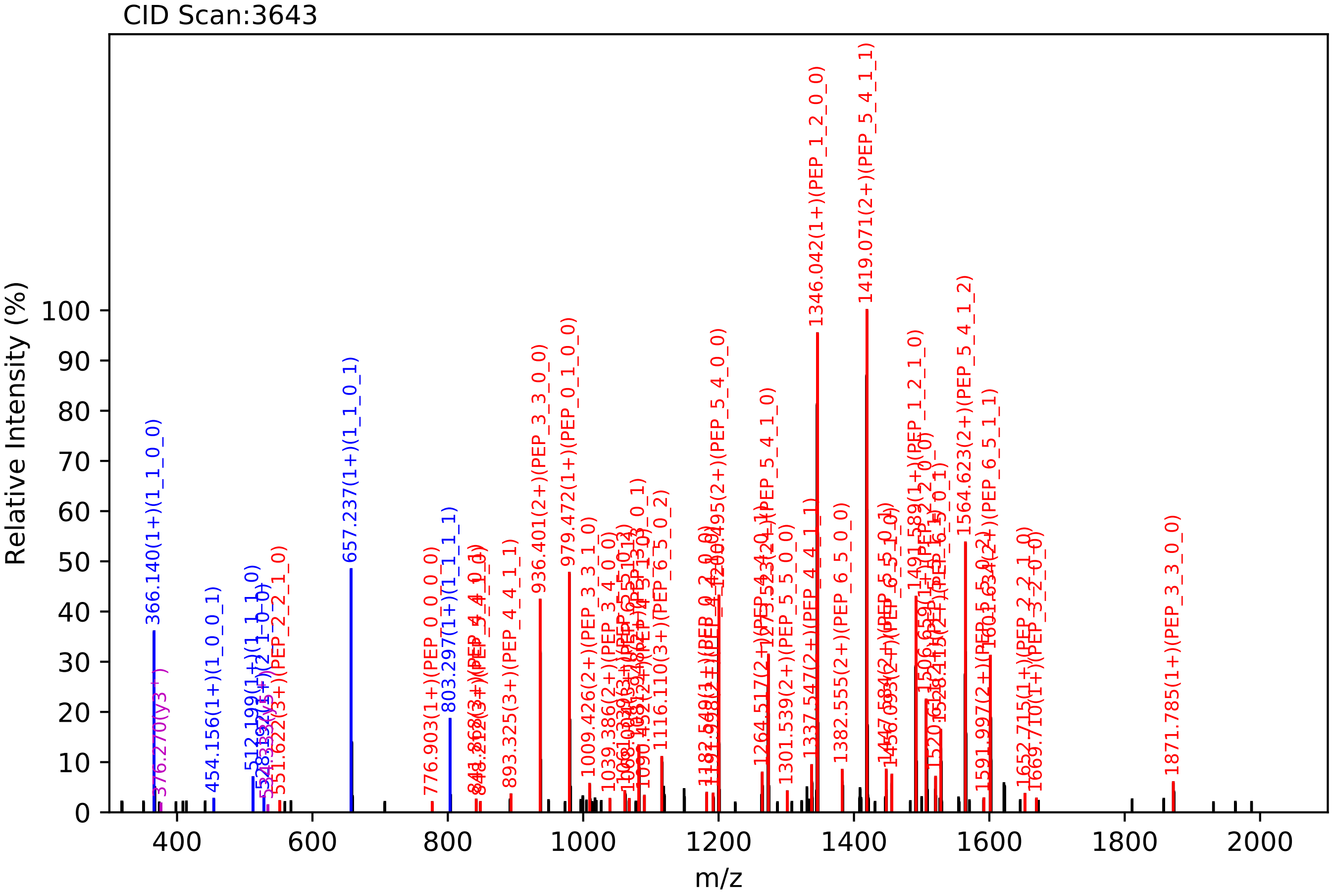

Training set no. 26, Experiment: AGP exp_1

ENGTISR(=PEP)_6_5_1_2, m/z:1164.79(3+), RT:21.11, Y-score:84.03

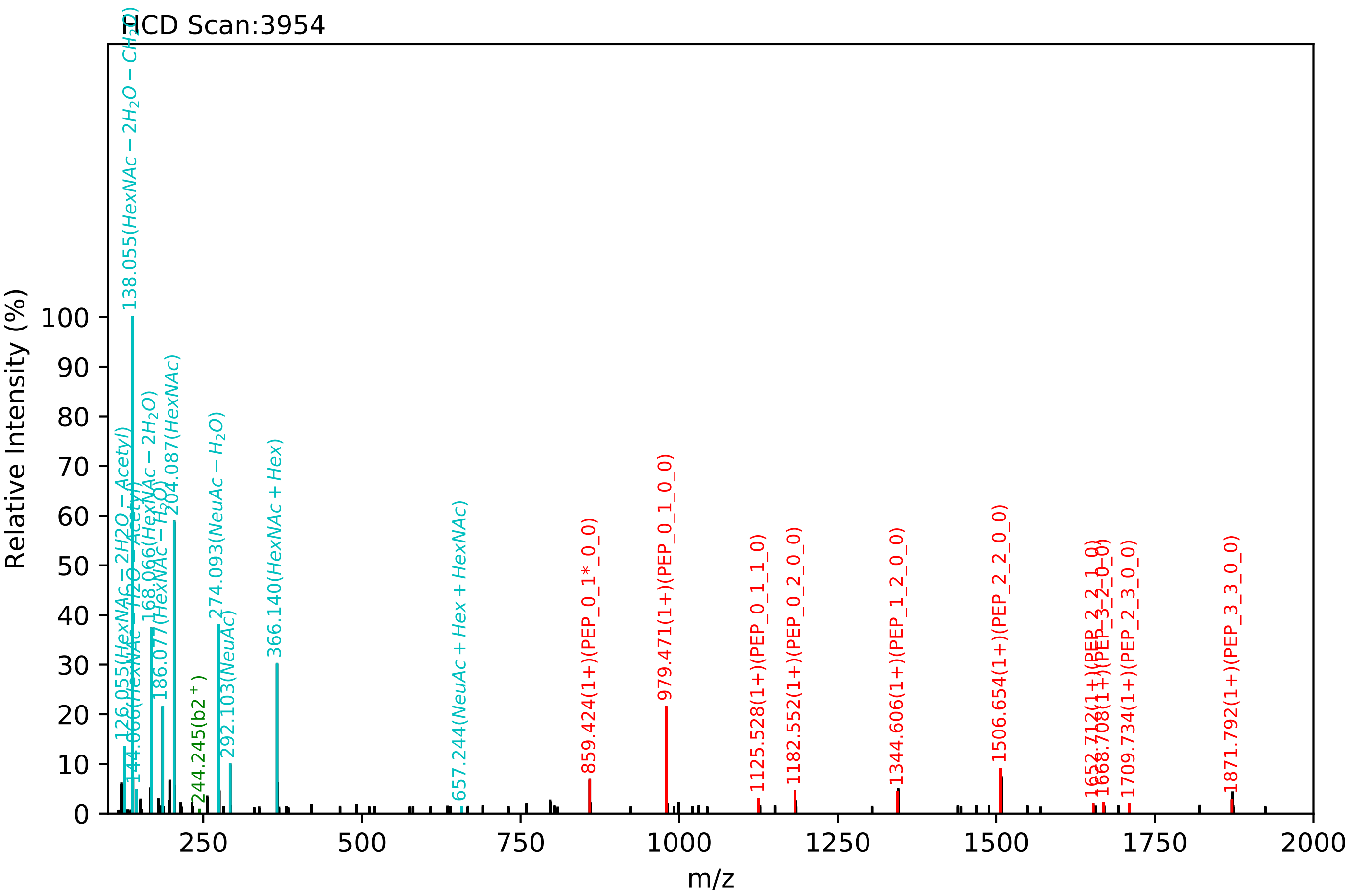

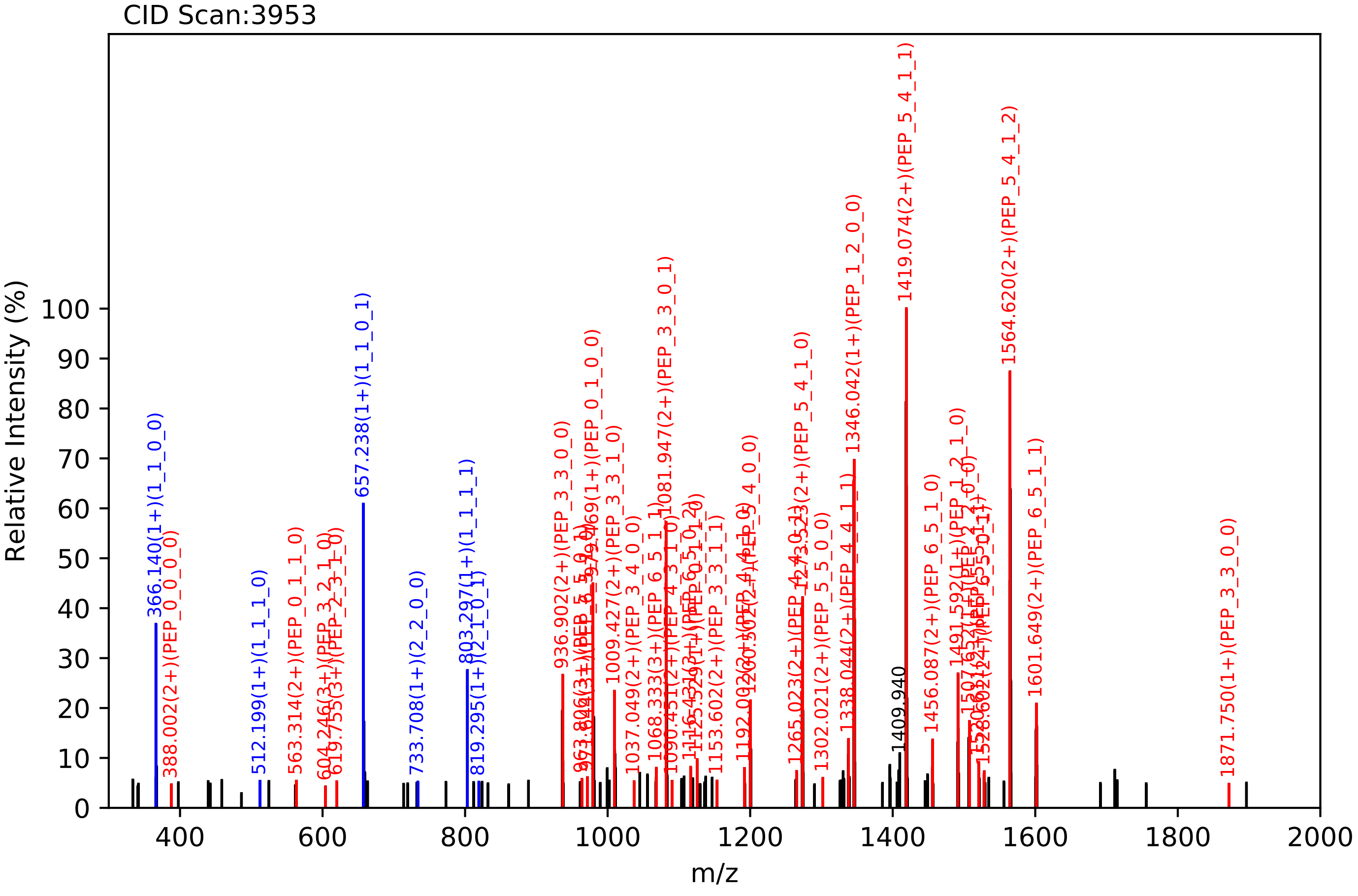

Training set no. 27, Experiment: AGP exp_2

ENGTISR(=PEP)_6_5_1_2, m/z:1164.79(3+), RT:20.35, Y-score:81.79

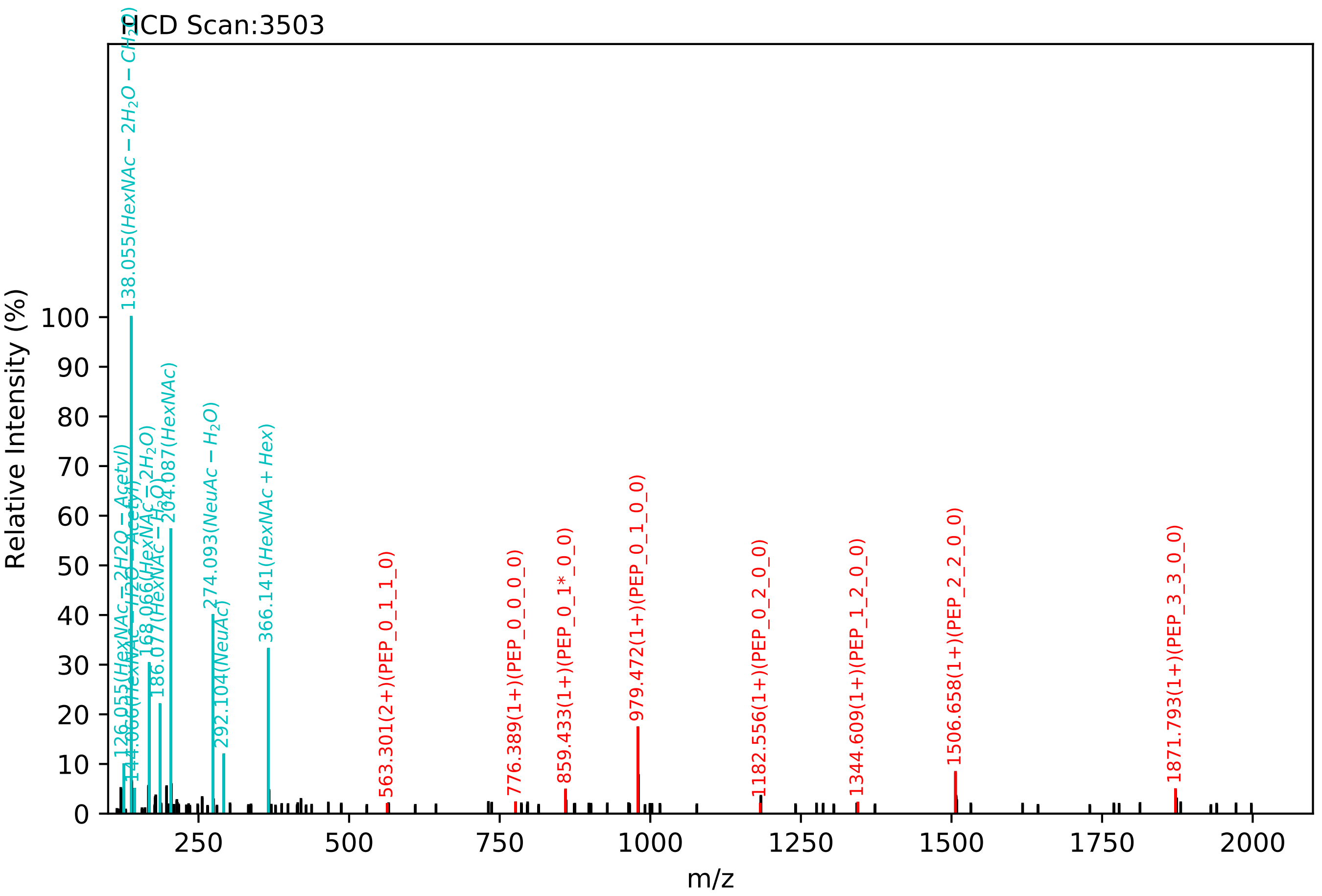

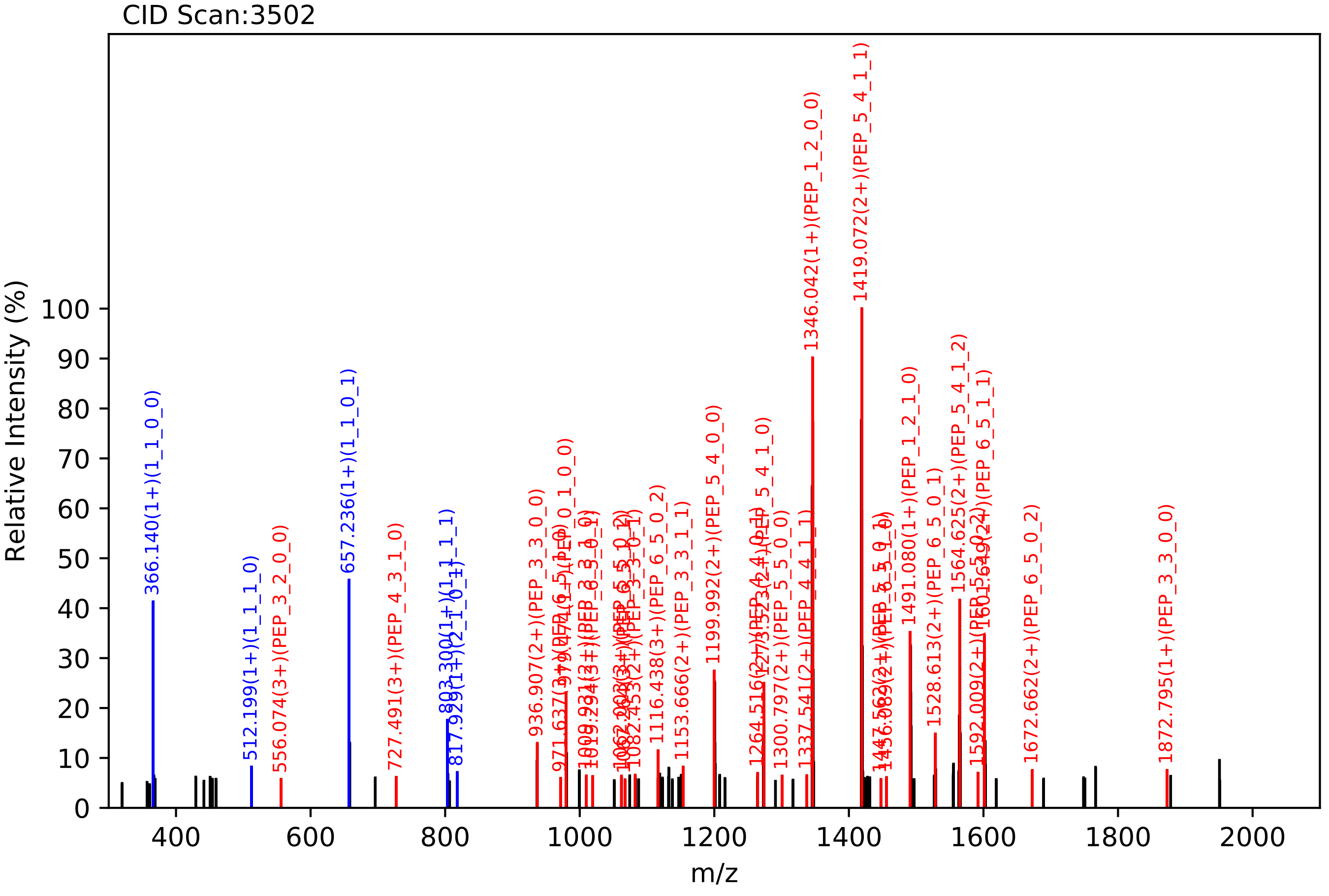

Training set no. 28, Experiment: AGP exp_2

ENGTISR(=PEP)_6_5_1_2, m/z:1164.79(3+), RT:20.91, Y-score:78.07

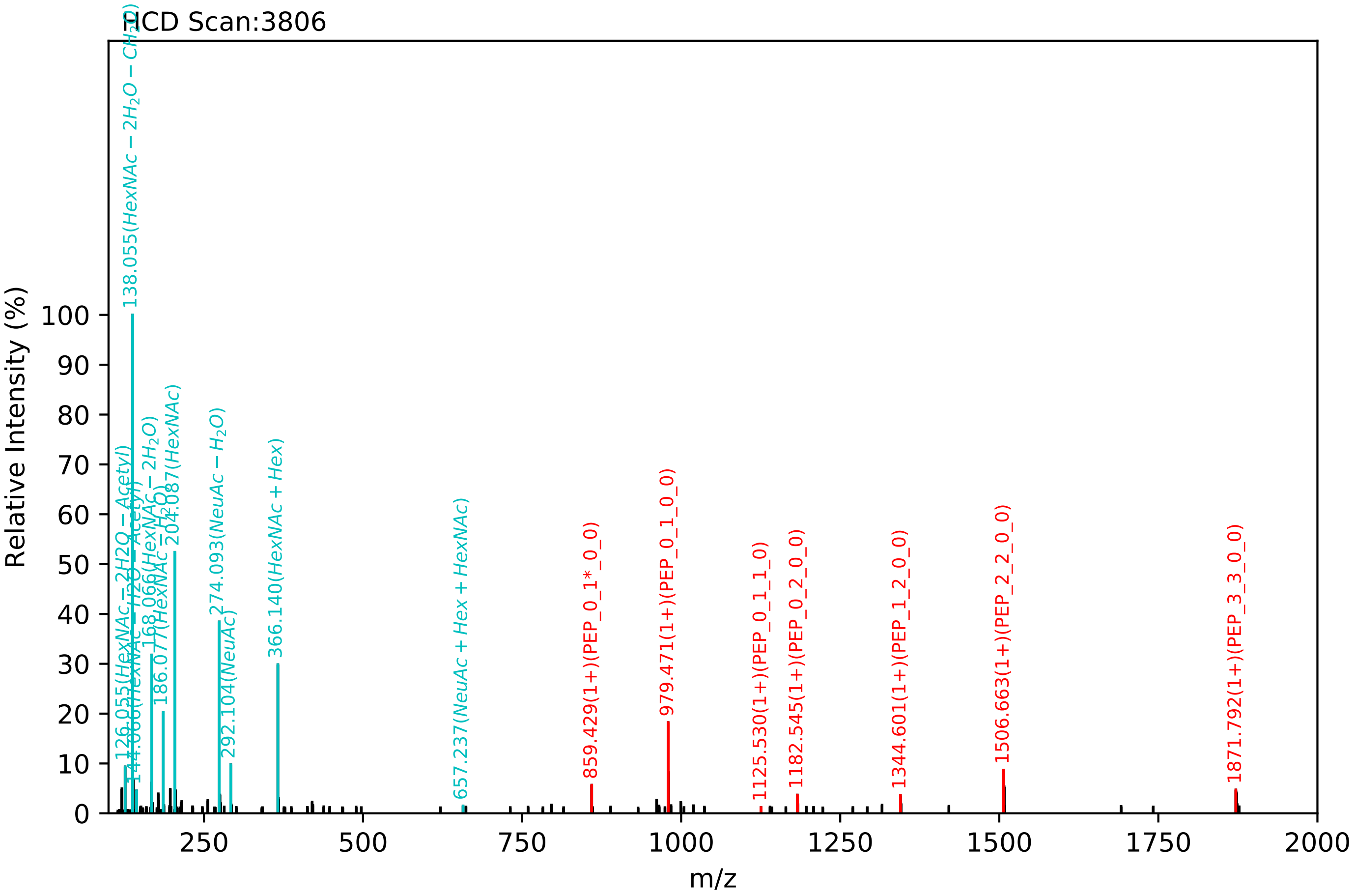

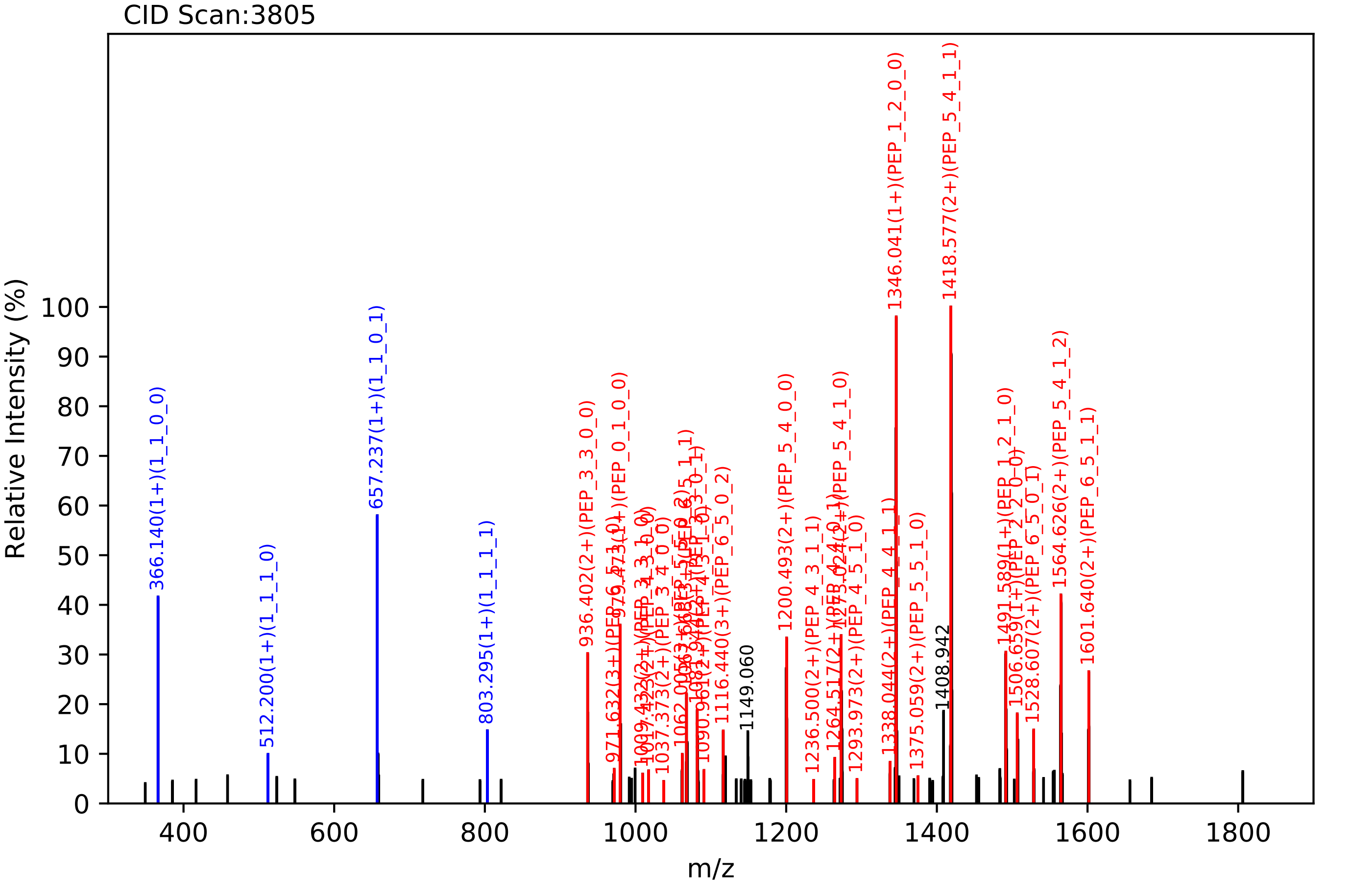

Training set no. 29, Experiment: AGP exp_2

ENGTISR(=PEP)_6_5_1_3, m/z:1261.82(3+), RT:22.65, Y-score:89.99

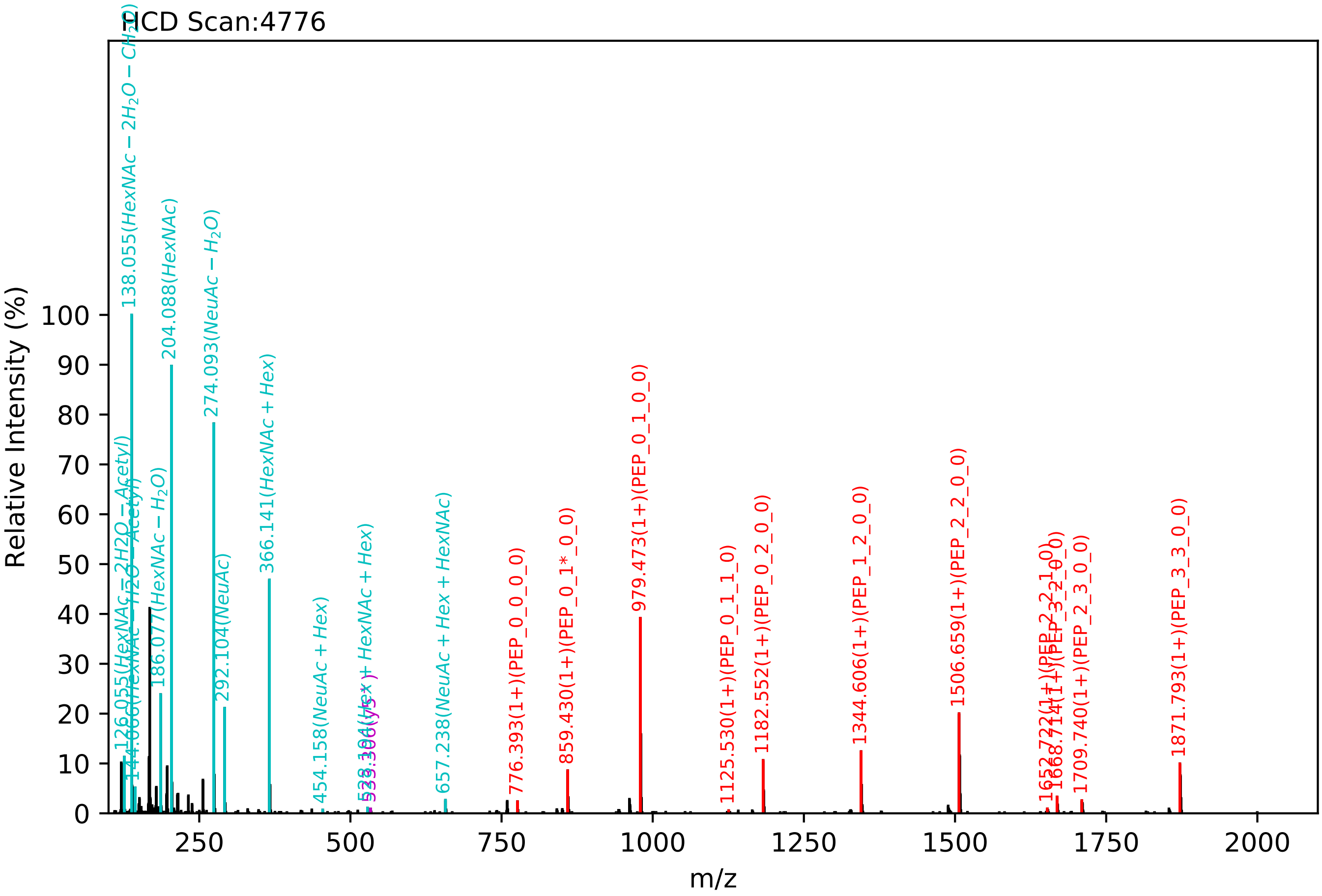

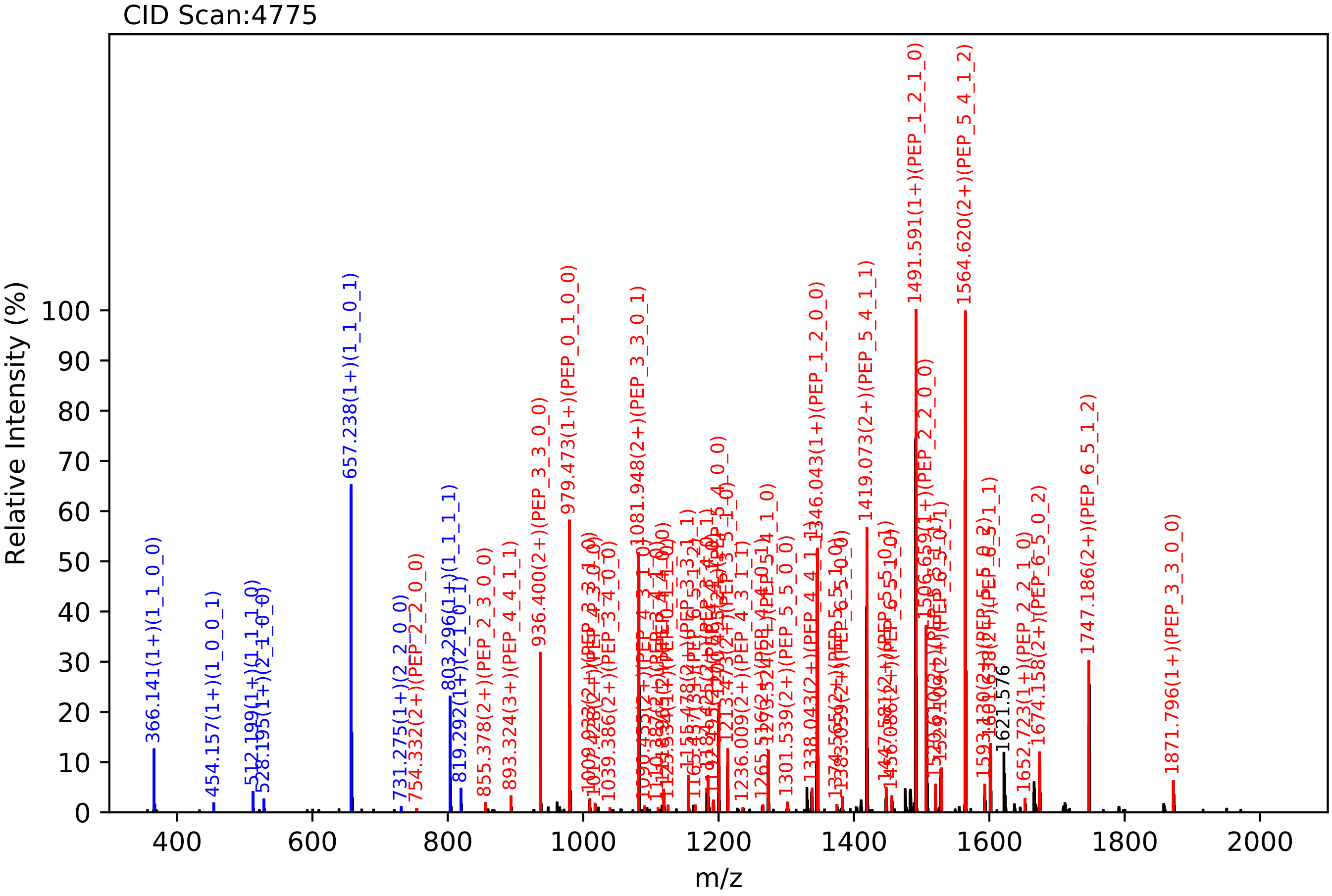

Training set no. 30, Experiment: AGP exp_1

ENGTISR(=PEP)_6_5_1_3, m/z:1261.83(3+), RT:22.44, Y-score:86.94

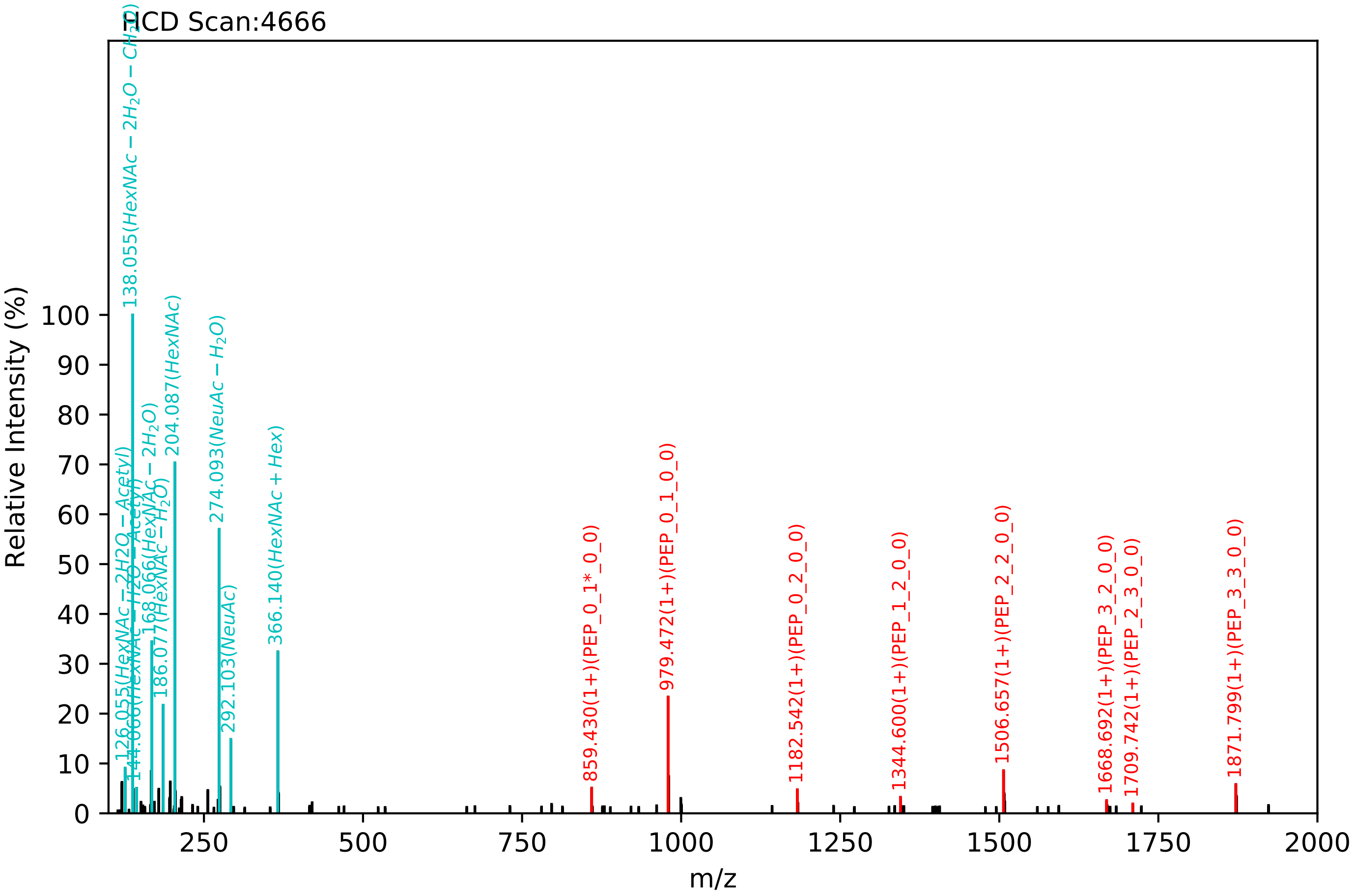

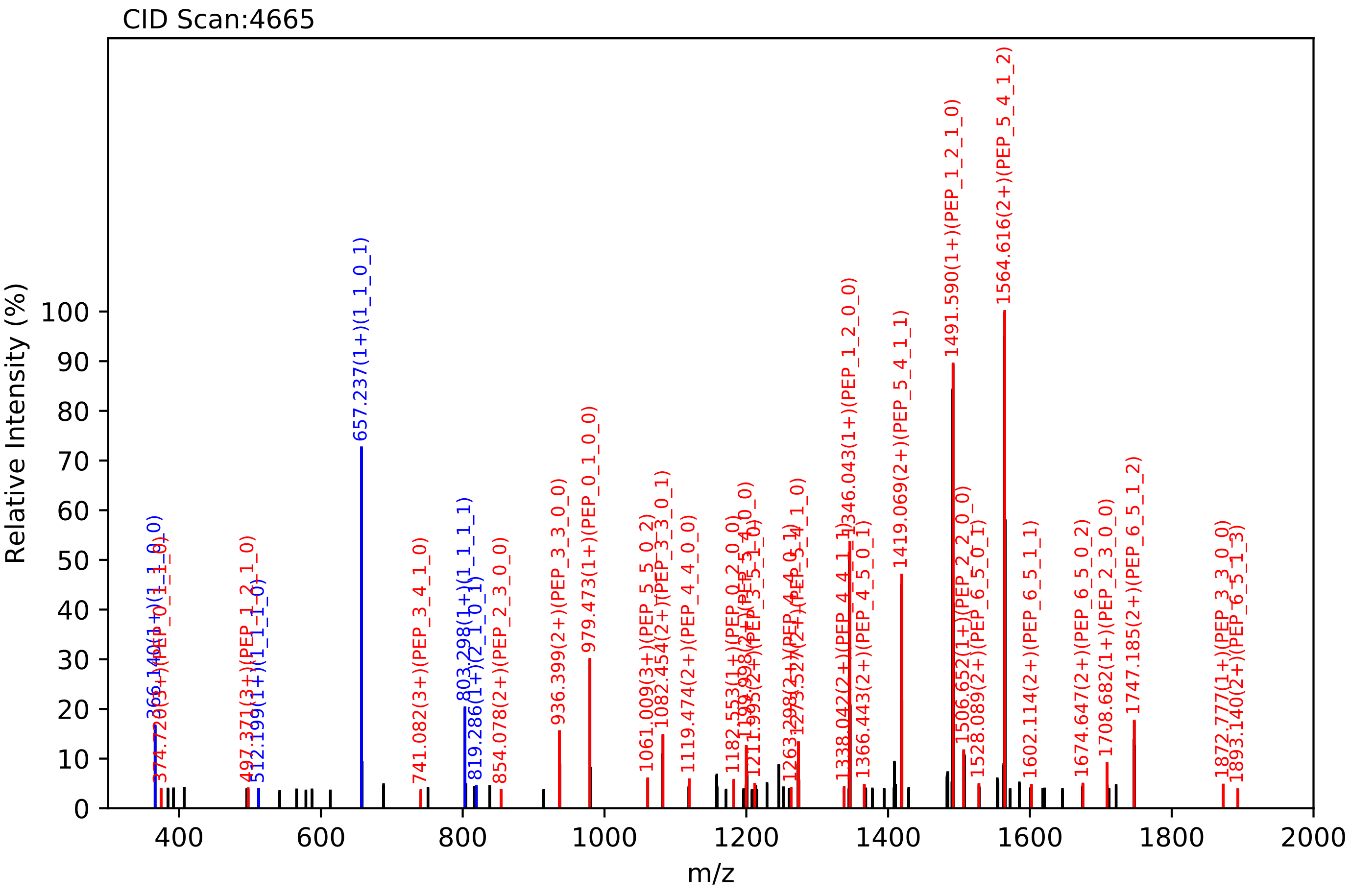

Training set no. 31, Experiment: AGP exp_45

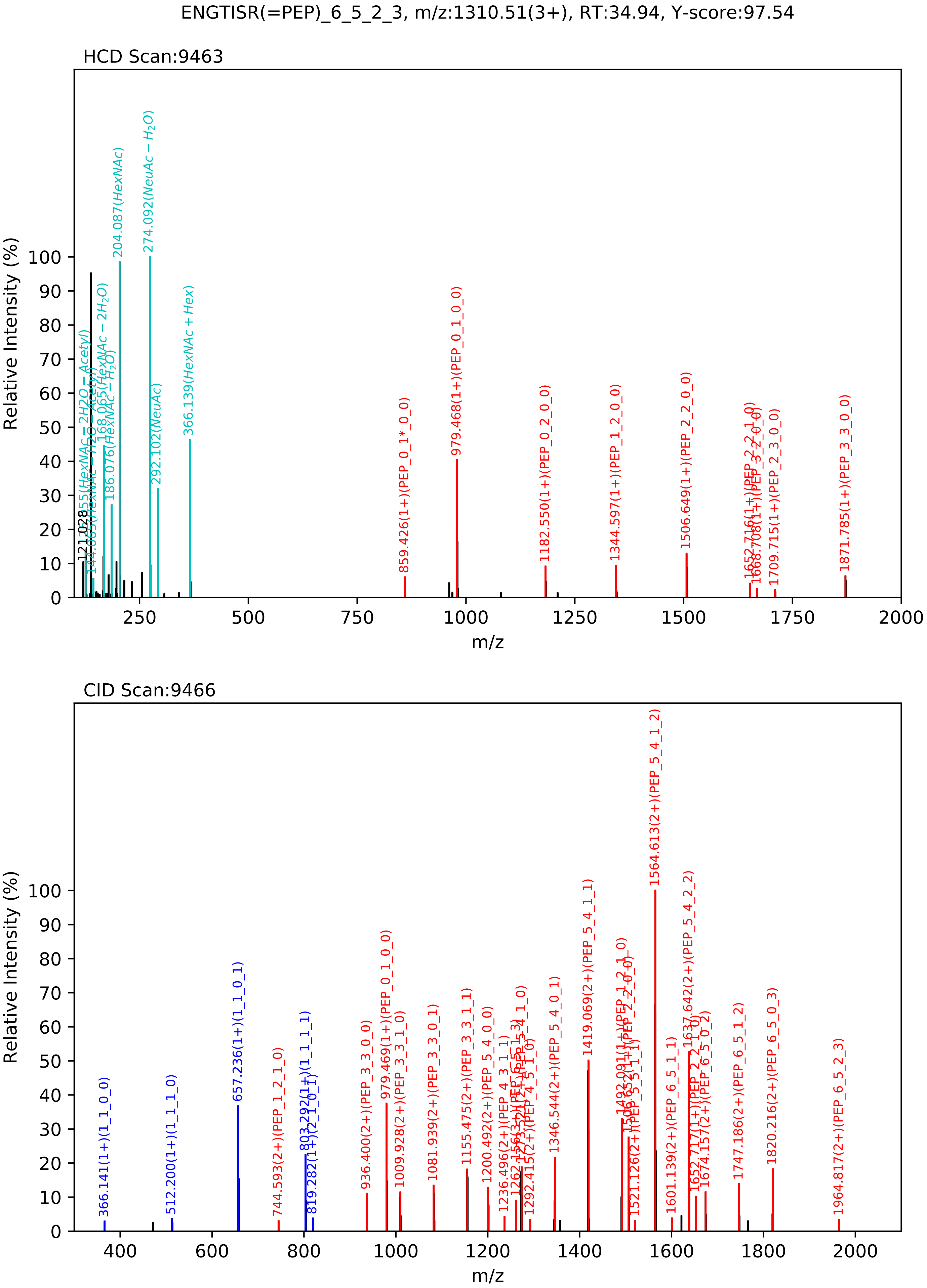

Training set no. 32, Experiment: AGP exp_23

ENGTISR(=PEP)_6_5_2_3, m/z:1310.51(3+), RT:34.35, Y-score:91.68

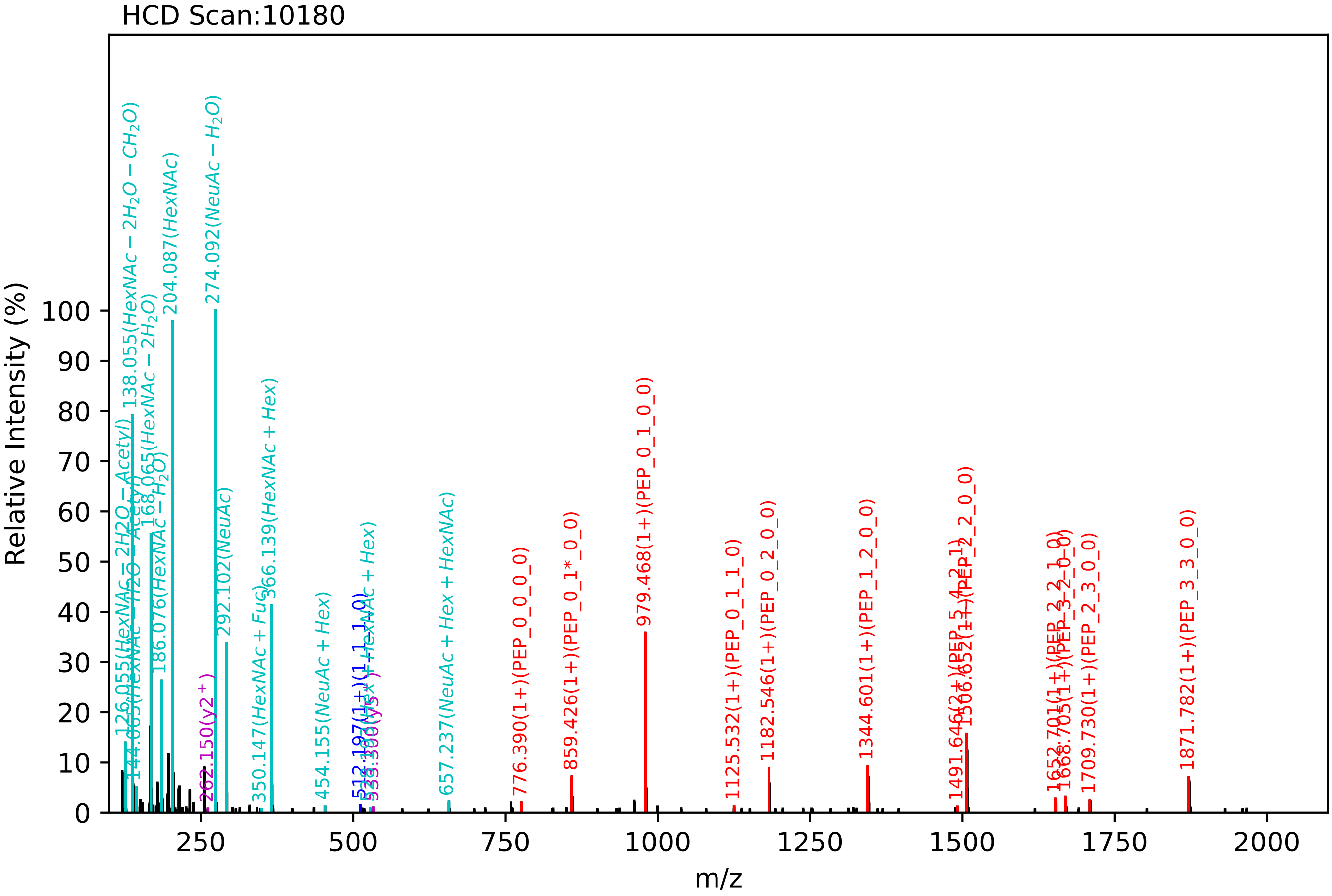

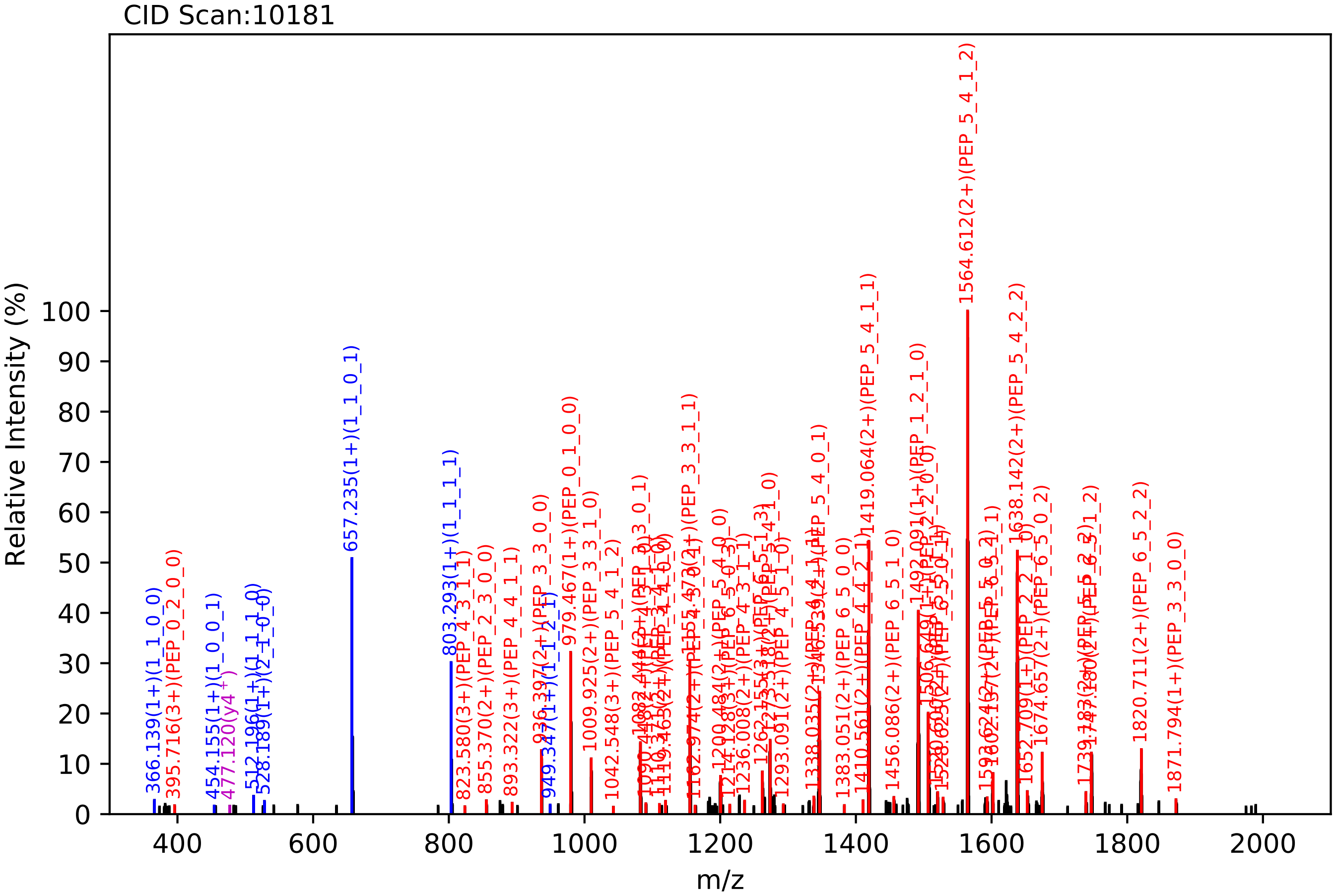

Training set no. 33, Experiment: AGP exp_29

ENGTISR(=PEP)_6_5_2_3, m/z:1310.51(3+), RT:39.89, Y-score:90.73

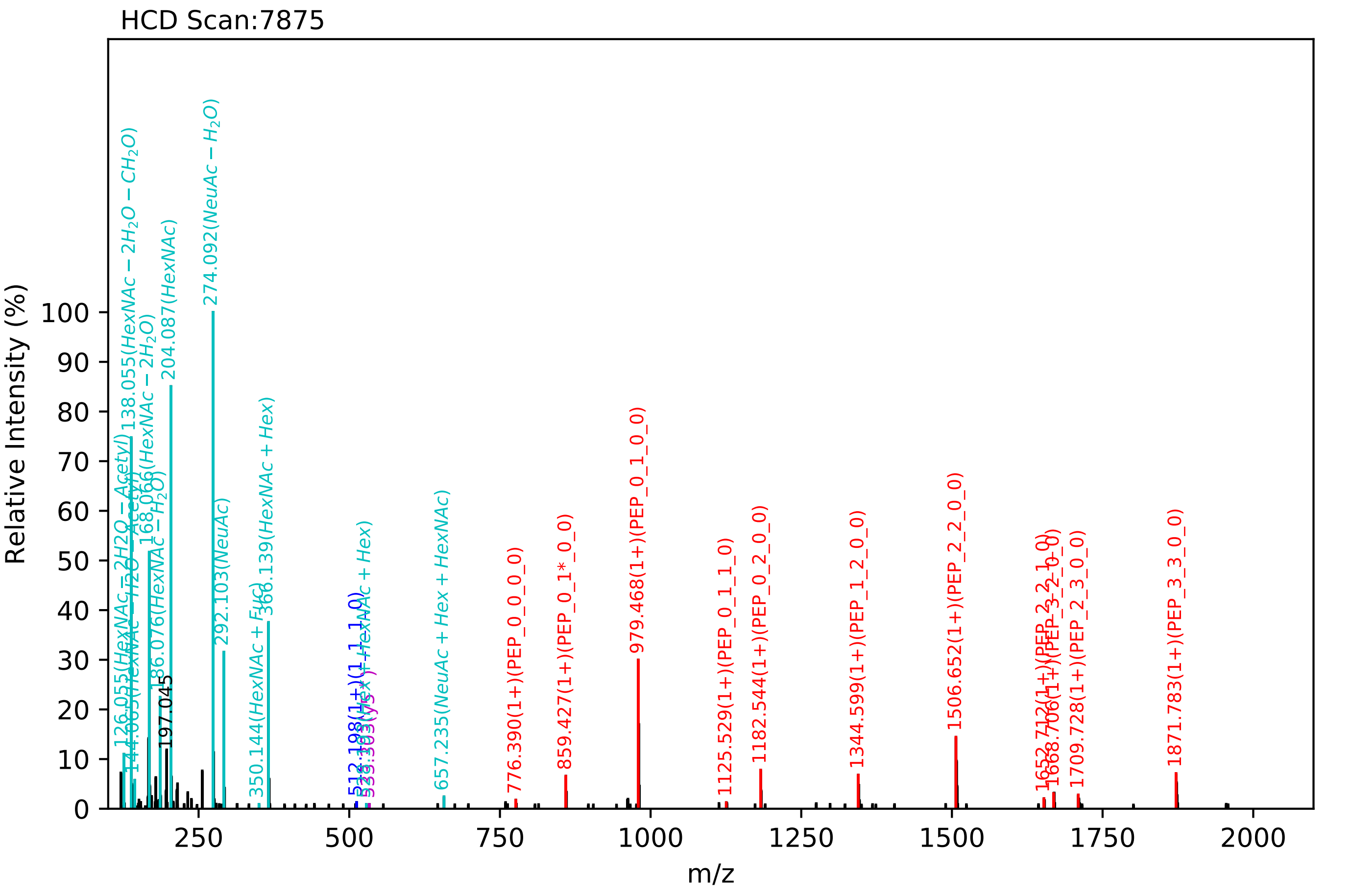

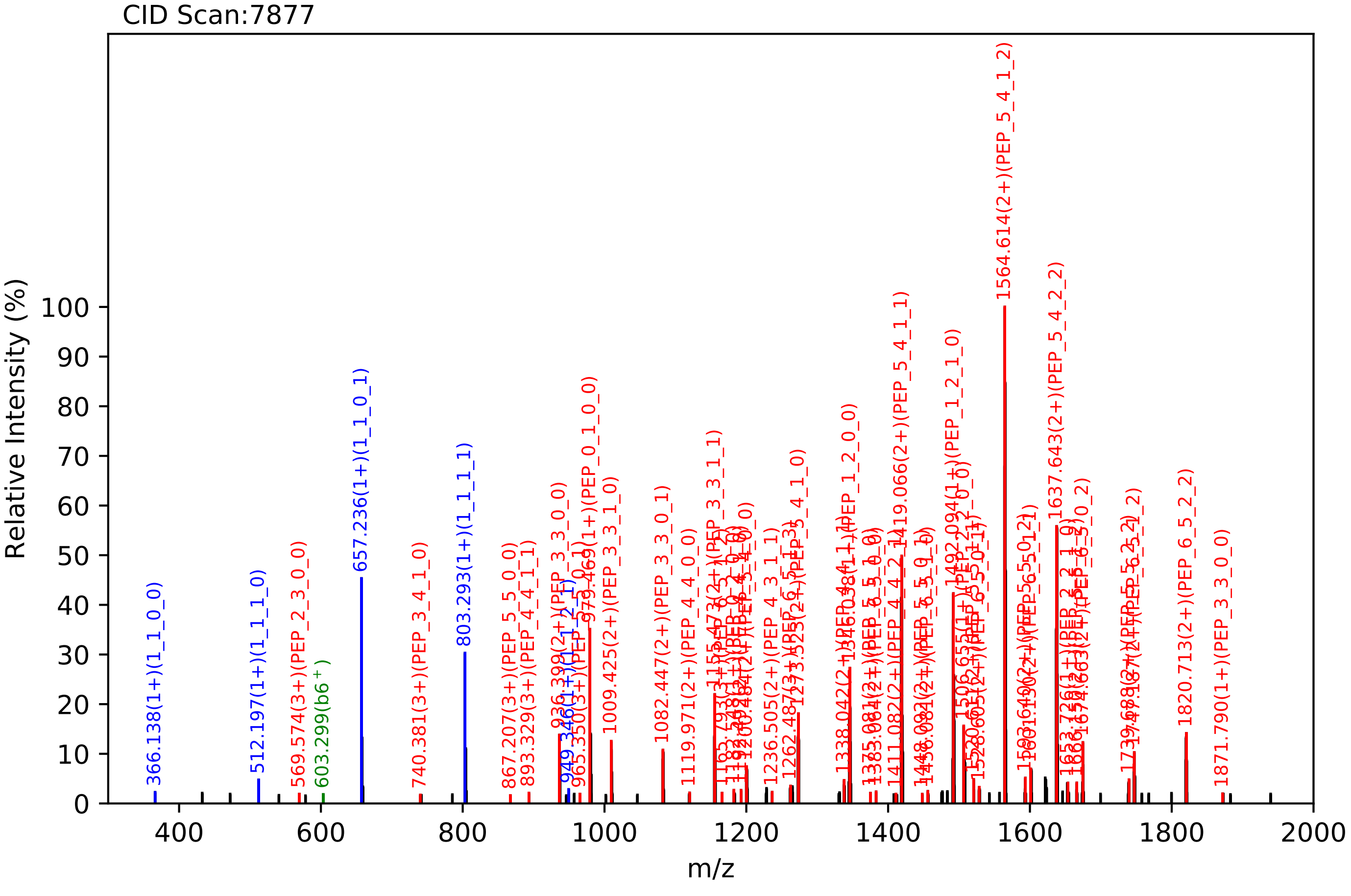

Training set no. 34, Experiment: AGP exp_21

ENGTISR(=PEP)_6_5_2_3, m/z:1310.51(3+), RT:33.90, Y-score:89.95

HCD Scan:9465

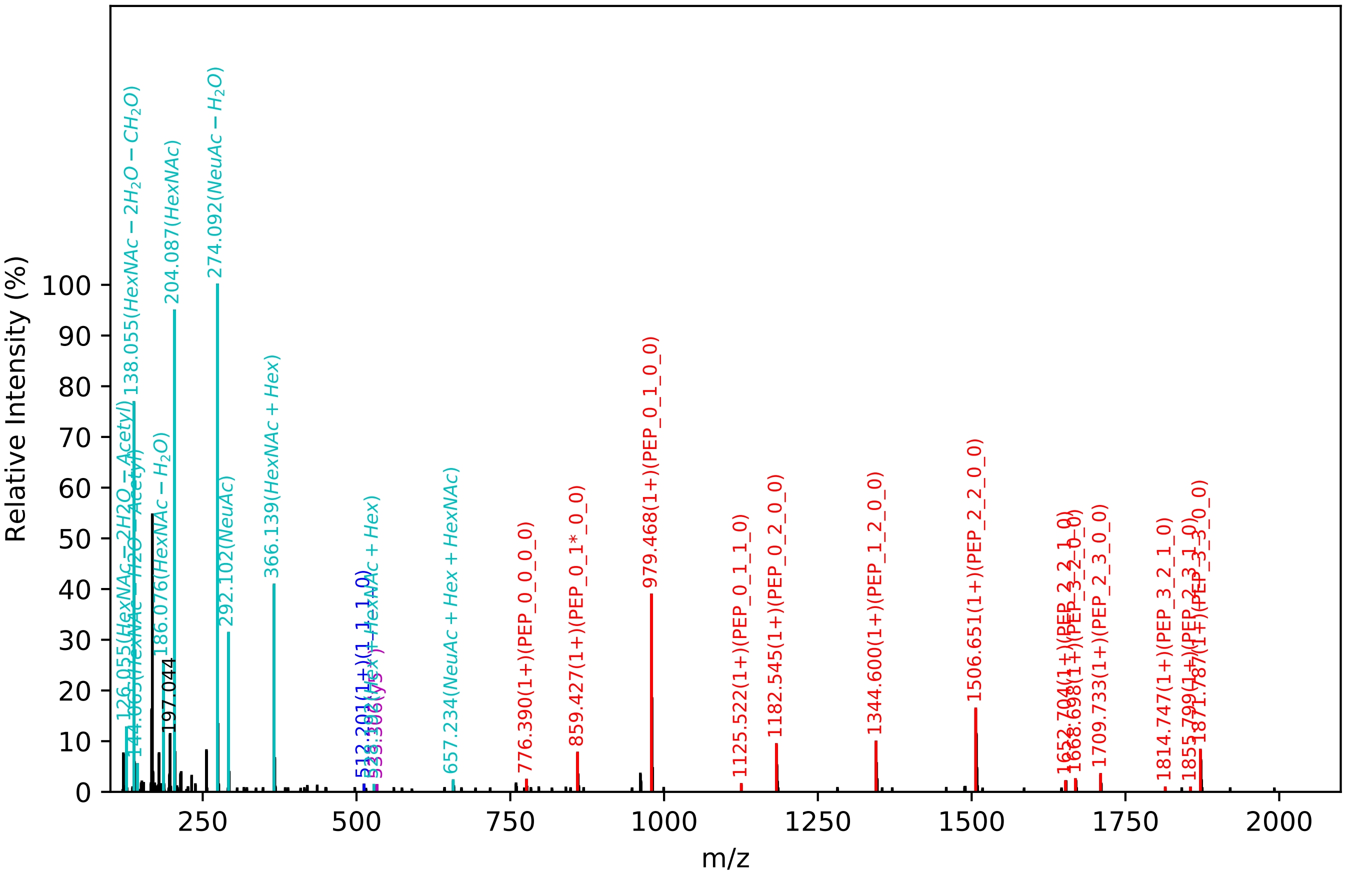

CID Scan:9470

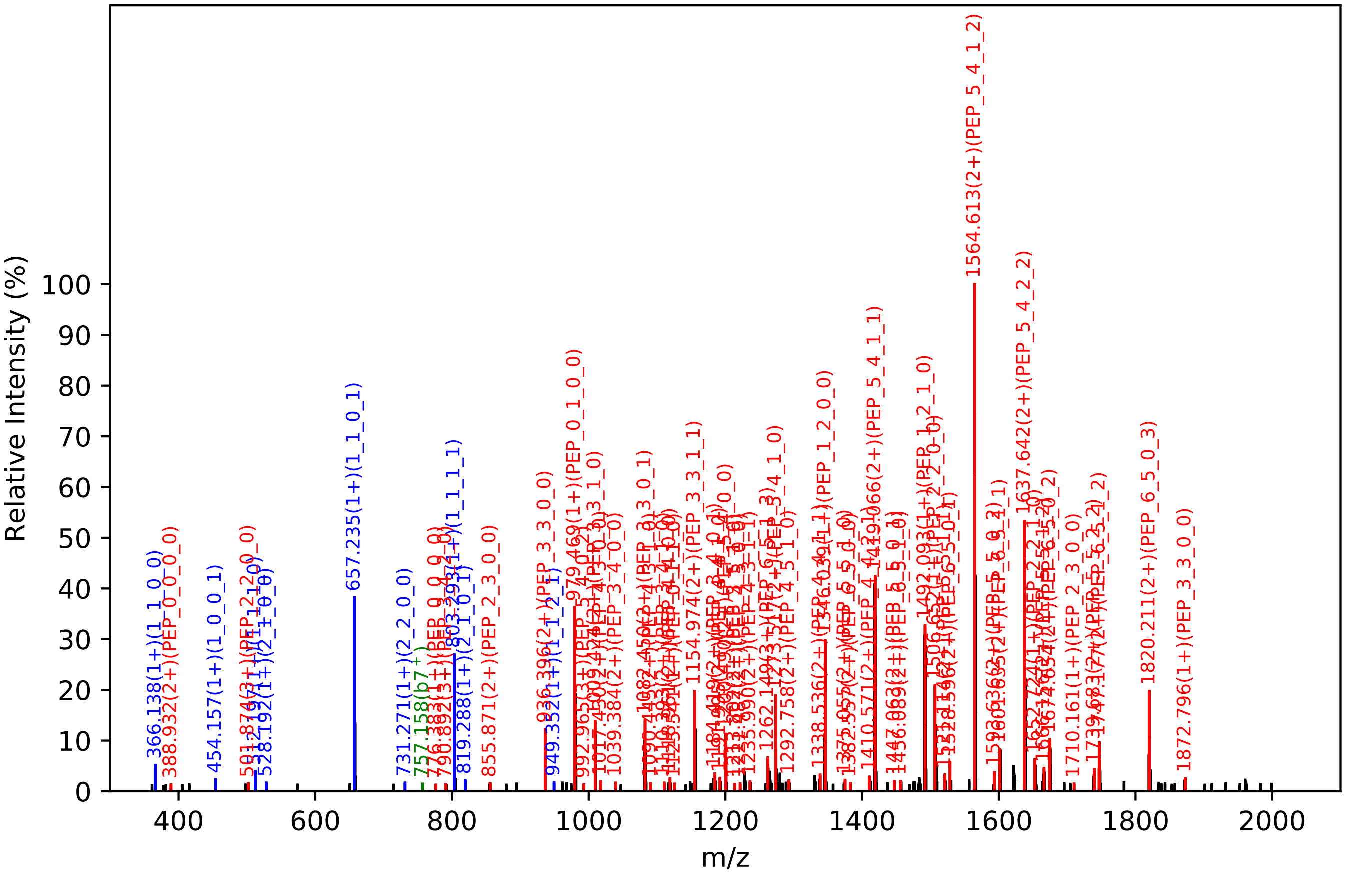

Training set no. 35, Experiment: AGP exp_8

ENGTISR(=PEP)_6_5_2_3, m/z:1310.51(3+), RT:33.86, Y-score:89.55

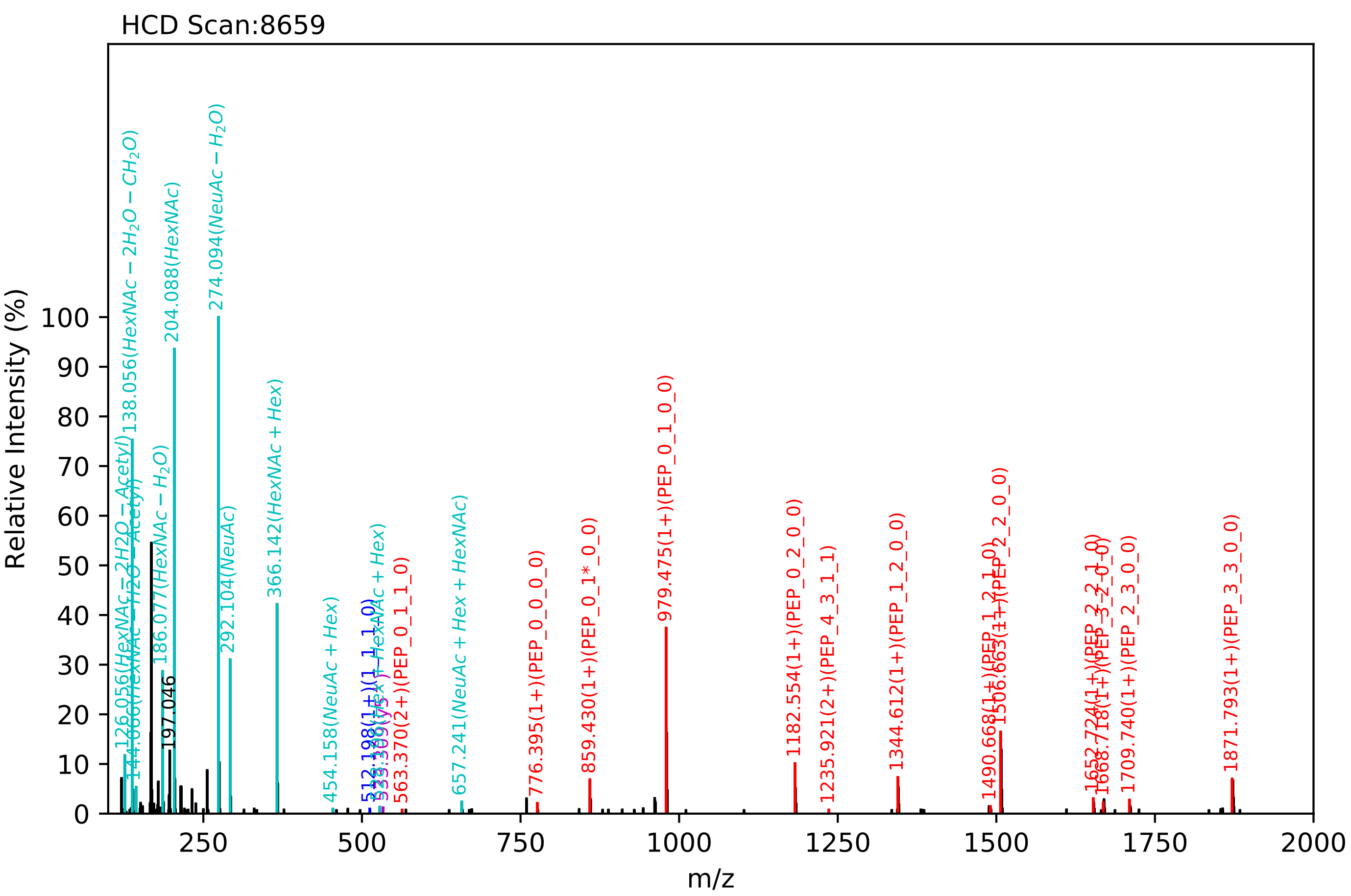

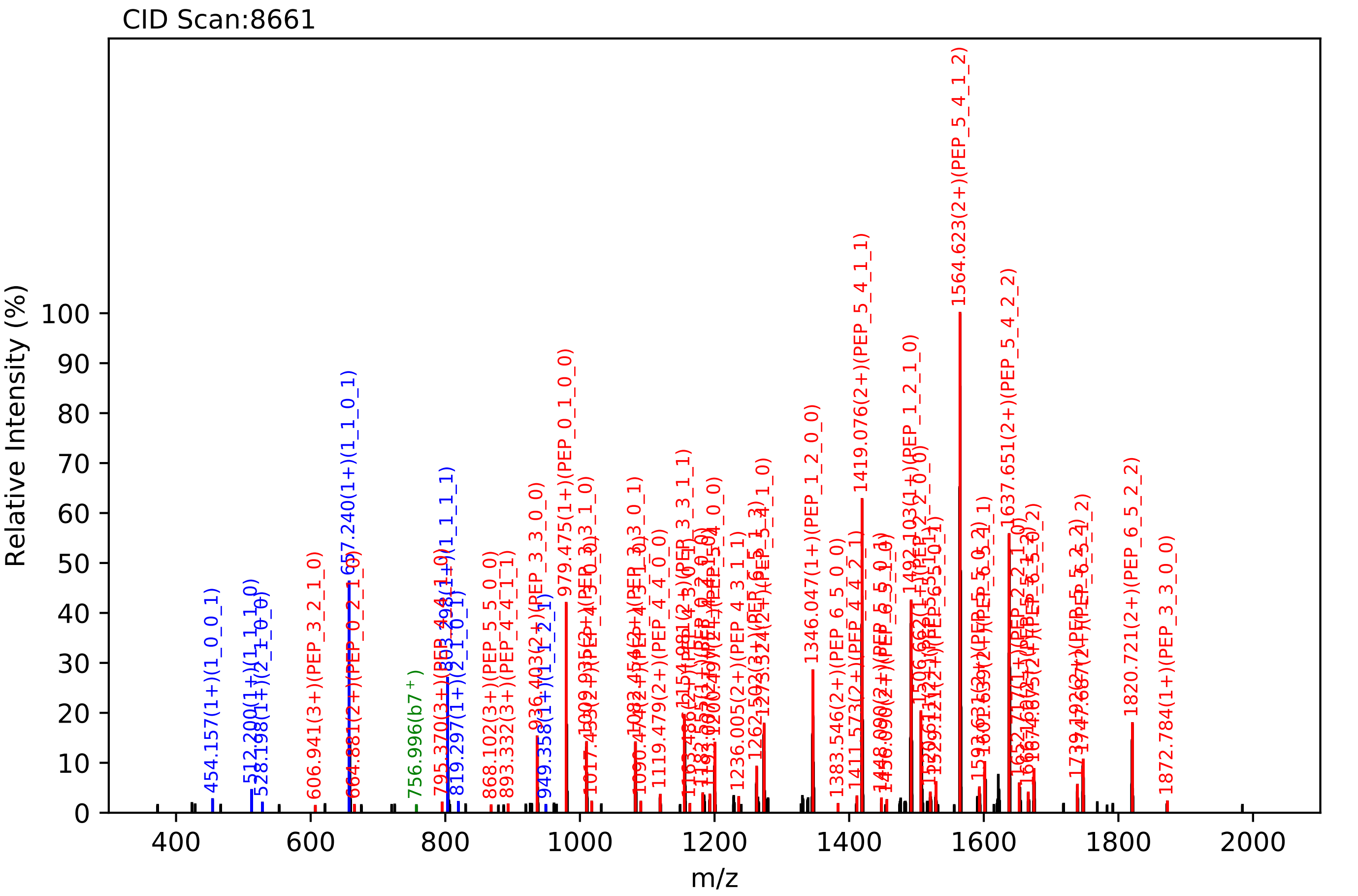

Training set no. 36, Experiment: AGP exp_25

ENGTISR(=PEP)_6_5_2_3, m/z:1310.51(3+), RT:34.67, Y-score:89.42

HCD Scan:10473

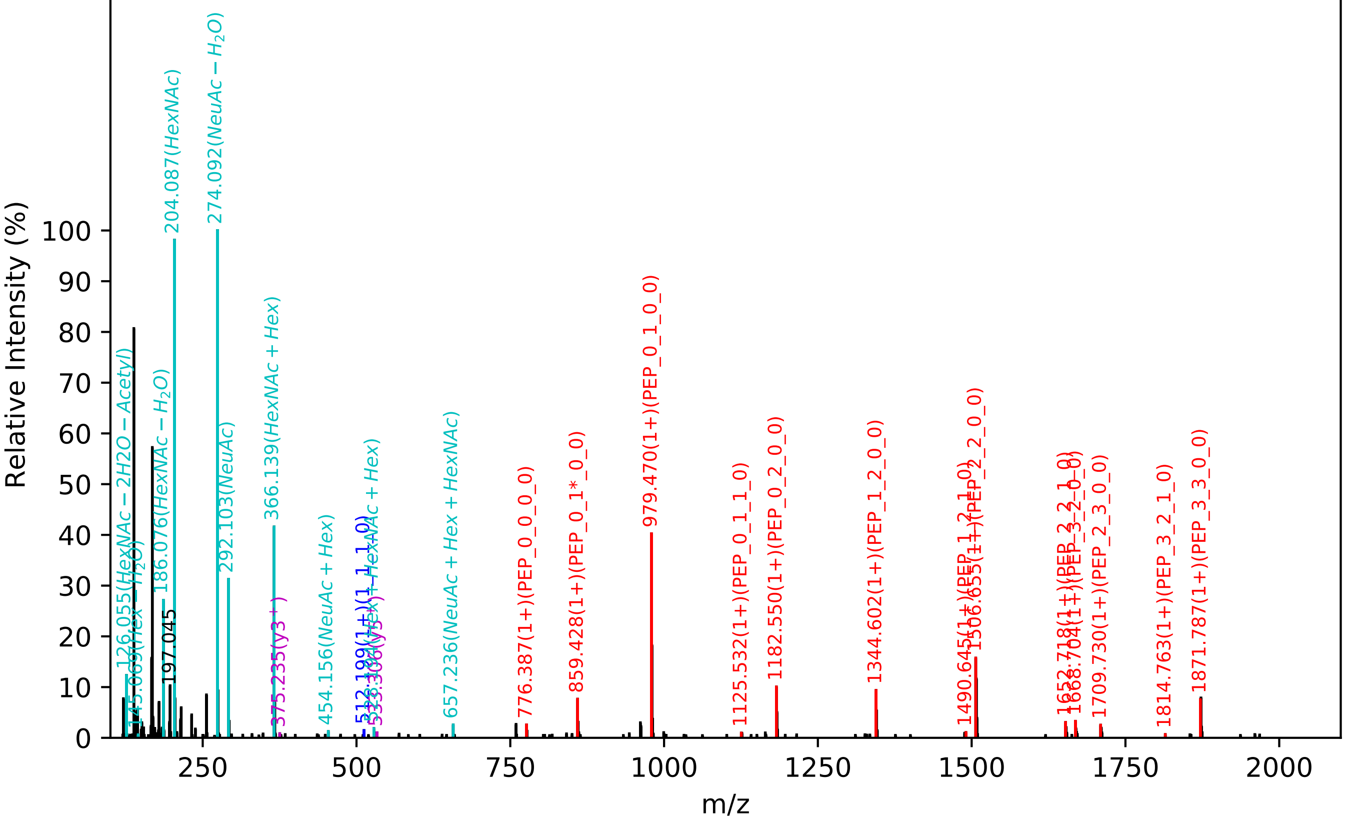

CID Scan:10476

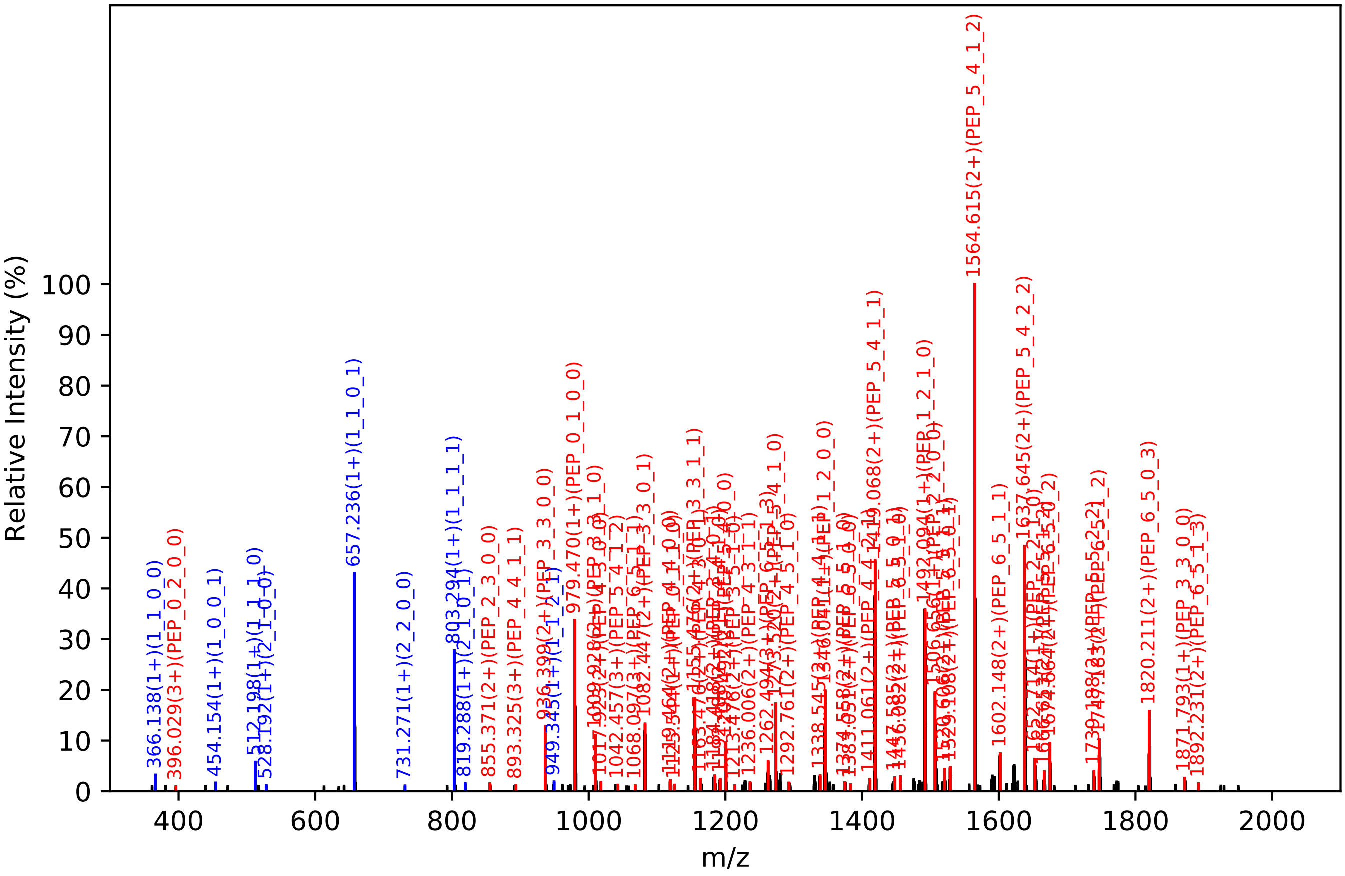

Training set no. 37, Experiment: AGP exp_33

ENGTISR(=PEP)_6_5_2_3, m/z:1310.51(3+), RT:45.79, Y-score:89.05

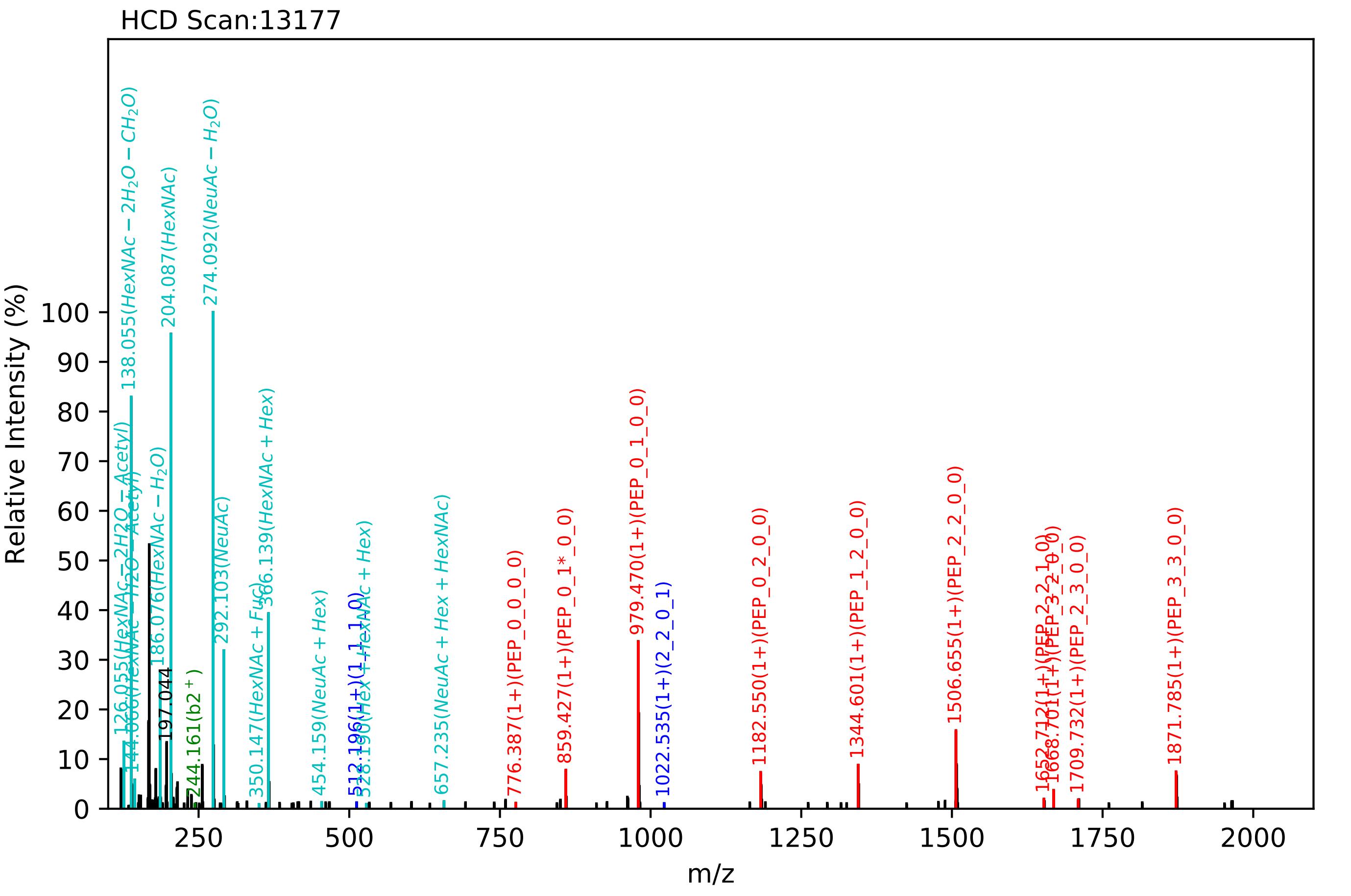

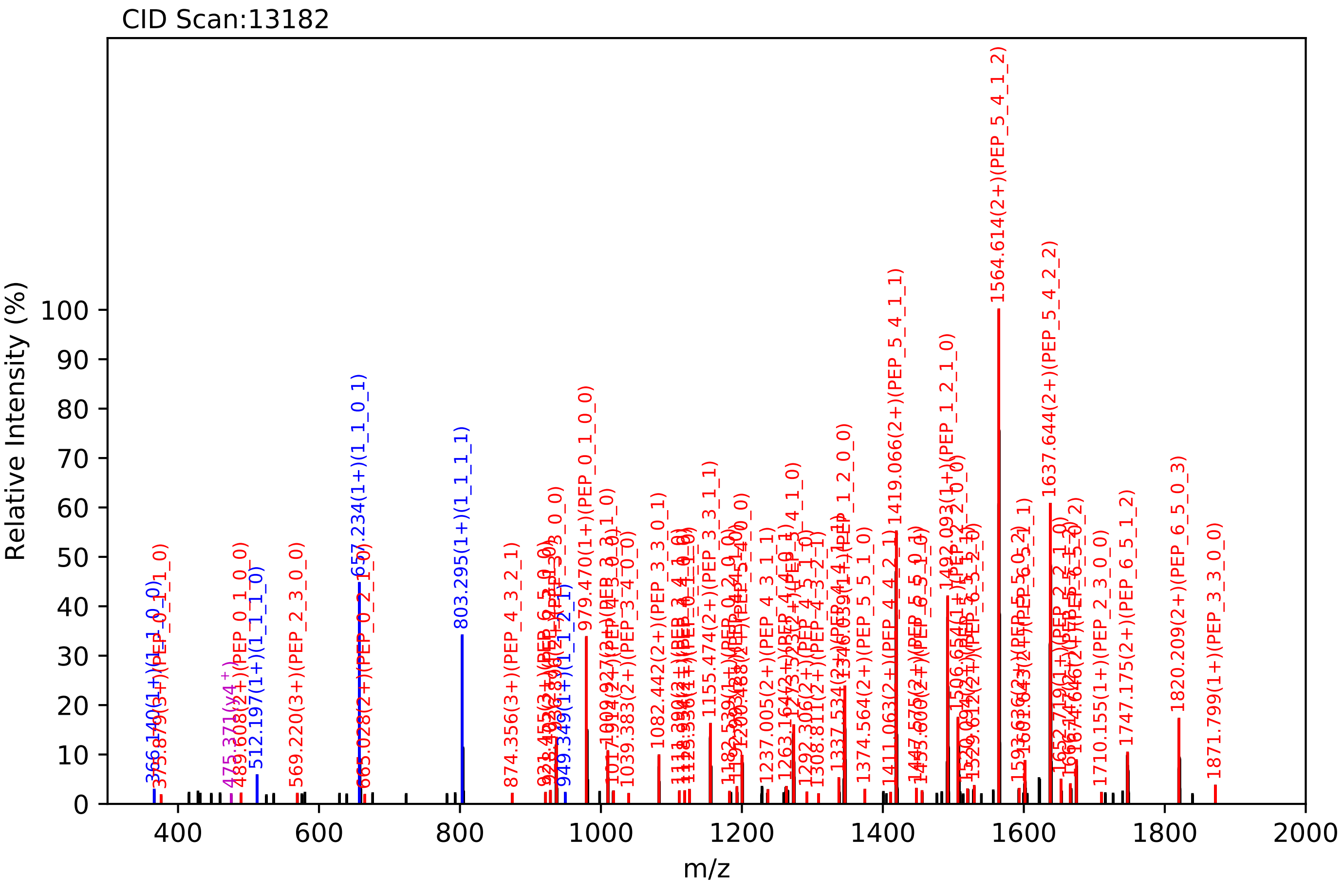

Training set no. 38, Experiment: AGP exp_42

ENGTISR(=PEP)_6_5_2_3, m/z:1310.51(3+), RT:33.79, Y-score:88.83

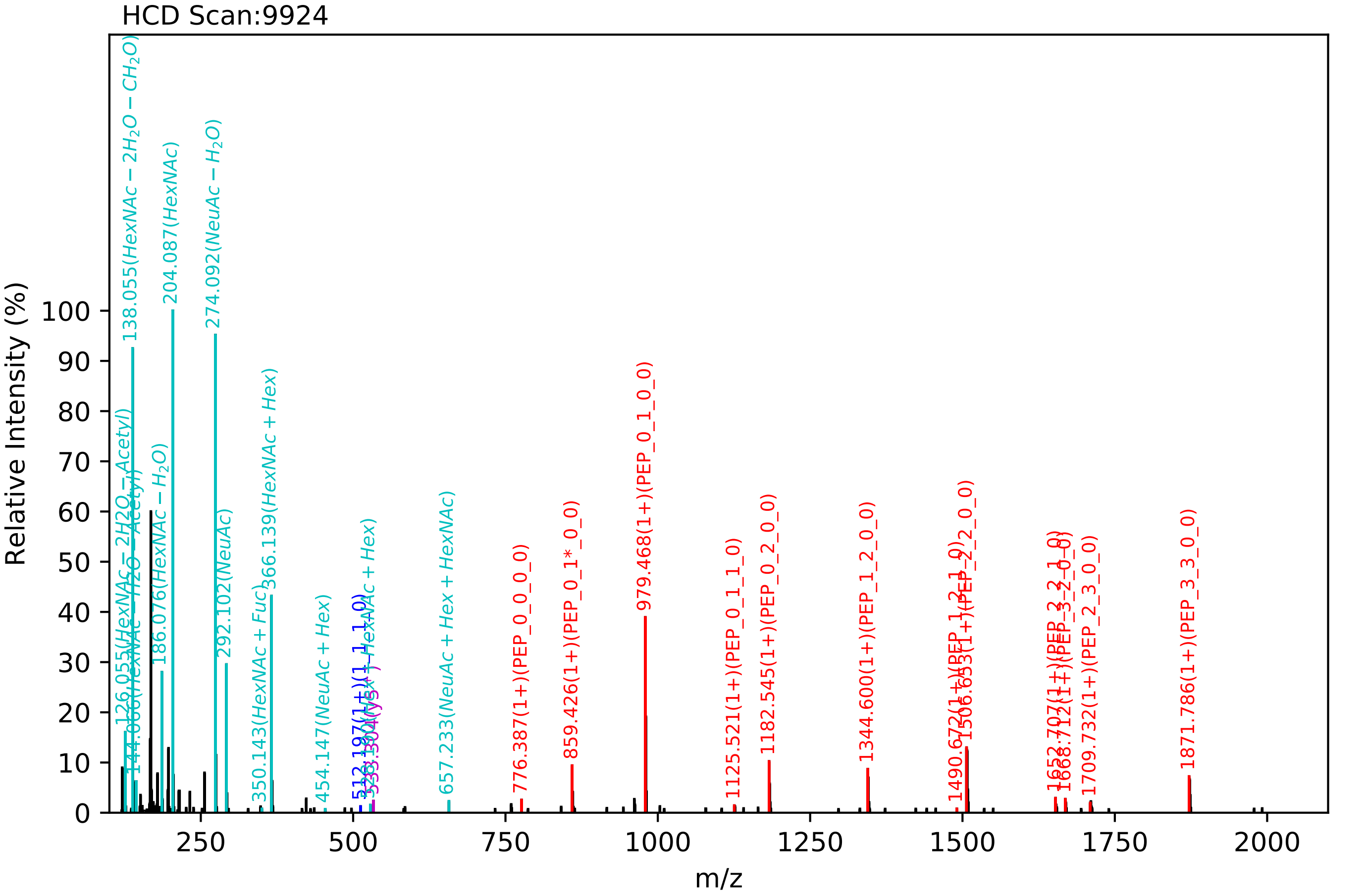

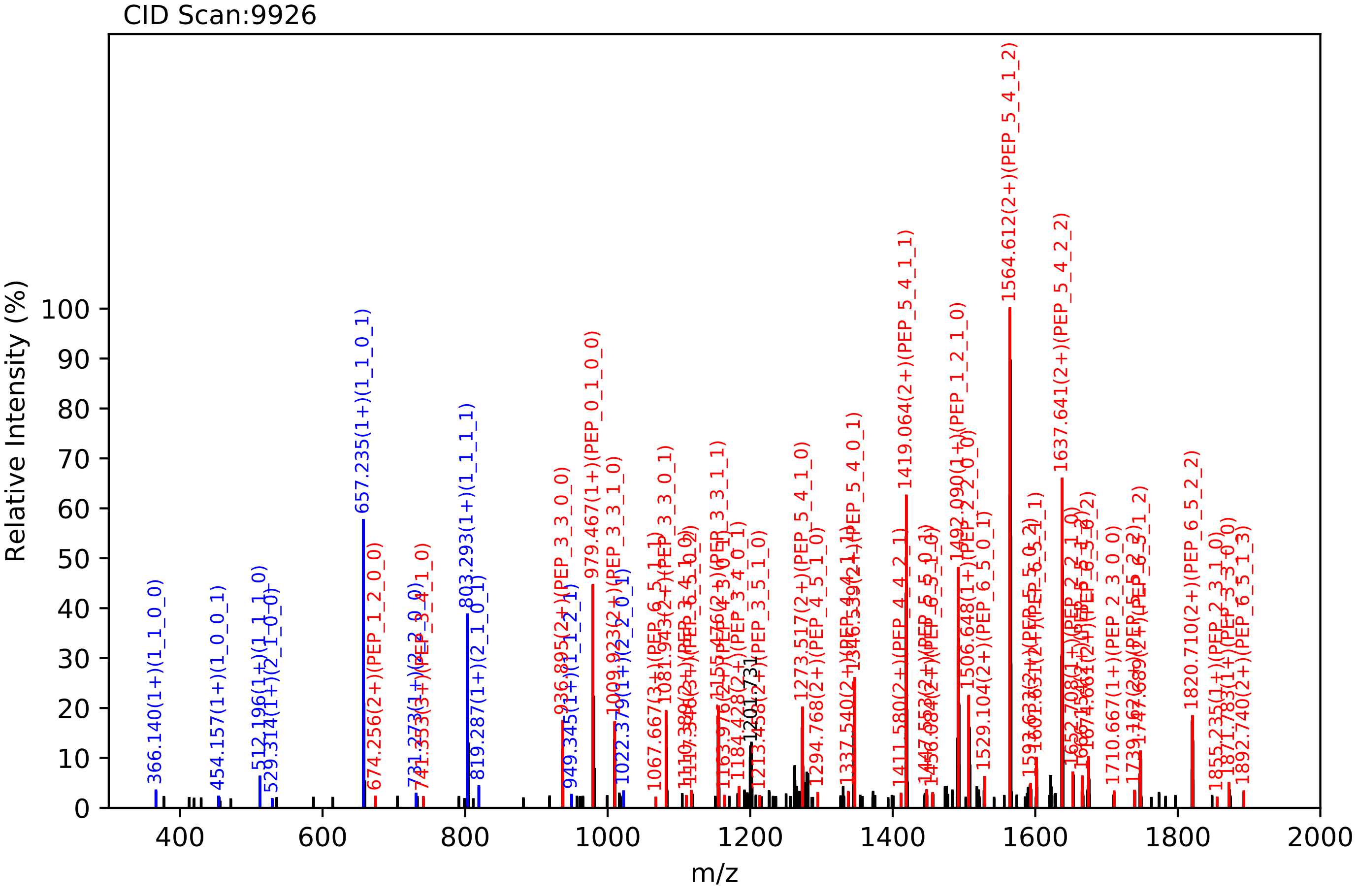

Training set no. 39, Experiment: AGP exp_19

ENGTISR(=PEP)_6_5_2_3, m/z:1310.51(3+), RT:33.89, Y-score:88.44

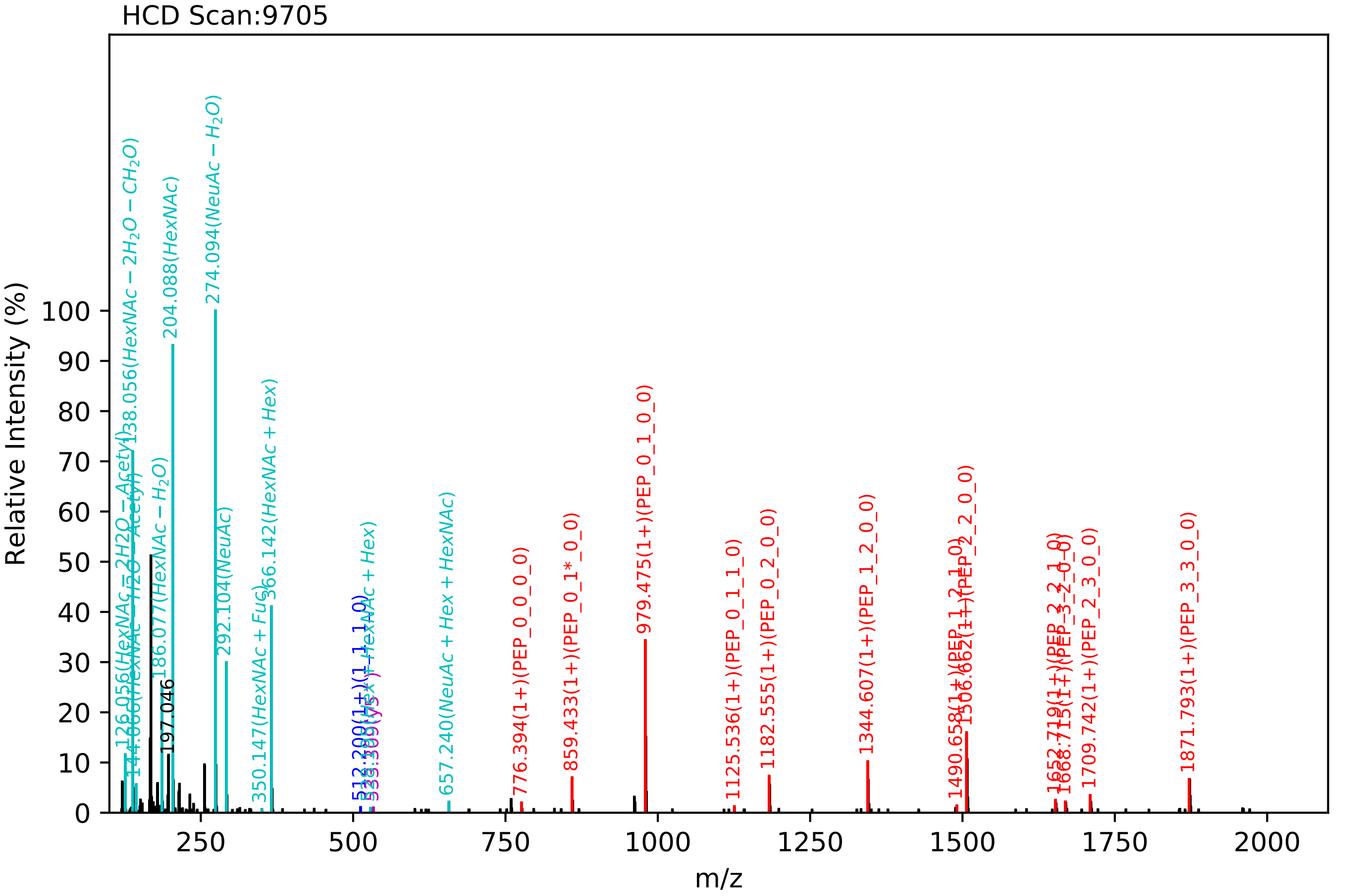

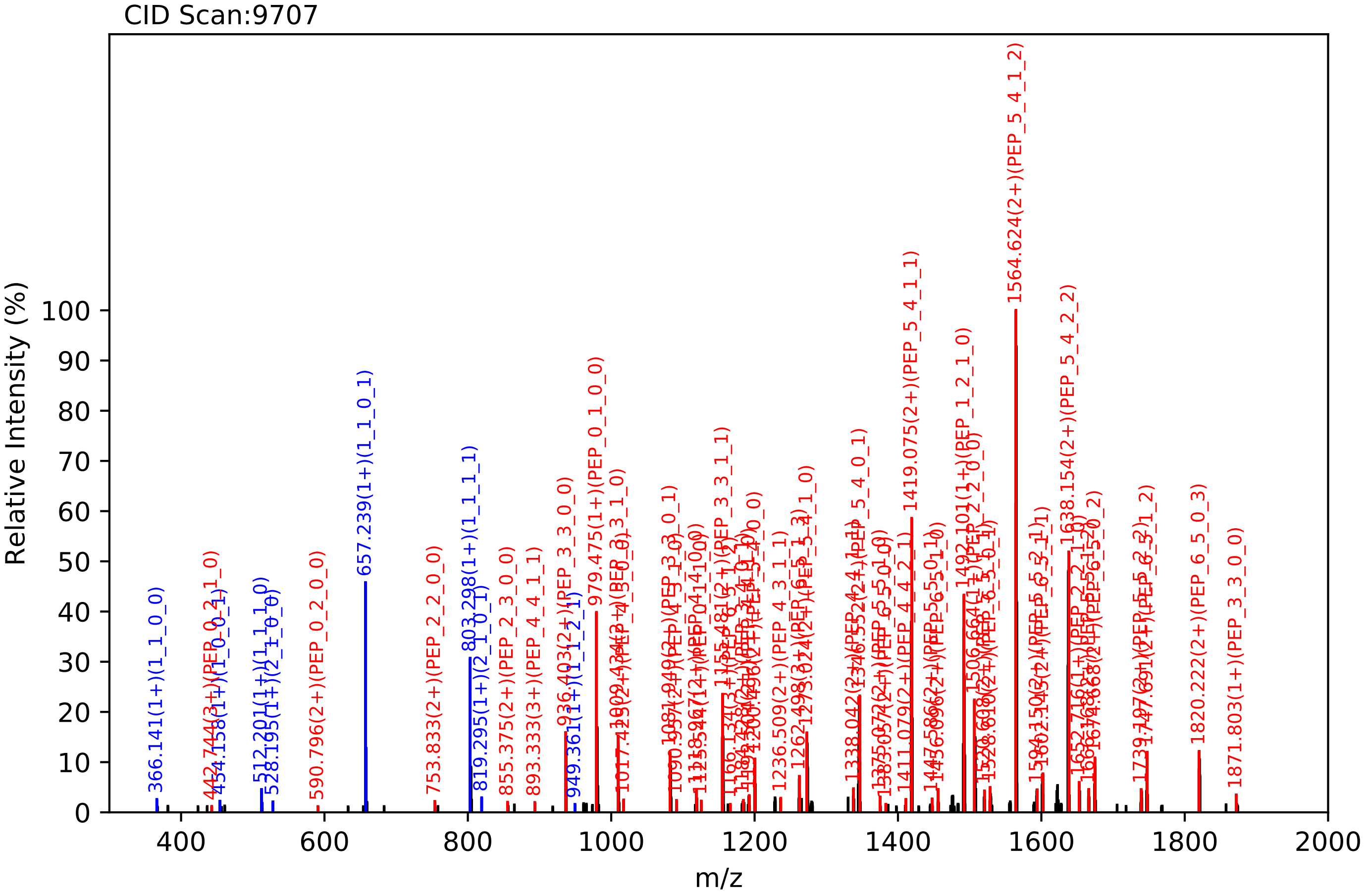

Training set no. 40, Experiment: AGP exp_17

ENGTISR(=PEP)_6_5_2_3, m/z:1310.51(3+), RT:33.79, Y-score:88.11

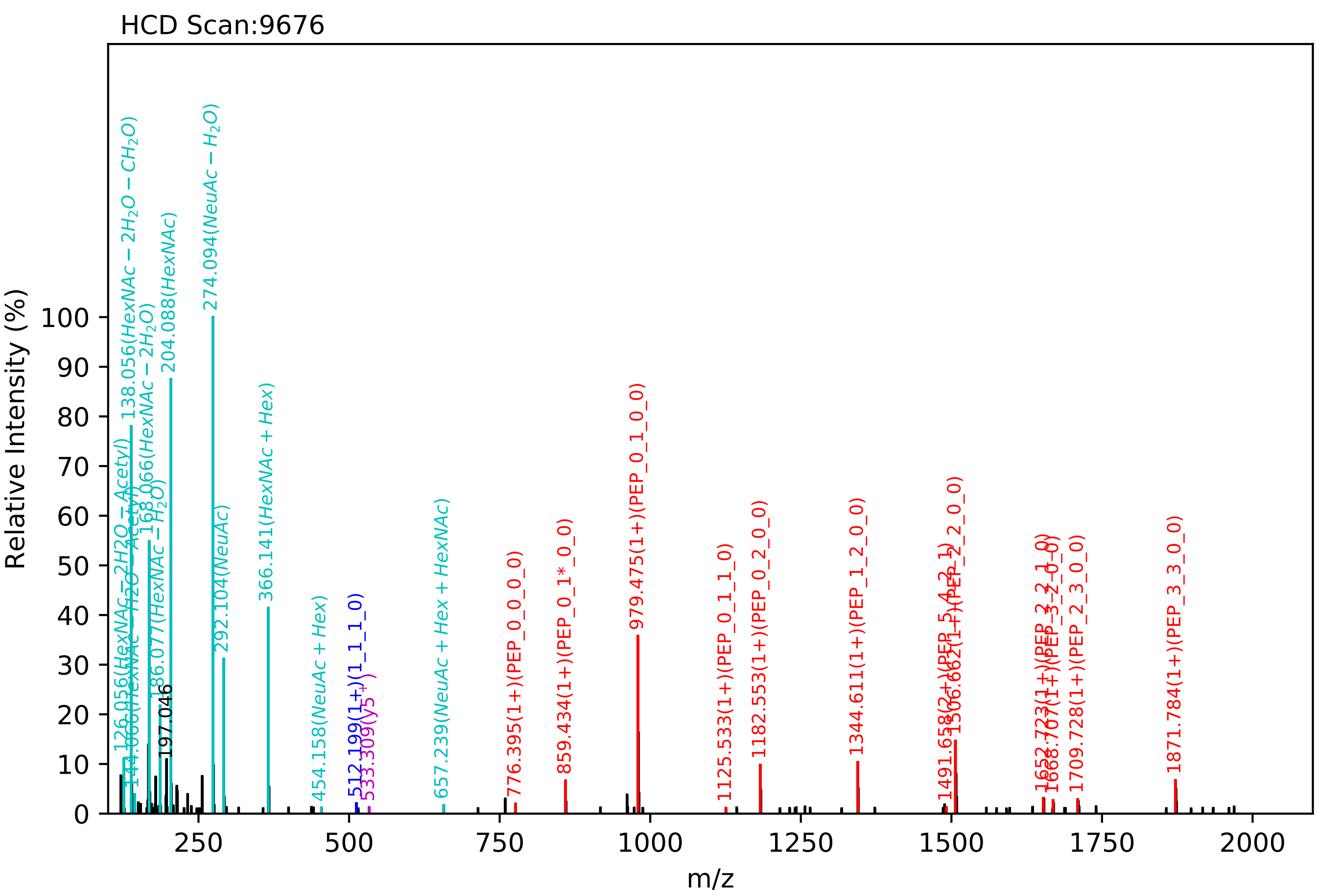

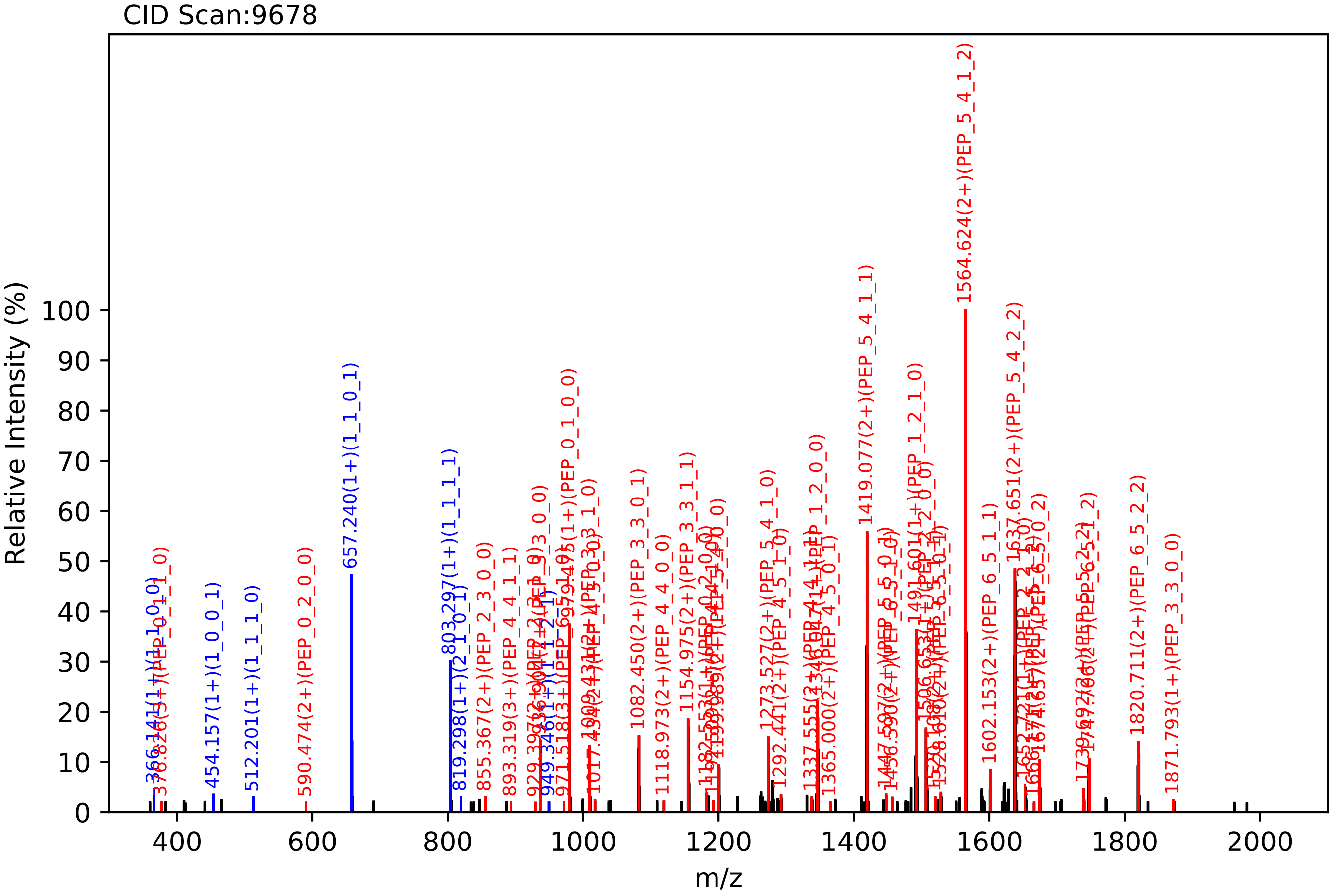

Training set no. 41, Experiment: AGP exp_18

ENGTISR(=PEP)_6_5_2_3, m/z:1310.51(3+), RT:34.02, Y-score:88.02

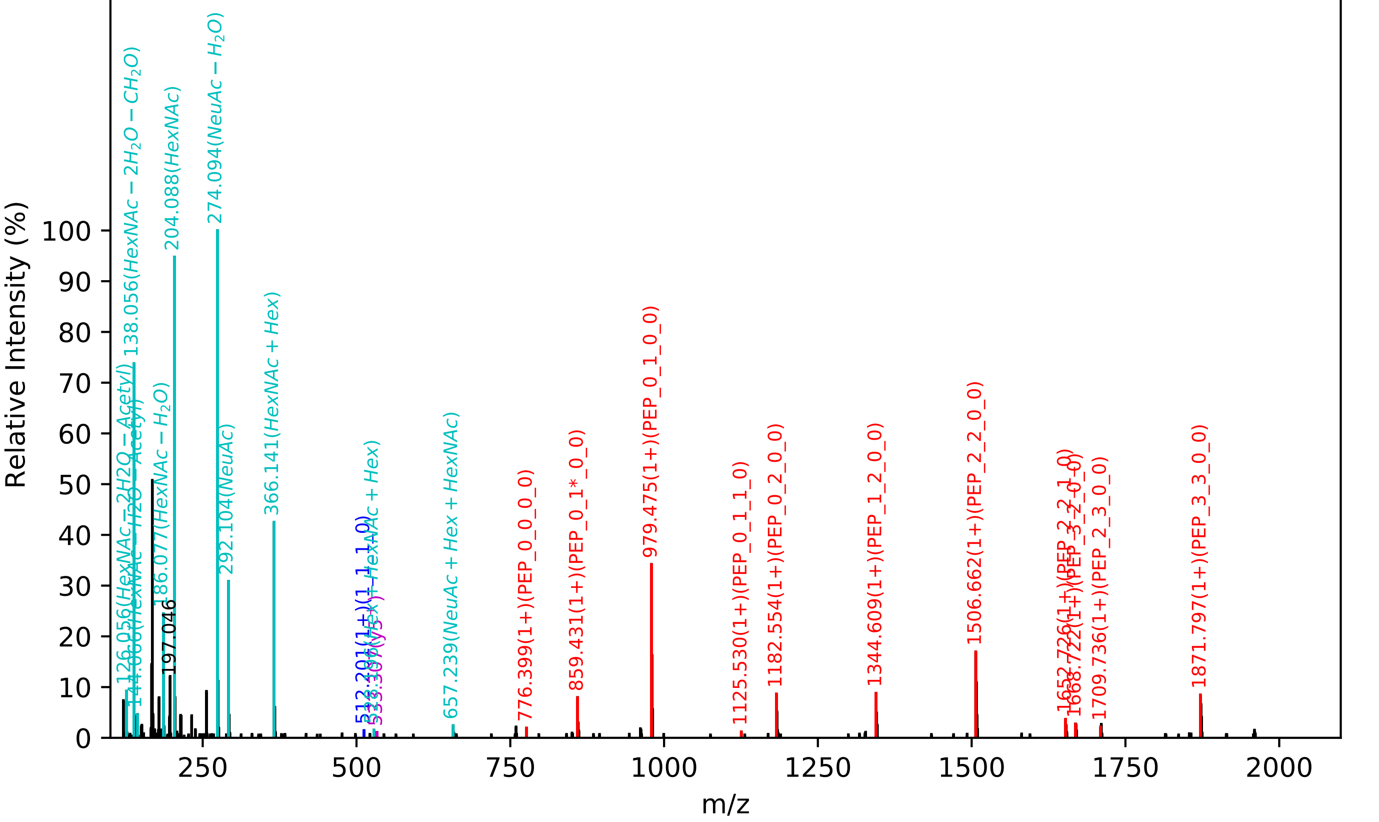

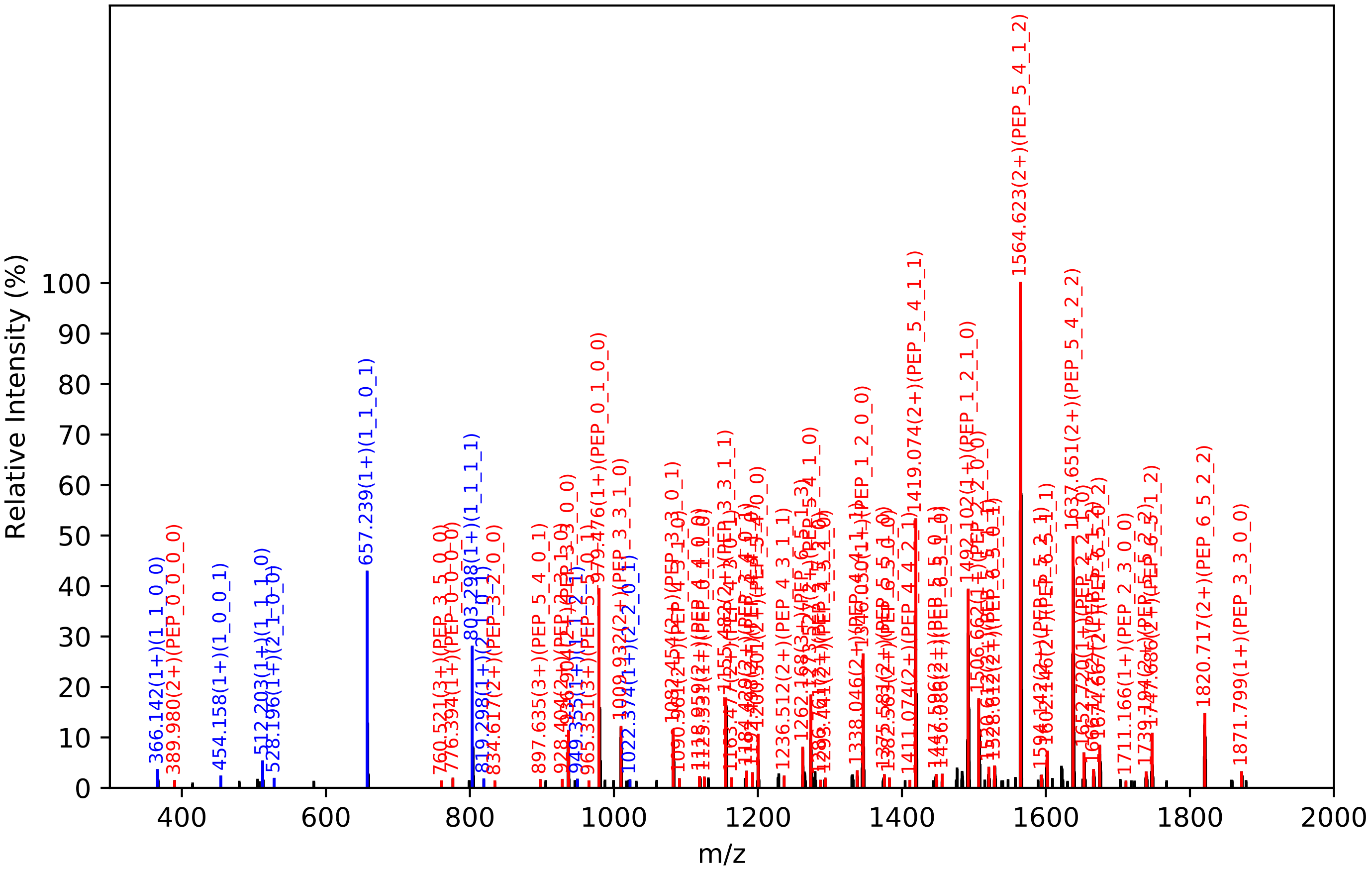

Training set no. 42, Experiment: AGP exp_38

ENGTISR(=PEP)_6_5_2_3, m/z:1310.51(3+), RT:33.70, Y-score:87.56

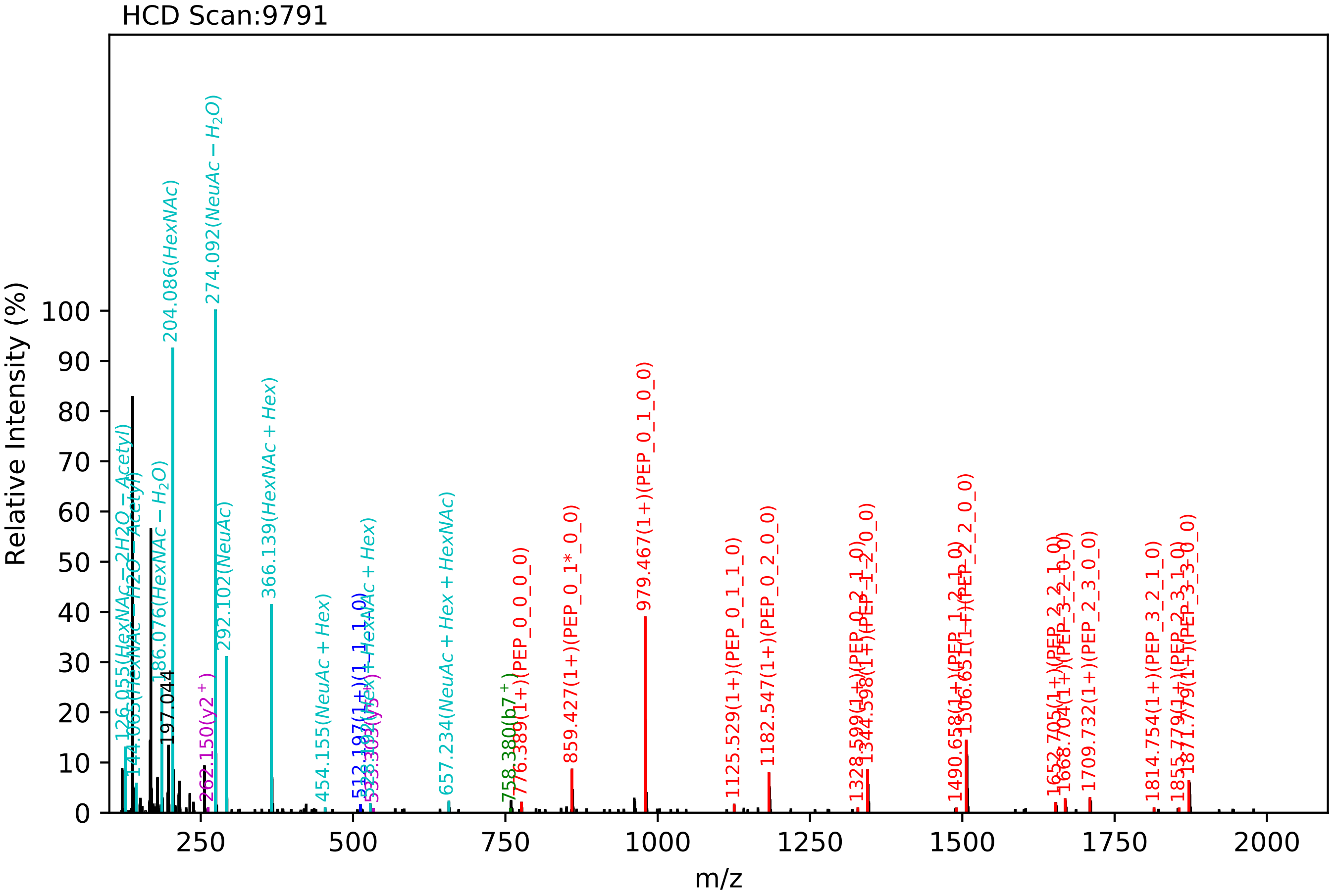

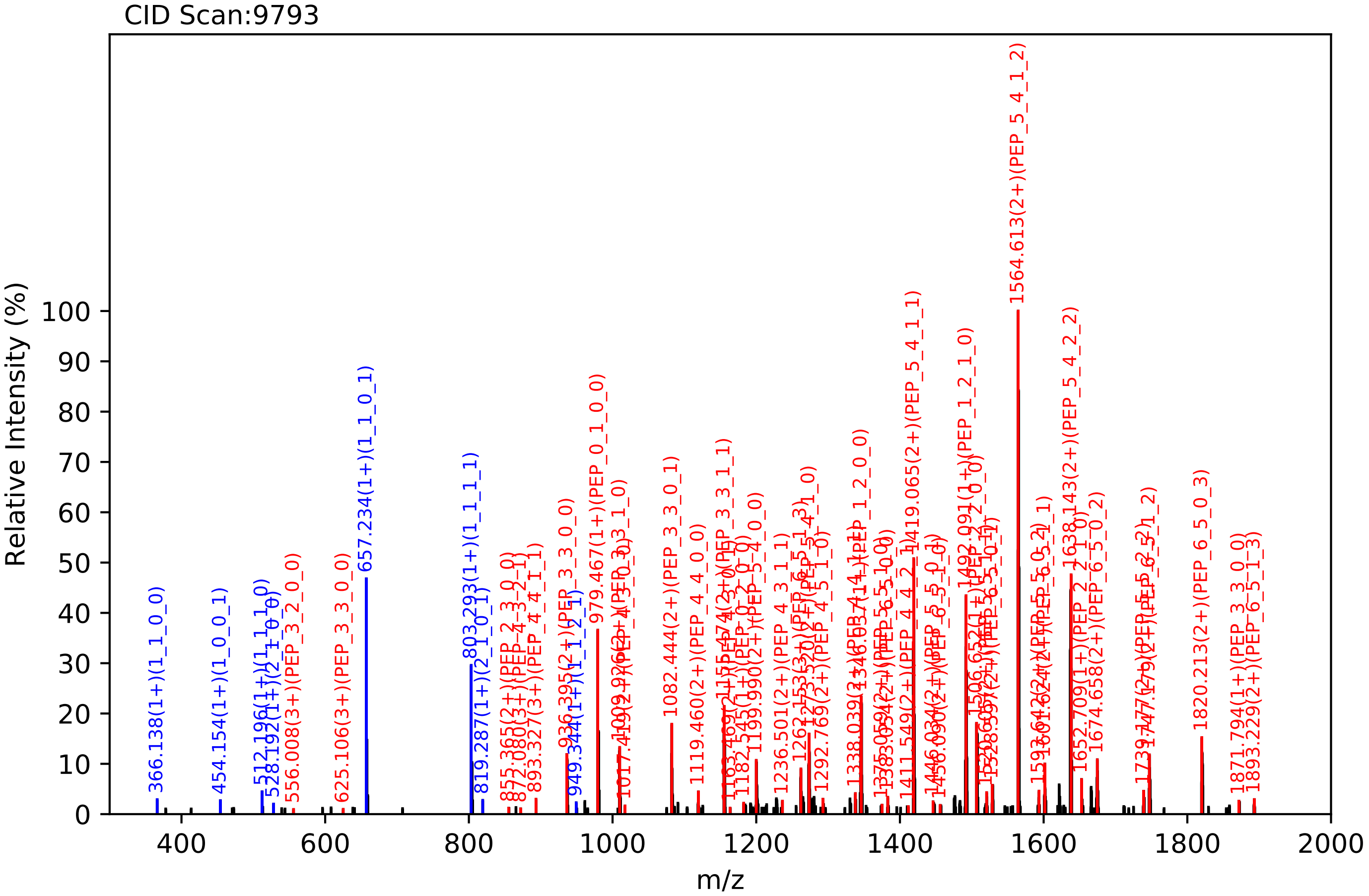

Training set no. 43, Experiment: AGP exp_26

ENGTISR(=PEP)_6_5_2_3, m/z:1310.51(3+), RT:34.55, Y-score:87.48

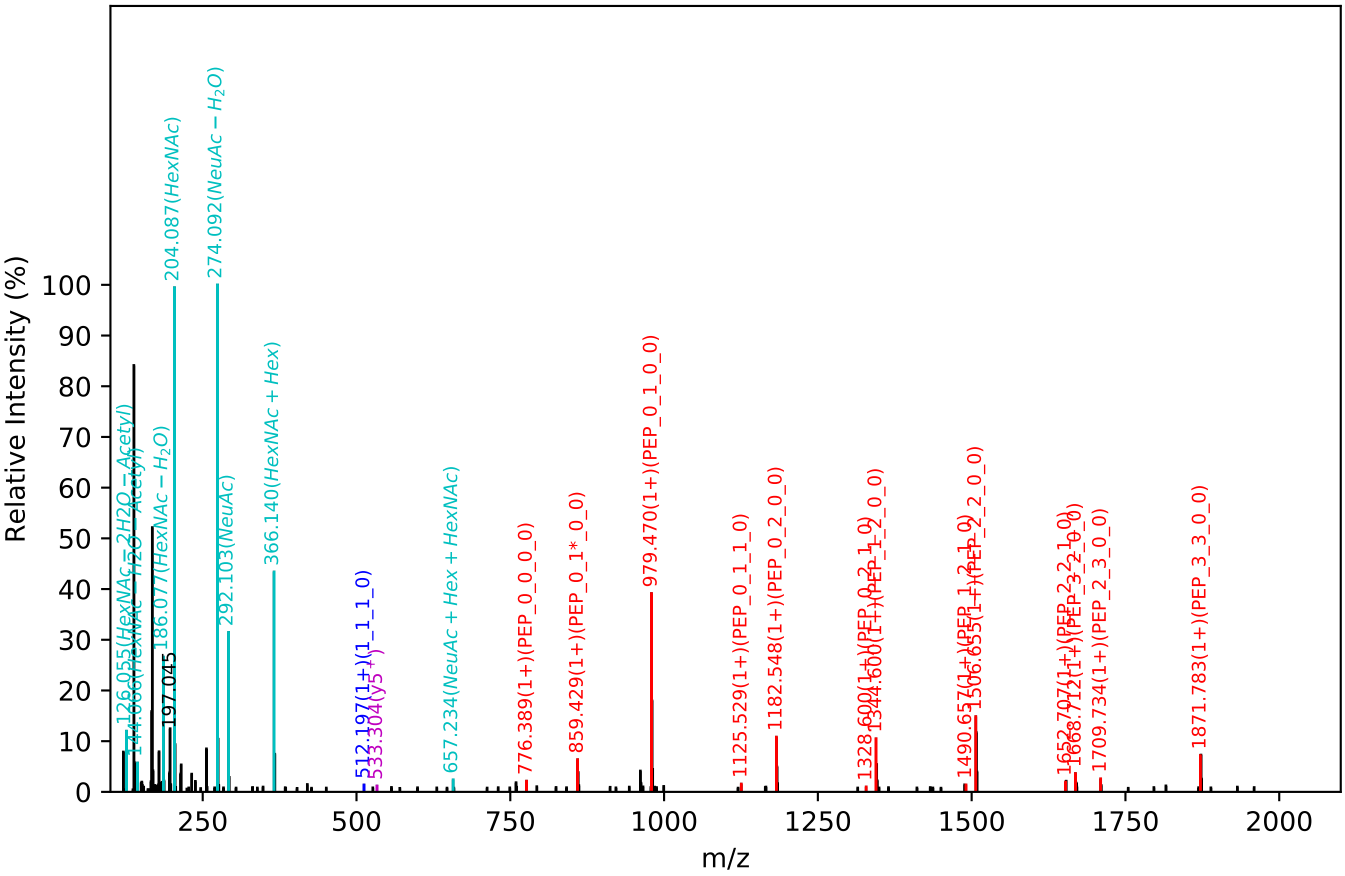

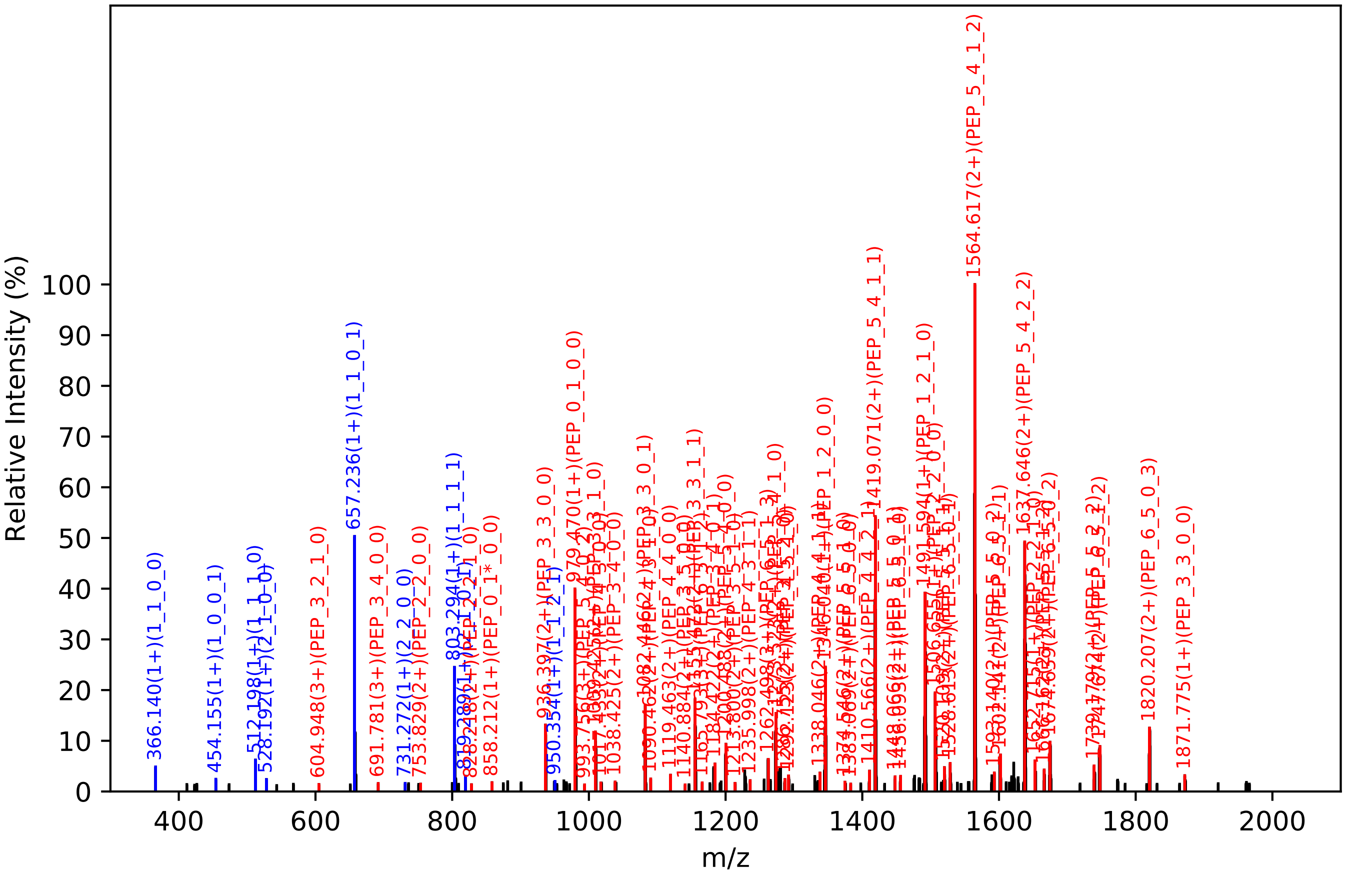

Training set no. 44, Experiment: AGP exp_31

ENGTISR(=PEP)_6_5_2_3, m/z:1310.51(3+), RT:38.59, Y-score:87.16

HCD Scan:6867

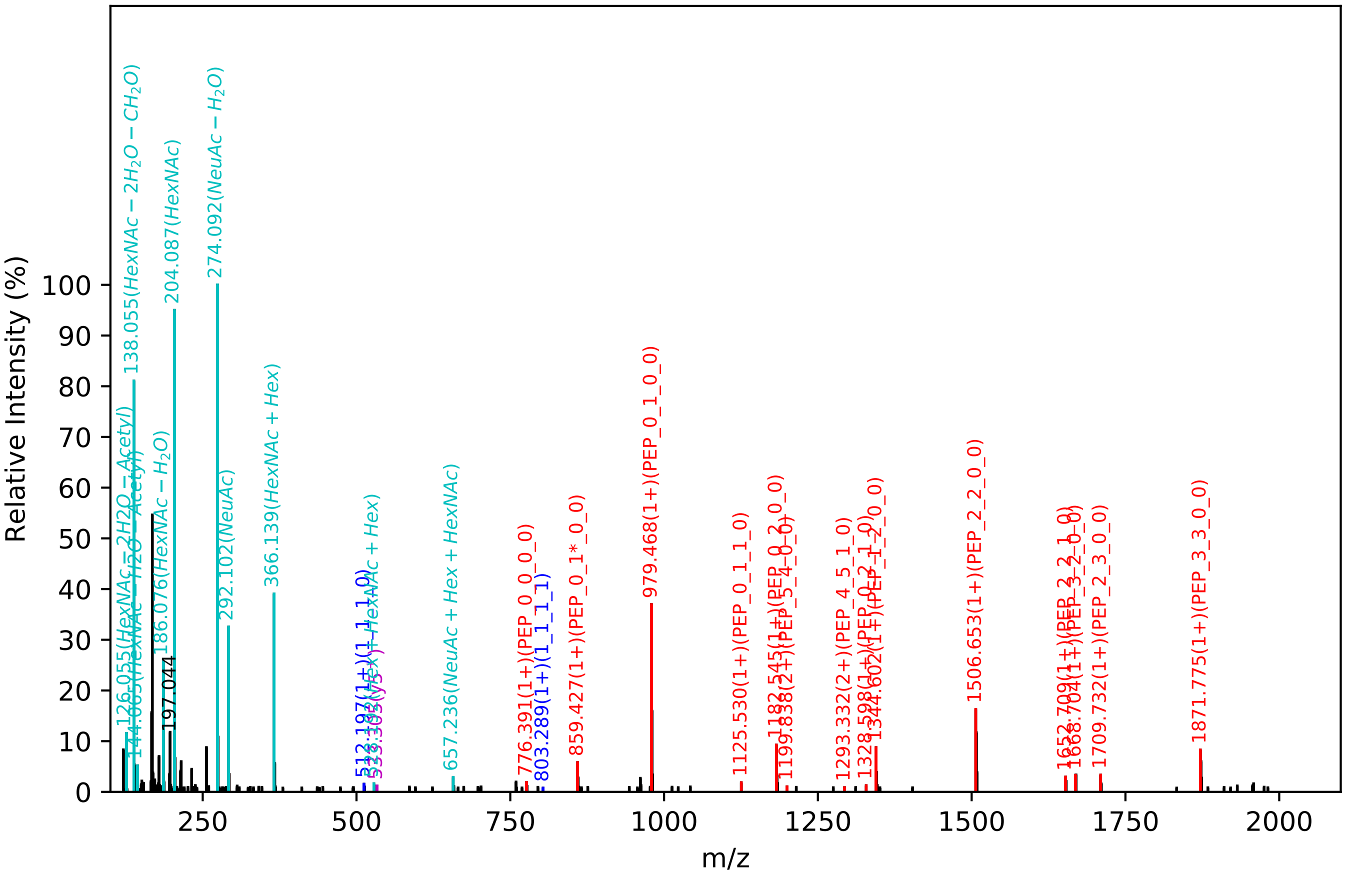

CID Scan:6869

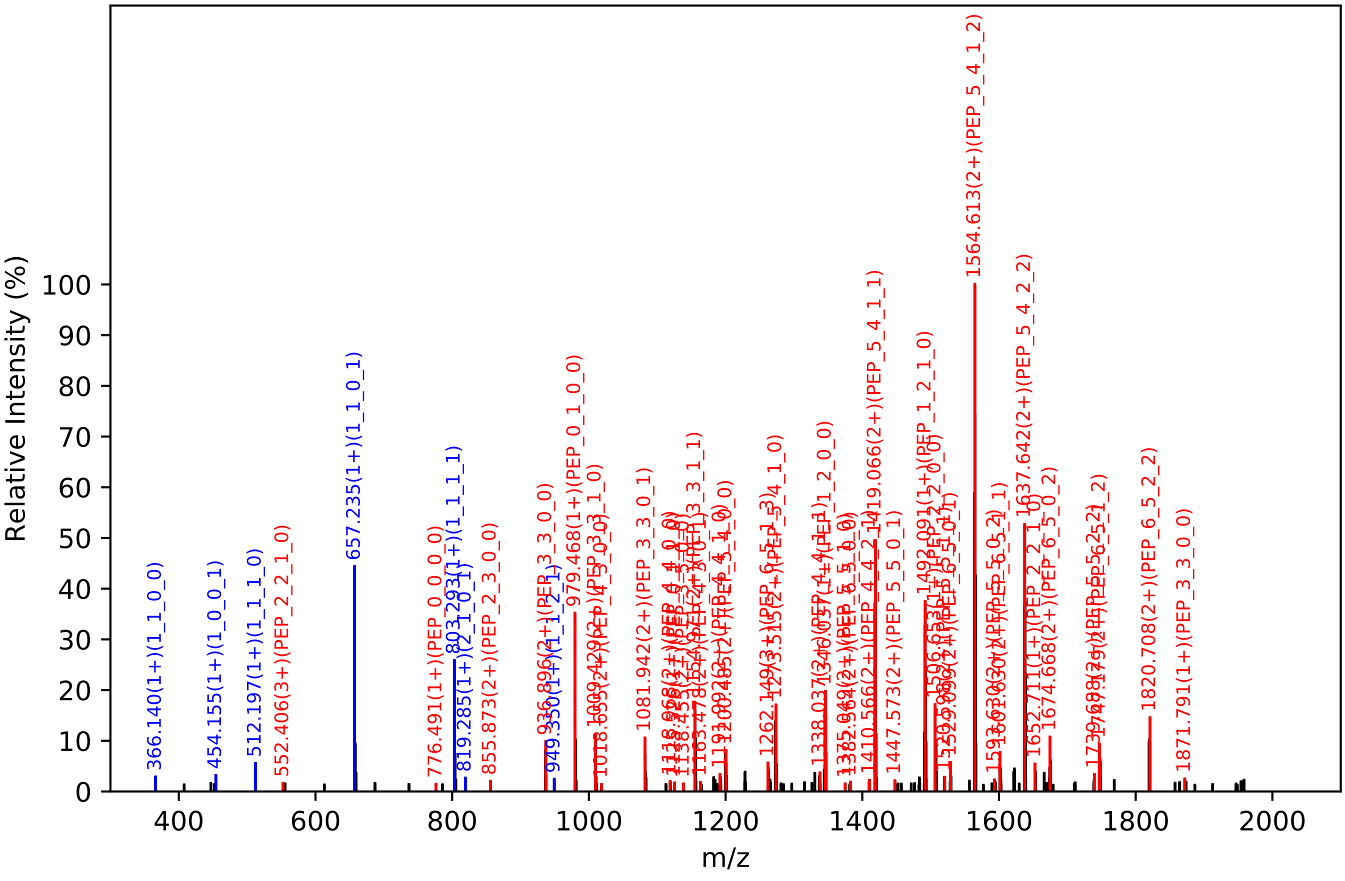

Training set no. 45, Experiment: AGP exp_37

ENGTISR(=PEP)_6_5_2_3, m/z:1310.51(3+), RT:34.08, Y-score:86.98

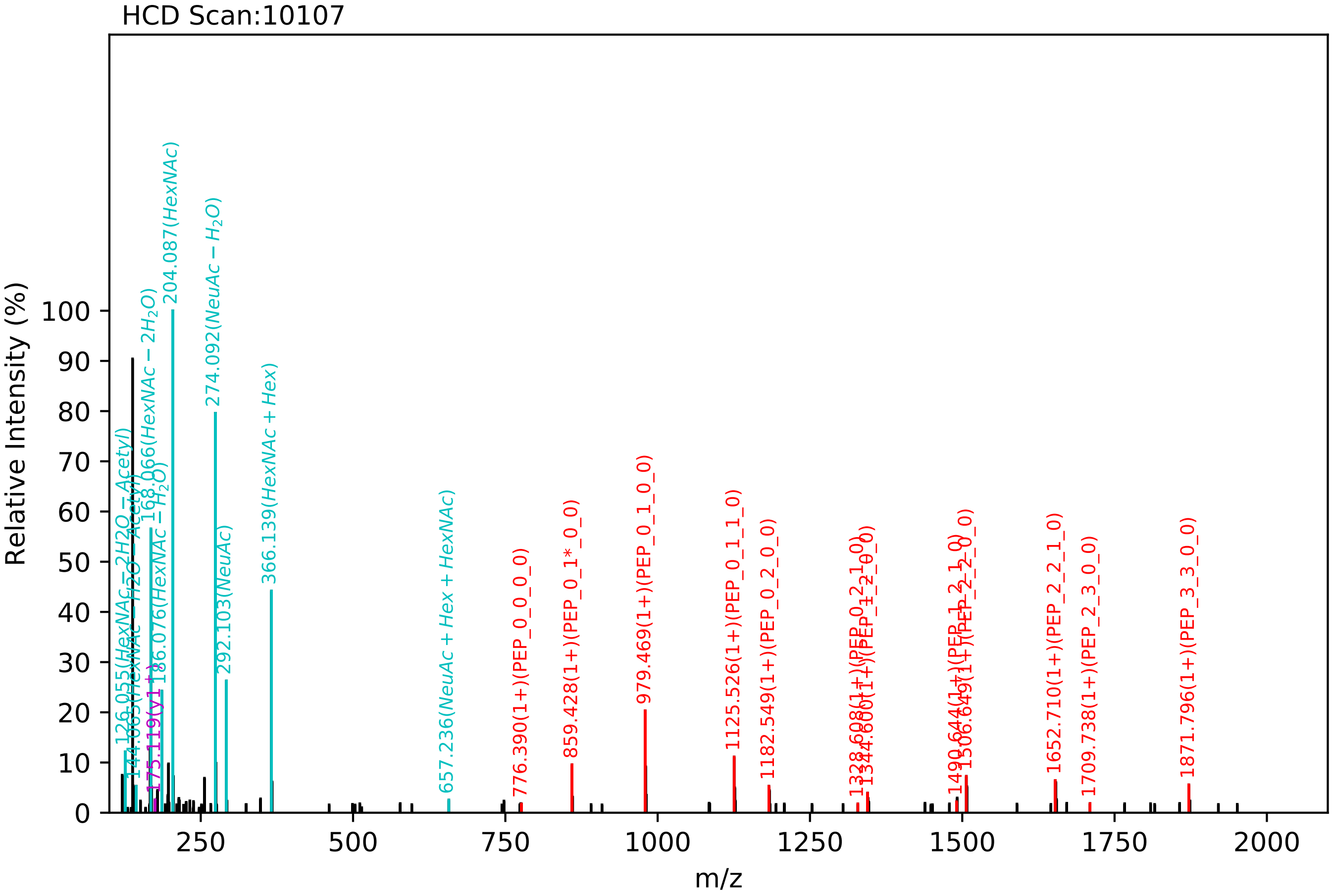

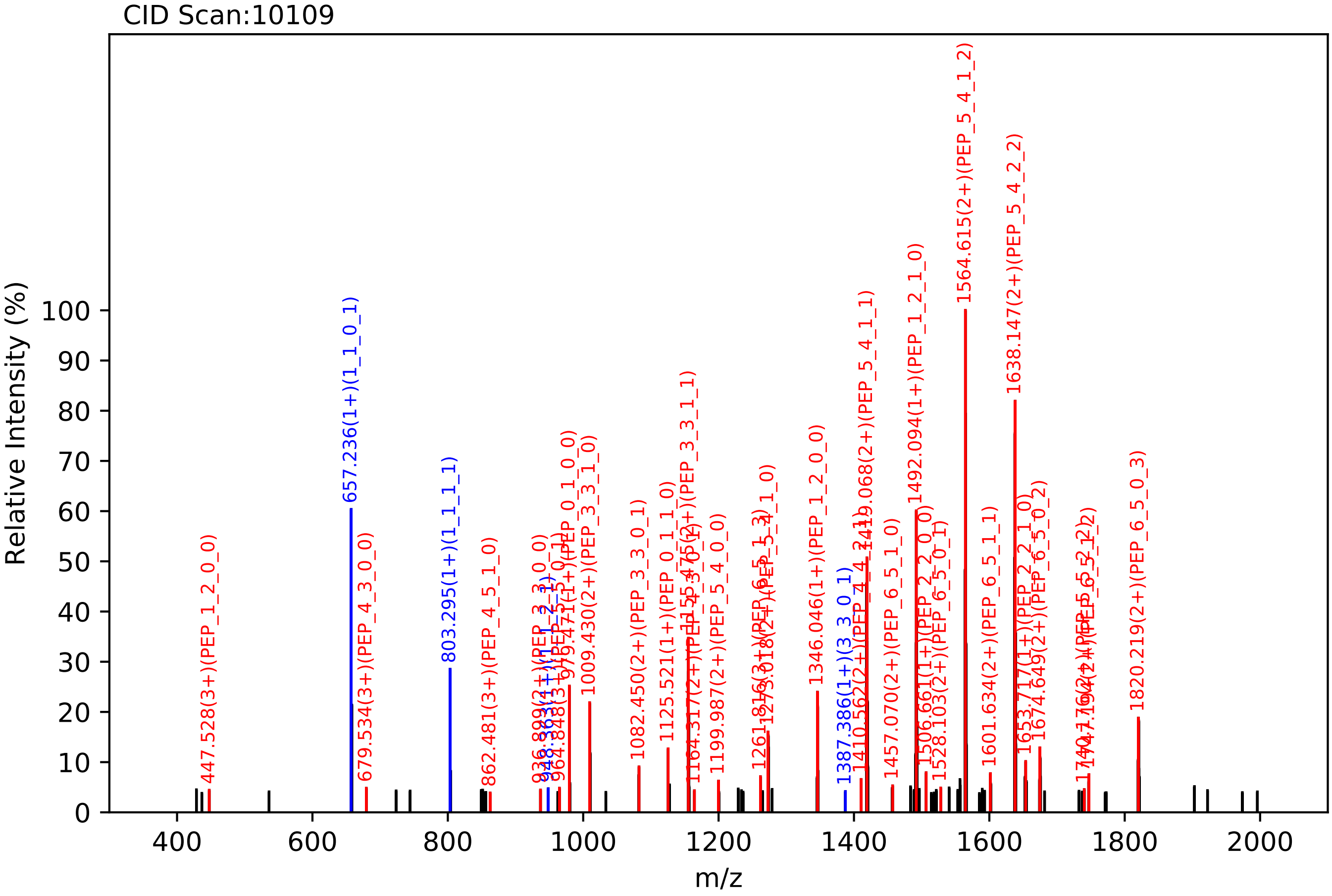

Training set no. 46, Experiment: AGP exp_13

ENGTISR(=PEP)_6_5_2_3, m/z:1310.51(3+), RT:41.77, Y-score:86.46

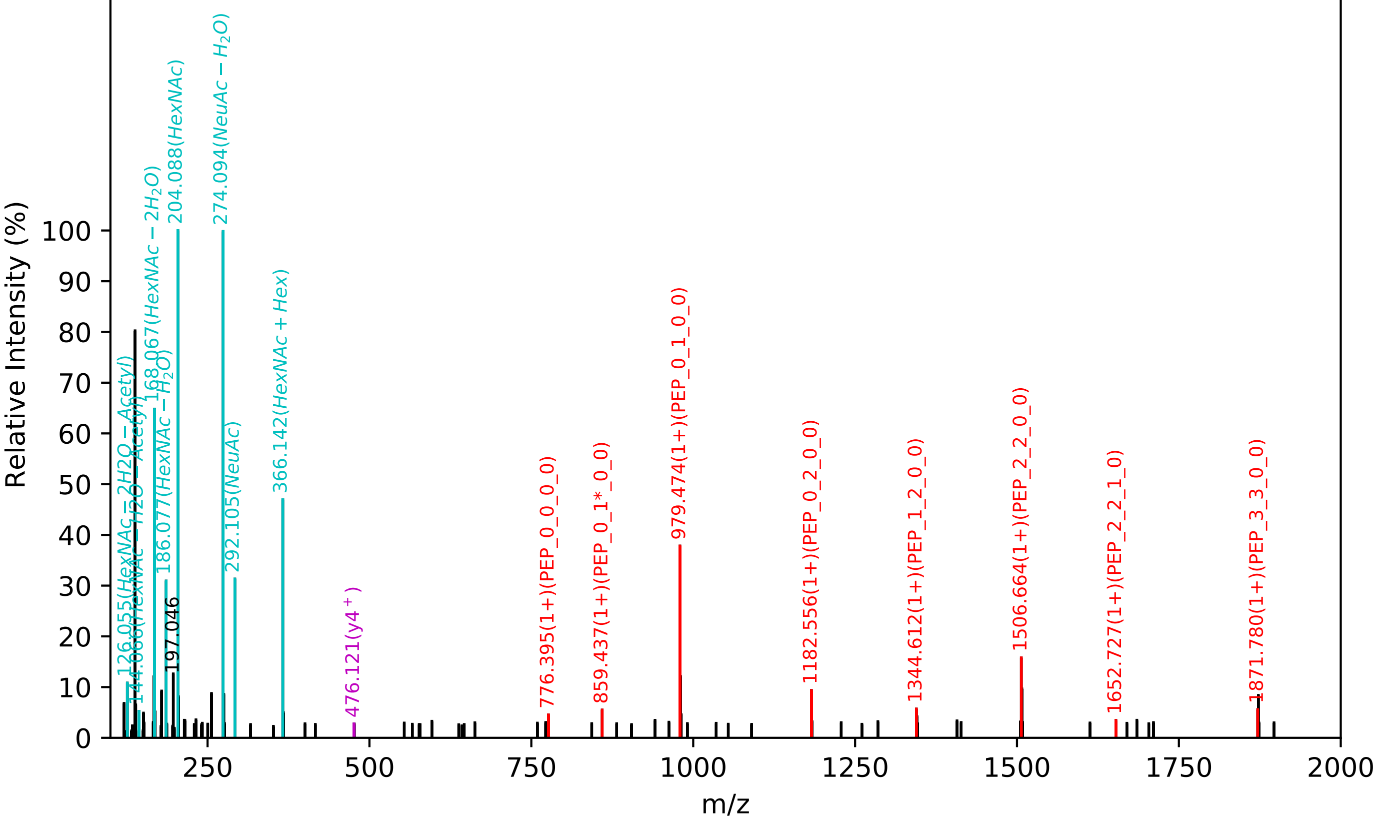

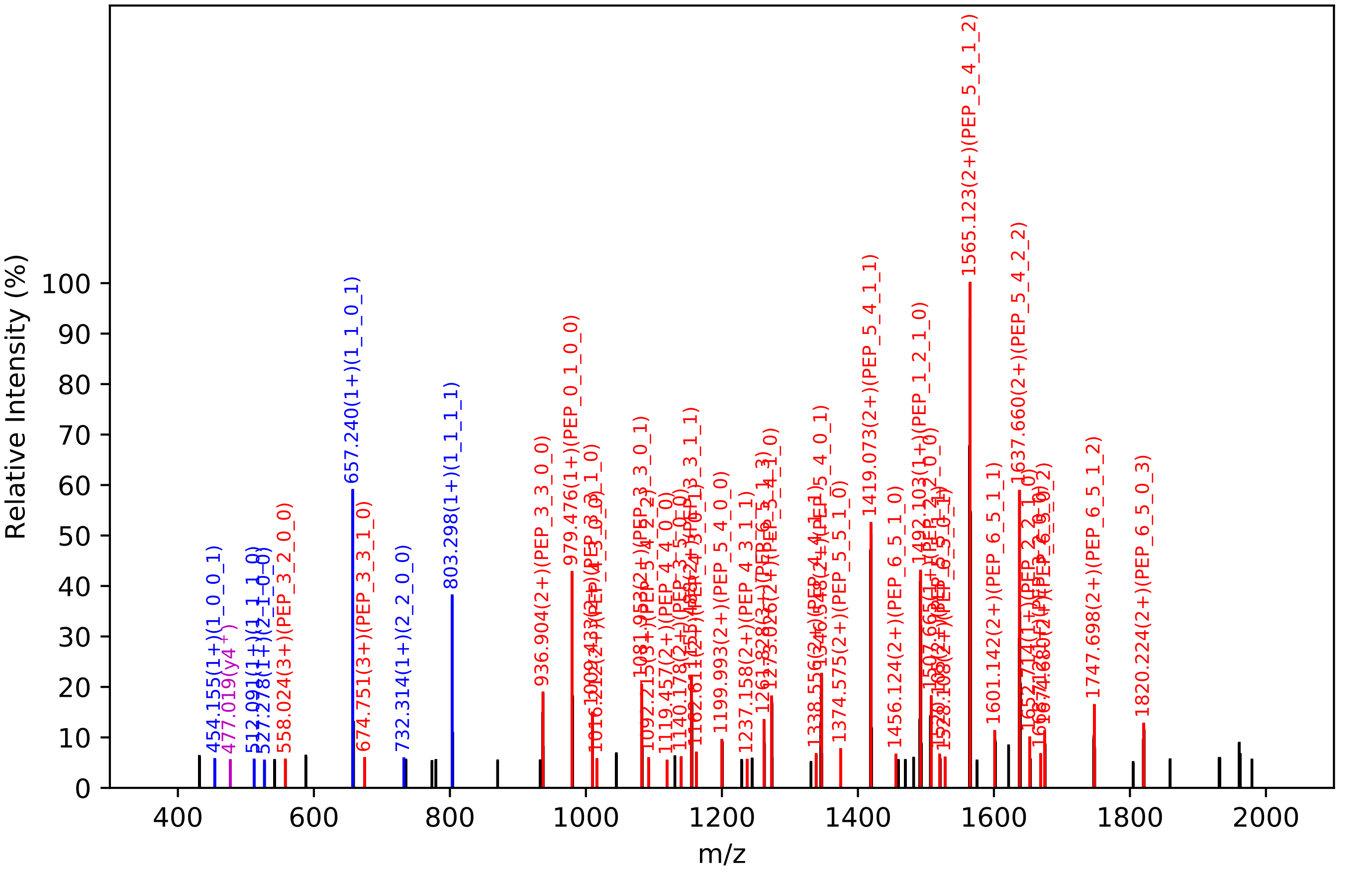

Training set no. 47, Experiment: AGP exp_1

ENGTISR(=PEP)_6_5_2_3, m/z:1310.51(3+), RT:22.60, Y-score:85.85

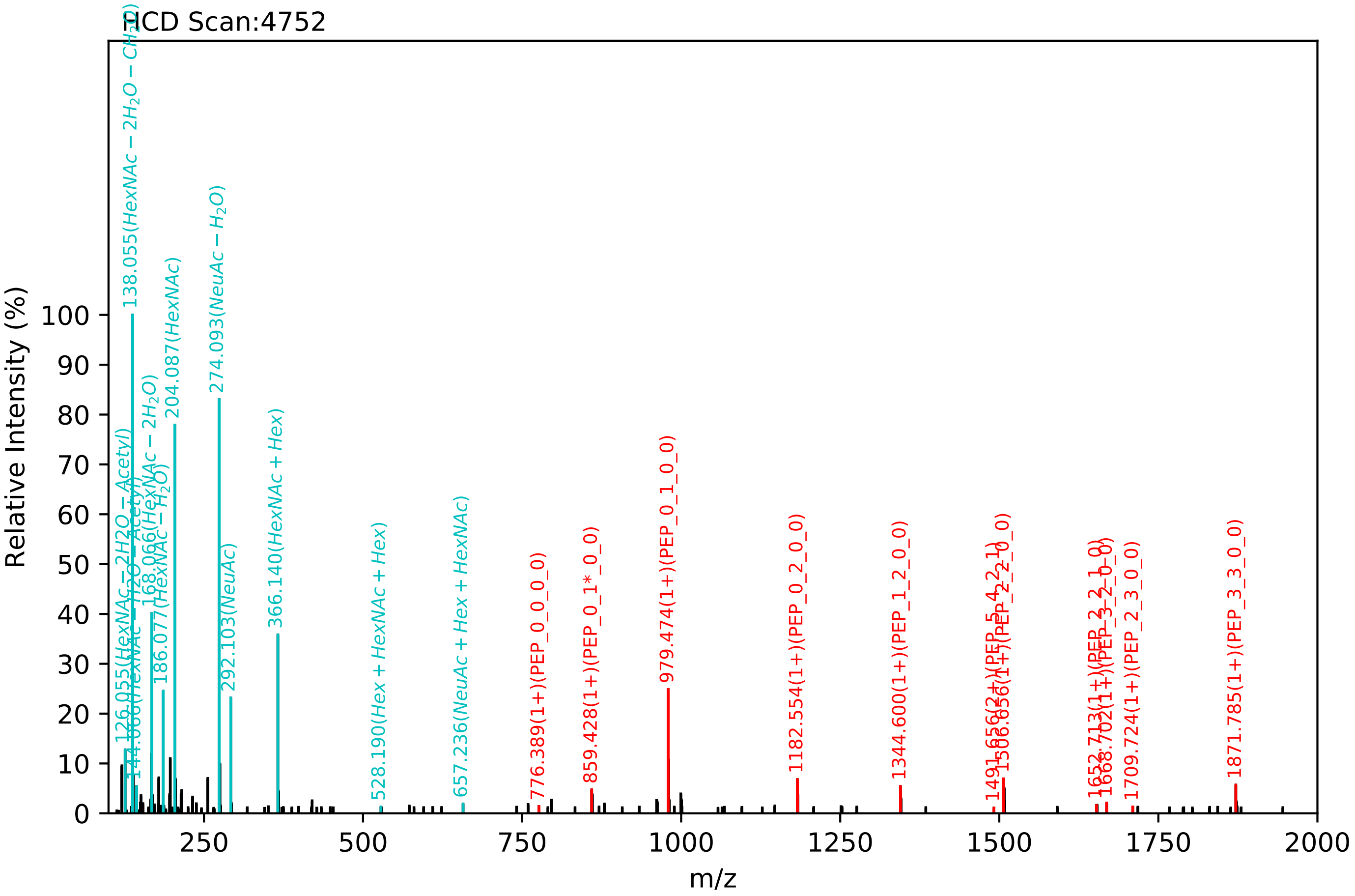

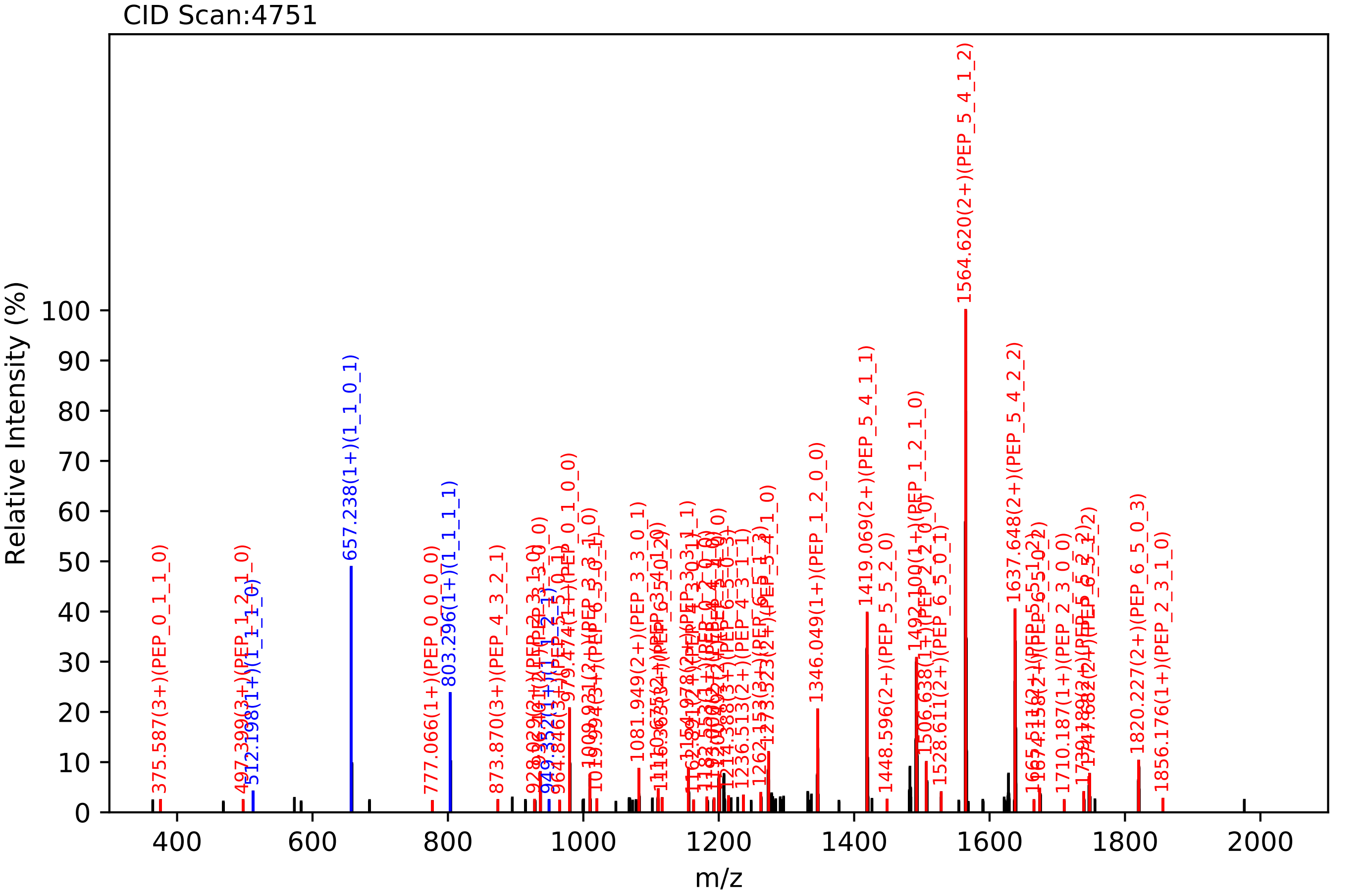

Training set no. 48, Experiment: AGP exp_9

ENGTISR(=PEP)_6_5_2_3, m/z:1310.51(3+), RT:33.70, Y-score:85.38

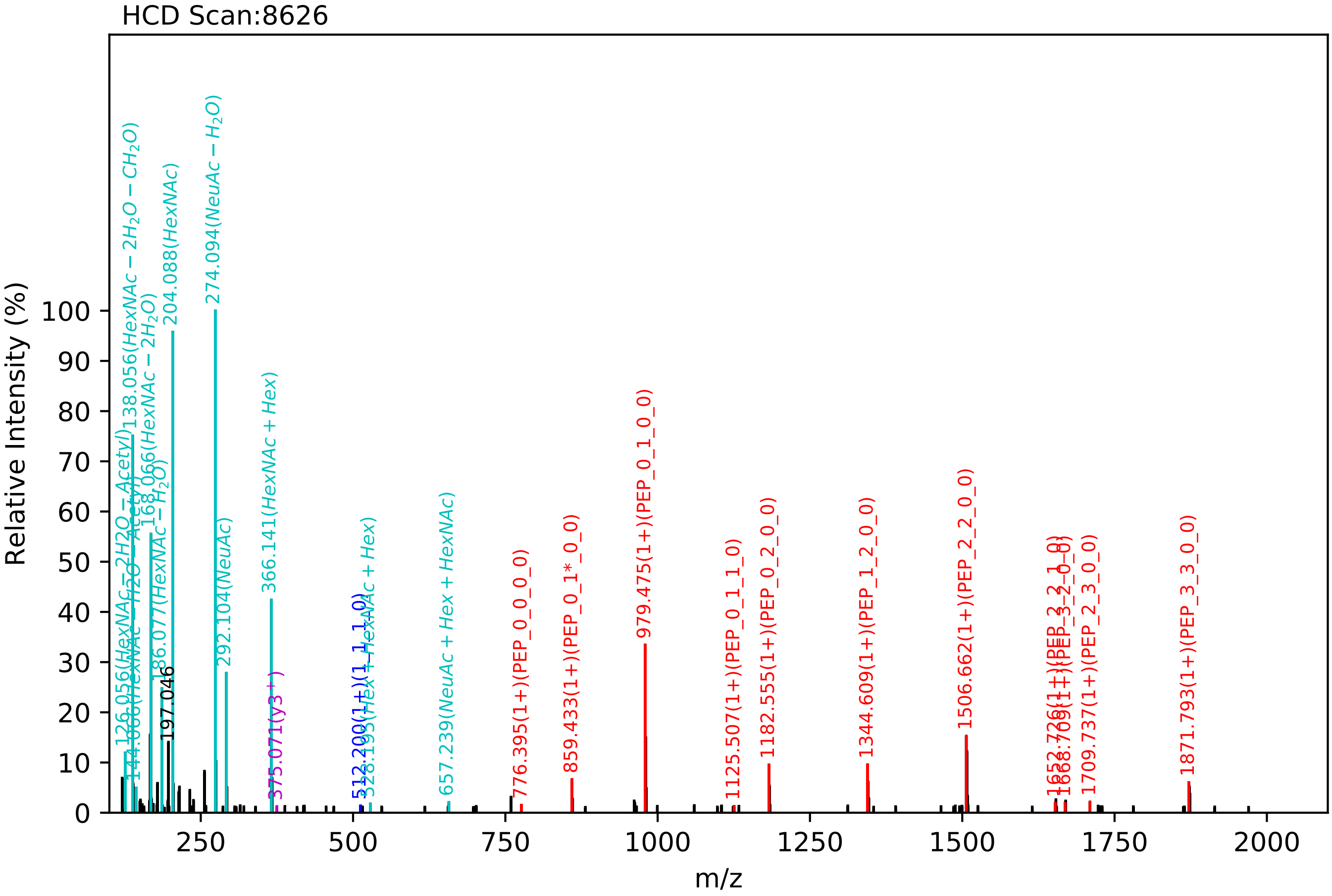

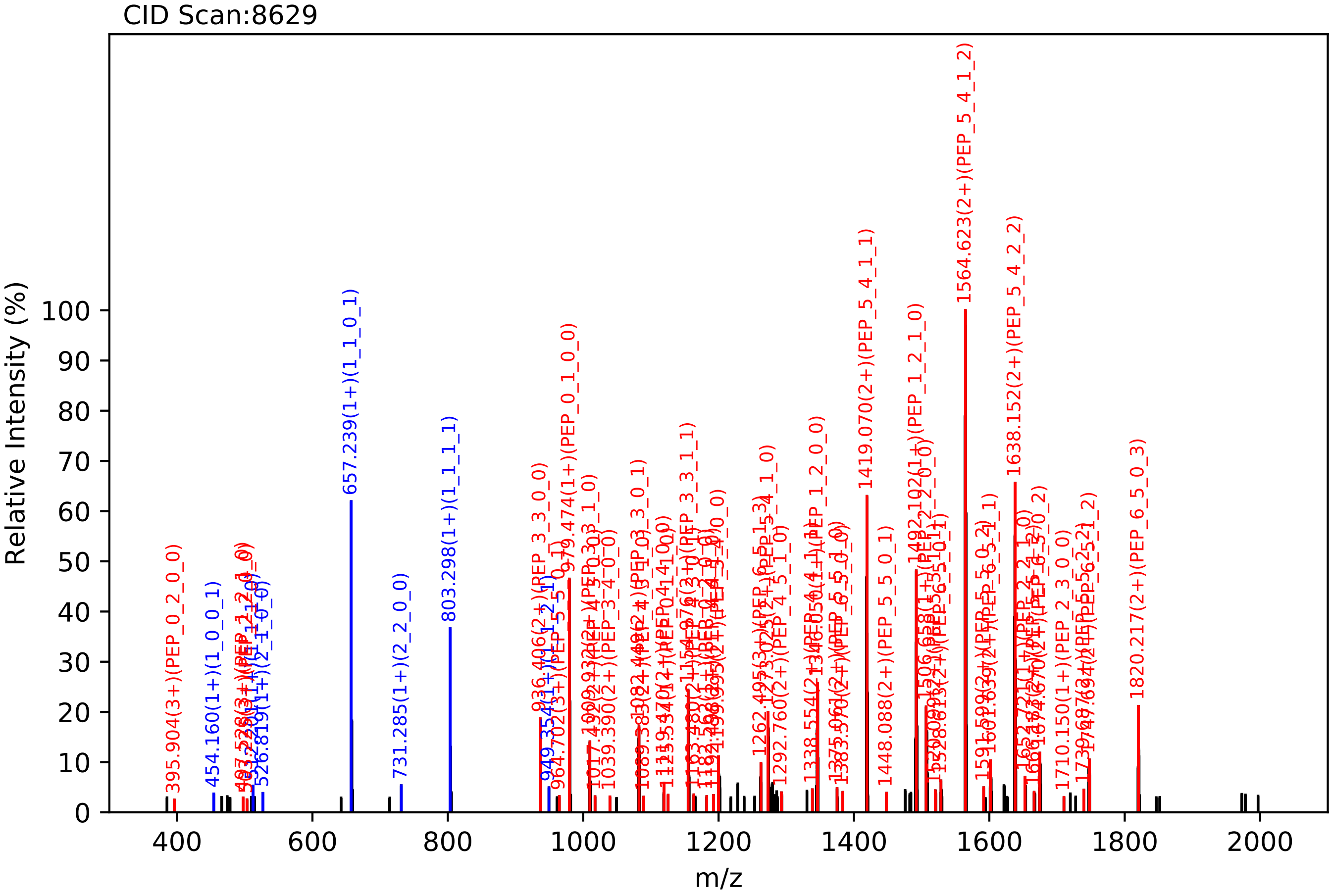

Training set no. 49, Experiment: AGP exp_33

ENGTISR(=PEP)_6_5_2_3, m/z:1310.51(3+), RT:34.41, Y-score:84.88

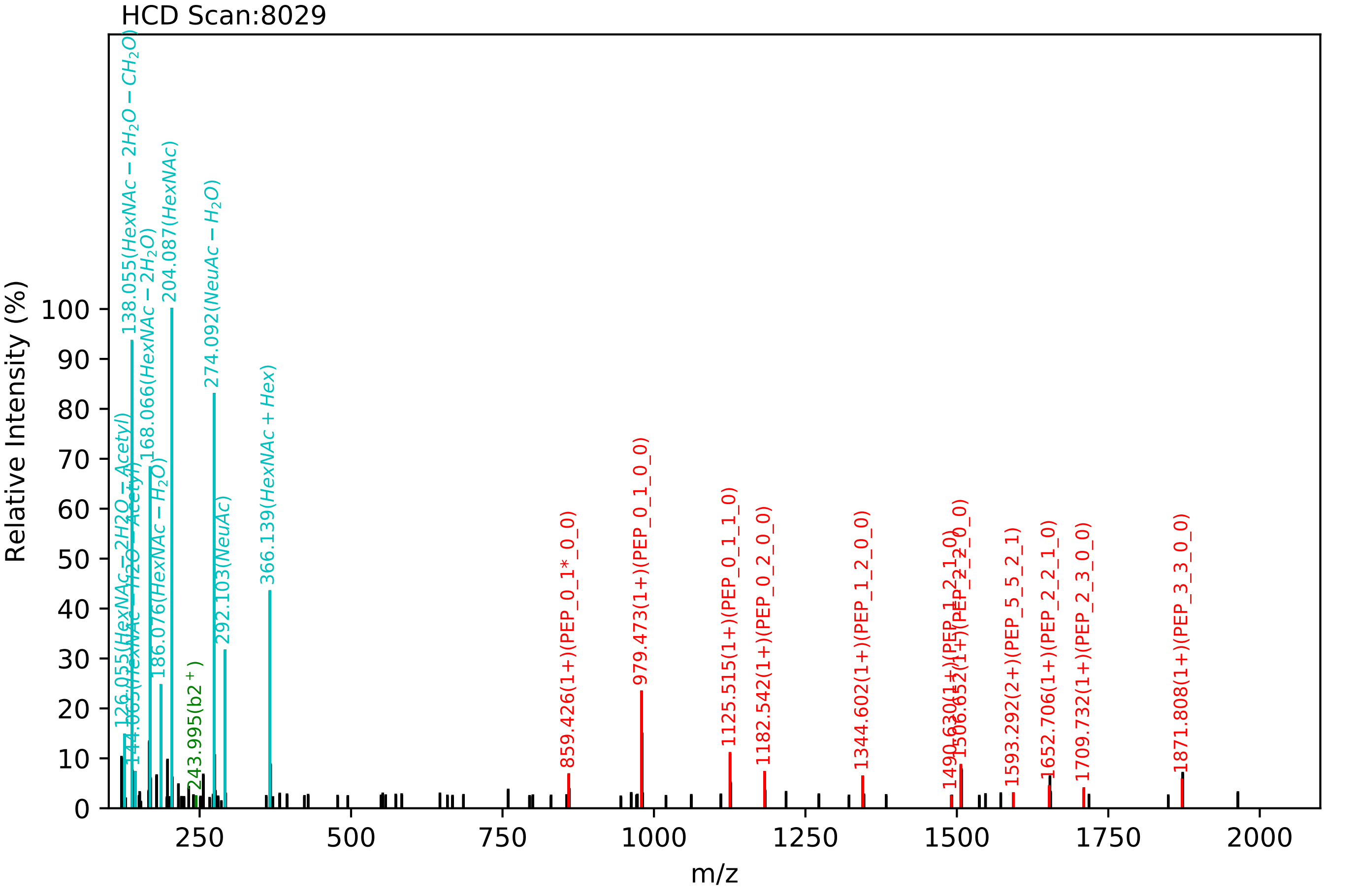

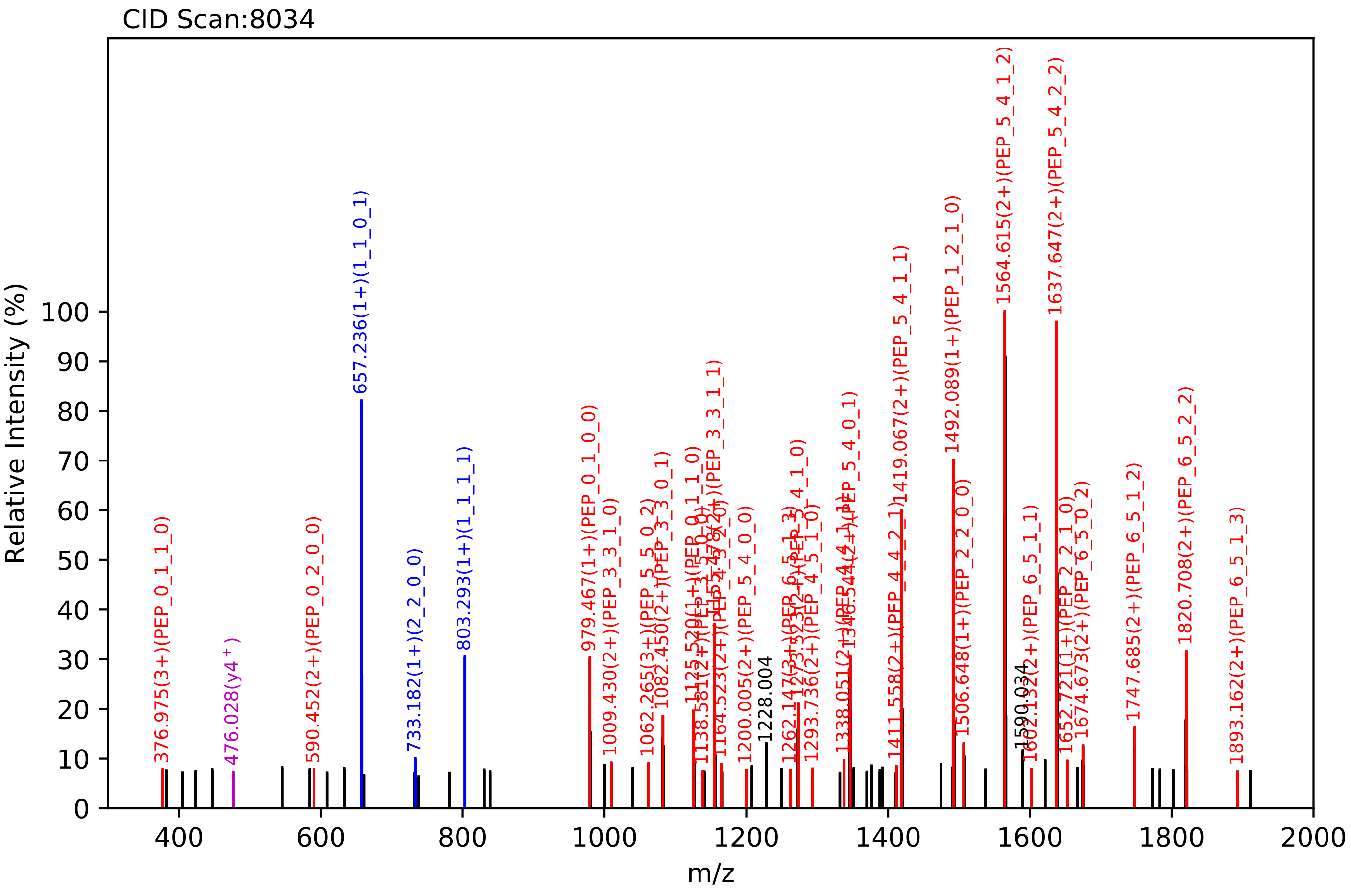

Training set no. 50, Experiment: AGP exp_12

ENGTISR(=PEP)_6_5_2_3, m/z:1310.84(3+), RT:41.85, Y-score:84.53

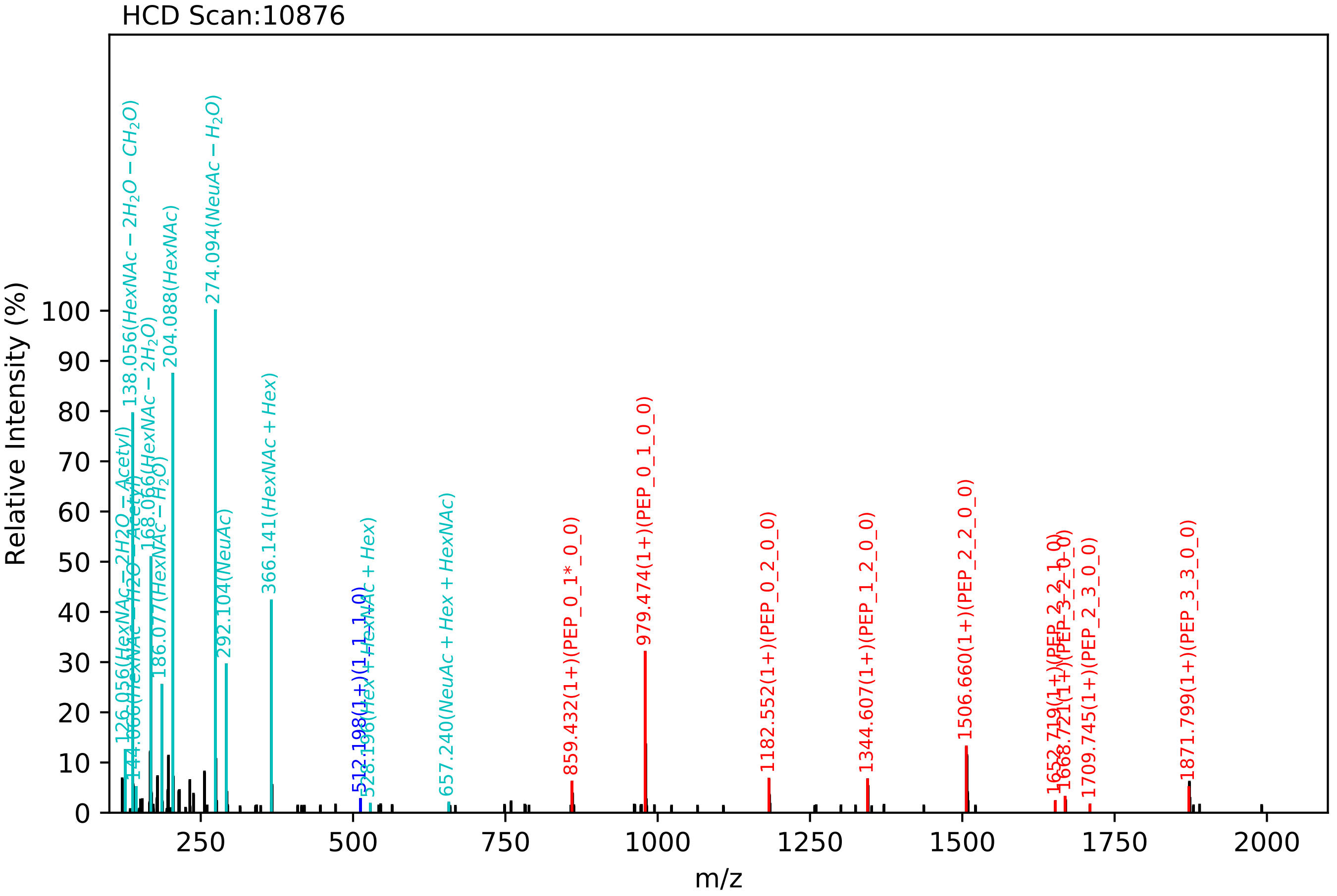

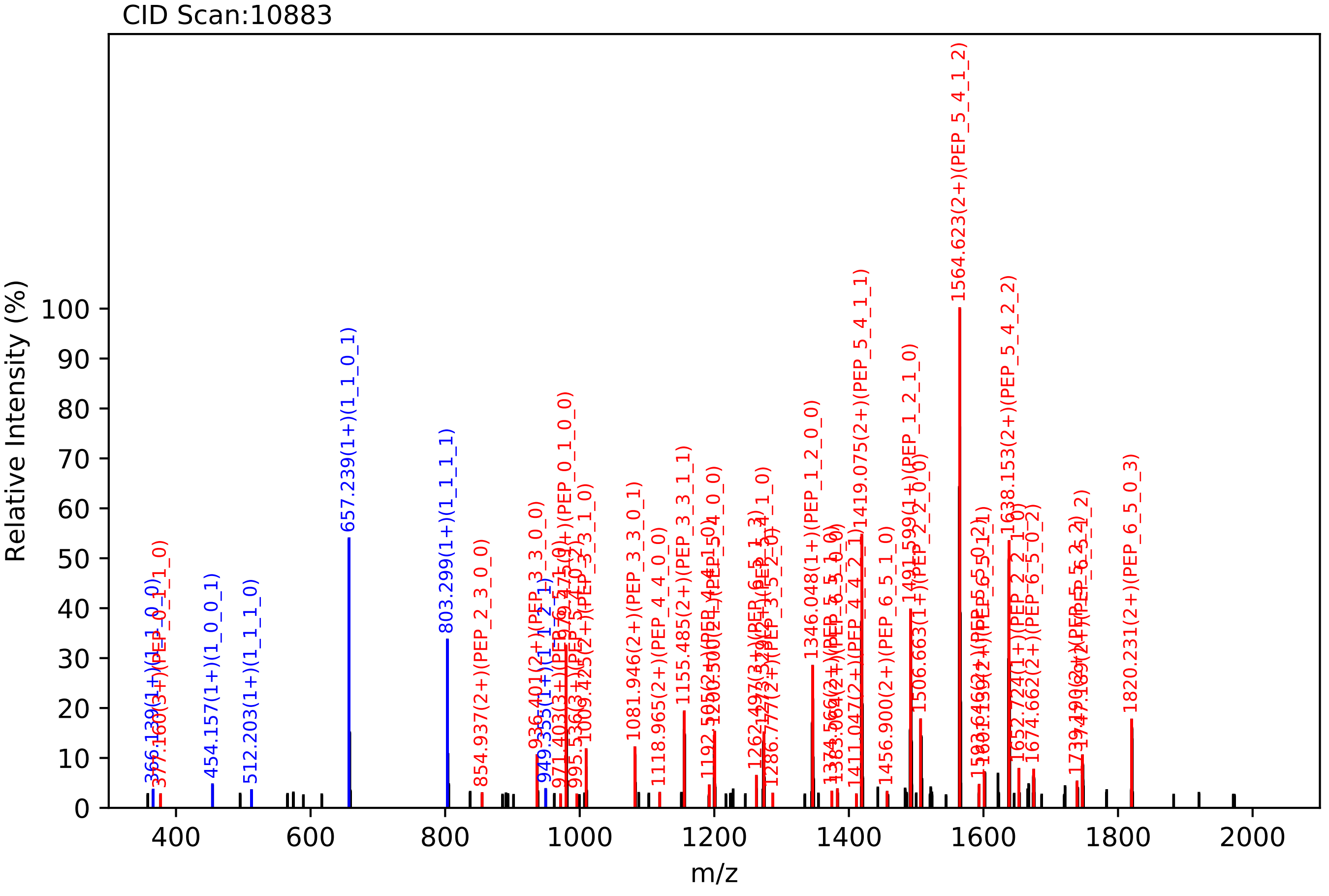

Training set no. 51, Experiment: AGP exp_26

ENGTISR(=PEP)_6_5_2_3, m/z:1310.51(3+), RT:35.24, Y-score:84.35

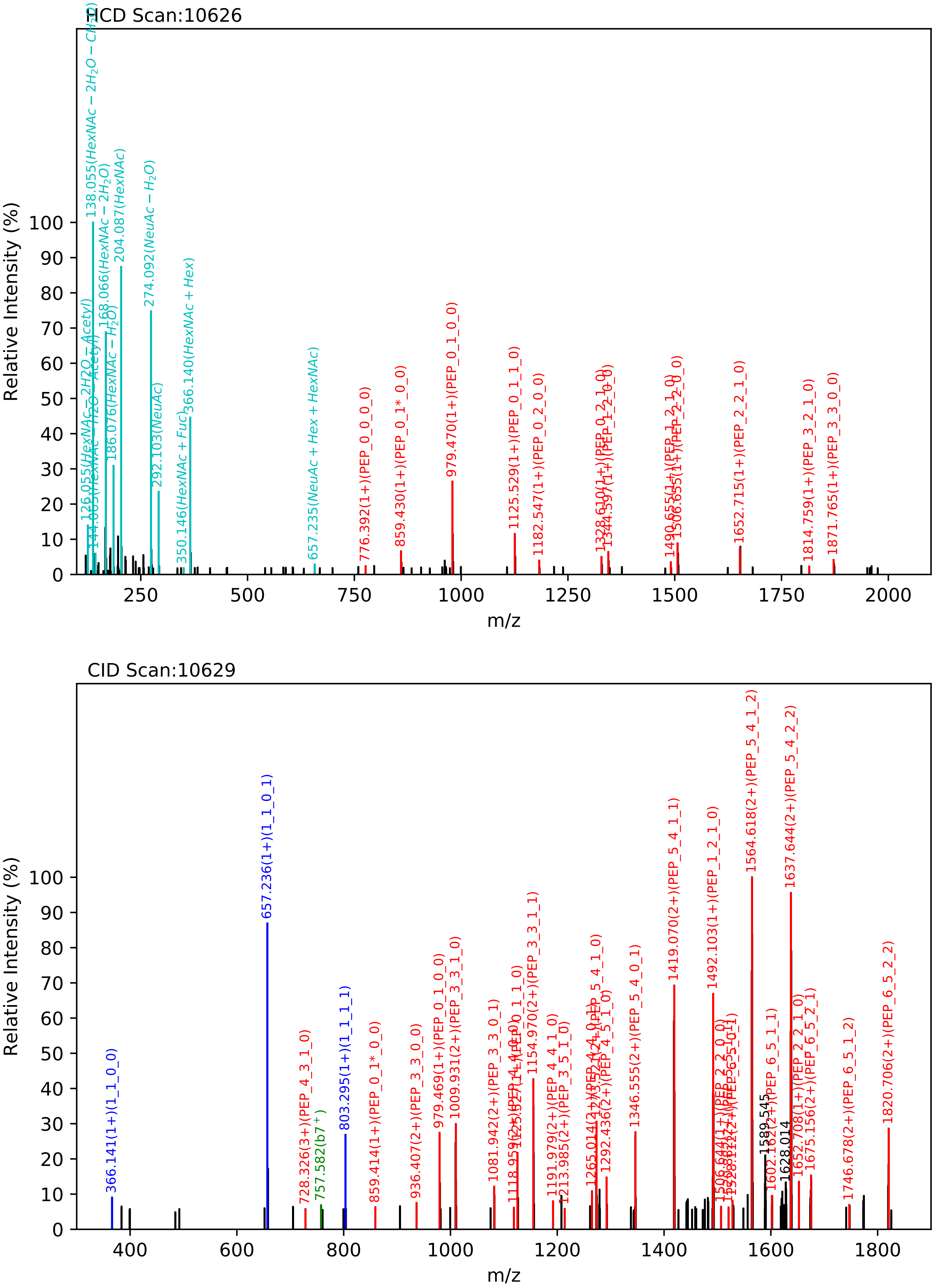

Training set no. 52, Experiment: AGP exp_42

ENGTISR(=PEP)_6_5_2_3, m/z:1310.51(3+), RT:34.39, Y-score:82.69

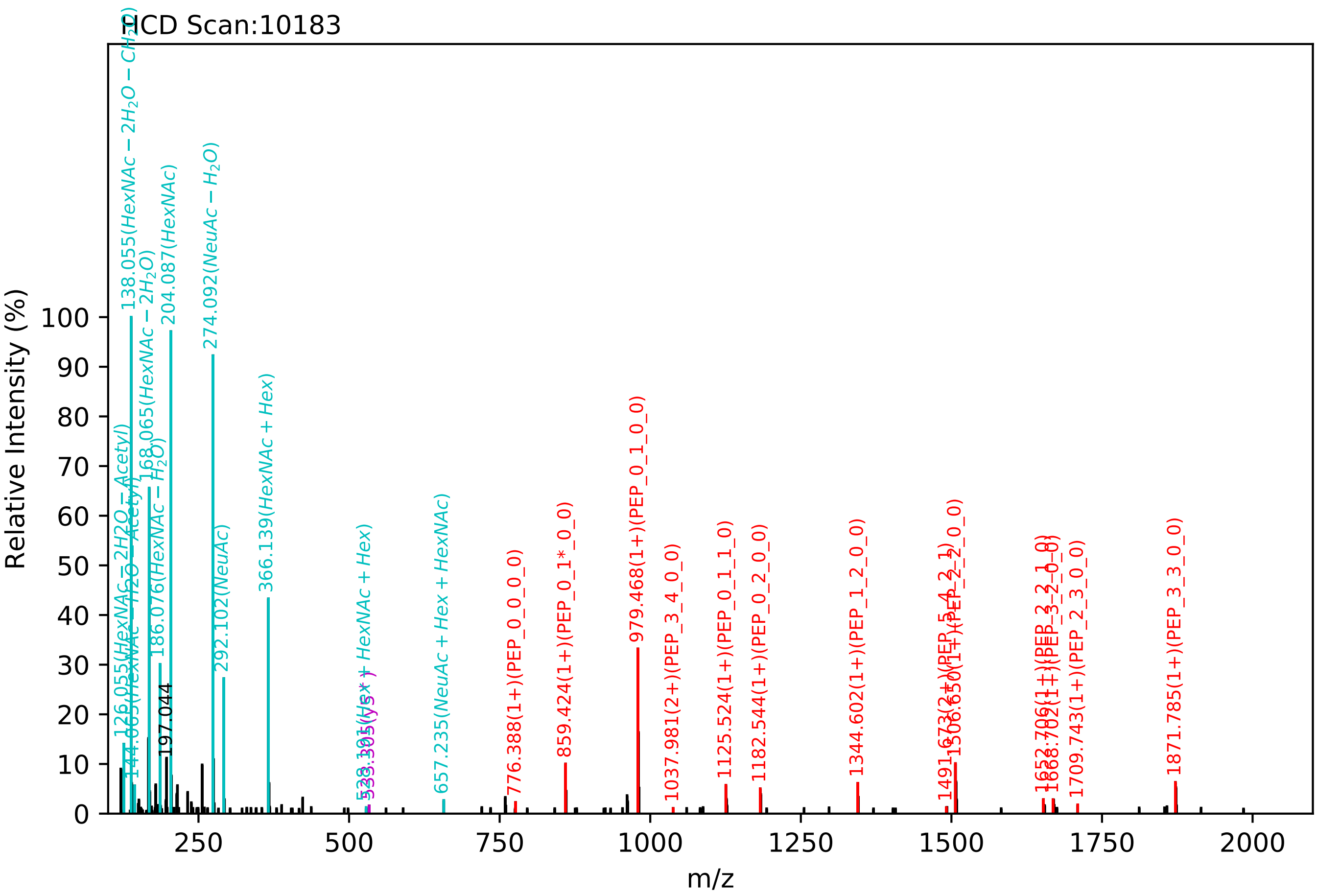

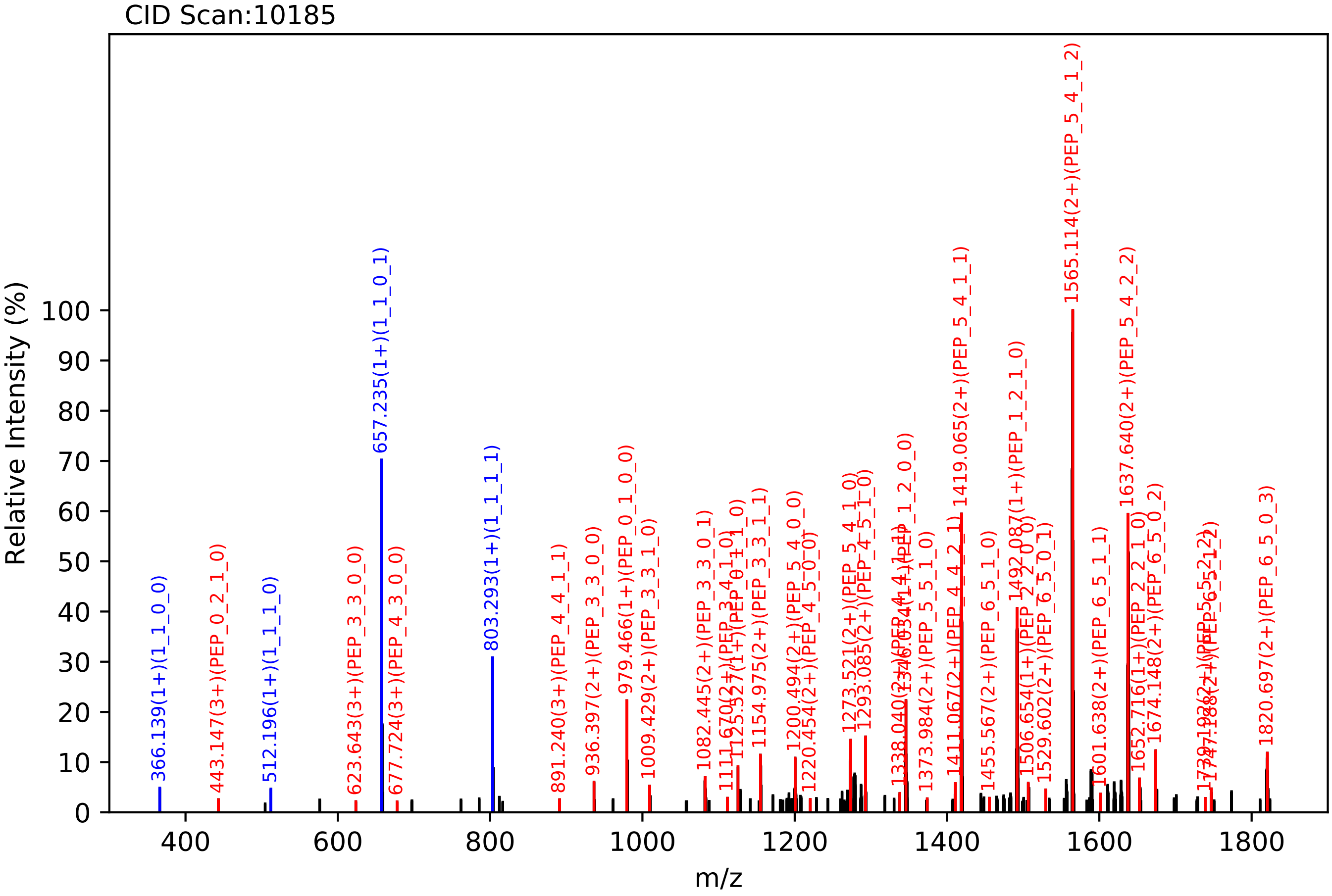

Training set no. 53, Experiment: AGP exp_16

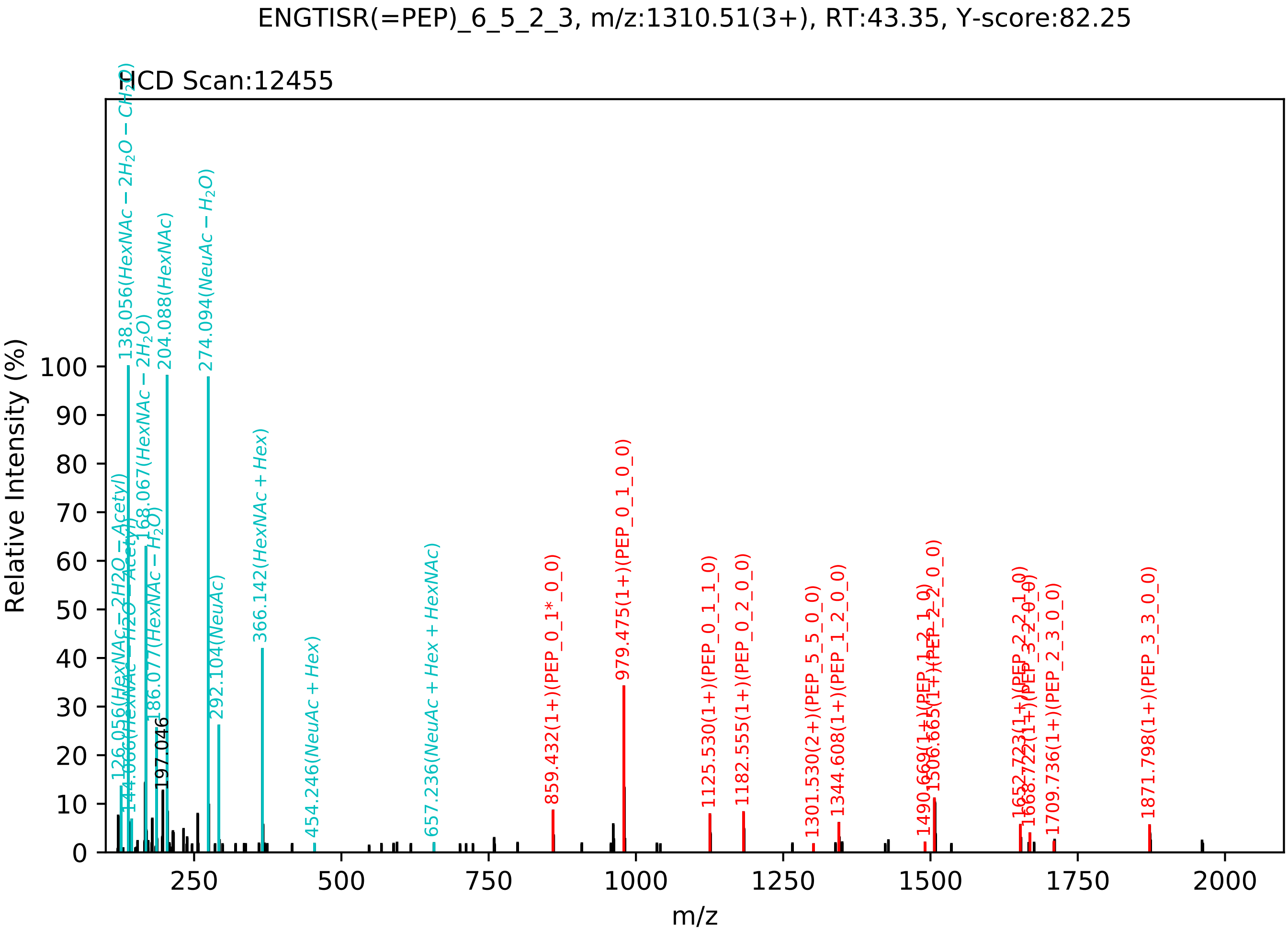

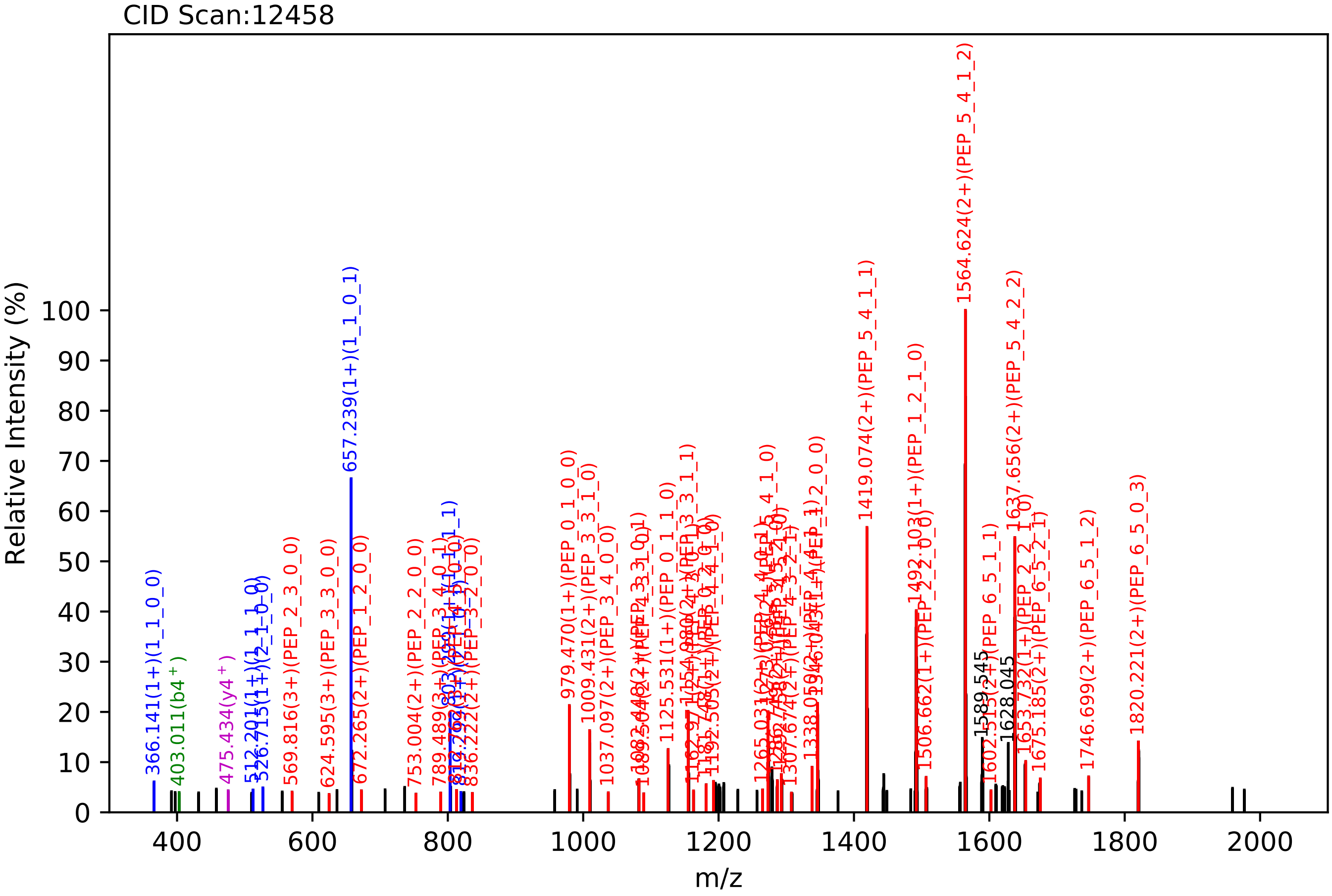

Training set no. 54, Experiment: AGP exp_43

ENGTISR(=PEP)_6_5_2_3, m/z:1310.51(3+), RT:34.11, Y-score:81.88

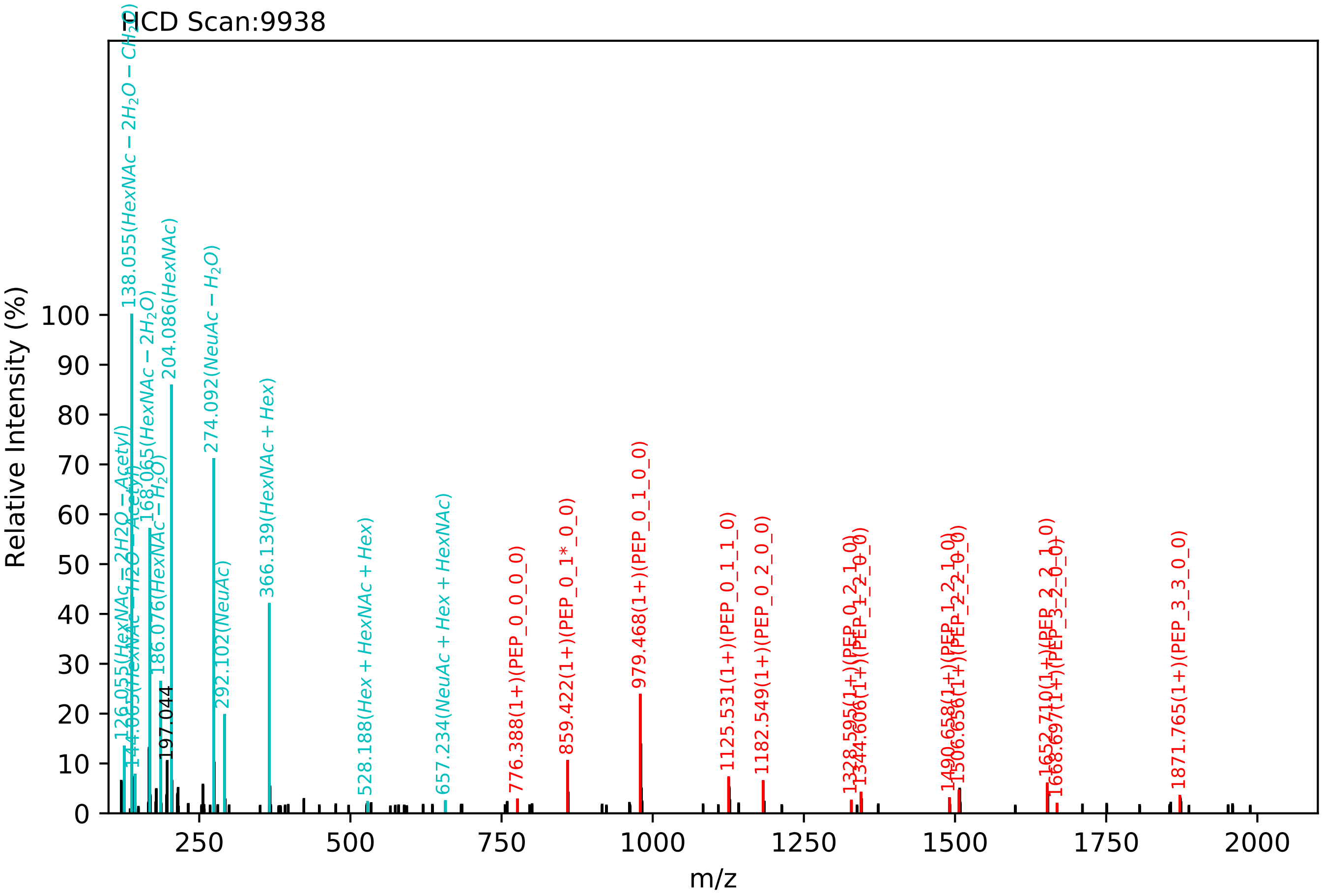

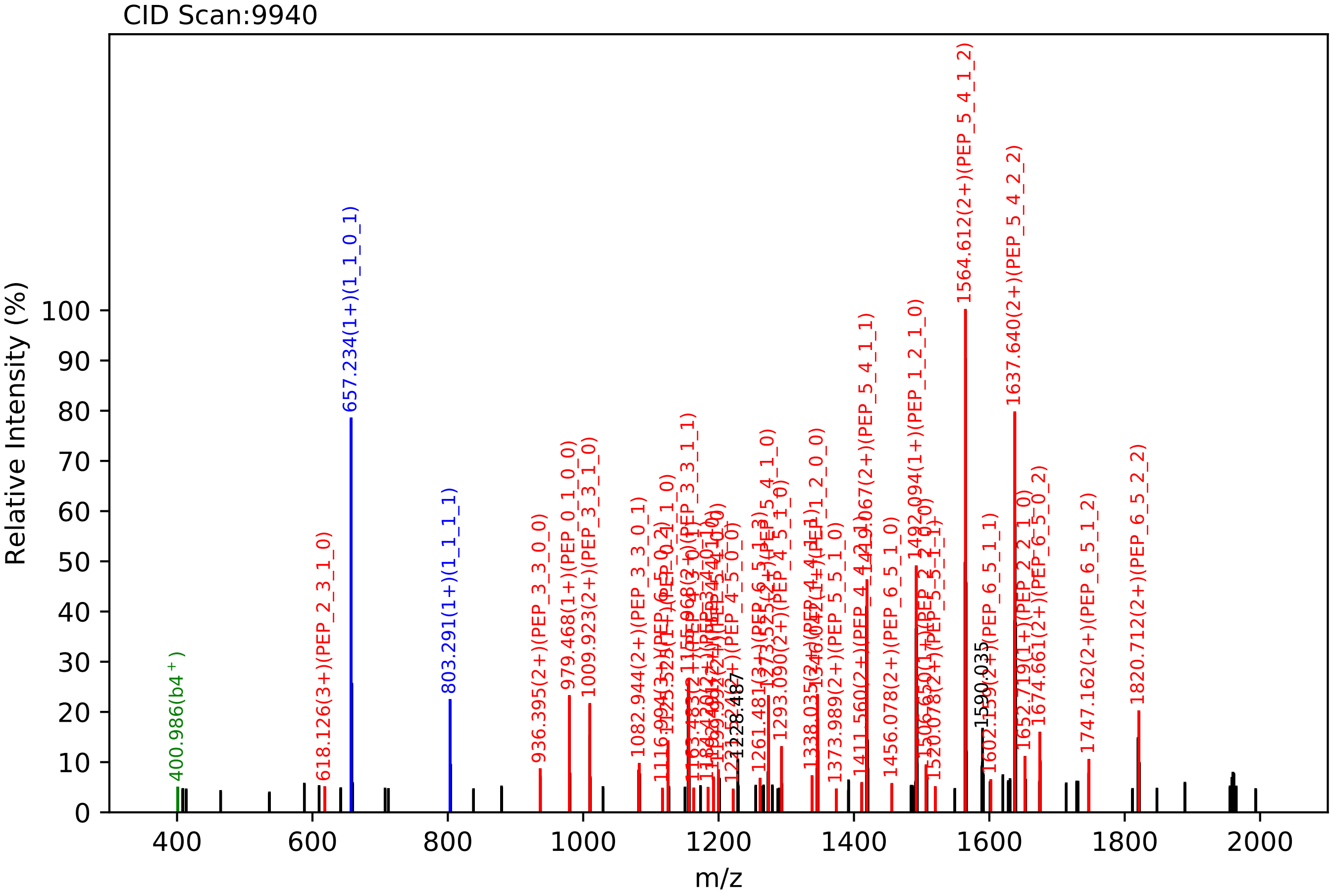

Training set no. 55, Experiment: AGP exp_20

ENGTISR(=PEP)_6_5_2_3, m/z:1310.51(3+), RT:34.65, Y-score:81.72

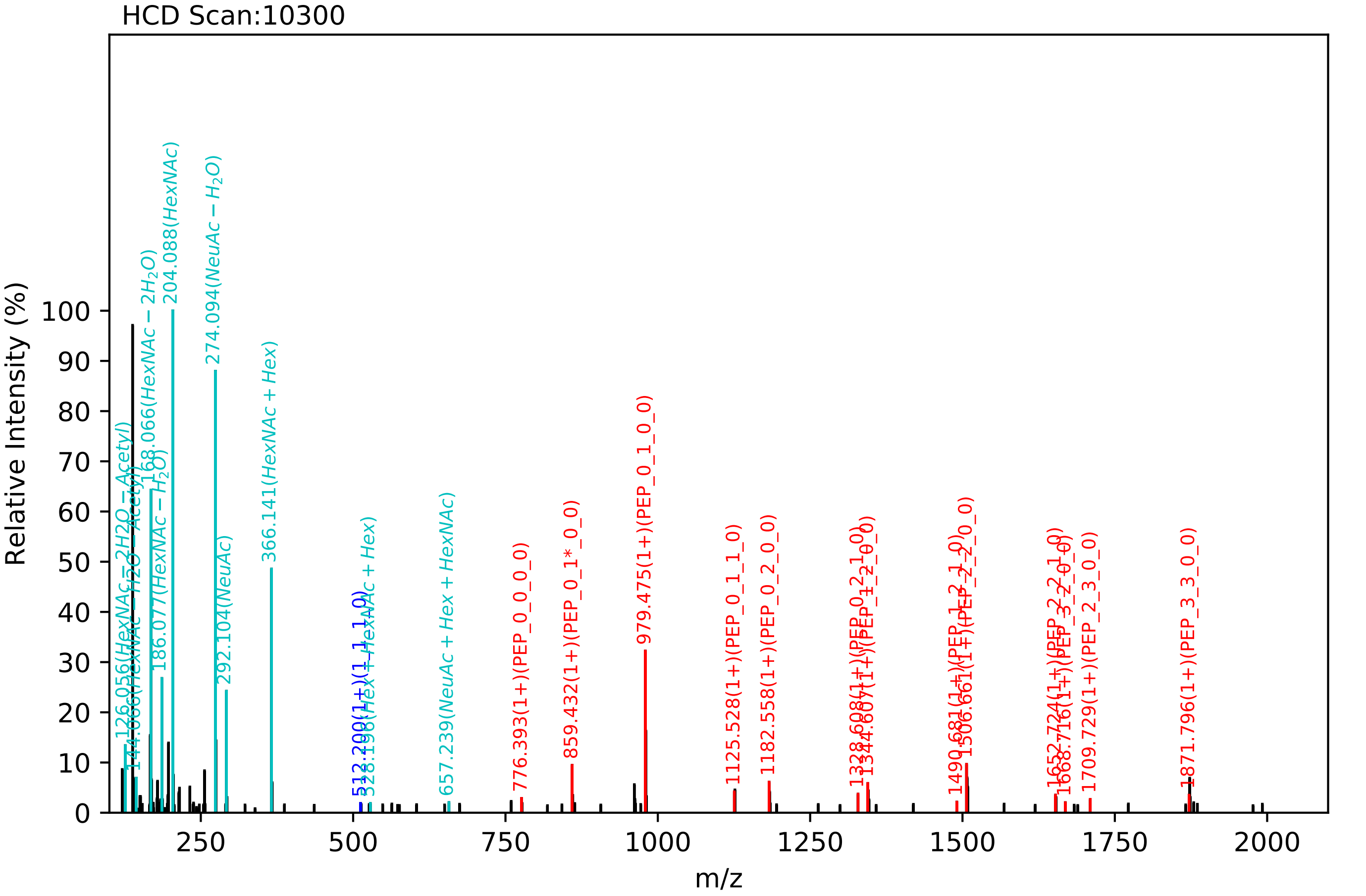

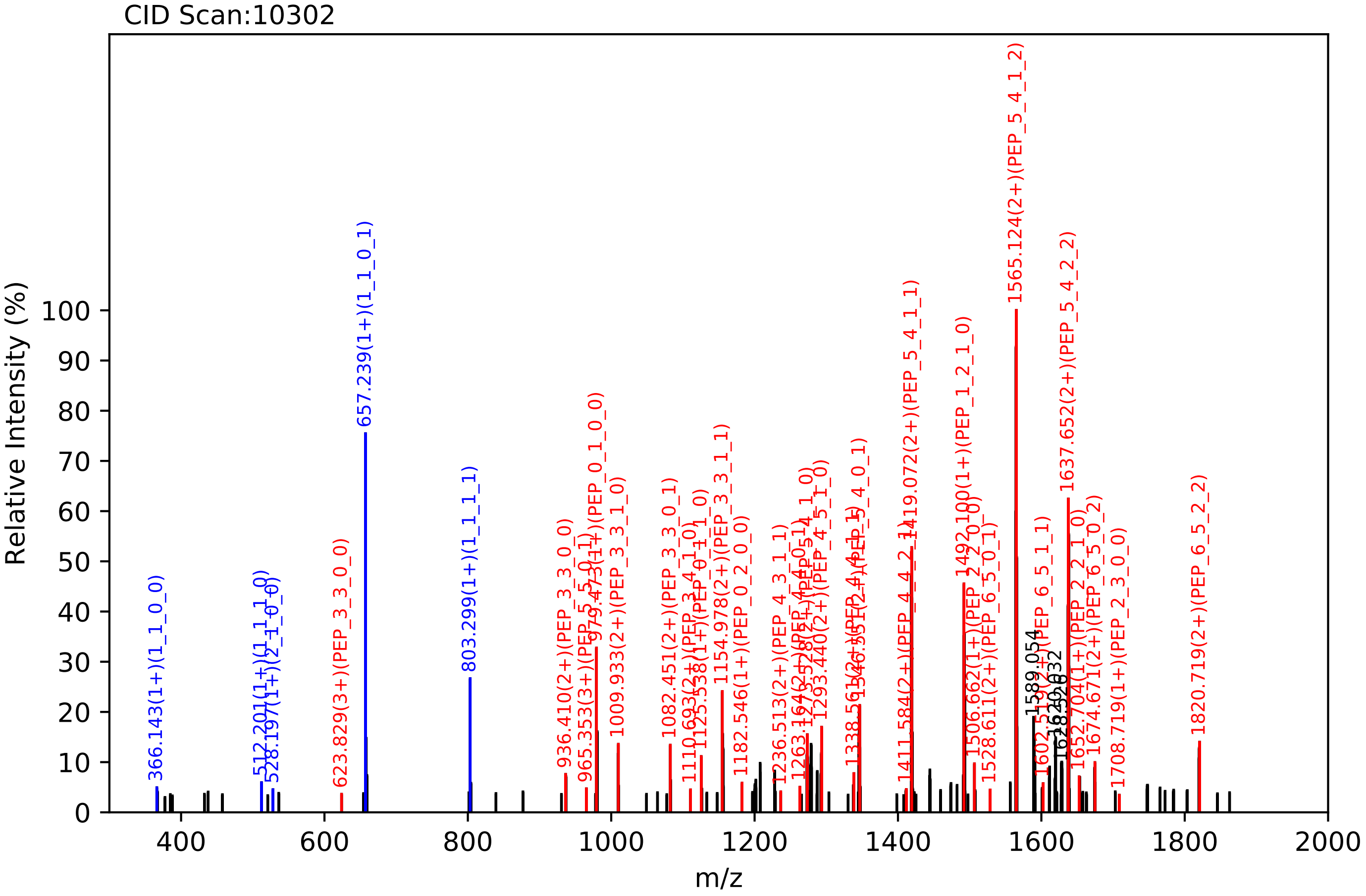

Training set no. 56, Experiment: AGP exp_15

ENGTISR(=PEP)_6_5_2_3, m/z:1310.51(3+), RT:43.44, Y-score:80.20

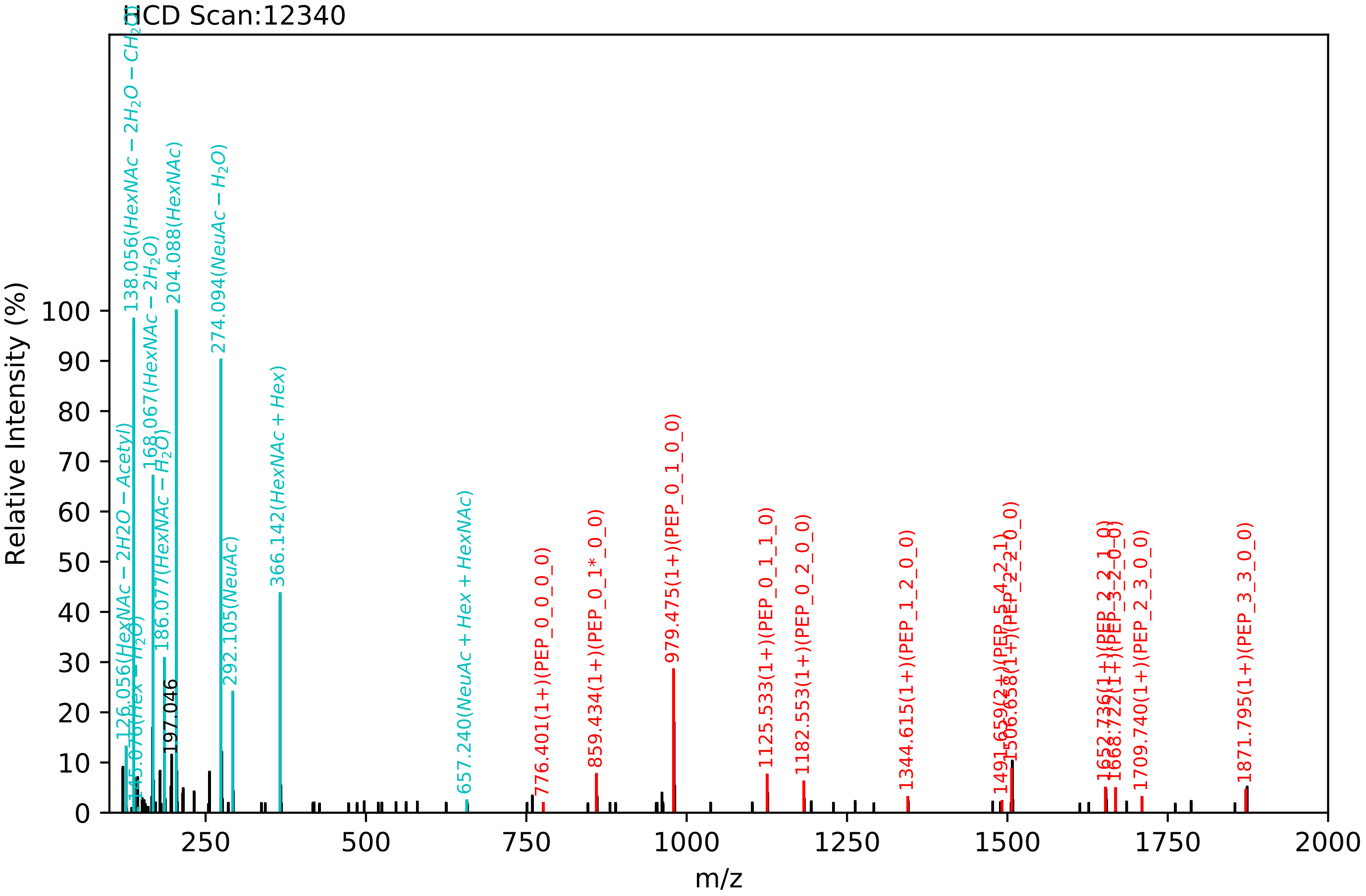

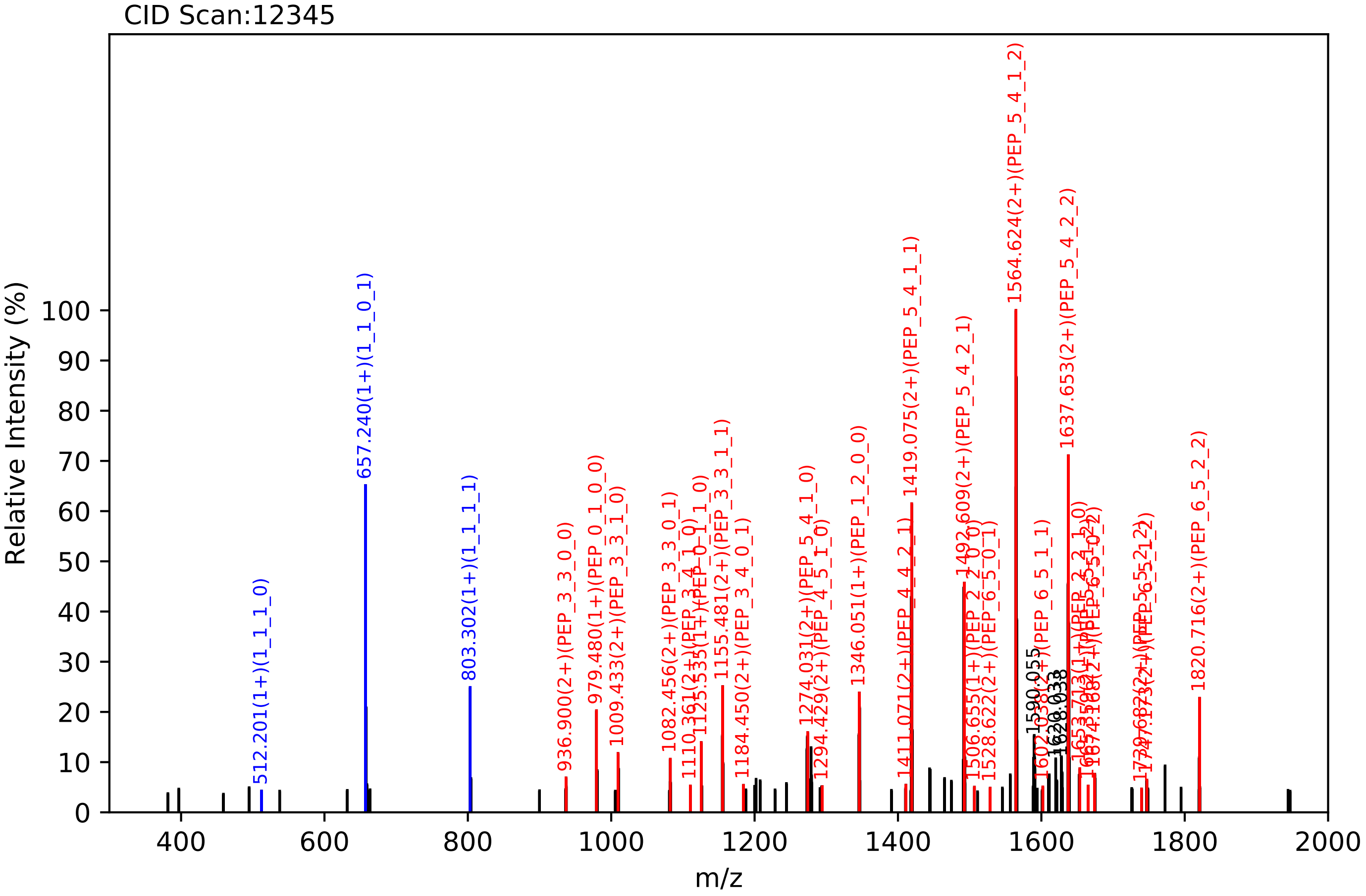

Training set no. 57, Experiment: AGP exp_2

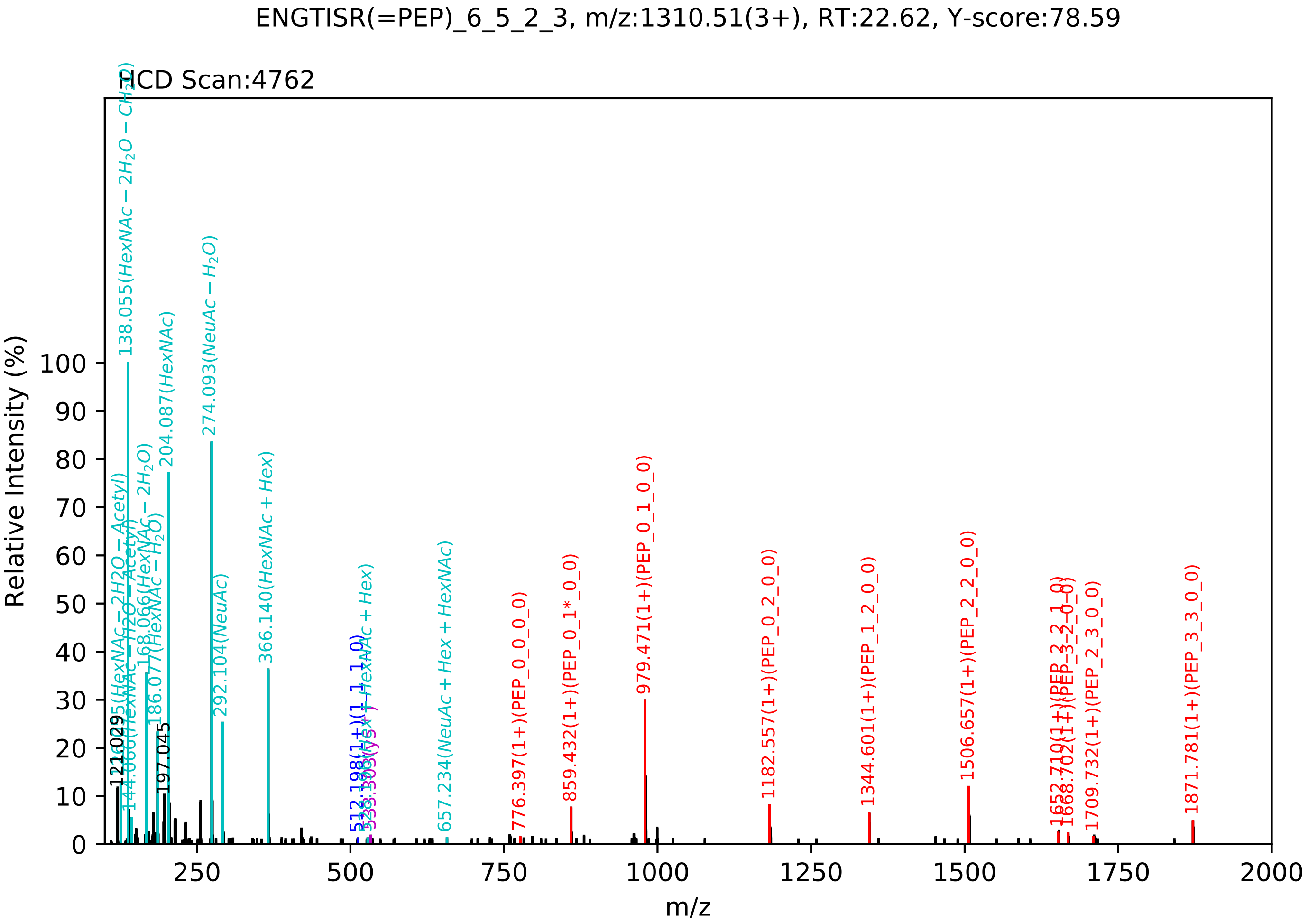

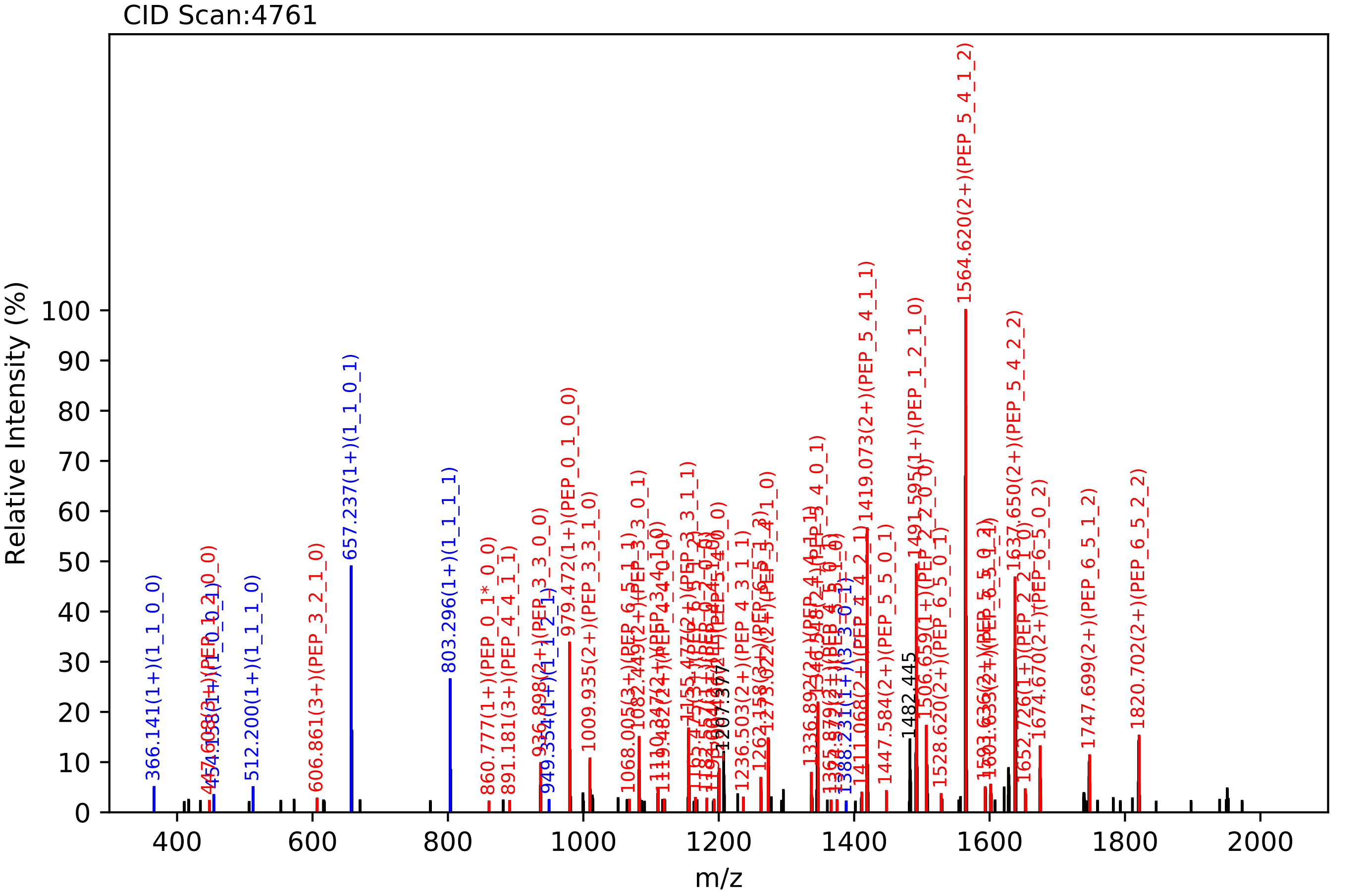

Training set no. 58, Experiment: AGP exp_2

ENGTISR(=PEP)_7_6_0_3, m/z:1001.39(4+), RT:22.41, Y-score:70.19

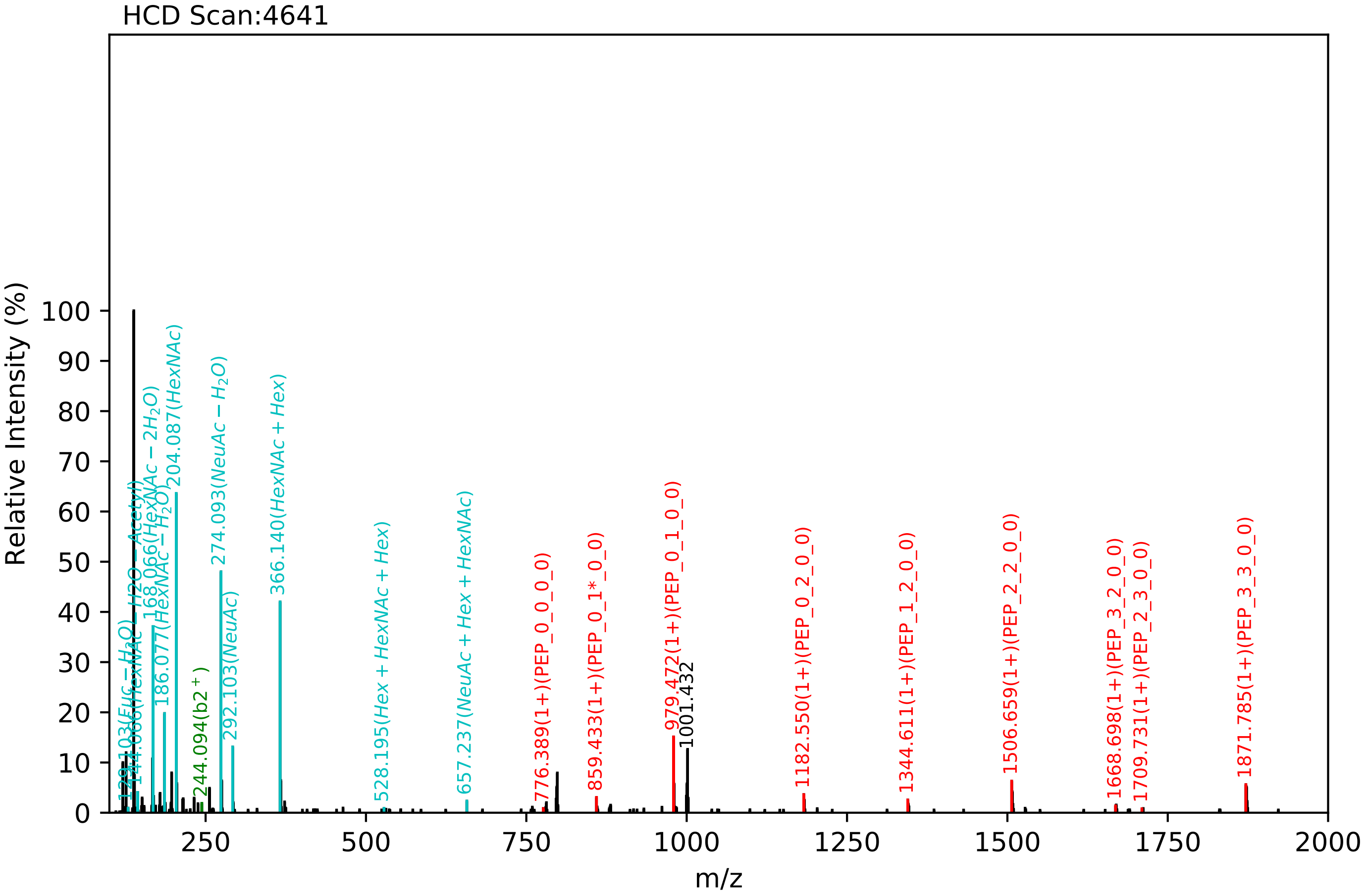

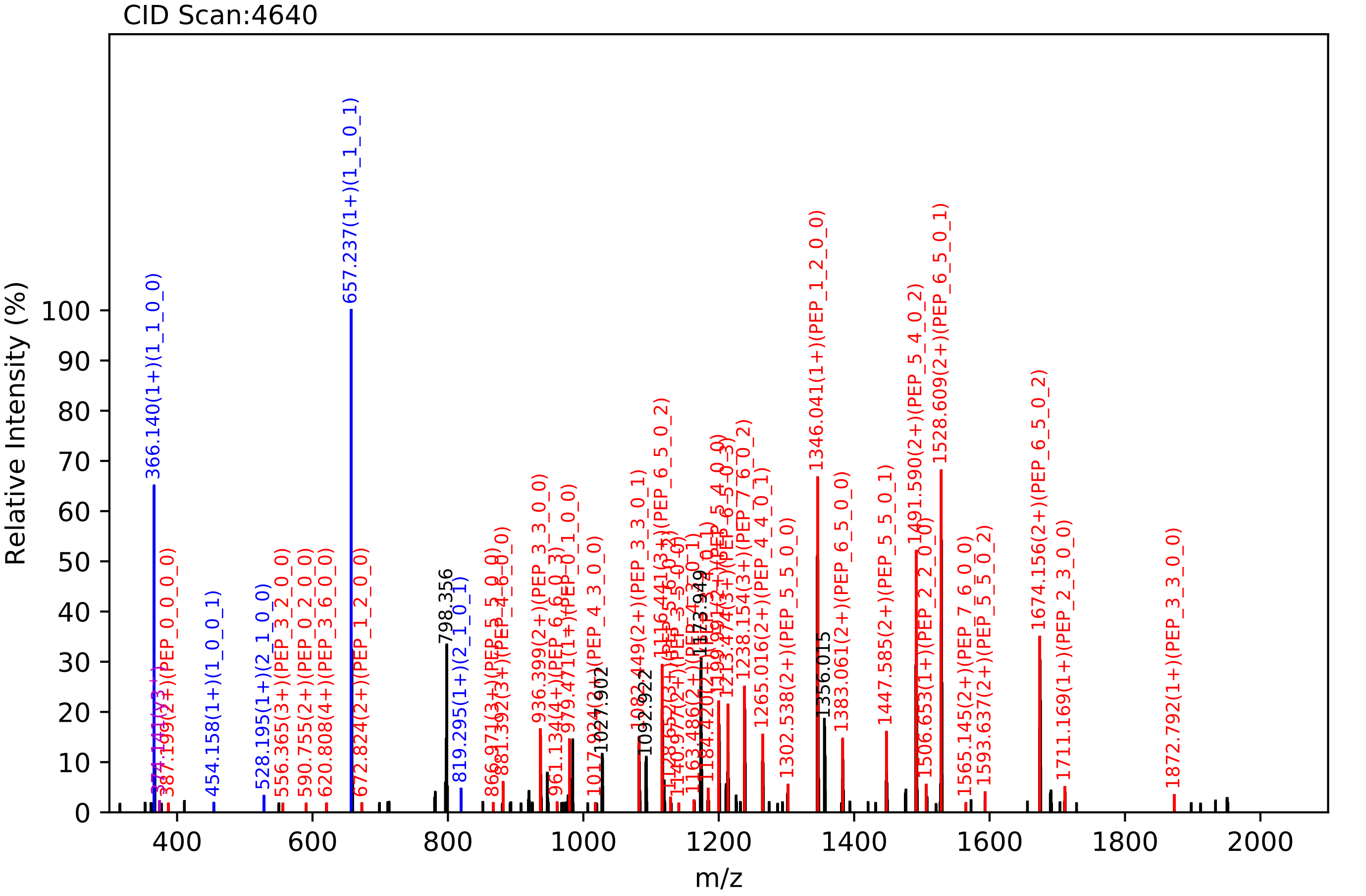

Training set no. 59, Experiment: AGP exp_2

ENGTISR(=PEP)_7_6_1_1, m/z:1189.47(3+), RT:17.02, Y-score:92.33

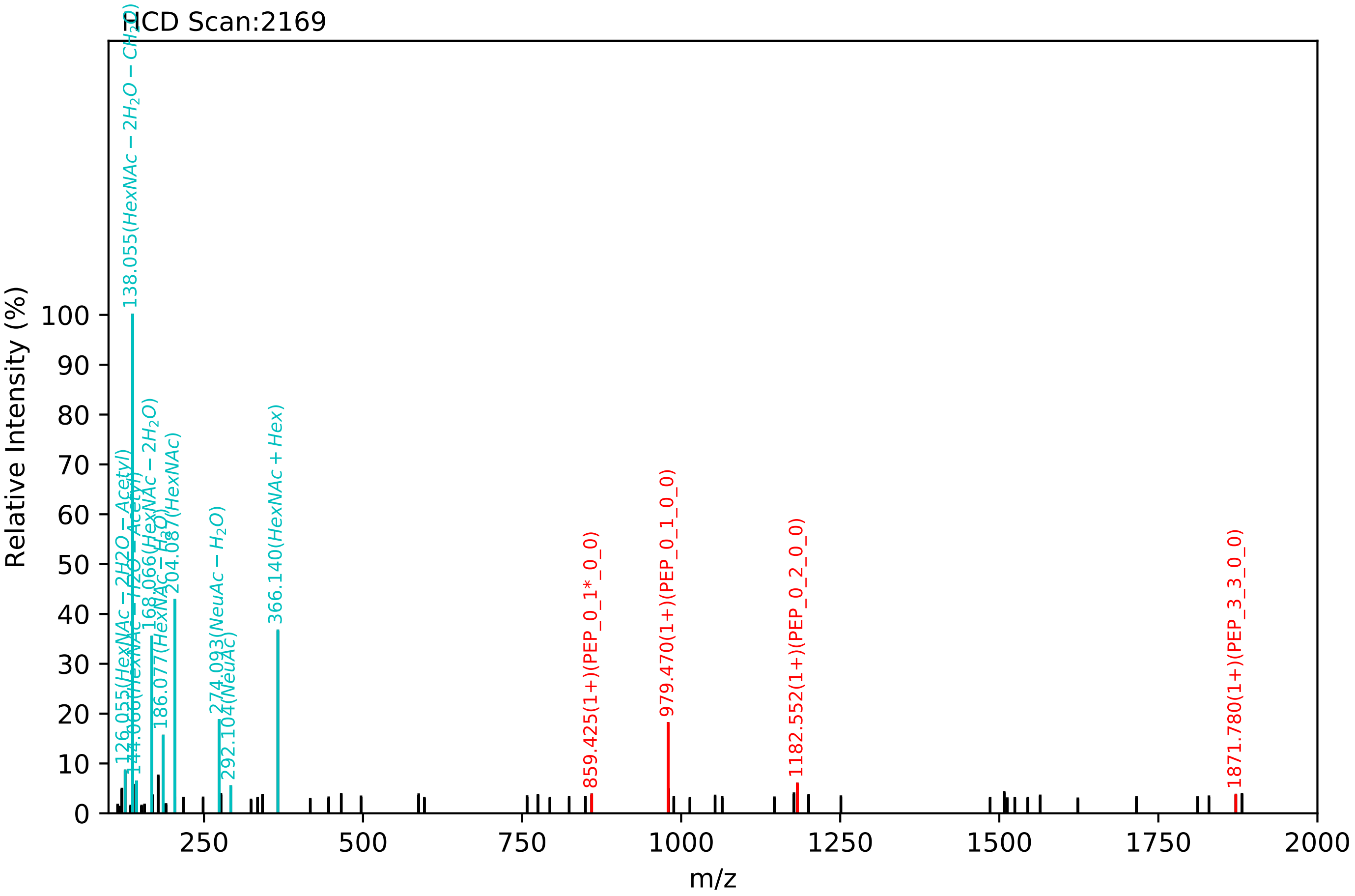

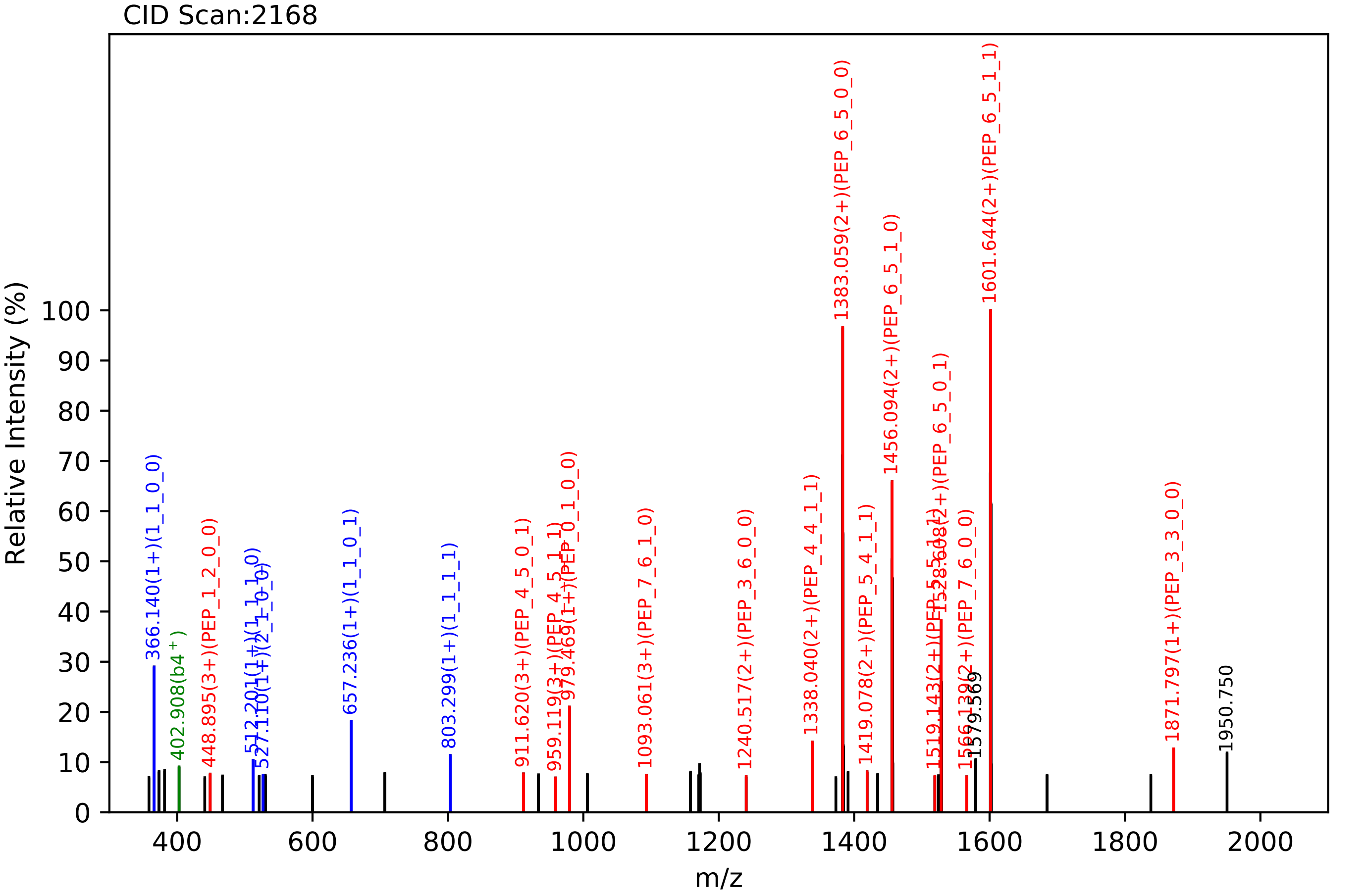

Training set no. 60, Experiment: AGP exp_1

ENGTISR(=PEP)_7_6_1_2, m/z:1286.50(3+), RT:20.60, Y-score:94.32

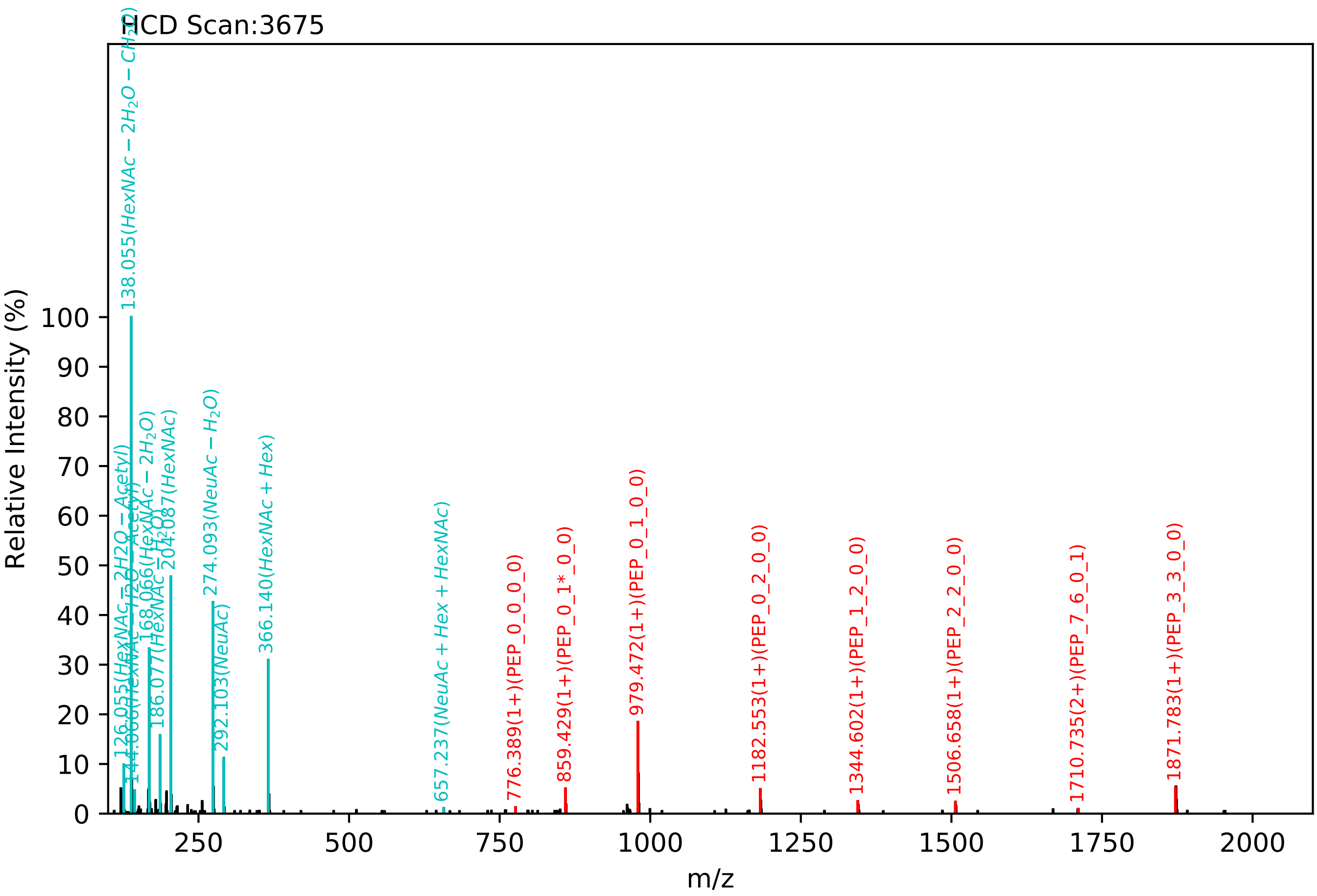

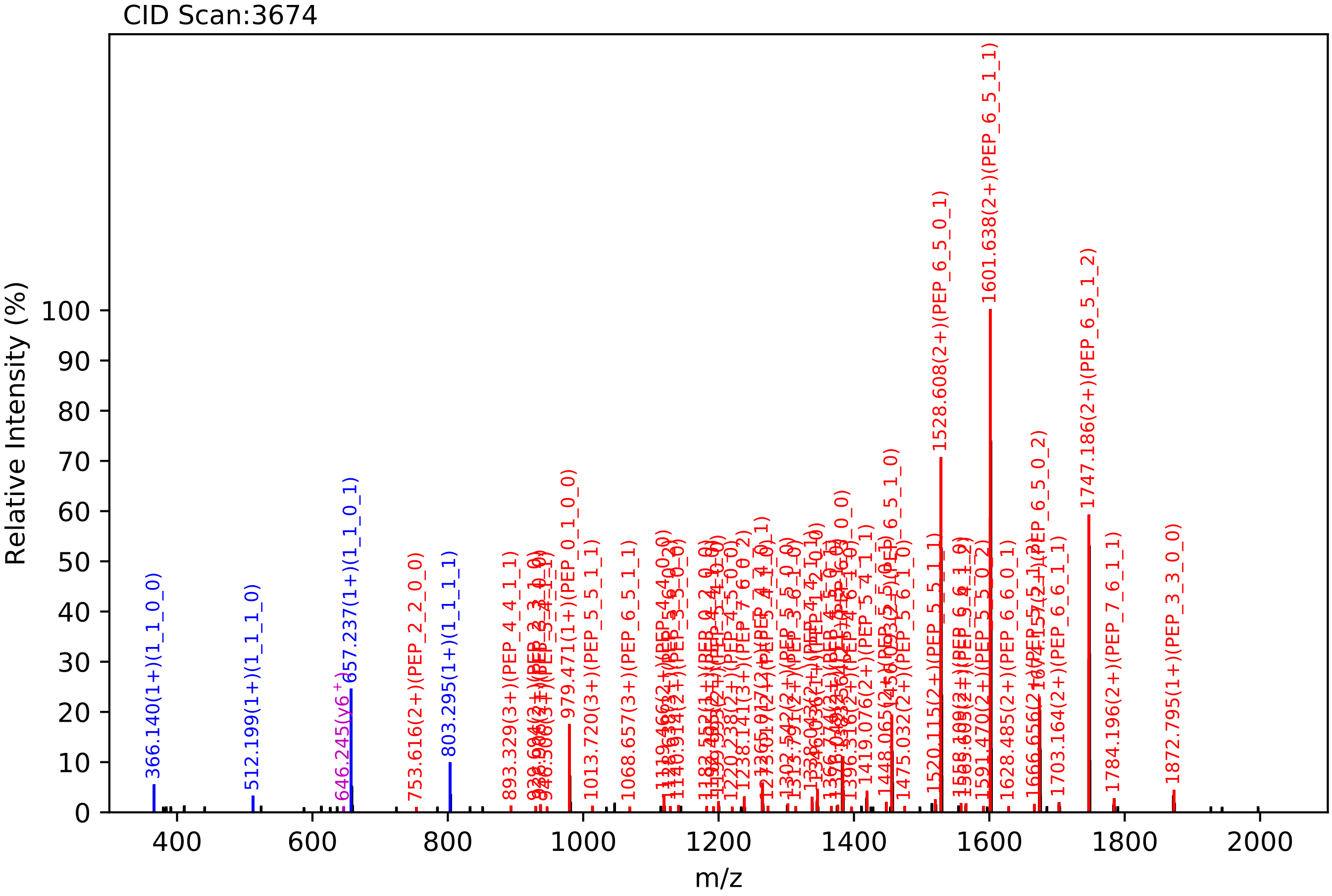

Training set no. 61, Experiment: AGP exp_2

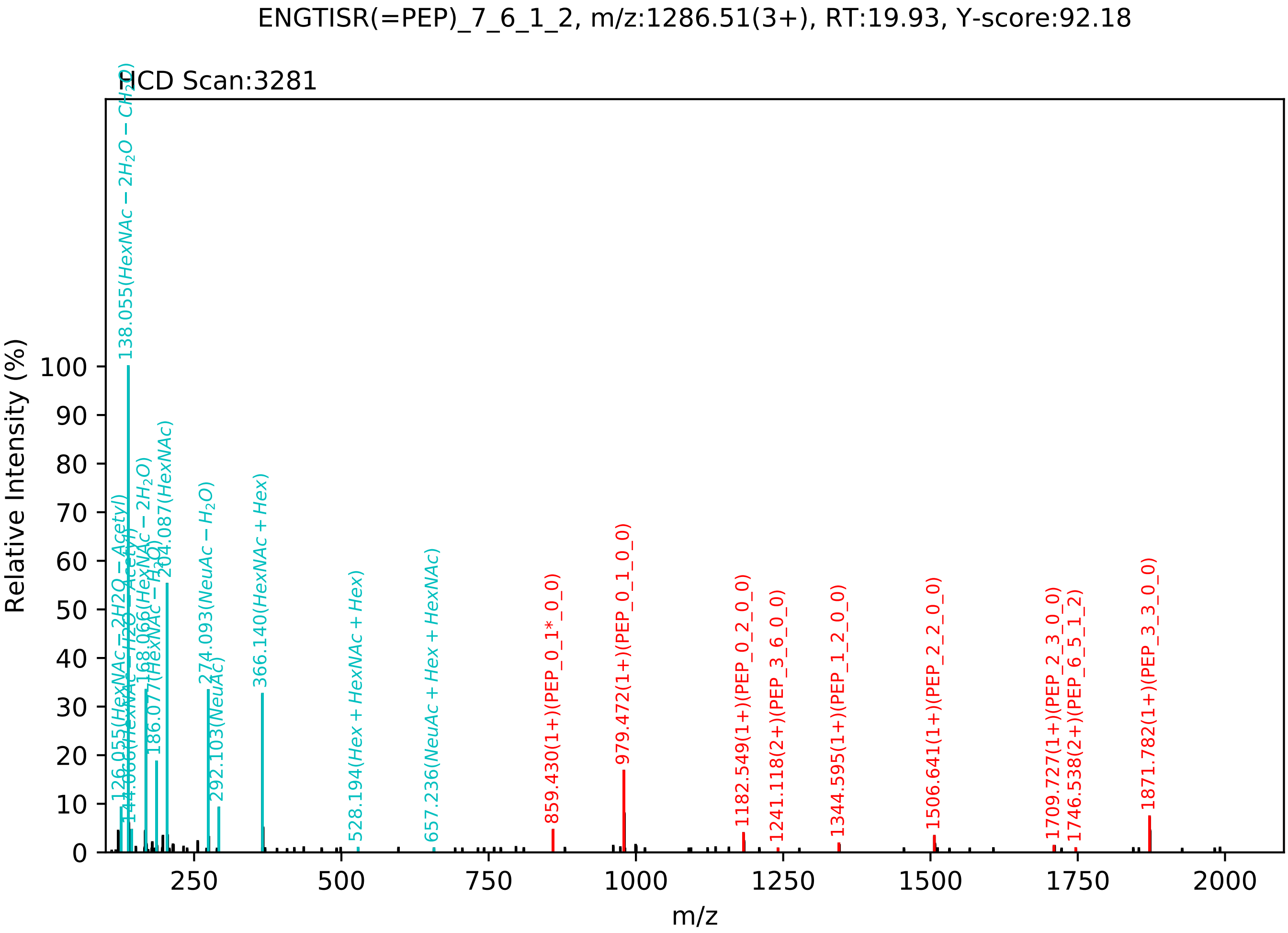

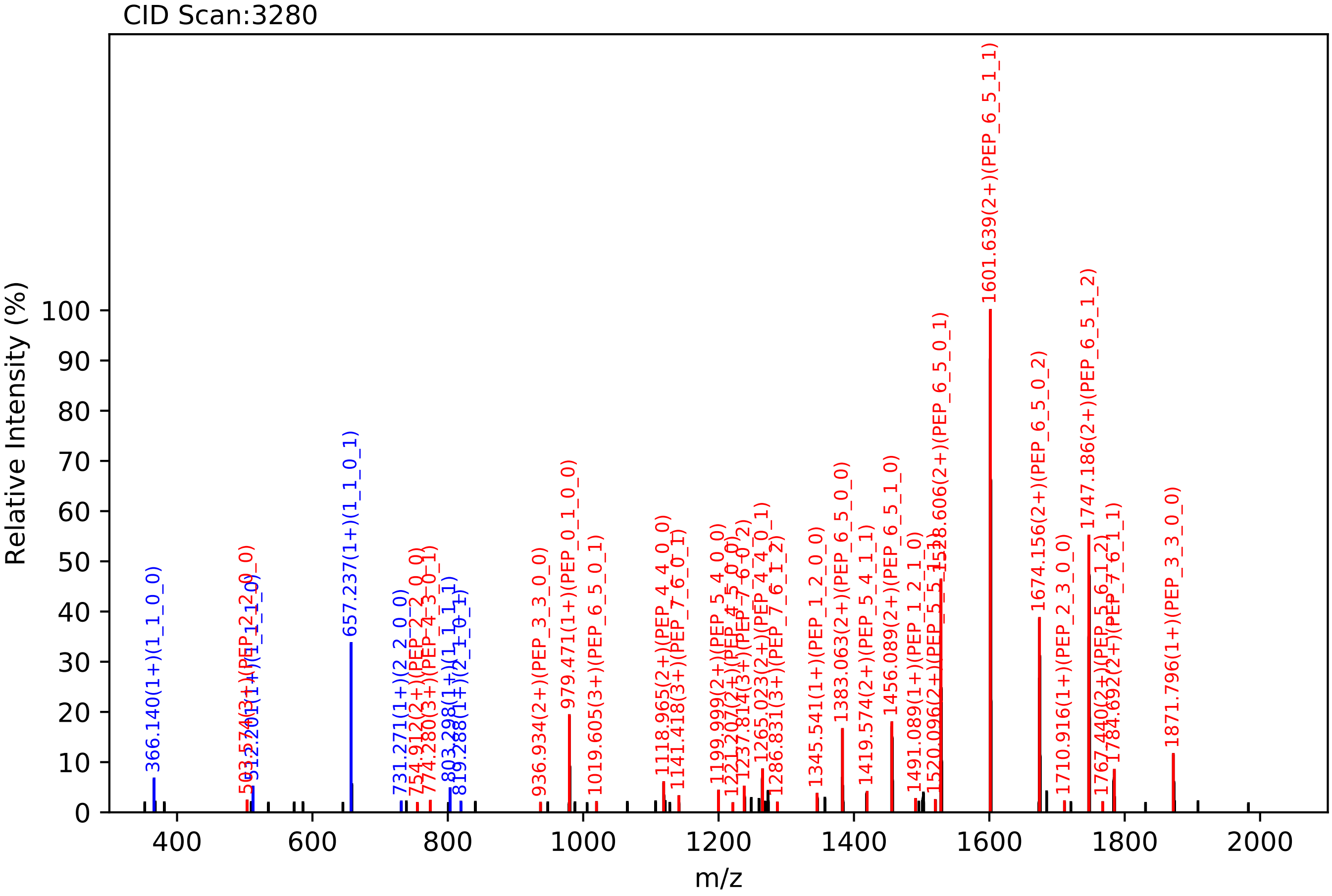

Training set no. 62, Experiment: AGP exp_2

ENGTISR(=PEP)_7_6_1_2, m/z:1286.50(3+), RT:20.49, Y-score:91.14

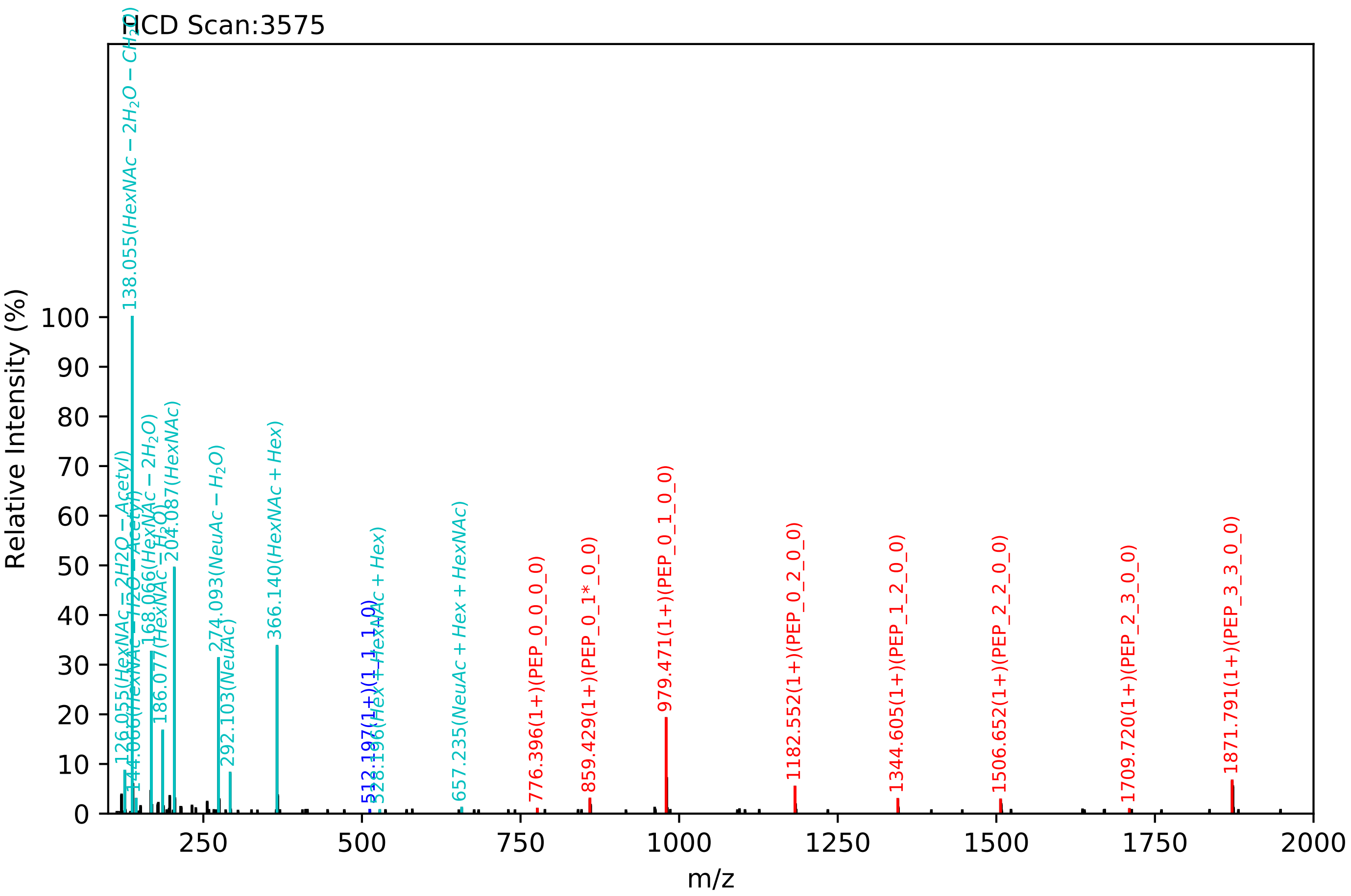

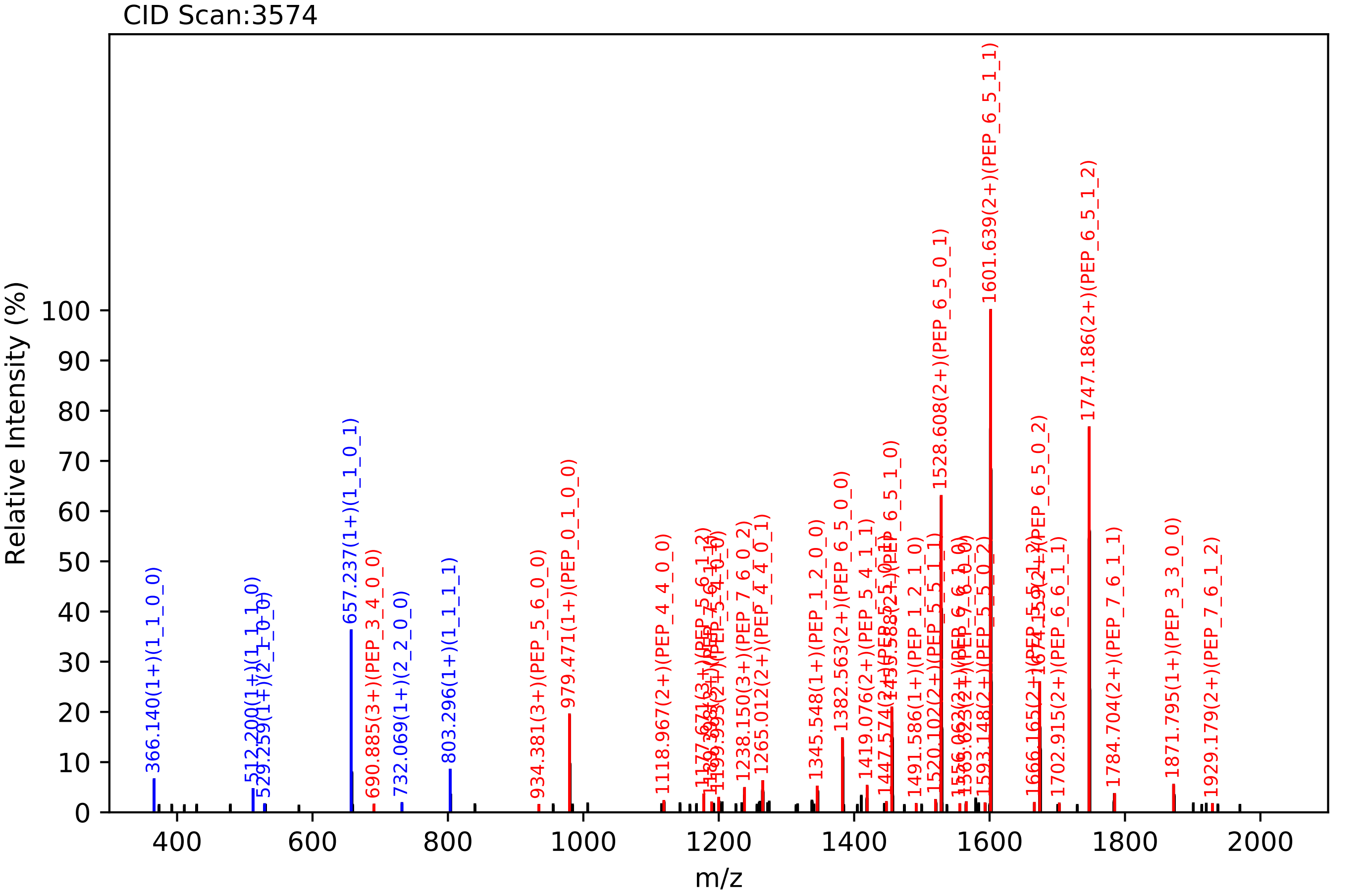

Training set no. 63, Experiment: AGP exp_2

ENGTISR(=PEP)_7_6_1_2, m/z:1286.50(3+), RT:19.31, Y-score:90.96

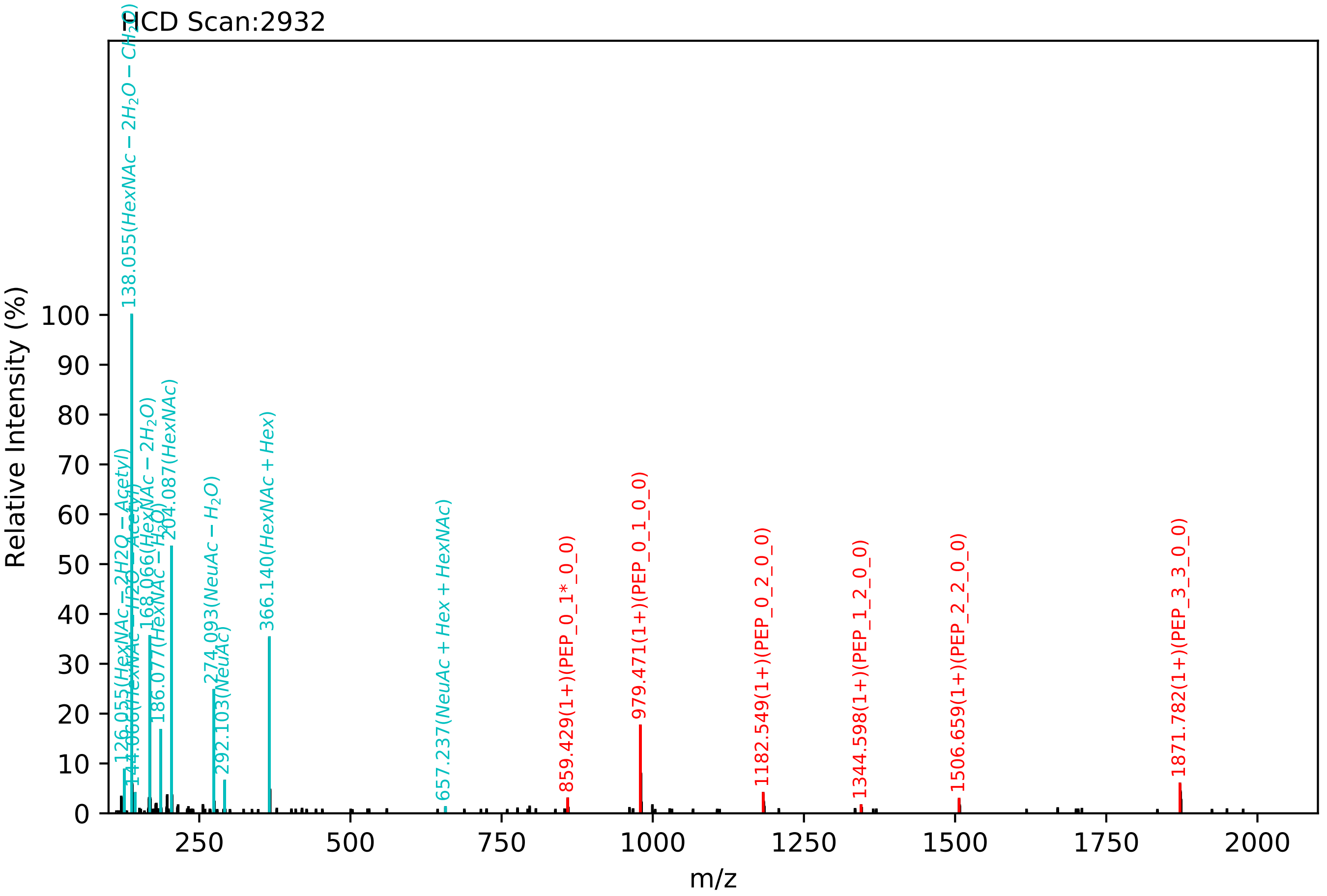

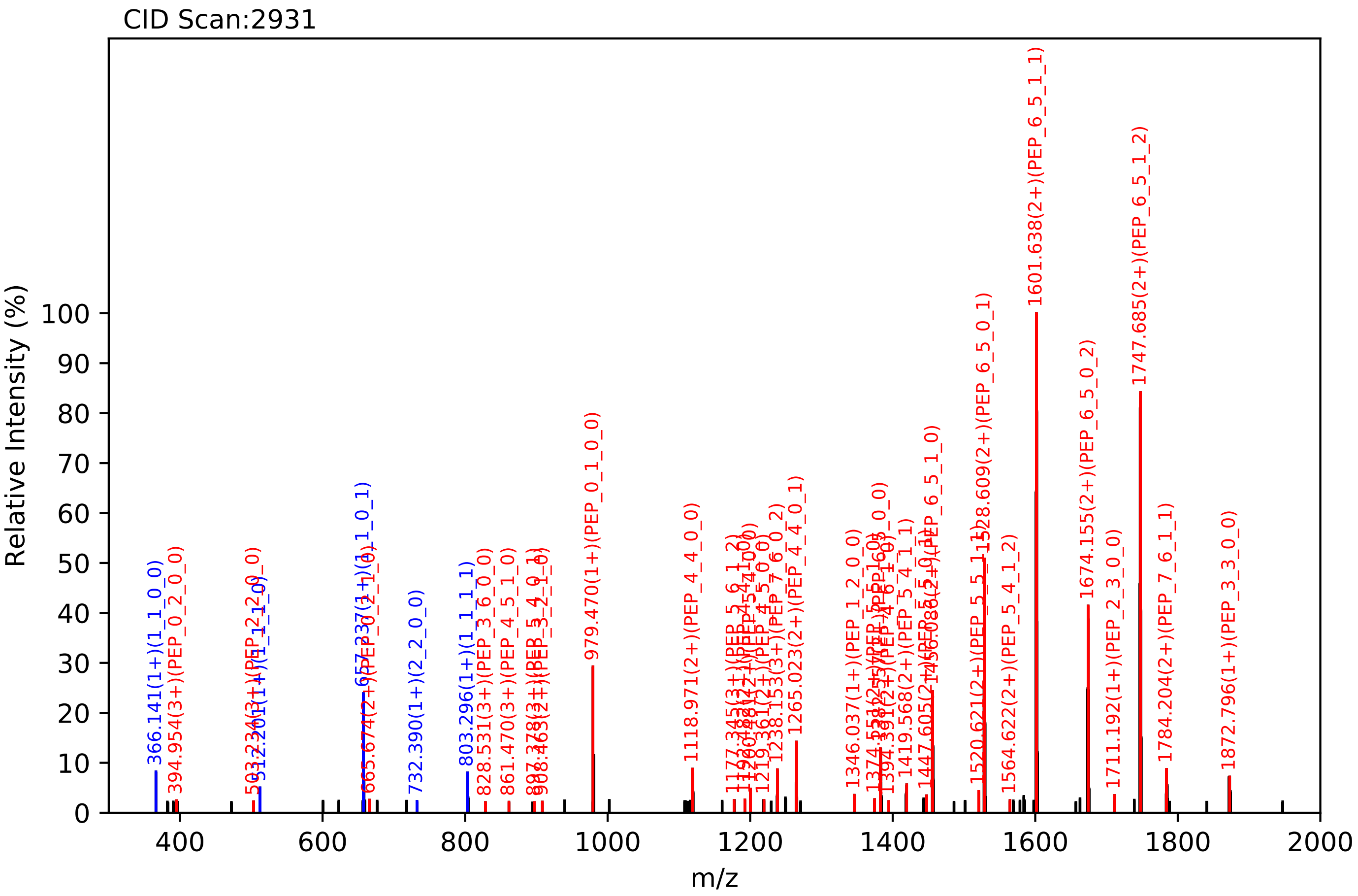

Training set no. 64, Experiment: AGP exp_1

ENGTISR(=PEP)_7_6_1_2, m/z:1286.50(3+), RT:20.01, Y-score:89.83

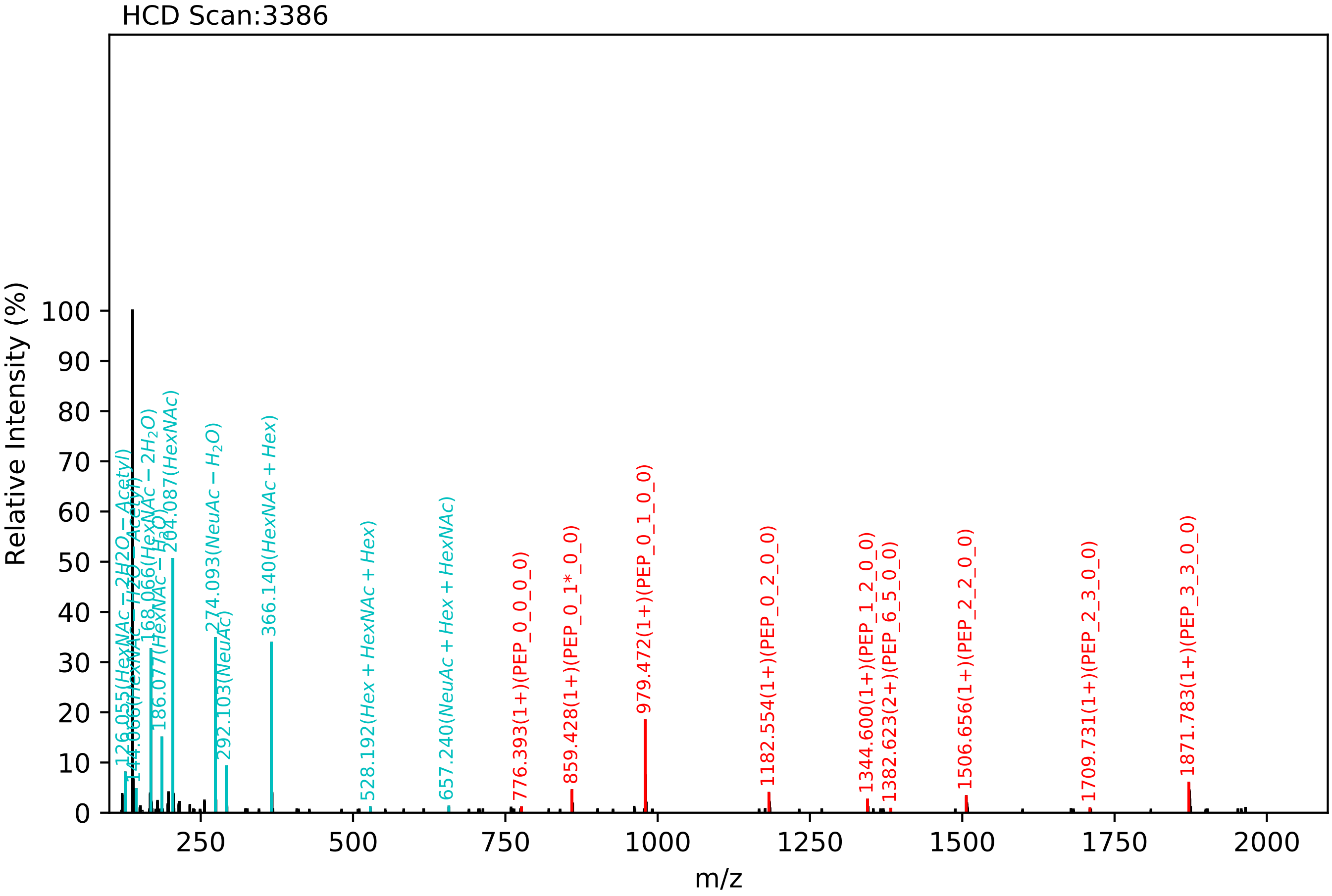

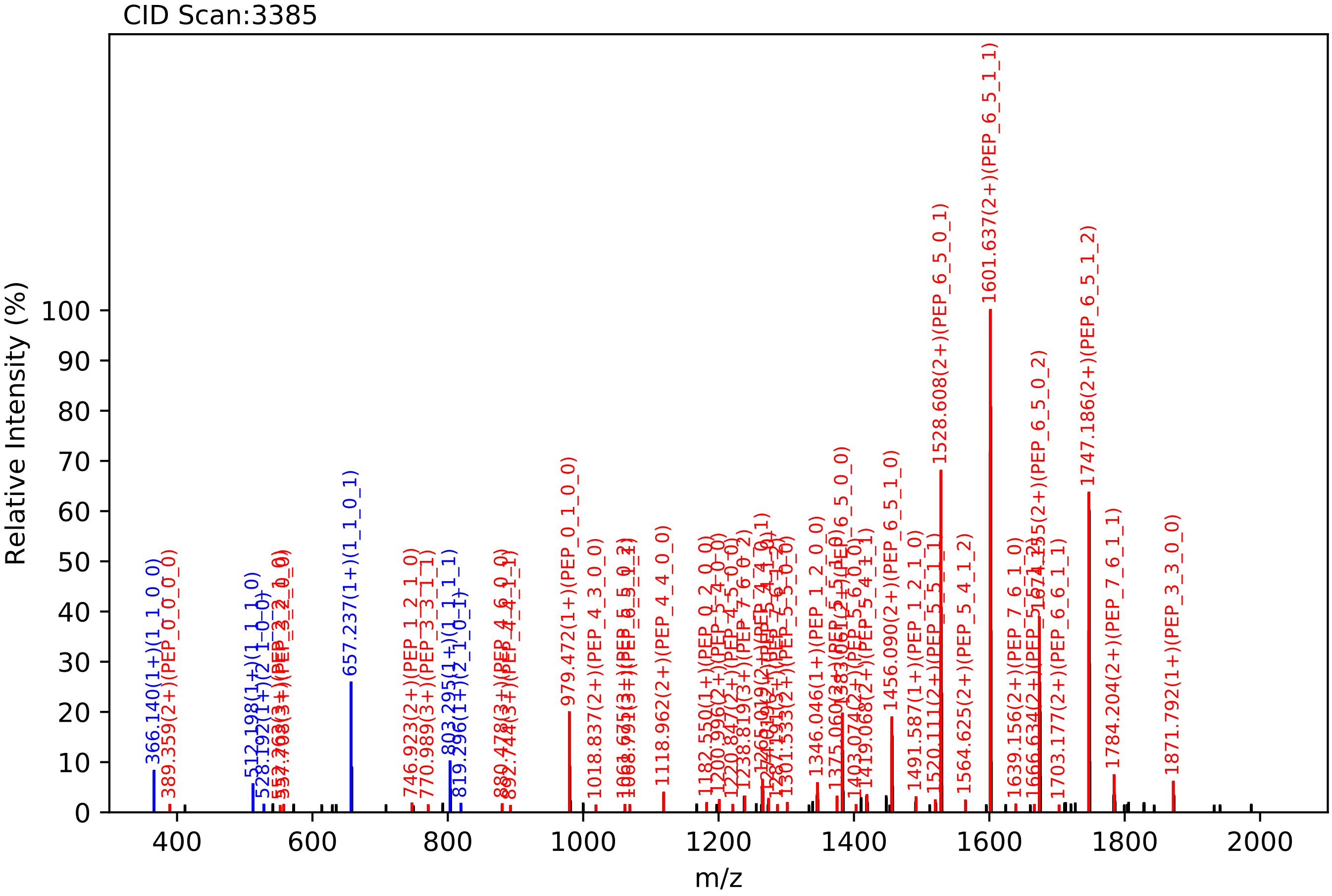

Training set no. 65, Experiment: AGP exp_1

ENGTISR(=PEP)_7_6_1_2, m/z:1286.50(3+), RT:18.88, Y-score:89.39

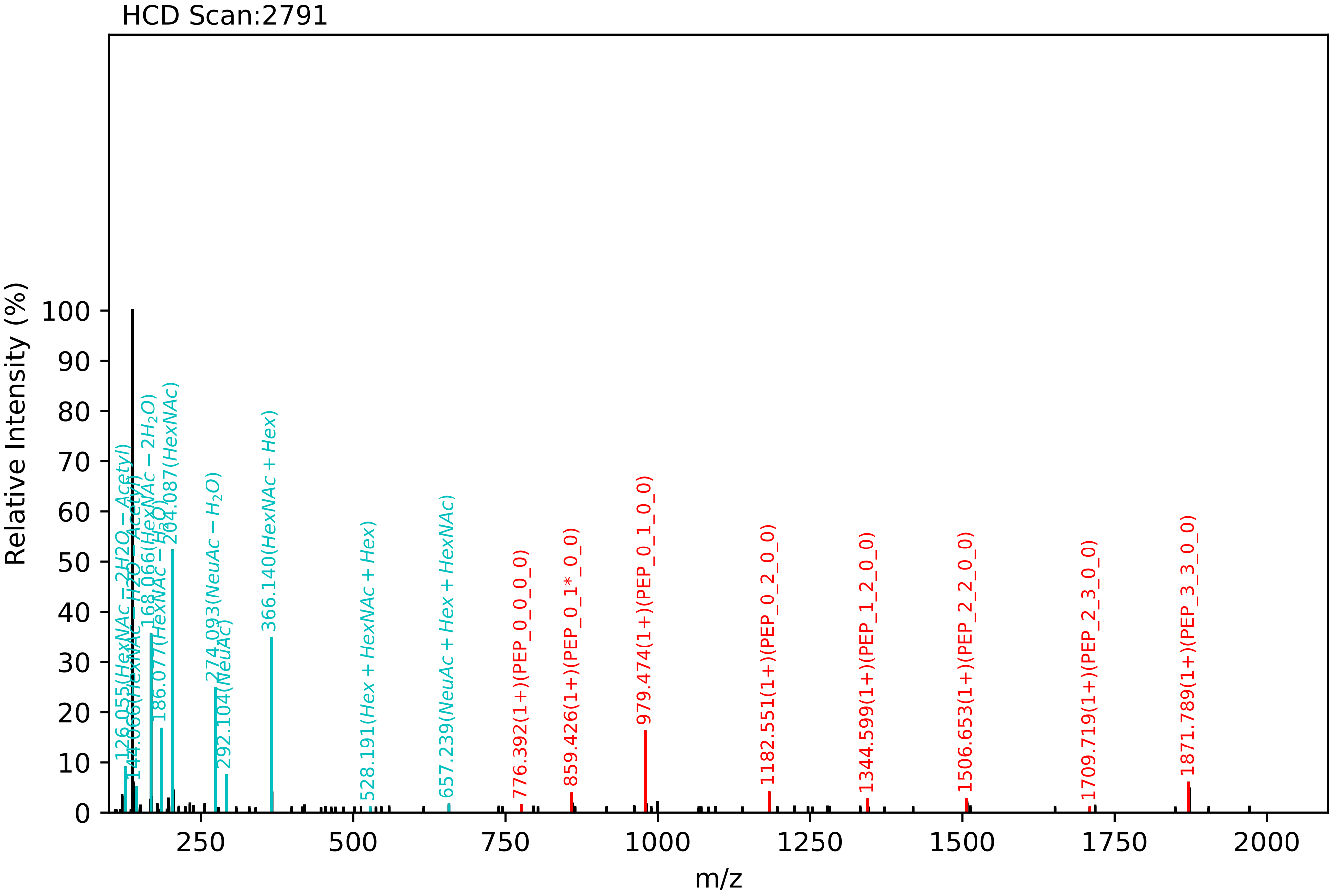

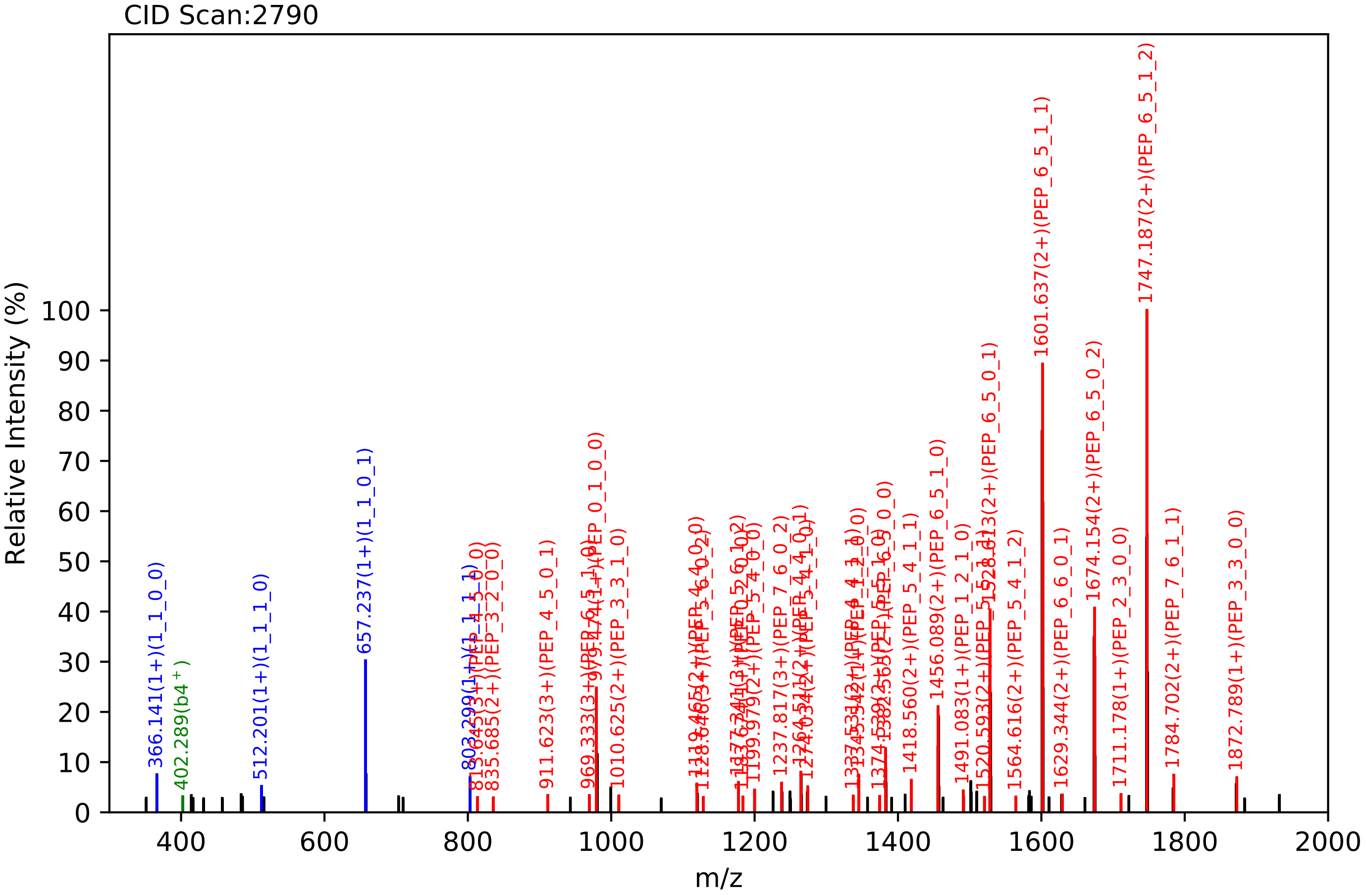

Training set no. 66, Experiment: AGP exp_2

ENGTISR(=PEP)_7_6_1_2, m/z:1286.50(3+), RT:21.07, Y-score:84.98

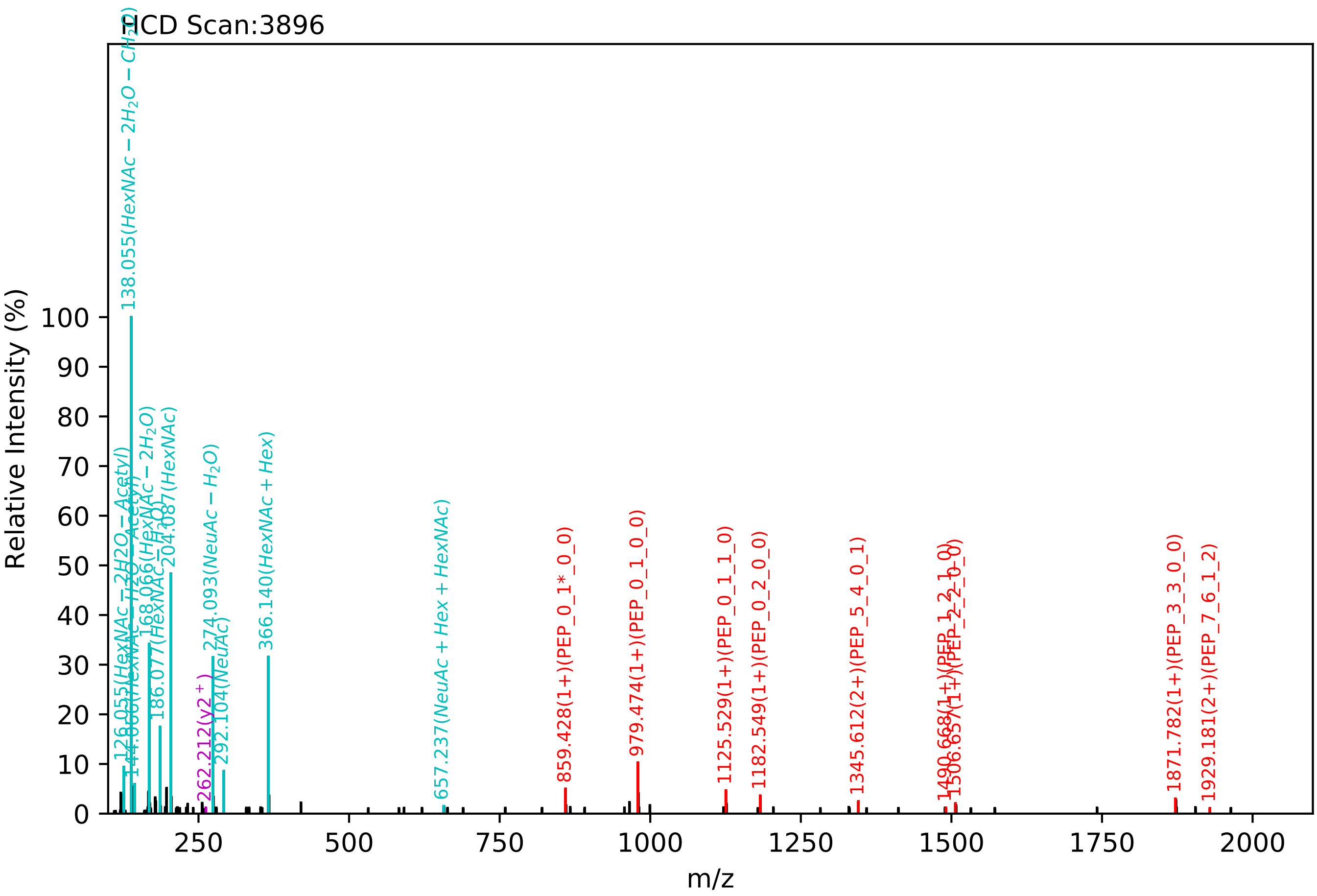

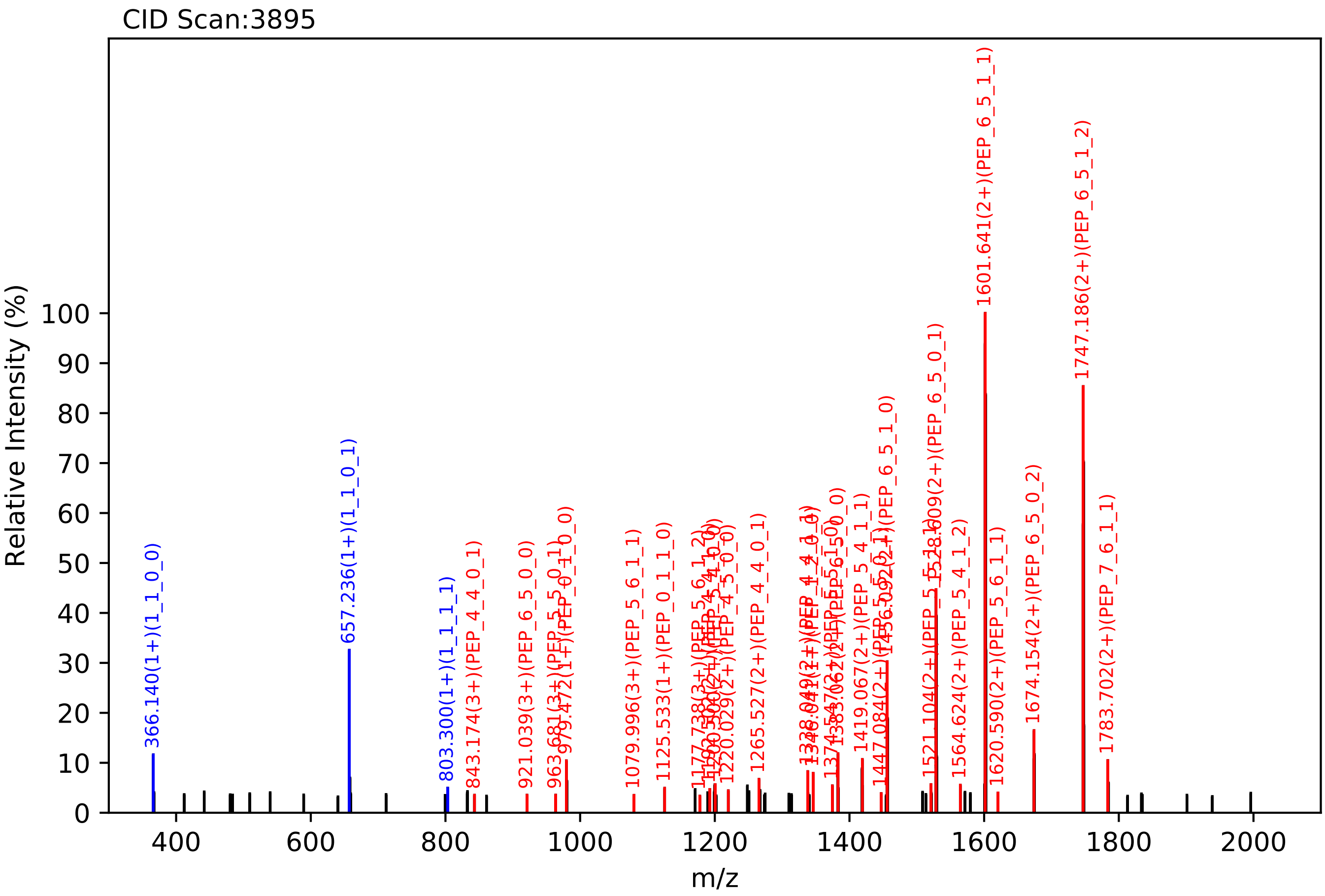

Training set no. 67, Experiment: AGP exp_1

ENGTISR(=PEP)_7_6_1_3, m/z:1037.90(4+), RT:22.48, Y-score:87.39

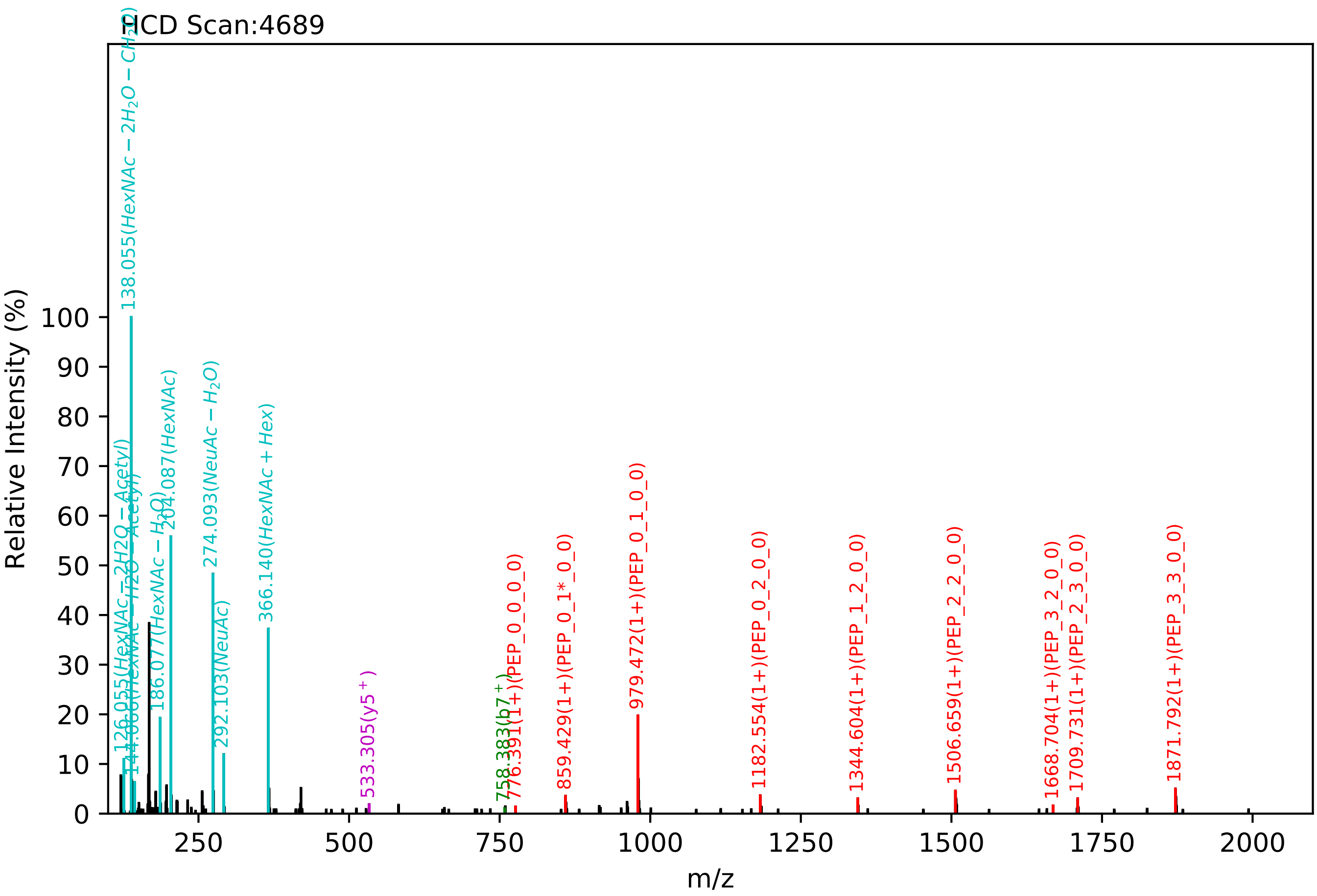

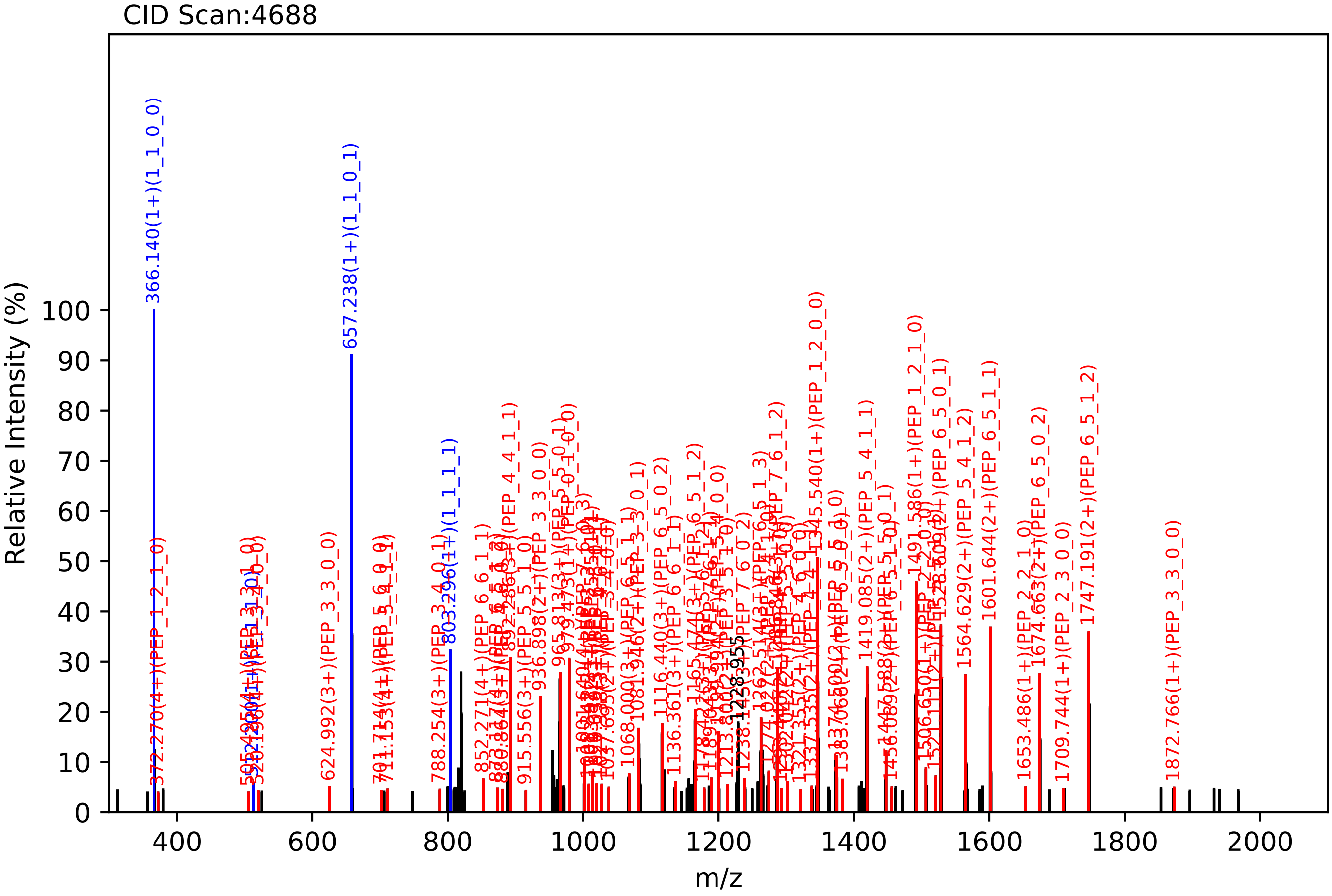

Training set no. 68, Experiment: AGP exp_2

ENGTISR(=PEP)_7_6_1_3, m/z:1037.90(4+), RT:22.51, Y-score:85.60

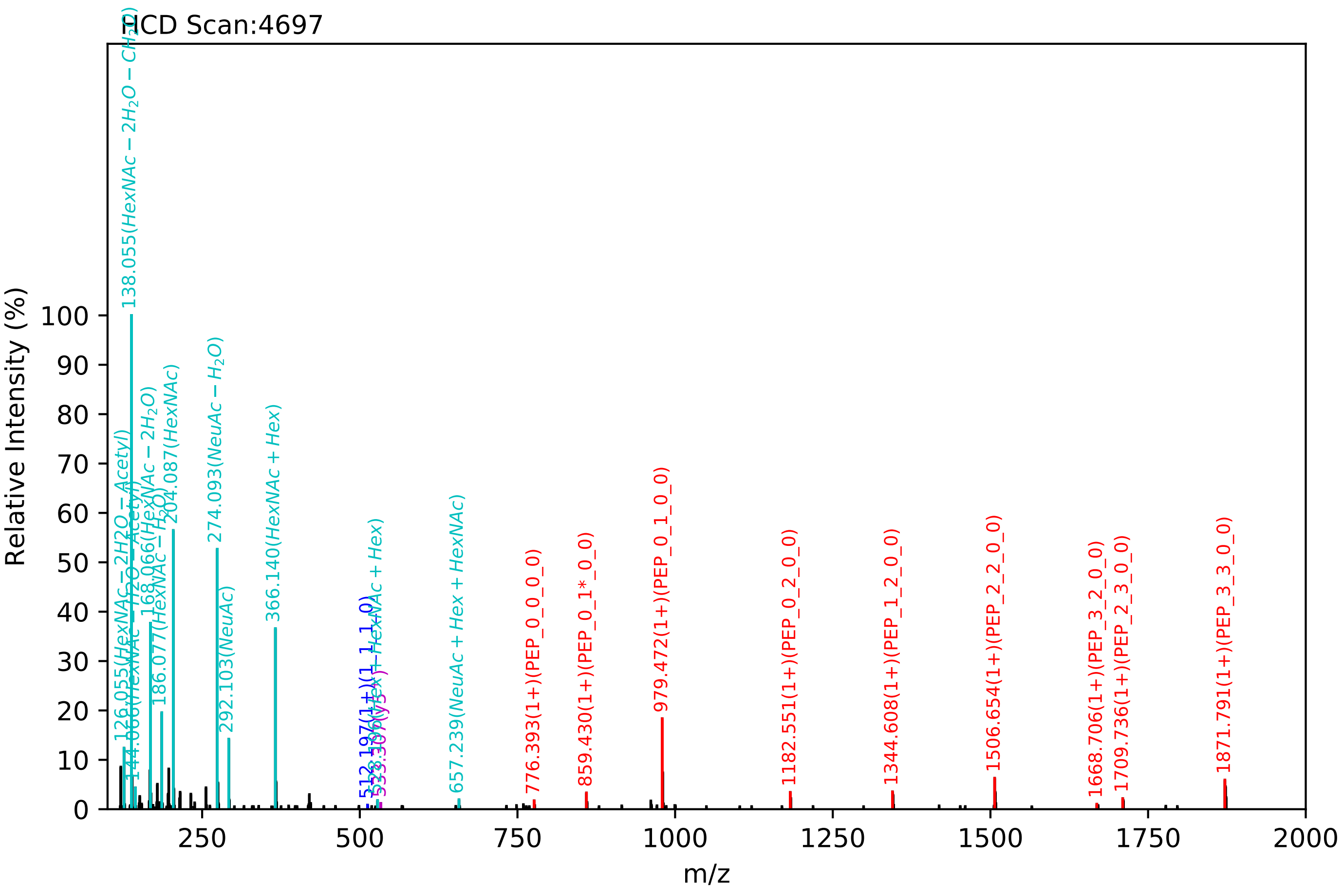

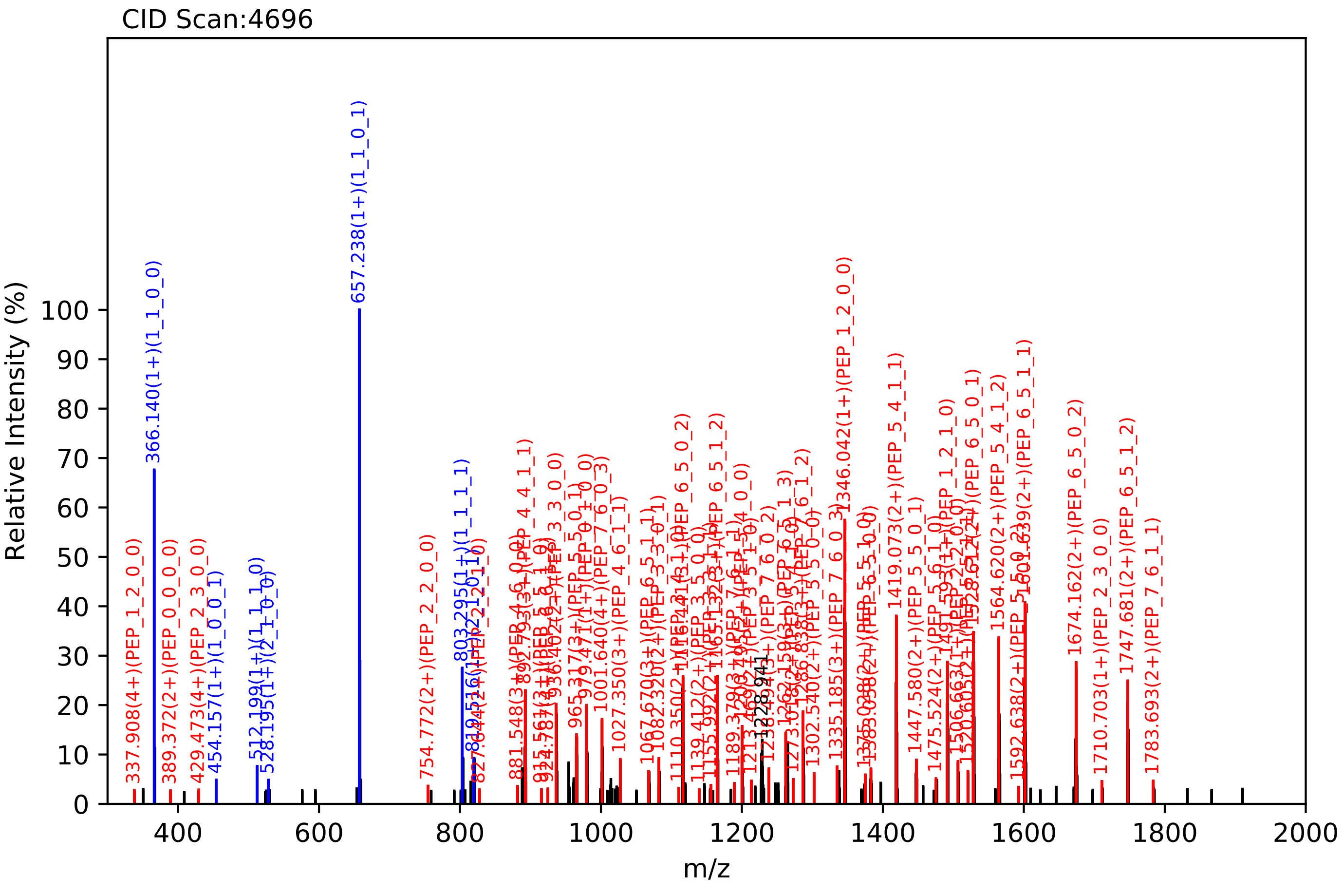

Training set no. 69, Experiment: AGP exp_1

ENGTISR(=PEP)_7_6_1_4, m/z:1110.68(4+), RT:24.93, Y-score:86.37

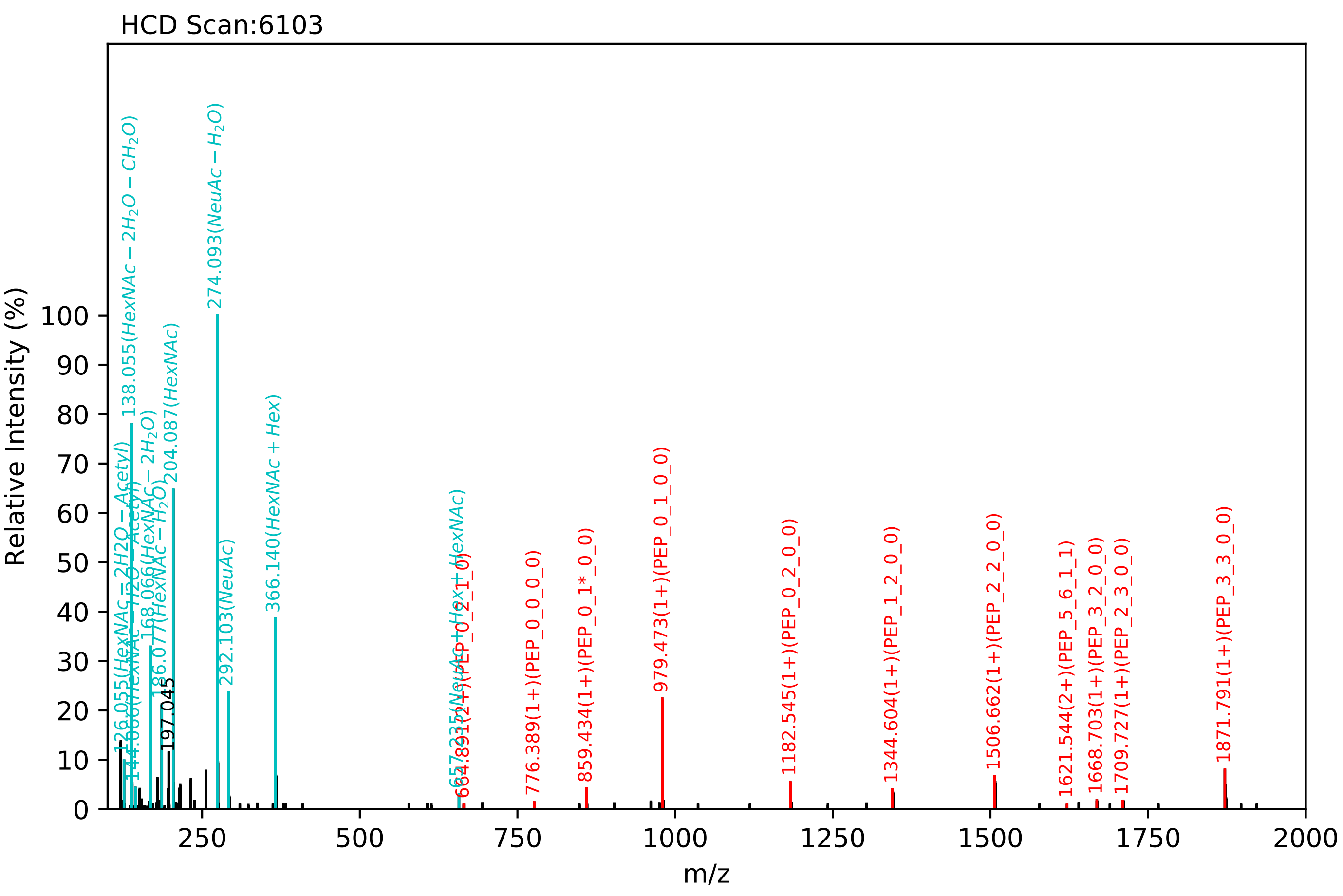

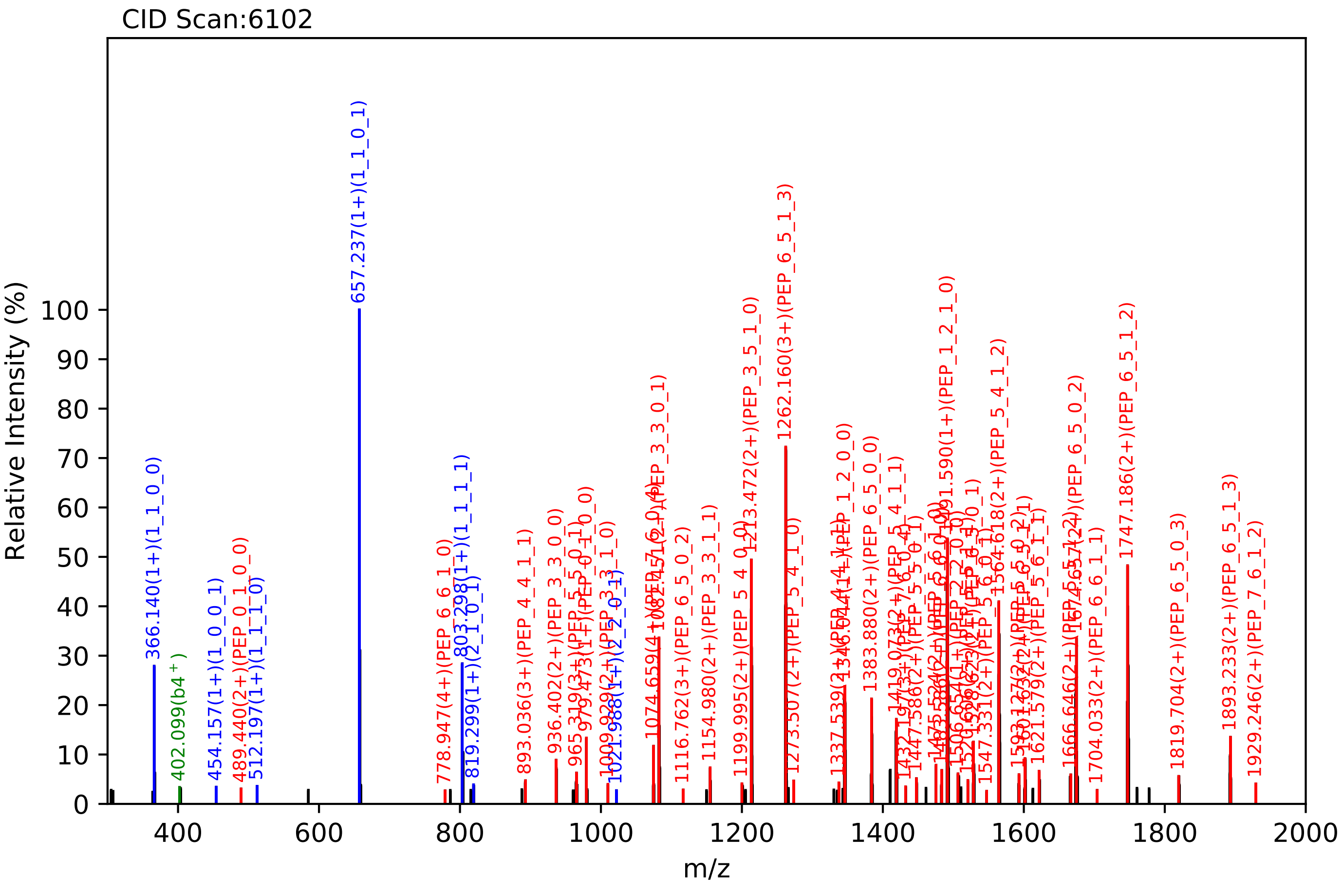

Training set no. 70, Experiment: AGP exp_2

ENGTISR(=PEP)_7_6_1_4, m/z:1110.68(4+), RT:24.99, Y-score:83.30

HCD Scan:6115

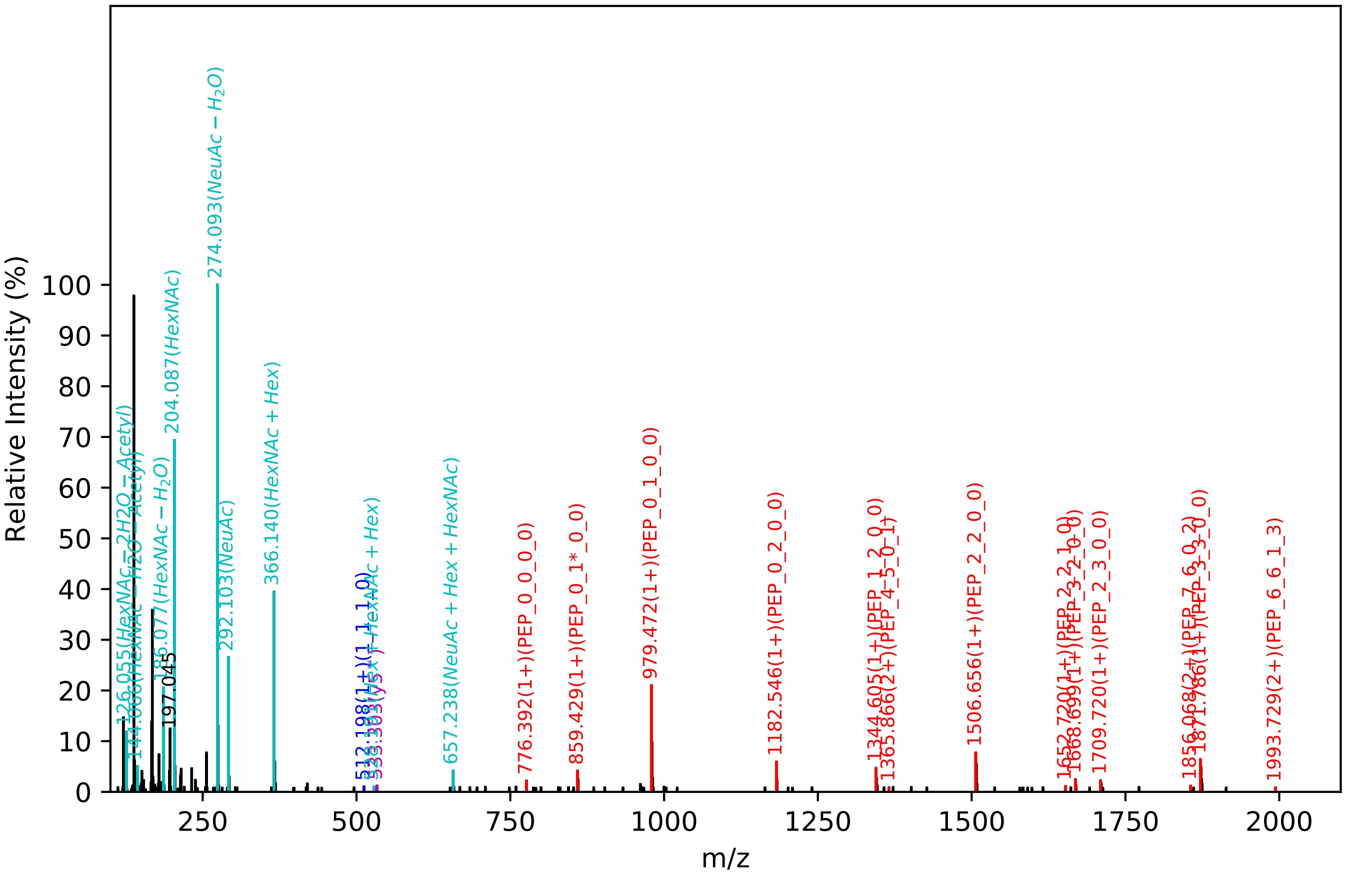

CID Scan:6114

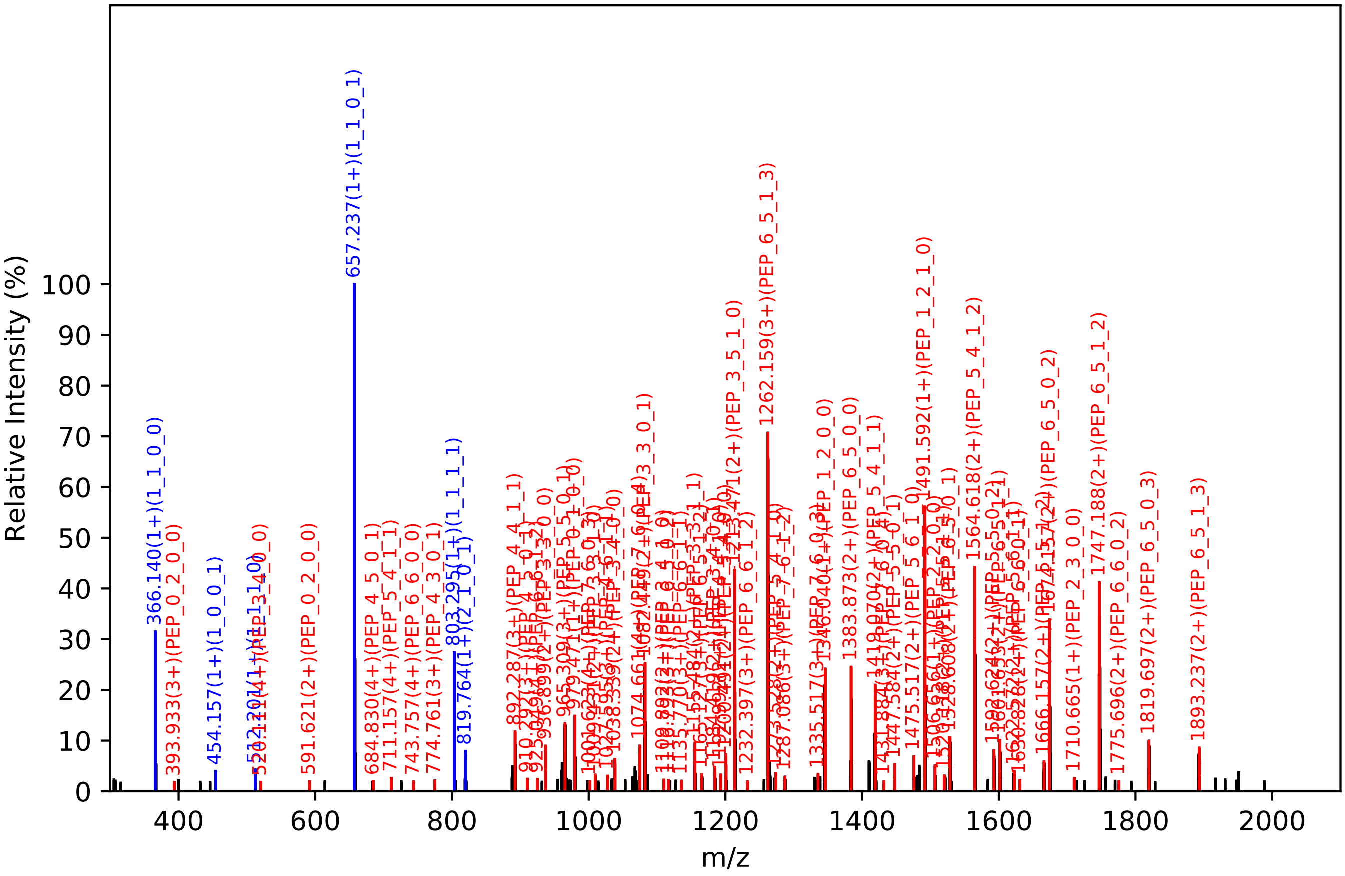

Training set no. 71, Experiment: AGP exp_1

ENGTISR(=PEP)_7_6_2_4, m/z:1147.19(4+), RT:24.59, Y-score:82.24

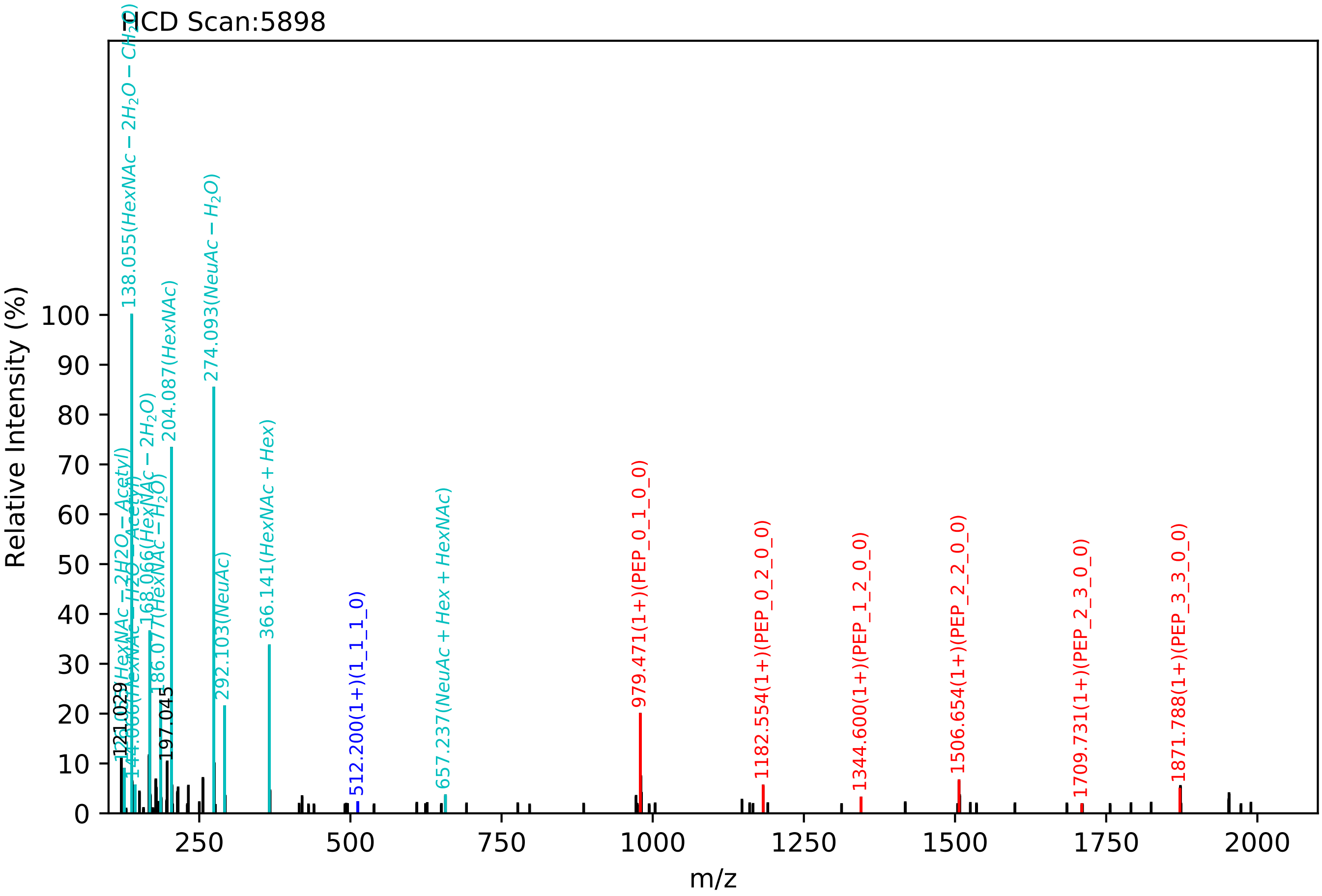

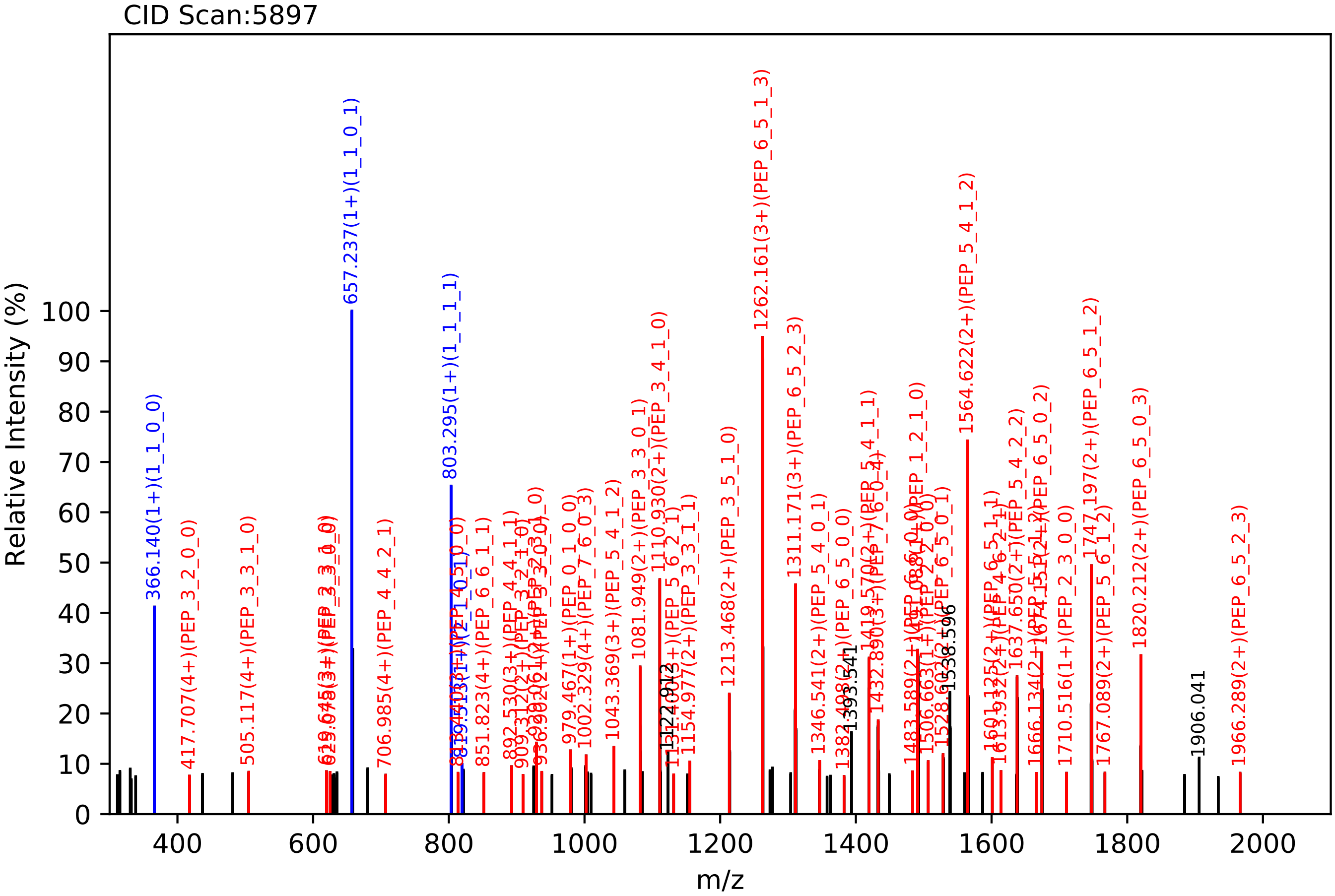

Training set no. 72, Experiment: AGP exp_2

LVPVPITNATLDQITGK(=PEP)_4_4_0_1, m/z:1177.89(3+), RT:49.25, Y-score:79.33

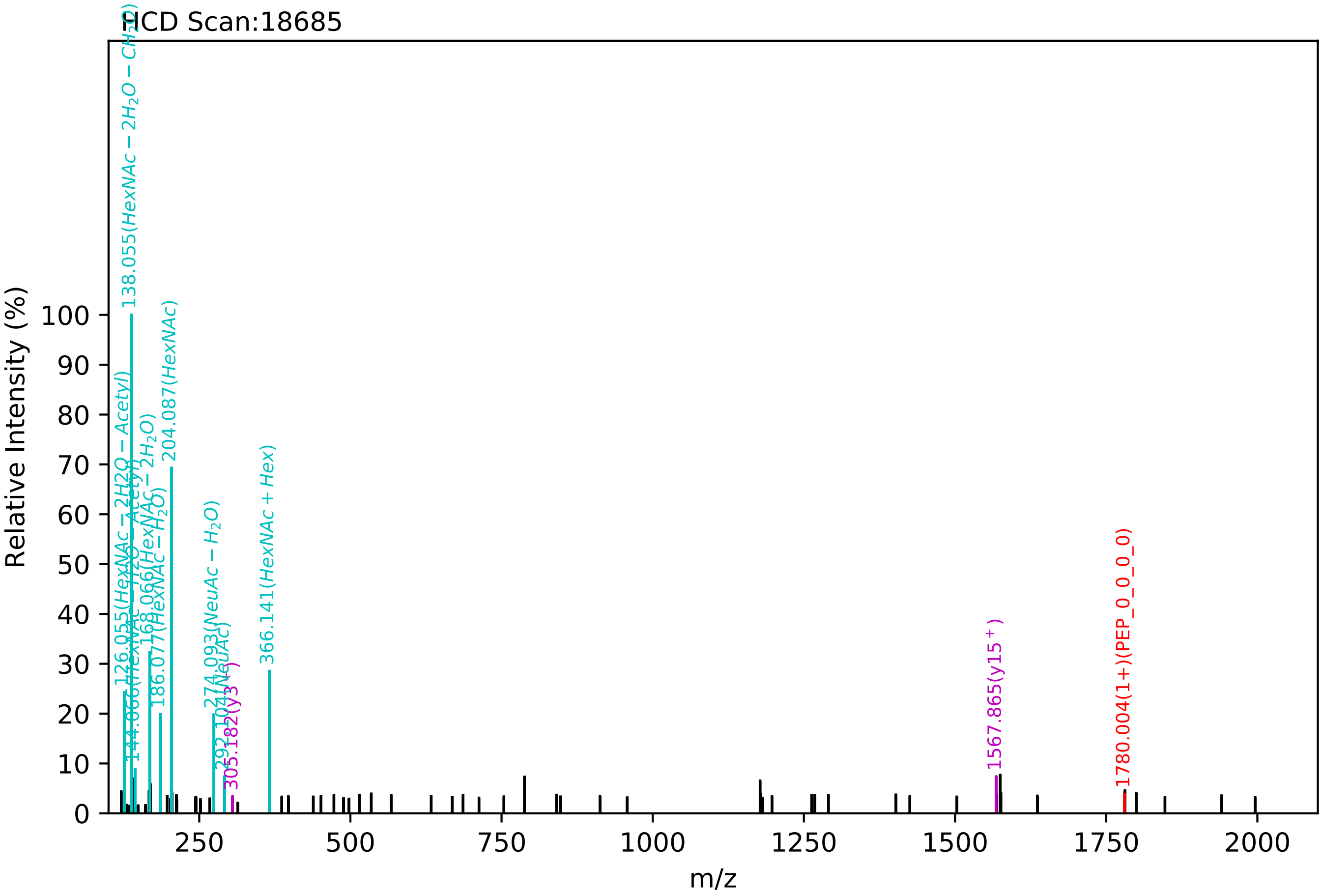

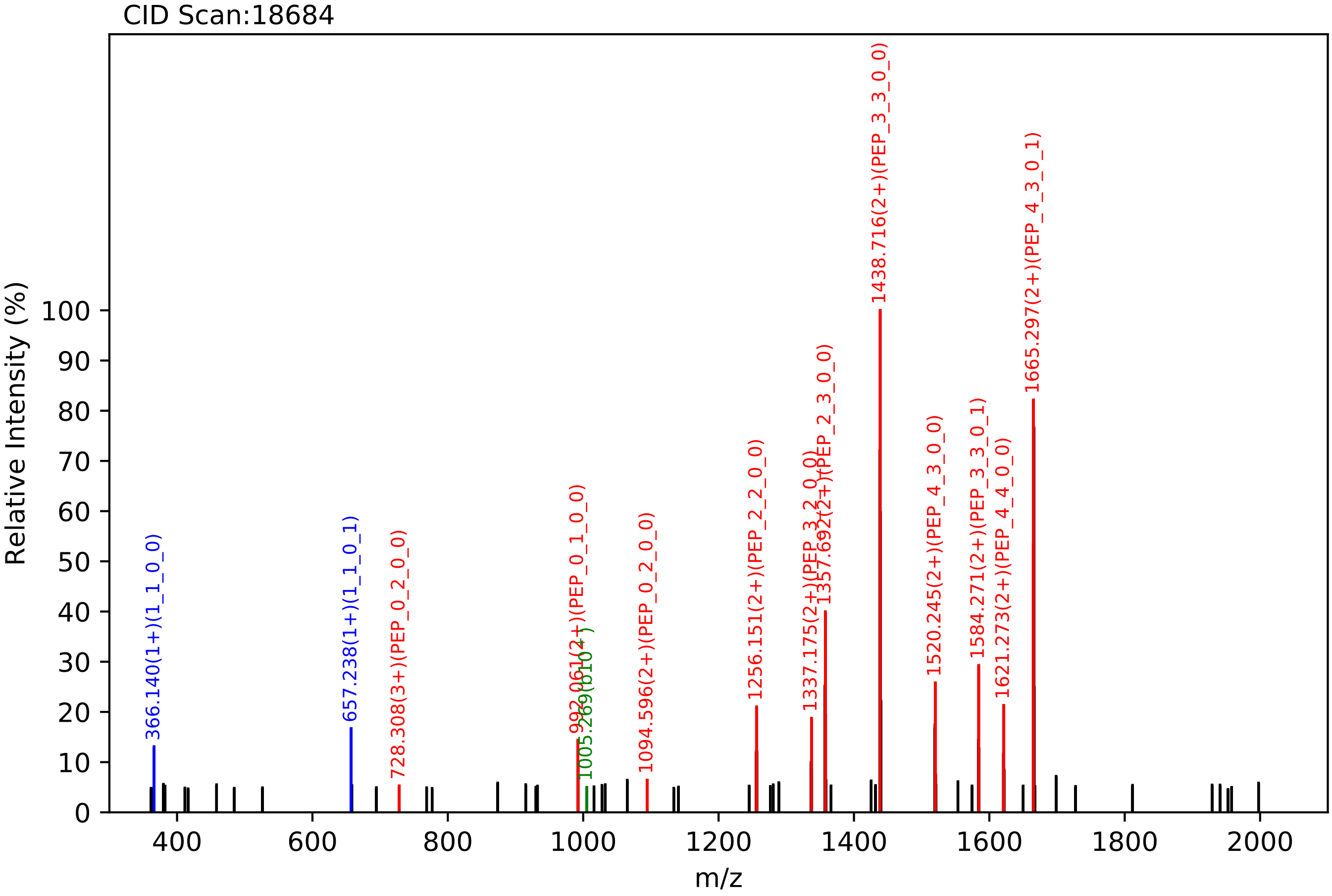

Training set no. 73, Experiment: AGP exp_2

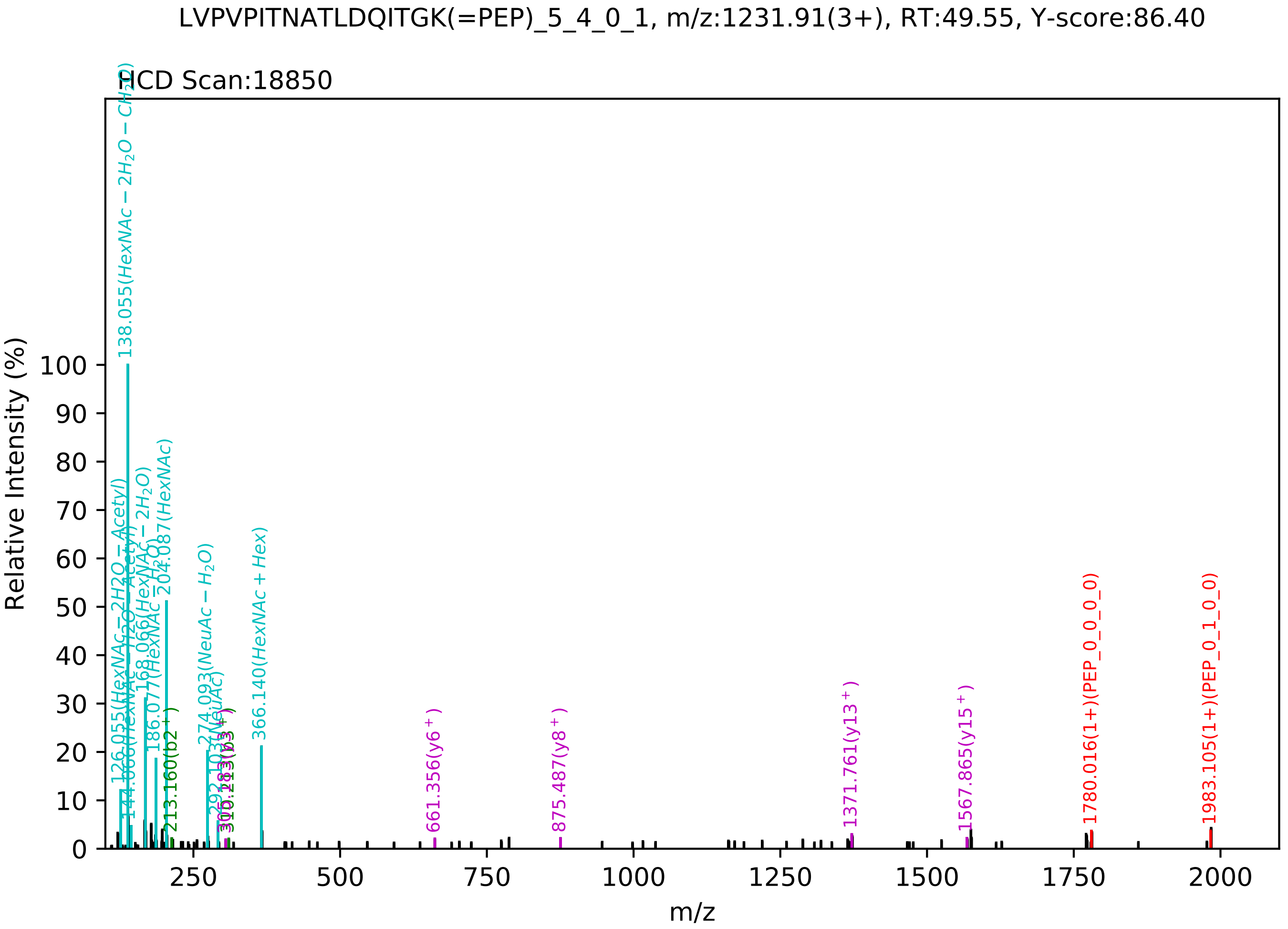

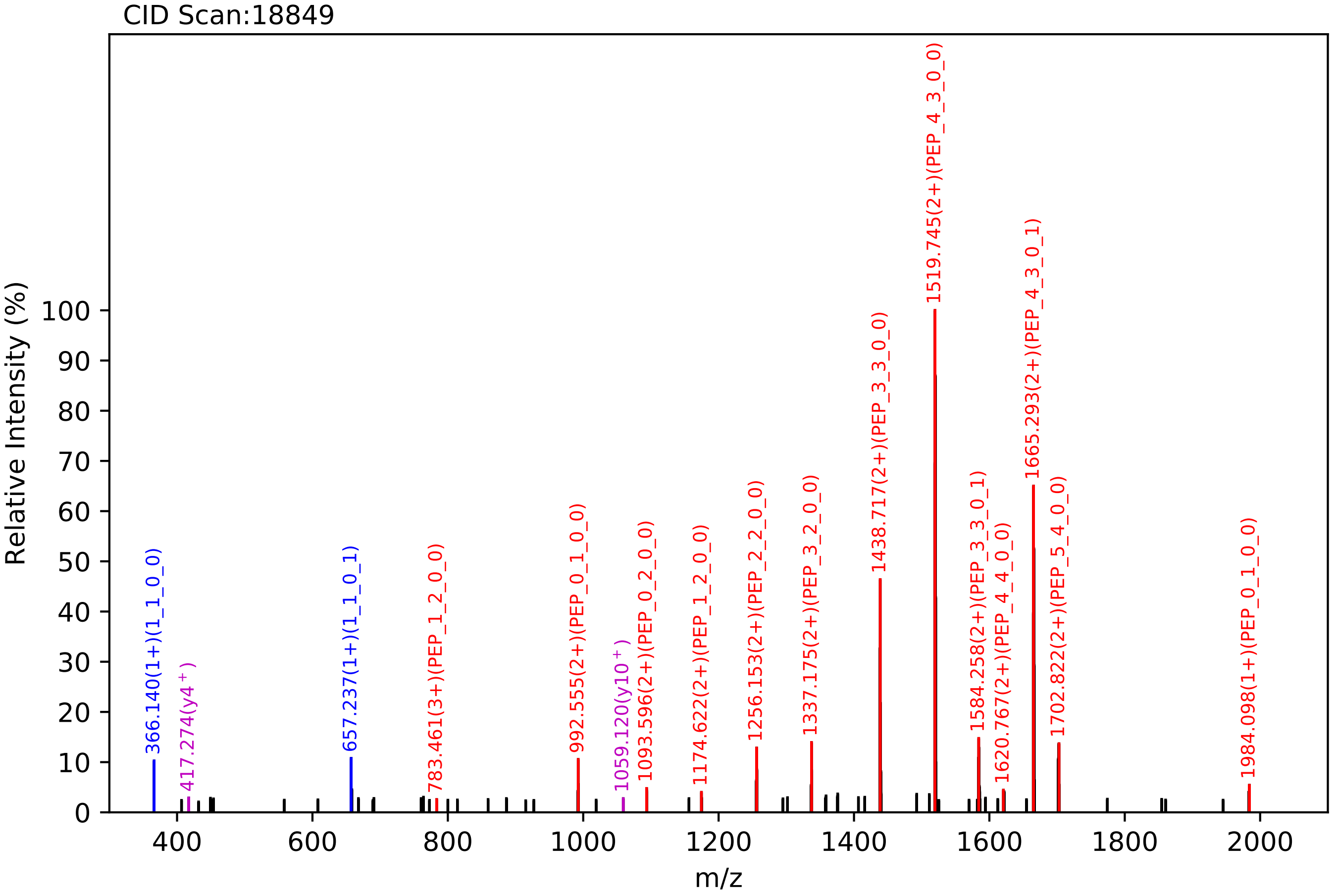

Training set no. 74, Experiment: AGP exp_2

LVPVPITNATLDQITGK(=PEP)_5_4_0_1, m/z:924.18(4+), RT:48.74, Y-score:74.67

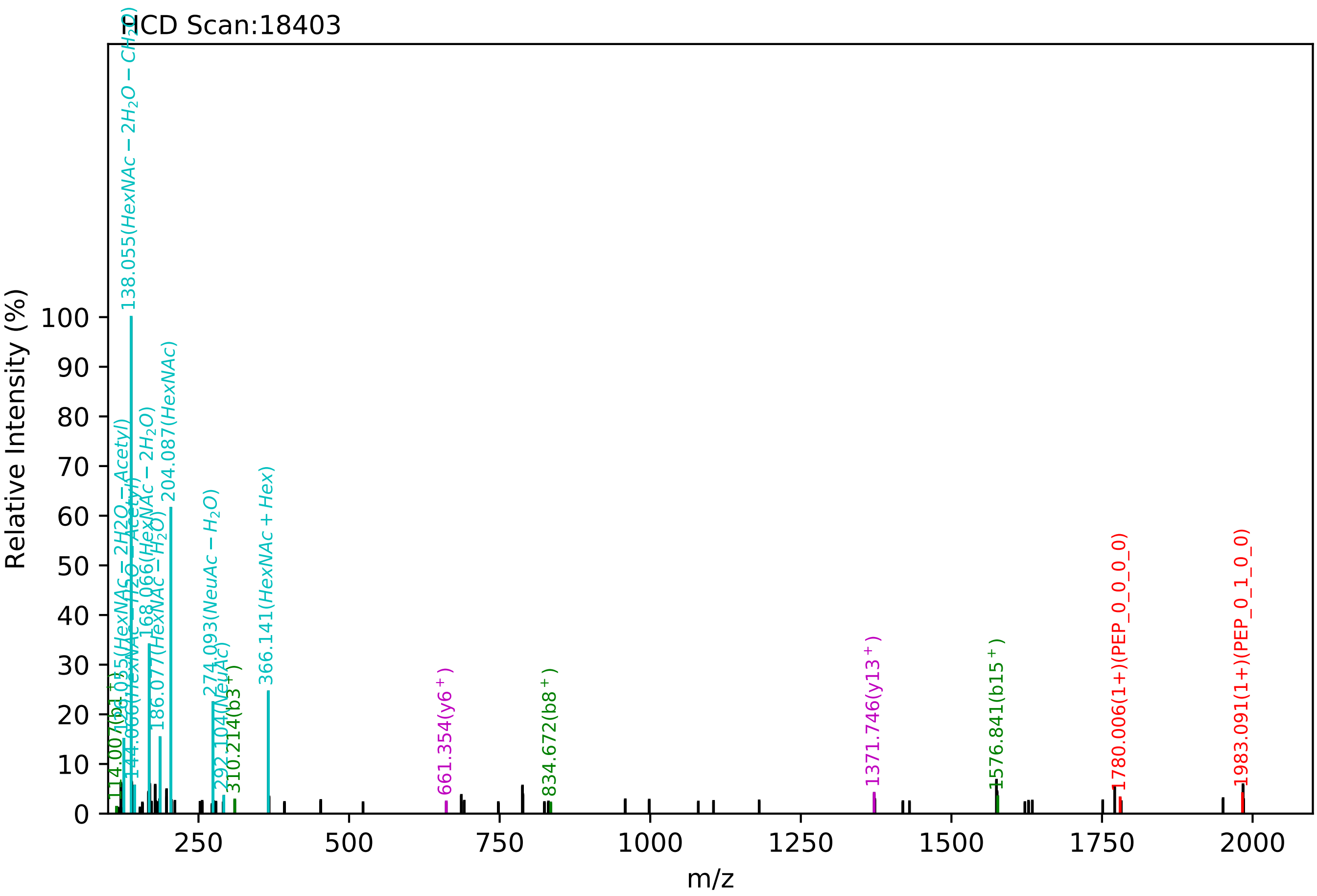

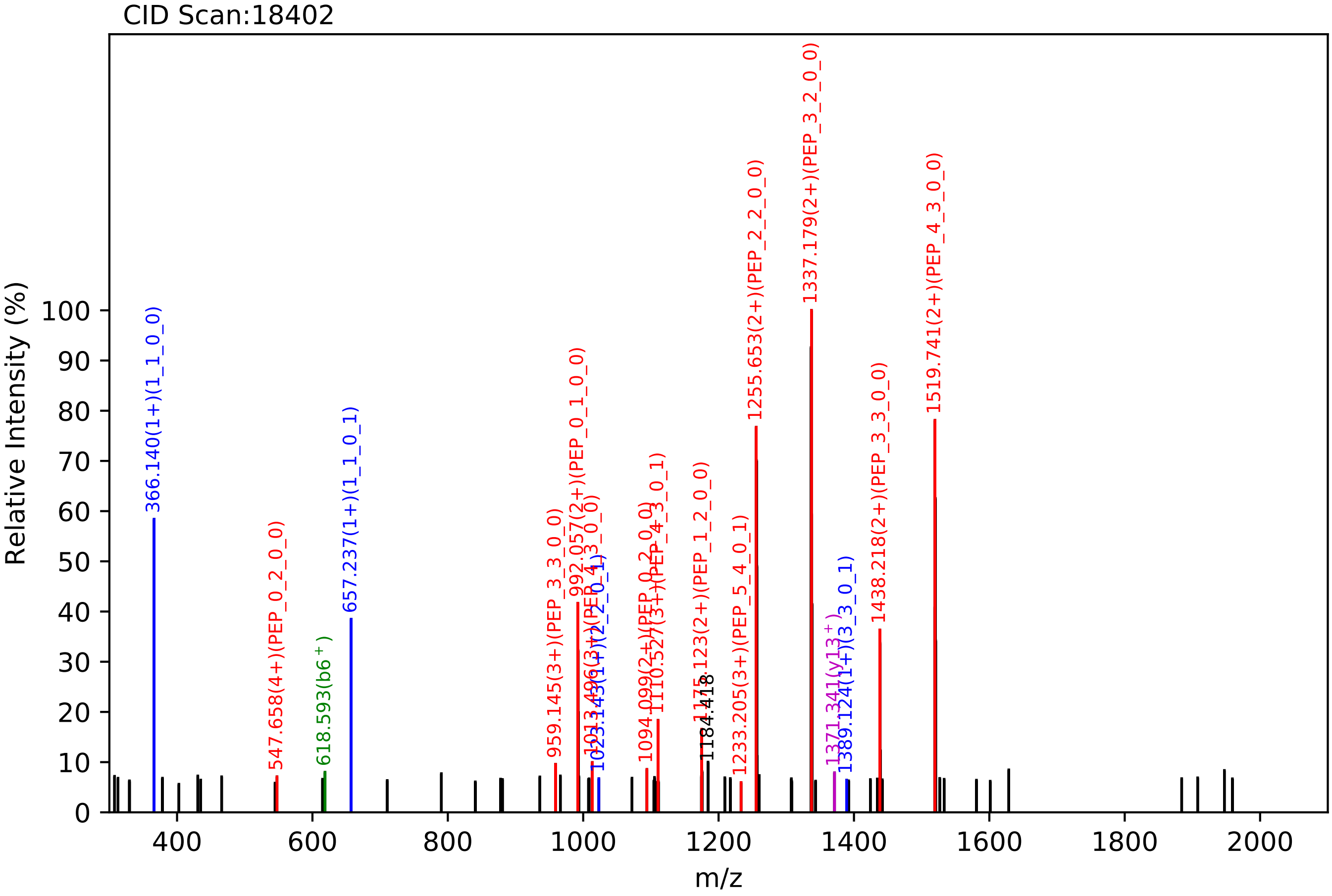

Training set no. 75, Experiment: AGP exp_1

LVPVPITNATLDQITGK(=PEP)_5_4_0_1, m/z:924.18(4+), RT:49.14, Y-score:73.45

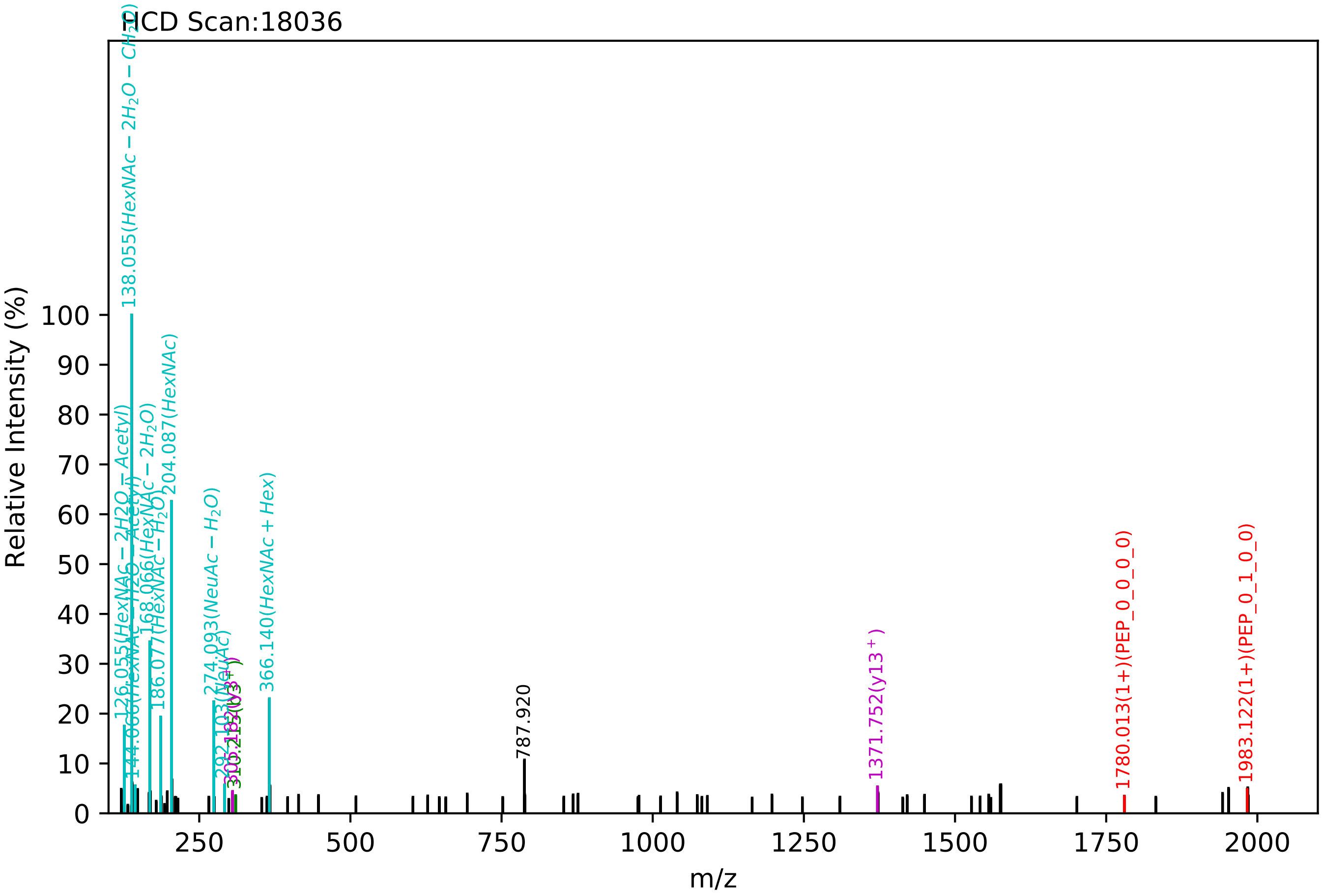

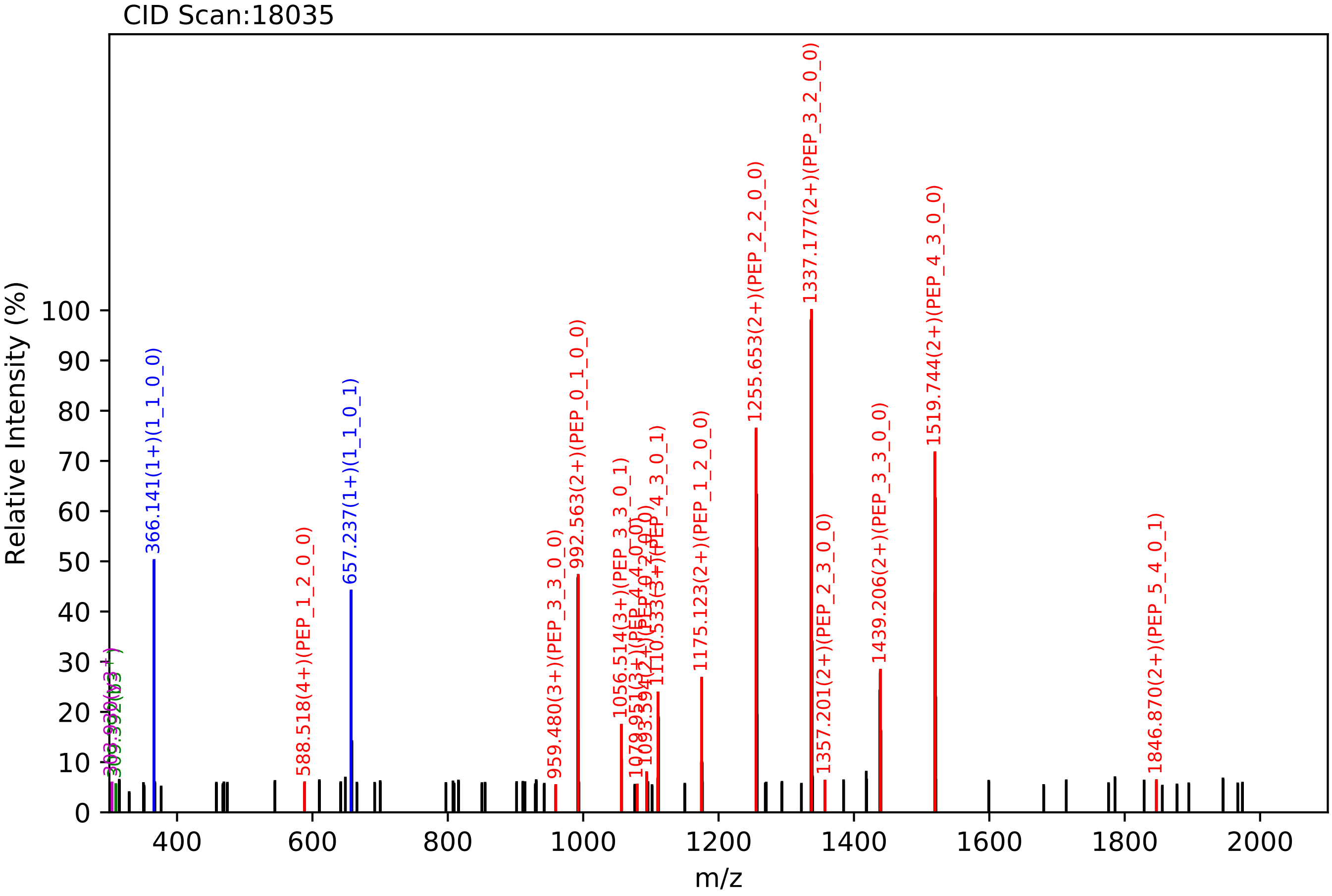

Training set no. 76, Experiment: AGP exp_1

LVPVPITNATLDQITGK(=PEP)_5_4_0_2, m/z:1328.94(3+), RT:55.54, Y-score:91.12

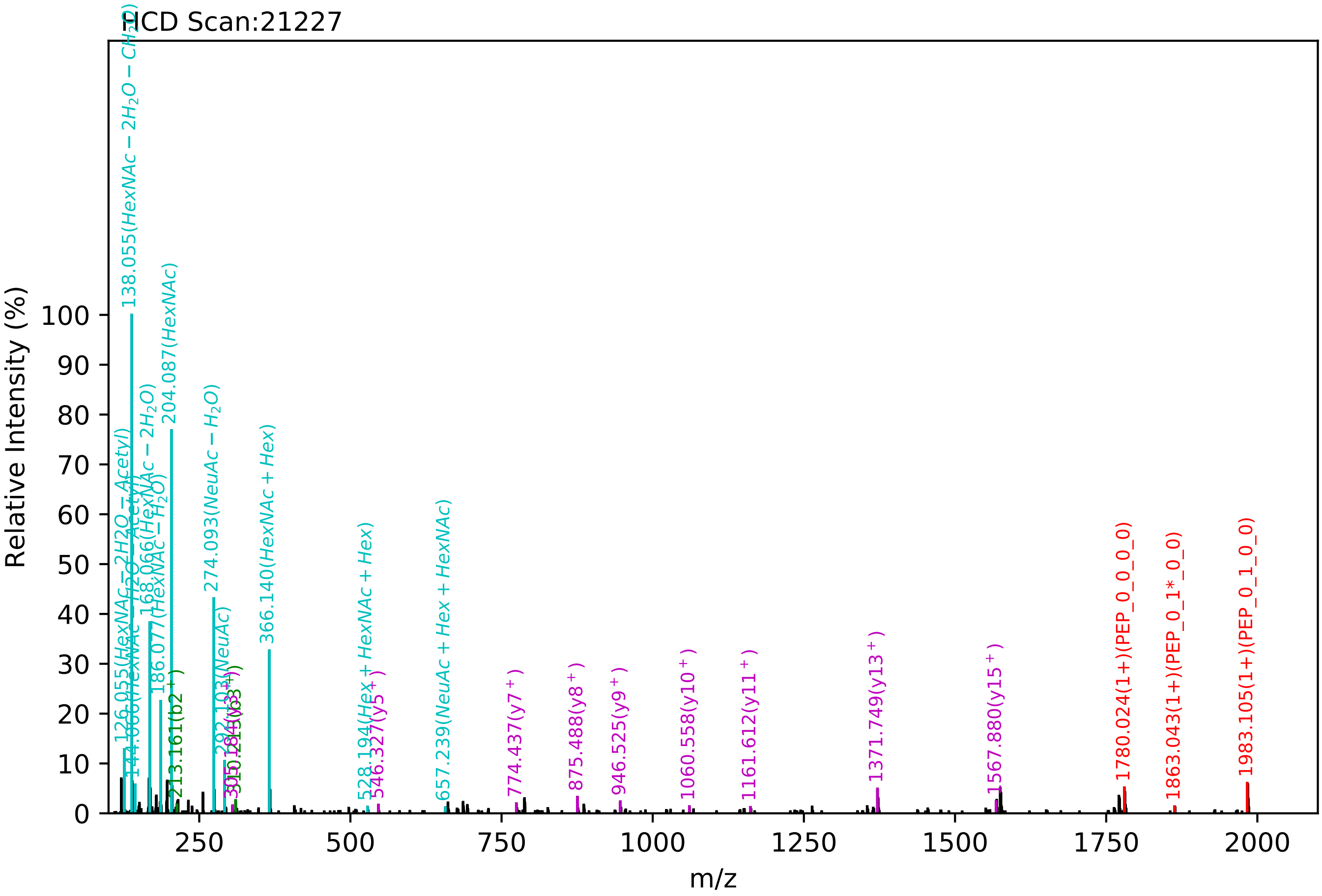

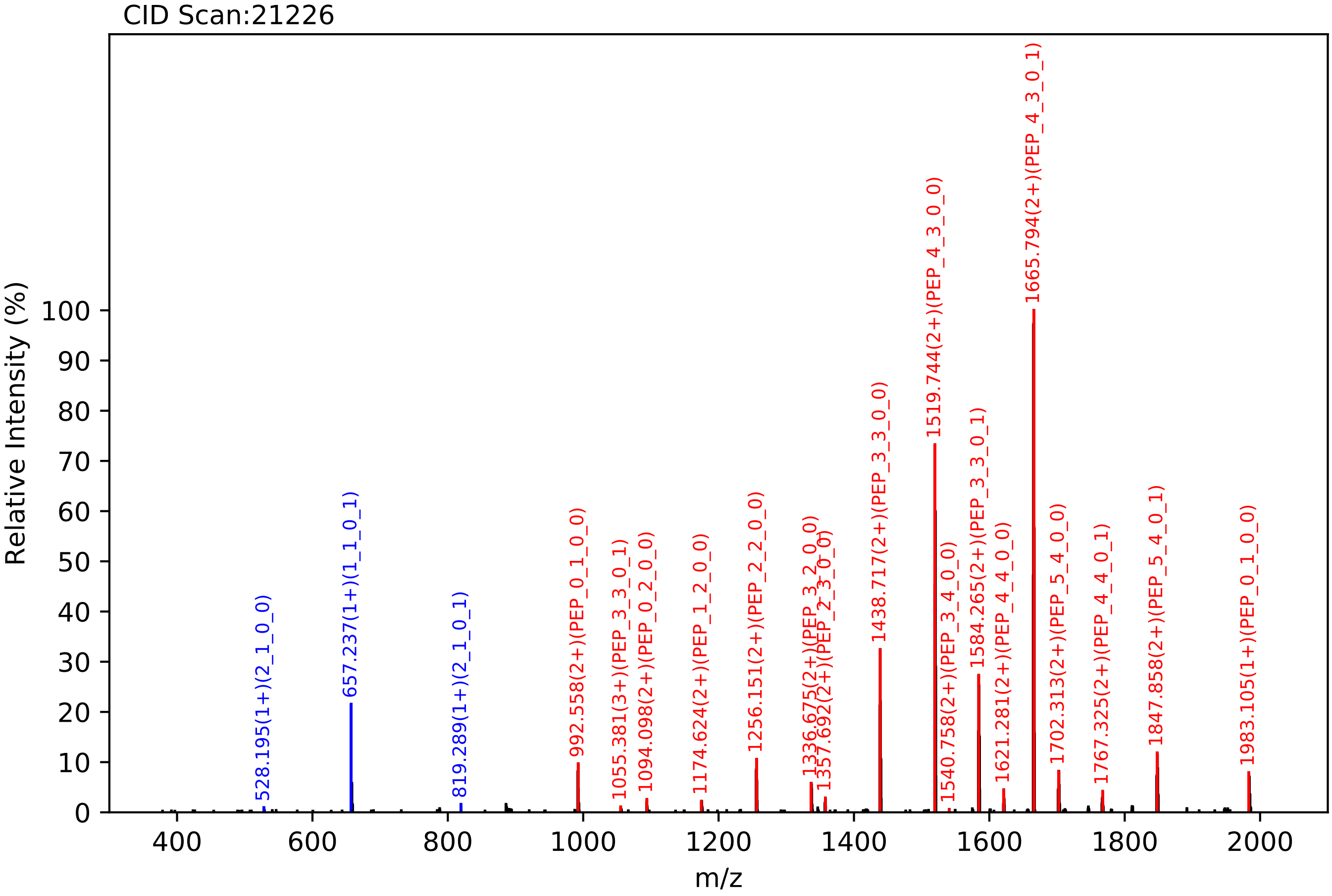

Training set no. 77, Experiment: AGP exp_2

LVPVPITNATLDQITGK(=PEP)_5_4_0_2, m/z:1328.94(3+), RT:55.11, Y-score:88.66

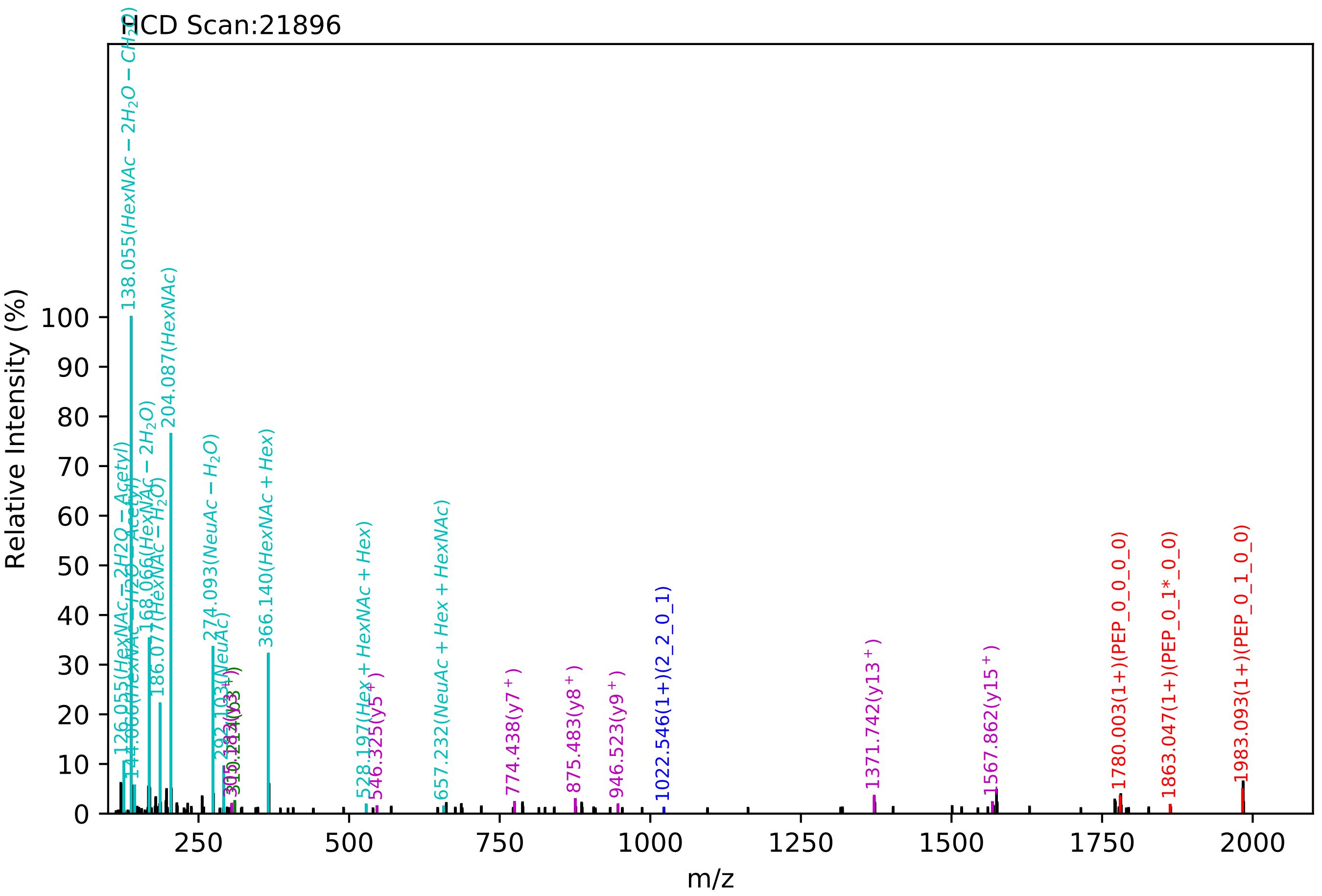

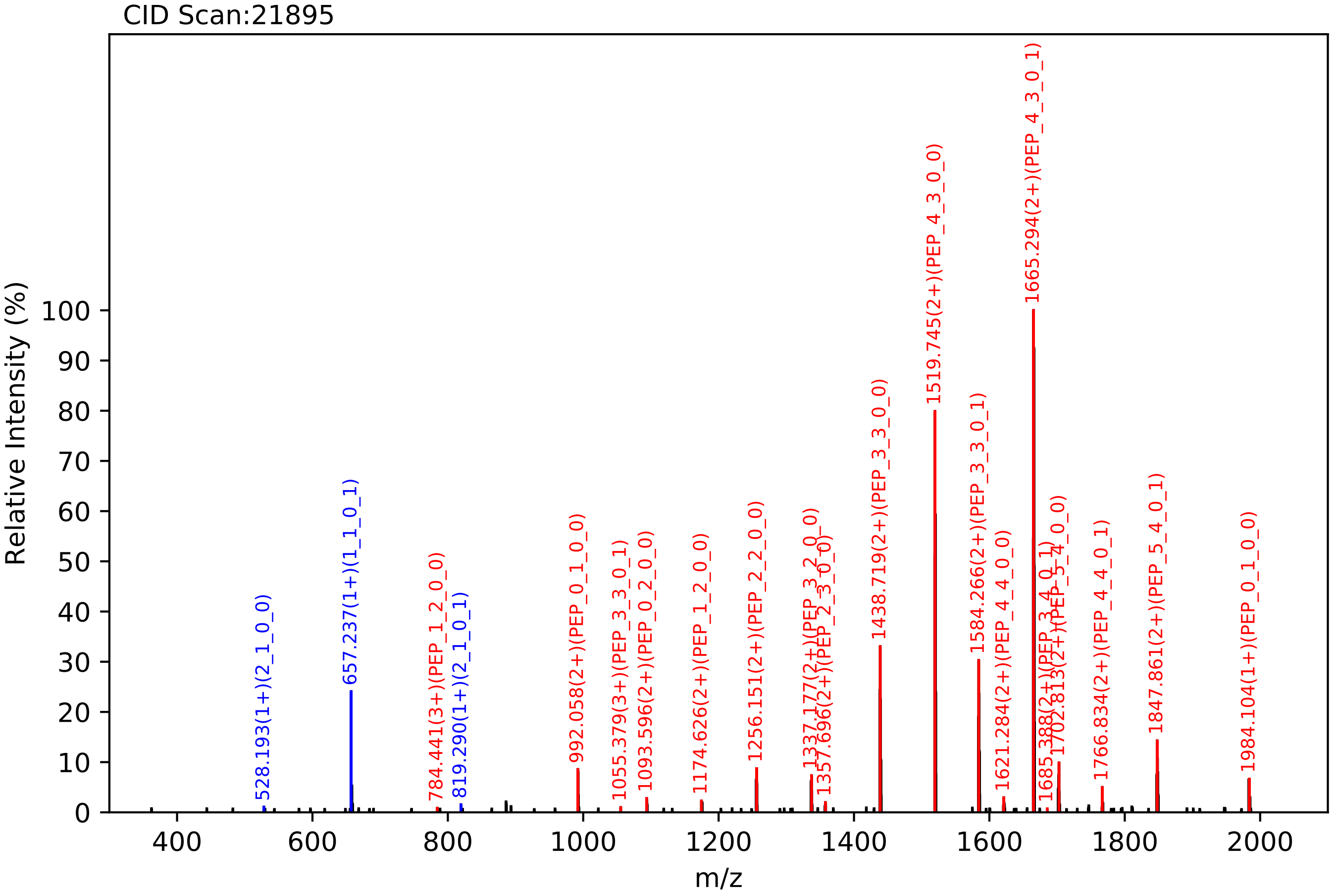

Training set no. 78, Experiment: AGP exp_1

LVPVPITNATLDQITGK(=PEP)_5_4_0_2, m/z:1329.28(3+), RT:54.99, Y-score:88.19

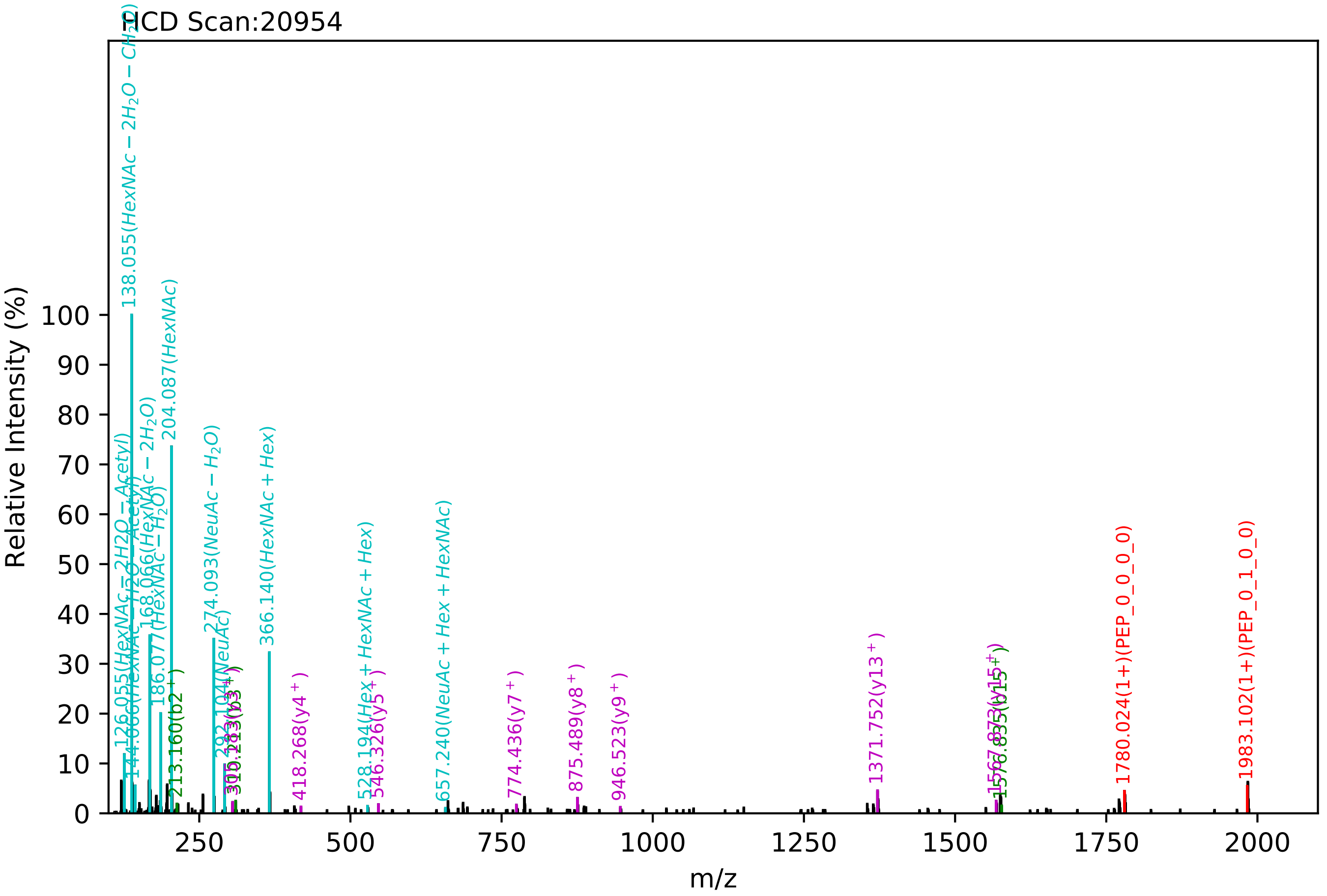

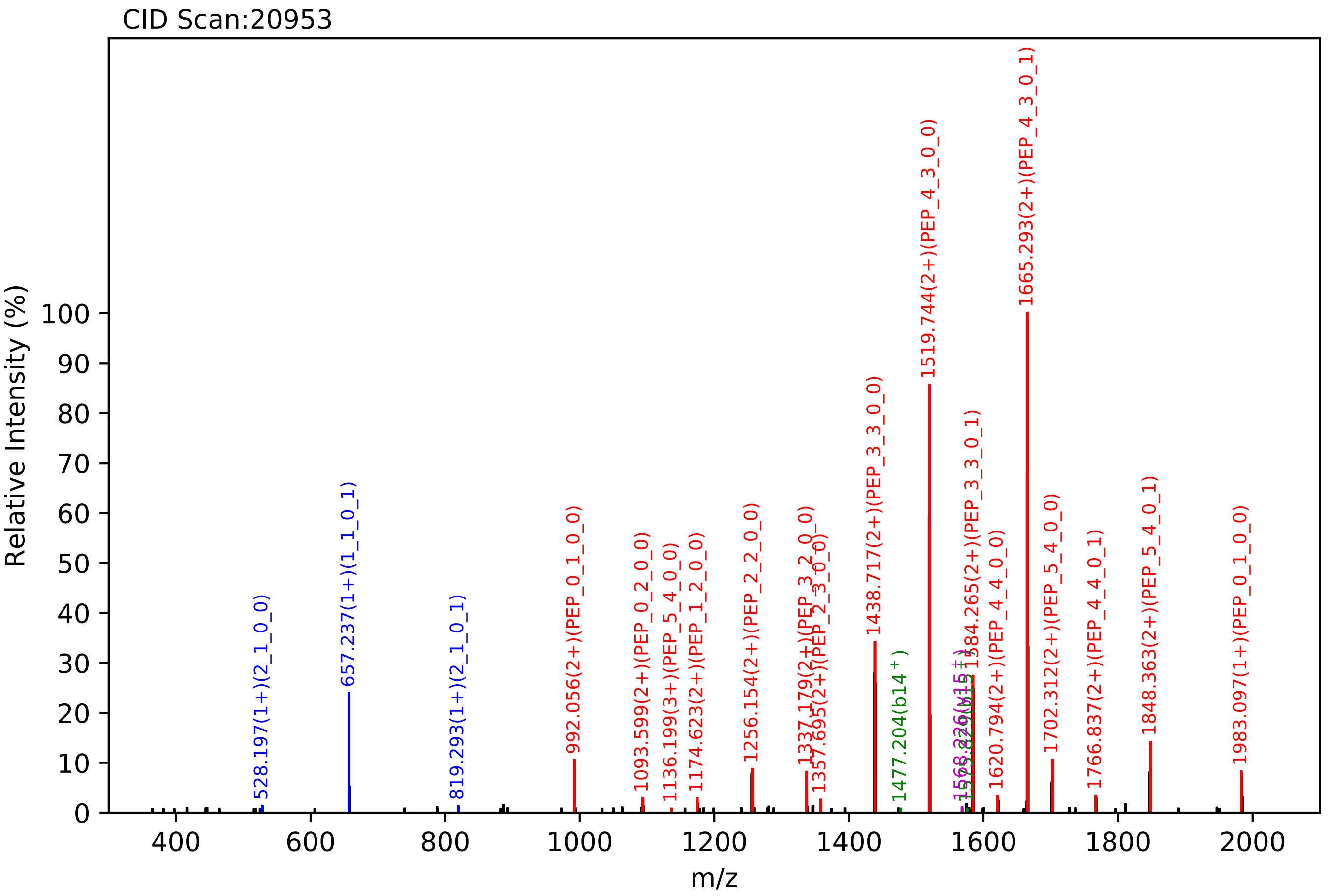

Training set no. 79, Experiment: AGP exp_2

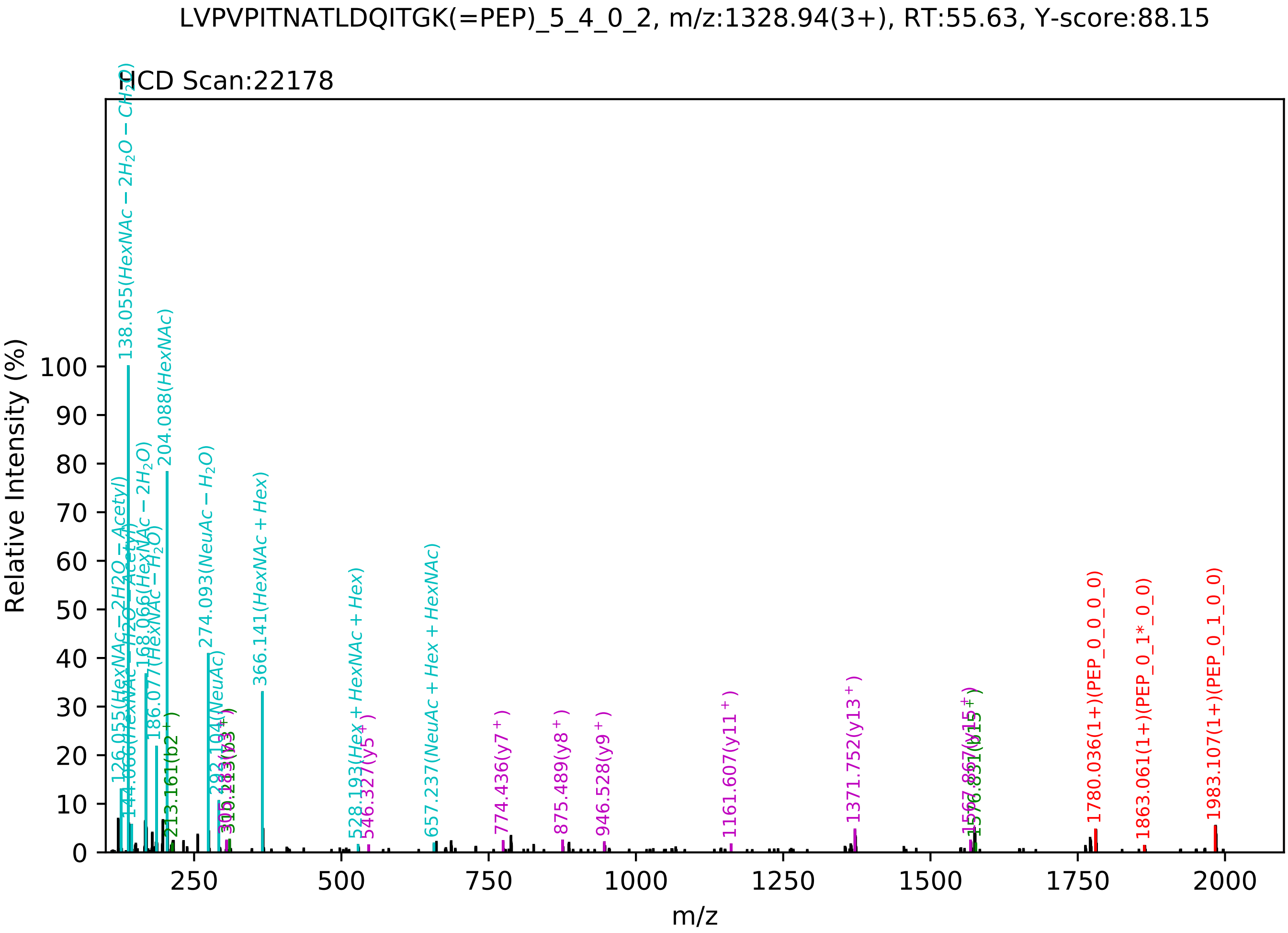

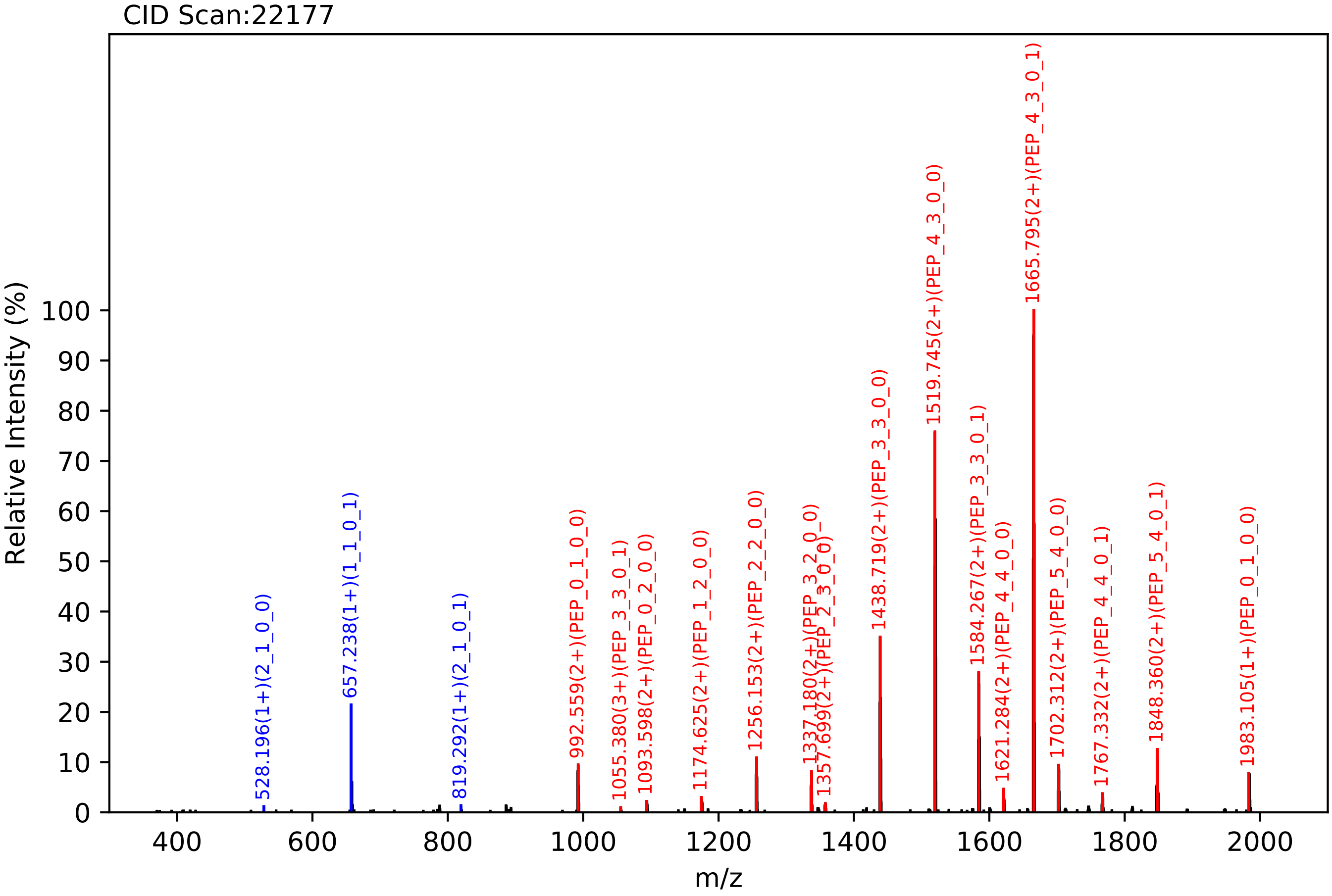

Training set no. 80, Experiment: AGP exp_2

LVPVPITNATLDQITGK(=PEP)_5_4_0_2, m/z:996.96(4+), RT:55.64, Y-score:83.43

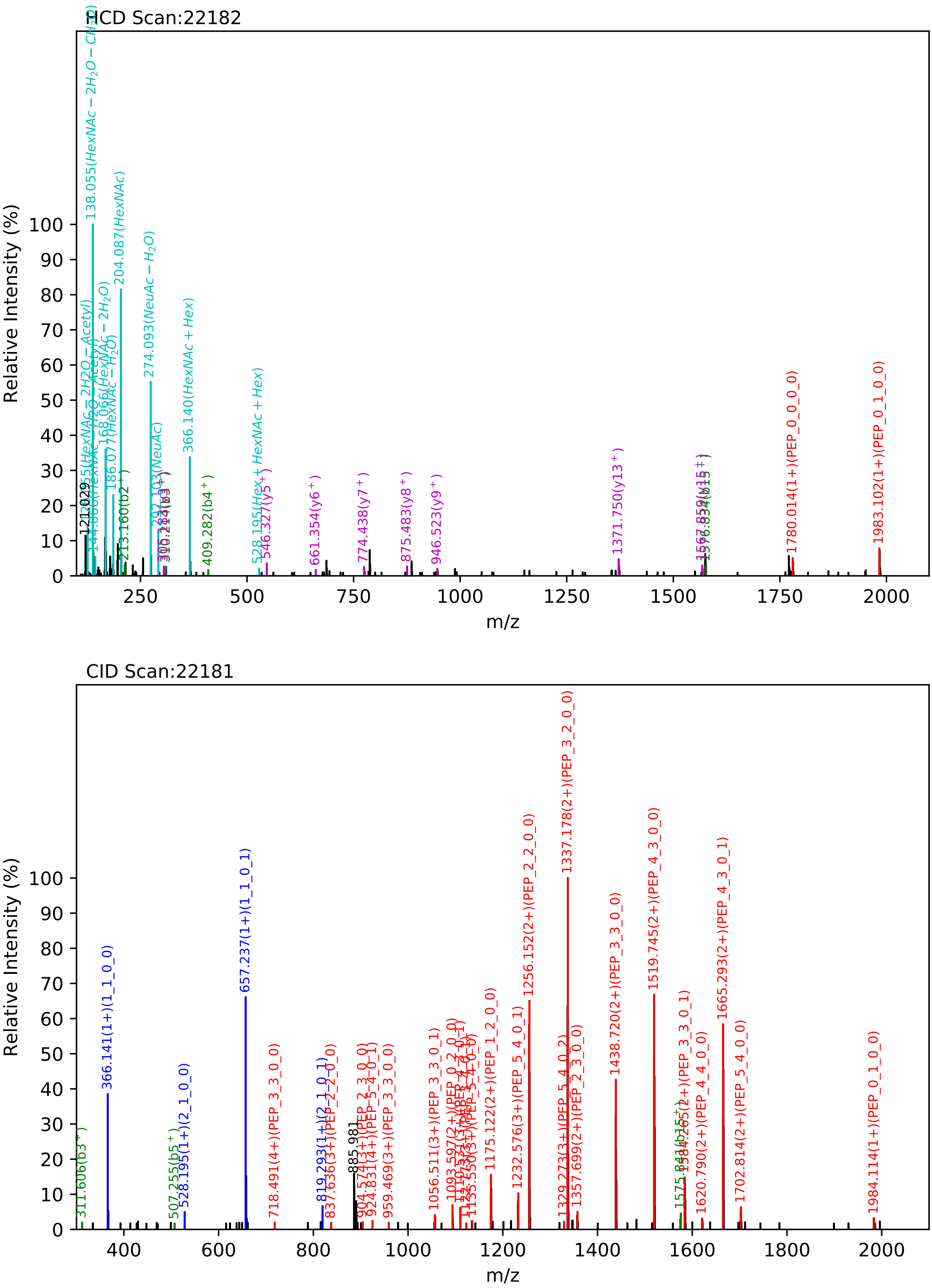

Training set no. 81, Experiment: AGP exp_1

LVPVPITNATLDQITGK(=PEP)_5_4_0_2, m/z:996.96(4+), RT:55.55, Y-score:82.20

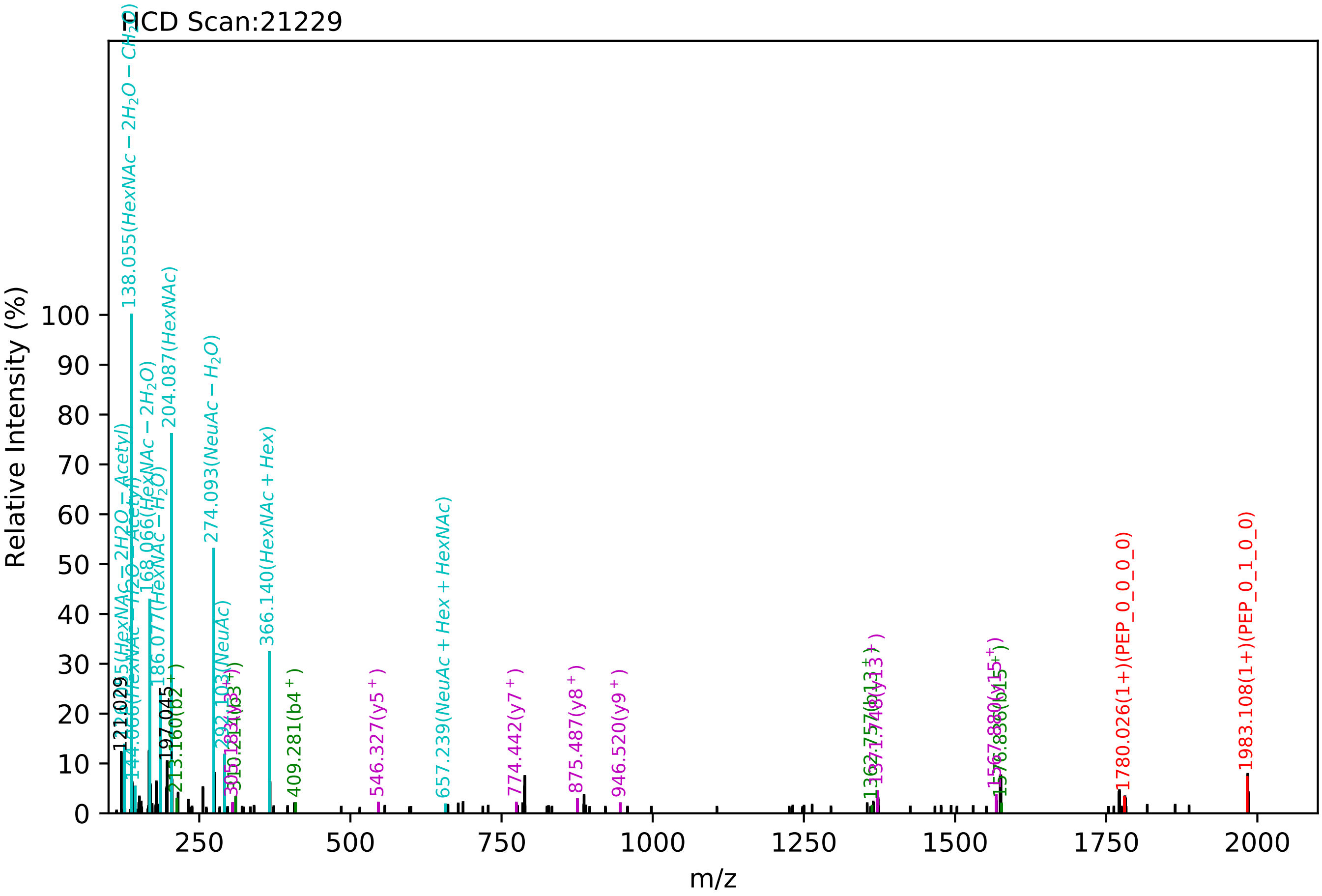

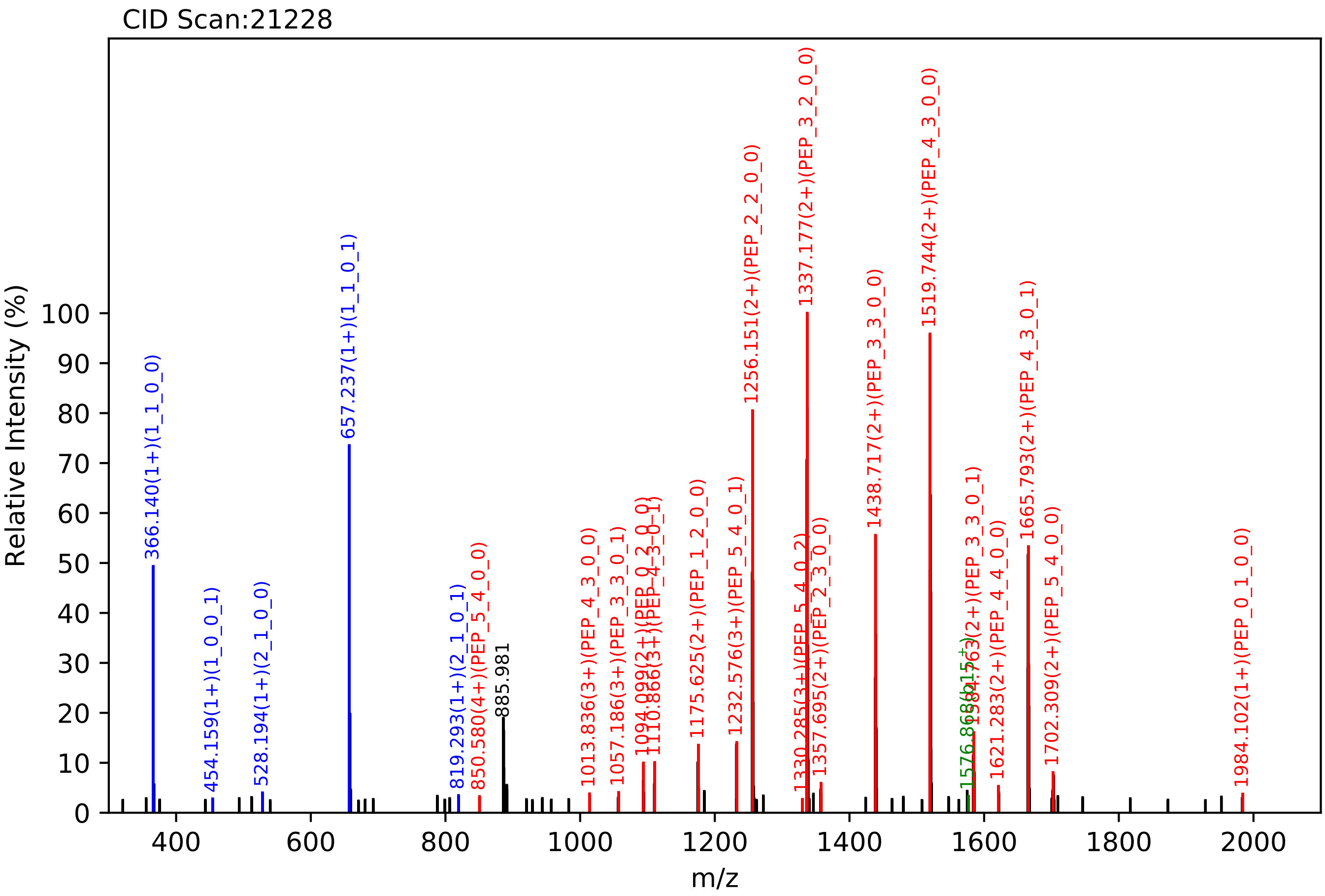

Training set no. 82, Experiment: AGP exp_1

LVPVPITNATLDQITGK(=PEP)_6_5_0_1, m/z:1015.46(4+), RT:48.59, Y-score:87.42

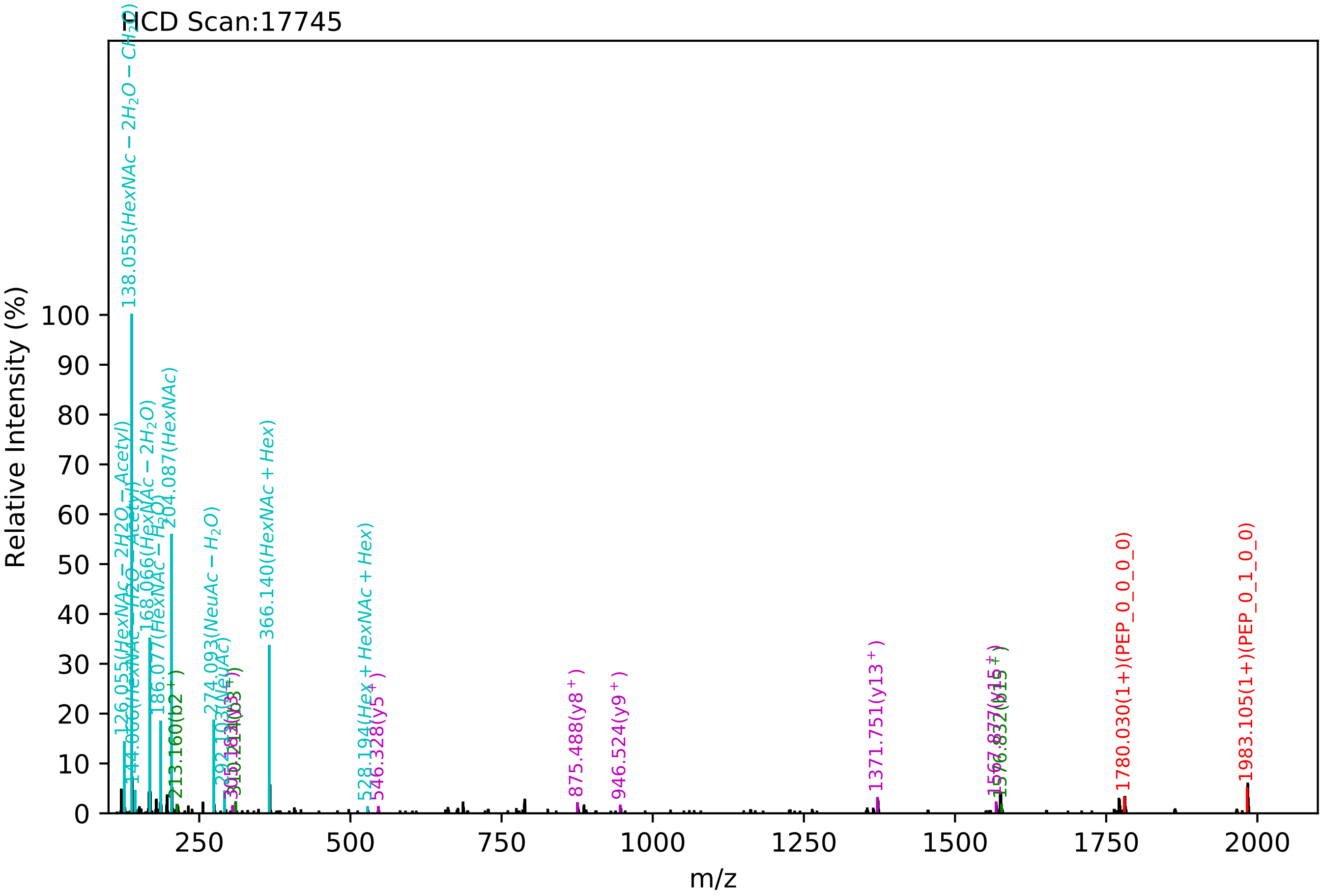

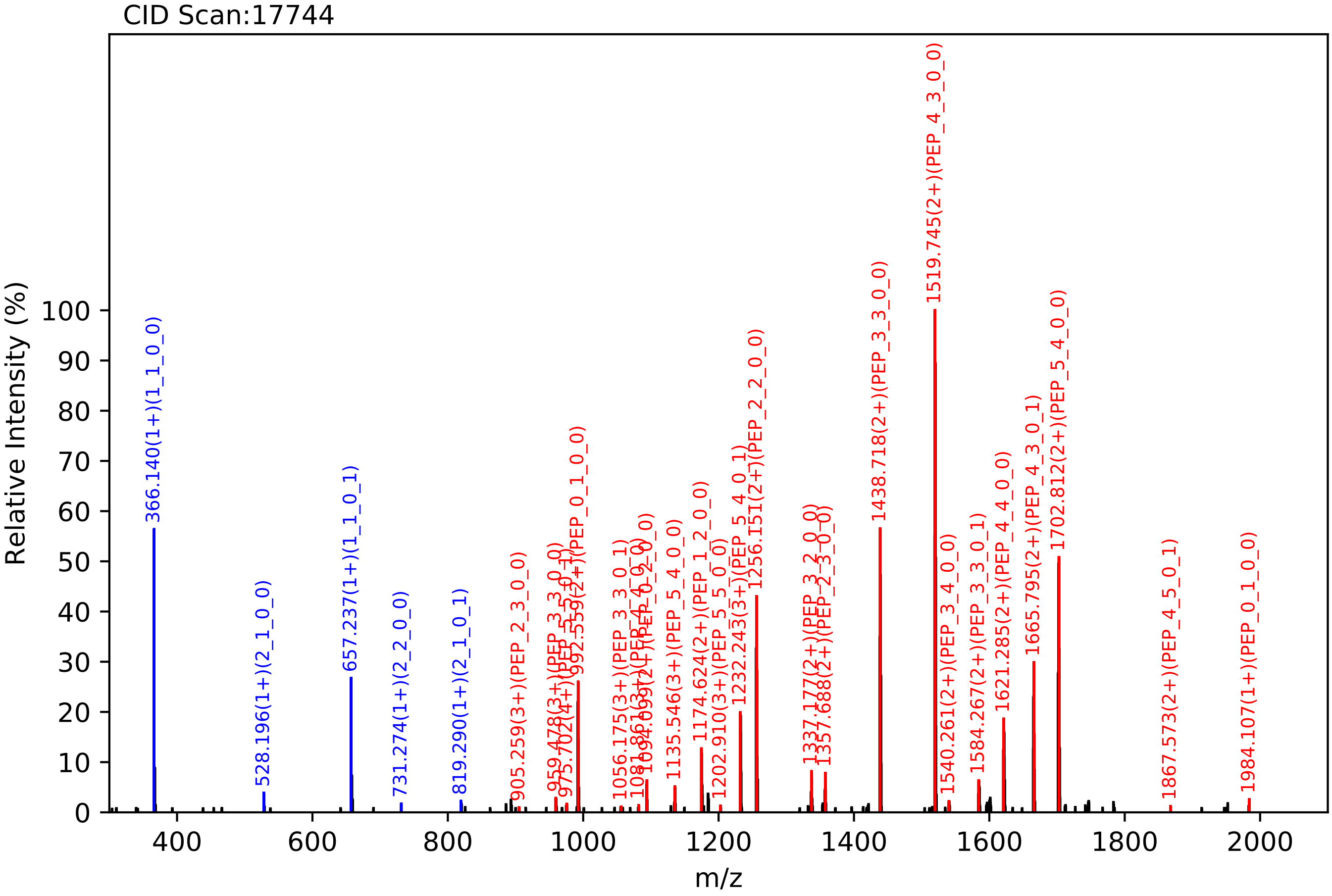

Training set no. 83, Experiment: AGP exp_2

LVPVPITNATLDQITGK(=PEP)_6_5_0_1, m/z:1015.46(4+), RT:48.77, Y-score:86.99

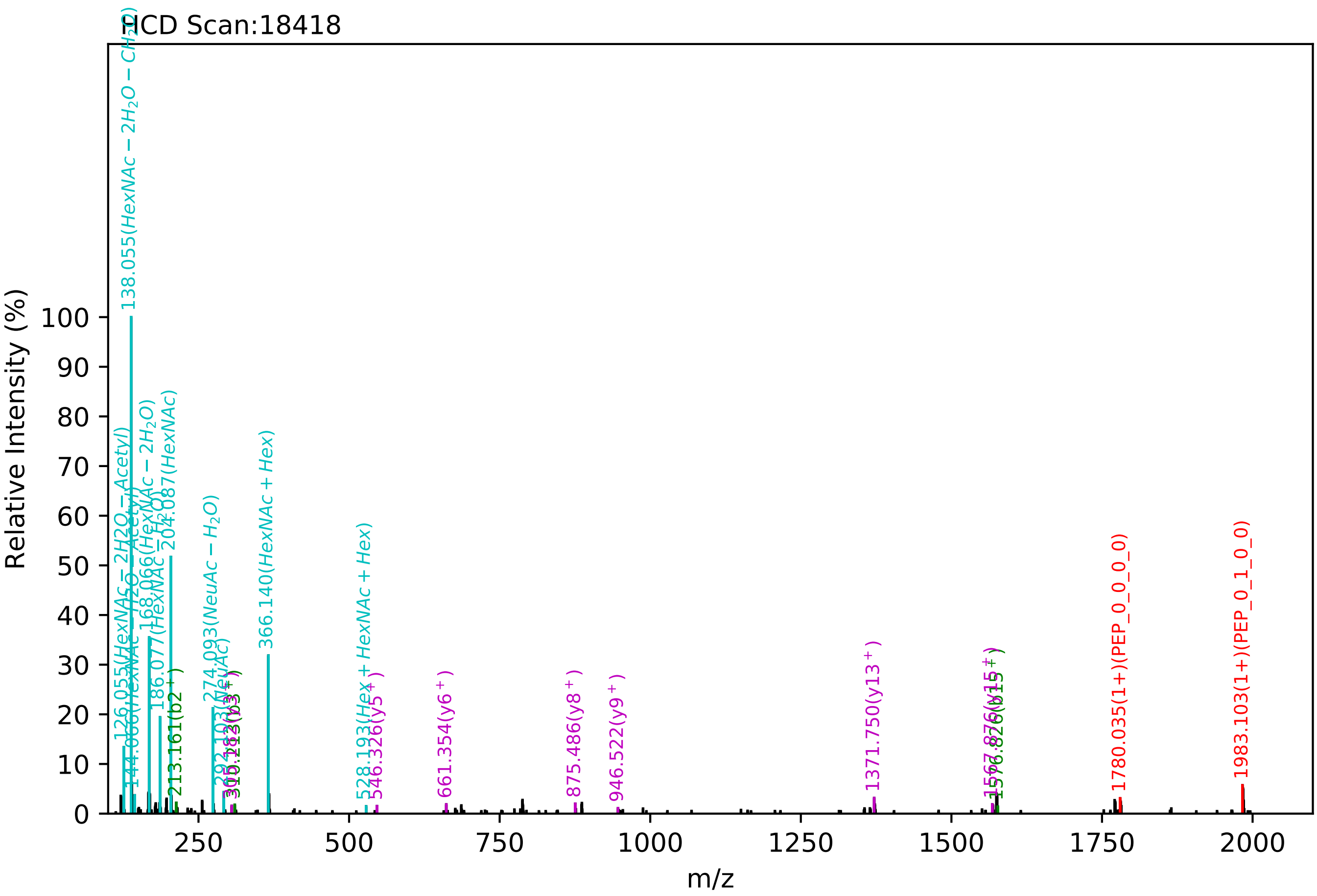

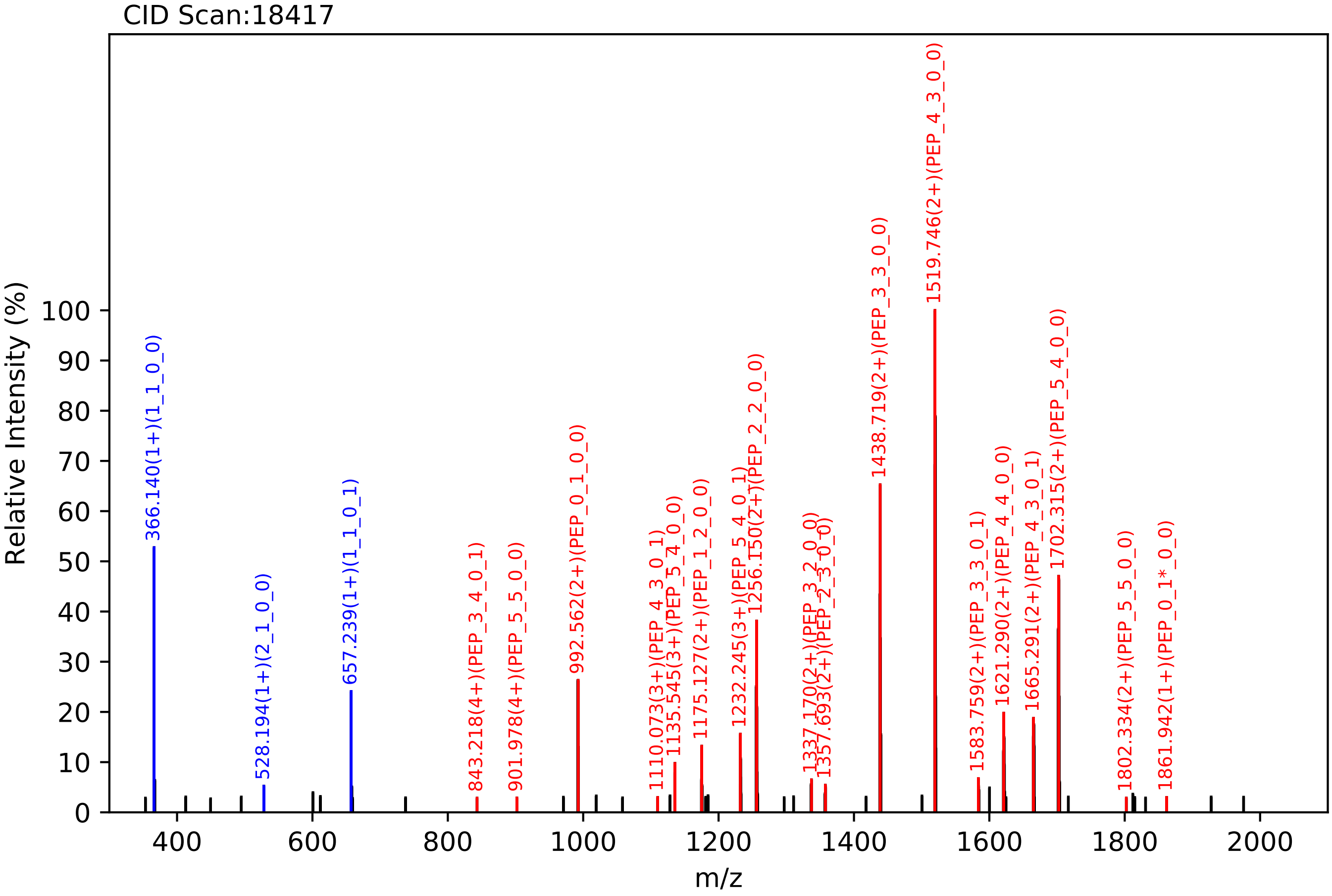

Training set no. 84, Experiment: AGP exp_2

LVPVPITNATLDQITGK(=PEP)_6_5_0_2, m/z:1450.65(3+), RT:54.68, Y-score:89.40

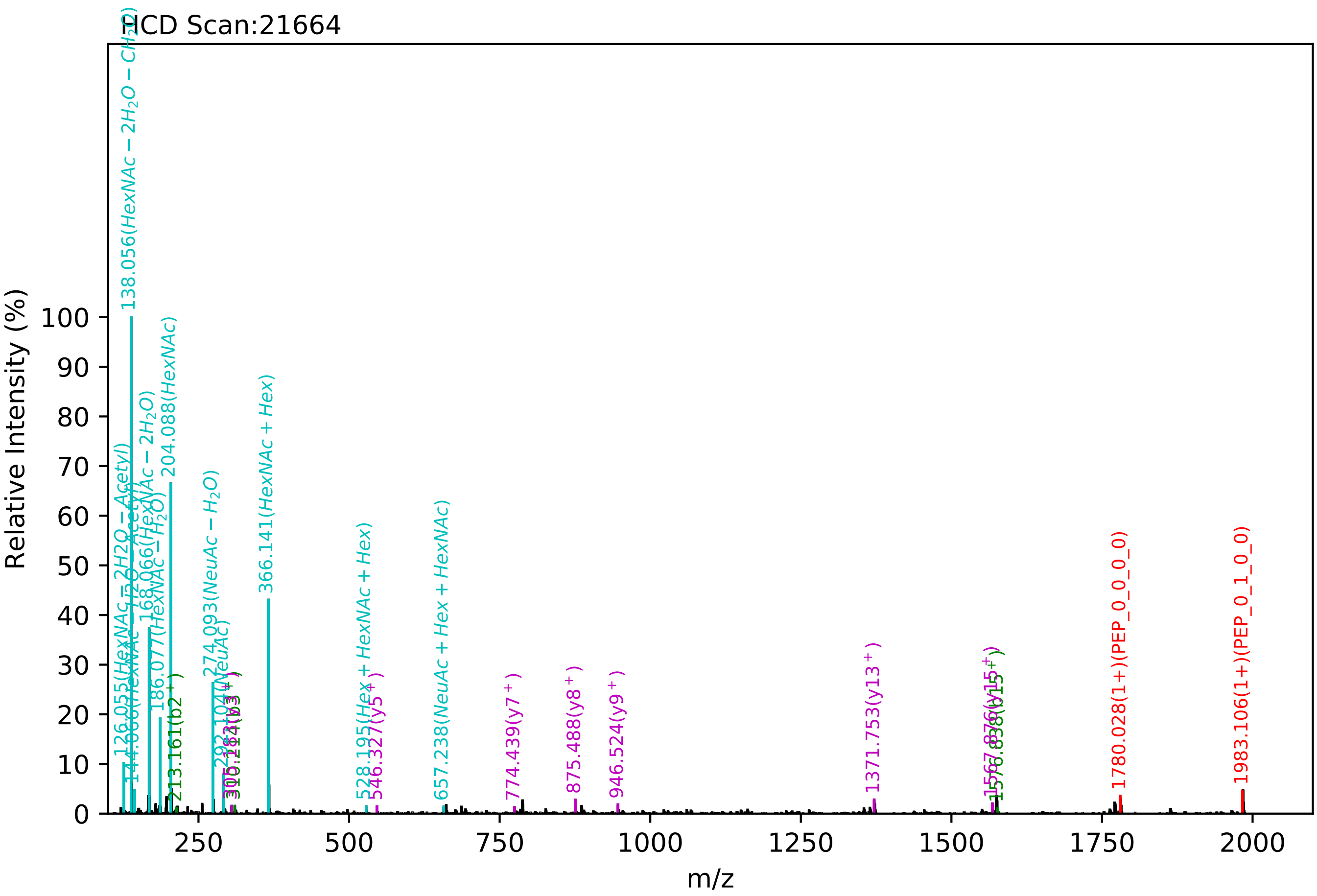

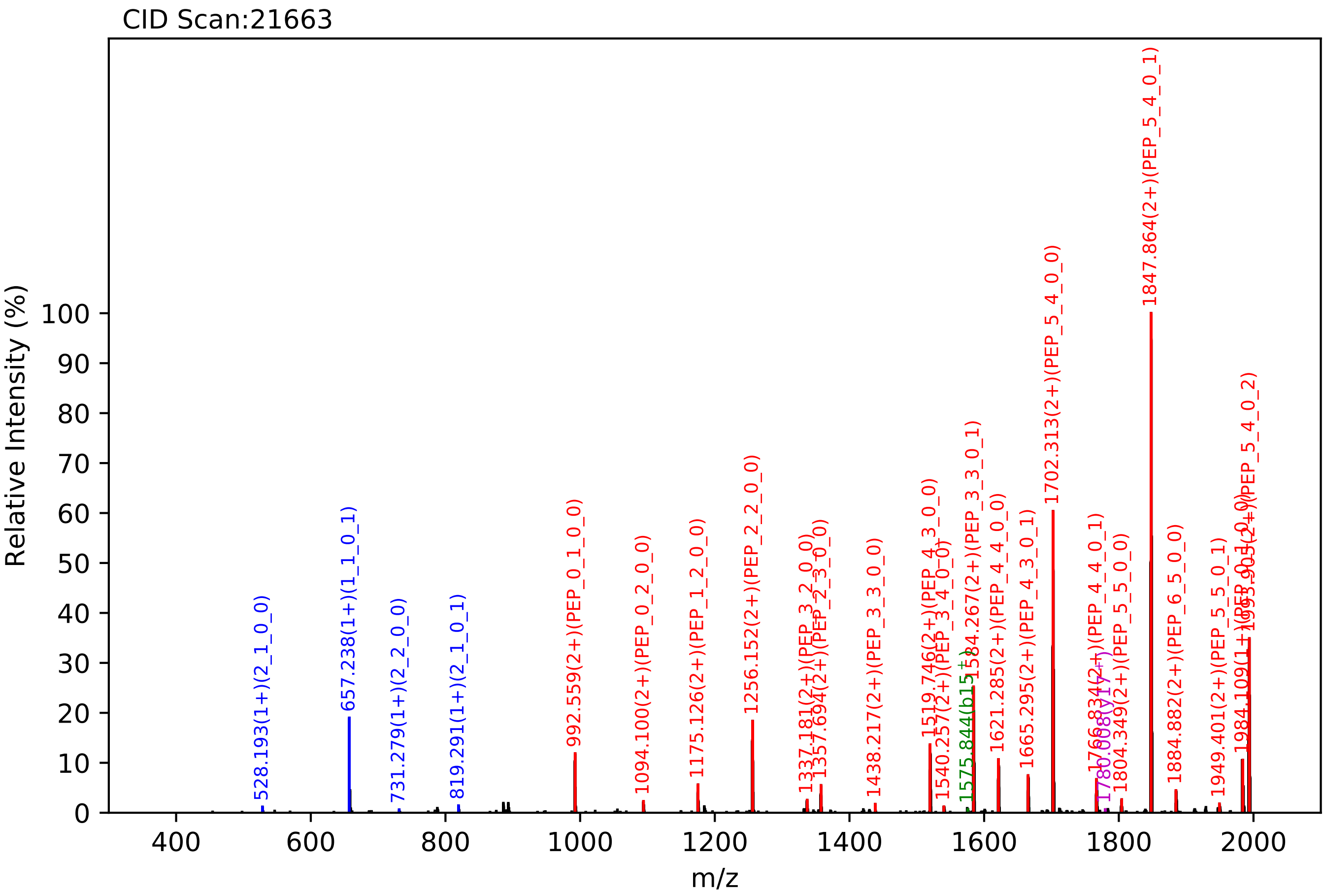

Training set no. 85, Experiment: AGP exp_2

LVPVPITNATLDQITGK(=PEP)_6_5_0_2, m/z:1450.65(3+), RT:55.22, Y-score:88.41

HCD Scan:21948

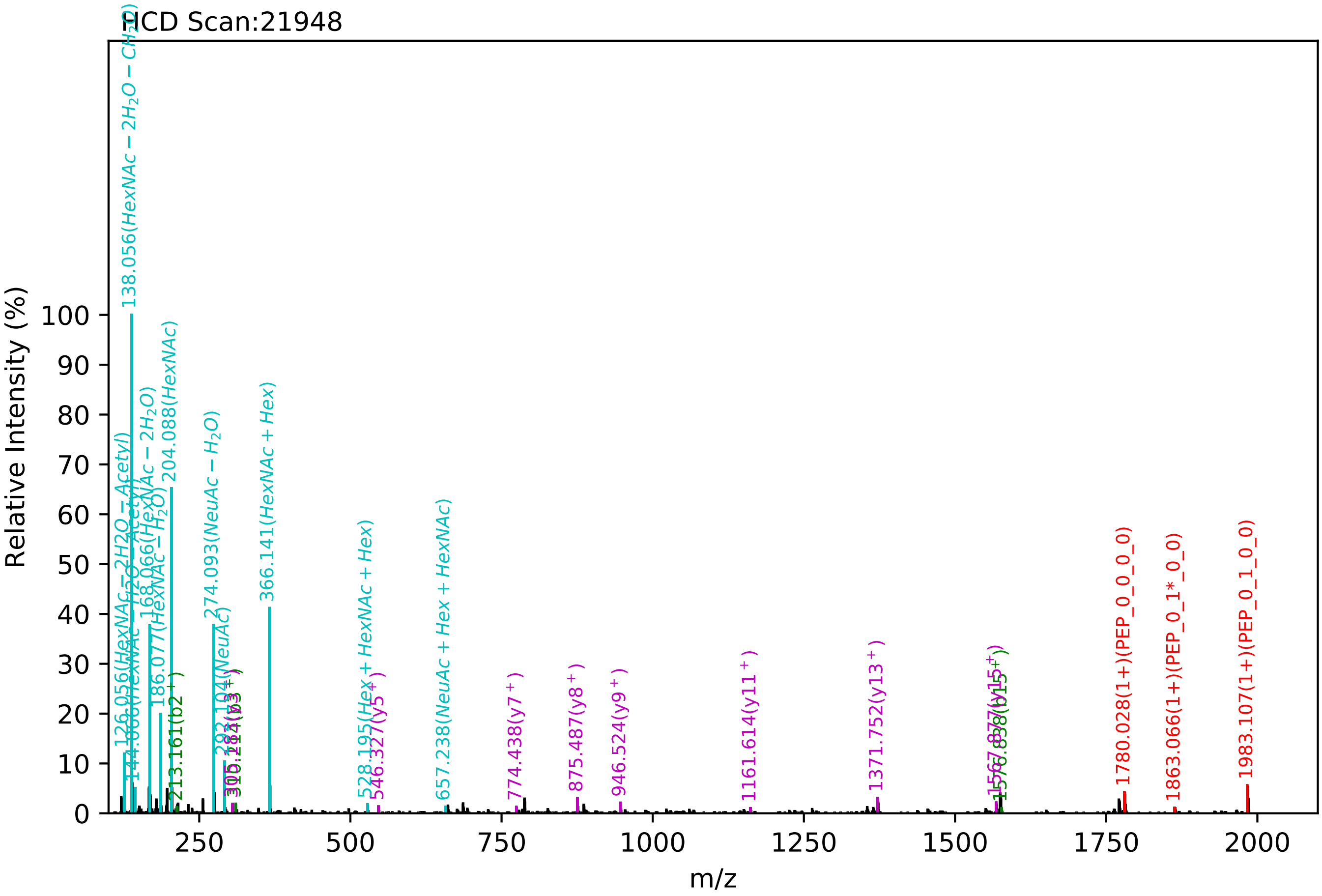

CID Scan:21947

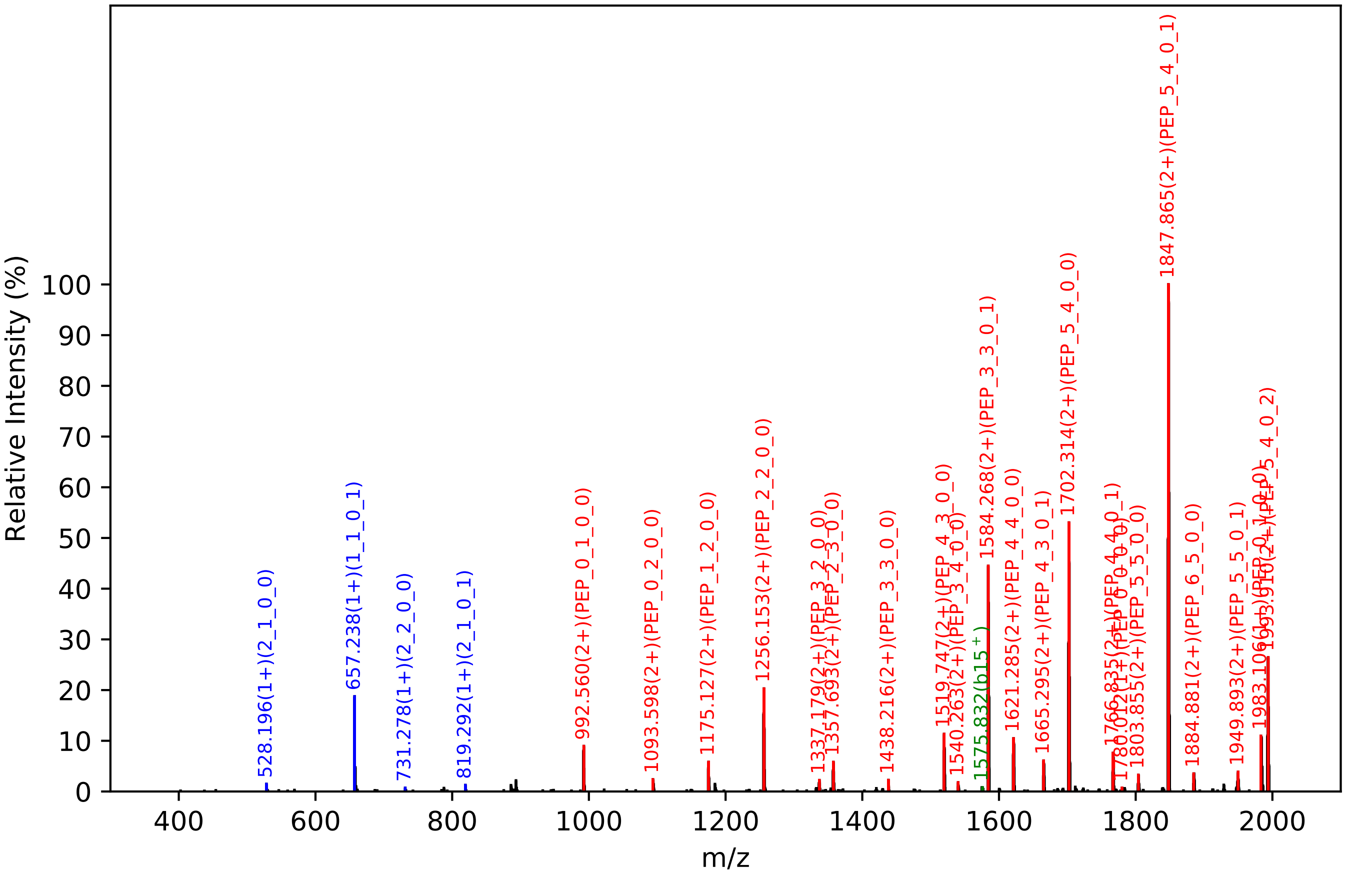

Training set no. 86, Experiment: AGP exp_2

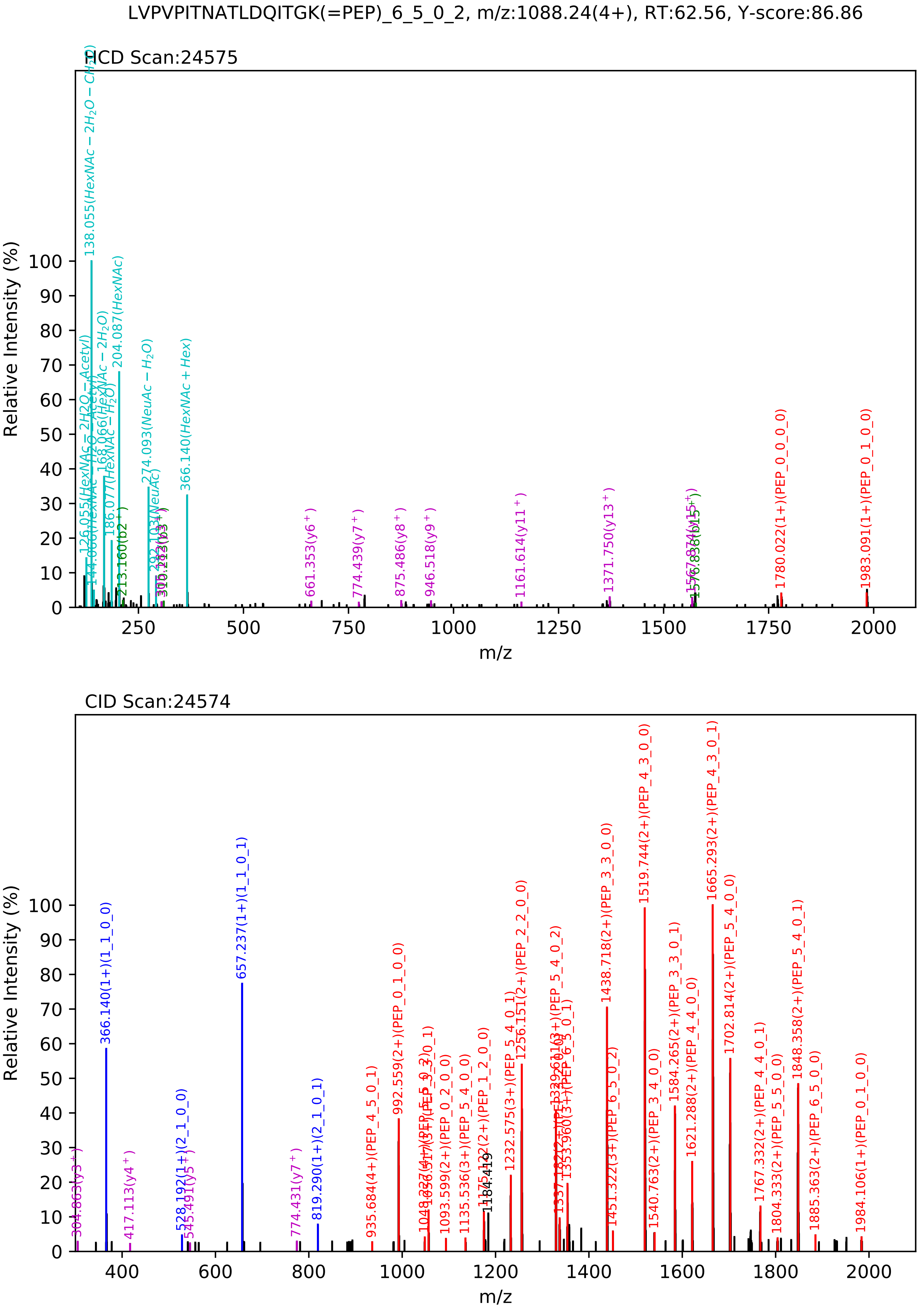

Training set no. 87, Experiment: AGP exp_1

LVPVPITNATLDQITGK(=PEP)_6_5_0_2, m/z:1088.24(4+), RT:55.40, Y-score:84.31

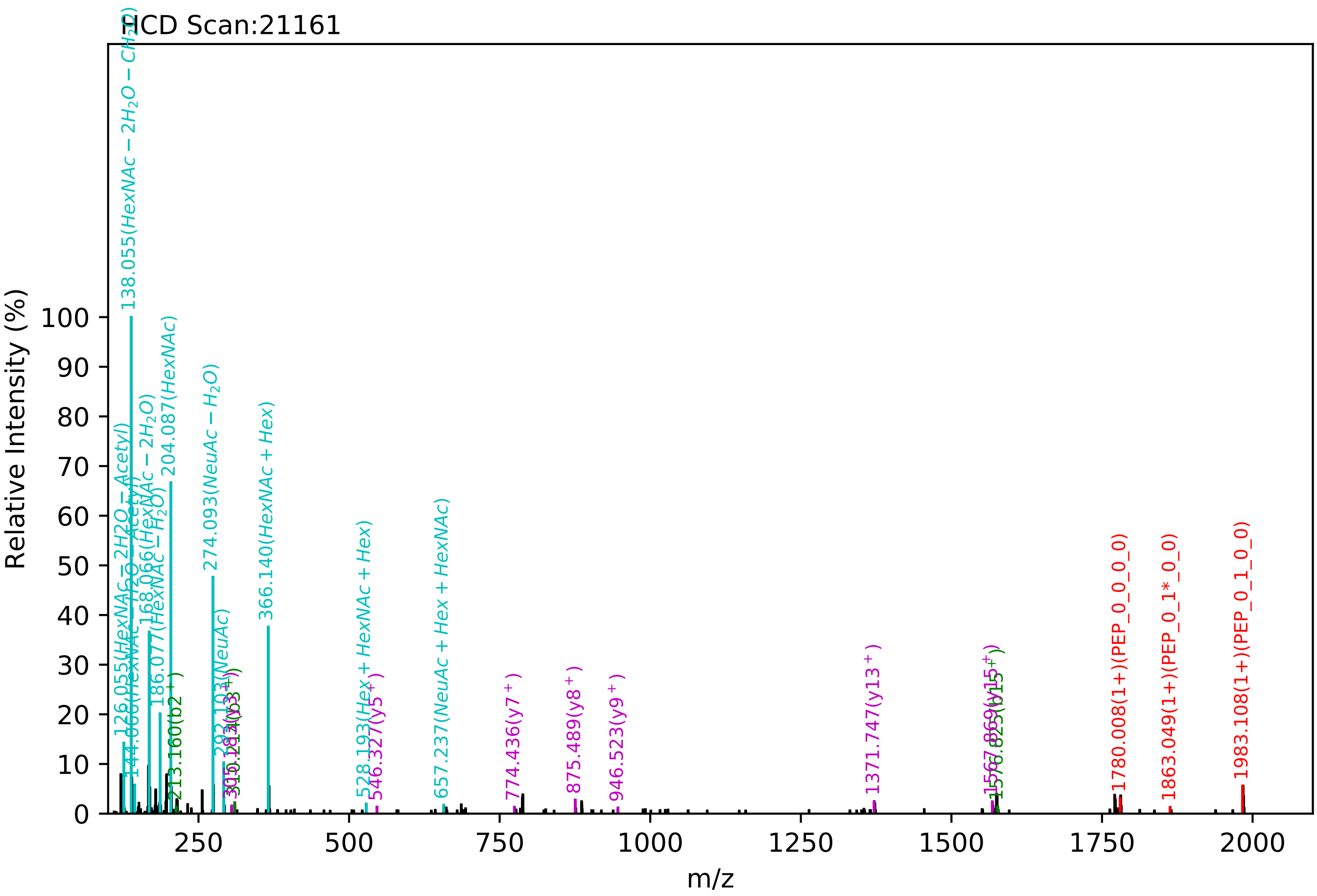

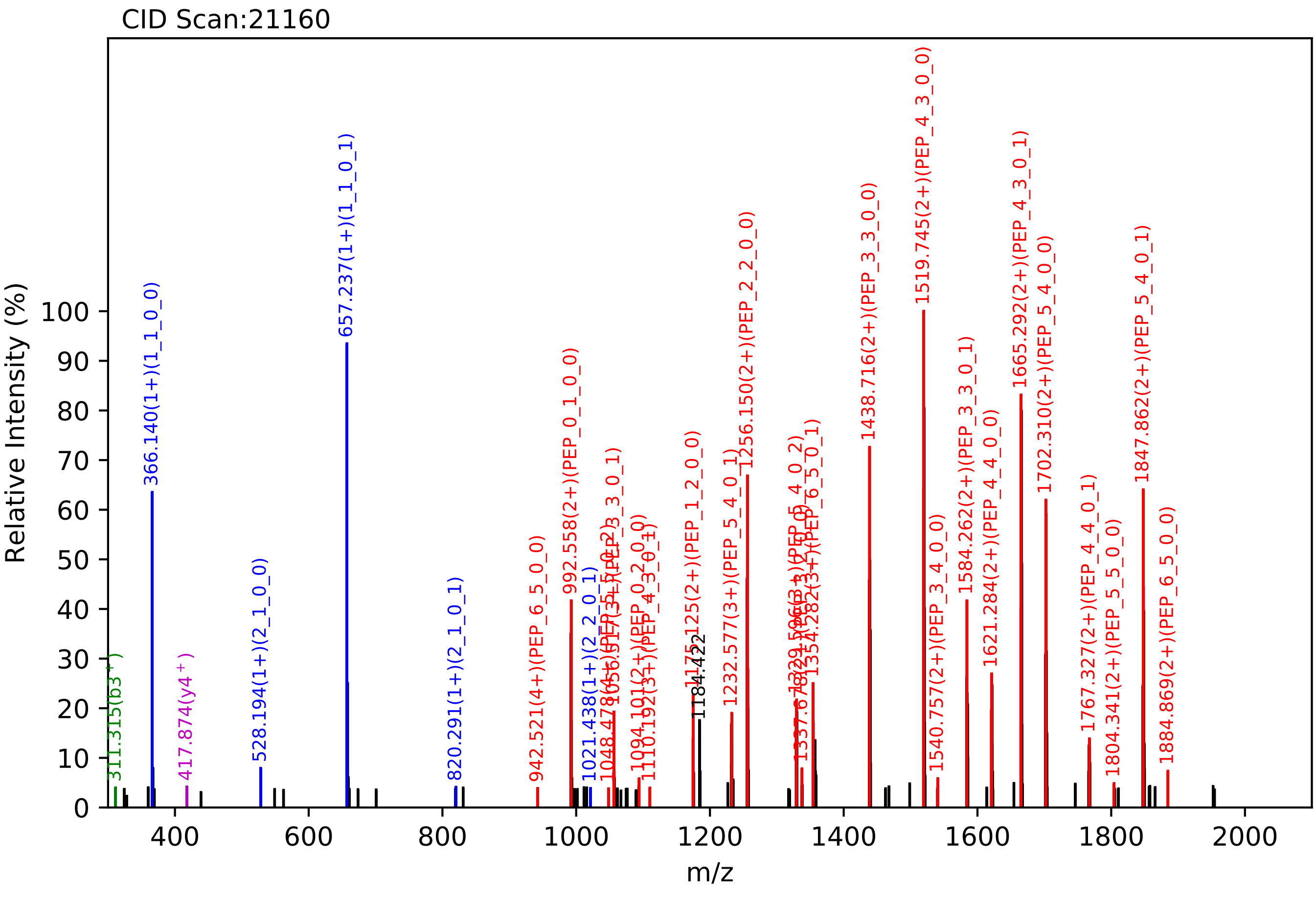

Training set no. 88, Experiment: AGP exp_1

LVPVPITNATLDQITGK(=PEP)_6_5_0_2, m/z:1088.24(4+), RT:54.70, Y-score:84.29

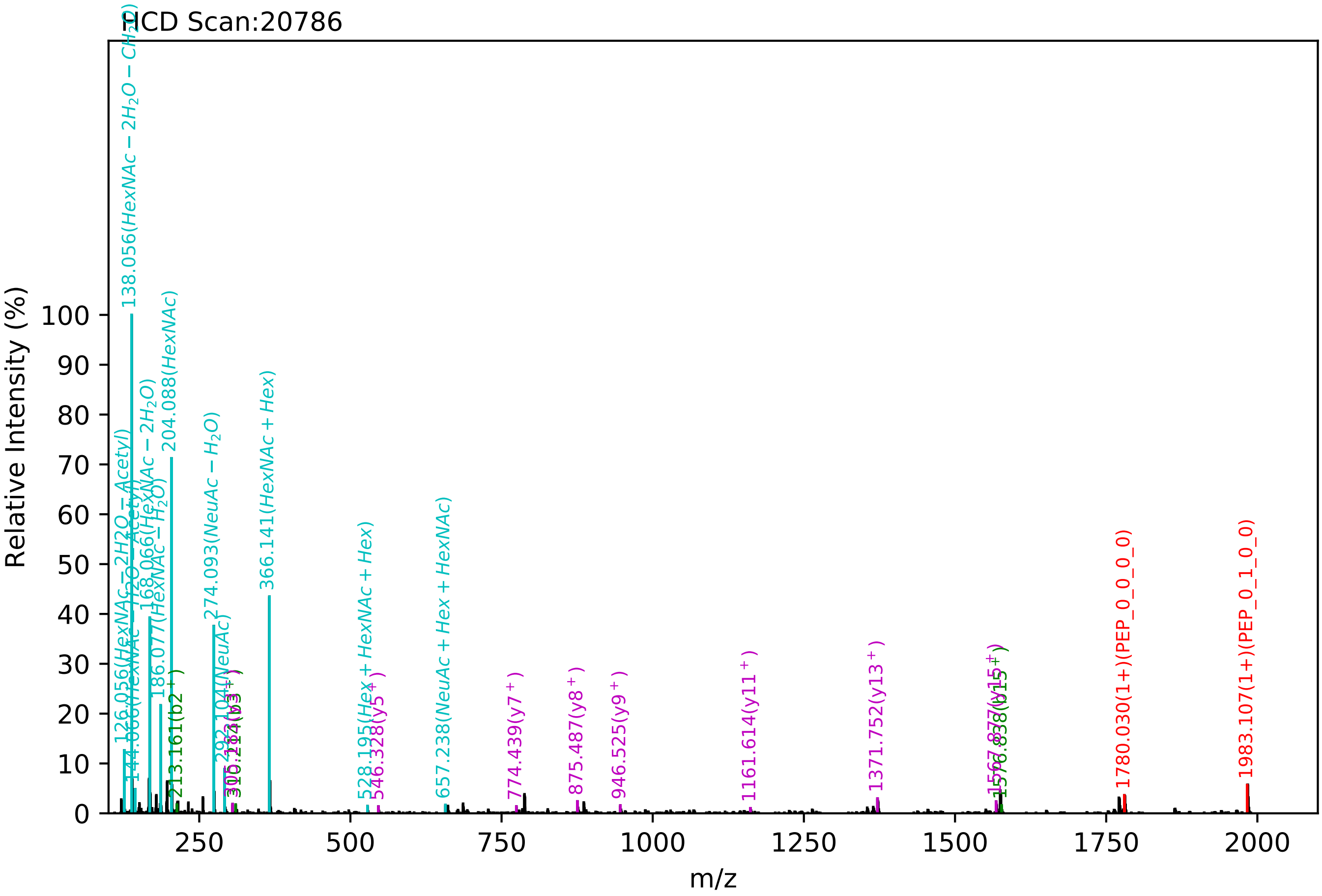

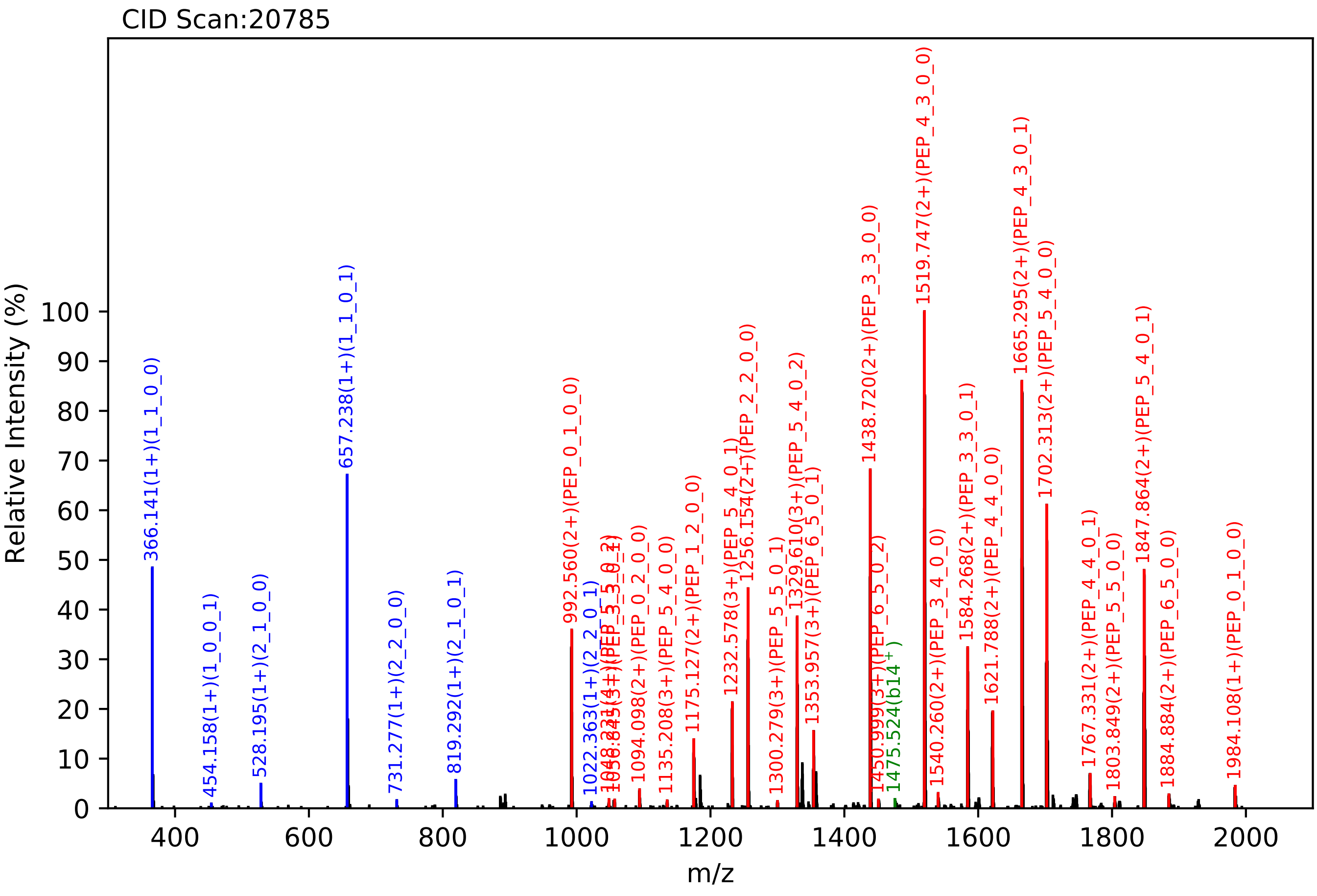

Training set no. 89, Experiment: AGP exp_1

LVPVPITNATLDQITGK(=PEP)_6_5_0_2, m/z:1088.24(4+), RT:62.30, Y-score:84.17

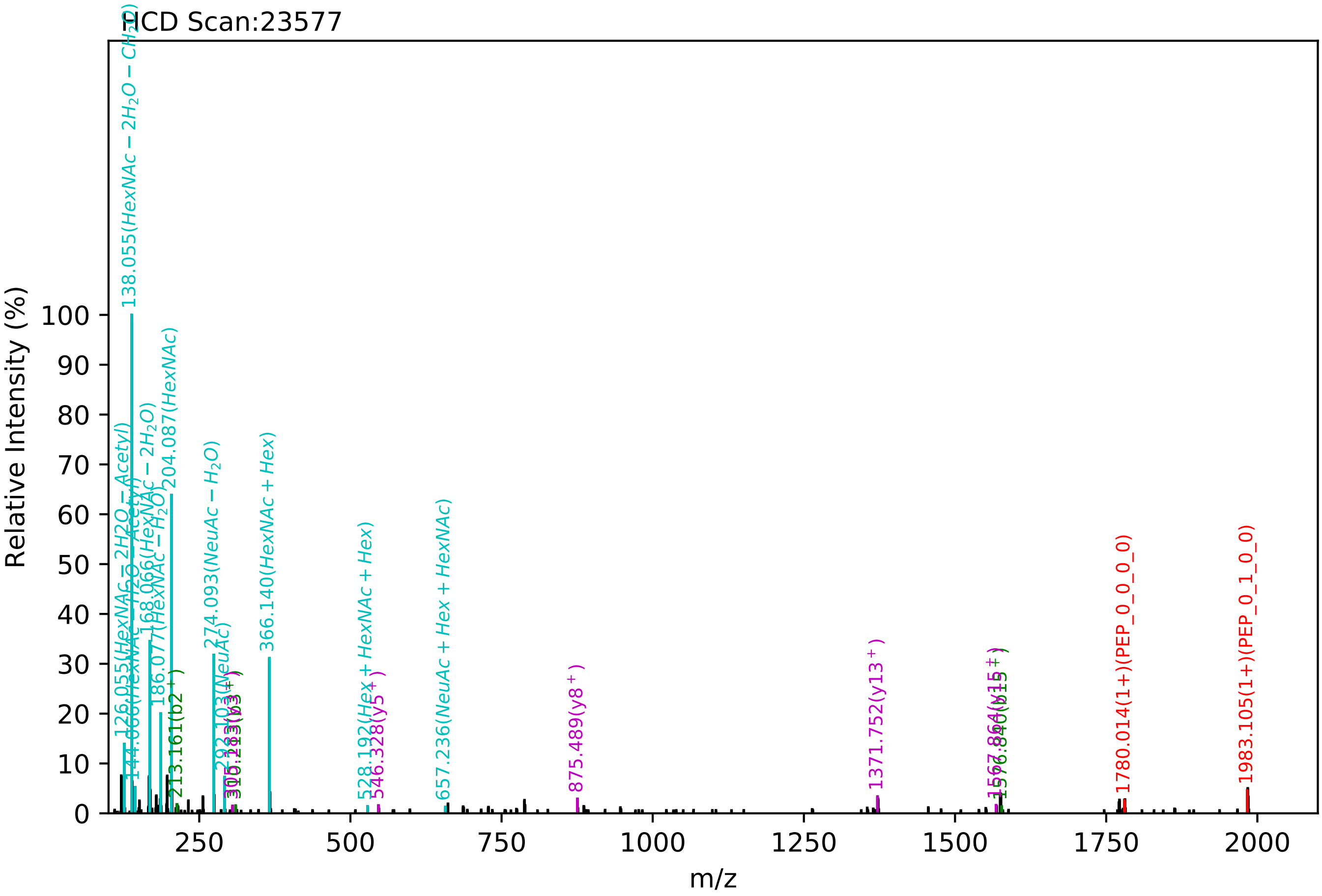

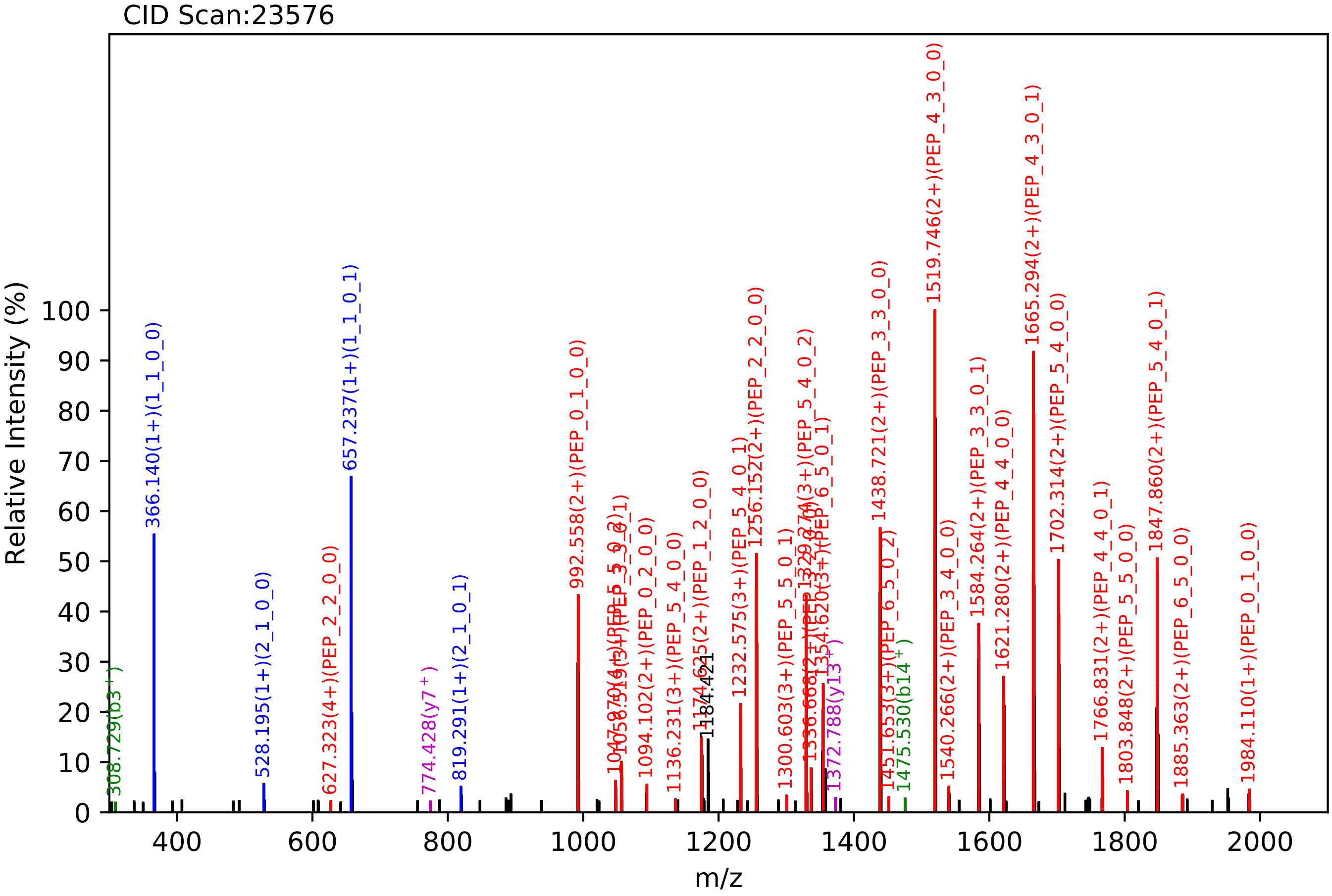

Training set no. 90, Experiment: AGP exp_2

LVPVPITNATLDQITGK(=PEP)_6_5_0_2, m/z:1088.24(4+), RT:55.61, Y-score:82.40

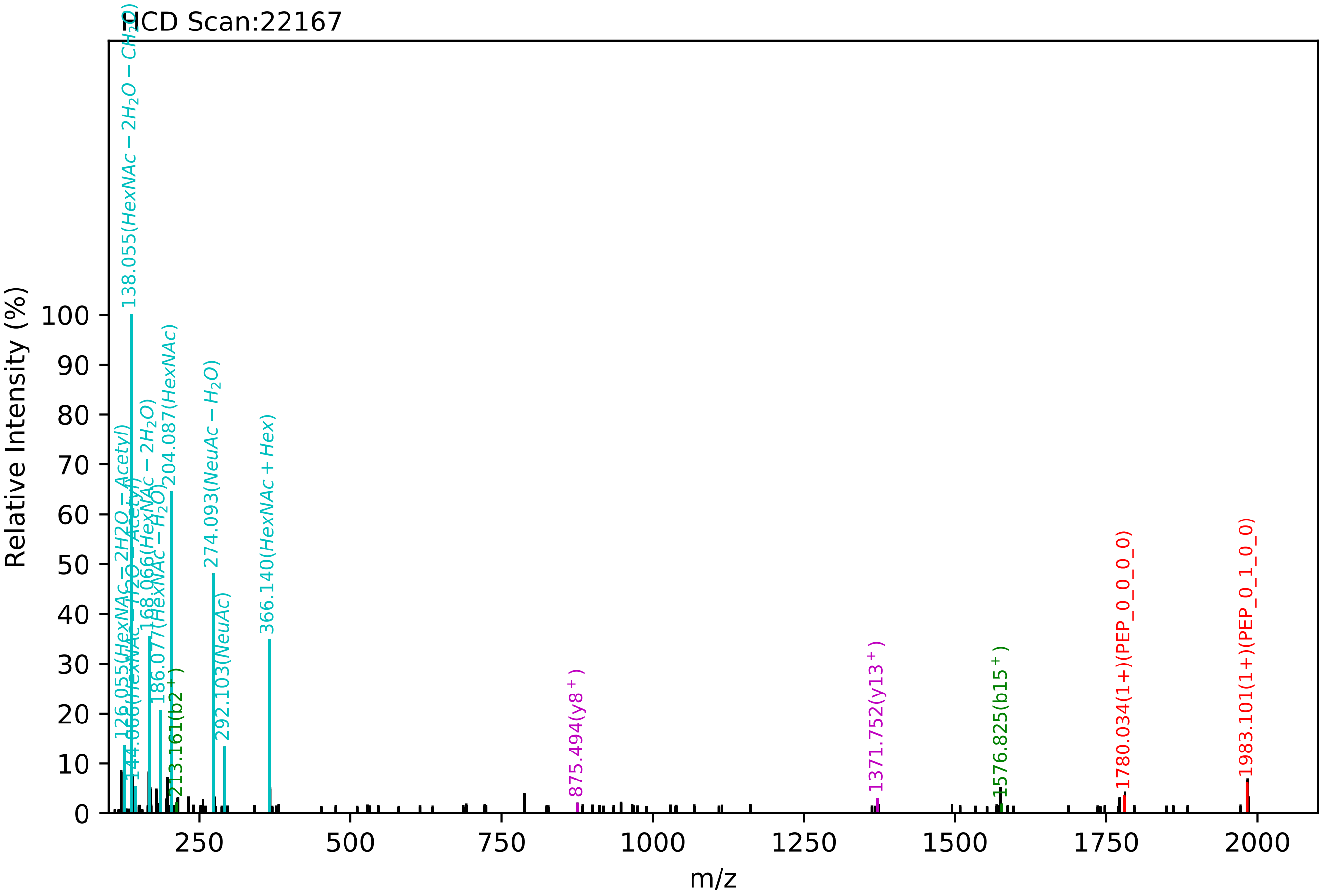

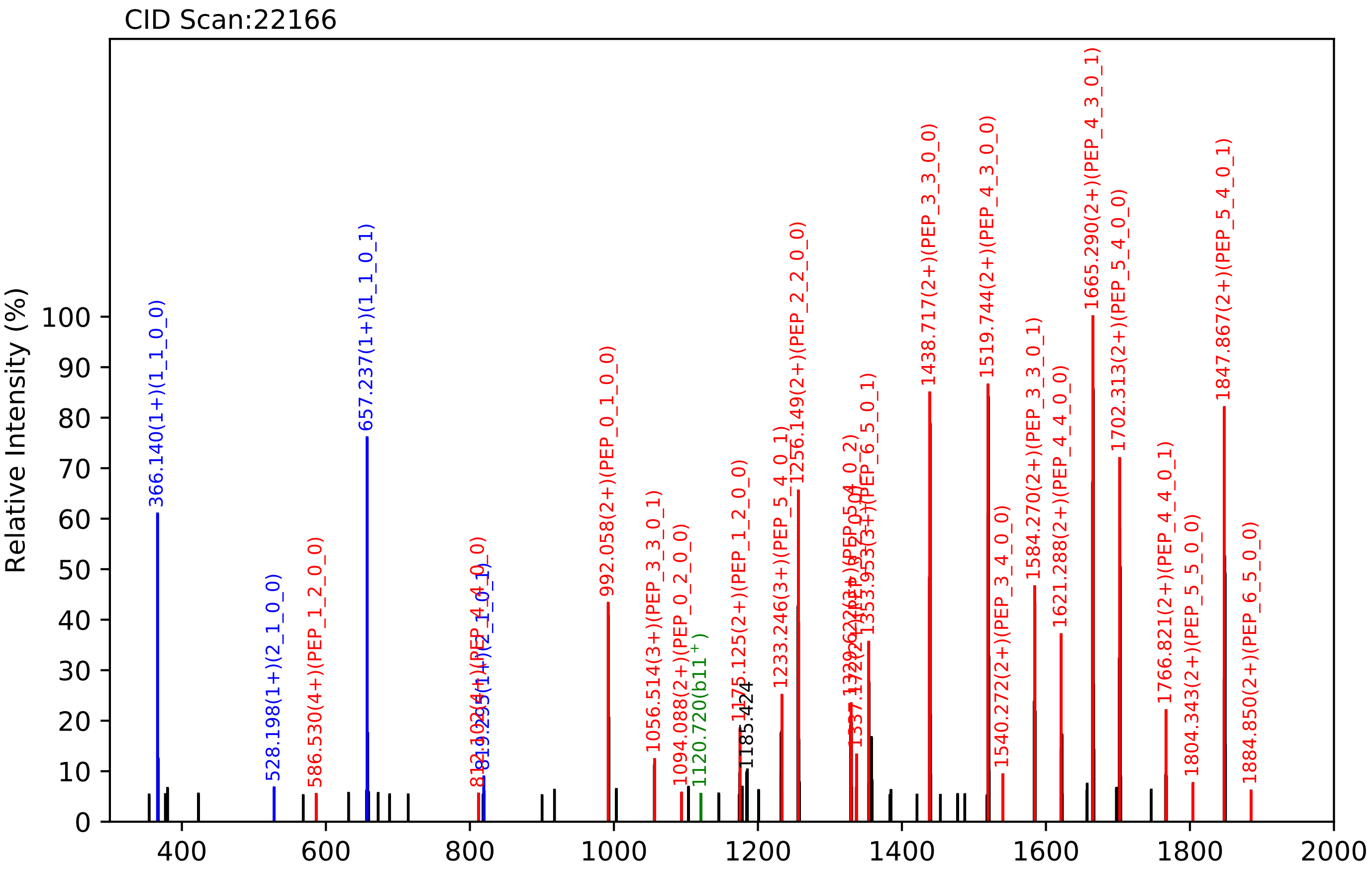

Training set no. 91, Experiment: AGP exp_2

LVPVPITNATLDQITGK(=PEP)_6_5_0_3, m/z:1547.69(3+), RT:62.75, Y-score:88.74

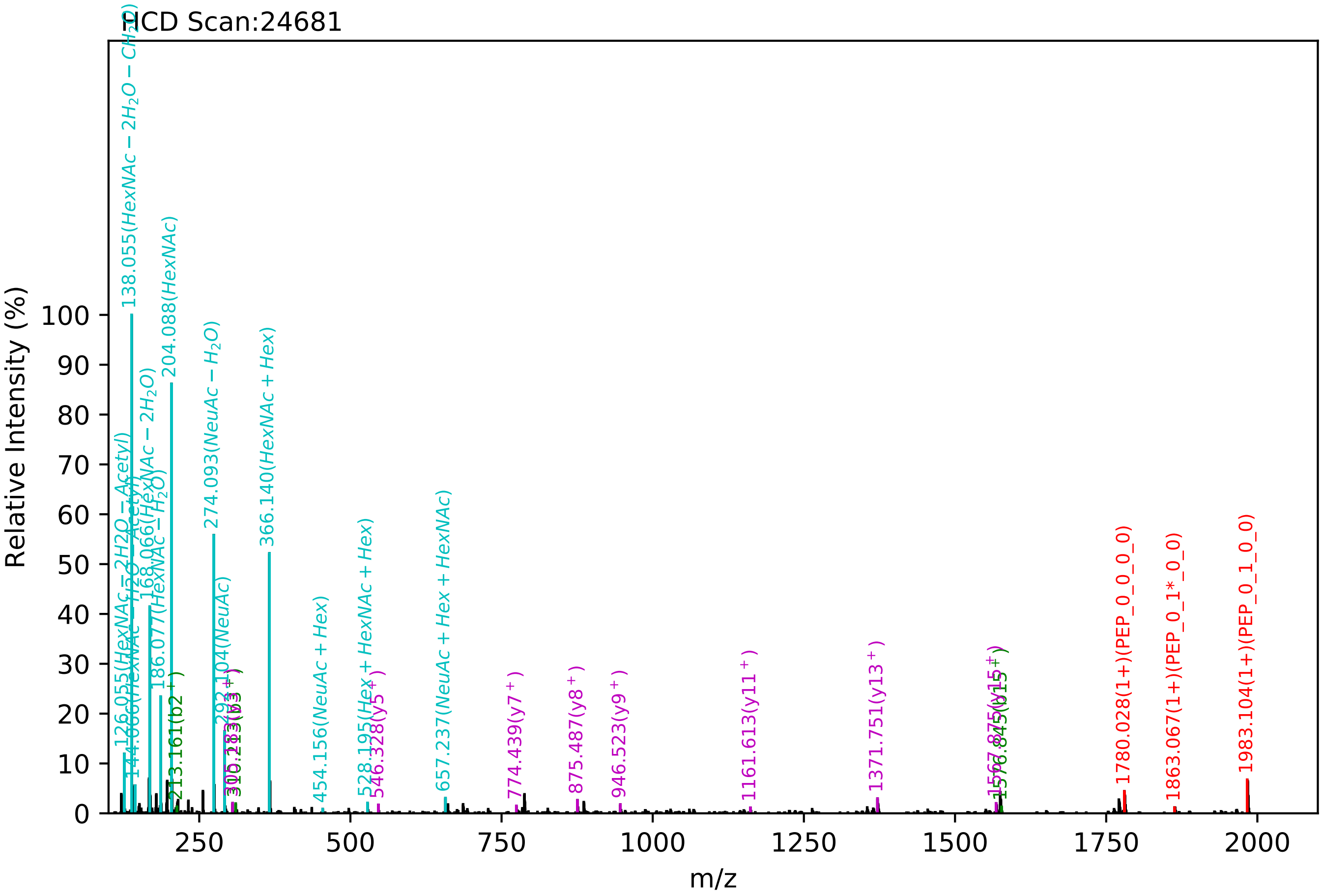

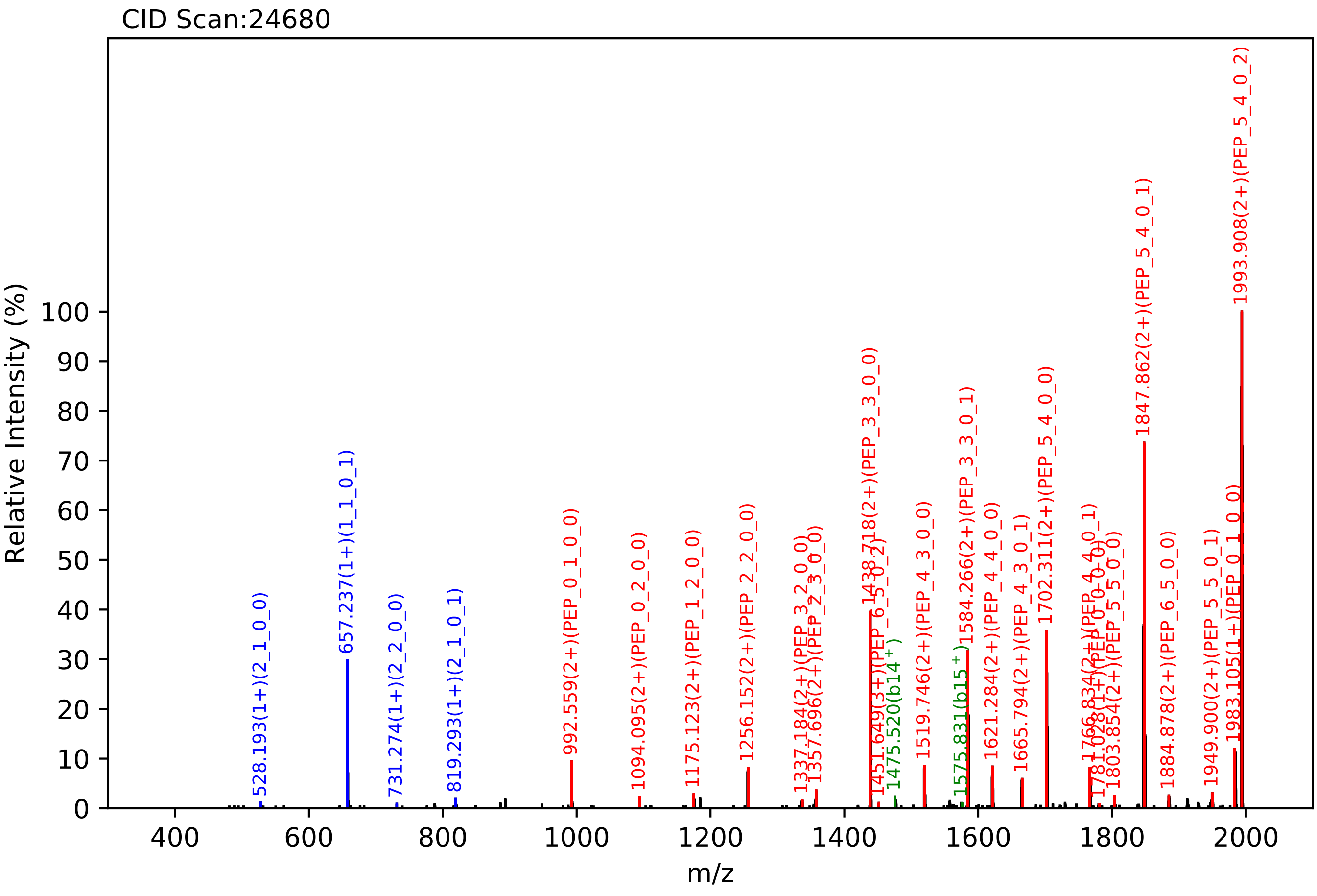

Training set no. 92, Experiment: AGP exp_1

LVPVPITNATLDQITGK(=PEP)_6_5_0_3, m/z:1160.55(4+), RT:62.43, Y-score:87.90

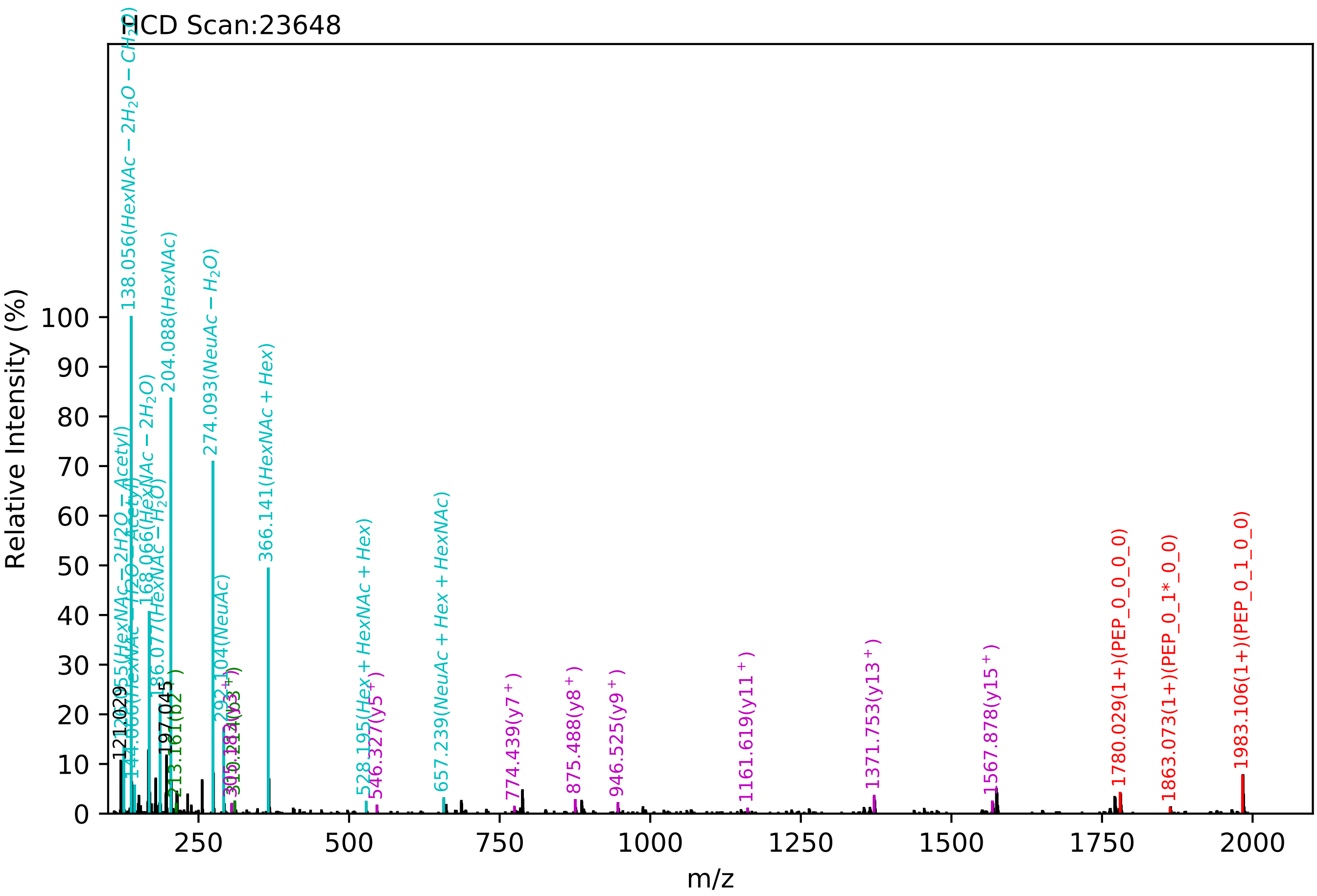

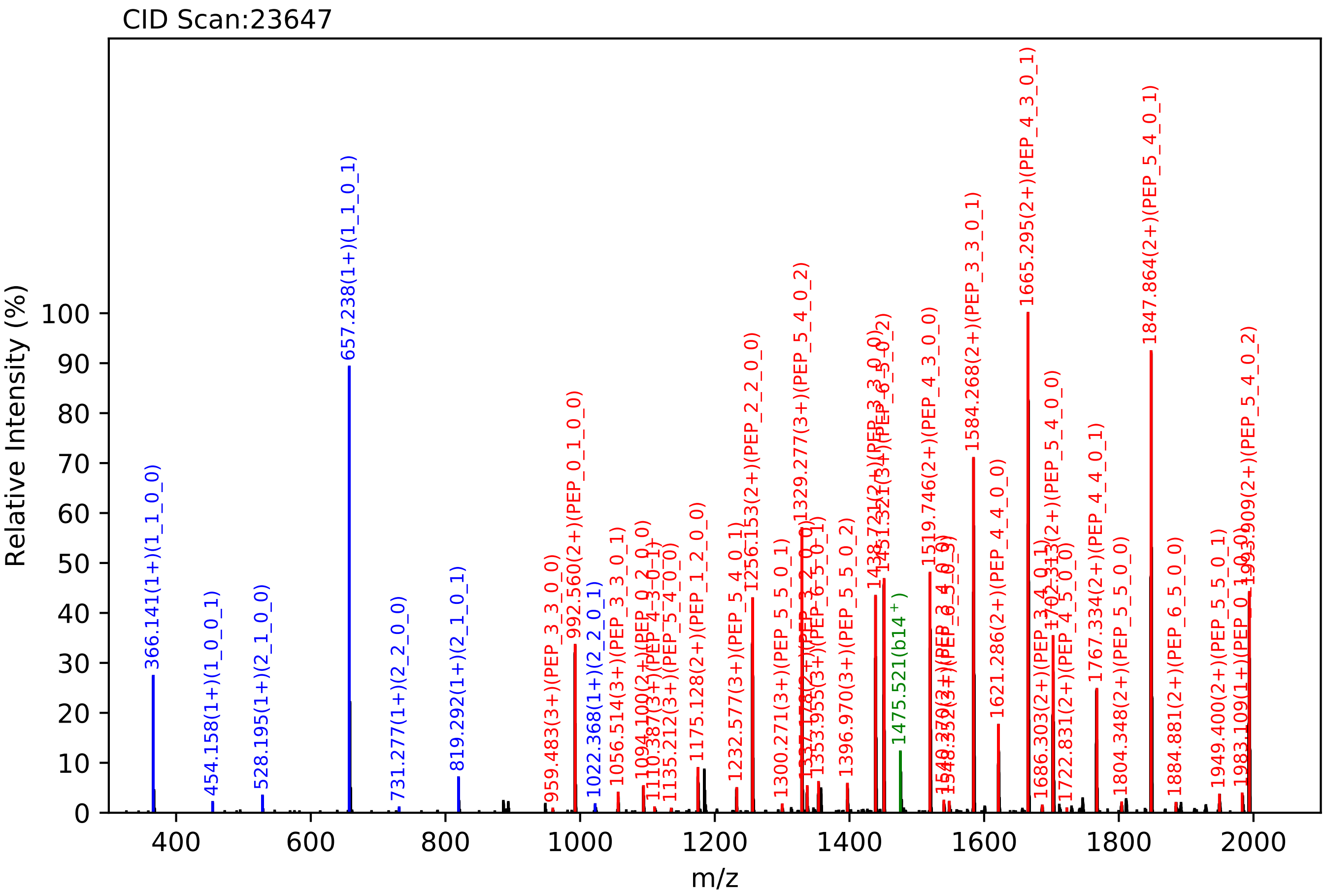

Training set no. 93, Experiment: AGP exp_1

LVPVPITNATLDQITGK(=PEP)_6_5_0_3, m/z:1547.69(3+), RT:62.07, Y-score:87.11

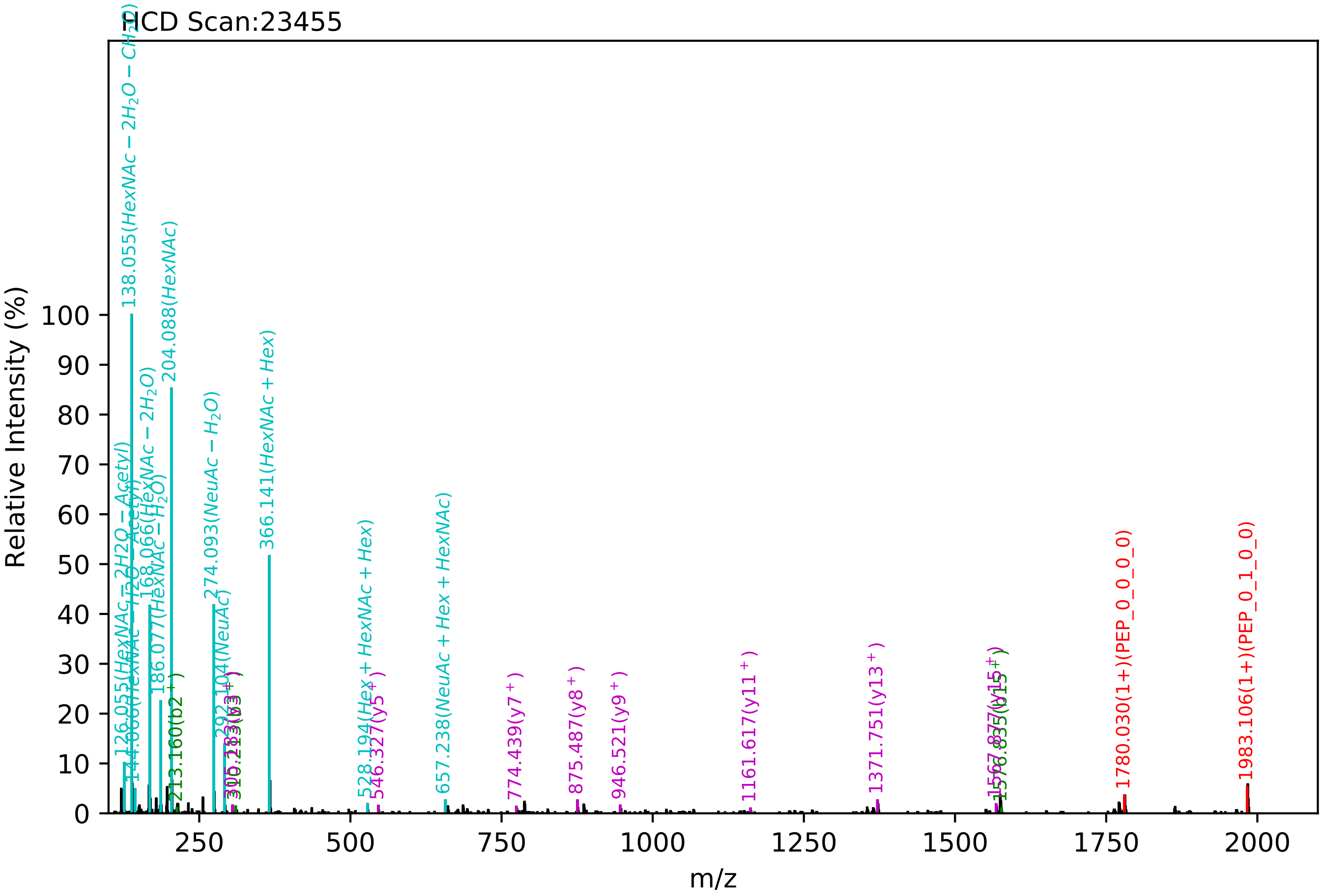

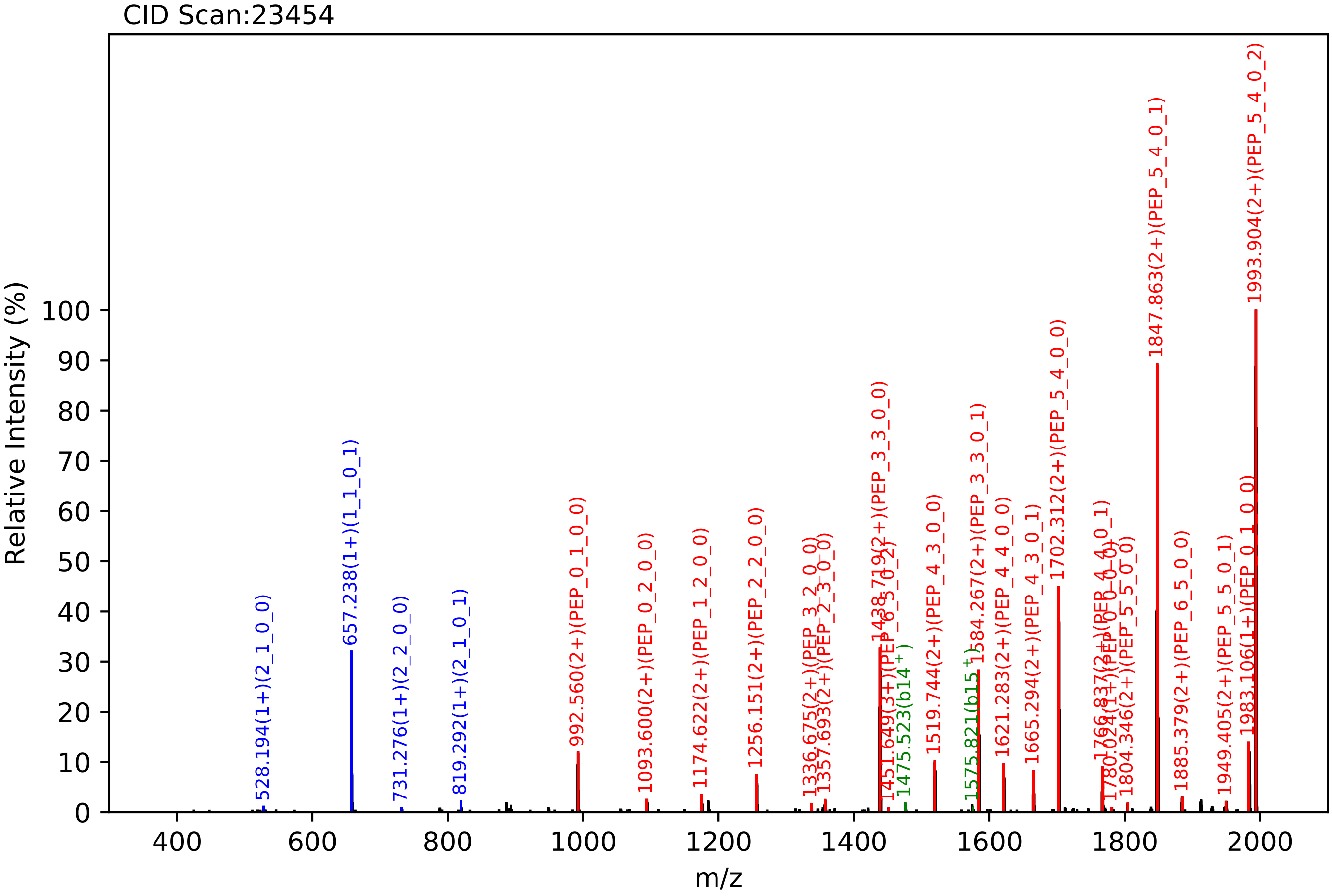

Training set no. 94, Experiment: AGP exp_2

LVPVPITNATLDQITGK(=PEP)_6_5_0_3, m/z:1160.76(4+), RT:62.05, Y-score:86.06

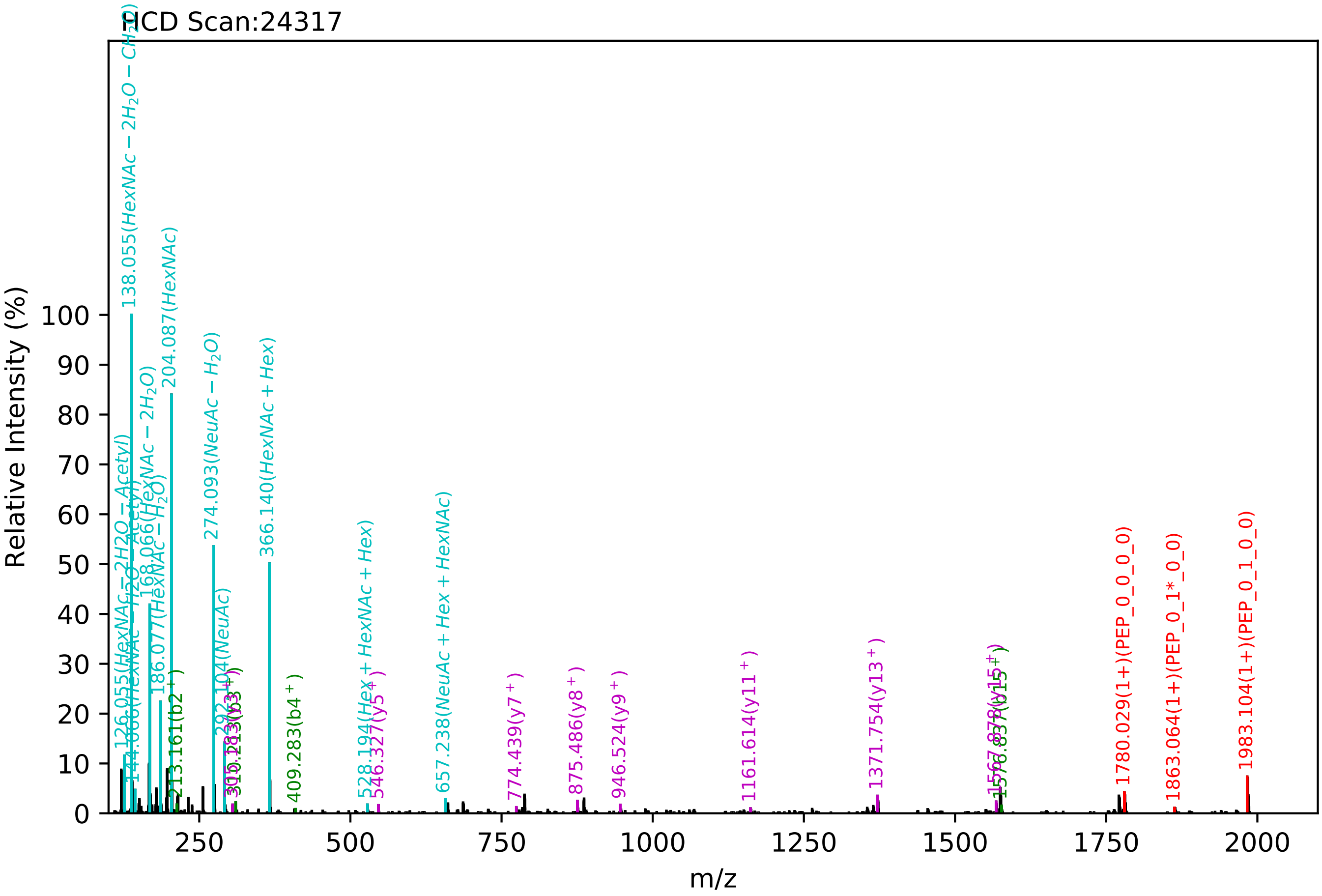

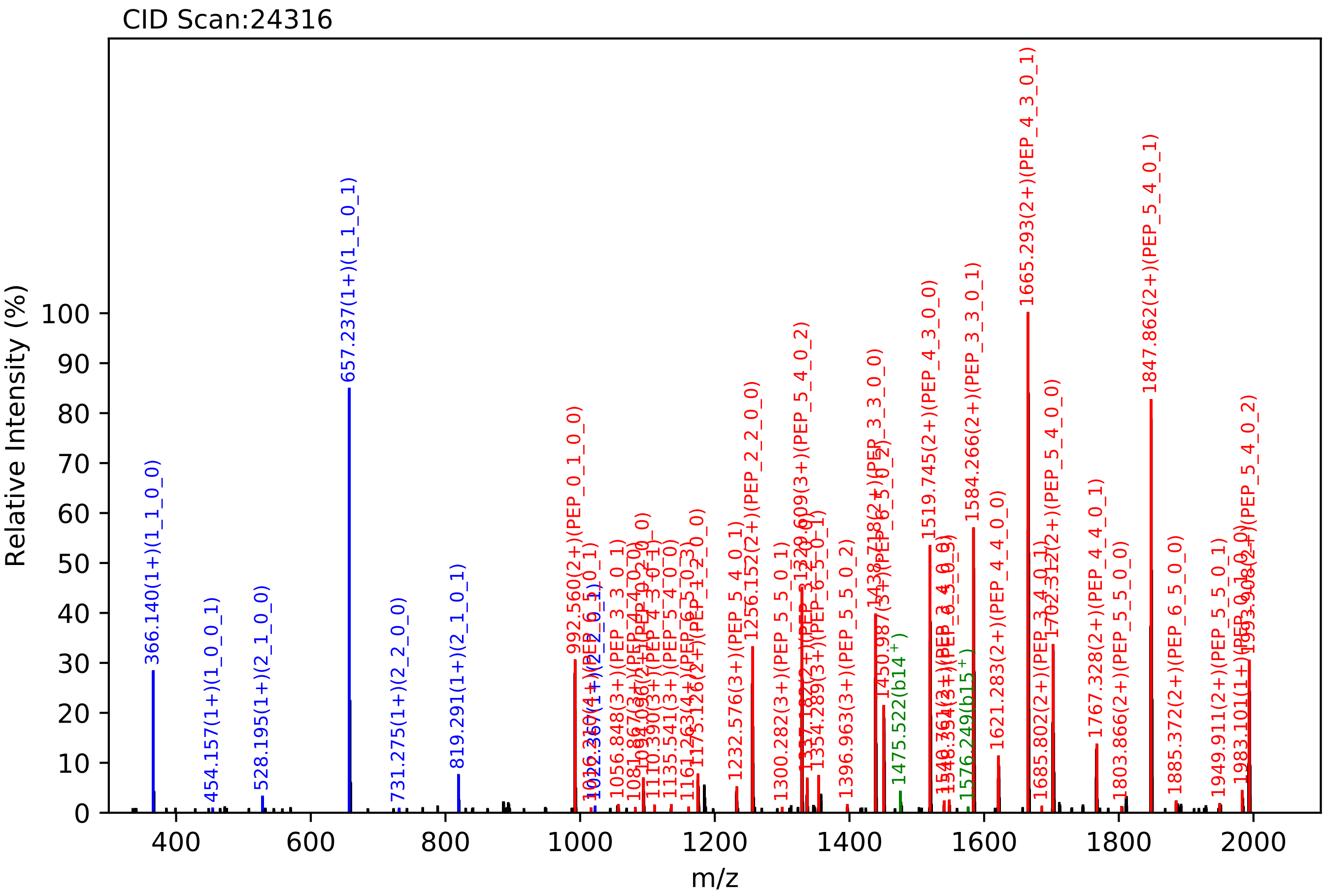

Training set no. 95, Experiment: AGP exp_1

LVPVPITNATLDQITGK(=PEP)_6_5_0_3, m/z:1161.01(4+), RT:62.70, Y-score:85.71

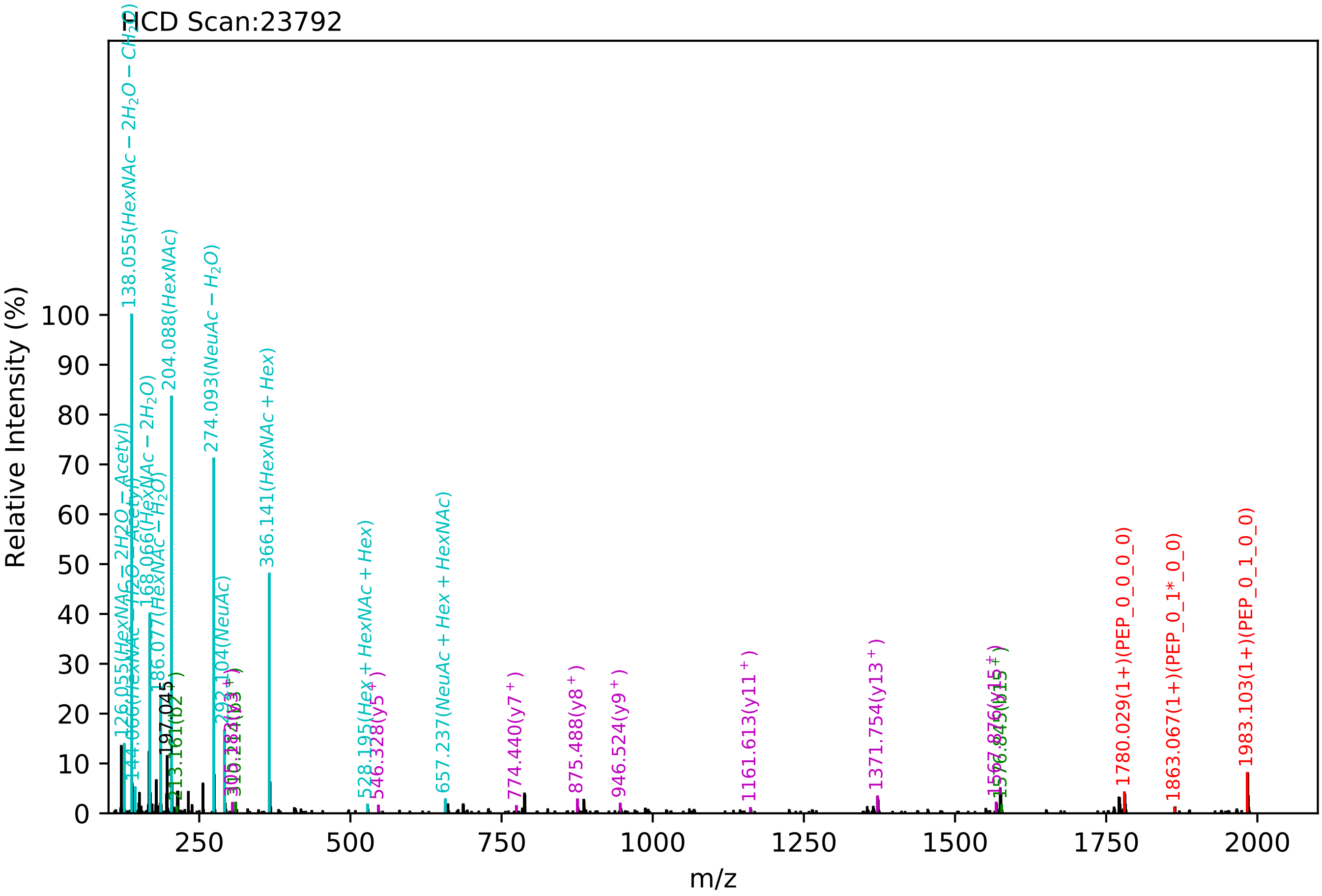

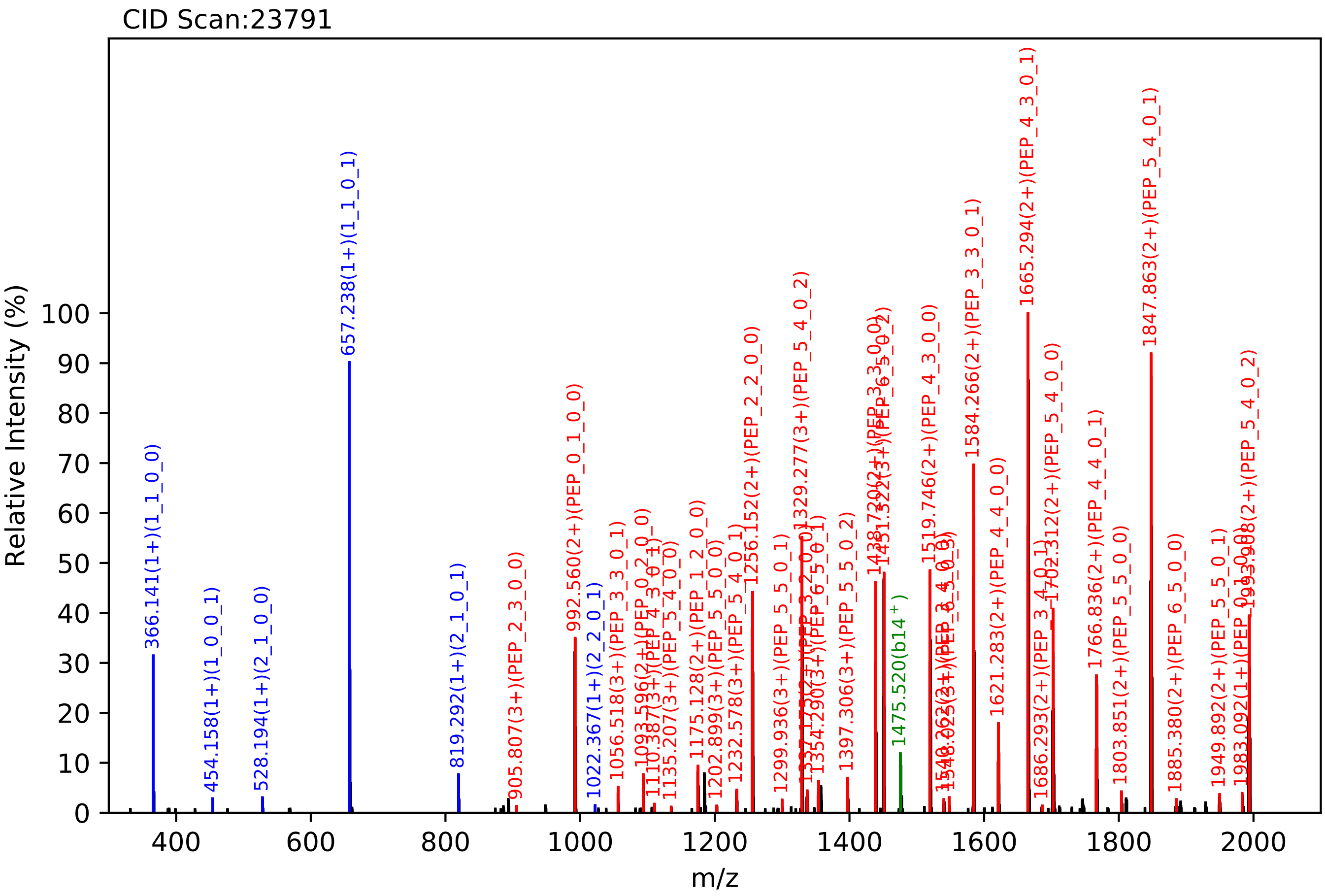

Training set no. 96, Experiment: AGP exp_1

LVPVPITNATLDQITGK(=PEP)_6_5_0_3, m/z:1161.03(4+), RT:62.15, Y-score:84.03

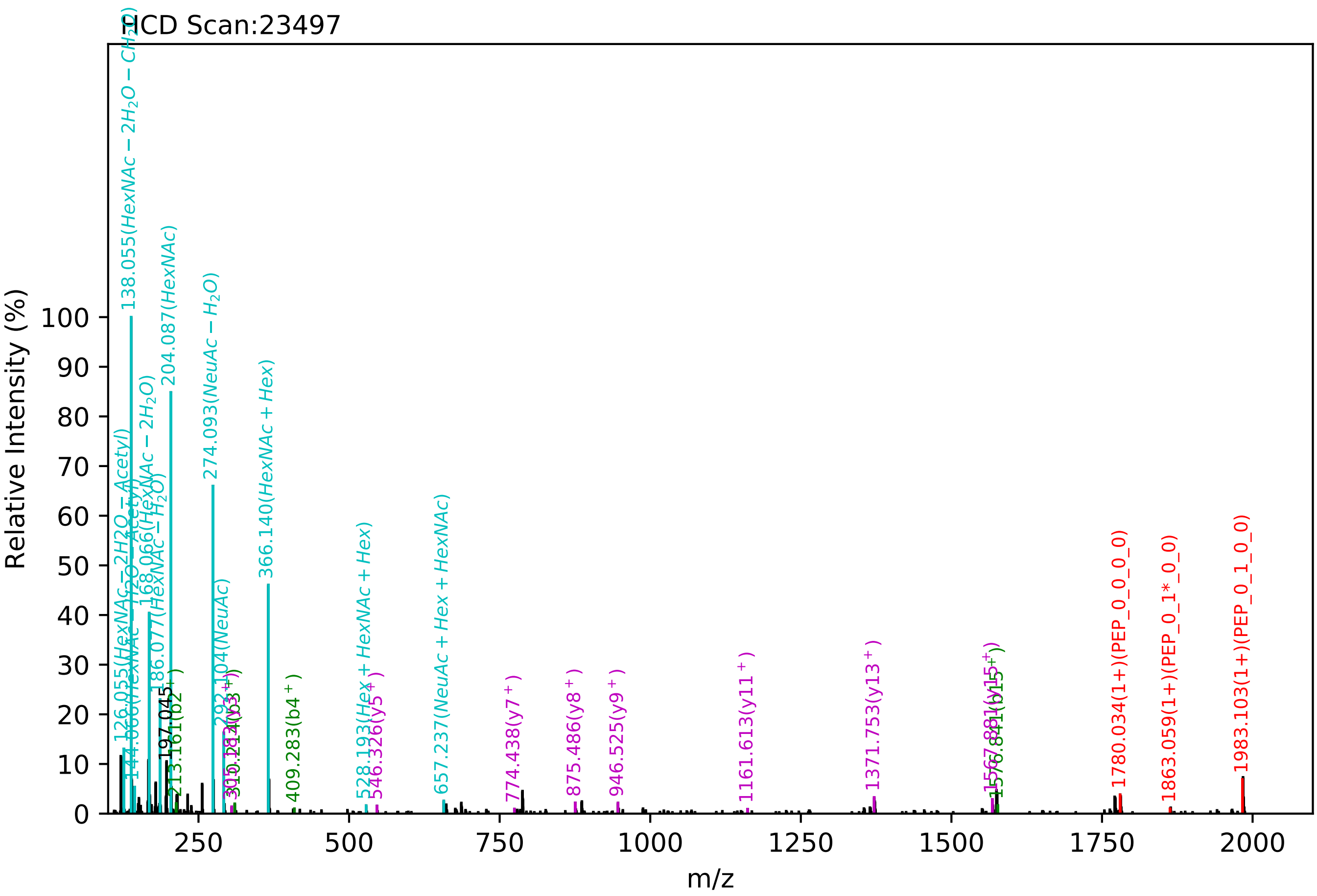

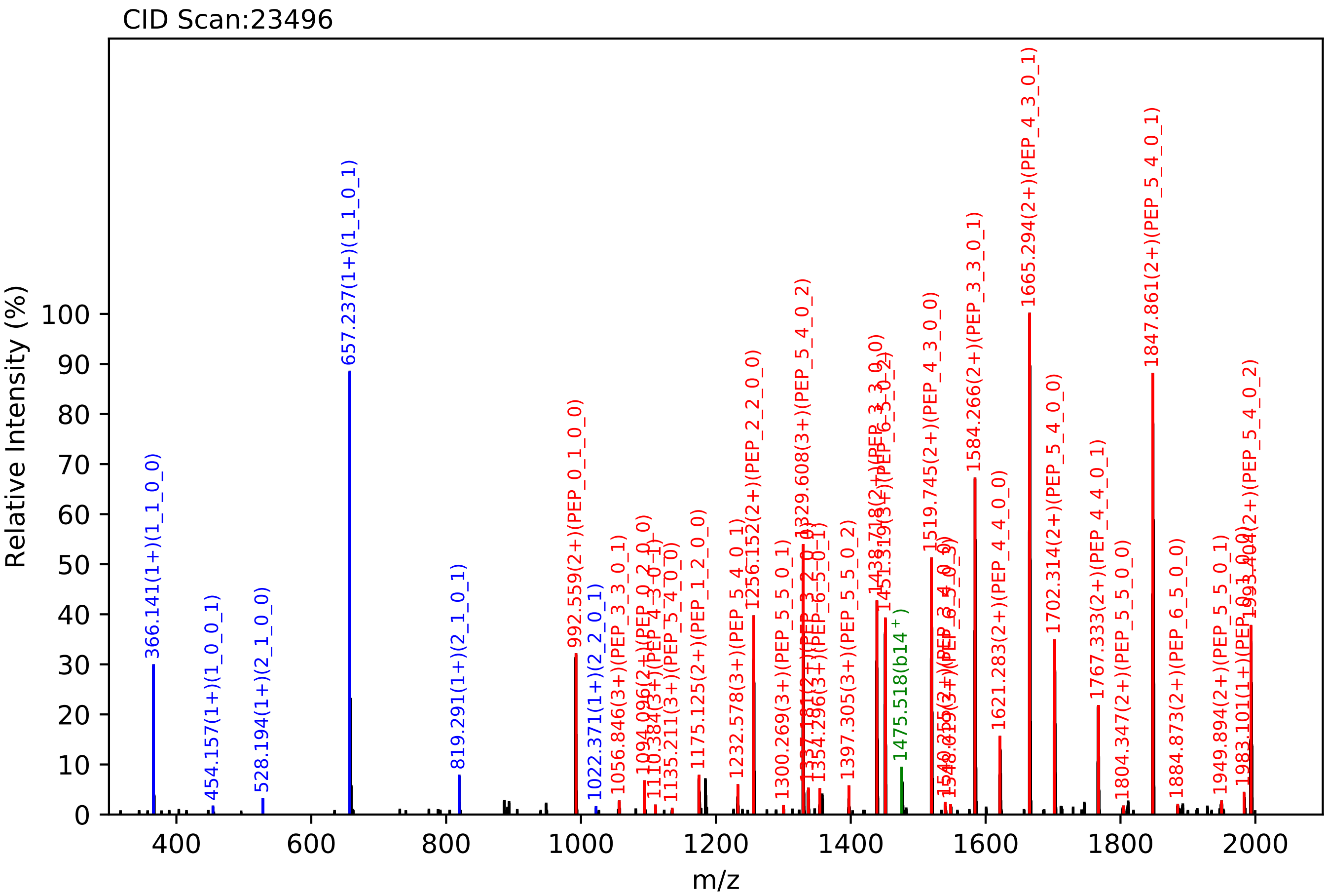

Training set no. 97, Experiment: AGP exp_2

LVPVPITNATLDQITGK(=PEP)_6_5_0_3, m/z:1161.01(4+), RT:63.11, Y-score:83.17

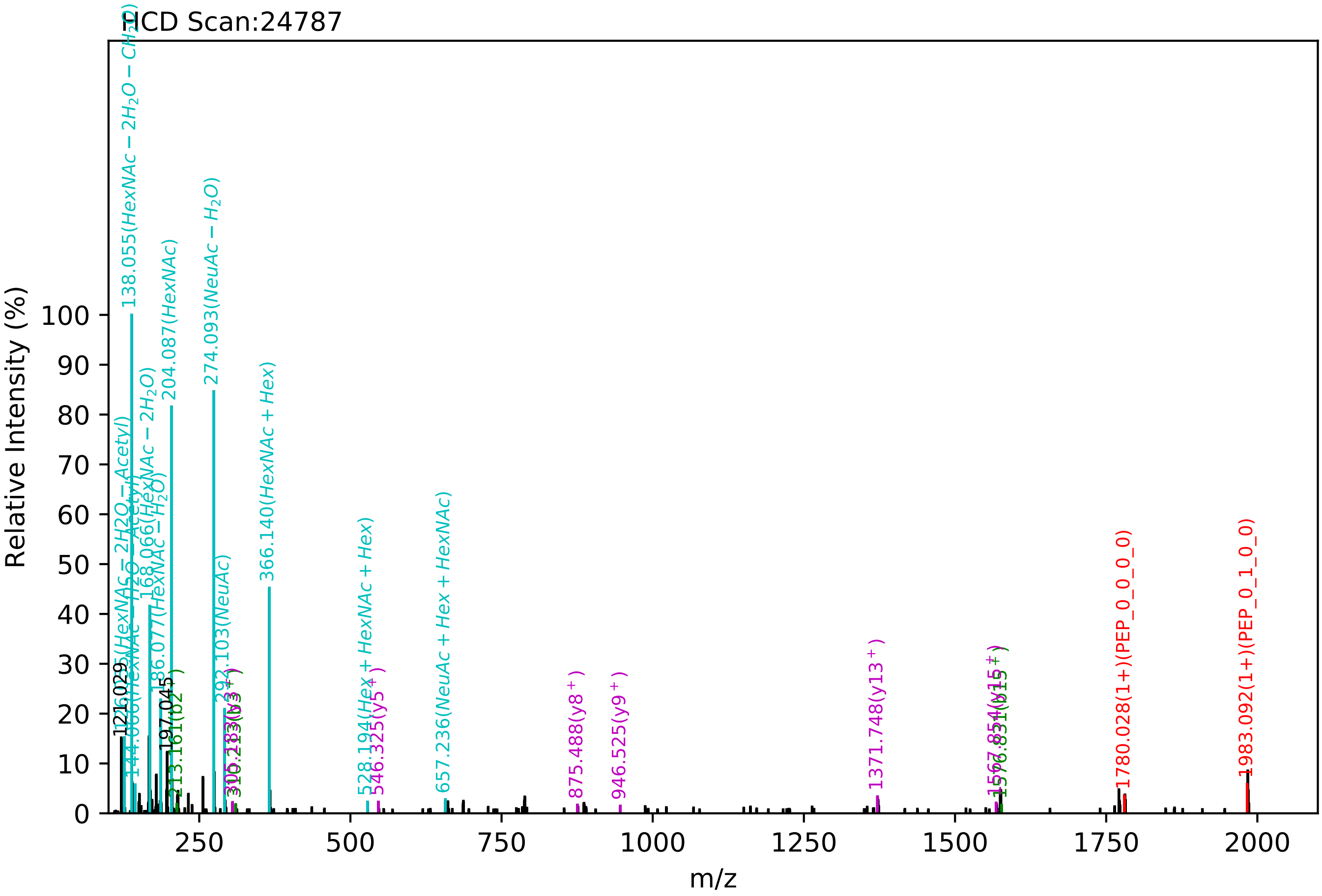

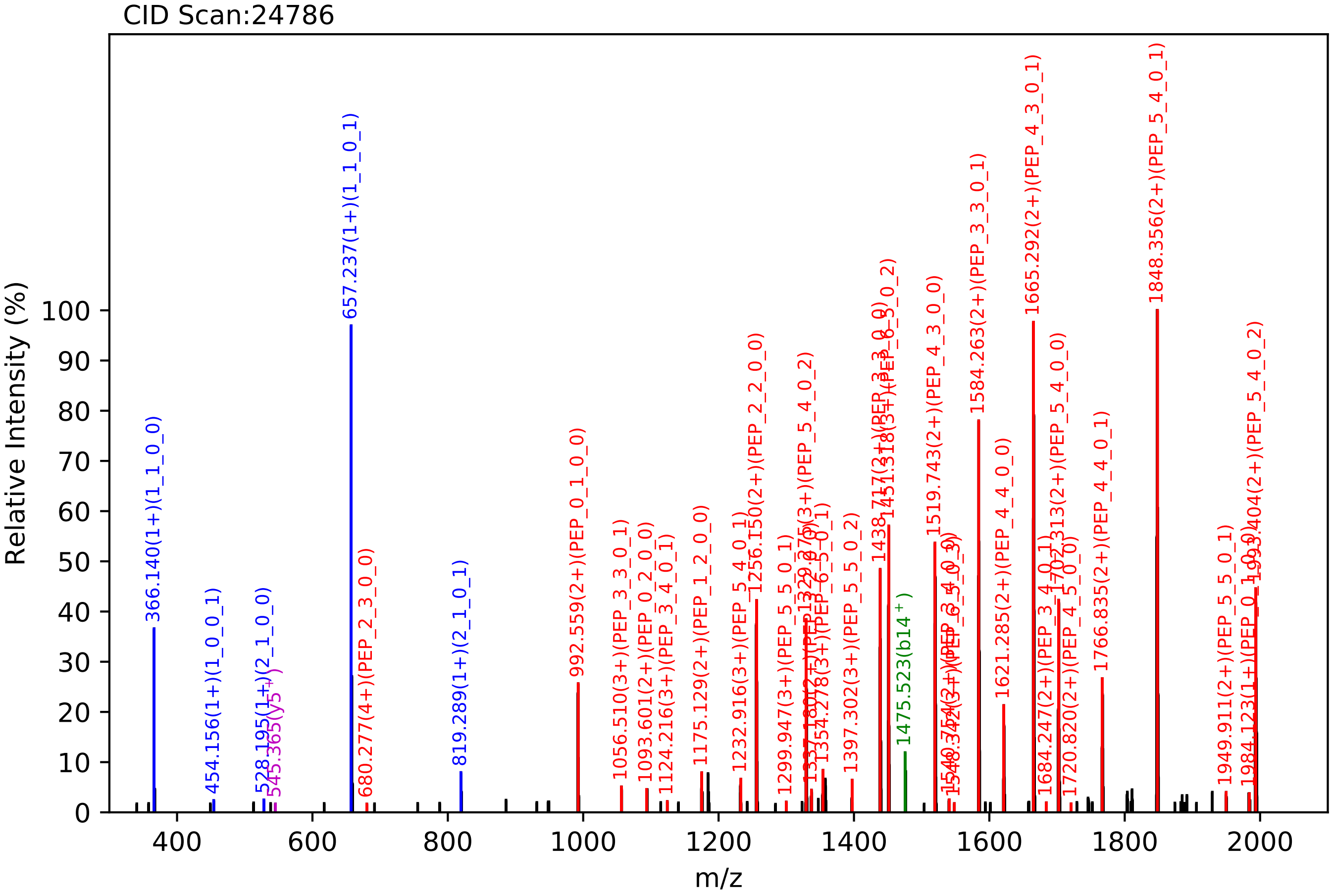

Training set no. 98, Experiment: AGP exp_2

LVPVPITNATLDQITGK(=PEP)_6_5_1_1, m/z:1402.30(3+), RT:48.63, Y-score:87.75

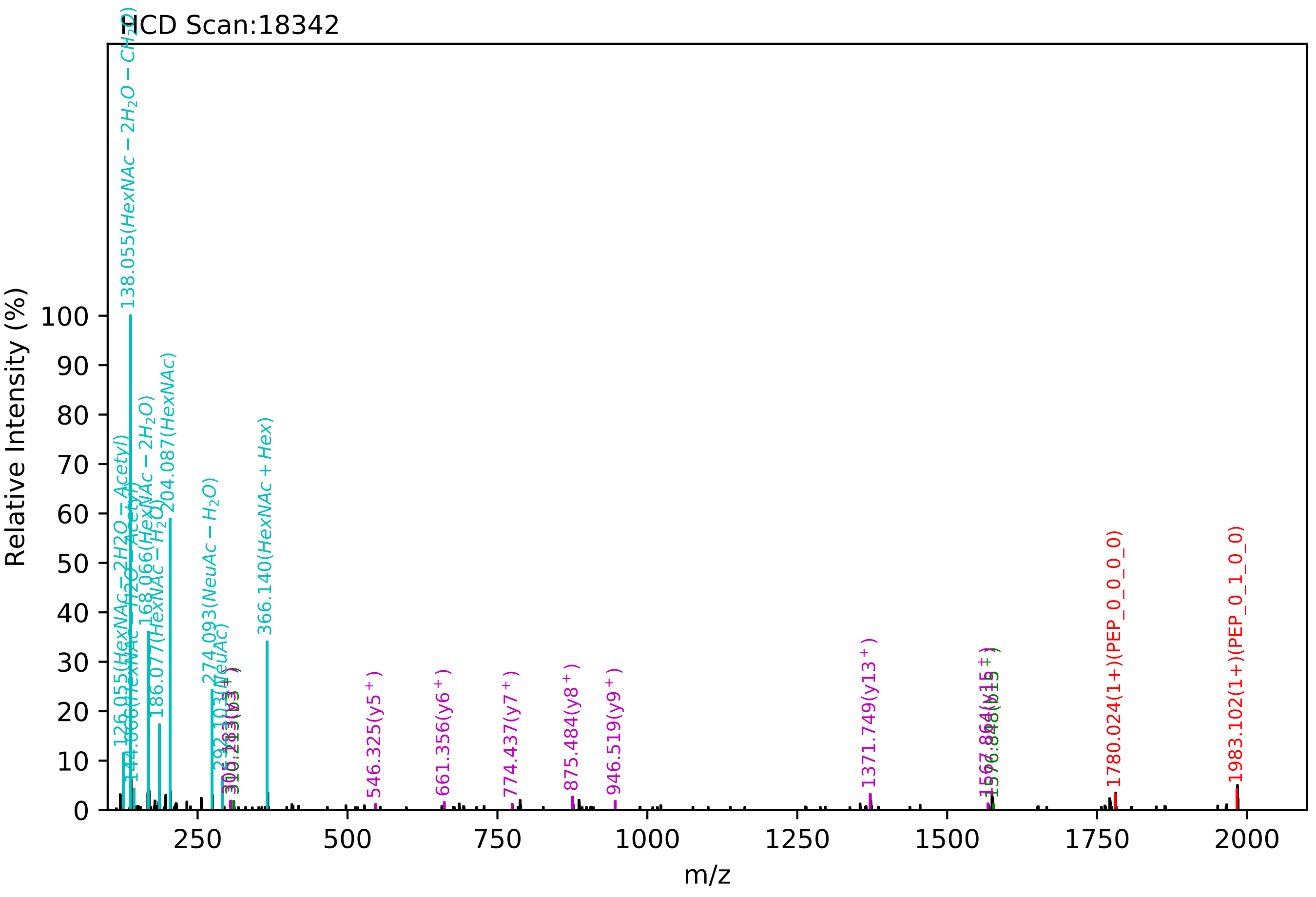

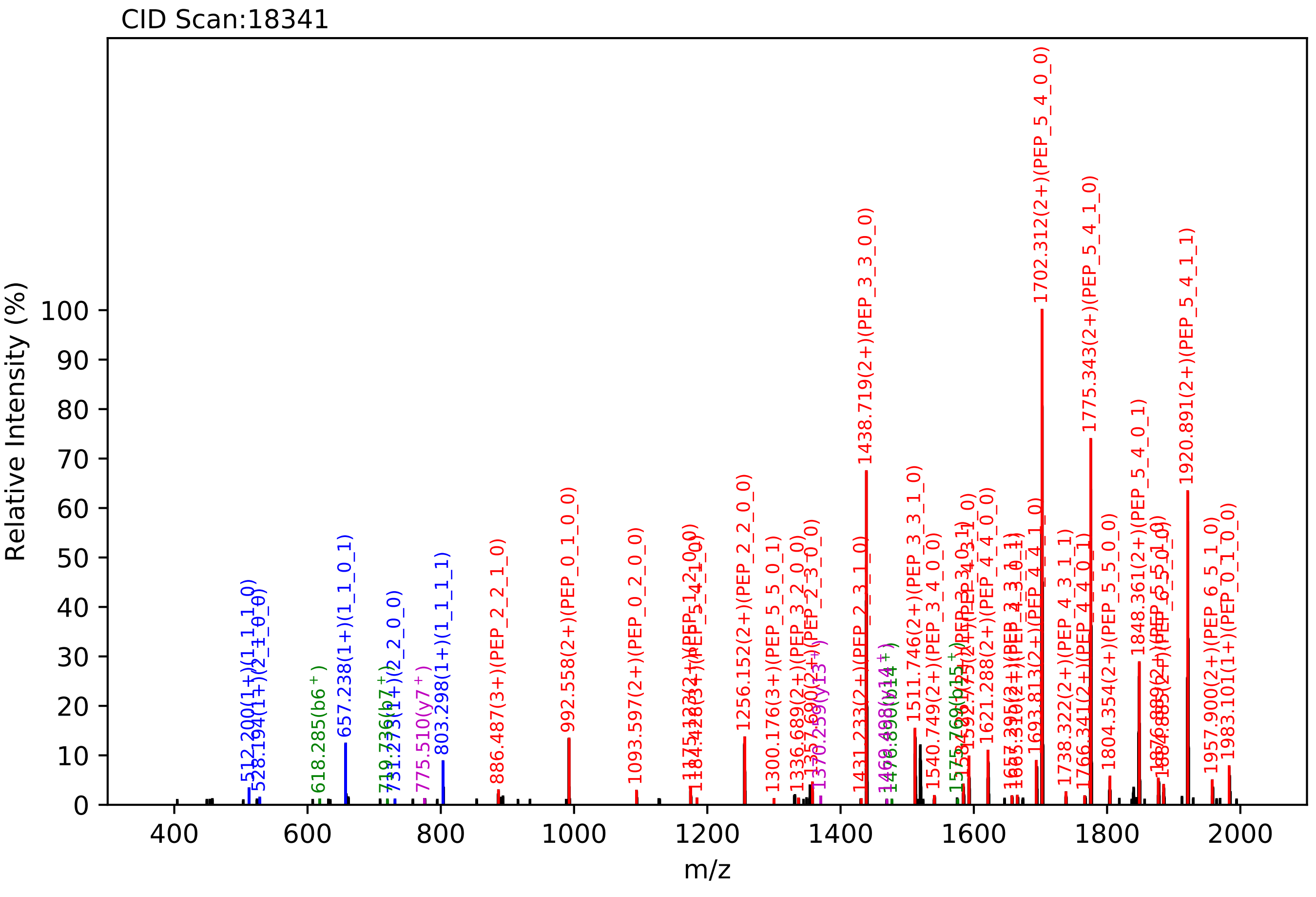

Training set no. 99, Experiment: AGP exp_1

LVPVPITNATLDQITGK(=PEP)_6_5_1_1, m/z:1402.30(3+), RT:48.47, Y-score:87.52

HCD Scan:17679

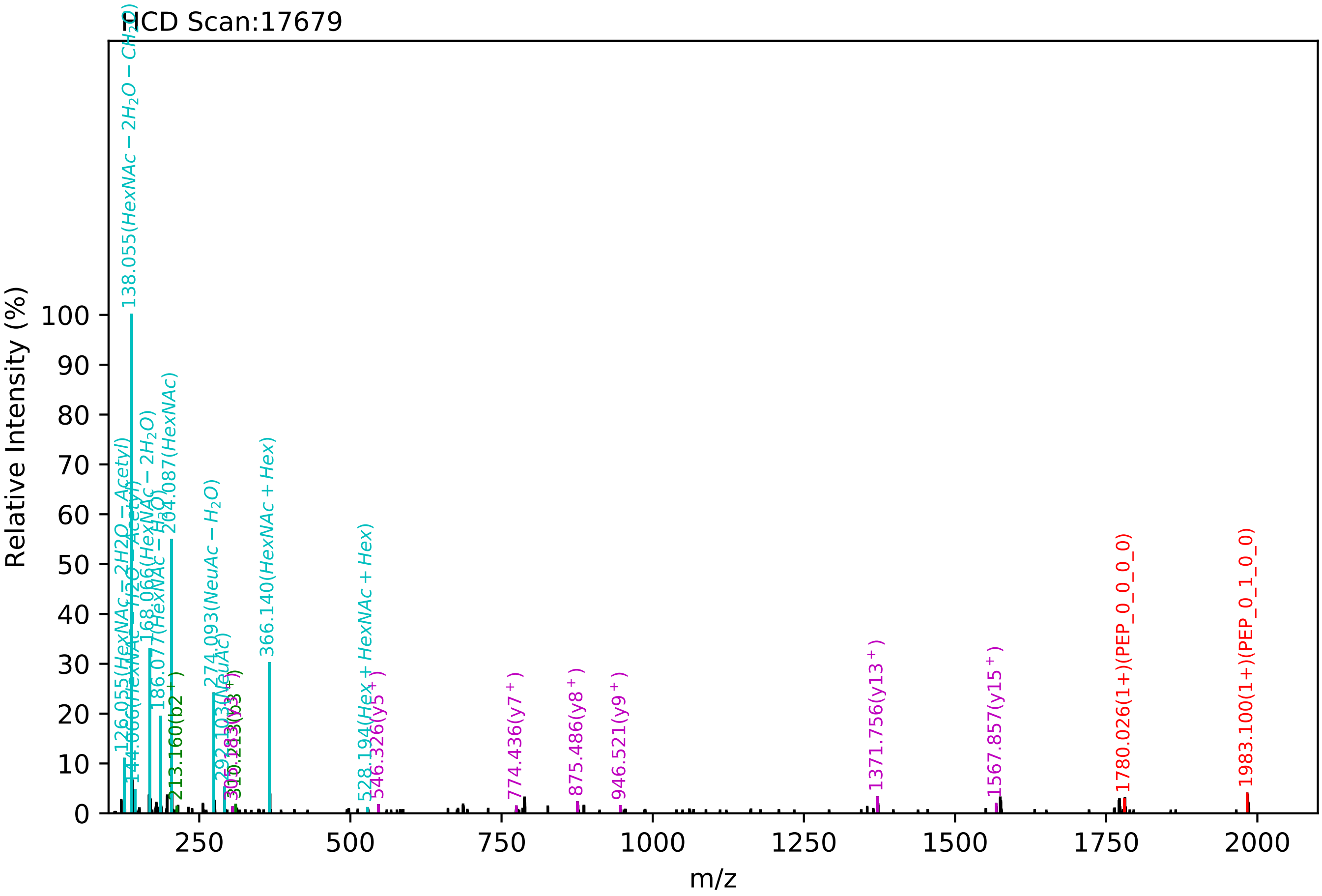

CID Scan:17678

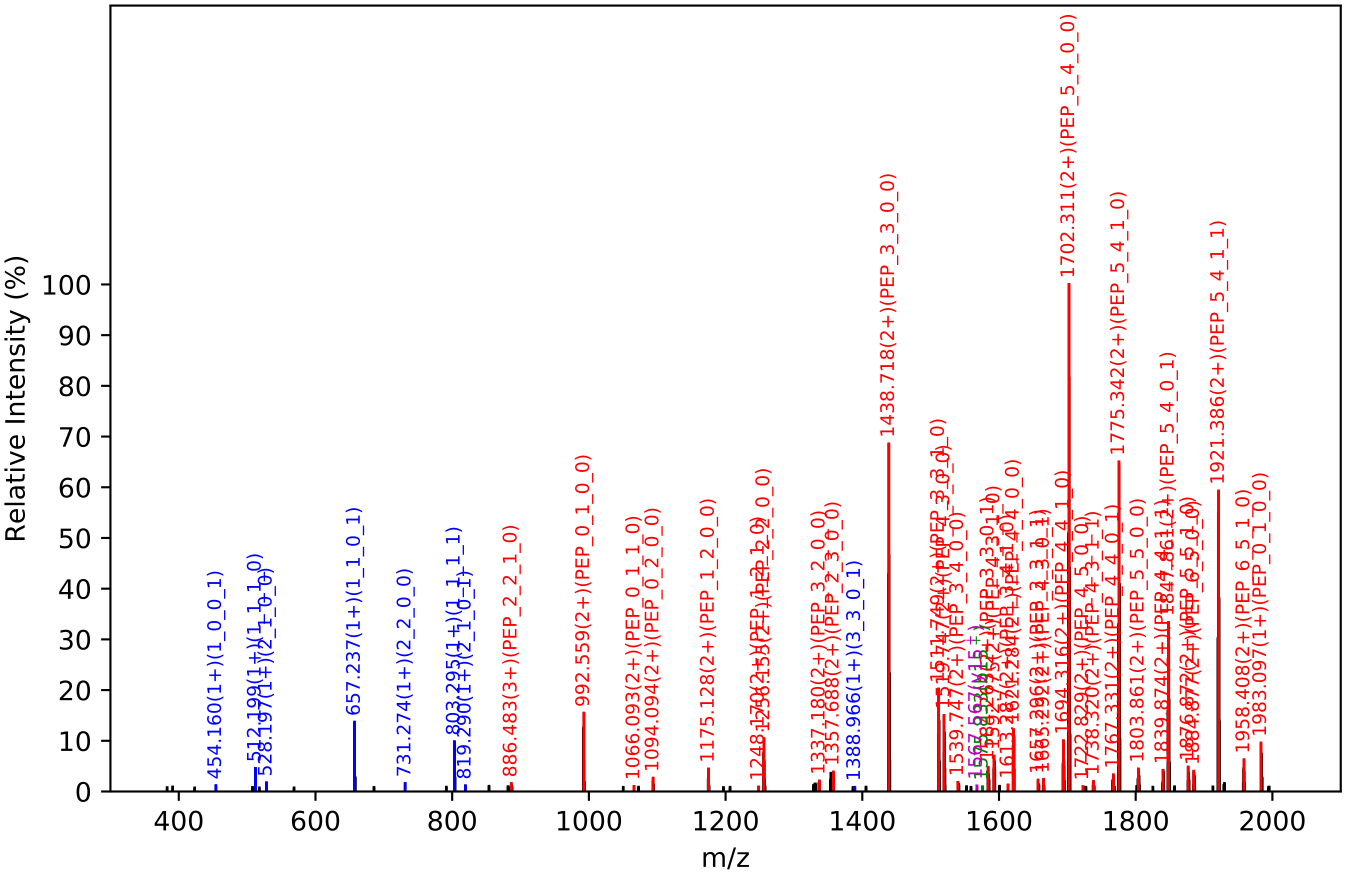

Training set no. 100, Experiment: AGP exp_2

LVPVPITNATLDQITGK(=PEP)_6_5_1_1, m/z:1051.98(4+), RT:48.65, Y-score:74.96

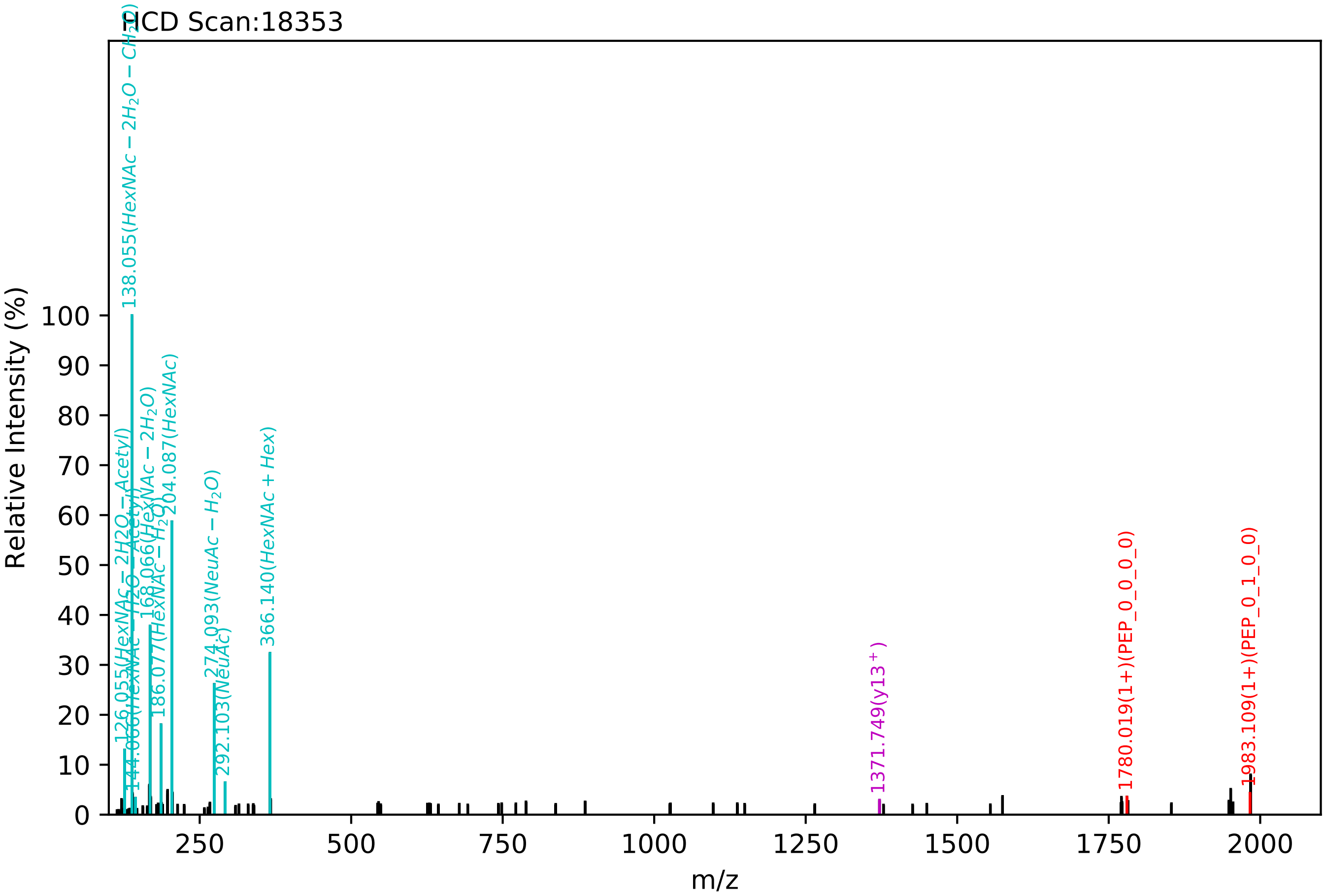

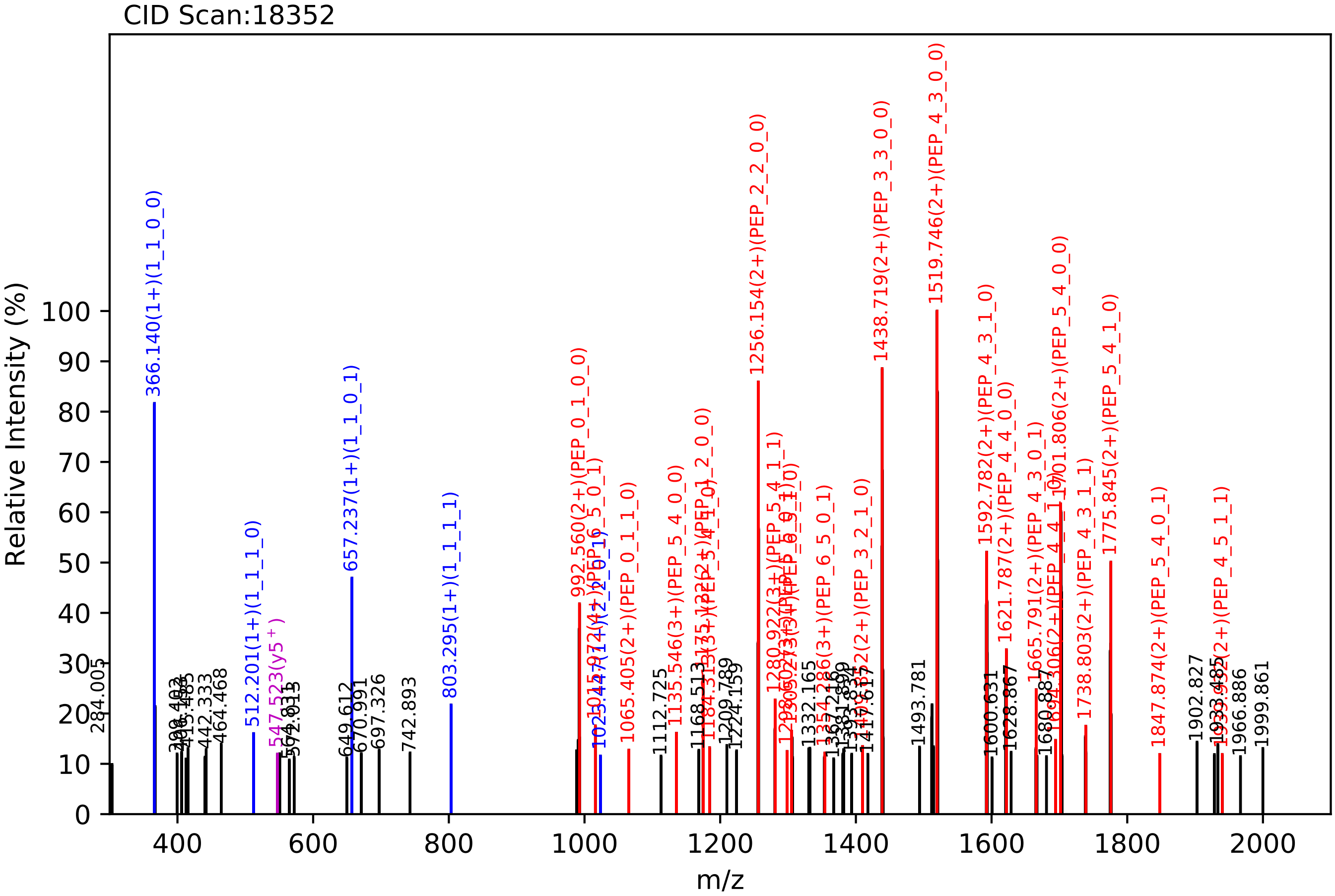

Training set no. 101, Experiment: AGP exp_1

LVPVPITNATLDQITGK(=PEP)_6_5_1_2, m/z:1499.34(3+), RT:54.78, Y-score:89.06

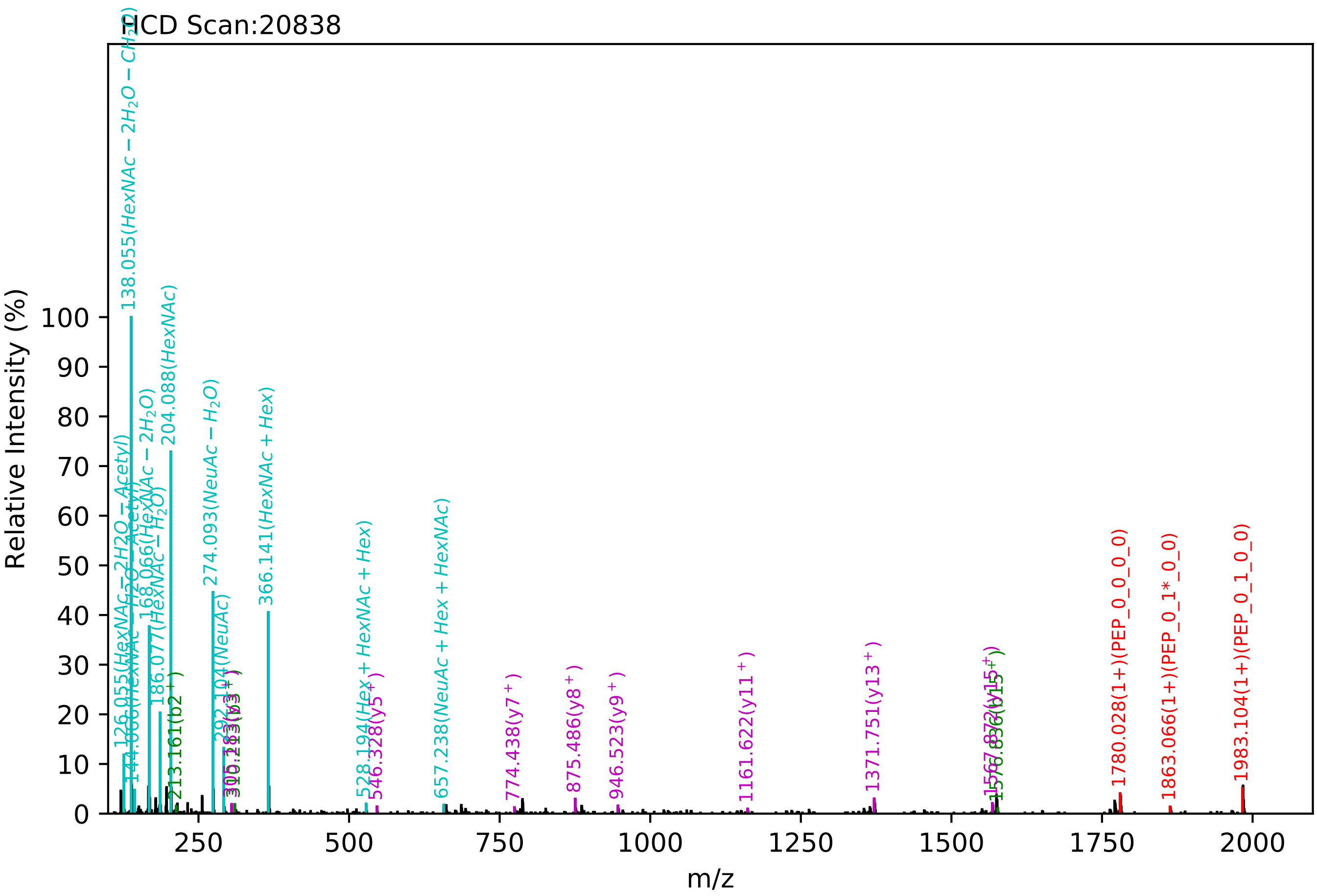

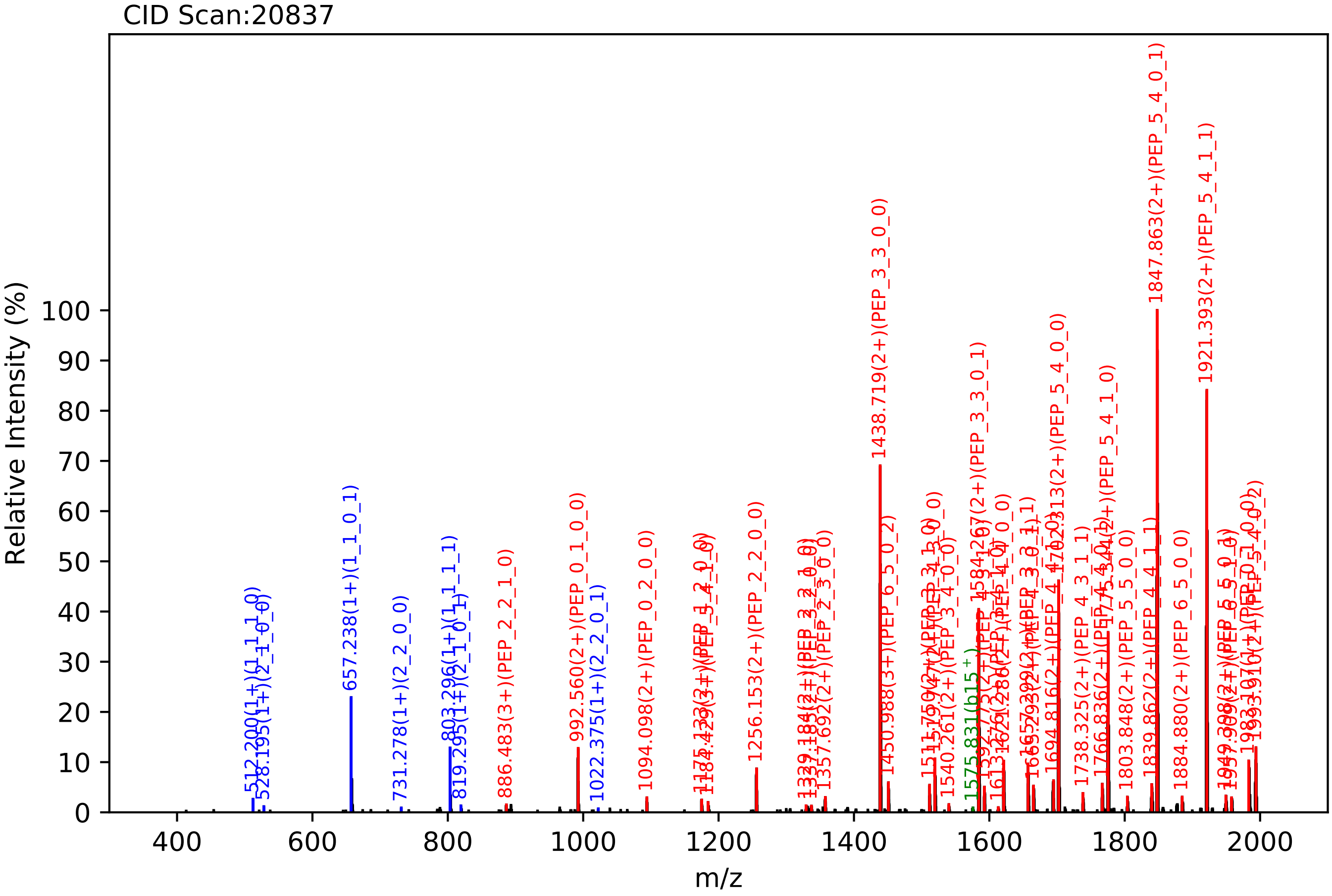

Training set no. 102, Experiment: AGP exp_2

LVPVPITNATLDQITGK(=PEP)_6_5_1_2, m/z:1499.33(3+), RT:54.39, Y-score:88.72

HCD Scan:21493

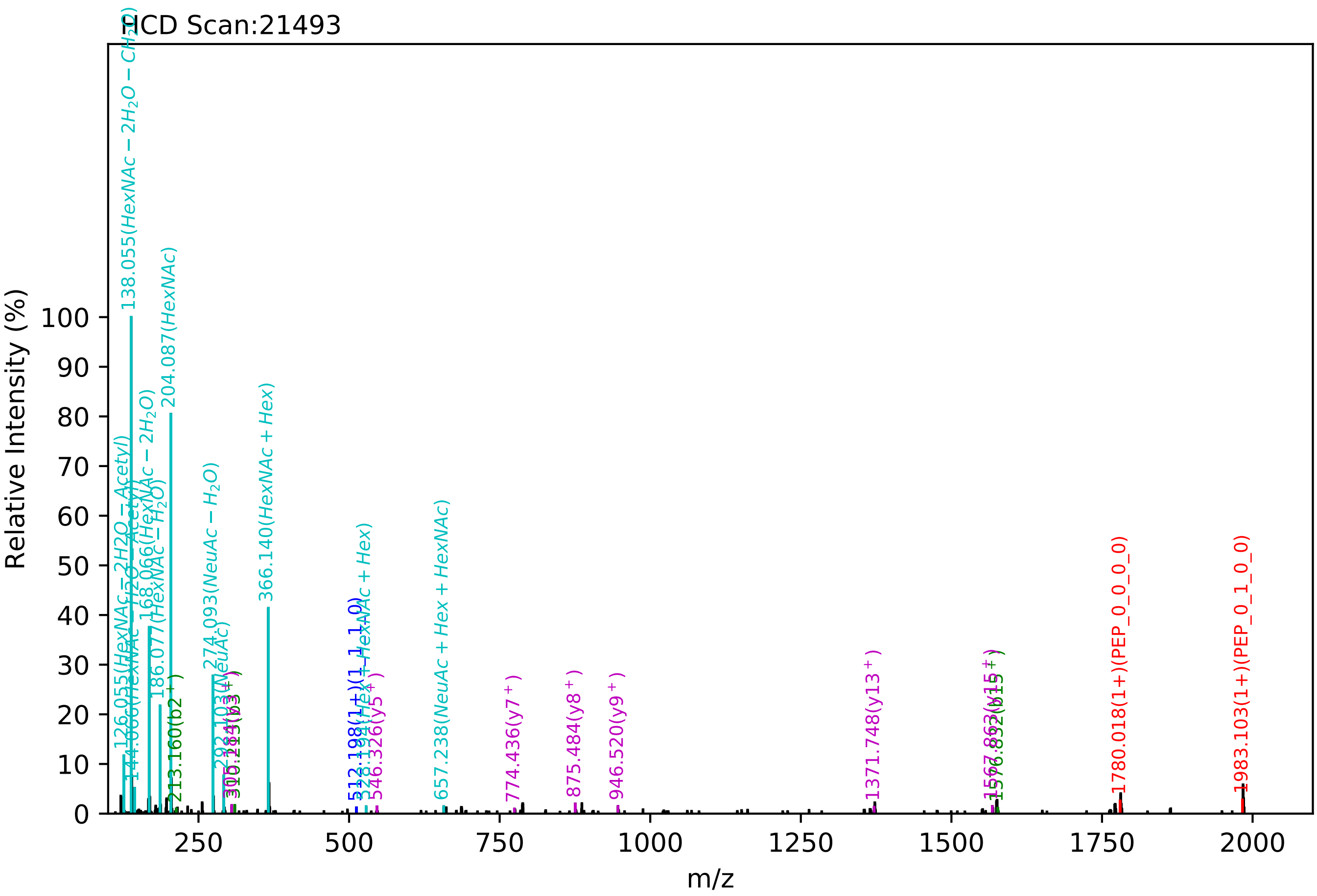

CID Scan:21492

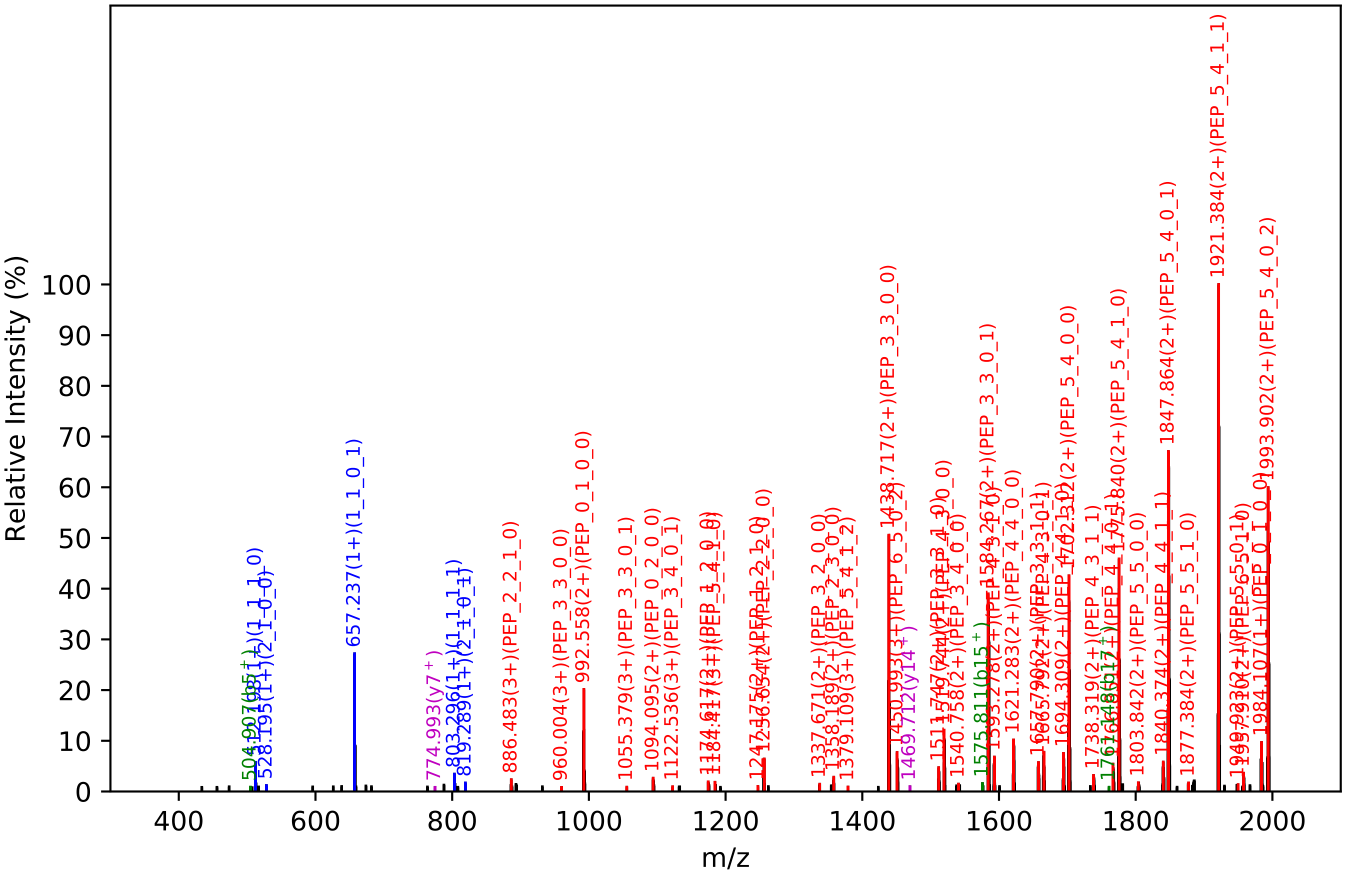

Training set no. 103, Experiment: AGP exp_2

LVPVPITNATLDQITGK(=PEP)_6_5_1_2, m/z:1499.33(3+), RT:54.94, Y-score:86.57

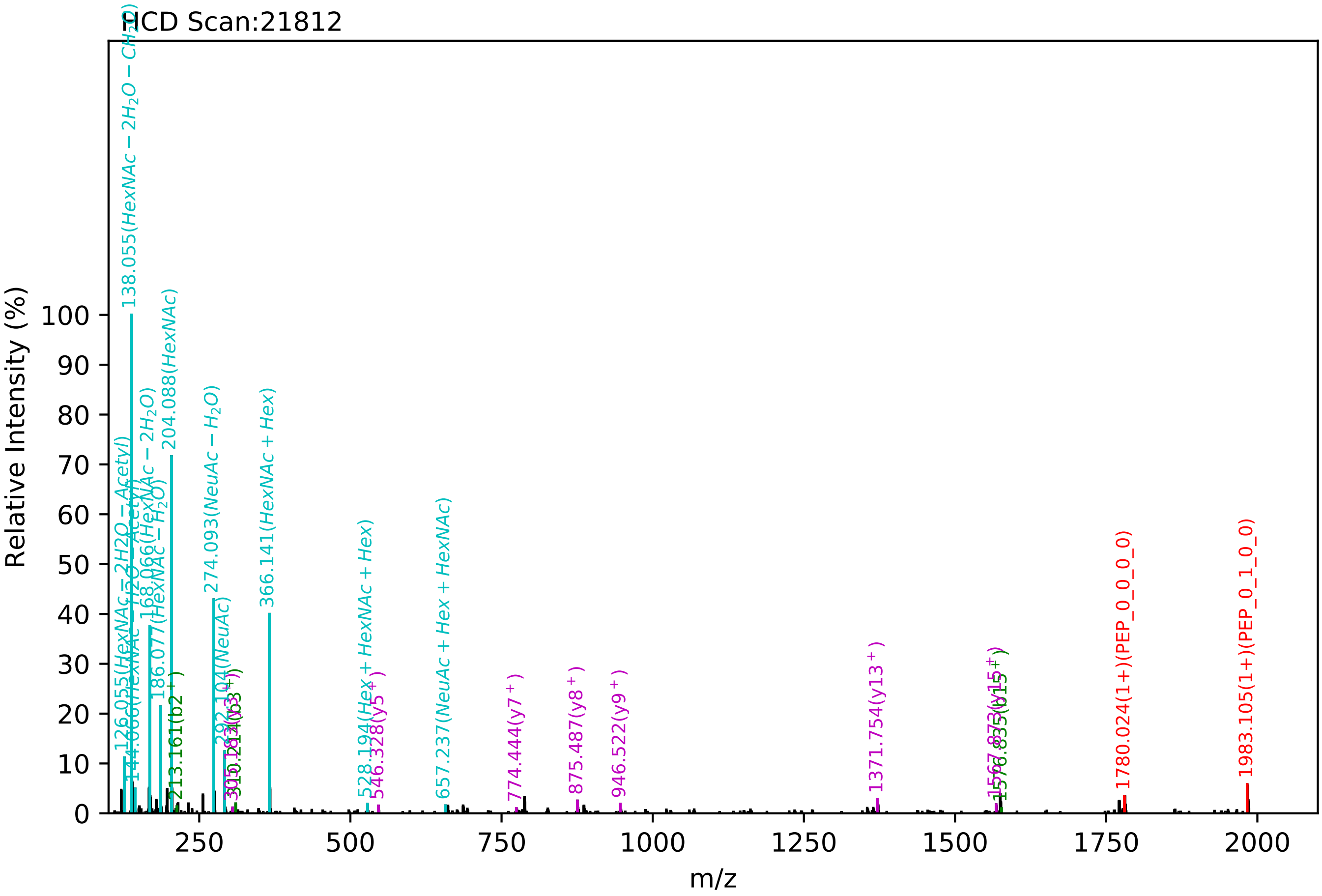

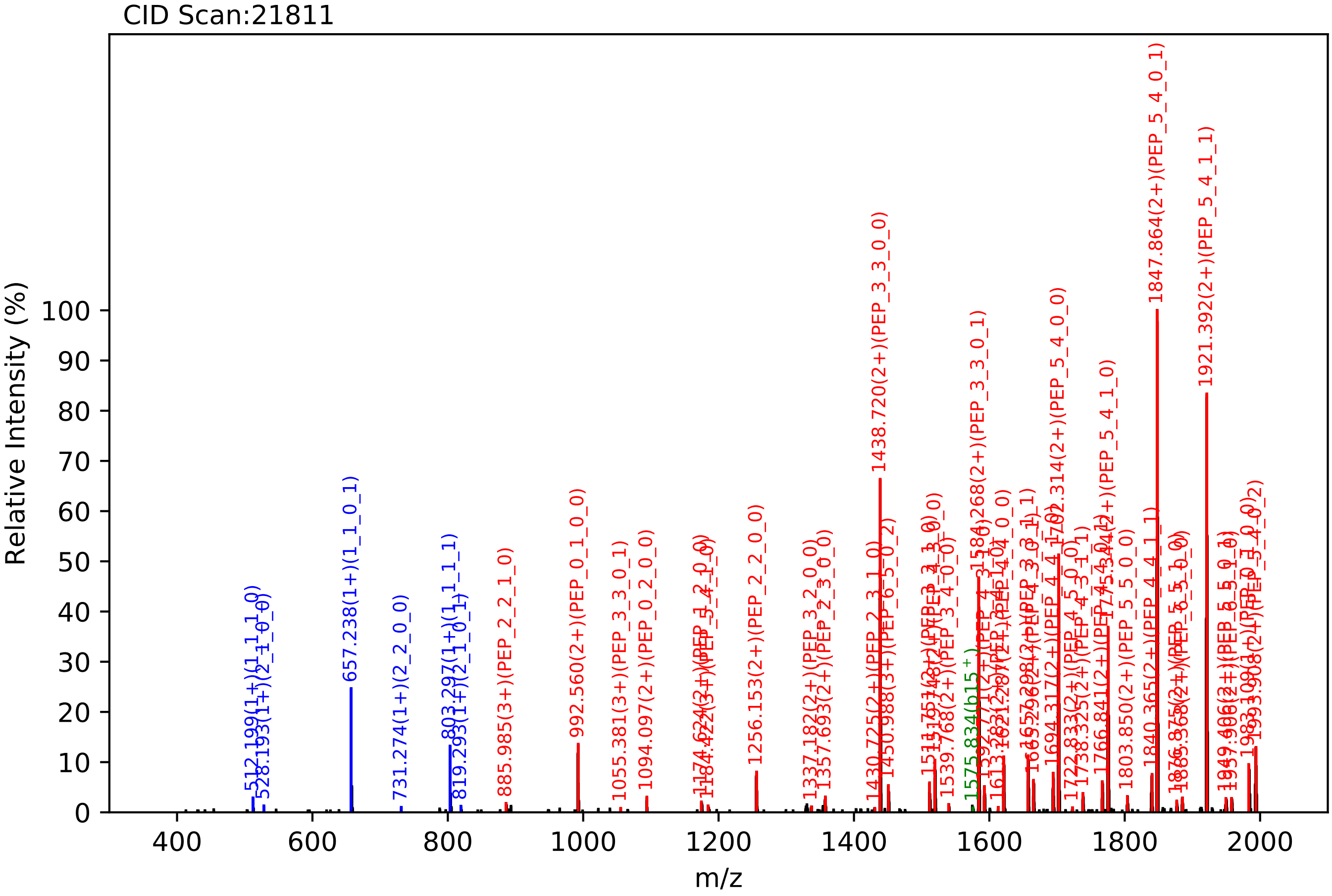

Training set no. 104, Experiment: AGP exp_2

LVPVPITNATLDQITGK(=PEP)_6_5_1_2, m/z:1124.76(4+), RT:54.77, Y-score:83.39

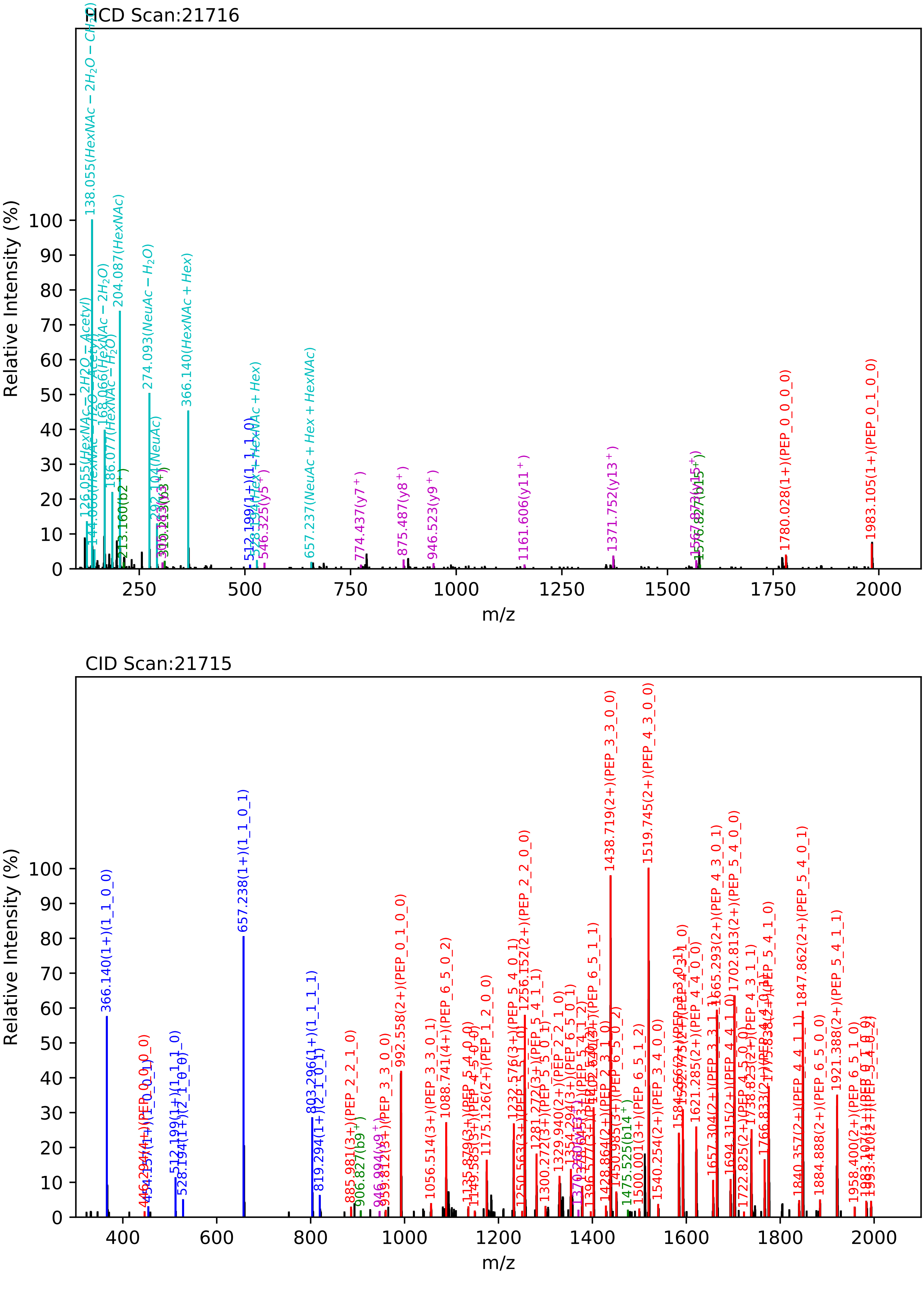

Training set no. 105, Experiment: AGP exp_1

LVPVPITNATLDQITGK(=PEP)_6_5_1_2, m/z:1124.75(4+), RT:54.81, Y-score:83.10

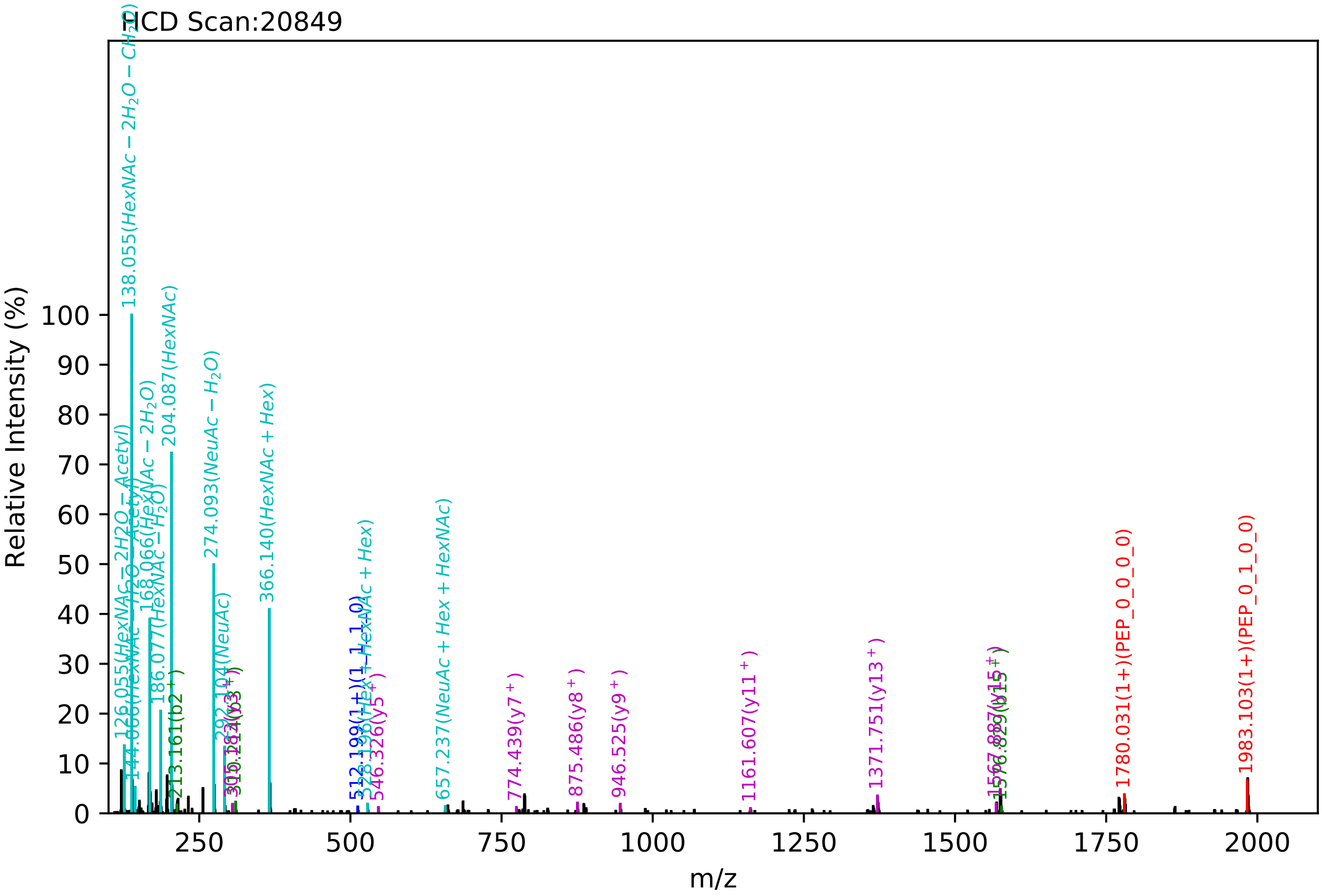

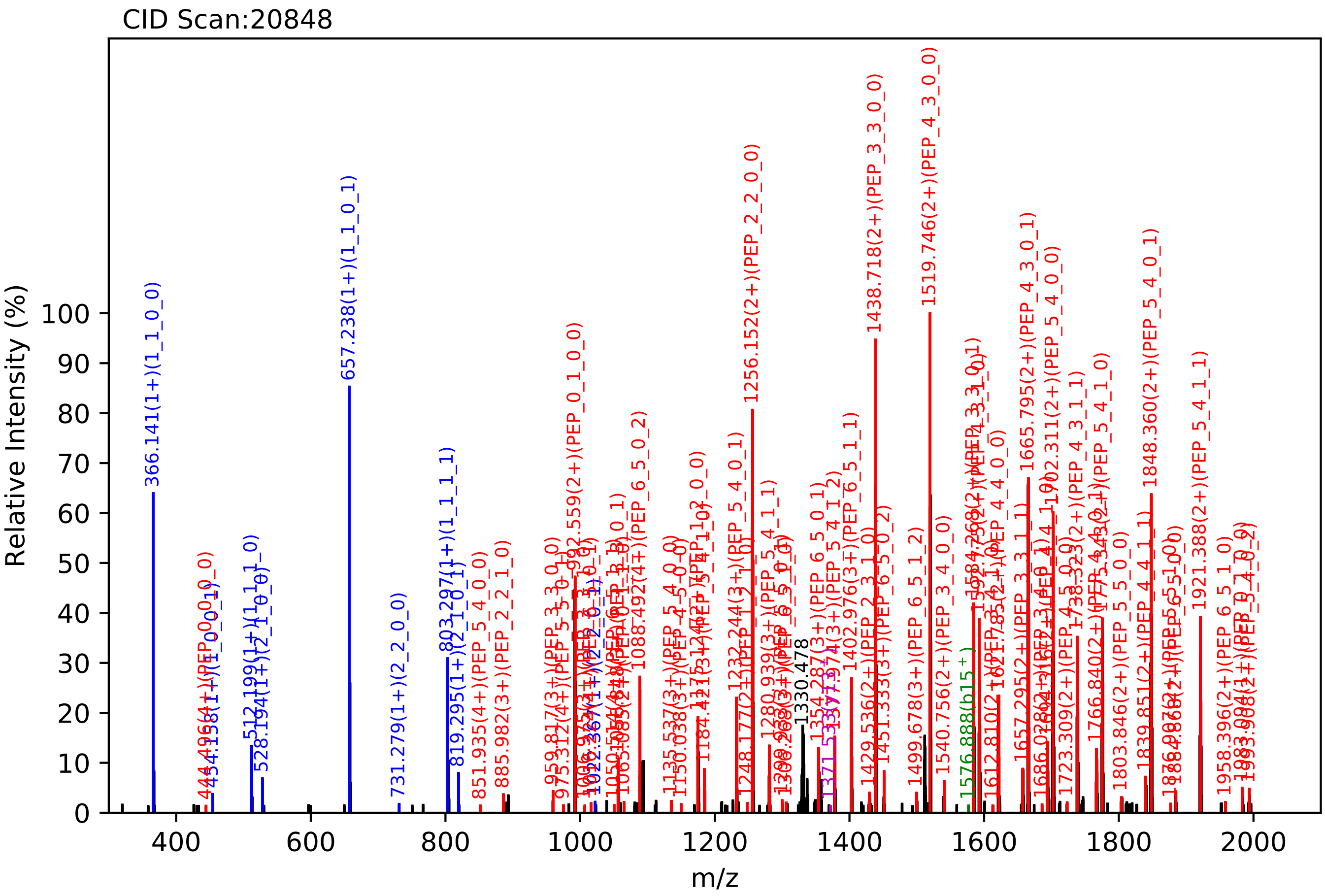

Training set no. 106, Experiment: AGP exp_1

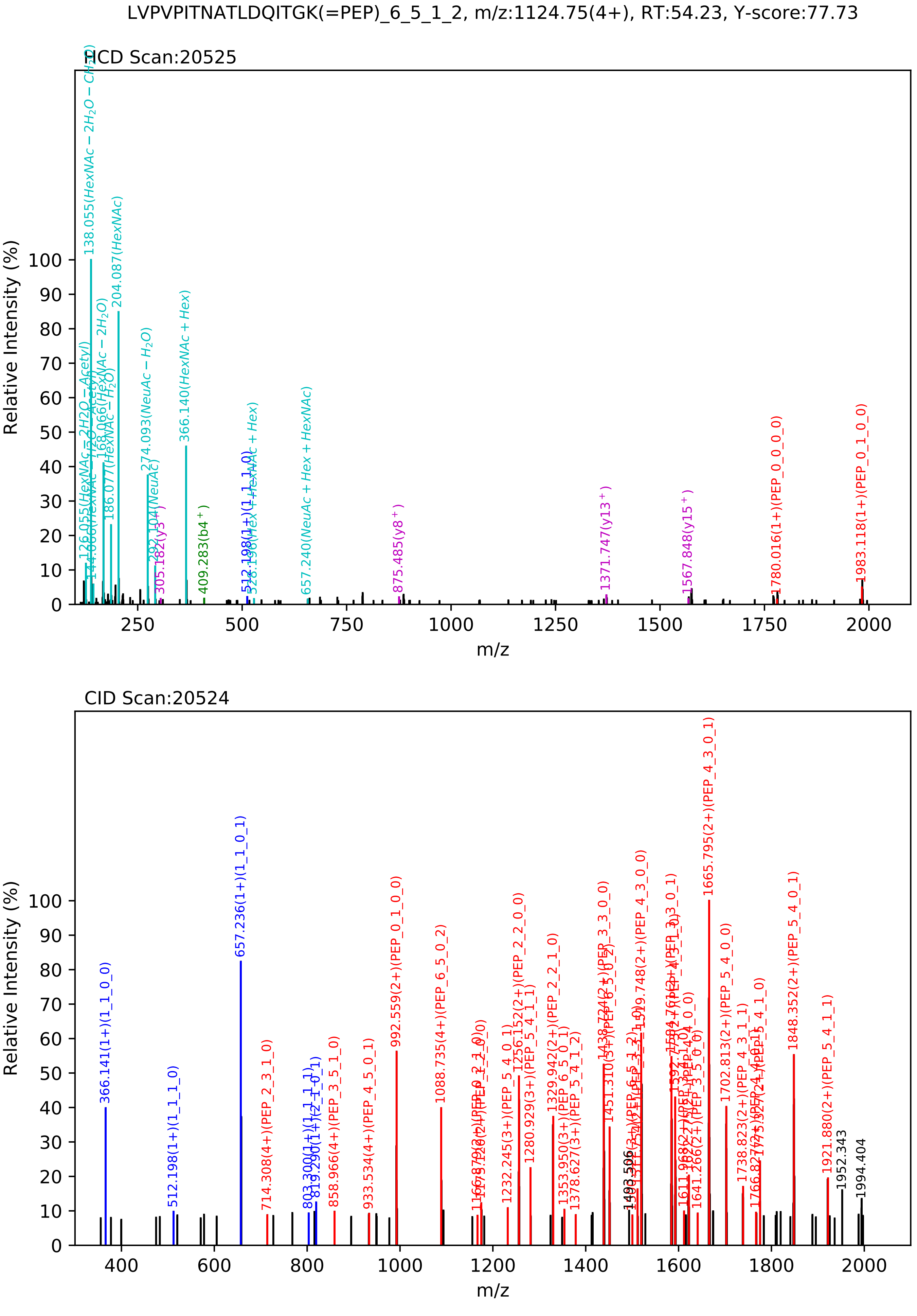

Training set no. 107, Experiment: AGP exp_1

LVPVPITNATLDQITGK(=PEP)_6_5_1_3, m/z:1596.37(3+), RT:62.80, Y-score:87.90

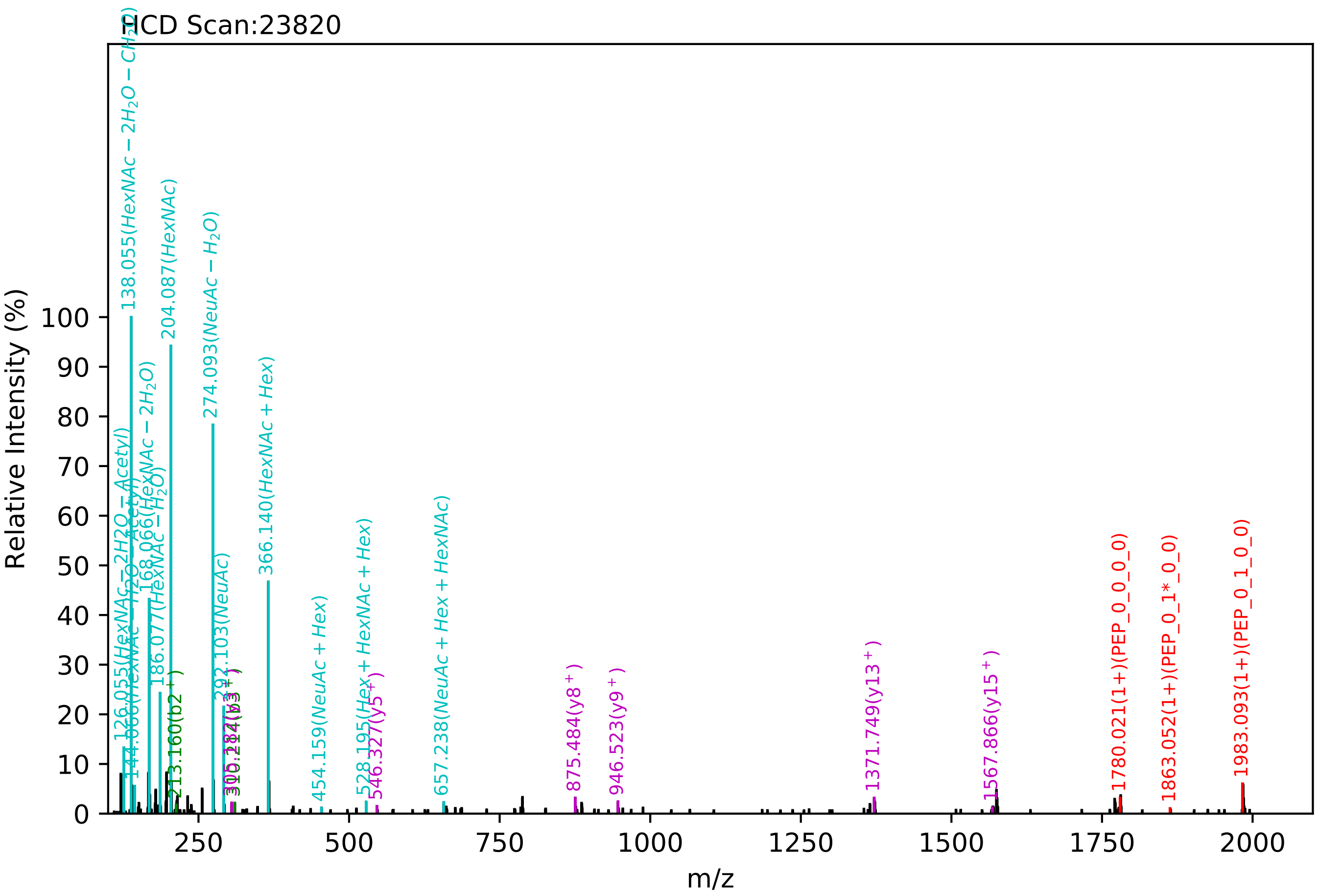

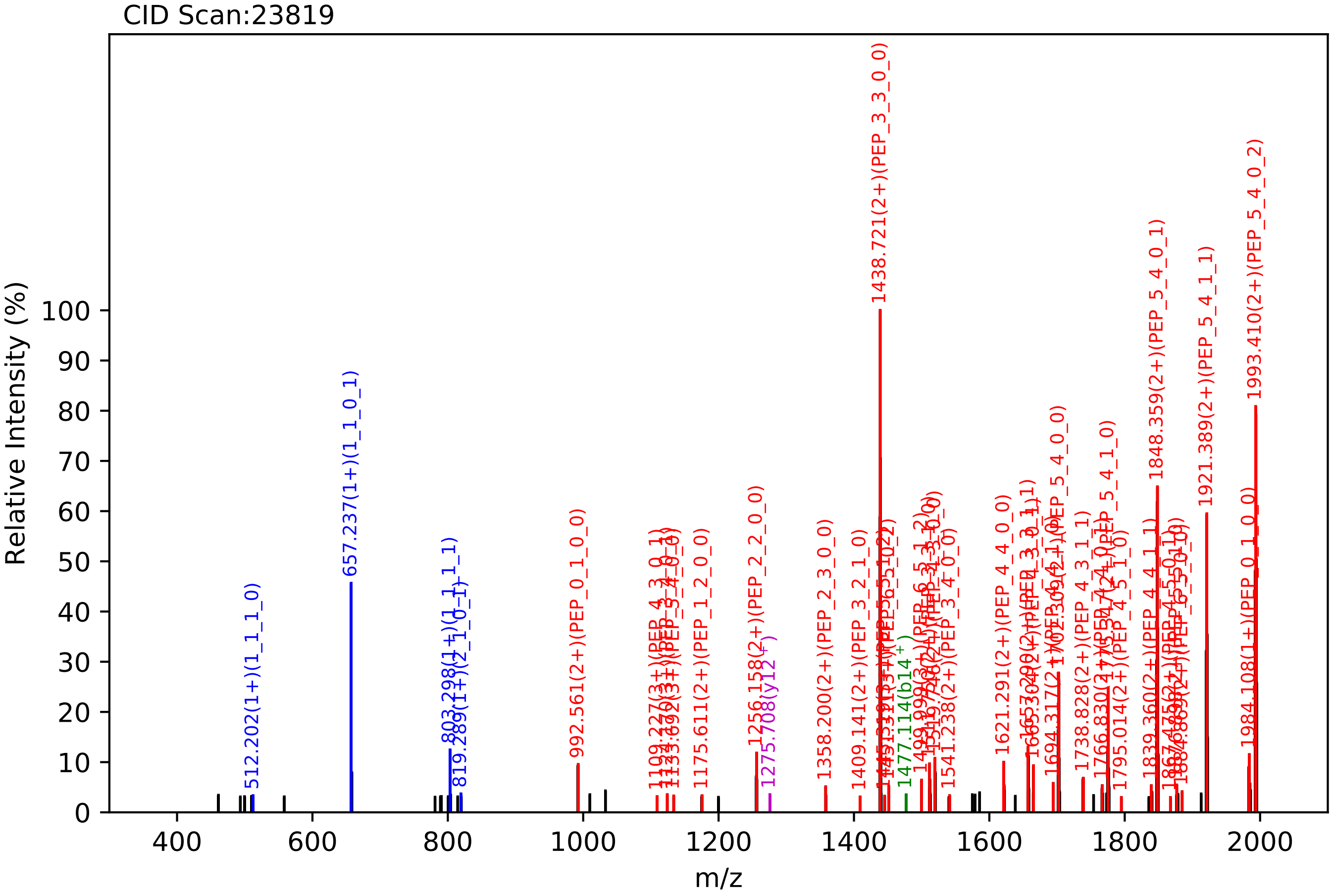

Training set no. 108, Experiment: AGP exp_2

LVPVPITNATLDQITGK(=PEP)_6_5_1_3, m/z:1596.37(3+), RT:62.39, Y-score:87.08

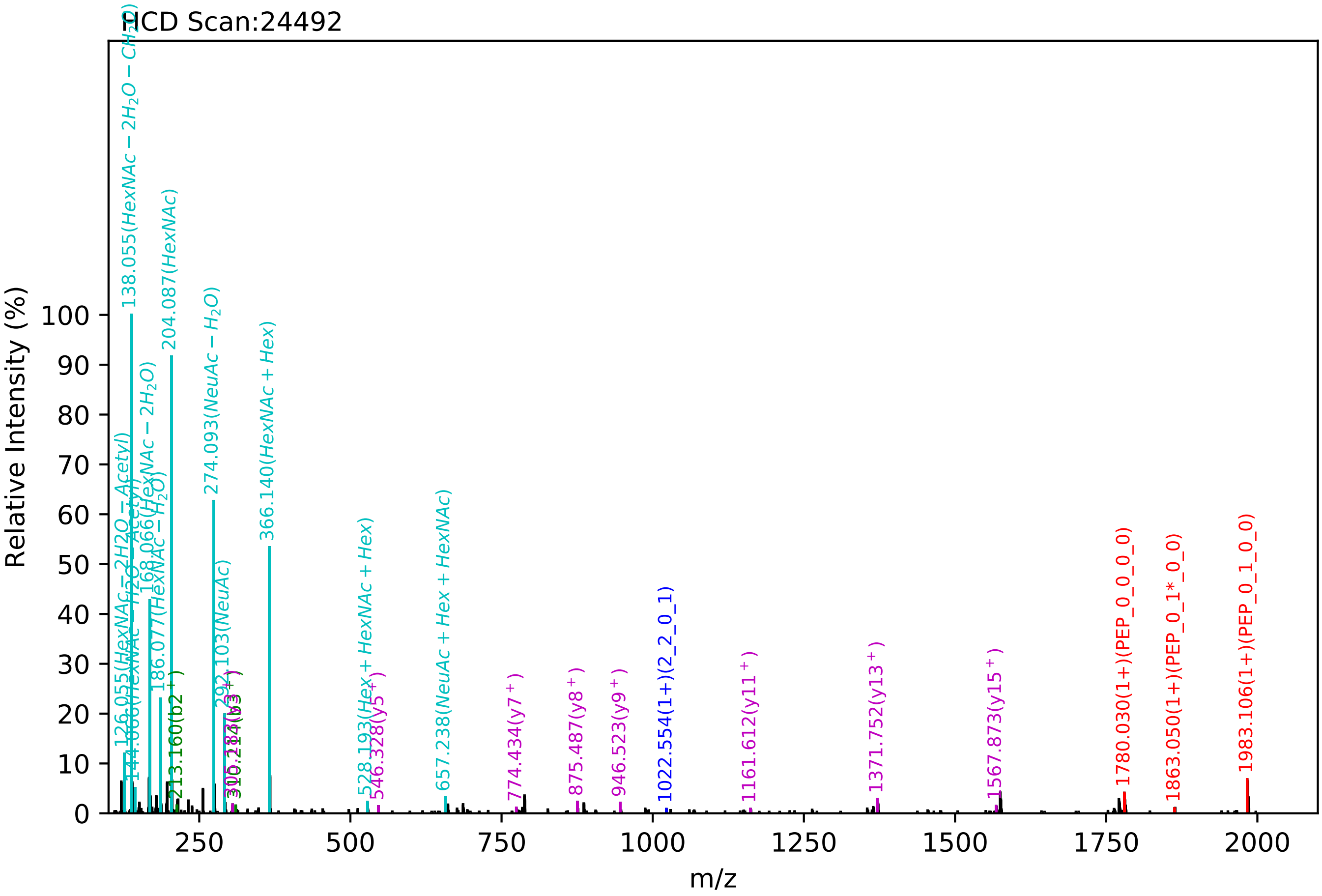

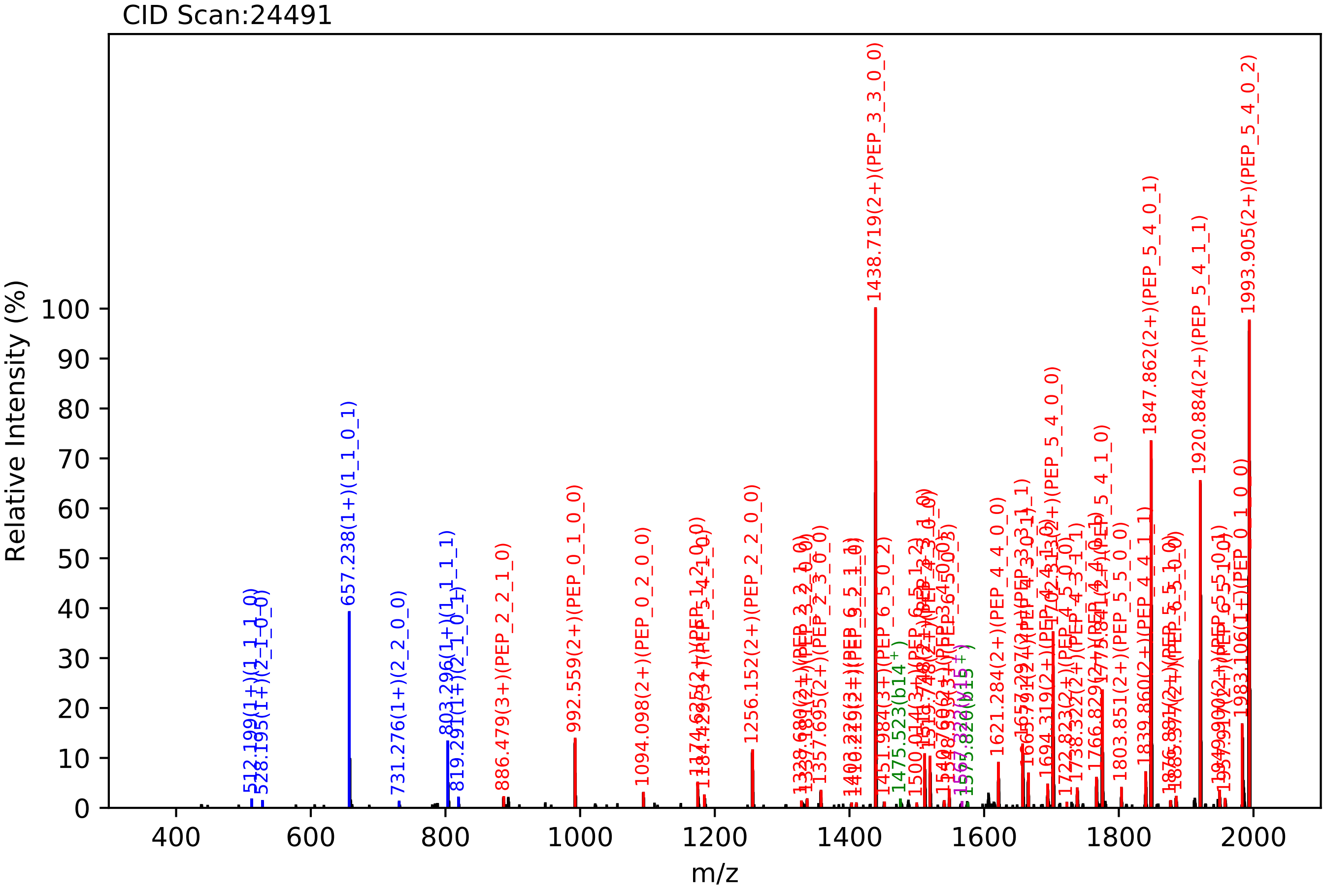

Training set no. 109, Experiment: AGP exp_2

LVPVPITNATLDQITGK(=PEP)_6_5_1_3, m/z:1596.37(3+), RT:62.91, Y-score:86.57

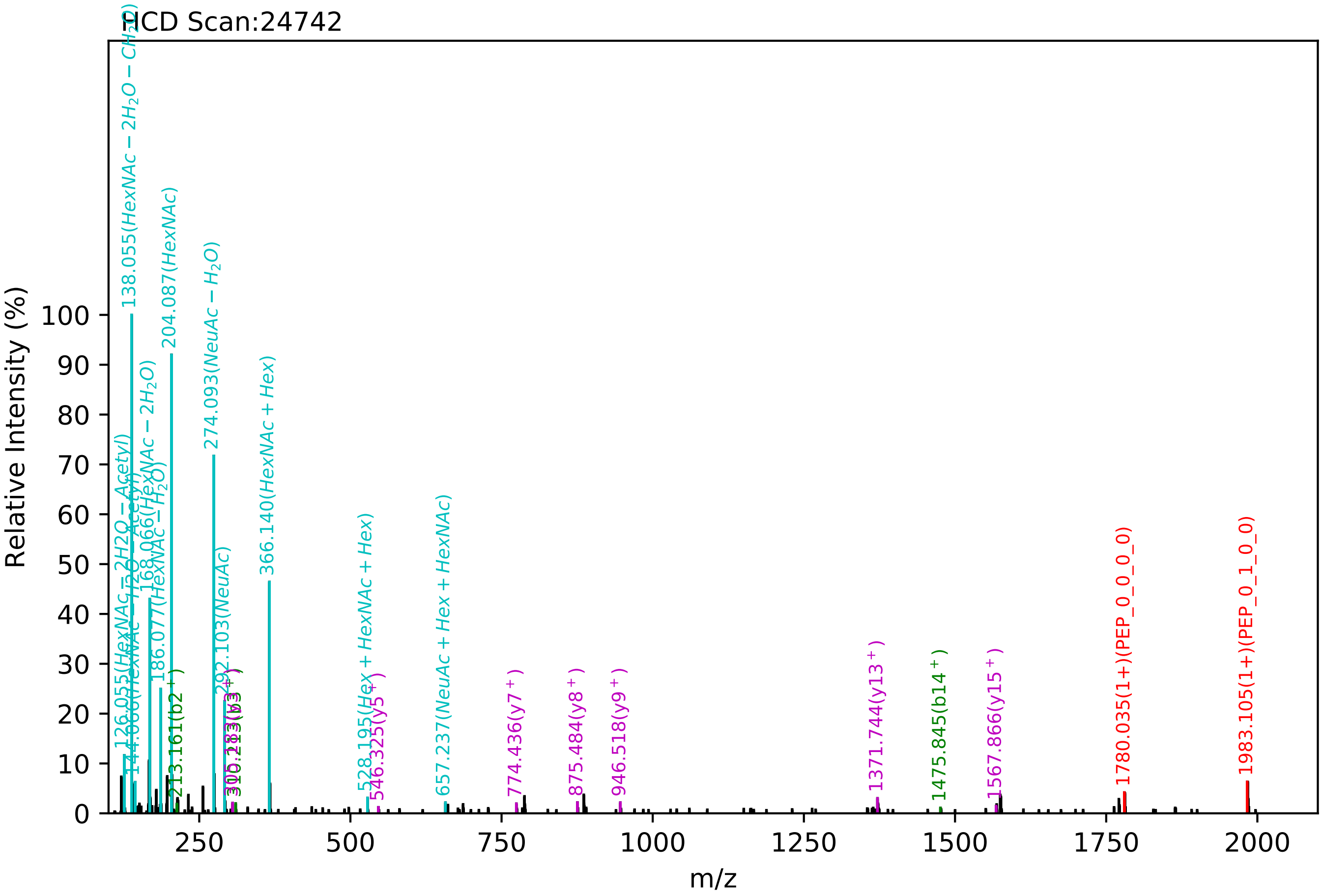

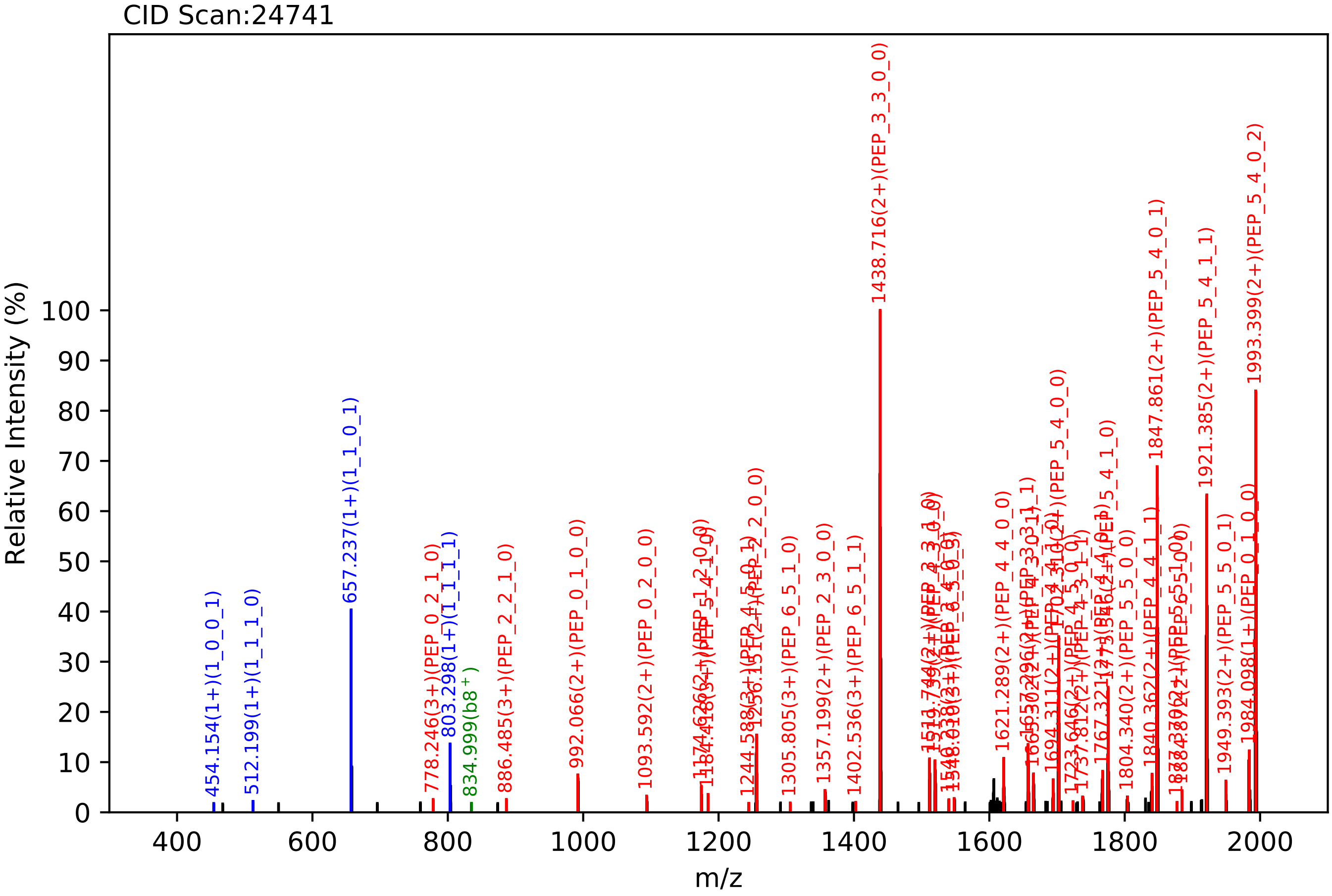

Training set no. 110, Experiment: AGP exp_1

LVPVPITNATLDQITGK(=PEP)_6_5_1_3, m/z:1596.37(3+), RT:62.28, Y-score:85.27

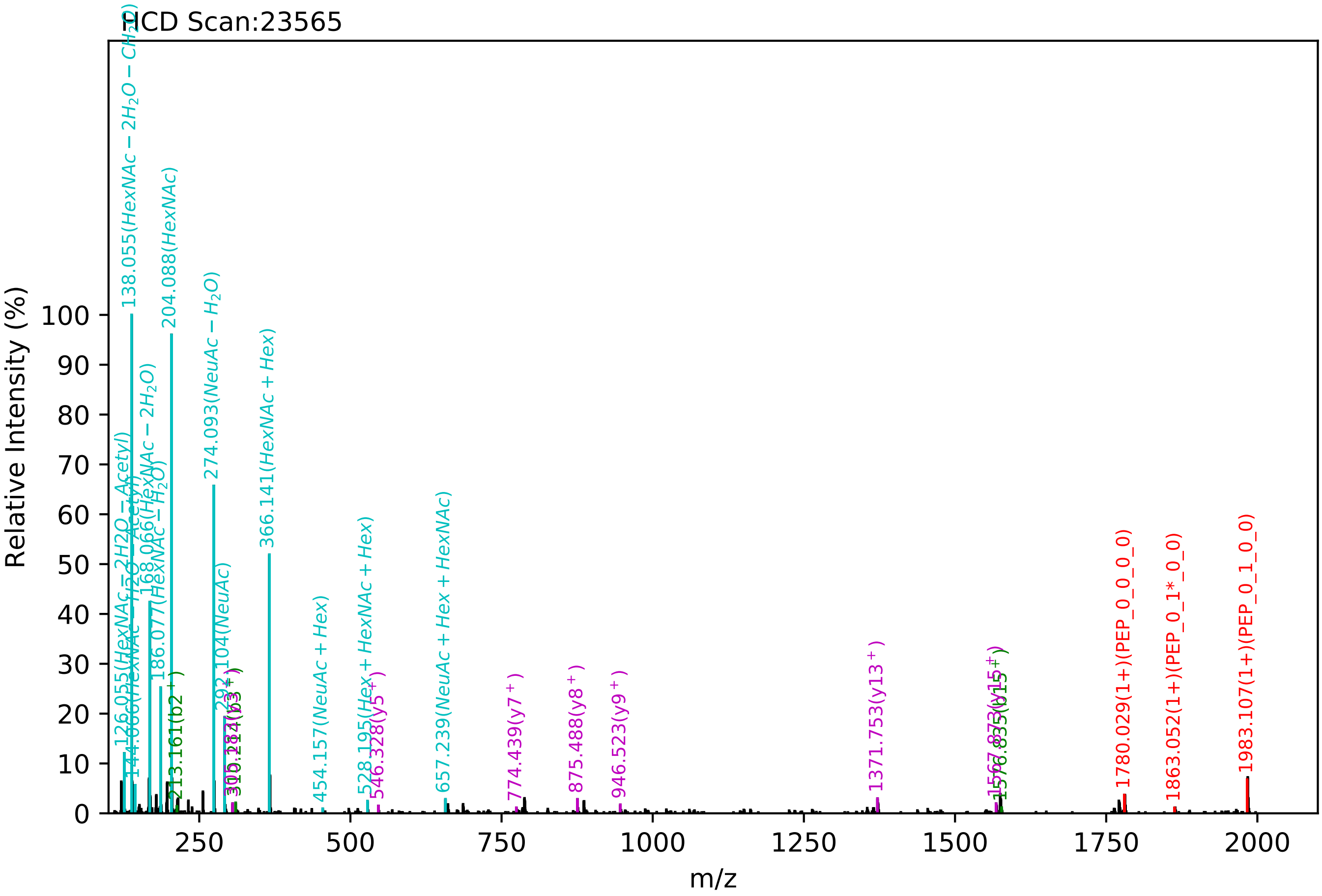

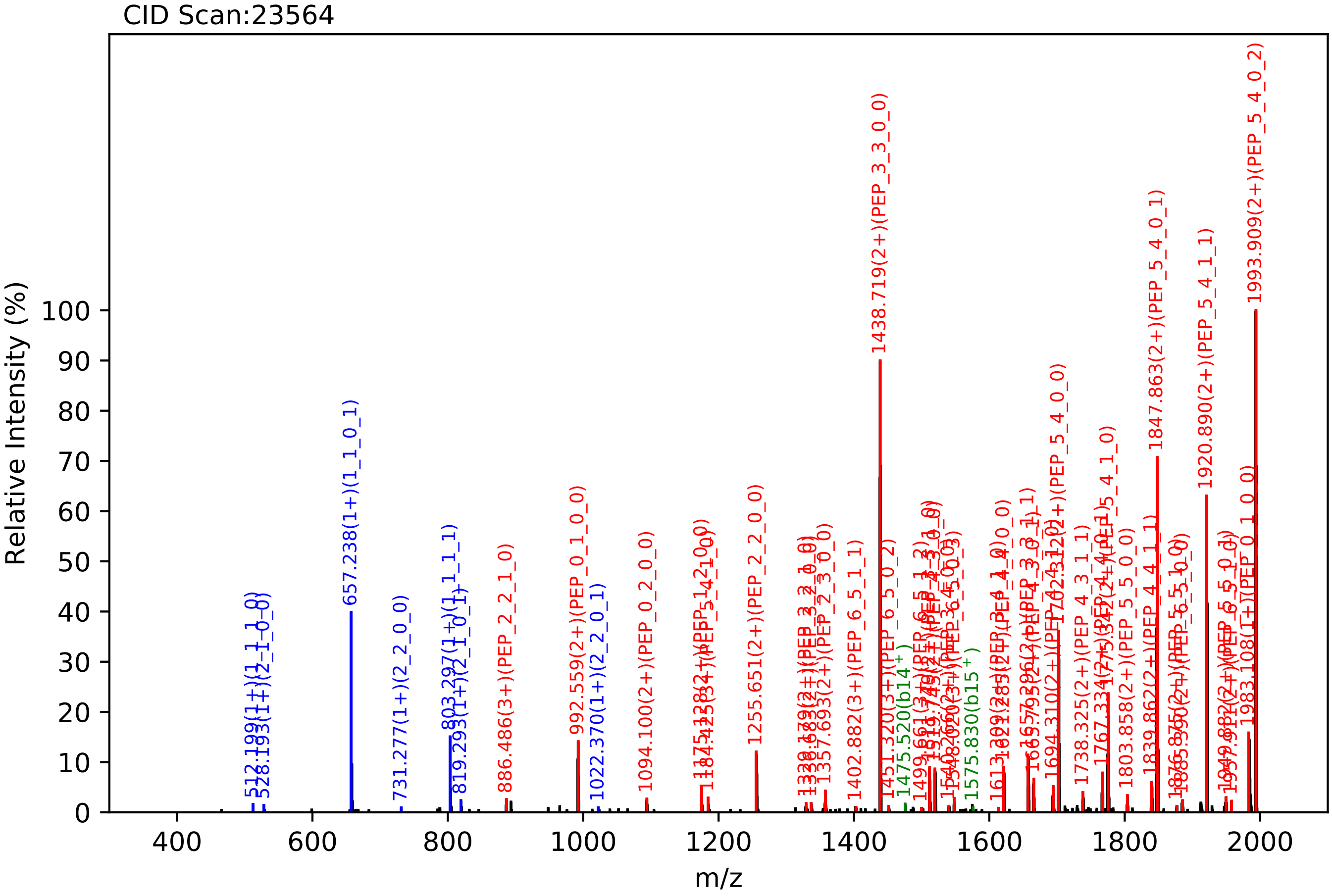

Training set no. 111, Experiment: AGP exp_1

LVPVPITNATLDQITGK(=PEP)_6_5_1_3, m/z:1198.28(4+), RT:62.05, Y-score:85.05

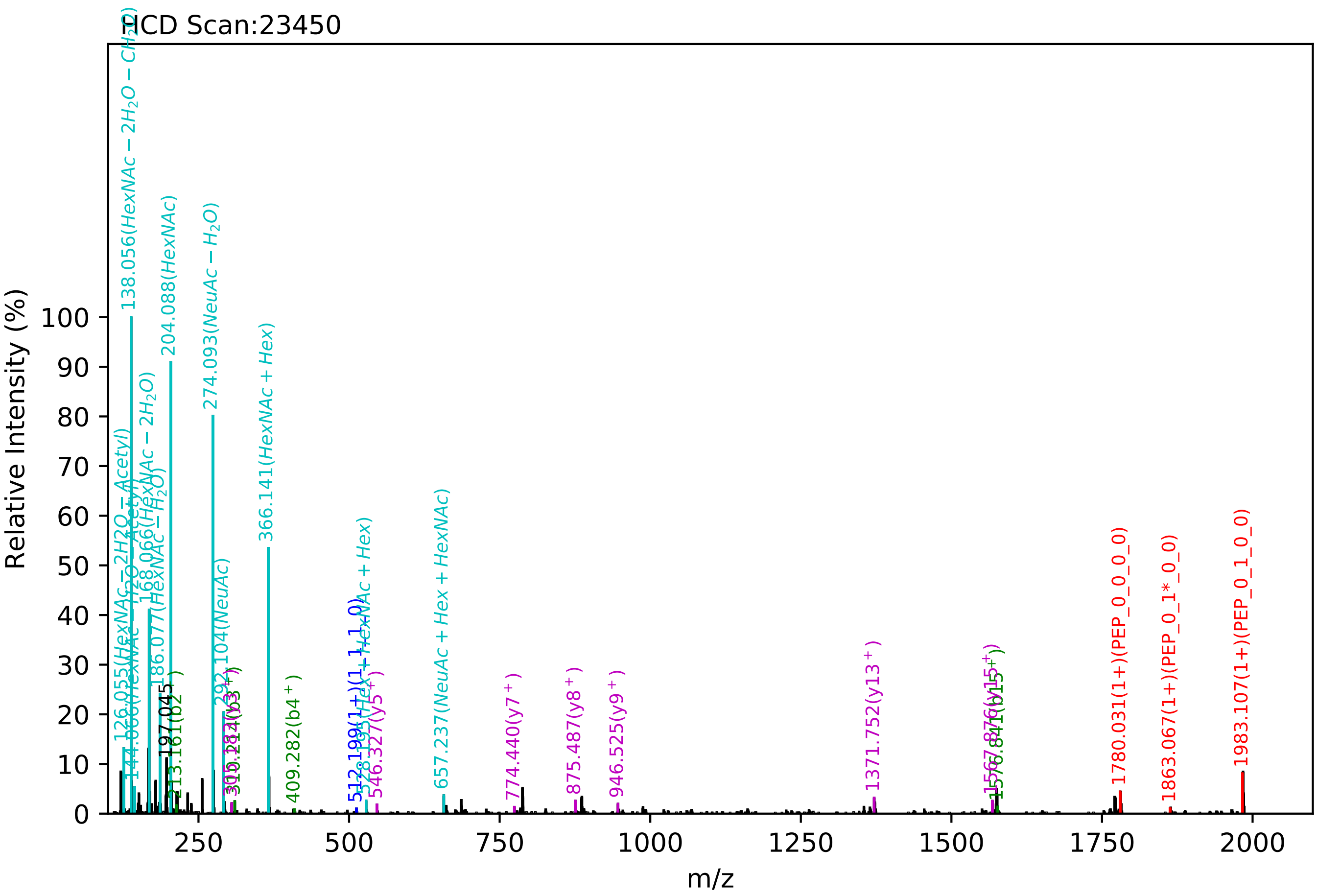

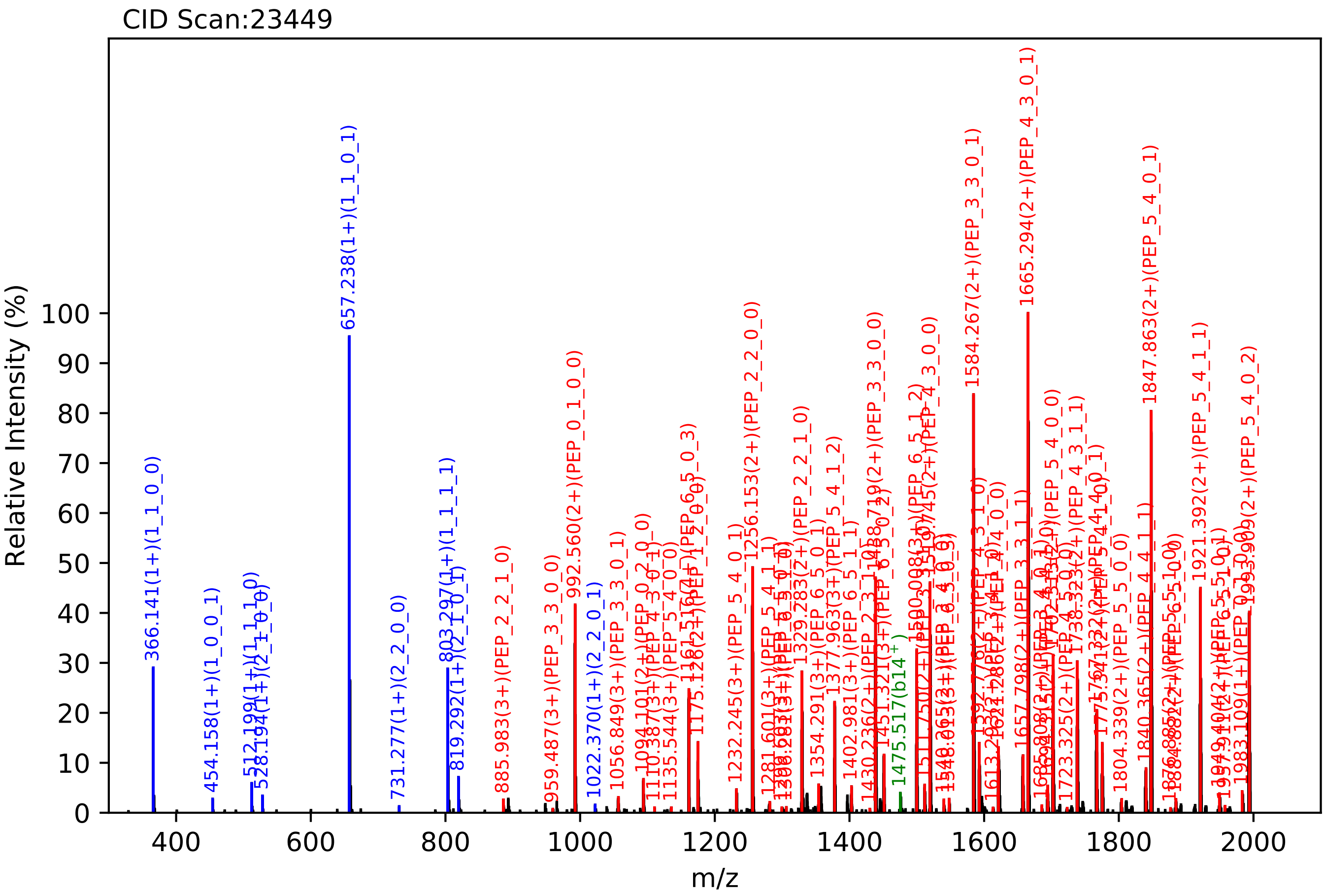

Training set no. 112, Experiment: AGP exp_2

LVPVPITNATLDQITGK(=PEP)_6_5_1_3, m/z:1197.55(4+), RT:62.30, Y-score:84.45

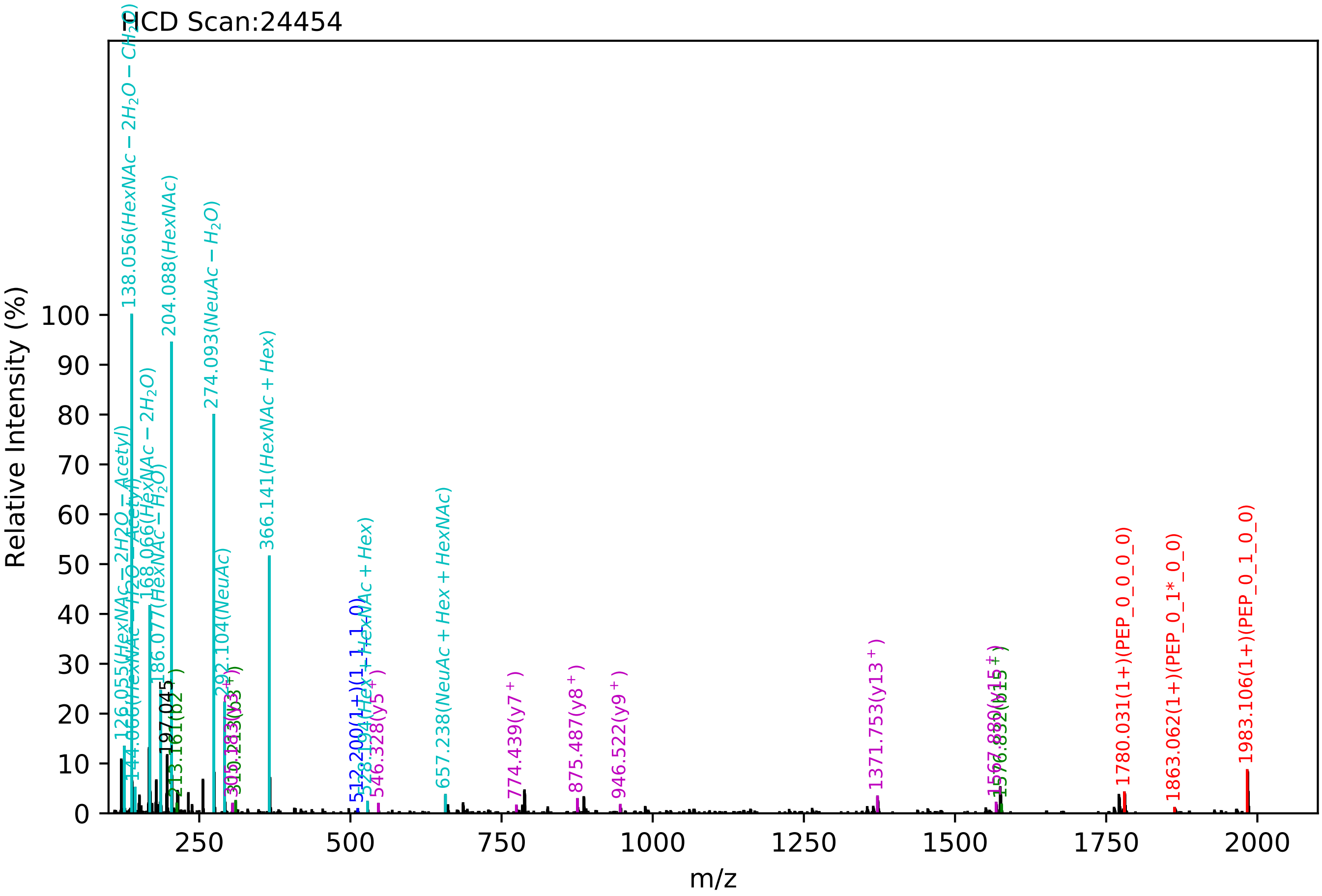

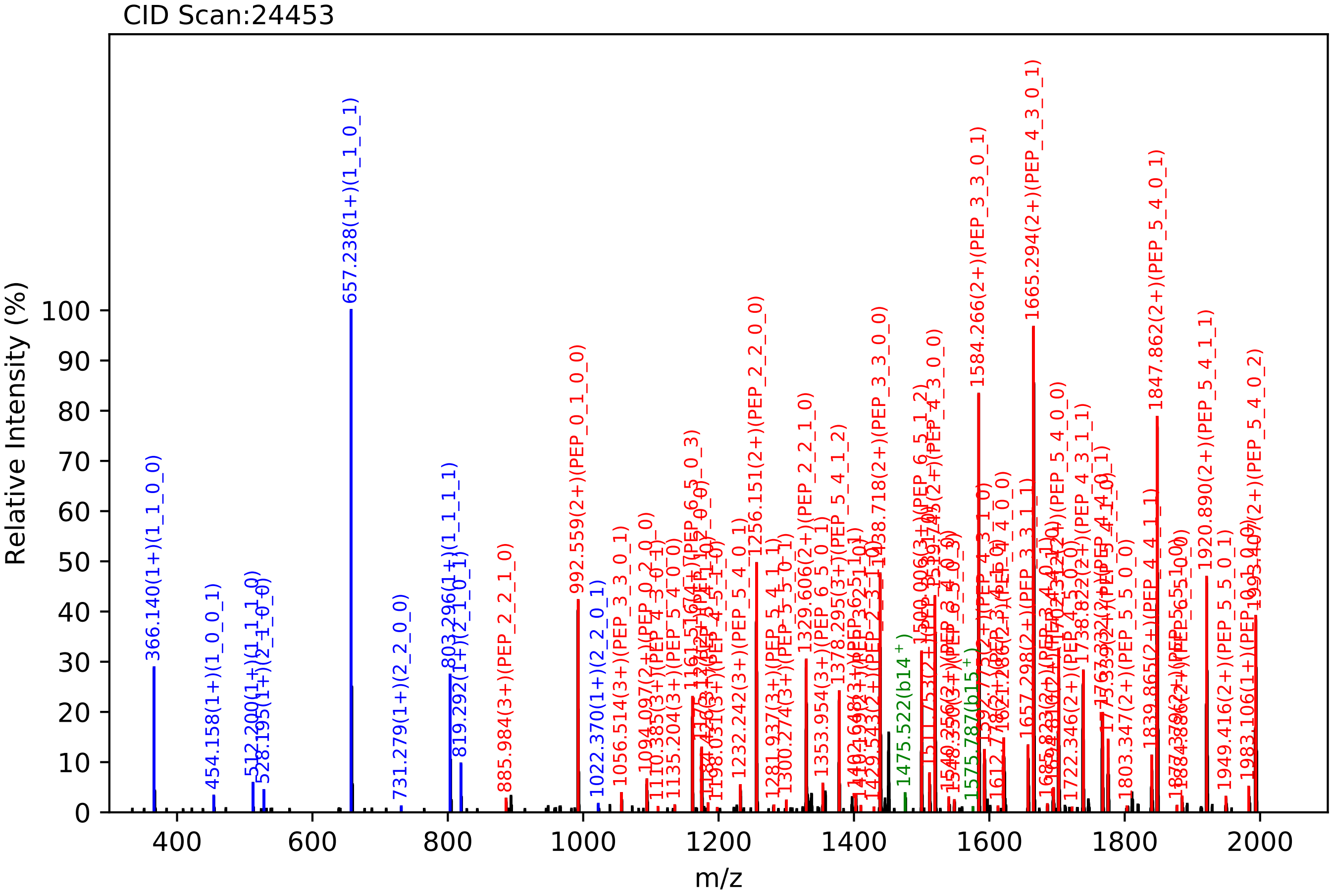

Training set no. 113, Experiment: AGP exp_2

LVPVPITNATLDQITGK(=PEP)_6_5_1_3, m/z:1197.53(4+), RT:62.47, Y-score:83.39

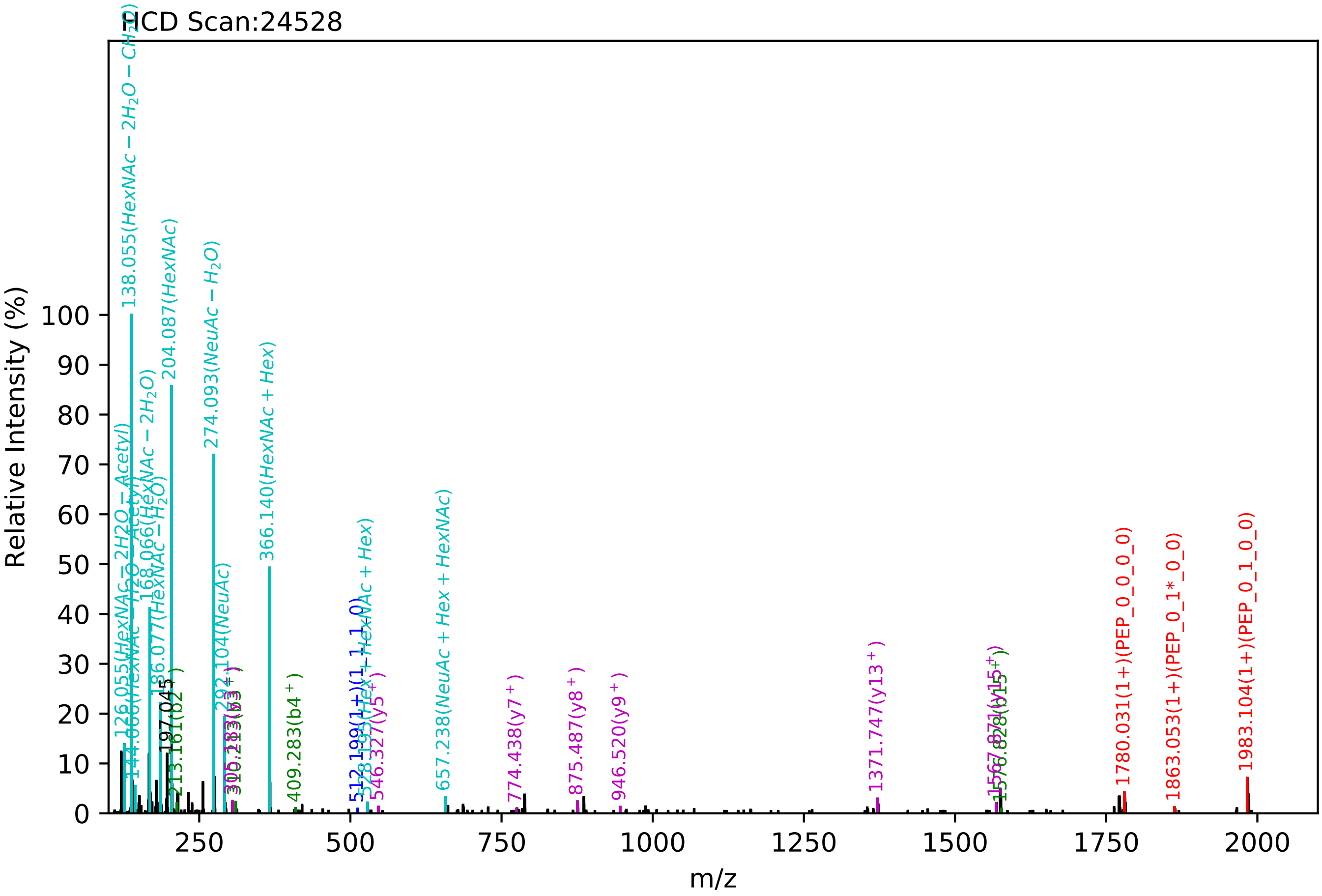

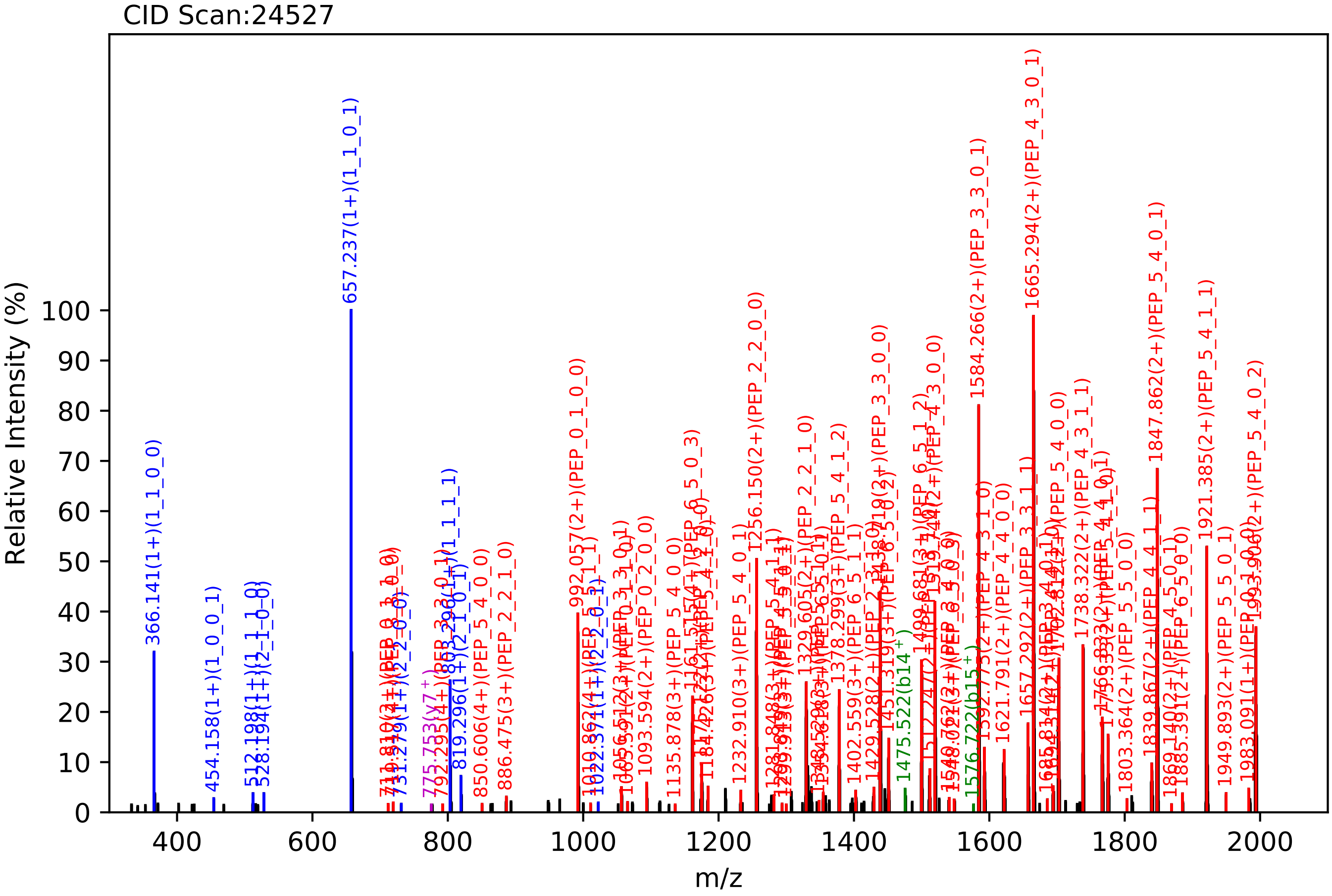

Training set no. 114, Experiment: AGP exp_1

LVPVPITNATLDQITGK(=PEP)_6_5_1_3, m/z:1197.53(4+), RT:62.43, Y-score:82.53

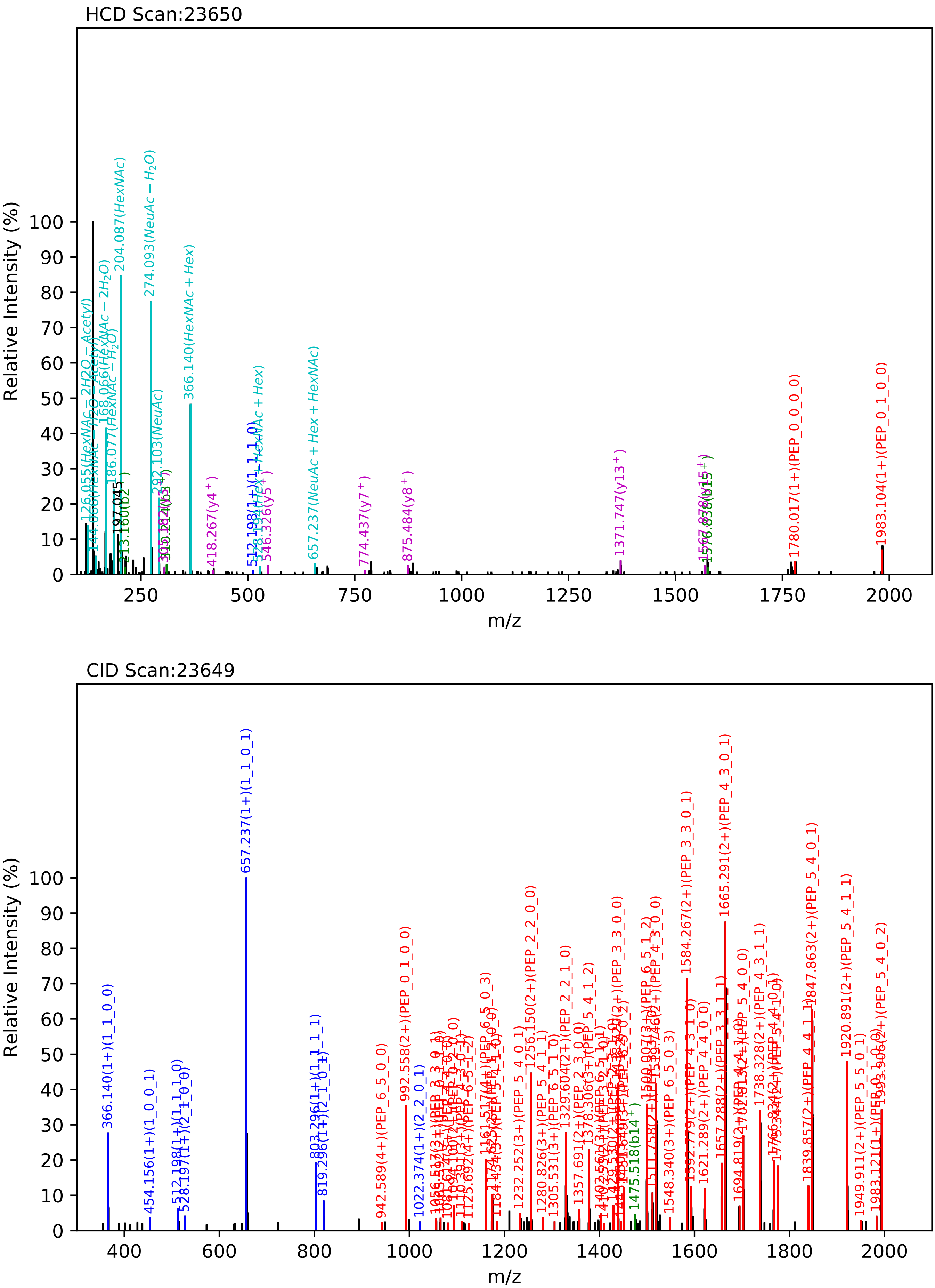

Training set no. 115, Experiment: AGP exp_2

LVPVPITNATLDQITGK(=PEP)_6_5_1_3, m/z:1197.53(4+), RT:62.98, Y-score:79.11

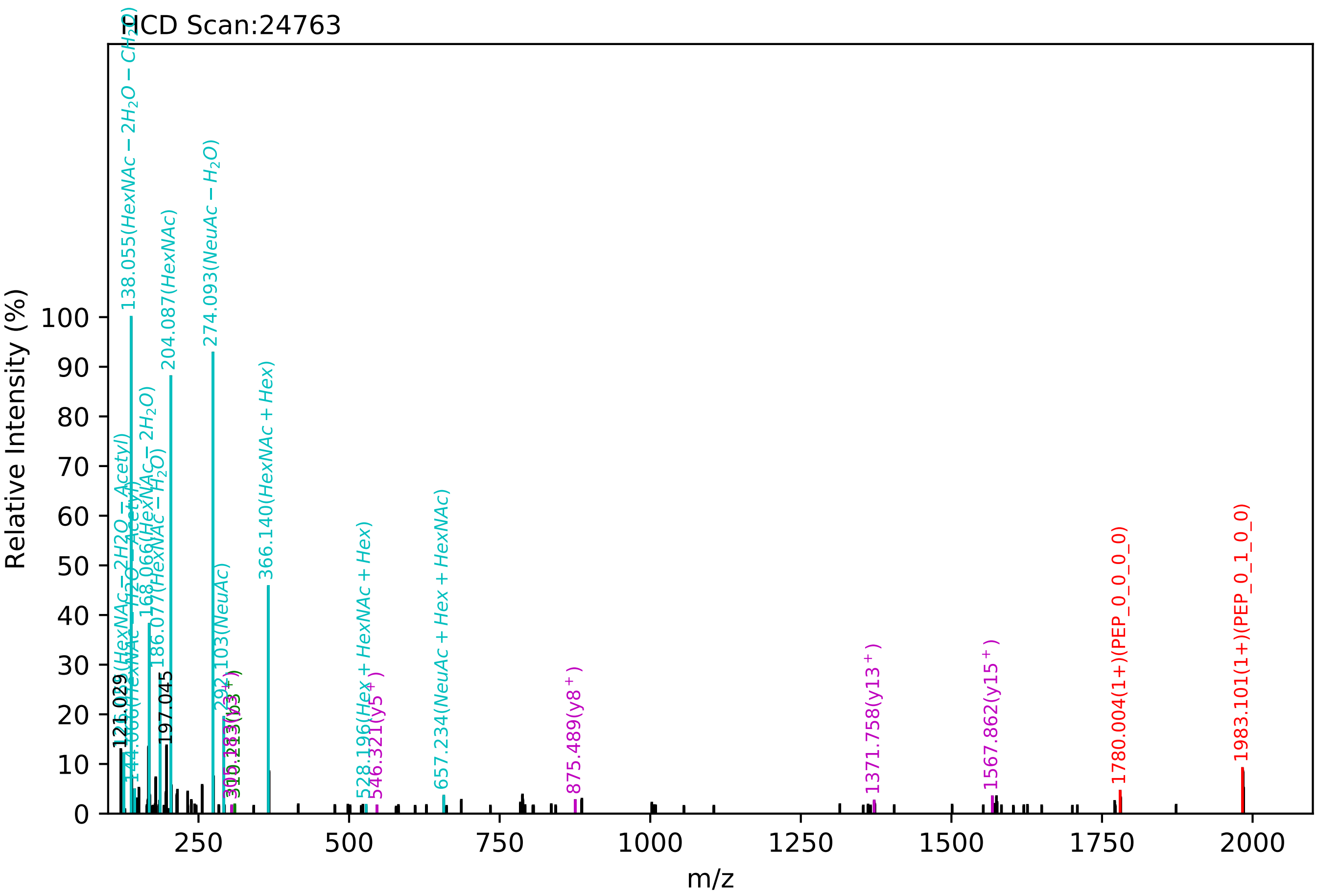

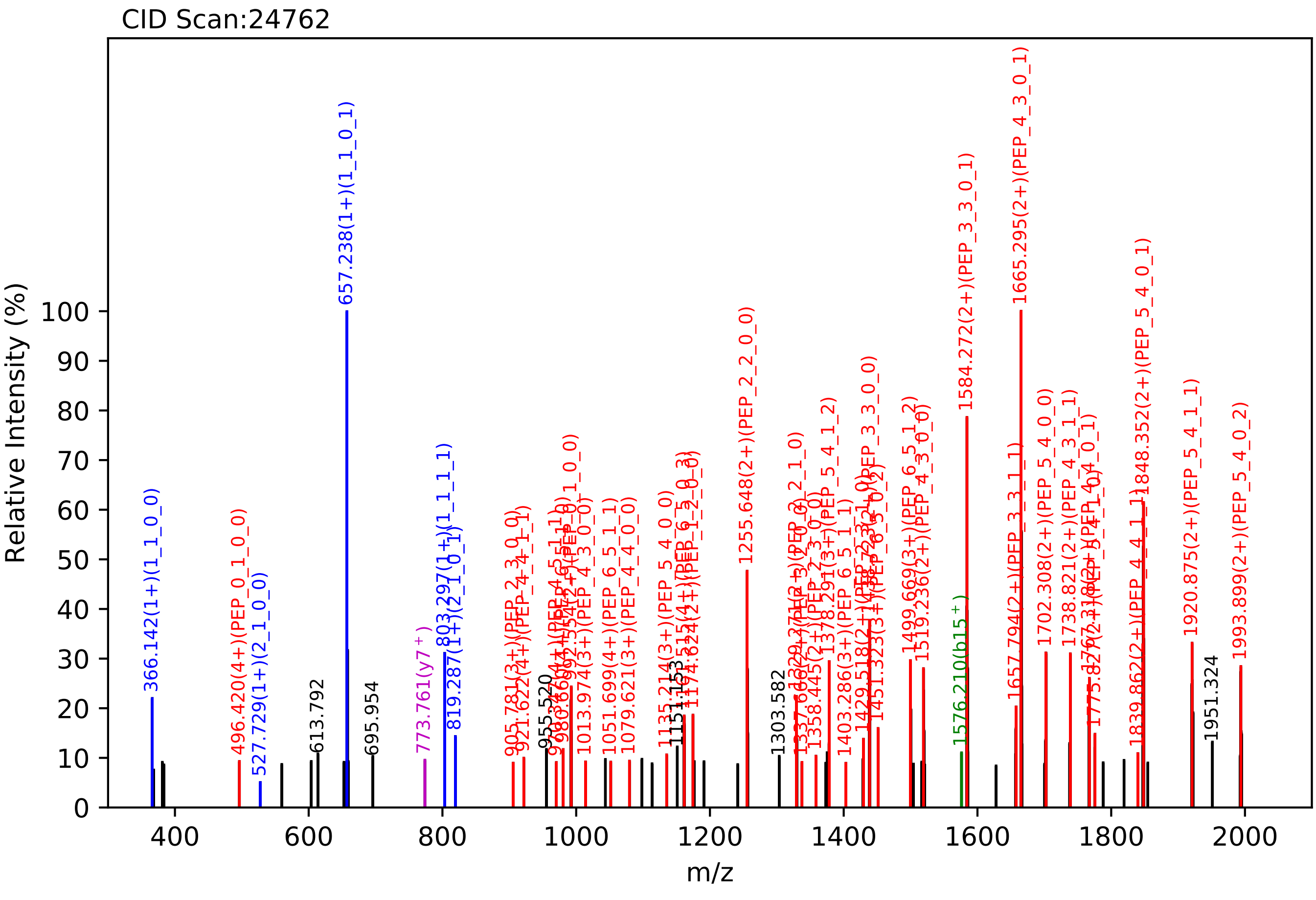

Training set no. 116, Experiment: AGP exp_19

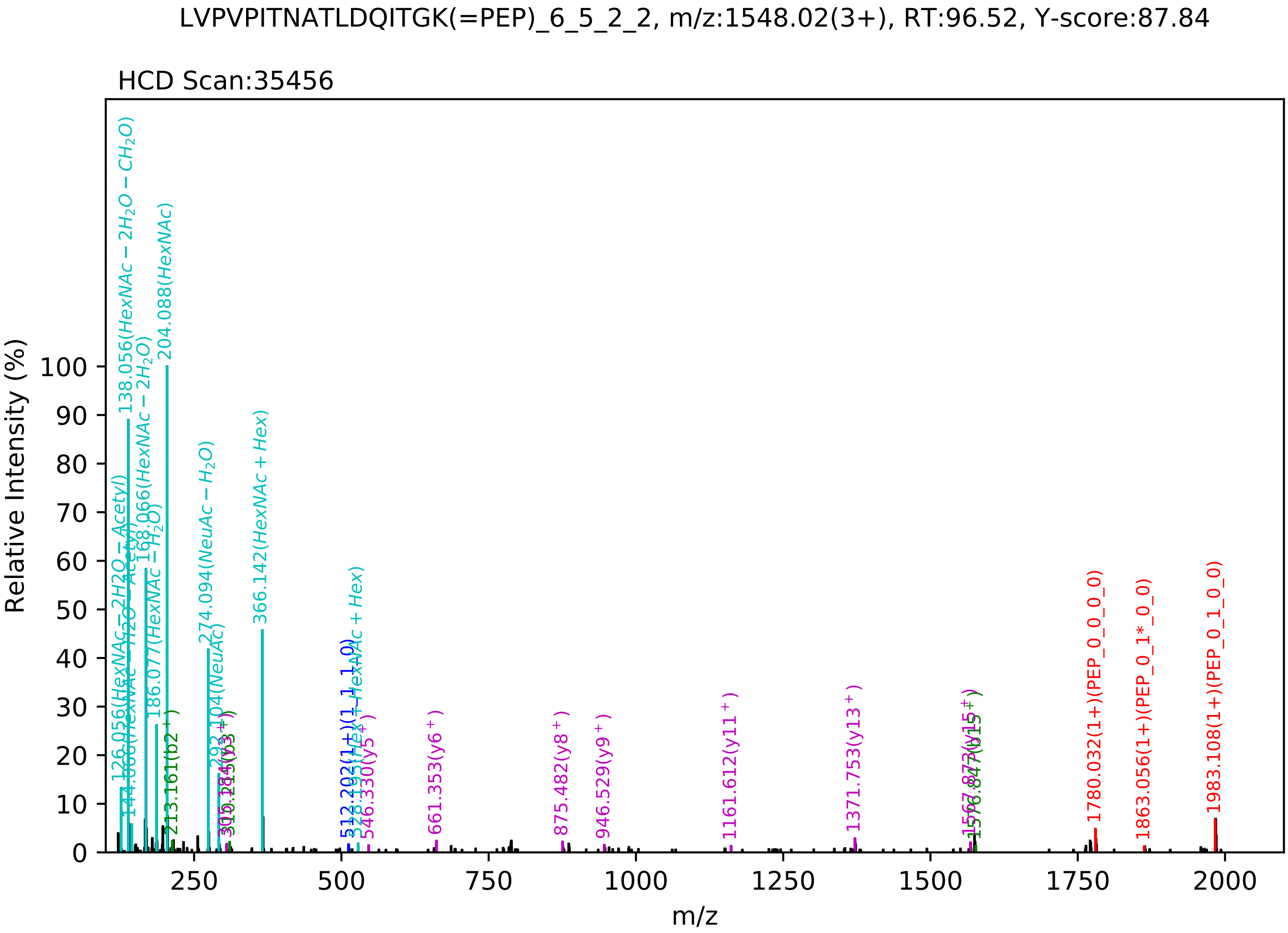

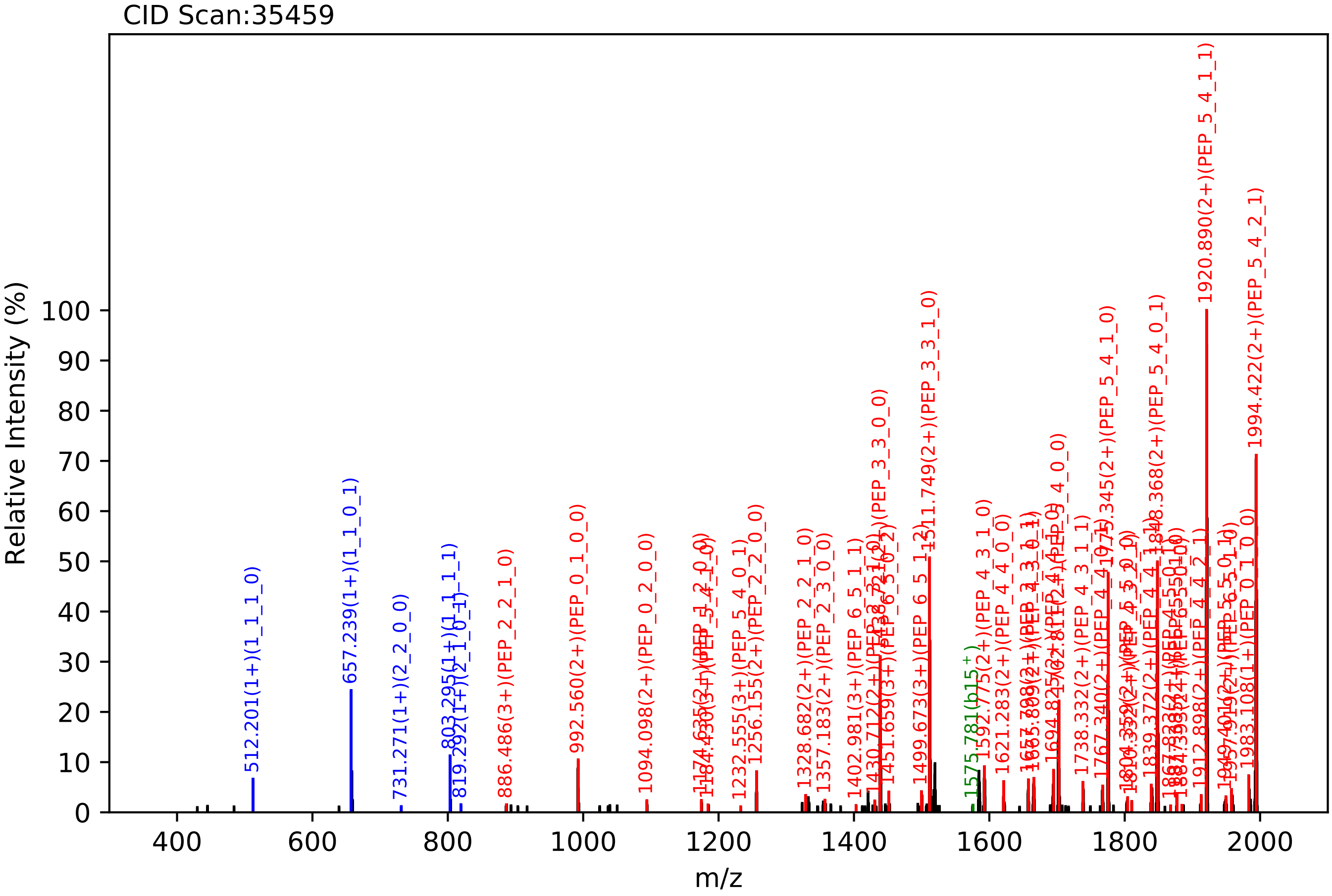

Training set no. 117, Experiment: AGP exp_11

LVPVPITNATLDQITGK(=PEP)_6_5_2_3, m/z:1234.04(4+), RT:82.88, Y-score:79.26

HCD Scan:29918

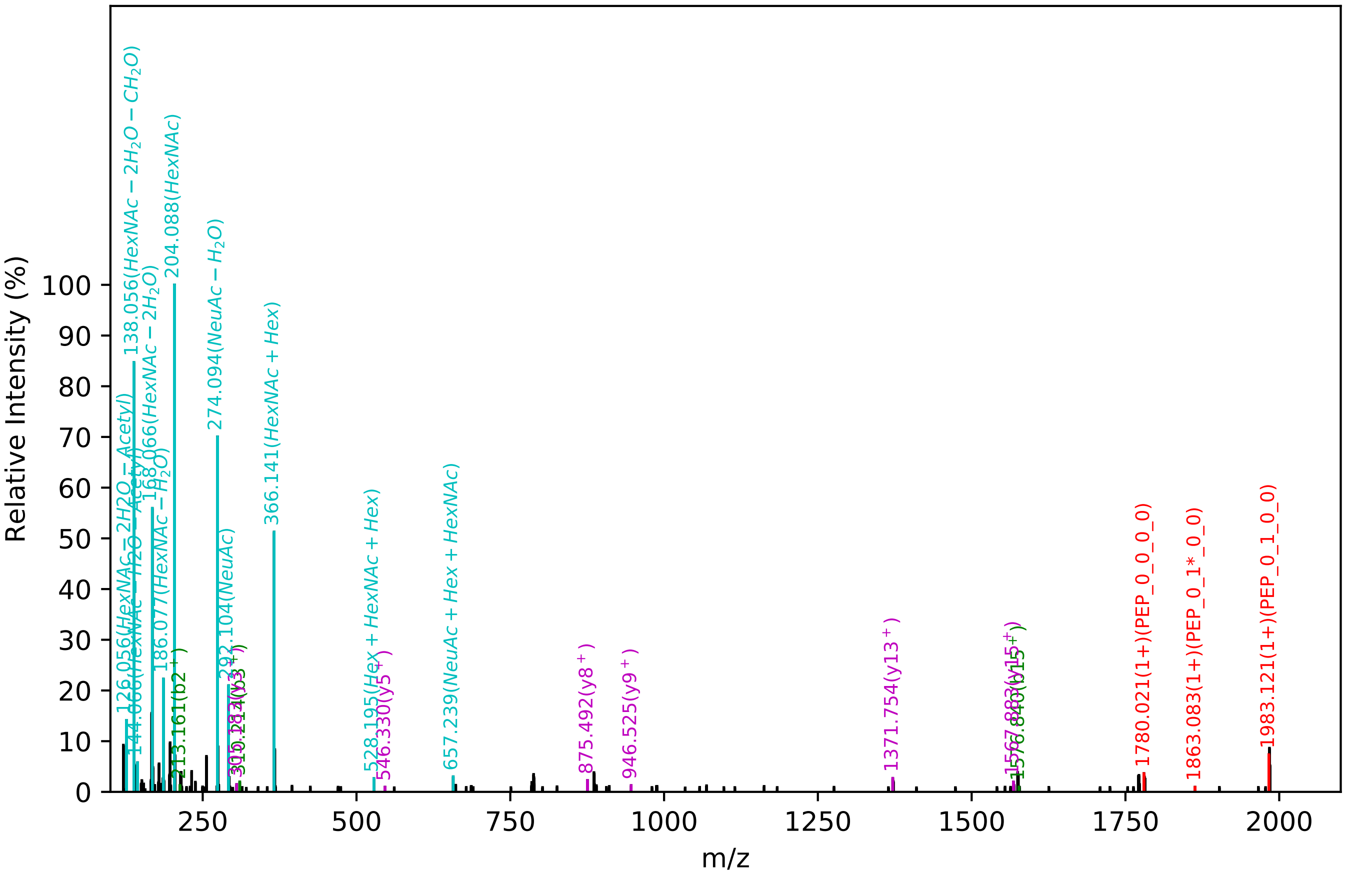

CID Scan:29920

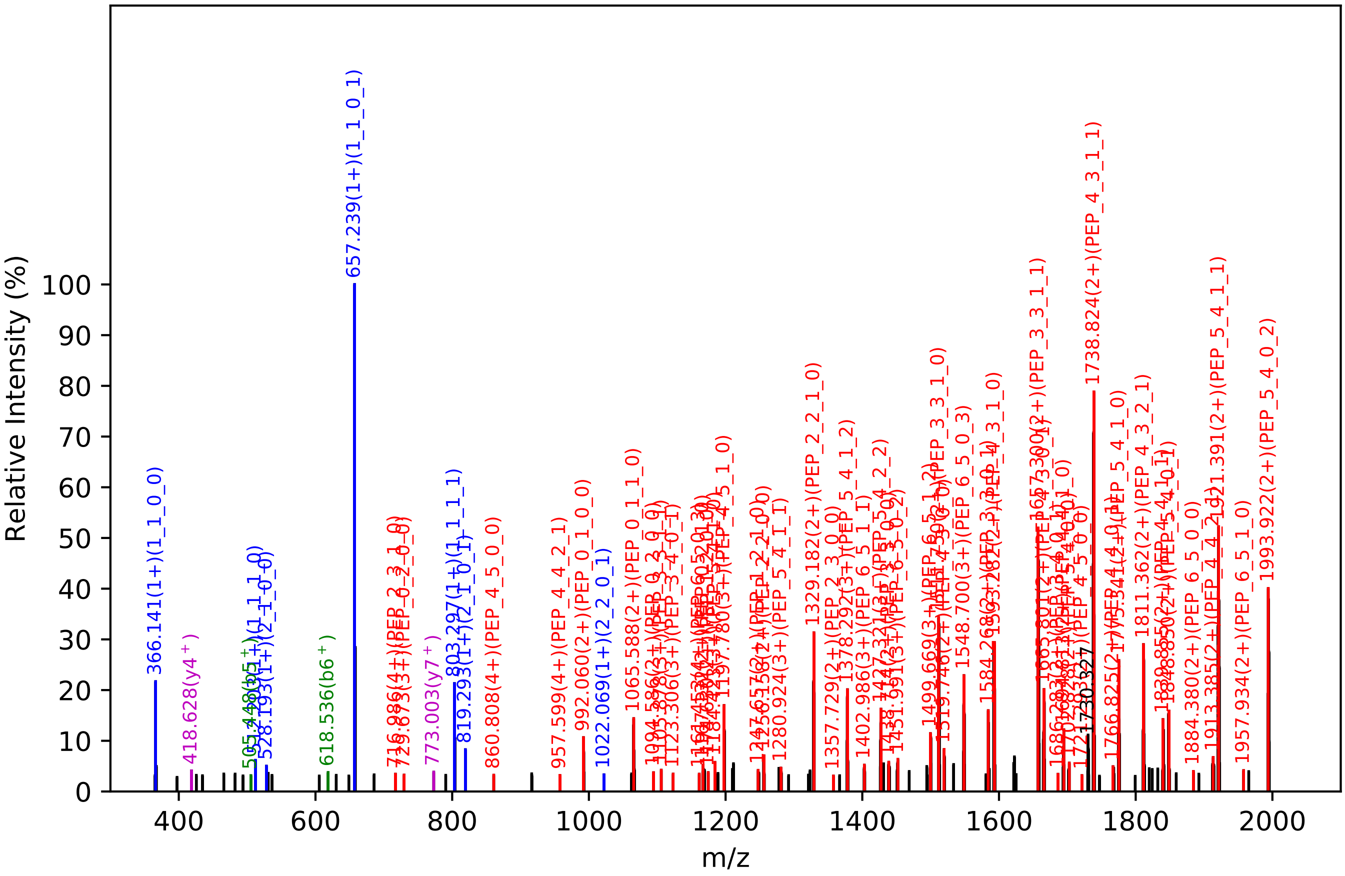

Training set no. 118, Experiment: AGP exp_1

NEEYNK(=PEP)_4_3_0_0, m/z:1027.40(2+), RT:15.42, Y-score:88.87

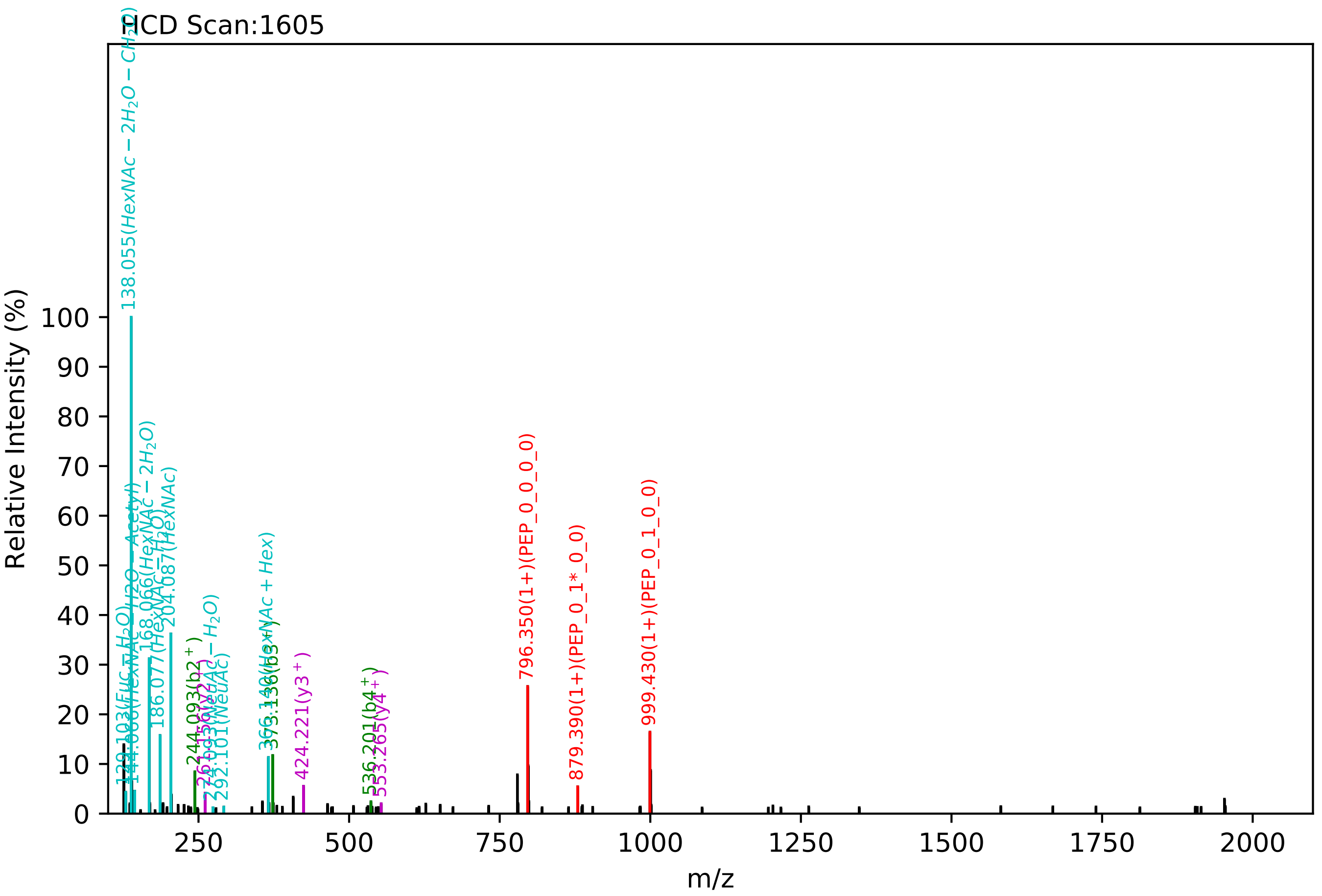

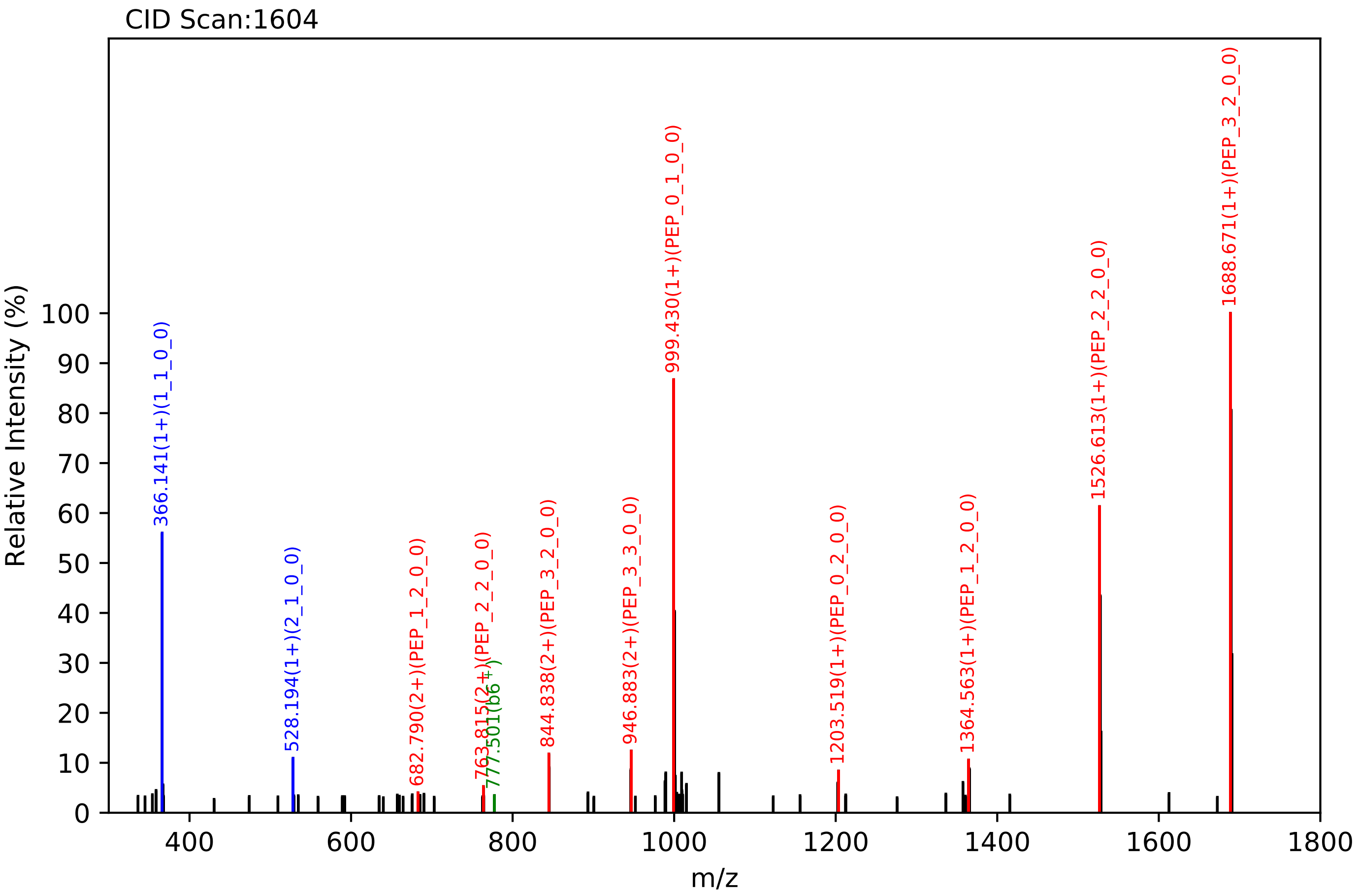

Training set no. 119, Experiment: AGP exp_2

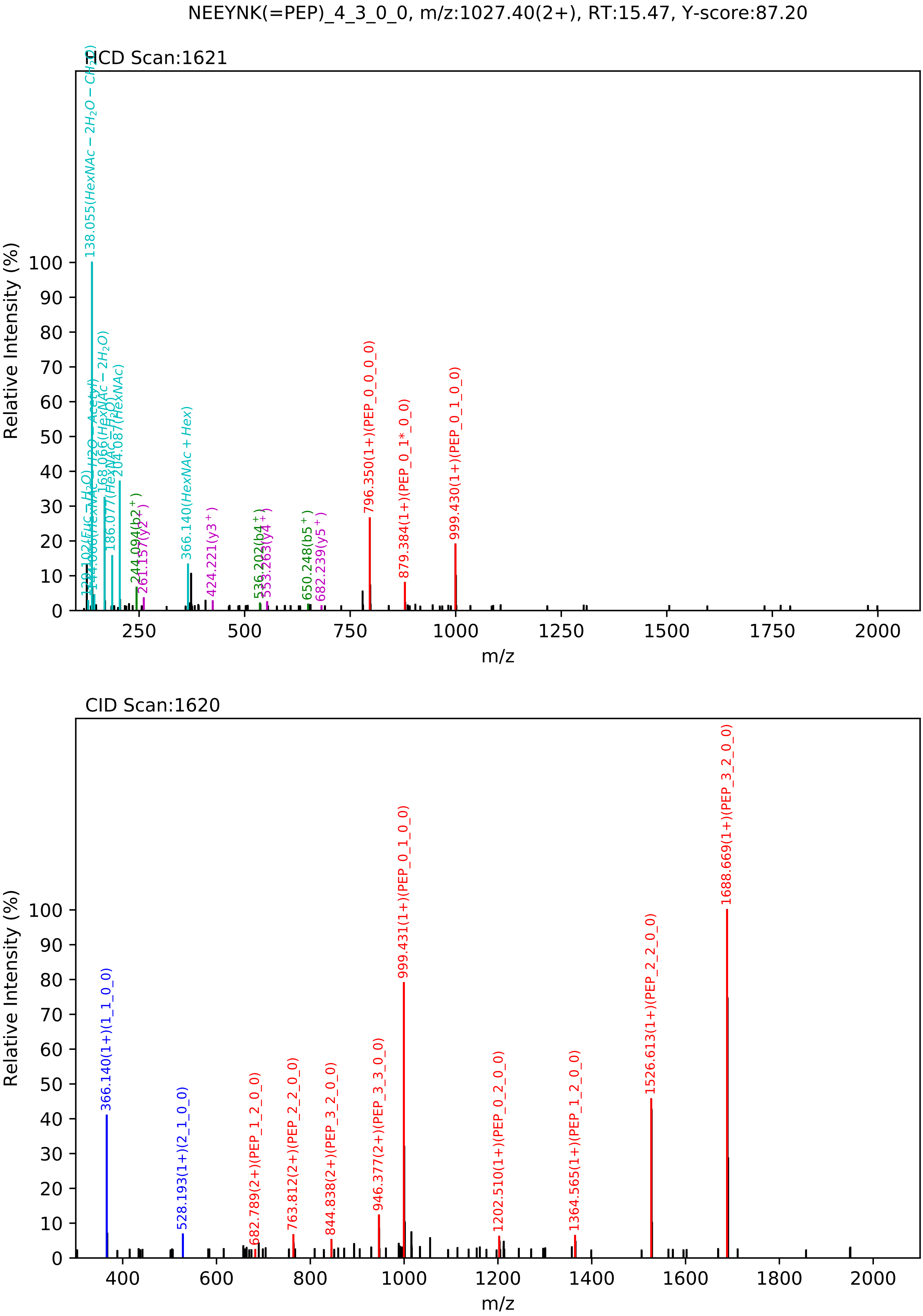

Training set no. 120, Experiment: AGP exp_2

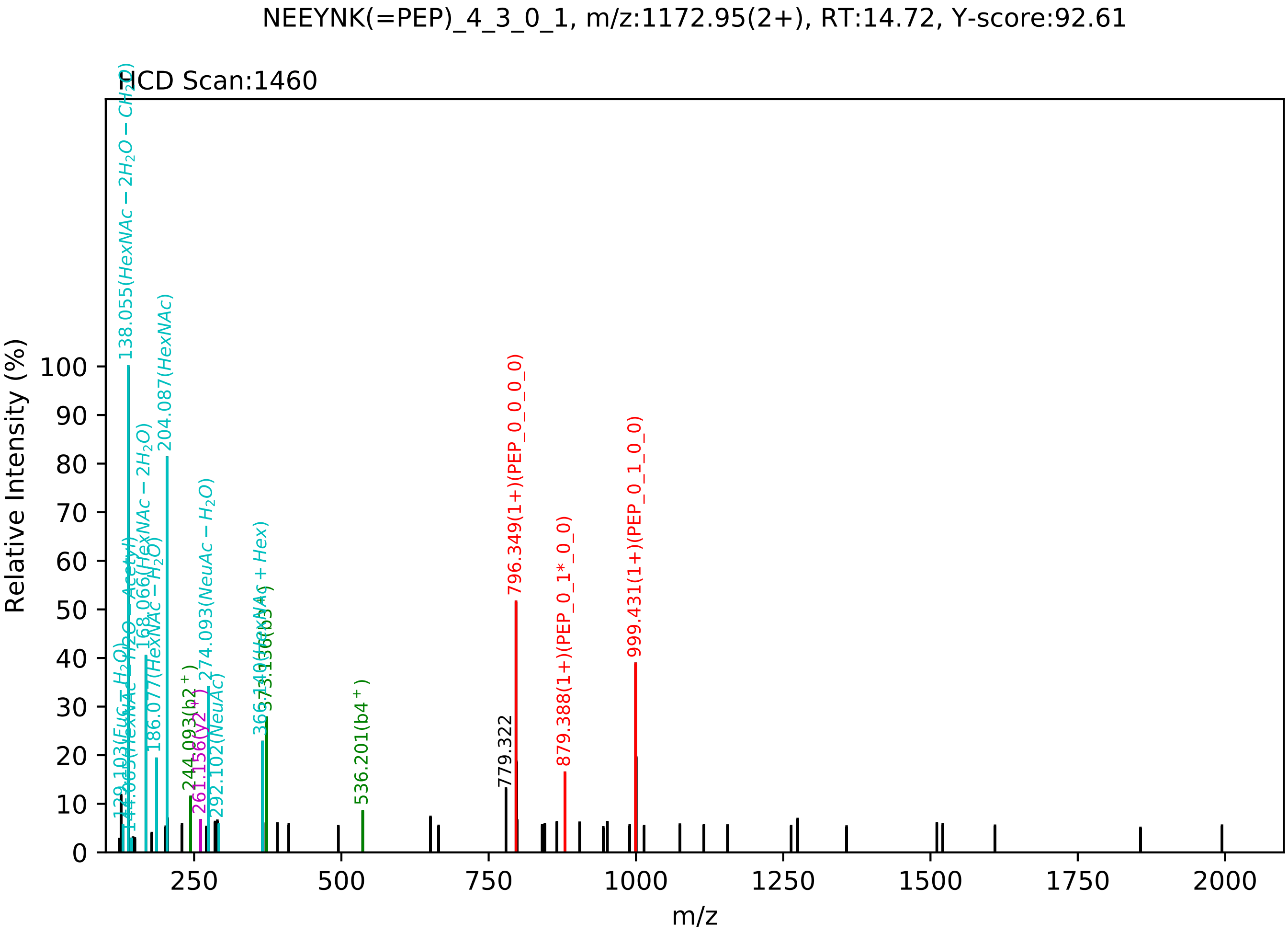

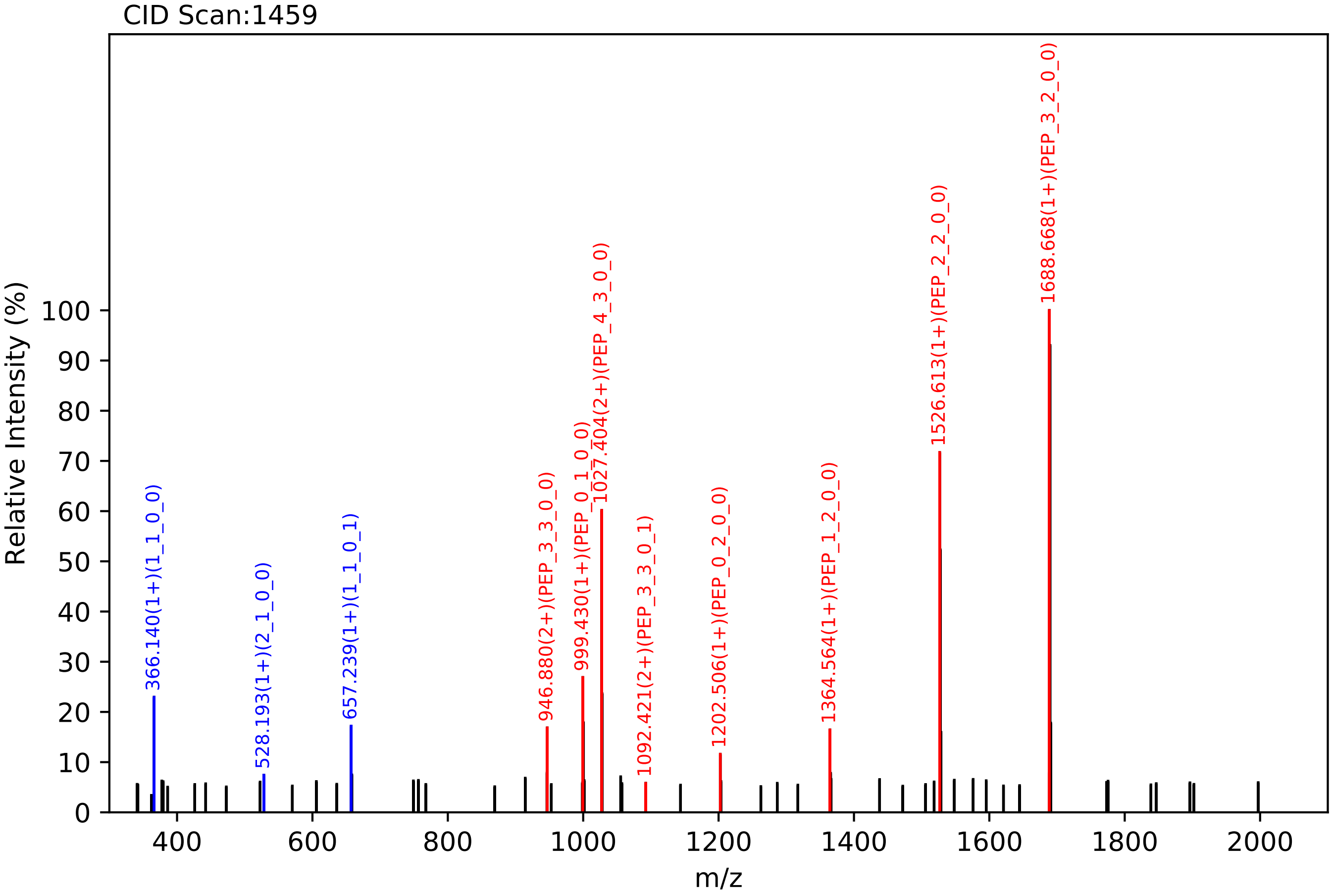

Training set no. 121, Experiment: AGP exp_2

NEEYNK(=PEP)_4_3_0_1, m/z:1172.95(2+), RT:14.71, Y-score:90.59

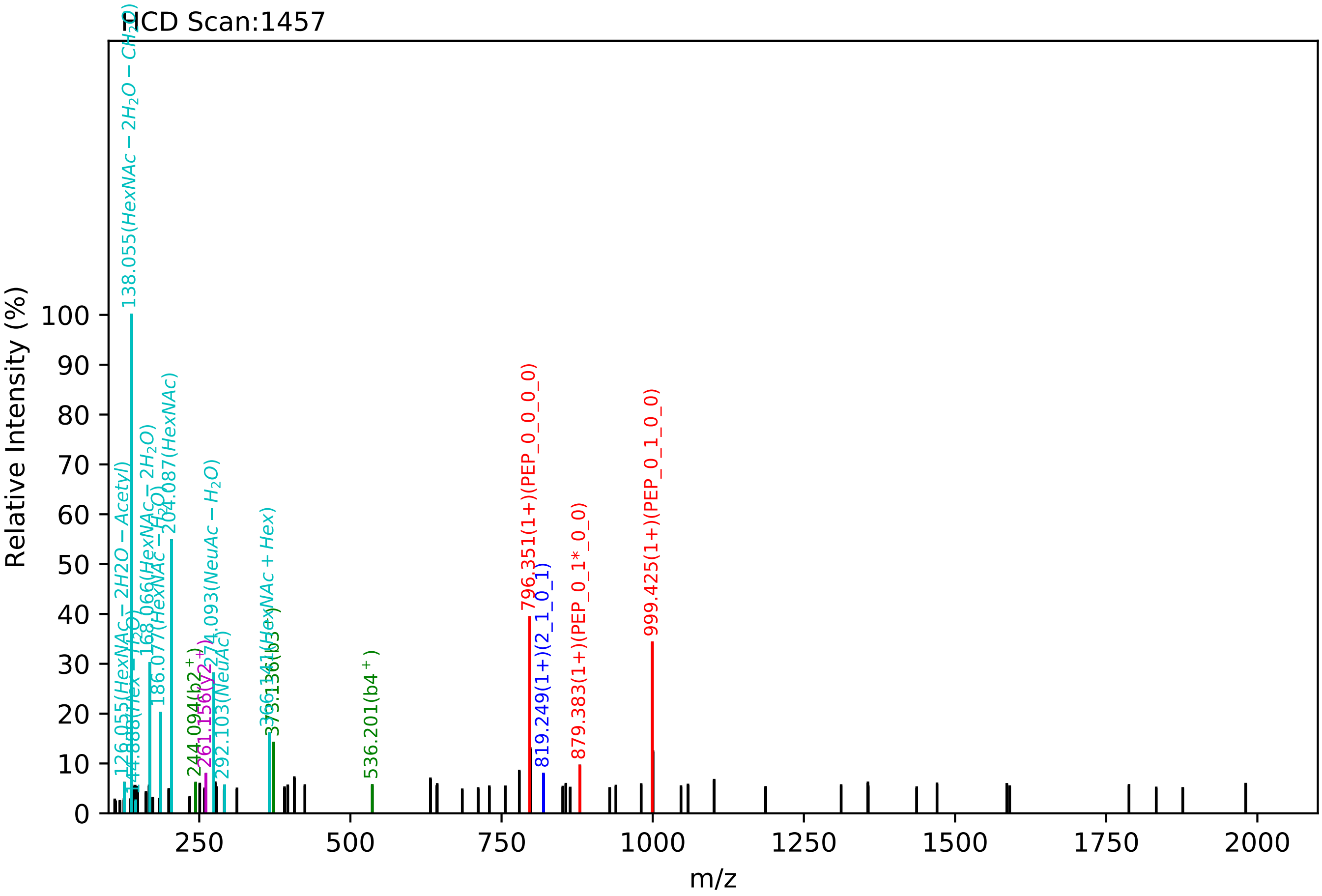

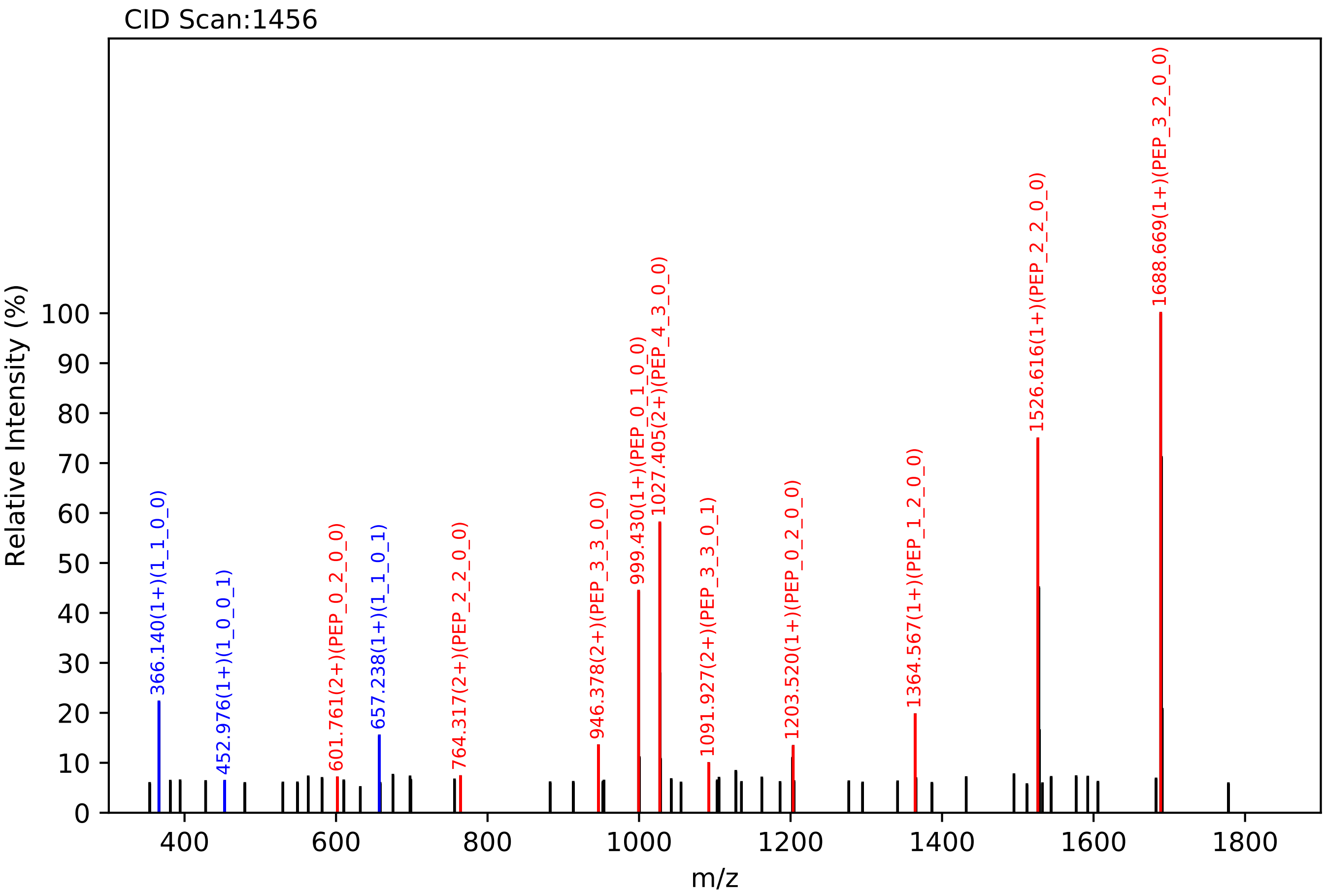

Training set no. 122, Experiment: AGP exp_2

NEEYNK(=PEP)_4_3_0_1, m/z:1172.95(2+), RT:16.35, Y-score:90.19

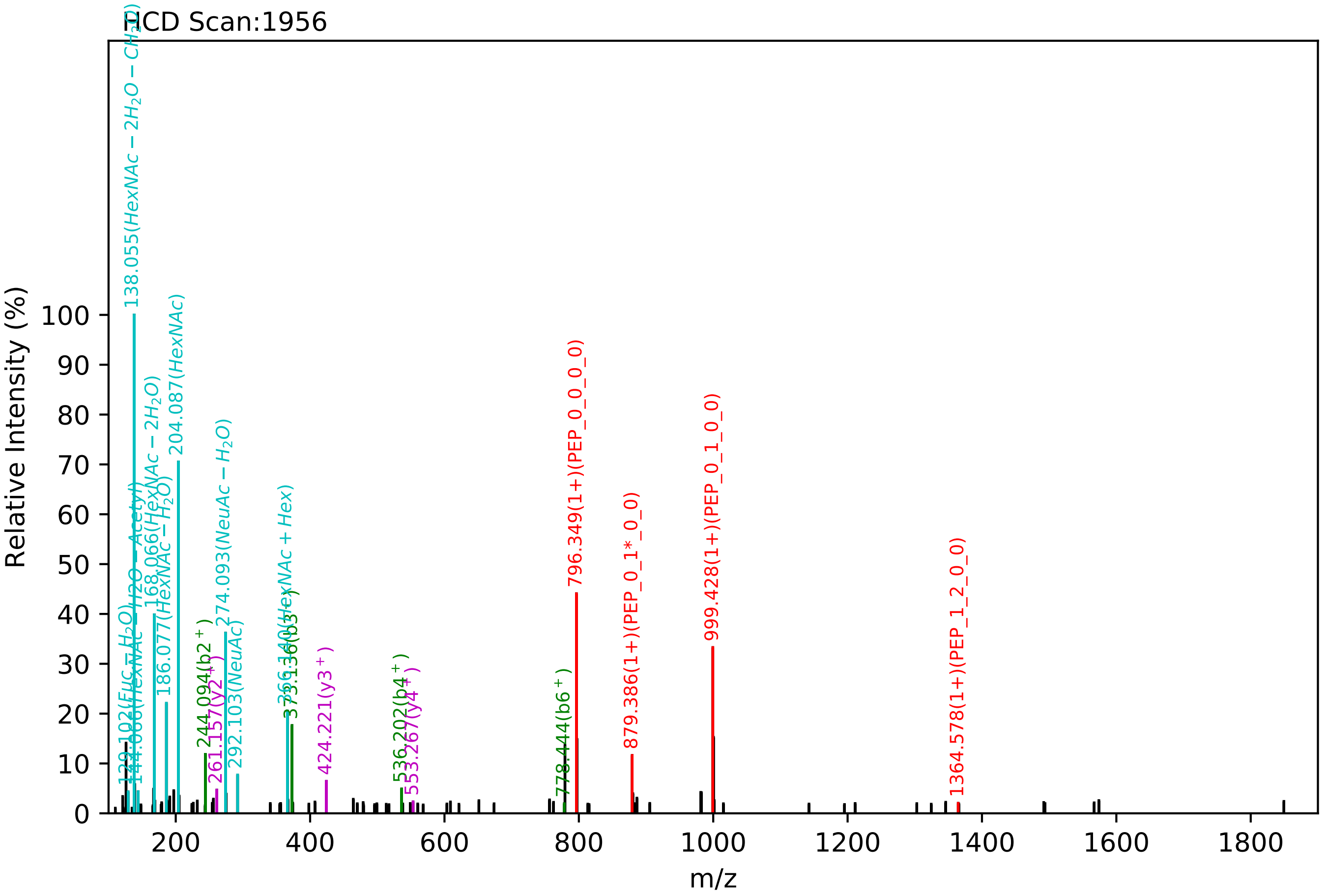

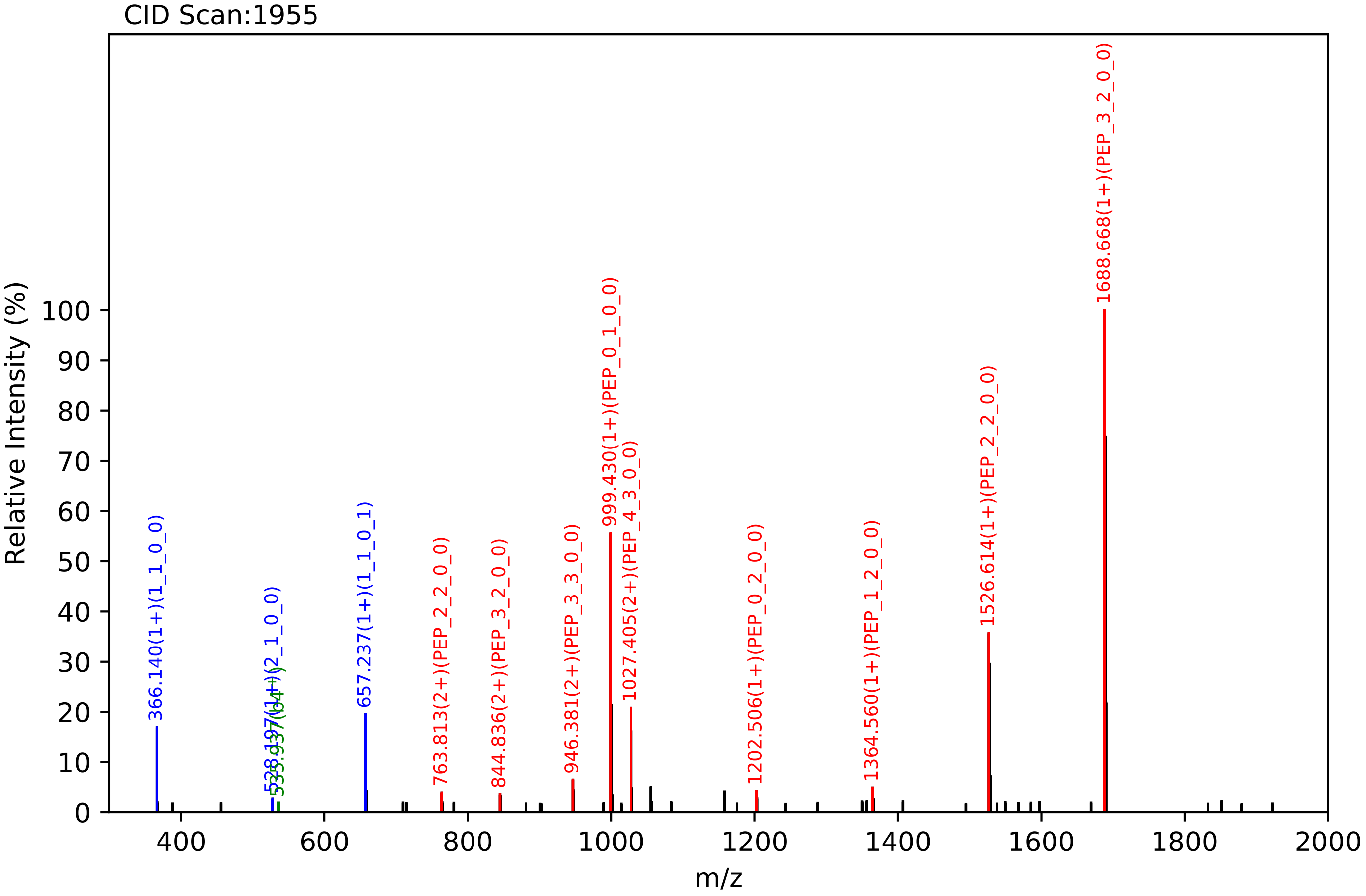

Training set no. 123, Experiment: AGP exp_1

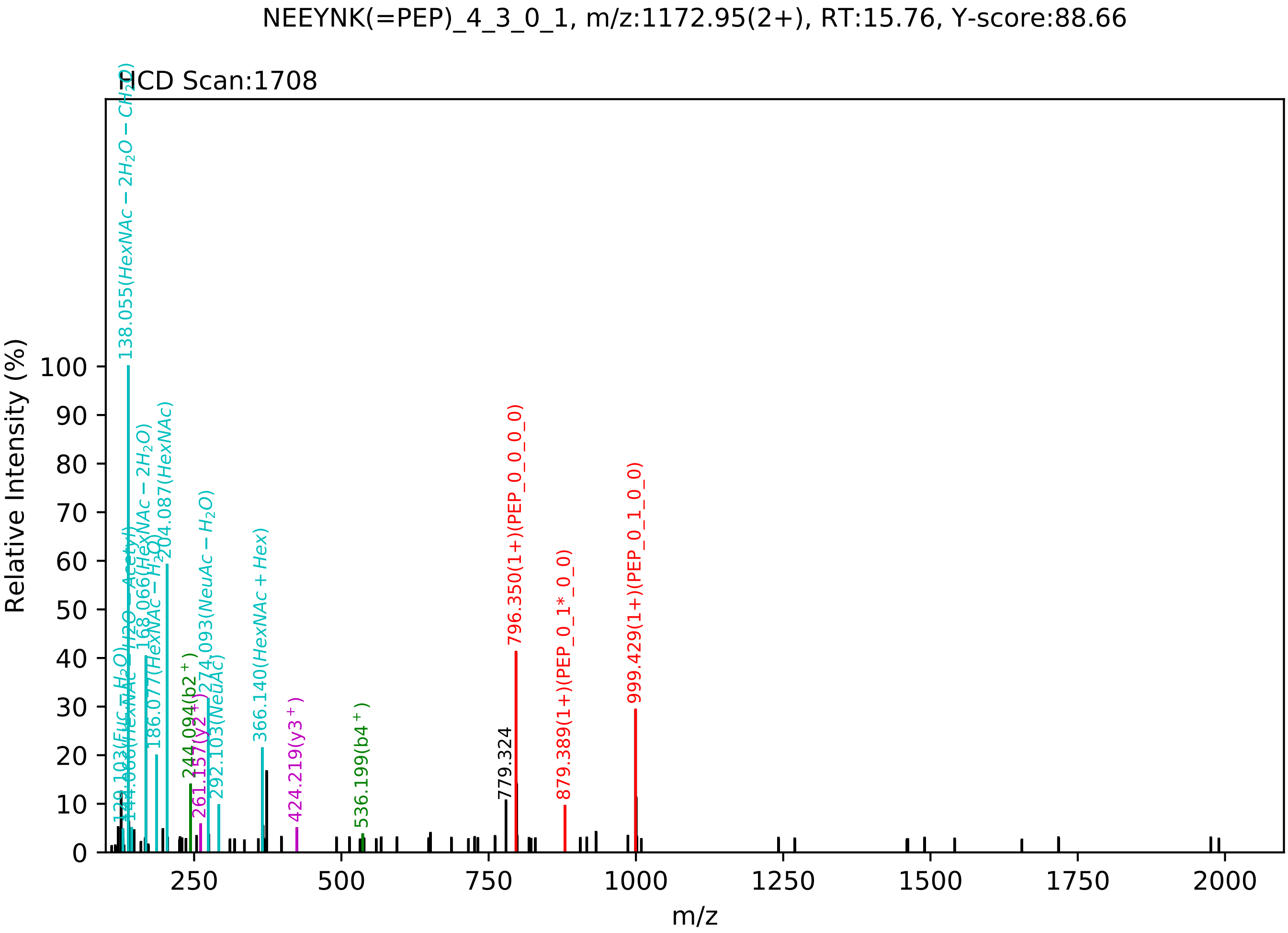

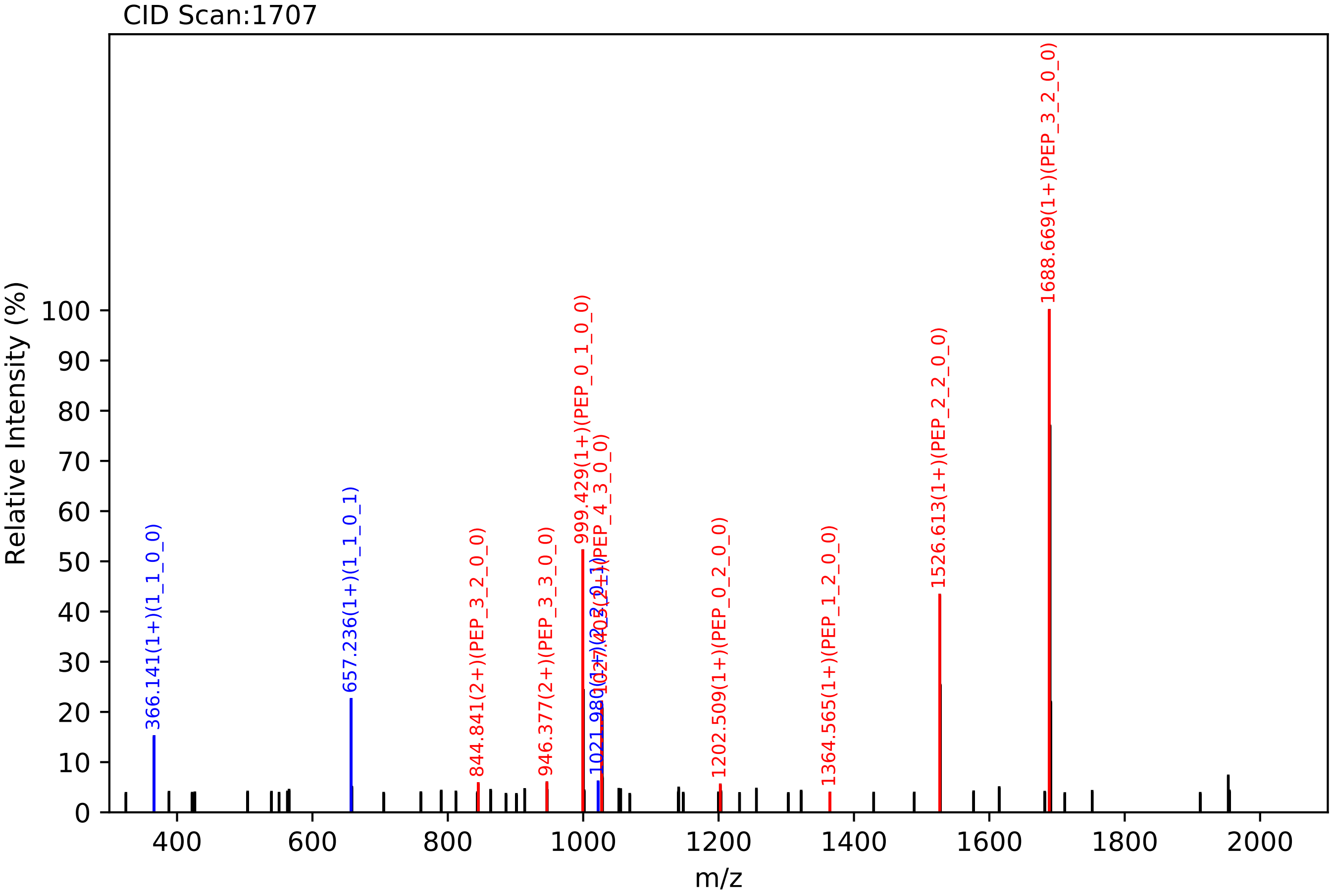

Training set no. 124, Experiment: AGP exp_1

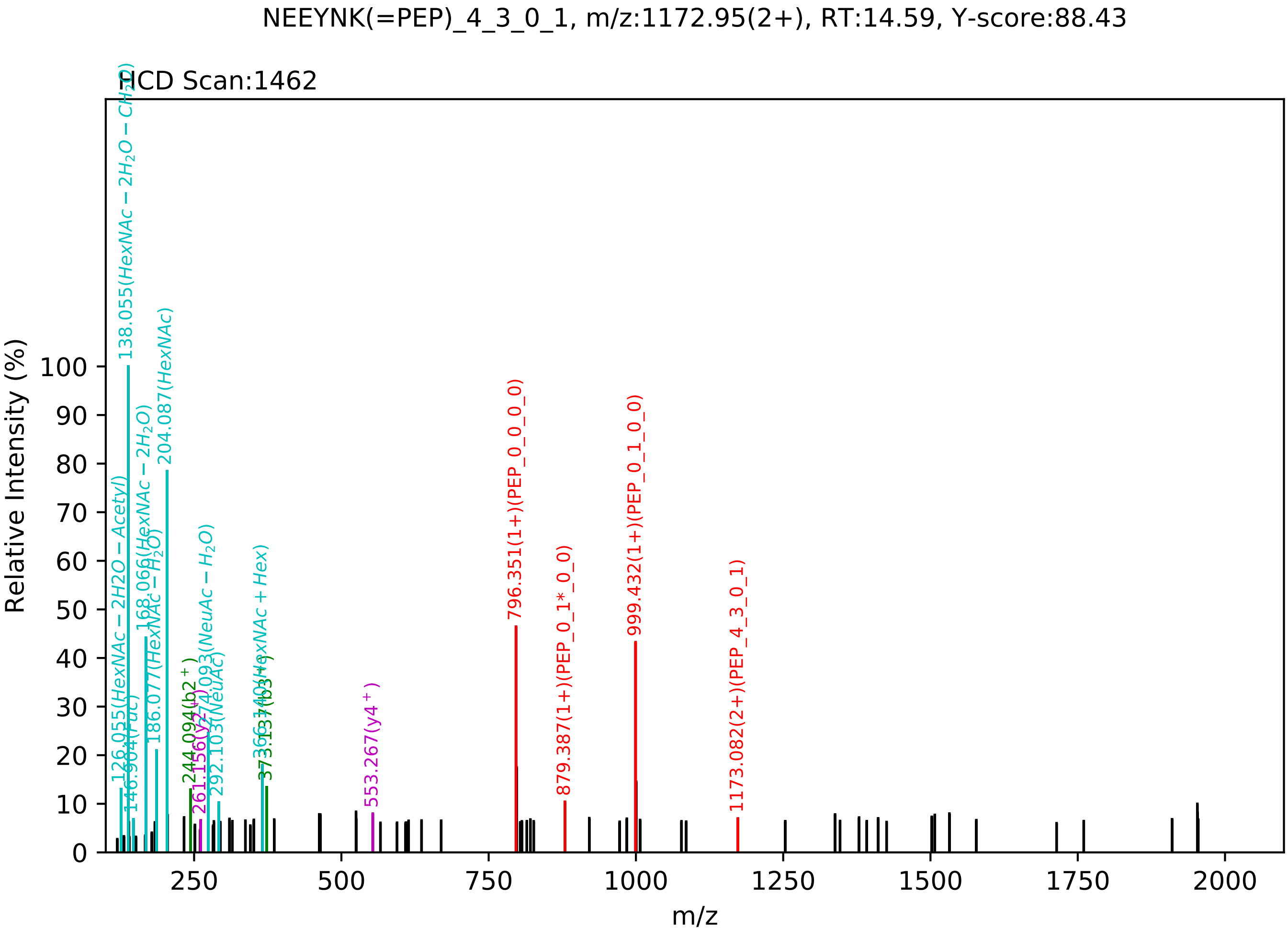

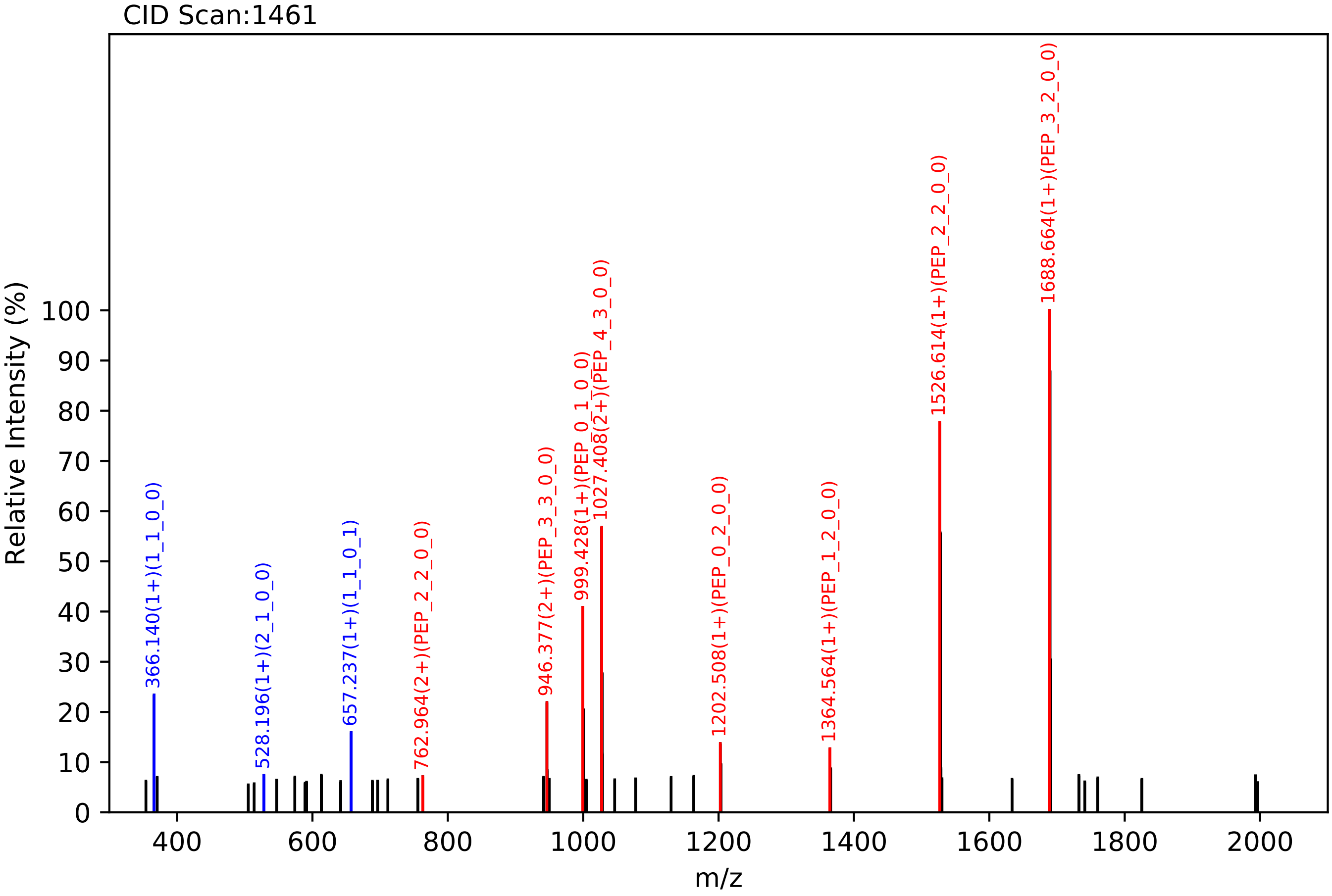

Training set no. 125, Experiment: AGP exp_2

NEEYNK(=PEP)_4_4_0_1, m/z:1274.49(2+), RT:15.37, Y-score:93.76

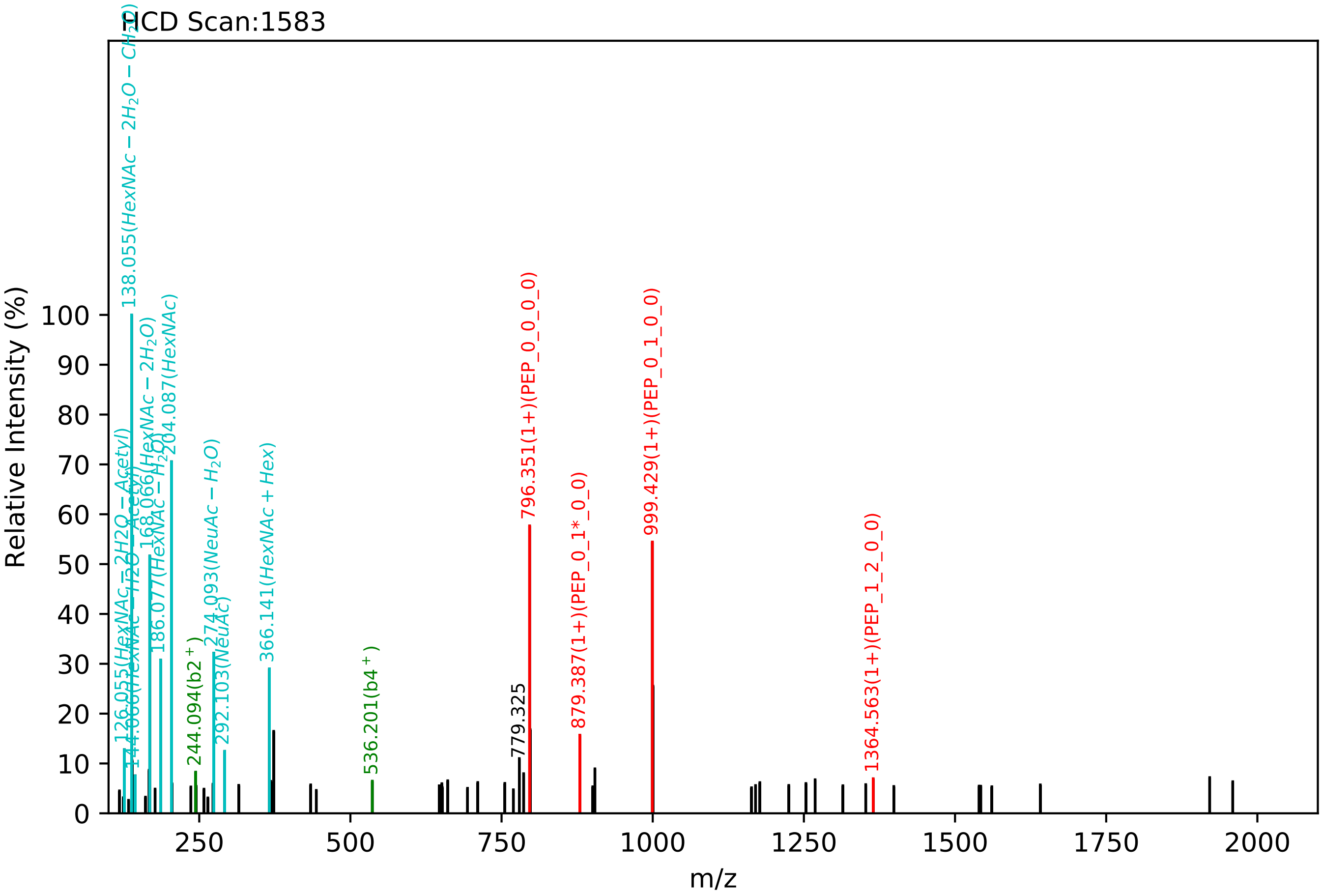

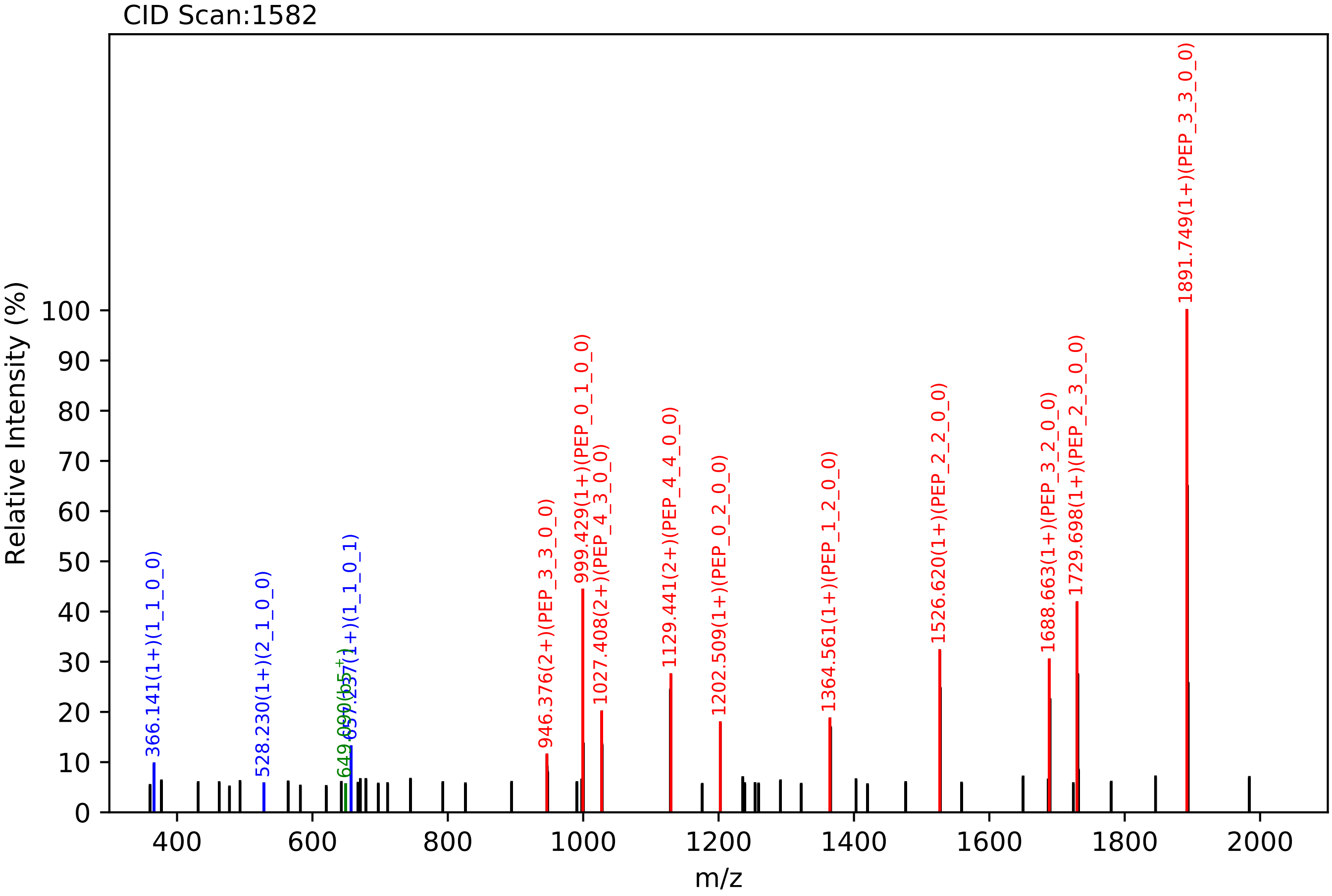

Training set no. 126, Experiment: AGP exp_2

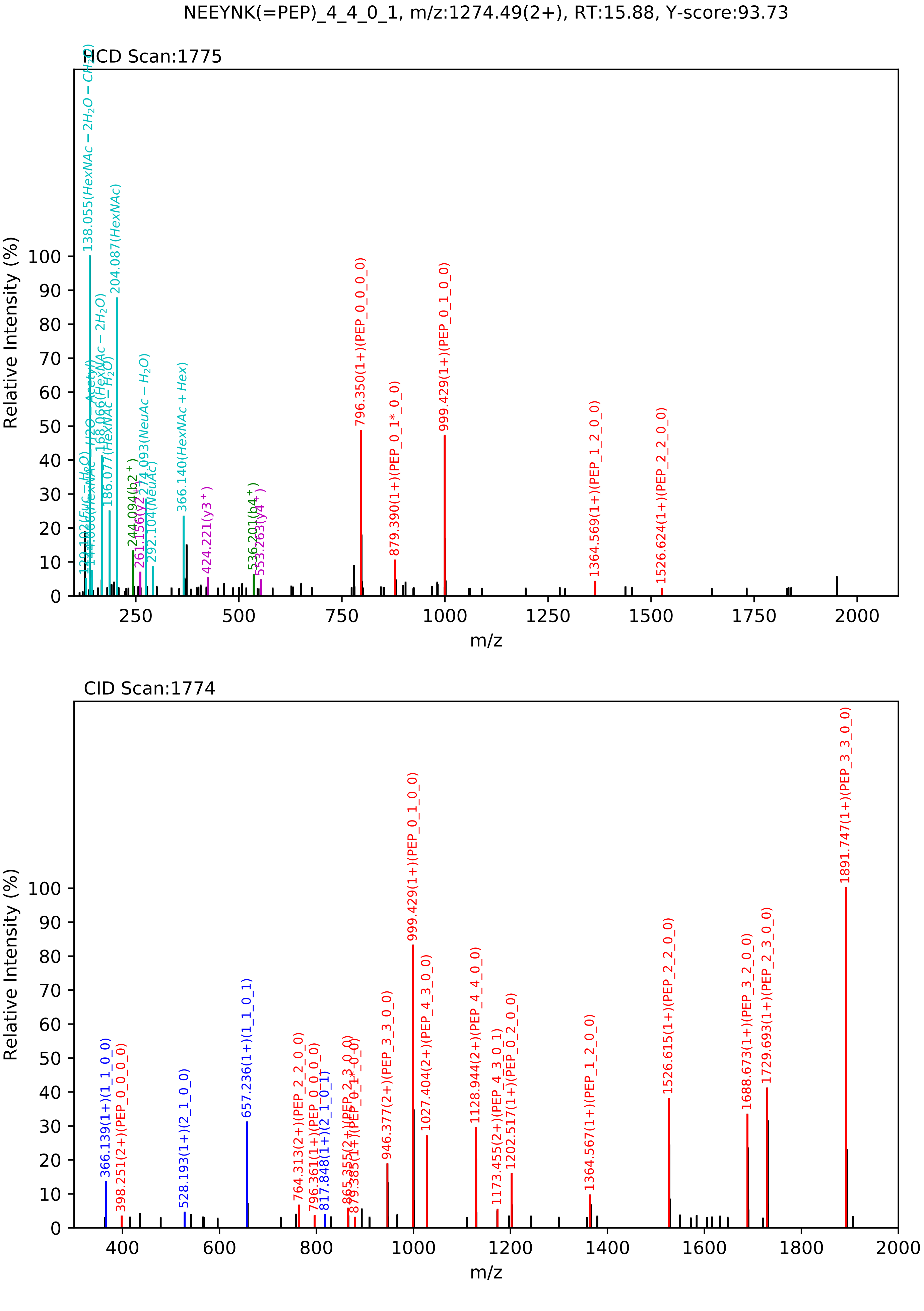

Training set no. 127, Experiment: AGP exp_1

NEEYNK(=PEP)_4_4_0_1, m/z:1274.49(2+), RT:15.82, Y-score:91.92

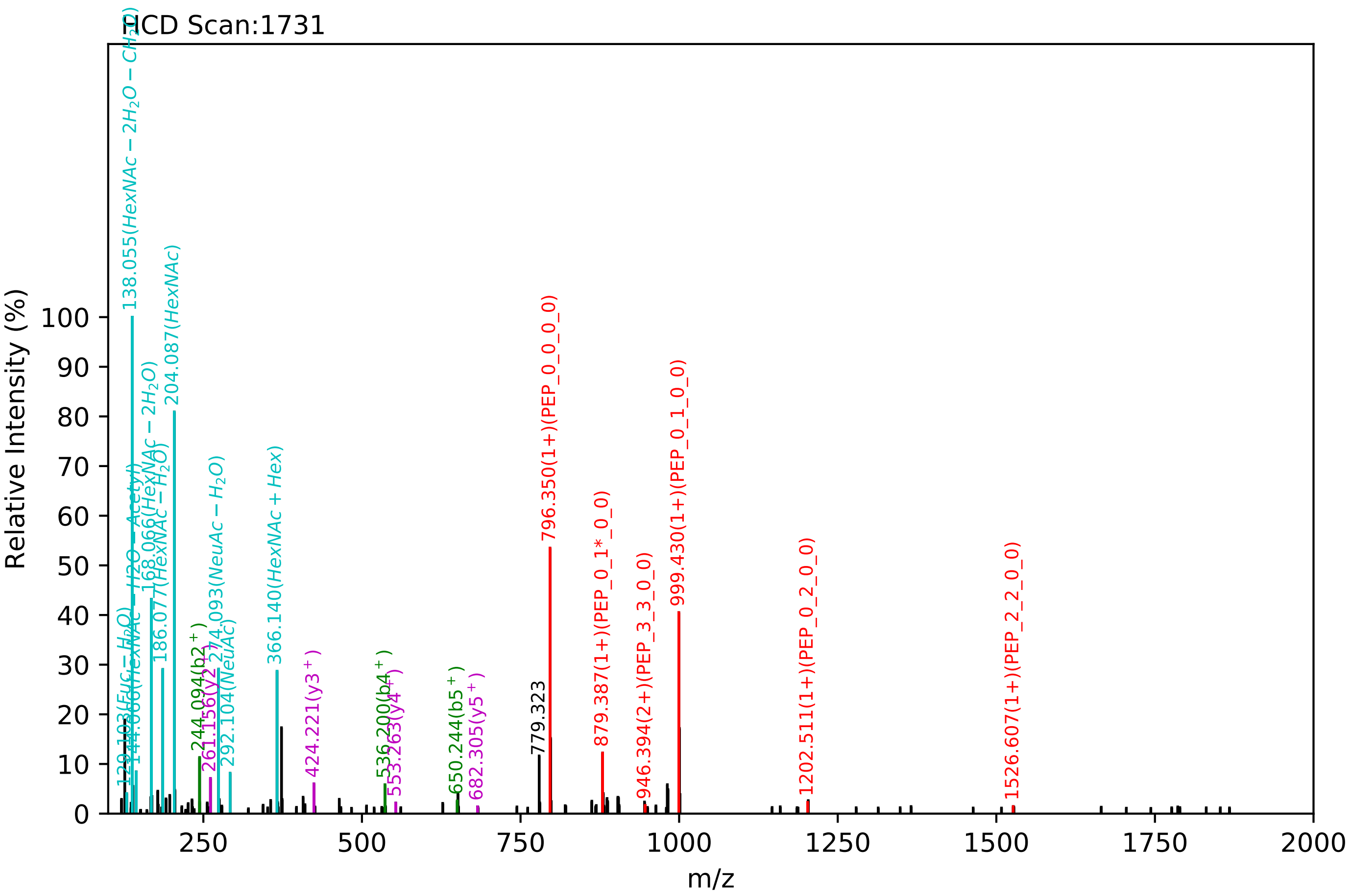

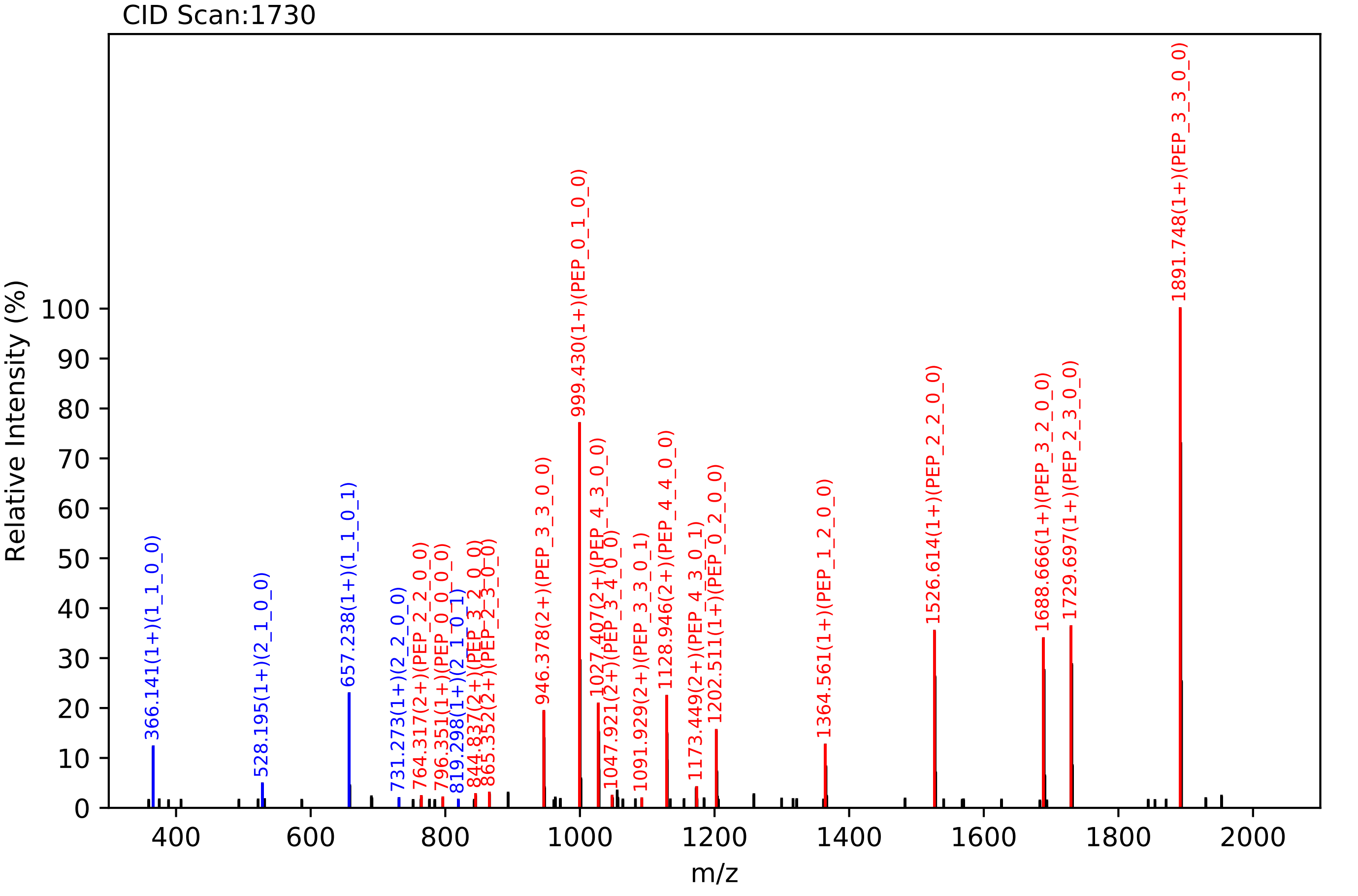

Training set no. 128, Experiment: AGP exp_1

NEEYNK(=PEP)_4_4_0_1, m/z:1274.49(2+), RT:15.31, Y-score:91.35

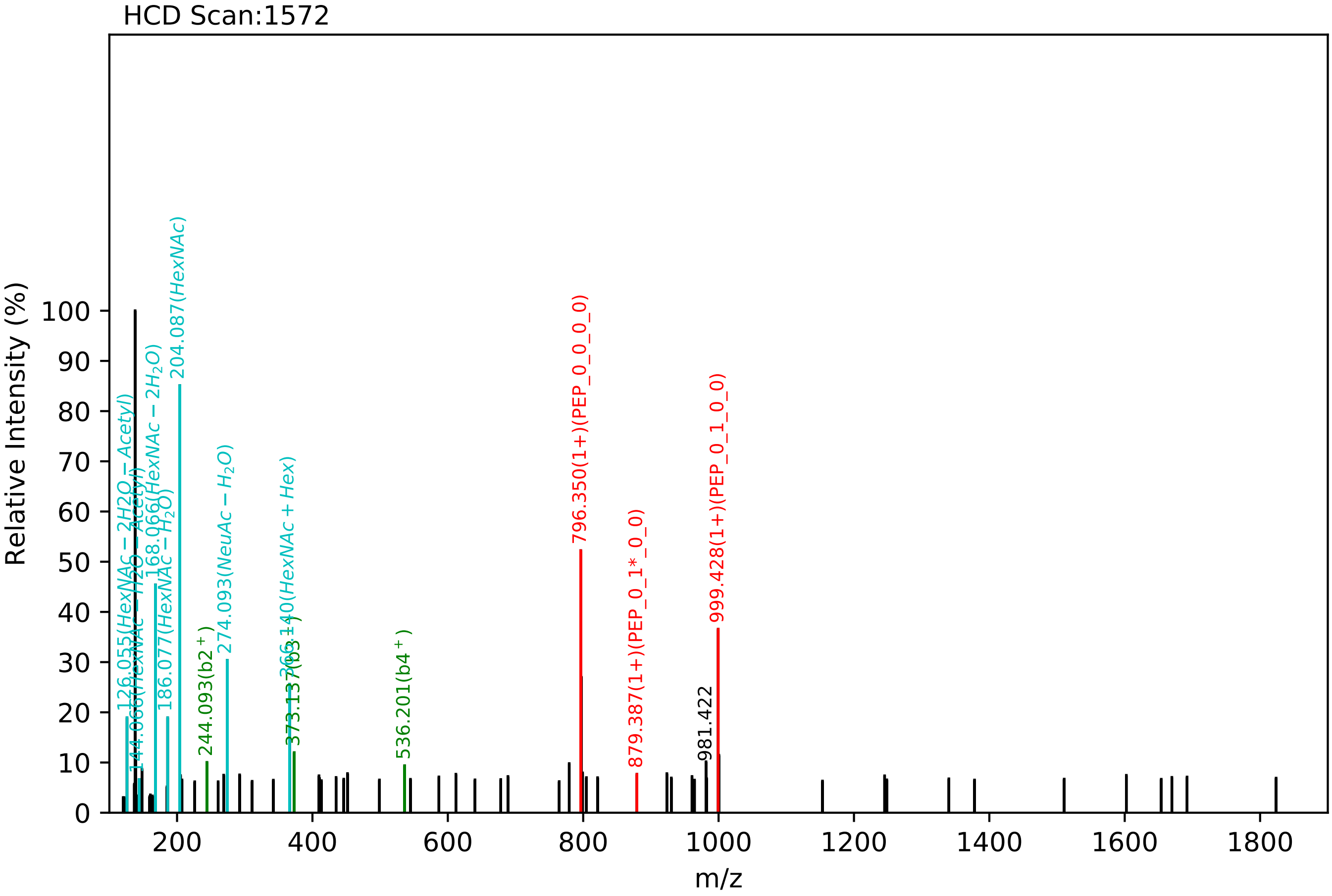

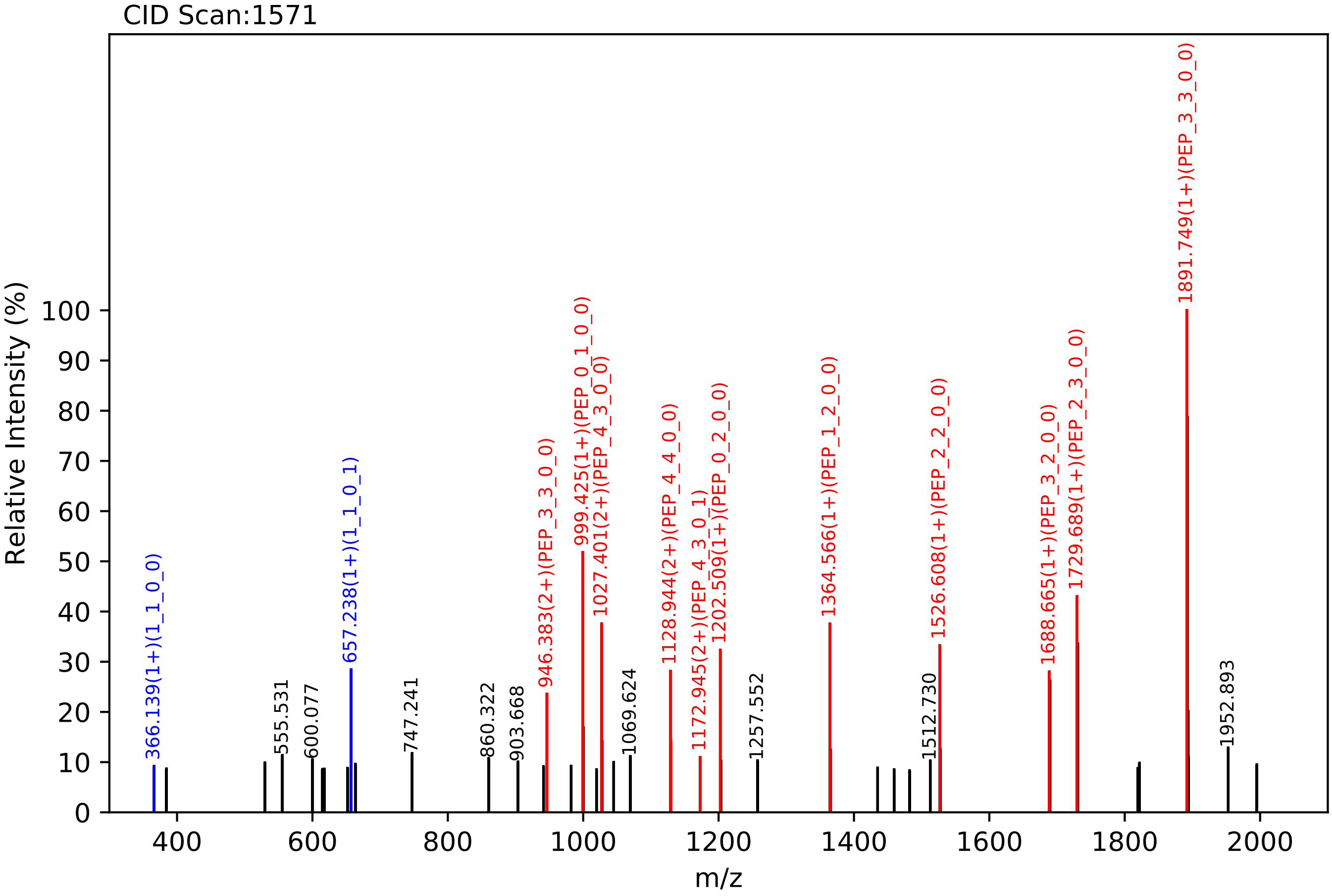

Training set no. 129, Experiment: AGP exp_1

NEEYNK(=PEP)_4_4_0_1, m/z:850.00(3+), RT:15.39, Y-score:85.10

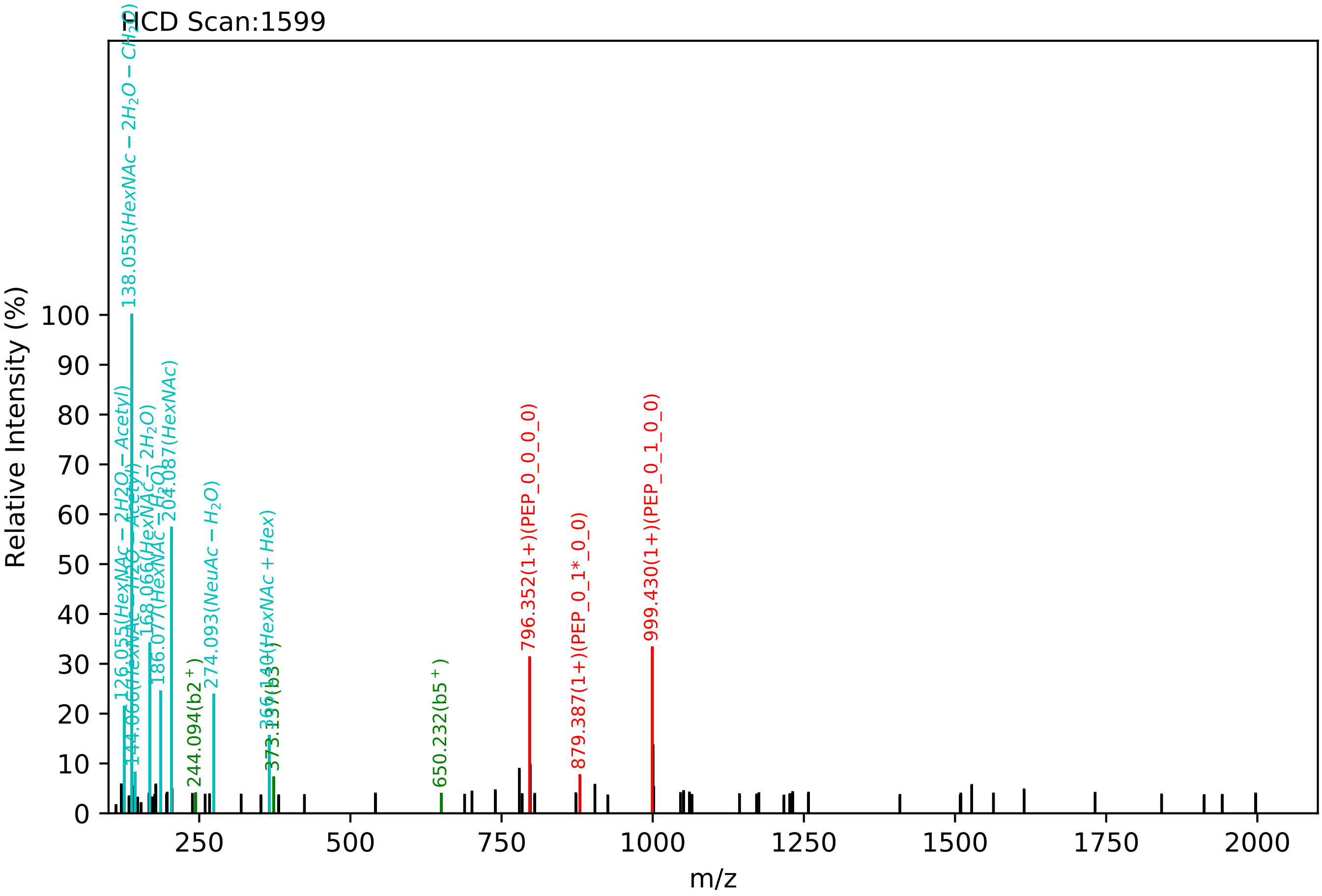

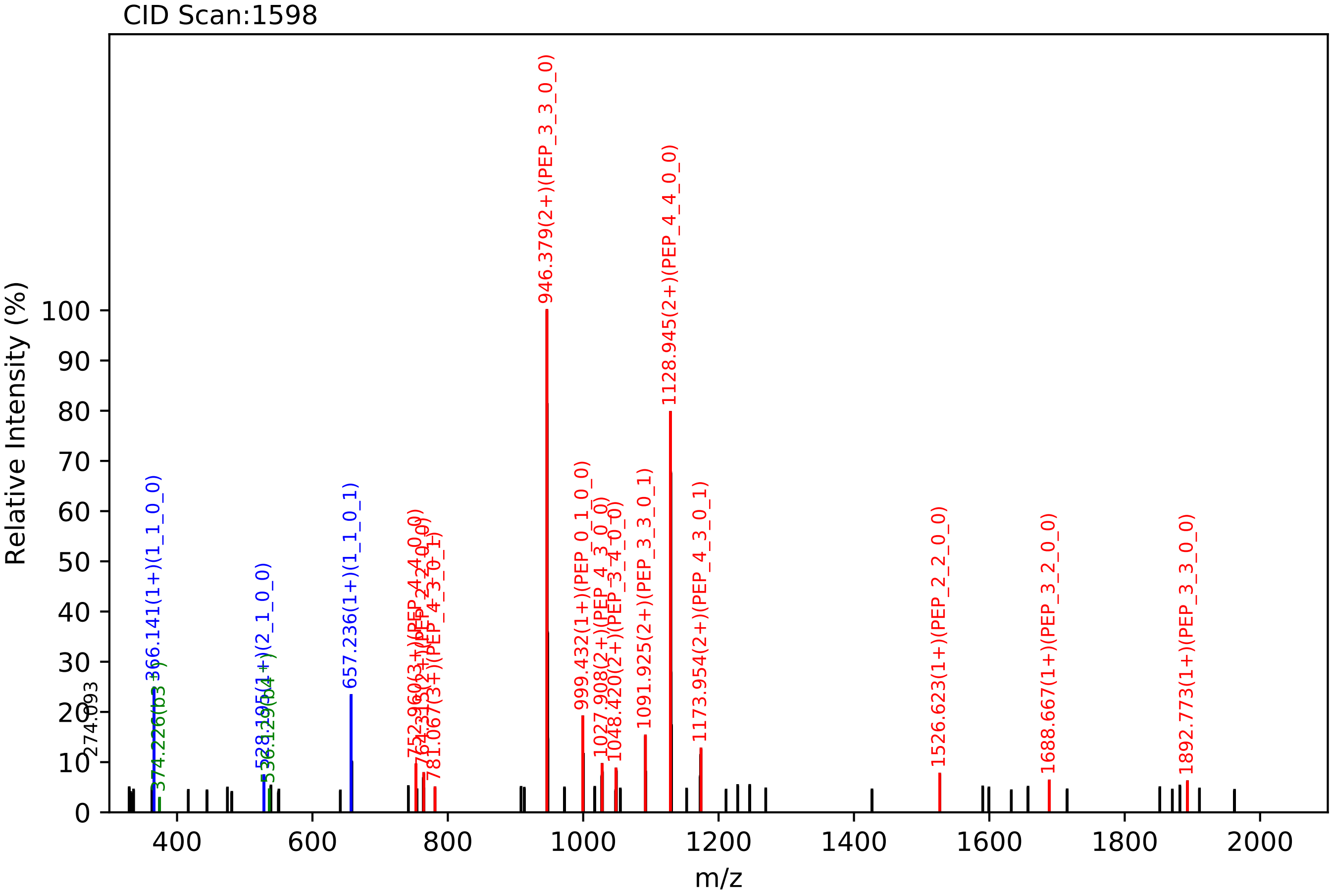

Training set no. 130, Experiment: AGP exp_1

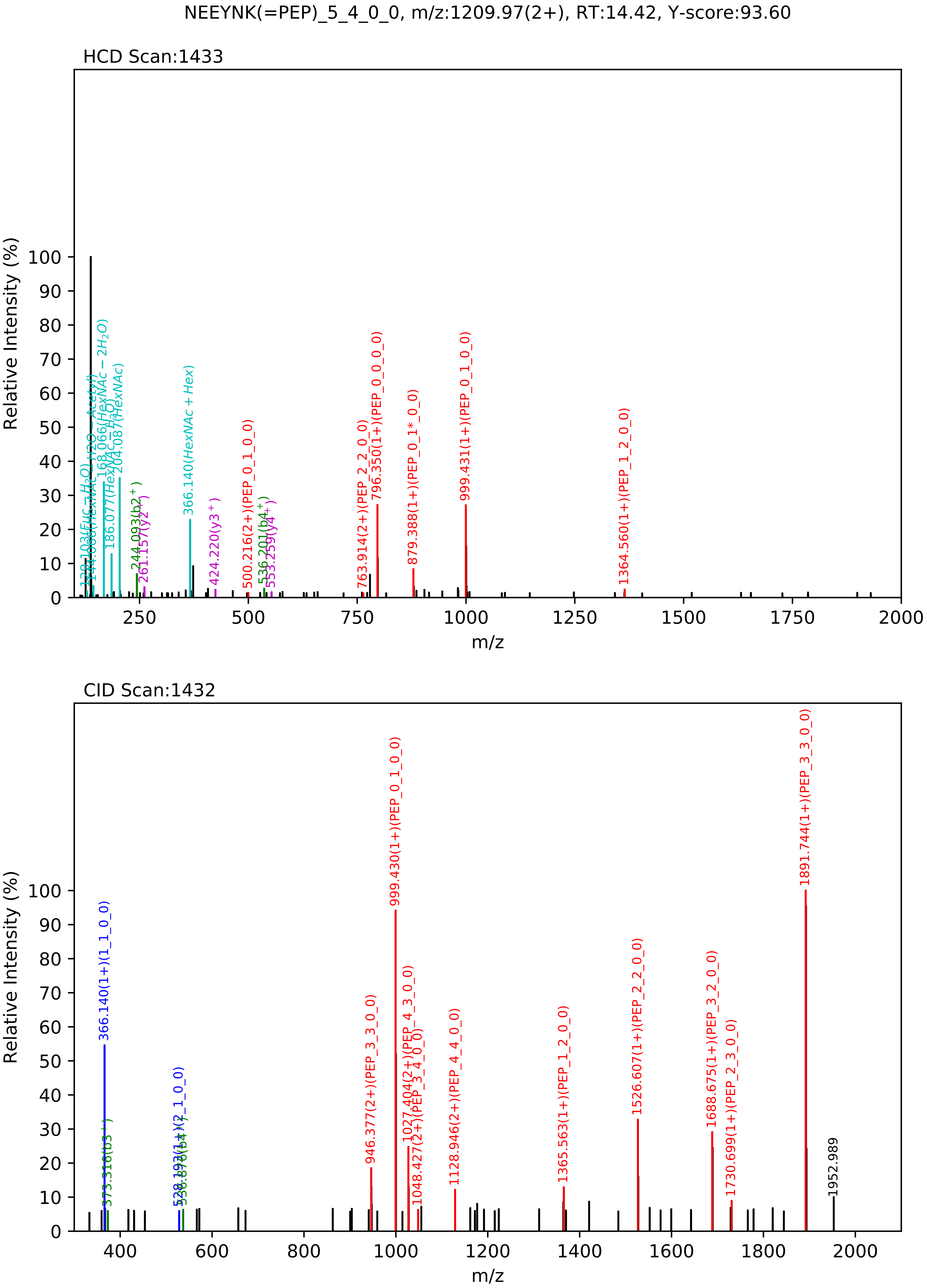

Training set no. 131, Experiment: AGP exp_2

NEEYNK(=PEP)_5_4_0_0, m/z:1209.97(2+), RT:14.41, Y-score:89.37

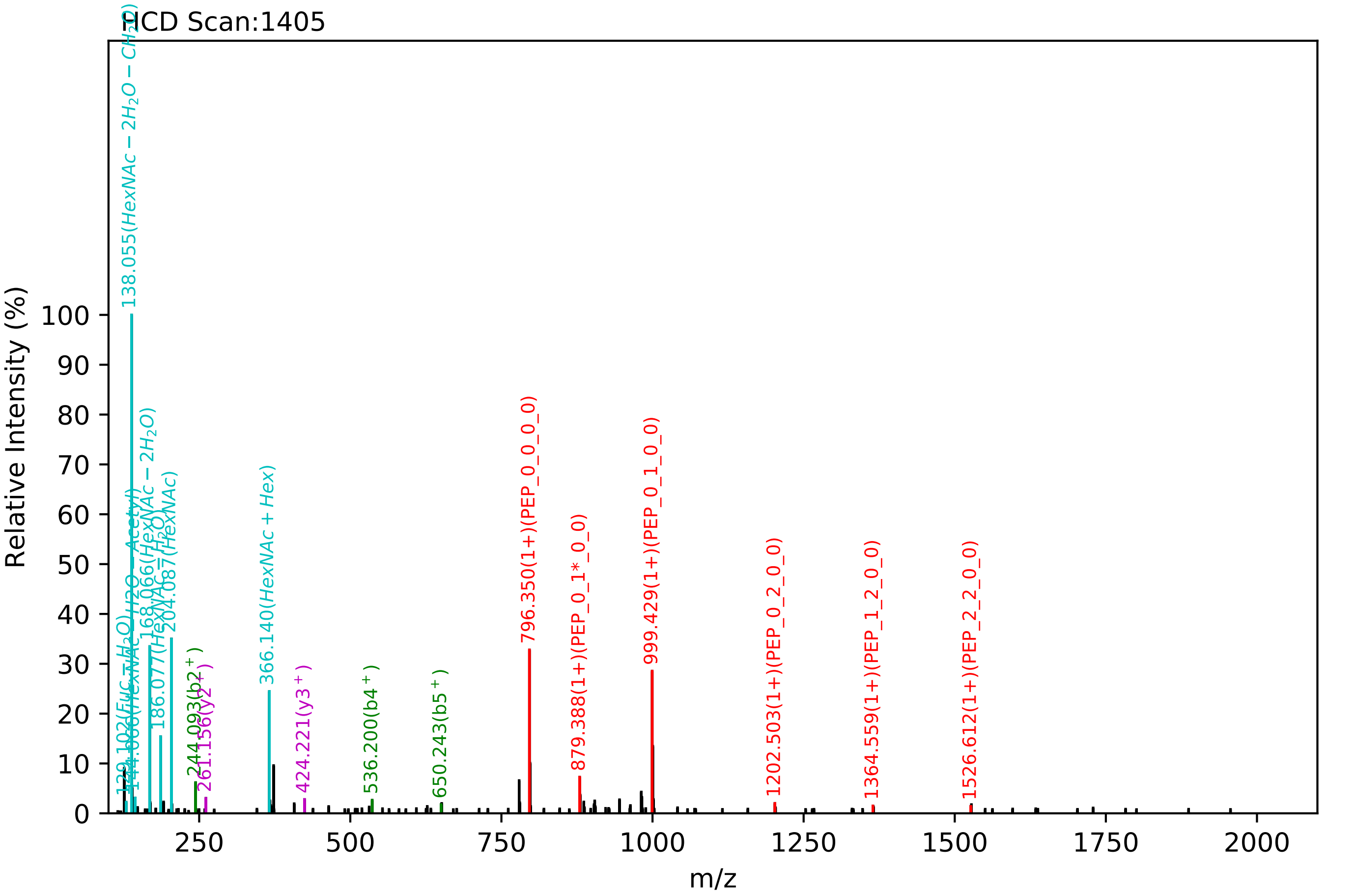

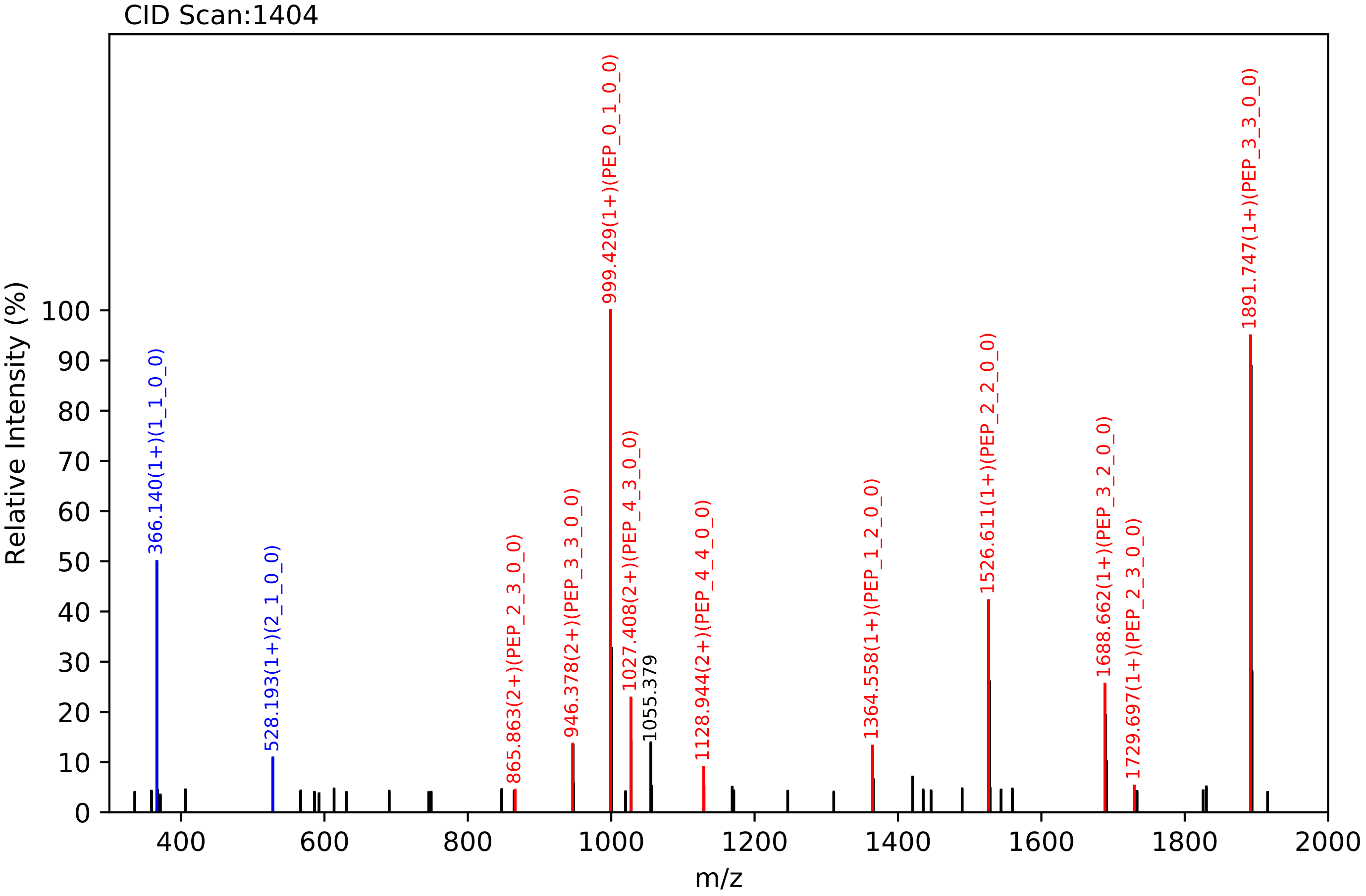

Training set no. 132, Experiment: AGP exp_2

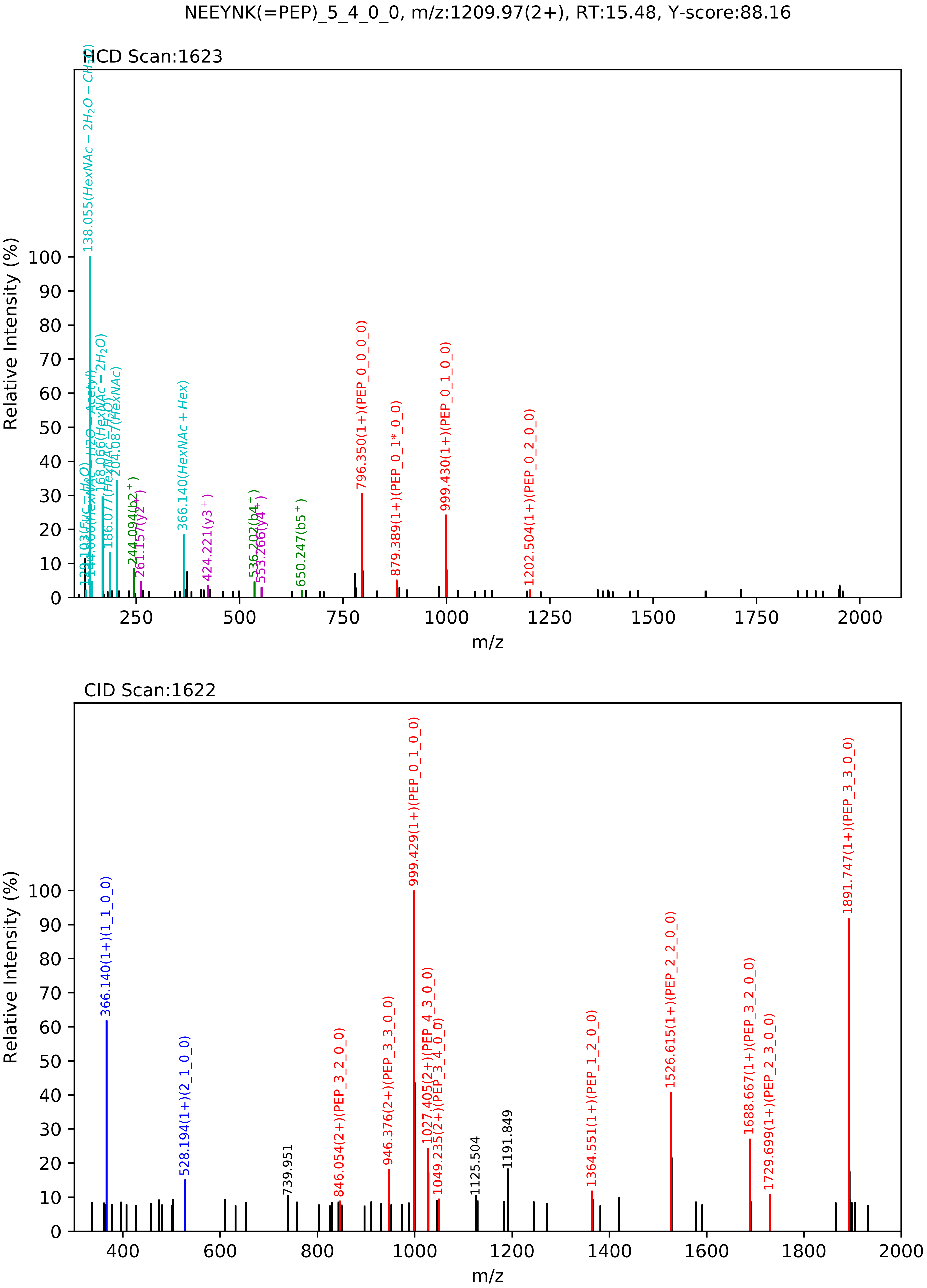

Training set no. 133, Experiment: AGP exp_2

NEEYNK(=PEP)_5_4_0_0, m/z:1209.97(2+), RT:16.07, Y-score:83.04

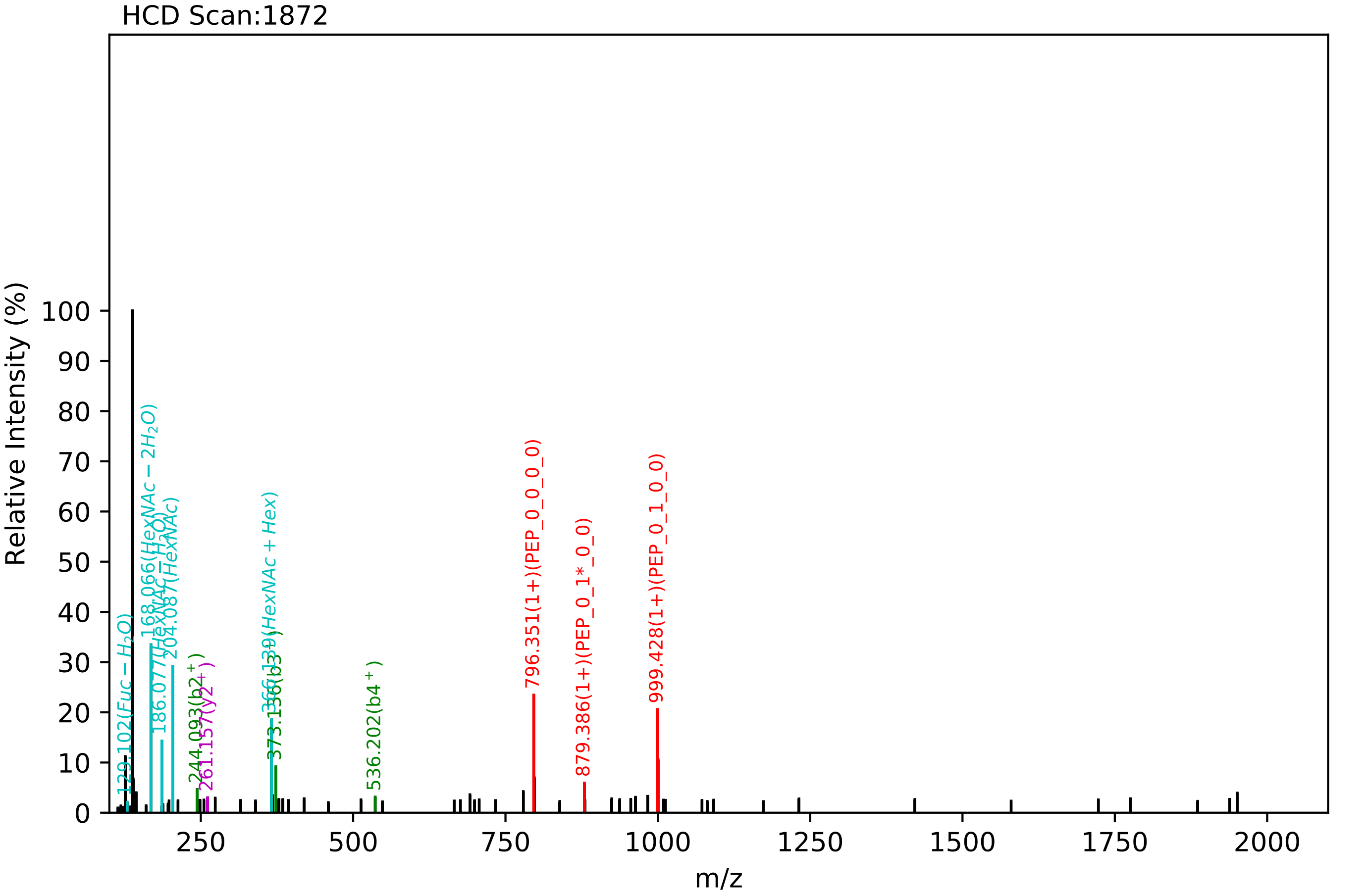

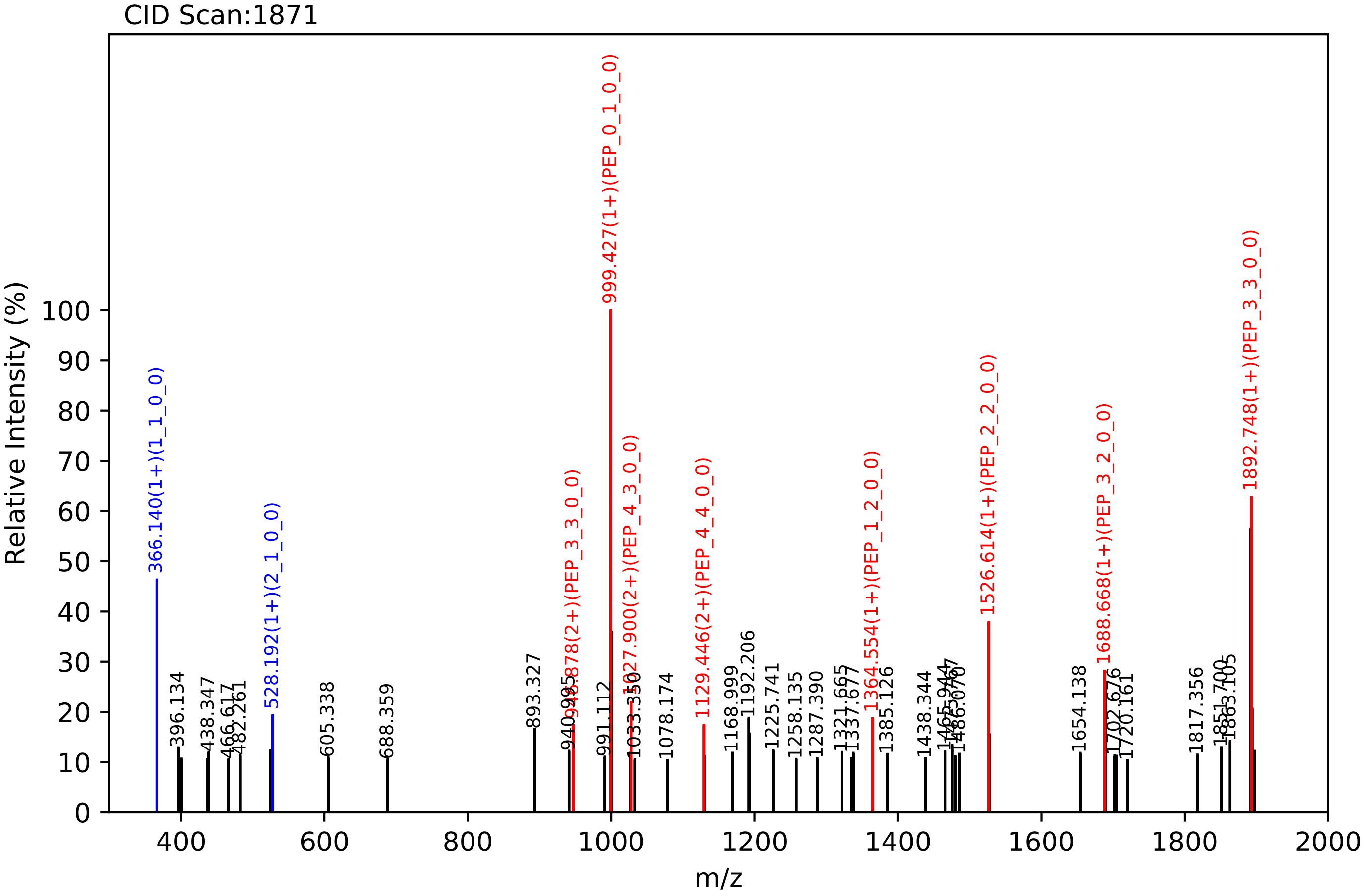

Training set no. 134, Experiment: AGP exp_2

NEEYNK(=PEP)_5_4_0_1, m/z:904.02(3+), RT:16.39, Y-score:94.18

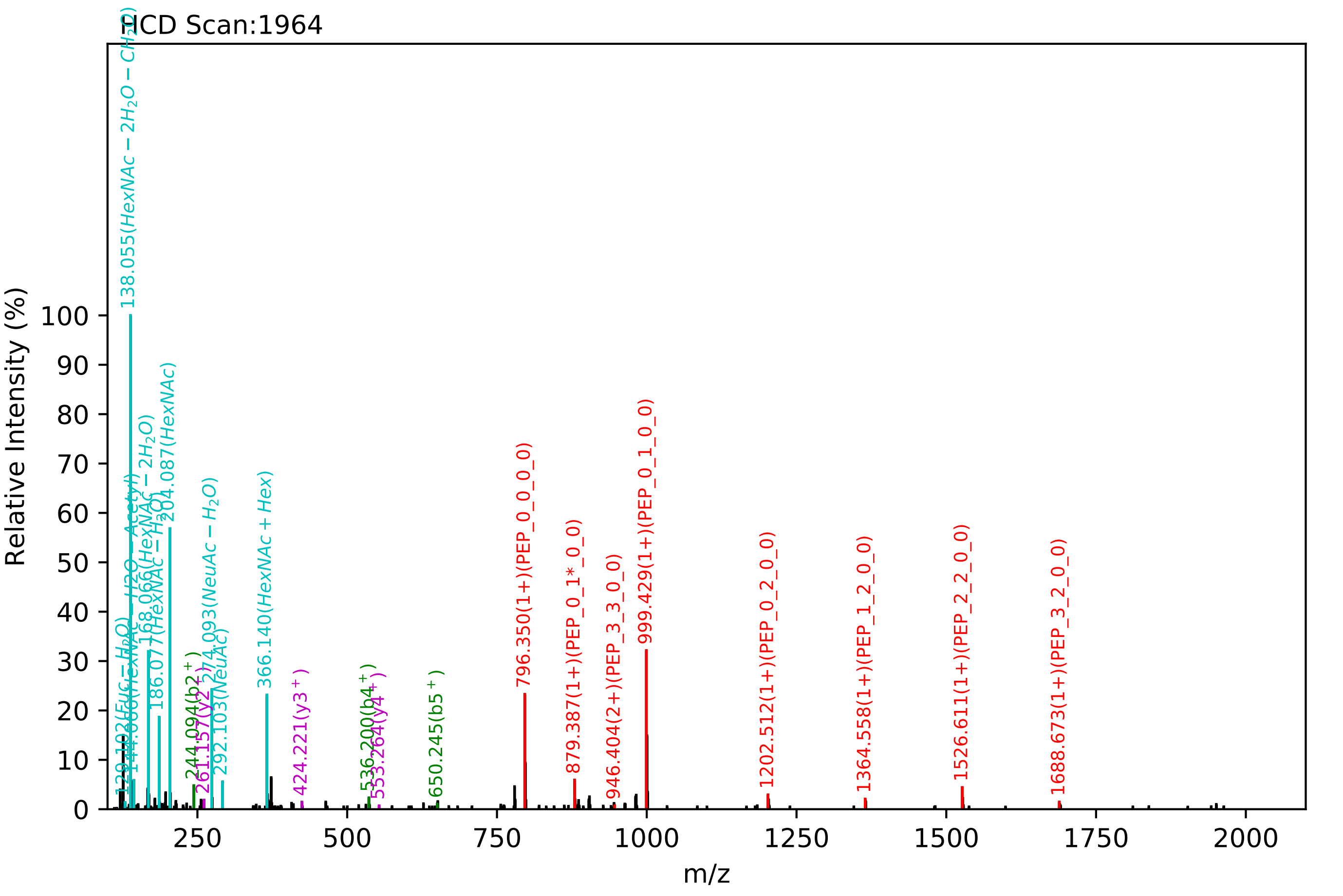

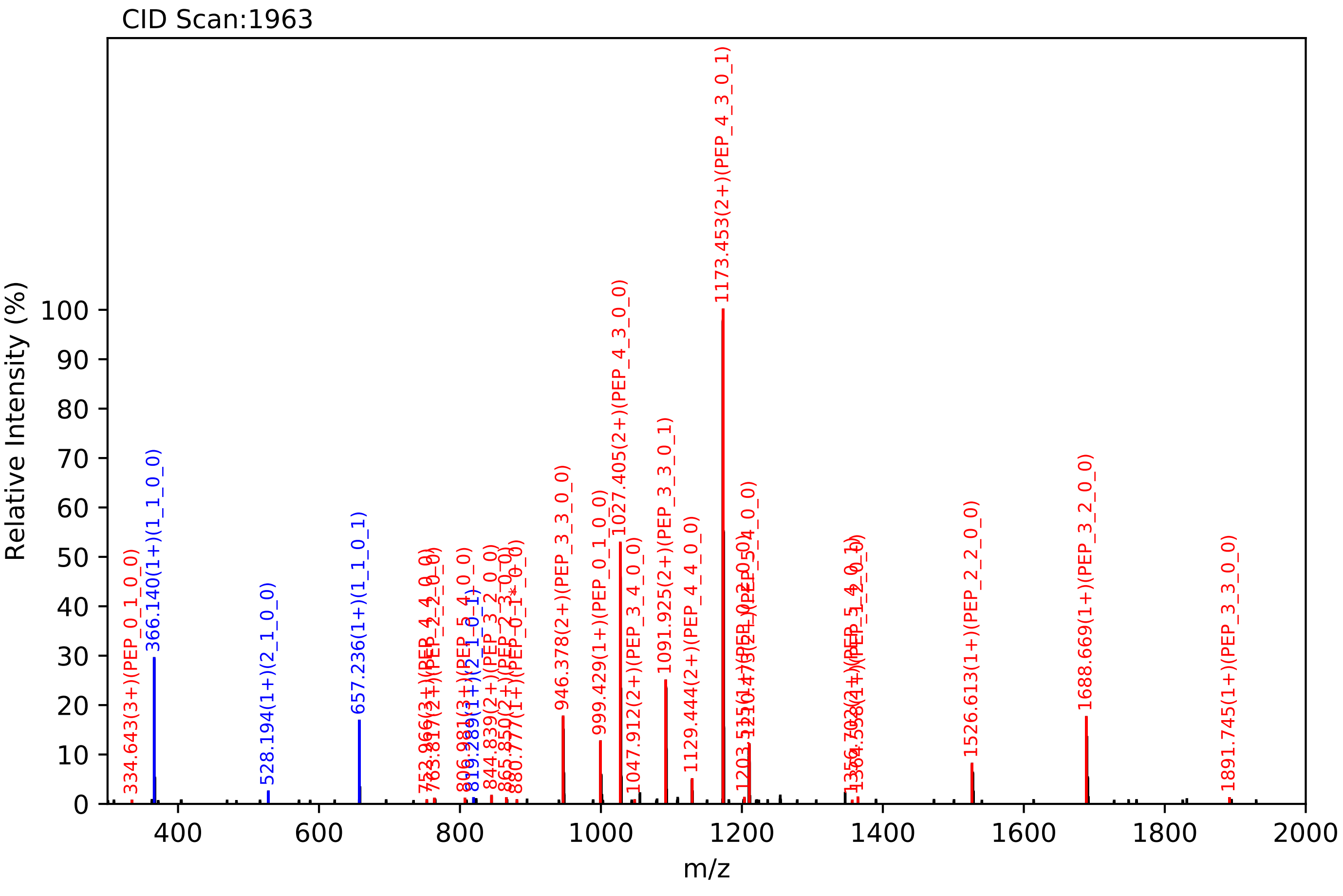

Training set no. 135, Experiment: AGP exp_2

NEEYNK(=PEP)_5_4_0_1, m/z:1355.52(2+), RT:16.38, Y-score:93.62

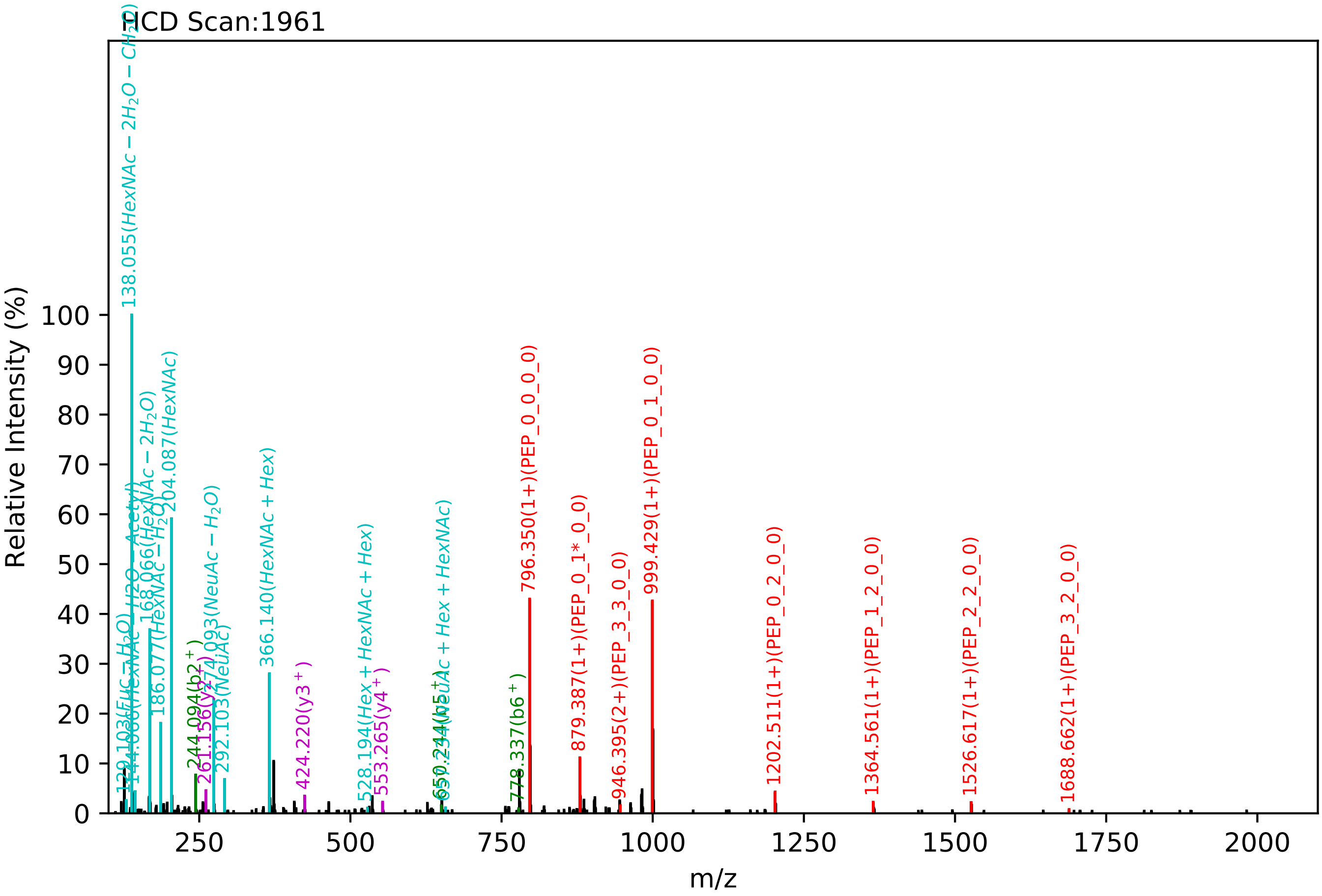

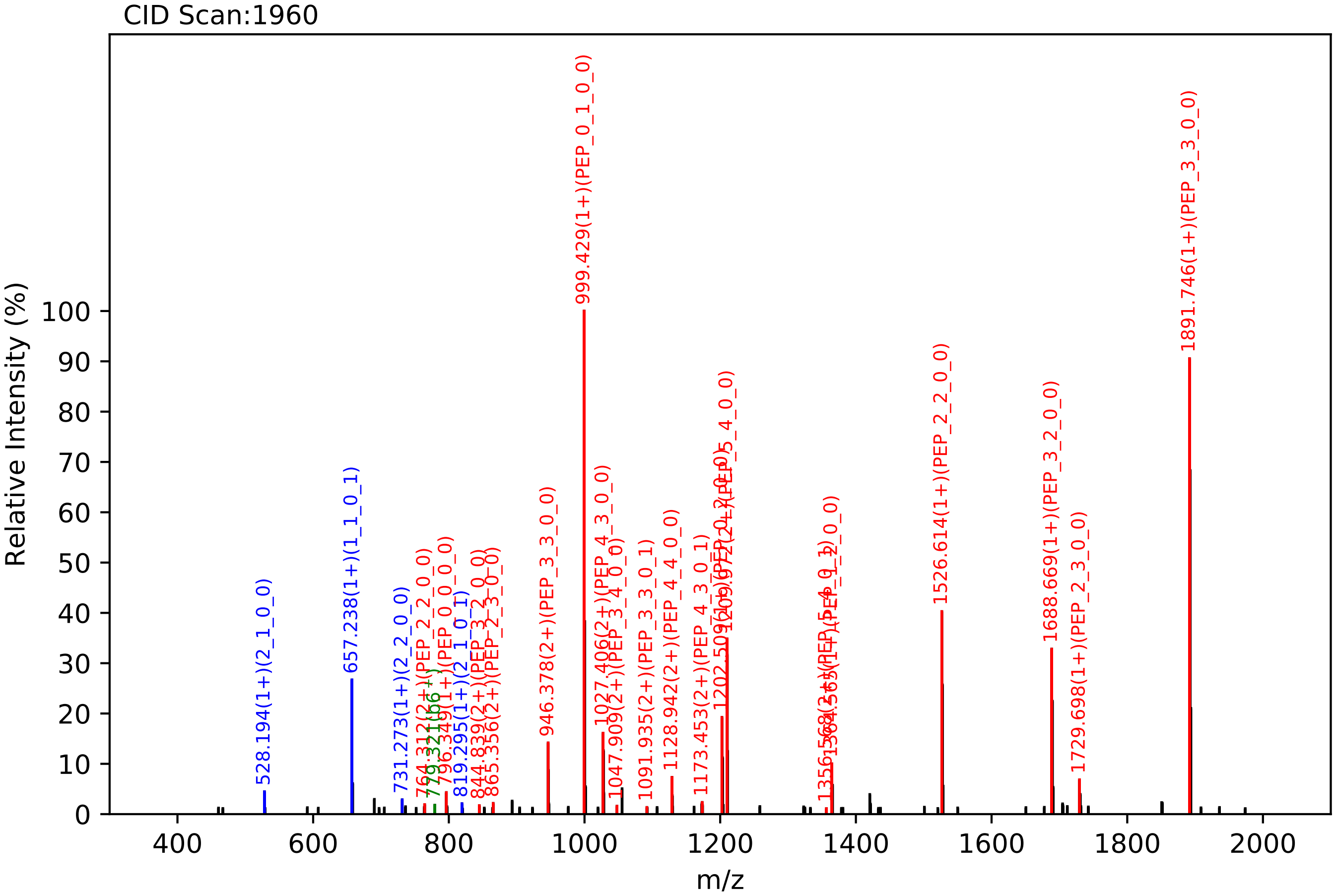

Training set no. 136, Experiment: AGP exp_2

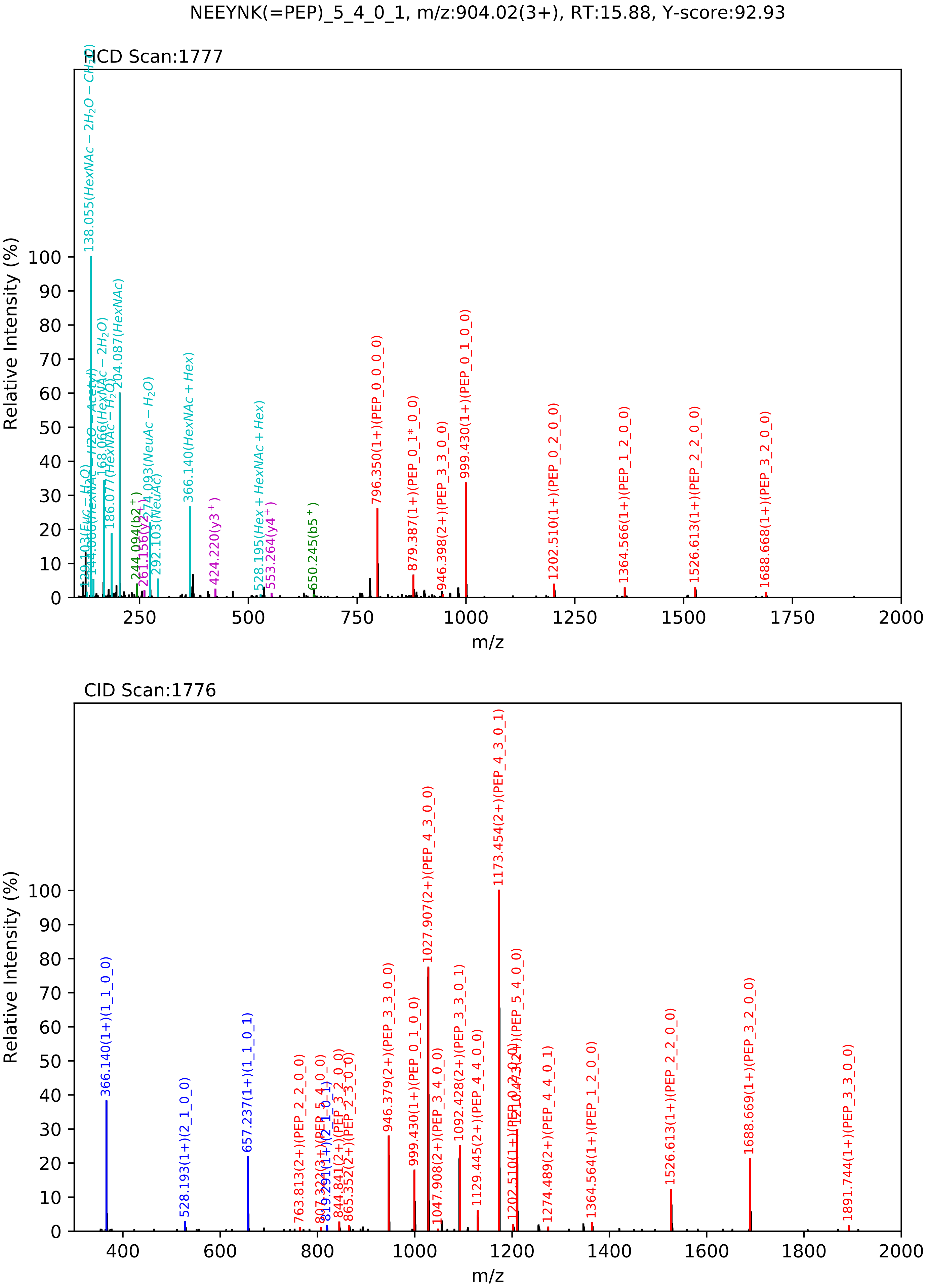

Training set no. 137, Experiment: AGP exp_2

NEEYNK(=PEP)_5_4_0_1, m/z:1356.02(2+), RT:15.88, Y-score:92.65

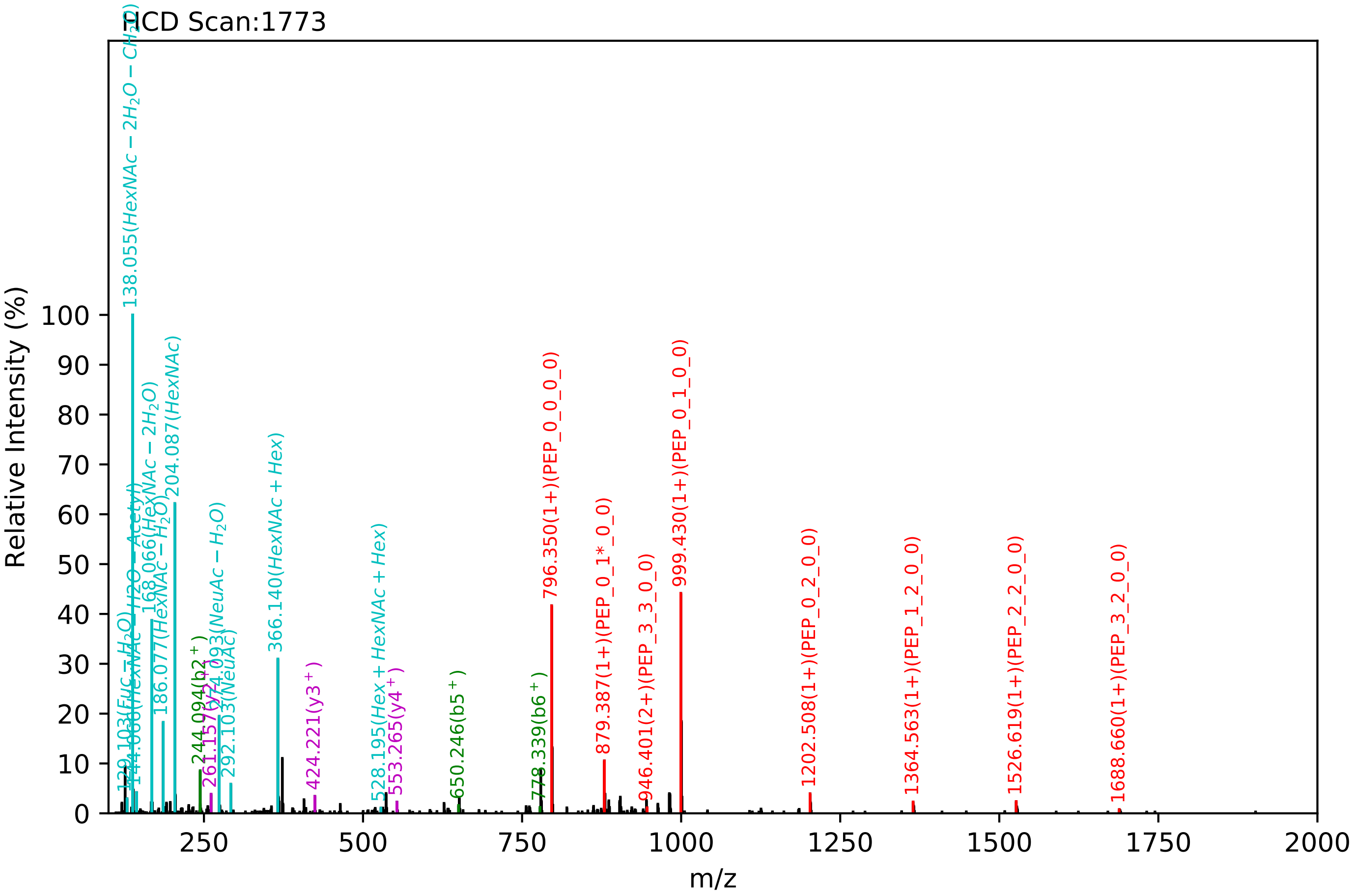

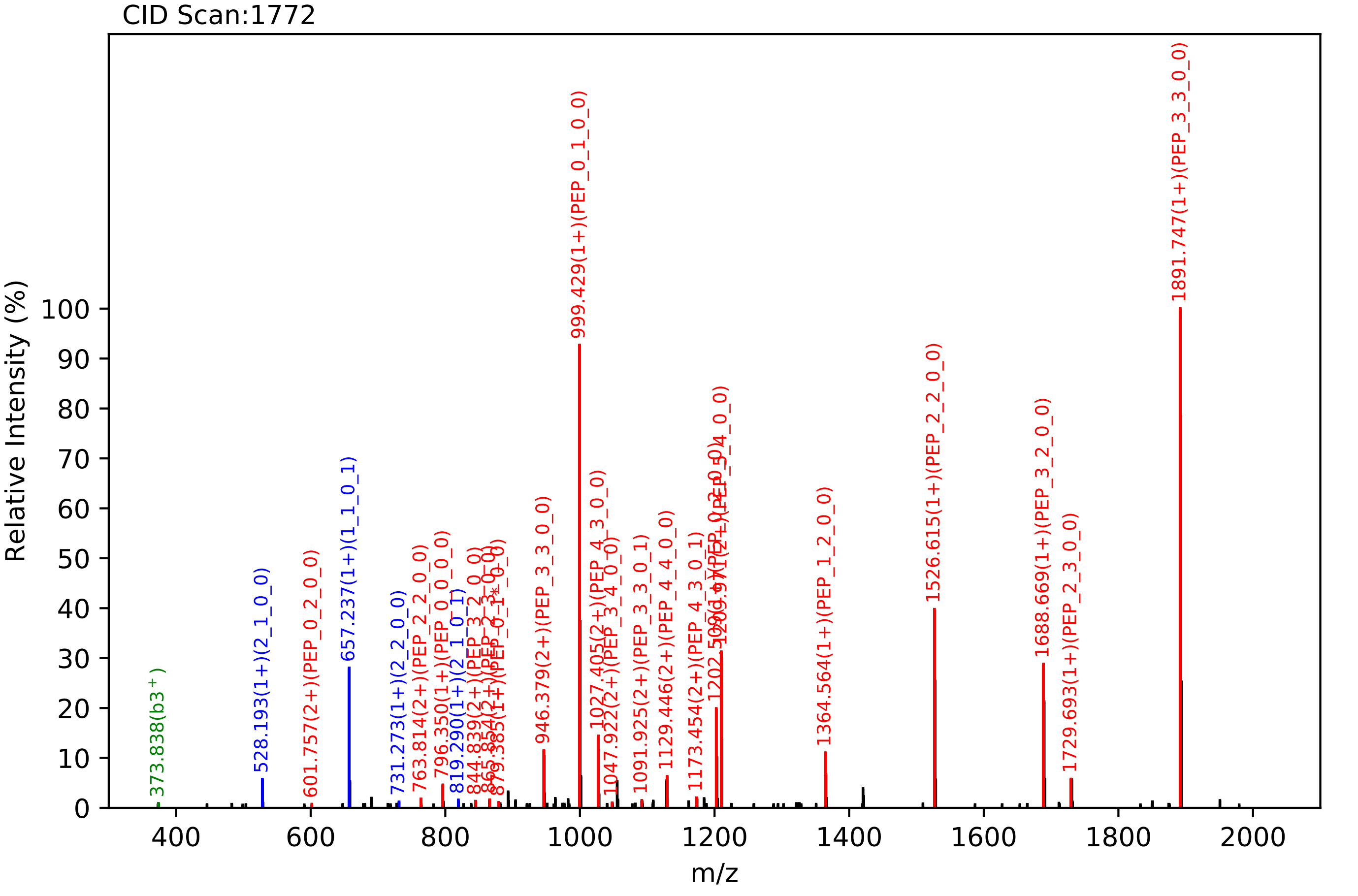

Training set no. 138, Experiment: AGP exp_1

NEEYNK(=PEP)_5_4_0_1, m/z:904.02(3+), RT:16.31, Y-score:92.64

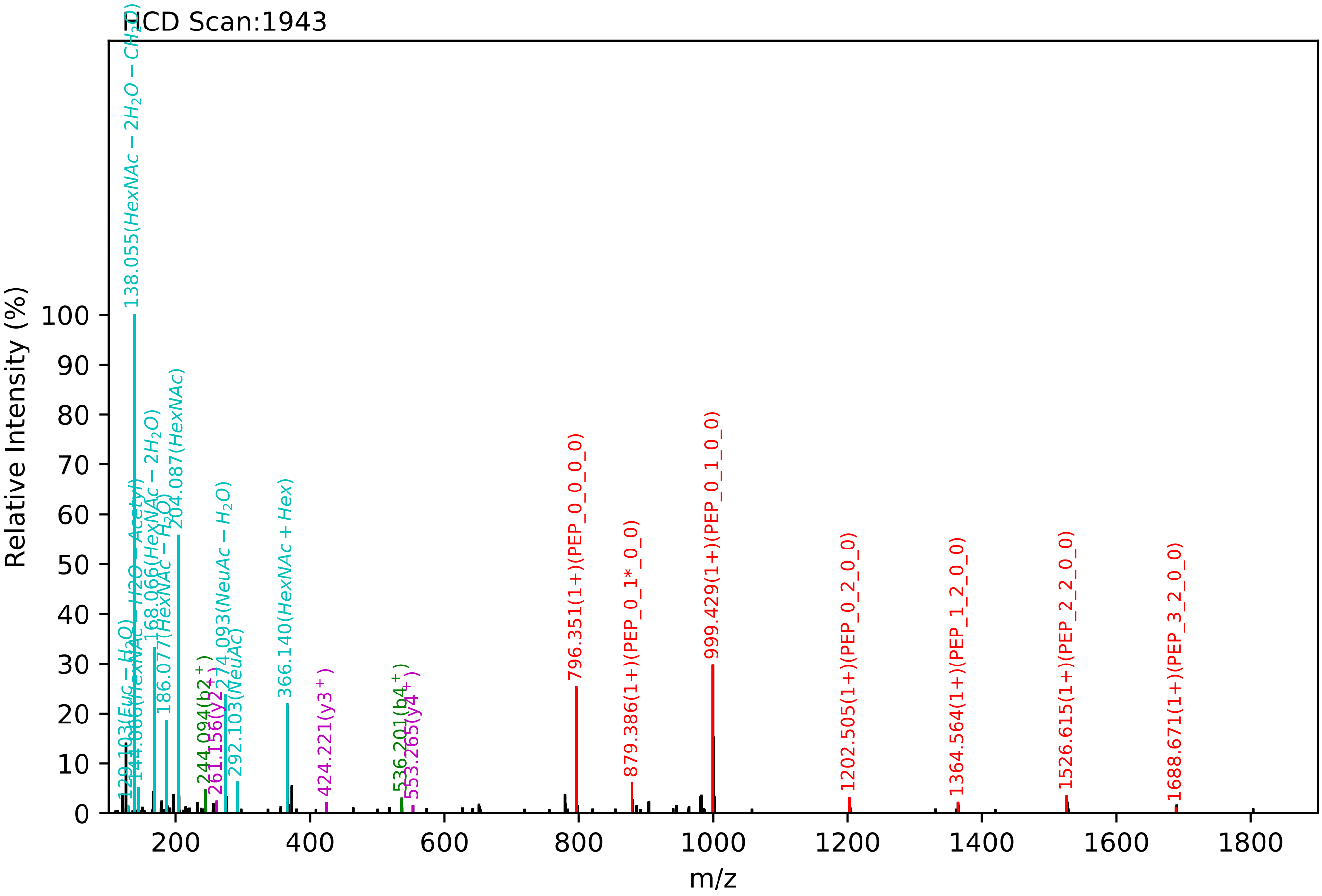

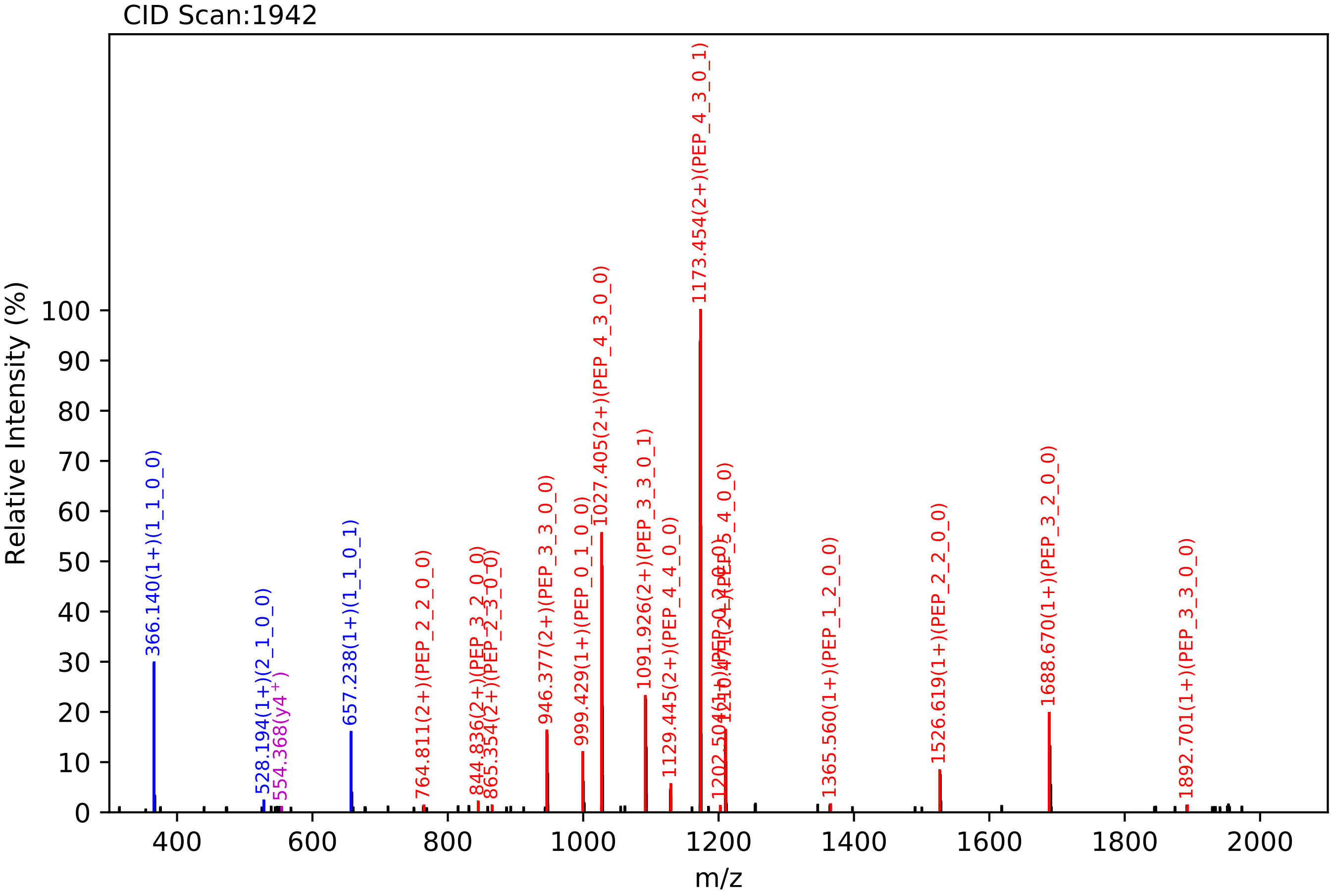

Training set no. 139, Experiment: AGP exp_1

NEEYNK(=PEP)_5_4_0_1, m/z:1356.02(2+), RT:15.79, Y-score:92.62

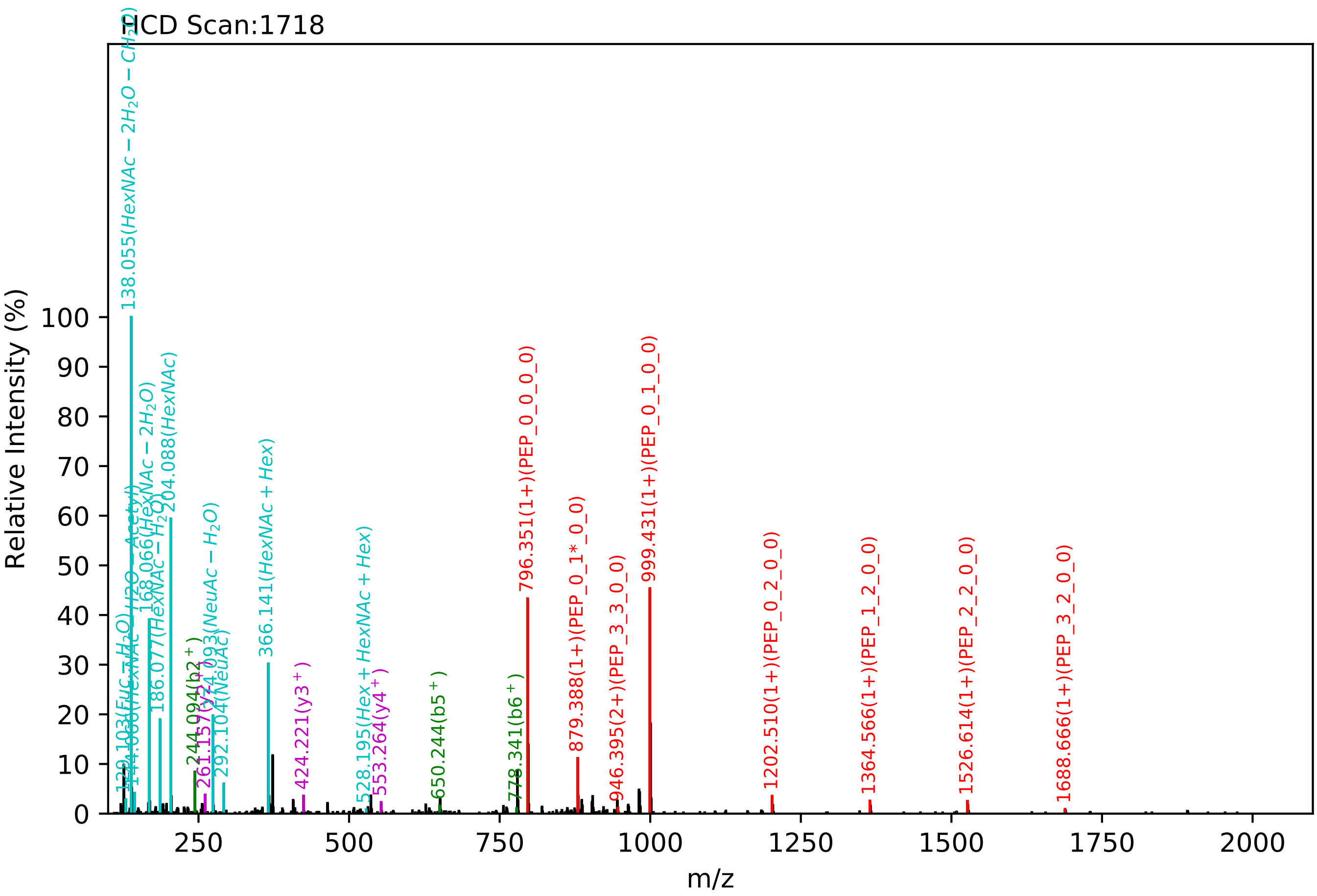

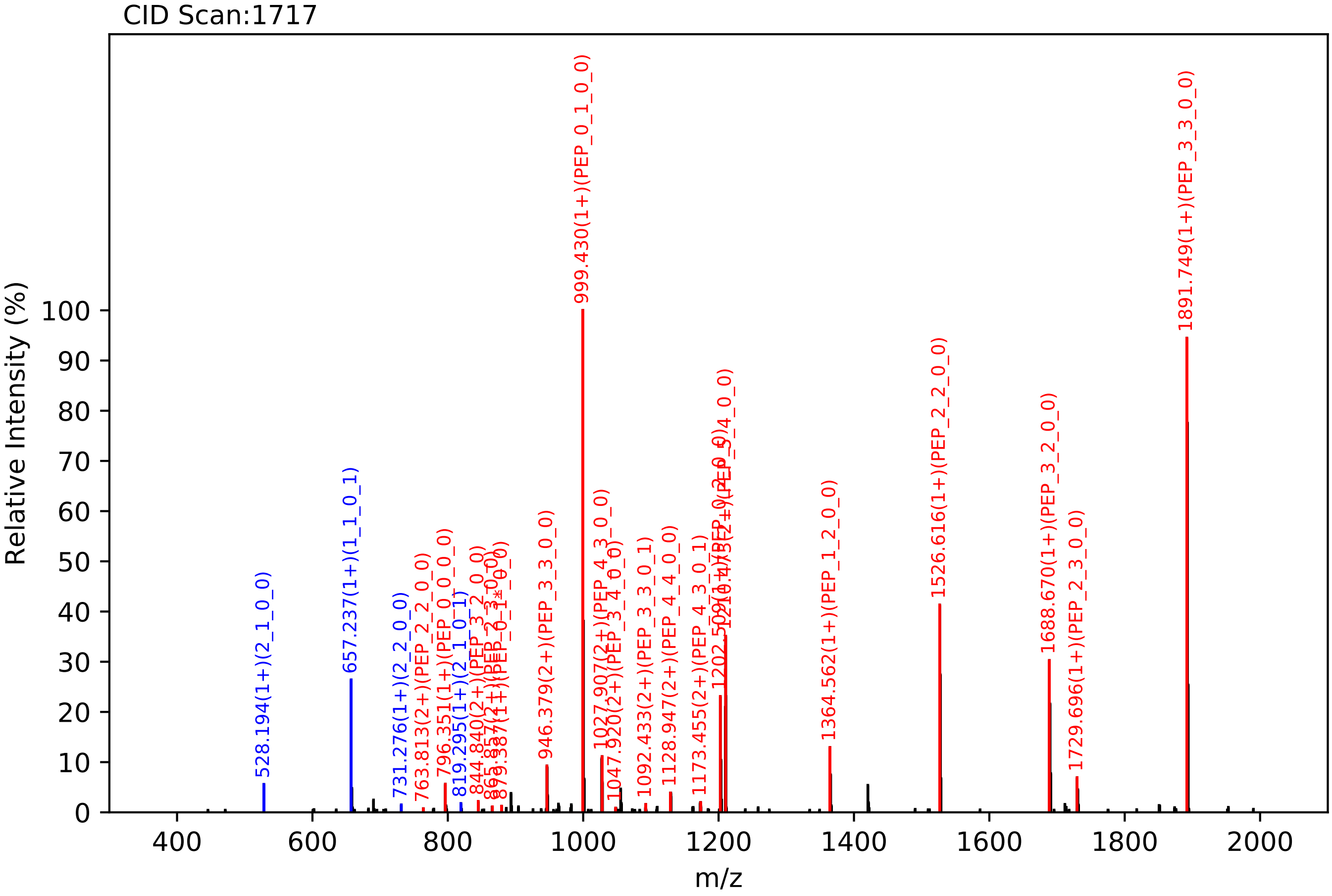

Training set no. 140, Experiment: AGP exp_2

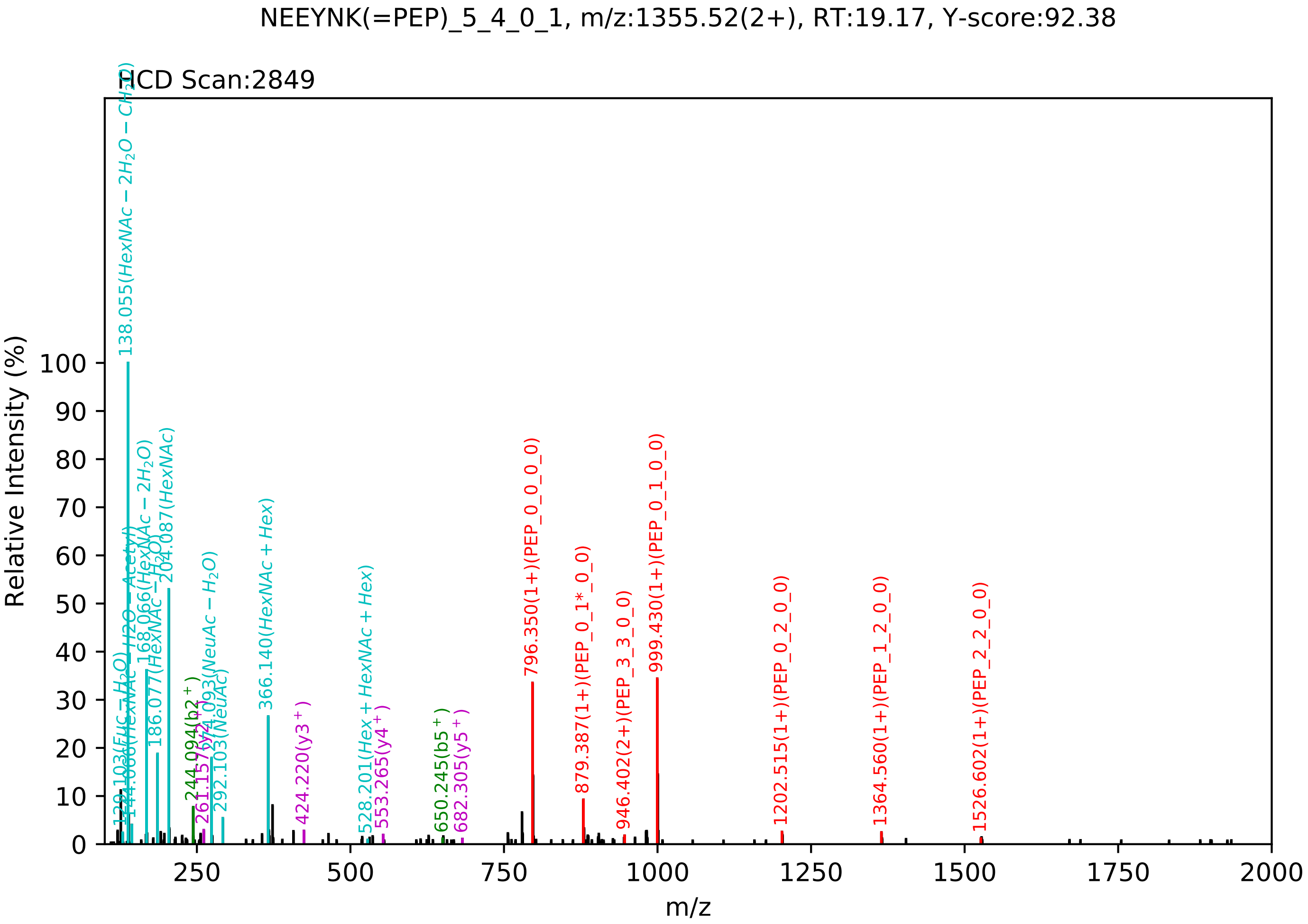

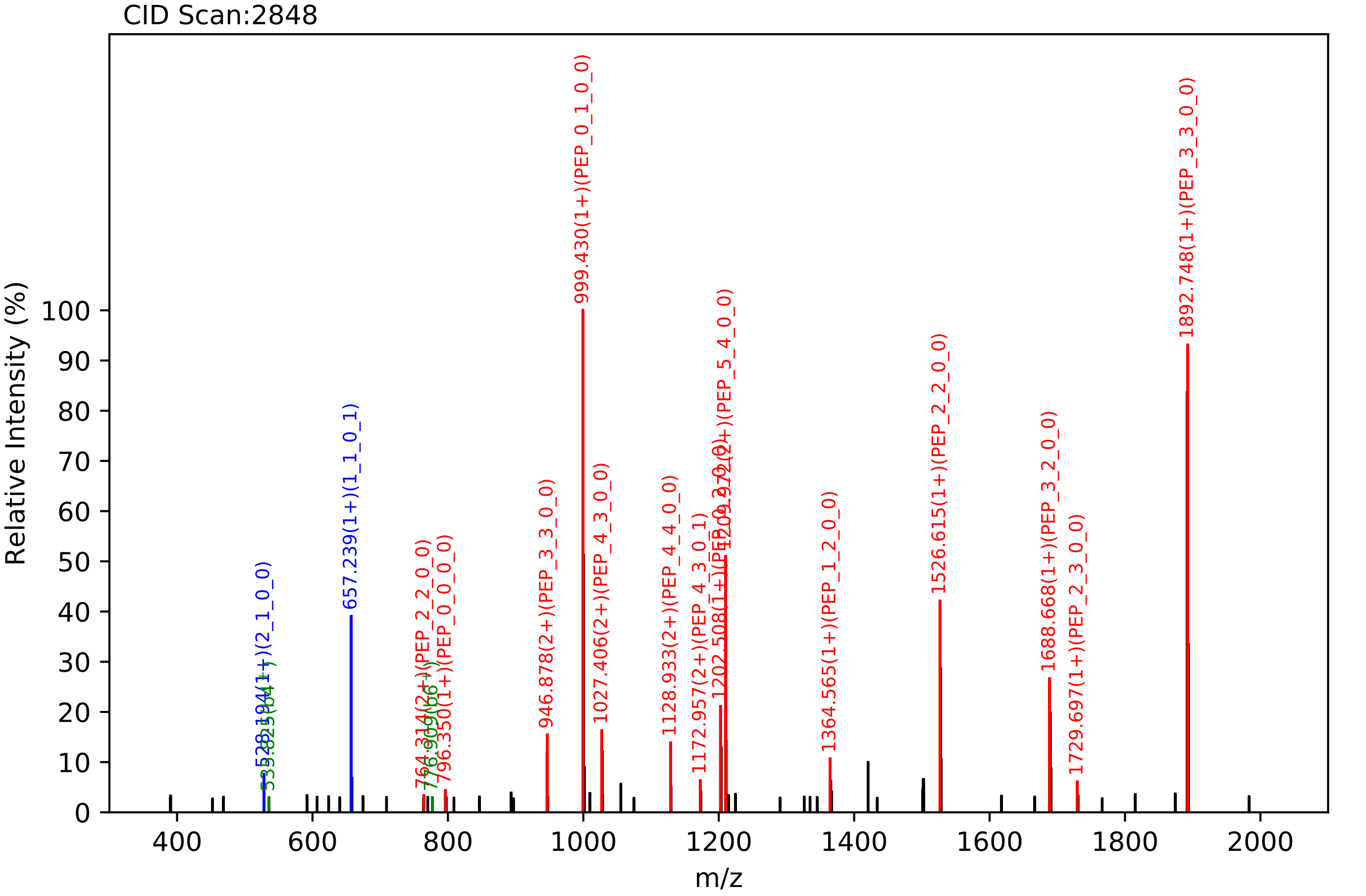

Training set no. 141, Experiment: AGP exp_1

NEEYNK(=PEP)_5_4_0_1, m/z:1355.52(2+), RT:18.94, Y-score:90.87

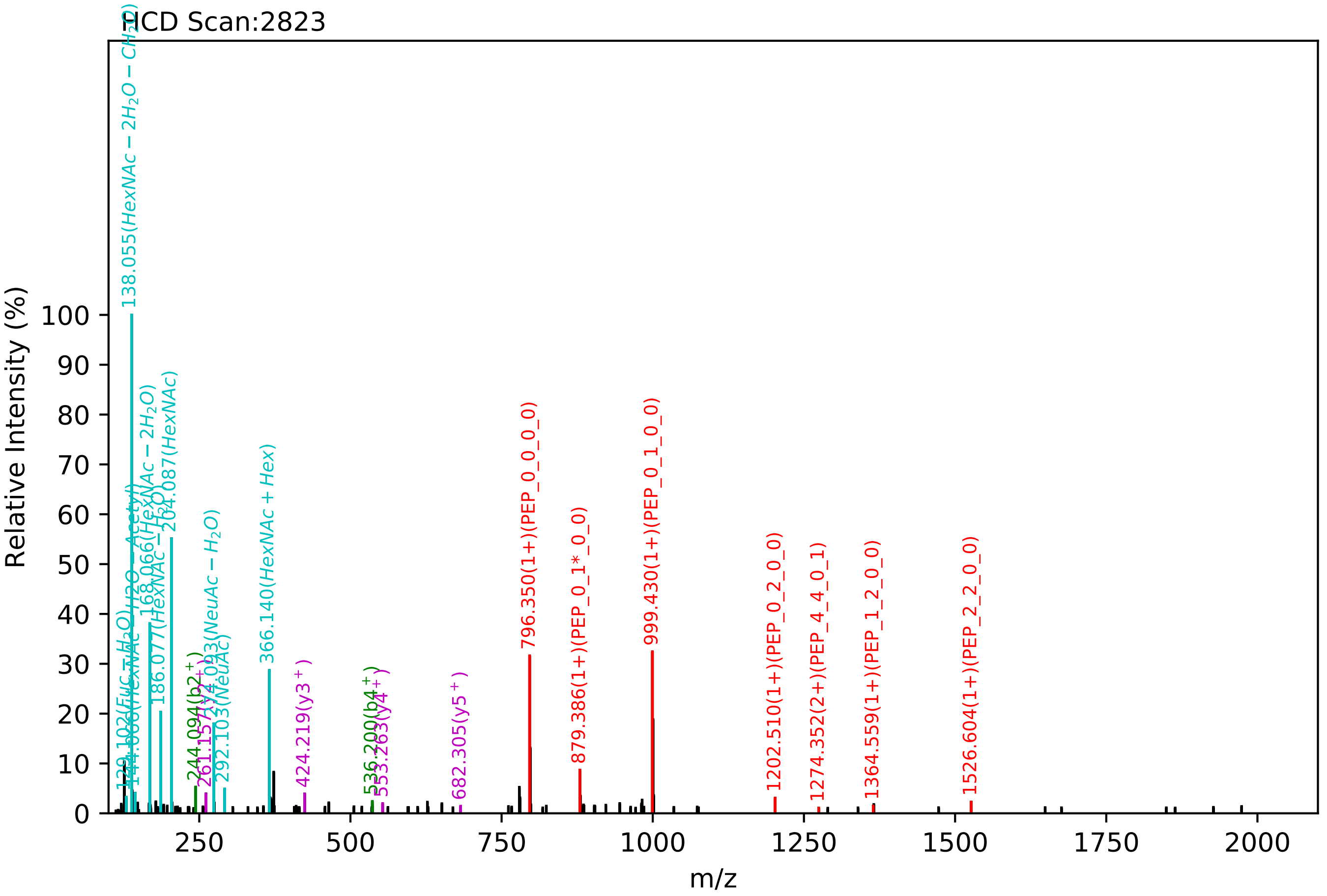

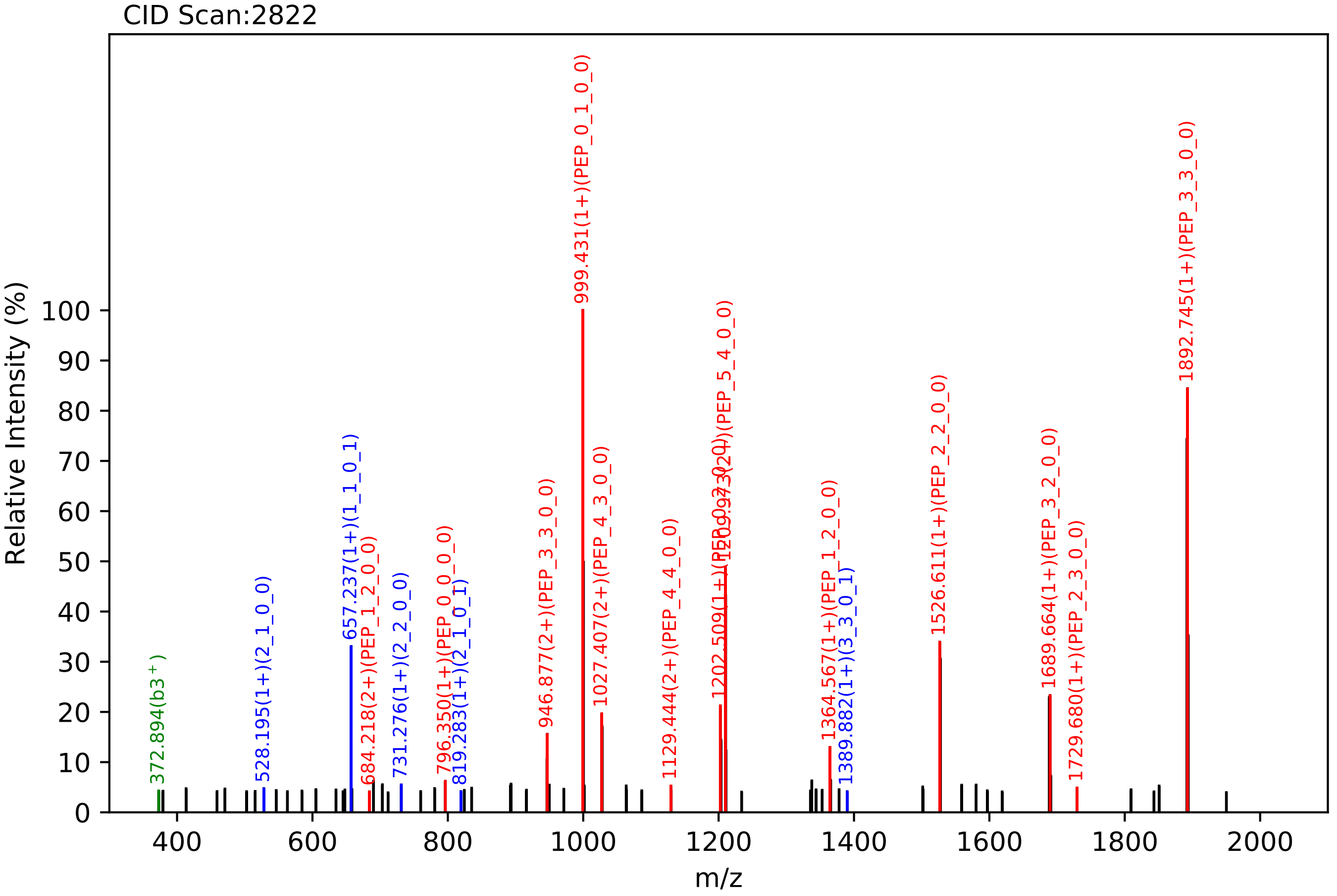

Training set no. 142, Experiment: AGP exp_1

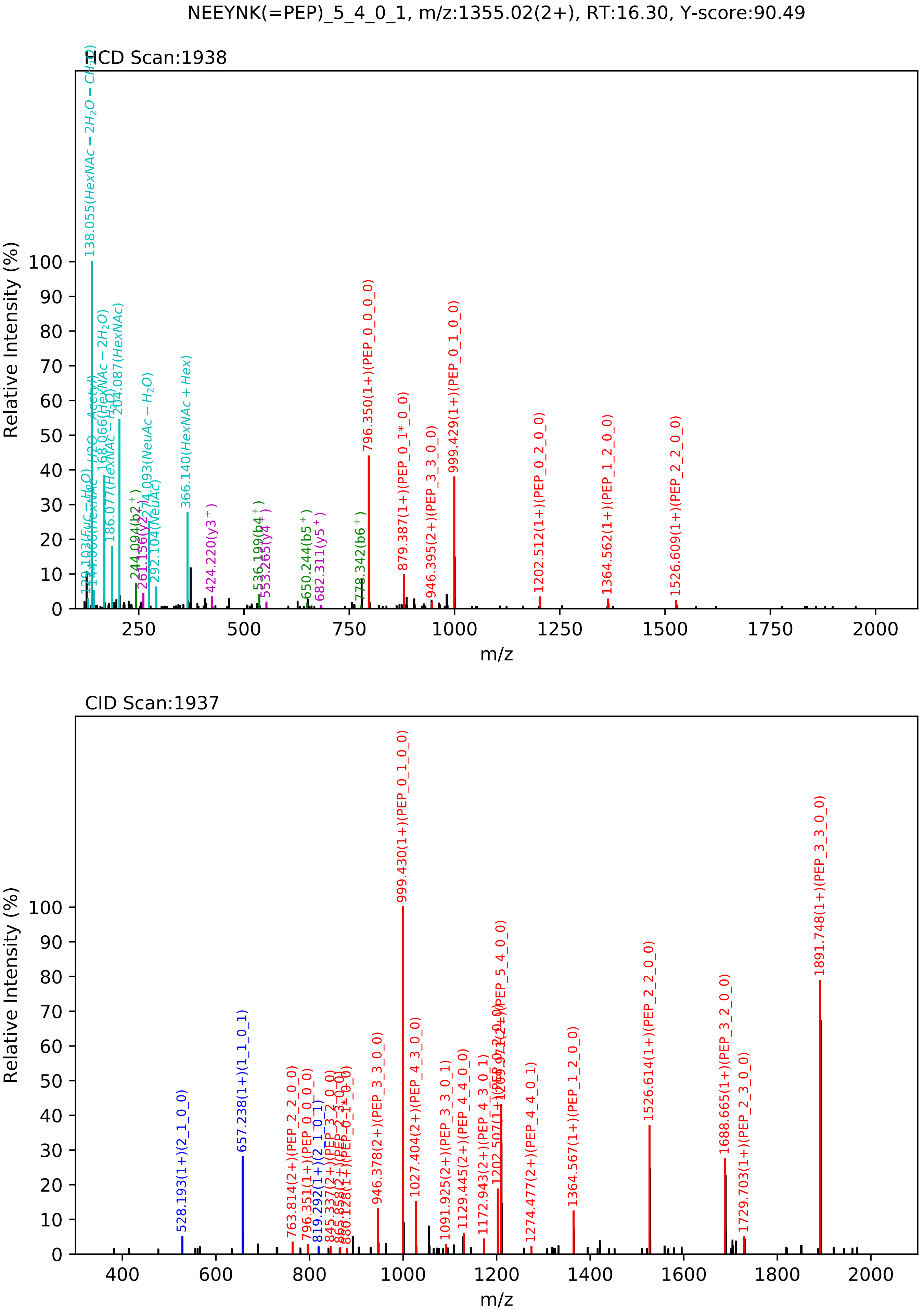

Training set no. 143, Experiment: AGP exp_1

NEEYNK(=PEP)_5_4_0_1, m/z:1355.52(2+), RT:16.81, Y-score:82.69

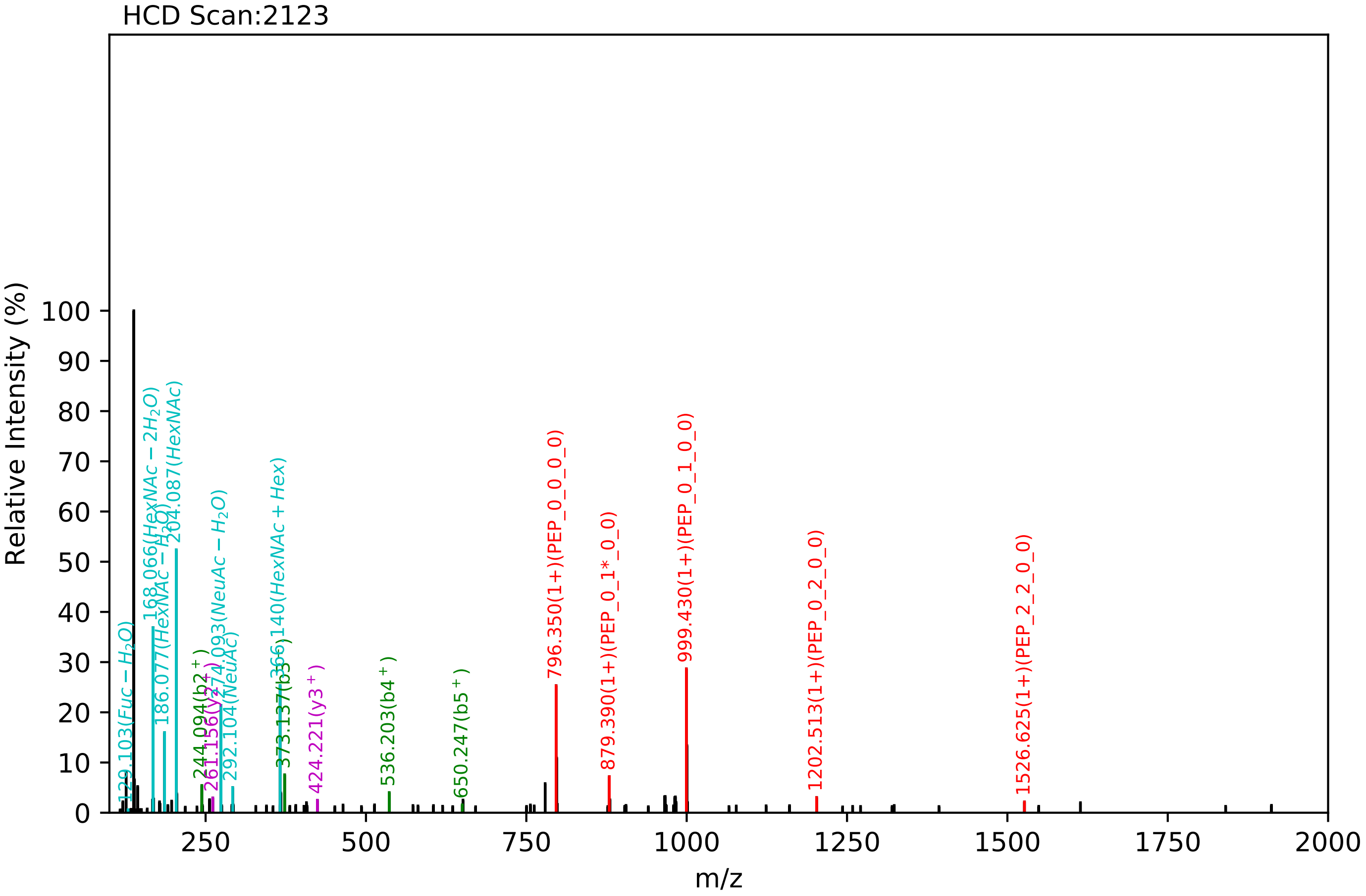

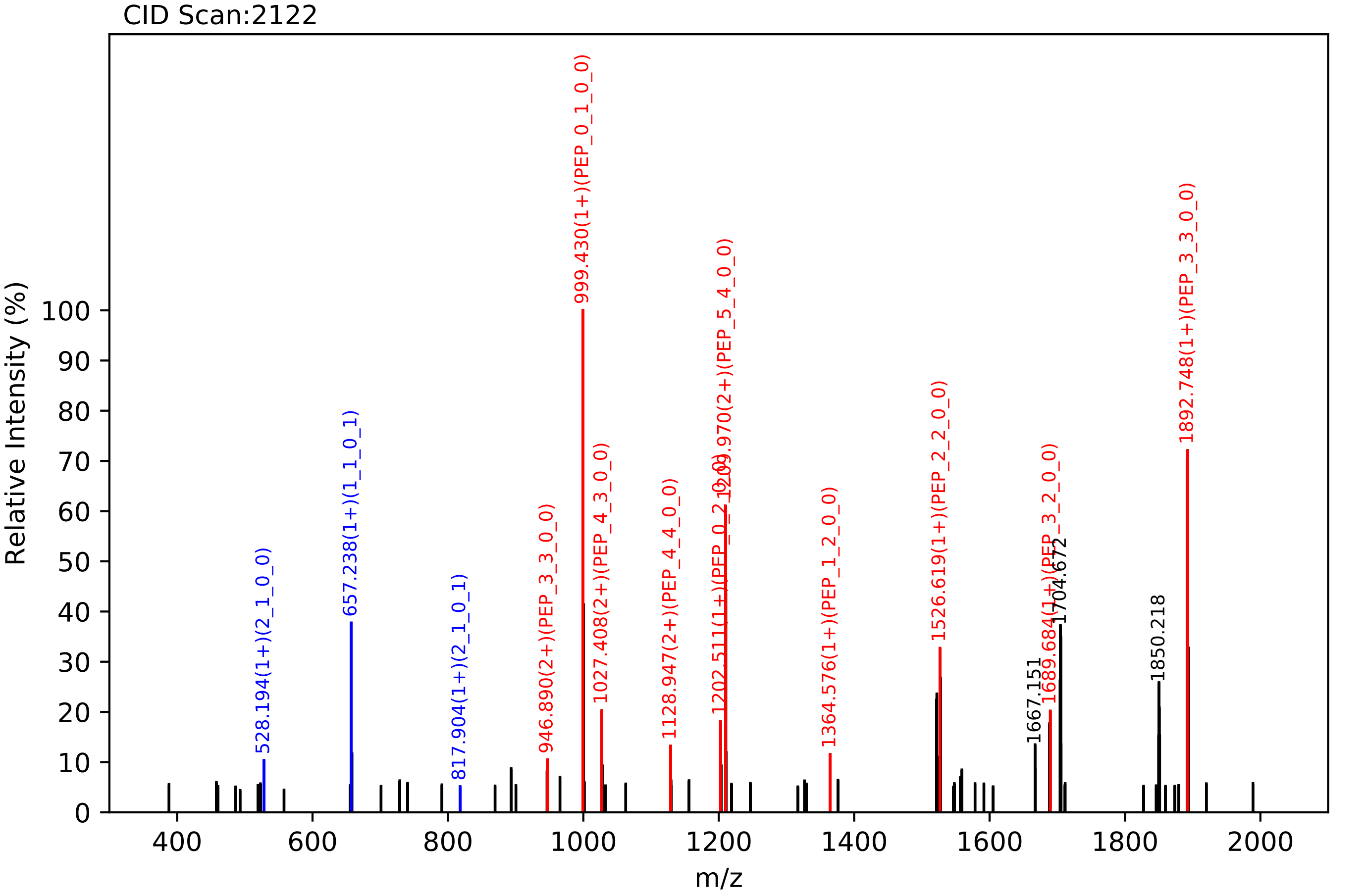

Training set no. 144, Experiment: AGP exp_2

NEEYNK(=PEP)_5_4_0_1, m/z:1355.52(2+), RT:16.90, Y-score:76.74

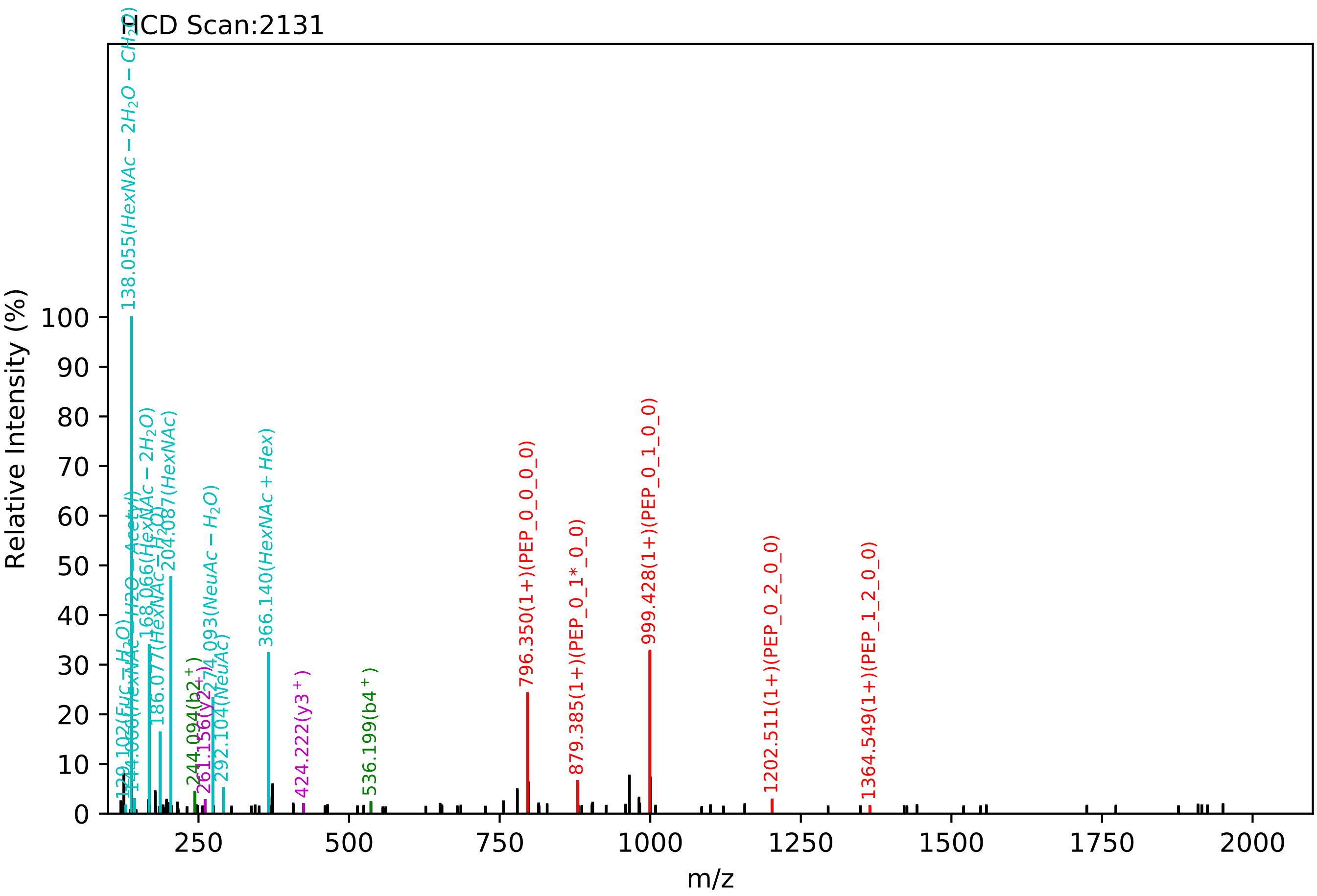

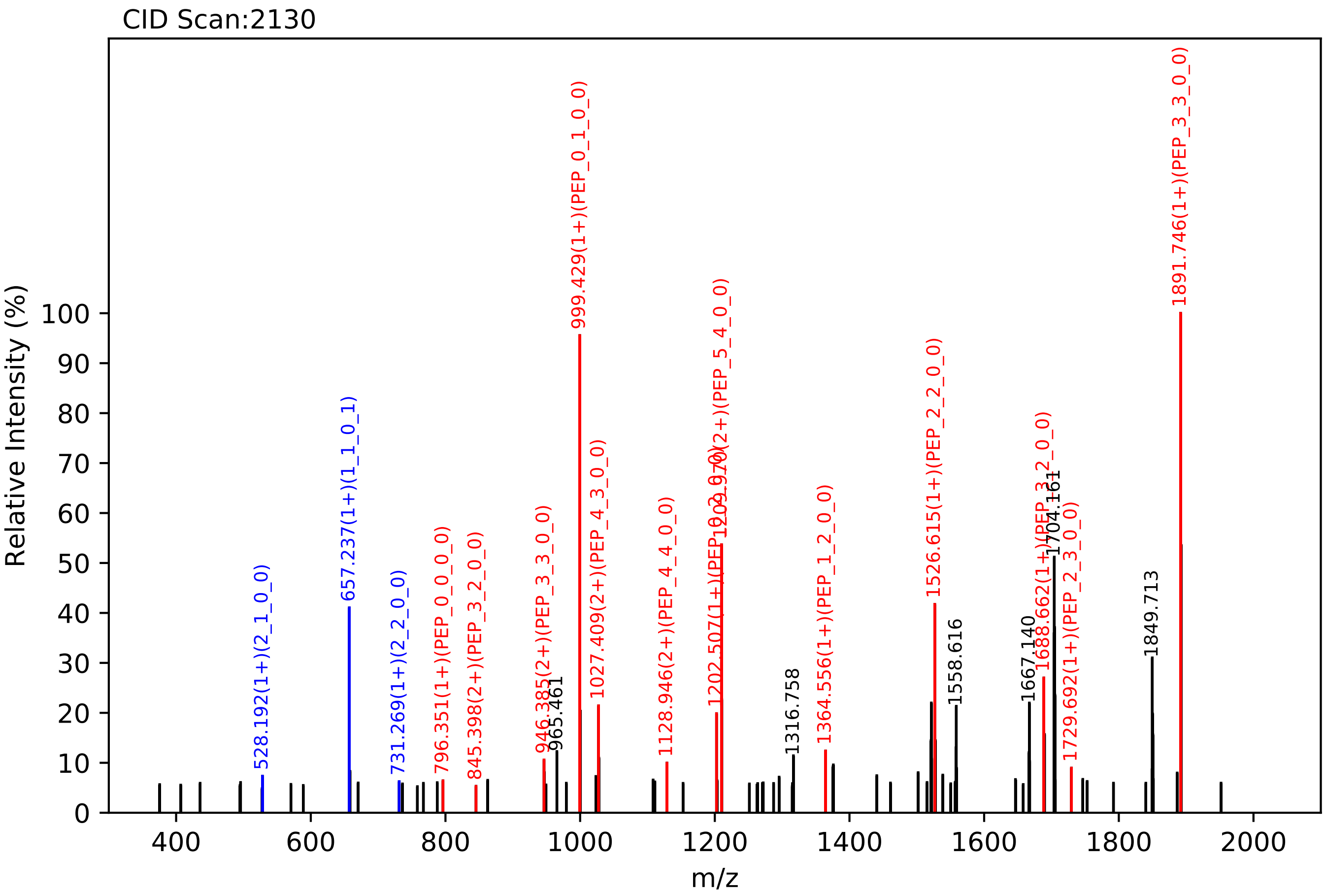

Training set no. 145, Experiment: AGP exp_2

NEEYNK(=PEP)_5_4_0_1, m/z:1356.02(2+), RT:17.40, Y-score:74.66

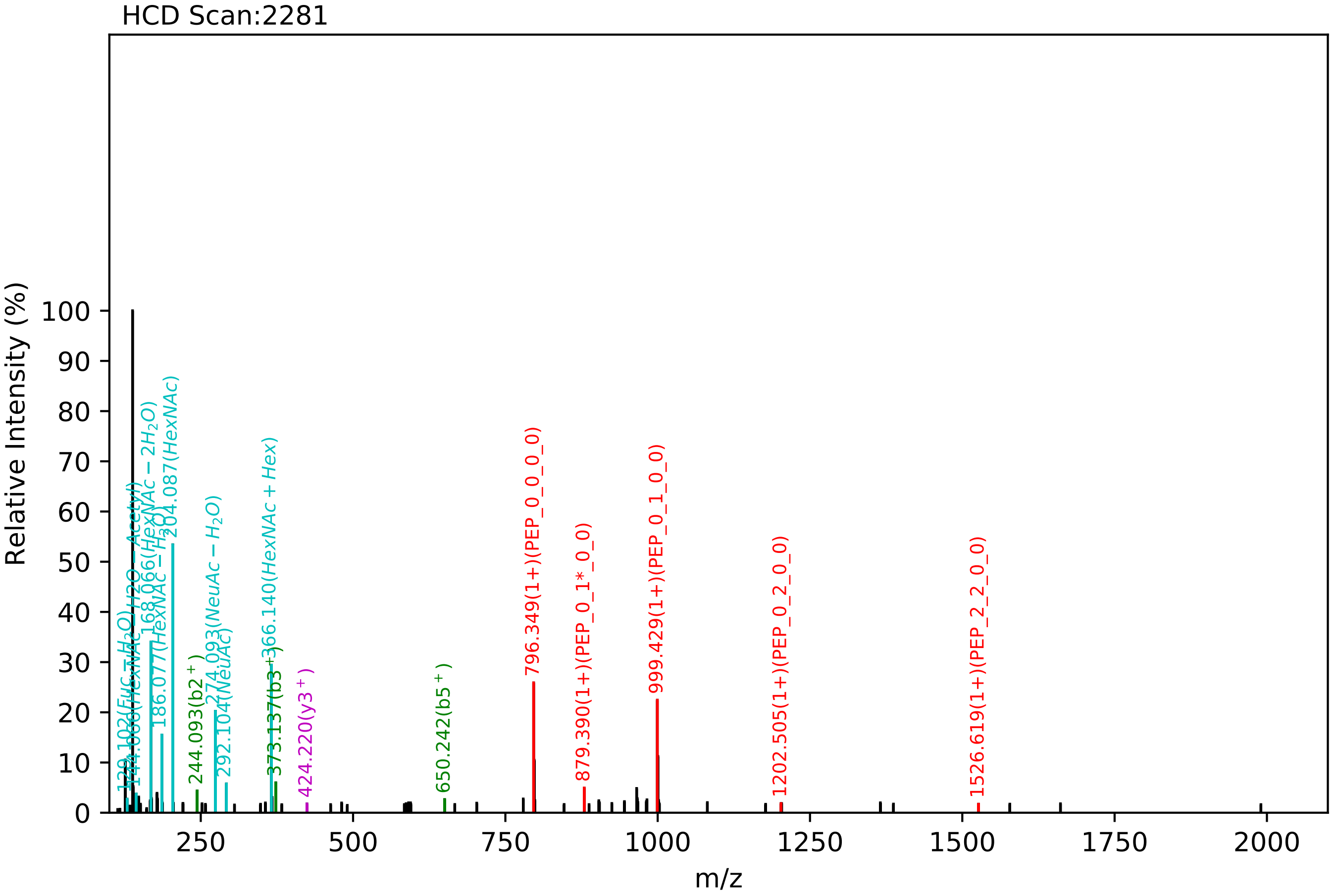

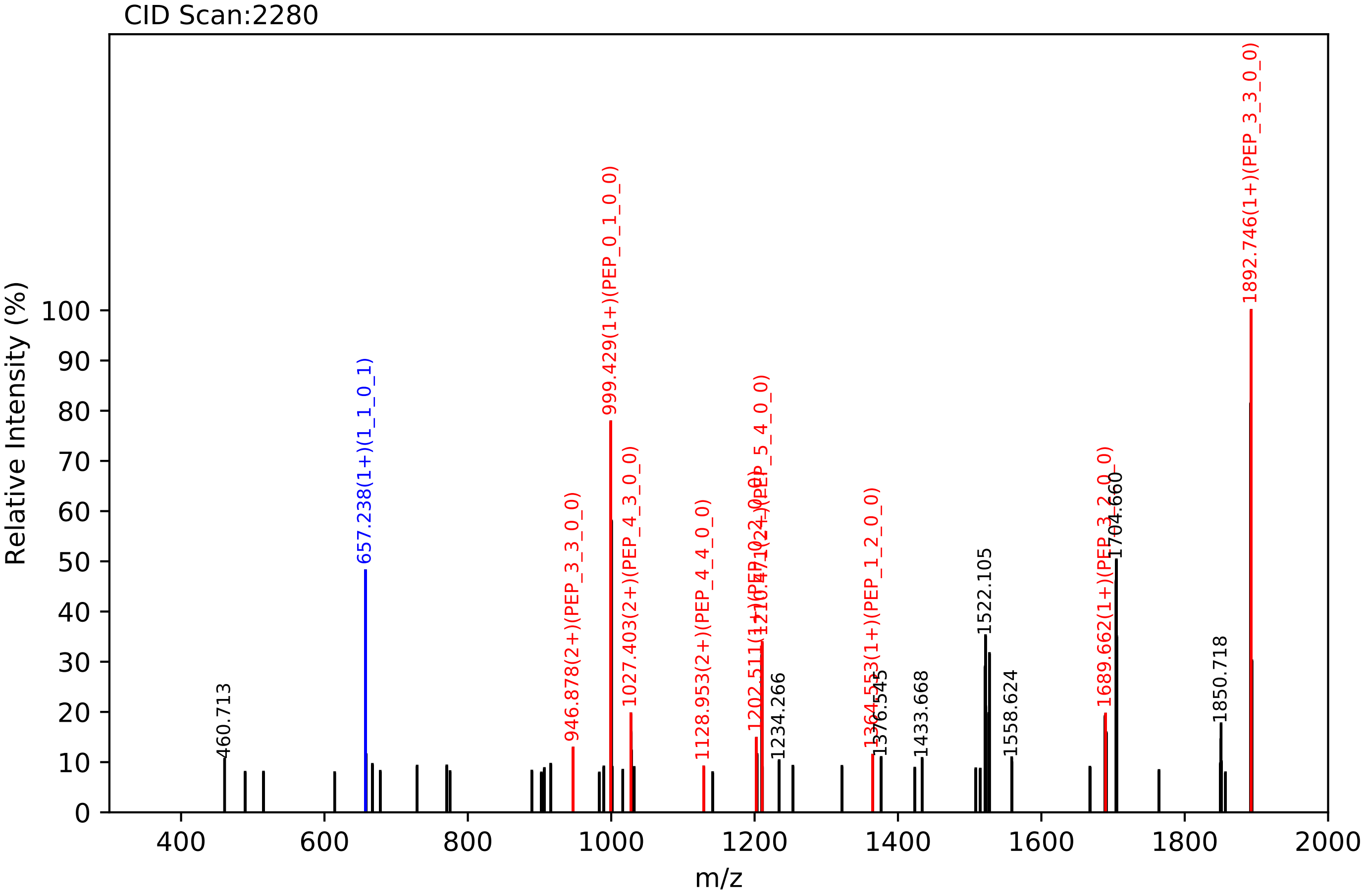

Training set no. 146, Experiment: AGP exp_1

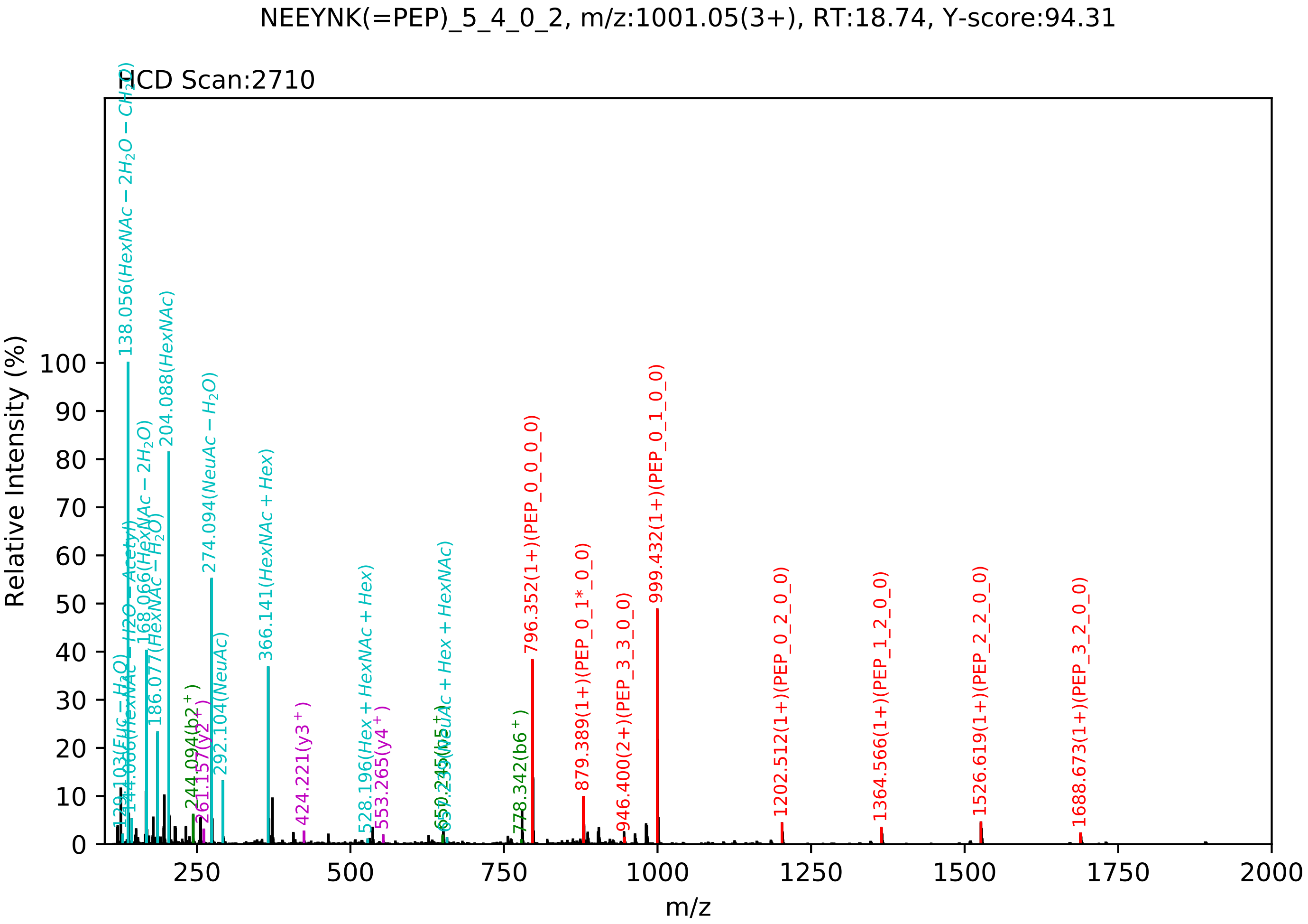

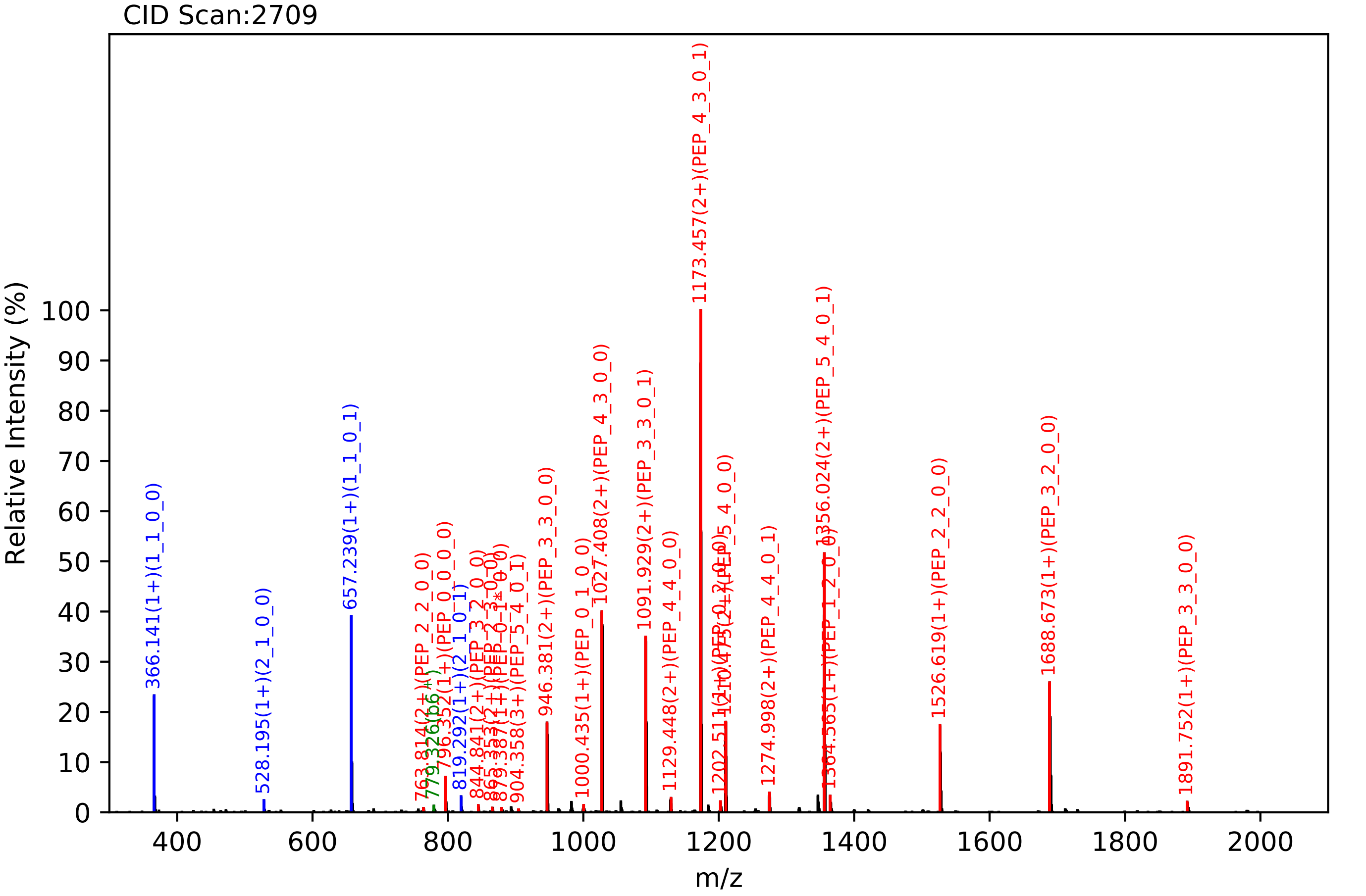

Training set no. 147, Experiment: AGP exp_2

NEEYNK(=PEP)_5_4_0_2, m/z:1001.05(3+), RT:20.24, Y-score:93.99

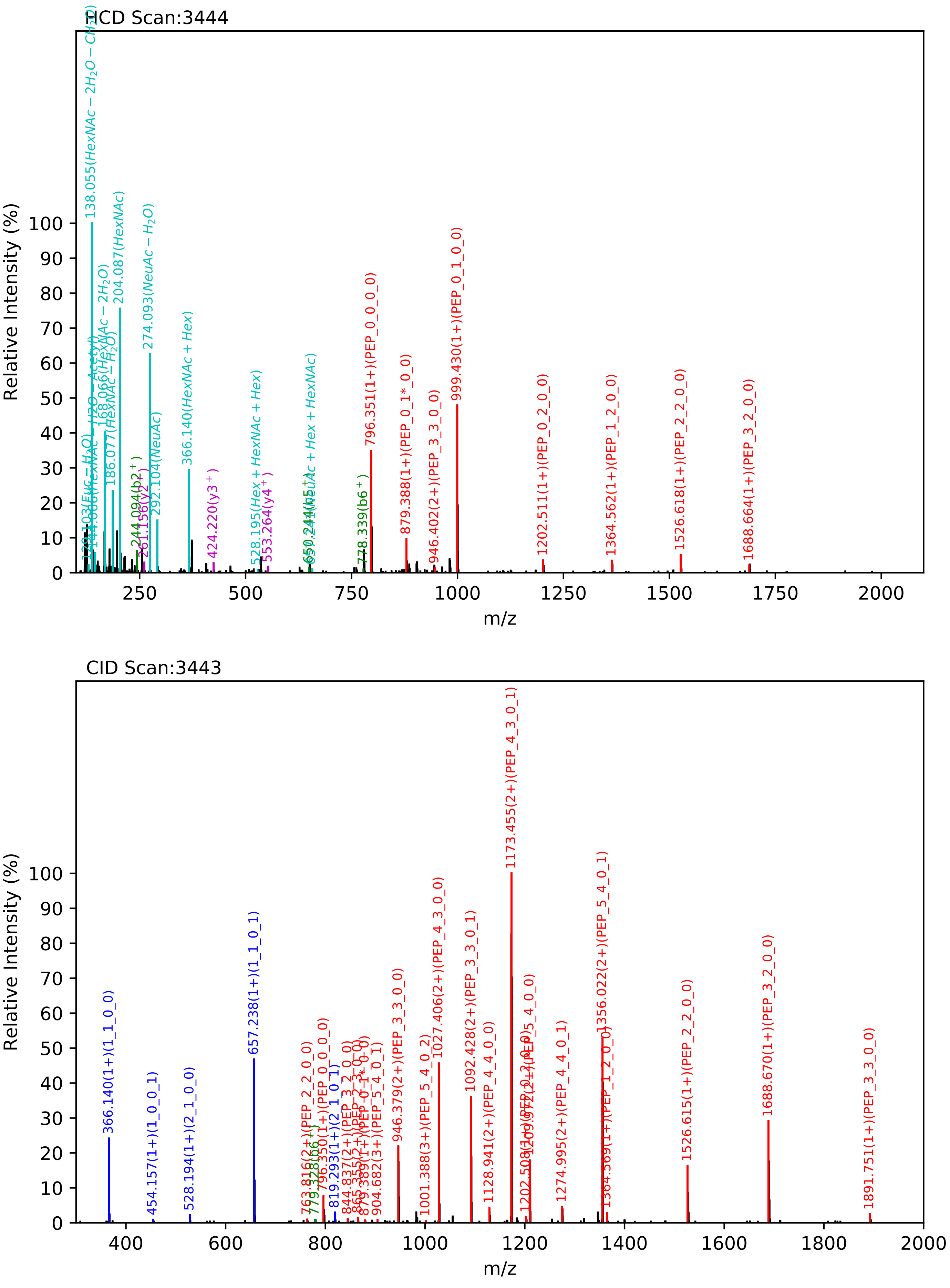

Training set no. 148, Experiment: AGP exp_2

NEEYNK(=PEP)_5_4_0_2, m/z:1002.05(3+), RT:18.92, Y-score:89.78

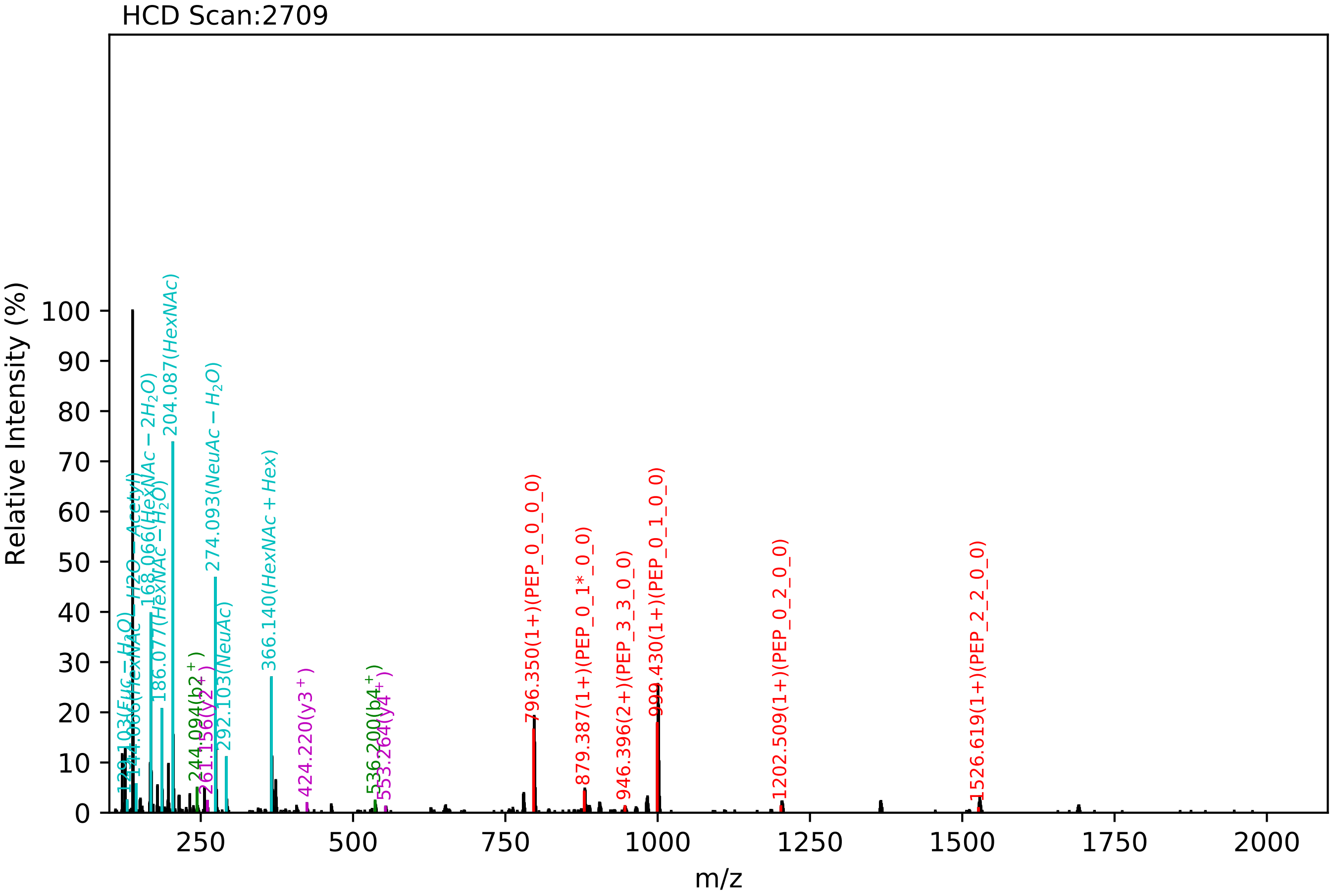

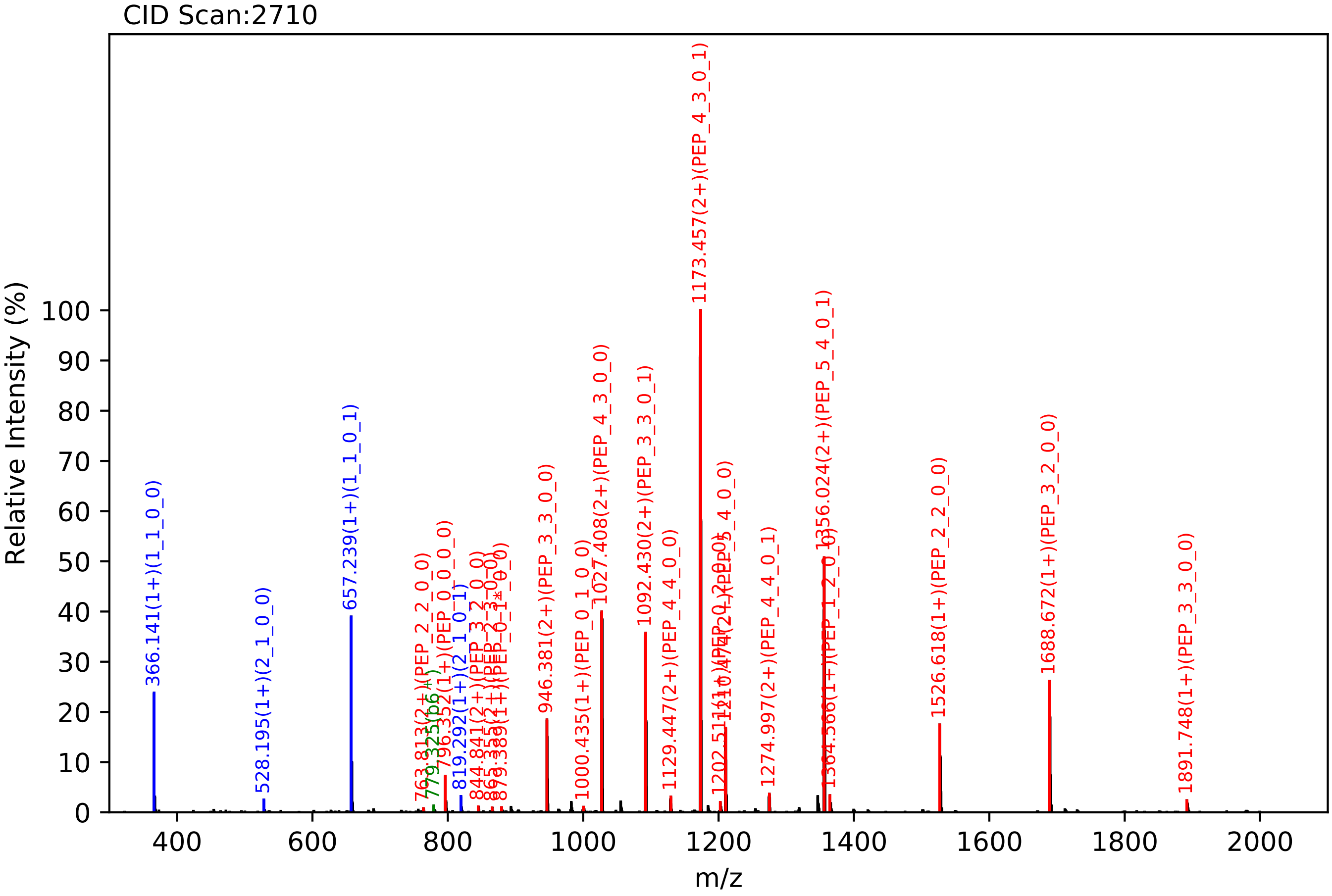

Training set no. 149, Experiment: AGP exp_1

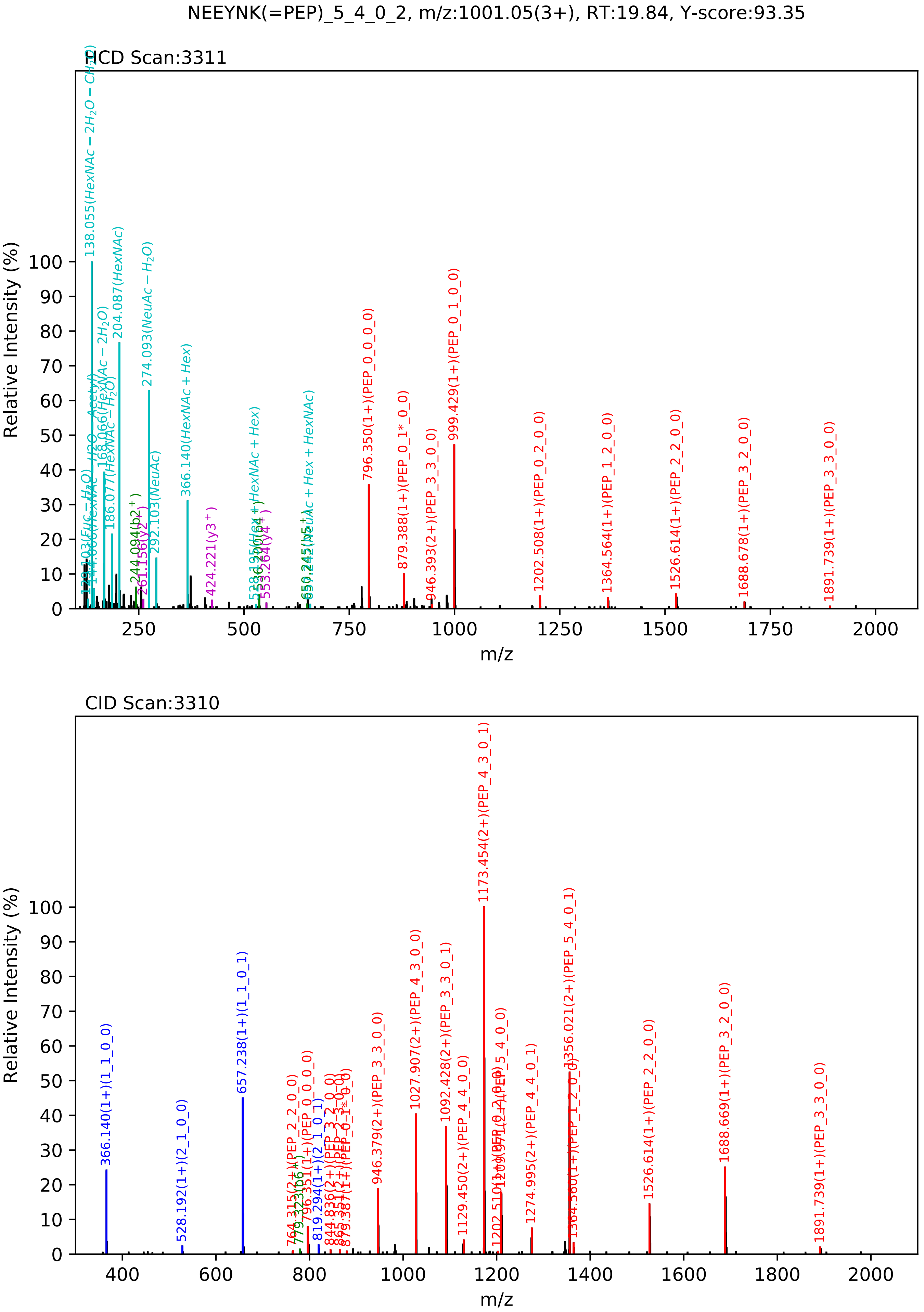

Training set no. 150, Experiment: AGP exp_2

NEEYNK(=PEP)_5_4_0_2, m/z:1001.05(3+), RT:19.09, Y-score:93.23

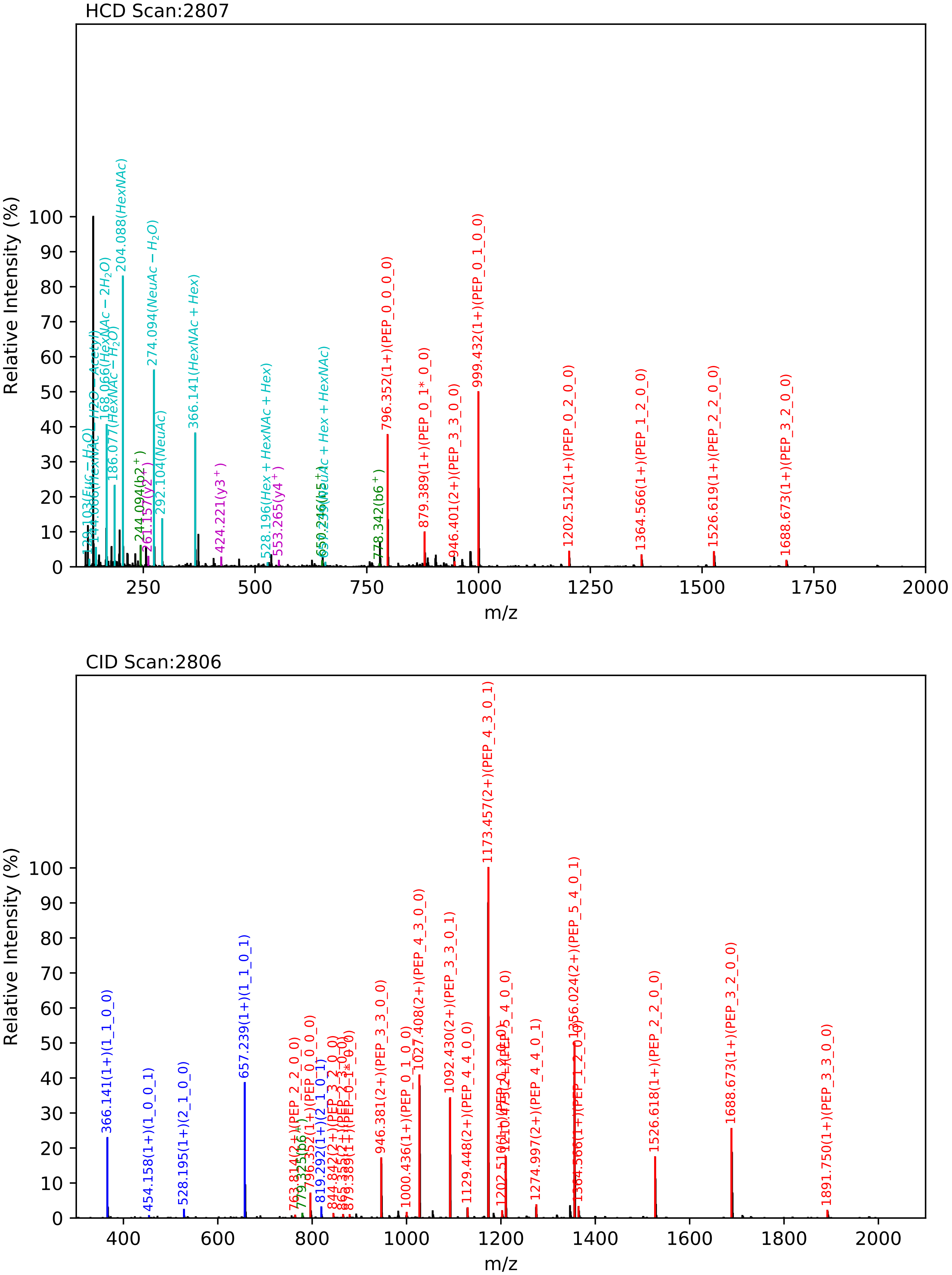

Training set no. 151, Experiment: AGP exp_1

NEEYNK(=PEP)_5_4_0_2, m/z:1001.05(3+), RT:19.33, Y-score:93.20

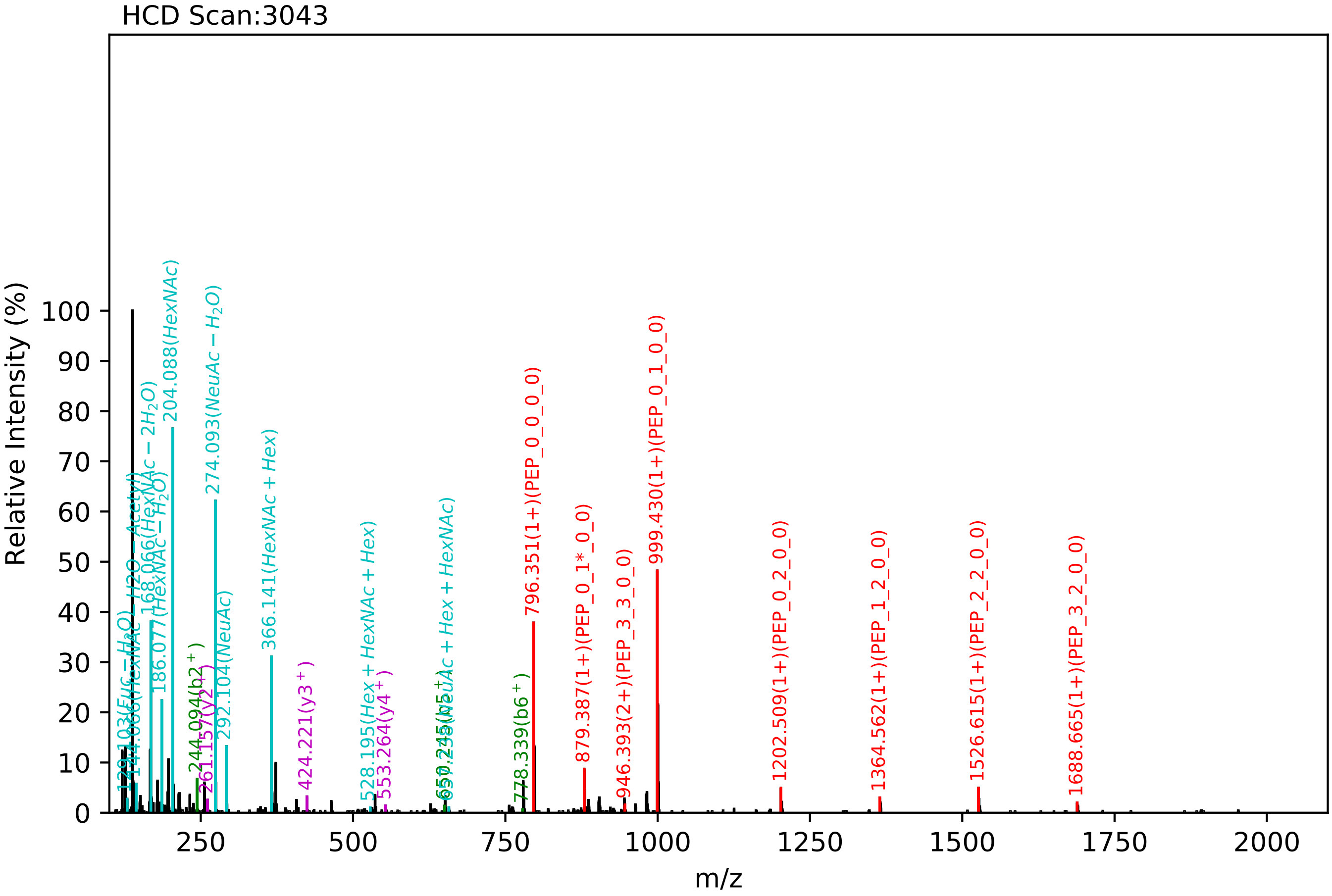

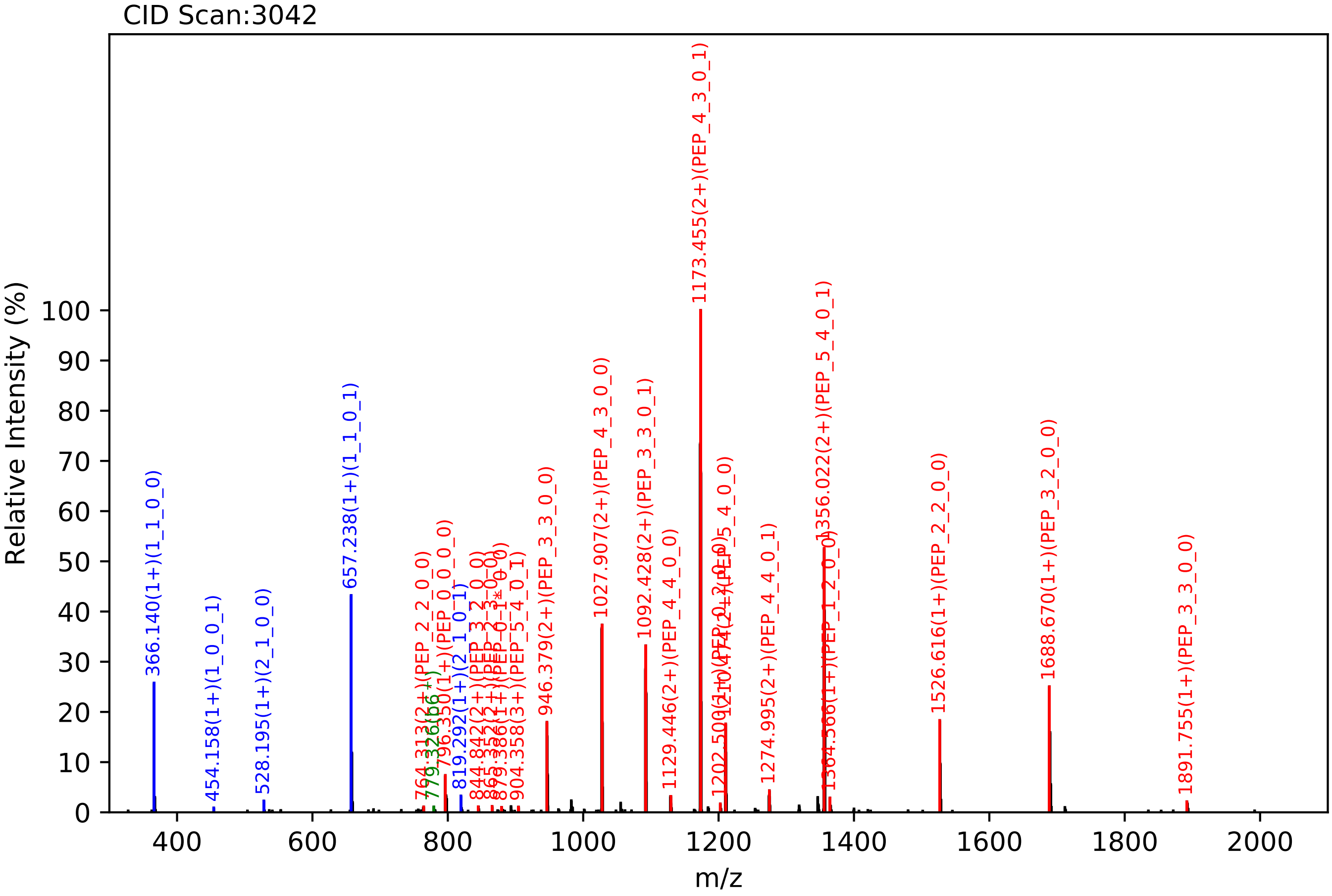

Training set no. 152, Experiment: AGP exp_2

NEEYNK(=PEP)_5_4_0_2, m/z:1001.05(3+), RT:19.69, Y-score:93.11

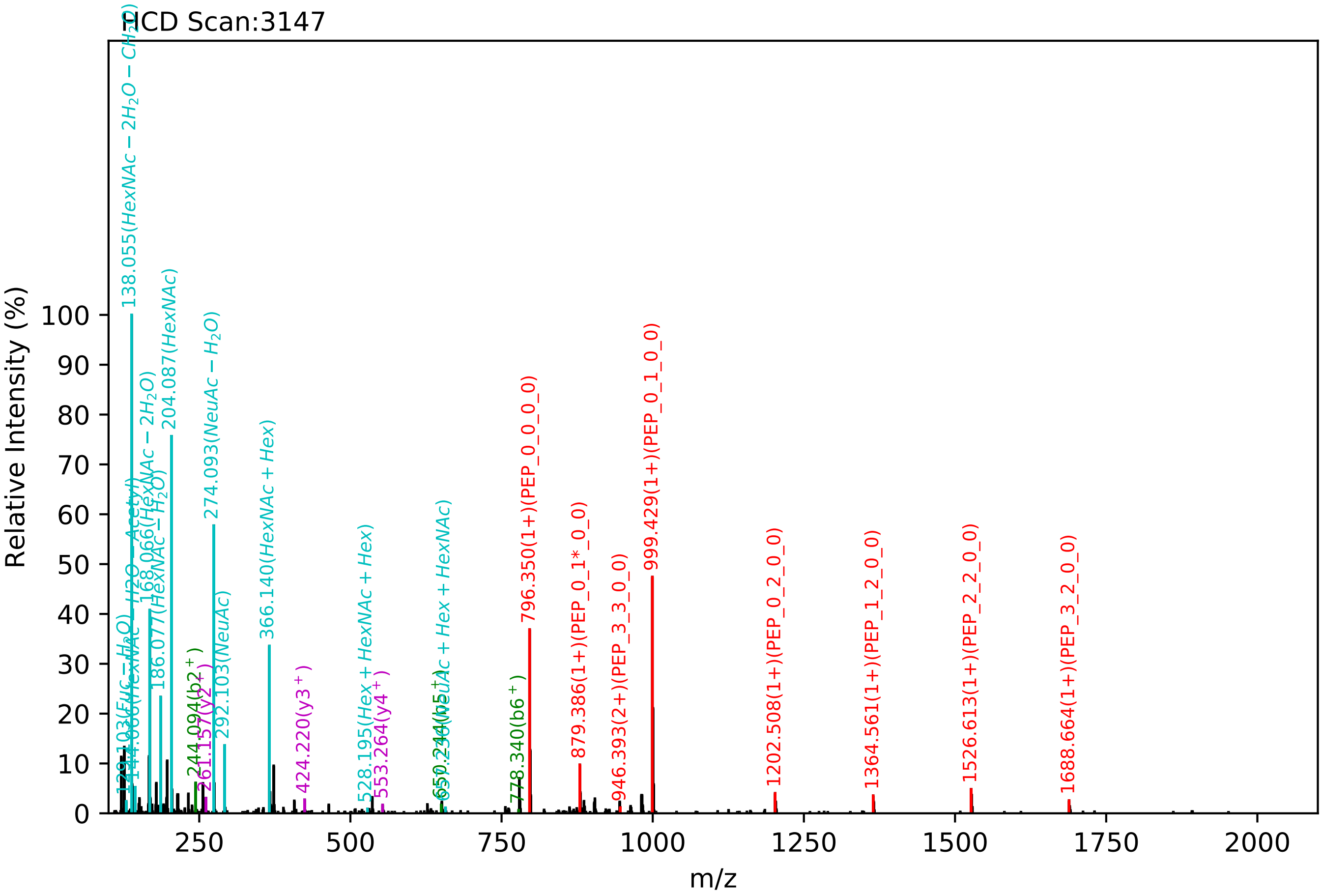

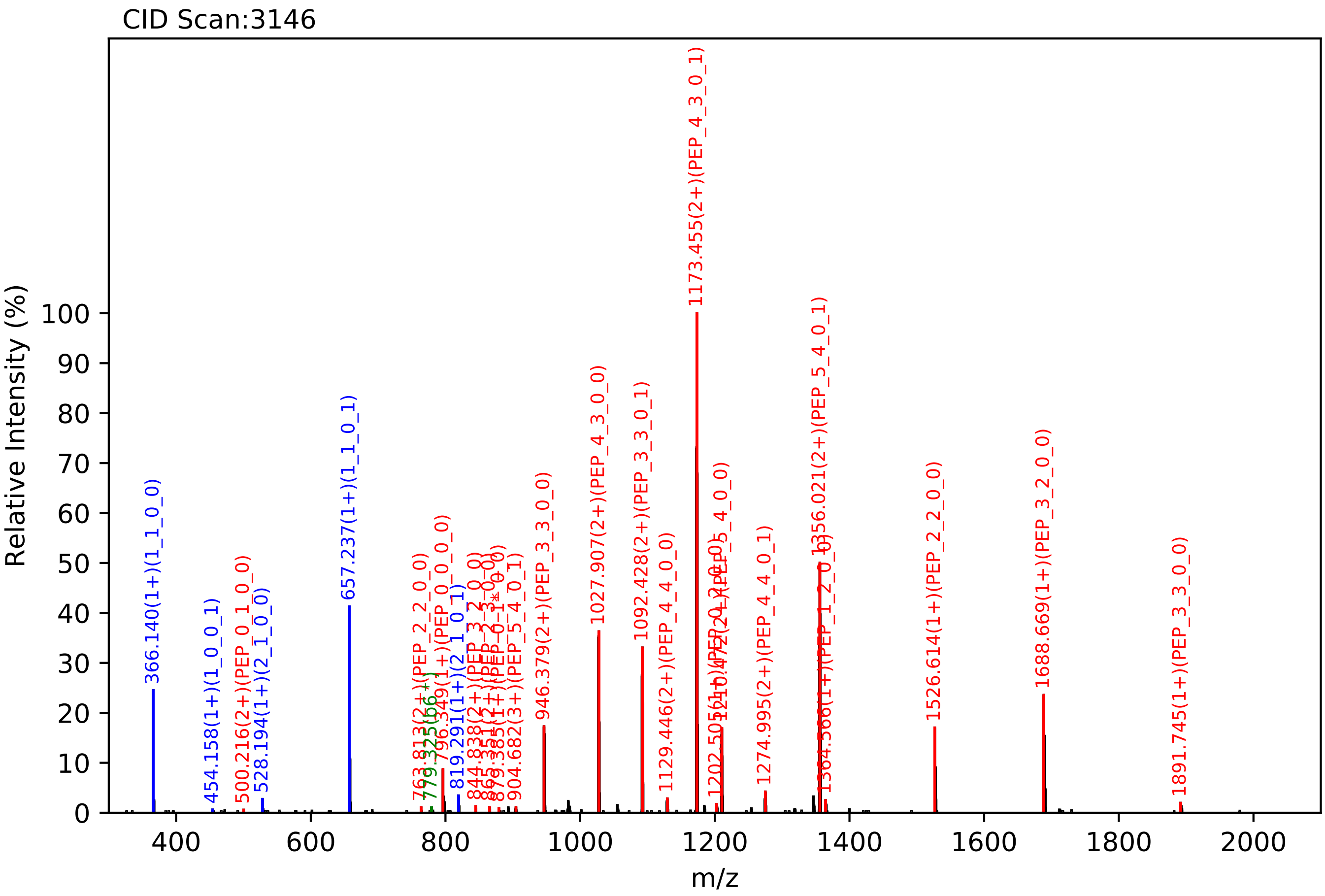

Training set no. 153, Experiment: AGP exp_2

NEEYNK(=PEP)_5_4_0_2, m/z:1501.57(2+), RT:18.61, Y-score:92.44

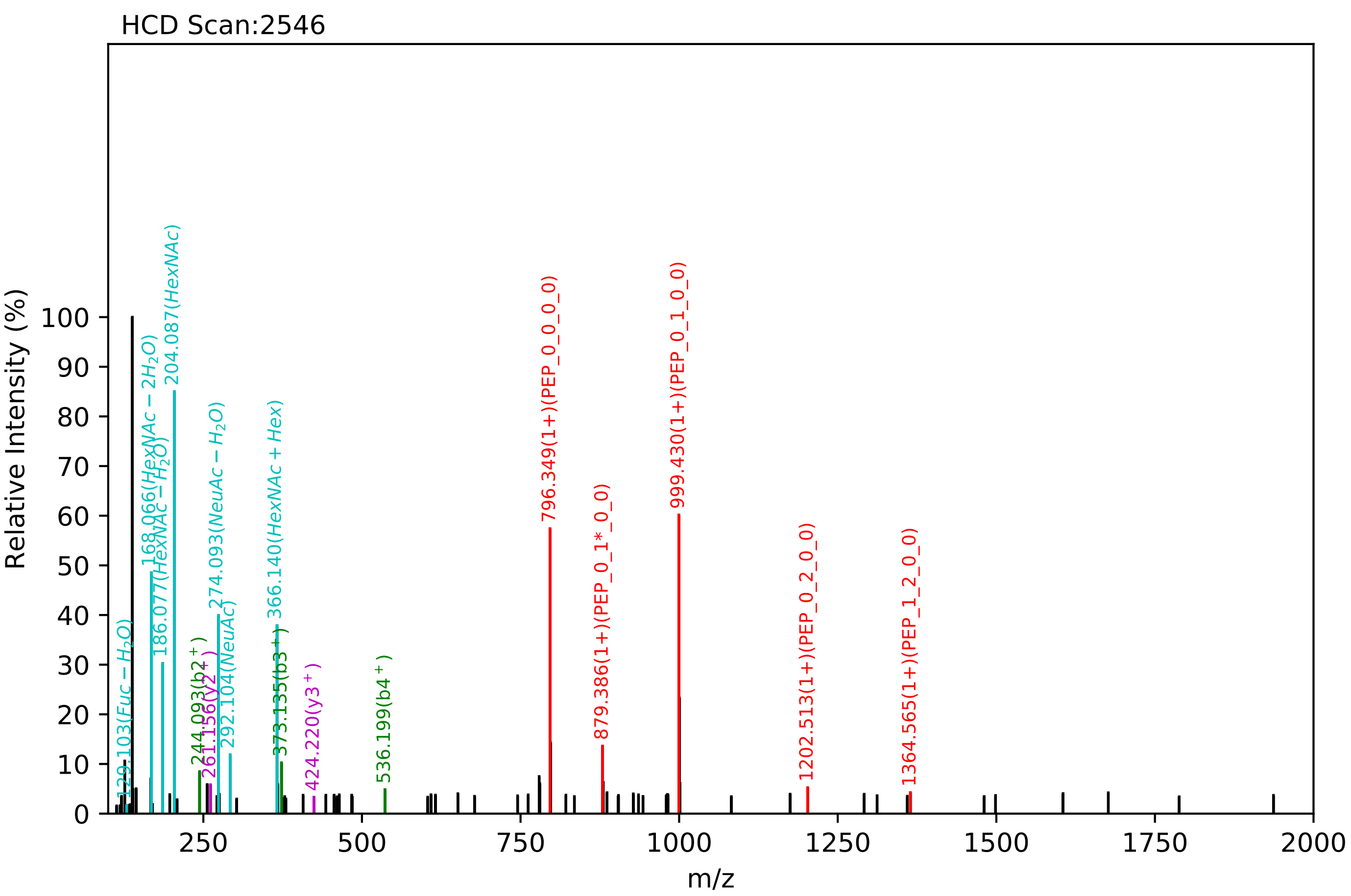

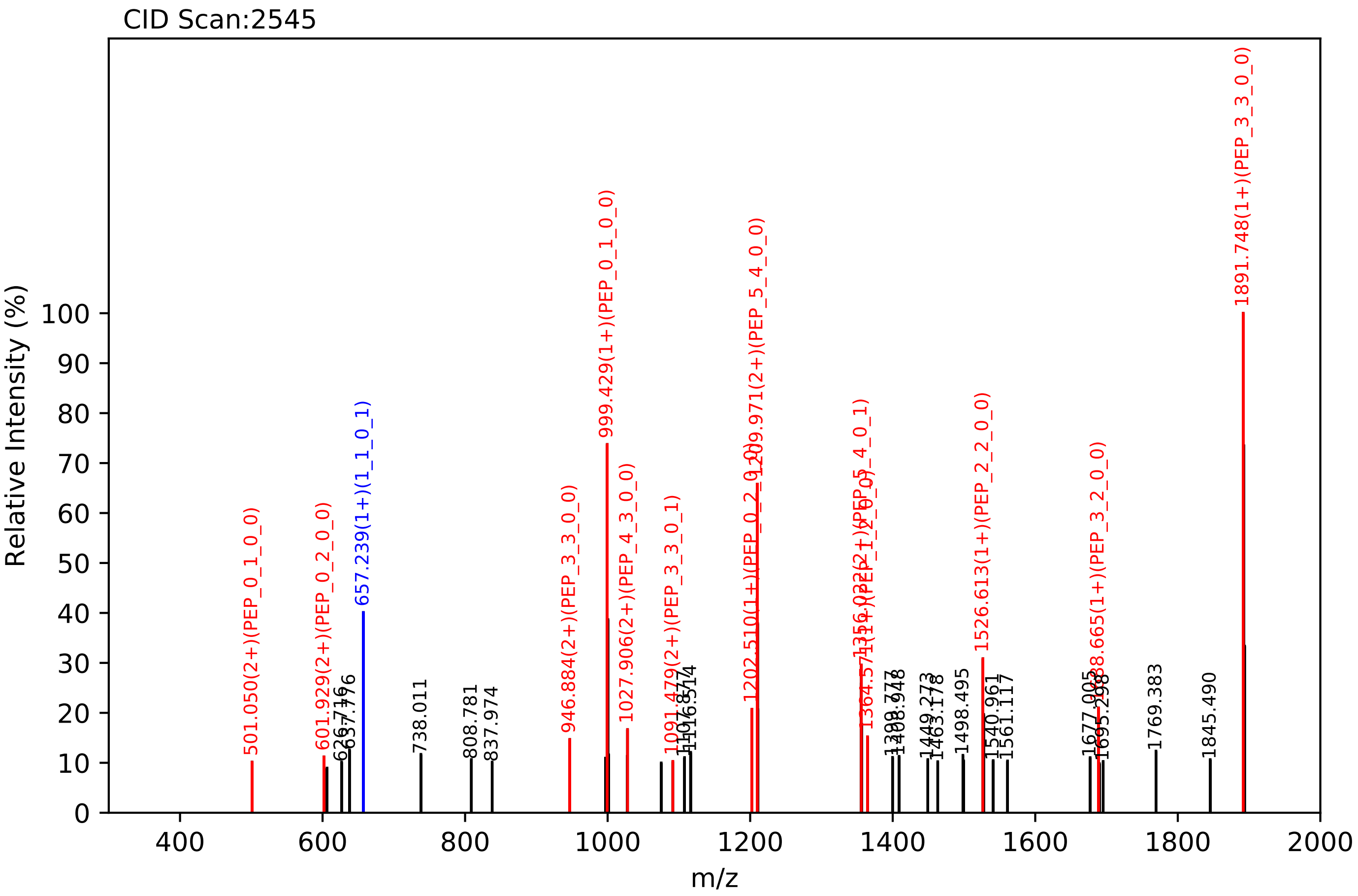

Training set no. 154, Experiment: AGP exp_2

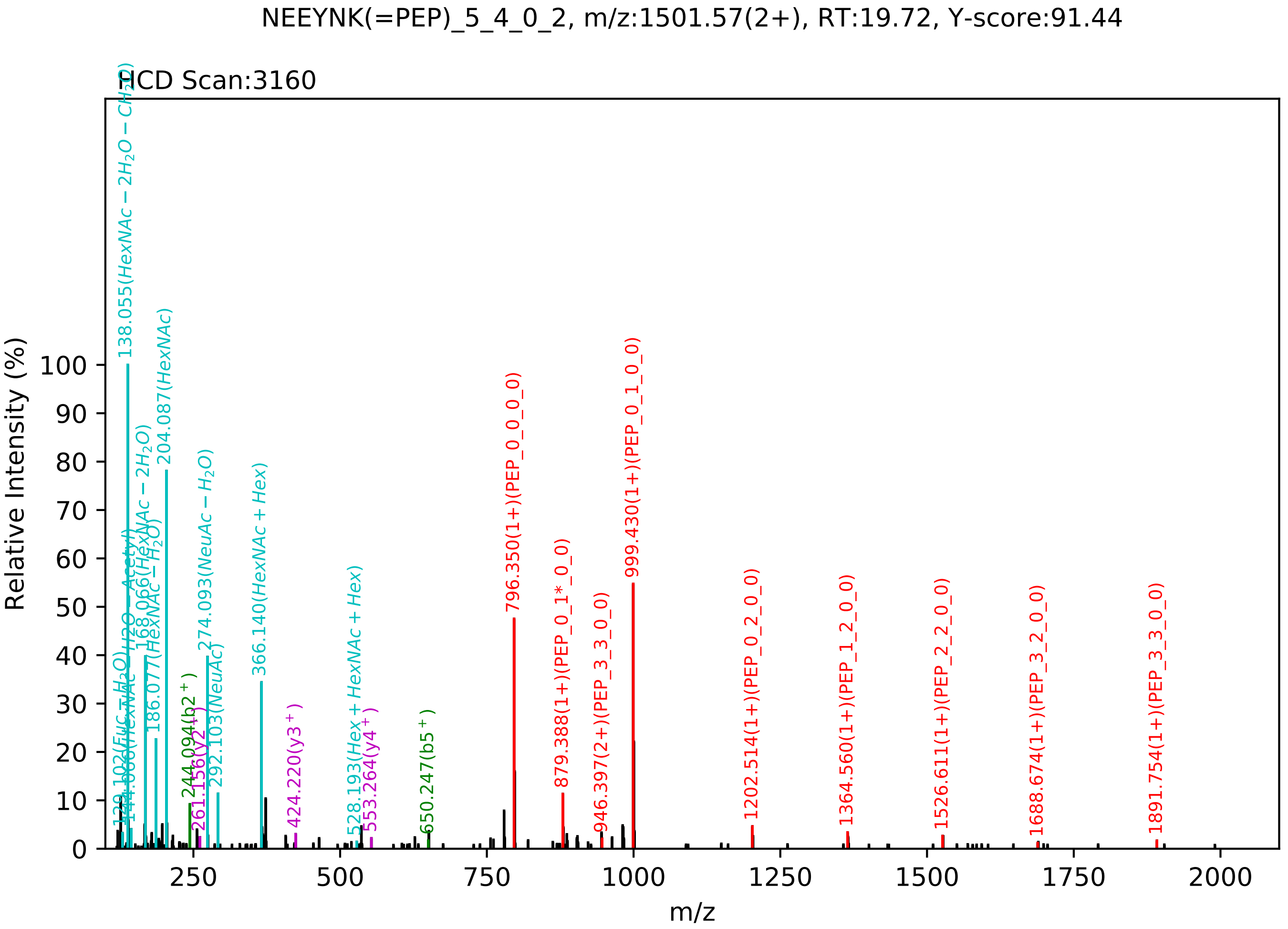

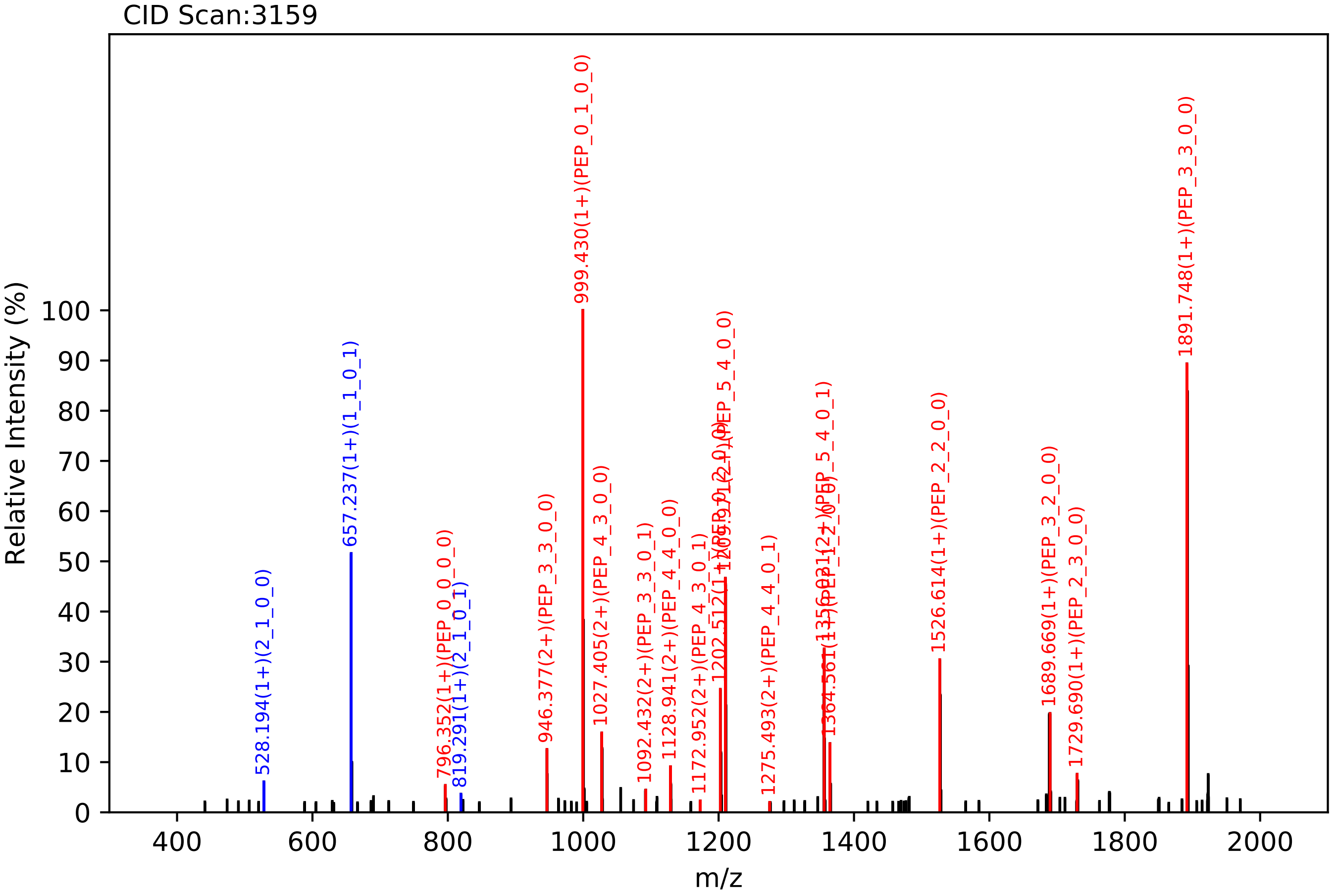

Training set no. 155, Experiment: AGP exp_2

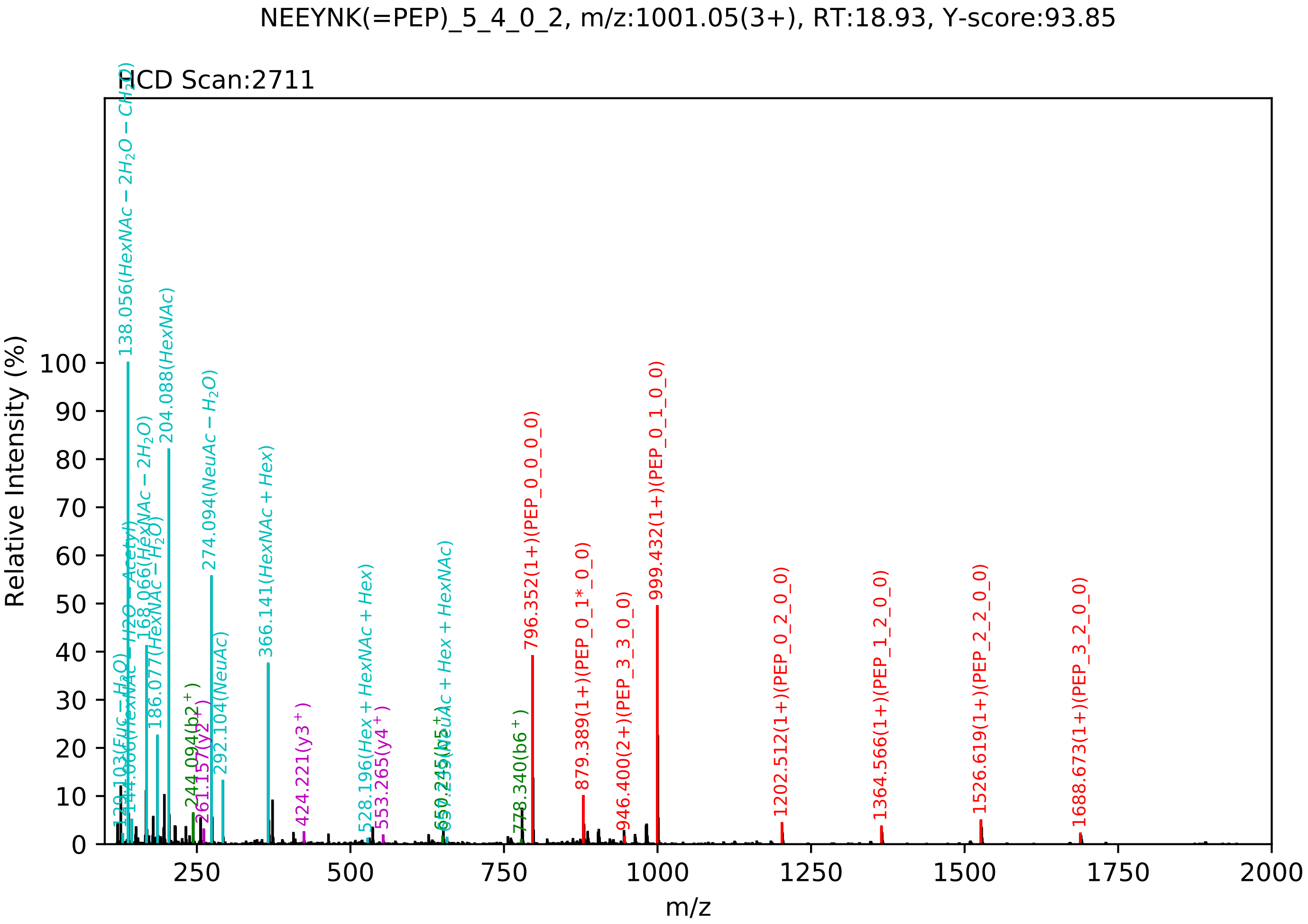

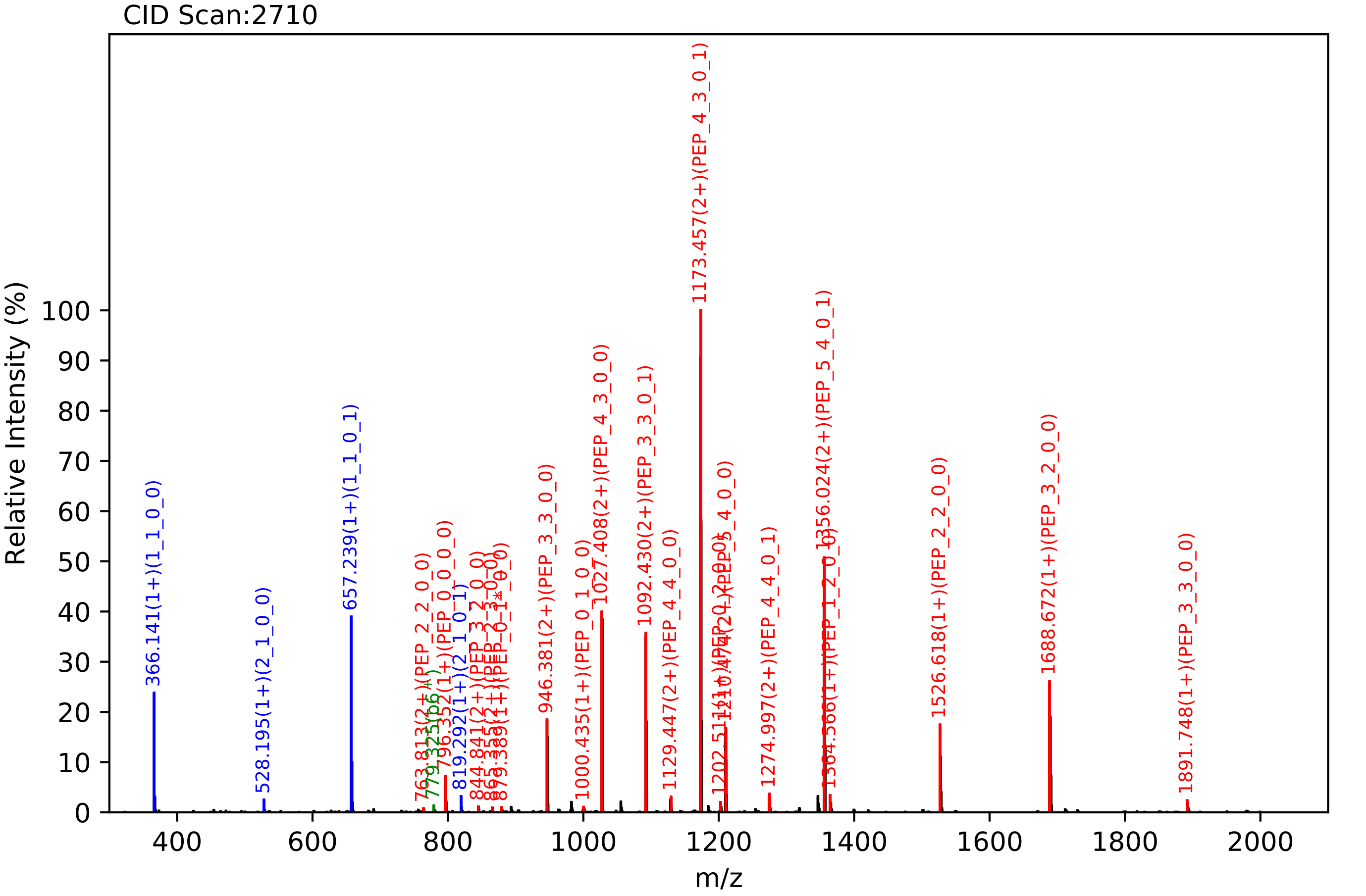

Training set no. 156, Experiment: AGP exp_1

NEEYNK(=PEP)_5_4_0_2, m/z:1501.57(2+), RT:19.46, Y-score:89.31

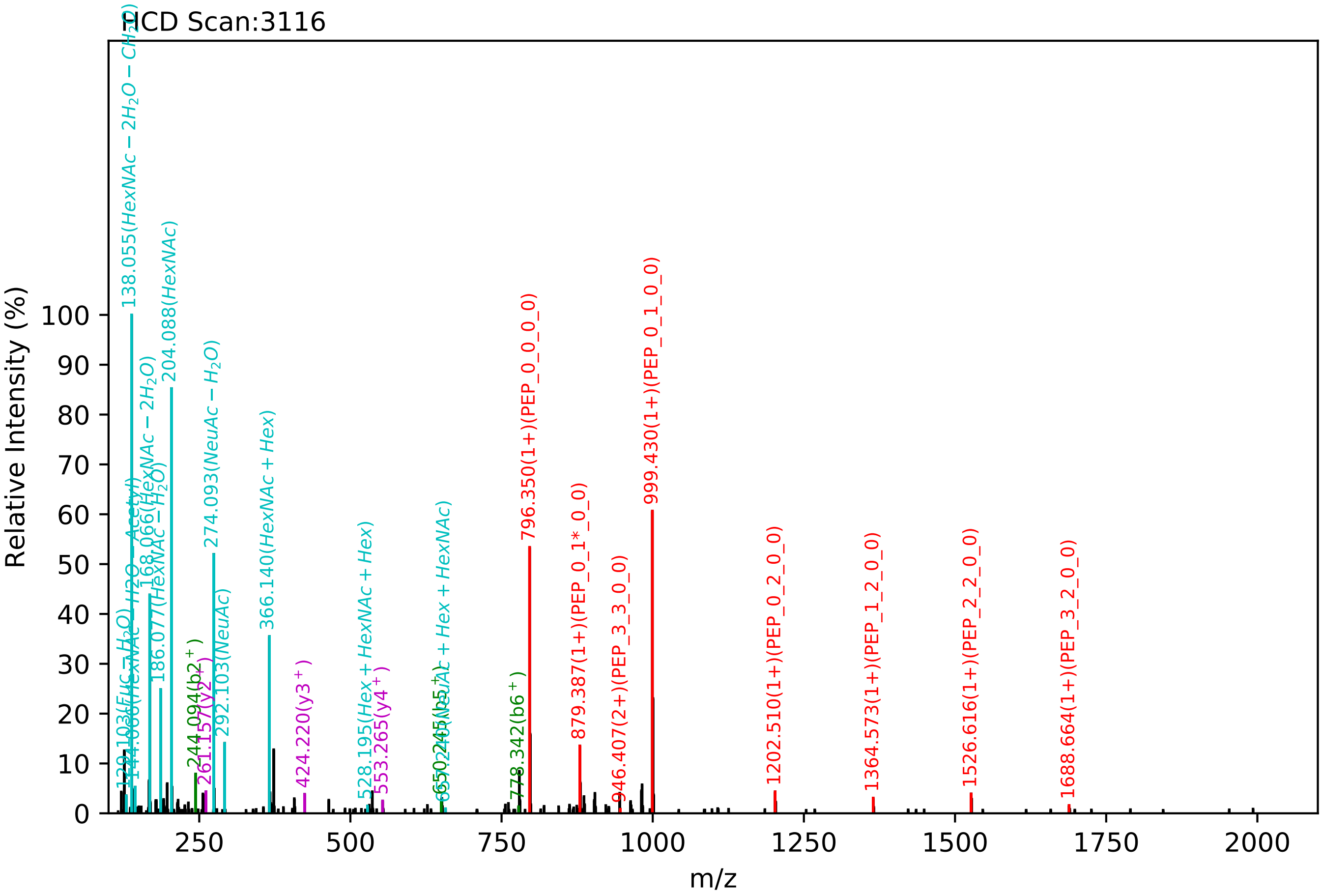

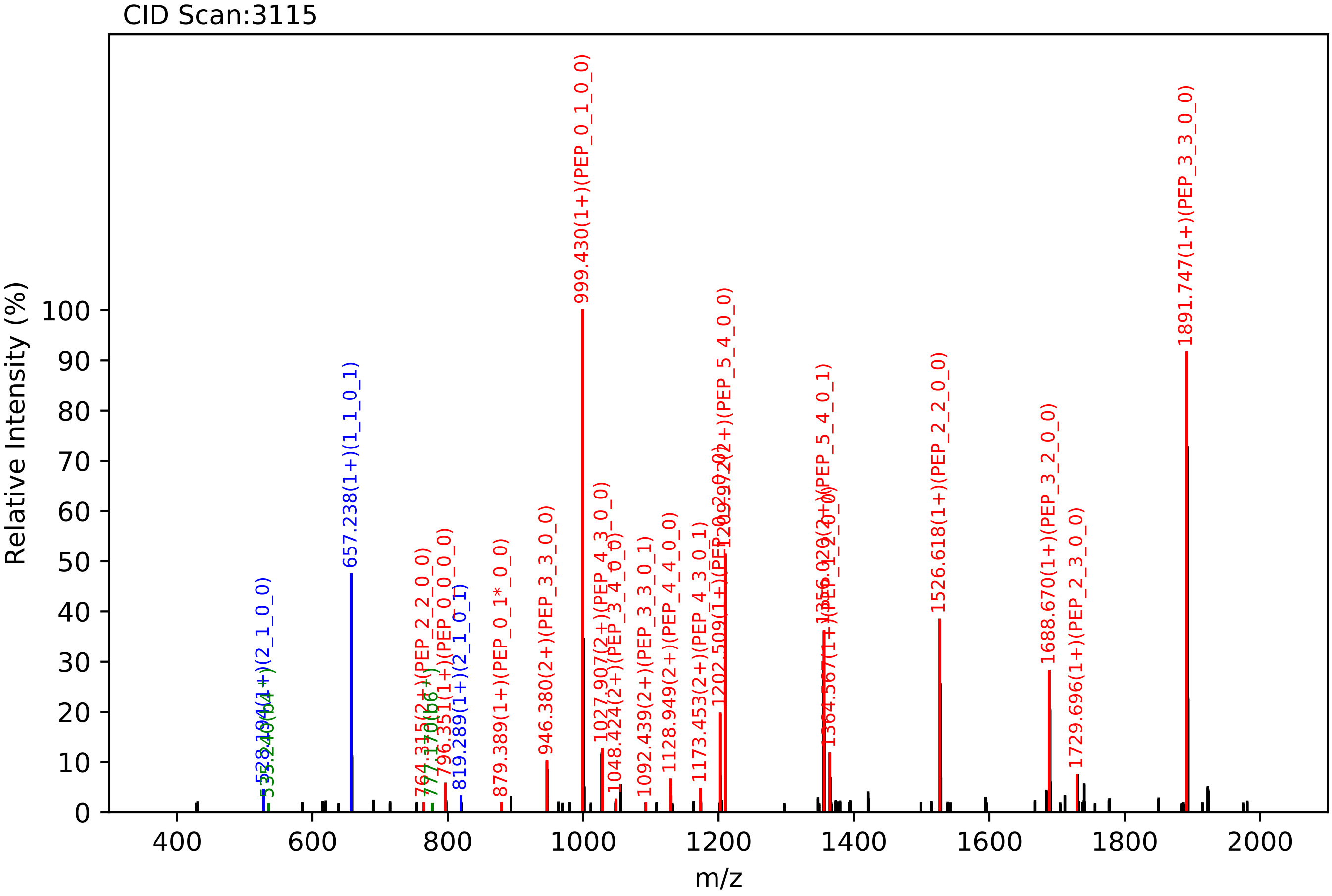

Training set no. 157, Experiment: AGP exp_2

NEEYNK(=PEP)_5_4_0_2, m/z:1501.57(2+), RT:22.17, Y-score:86.21

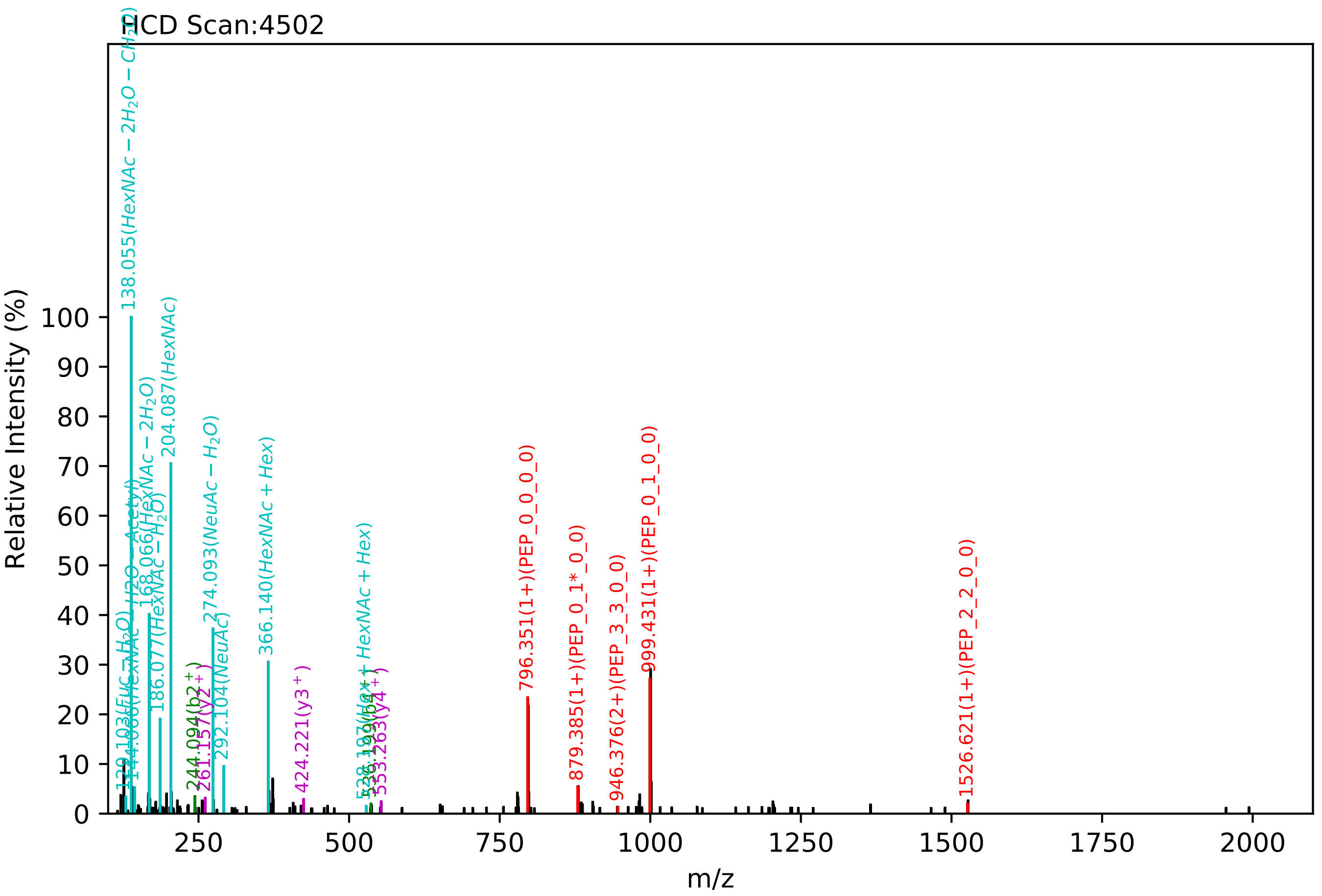

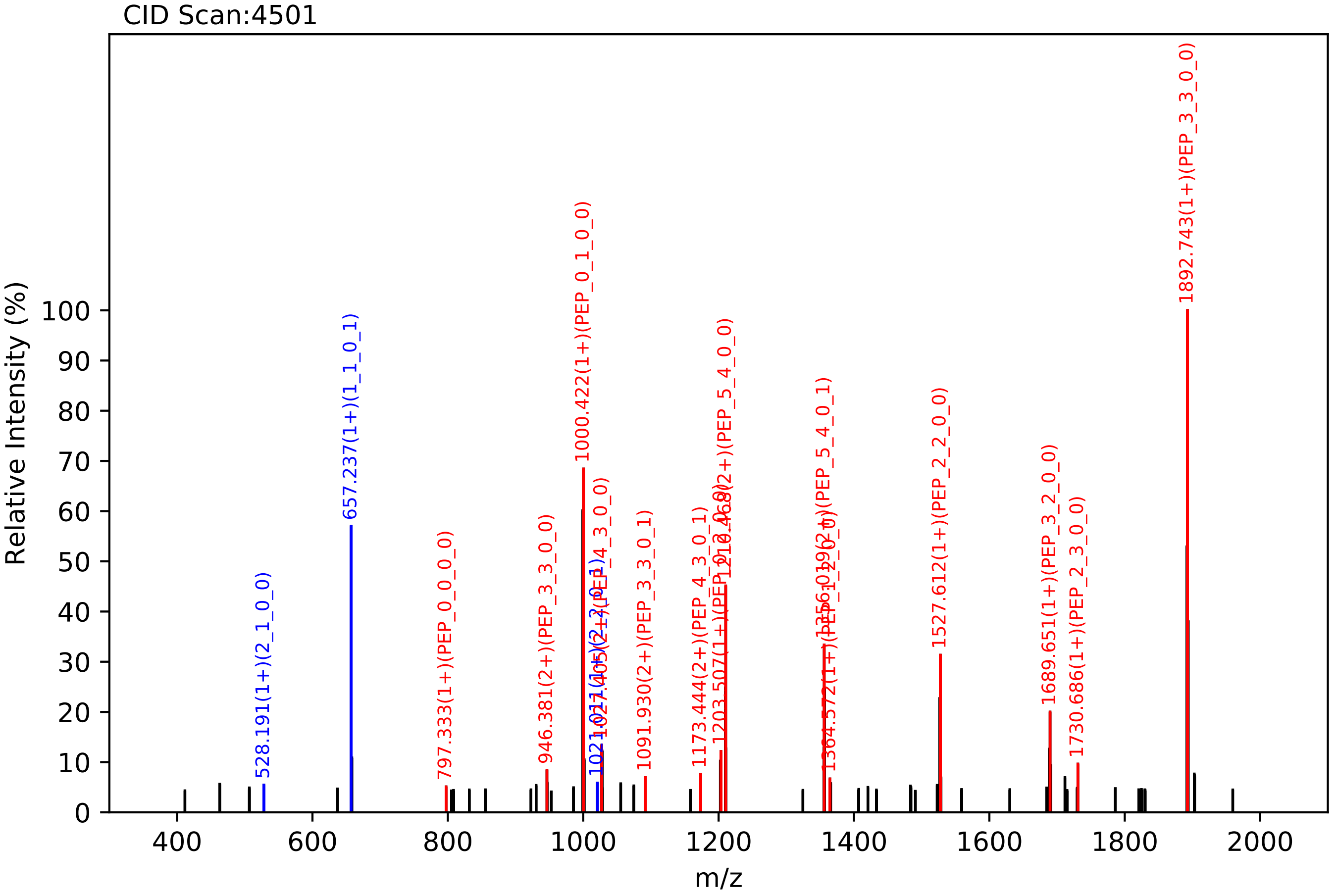

Training set no. 158, Experiment: AGP exp_2

NEEYNK(=PEP)_5_4_0_2, m/z:1001.05(3+), RT:20.79, Y-score:82.86

HCD Scan:3739

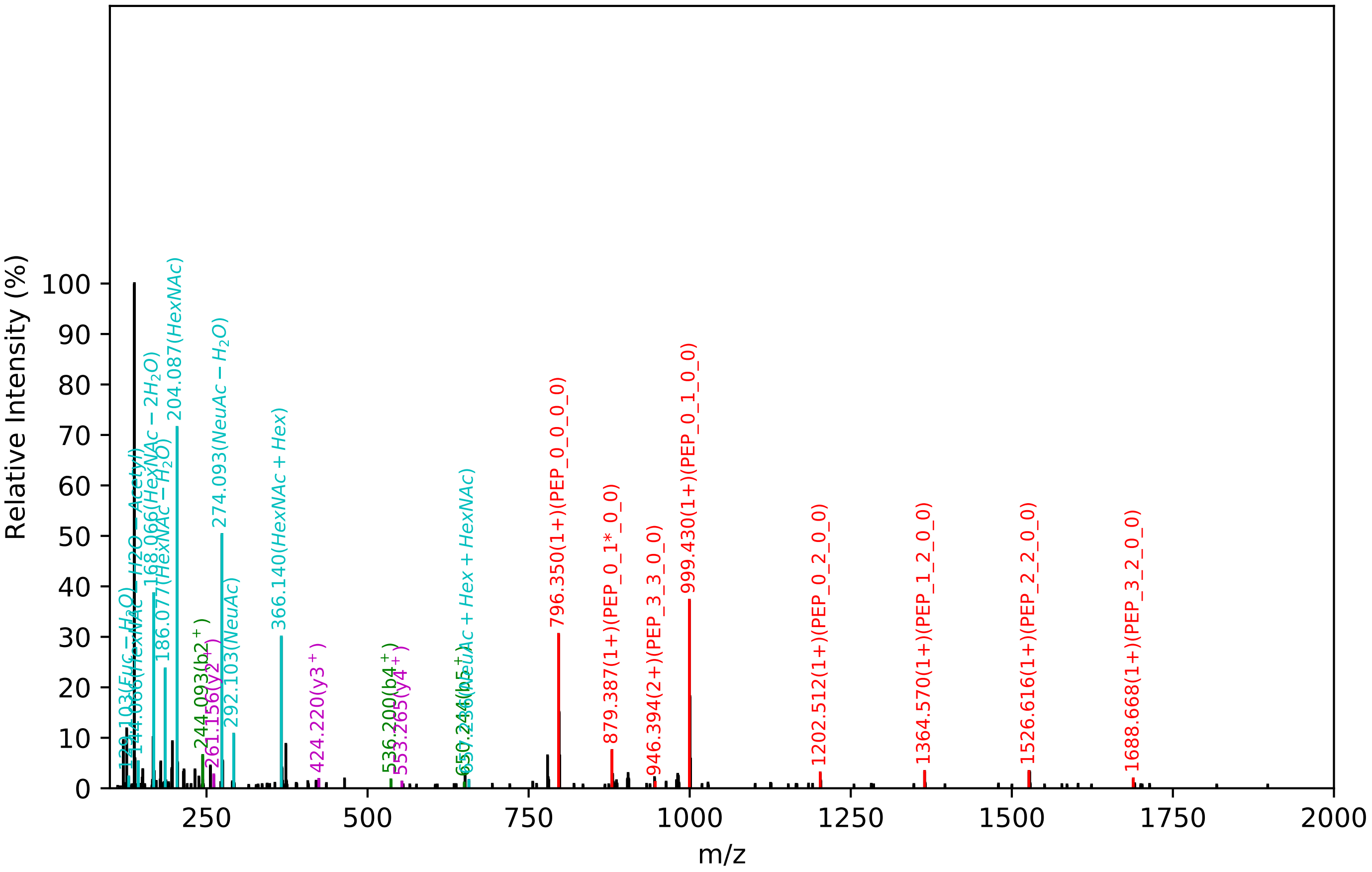

CID Scan:3738

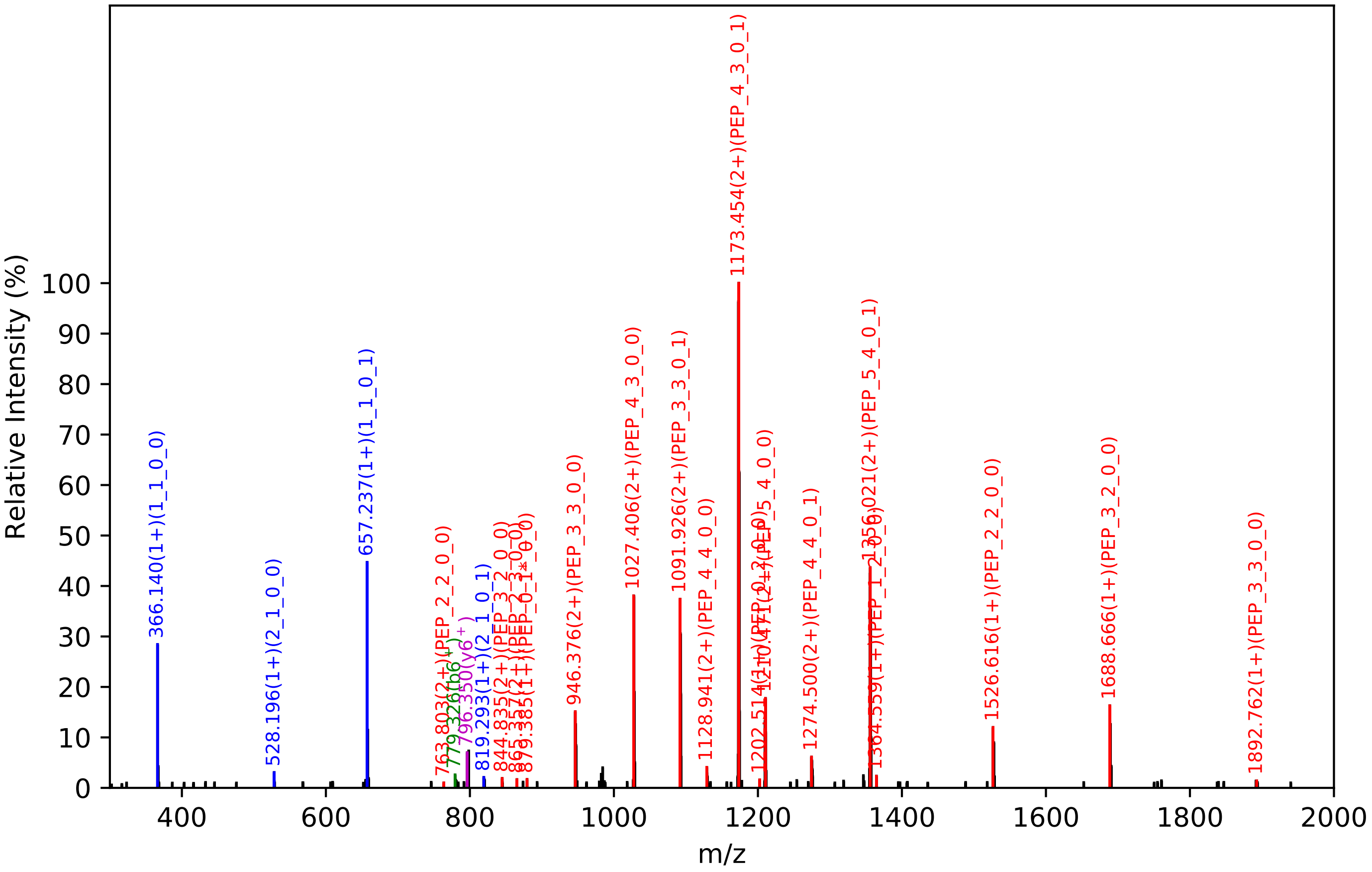

Training set no. 159, Experiment: AGP exp_2

NEEYNK(=PEP)_5_4_0_2, m/z:1501.06(2+), RT:20.29, Y-score:82.07

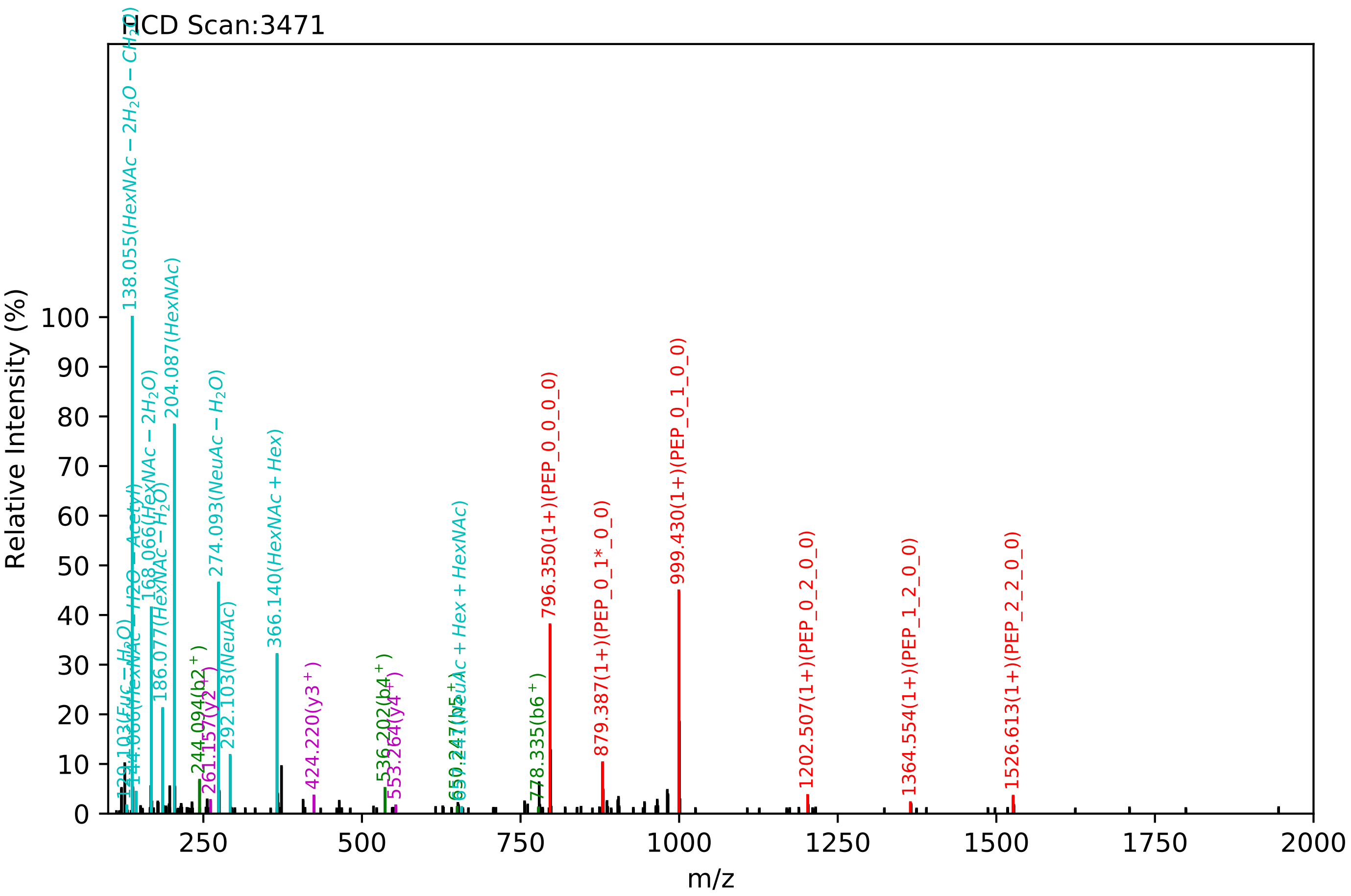

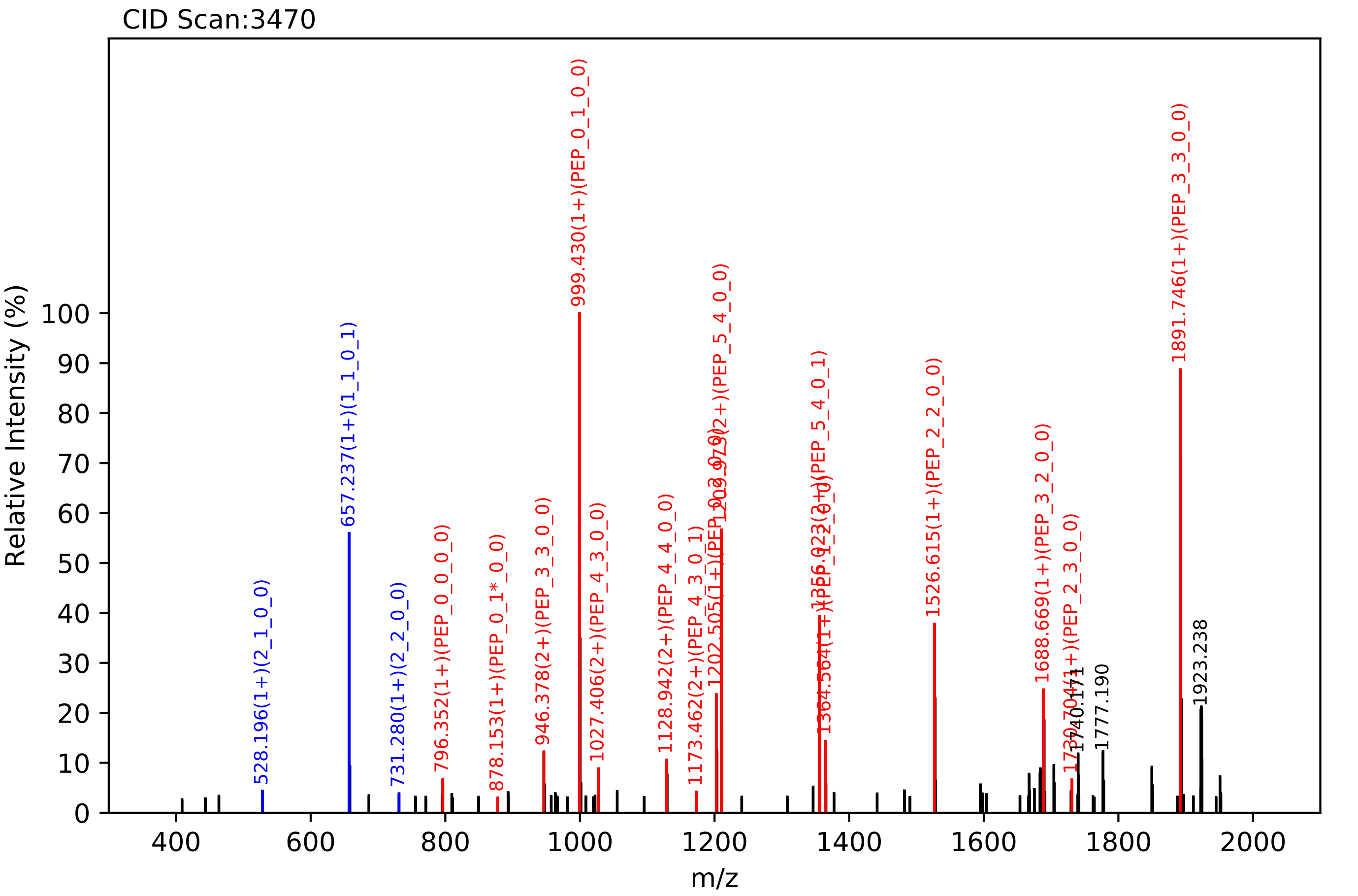

Training set no. 160, Experiment: AGP exp_1

NEEYNK(=PEP)_5_4_0_2, m/z:1501.57(2+), RT:20.01, Y-score:80.64

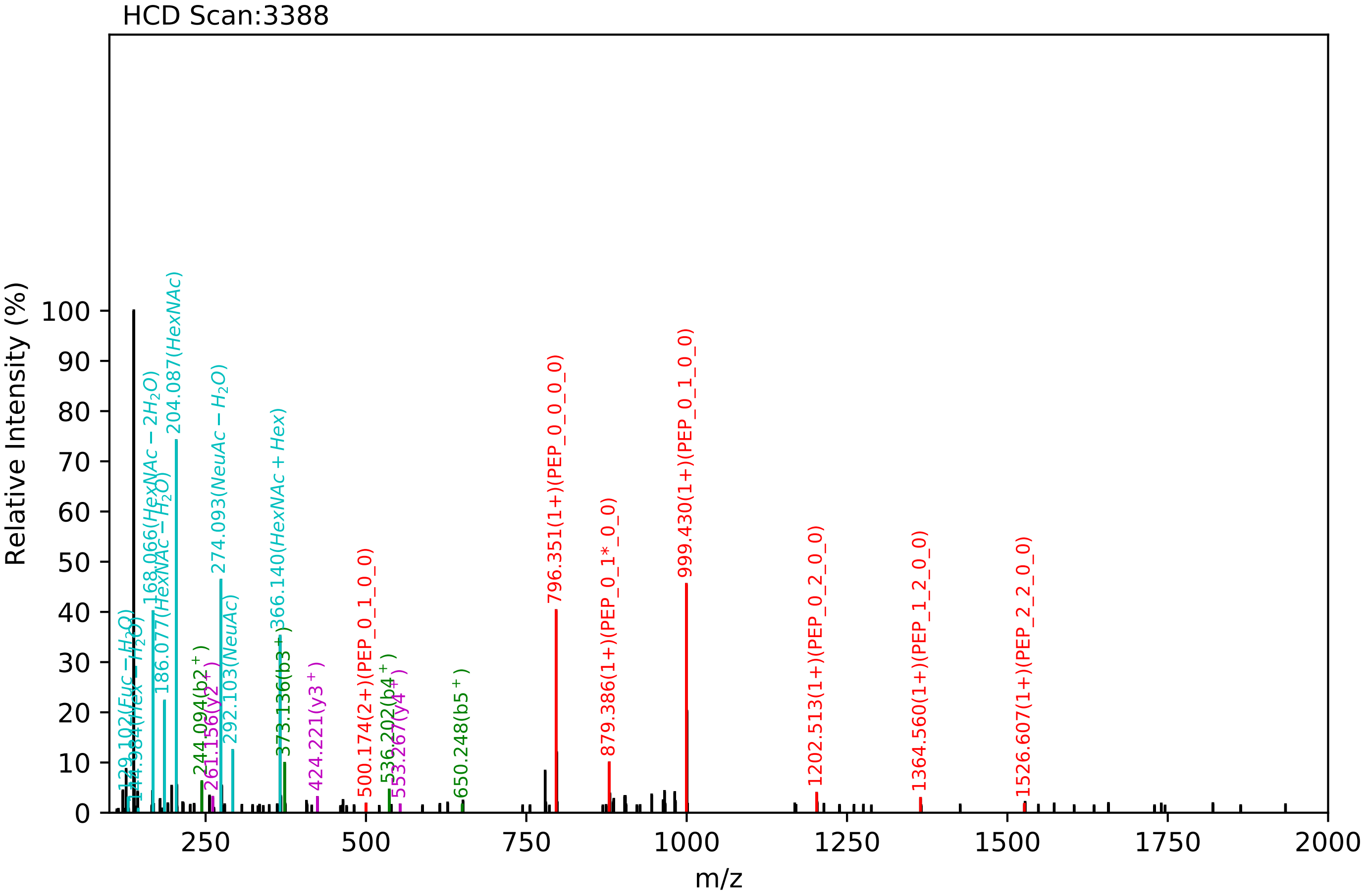

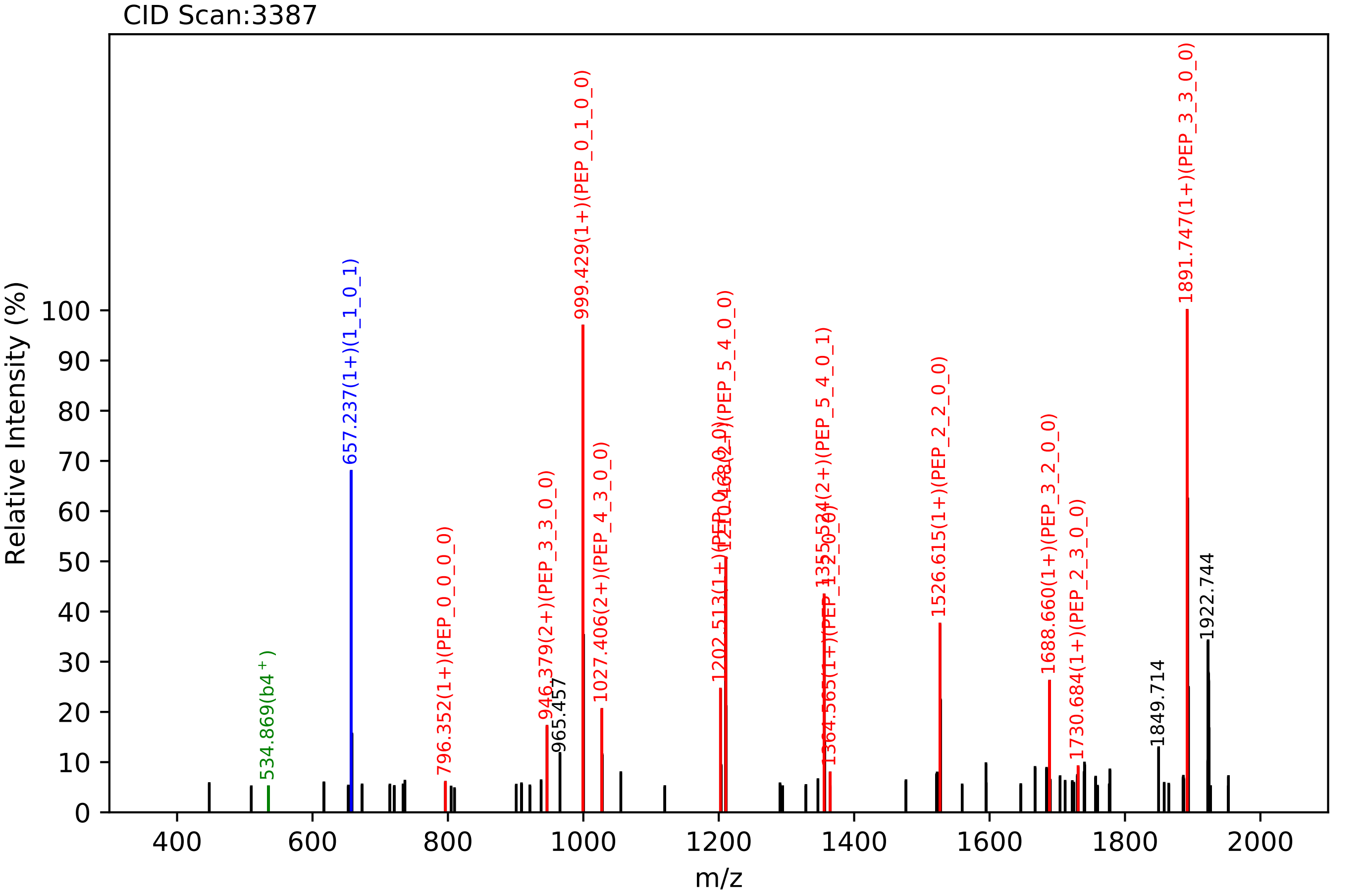

Training set no. 161, Experiment: AGP exp_2

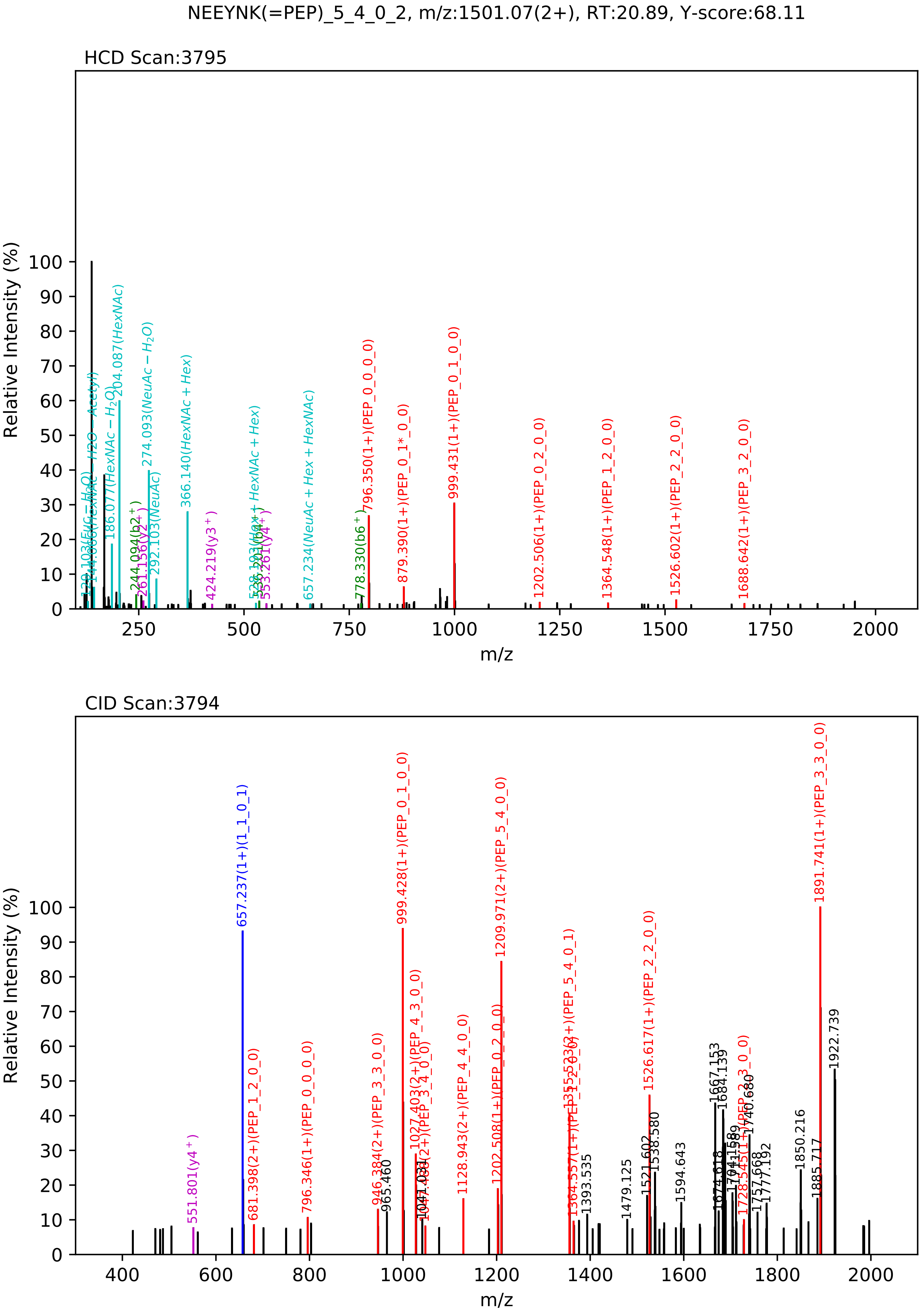

Training set no. 162, Experiment: AGP exp_1

NEEYNK(=PEP)_5_4_1_2, m/z:1049.73(3+), RT:19.21, Y-score:93.39

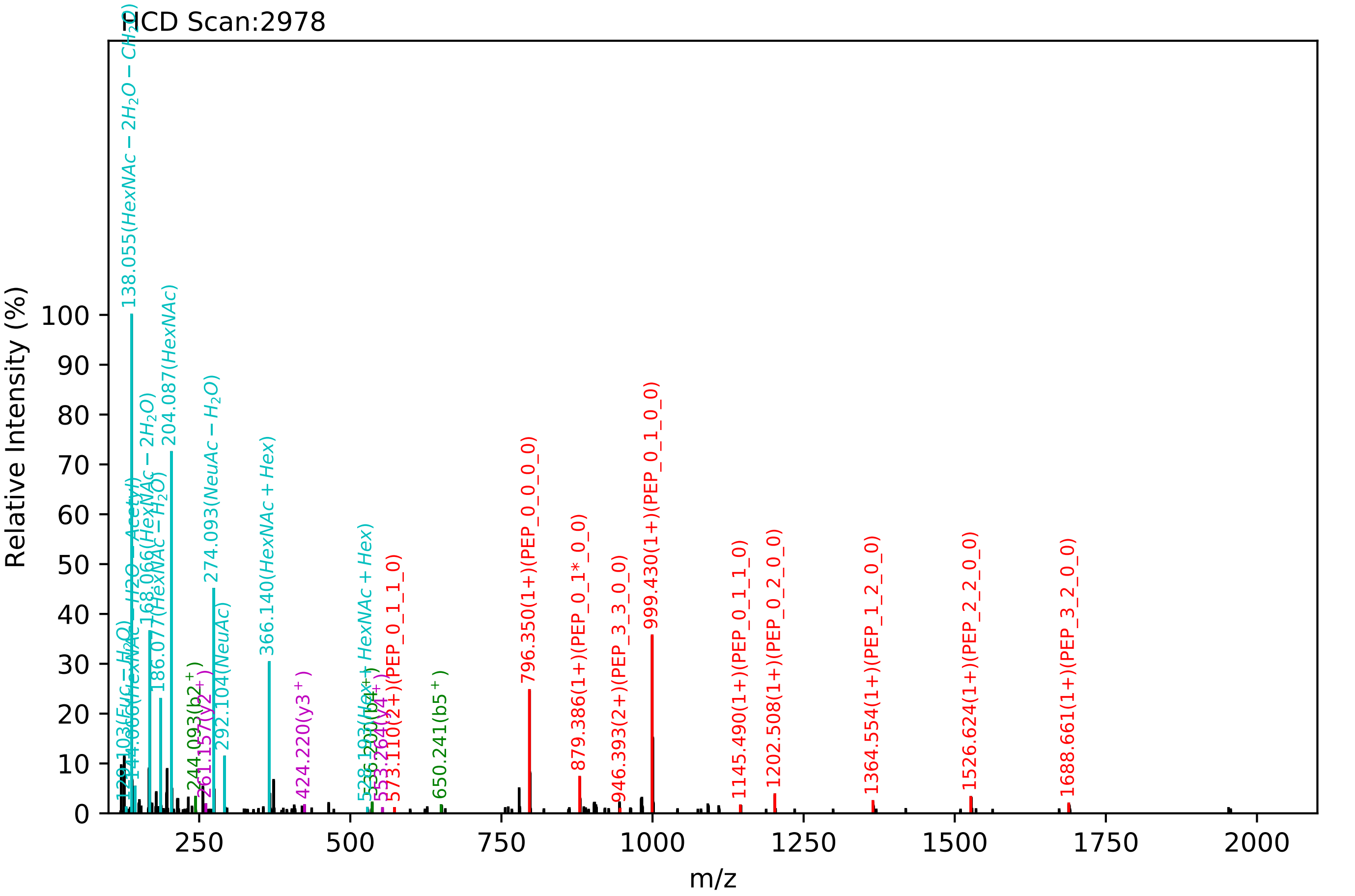

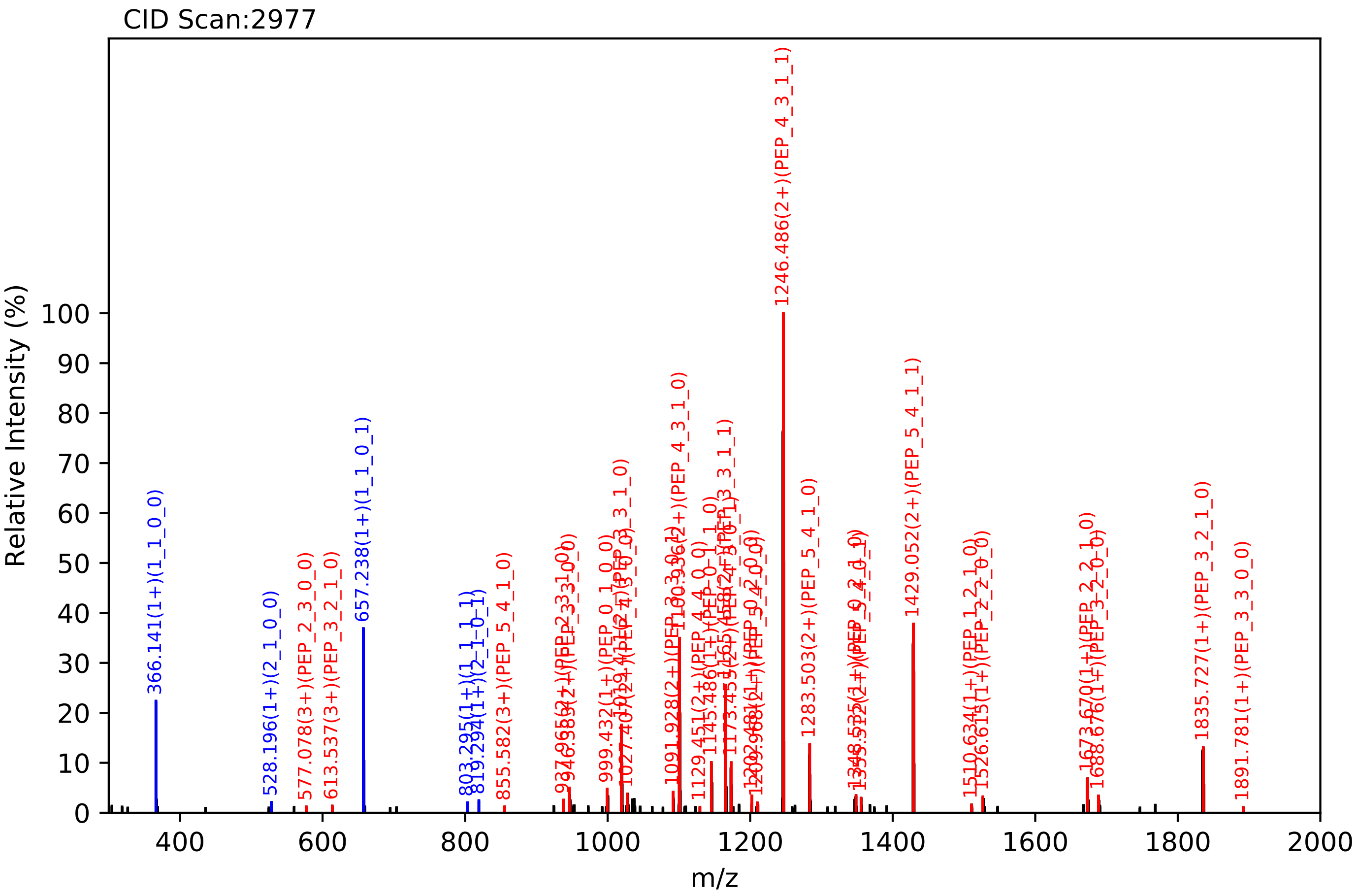

Training set no. 163, Experiment: AGP exp_2

NEEYNK(=PEP)_5_4_1_2, m/z:1049.73(3+), RT:19.65, Y-score:91.41

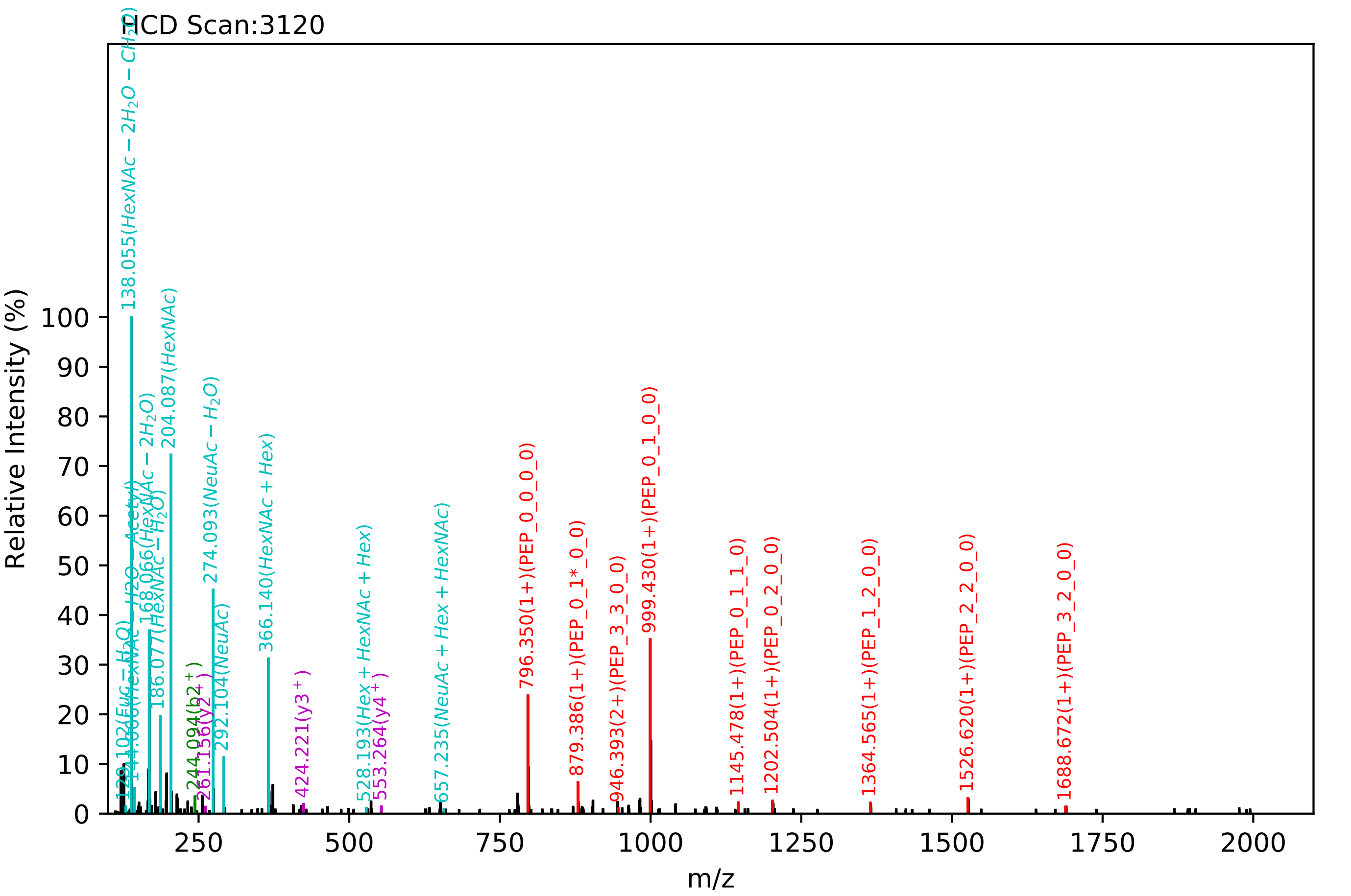

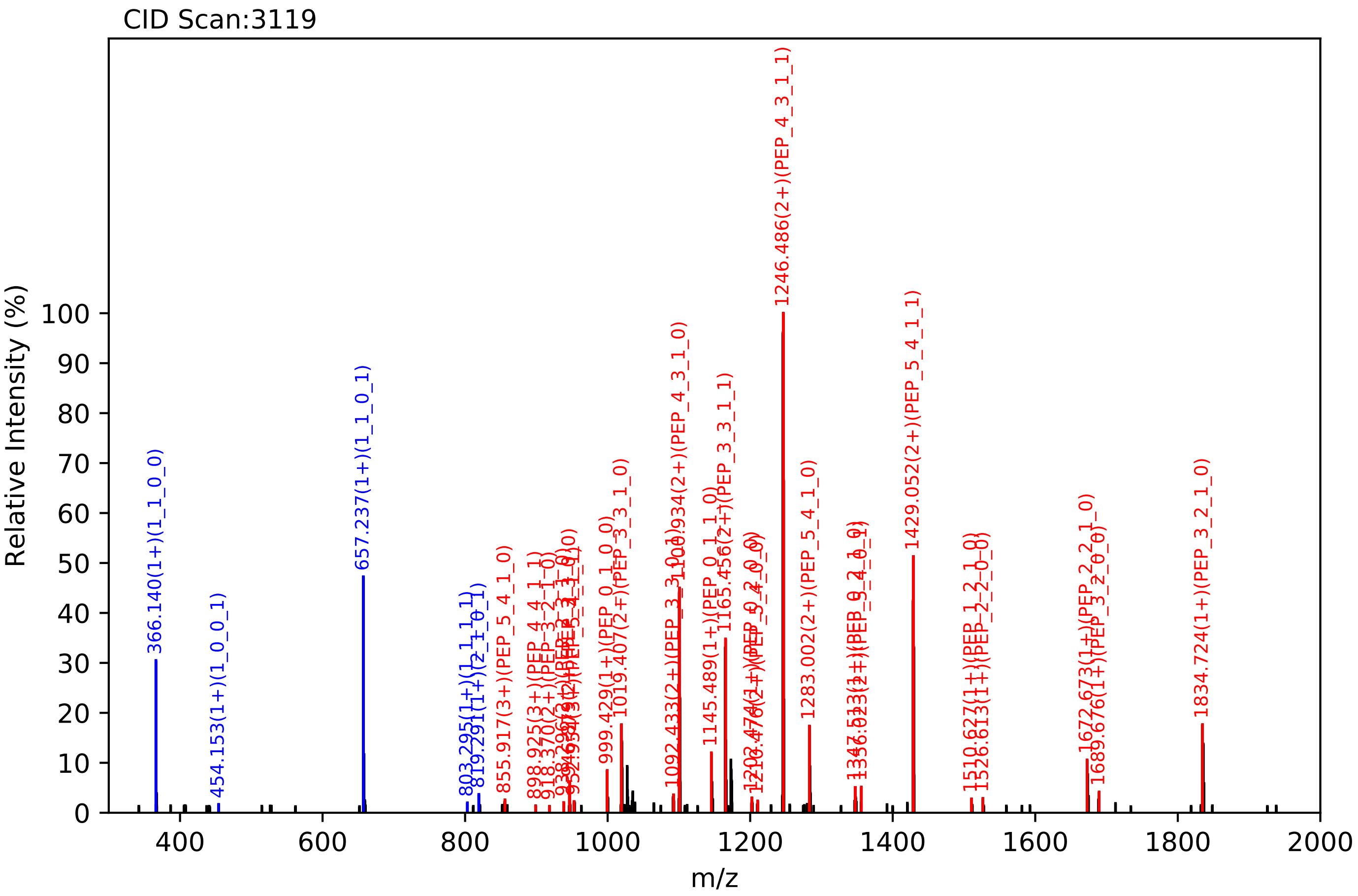

Training set no. 164, Experiment: AGP exp_1

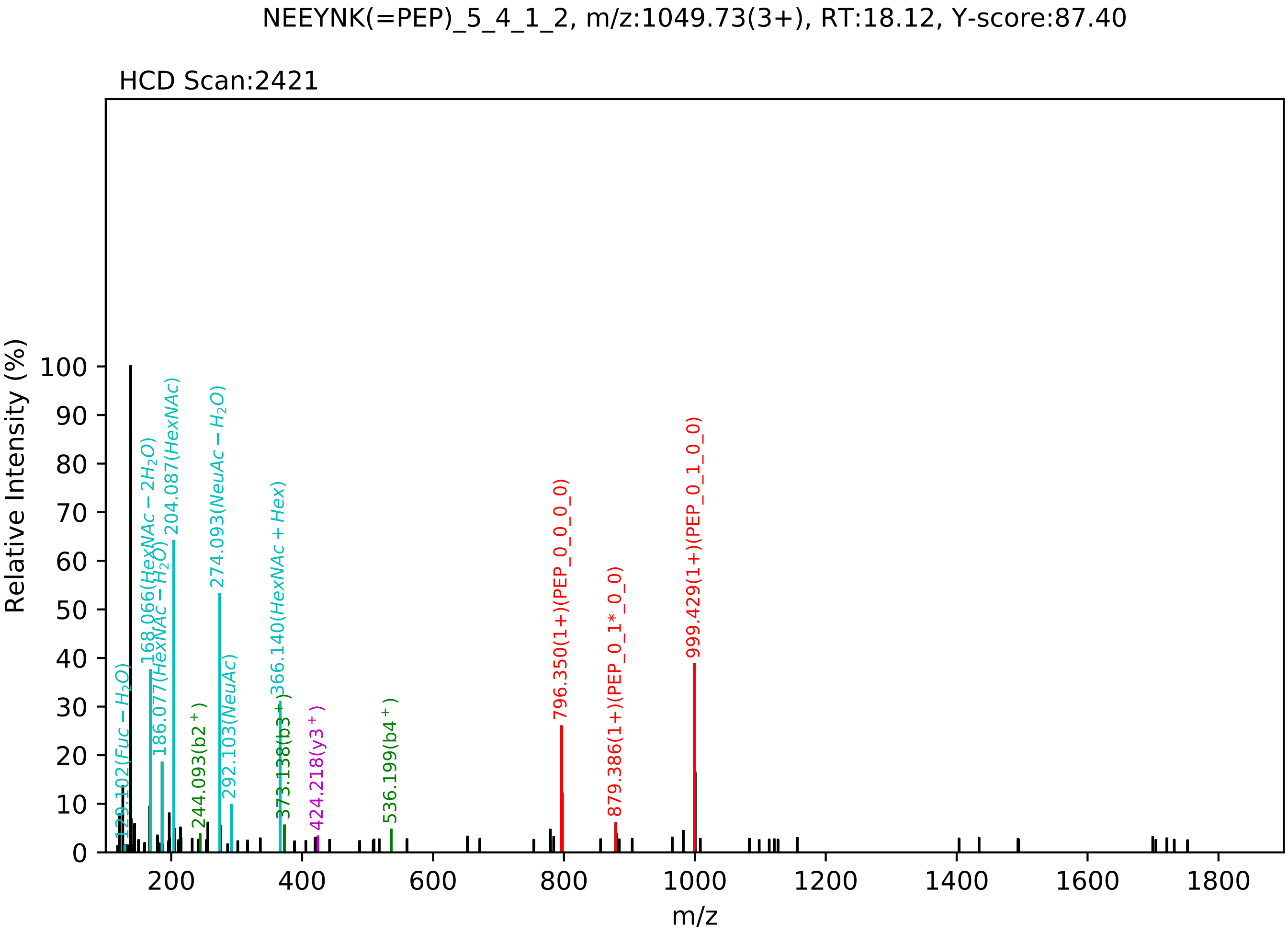

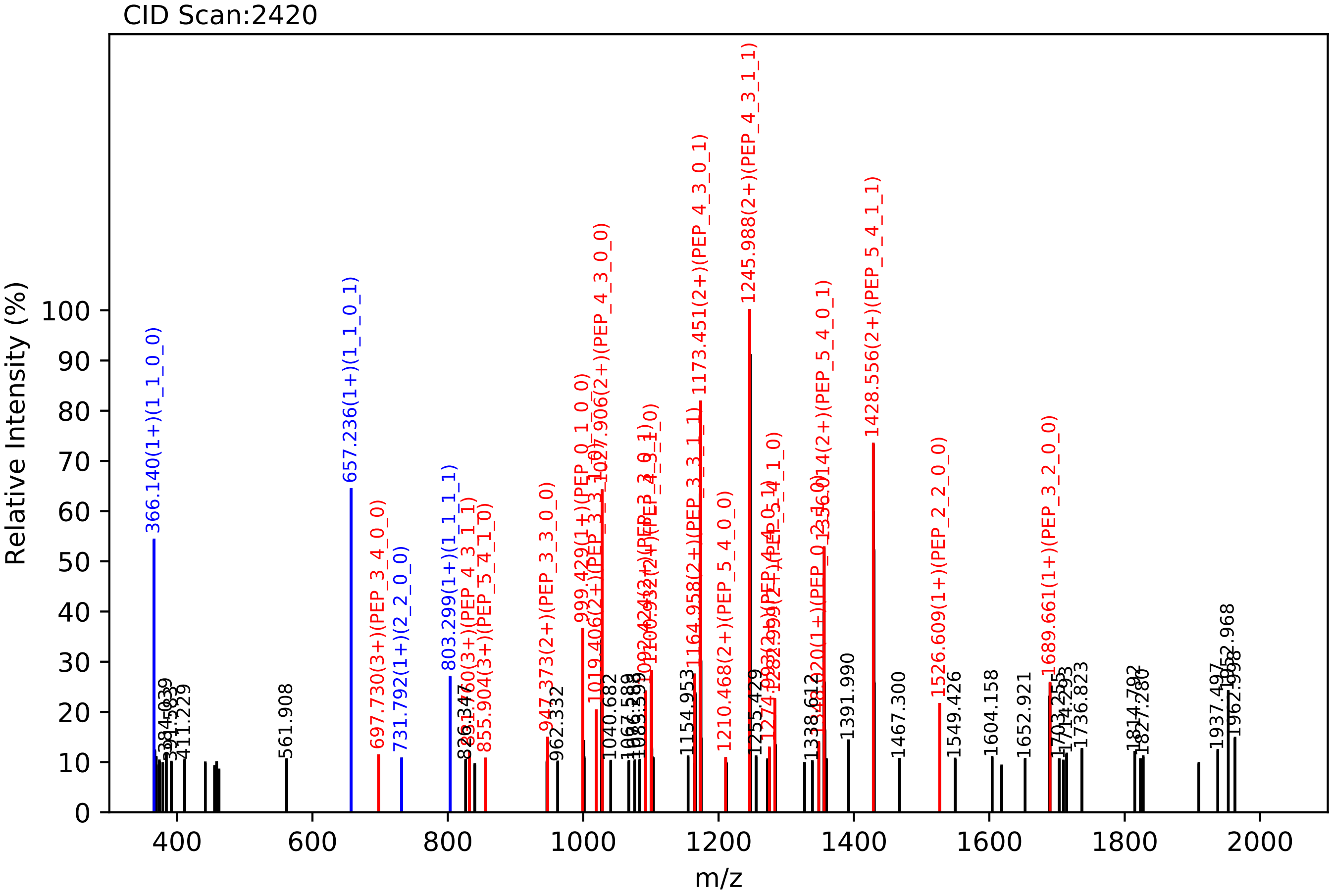

Training set no. 165, Experiment: AGP exp_2

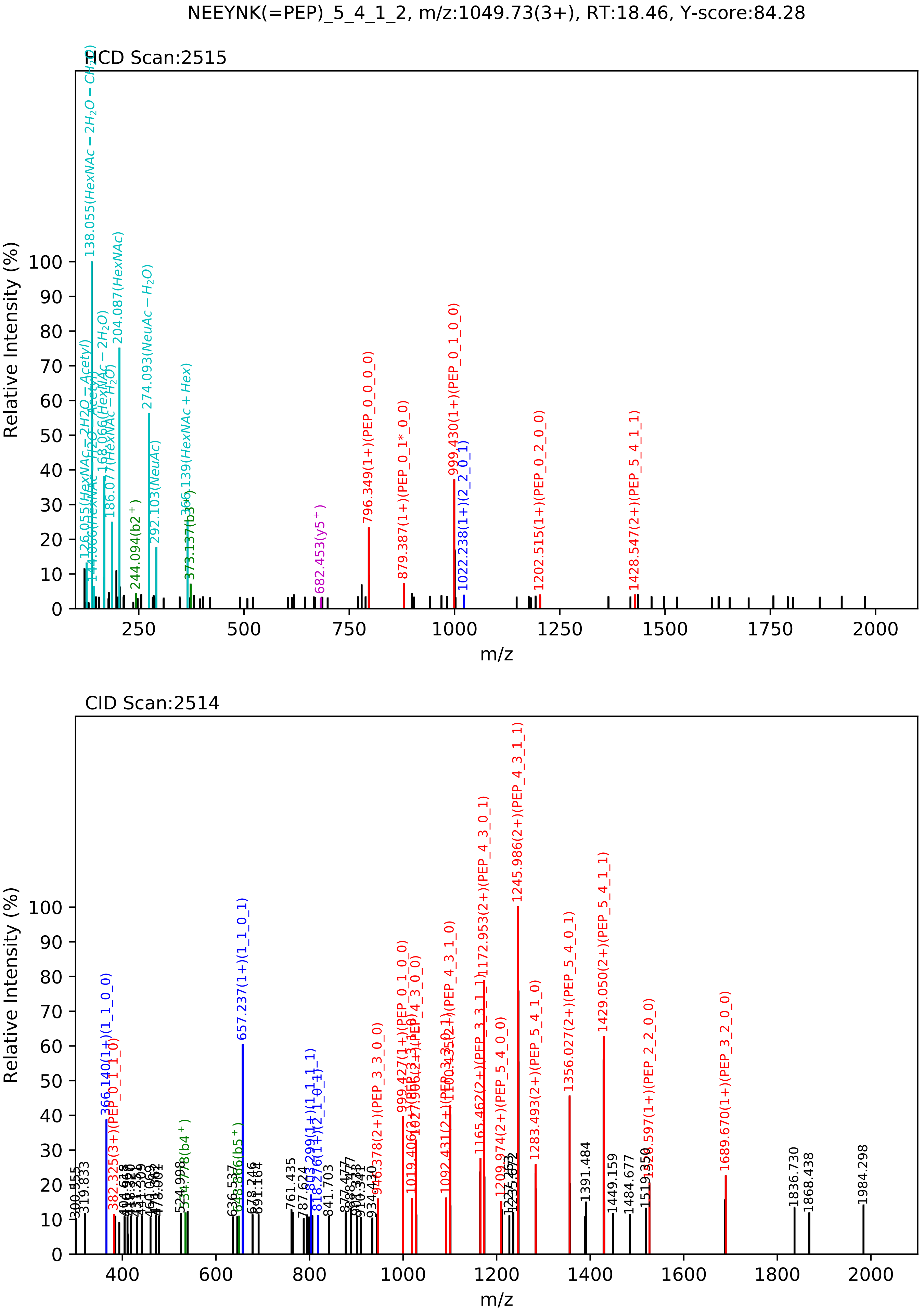

Training set no. 166, Experiment: AGP exp_1

NEEYNK(=PEP)_5_4_1_2, m/z:1049.73(3+), RT:18.64, Y-score:74.53

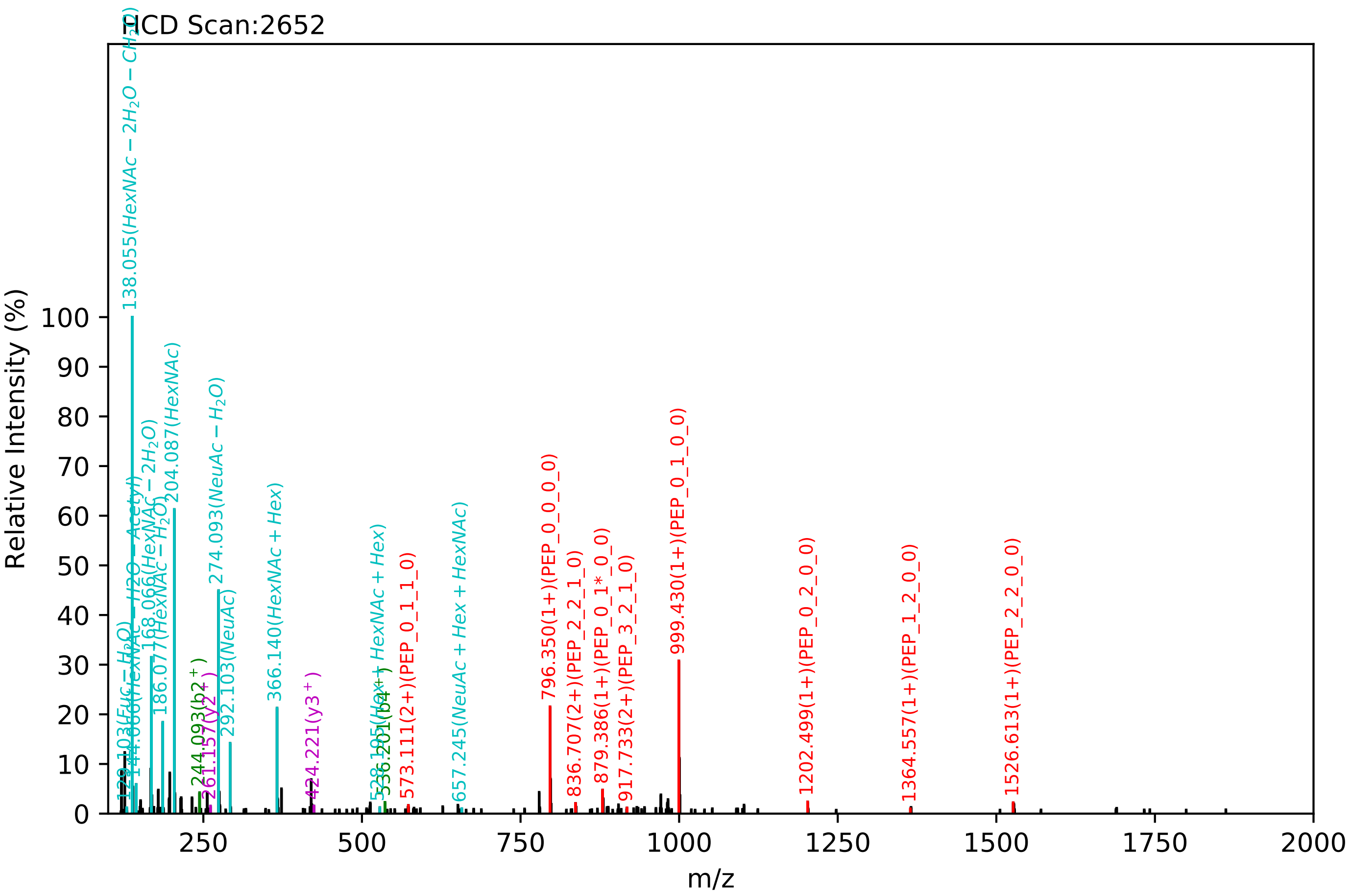

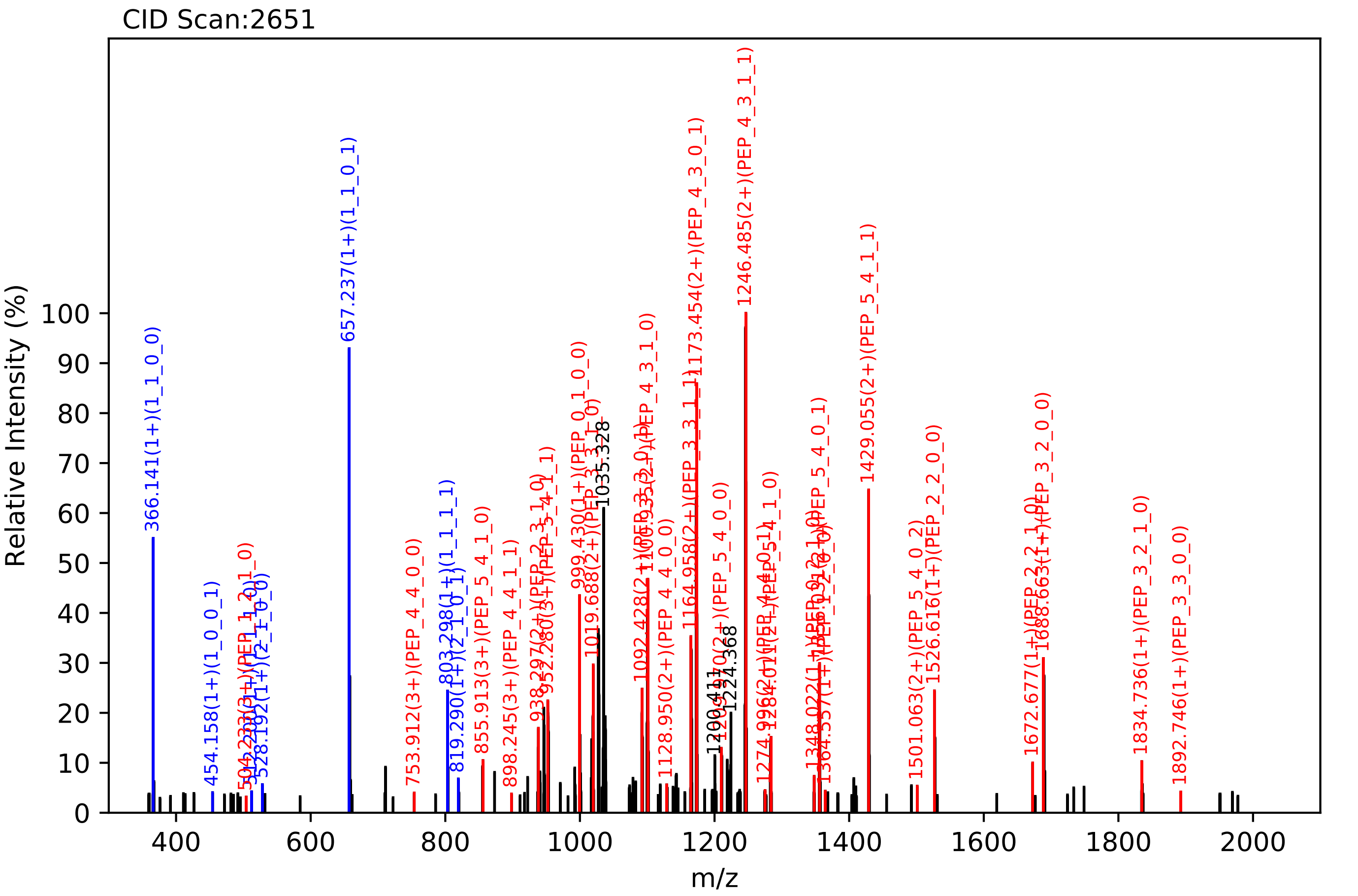

Training set no. 167, Experiment: AGP exp_2

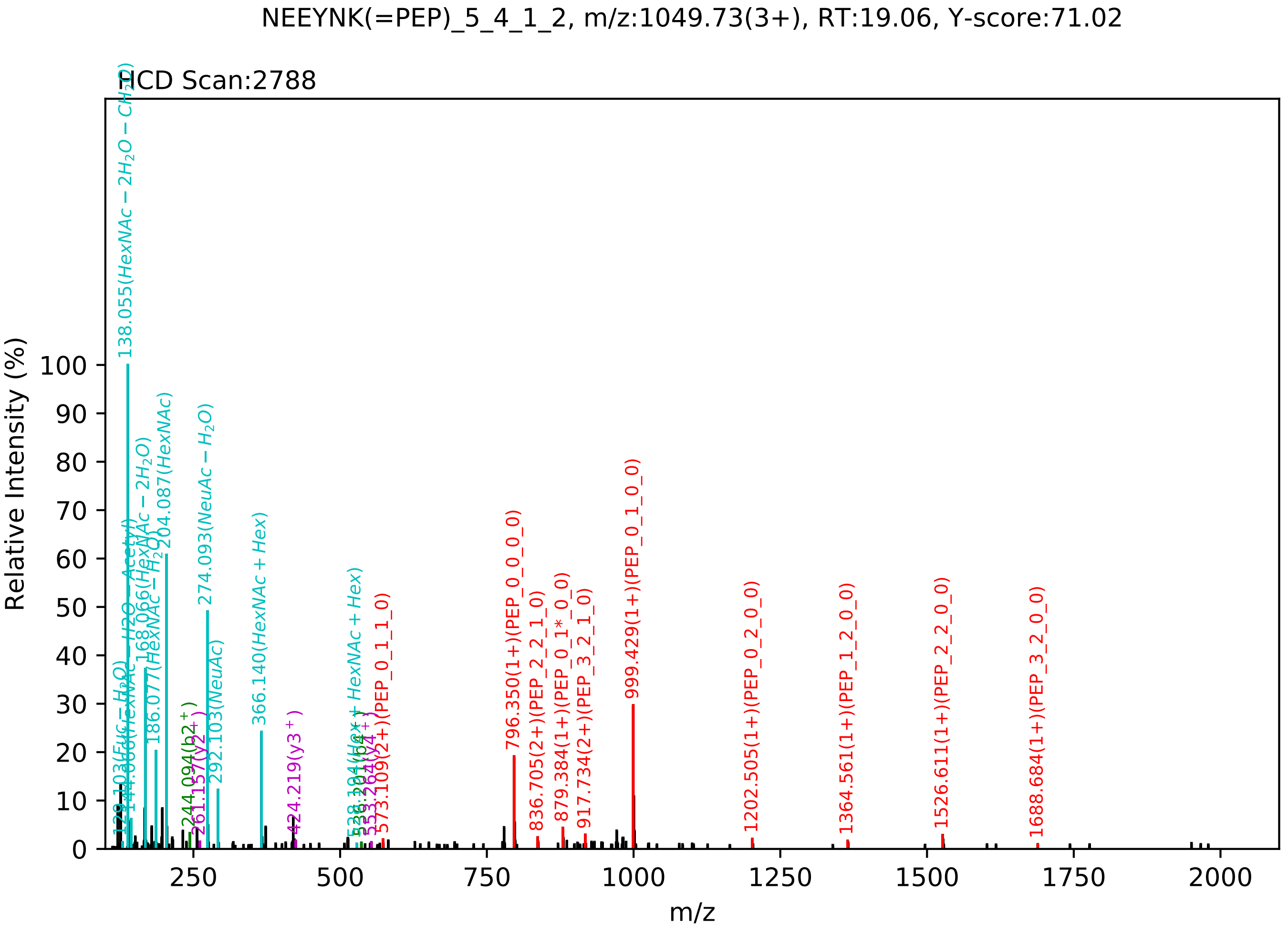

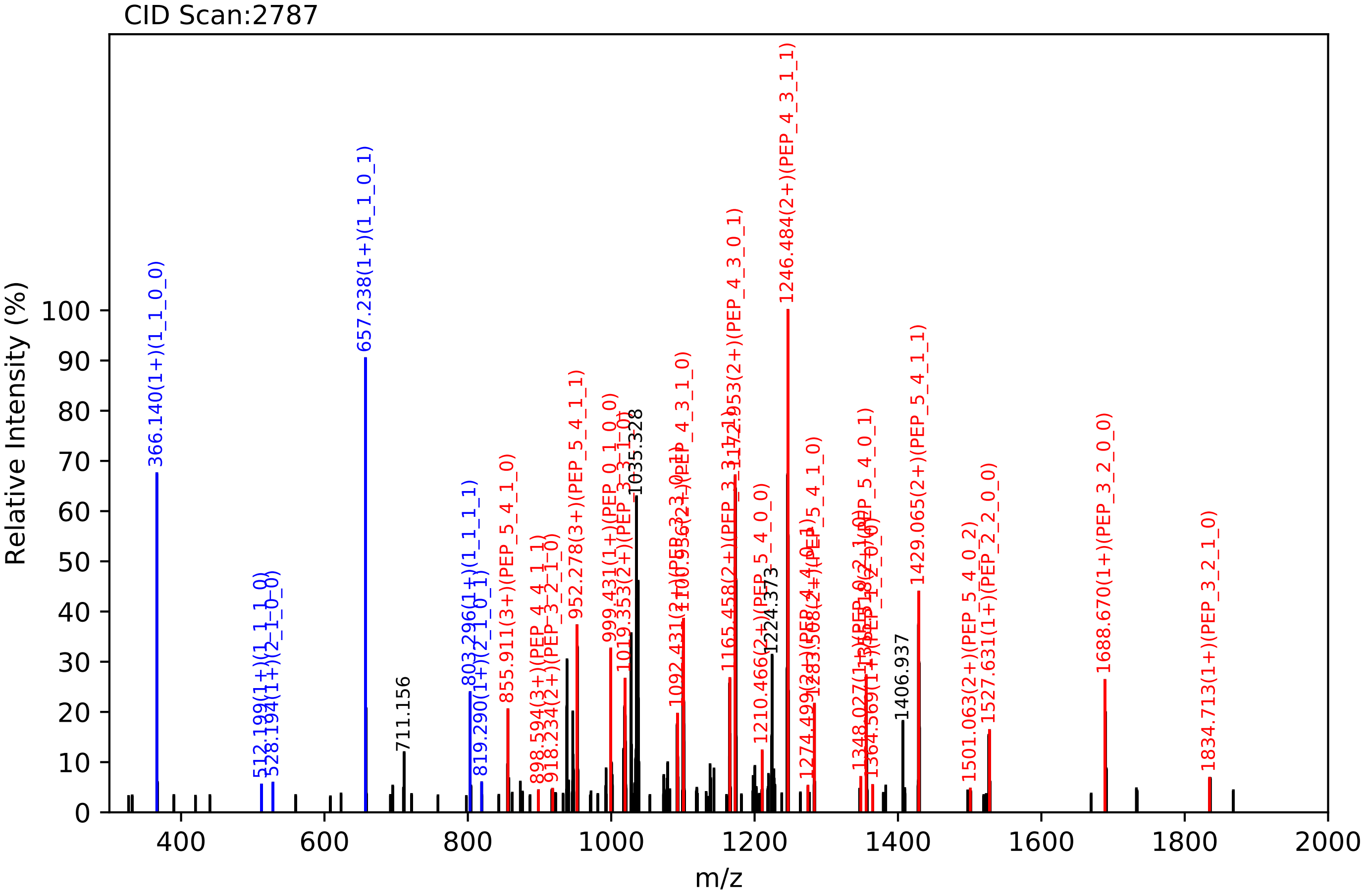

Training set no. 168, Experiment: AGP exp_19

NEEYNK(=PEP)_5_4_2_2, m/z:1098.42(3+), RT:21.11, Y-score:90.34

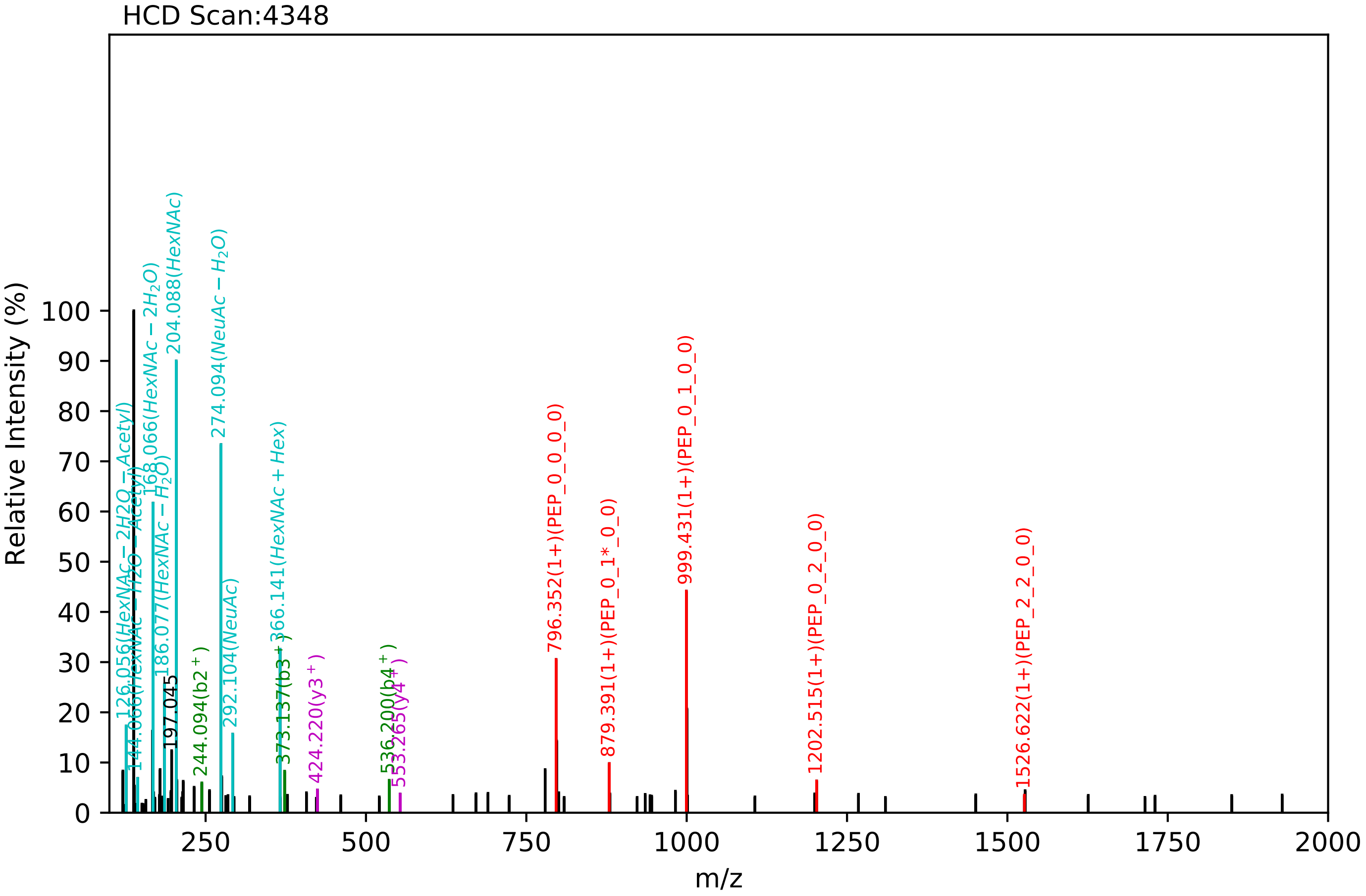

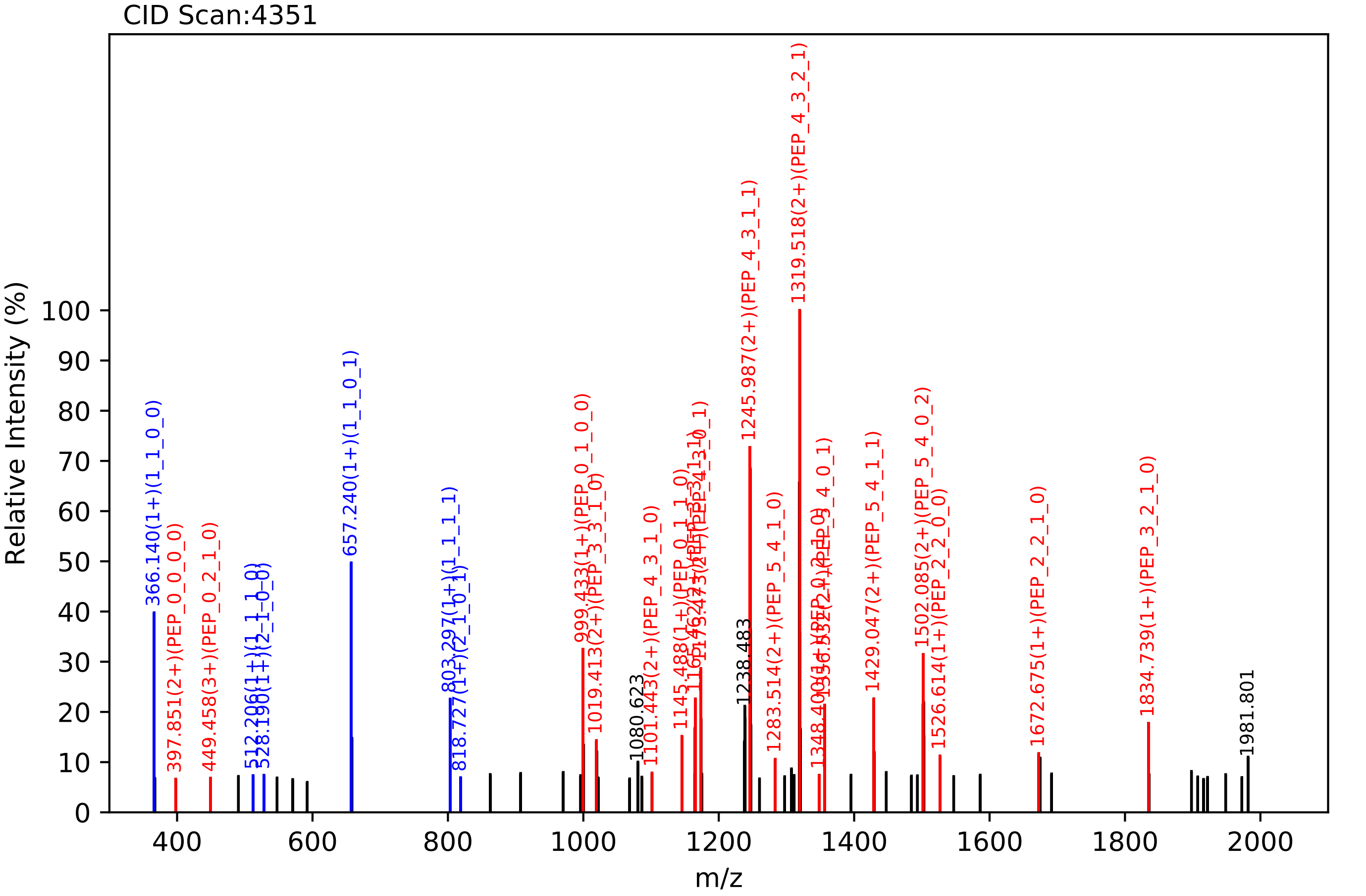

Training set no. 169, Experiment: AGP exp_9

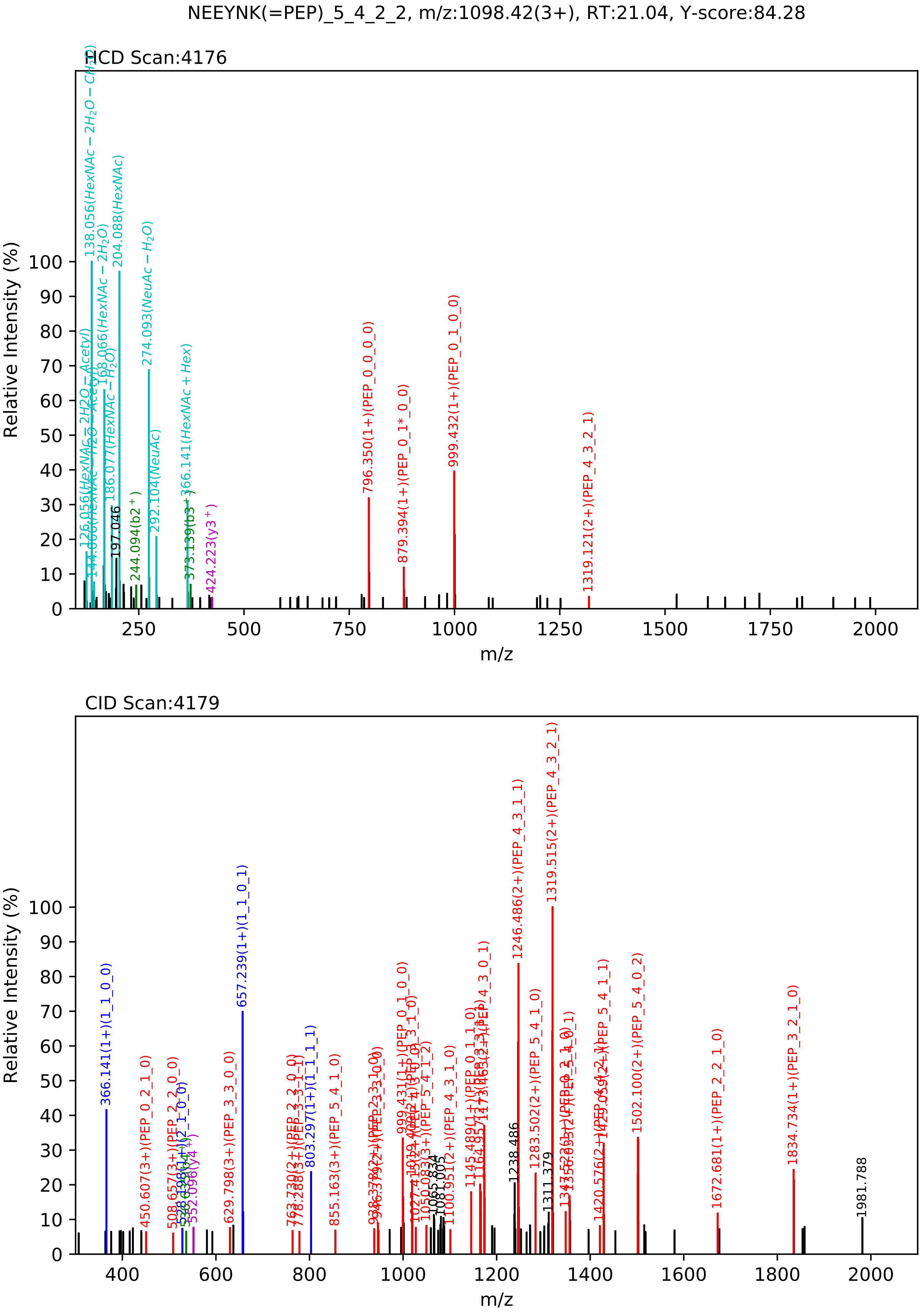

Training set no. 170, Experiment: AGP exp_36

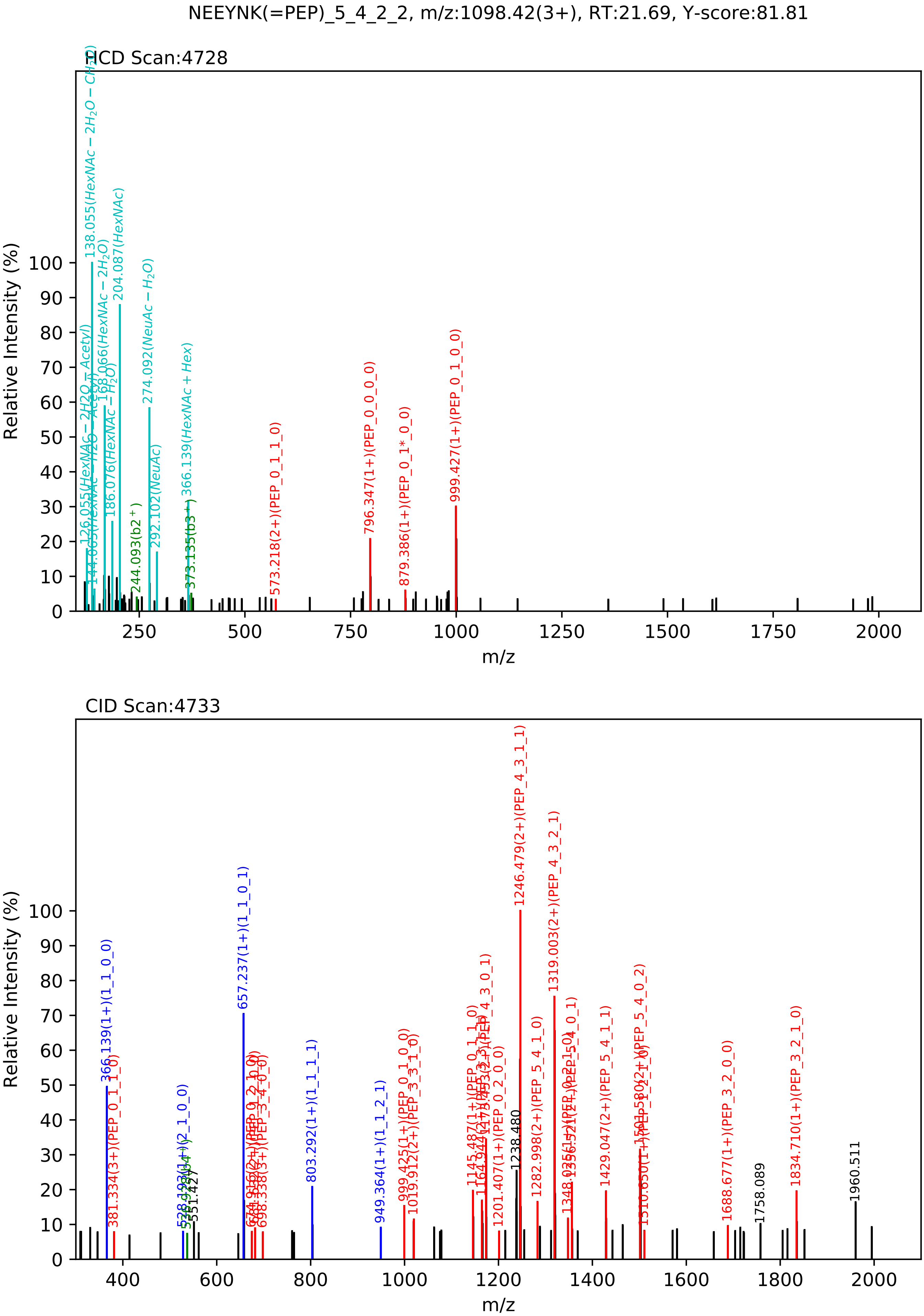

Training set no. 171, Experiment: AGP exp_22

NEEYNK(=PEP)_5_4_2_2, m/z:1098.42(3+), RT:21.05, Y-score:81.35

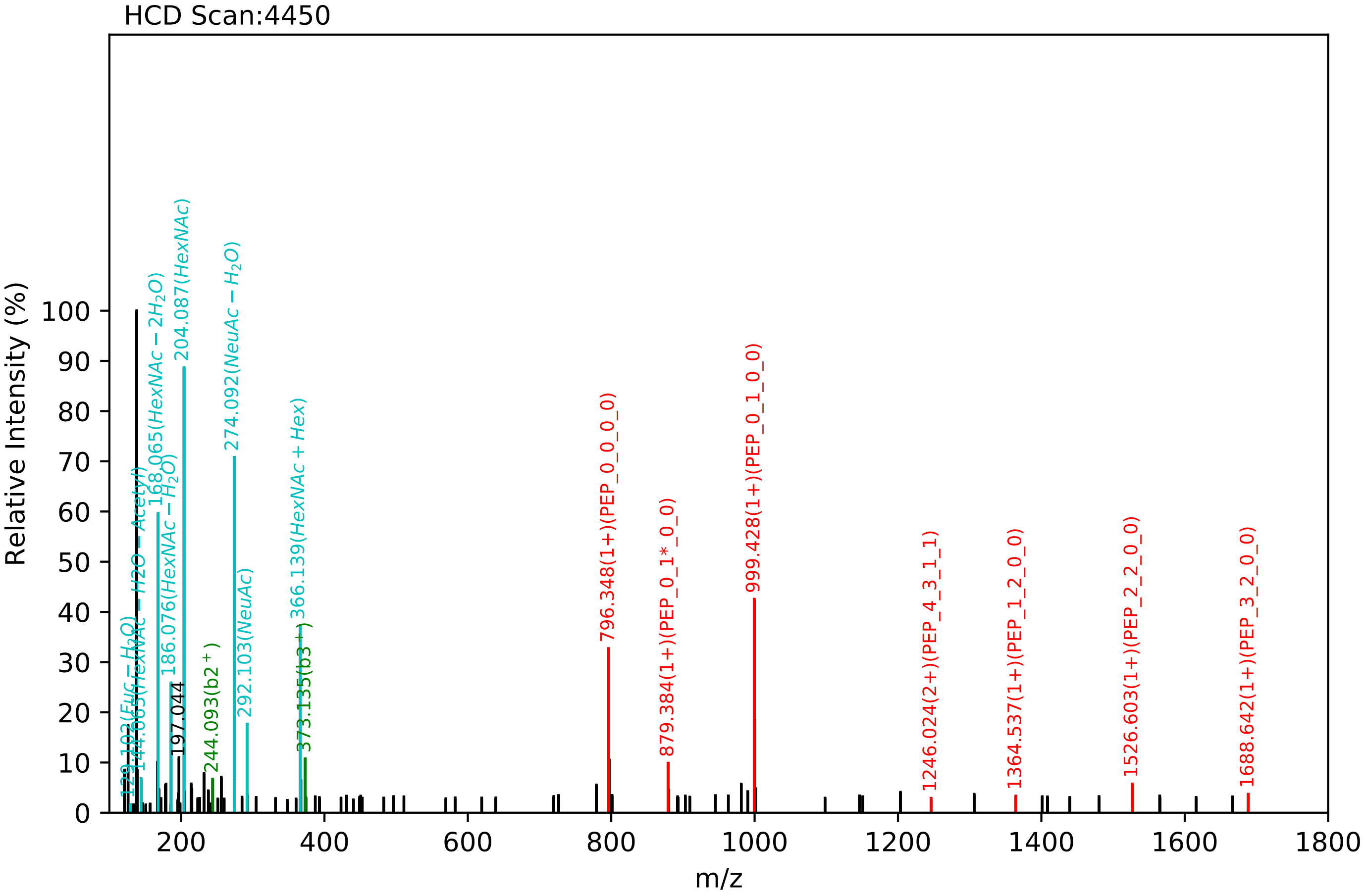

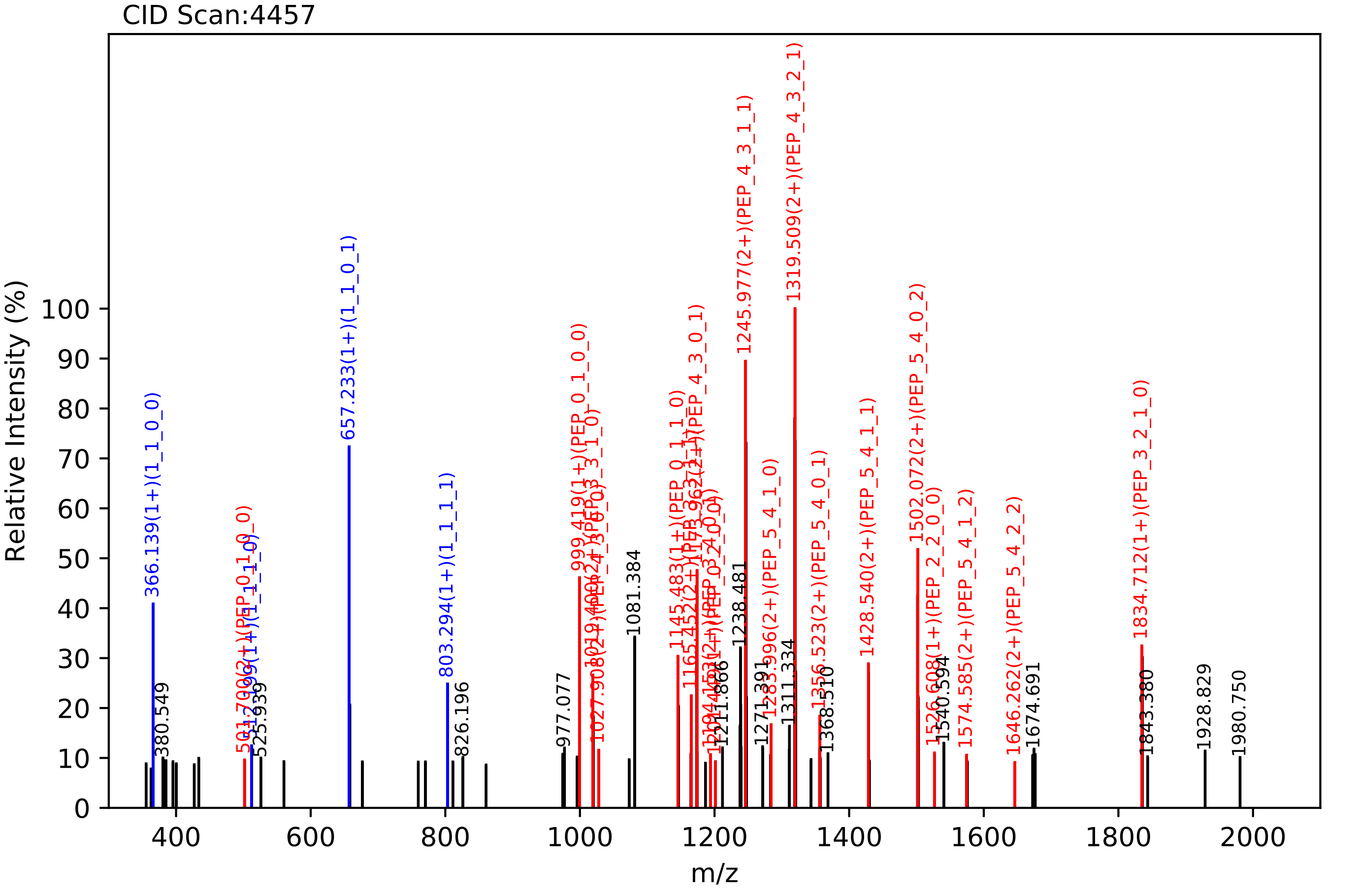

Training set no. 172, Experiment: AGP exp_13

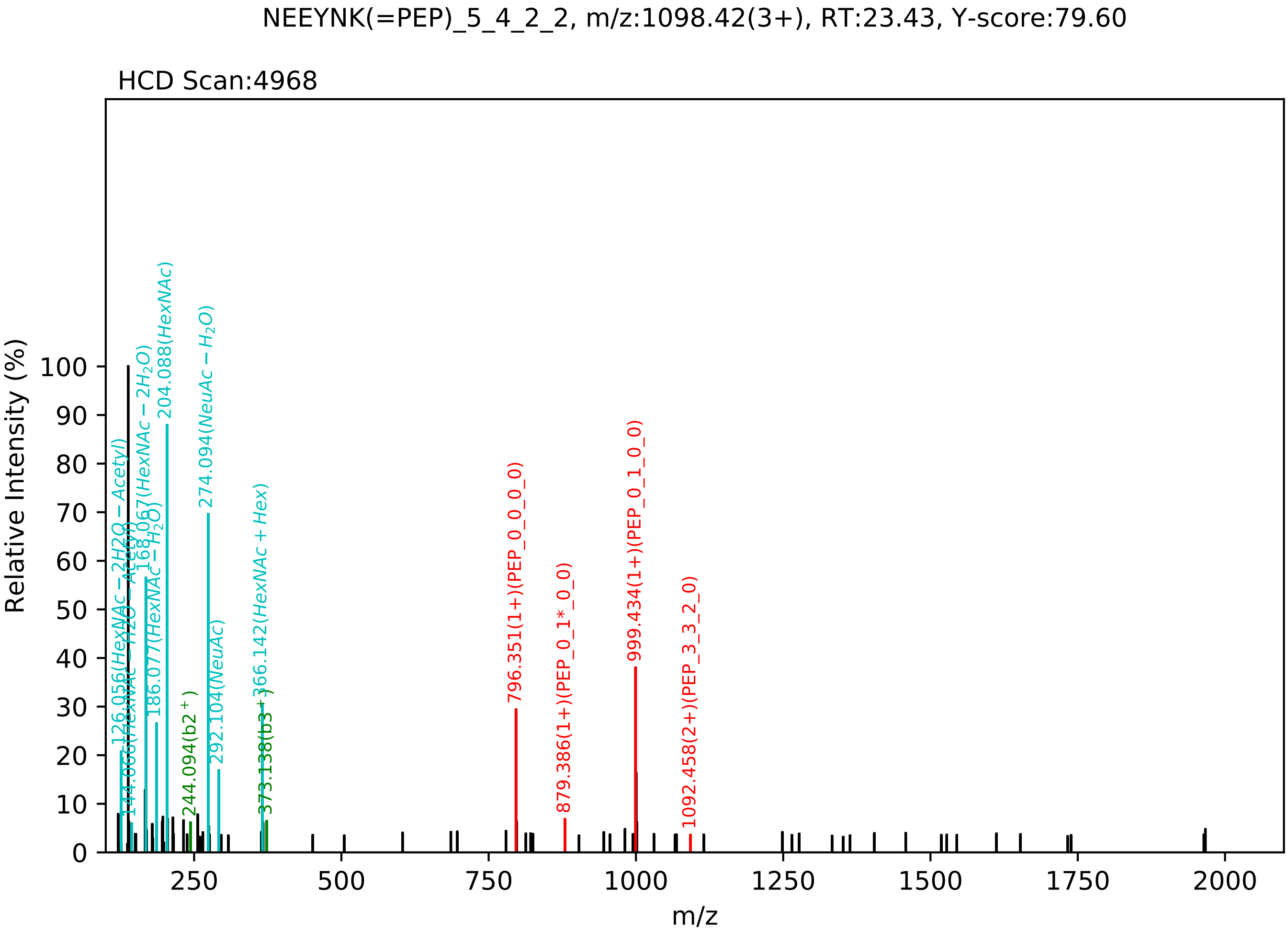

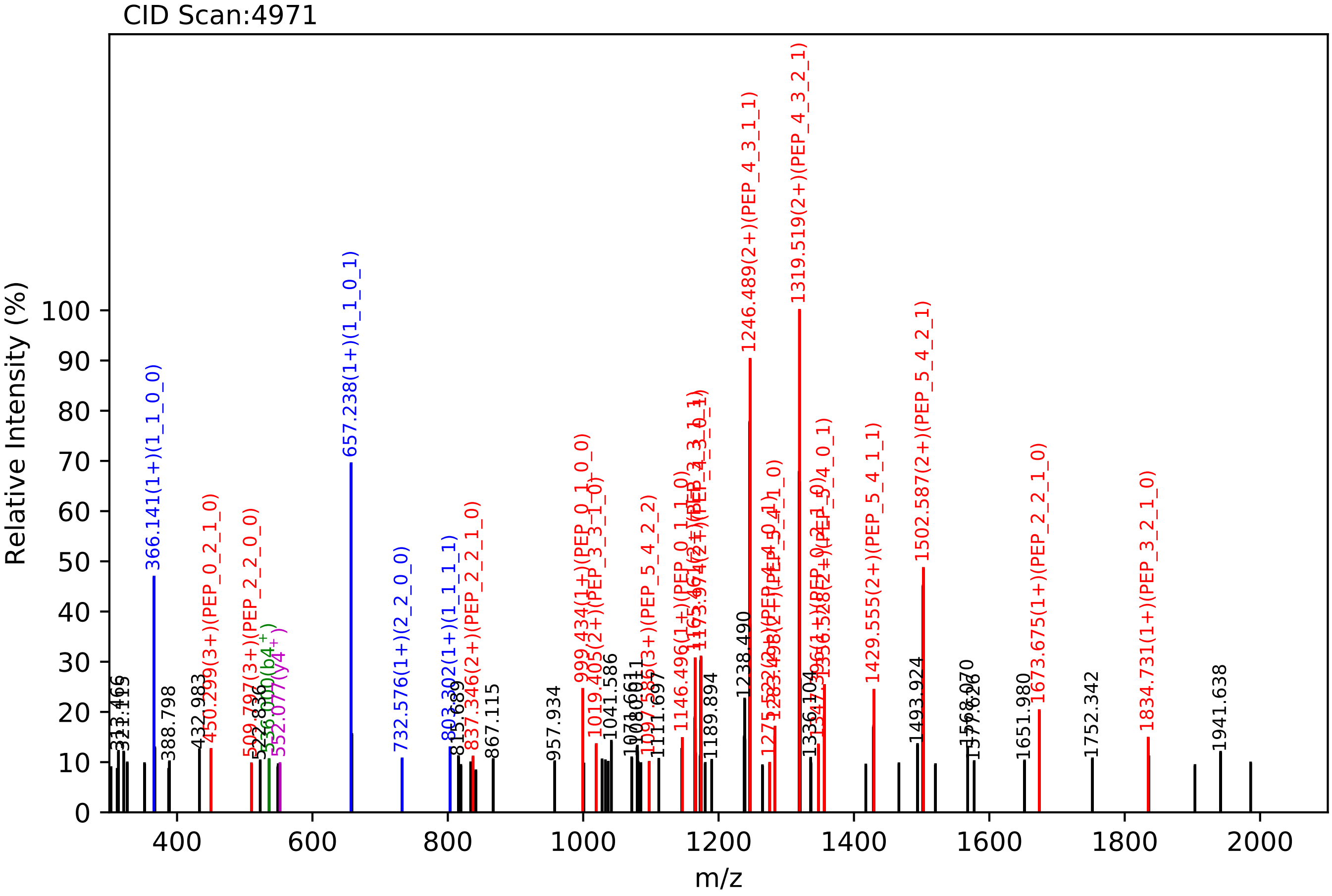

Training set no. 173, Experiment: AGP exp_15

NEEYNK(=PEP)_5_4_2_2, m/z:1098.42(3+), RT:23.37, Y-score:77.97

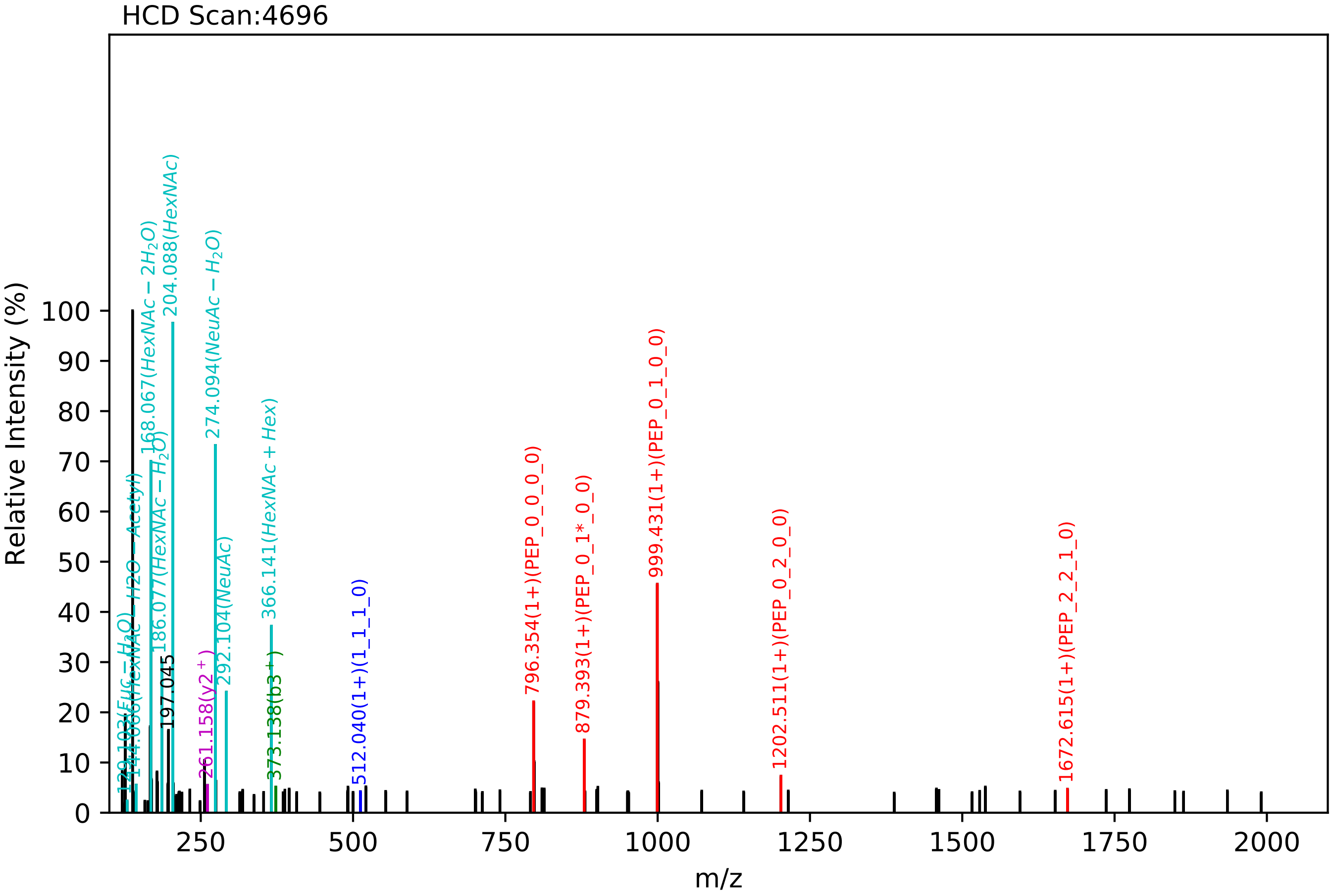

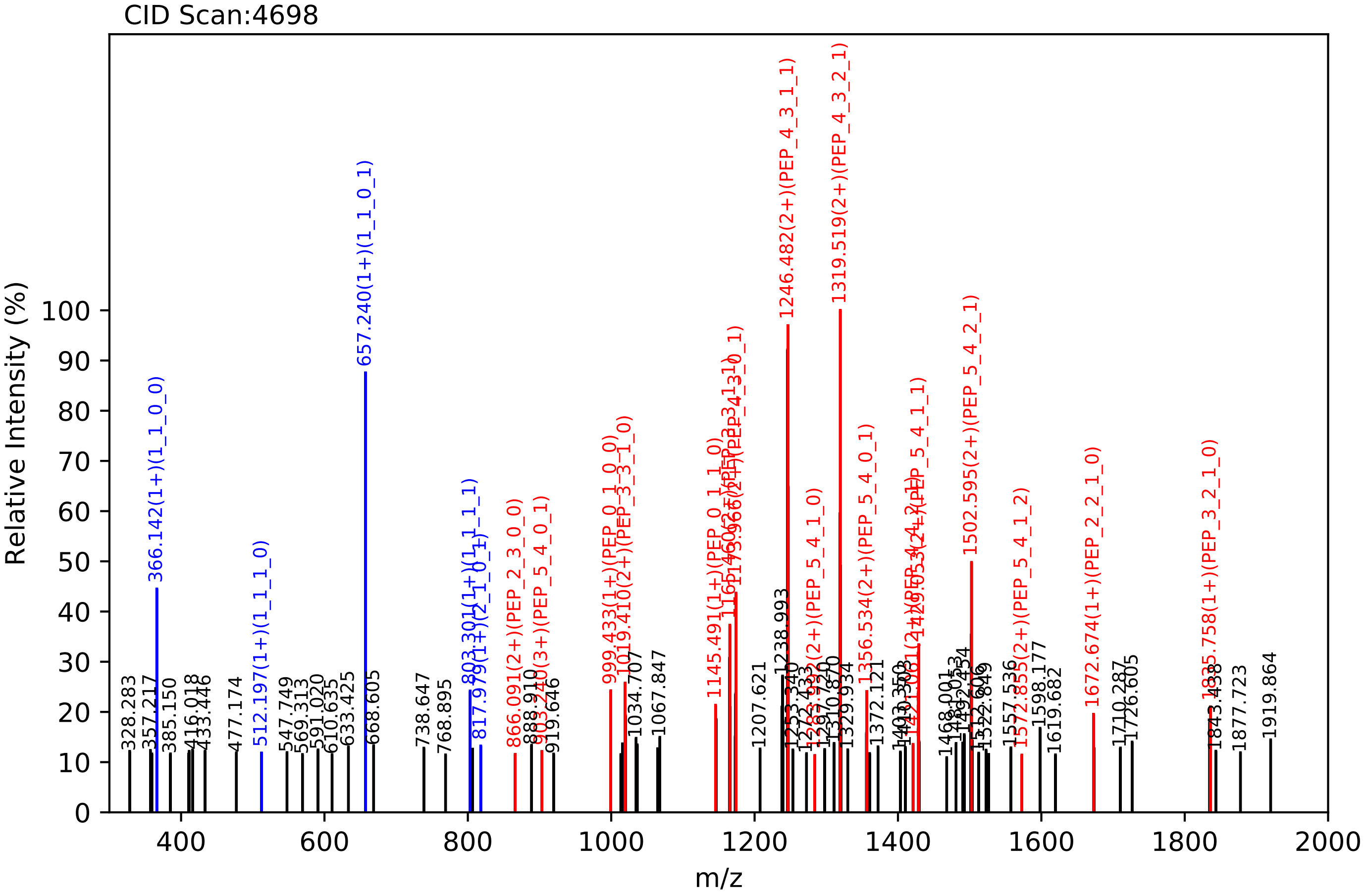

Training set no. 174, Experiment: AGP exp_20

NEEYNK(=PEP)_5_4_2_2, m/z:1098.42(3+), RT:21.02, Y-score:77.21

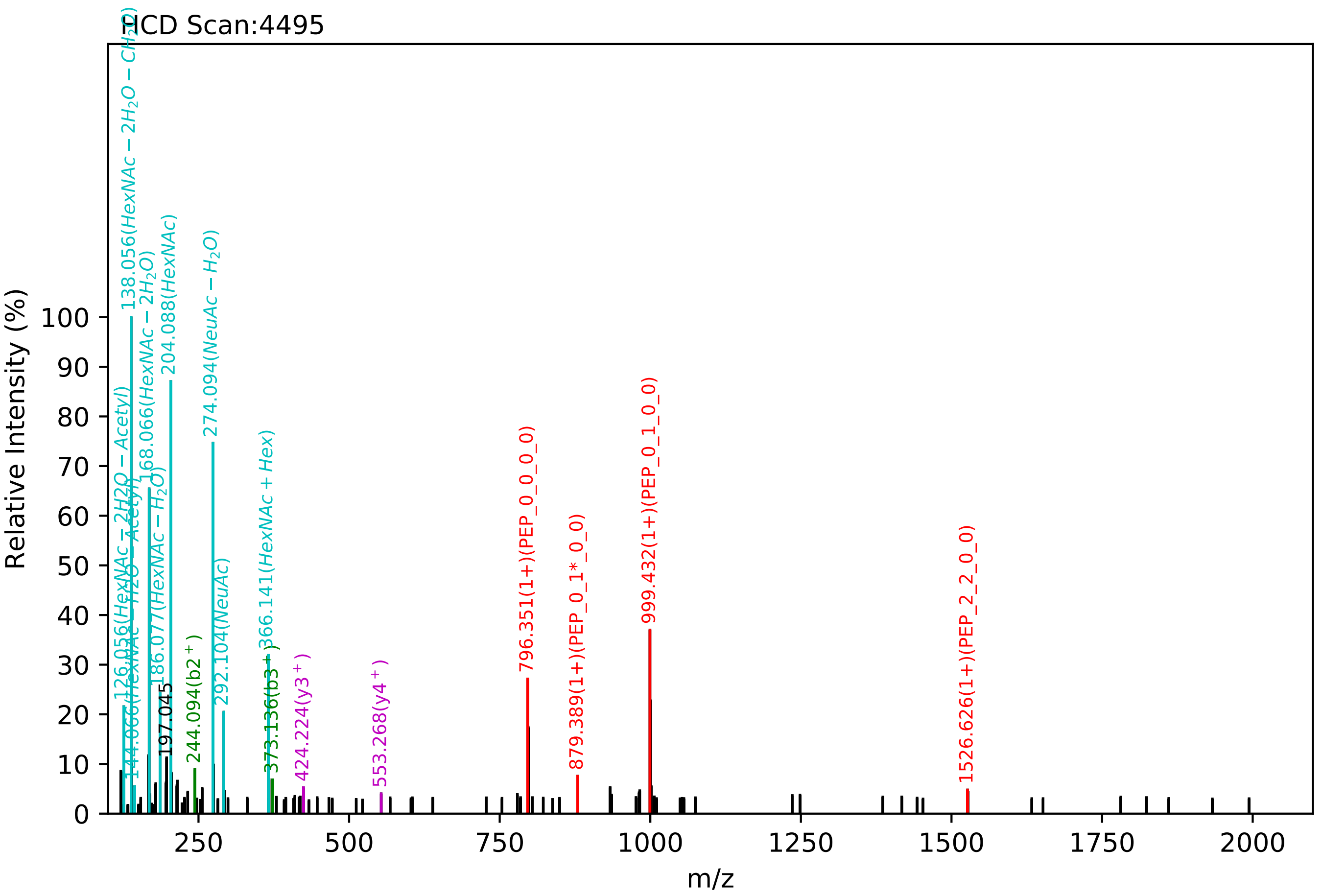

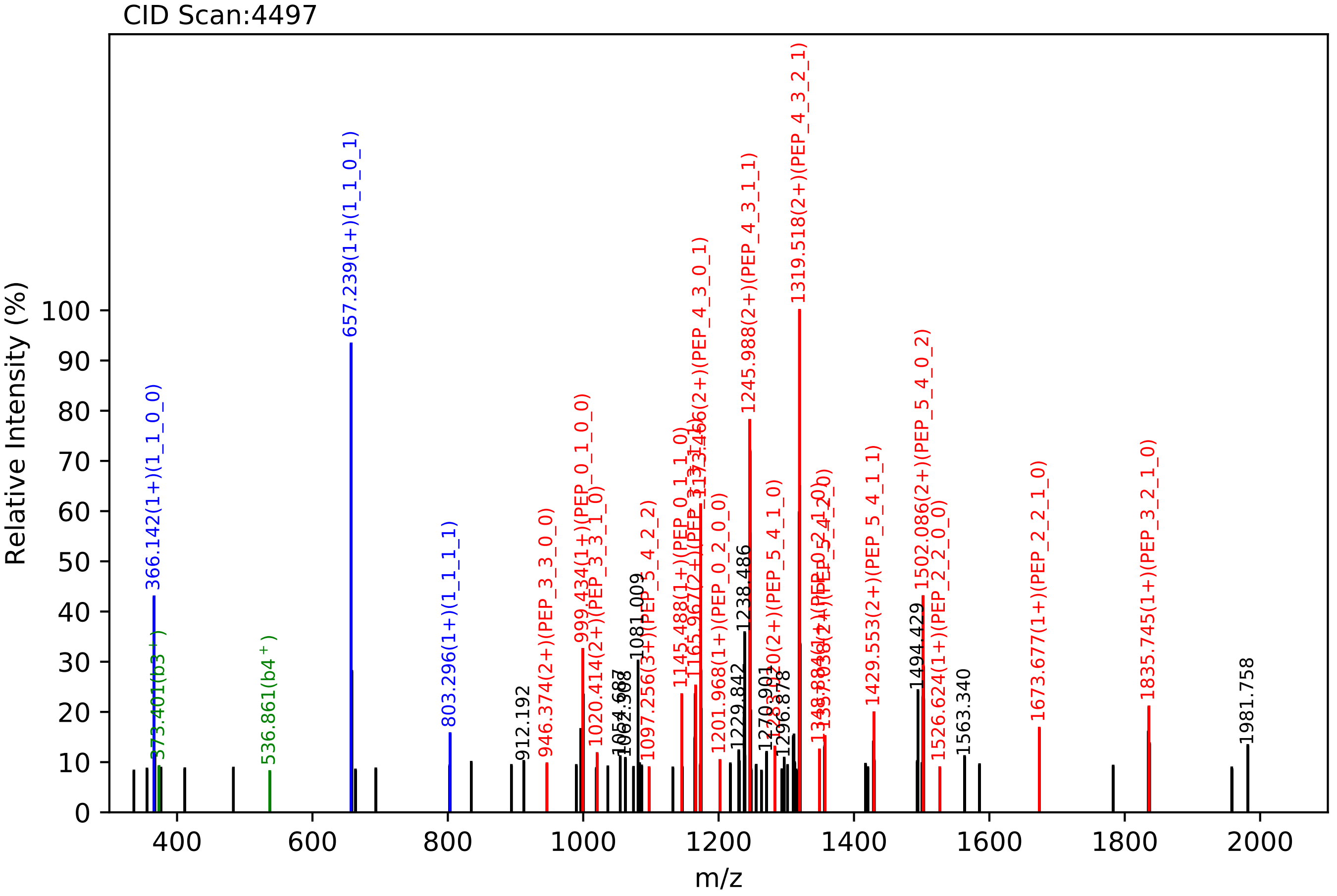

Training set no. 175, Experiment: AGP exp_23

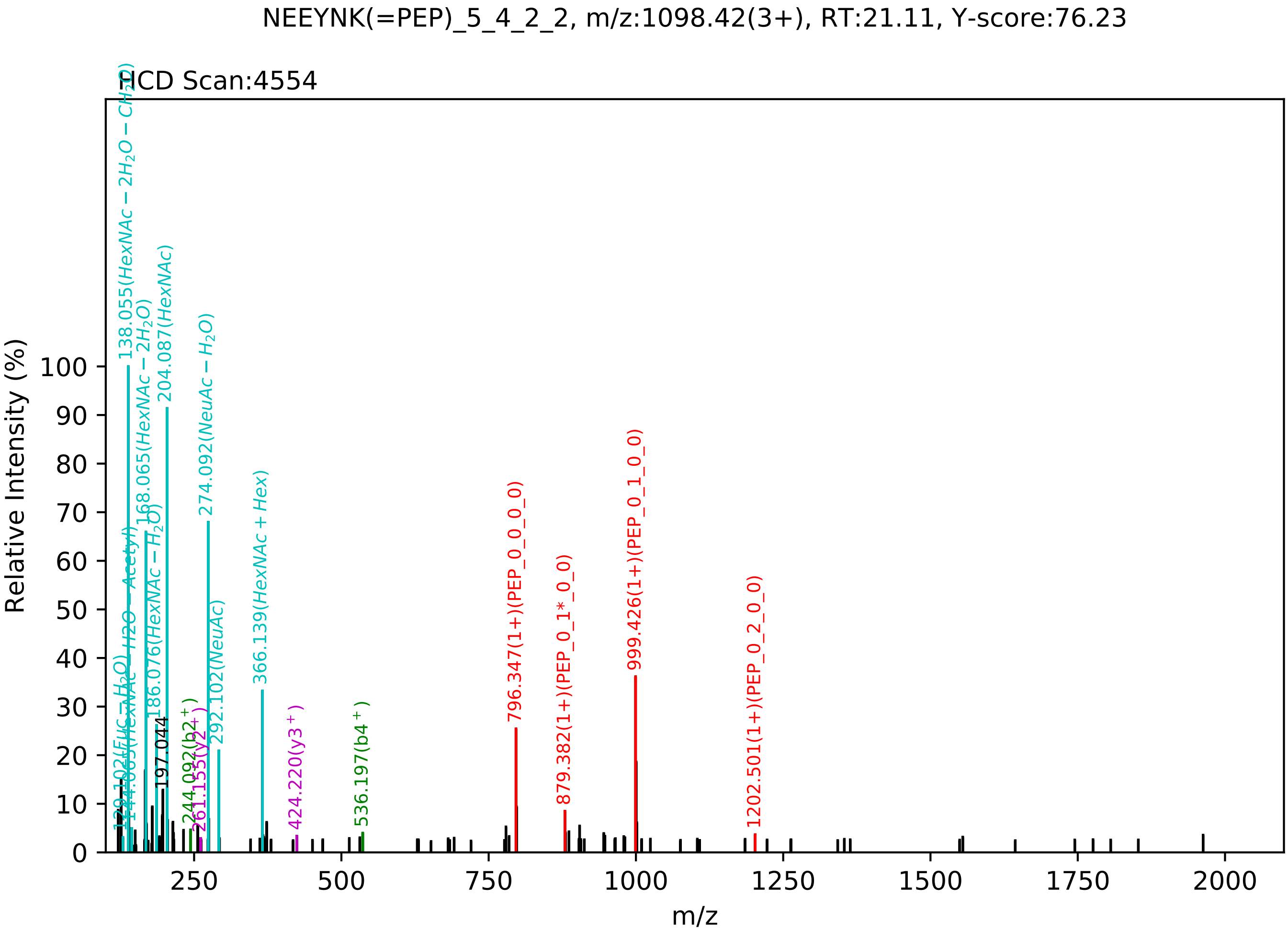

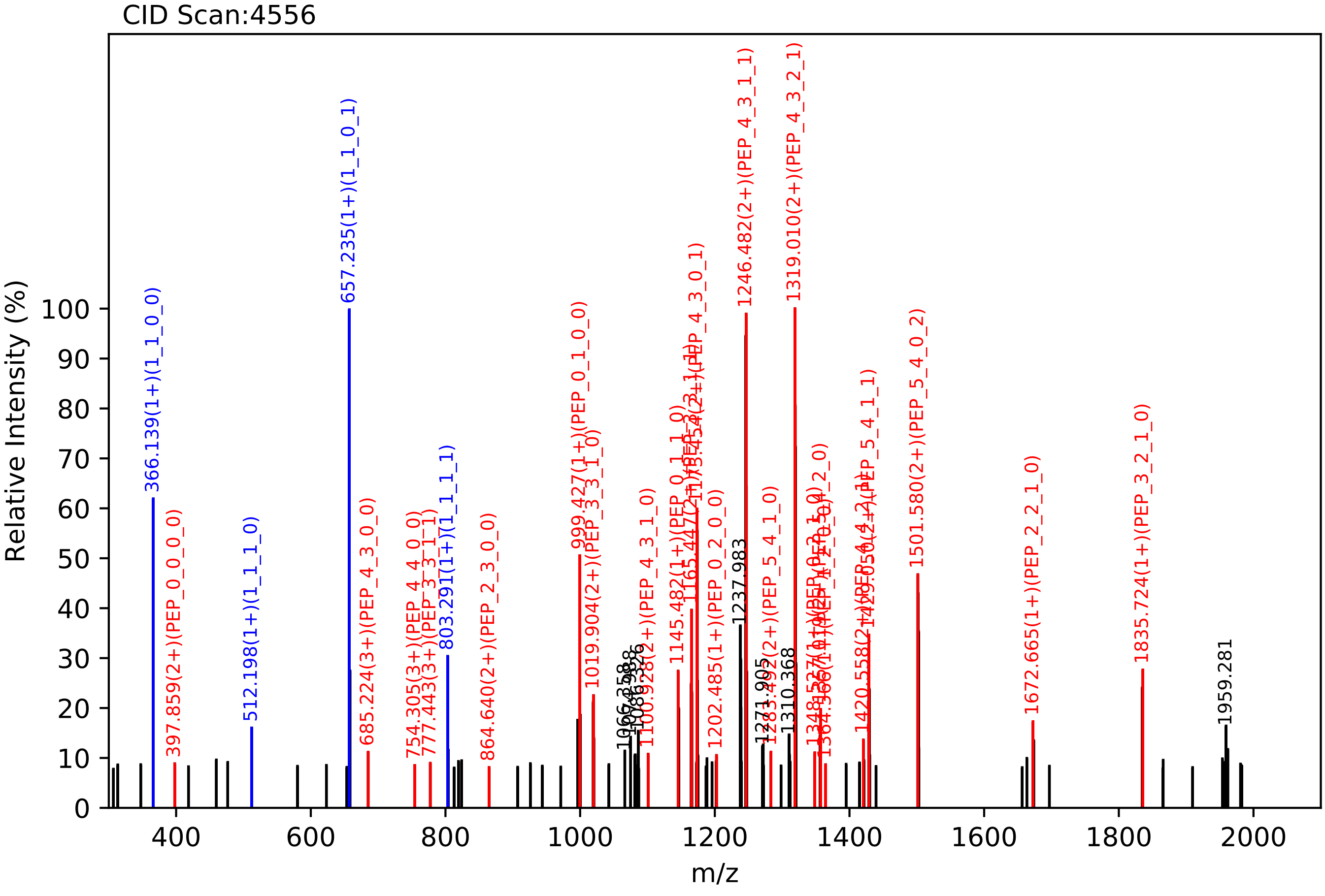

Training set no. 176, Experiment: AGP exp_1

NEEYNK(=PEP)_5_5_0_2, m/z:1068.74(3+), RT:20.53, Y-score:85.04

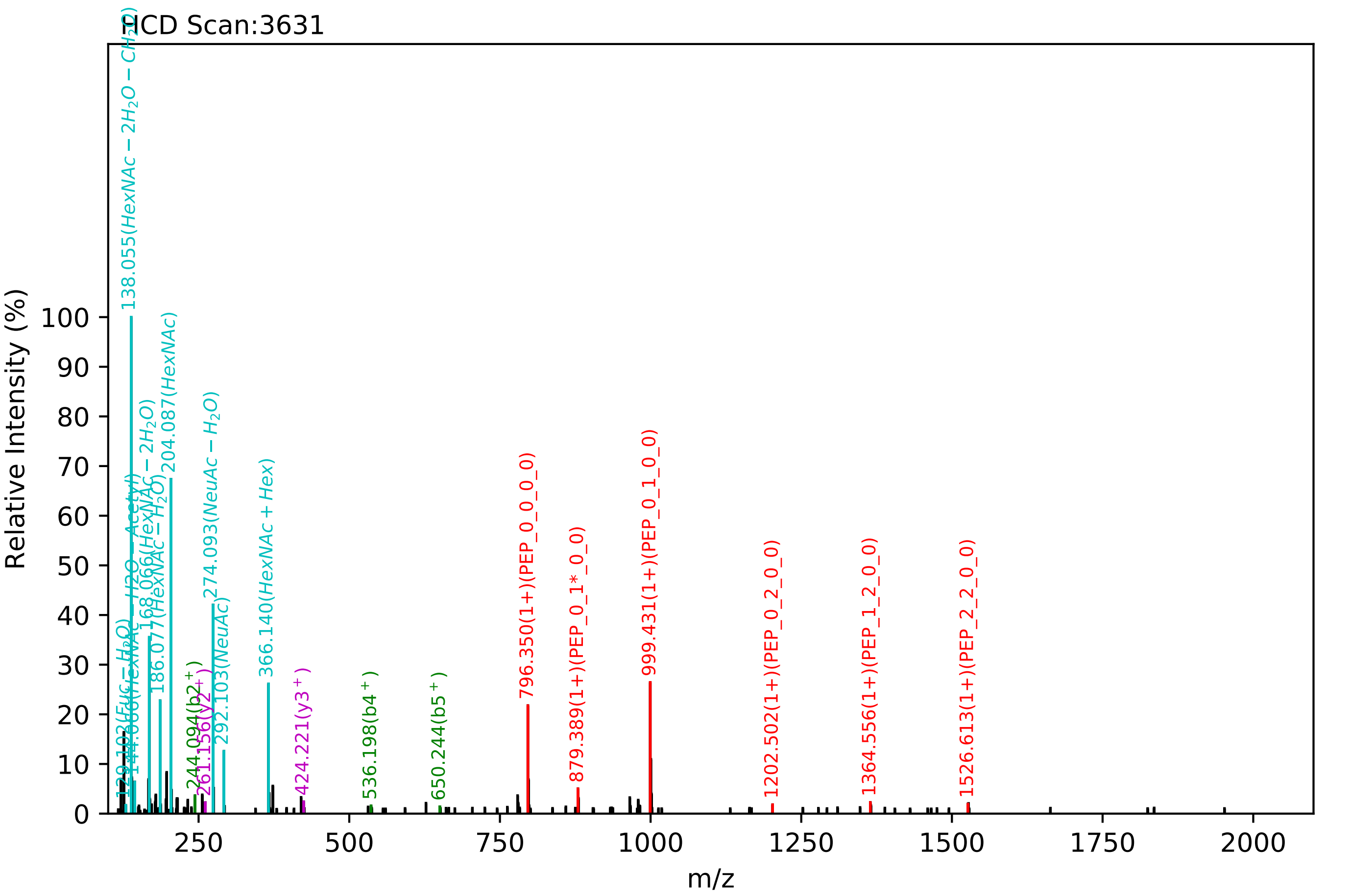

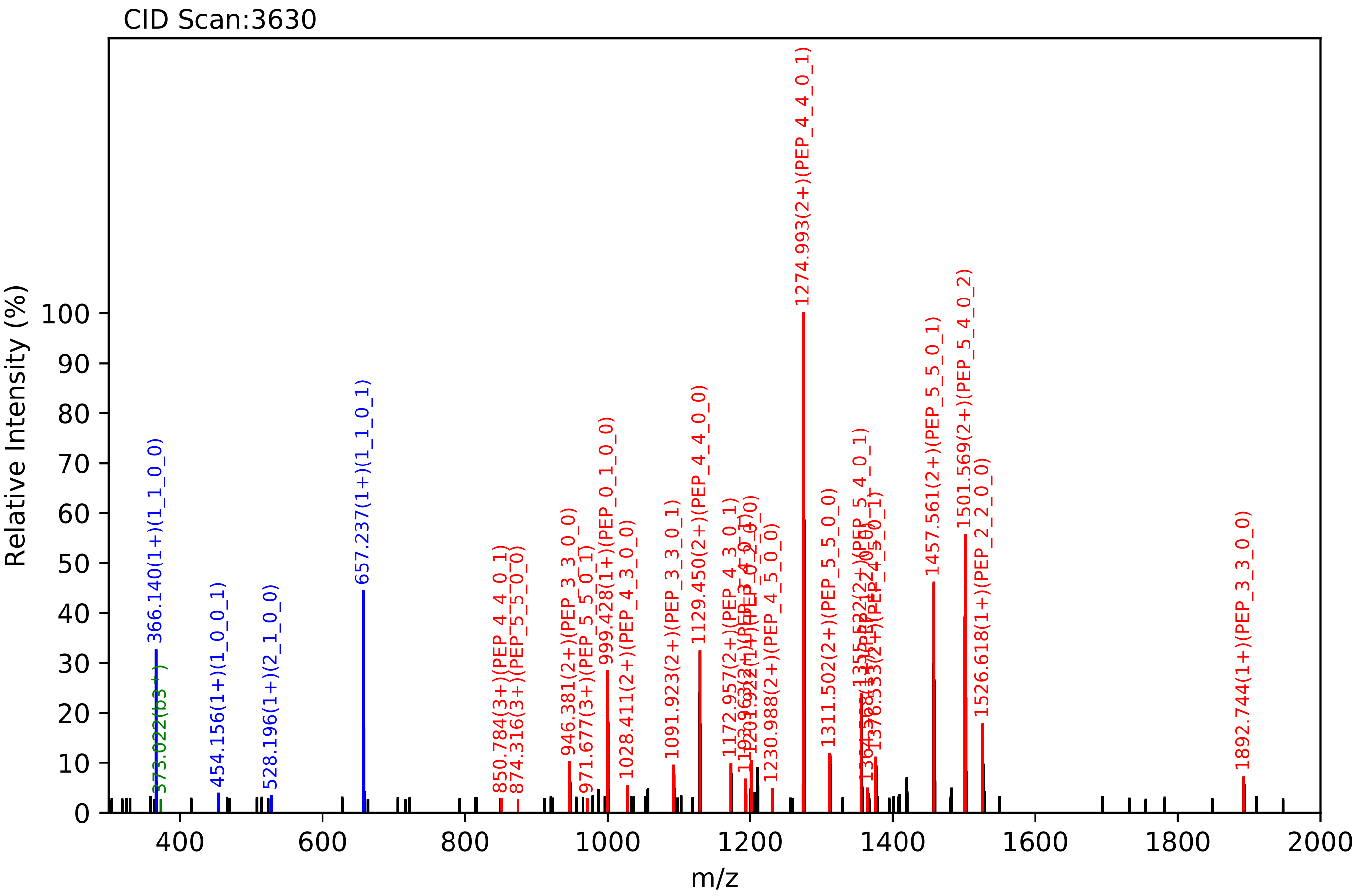

Training set no. 177, Experiment: AGP exp_2

NEEYNK(=PEP)_6_5_0_1, m/z:1025.73(3+), RT:16.92, Y-score:94.32

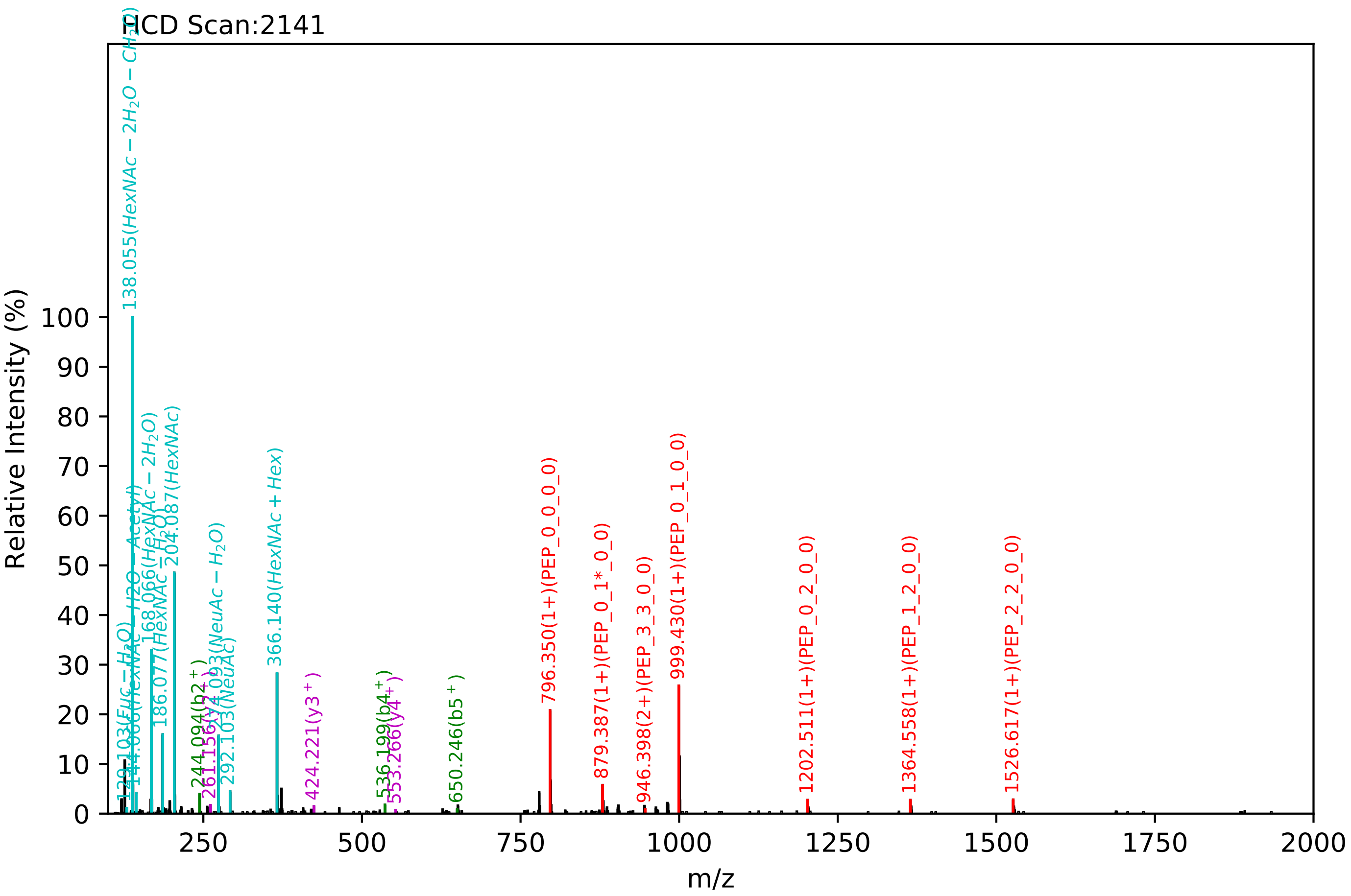

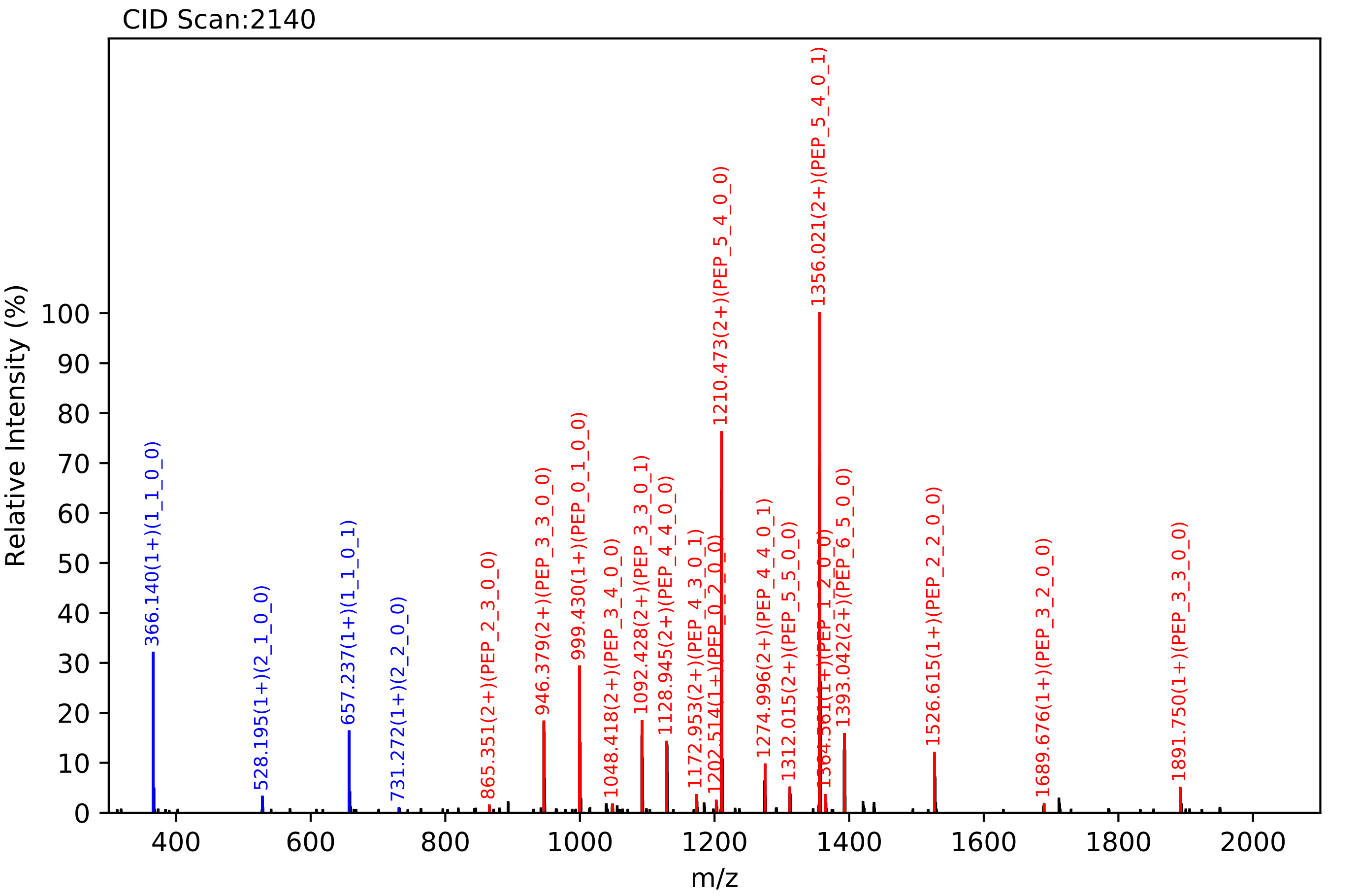

Training set no. 178, Experiment: AGP exp_2

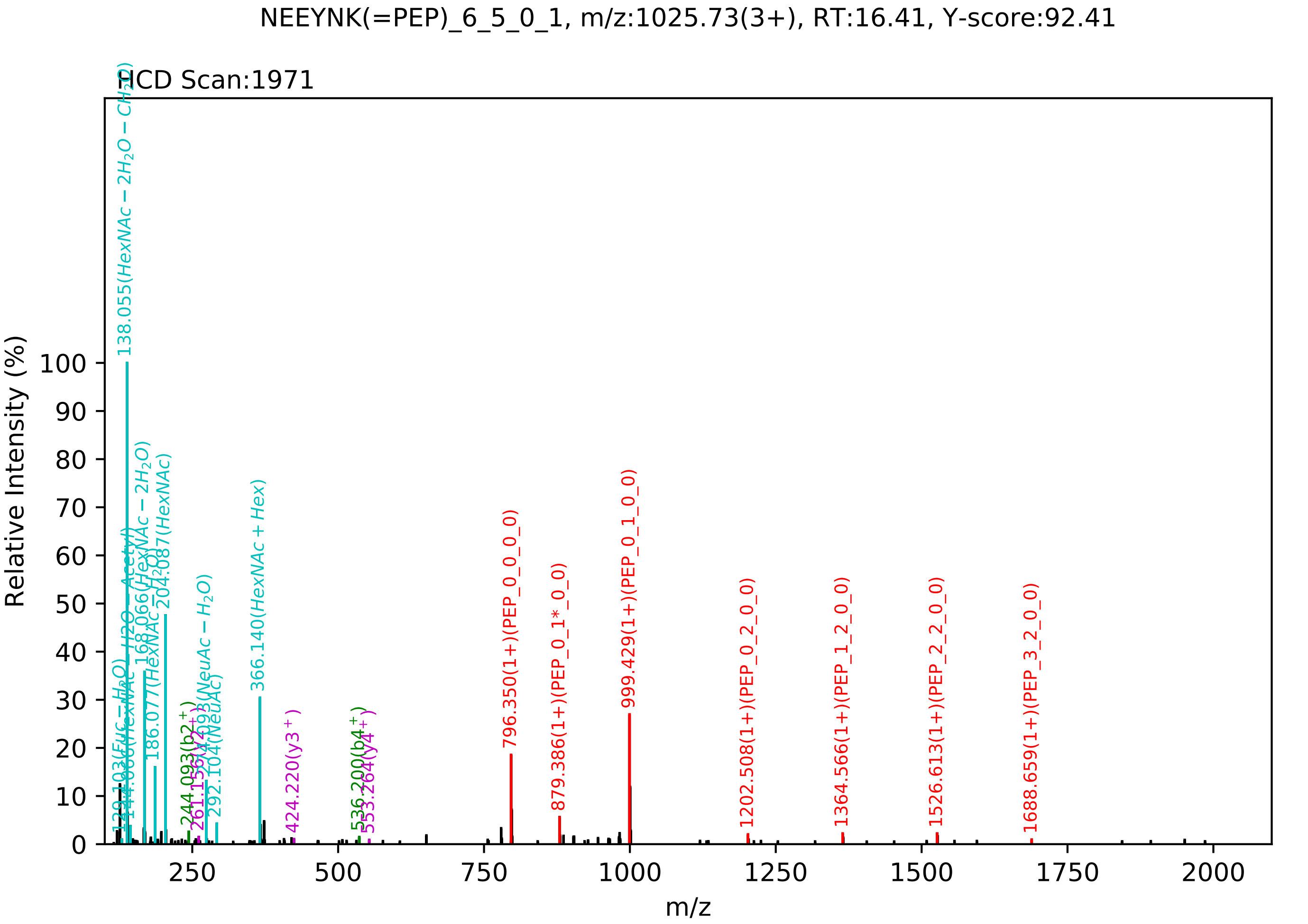

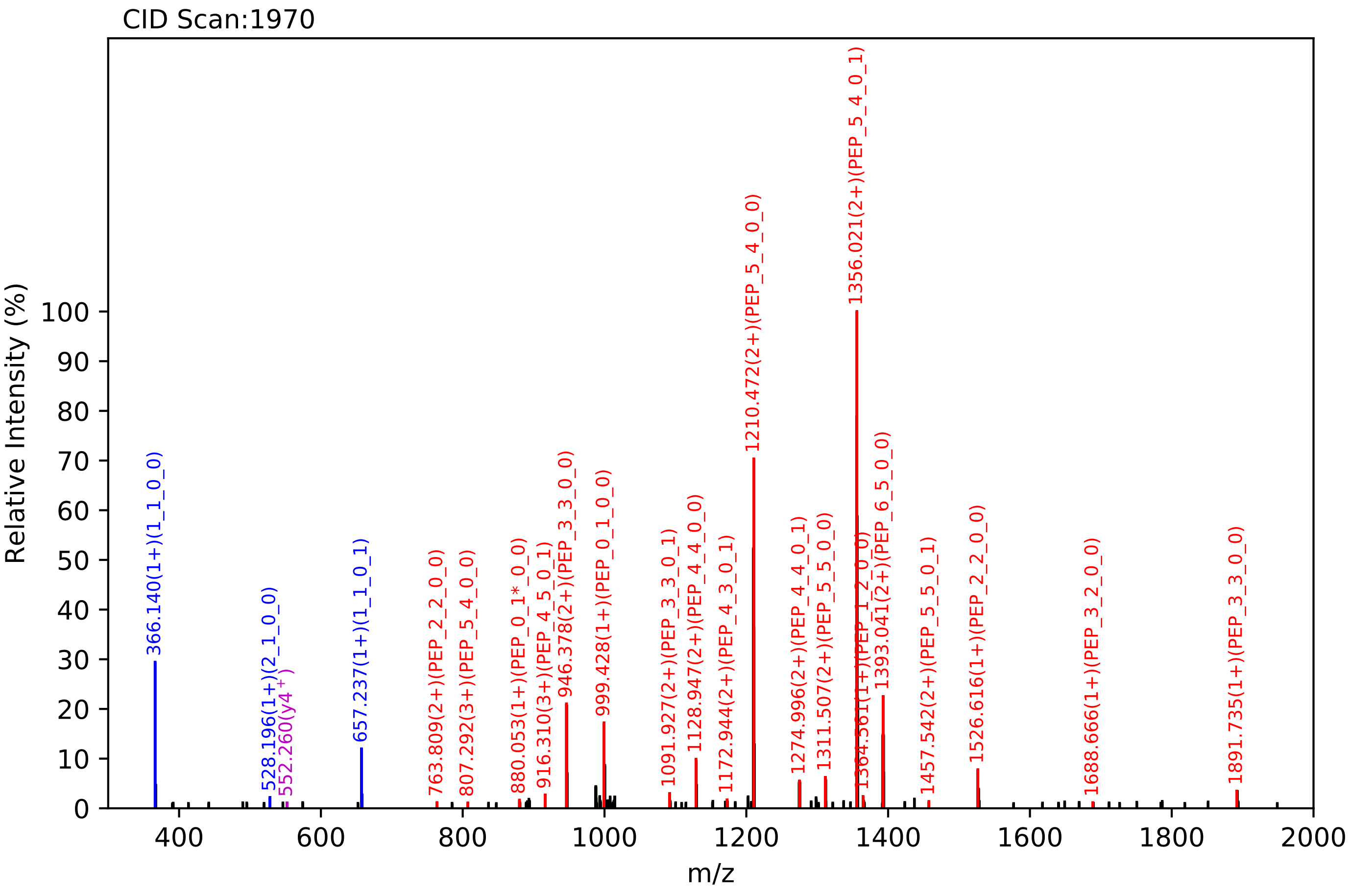

Training set no. 179, Experiment: AGP exp_1

NEEYNK(=PEP)_6_5_0_1, m/z:1025.73(3+), RT:16.25, Y-score:91.91

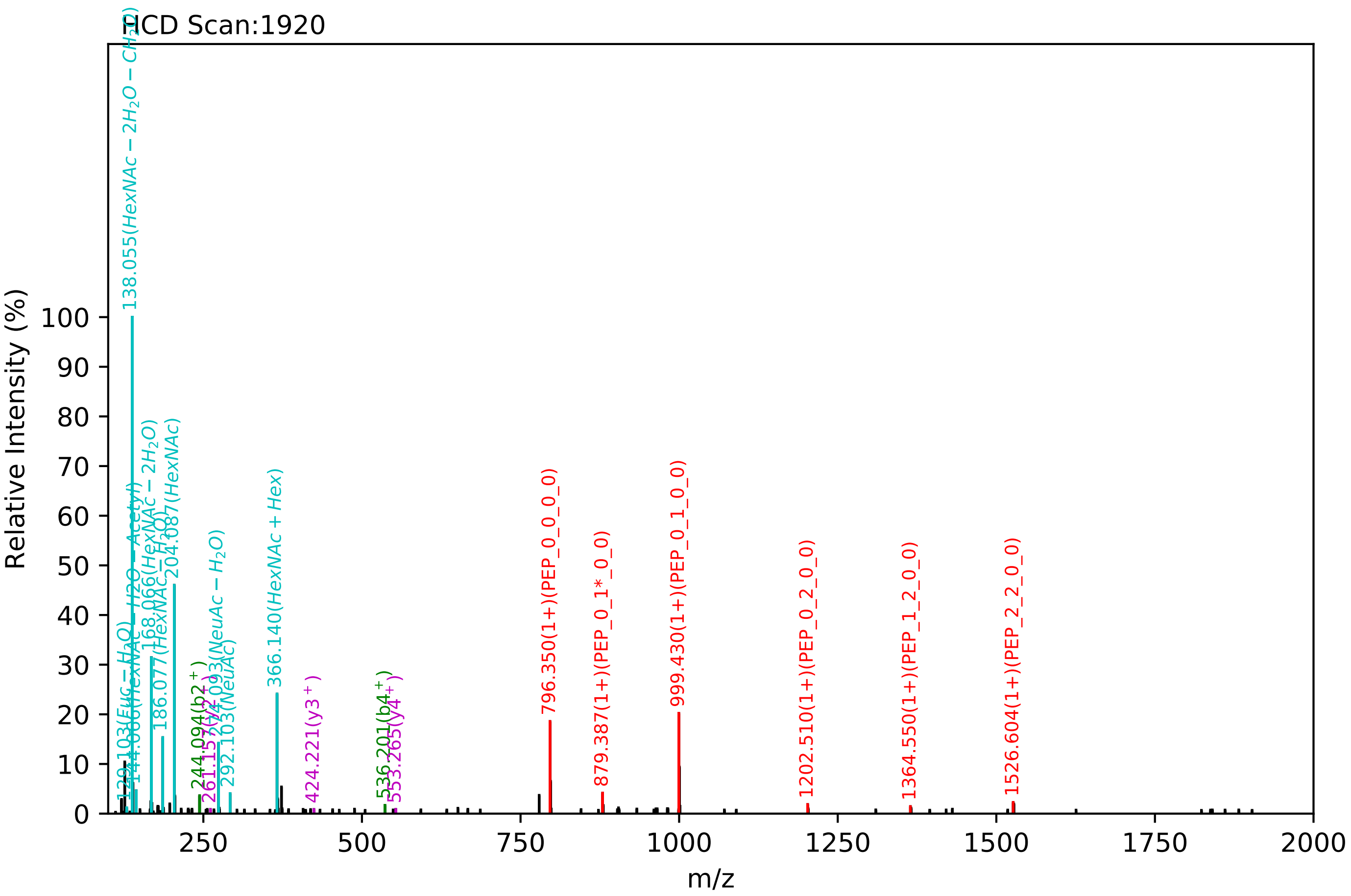

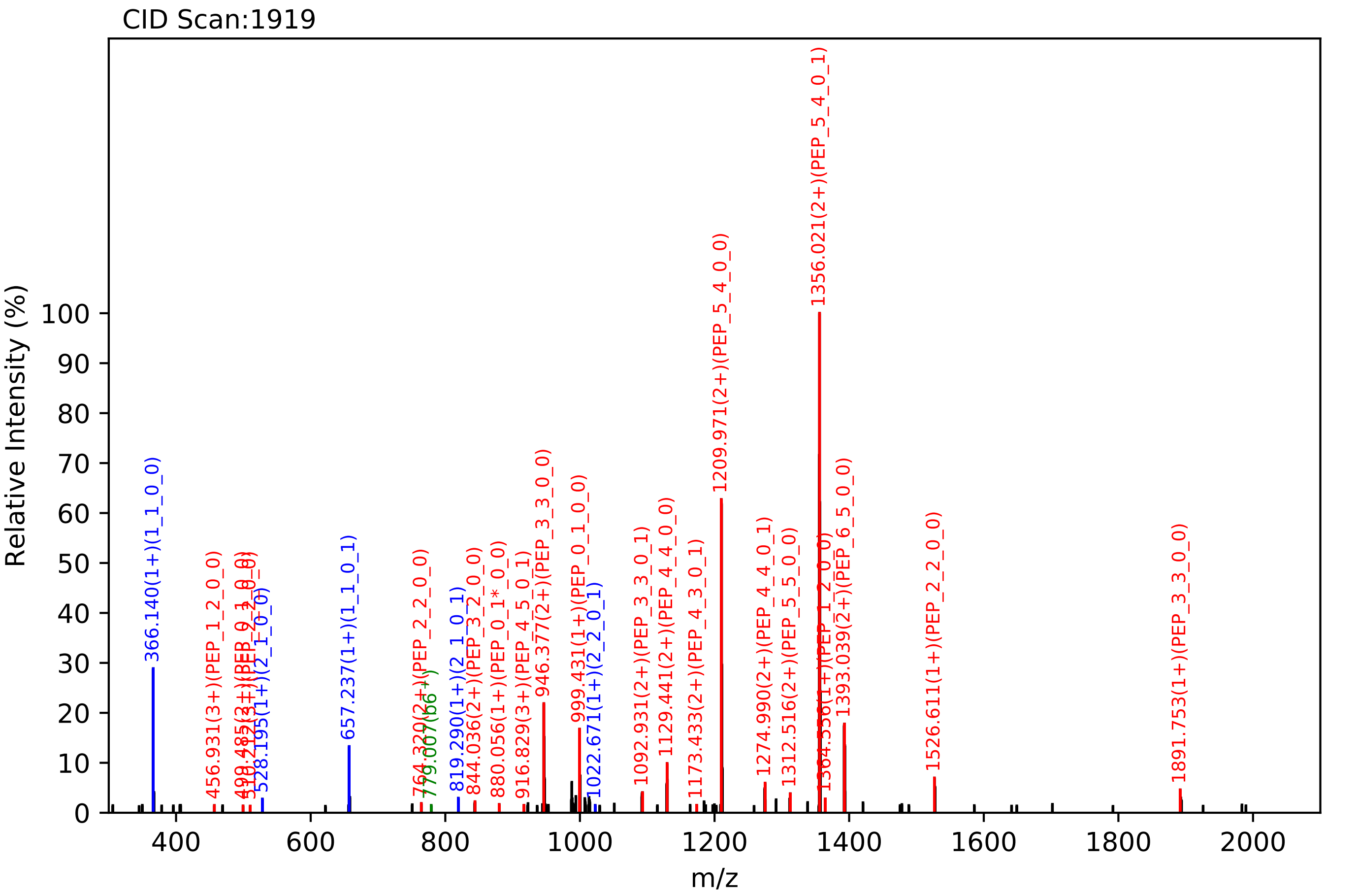

Training set no. 180, Experiment: AGP exp_2

NEEYNK(=PEP)_6_5_0_1, m/z:1025.73(3+), RT:17.43, Y-score:91.57

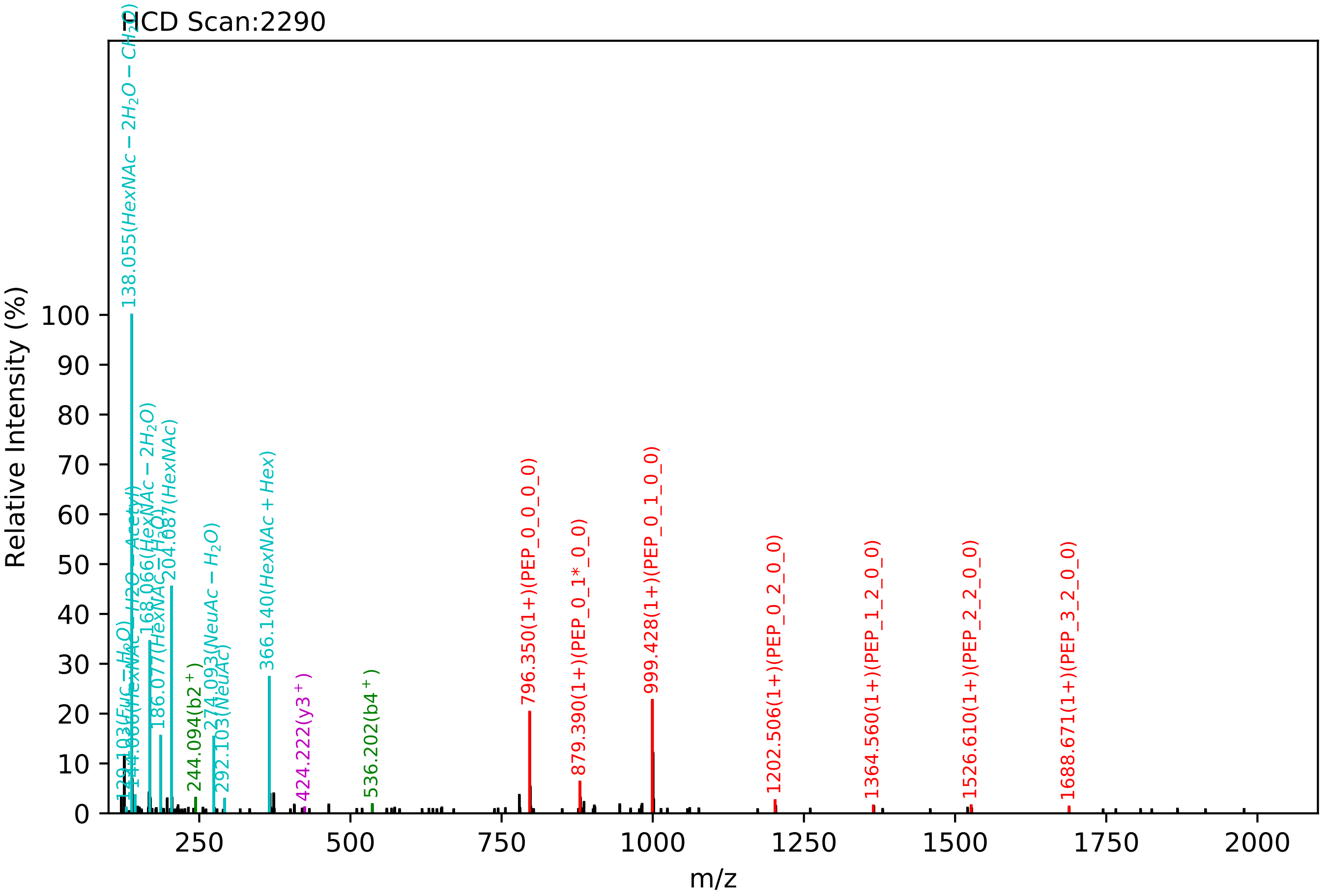

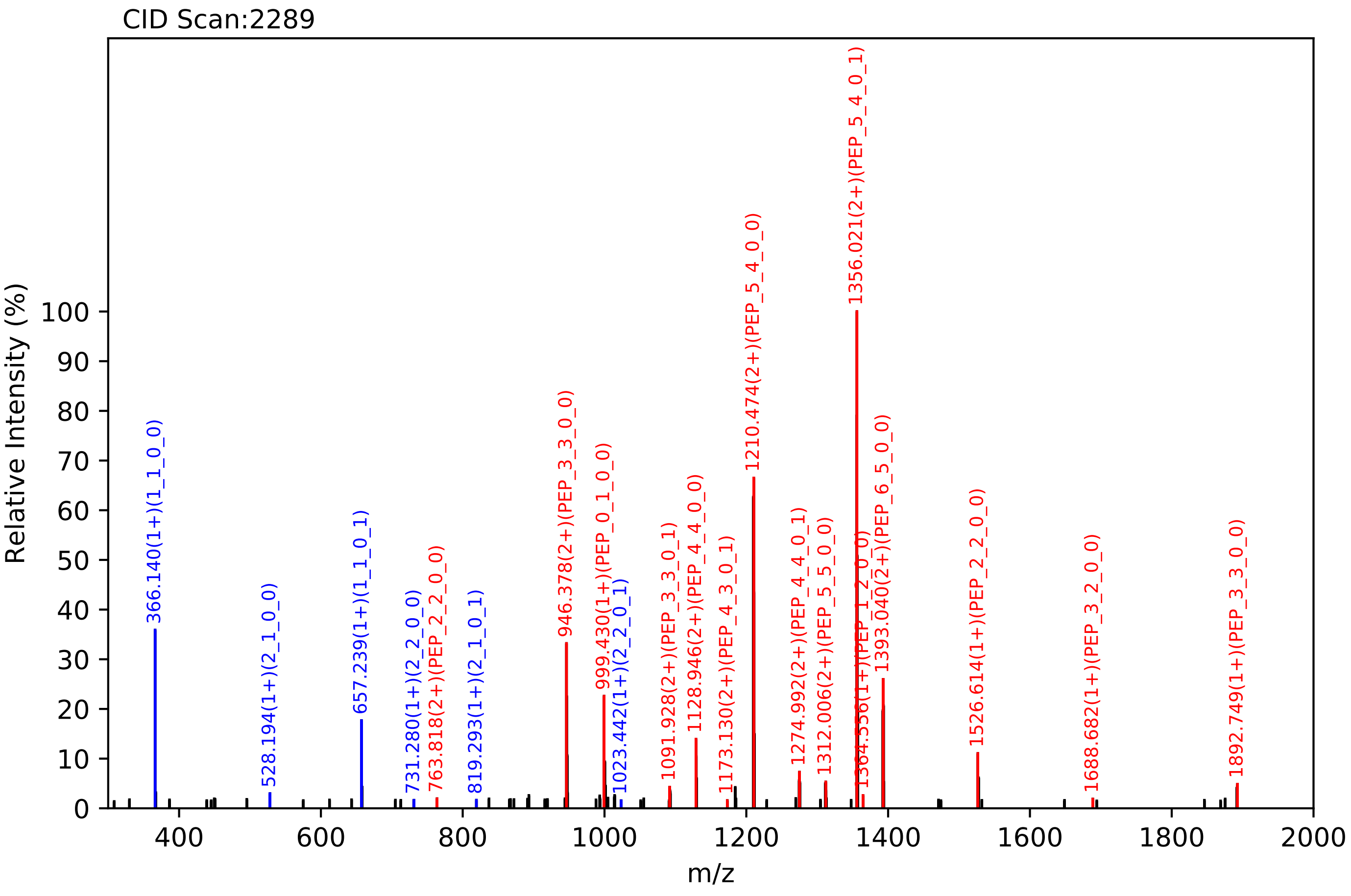

Training set no. 181, Experiment: AGP exp_1

NEEYNK(=PEP)_6_5_0_1, m/z:1025.73(3+), RT:16.76, Y-score:91.19

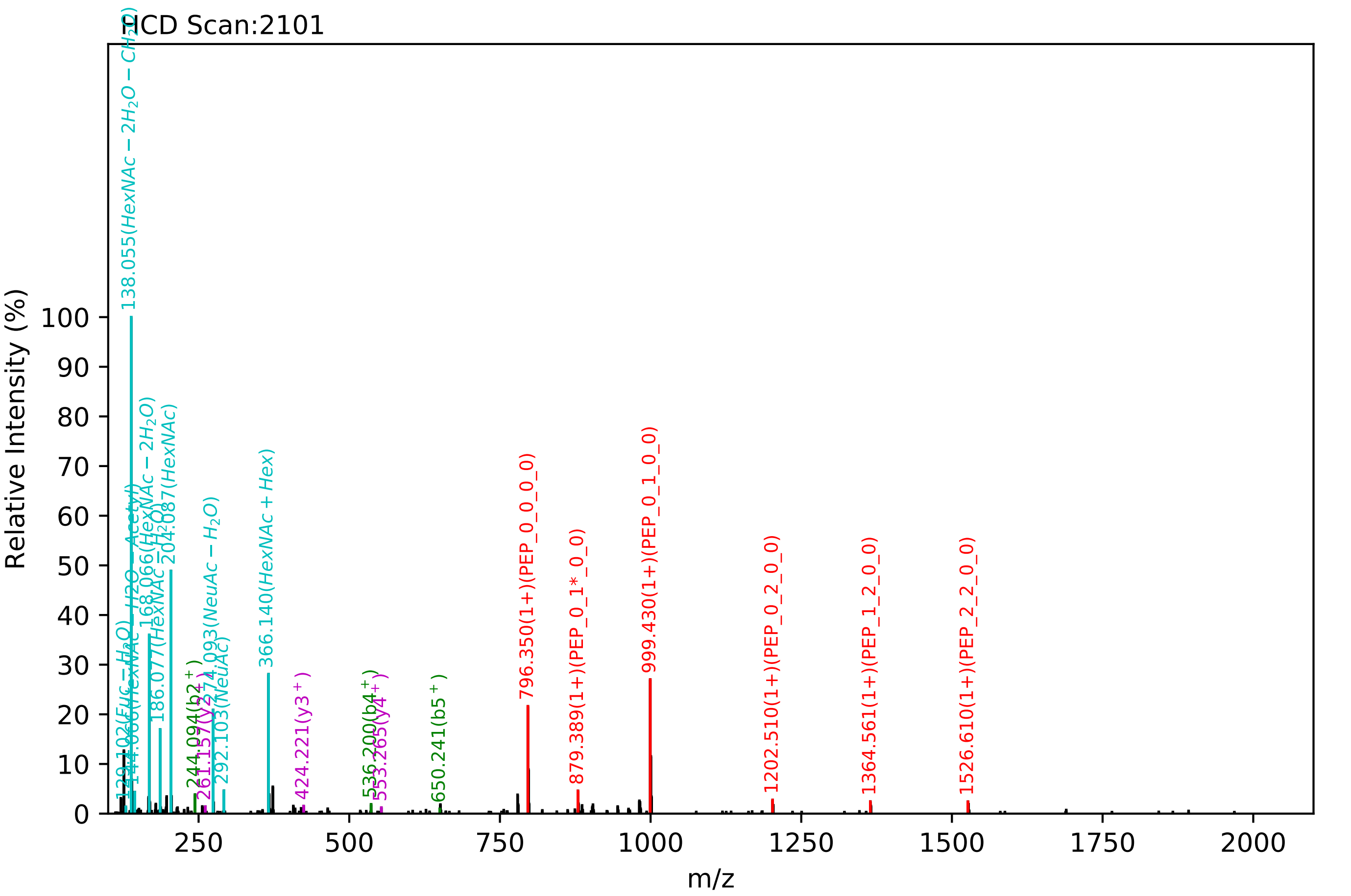

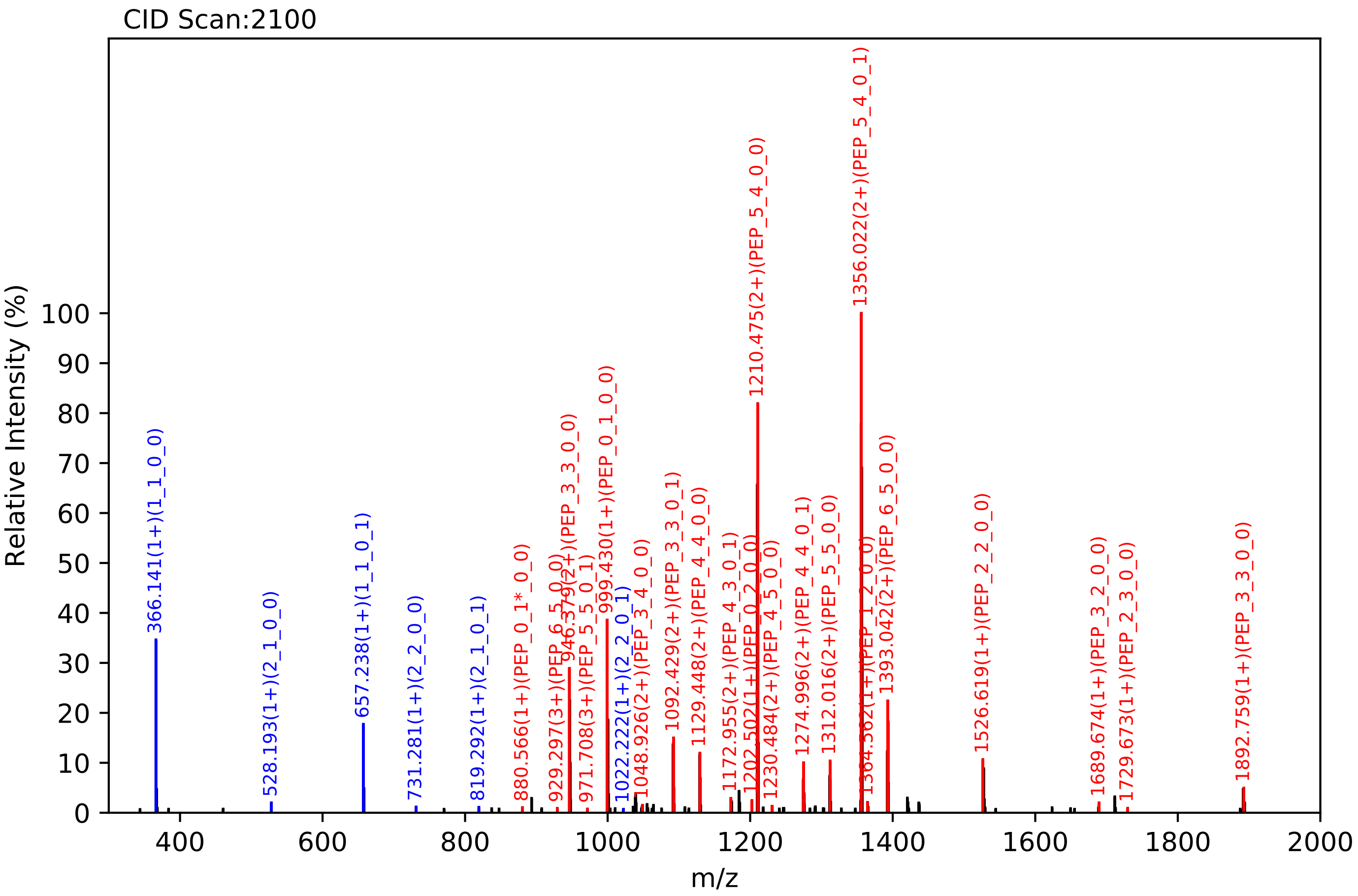

Training set no. 182, Experiment: AGP exp_1

NEEYNK(=PEP)_6_5_0_1, m/z:1025.73(3+), RT:17.27, Y-score:90.41

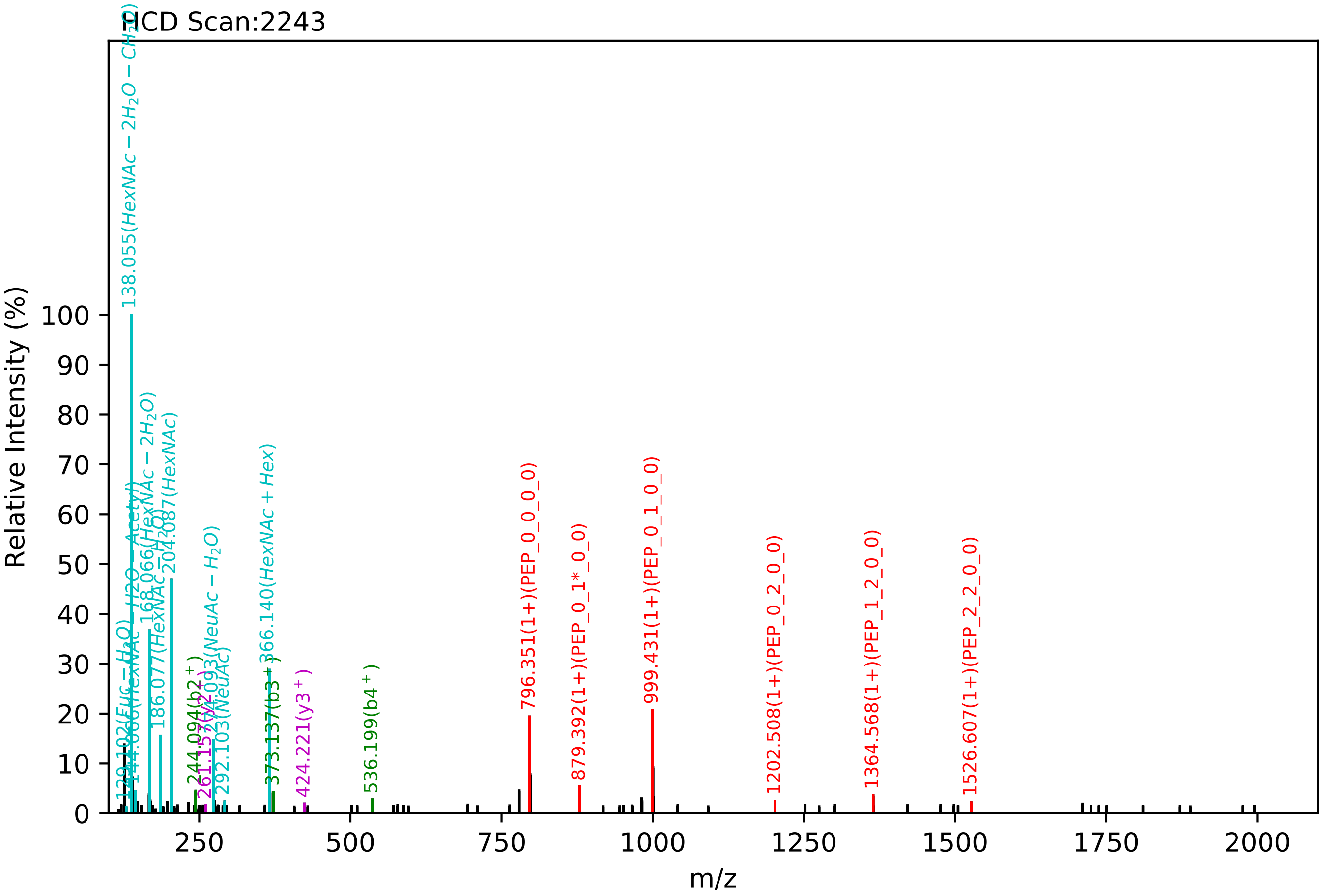

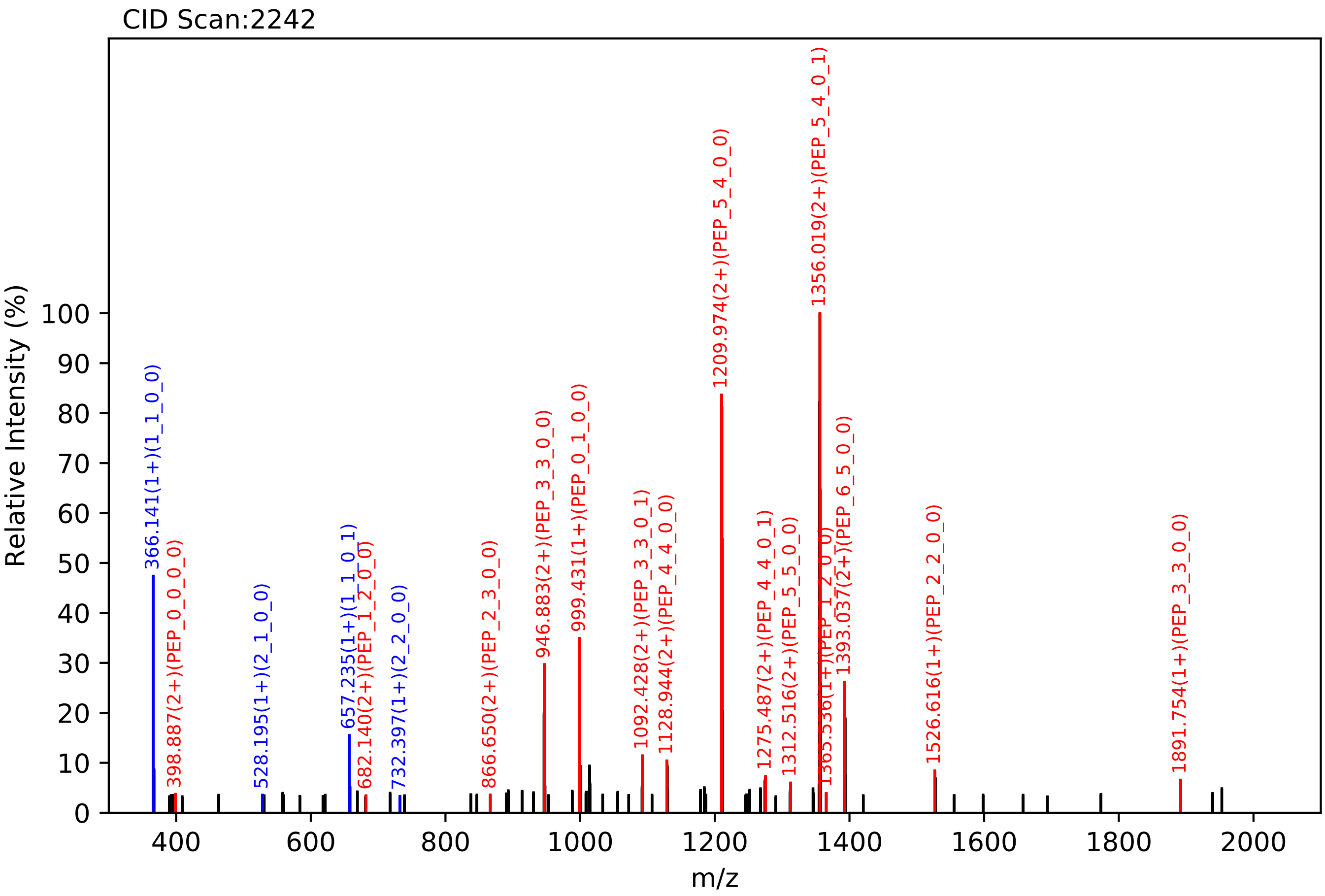

Training set no. 183, Experiment: AGP exp_2

NEEYNK(=PEP)_6_5_0_1, m/z:1025.73(3+), RT:16.40, Y-score:89.88

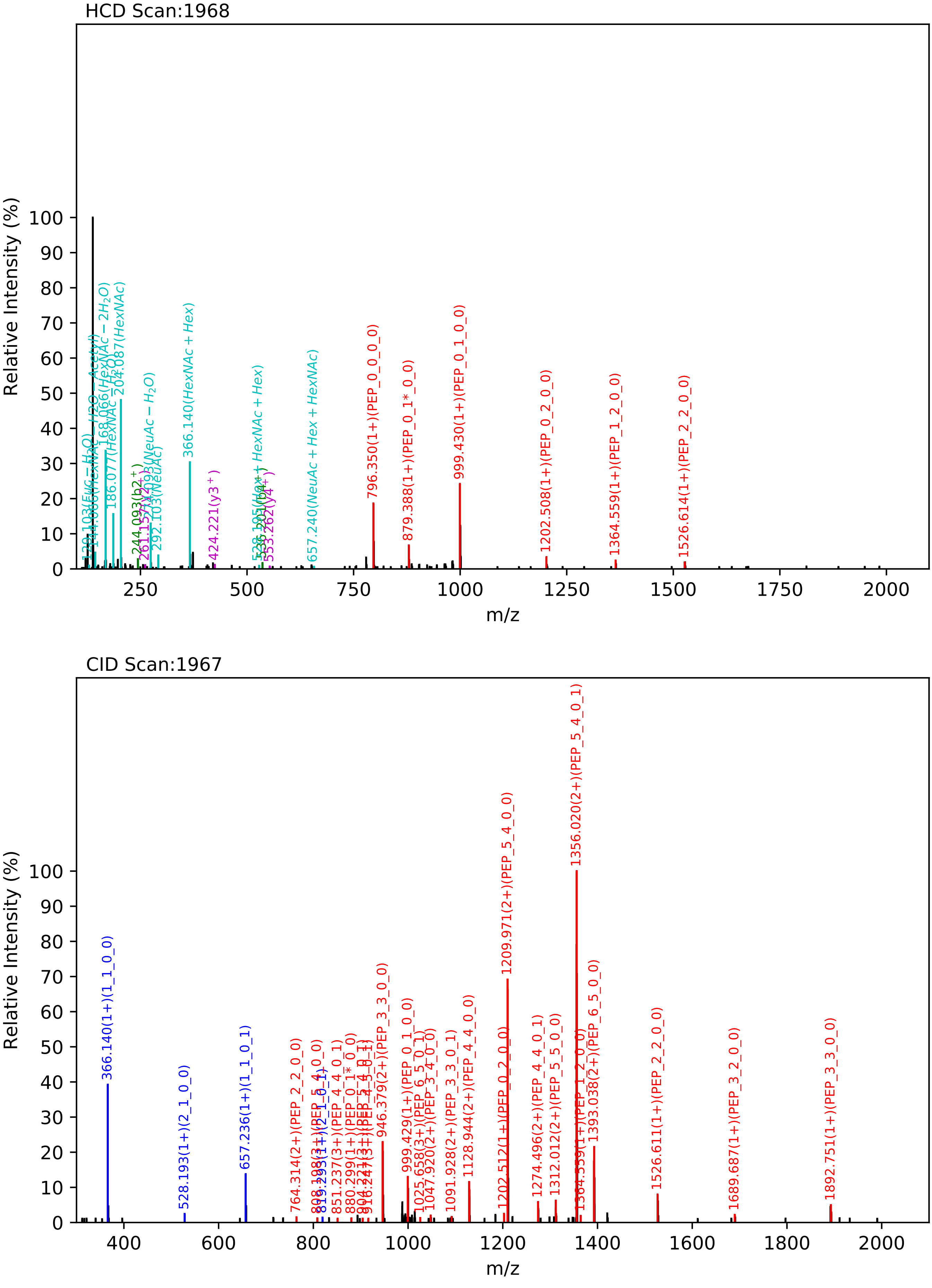

Training set no. 184, Experiment: AGP exp_2

NEEYNK(=PEP)_6_5_0_1, m/z:1538.09(2+), RT:17.14, Y-score:89.47

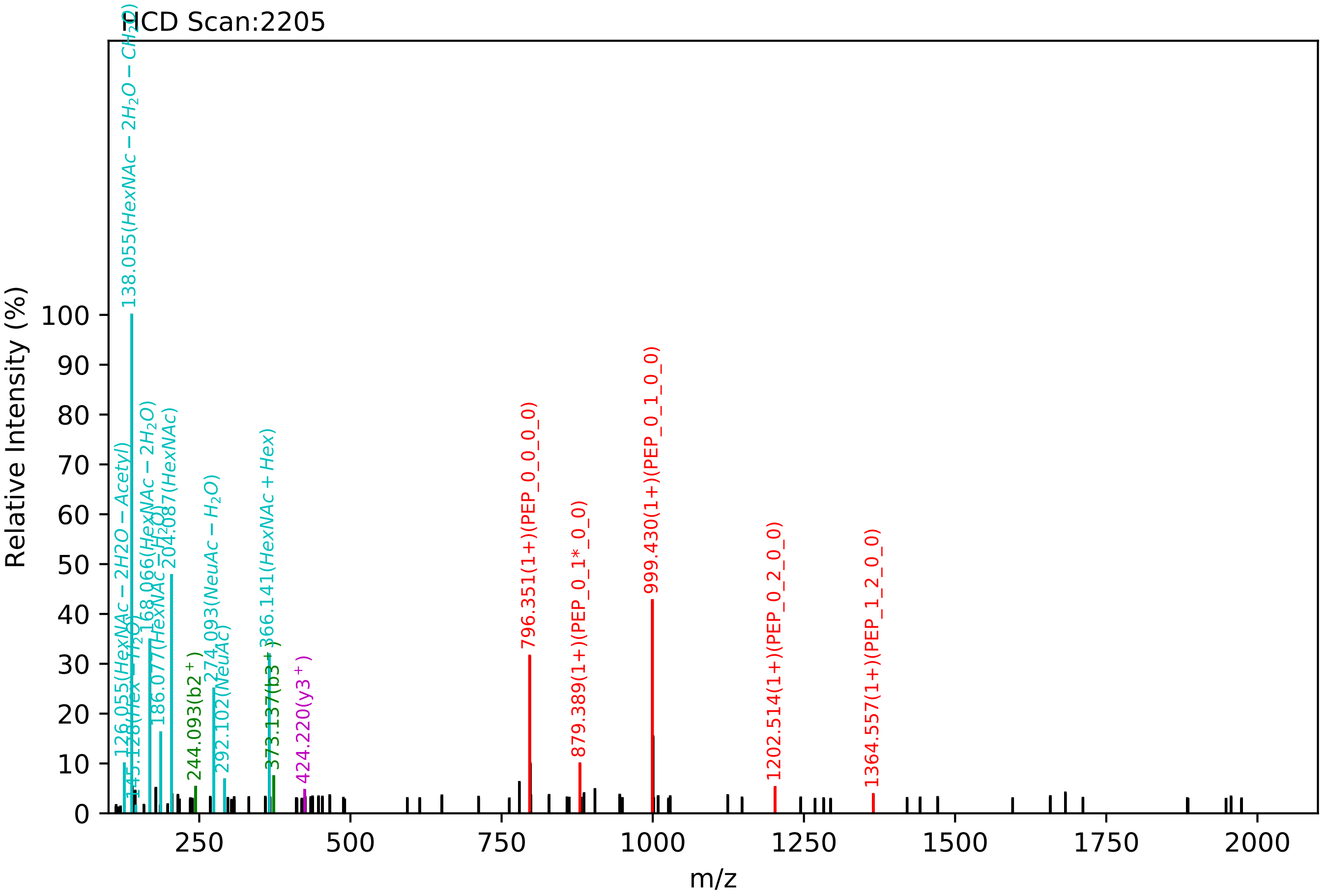

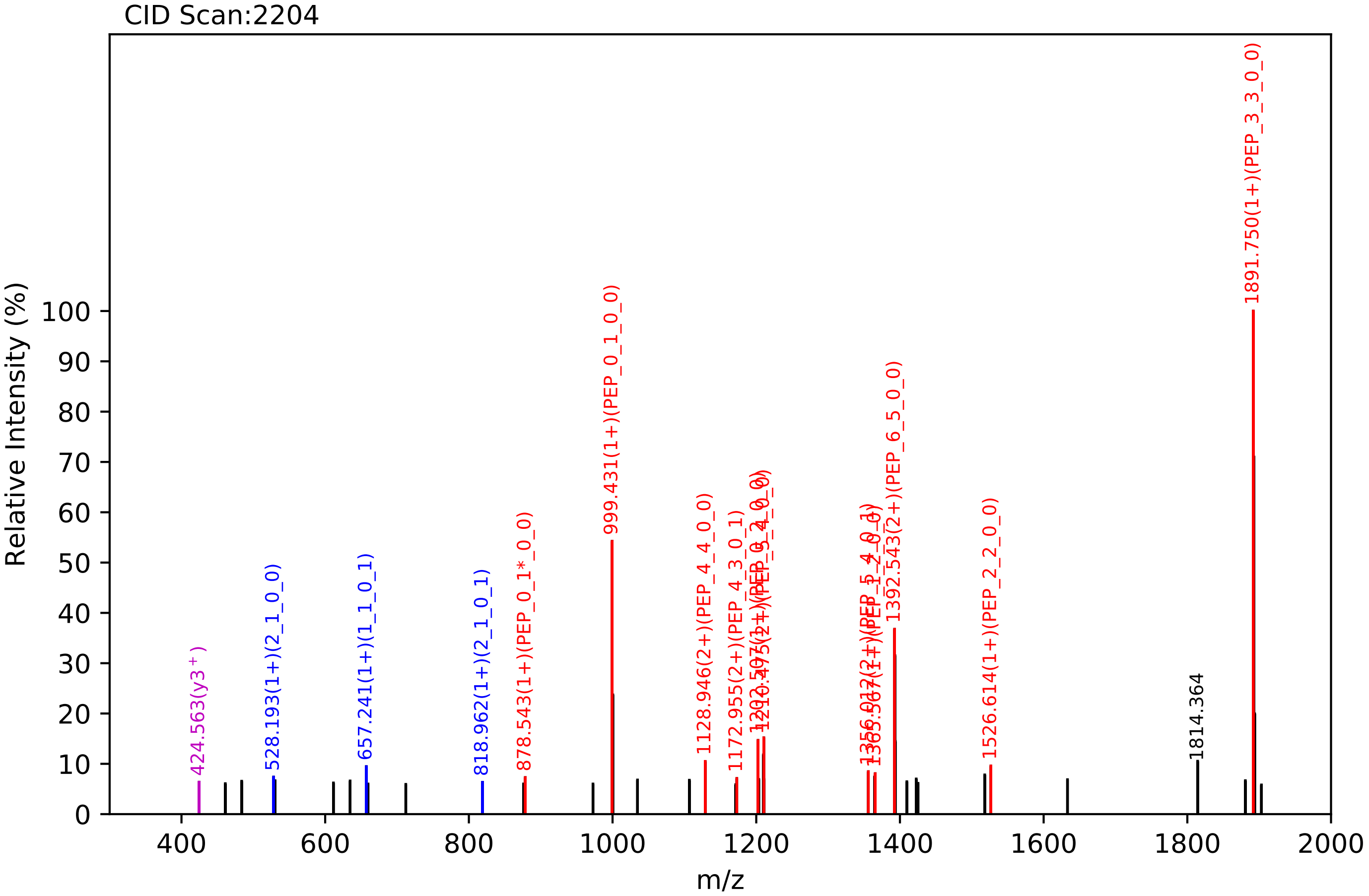

Training set no. 185, Experiment: AGP exp_1

NEEYNK(=PEP)_6_5_0_1, m/z:1538.08(2+), RT:16.93, Y-score:89.07

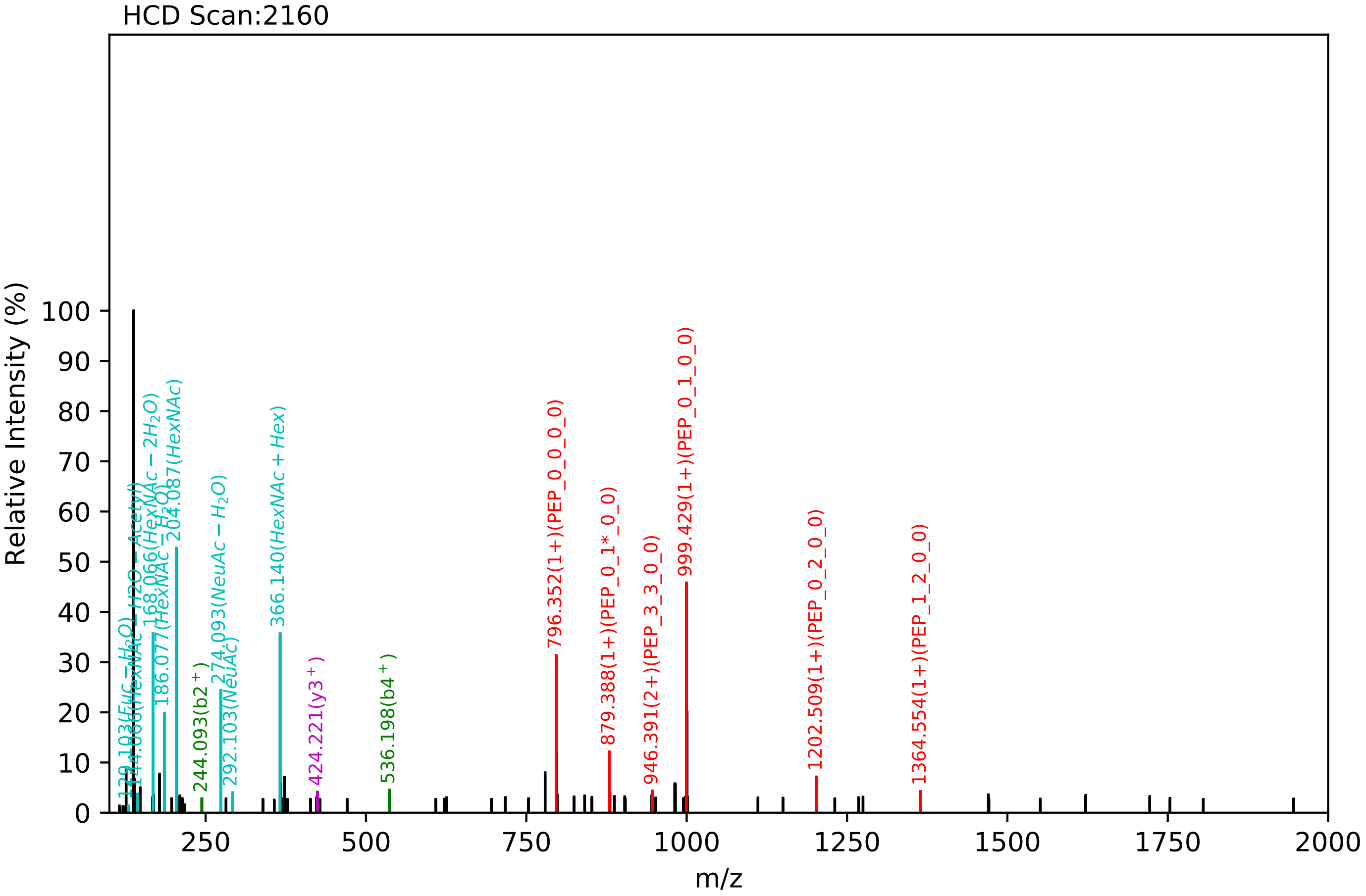

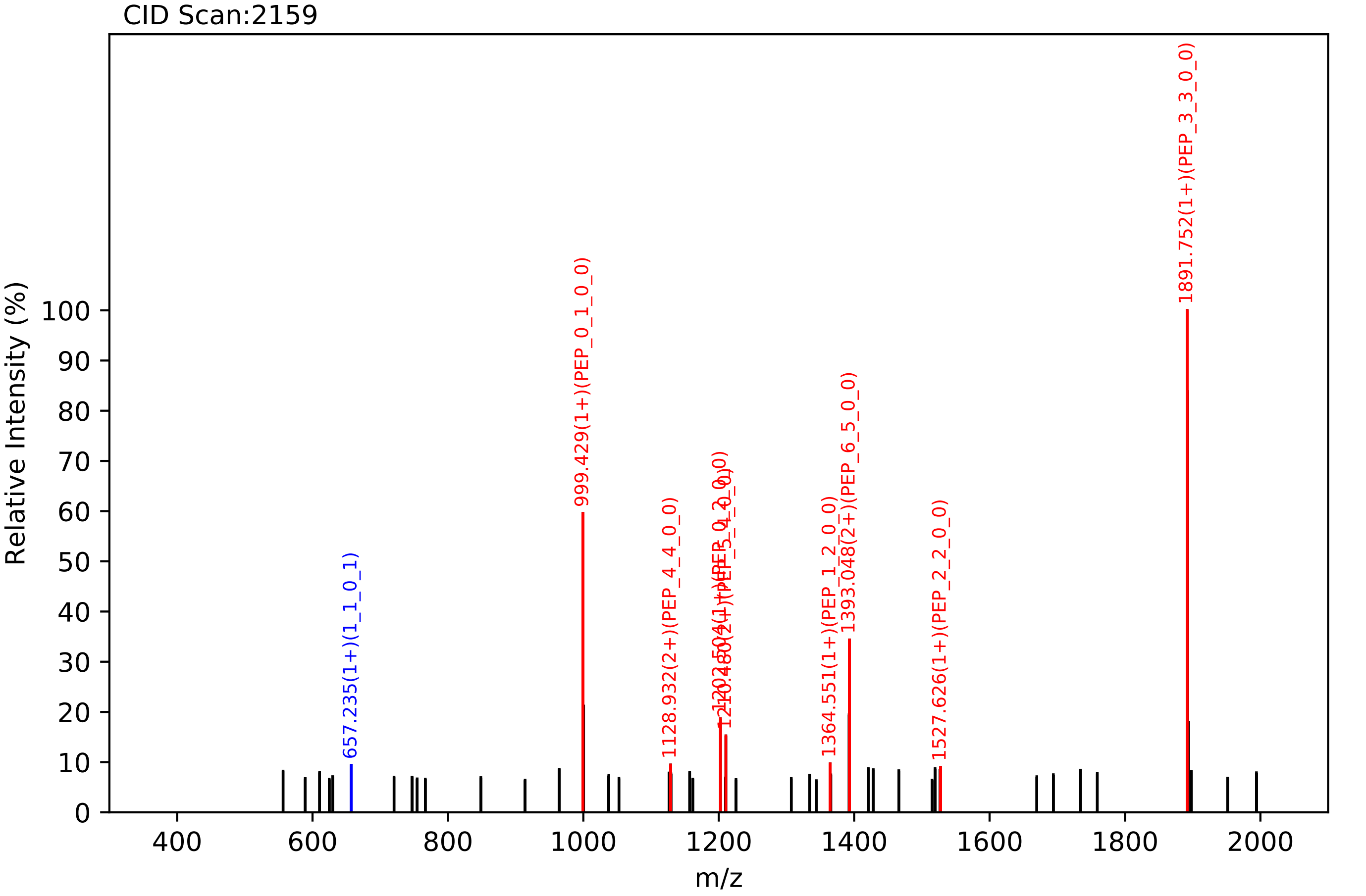

Training set no. 186, Experiment: AGP exp_1

NEEYNK(=PEP)_6_5_0_1, m/z:1025.73(3+), RT:15.74, Y-score:88.55

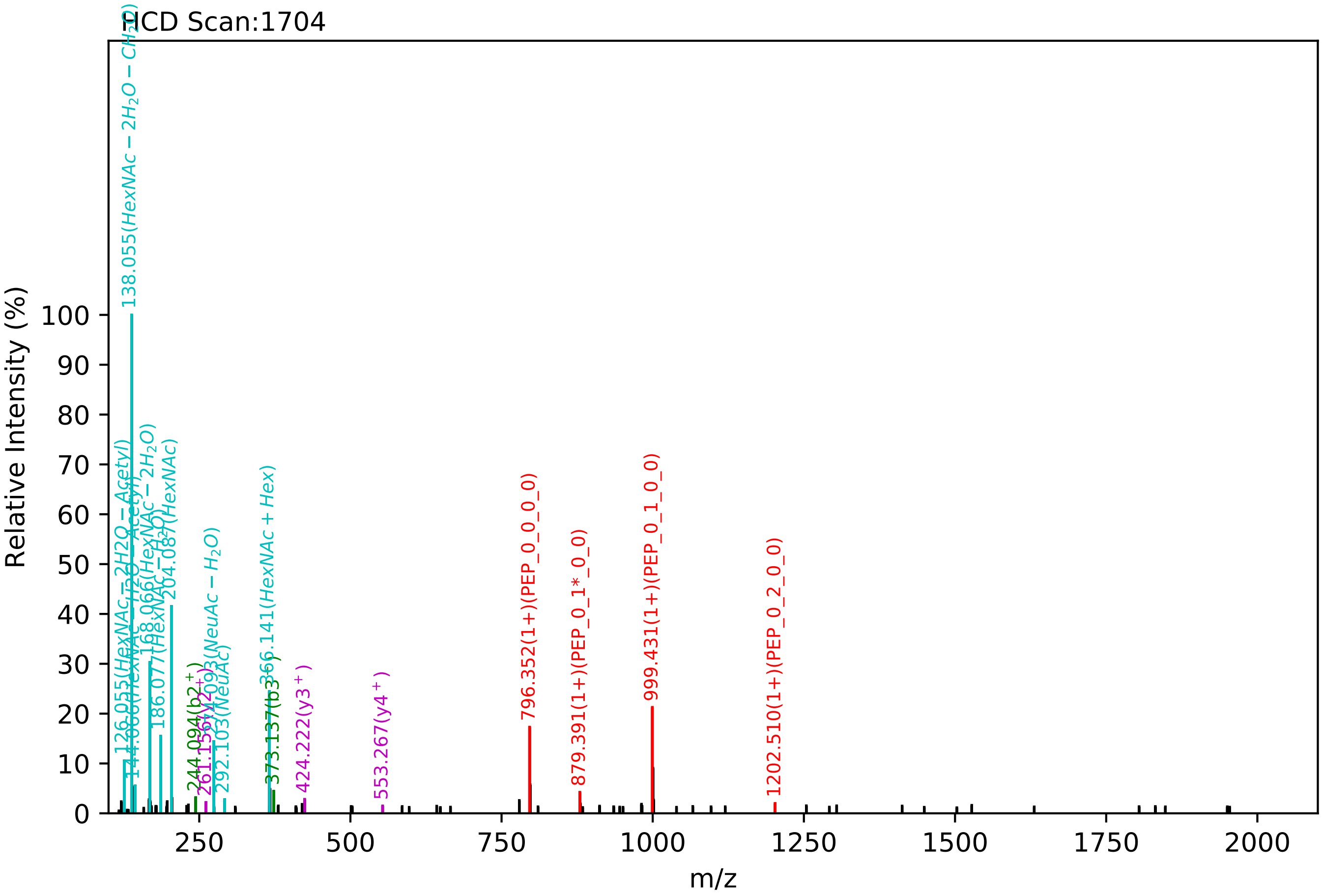

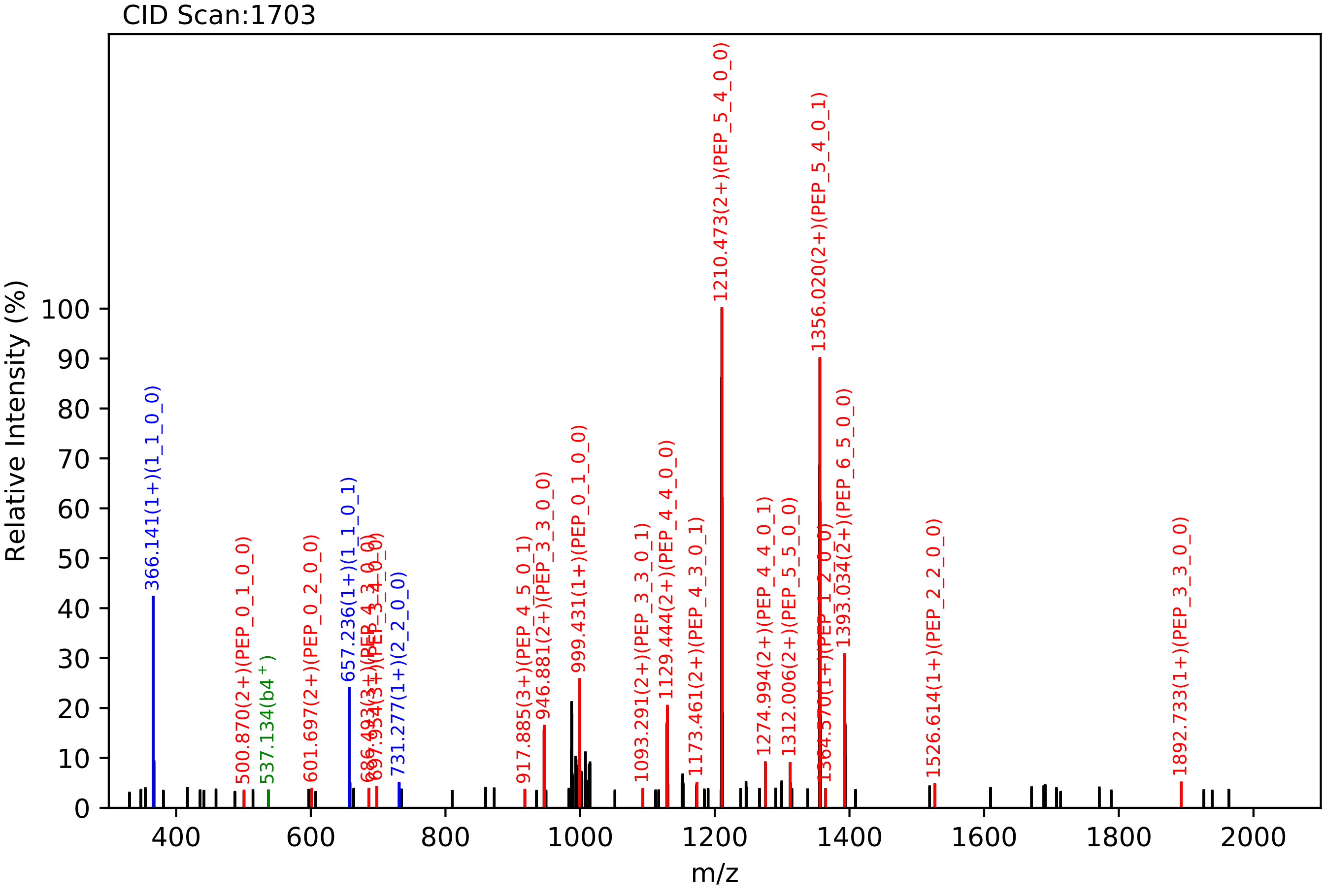

Training set no. 187, Experiment: AGP exp_2

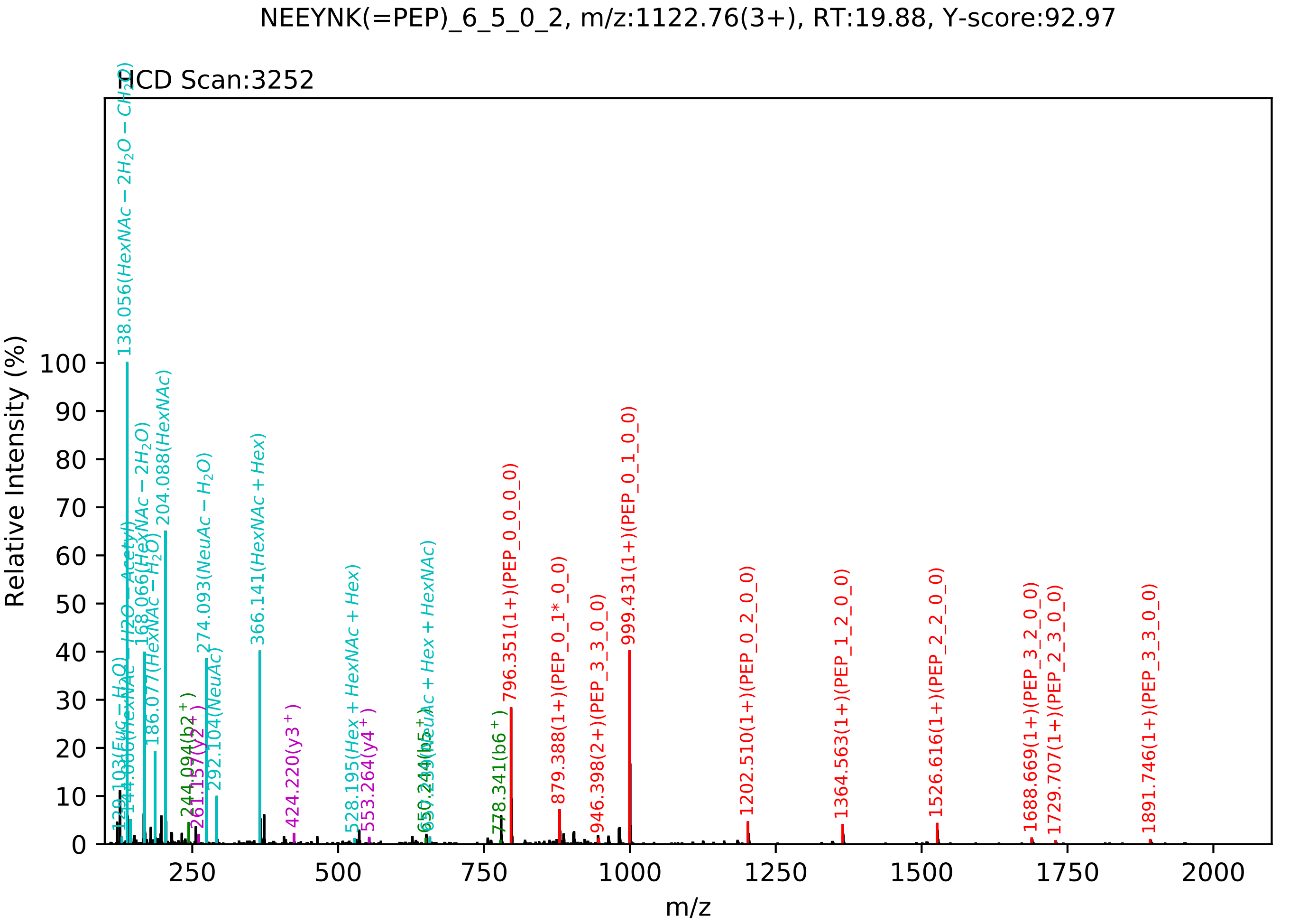

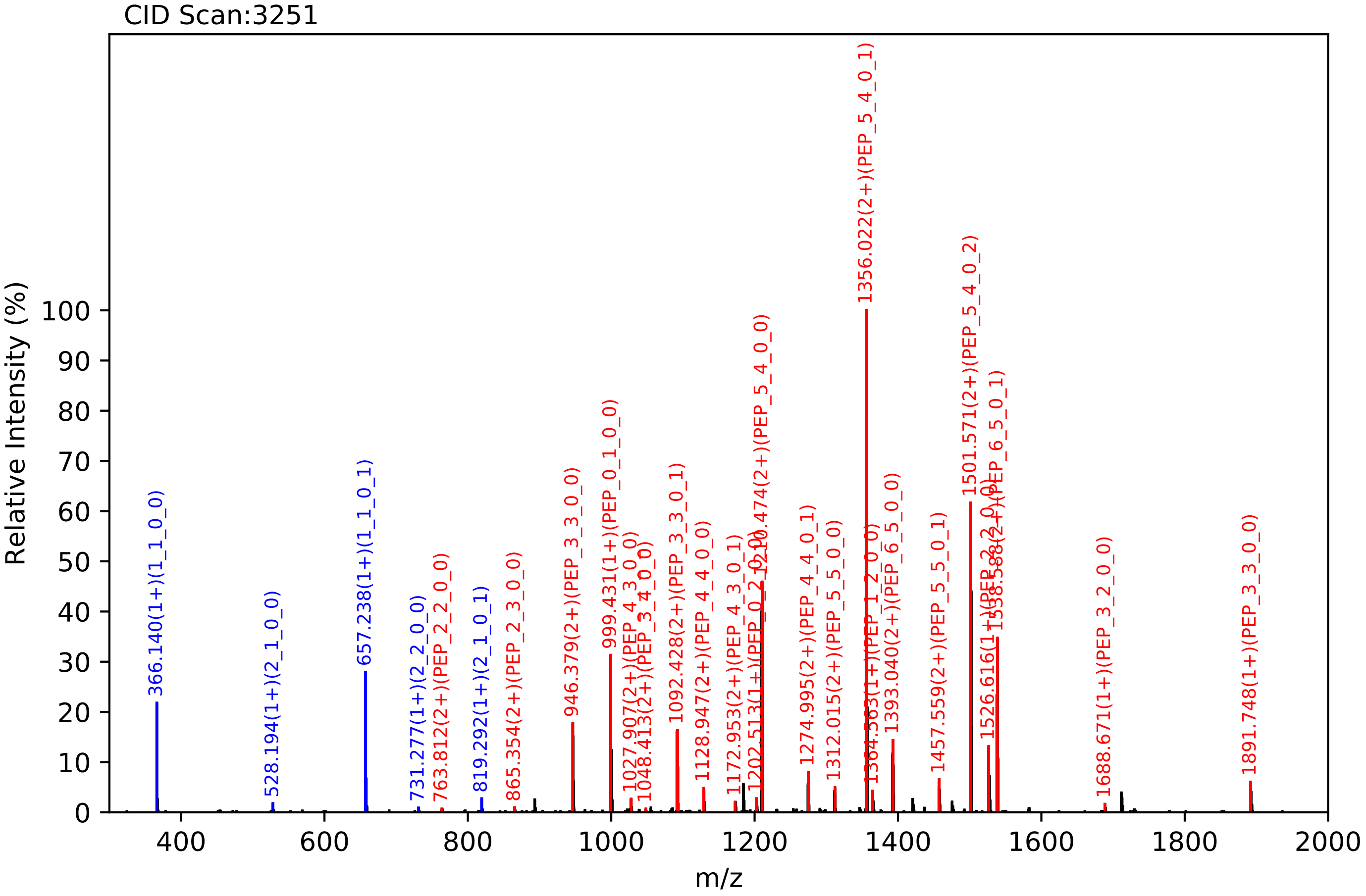

Training set no. 188, Experiment: AGP exp_1

NEEYNK(=PEP)_6_5_0_2, m/z:1122.76(3+), RT:20.05, Y-score:92.96

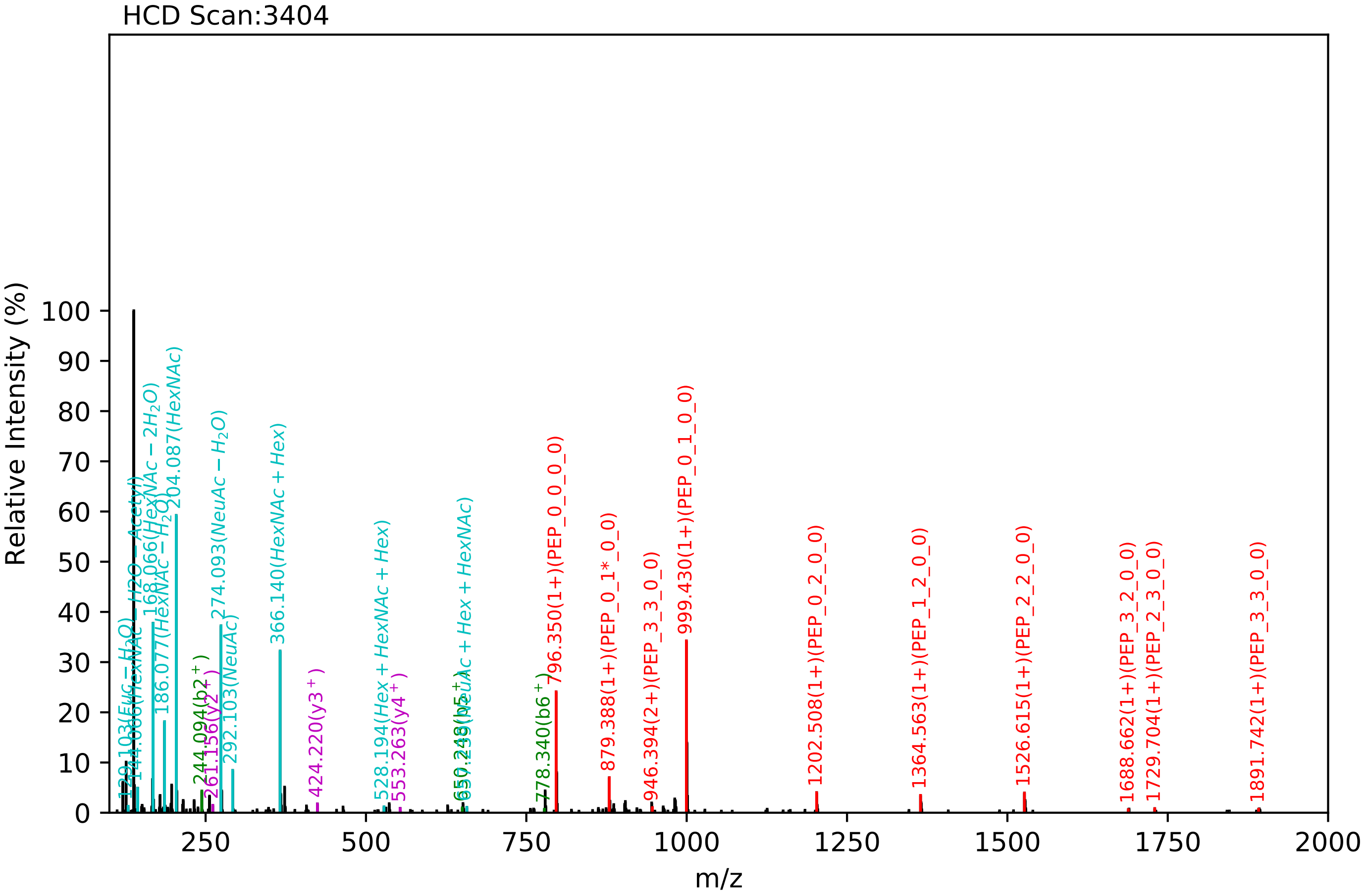

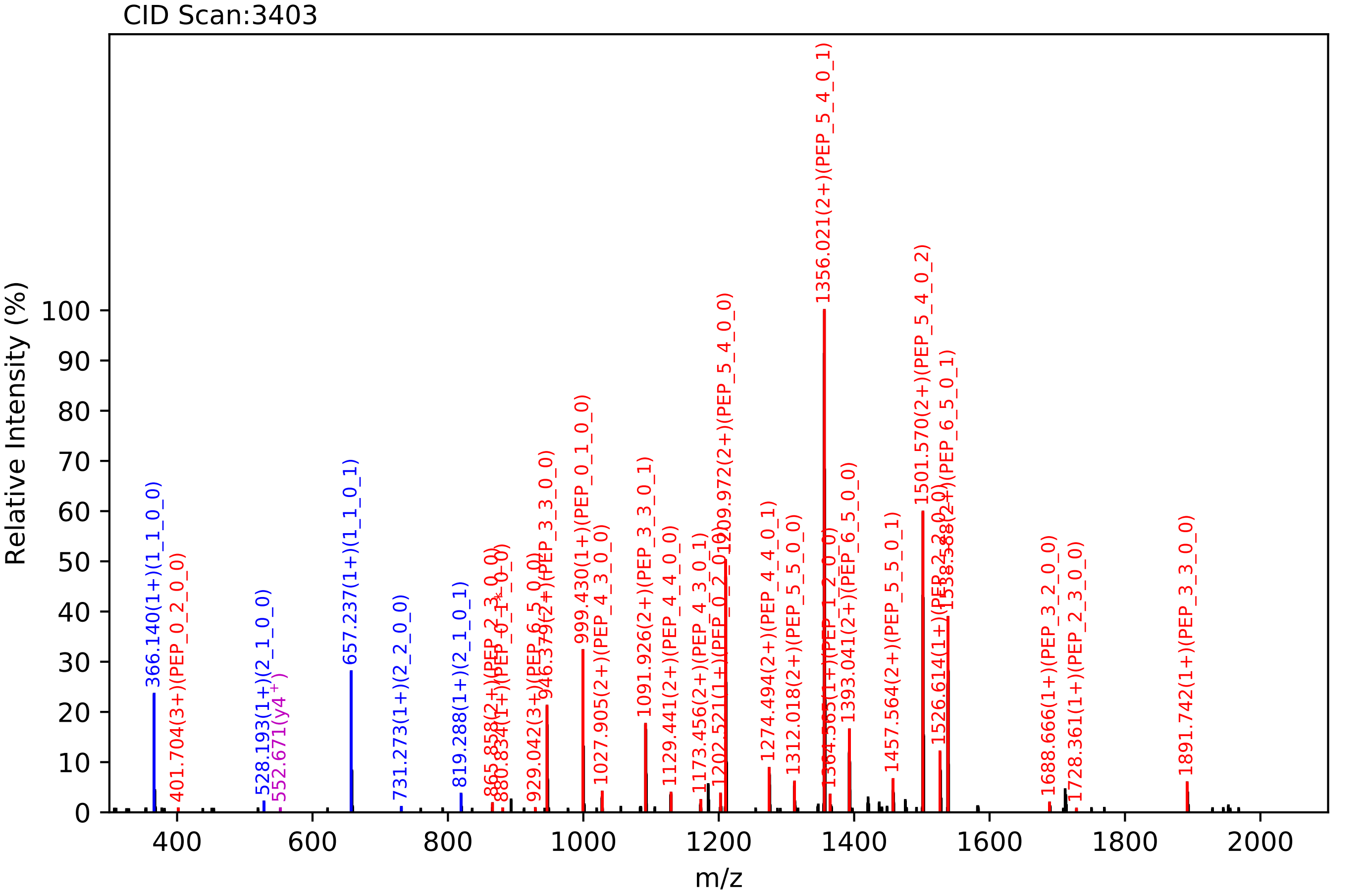

Training set no. 189, Experiment: AGP exp_2

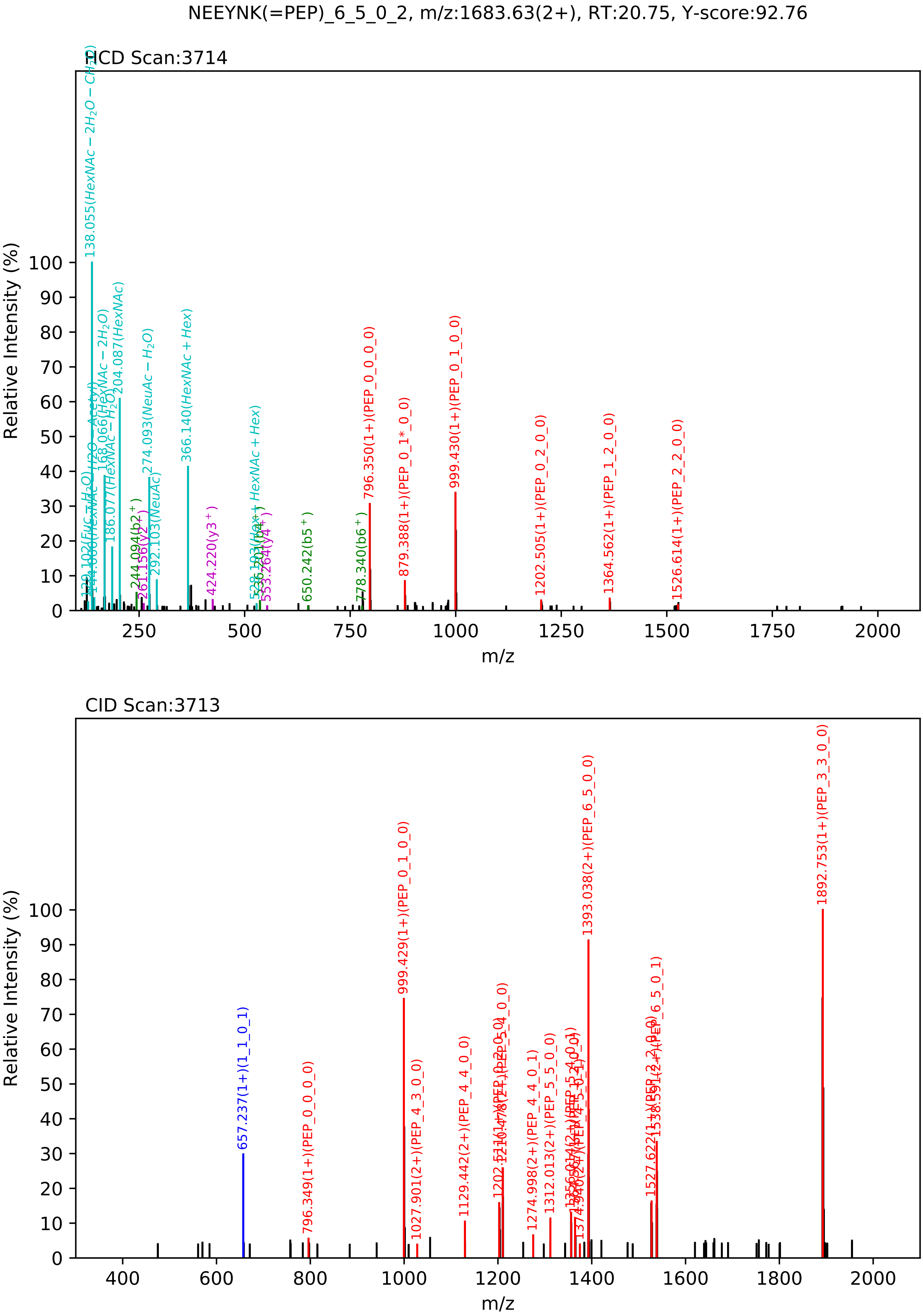

Training set no. 190, Experiment: AGP exp_2

NEEYNK(=PEP)_6_5_0_2, m/z:1122.76(3+), RT:20.49, Y-score:91.93

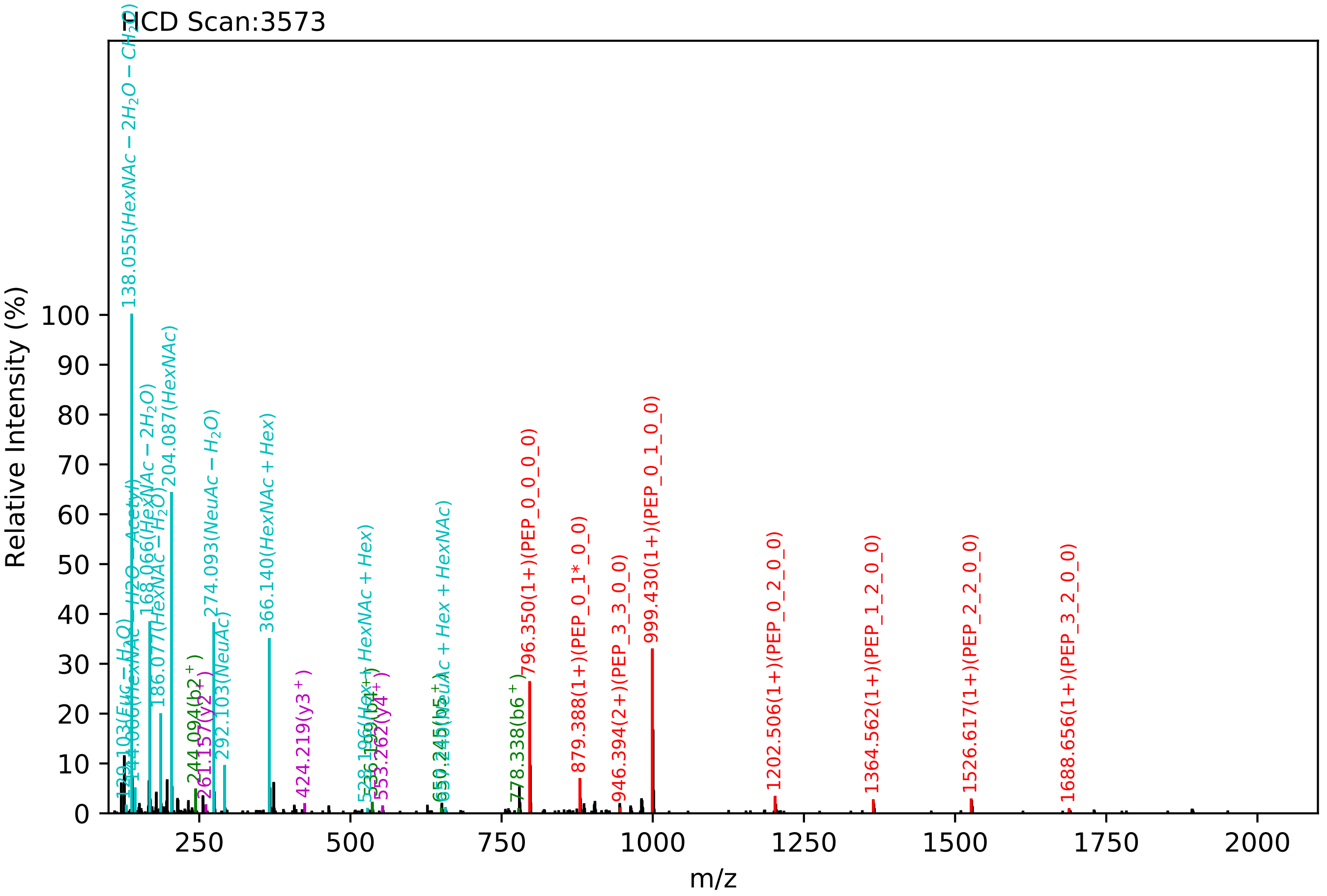

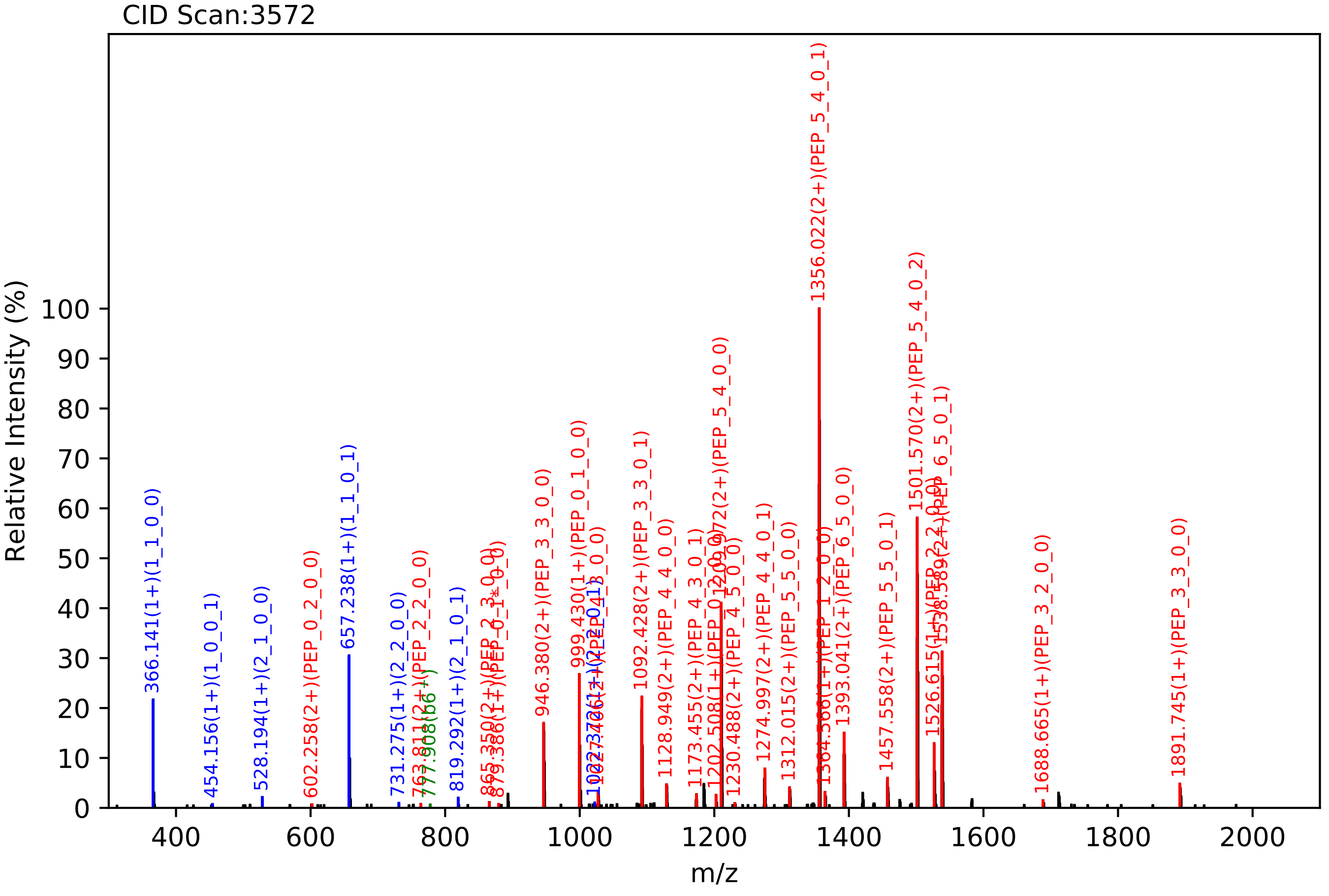

Training set no. 191, Experiment: AGP exp_1

NEEYNK(=PEP)_6_5_0_2, m/z:1122.42(3+), RT:20.60, Y-score:91.61

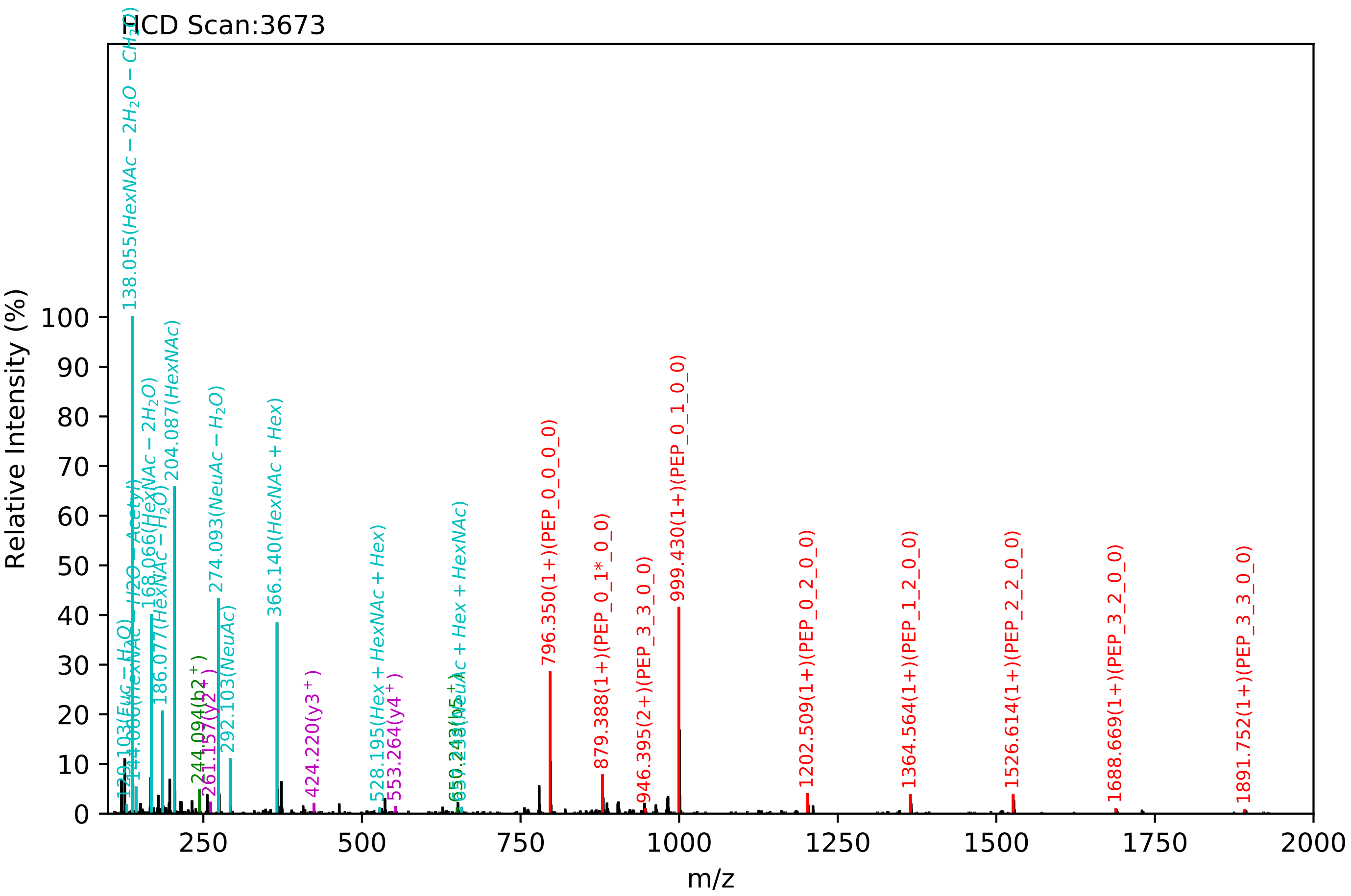

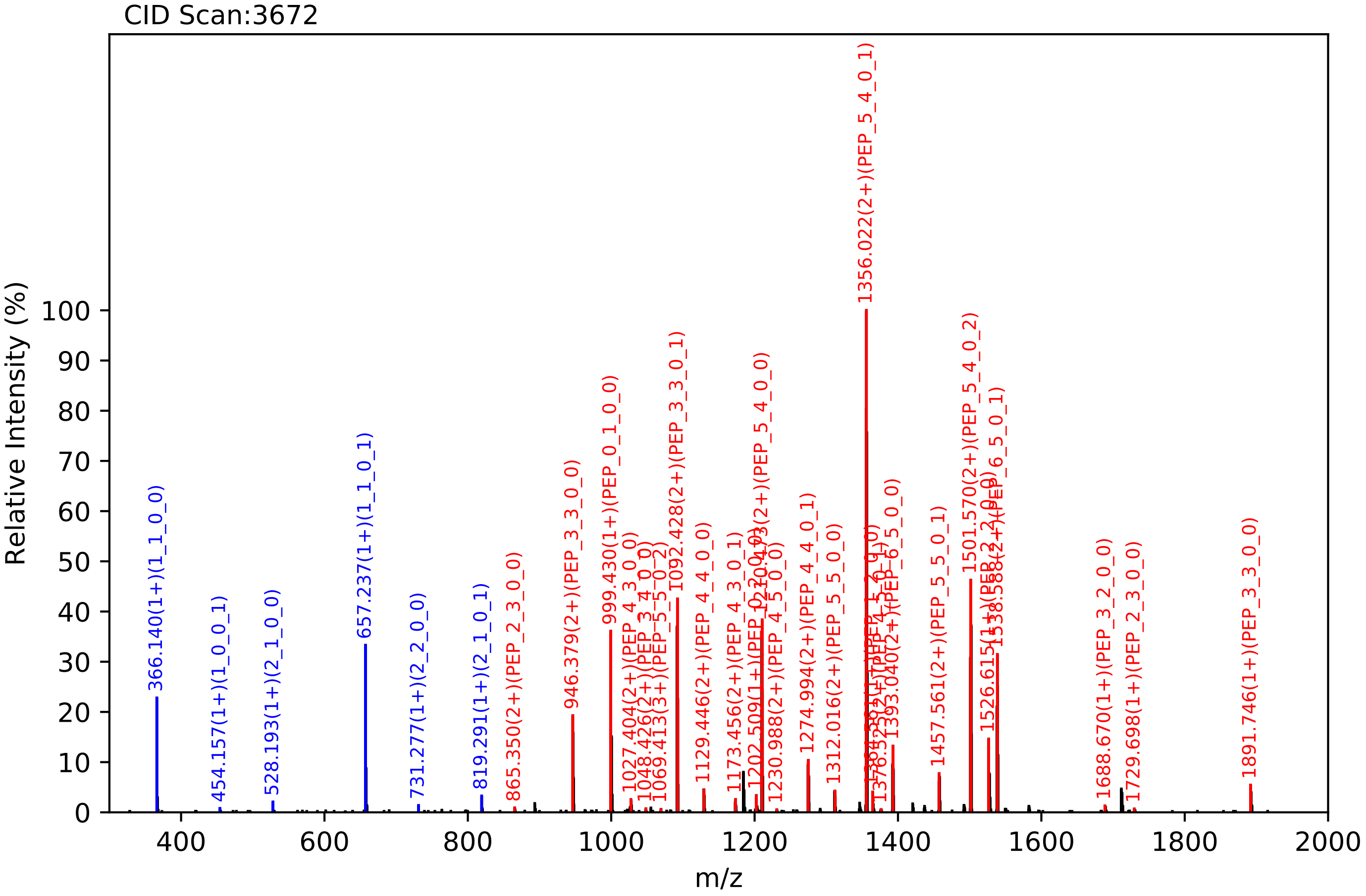

Training set no. 192, Experiment: AGP exp_1

NEEYNK(=PEP)_6_5_0_2, m/z:1122.76(3+), RT:19.53, Y-score:91.50

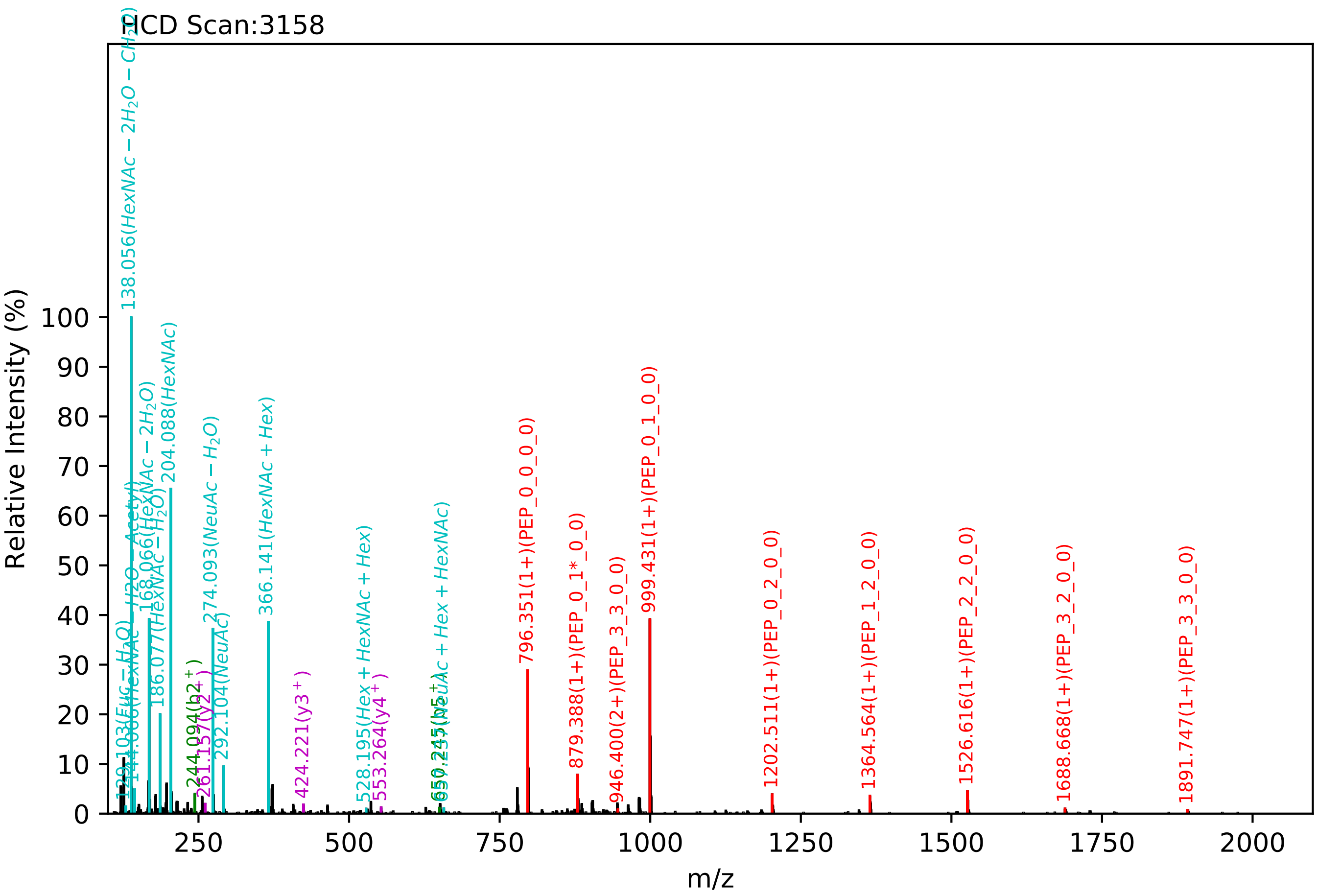

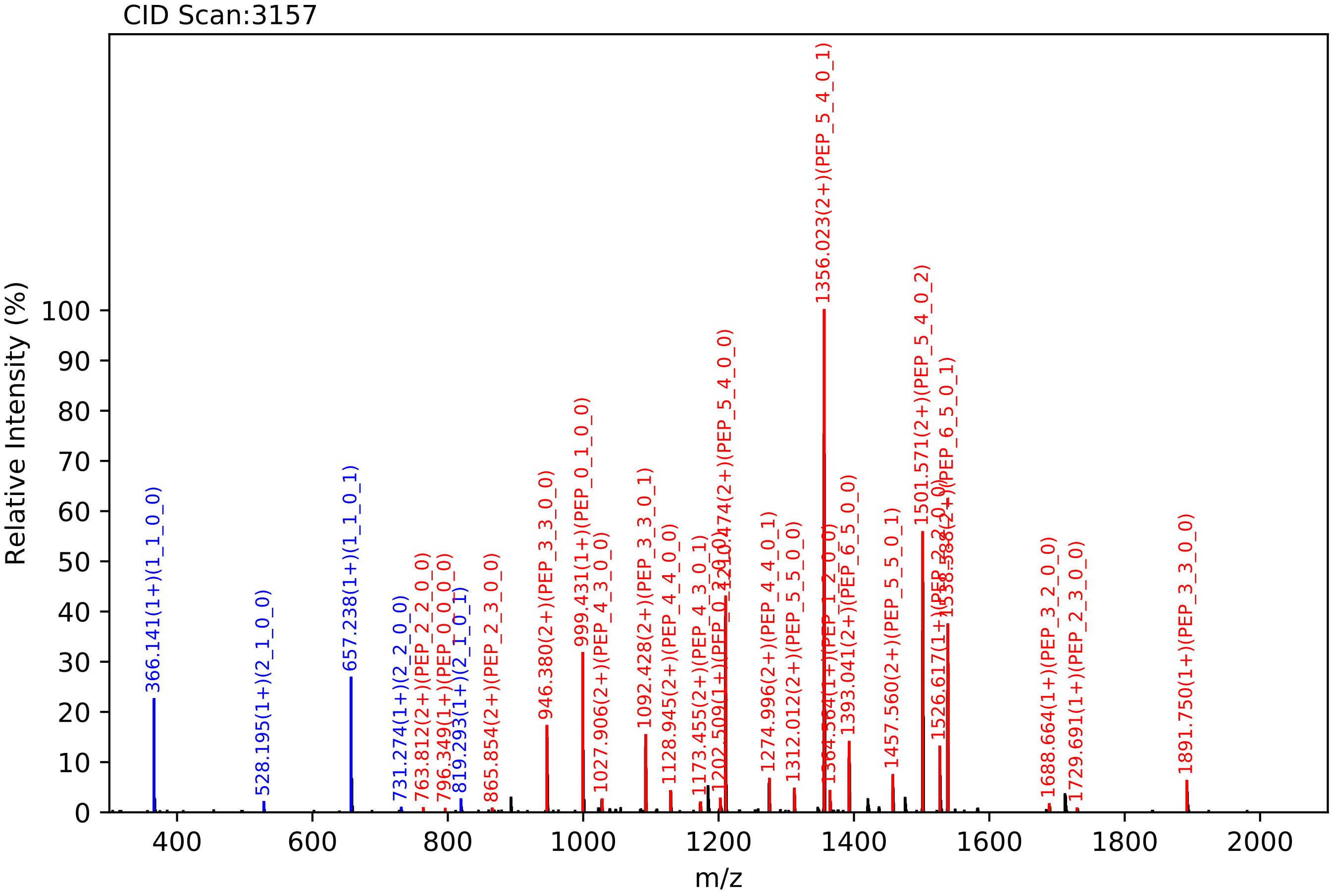

Training set no. 193, Experiment: AGP exp_1

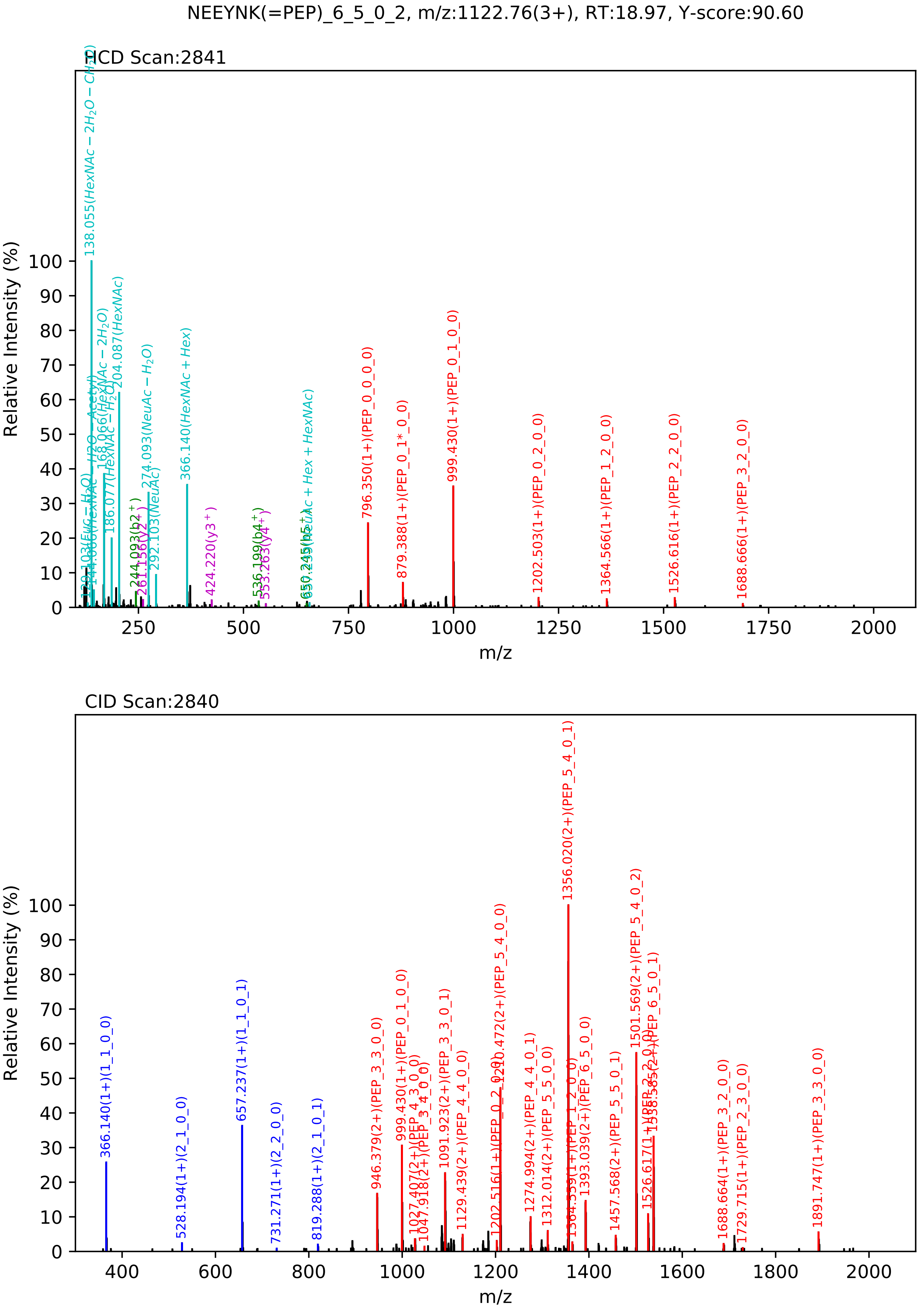

Training set no. 194, Experiment: AGP exp_2

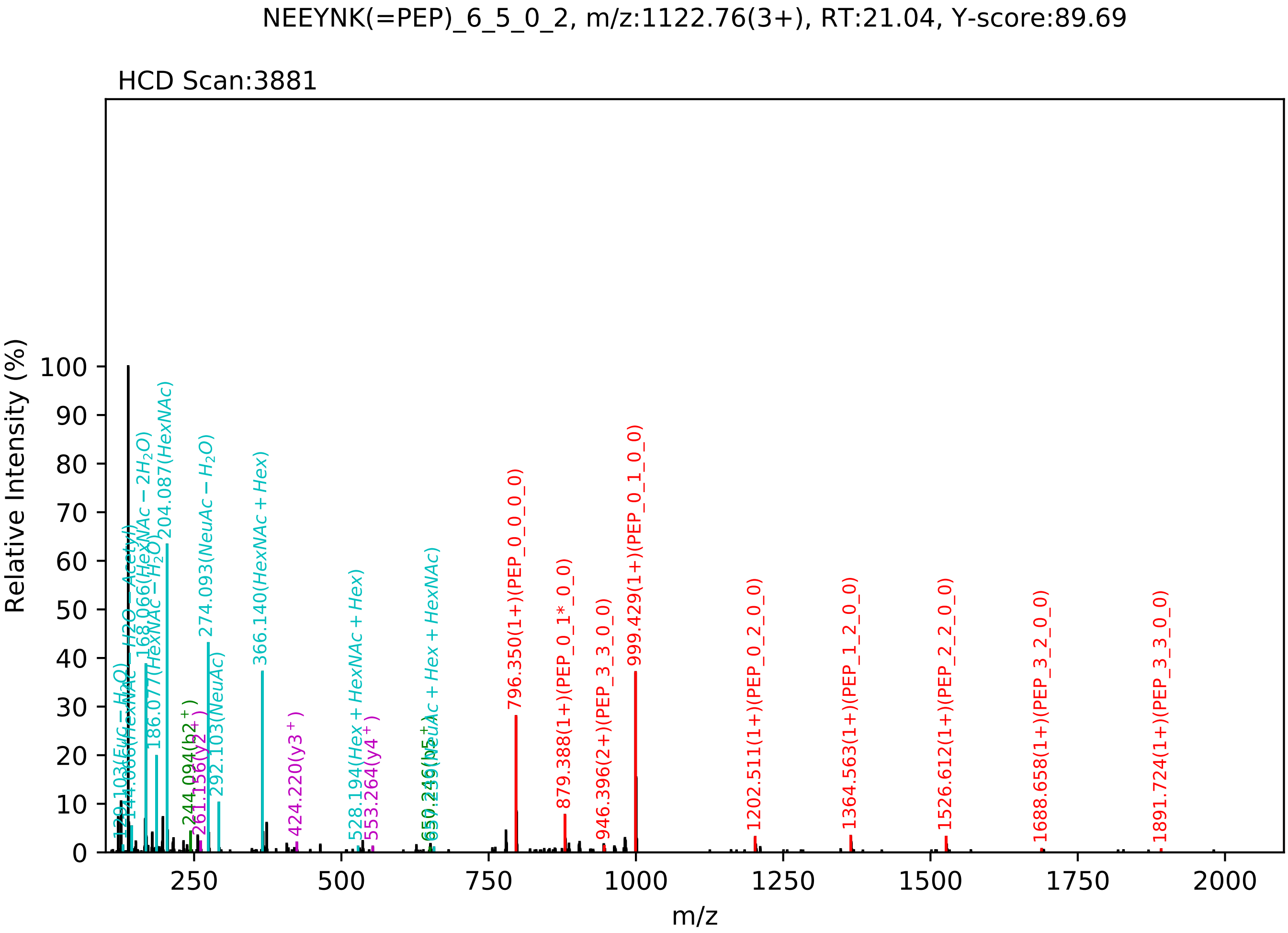

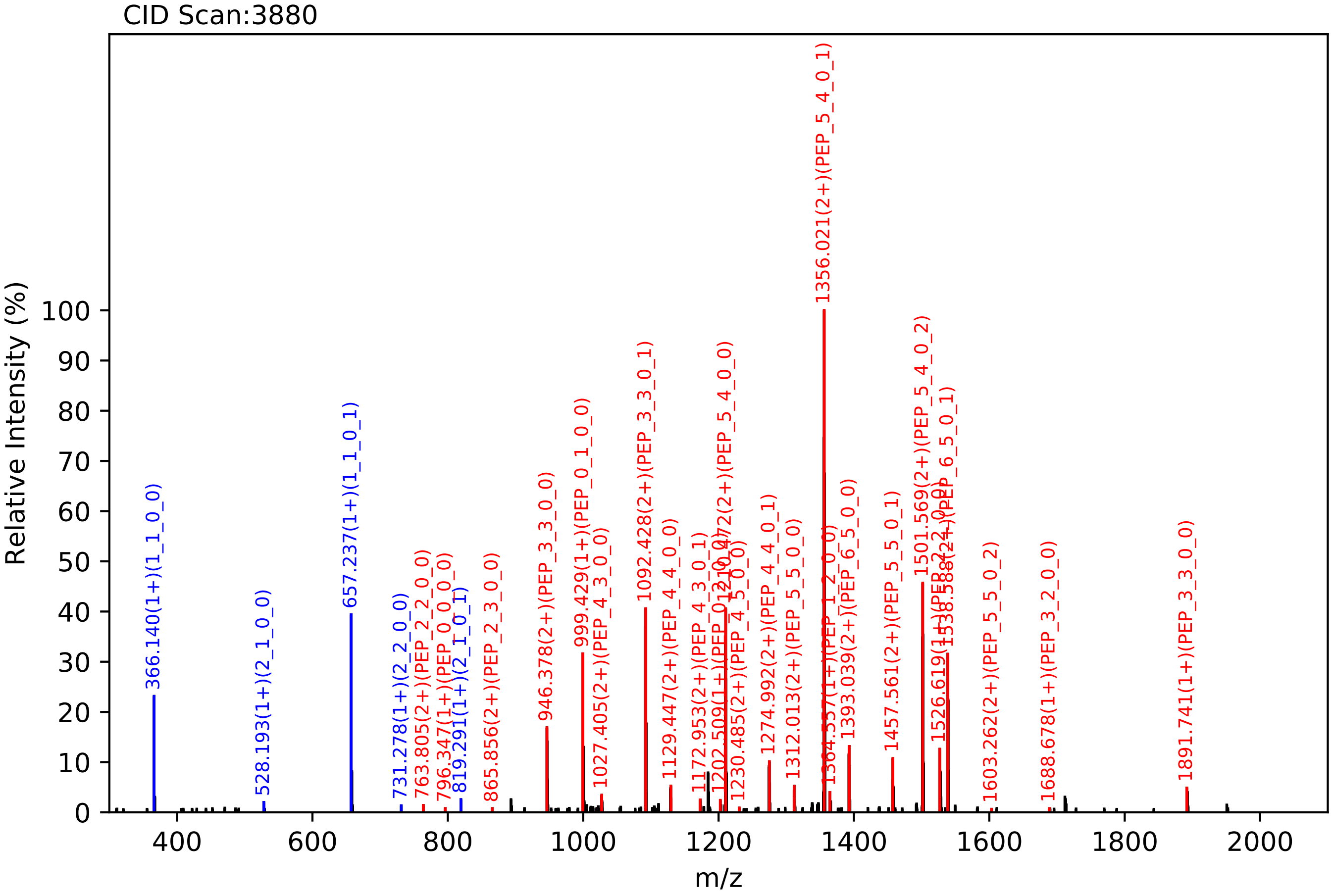

Training set no. 195, Experiment: AGP exp_2

NEEYNK(=PEP)_6_5_0_2, m/z:1122.76(3+), RT:19.33, Y-score:87.23

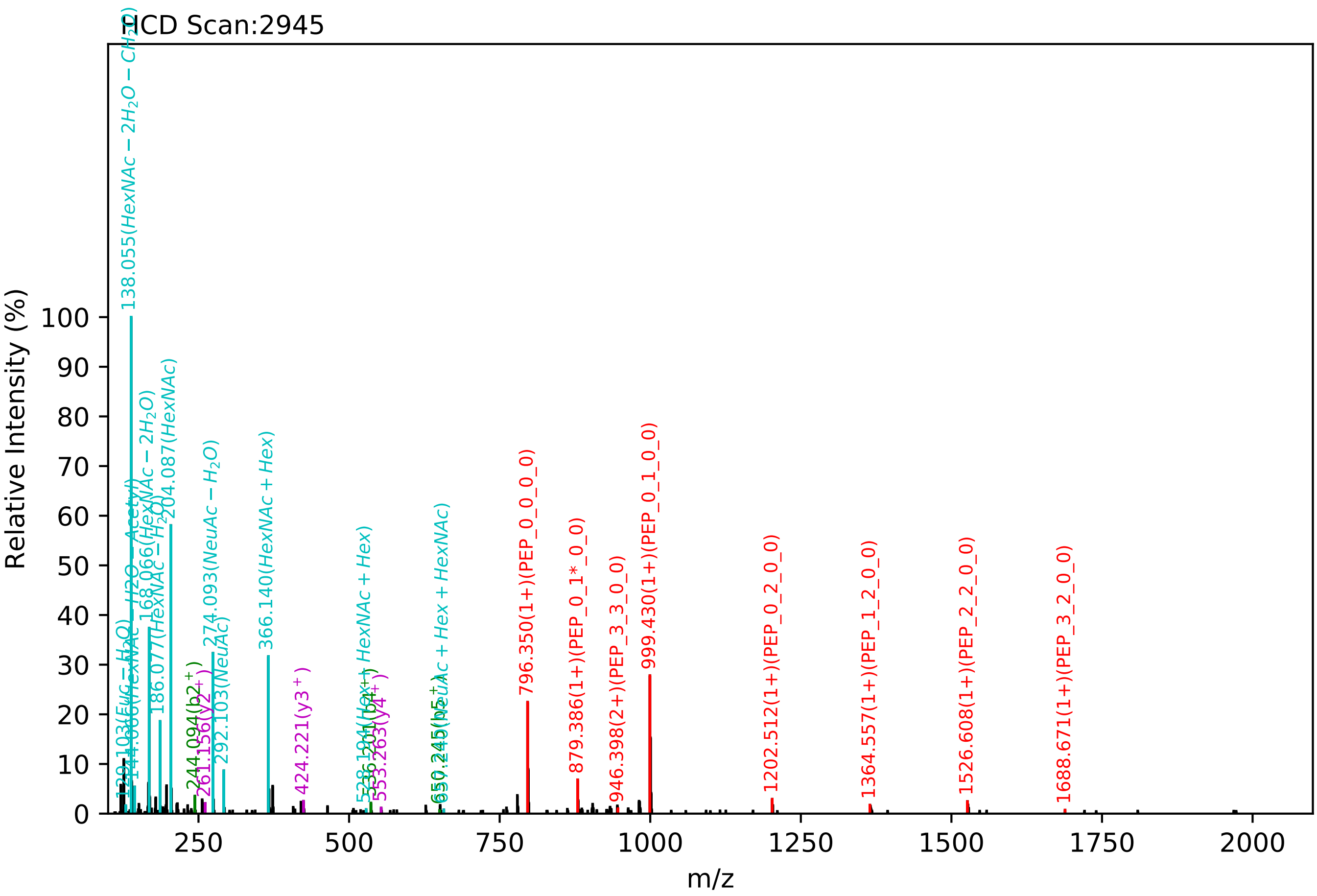

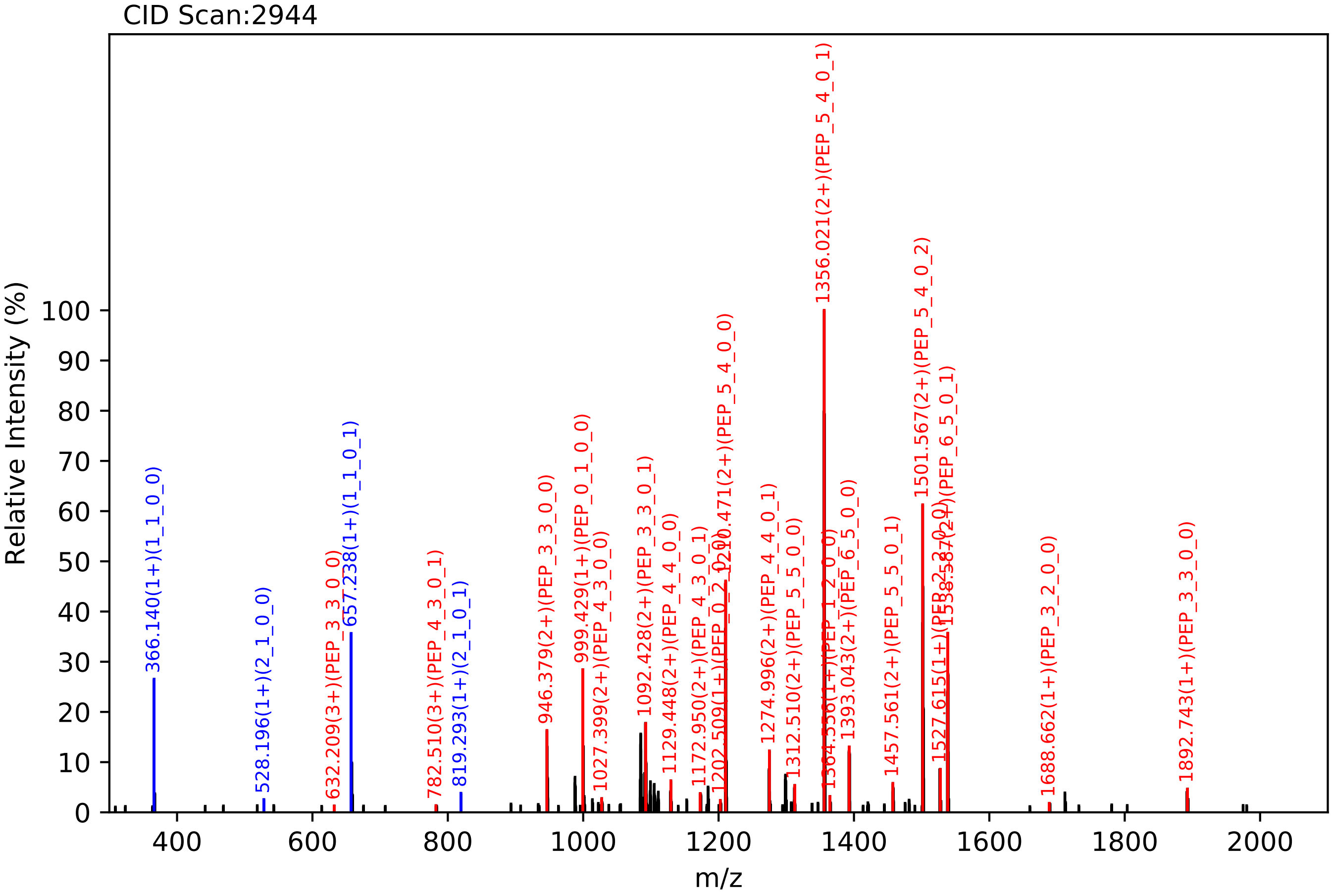

Training set no. 196, Experiment: AGP exp_2

NEEYNK(=PEP)_6_5_0_2, m/z:1683.63(2+), RT:19.54, Y-score:83.36

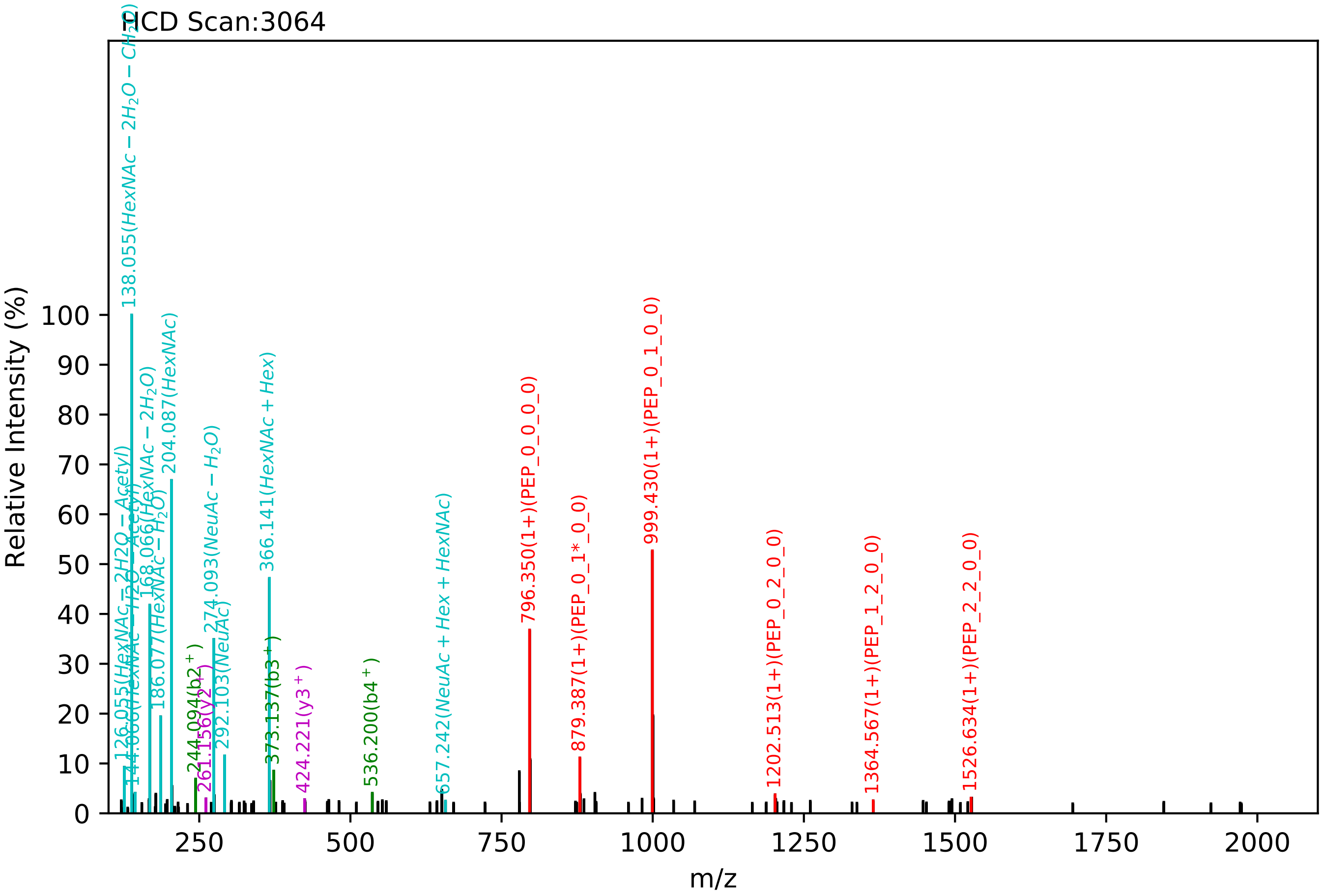

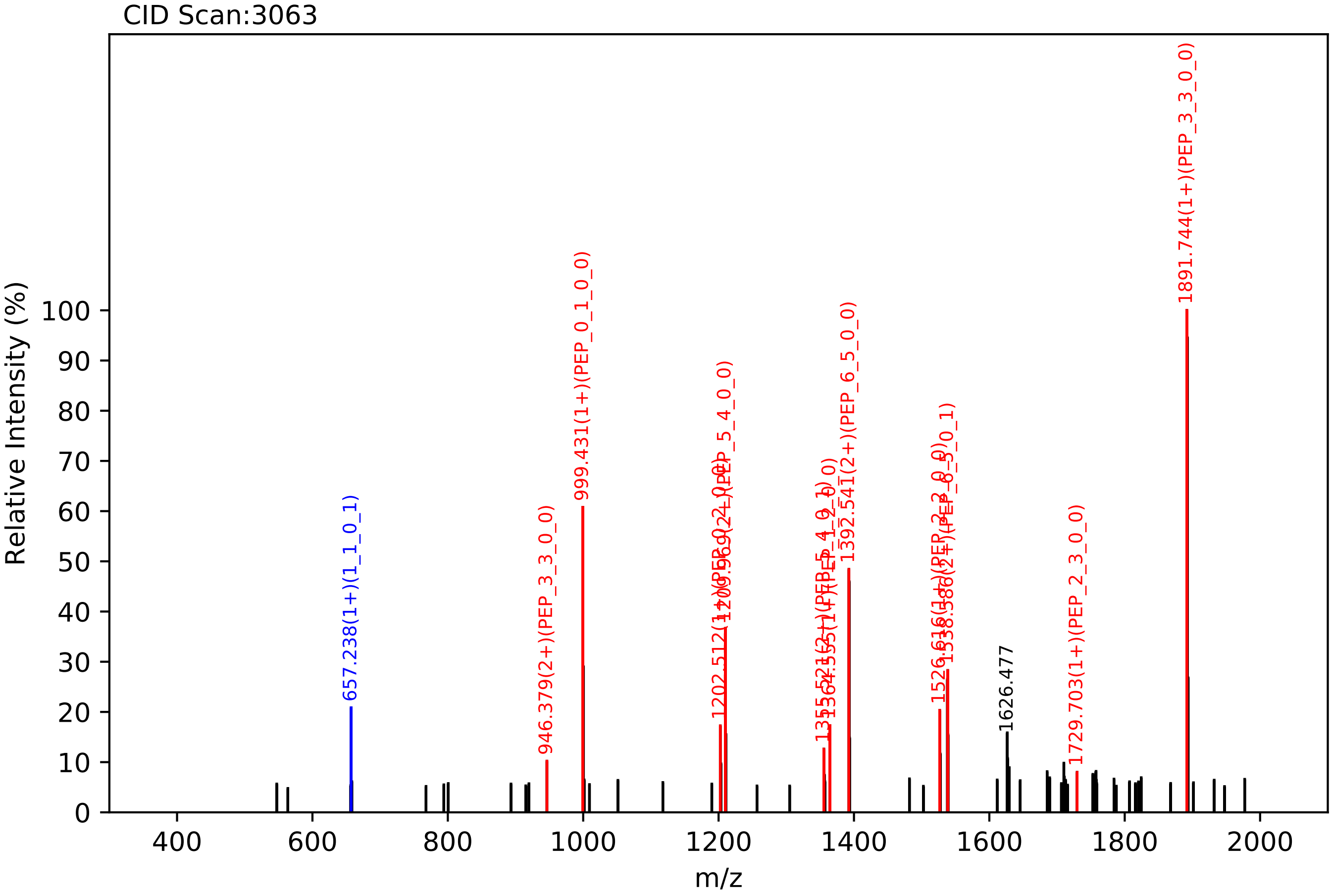

Training set no. 197, Experiment: AGP exp_1

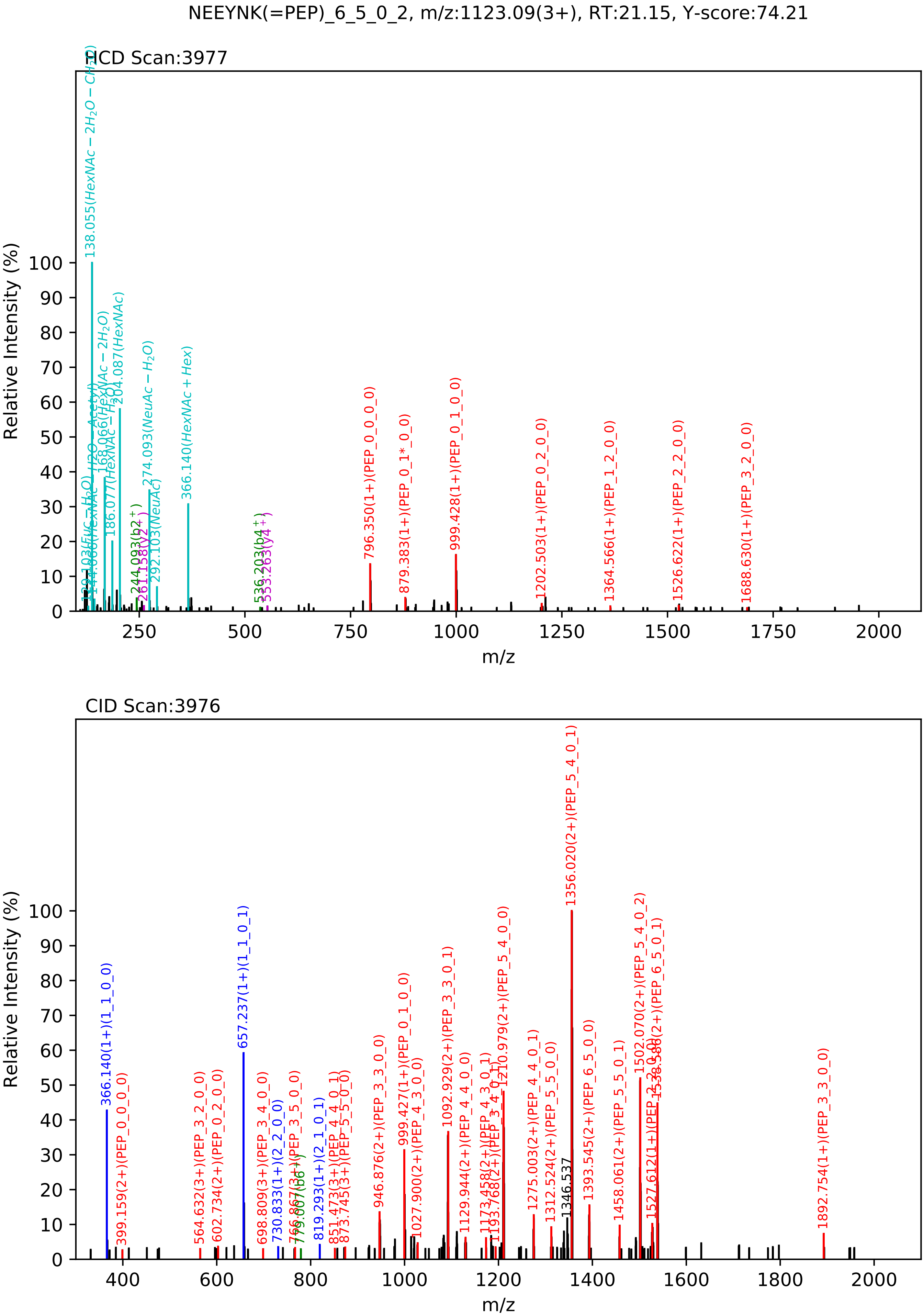

Training set no. 198, Experiment: AGP exp_2

NEEYNK(=PEP)_6_5_0_3, m/z:1219.79(3+), RT:22.31, Y-score:91.73

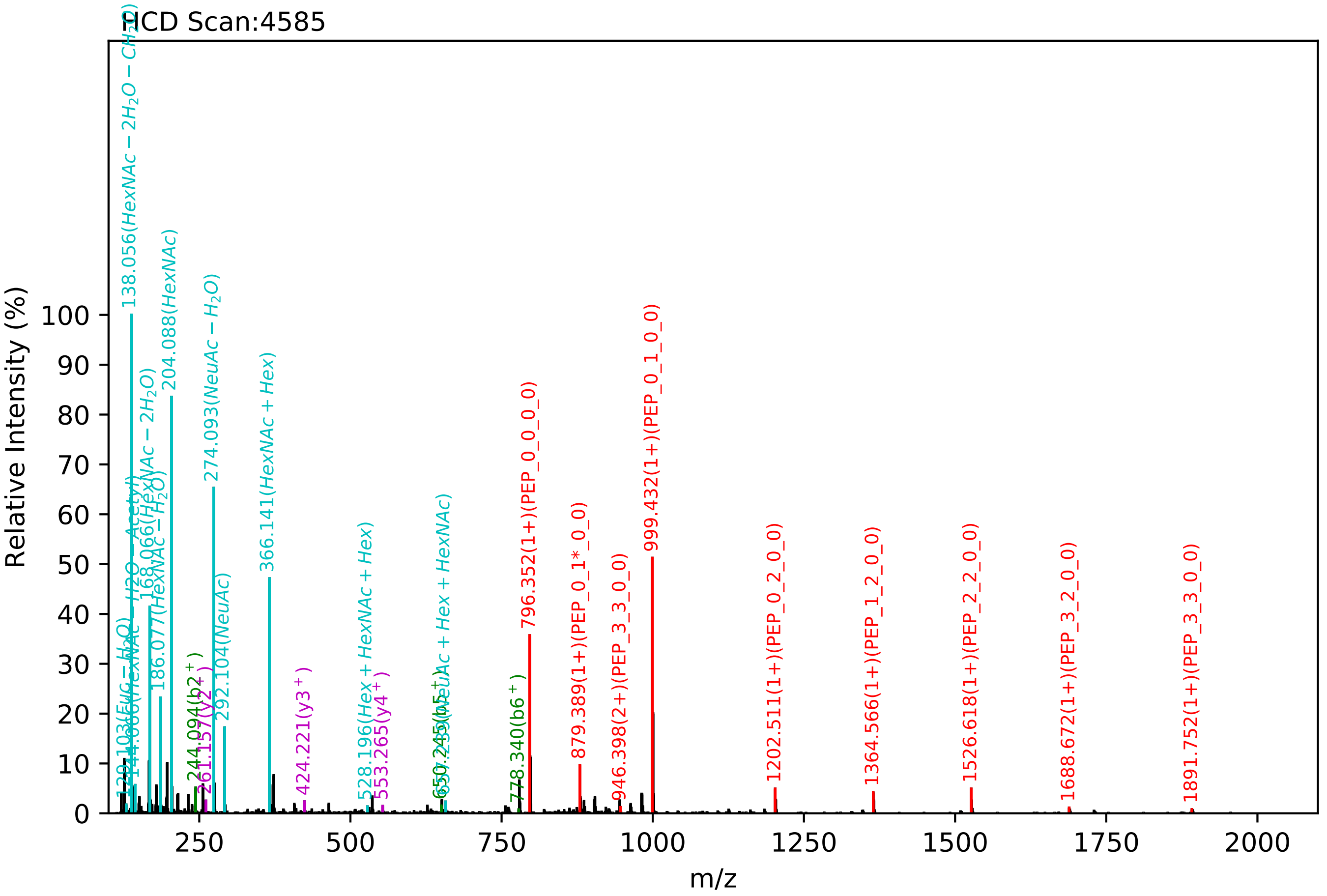

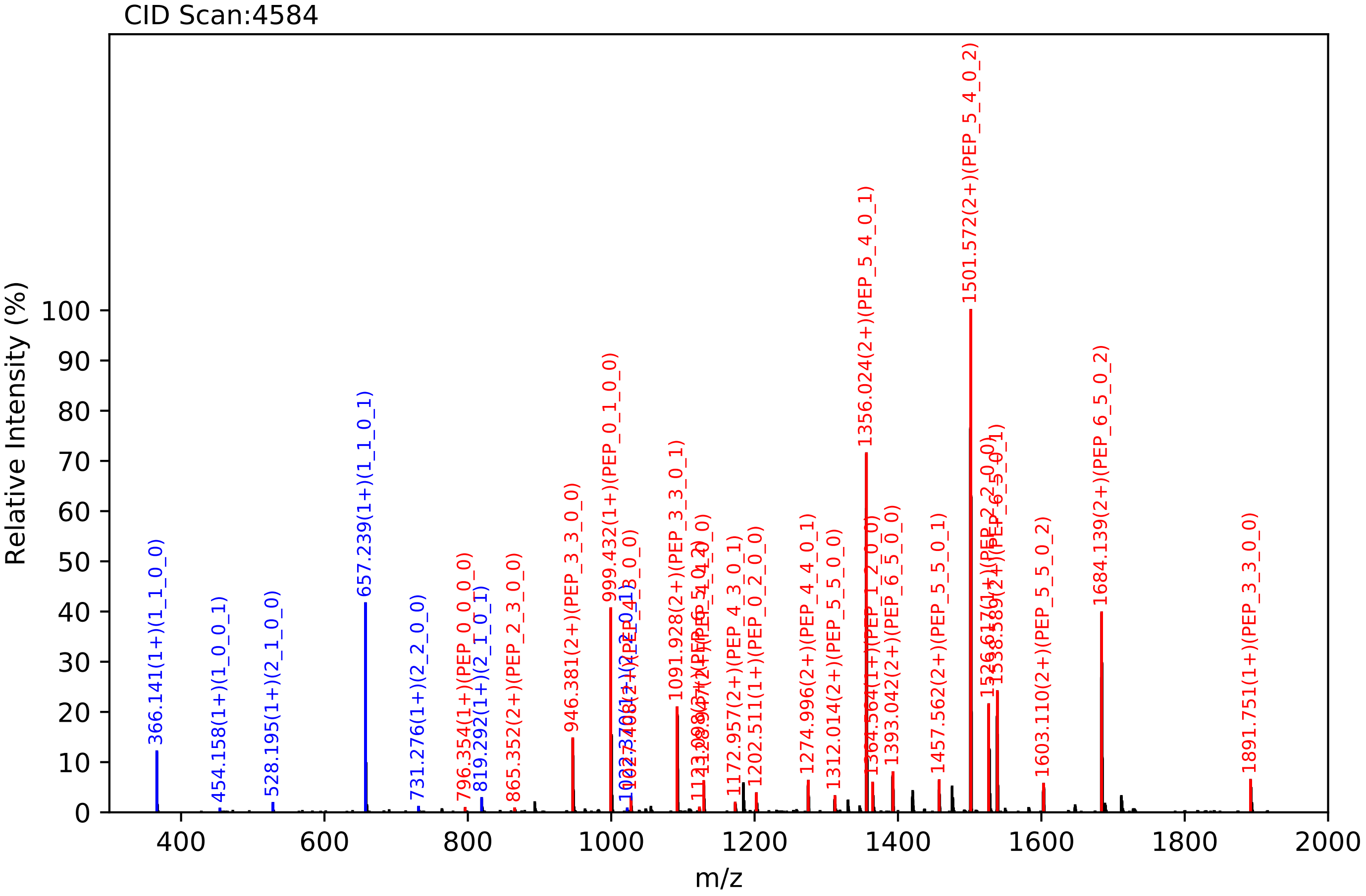

Training set no. 199, Experiment: AGP exp_1

NEEYNK(=PEP)_6_5_0_3, m/z:1219.45(3+), RT:22.14, Y-score:90.80

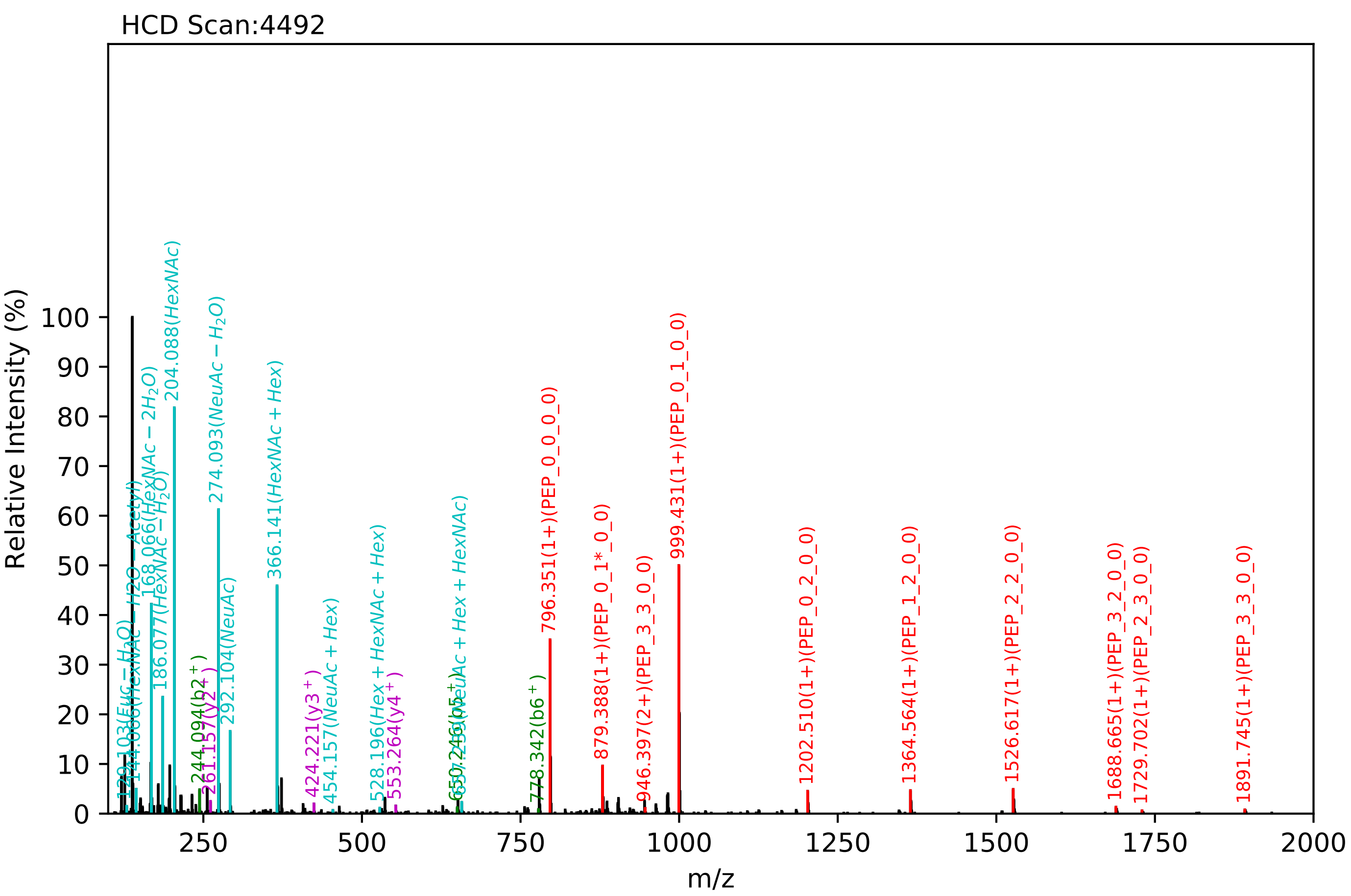

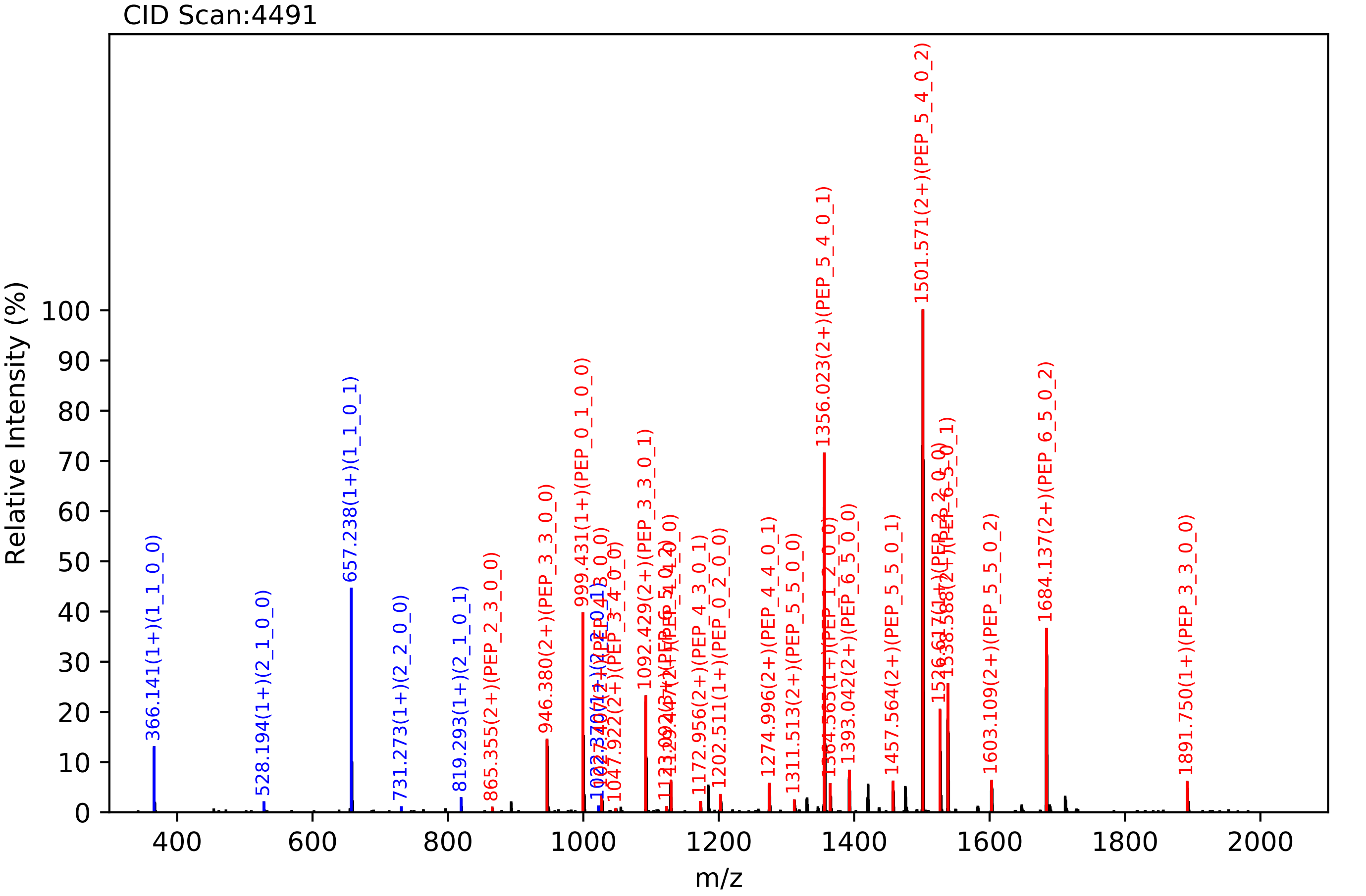

Training set no. 200, Experiment: AGP exp_2

NEEYNK(=PEP)_6_5_1_1, m/z:1074.41(3+), RT:16.70, Y-score:92.33

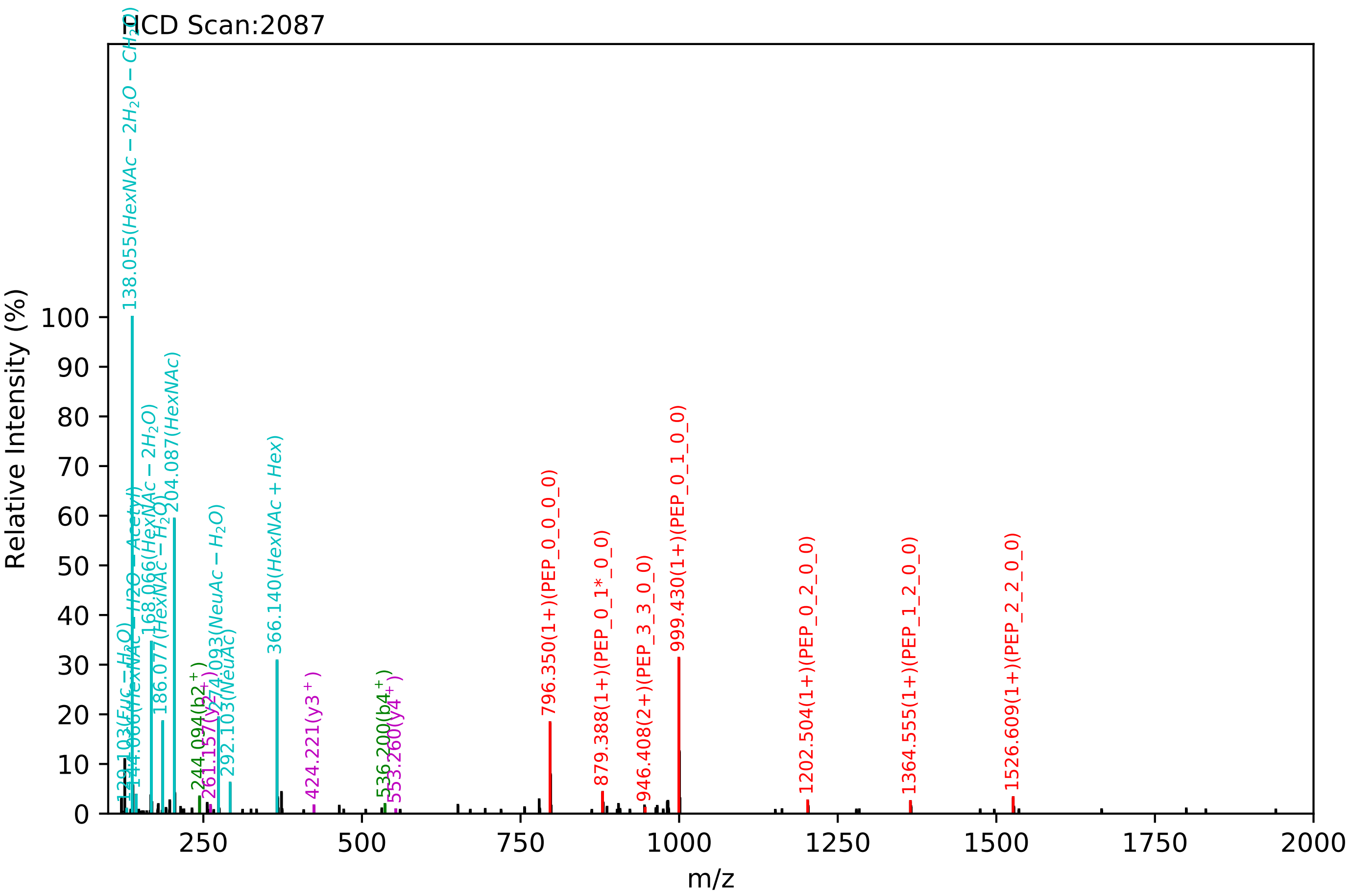

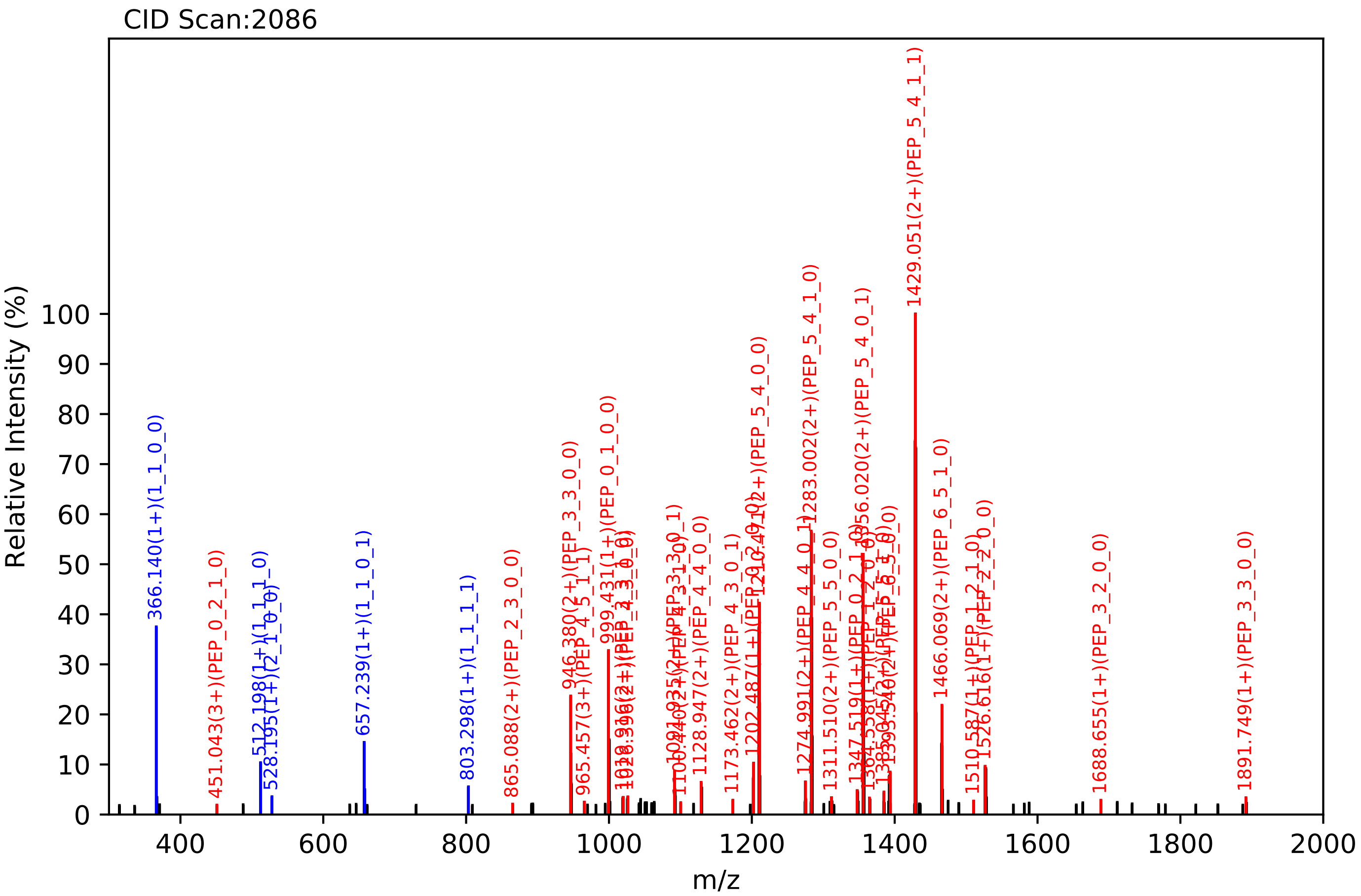

Training set no. 201, Experiment: AGP exp_1

NEEYNK(=PEP)_6_5_1_1, m/z:1074.41(3+), RT:16.66, Y-score:91.74

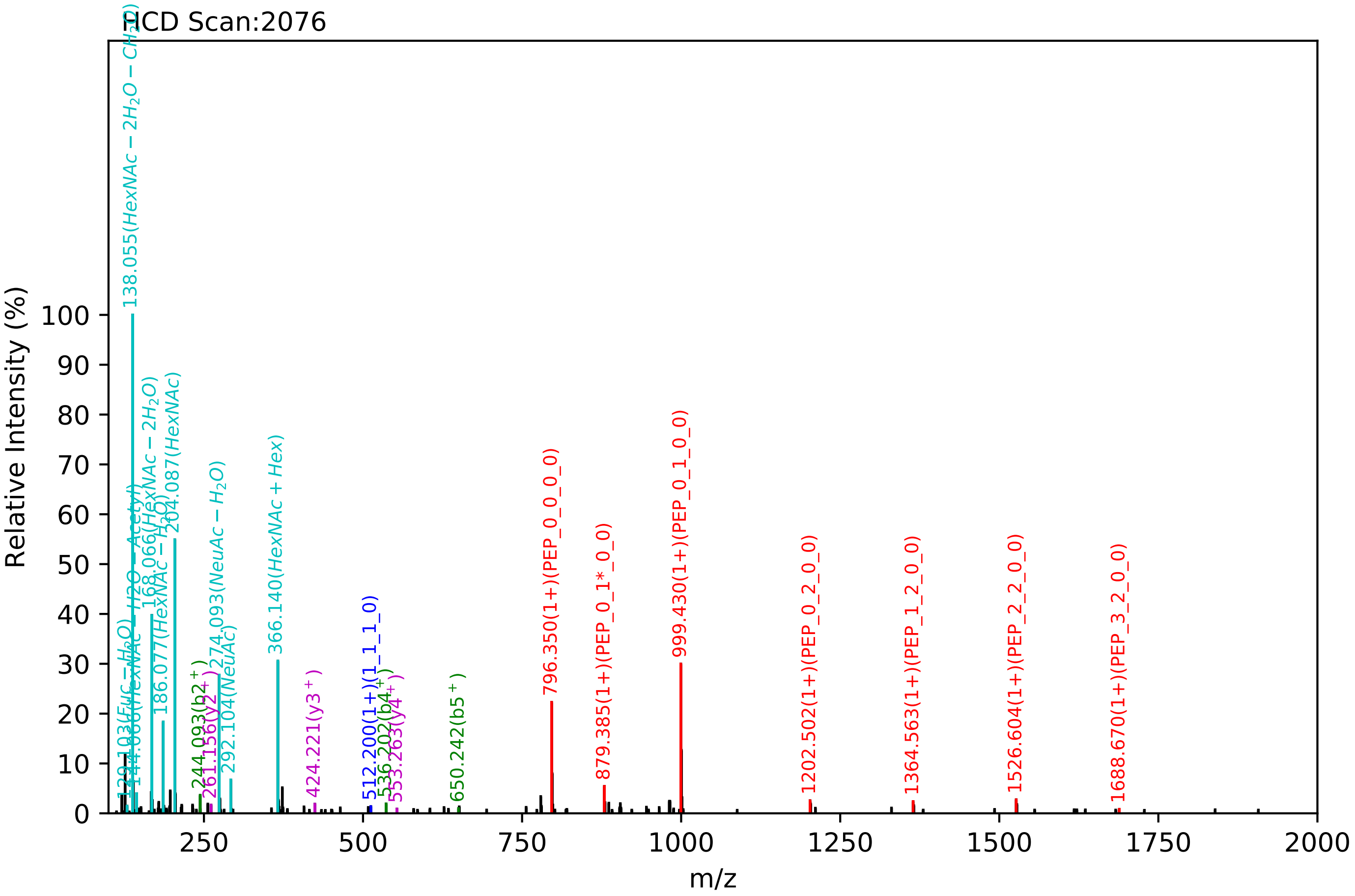

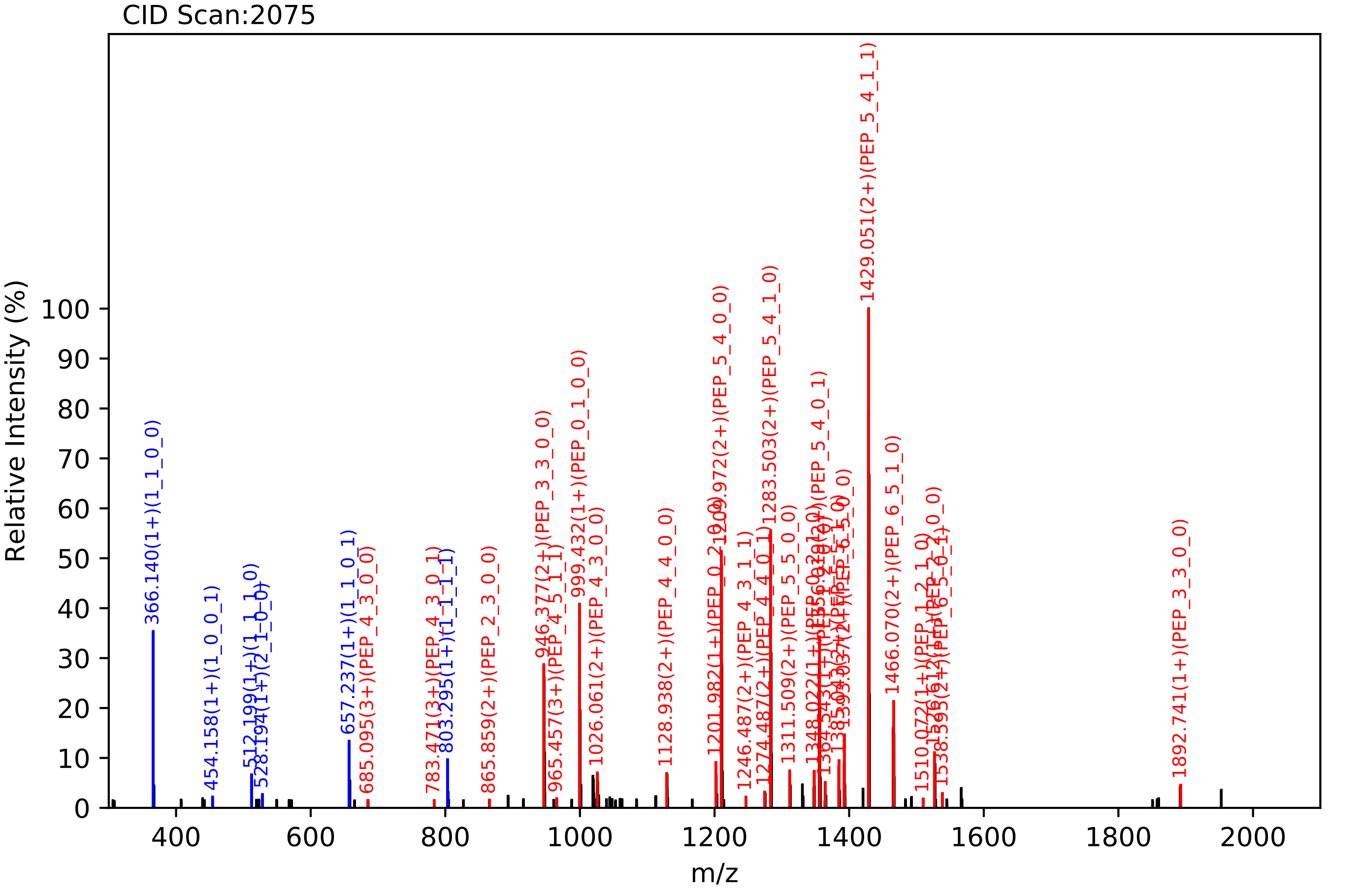

Training set no. 202, Experiment: AGP exp_1

NEEYNK(=PEP)_6_5_1_1, m/z:1074.41(3+), RT:16.15, Y-score:91.19

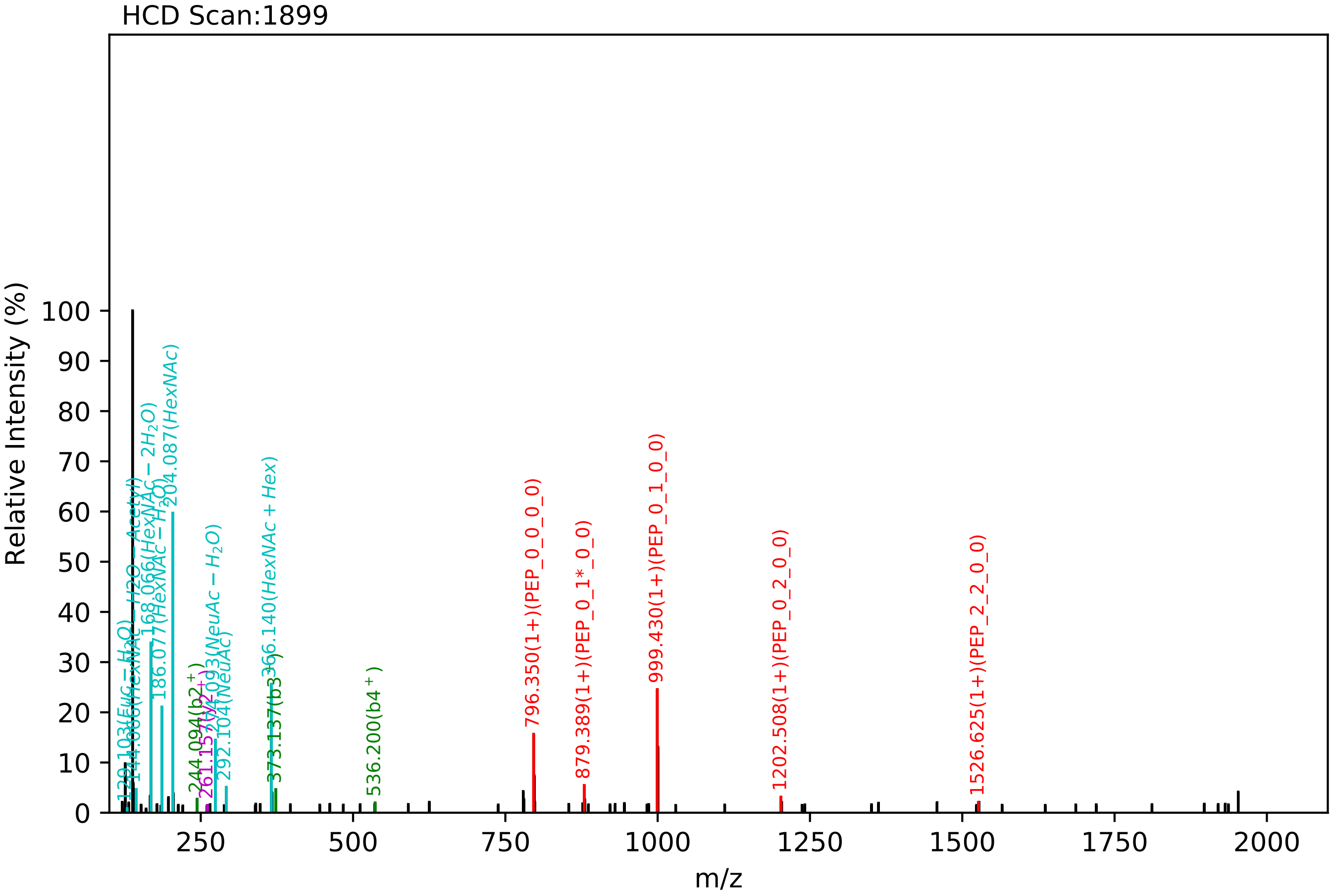

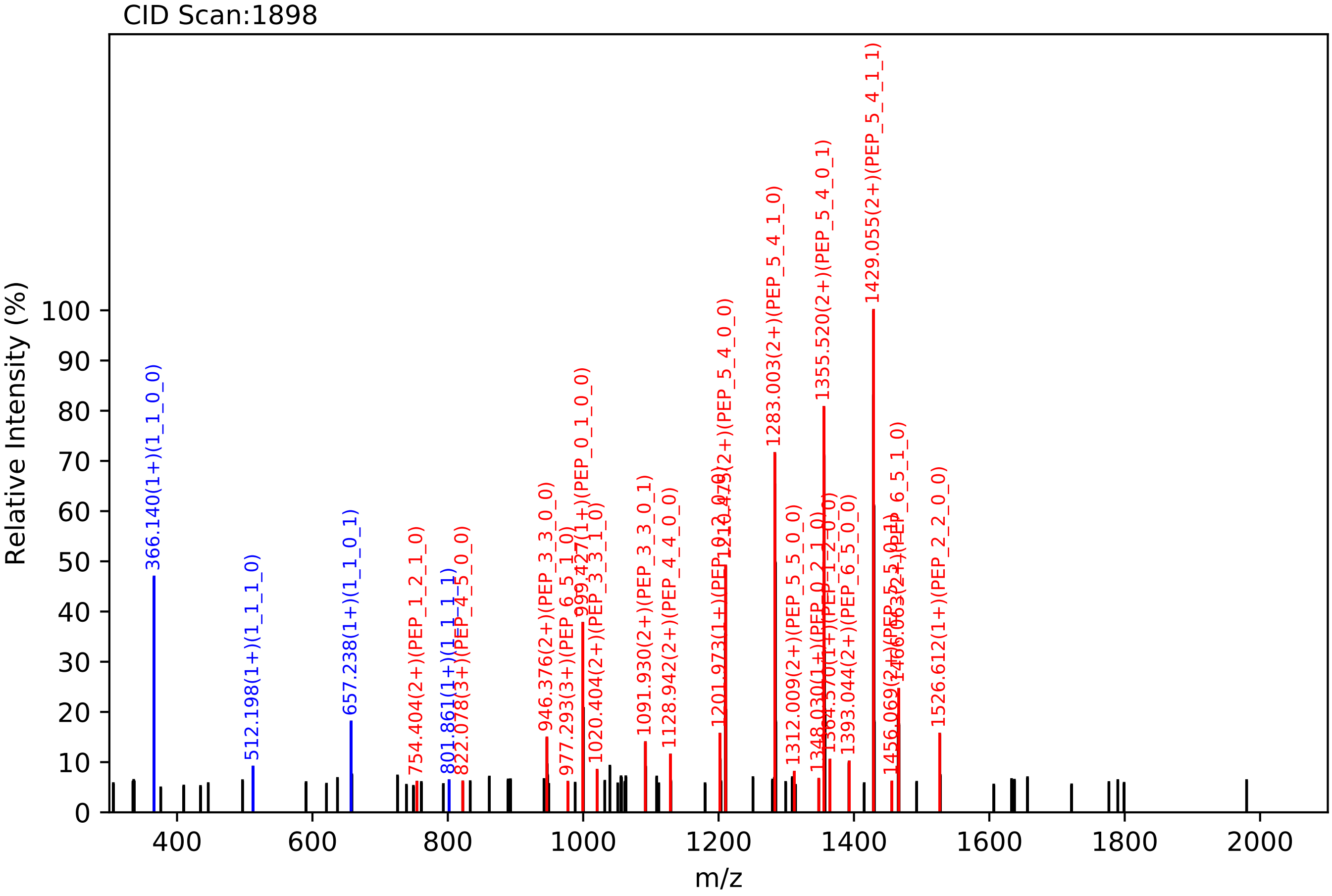

Training set no. 203, Experiment: AGP exp_1

NEEYNK(=PEP)_6_5_1_1, m/z:1074.41(3+), RT:15.62, Y-score:89.57

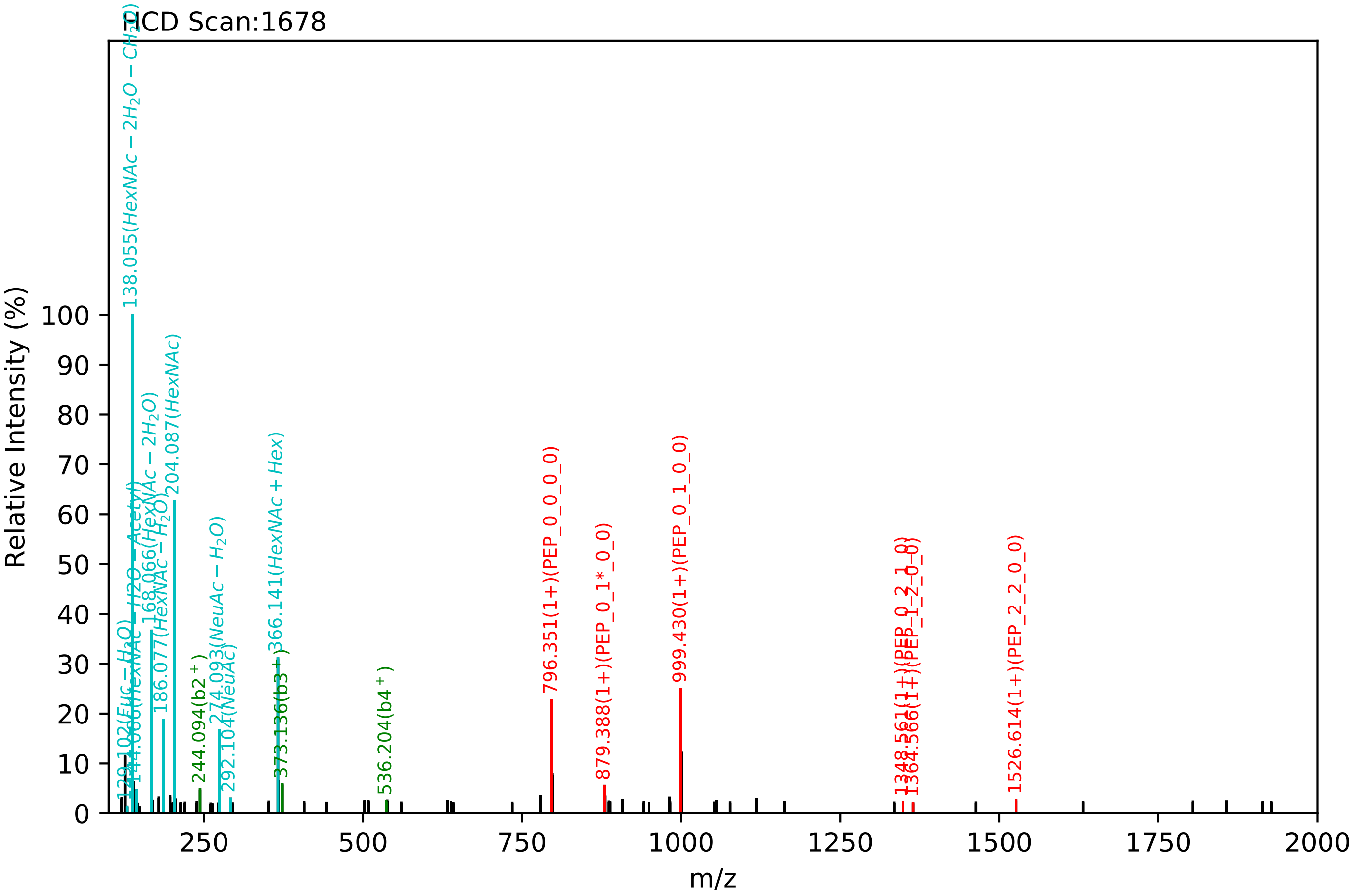

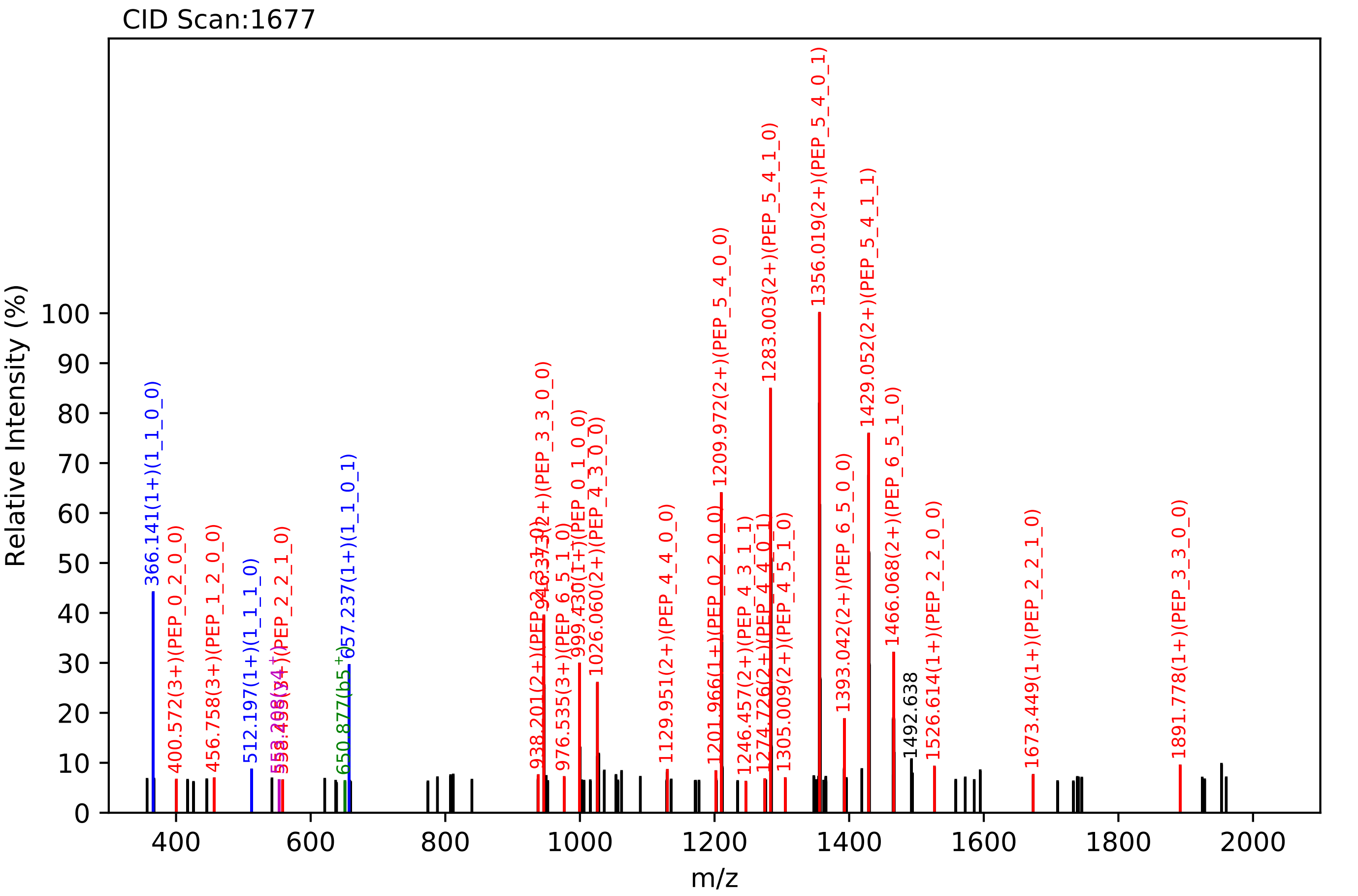

Training set no. 204, Experiment: AGP exp_2

NEEYNK(=PEP)_6_5_1_1, m/z:1074.41(3+), RT:15.66, Y-score:82.33

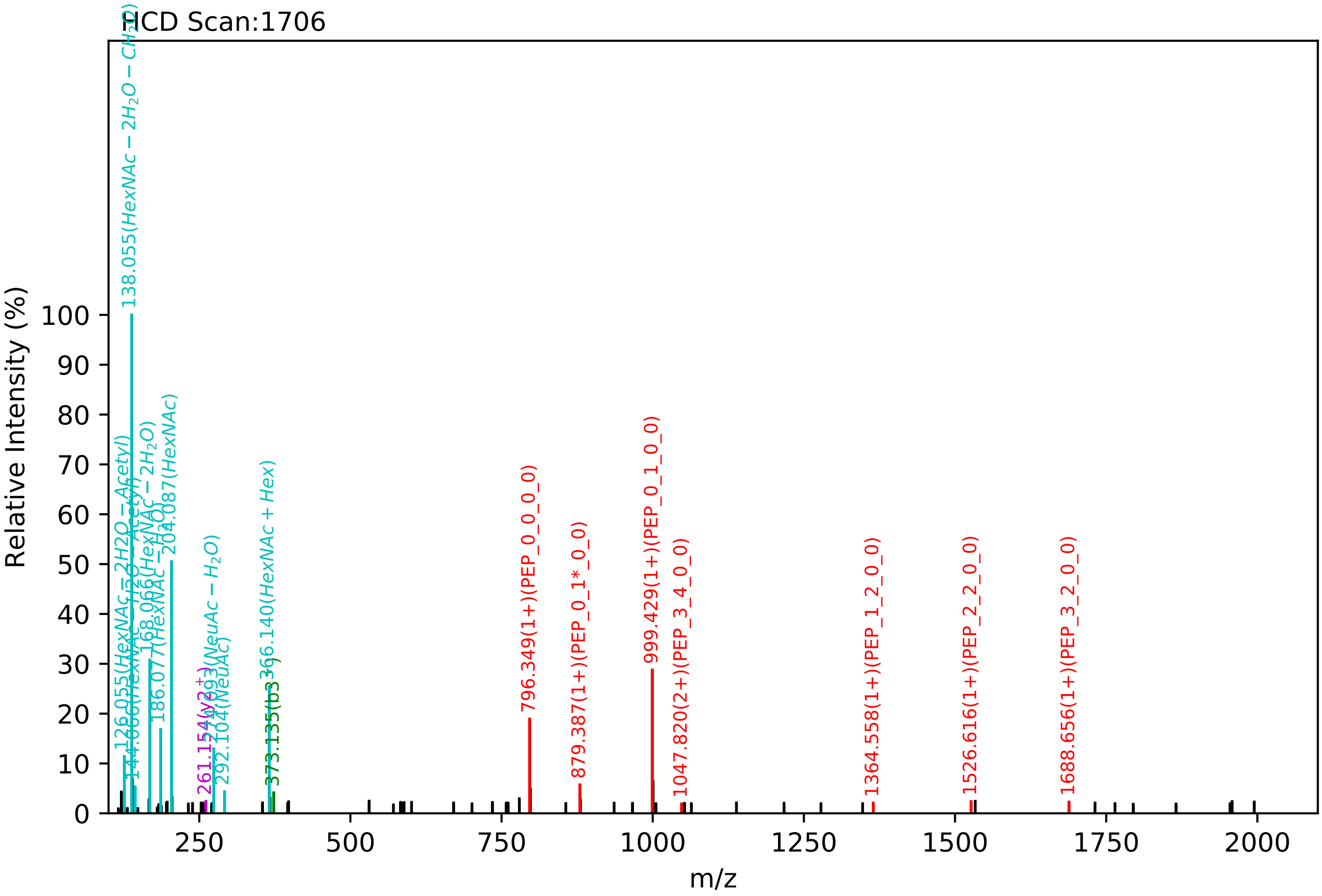

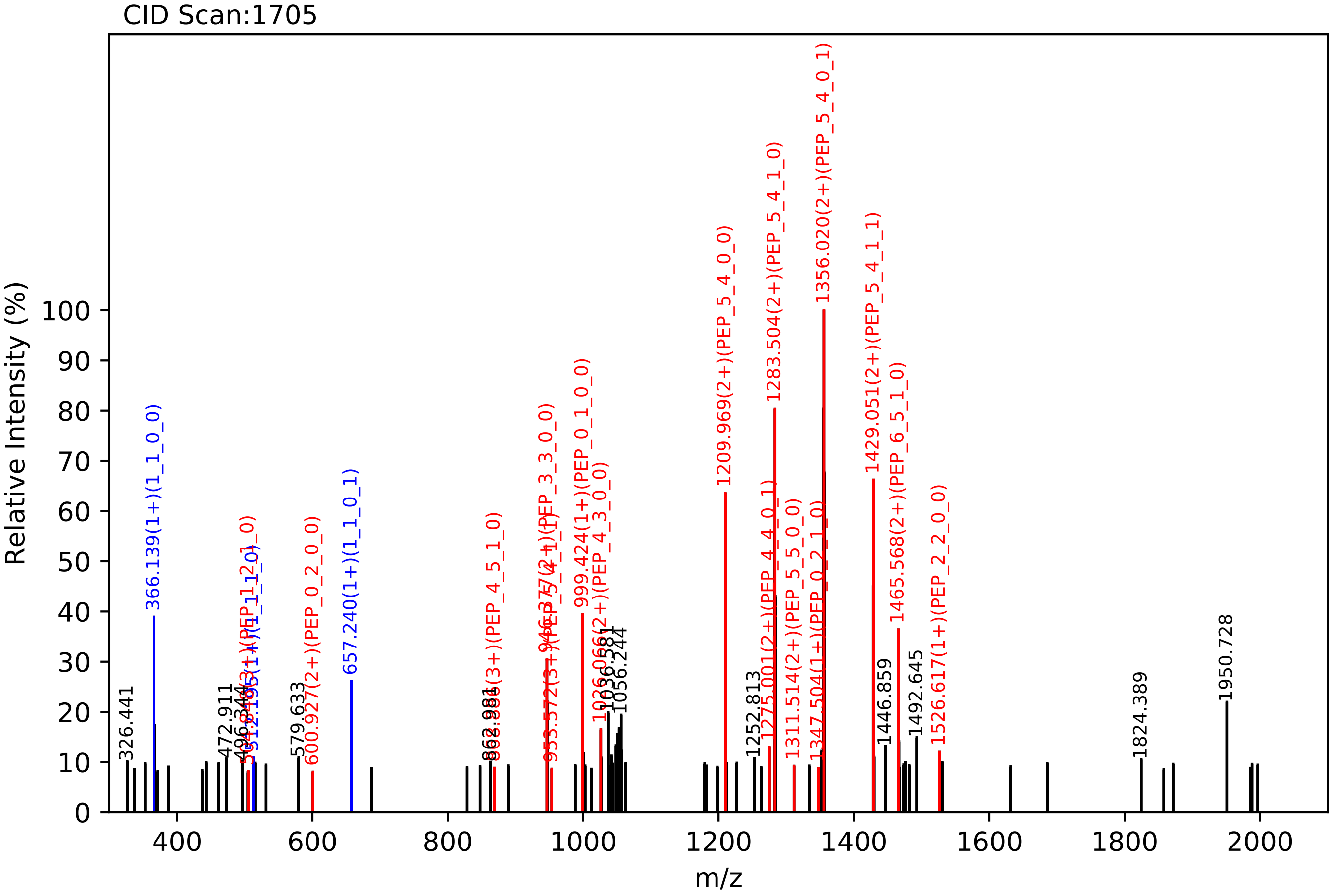

Training set no. 205, Experiment: AGP exp_1

NEEYNK(=PEP)_6_5_1_2, m/z:1171.45(3+), RT:18.87, Y-score:91.30

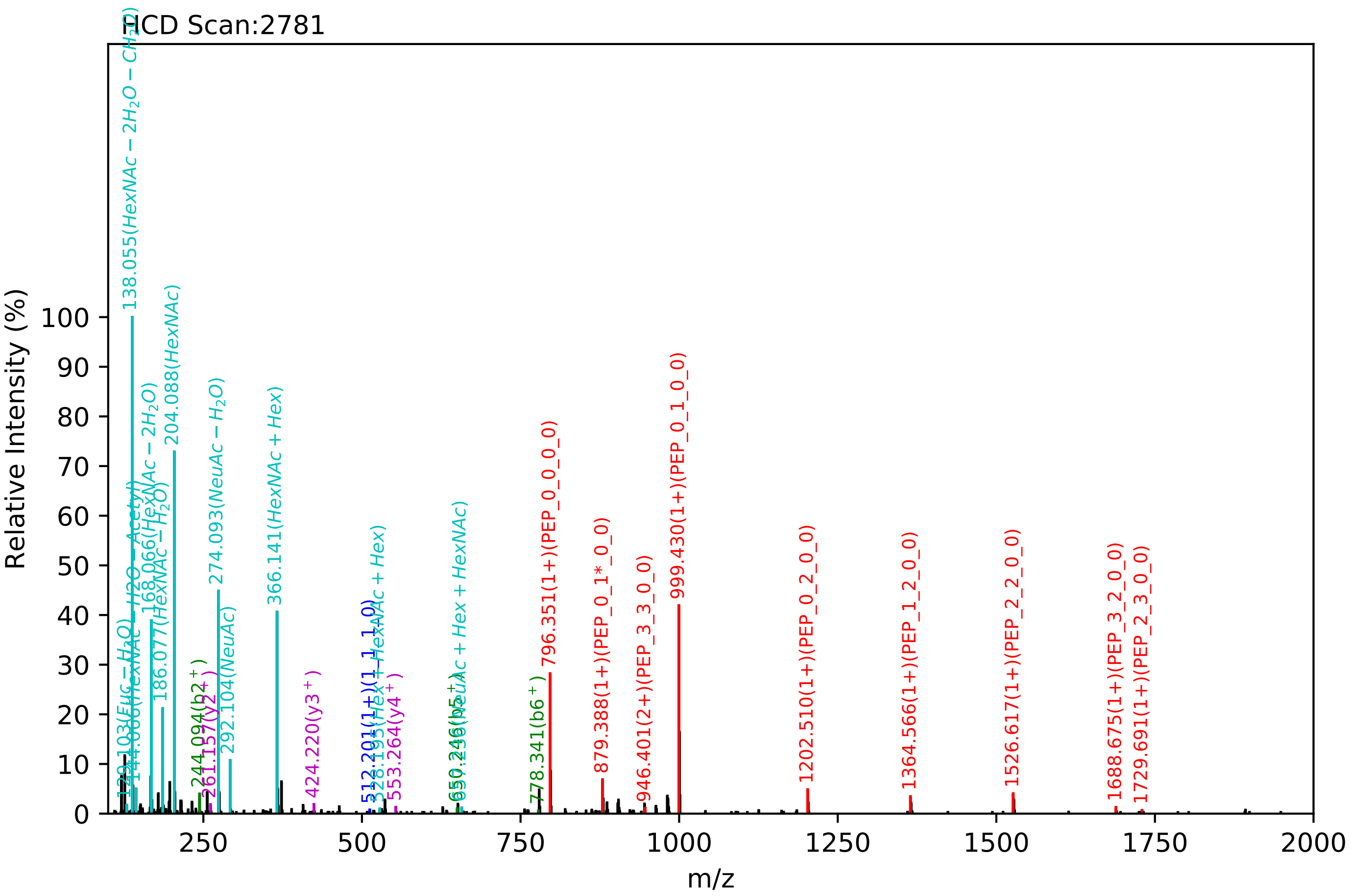

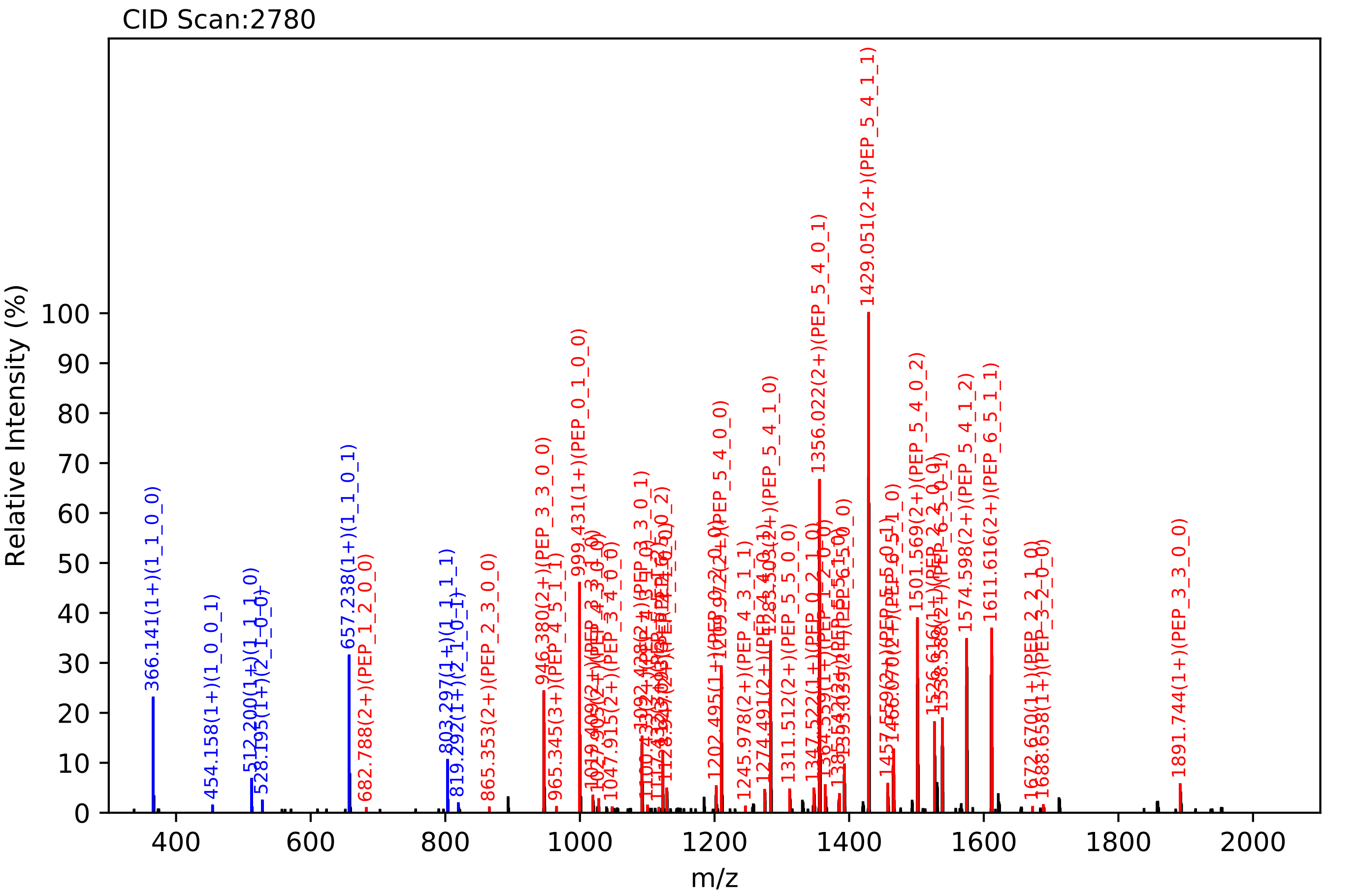

Training set no. 206, Experiment: AGP exp_1

NEEYNK(=PEP)_6_5_1_2, m/z:1171.44(3+), RT:18.27, Y-score:90.85

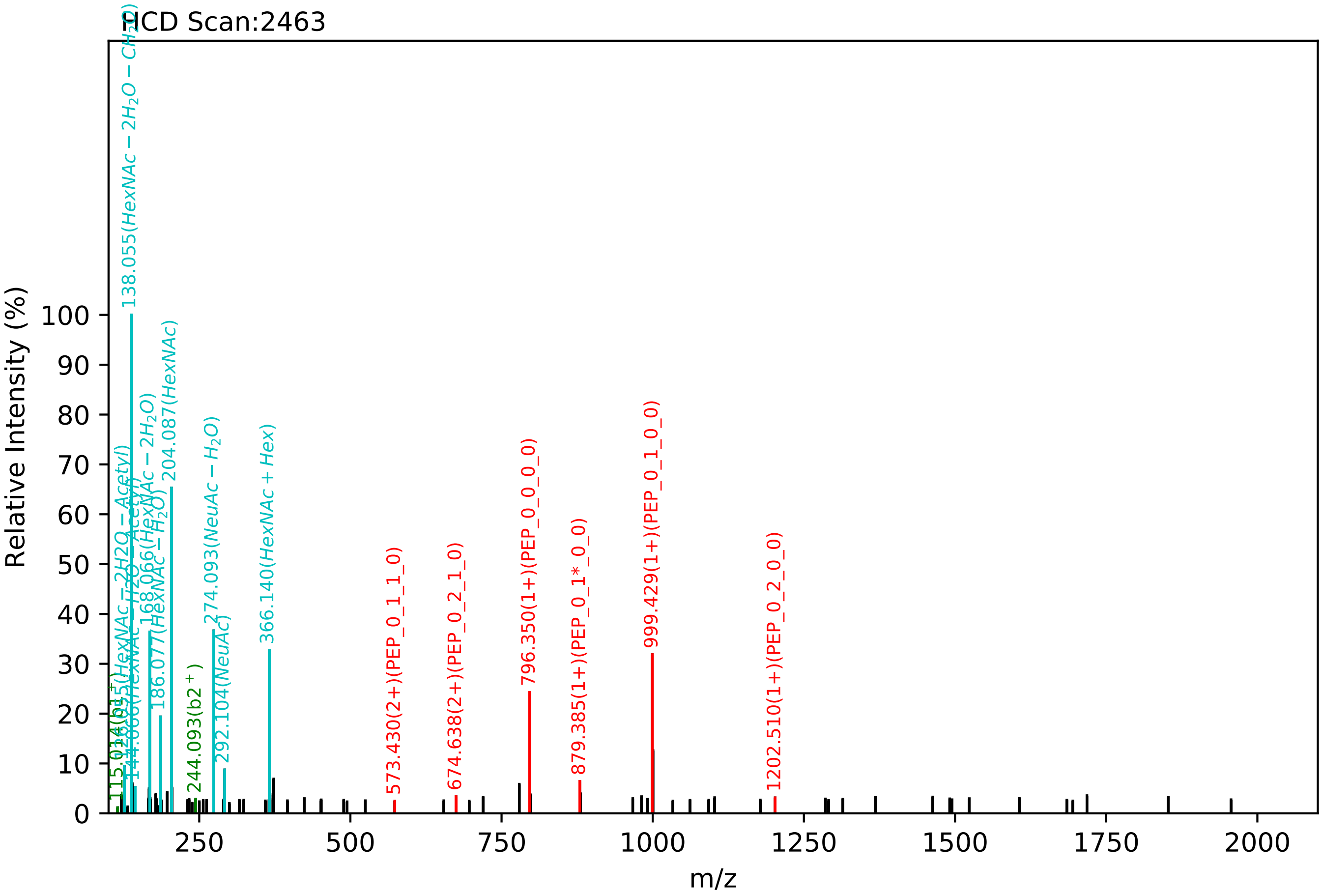

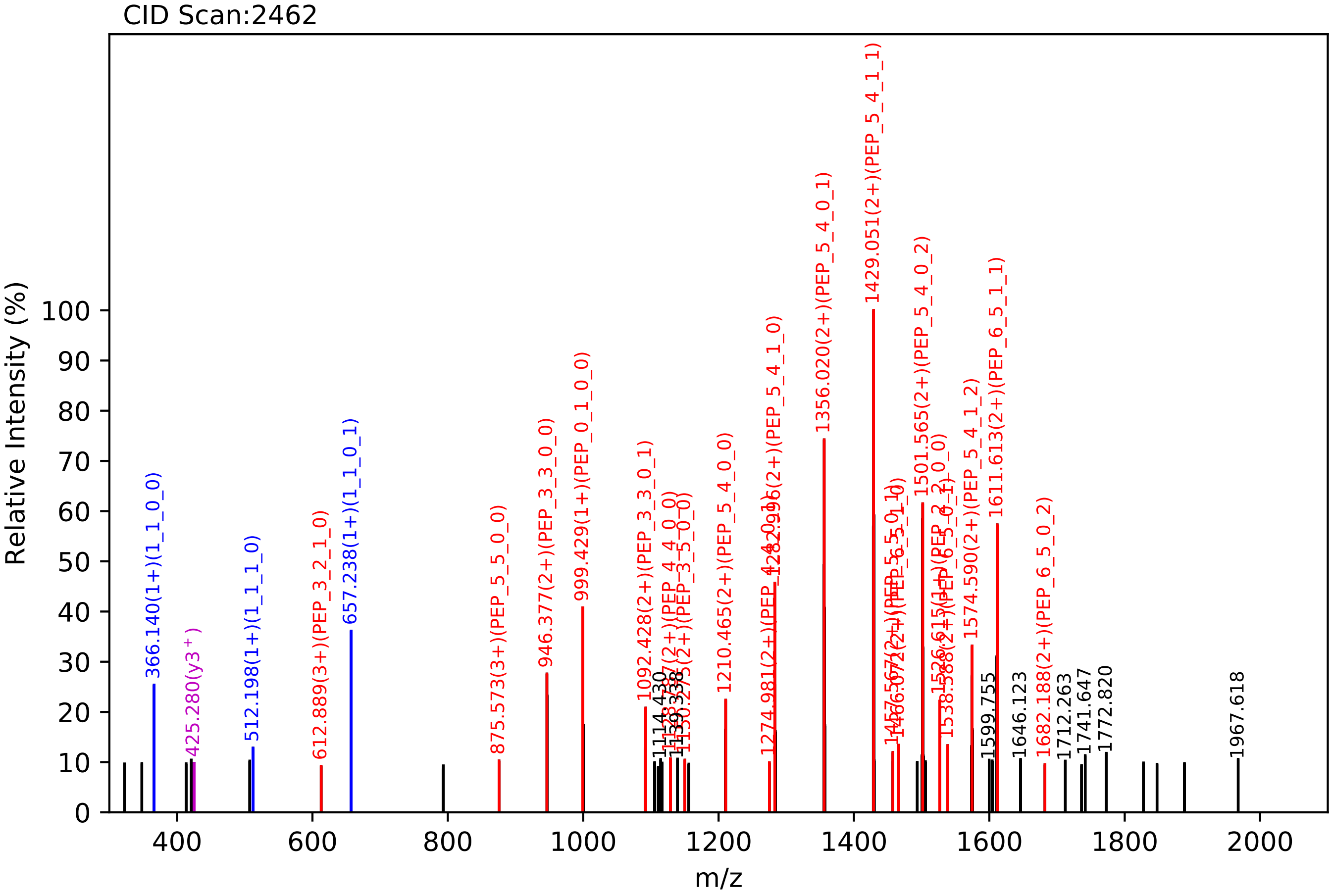

Training set no. 207, Experiment: AGP exp_1

NEEYNK(=PEP)_6_5_1_2, m/z:1171.44(3+), RT:20.43, Y-score:90.42

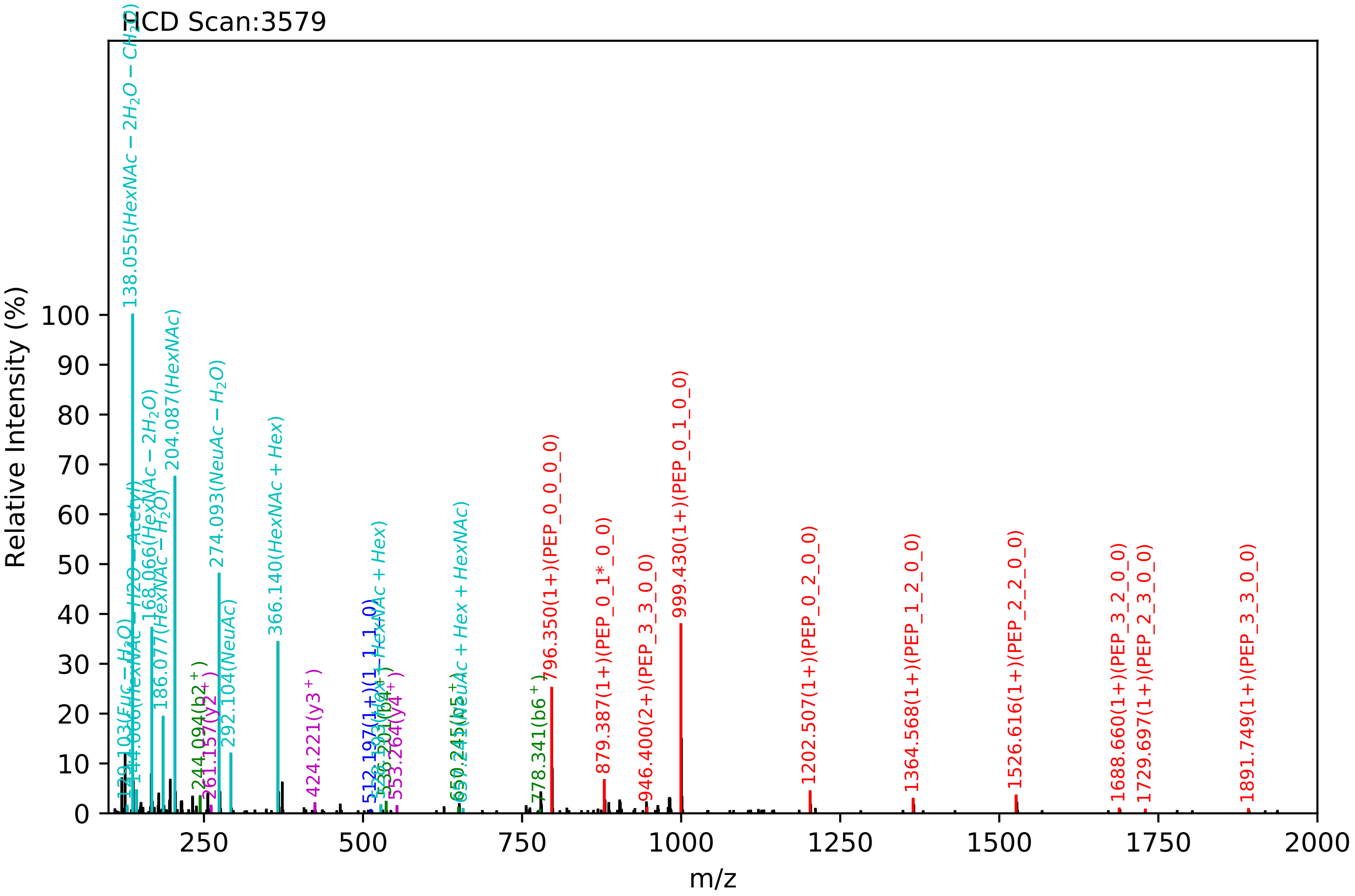

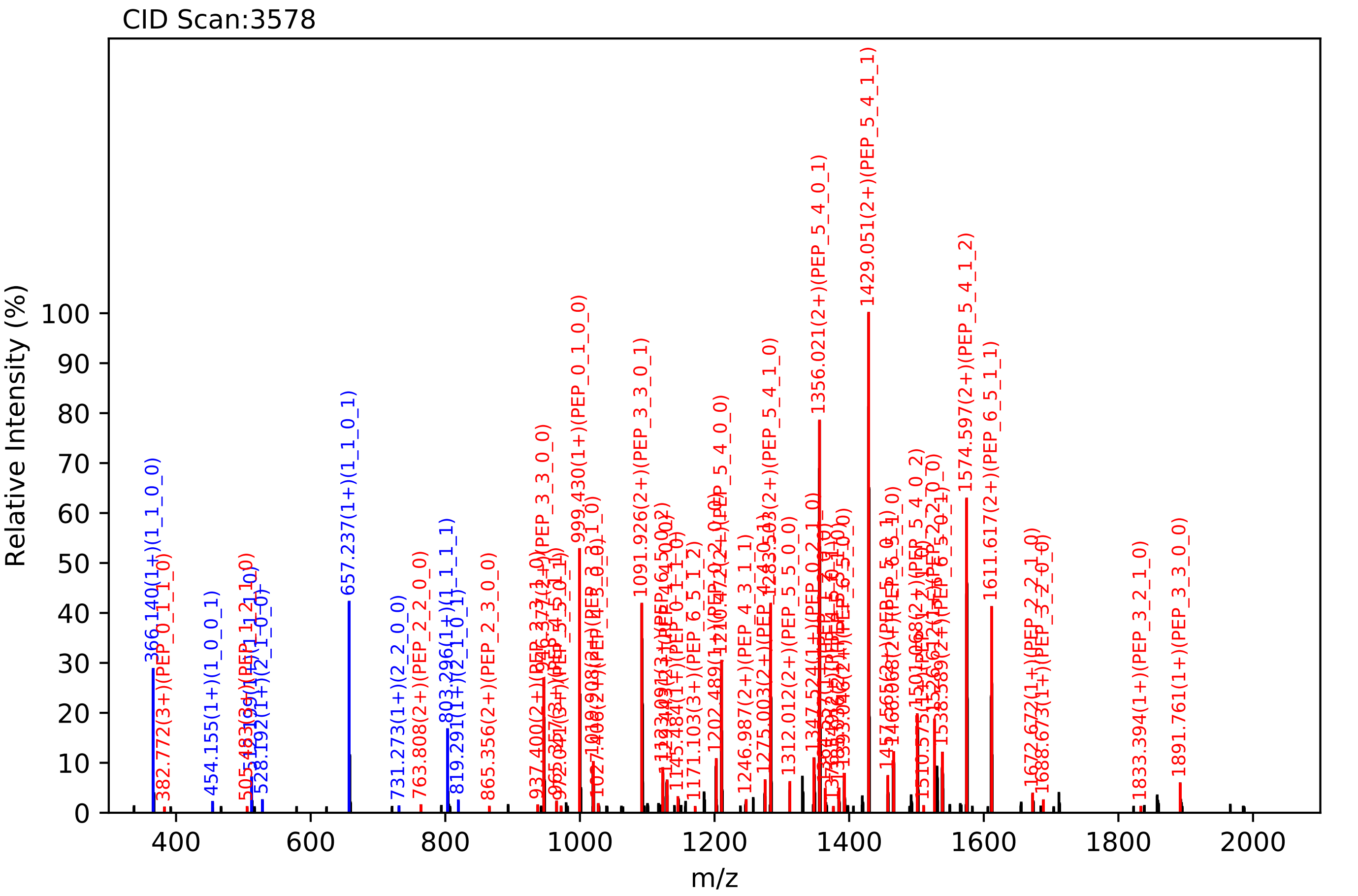

Training set no. 208, Experiment: AGP exp_2

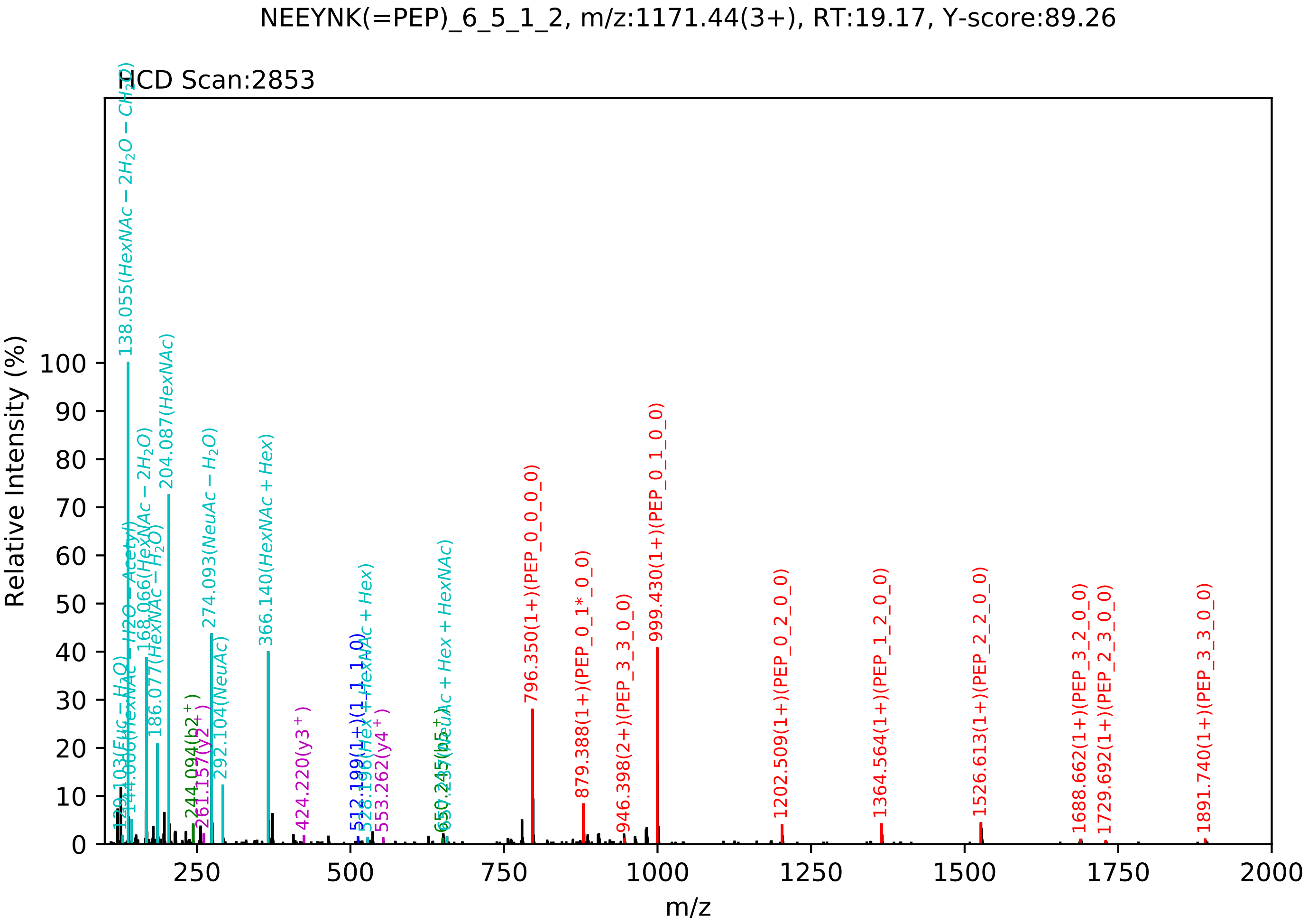

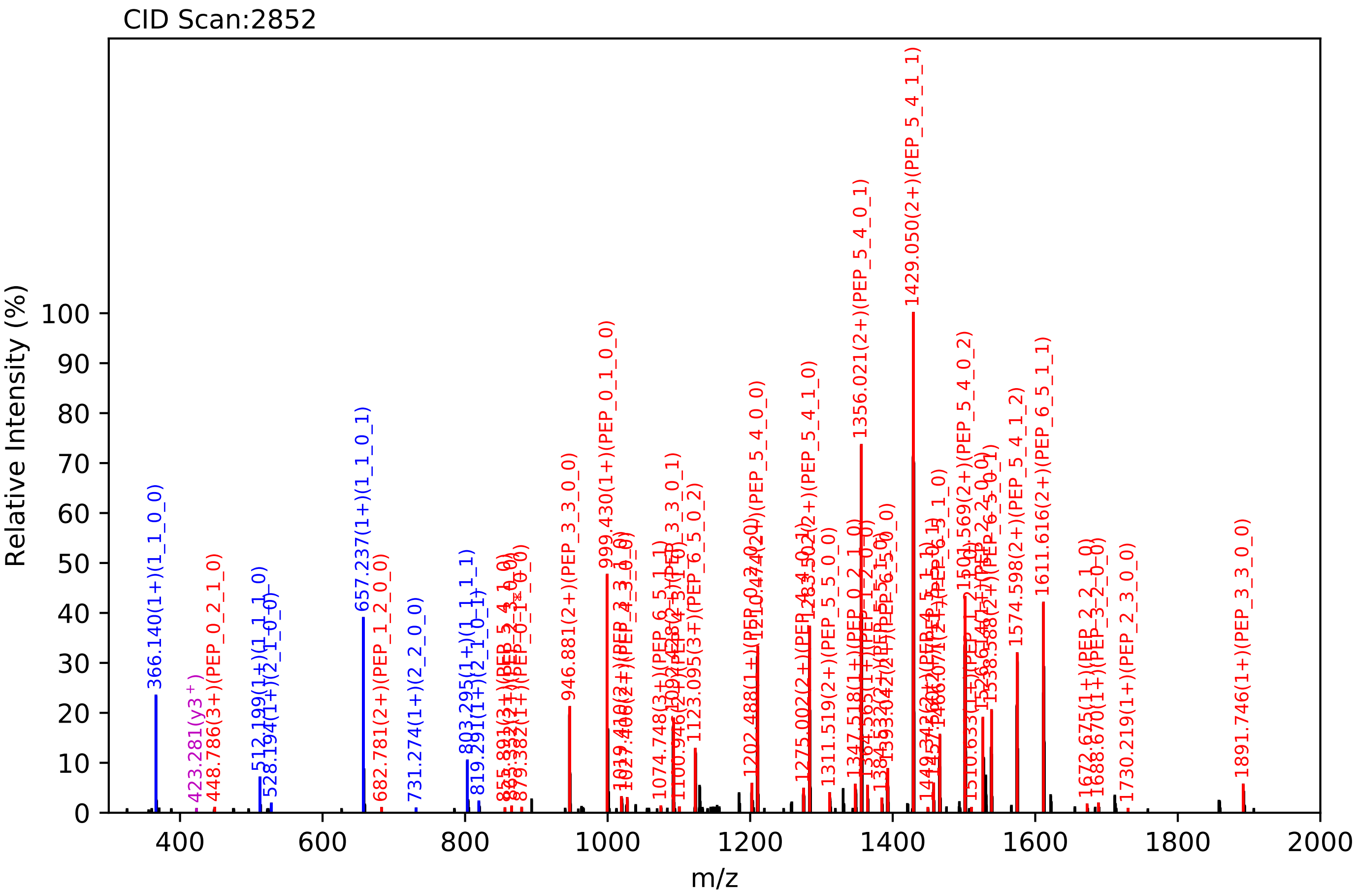

Training set no. 209, Experiment: AGP exp_2

NEEYNK(=PEP)_6_5_1_2, m/z:1171.44(3+), RT:19.73, Y-score:88.71

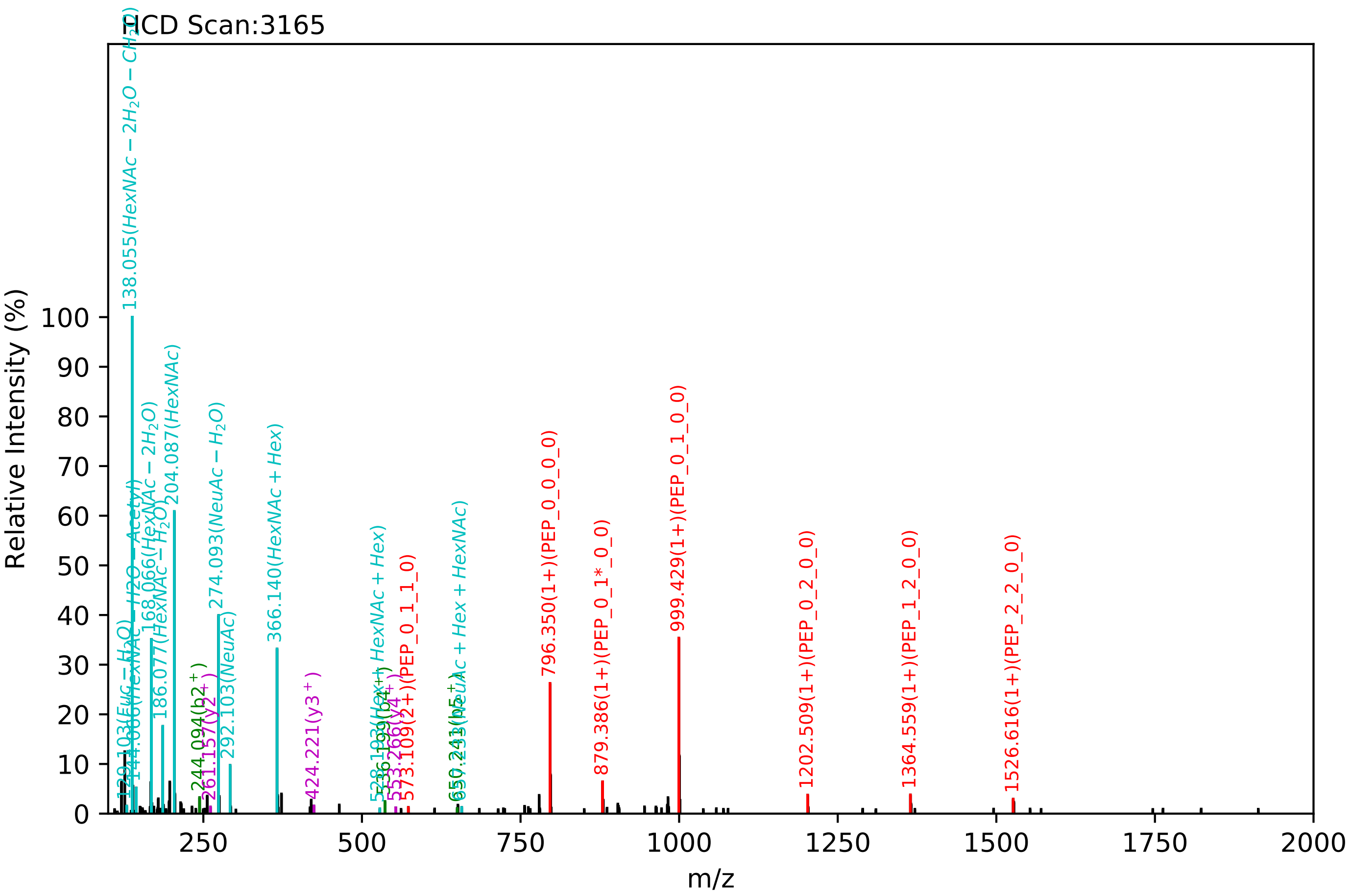

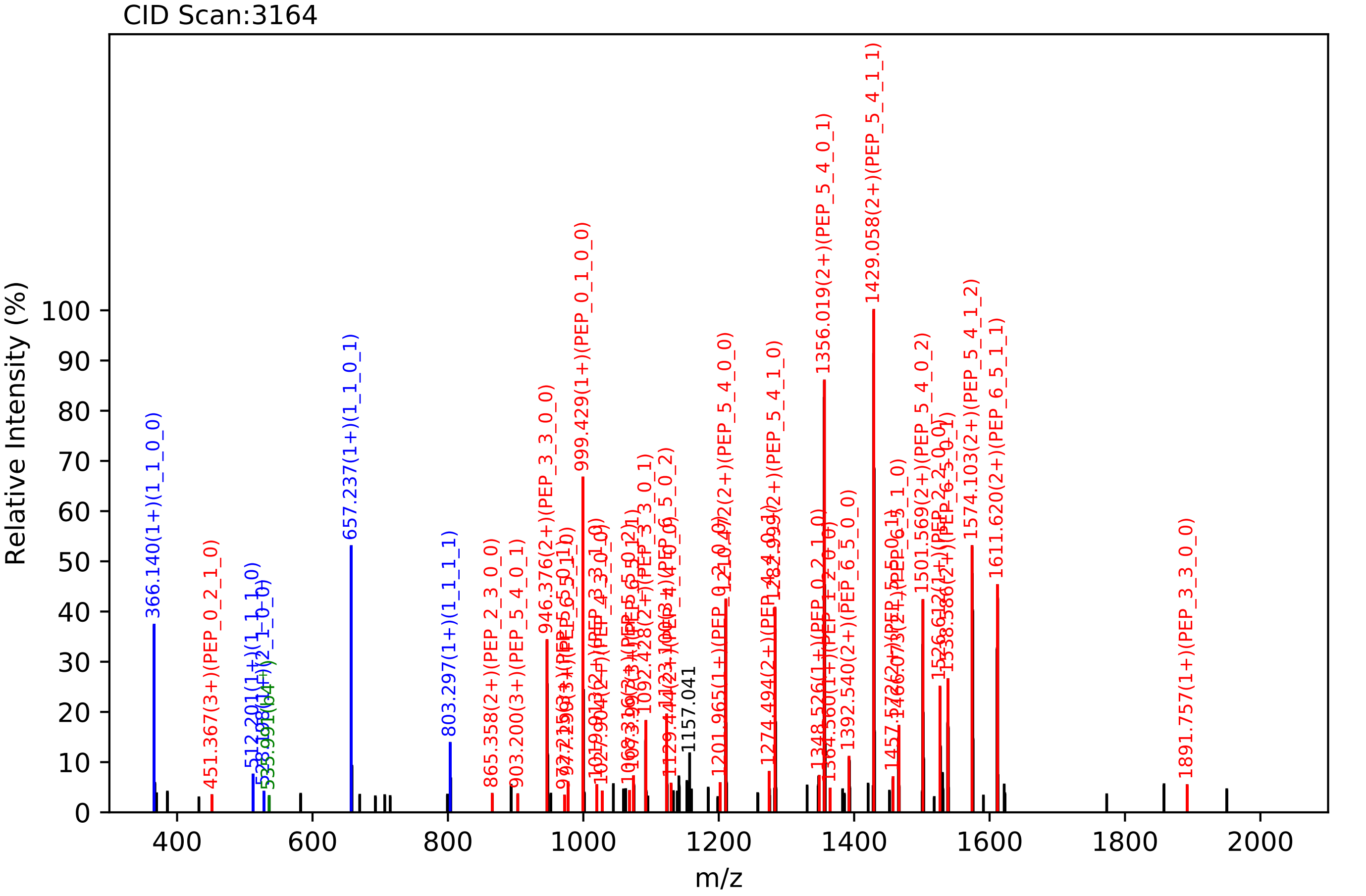

Training set no. 210, Experiment: AGP exp_1

NEEYNK(=PEP)_6_5_1_2, m/z:1171.44(3+), RT:19.38, Y-score:85.07

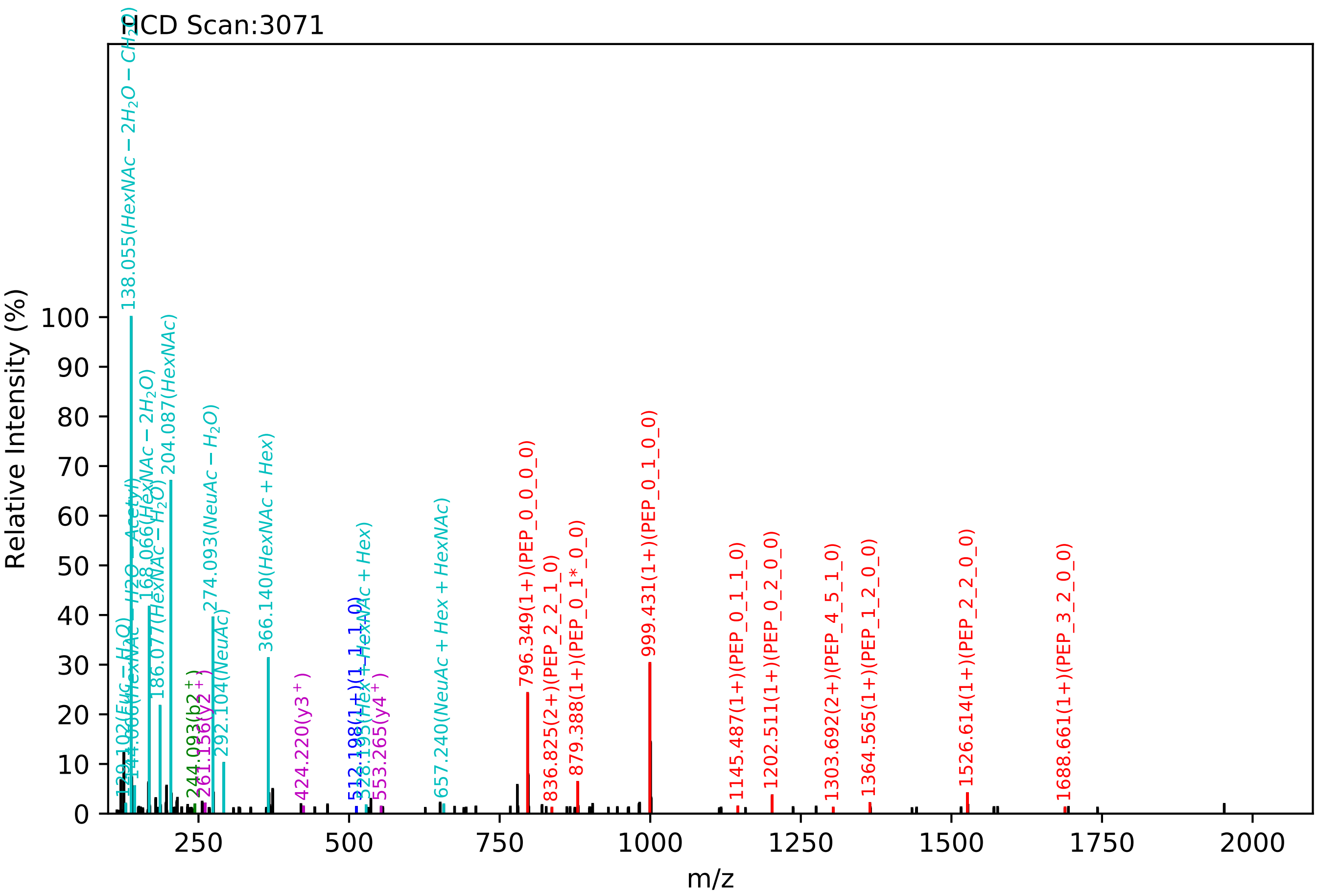

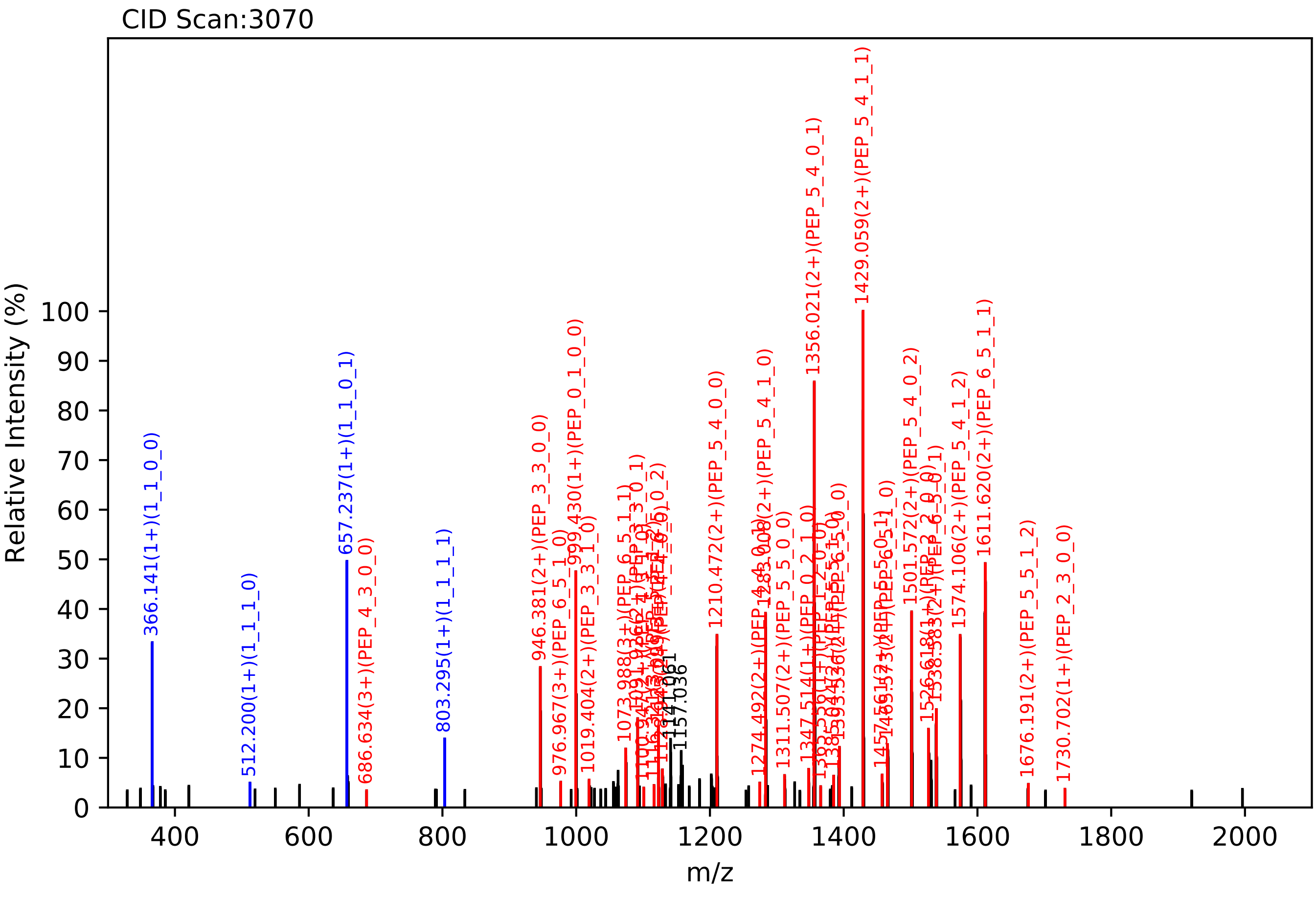

Training set no. 211, Experiment: AGP exp_2

NEEYNK(=PEP)_6_5_1_2, m/z:1171.44(3+), RT:20.88, Y-score:83.44

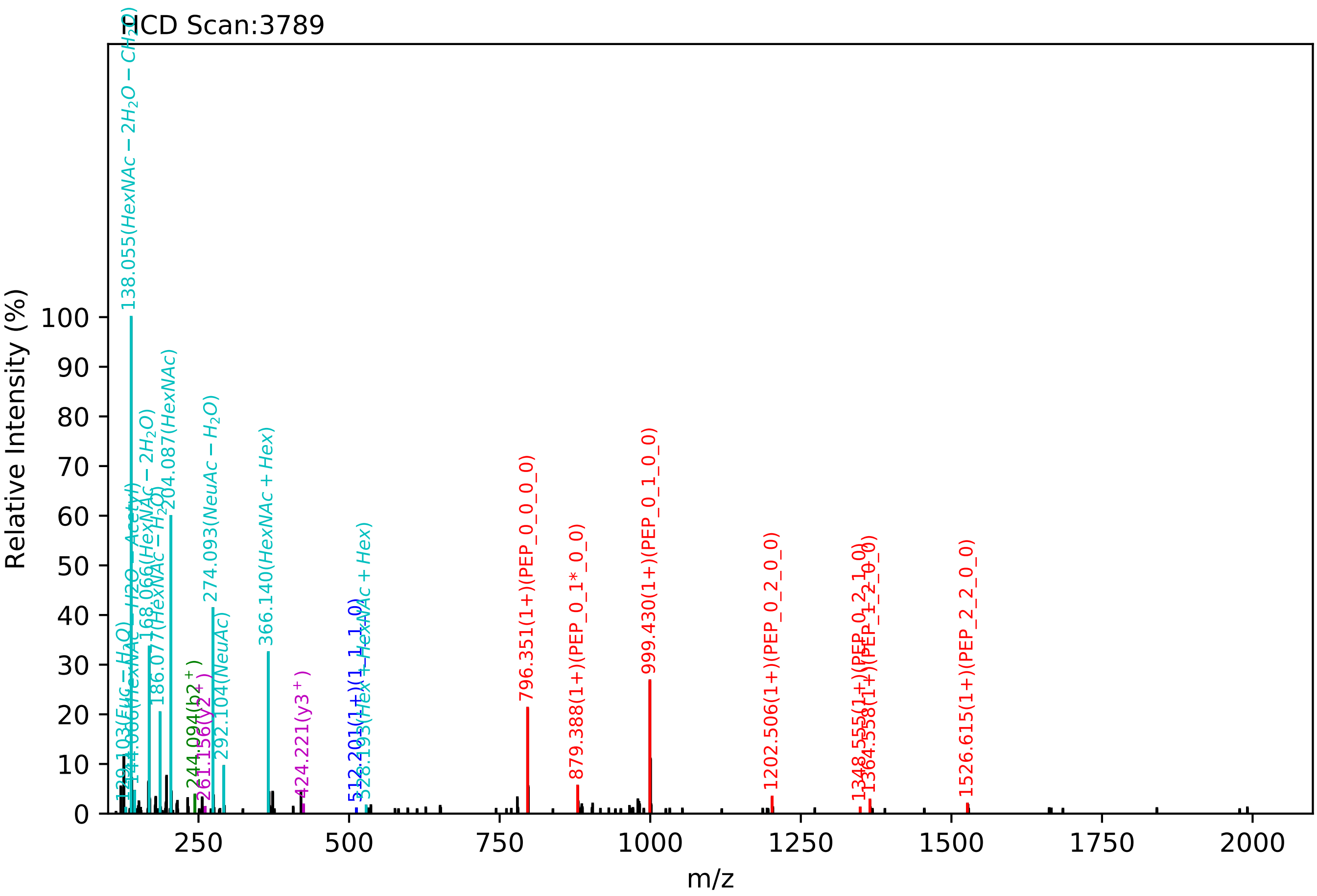

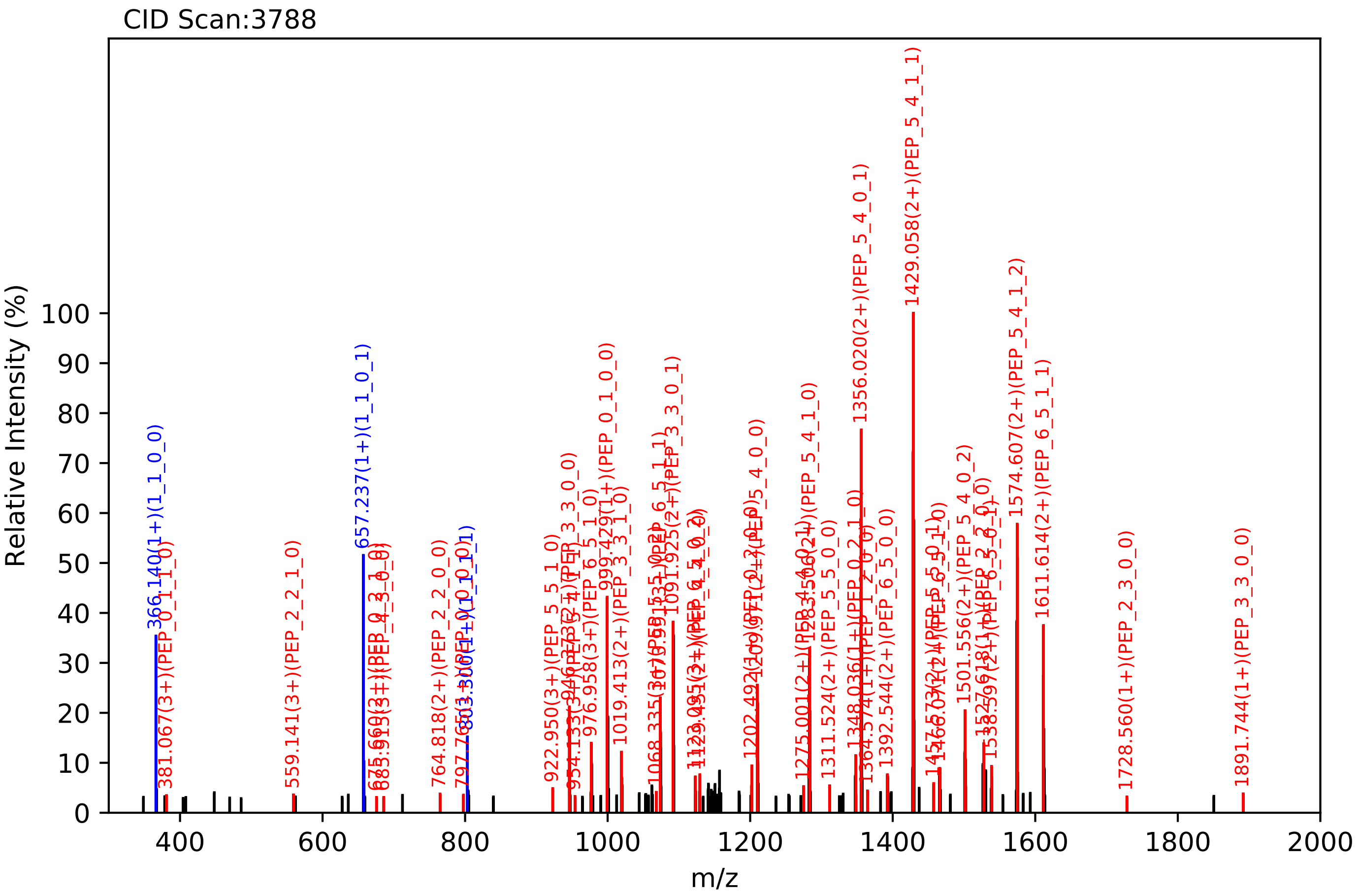

Training set no. 212, Experiment: AGP exp_1

NEEYNK(=PEP)_6_5_1_3, m/z:1268.14(3+), RT:21.96, Y-score:90.35

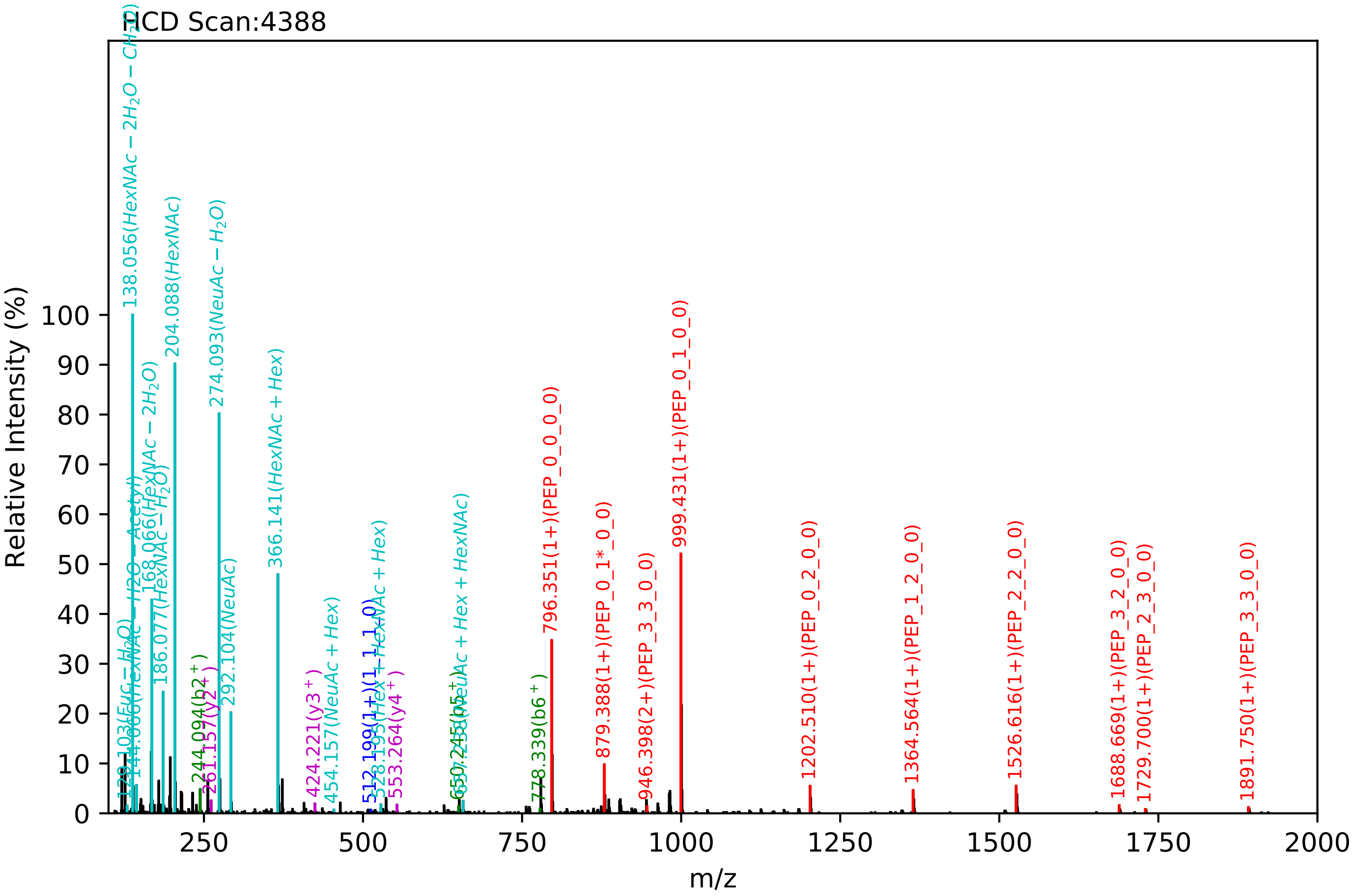

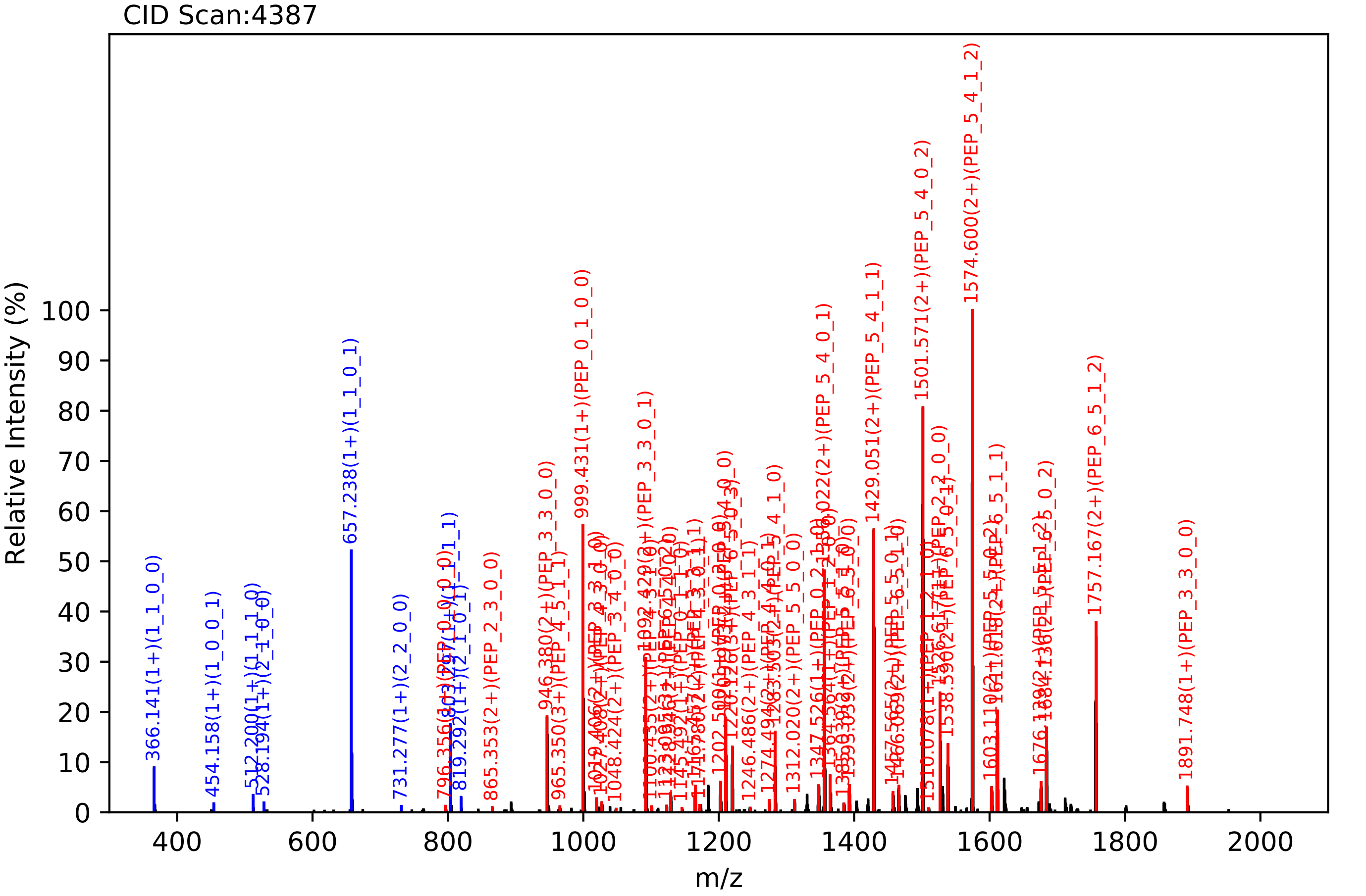

Training set no. 213, Experiment: AGP exp_2

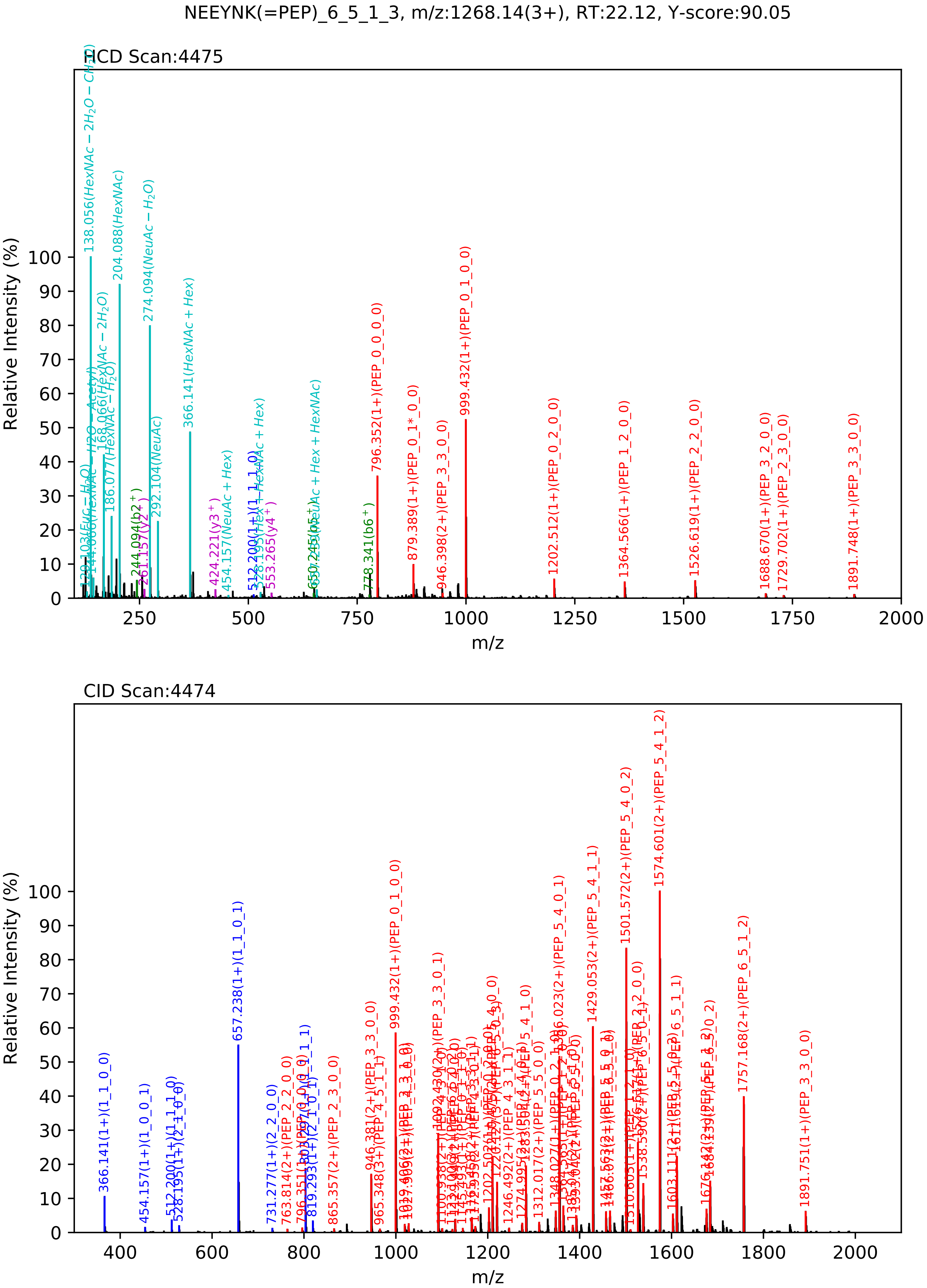

Training set no. 214, Experiment: AGP exp_25

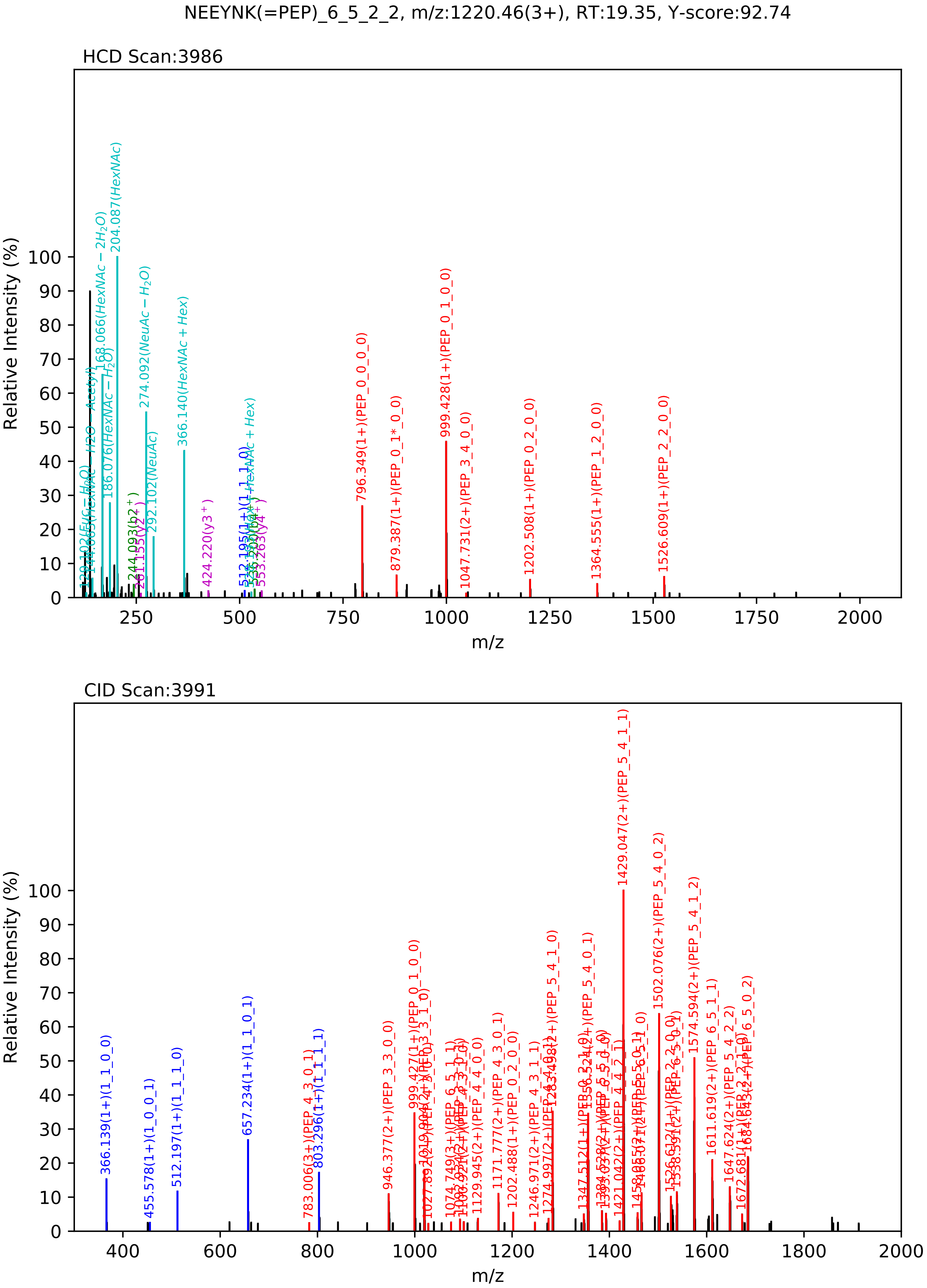

Training set no. 215, Experiment: AGP exp_34

NEEYNK(=PEP)_6_5_2_2, m/z:1220.13(3+), RT:24.16, Y-score:89.71

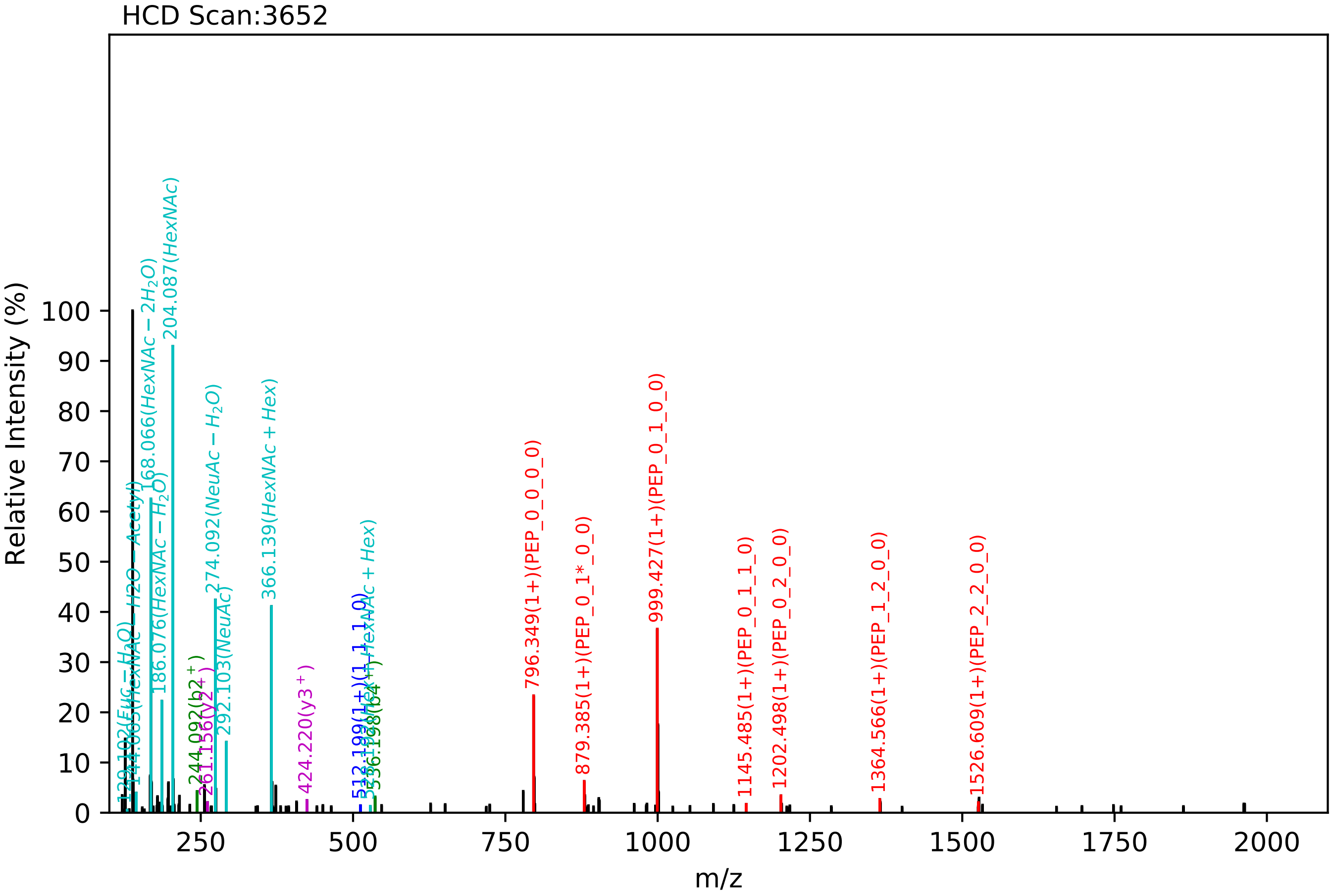

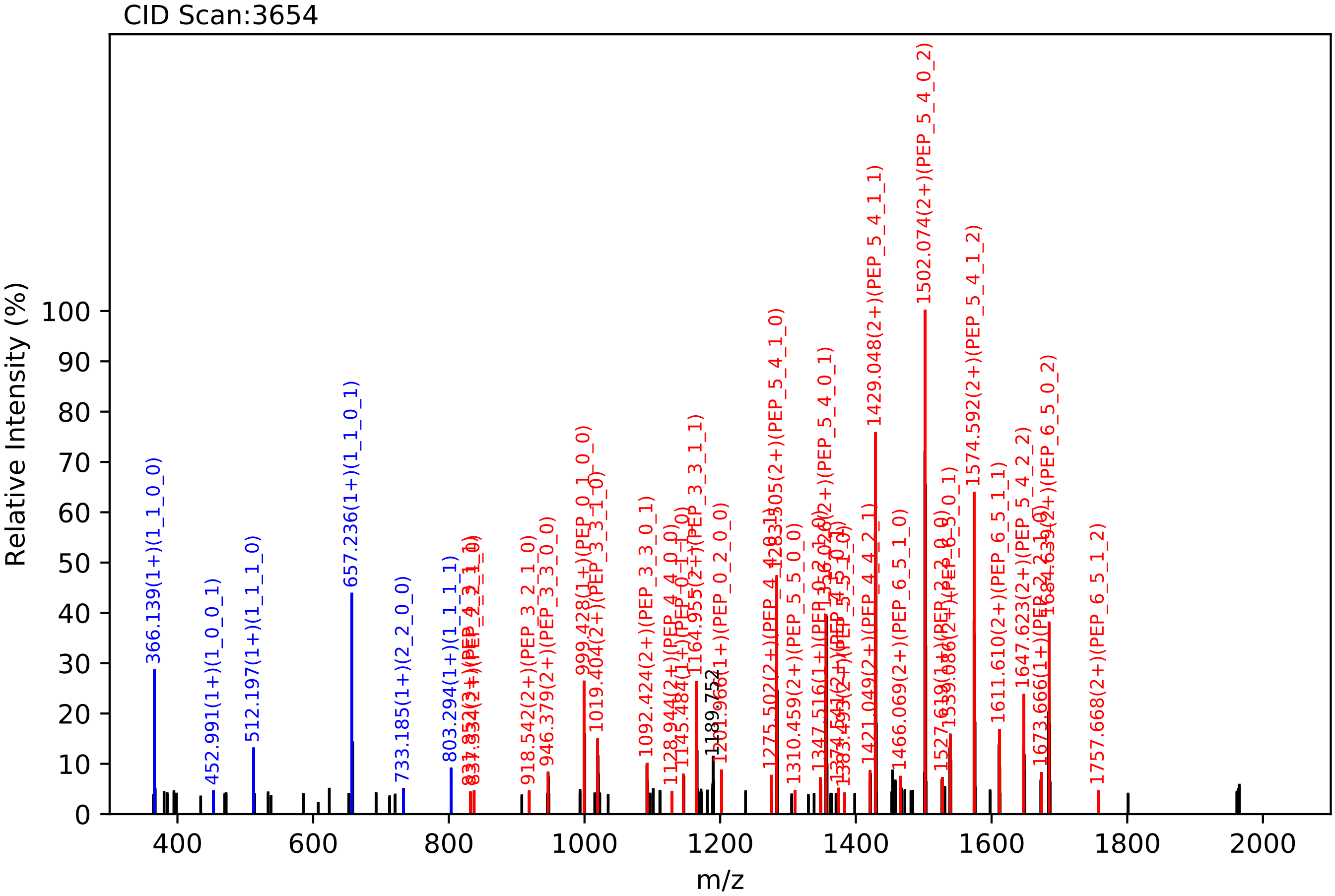

Training set no. 216, Experiment: AGP exp_27

NEEYNK(=PEP)_6_5_2_2, m/z:1220.13(3+), RT:21.42, Y-score:89.13

HCD Scan:4316

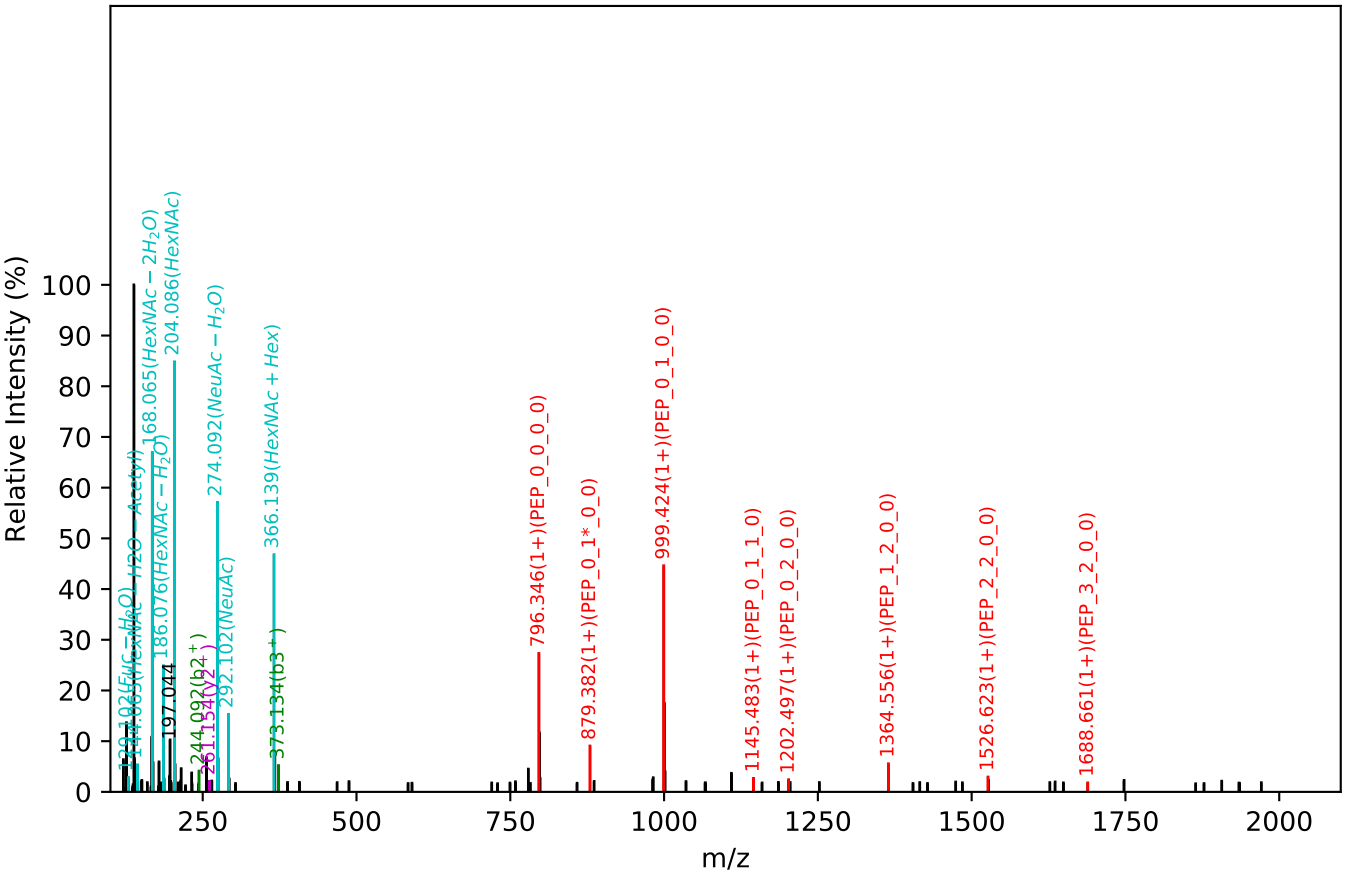

CID Scan:4318

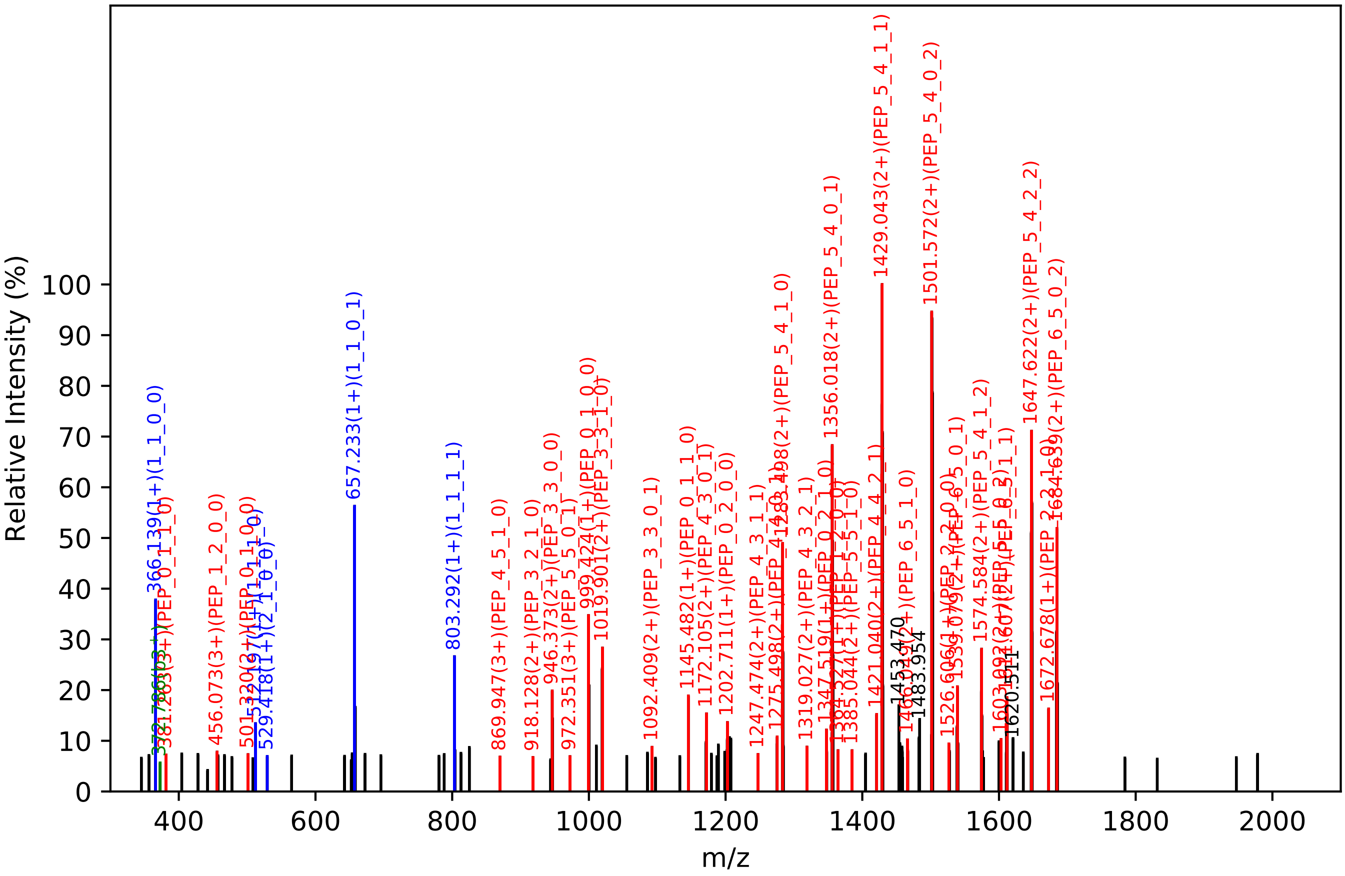

Training set no. 217, Experiment: AGP exp_45

NEEYNK(=PEP)_6_5_2_2, m/z:1220.13(3+), RT:24.41, Y-score:88.34

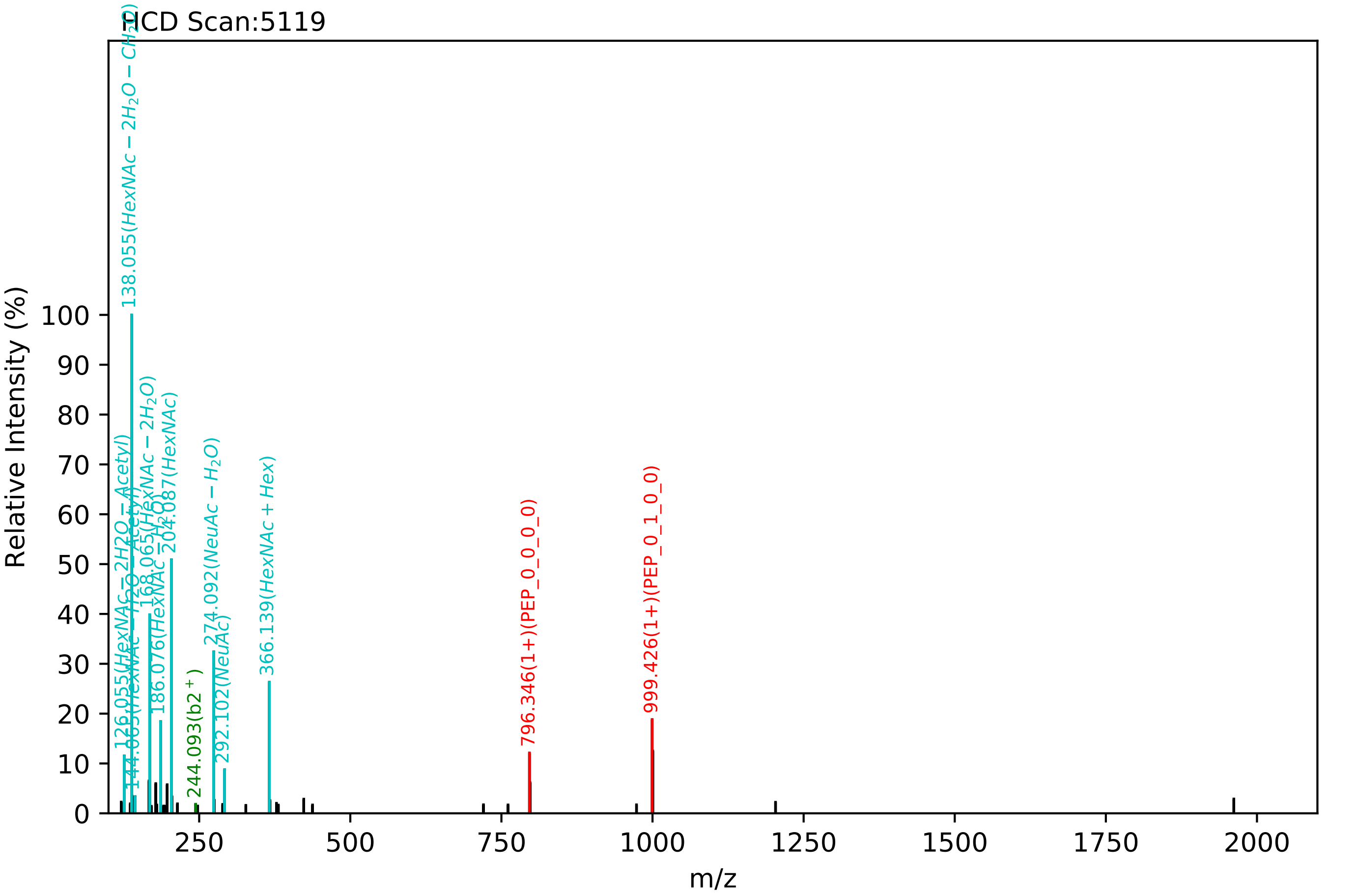

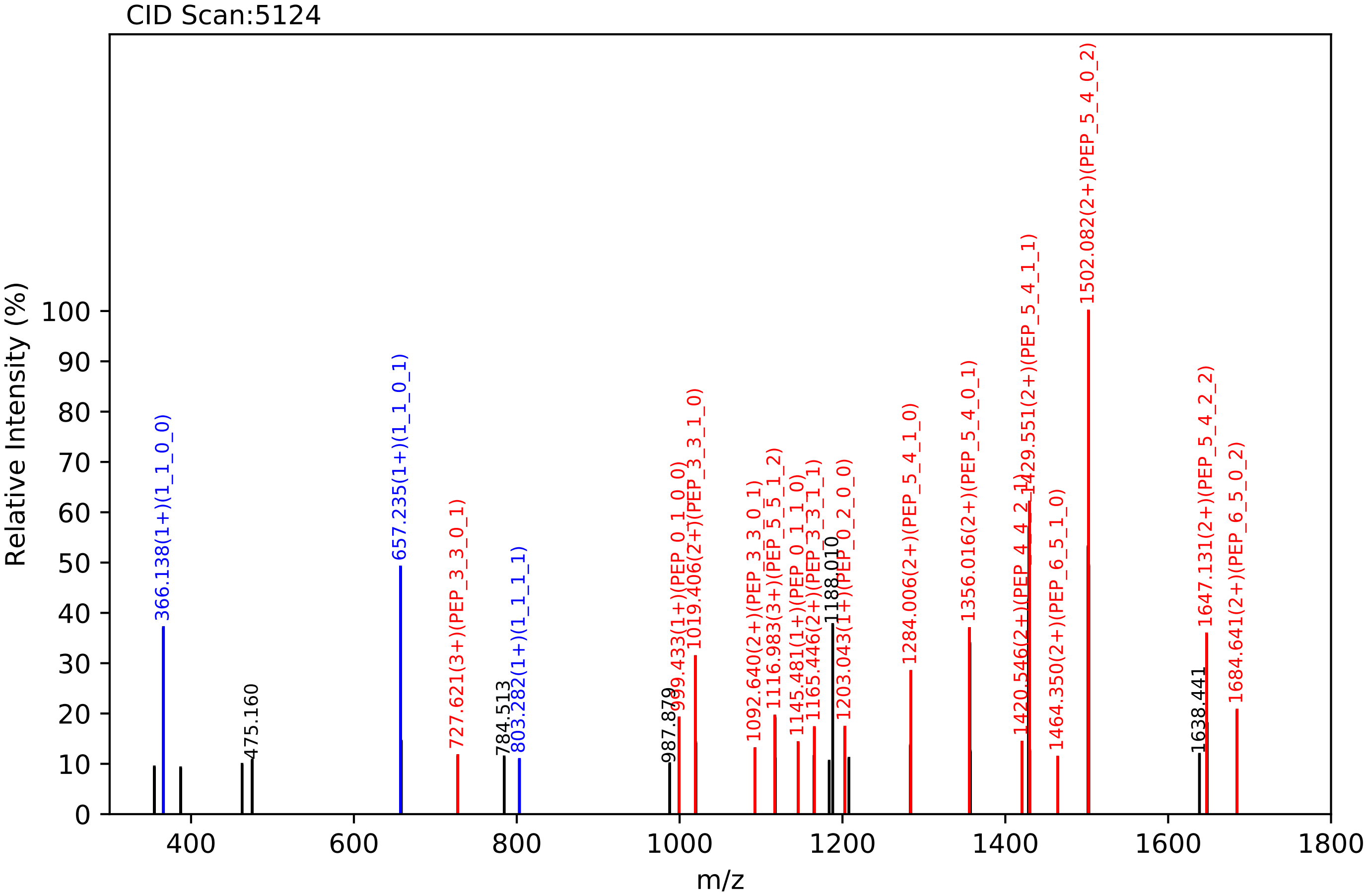

Training set no. 218, Experiment: AGP exp_9

NEEYNK(=PEP)_6_5_2_2, m/z:1220.13(3+), RT:20.16, Y-score:85.19

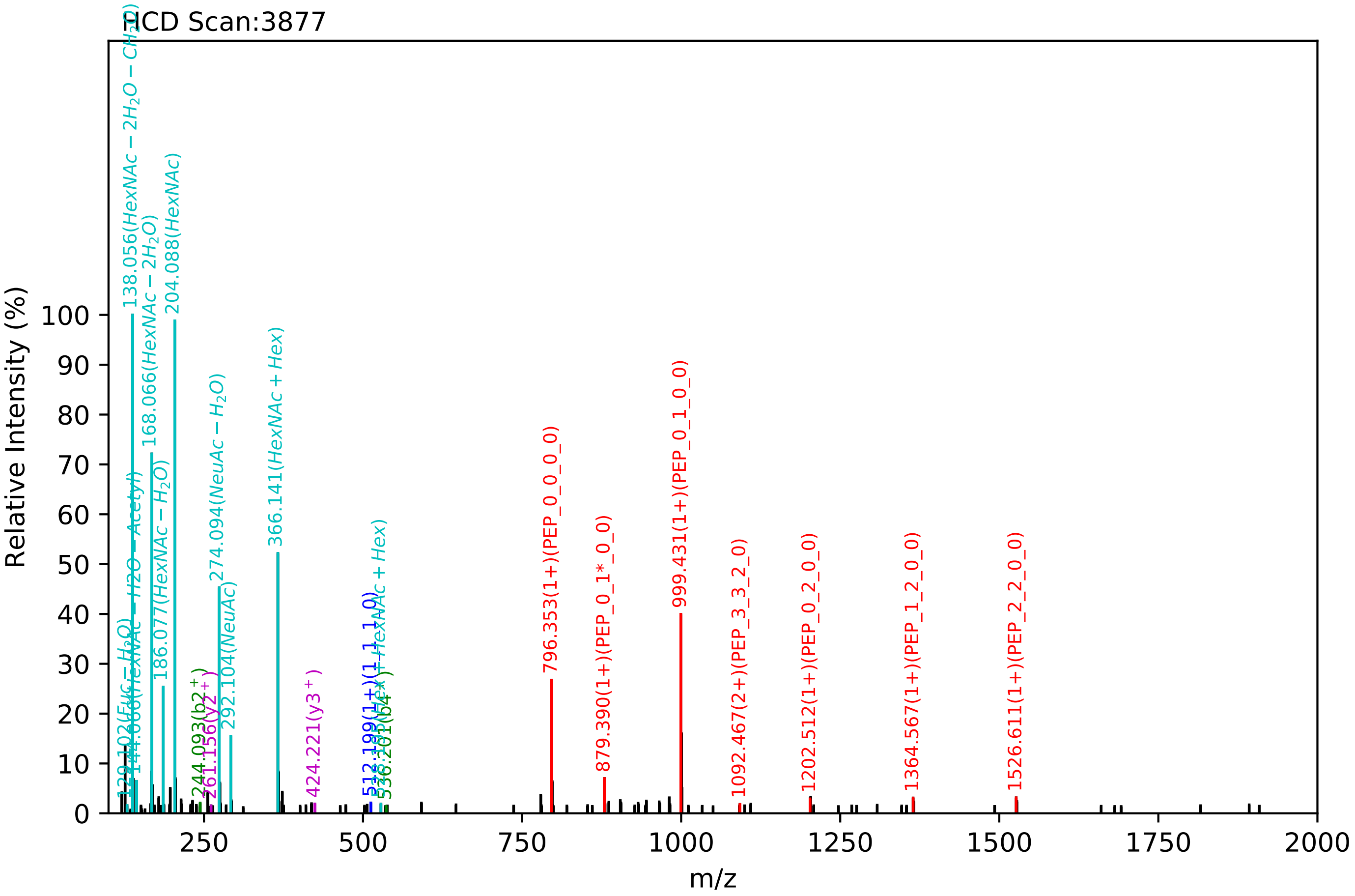

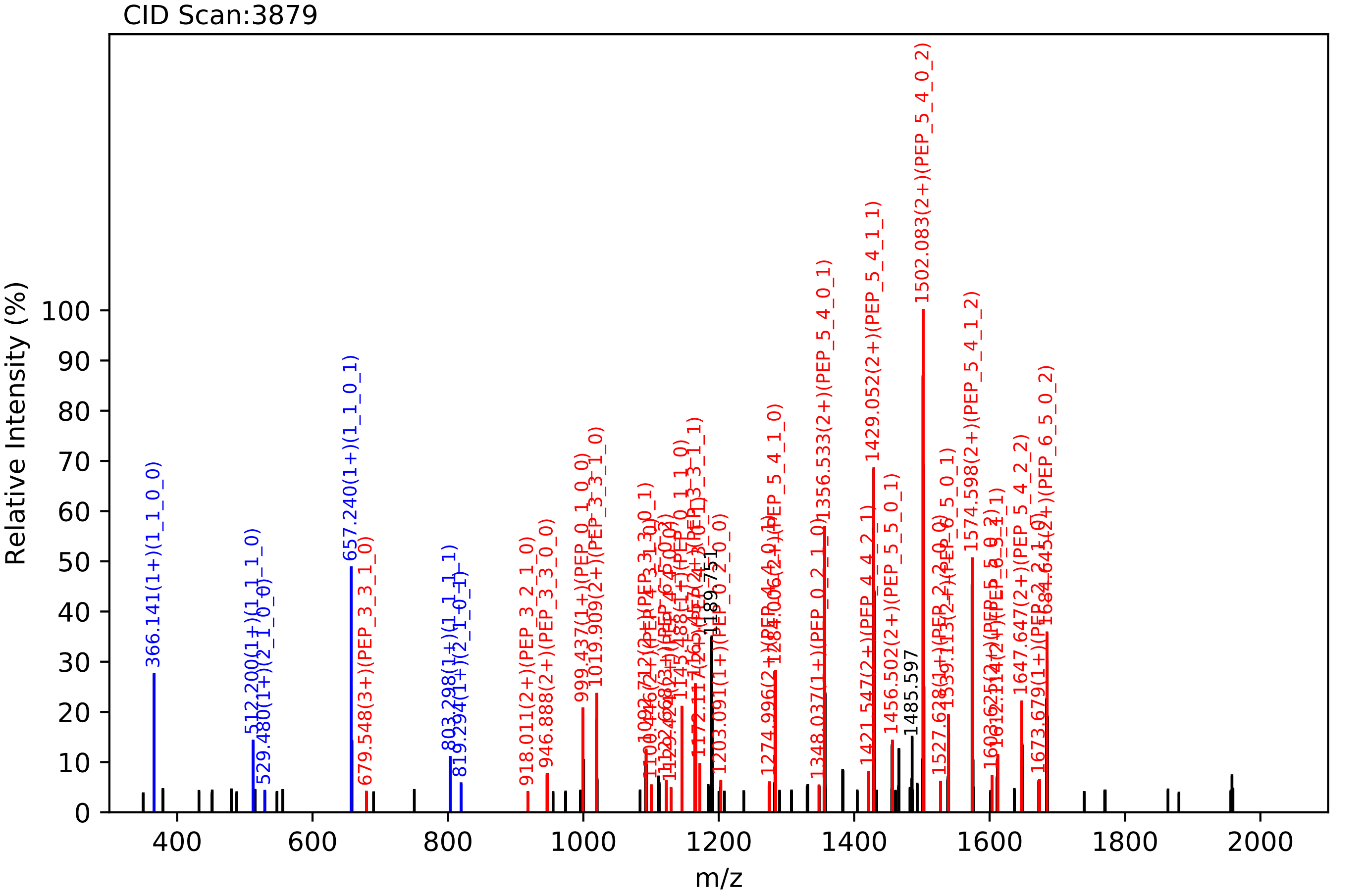

Training set no. 219, Experiment: AGP exp_28

NEEYNK(=PEP)_6_5_2_2, m/z:1220.13(3+), RT:21.43, Y-score:85.04

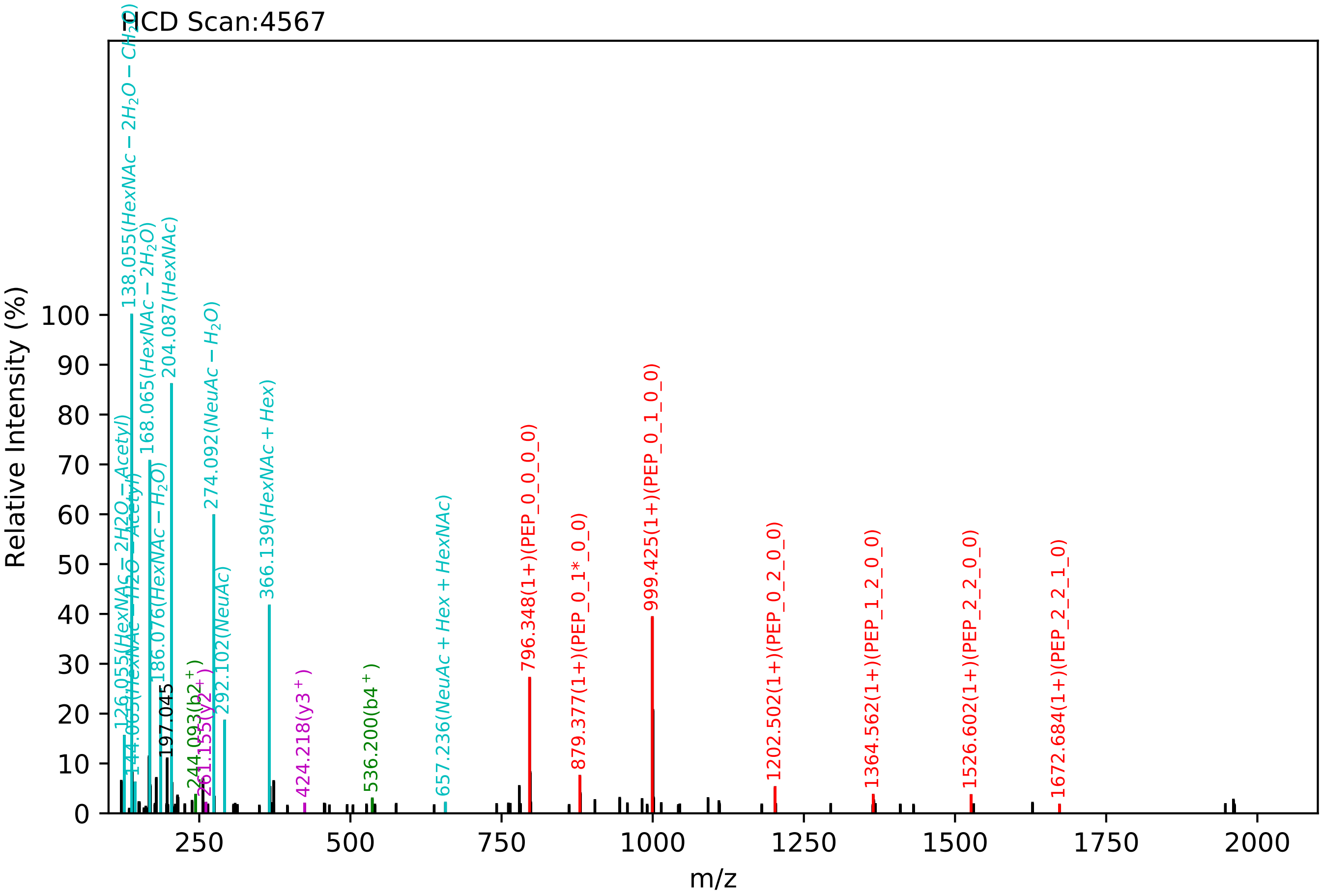

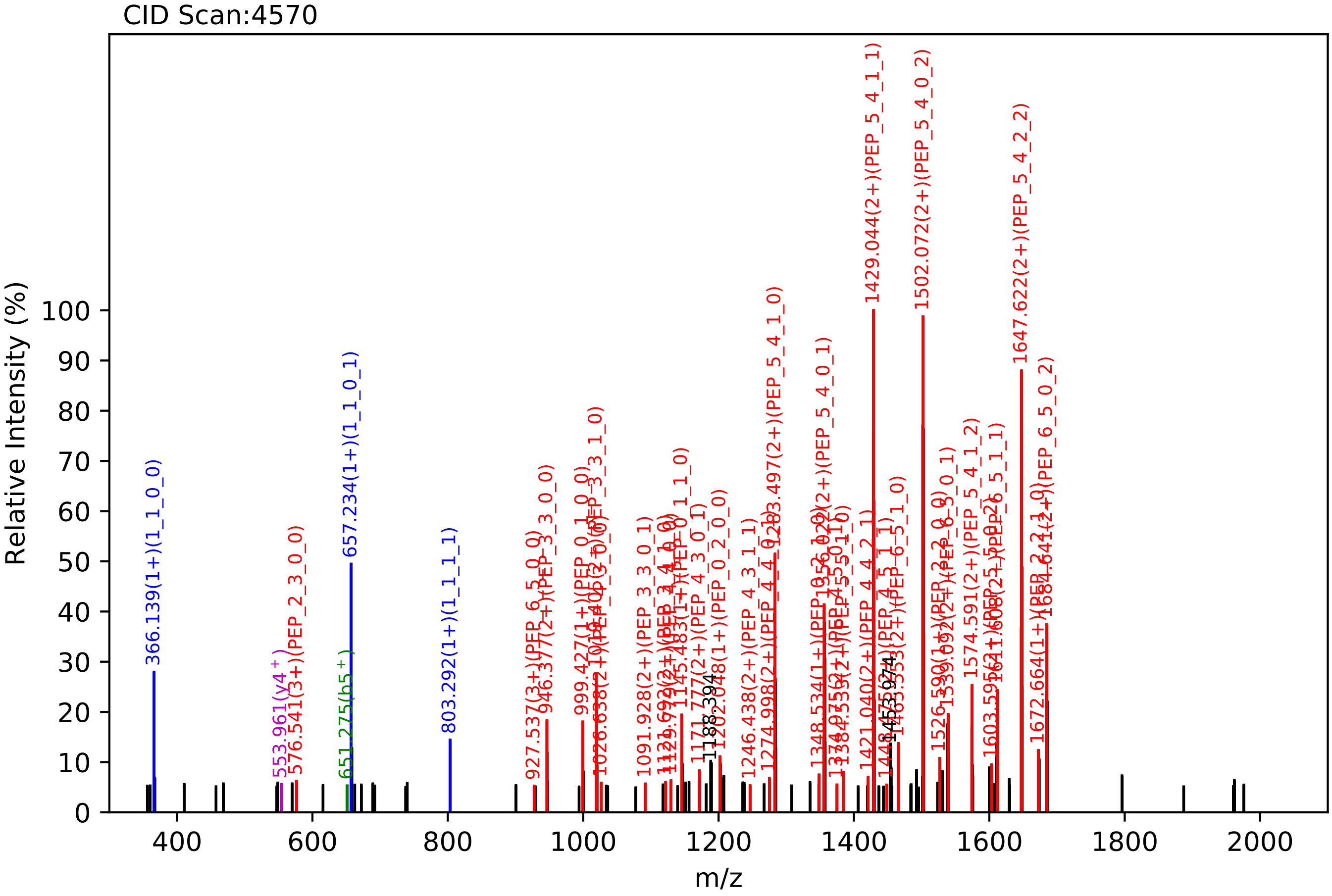

Training set no. 220, Experiment: AGP exp_37

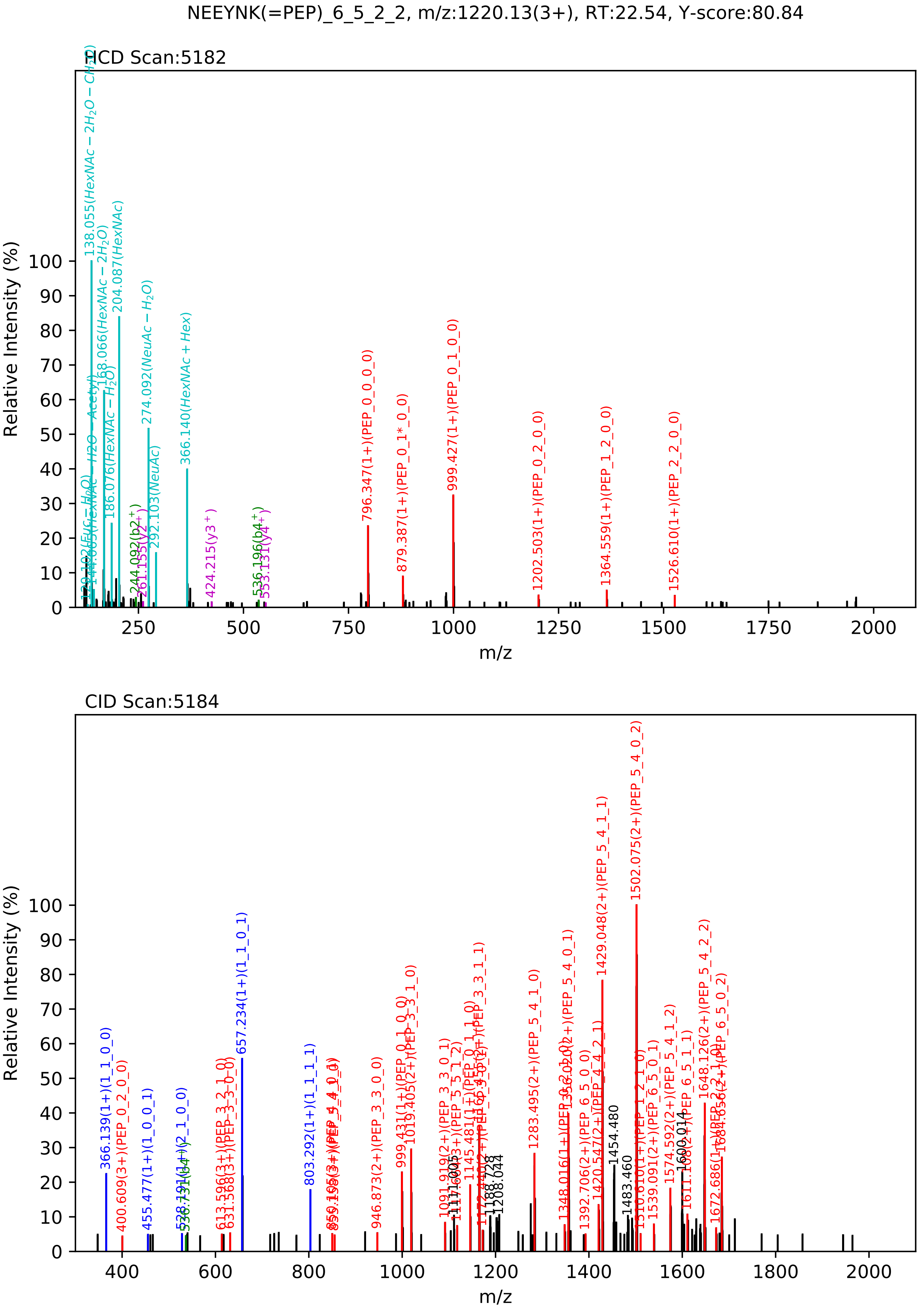

Training set no. 221, Experiment: AGP exp_37

NEEYNK(=PEP)_6_5_2_3, m/z:1317.16(3+), RT:31.40, Y-score:90.91

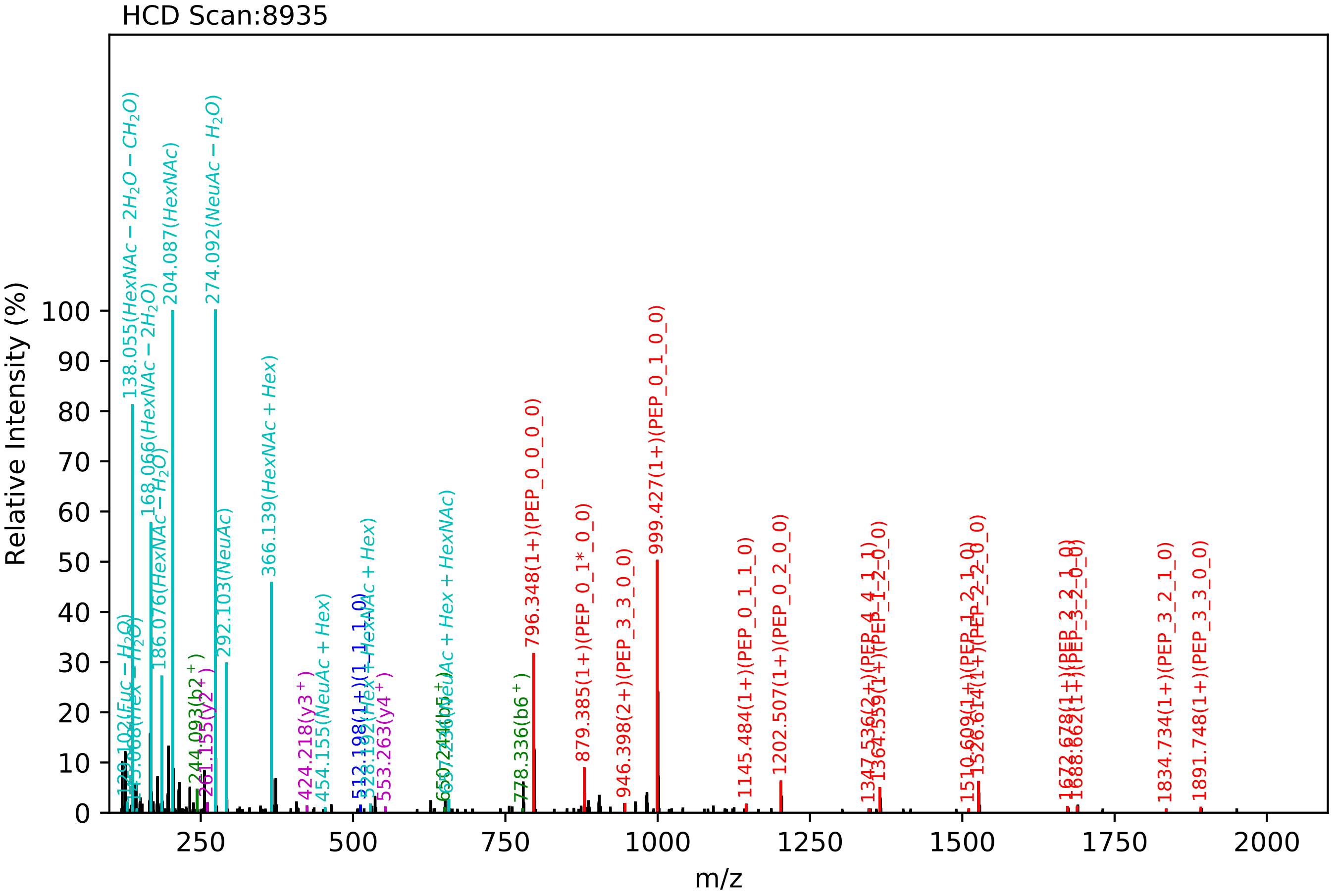

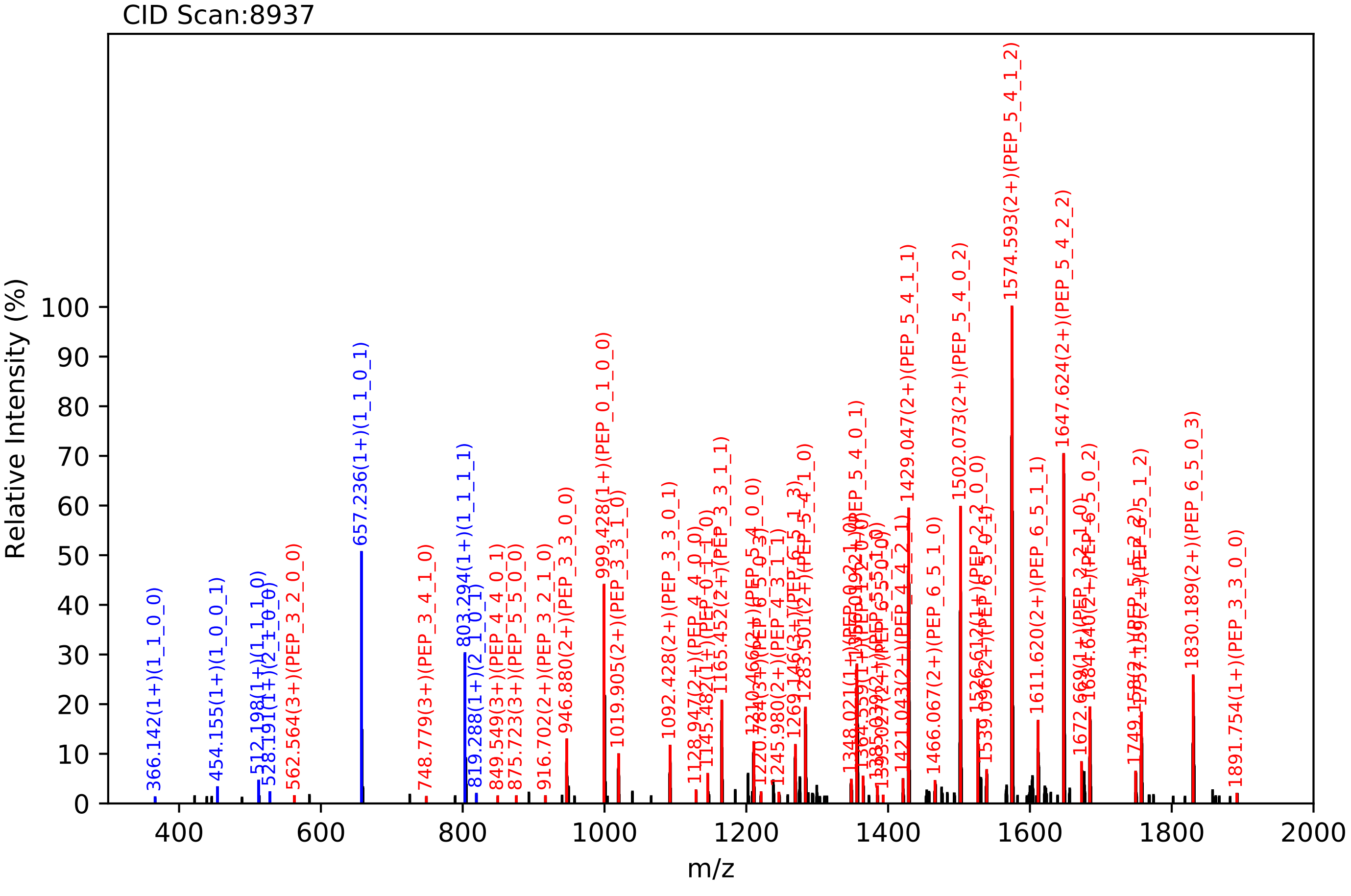

Training set no. 222, Experiment: AGP exp_8

NEEYNK(=PEP)_6_5_2_3, m/z:1317.16(3+), RT:30.43, Y-score:90.60

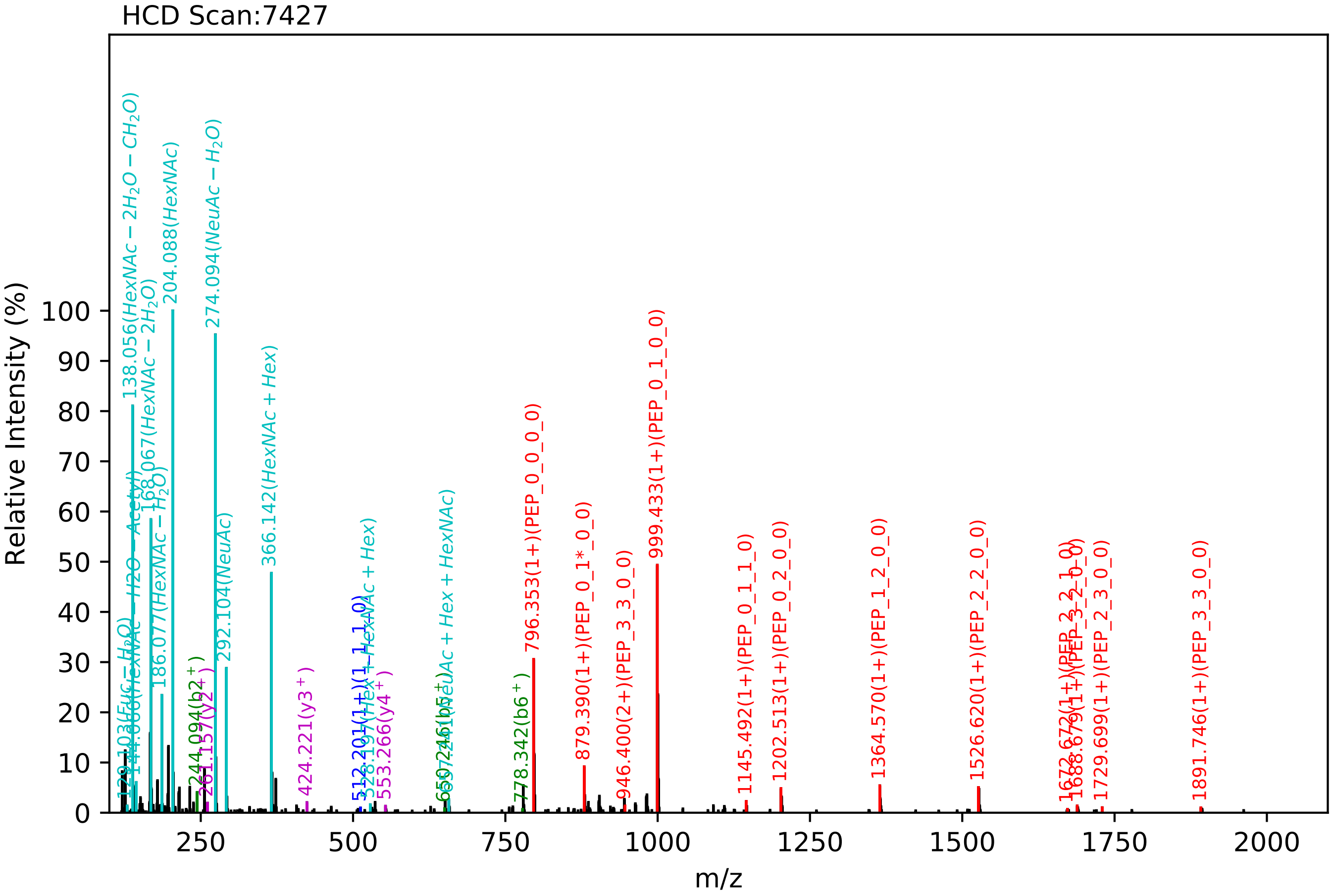

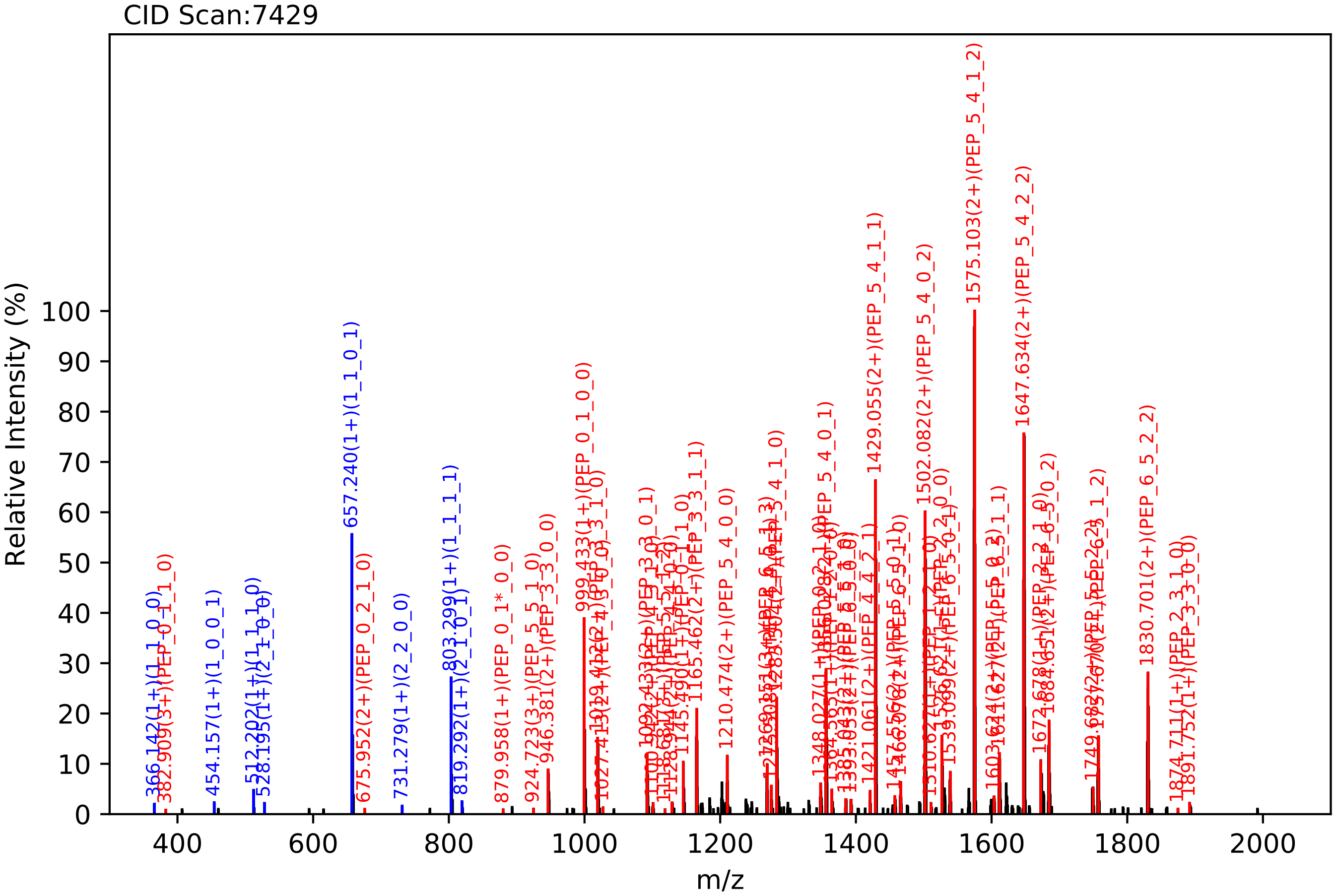

Training set no. 223, Experiment: AGP exp_44

NEEYNK(=PEP)_6_5_2_3, m/z:1317.16(3+), RT:32.91, Y-score:90.53

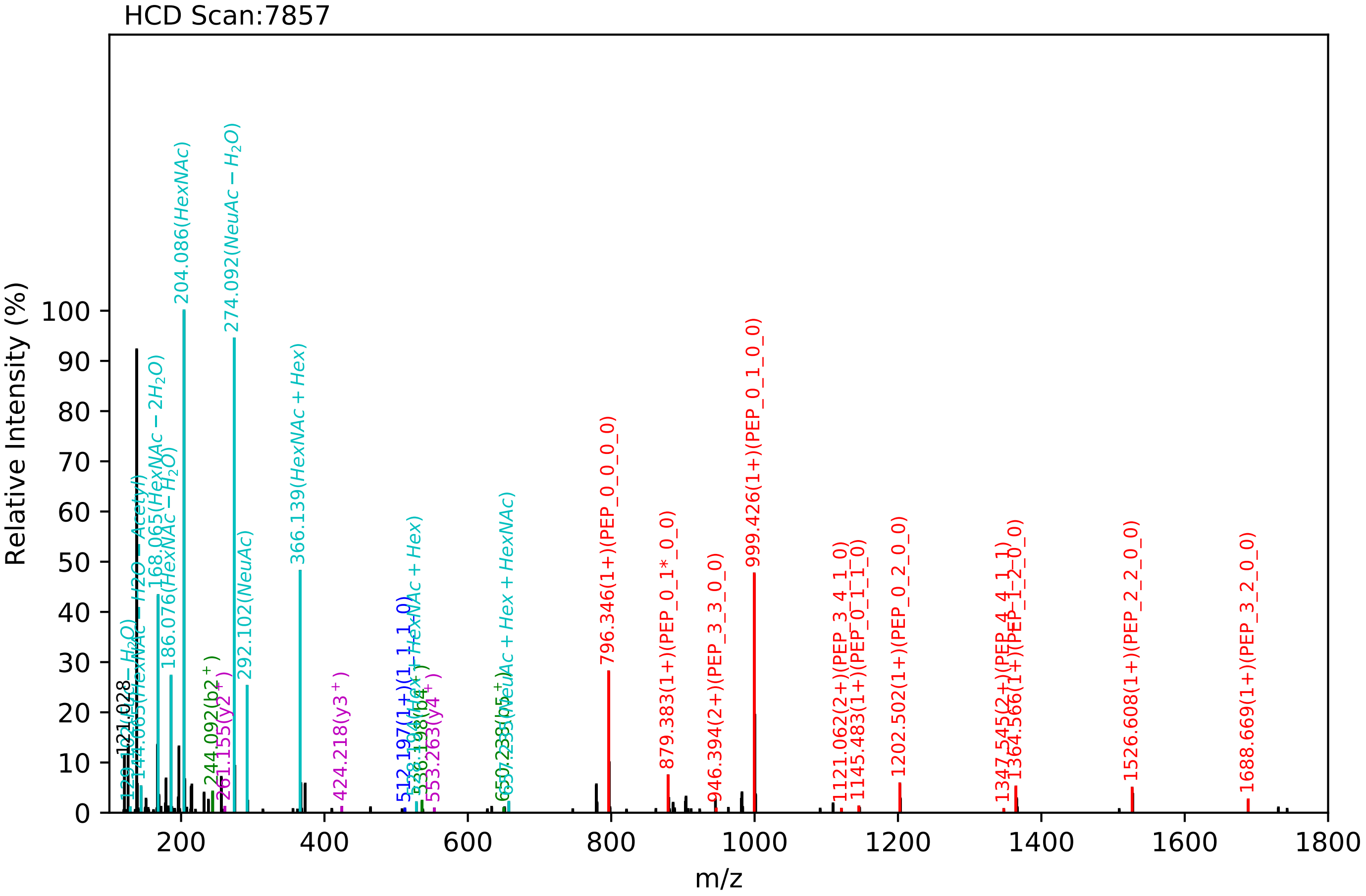

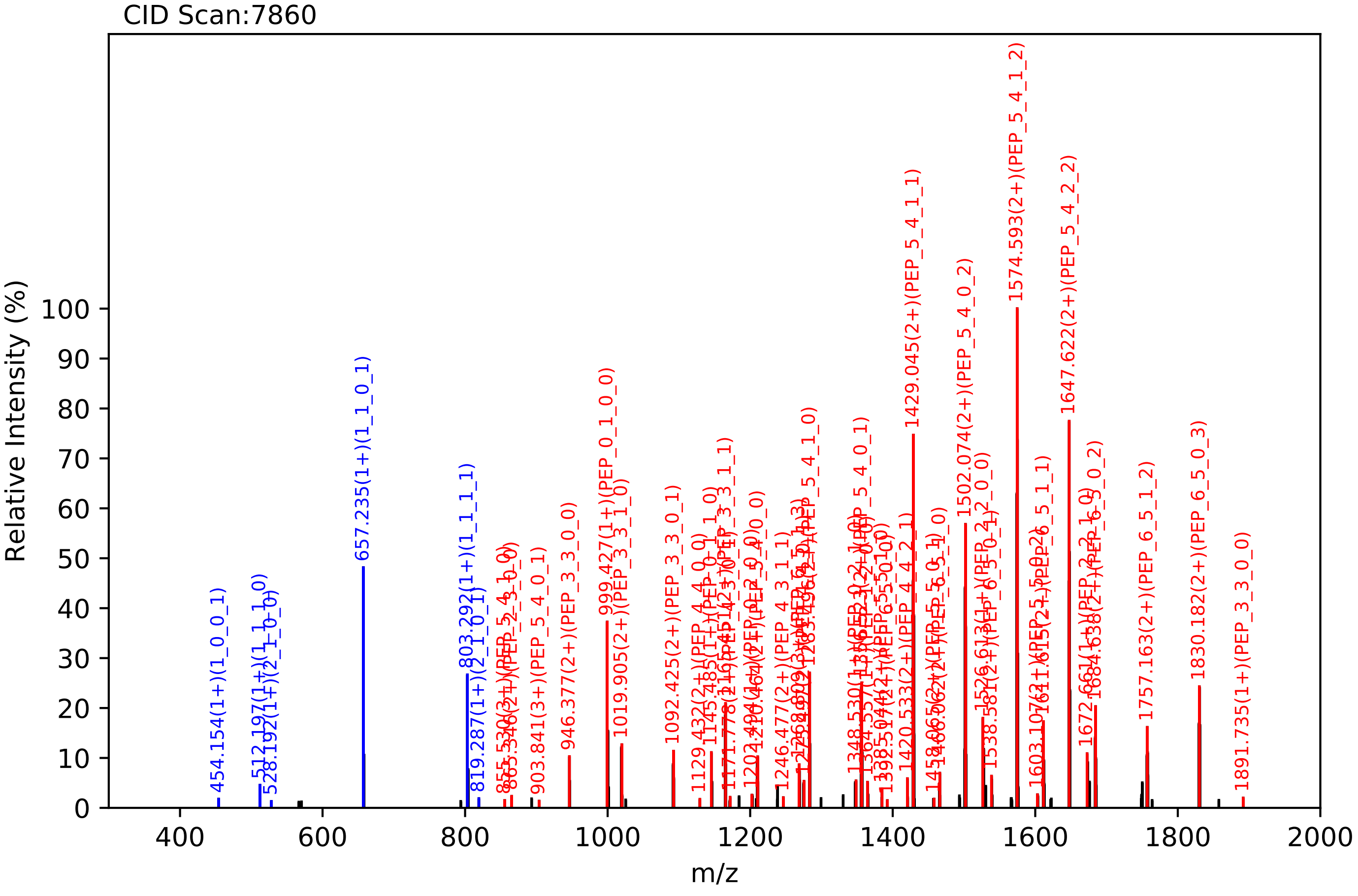

Training set no. 224, Experiment: AGP exp_7

NEEYNK(=PEP)_6_5_2_3, m/z:1317.16(3+), RT:29.52, Y-score:90.20

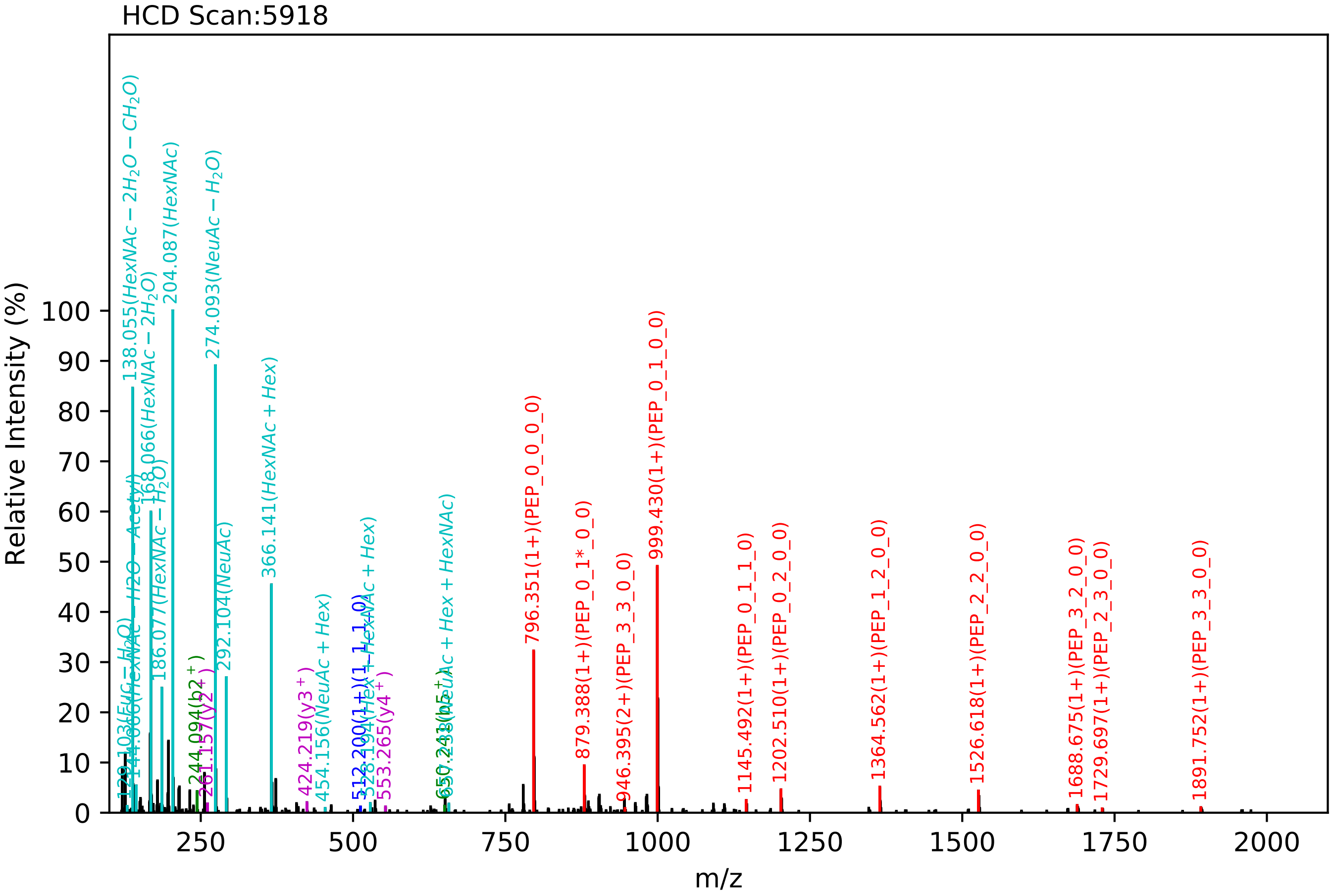

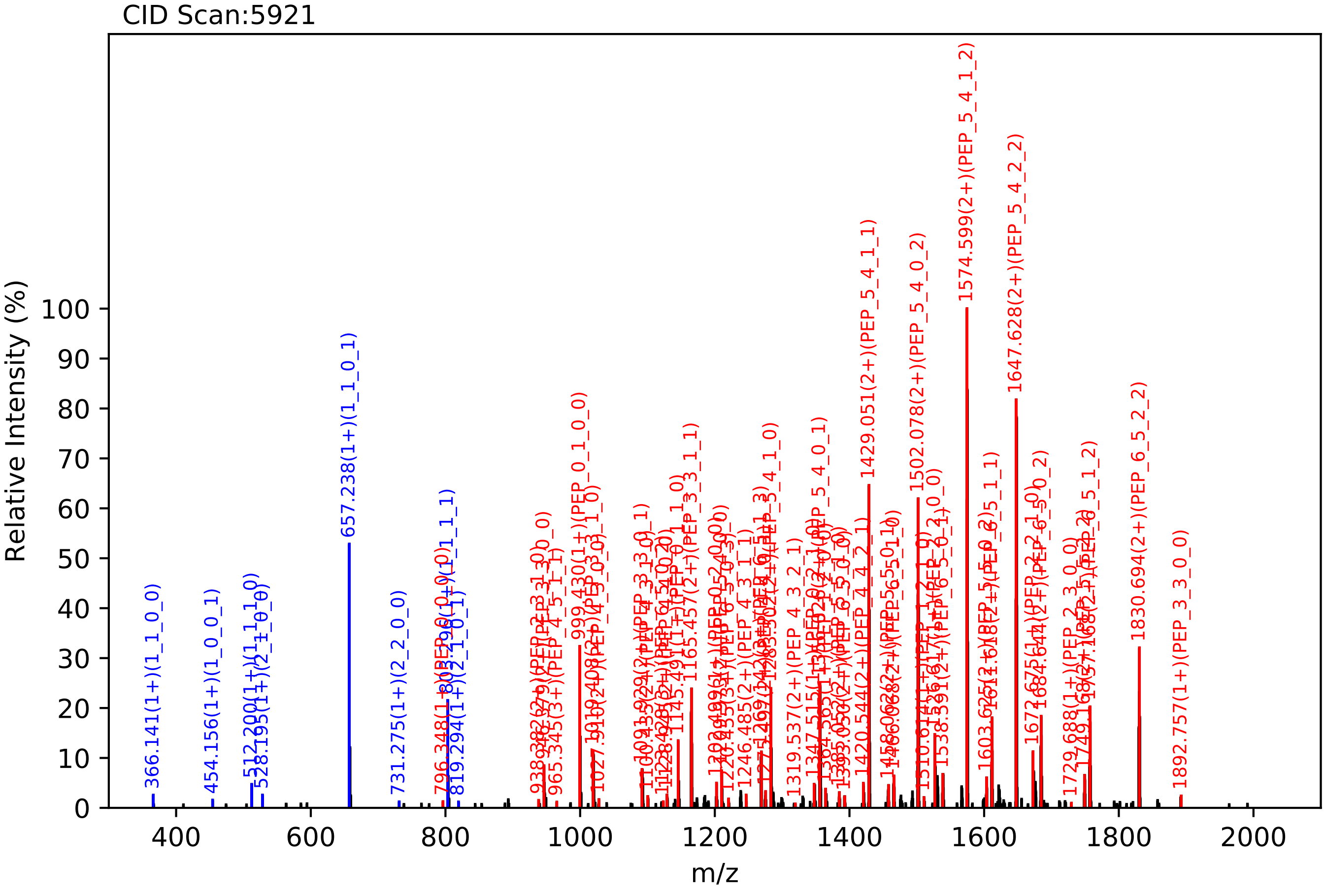

Training set no. 225, Experiment: AGP exp_20

NEEYNK(=PEP)_6_5_2_3, m/z:1317.16(3+), RT:30.45, Y-score:90.09

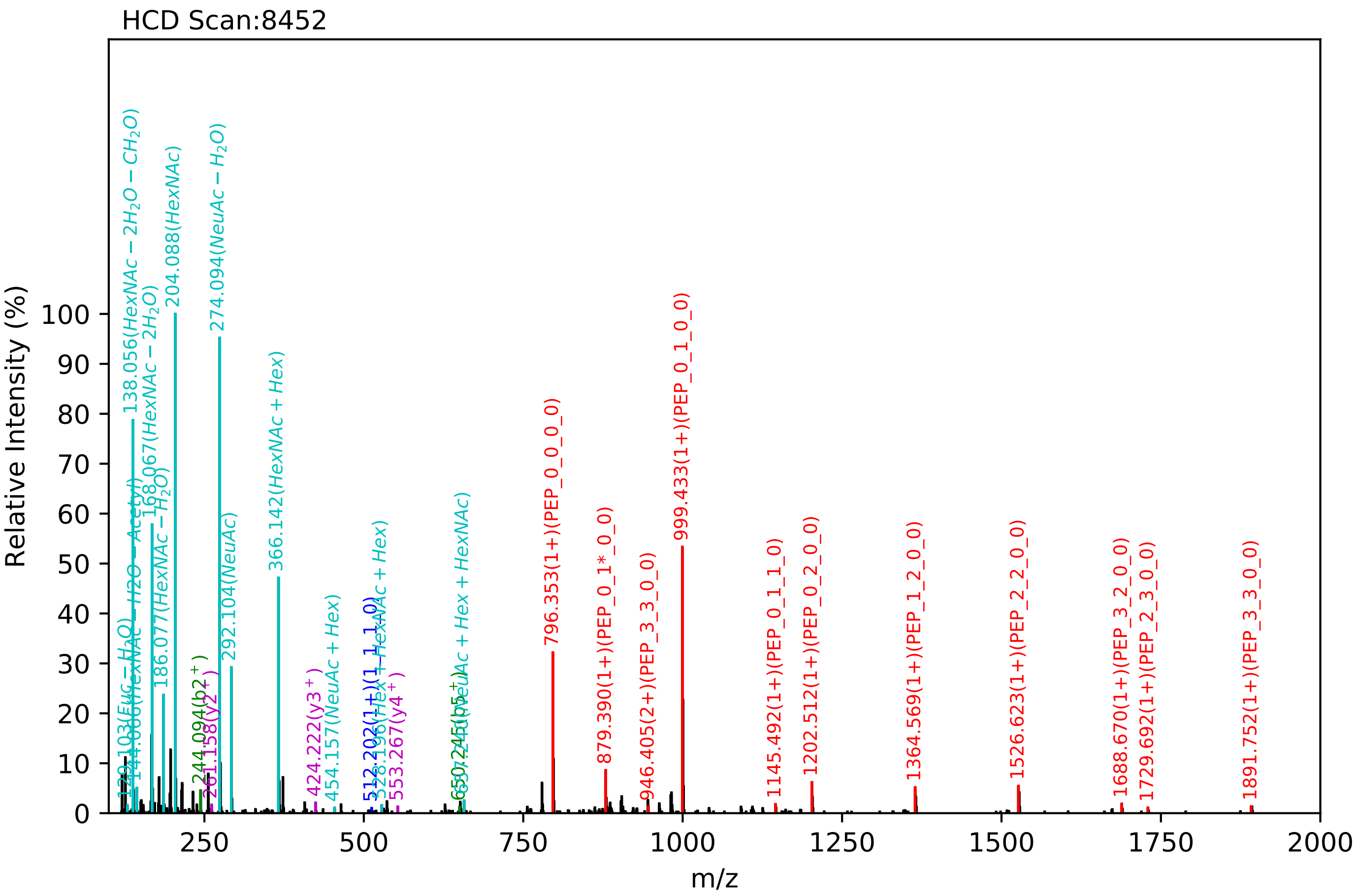

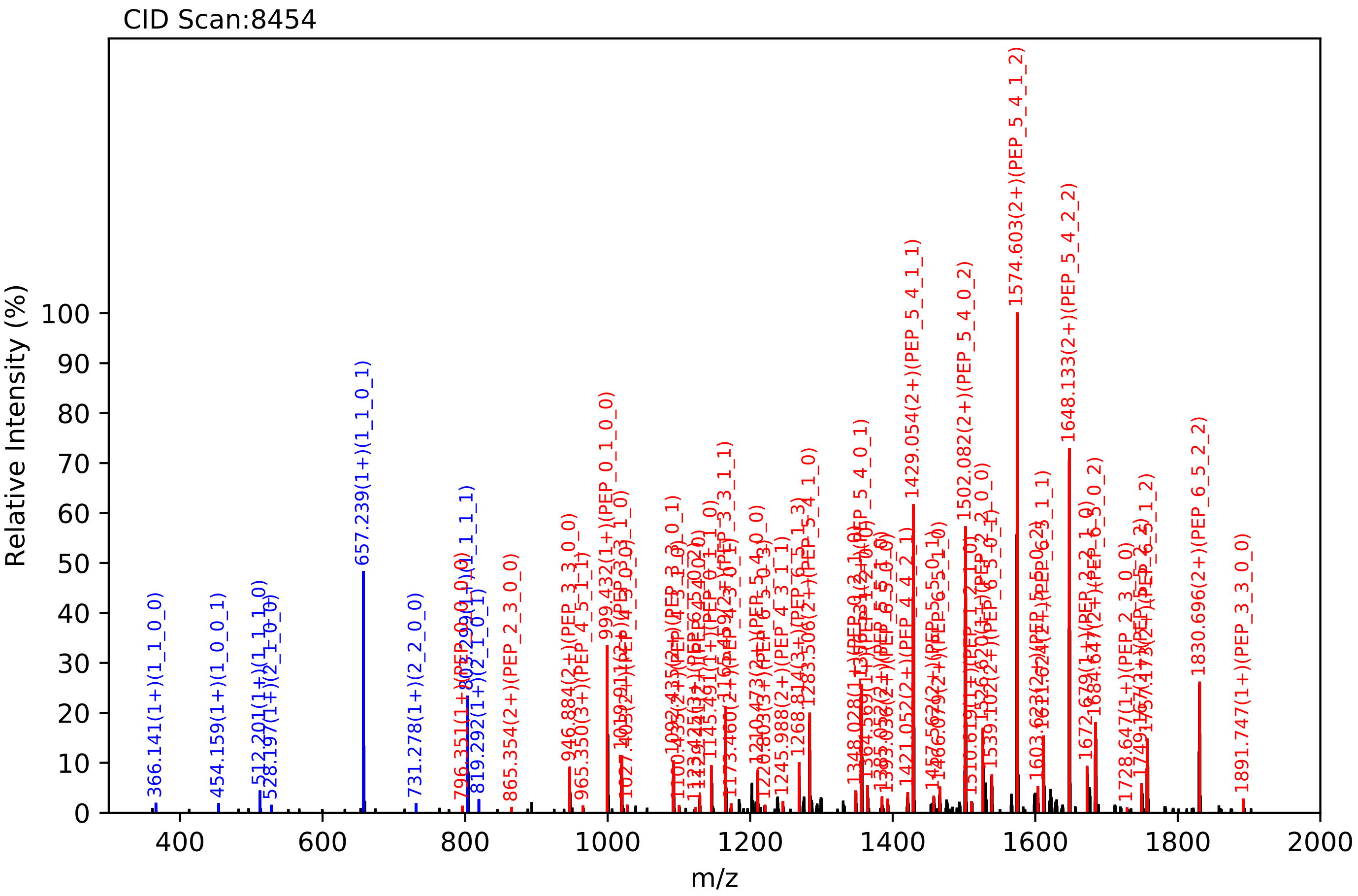

Training set no. 226, Experiment: AGP exp_1

NEEYNK(=PEP)_6_5_2_3, m/z:1317.16(3+), RT:22.03, Y-score:89.62

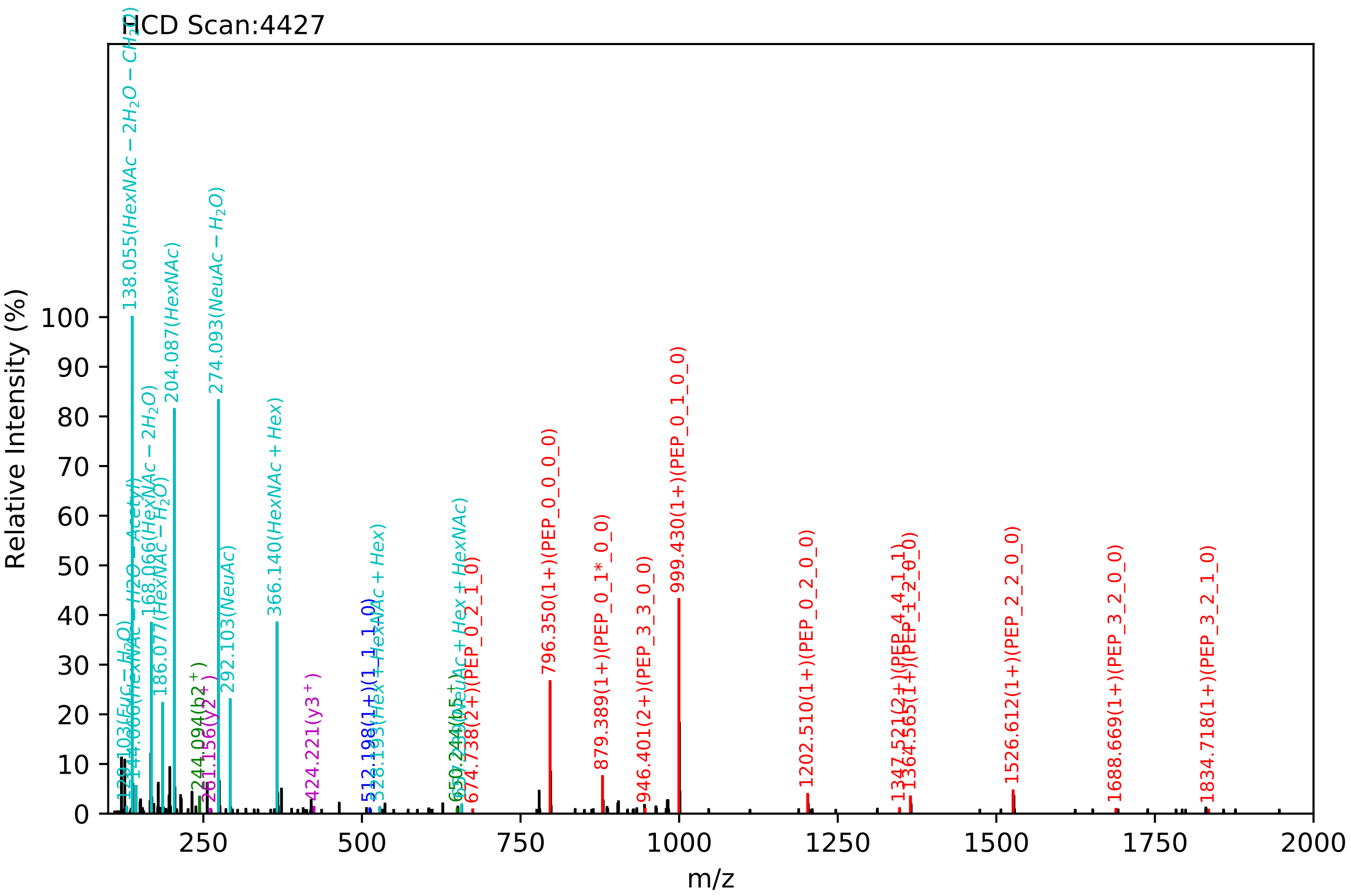

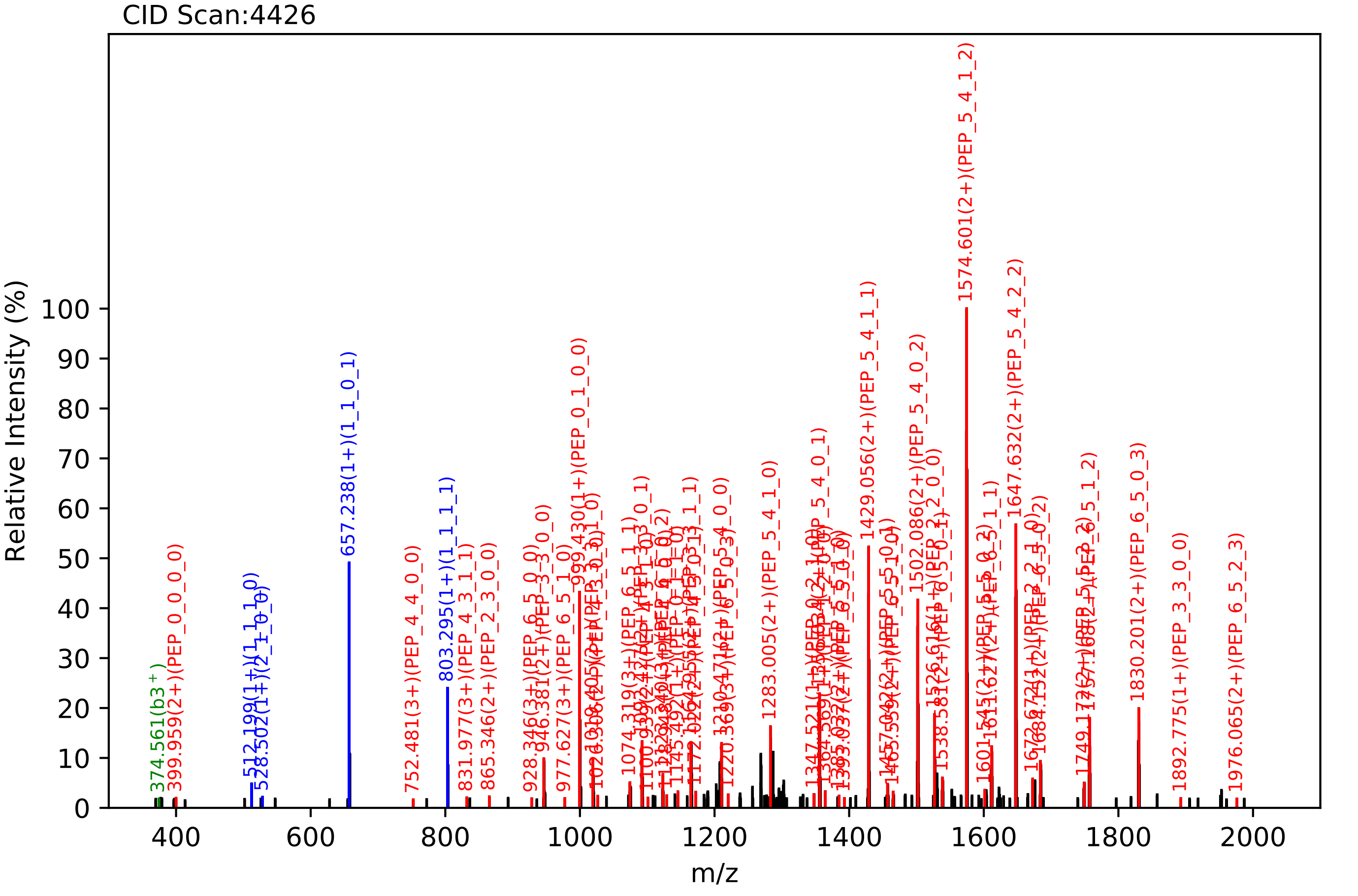

Training set no. 227, Experiment: AGP exp_34

NEEYNK(=PEP)_6_5_2_3, m/z:1317.16(3+), RT:31.93, Y-score:89.33

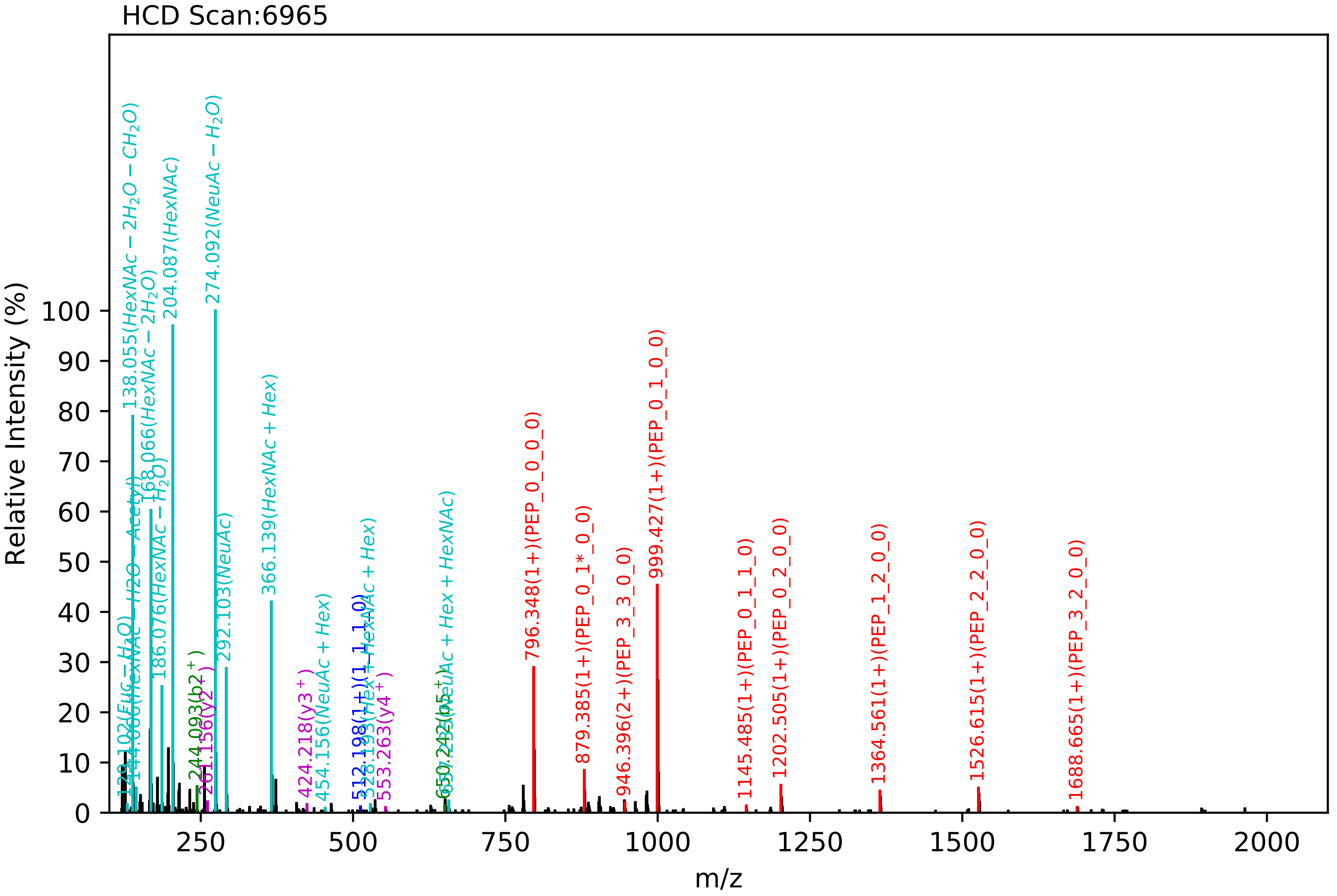

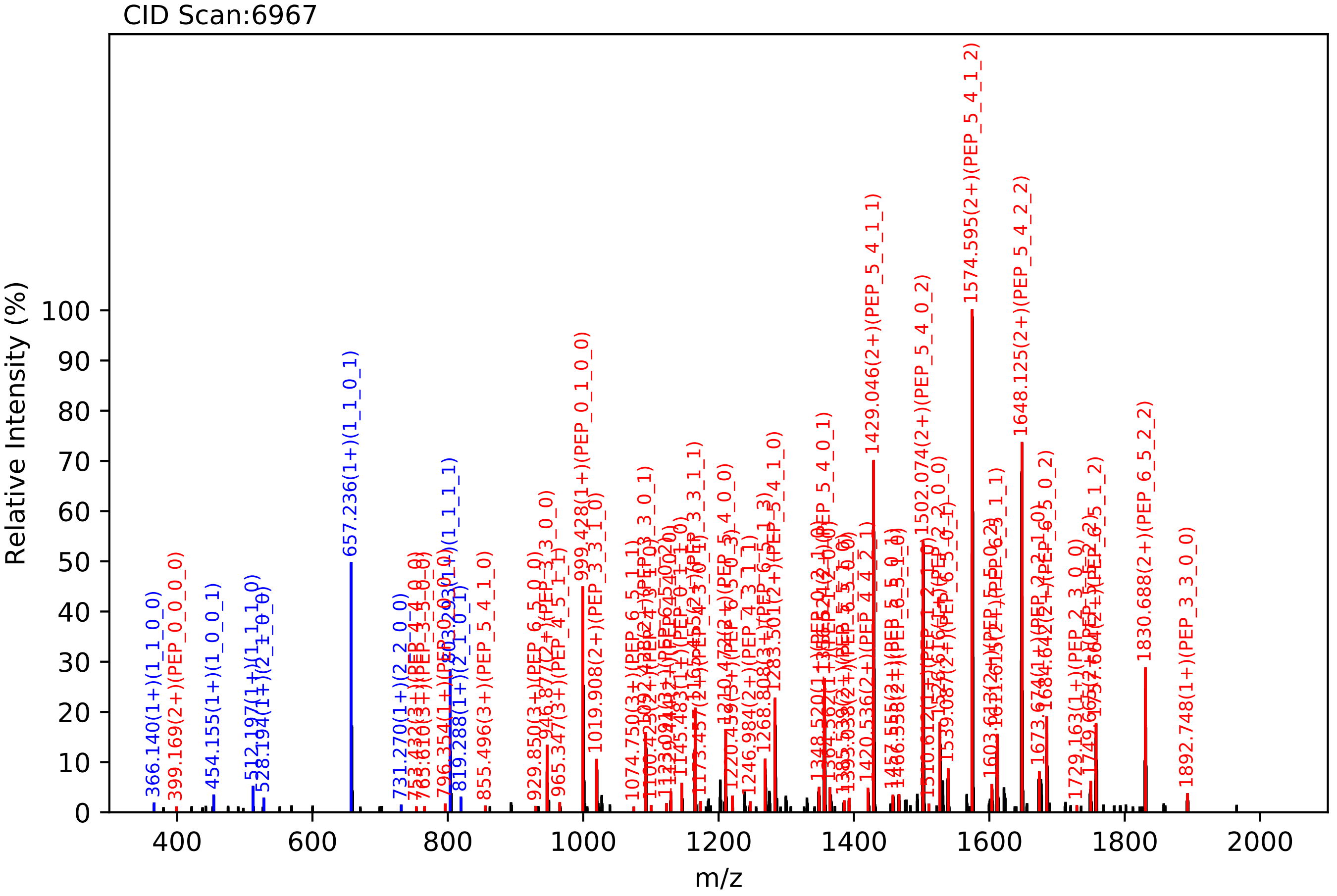

Training set no. 228, Experiment: AGP exp_36

NEEYNK(=PEP)_6_5_2_3, m/z:1317.16(3+), RT:31.10, Y-score:89.11

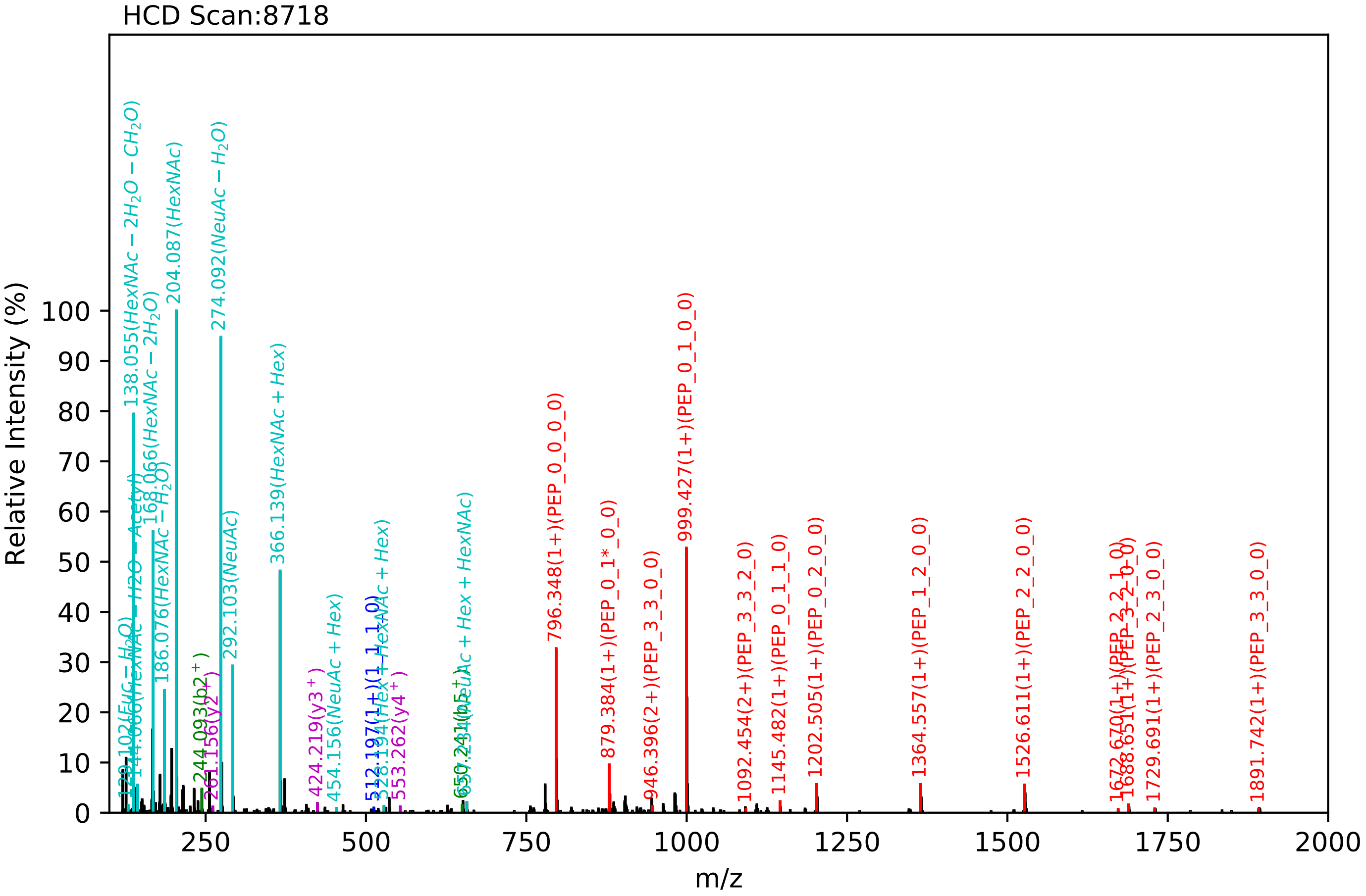

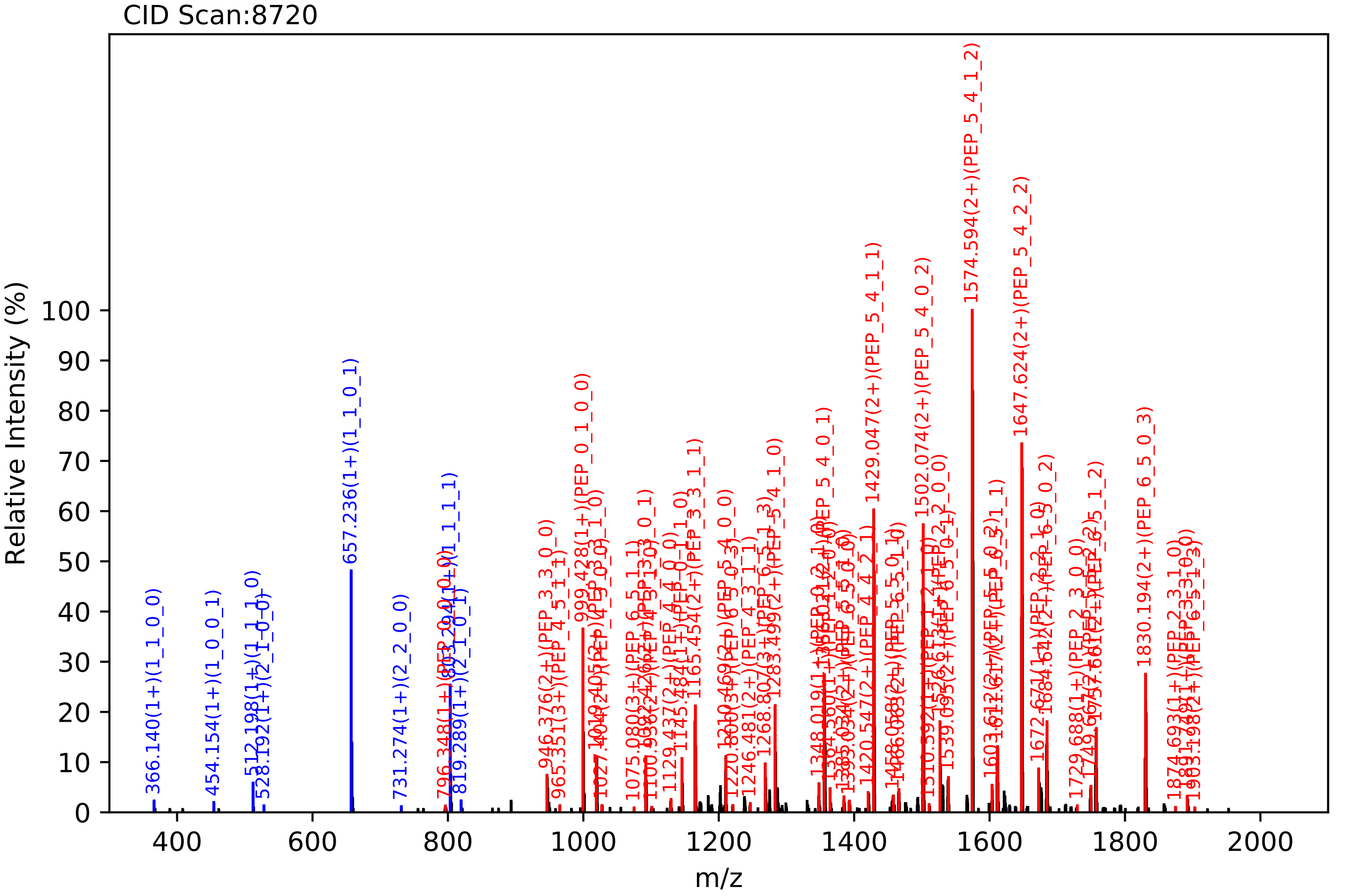

Training set no. 229, Experiment: AGP exp_33

NEEYNK(=PEP)_6_5_2_3, m/z:1317.16(3+), RT:31.97, Y-score:89.04

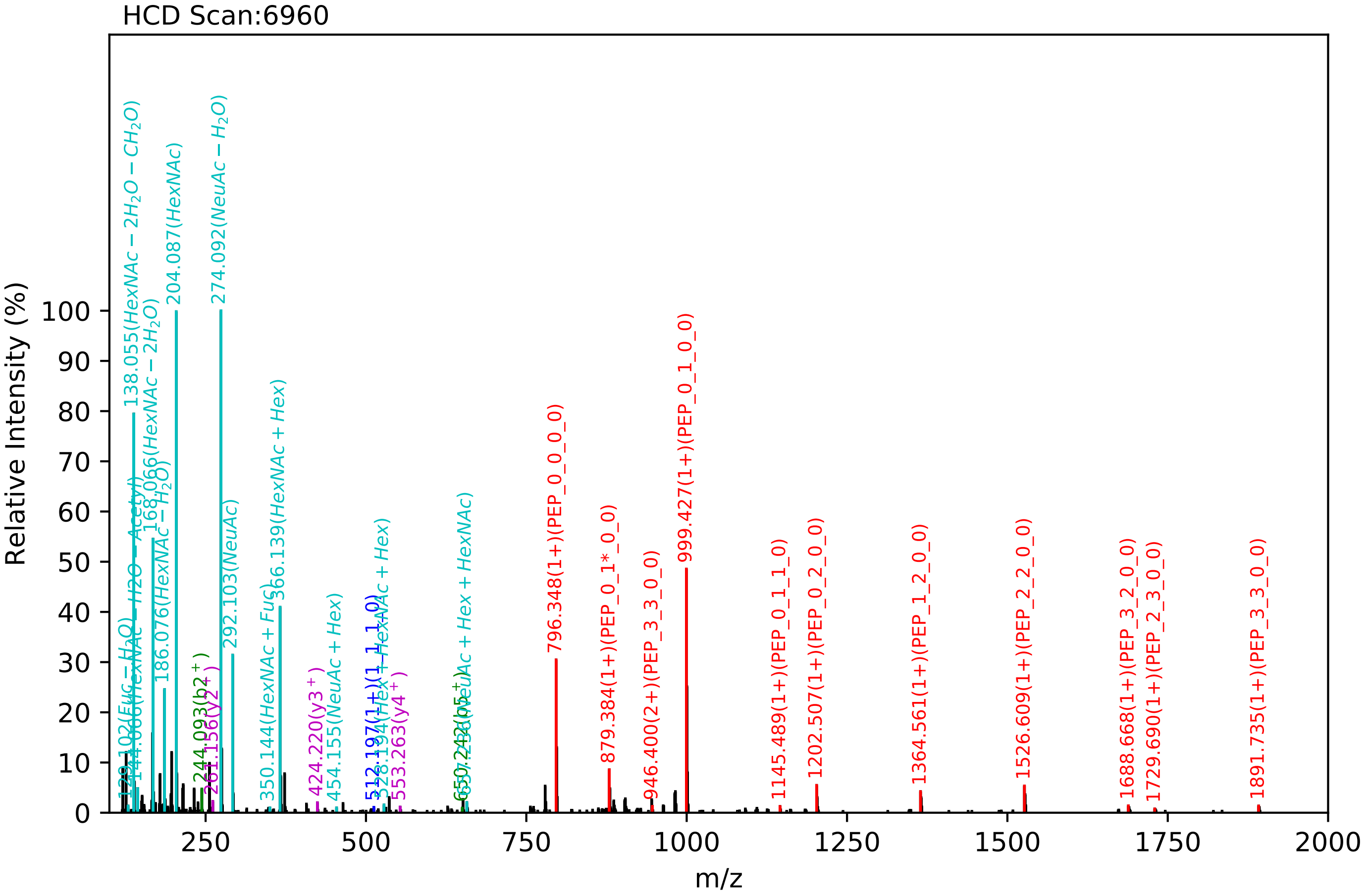

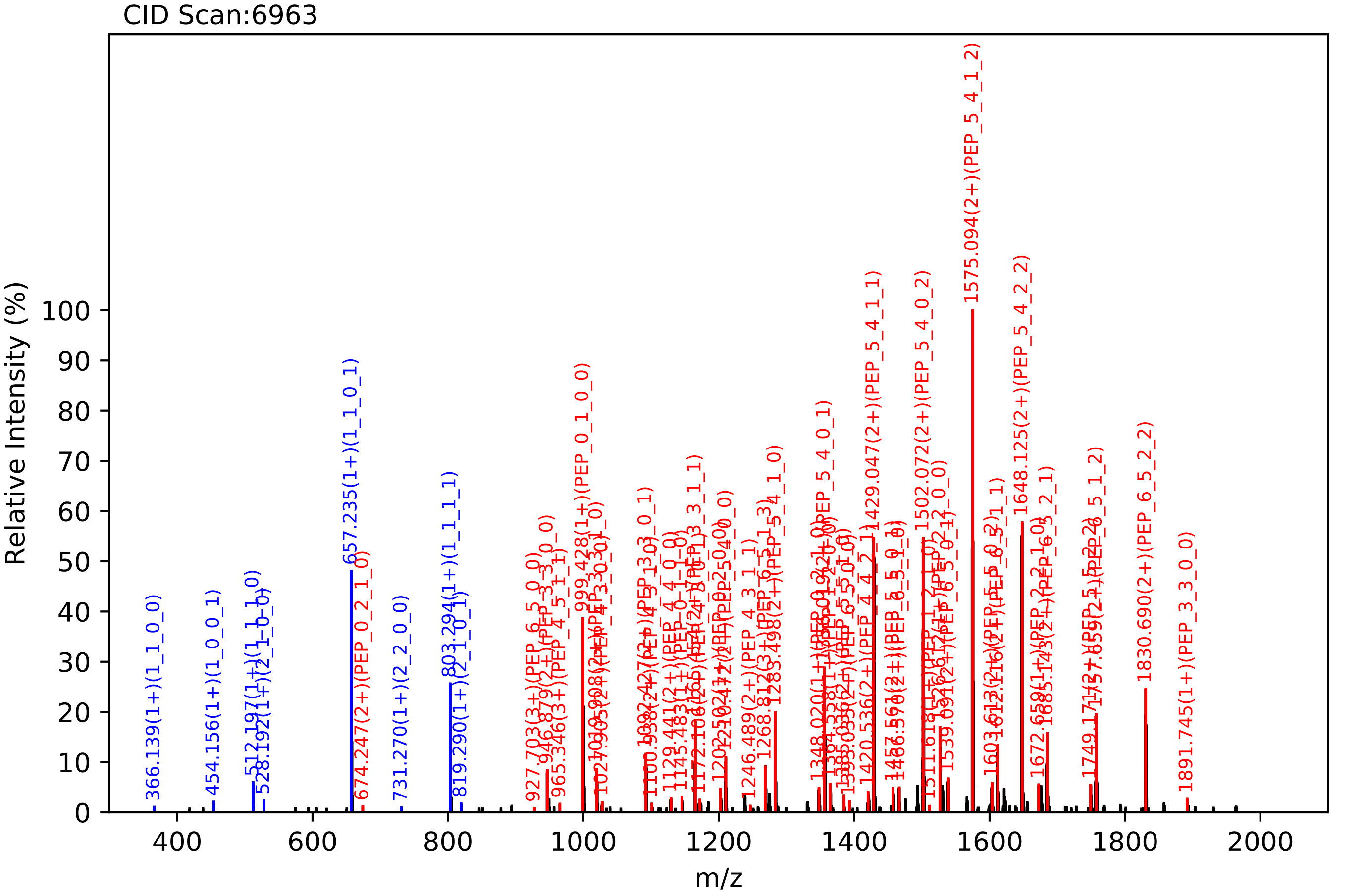

Training set no. 230, Experiment: AGP exp_17

NEEYNK(=PEP)_6_5_2_3, m/z:1317.16(3+), RT:30.62, Y-score:88.98

HCD Scan:8291

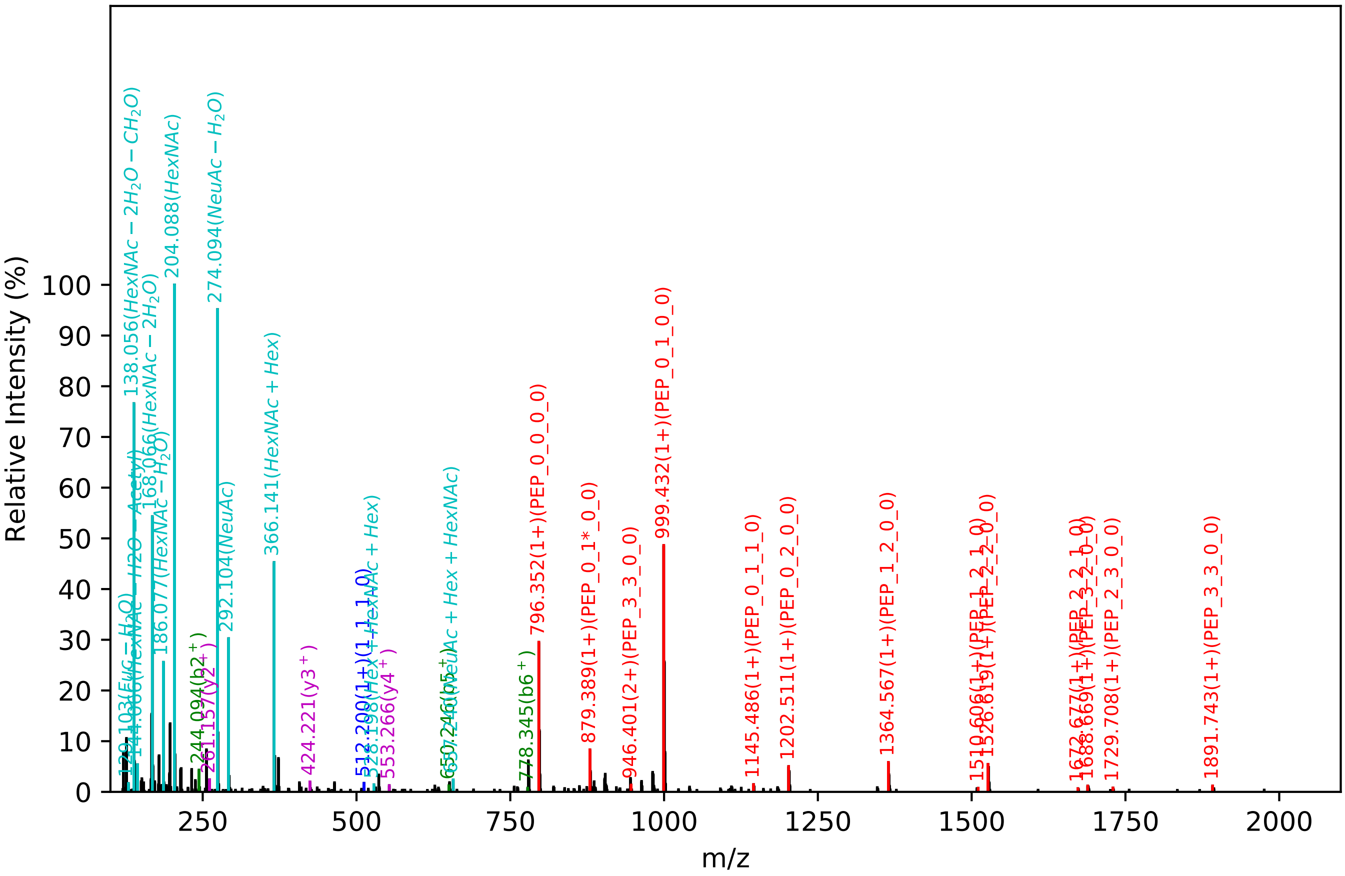

CID Scan:8293

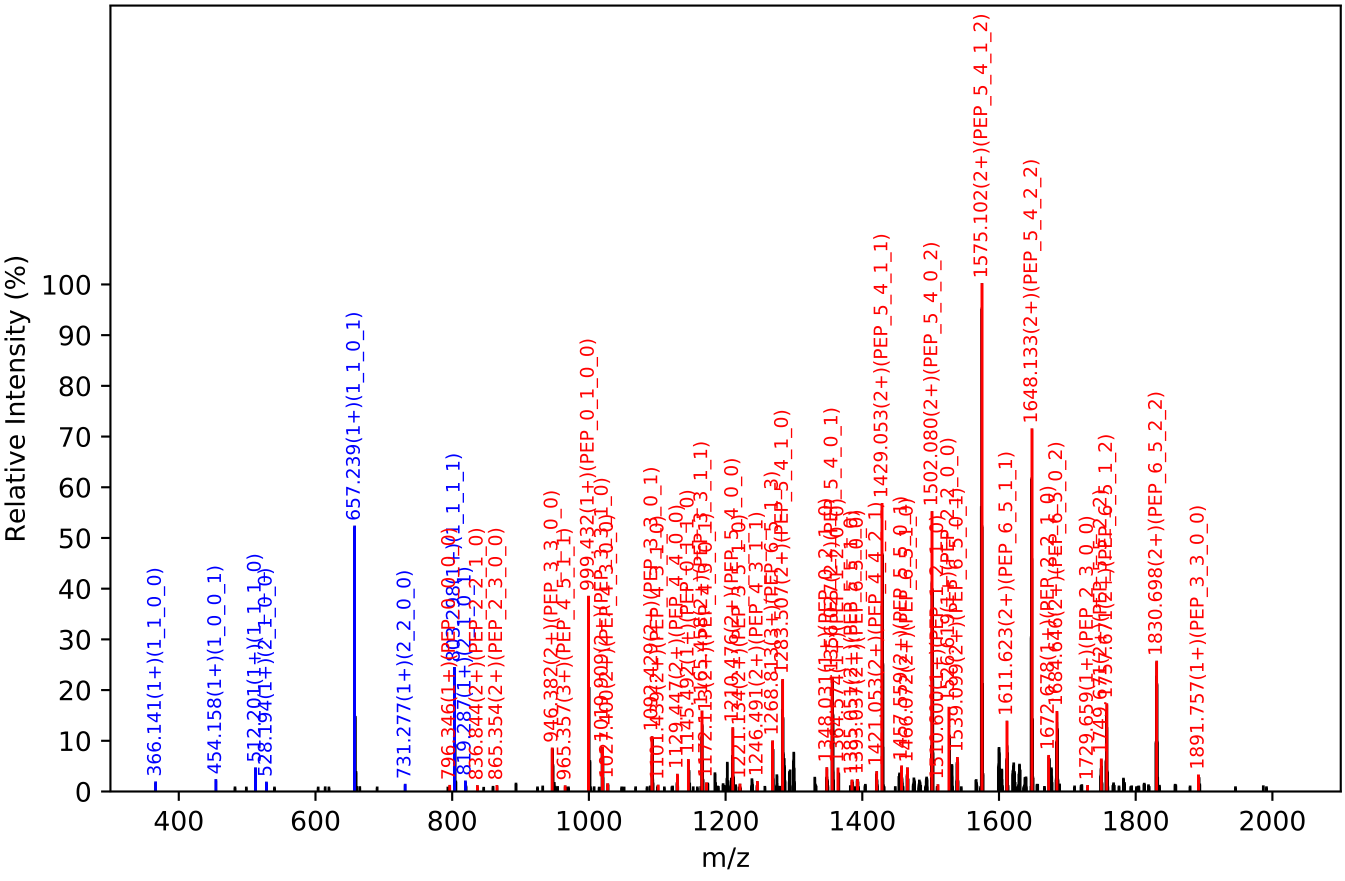

Training set no. 231, Experiment: AGP exp_9

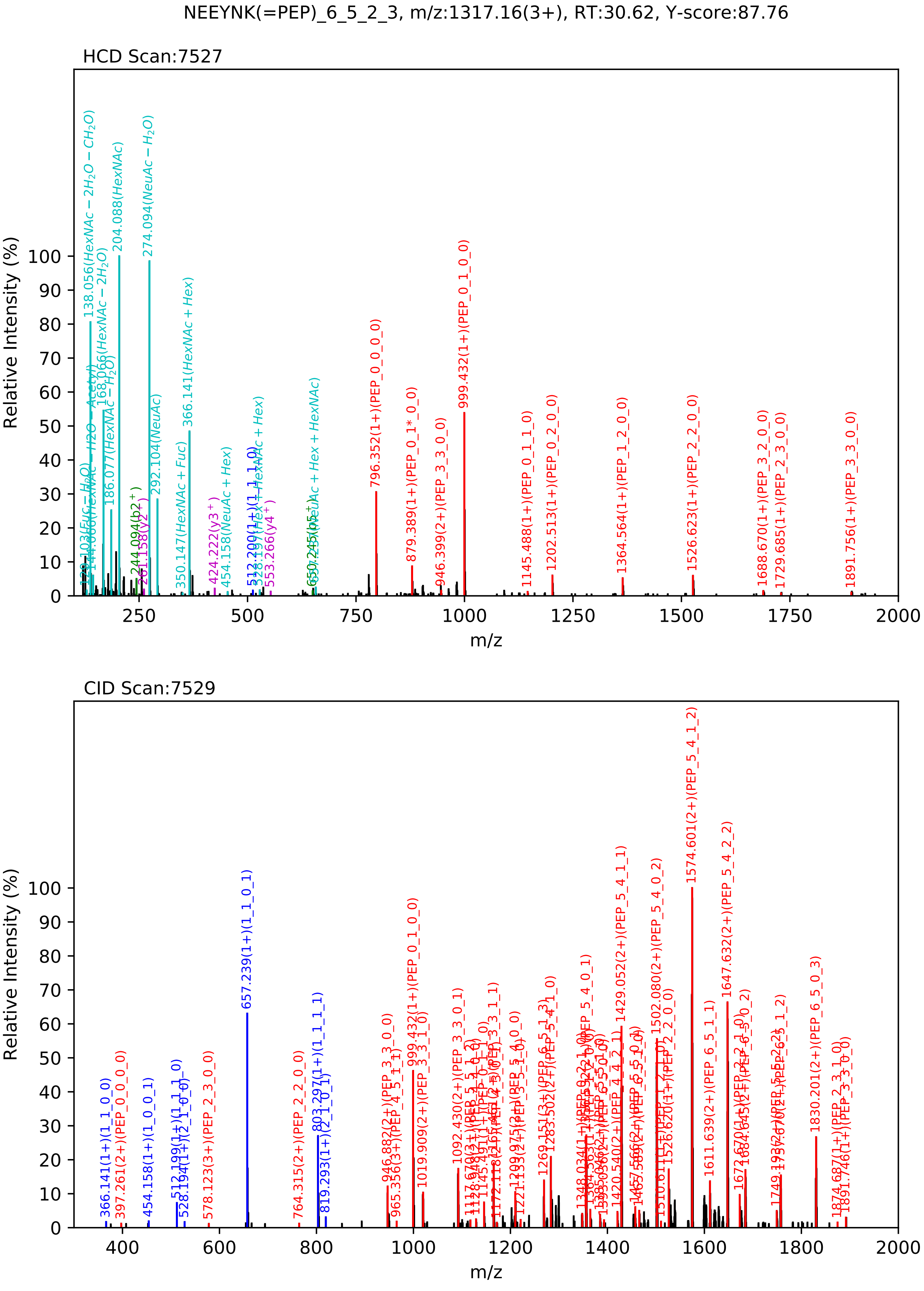

Training set no. 232, Experiment: AGP exp_42

NEEYNK(=PEP)_6_5_2_3, m/z:1317.16(3+), RT:31.55, Y-score:87.62

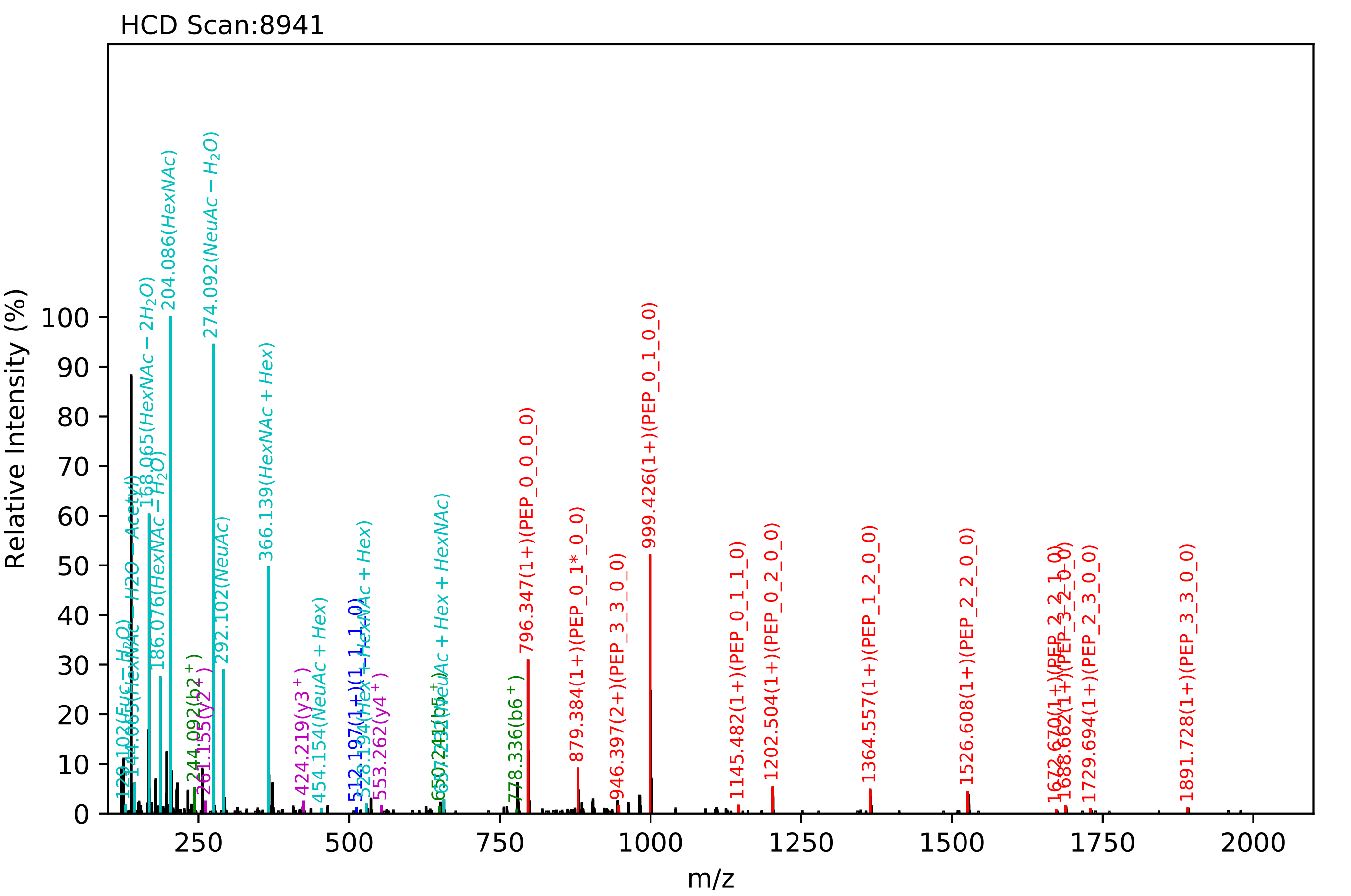

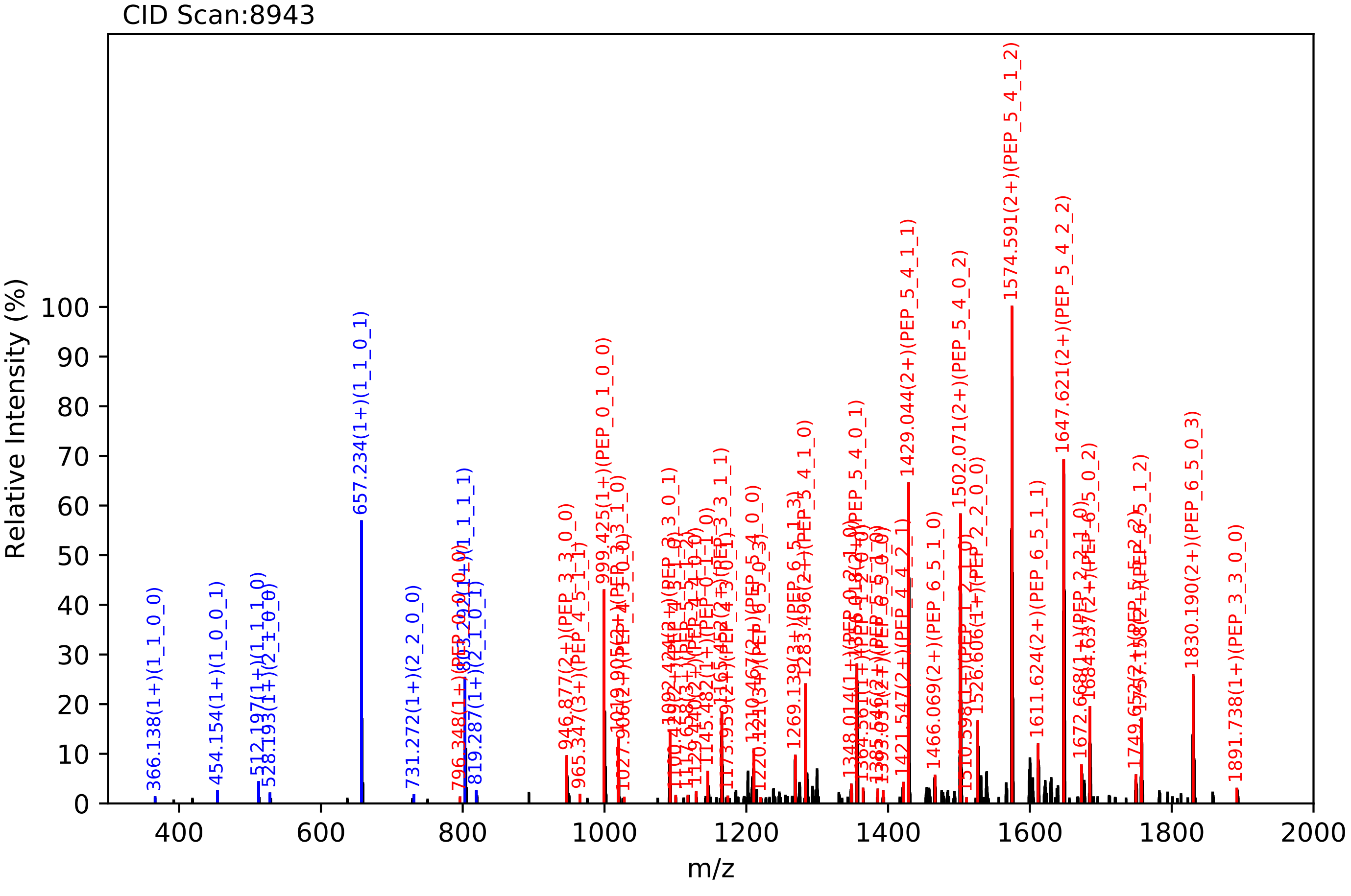

Training set no. 233, Experiment: AGP exp_28

NEEYNK(=PEP)_6_5_2_3, m/z:1317.16(3+), RT:31.66, Y-score:86.70

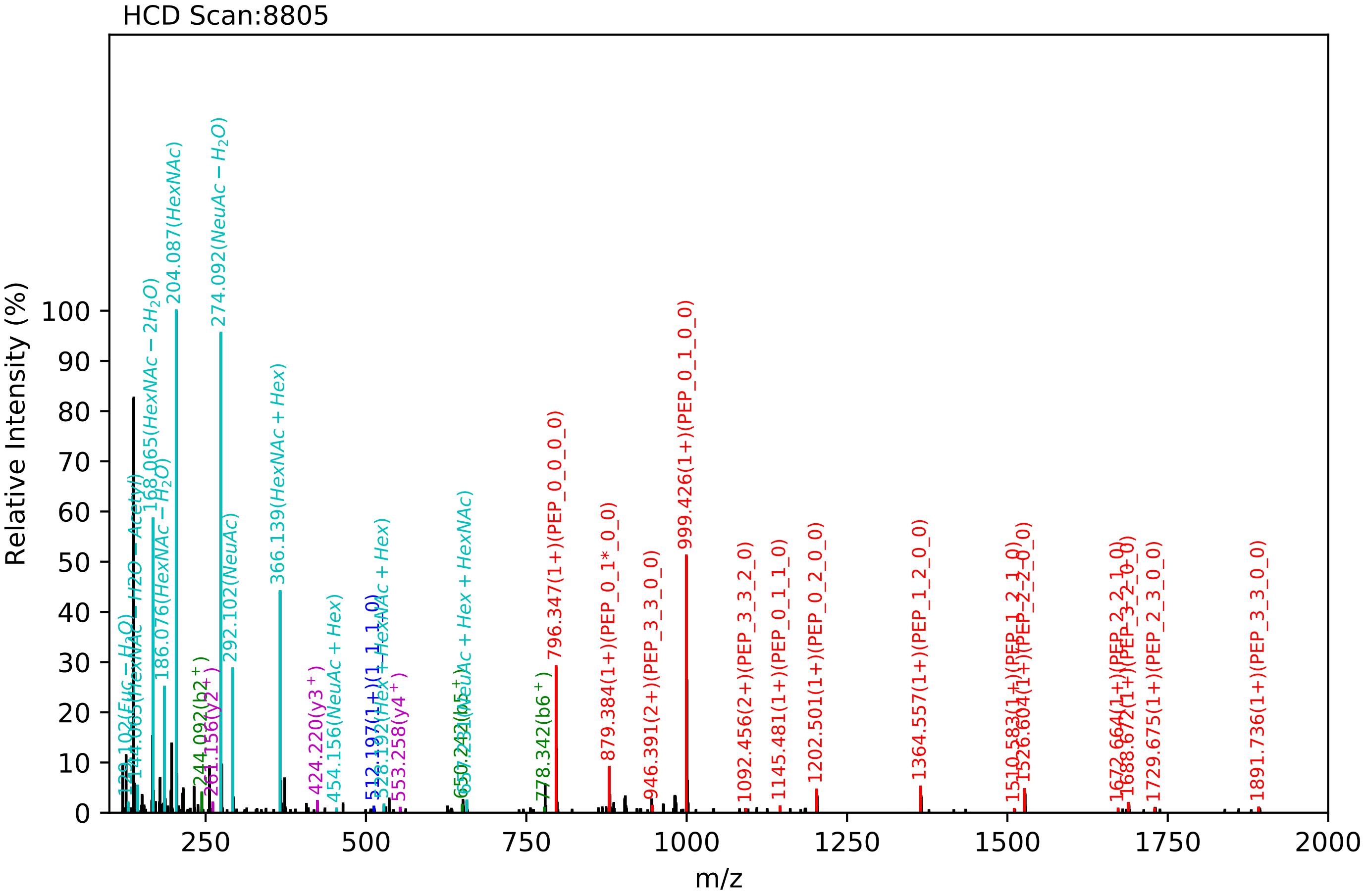

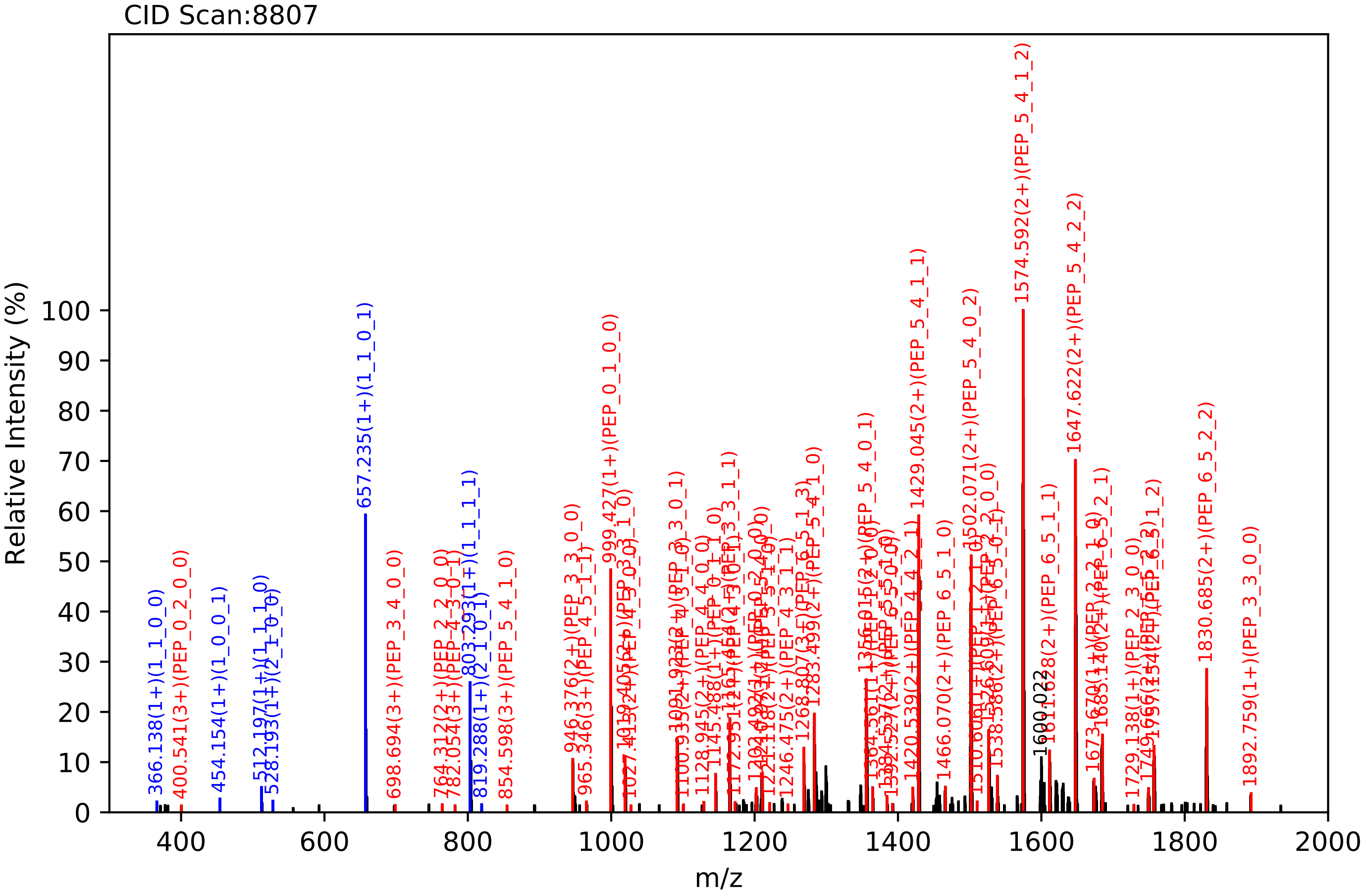

Training set no. 234, Experiment: AGP exp_2

NEEYNK(=PEP)_6_5_2_3, m/z:1317.16(3+), RT:22.07, Y-score:86.24

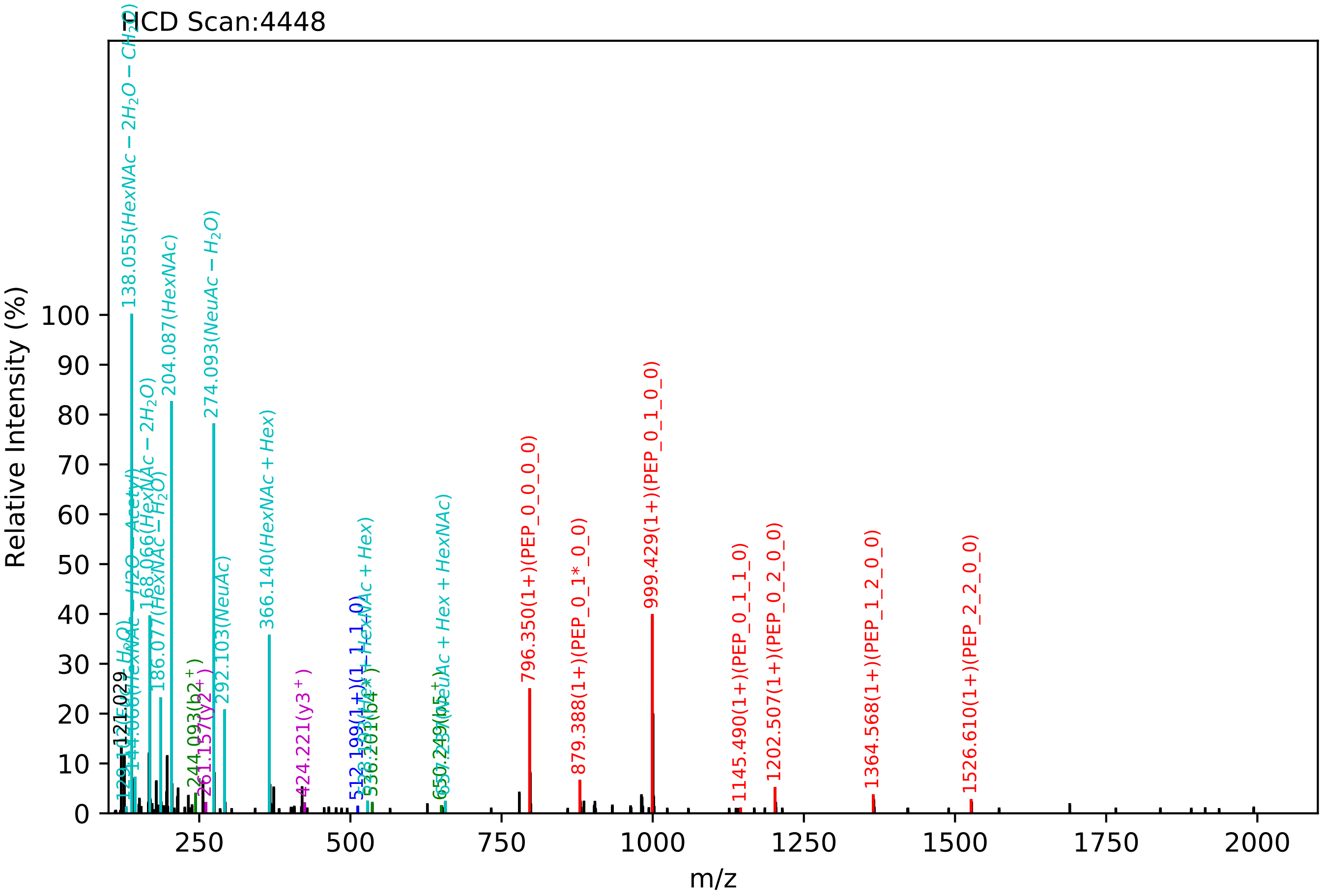

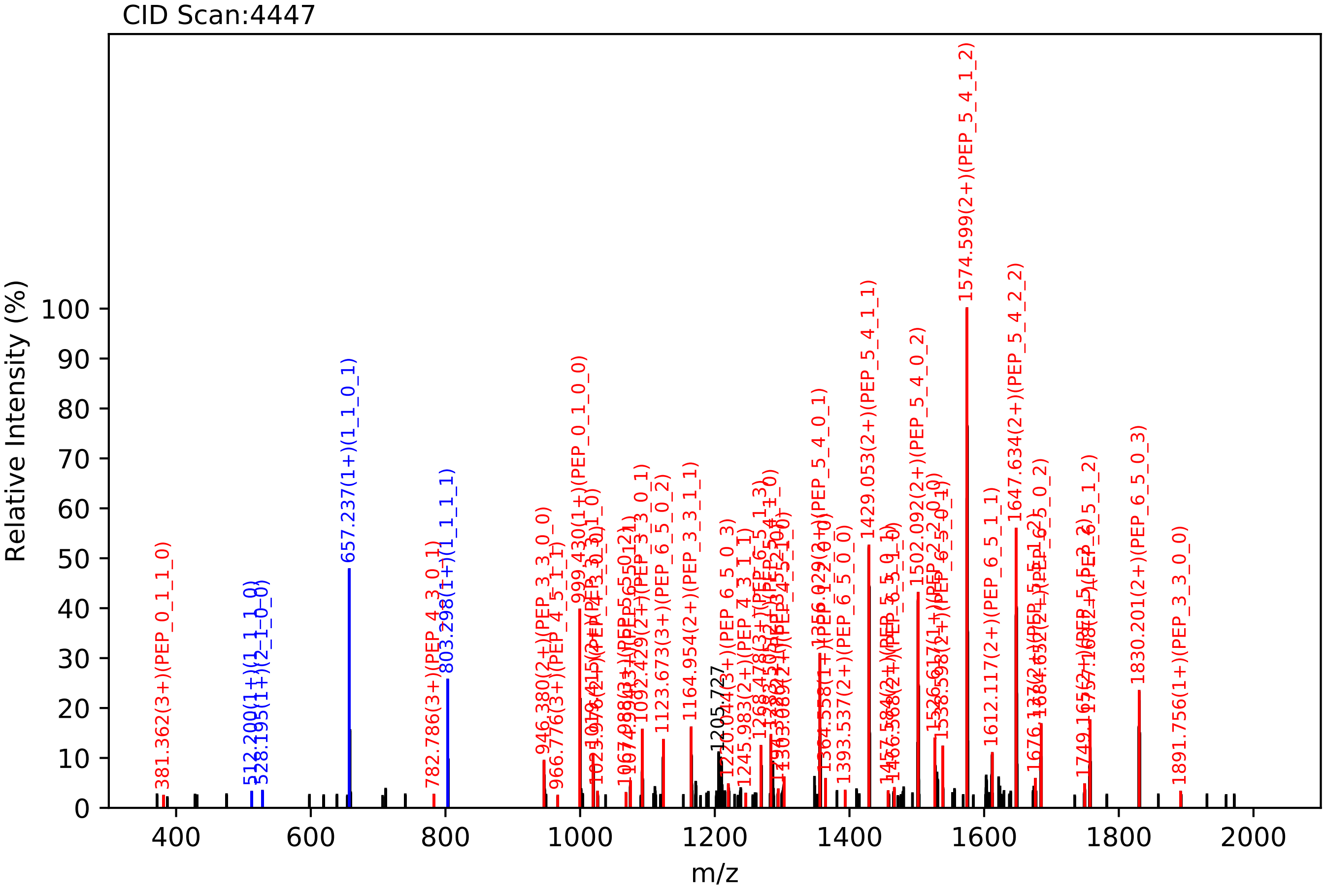

Training set no. 235, Experiment: AGP exp_28

NEEYNK(=PEP)_6_5_2_3, m/z:1315.77(3+), RT:31.30, Y-score:83.60

HCD Scan:8648

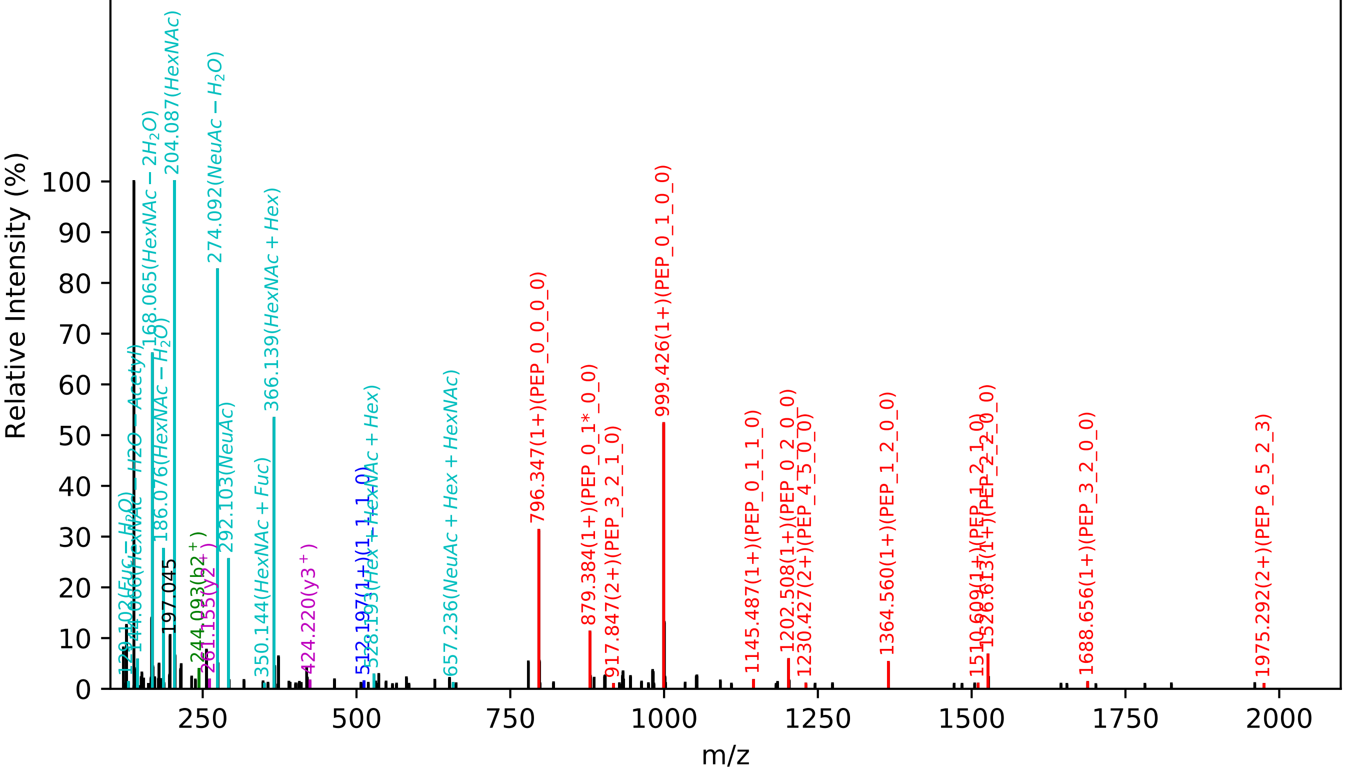

CID Scan:8651

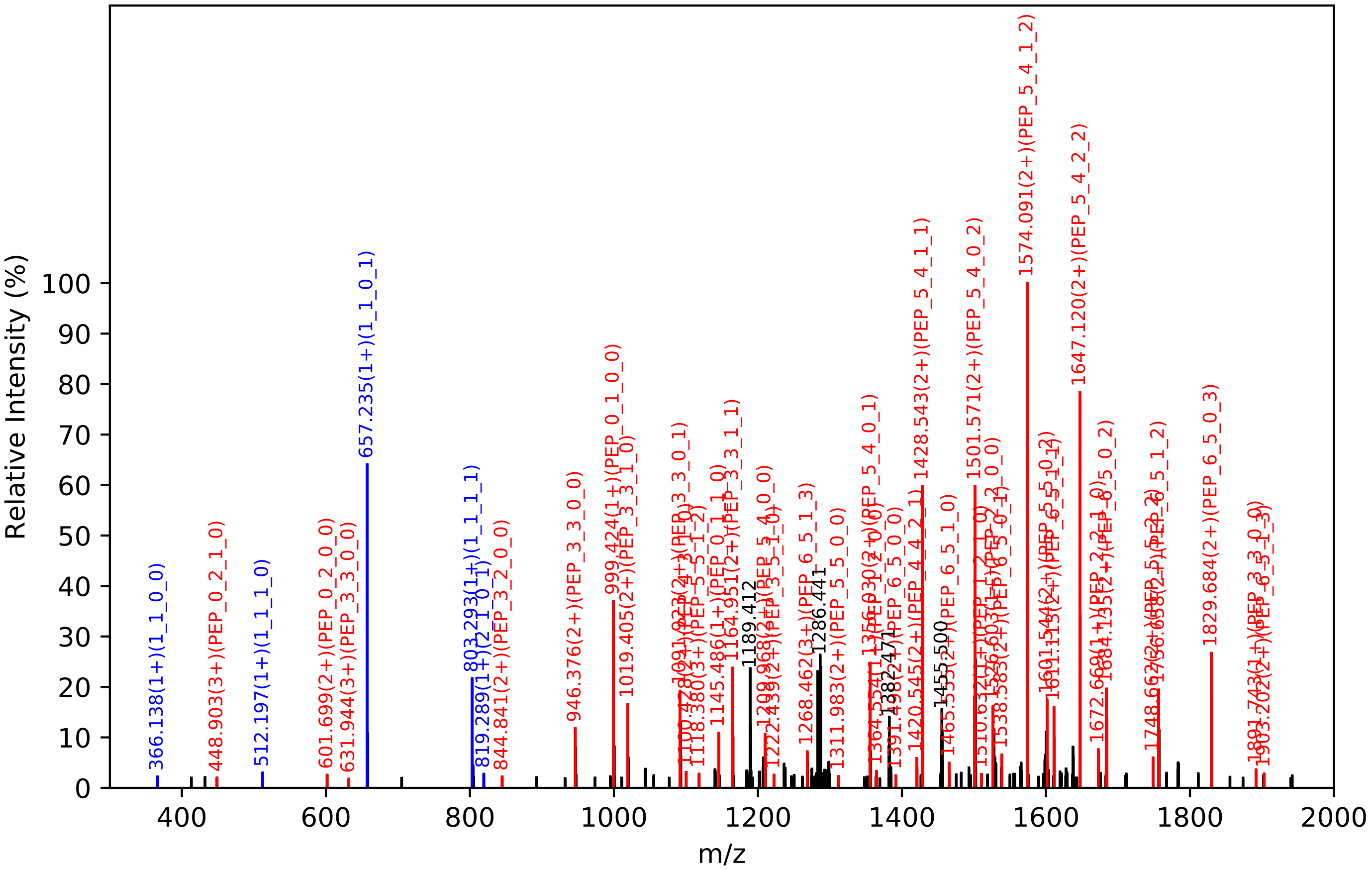

Training set no. 236, Experiment: AGP exp_15

NEEYNK(=PEP)_6_5_2_3, m/z:1317.16(3+), RT:37.28, Y-score:75.86

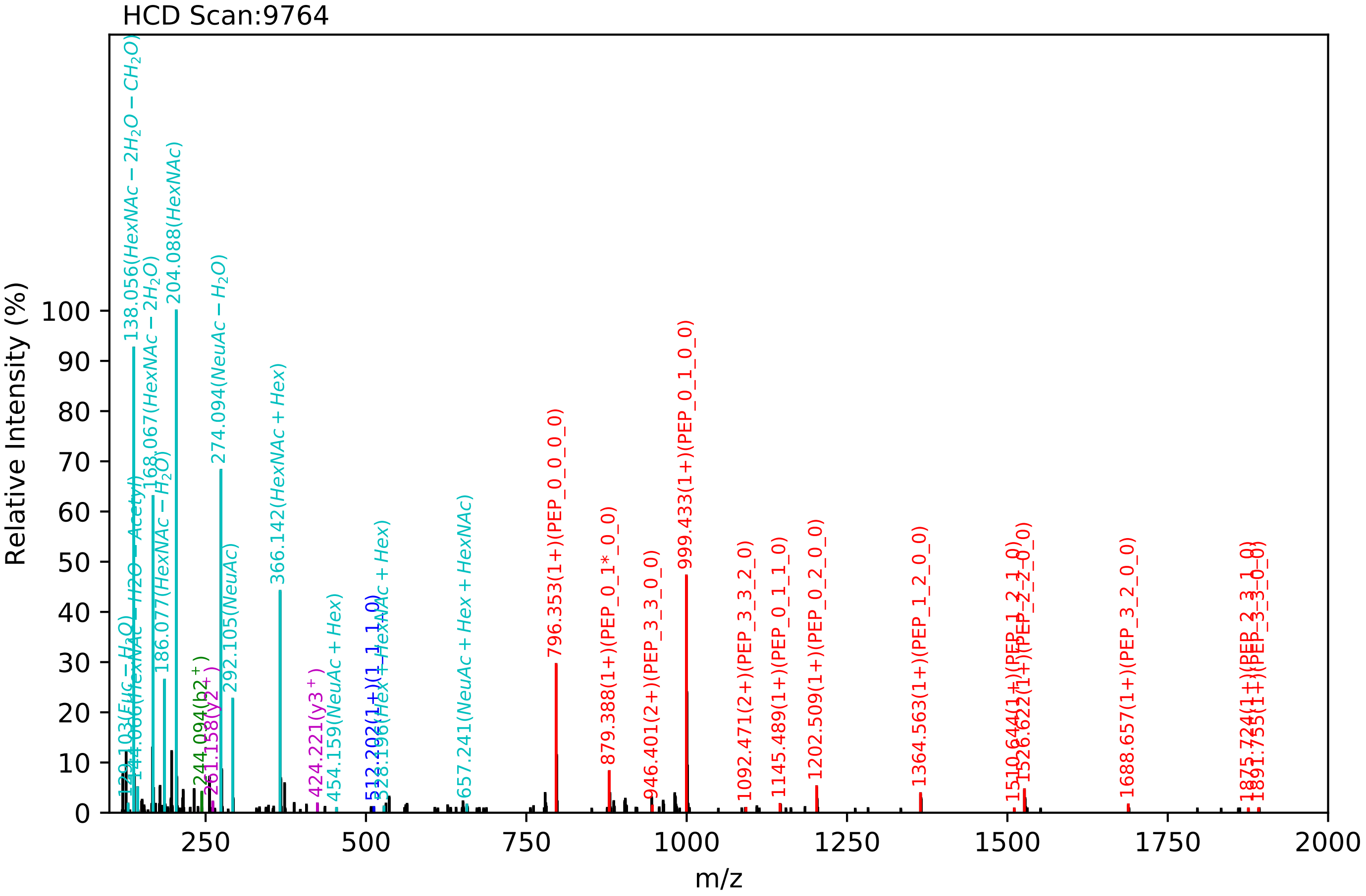

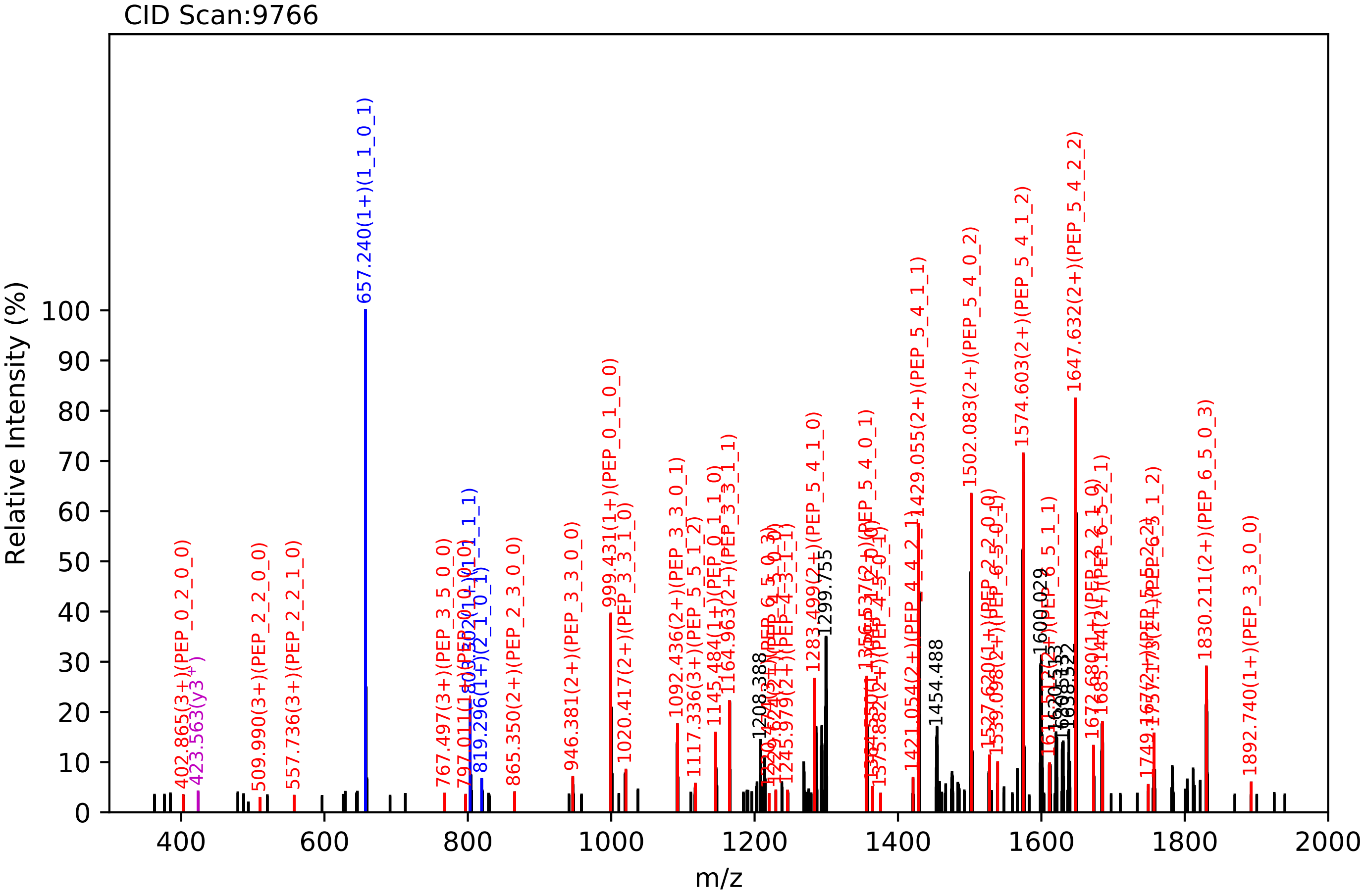

Training set no. 237, Experiment: AGP exp_14

NEEYNK(=PEP)_6_5_2_3, m/z:1317.16(3+), RT:37.37, Y-score:75.15

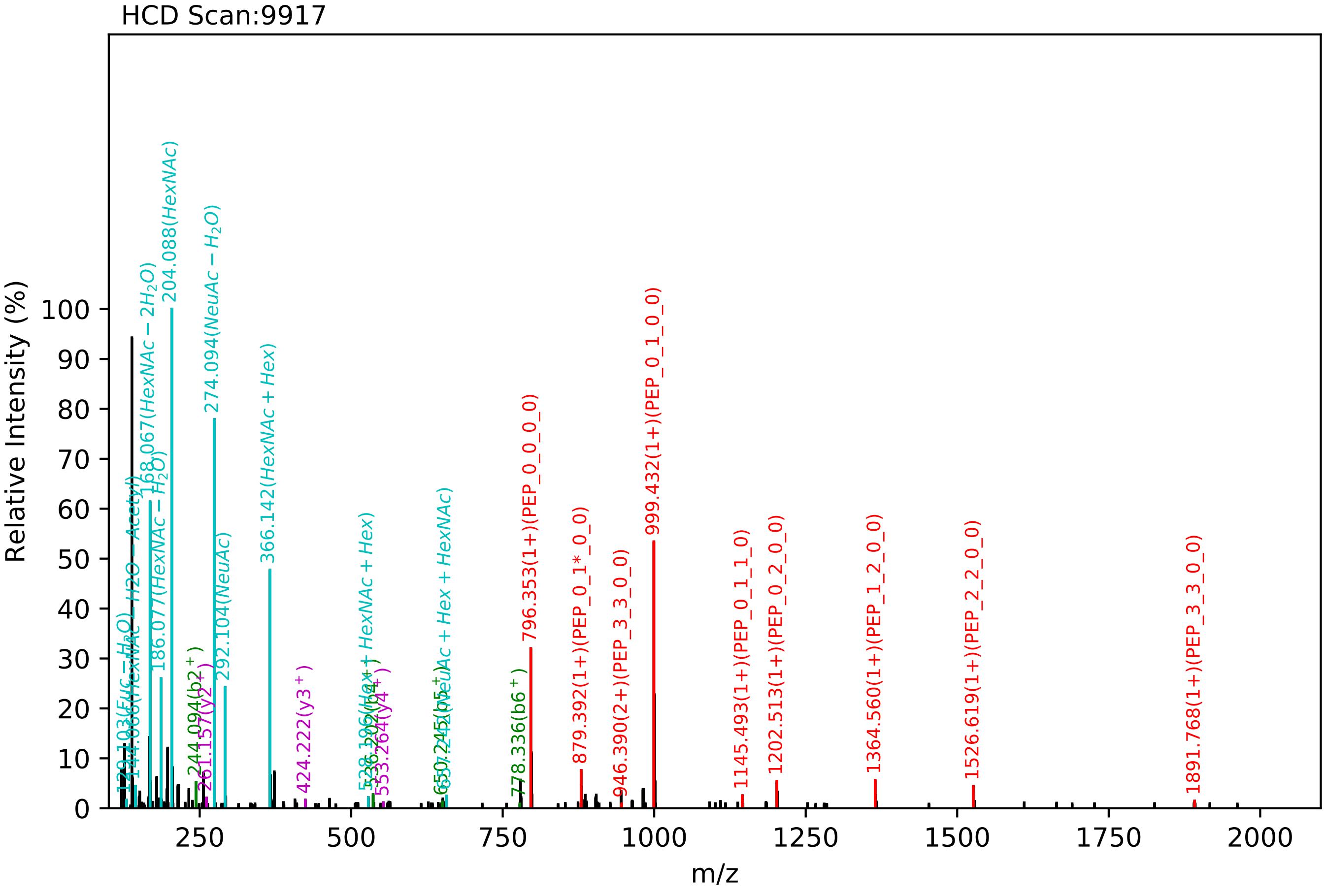

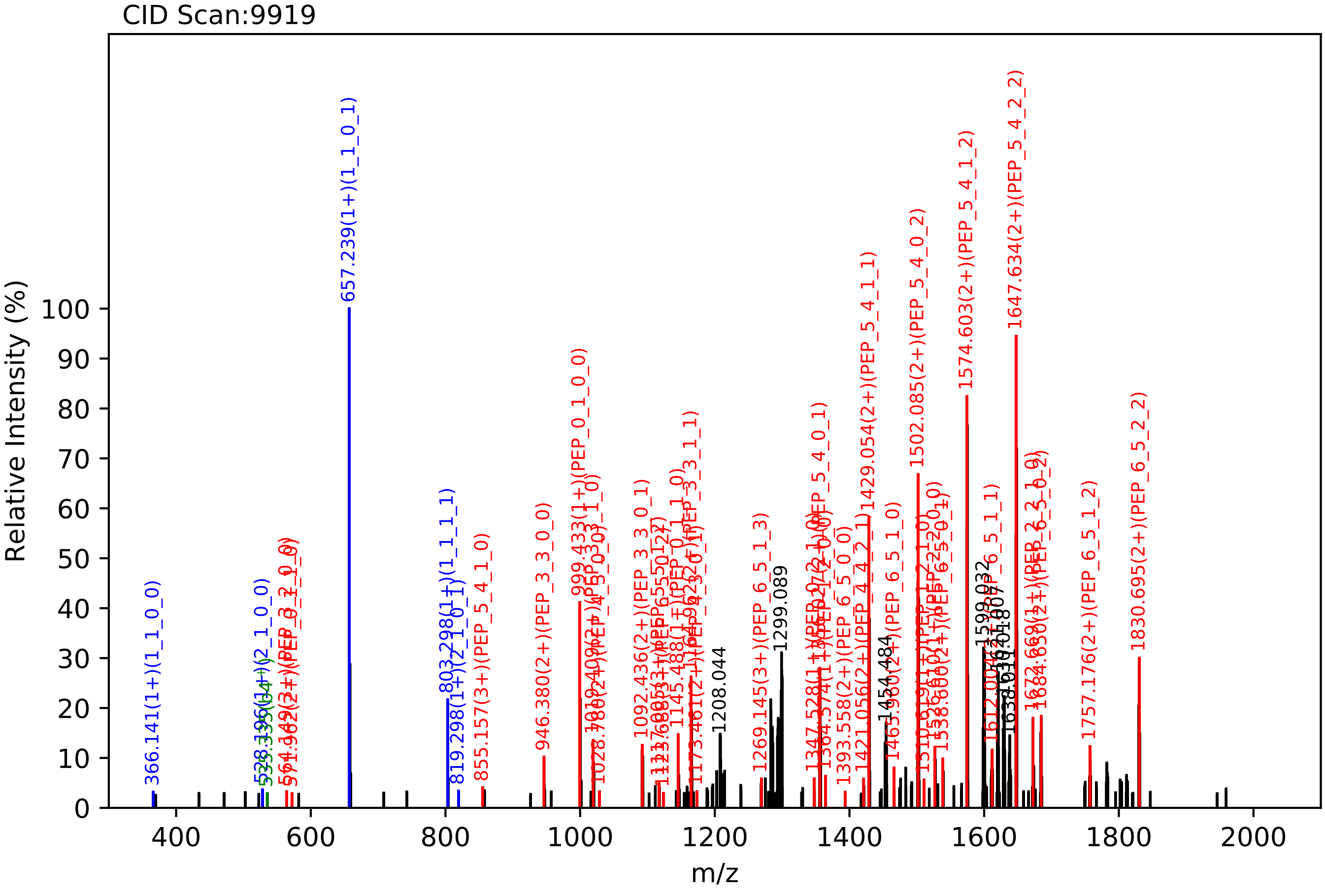

Training set no. 238, Experiment: AGP exp_16

NEEYNK(=PEP)_6_5_2_3, m/z:1317.16(3+), RT:37.30, Y-score:67.70

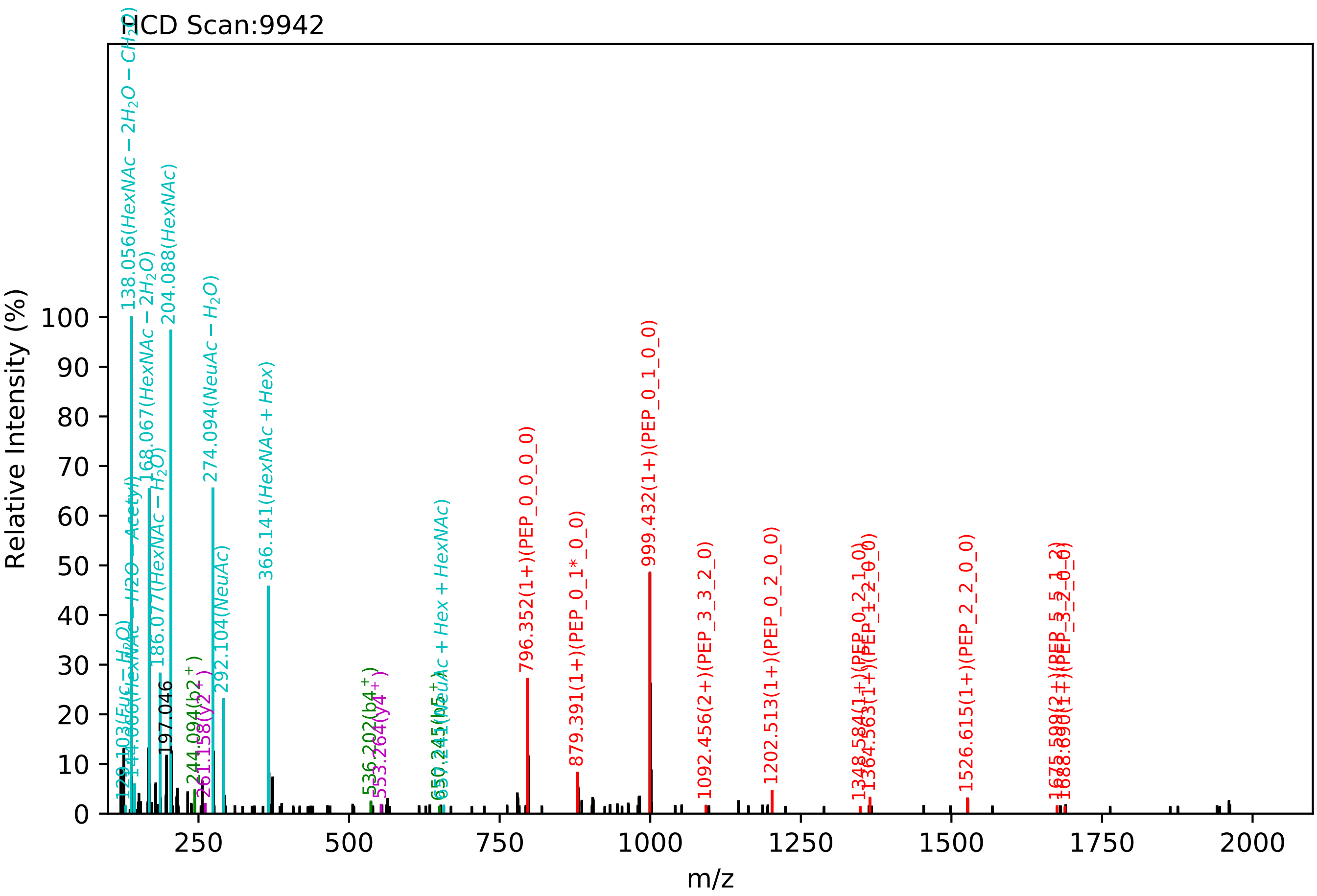

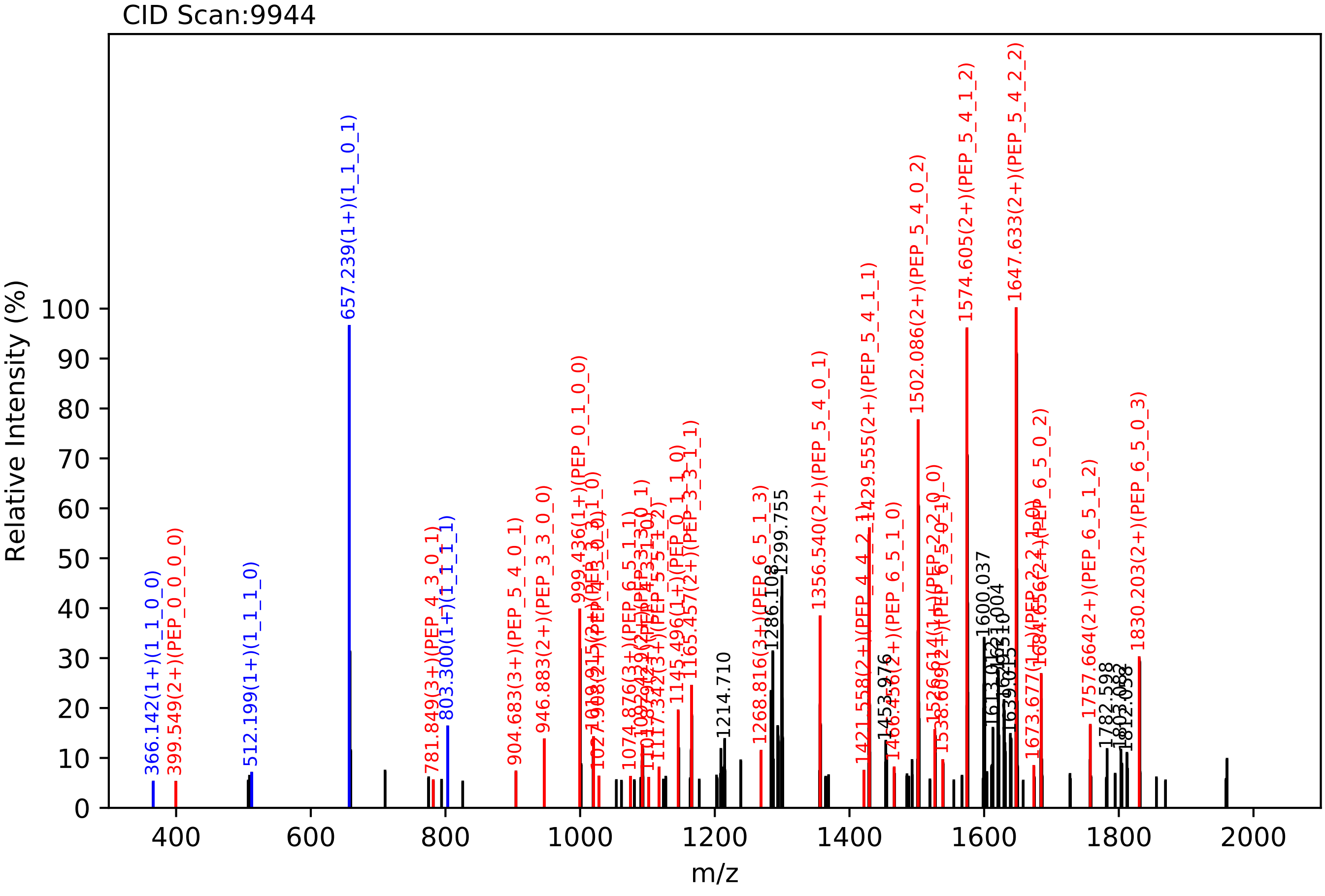

Training set no. 239, Experiment: AGP exp_1

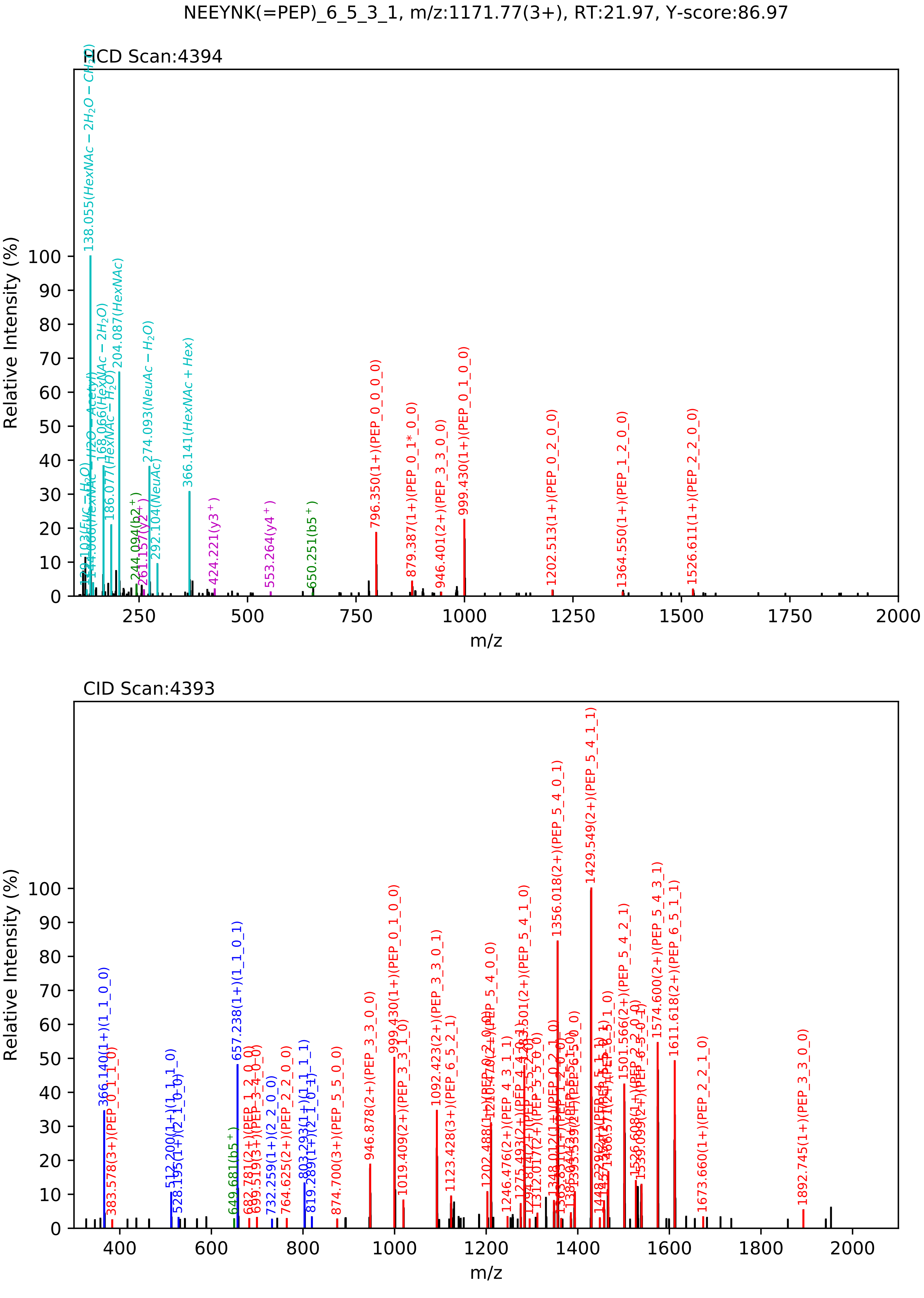

Training set no. 240, Experiment: AGP exp_1

SVQEIQATFFYFTPNK(=PEP)_6_5_0_3, m/z:1594.33(3+), RT:70.25, Y-score:77.47

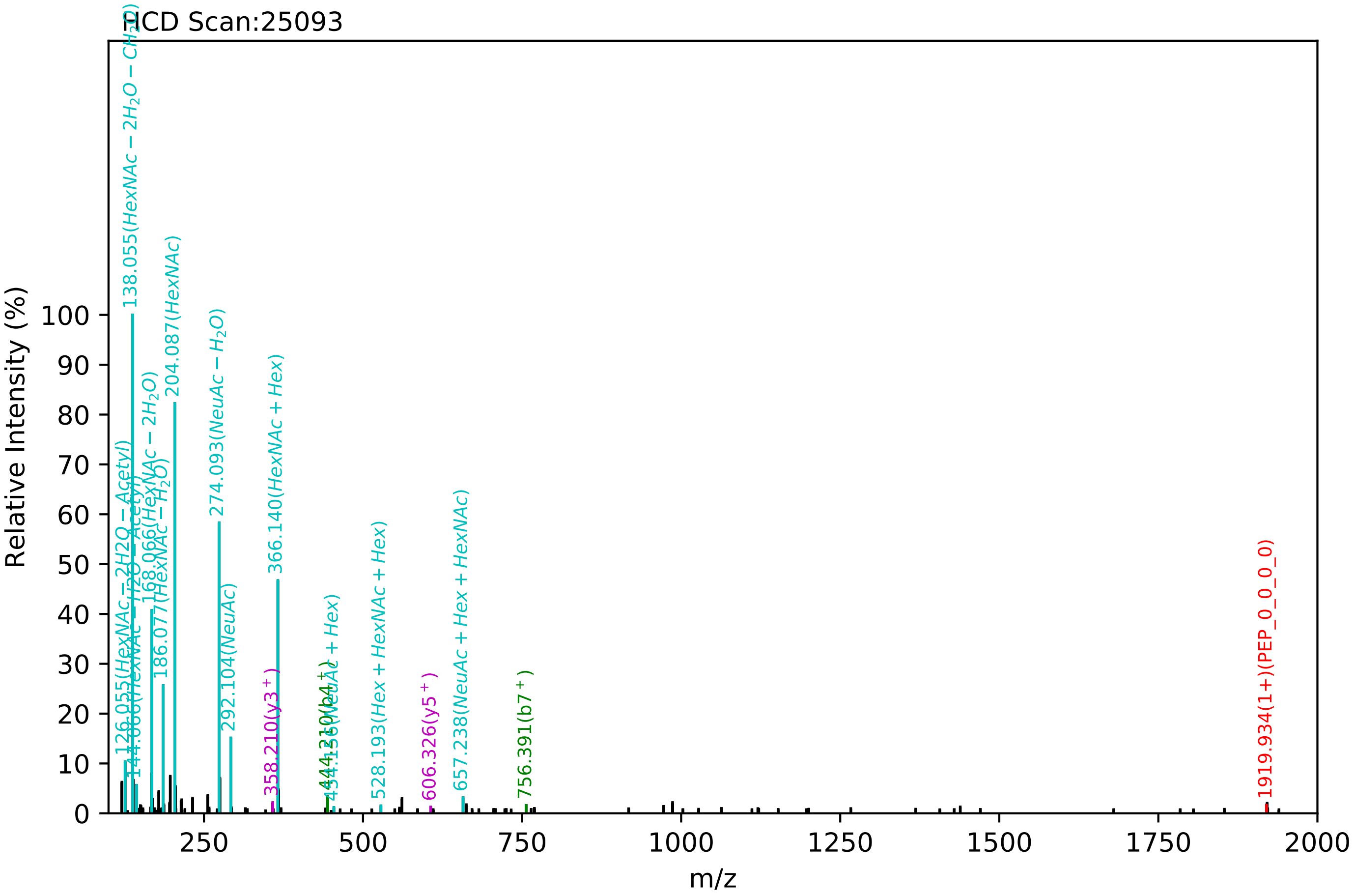

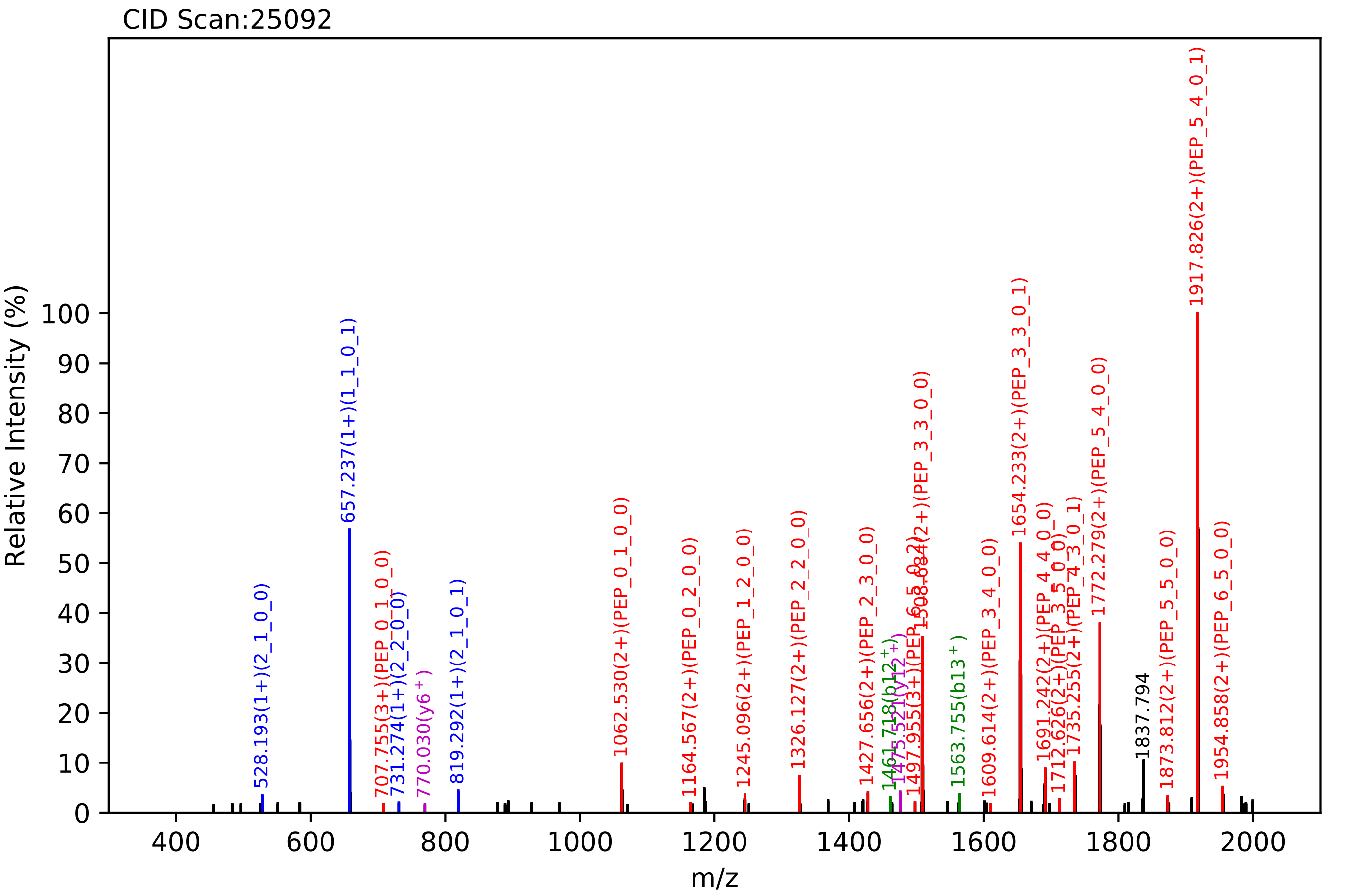

Training set no. 241, Experiment: AGP exp_1

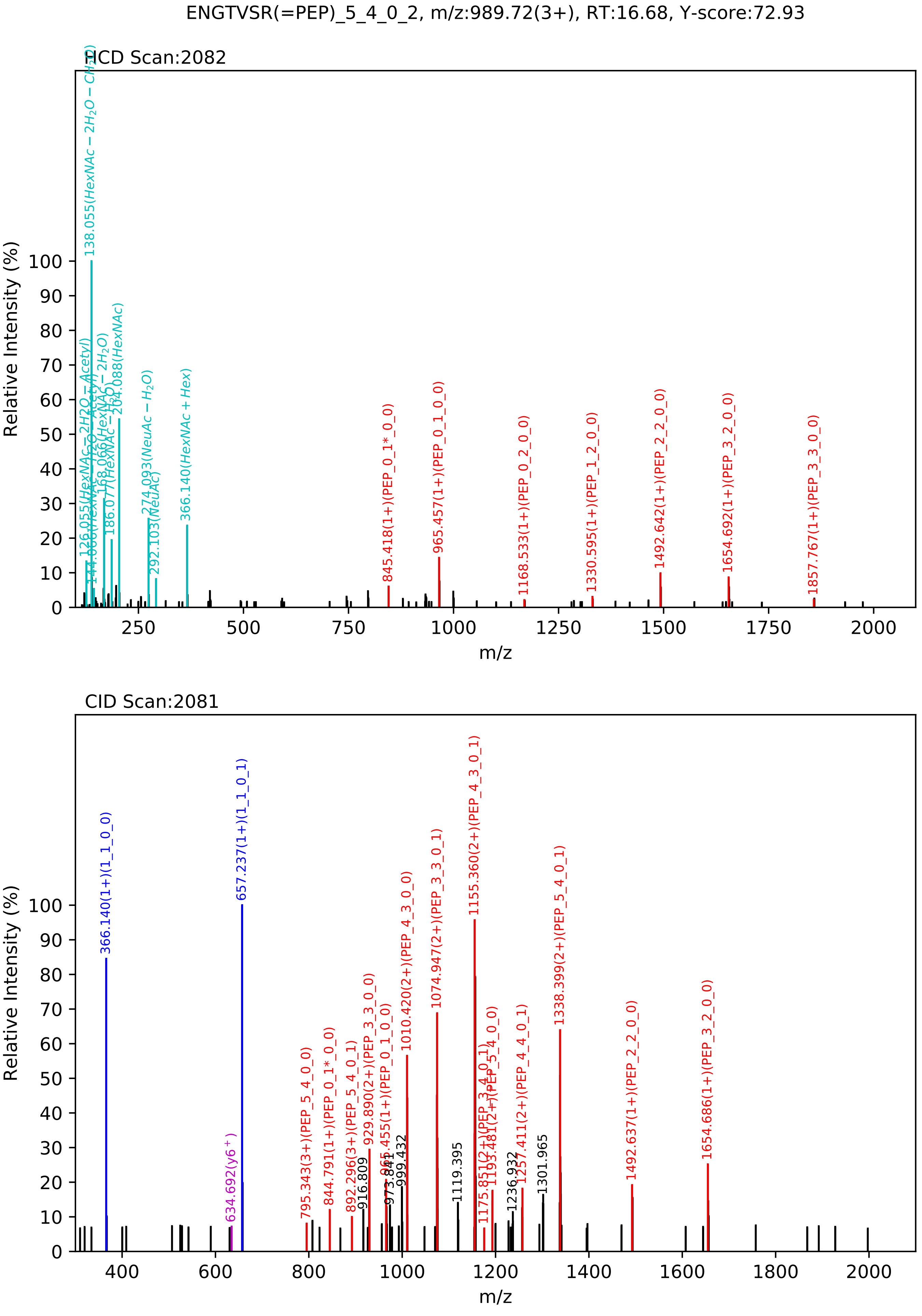

Training set no. 242, Experiment: AGP exp_2

ENGTVSR(=PEP)_6_5_0_1, m/z:1014.40(3+), RT:15.50, Y-score:87.13

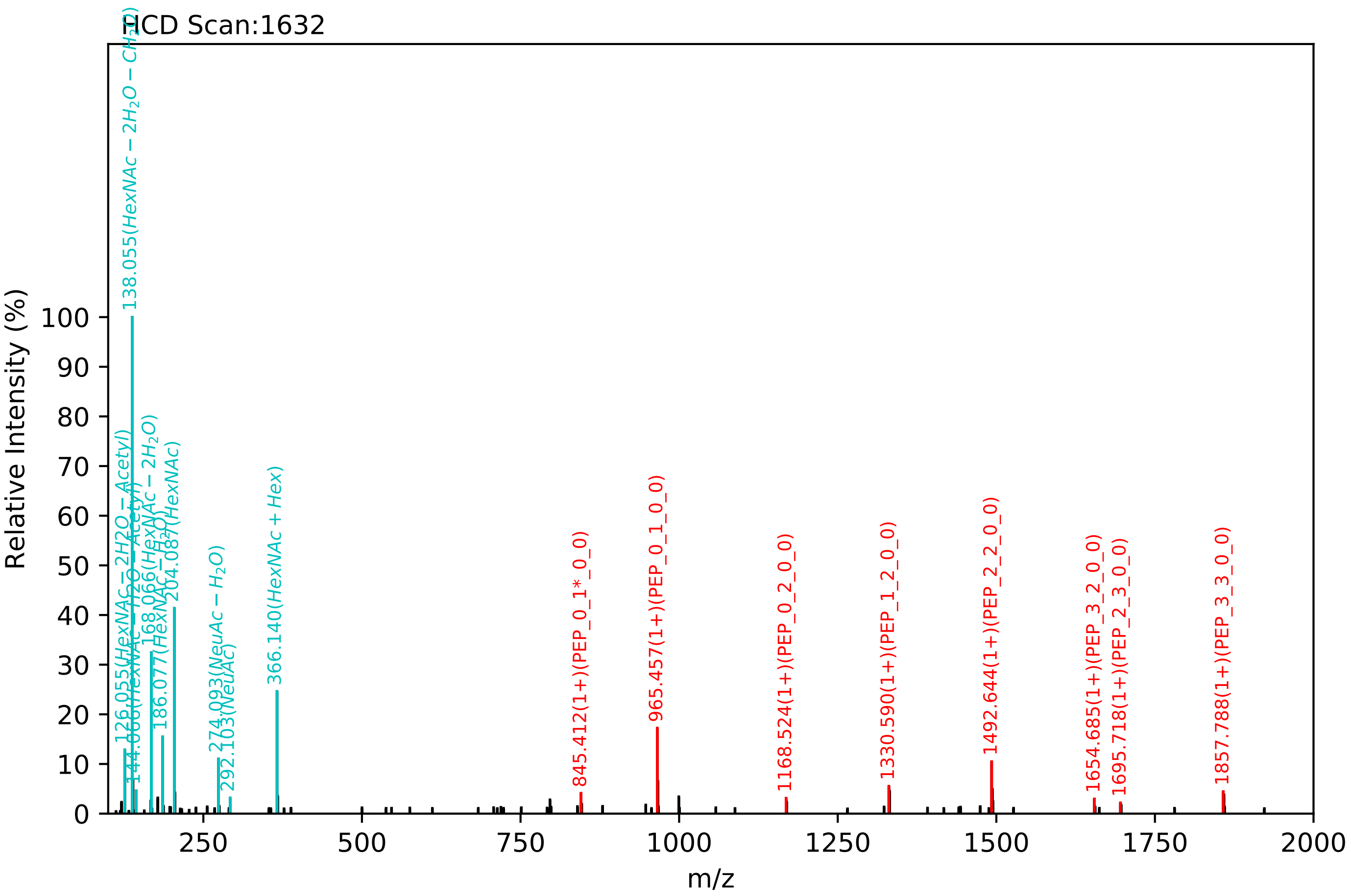

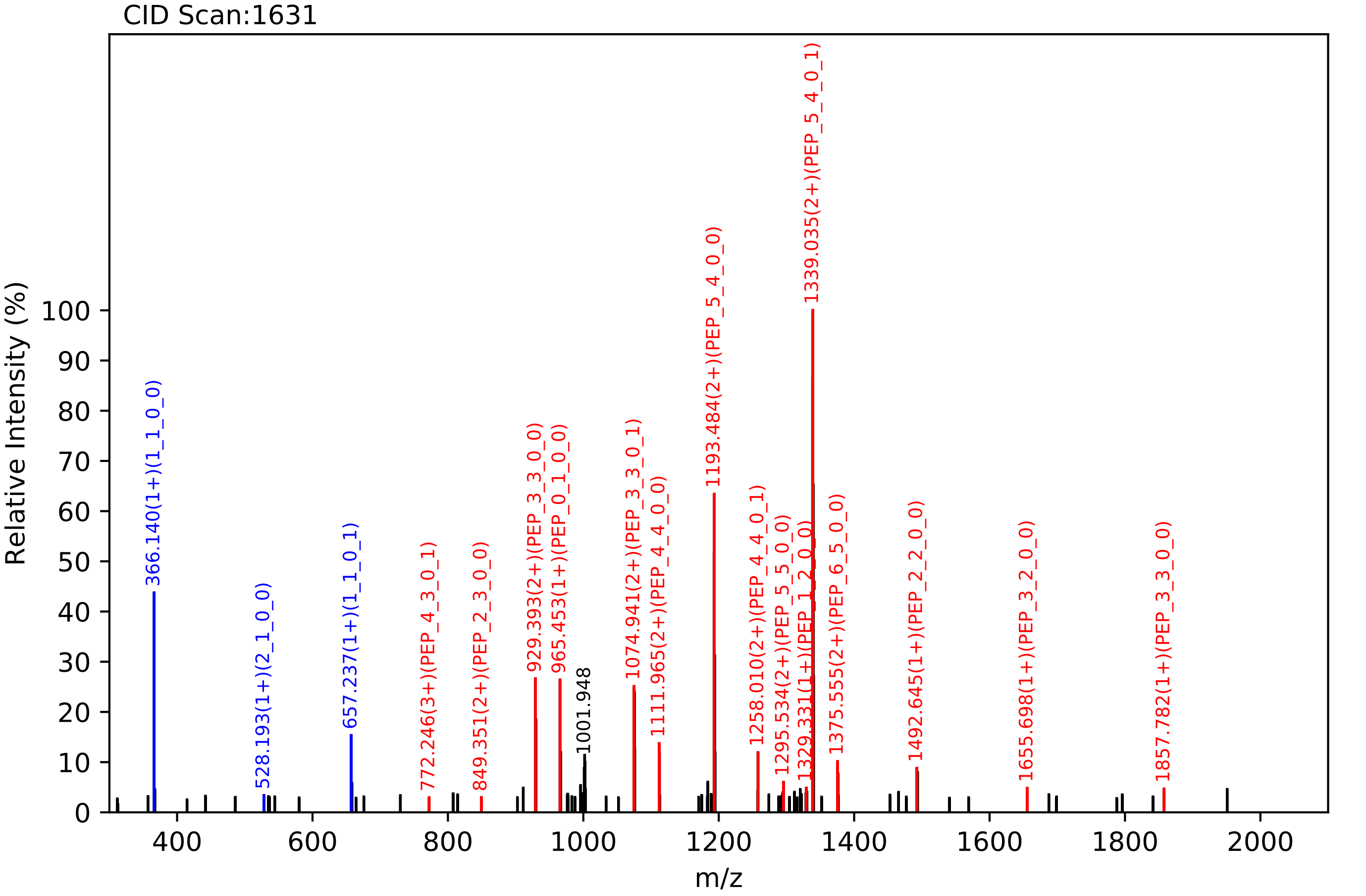

Training set no. 243, Experiment: AGP exp_1

ENGTVSR(=PEP)_6_5_0_2, m/z:1111.43(3+), RT:17.60, Y-score:94.58

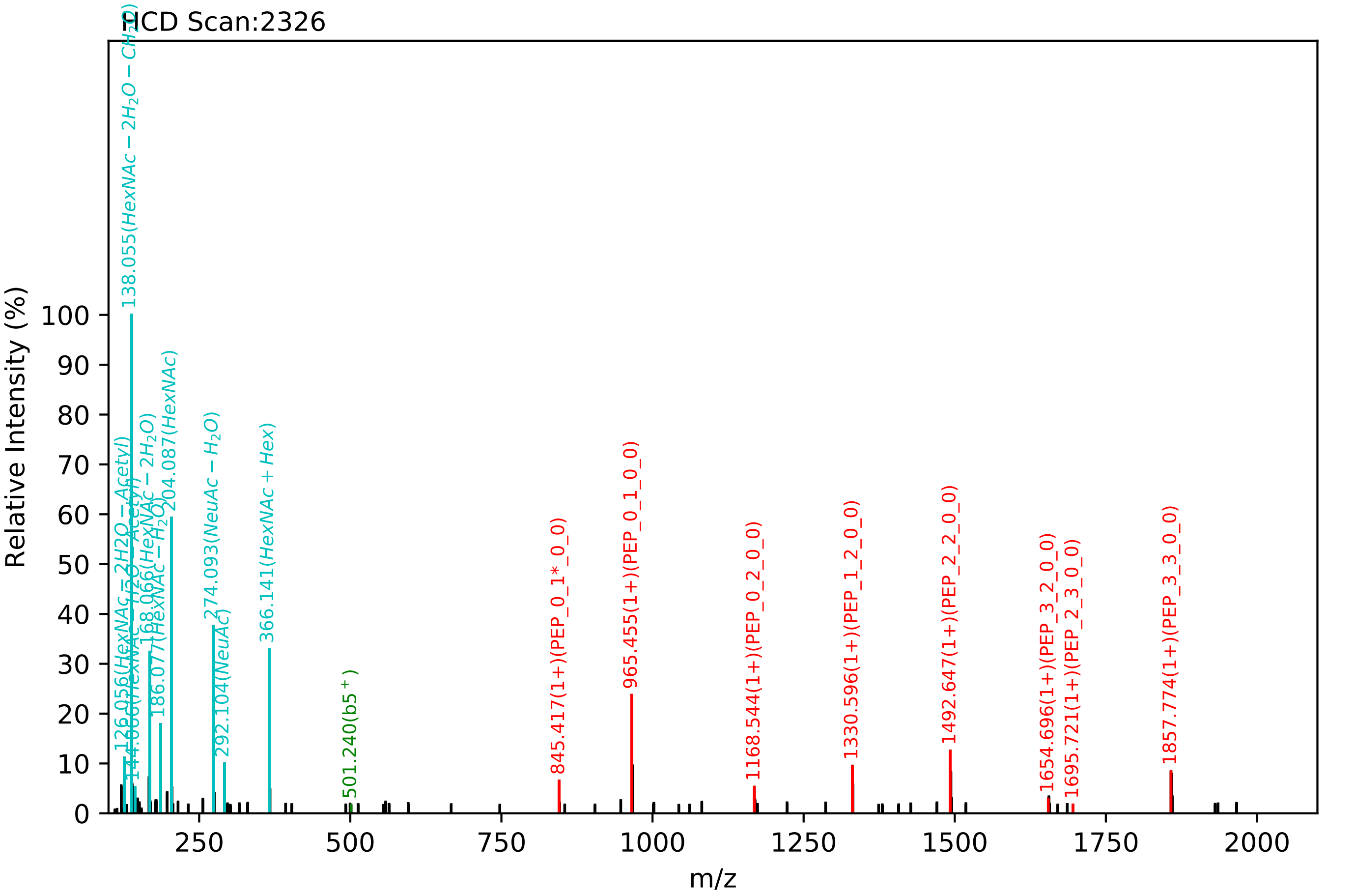

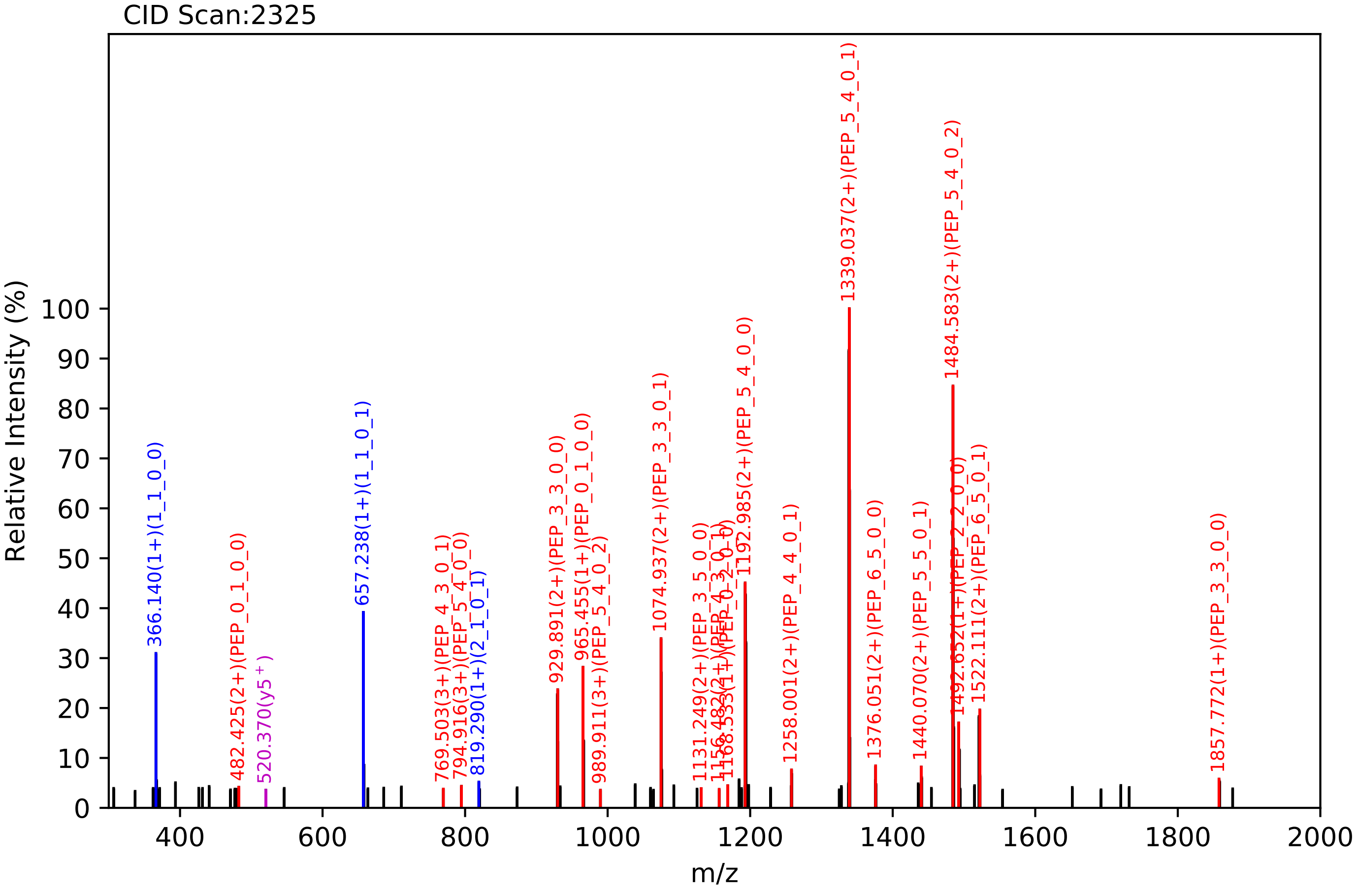

Training set no. 244, Experiment: AGP exp_2

ENGTVSR(=PEP)_6_5_0_2, m/z:1111.43(3+), RT:17.69, Y-score:93.49

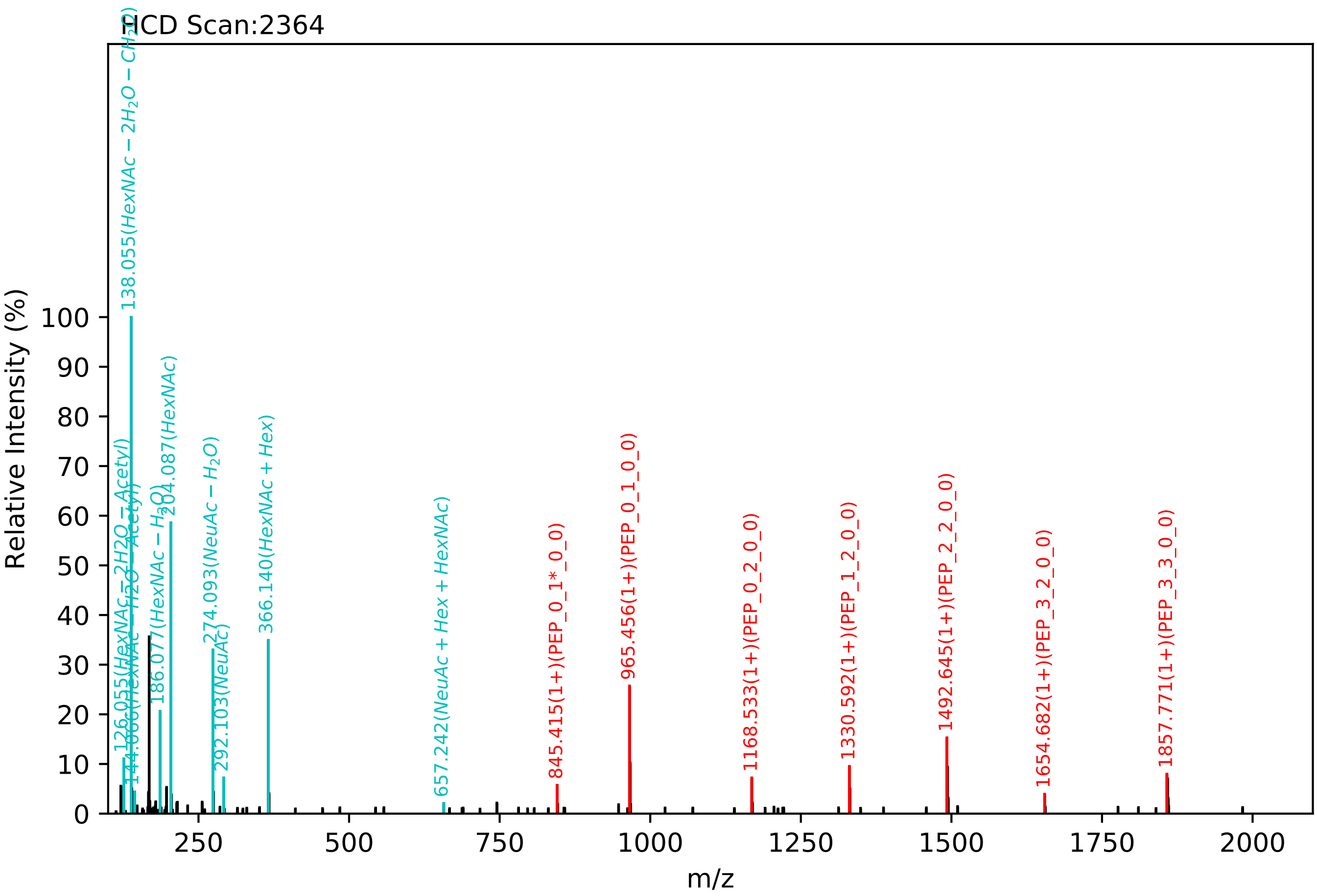

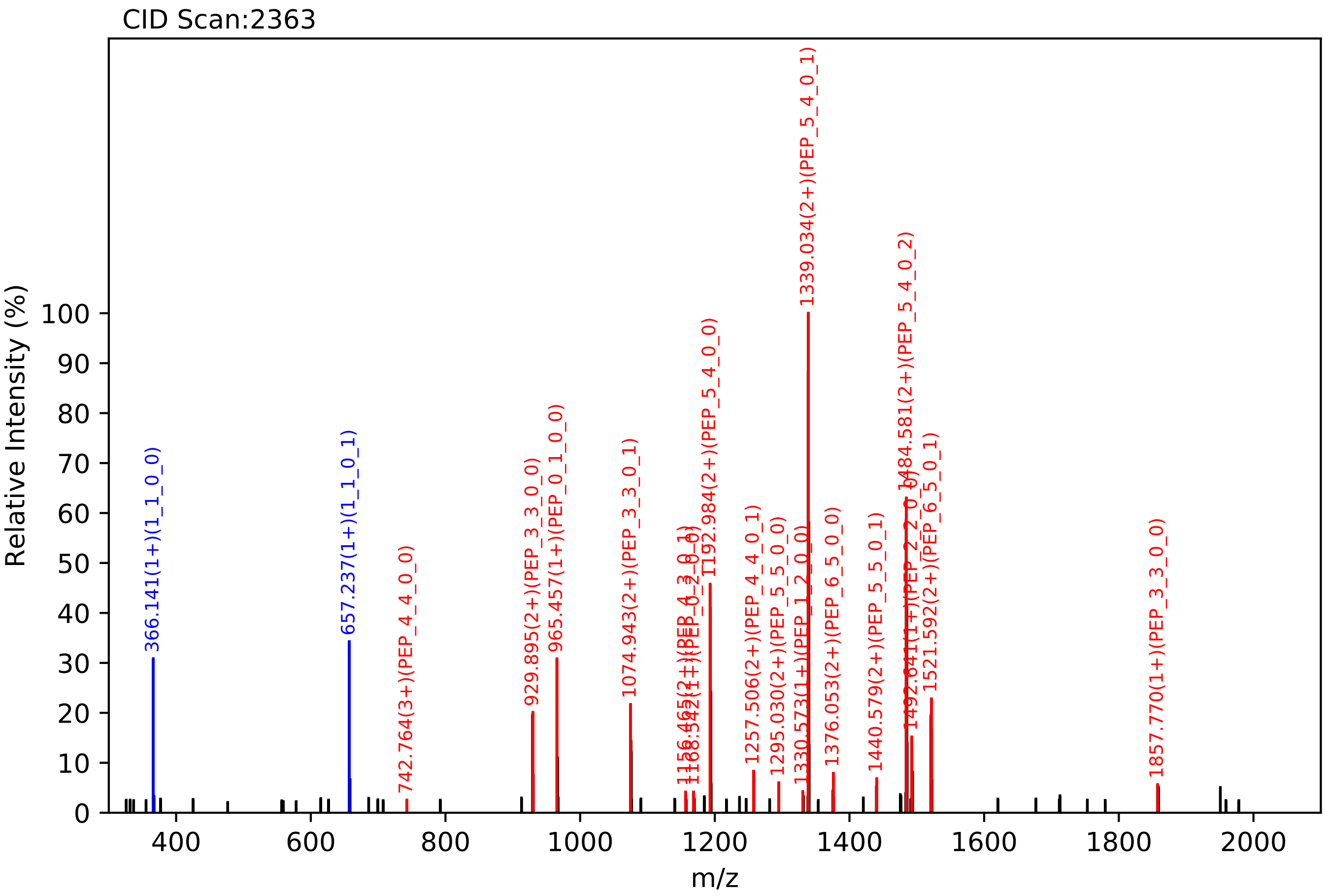

Training set no. 245, Experiment: AGP exp_1

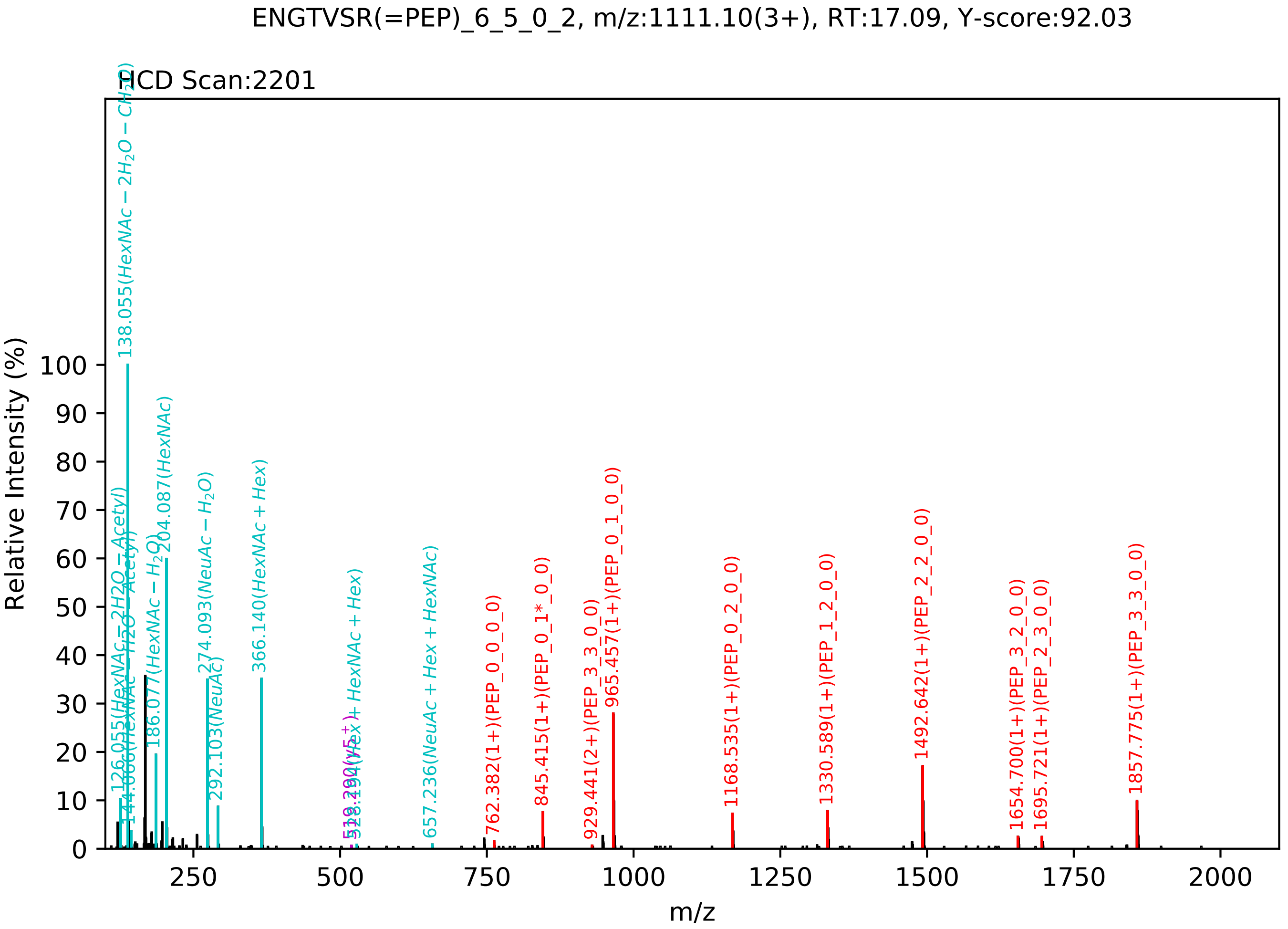

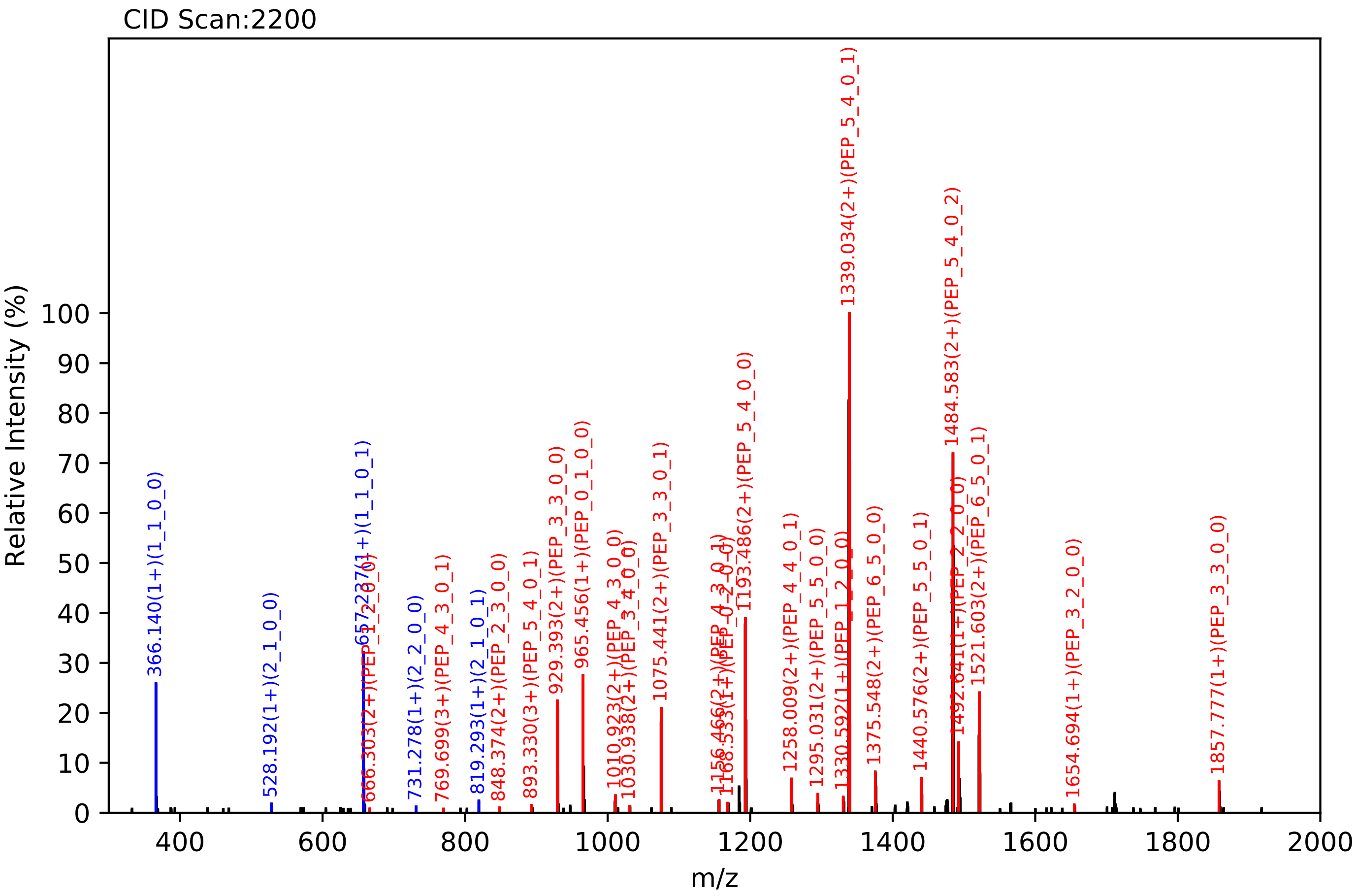

Training set no. 246, Experiment: AGP exp_1

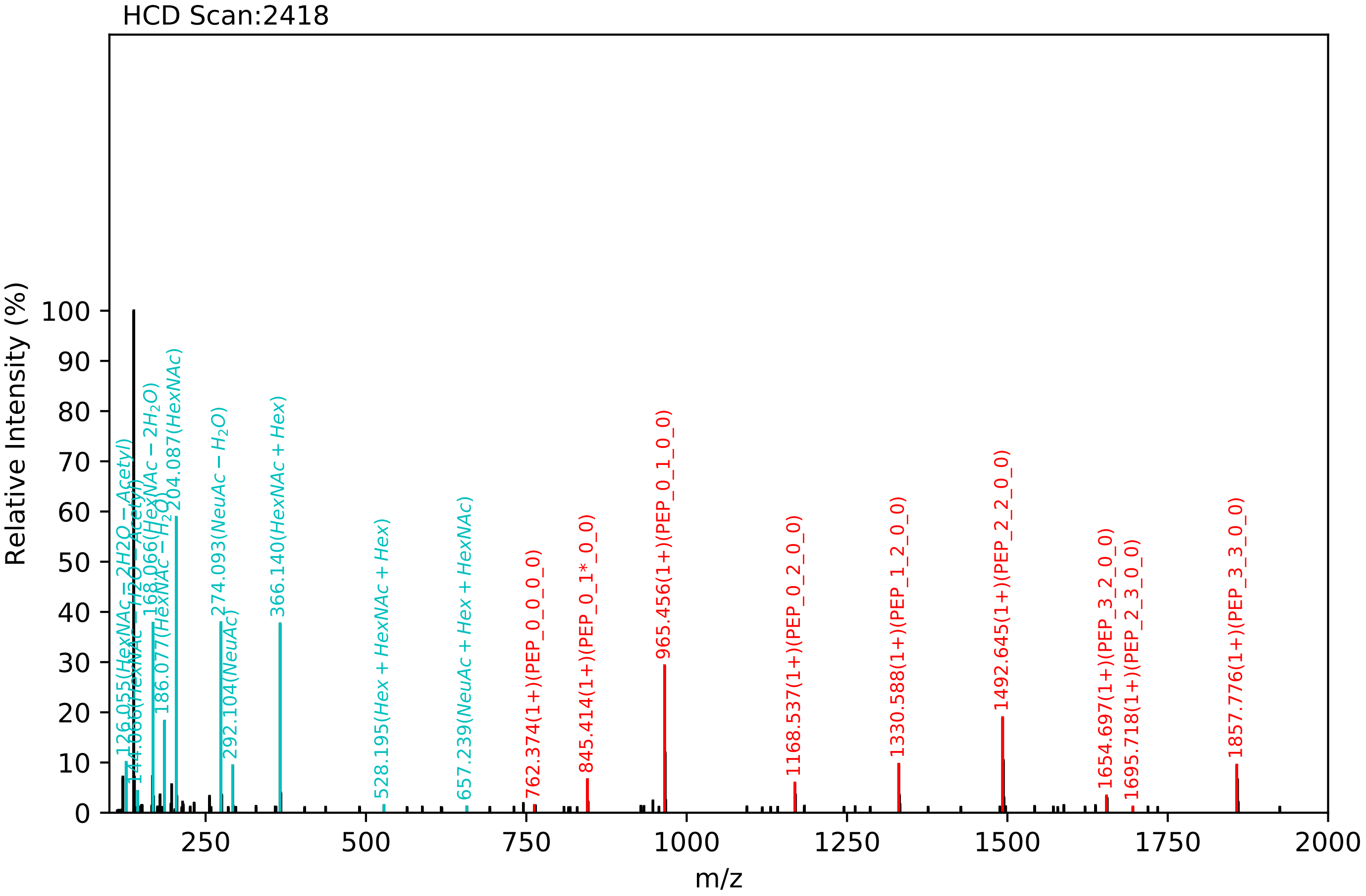

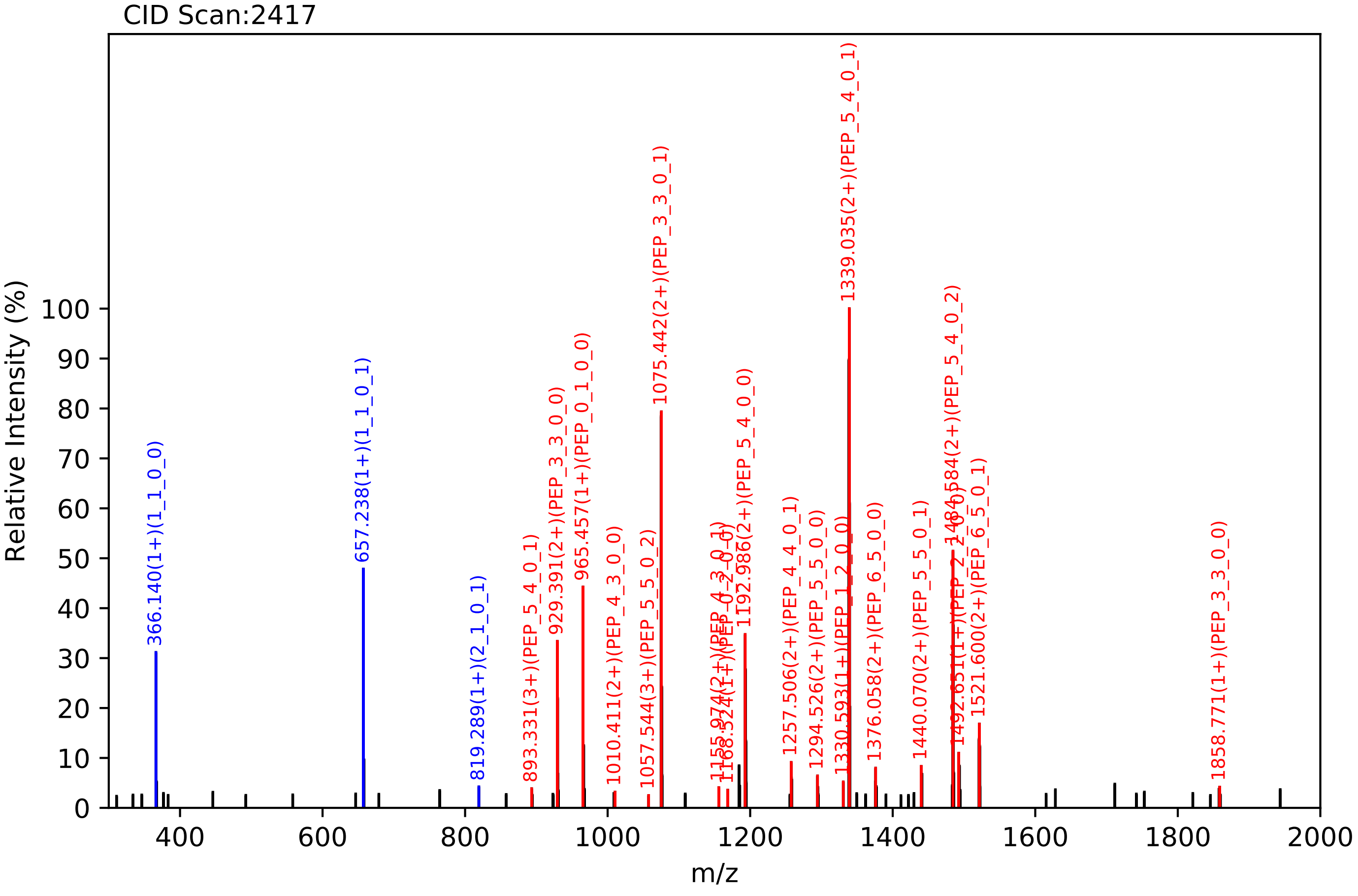

Training set no. 247, Experiment: AGP exp_2

ENGTVSR(=PEP)_6_5_0_3, m/z:1208.46(3+), RT:21.29, Y-score:90.22

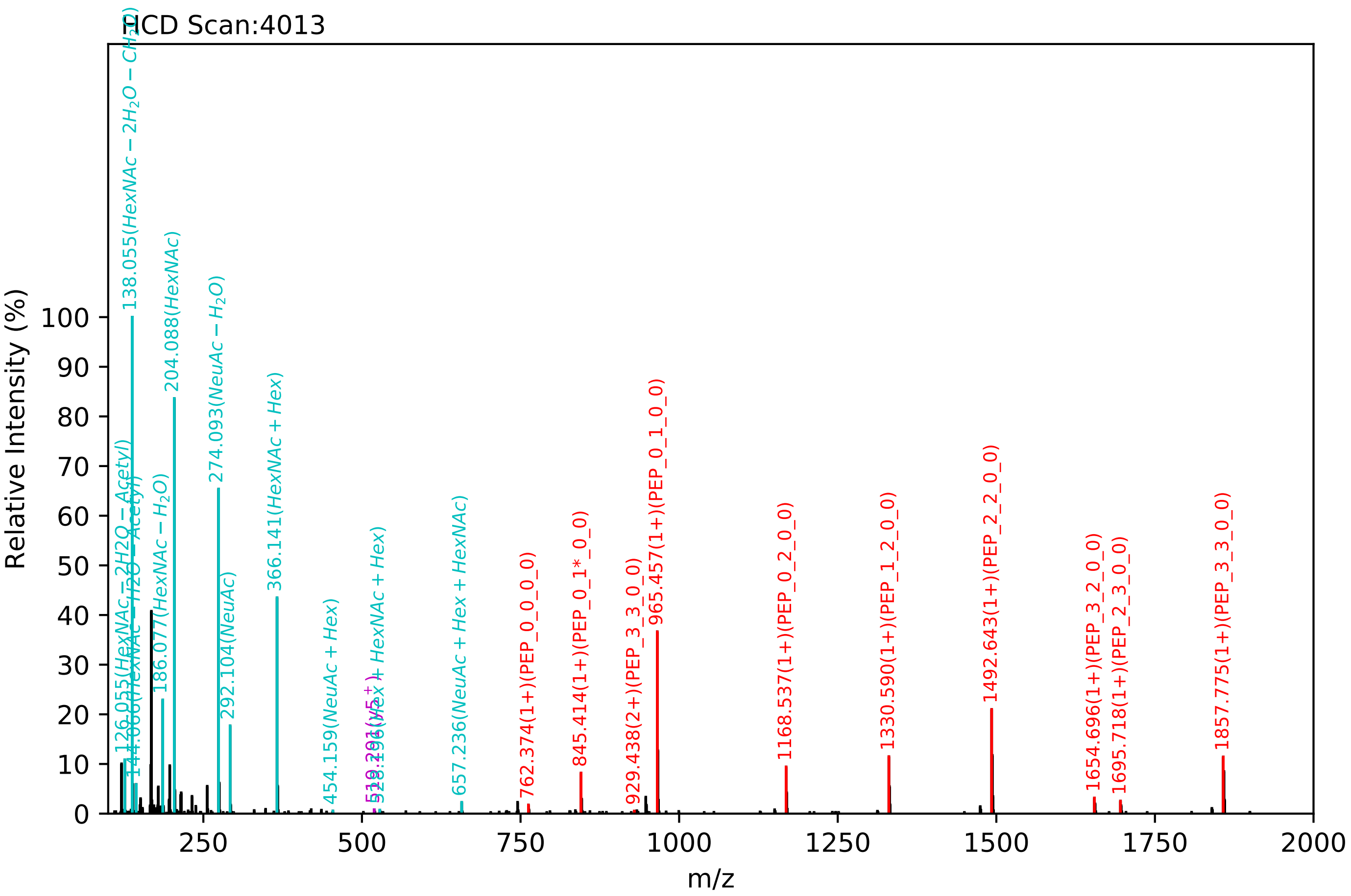

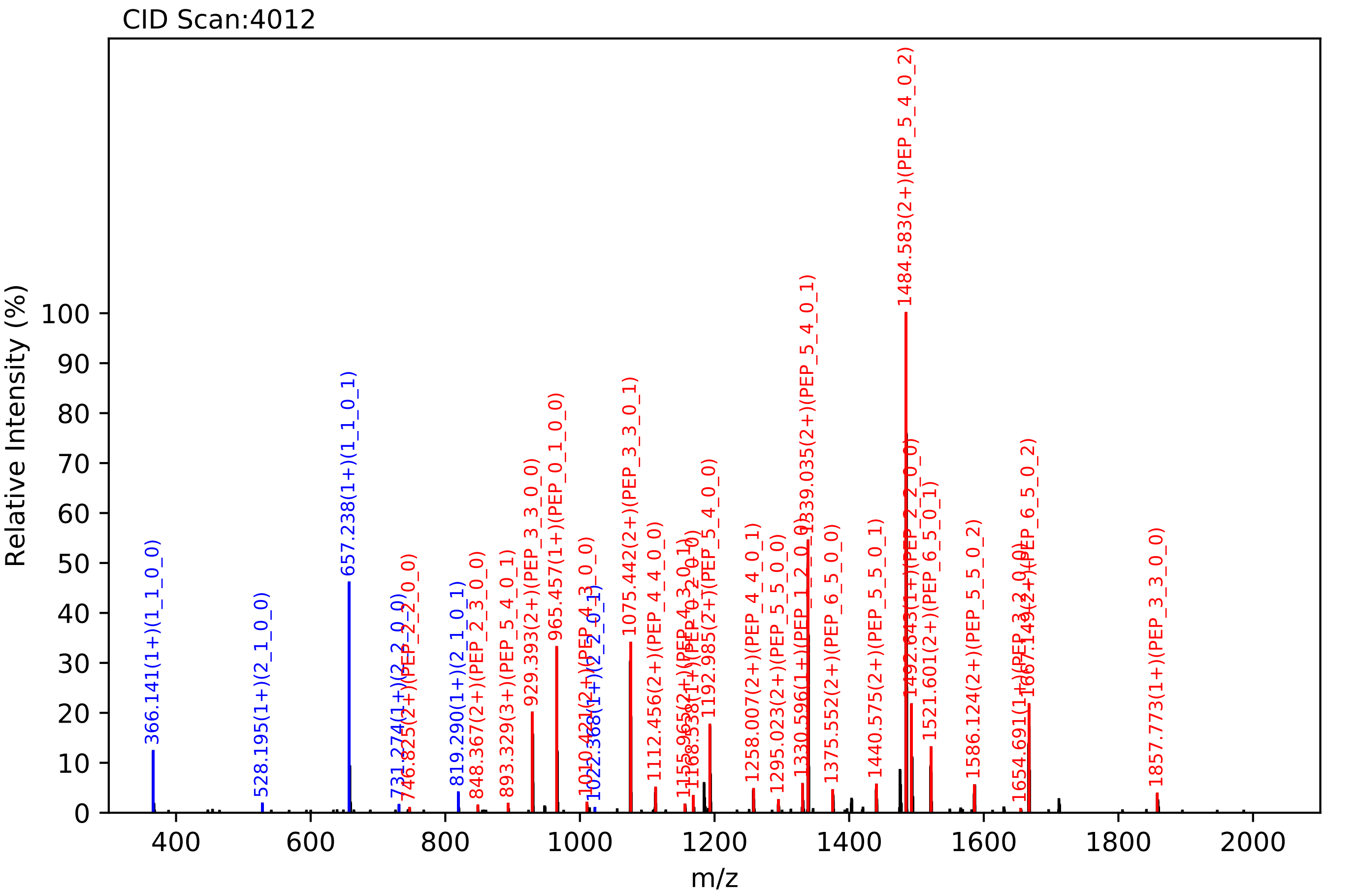

Training set no. 248, Experiment: AGP exp_1

ENGTVSR(=PEP)_6_5_0_3, m/z:1208.13(3+), RT:20.96, Y-score:90.16

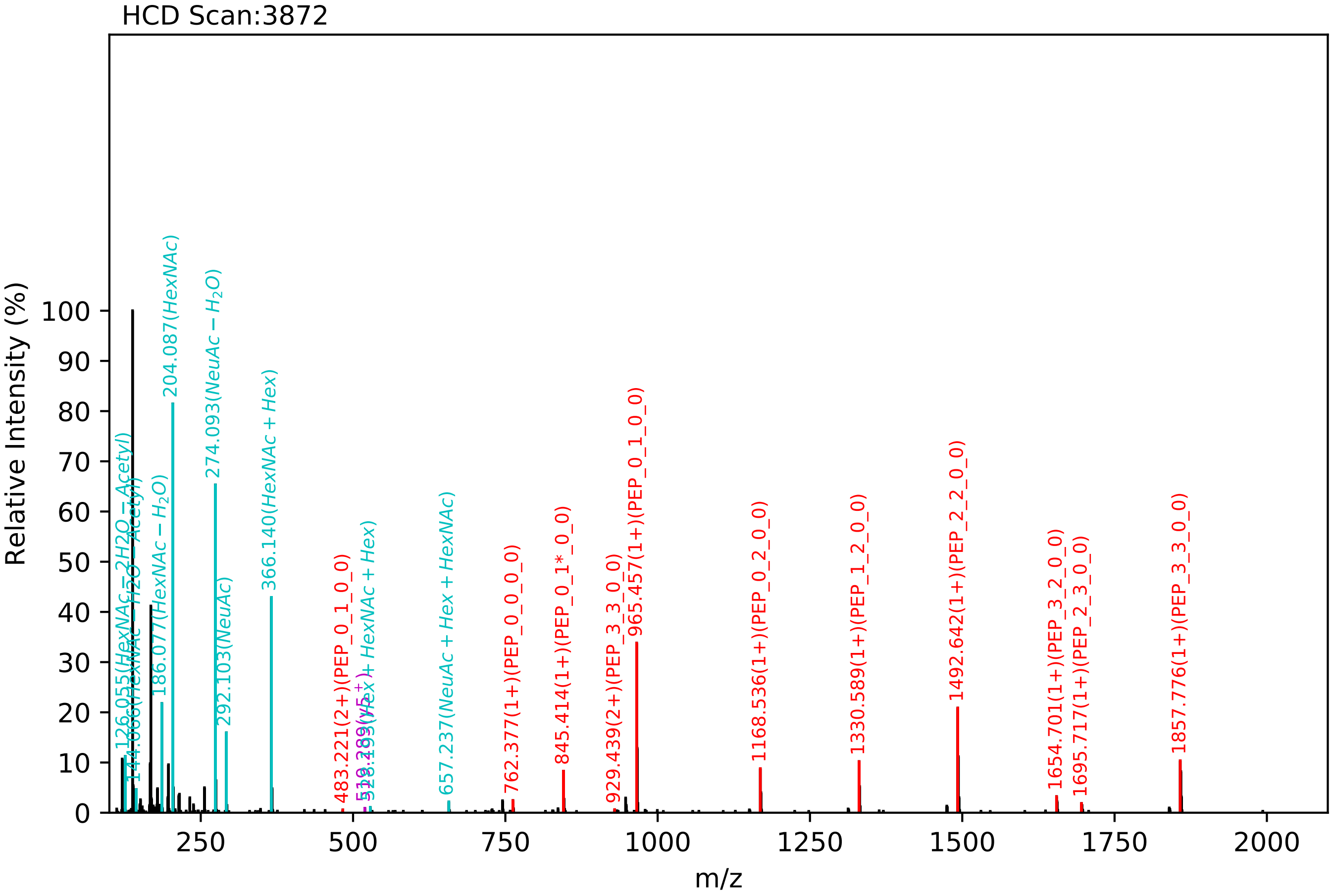

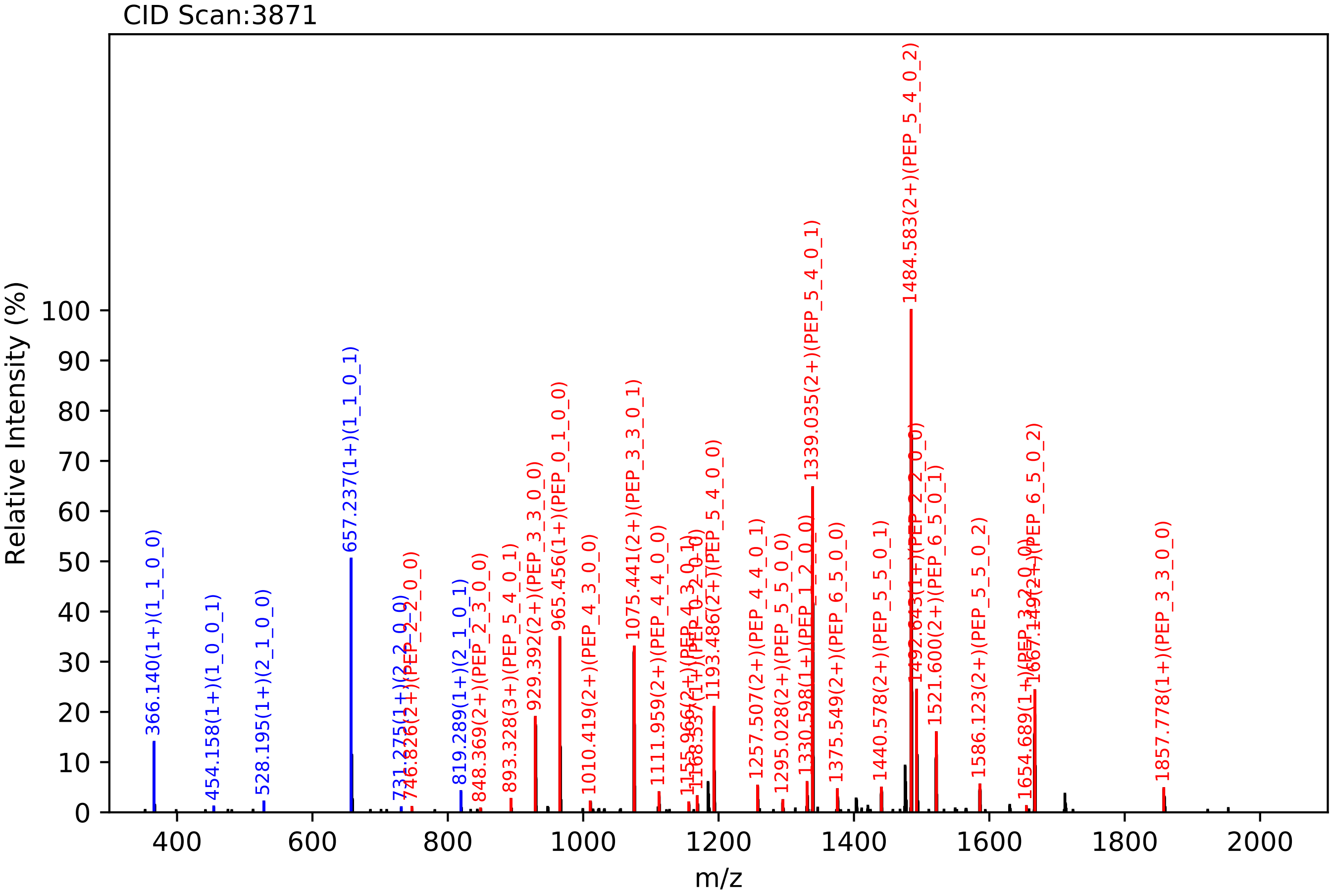

Training set no. 249, Experiment: AGP exp_1

ENGTVSR(=PEP)_6_5_0_3, m/z:1208.47(3+), RT:21.49, Y-score:86.19

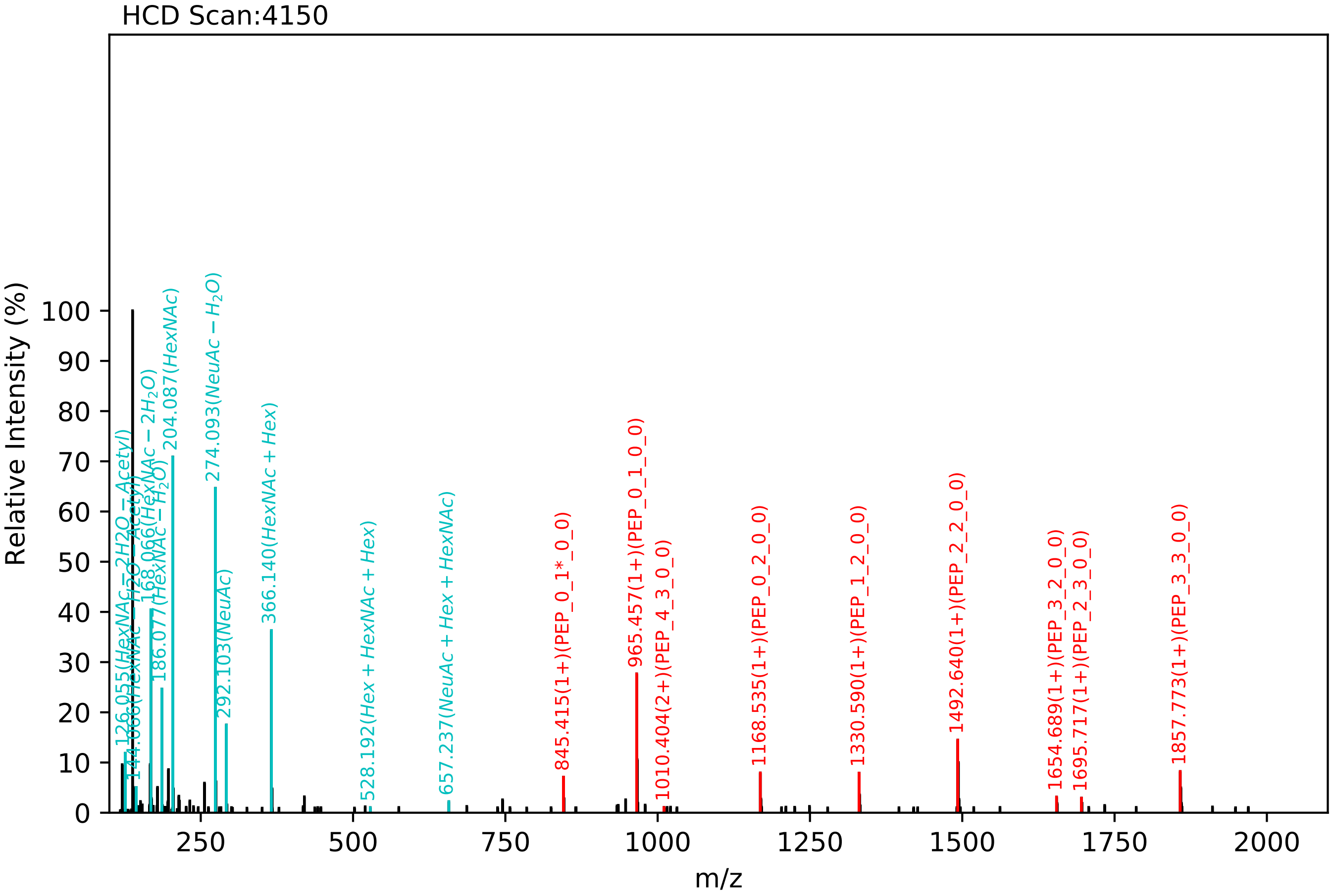

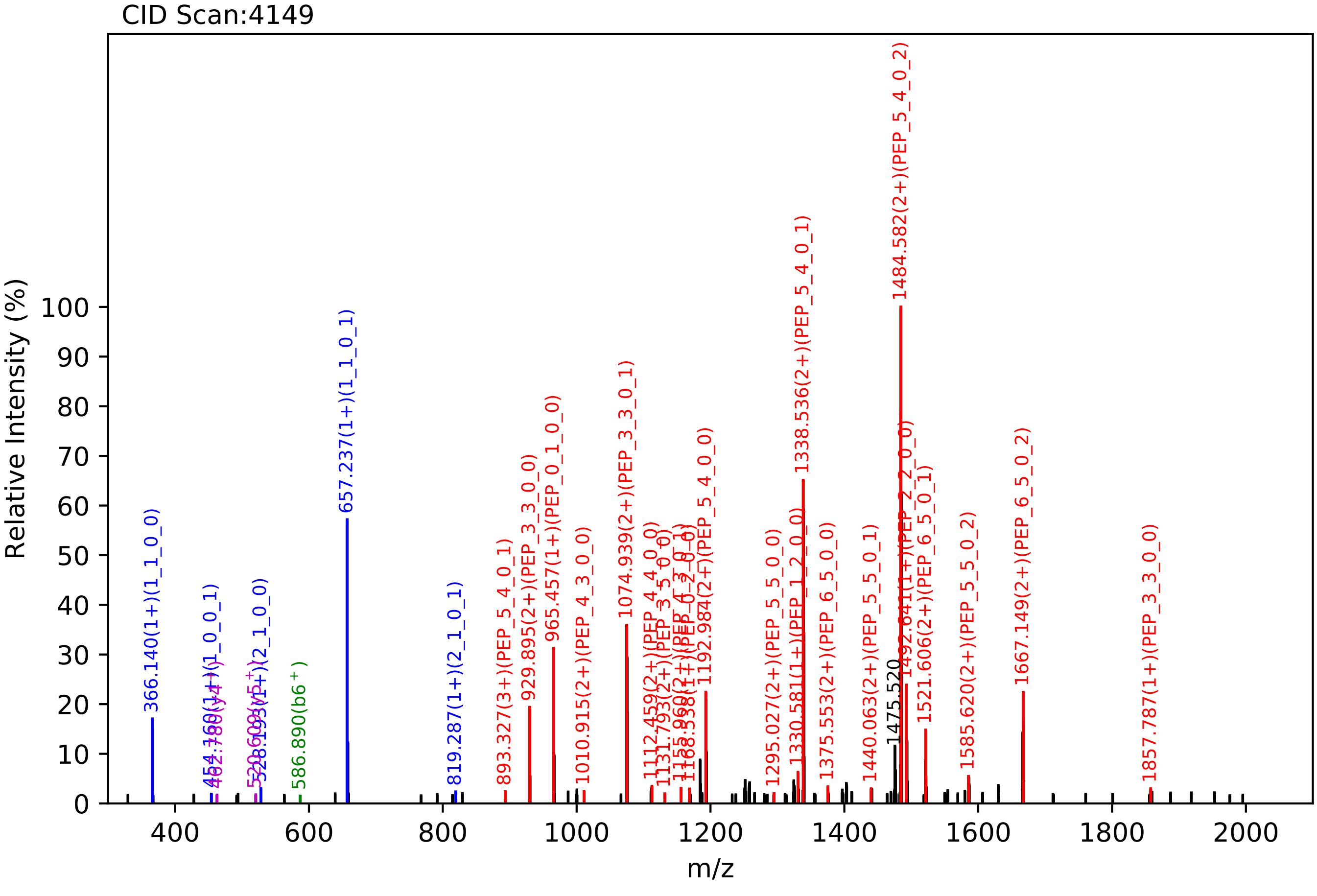

Training set no. 250, Experiment: AGP exp_2

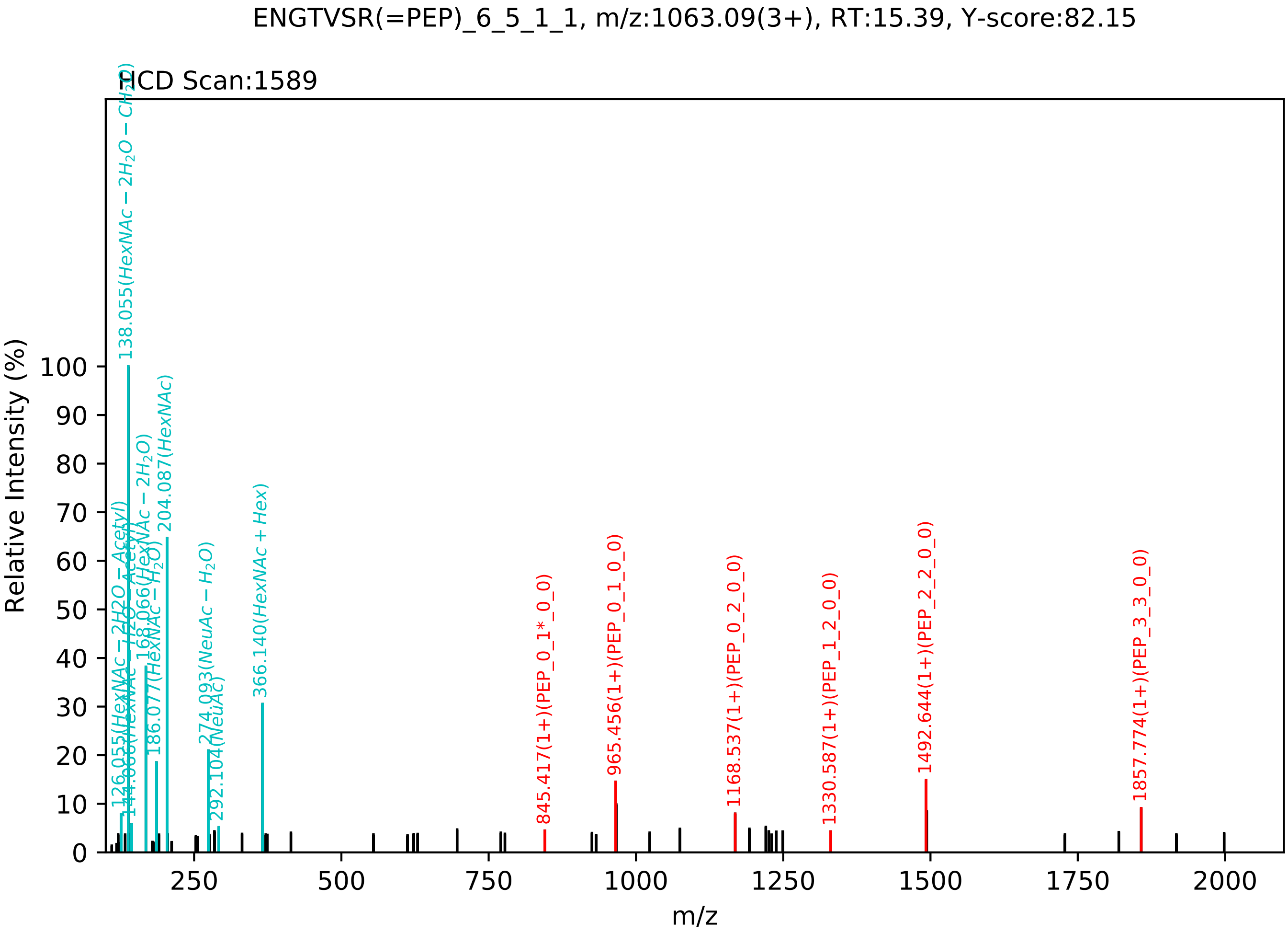

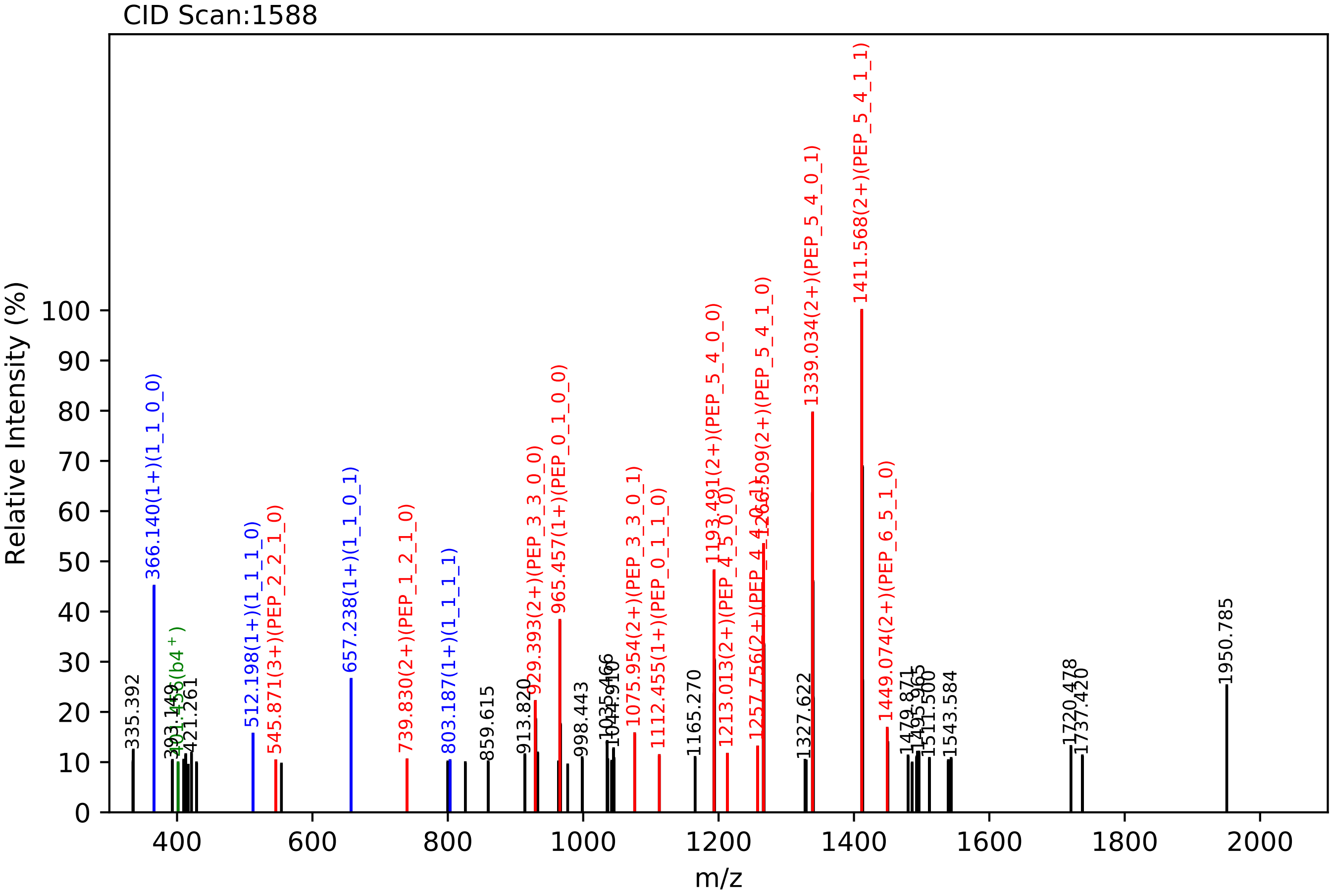

Training set no. 251, Experiment: AGP exp_2

ENGTVSR(=PEP)_6_5_1_2, m/z:1159.79(3+), RT:18.00, Y-score:89.09

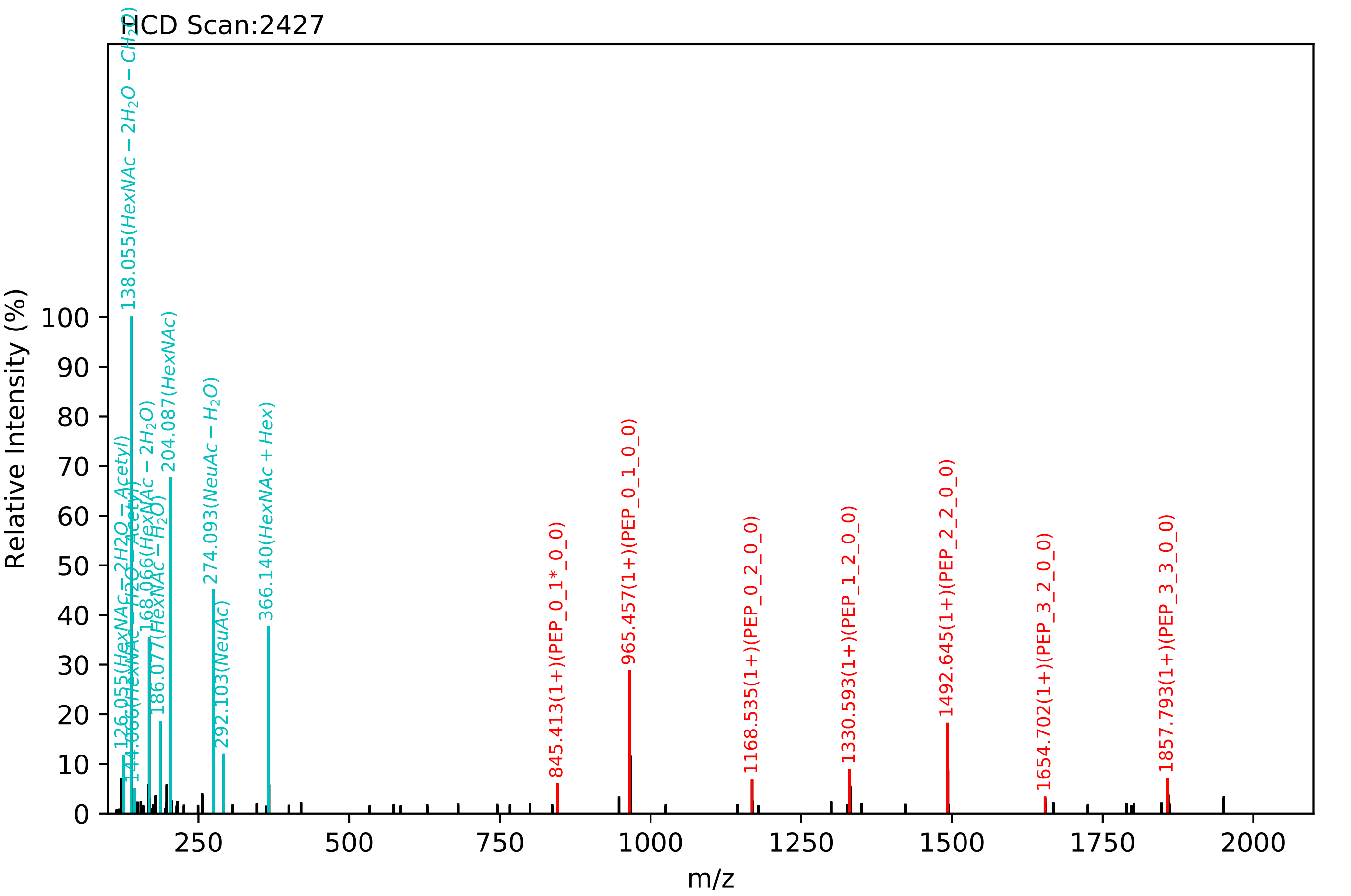

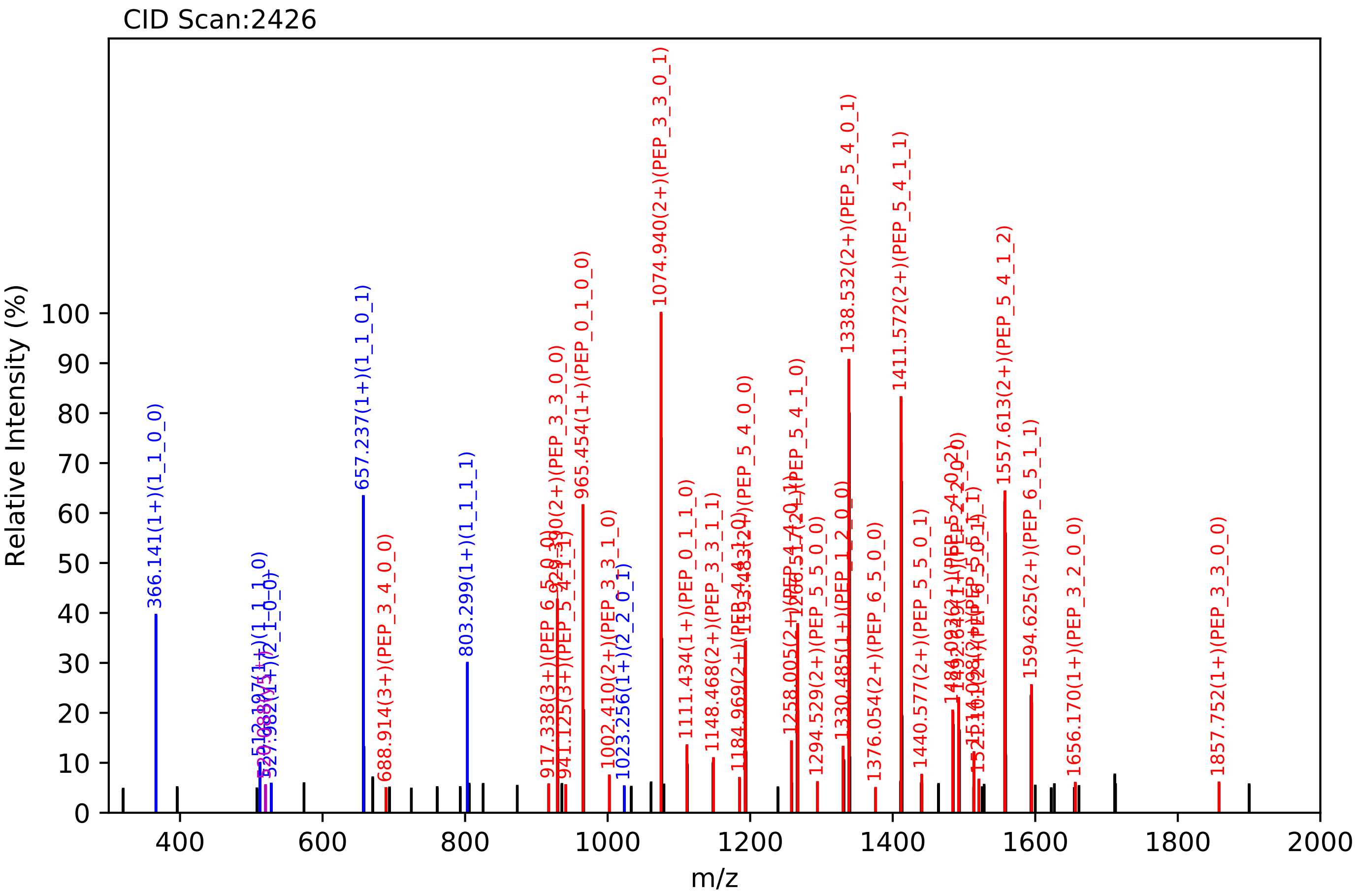

Training set no. 252, Experiment: AGP exp_1

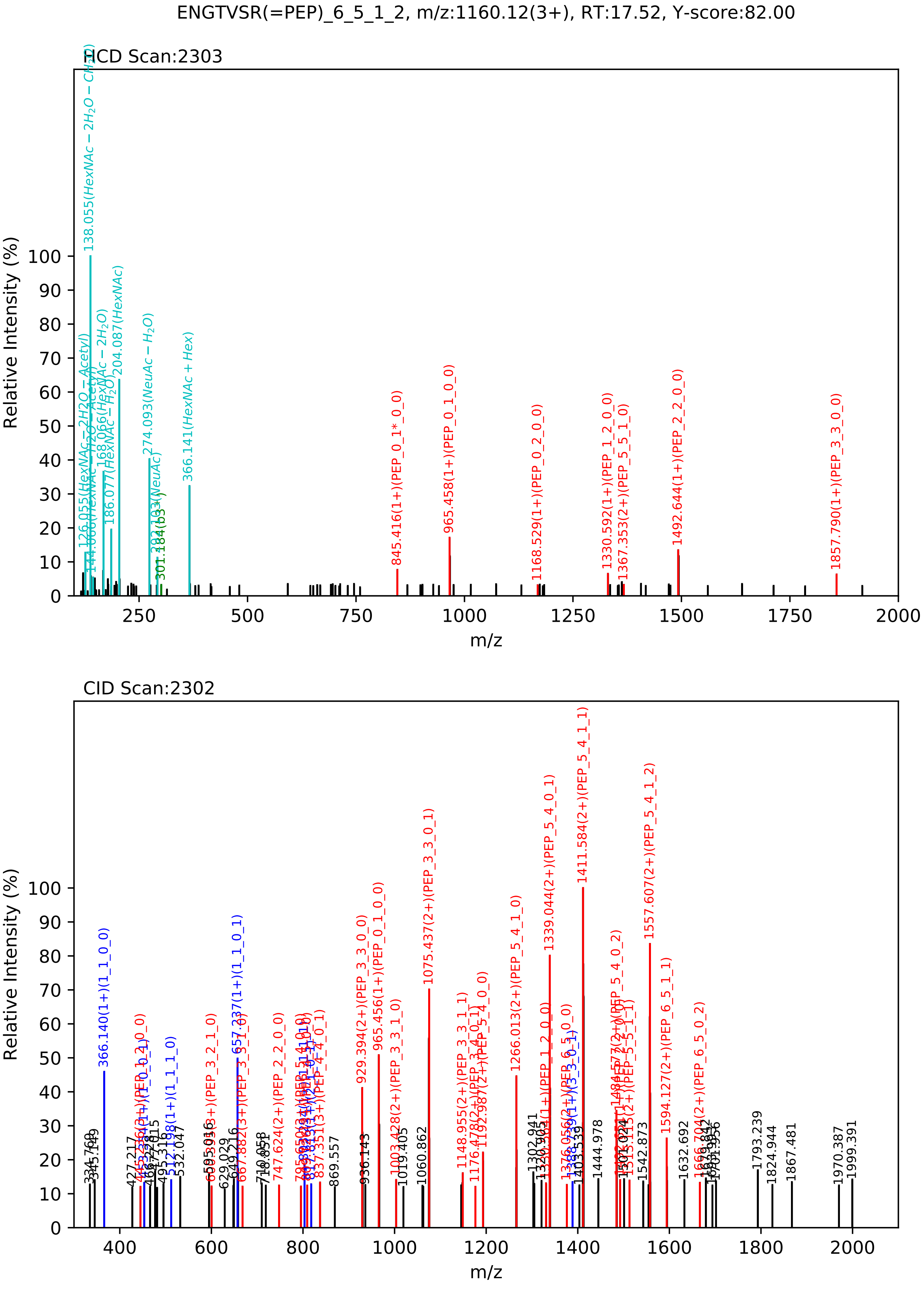

Training set no. 253, Experiment: AGP exp_2

ENGTVSR(=PEP)_6_5_1_3, m/z:1257.15(3+), RT:20.99, Y-score:89.77

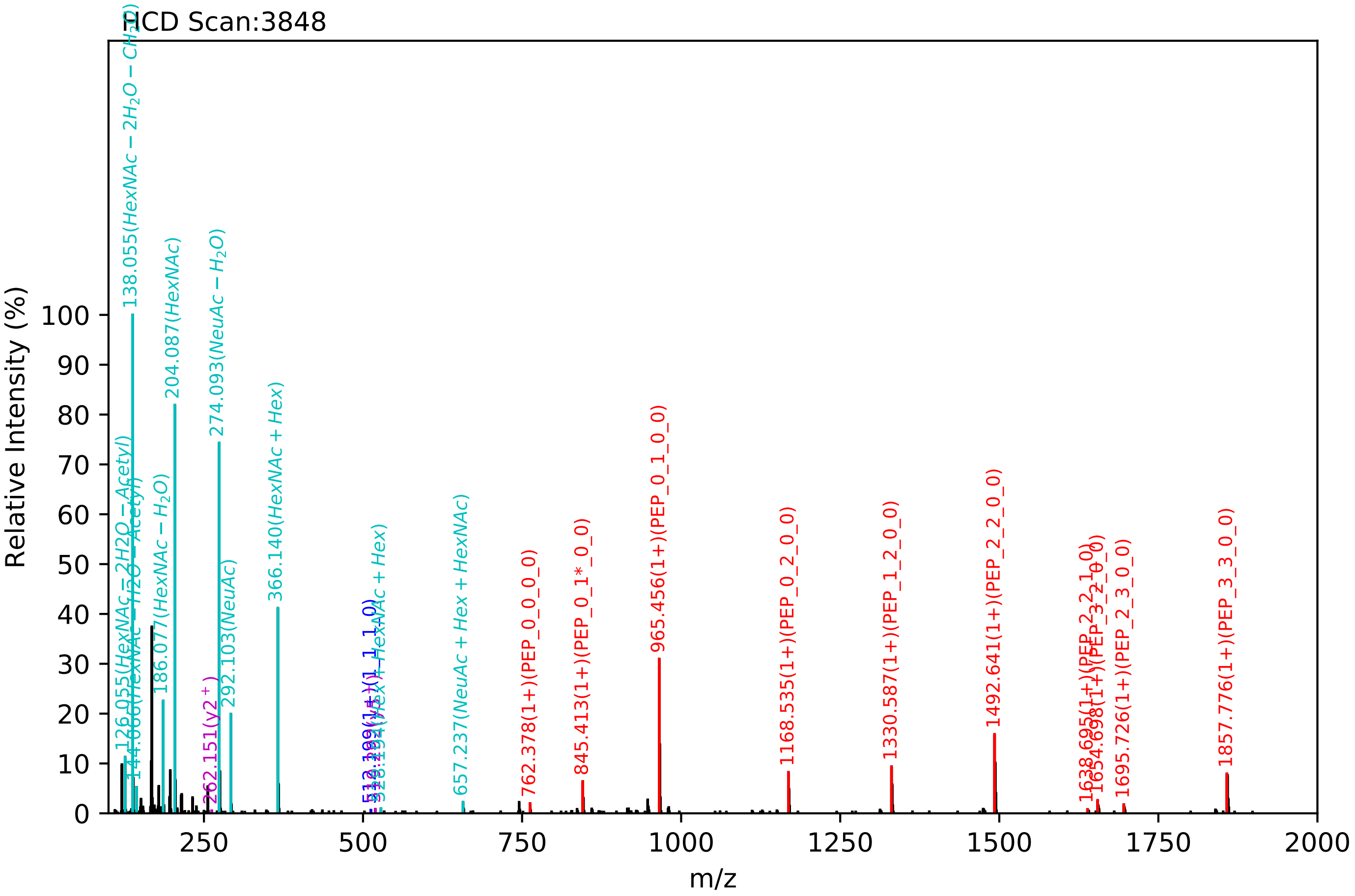

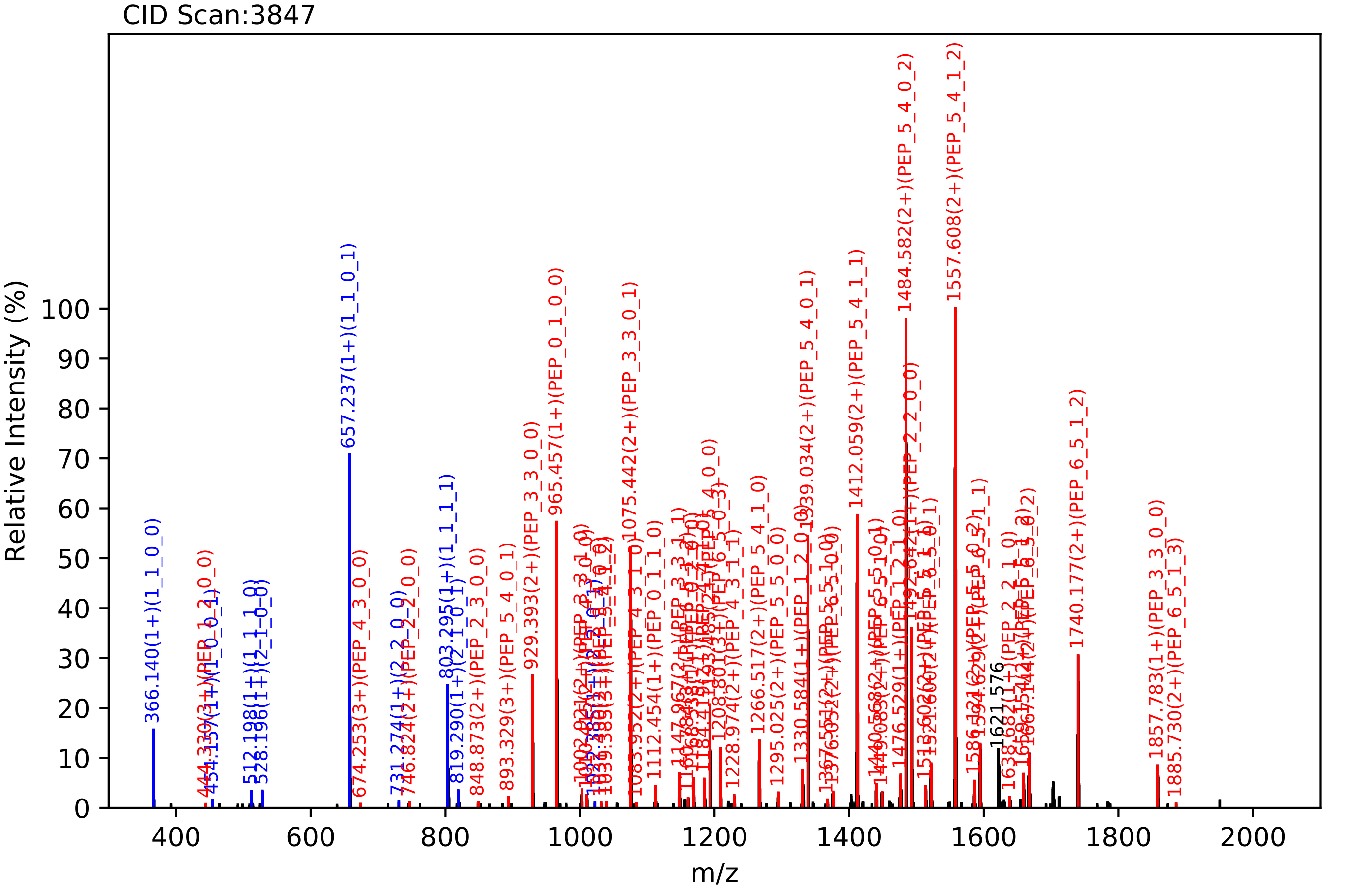

Training set no. 254, Experiment: AGP exp_1

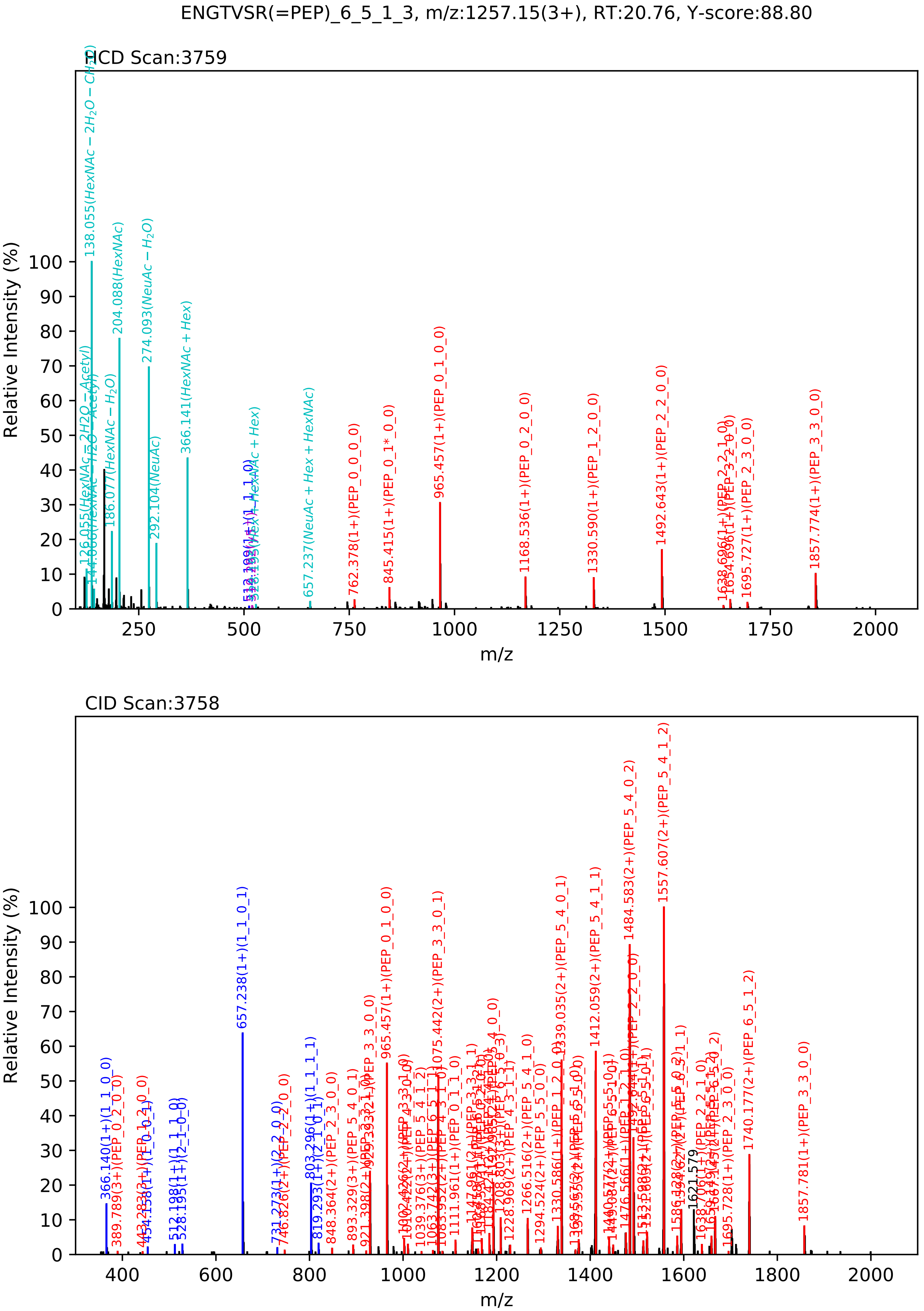

Training set no. 255, Experiment: AGP exp_1

ENGTVSR(=PEP)_6_5_1_3, m/z:1256.82(3+), RT:21.28, Y-score:78.08

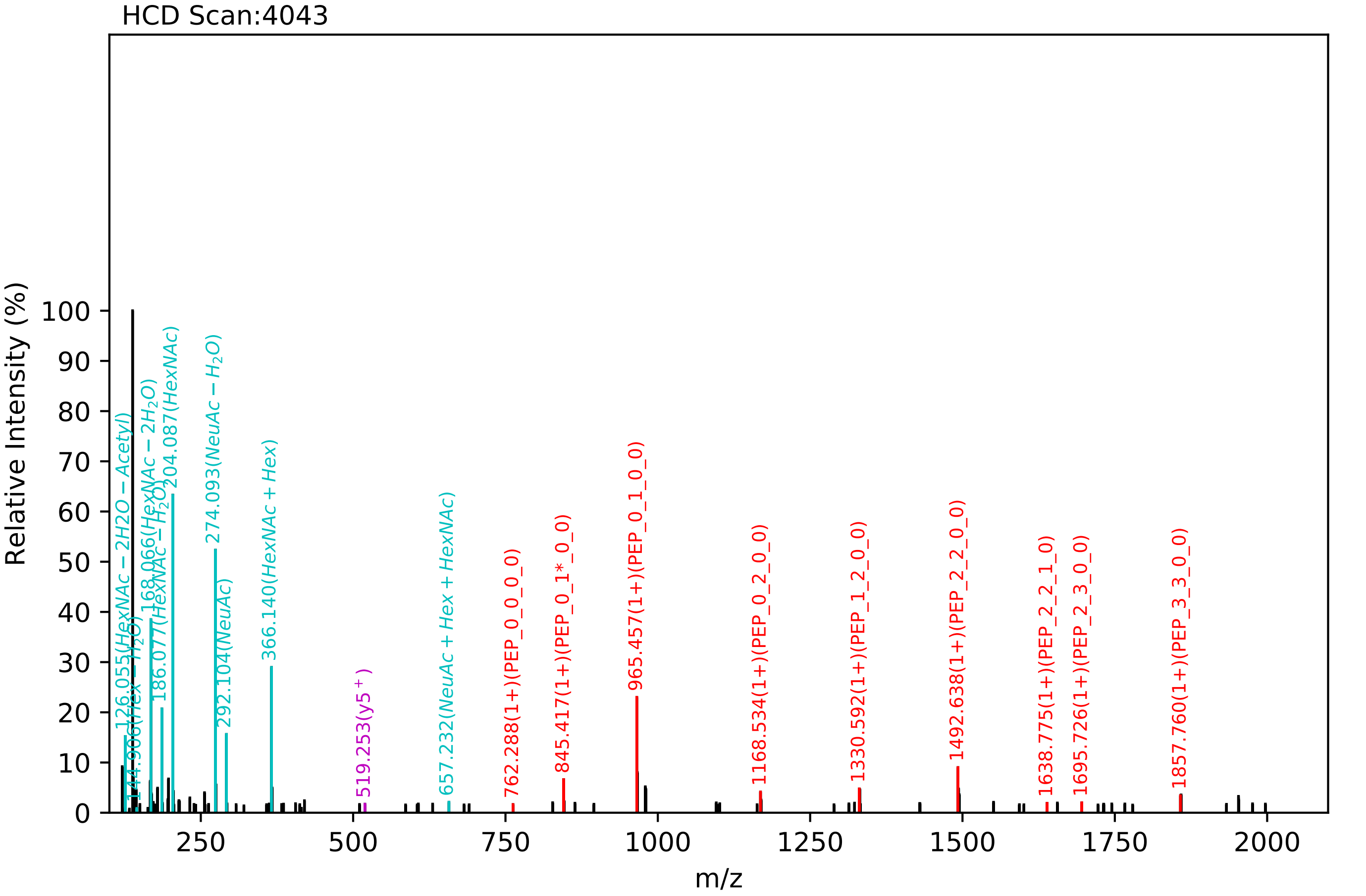

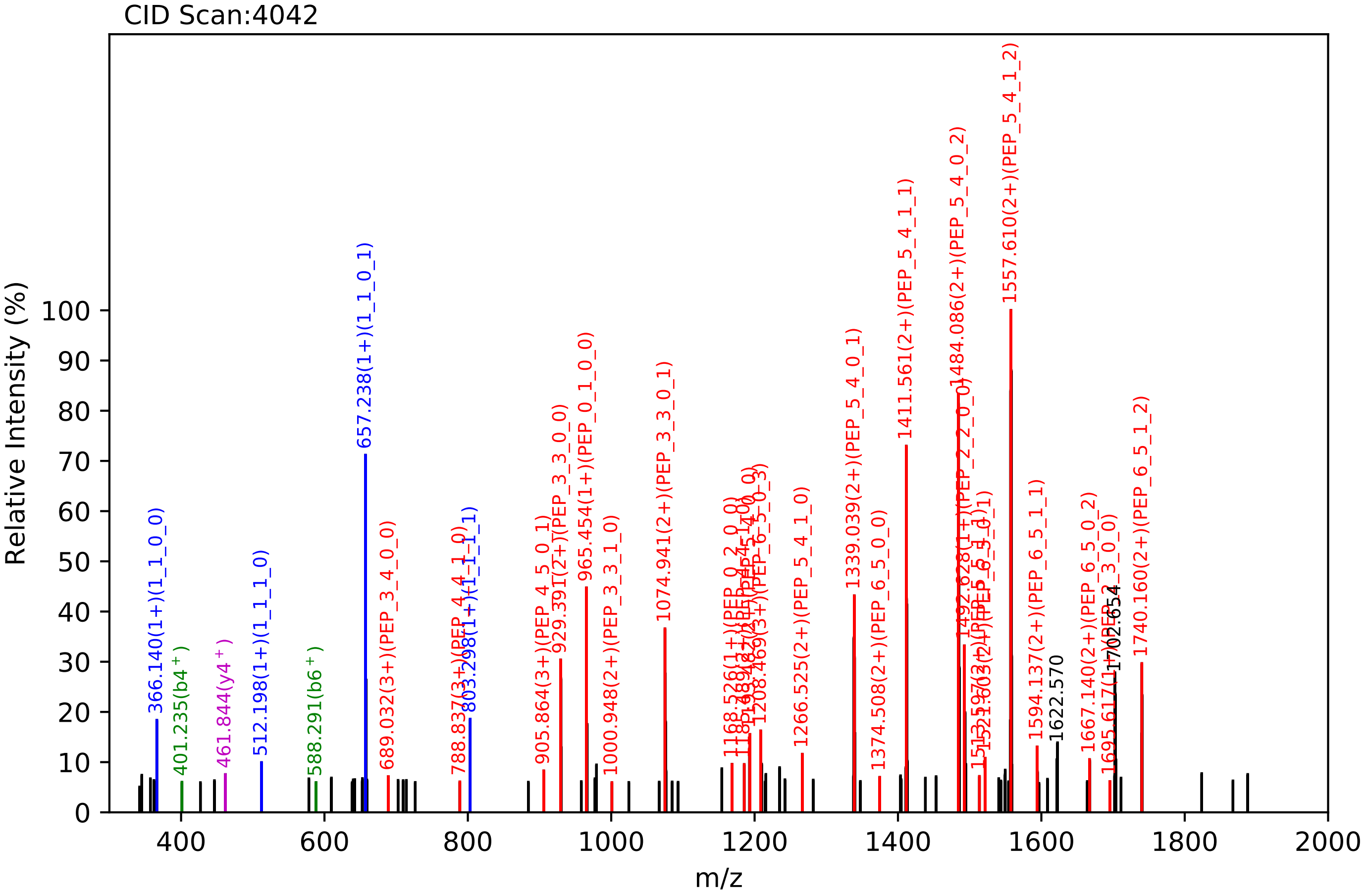

Training set no. 256, Experiment: AGP exp_28

ENGTVSR(=PEP)_6_5_2_3, m/z:1305.84(3+), RT:26.94, Y-score:92.58

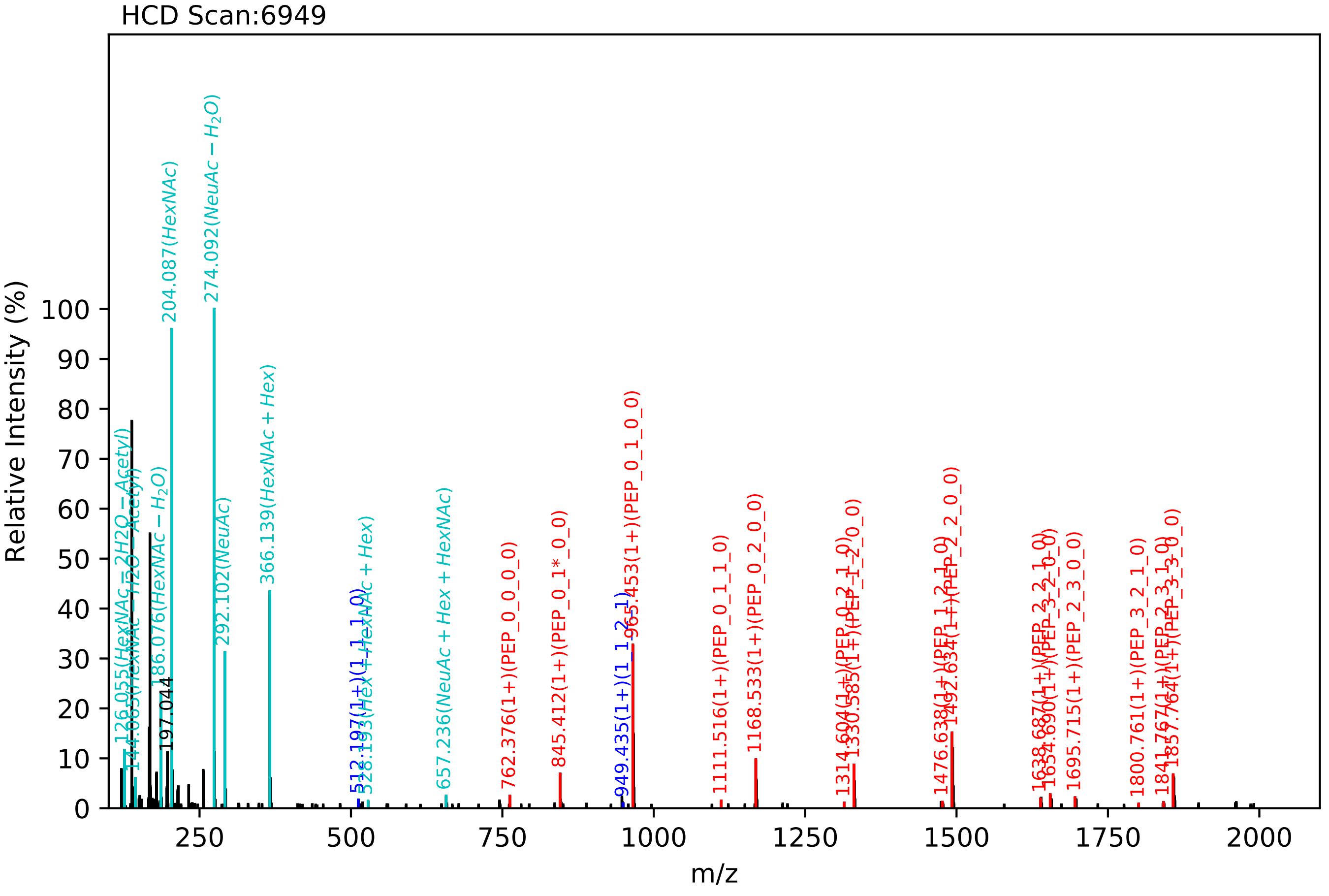

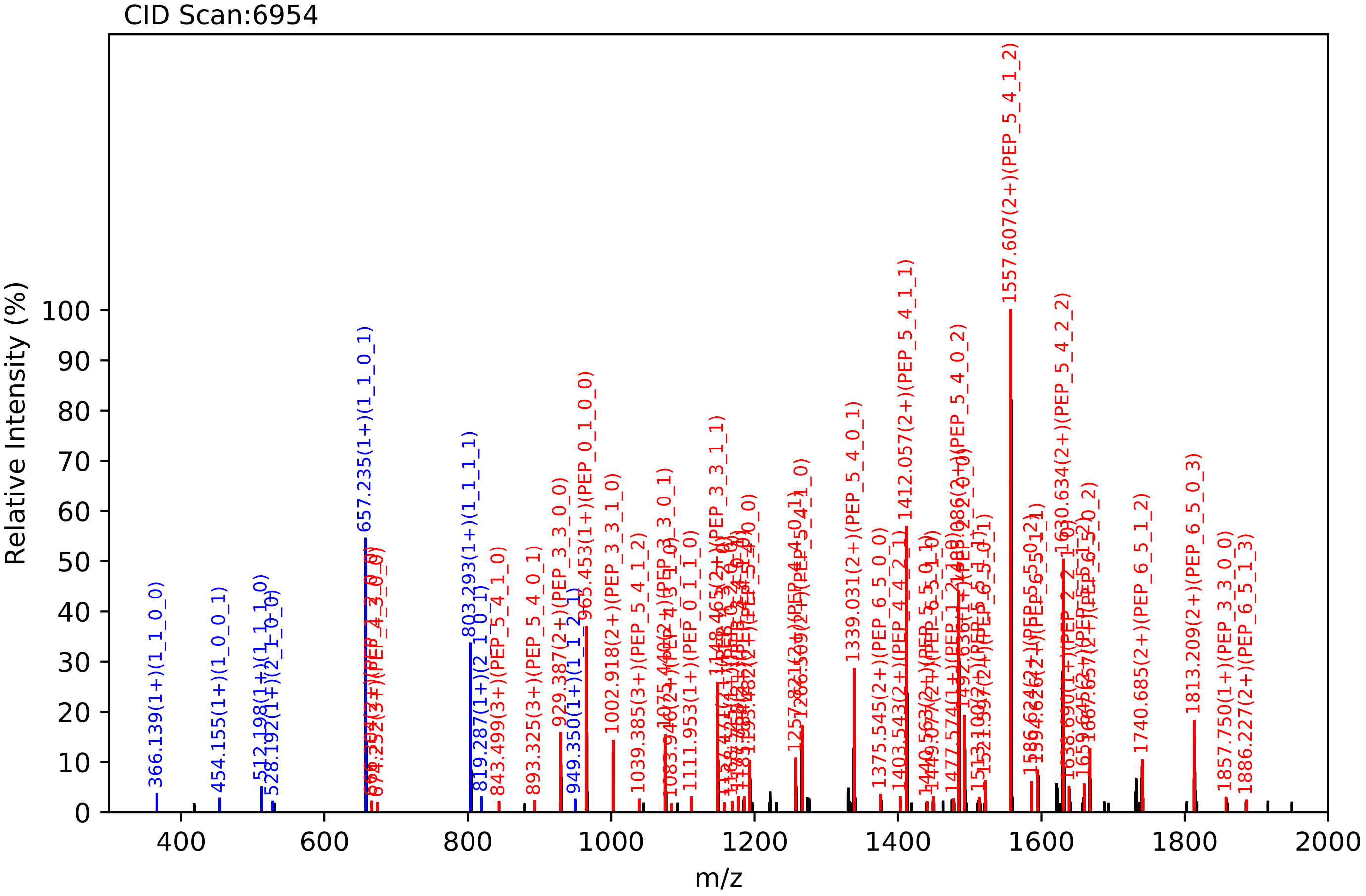

Training set no. 257, Experiment: AGP exp_26

ENGTVSR(=PEP)_6_5_2_3, m/z:1305.84(3+), RT:26.76, Y-score:91.79

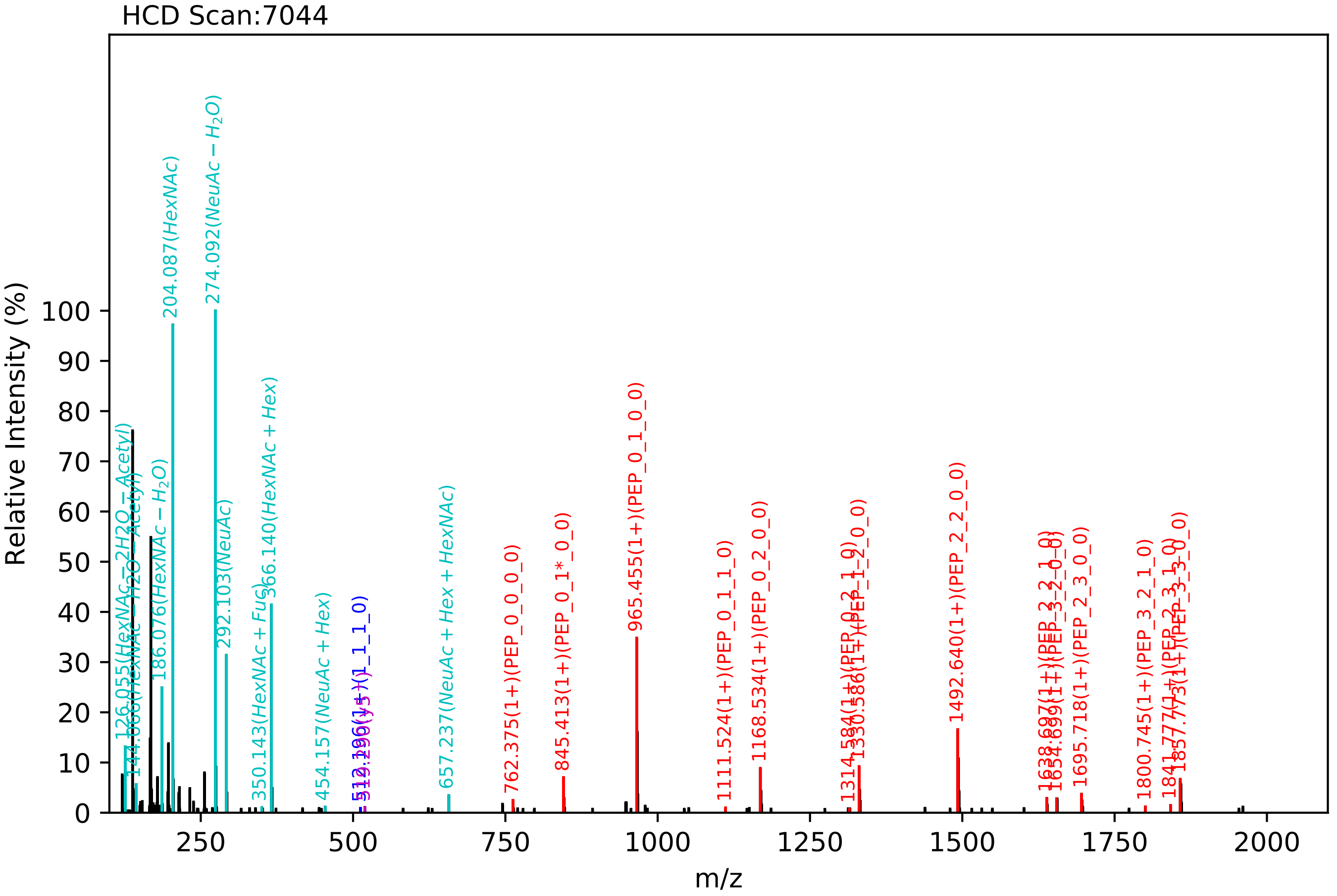

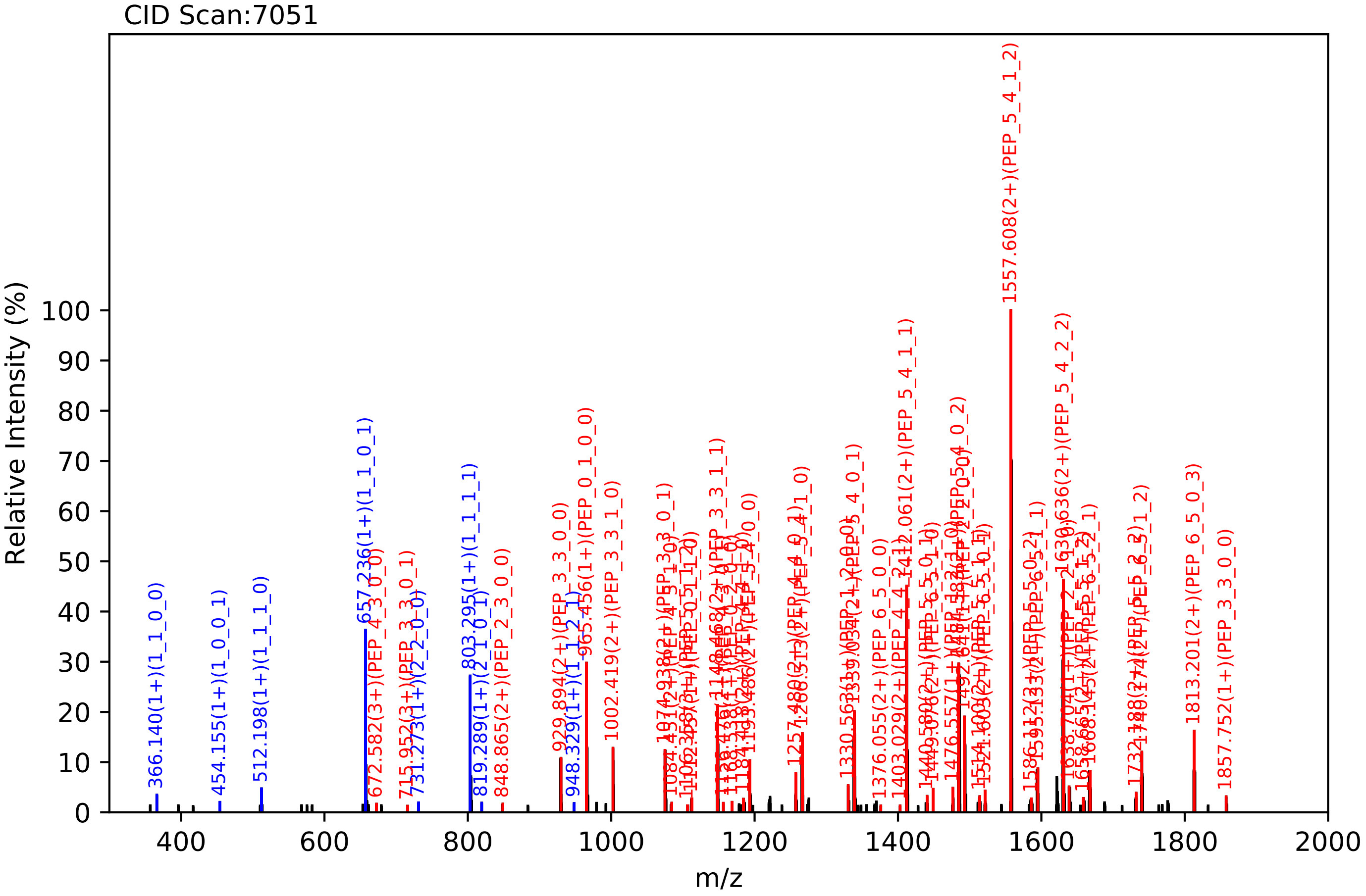

Training set no. 258, Experiment: AGP exp_36

ENGTVSR(=PEP)_6_5_2_3, m/z:1305.84(3+), RT:27.16, Y-score:91.27

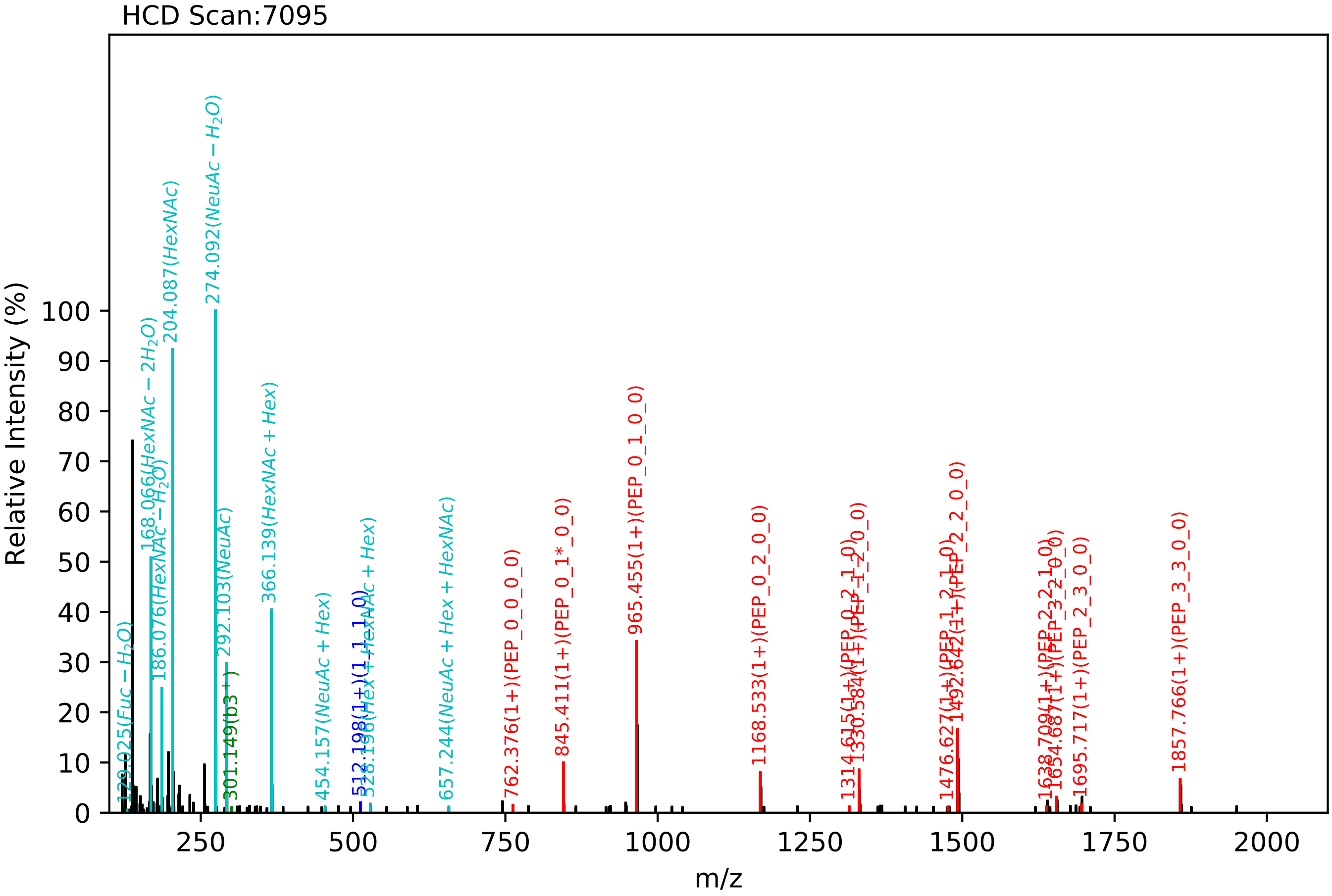

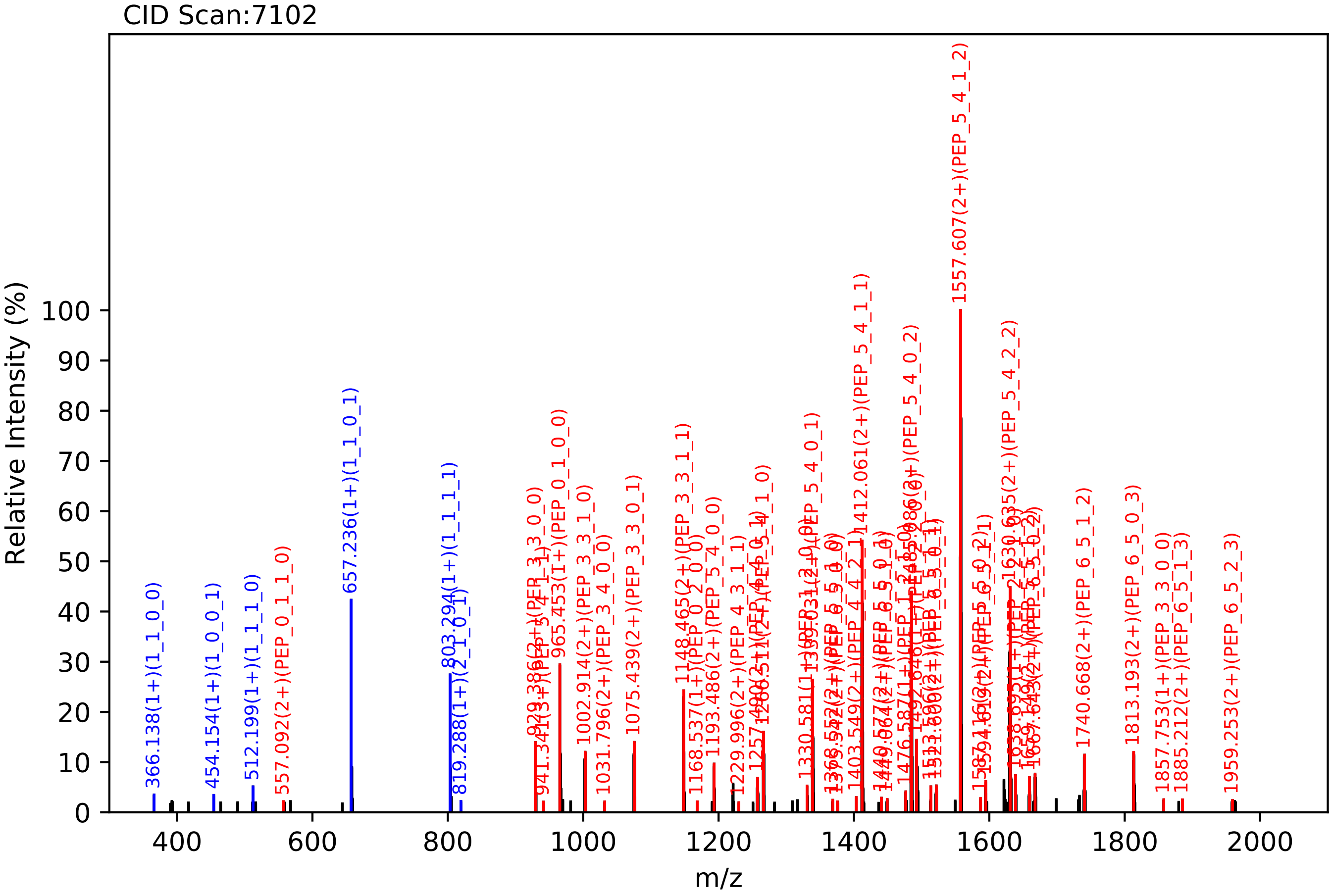

Training set no. 259, Experiment: AGP exp_27

ENGTVSR(=PEP)_6_5_2_3, m/z:1305.84(3+), RT:26.80, Y-score:88.57

HCD Scan:6627

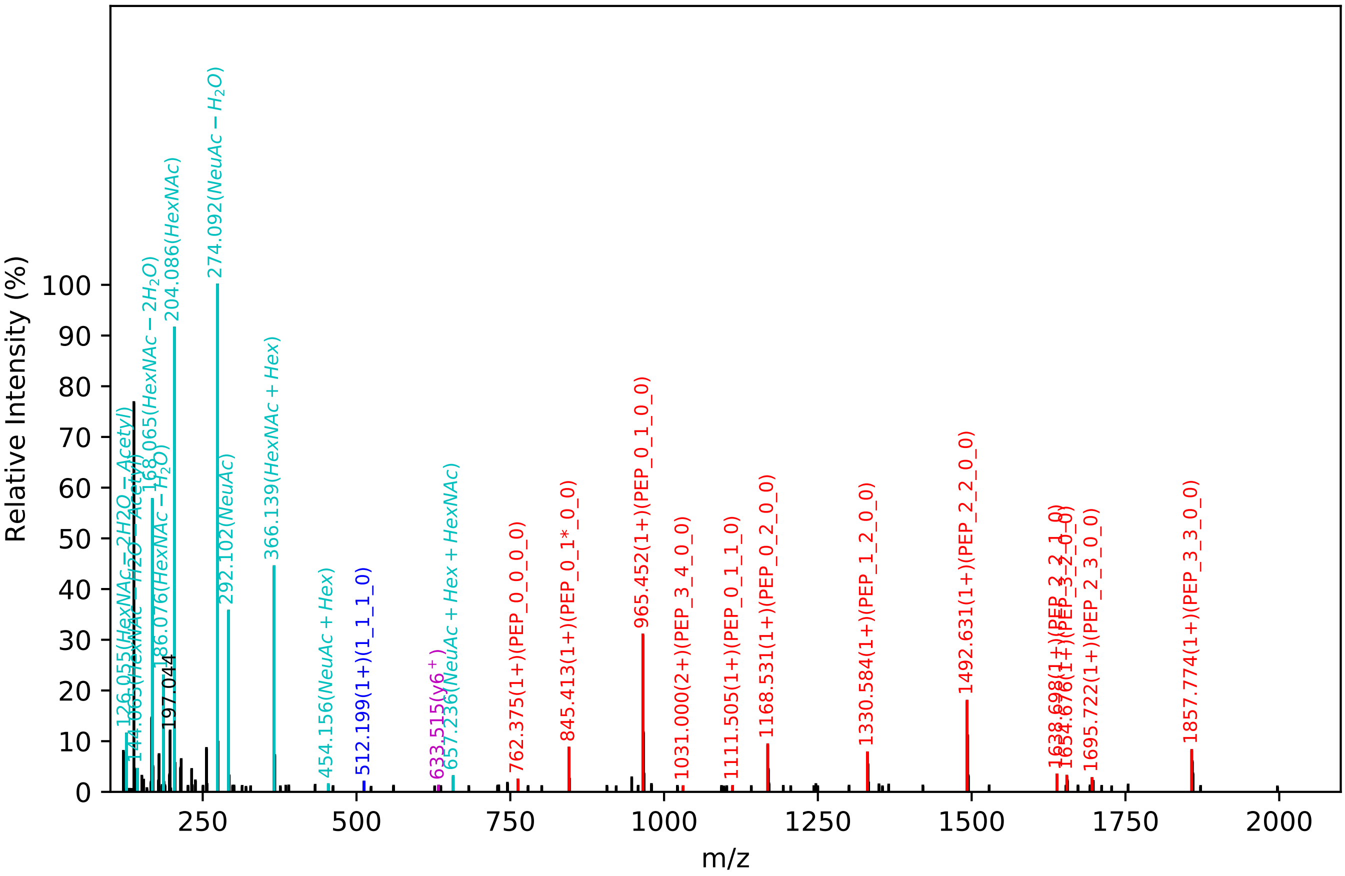

CID Scan:6629

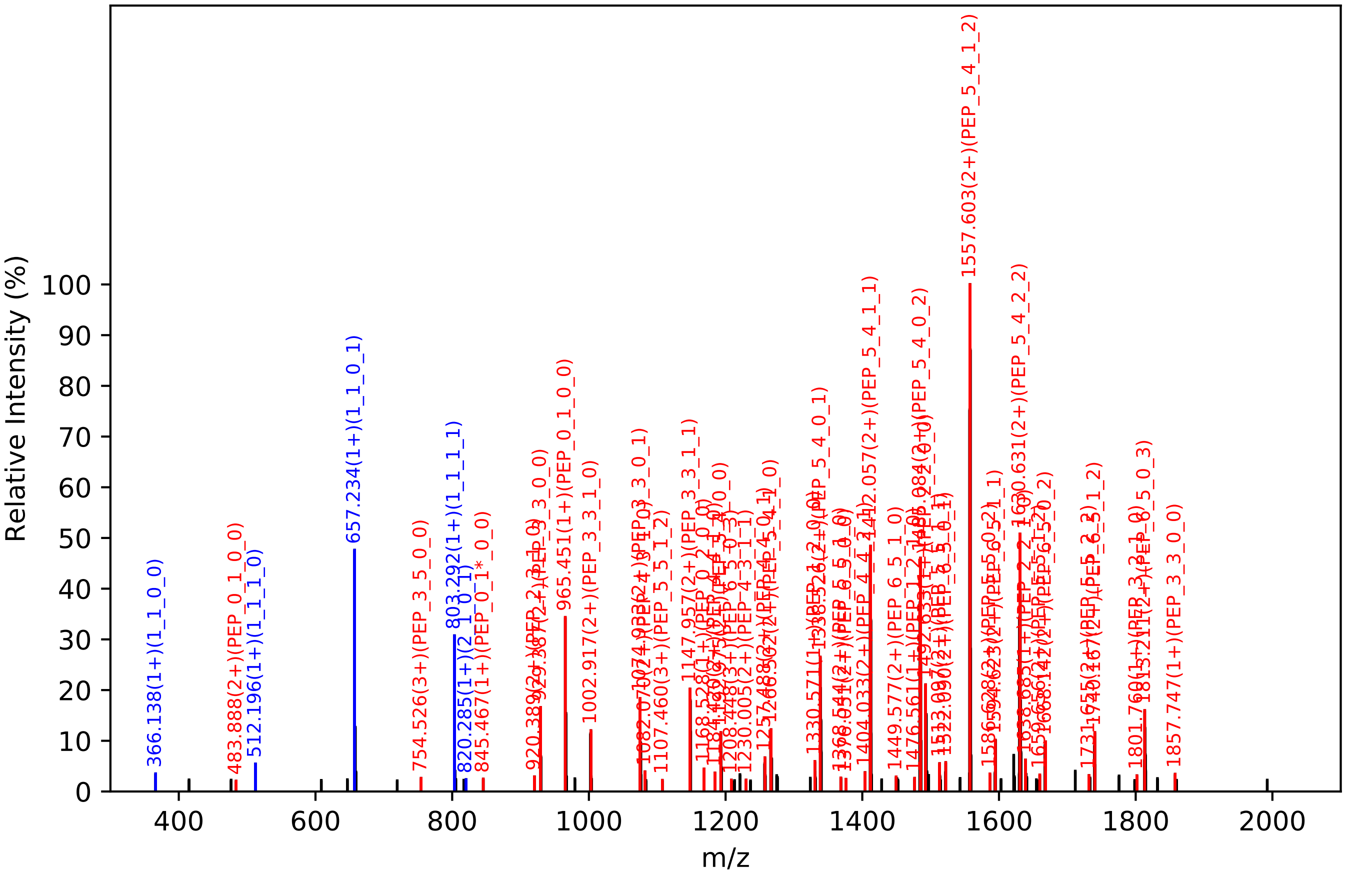

Training set no. 260, Experiment: AGP exp_22

ENGTVSR(=PEP)_6_5_2_3, m/z:1305.84(3+), RT:26.19, Y-score:87.72

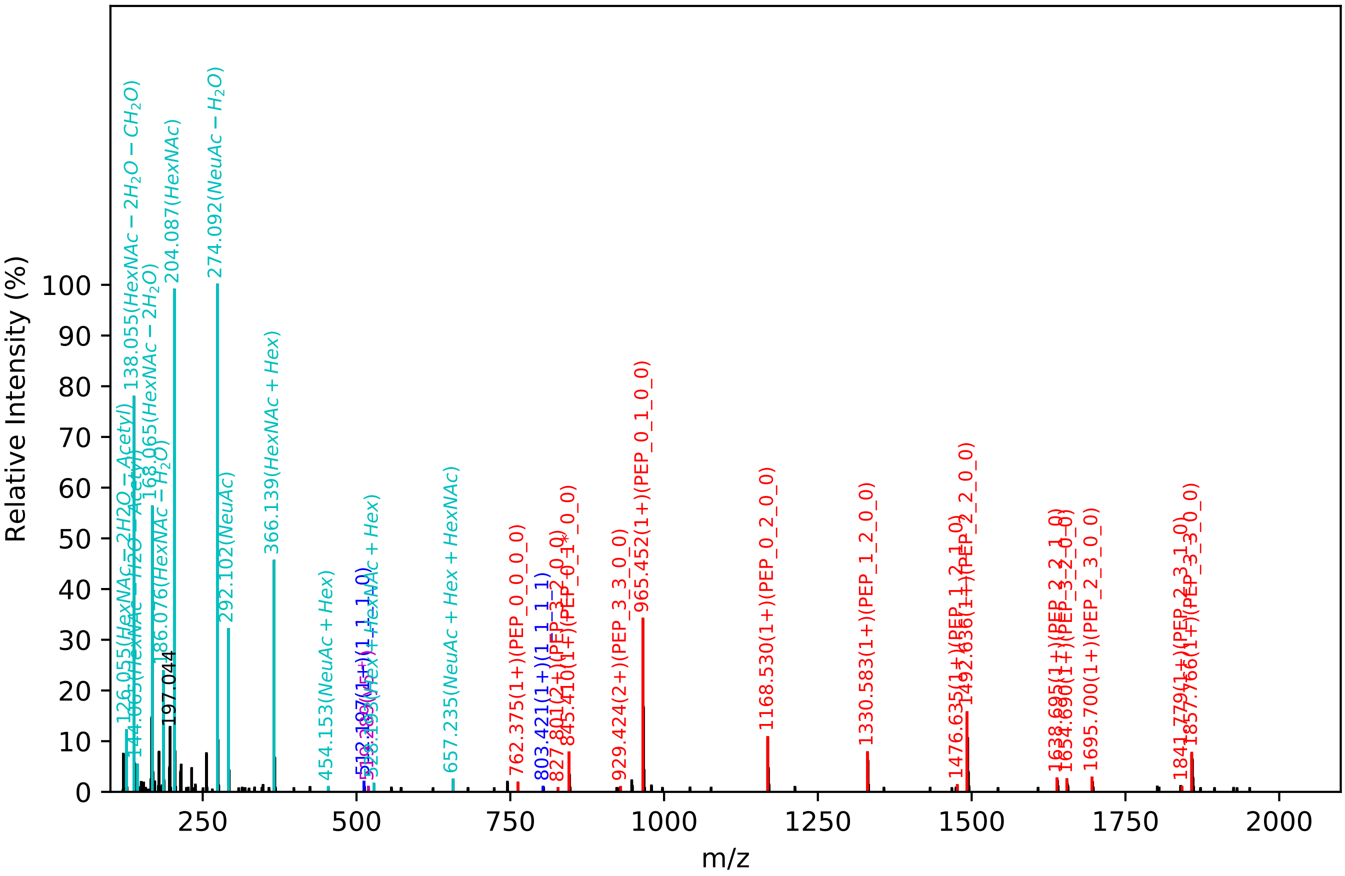

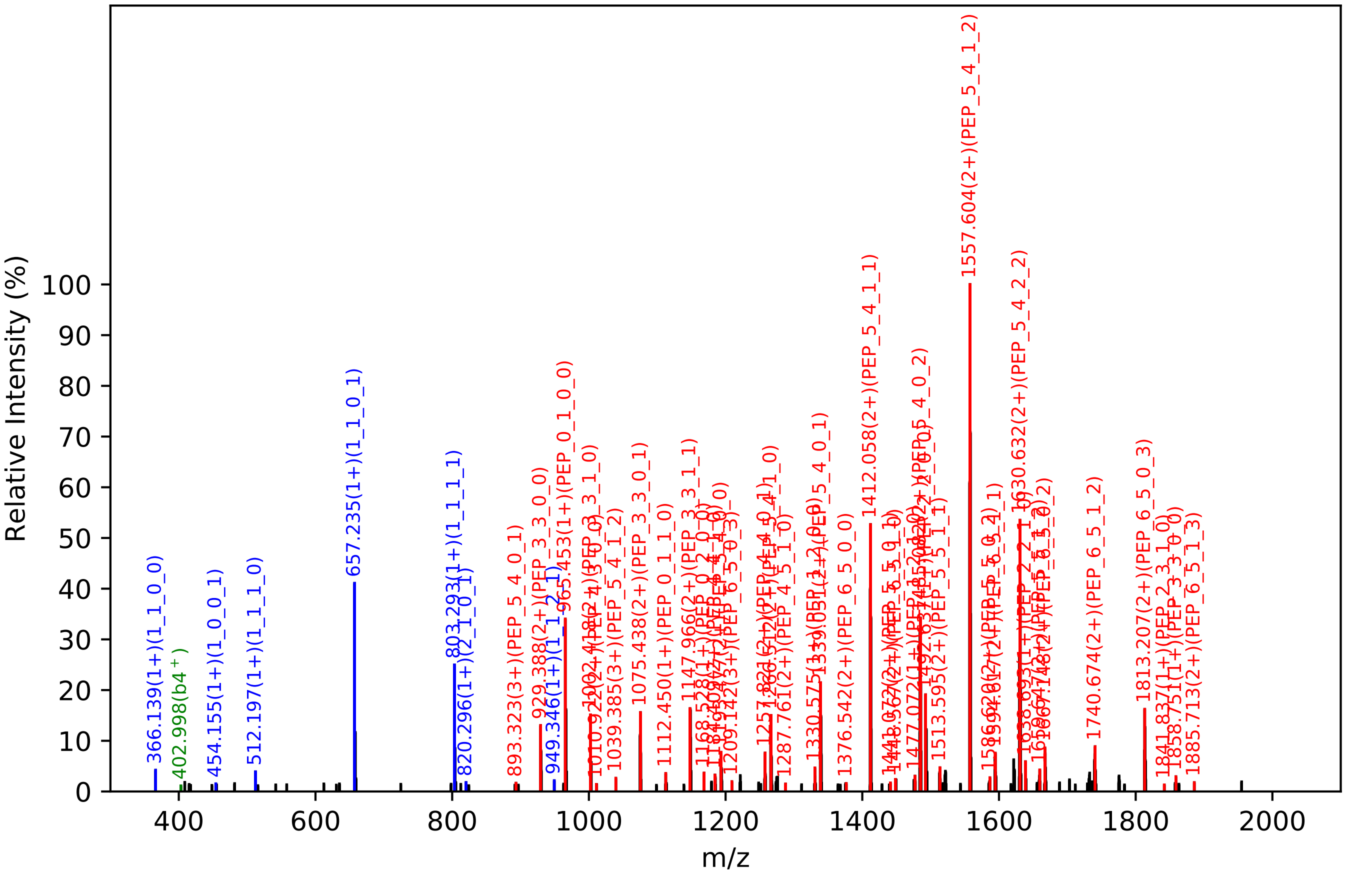

Training set no. 261, Experiment: AGP exp_14

ENGTVSR(=PEP)_6_5_2_3, m/z:1305.84(3+), RT:30.01, Y-score:83.96

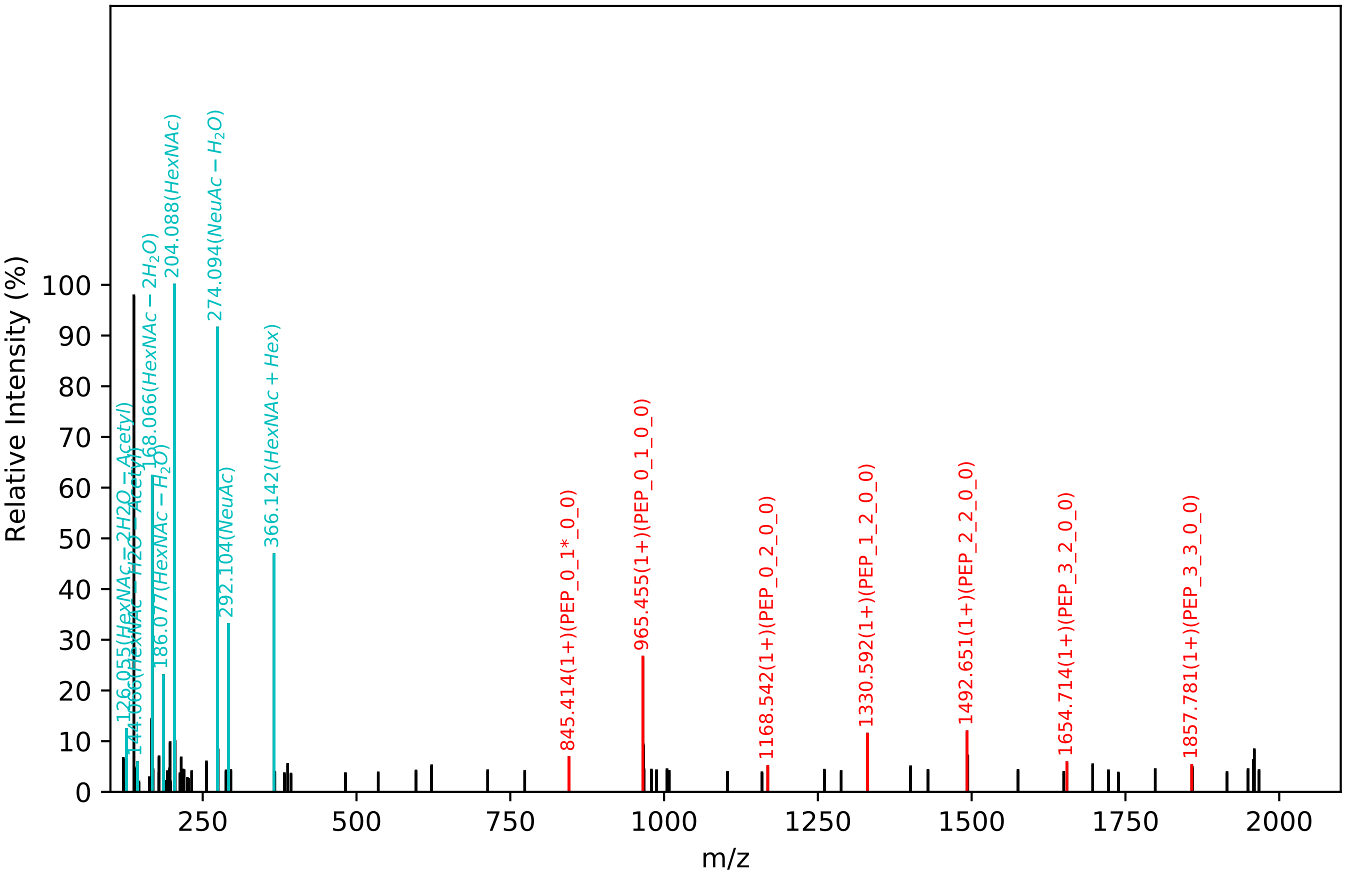

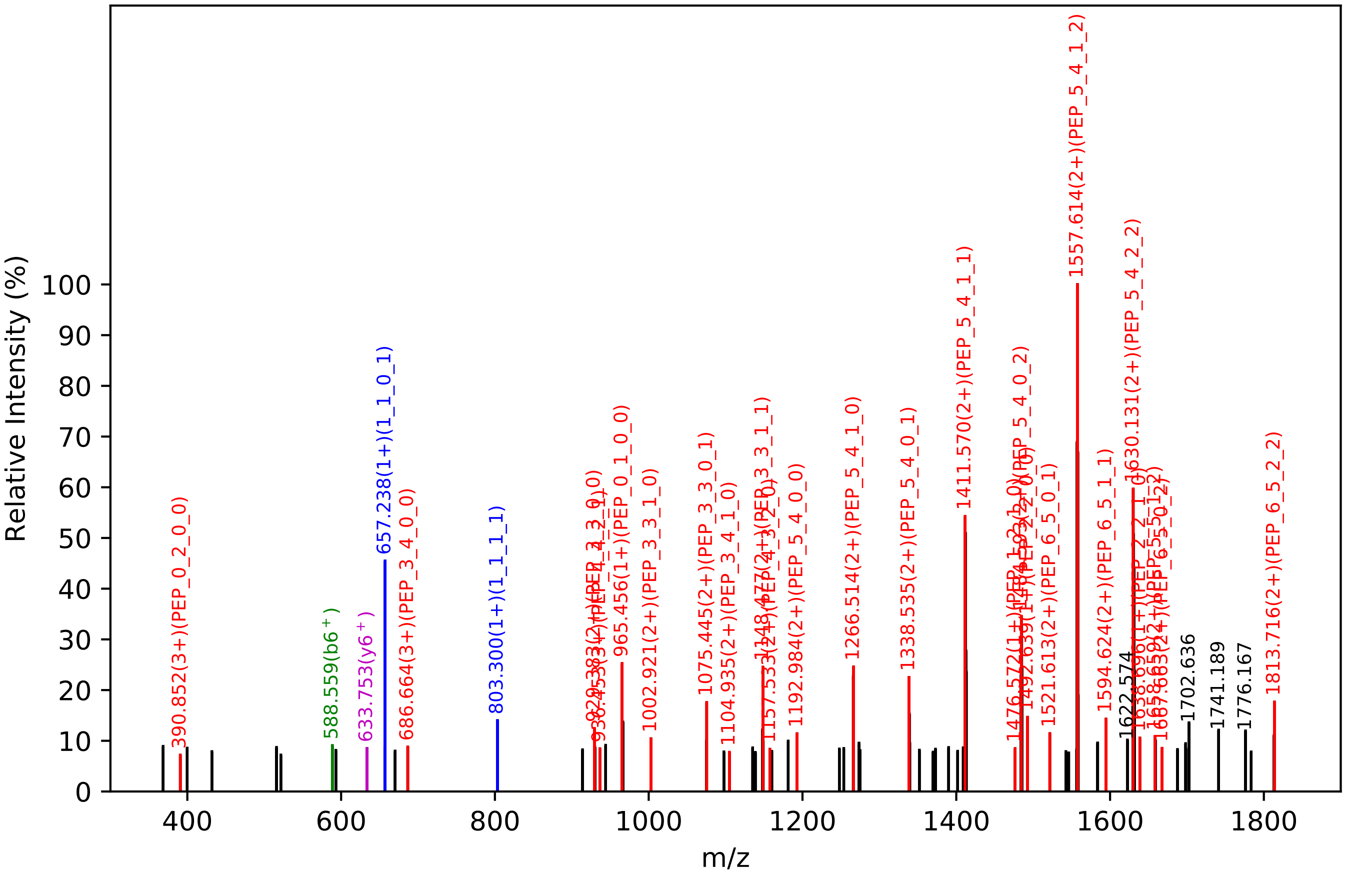

Training set no. 262, Experiment: AGP exp_5

ENGTVSR(=PEP)_6_5_2_3, m/z:1305.84(3+), RT:28.86, Y-score:83.01

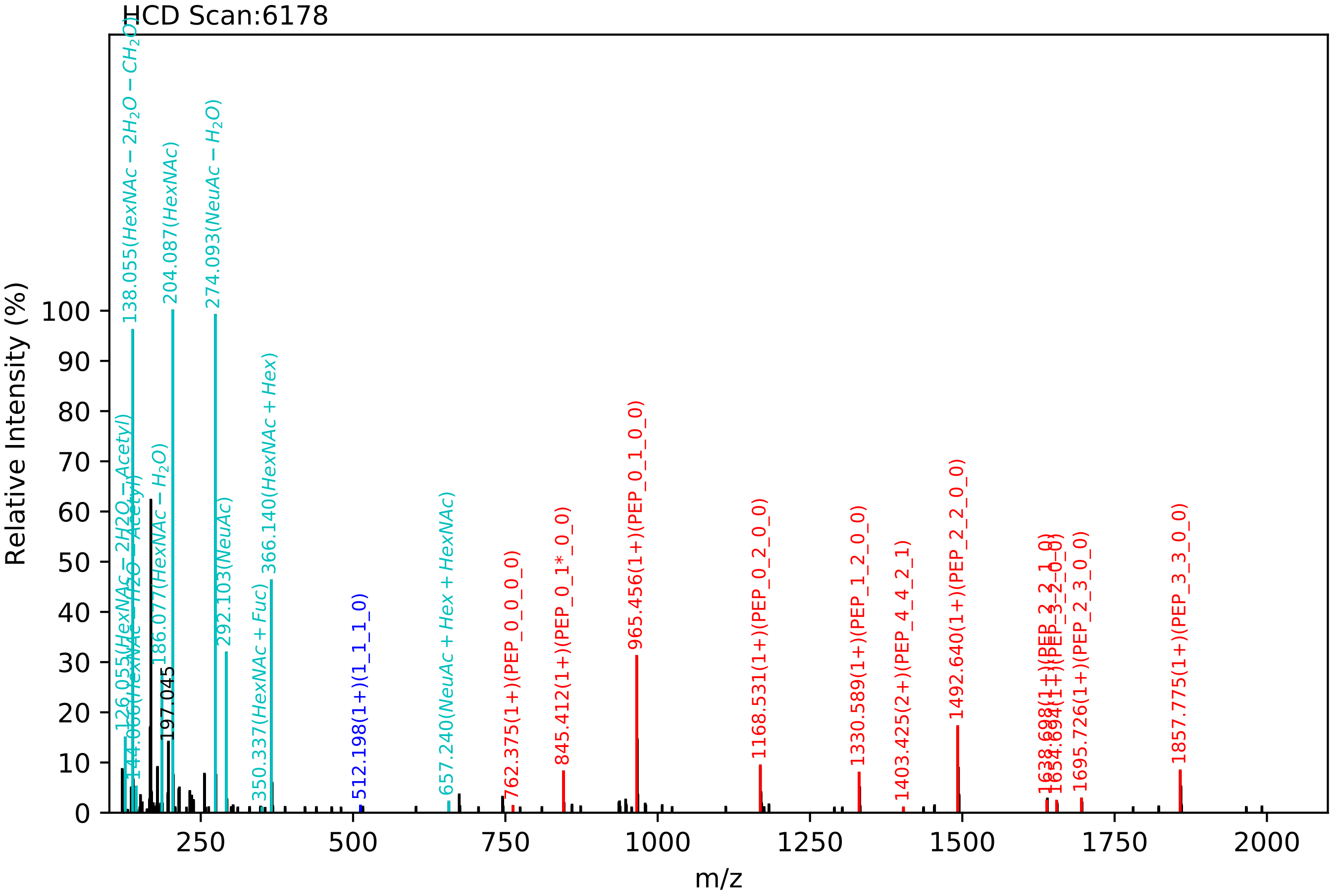

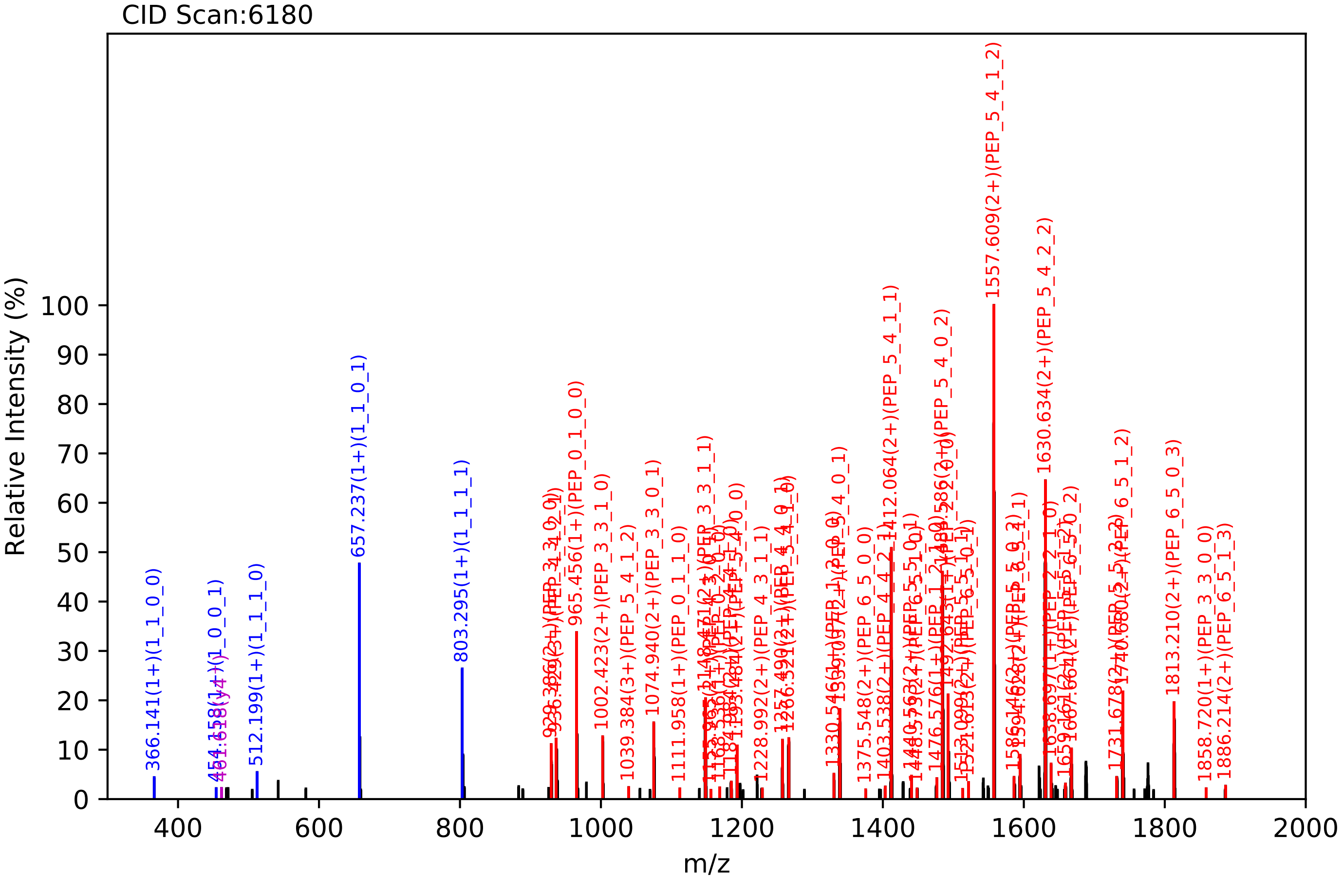

Training set no. 263, Experiment: AGP exp_2

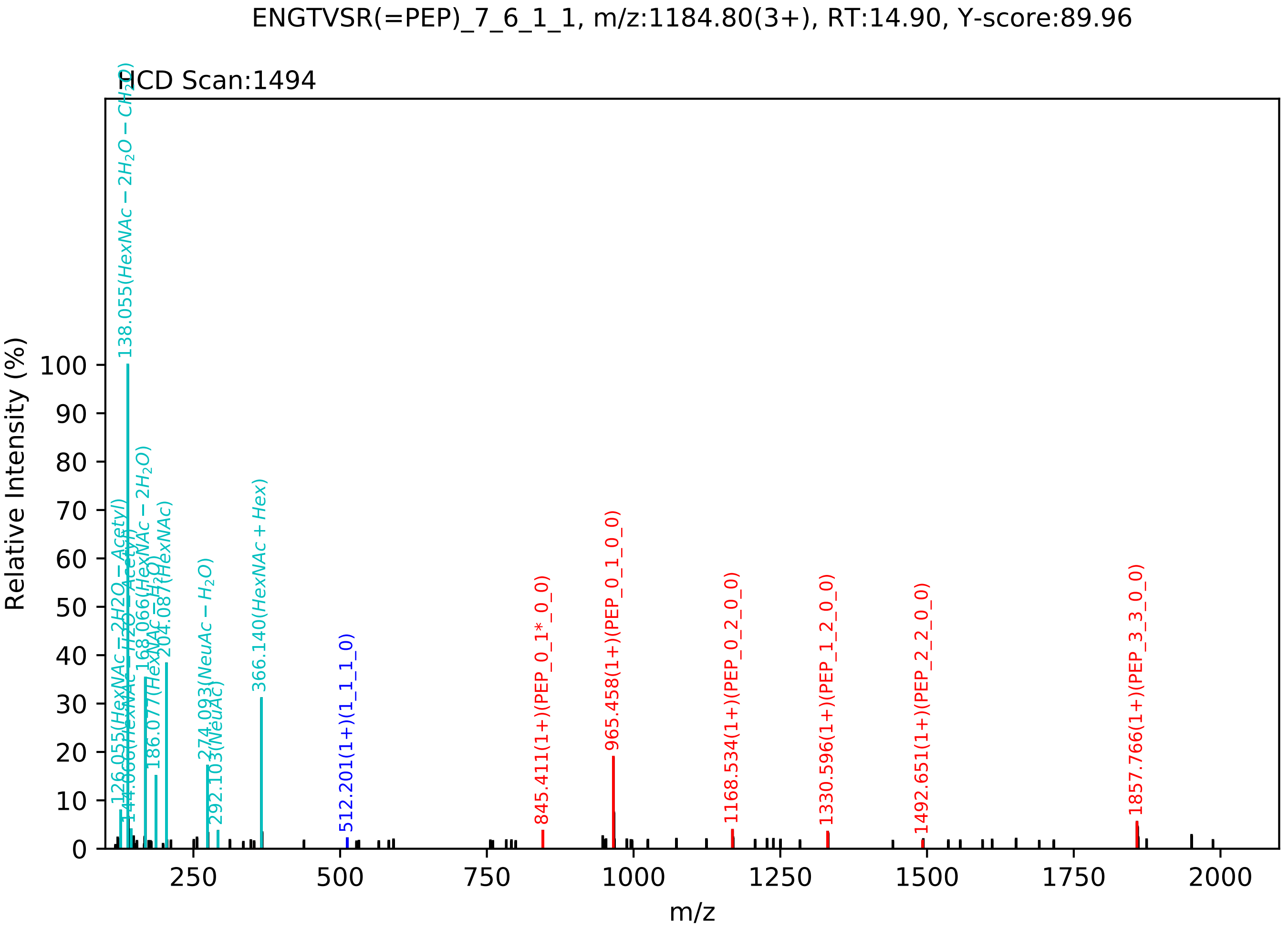

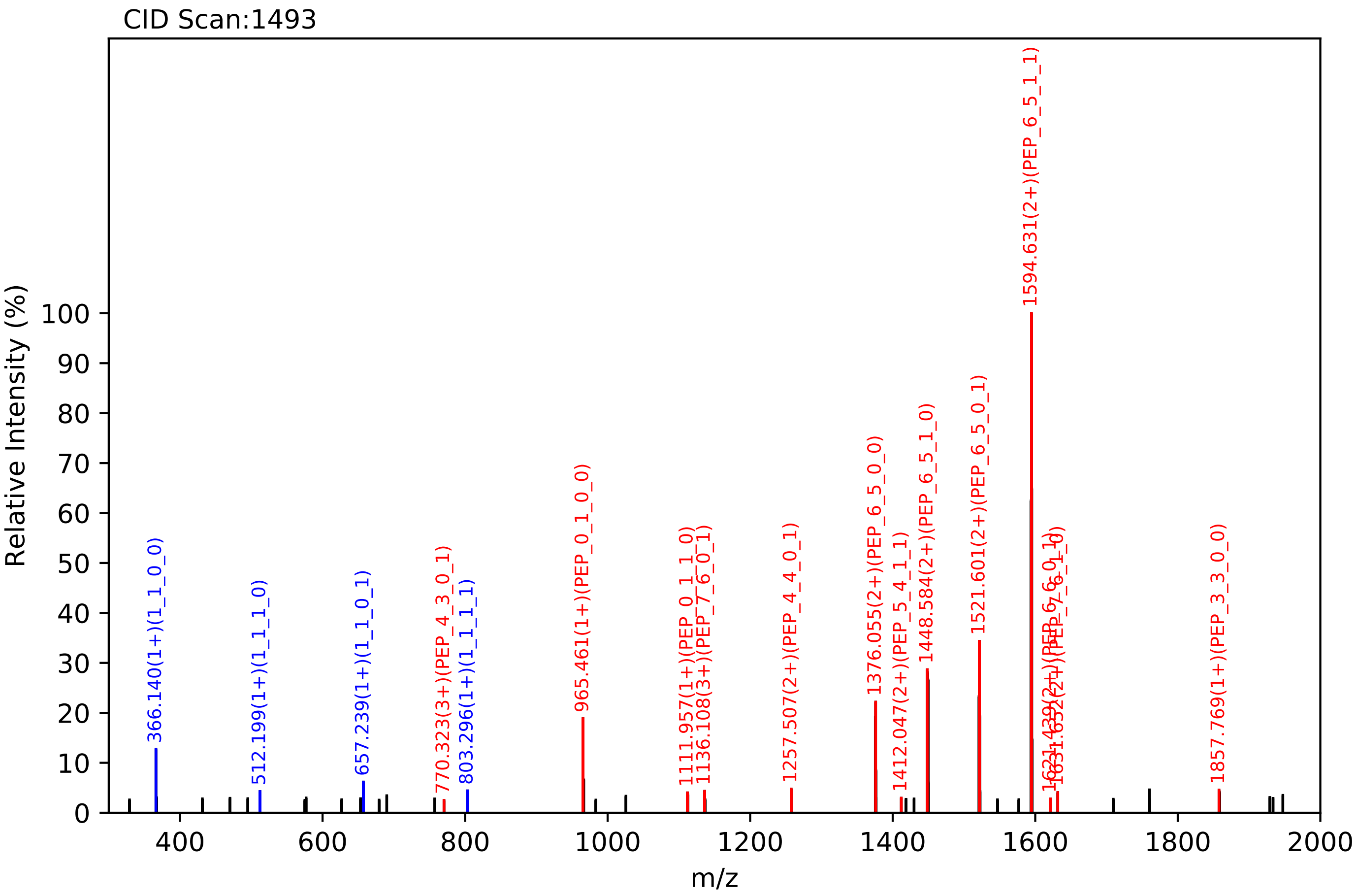

Training set no. 264, Experiment: AGP exp_1

ENGTVSR(=PEP)_7_6_1_2, m/z:1281.83(3+), RT:16.51, Y-score:91.29

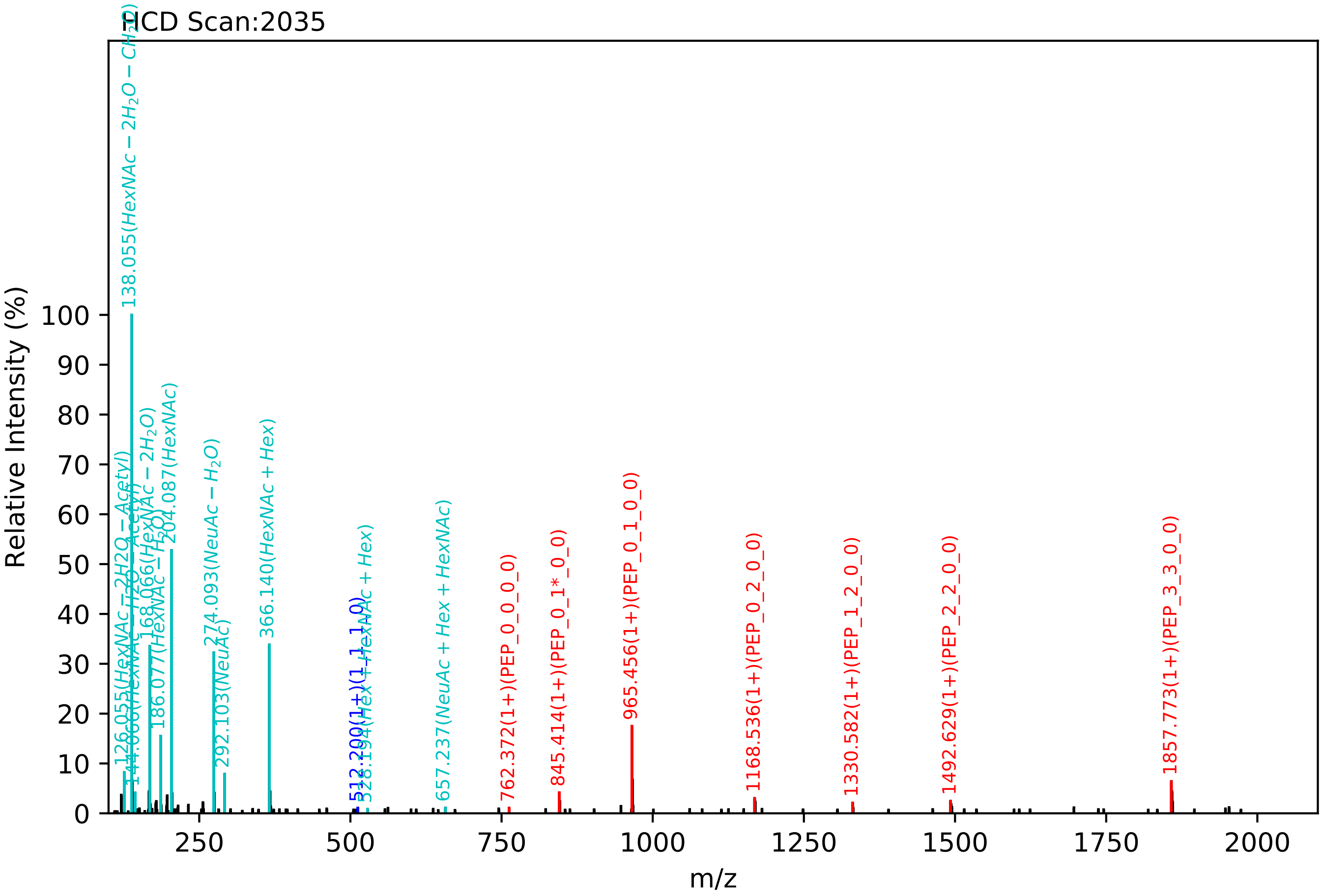

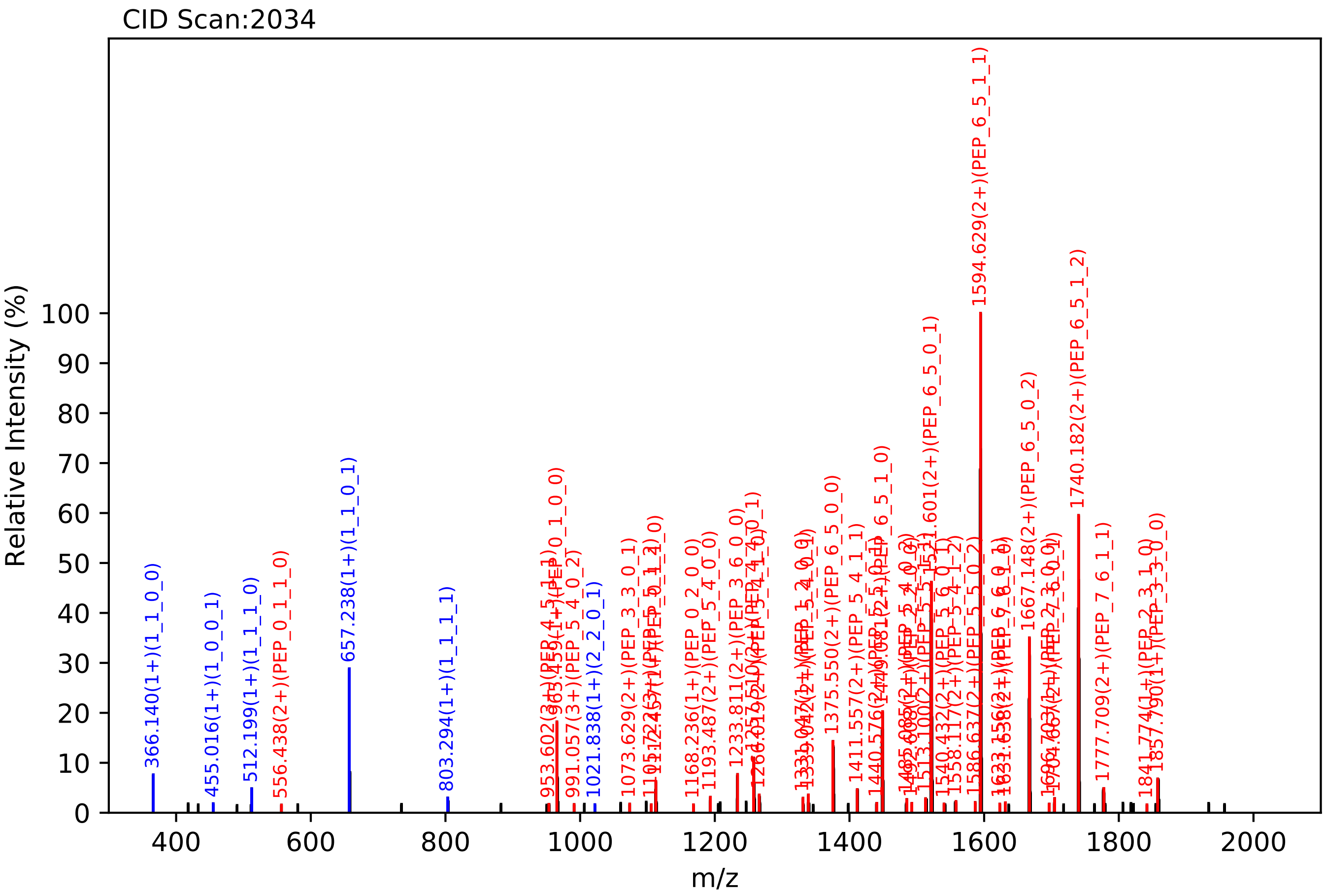

Training set no. 265, Experiment: AGP exp_2

ENGTVSR(=PEP)_7_6_1_2, m/z:1281.83(3+), RT:16.67, Y-score:90.75

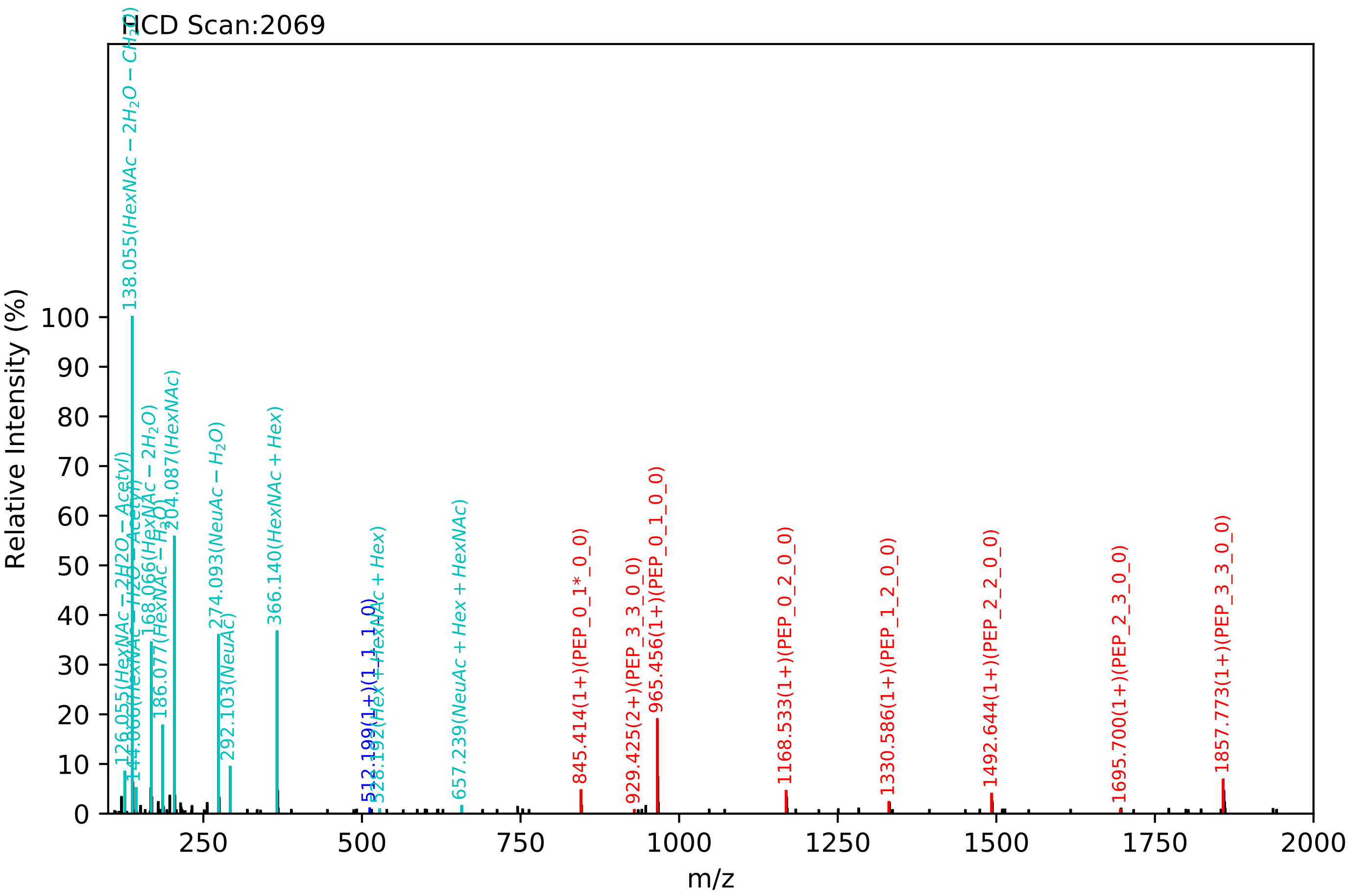

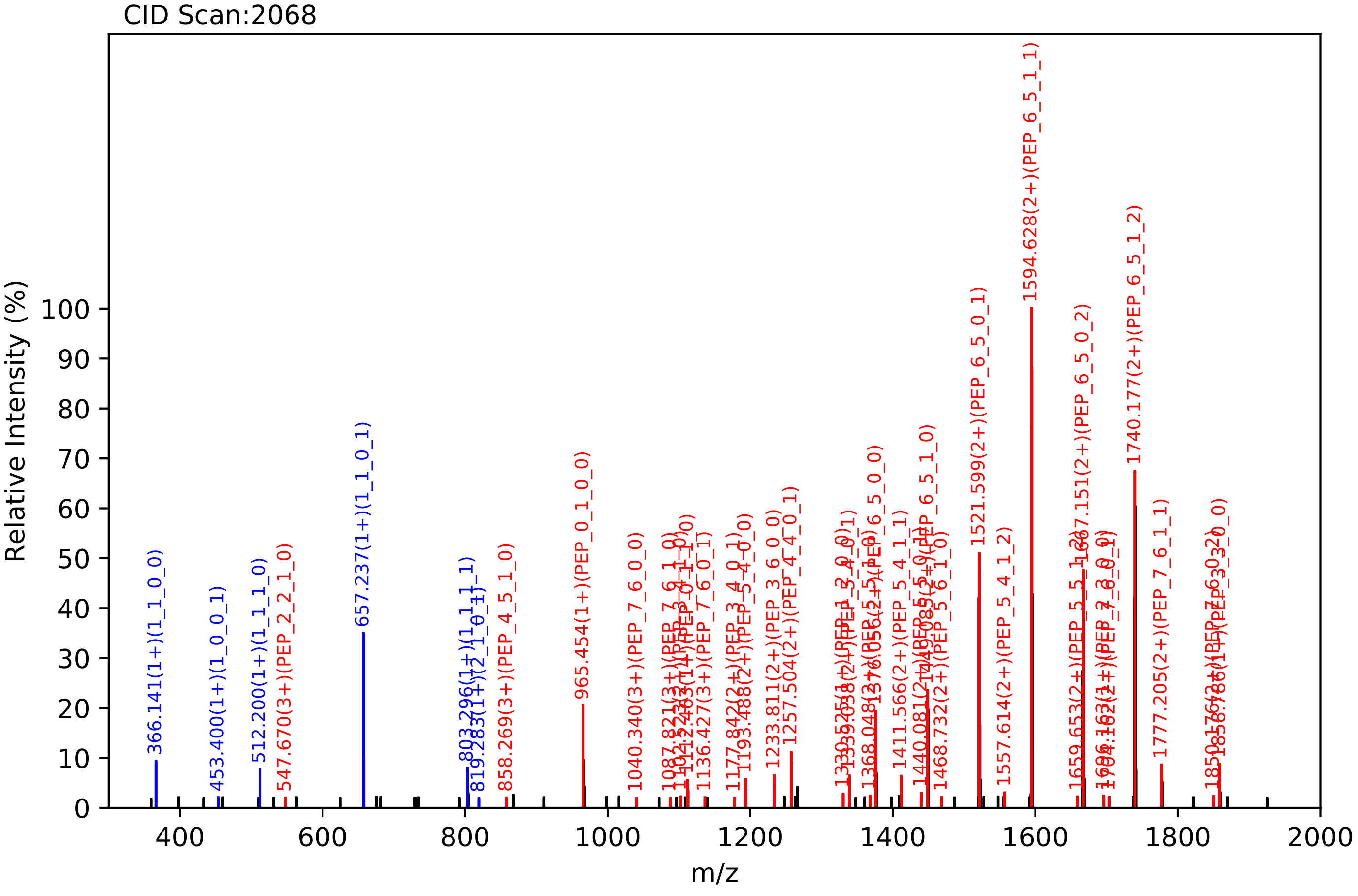

Training set no. 266, Experiment: AGP exp_1

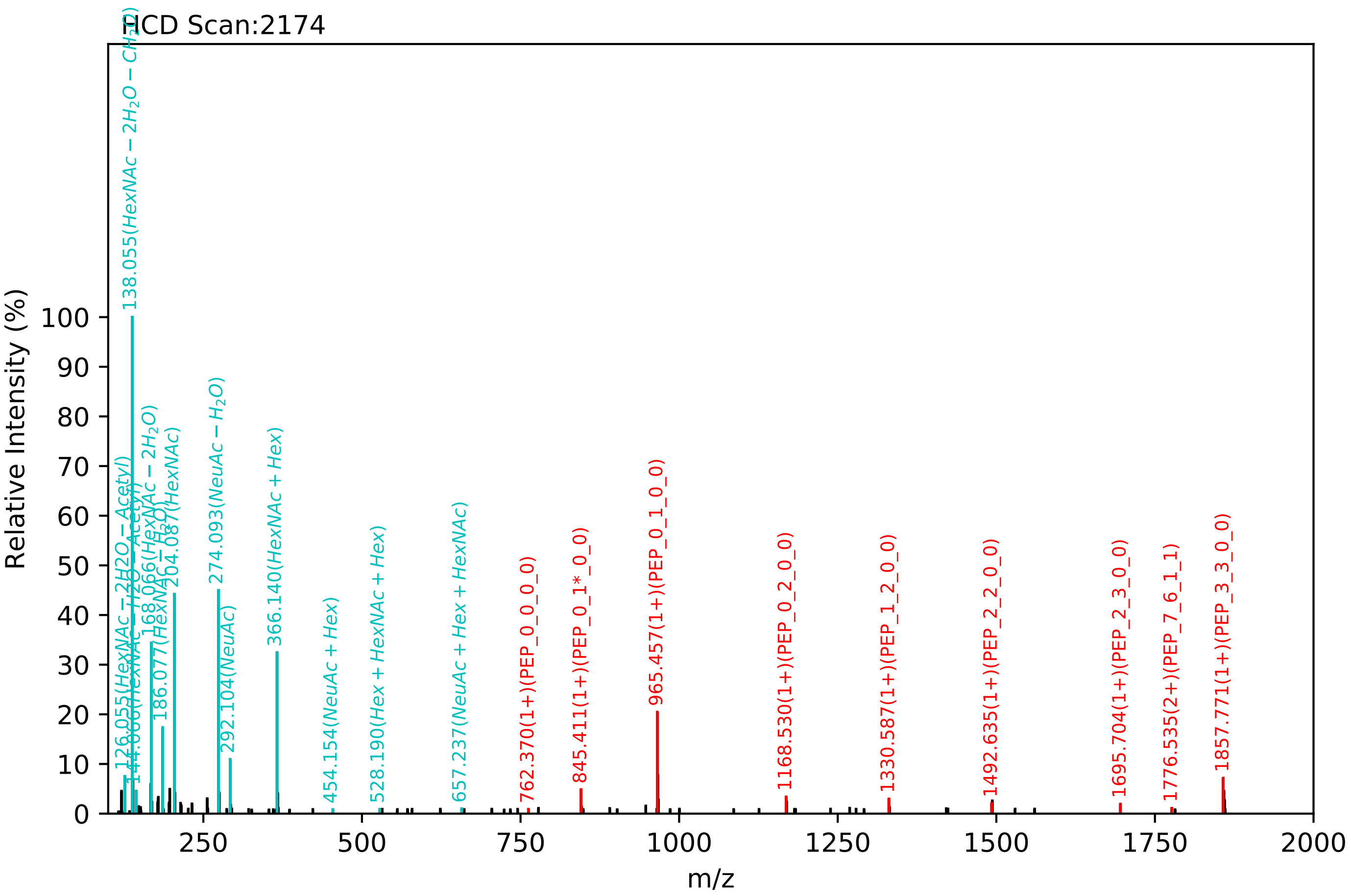

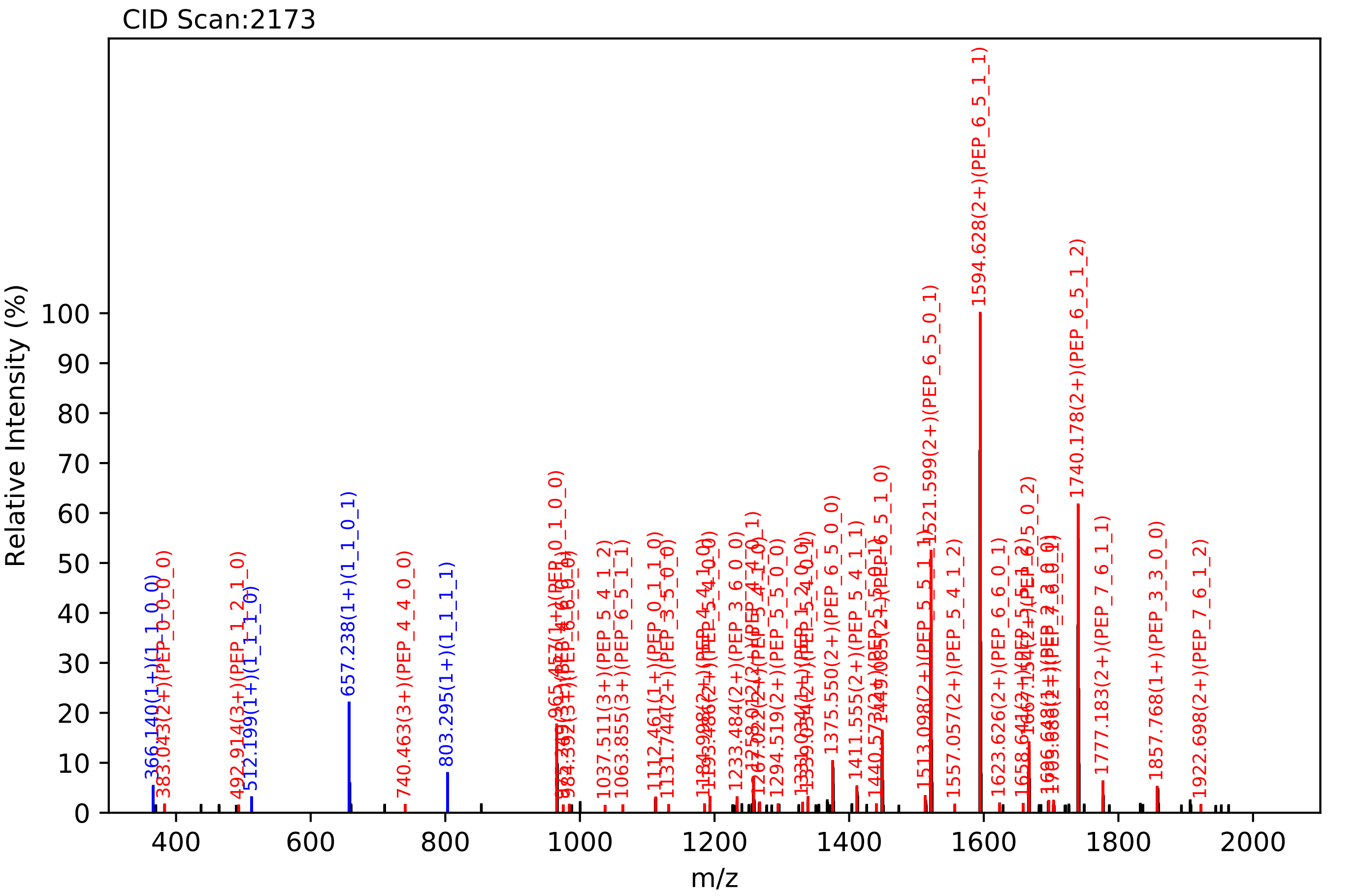

Training set no. 267, Experiment: AGP exp_2

ENGTVSR(=PEP)_7_6_1_4, m/z:1107.17(4+), RT:23.06, Y-score:82.44

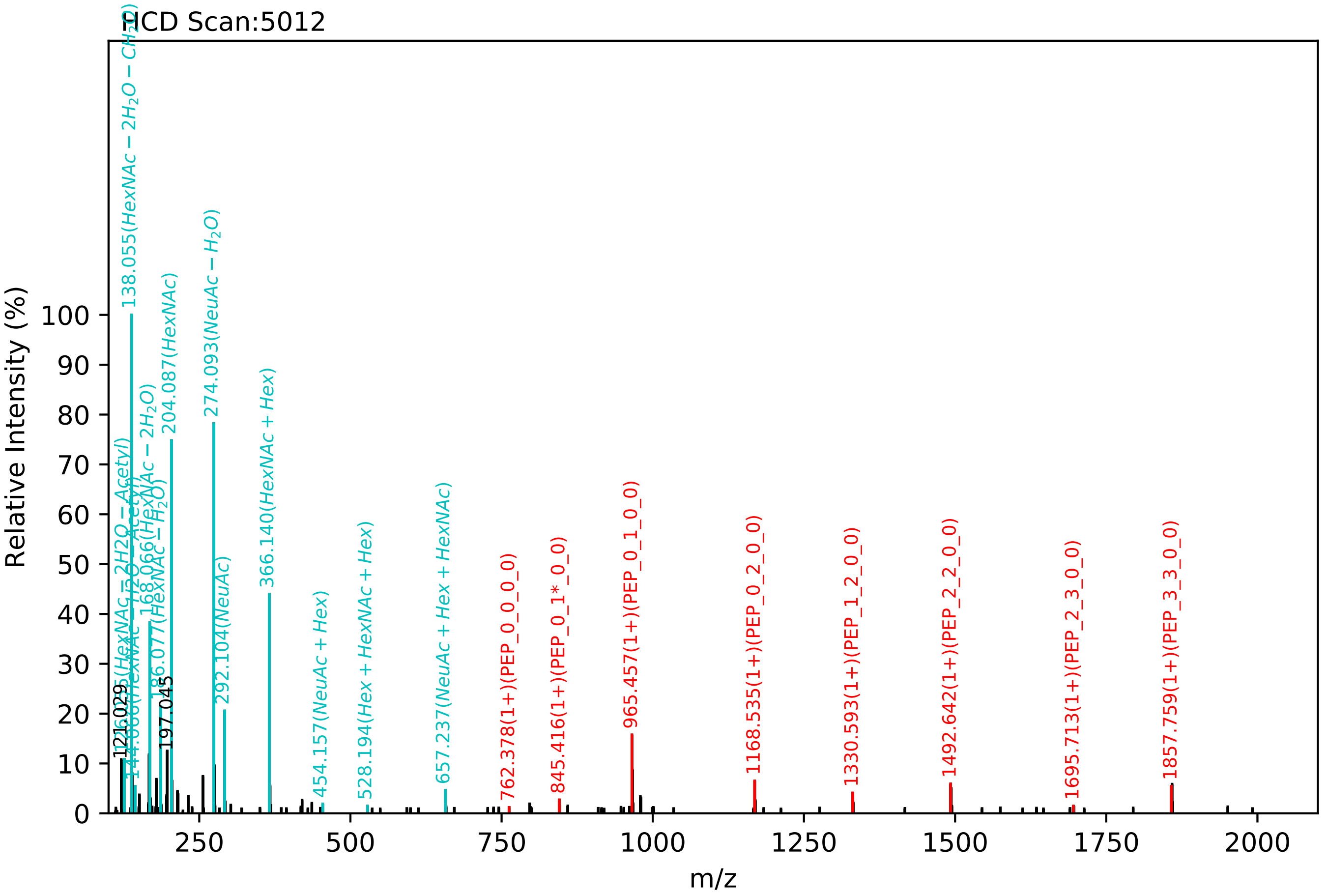

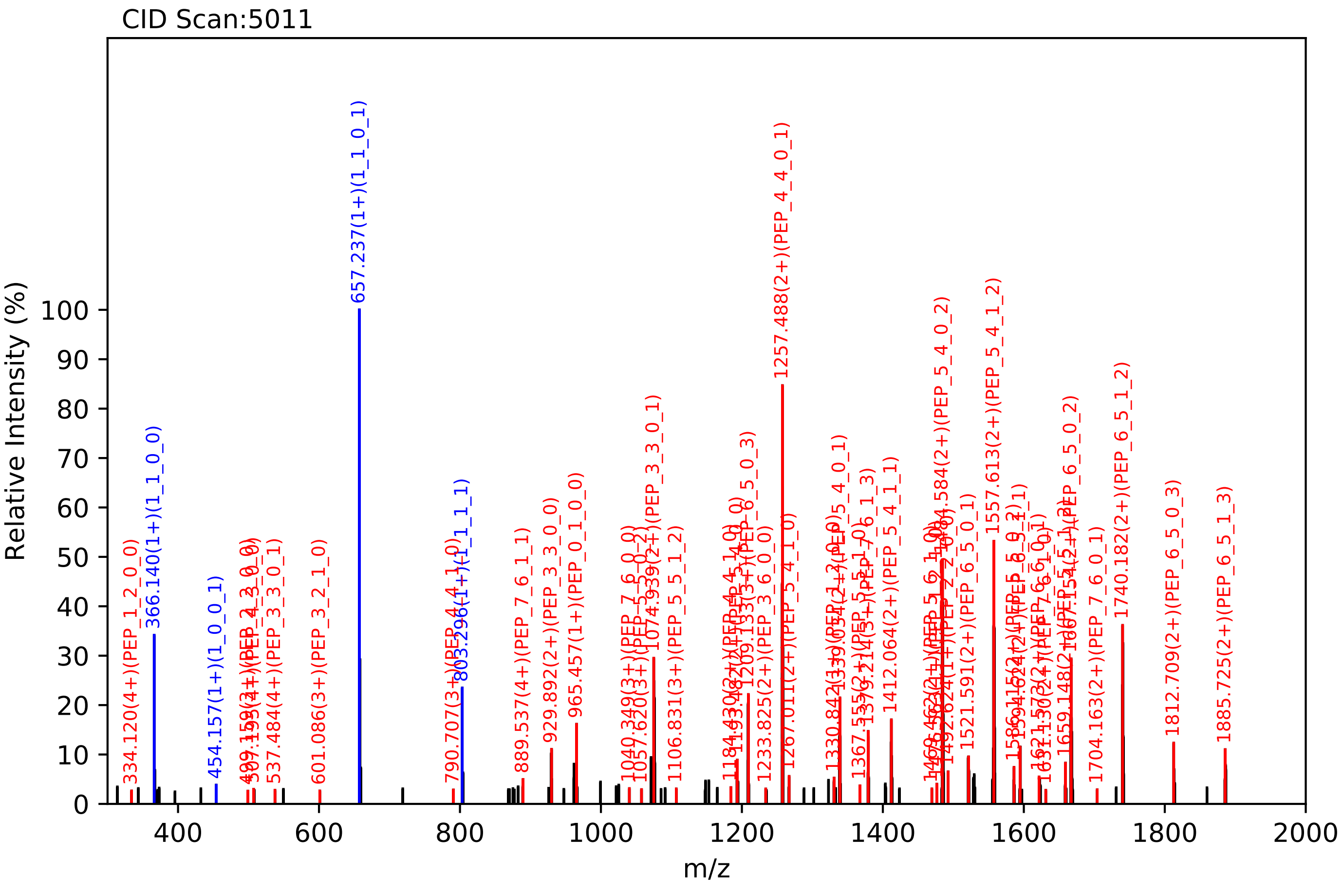

Training set no. 268, Experiment: AGP exp_1

ENGTVSR(=PEP)_7_6_1_4, m/z:1106.92(4+), RT:23.22, Y-score:80.37

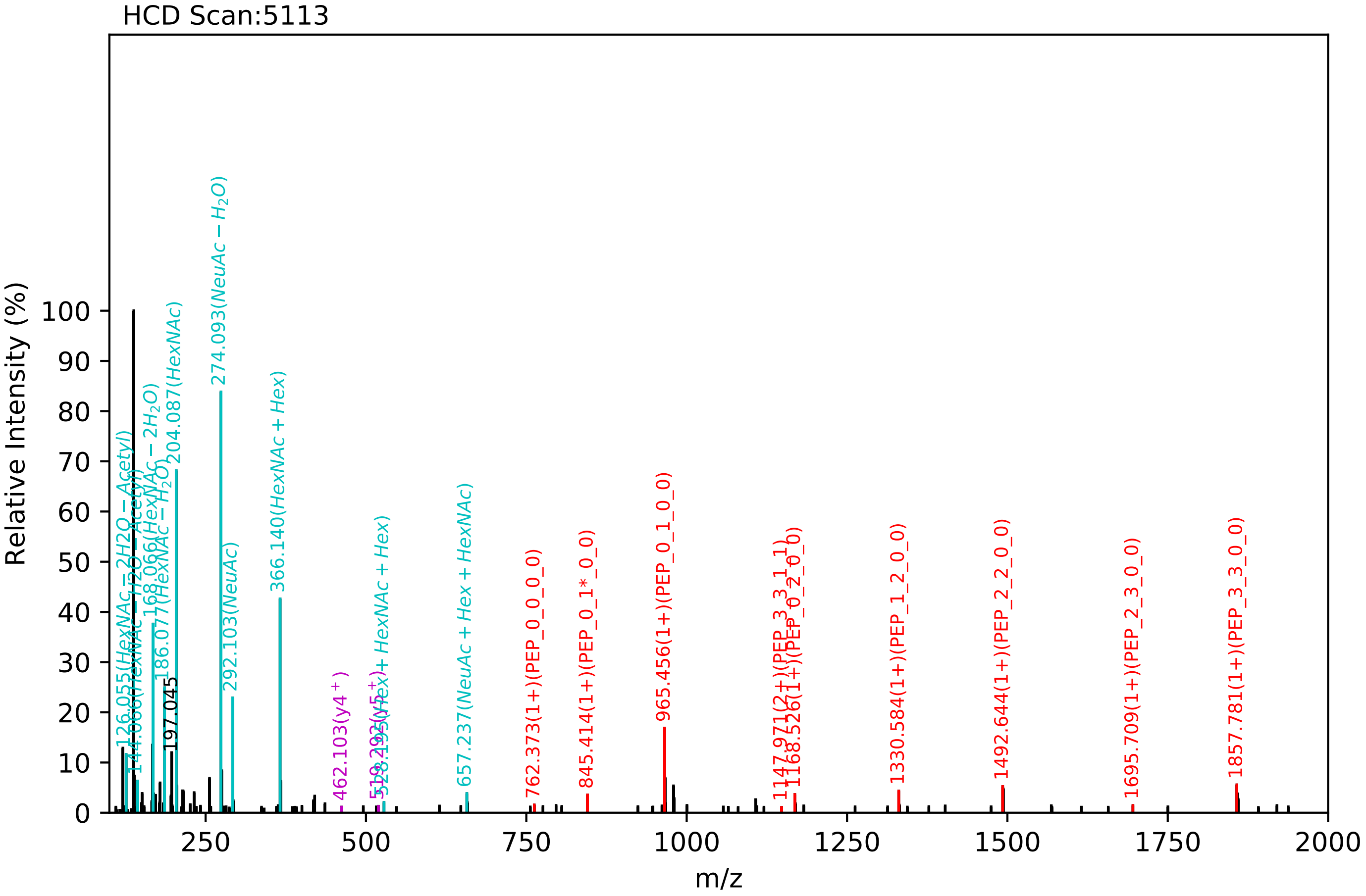

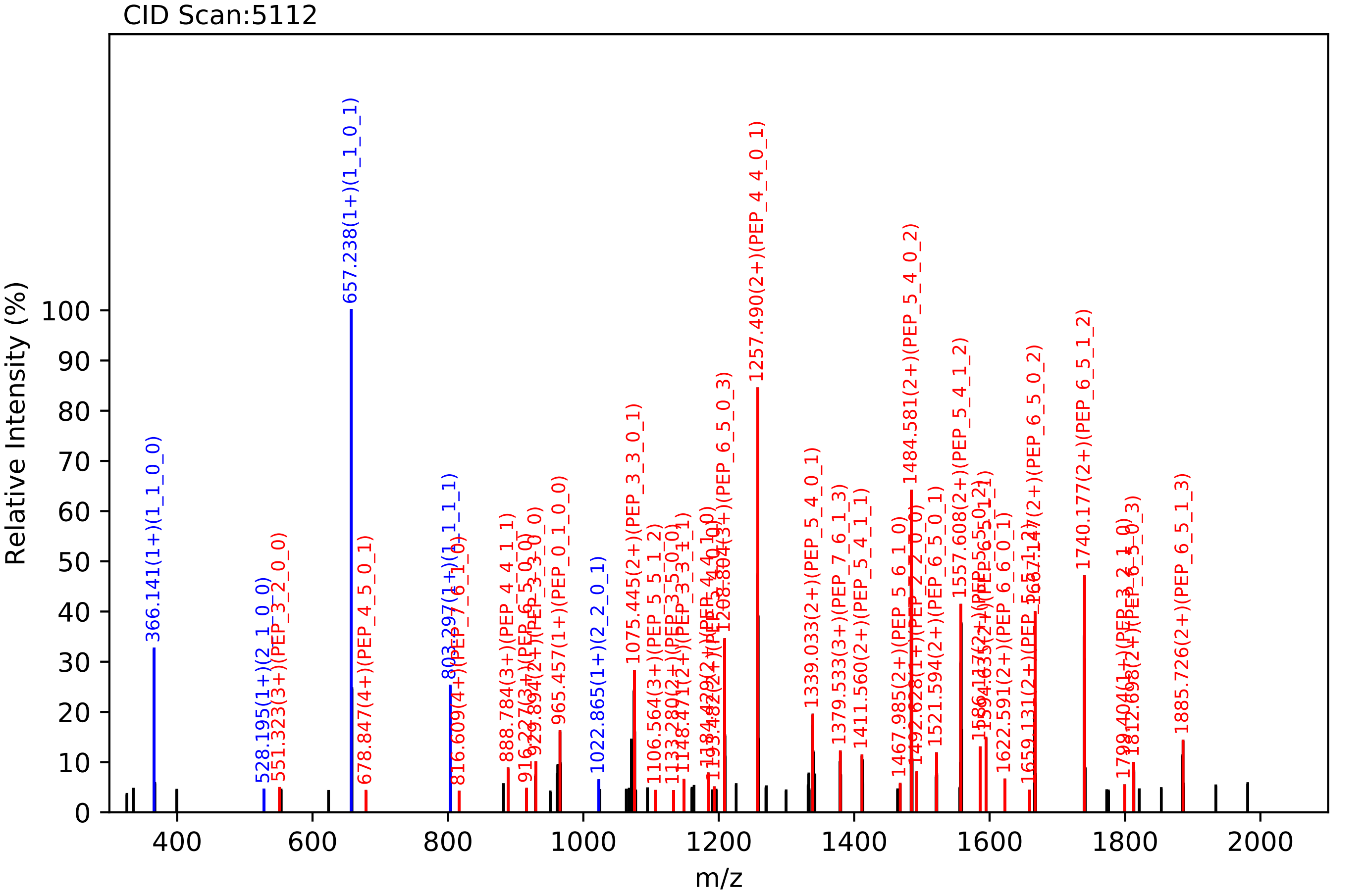

Training set no. 269, Experiment: AGP exp_1

LVPVPITNATLDR(=PEP)_3_3_0_0, m/z:1252.61(2+), RT:42.40, Y-score:76.61

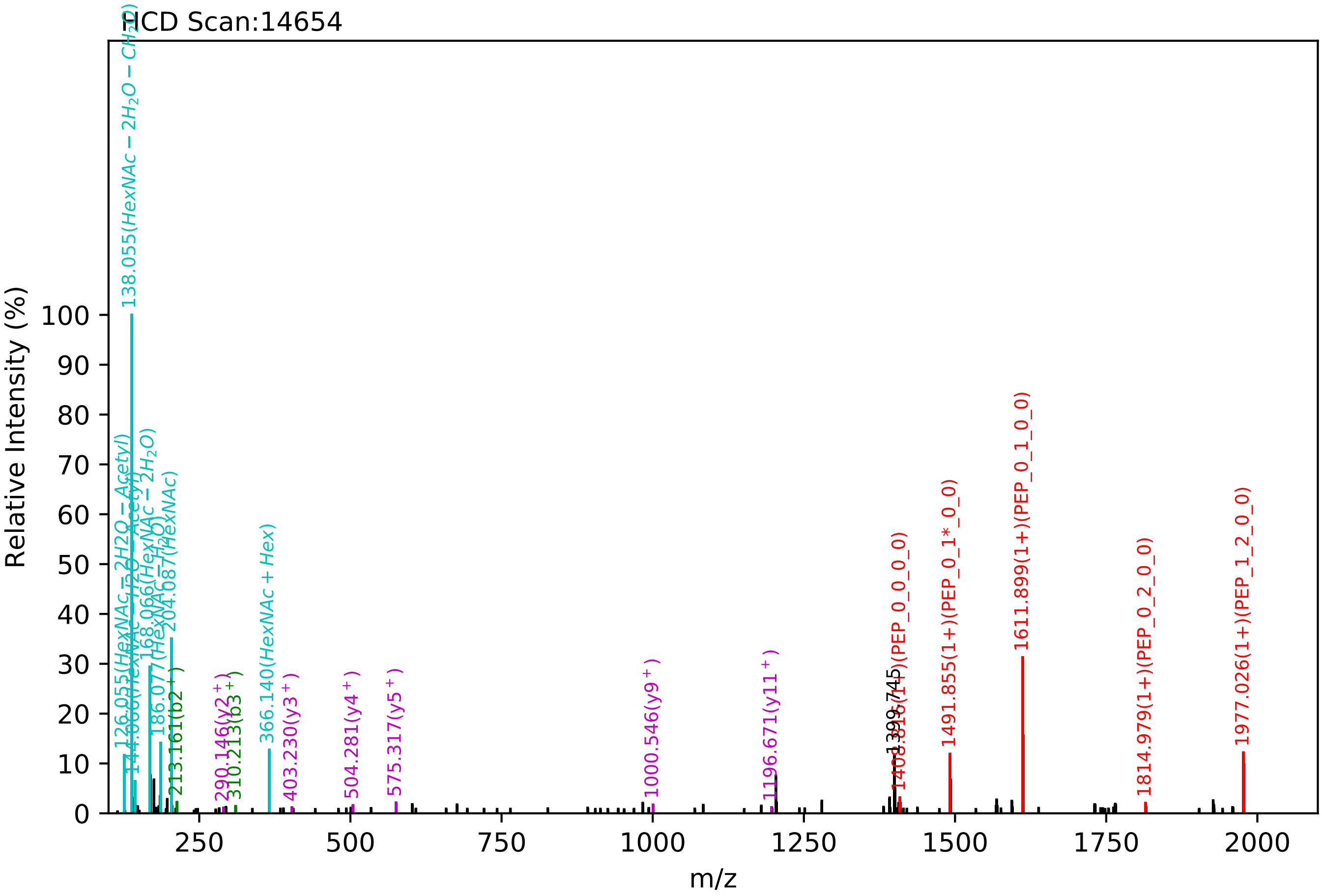

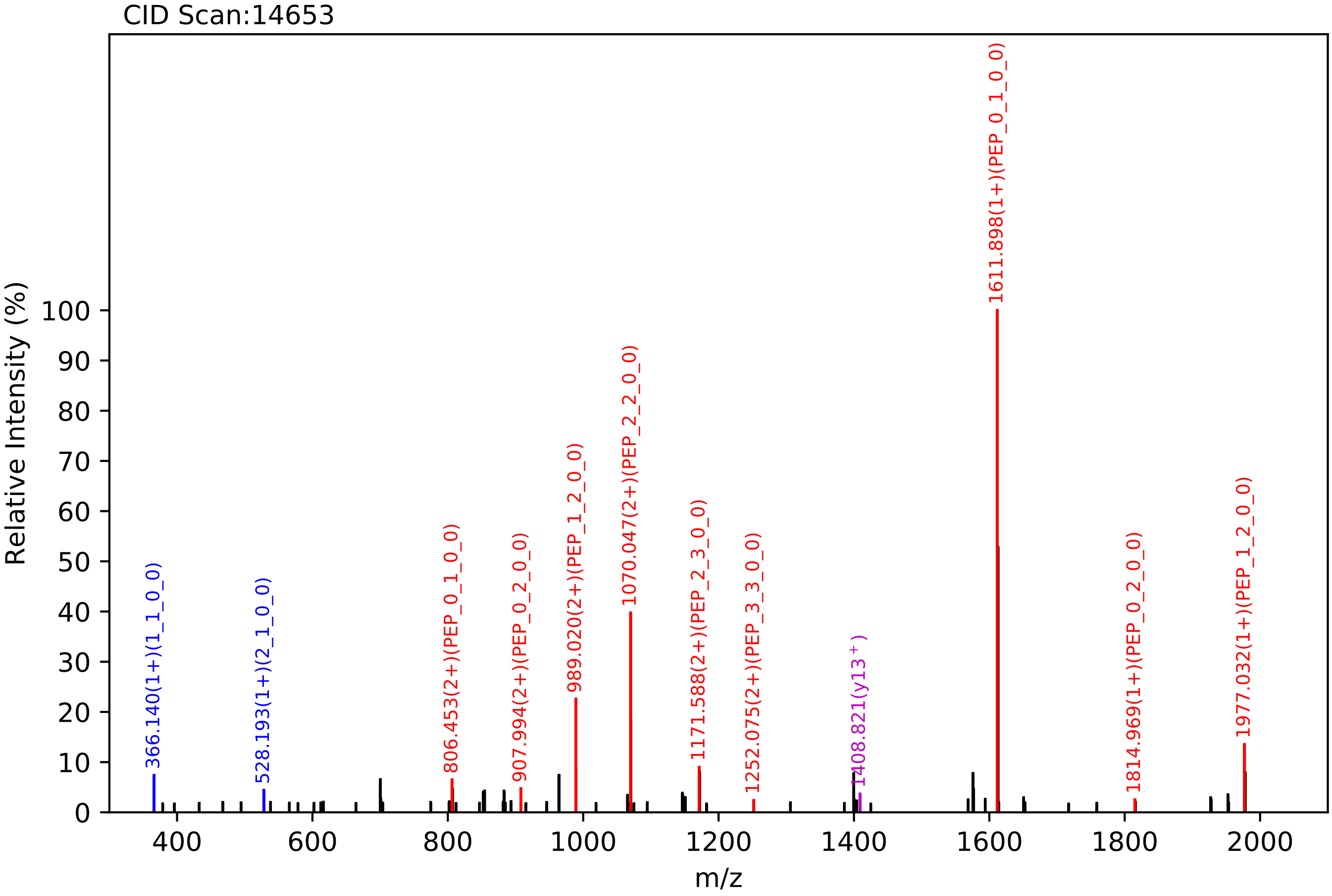

Training set no. 270, Experiment: AGP exp_2

LVPVPITNATLDR(=PEP)_3_3_0_0, m/z:1252.11(2+), RT:42.85, Y-score:76.39

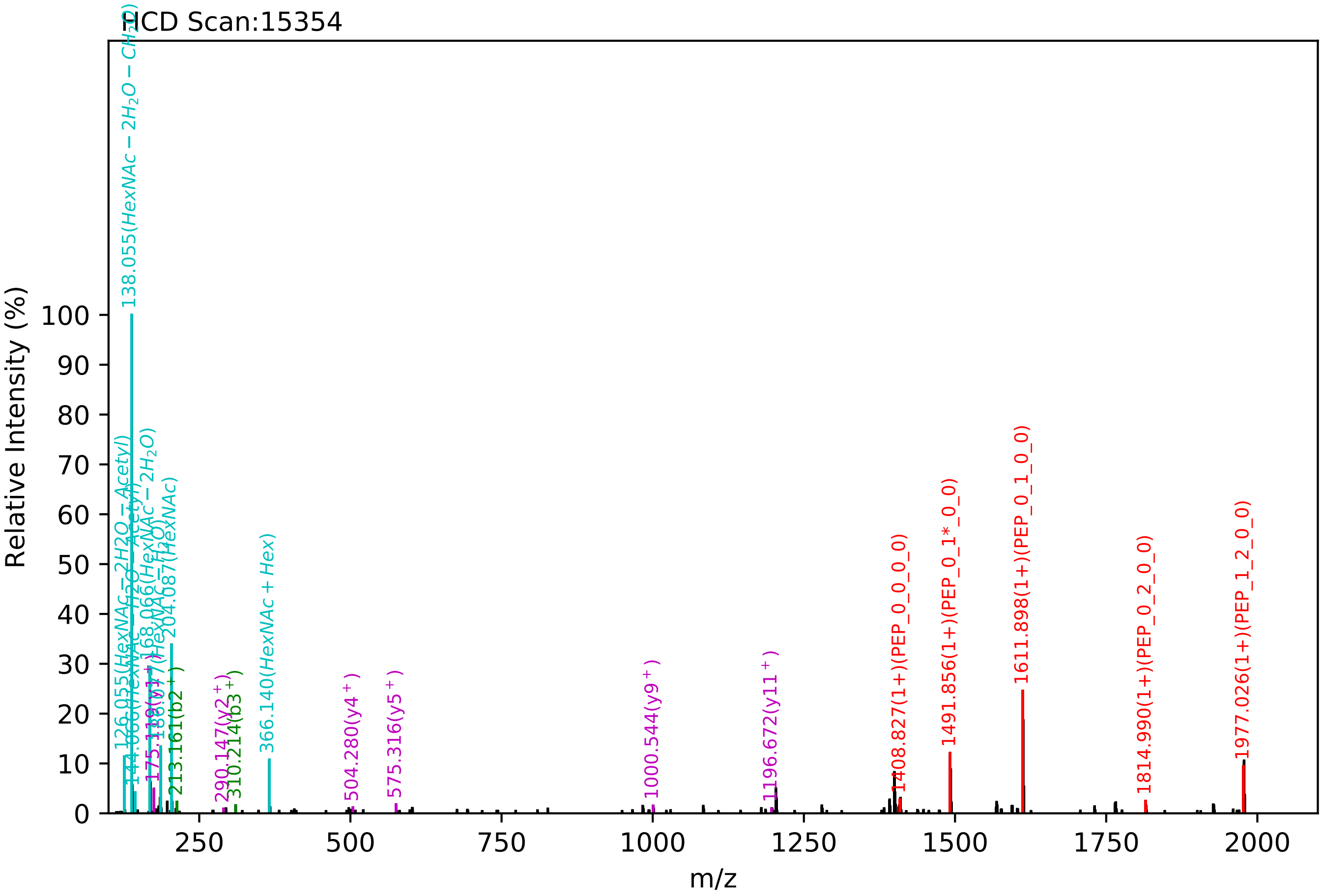

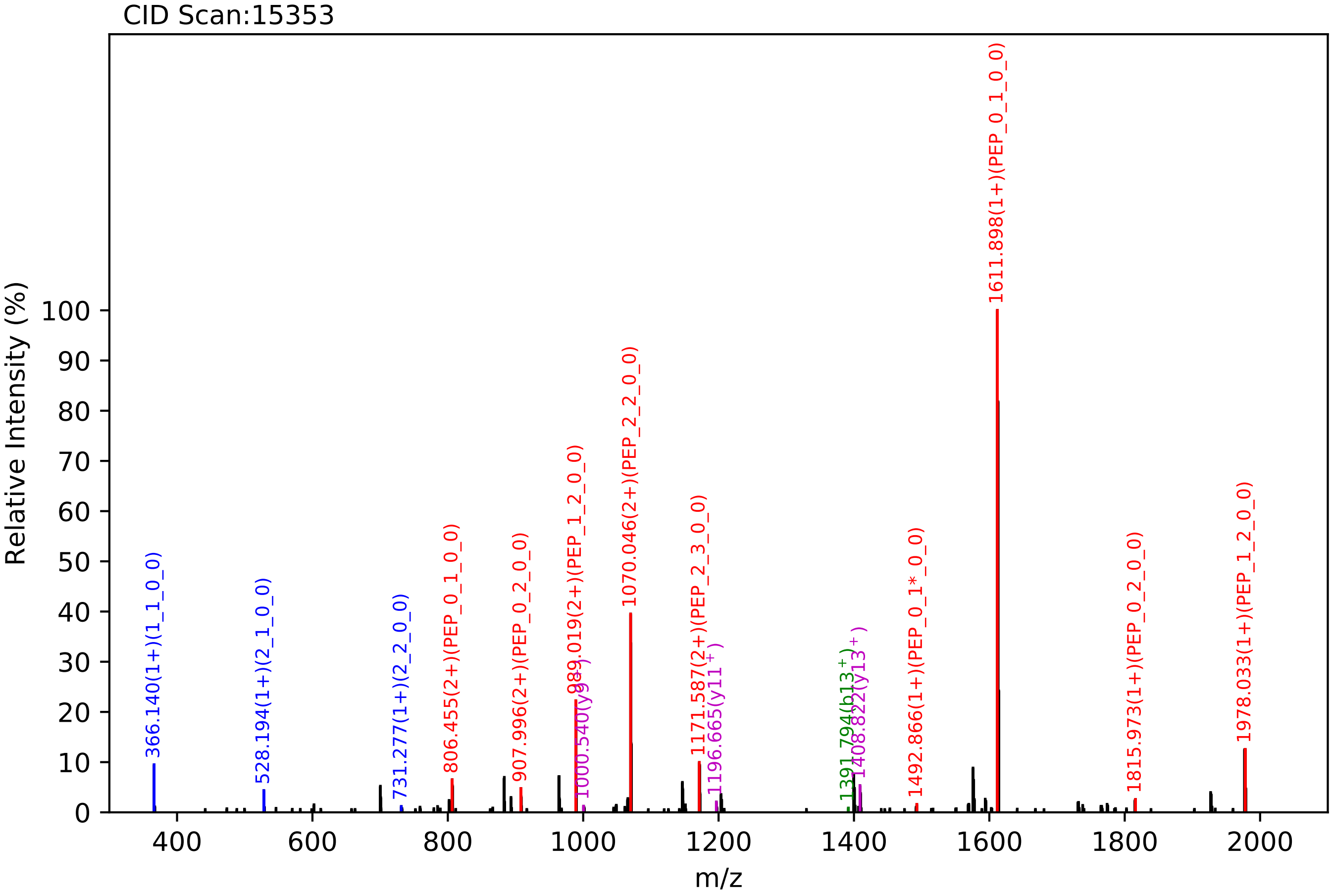

Training set no. 271, Experiment: AGP exp_2

LVPVPITNATLDR(=PEP)_3_3_0_0, m/z:1252.61(2+), RT:42.99, Y-score:75.33

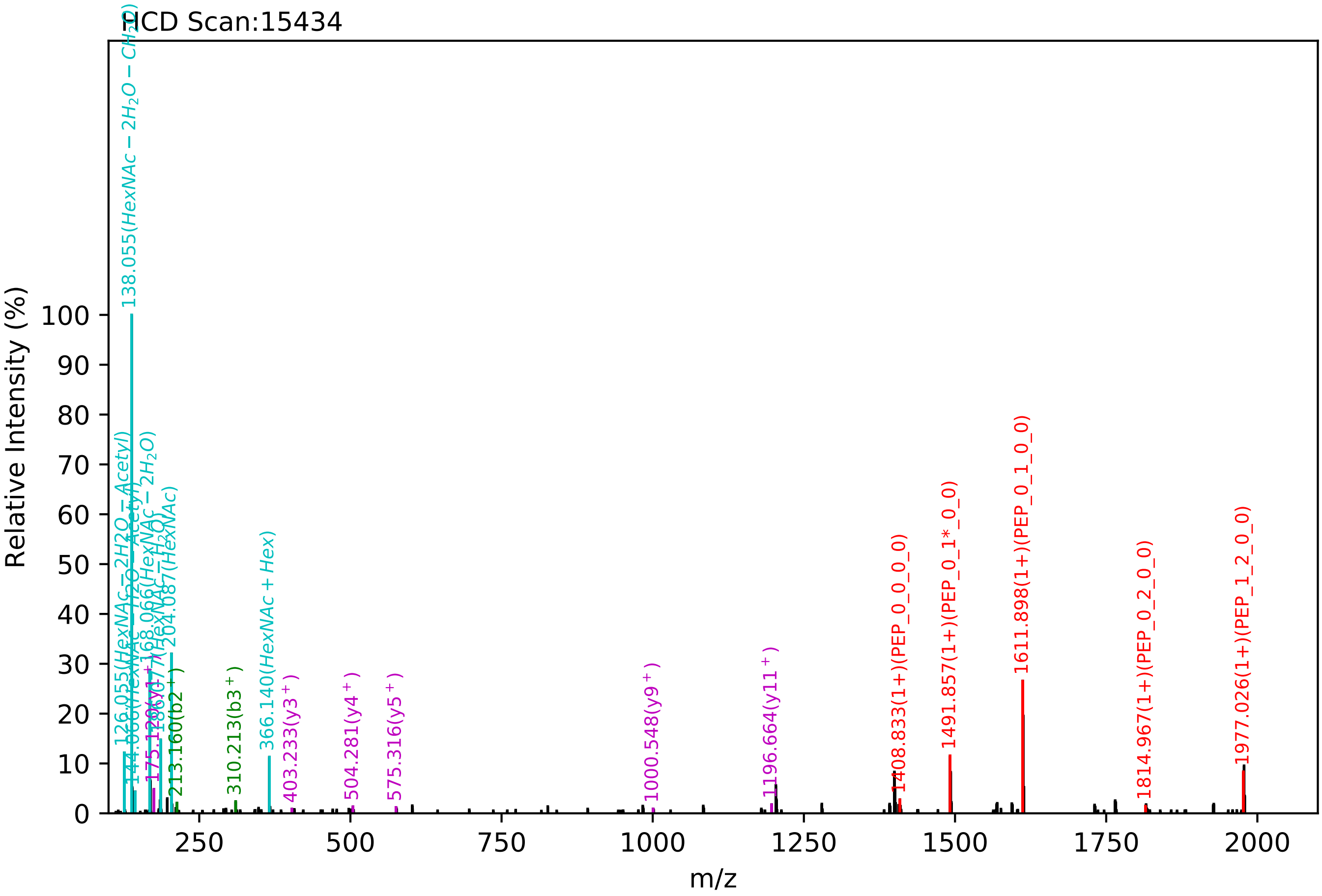

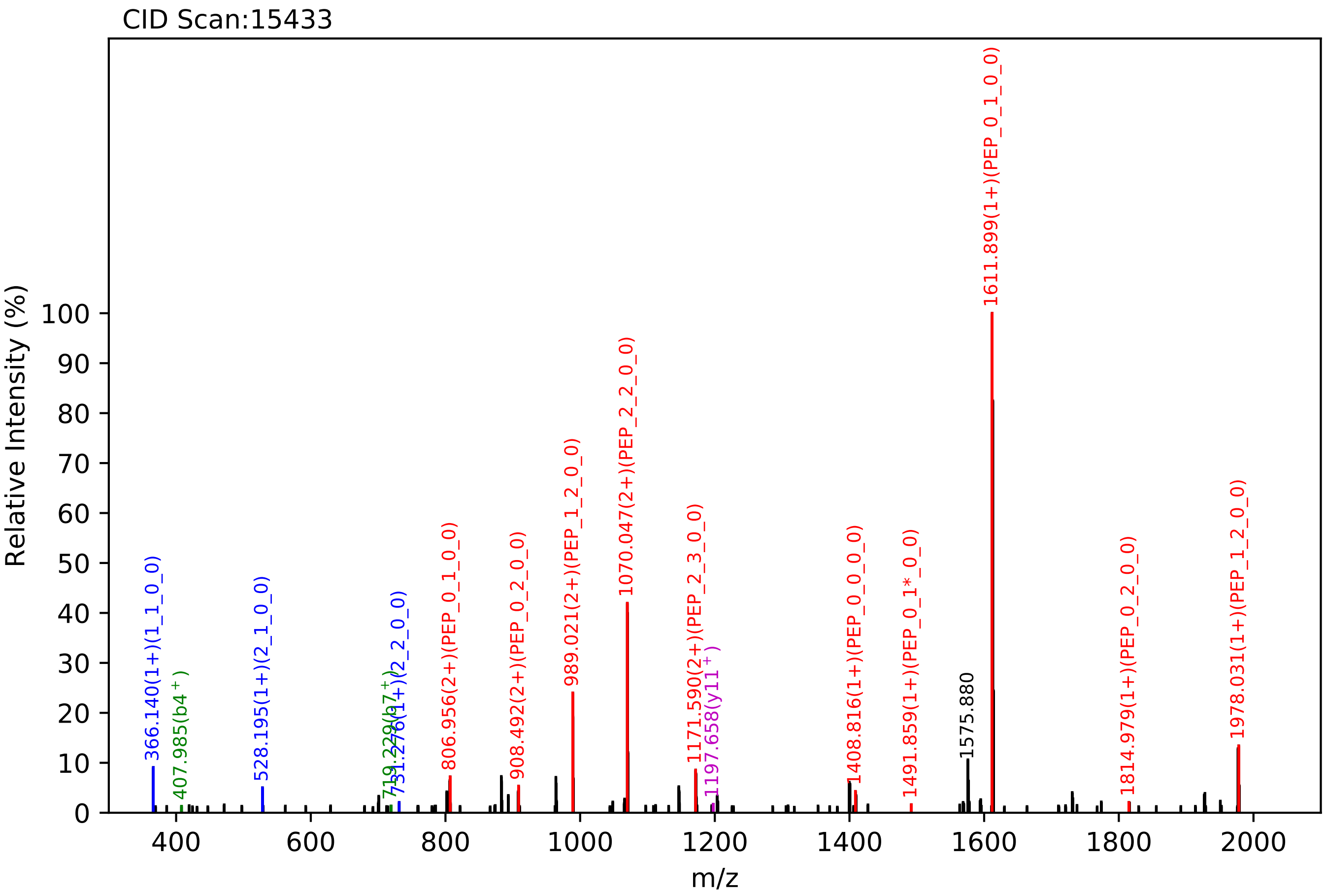

Training set no. 272, Experiment: AGP exp_1

LVPVPITNATLDR(=PEP)_3_3_0_0, m/z:1252.61(2+), RT:42.54, Y-score:74.82

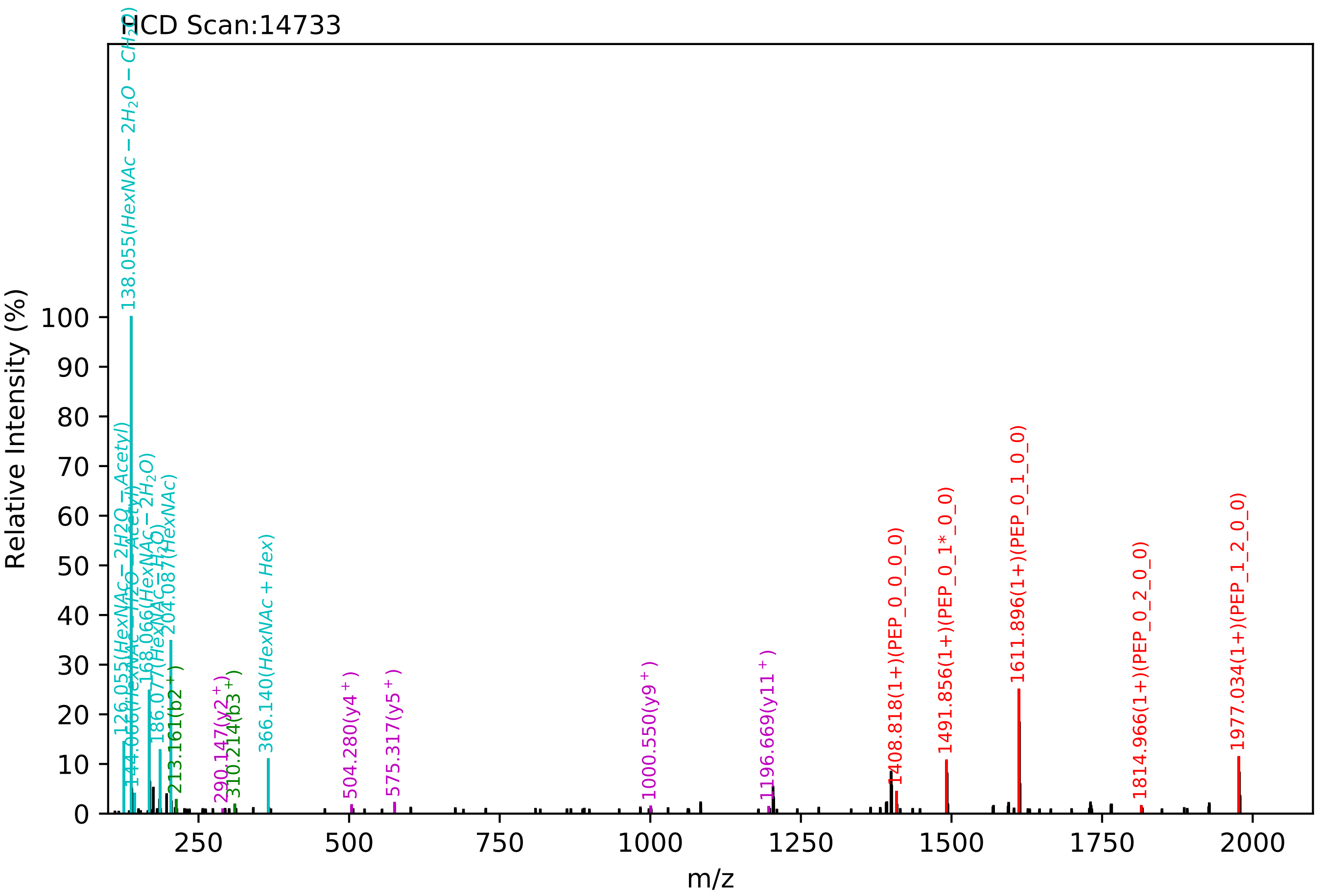

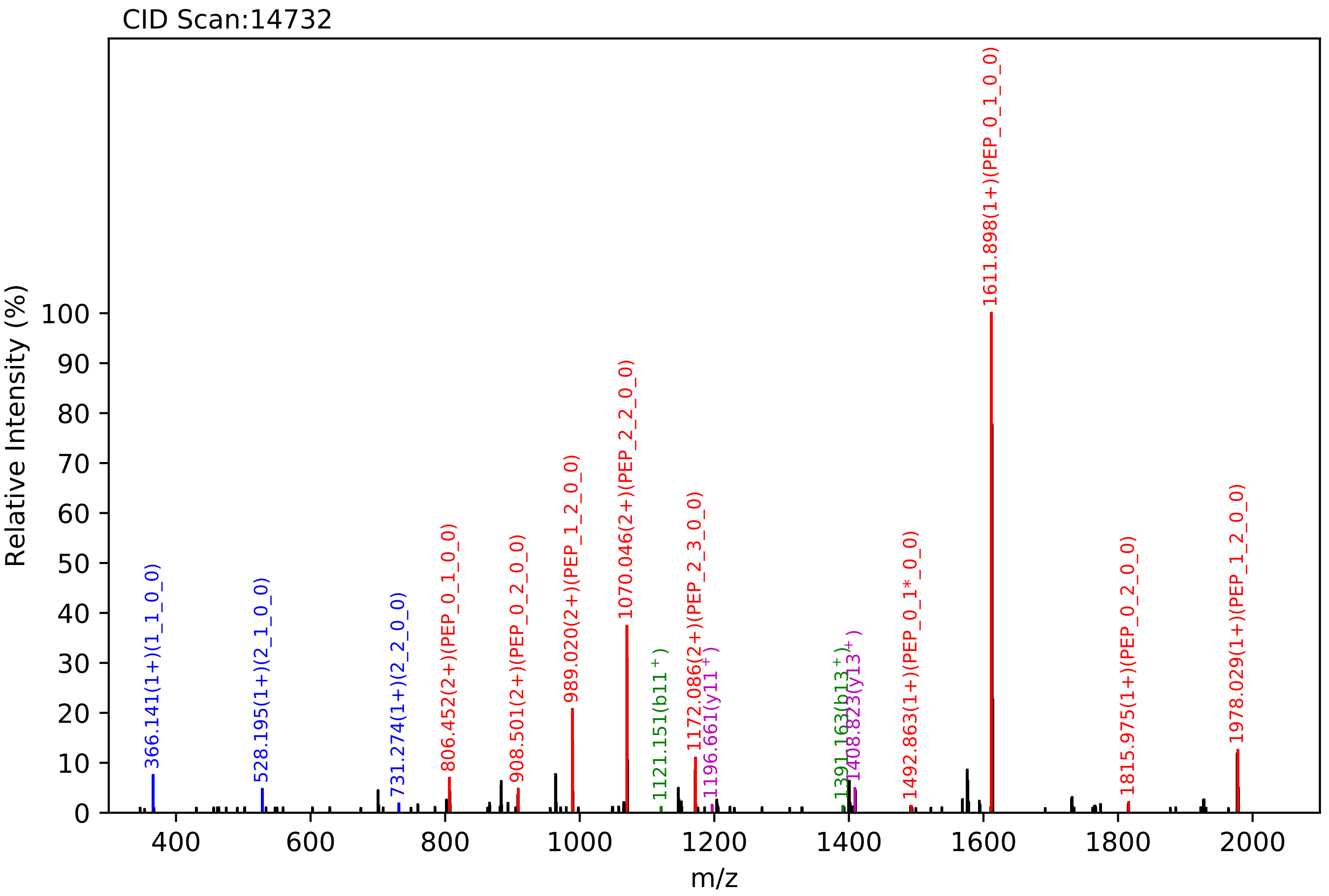

Training set no. 273, Experiment: AGP exp_2

LVPVPITNATLDR(=PEP)_4_4_0_1, m/z:1054.16(3+), RT:37.23, Y-score:80.74

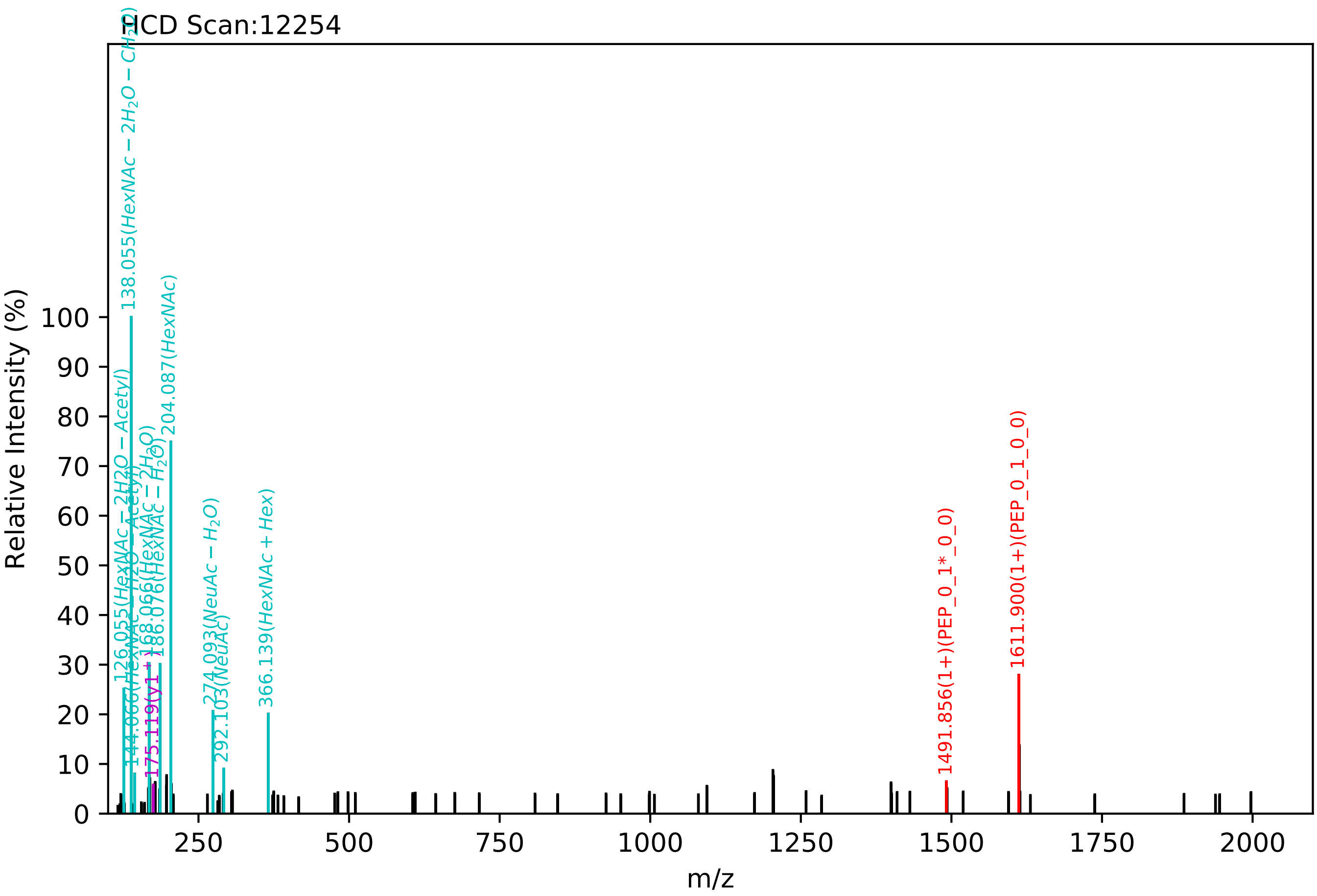

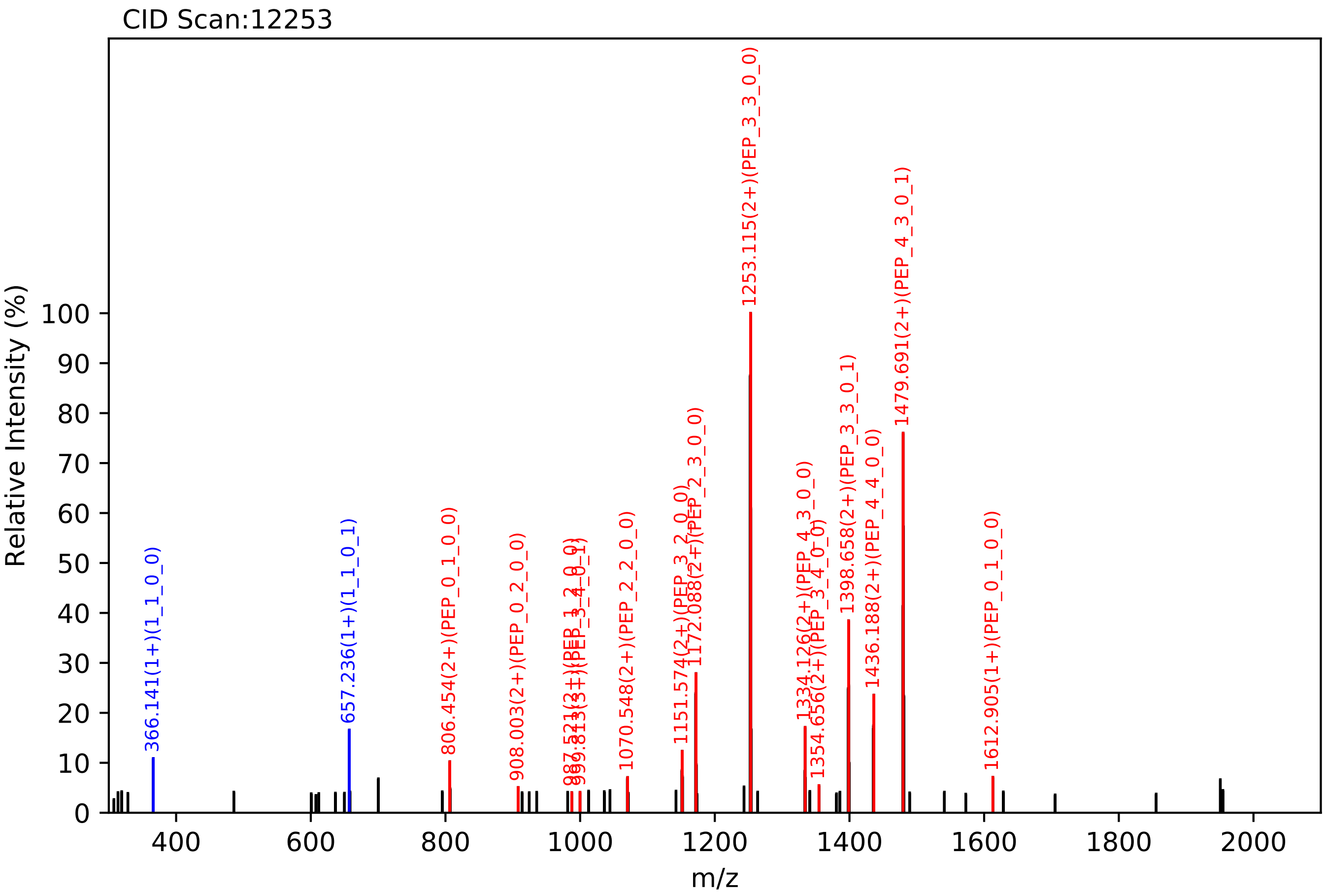

Training set no. 274, Experiment: AGP exp_1

LVPVPITNATLDR(=PEP)_5_4_0_1, m/z:1661.75(2+), RT:42.47, Y-score:86.58

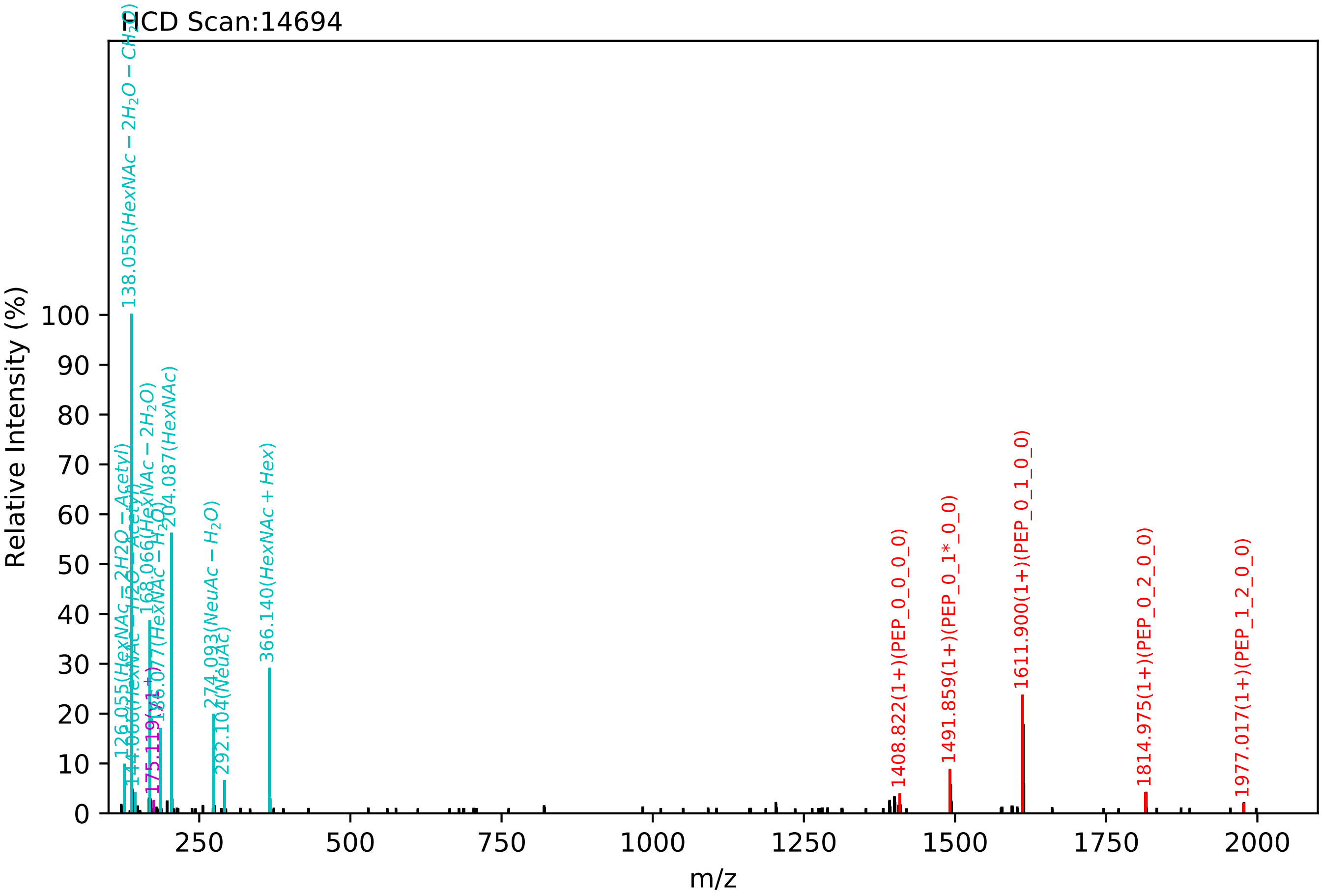

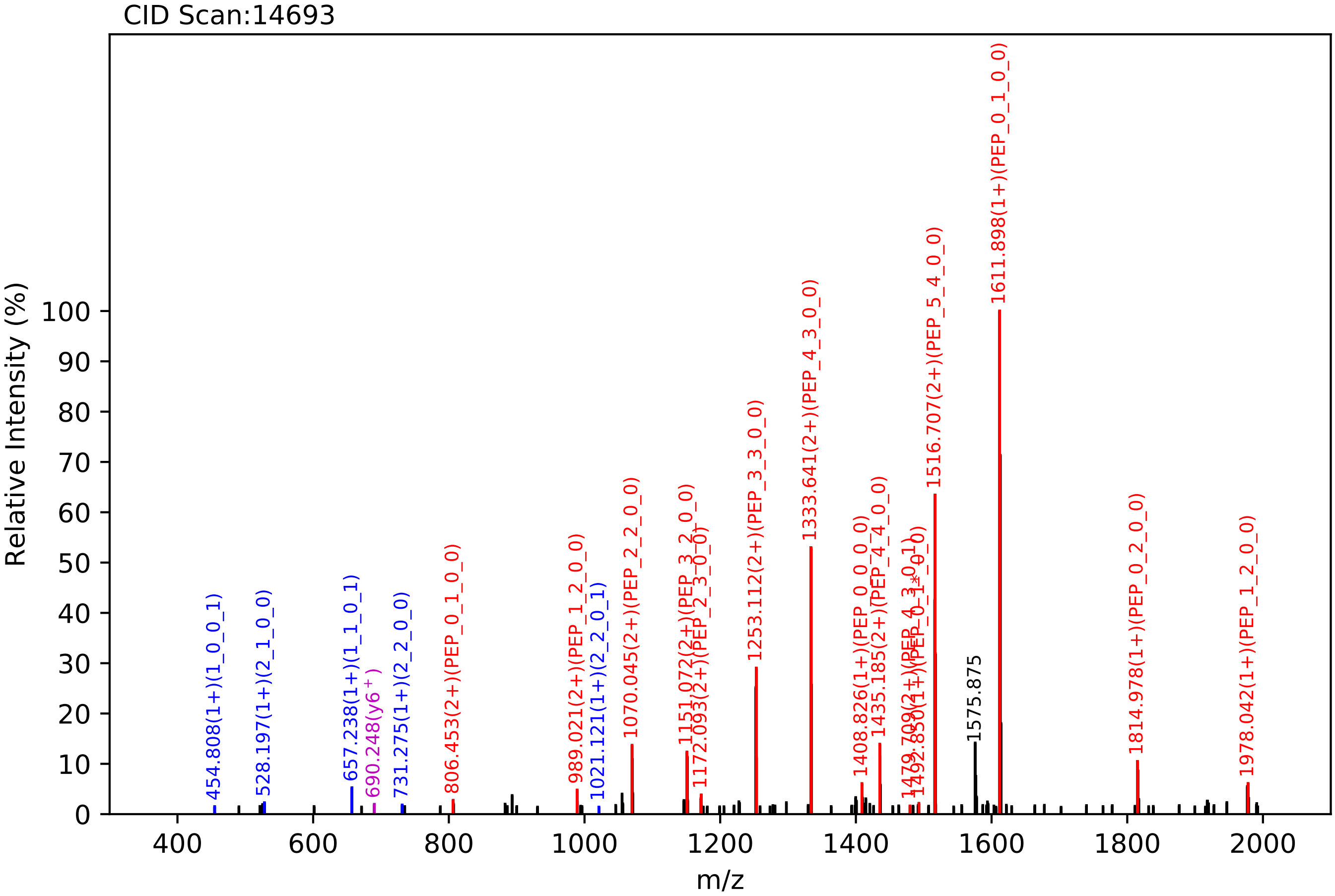

Training set no. 275, Experiment: AGP exp_2

LVPVPITNATLDR(=PEP)_5_4_0_1, m/z:1661.75(2+), RT:43.11, Y-score:84.27

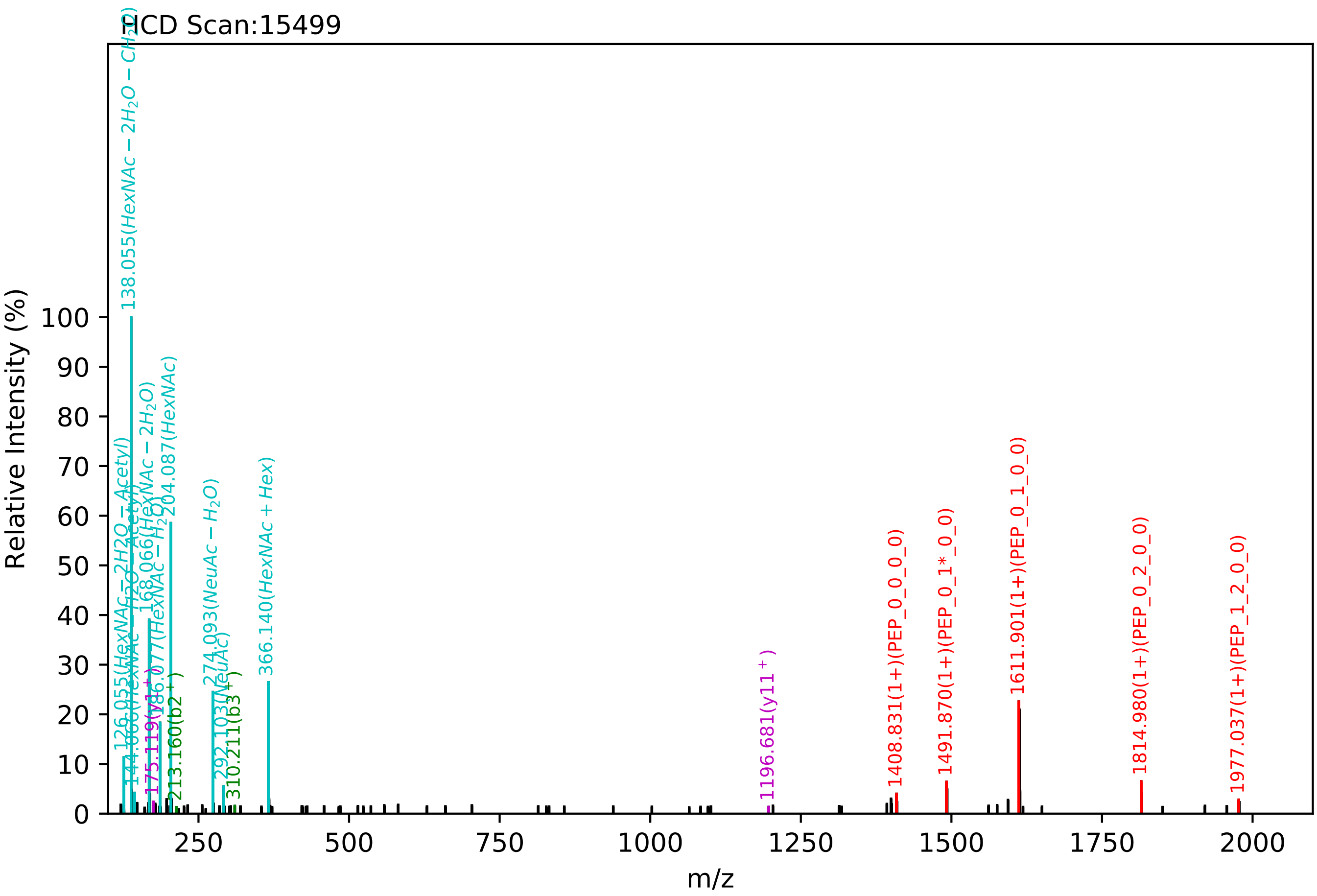

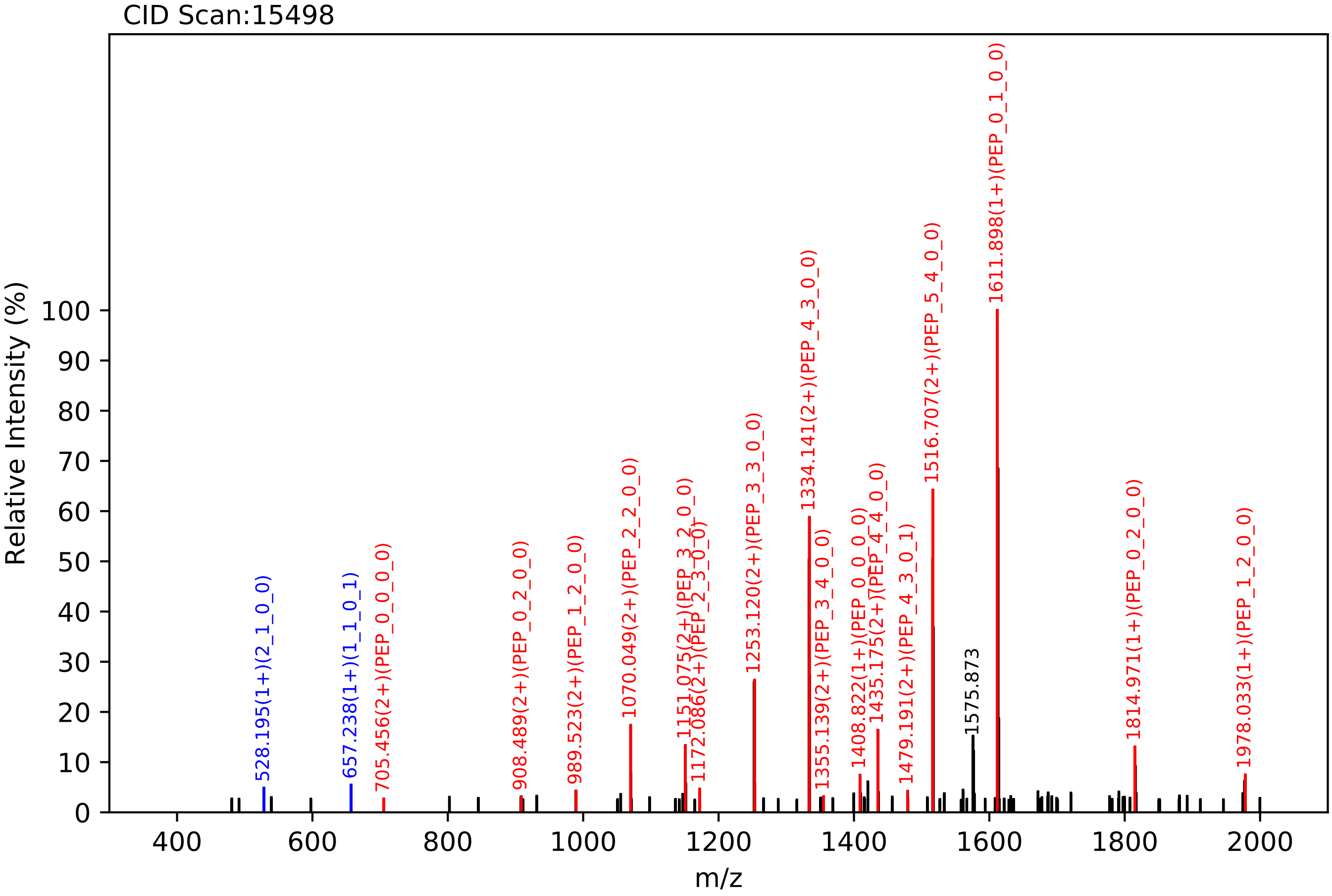

Training set no. 276, Experiment: AGP exp_2

LVPVPITNATLDR(=PEP)_5_4_0_1, m/z:1108.17(3+), RT:42.84, Y-score:79.52

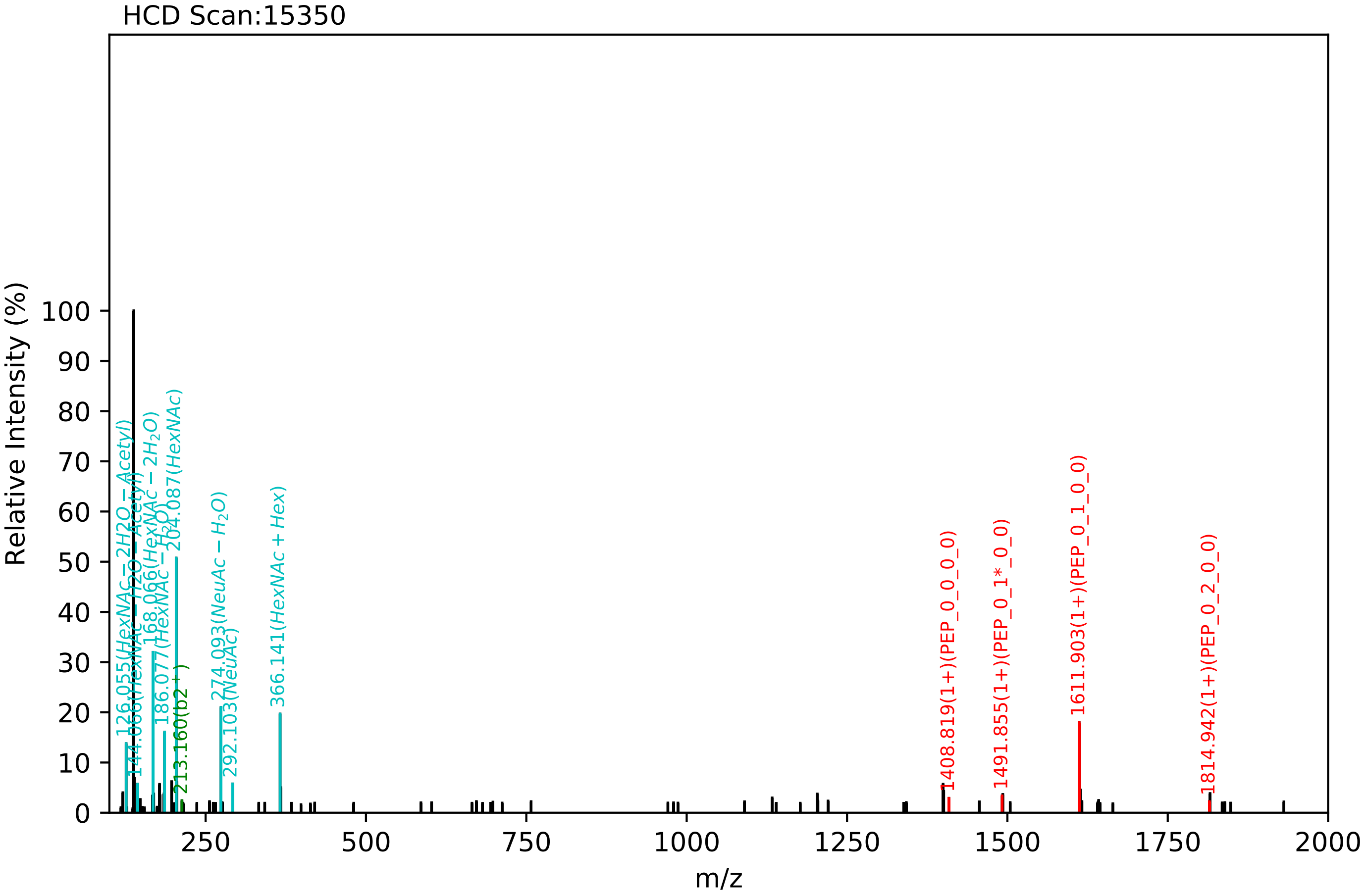

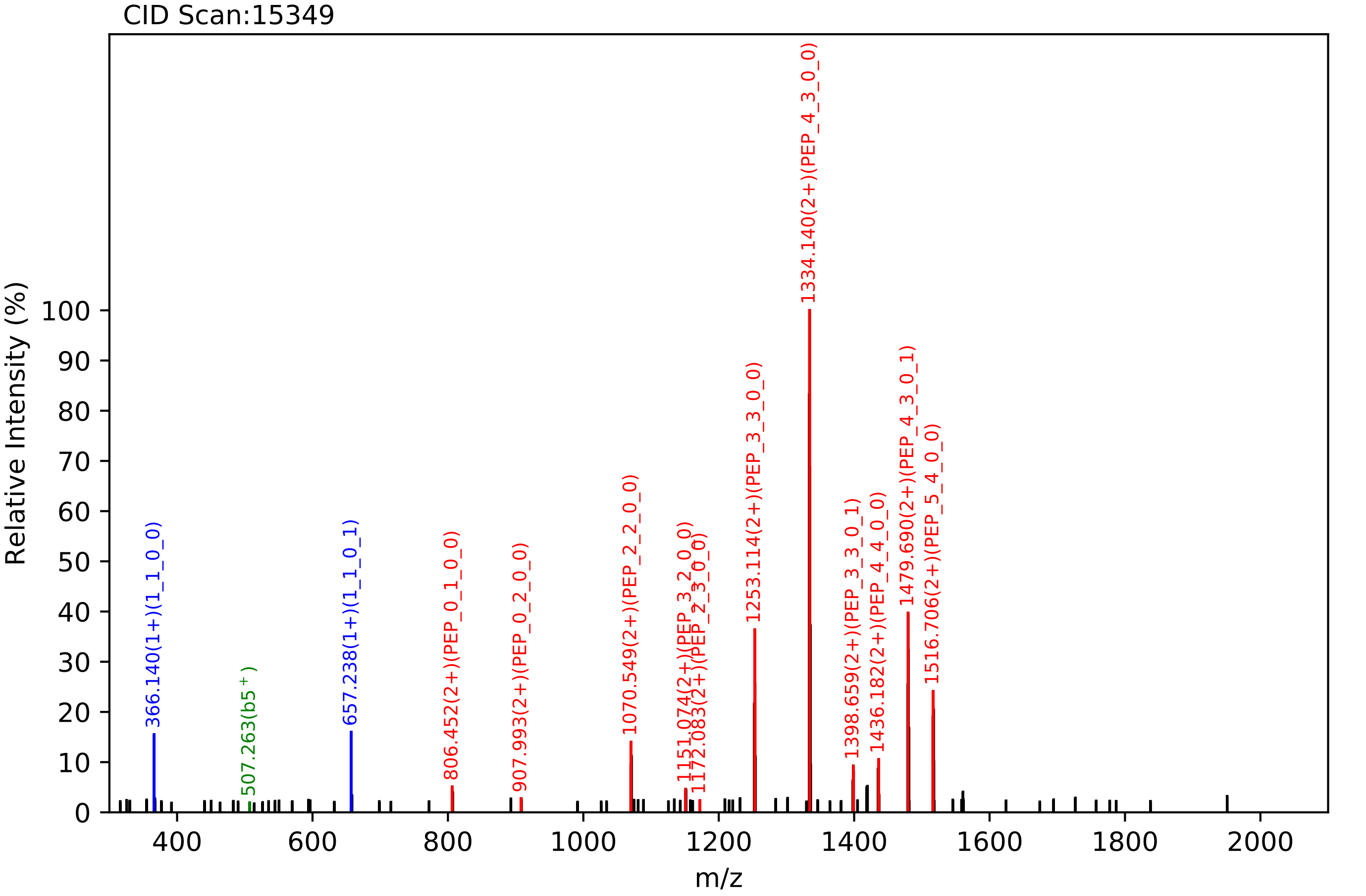

Training set no. 277, Experiment: AGP exp_2

LVPVPITNATLDR(=PEP)_5_4_0_1, m/z:831.38(4+), RT:36.89, Y-score:76.90

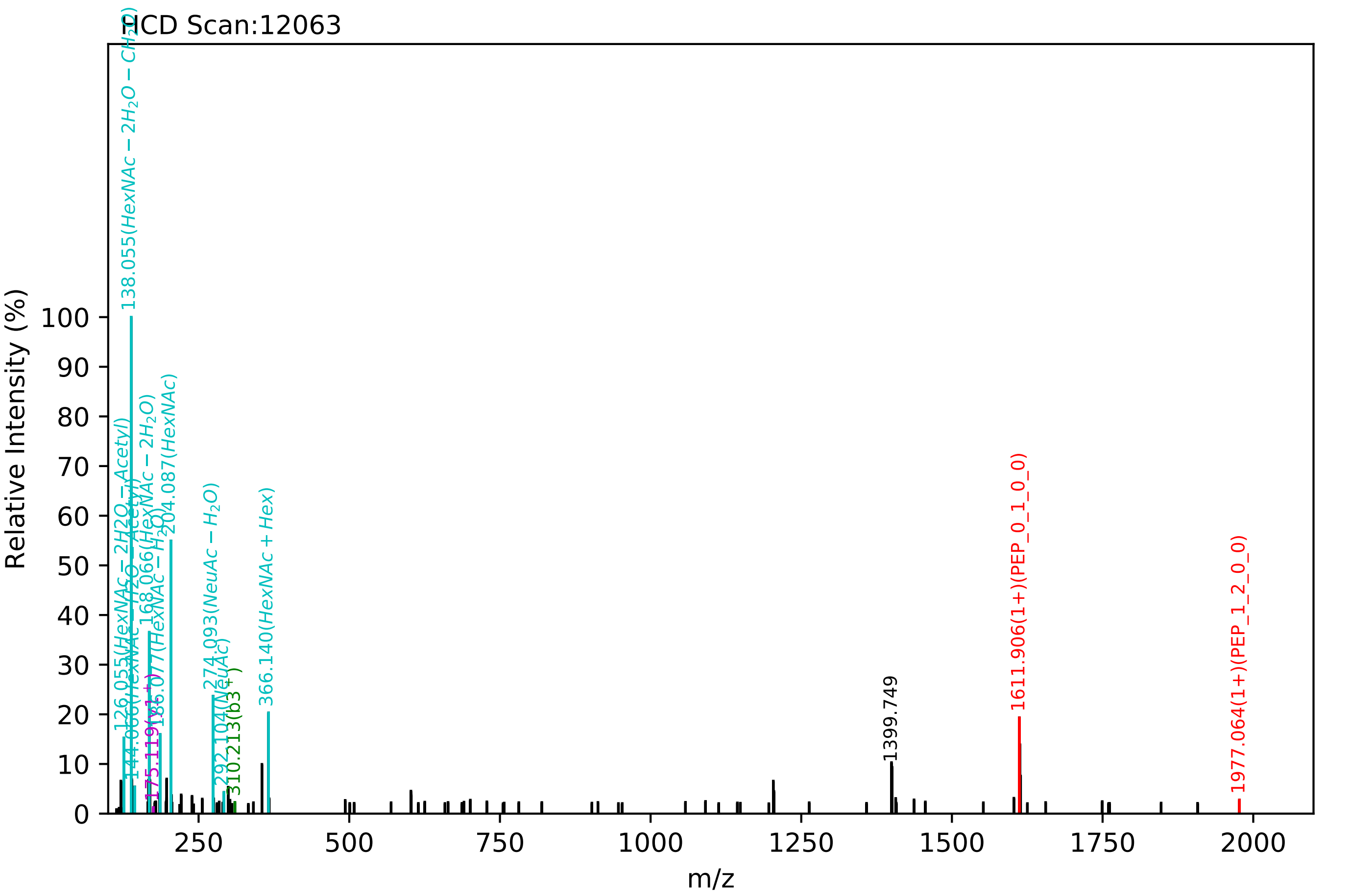

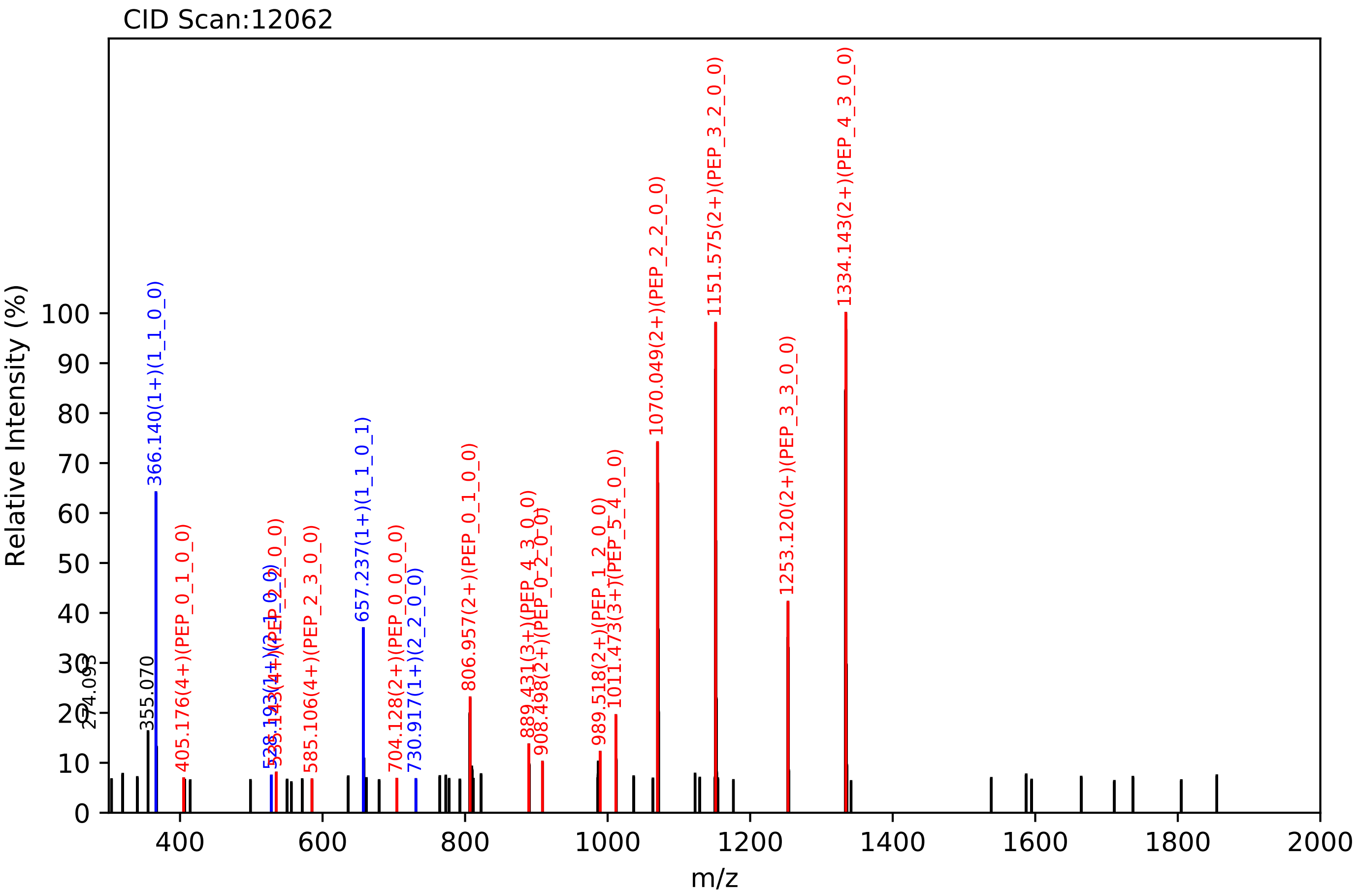

Training set no. 278, Experiment: AGP exp_1

LVPVPITNATLDR(=PEP)_5_4_0_2, m/z:1205.20(3+), RT:43.68, Y-score:91.18

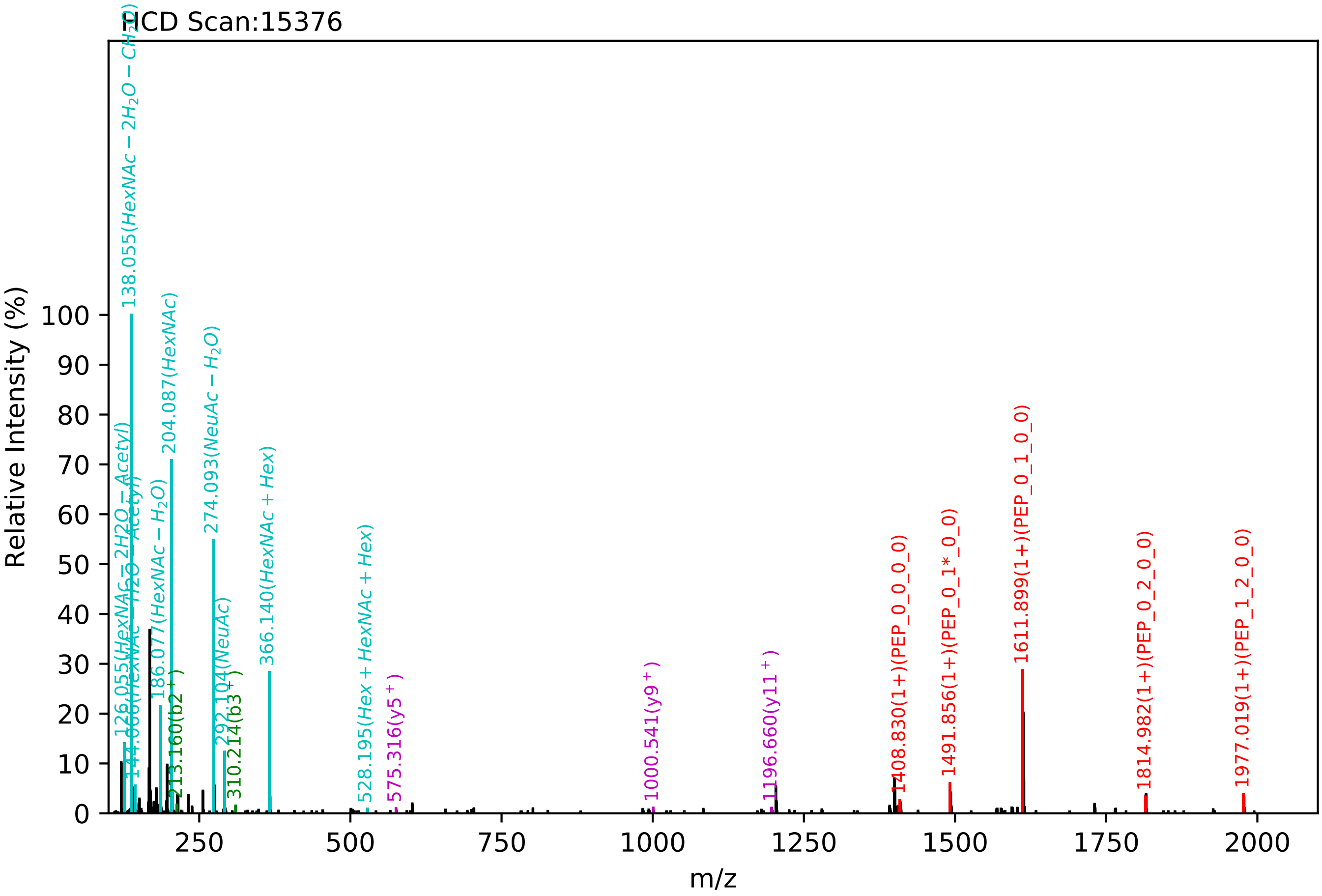

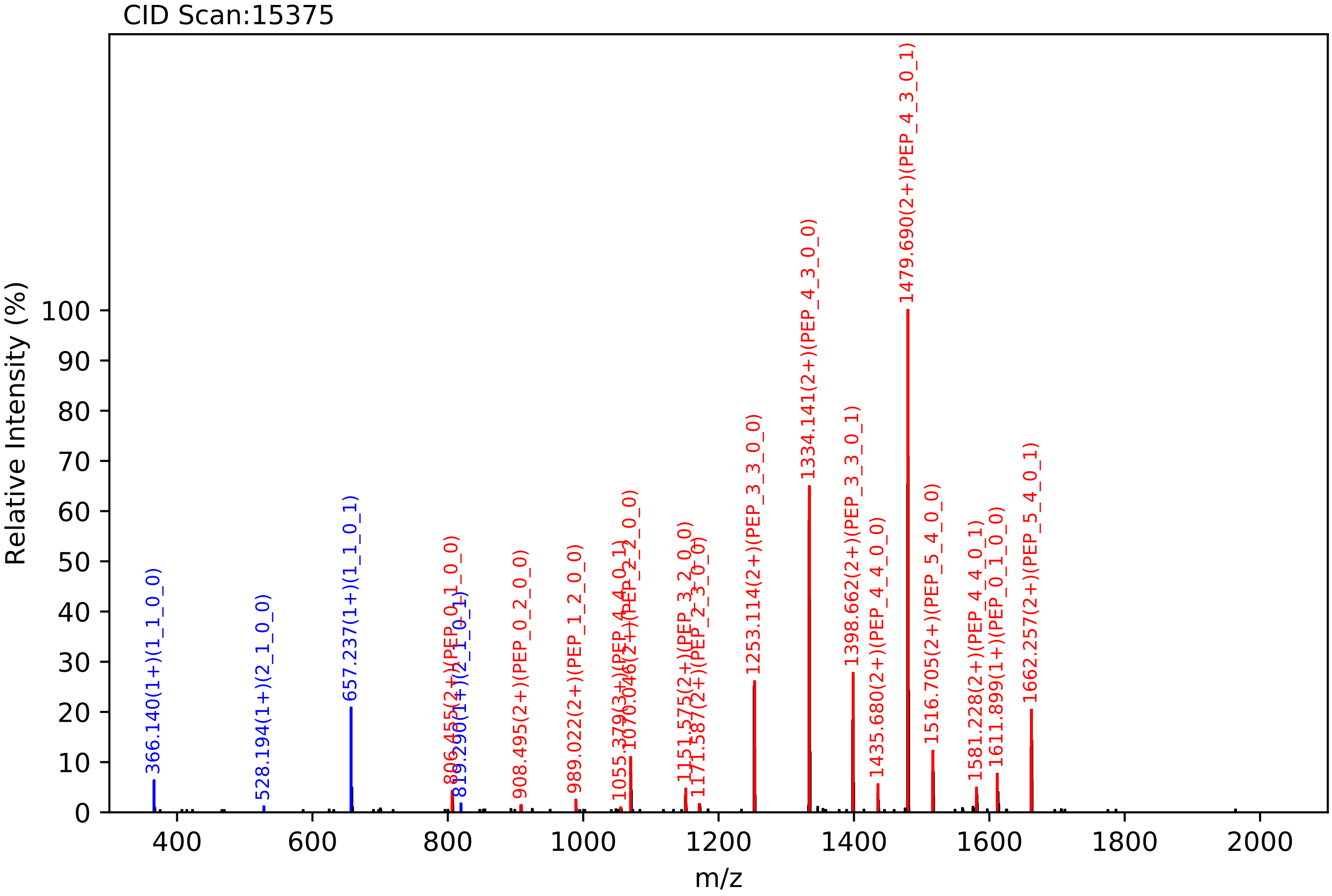

Training set no. 279, Experiment: AGP exp_2

LVPVPITNATLDR(=PEP)_5_4_0_2, m/z:1807.30(2+), RT:43.28, Y-score:87.32

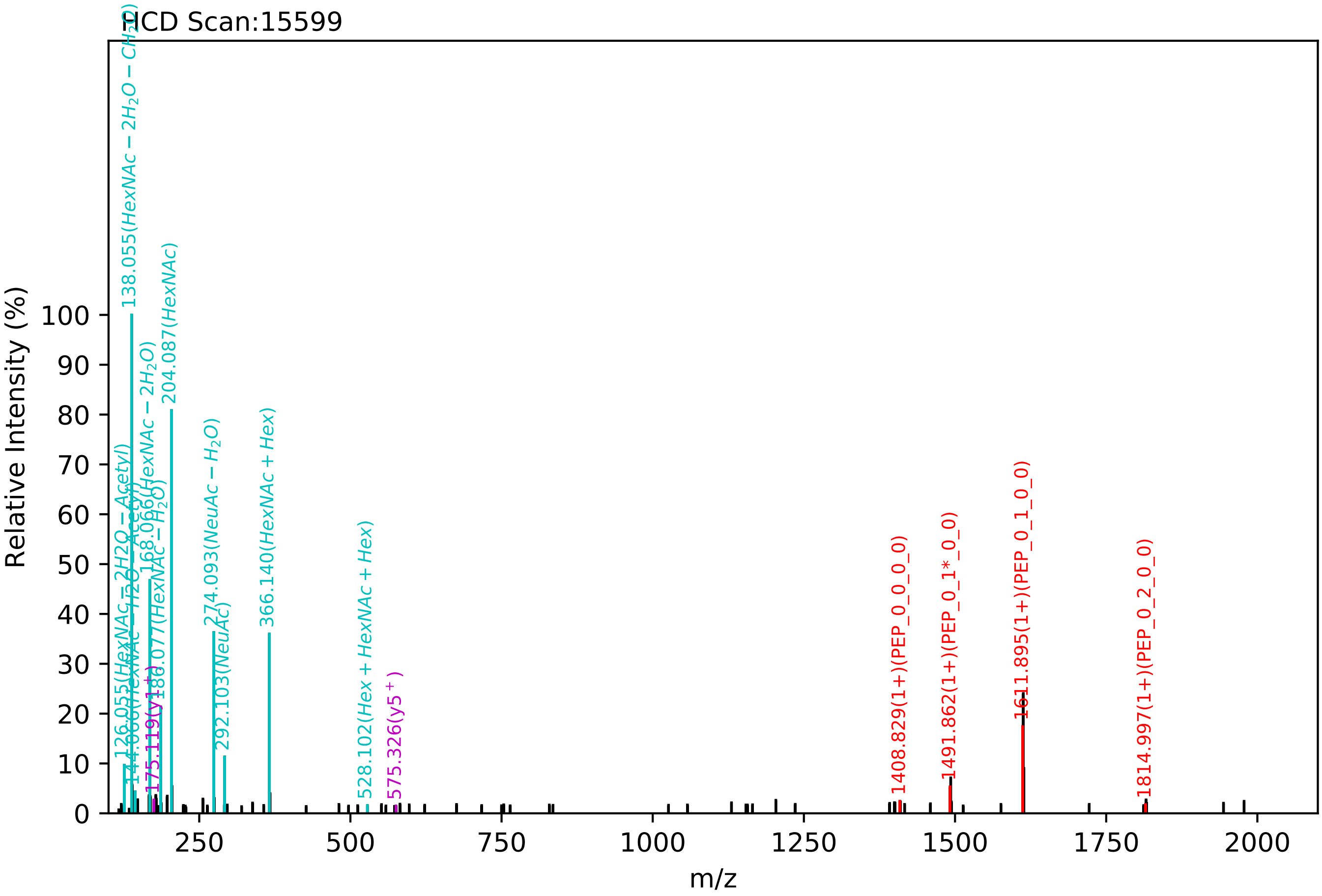

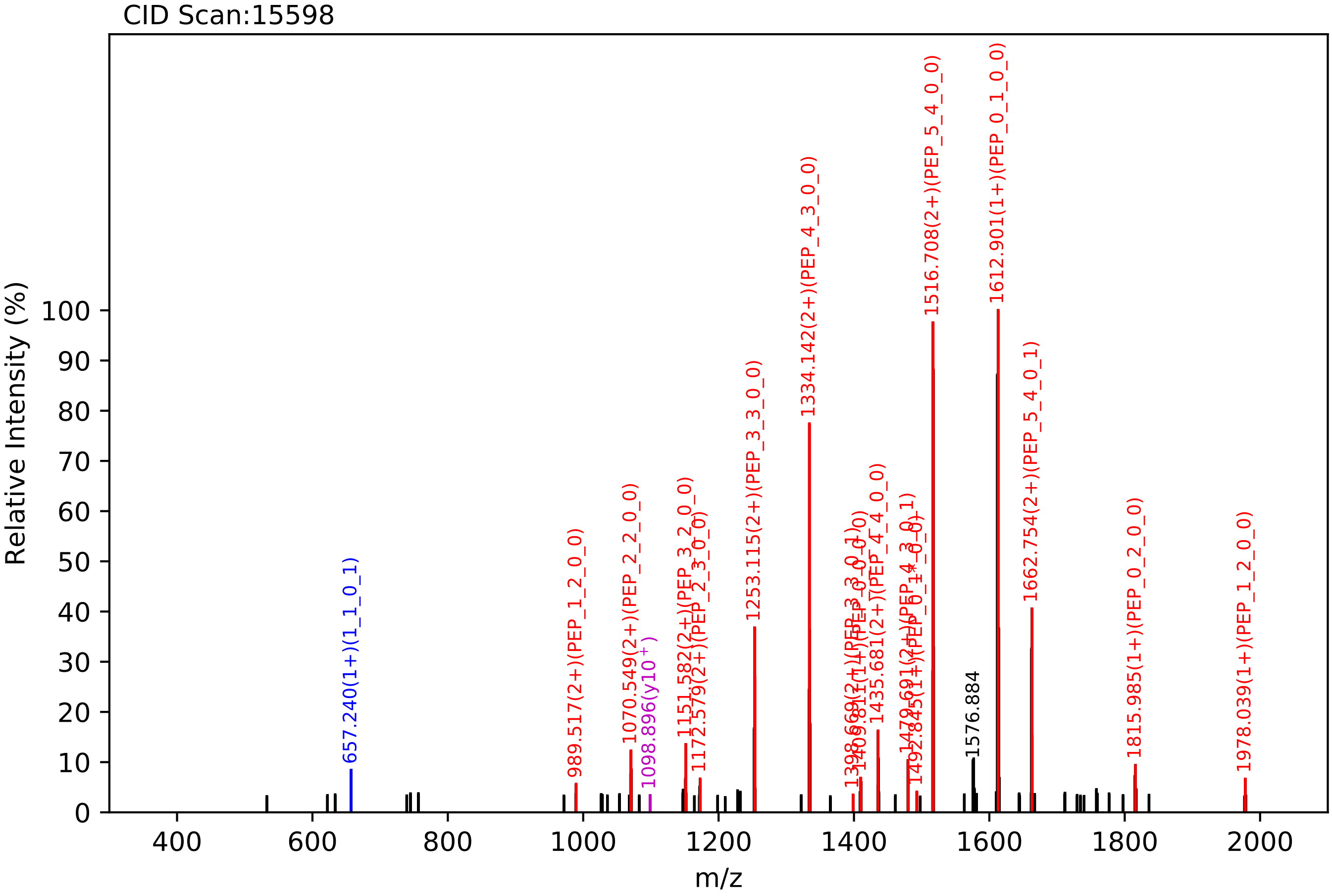

Training set no. 280, Experiment: AGP exp_1

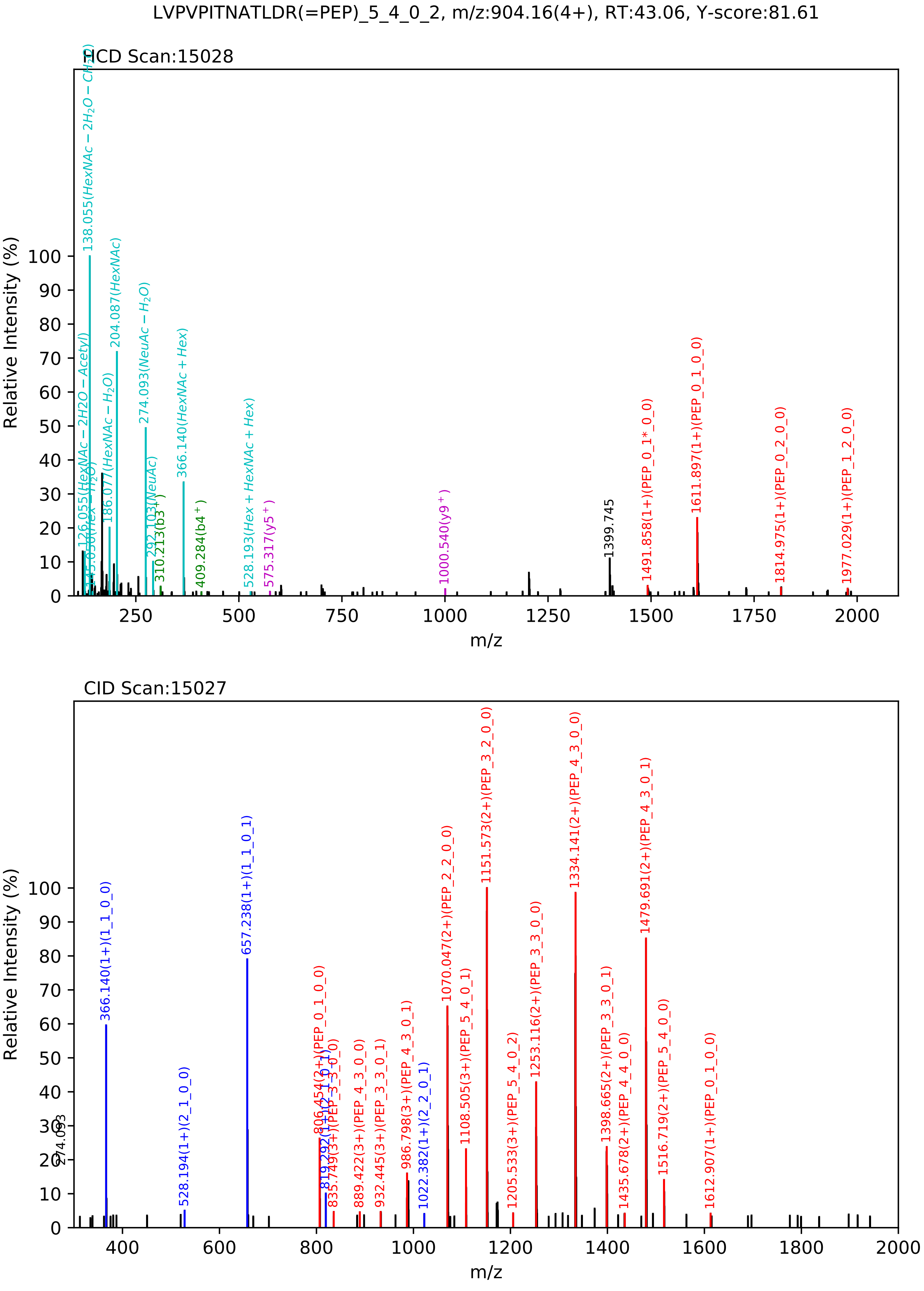

Training set no. 281, Experiment: AGP exp_1

LVPVPITNATLDR(=PEP)_5_4_1_2, m/z:1253.89(3+), RT:43.12, Y-score:77.44

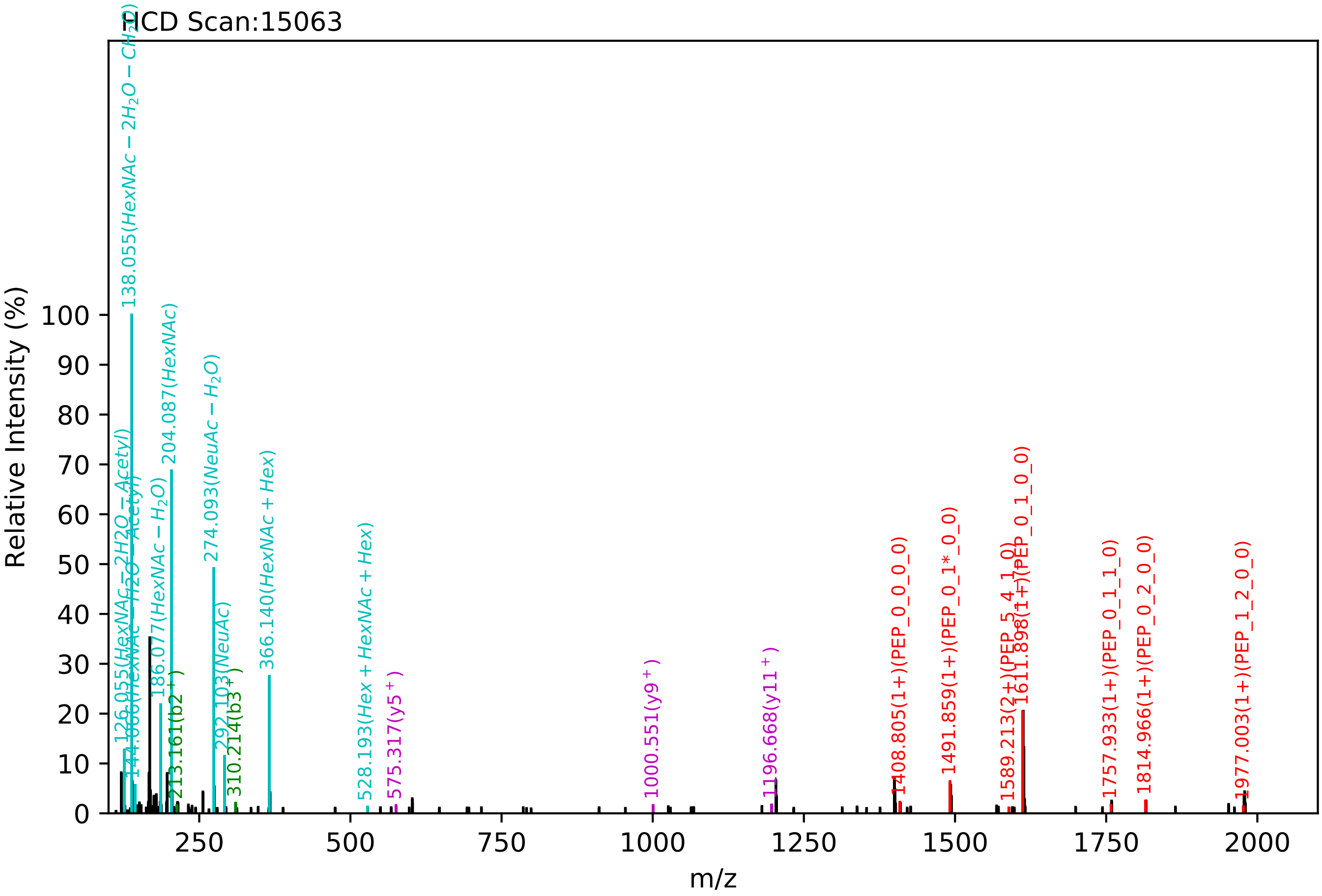

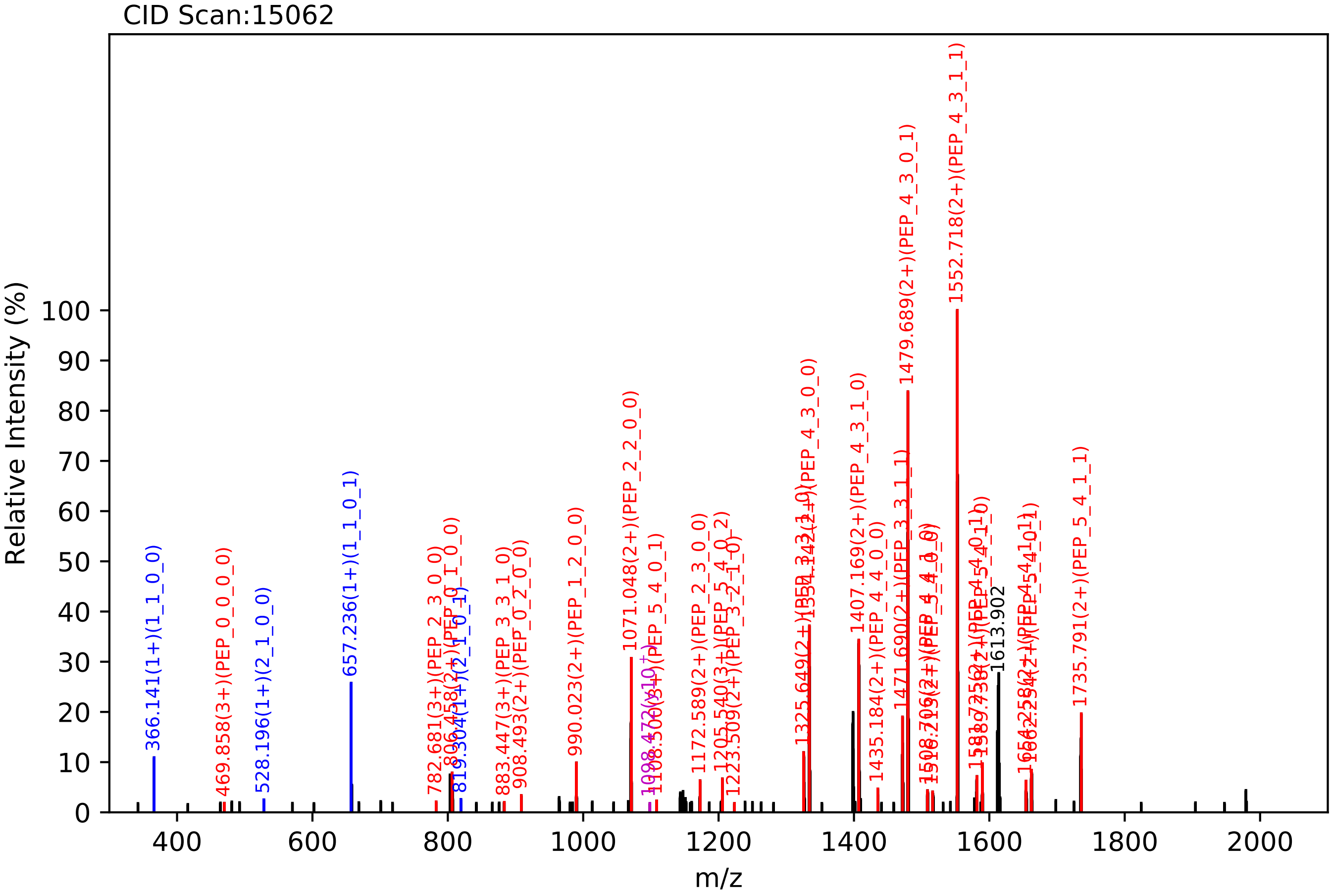

Training set no. 282, Experiment: AGP exp_2

LVPVPITNATLDR(=PEP)_5_4_1_2, m/z:1253.89(3+), RT:43.18, Y-score:74.07

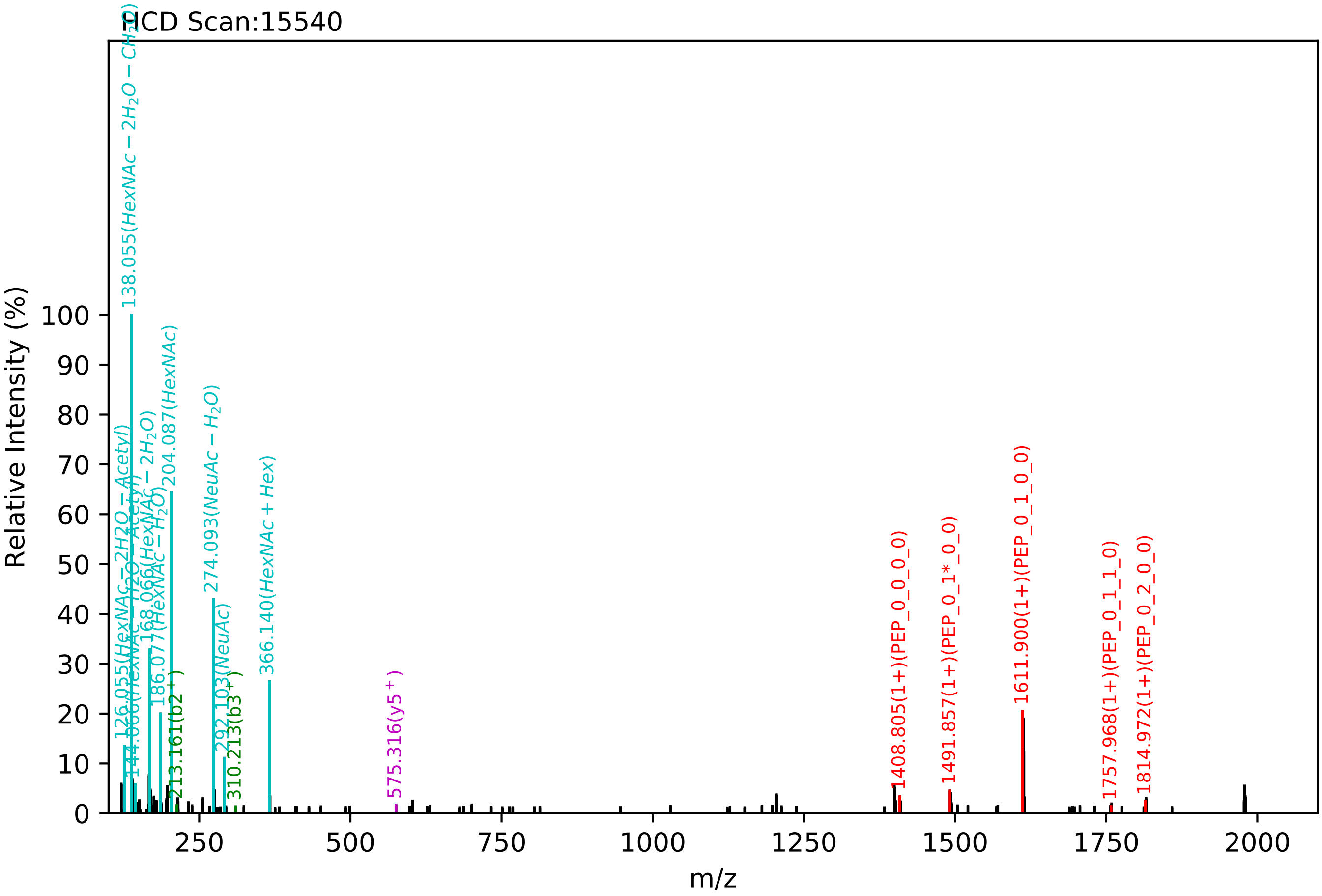

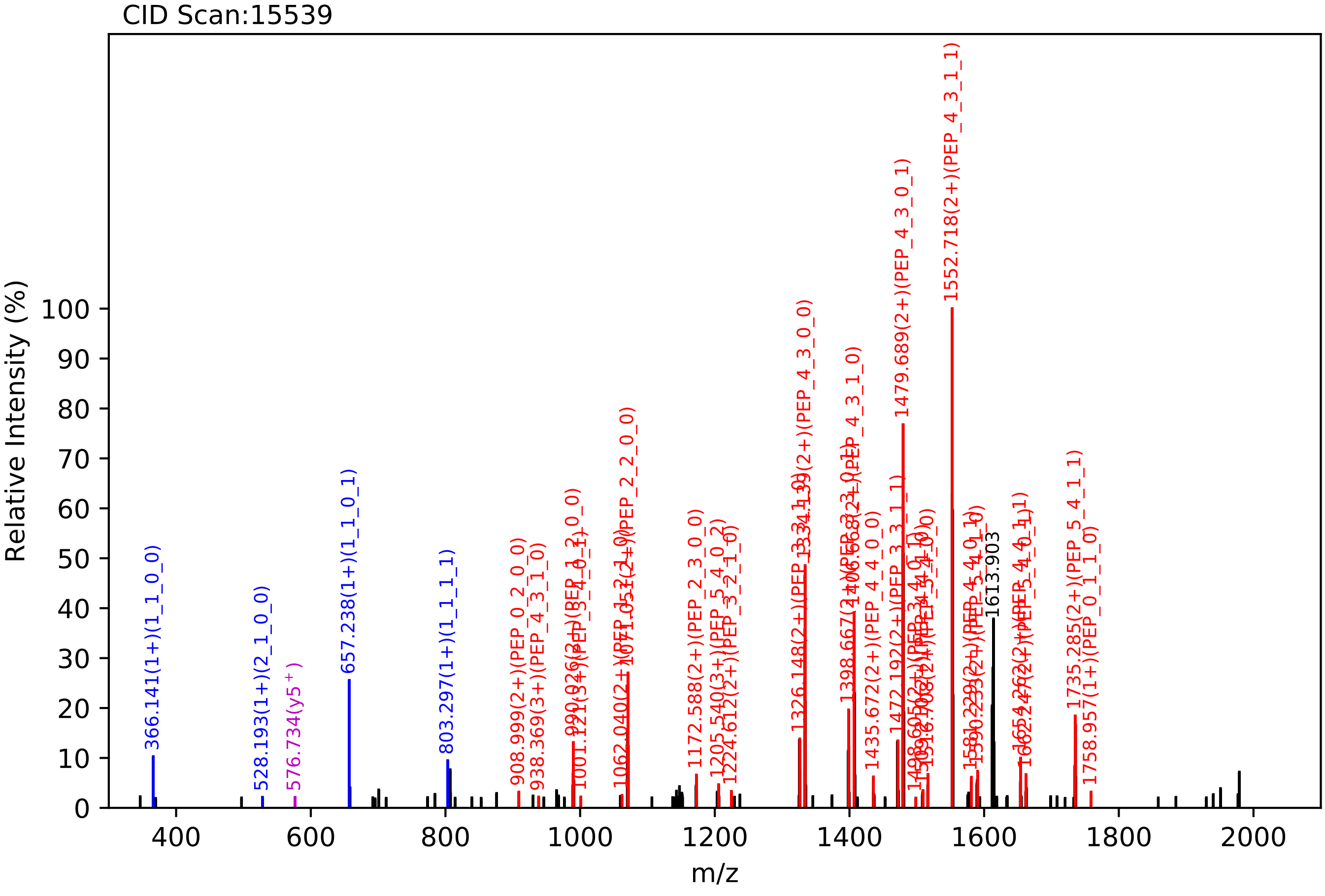

Training set no. 283, Experiment: AGP exp_2

LVPVPITNATLDR(=PEP)_5_5_0_1, m/z:1175.86(3+), RT:37.06, Y-score:87.48

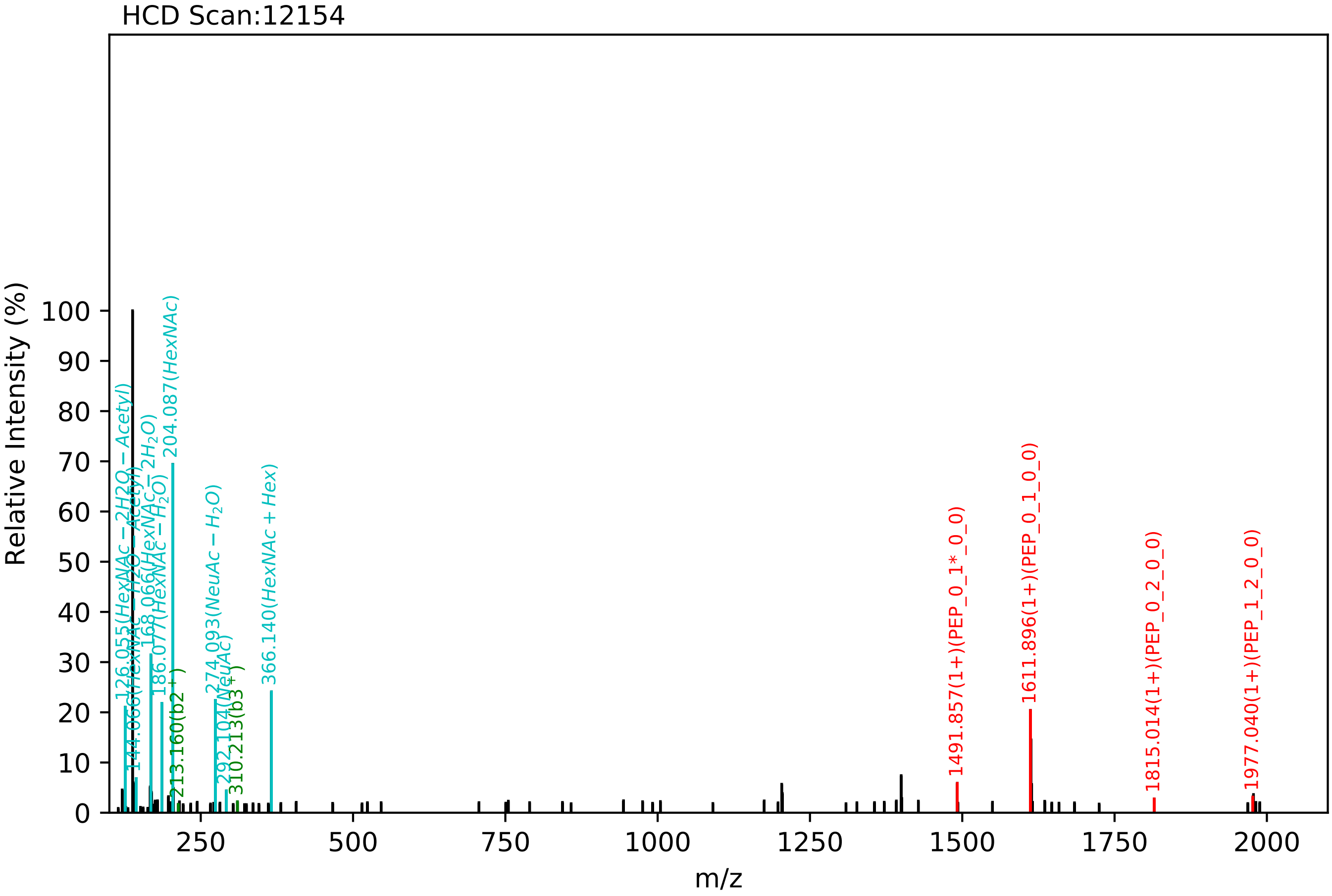

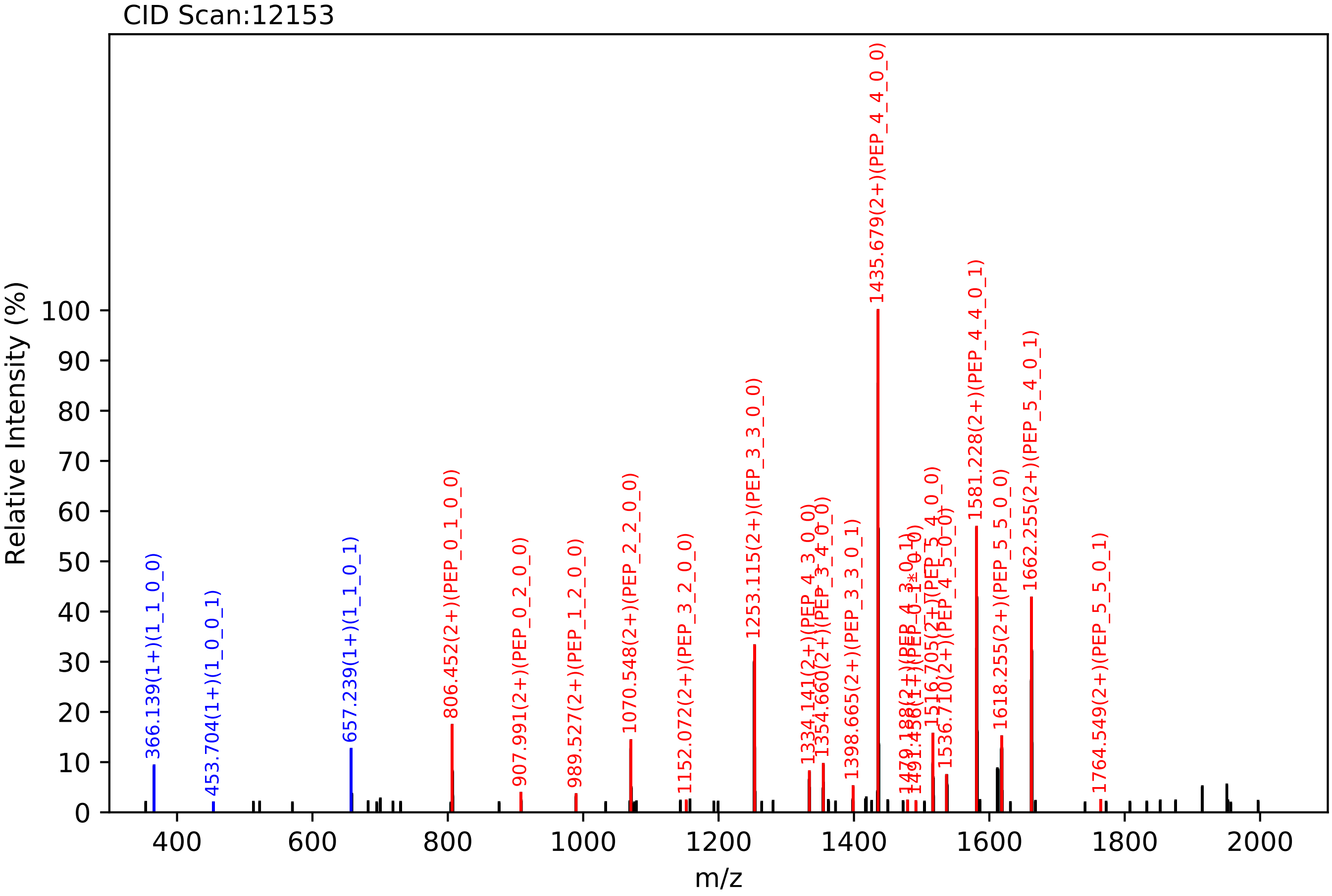

Training set no. 284, Experiment: AGP exp_1

LVPVPITNATLDR(=PEP)_5_5_0_1, m/z:1175.86(3+), RT:36.44, Y-score:86.42

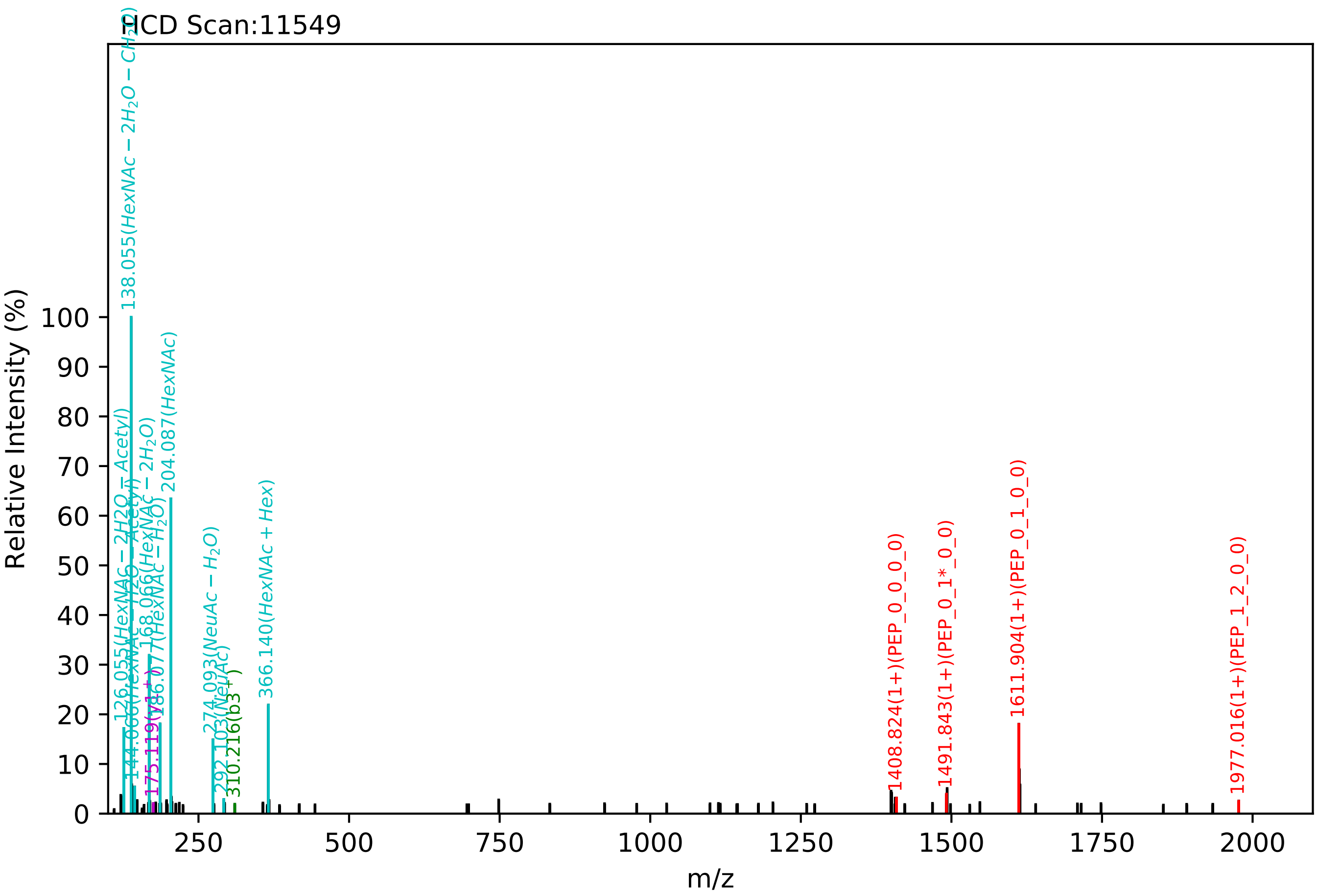

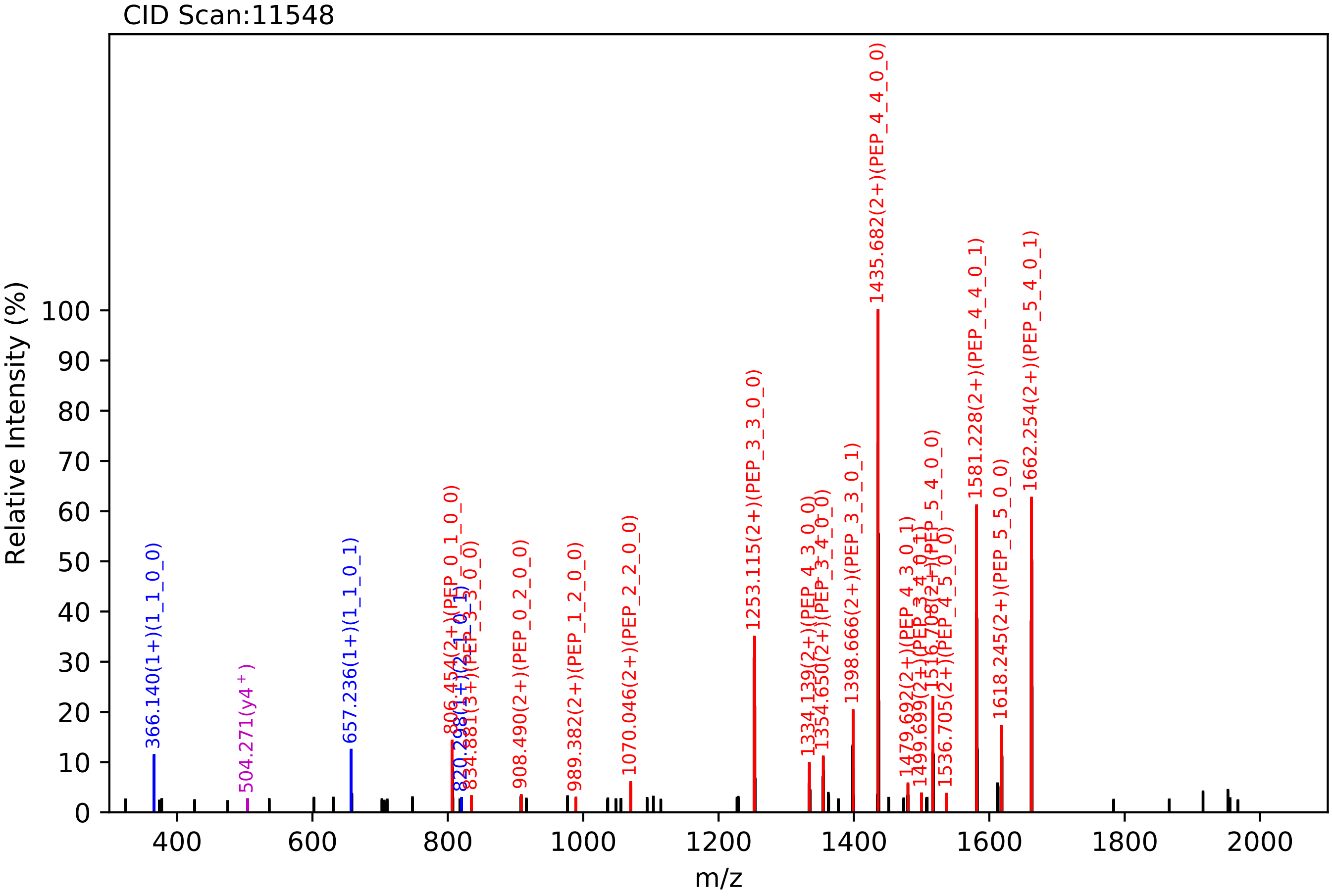

Training set no. 285, Experiment: AGP exp_1

LVPVPITNATLDR(=PEP)_5_5_0_1, m/z:1175.86(3+), RT:36.98, Y-score:86.13

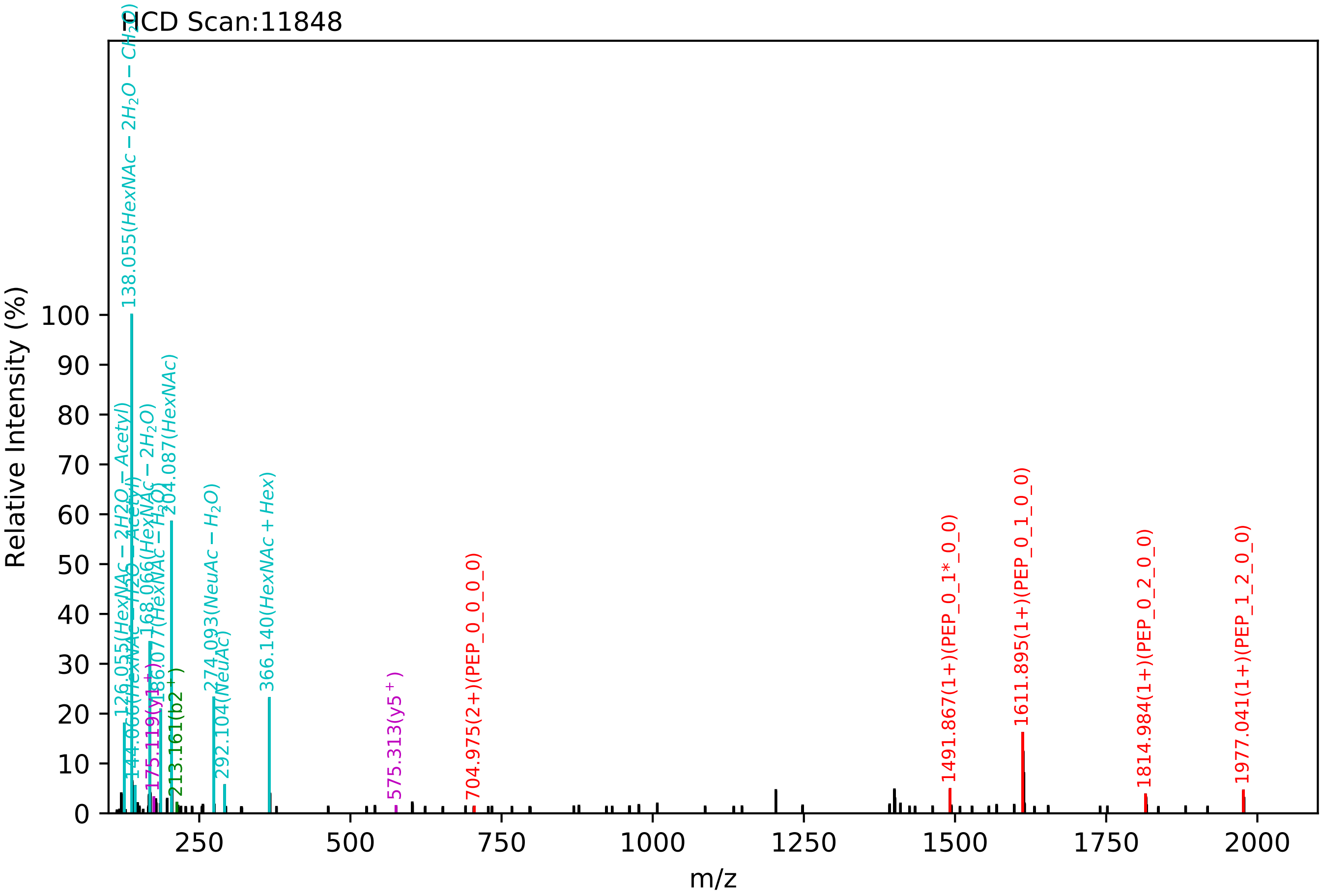

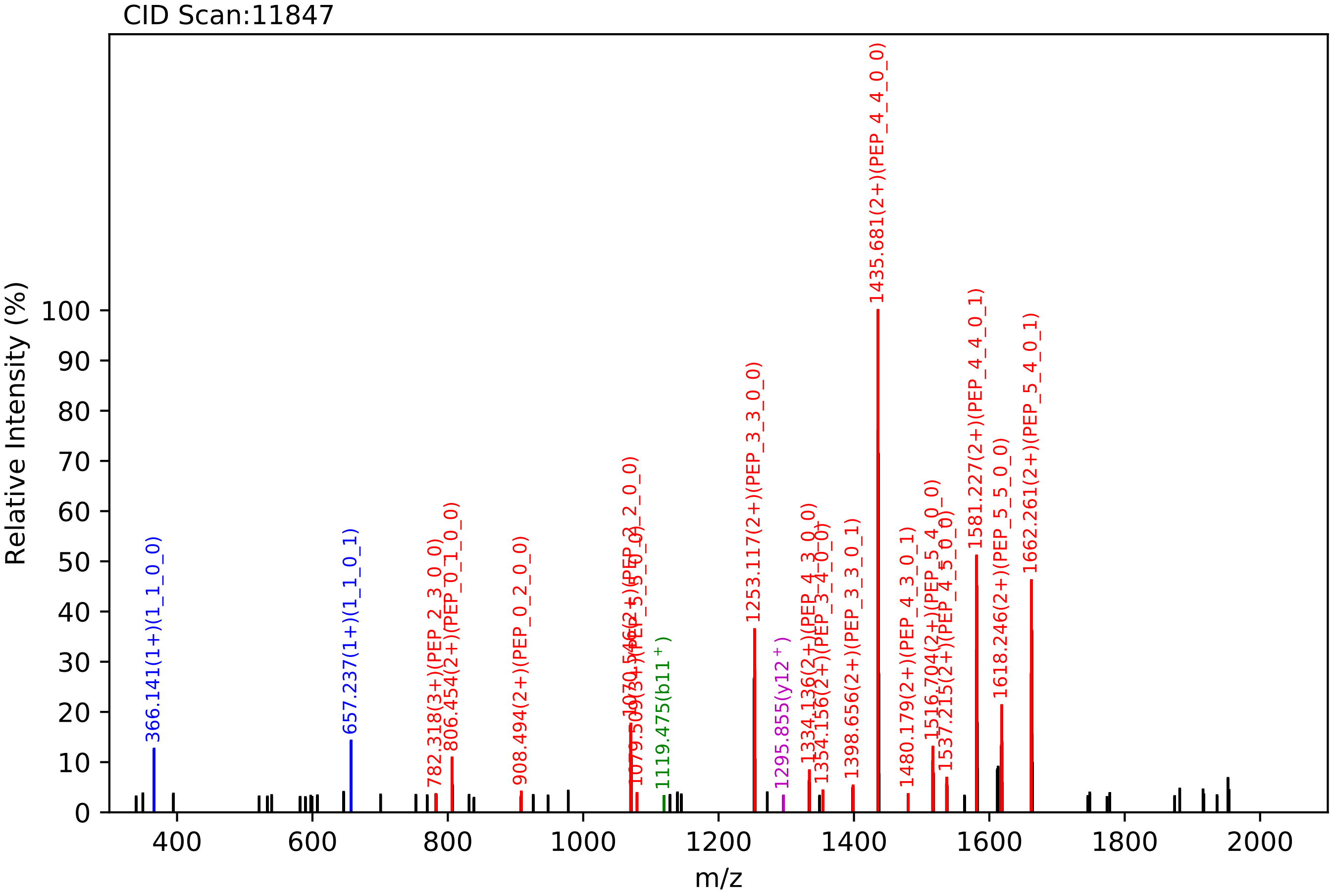

Training set no. 286, Experiment: AGP exp_2

LVPVPITNATLDR(=PEP)_5_5_0_2, m/z:1272.89(3+), RT:43.47, Y-score:88.83

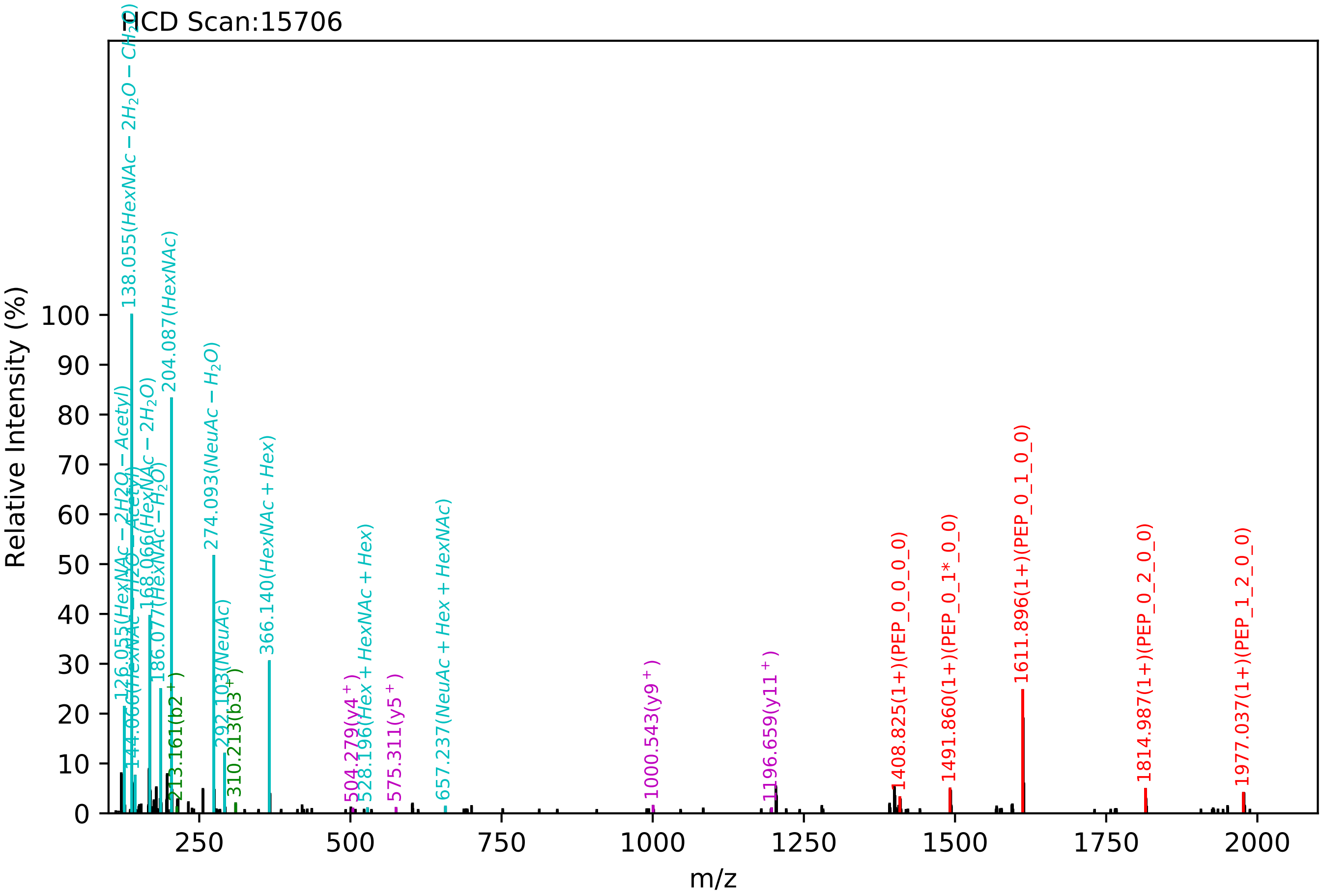

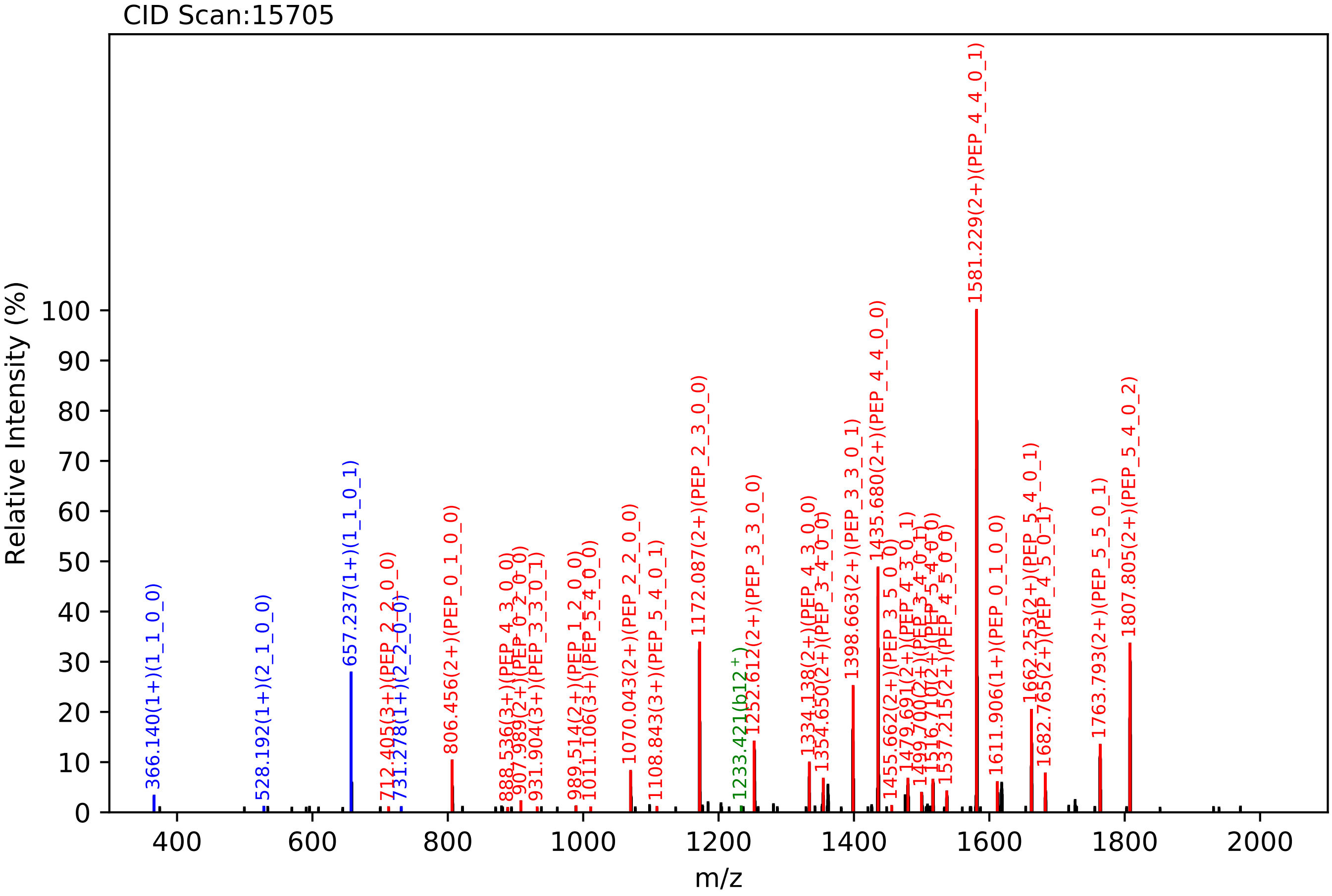

Training set no. 287, Experiment: AGP exp_2

LVPVPITNATLDR(=PEP)_6_5_0_1, m/z:1229.54(3+), RT:36.98, Y-score:91.79

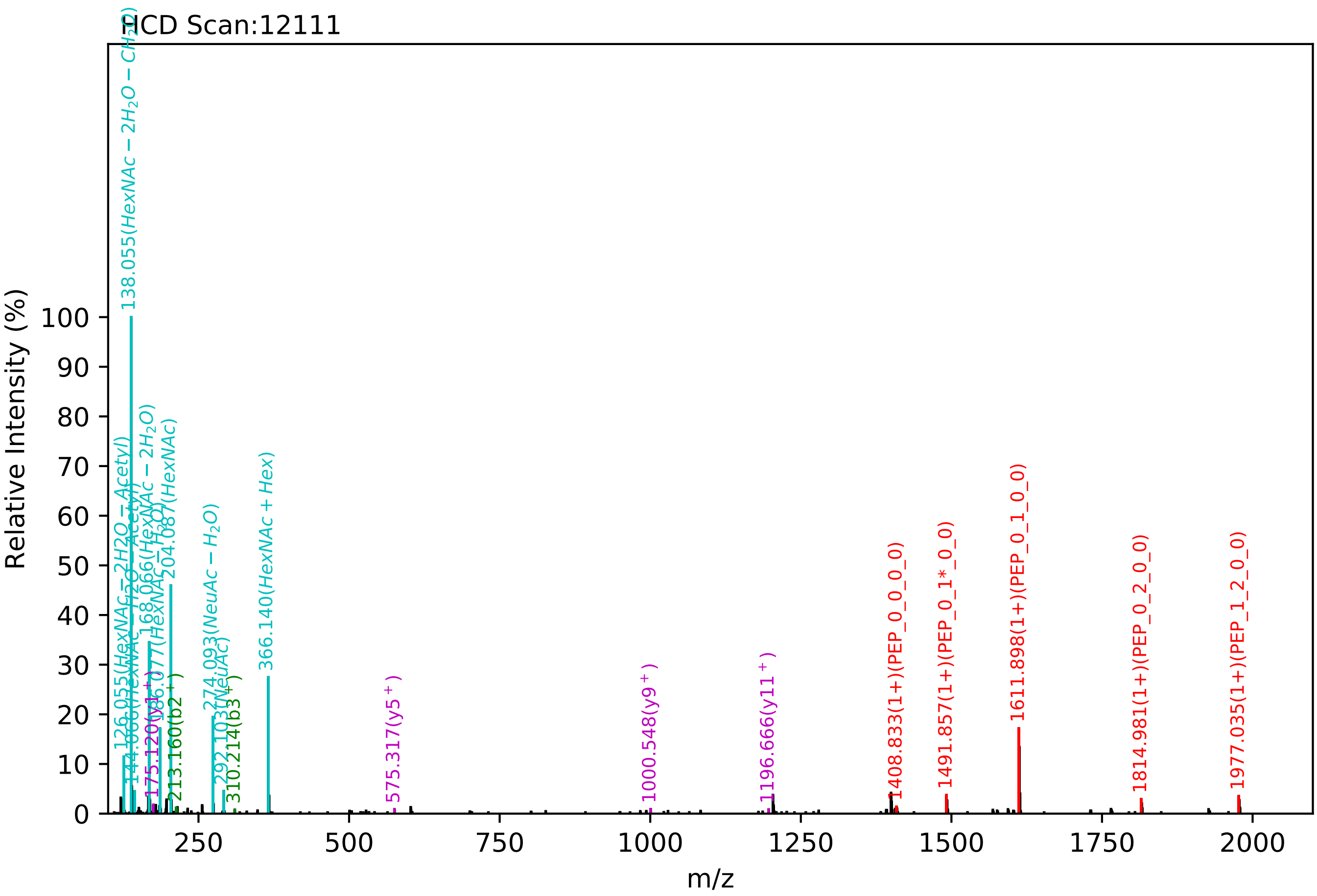

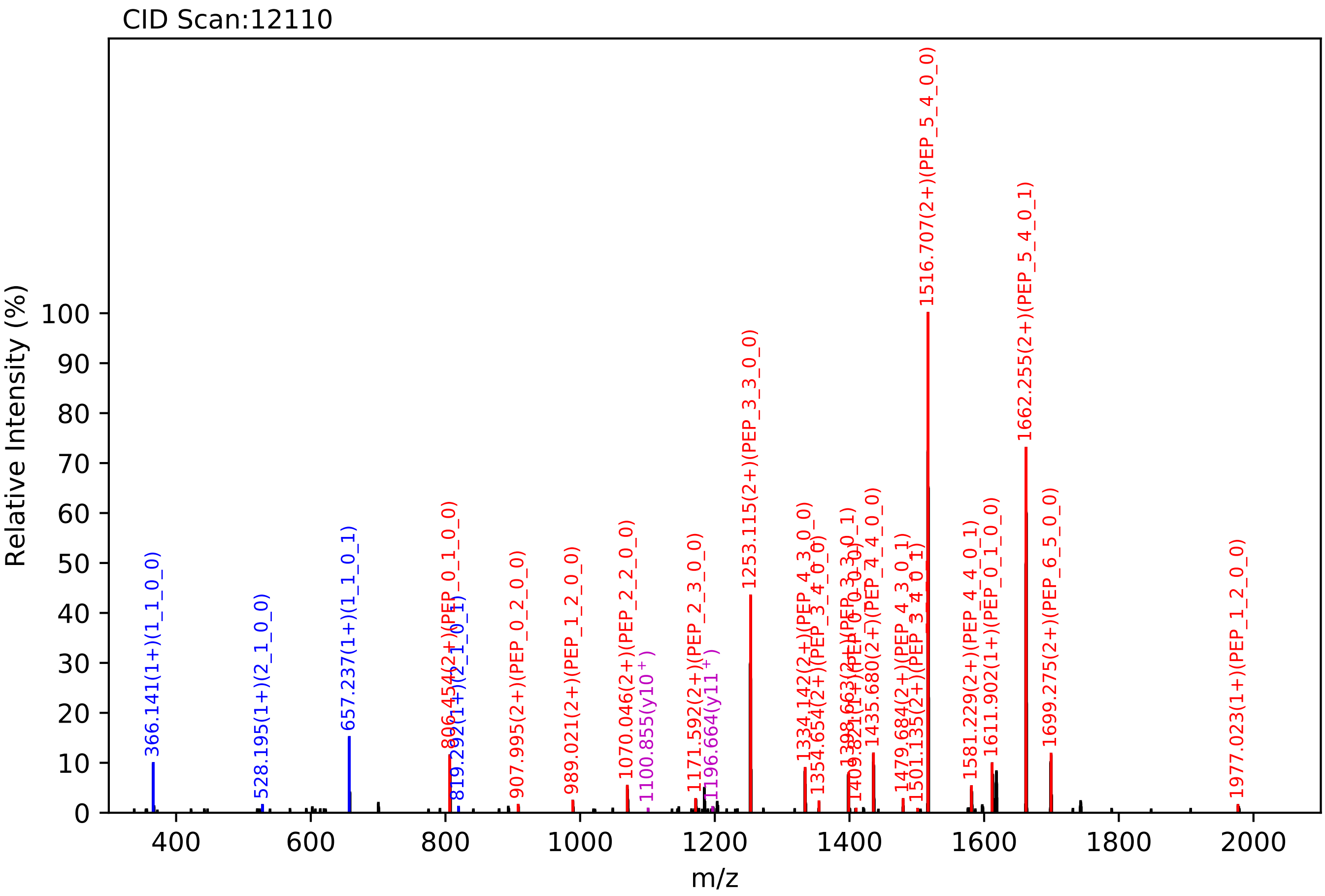

Training set no. 288, Experiment: AGP exp_2

LVPVPITNATLDR(=PEP)_6_5_0_1, m/z:922.66(4+), RT:36.67, Y-score:88.09

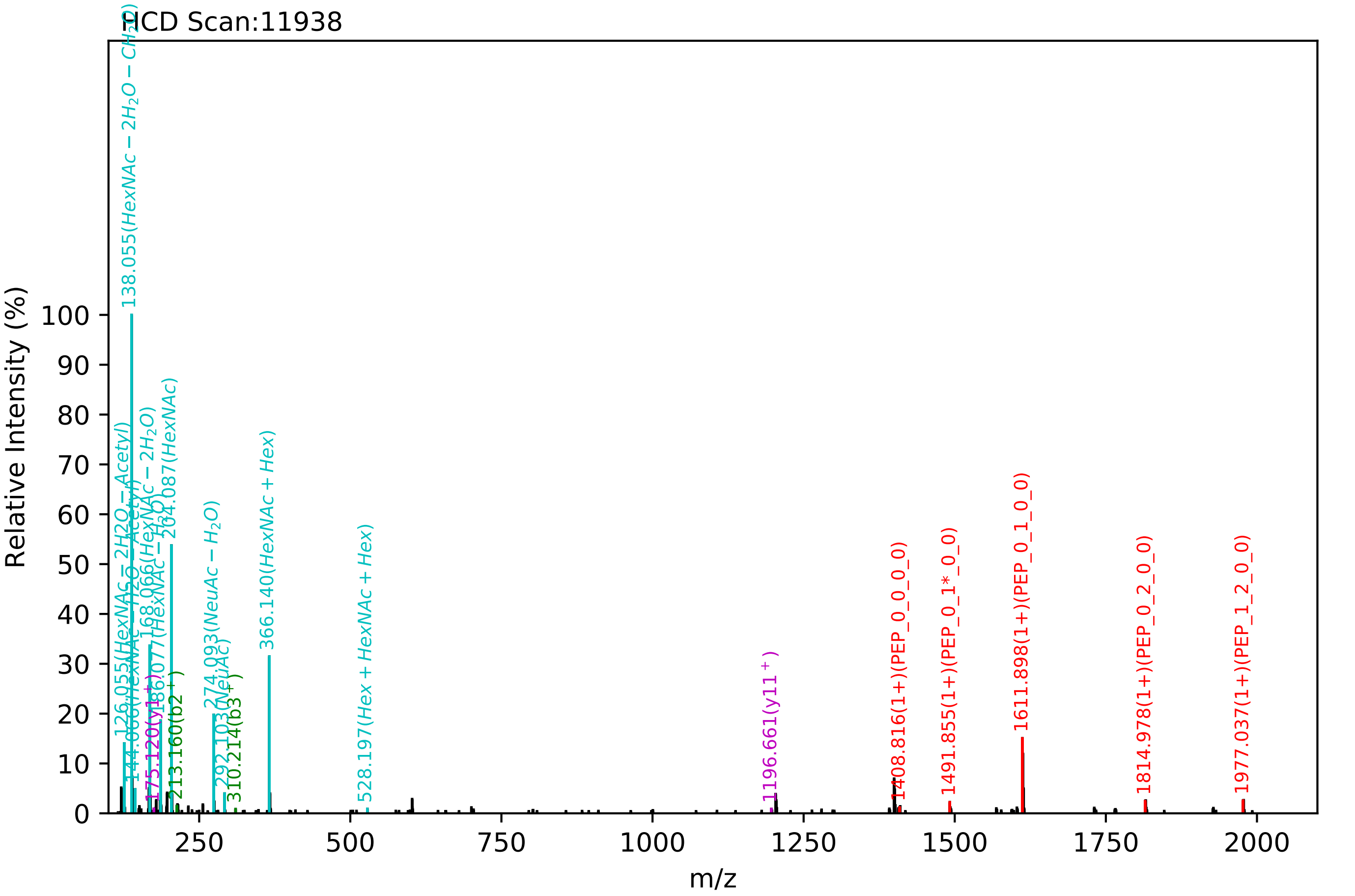

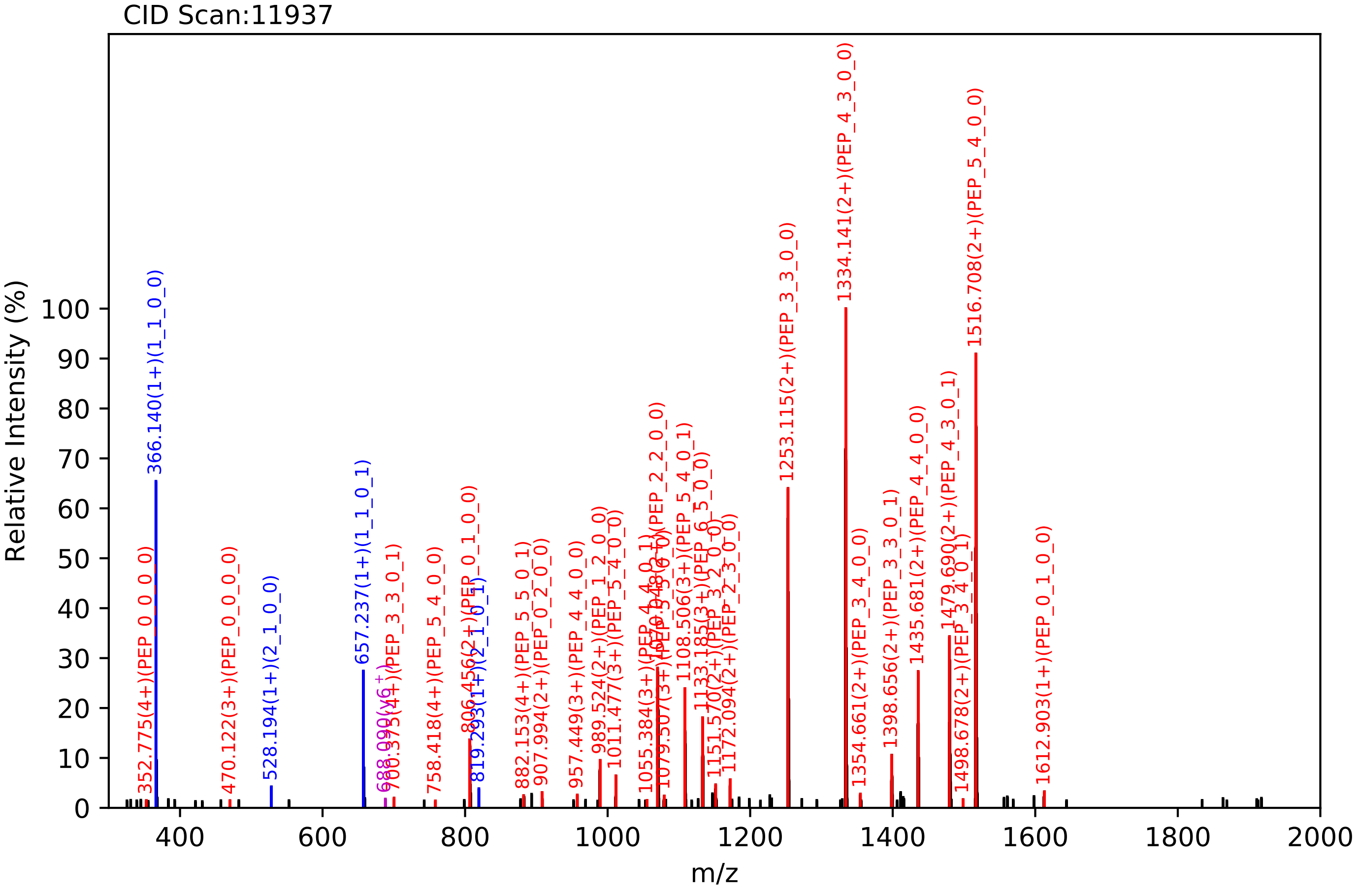

Training set no. 289, Experiment: AGP exp_1

LVPVPITNATLDR(=PEP)_6_5_0_1, m/z:922.66(4+), RT:36.55, Y-score:87.64

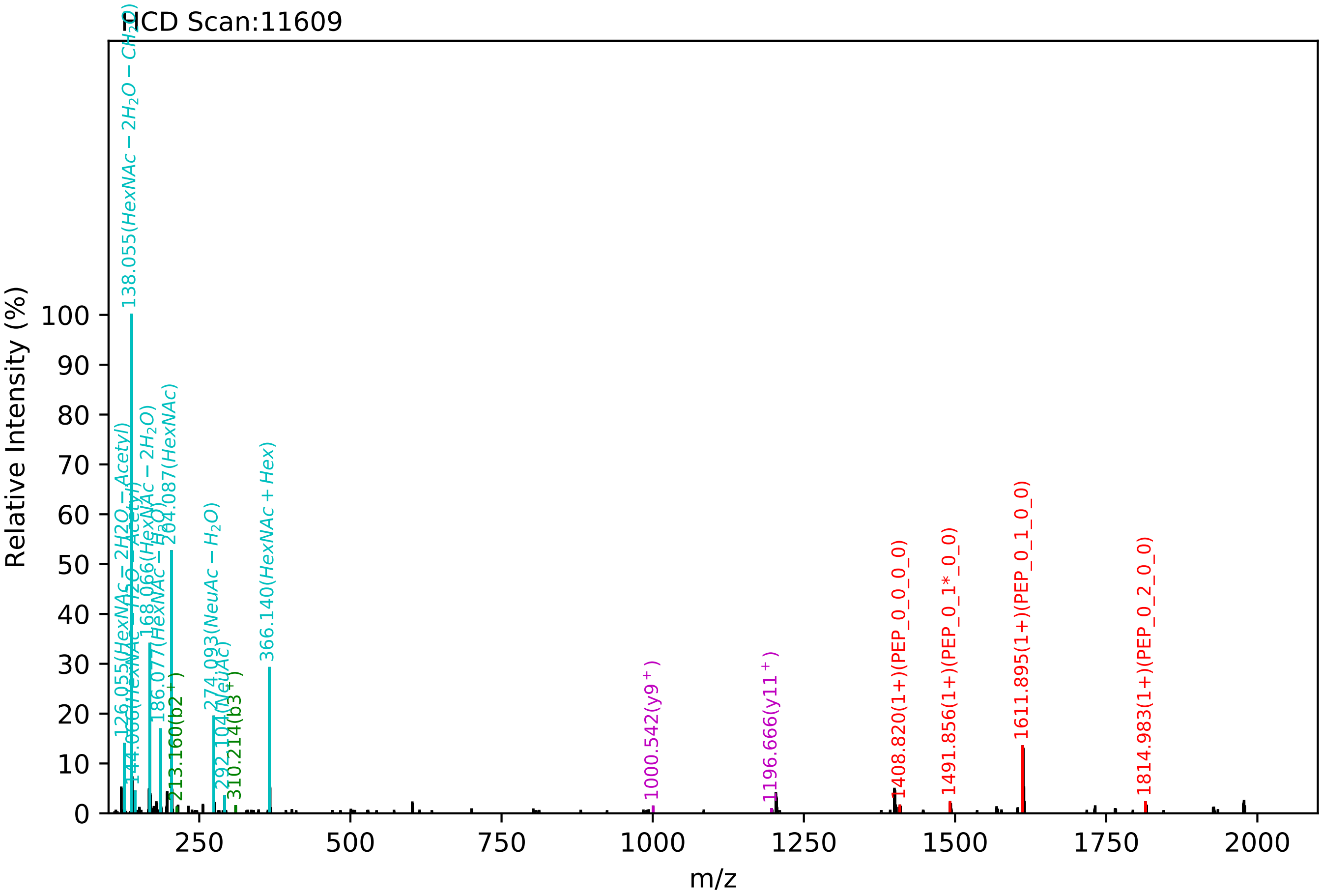

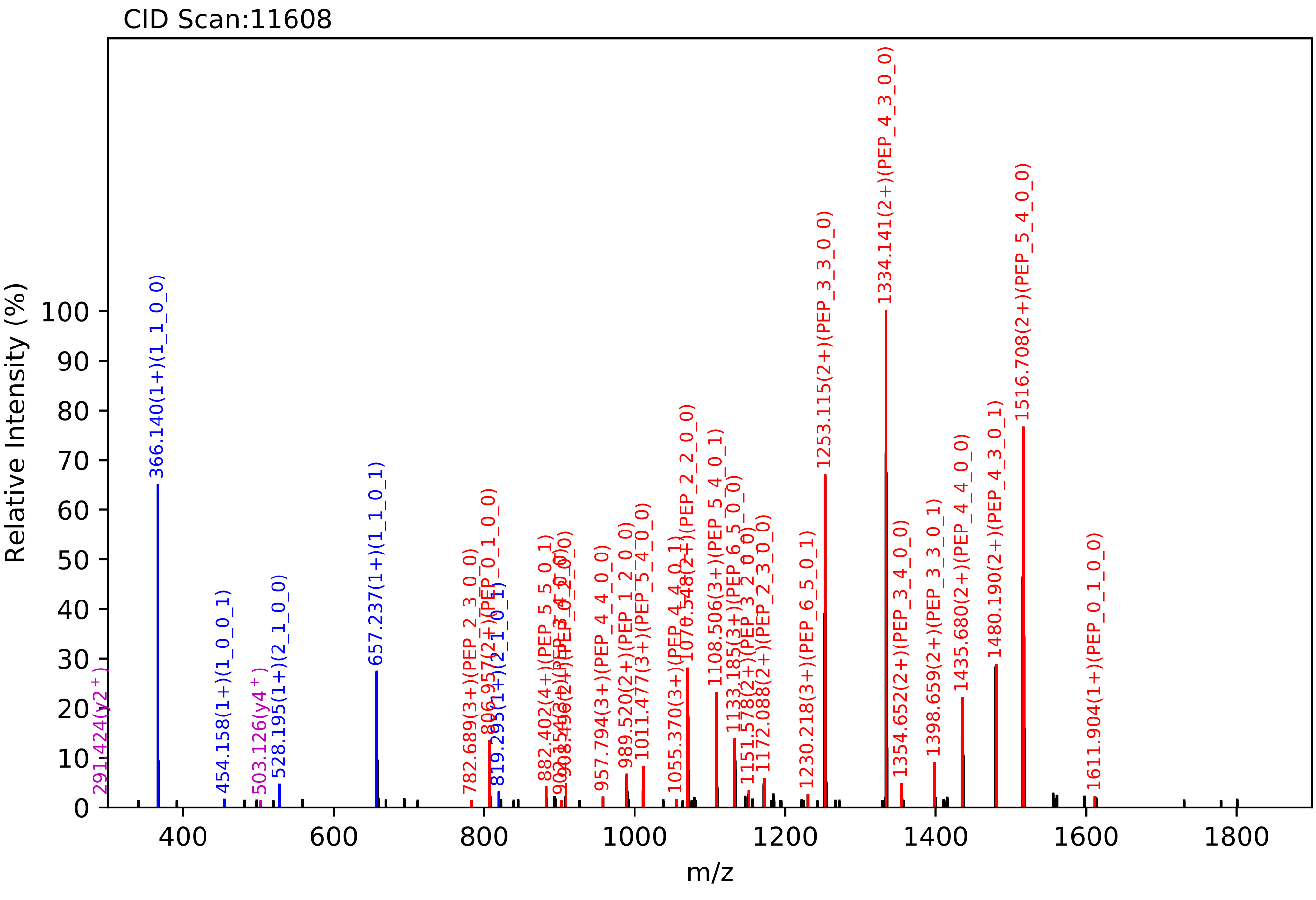

Training set no. 290, Experiment: AGP exp_1

LVPVPITNATLDR(=PEP)_6_5_0_2, m/z:1989.86(2+), RT:42.46, Y-score:94.83

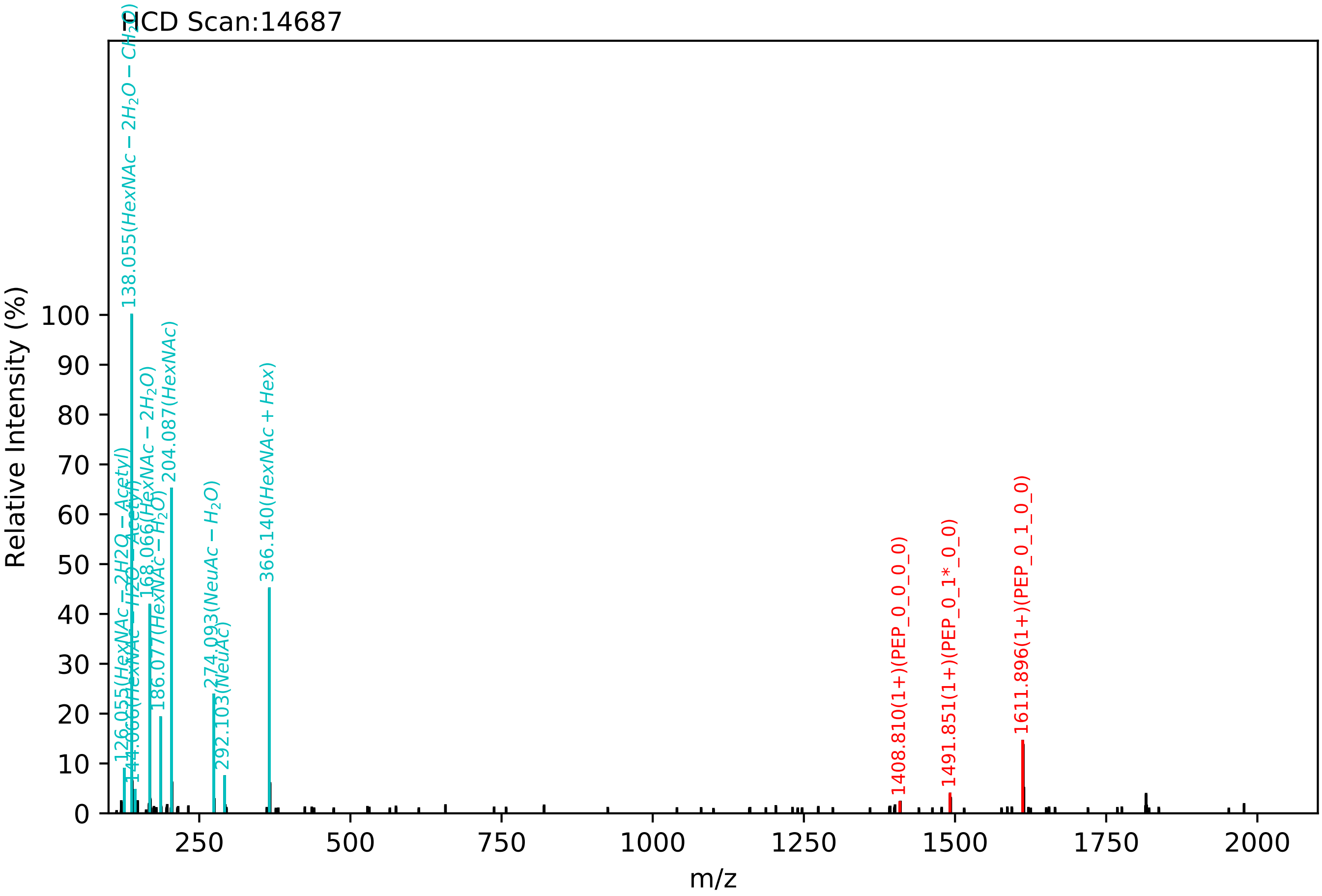

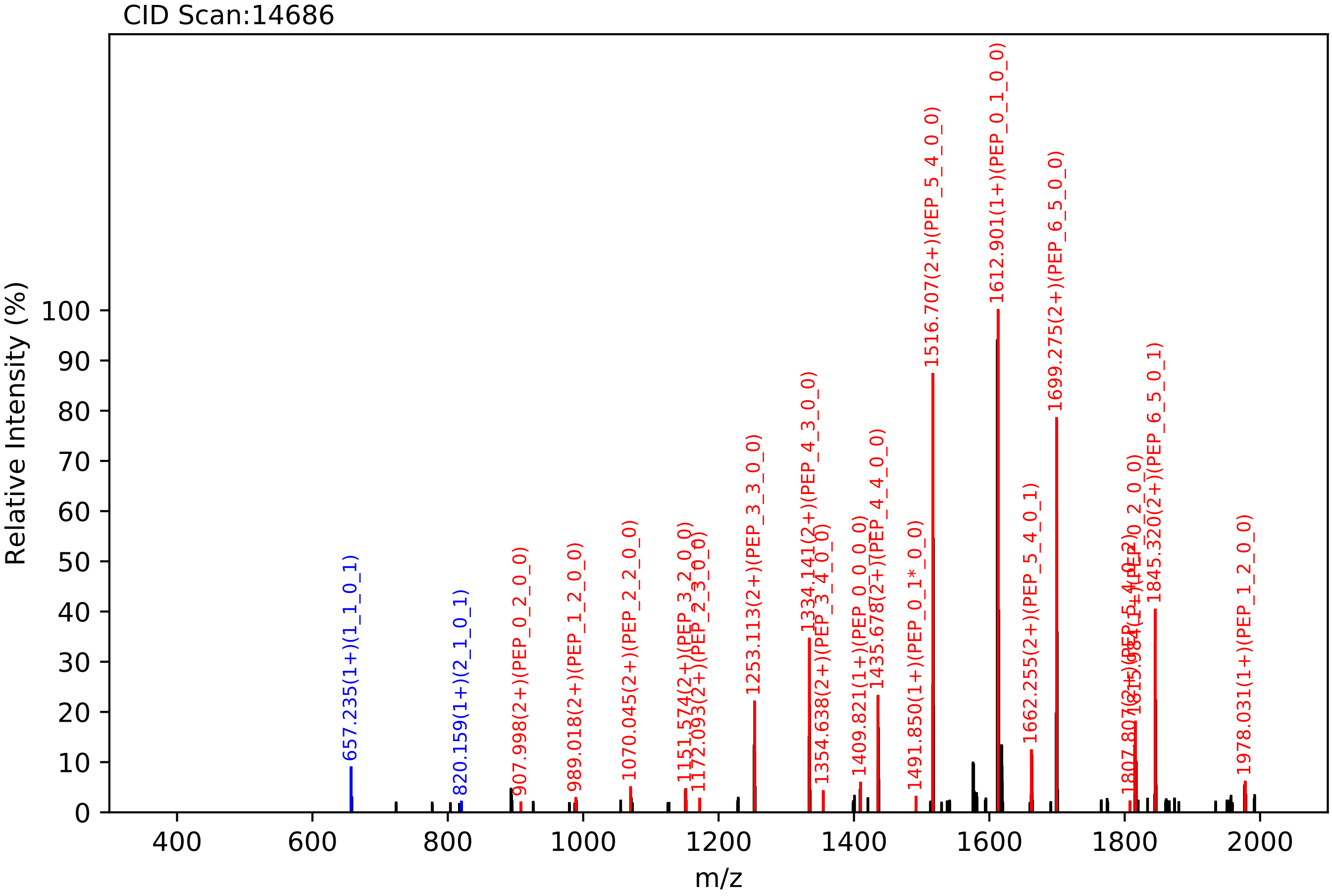

Training set no. 291, Experiment: AGP exp_1

LVPVPITNATLDR(=PEP)_6_5_0_2, m/z:995.44(4+), RT:42.92, Y-score:86.69

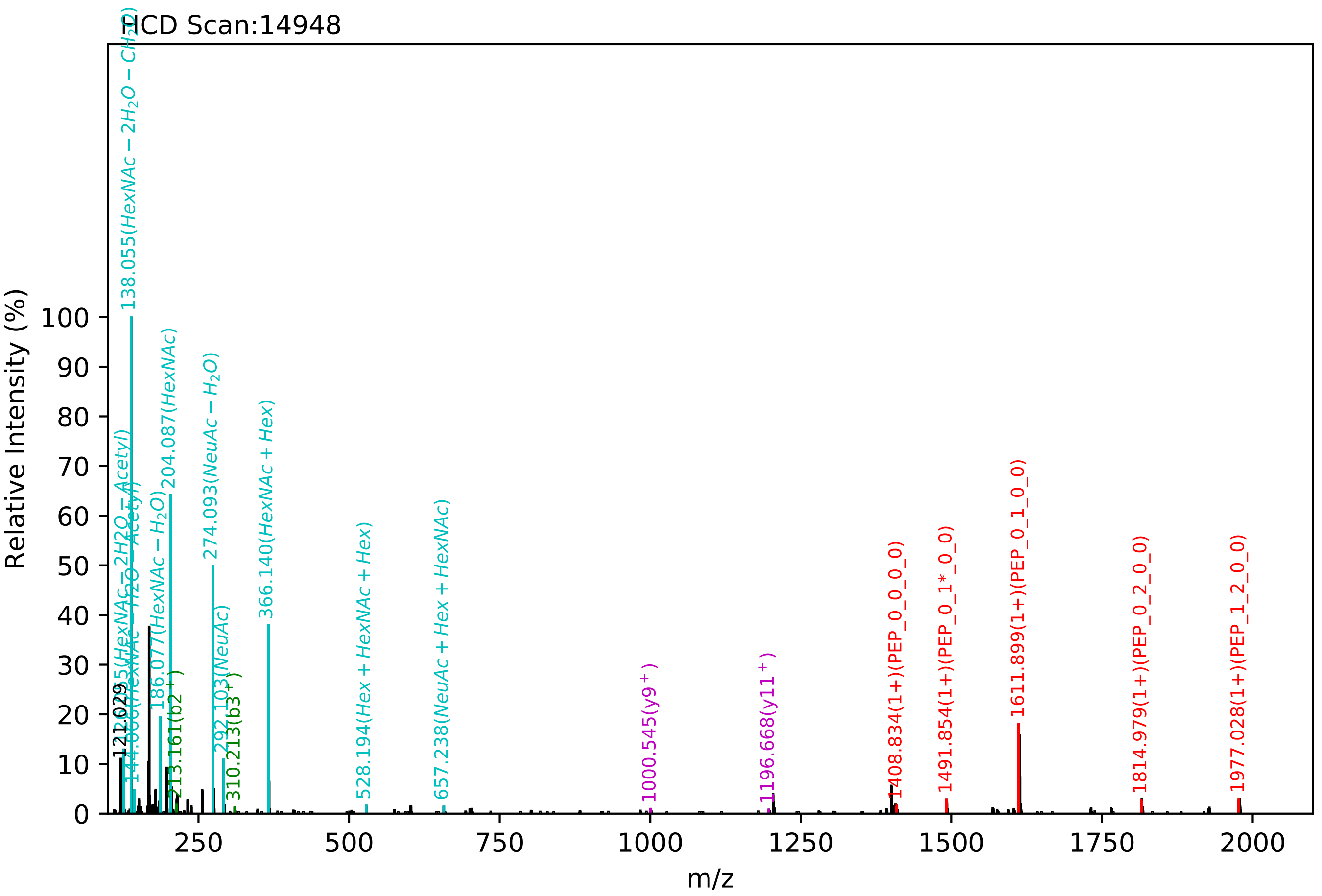

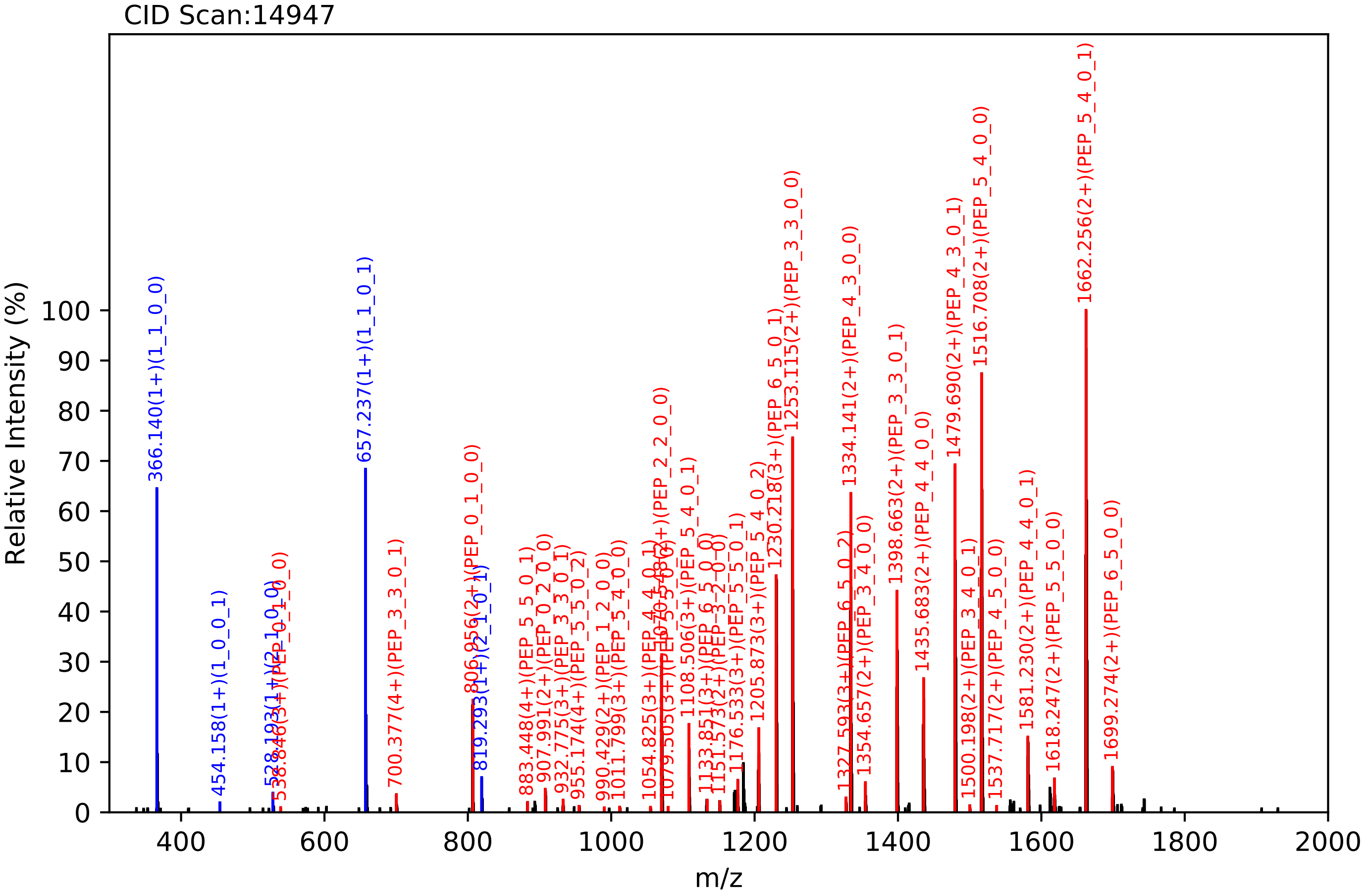

Training set no. 292, Experiment: AGP exp_2

LVPVPITNATLDR(=PEP)_6_5_0_2, m/z:995.44(4+), RT:43.04, Y-score:85.93

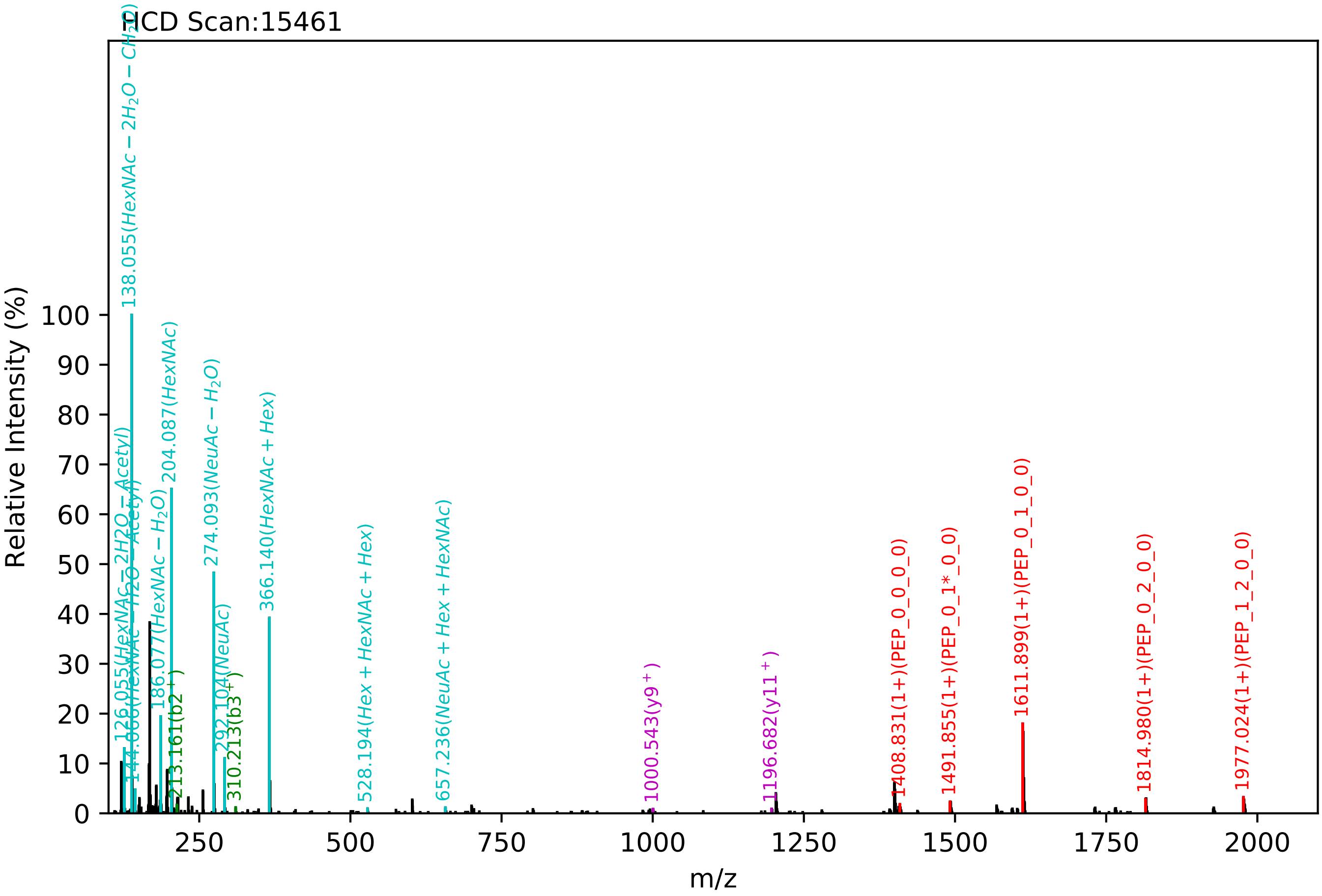

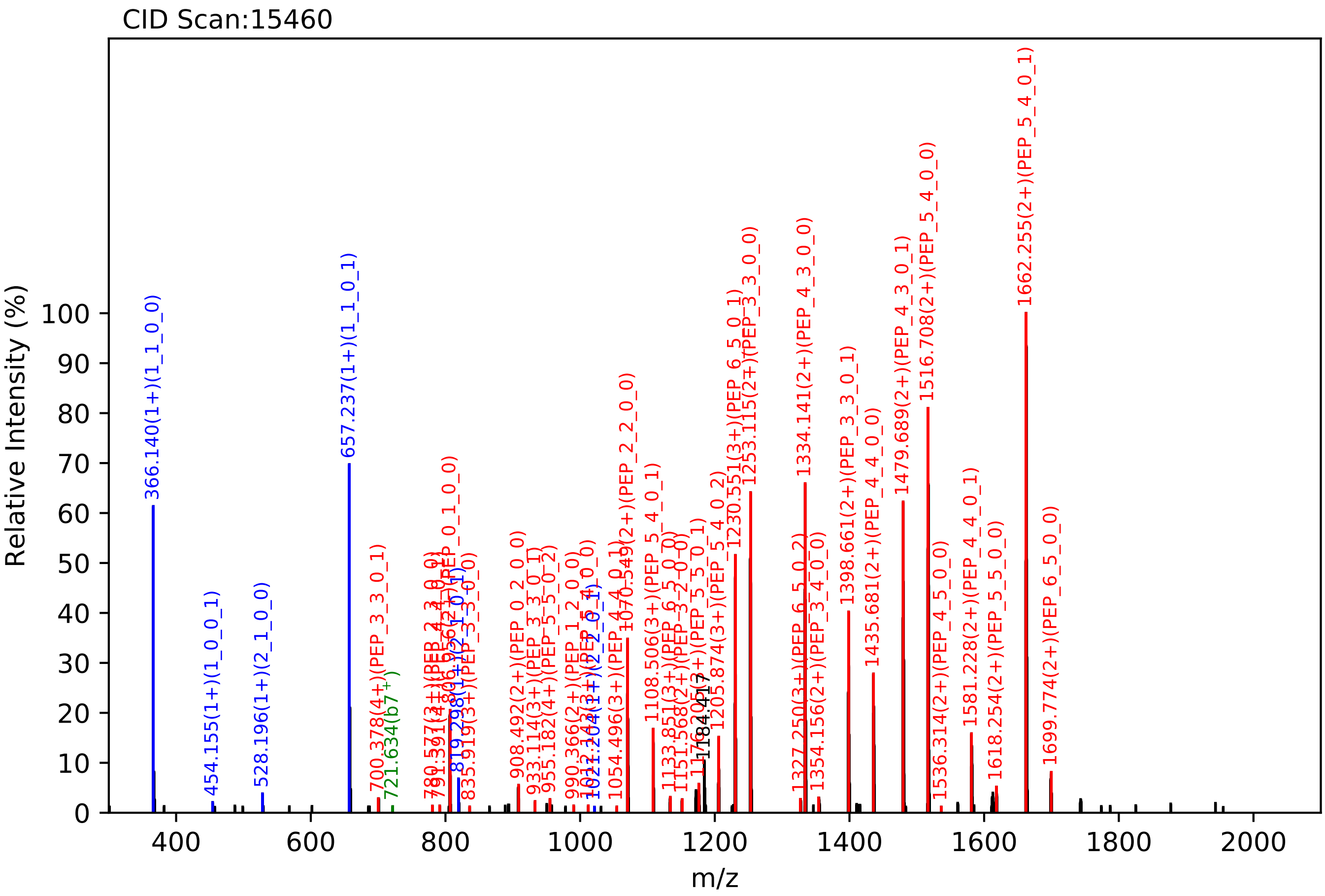

Training set no. 293, Experiment: AGP exp_1

LVPVPITNATLDR(=PEP)_6_5_0_2, m/z:995.44(4+), RT:42.26, Y-score:84.58

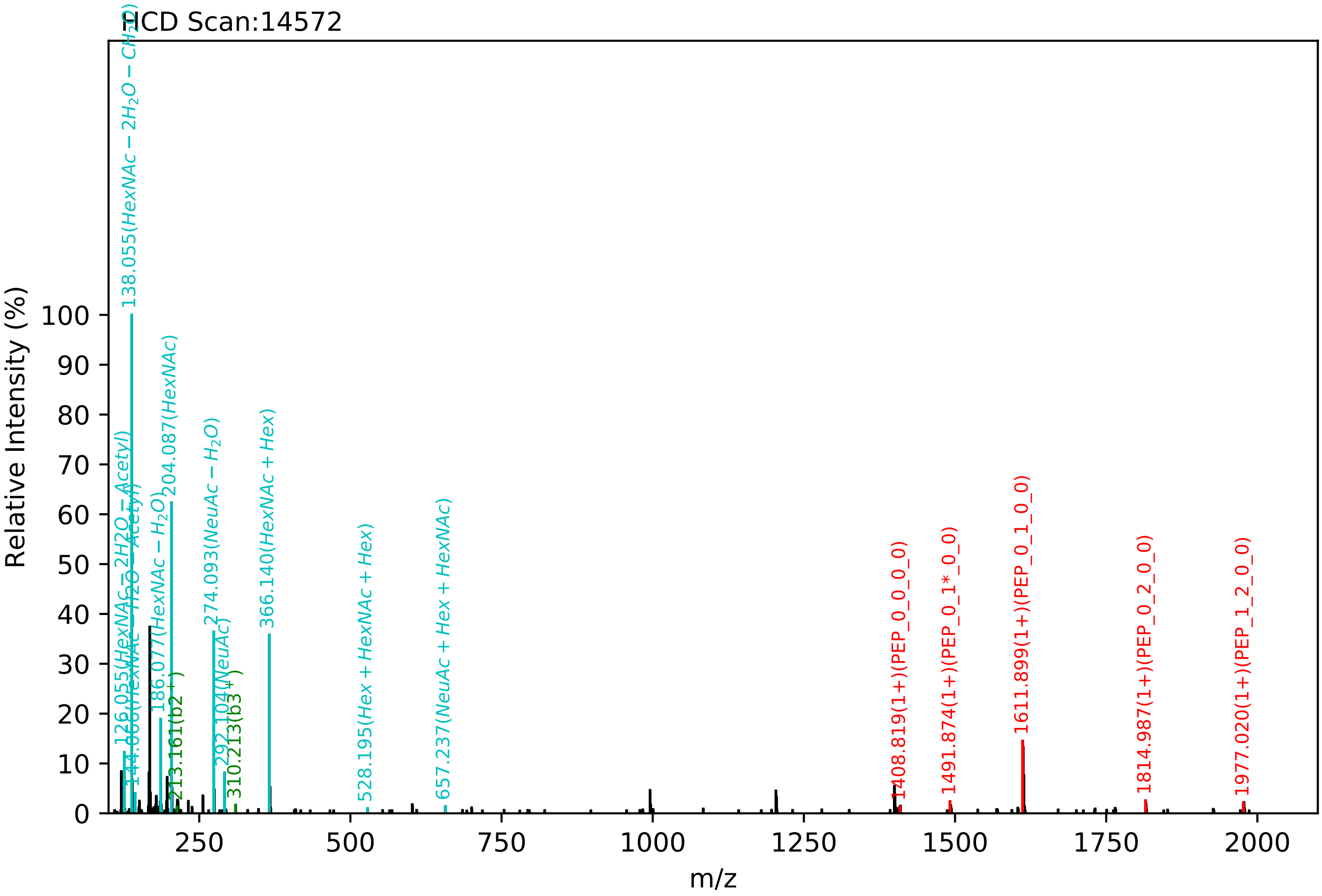

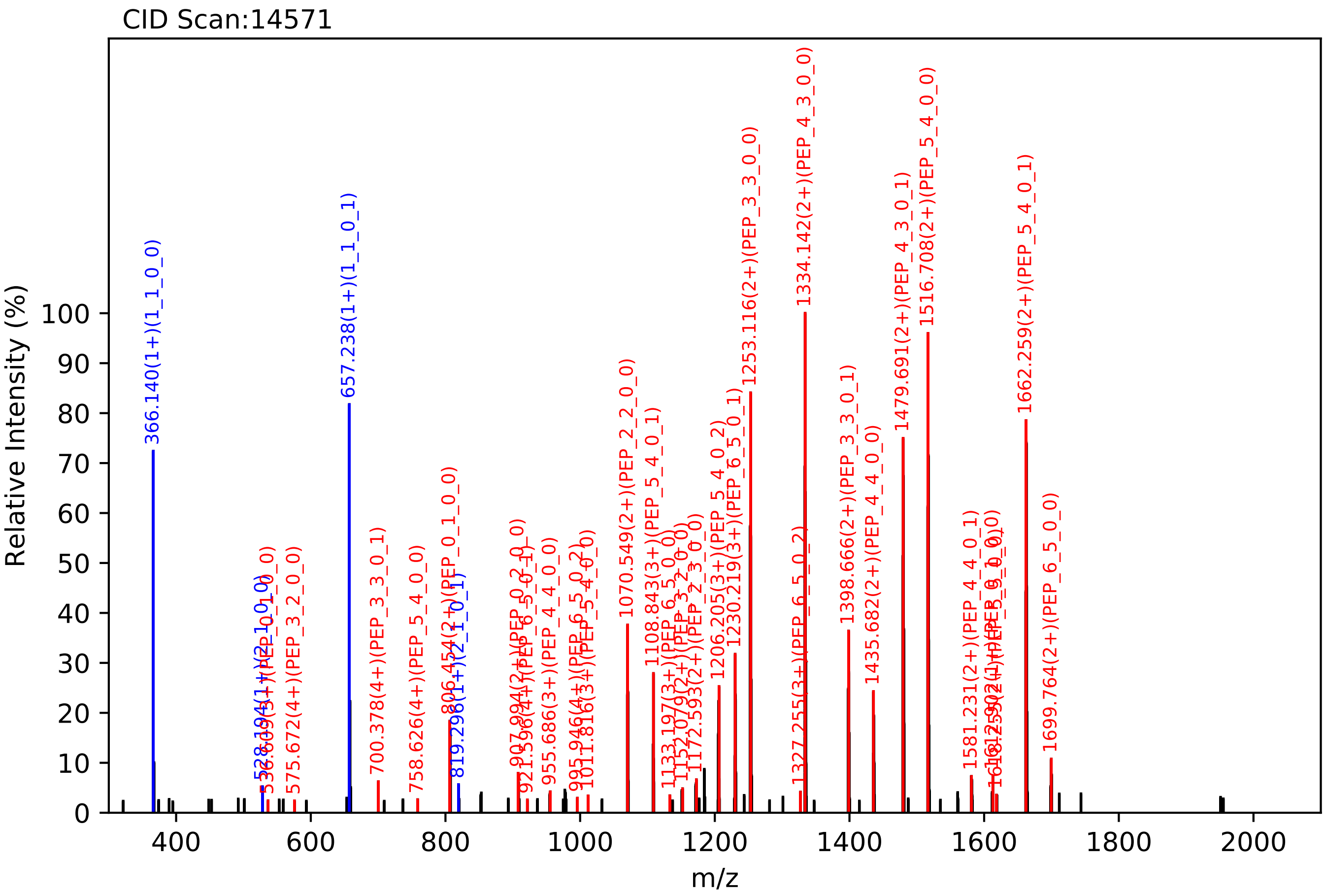

Training set no. 294, Experiment: AGP exp_2

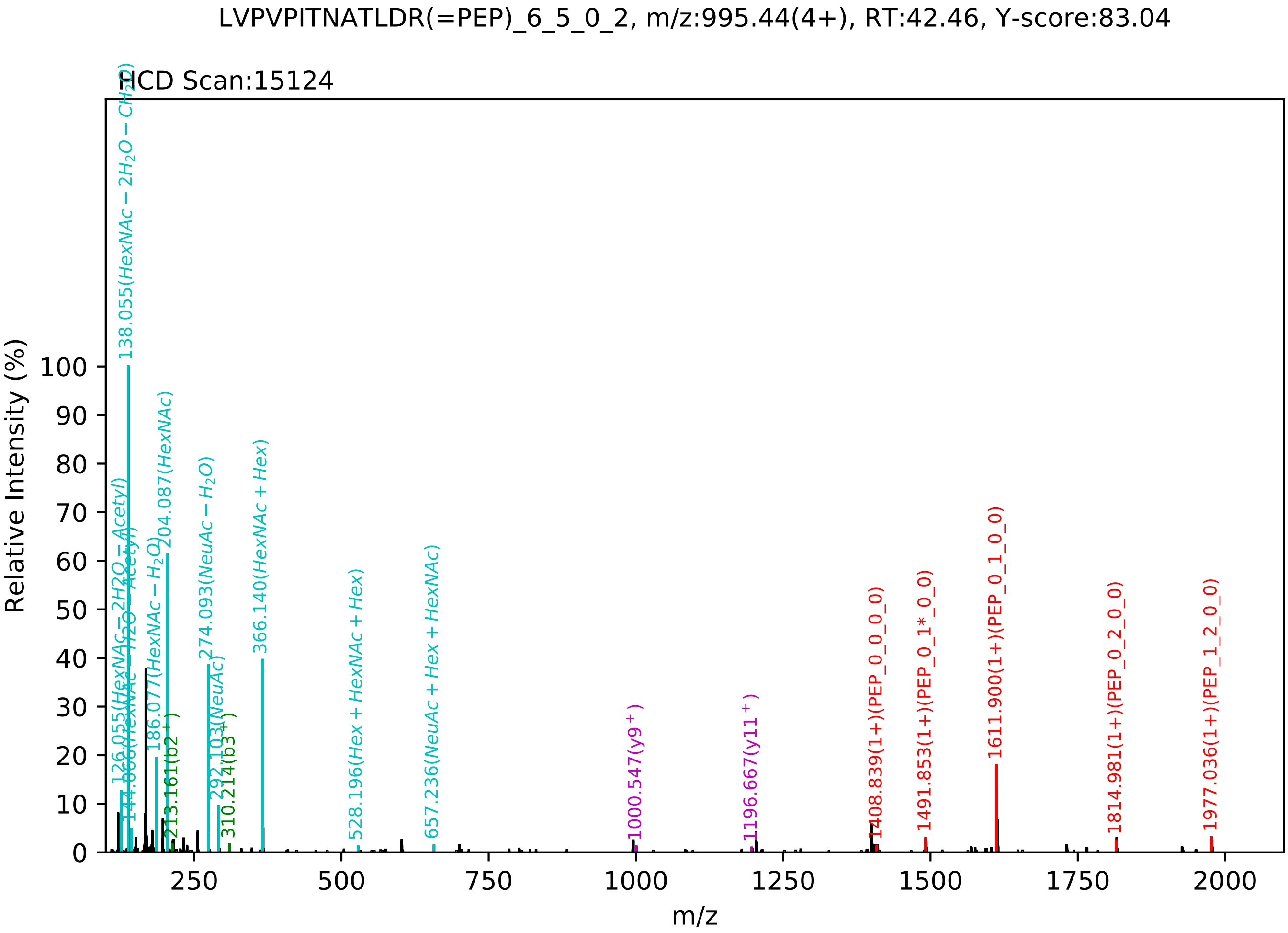

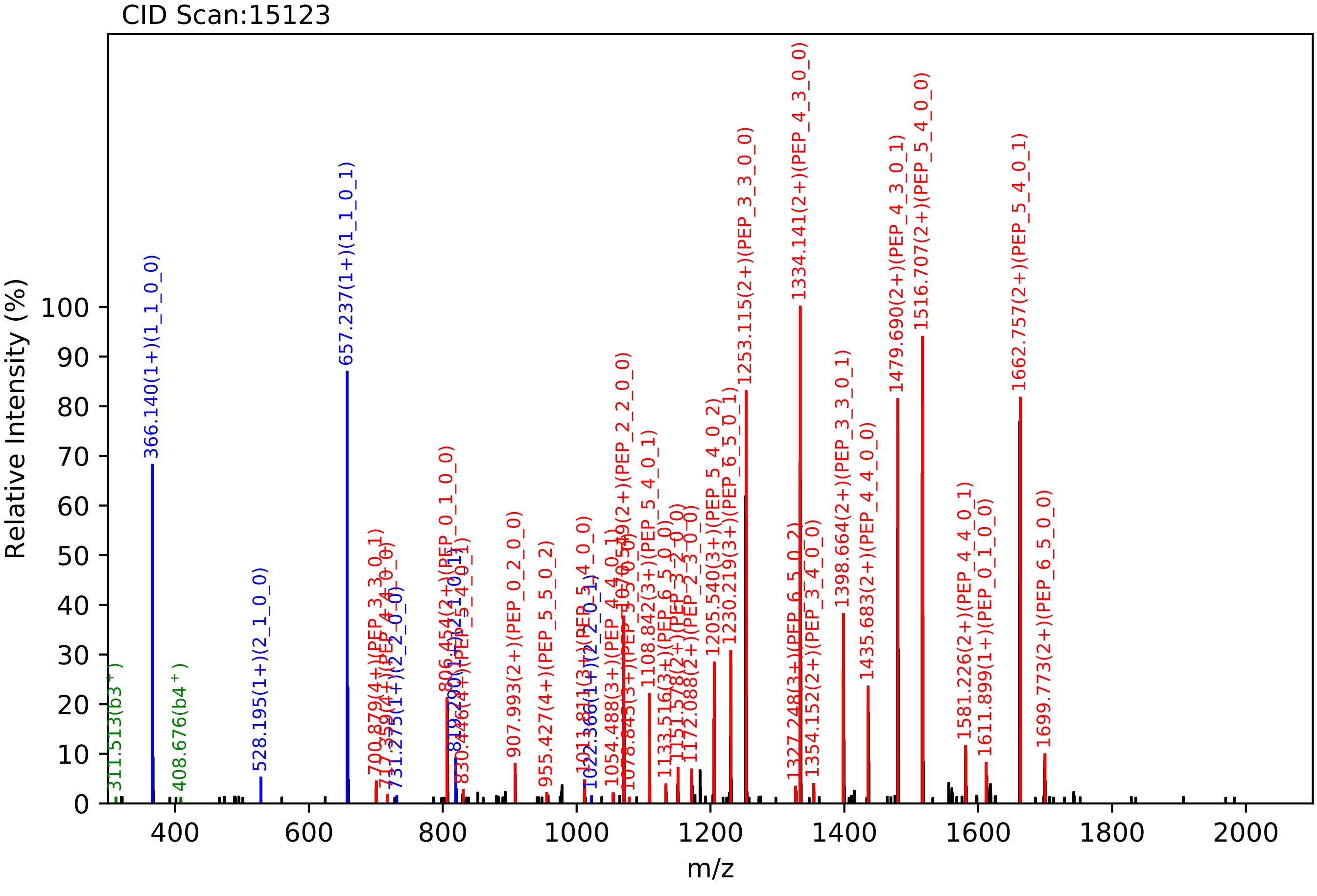

Training set no. 295, Experiment: AGP exp_1

LVPVPITNATLDR(=PEP)_6_5_0_3, m/z:1068.21(4+), RT:49.64, Y-score:84.82

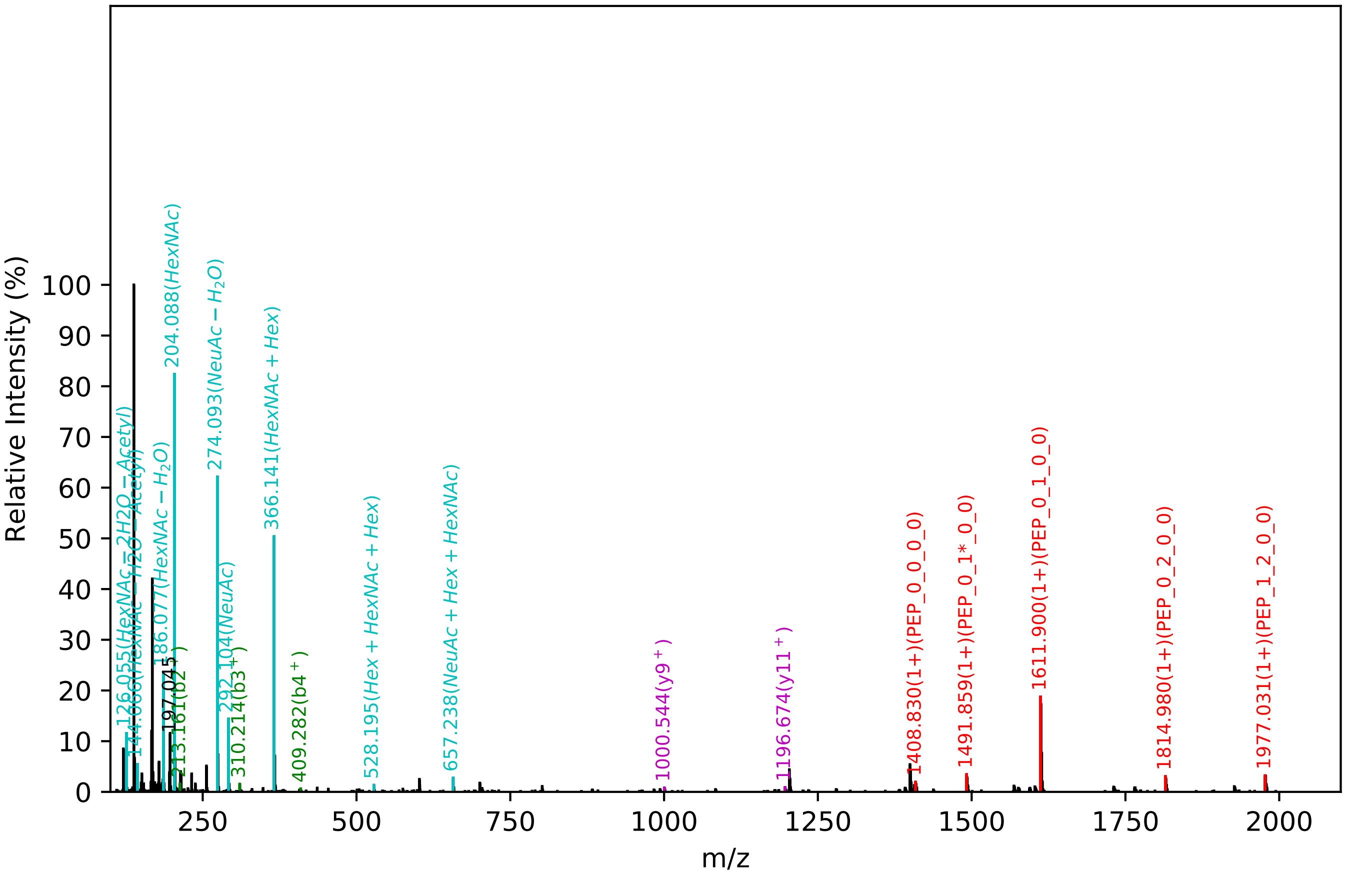

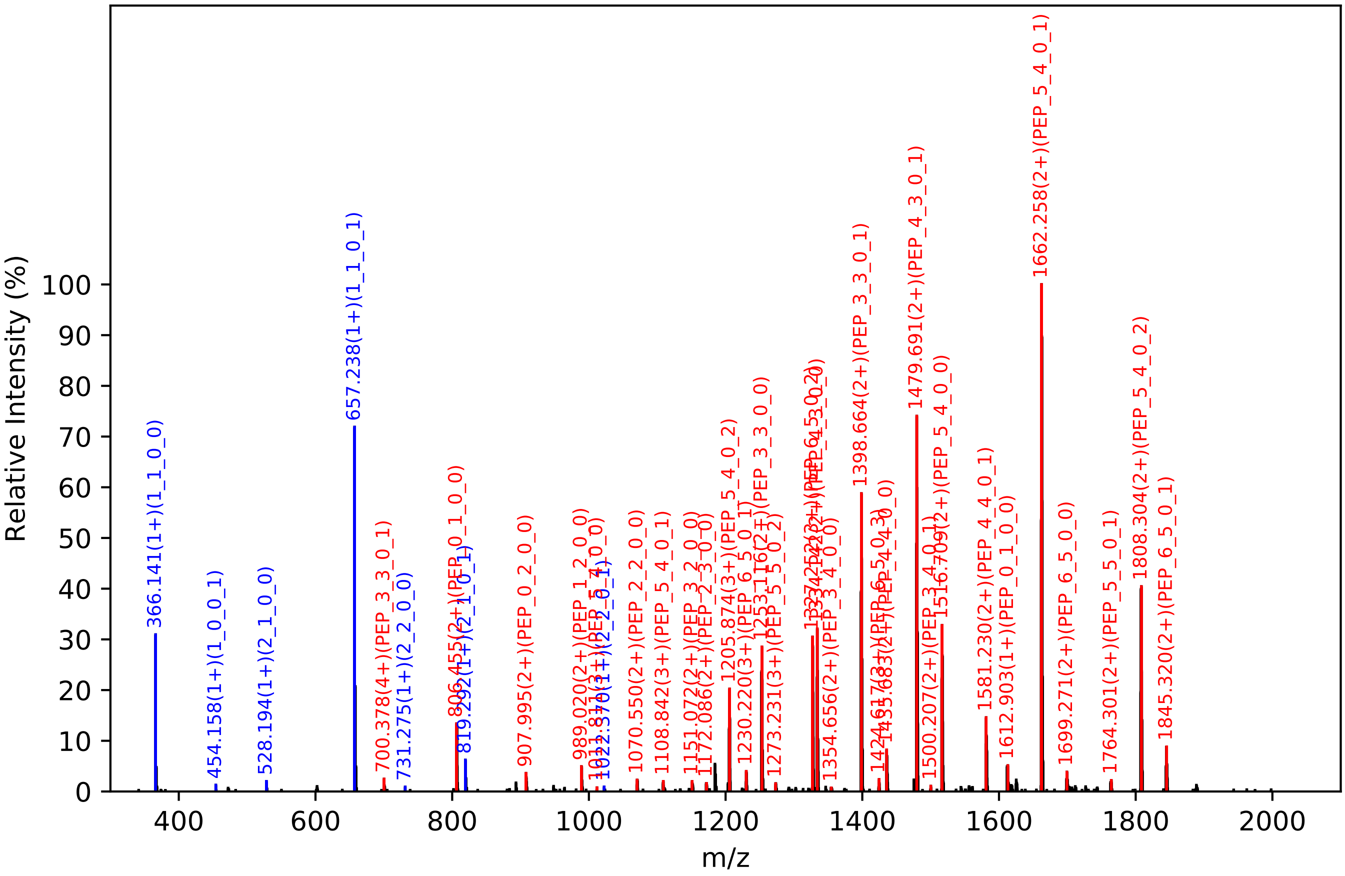

Training set no. 296, Experiment: AGP exp_1

LVPVPITNATLDR(=PEP)_6_5_0_3, m/z:1068.22(4+), RT:50.22, Y-score:84.54

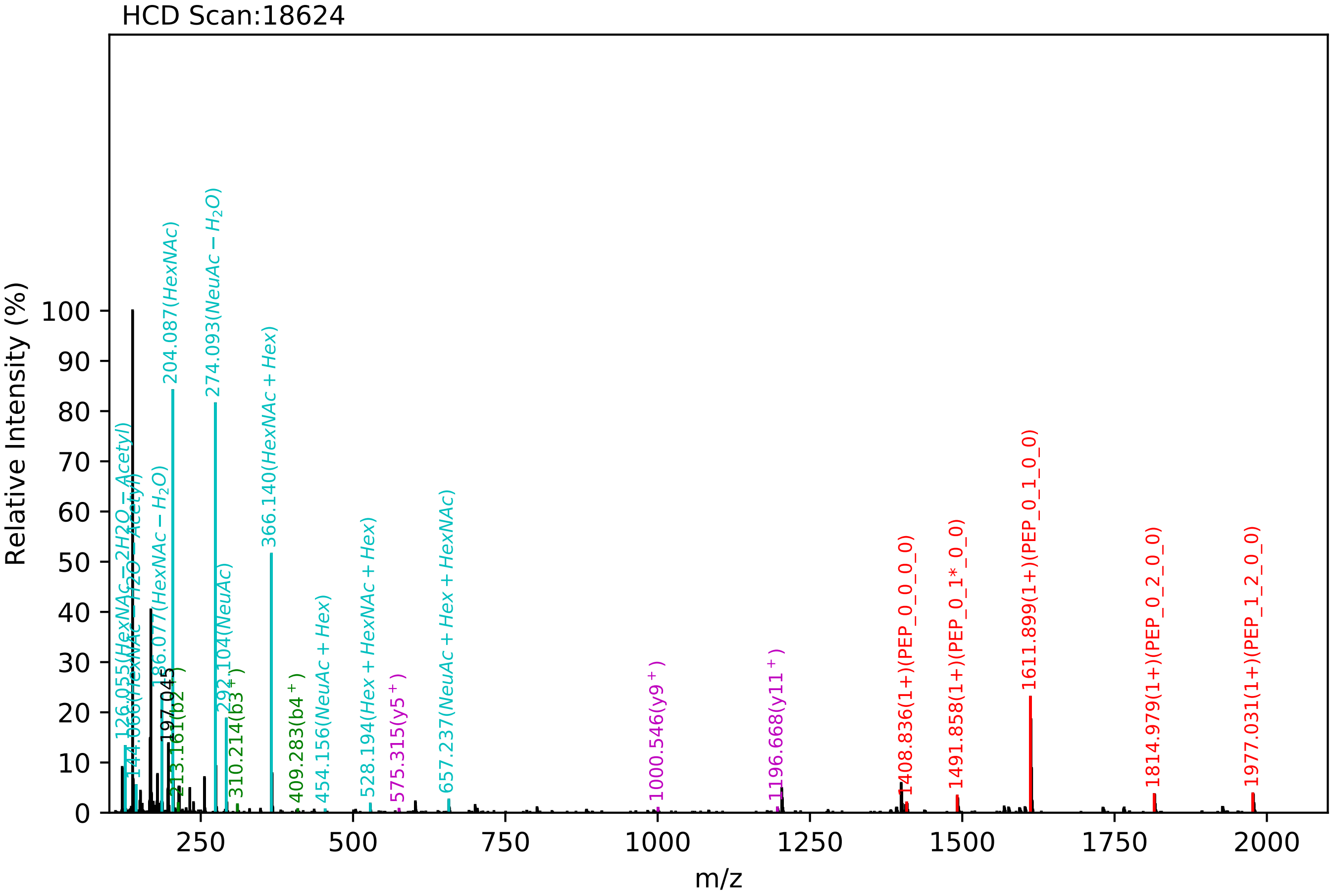

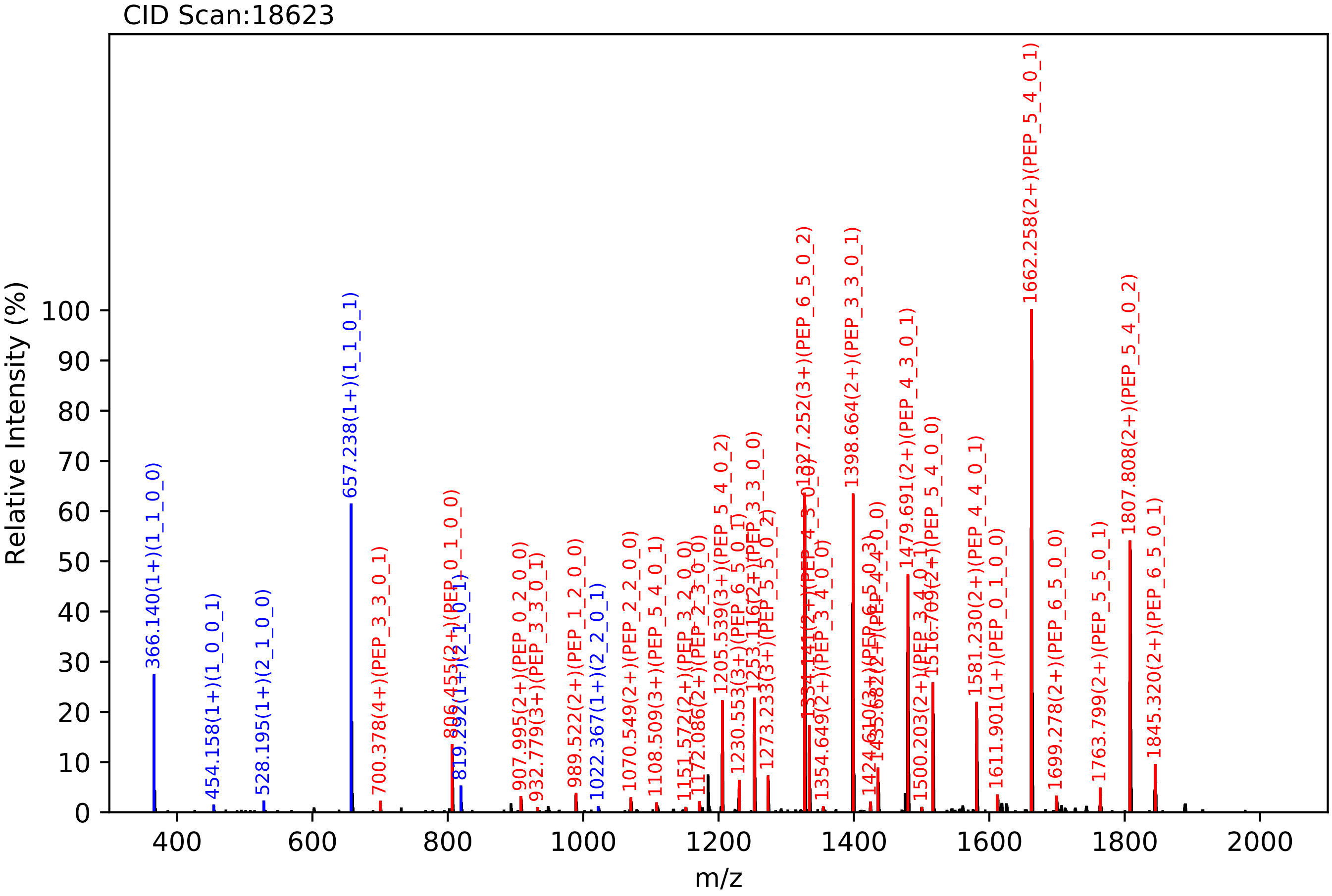

Training set no. 297, Experiment: AGP exp_2

LVPVPITNATLDR(=PEP)_6_5_0_3, m/z:1068.21(4+), RT:50.33, Y-score:84.38

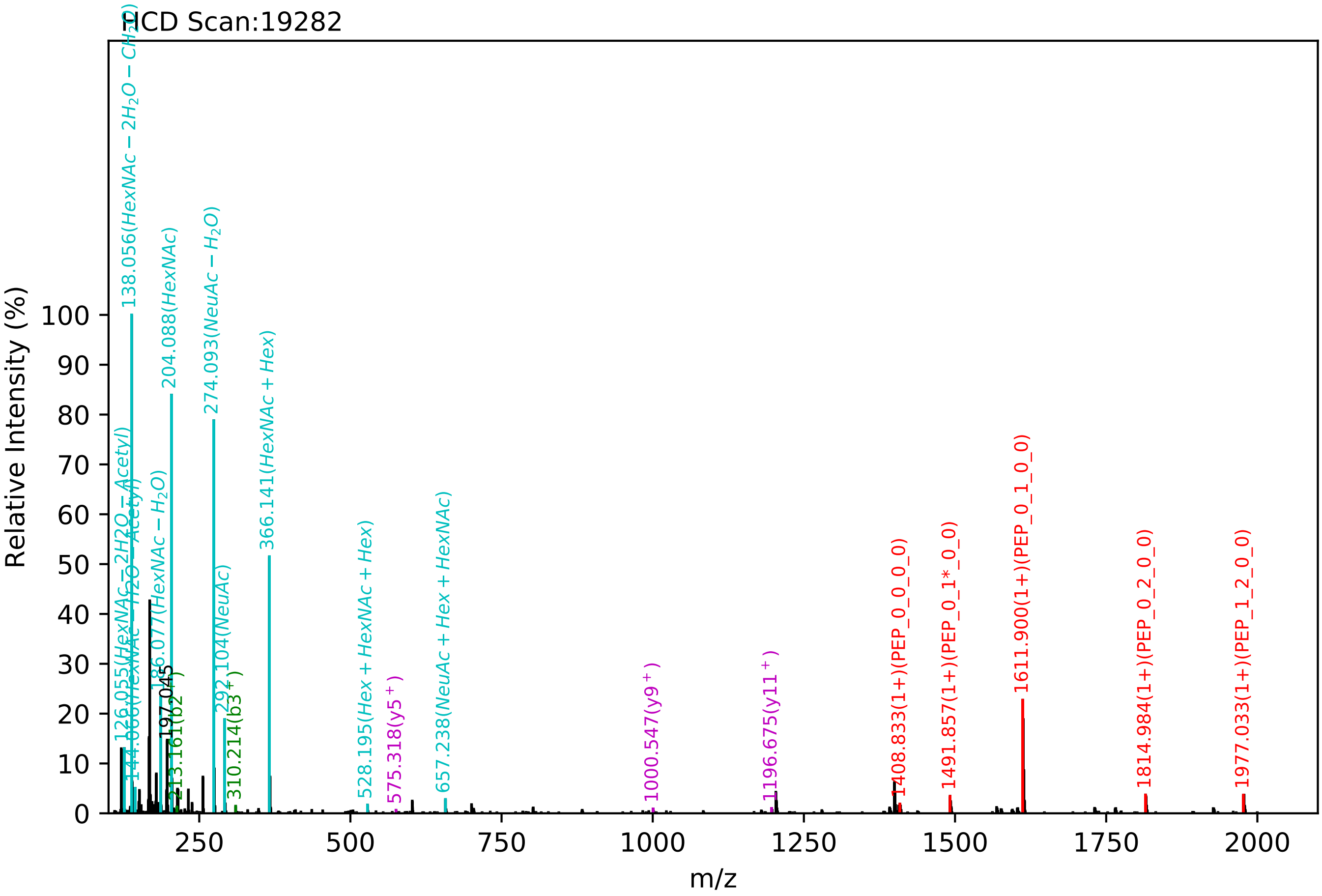

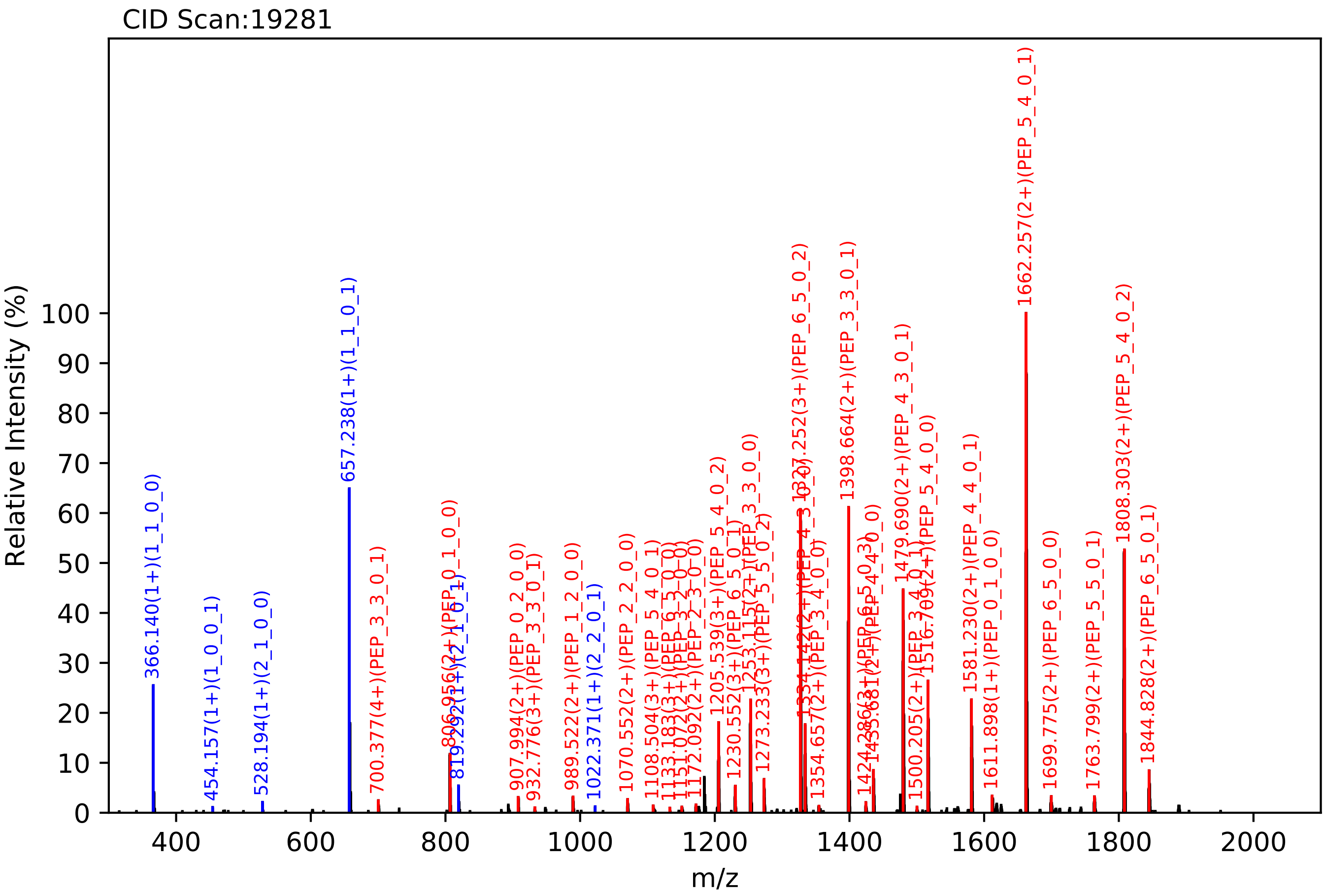

Training set no. 298, Experiment: AGP exp_2

LVPVPITNATLDR(=PEP)_6_5_0_3, m/z:1068.21(4+), RT:50.89, Y-score:84.17

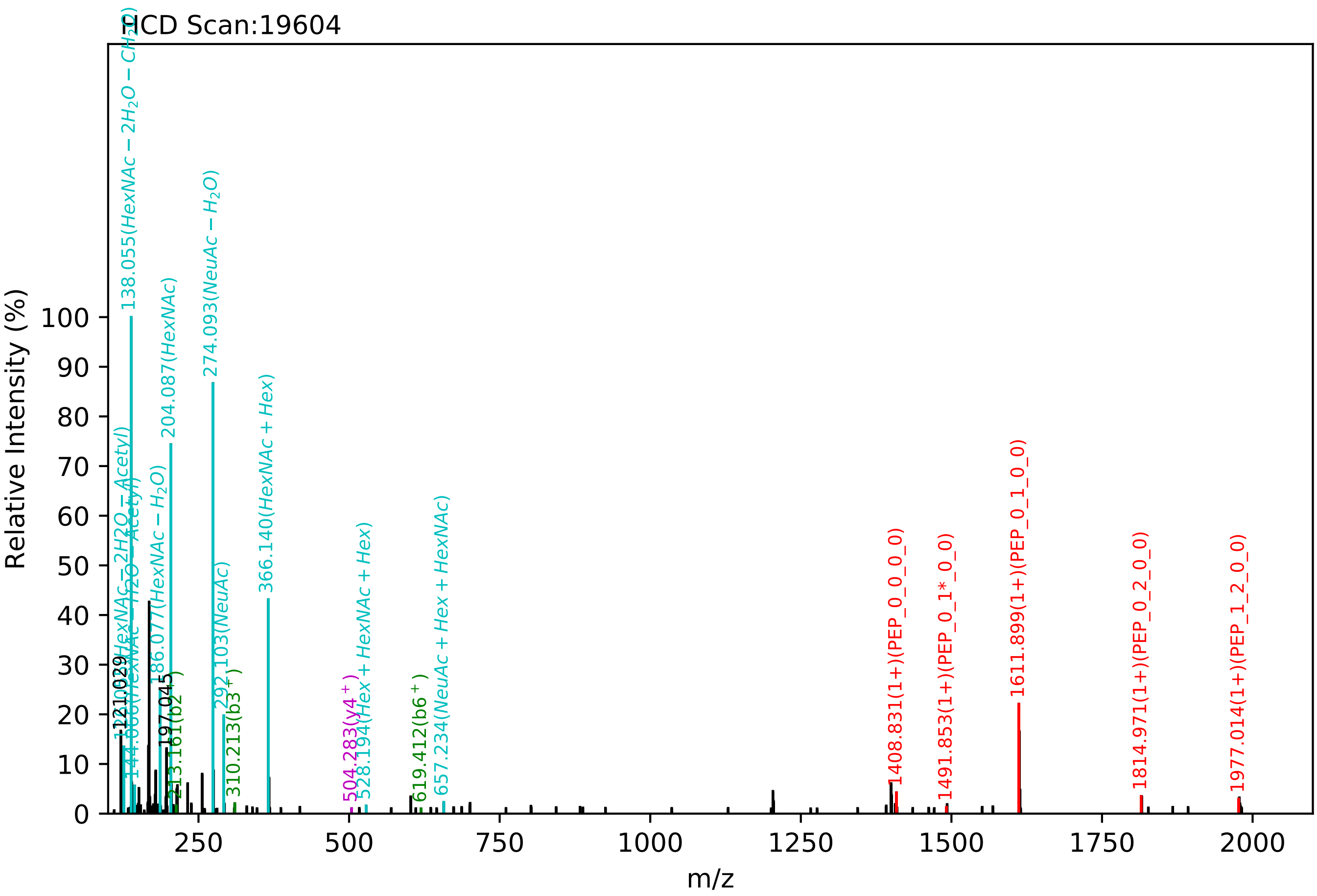

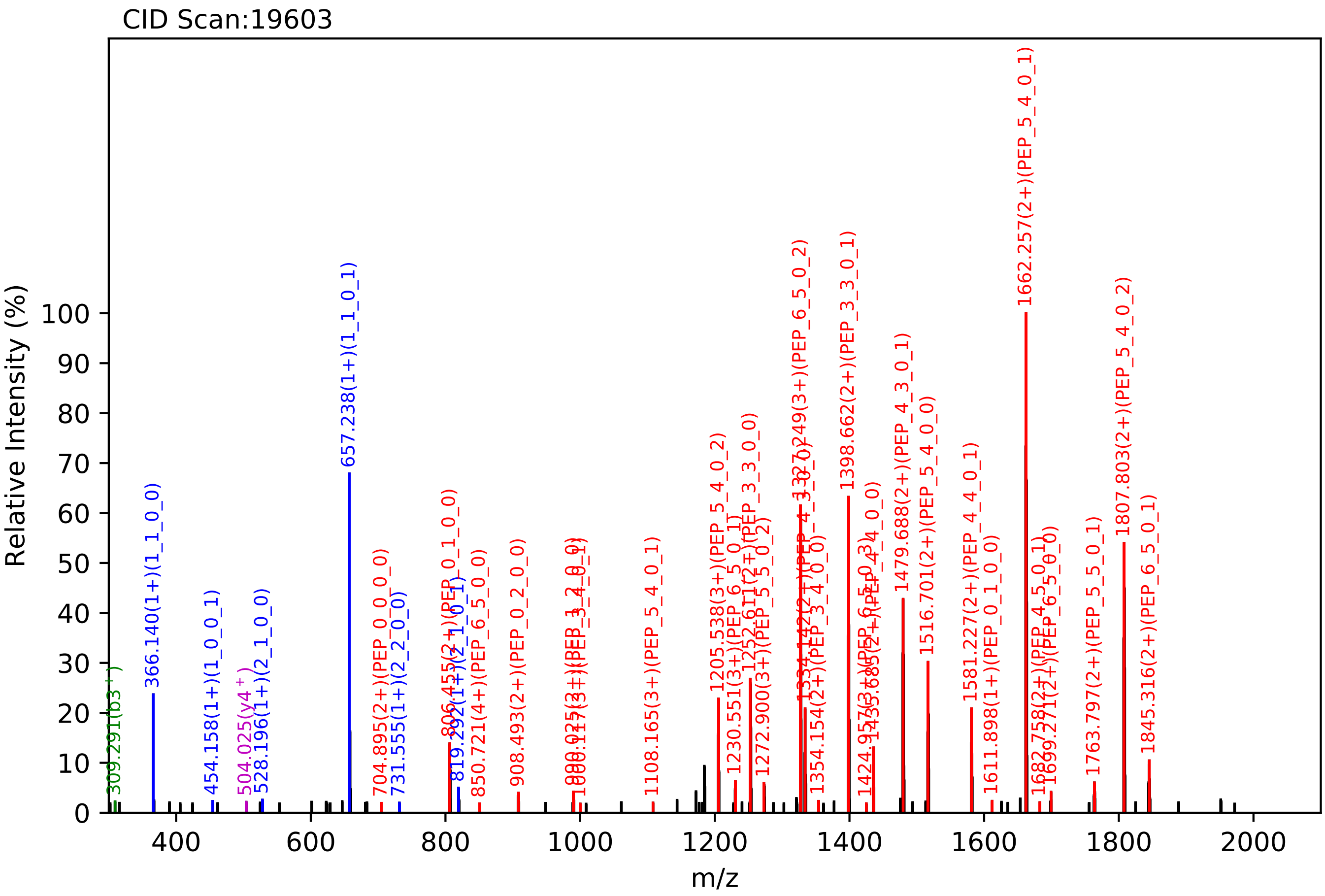

Training set no. 299, Experiment: AGP exp_2

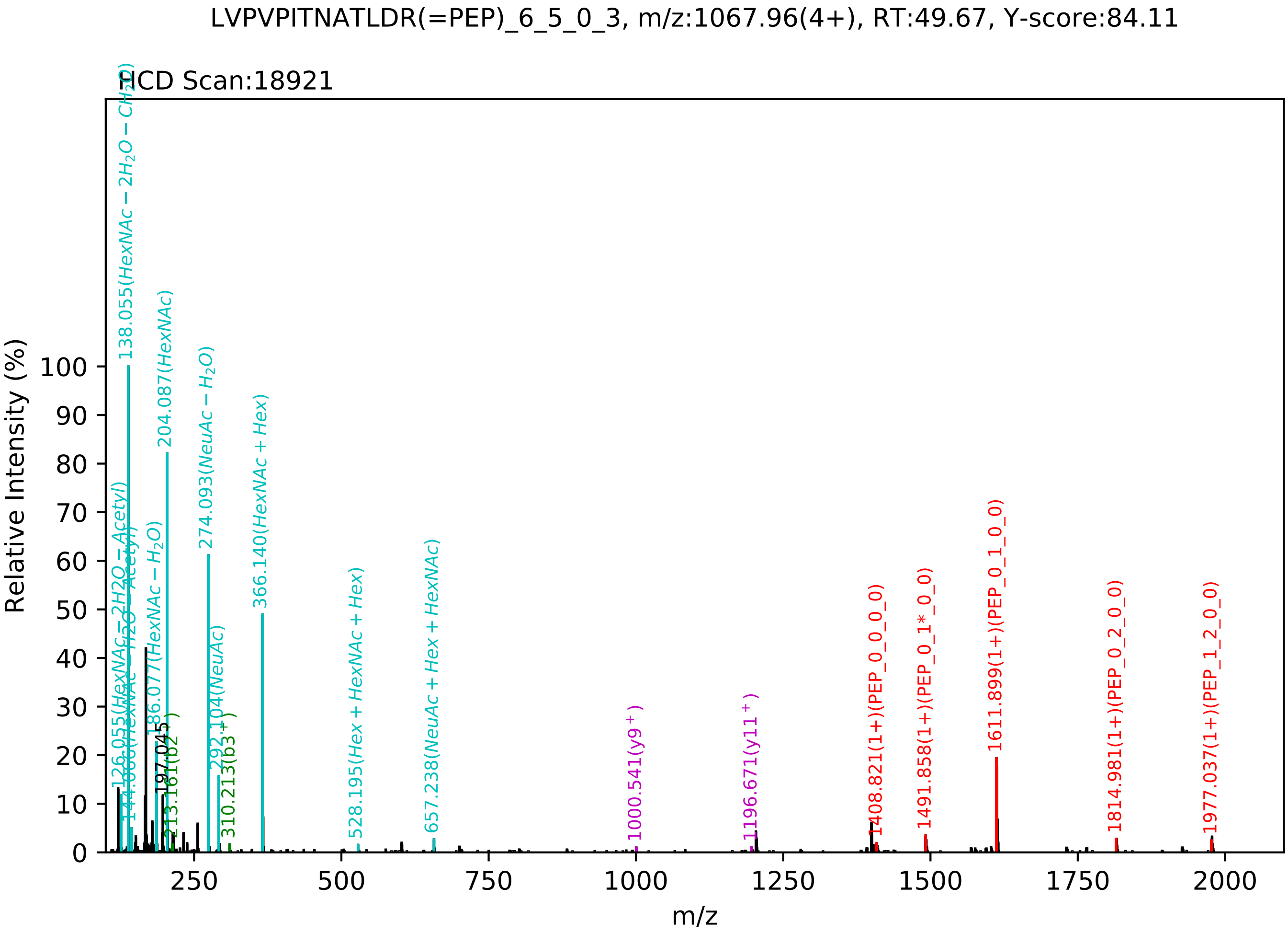

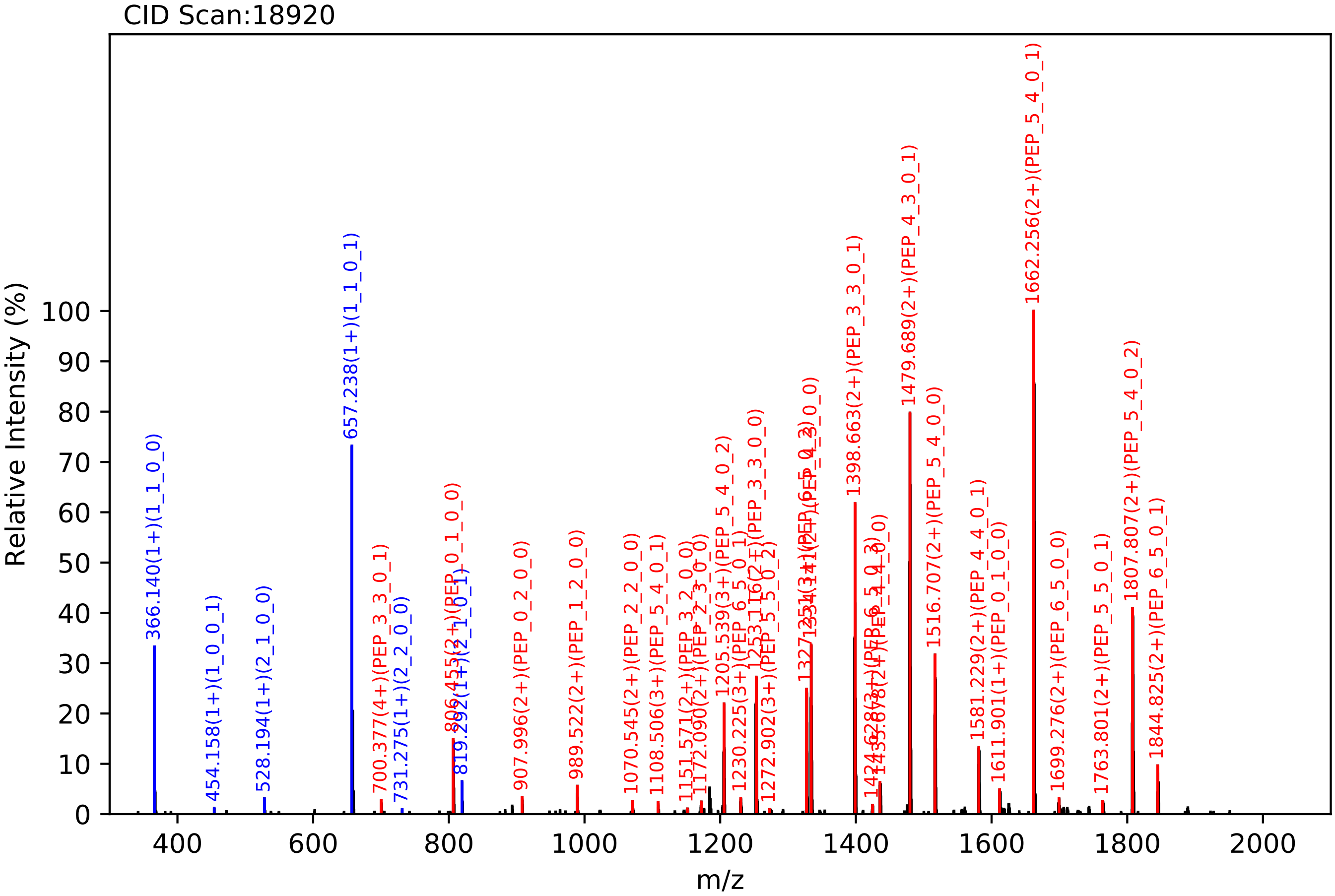

Training set no. 300, Experiment: AGP exp_2

LVPVPITNATLDR(=PEP)_6_5_1_1, m/z:1278.57(3+), RT:36.42, Y-score:92.84

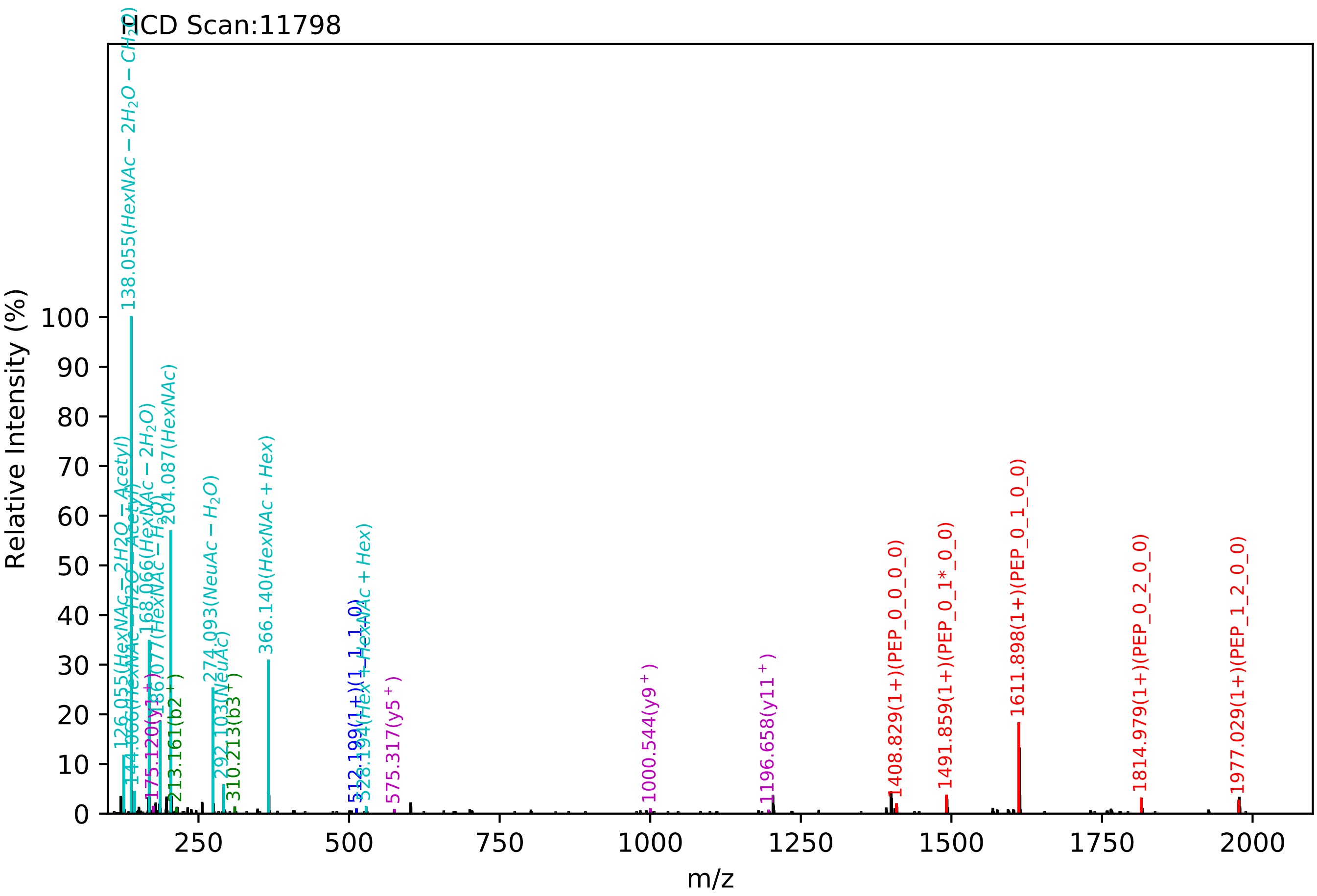

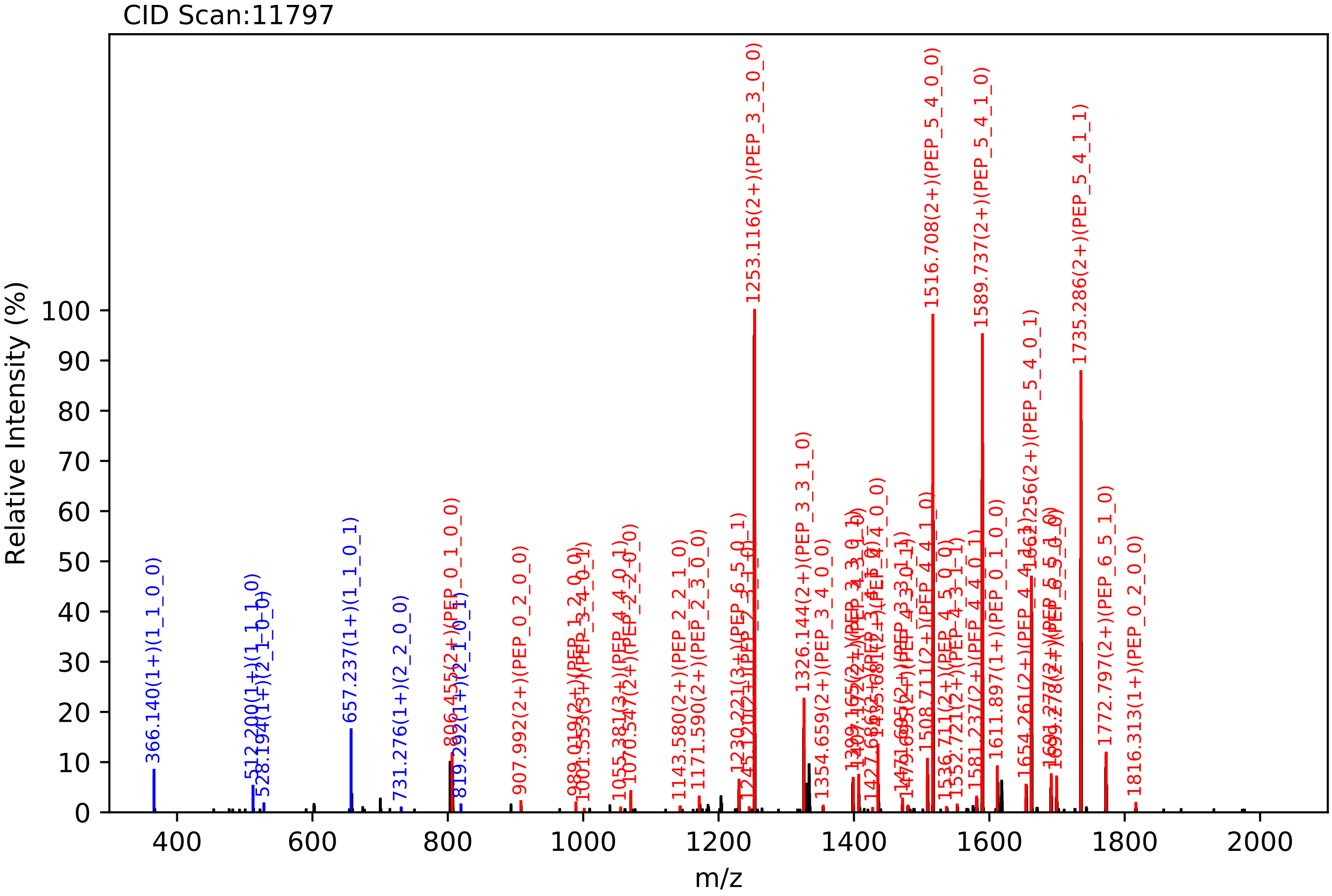

Training set no. 301, Experiment: AGP exp_1

LVPVPITNATLDR(=PEP)_6_5_1_1, m/z:1278.57(3+), RT:36.17, Y-score:89.33

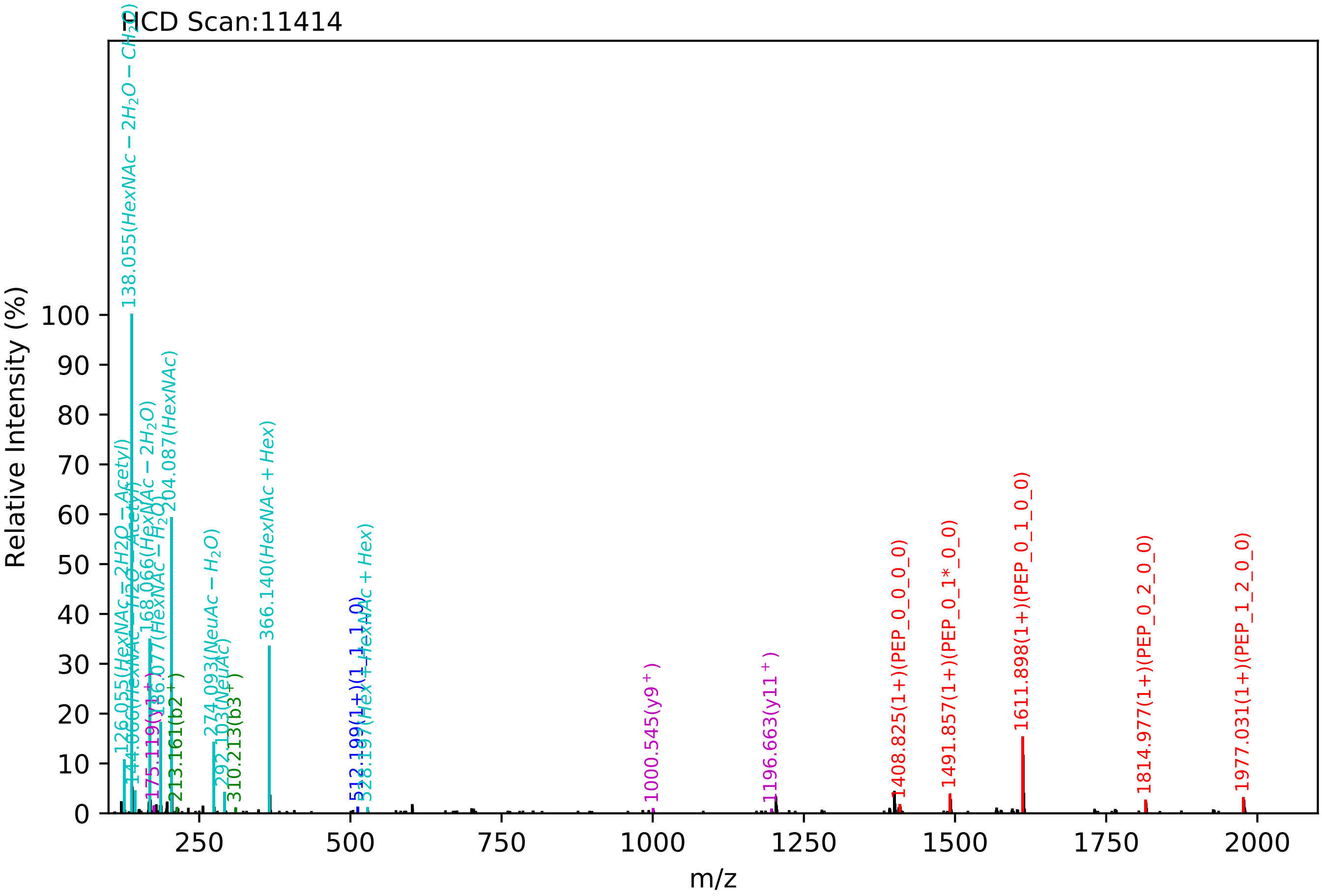

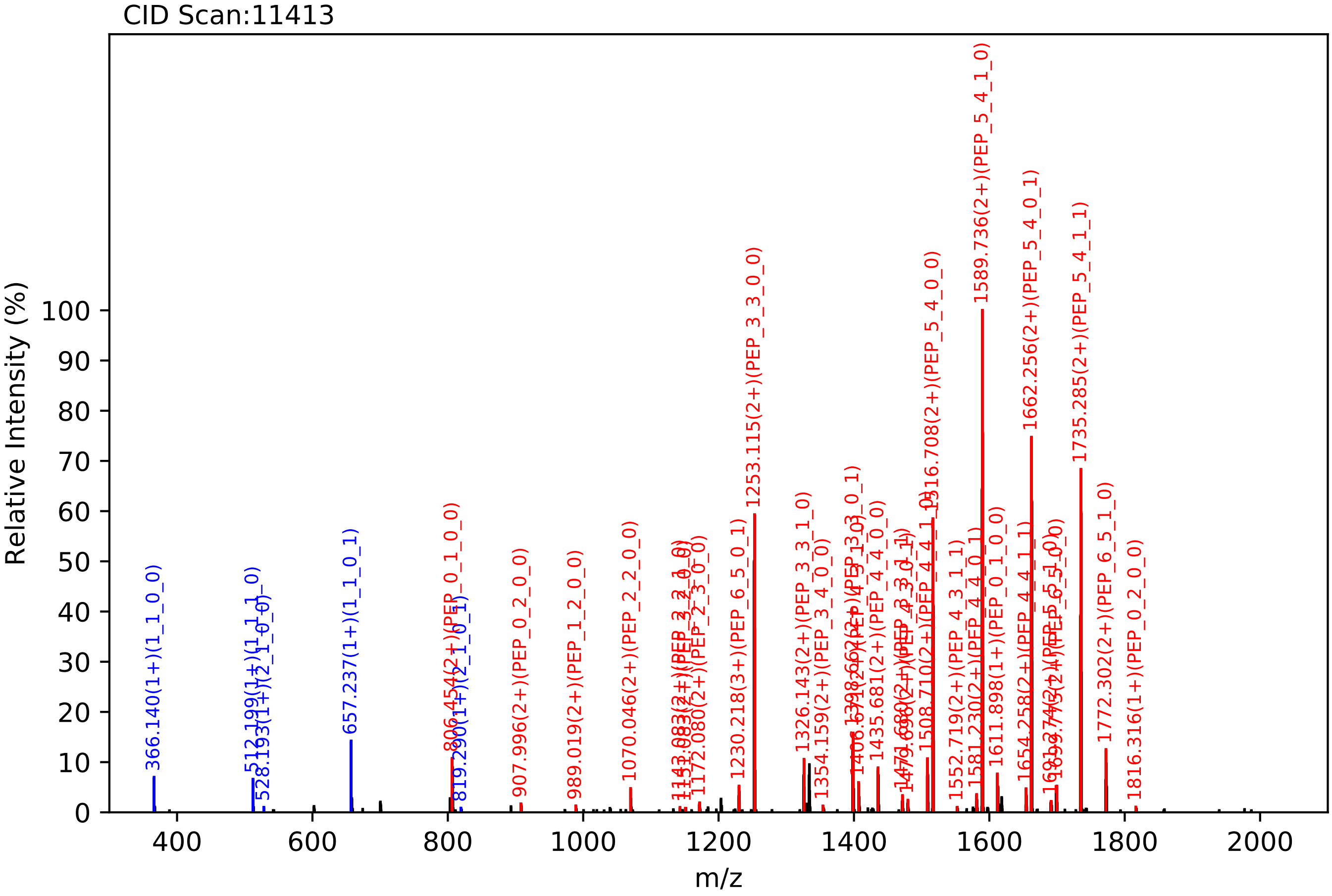

Training set no. 302, Experiment: AGP exp_1

LVPVPITNATLDR(=PEP)_6_5_1_2, m/z:1375.60(3+), RT:42.44, Y-score:90.84

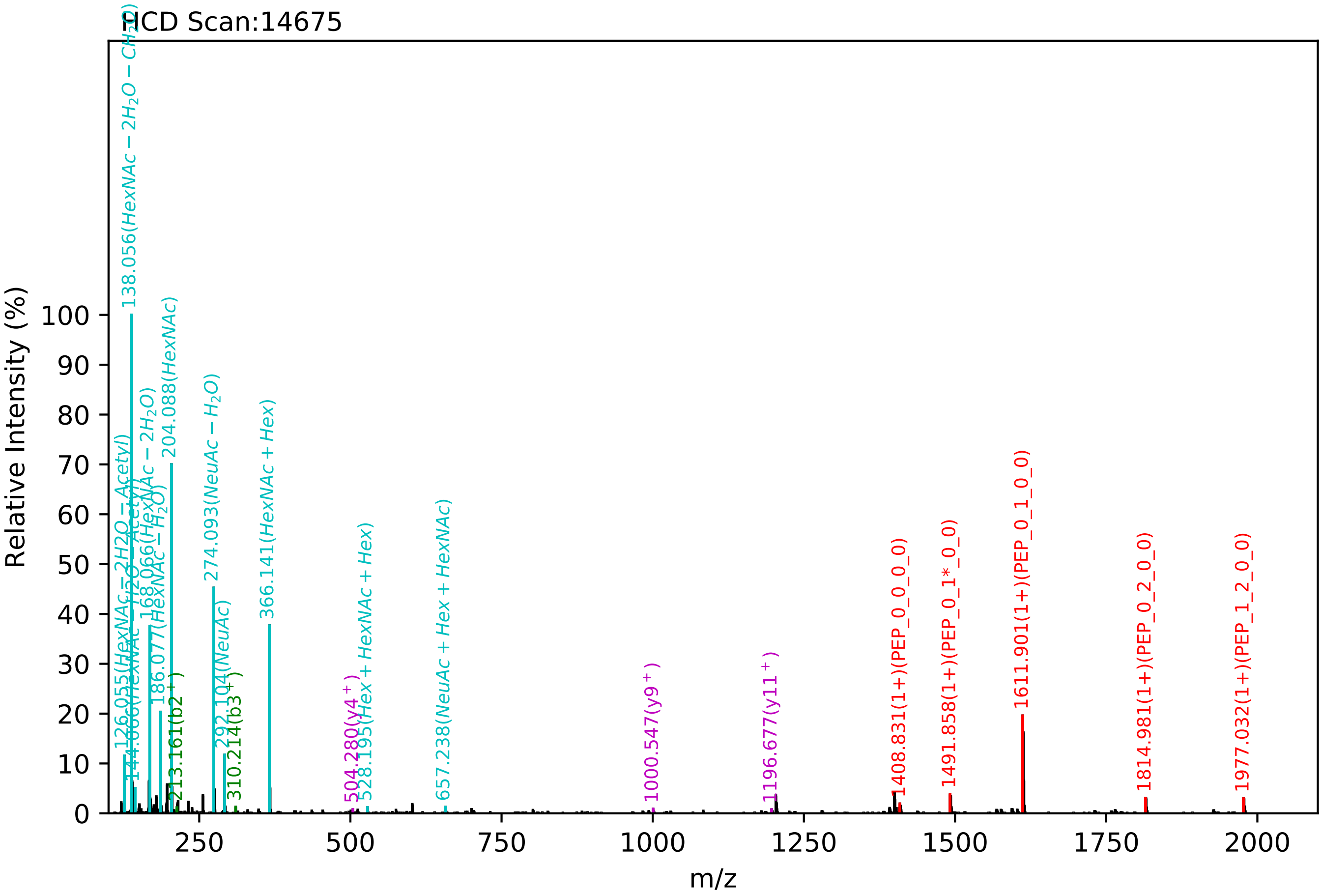

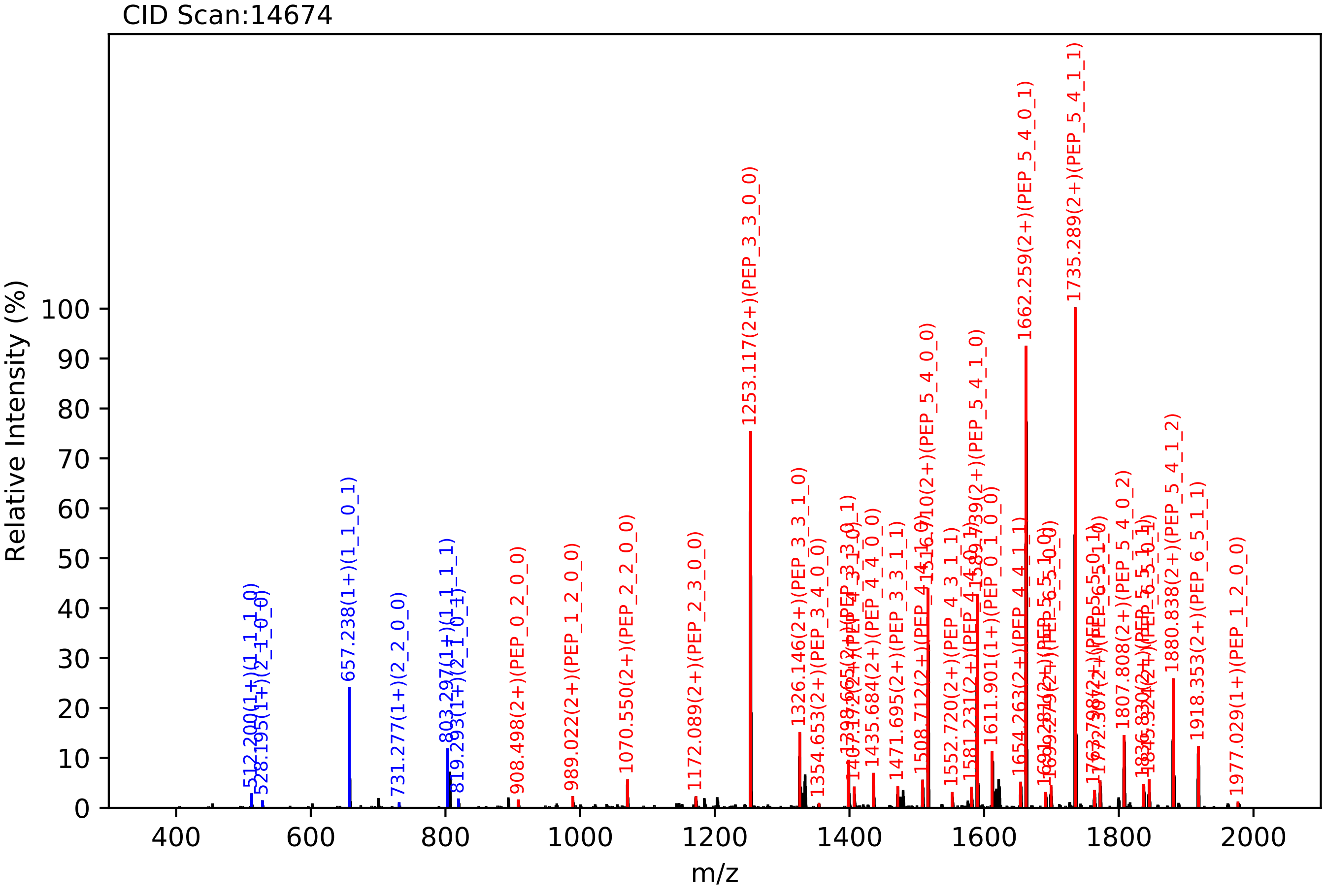

Training set no. 303, Experiment: AGP exp_2

LVPVPITNATLDR(=PEP)_6_5_1_2, m/z:1375.60(3+), RT:42.66, Y-score:90.41

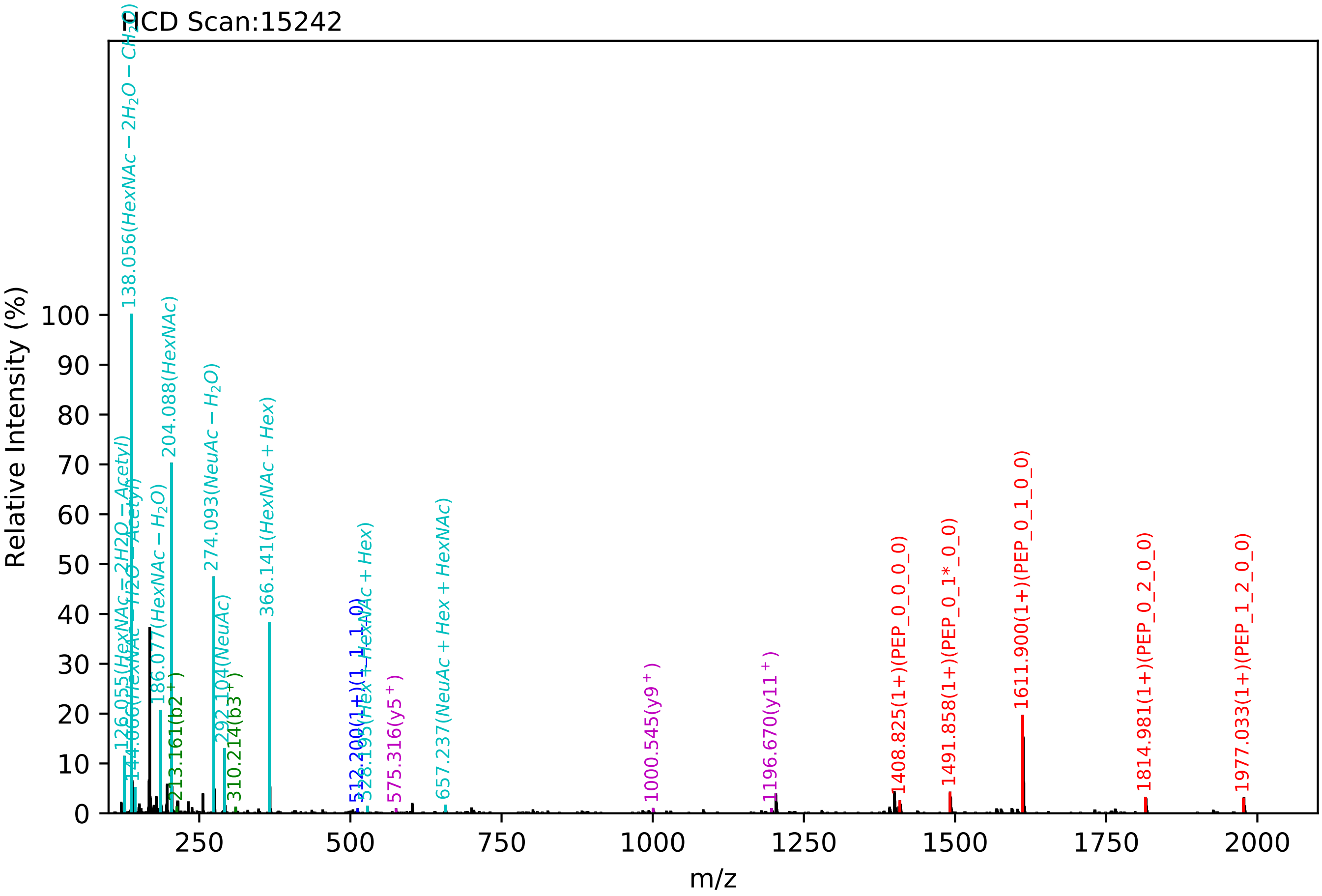

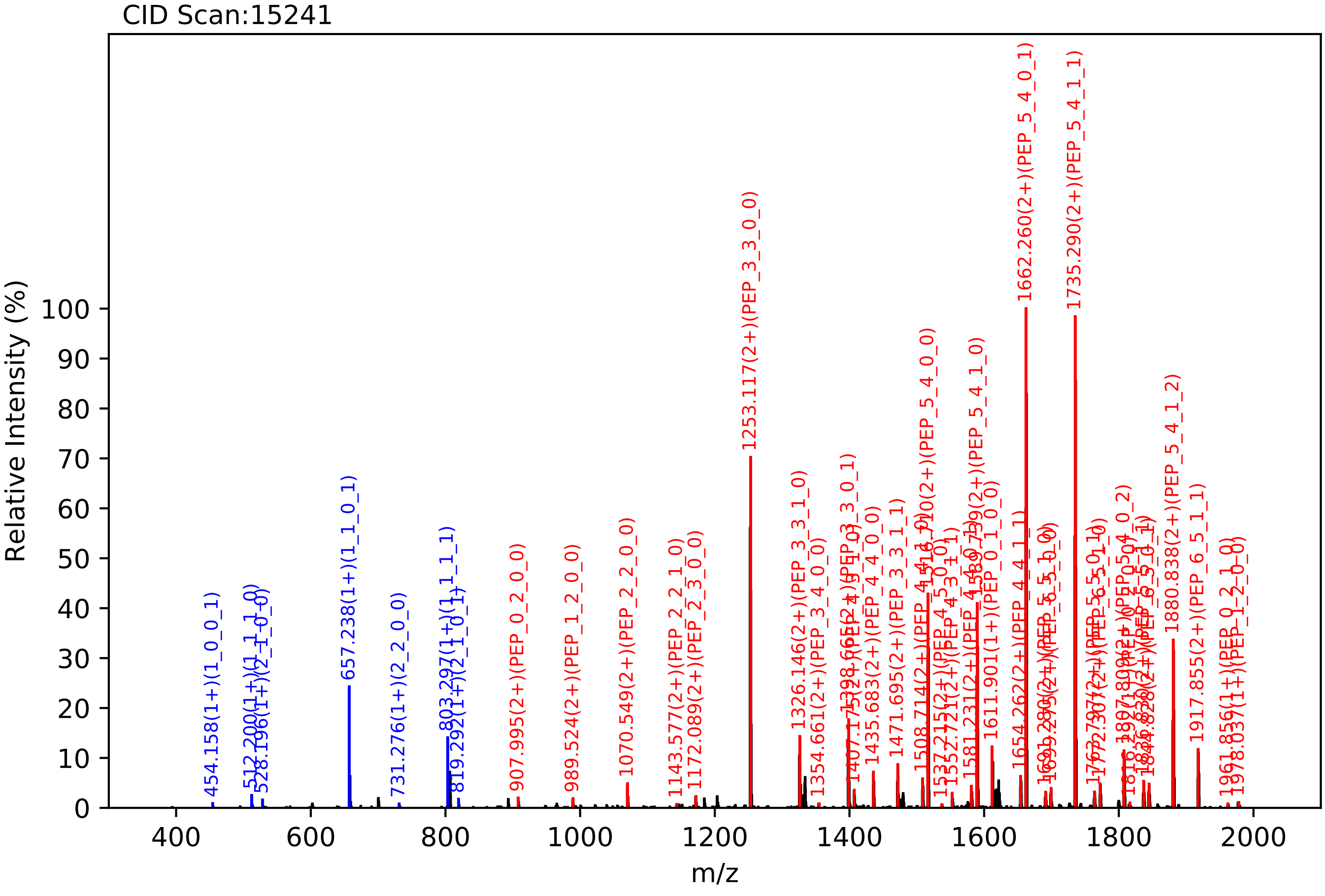

Training set no. 304, Experiment: AGP exp_1

LVPVPITNATLDR(=PEP)_6_5_1_2, m/z:1375.93(3+), RT:43.03, Y-score:90.37

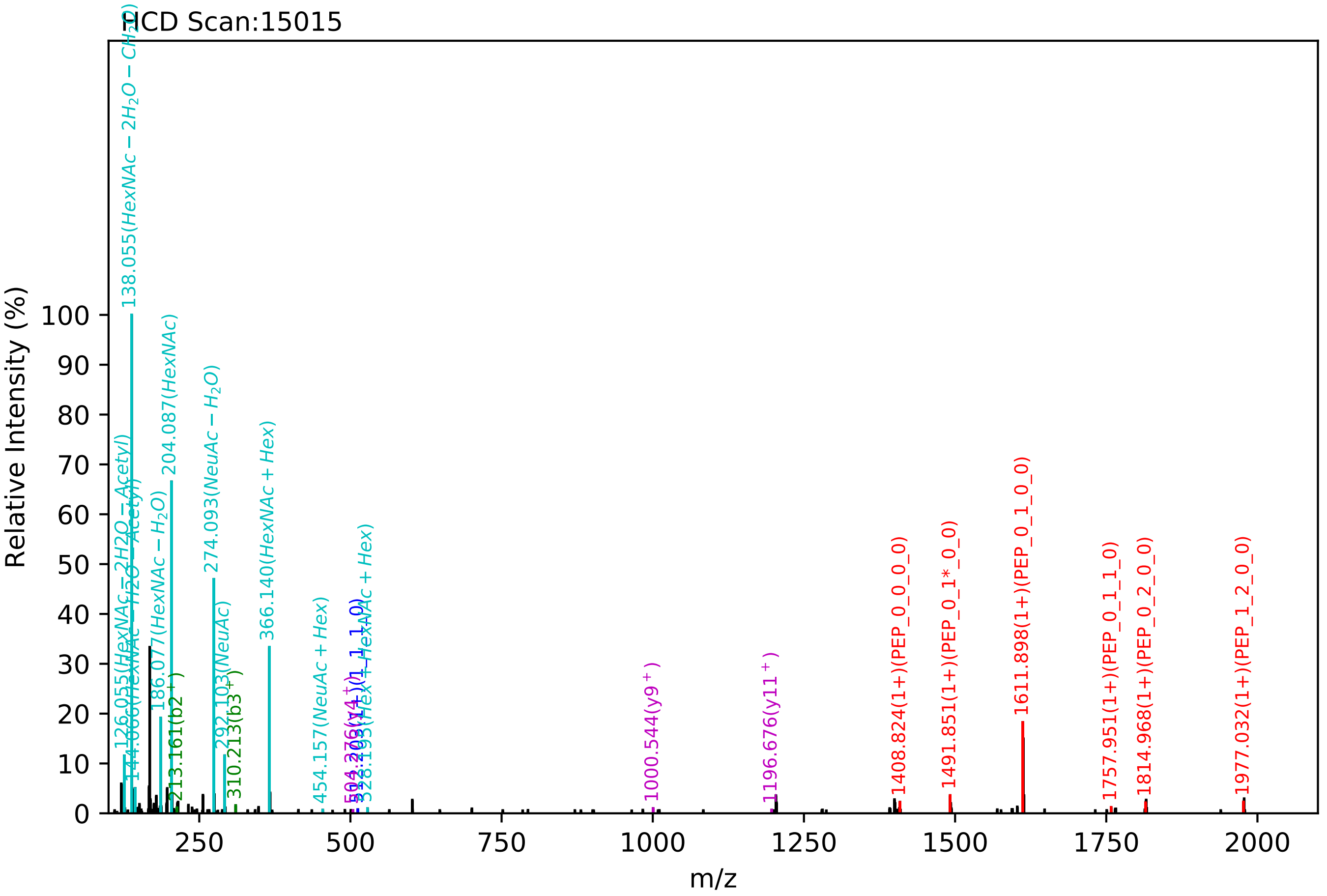

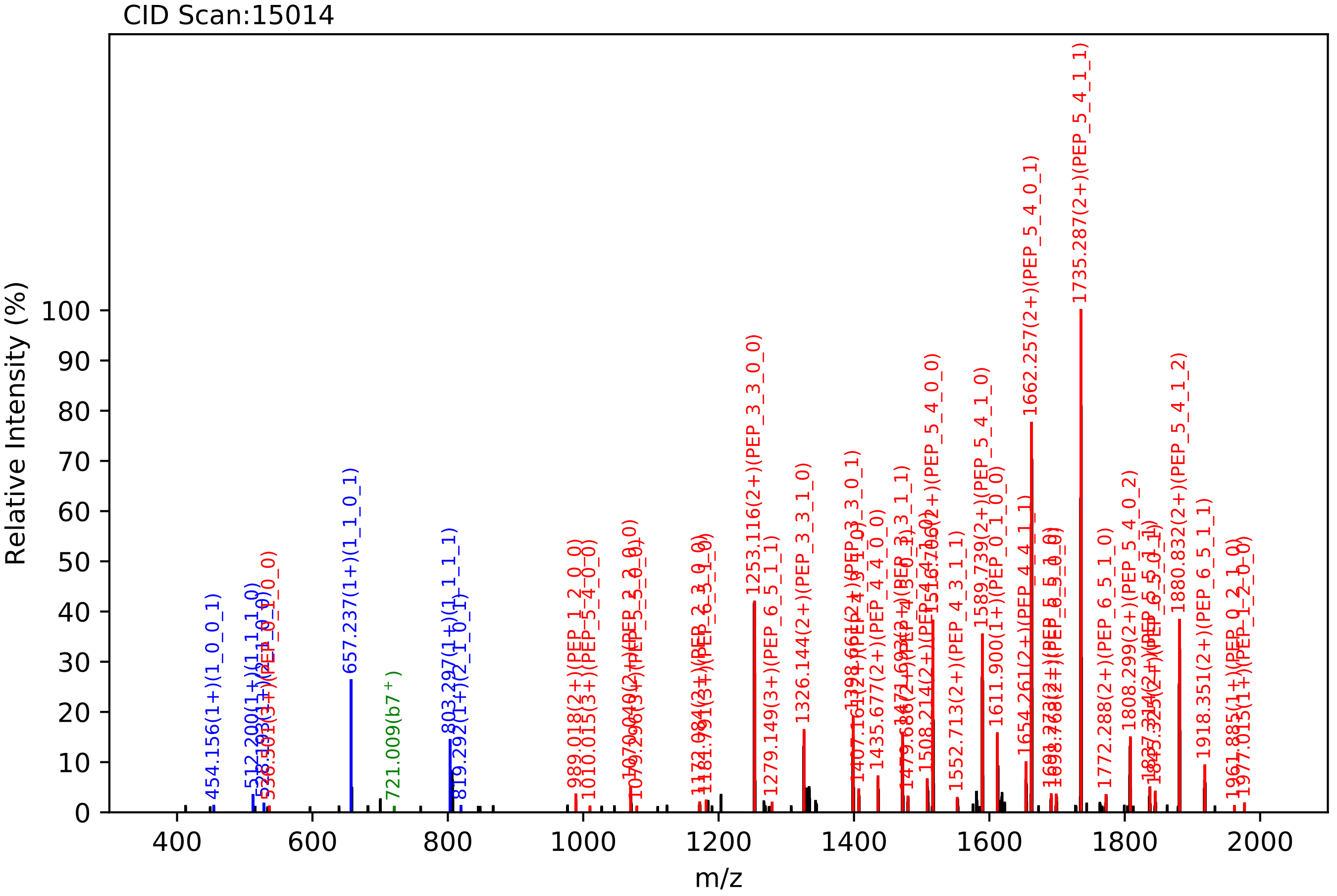

Training set no. 305, Experiment: AGP exp_2

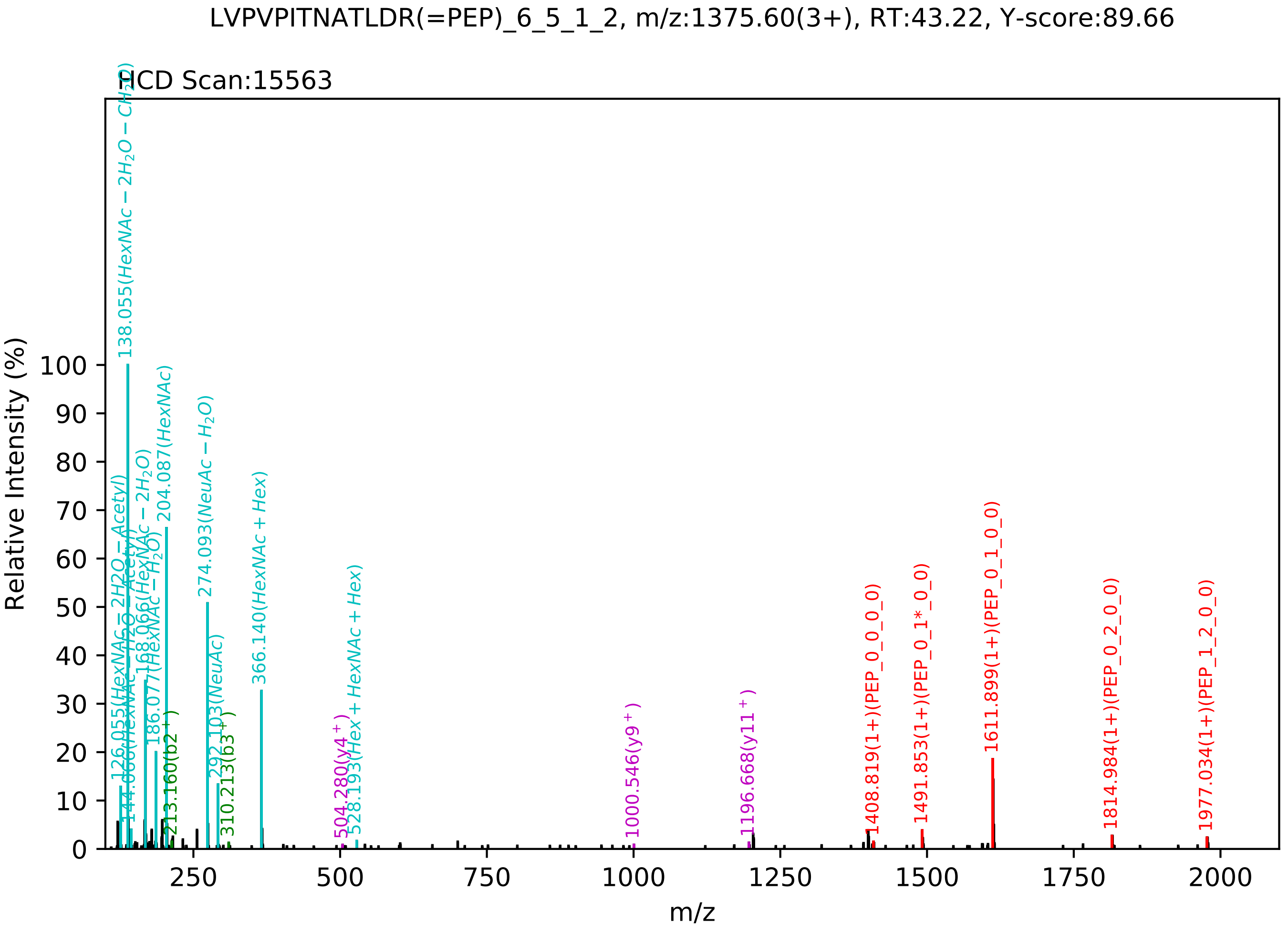

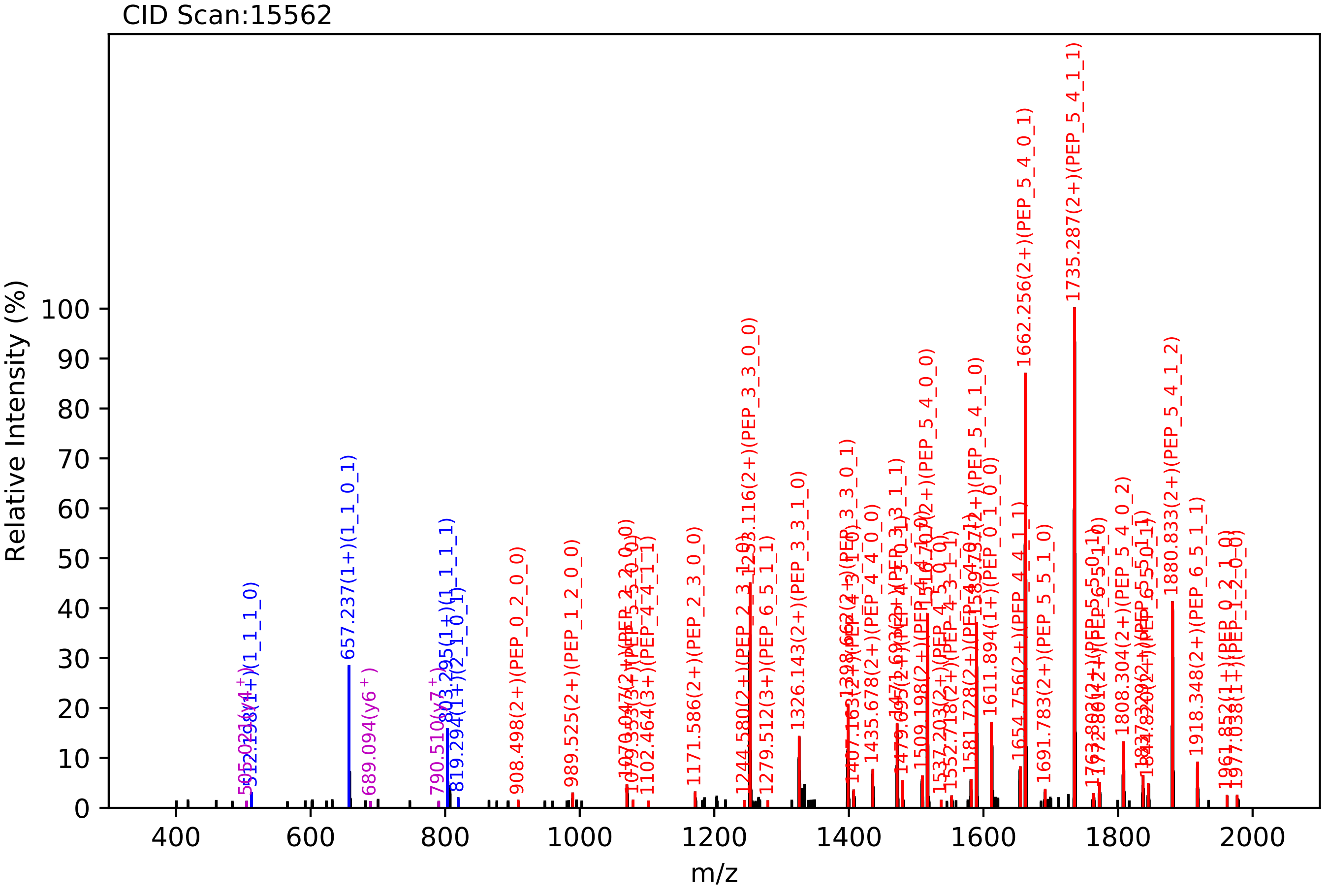

Training set no. 306, Experiment: AGP exp_2

LVPVPITNATLDR(=PEP)_6_5_1_2, m/z:1375.60(3+), RT:42.03, Y-score:85.14

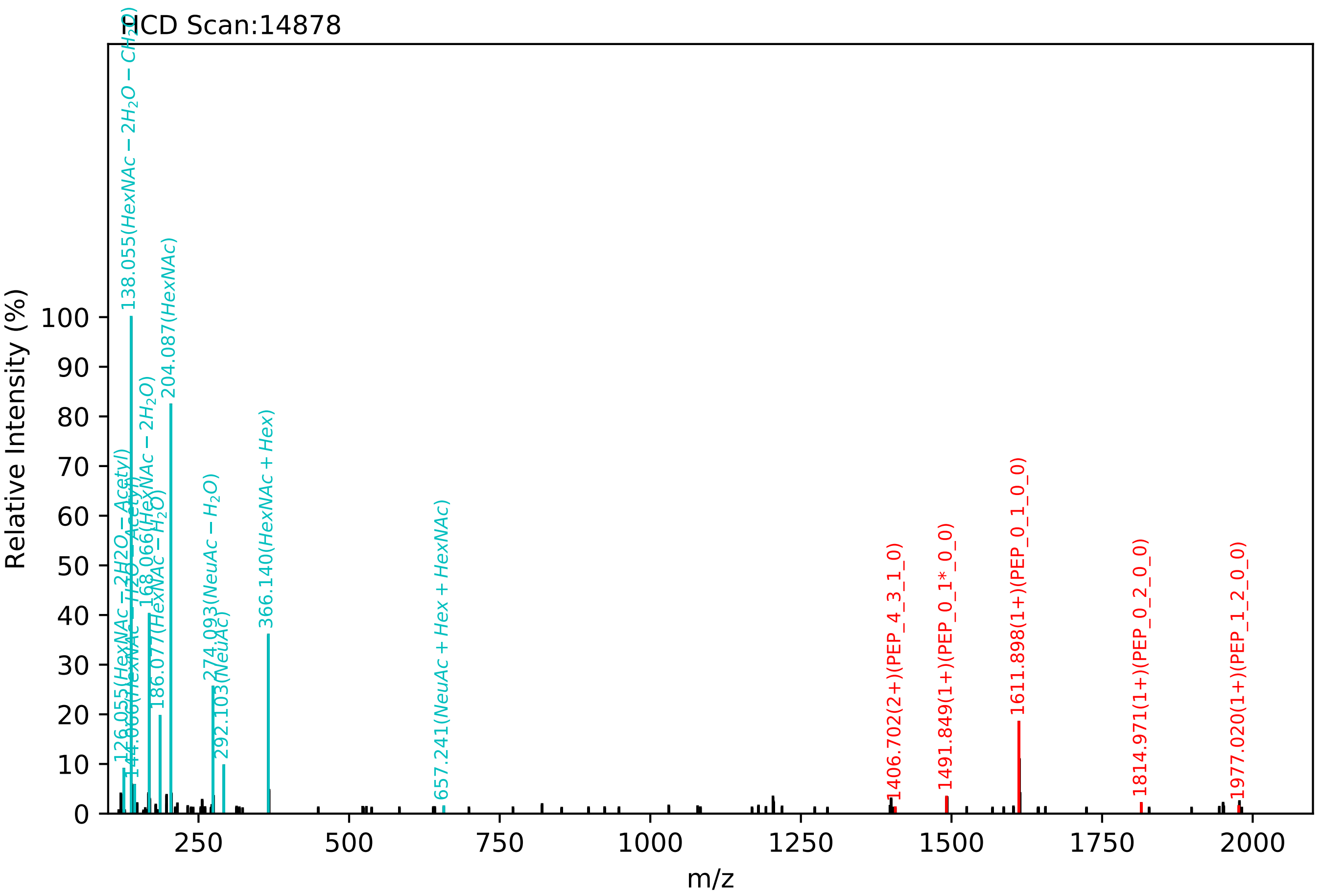

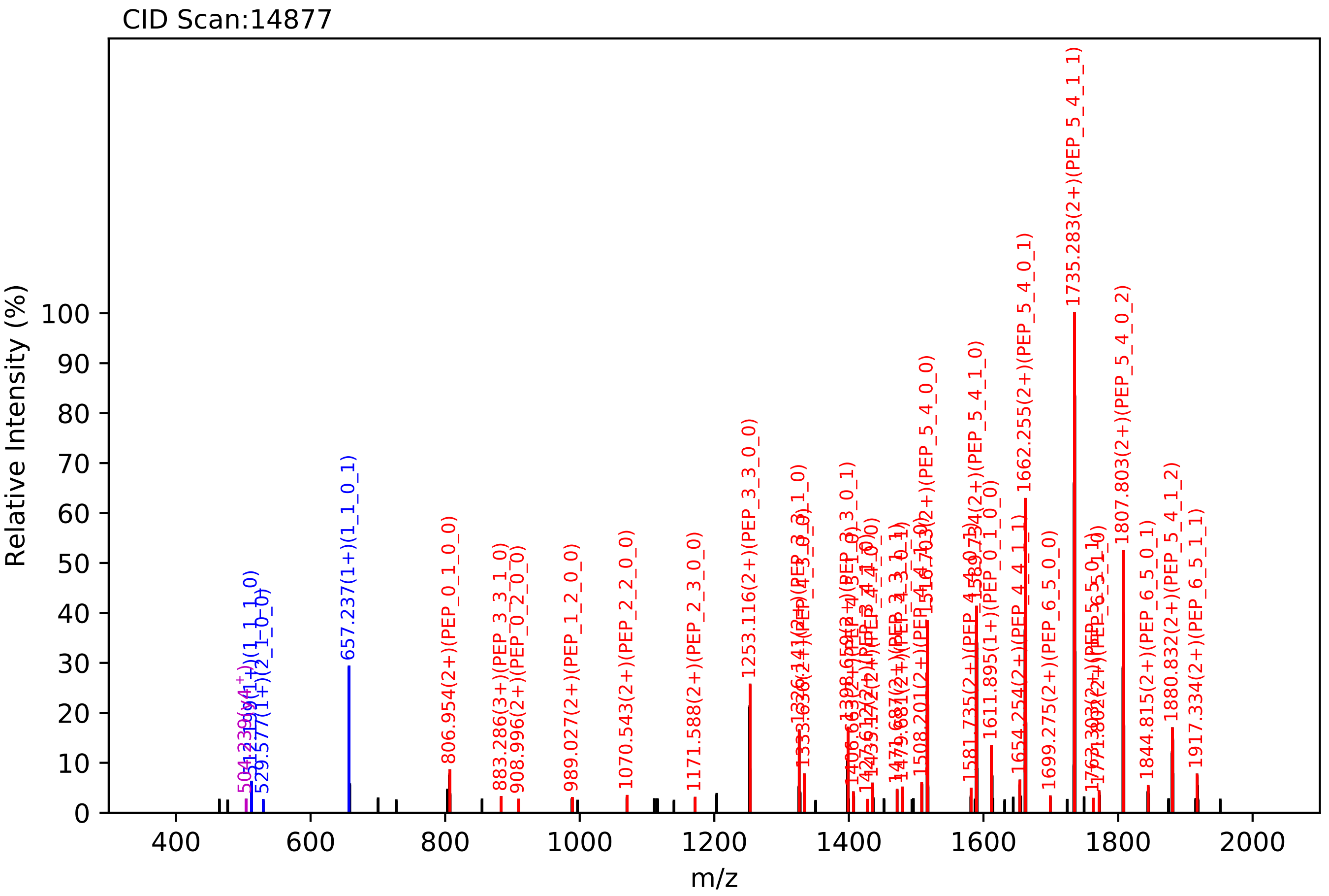

Training set no. 307, Experiment: AGP exp_1

LVPVPITNATLDR(=PEP)_6_5_1_2, m/z:1031.95(4+), RT:42.63, Y-score:82.43

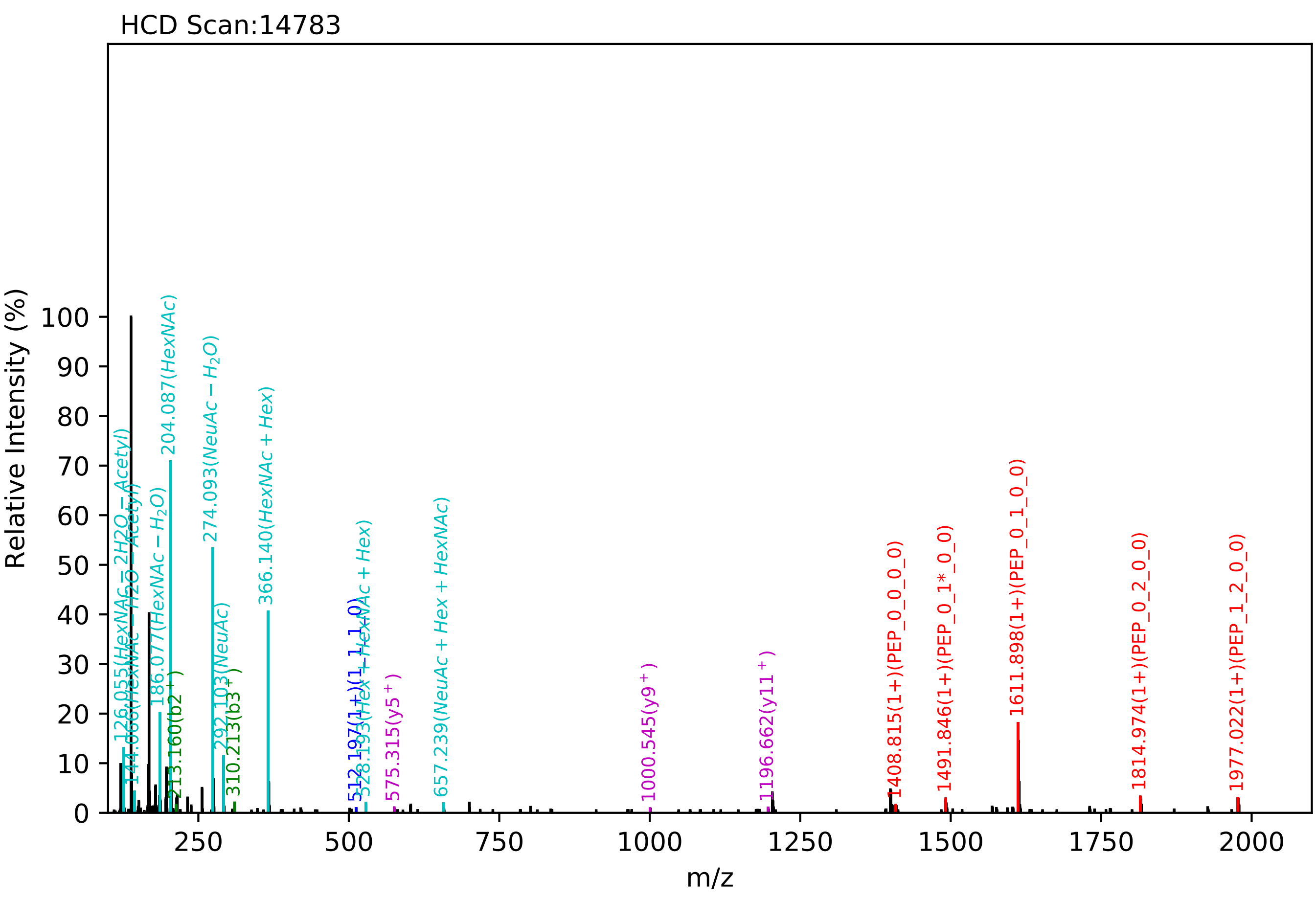

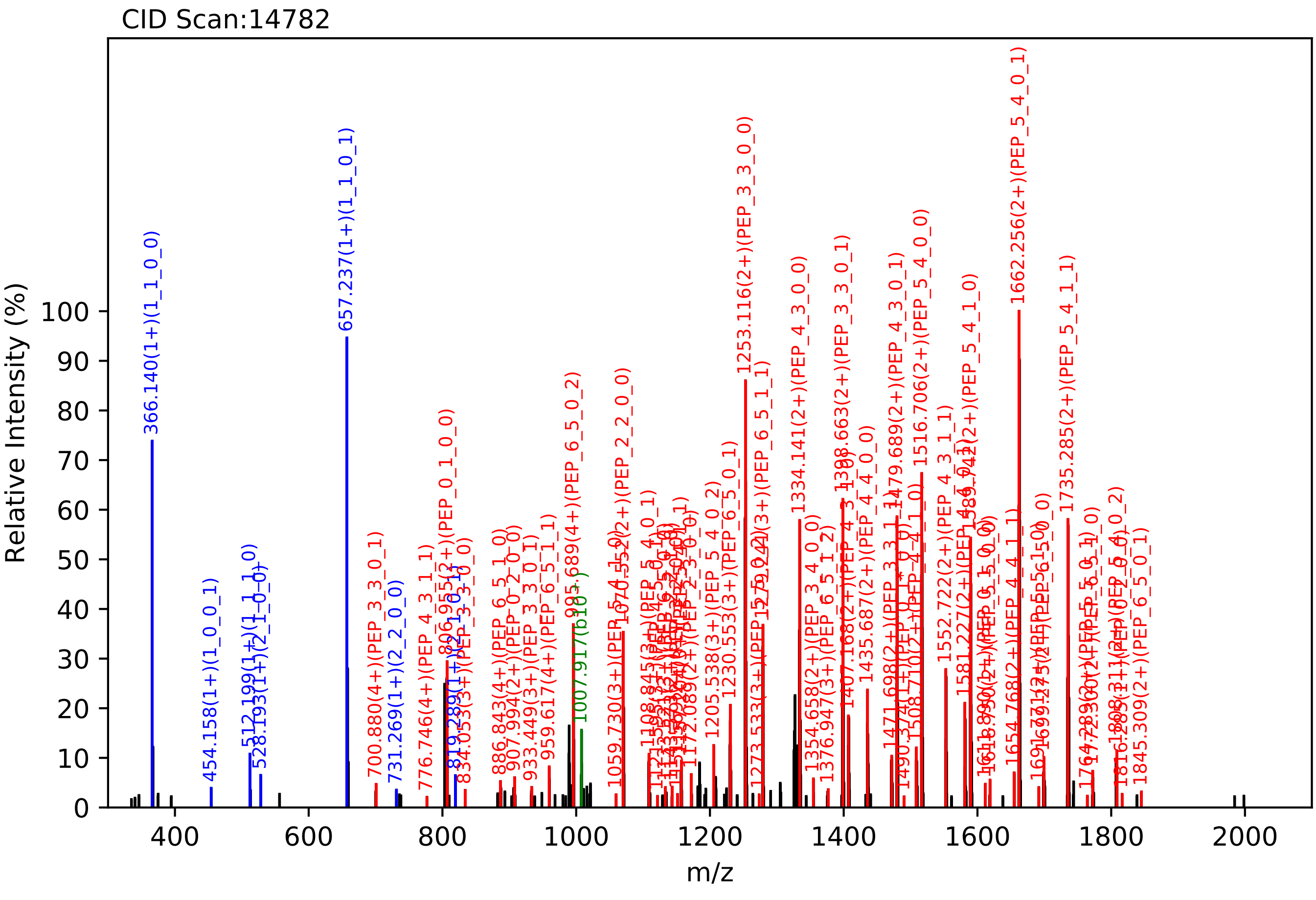

Training set no. 308, Experiment: AGP exp_2

LVPVPITNATLDR(=PEP)_6_5_1_2, m/z:1031.95(4+), RT:42.74, Y-score:79.16

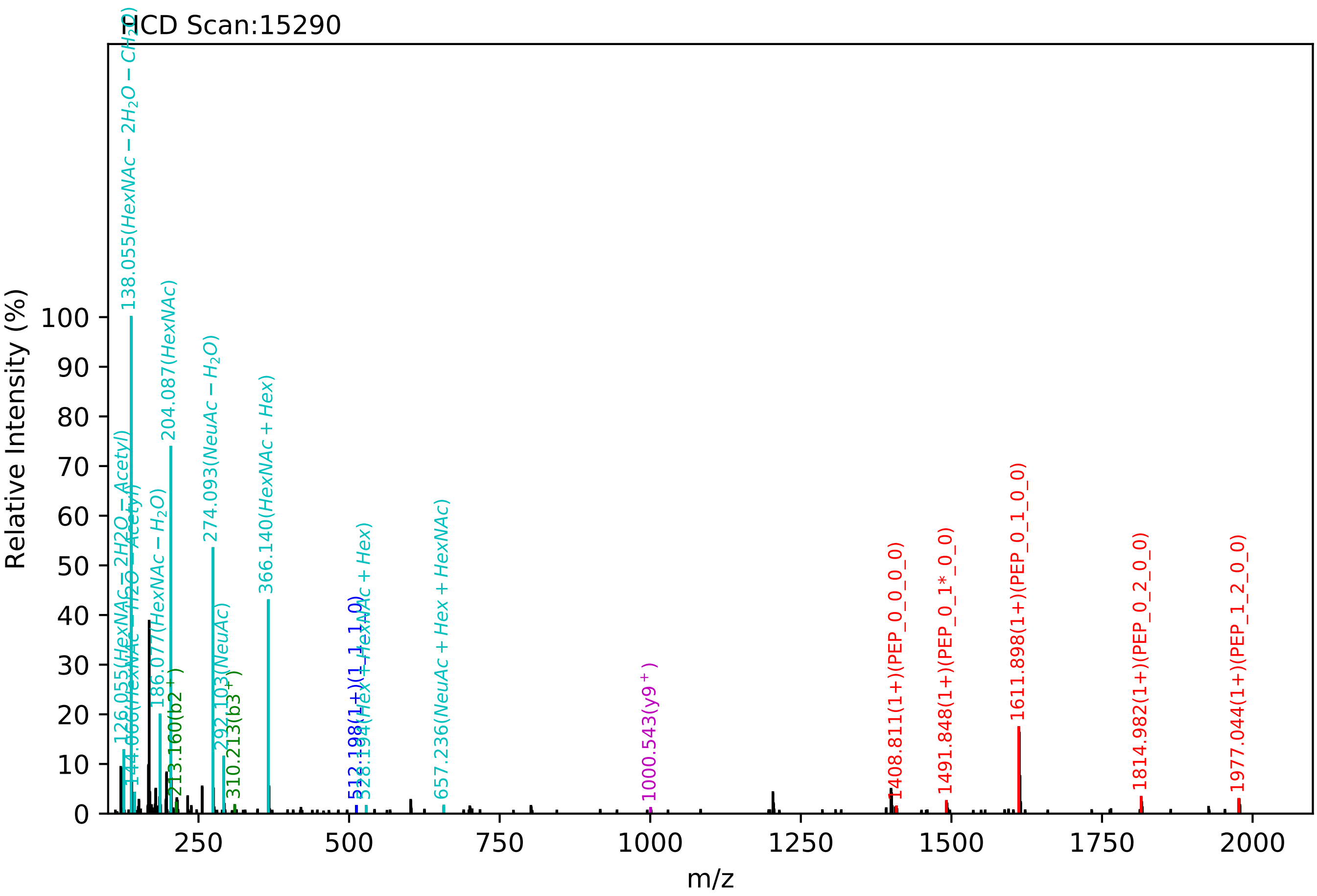

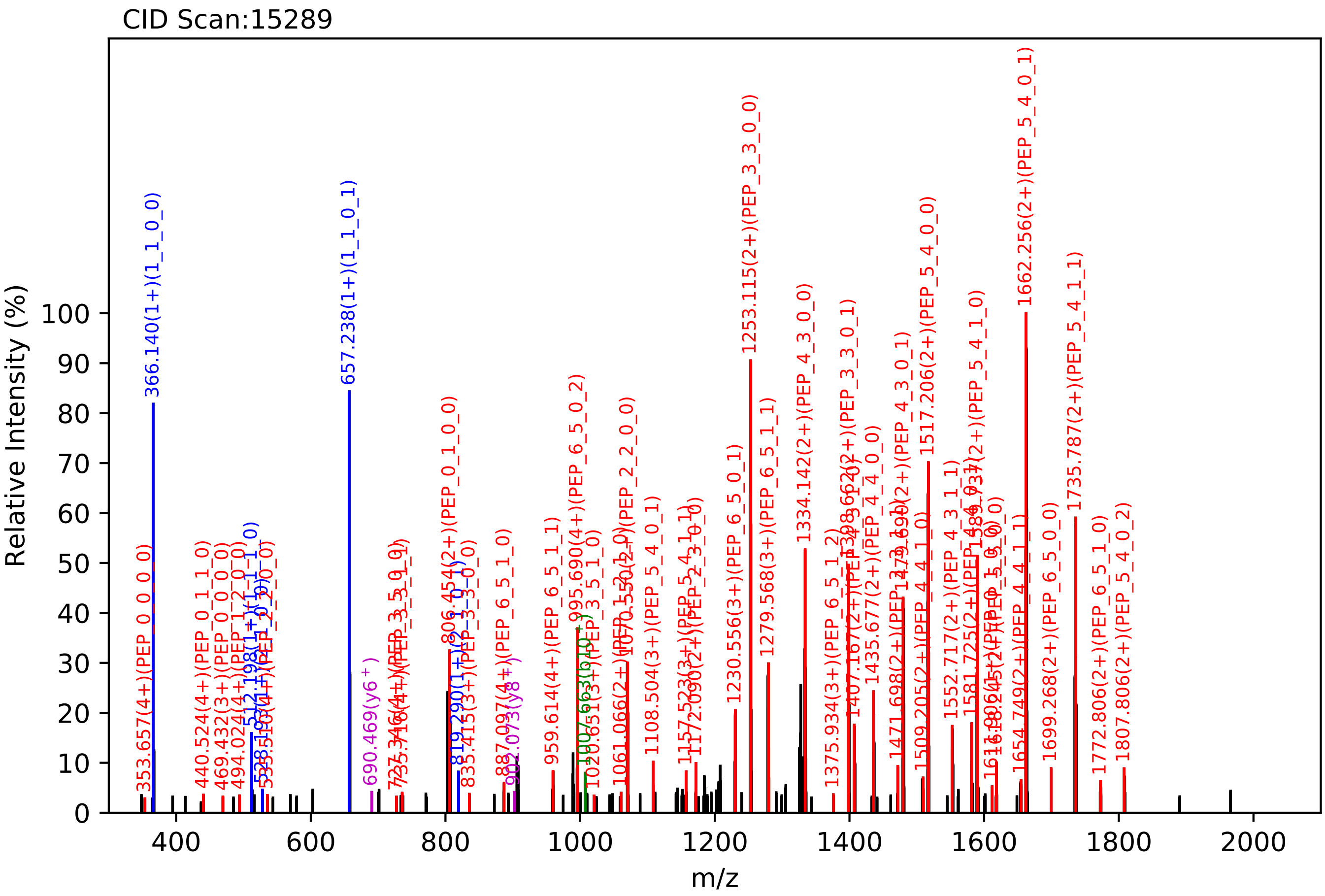

Training set no. 309, Experiment: AGP exp_1

LVPVPITNATLDR(=PEP)_6_5_1_2, m/z:1031.95(4+), RT:42.02, Y-score:67.80

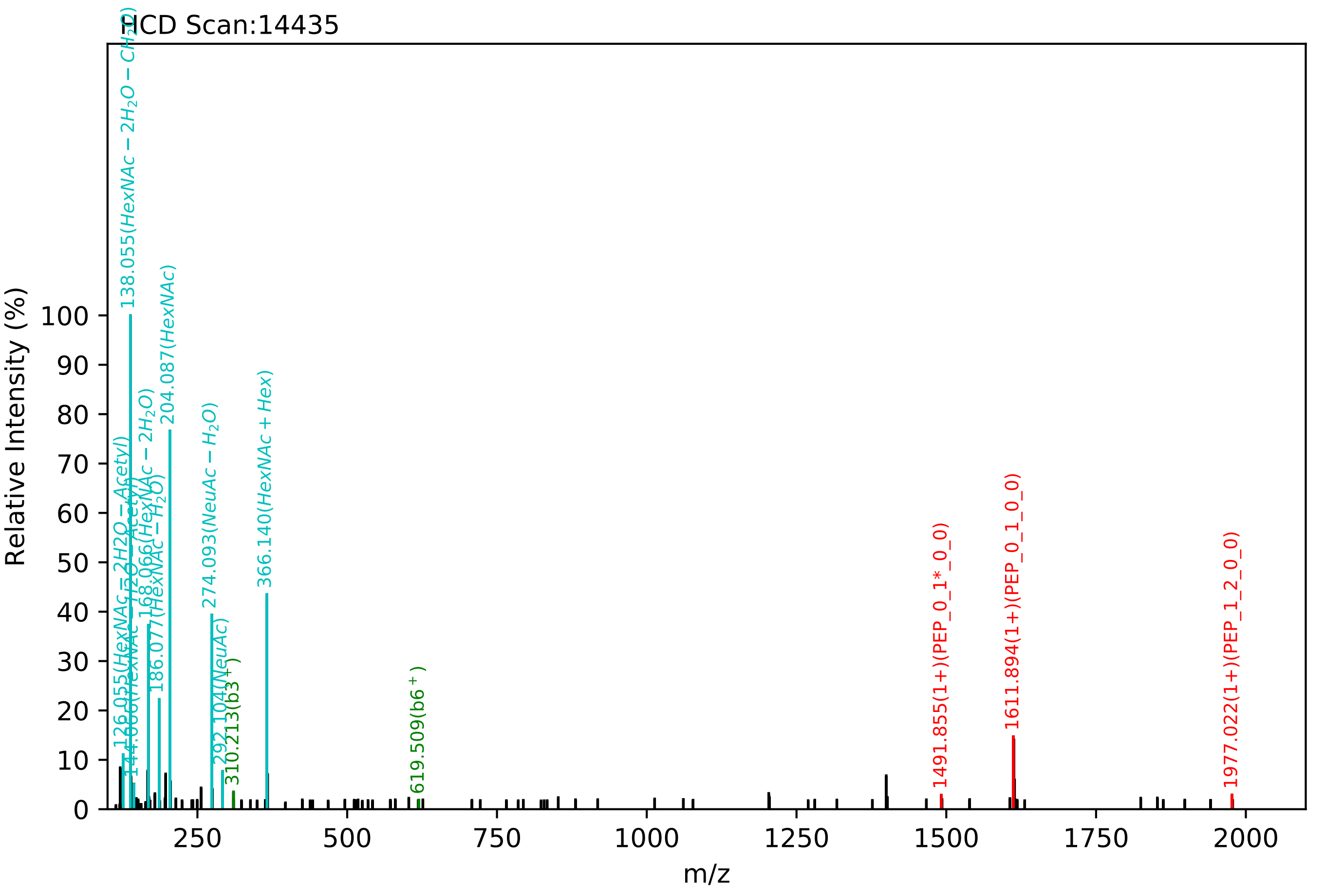

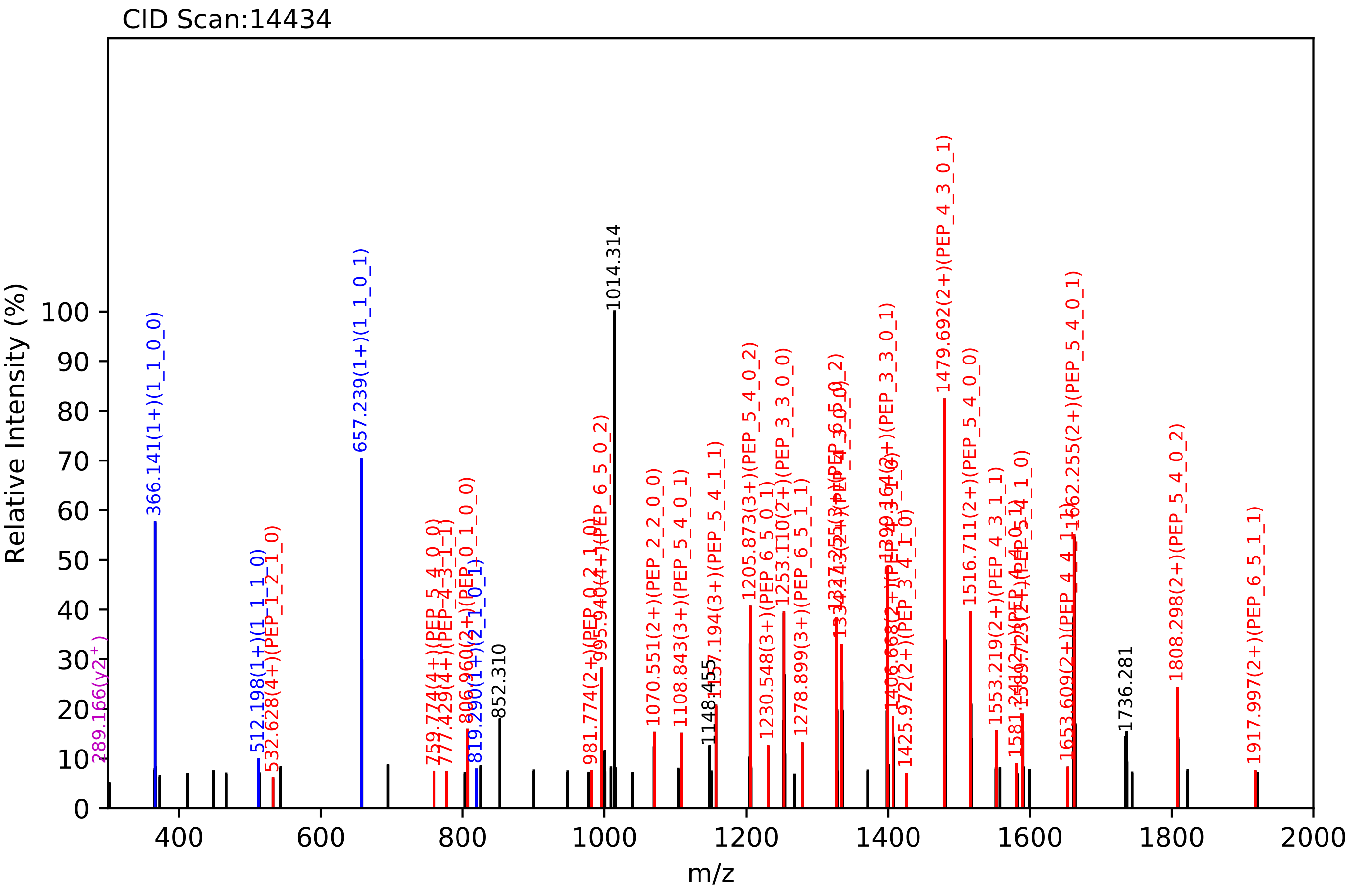

Training set no. 310, Experiment: AGP exp_1

LVPVPITNATLDR(=PEP)_6_5_1_2, m/z:1031.95(4+), RT:42.03, Y-score:67.45

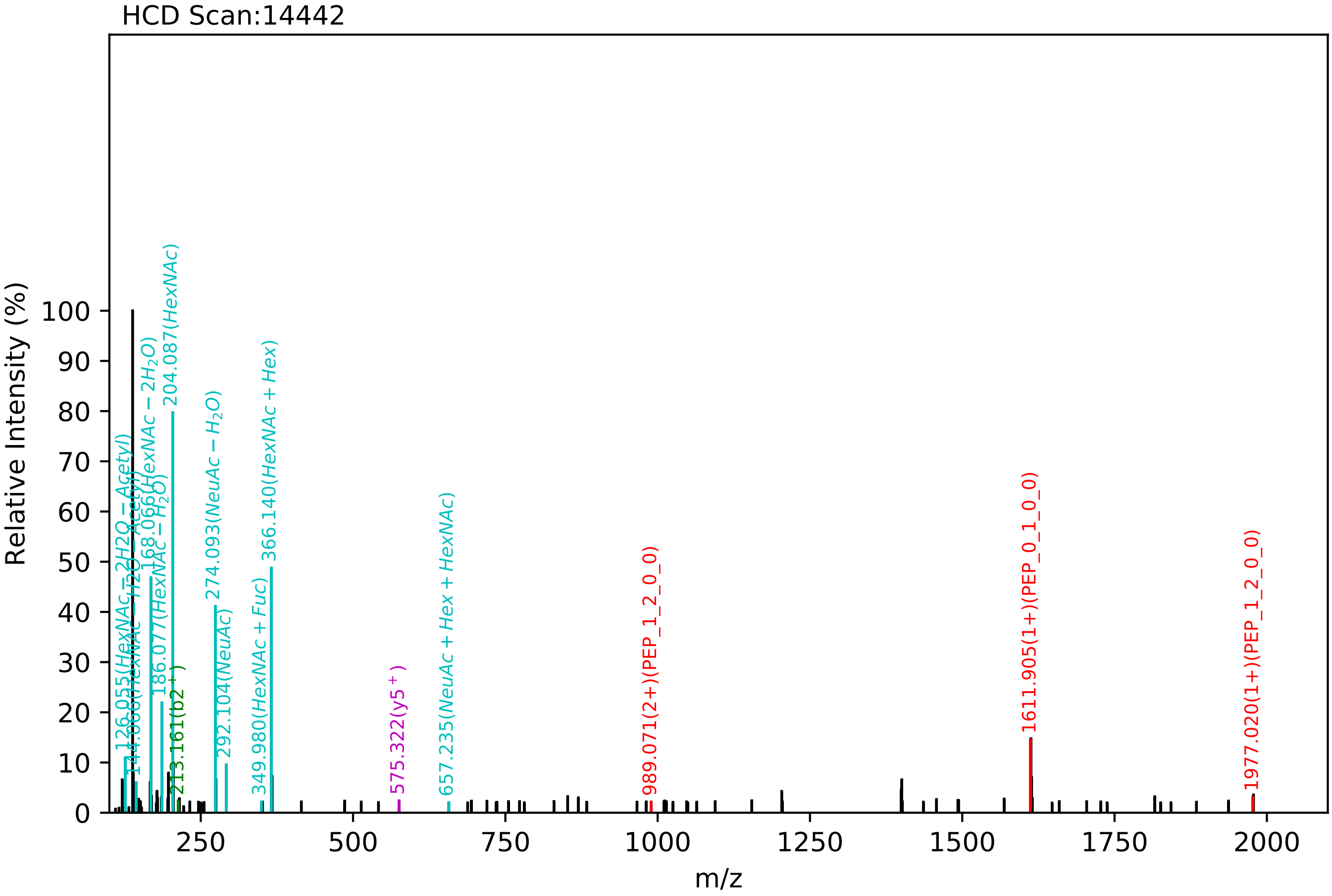

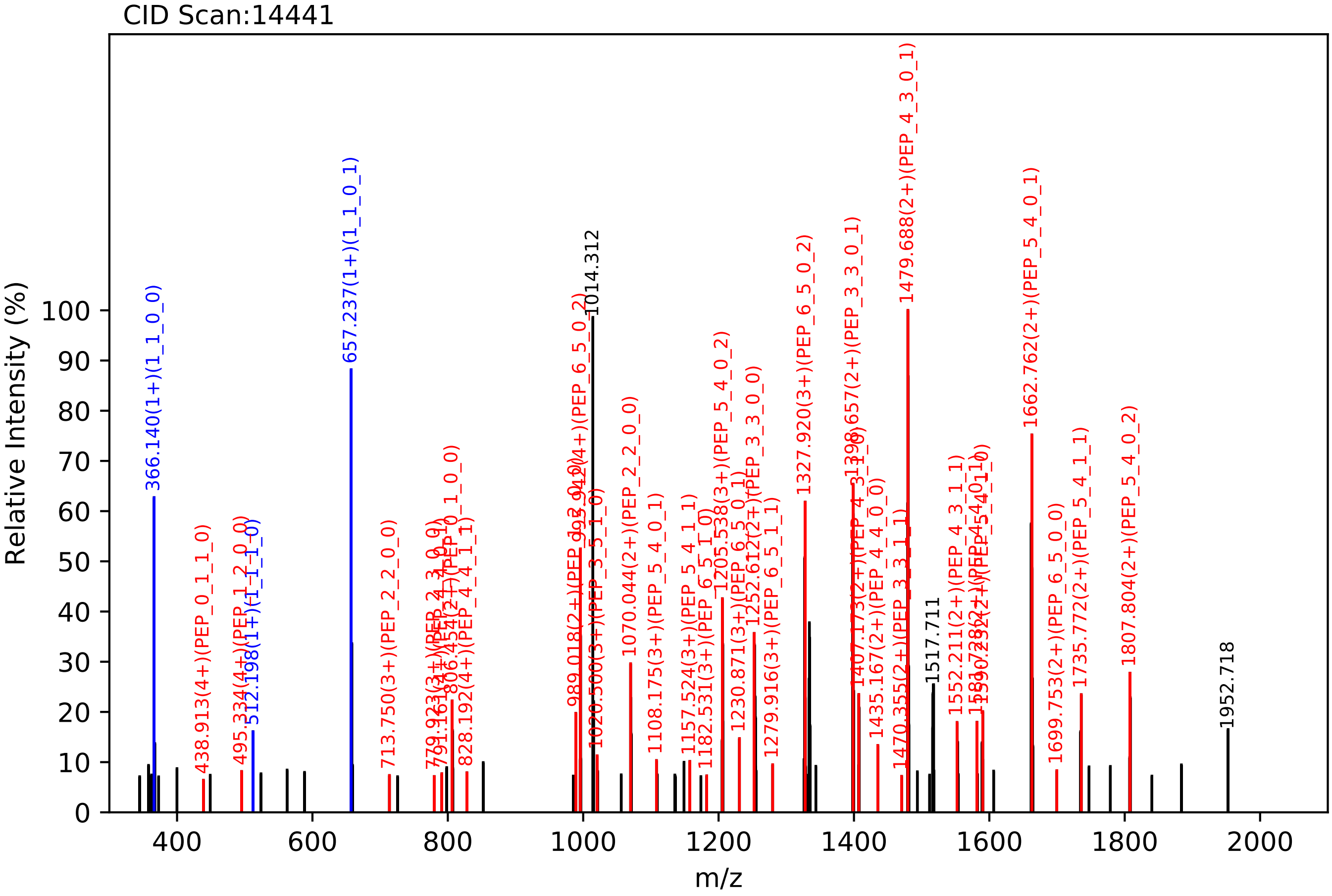

Training set no. 311, Experiment: AGP exp_1

LVPVPITNATLDR(=PEP)_6_5_1_3, m/z:1472.63(3+), RT:49.89, Y-score:89.54

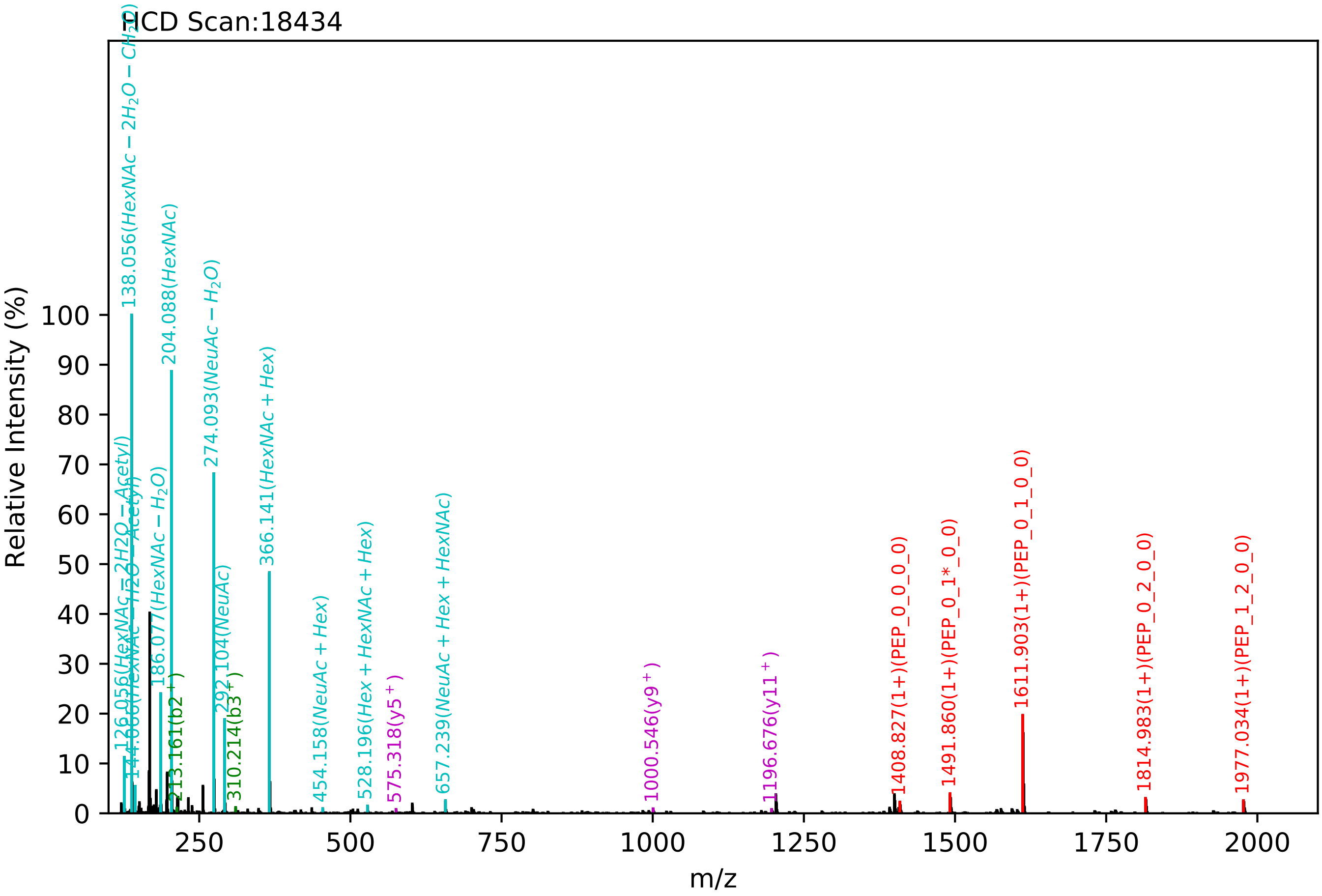

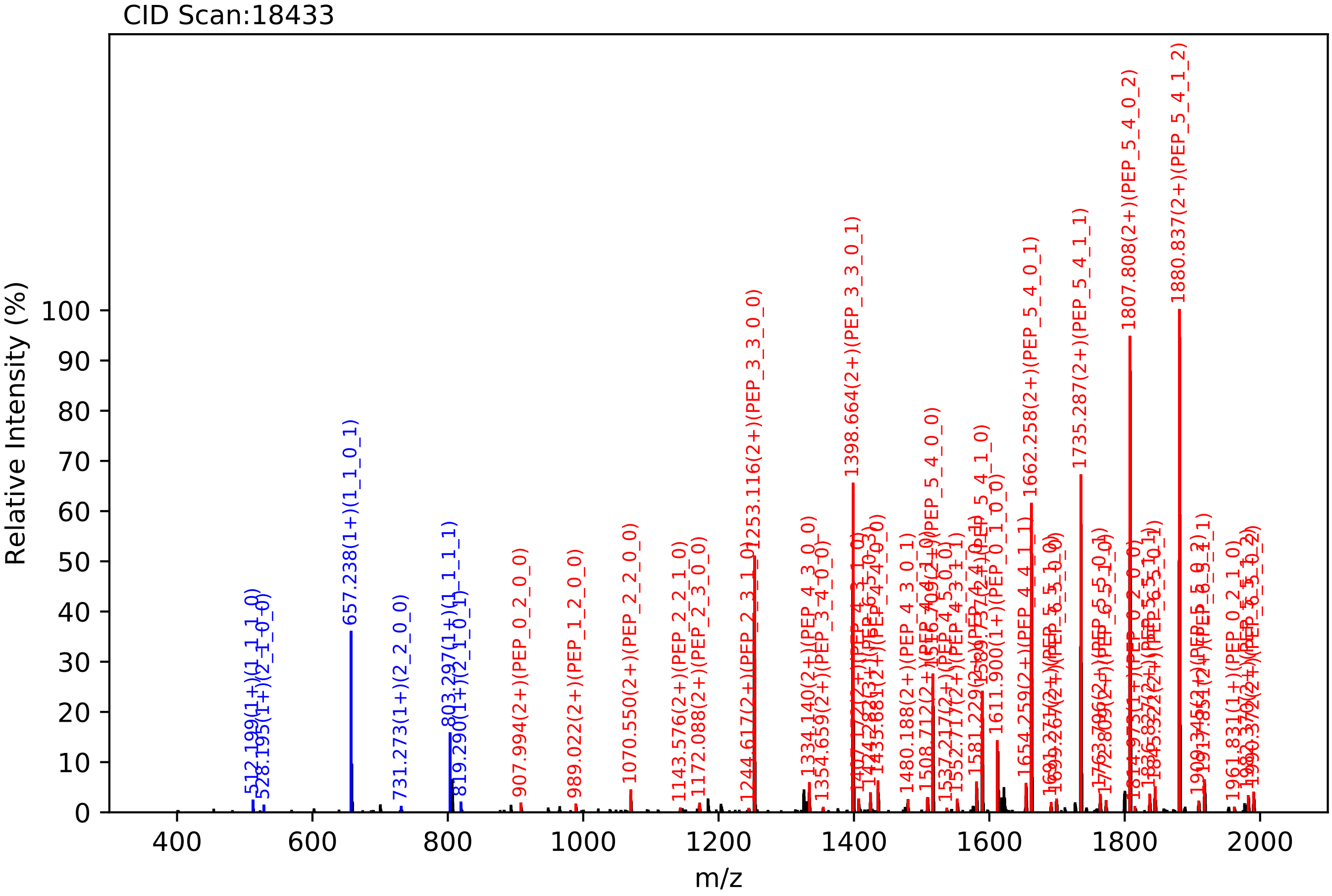

Training set no. 312, Experiment: AGP exp_1

LVPVPITNATLDR(=PEP)_6_5_1_3, m/z:1104.73(4+), RT:50.10, Y-score:78.63

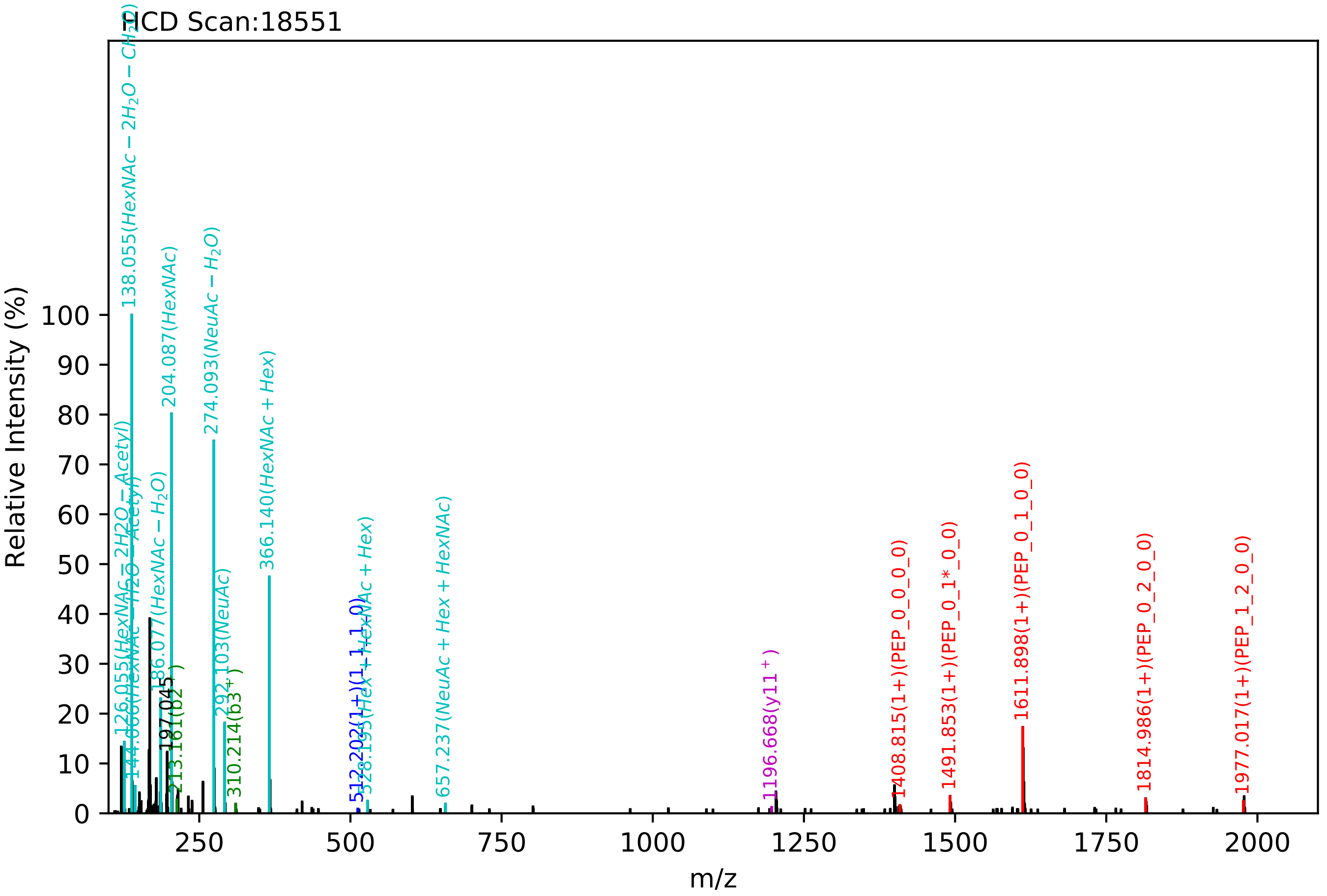

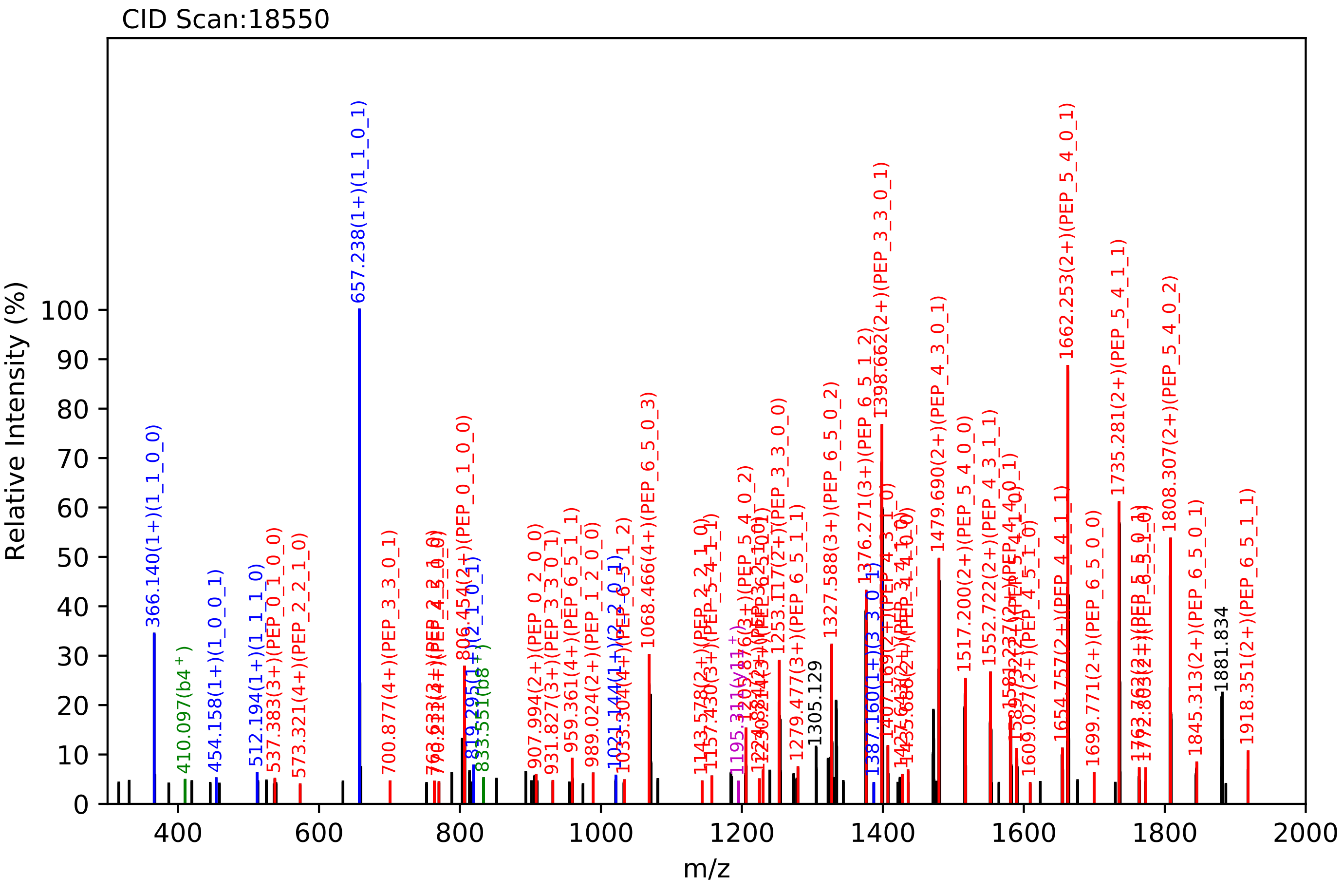

Training set no. 313, Experiment: AGP exp_2

LVPVPITNATLDR(=PEP)_6_5_1_3, m/z:1104.72(4+), RT:50.14, Y-score:77.19

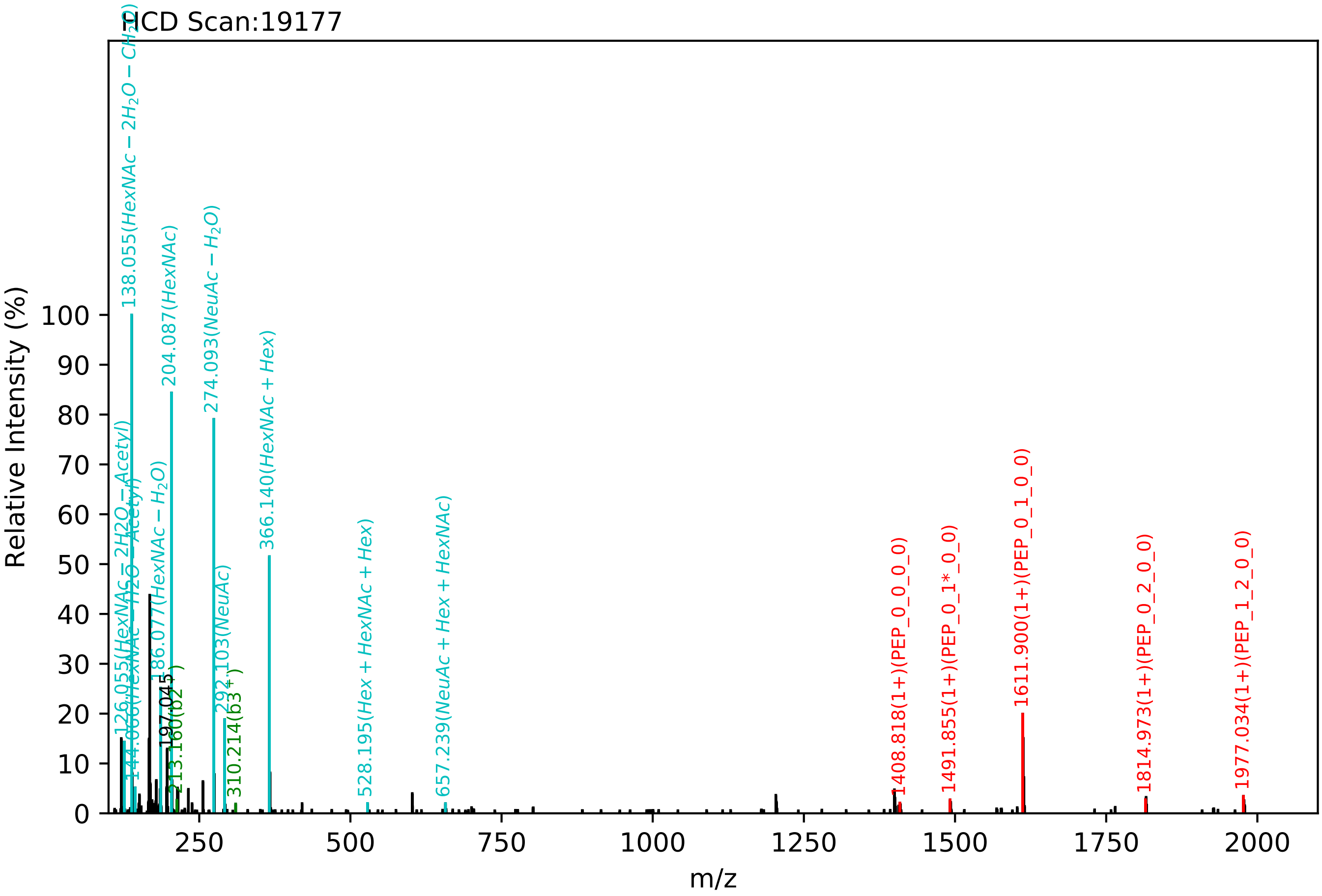

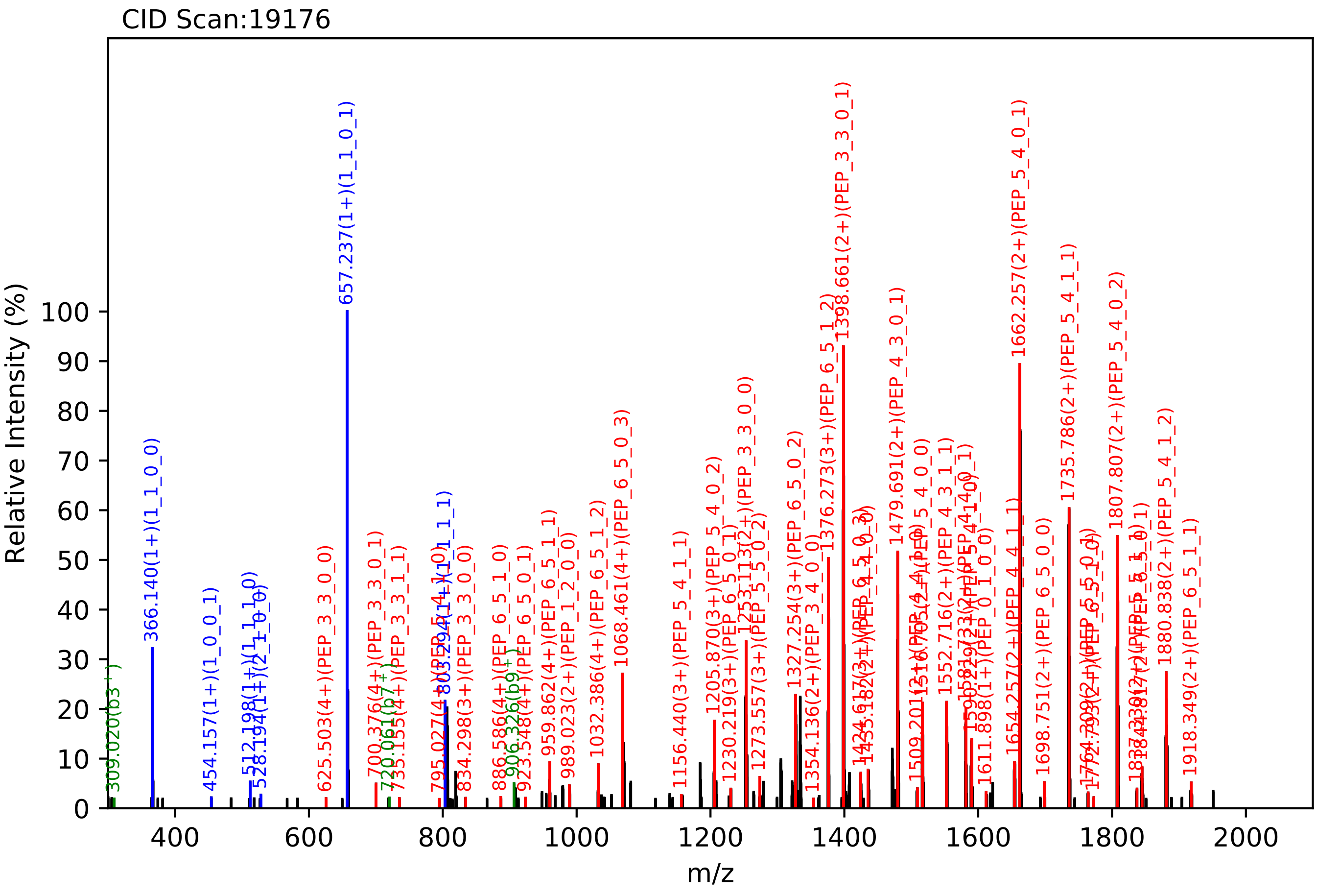

Training set no. 314, Experiment: IgG exp_4

EEQYNSTYR(=PEP)_3_3_0_0, m/z:1142.96(2+), RT:21.05, Y-score:84.41

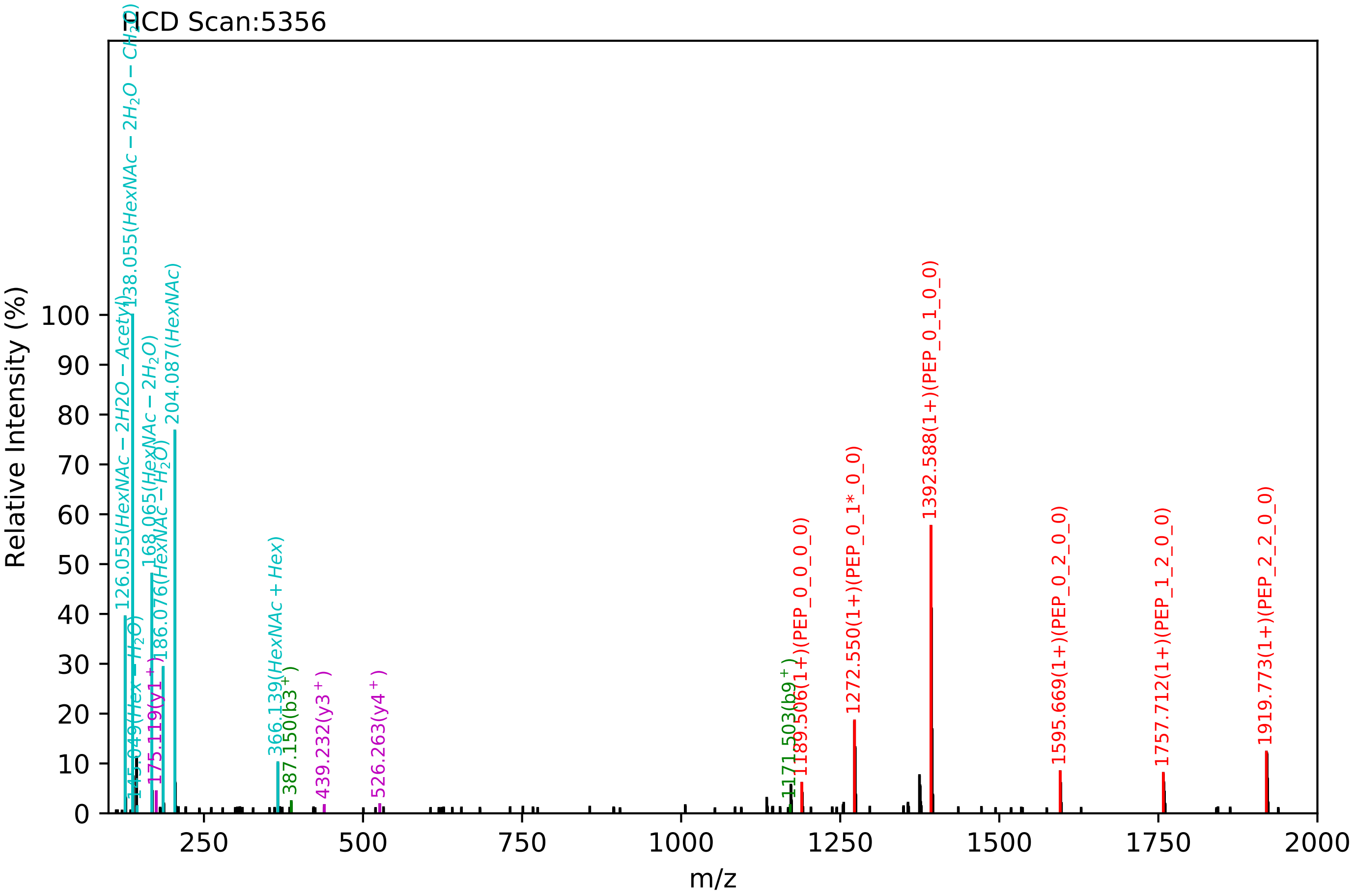

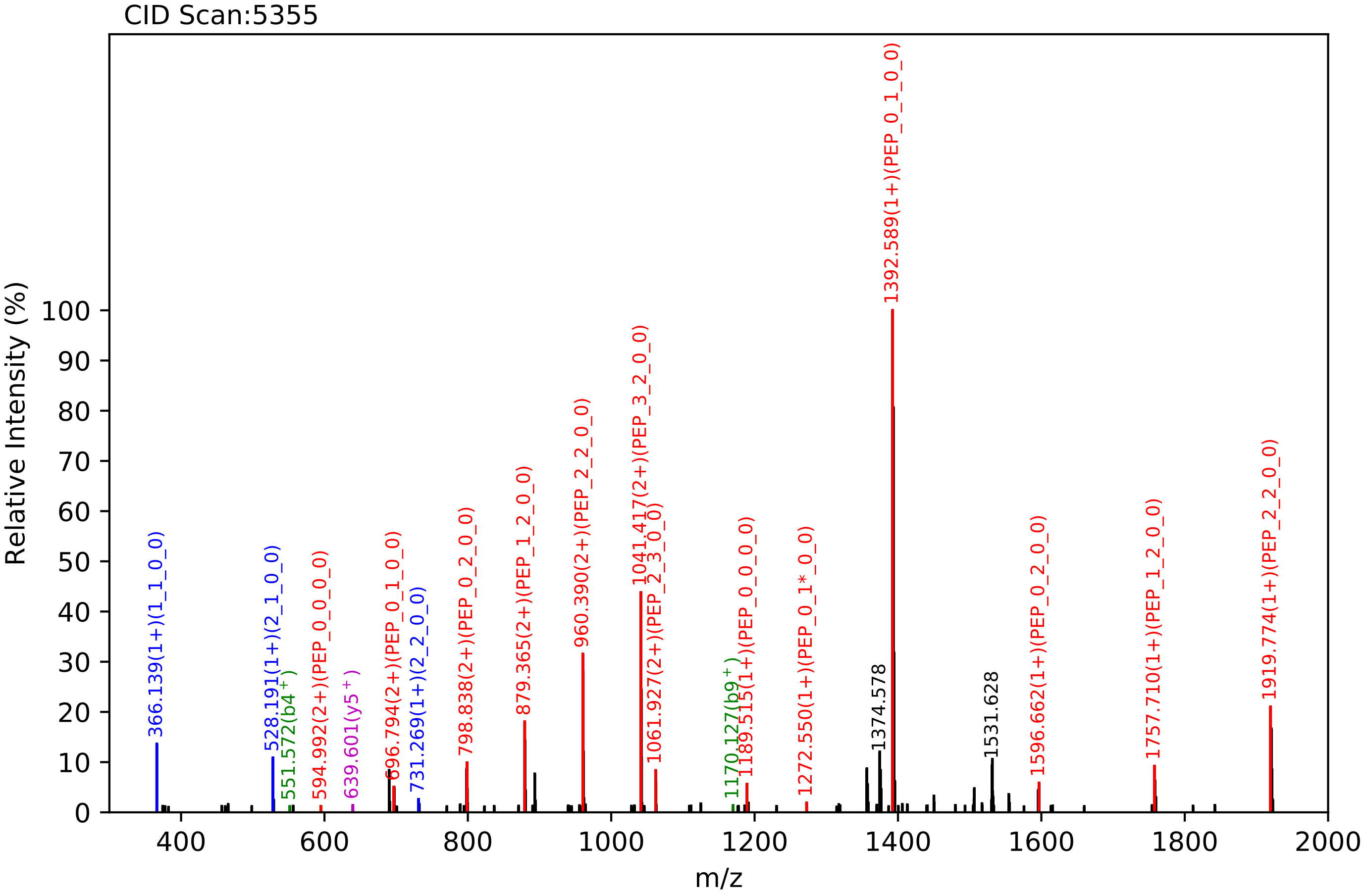

Training set no. 315, Experiment: IgG exp_3

EEQYNSTYR(=PEP)_3_3_1_0, m/z:1215.98(2+), RT:23.25, Y-score:88.67

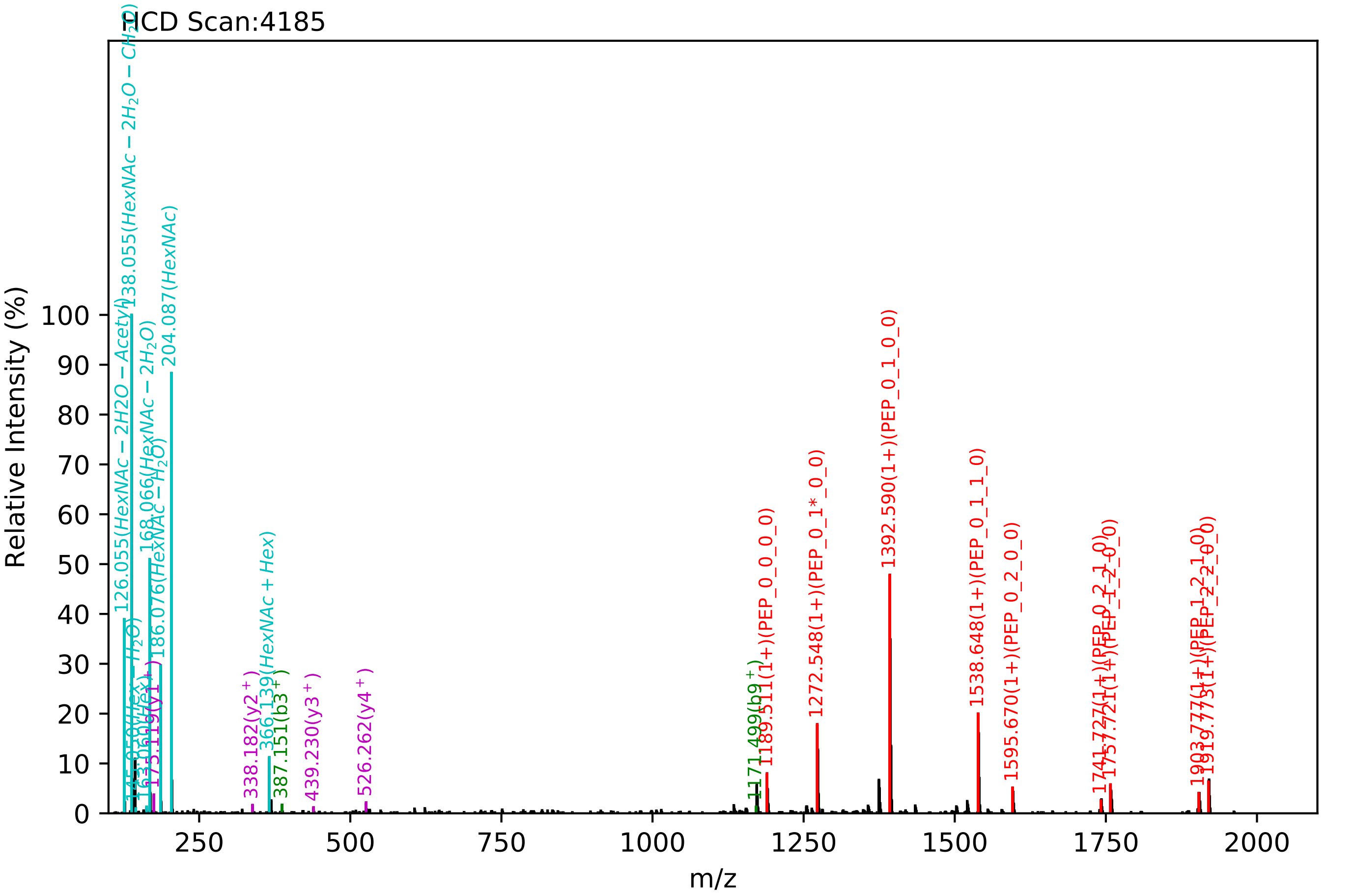

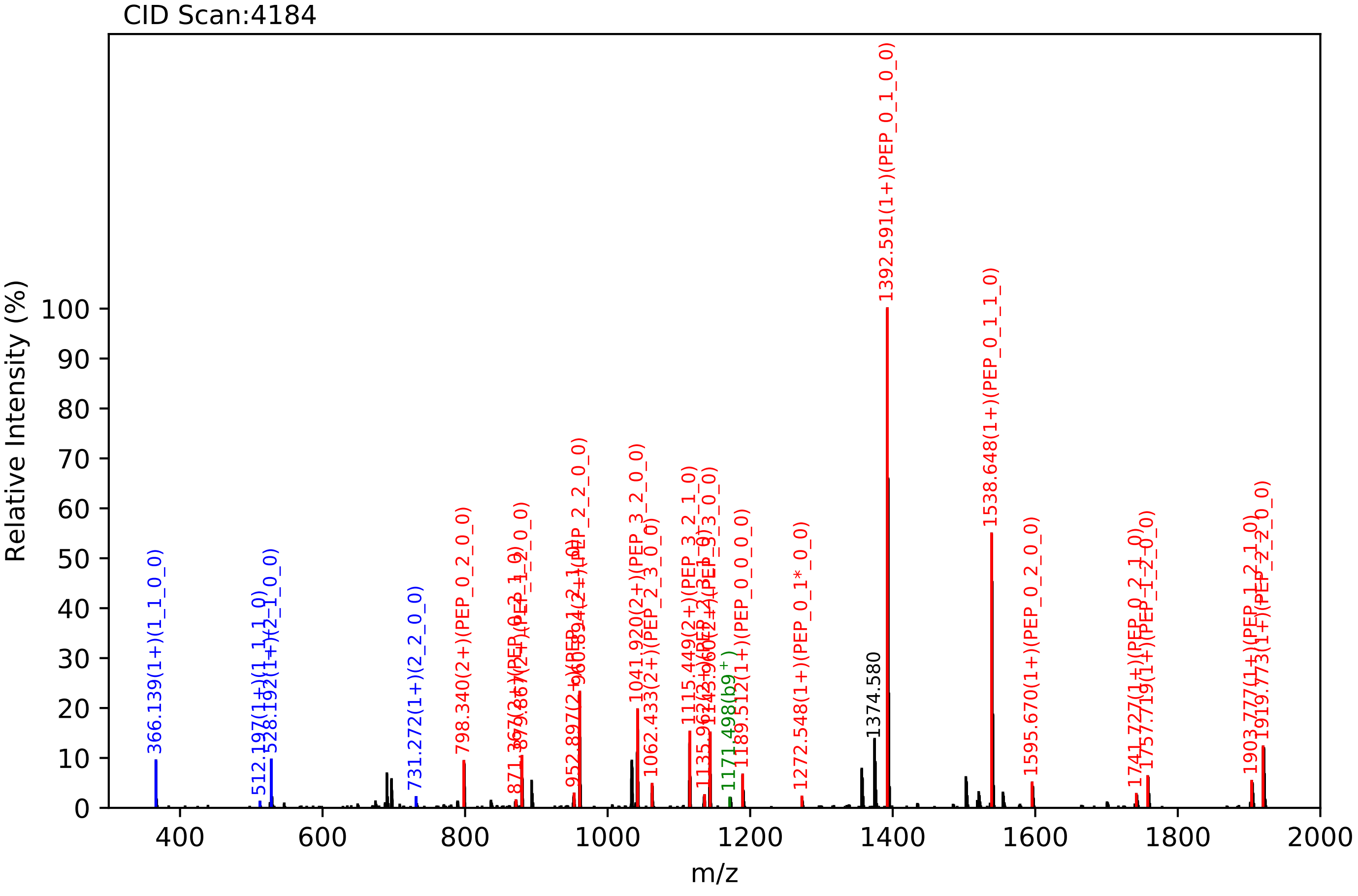

Training set no. 316, Experiment: IgG exp_1

EEQYNSTYR(=PEP)_3_3_1_0, m/z:1215.99(2+), RT:22.10, Y-score:88.30

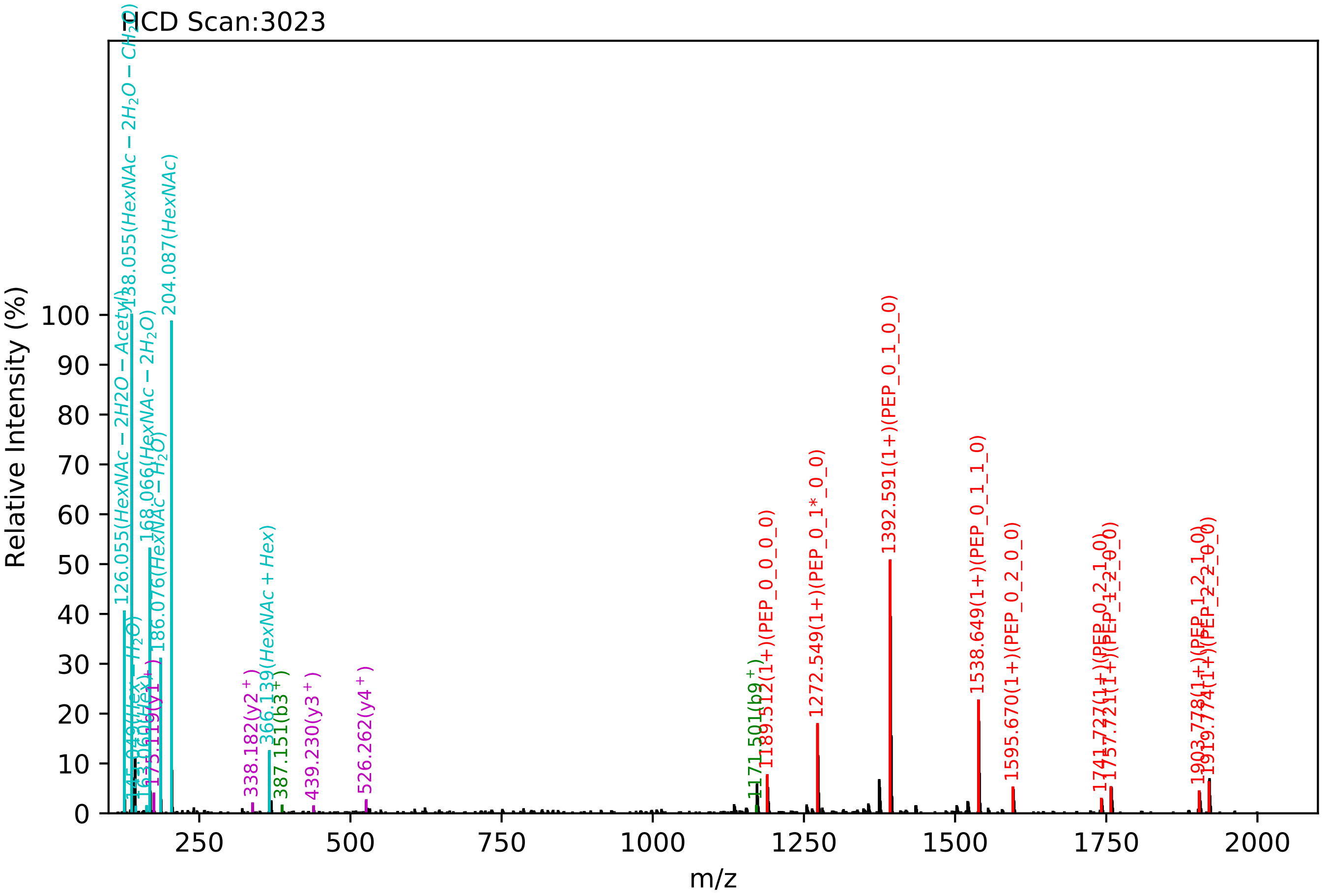

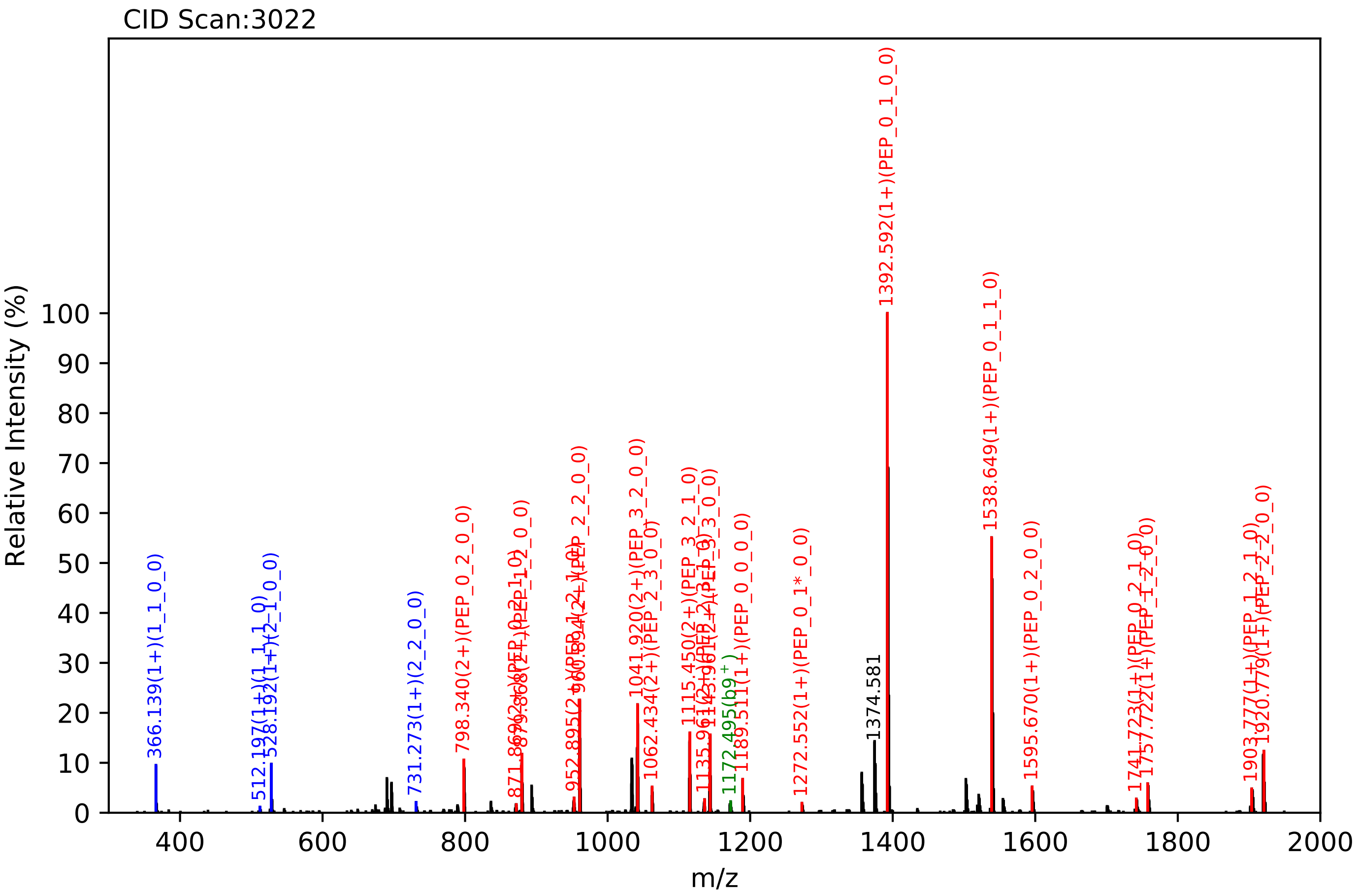

Training set no. 317, Experiment: IgG exp_4

EEQYNSTYR(=PEP)_3_3_1_0, m/z:1215.99(2+), RT:20.99, Y-score:87.59

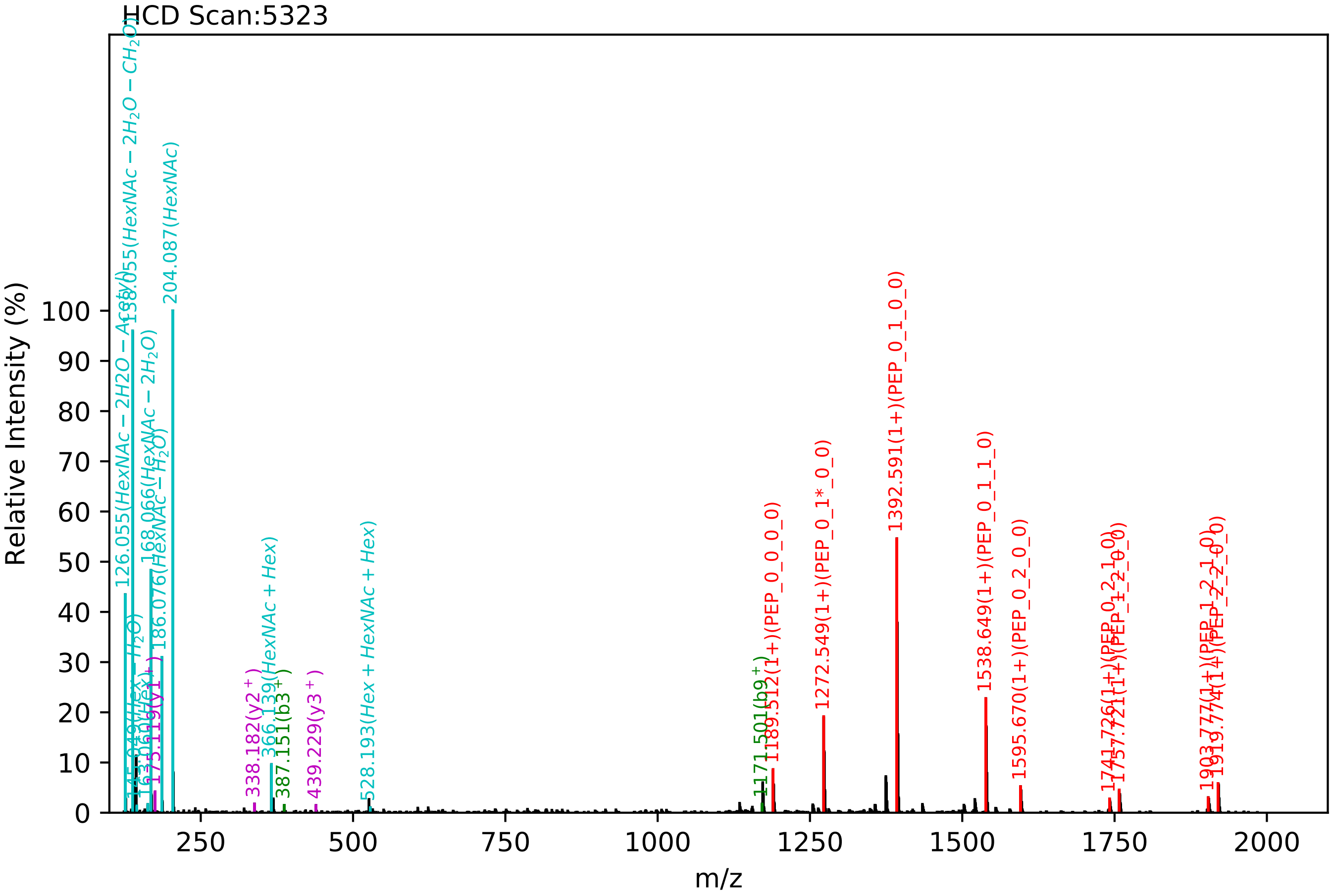

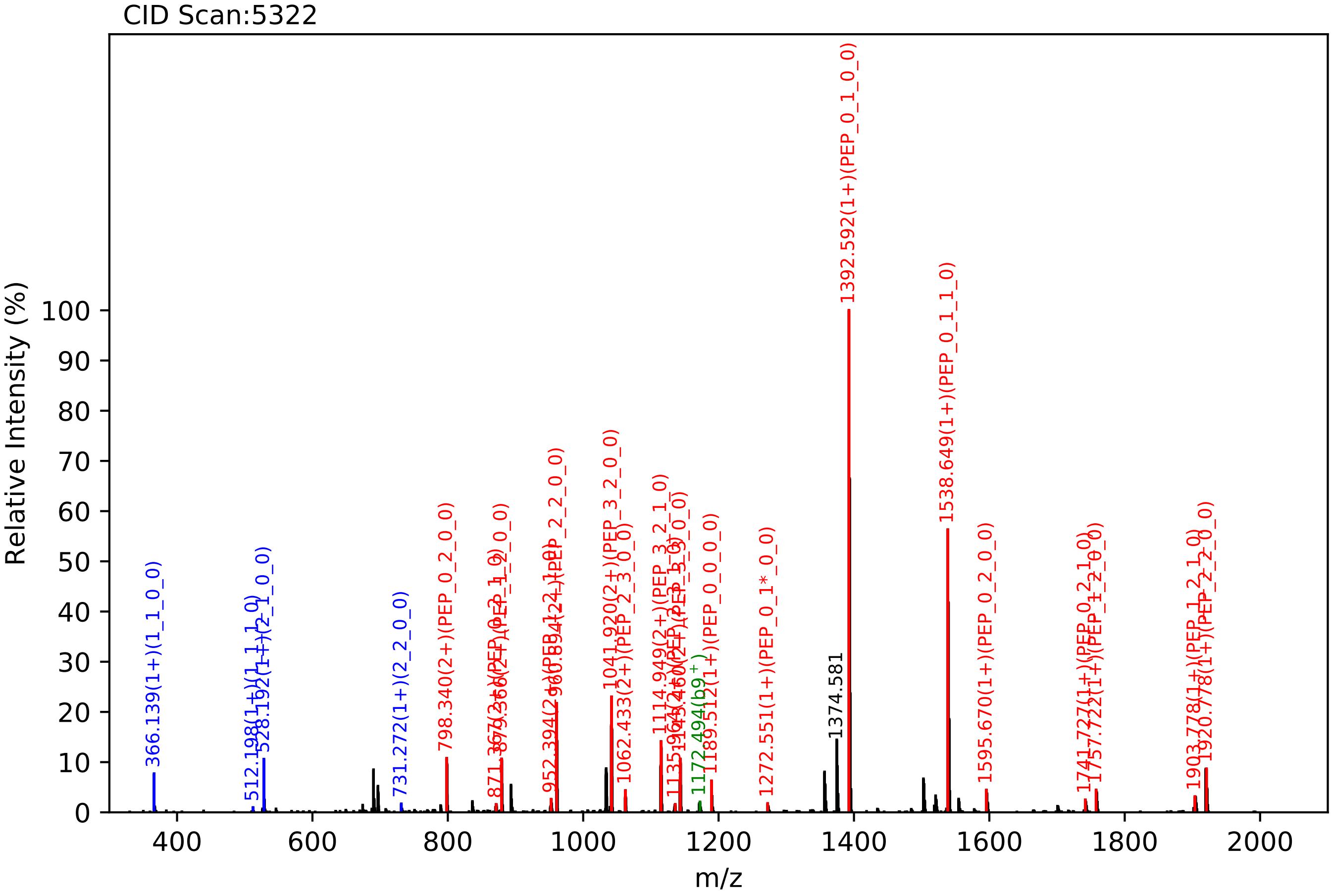

Training set no. 318, Experiment: IgG exp_2

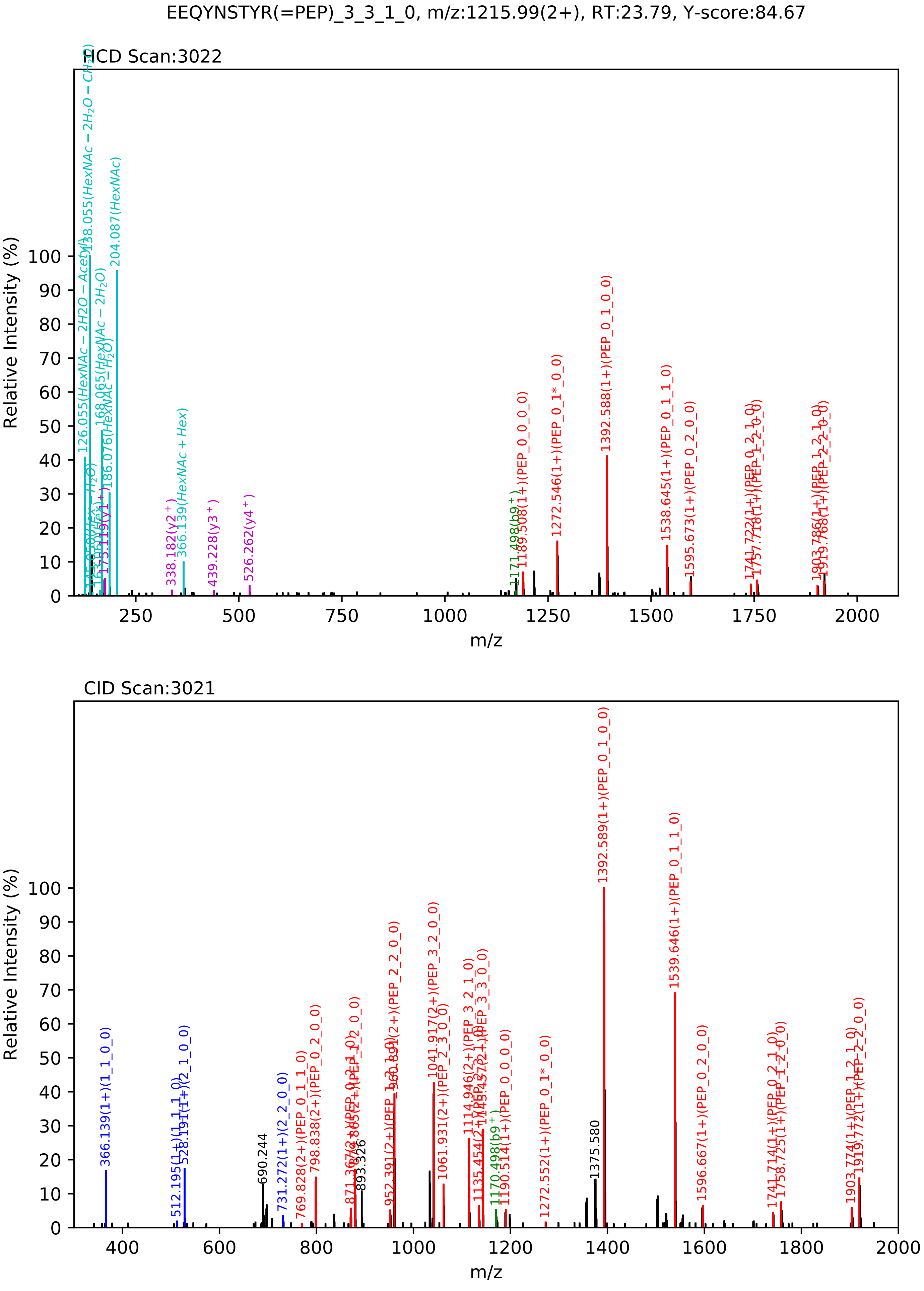

Training set no. 319, Experiment: IgG exp_4

EEQYNSTYR(=PEP)_3_4_0_0, m/z:1244.50(2+), RT:21.01, Y-score:88.76

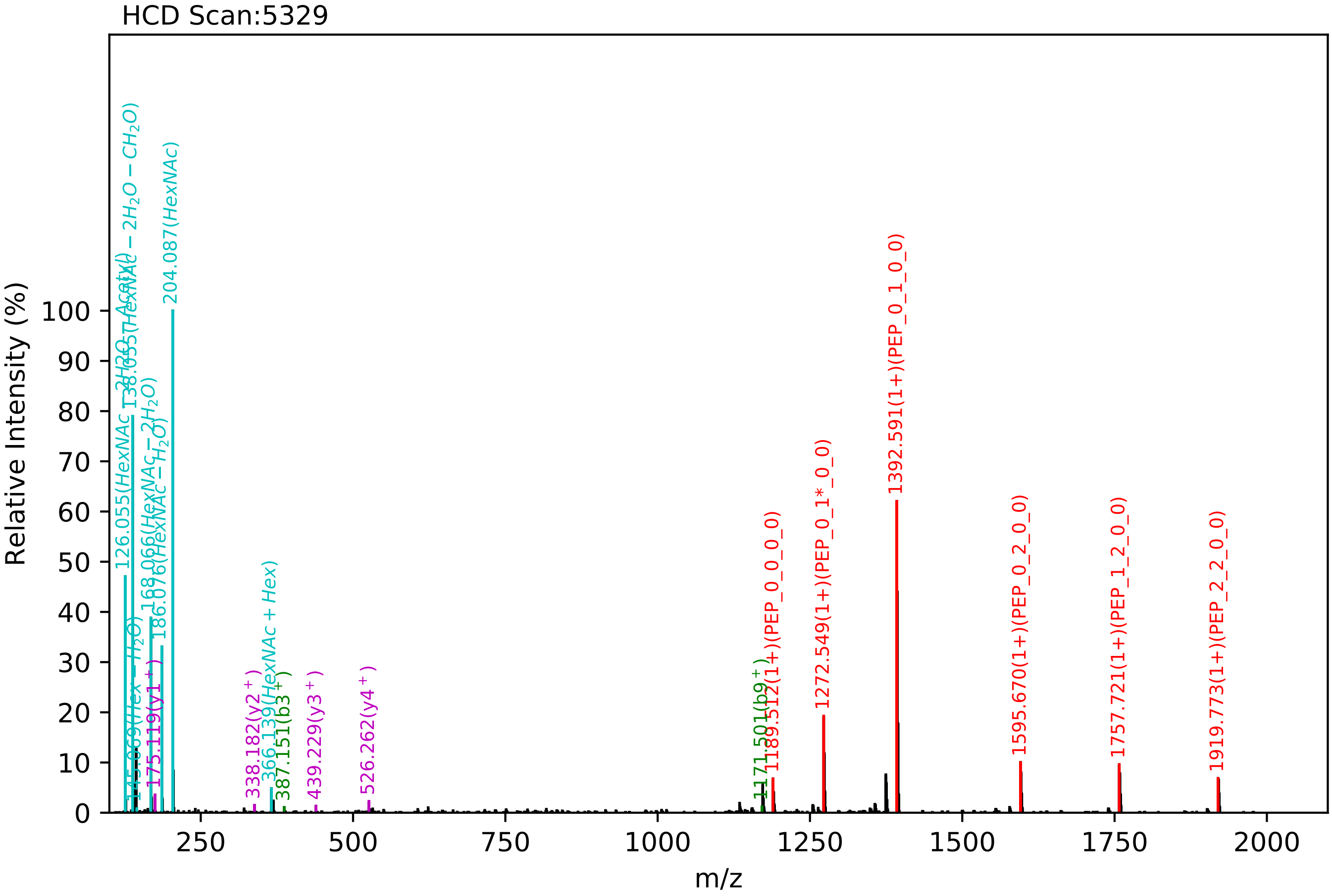

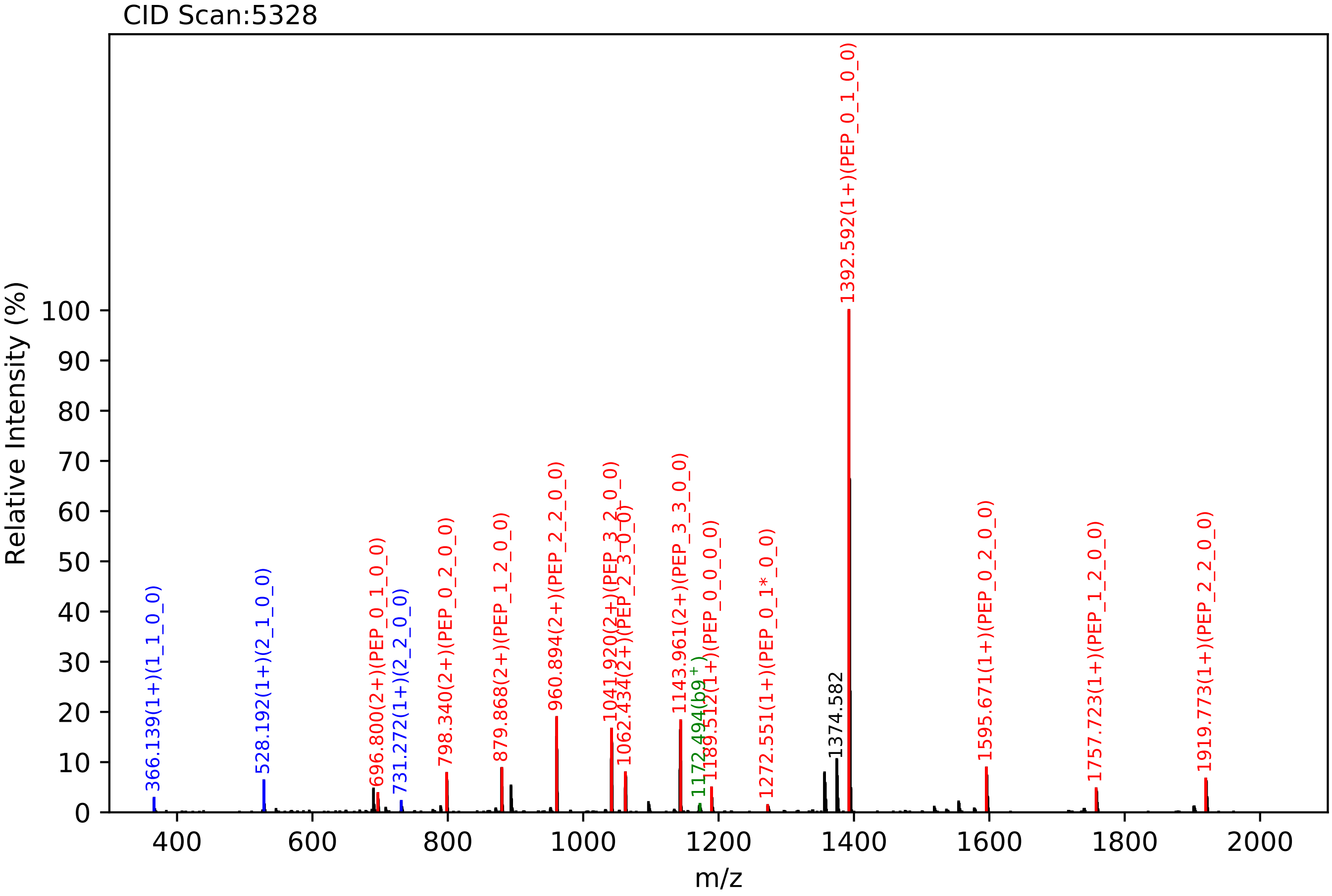

Training set no. 320, Experiment: IgG exp_3

EEQYNSTYR(=PEP)_3_4_0_0, m/z:1244.49(2+), RT:23.32, Y-score:87.76

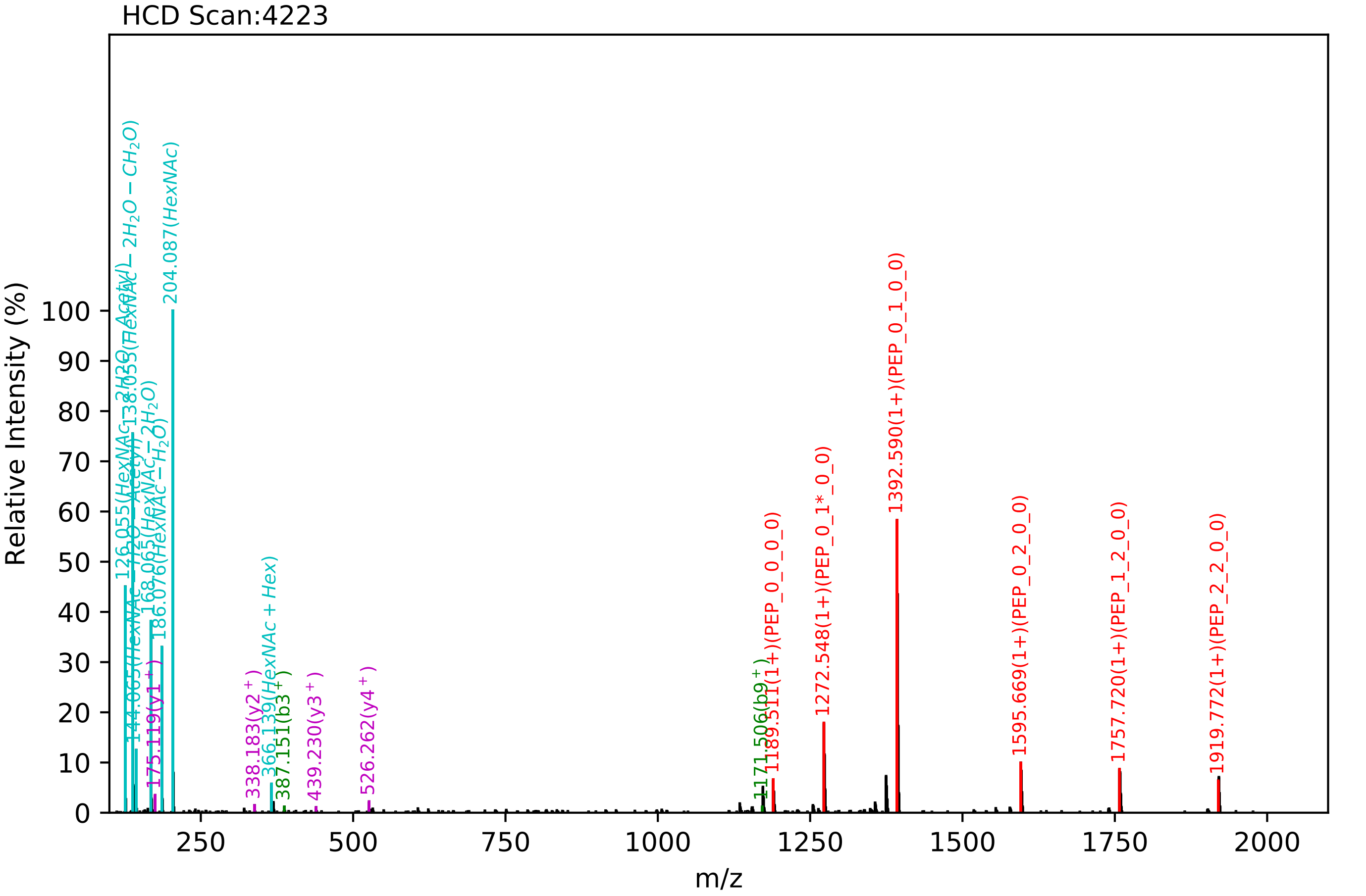

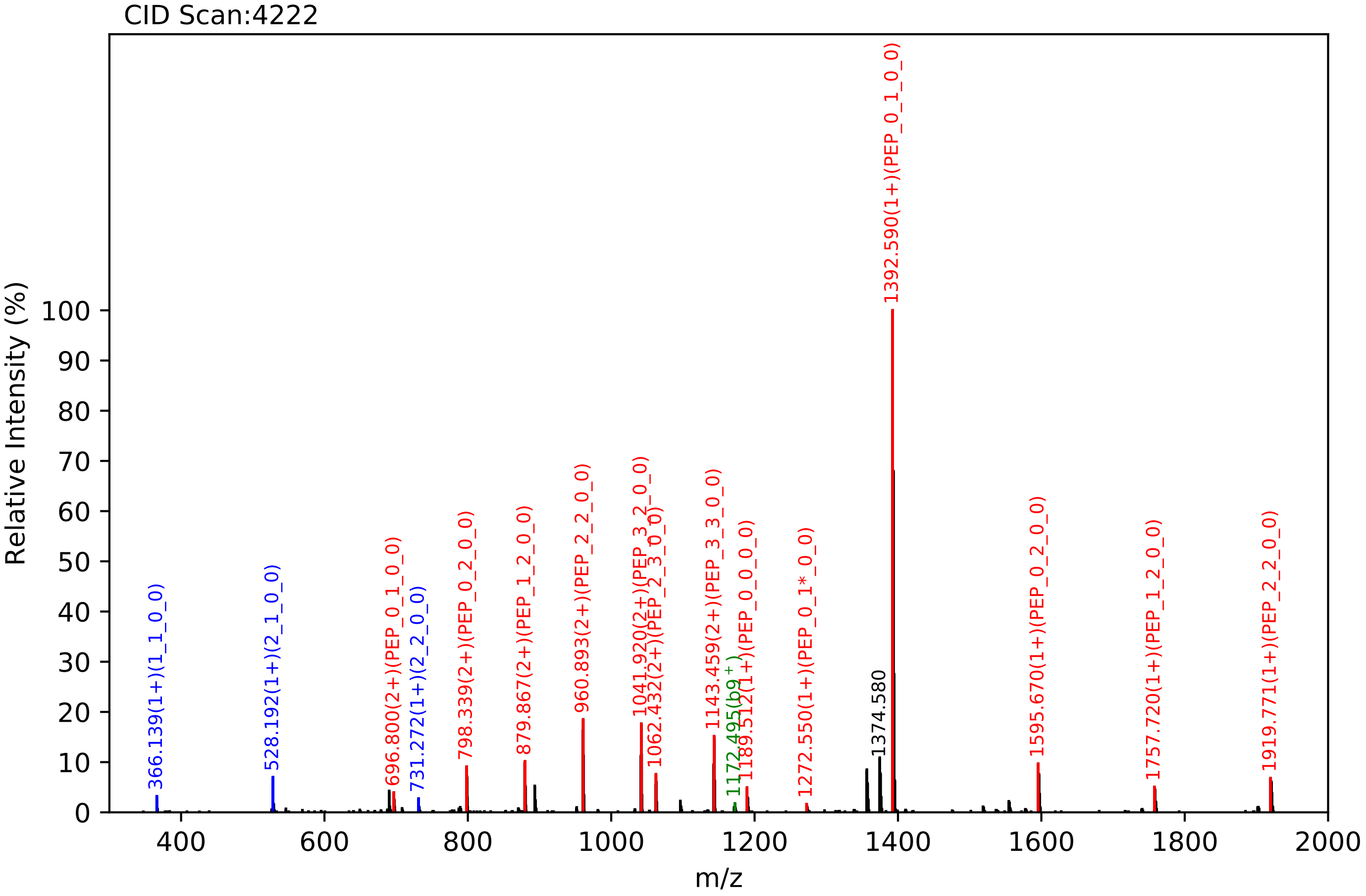

Training set no. 321, Experiment: IgG exp_1

EEQYNSTYR(=PEP)_3_4_0_0, m/z:1244.50(2+), RT:22.28, Y-score:86.45

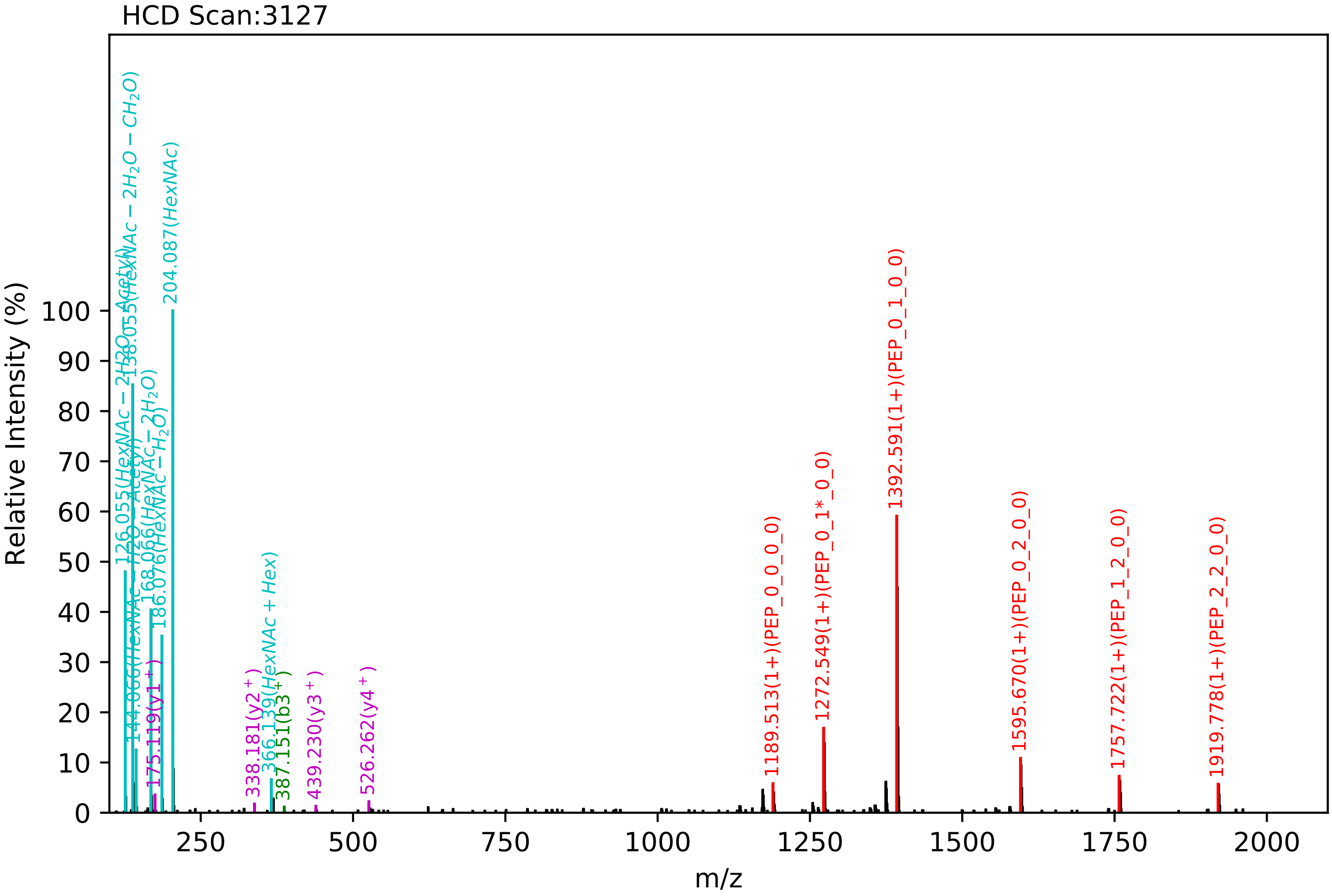

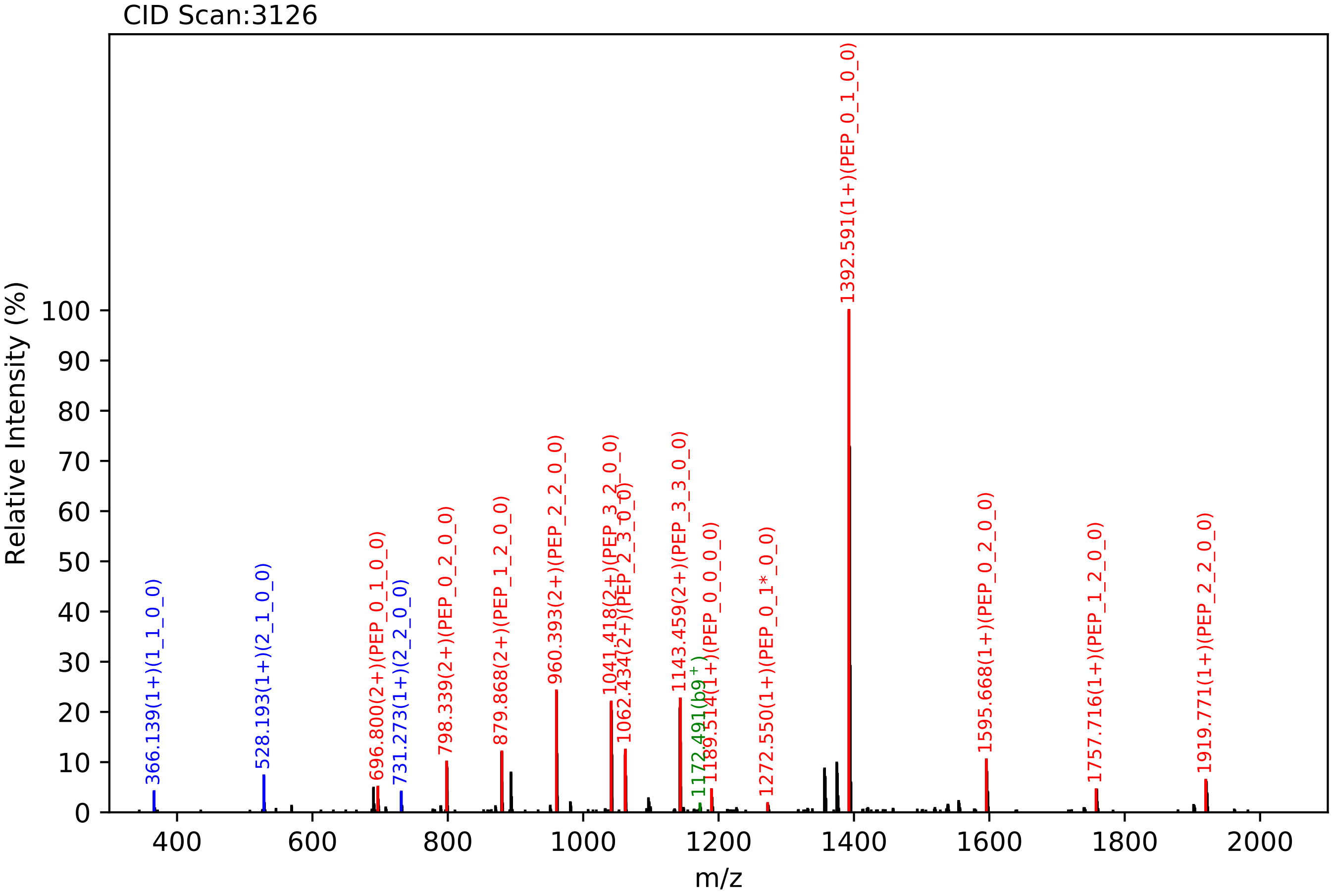

Training set no. 322, Experiment: IgG exp_3

EEQYNSTYR(=PEP)_3_4_1_0, m/z:1317.53(2+), RT:23.09, Y-score:89.03

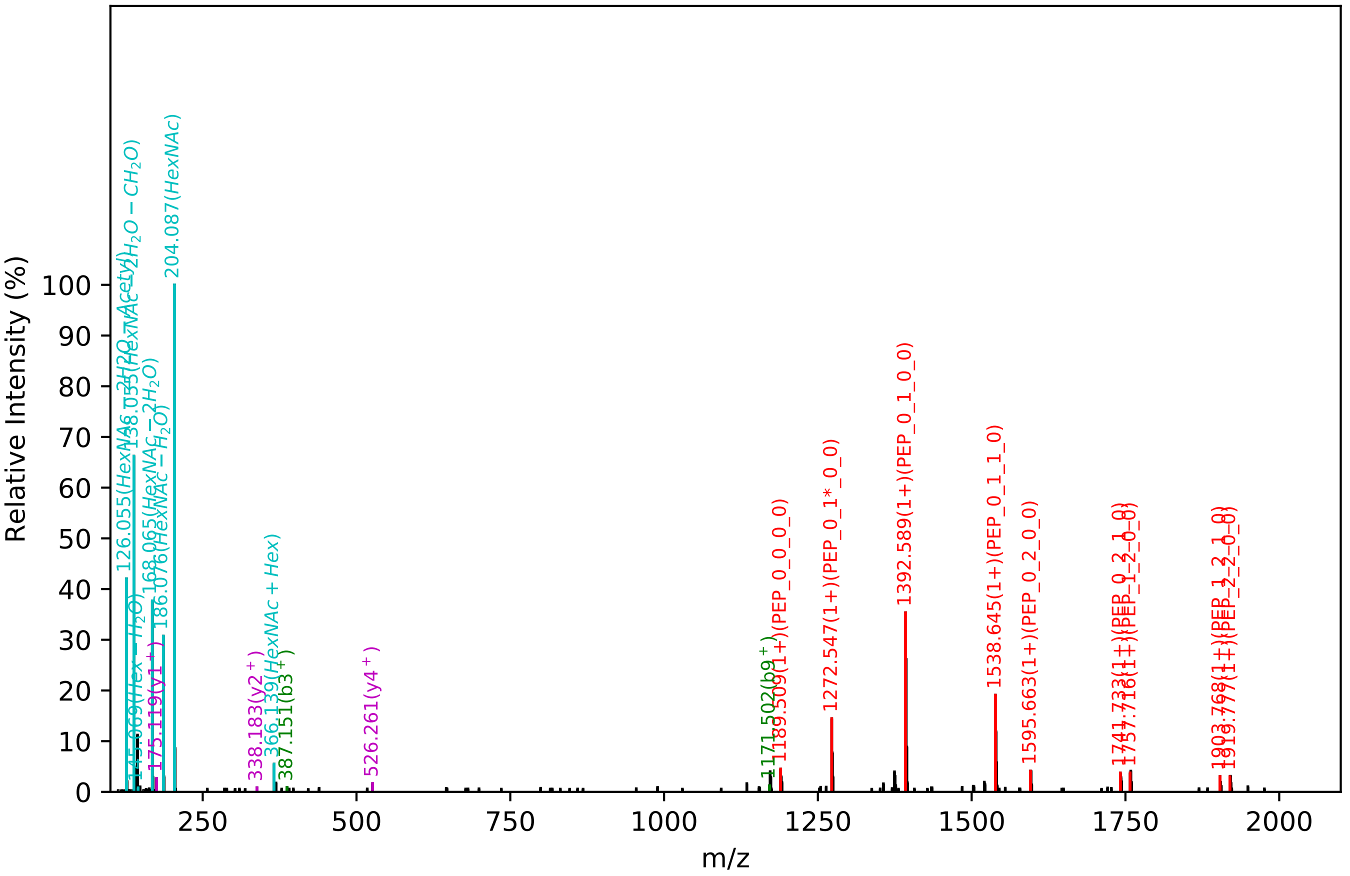

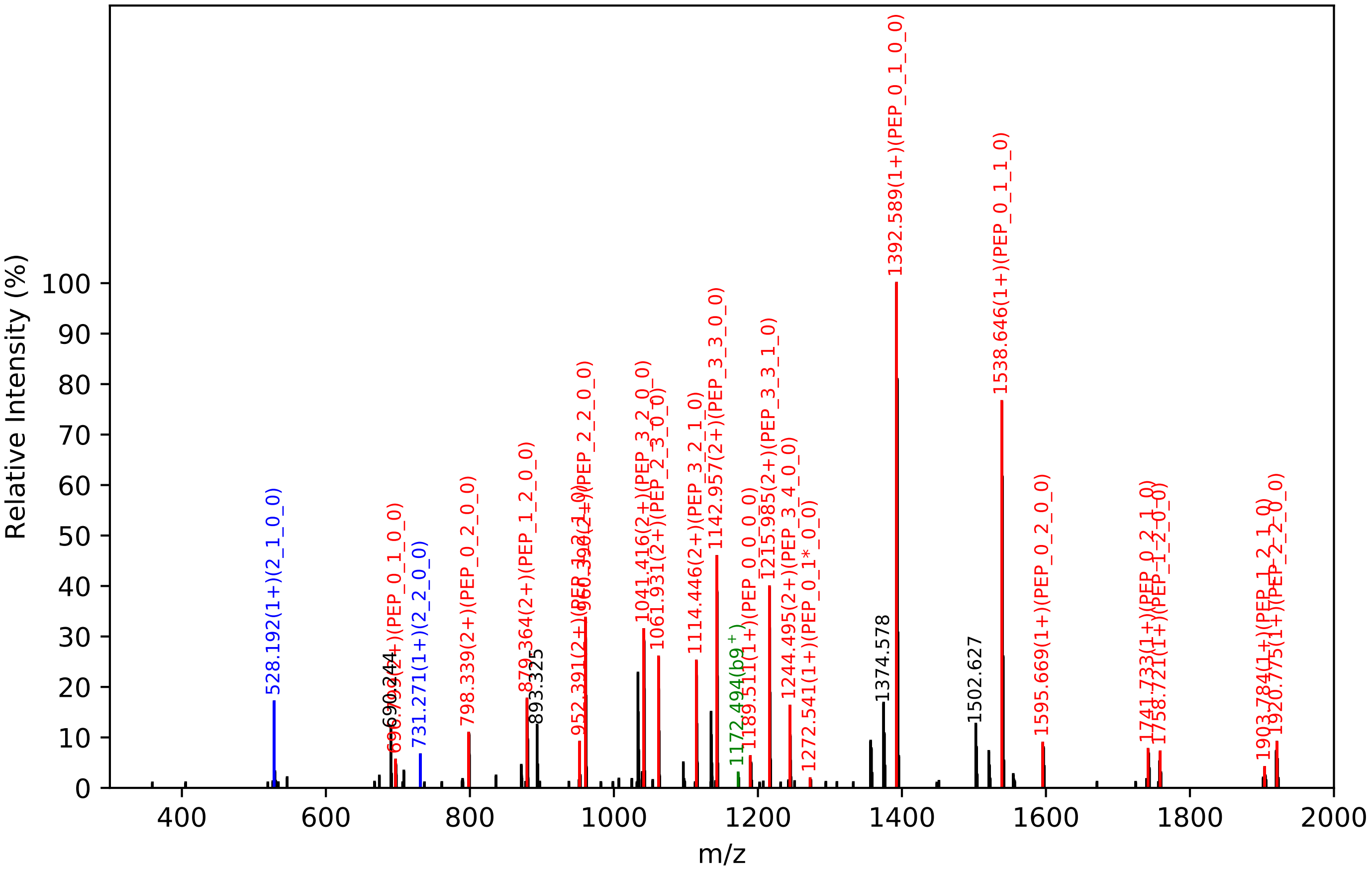

Training set no. 323, Experiment: IgG exp_3

EEQYNSTYR(=PEP)_3_4_1_0, m/z:1317.53(2+), RT:23.63, Y-score:88.86

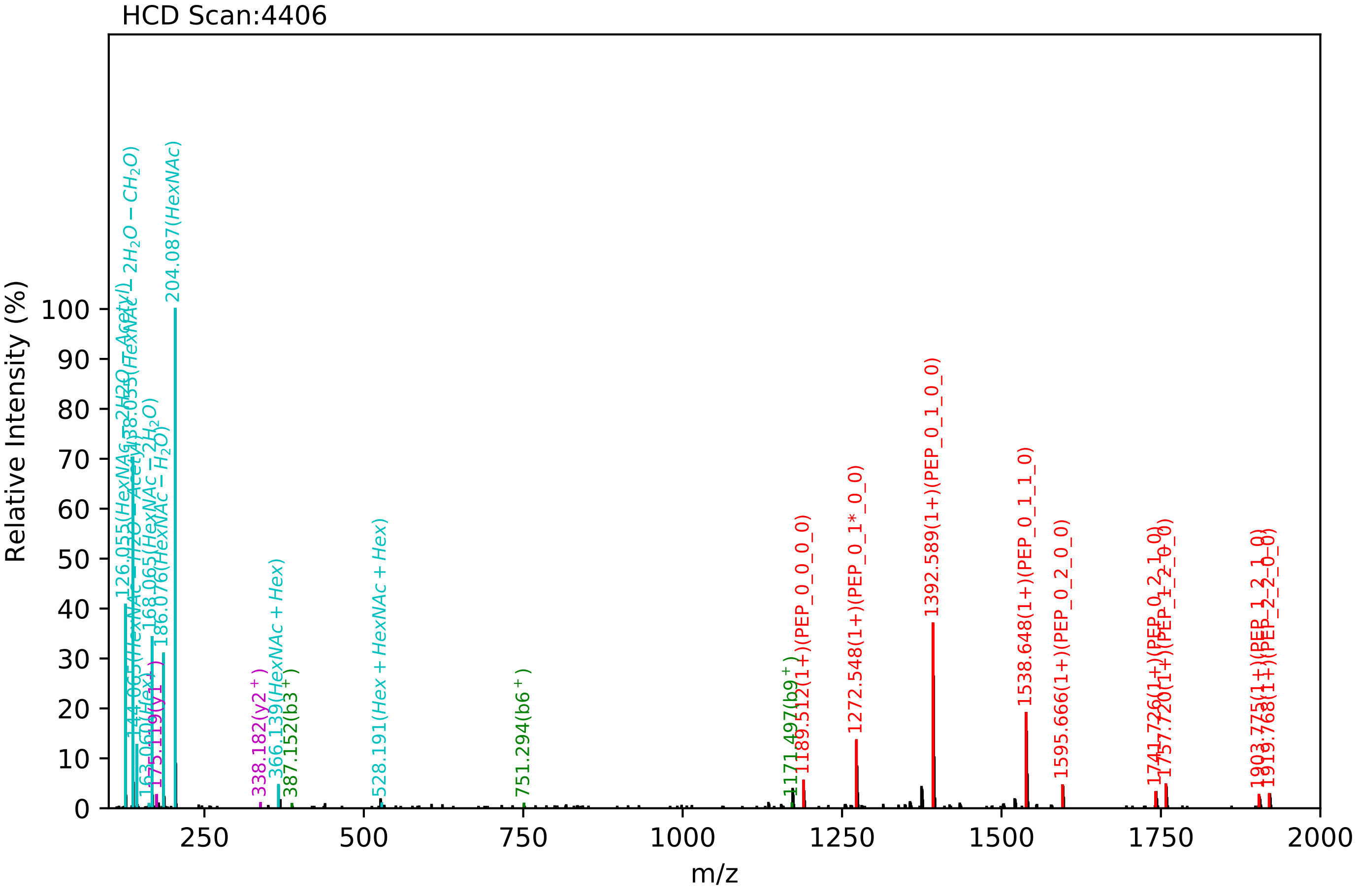

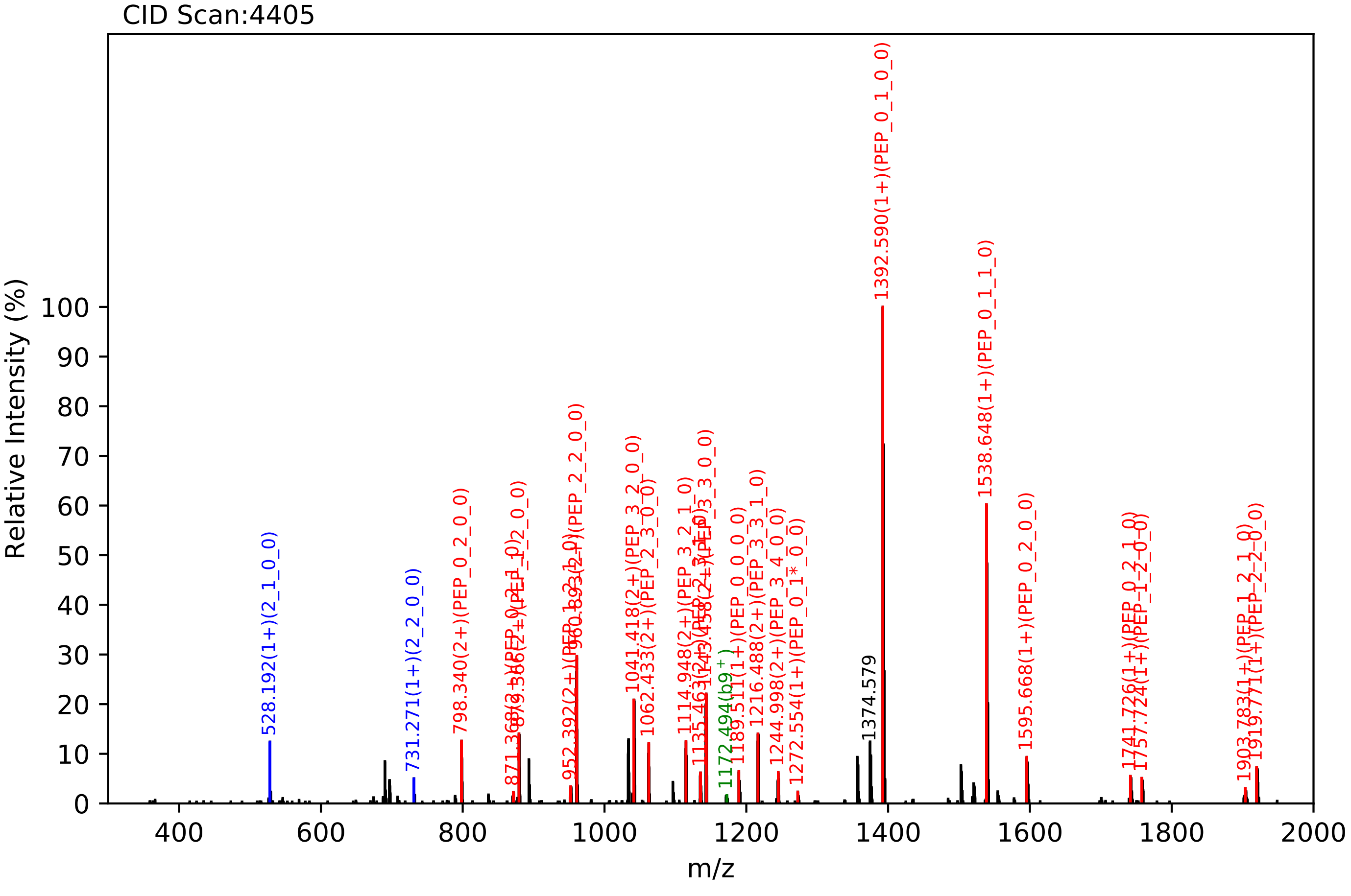

Training set no. 324, Experiment: IgG exp_4

EEQYNSTYR(=PEP)_3_4_1_0, m/z:1317.52(2+), RT:20.76, Y-score:88.44

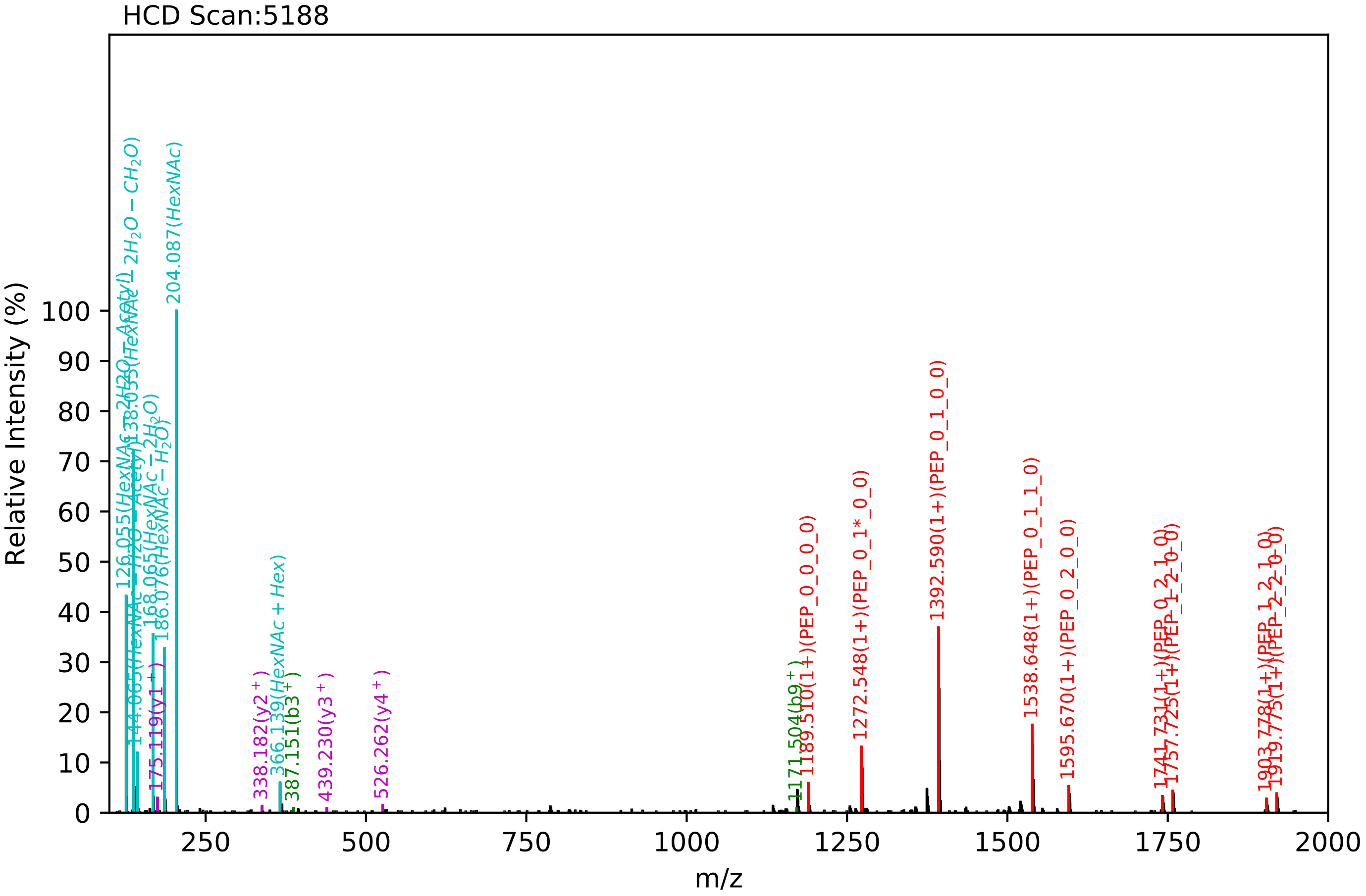

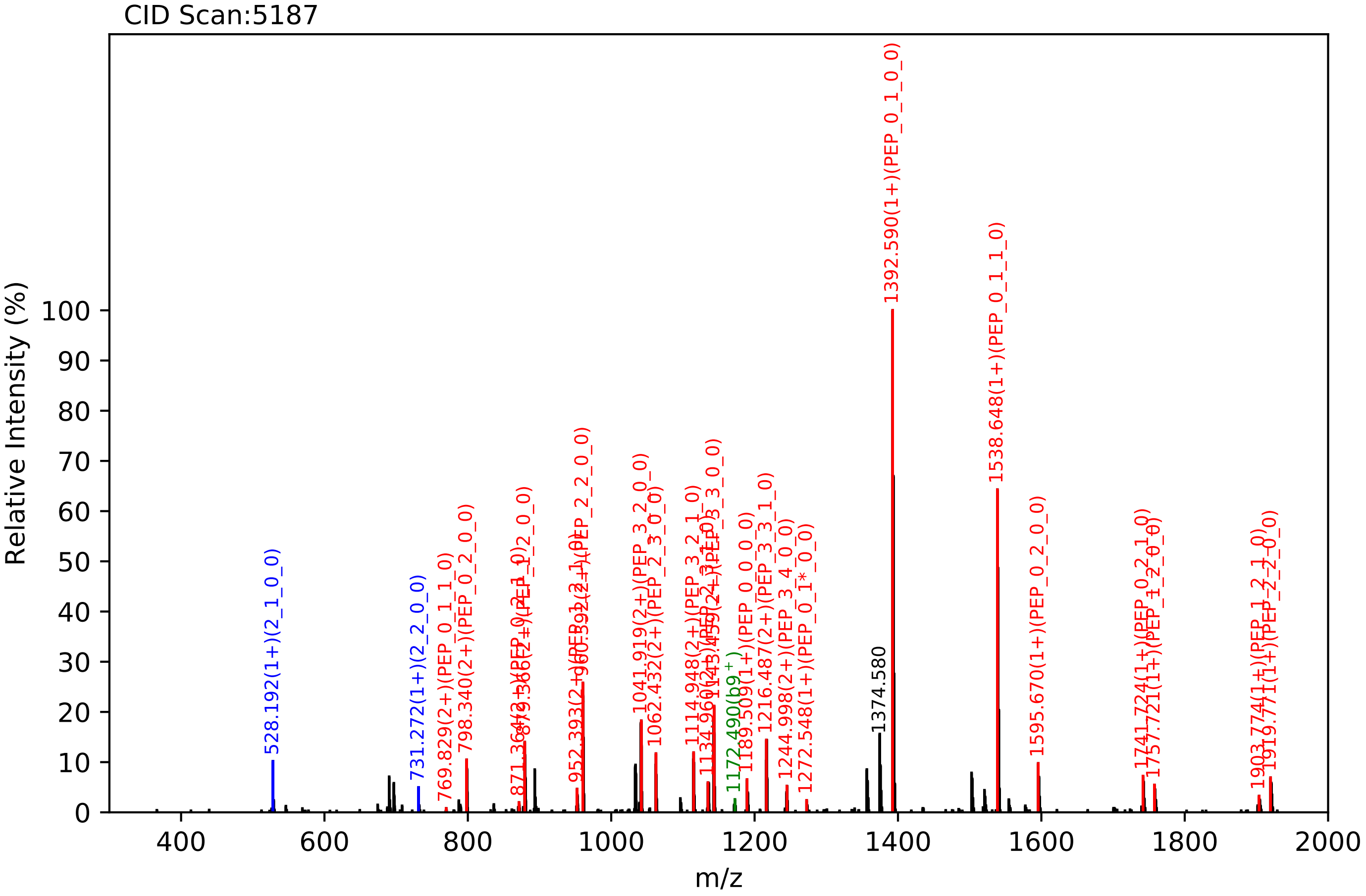

Training set no. 325, Experiment: IgG exp_2

EEQYNSTYR(=PEP)_3_4_1_0, m/z:1317.53(2+), RT:23.75, Y-score:88.34

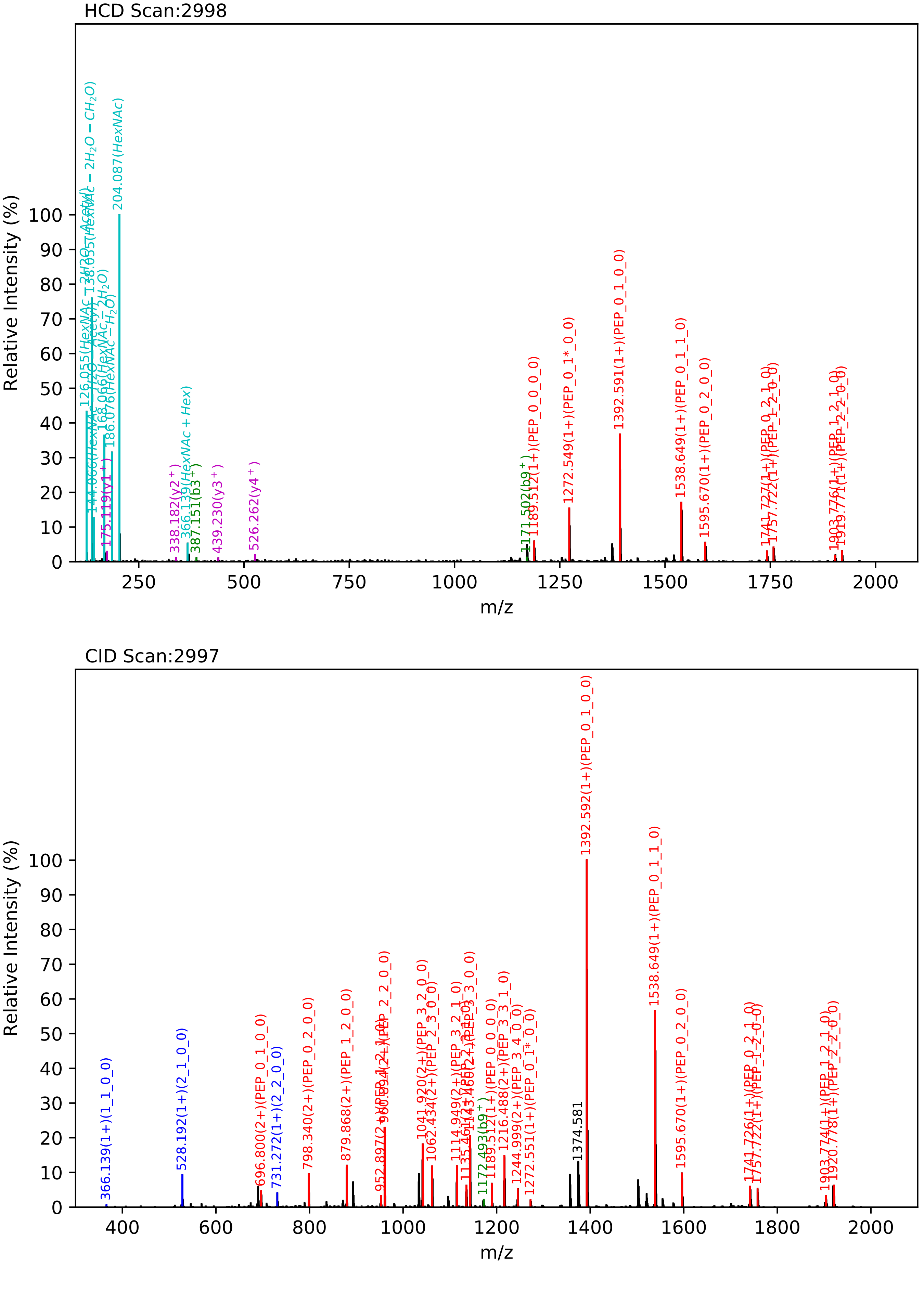

Training set no. 326, Experiment: IgG exp_1

EEQYNSTYR(=PEP)_3_4_1_0, m/z:1317.53(2+), RT:21.95, Y-score:83.86

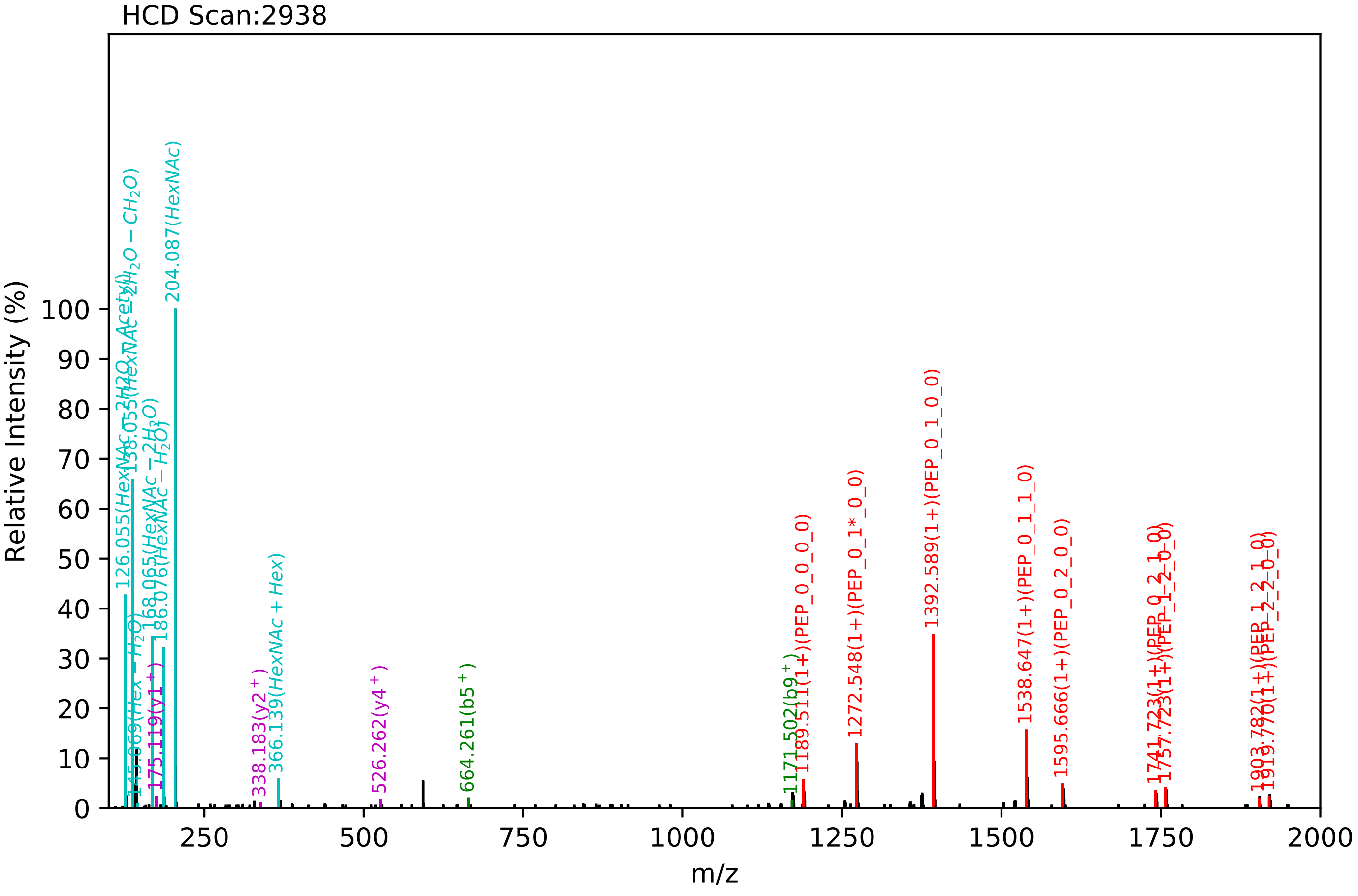

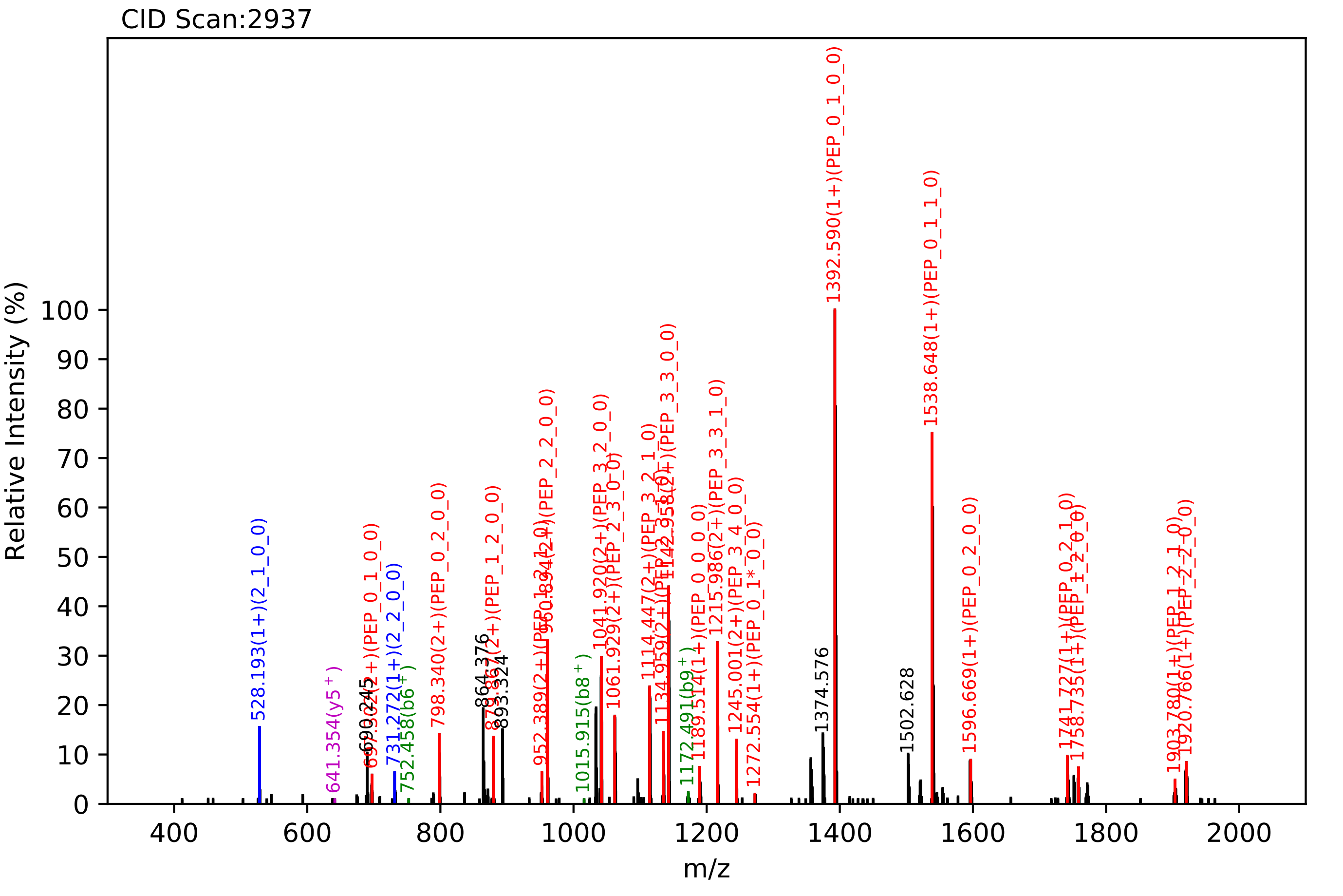

Training set no. 327, Experiment: IgG exp_4

EEQYNSTYR(=PEP)_3_5_0_0, m/z:1346.03(2+), RT:21.25, Y-score:78.06

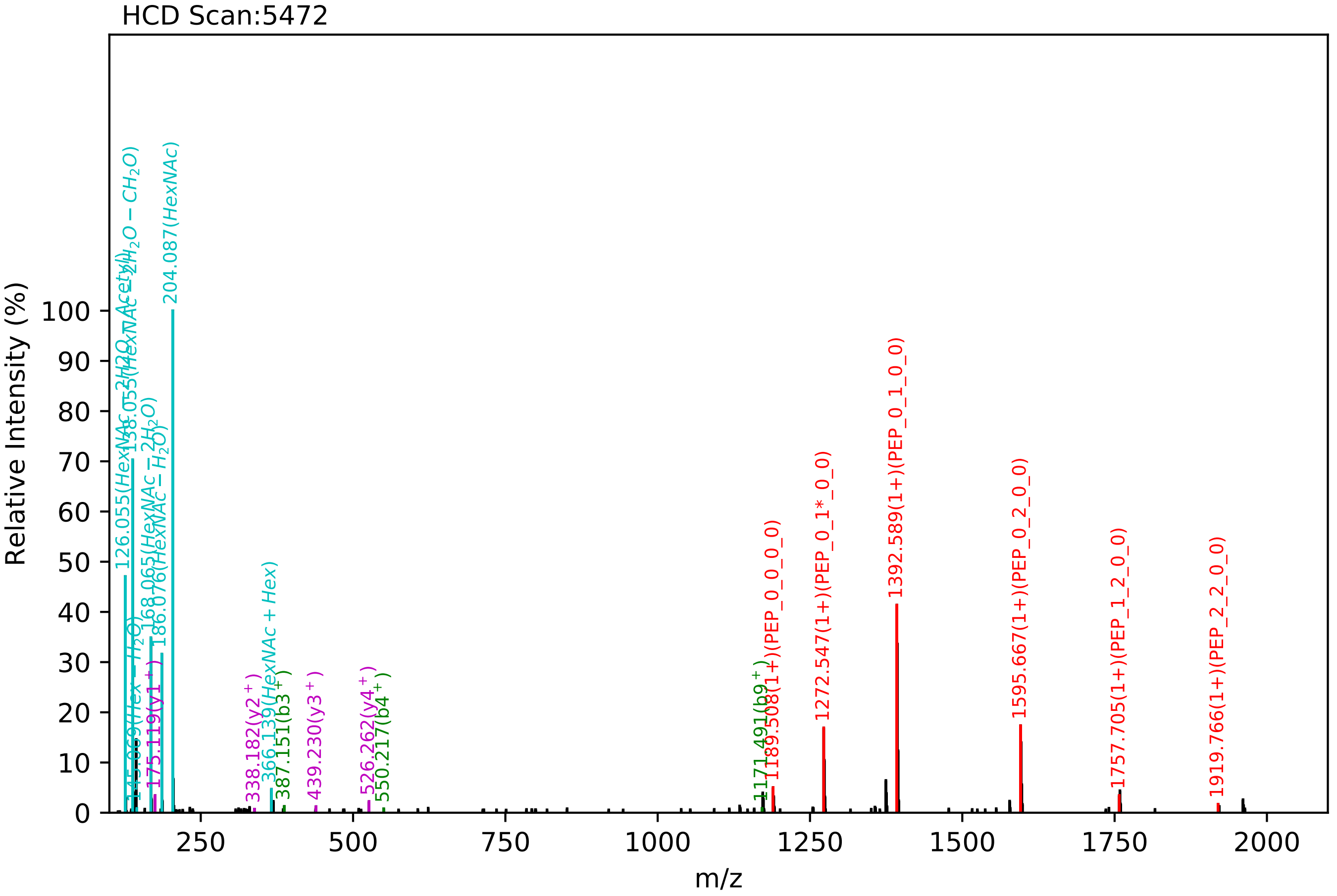

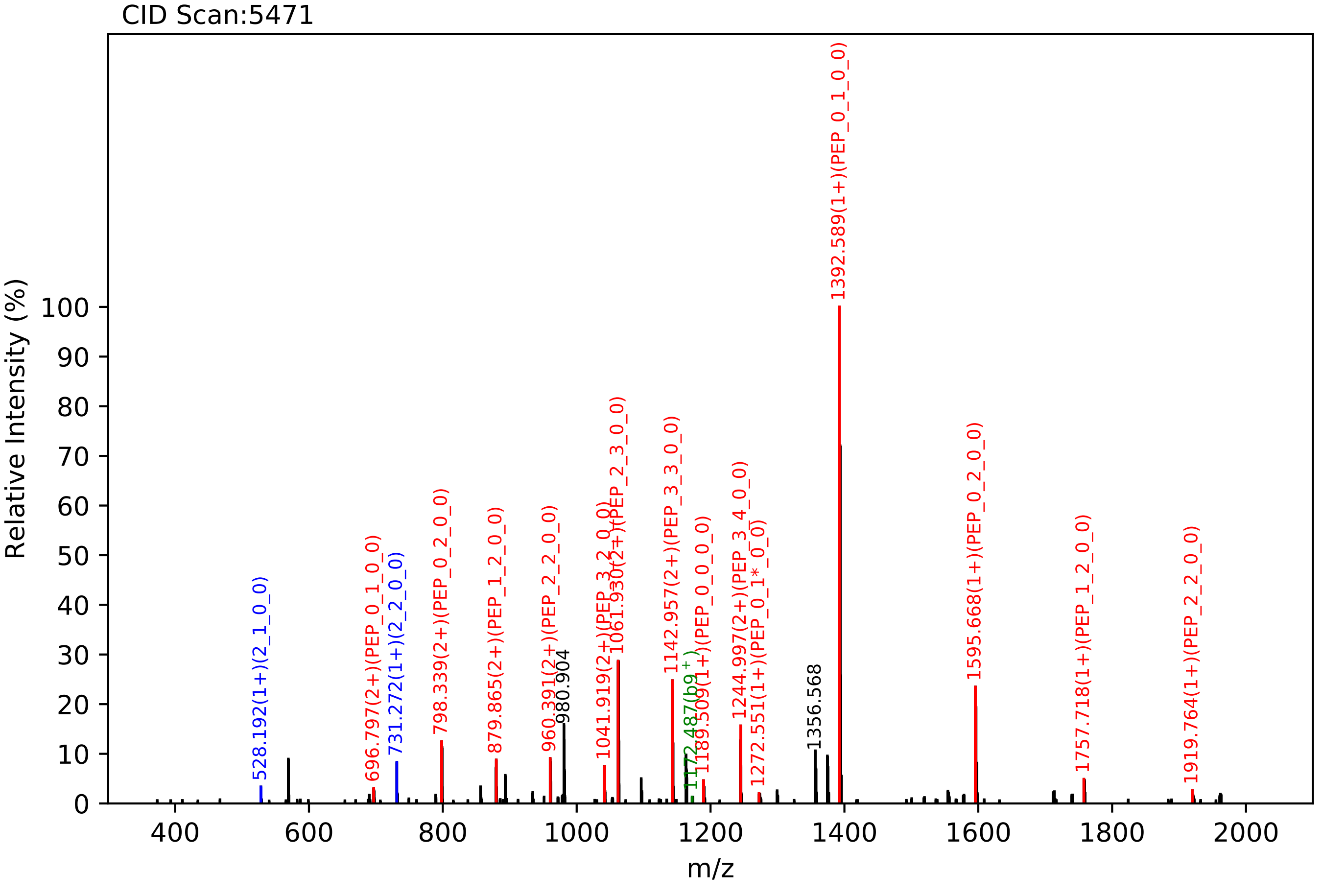

Training set no. 328, Experiment: IgG exp_3

EEQYNSTYR(=PEP)_3_5_1_0, m/z:1419.06(2+), RT:24.64, Y-score:83.98

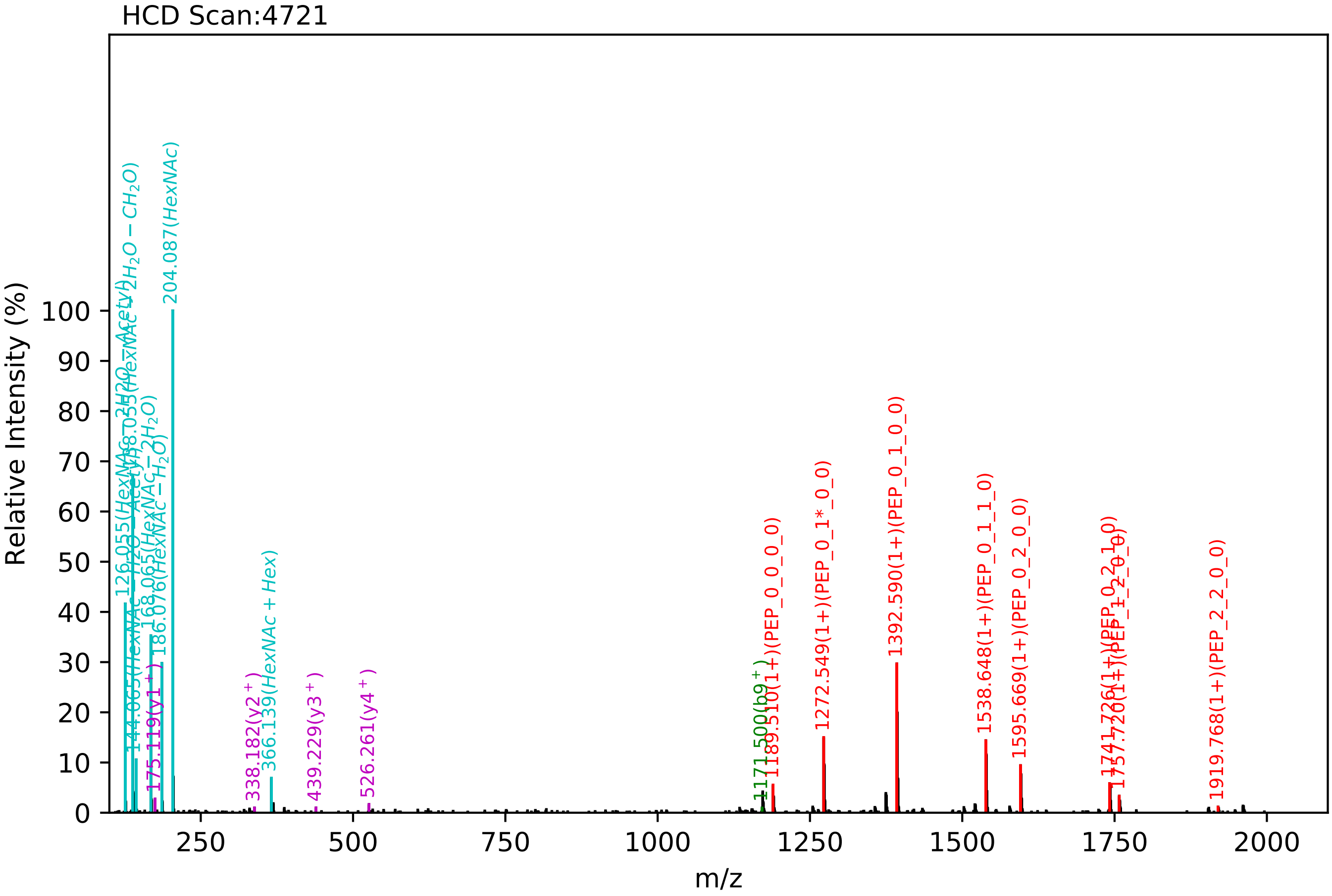

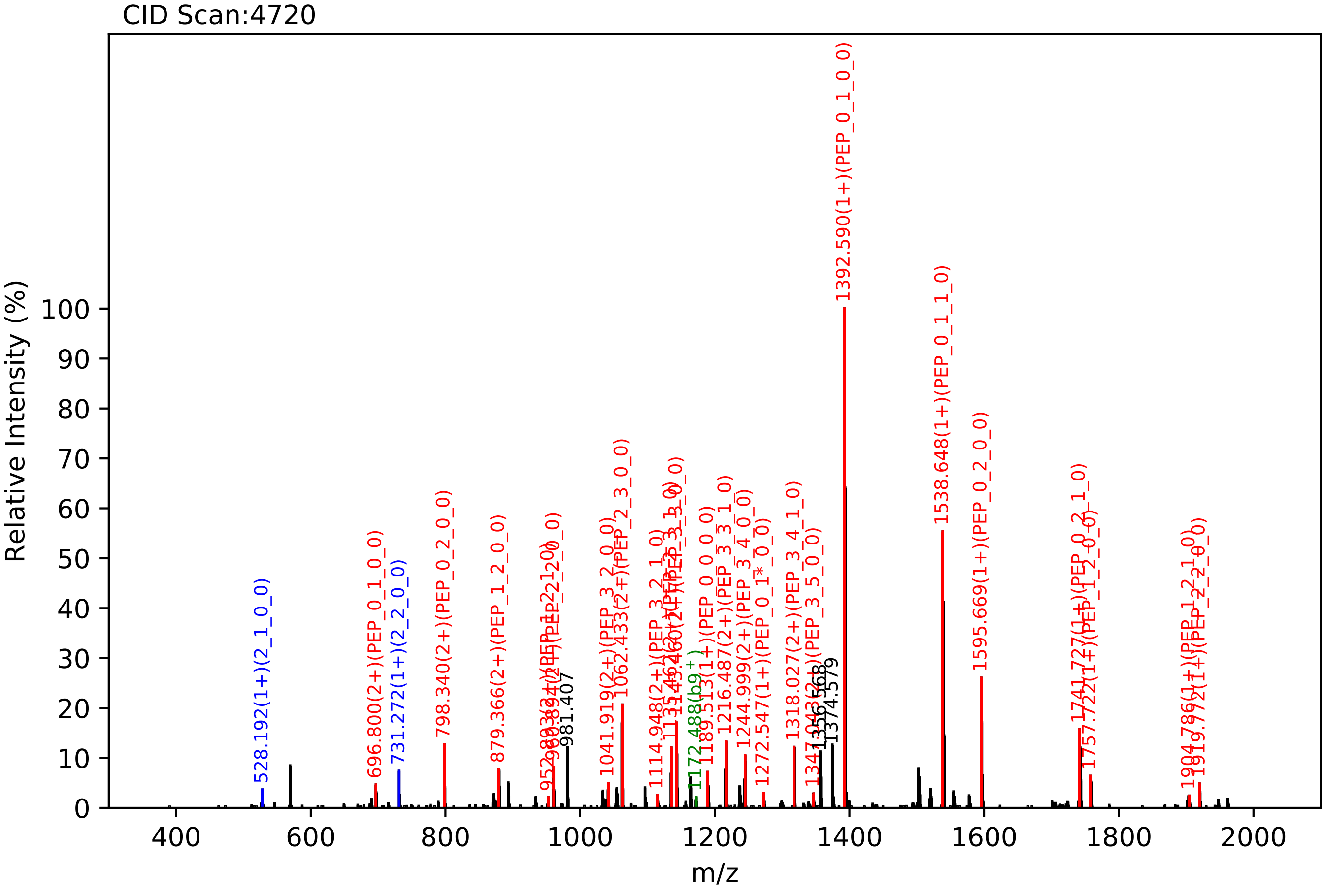

Training set no. 329, Experiment: IgG exp_4

EEQYNSTYR(=PEP)_3_5_1_0, m/z:1419.07(2+), RT:21.19, Y-score:82.11

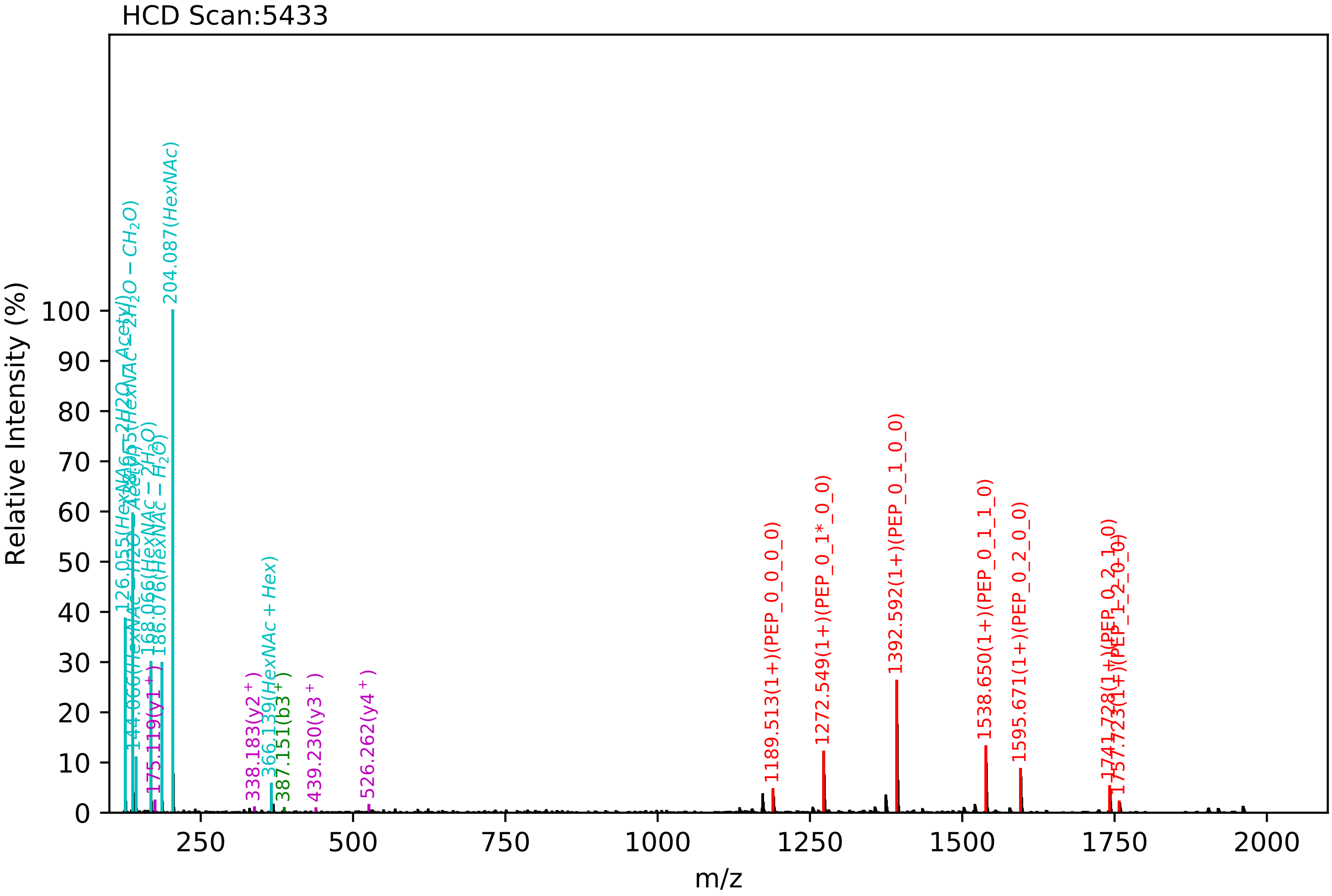

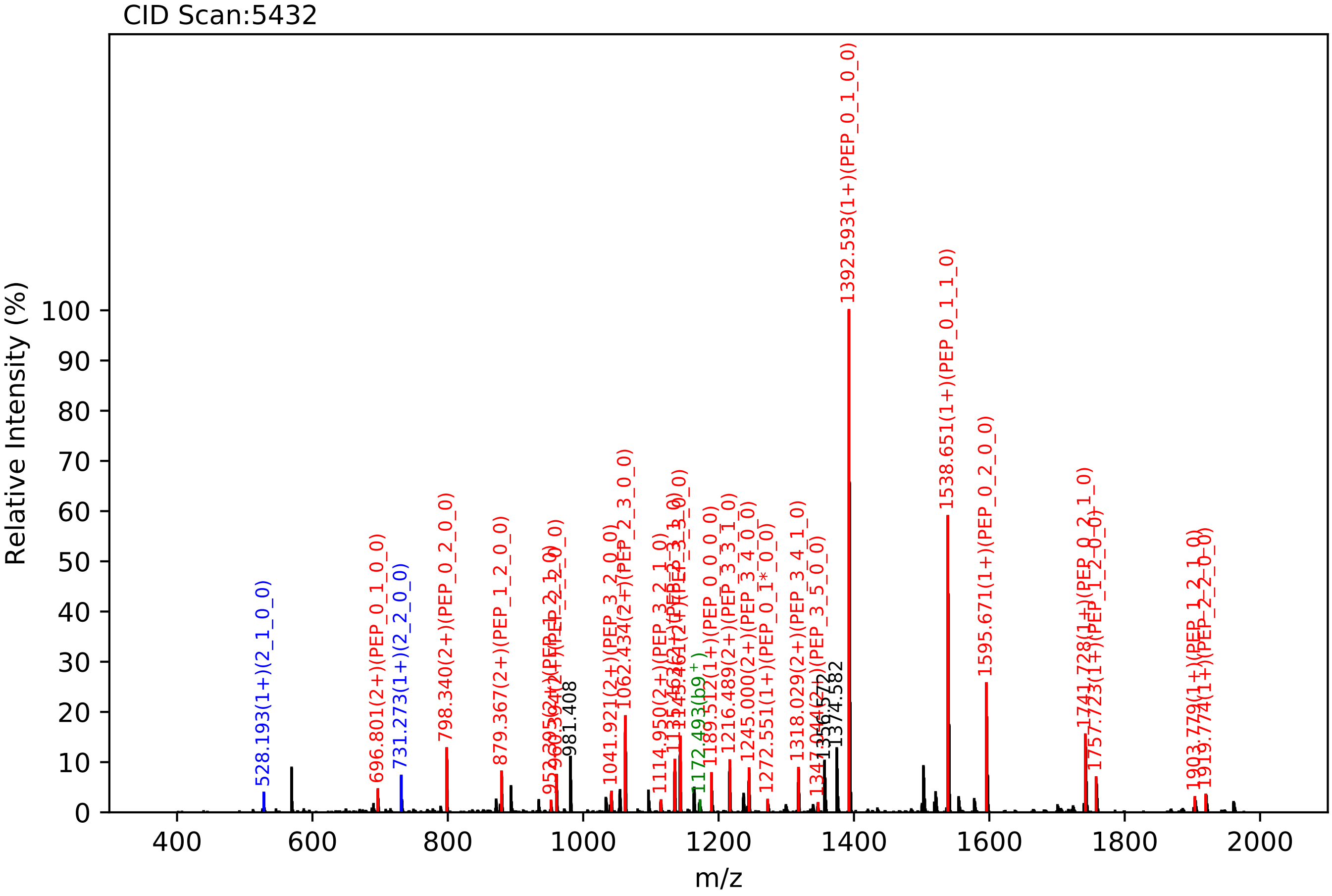

Training set no. 330, Experiment: IgG exp_1

EEQYNSTYR(=PEP)_3_5_1_0, m/z:1419.07(2+), RT:22.32, Y-score:81.08

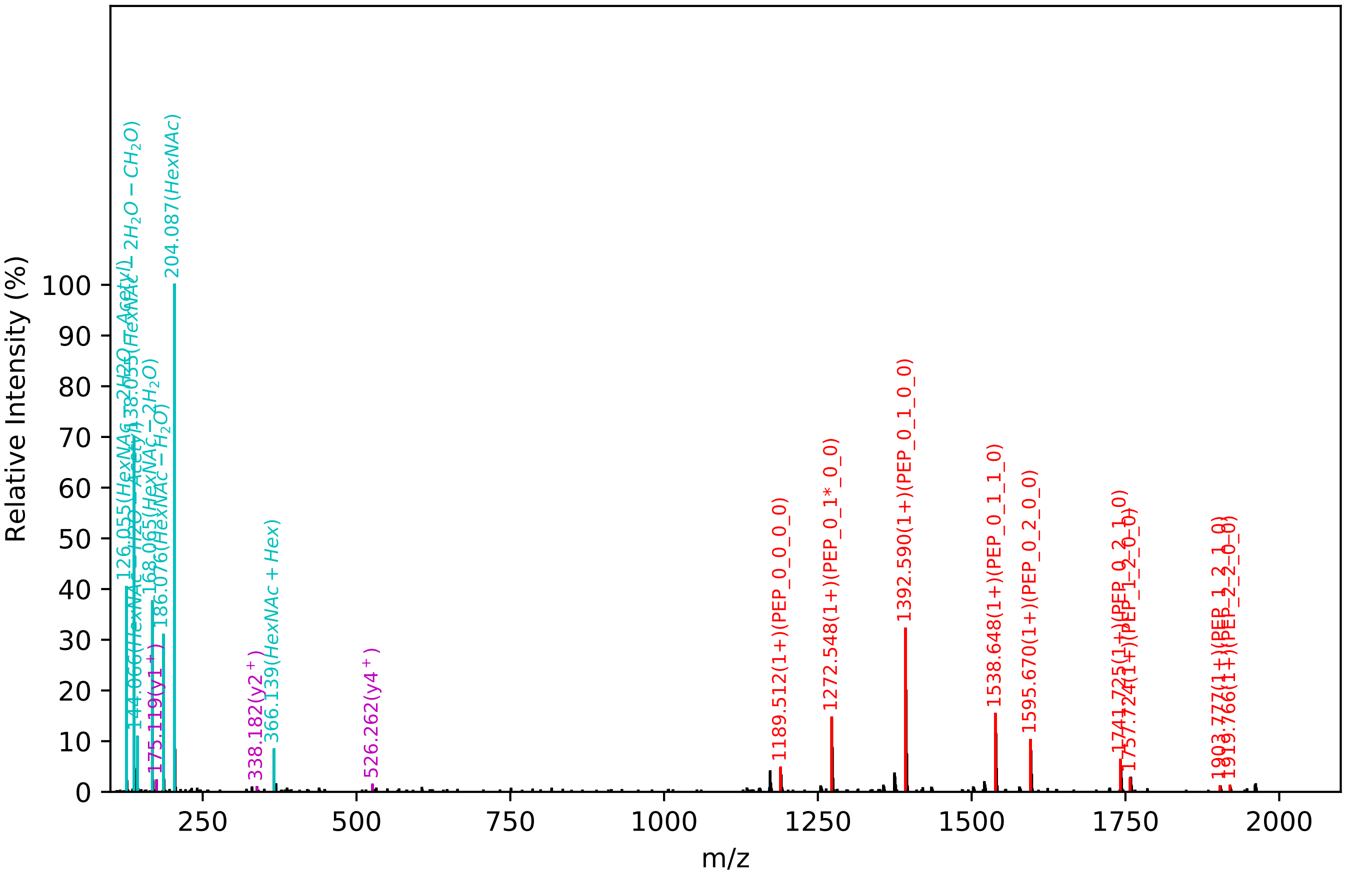

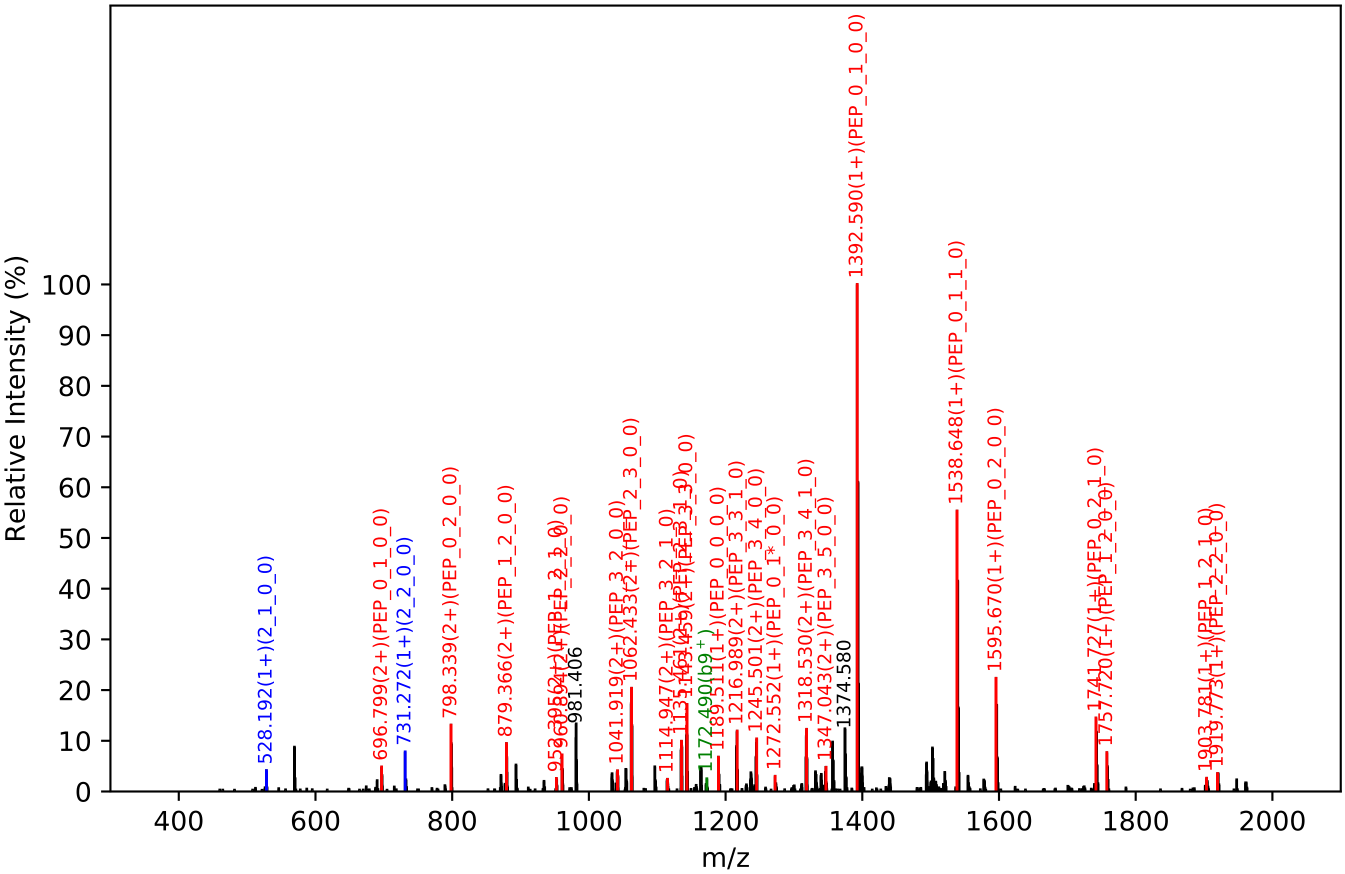

Training set no. 331, Experiment: IgG exp_1

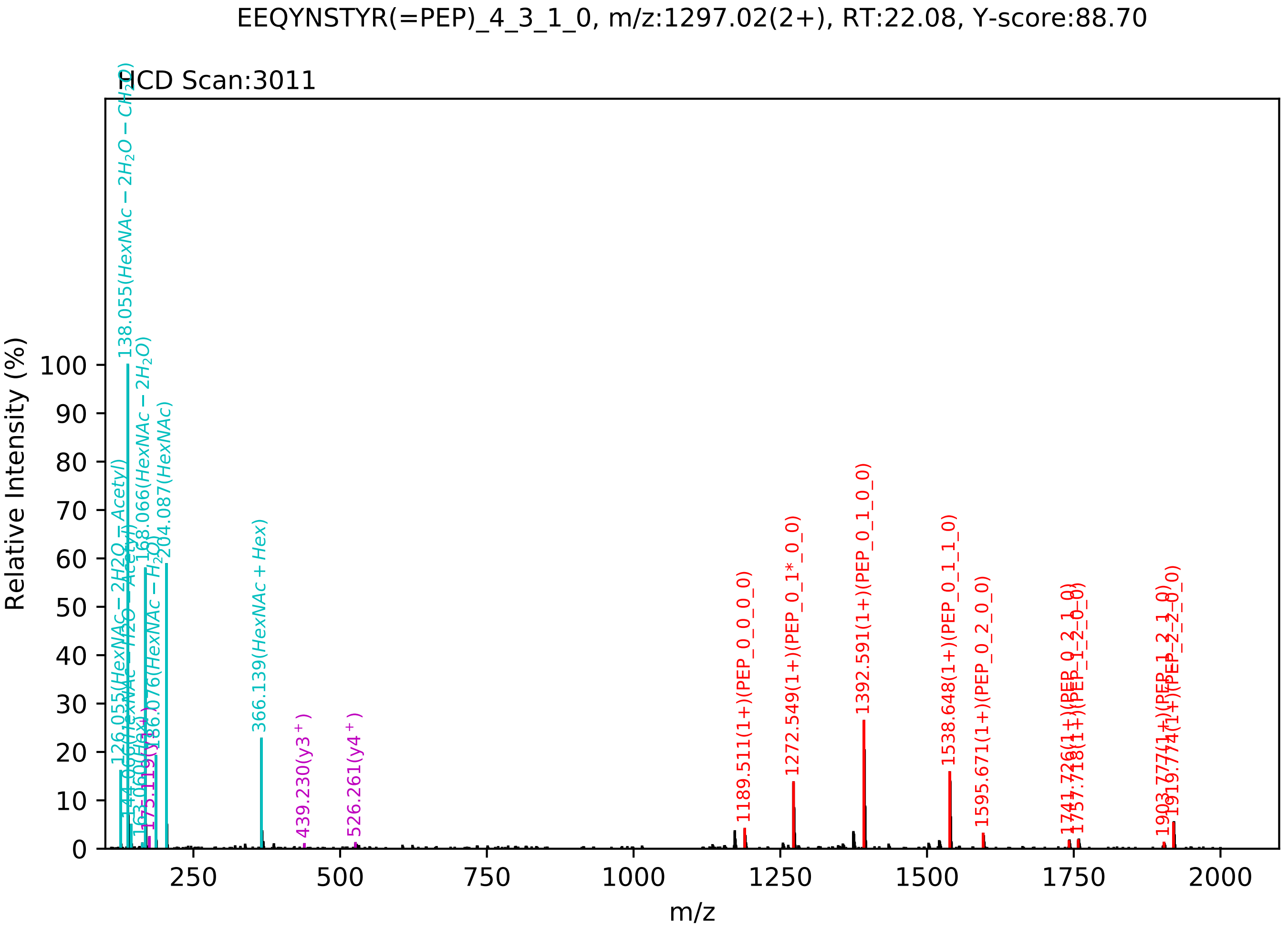

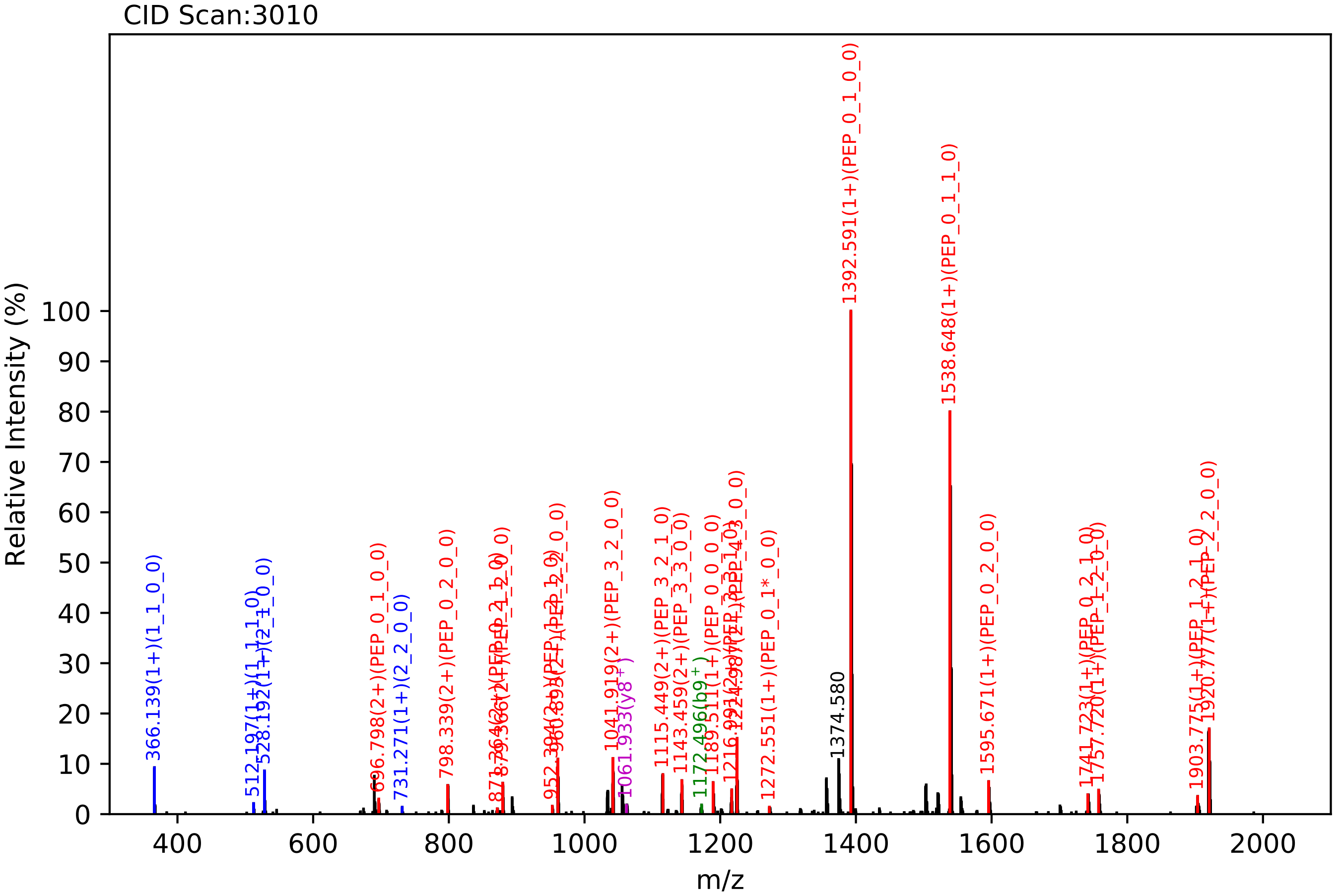

Training set no. 332, Experiment: IgG exp_4

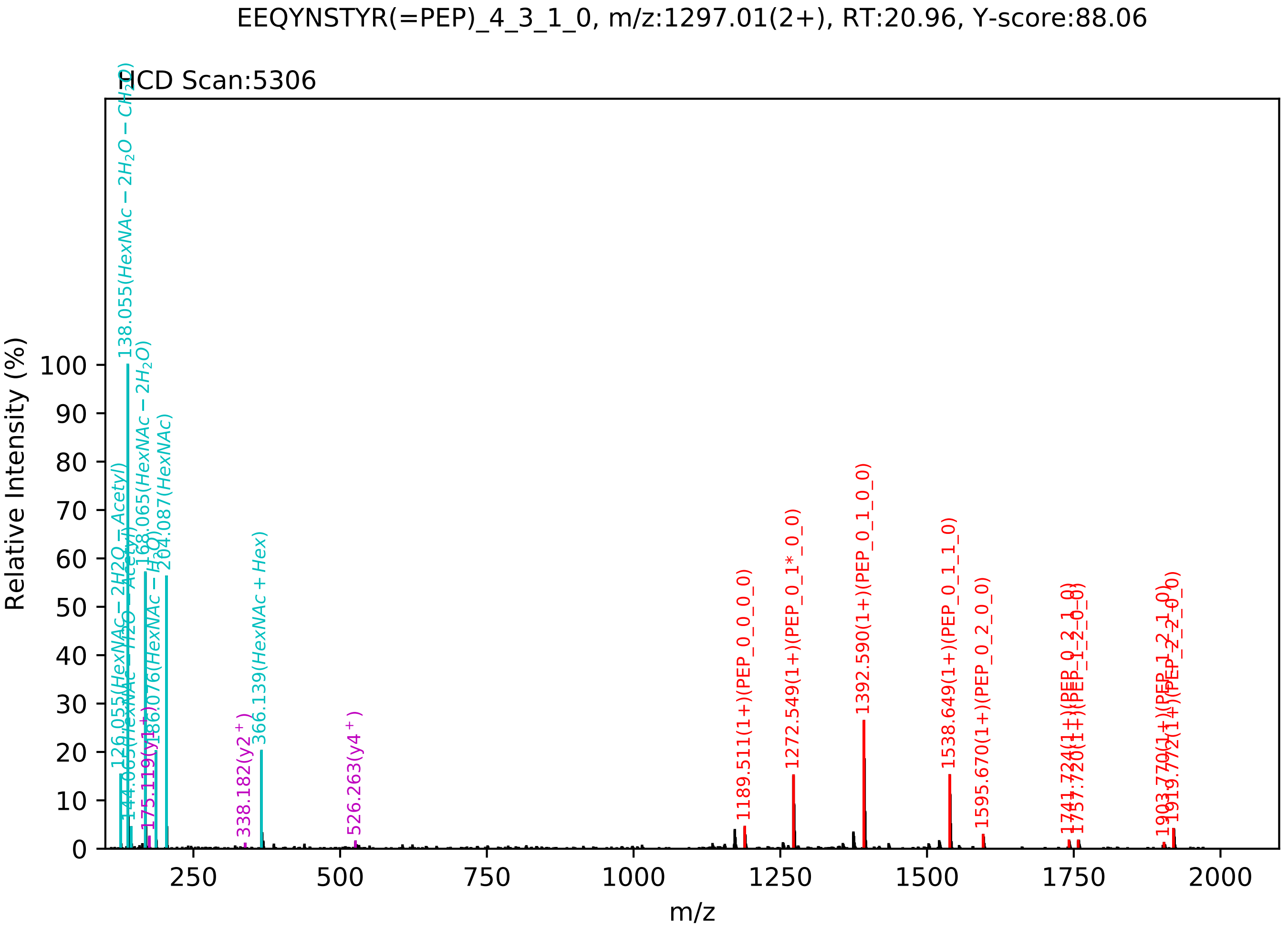

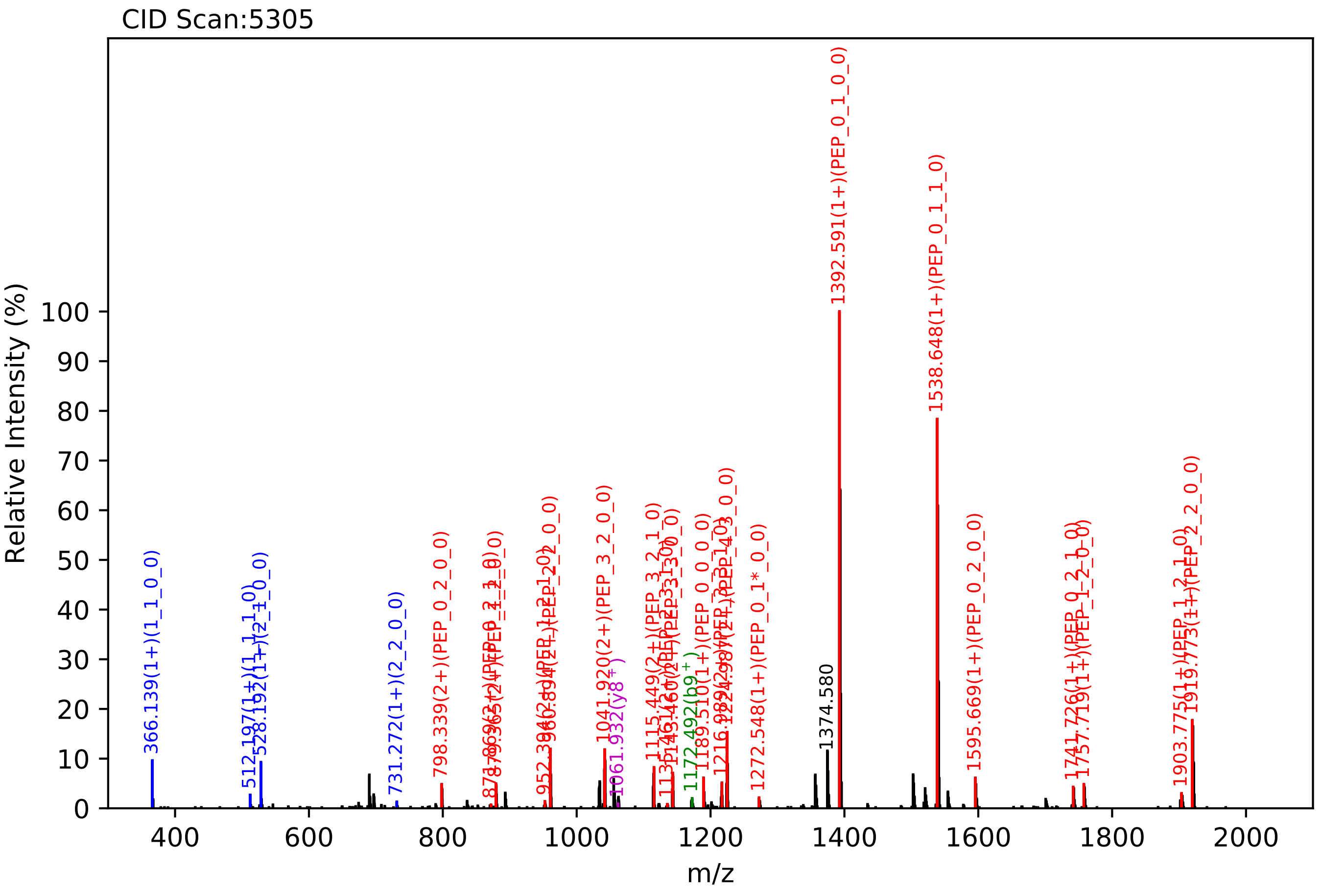

Training set no. 333, Experiment: IgG exp_3

EEQYNSTYR(=PEP)_4_3_1_0, m/z:1297.01(2+), RT:23.23, Y-score:87.86

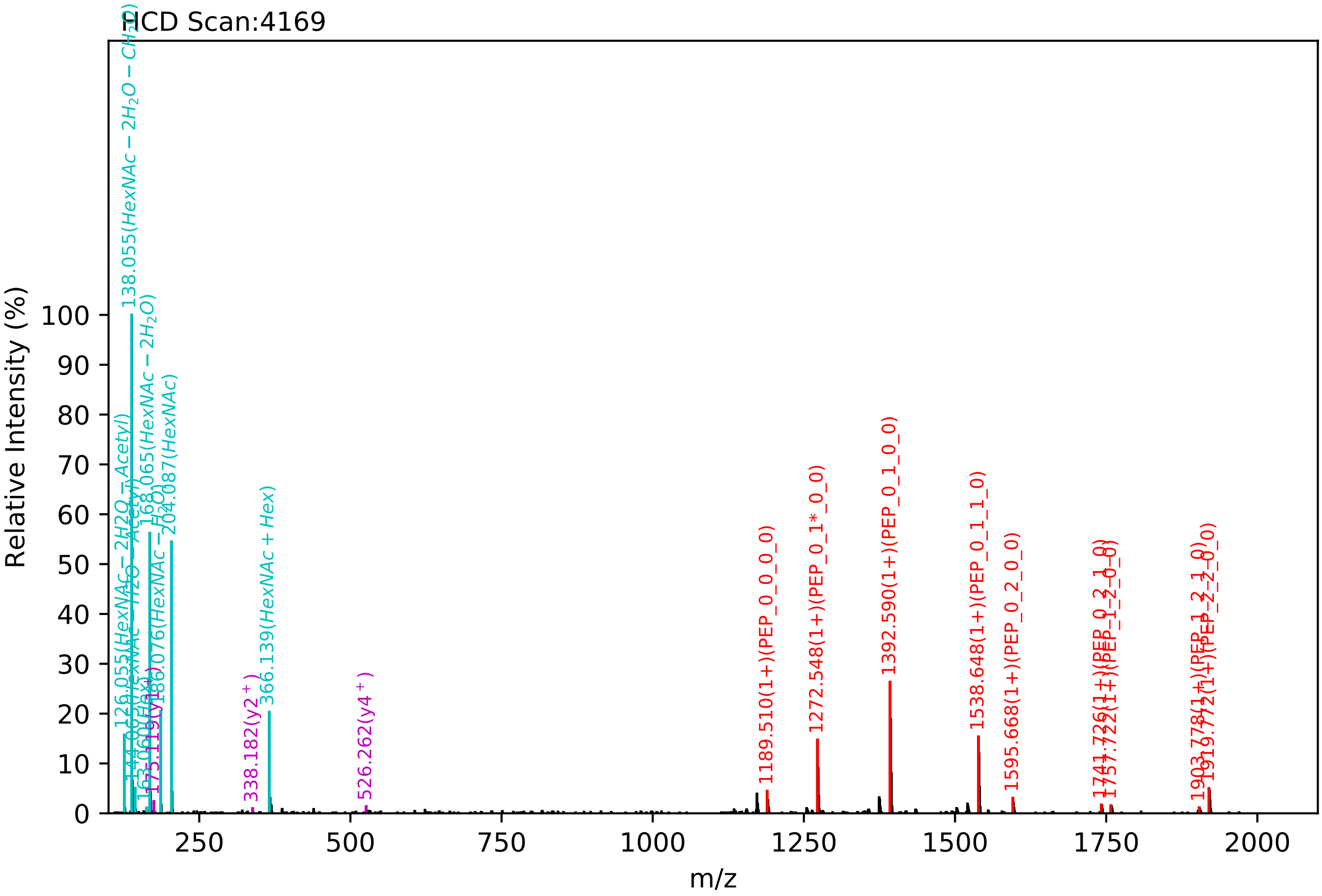

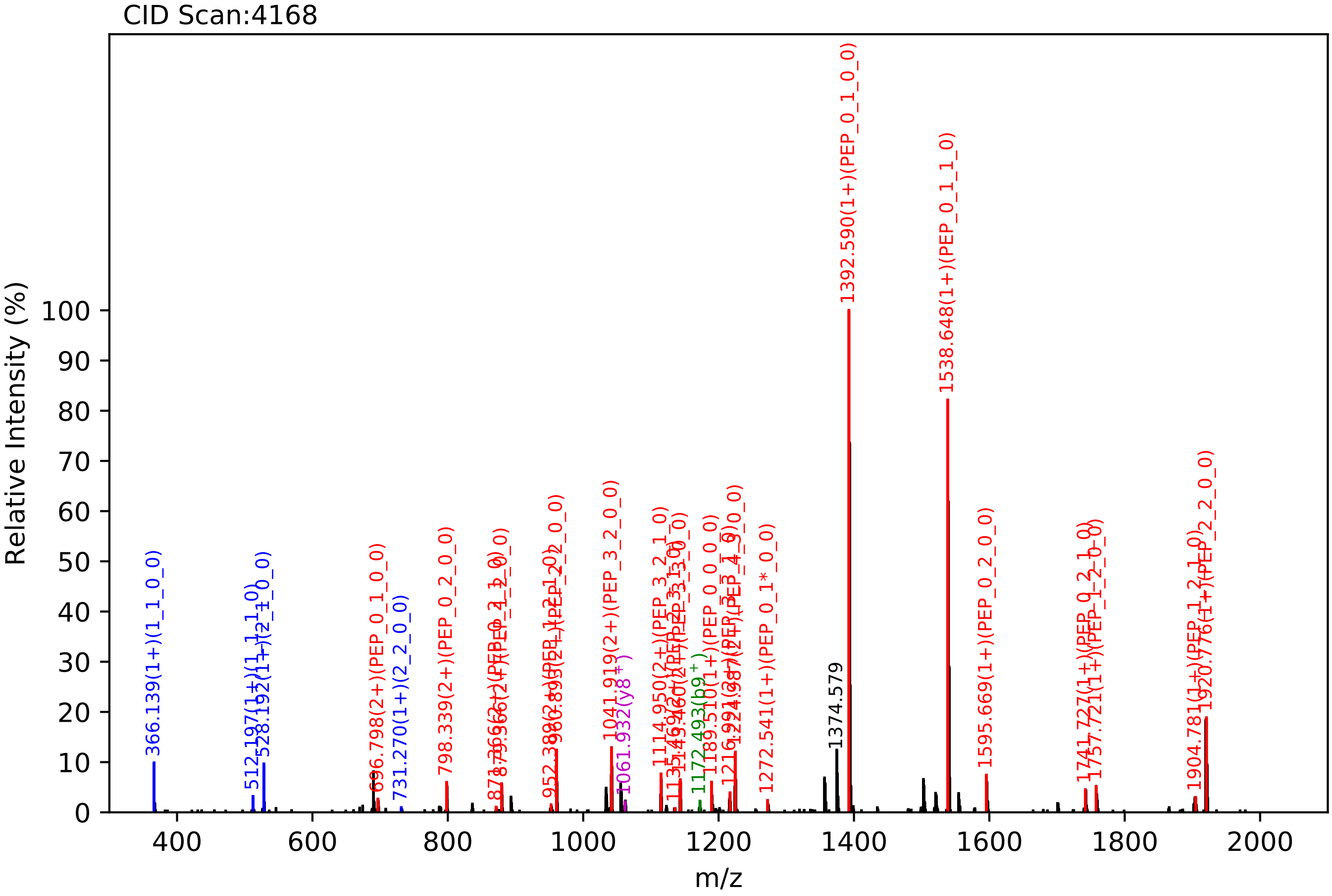

Training set no. 334, Experiment: IgG exp_3

EEQYNSTYR(=PEP)_4_4_0_0, m/z:1325.52(2+), RT:23.28, Y-score:87.85

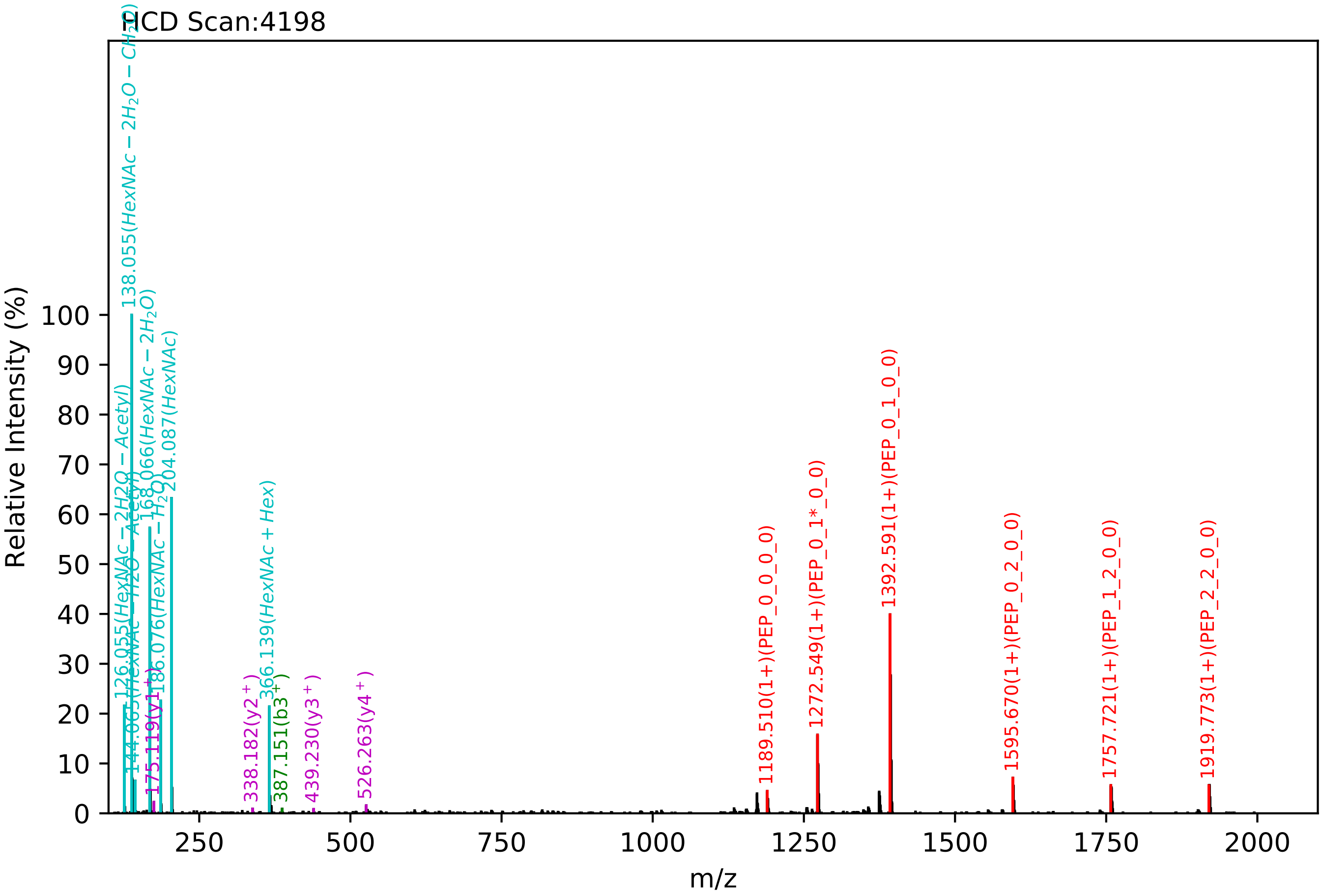

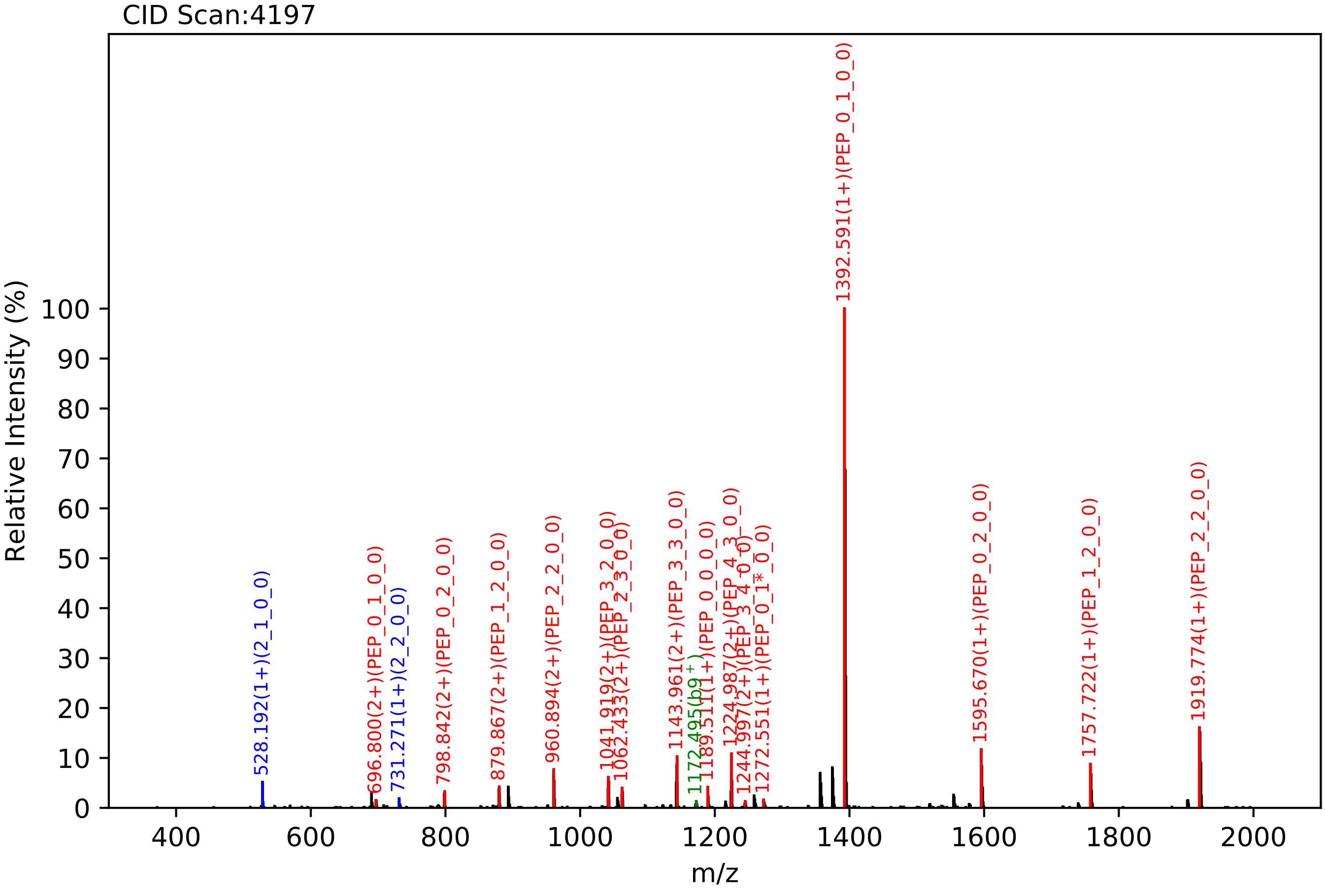

Training set no. 335, Experiment: IgG exp_1

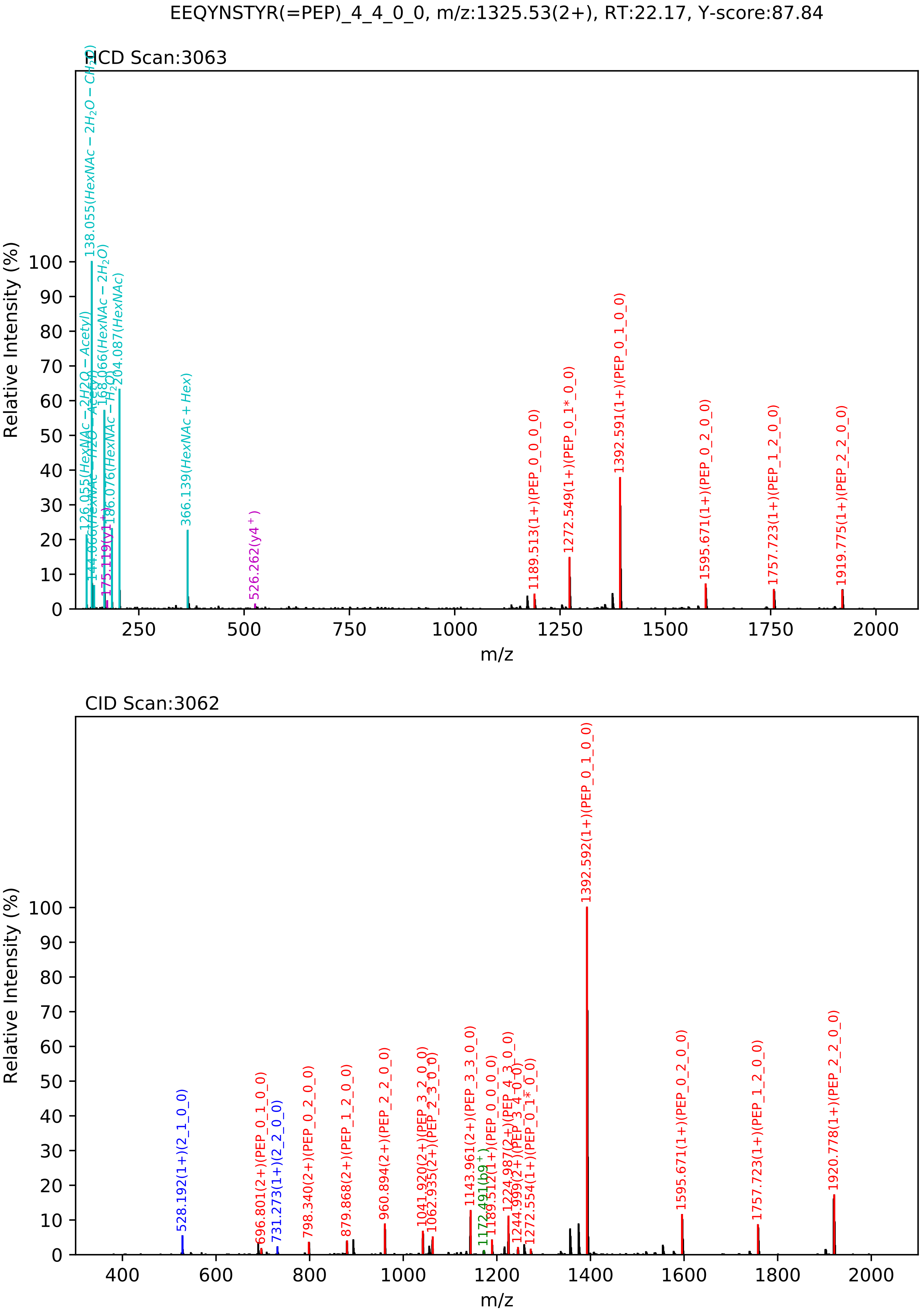

Training set no. 336, Experiment: IgG exp_4

EEQYNSTYR(=PEP)_4_4_0_0, m/z:1325.52(2+), RT:20.99, Y-score:87.66

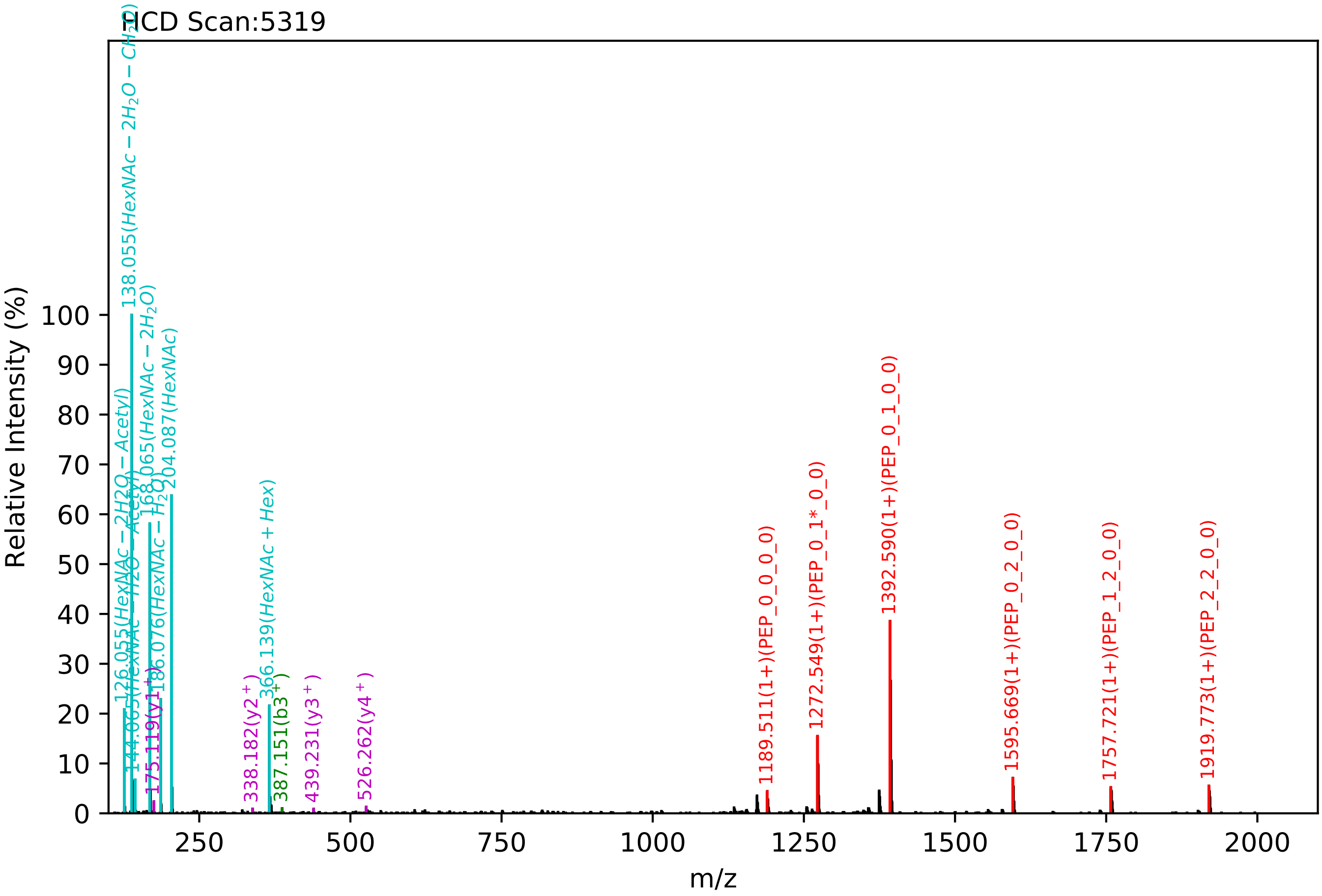

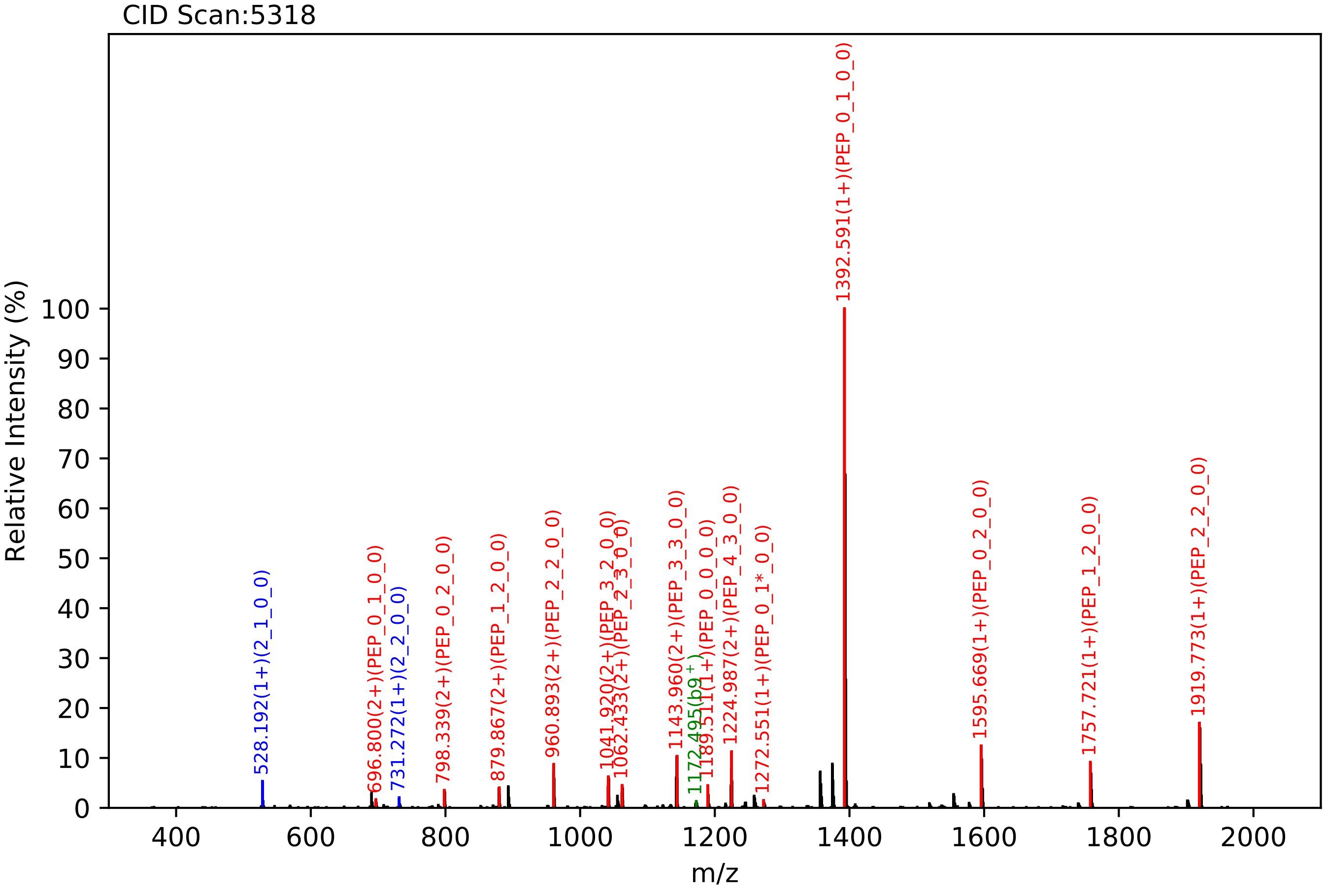

Training set no. 337, Experiment: IgG exp_4

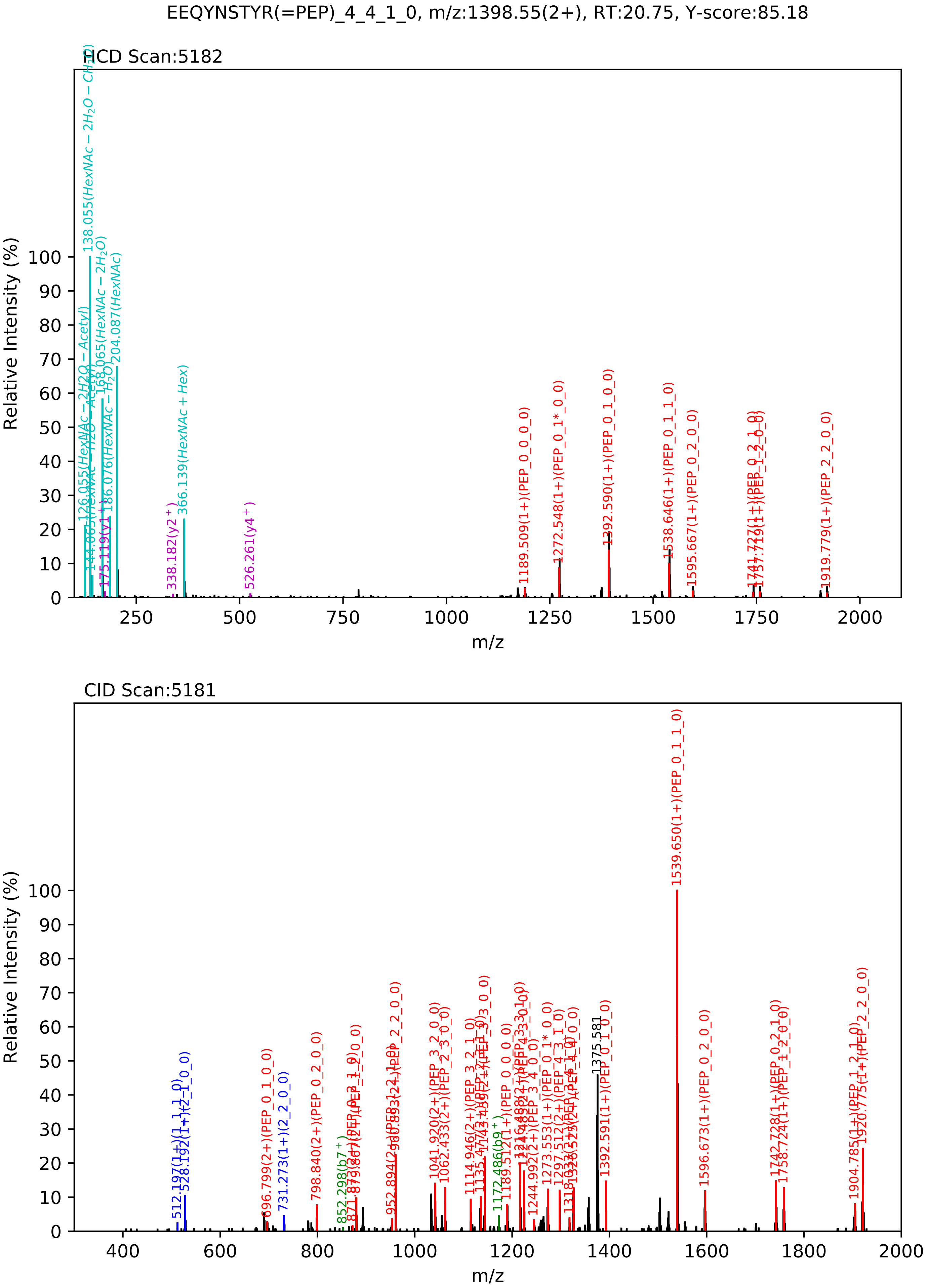

Training set no. 338, Experiment: IgG exp_2

EEQYNSTYR(=PEP)_4_4_1_0, m/z:1398.56(2+), RT:23.83, Y-score:84.36

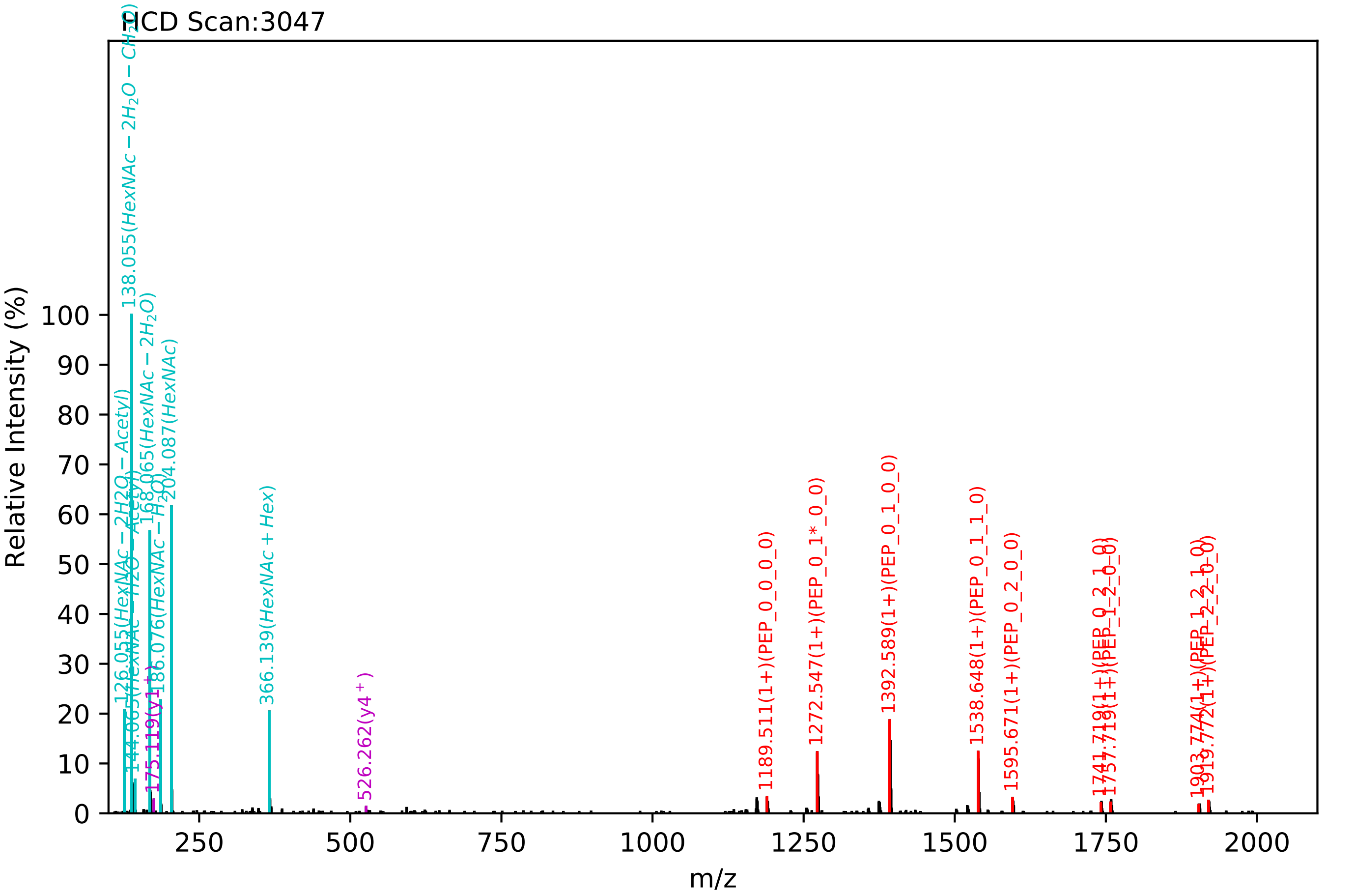

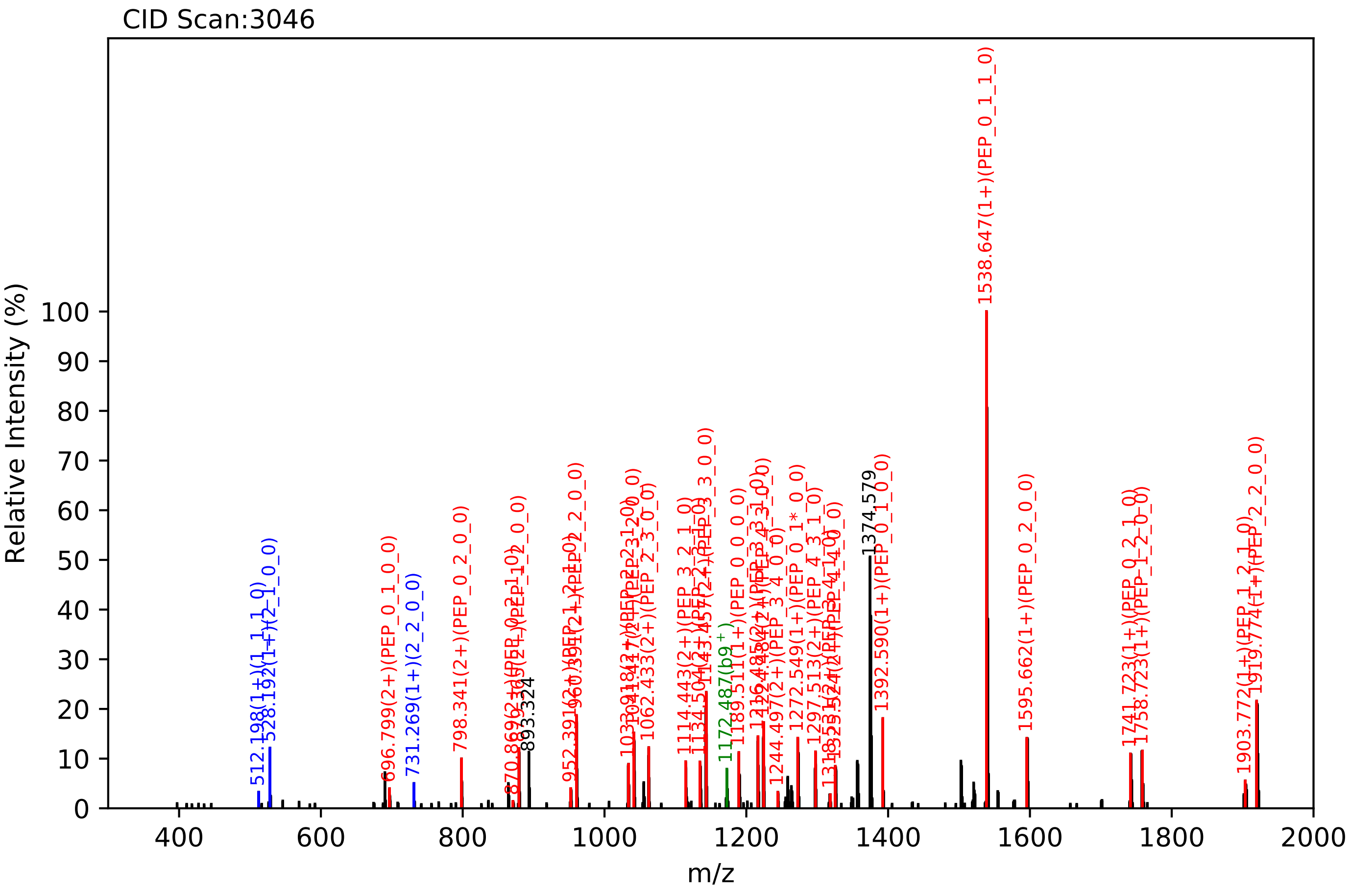

Training set no. 339, Experiment: IgG exp_3

EEQYNSTYR(=PEP)_4_4_1_0, m/z:1398.55(2+), RT:23.64, Y-score:84.35

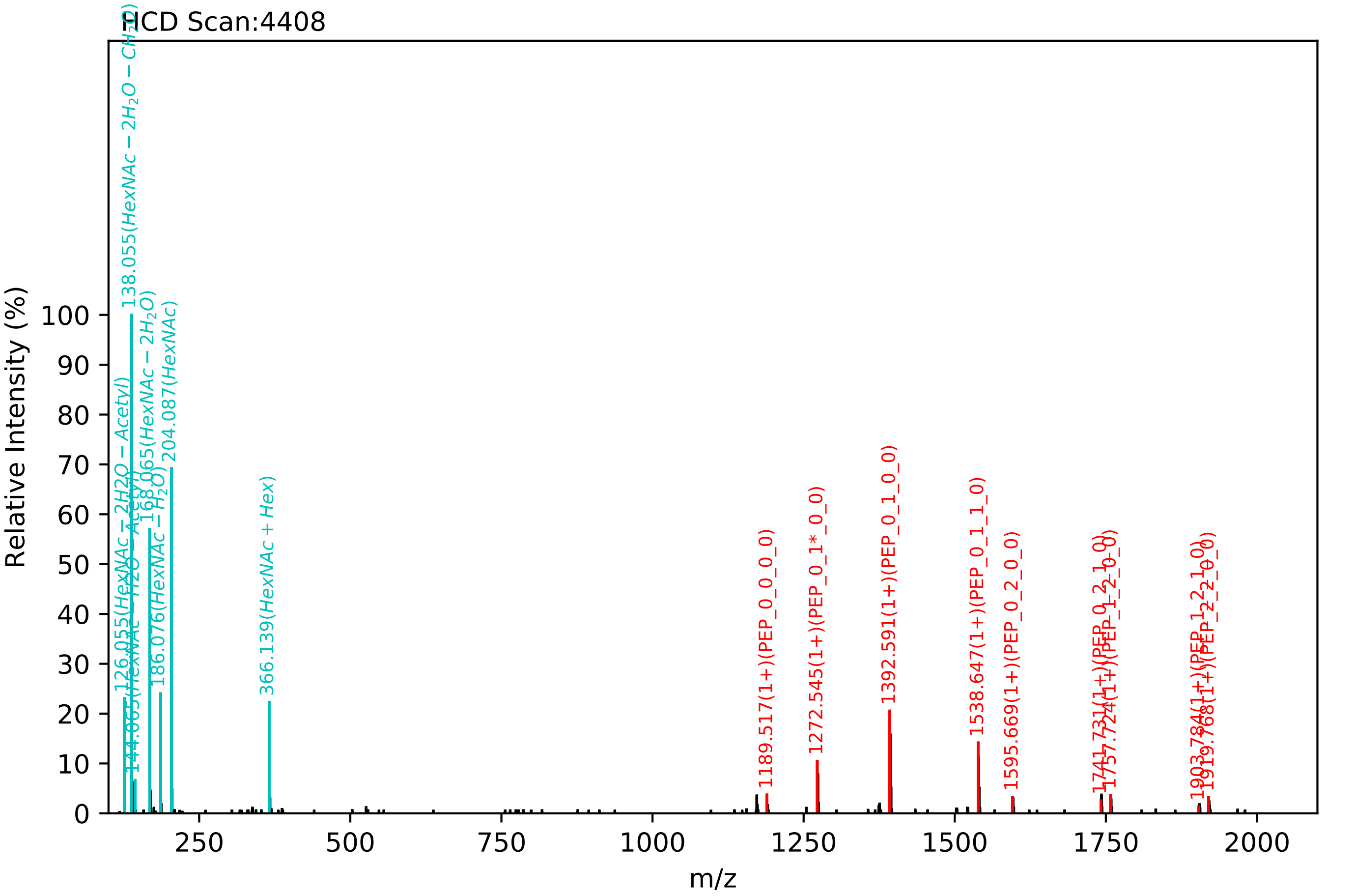

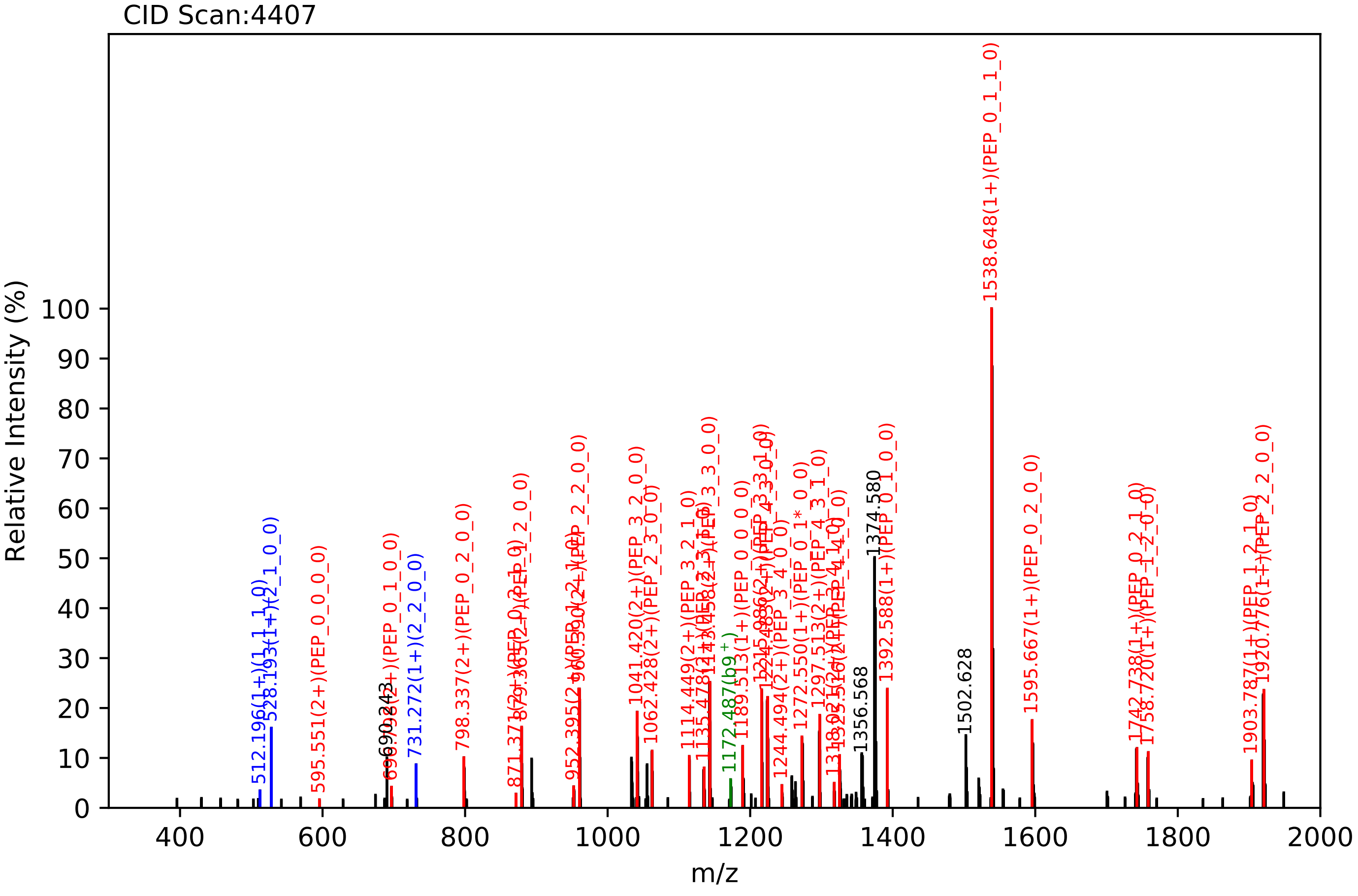

Training set no. 340, Experiment: IgG exp_1

EEQYNSTYR(=PEP)_4_4_1_0, m/z:1398.55(2+), RT:21.88, Y-score:84.25

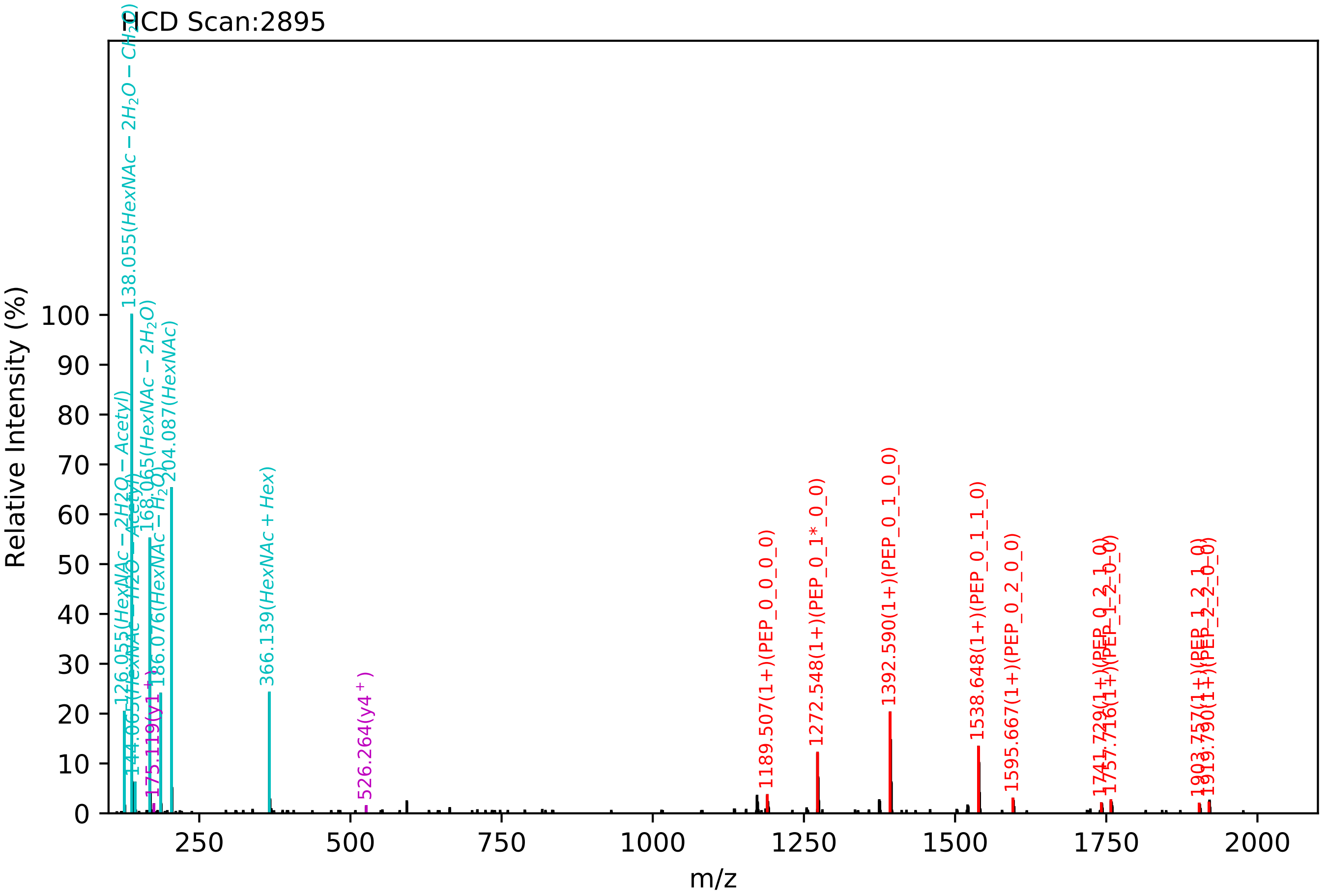

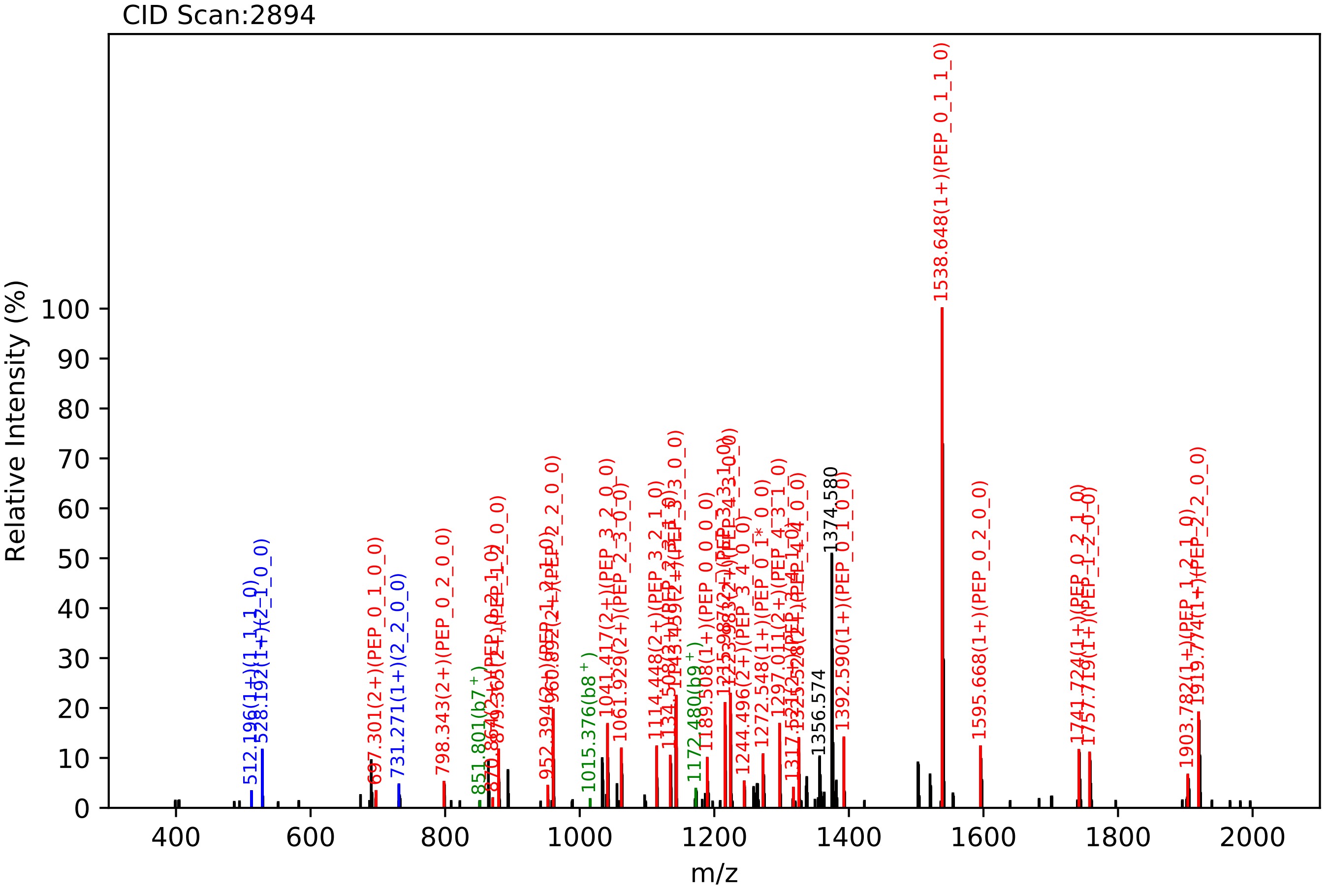

Training set no. 341, Experiment: IgG exp_3

EEQYNSTYR(=PEP)_4_4_1_0, m/z:1398.55(2+), RT:23.07, Y-score:84.18

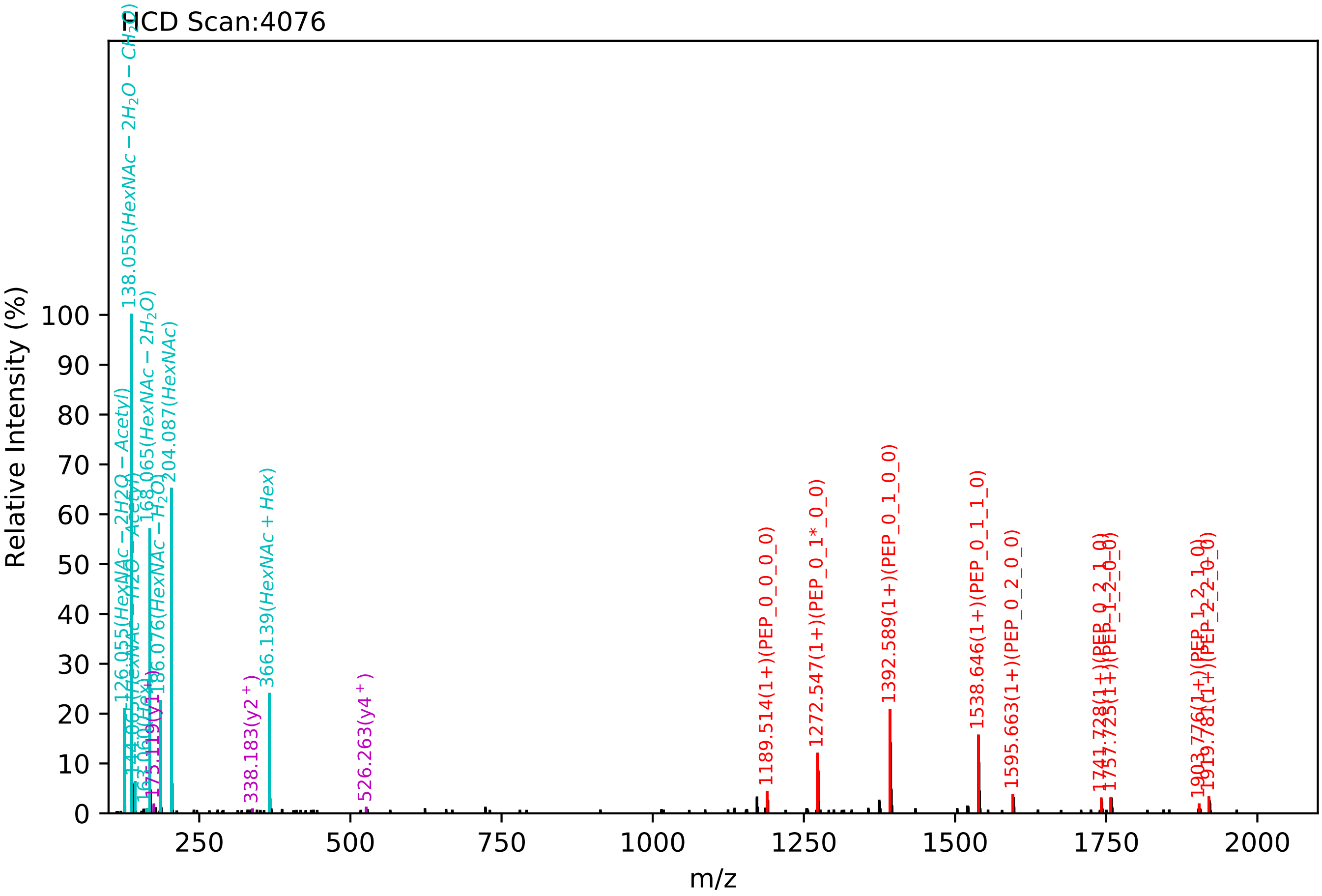

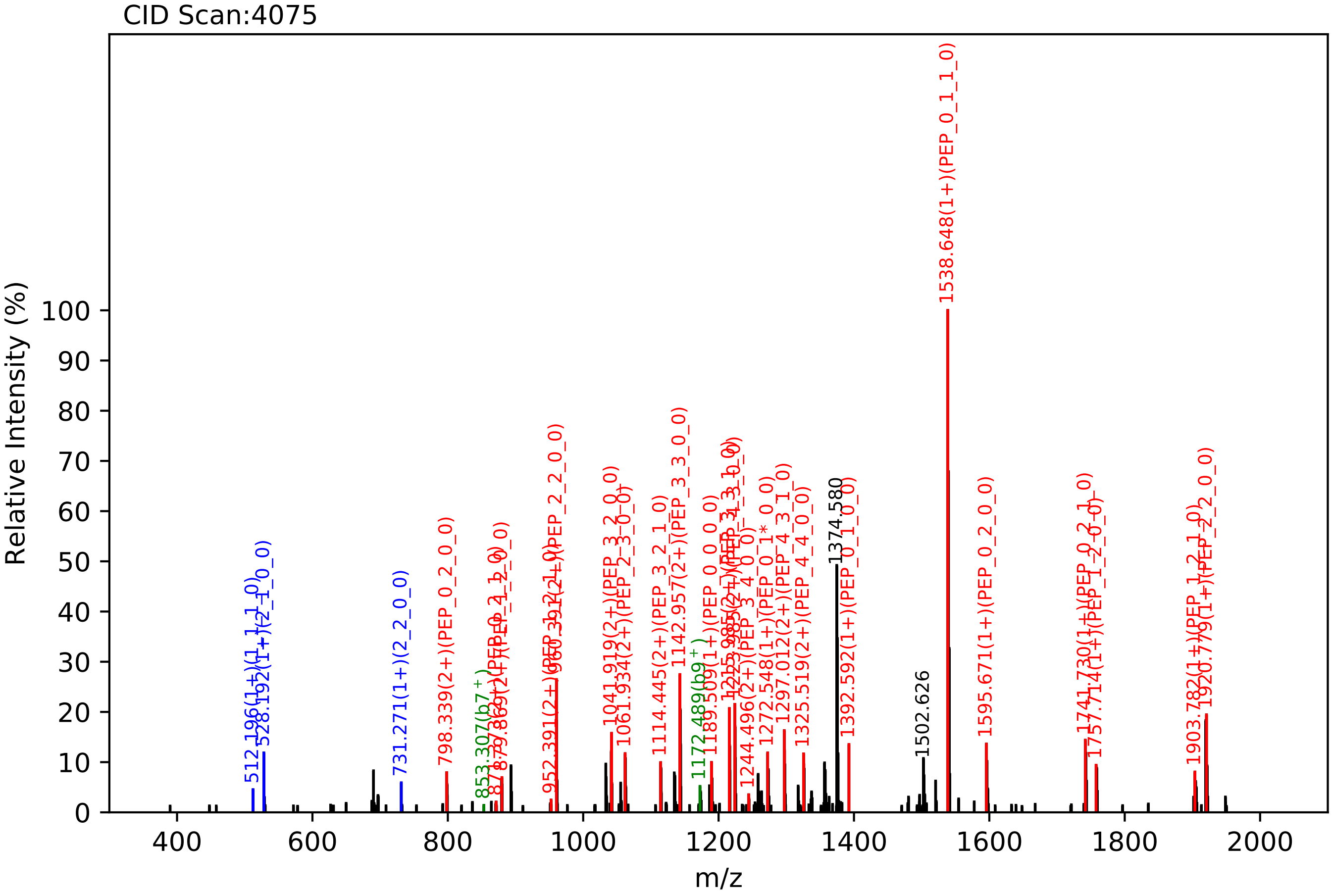

Training set no. 342, Experiment: IgG exp_1

EEQYNSTYR(=PEP)_4_4_1_0, m/z:1398.55(2+), RT:22.54, Y-score:83.56

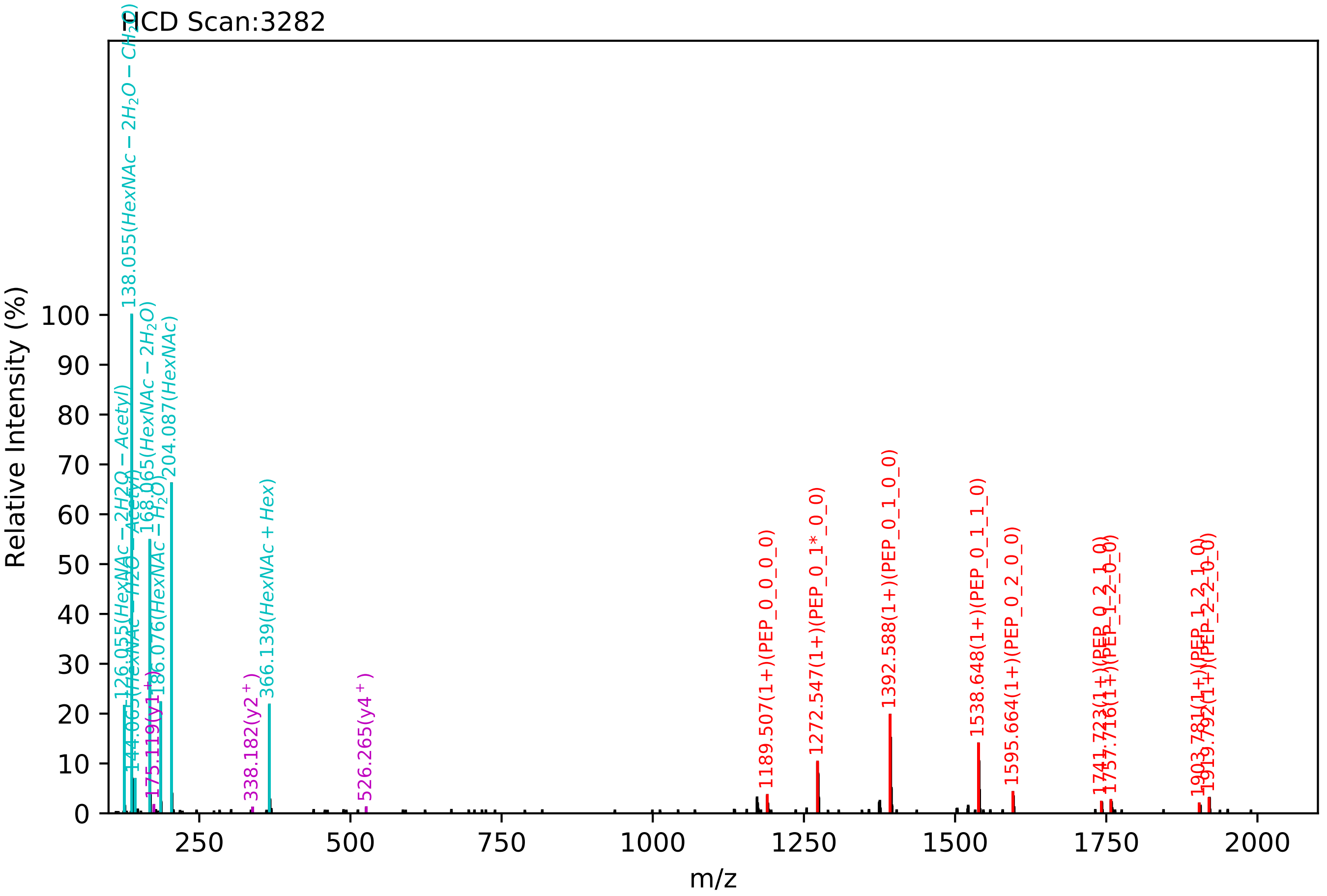

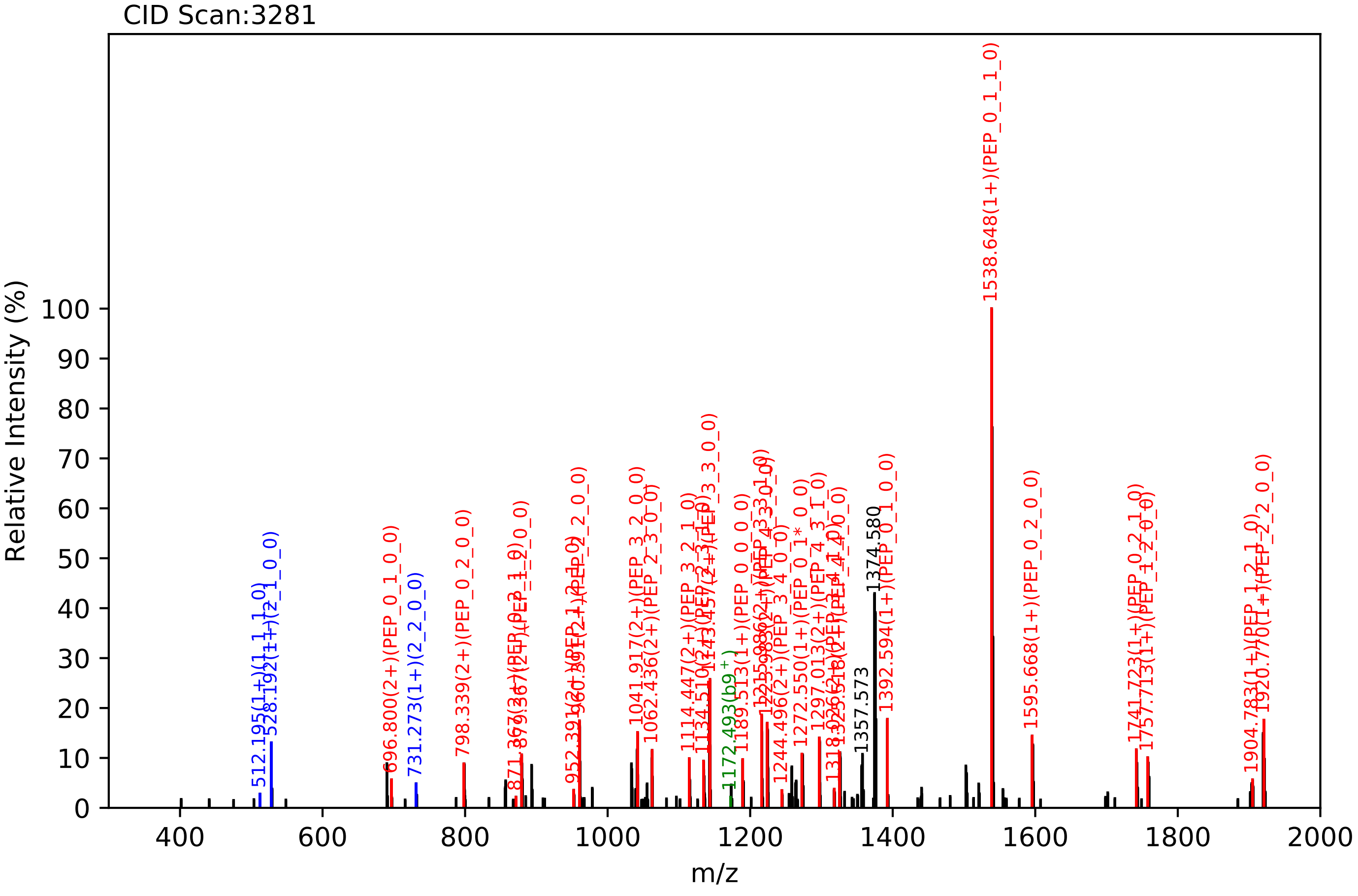

Training set no. 343, Experiment: IgG exp_2

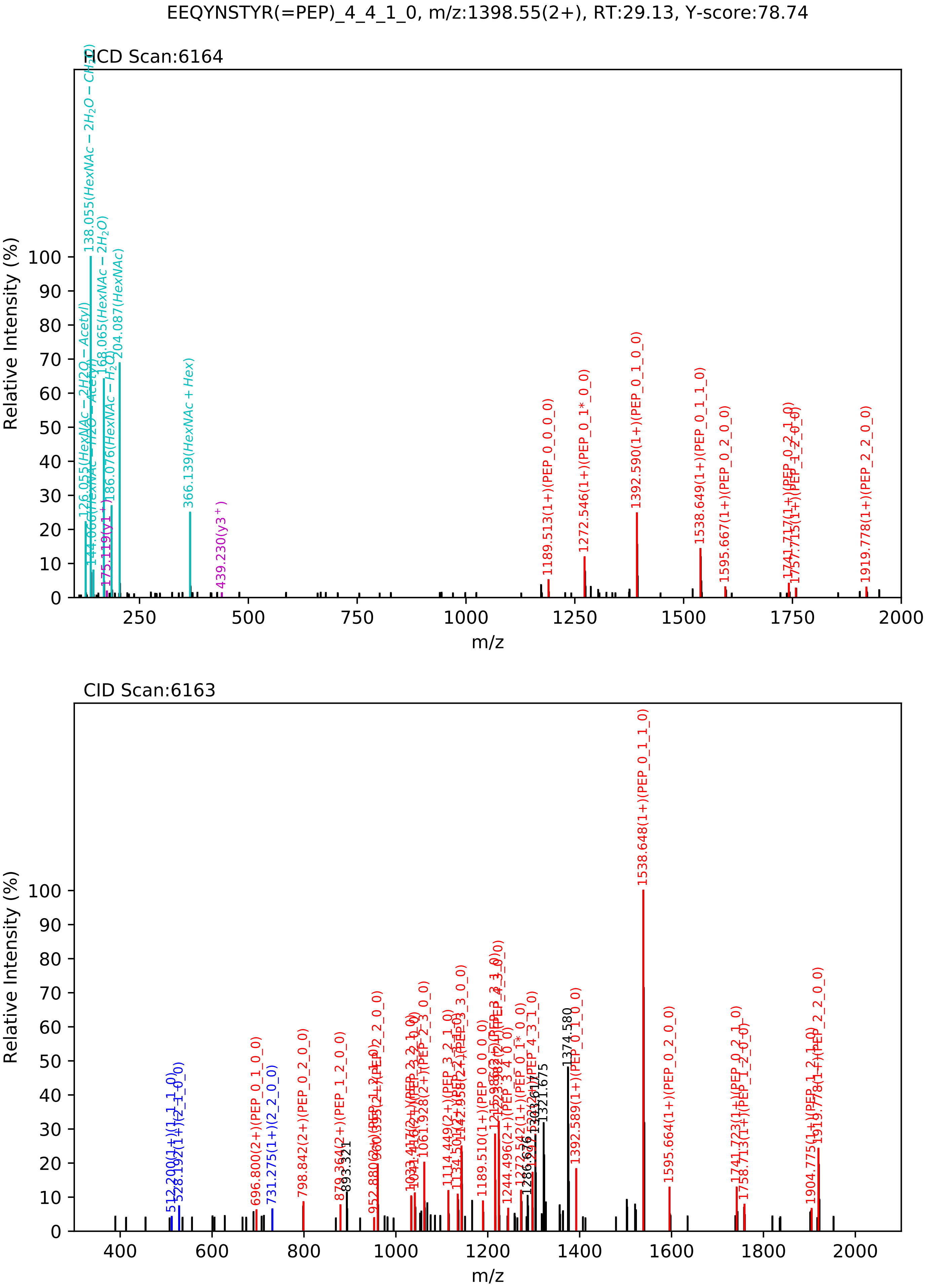

Training set no. 344, Experiment: IgG exp_3

EEQYNSTYR(=PEP)_4_4_1_1, m/z:1029.74(3+), RT:25.42, Y-score:94.45

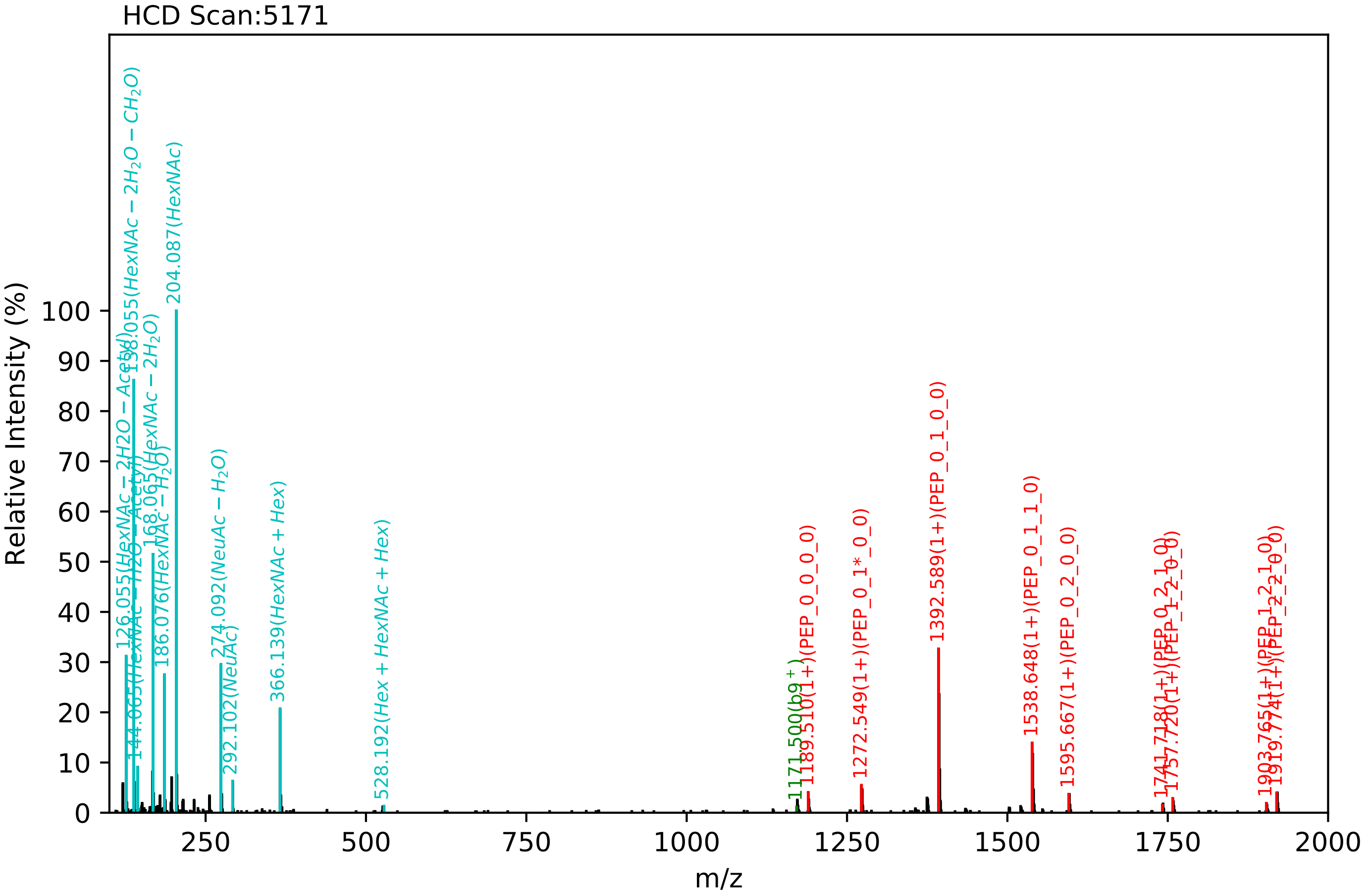

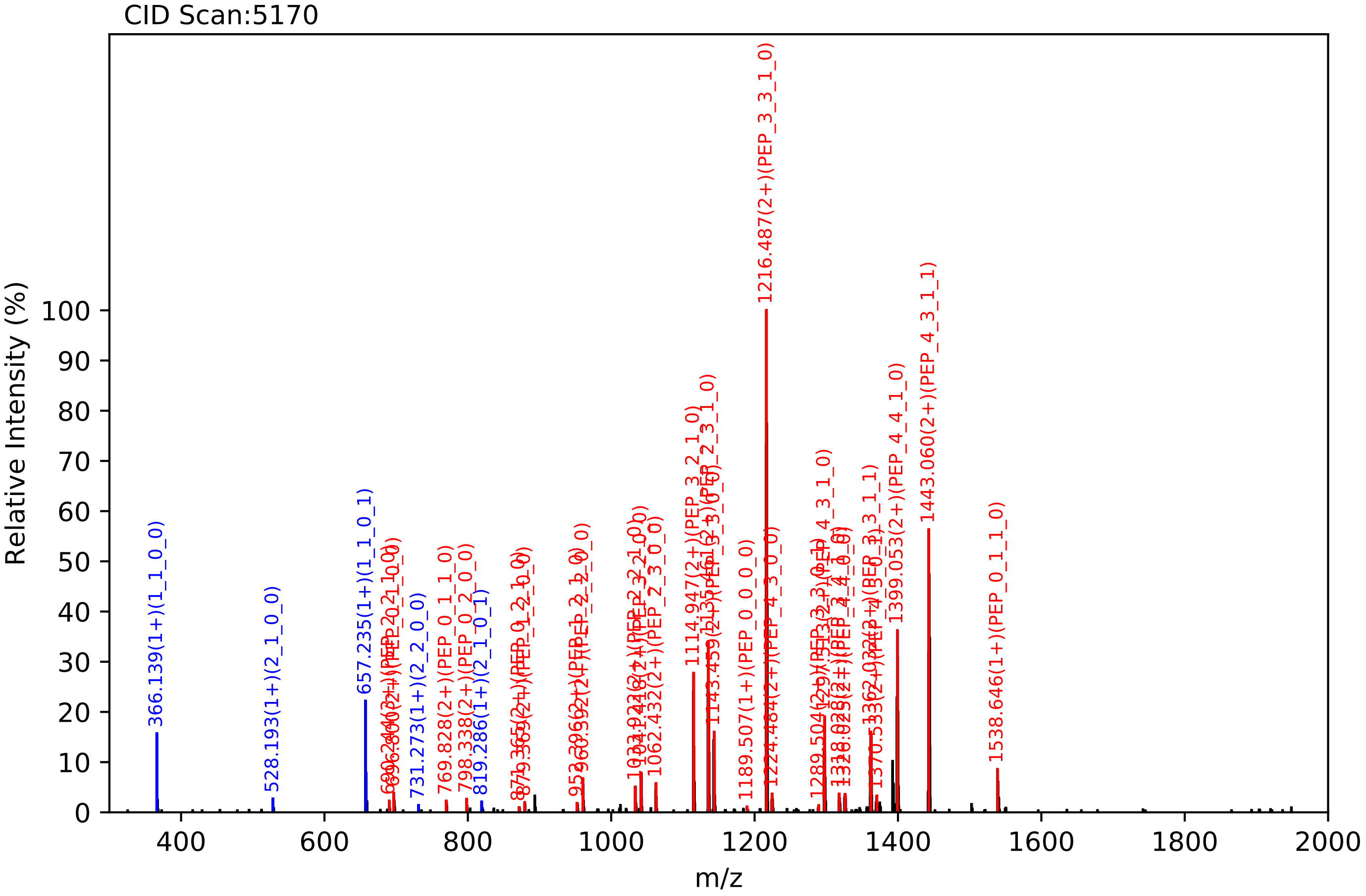

Training set no. 345, Experiment: IgG exp_4

EEQYNSTYR(=PEP)_4_4_1_1, m/z:1029.74(3+), RT:22.38, Y-score:92.54

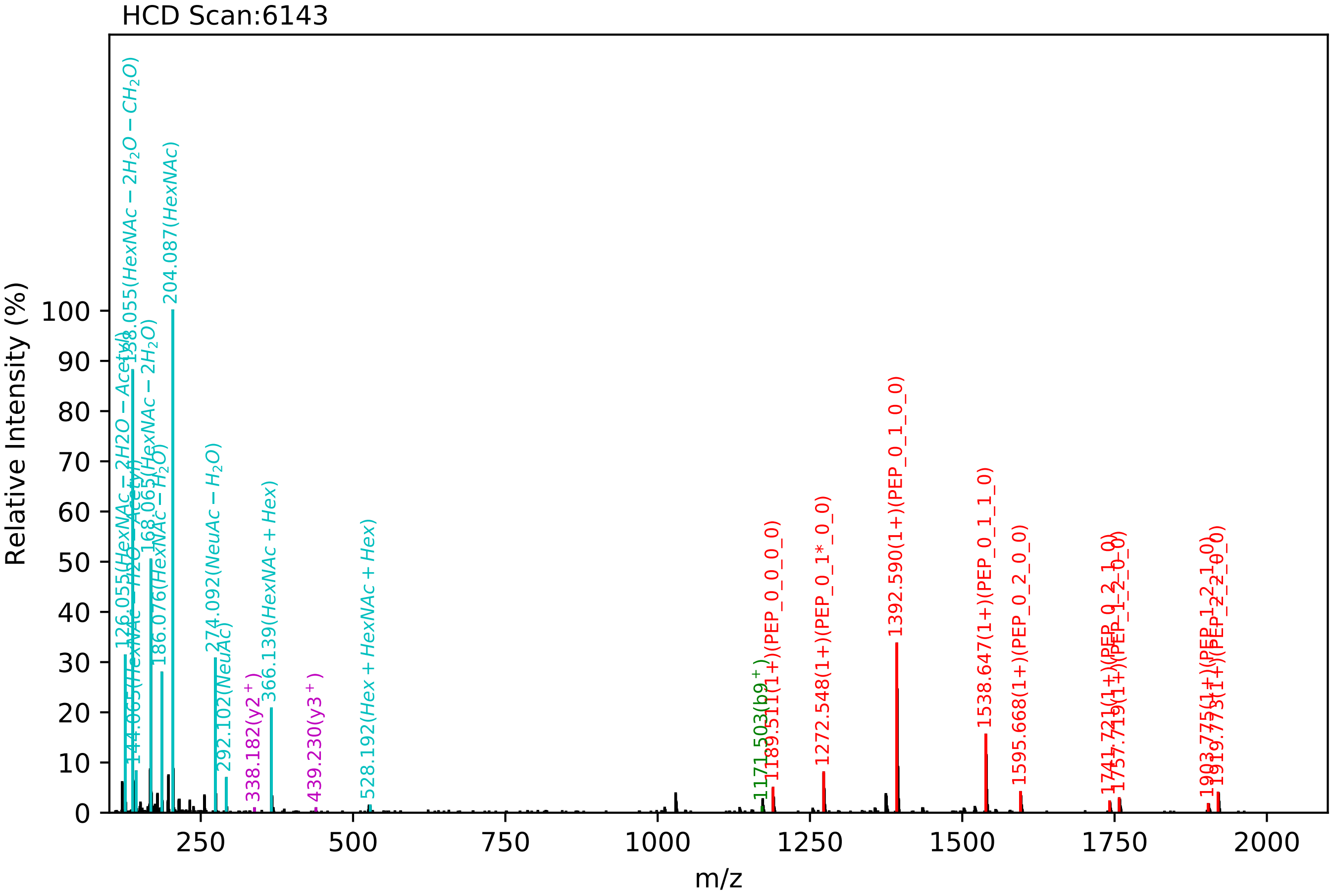

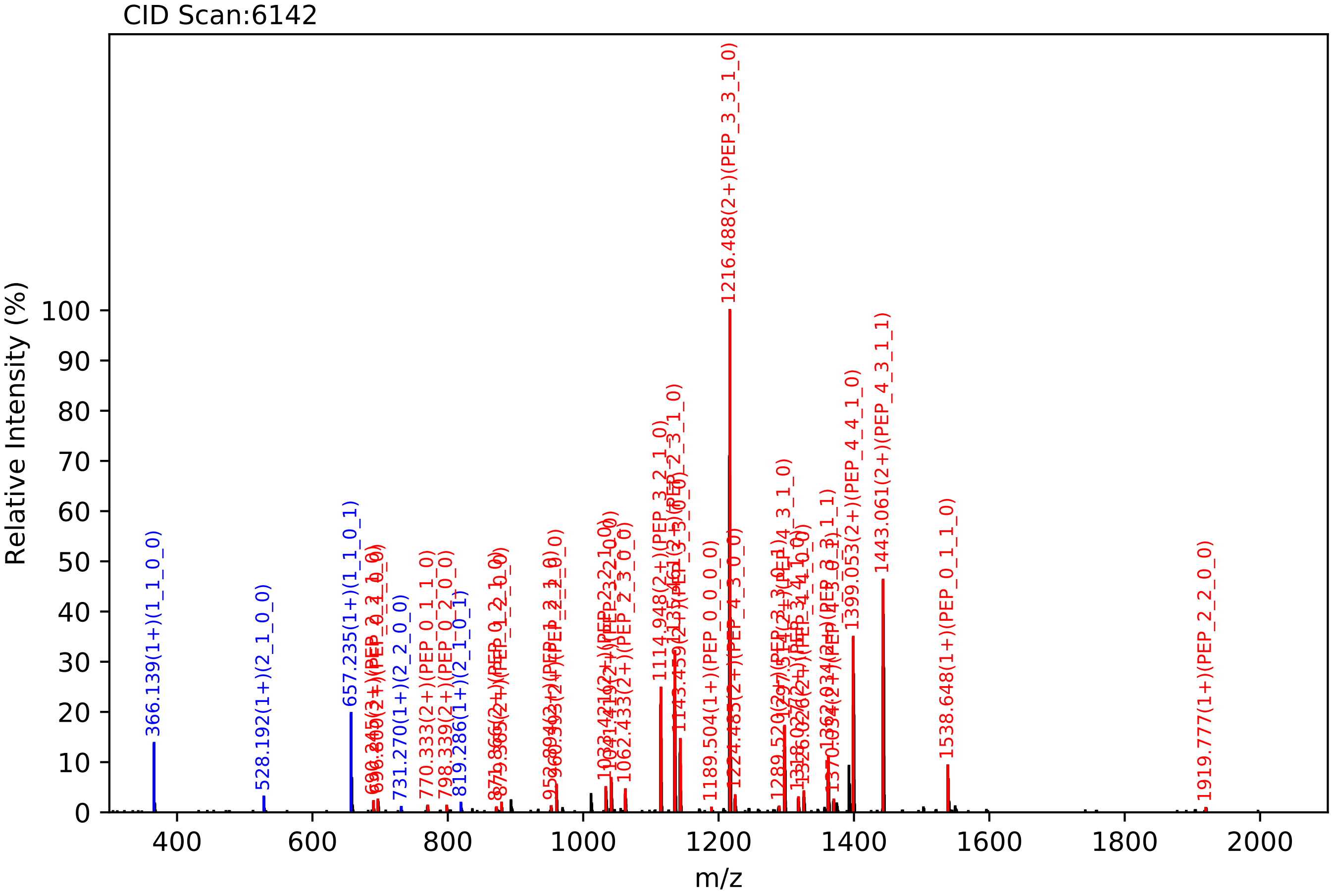

Training set no. 346, Experiment: IgG exp_1

EEQYNSTYR(=PEP)_4_4_1_1, m/z:1029.74(3+), RT:23.40, Y-score:89.33

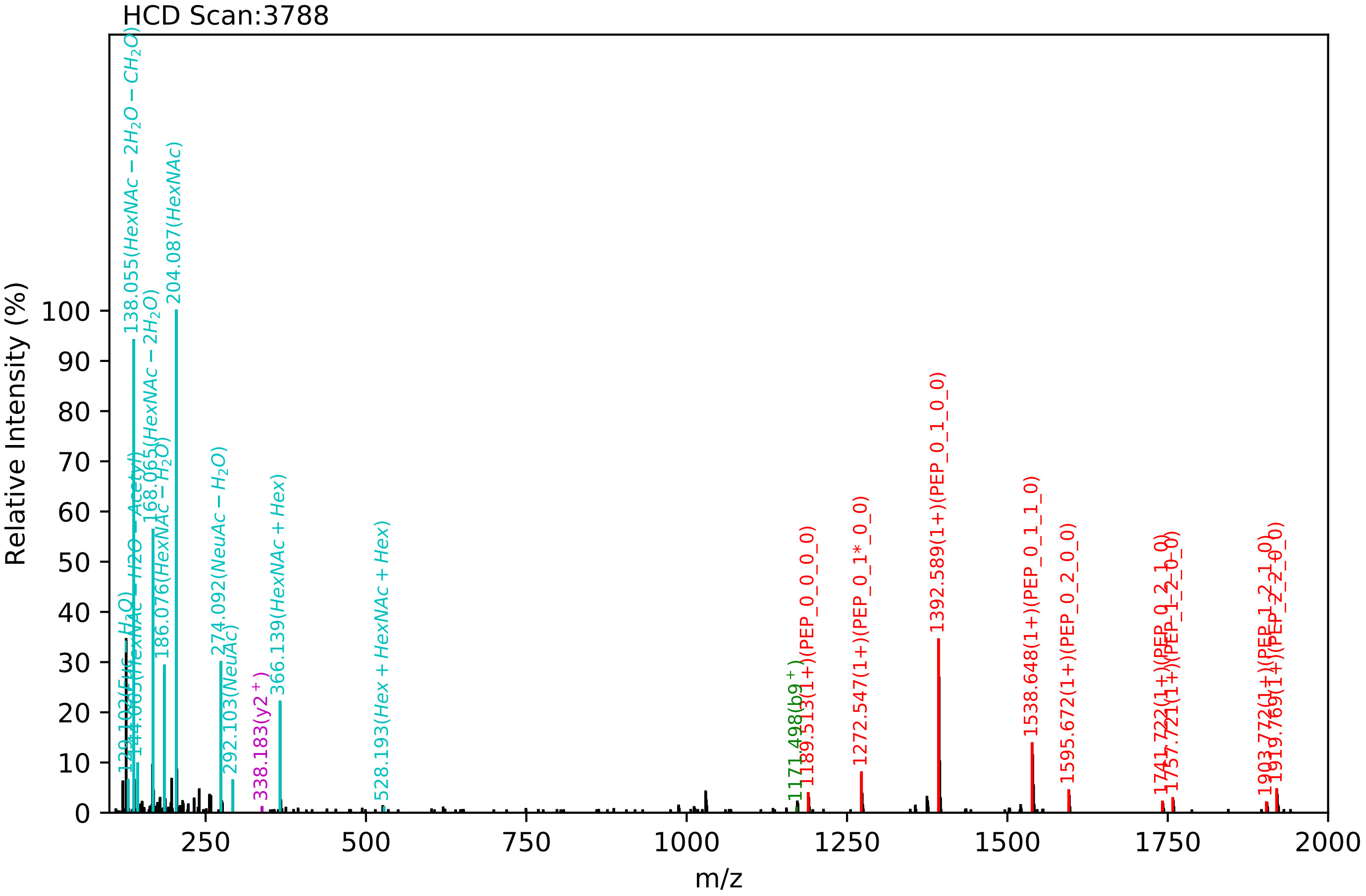

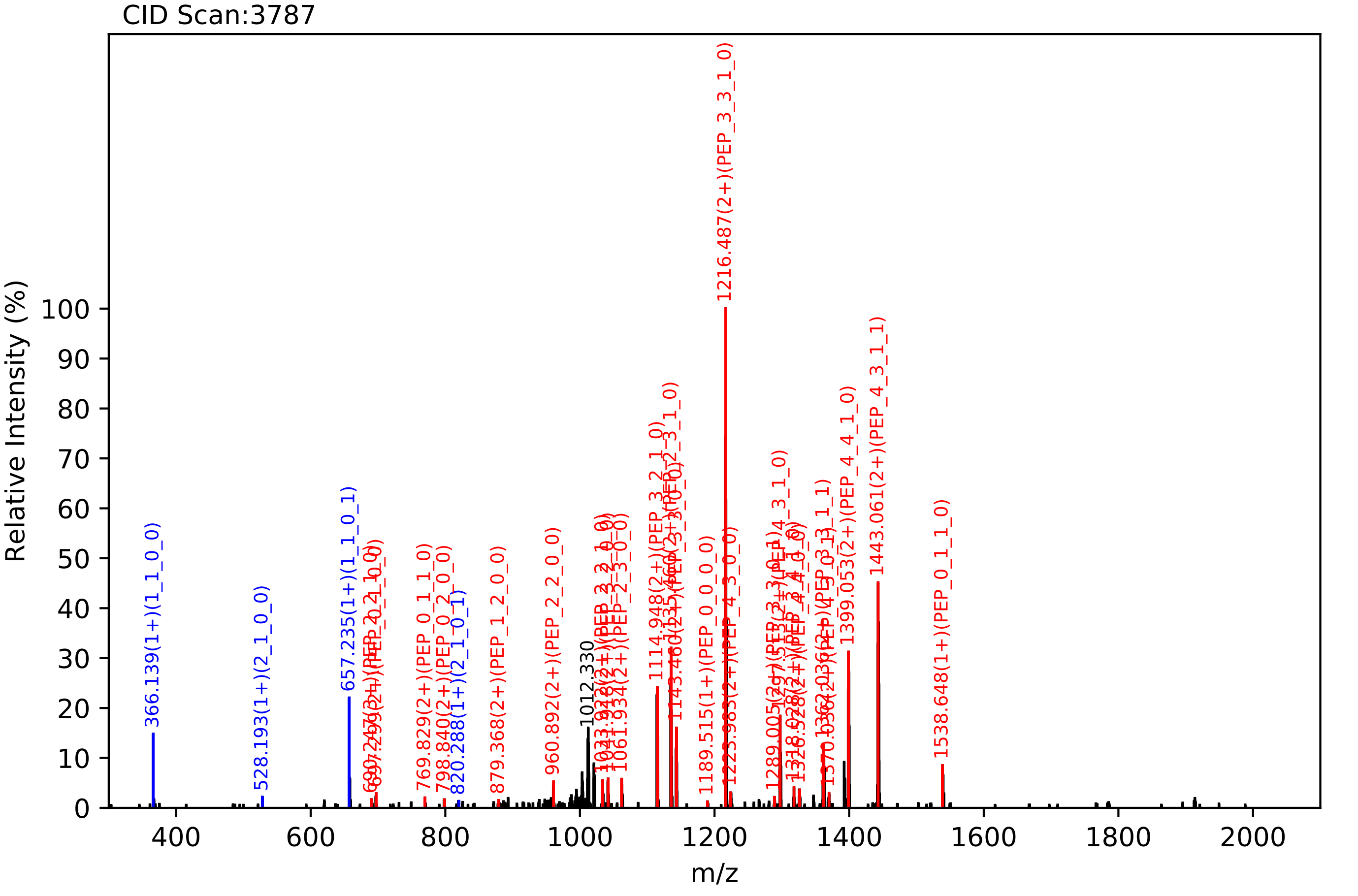

Training set no. 347, Experiment: IgG exp_3

EEQYNSTYR(=PEP)_4_4_1_1, m/z:1544.10(2+), RT:25.46, Y-score:84.75

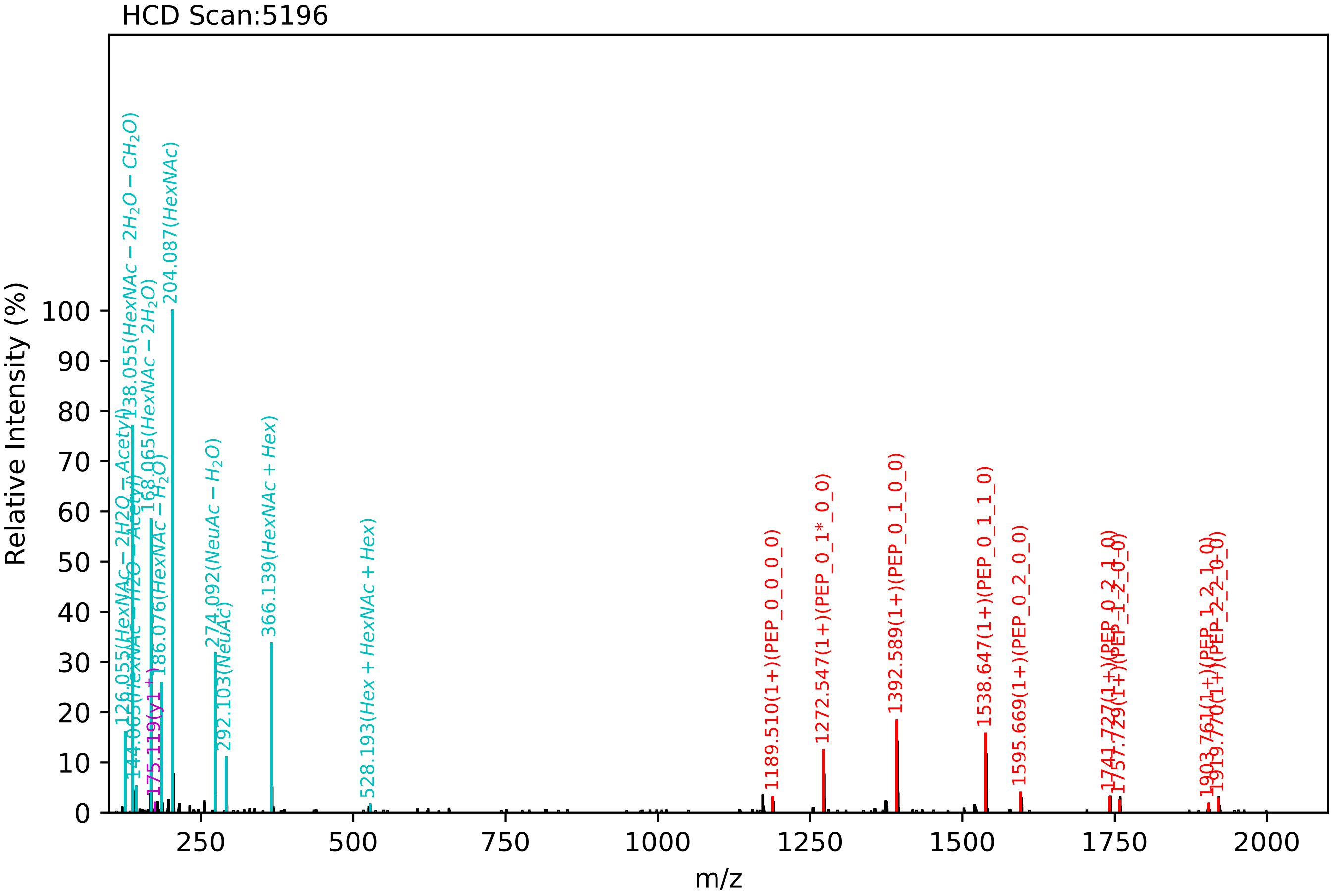

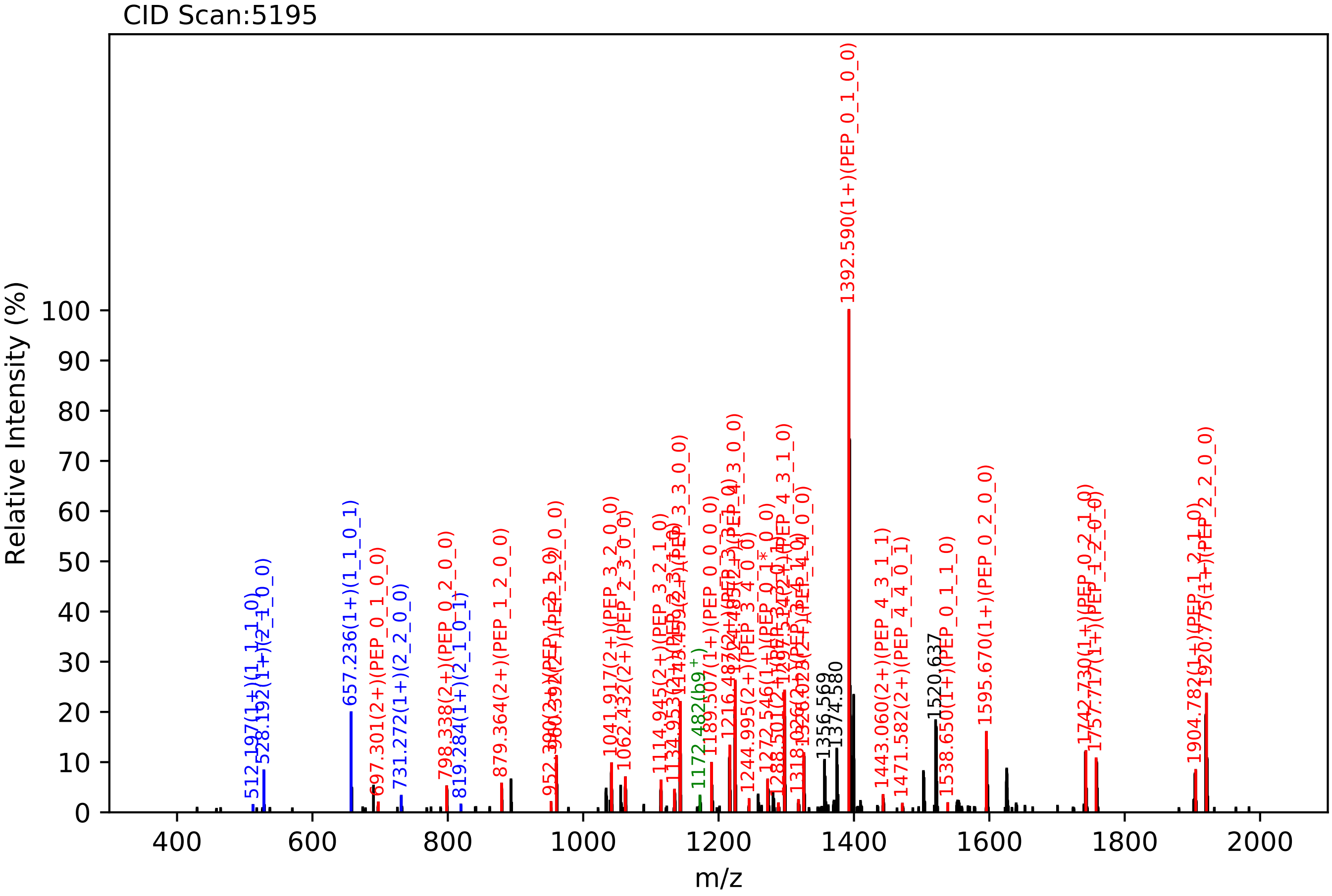

Training set no. 348, Experiment: IgG exp_1

EEQYNSTYR(=PEP)_4_4_1_1, m/z:1544.10(2+), RT:23.38, Y-score:82.19

HCD Scan:3775

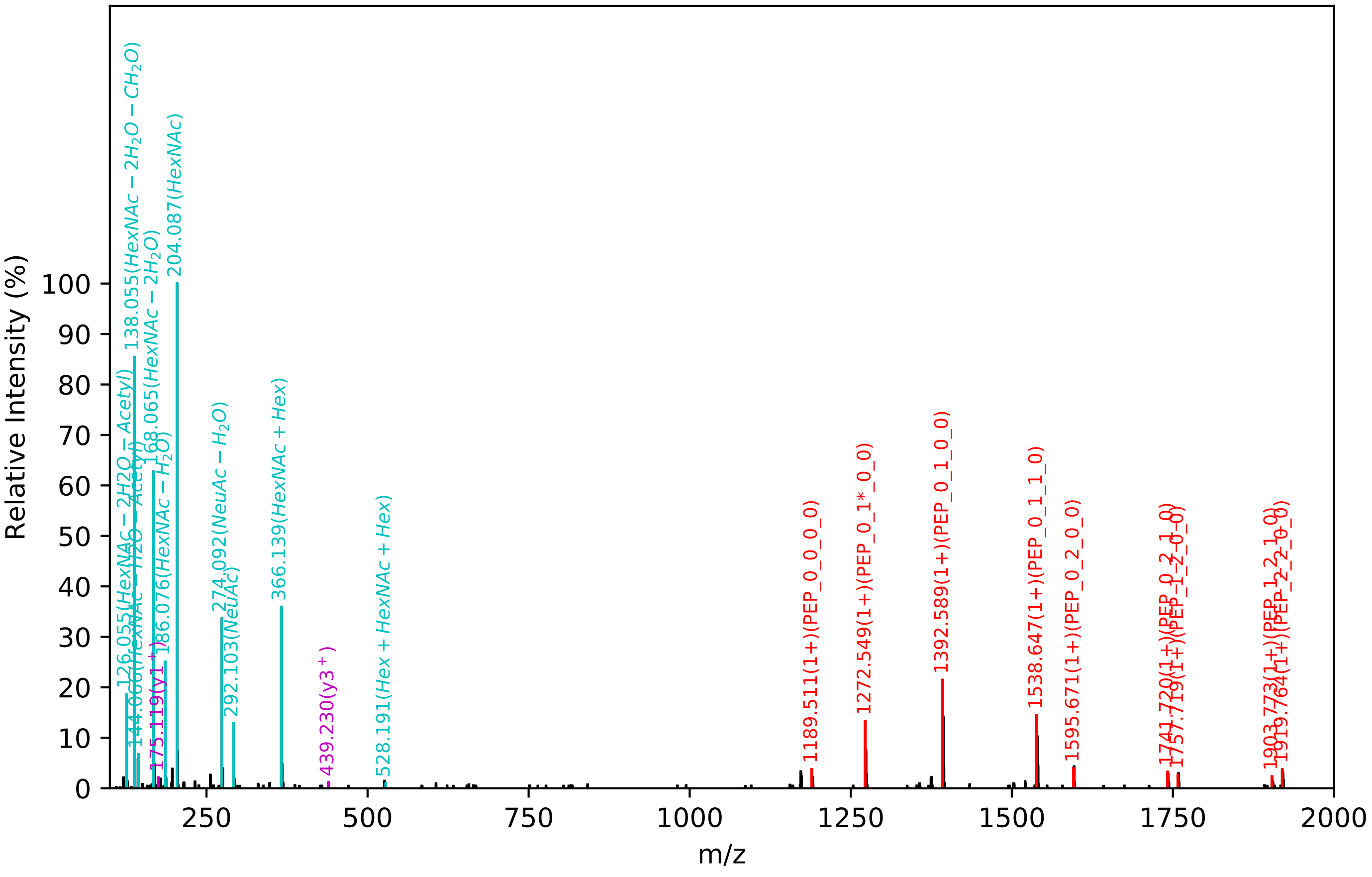

CID Scan:3774

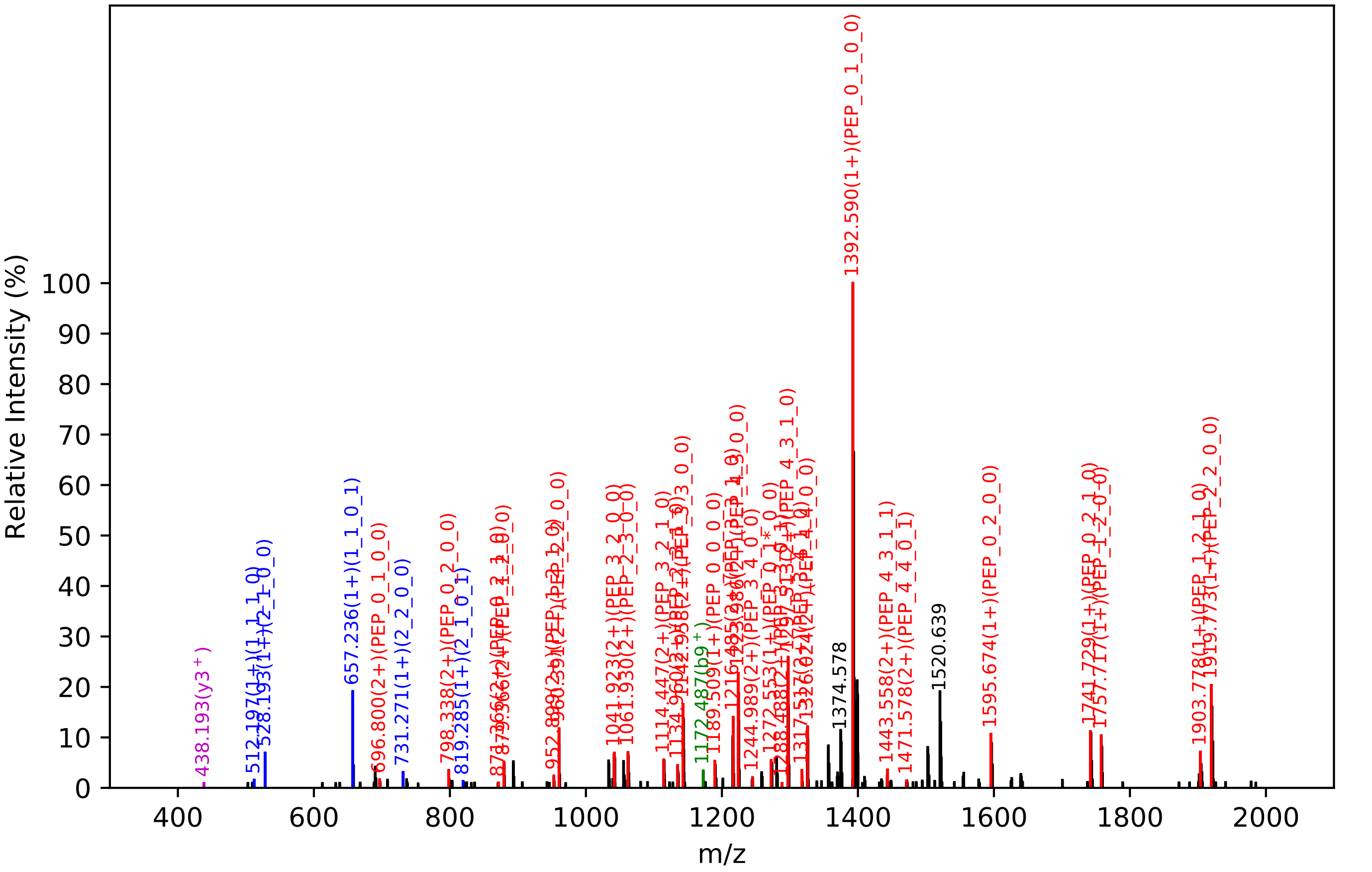

Training set no. 349, Experiment: IgG exp_4

EEQYNSTYR(=PEP)_4_4_1_1, m/z:1544.10(2+), RT:22.39, Y-score:82.11

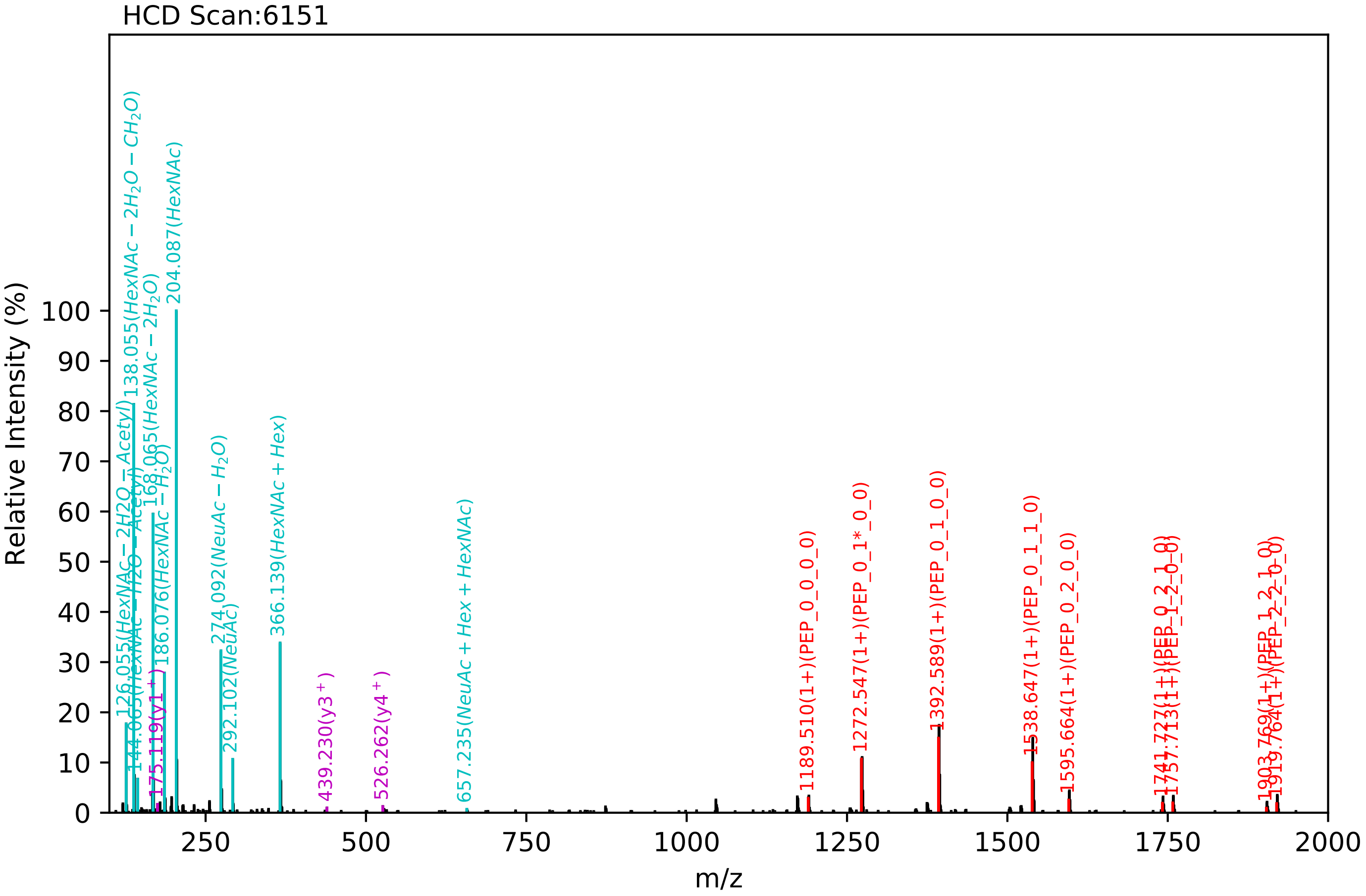

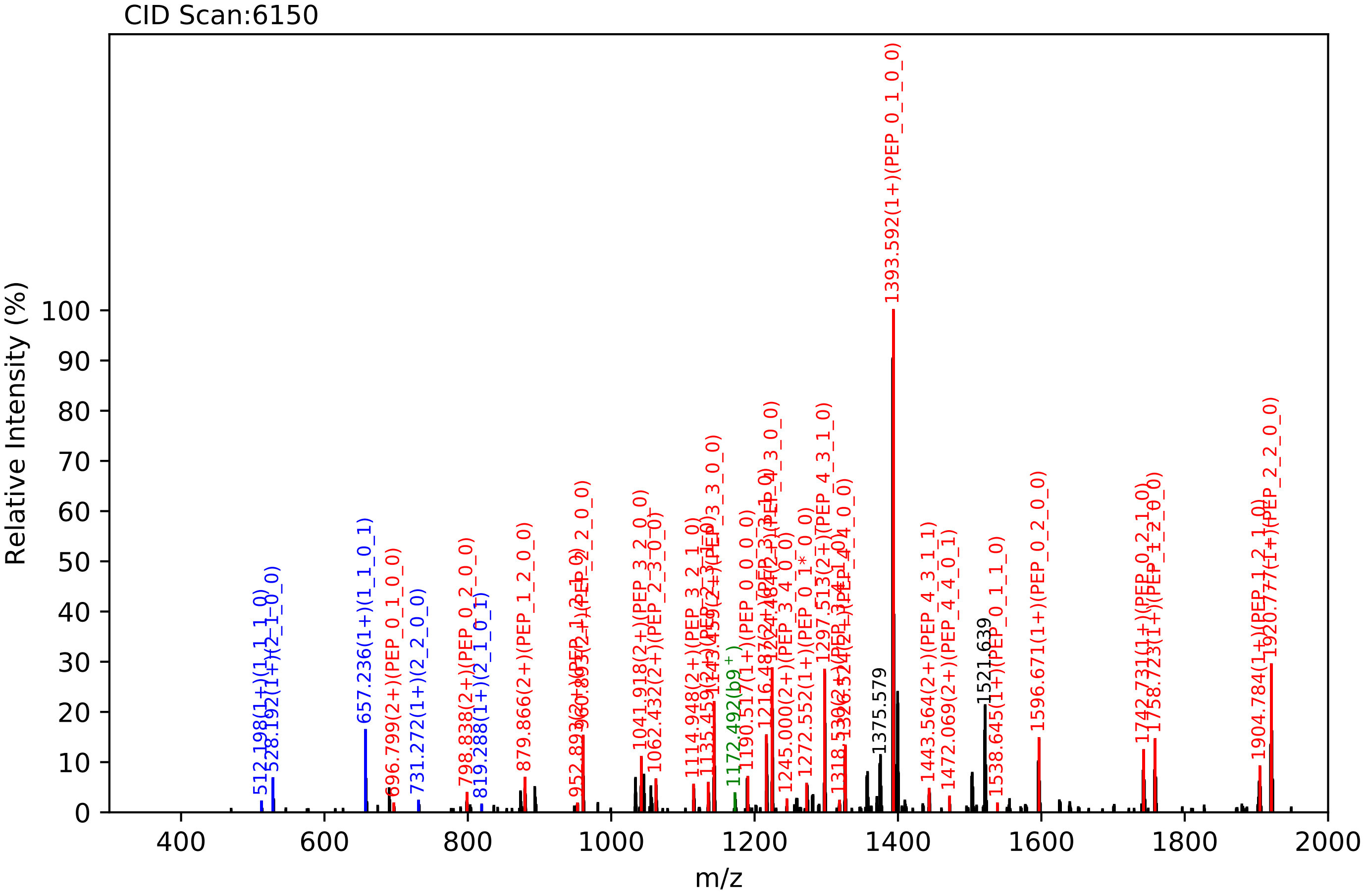

Training set no. 350, Experiment: IgG exp_2

EEQYNSTYR(=PEP)_4_4_1_1, m/z:1029.74(3+), RT:24.13, Y-score:71.94

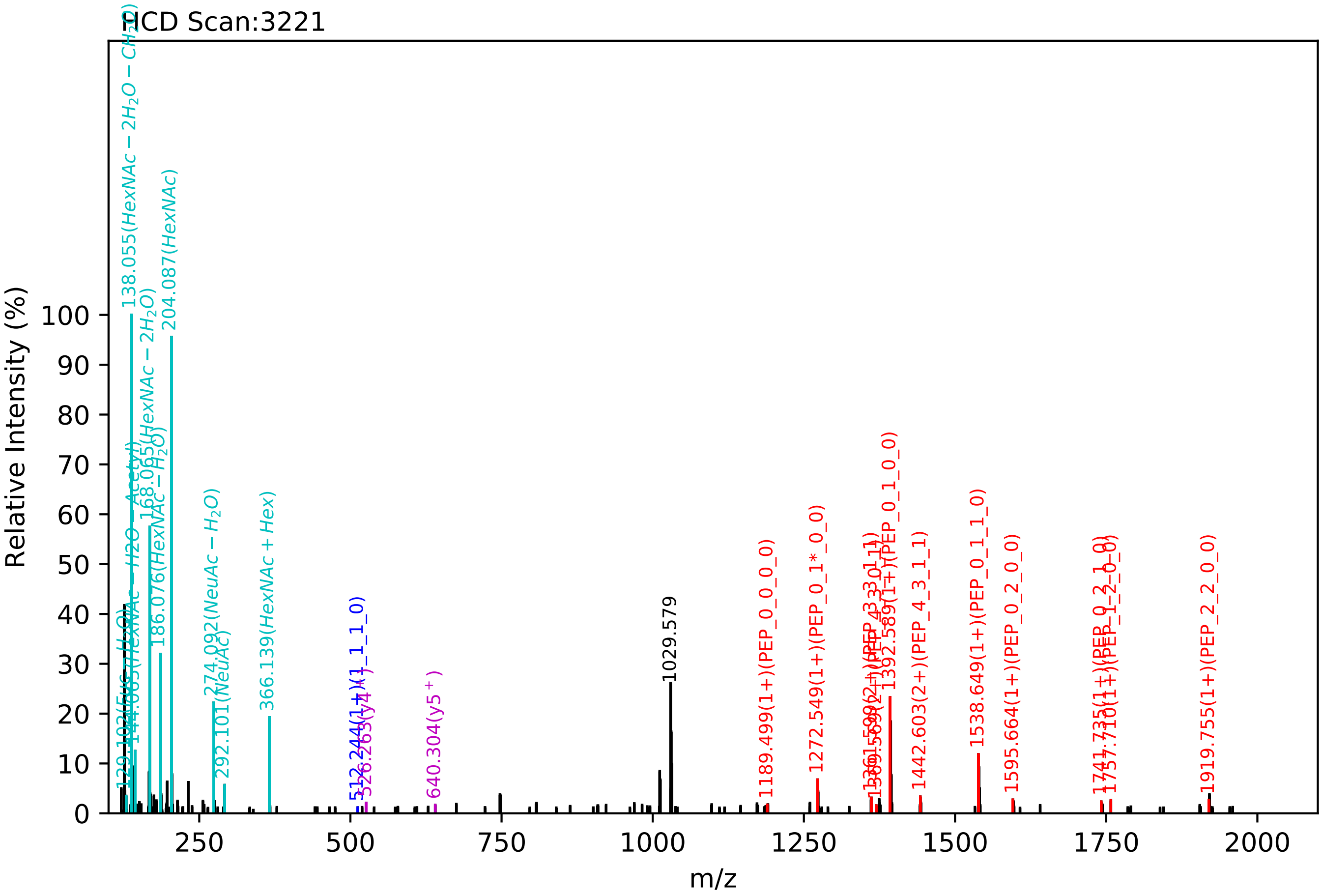

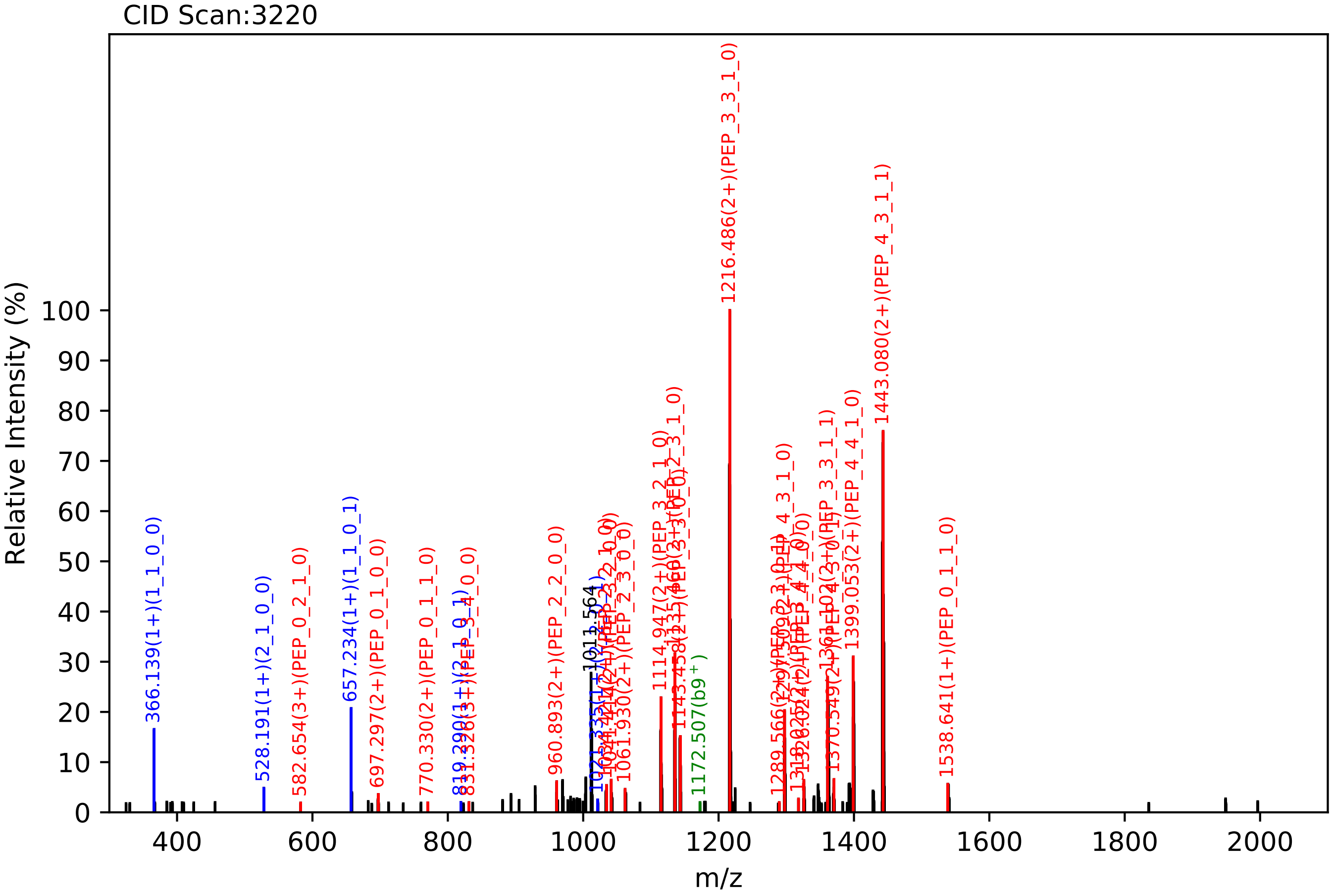

Training set no. 351, Experiment: IgG exp_2

EEQYNSTYR(=PEP)_4_4_1_1, m/z:1544.10(2+), RT:24.14, Y-score:67.77

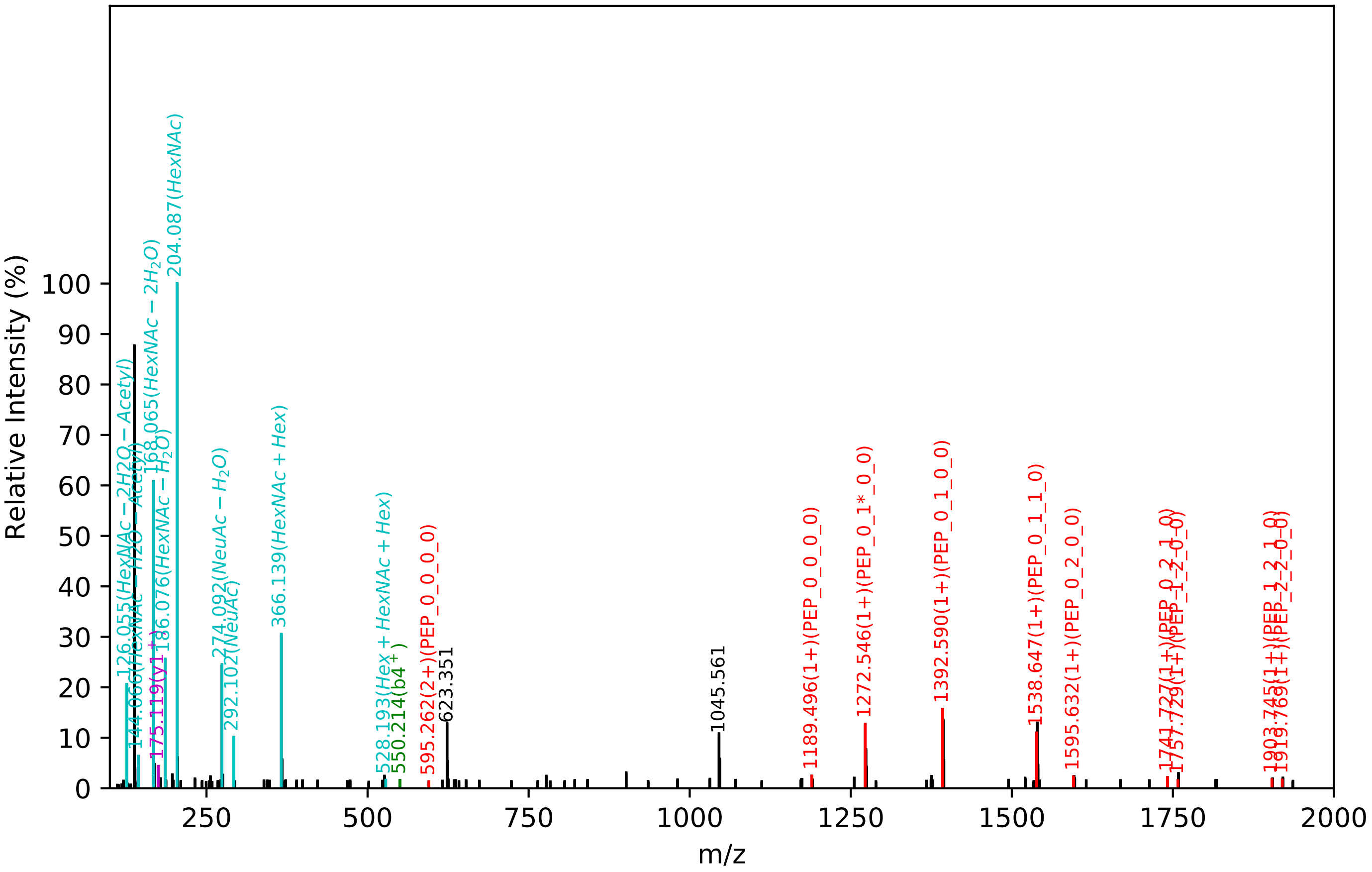

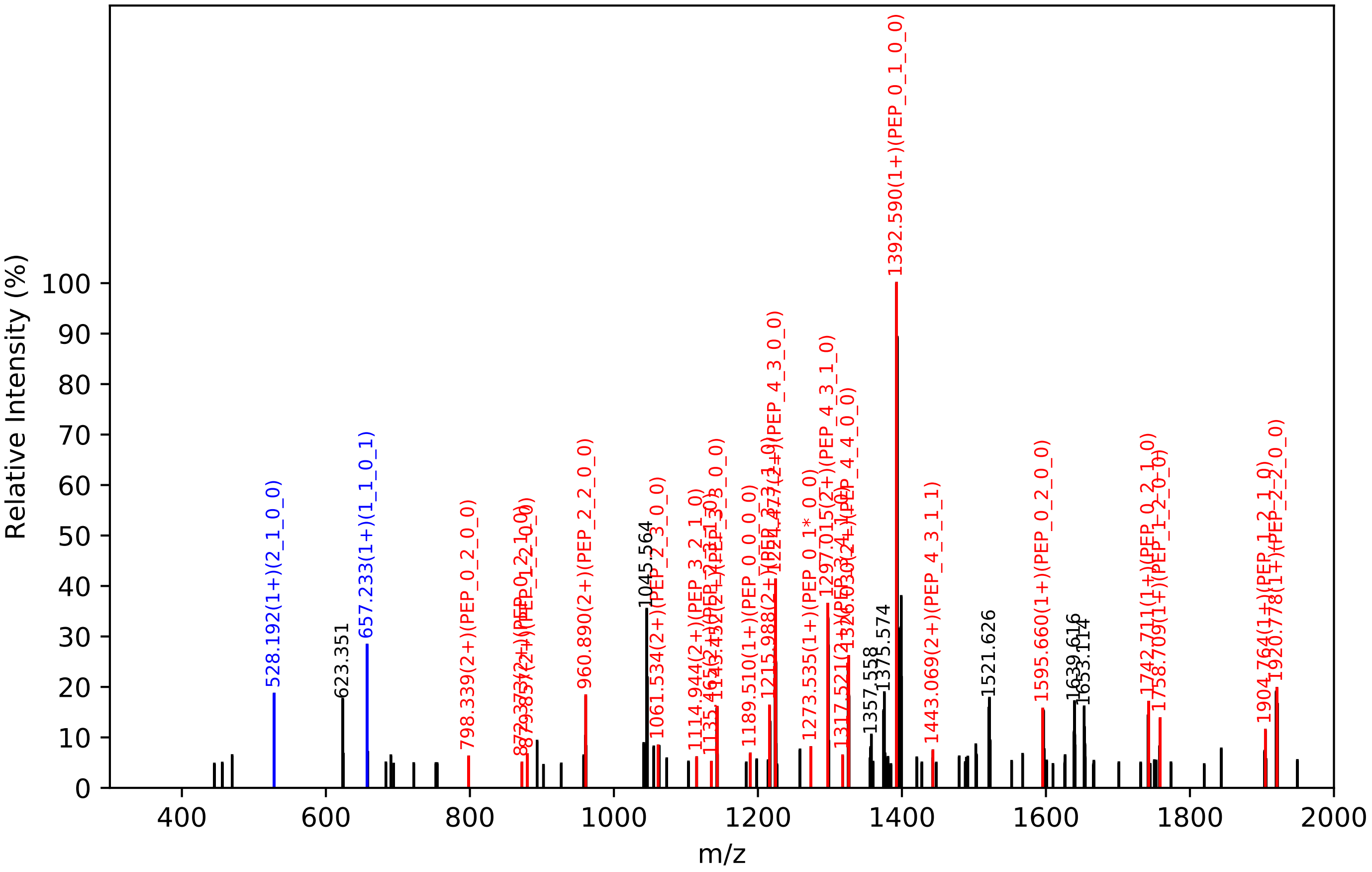

Training set no. 352, Experiment: IgG exp_1

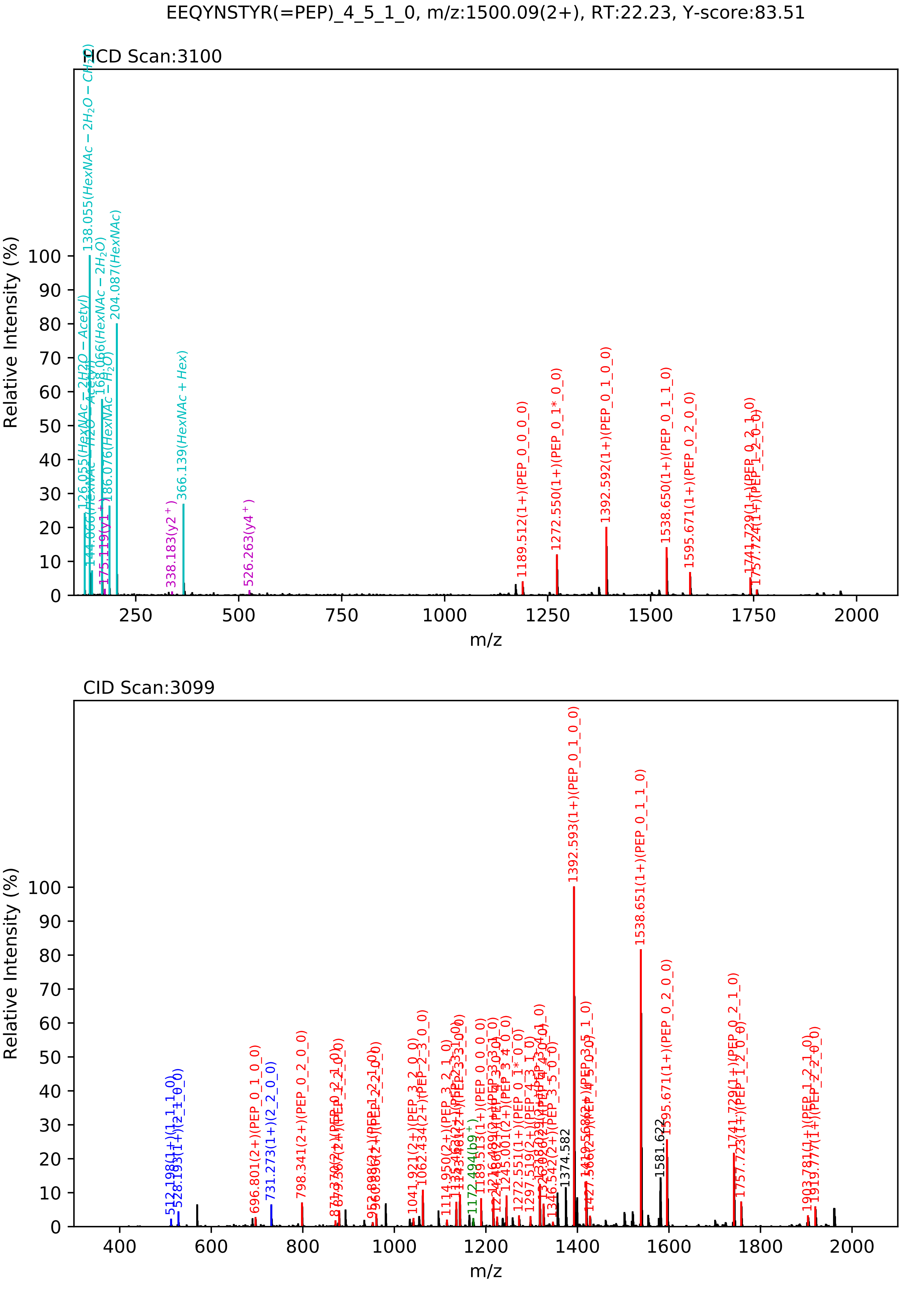

Training set no. 353, Experiment: IgG exp_4

EEQYNSTYR(=PEP)_4_5_1_0, m/z:1500.09(2+), RT:21.18, Y-score:83.47

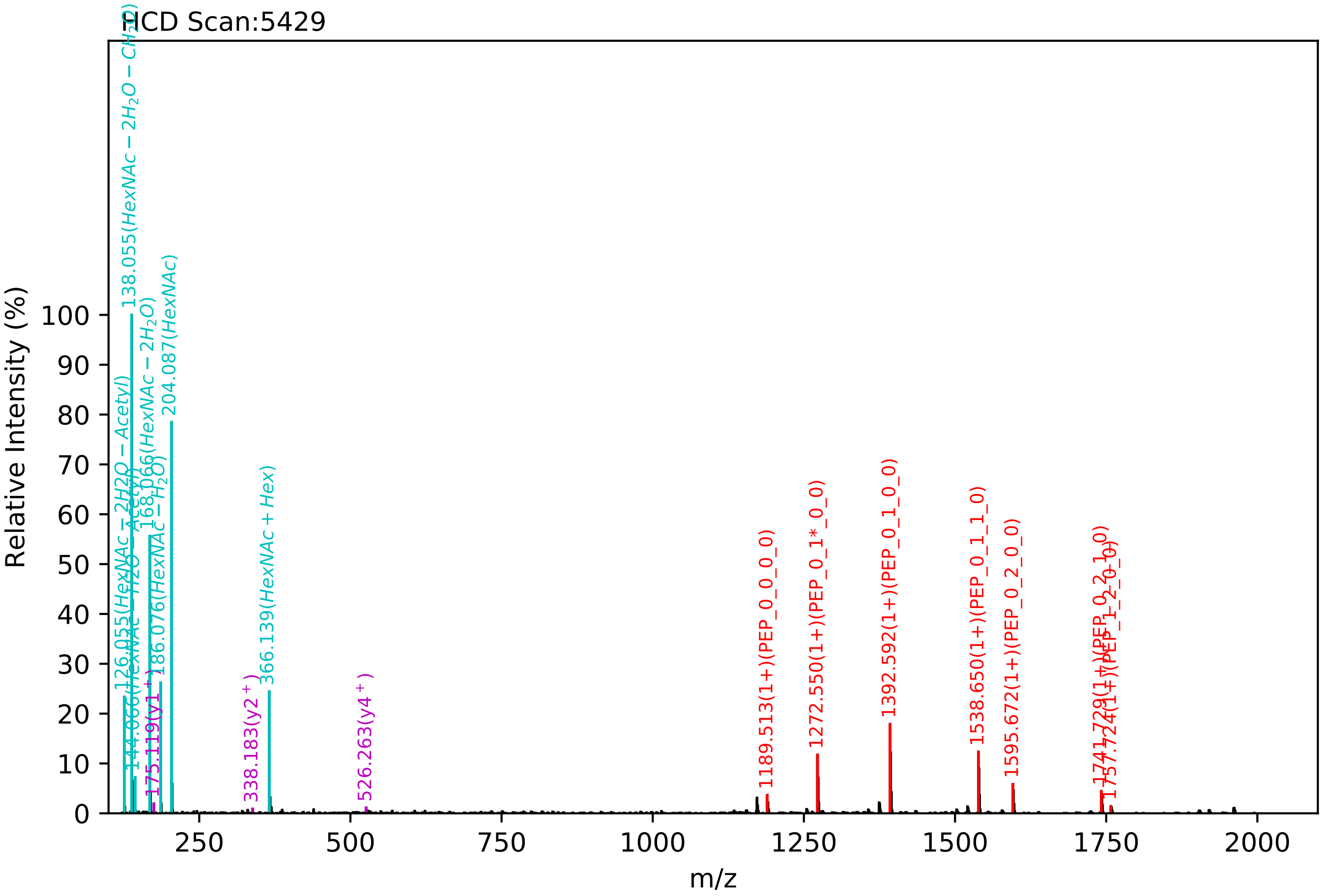

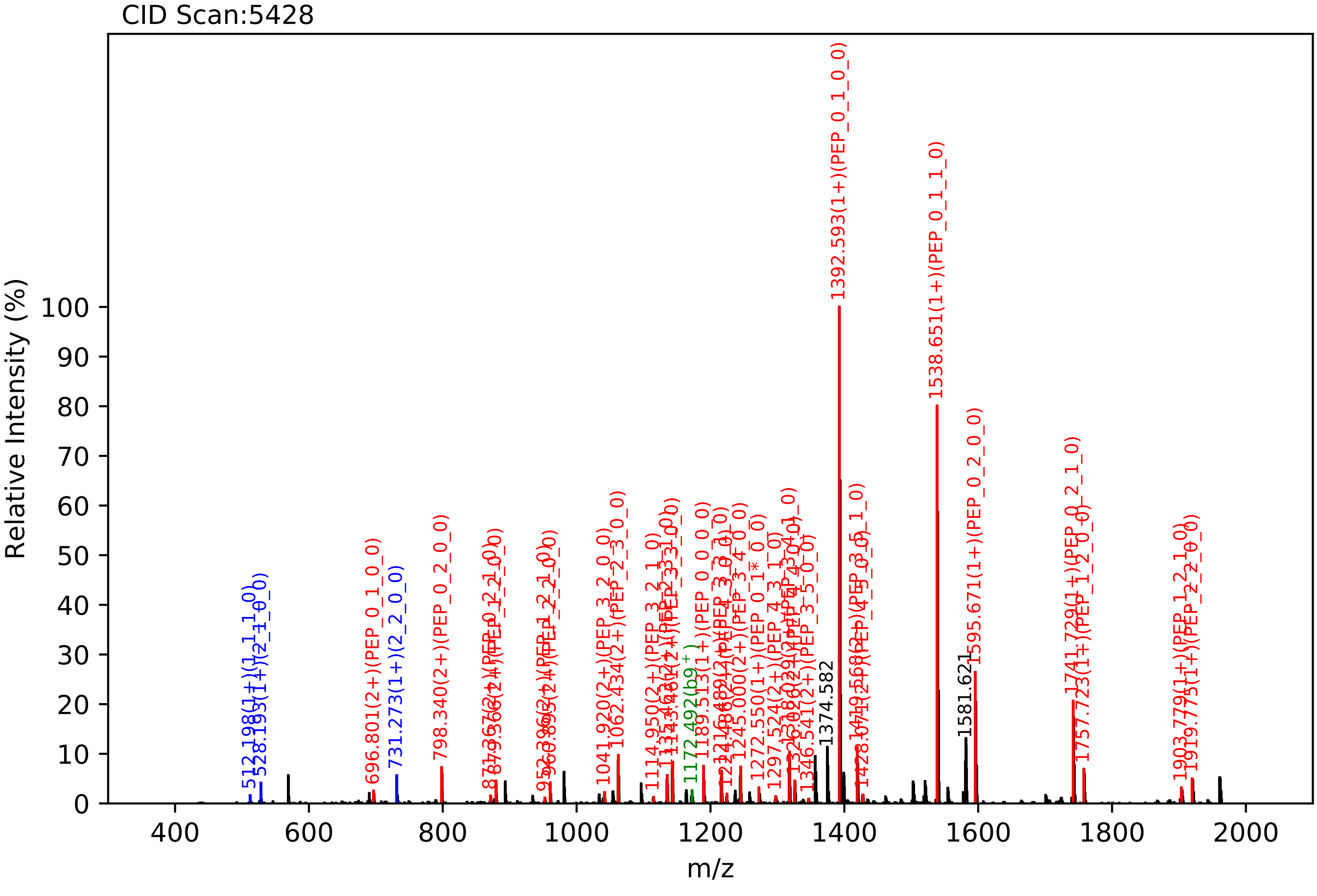

Training set no. 354, Experiment: IgG exp_3

EEQYNSTYR(=PEP)_4_5_1_0, m/z:1500.09(2+), RT:24.63, Y-score:82.93

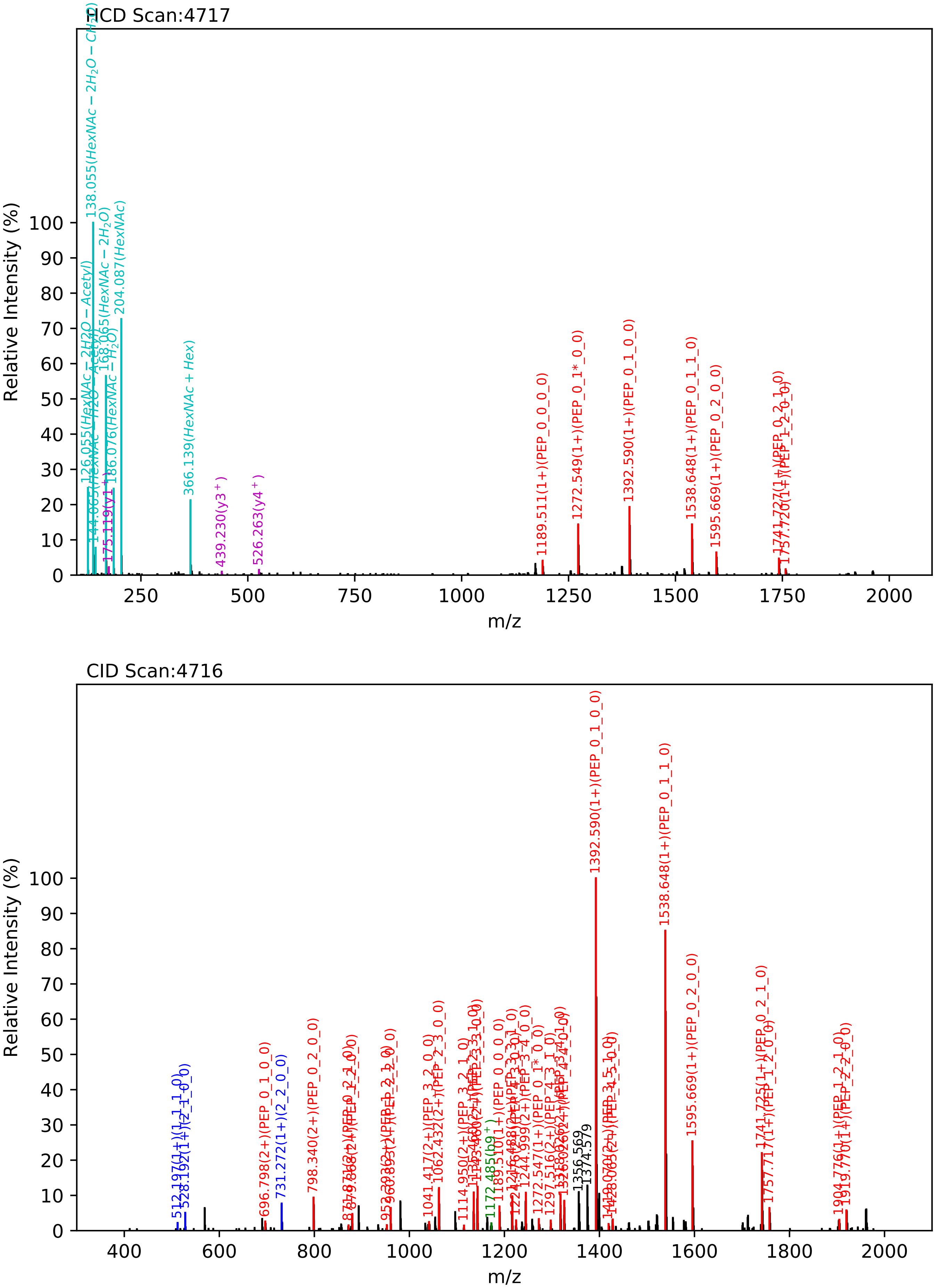

Training set no. 355, Experiment: IgG exp_2

EEQYNSTYR(=PEP)_4_5_1_0, m/z:1500.10(2+), RT:23.84, Y-score:75.90

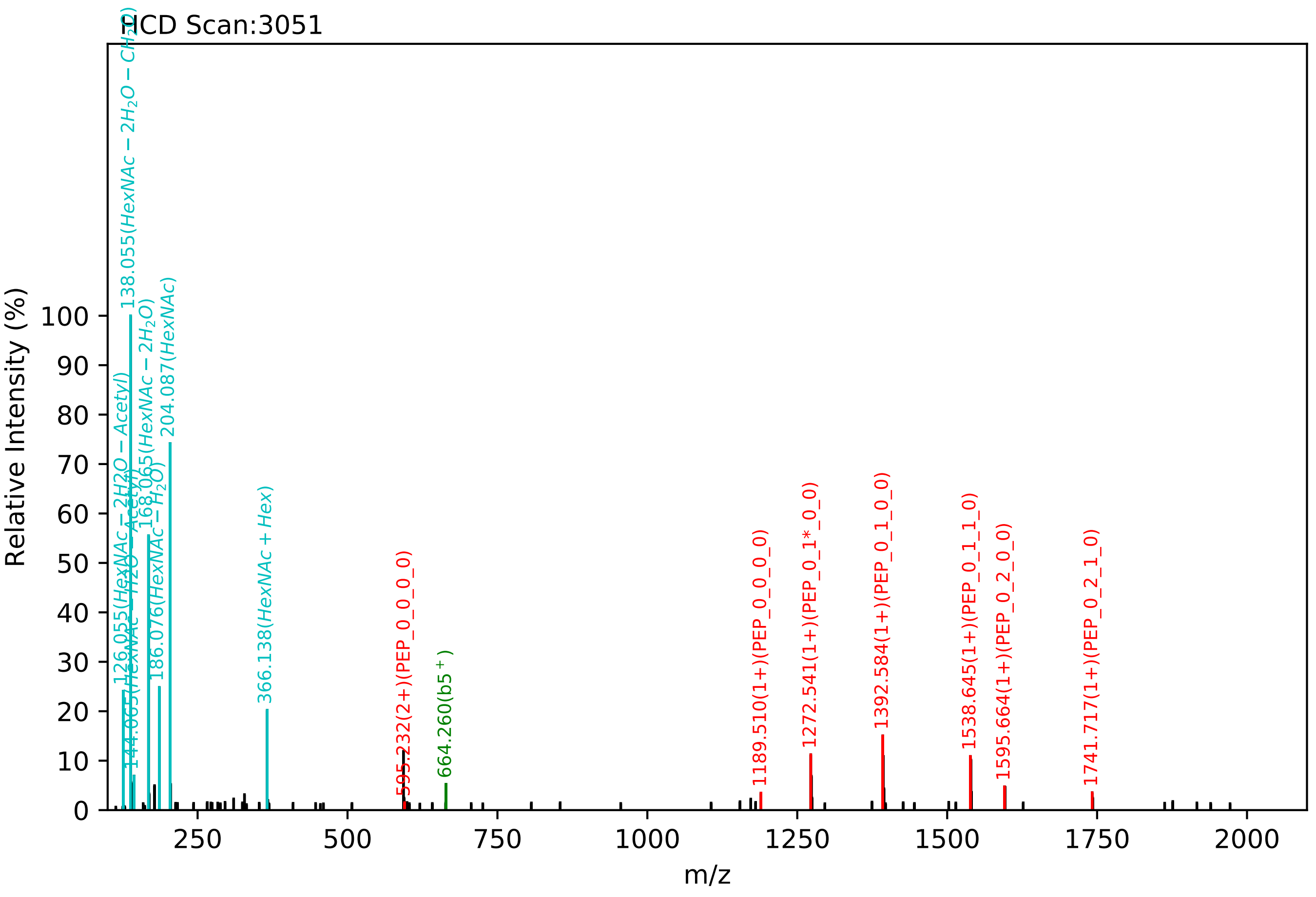

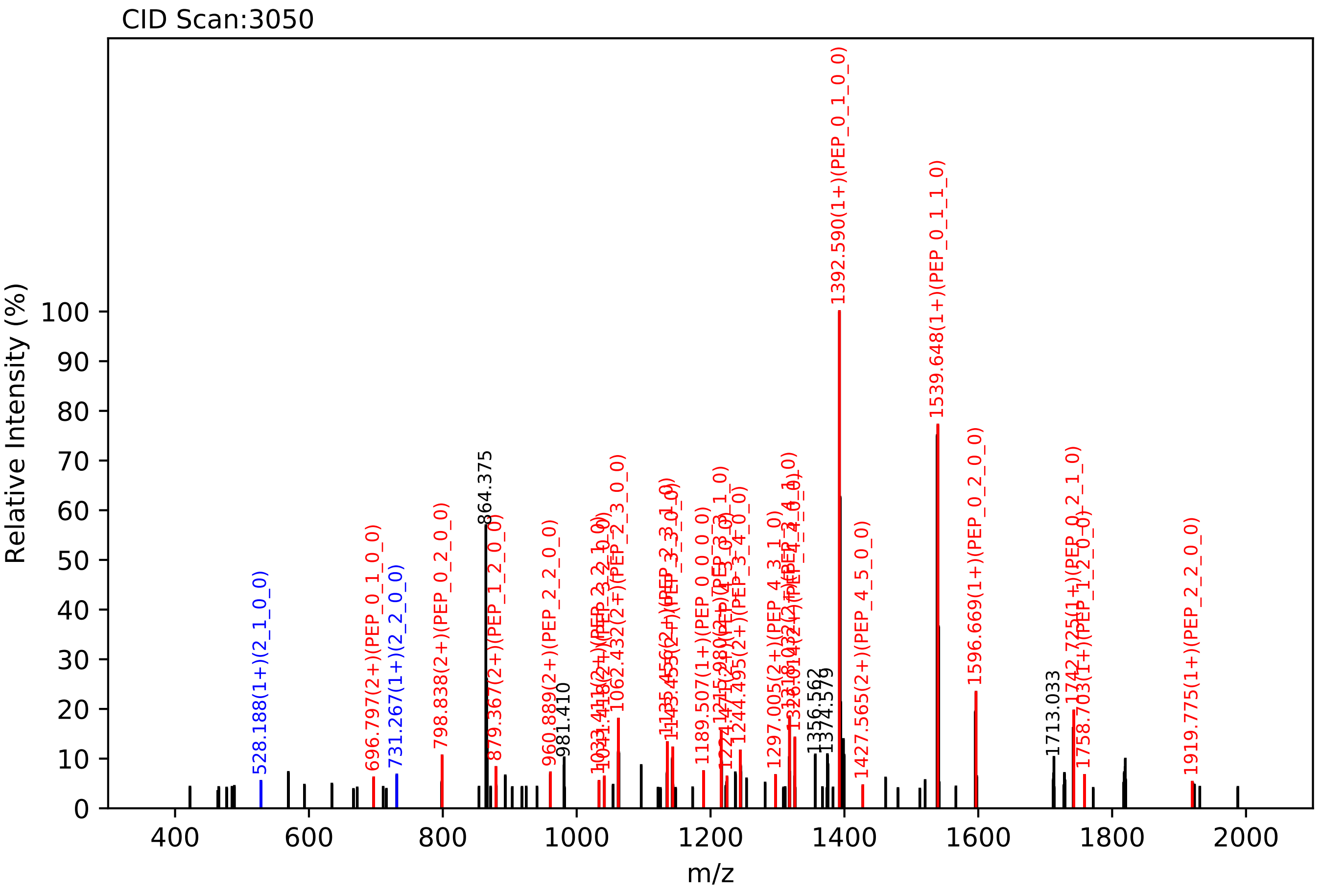

Training set no. 356, Experiment: IgG exp_4

EEQYNSTYR(=PEP)_5_4_0_0, m/z:1406.55(2+), RT:20.95, Y-score:87.59

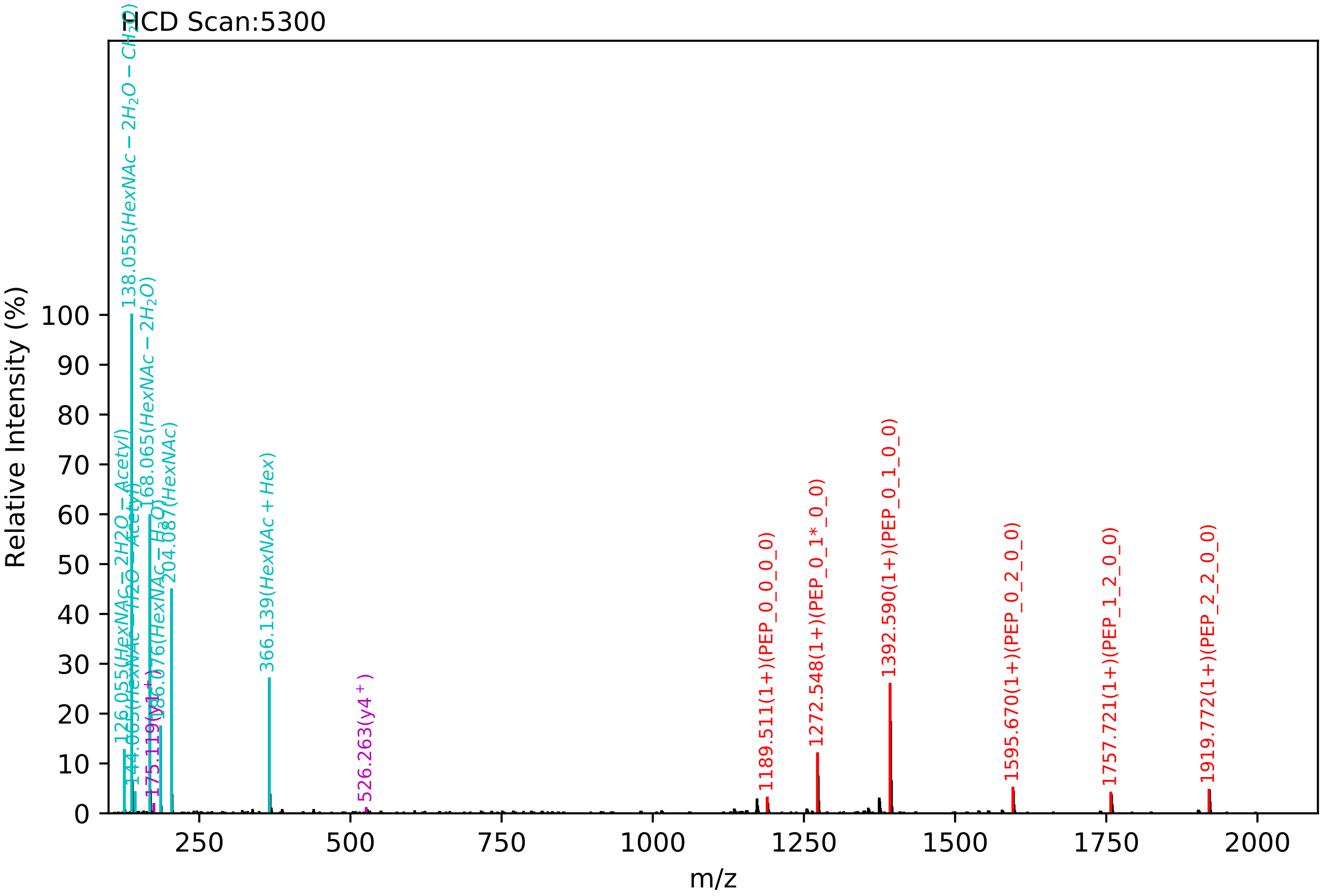

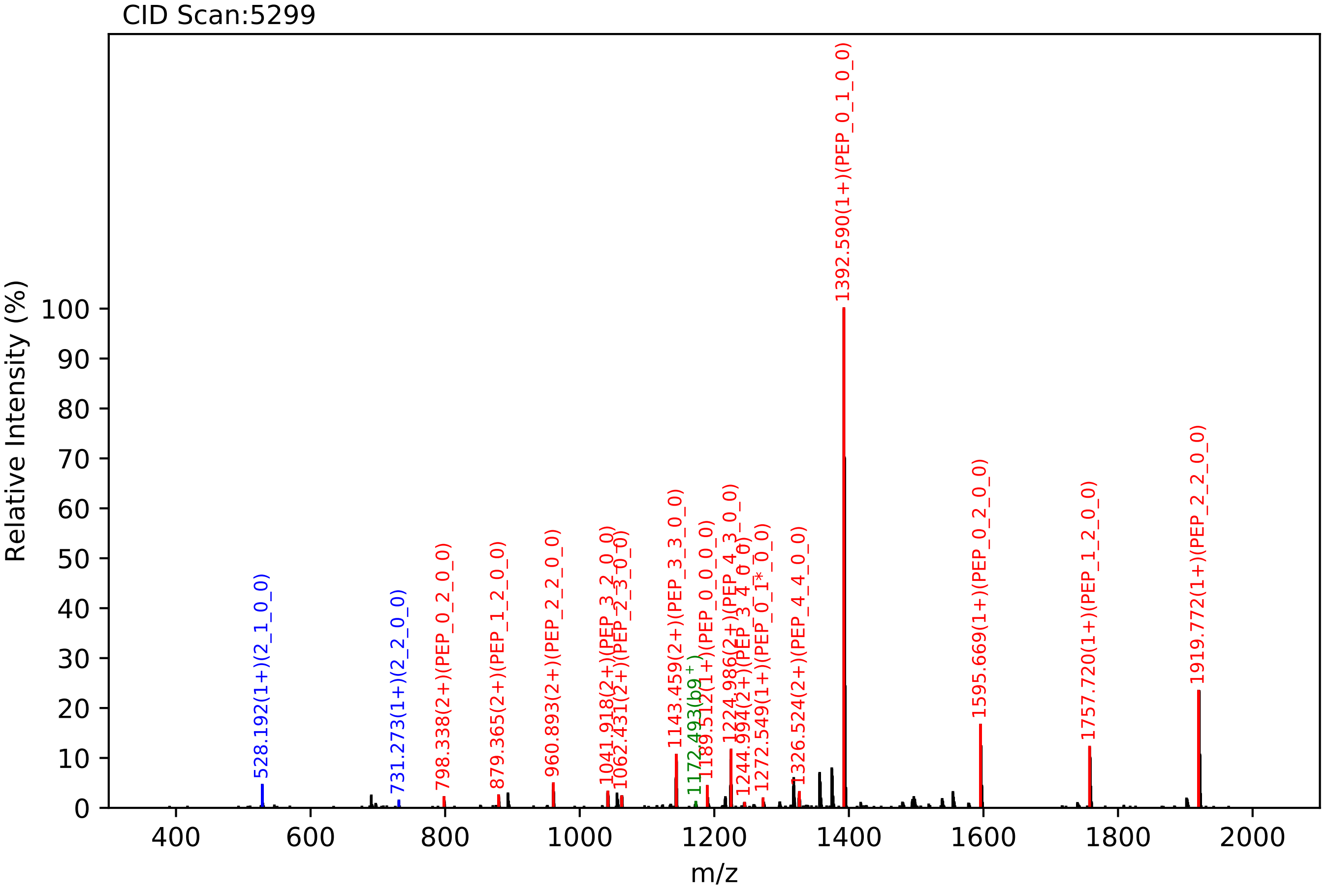

Training set no. 357, Experiment: IgG exp_3

EEQYNSTYR(=PEP)_5_4_0_0, m/z:1407.05(2+), RT:23.28, Y-score:85.12

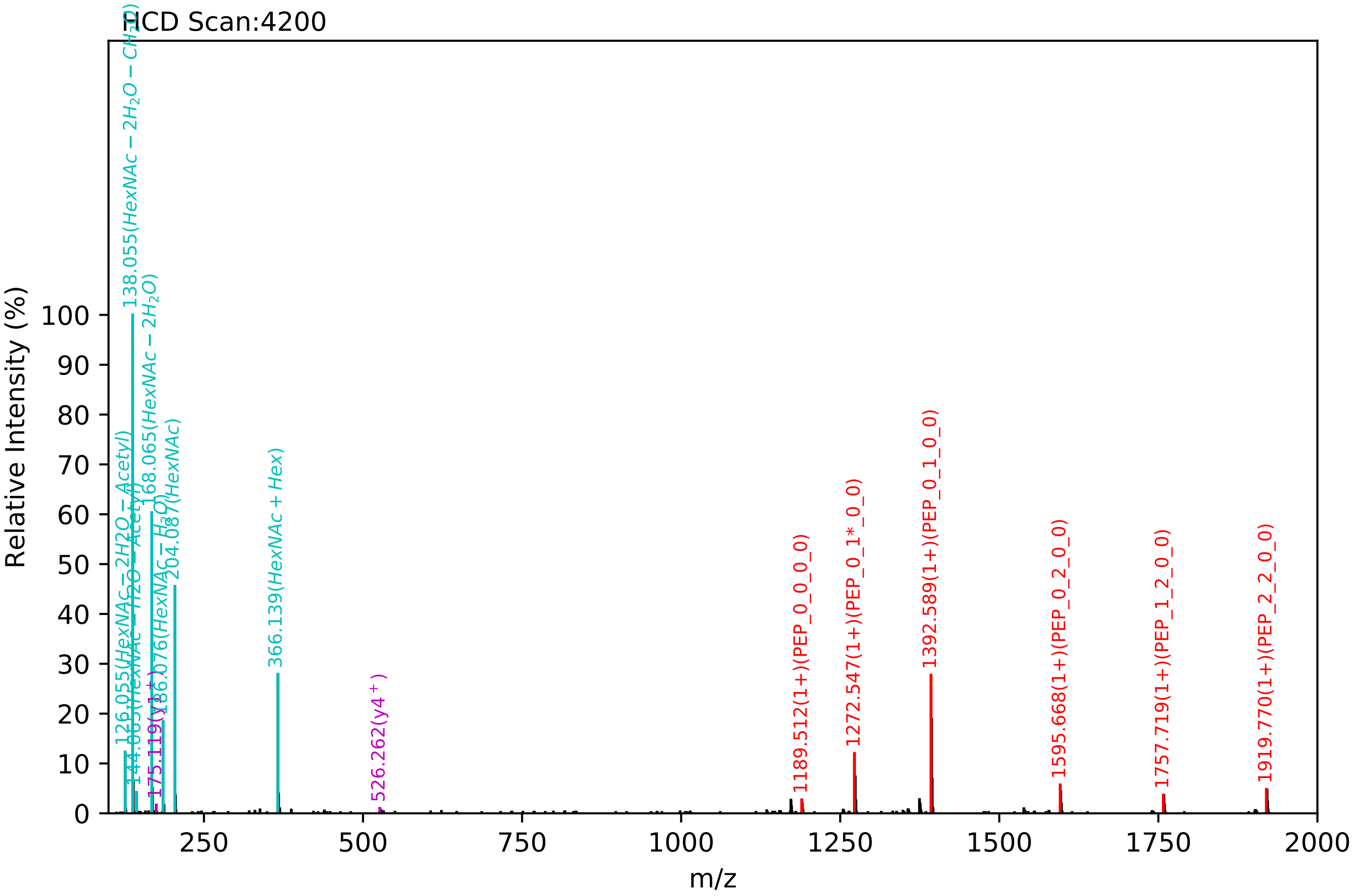

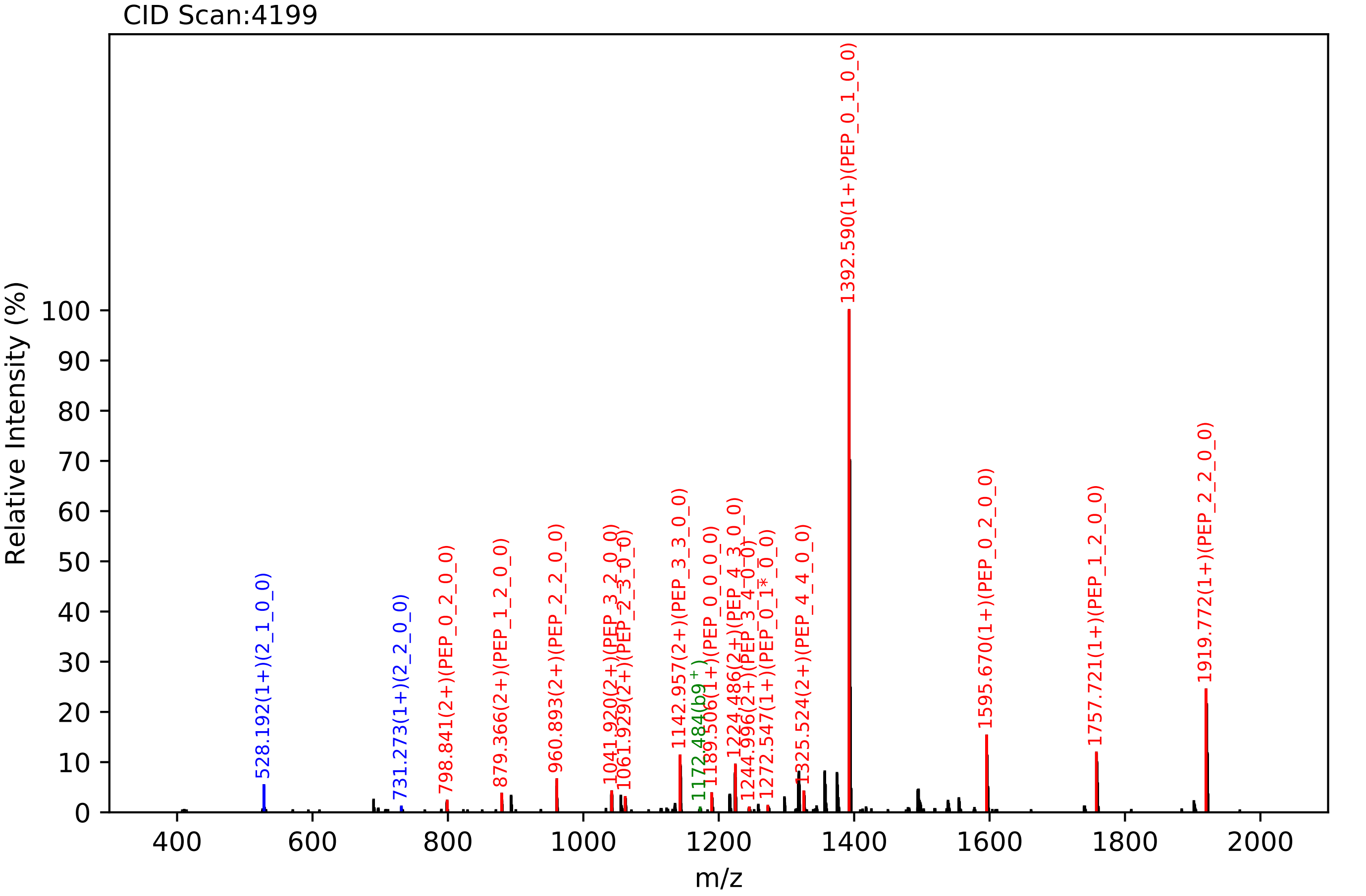

Training set no. 358, Experiment: IgG exp_3

EEQYNSTYR(=PEP)_5_4_0_1, m/z:1552.10(2+), RT:25.57, Y-score:88.12

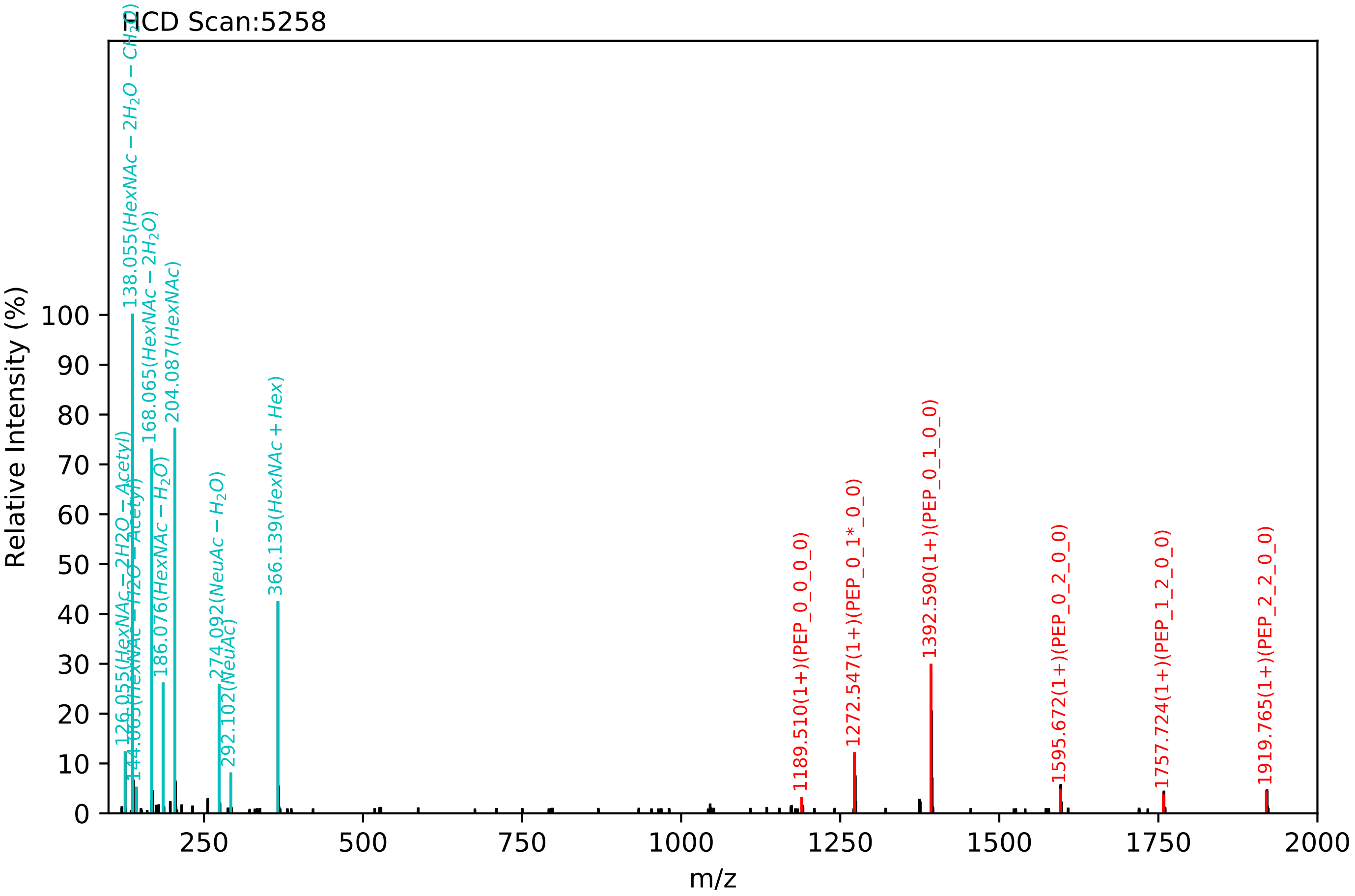

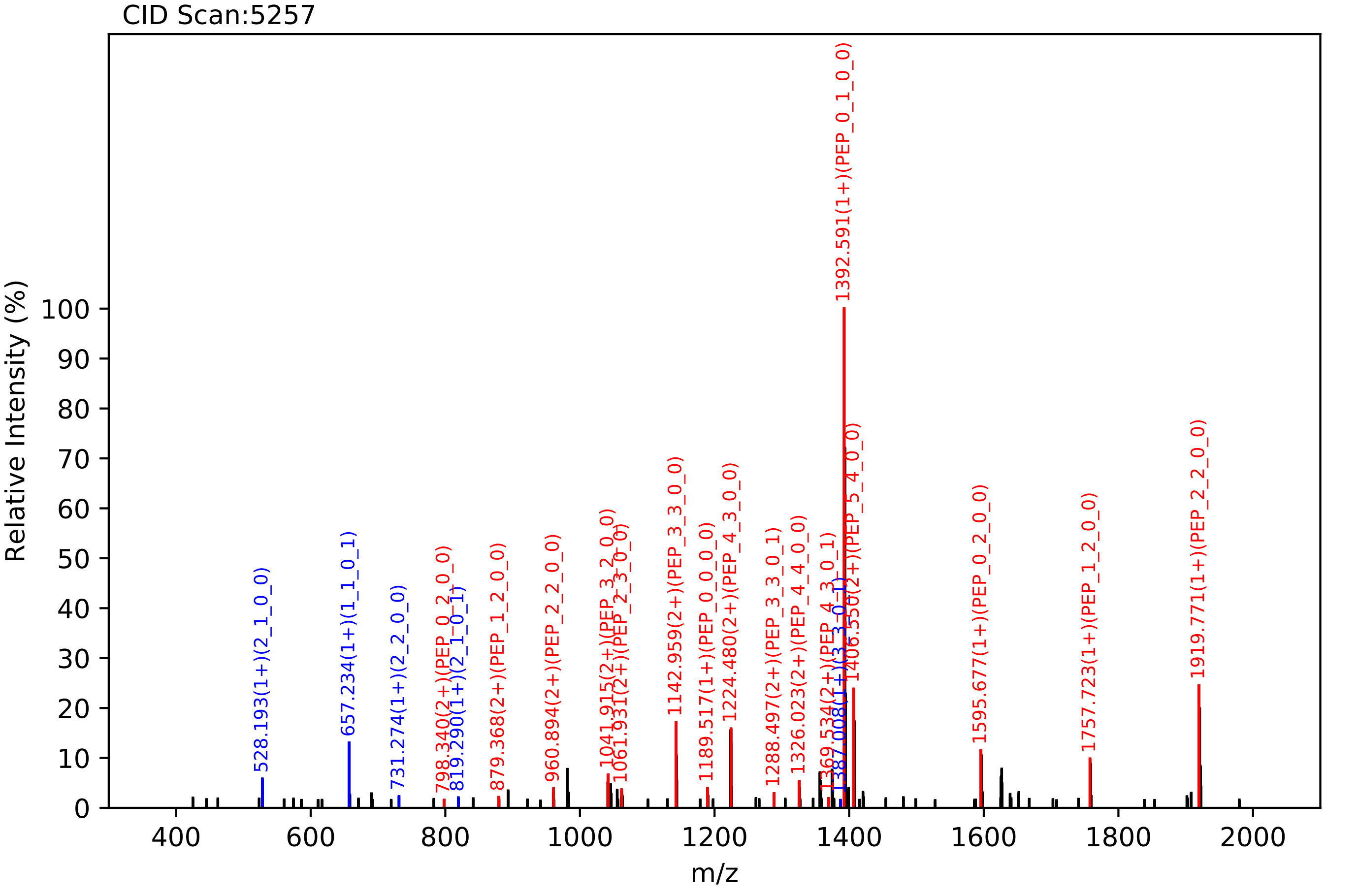

Training set no. 359, Experiment: IgG exp_1

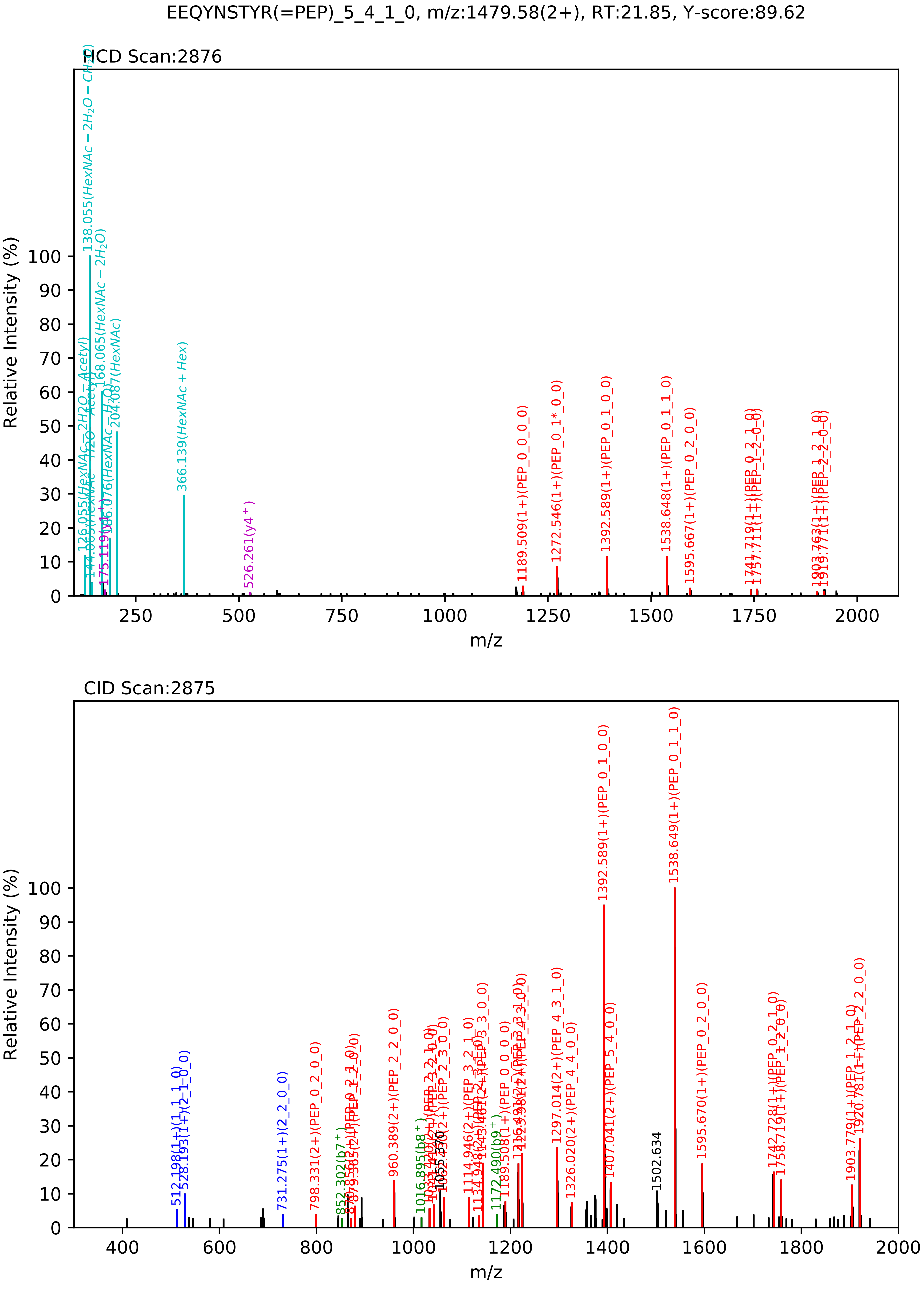

Training set no. 360, Experiment: IgG exp_3

EEQYNSTYR(=PEP)_5_4_1_0, m/z:1479.58(2+), RT:23.08, Y-score:88.56

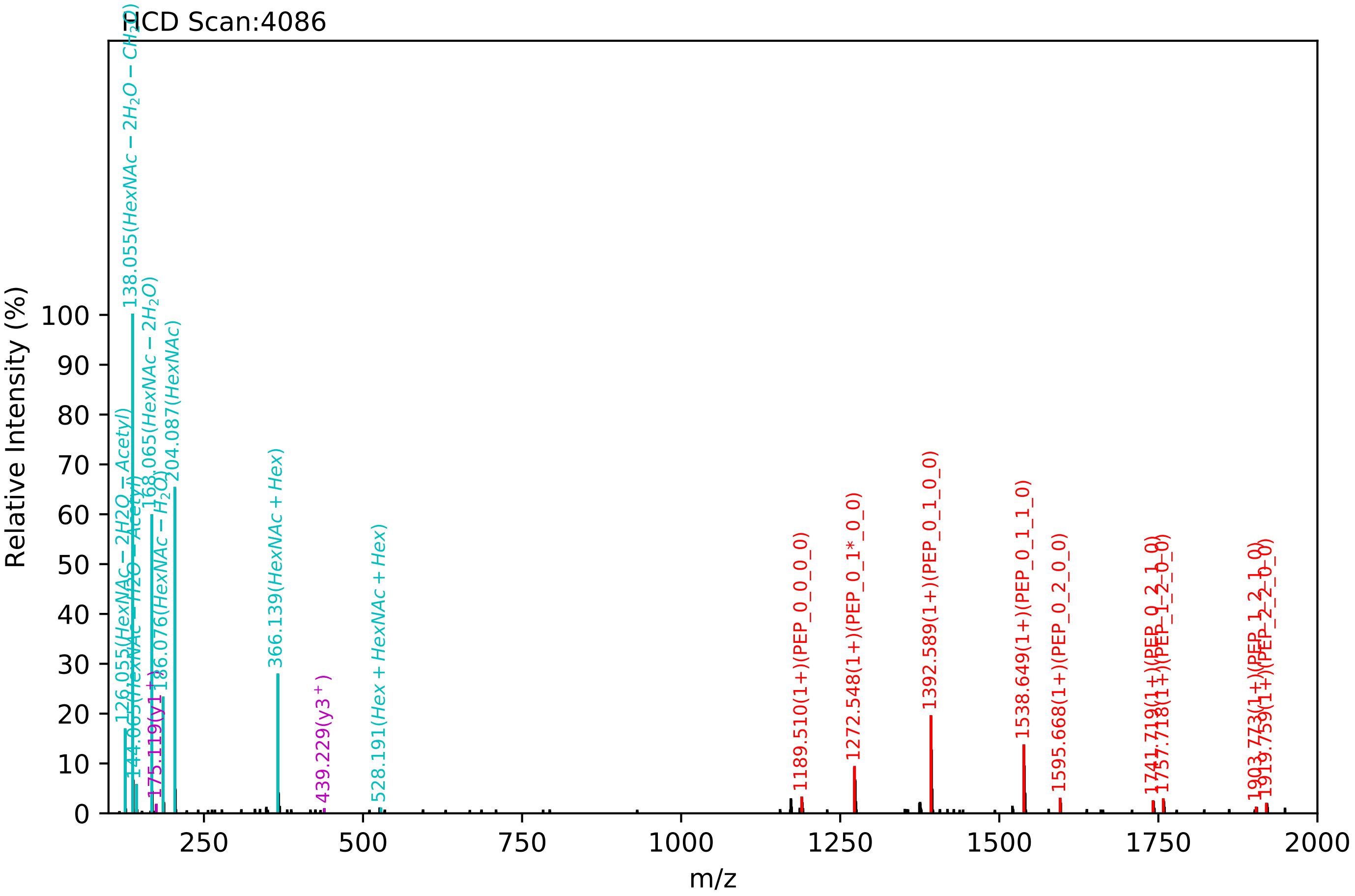

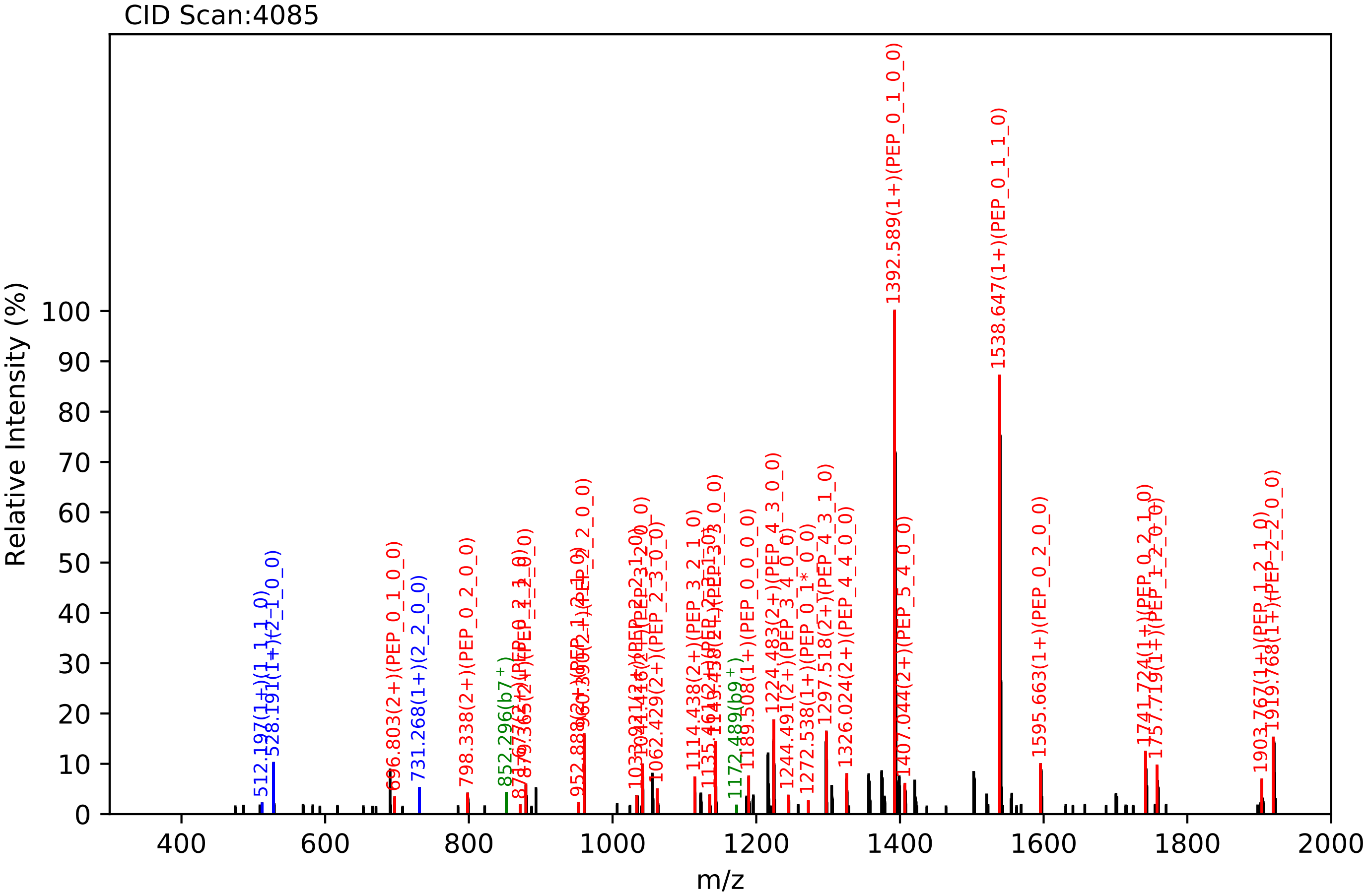

Training set no. 361, Experiment: IgG exp_2

EEQYNSTYR(=PEP)_5_4_1_0, m/z:1480.08(2+), RT:23.76, Y-score:85.29

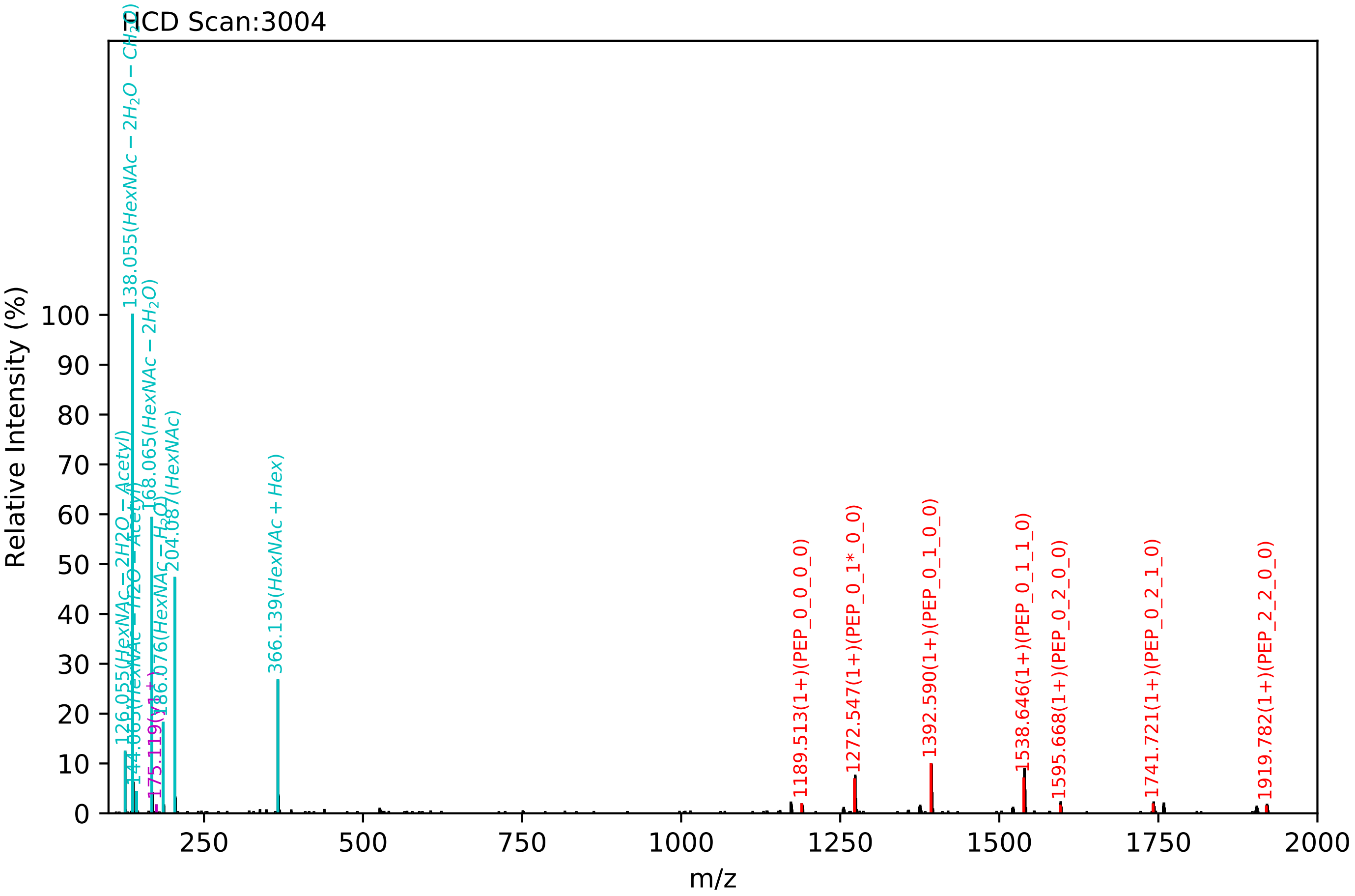

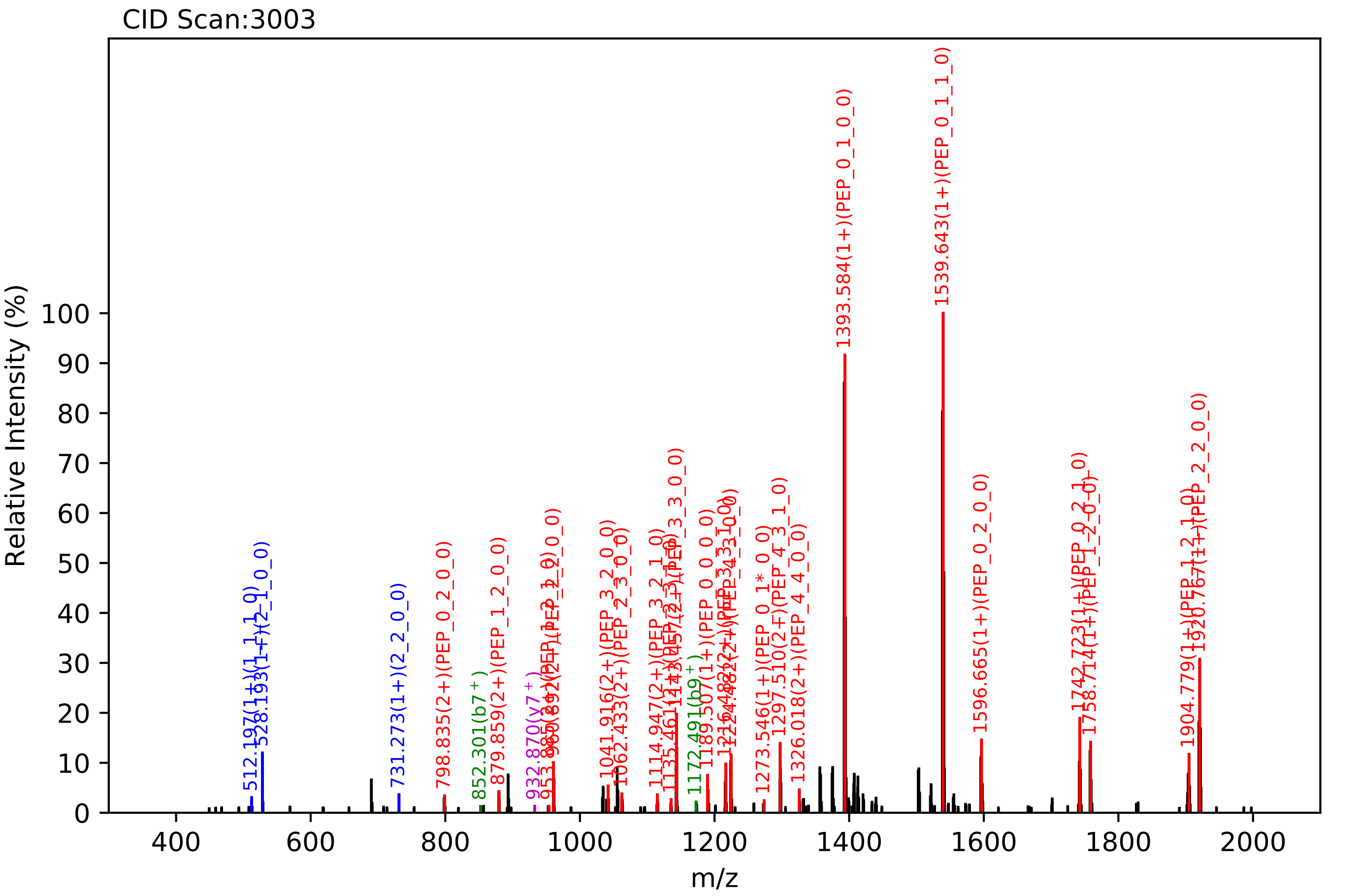

Training set no. 362, Experiment: IgG exp_4

EEQYNSTYR(=PEP)_5_4_1_0, m/z:1479.58(2+), RT:20.75, Y-score:83.96

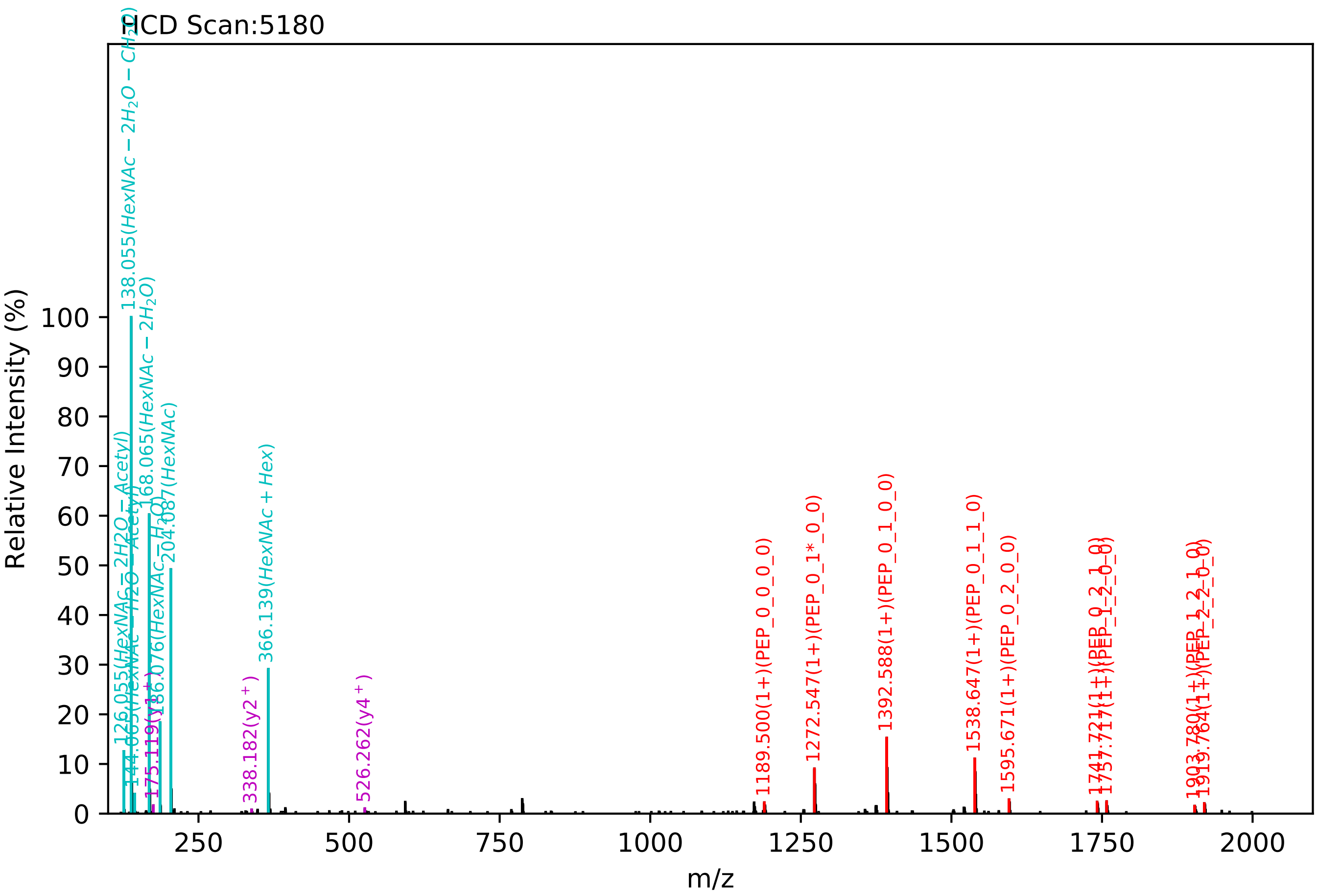

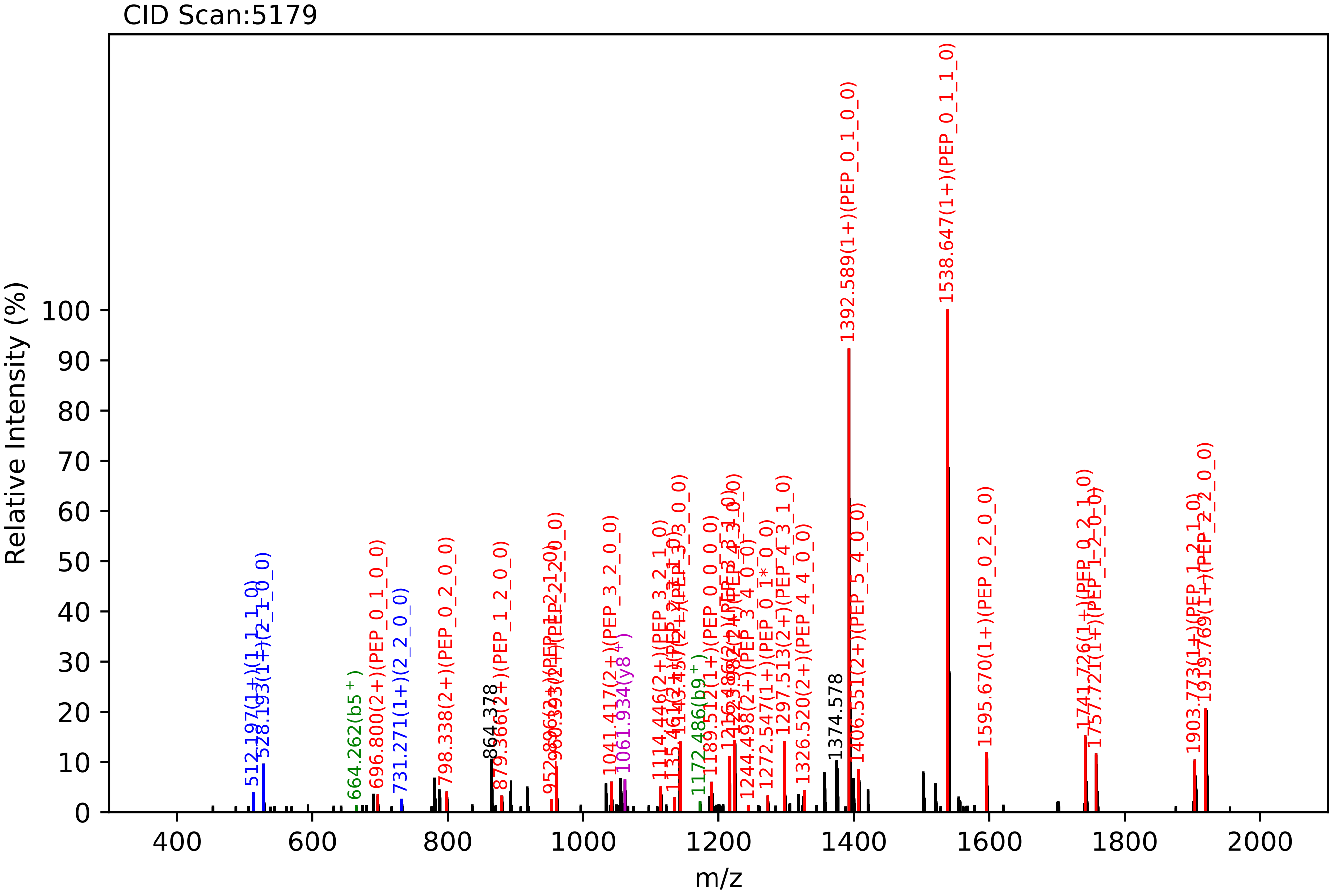

Training set no. 363, Experiment: IgG exp_3

EEQYNSTYR(=PEP)_5_4_1_0, m/z:1479.58(2+), RT:23.64, Y-score:83.27

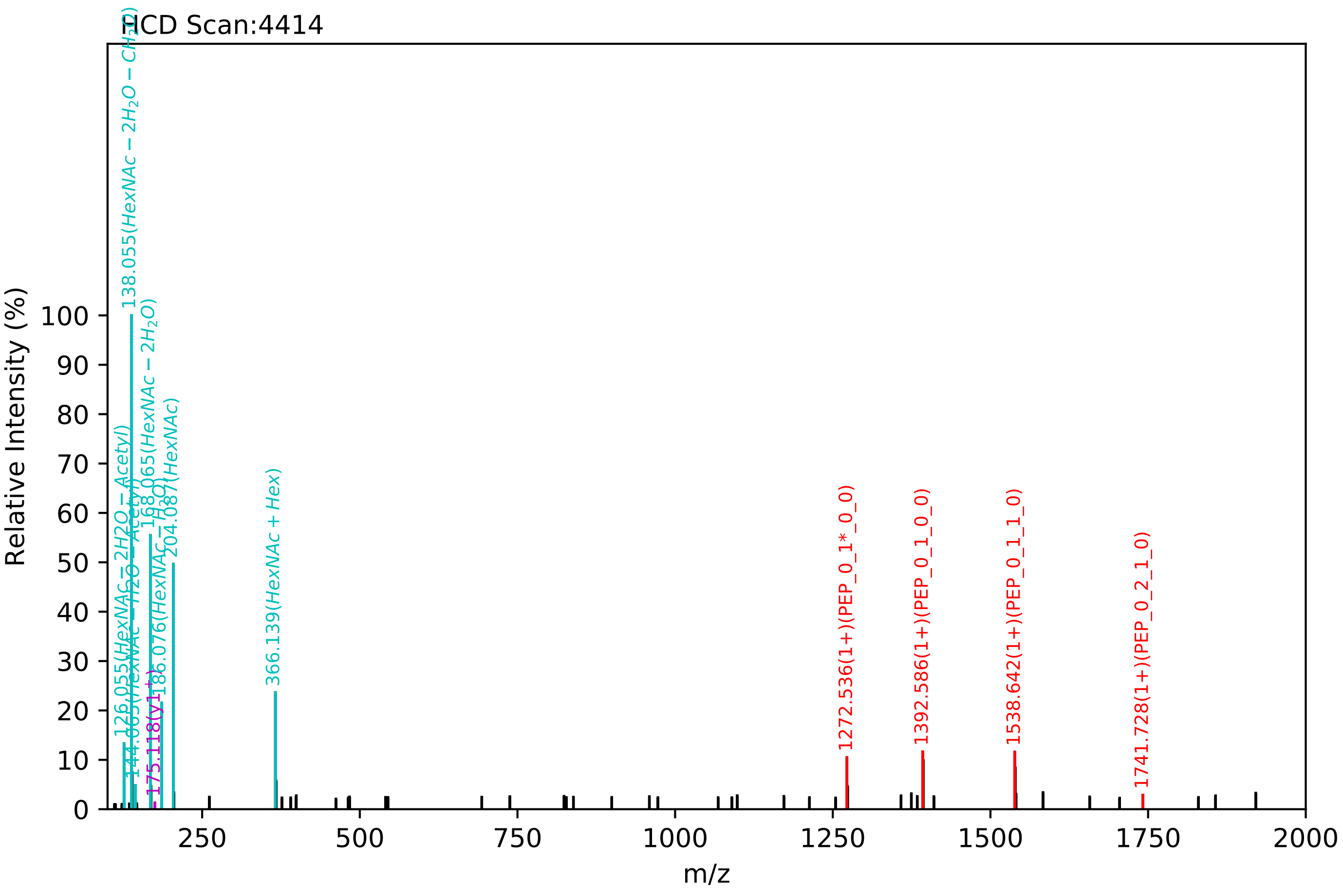

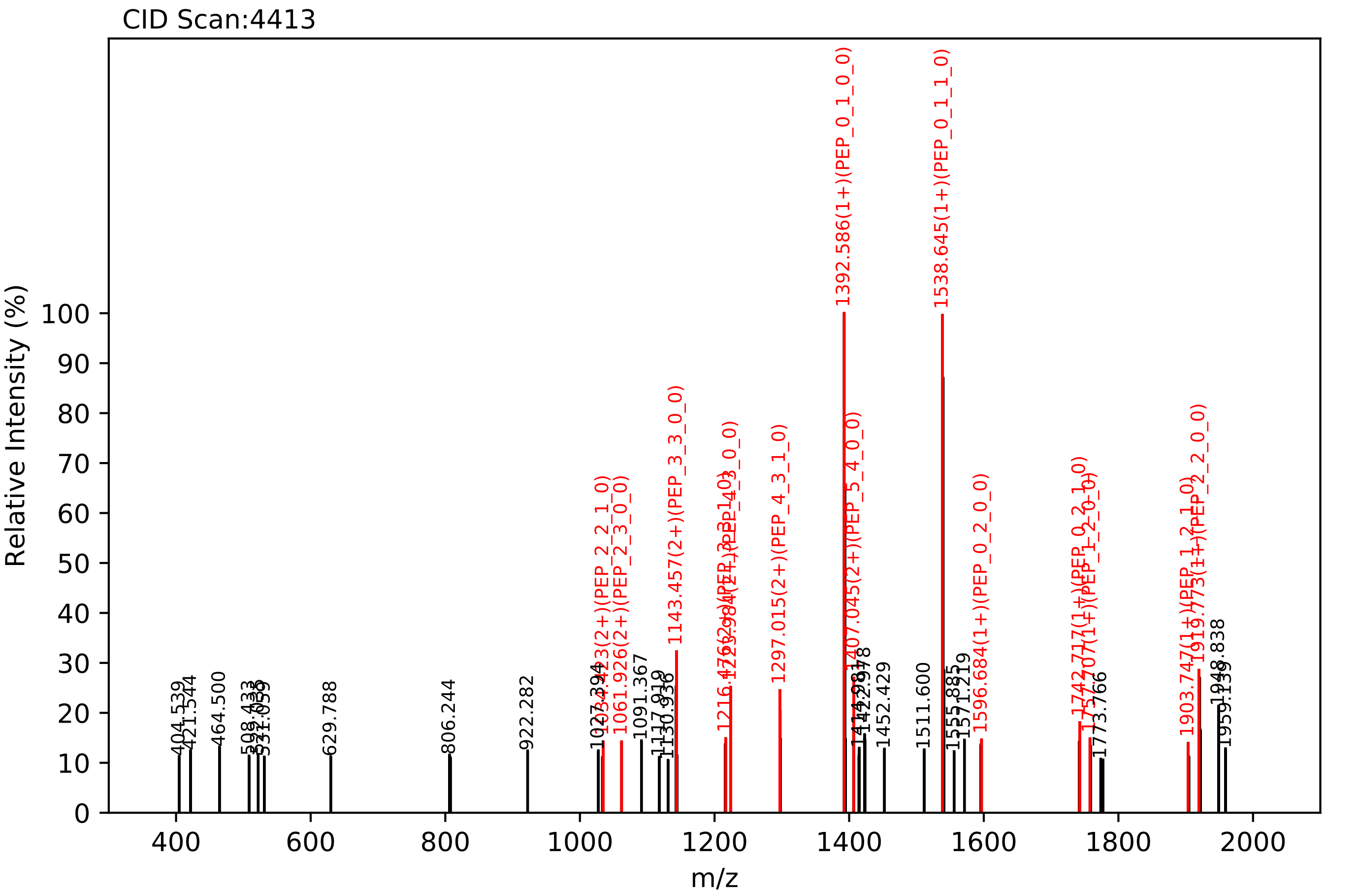

Training set no. 364, Experiment: IgG exp_3

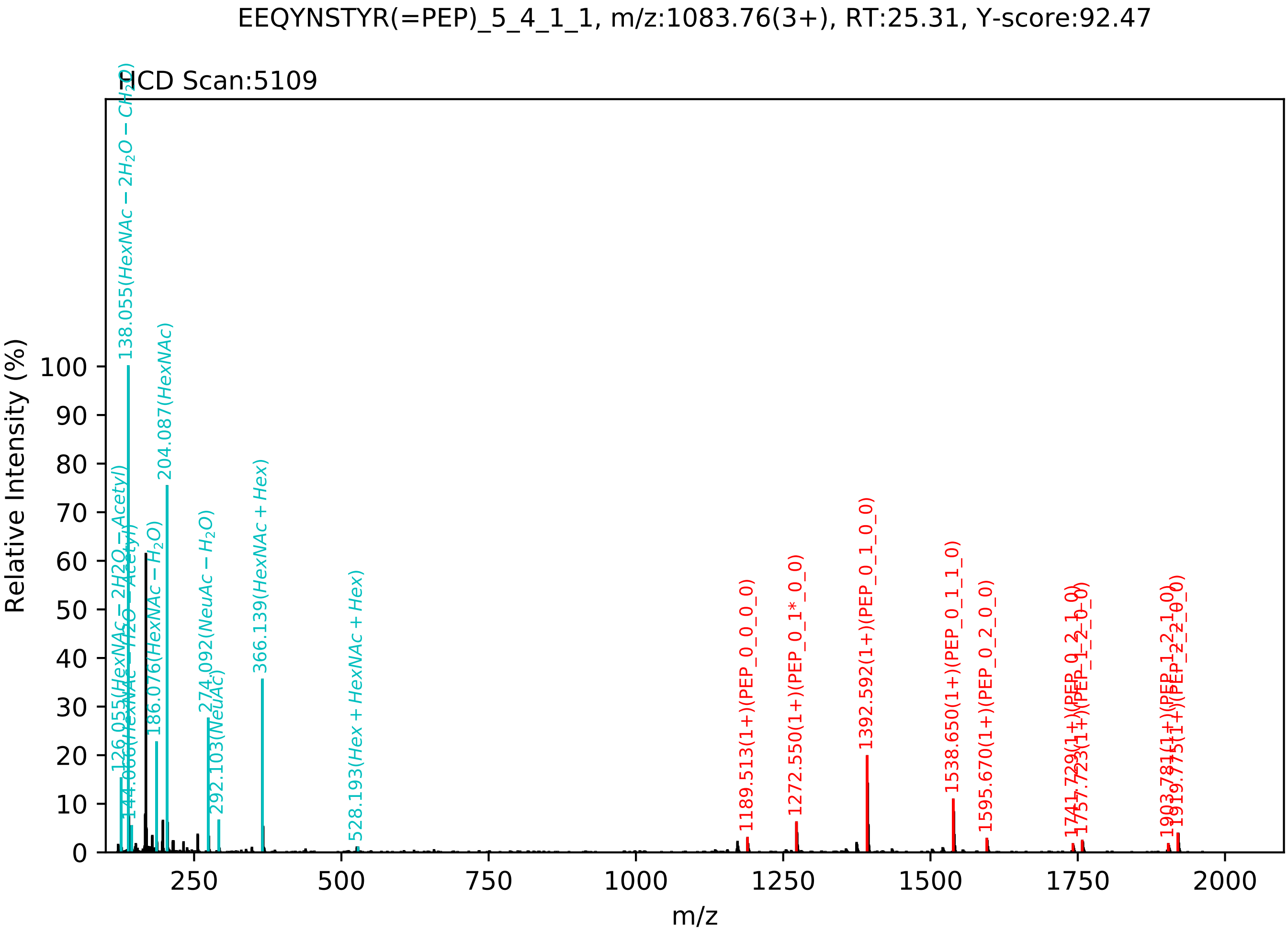

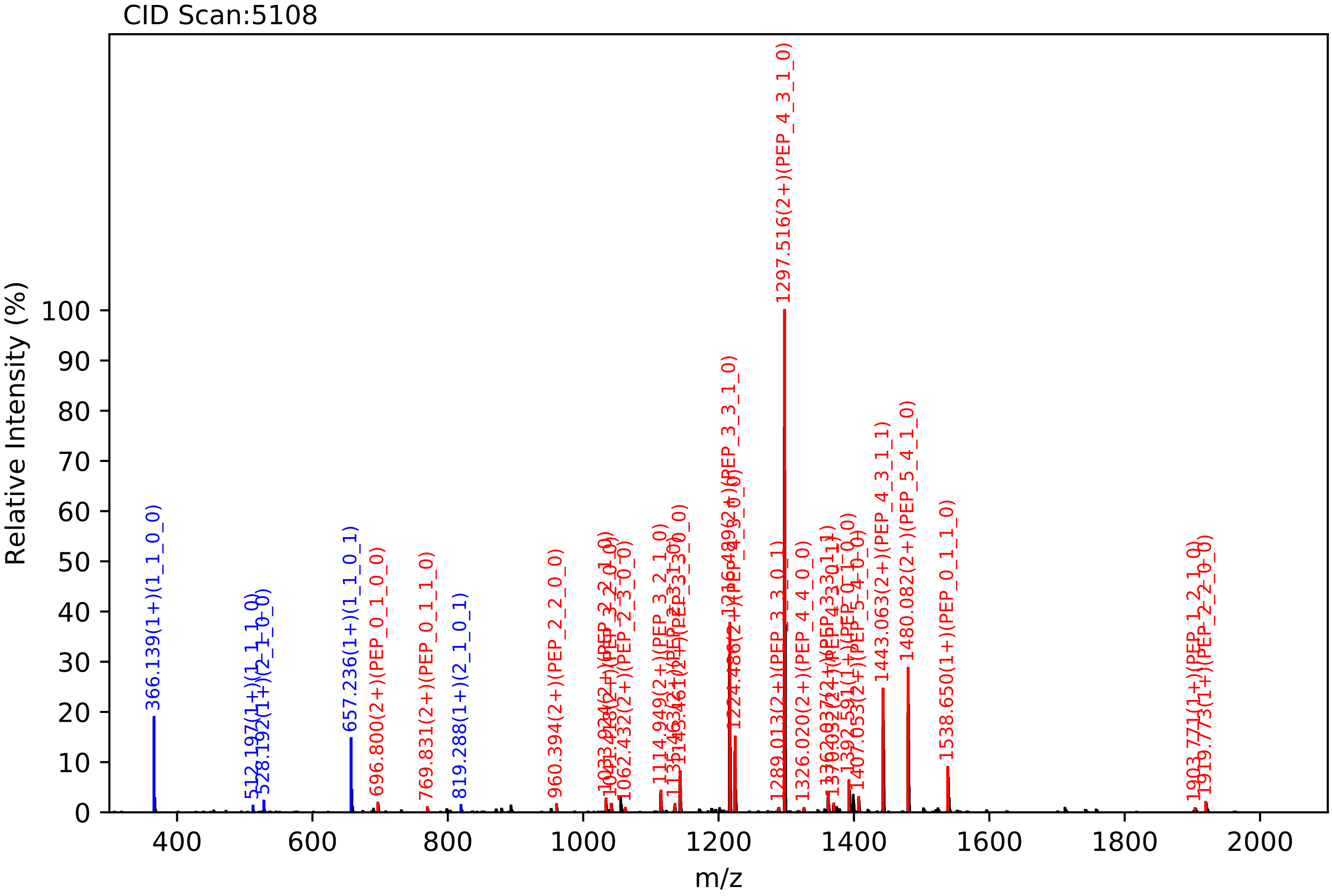

Training set no. 365, Experiment: IgG exp_1

EEQYNSTYR(=PEP)_5_4_1_1, m/z:1083.76(3+), RT:23.20, Y-score:92.23

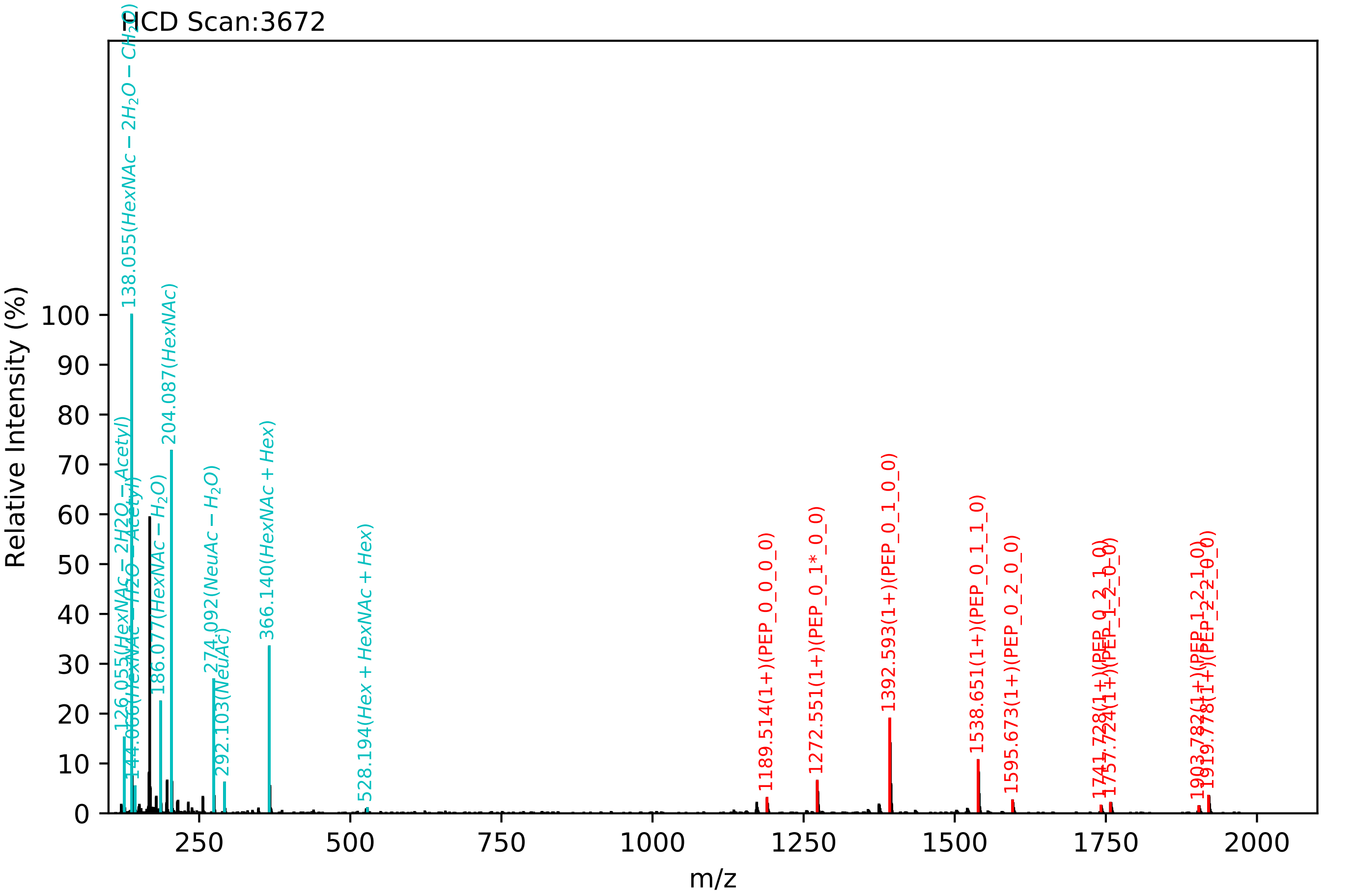

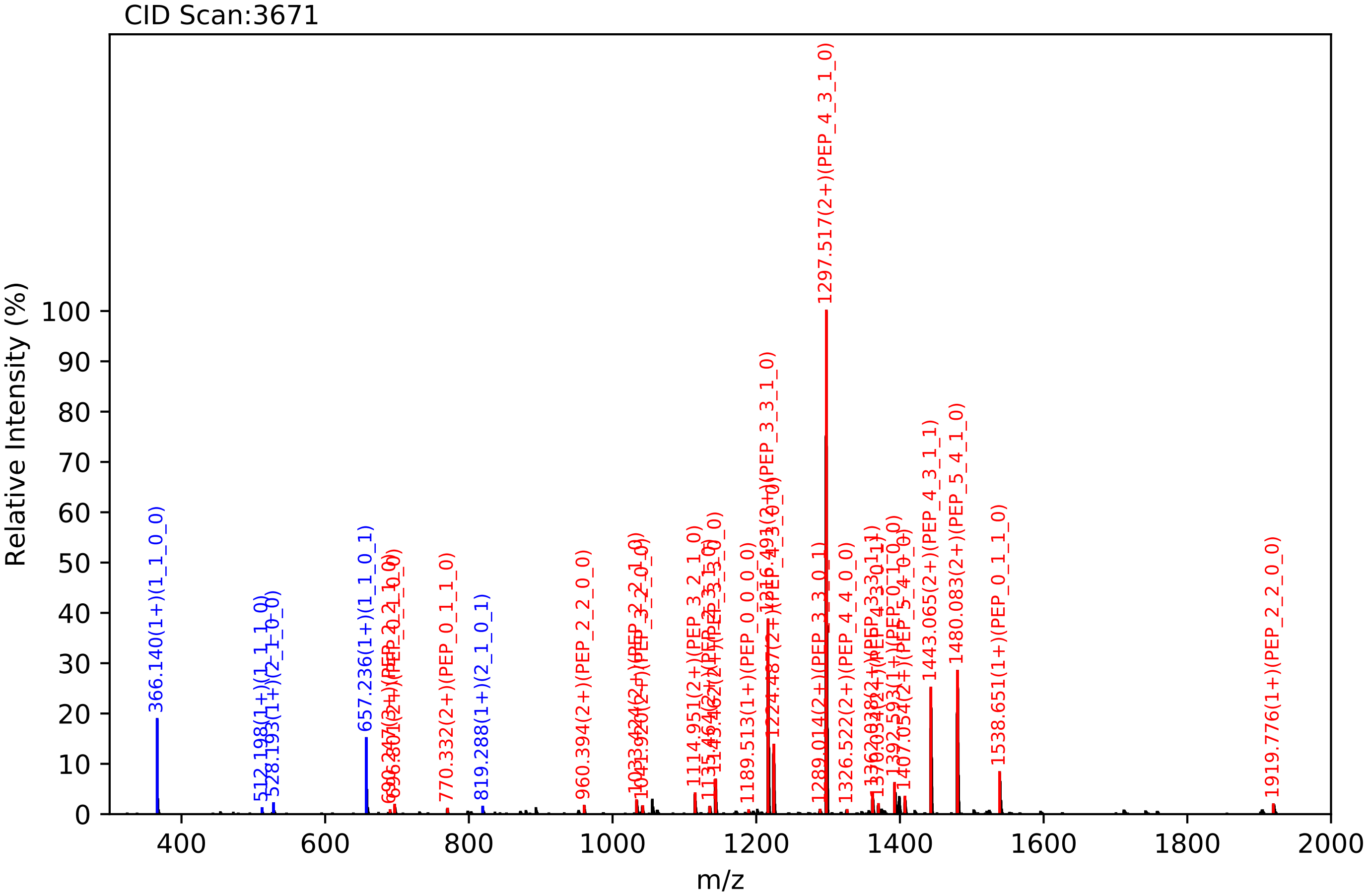

Training set no. 366, Experiment: IgG exp_4

EEQYNSTYR(=PEP)_5_4_1_1, m/z:1083.75(3+), RT:22.32, Y-score:92.15

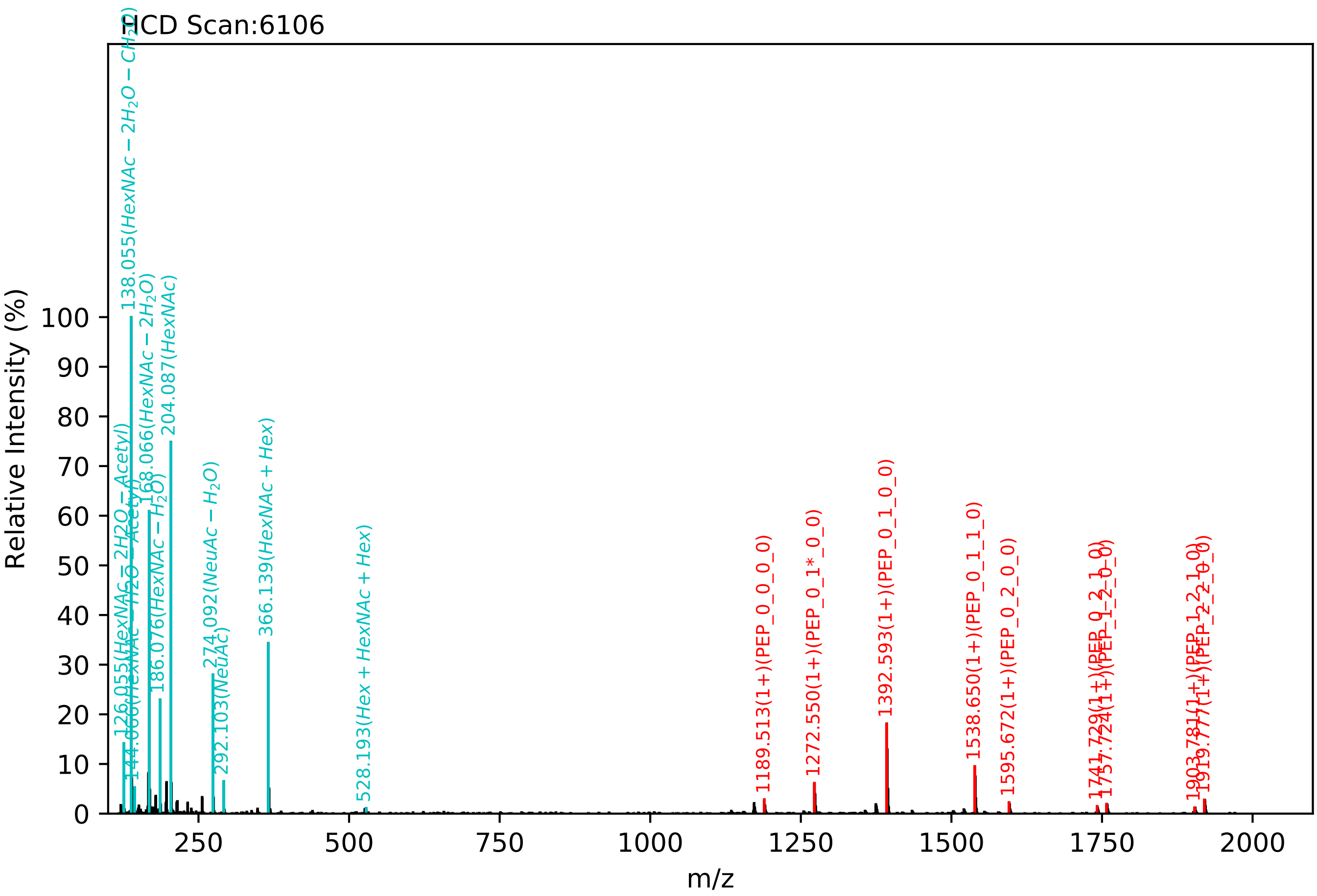

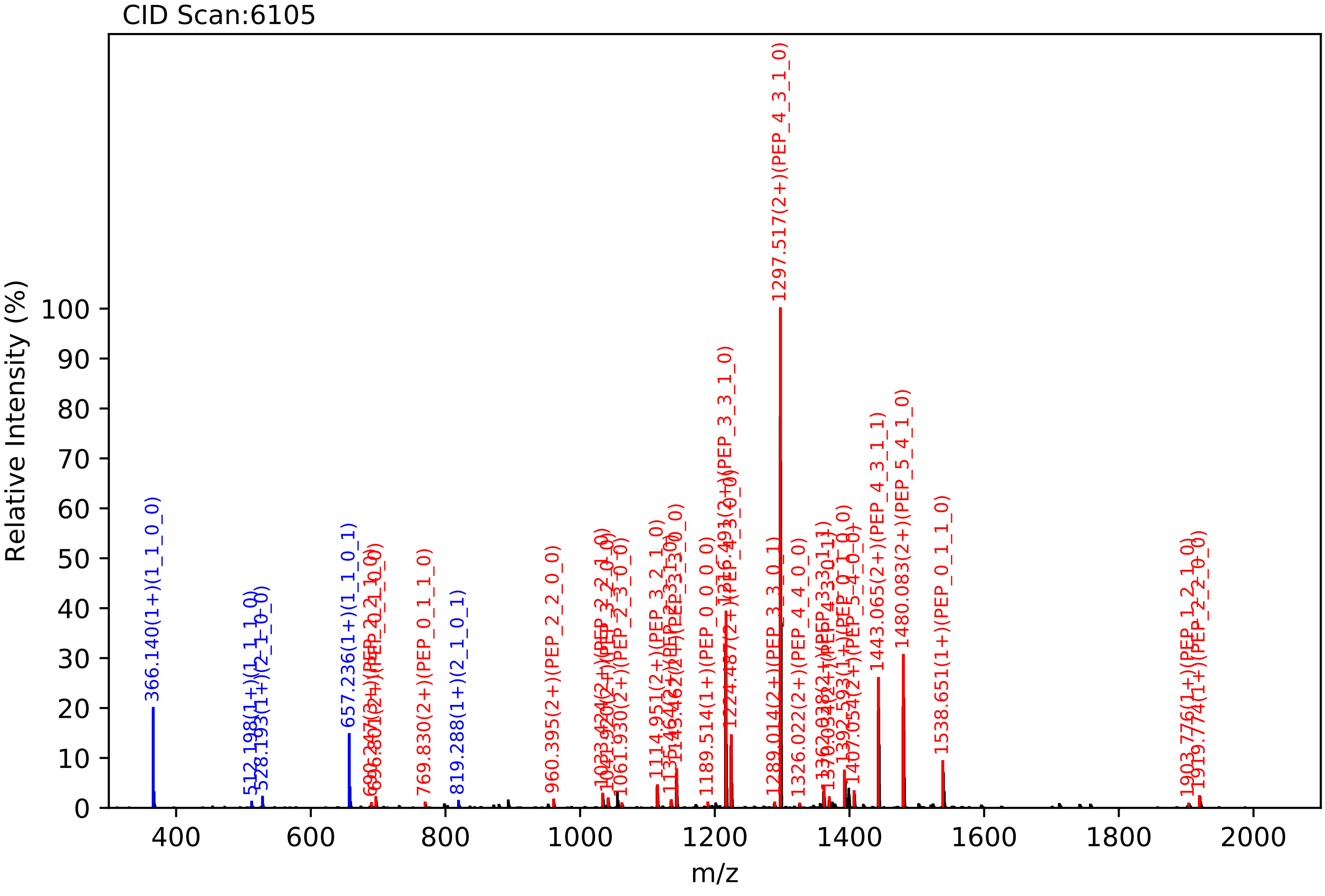

Training set no. 367, Experiment: IgG exp_3

EEQYNSTYR(=PEP)_5_4_1_1, m/z:1625.13(2+), RT:25.32, Y-score:89.58

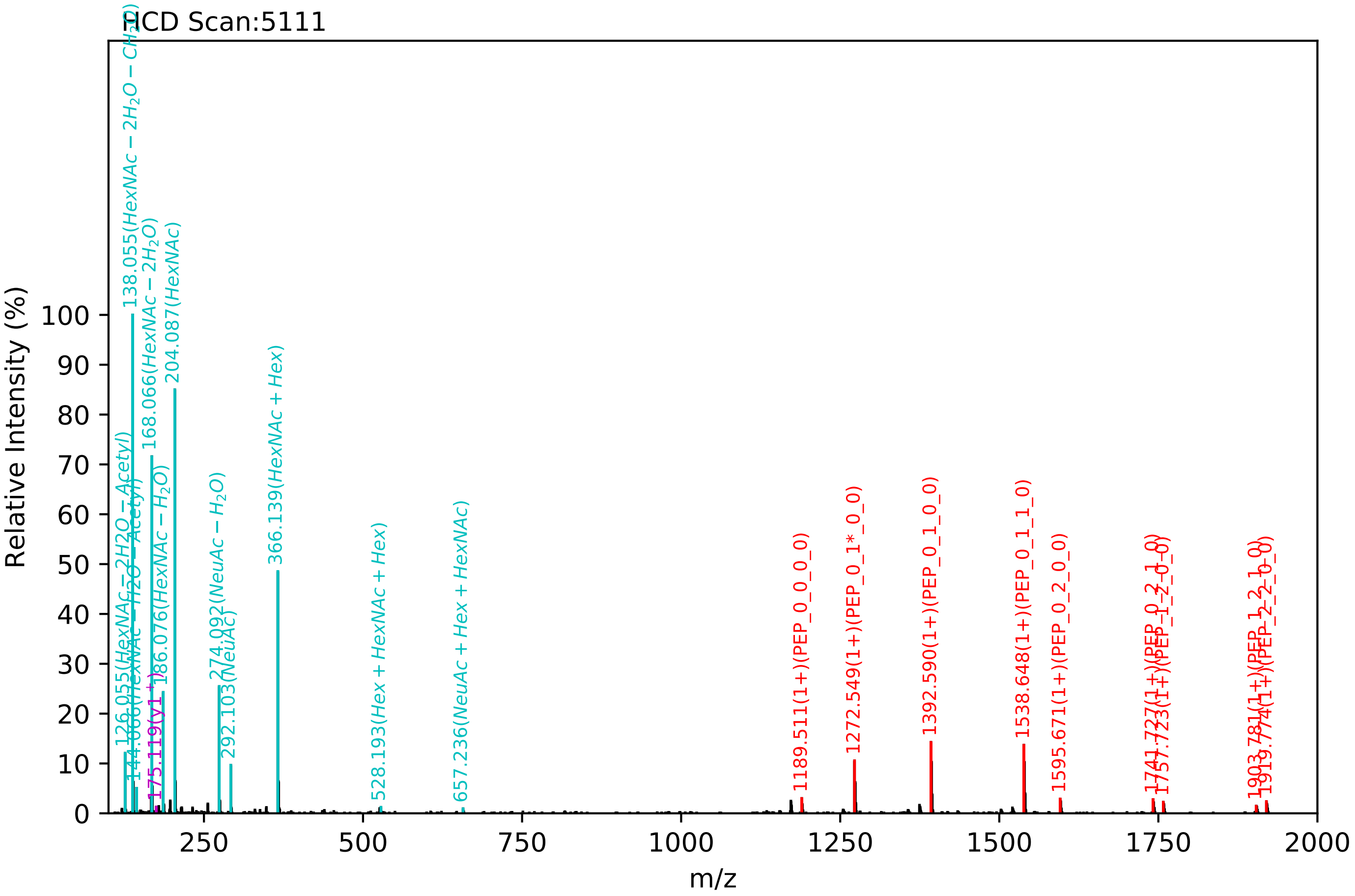

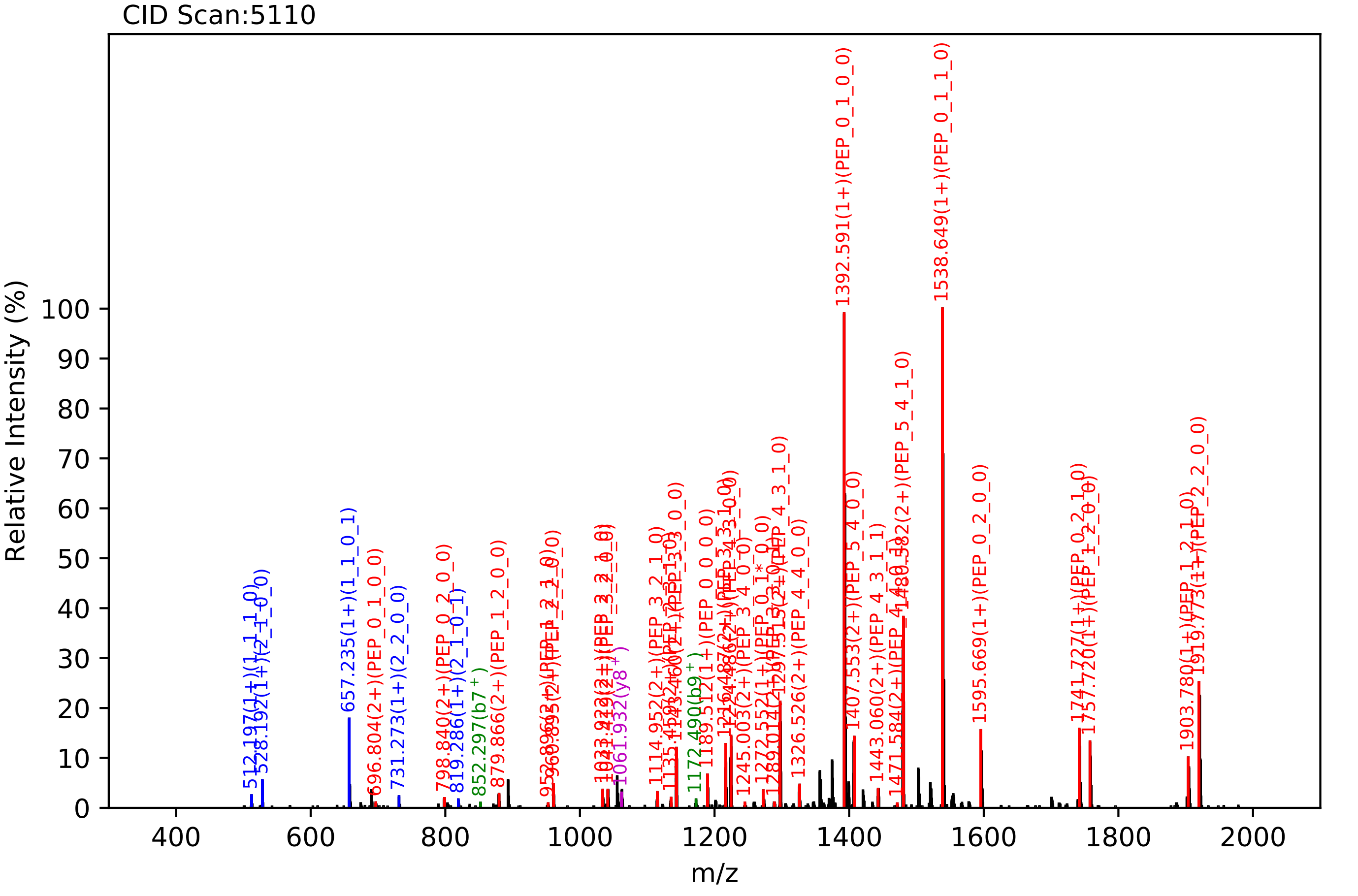

Training set no. 368, Experiment: IgG exp_4

EEQYNSTYR(=PEP)_5_4_1_1, m/z:1625.13(2+), RT:22.32, Y-score:89.36

HCD Scan:6110

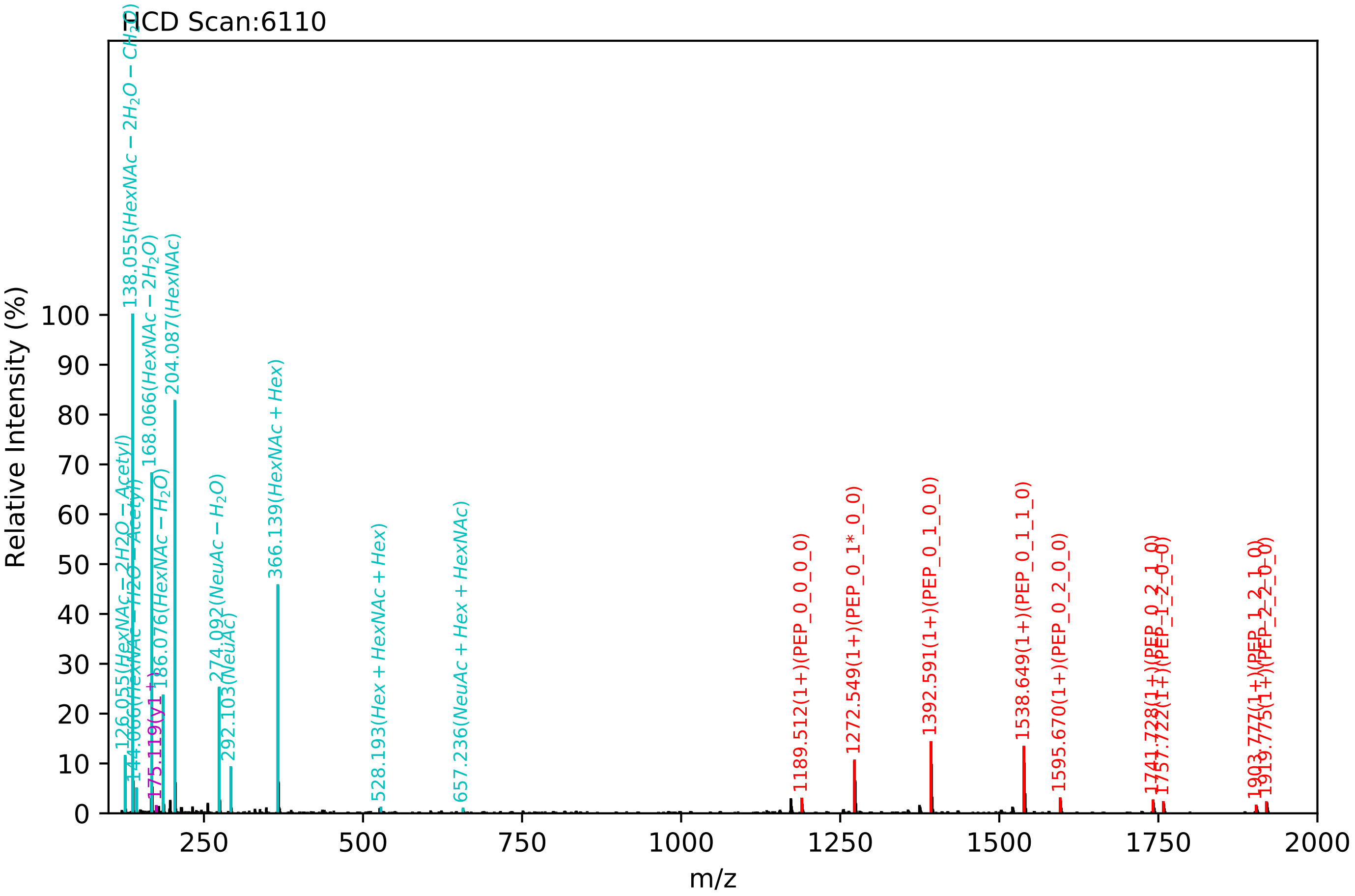

CID Scan:6109

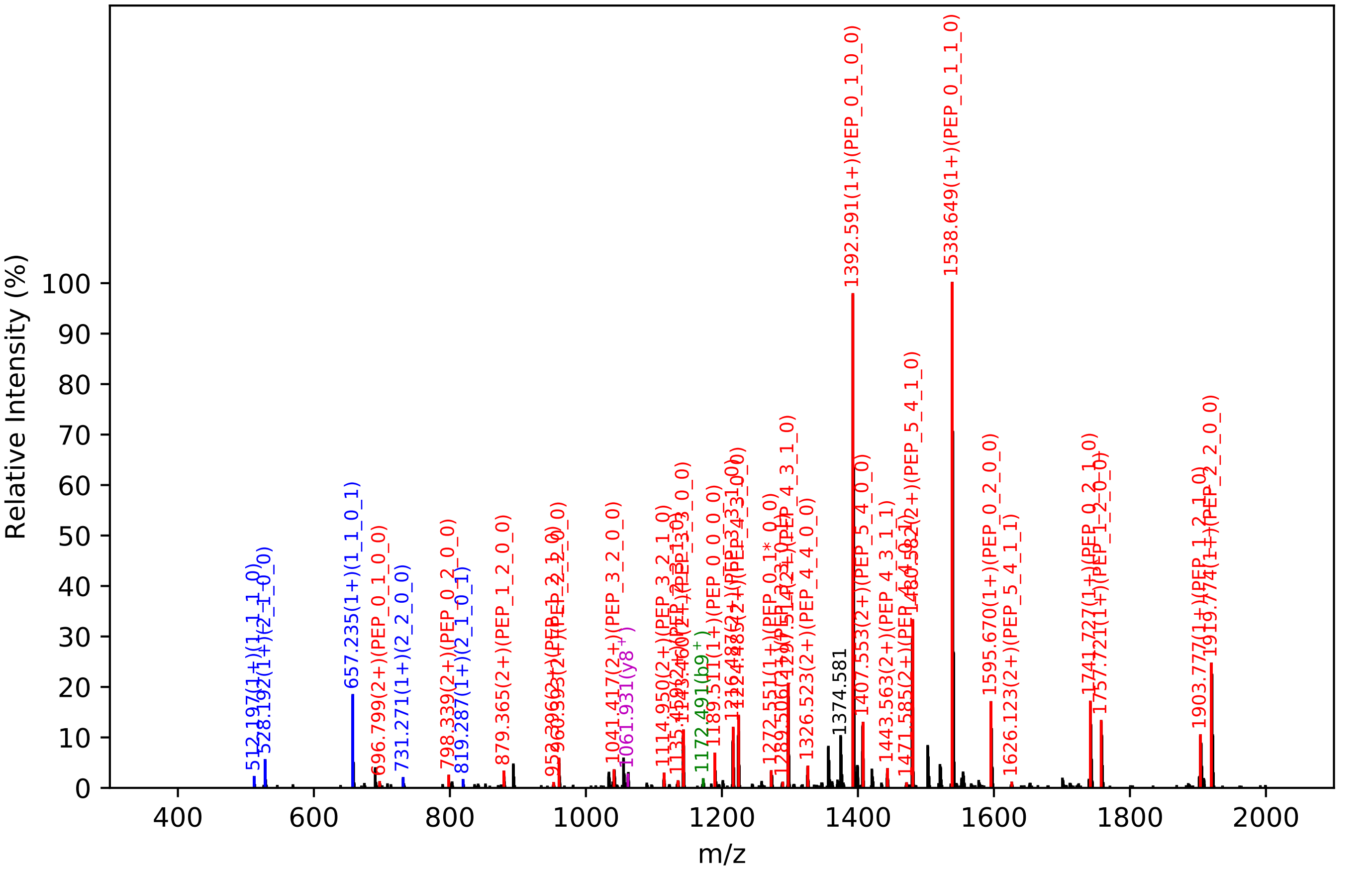

Training set no. 369, Experiment: IgG exp_1

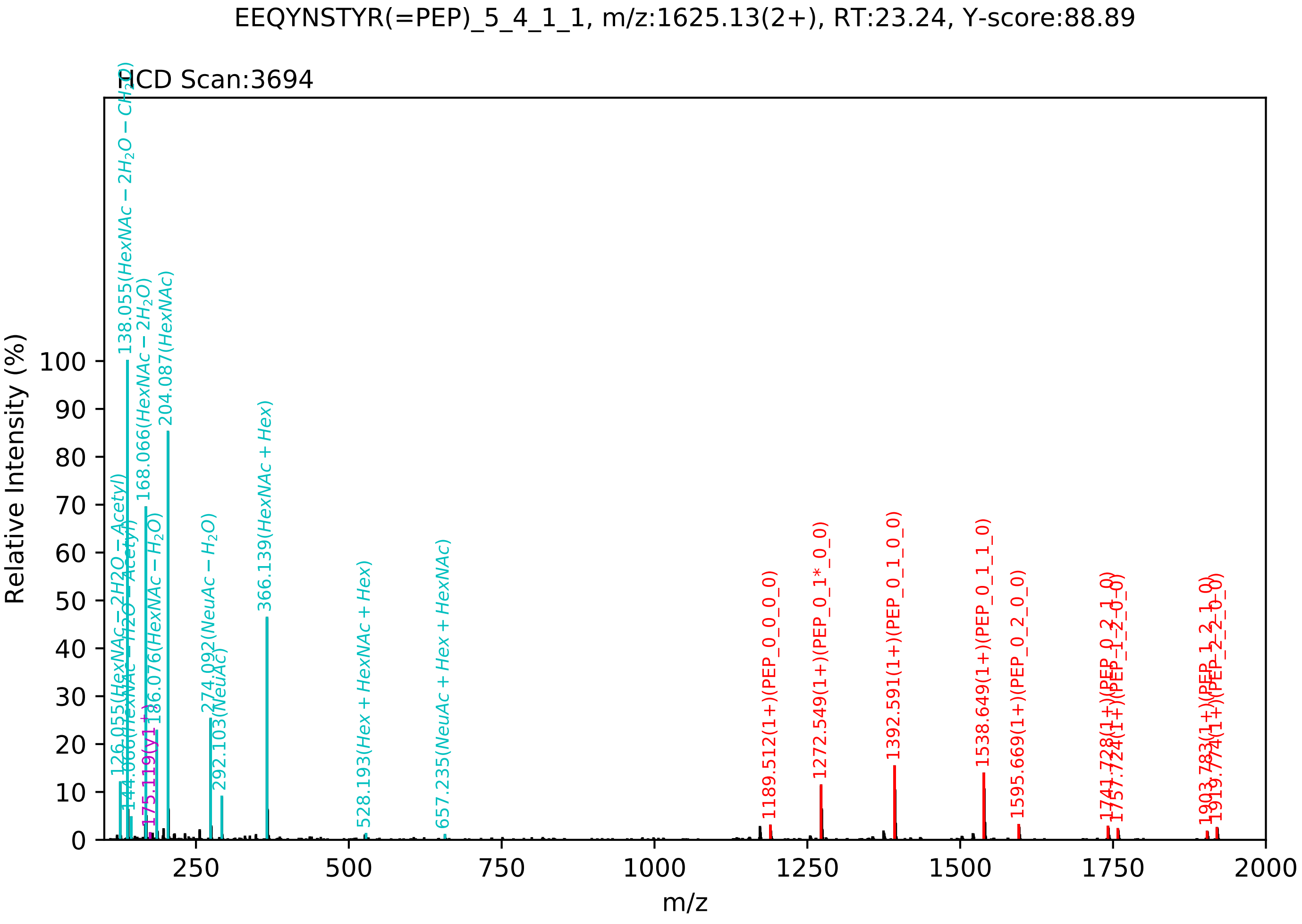

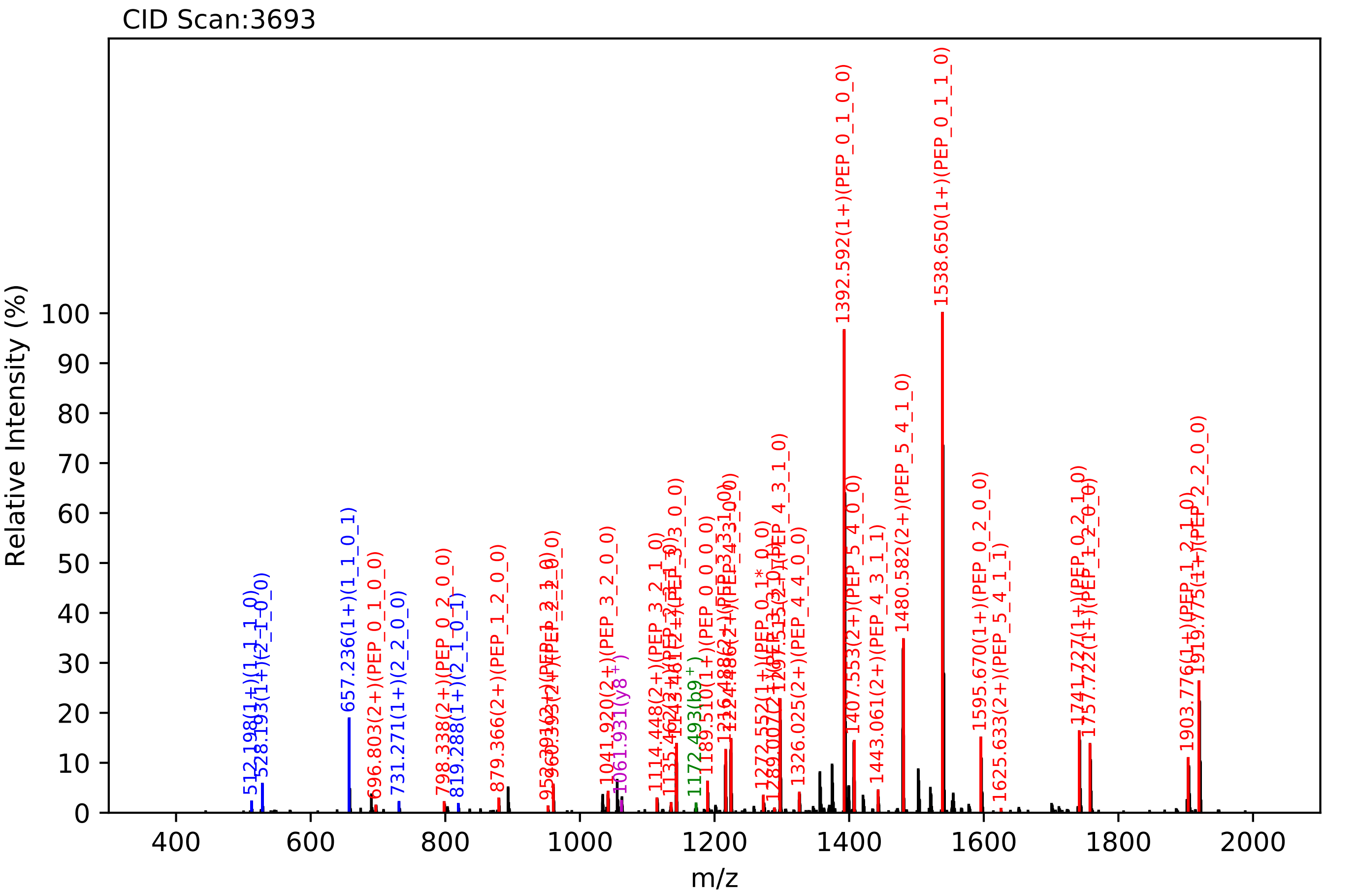

Training set no. 370, Experiment: IgG exp_2

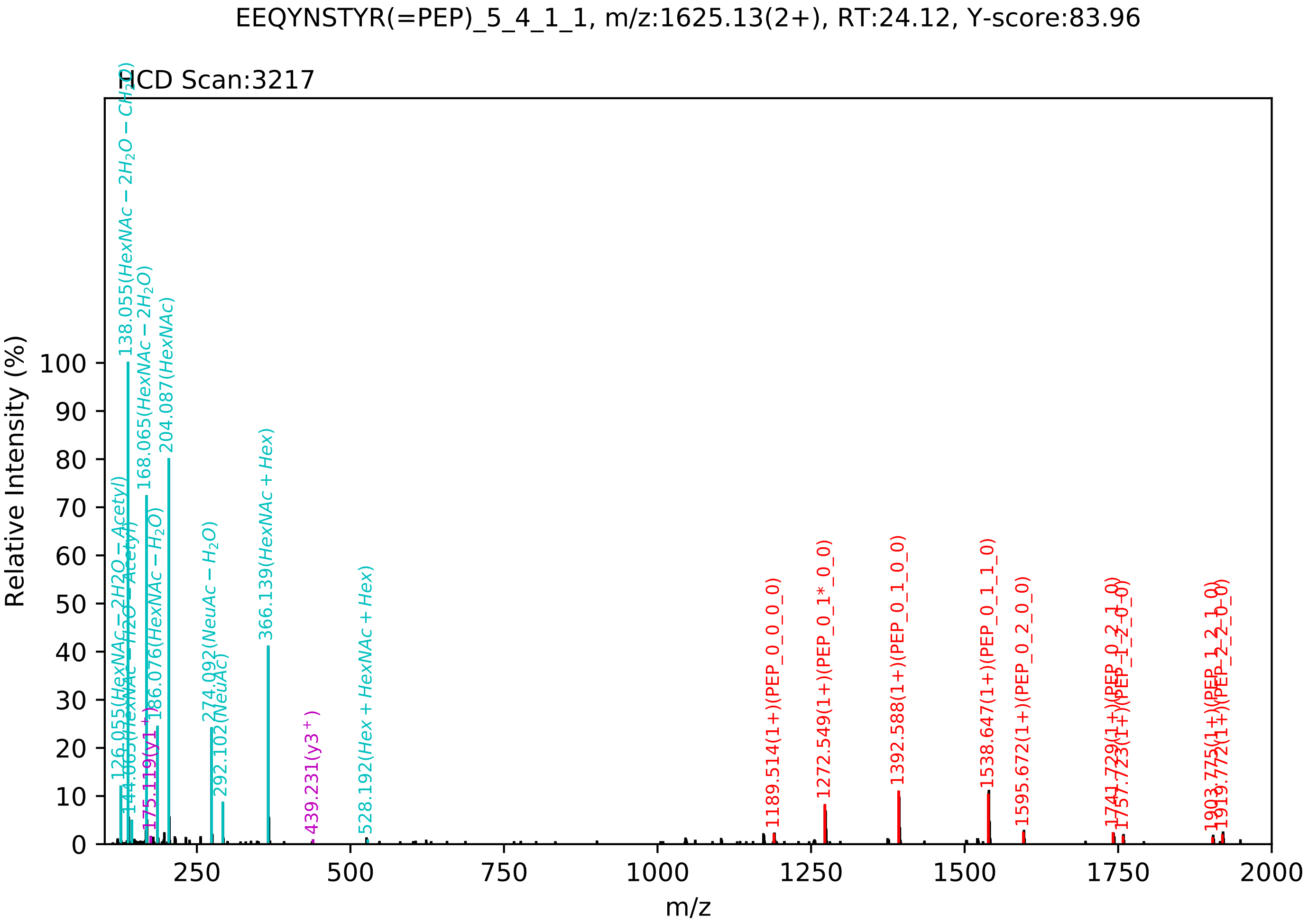

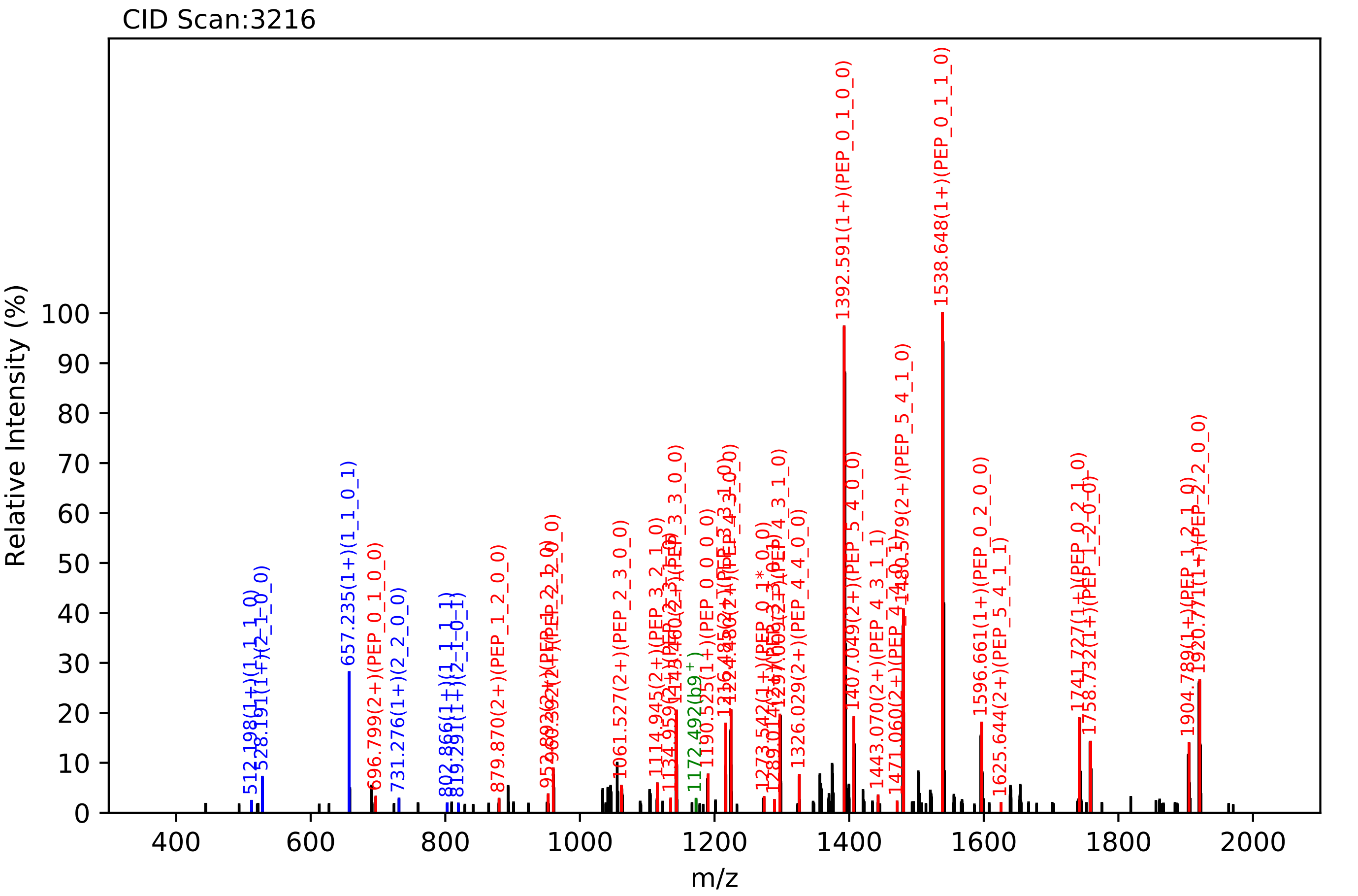

Training set no. 371, Experiment: IgG exp_4

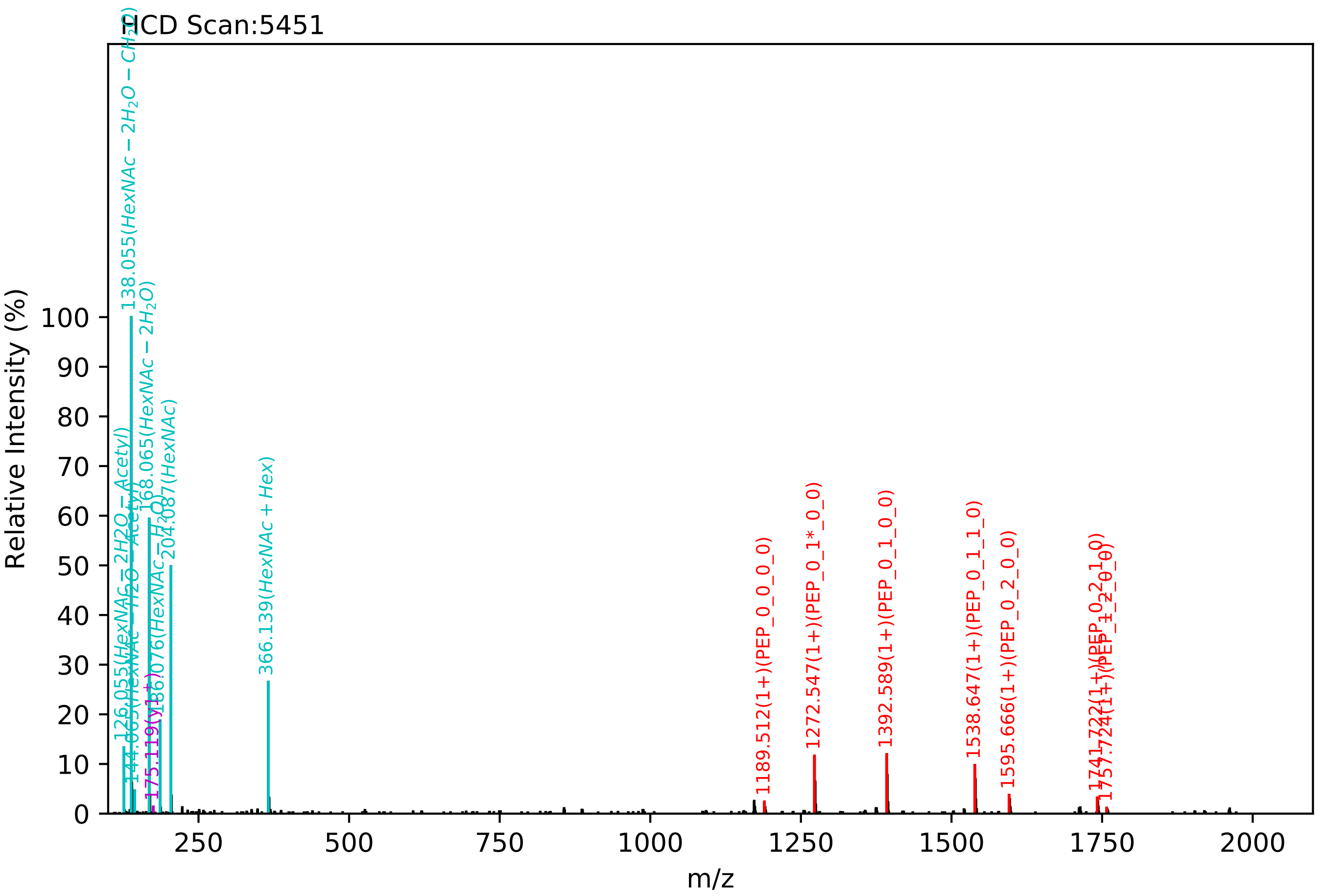

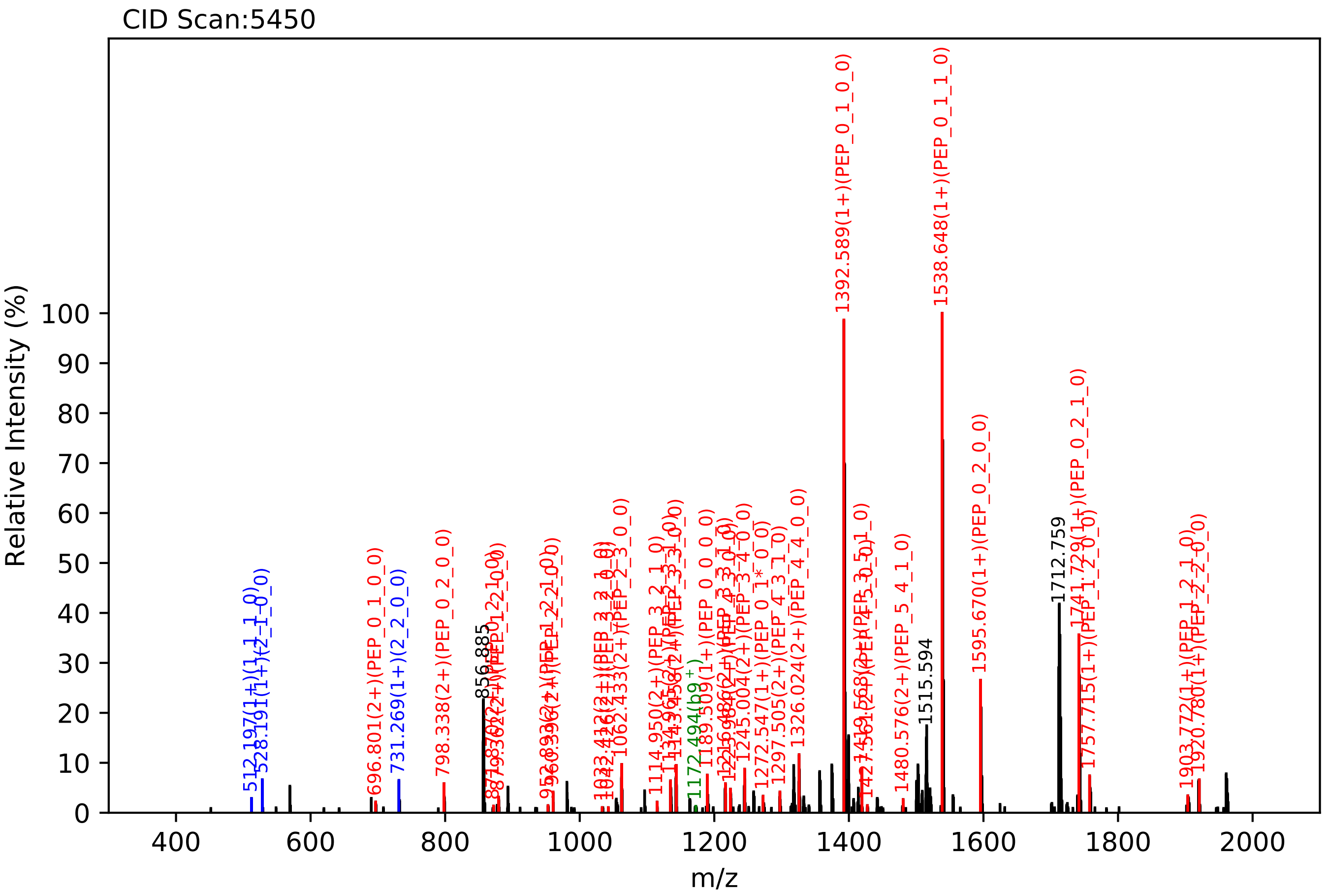

Training set no. 372, Experiment: IgG exp_4

EEQFNSTFR(=PEP)_3_3_1_0, m/z:1199.99(2+), RT:24.16, Y-score:89.03

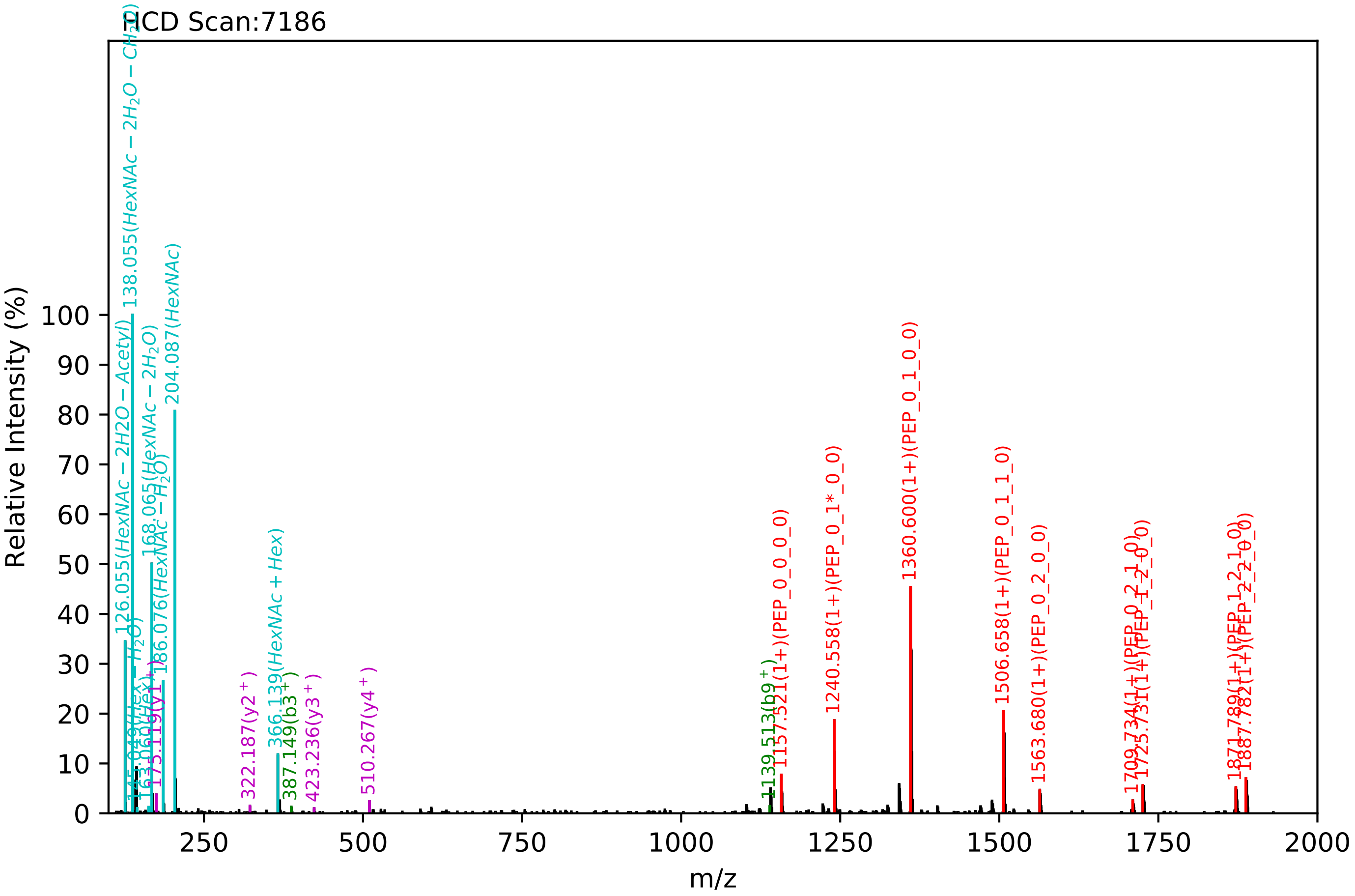

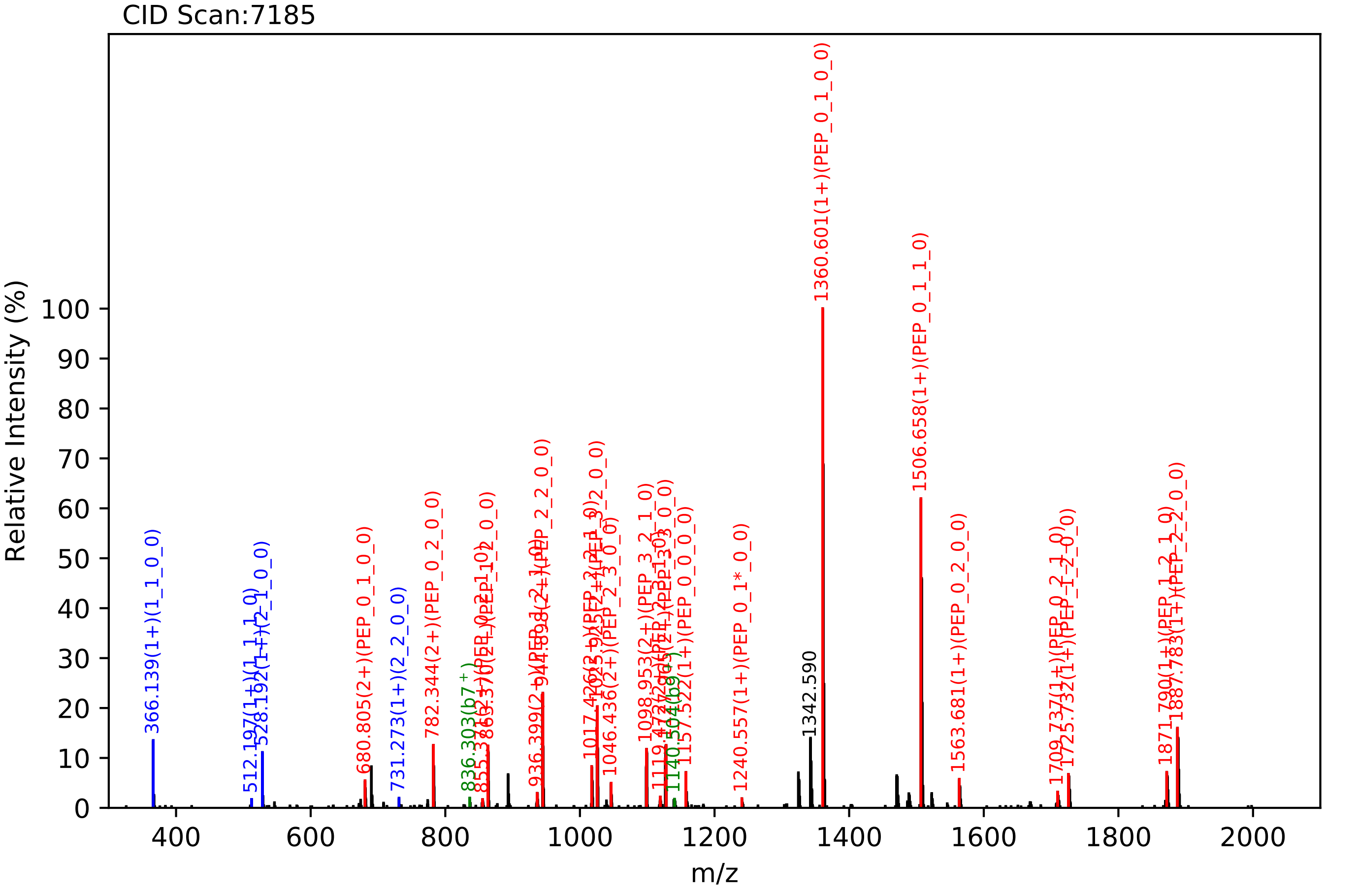

Training set no. 373, Experiment: IgG exp_1

EEQFNSTFR(=PEP)_3_3_1_0, m/z:1199.99(2+), RT:24.51, Y-score:88.12

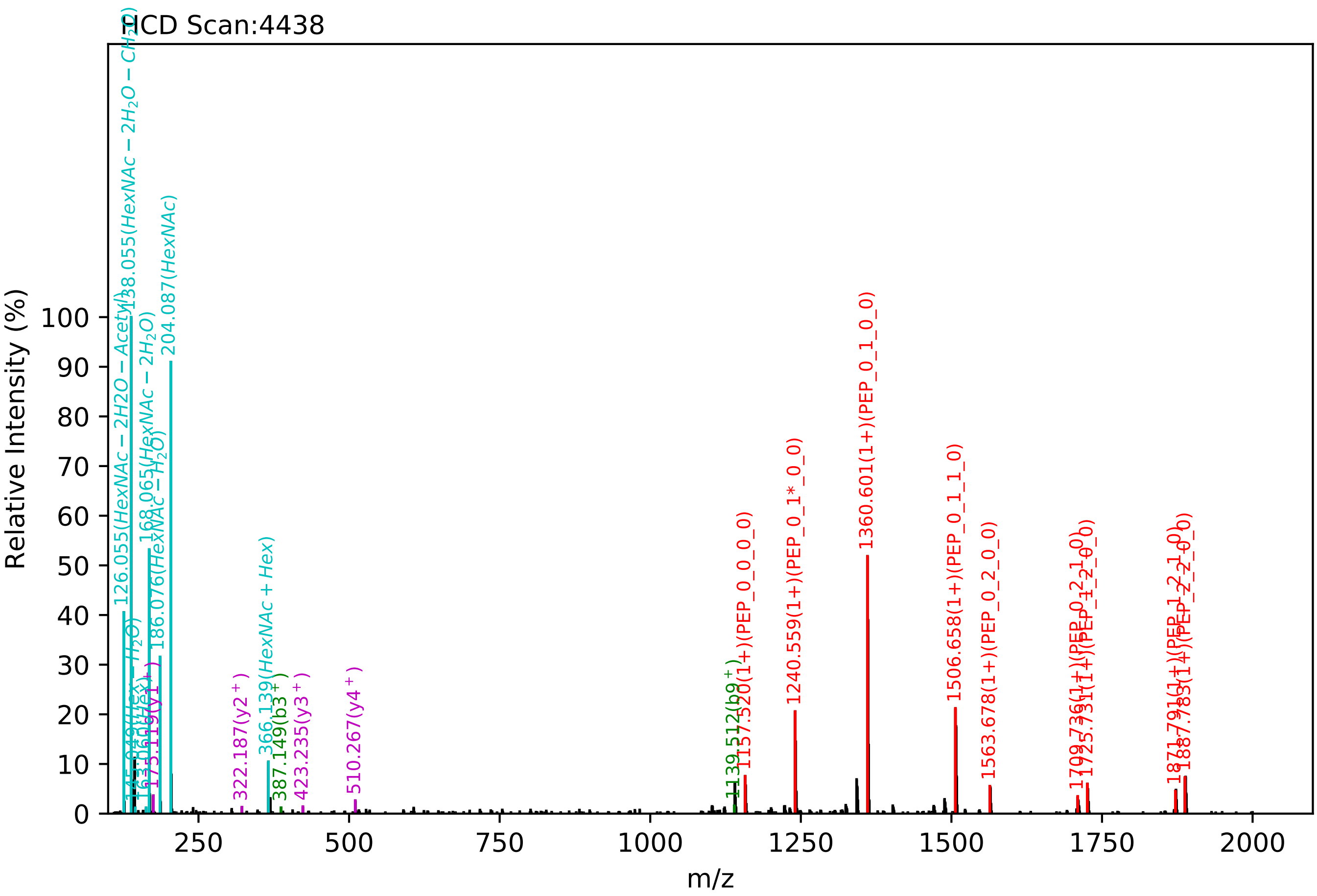

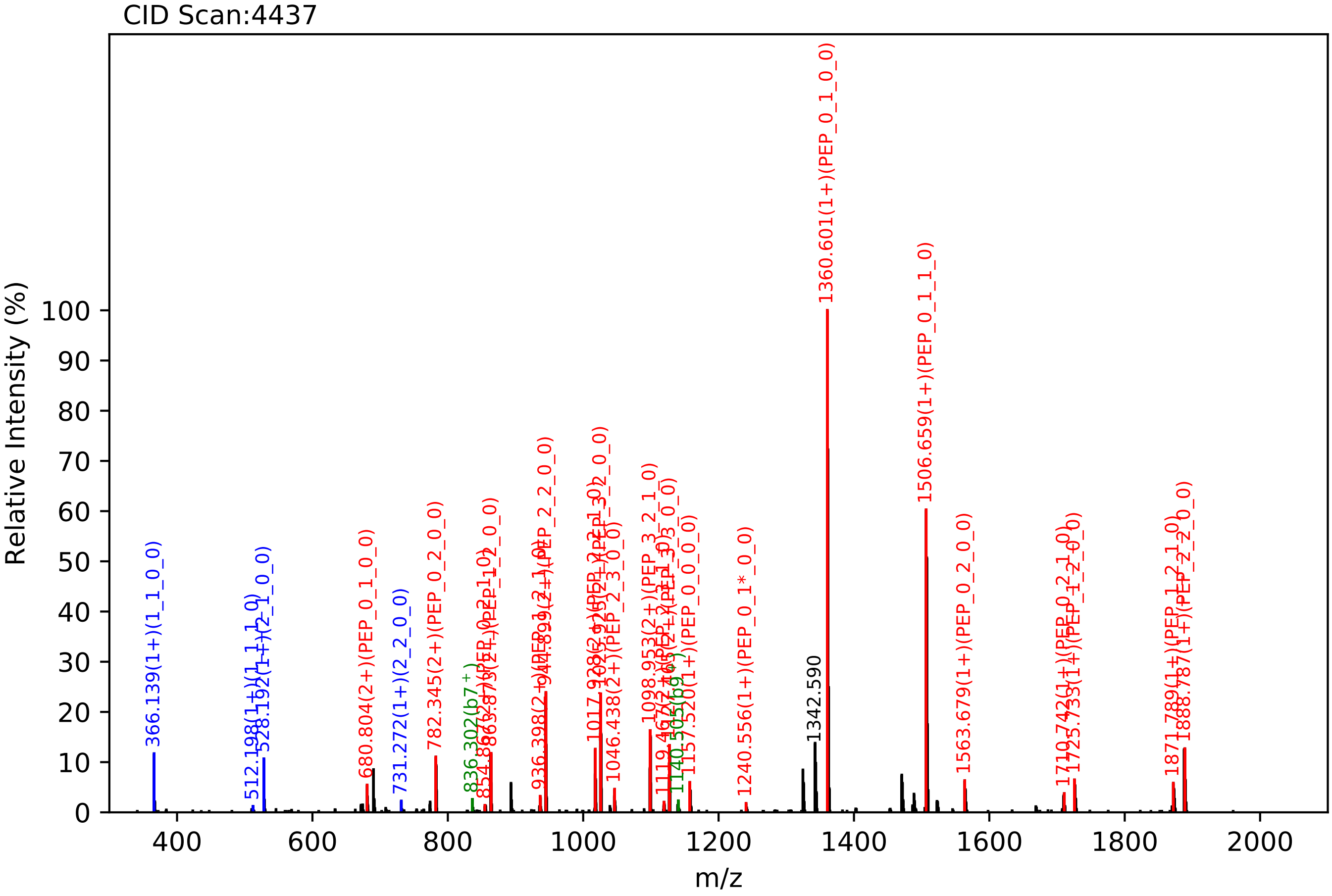

Training set no. 374, Experiment: IgG exp_3

EEQFNSTFR(=PEP)_3_3_1_0, m/z:1199.99(2+), RT:26.39, Y-score:87.48

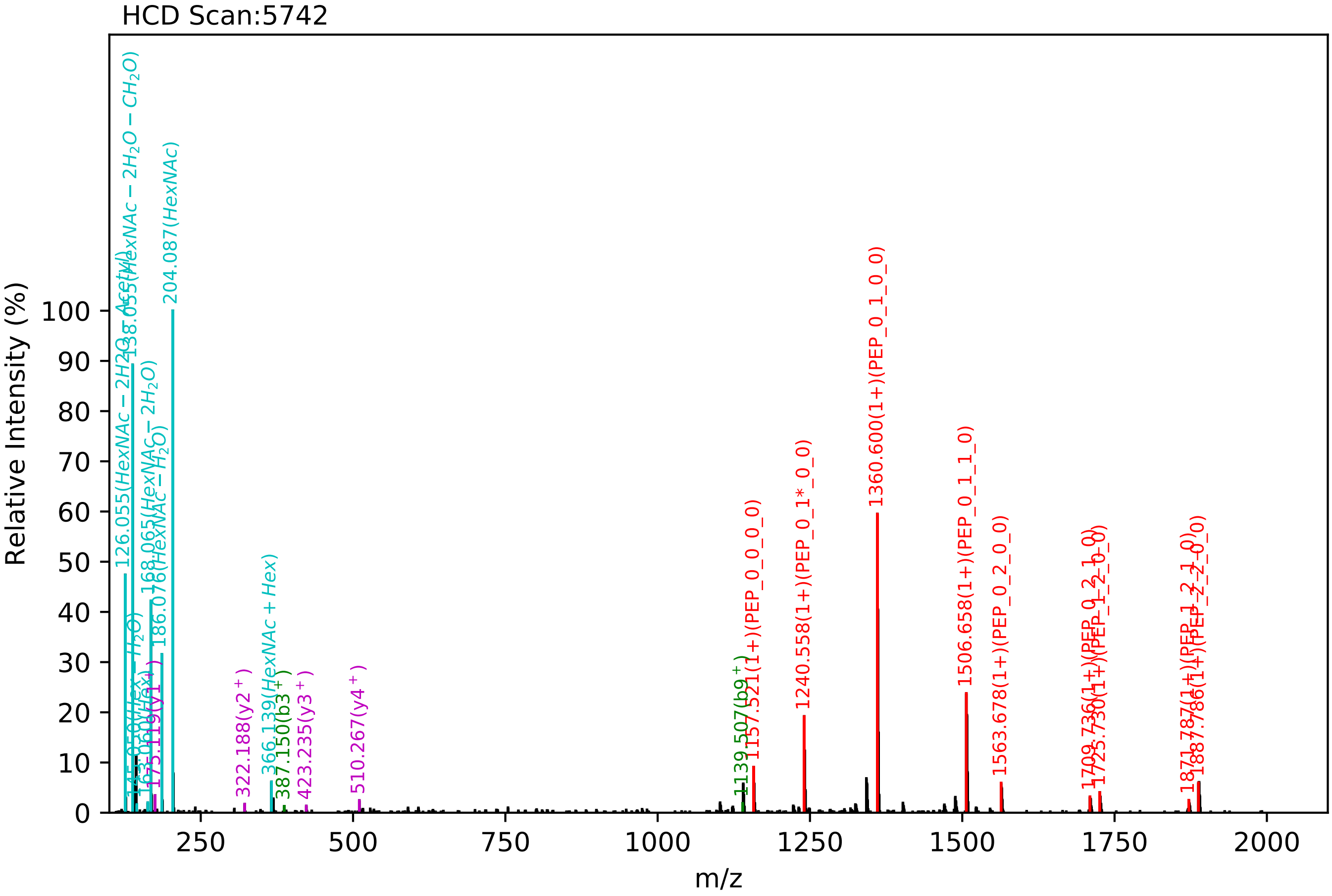

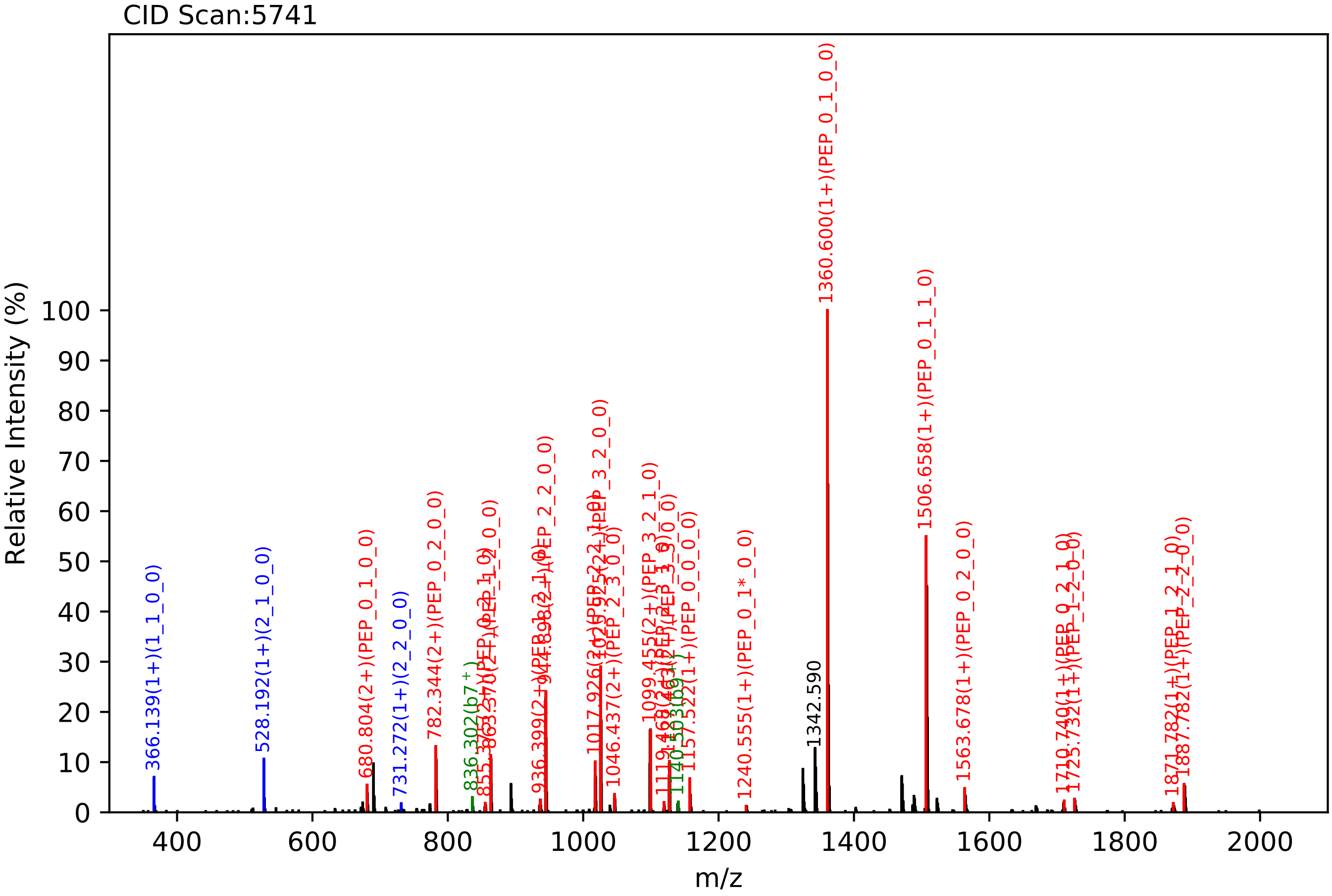

Training set no. 375, Experiment: IgG exp_2

EEQFNSTFR(=PEP)_3_3_1_0, m/z:1199.99(2+), RT:24.62, Y-score:86.83

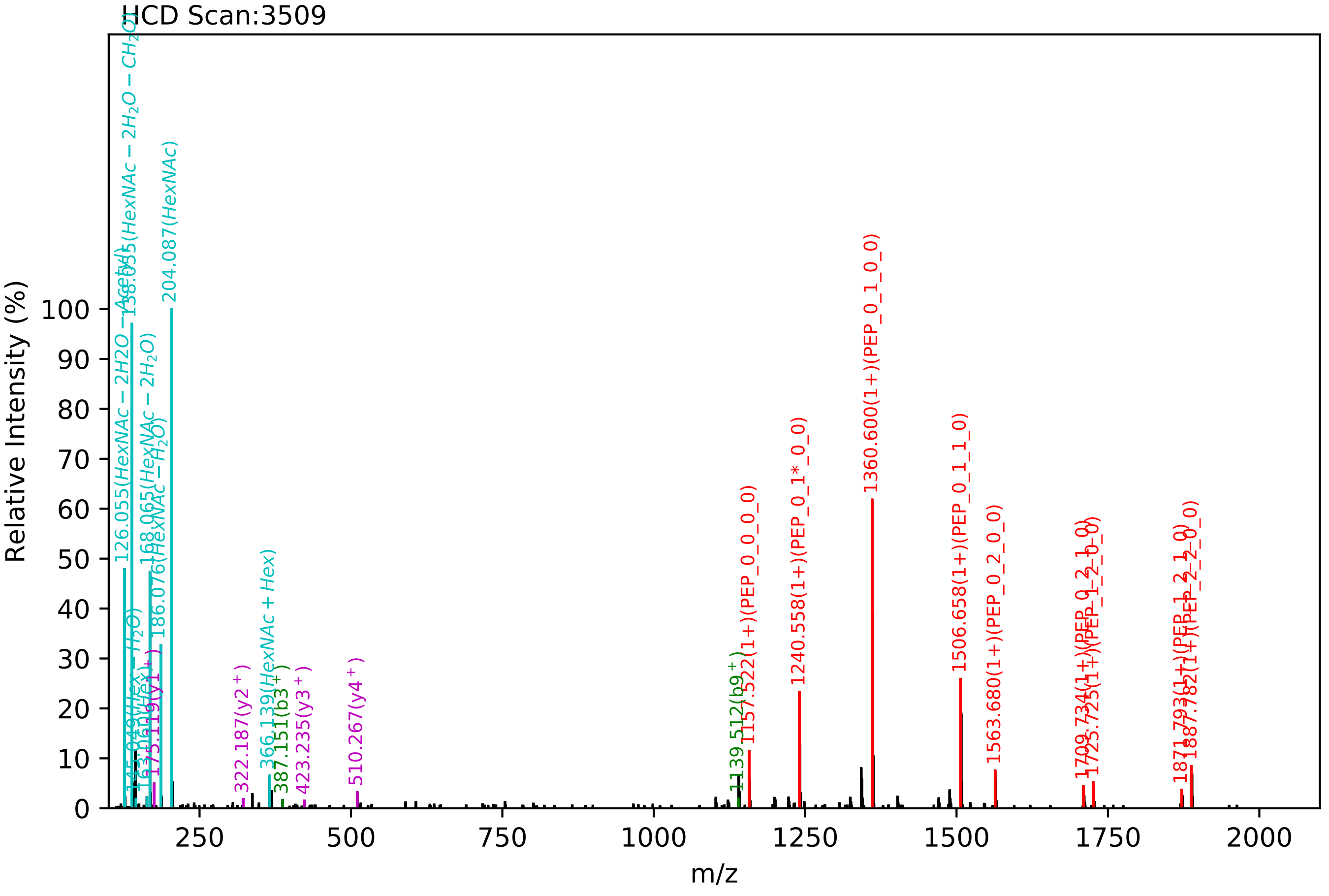

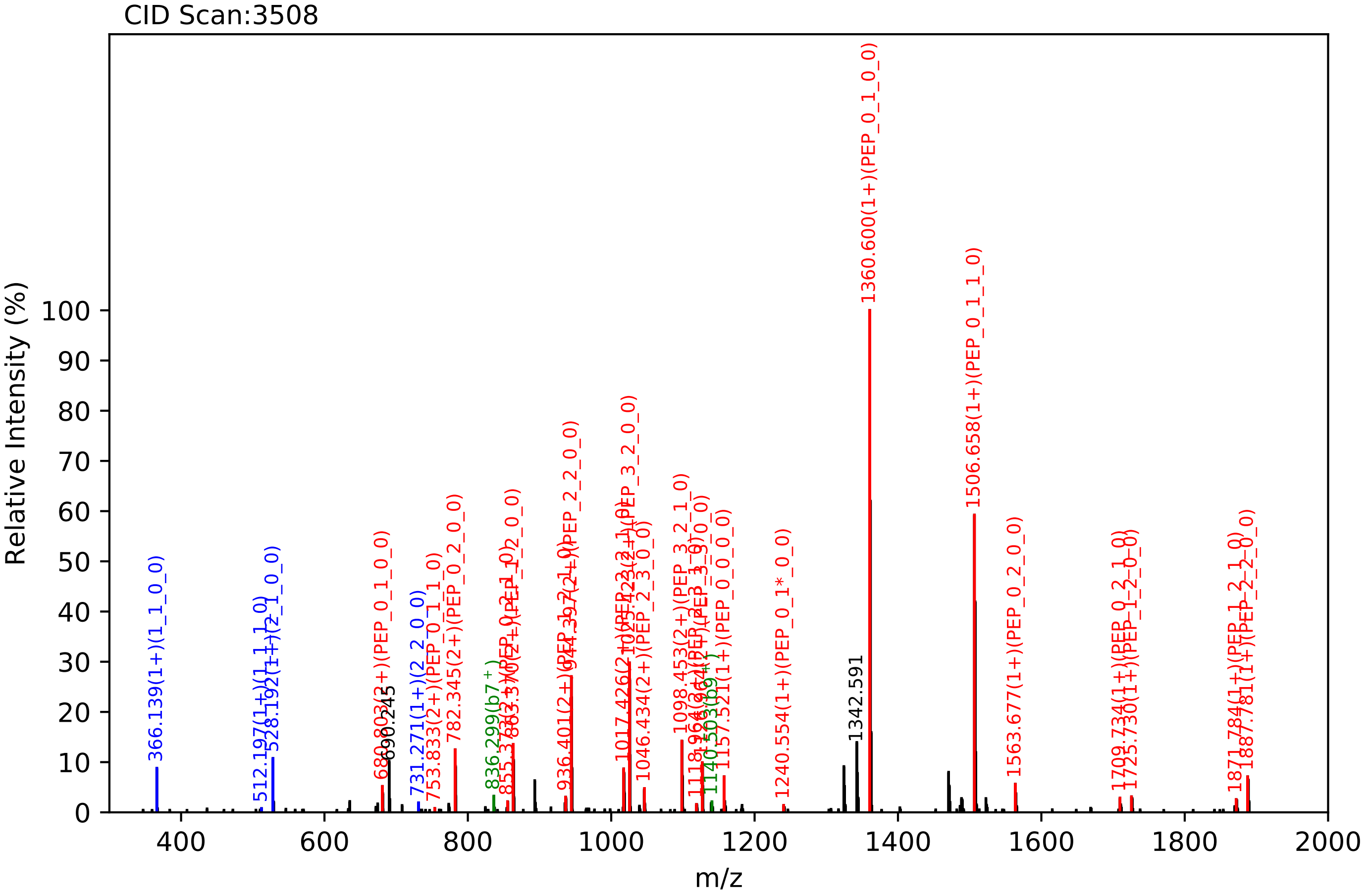

Training set no. 376, Experiment: IgG exp_3

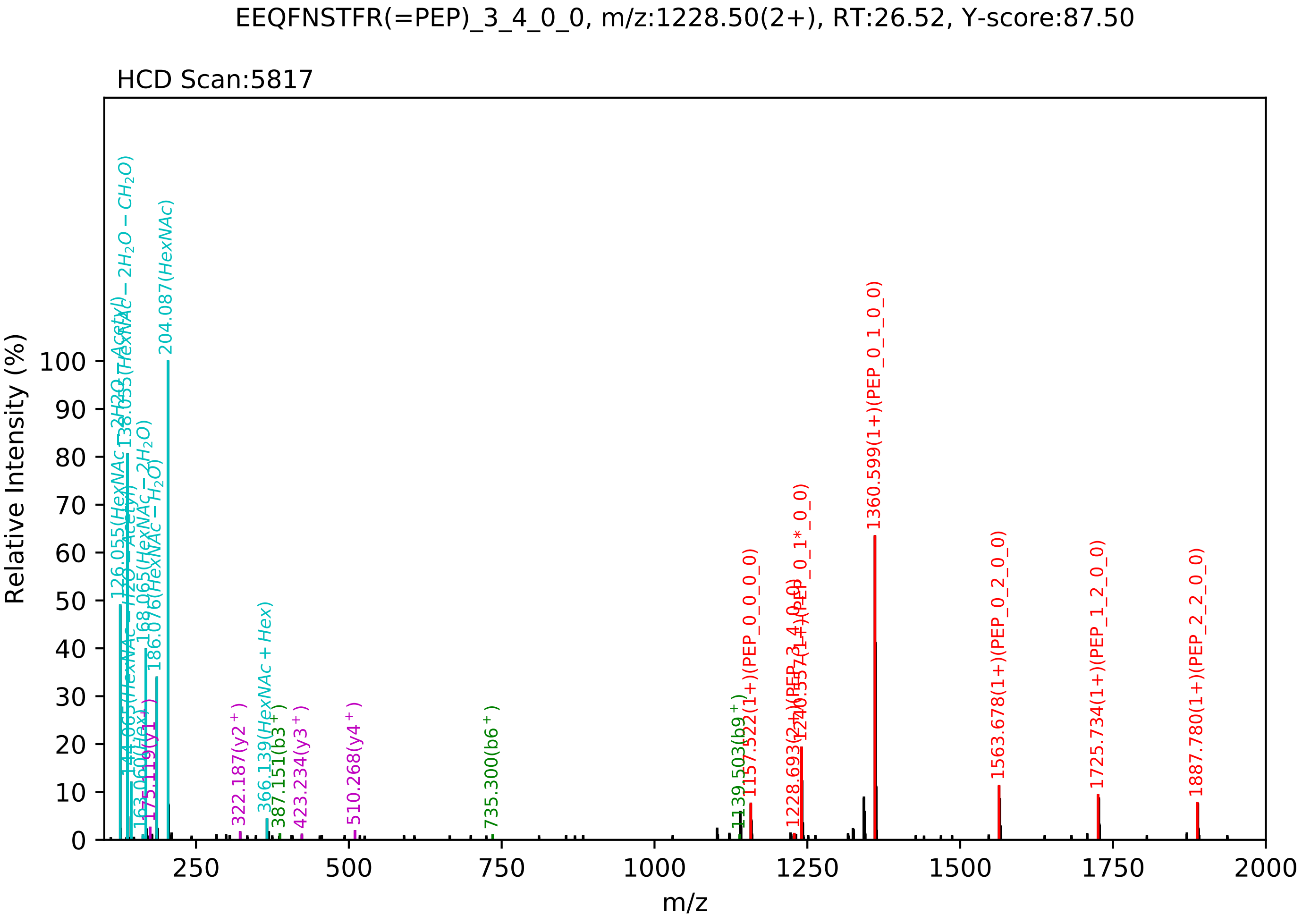

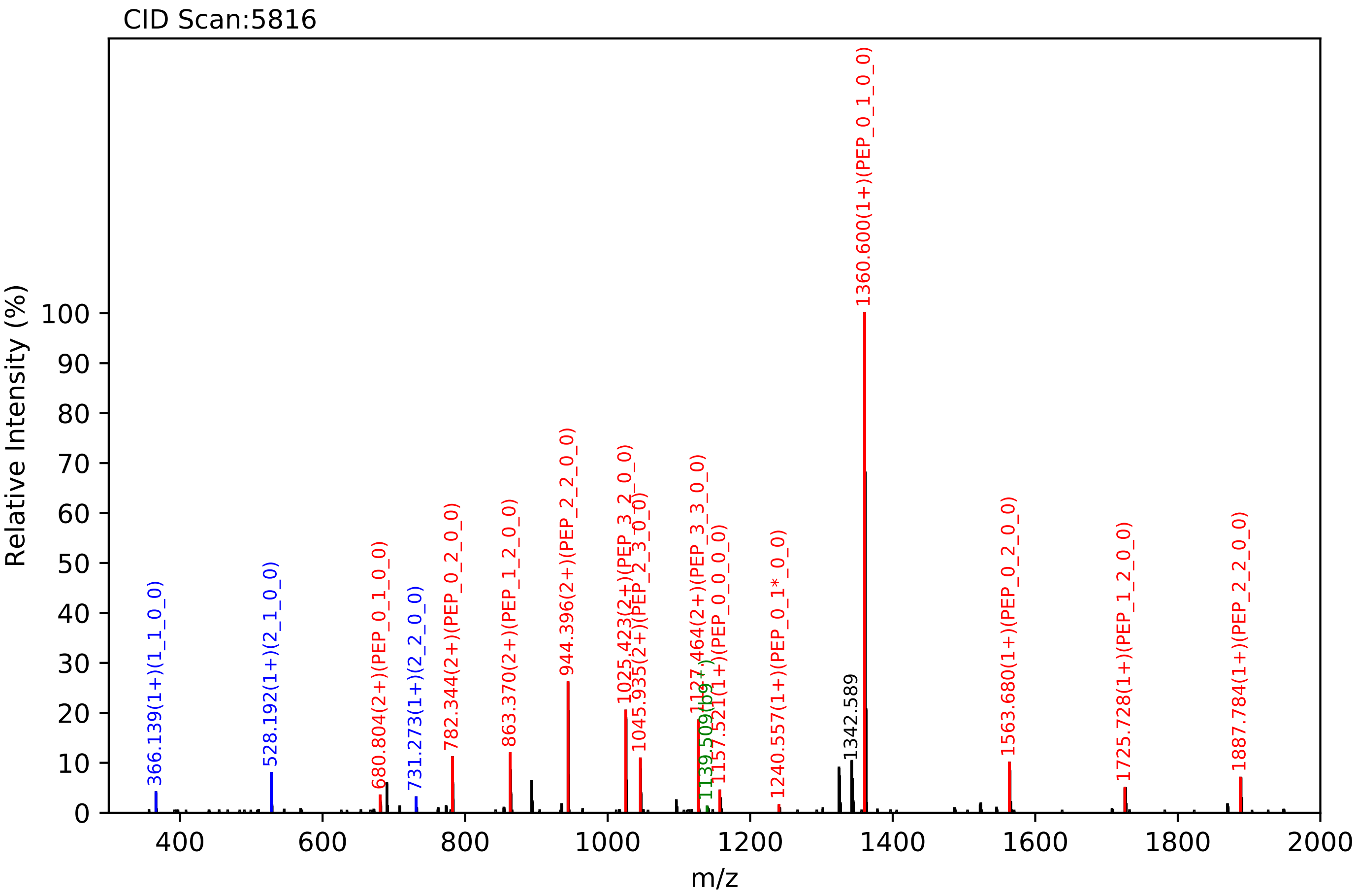

Training set no. 377, Experiment: IgG exp_4

EEQFNSTFR(=PEP)_3_4_0_0, m/z:1228.51(2+), RT:24.37, Y-score:87.47

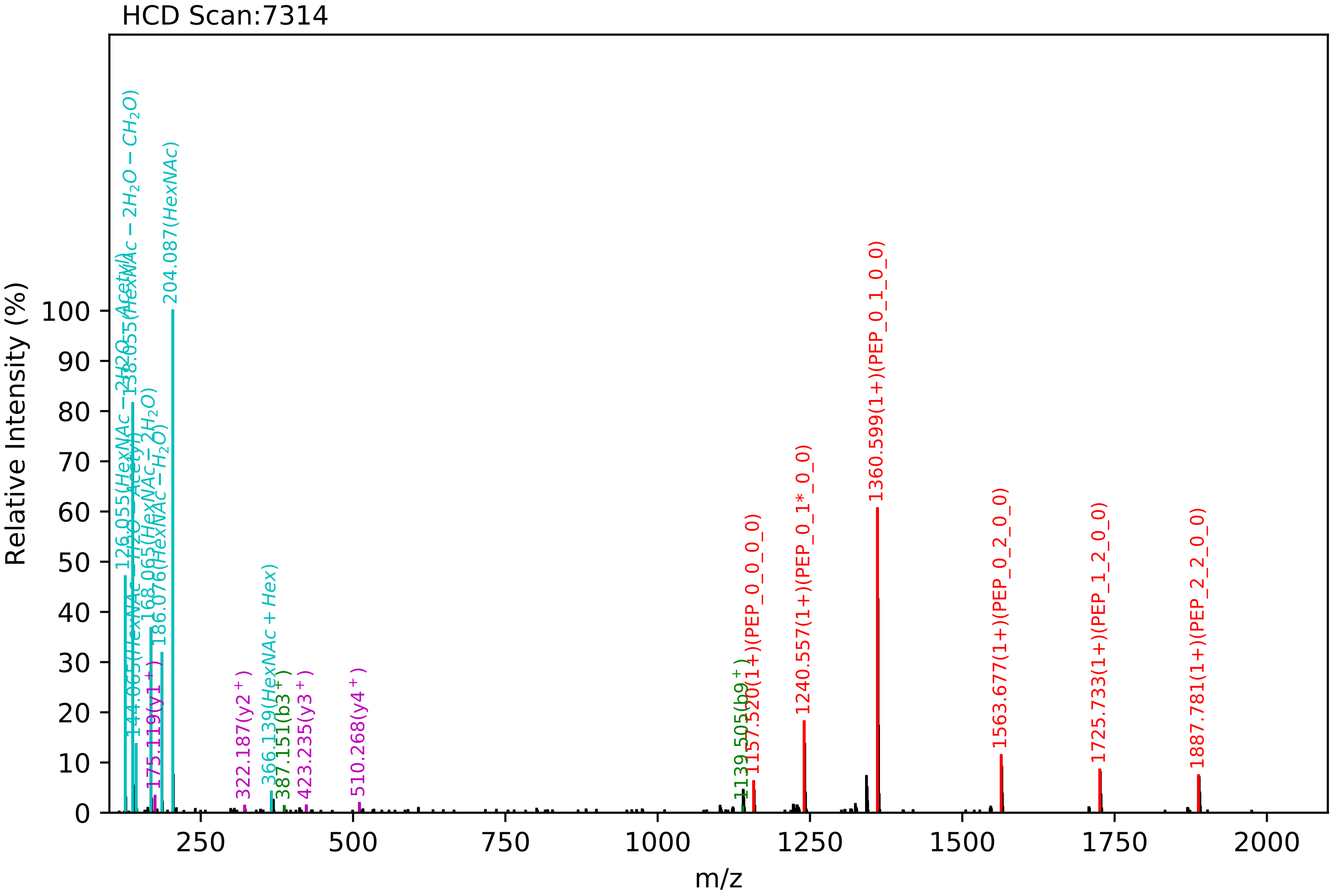

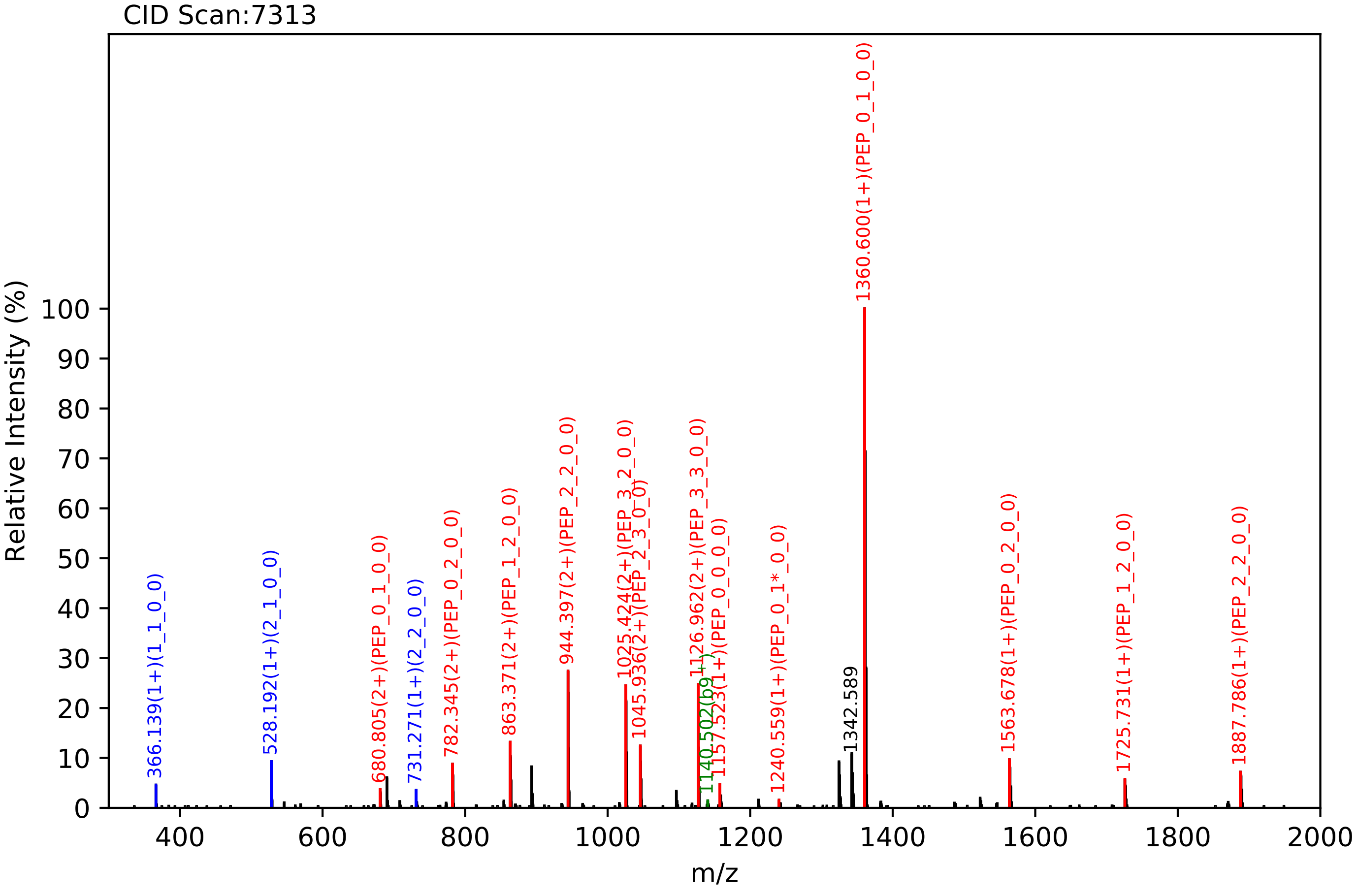

Training set no. 378, Experiment: IgG exp_1

EEQFNSTFR(=PEP)_3_4_0_0, m/z:1228.50(2+), RT:24.71, Y-score:85.68

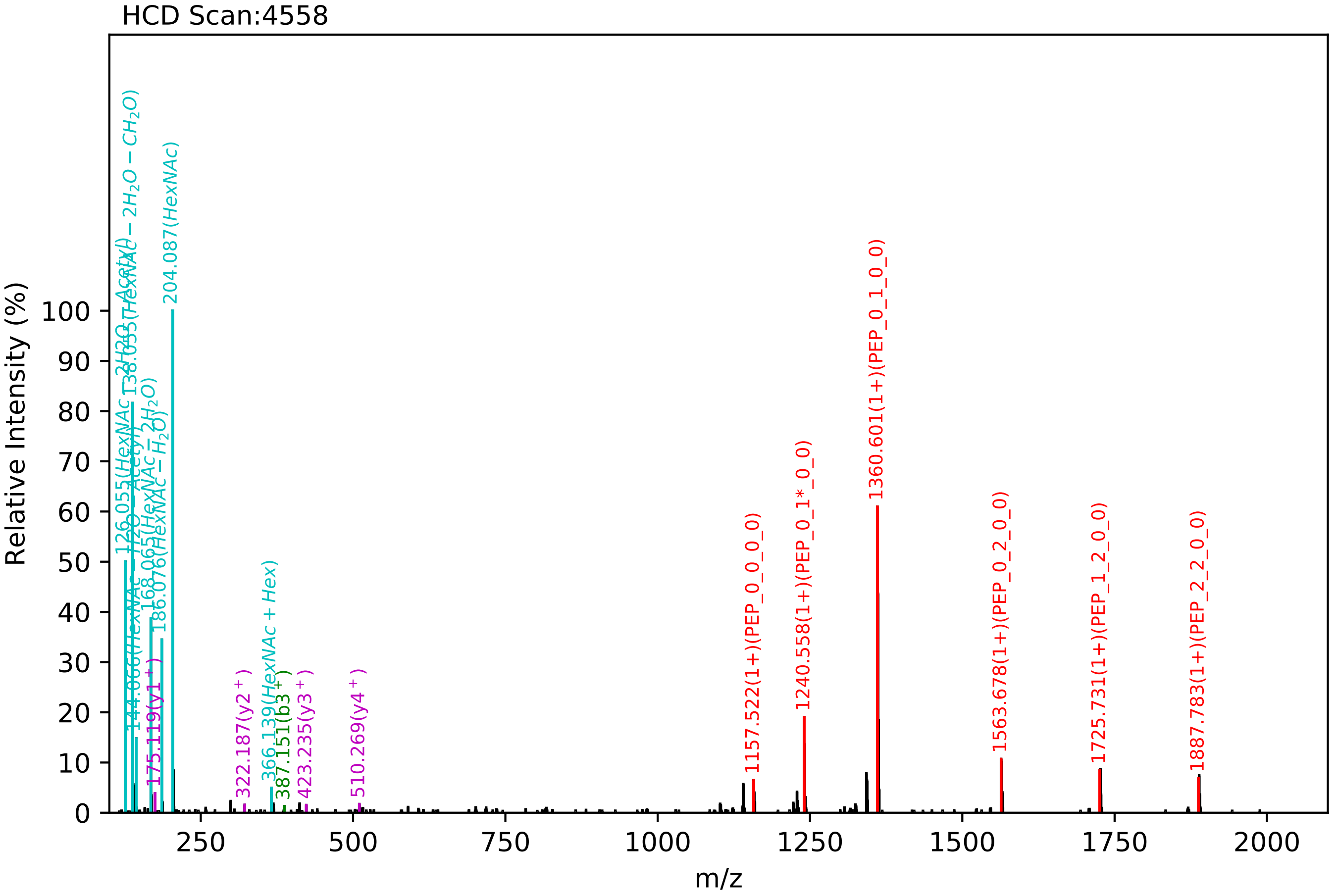

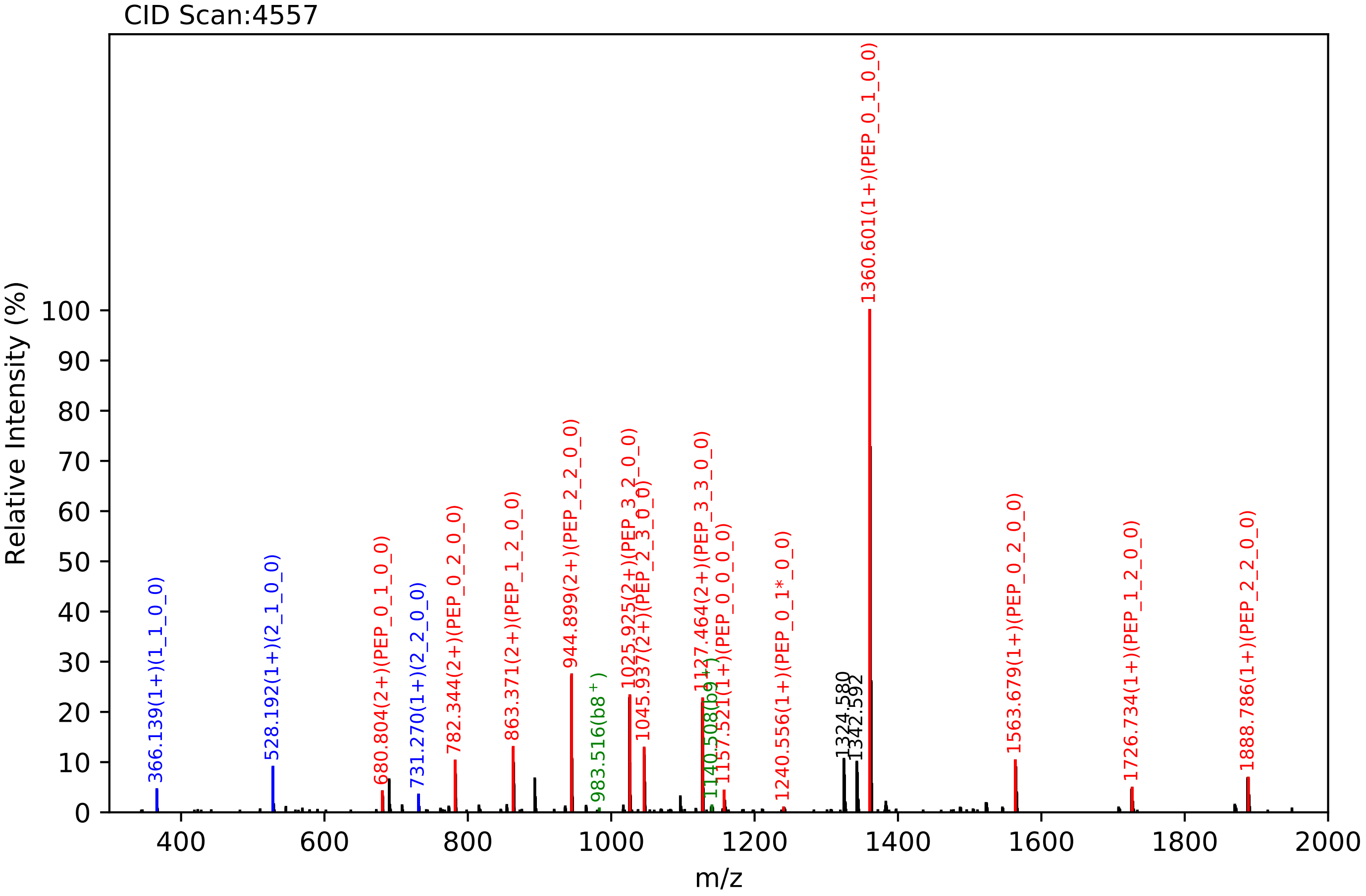

Training set no. 379, Experiment: IgG exp_1

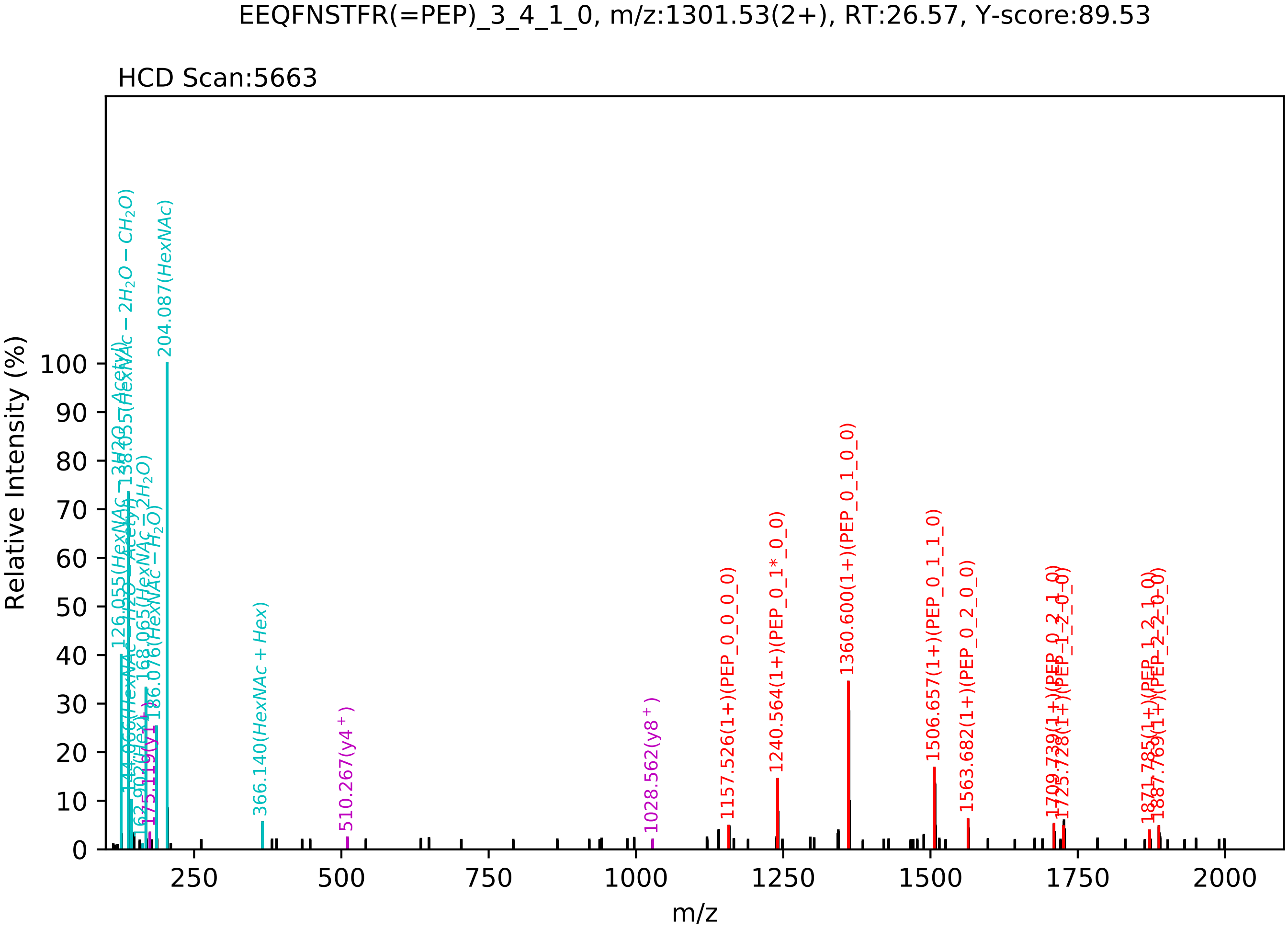

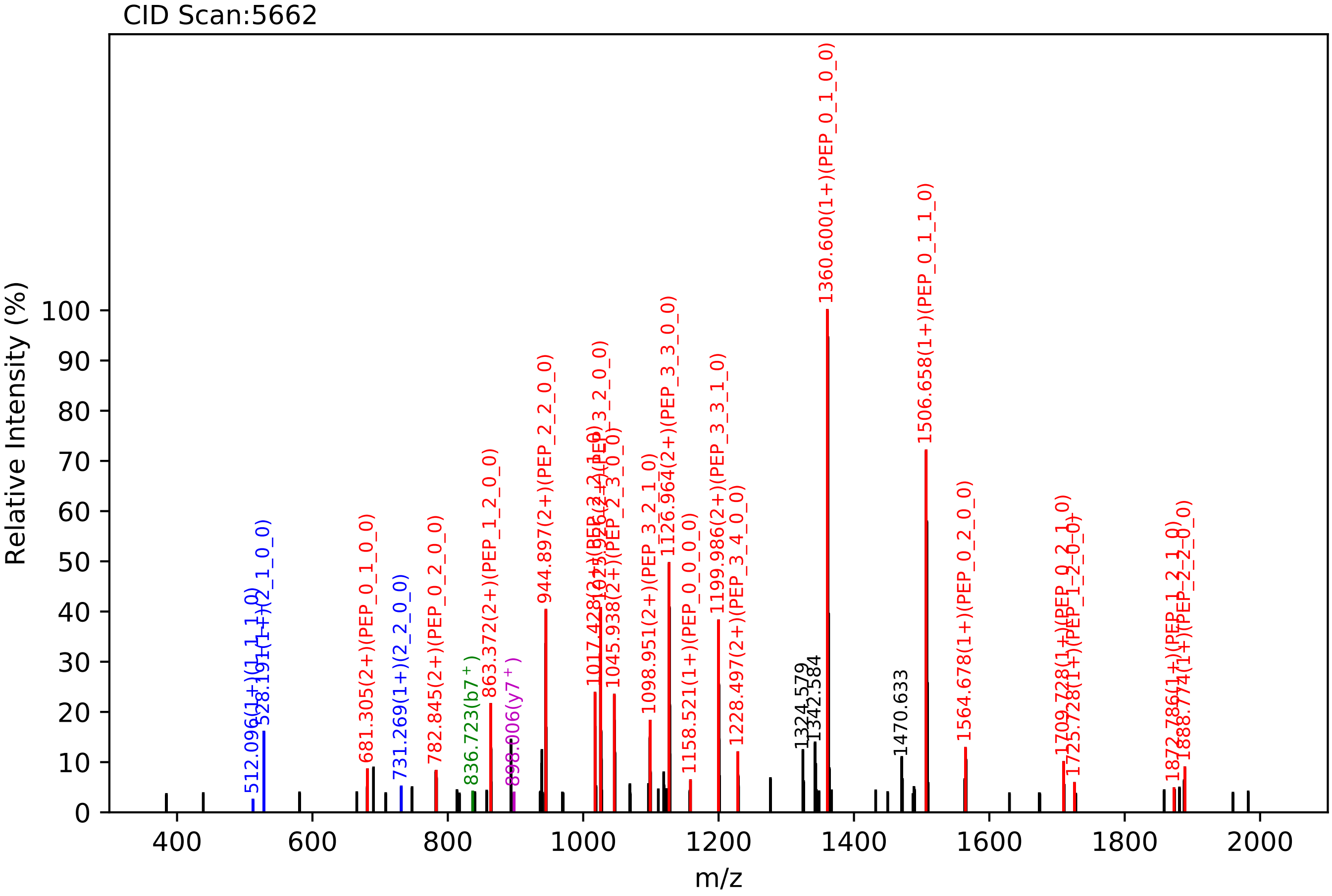

Training set no. 380, Experiment: IgG exp_2

EEQFNSTFR(=PEP)_3_4_1_0, m/z:1301.53(2+), RT:24.56, Y-score:88.91

HCD Scan:3470

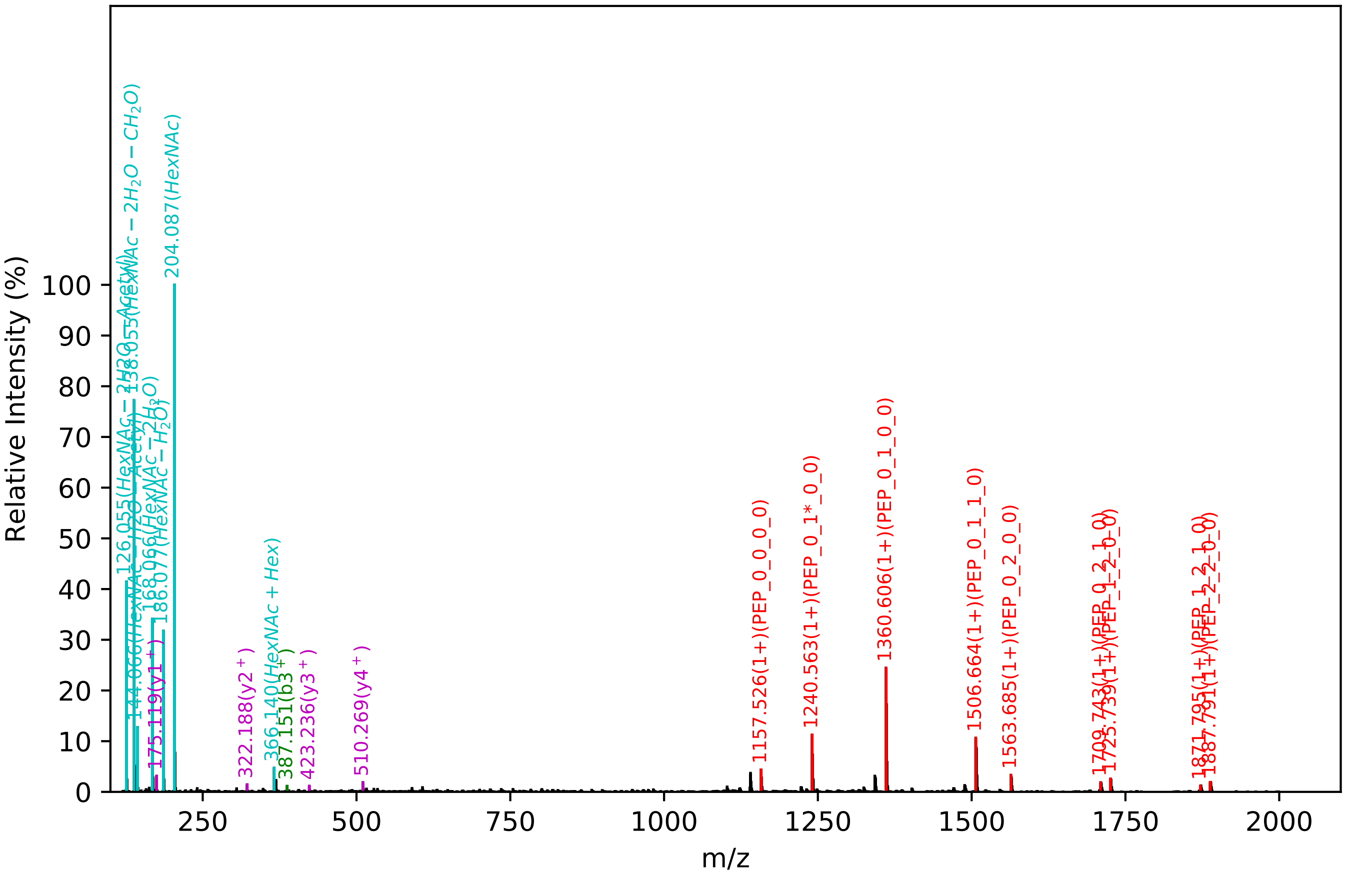

CID Scan:3469

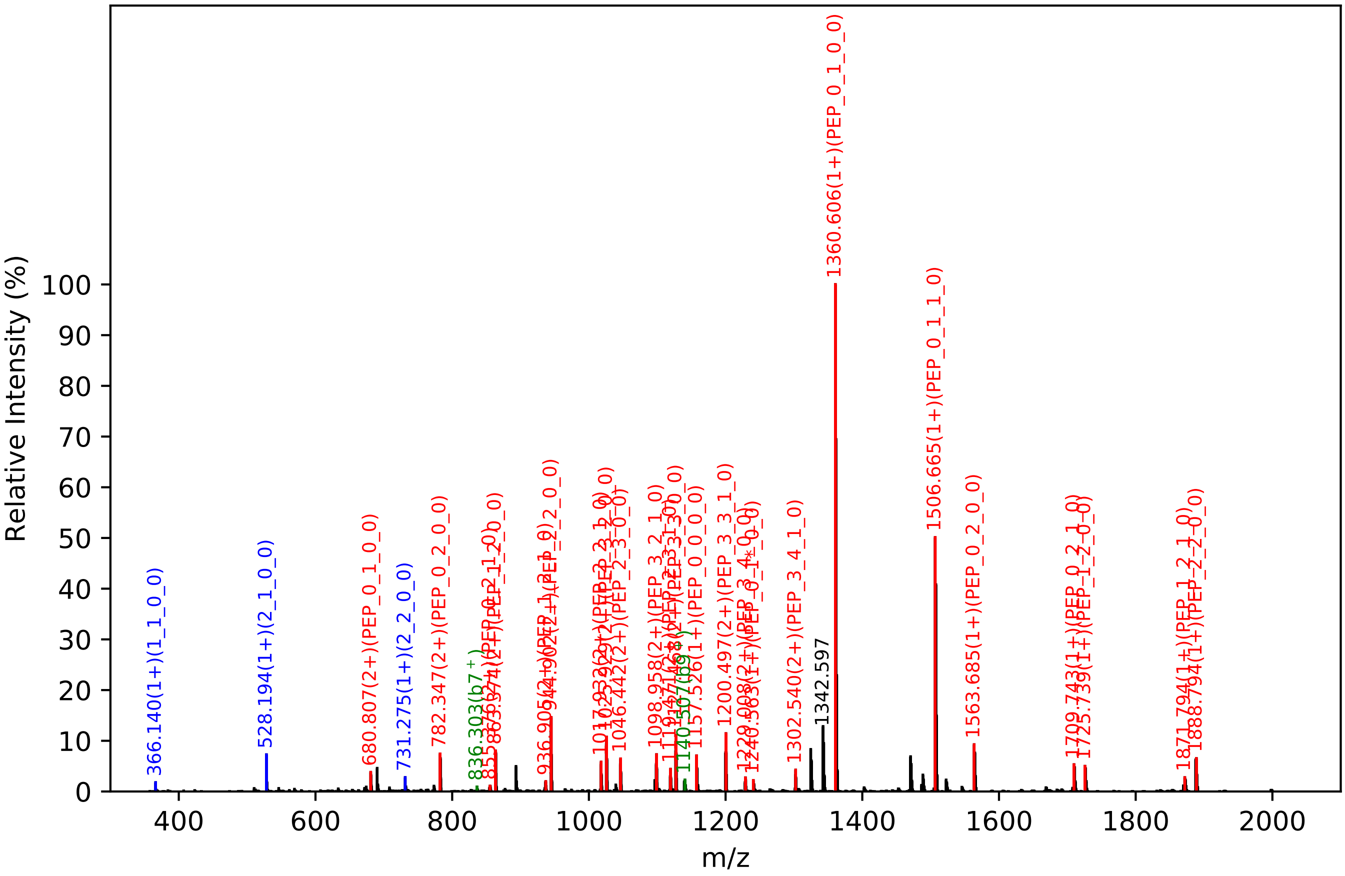

Training set no. 381, Experiment: IgG exp_3

EEQFNSTFR(=PEP)_3_4_1_0, m/z:1301.53(2+), RT:26.35, Y-score:88.70

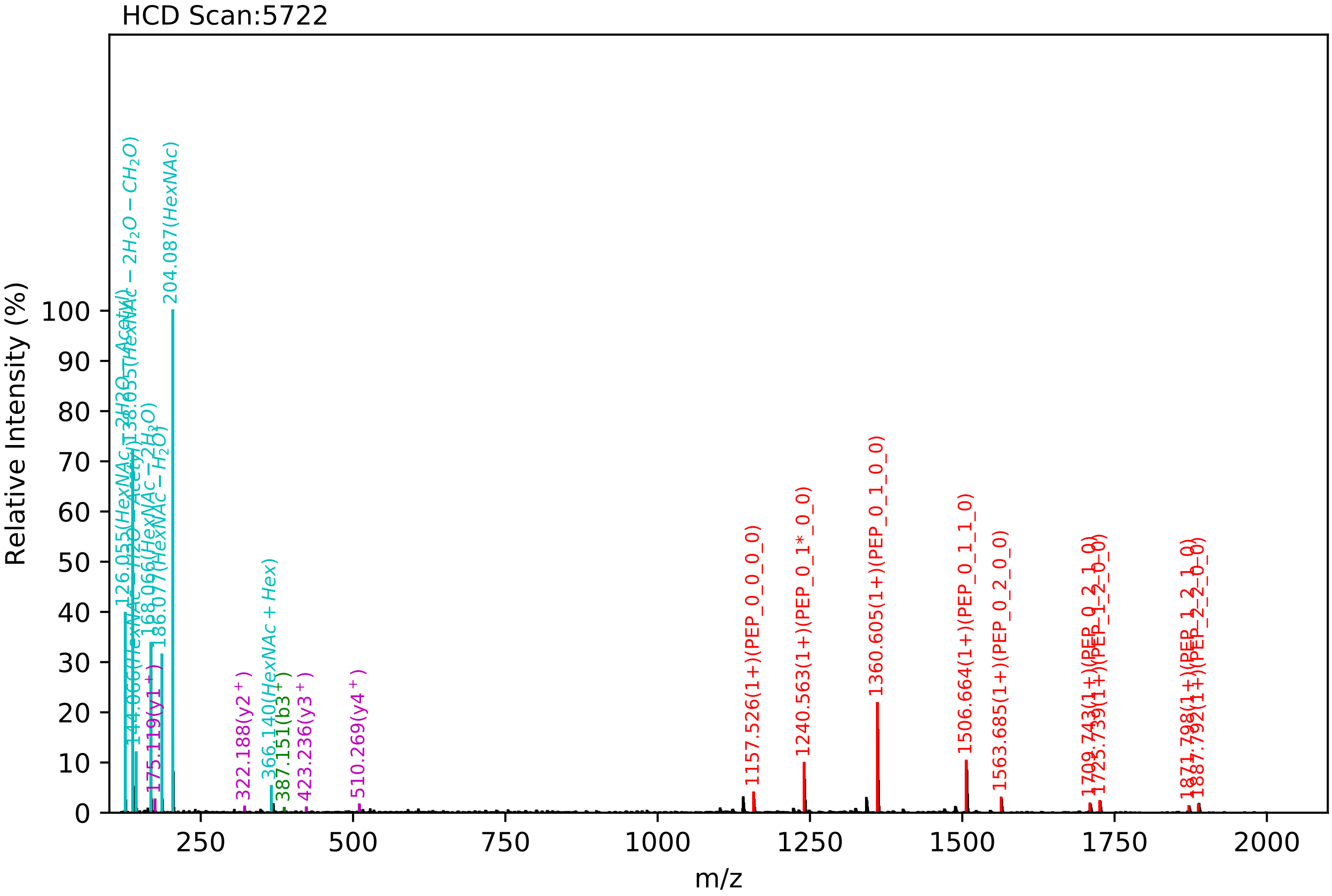

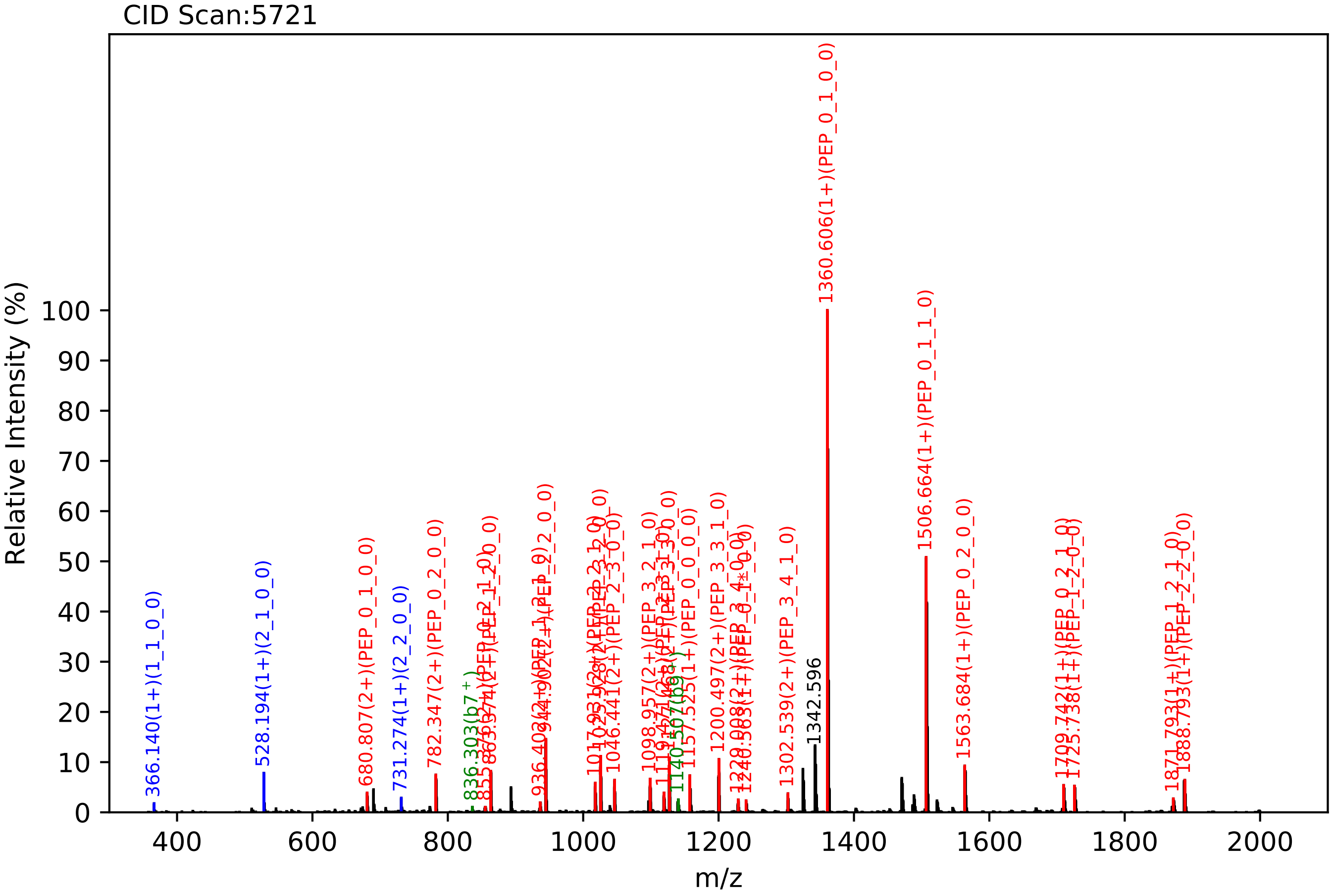

Training set no. 382, Experiment: IgG exp_4

EEQFNSTFR(=PEP)_3_4_1_0, m/z:1301.53(2+), RT:24.66, Y-score:88.33

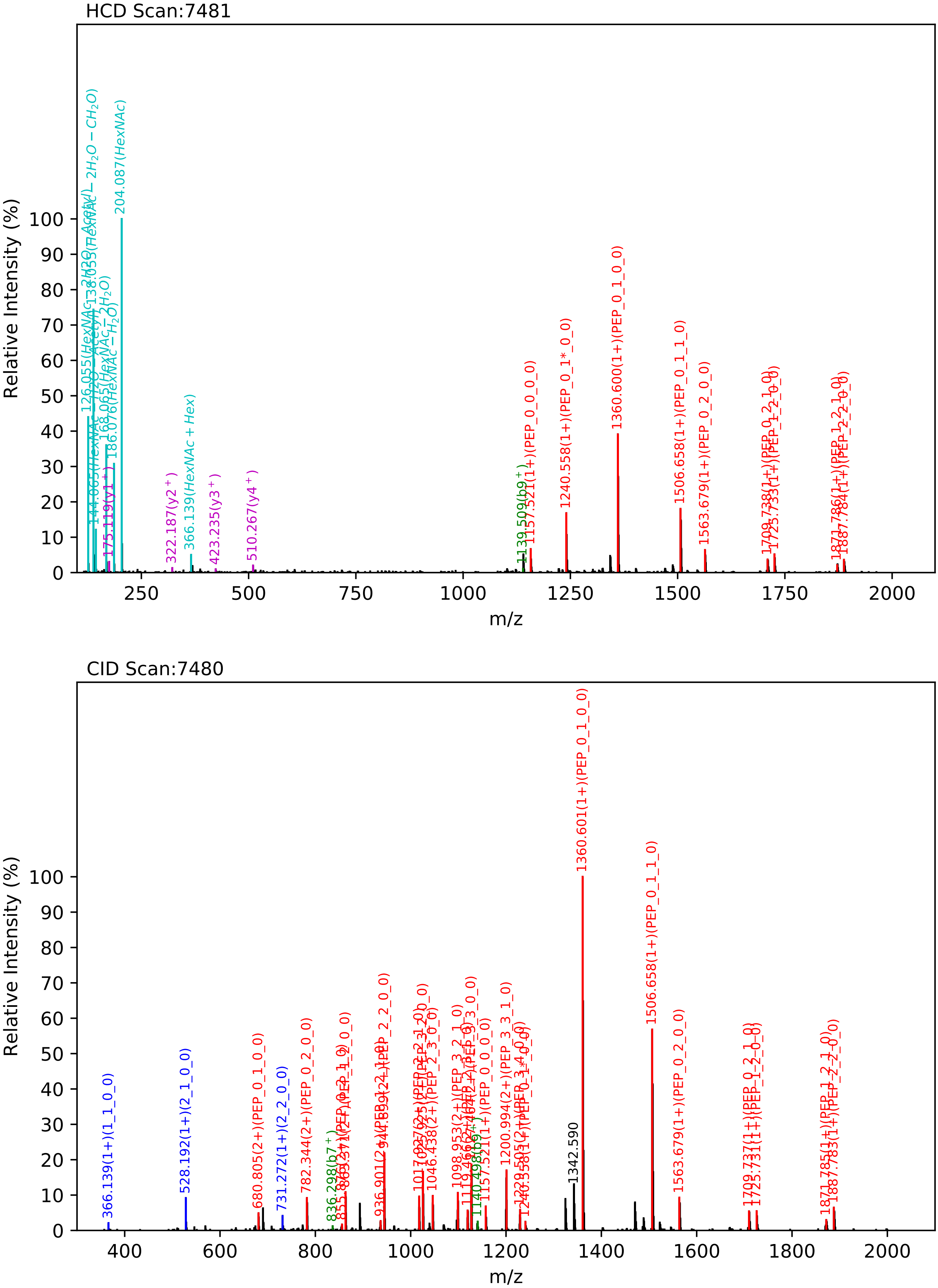

Training set no. 383, Experiment: IgG exp_1

EEQFNSTFR(=PEP)_3_4_1_0, m/z:1301.53(2+), RT:24.89, Y-score:88.27

HCD Scan:4662

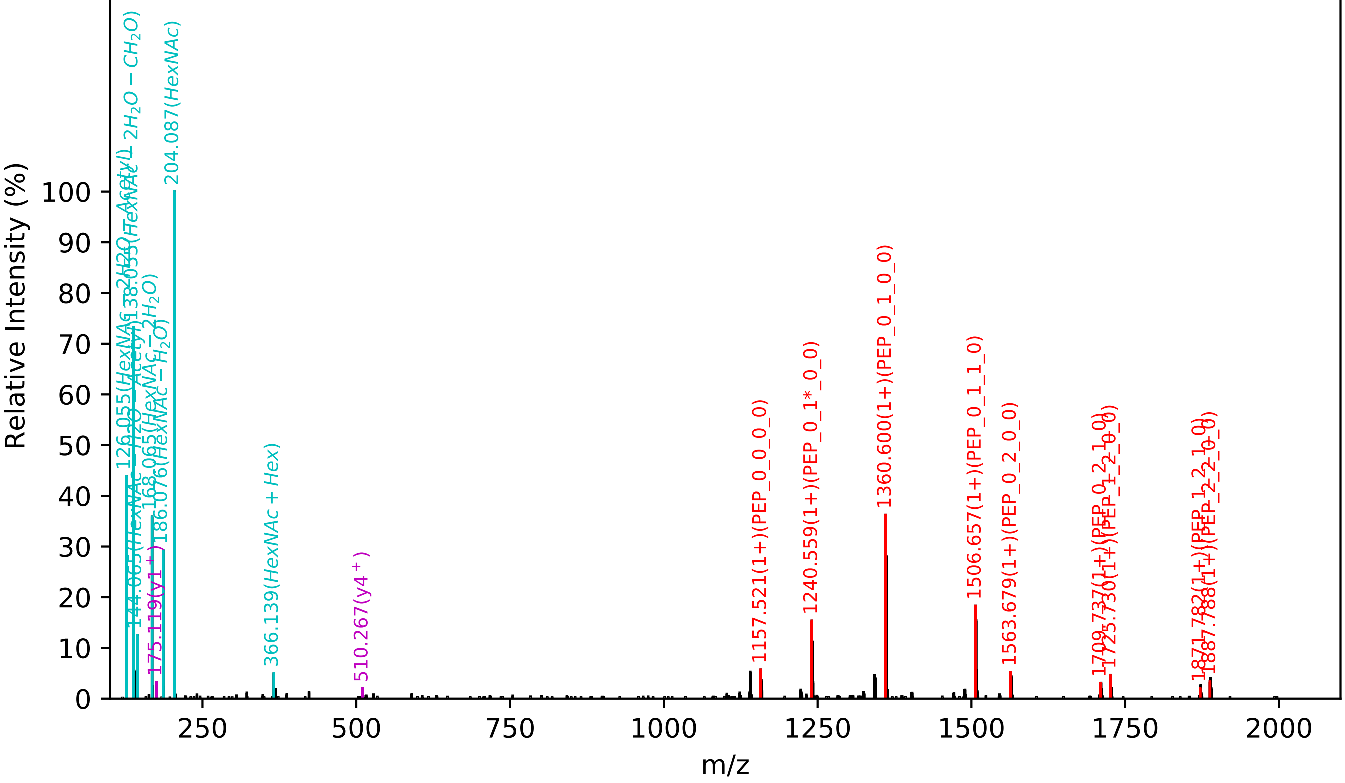

CID Scan:4661

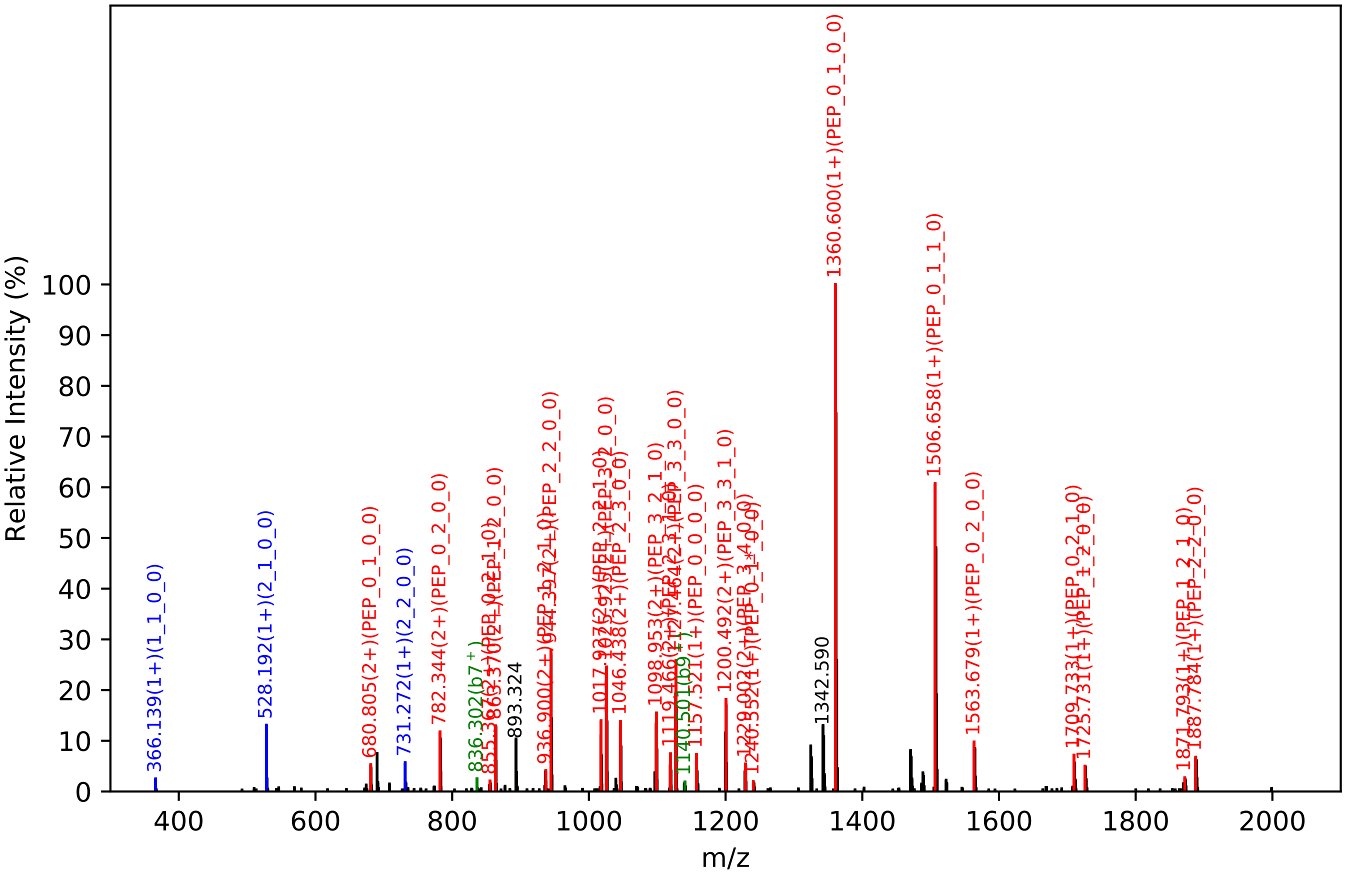

Training set no. 384, Experiment: IgG exp_3

EEQFNSTFR(=PEP)_3_4_1_0, m/z:1301.53(2+), RT:26.95, Y-score:88.21

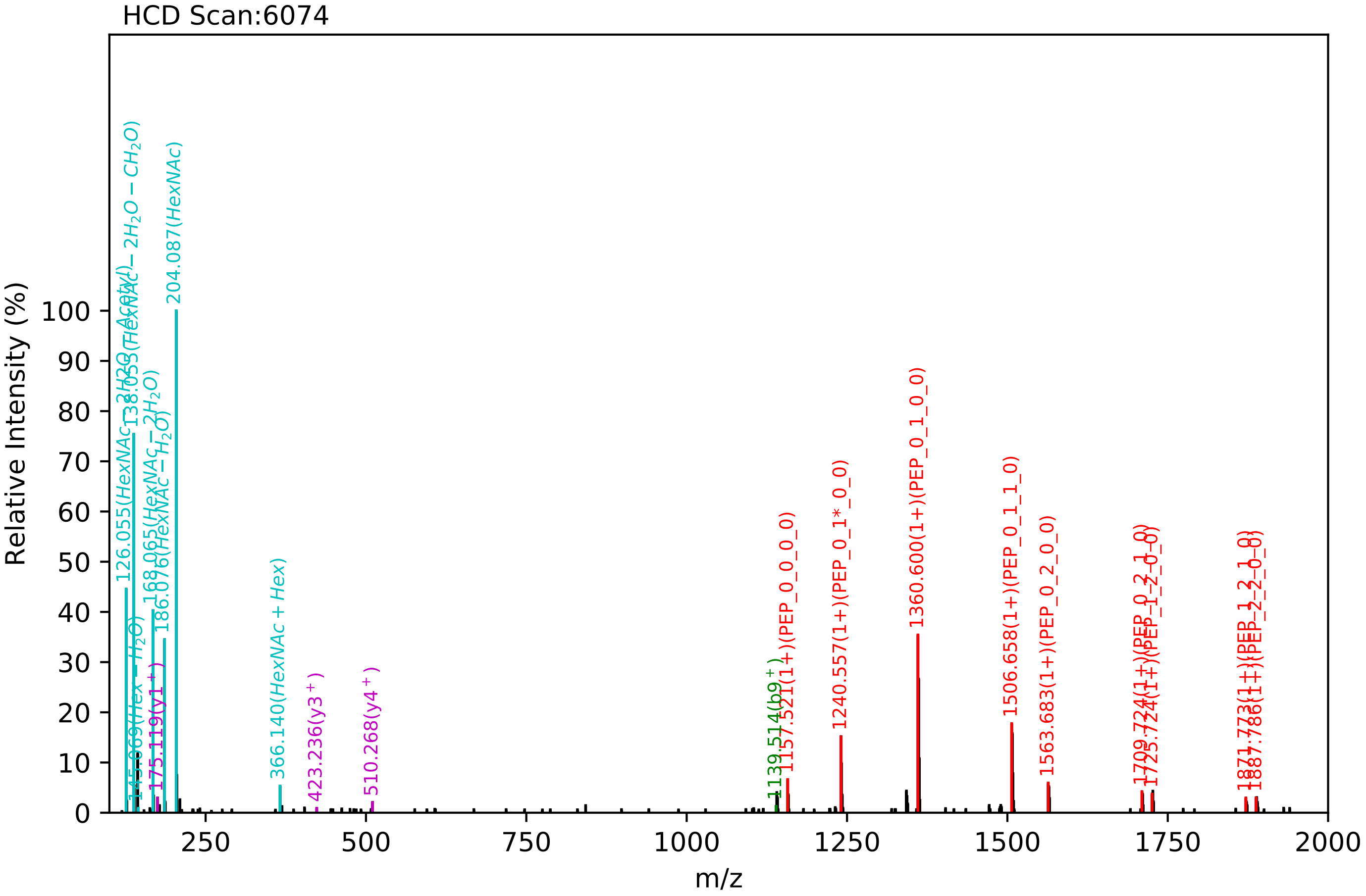

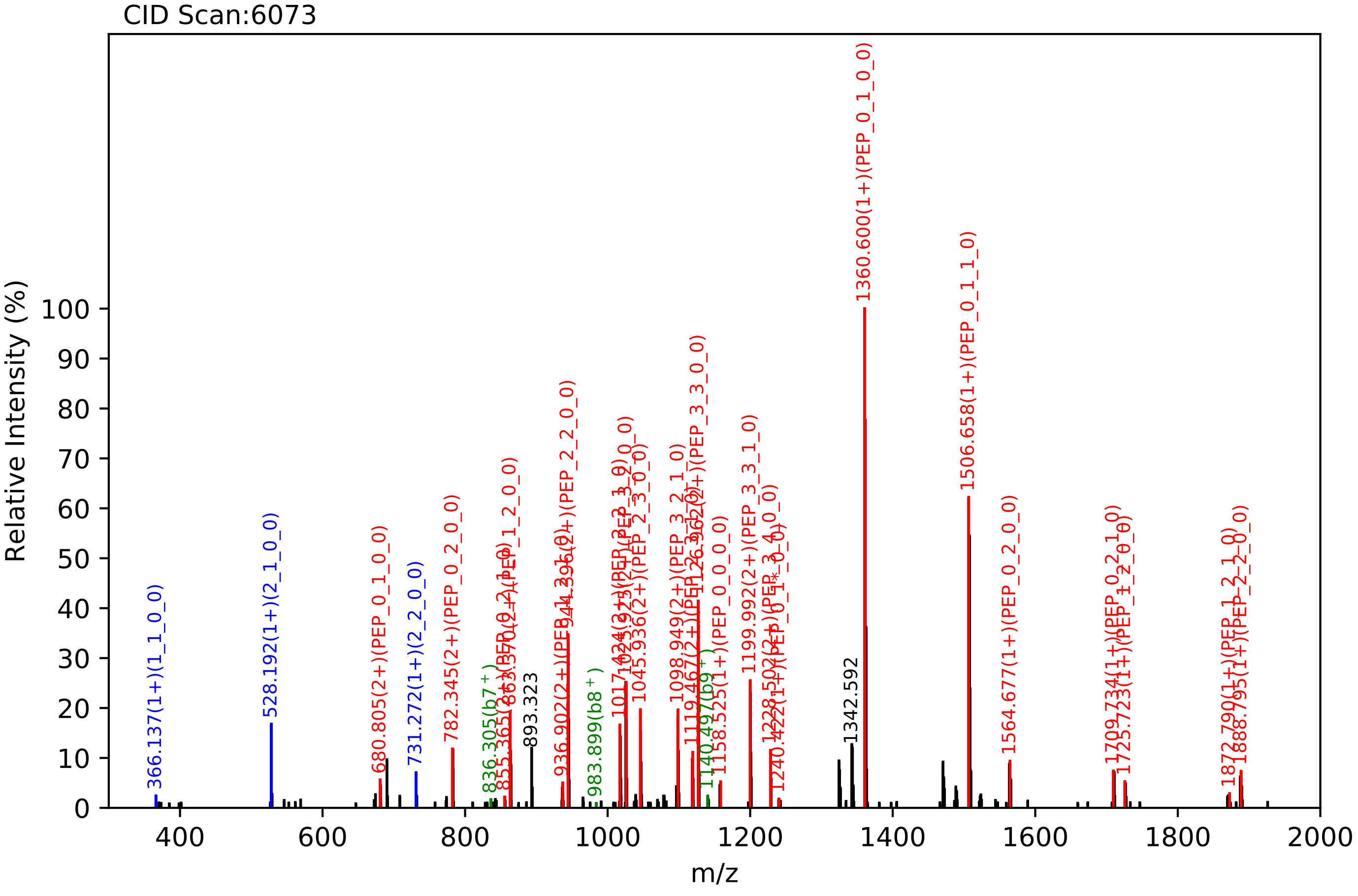

Training set no. 385, Experiment: IgG exp_4

EEQFNSTFR(=PEP)_3_4_1_0, m/z:1301.53(2+), RT:23.80, Y-score:86.50

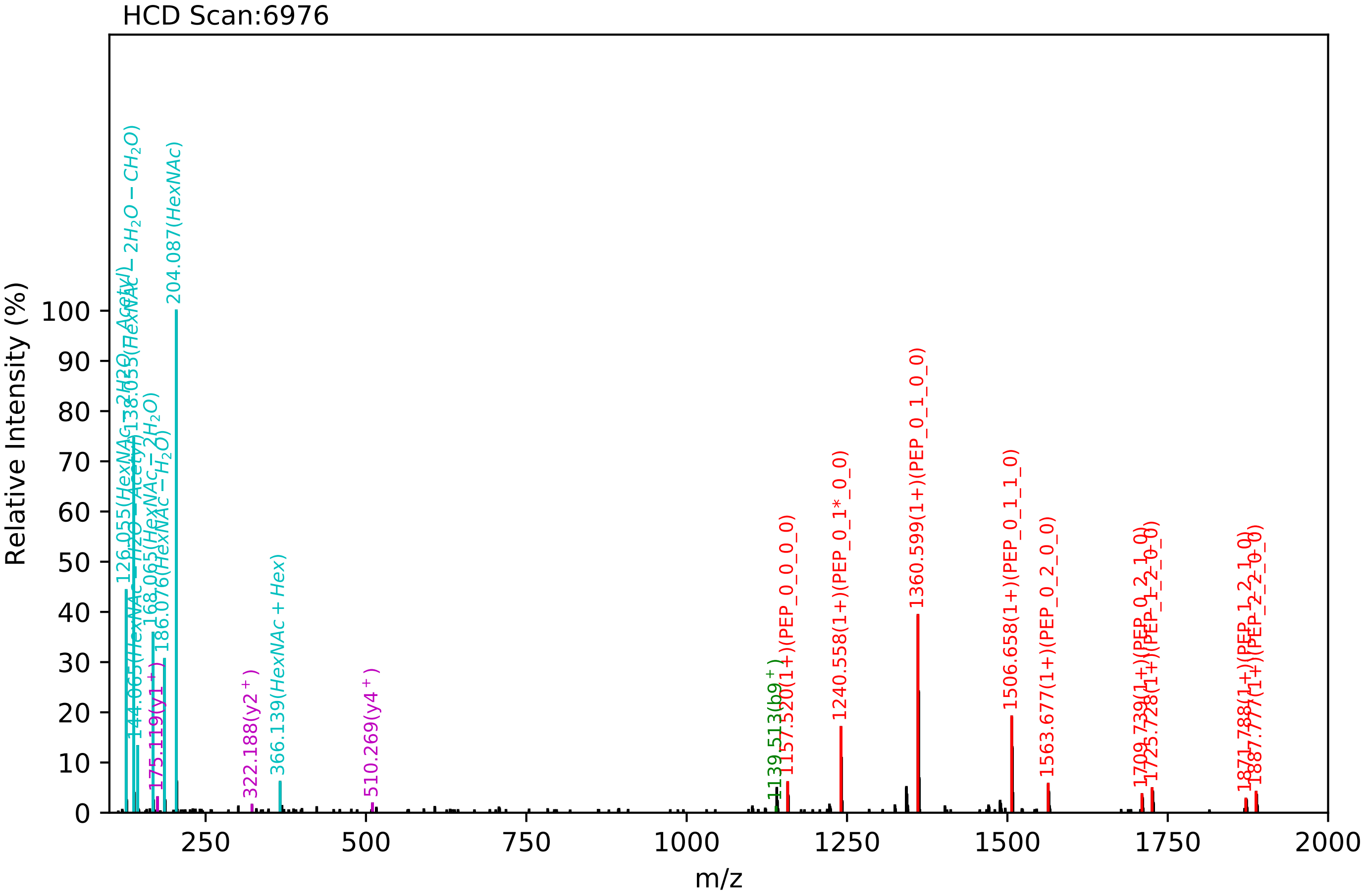

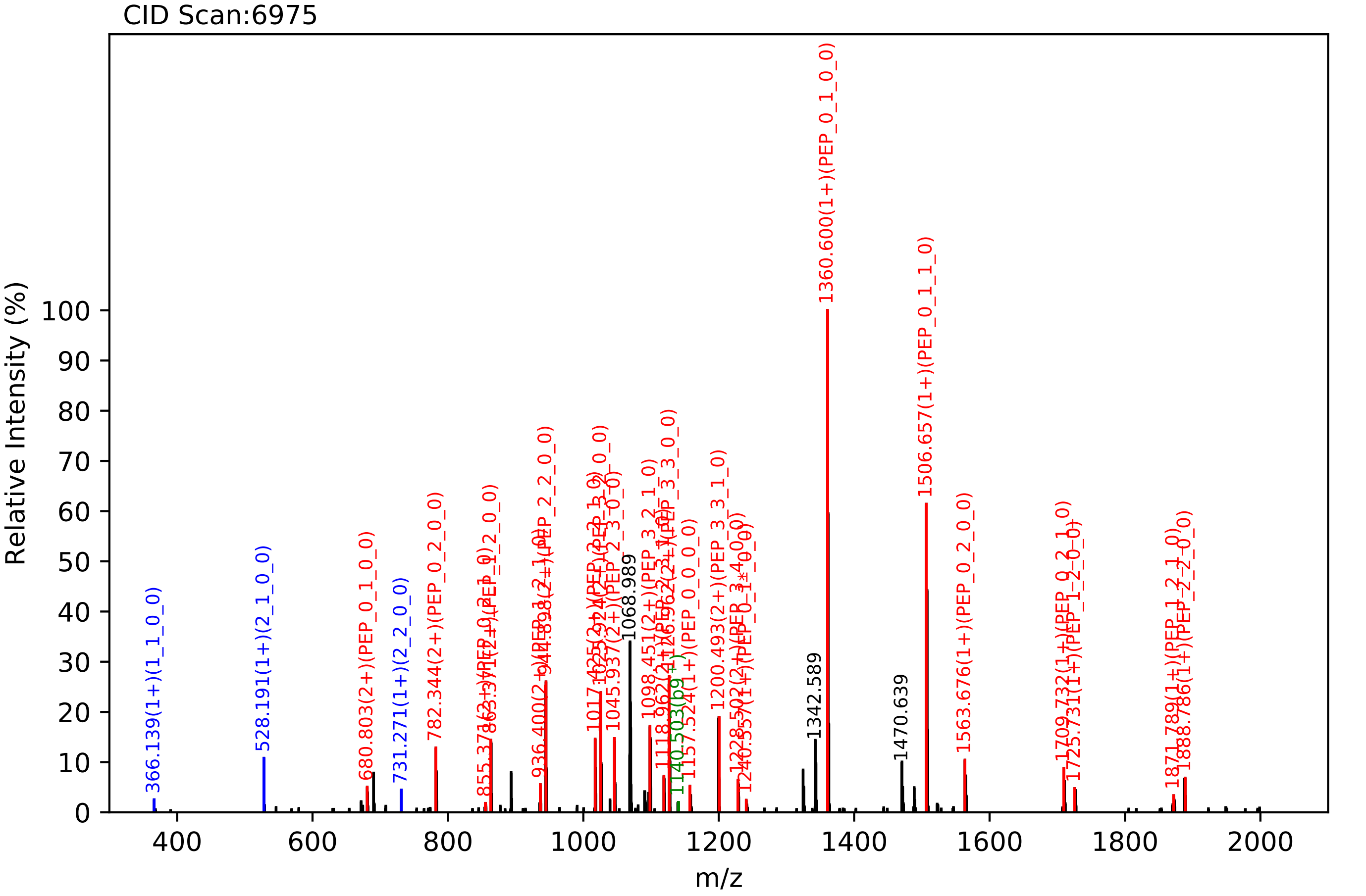

Training set no. 386, Experiment: IgG exp_1

EEQFNSTFR(=PEP)_3_4_1_0, m/z:1301.53(2+), RT:24.32, Y-score:85.97

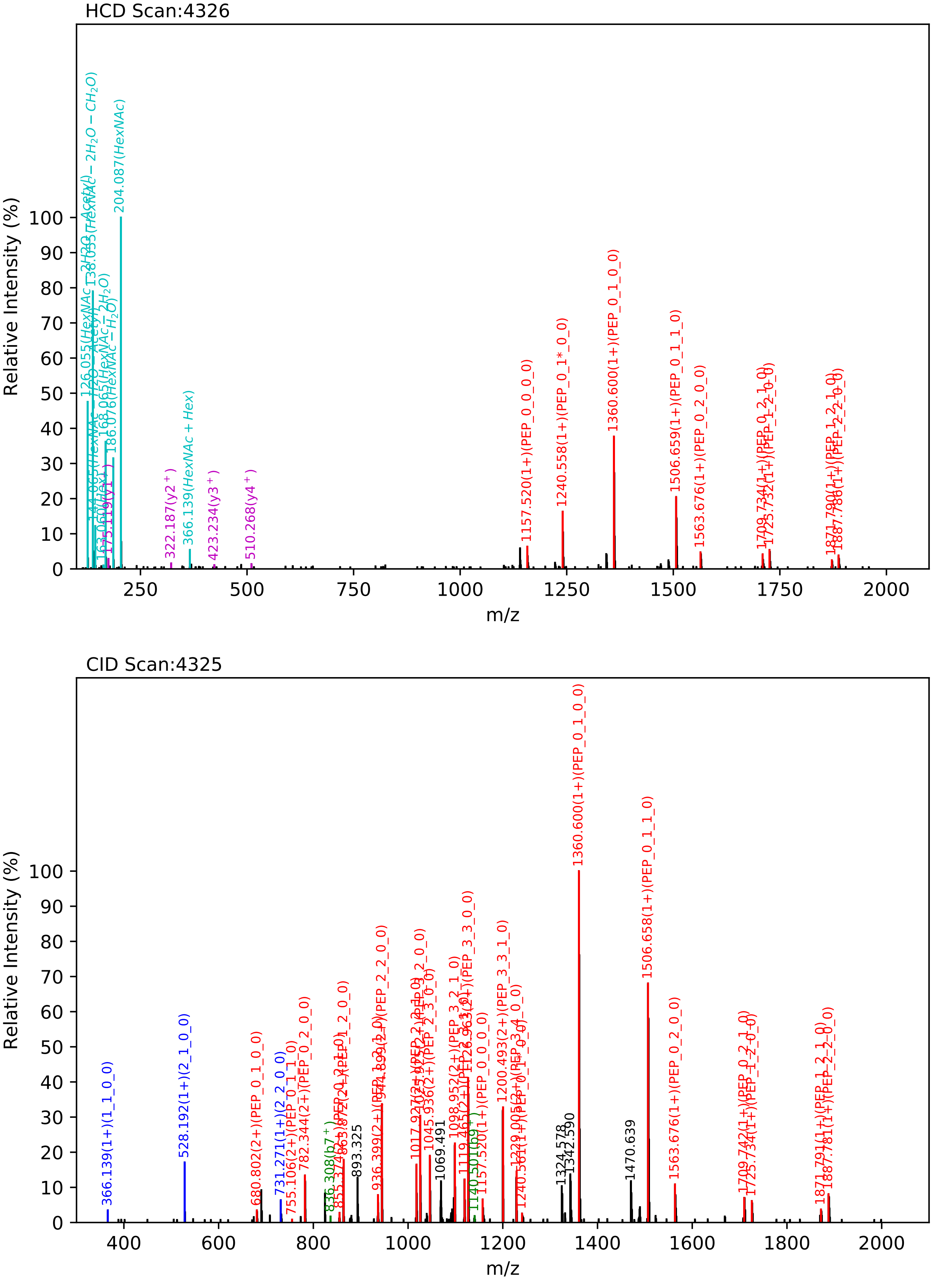

Training set no. 387, Experiment: IgG exp_2

EEQFNSTFR(=PEP)_3_4_1_0, m/z:1301.53(2+), RT:25.83, Y-score:74.65

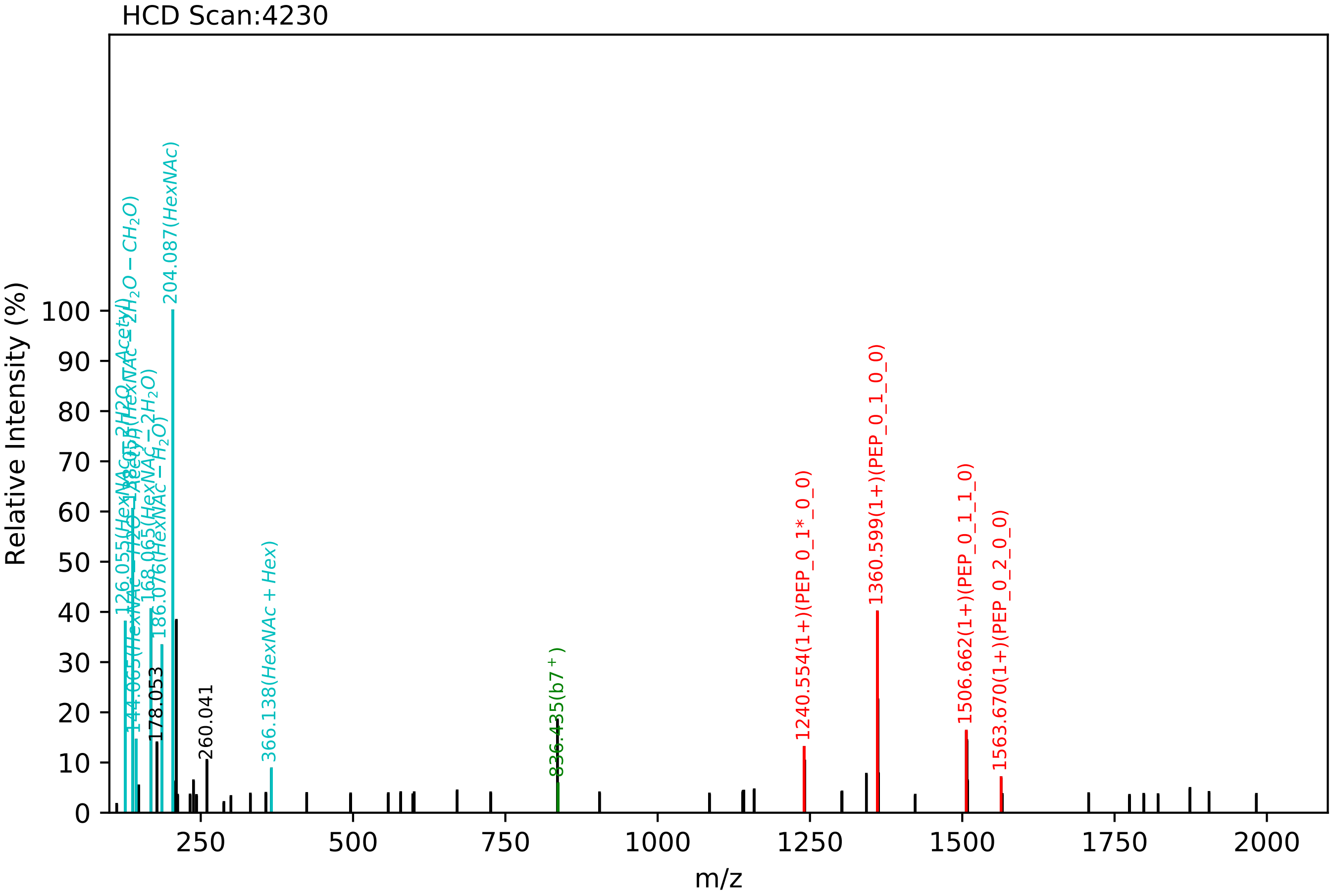

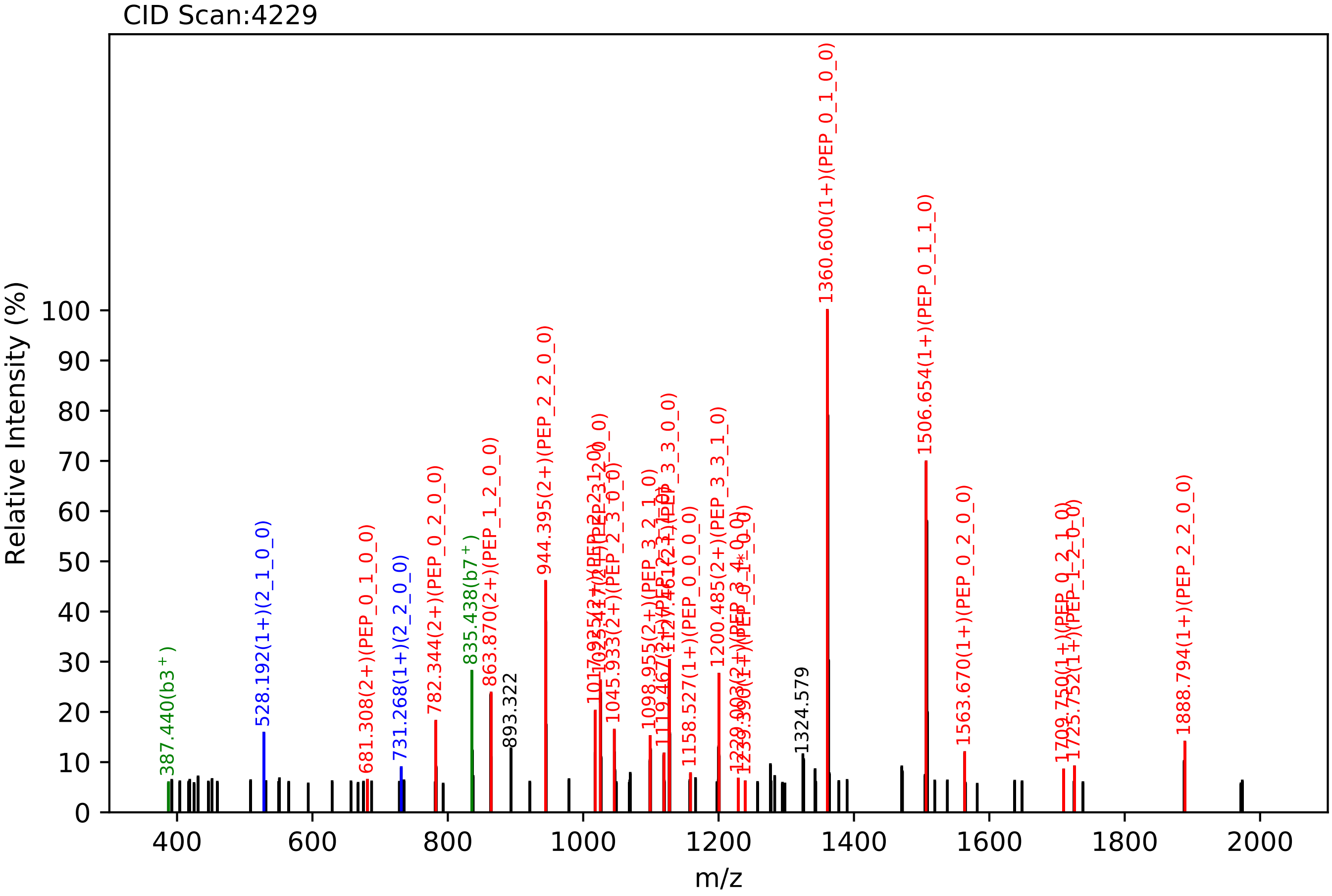

Training set no. 388, Experiment: IgG exp_2

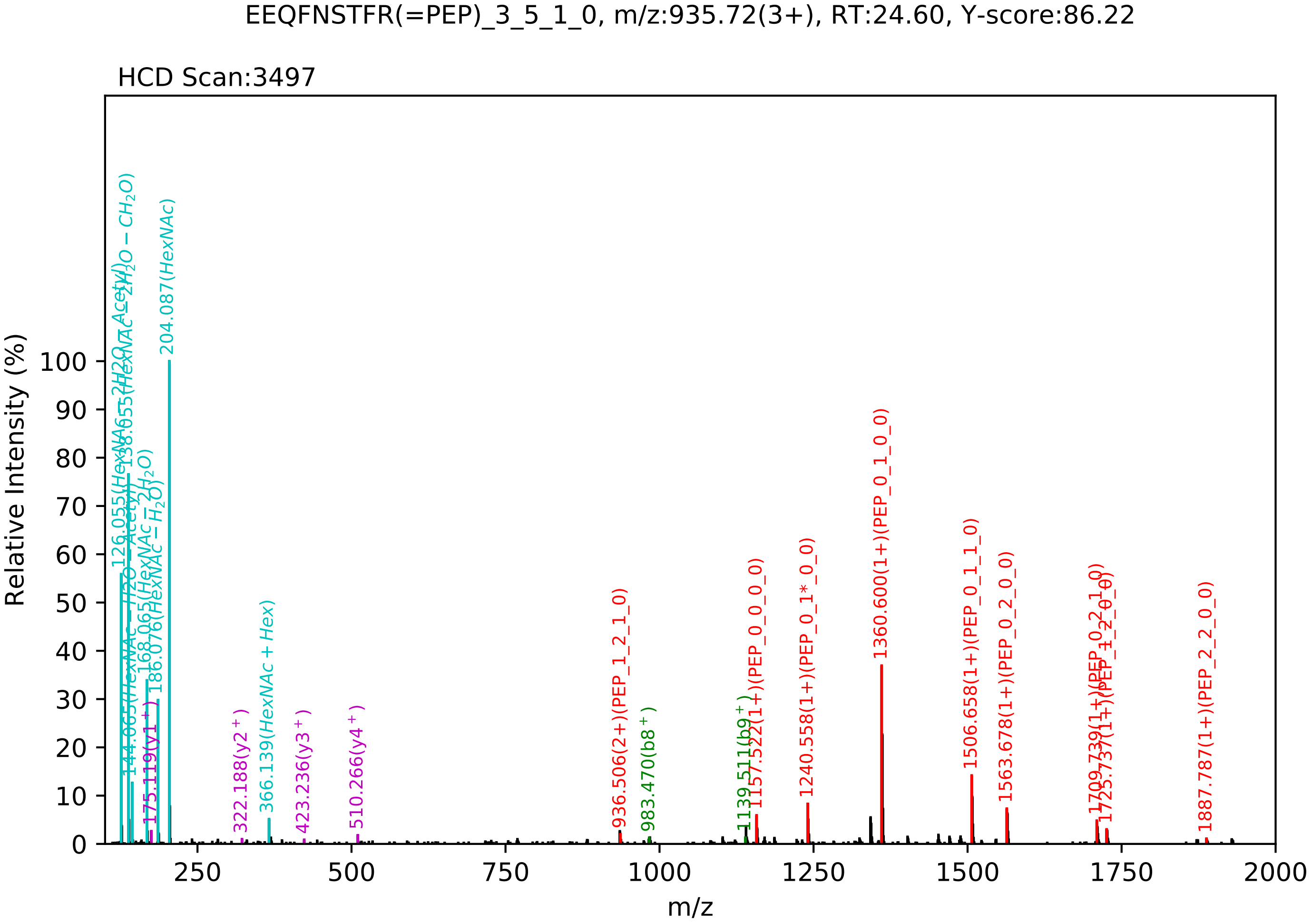

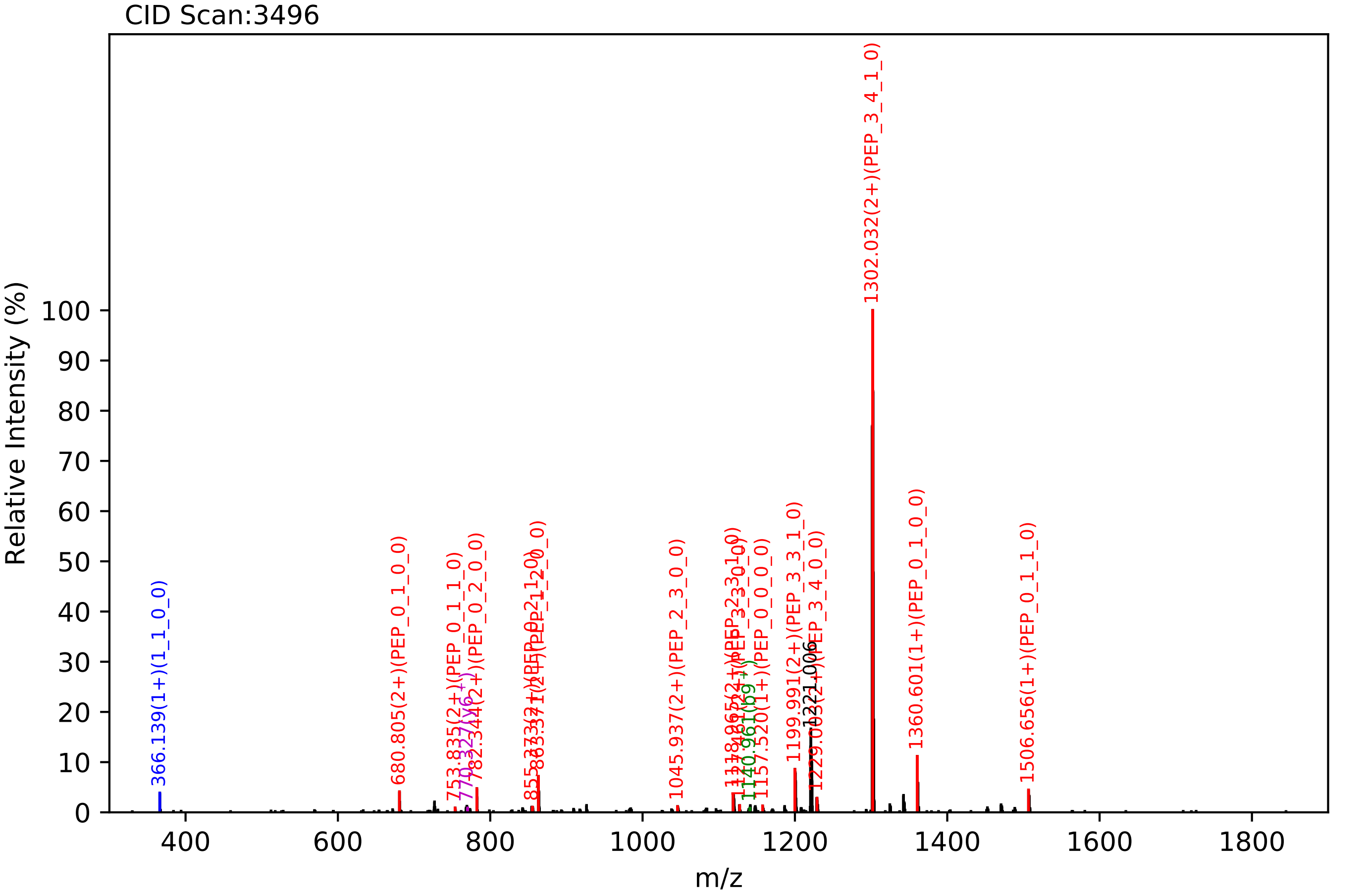

Training set no. 389, Experiment: IgG exp_2

EEQFNSTFR(=PEP)_3_5_1_0, m/z:1403.07(2+), RT:24.61, Y-score:83.19

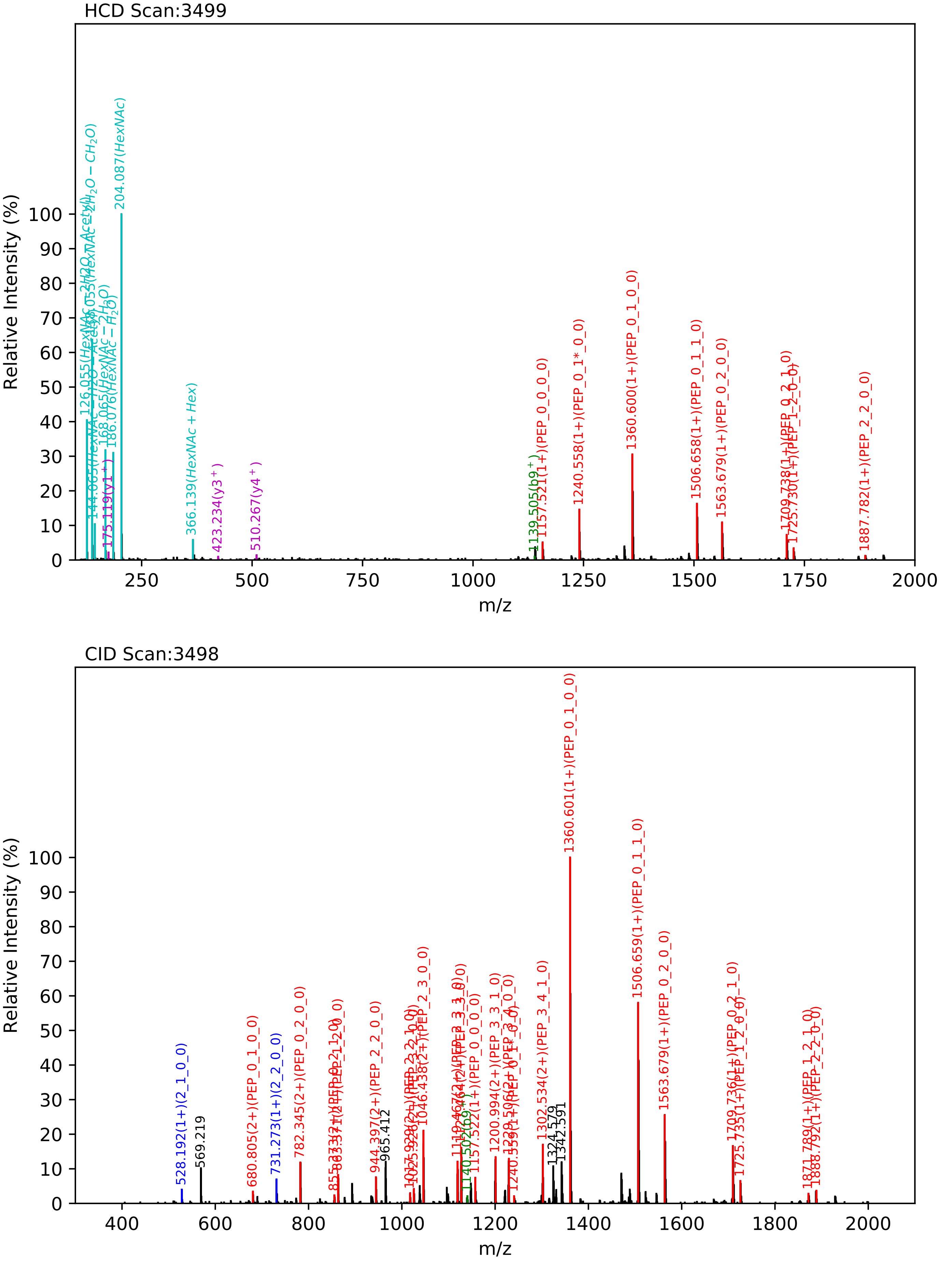

Training set no. 390, Experiment: IgG exp_4

EEQFNSTFR(=PEP)_3_5_1_0, m/z:1403.07(2+), RT:24.37, Y-score:82.86

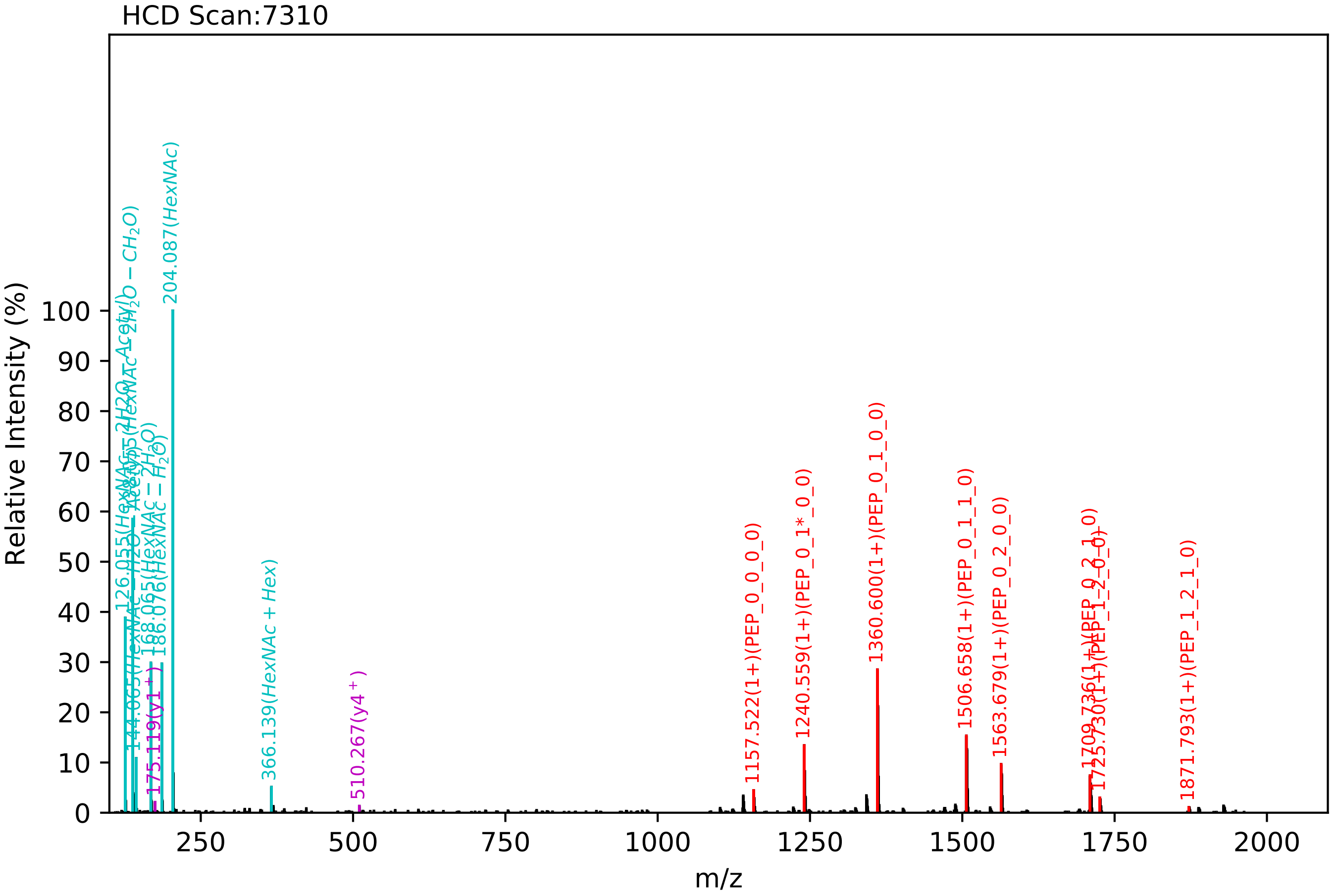

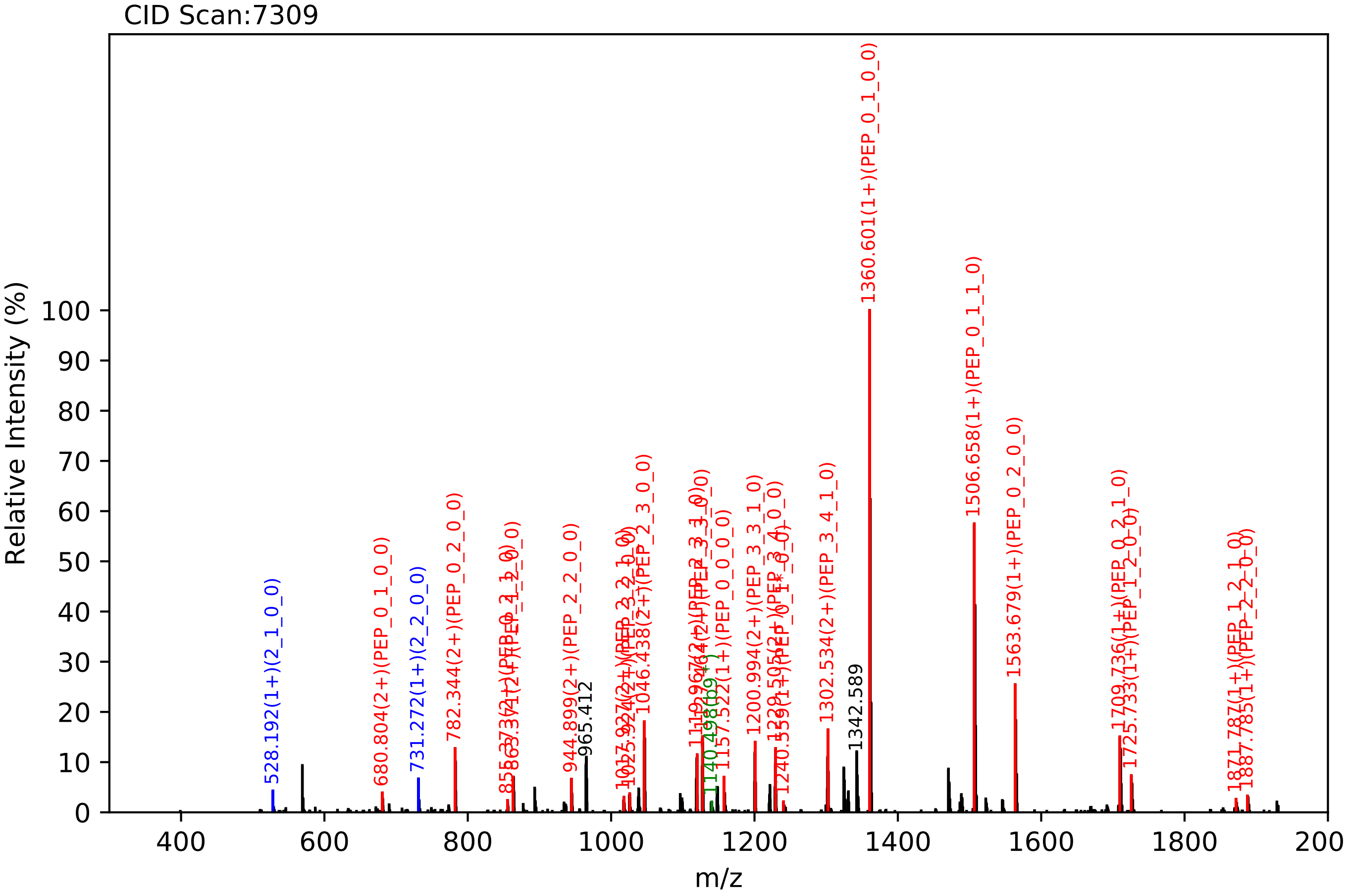

Training set no. 391, Experiment: IgG exp_3

EEQFNSTFR(=PEP)_3_5_1_0, m/z:1403.07(2+), RT:26.43, Y-score:82.82

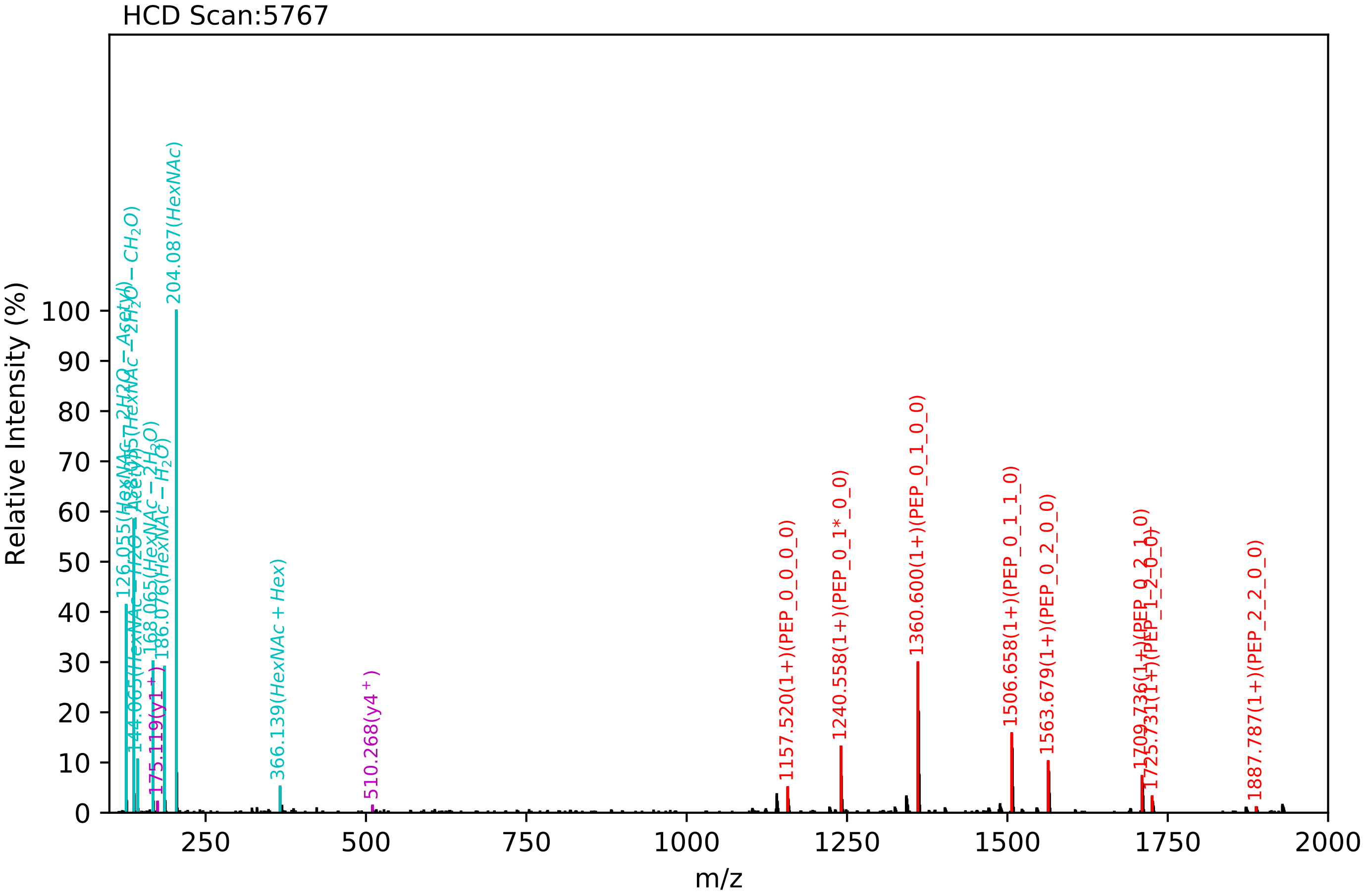

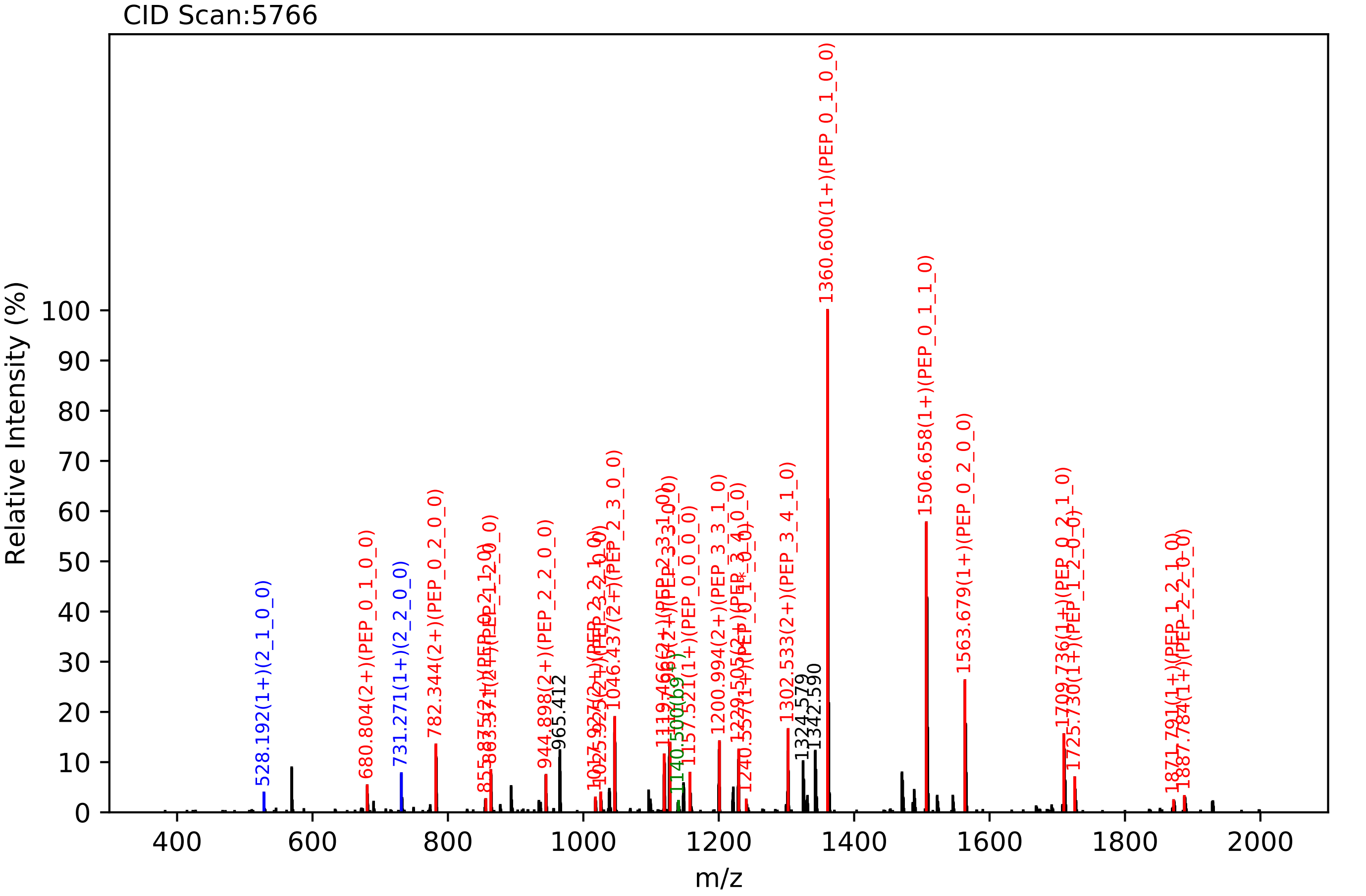

Training set no. 392, Experiment: IgG exp_1

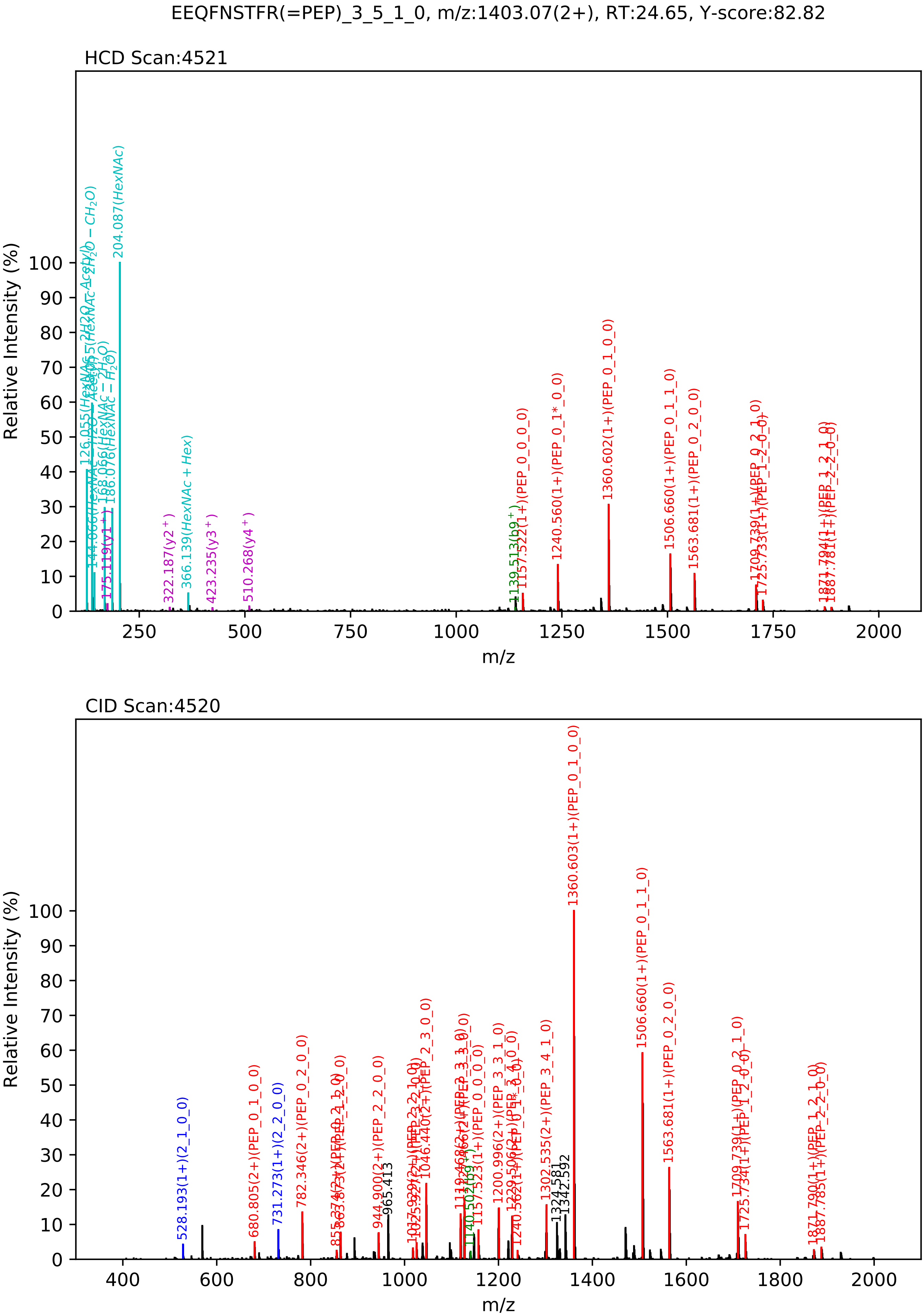

Training set no. 393, Experiment: IgG exp_4

EEQFNSTFR(=PEP)_4_3_1_0, m/z:1281.02(2+), RT:24.22, Y-score:86.96

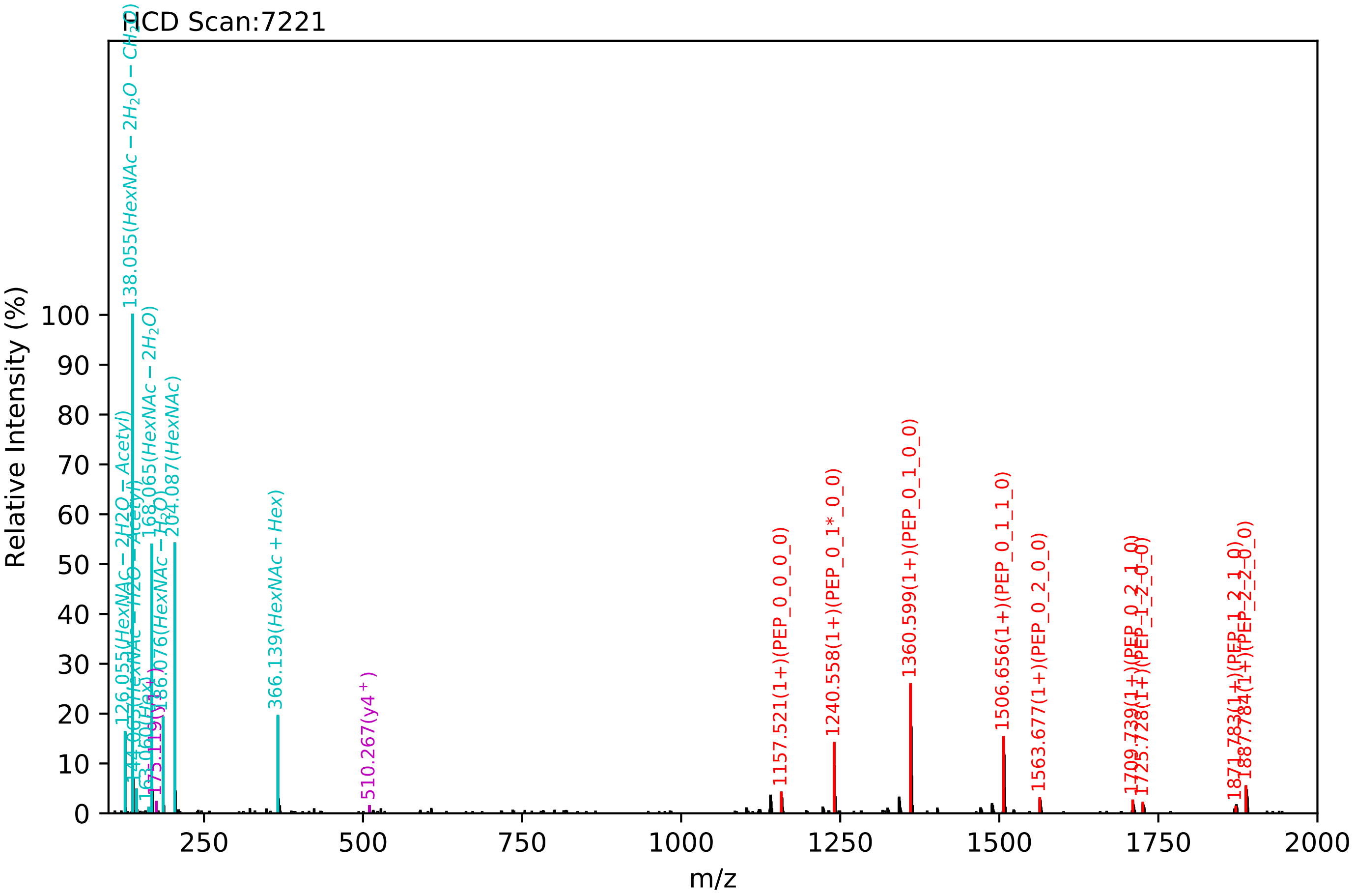

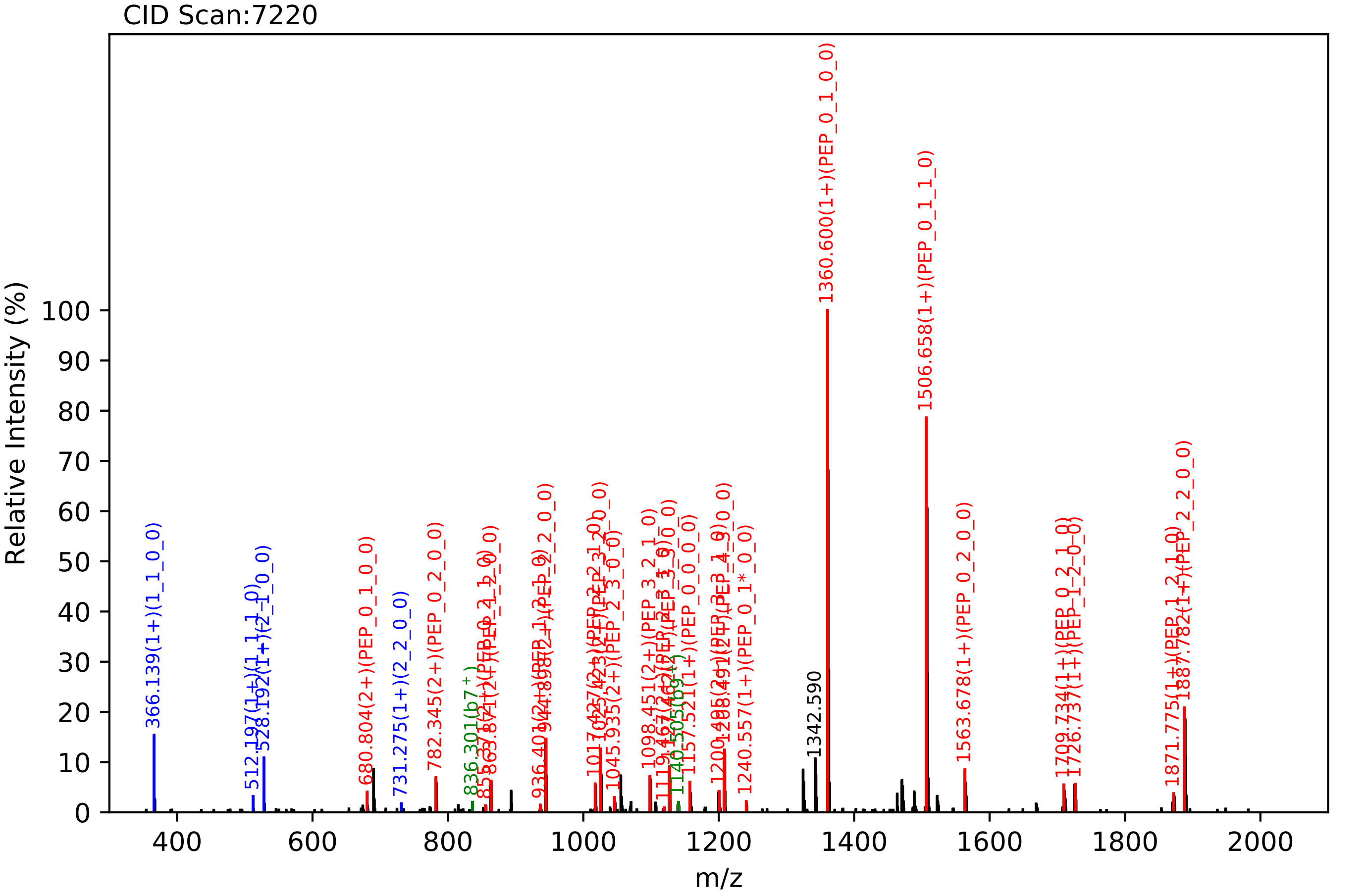

Training set no. 394, Experiment: IgG exp_4

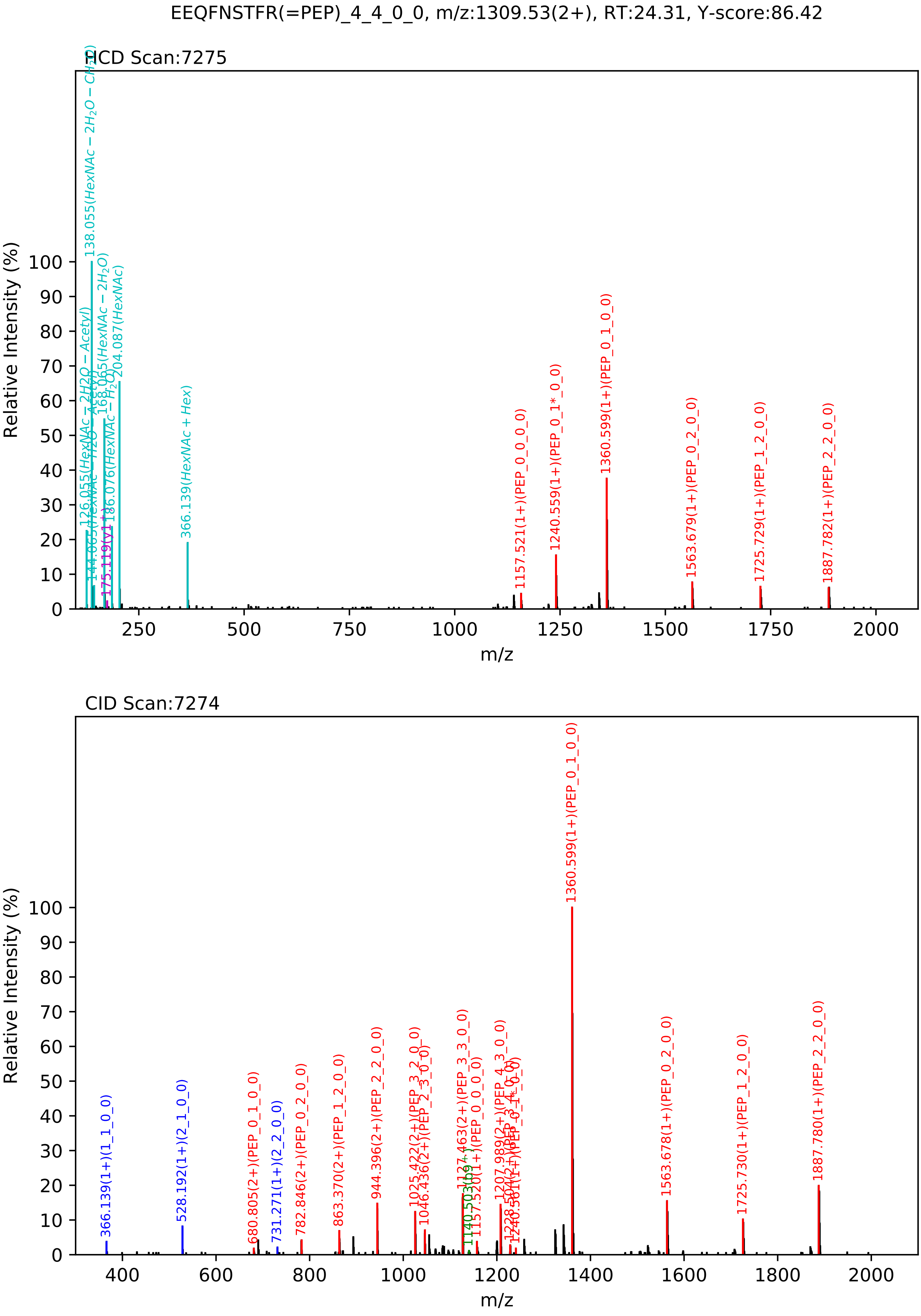

Training set no. 395, Experiment: IgG exp_4

EEQFNSTFR(=PEP)_4_4_1_0, m/z:922.04(3+), RT:24.30, Y-score:98.03

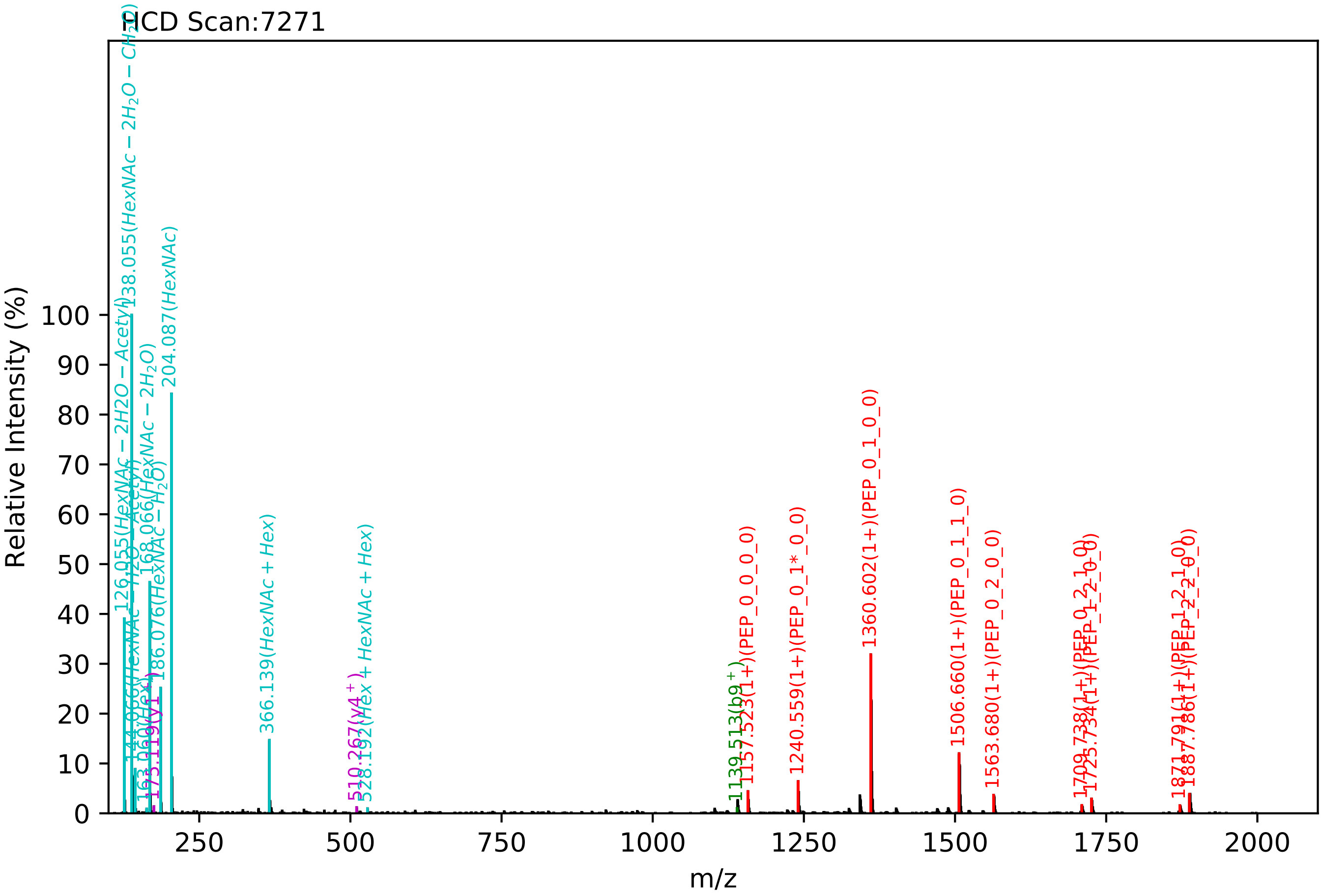

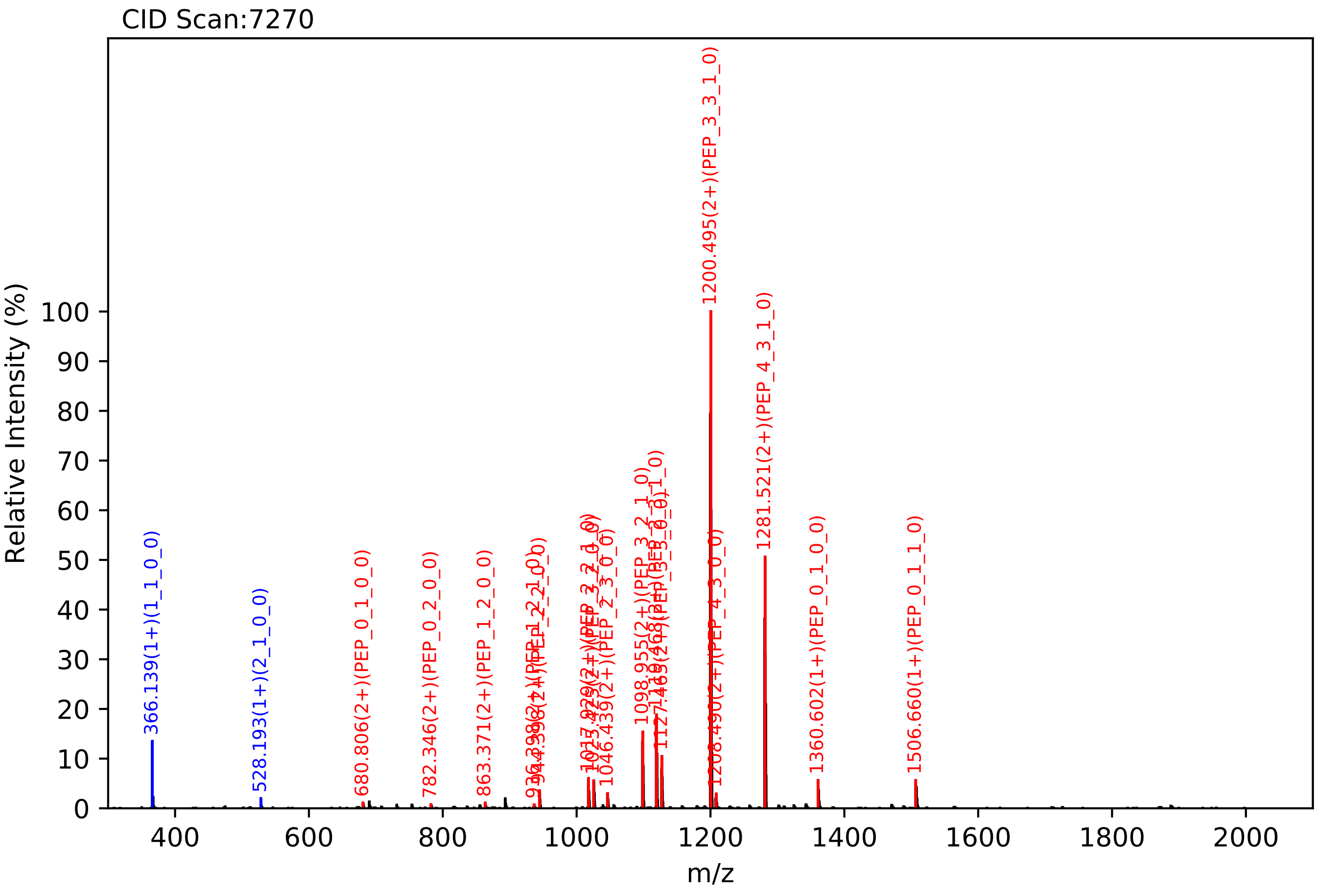

Training set no. 396, Experiment: IgG exp_1

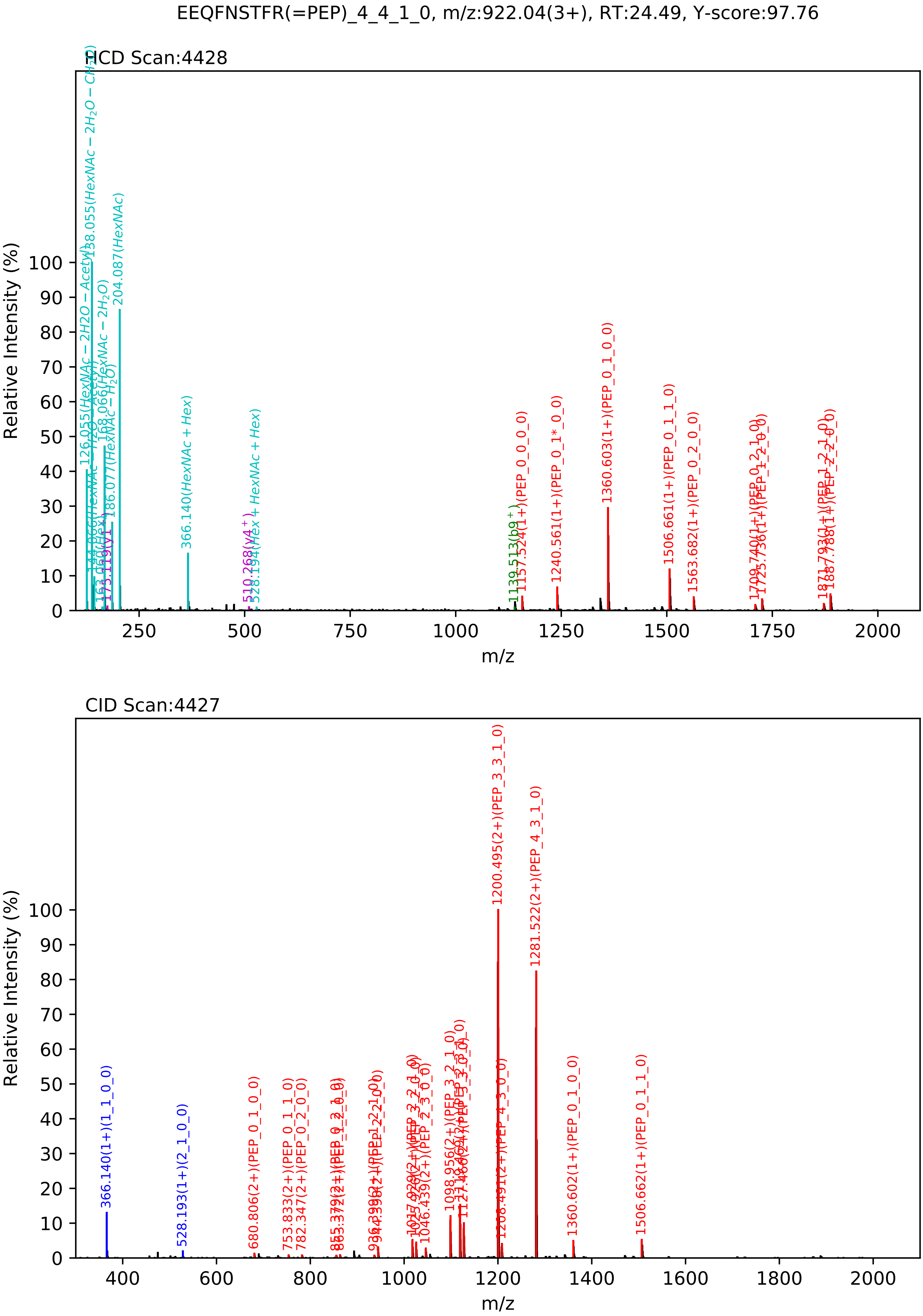

Training set no. 397, Experiment: IgG exp_3

EEQFNSTFR(=PEP)_4_4_1_0, m/z:922.04(3+), RT:26.31, Y-score:97.00

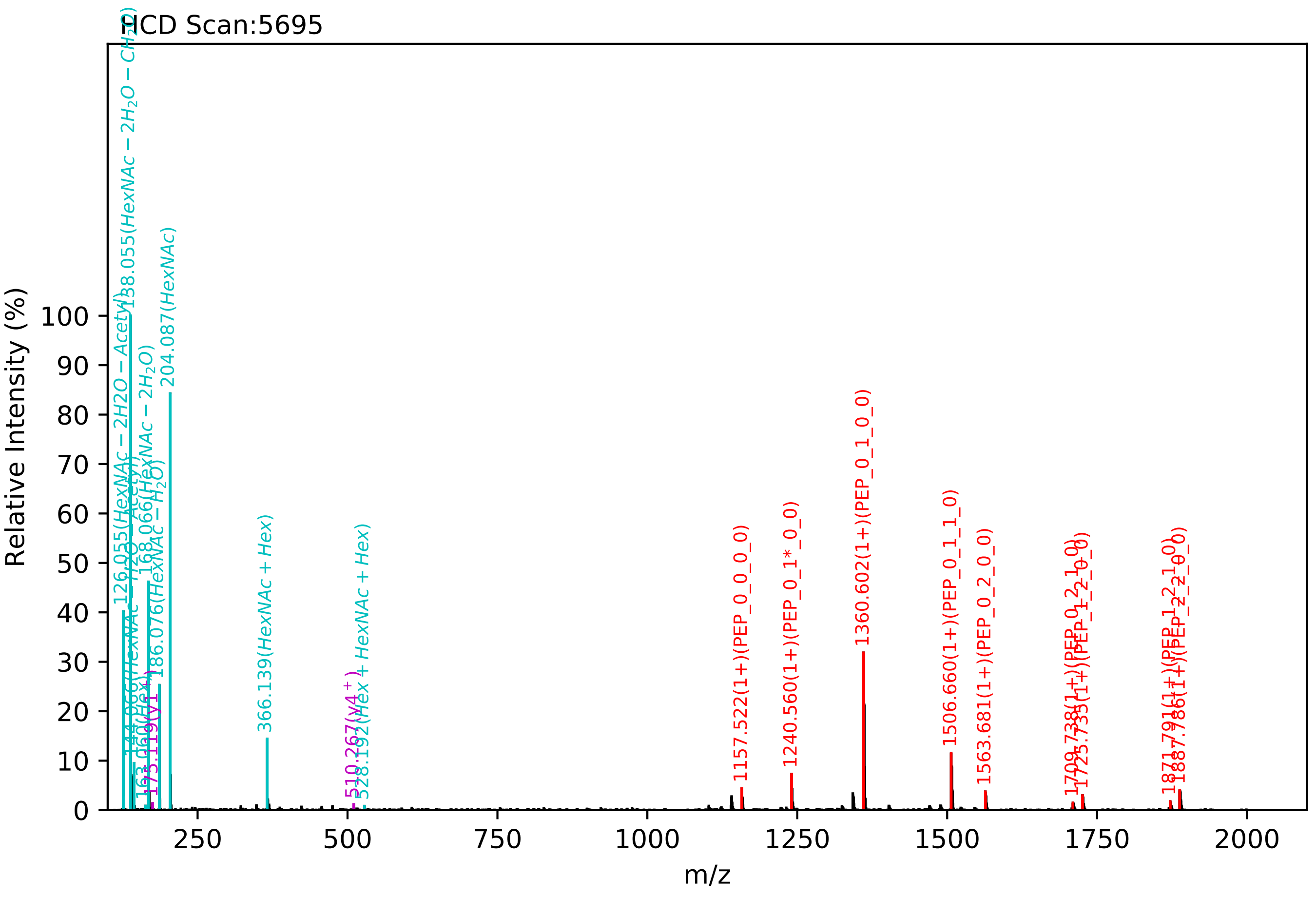

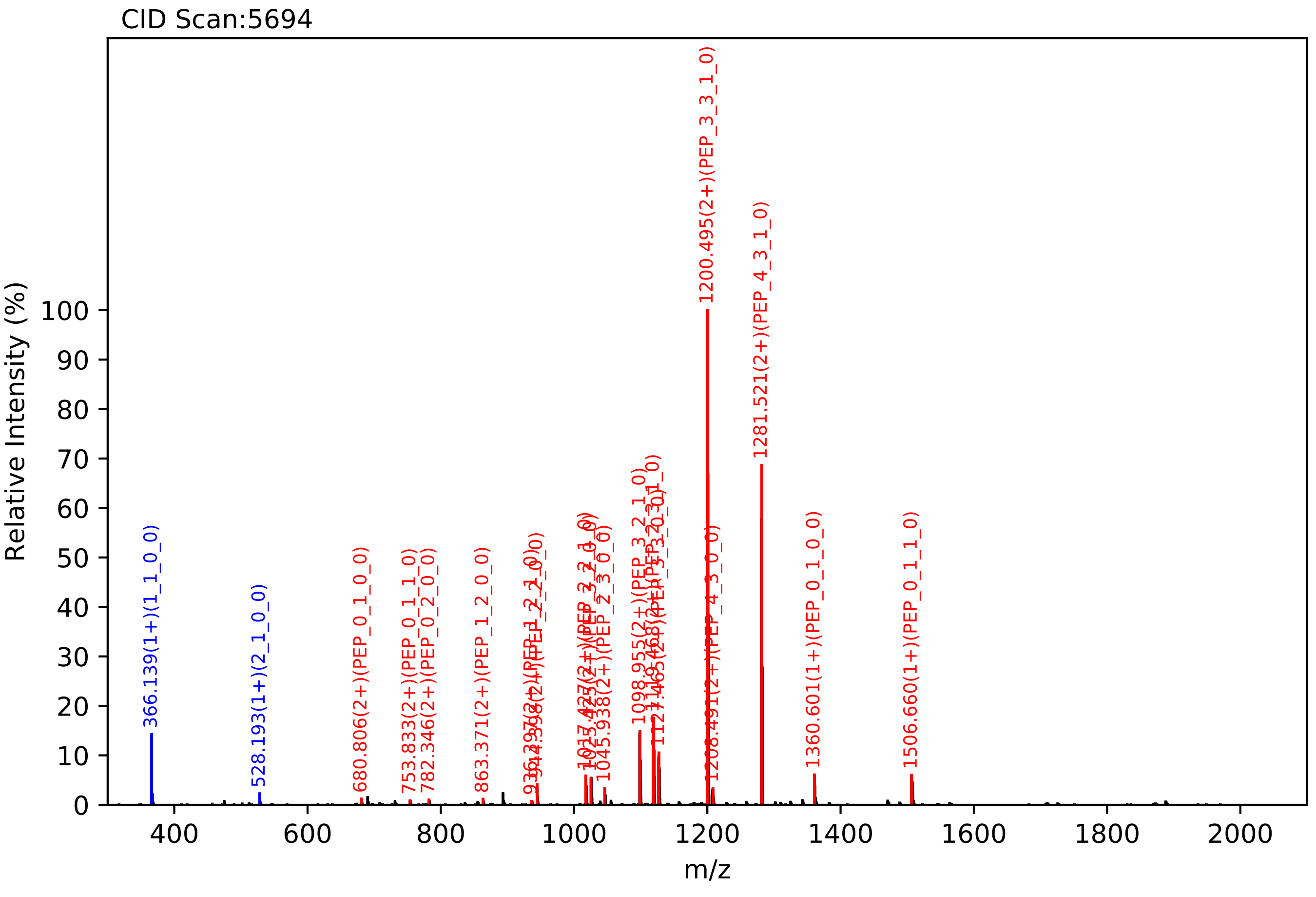

Training set no. 398, Experiment: IgG exp_2

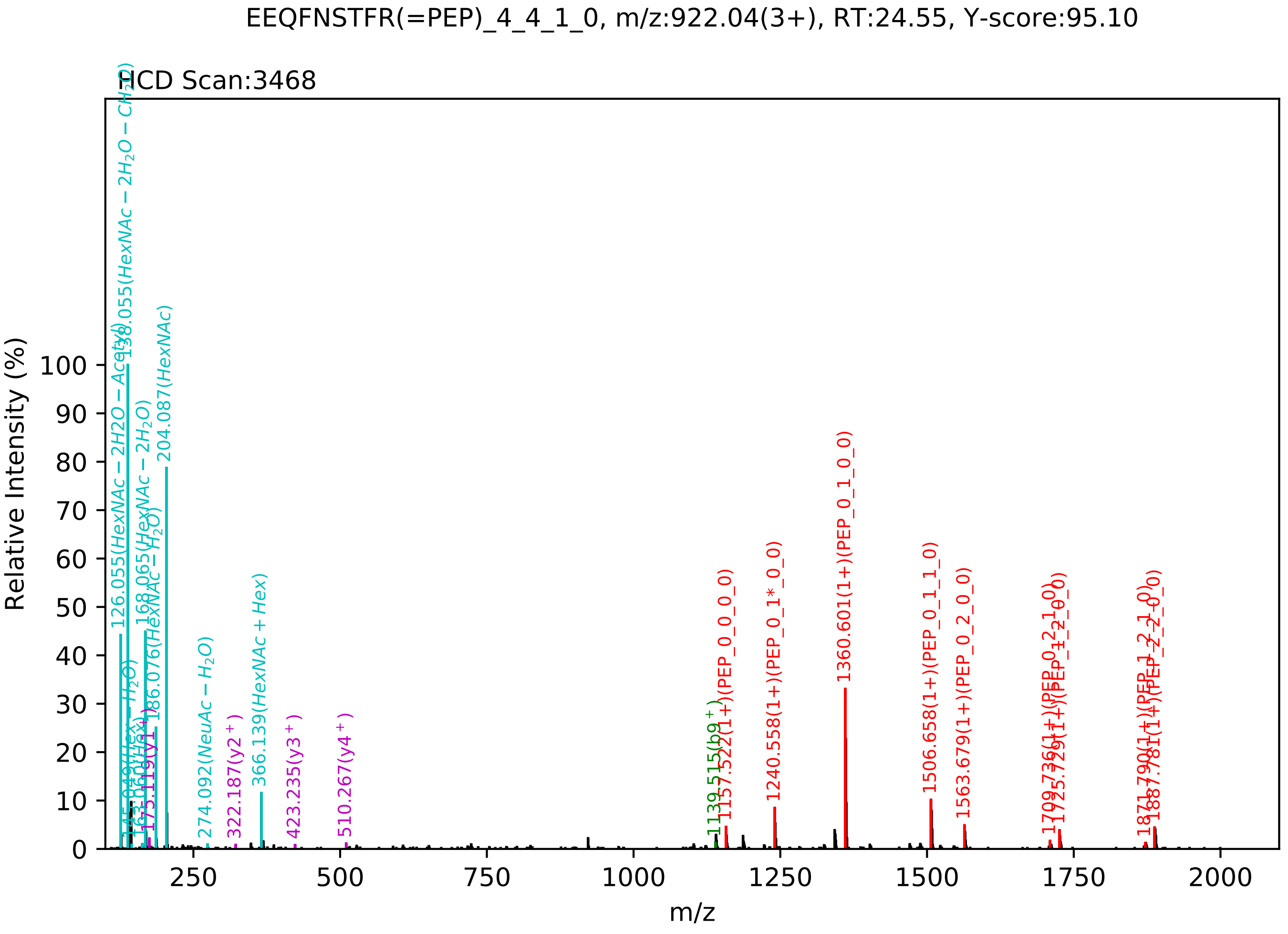

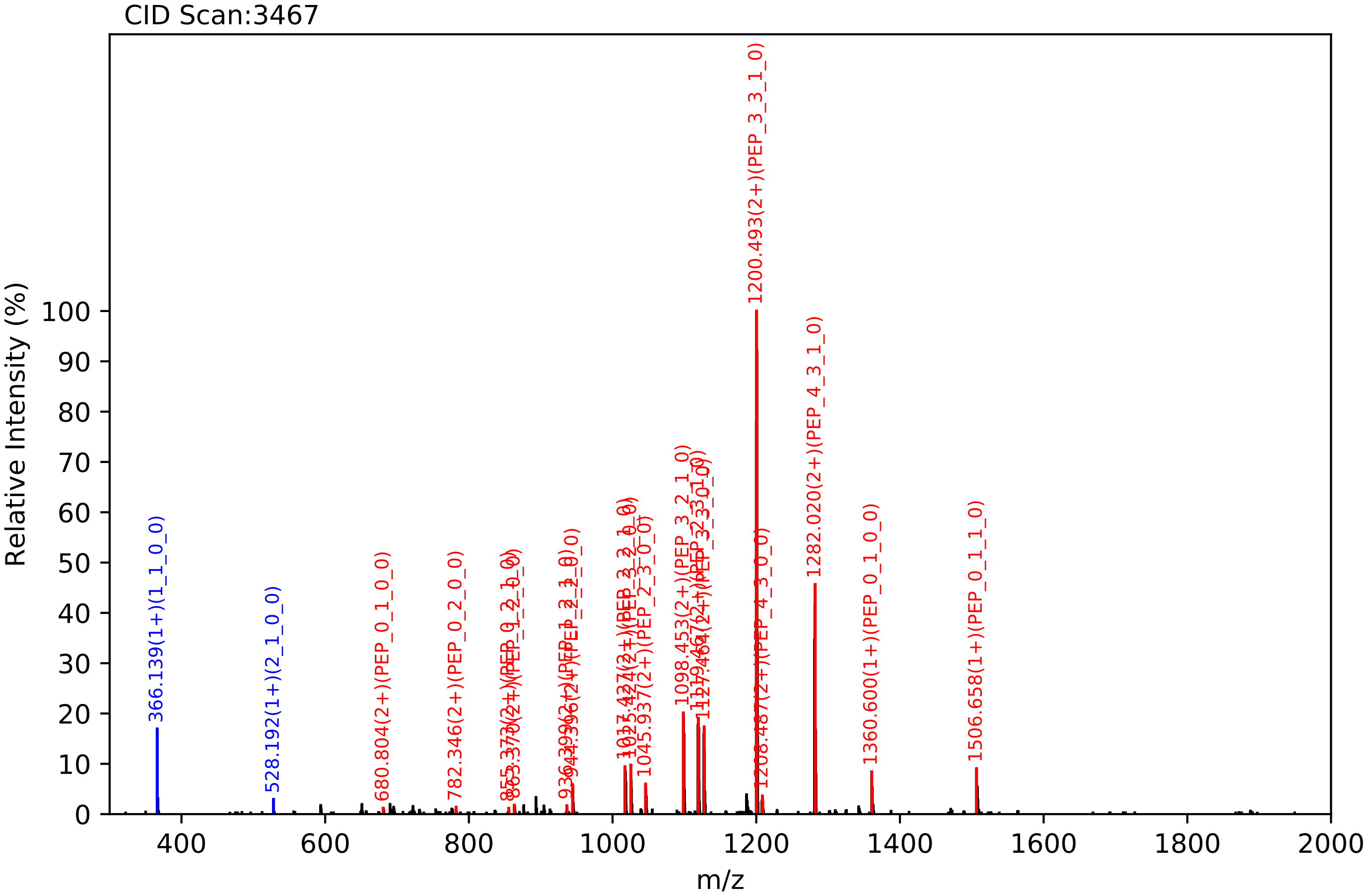

Training set no. 399, Experiment: IgG exp_4

EEQFNSTFR(=PEP)_4_4_1_0, m/z:1382.56(2+), RT:23.72, Y-score:90.21

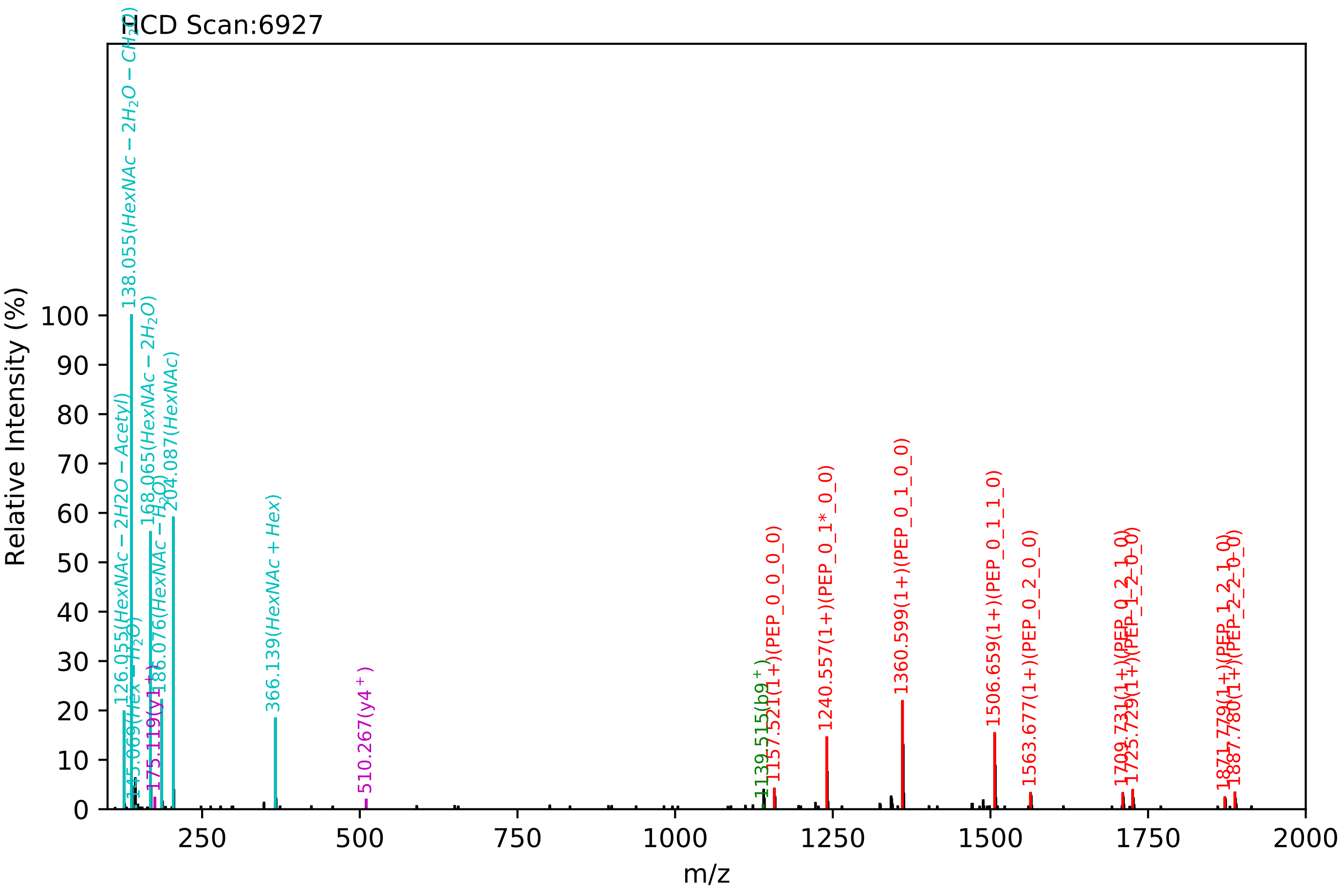

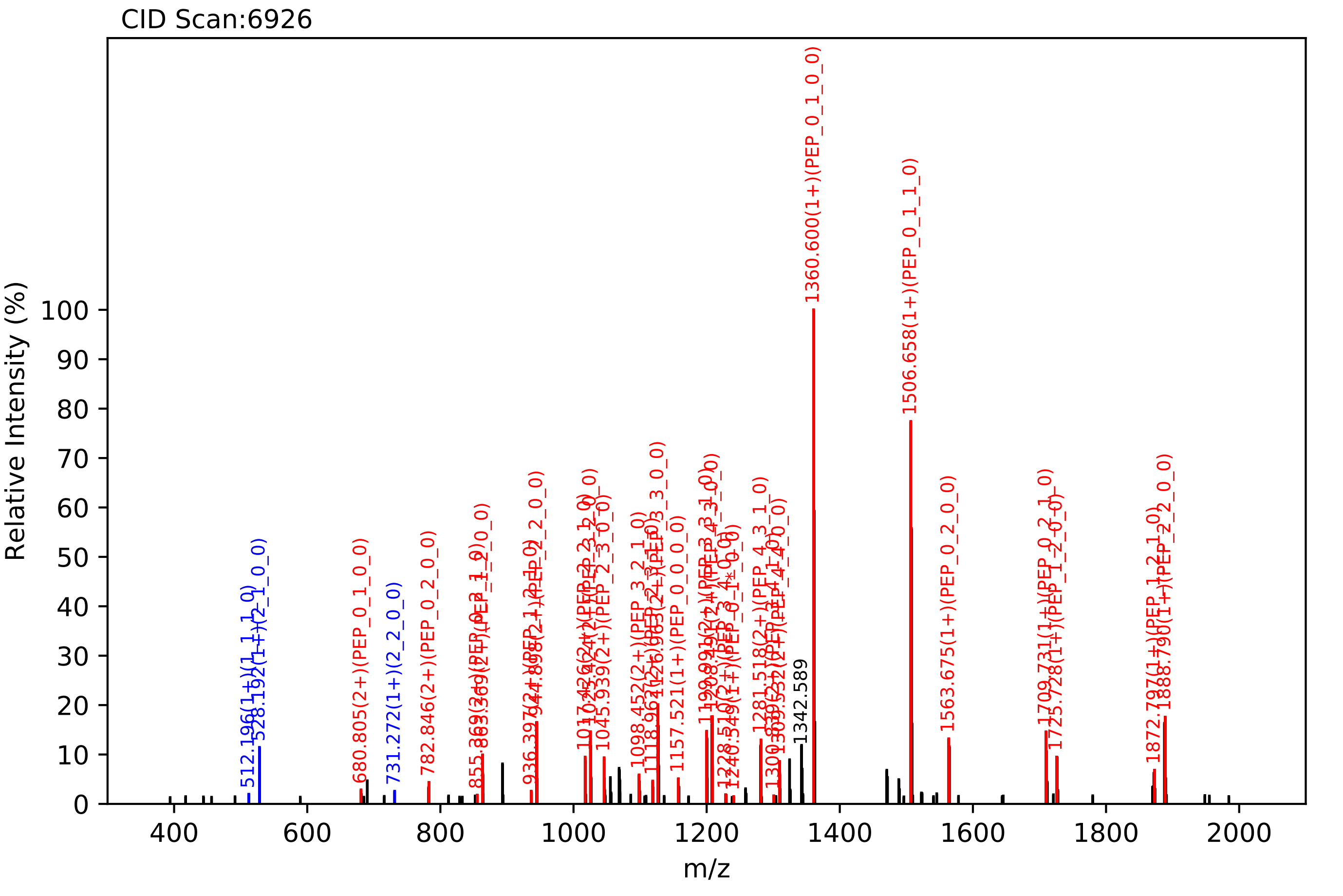

Training set no. 400, Experiment: IgG exp_3

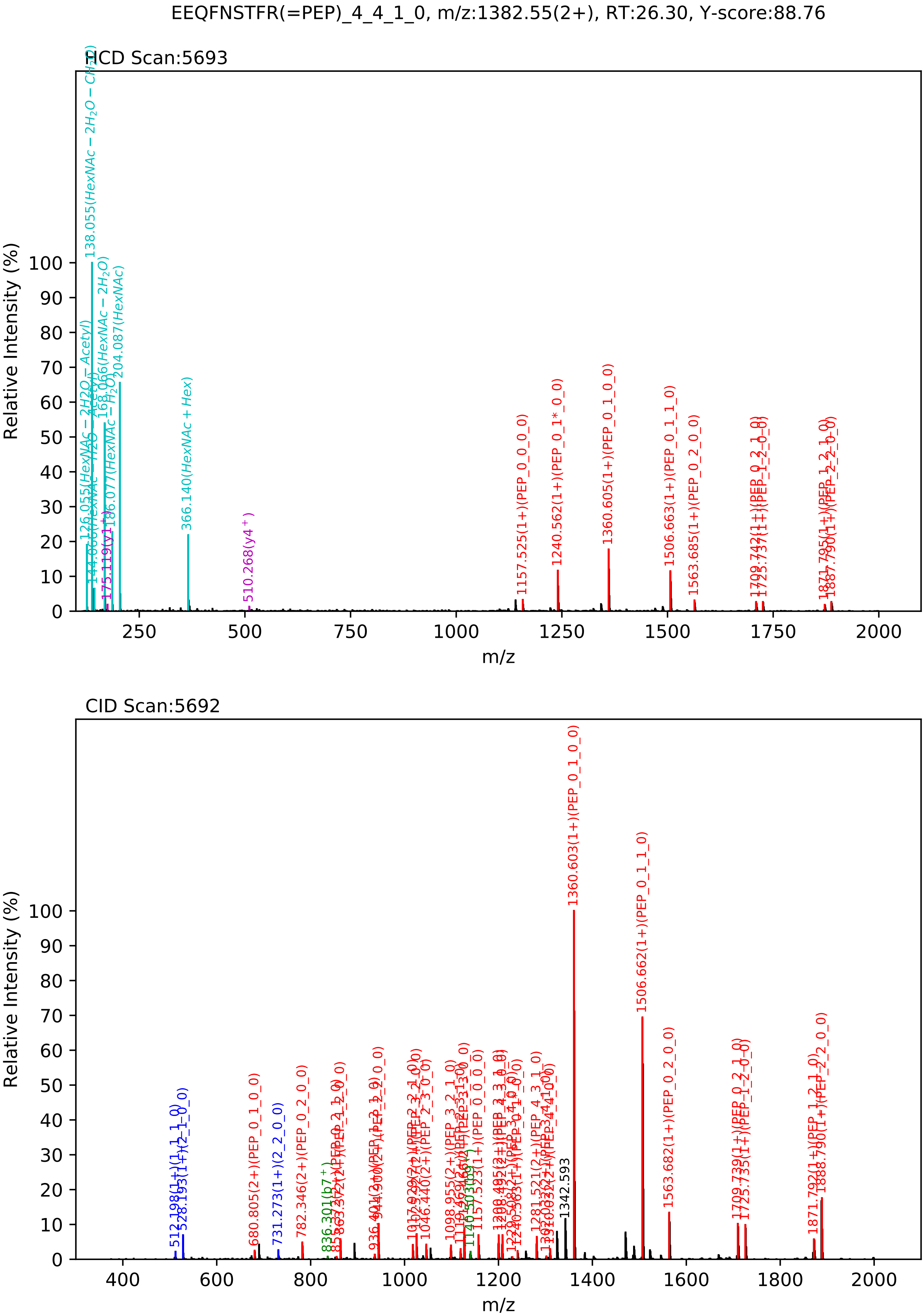

Training set no. 401, Experiment: IgG exp_2

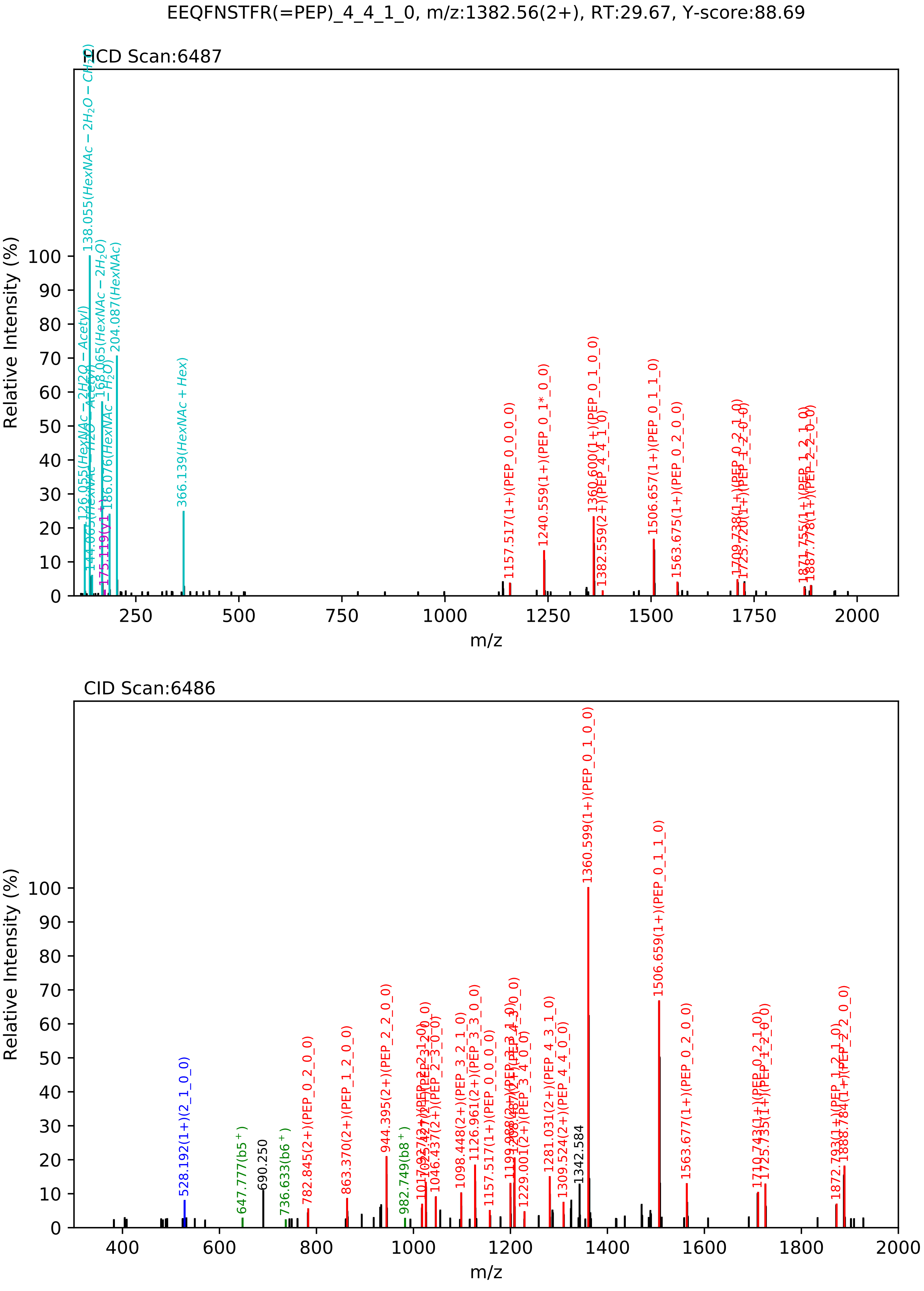

Training set no. 402, Experiment: IgG exp_4

EEQFNSTFR(=PEP)_4_4_1_0, m/z:1382.56(2+), RT:24.30, Y-score:88.69

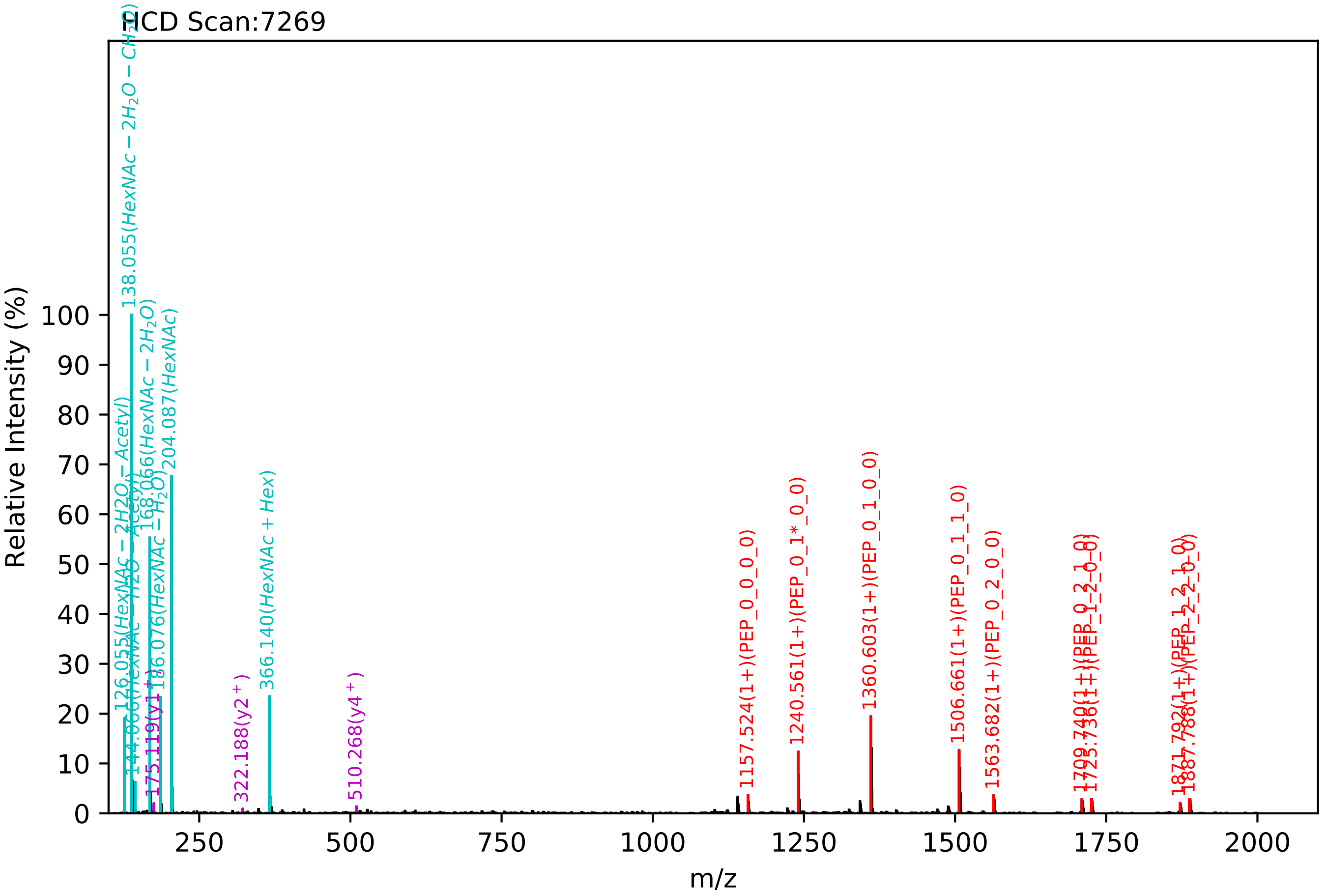

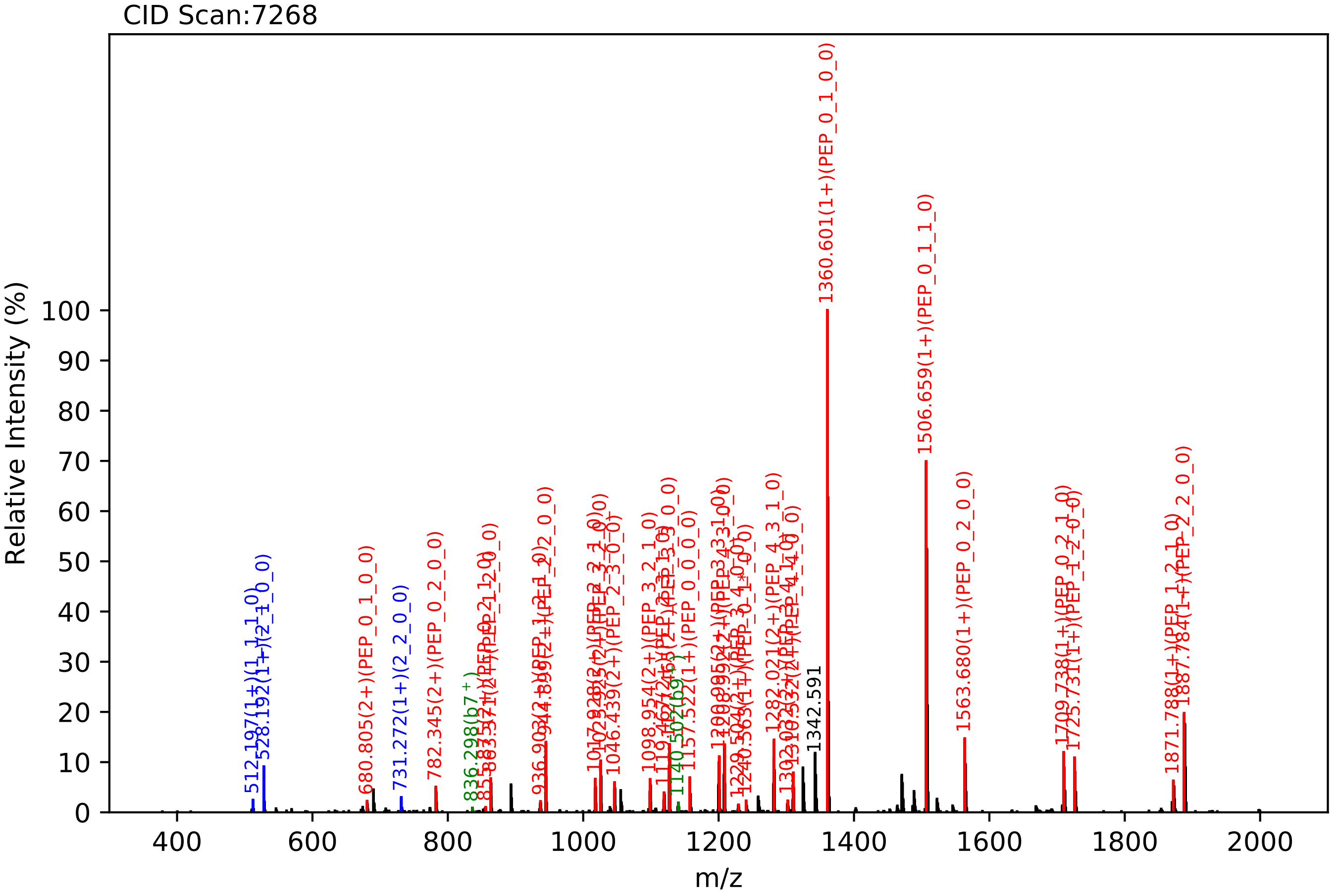

Training set no. 403, Experiment: IgG exp_2

EEQFNSTFR(=PEP)_4_4_1_0, m/z:1382.56(2+), RT:24.43, Y-score:87.65

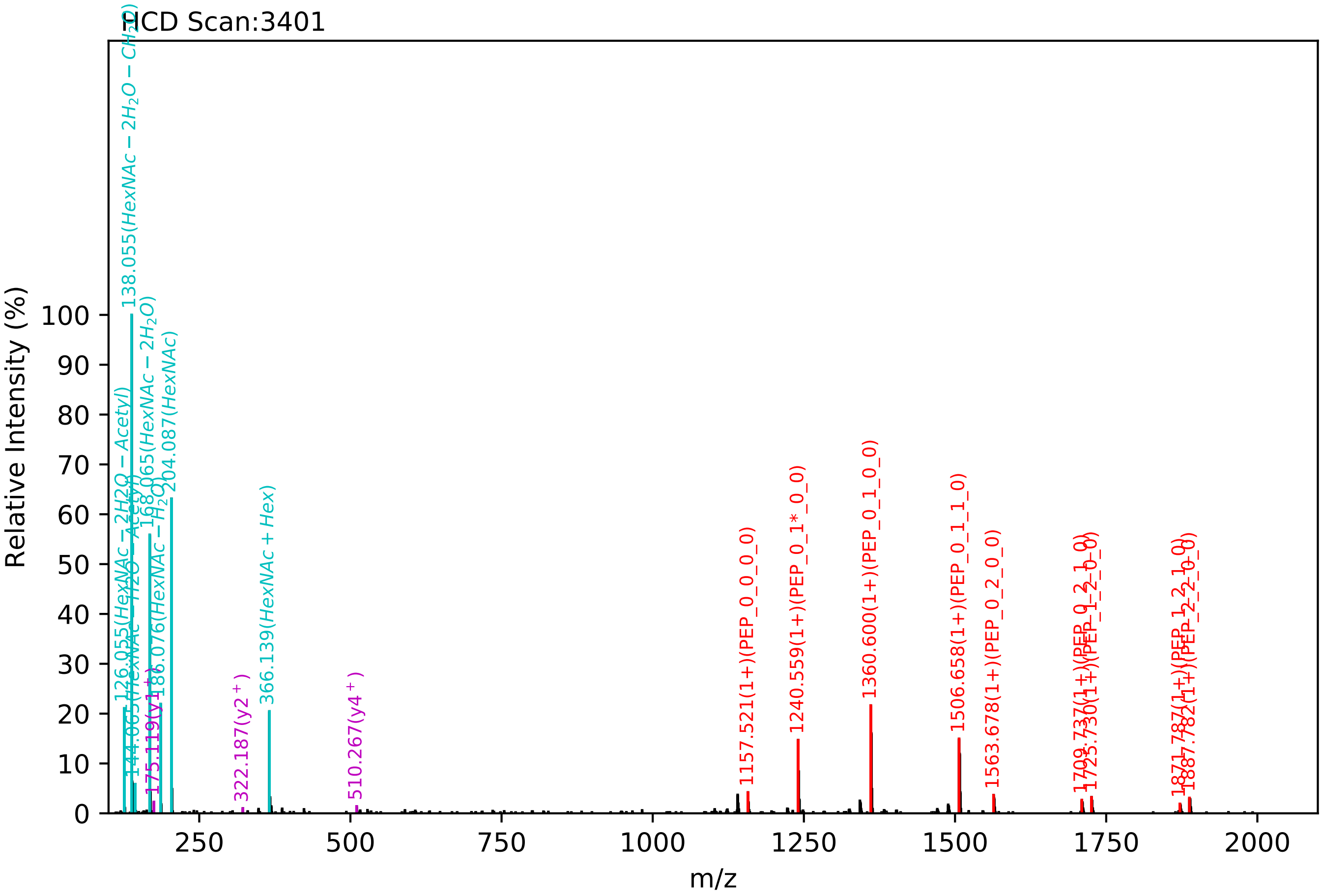

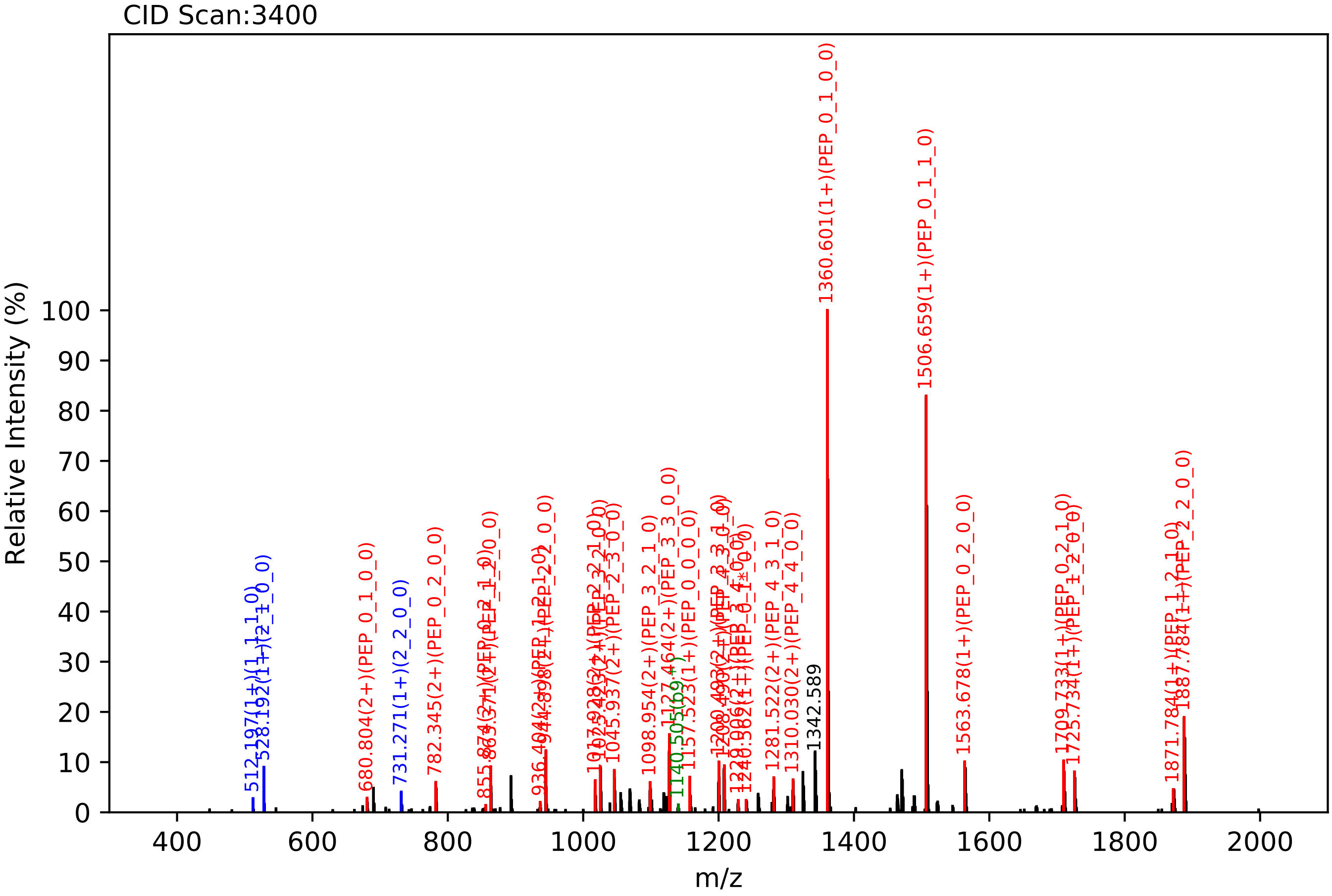

Training set no. 404, Experiment: IgG exp_1

EEQFNSTFR(=PEP)_4_4_1_0, m/z:1382.56(2+), RT:24.98, Y-score:86.20

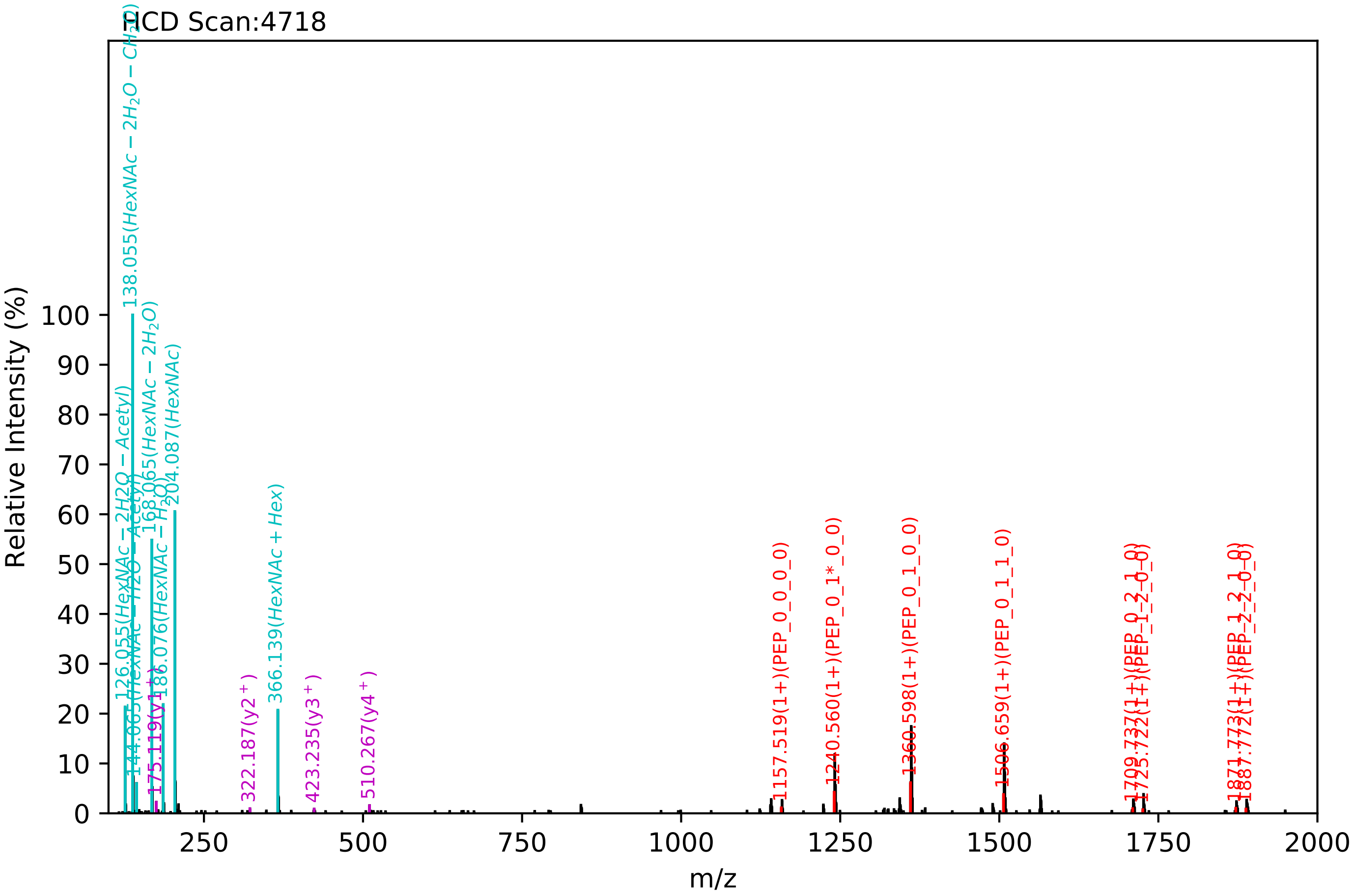

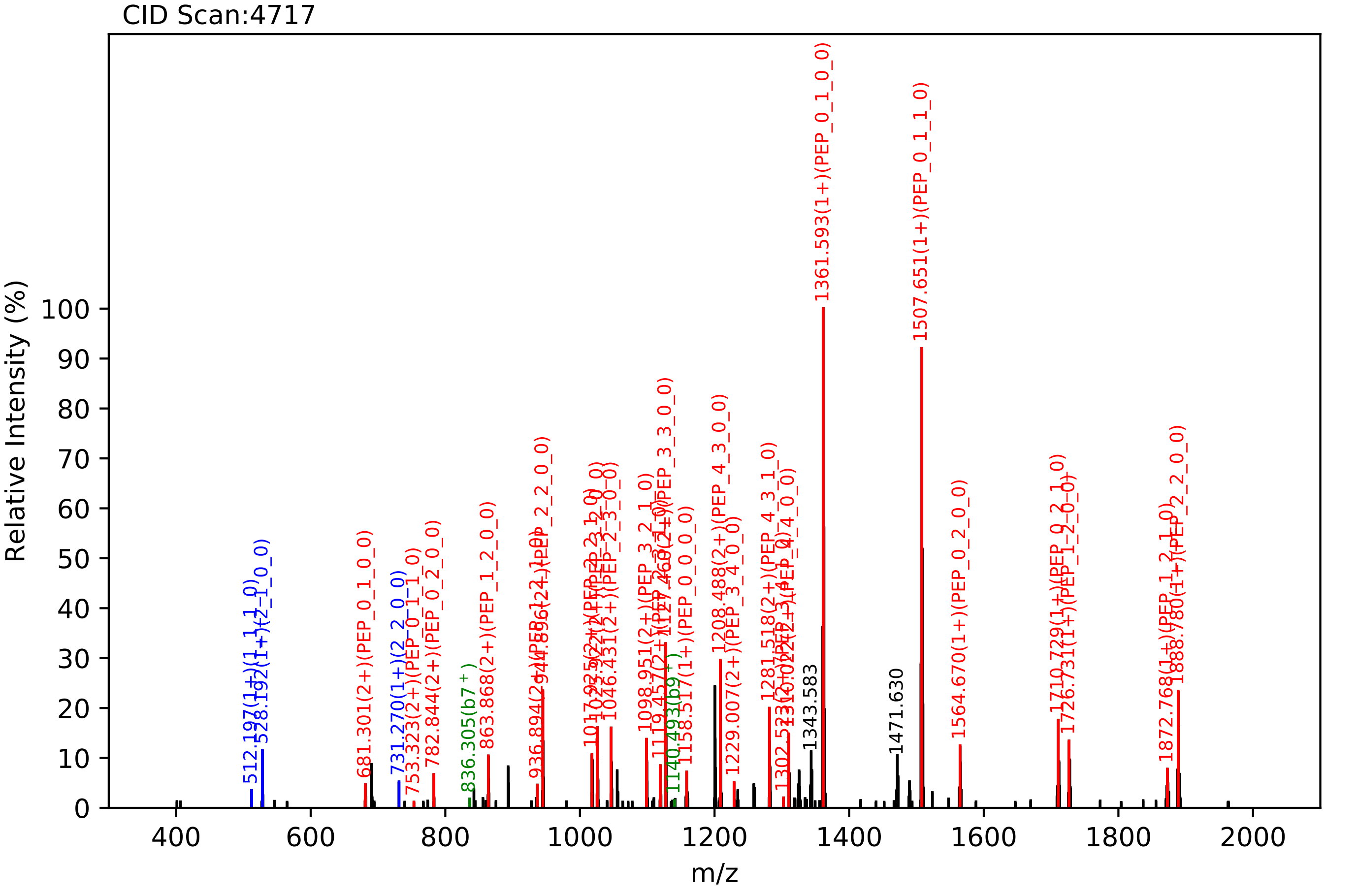

Training set no. 405, Experiment: IgG exp_1

EEQFNSTFR(=PEP)_4_4_1_0, m/z:1382.56(2+), RT:25.65, Y-score:82.48

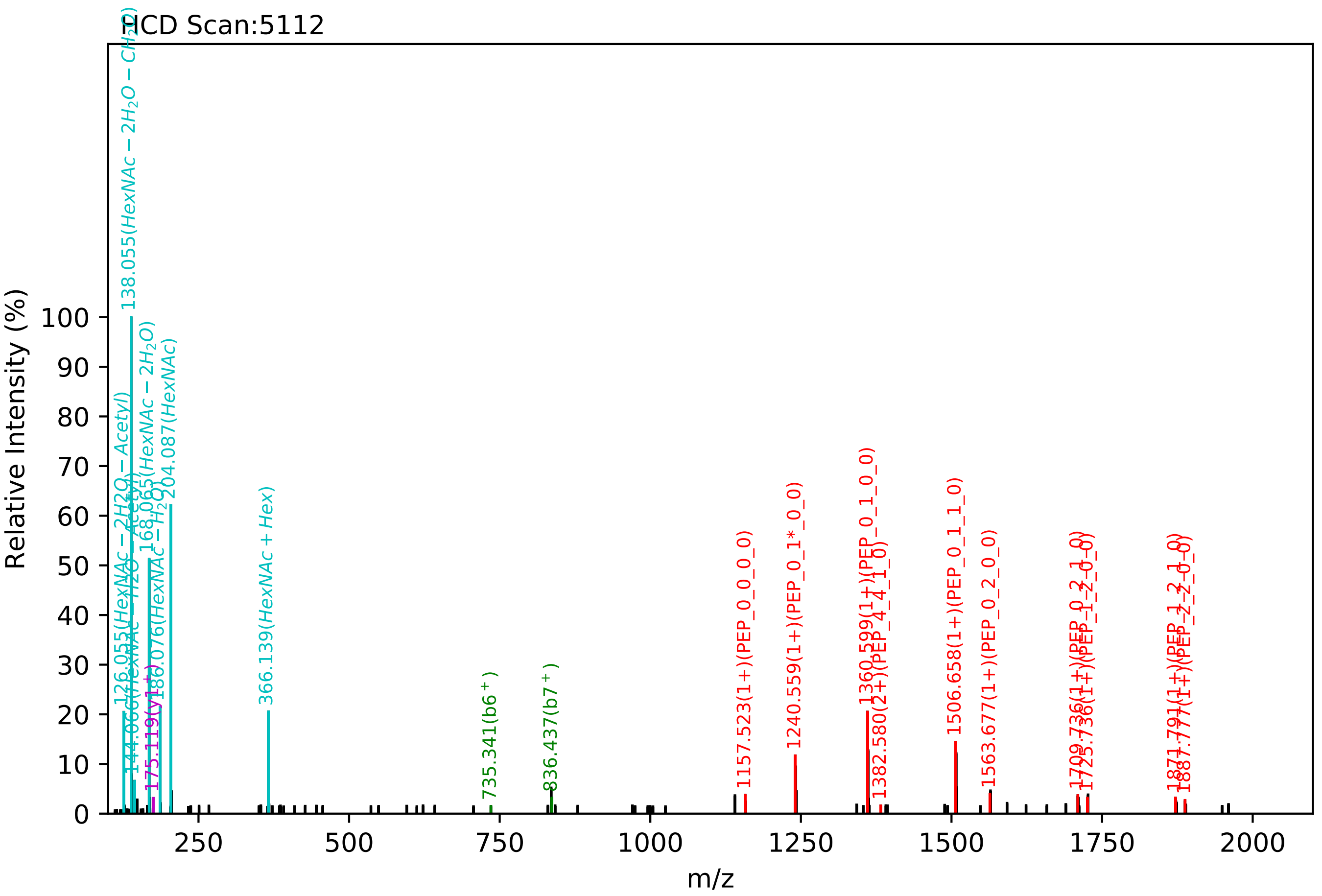

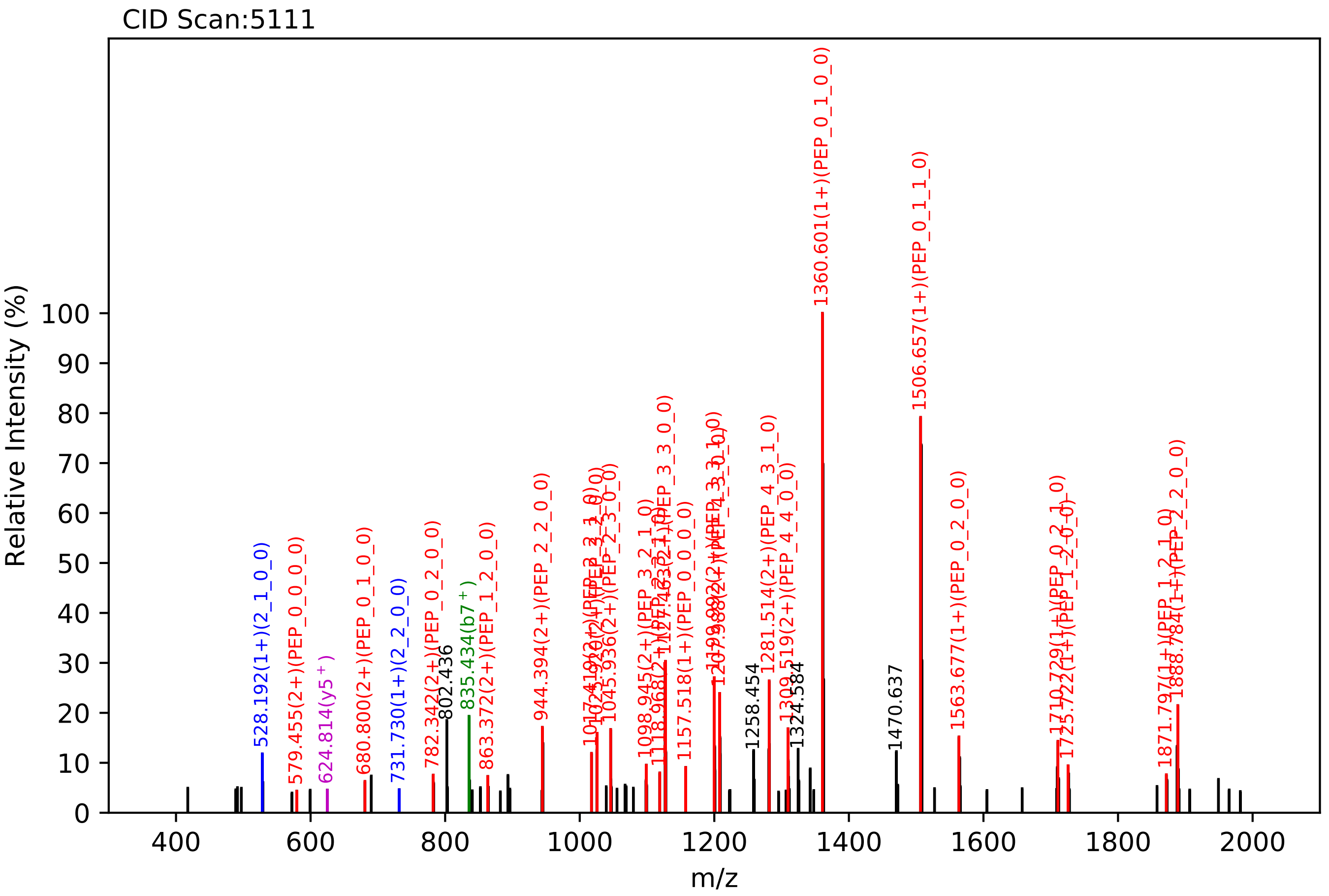

Training set no. 406, Experiment: IgG exp_4

EEQFNSTFR(=PEP)_4_4_1_0, m/z:1382.56(2+), RT:24.90, Y-score:78.21

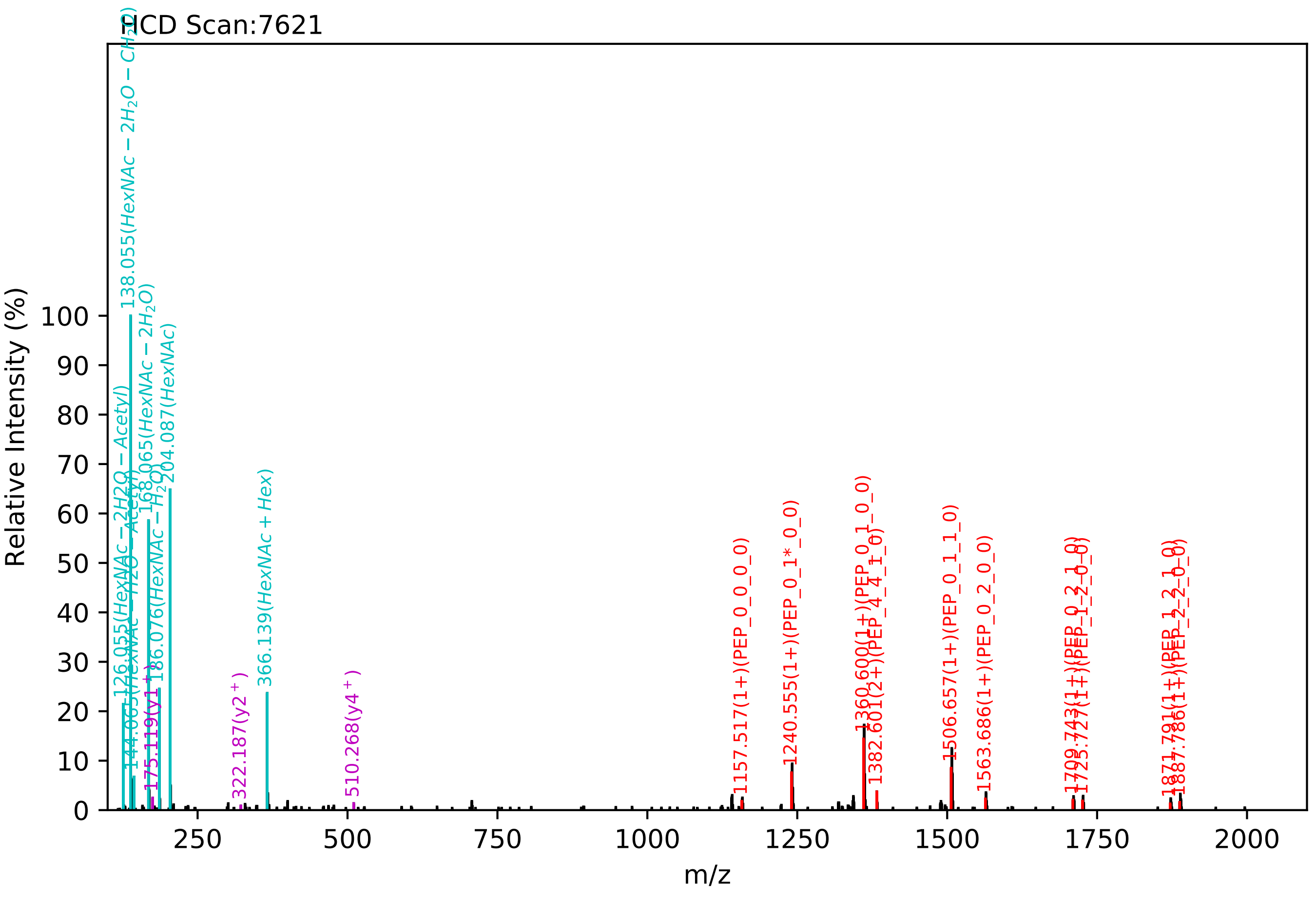

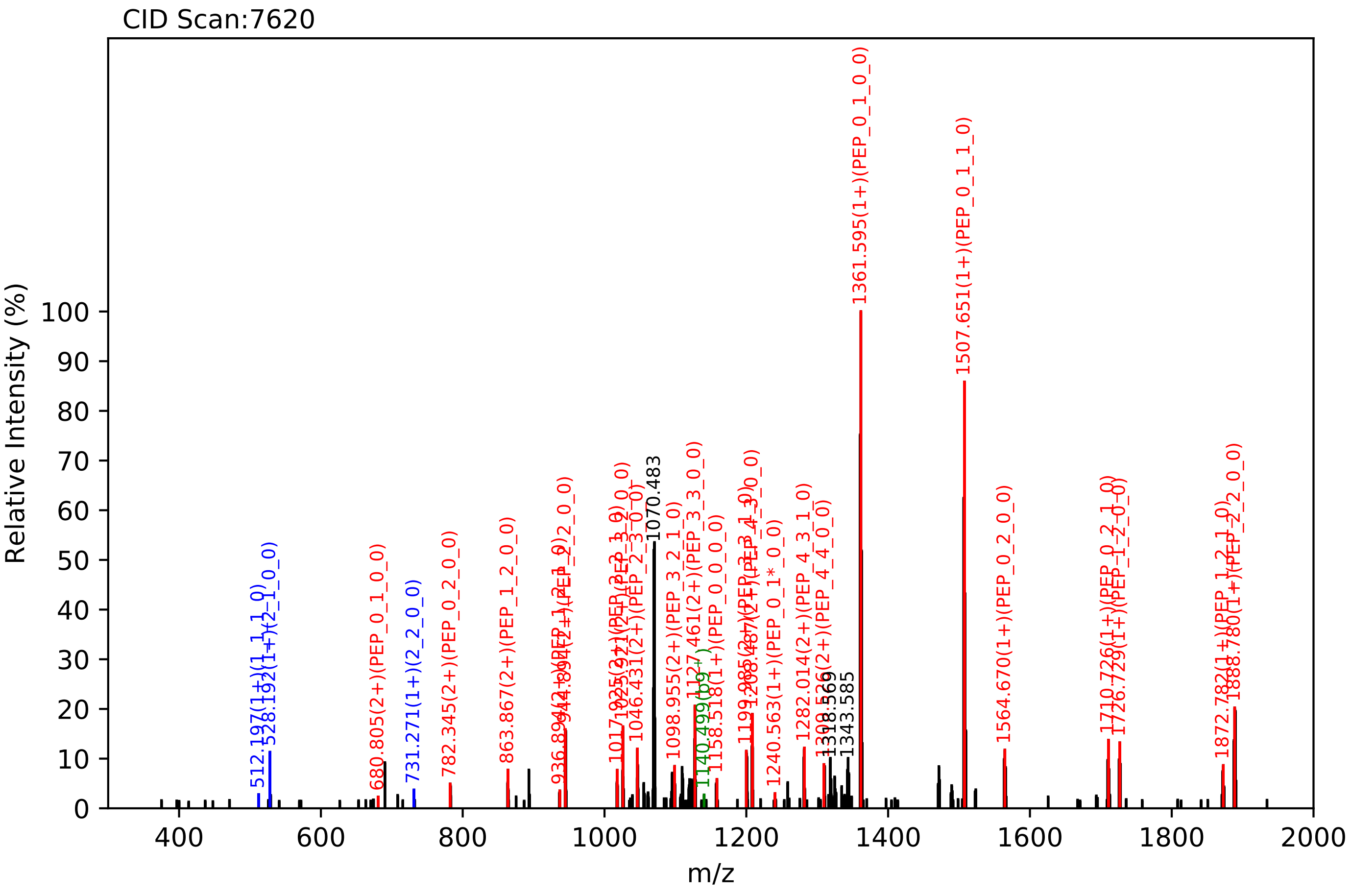

Training set no. 407, Experiment: IgG exp_1

EEQFNSTFR(=PEP)_4_4_1_1, m/z:1019.07(3+), RT:27.29, Y-score:92.94

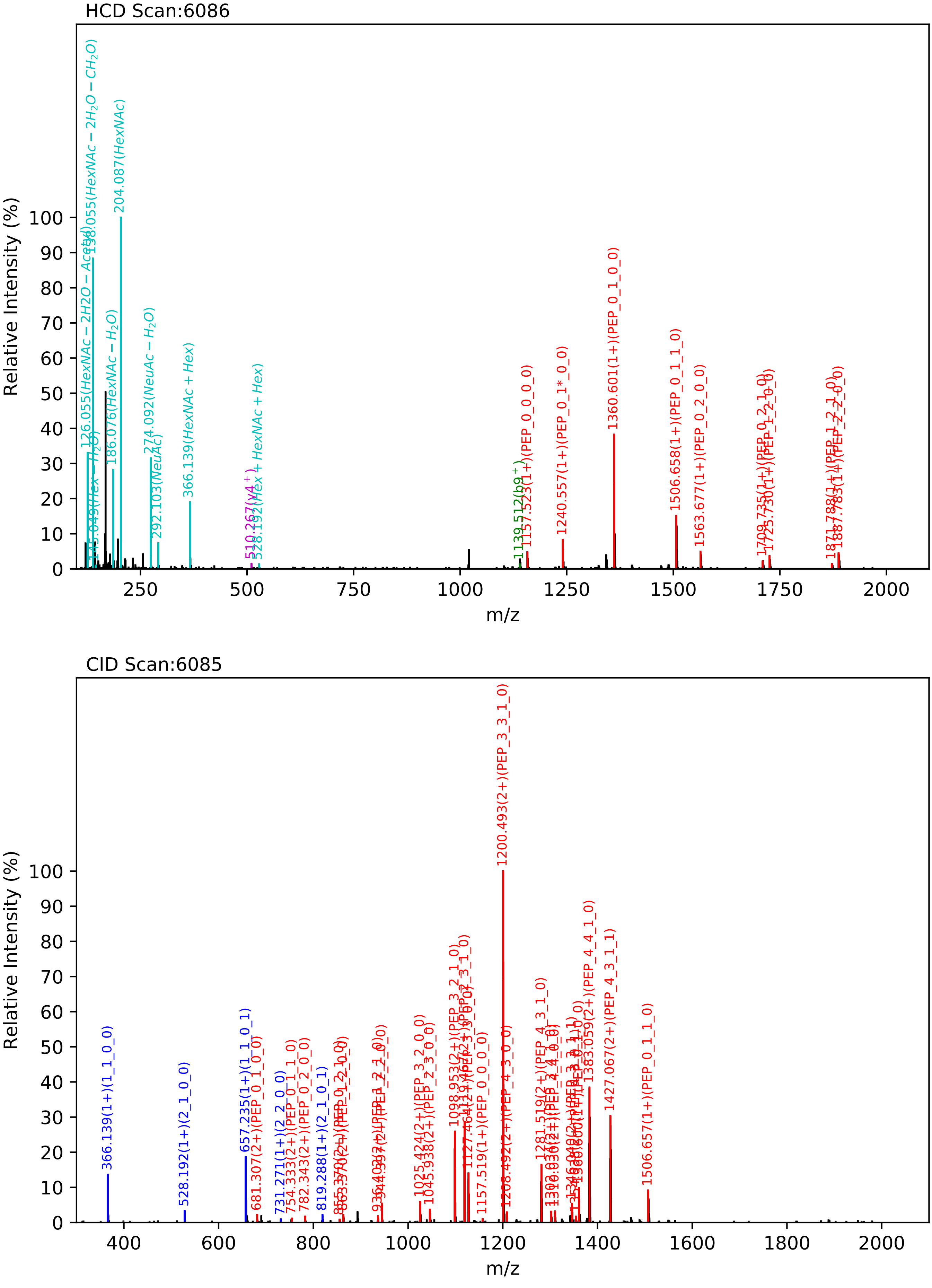

Training set no. 408, Experiment: IgG exp_2

EEQFNSTFR(=PEP)_4_4_1_1, m/z:1019.07(3+), RT:27.29, Y-score:92.34

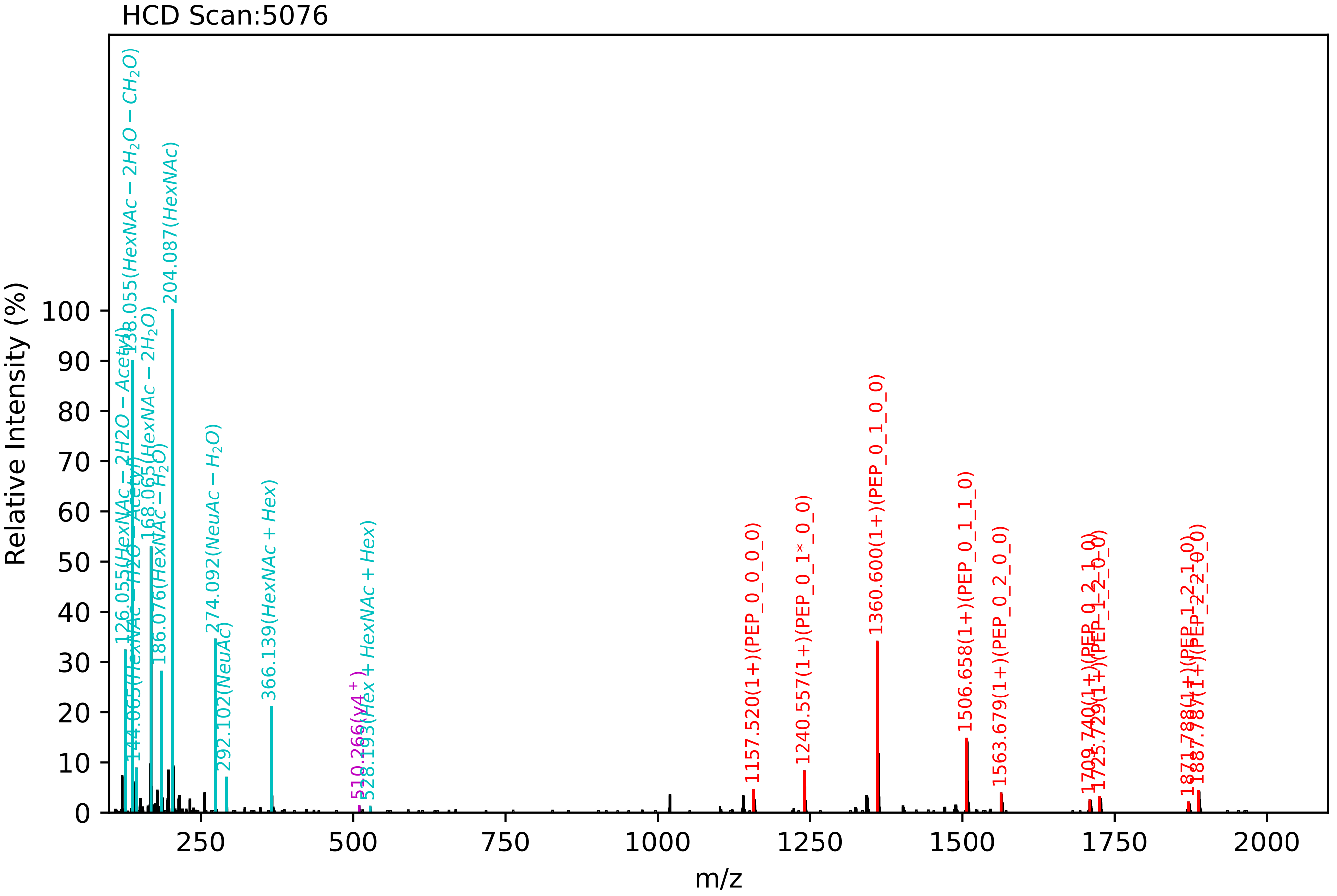

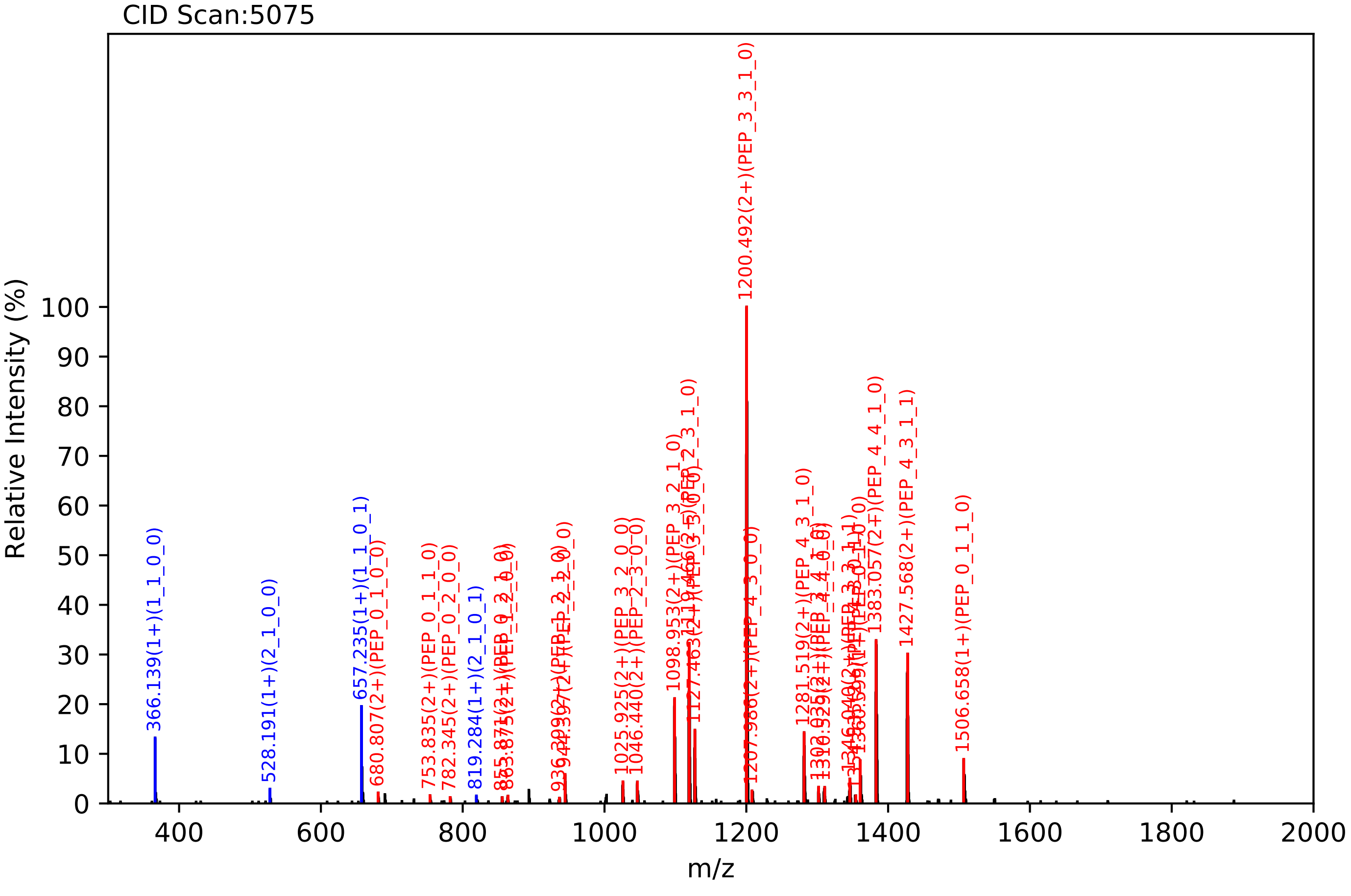

Training set no. 409, Experiment: IgG exp_2

EEQFNSTFR(=PEP)_4_4_1_1, m/z:1528.61(2+), RT:26.77, Y-score:88.90

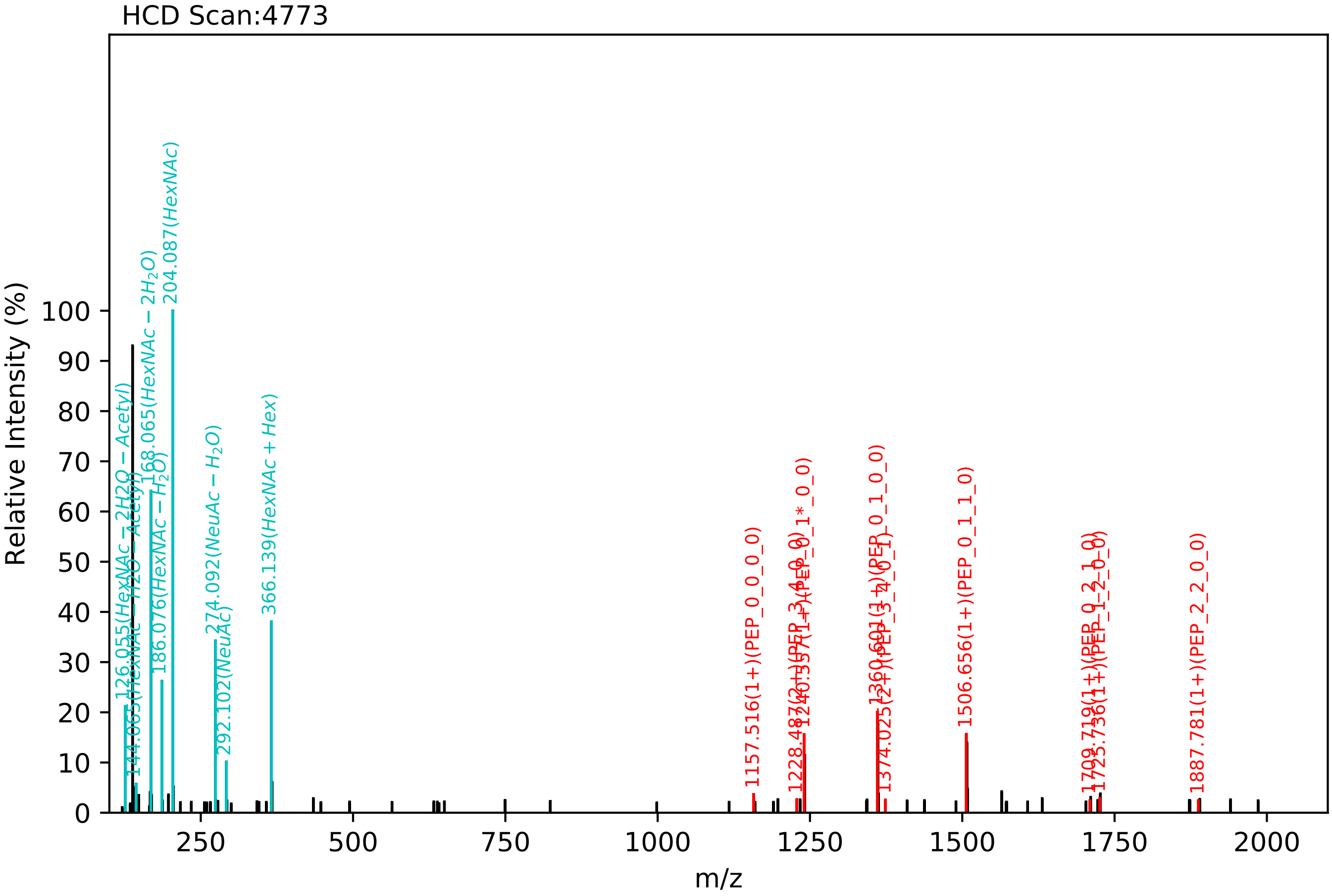

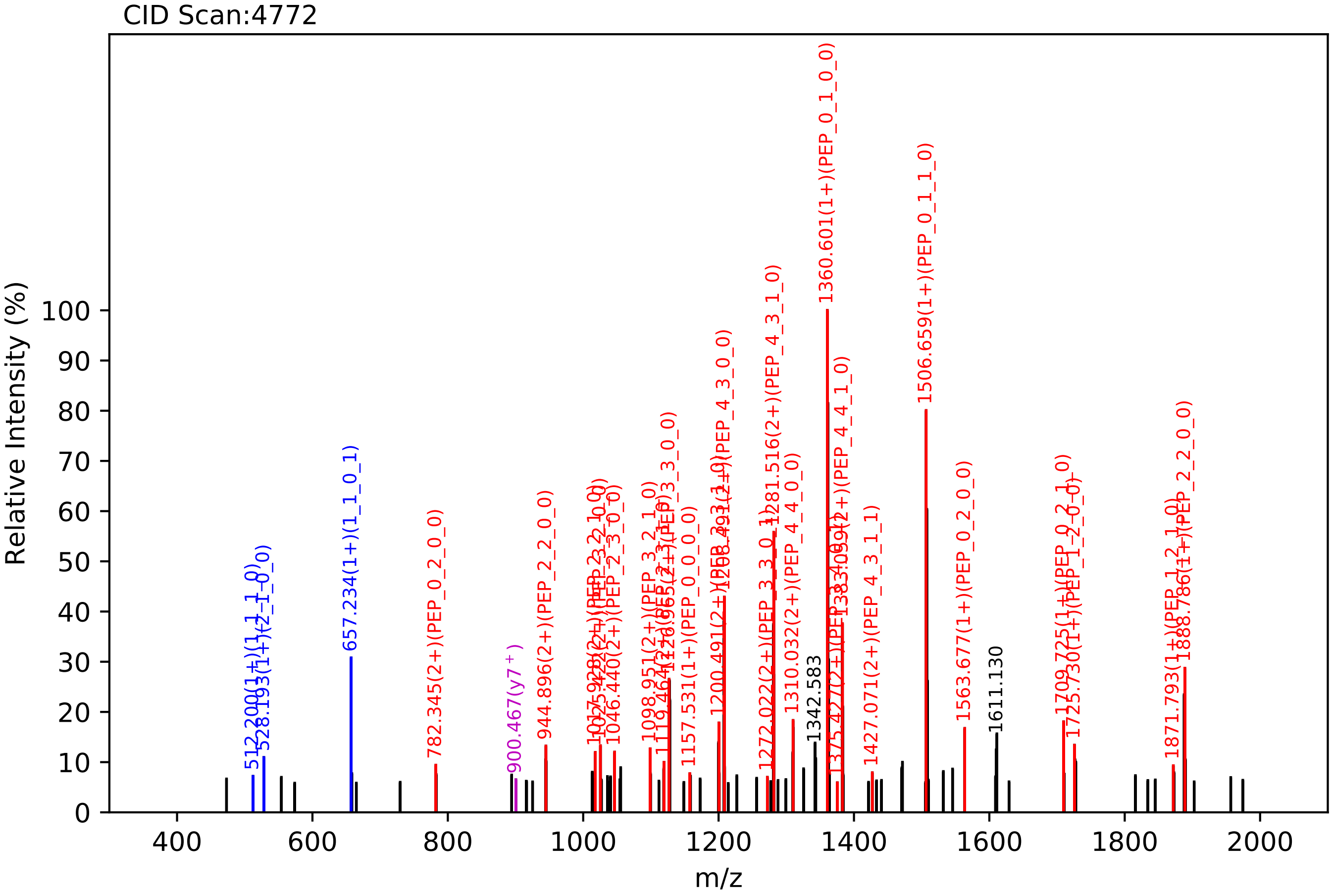

Training set no. 410, Experiment: IgG exp_1

EEQFNSTFR(=PEP)_4_4_1_1, m/z:1528.11(2+), RT:27.29, Y-score:88.89

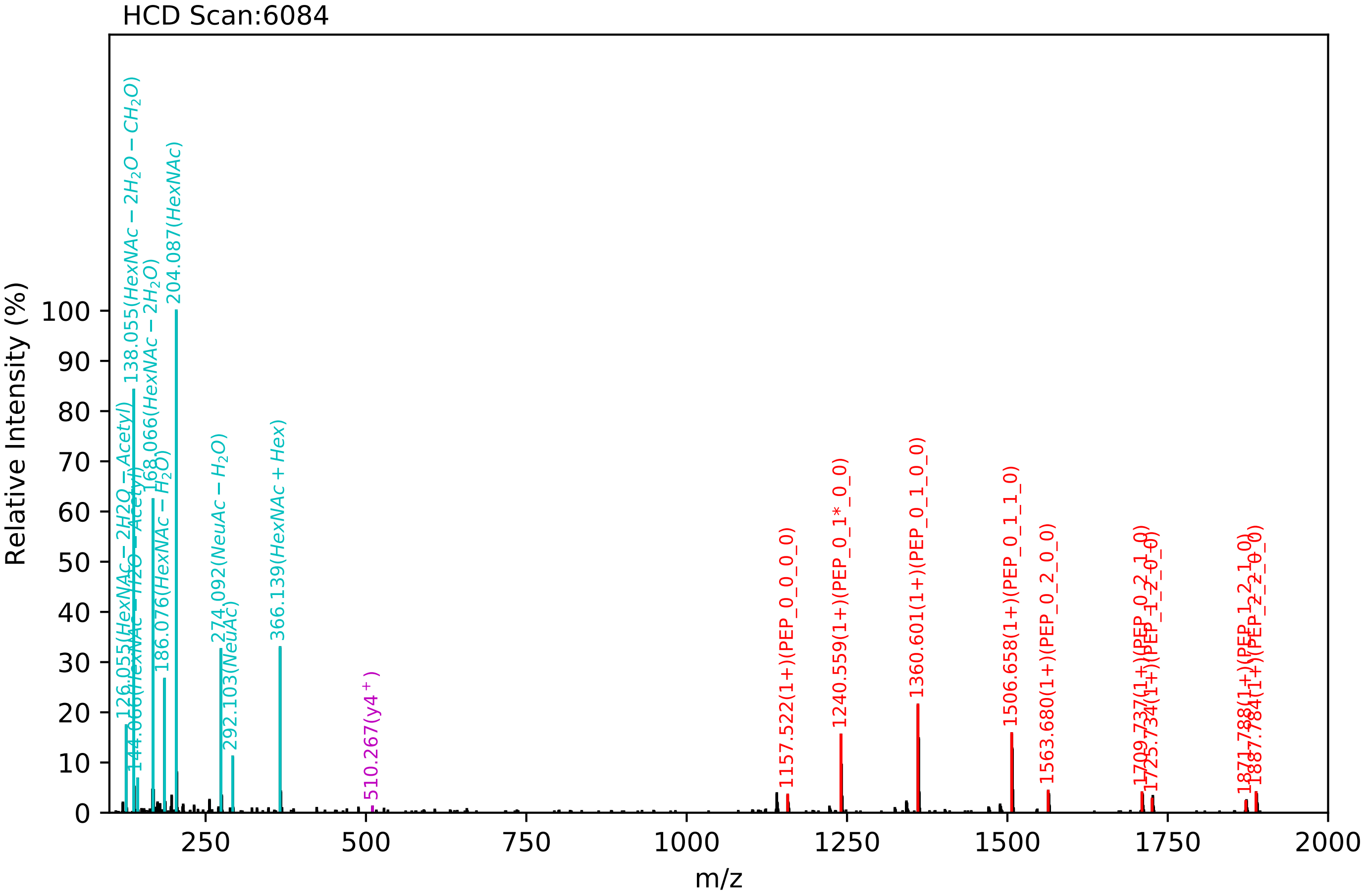

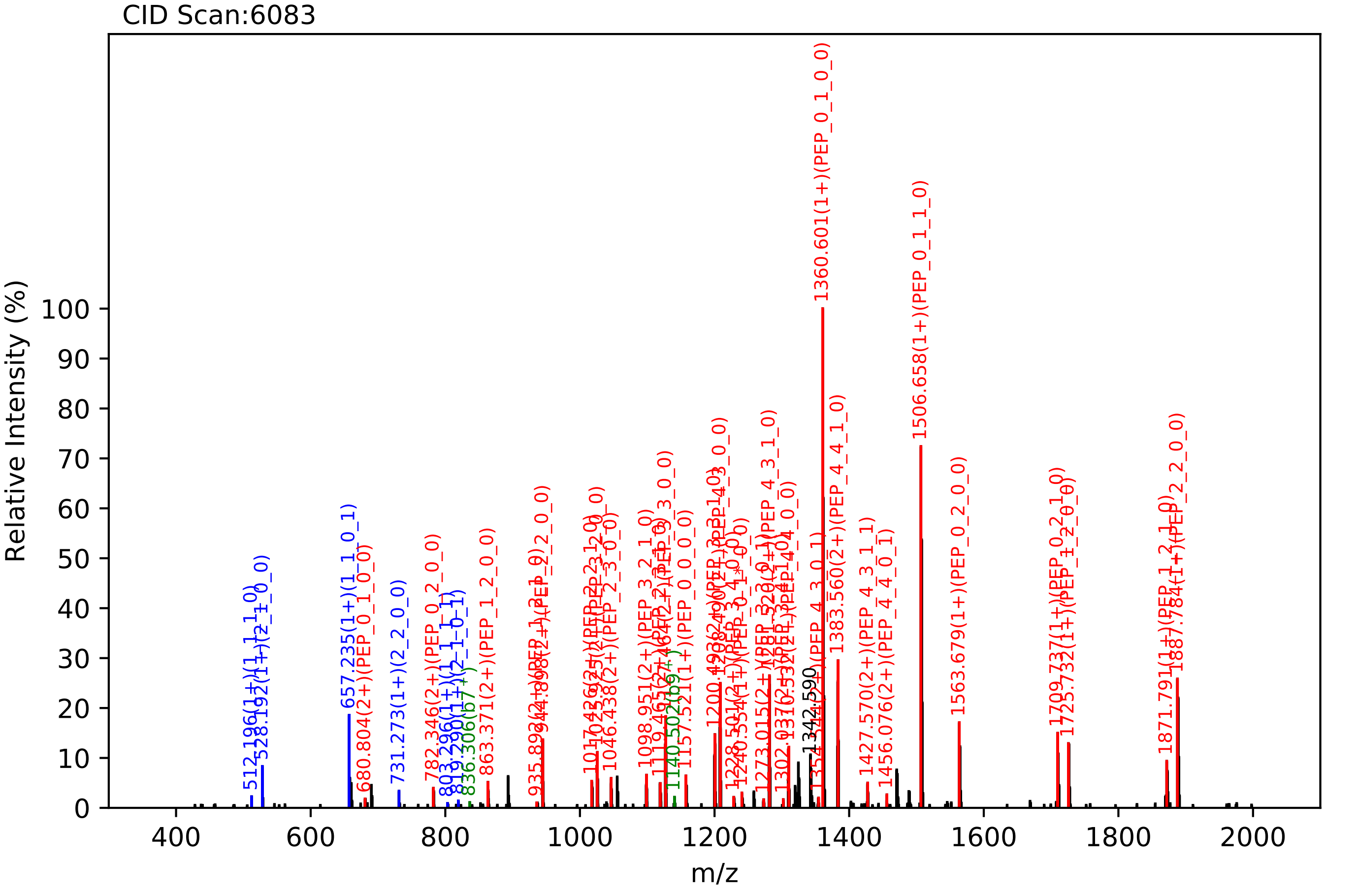

Training set no. 411, Experiment: IgG exp_2

EEQFNSTFR(=PEP)_4_4_1_1, m/z:1019.07(3+), RT:26.72, Y-score:84.96

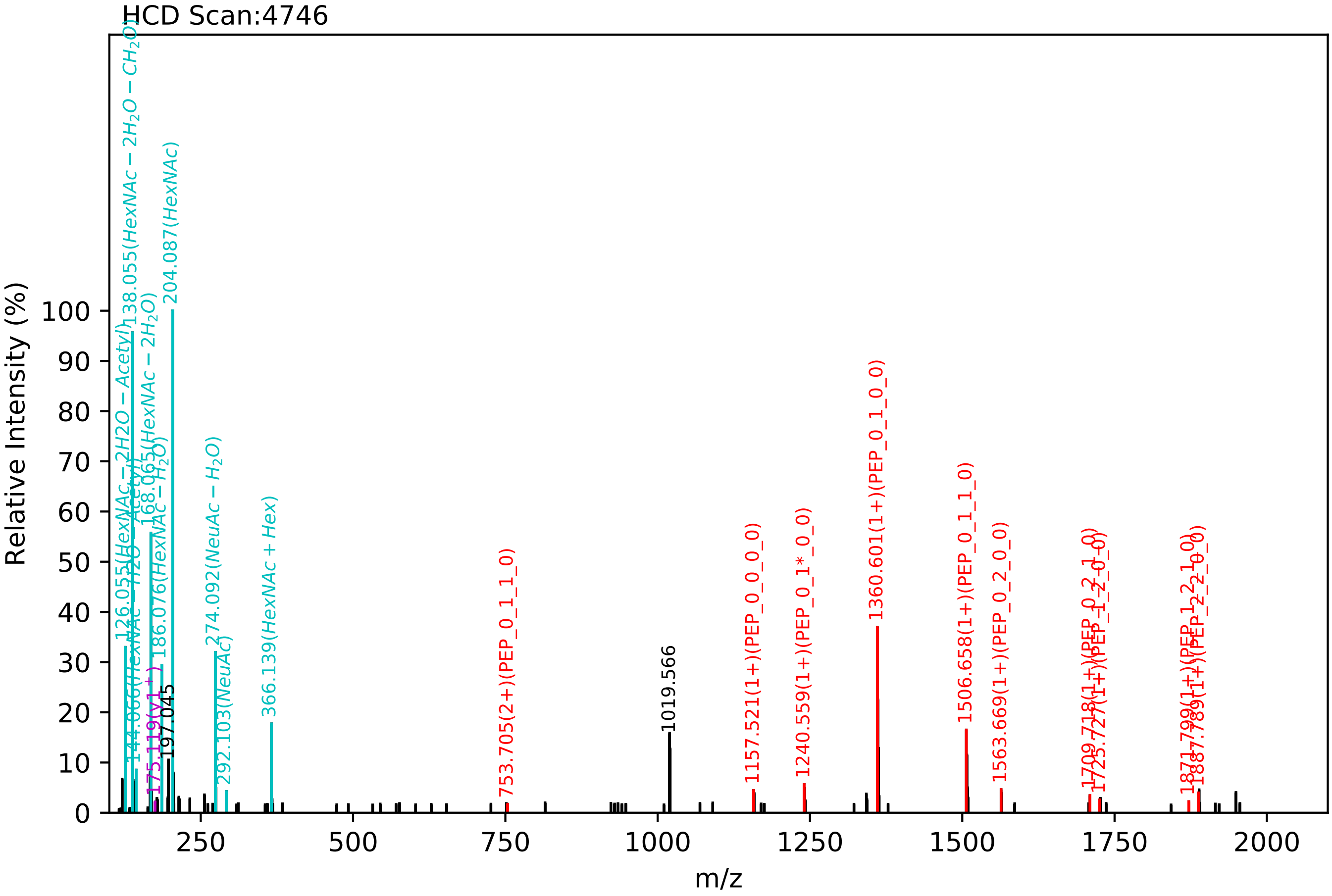

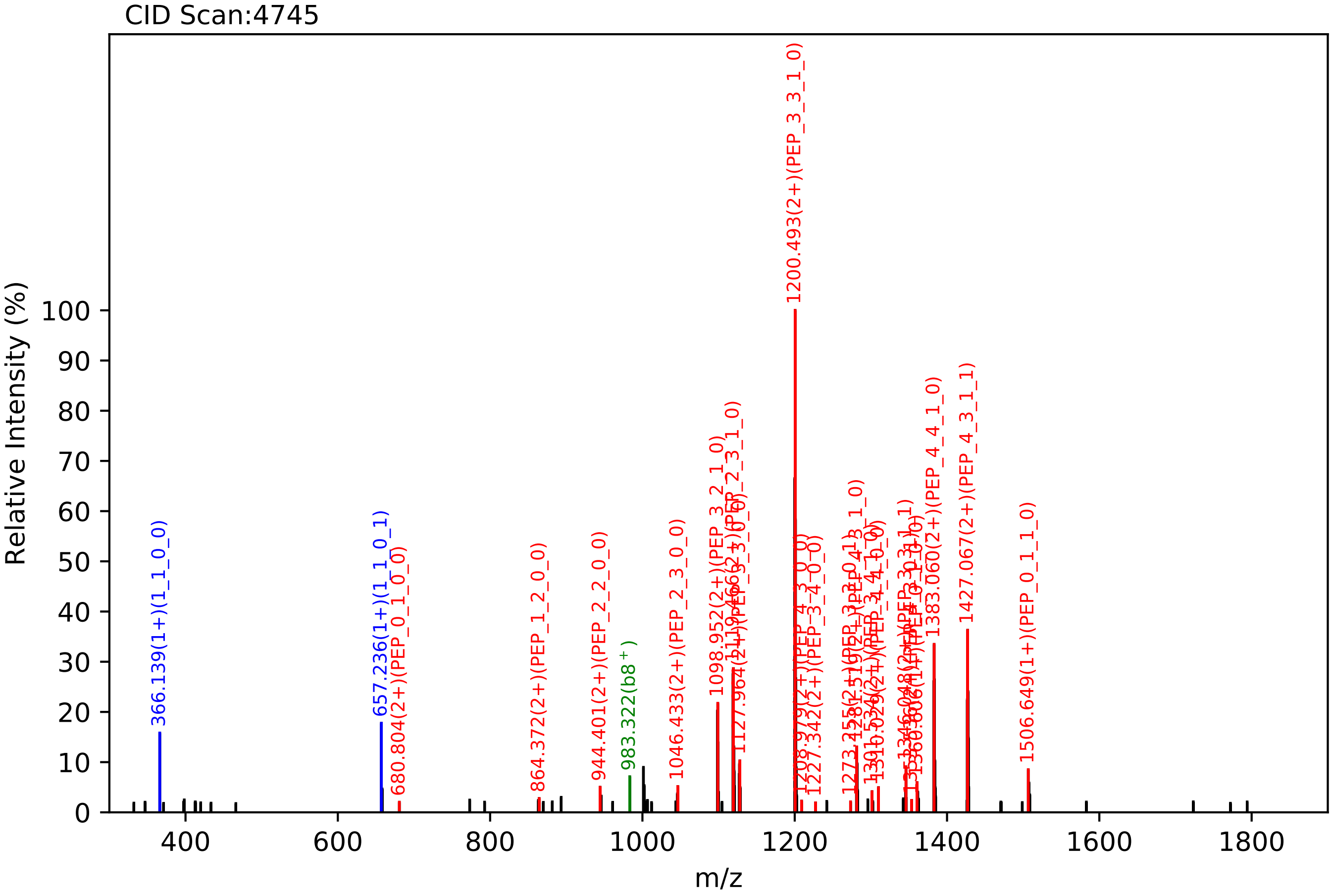

Training set no. 412, Experiment: IgG exp_2

EEQFNSTFR(=PEP)_4_4_1_1, m/z:1528.11(2+), RT:27.35, Y-score:80.44

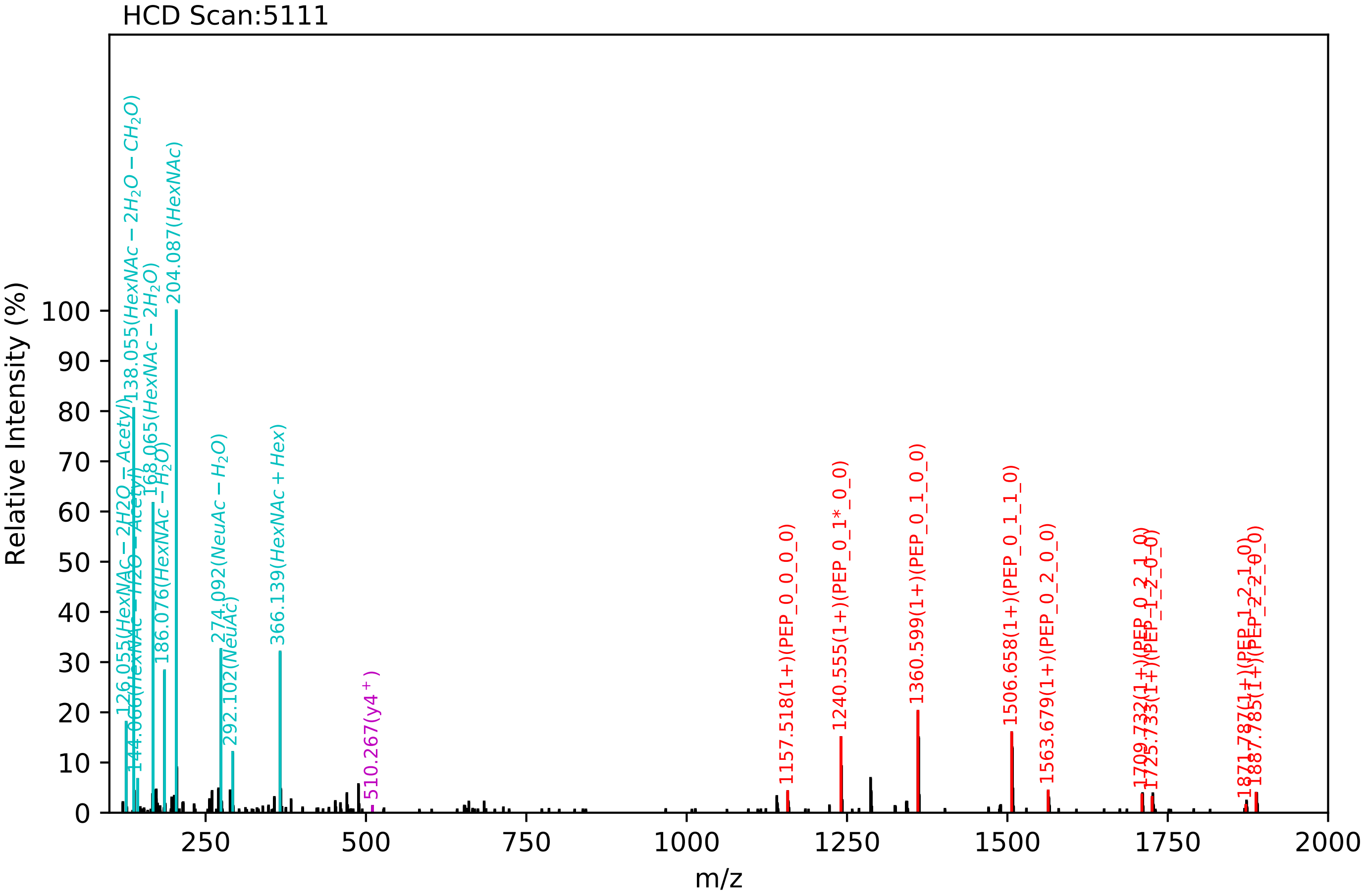

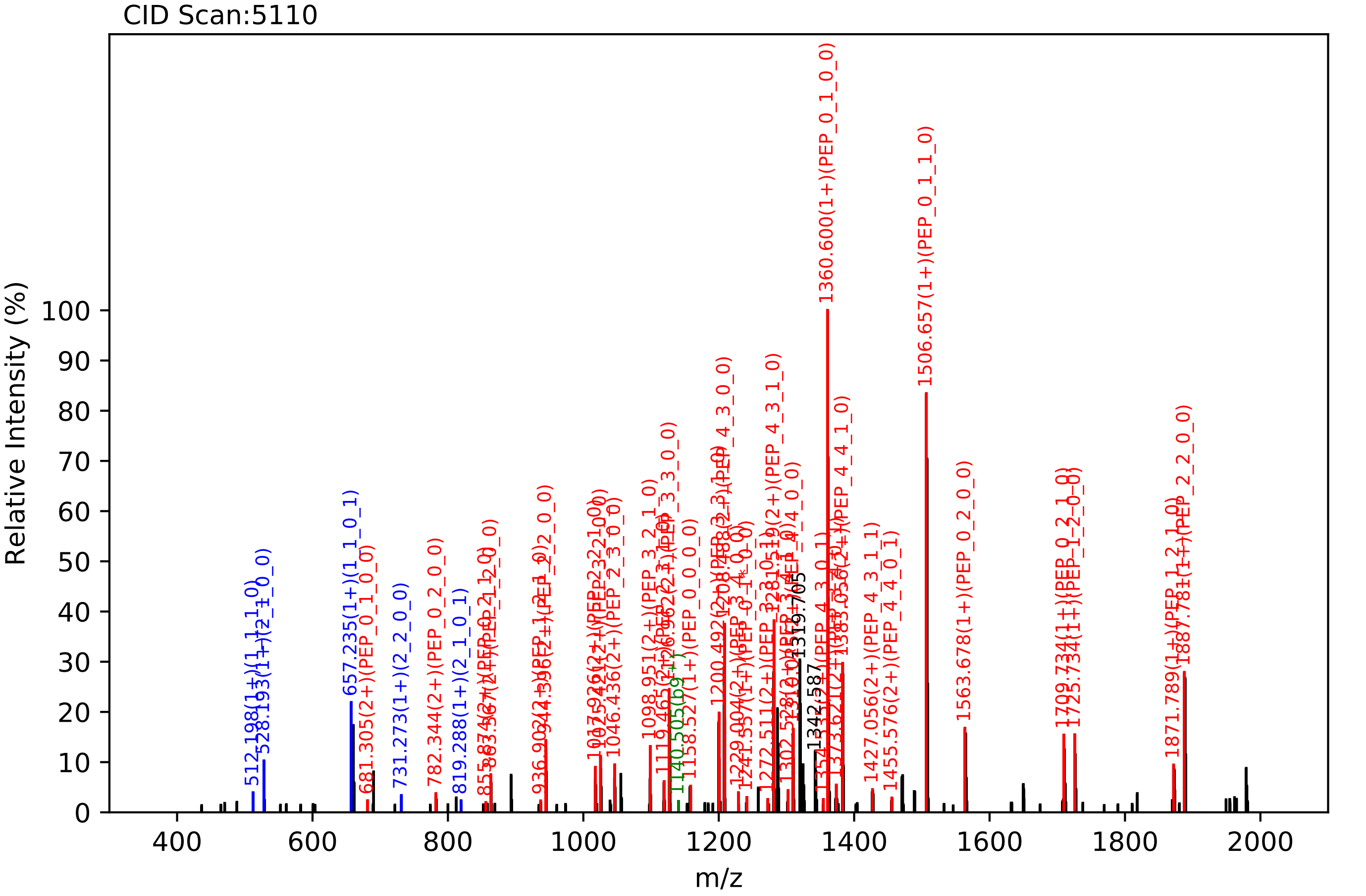

Training set no. 413, Experiment: IgG exp_4

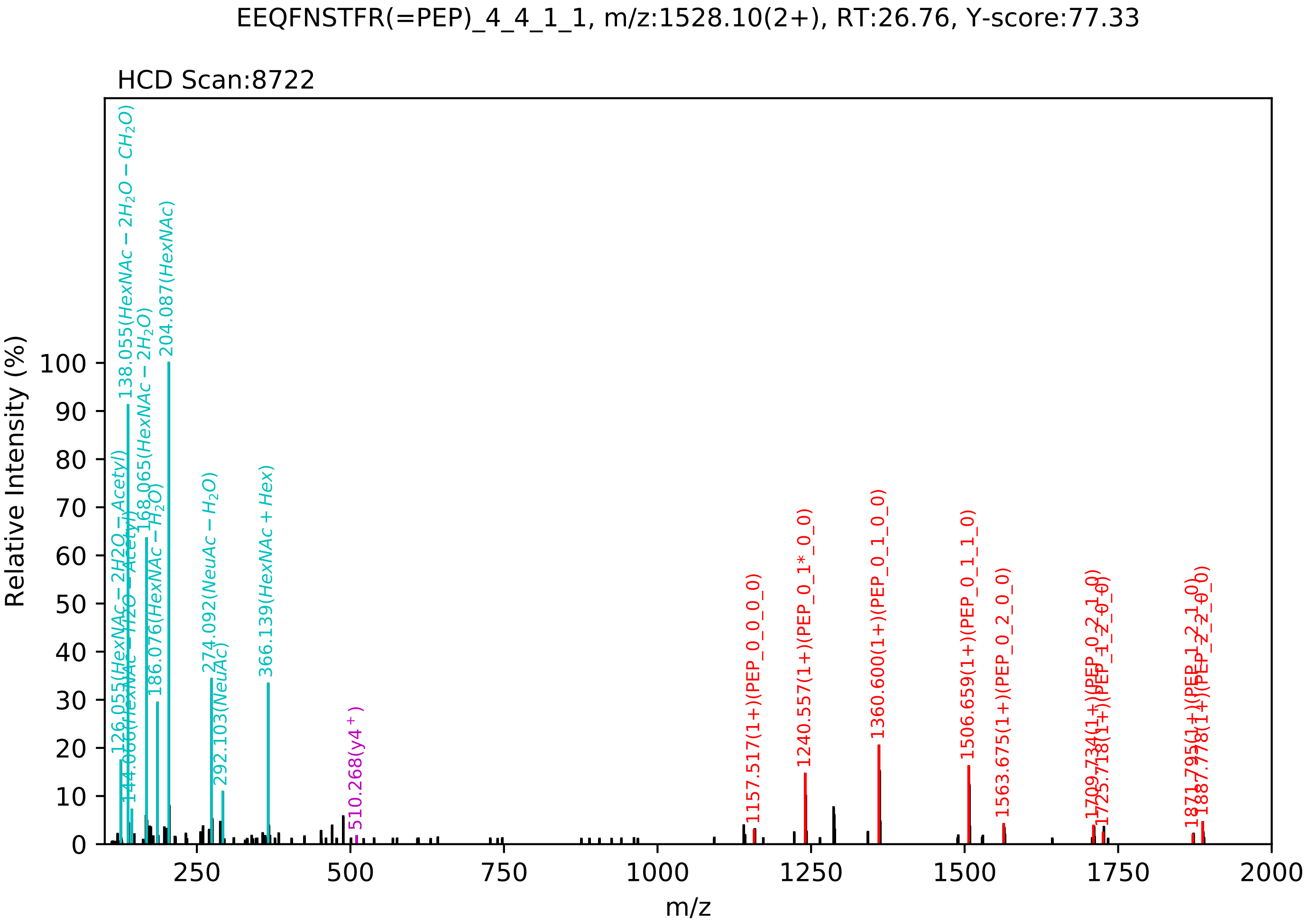

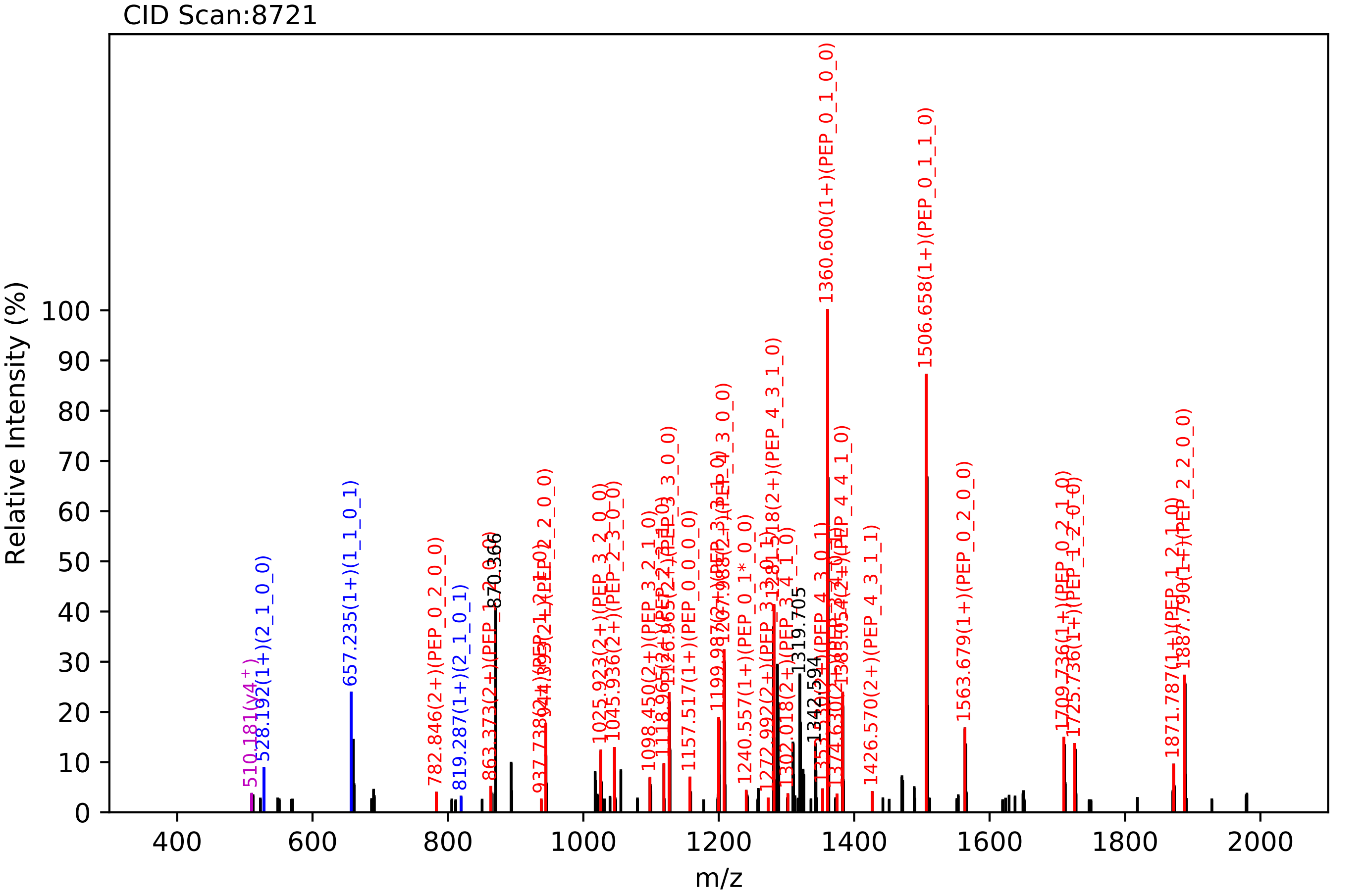

Training set no. 414, Experiment: IgG exp_1

EEQFNSTFR(=PEP)_4_5_1_0, m/z:1484.10(2+), RT:24.56, Y-score:83.28

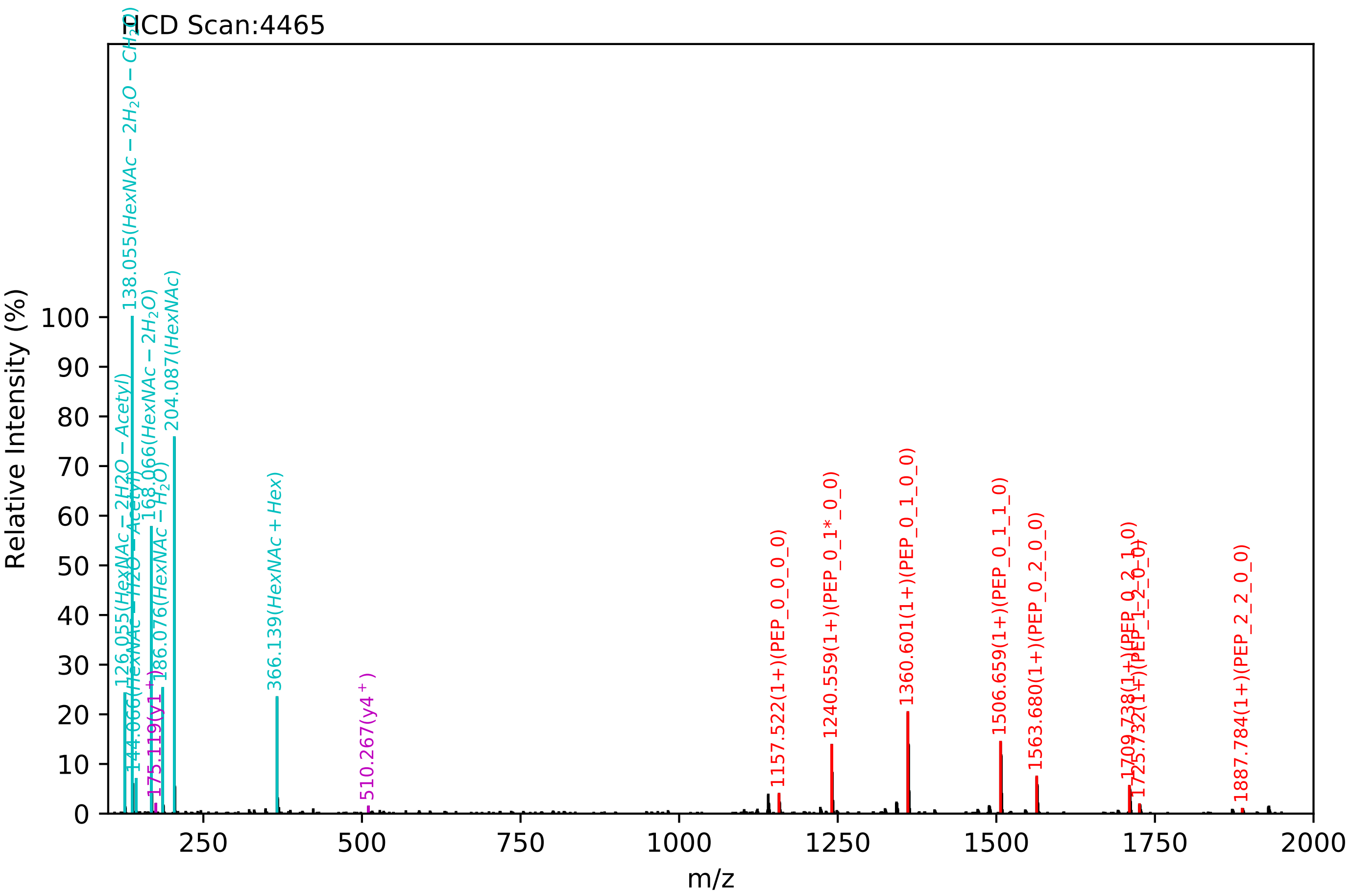

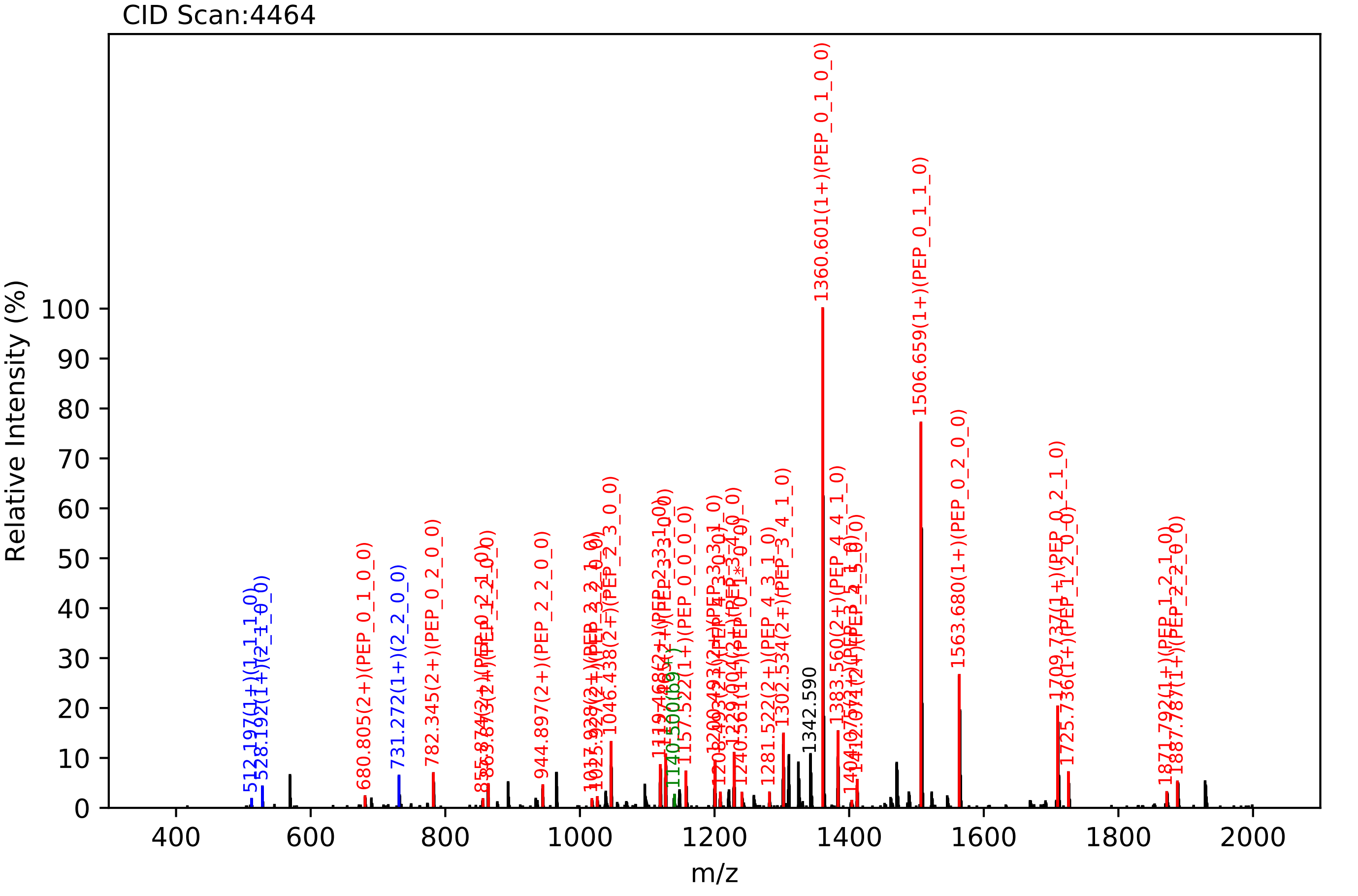

Training set no. 415, Experiment: IgG exp_3

EEQFNSTFR(=PEP)_4_5_1_0, m/z:1484.10(2+), RT:26.39, Y-score:83.16

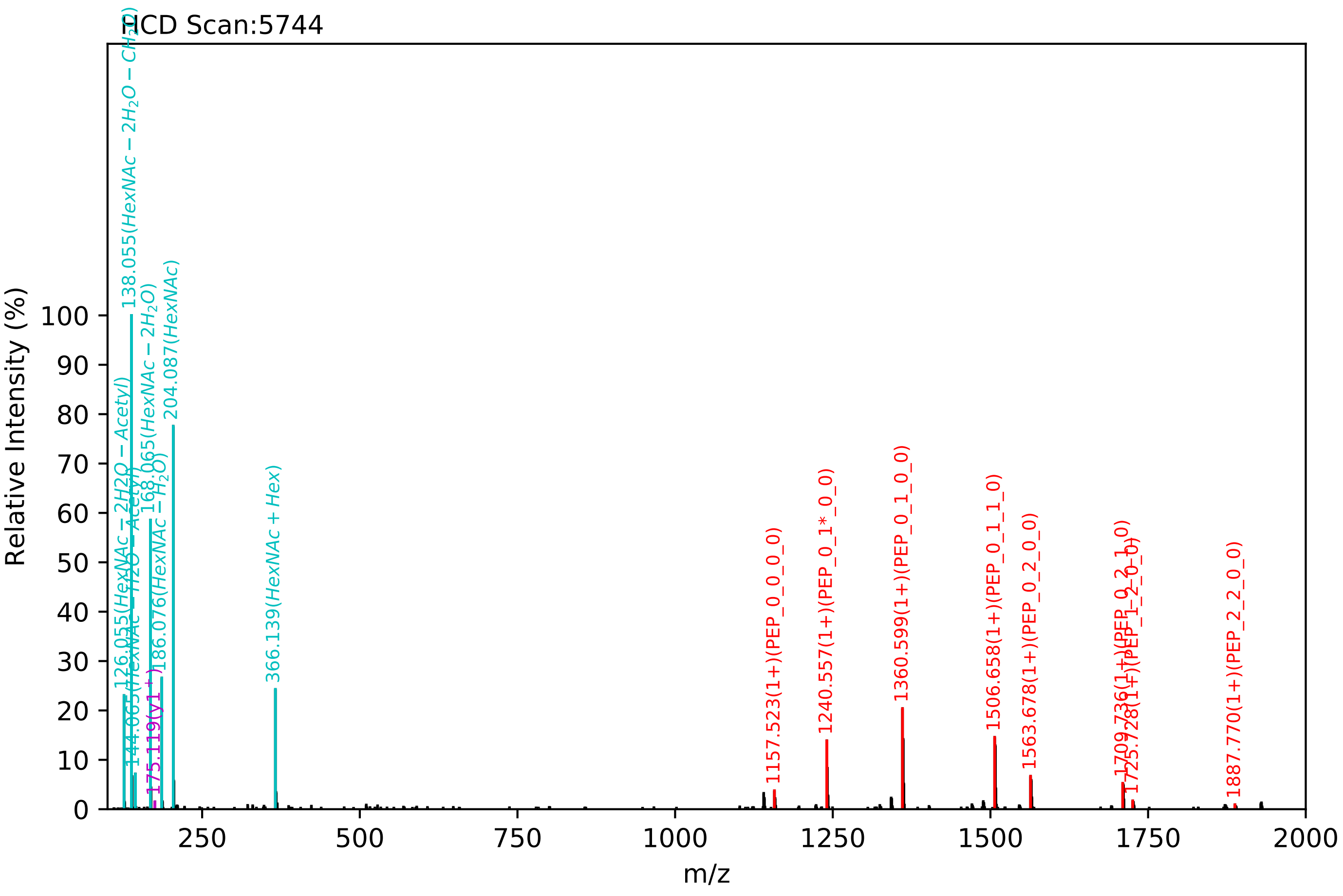

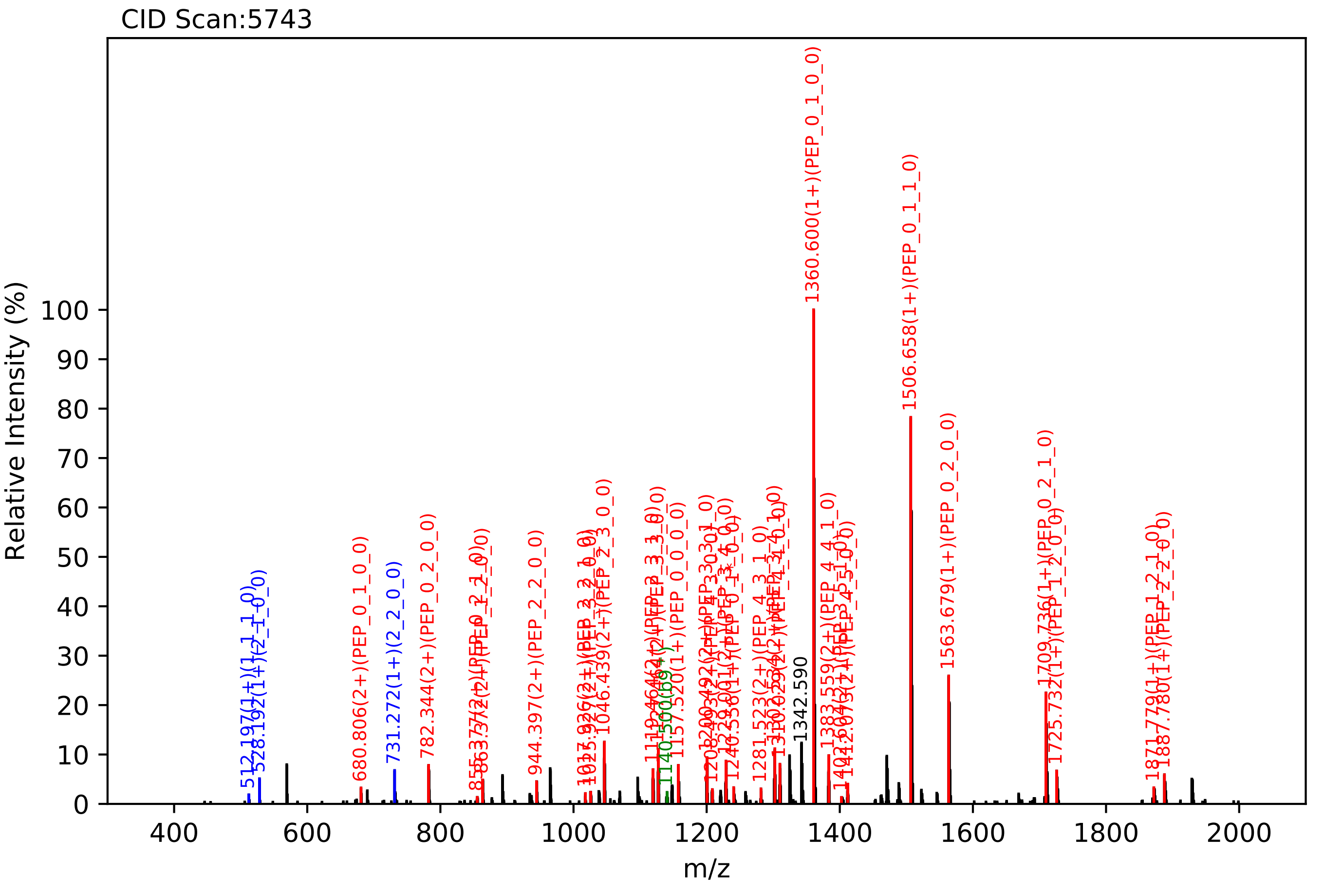

Training set no. 416, Experiment: IgG exp_4

EEQFNSTFR(=PEP)_4_5_1_0, m/z:1484.10(2+), RT:24.28, Y-score:82.93

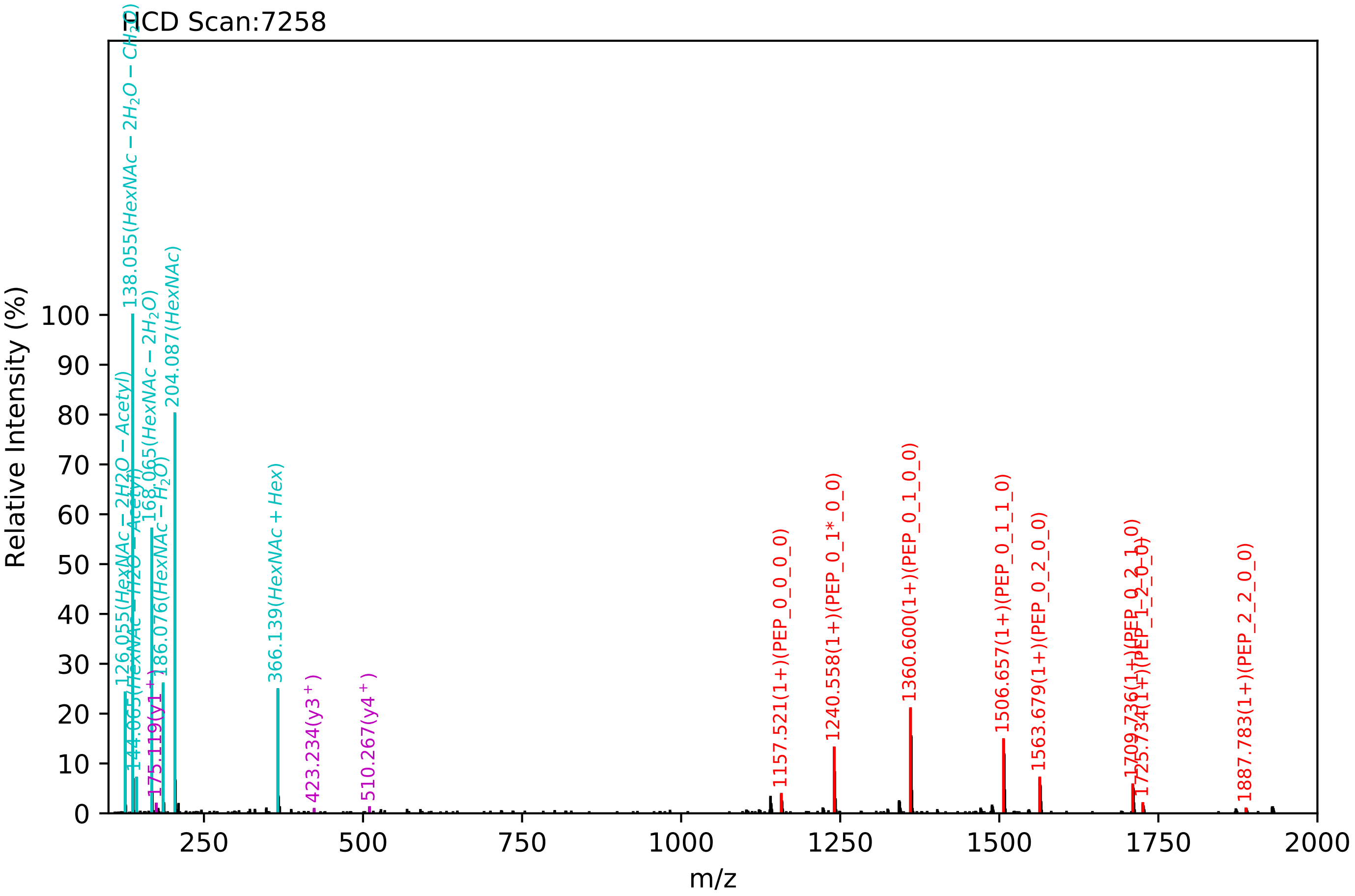

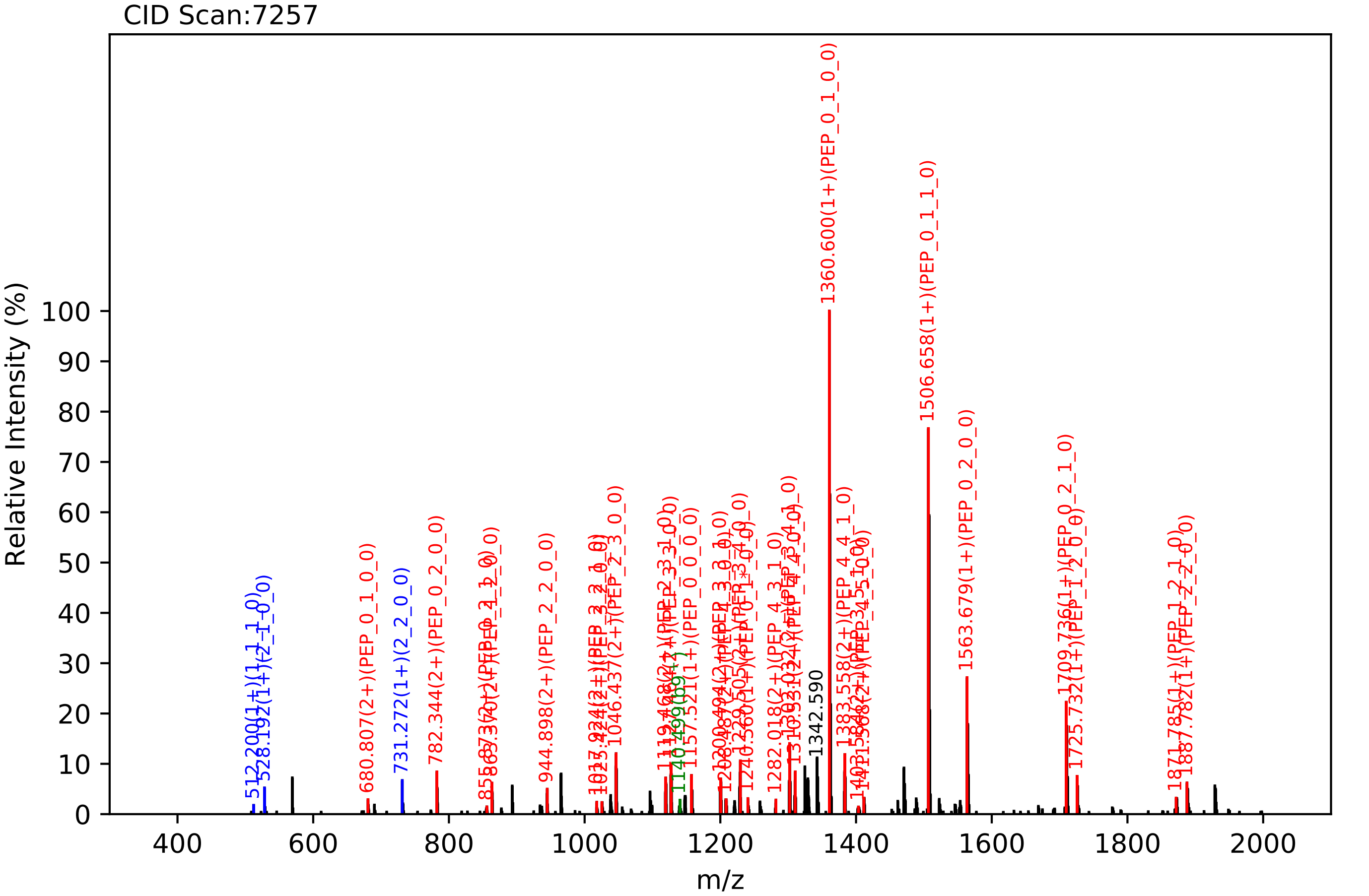

Training set no. 417, Experiment: IgG exp_2

EEQFNSTFR(=PEP)_4_5_1_0, m/z:1484.10(2+), RT:24.63, Y-score:80.79

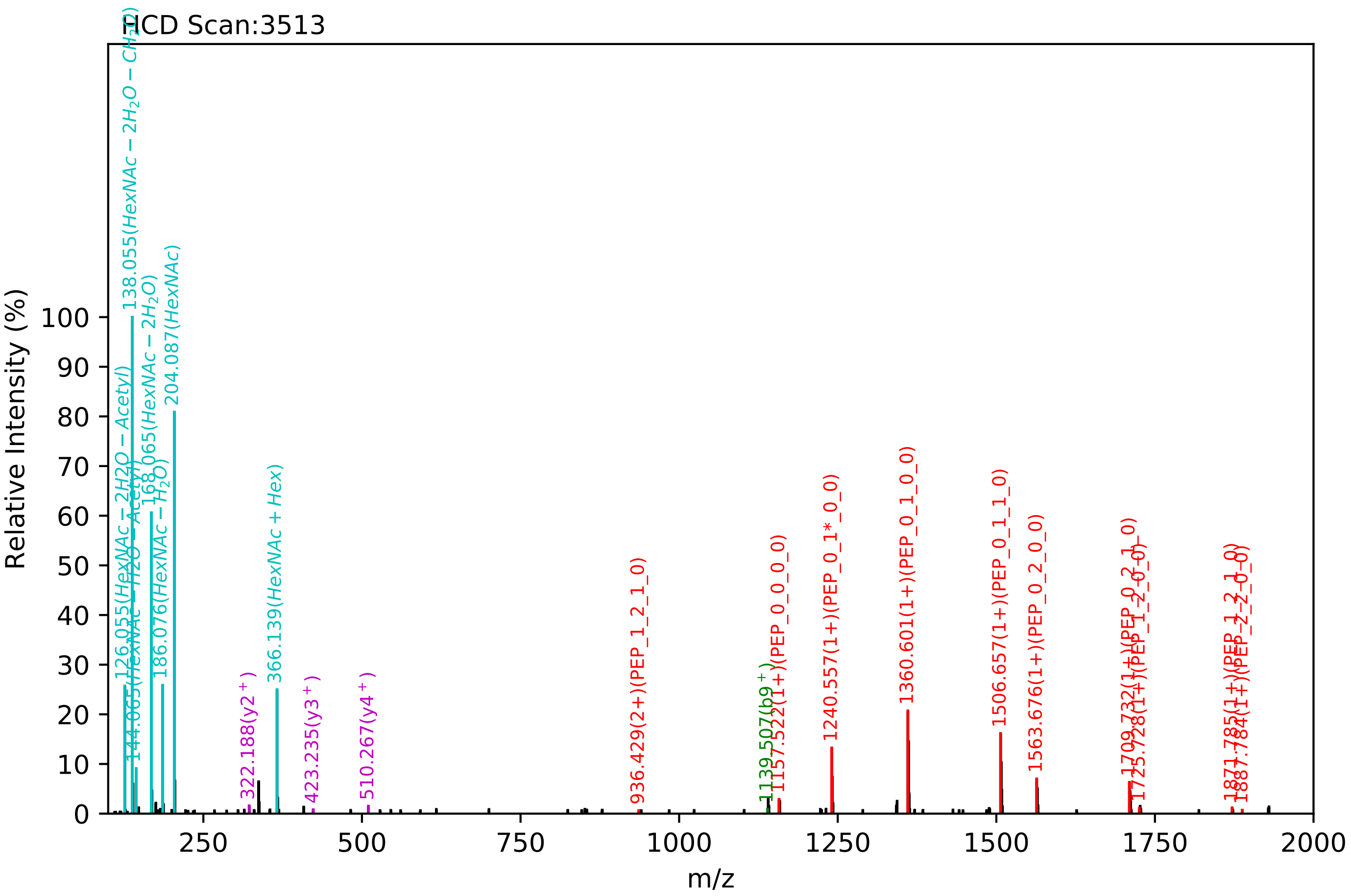

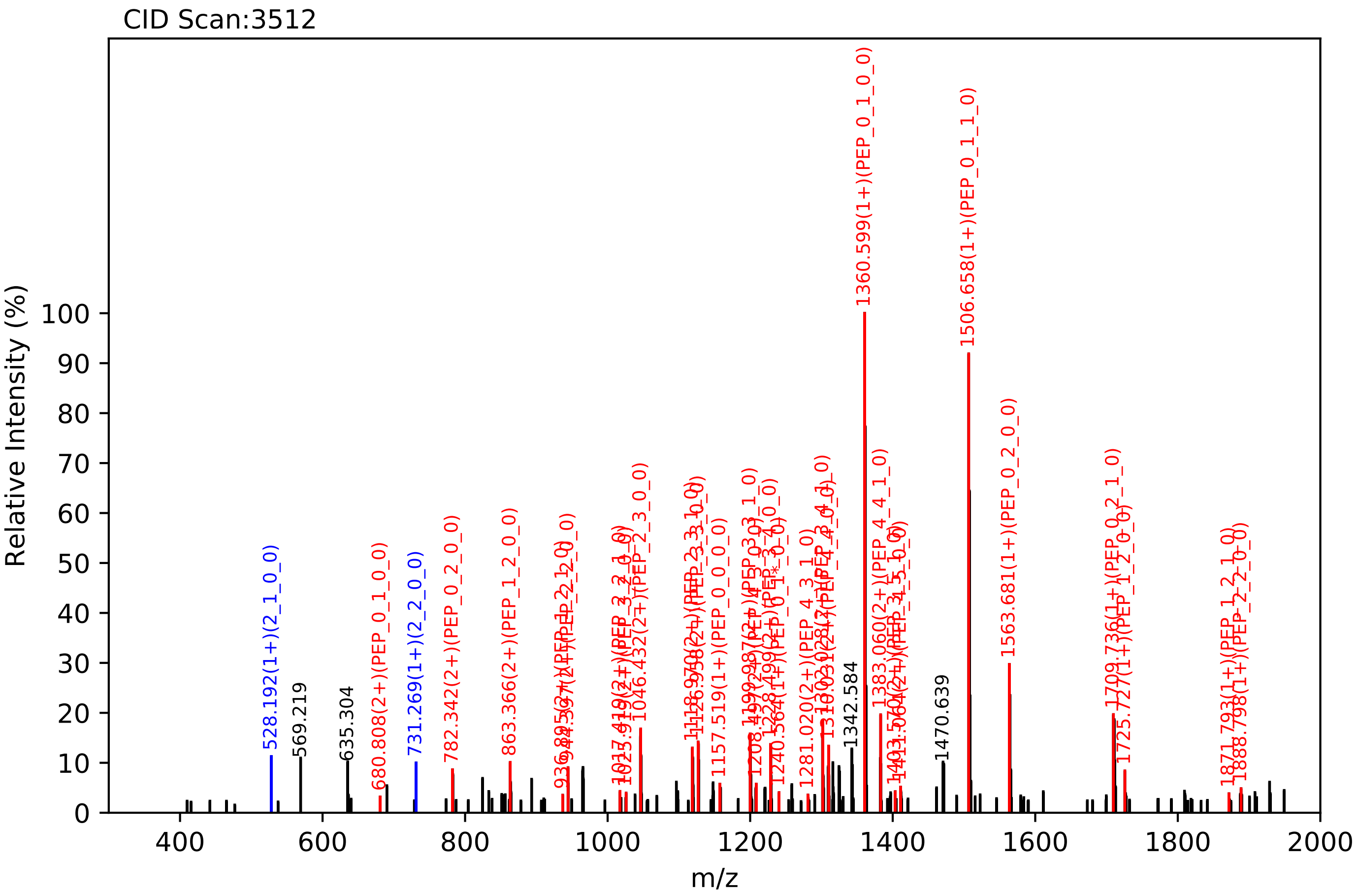

Training set no. 418, Experiment: IgG exp_4

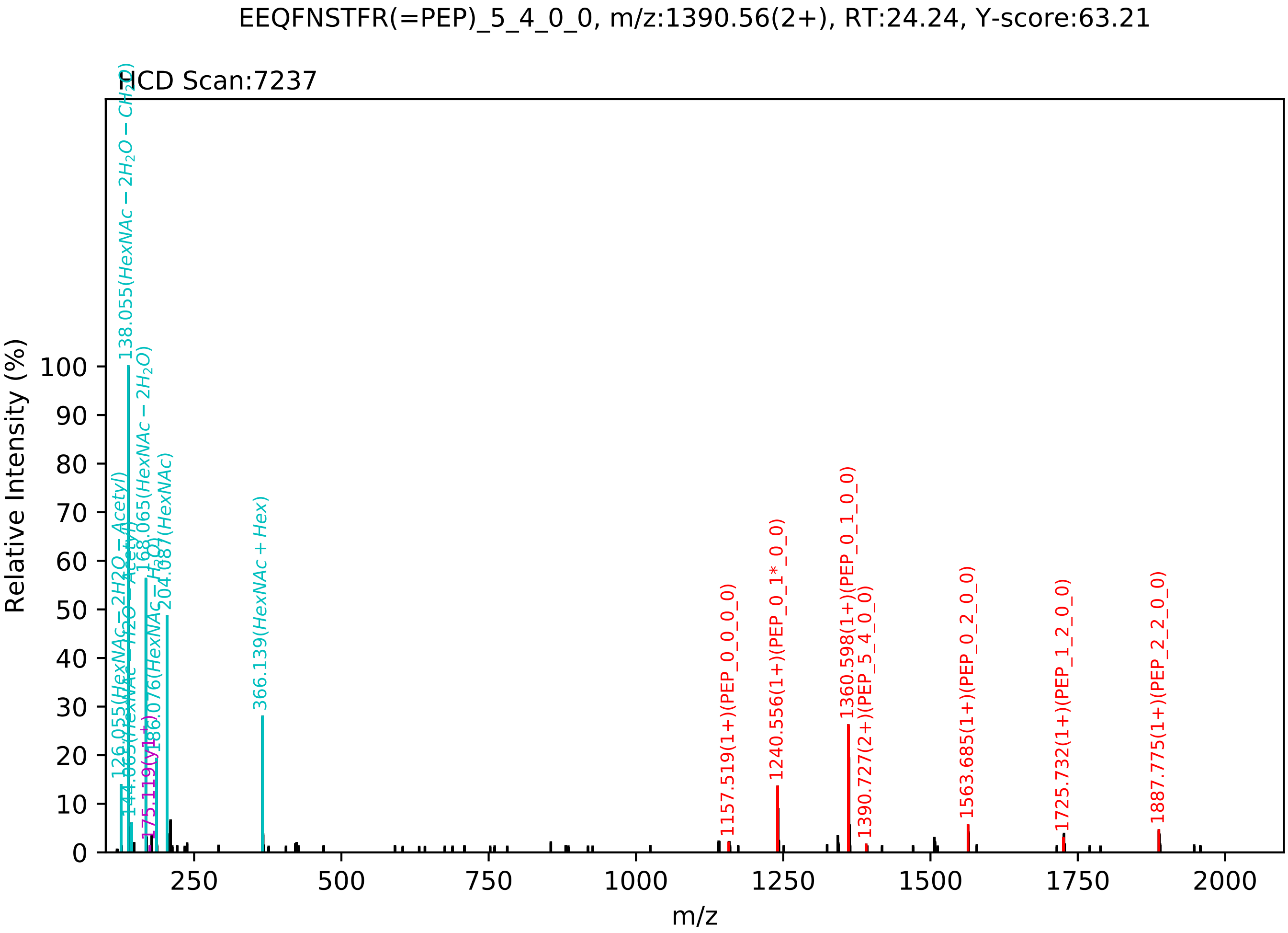

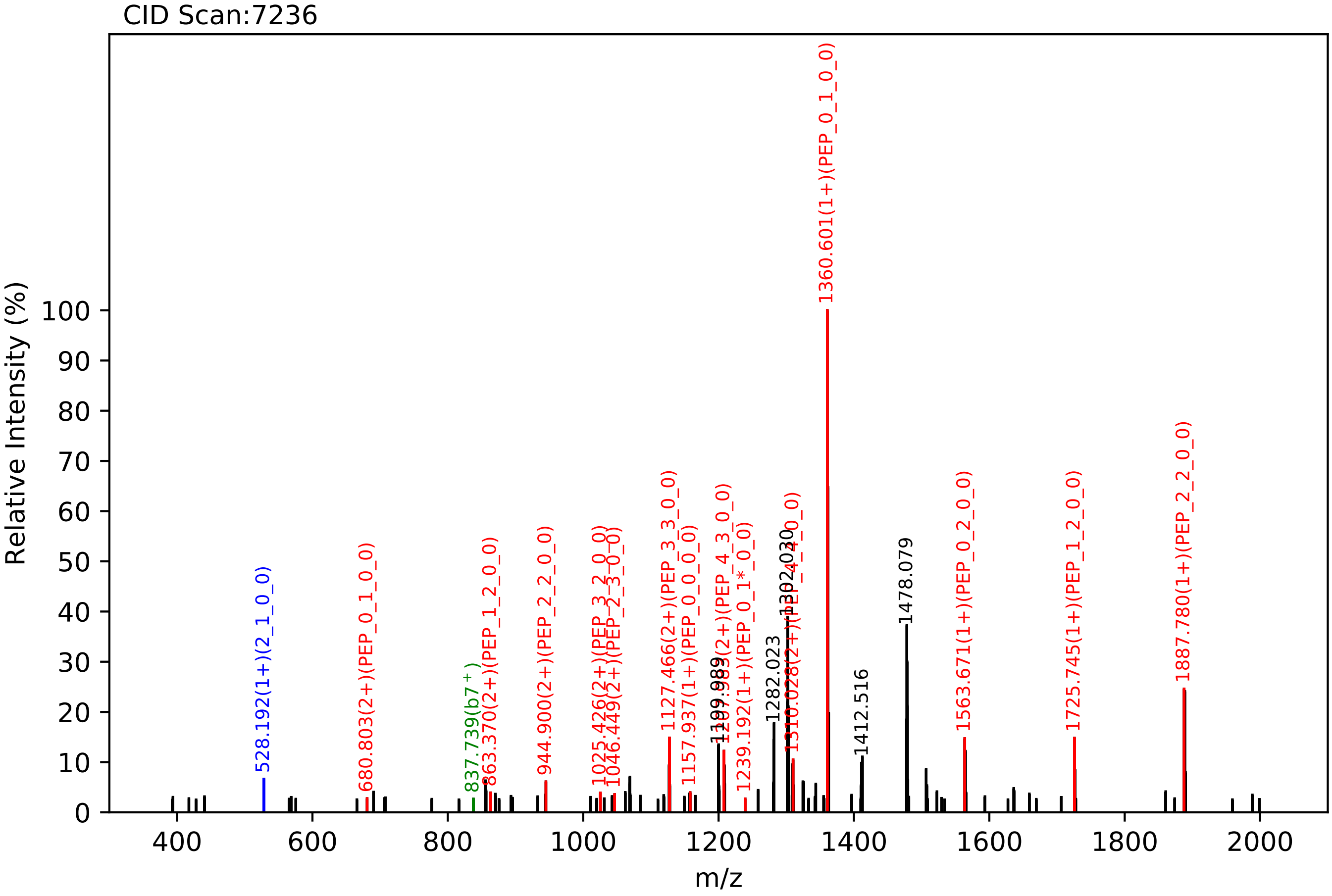

Training set no. 419, Experiment: IgG exp_4

EEQFNSTFR(=PEP)_5_4_1_0, m/z:1463.59(2+), RT:24.21, Y-score:89.50

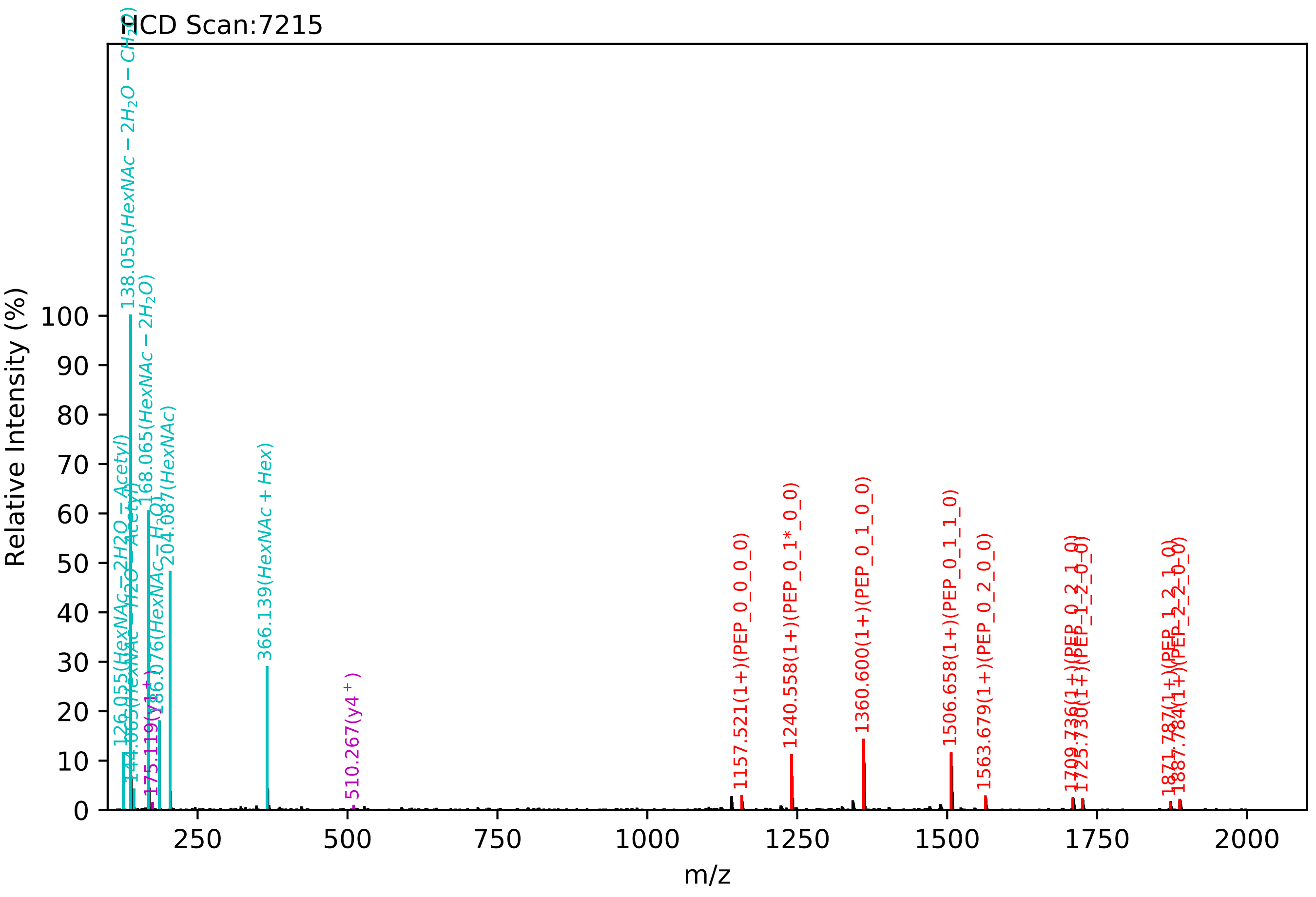

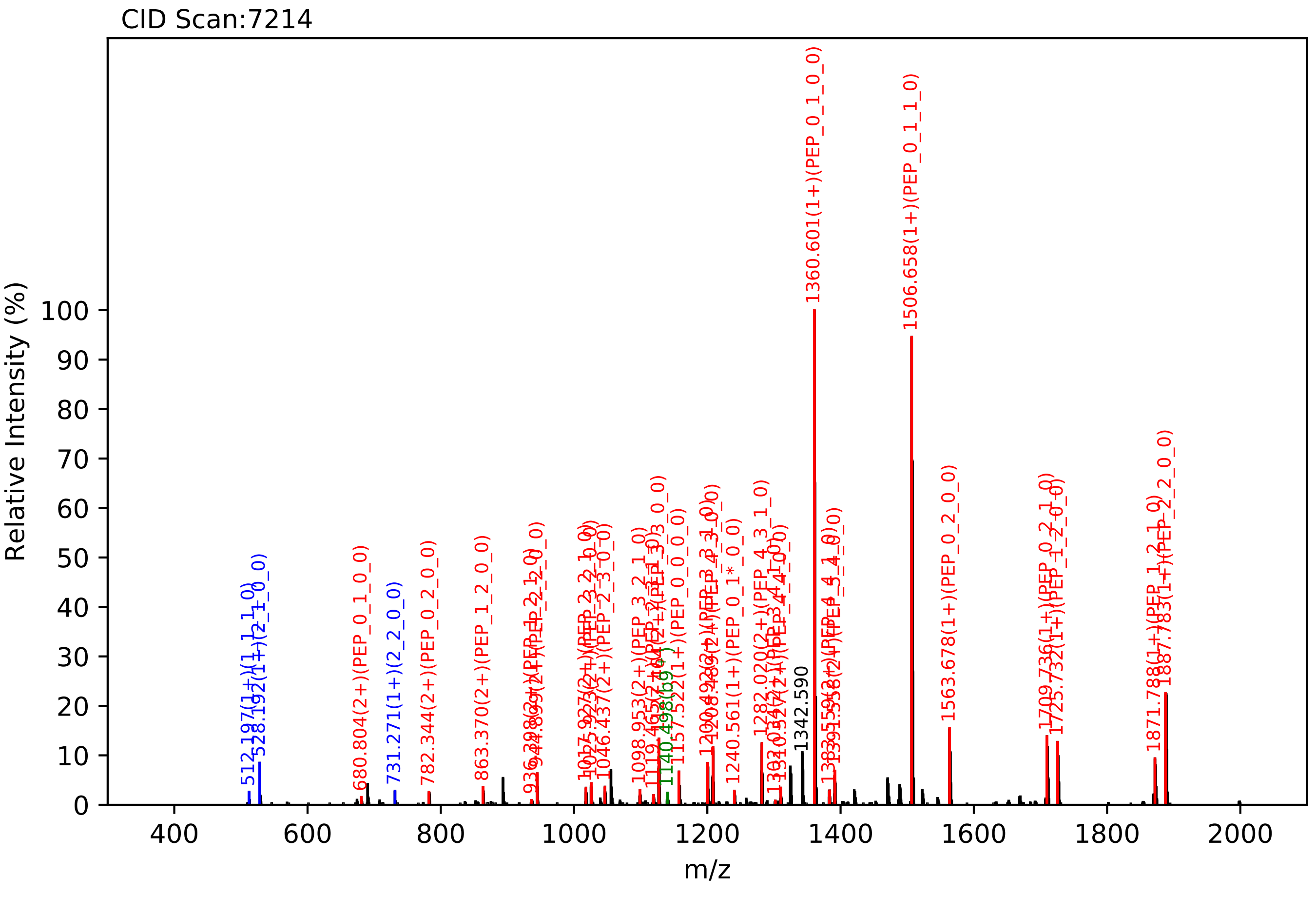

Training set no. 420, Experiment: IgG exp_3

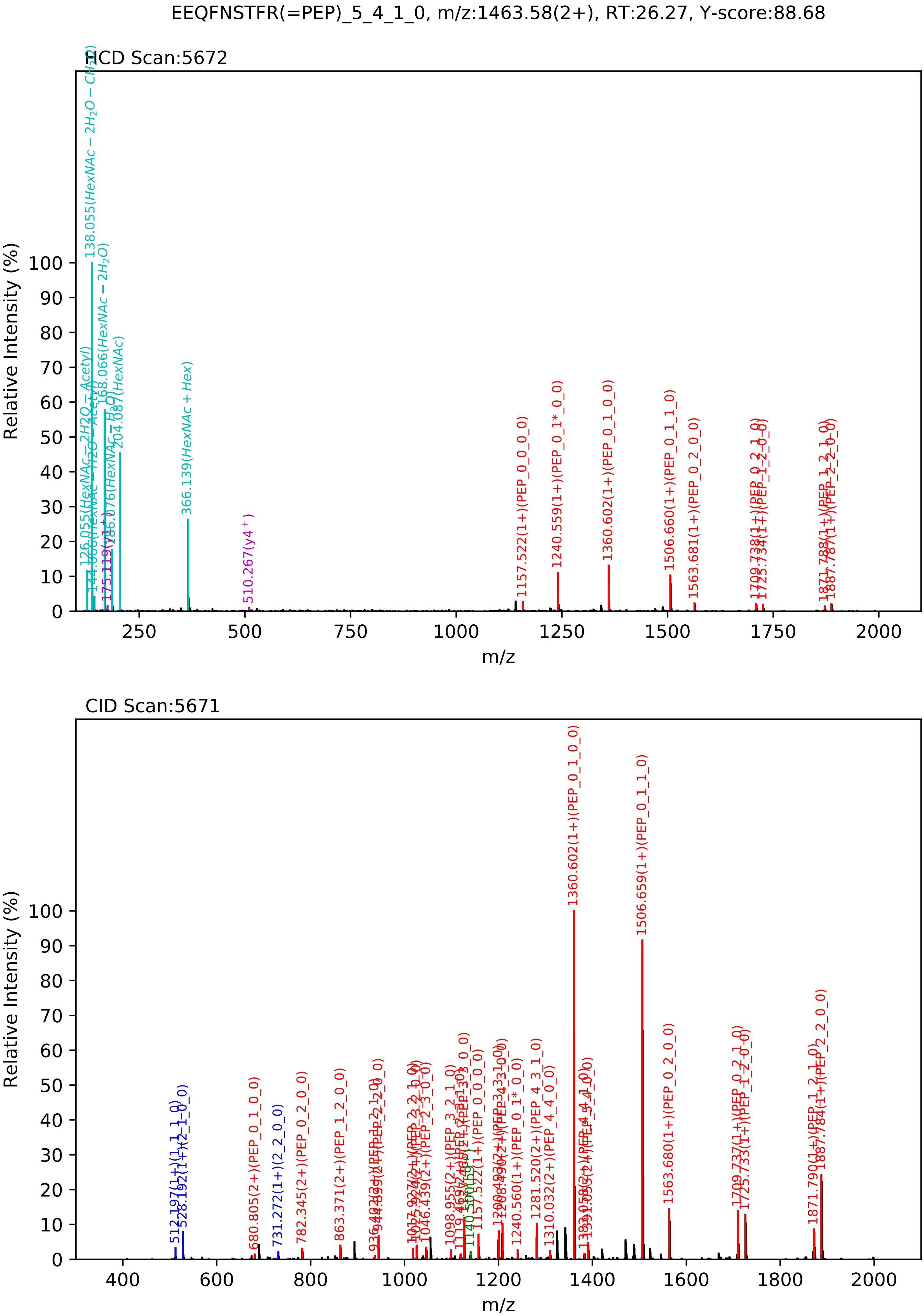

Training set no. 421, Experiment: IgG exp_1

EEQFNSTFR(=PEP)_5_4_1_0, m/z:1463.59(2+), RT:24.40, Y-score:88.21

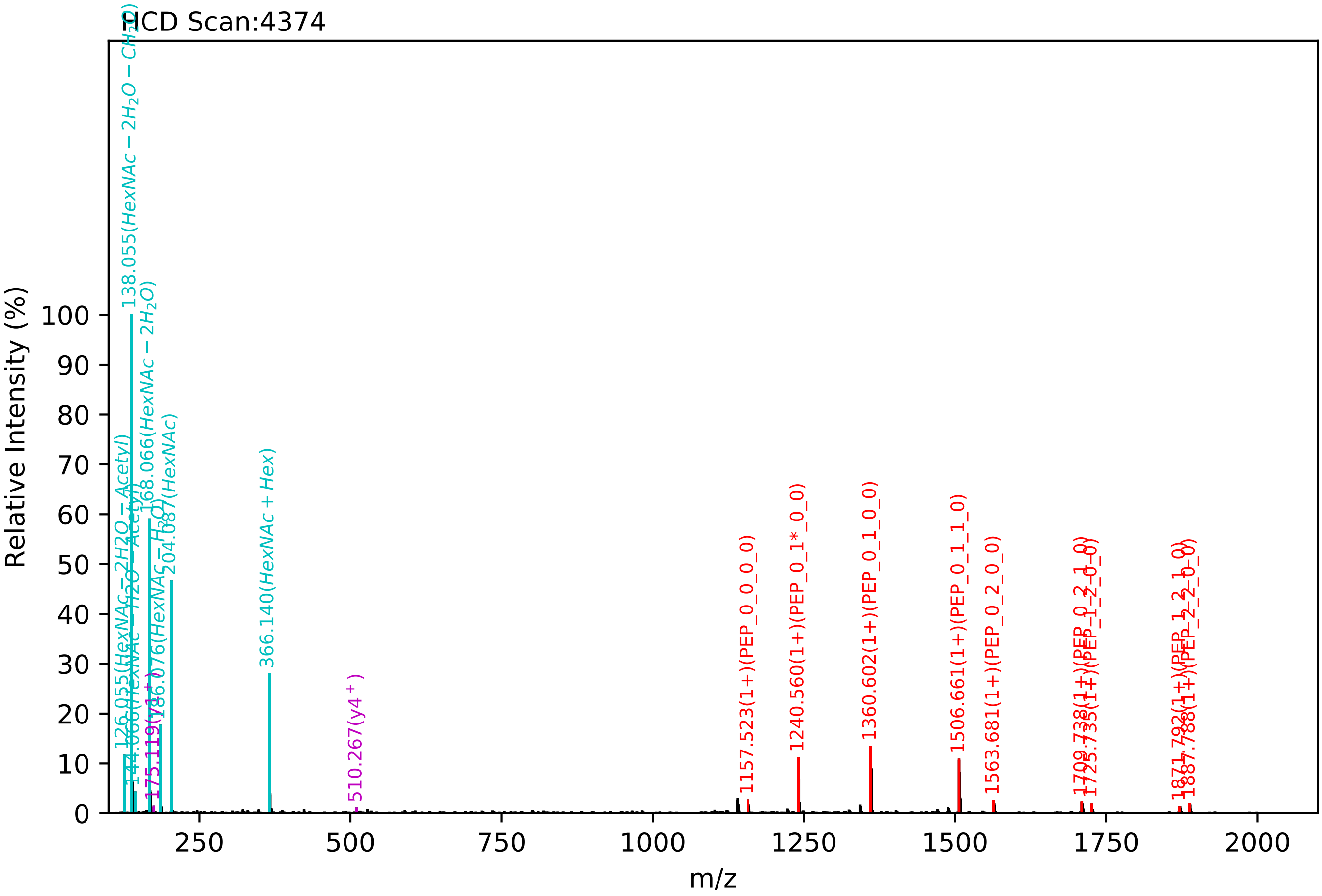

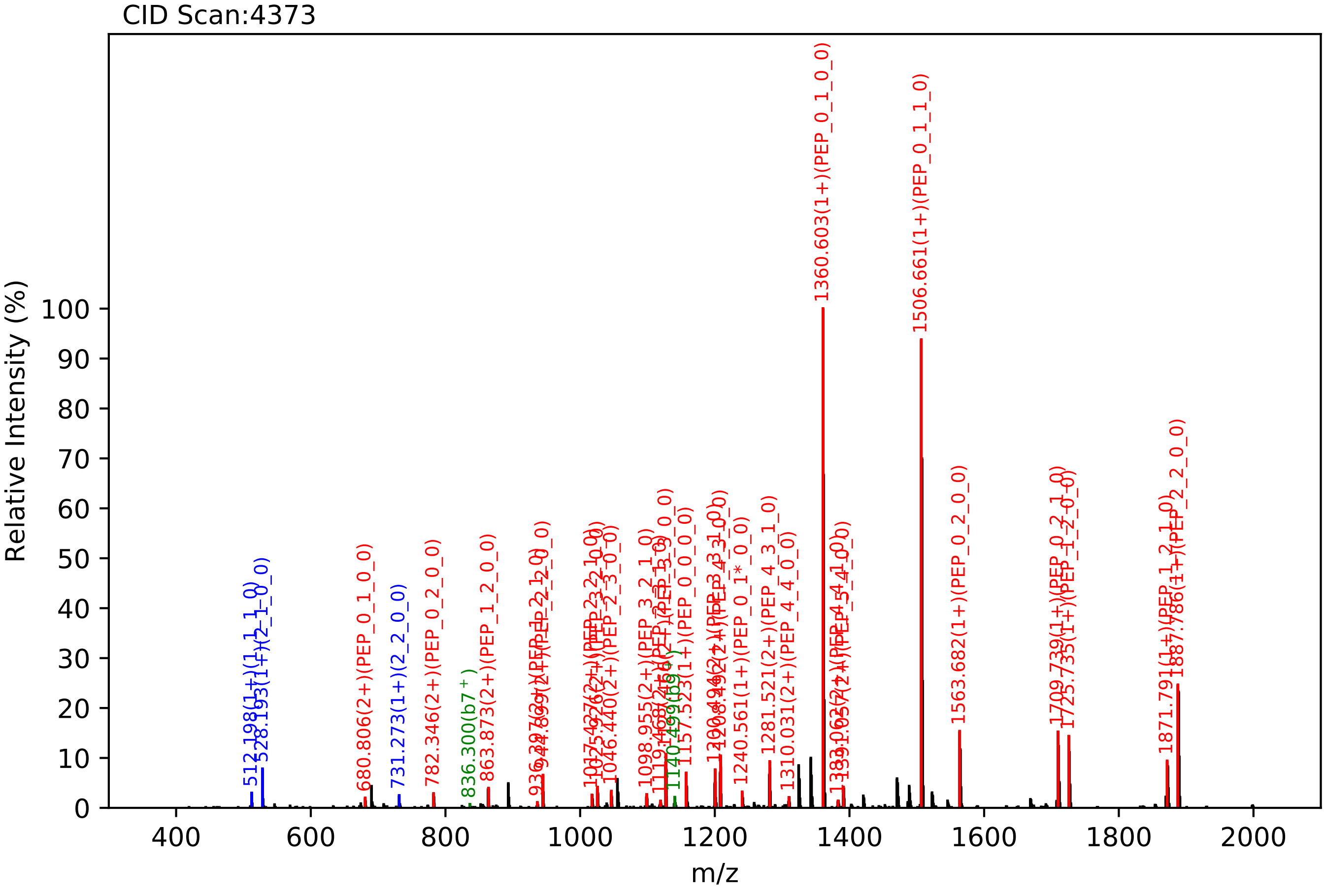

Training set no. 422, Experiment: IgG exp_2

EEQFNSTFR(=PEP)_5_4_1_0, m/z:1463.58(2+), RT:24.50, Y-score:88.14

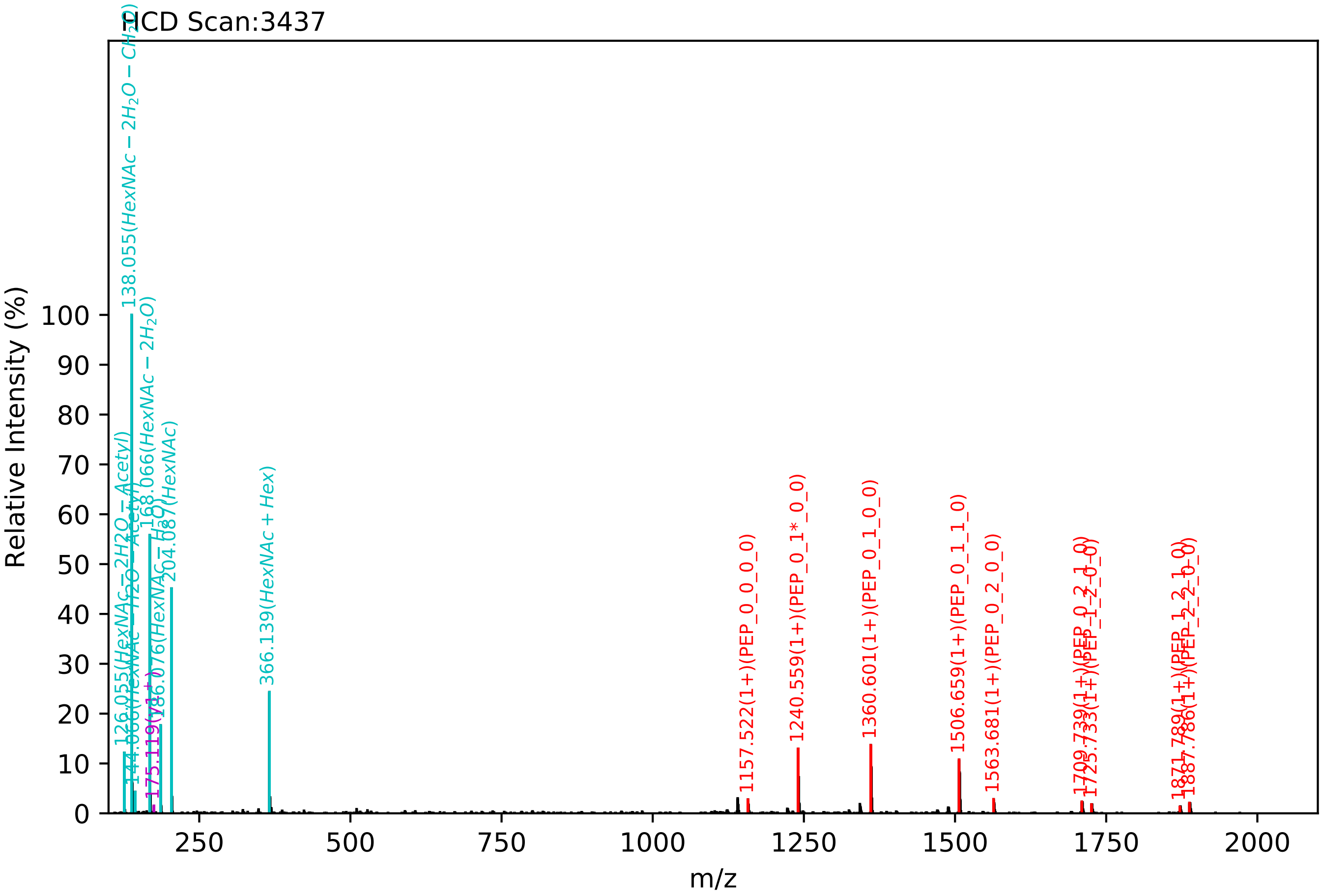

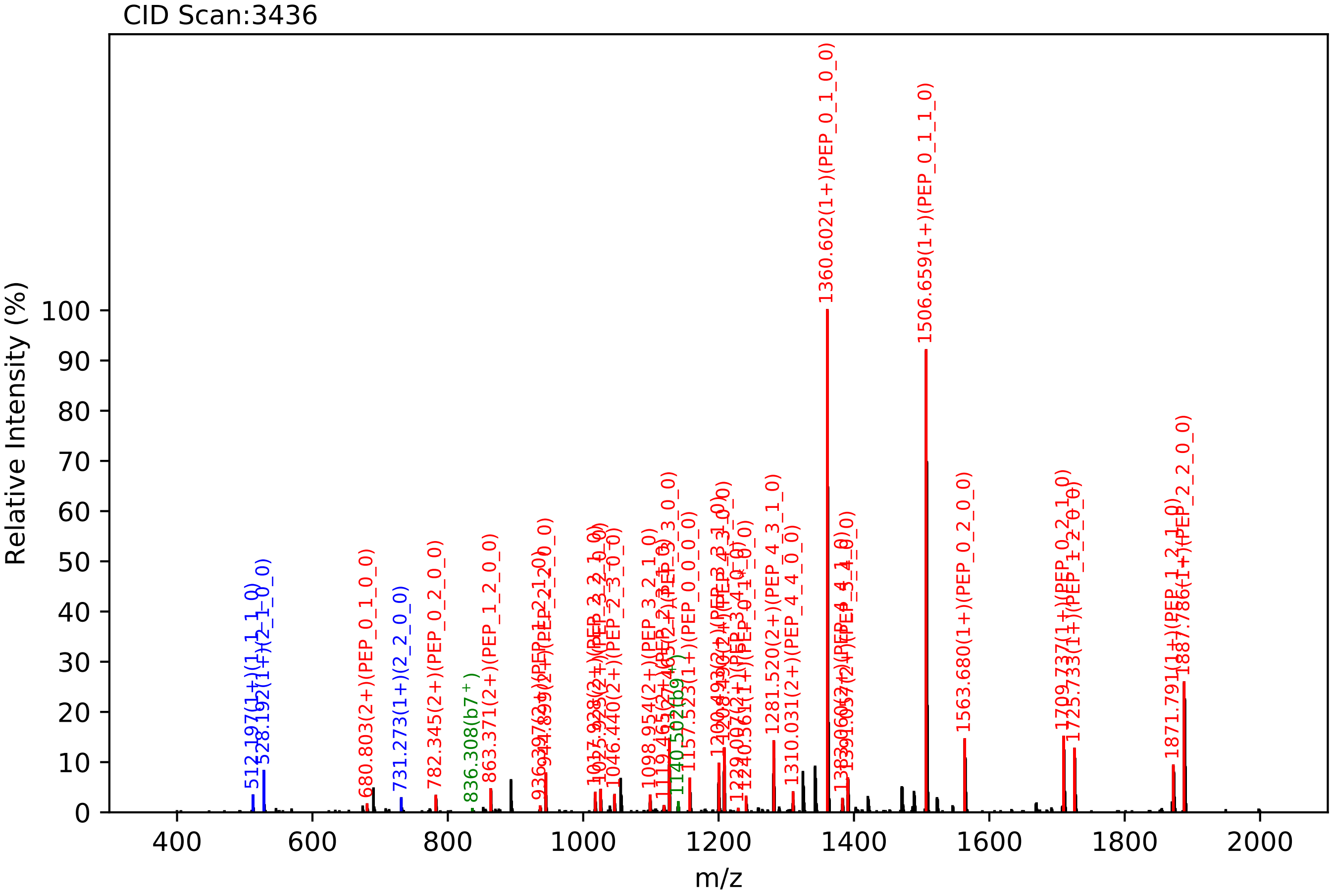

Training set no. 423, Experiment: IgG exp_4

EEQFNSTFR(=PEP)_5_4_1_1, m/z:1609.13(2+), RT:26.67, Y-score:92.37

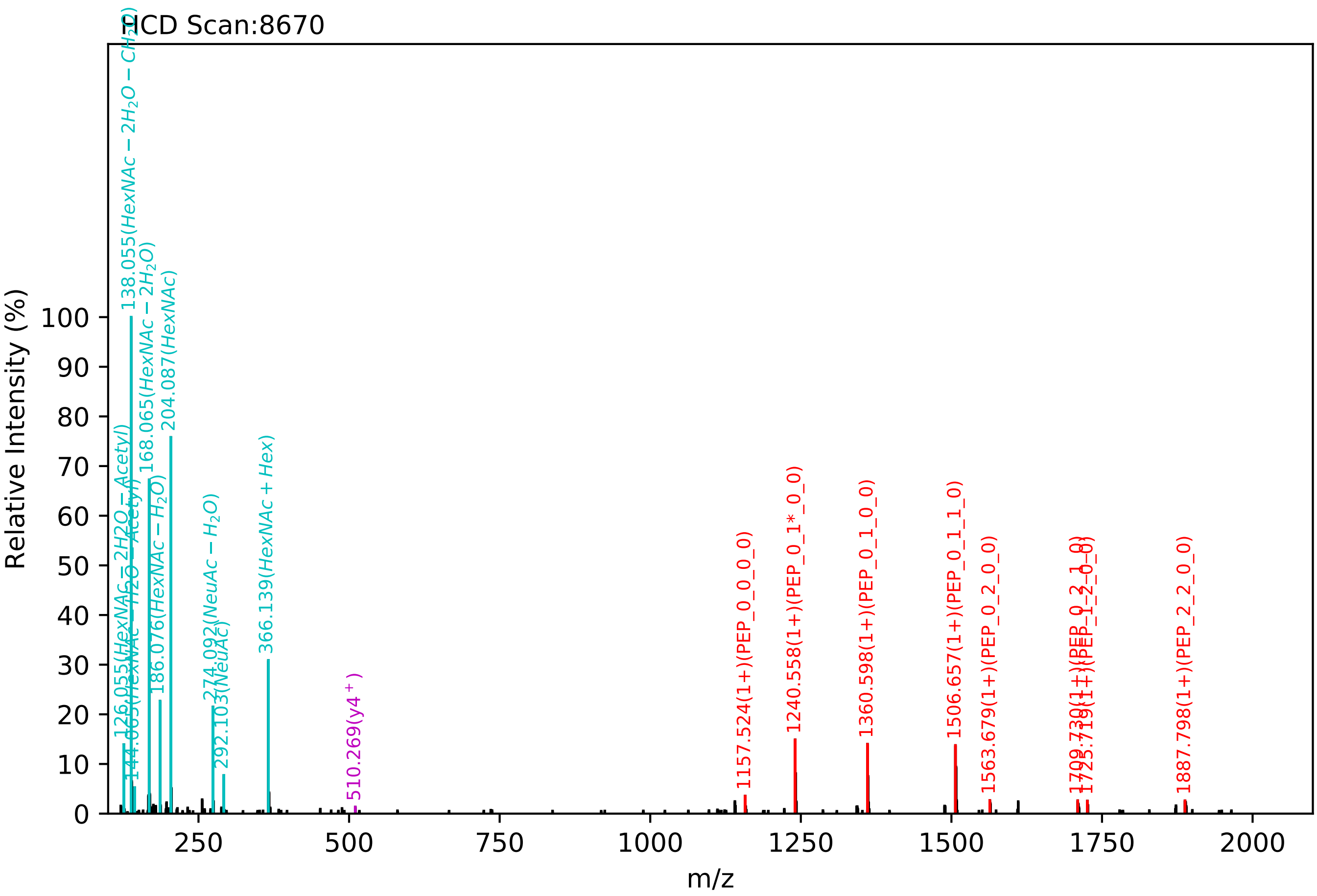

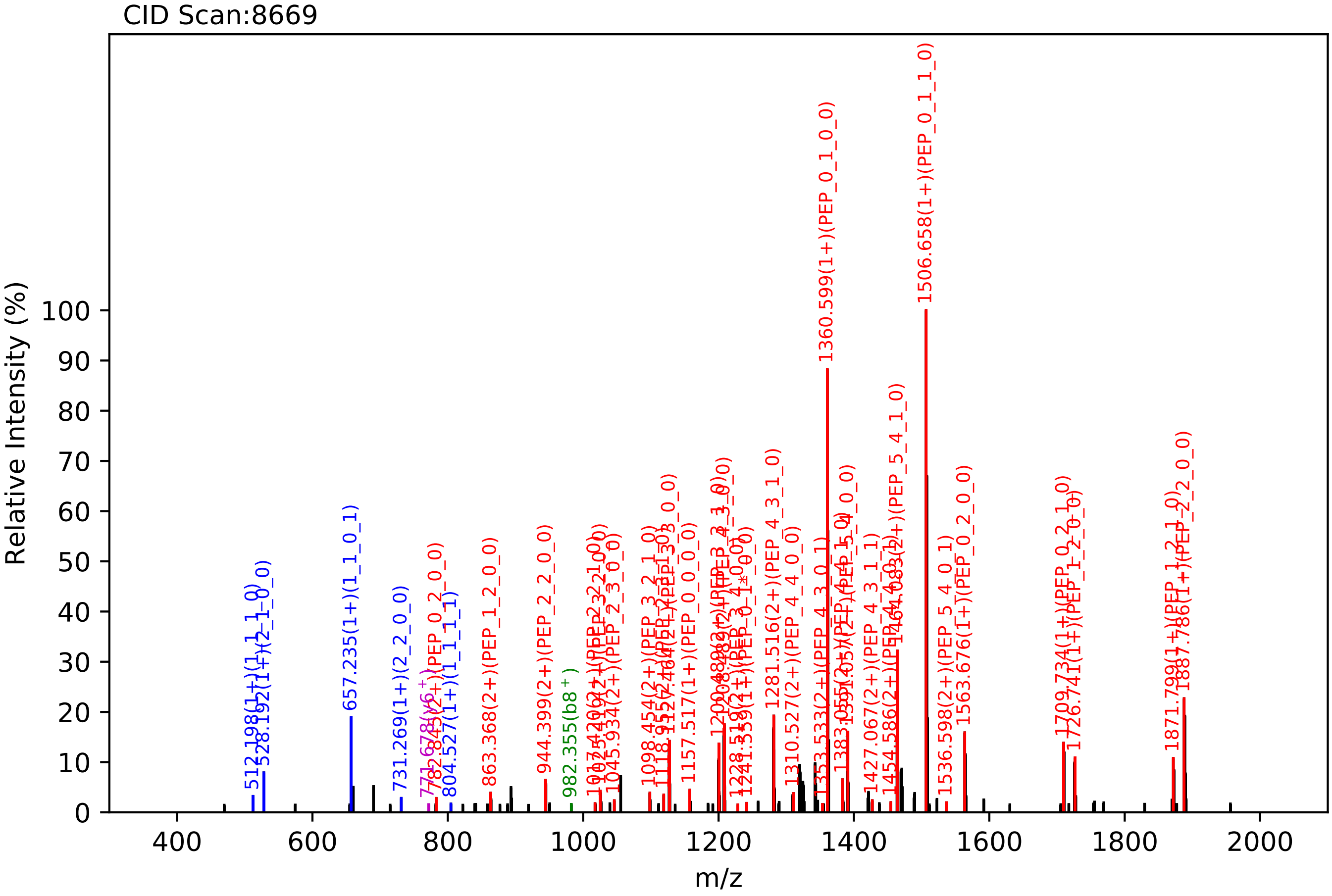

Training set no. 424, Experiment: IgG exp_1

EEQFNSTFR(=PEP)_5_4_1_1, m/z:1609.13(2+), RT:27.05, Y-score:90.76

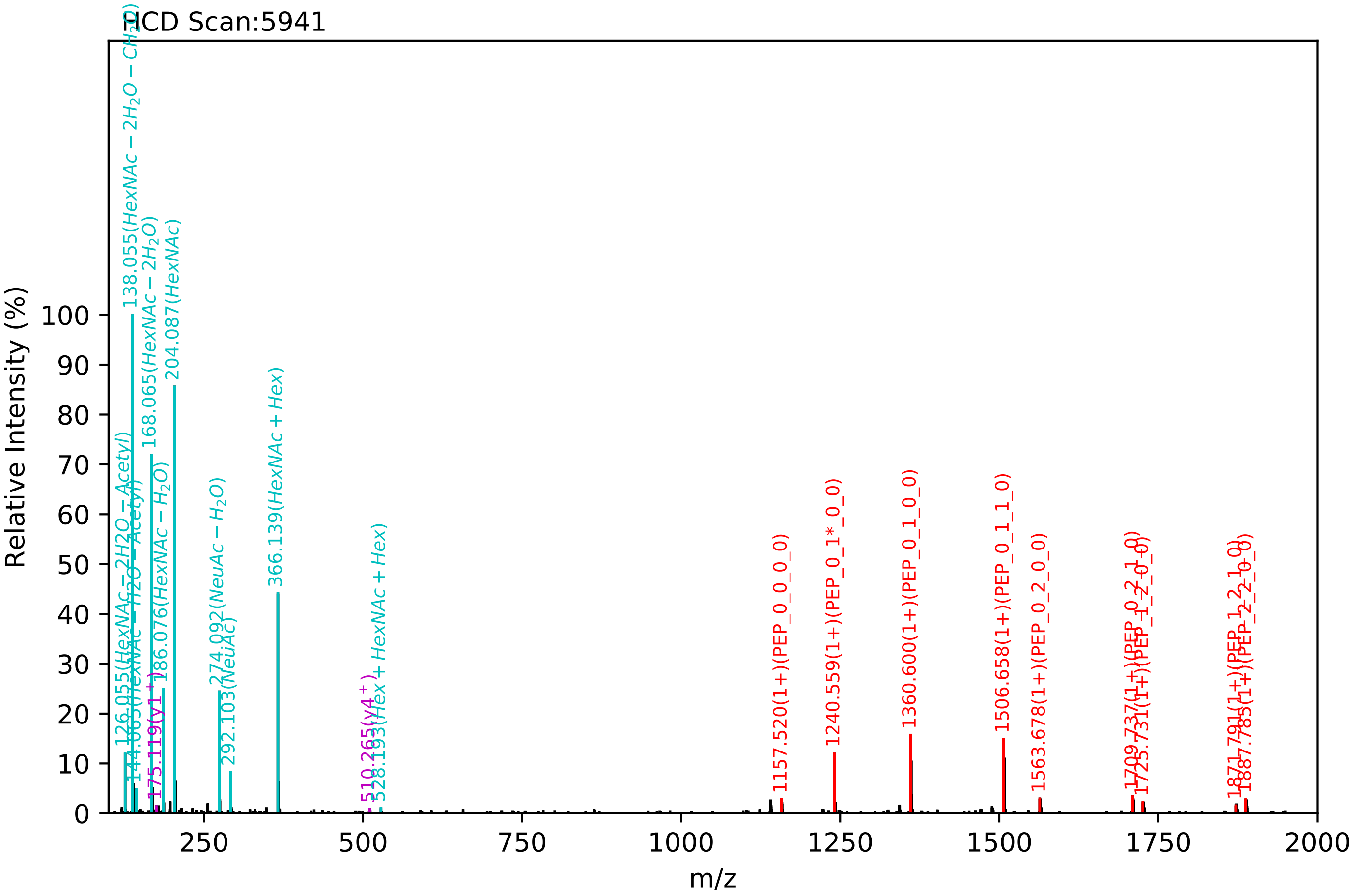

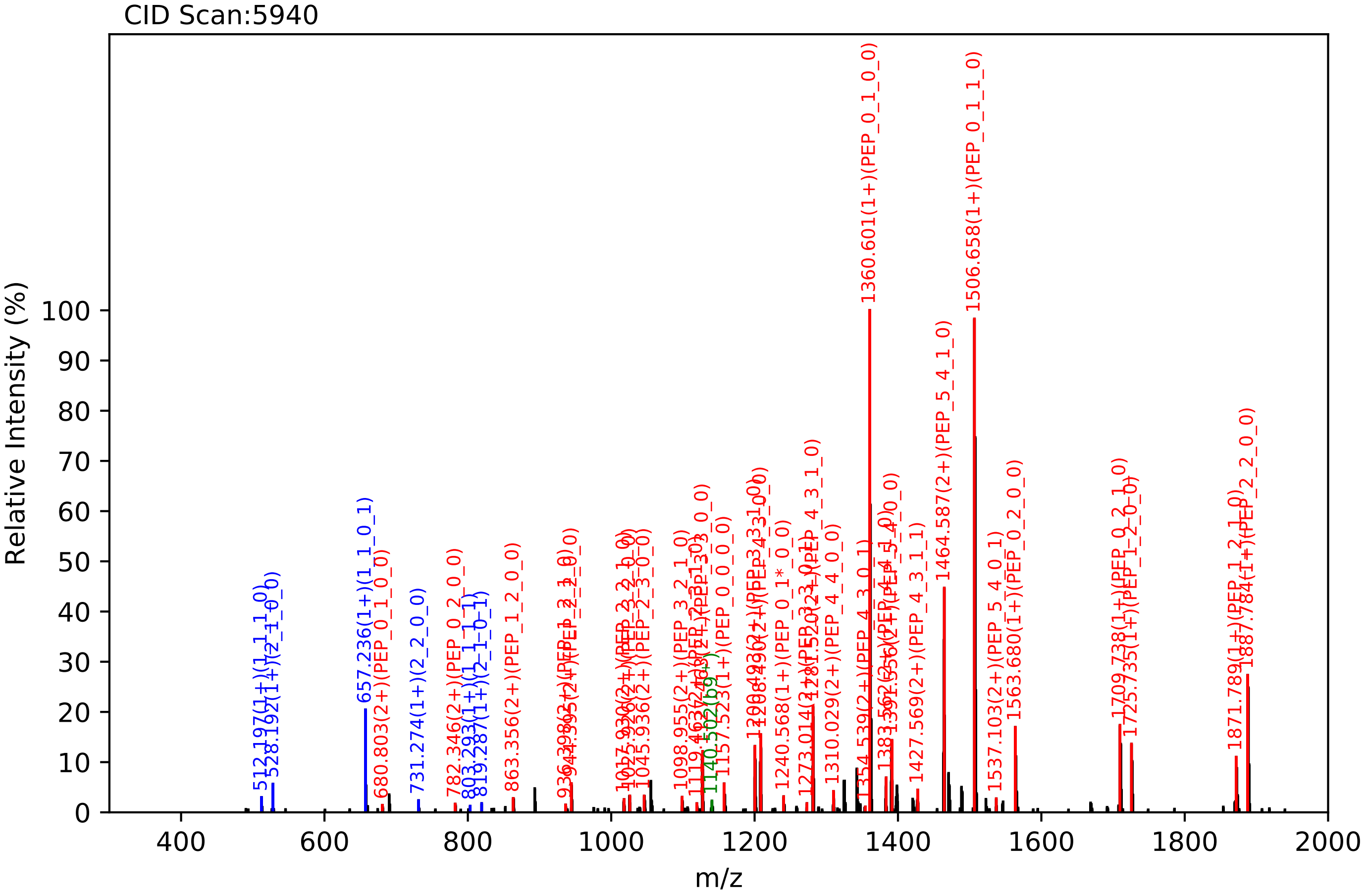

Training set no. 425, Experiment: IgG exp_2

EEQFNSTFR(=PEP)_5_4_1_1, m/z:1609.13(2+), RT:26.94, Y-score:89.74

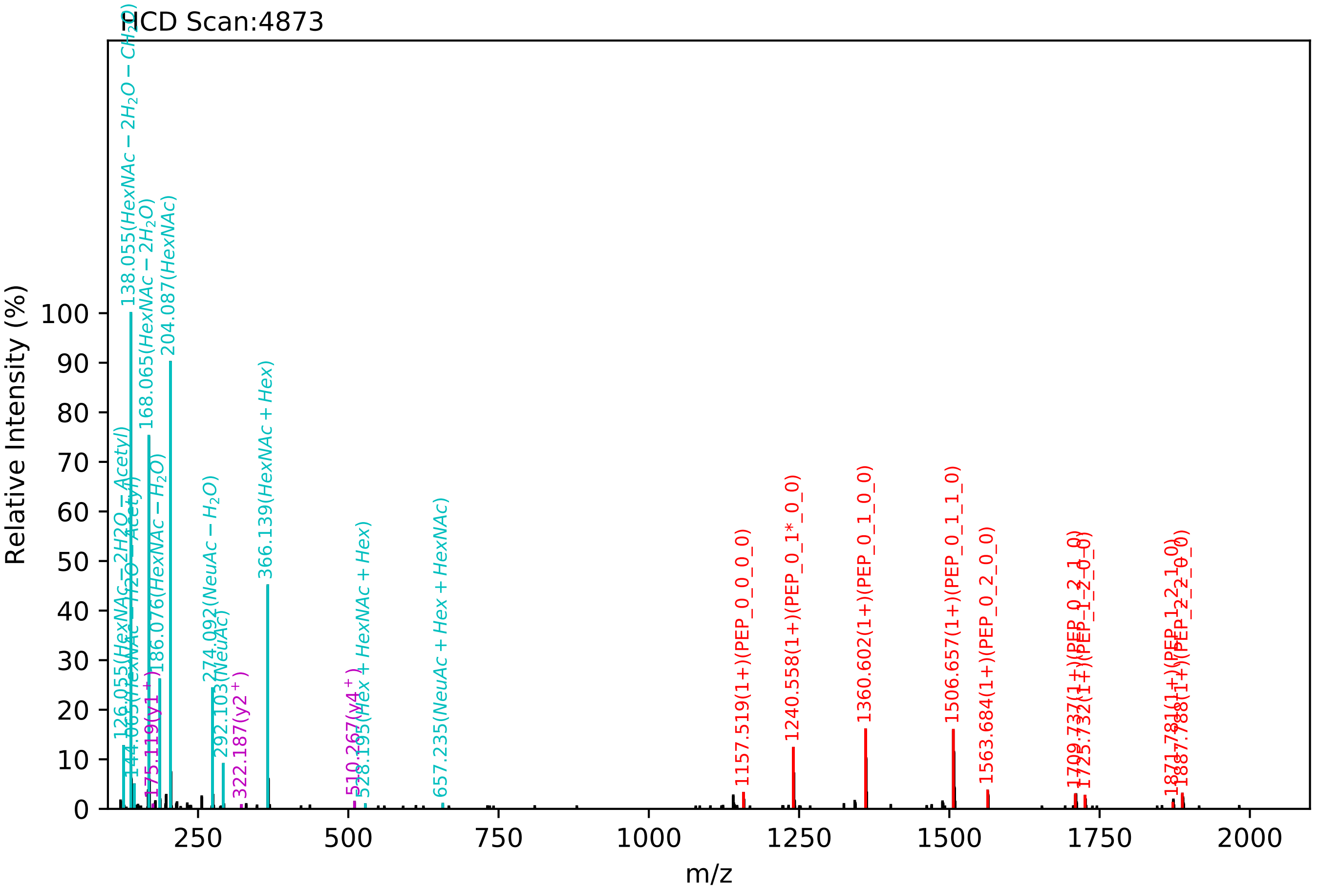

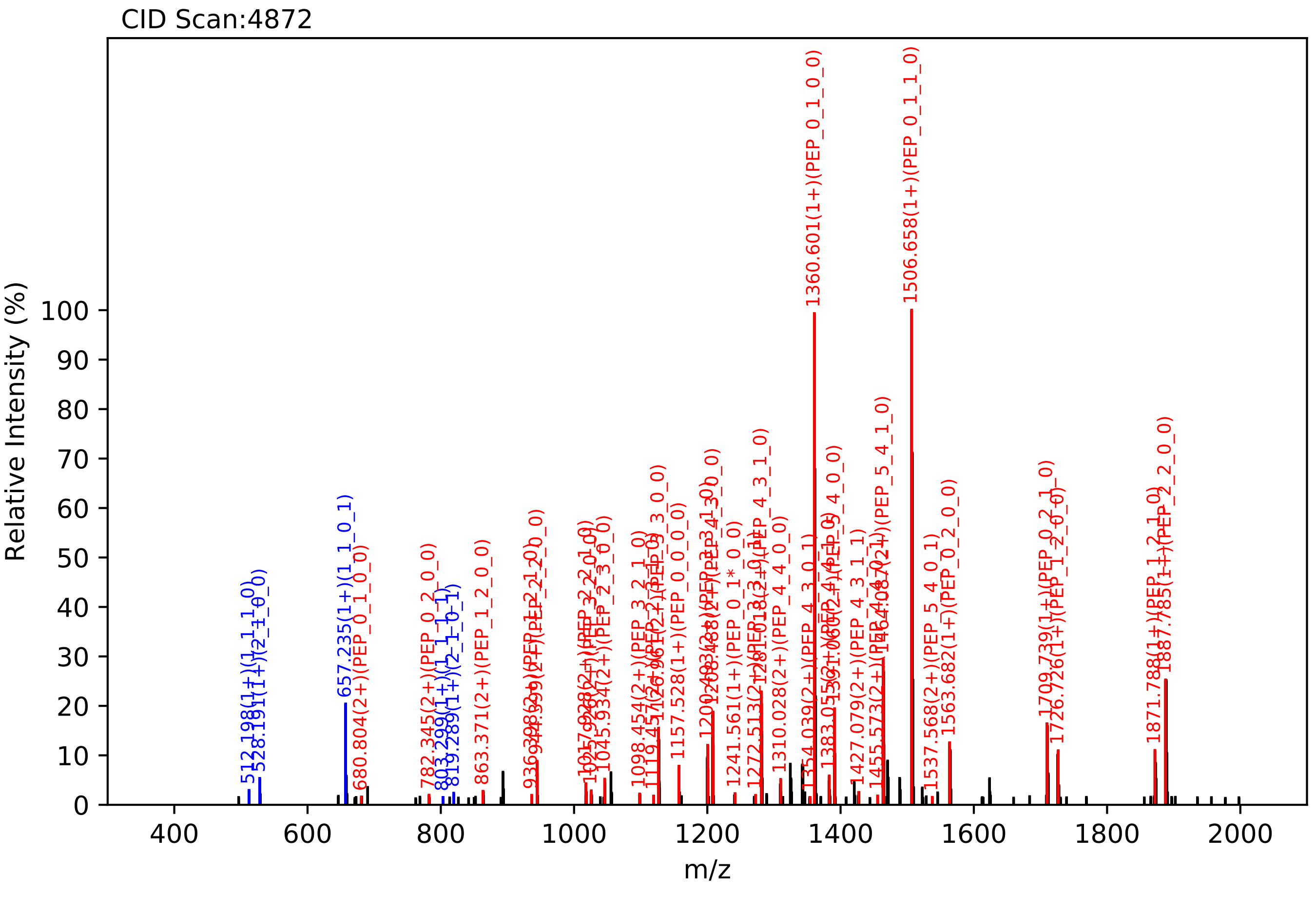

Training set no. 426, Experiment: IgG exp_2

EEQFNSTFR(=PEP)_5_4_1_1, m/z:1609.14(2+), RT:26.38, Y-score:89.27

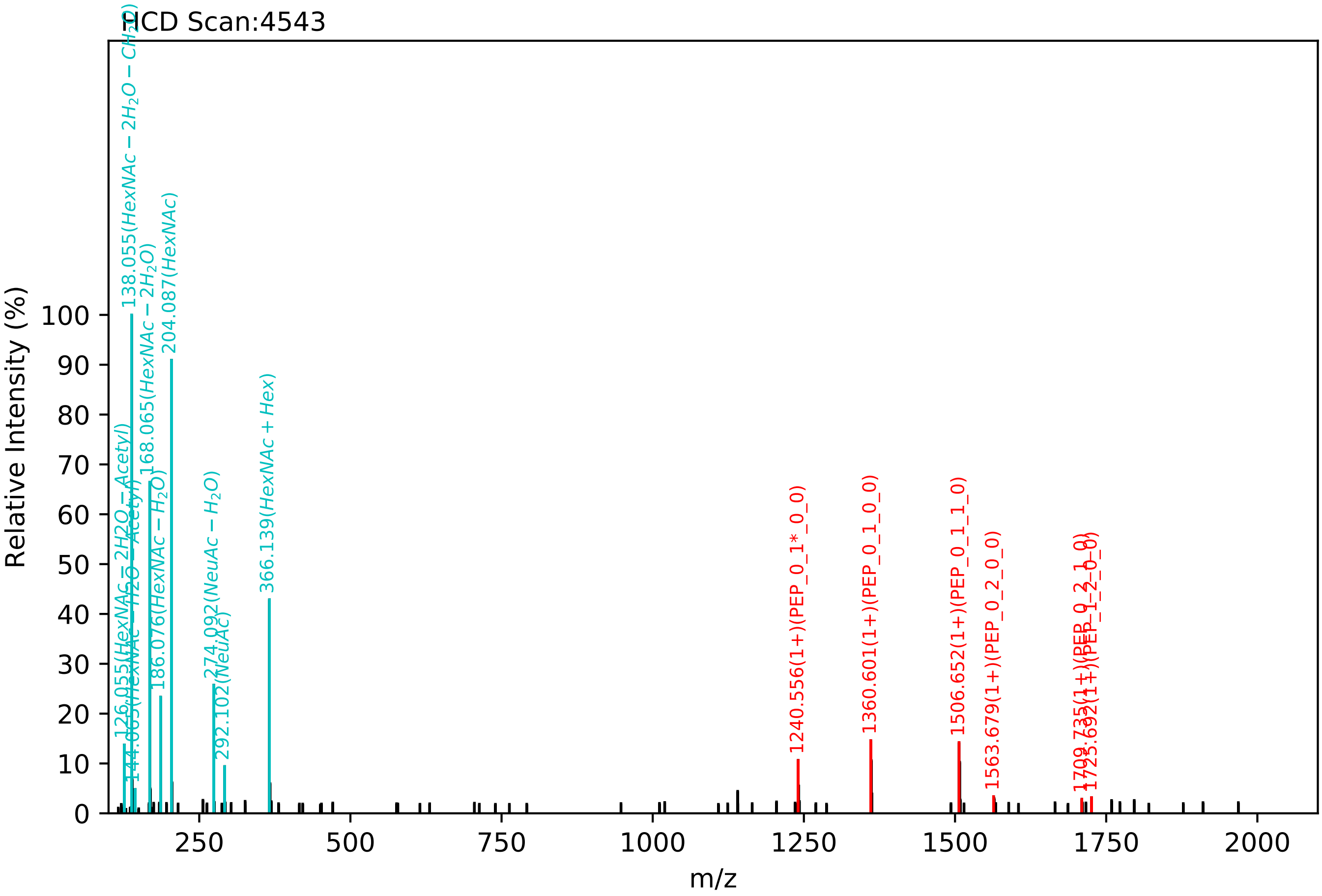

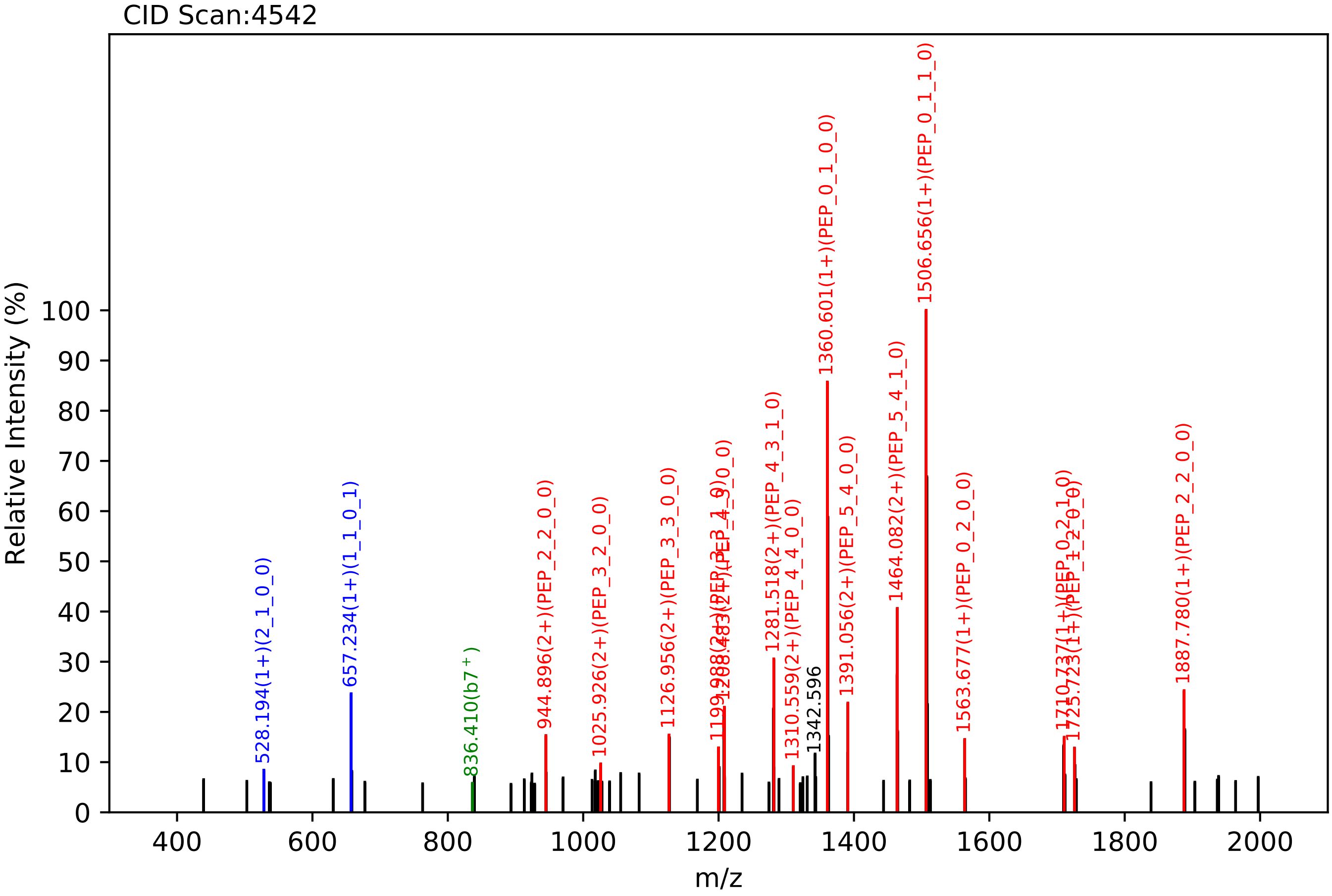

Training set no. 427, Experiment: IgG exp_1

EEQFNSTFR(=PEP)_5_5_1_0, m/z:1043.75(3+), RT:24.52, Y-score:95.34

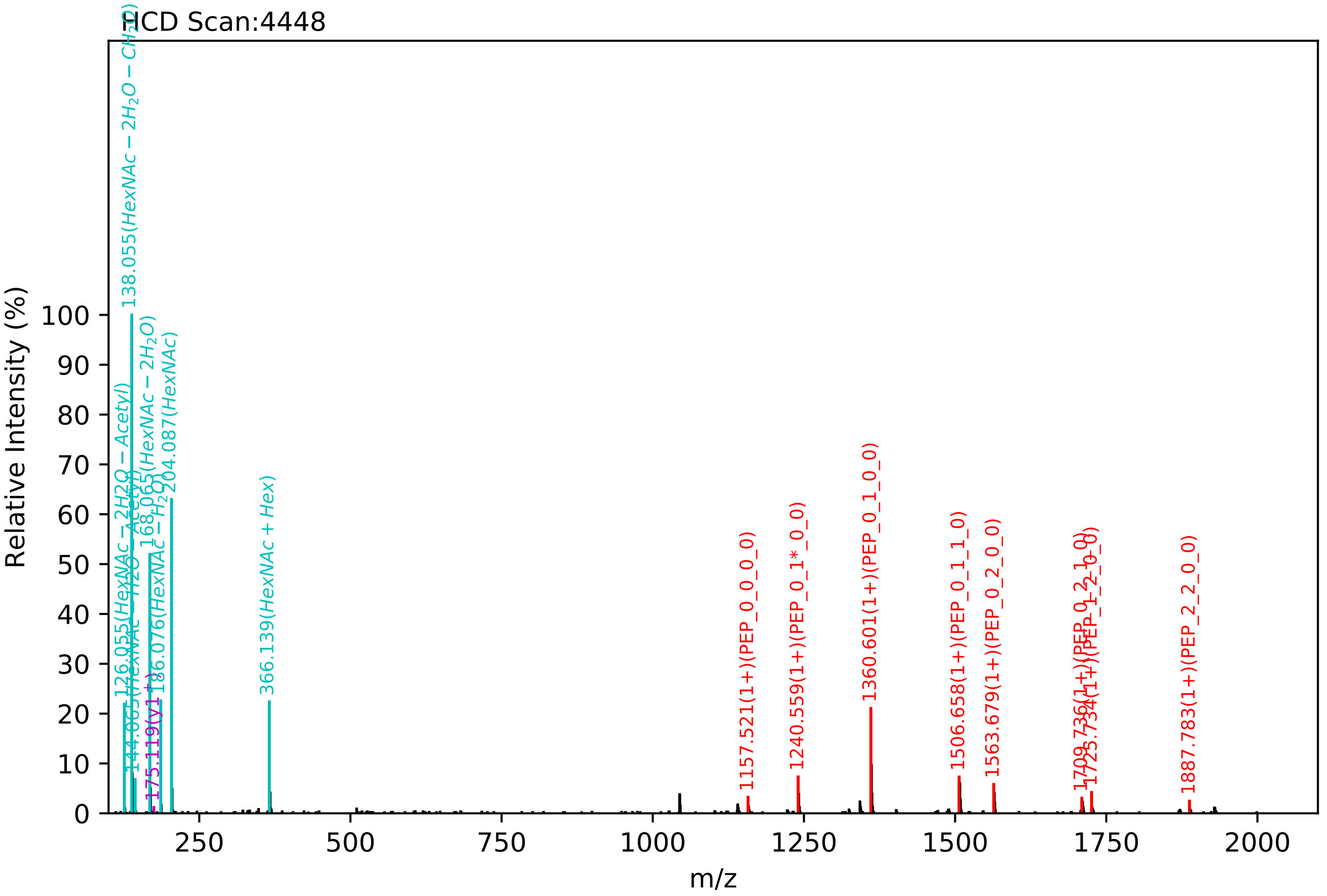

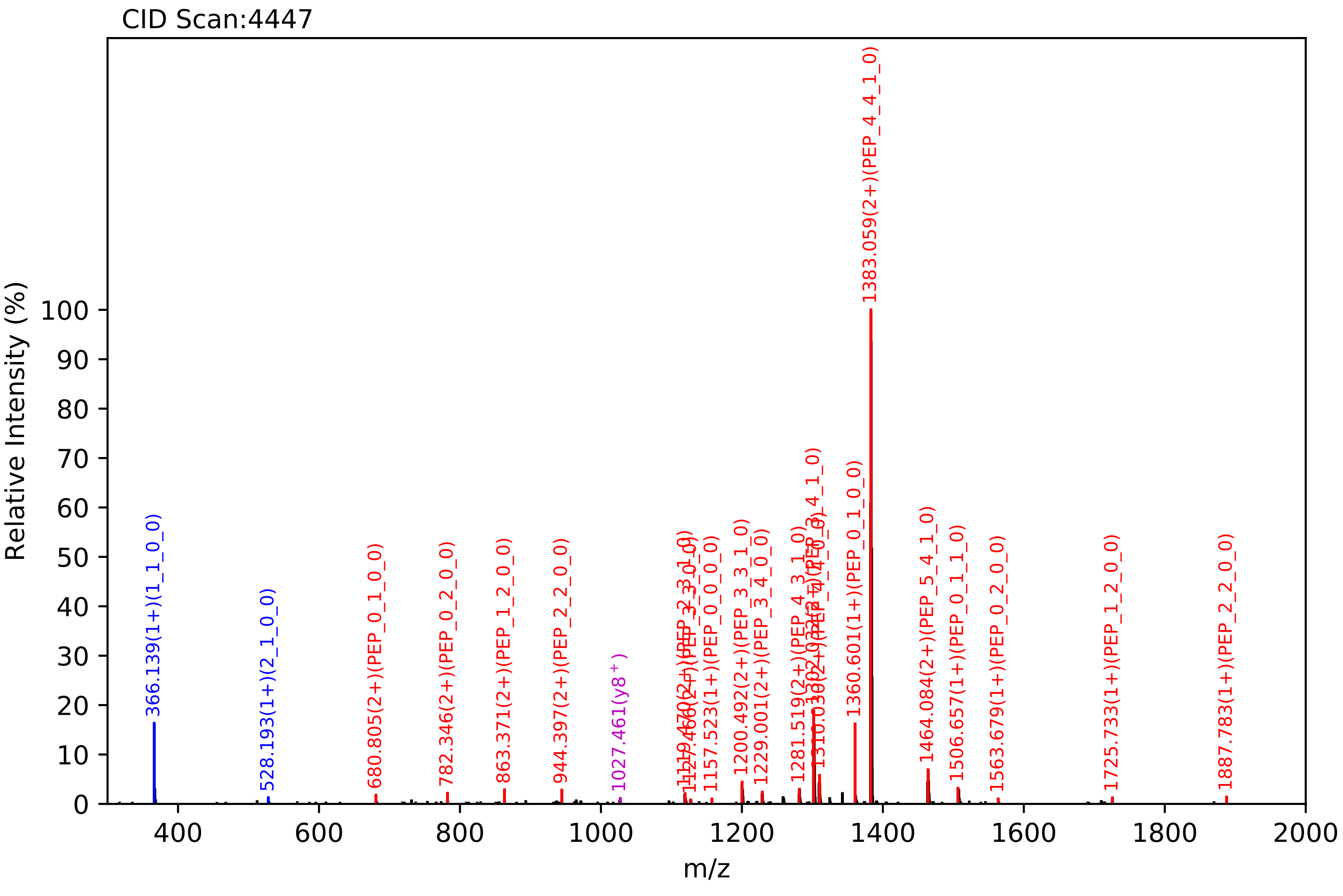

Training set no. 428, Experiment: IgG exp_1

EEQFNSTFR(=PEP)_5_5_1_0, m/z:1565.13(2+), RT:24.56, Y-score:69.35

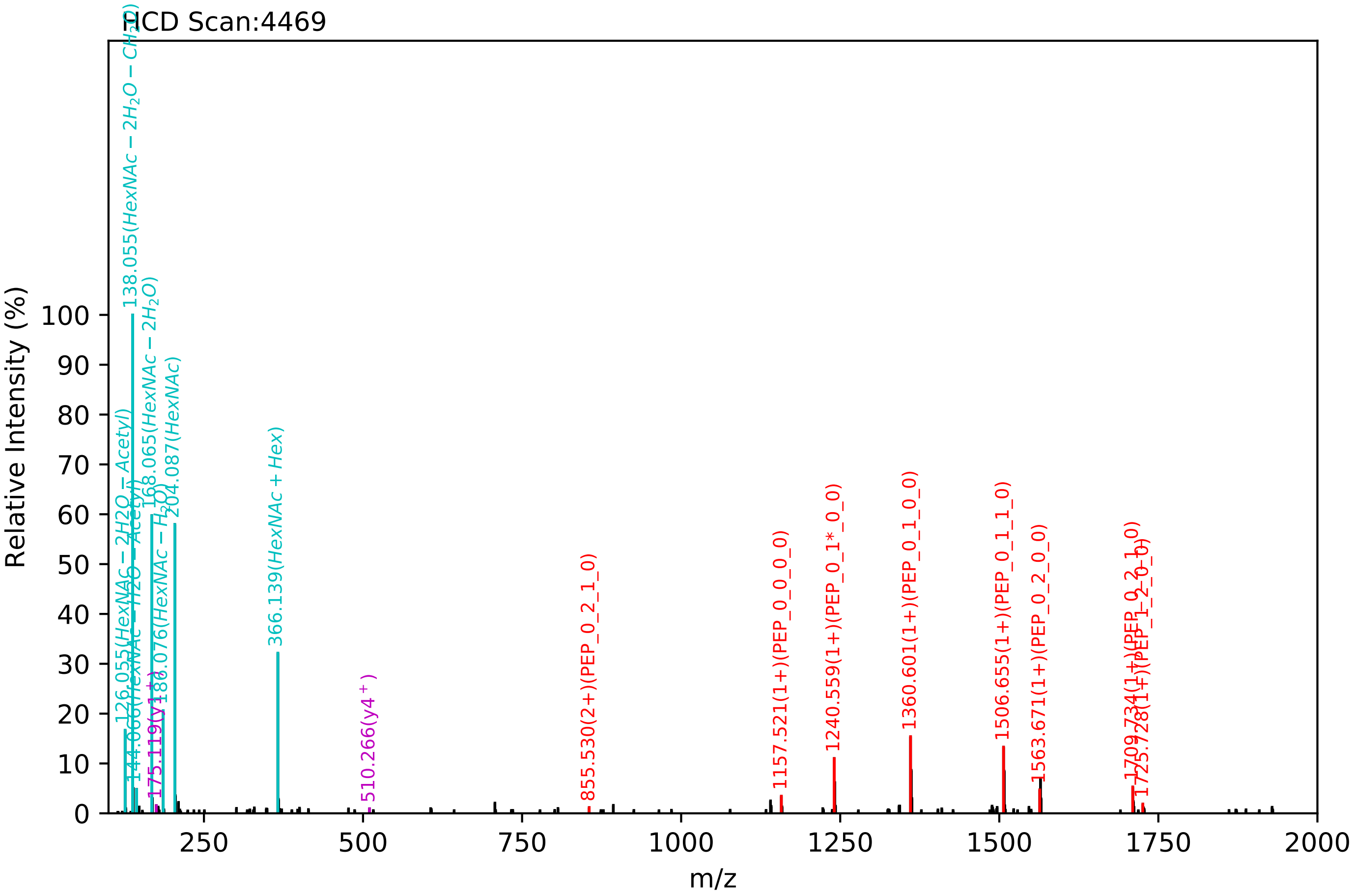

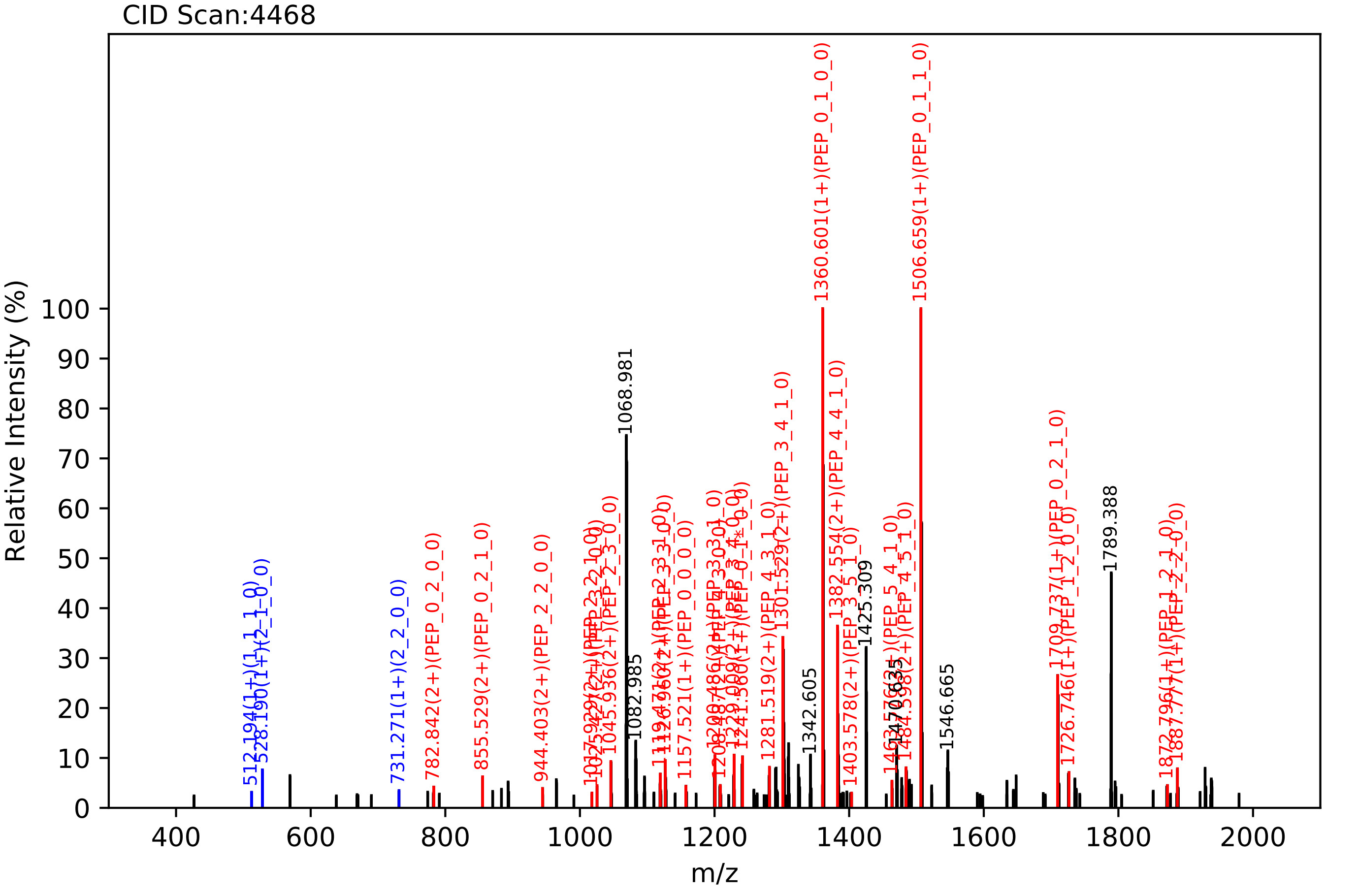

Training set no. 429, Experiment: IgG exp_3

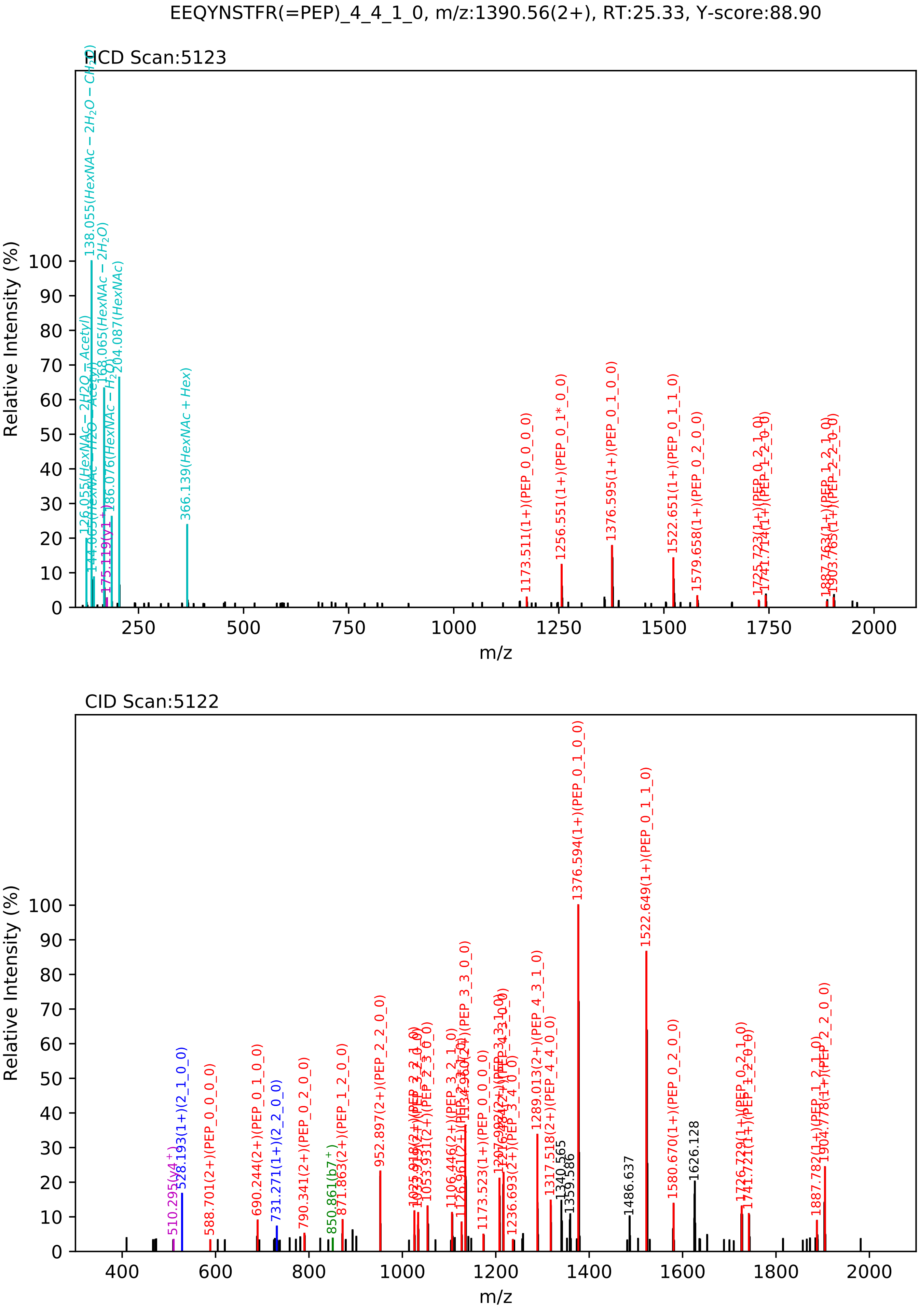

Training set no. 430, Experiment: IgG exp_3

EEQFNSTYR(=PEP)_3_4_1_0, m/z:1309.53(2+), RT:25.35, Y-score:89.31

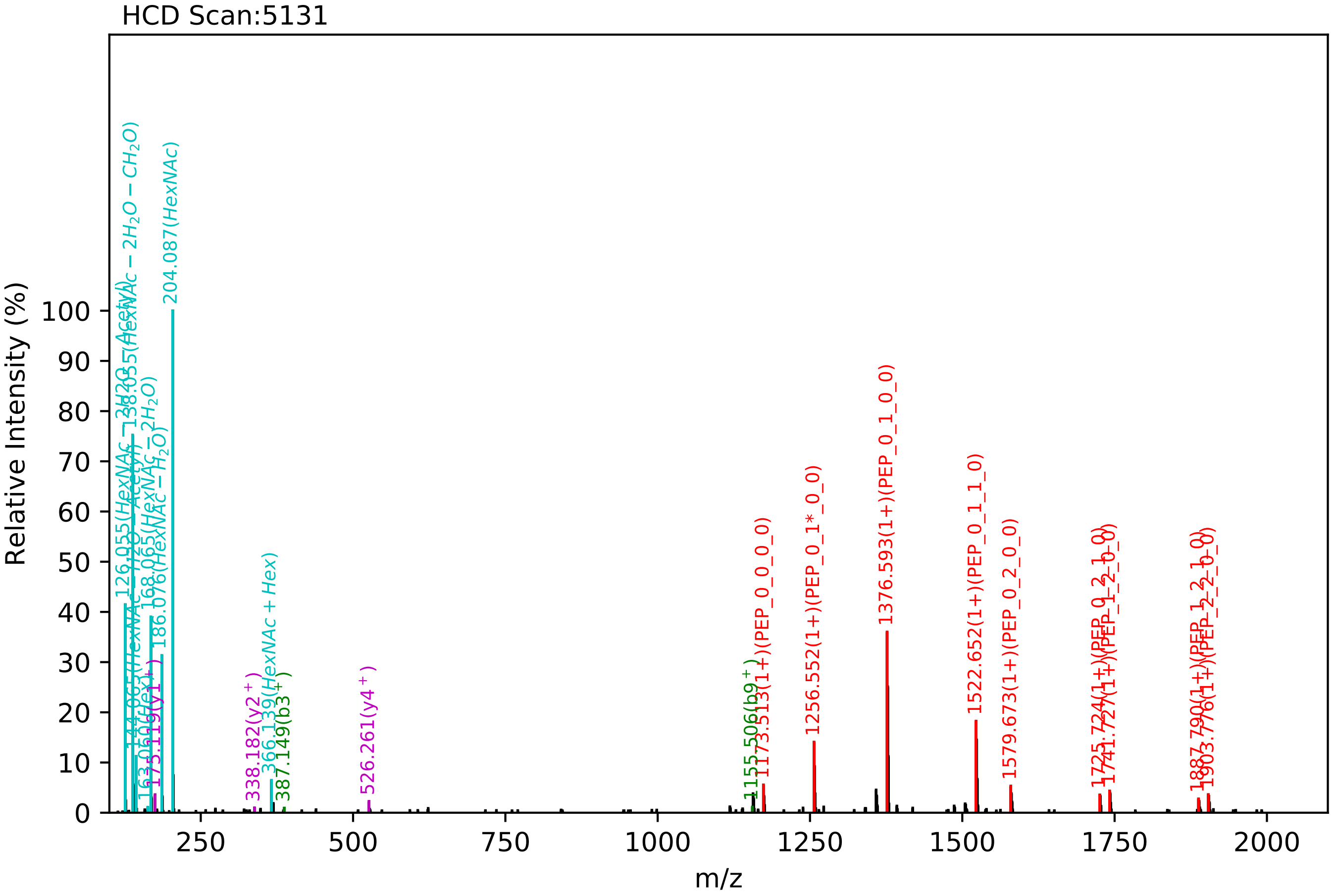

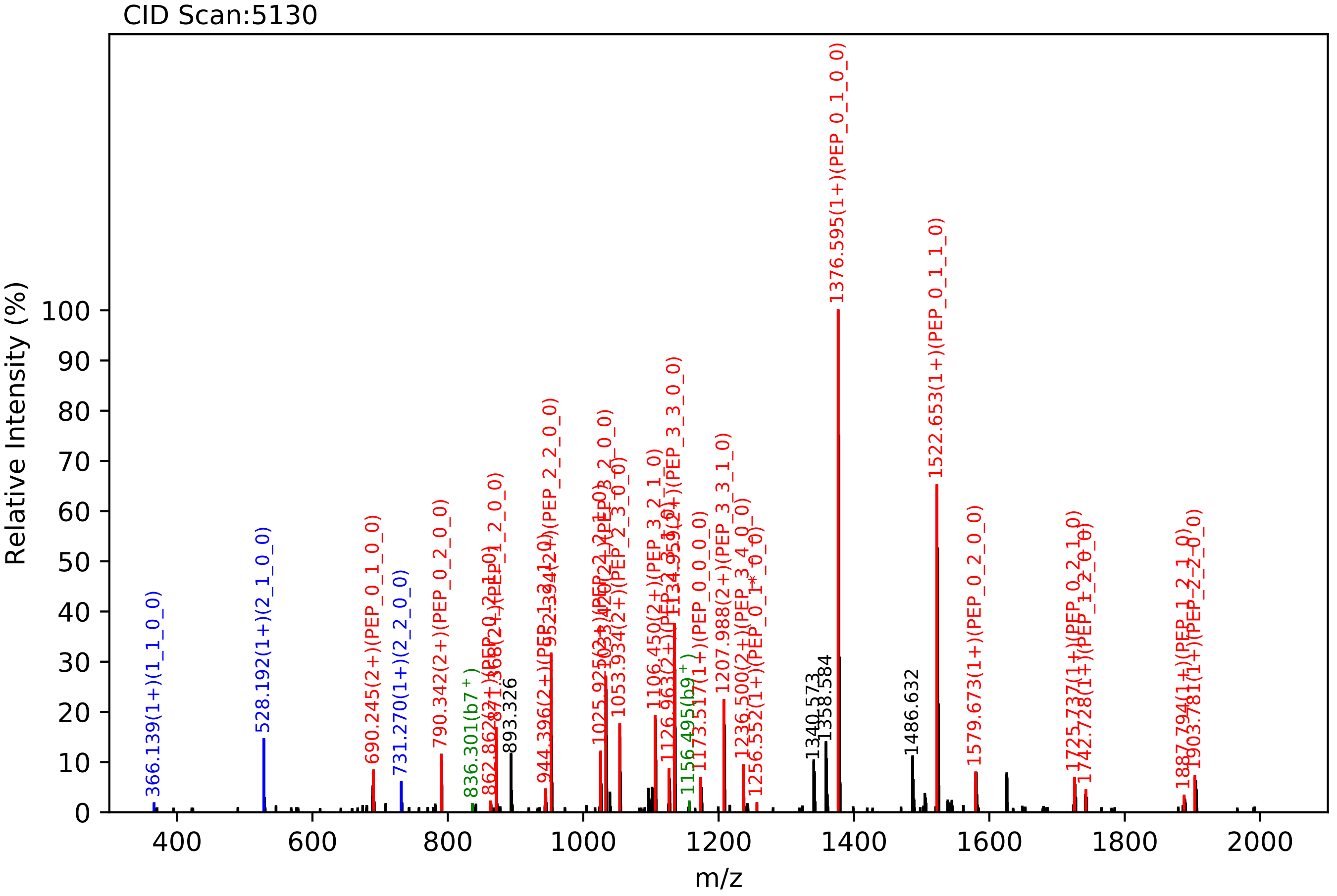

Training set no. 431, Experiment: IgG exp_1

EEQFNSTYR(=PEP)_3_4_1_0, m/z:1309.53(2+), RT:23.32, Y-score:88.19

HCD Scan:3740

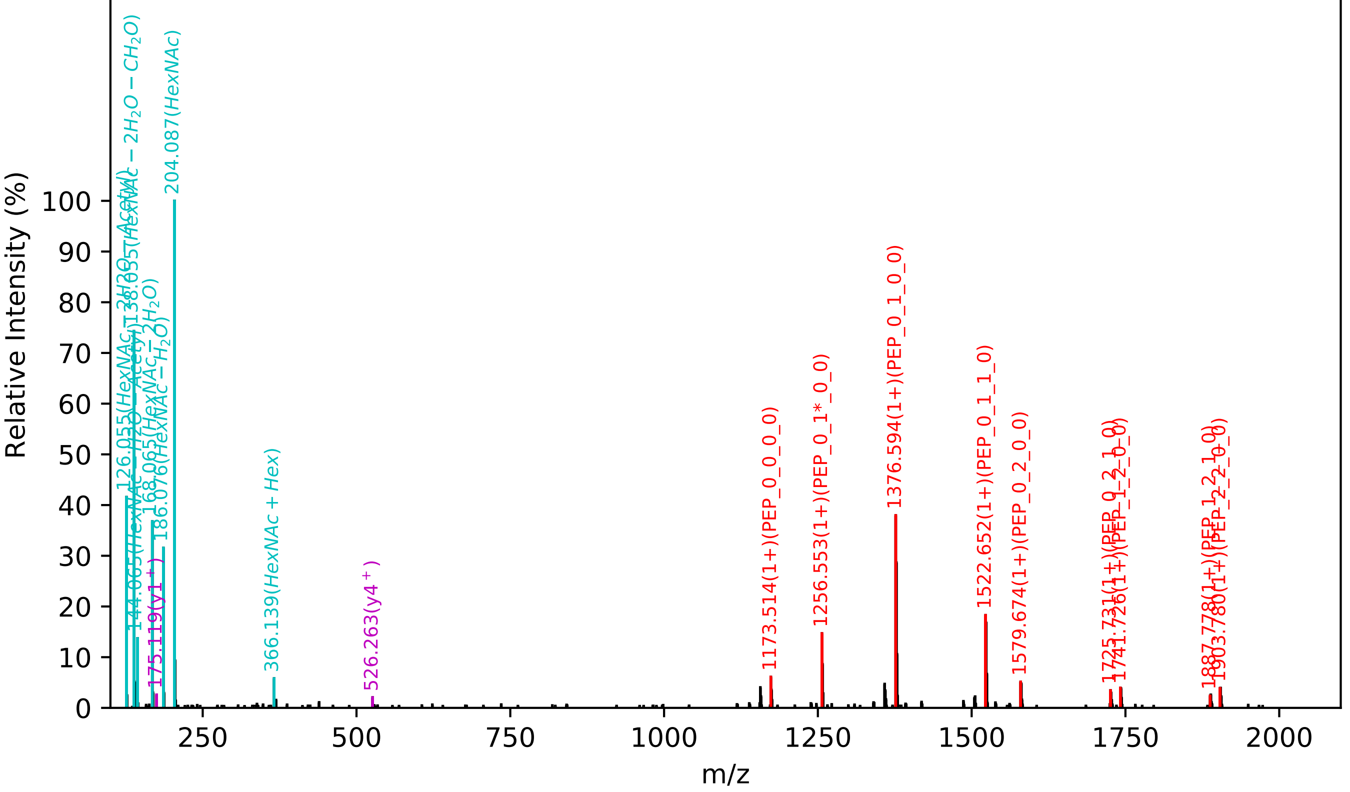

CID Scan:3739

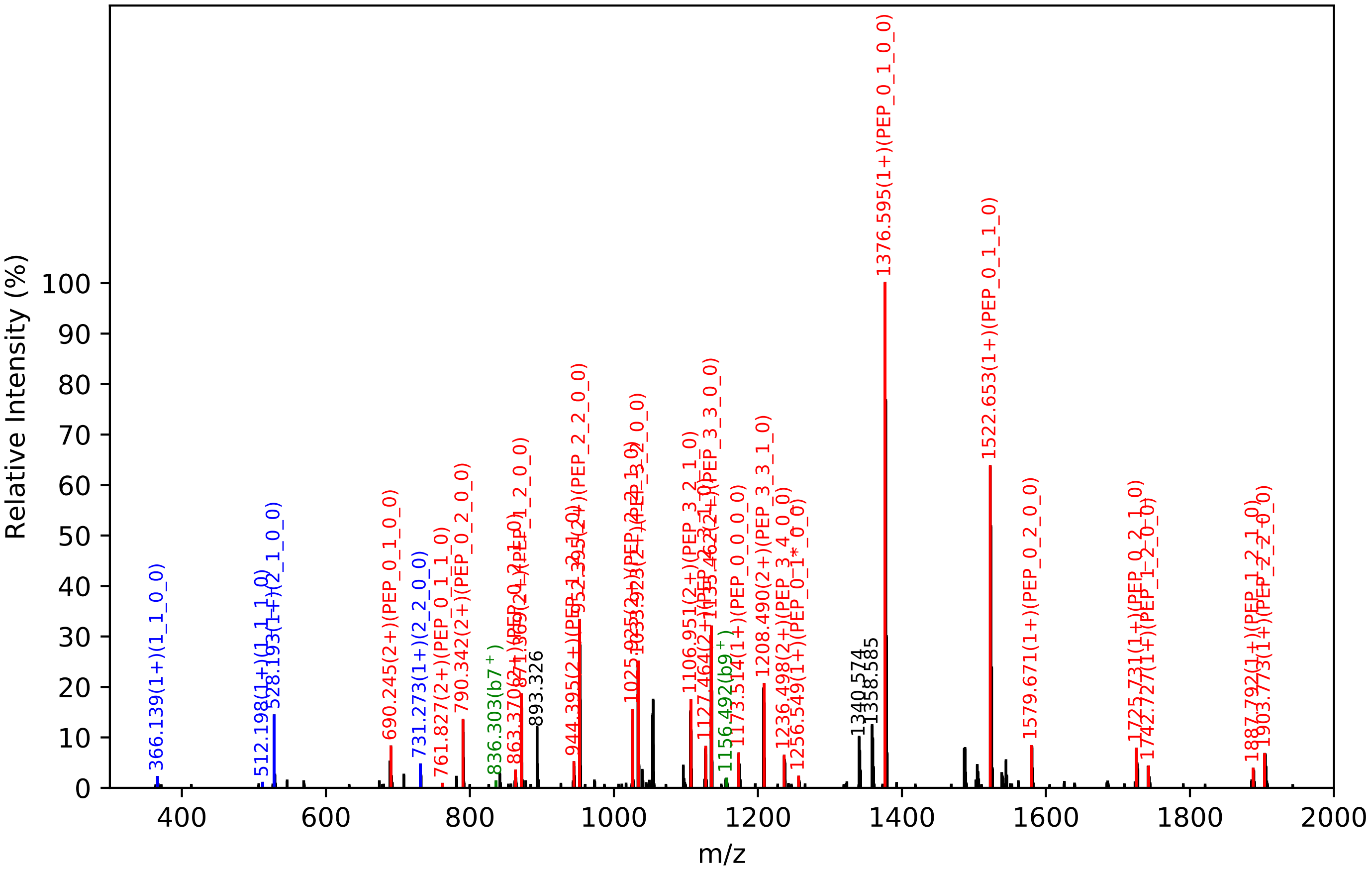

Training set no. 432, Experiment: IgG exp_4

EEQFNSTYR(=PEP)_3_4_1_0, m/z:1309.53(2+), RT:22.30, Y-score:77.12

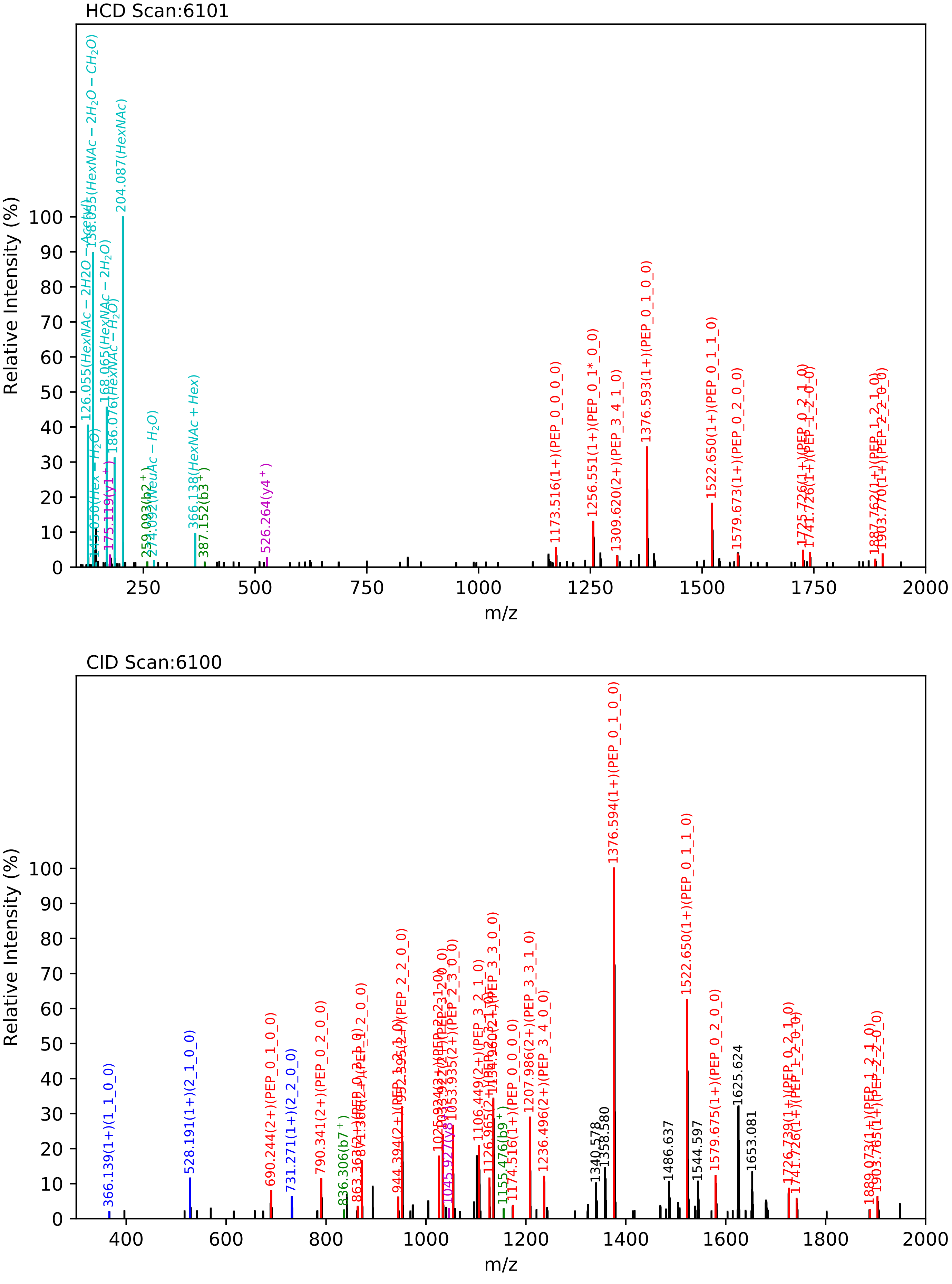

Training set no. 433, Experiment: IgG exp_1

EEQFNSTYR(=PEP)_4_4_1_0, m/z:1390.56(2+), RT:23.27, Y-score:87.86

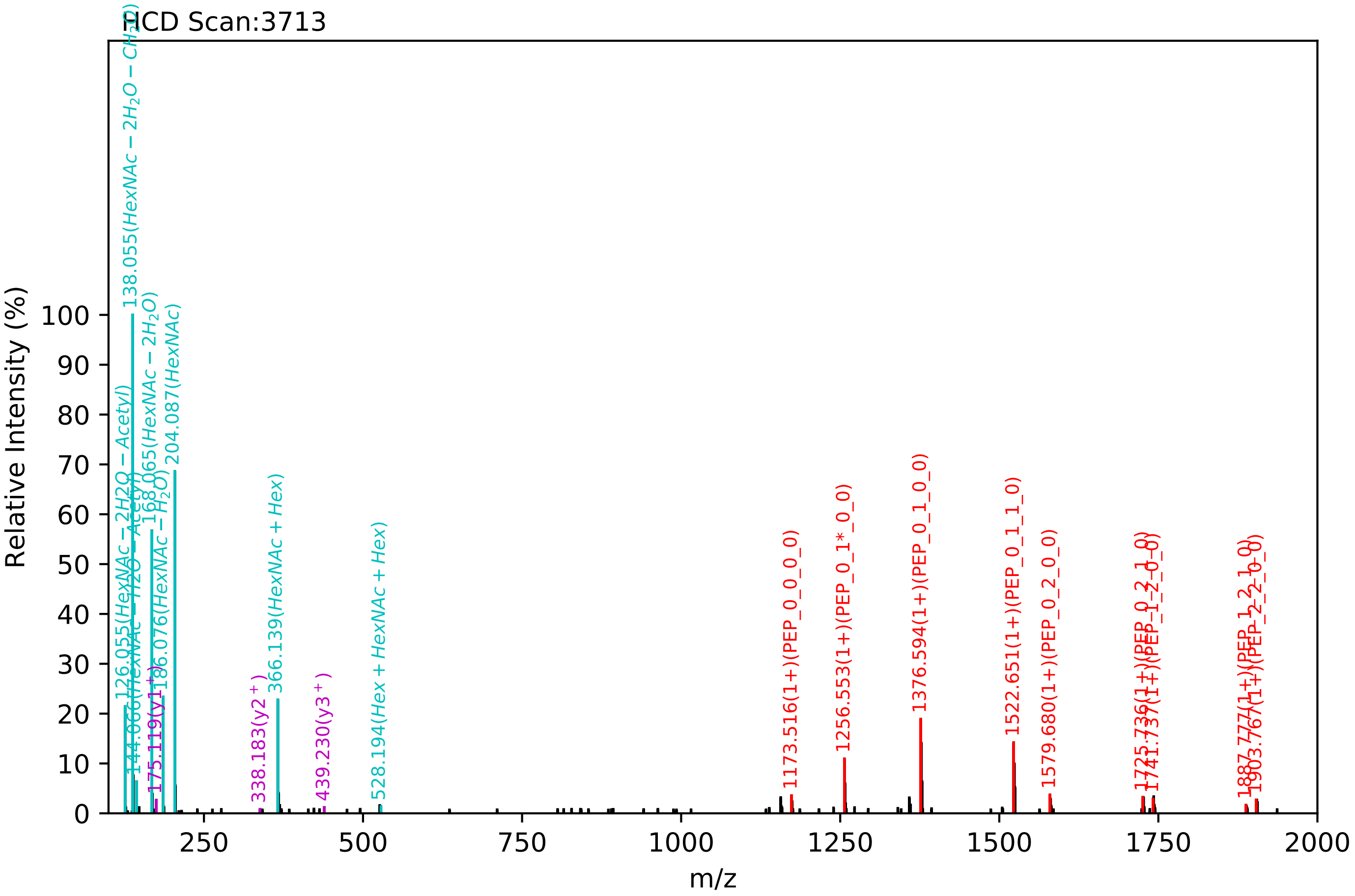

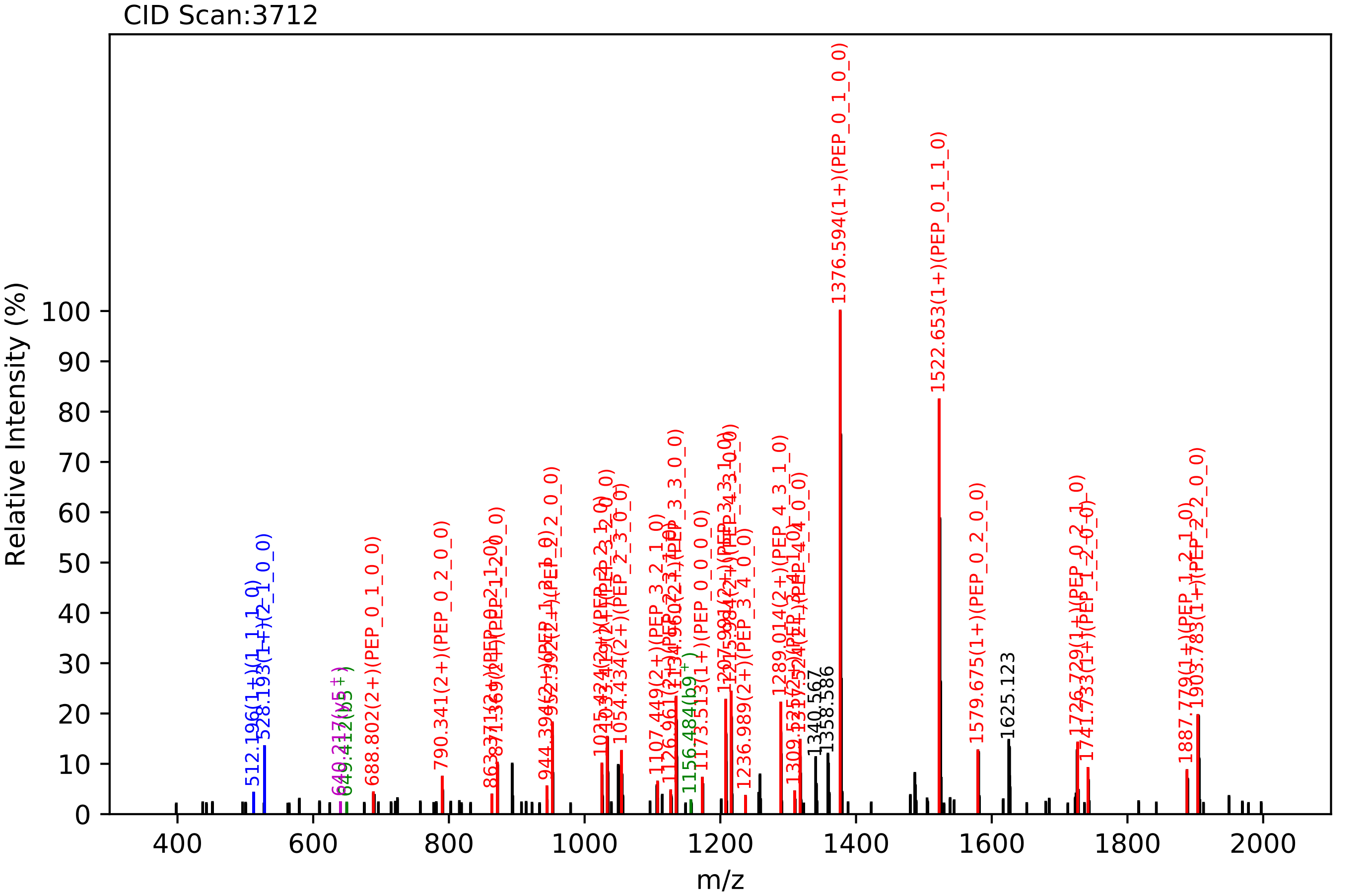

Supplementary Figure 3. MS/MS spectra of N-glycopeptides identified from alpha-1-acid glycoprotein and immunoglobulin gamma for test set.

Test set no. 1, Experiment: AGP exp_3

ENGTISR(=PEP)_4_4_0_1, m/z:1264.51(2+), RT:16.69, Y-score:82.52

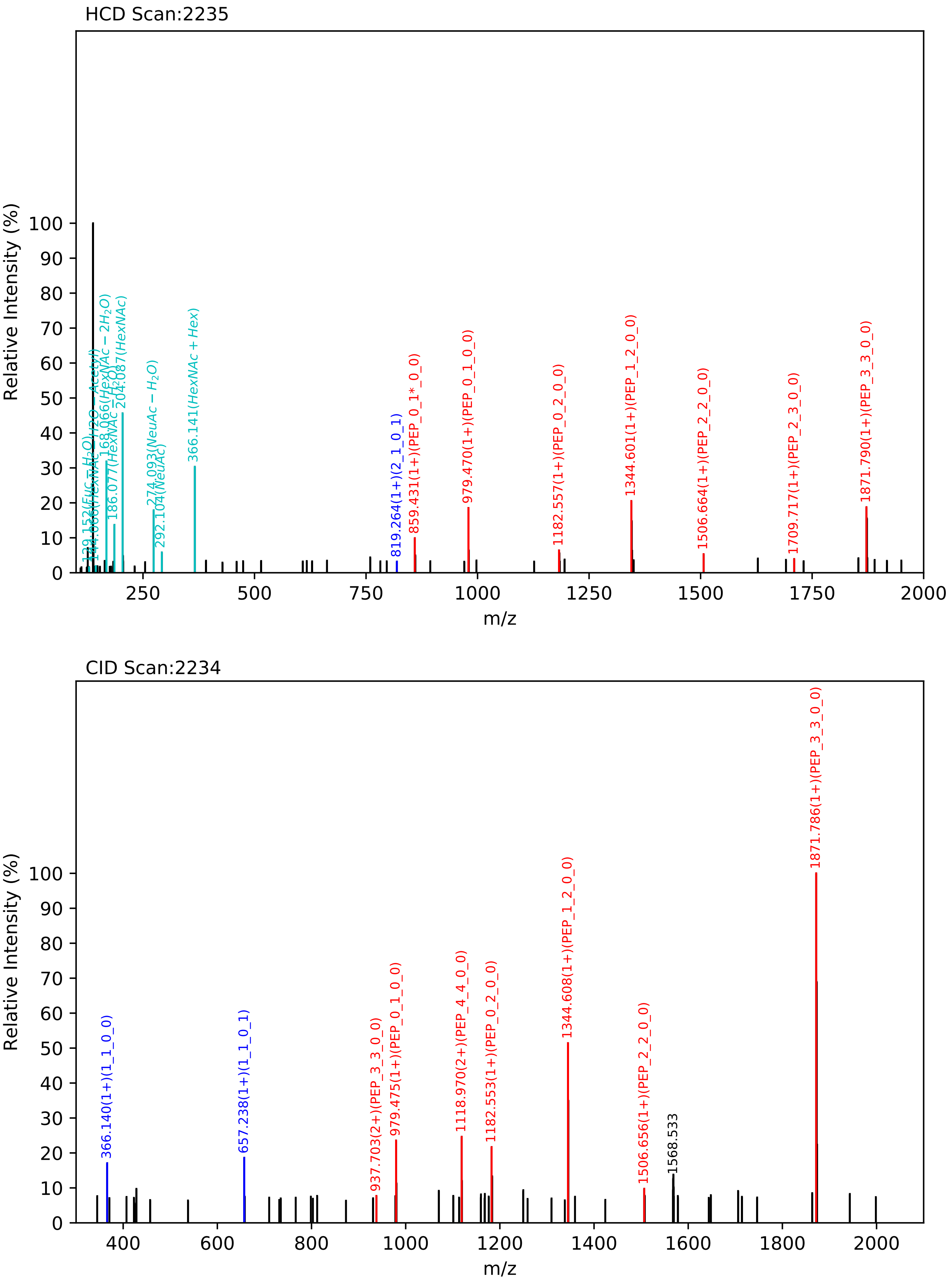

Test set no. 2, Experiment: AGP exp_4

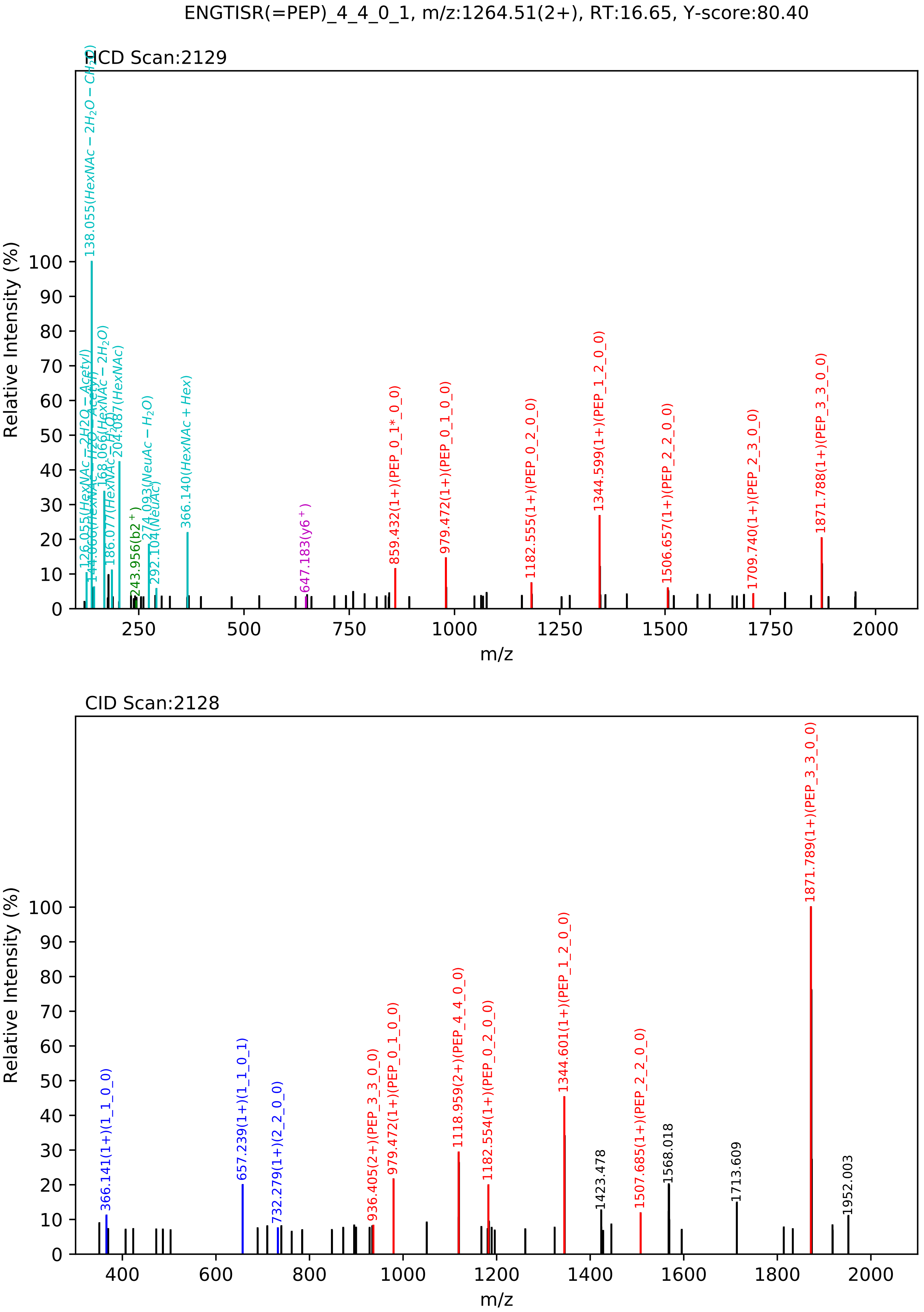

Test set no. 3, Experiment: AGP exp_3

ENGTISR(=PEP)_5_4_0_2, m/z:994.39(3+), RT:20.45, Y-score:89.73

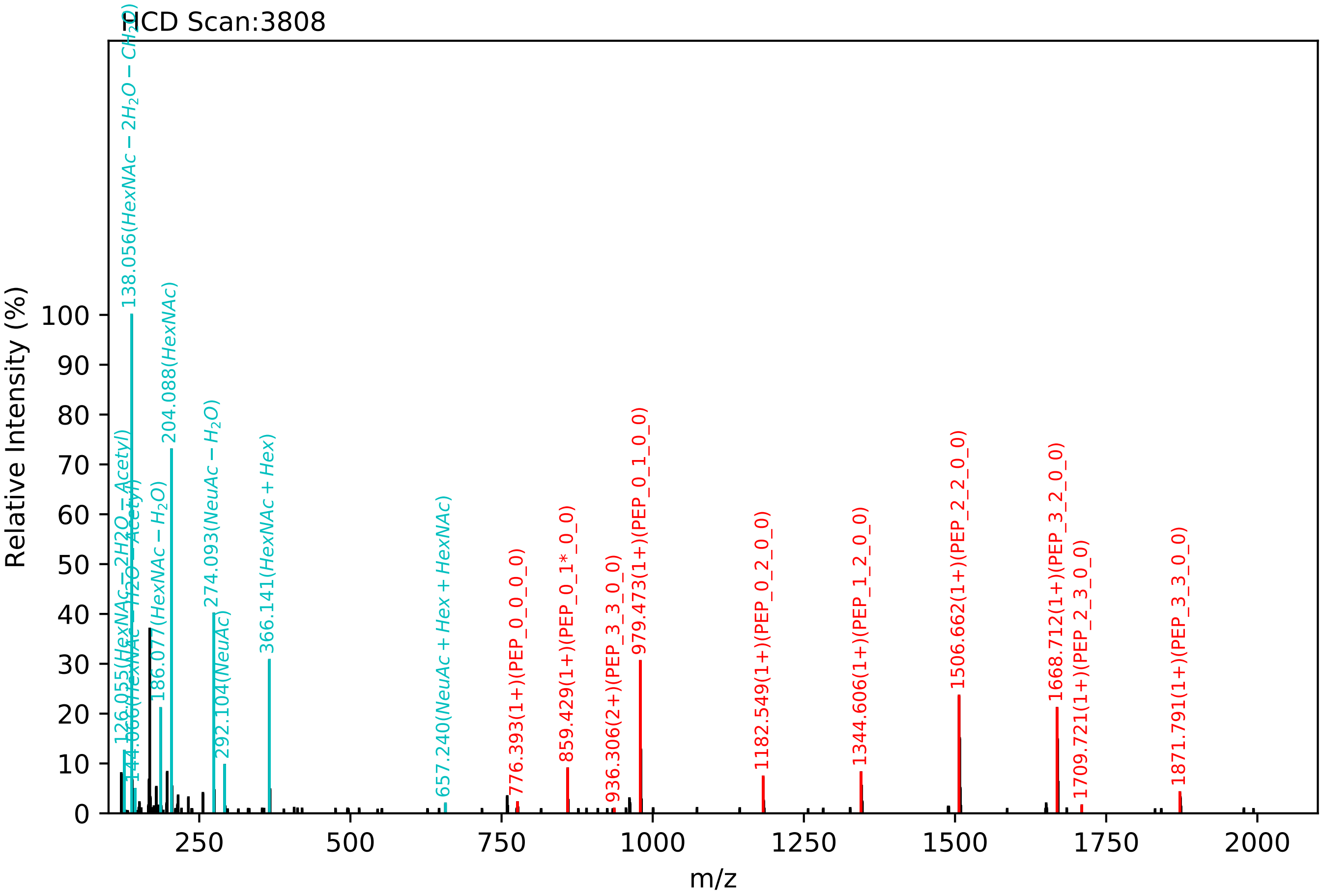

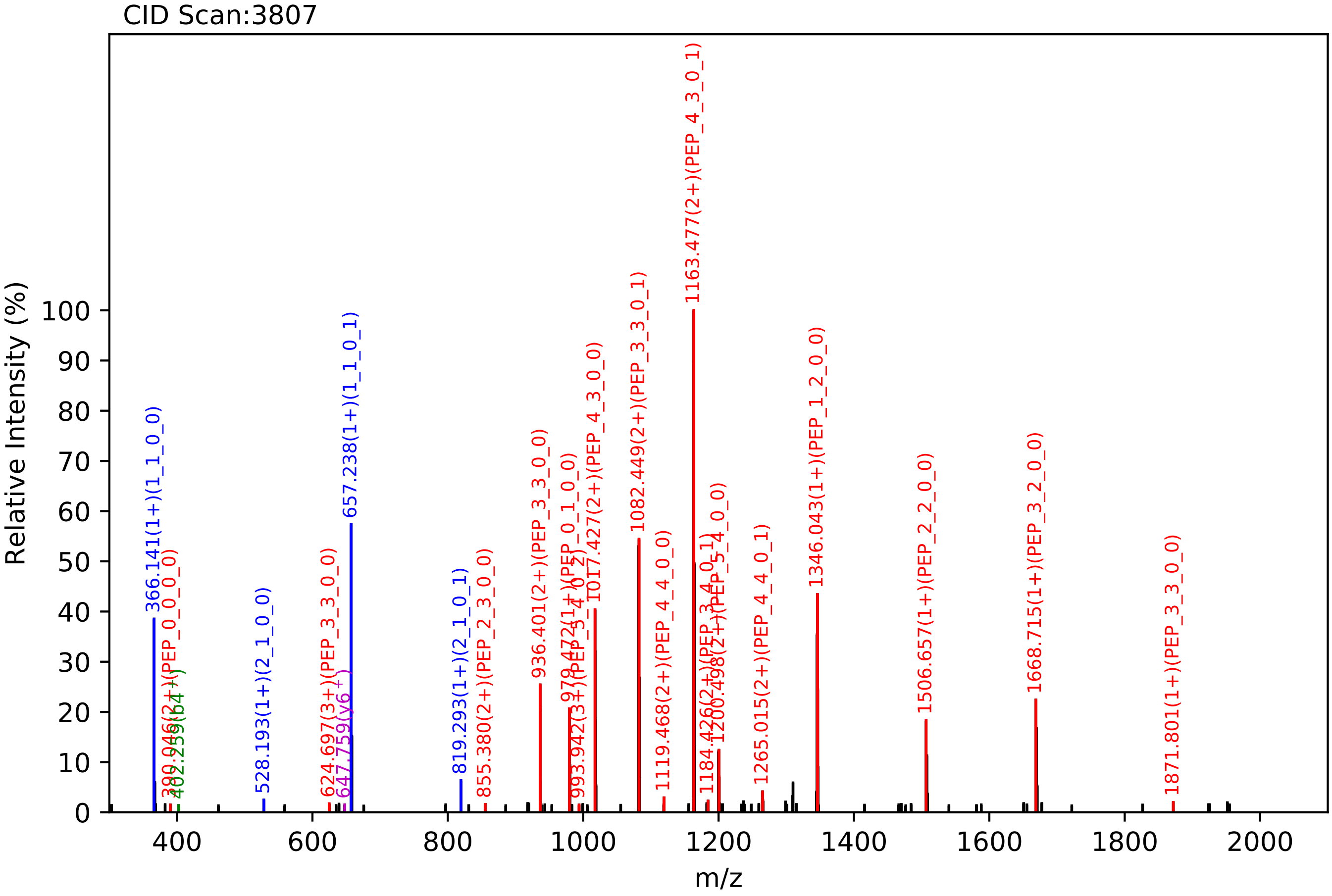

Test set no. 4, Experiment: AGP exp_4

ENGTISR(=PEP)_5_4_0_2, m/z:1491.08(2+), RT:20.51, Y-score:85.93

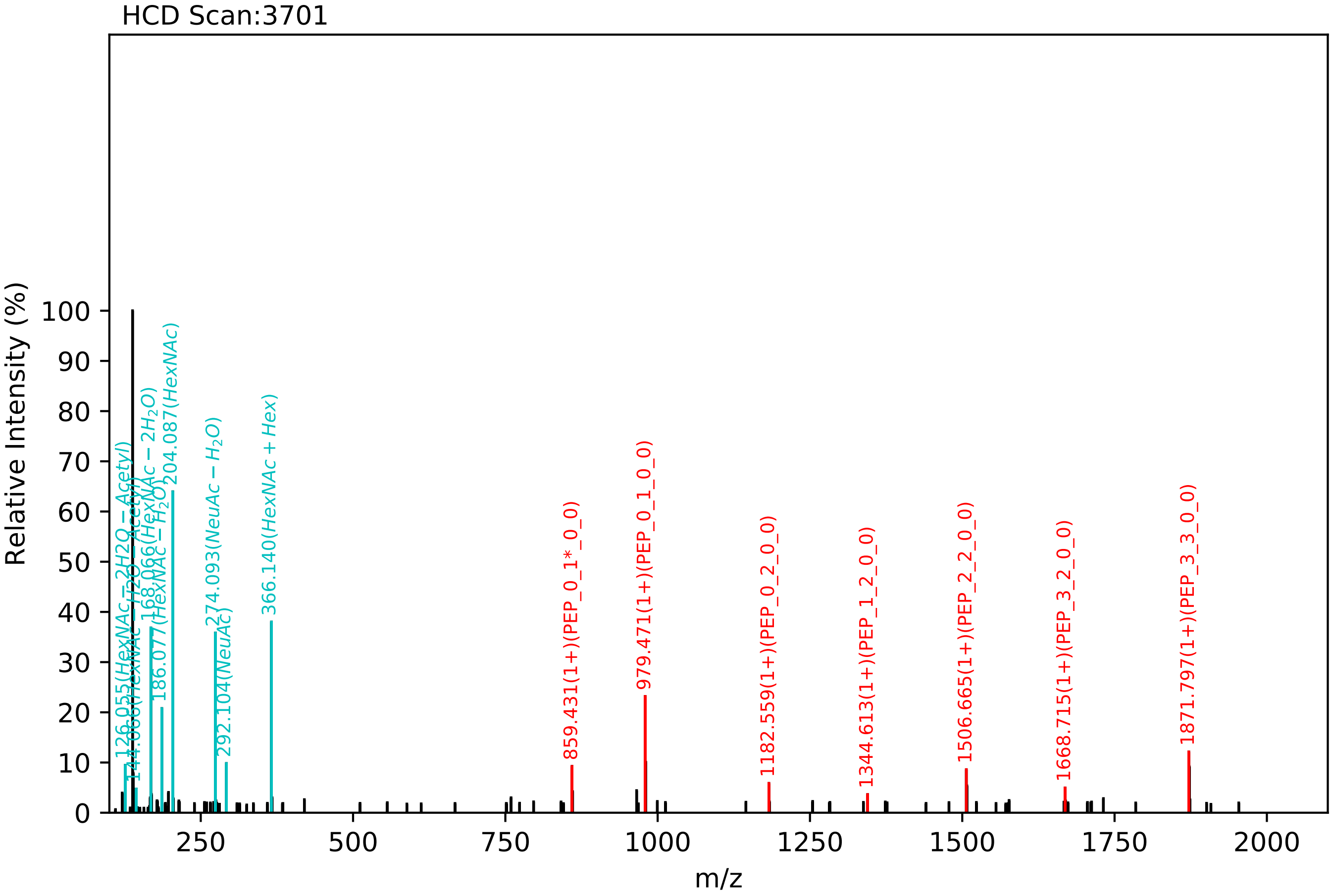

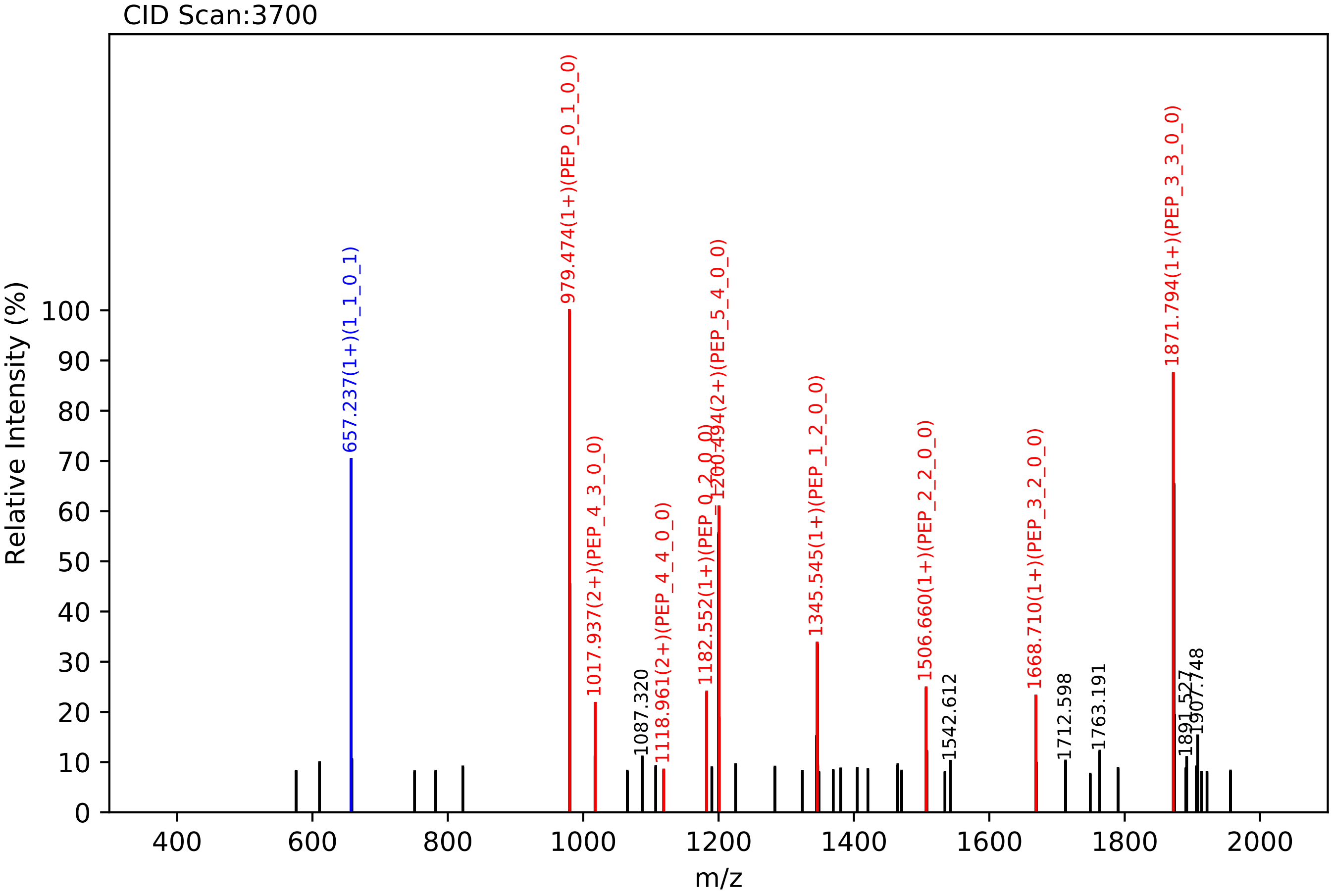

Test set no. 5, Experiment: AGP exp_3

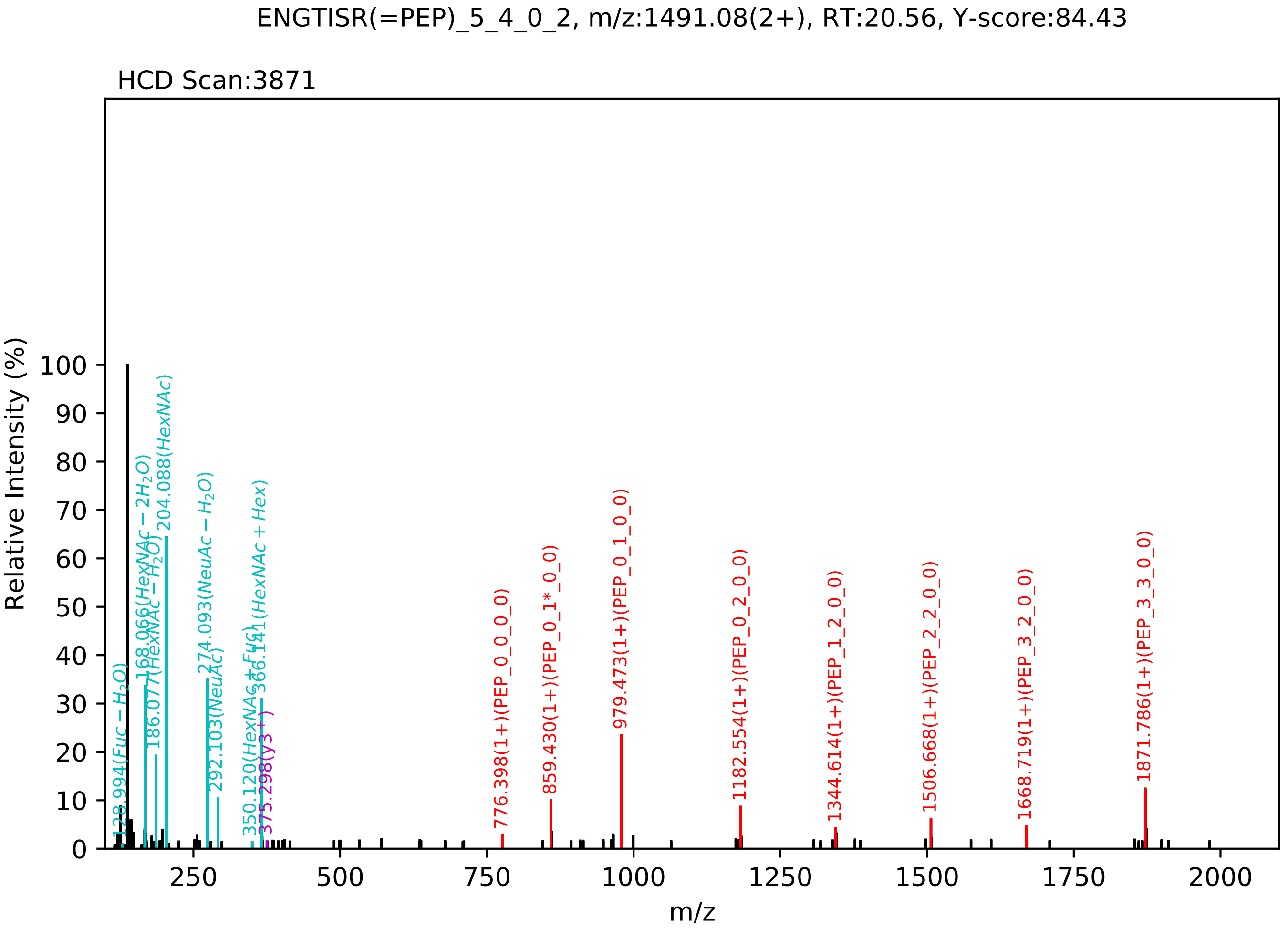

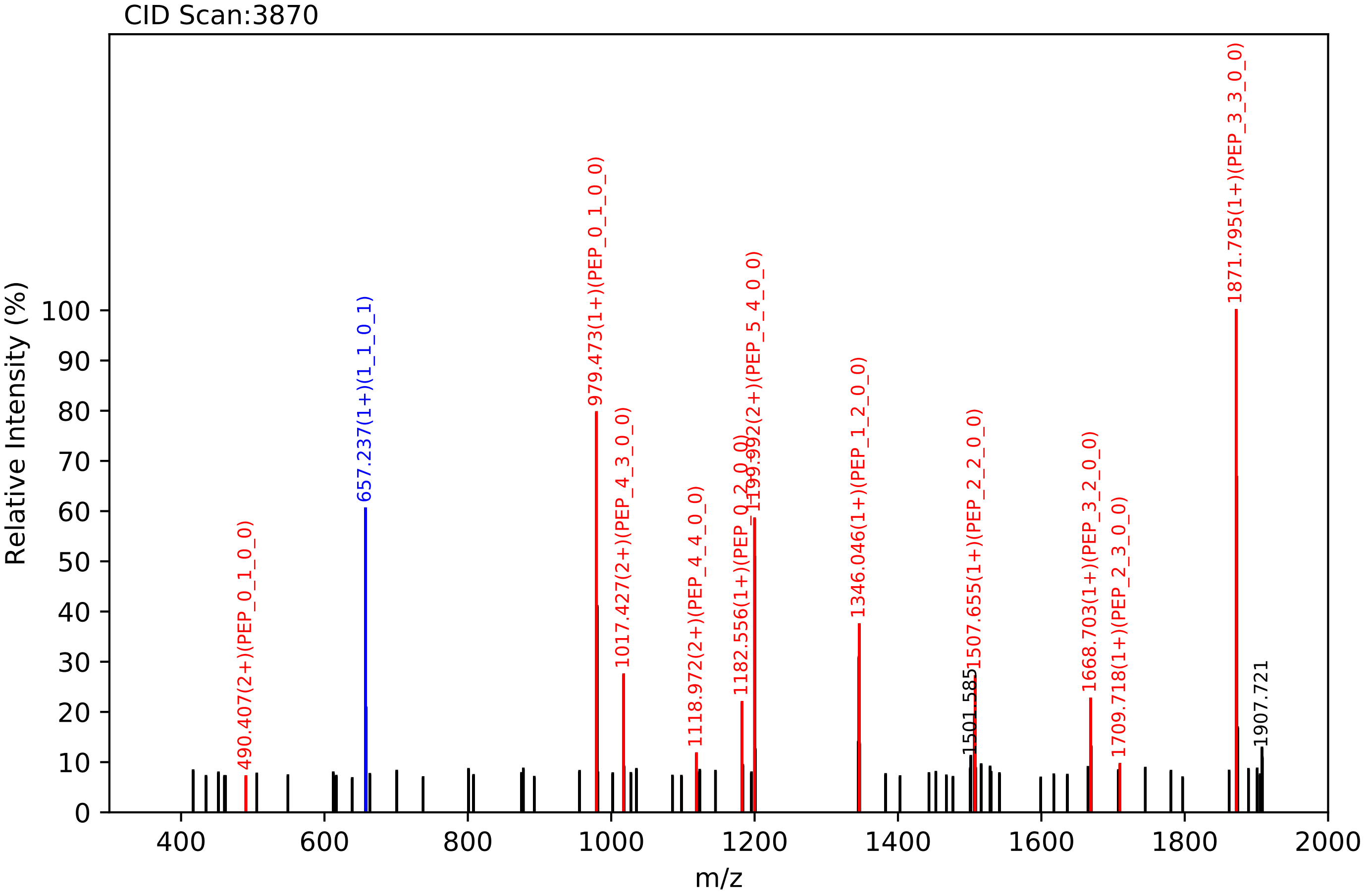

Test set no. 6, Experiment: AGP exp_4

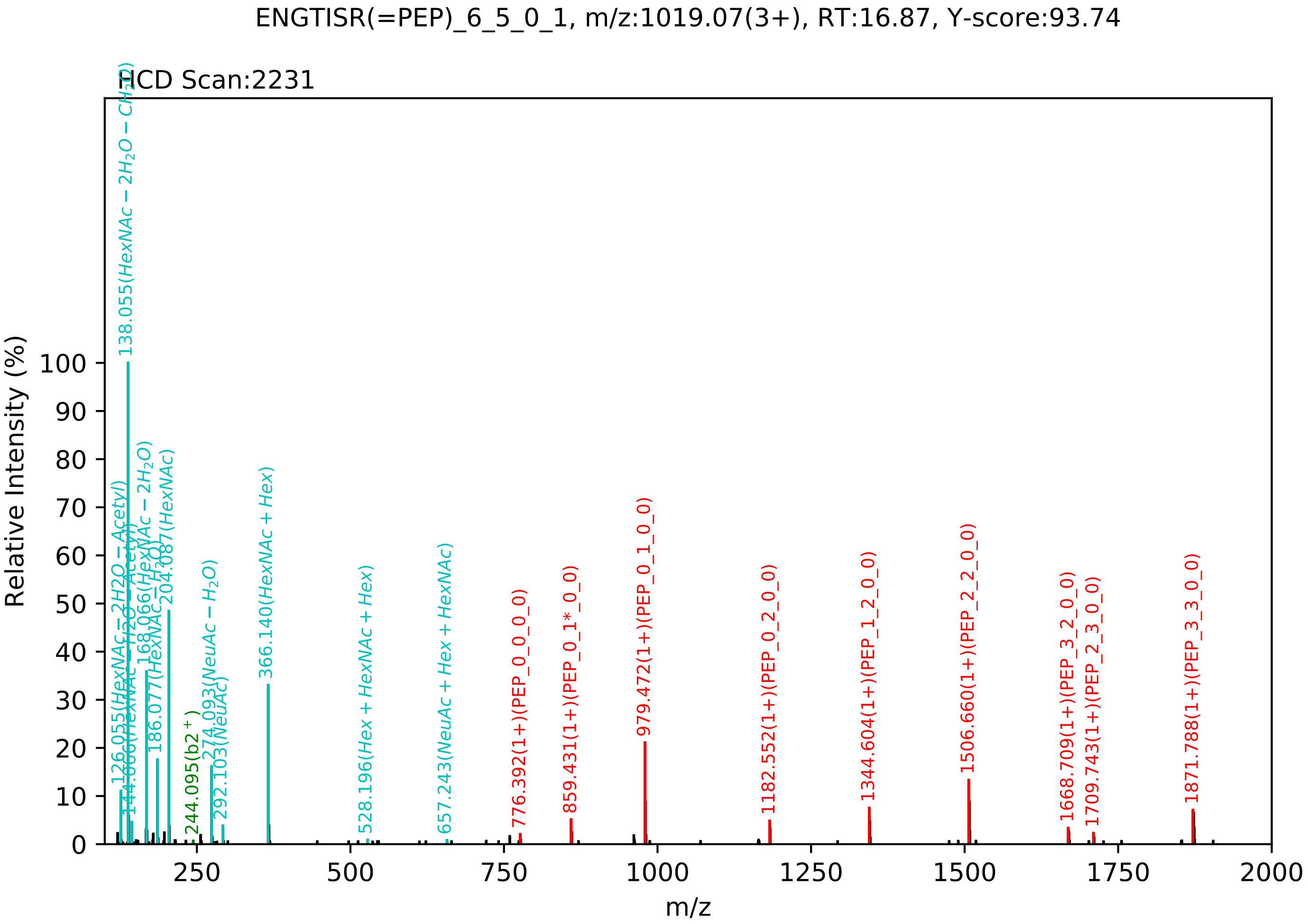

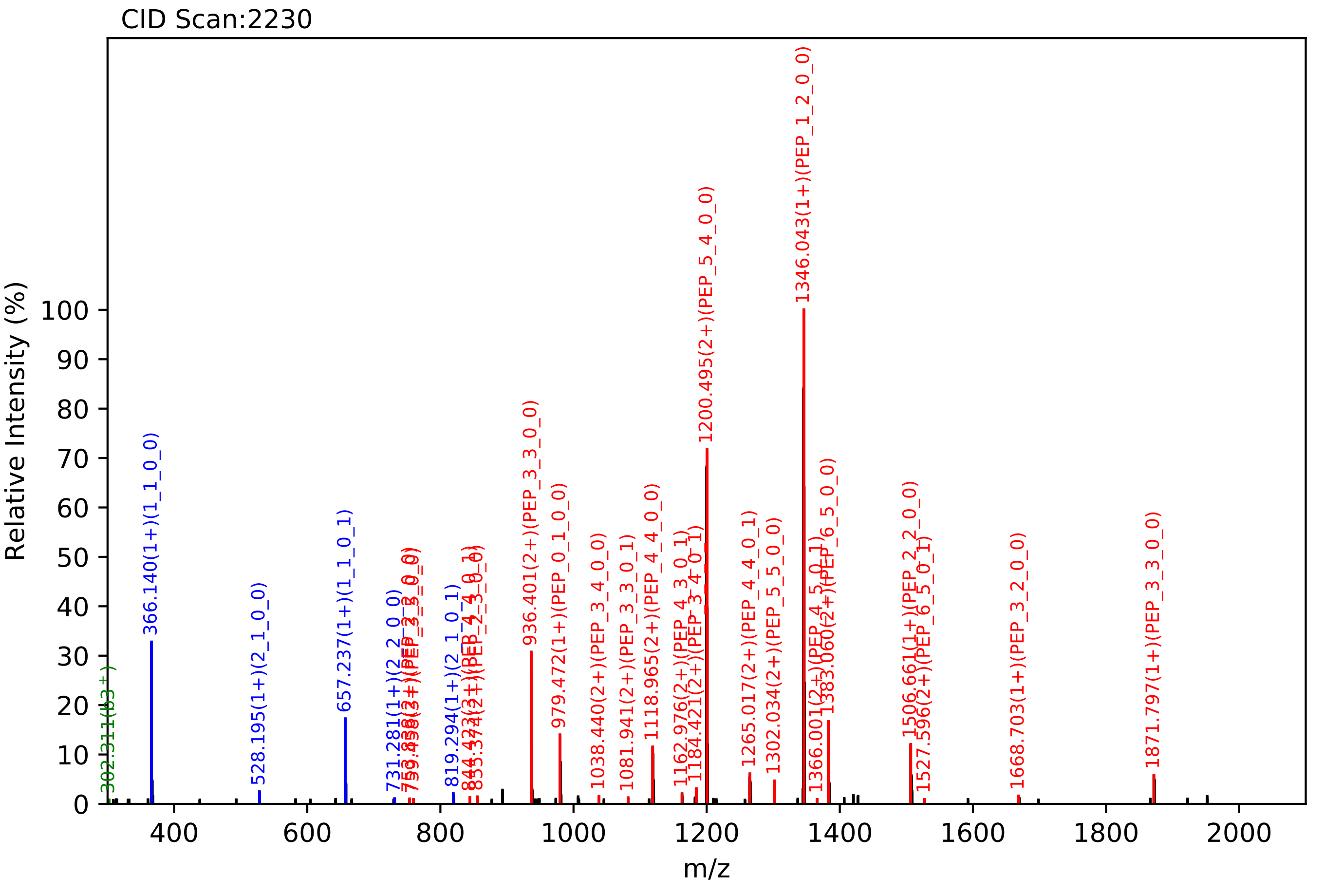

Test set no. 7, Experiment: AGP exp_3

ENGTISR(=PEP)_6_5_0_1, m/z:1019.07(3+), RT:17.88, Y-score:93.50

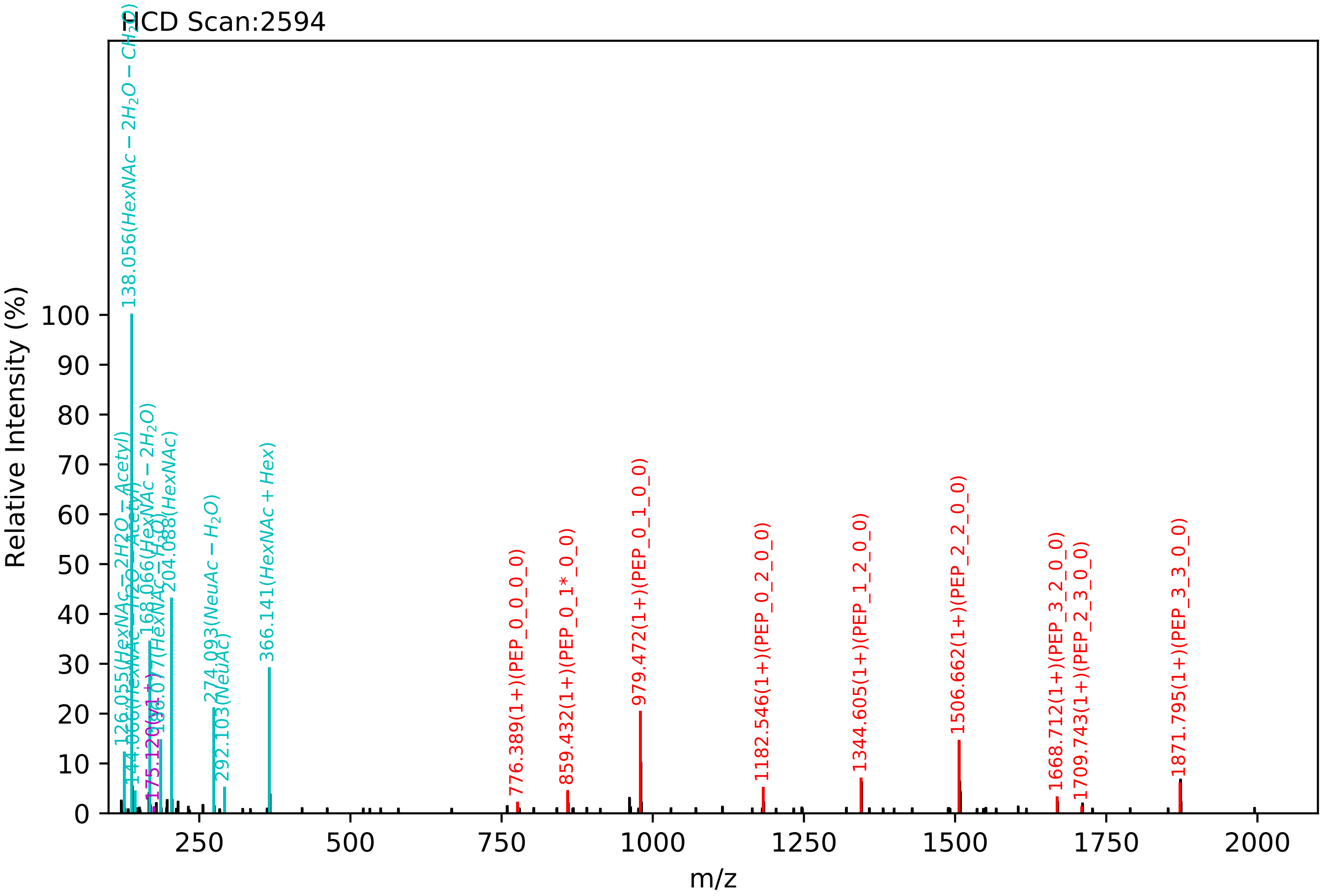

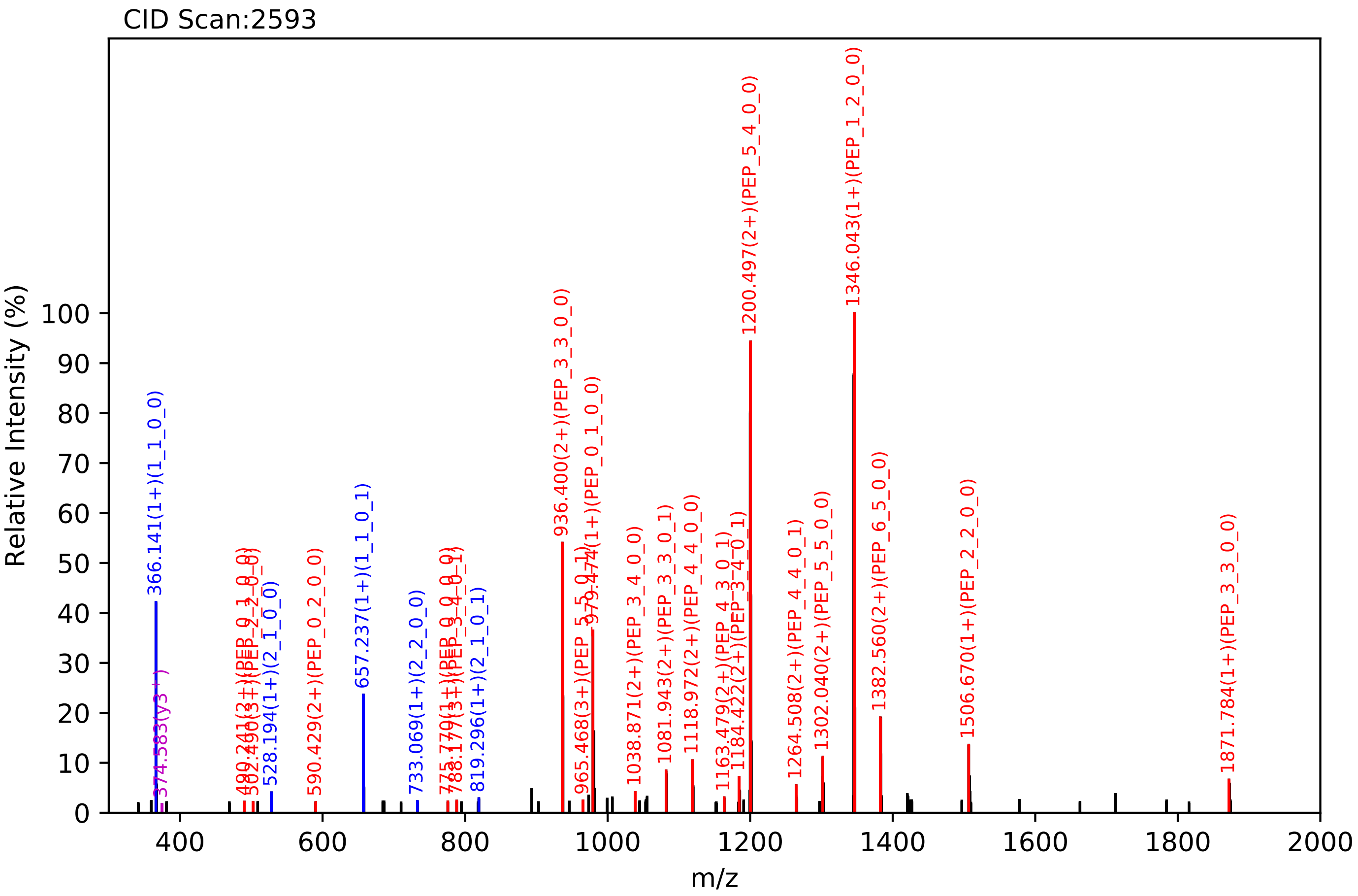

Test set no. 8, Experiment: AGP exp_4

ENGTISR(=PEP)_6_5_0_1, m/z:1019.07(3+), RT:17.89, Y-score:91.98

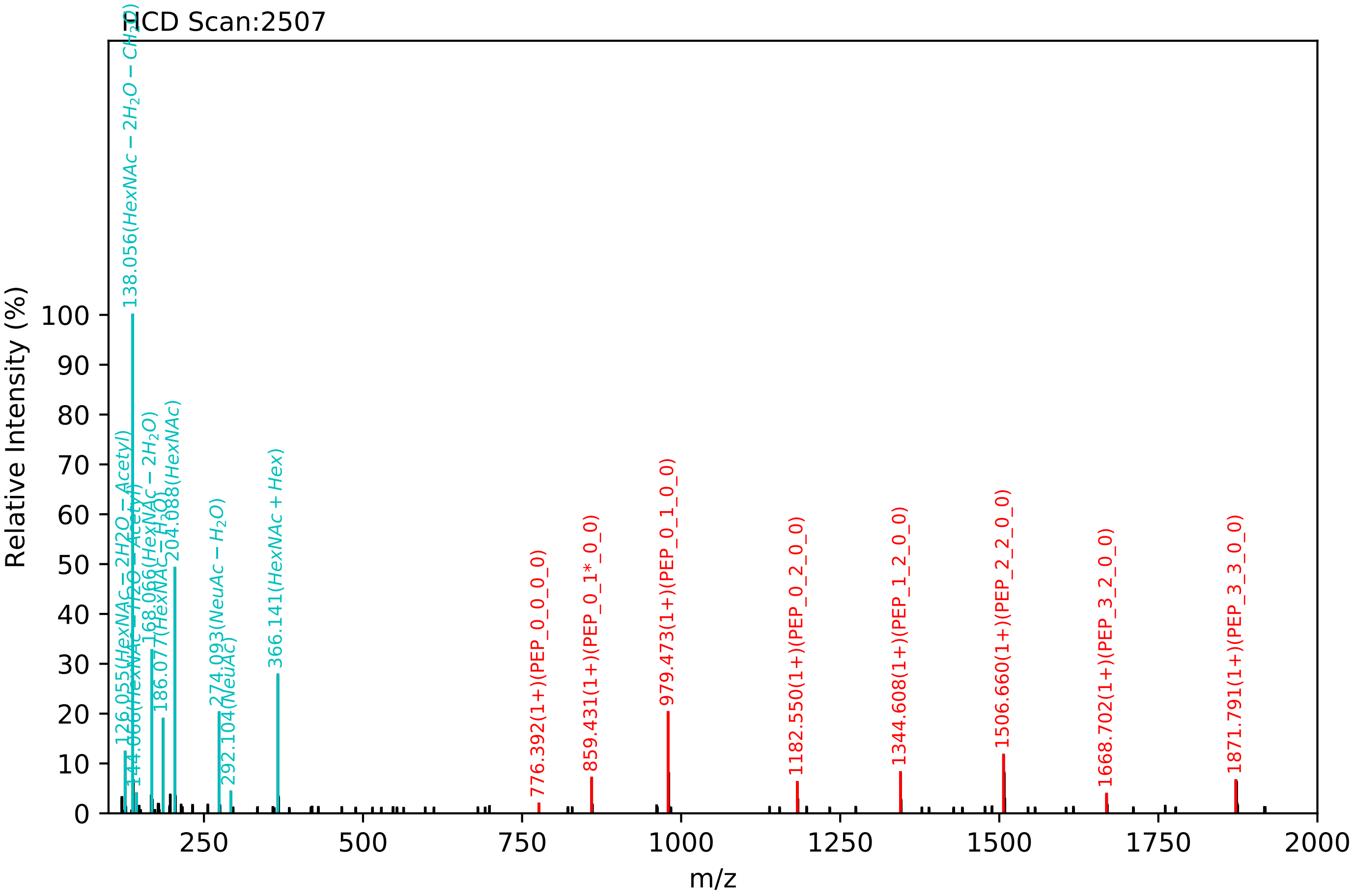

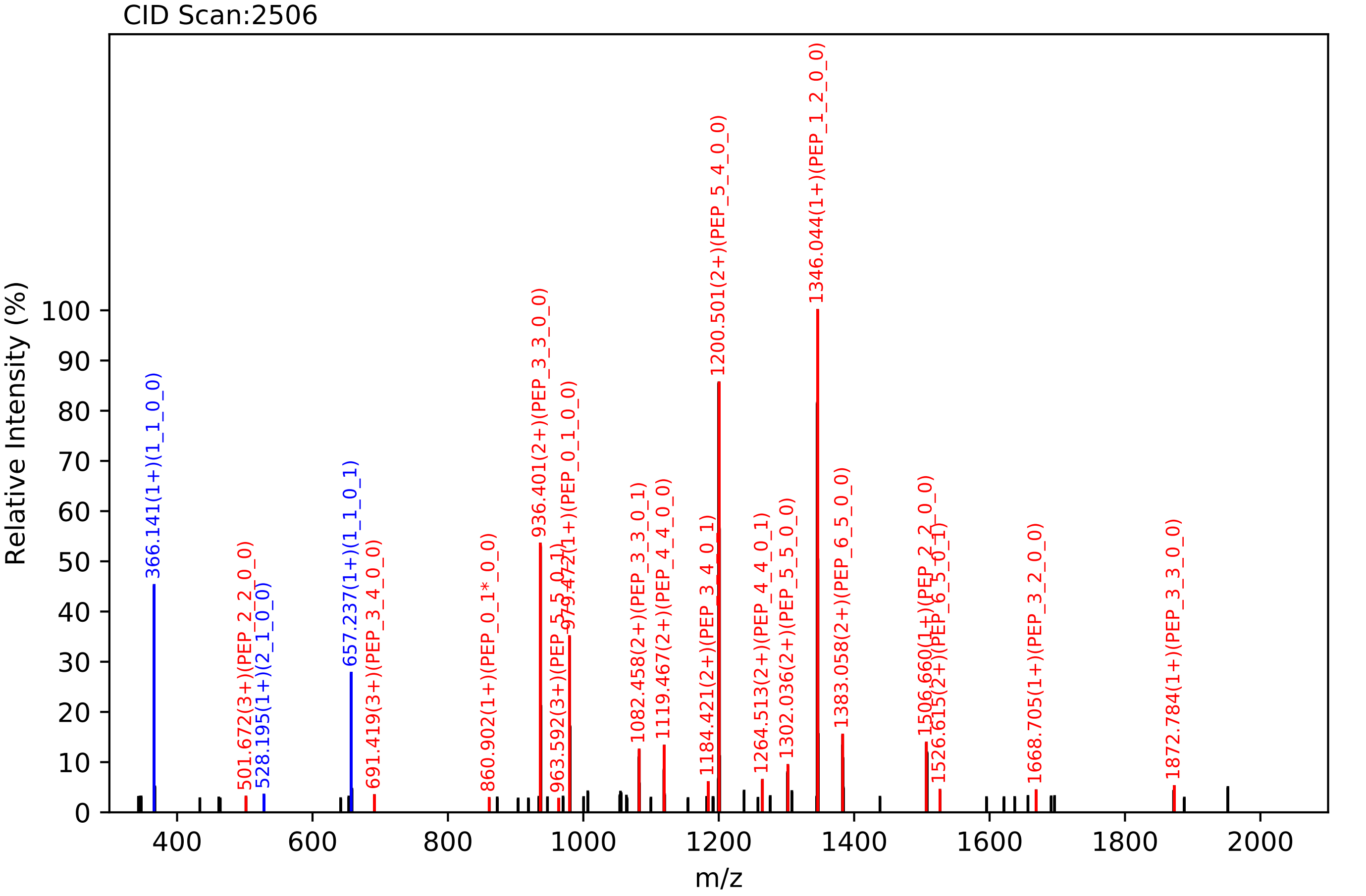

Test set no. 9, Experiment: AGP exp_3

ENGTISR(=PEP)_6_5_0_2, m/z:1116.10(3+), RT:20.68, Y-score:94.68

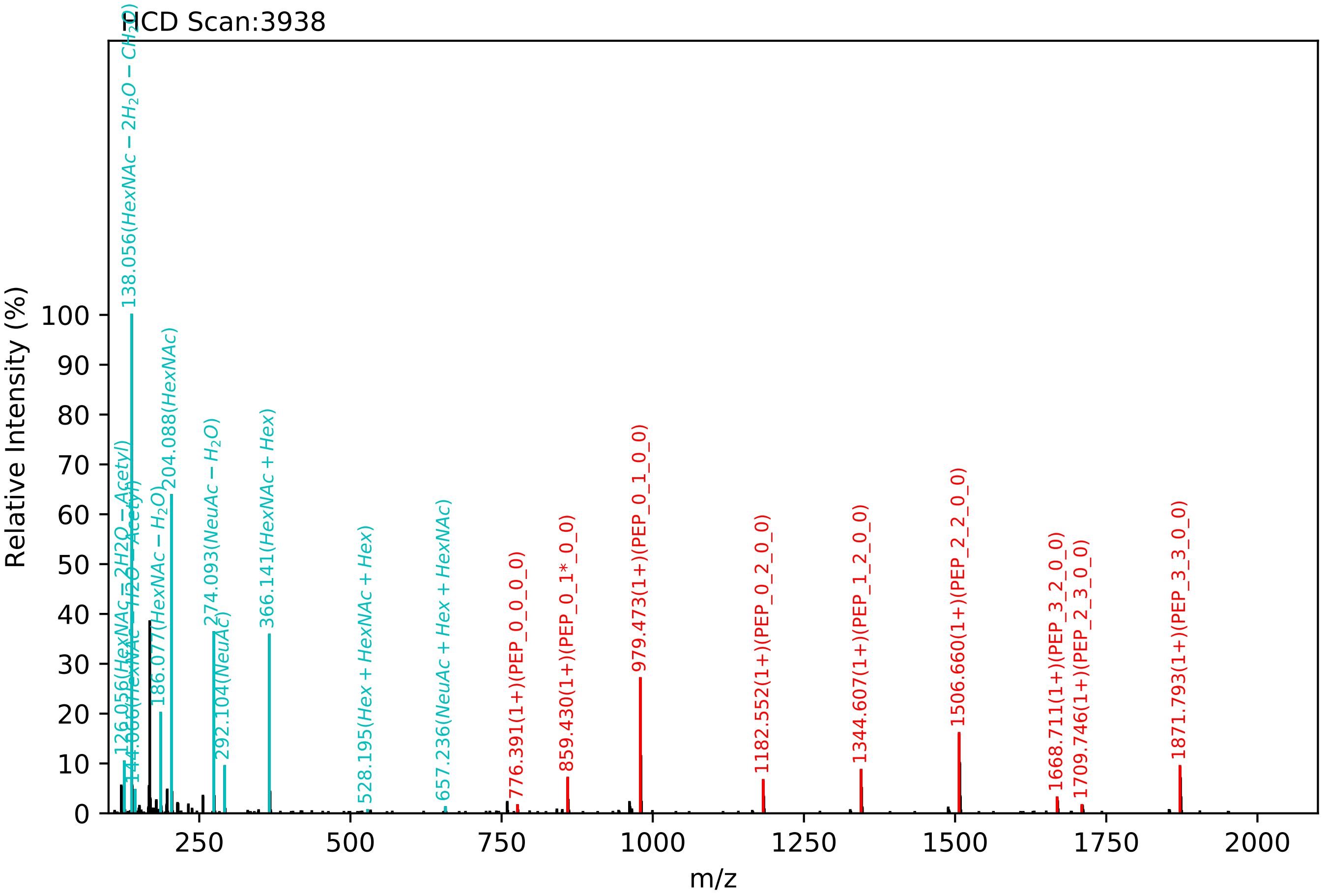

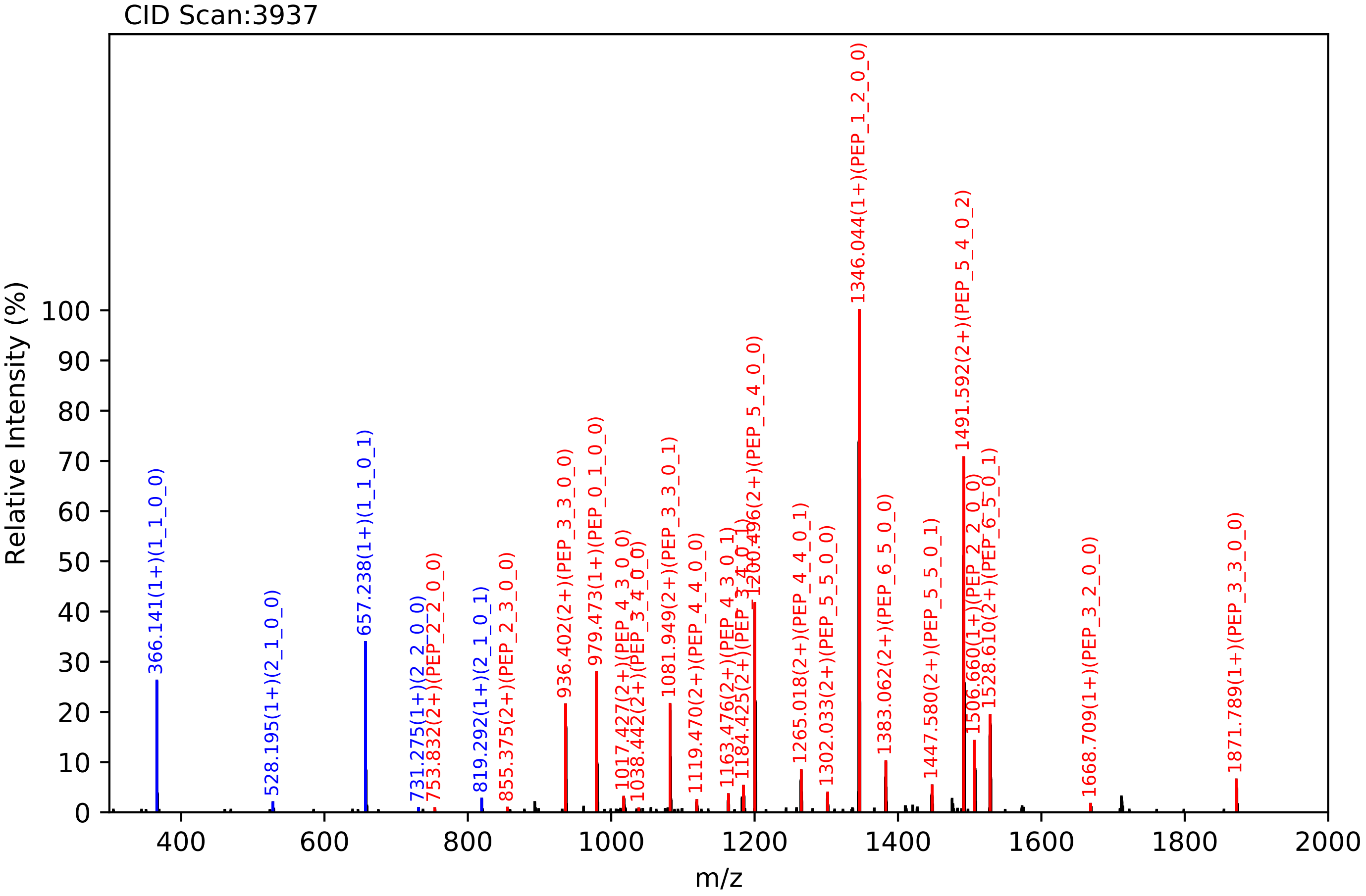

Test set no. 10, Experiment: AGP exp_3

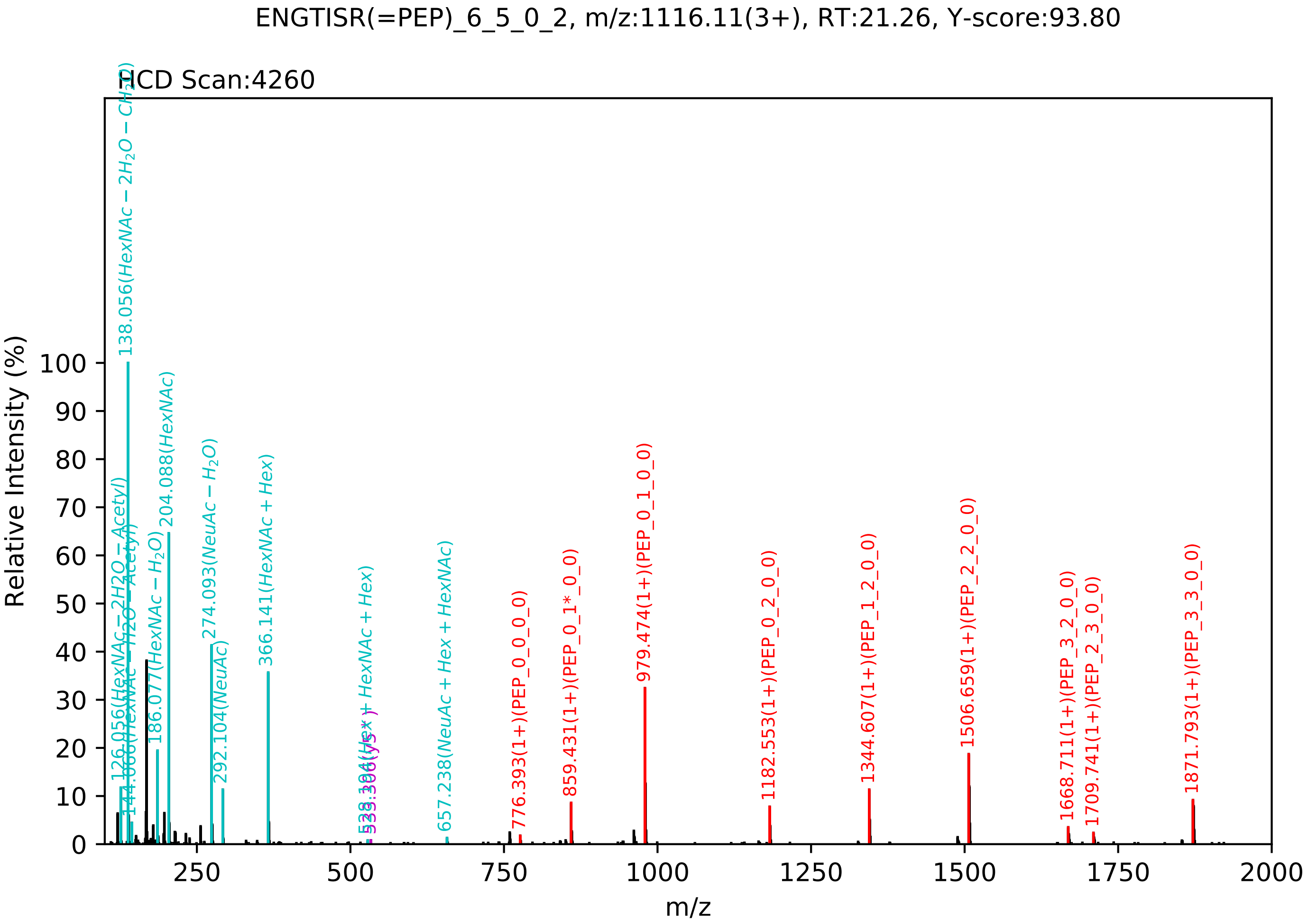

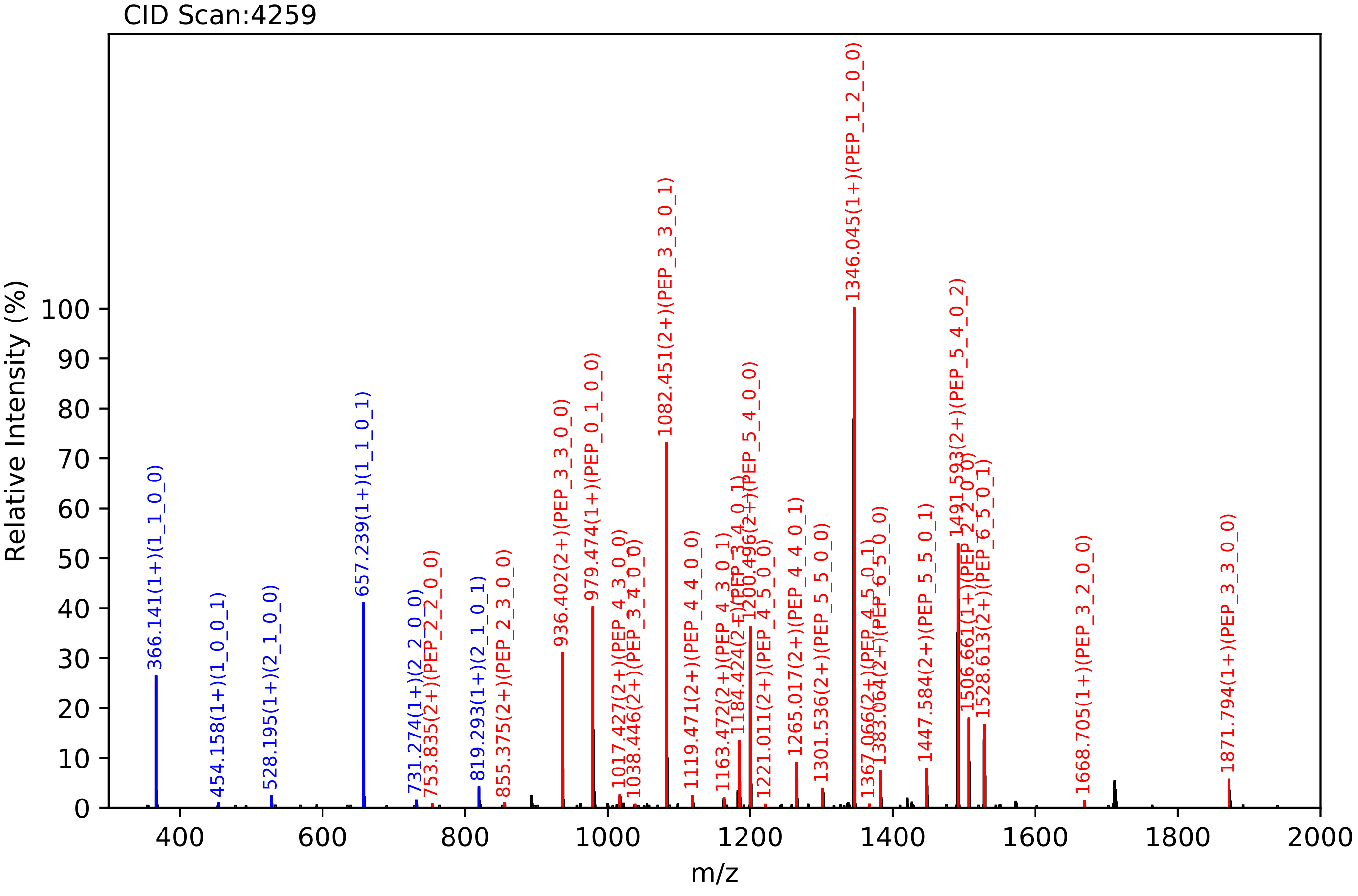

Test set no. 11, Experiment: AGP exp_4

ENGTISR(=PEP)_6_5_0_2, m/z:1116.11(3+), RT:20.96, Y-score:91.96

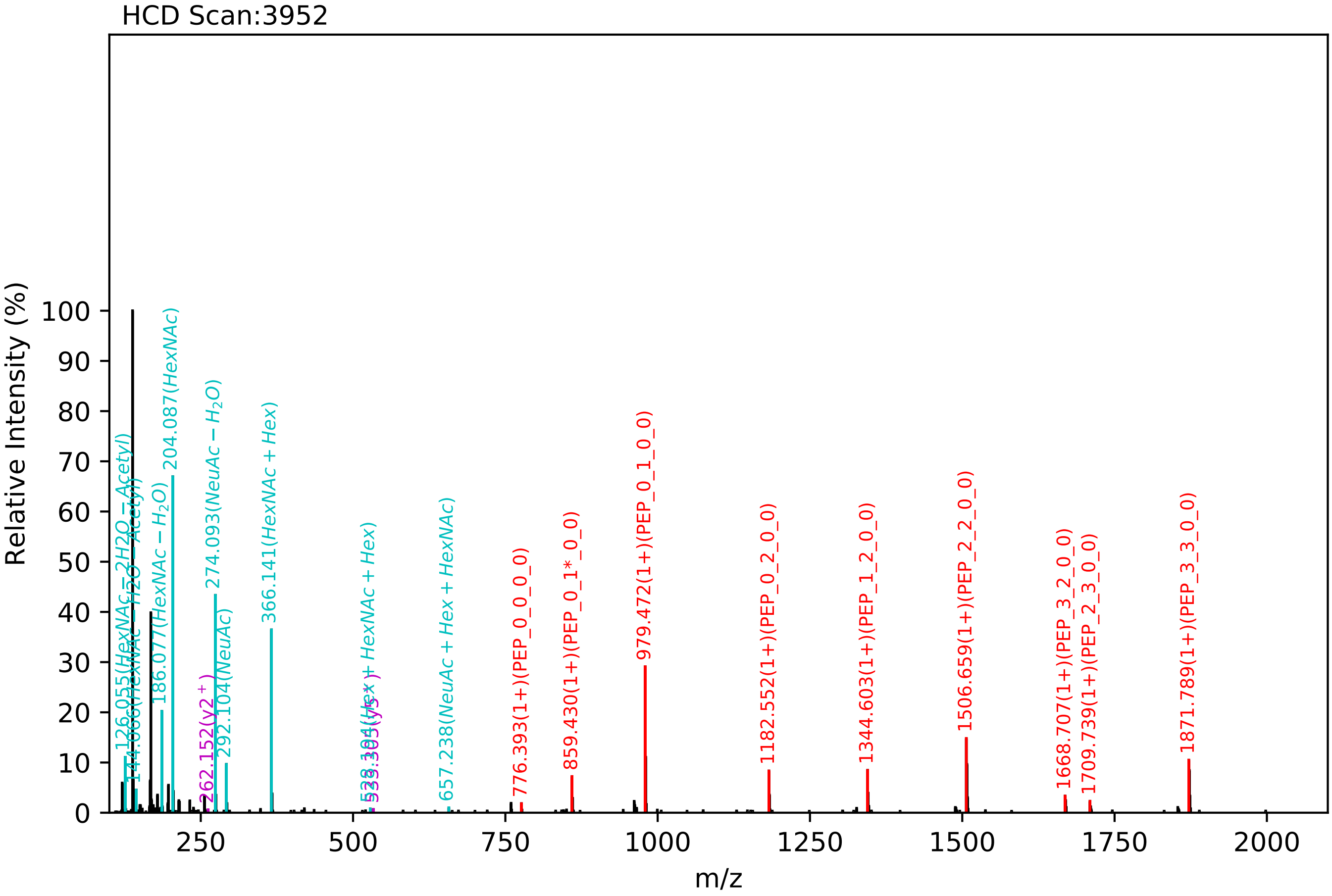

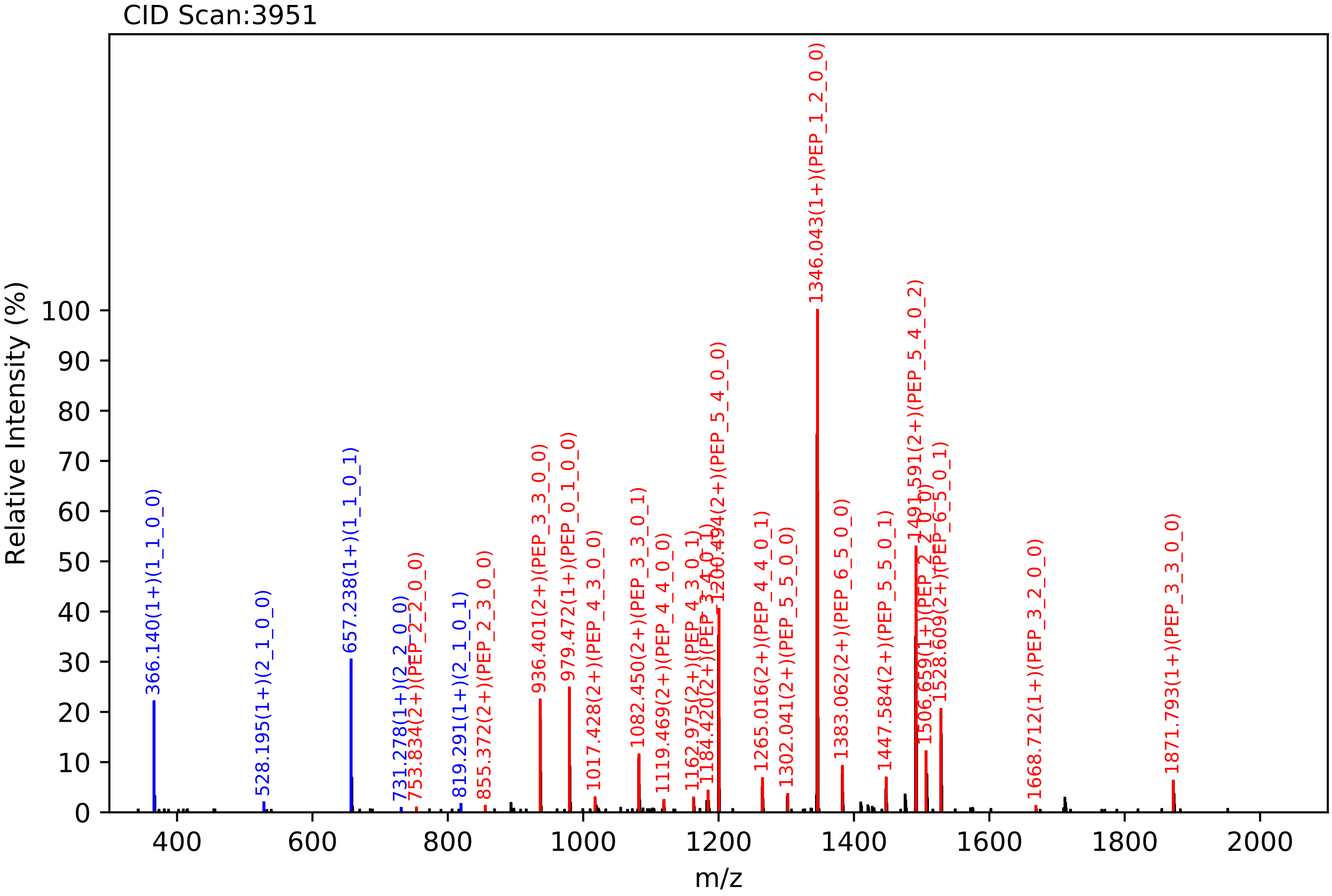

Test set no. 12, Experiment: AGP exp_3

ENGTISR(=PEP)_6_5_0_2, m/z:1673.65(2+), RT:20.91, Y-score:88.57

HCD Scan:4071

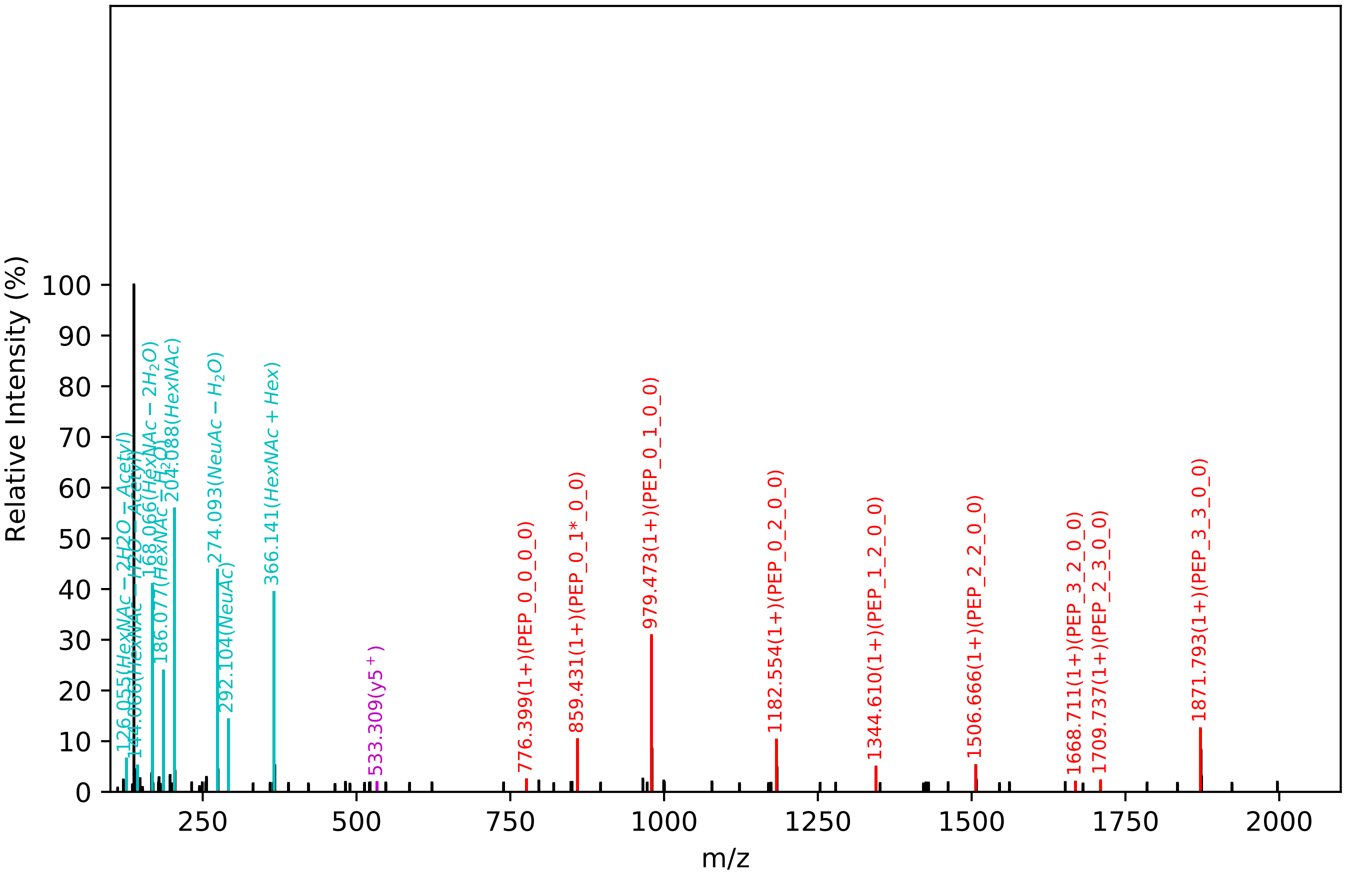

CID Scan:4070

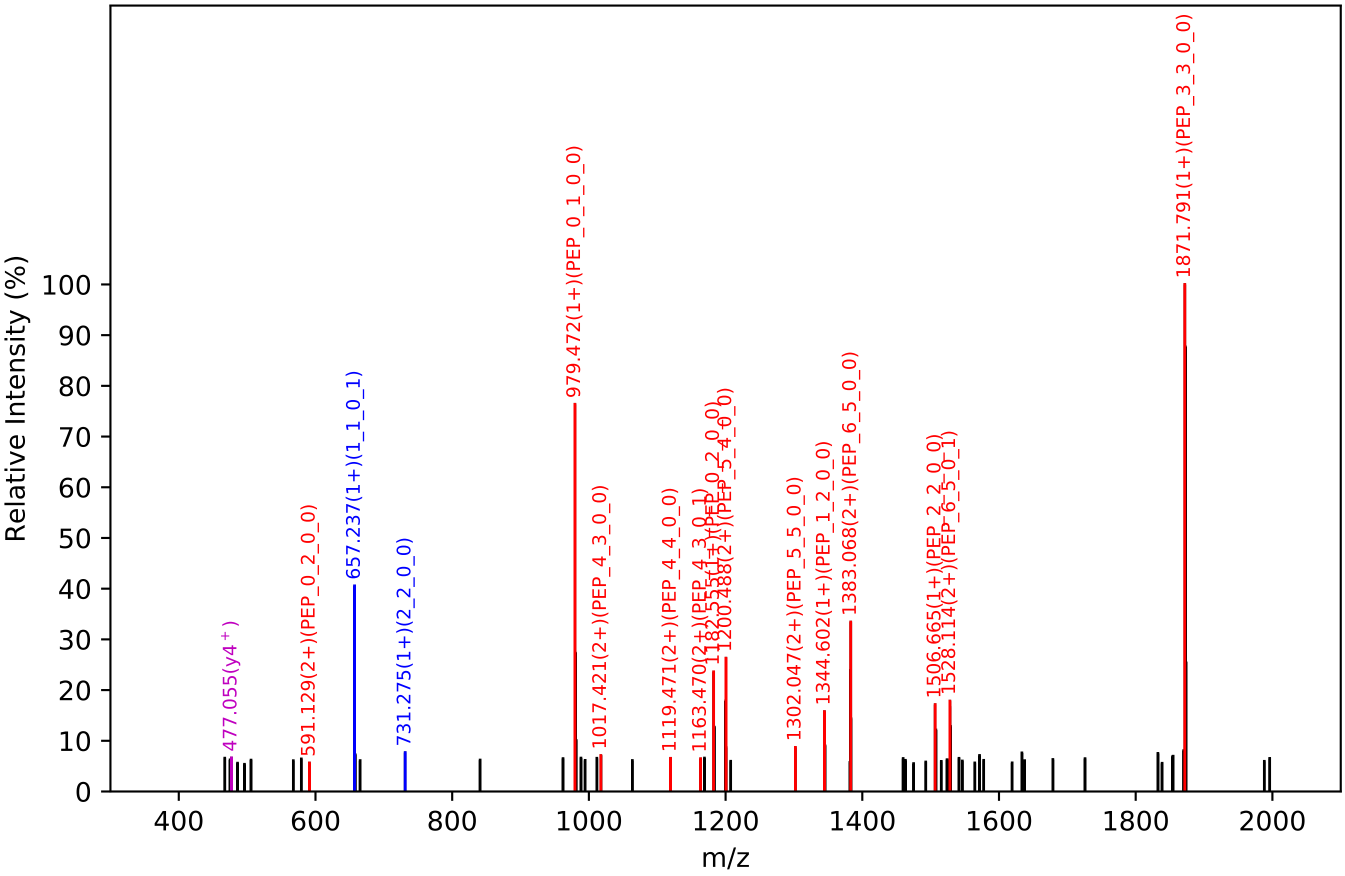

Test set no. 13, Experiment: AGP exp_4

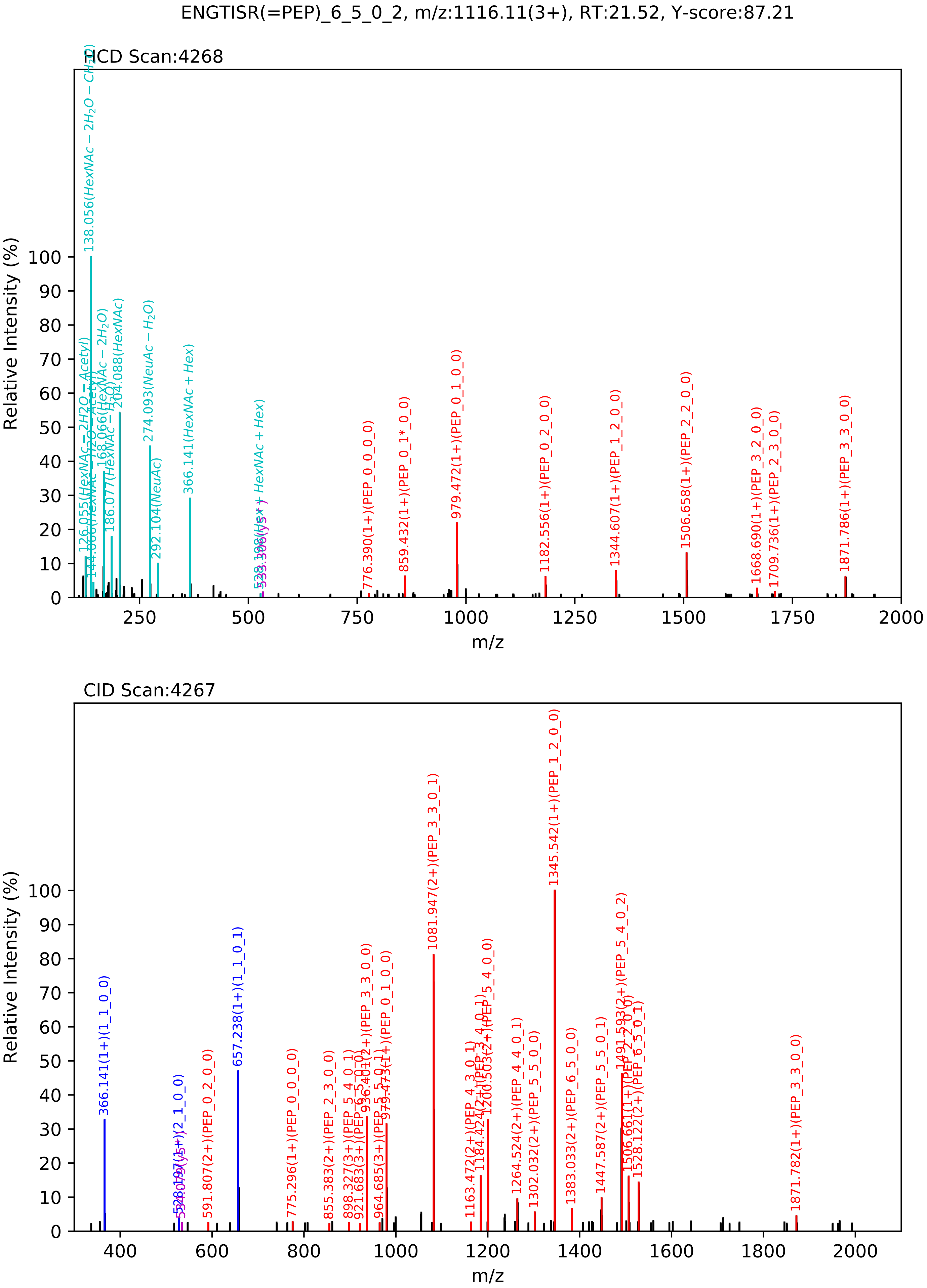

Test set no. 14, Experiment: AGP exp_4

ENGTISR(=PEP)_6_5_0_2, m/z:1673.65(2+), RT:22.51, Y-score:83.42

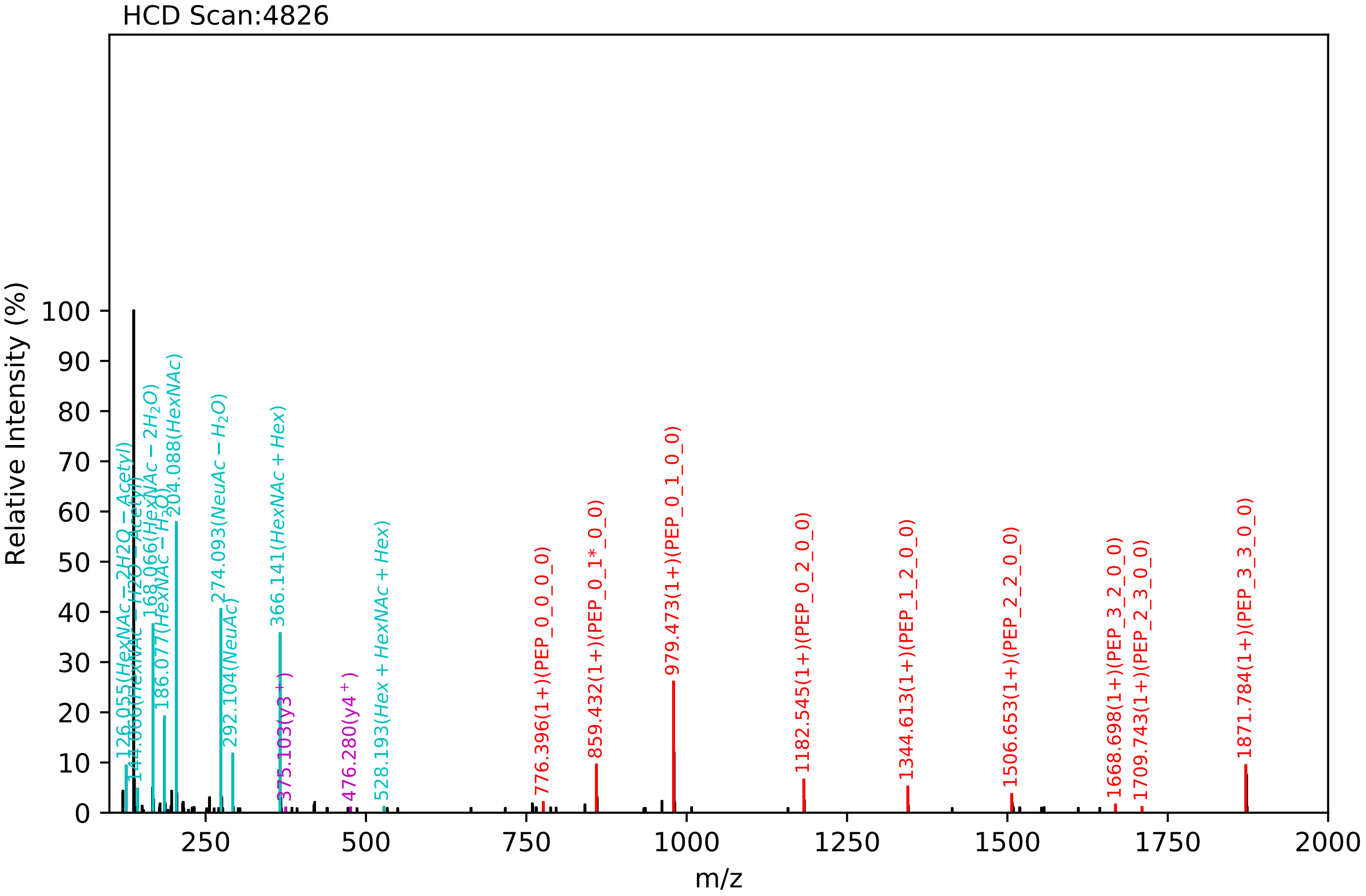

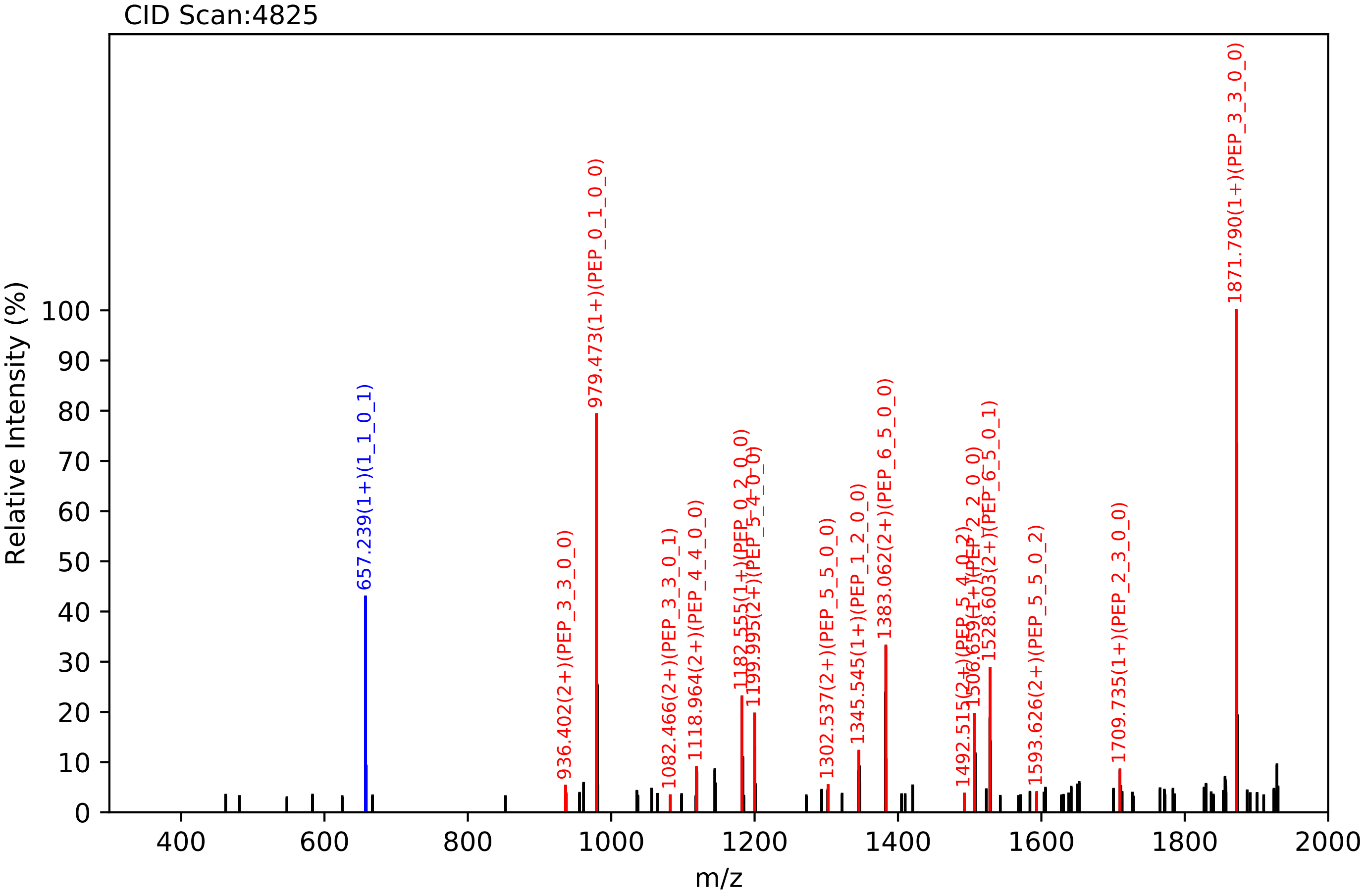

Test set no. 15, Experiment: AGP exp_3

ENGTISR(=PEP)_6_5_0_2, m/z:1116.10(3+), RT:22.67, Y-score:81.87

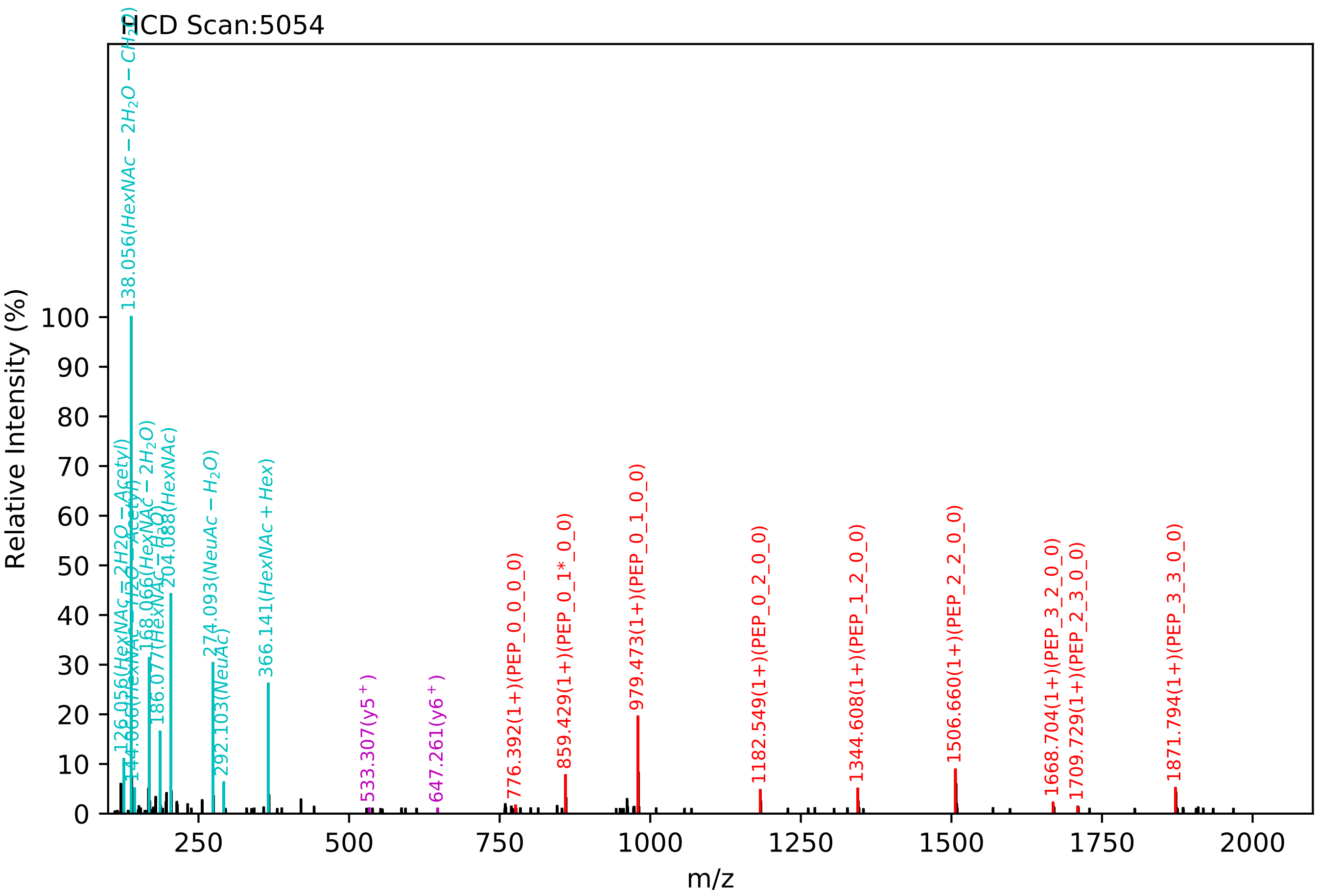

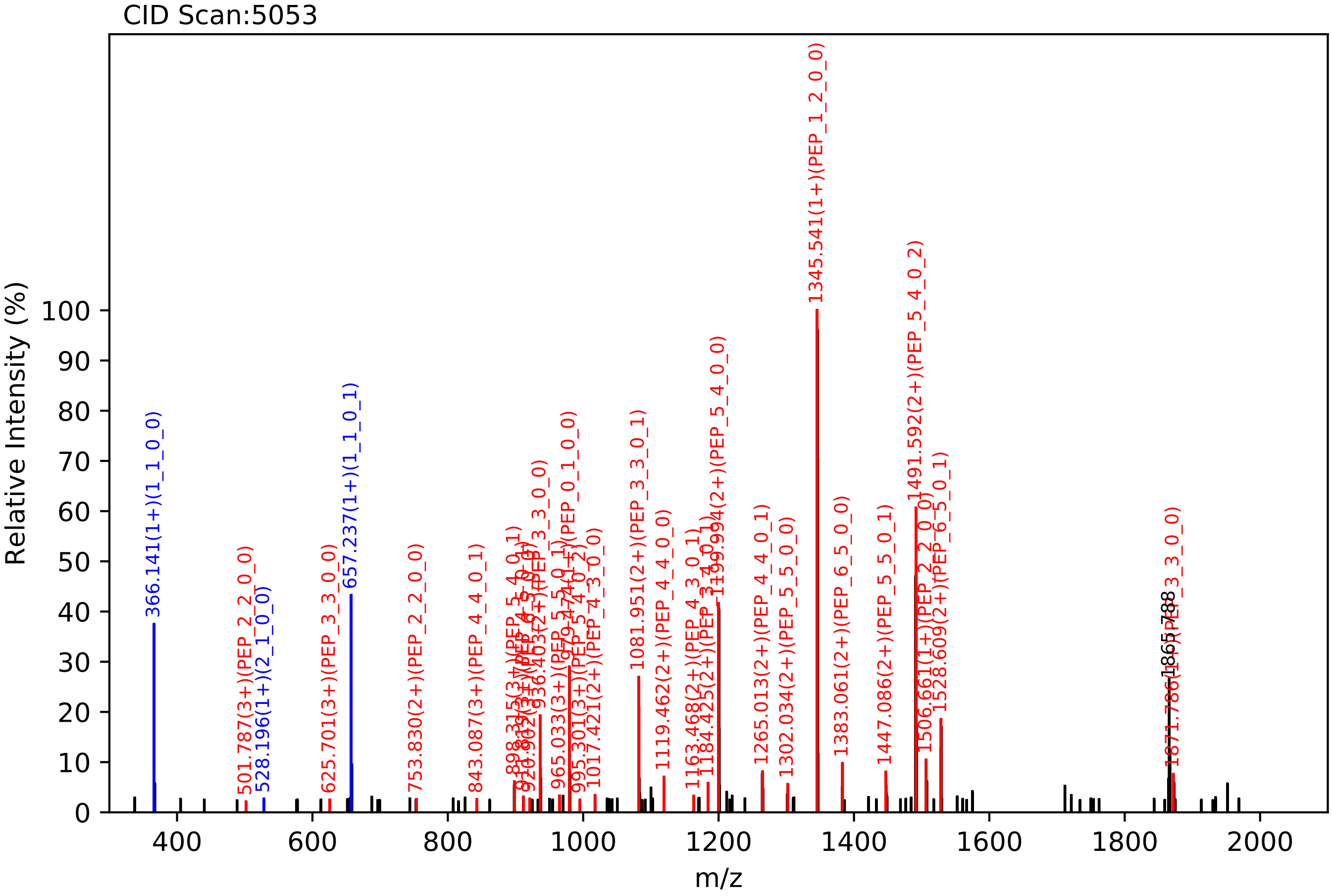

Test set no. 16, Experiment: AGP exp_4

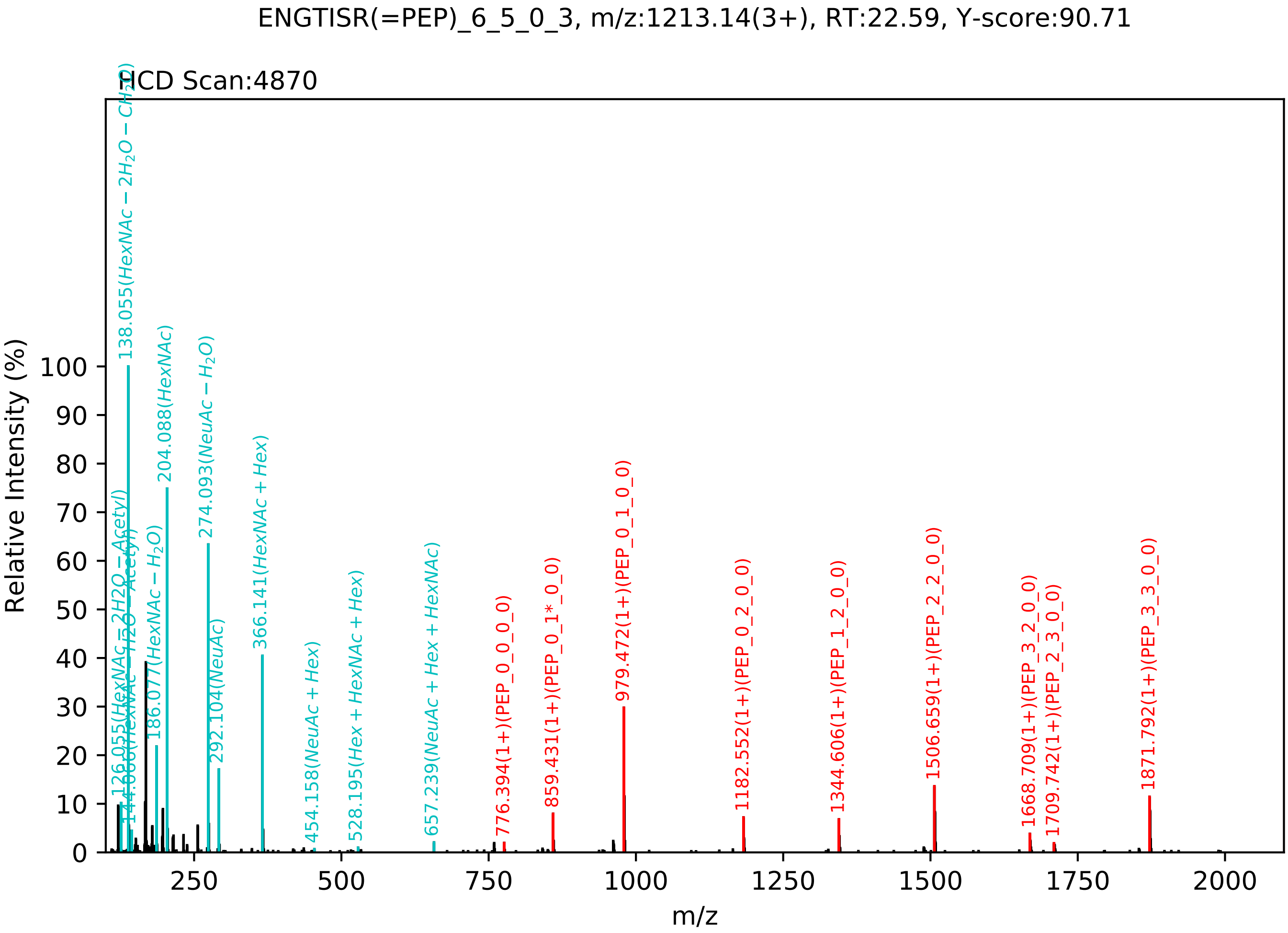

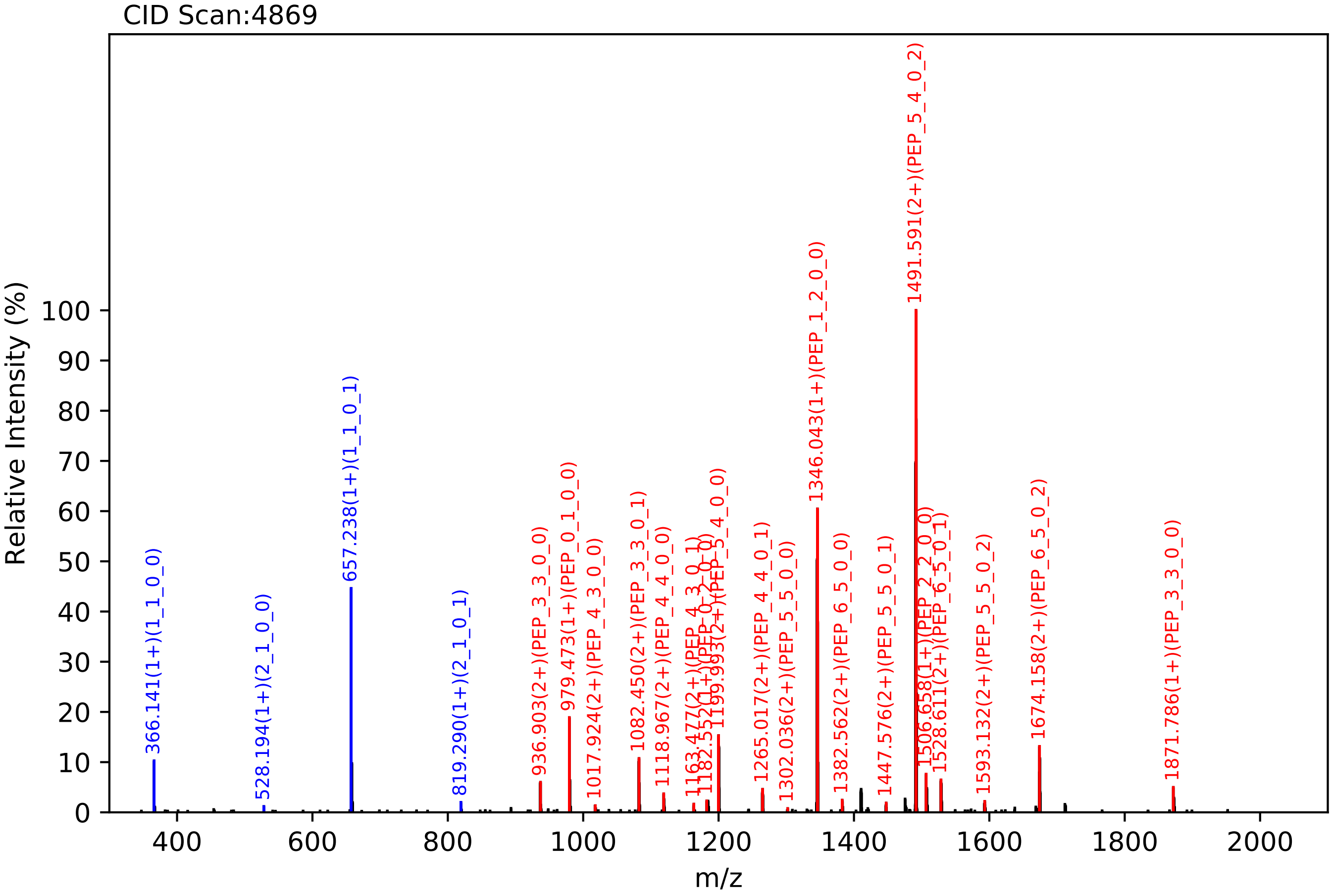

Test set no. 17, Experiment: AGP exp_4

ENGTISR(=PEP)_6_5_0_3, m/z:1819.21(2+), RT:22.62, Y-score:88.99

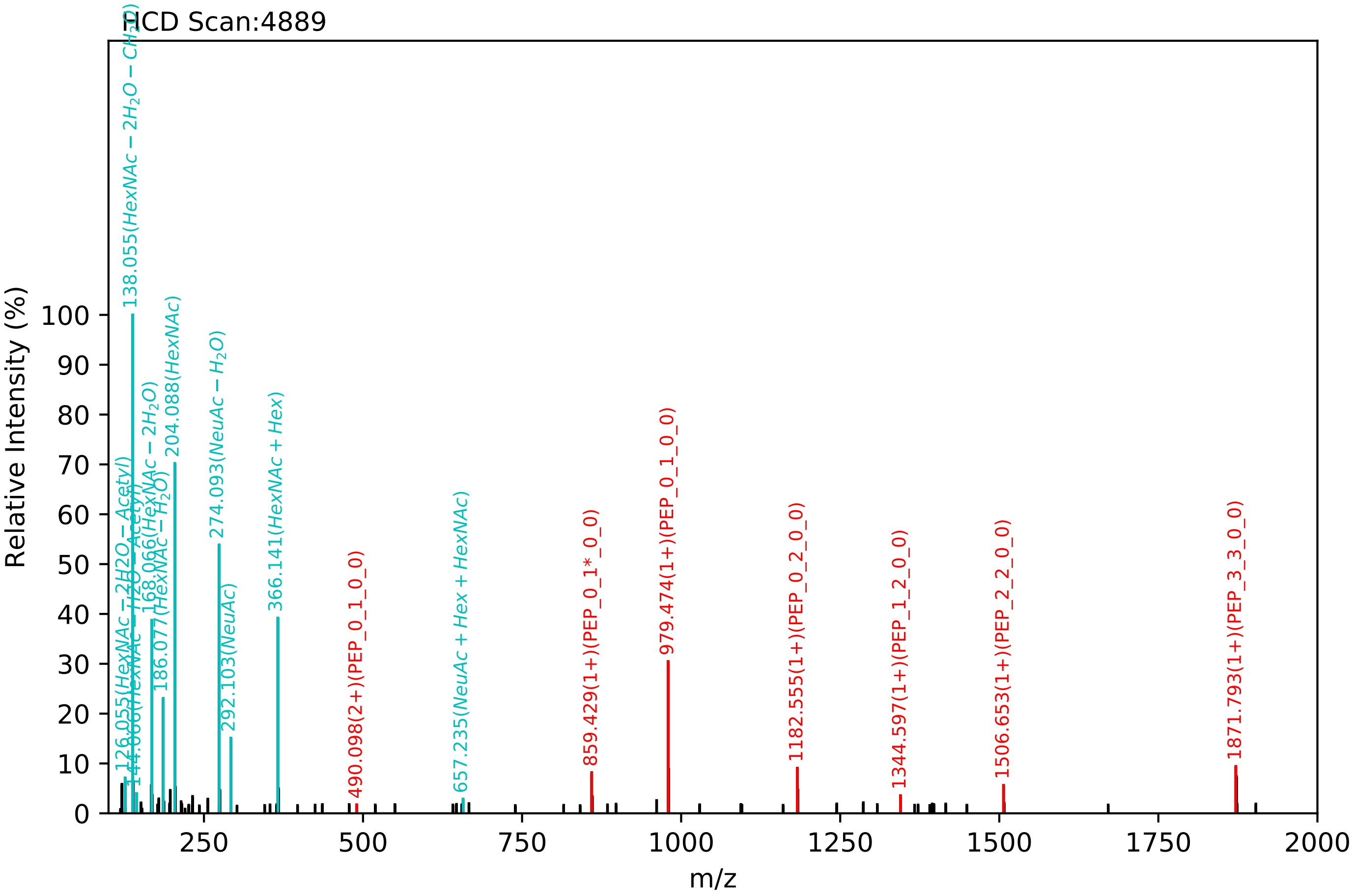

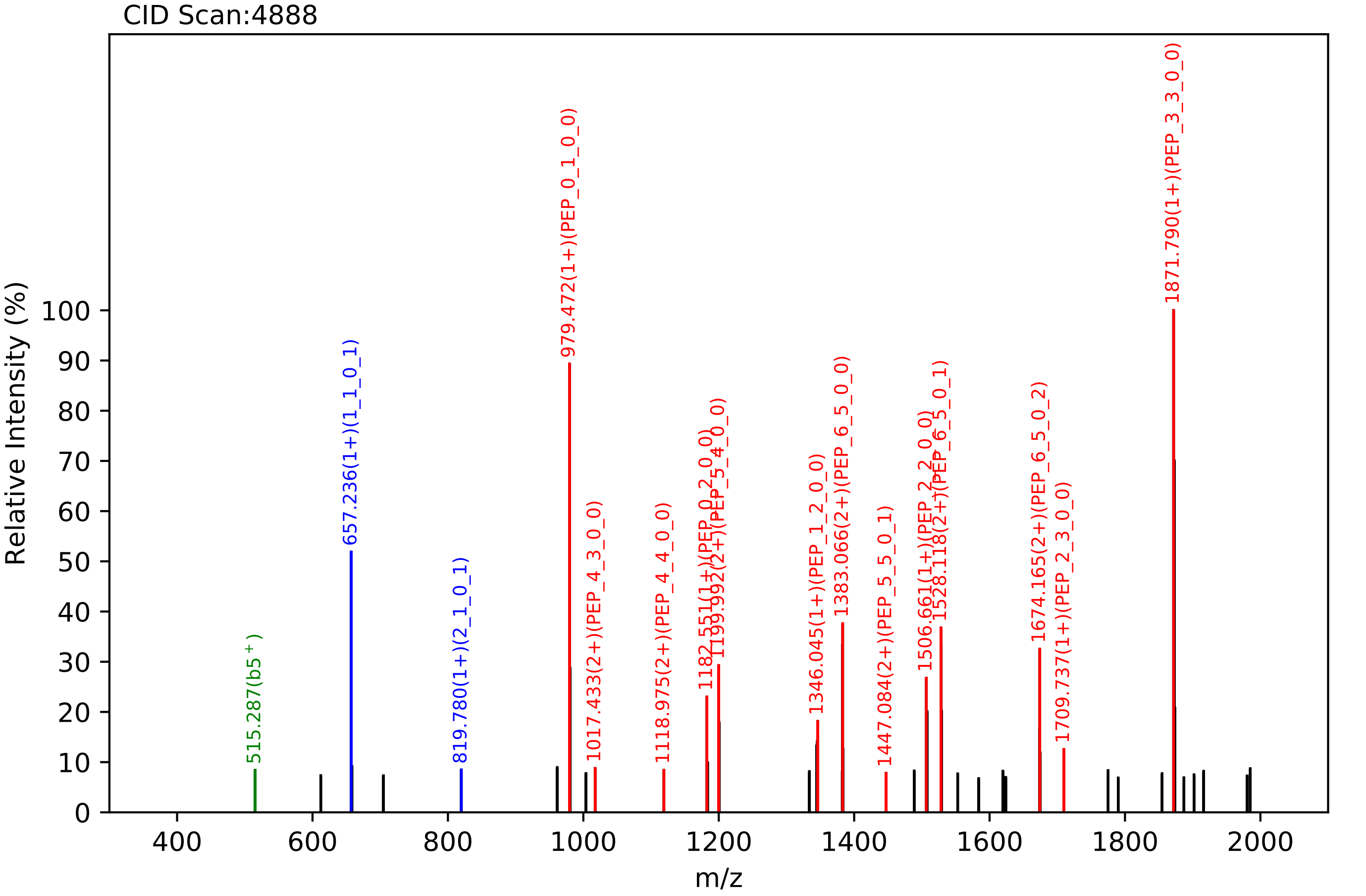

Test set no. 18, Experiment: AGP exp_3

ENGTISR(=PEP)_6_5_0_3, m/z:1819.71(2+), RT:22.73, Y-score:86.30

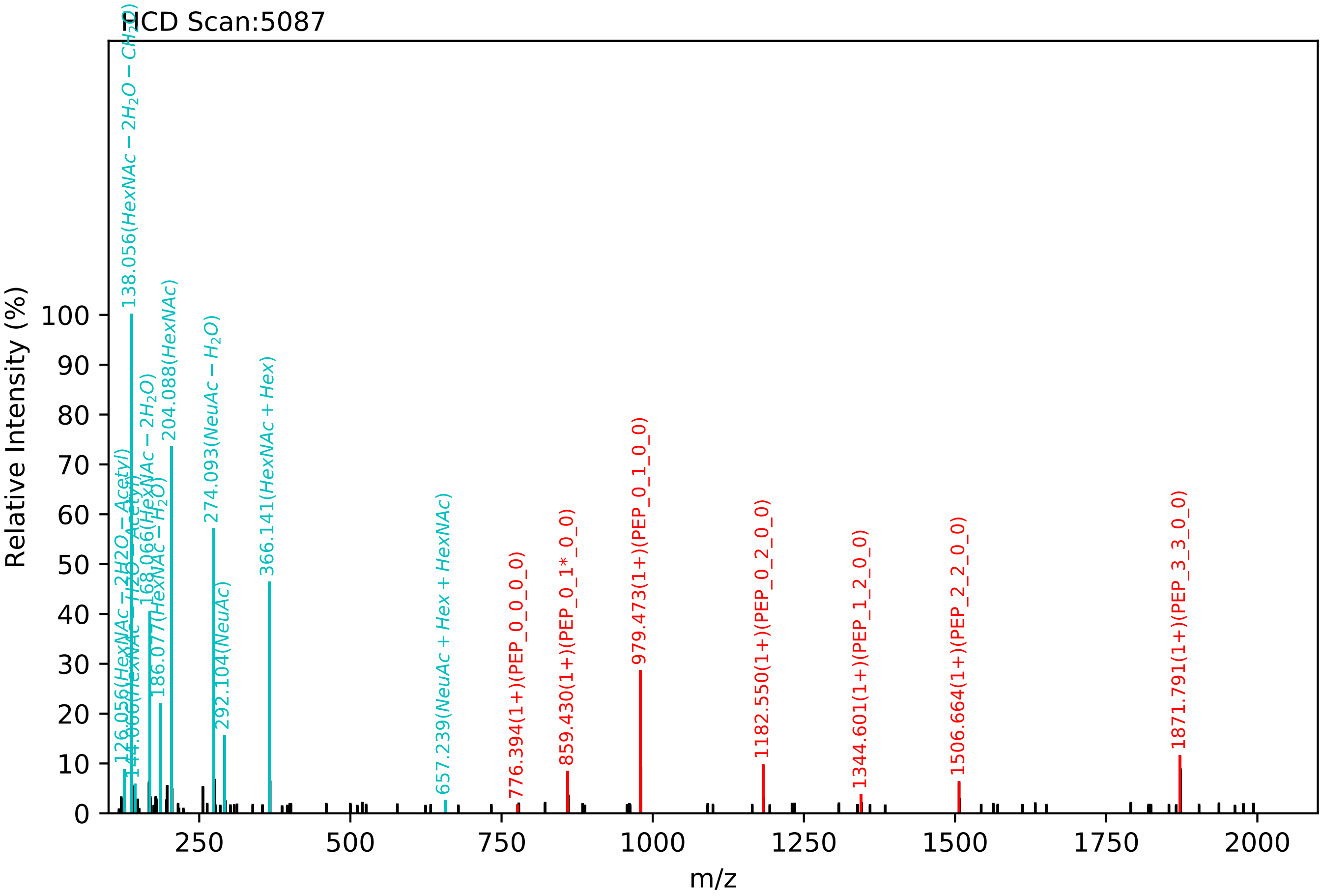

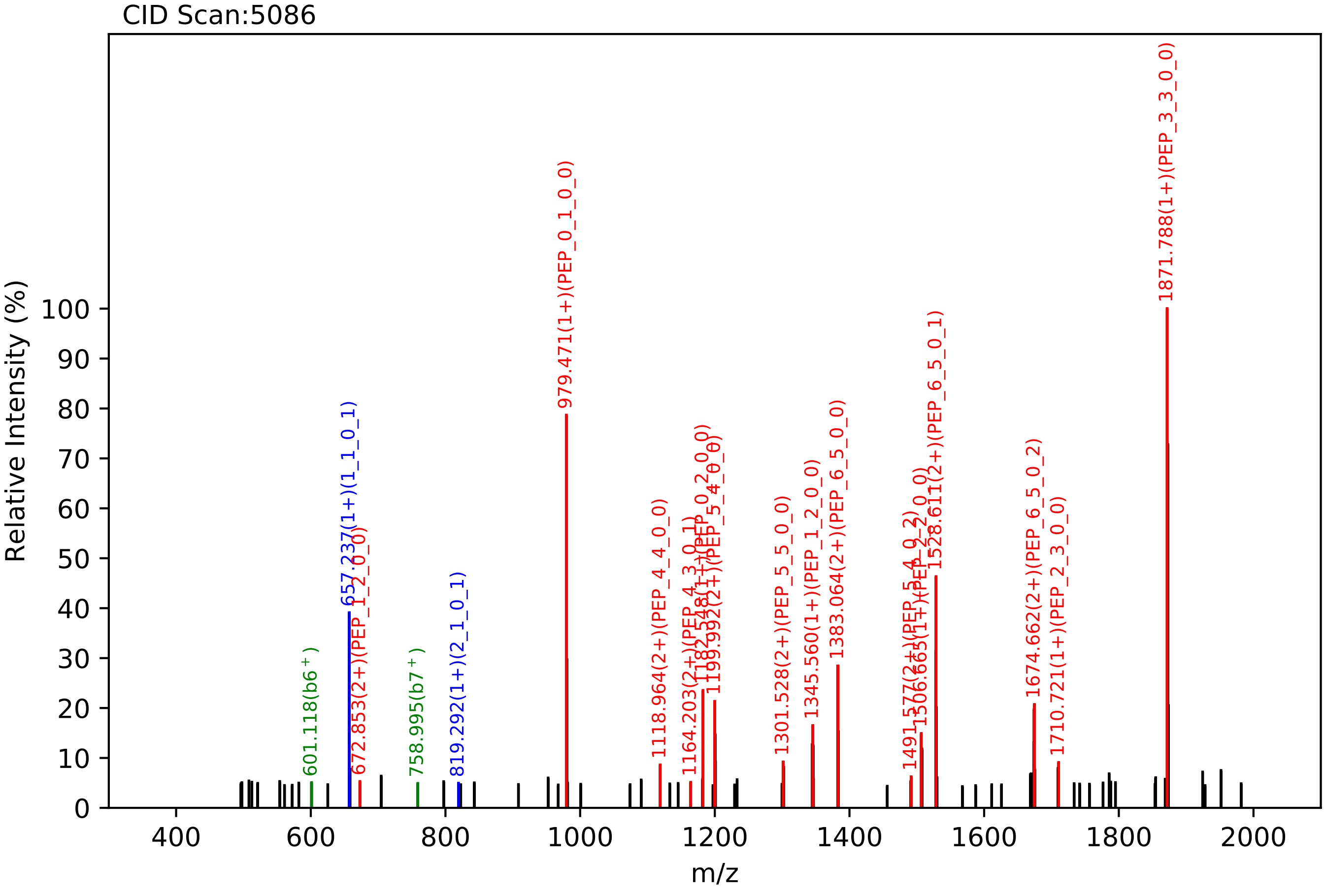

Test set no. 19, Experiment: AGP exp_4

ENGTISR(=PEP)_6_5_0_3, m/z:1213.14(3+), RT:25.16, Y-score:84.00

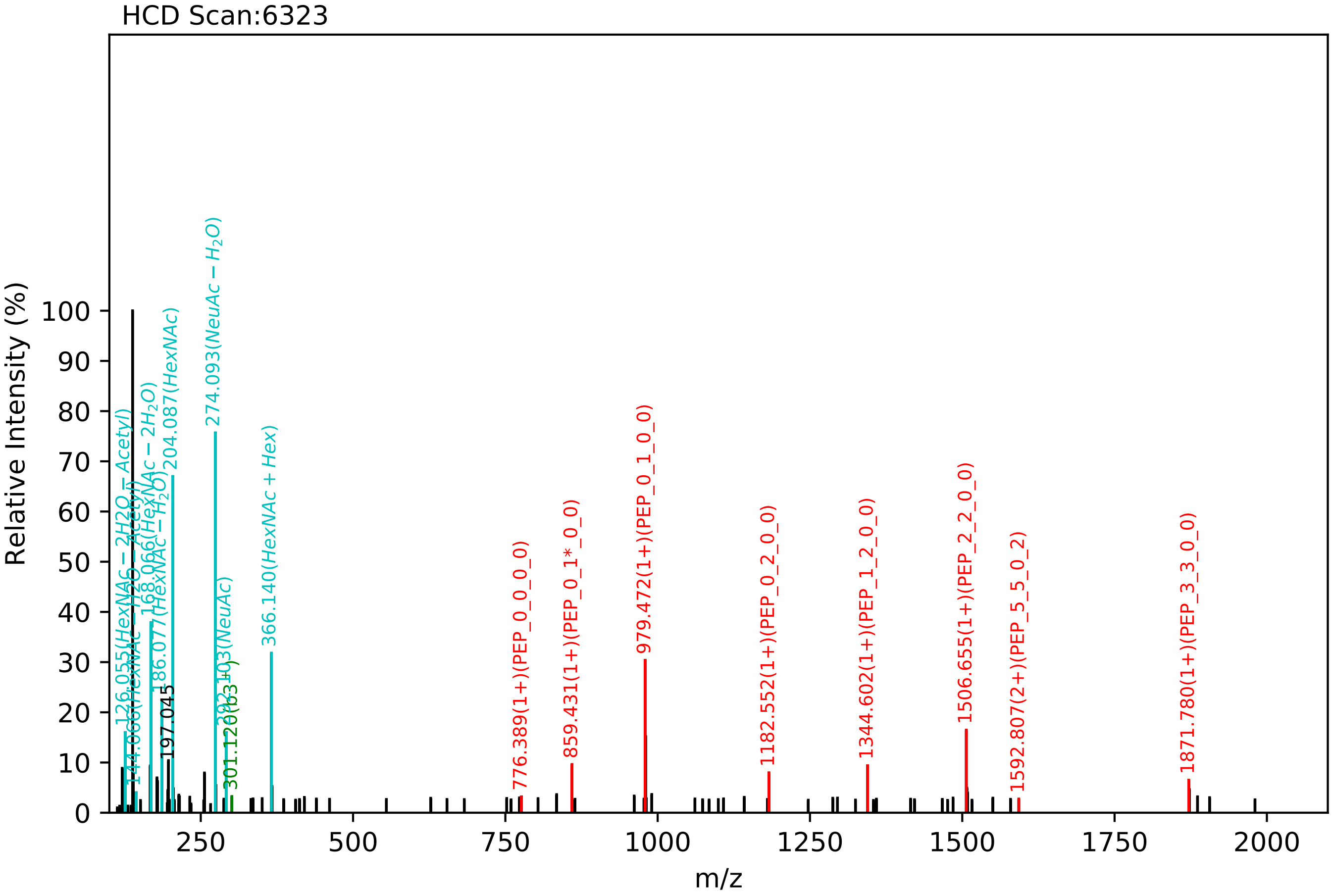

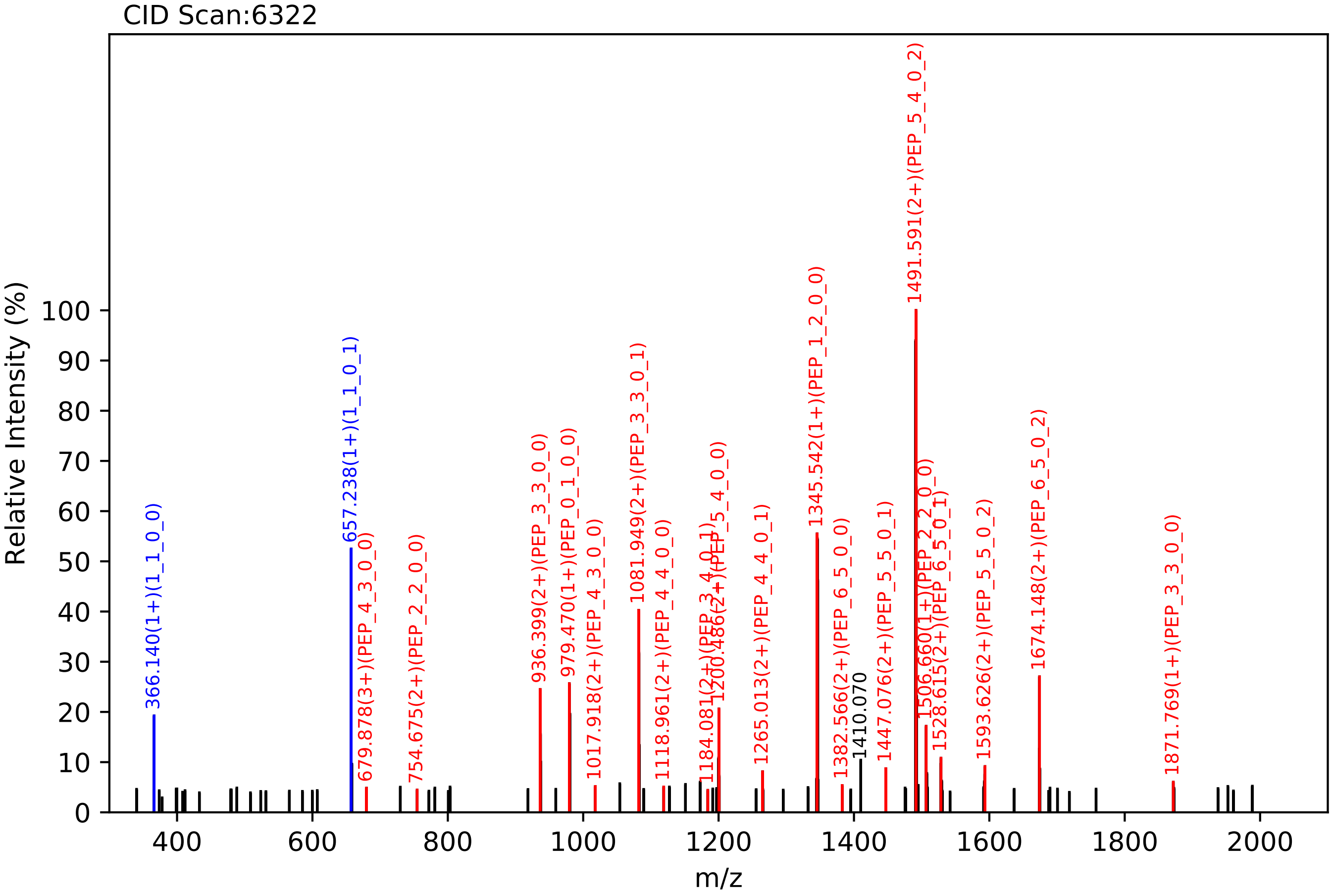

Test set no. 20, Experiment: AGP exp_4

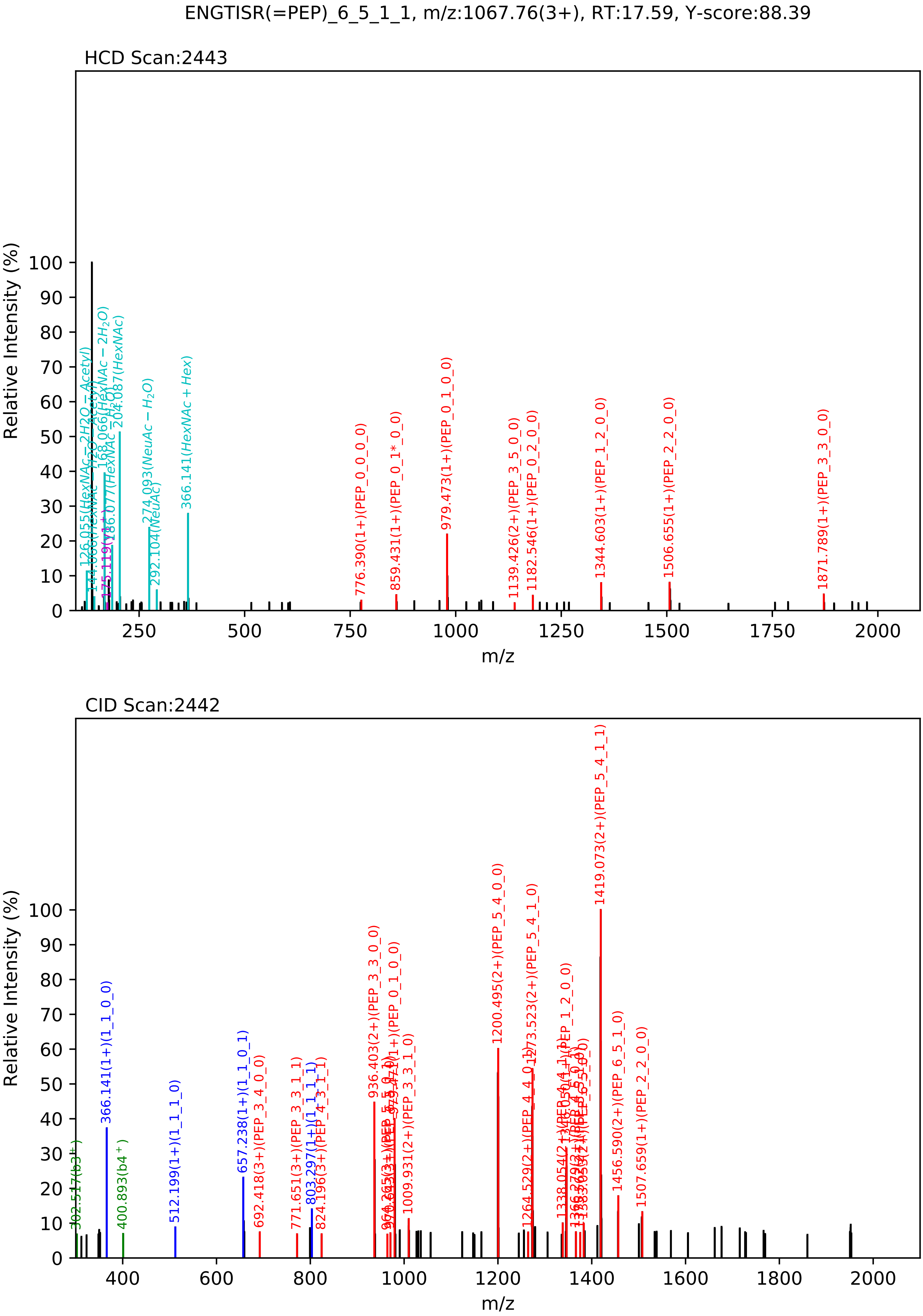

Test set no. 21, Experiment: AGP exp_3

ENGTISR(=PEP)_6_5_1_1, m/z:1067.76(3+), RT:17.76, Y-score:84.30

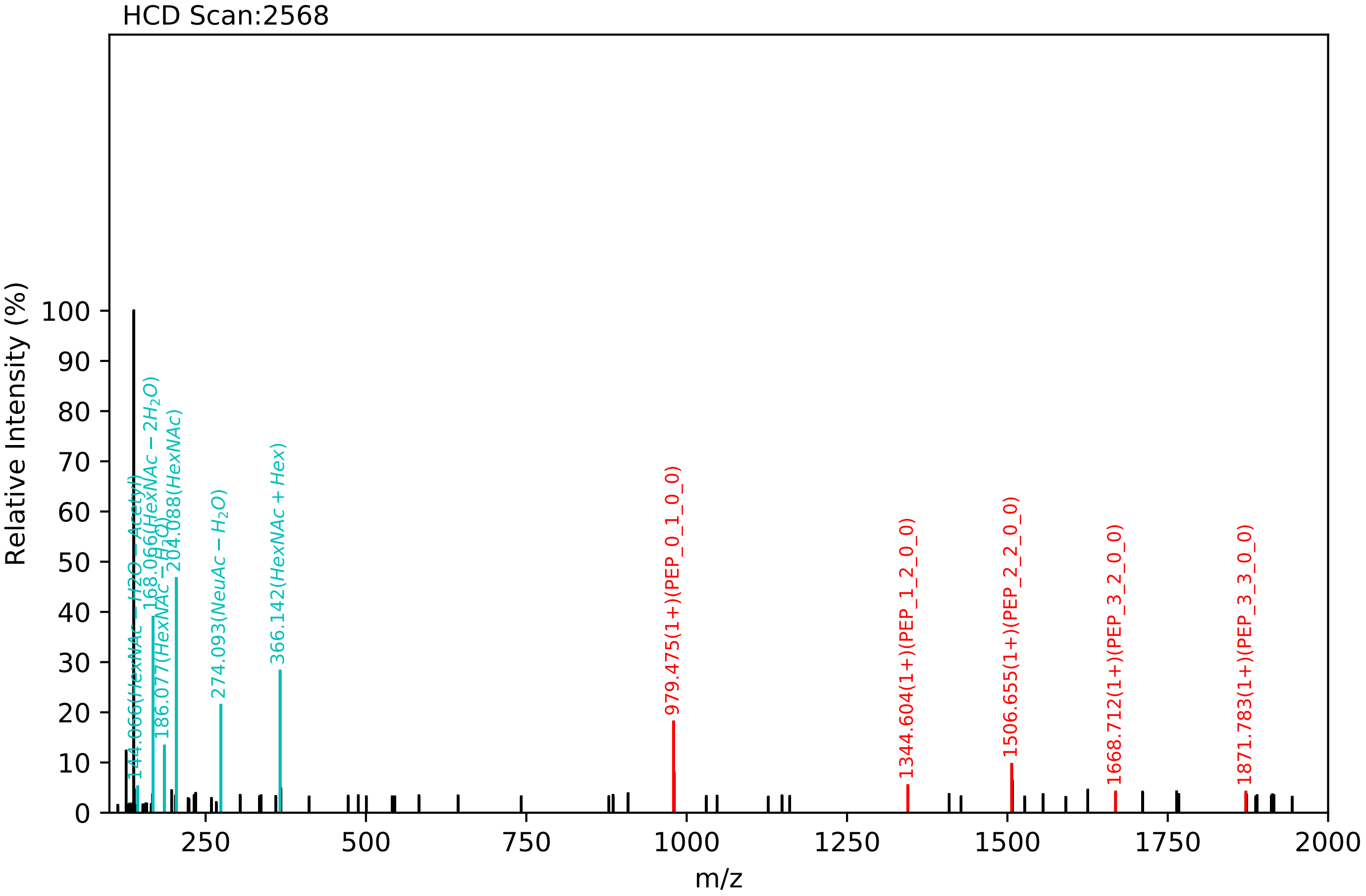

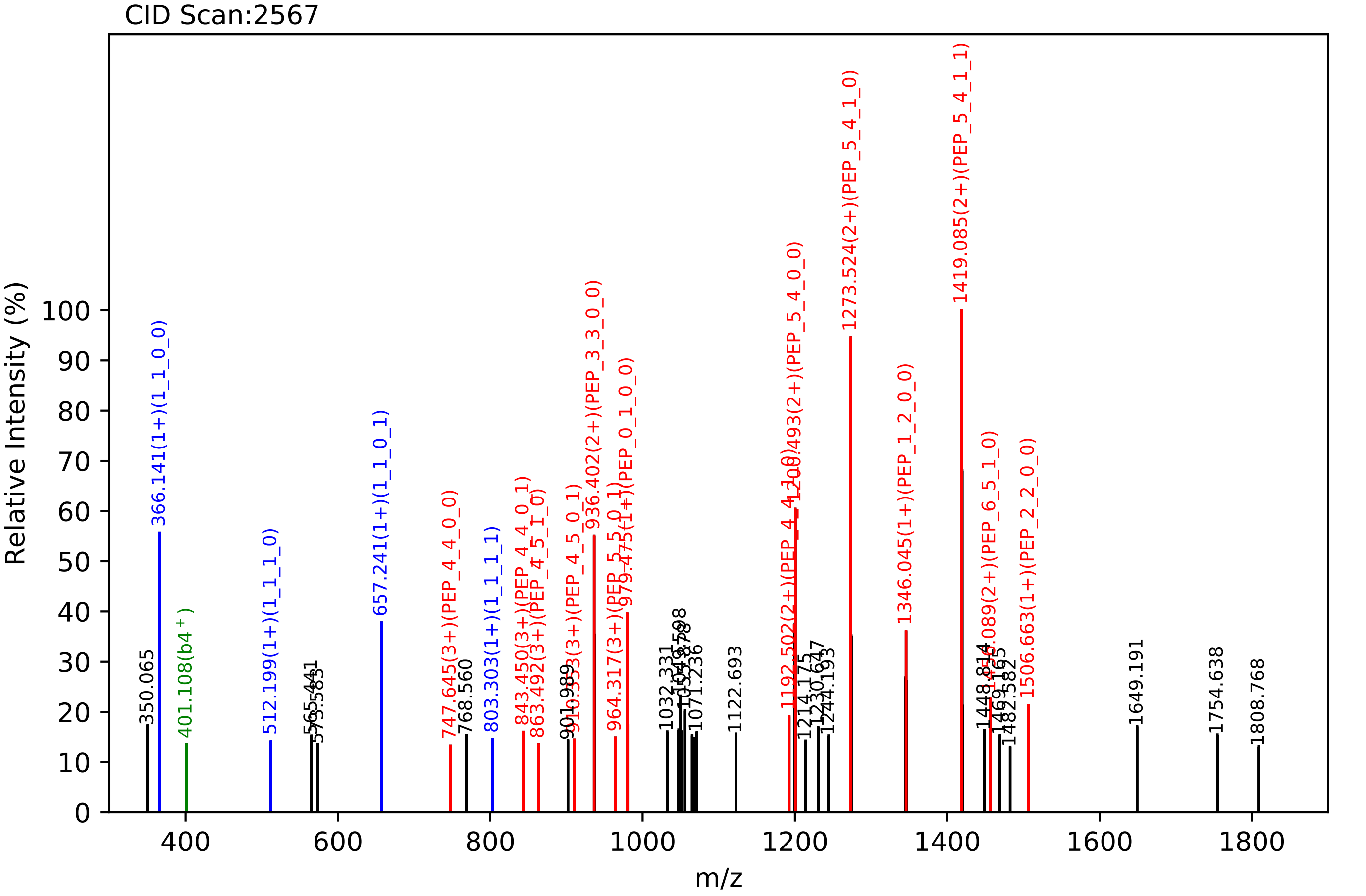

Test set no. 22, Experiment: AGP exp_4

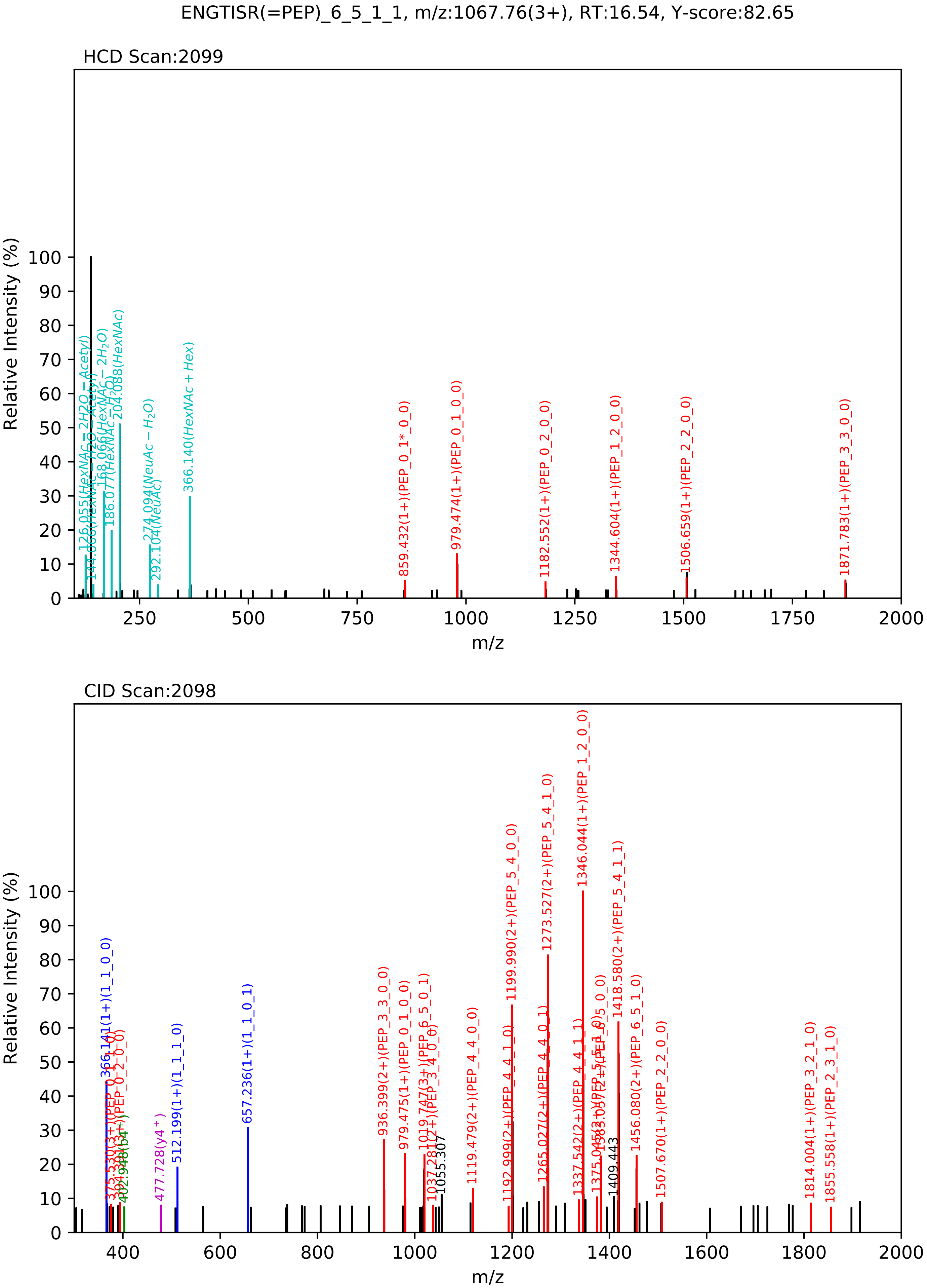

Test set no. 23, Experiment: AGP exp_3

ENGTISR(=PEP)_6_5_1_1, m/z:1067.76(3+), RT:16.76, Y-score:81.67

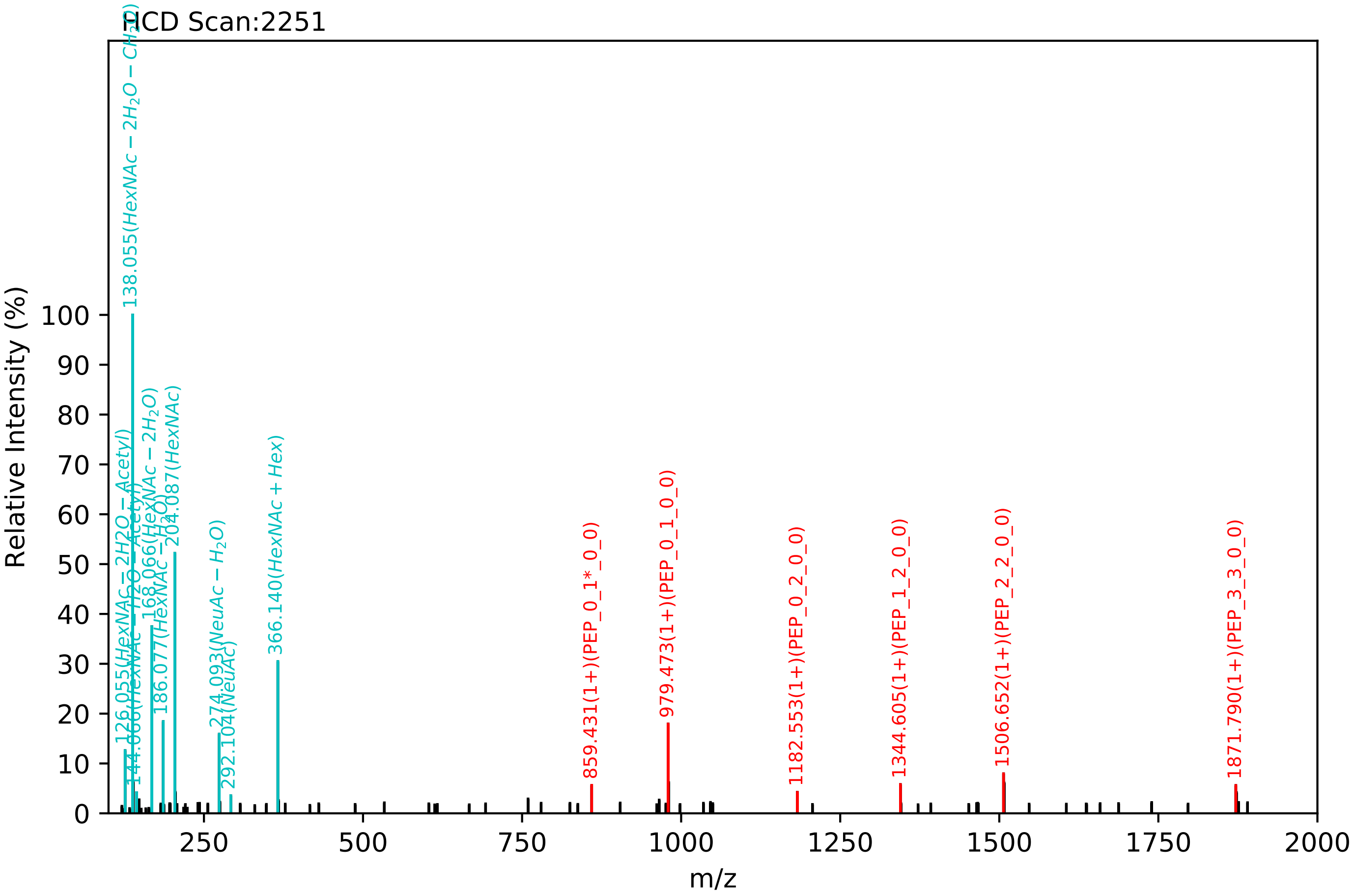

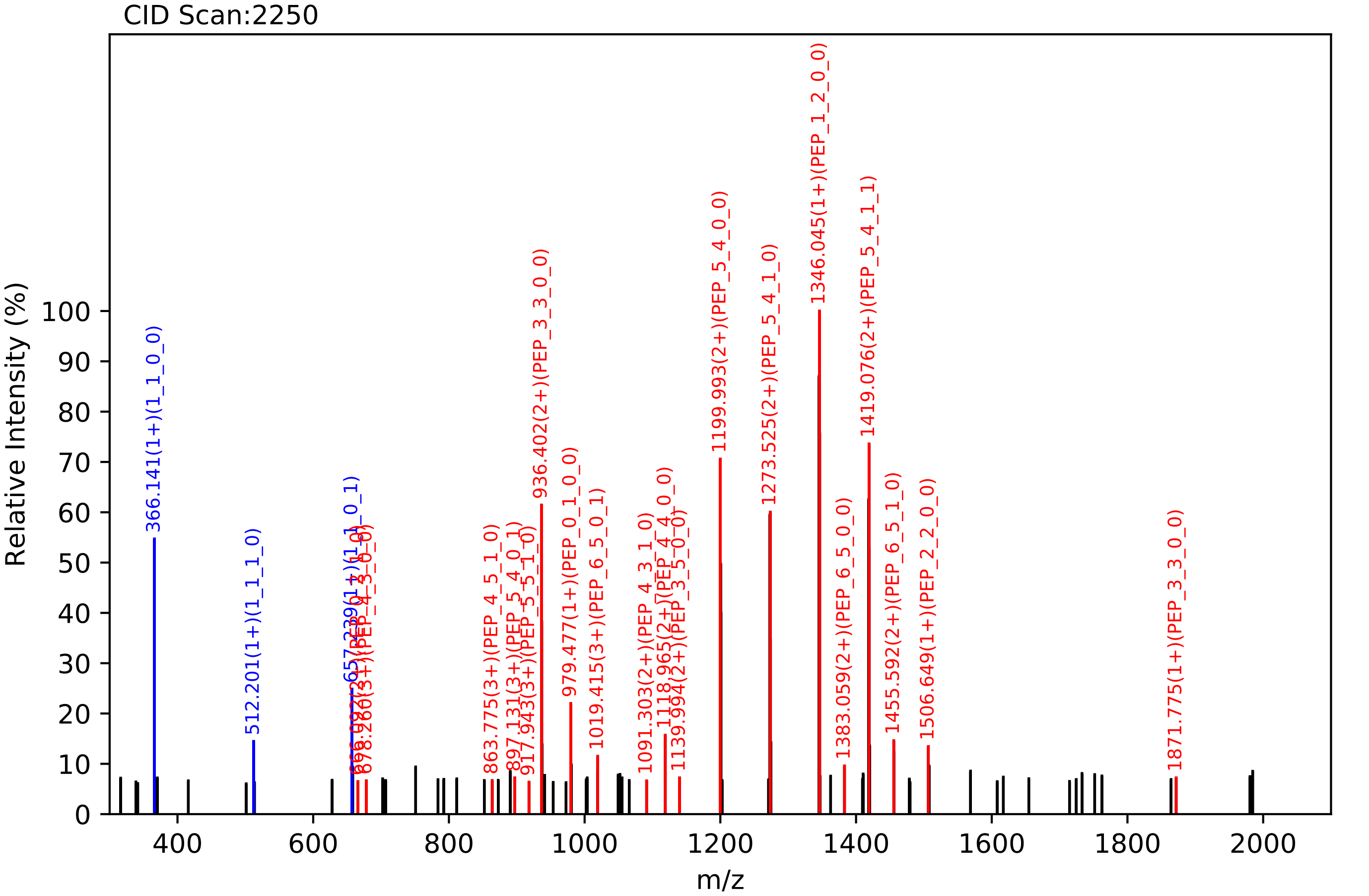

Test set no. 24, Experiment: AGP exp_3

ENGTISR(=PEP)_6_5_1_2, m/z:1164.79(3+), RT:20.28, Y-score:89.90

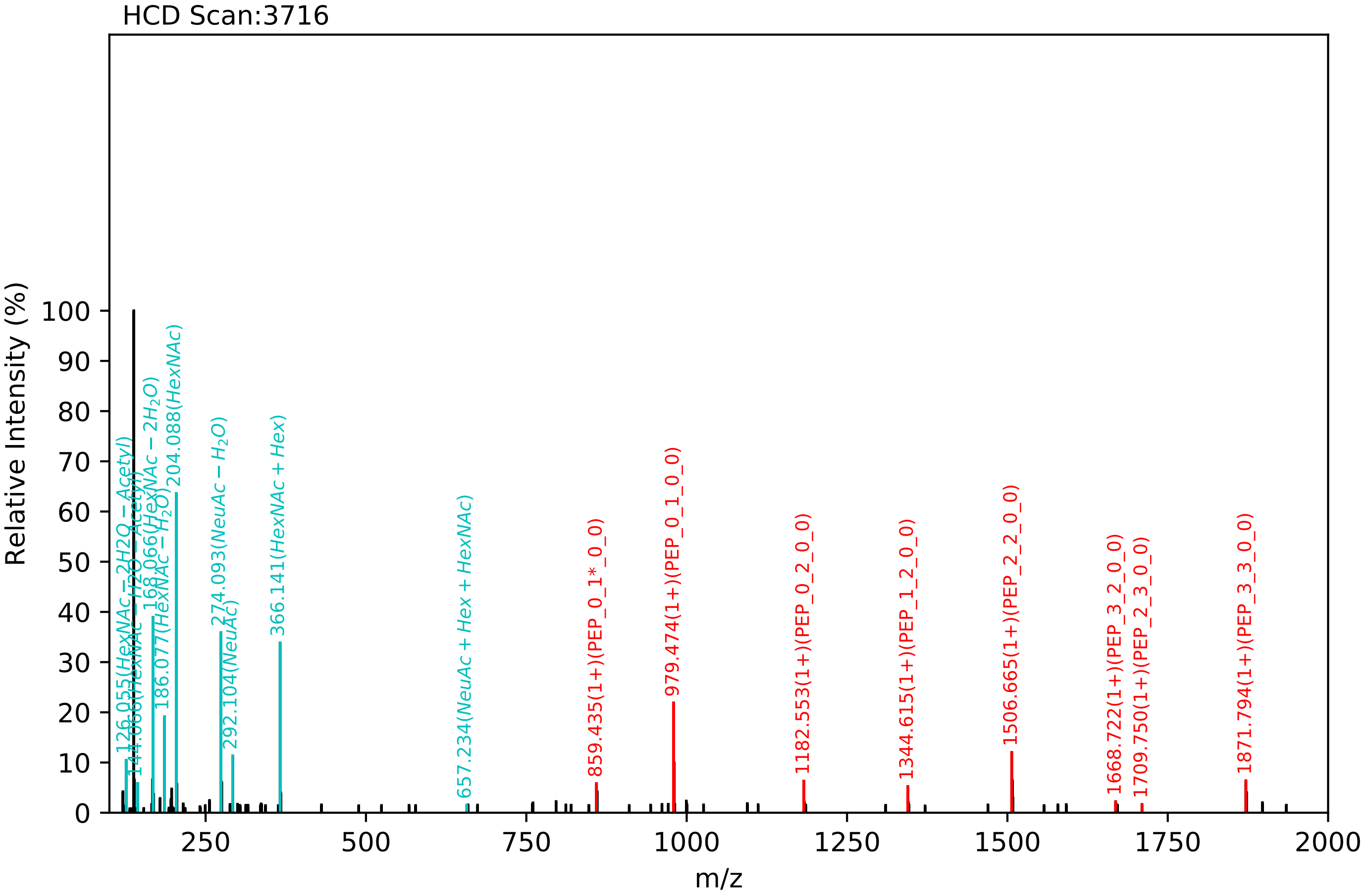

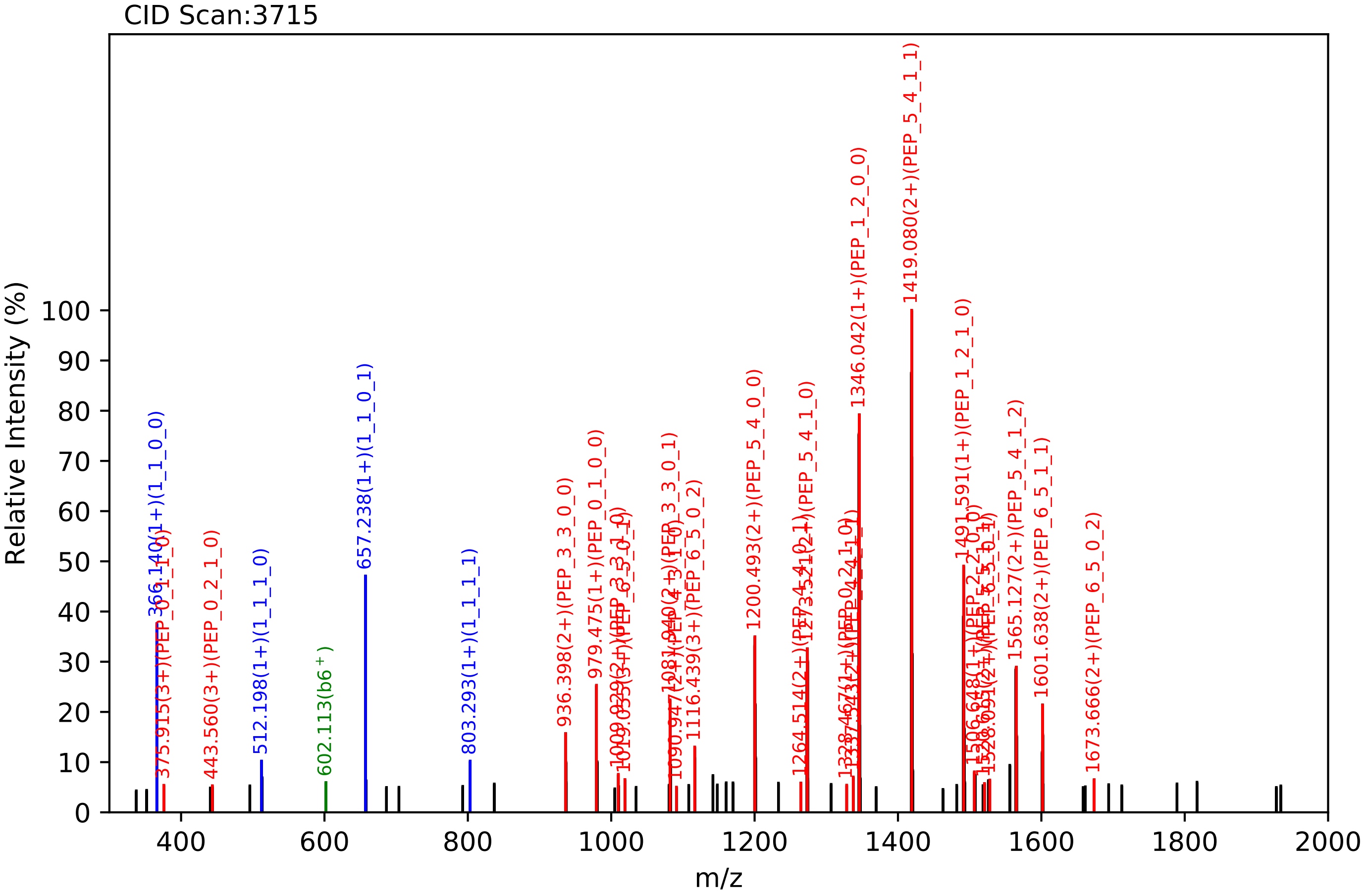

Test set no. 25, Experiment: AGP exp_4

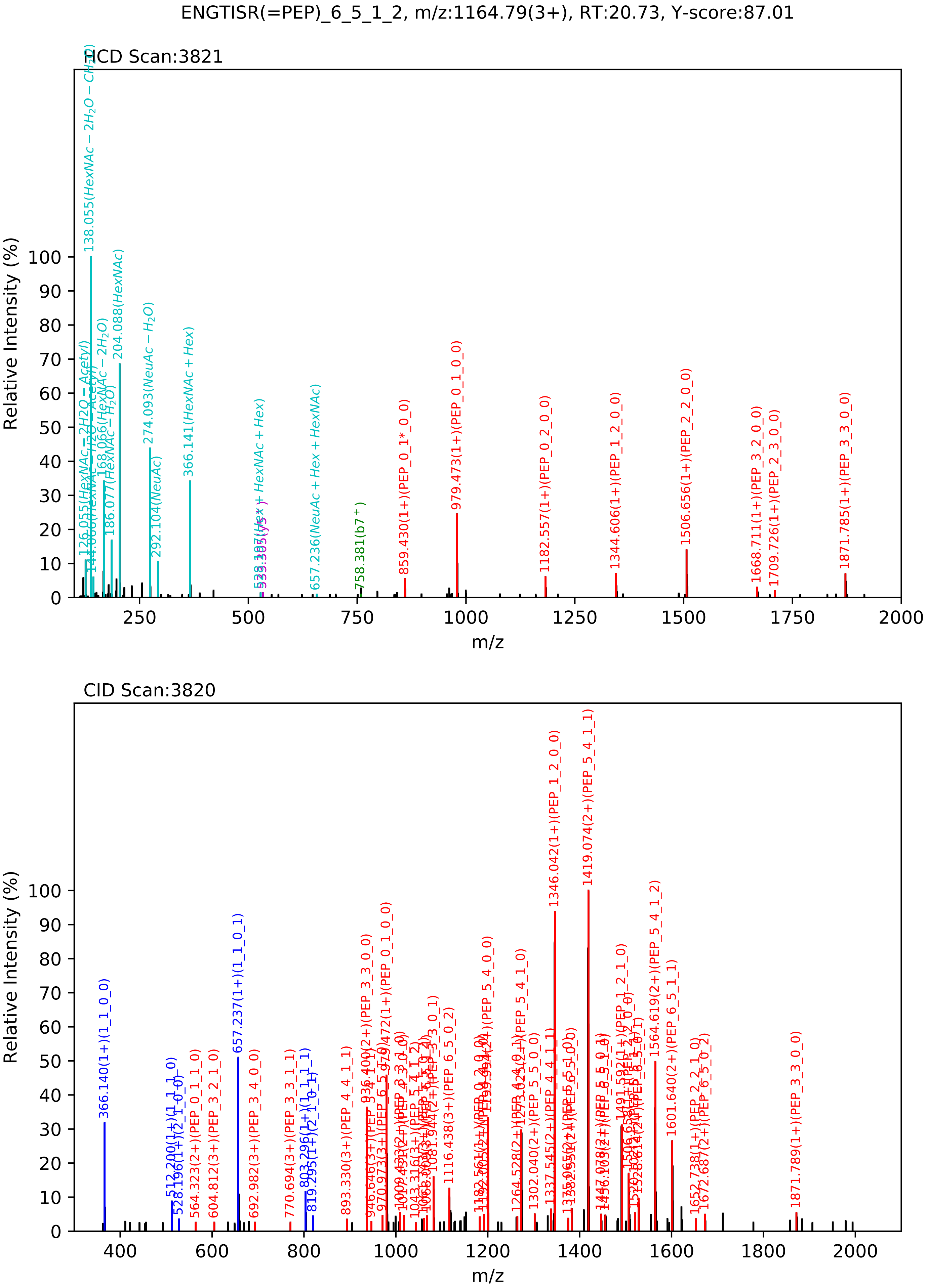

Test set no. 26, Experiment: AGP exp_3

ENGTISR(=PEP)_6_5_1_2, m/z:1164.79(3+), RT:20.92, Y-score:83.95

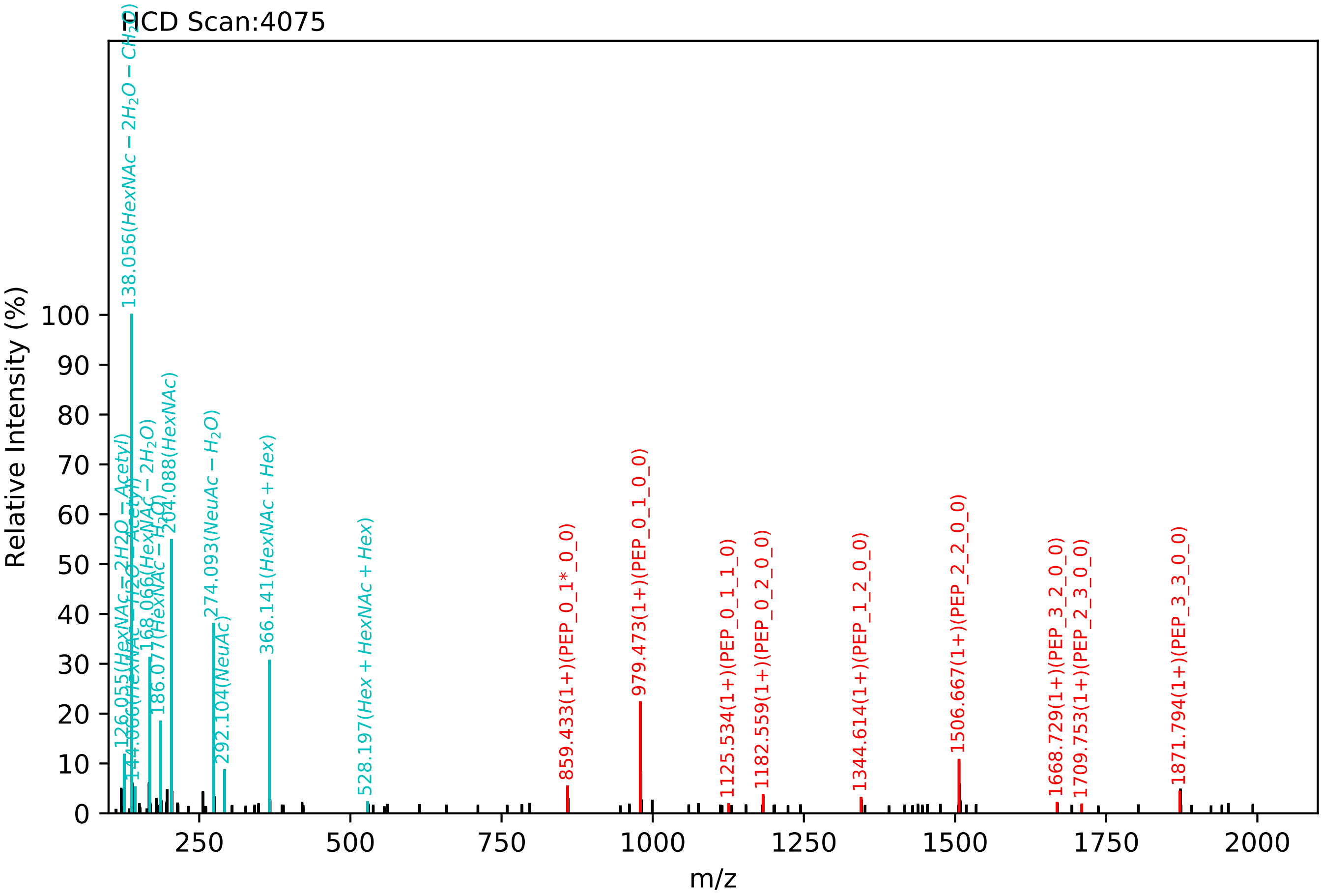

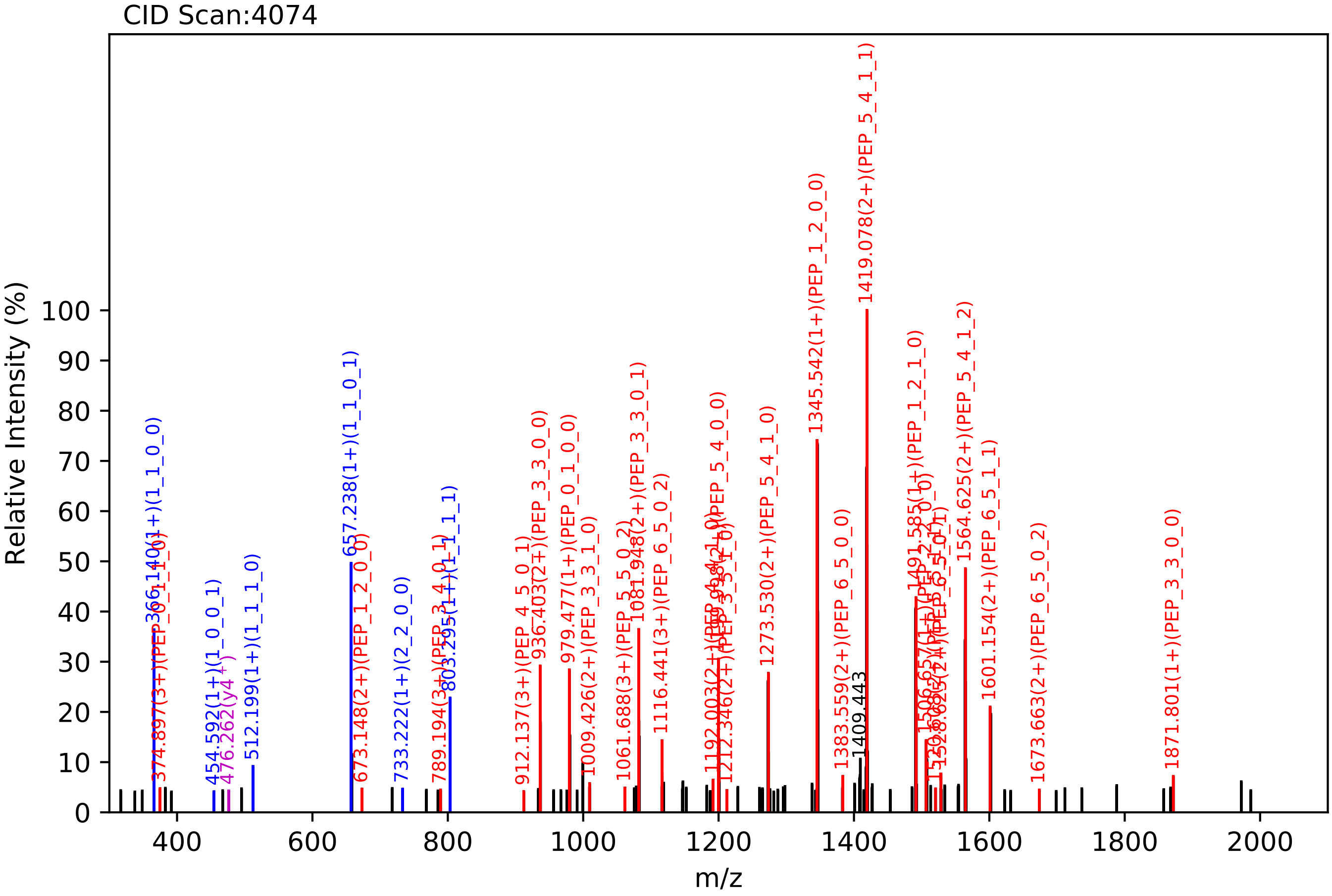

Test set no. 27, Experiment: AGP exp_4

ENGTISR(=PEP)_6_5_1_2, m/z:1164.79(3+), RT:21.33, Y-score:70.96

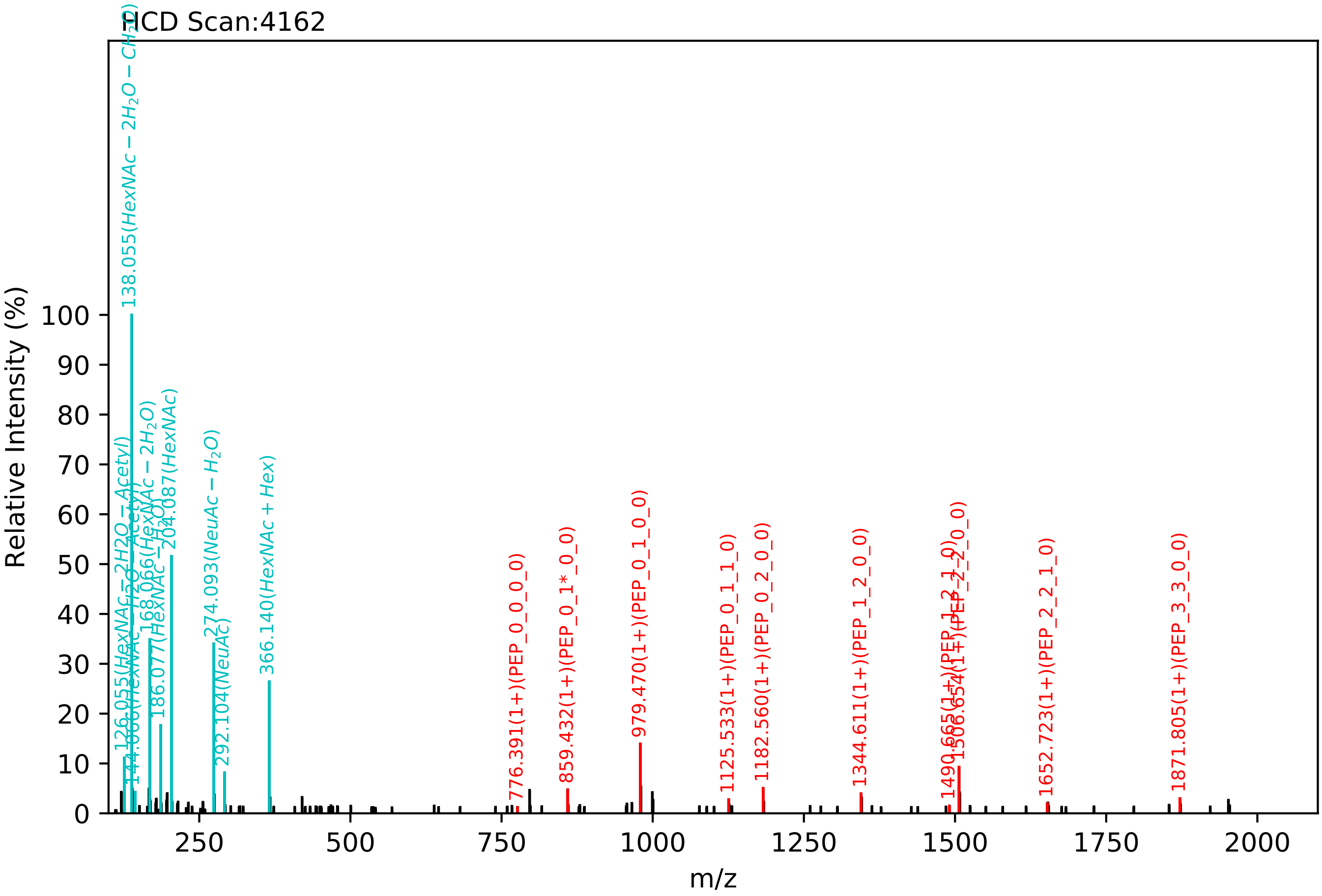

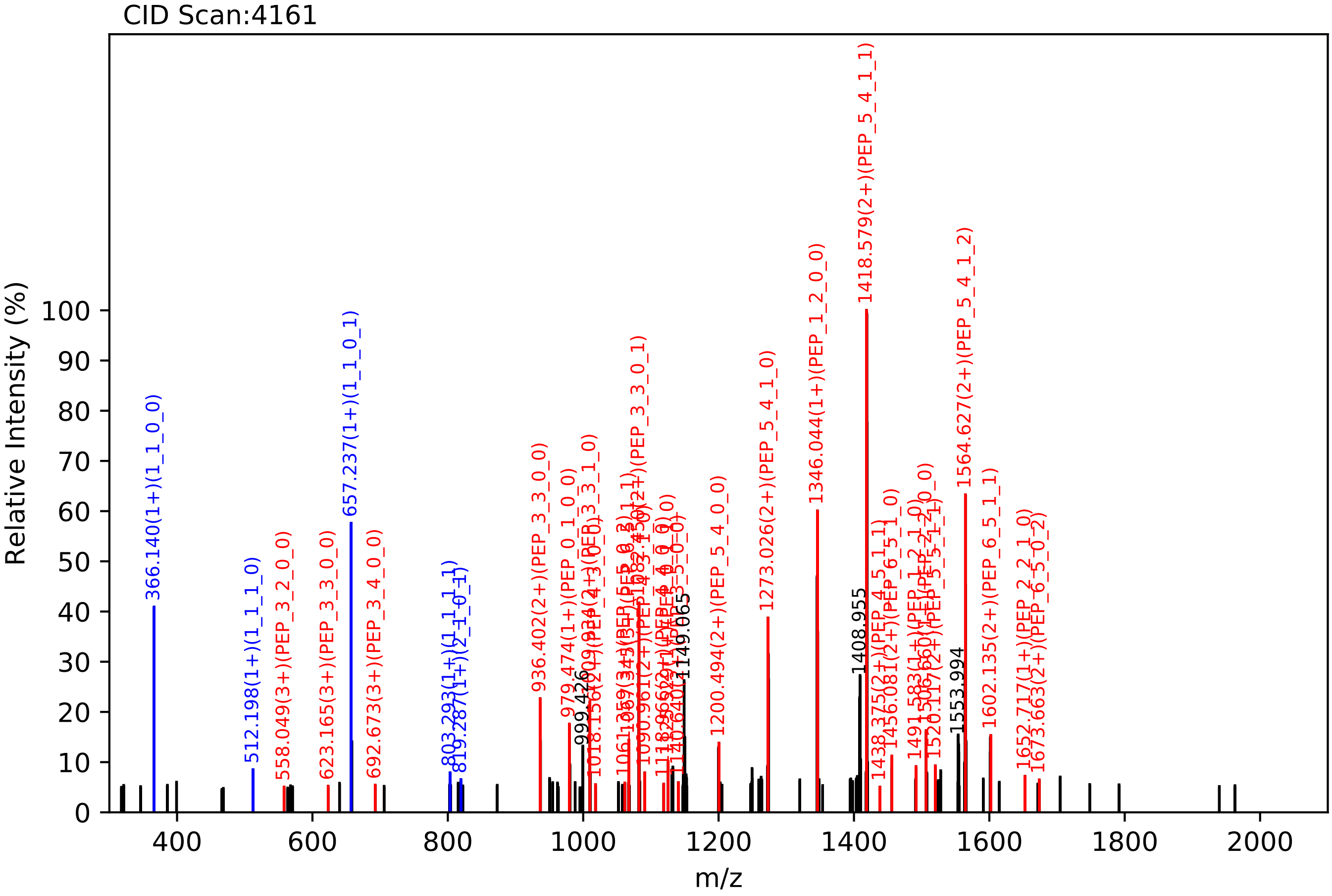

Test set no. 28, Experiment: AGP exp_3

ENGTISR(=PEP)_6_5_1_3, m/z:1261.83(3+), RT:22.59, Y-score:92.48

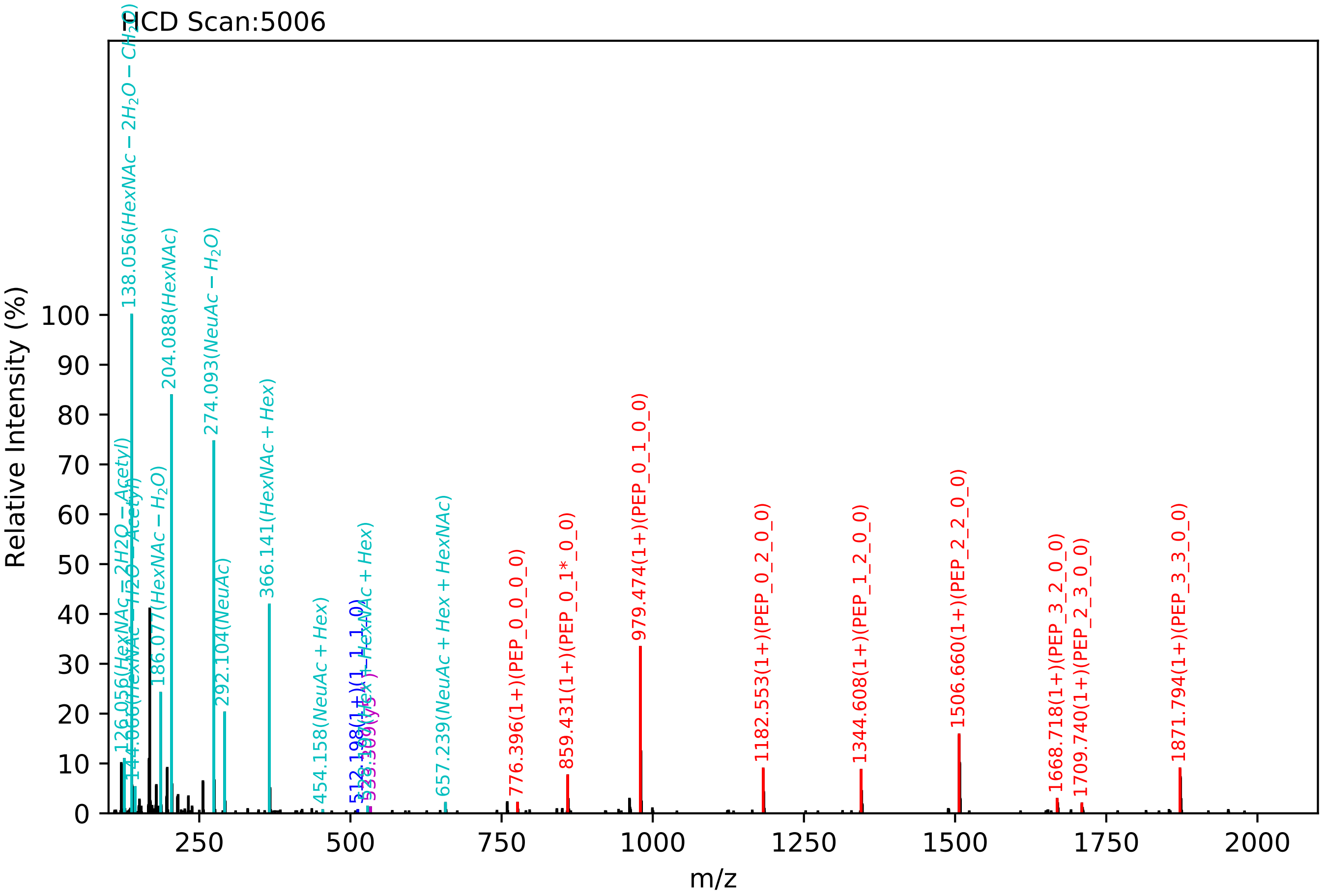

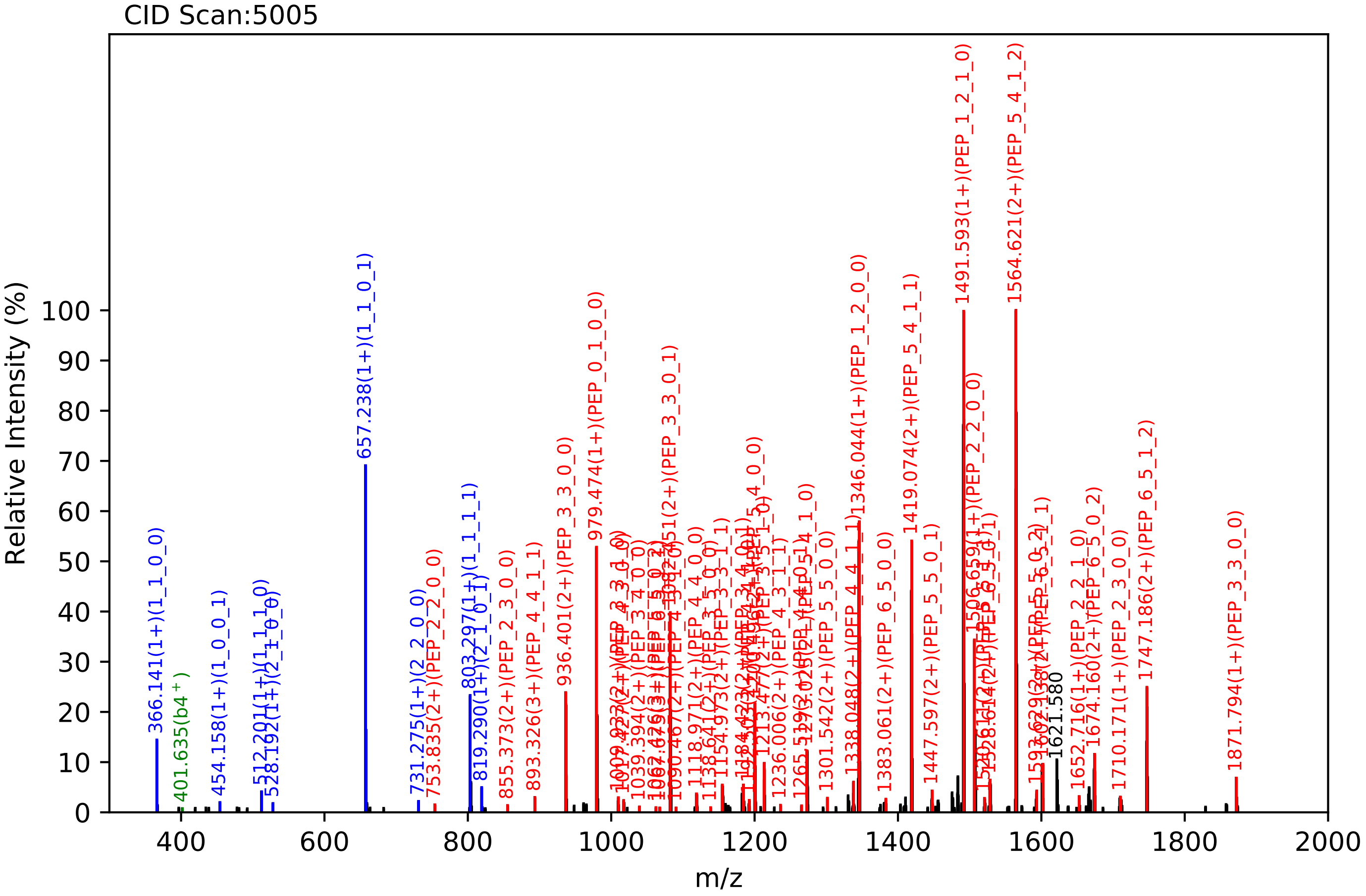

Test set no. 29, Experiment: AGP exp_4

ENGTISR(=PEP)_6_5_1_3, m/z:1261.82(3+), RT:22.52, Y-score:91.50

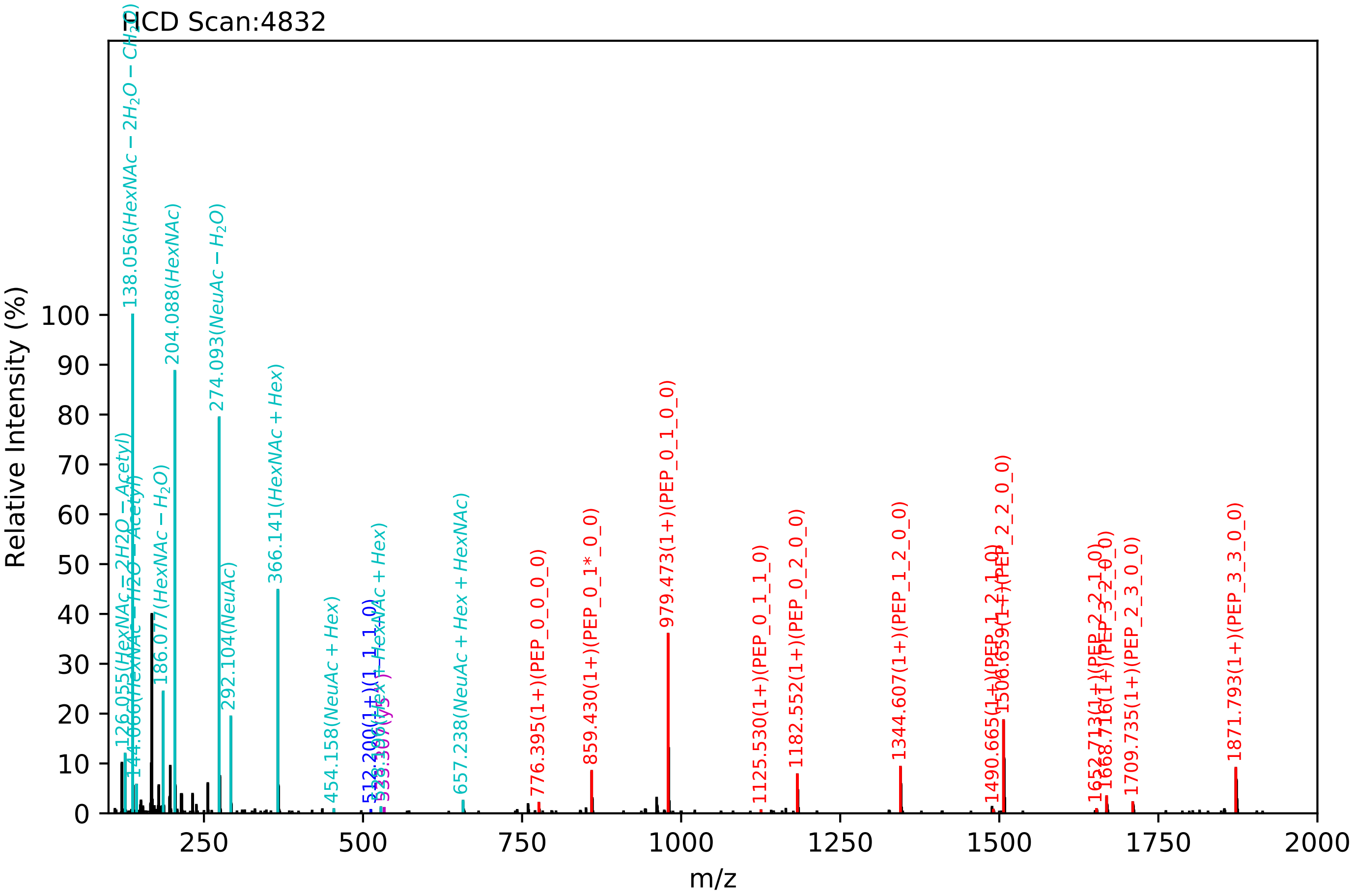

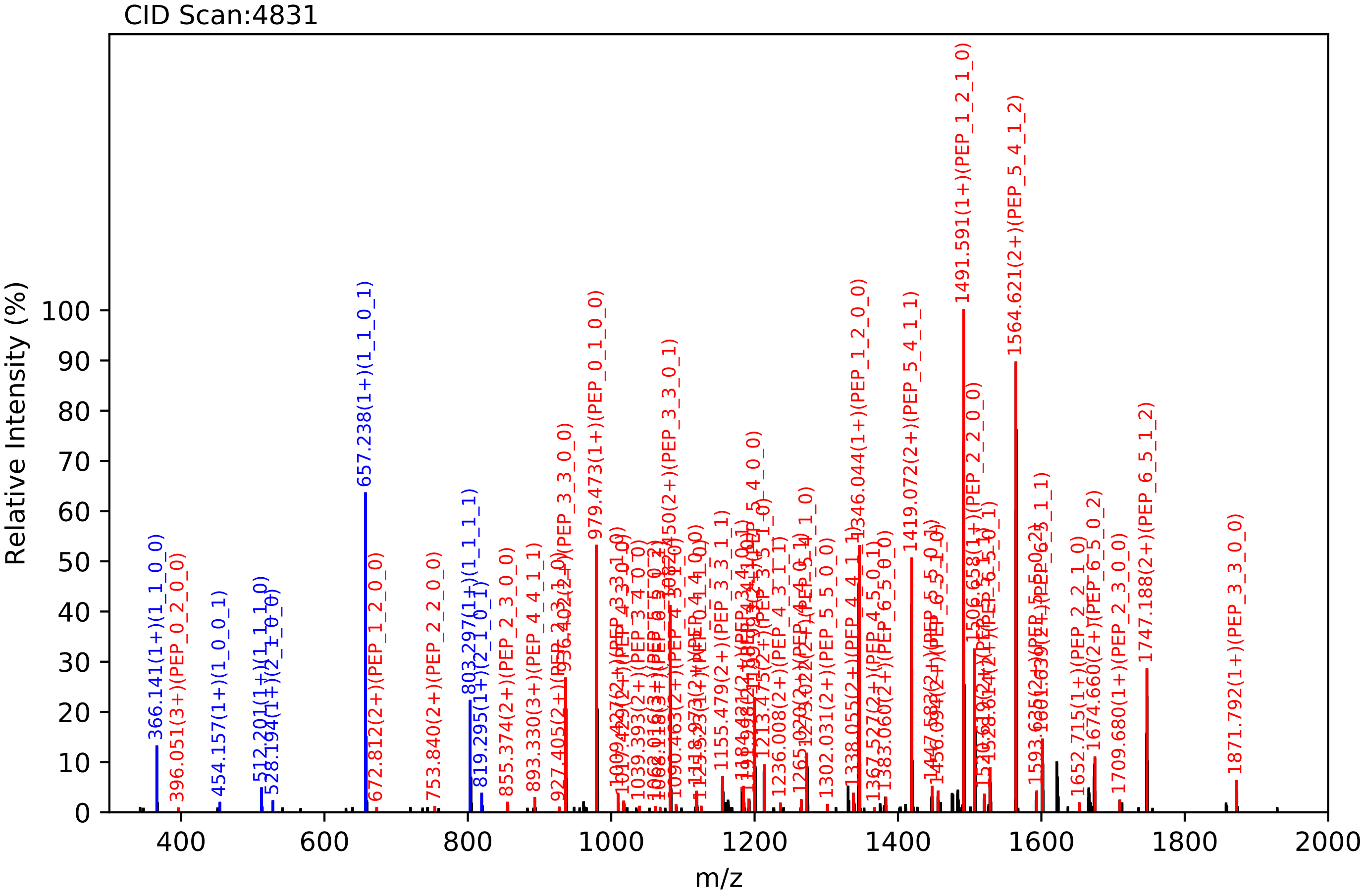

Test set no. 30, Experiment: AGP exp_40

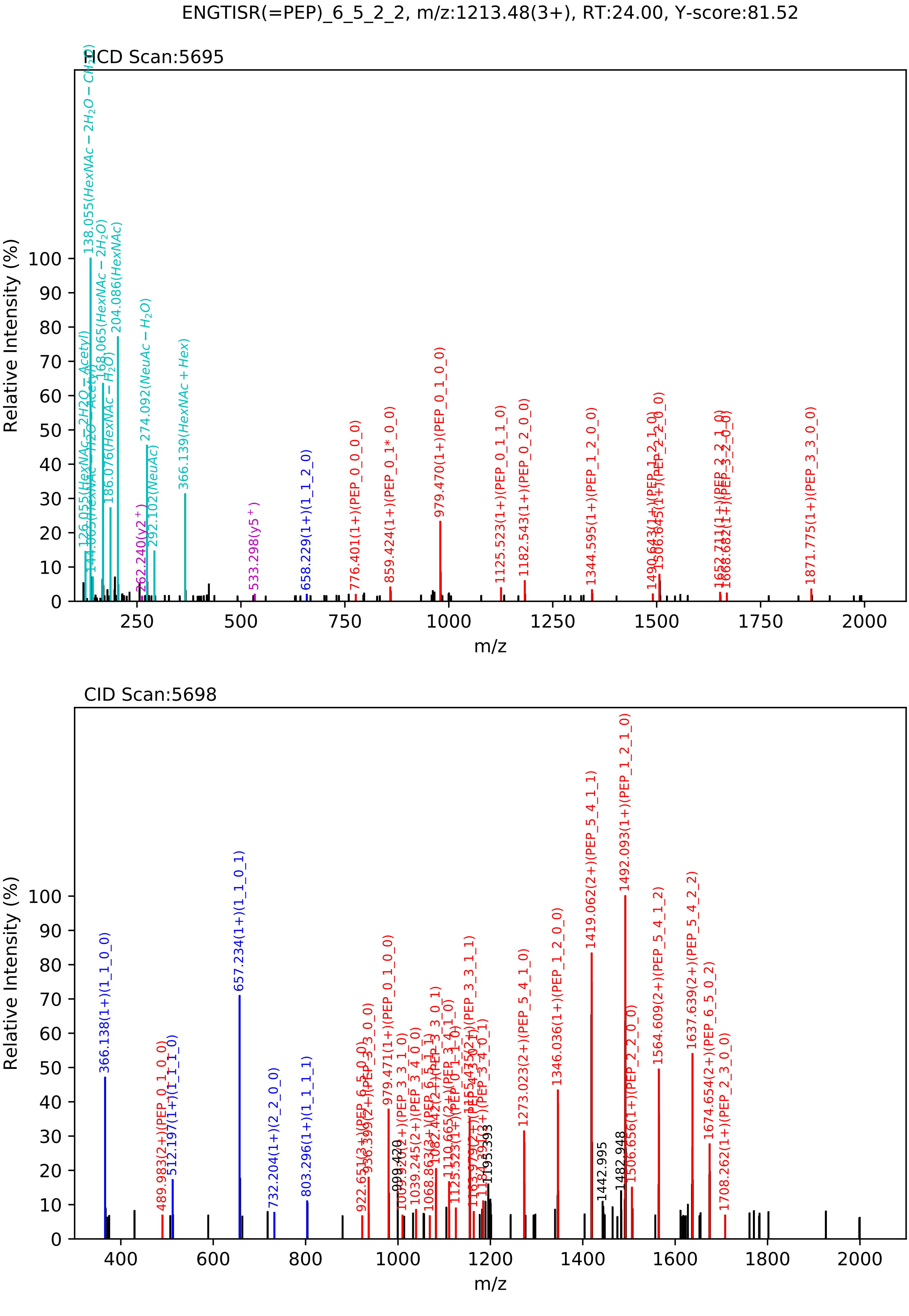

Test set no. 31, Experiment: AGP exp_30

ENGTISR(=PEP)_6_5_2_3, m/z:1310.51(3+), RT:39.94, Y-score:91.71

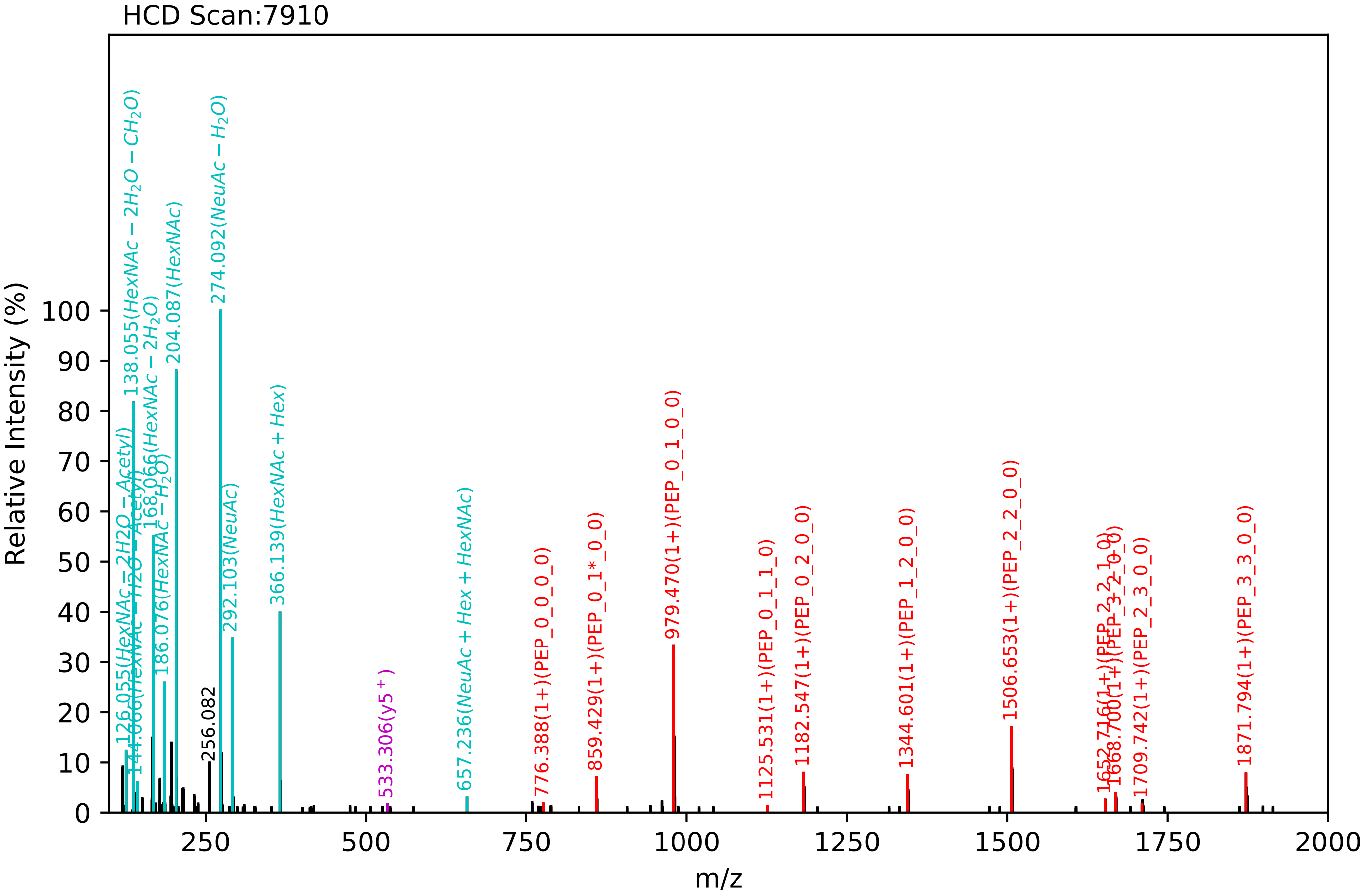

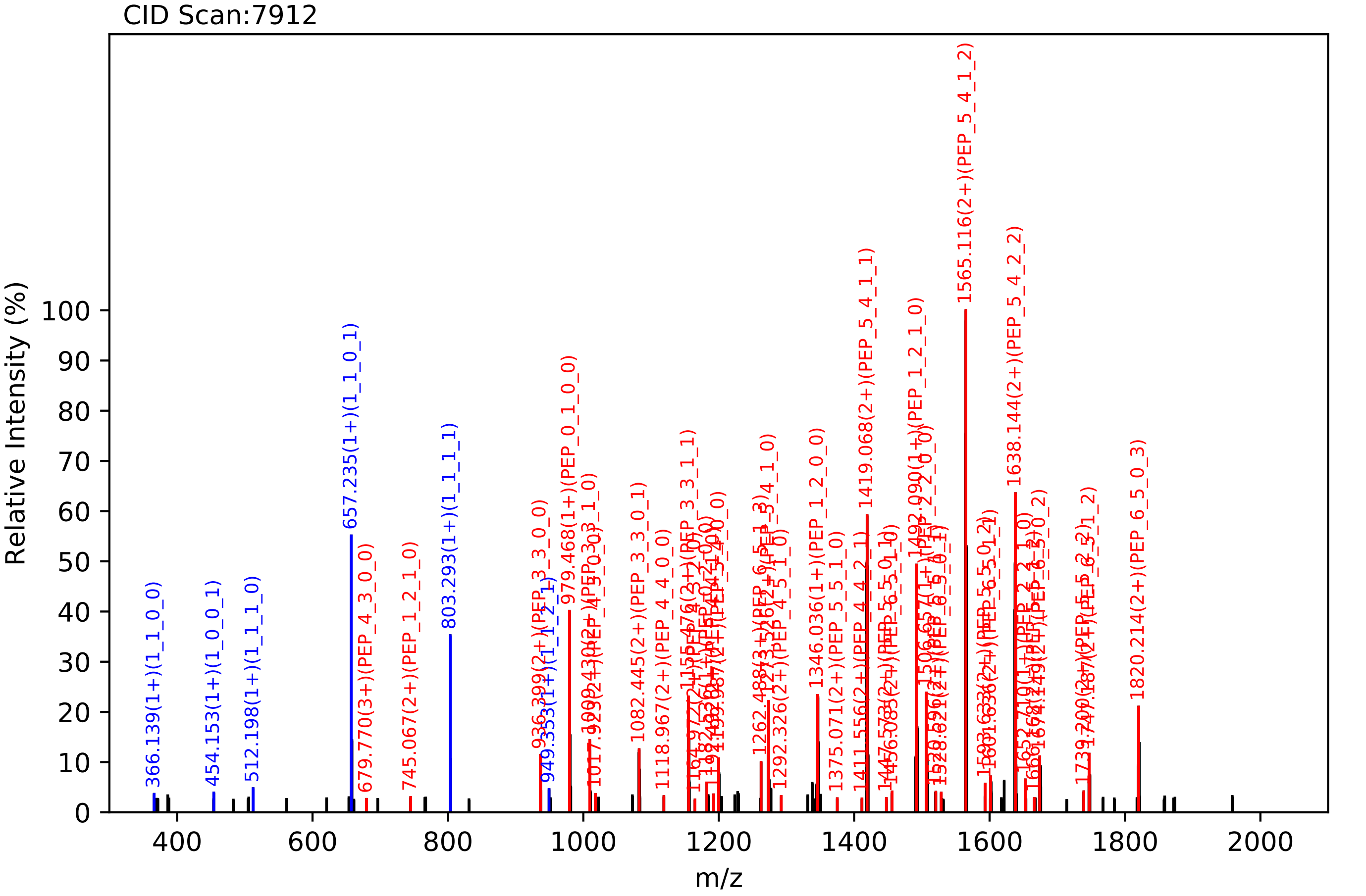

Test set no. 32, Experiment: AGP exp_36

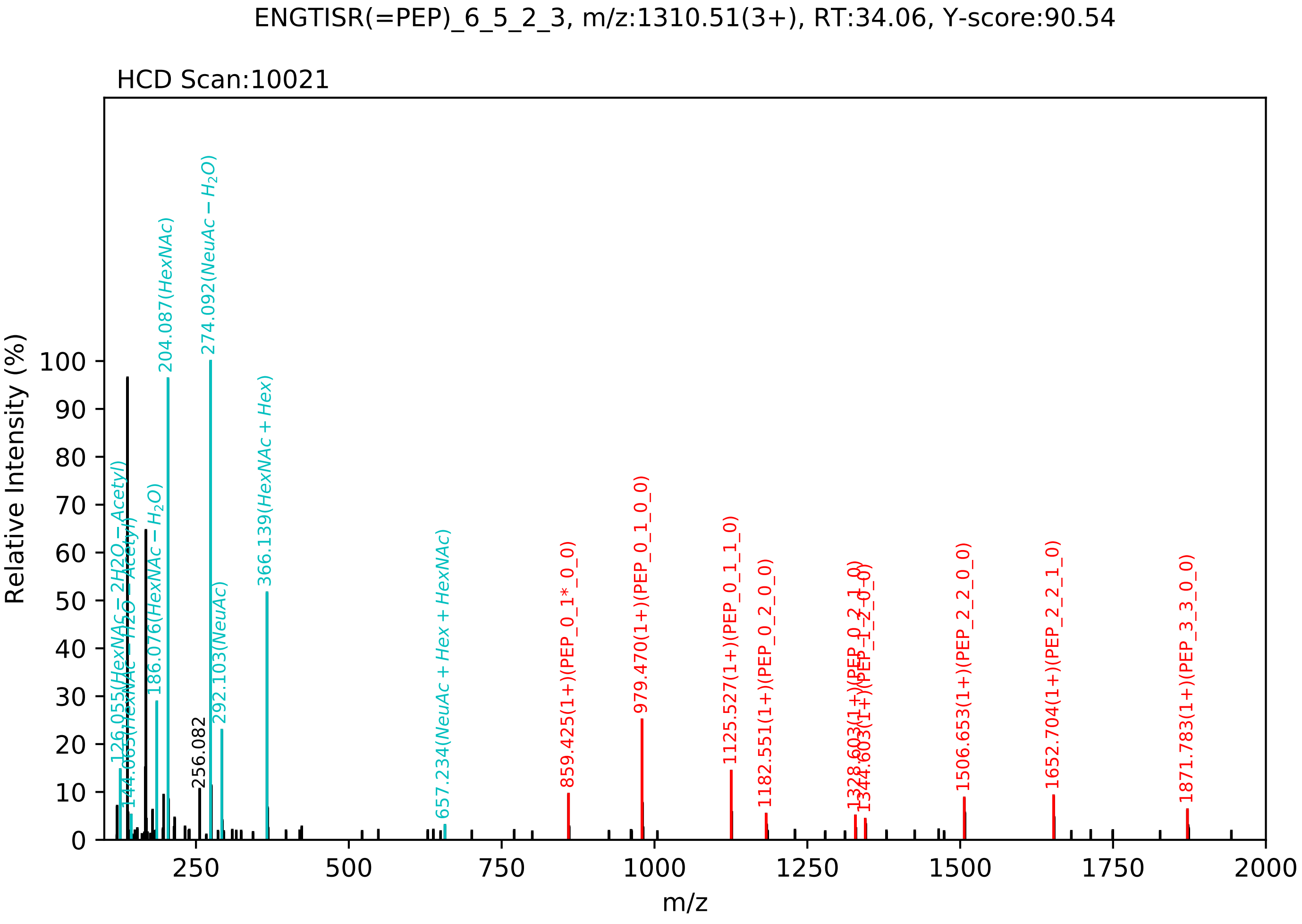

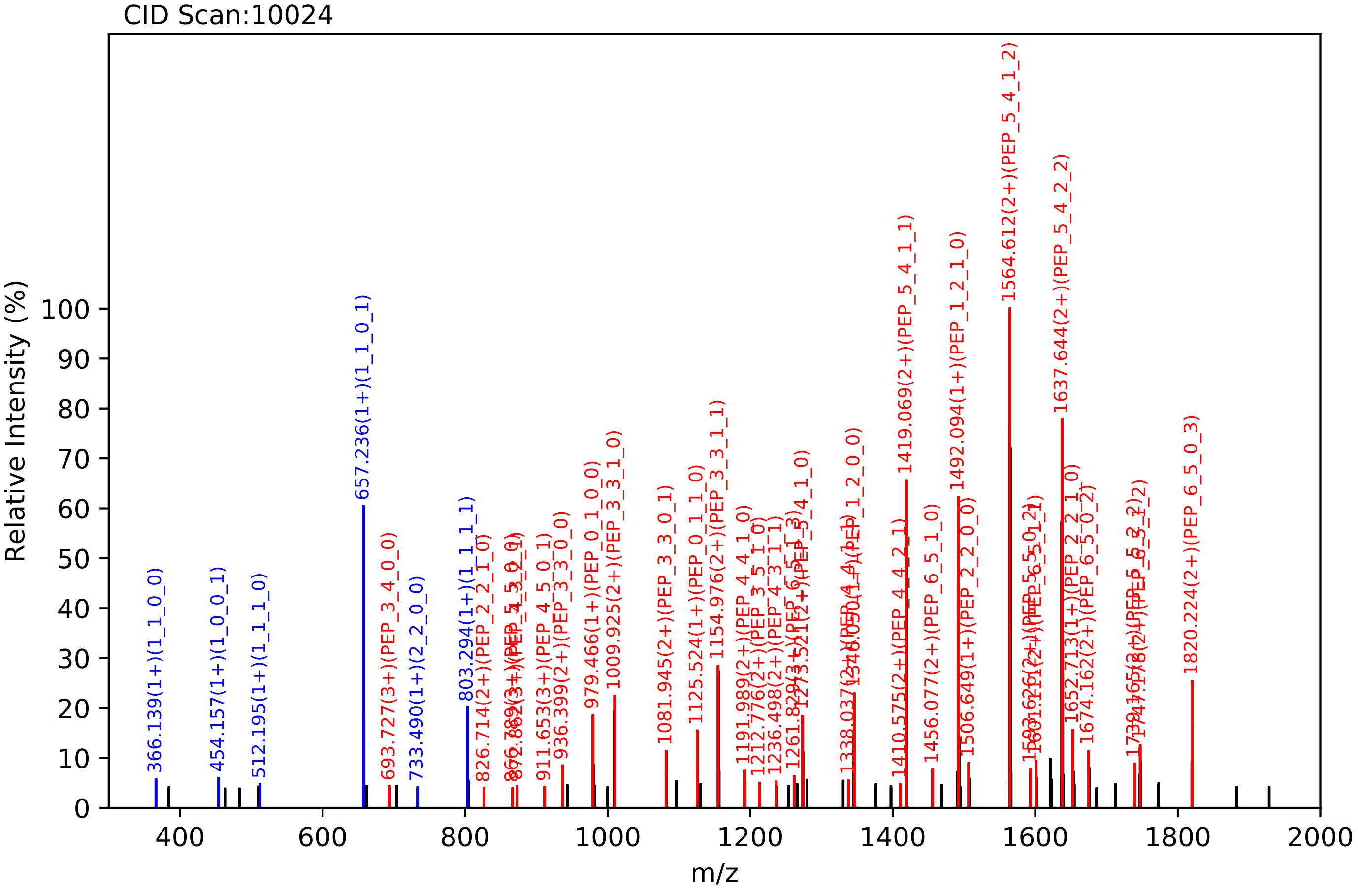

Test set no. 33, Experiment: AGP exp_37

ENGTISR(=PEP)_6_5_2_3, m/z:1310.51(3+), RT:33.53, Y-score:90.23

HCD Scan:9865

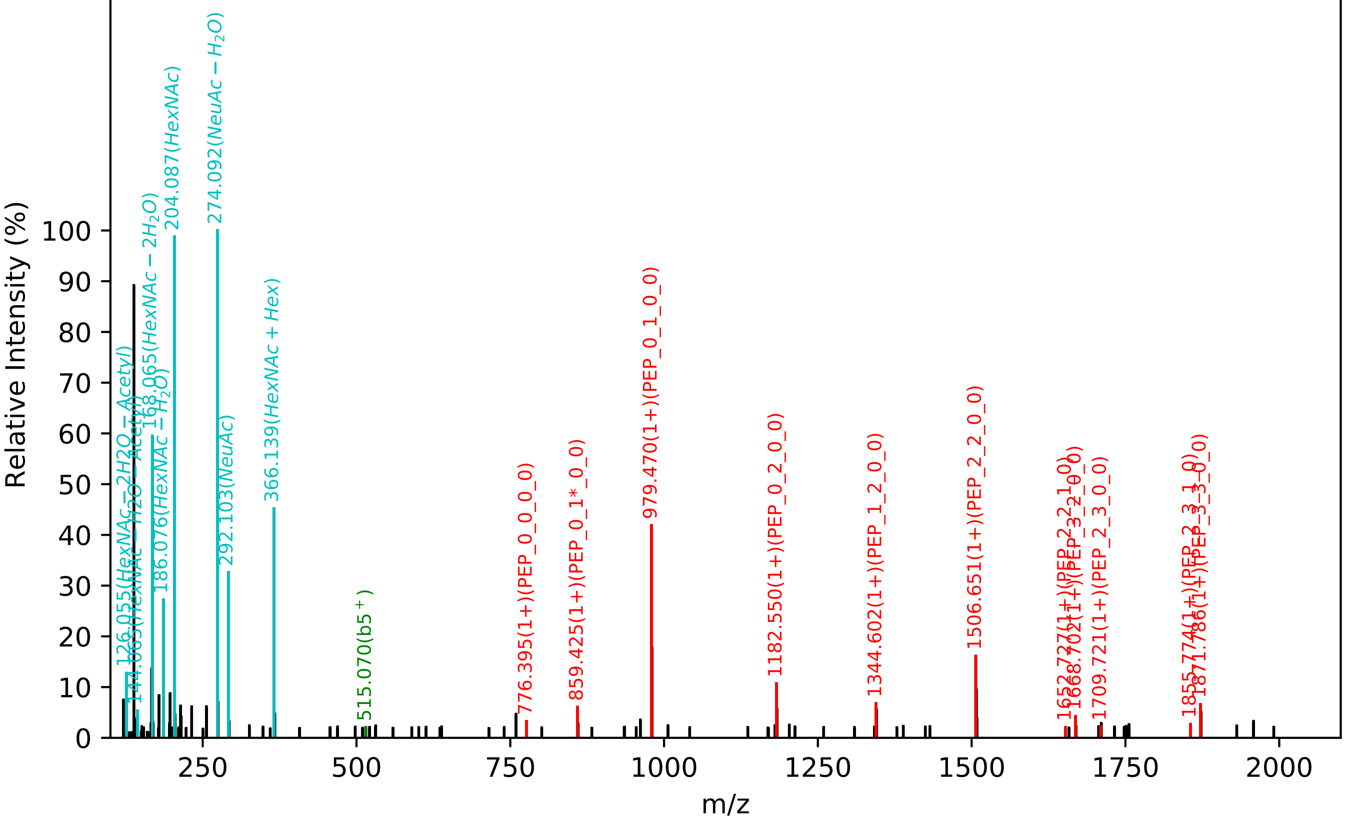

CID Scan:9868

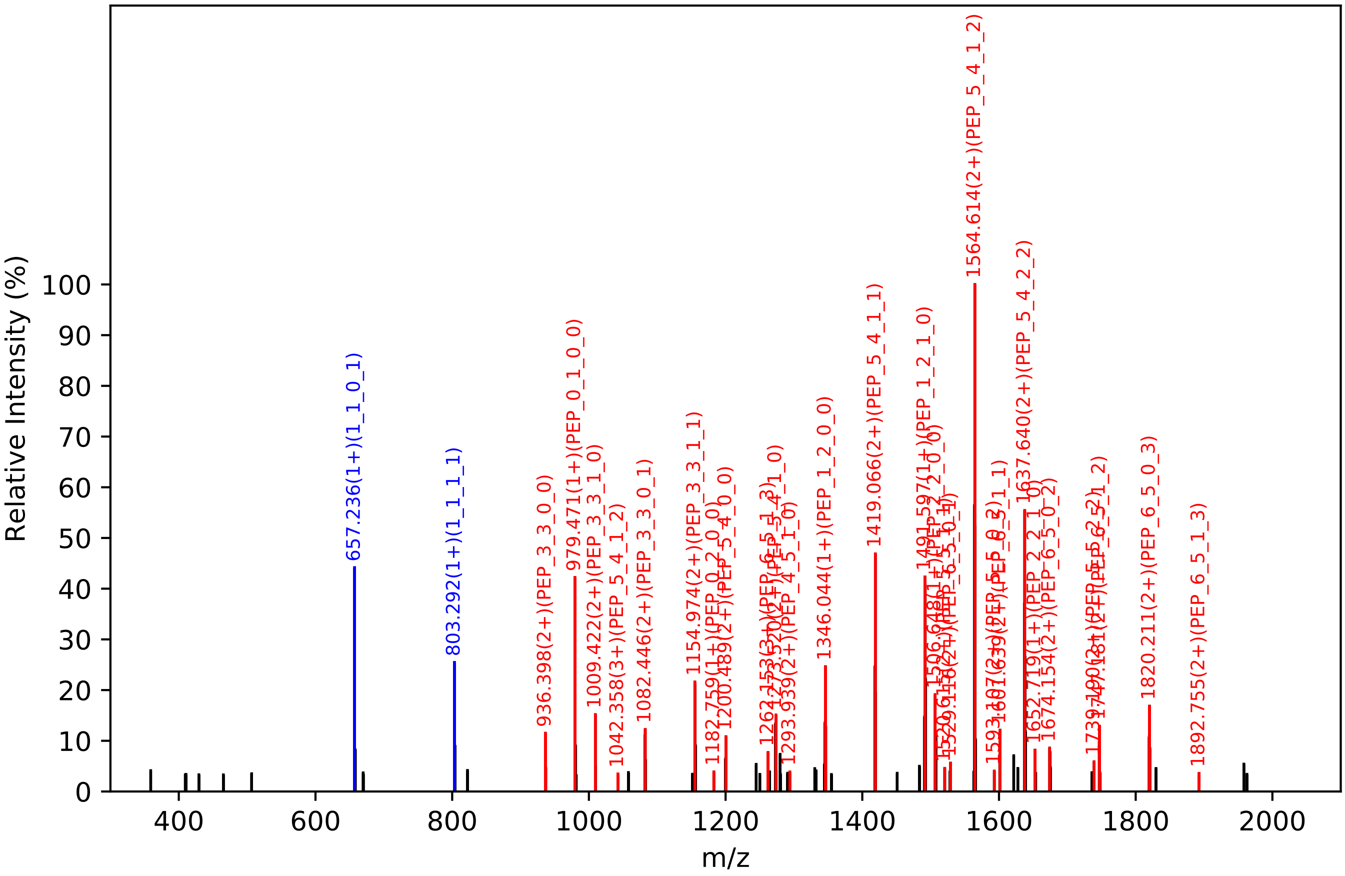

Test set no. 34, Experiment: AGP exp_35

ENGTISR(=PEP)_6_5_2_3, m/z:1310.51(3+), RT:33.60, Y-score:89.94

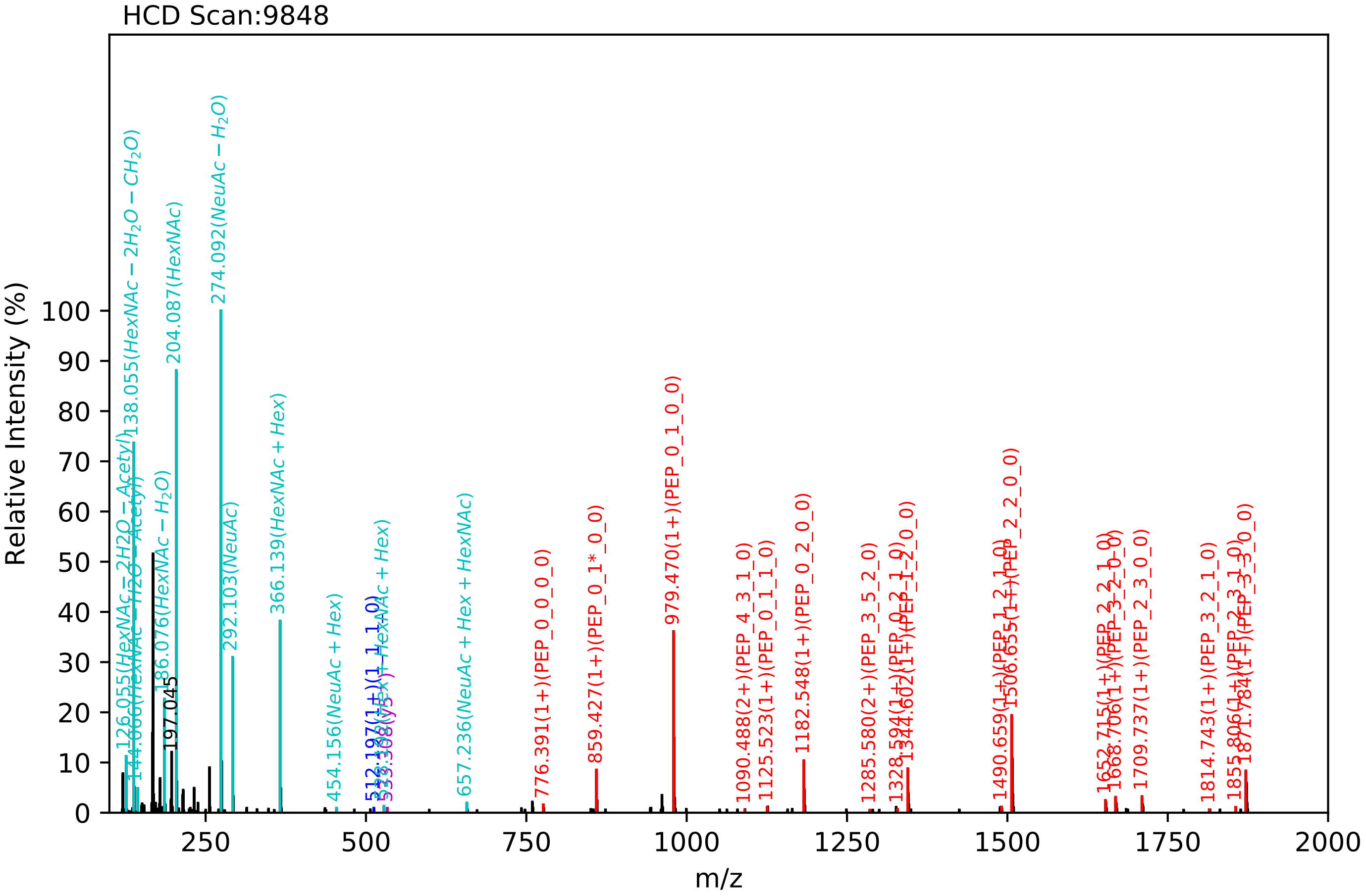

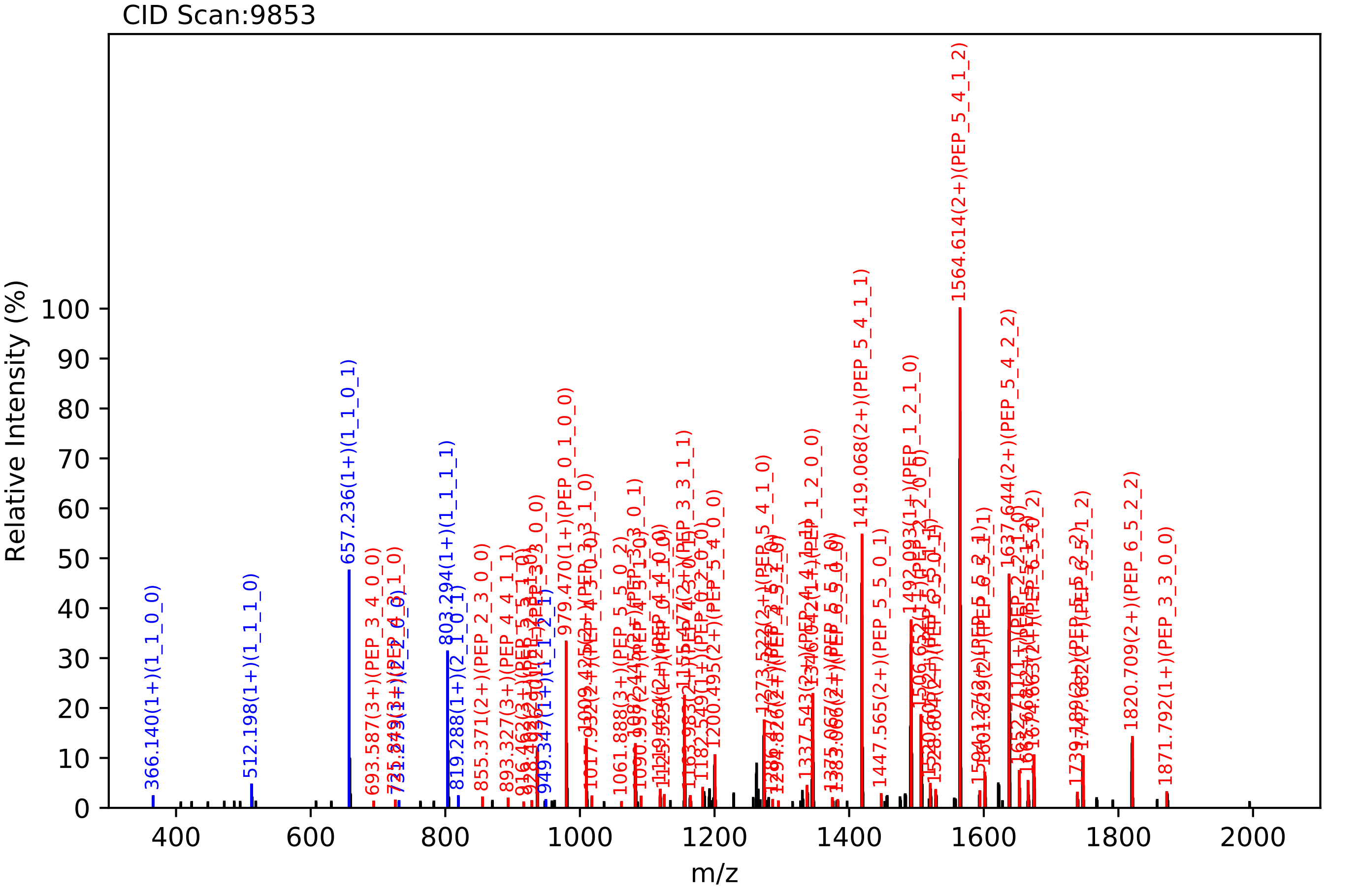

Test set no. 35, Experiment: AGP exp_34

ENGTISR(=PEP)_6_5_2_3, m/z:1310.51(2+), RT:34.27, Y-score:89.04

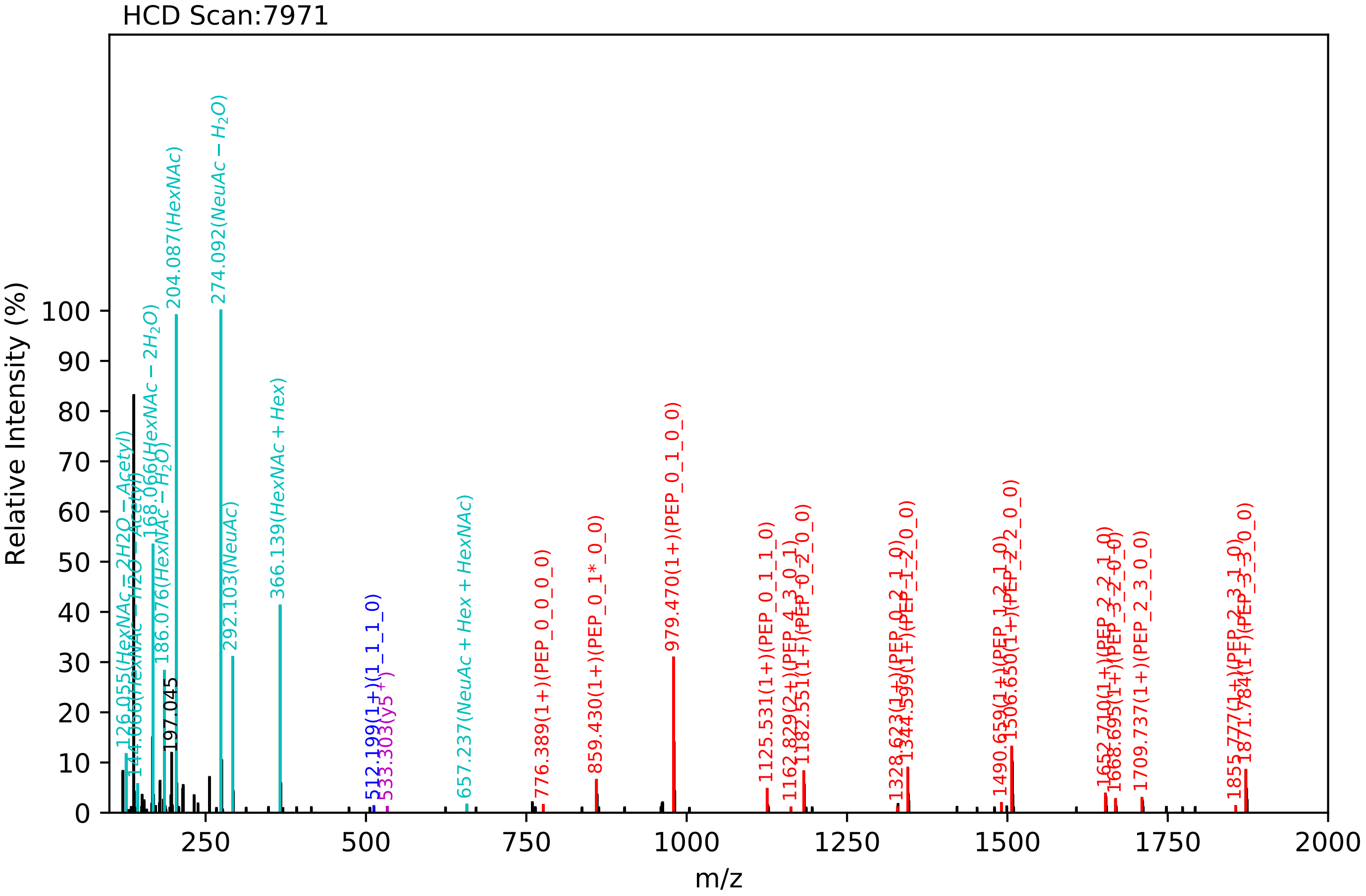

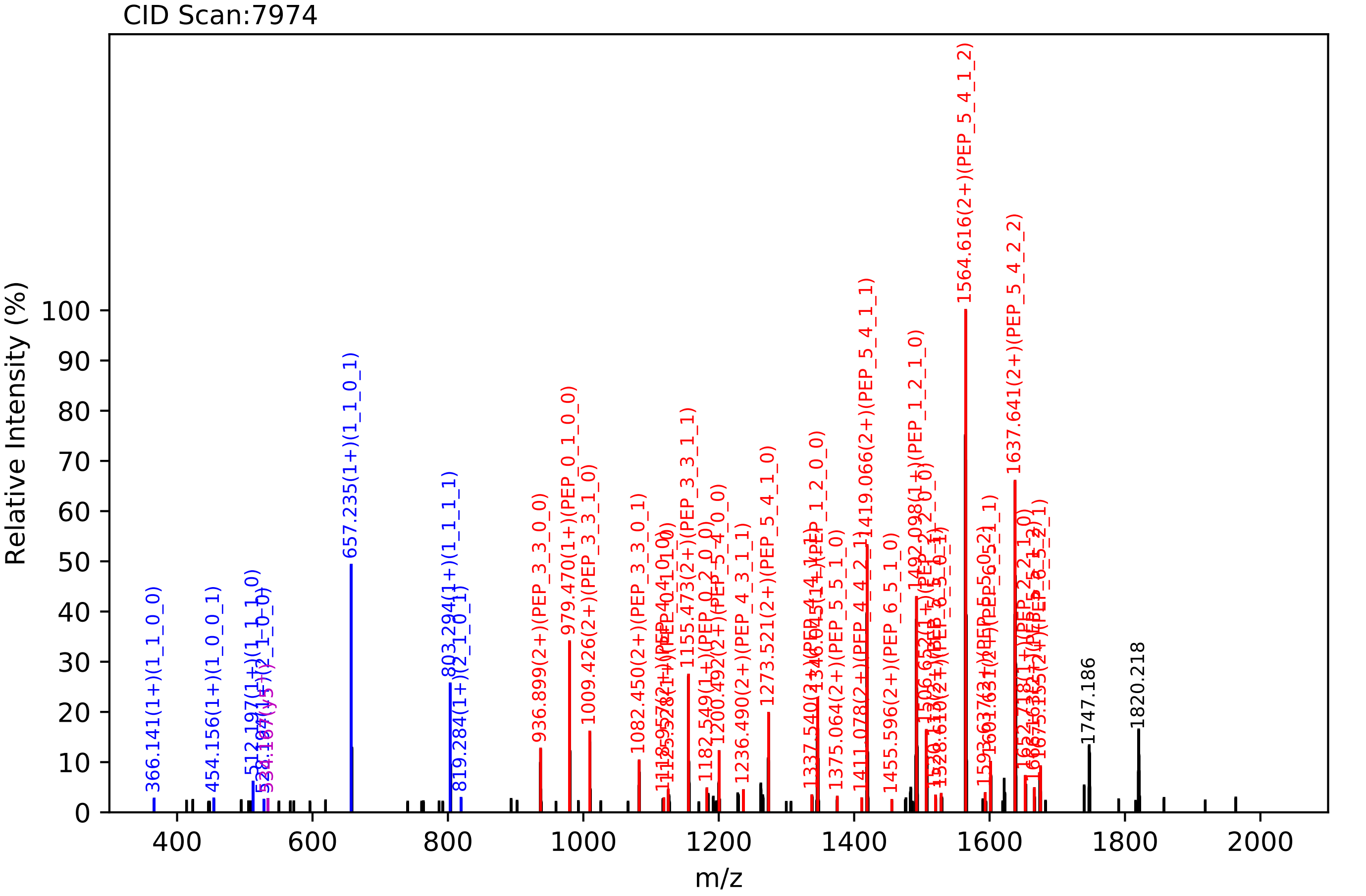

Test set no. 36, Experiment: AGP exp_22

ENGTISR(=PEP)_6_5_2_3, m/z:1310.51(3+), RT:33.93, Y-score:88.45

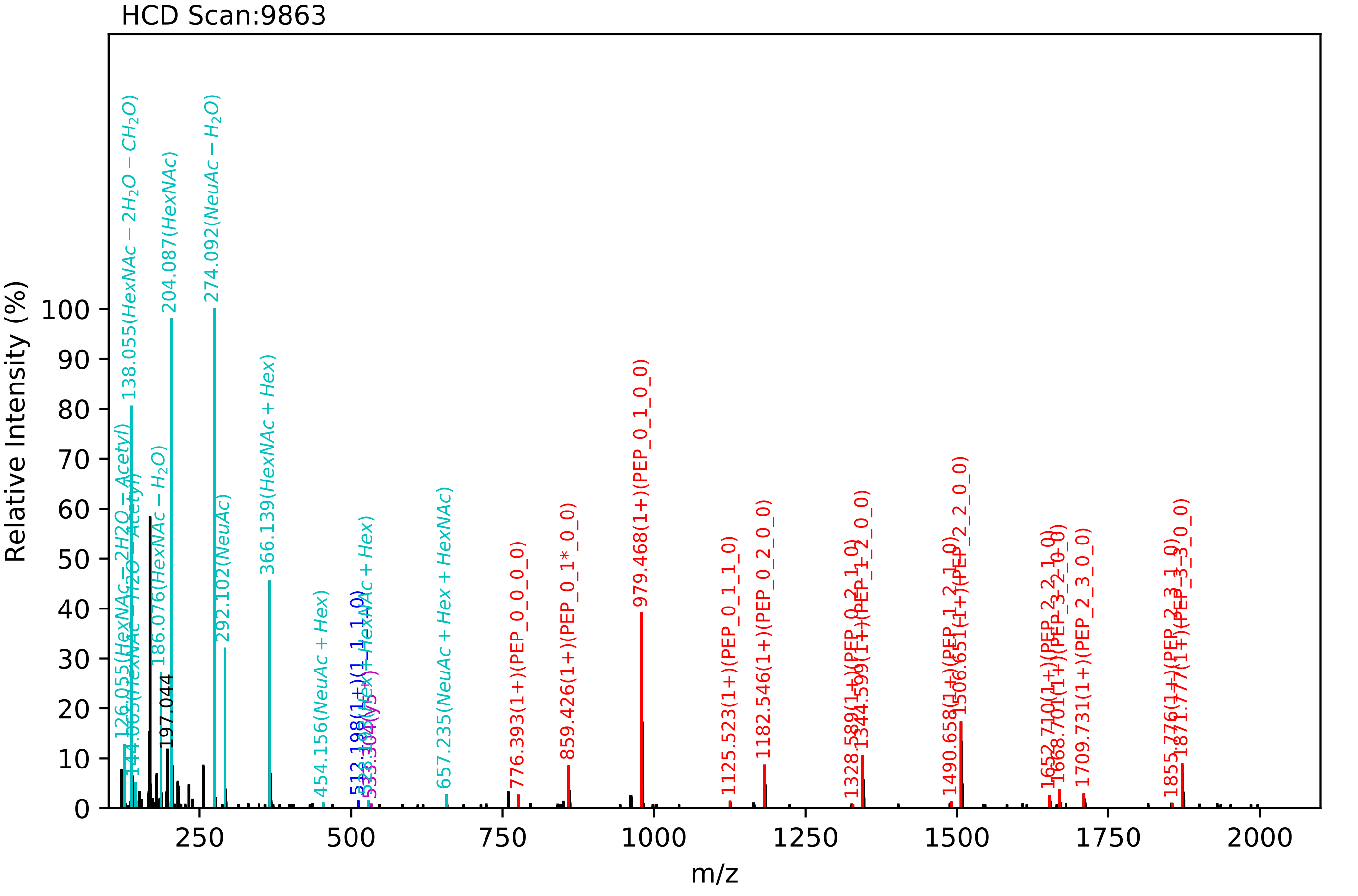

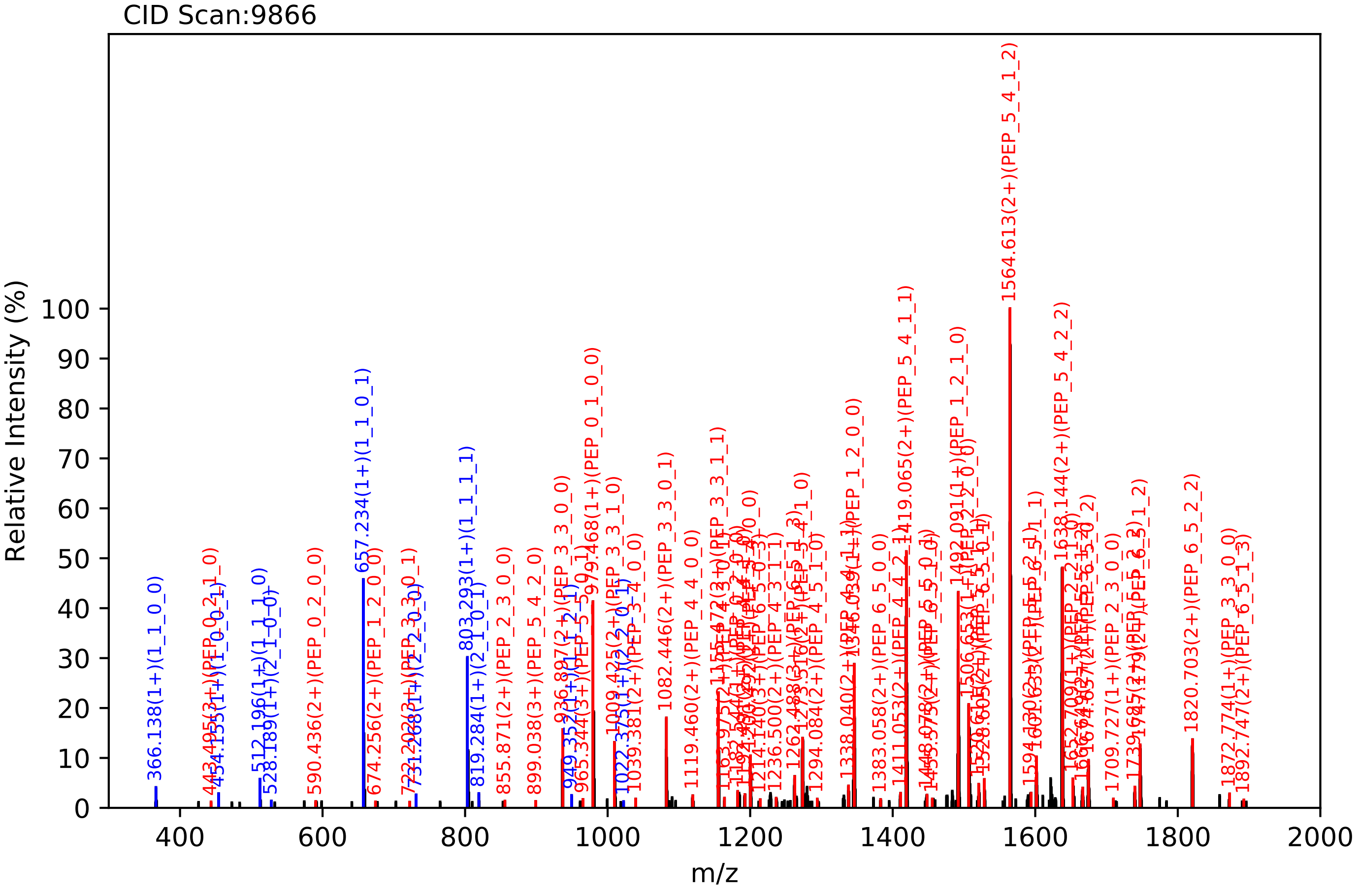

Test set no. 37, Experiment: AGP exp_28

ENGTISR(=PEP)_6_5_2_3, m/z:1310.51(3+), RT:34.79, Y-score:87.89

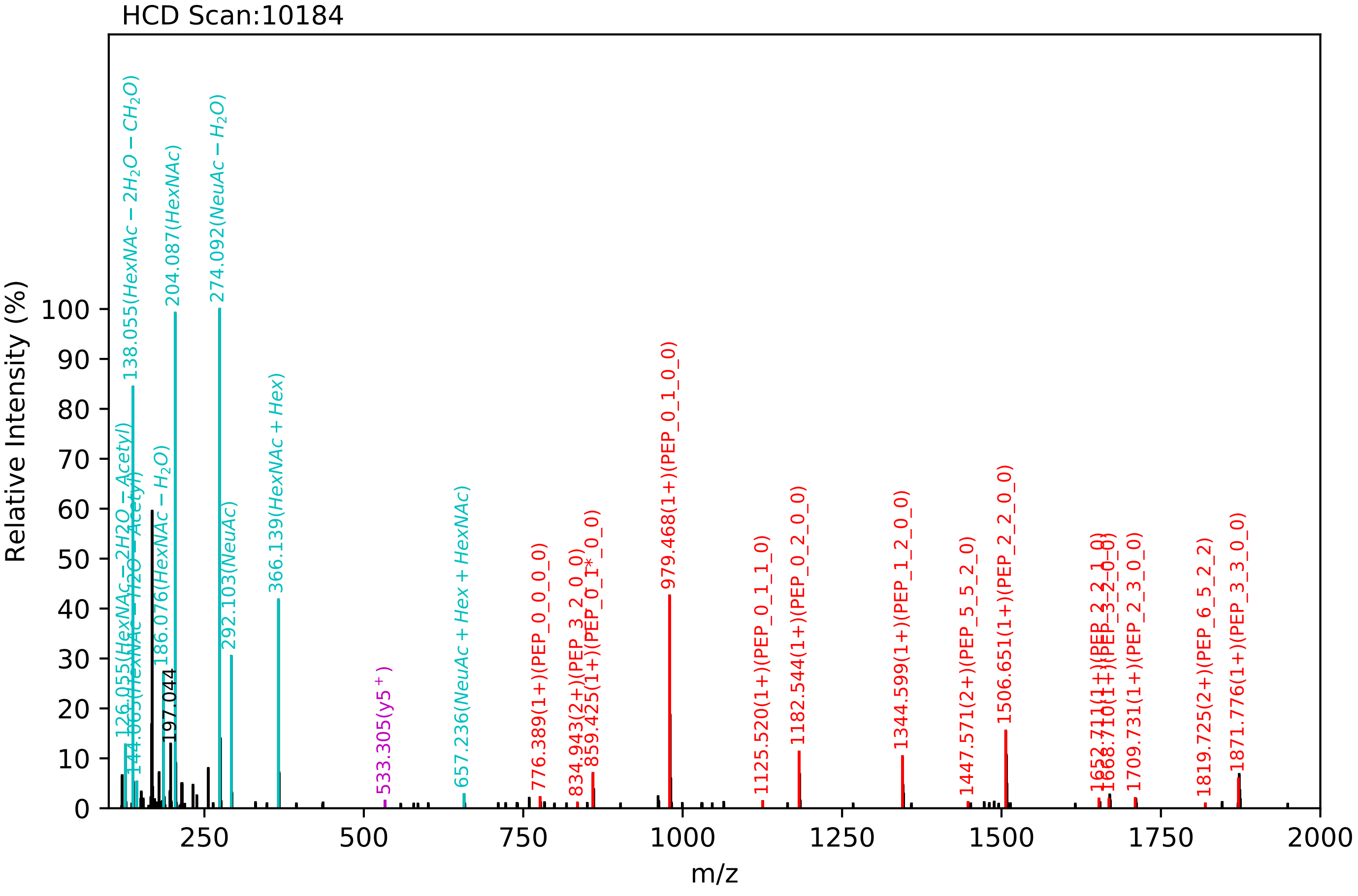

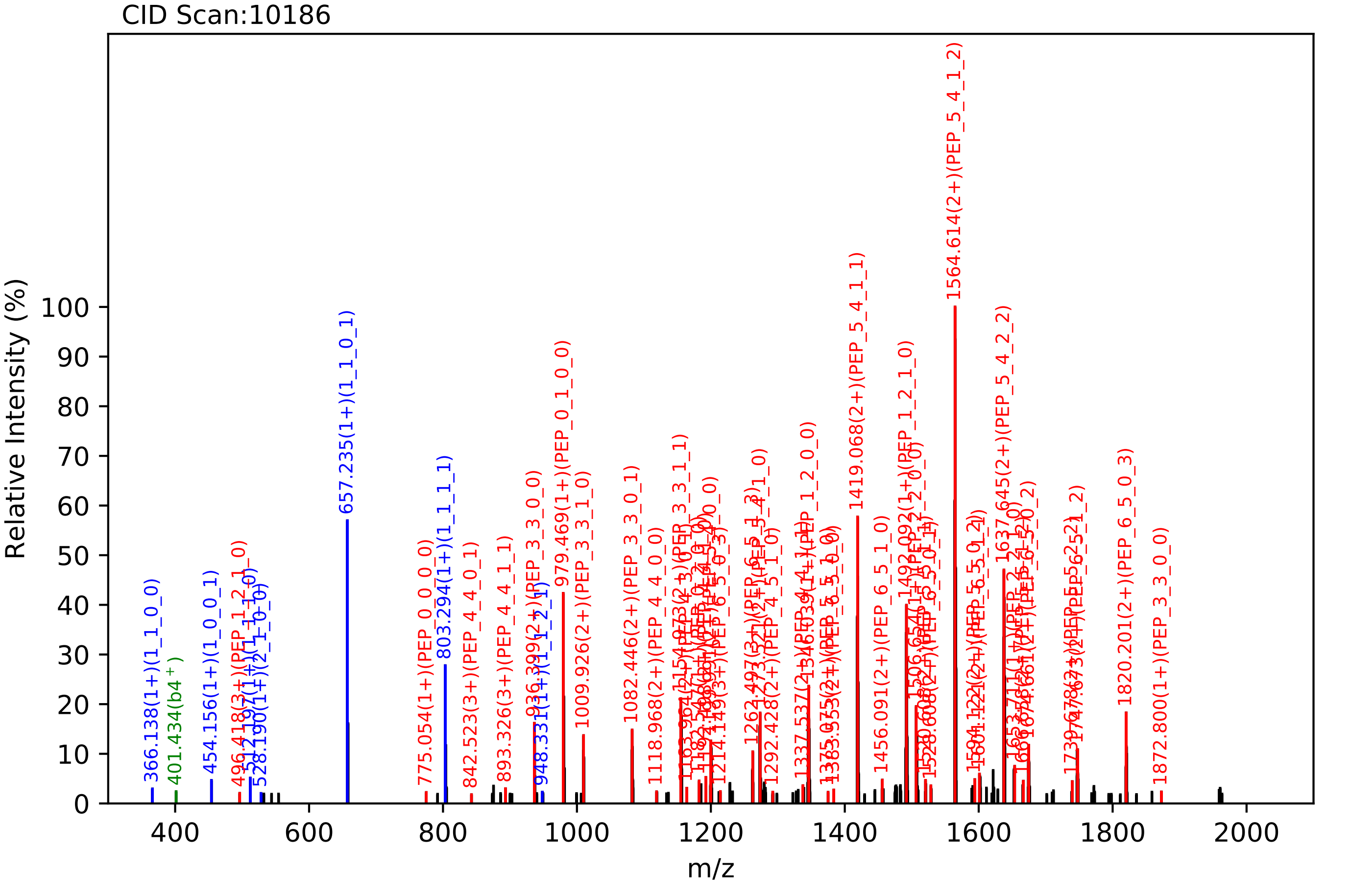

Test set no. 38, Experiment: AGP exp_20

ENGTISR(=PEP)_6_5_2_3, m/z:1310.51(3+), RT:33.96, Y-score:87.69

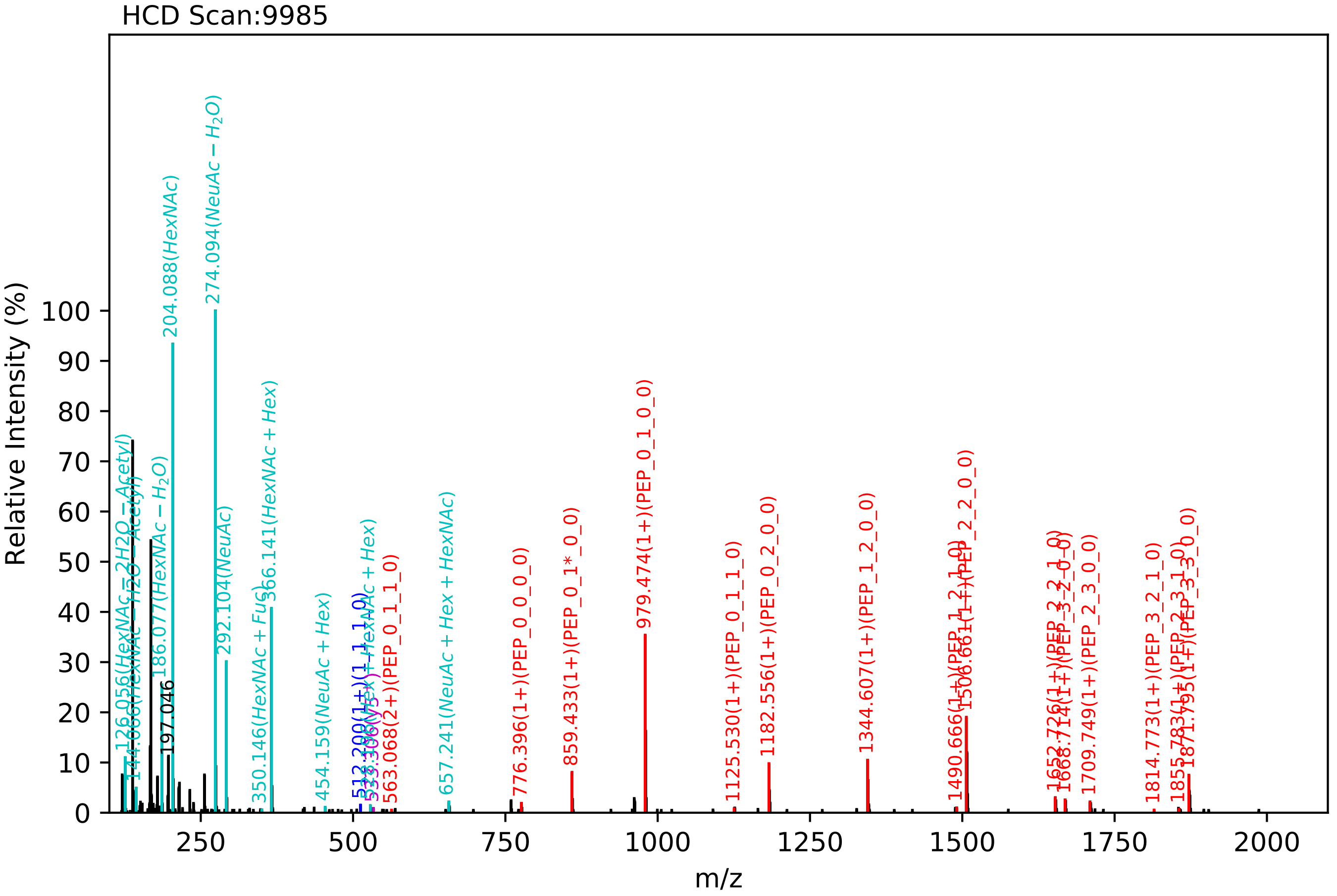

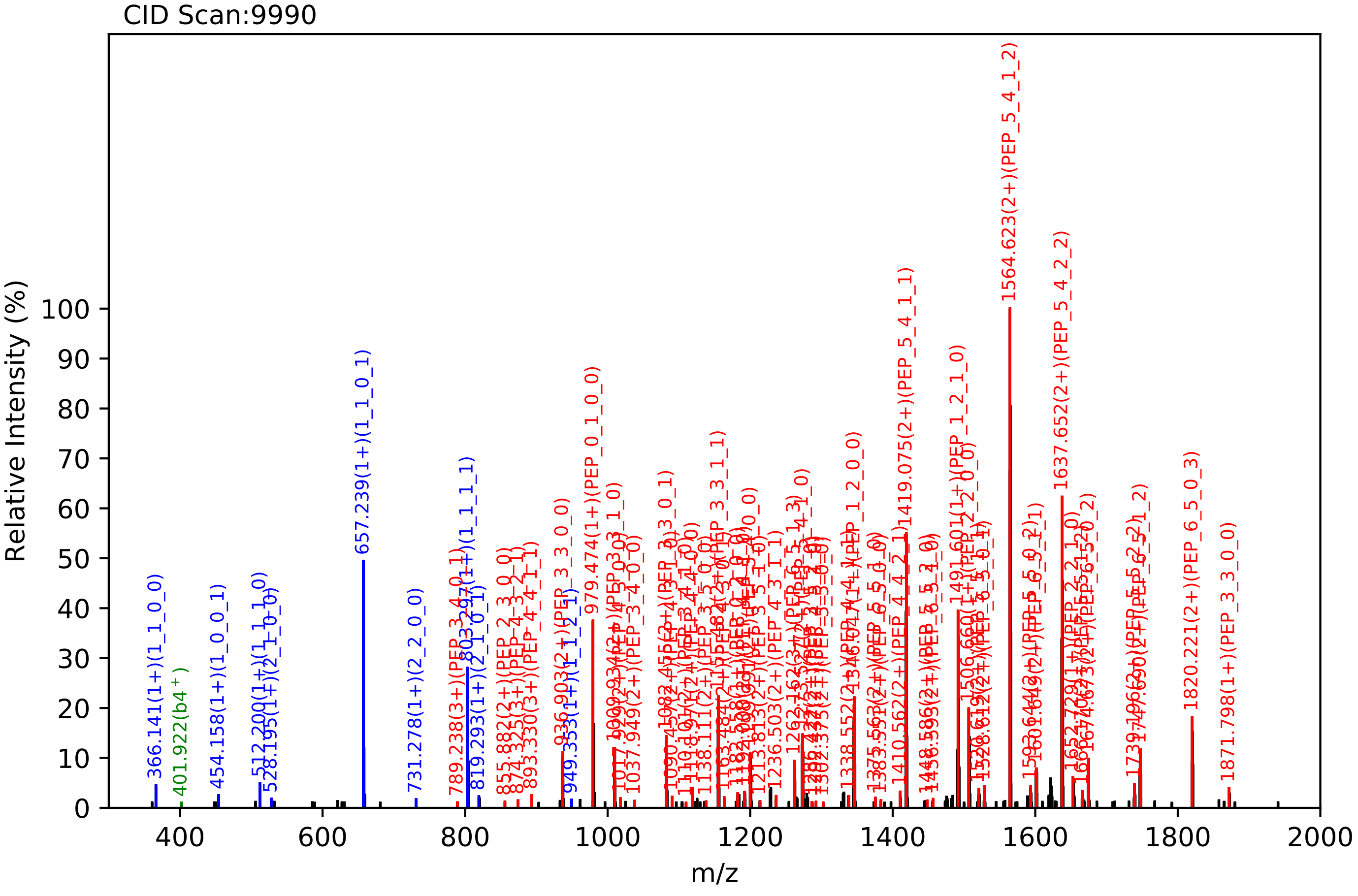

Test set no. 39, Experiment: AGP exp_32

ENGTISR(=PEP)_6_5_2_3, m/z:1310.51(3+), RT:34.19, Y-score:87.52

HCD Scan:8075

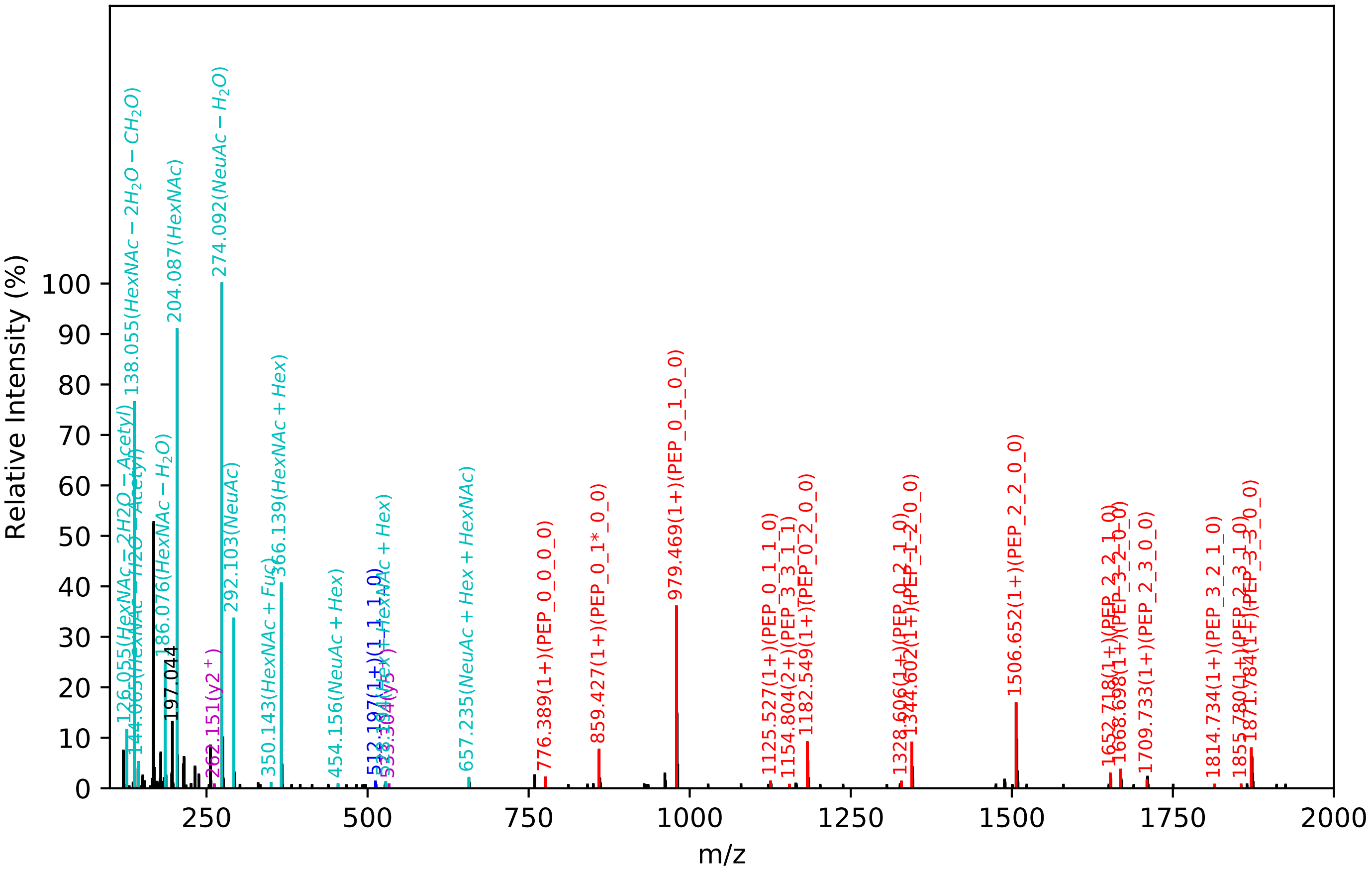

CID Scan:8077

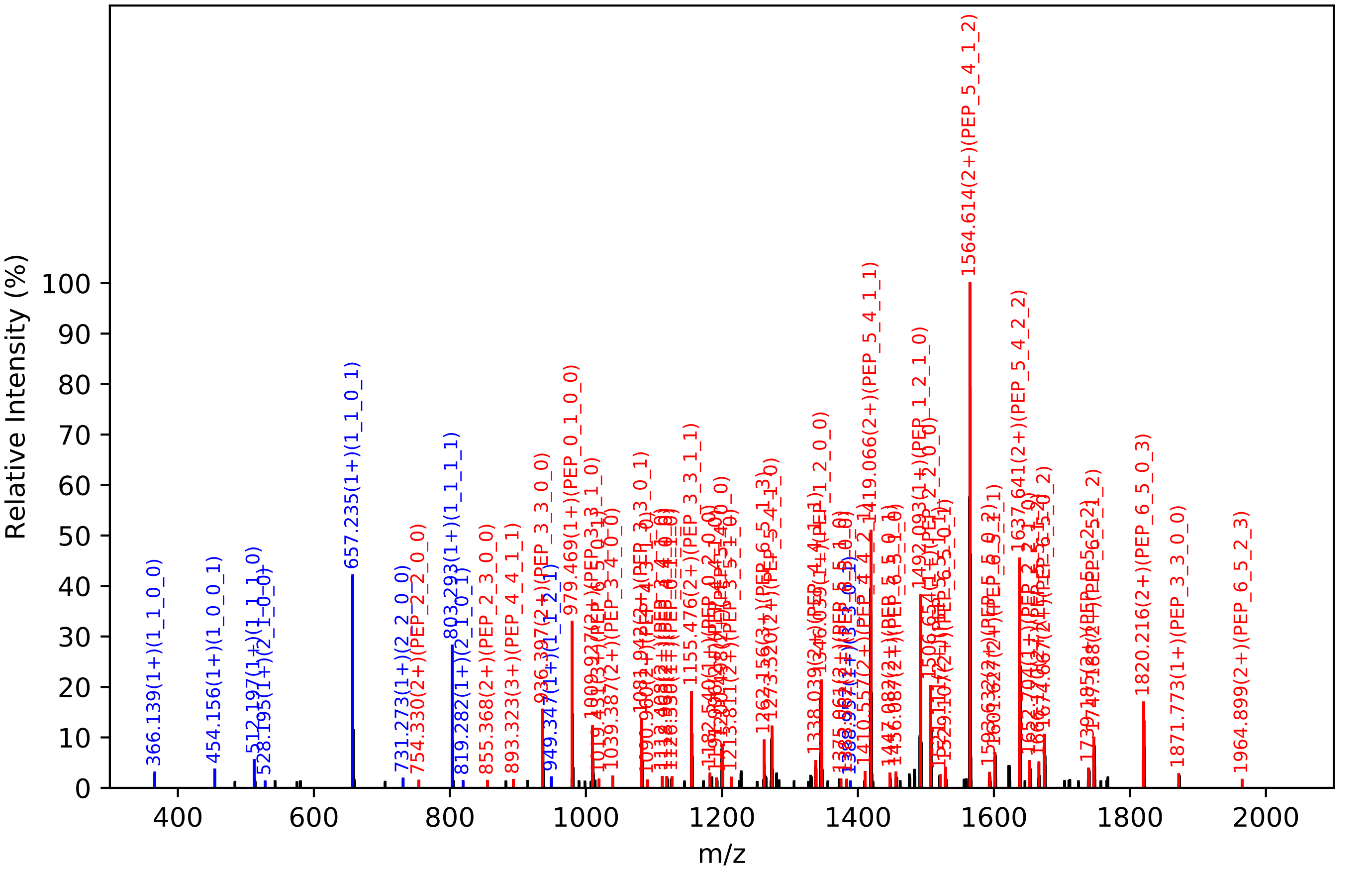

Test set no. 40, Experiment: AGP exp_18

ENGTISR(=PEP)_6_5_2_3, m/z:1310.51(3+), RT:34.64, Y-score:84.64

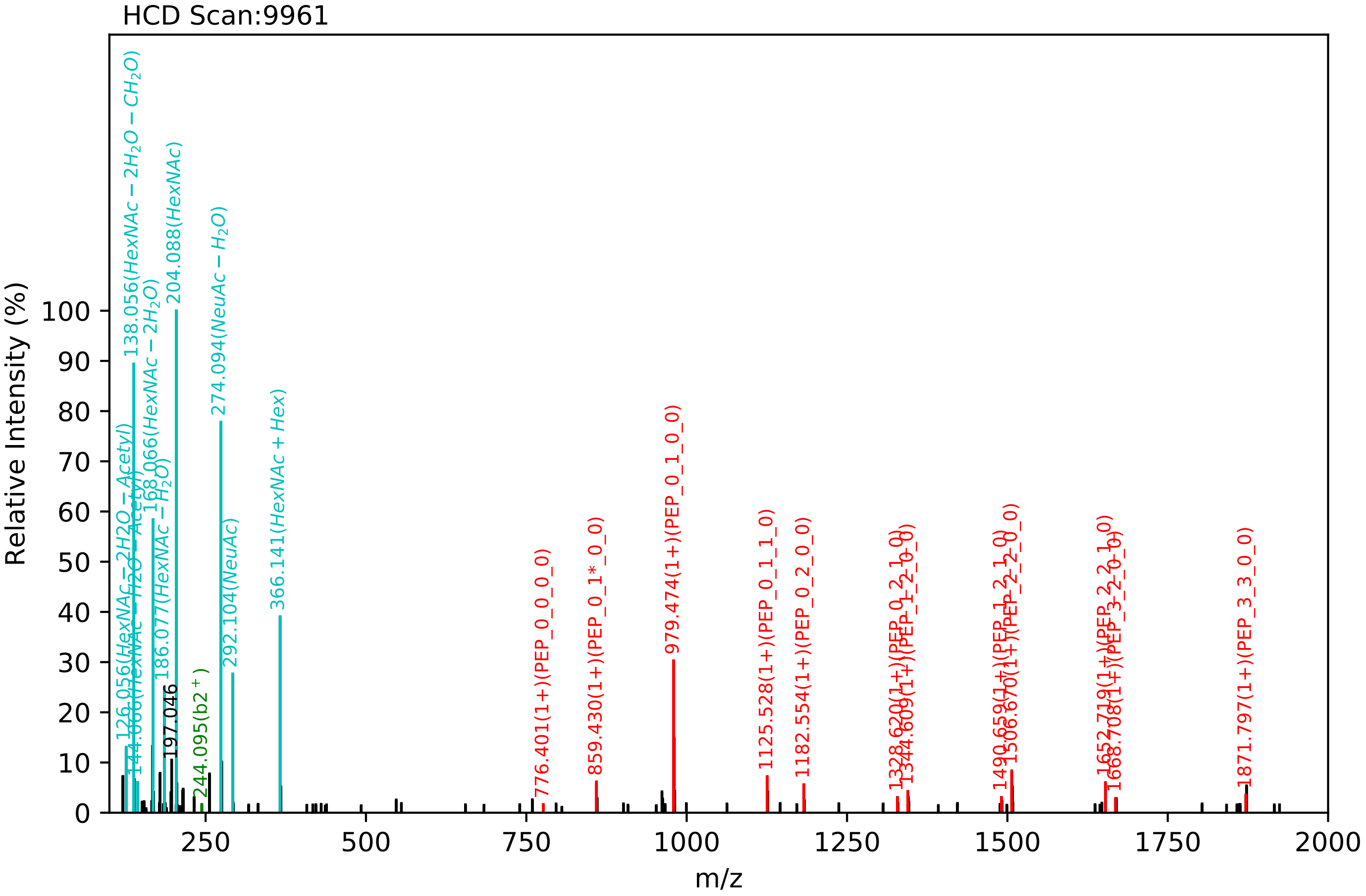

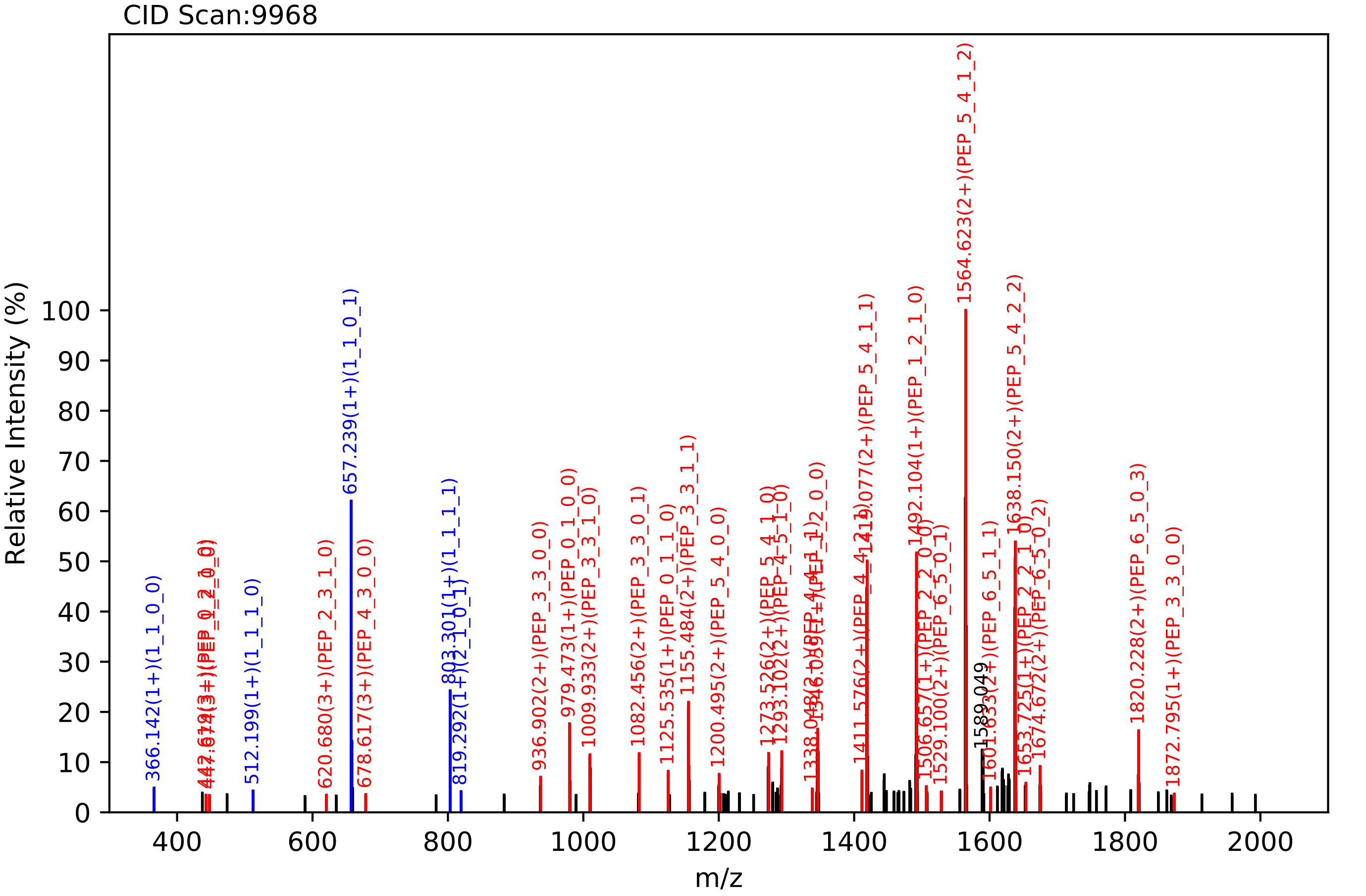

Test set no. 41, Experiment: AGP exp_19

ENGTISR(=PEP)_6_5_2_3, m/z:1310.51(3+), RT:34.65, Y-score:83.75

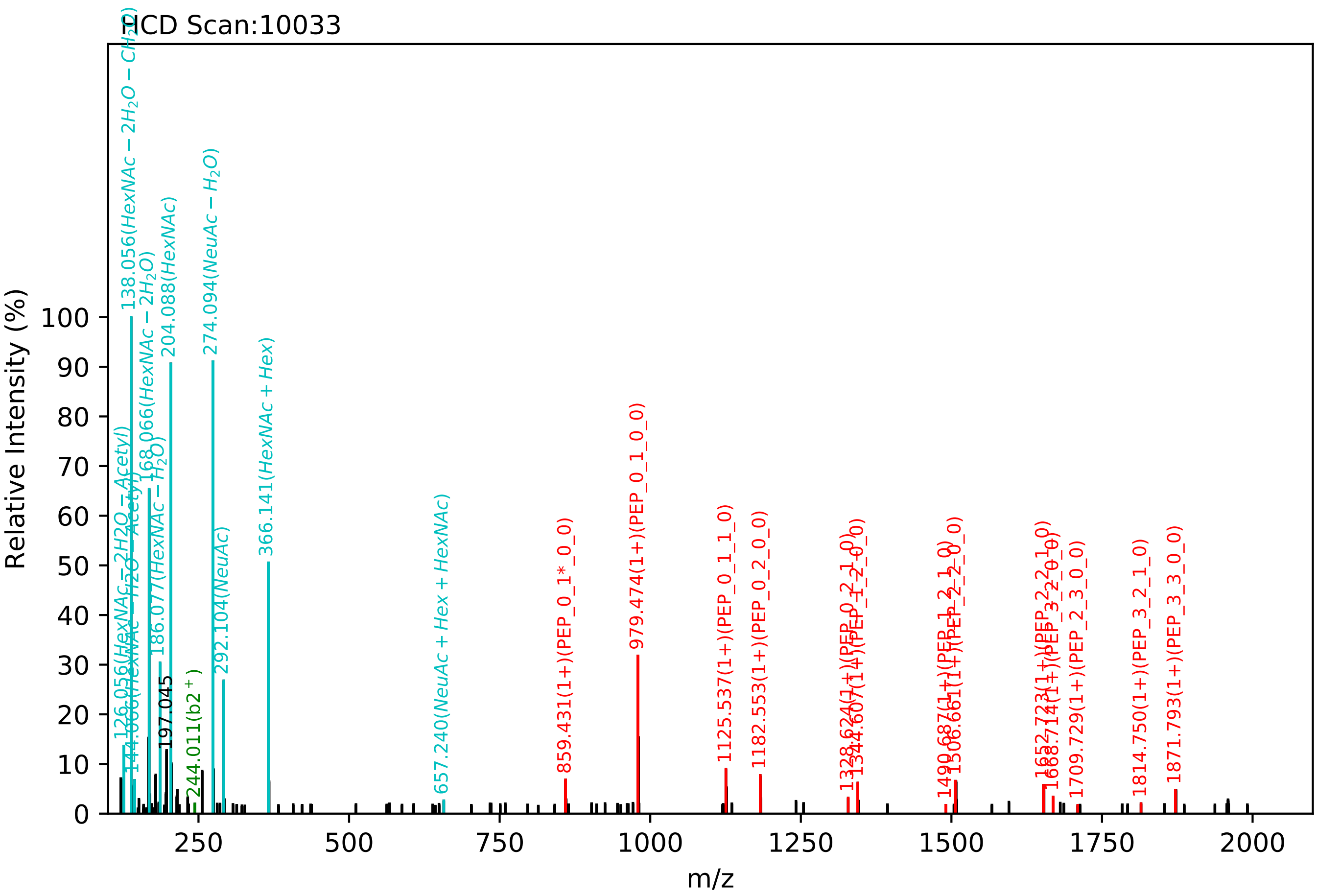

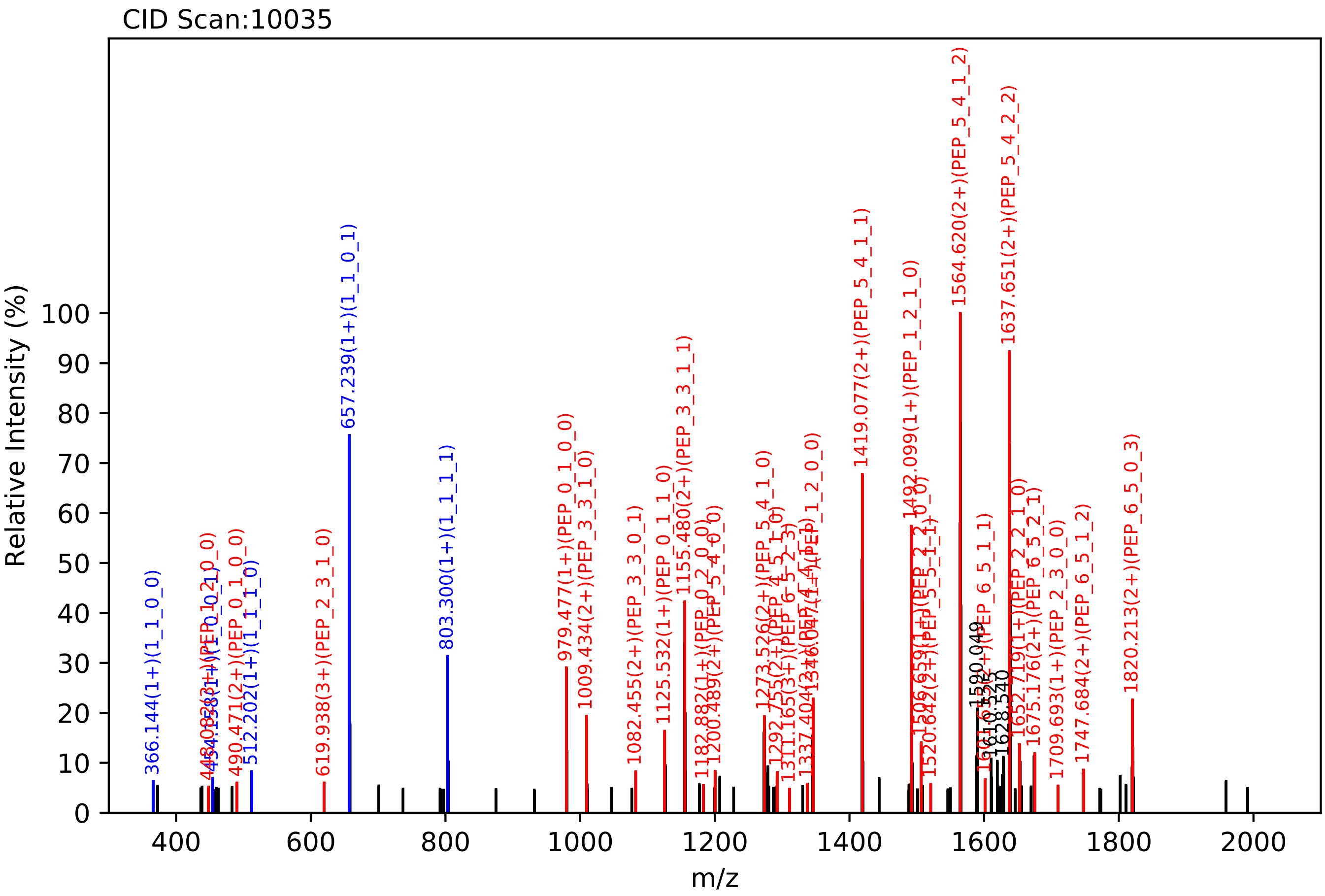

Test set no. 42, Experiment: AGP exp_25

ENGTISR(=PEP)_6_5_2_3, m/z:1310.51(3+), RT:35.34, Y-score:82.00

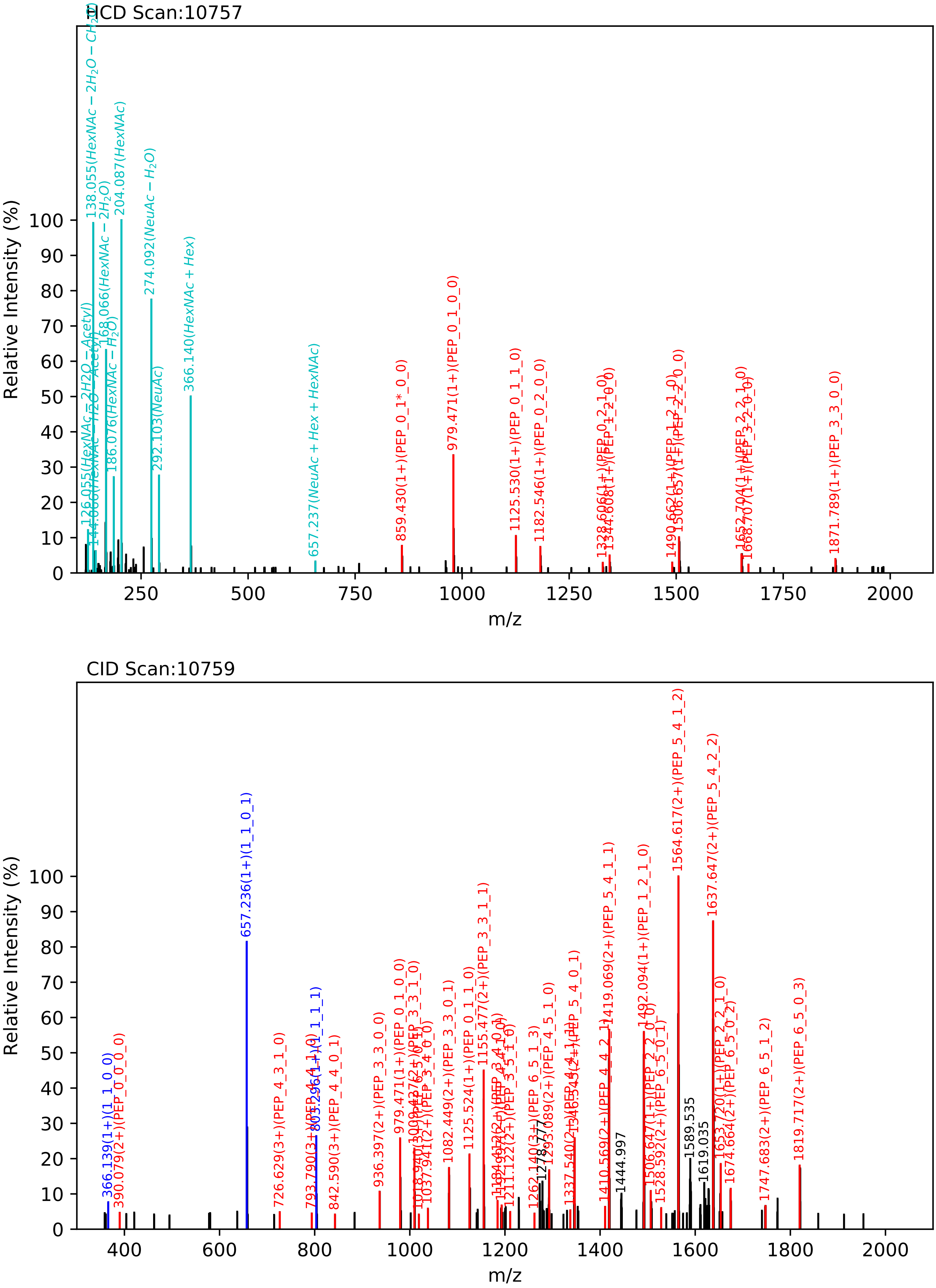

Test set no. 43, Experiment: AGP exp_3

ENGTISR(=PEP)_6_5_2_3, m/z:1310.51(3+), RT:22.68, Y-score:81.15

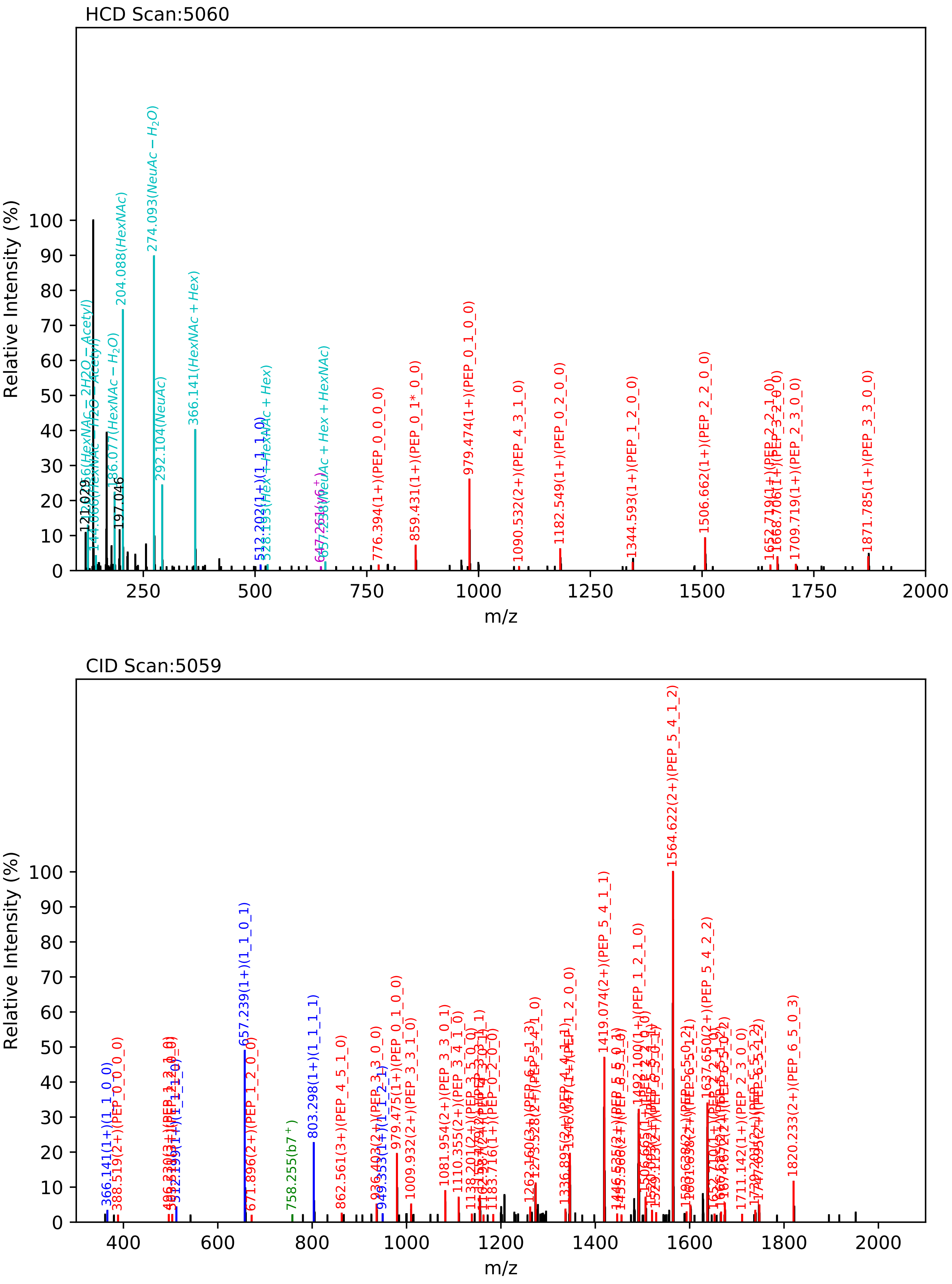

Test set no. 44, Experiment: AGP exp_3

ENGTISR(=PEP)_7_6_1_1, m/z:1189.47(3+), RT:16.86, Y-score:85.17

HCD Scan:2284

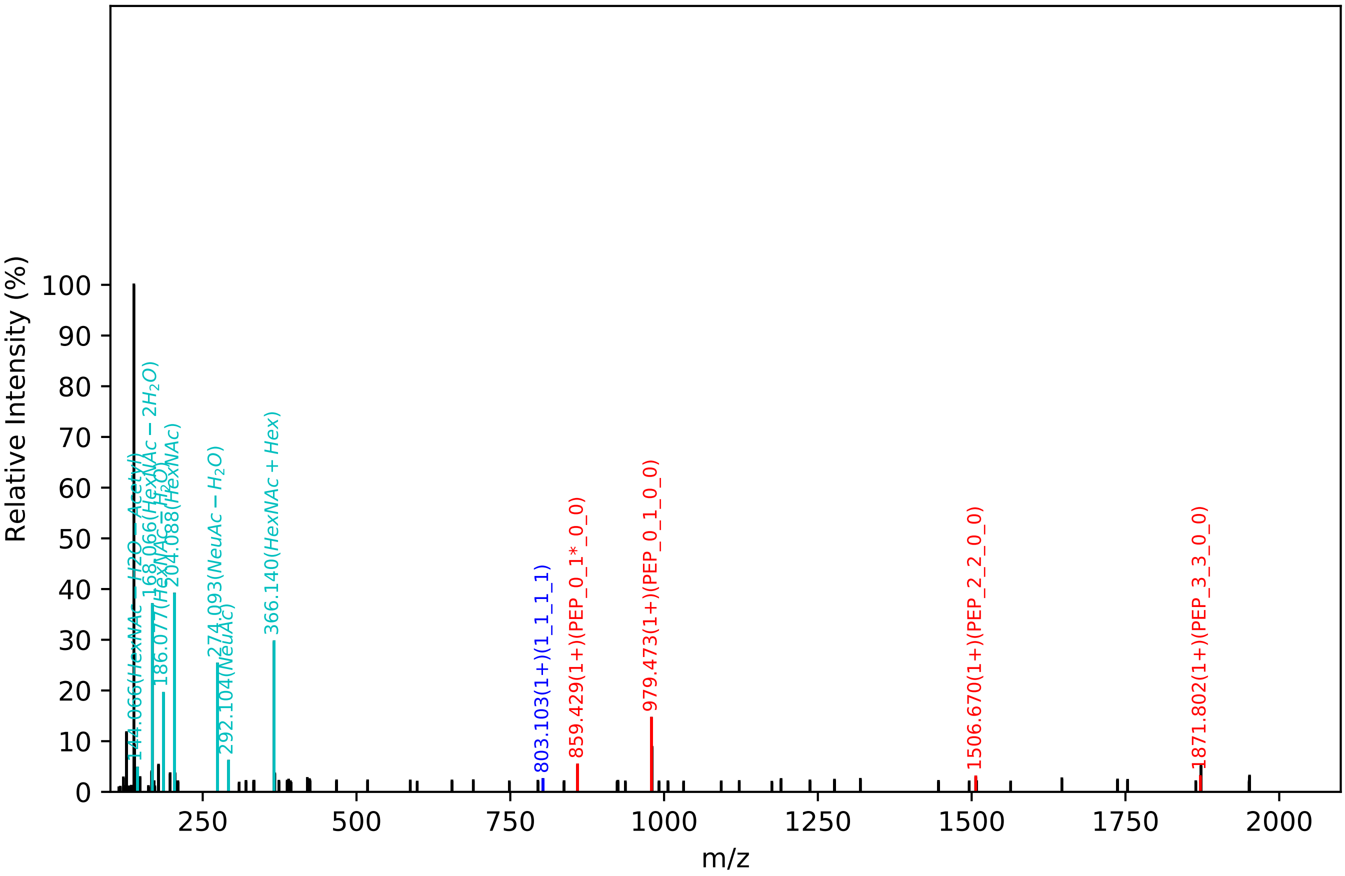

CID Scan:2283

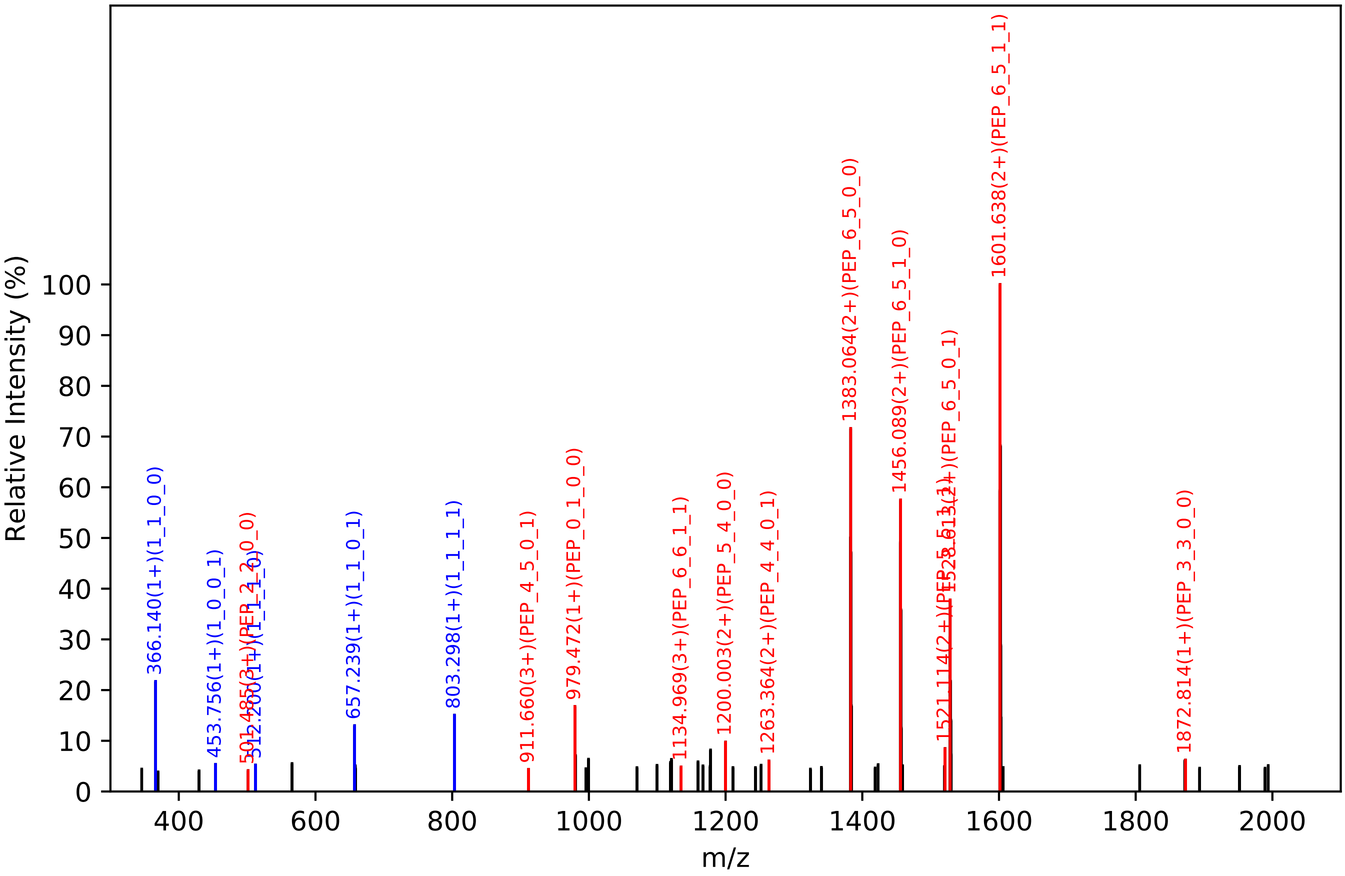

Test set no. 45, Experiment: AGP exp_4

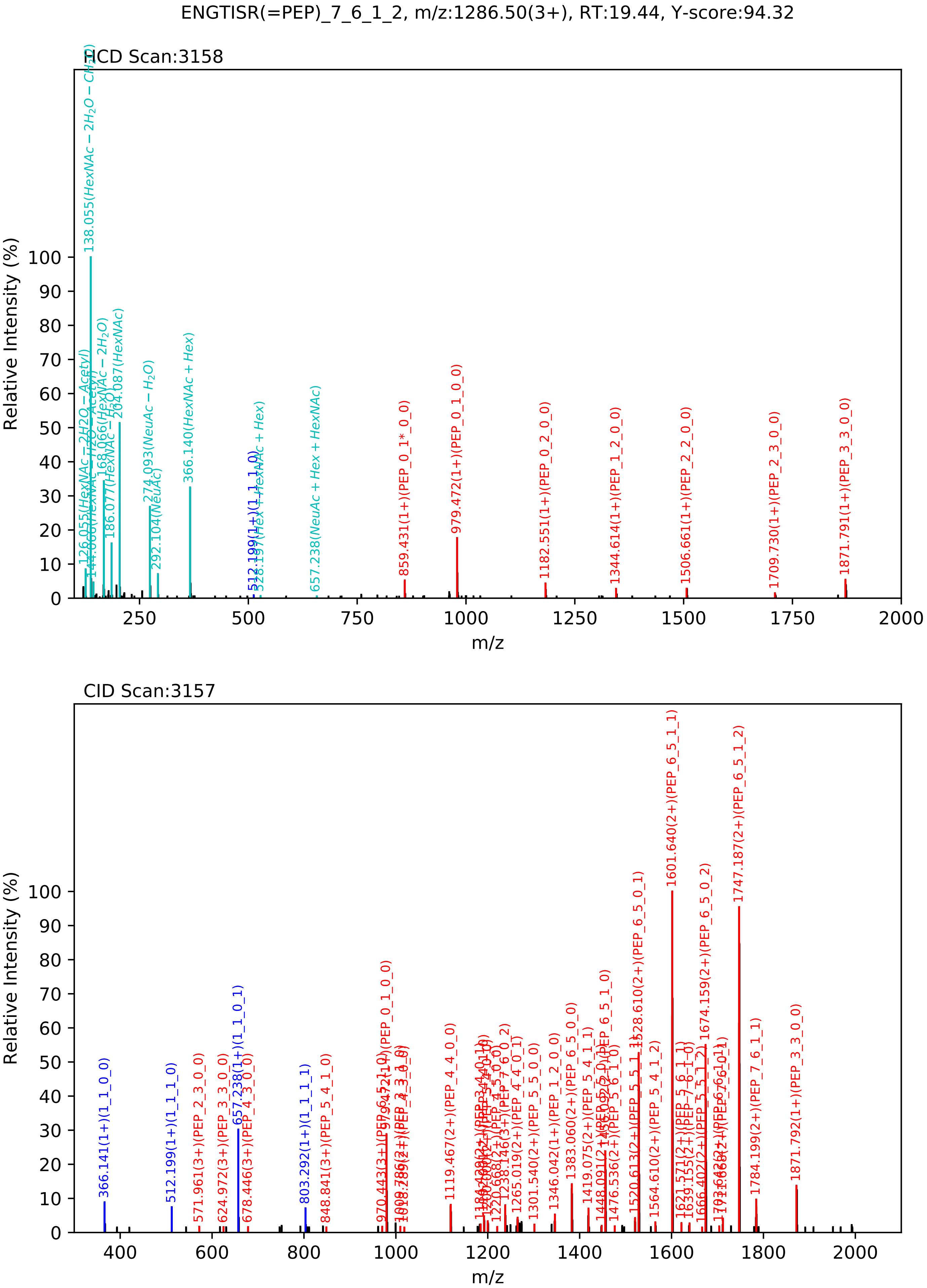

Test set no. 46, Experiment: AGP exp_4

ENGTISR(=PEP)_7_6_1_2, m/z:1286.50(3+), RT:20.00, Y-score:93.76

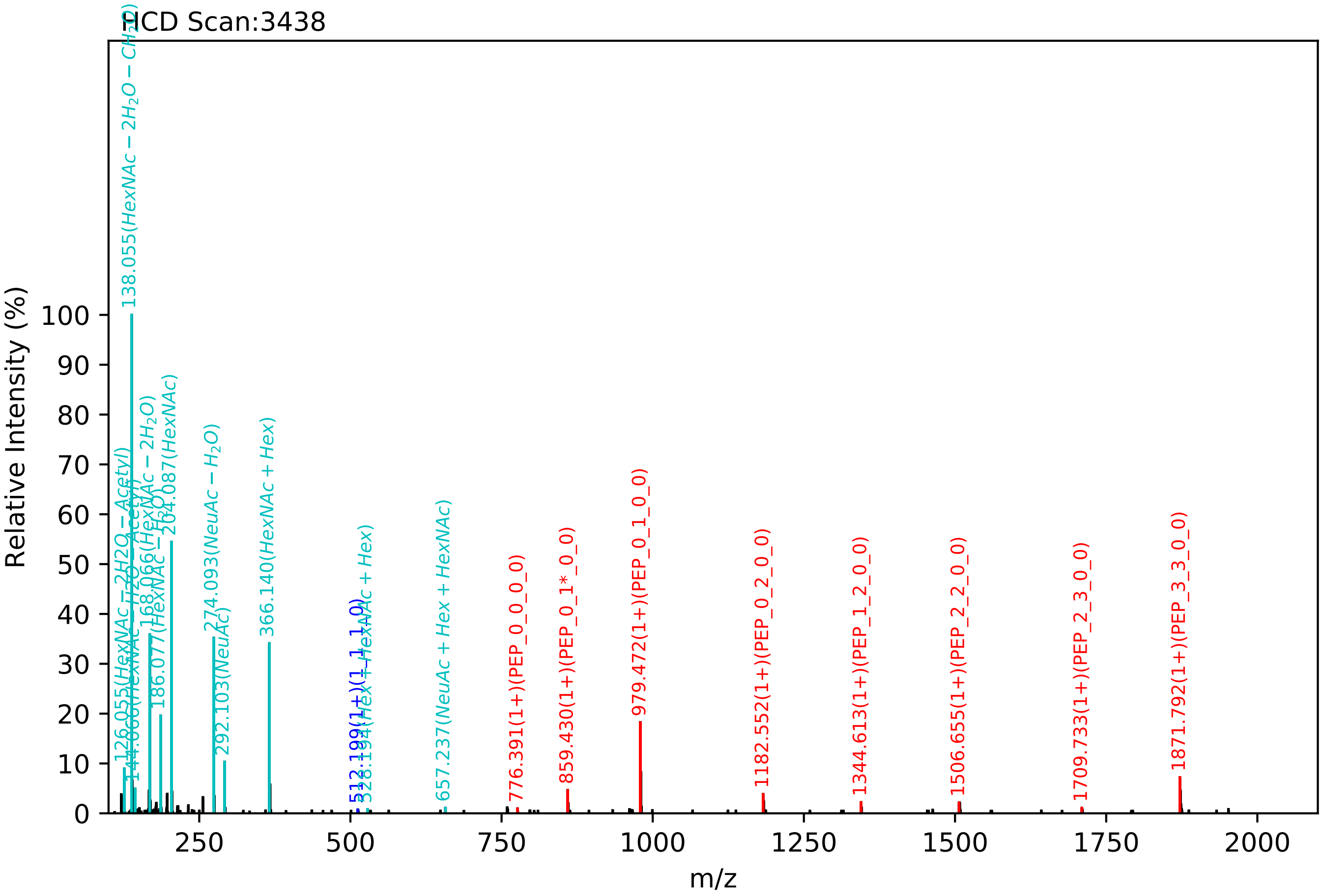

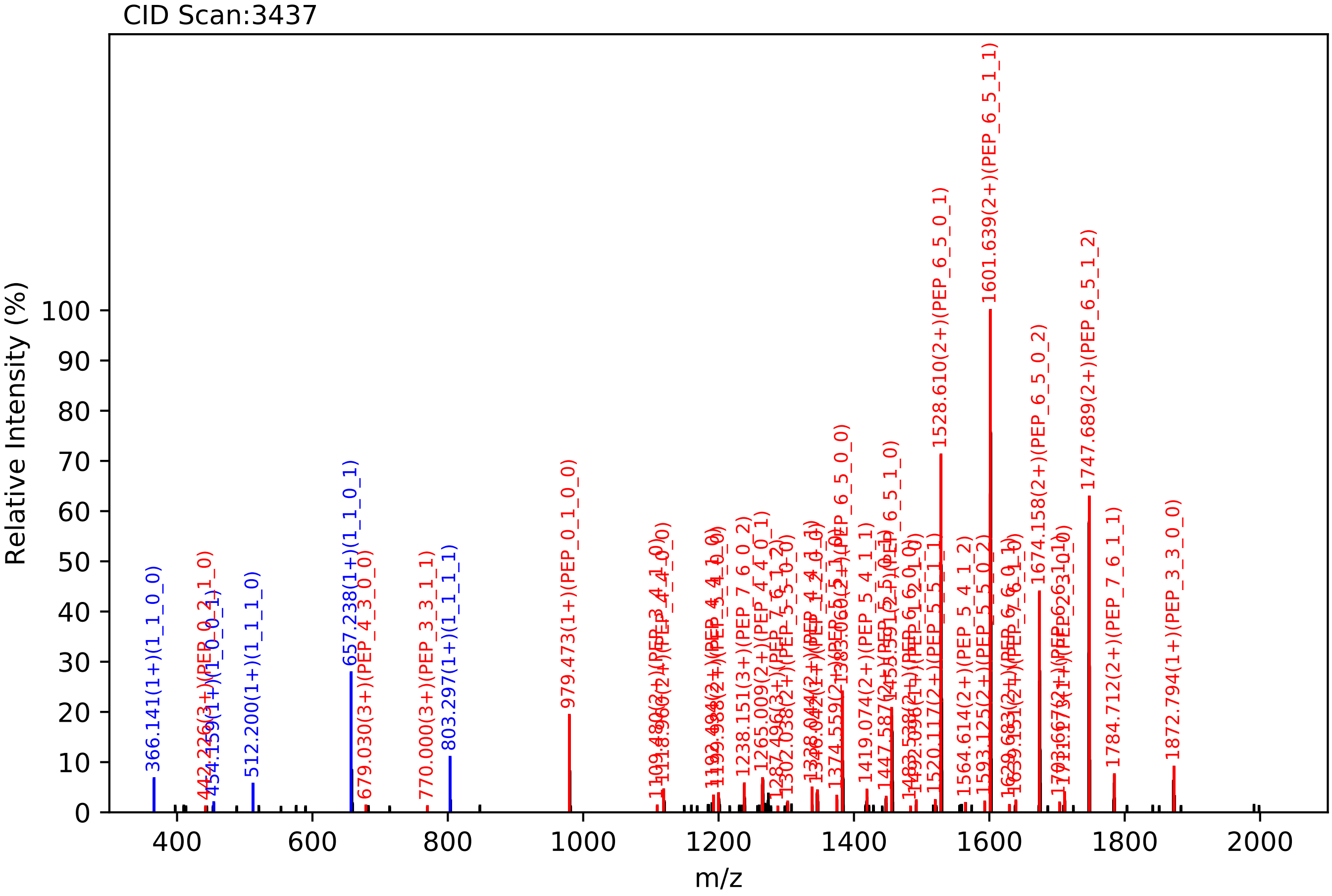

Test set no. 47, Experiment: AGP exp_3

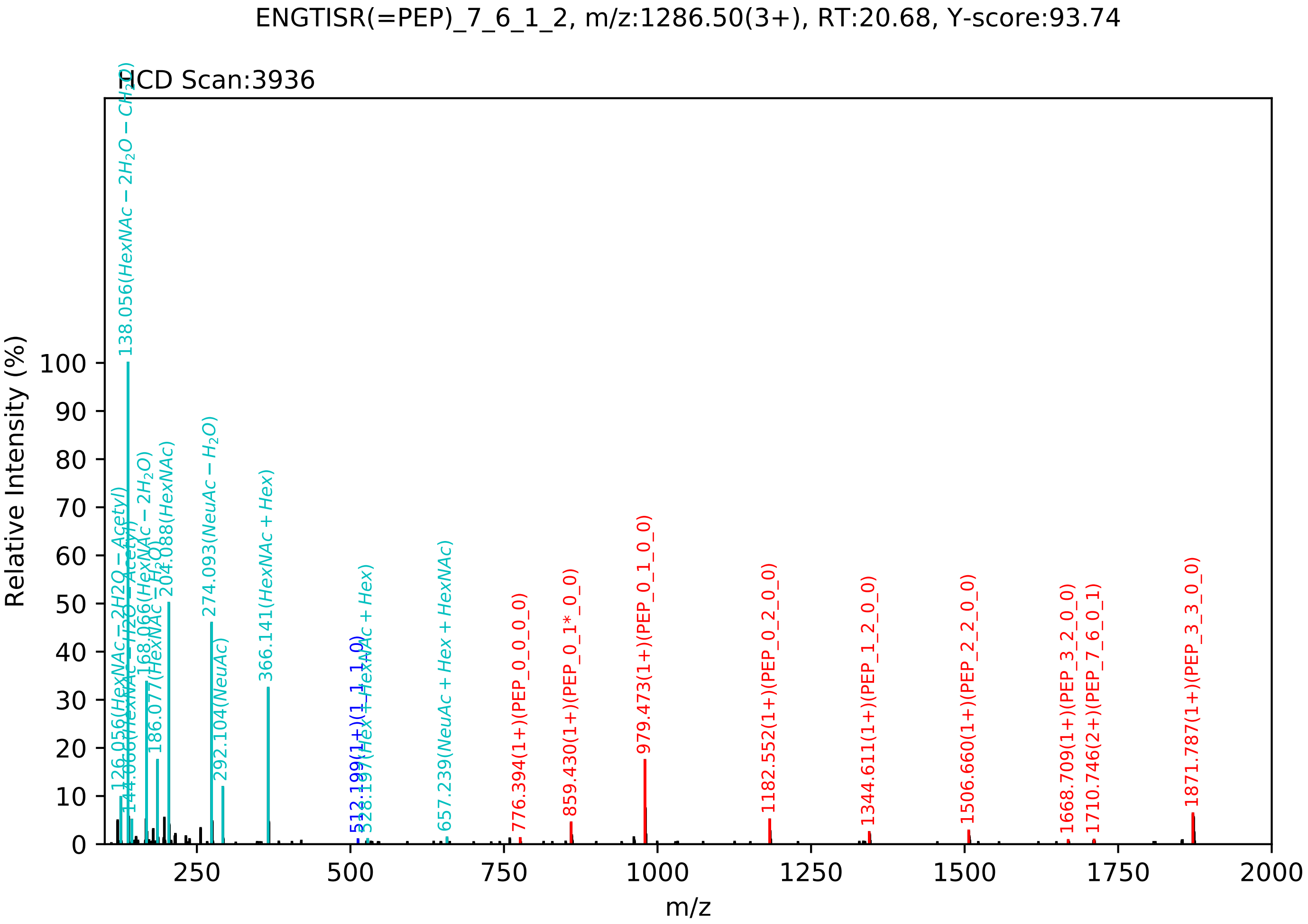

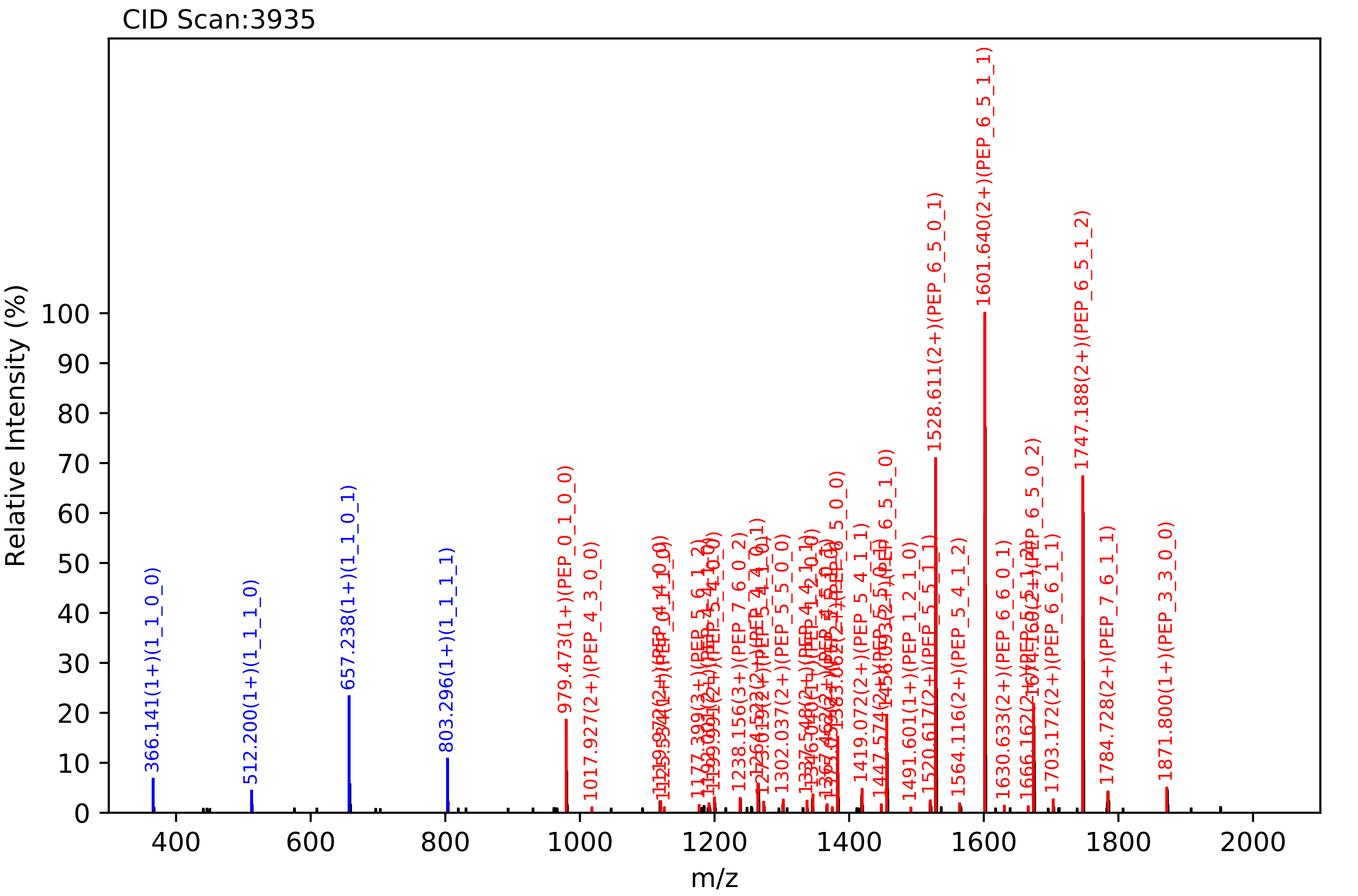

Test set no. 48, Experiment: AGP exp_3

ENGTISR(=PEP)_7_6_1_2, m/z:1286.51(3+), RT:20.09, Y-score:93.32

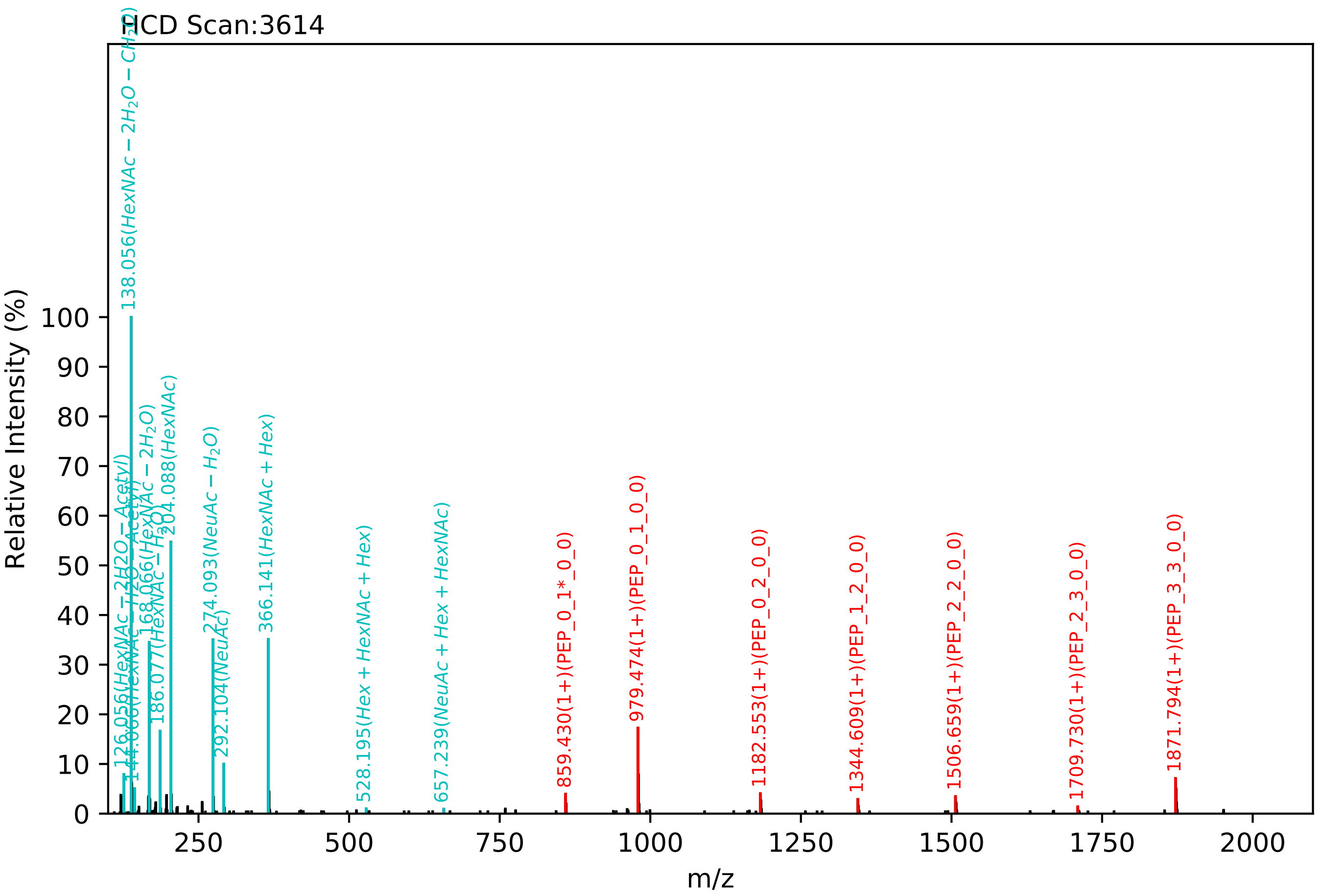

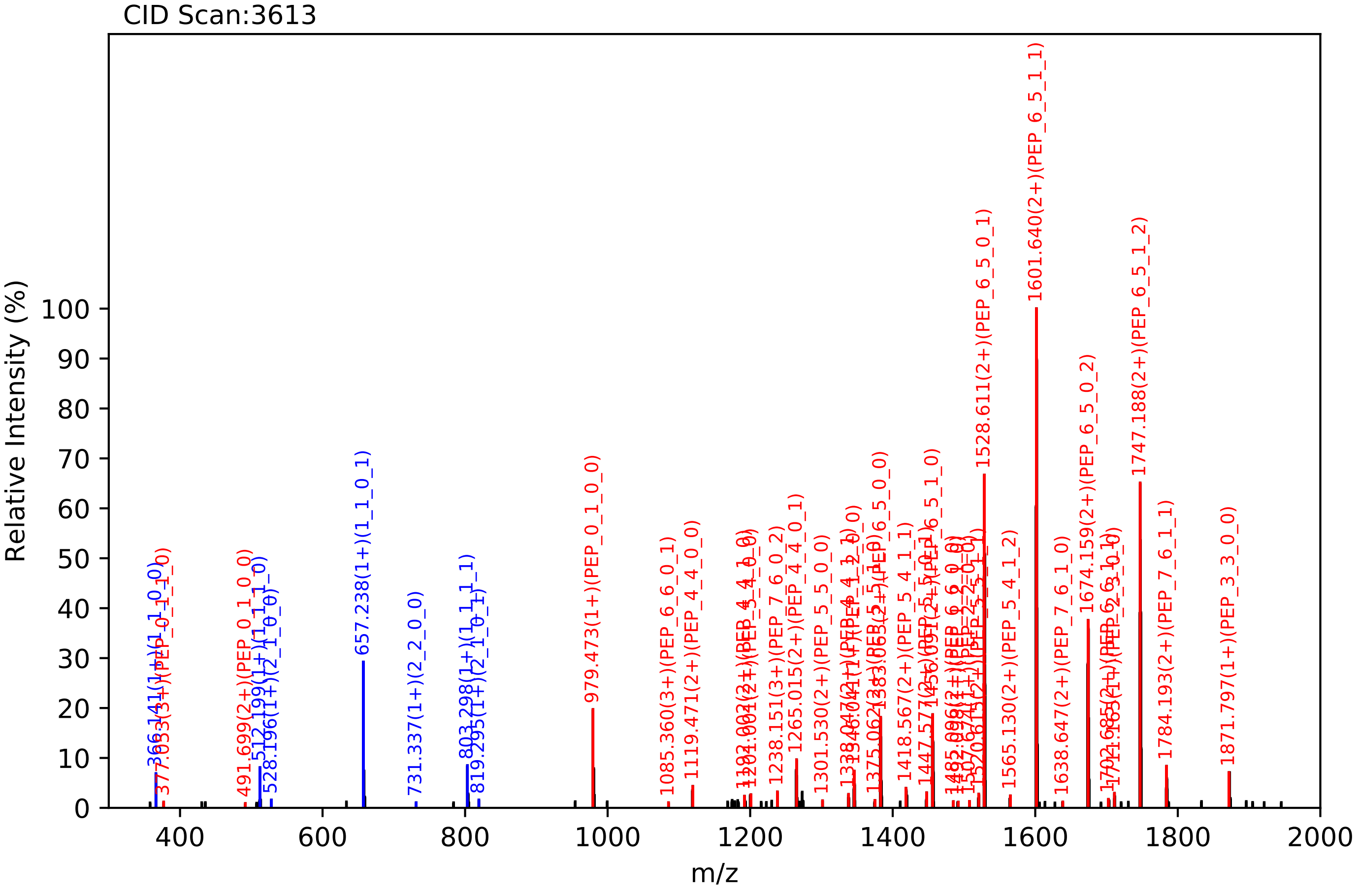

Test set no. 49, Experiment: AGP exp_4

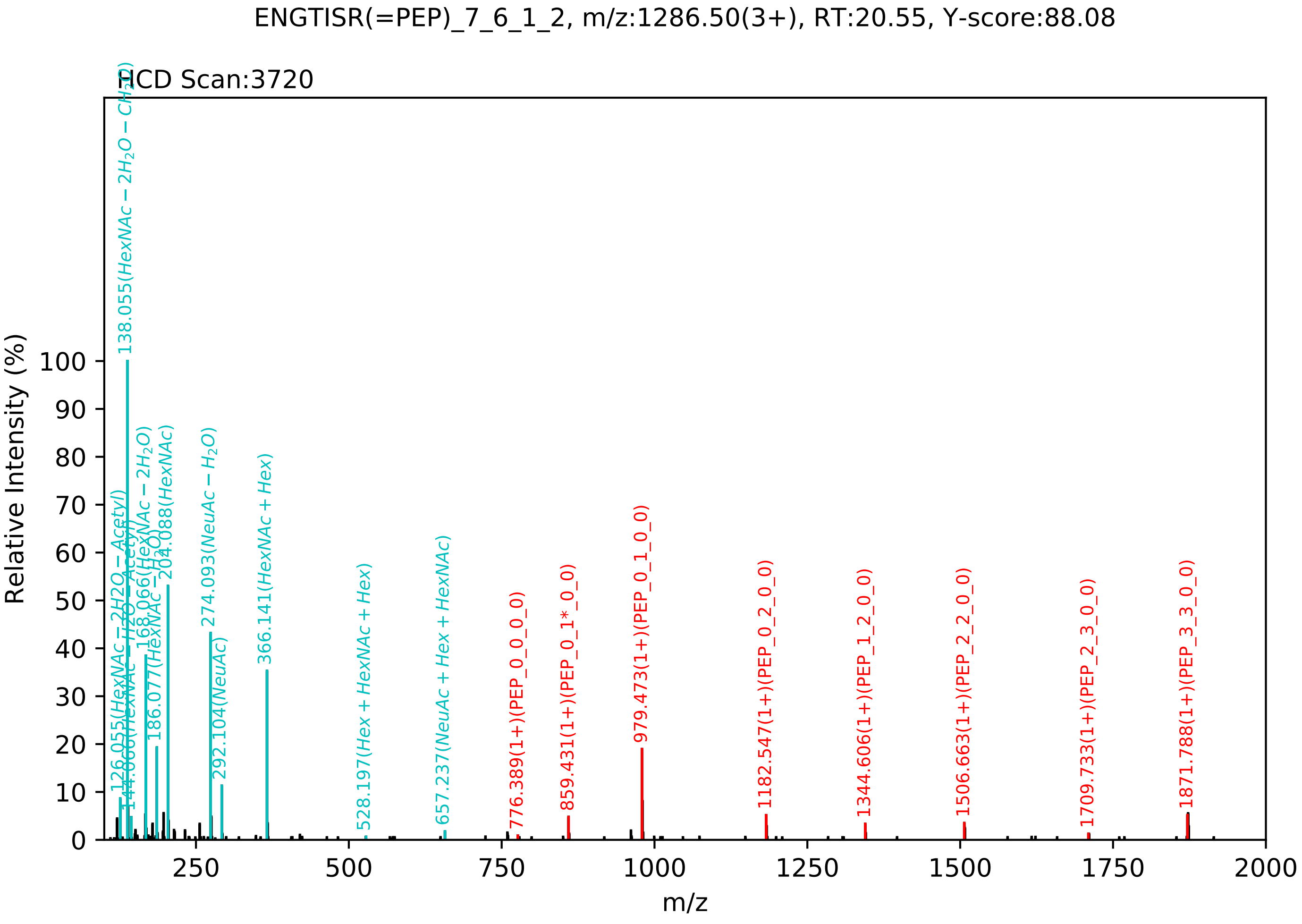

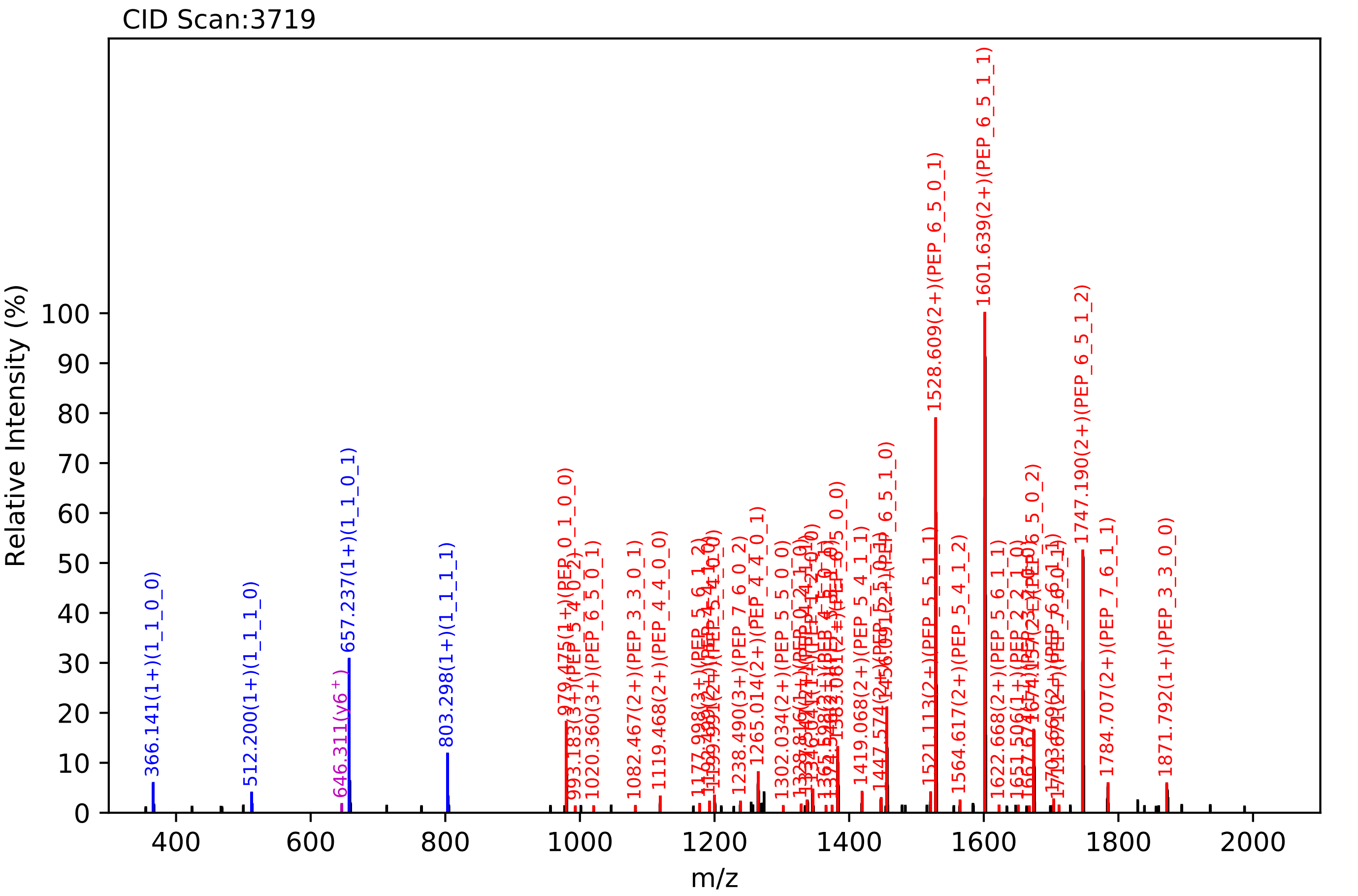

Test set no. 50, Experiment: AGP exp_3

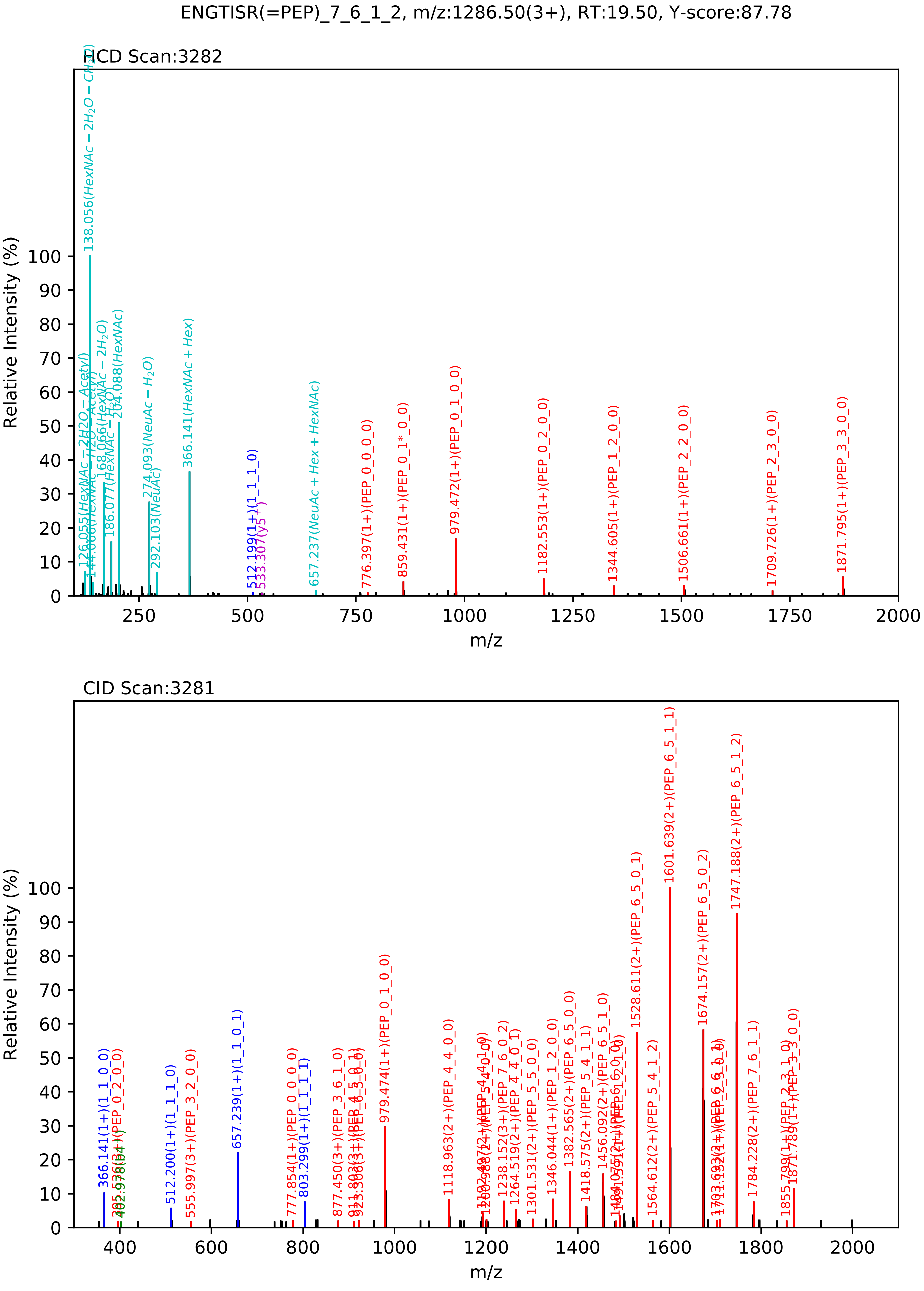

Test set no. 51, Experiment: AGP exp_3

ENGTISR(=PEP)_7_6_1_3, m/z:1037.90(4+), RT:22.47, Y-score:86.25

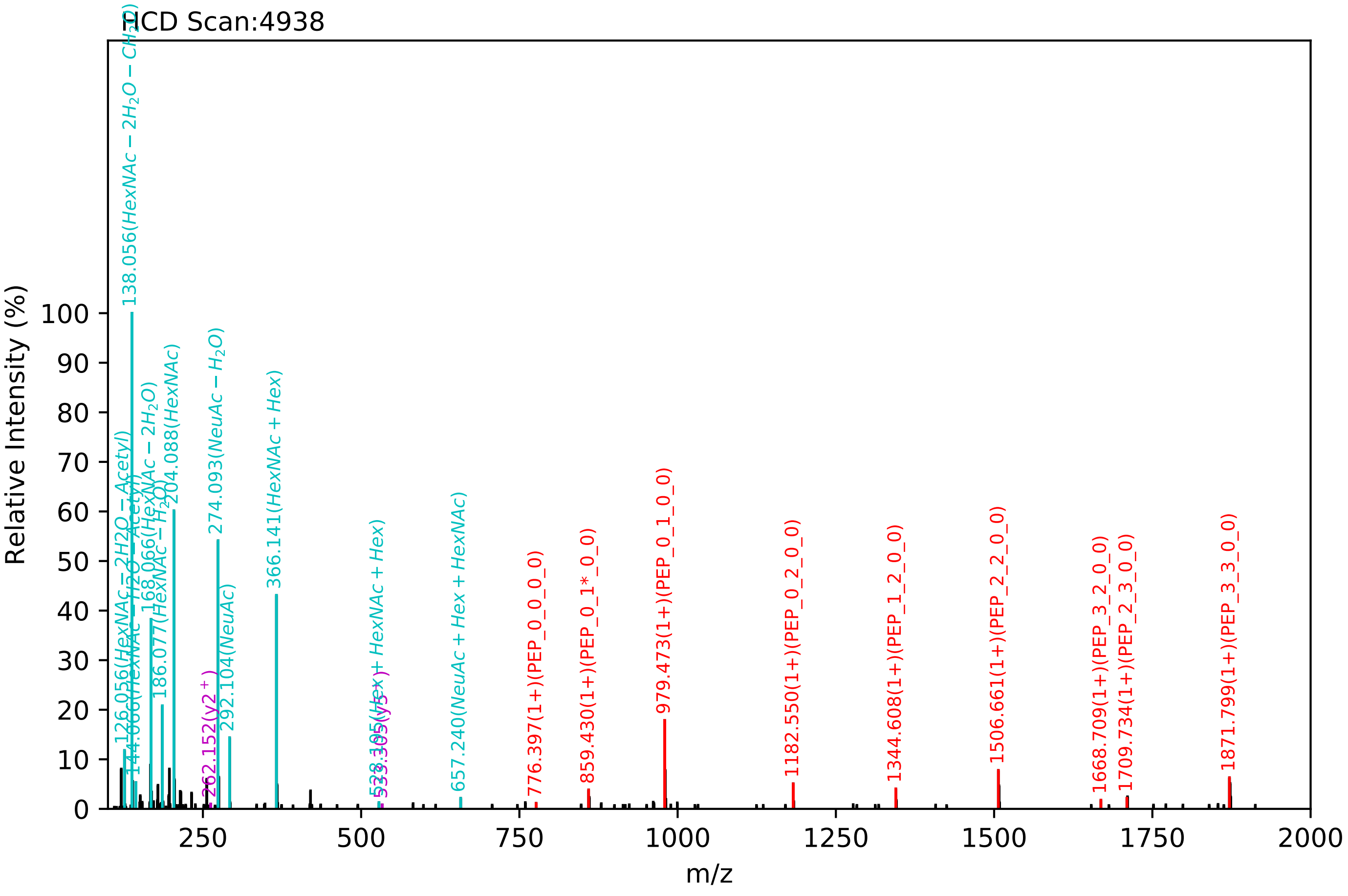

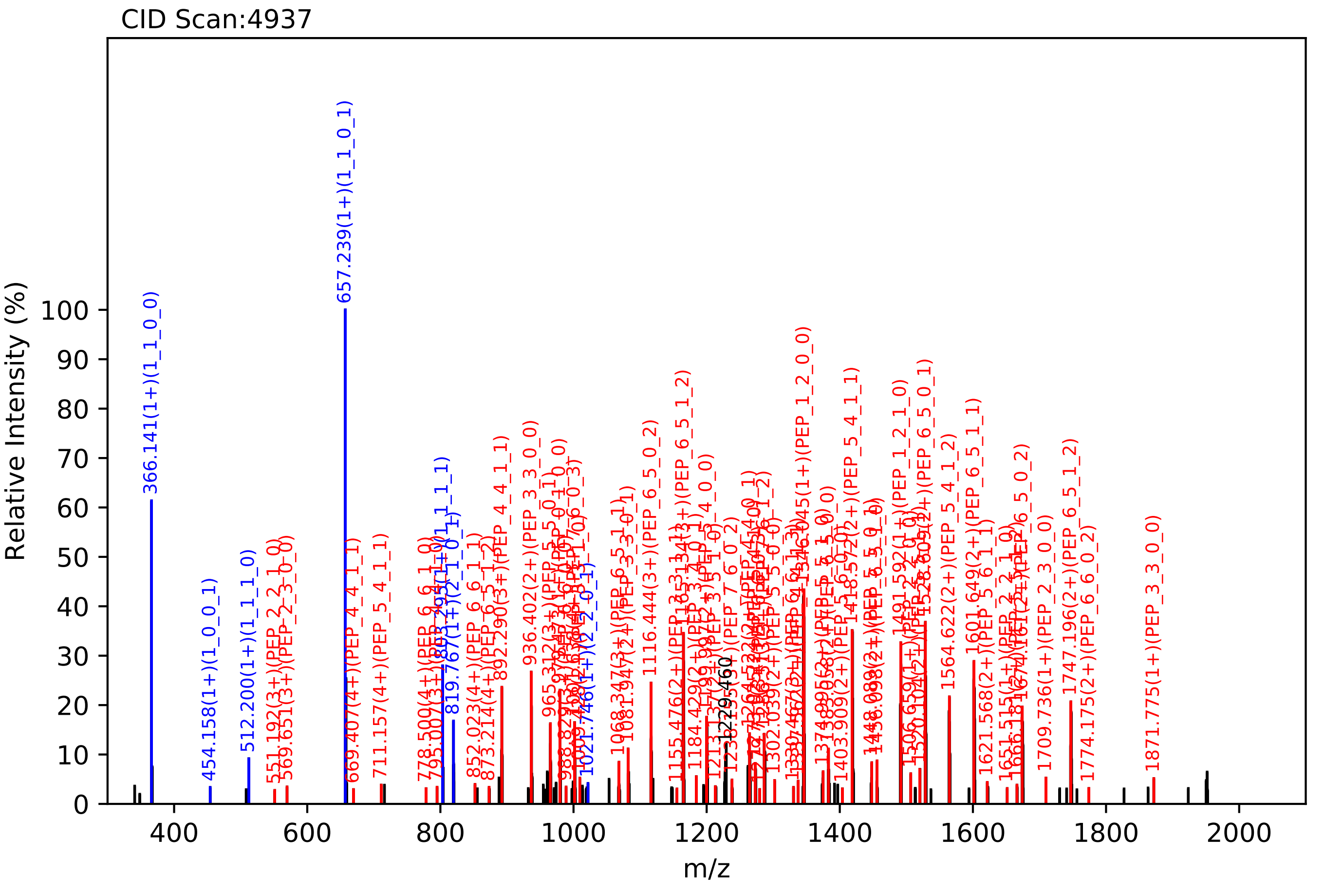

Test set no. 52, Experiment: AGP exp_3

ENGTISR(=PEP)_7_6_1_4, m/z:1110.68(4+), RT:25.04, Y-score:86.75

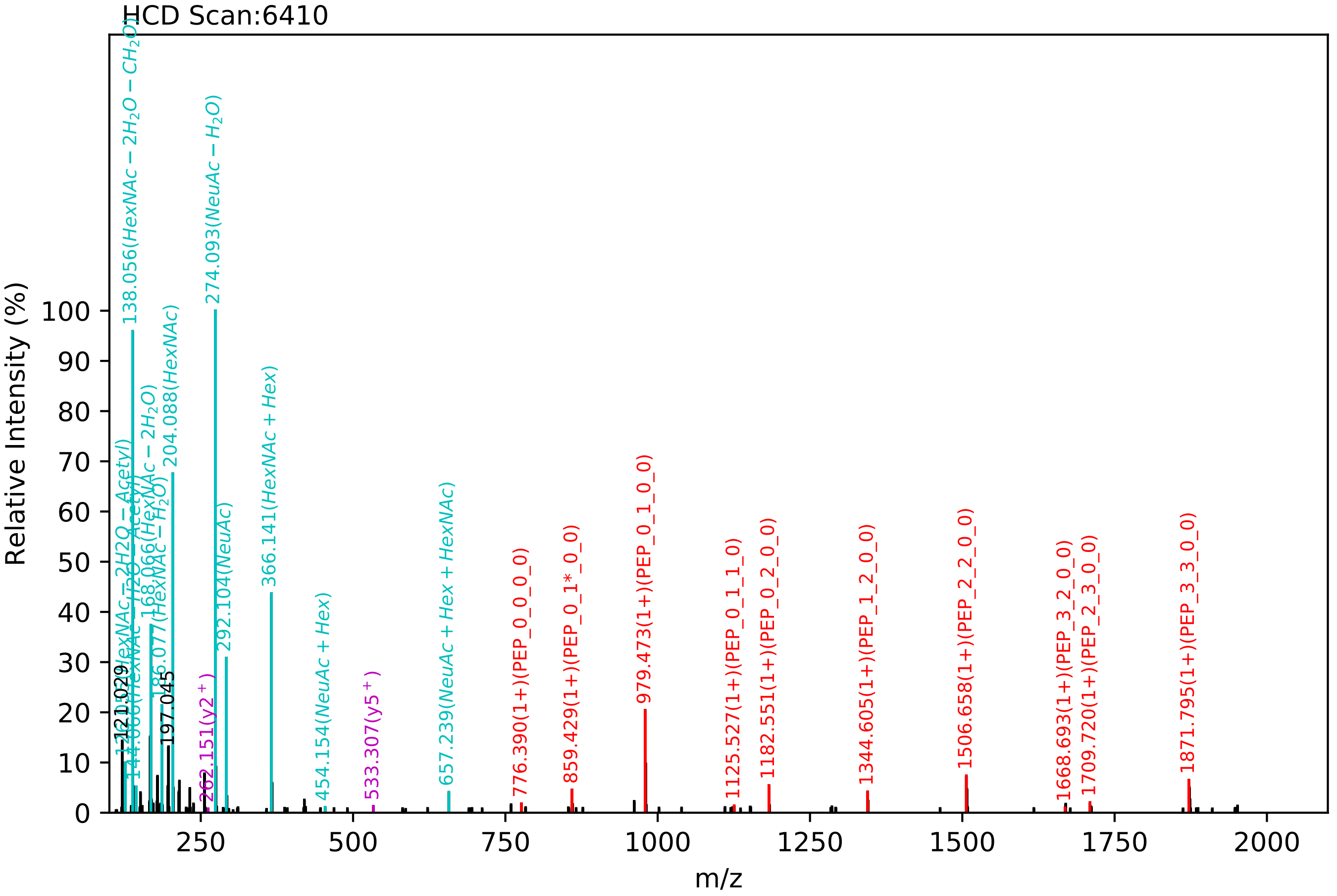

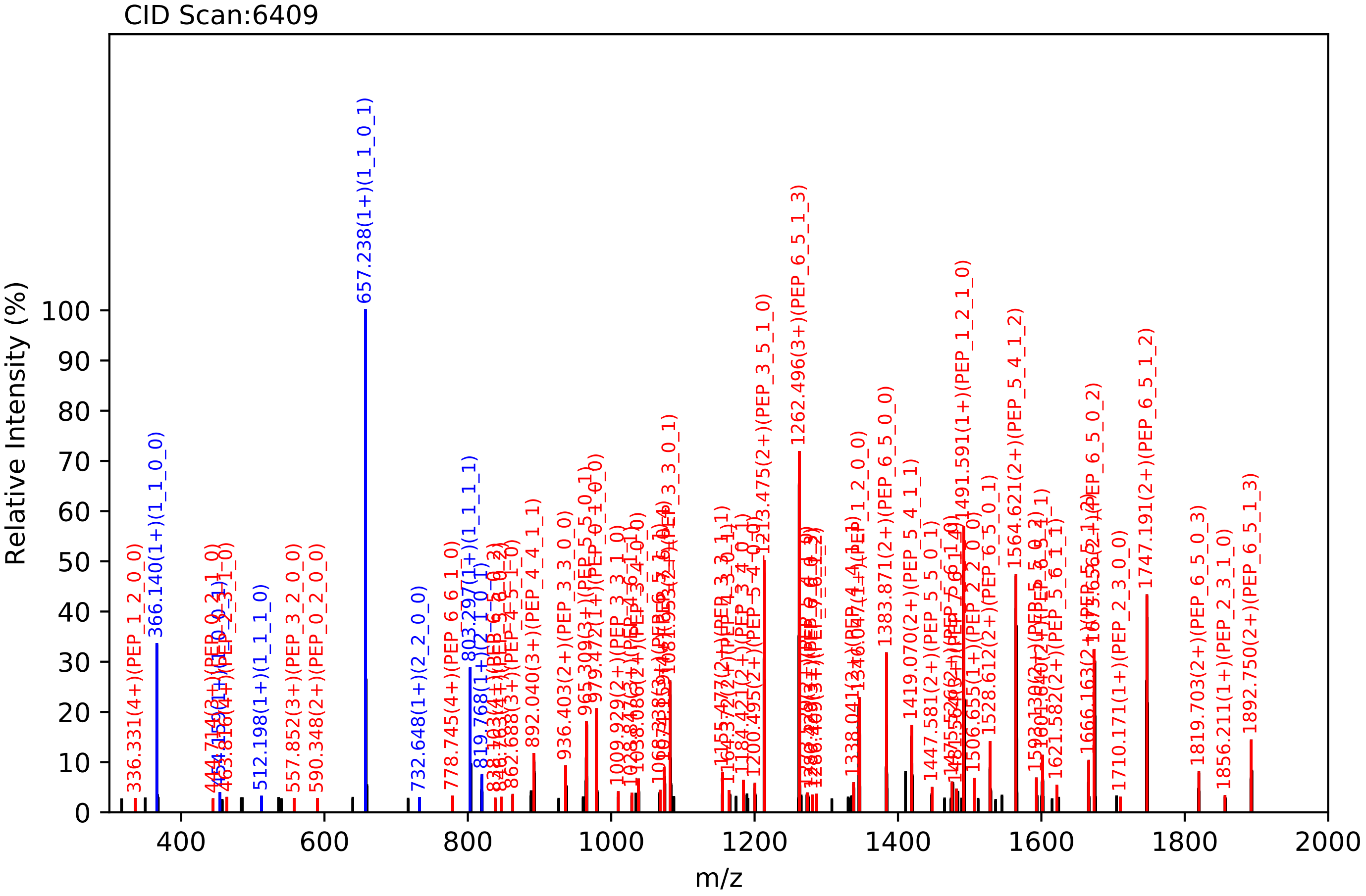

Test set no. 53, Experiment: AGP exp_4

ENGTISR(=PEP)_7_6_1_4, m/z:1110.68(4+), RT:24.64, Y-score:84.51

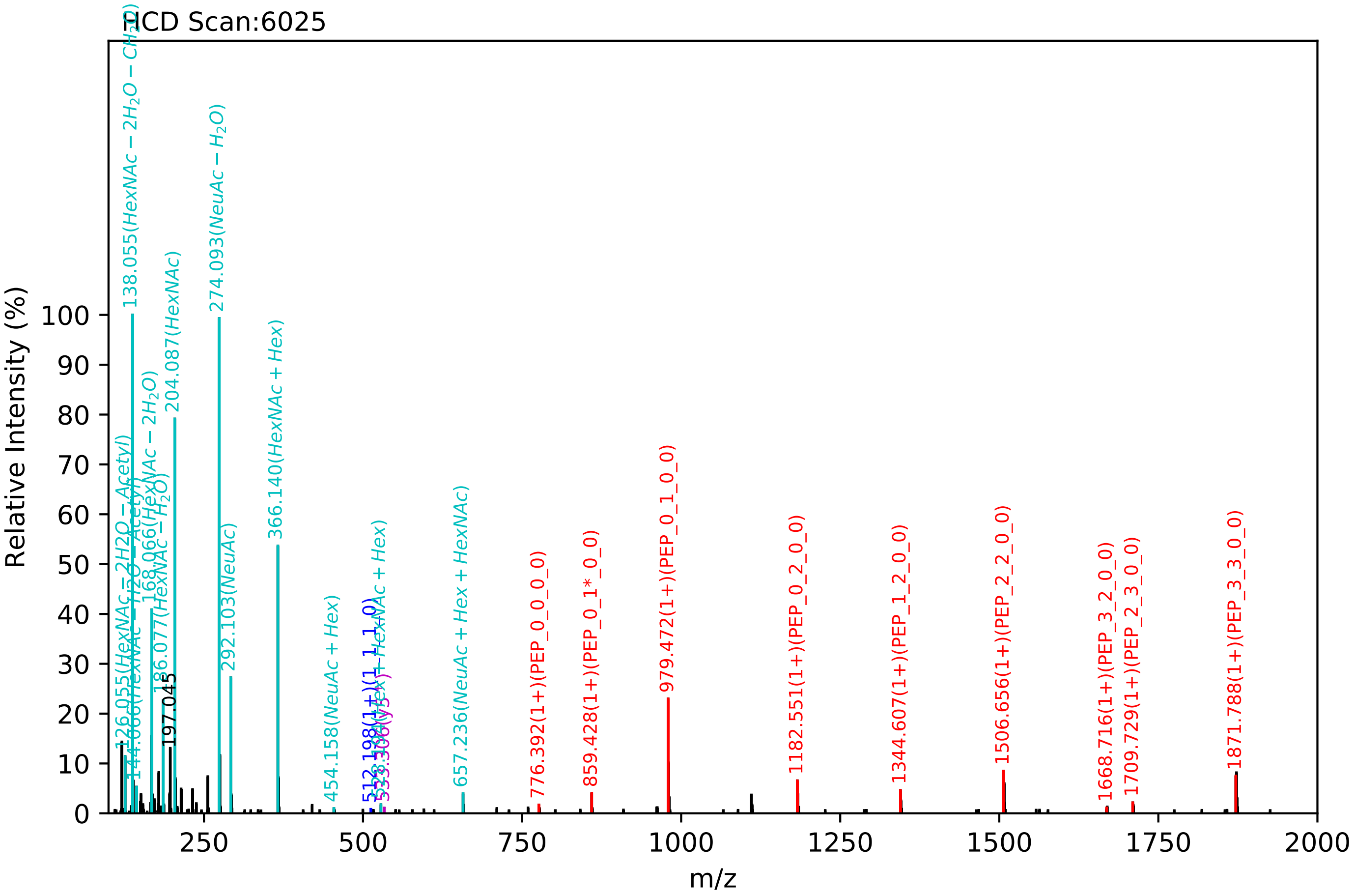

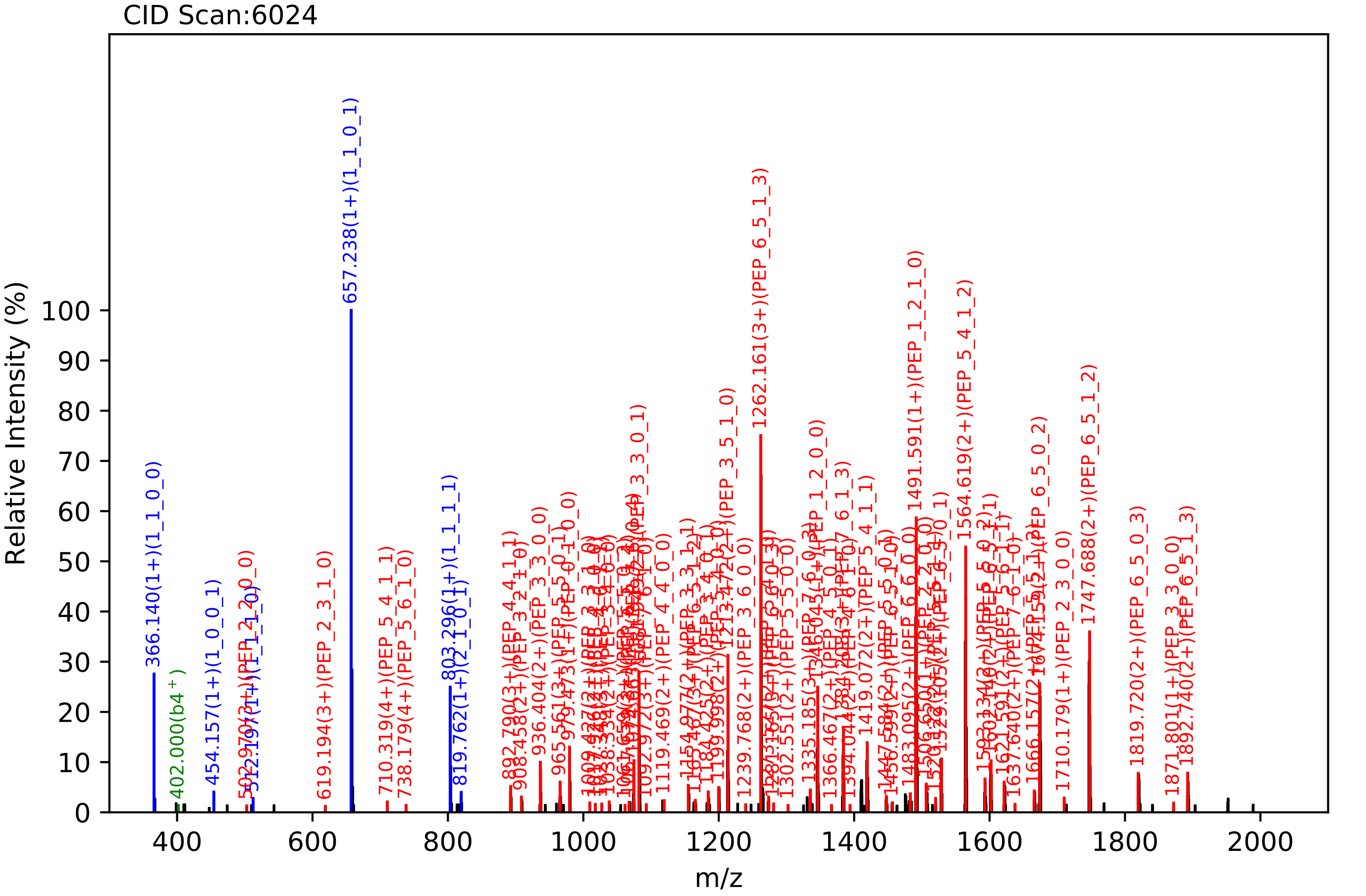

Test set no. 54, Experiment: AGP exp_3

ENGTISR(=PEP)_7_6_2_4, m/z:1147.19(4+), RT:24.60, Y-score:79.62

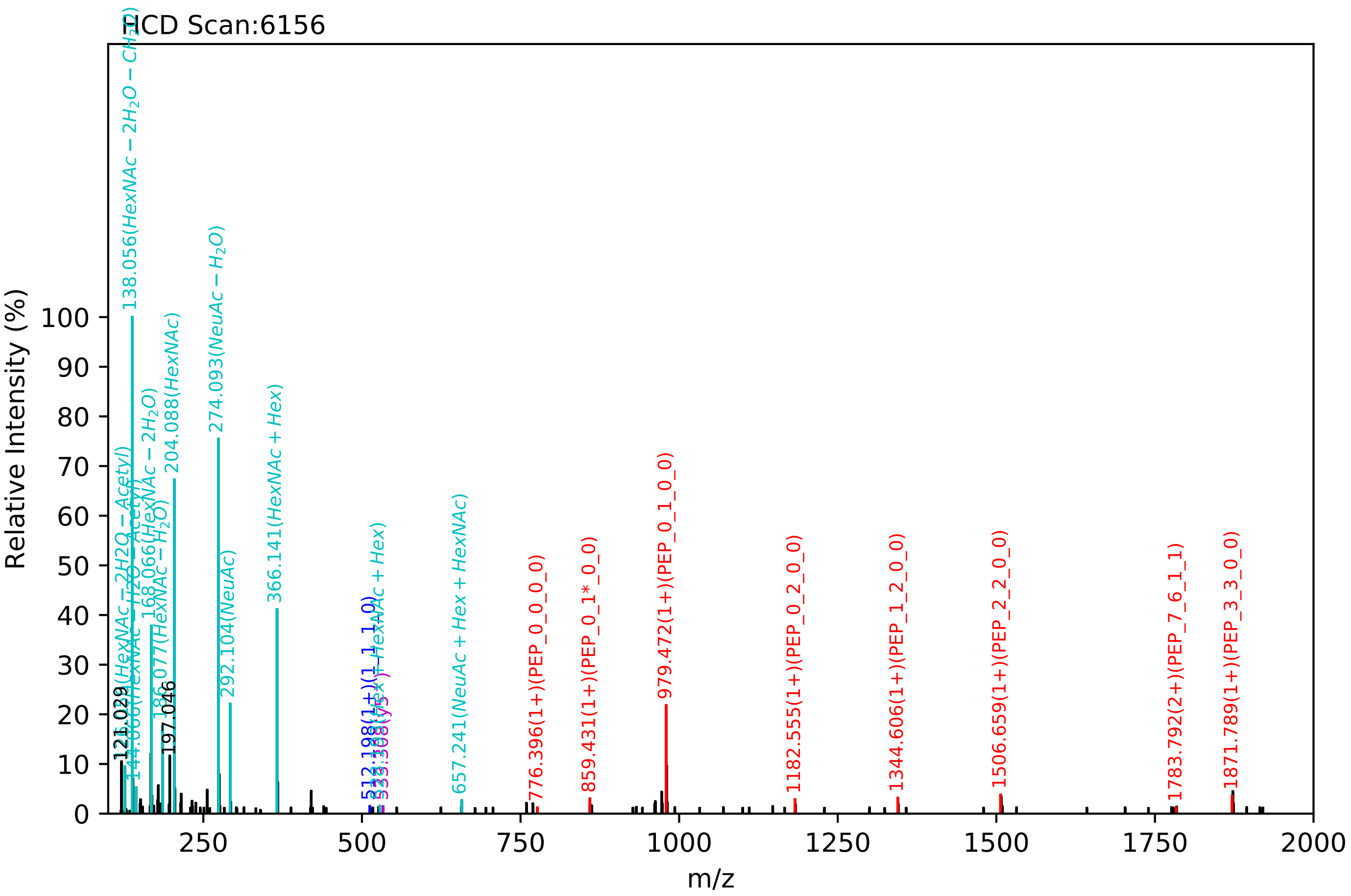

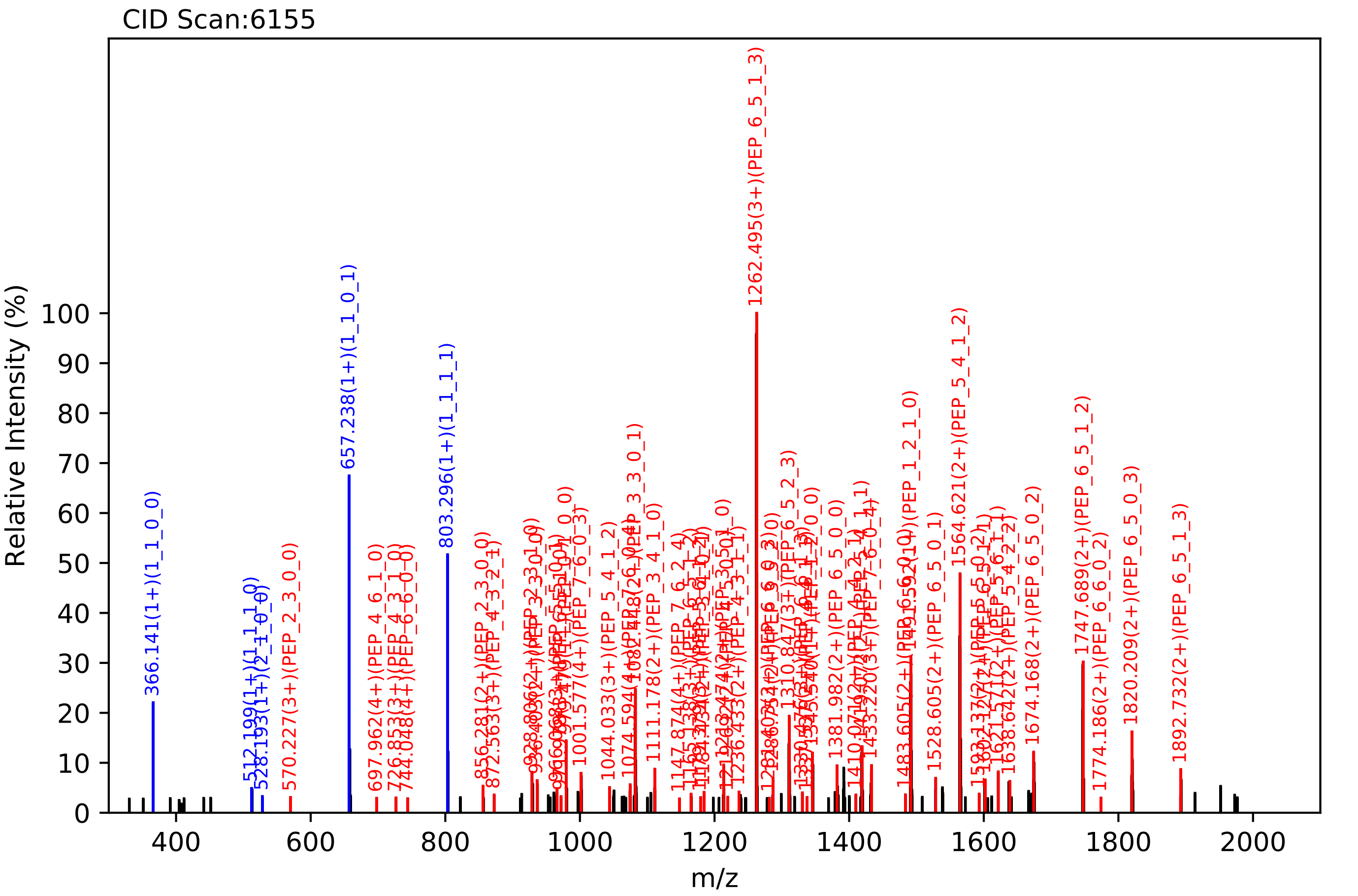

Test set no. 55, Experiment: AGP exp_4

LVPVPITNATLDQITGK(=PEP)_5_4_0_2, m/z:1328.61(3+), RT:55.29, Y-score:90.23

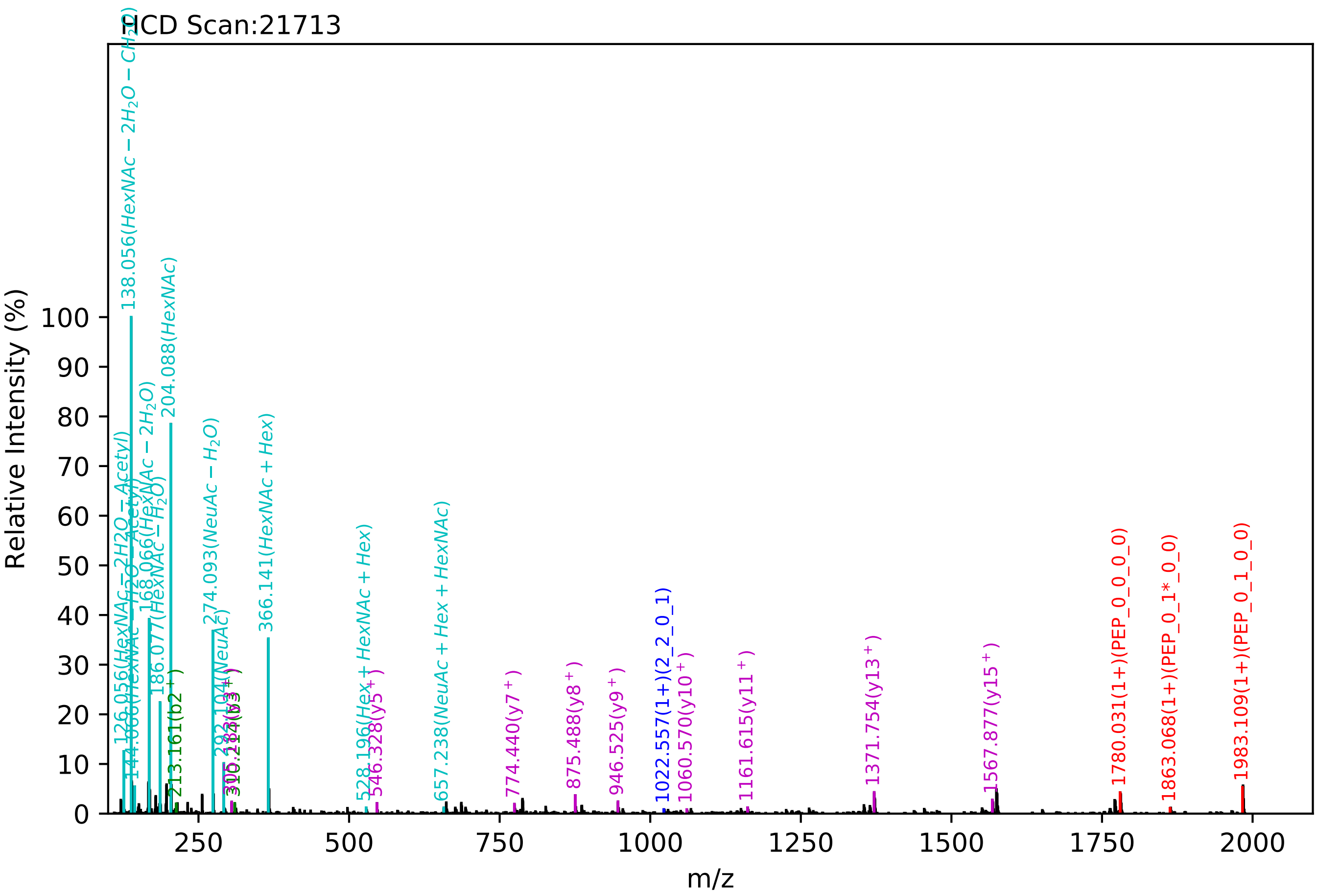

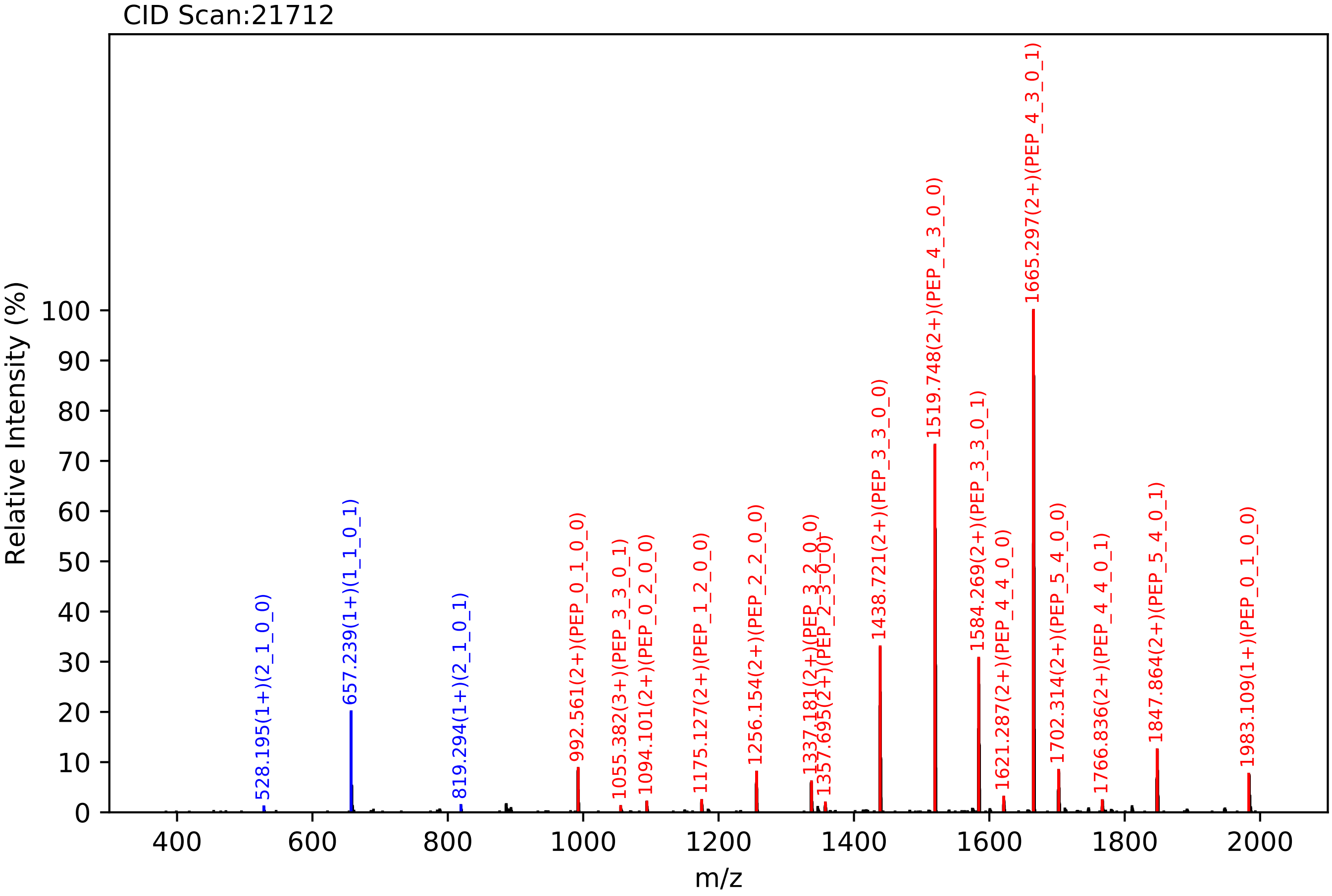

Test set no. 56, Experiment: AGP exp_3

LVPVPITNATLDQITGK(=PEP)_5_4_0_2, m/z:1328.94(3+), RT:56.01, Y-score:89.57

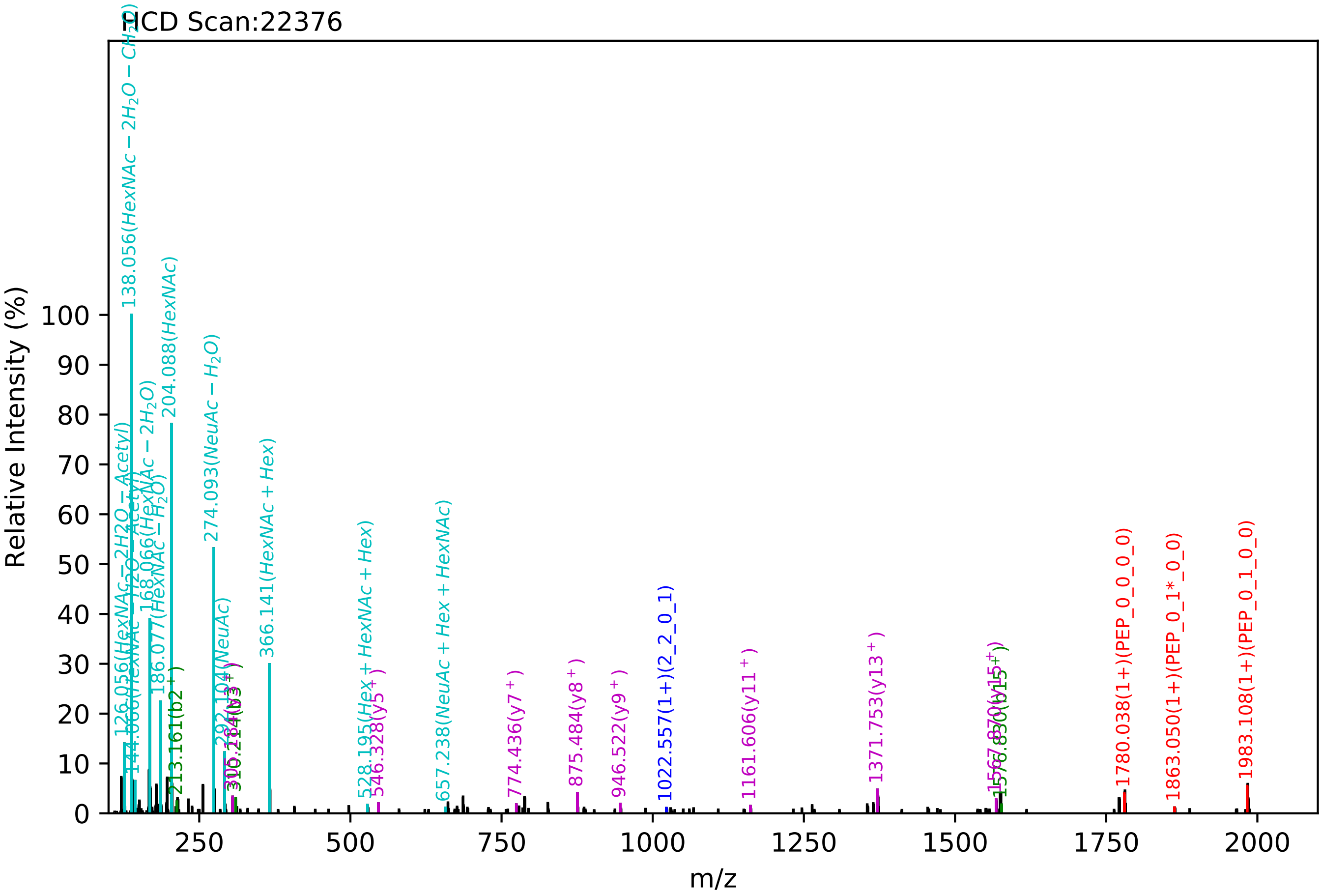

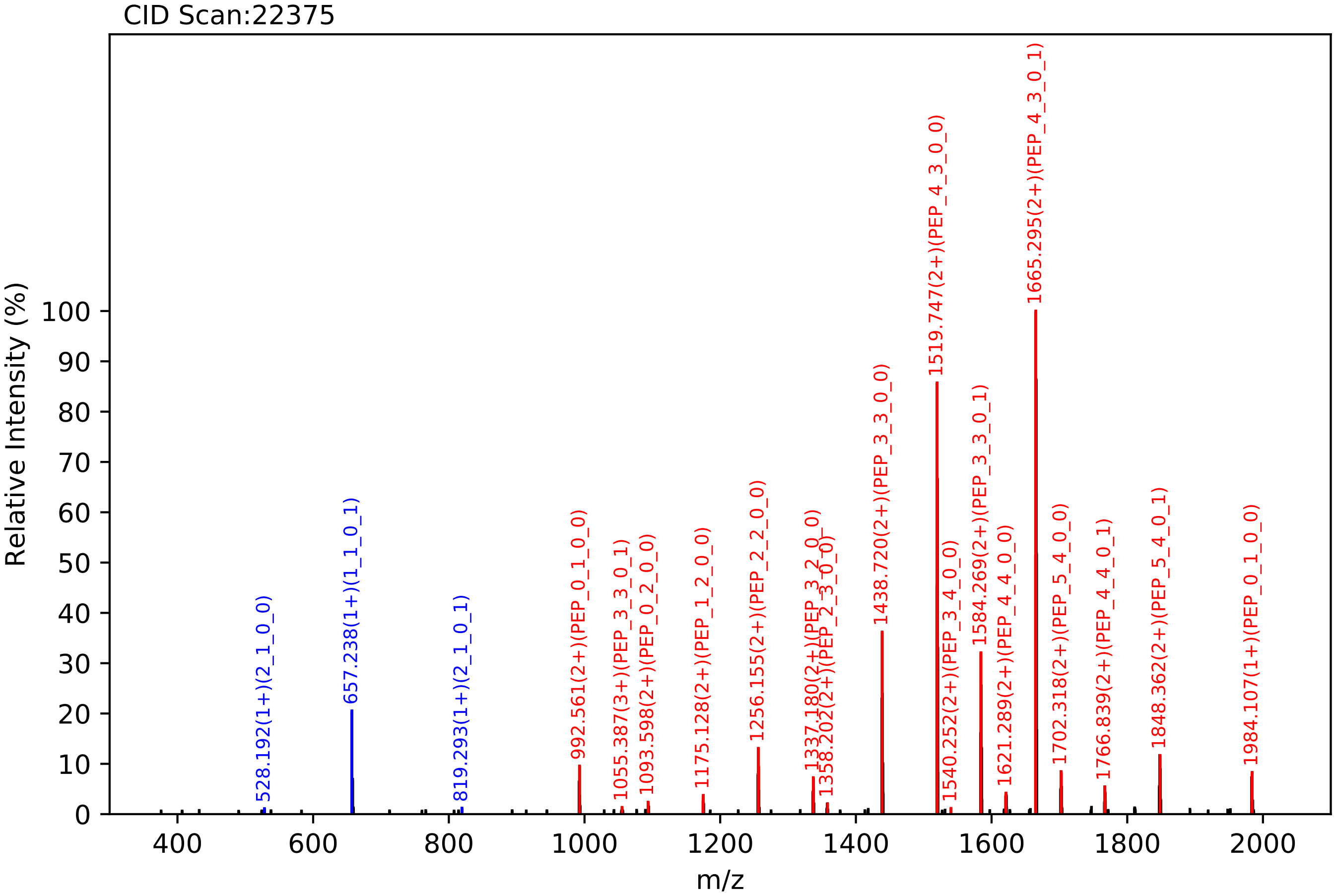

Test set no. 57, Experiment: AGP exp_4

LVPVPITNATLDQITGK(=PEP)_5_4_0_2, m/z:1328.94(3+), RT:55.85, Y-score:88.18

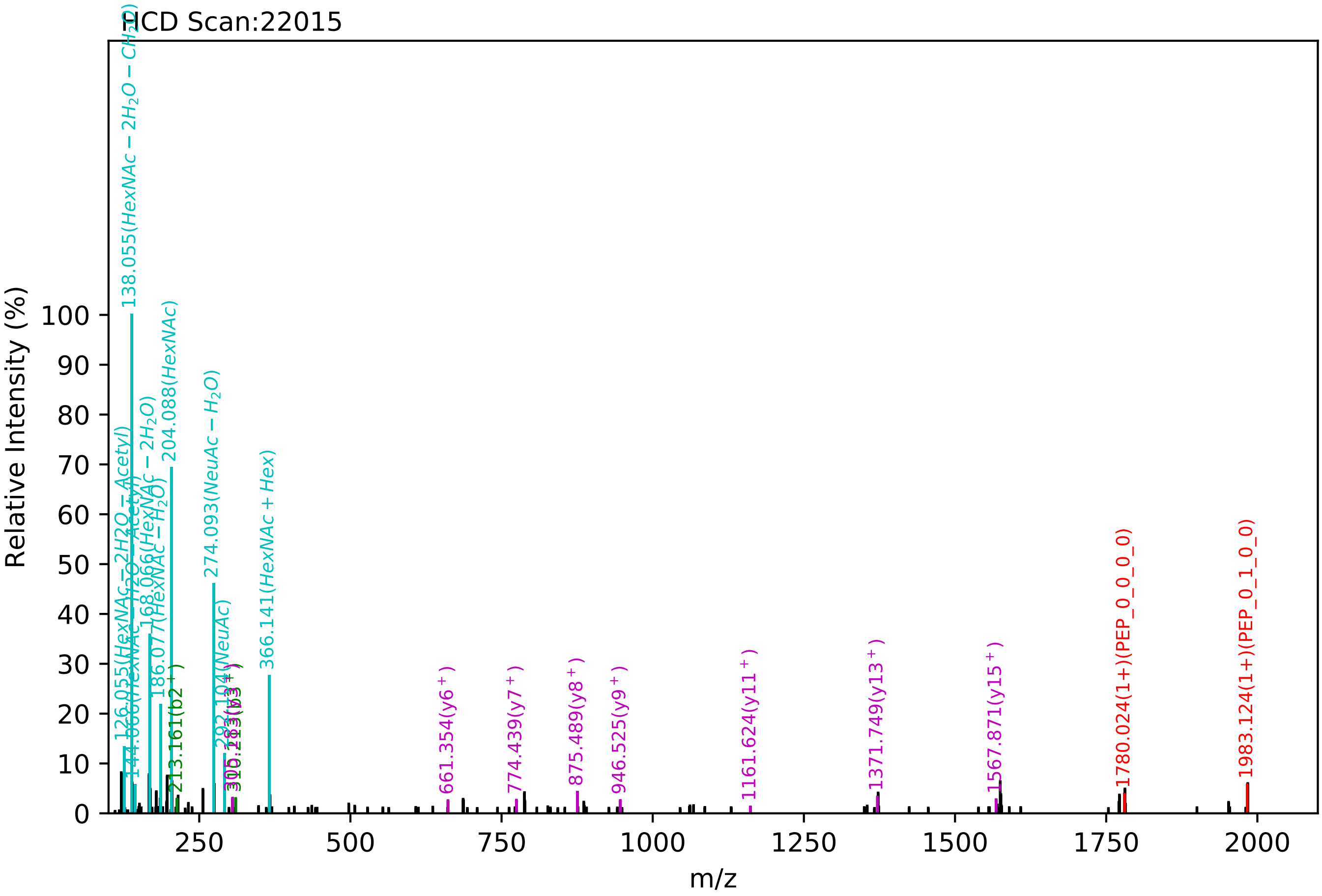

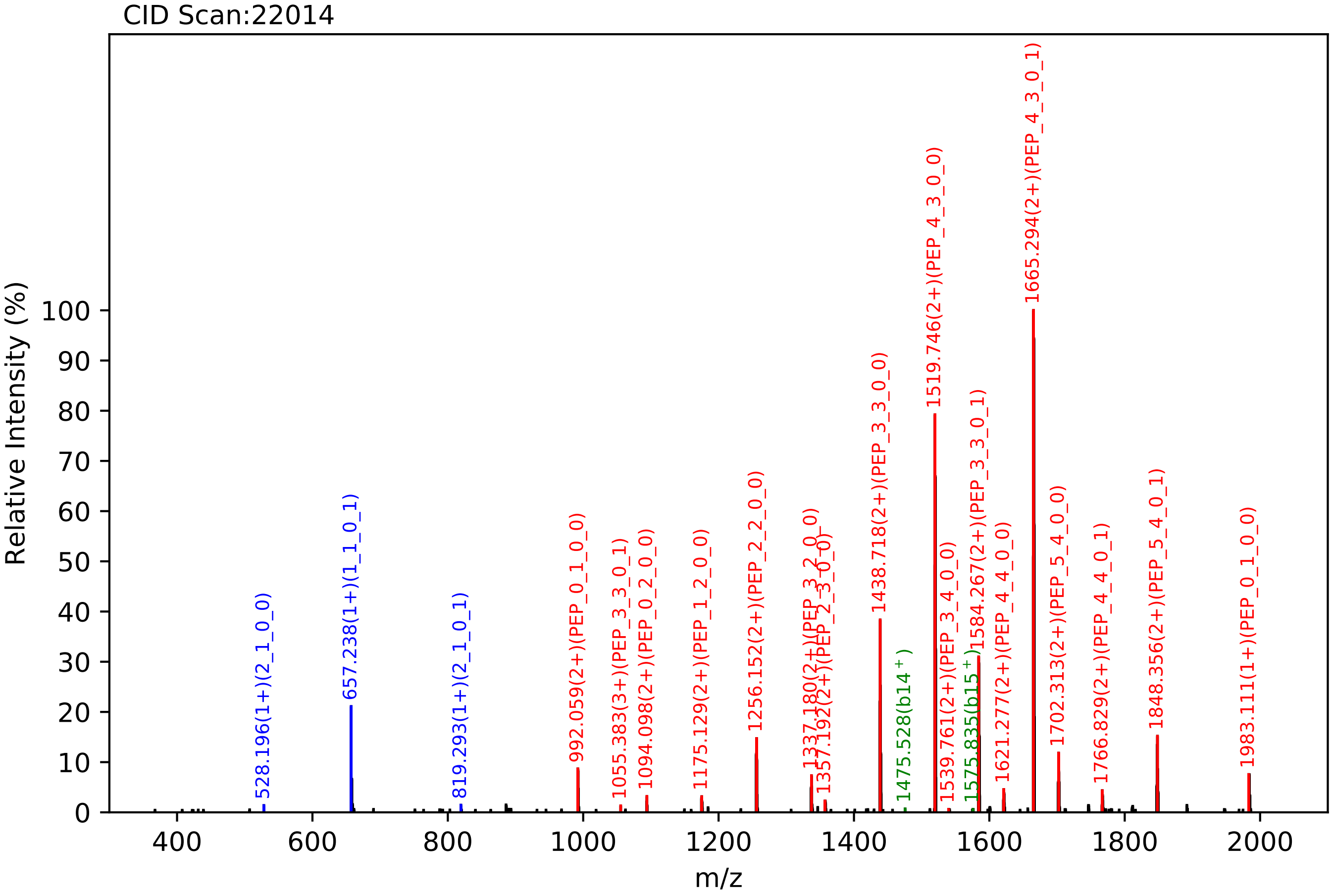

Test set no. 58, Experiment: AGP exp_4

LVPVPITNATLDQITGK(=PEP)_5_4_0_2, m/z:996.95(4+), RT:55.63, Y-score:84.21

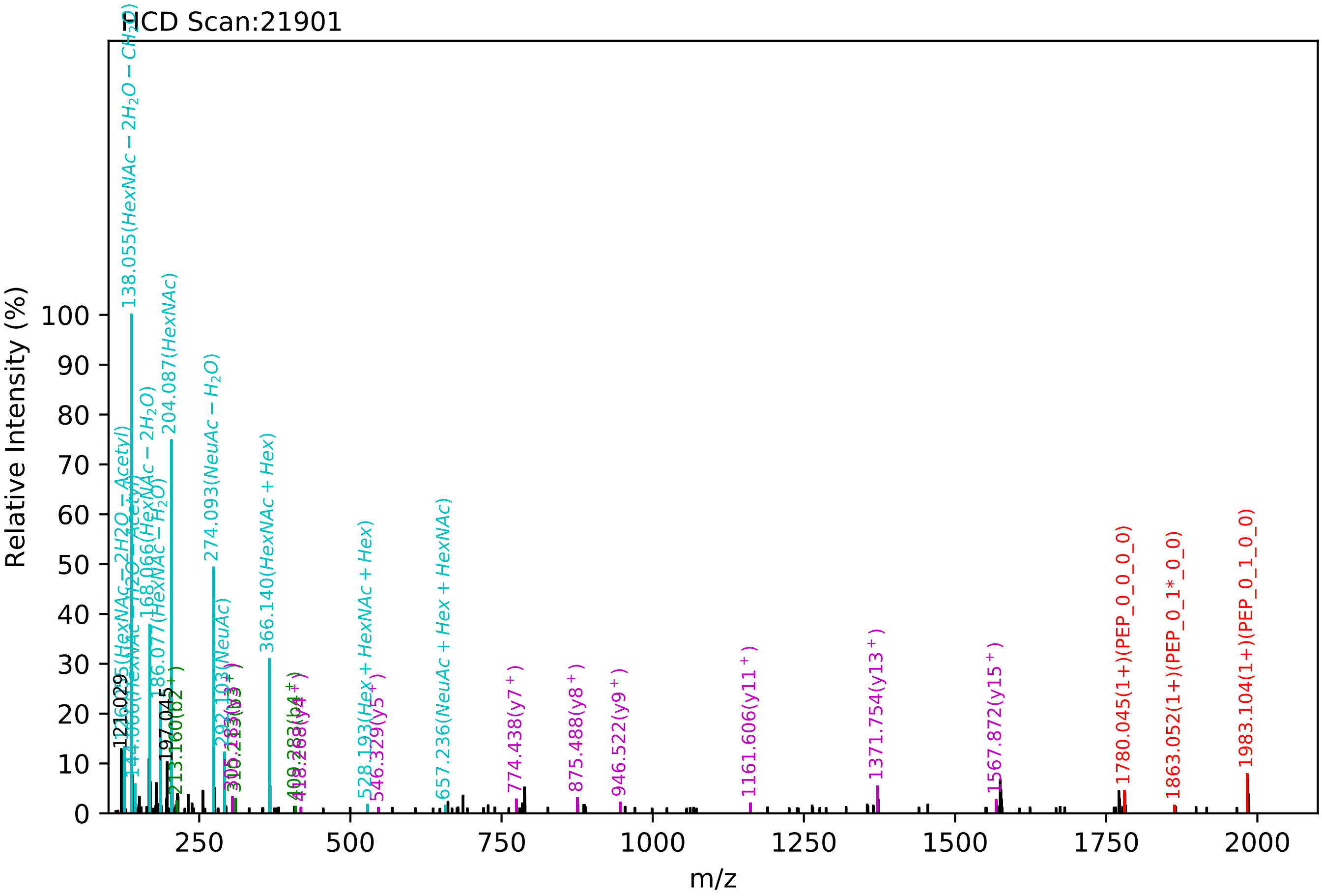

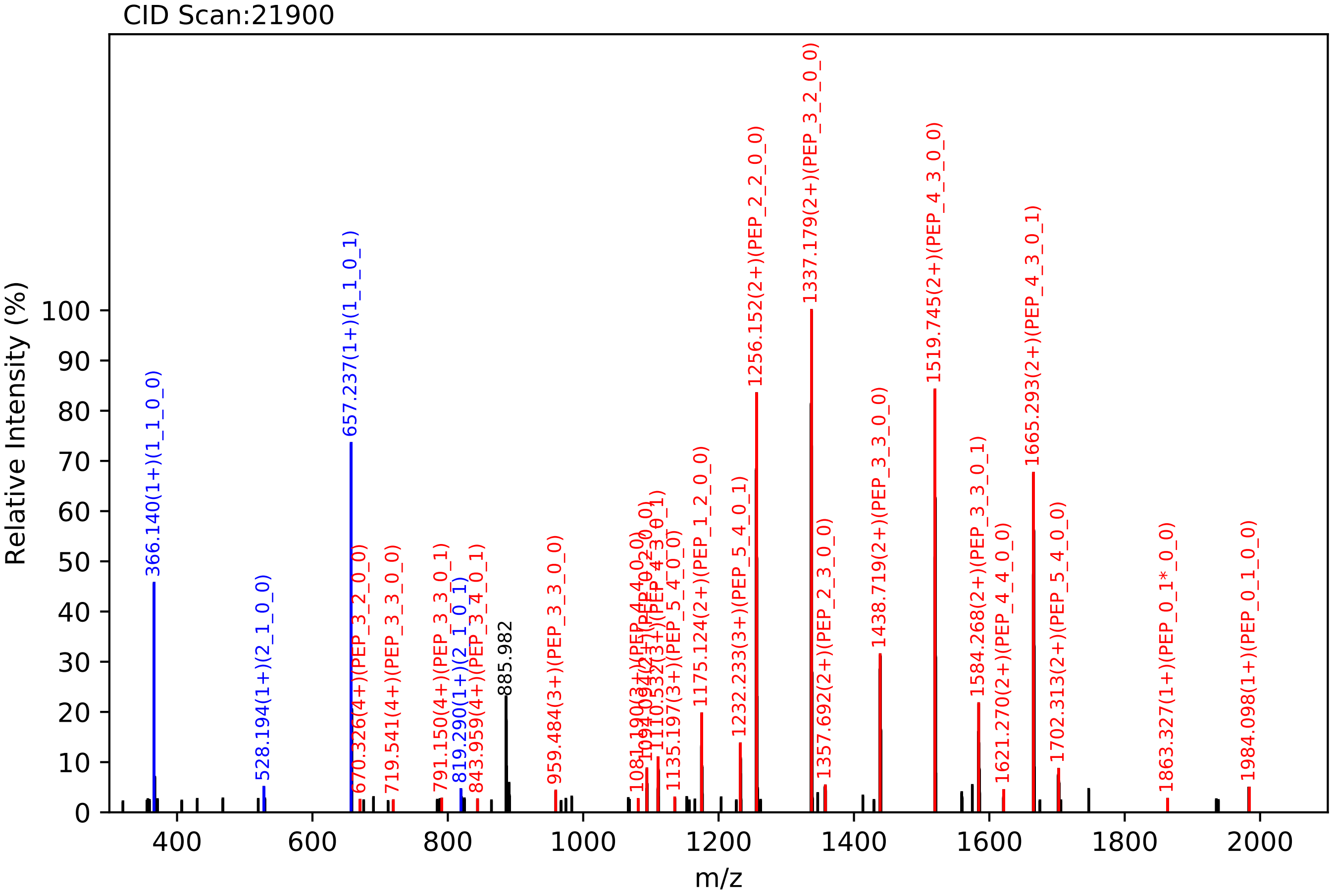

Test set no. 59, Experiment: AGP exp_3

LVPVPITNATLDQITGK(=PEP)_5_4_0_2, m/z:996.96(4+), RT:55.16, Y-score:76.37

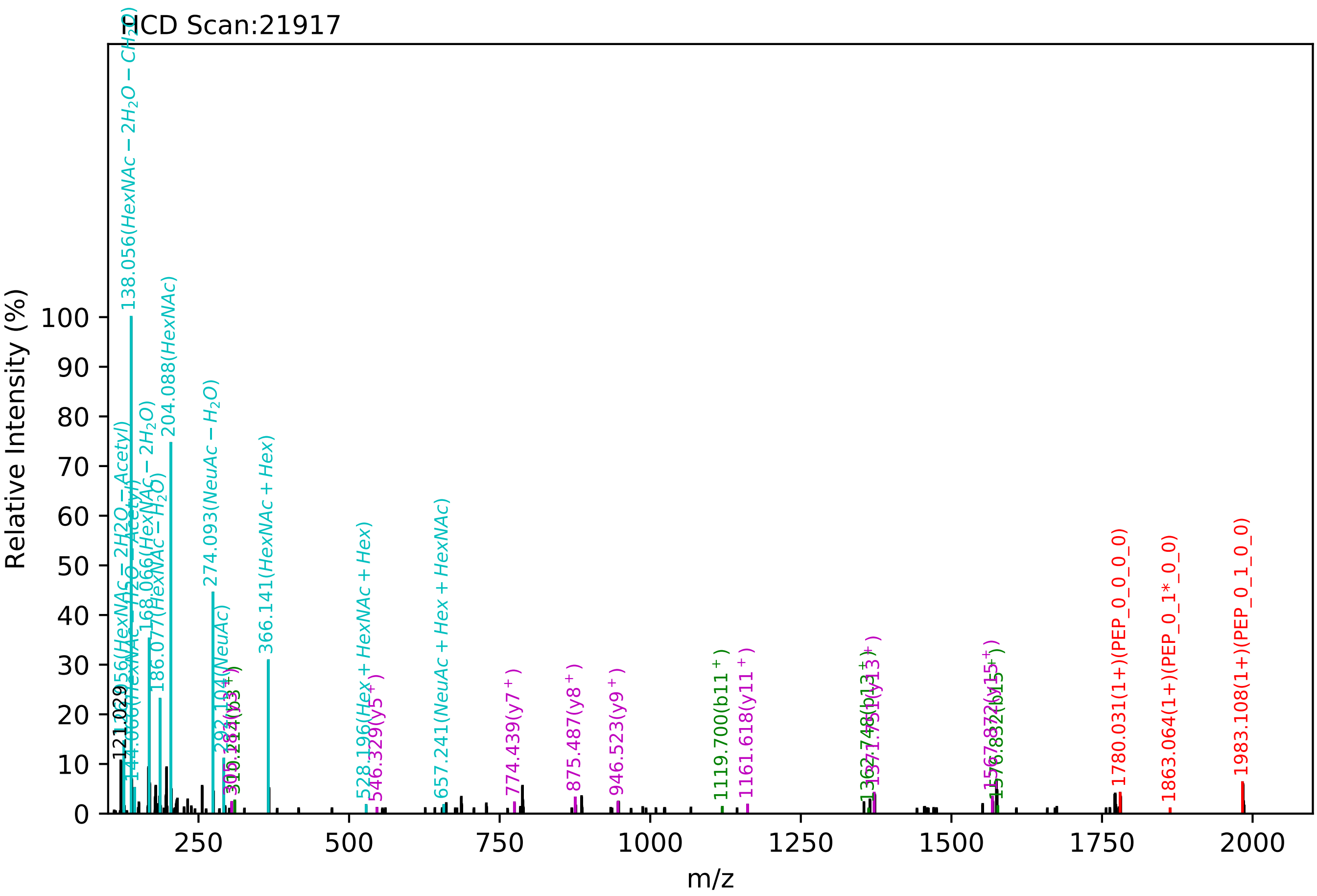

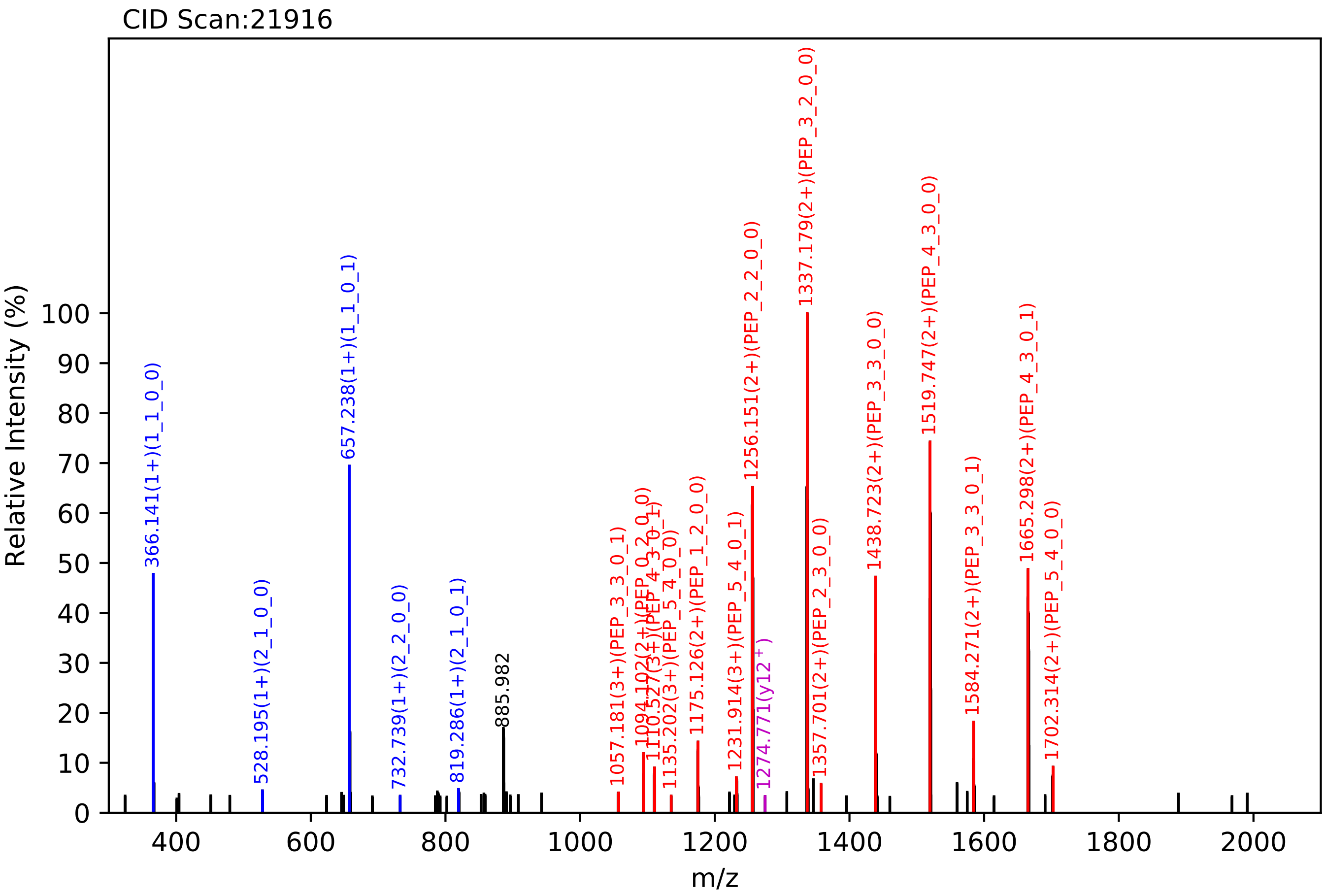

Test set no. 60, Experiment: AGP exp_3

LVPVPITNATLDQITGK(=PEP)_5_4_0_2, m/z:996.96(4+), RT:55.69, Y-score:75.24

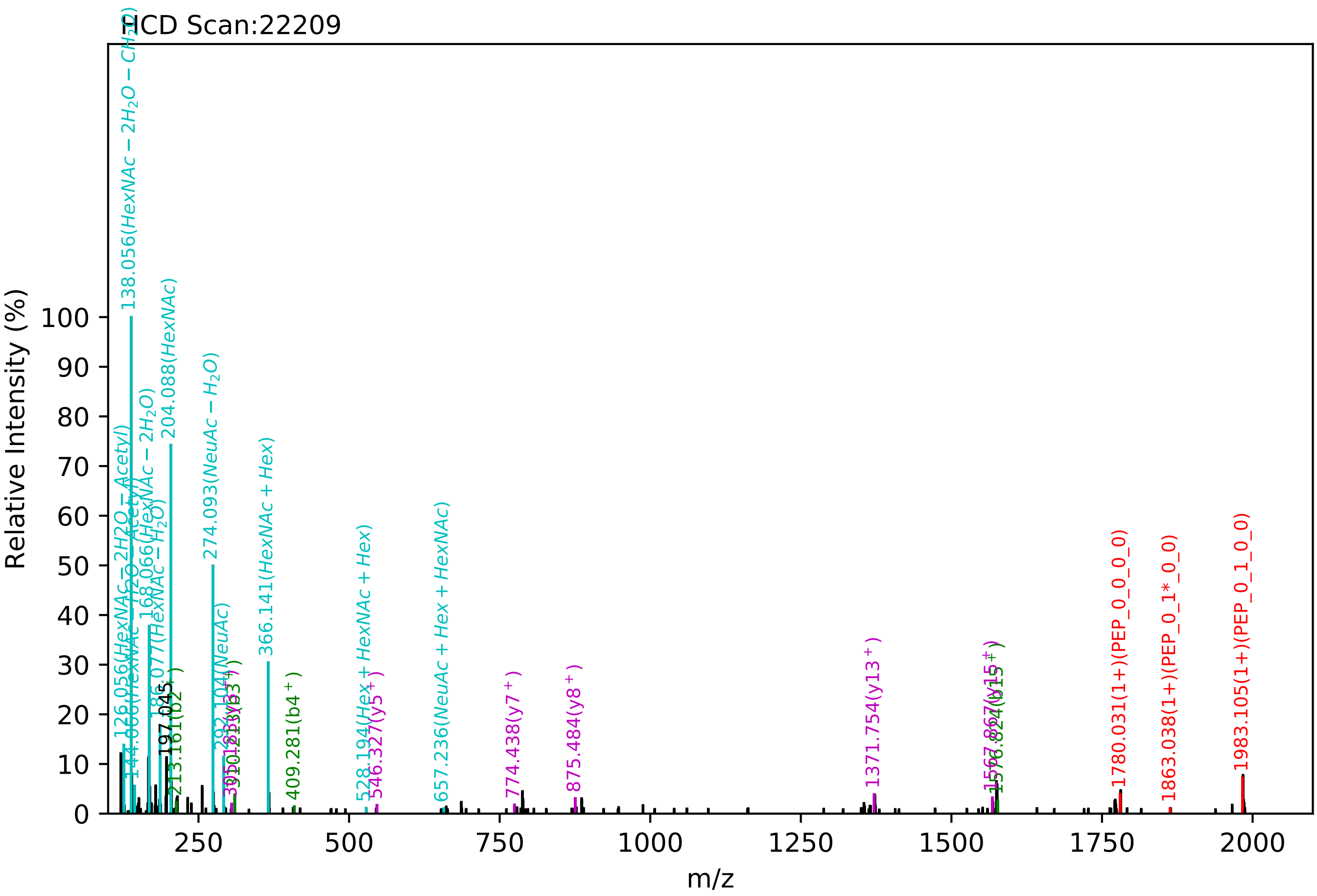

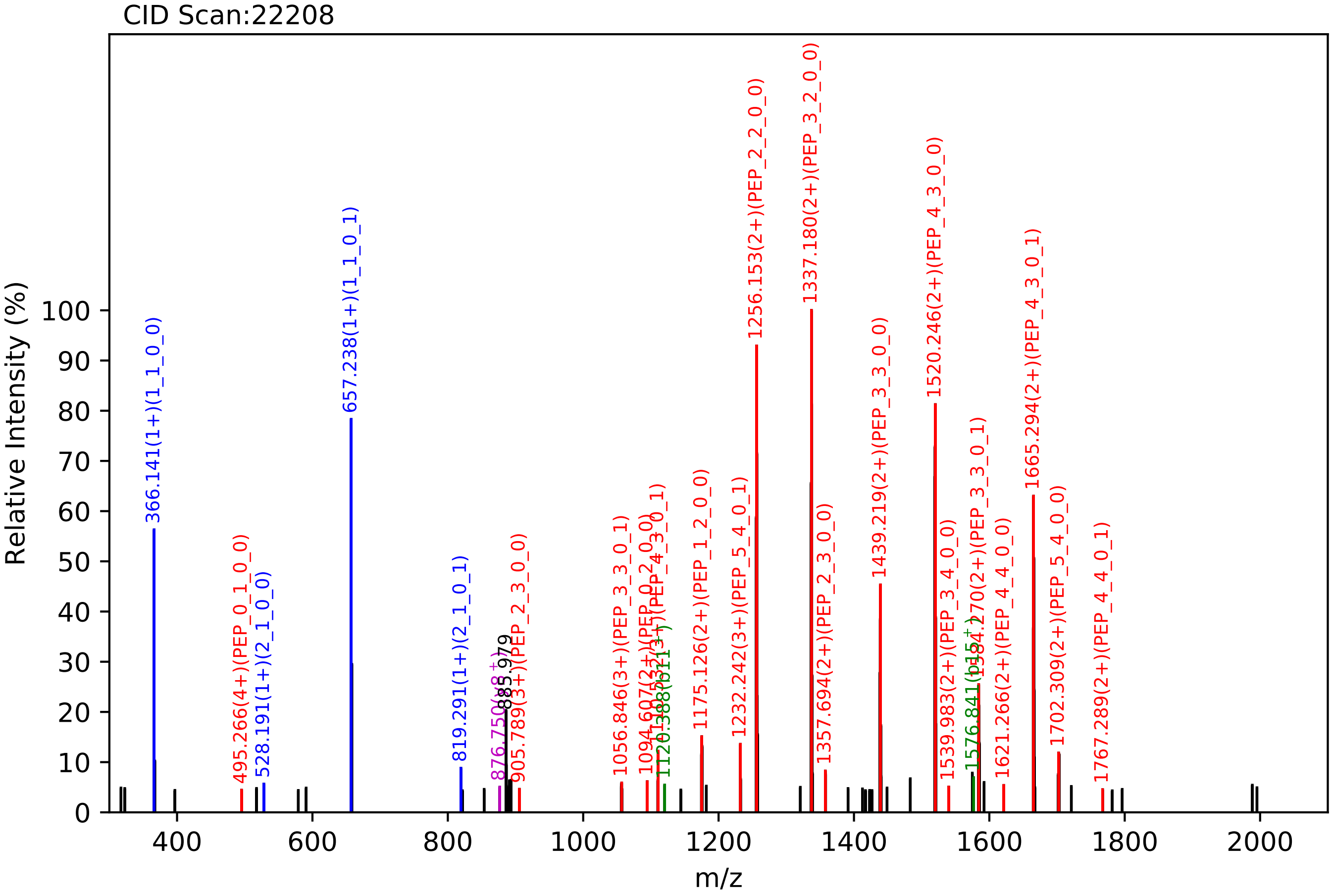

Test set no. 61, Experiment: AGP exp_4

LVPVPITNATLDQITGK(=PEP)_6_5_0_1, m/z:1353.62(3+), RT:48.25, Y-score:89.89

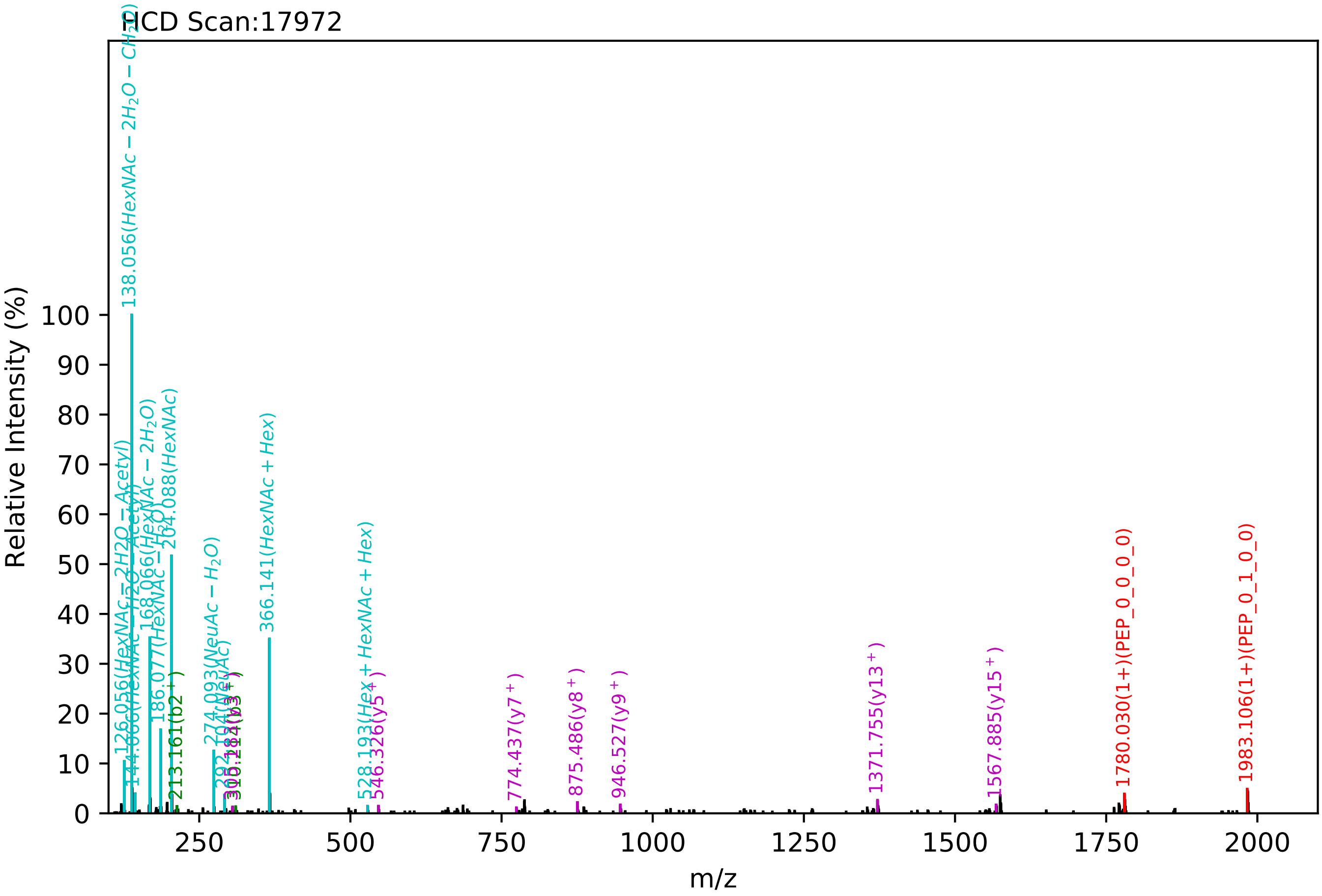

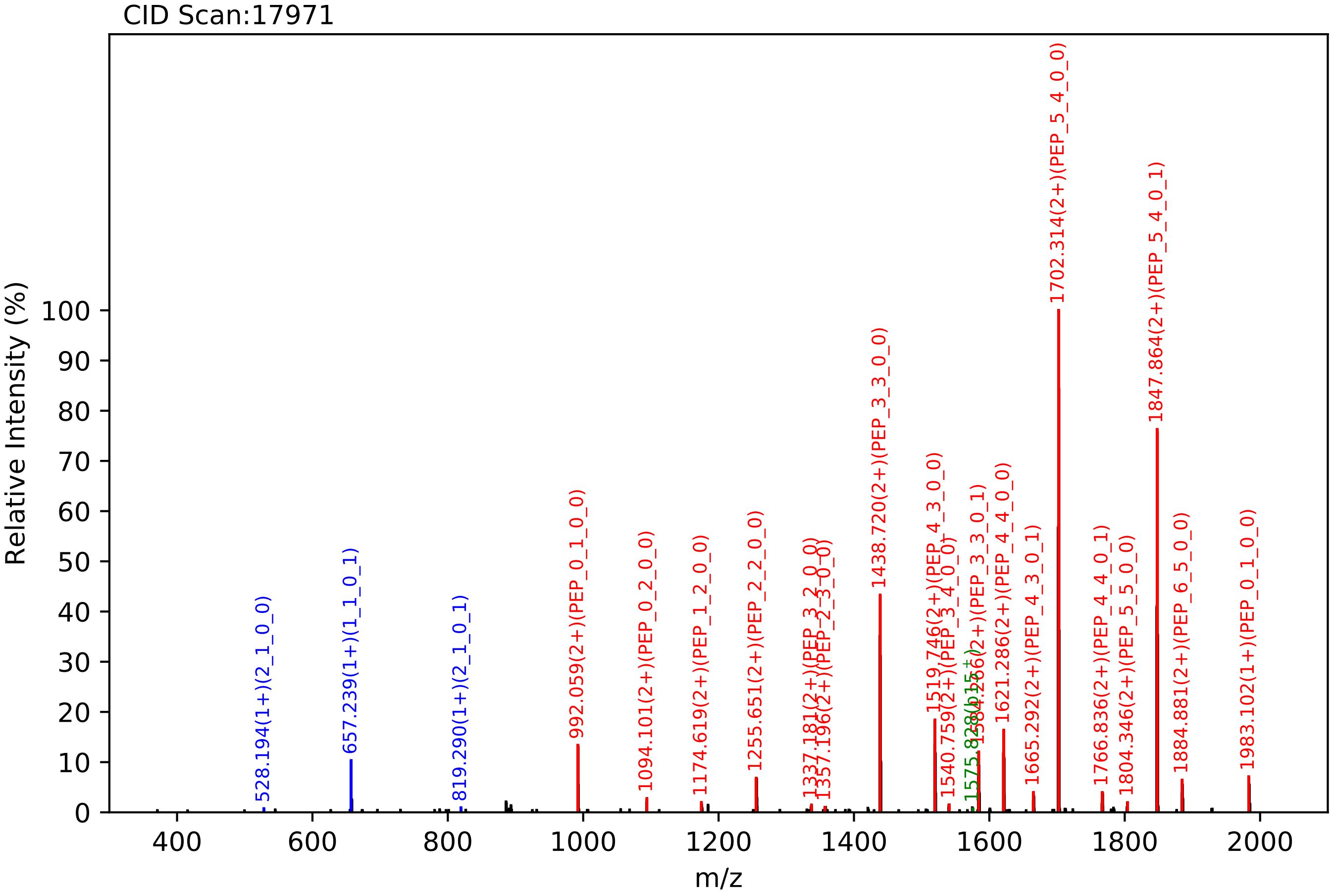

Test set no. 62, Experiment: AGP exp_4

LVPVPITNATLDQITGK(=PEP)_6_5_0_1, m/z:1015.46(4+), RT:48.64, Y-score:88.95

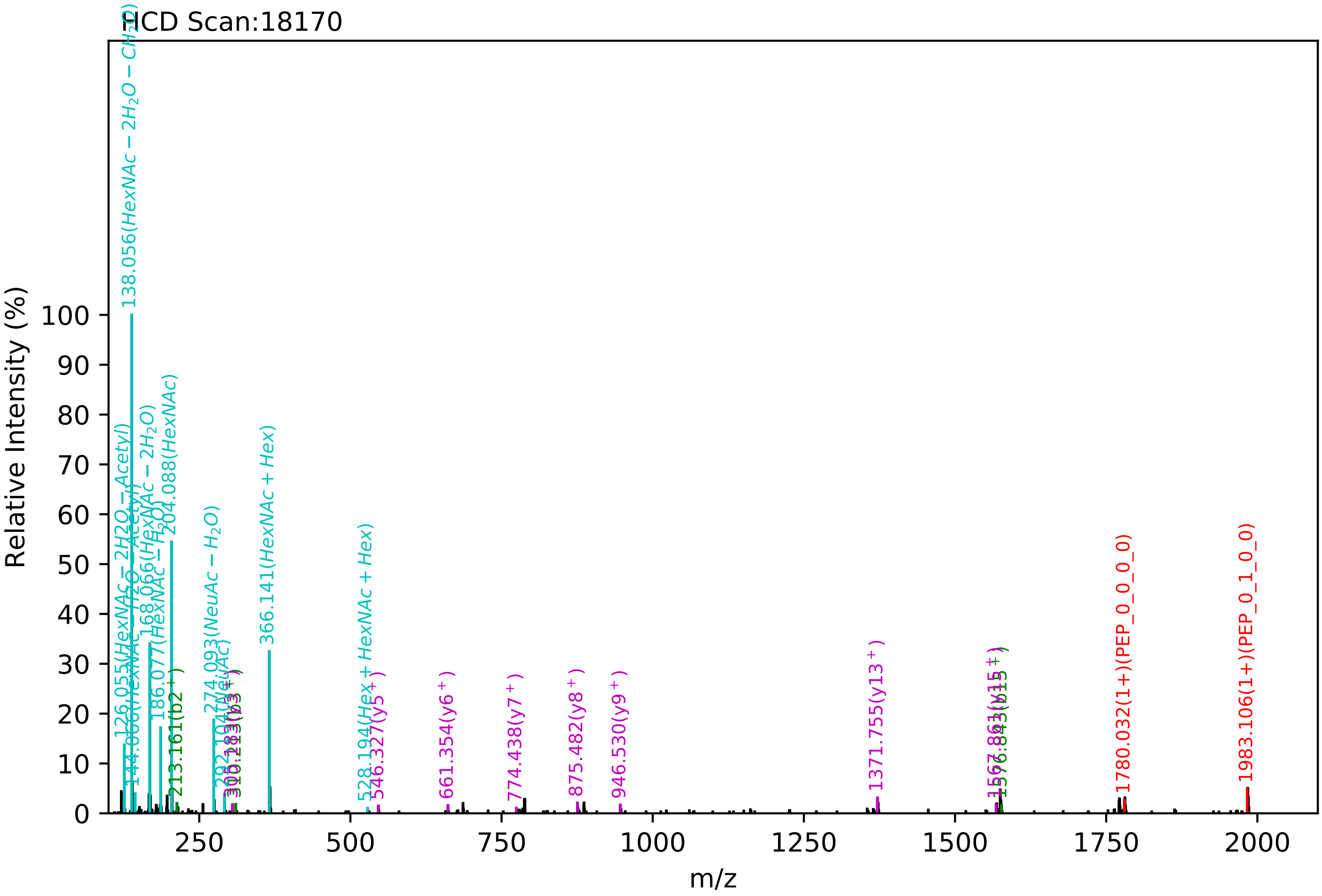

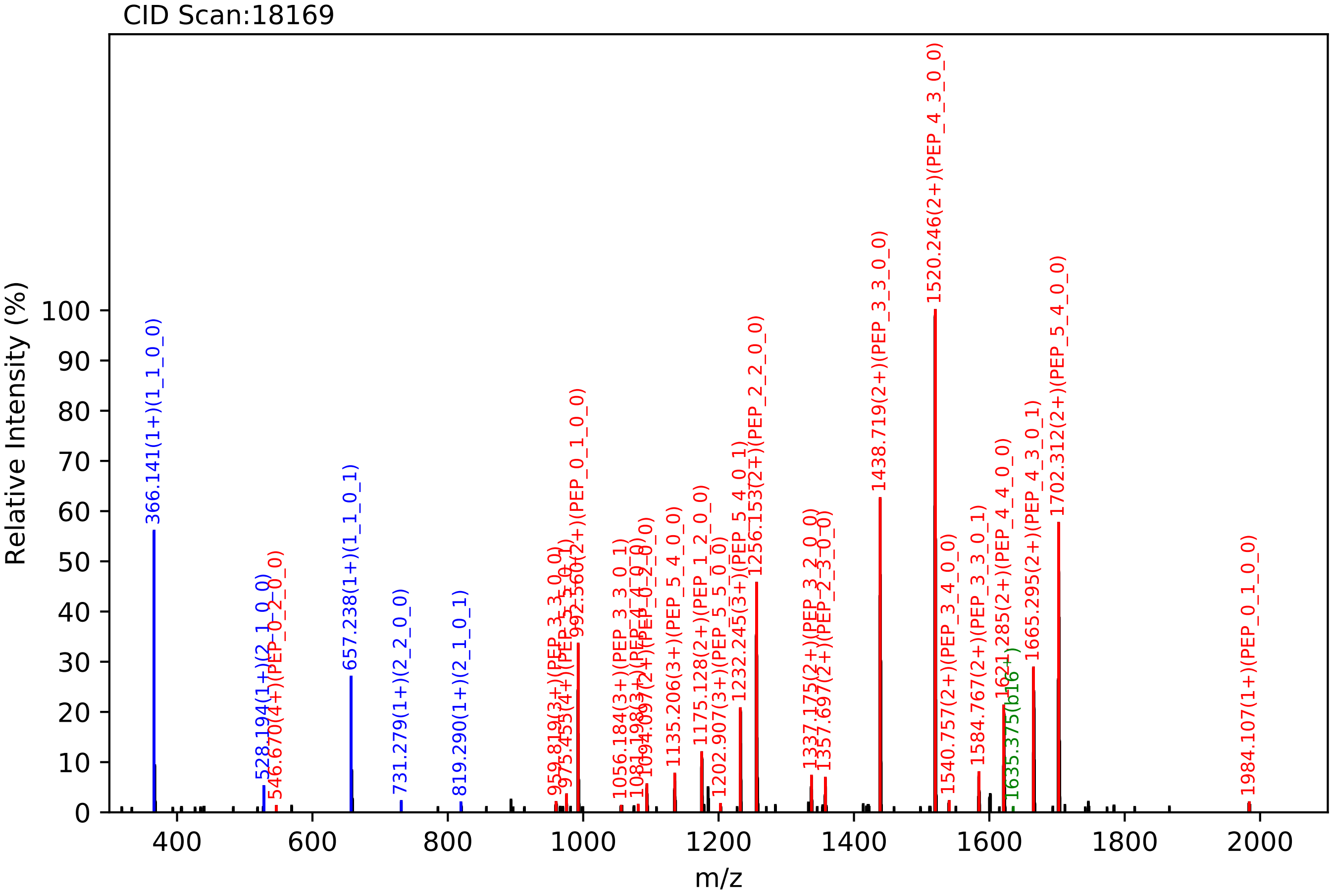

Test set no. 63, Experiment: AGP exp_3

LVPVPITNATLDQITGK(=PEP)_6_5_0_1, m/z:1015.46(4+), RT:48.24, Y-score:88.64

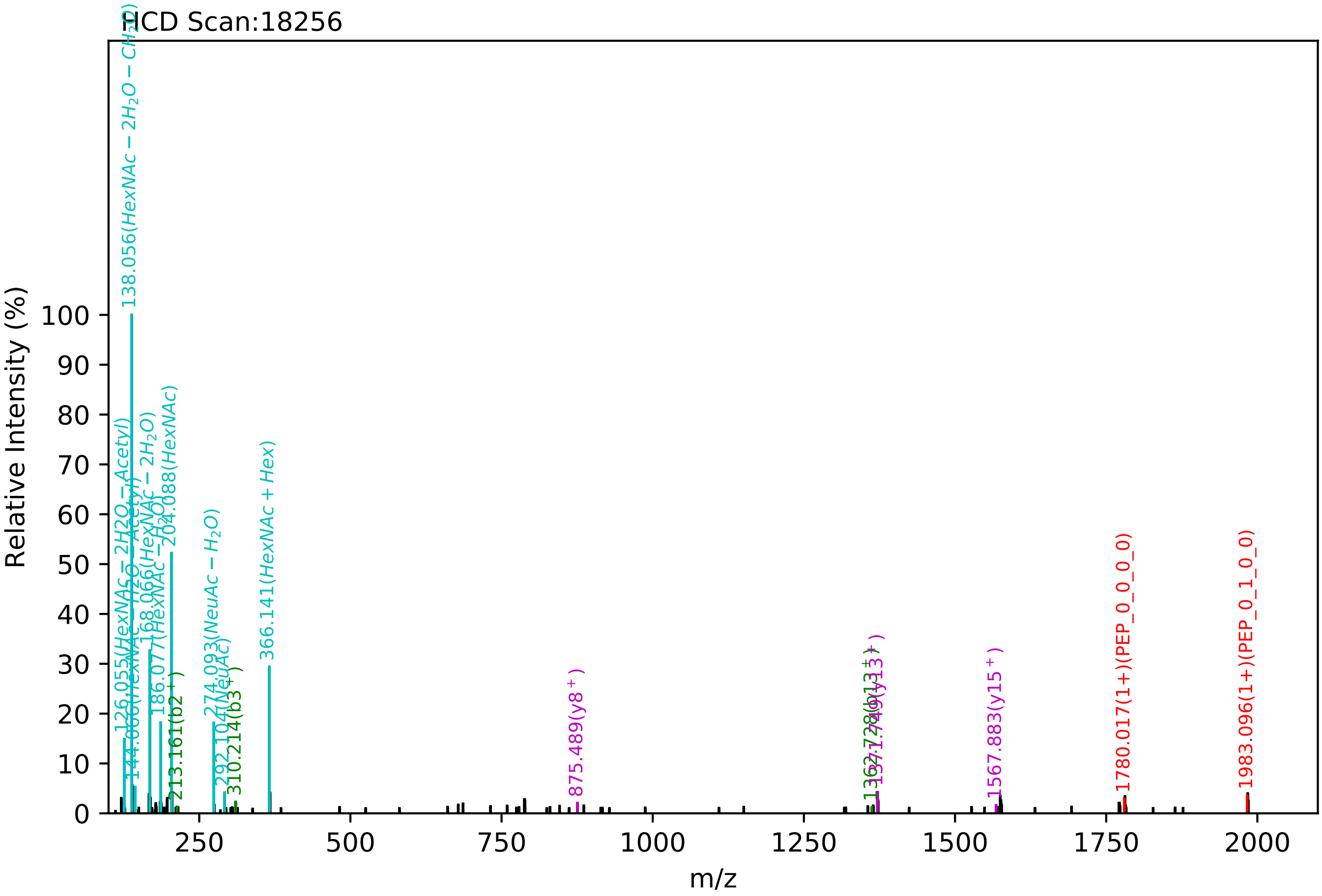

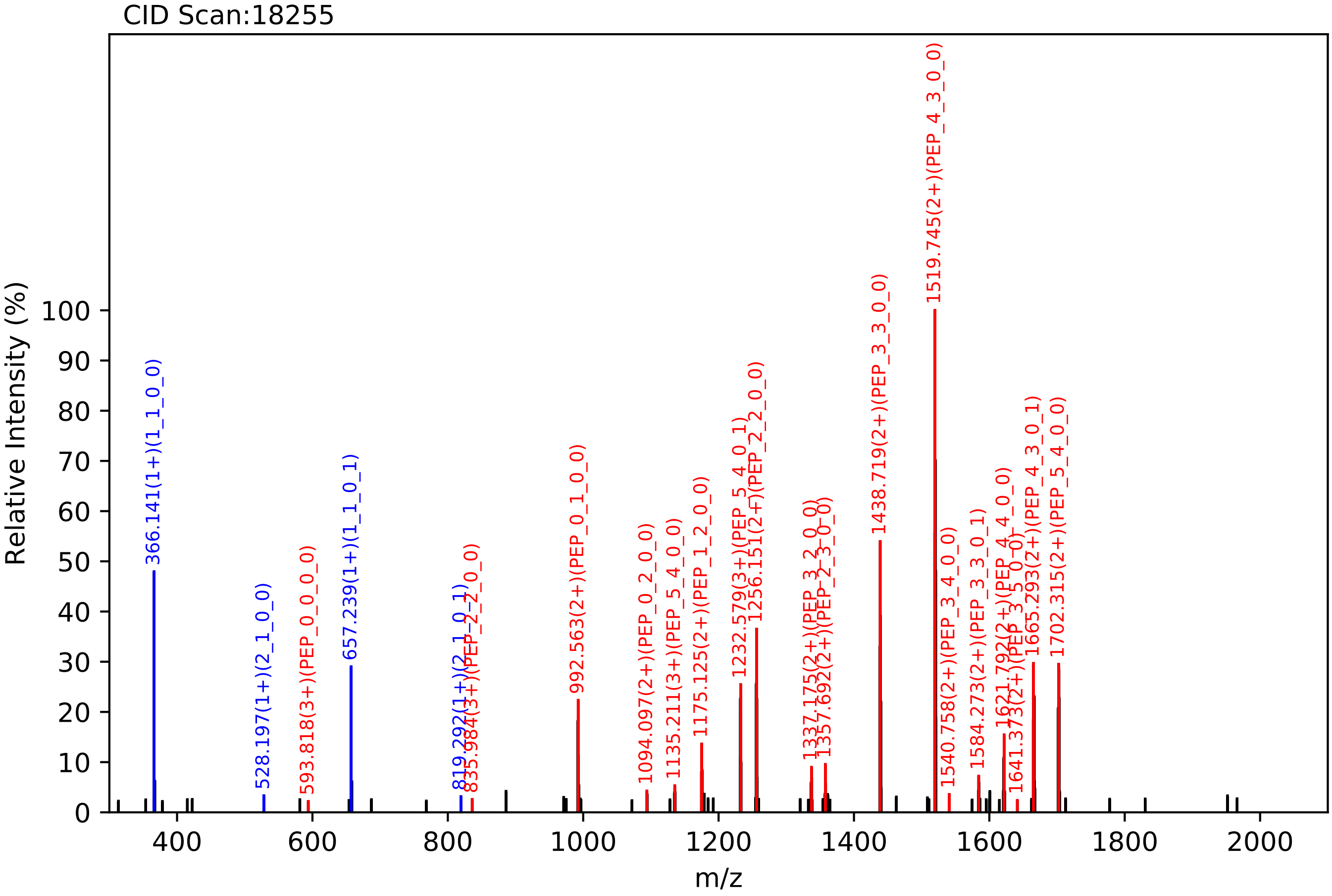

Test set no. 64, Experiment: AGP exp_3

LVPVPITNATLDQITGK(=PEP)_6_5_0_1, m/z:1015.46(4+), RT:48.80, Y-score:88.43

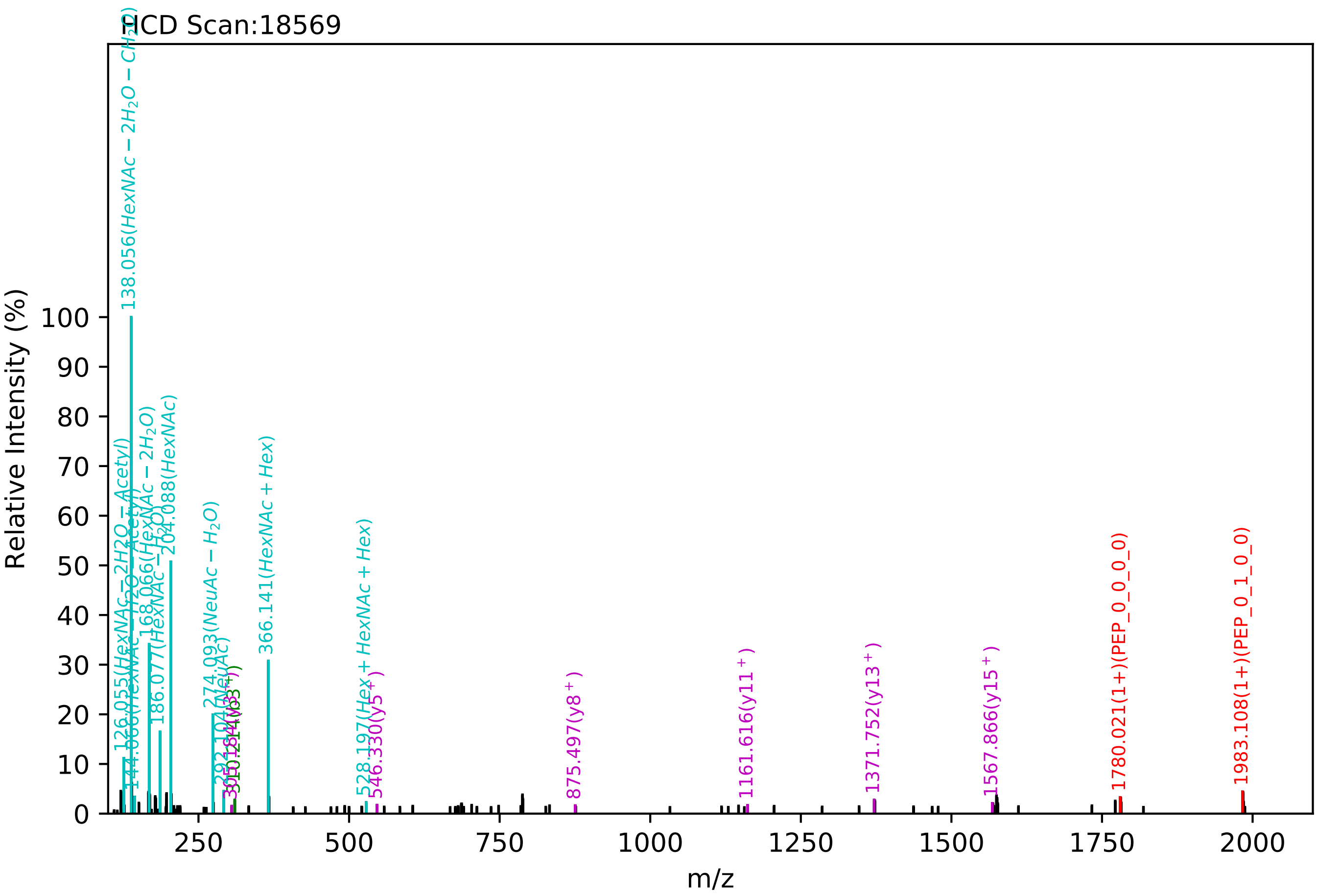

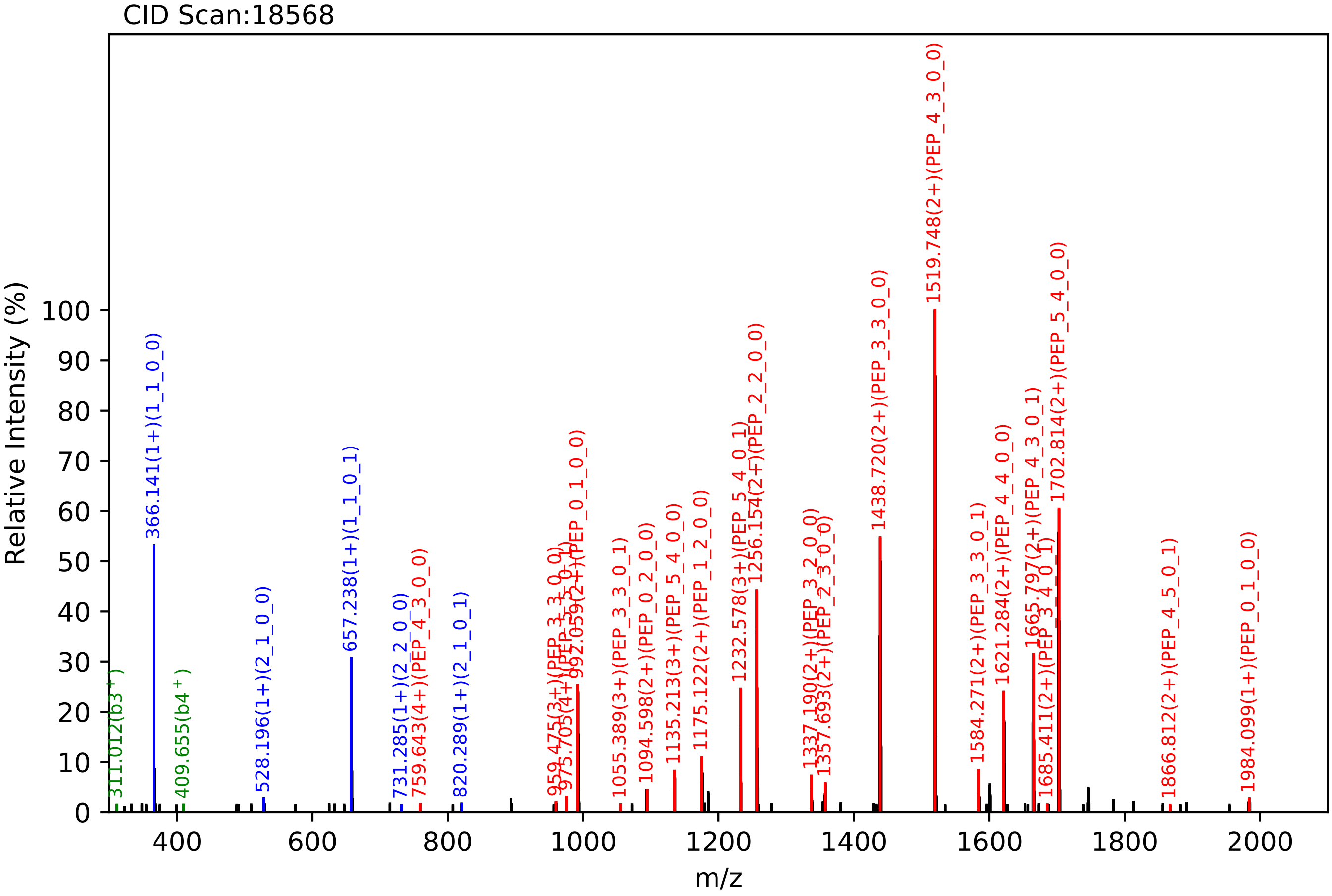

Test set no. 65, Experiment: AGP exp_3

LVPVPITNATLDQITGK(=PEP)_6_5_0_2, m/z:1450.65(3+), RT:54.88, Y-score:89.21

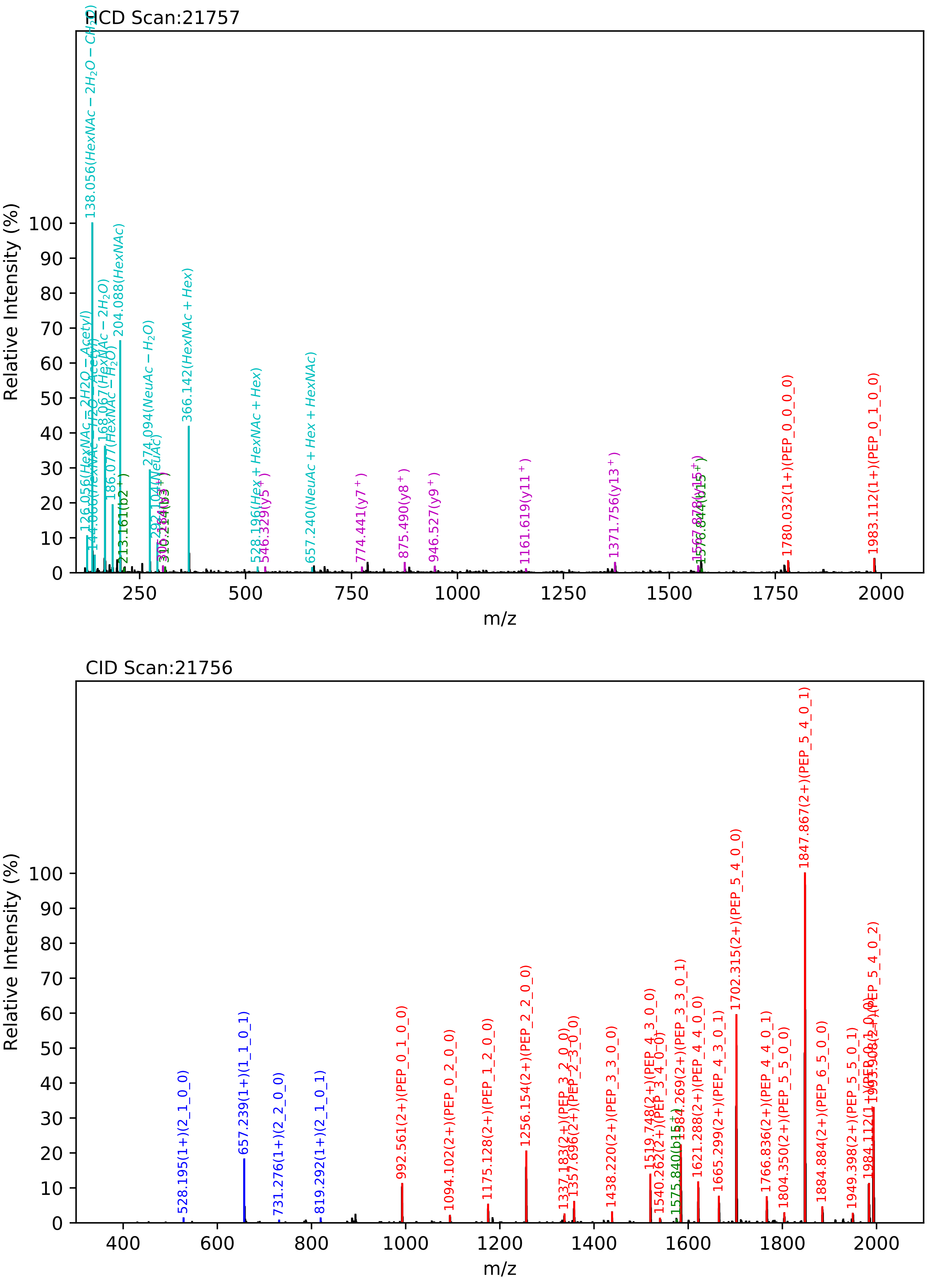

Test set no. 66, Experiment: AGP exp_3

LVPVPITNATLDQITGK(=PEP)_6_5_0_2, m/z:1087.99(4+), RT:54.96, Y-score:87.26

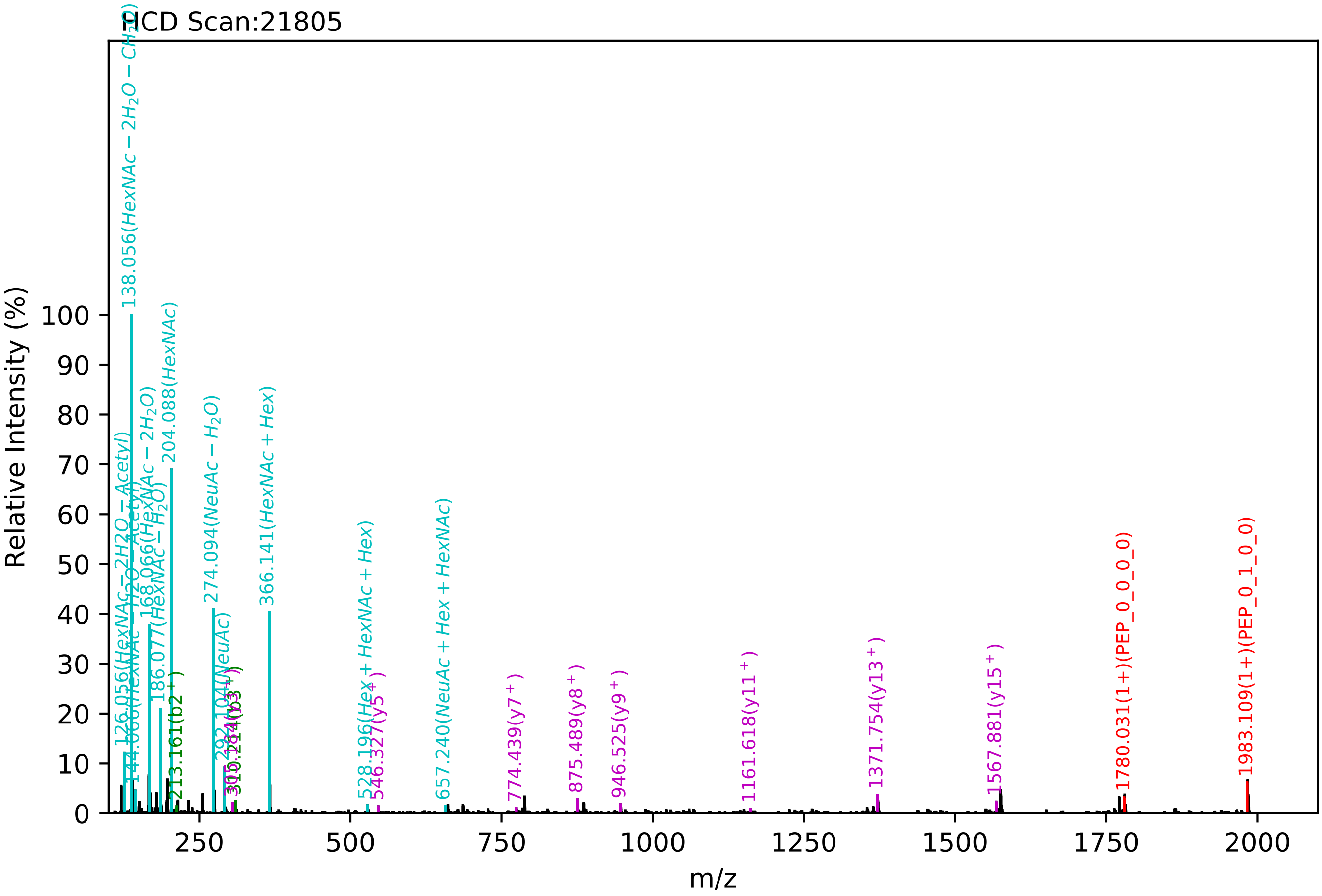

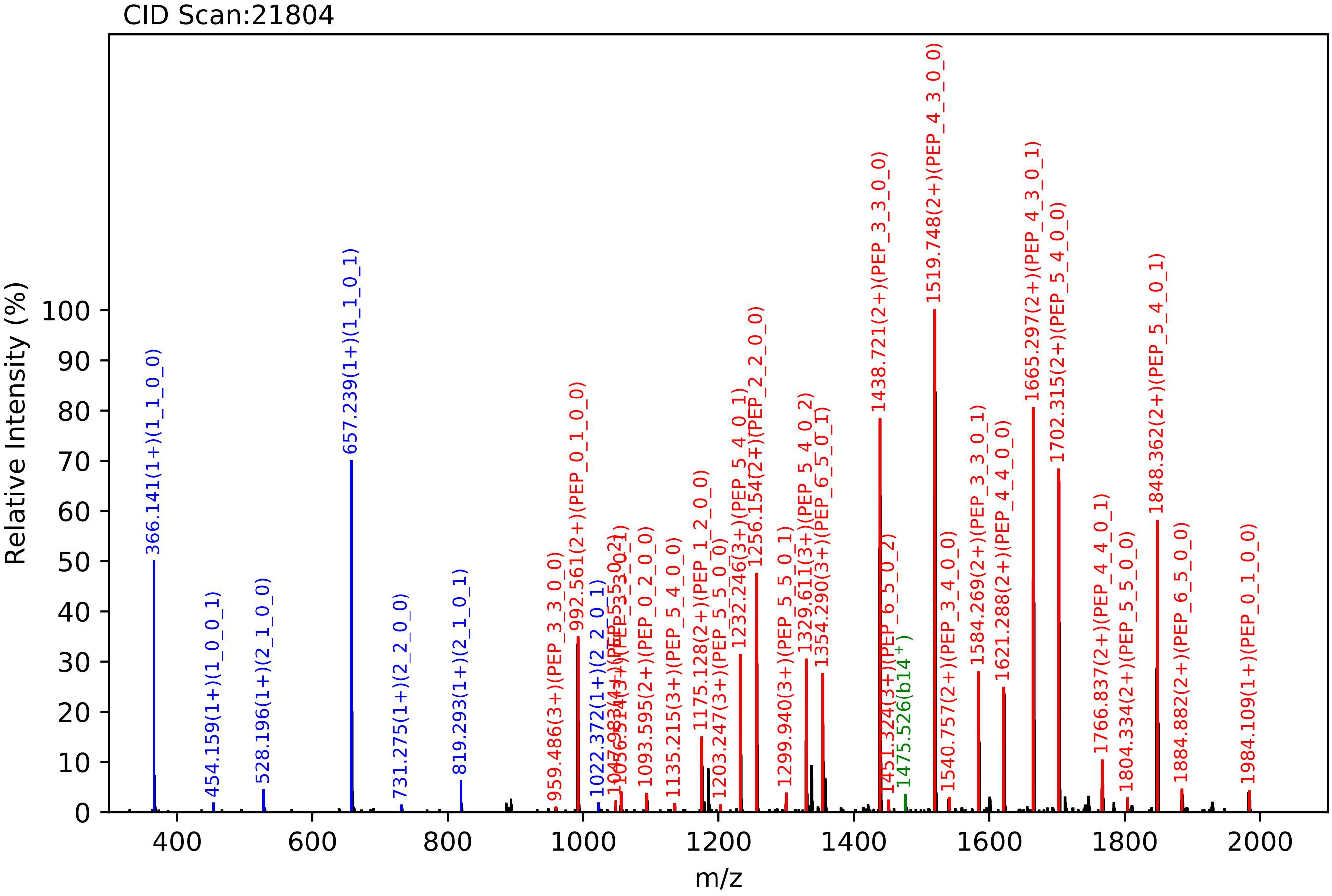

Test set no. 67, Experiment: AGP exp_3

LVPVPITNATLDQITGK(=PEP)_6_5_0_2, m/z:1088.24(4+), RT:54.55, Y-score:85.86

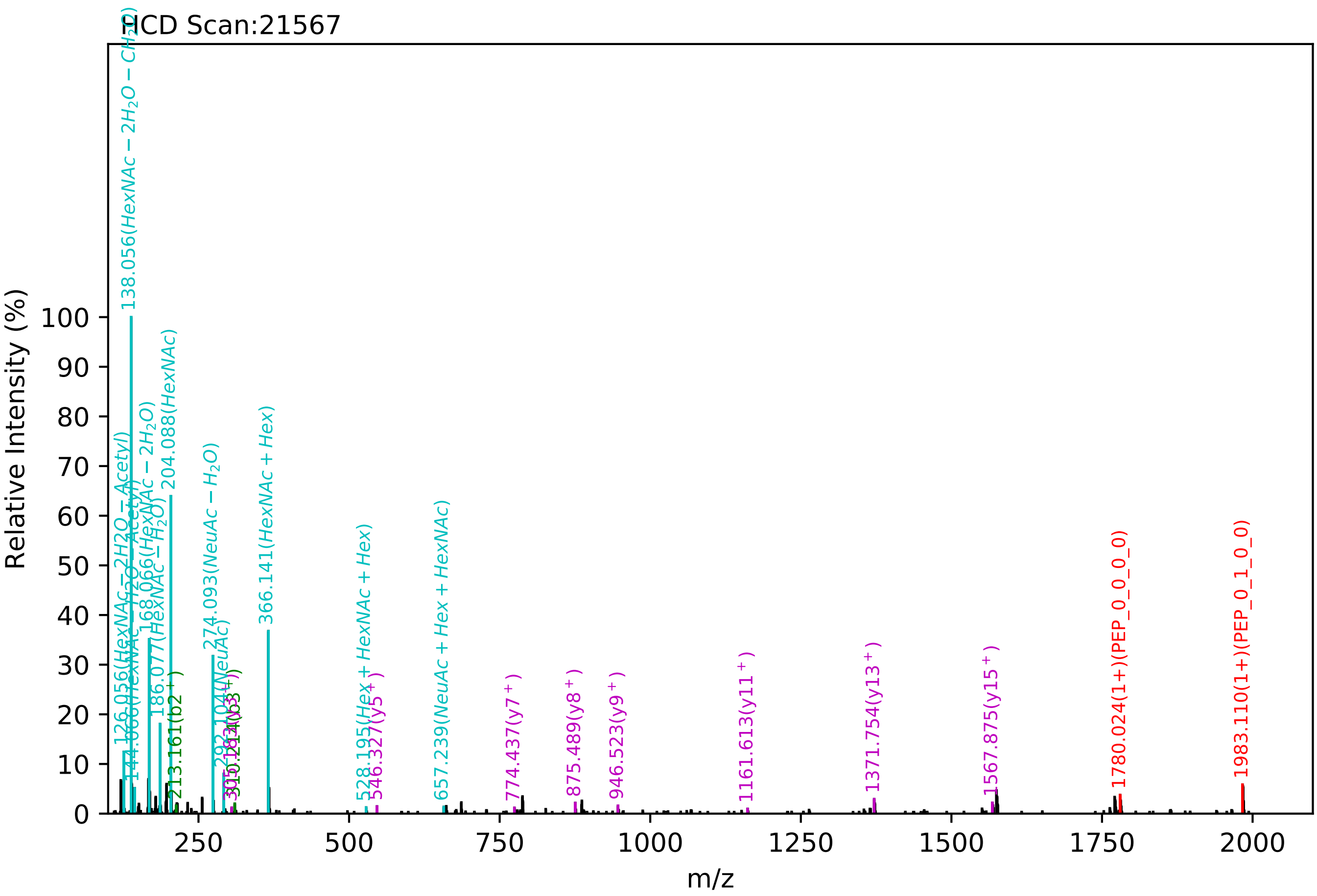

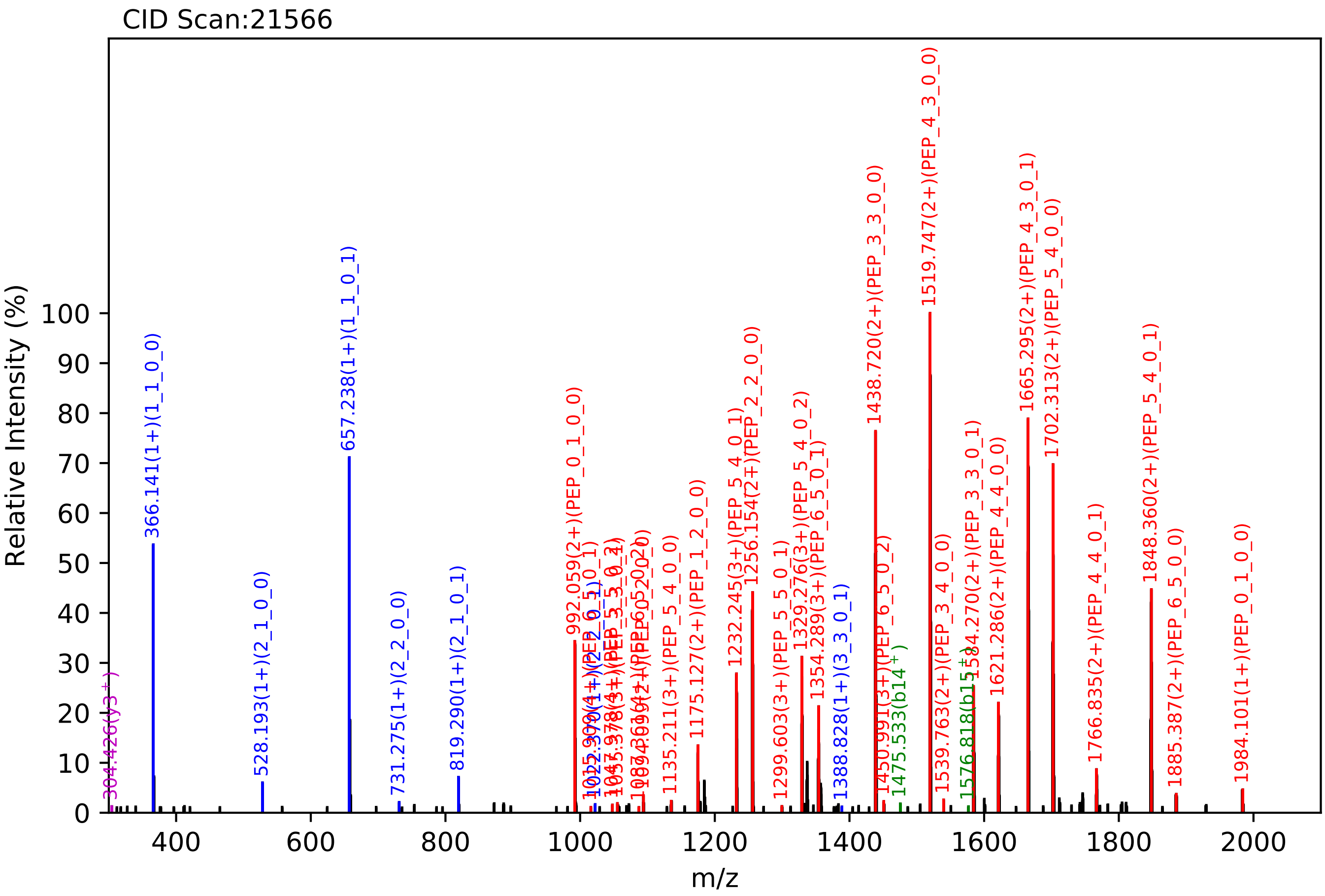

Test set no. 68, Experiment: AGP exp_4

LVPVPITNATLDQITGK(=PEP)_6_5_0_2, m/z:1088.24(4+), RT:55.00, Y-score:85.07

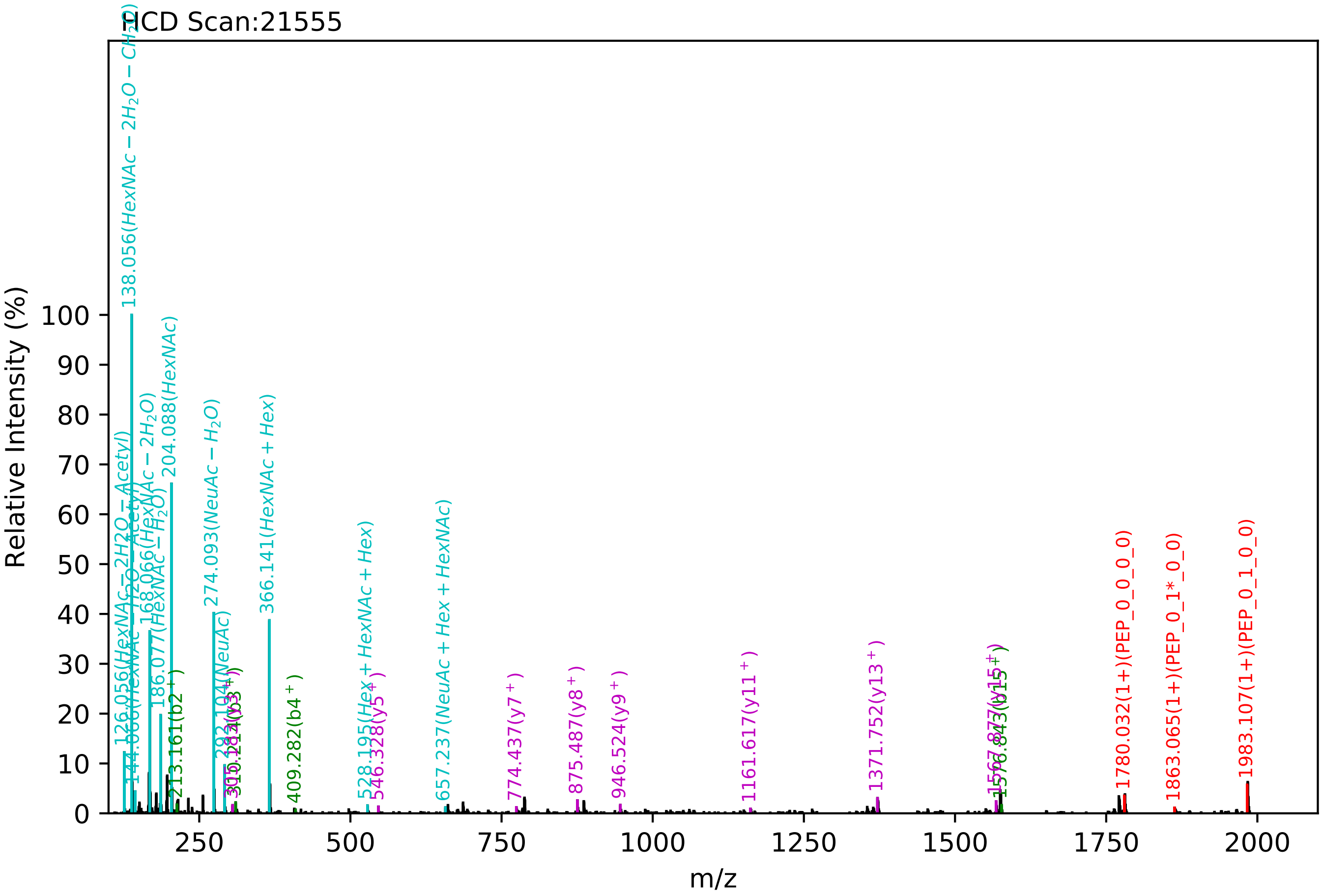

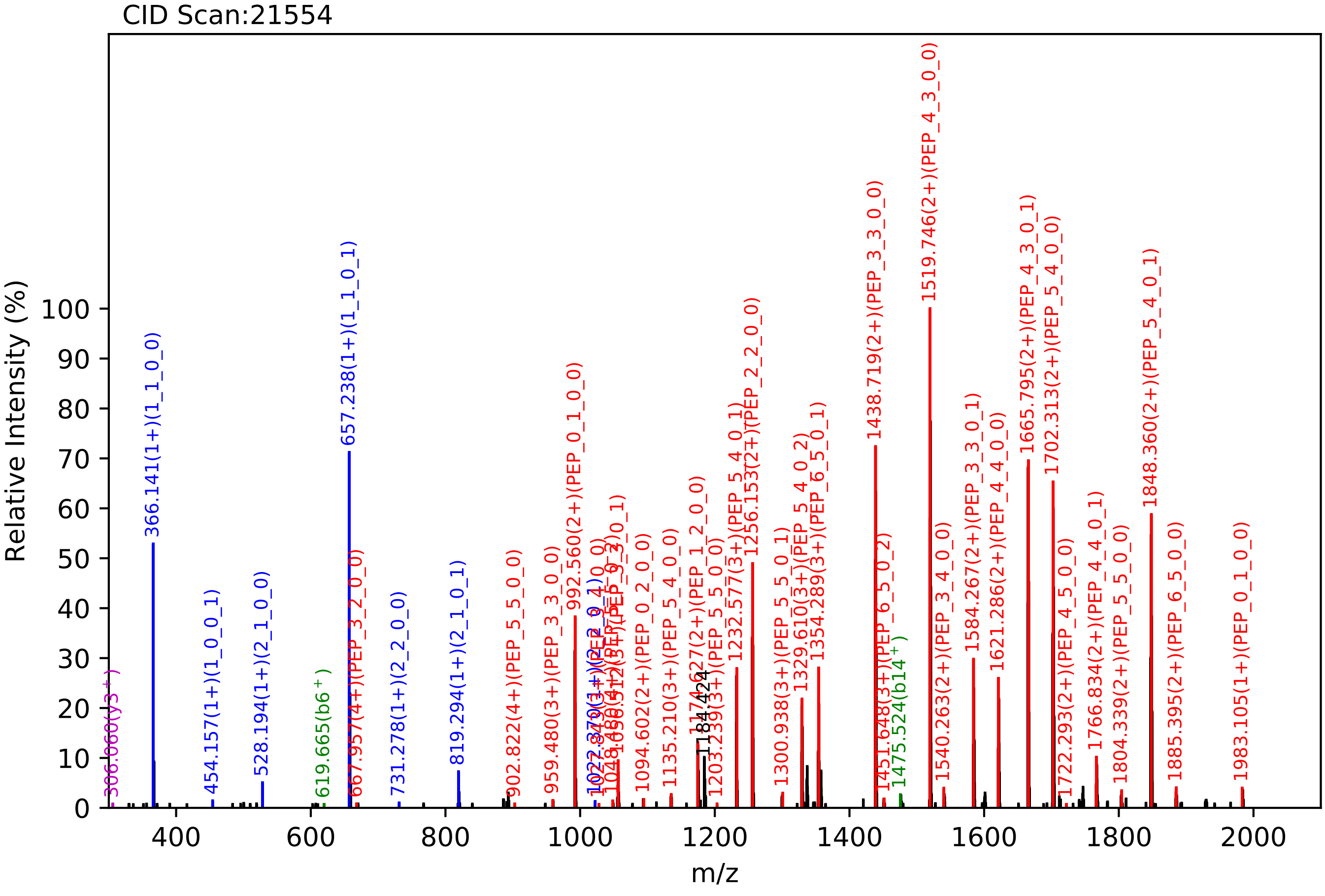

Test set no. 69, Experiment: AGP exp_3

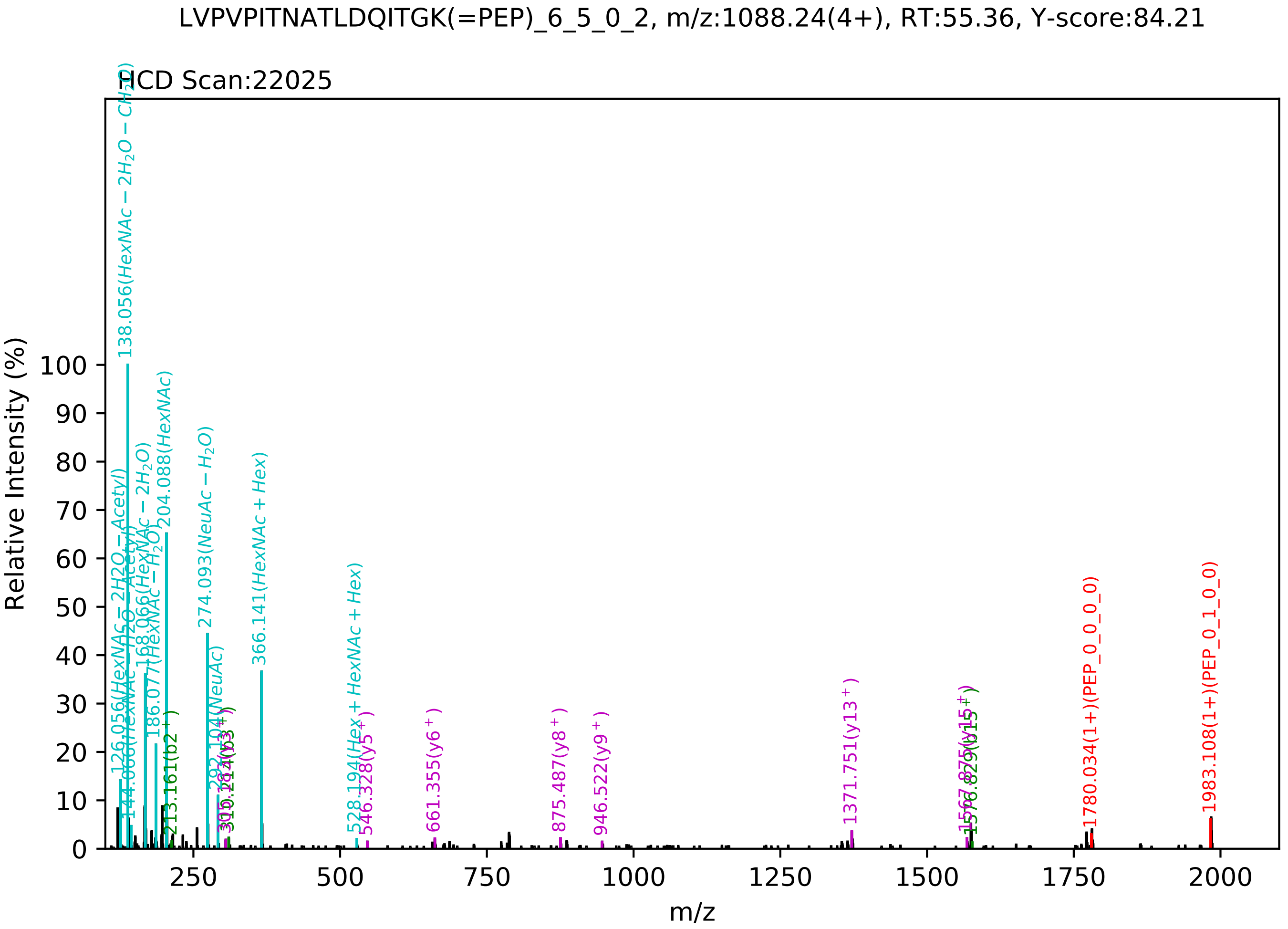

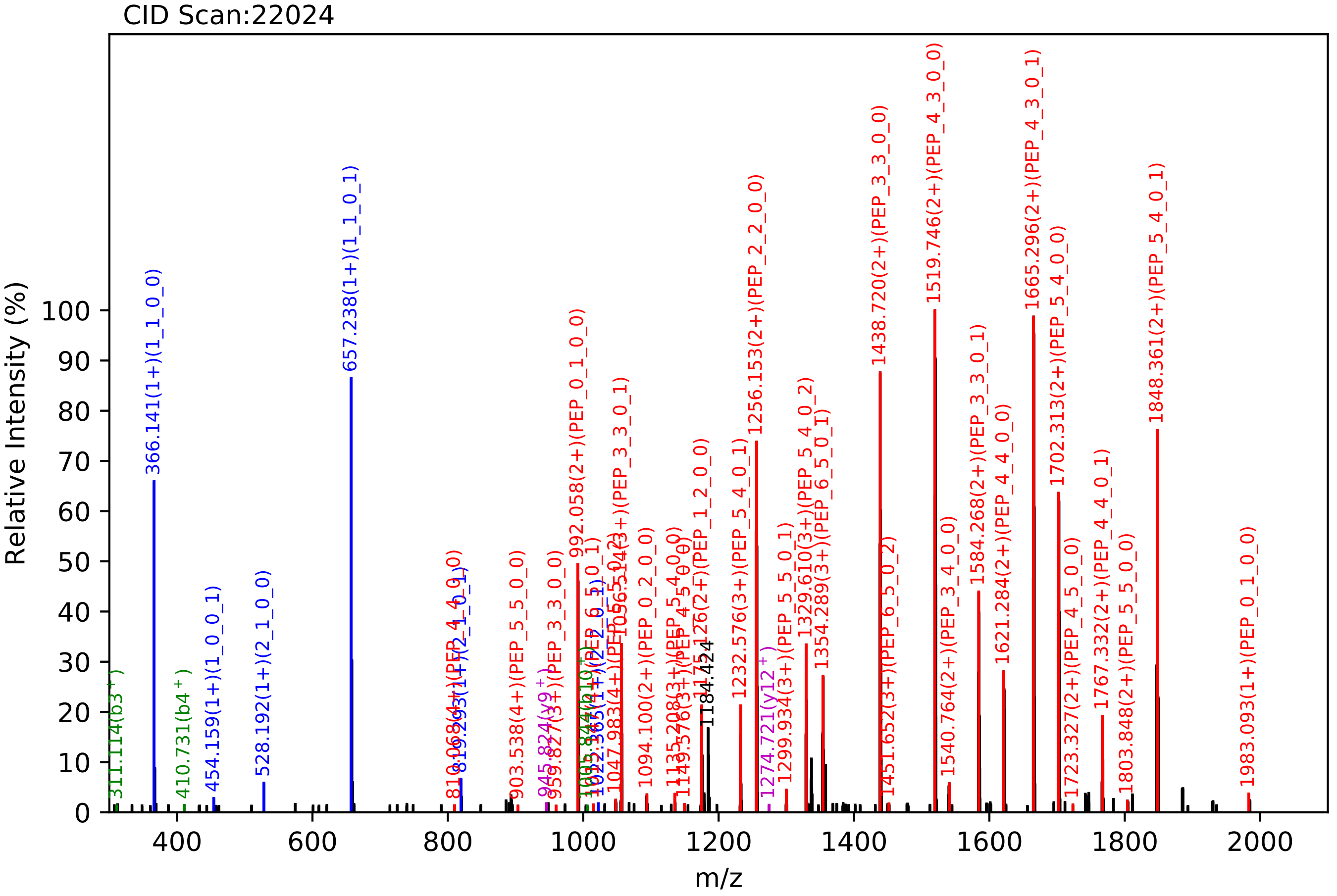

Test set no. 70, Experiment: AGP exp_4

LVPVPITNATLDQITGK(=PEP)_6_5_0_2, m/z:1088.24(4+), RT:55.57, Y-score:81.01

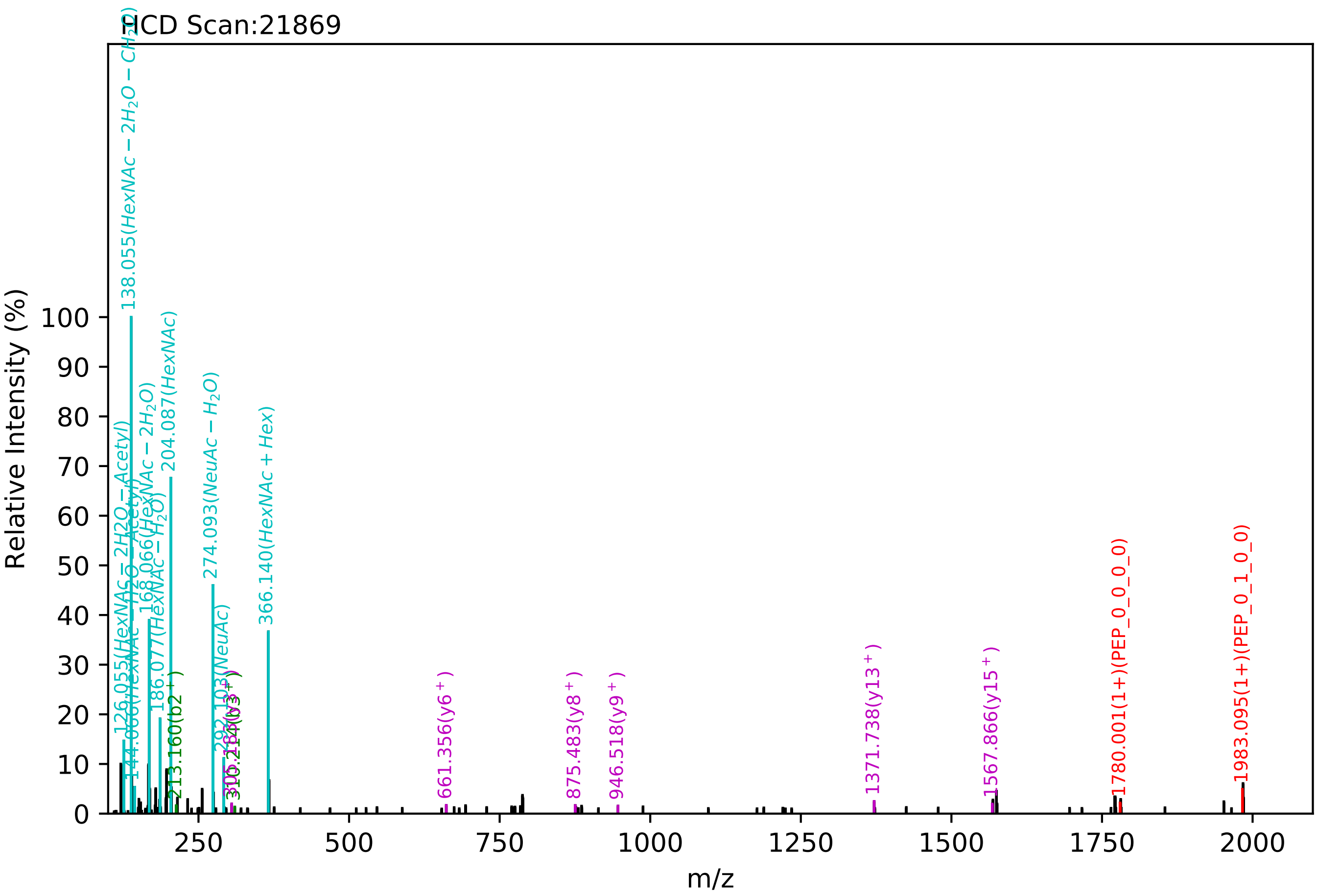

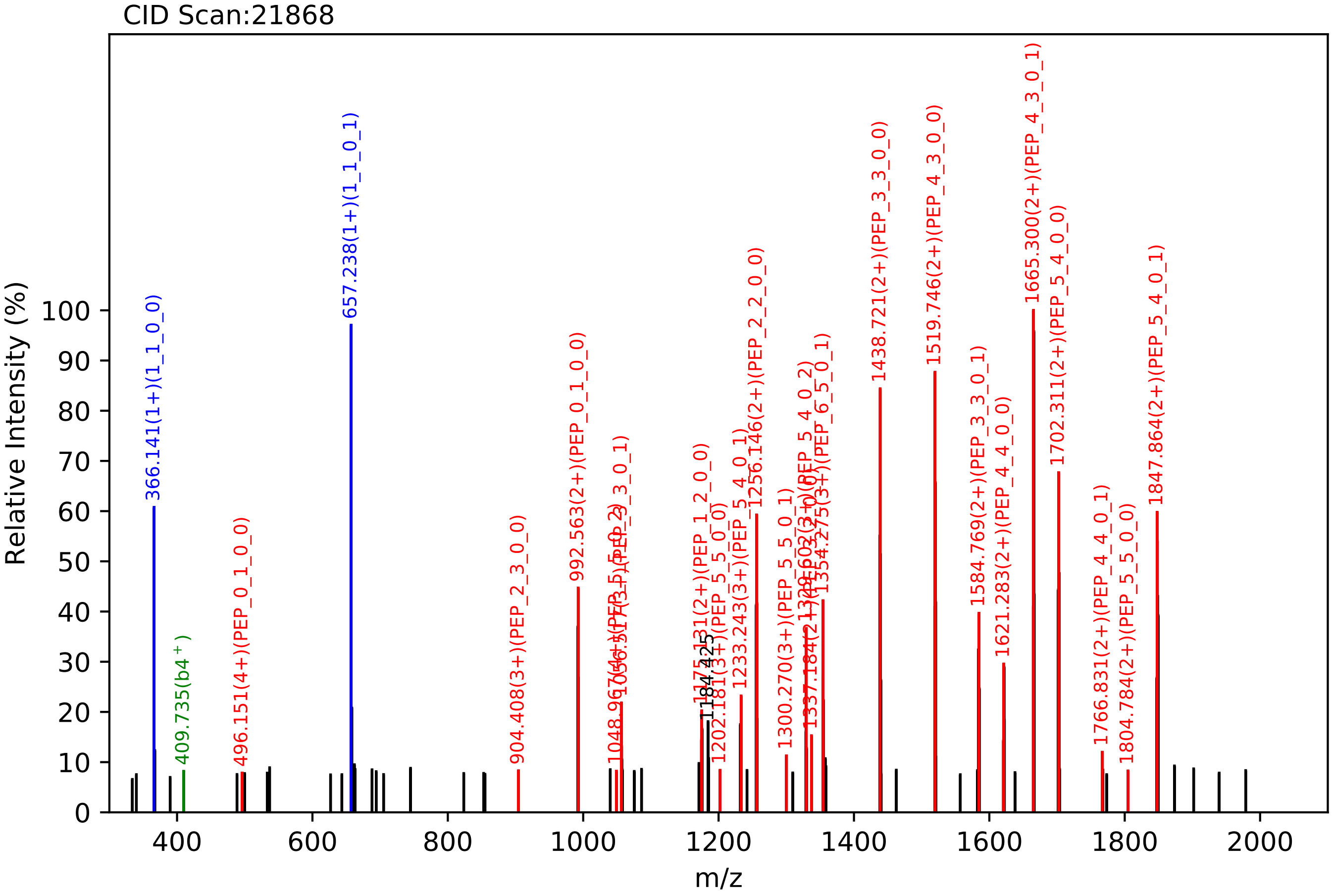

Test set no. 71, Experiment: AGP exp_4

LVPVPITNATLDQITGK(=PEP)_6_5_0_3, m/z:1547.68(3+), RT:62.33, Y-score:86.39

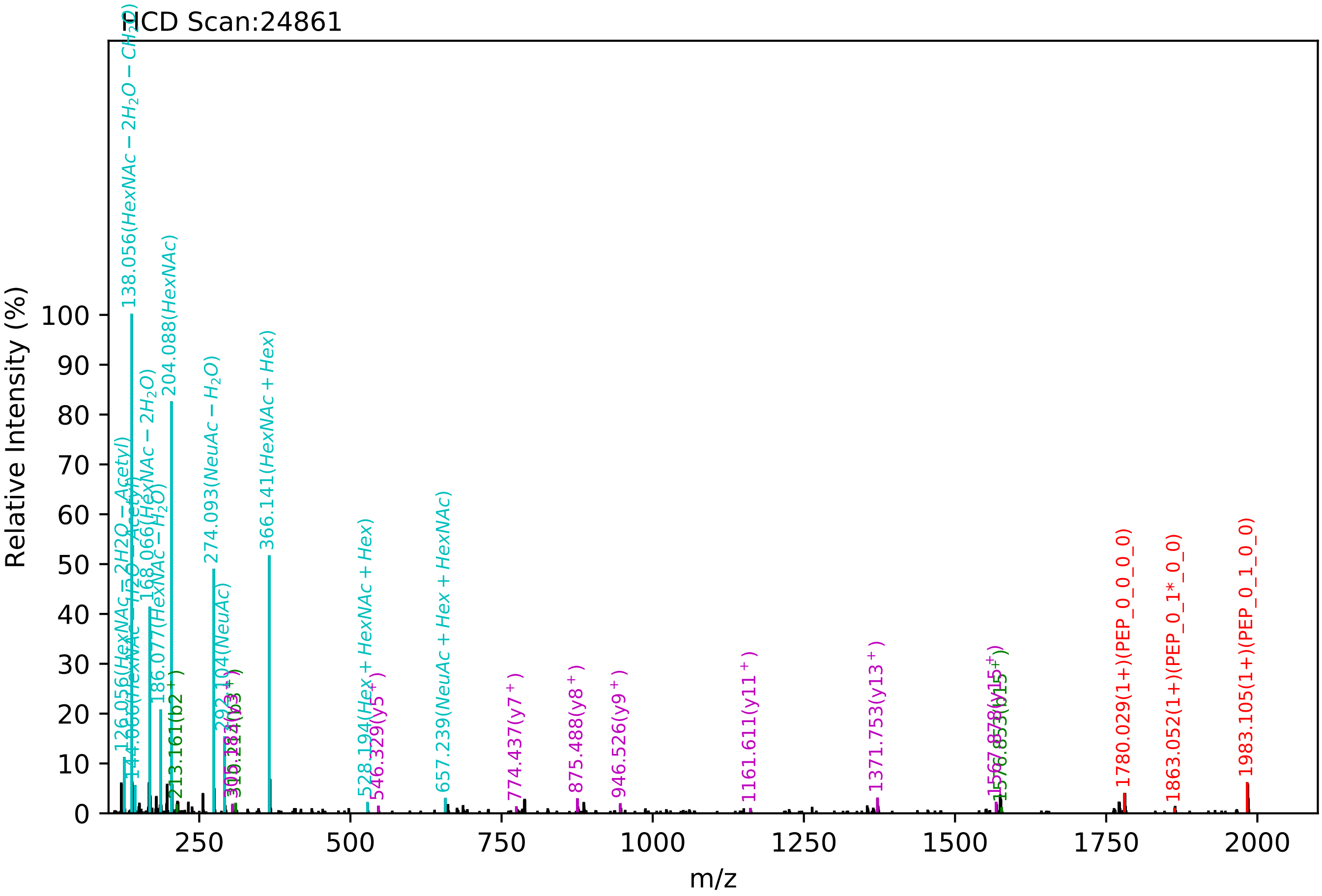

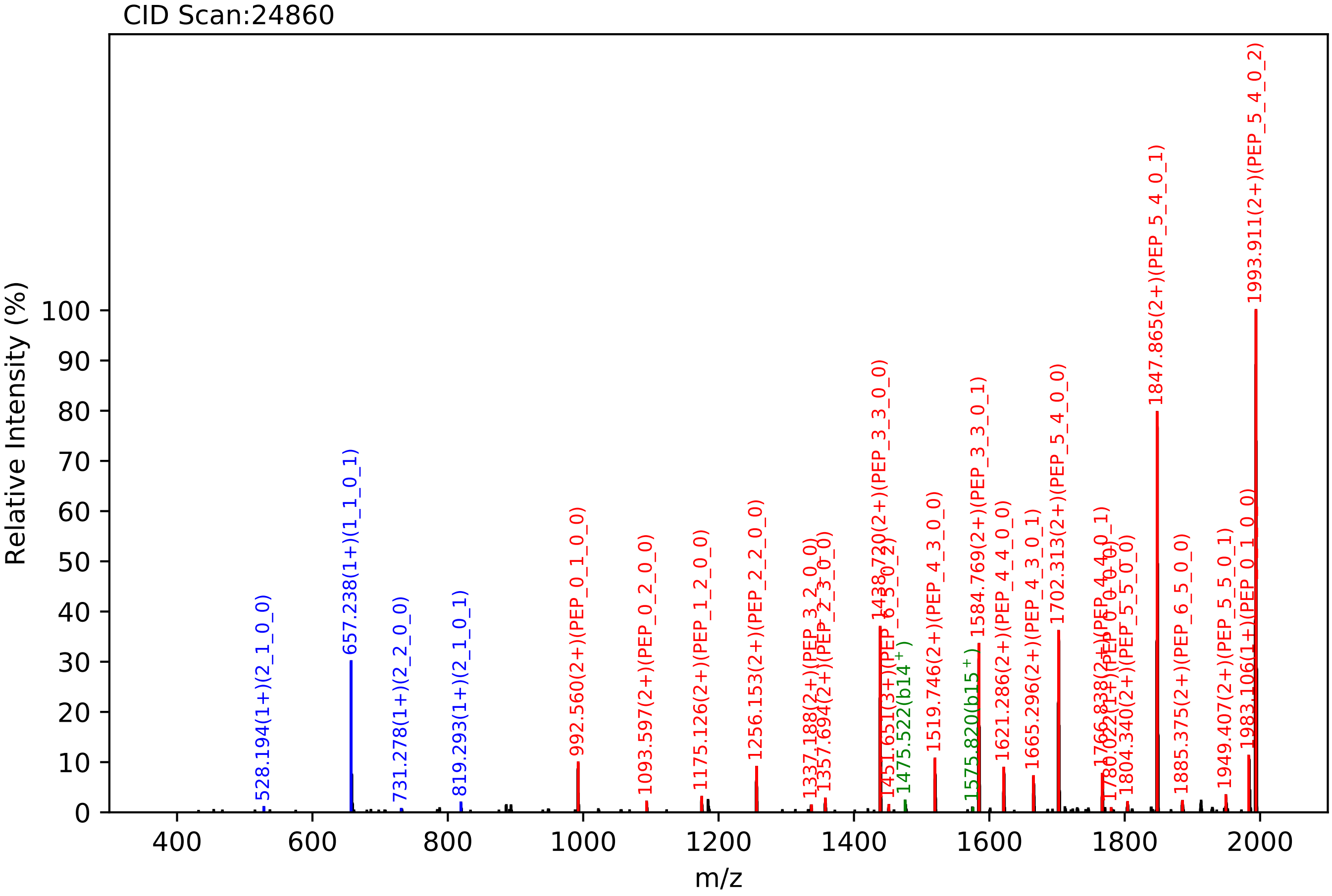

Test set no. 72, Experiment: AGP exp_3

LVPVPITNATLDQITGK(=PEP)_6_5_0_3, m/z:1547.68(3+), RT:63.37, Y-score:85.37

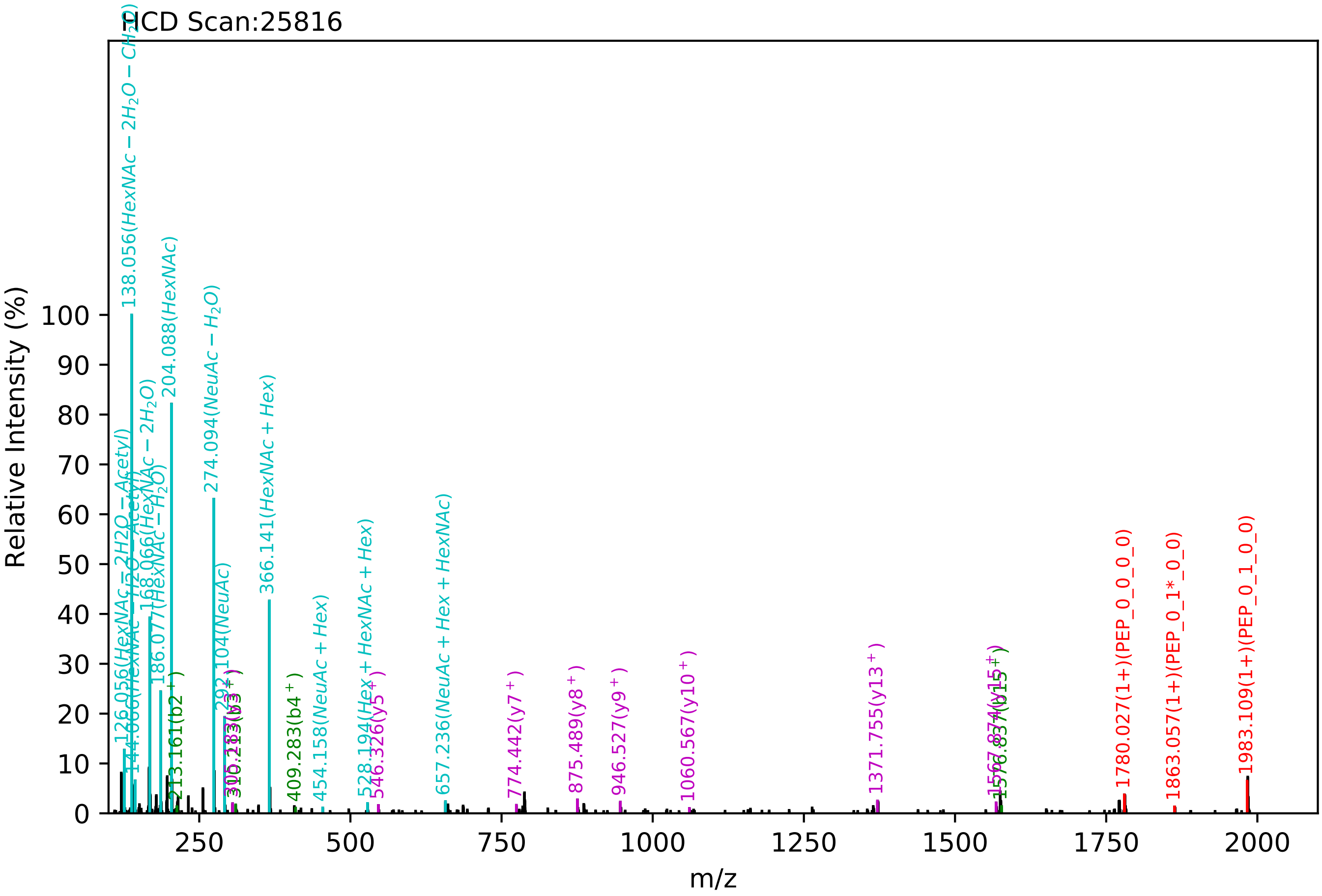

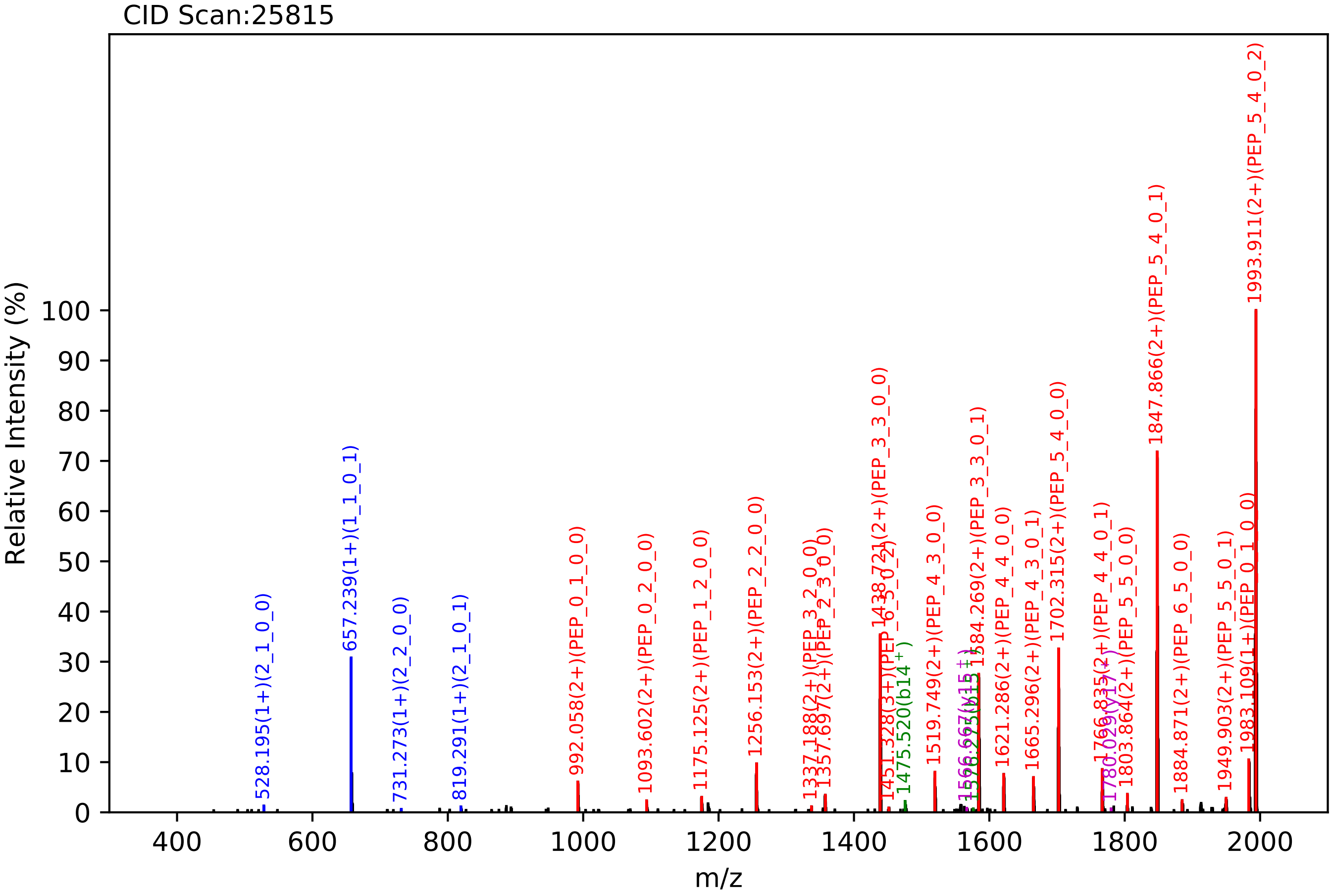

Test set no. 73, Experiment: AGP exp_3

LVPVPITNATLDQITGK(=PEP)_6_5_0_3, m/z:1160.77(4+), RT:62.84, Y-score:85.17

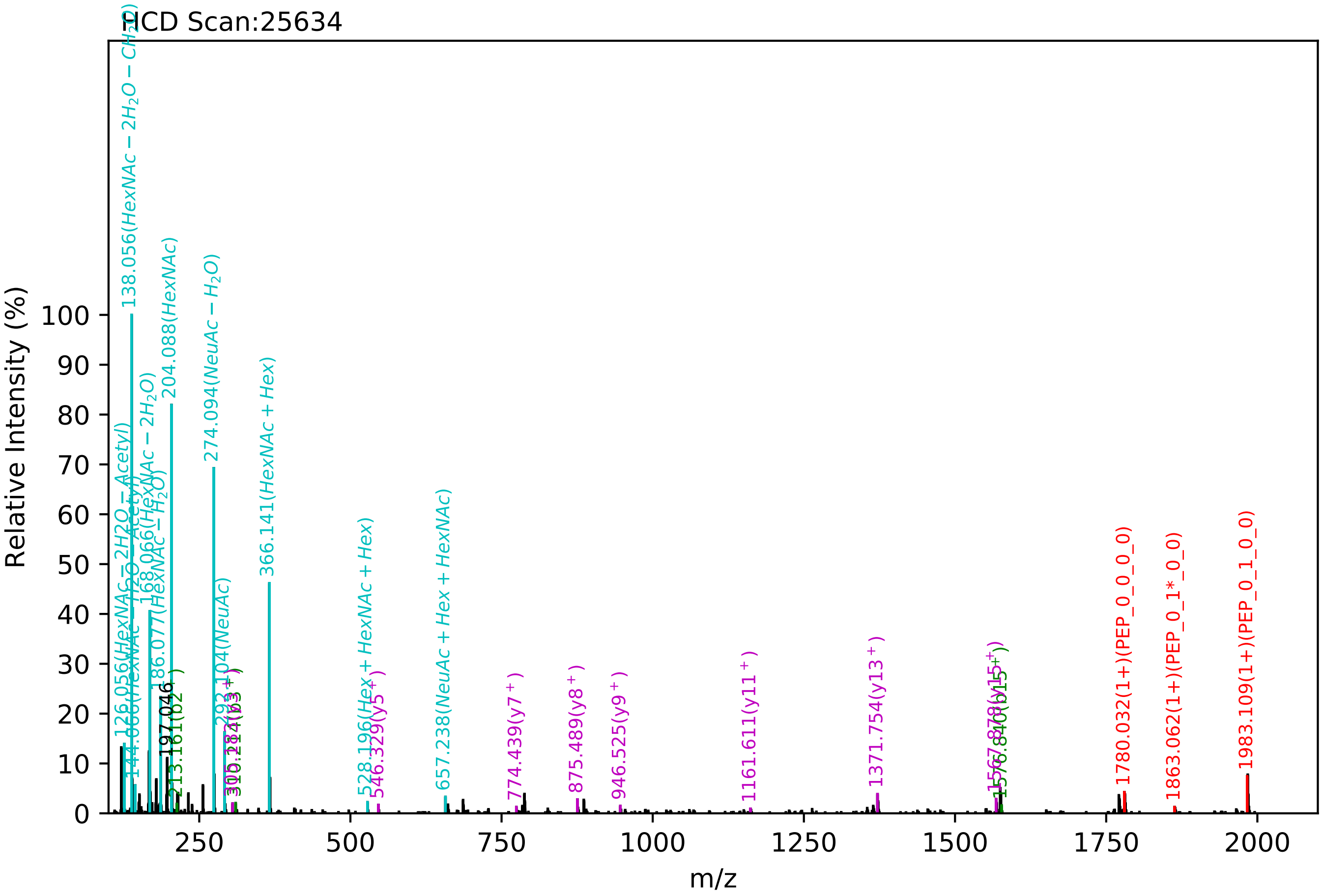

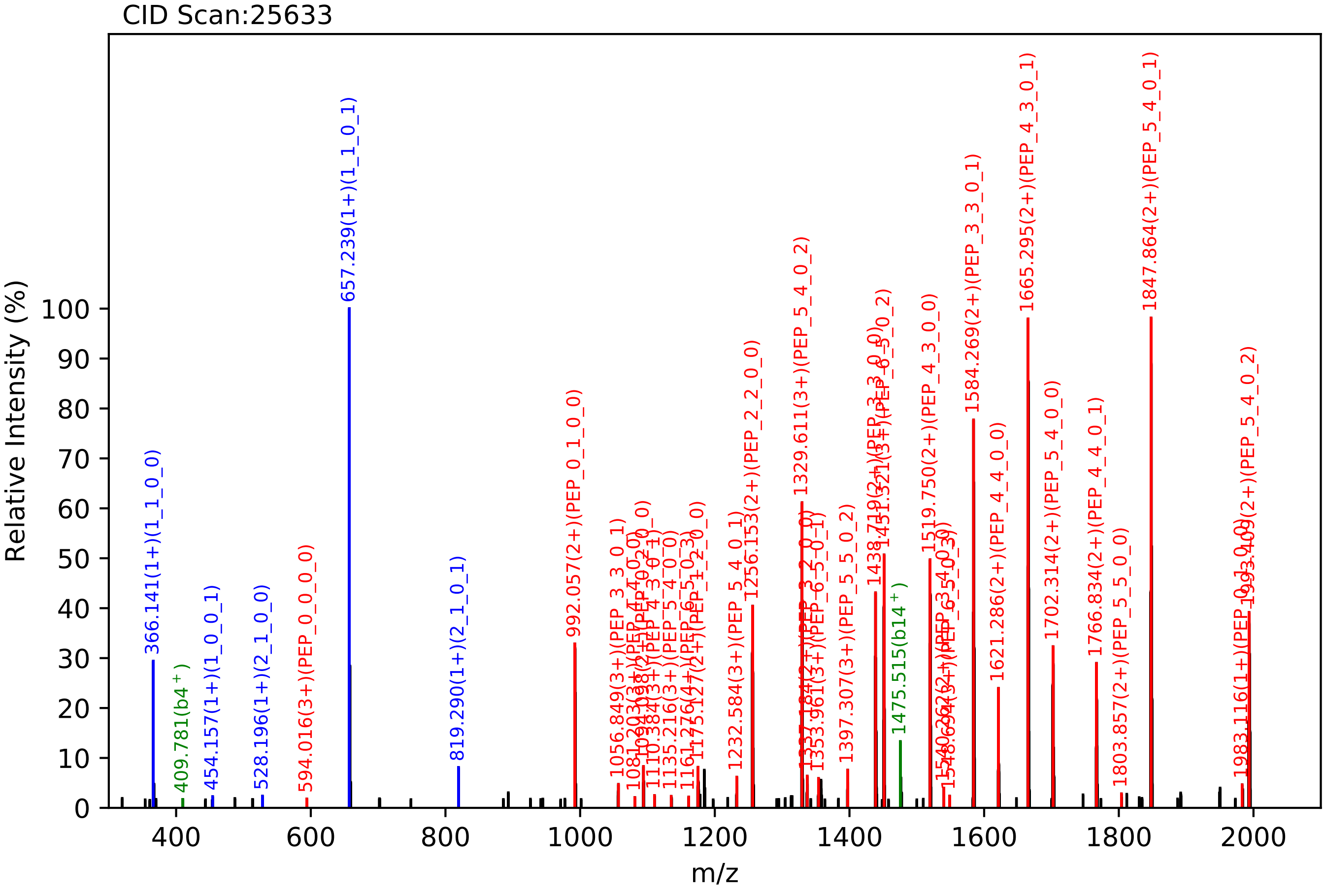

Test set no. 74, Experiment: AGP exp_3

LVPVPITNATLDQITGK(=PEP)_6_5_0_3, m/z:1160.76(4+), RT:62.24, Y-score:84.25

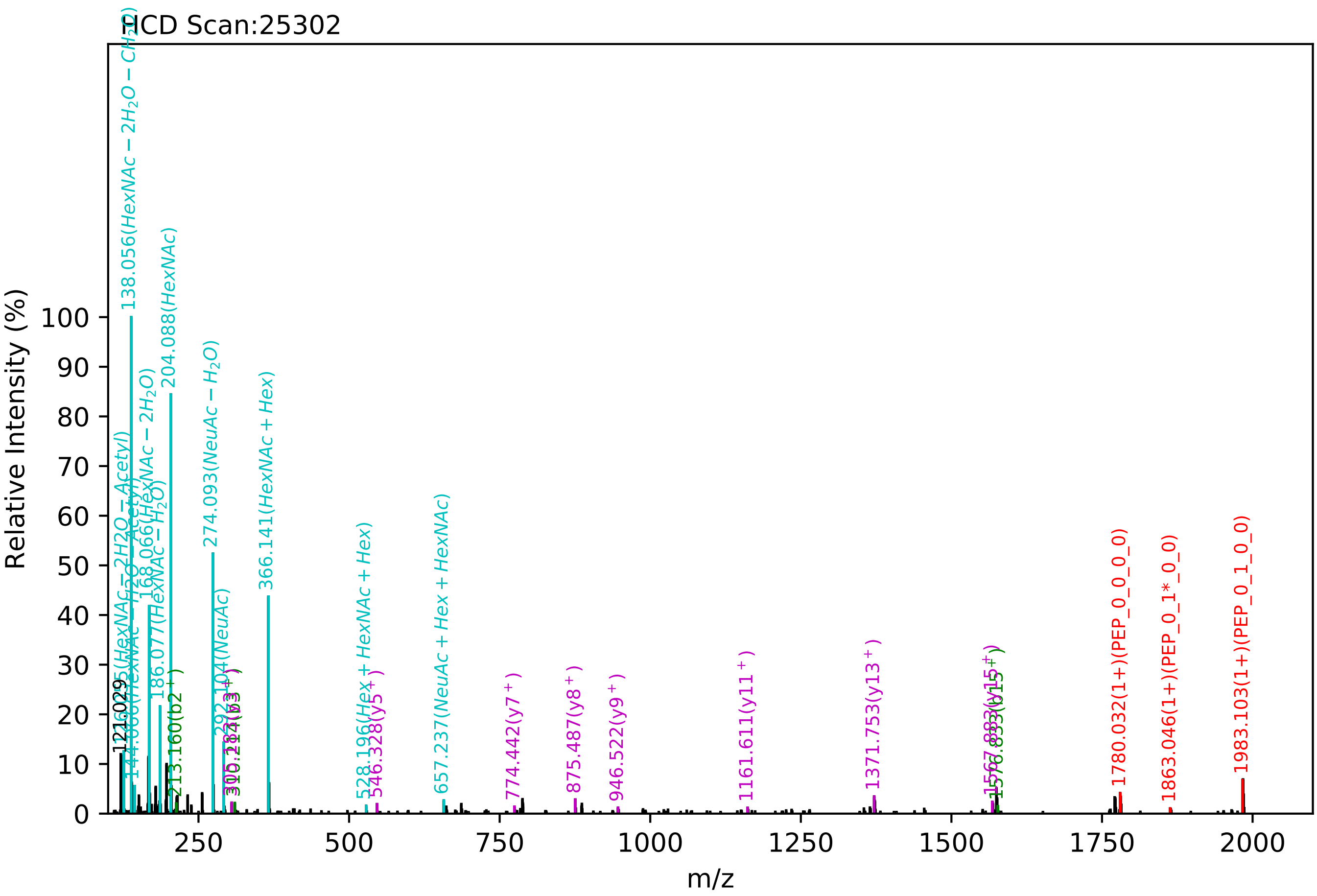

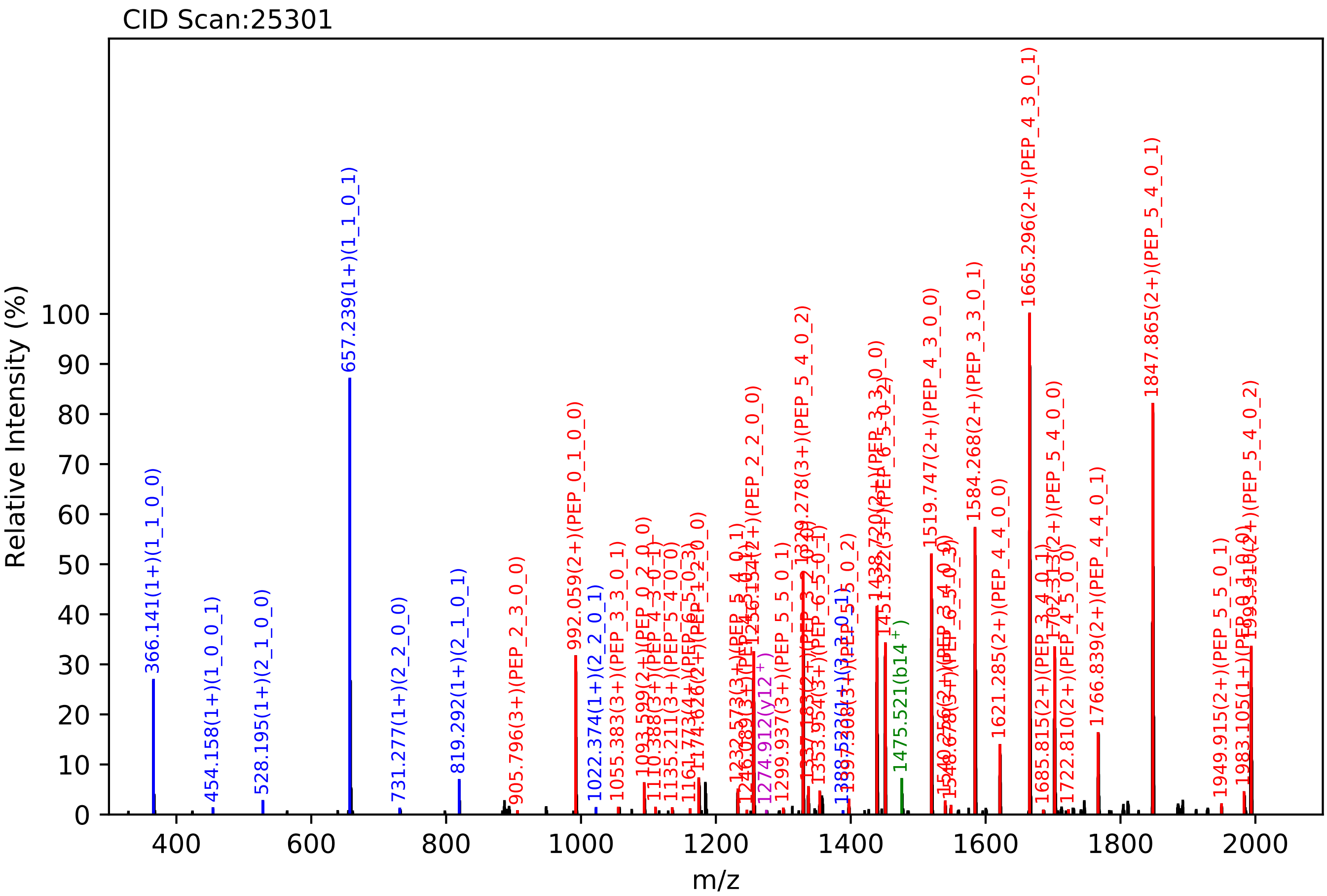

Test set no. 75, Experiment: AGP exp_3

LVPVPITNATLDQITGK(=PEP)_6_5_0_3, m/z:1162.01(4+), RT:62.76, Y-score:84.14

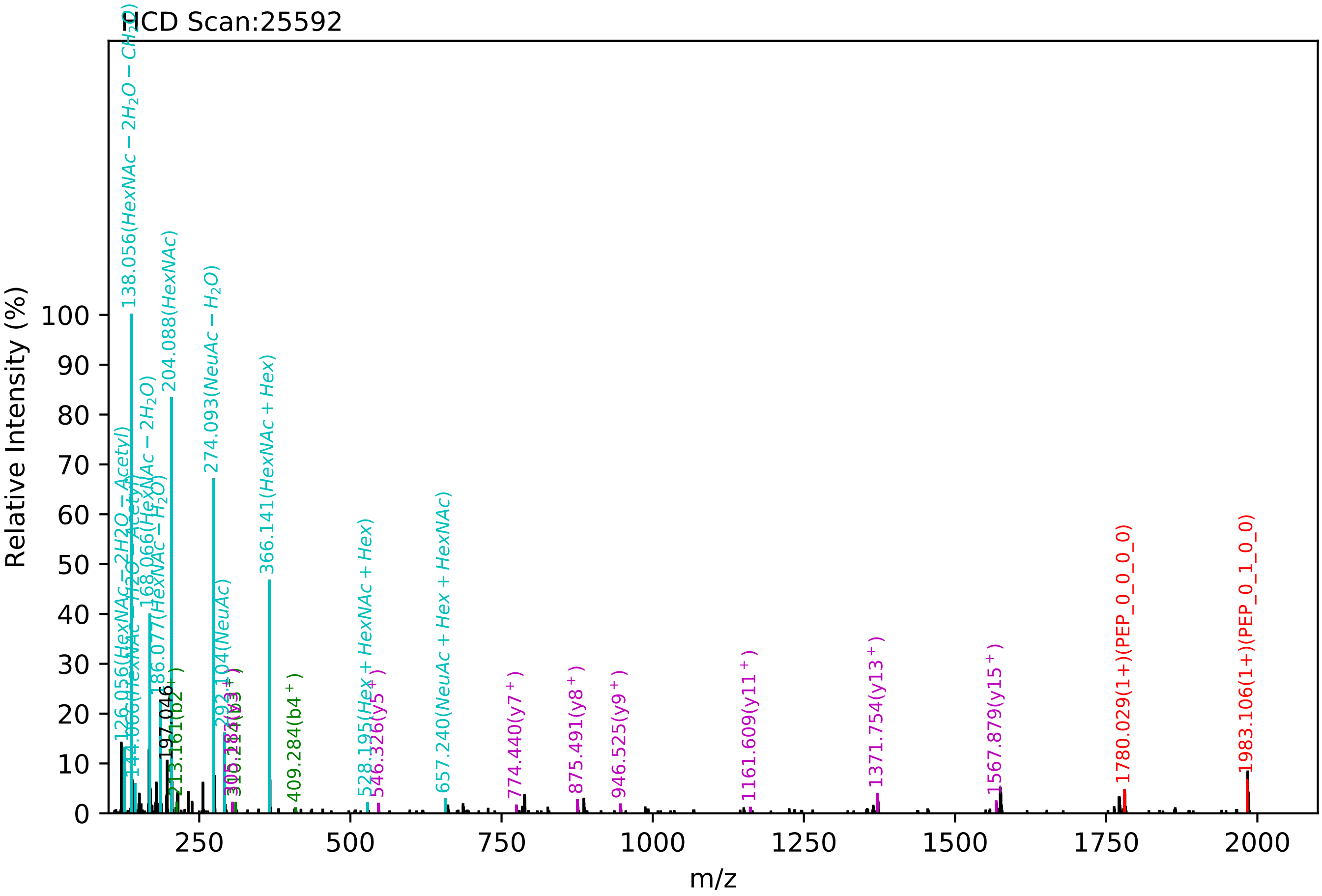

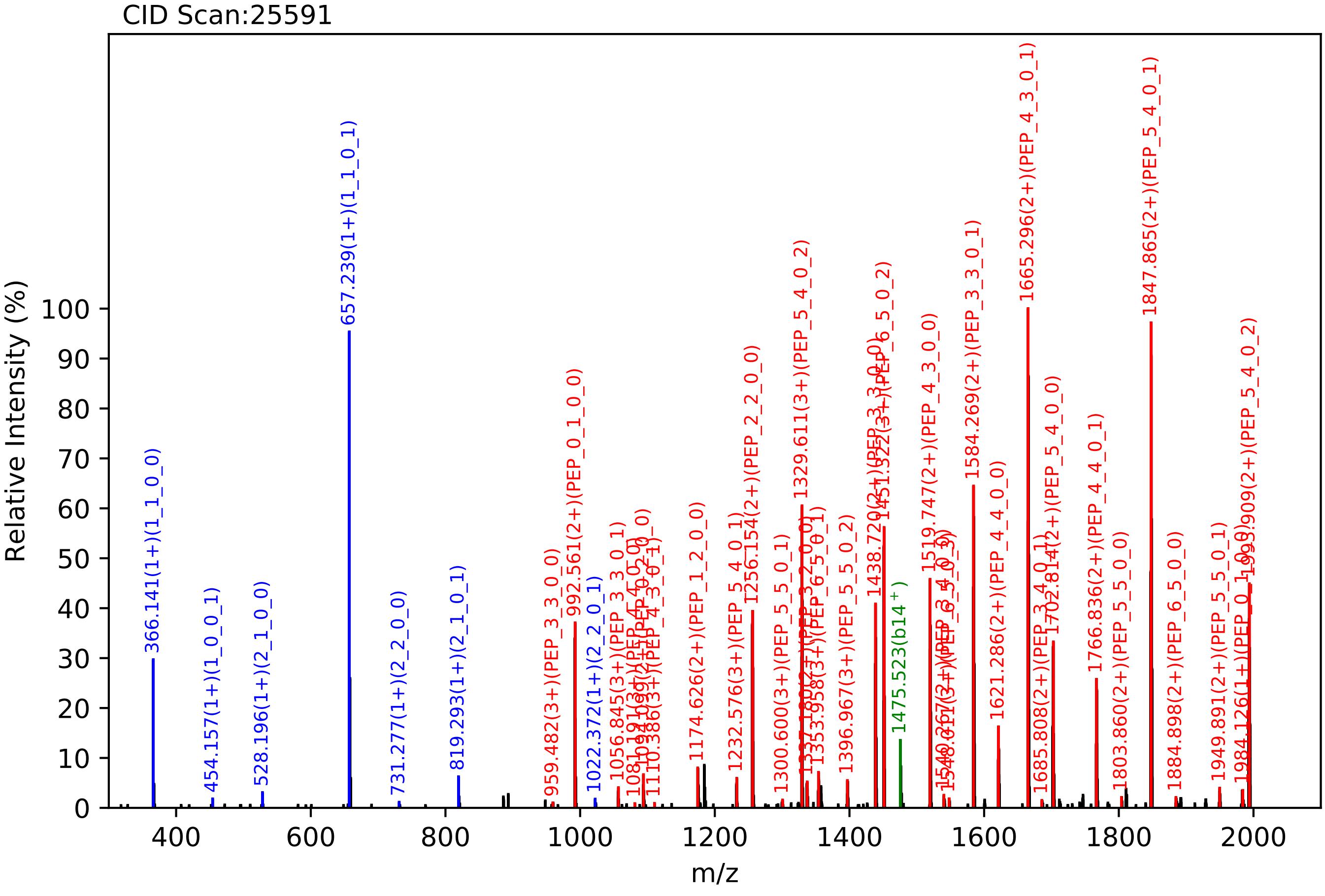

Test set no. 76, Experiment: AGP exp_4

LVPVPITNATLDQITGK(=PEP)_6_5_0_3, m/z:1547.69(3+), RT:61.80, Y-score:83.56

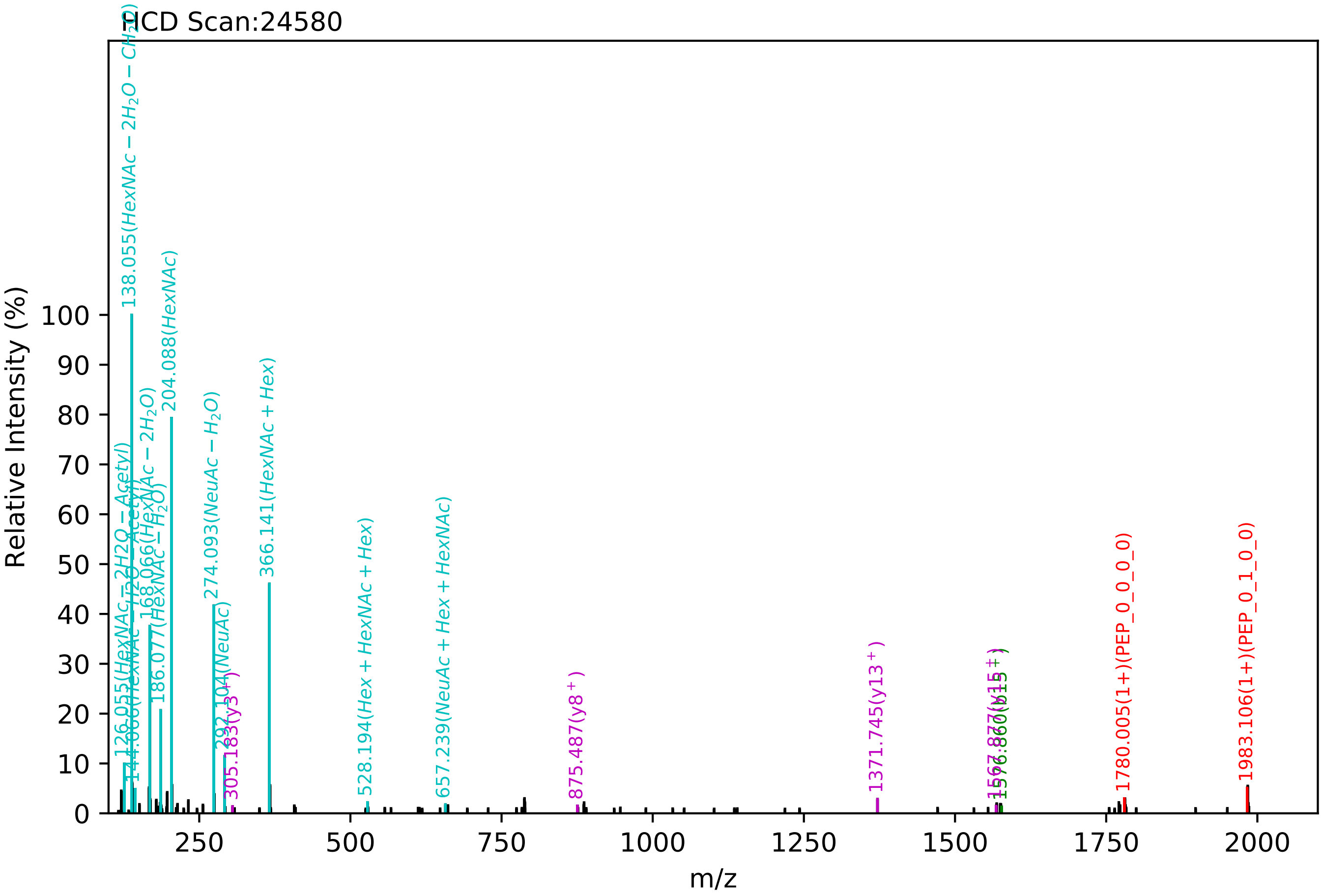

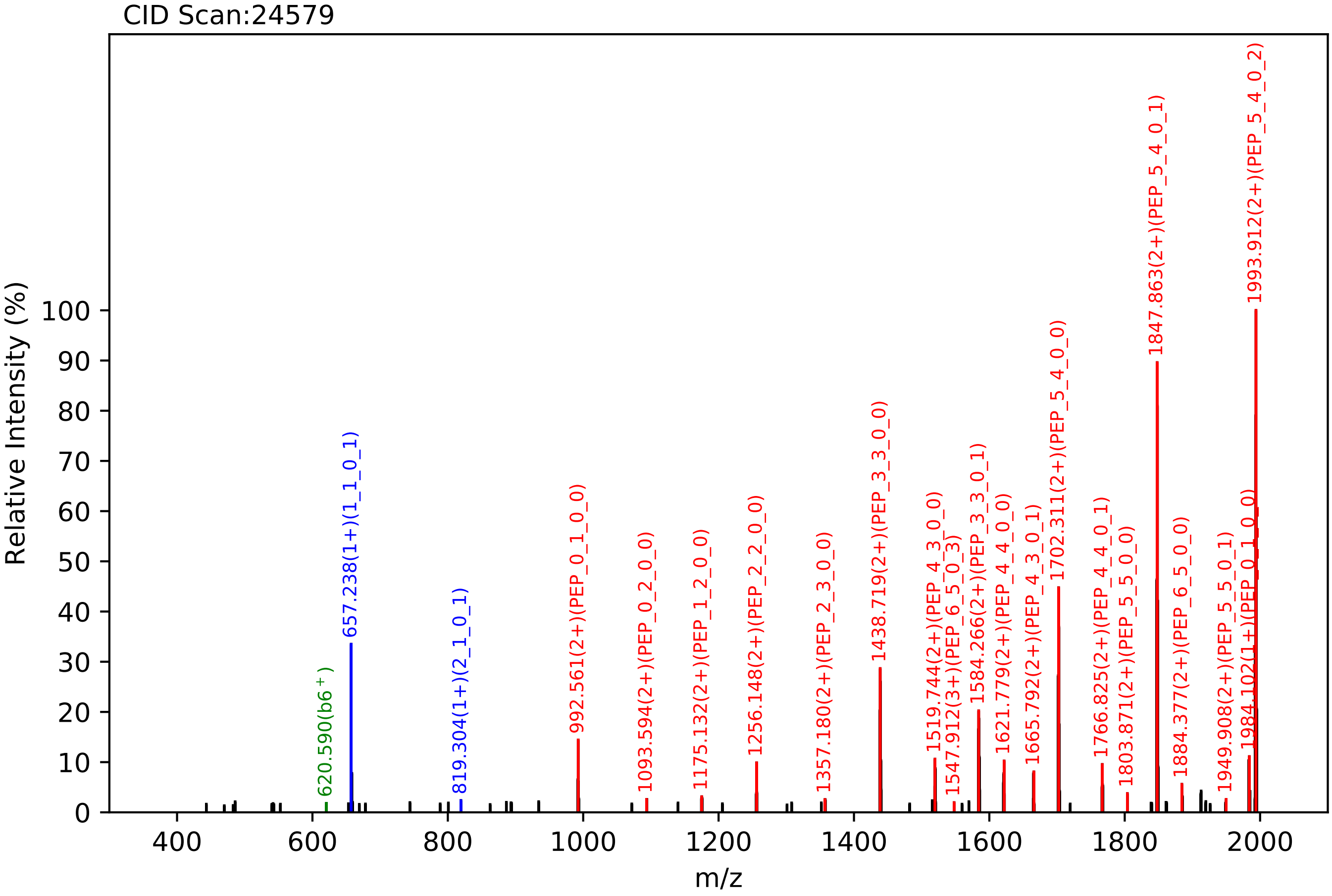

Test set no. 77, Experiment: AGP exp_4

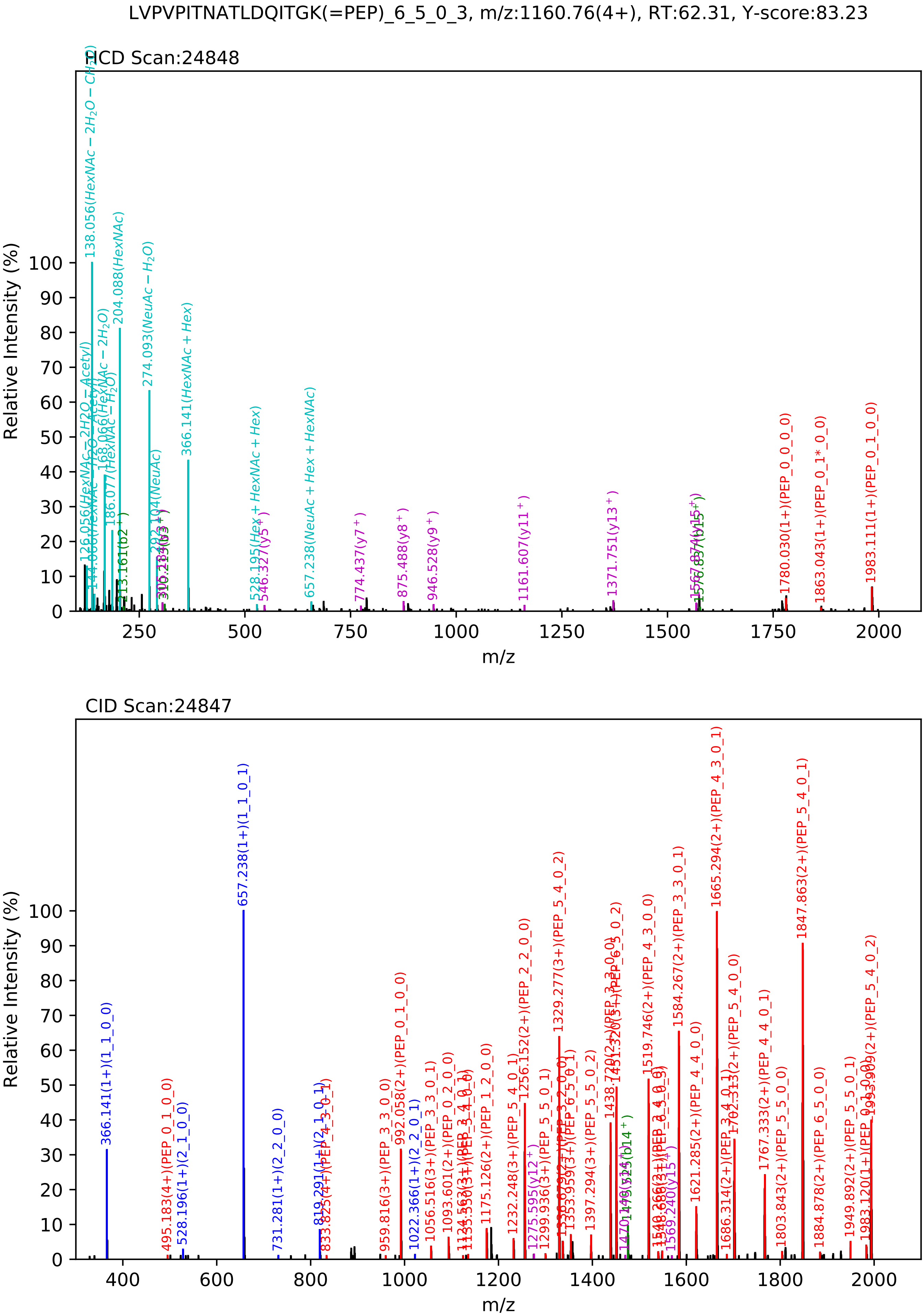

Test set no. 78, Experiment: AGP exp_4

LVPVPITNATLDQITGK(=PEP)_6_5_0_3, m/z:1160.77(4+), RT:63.37, Y-score:82.16

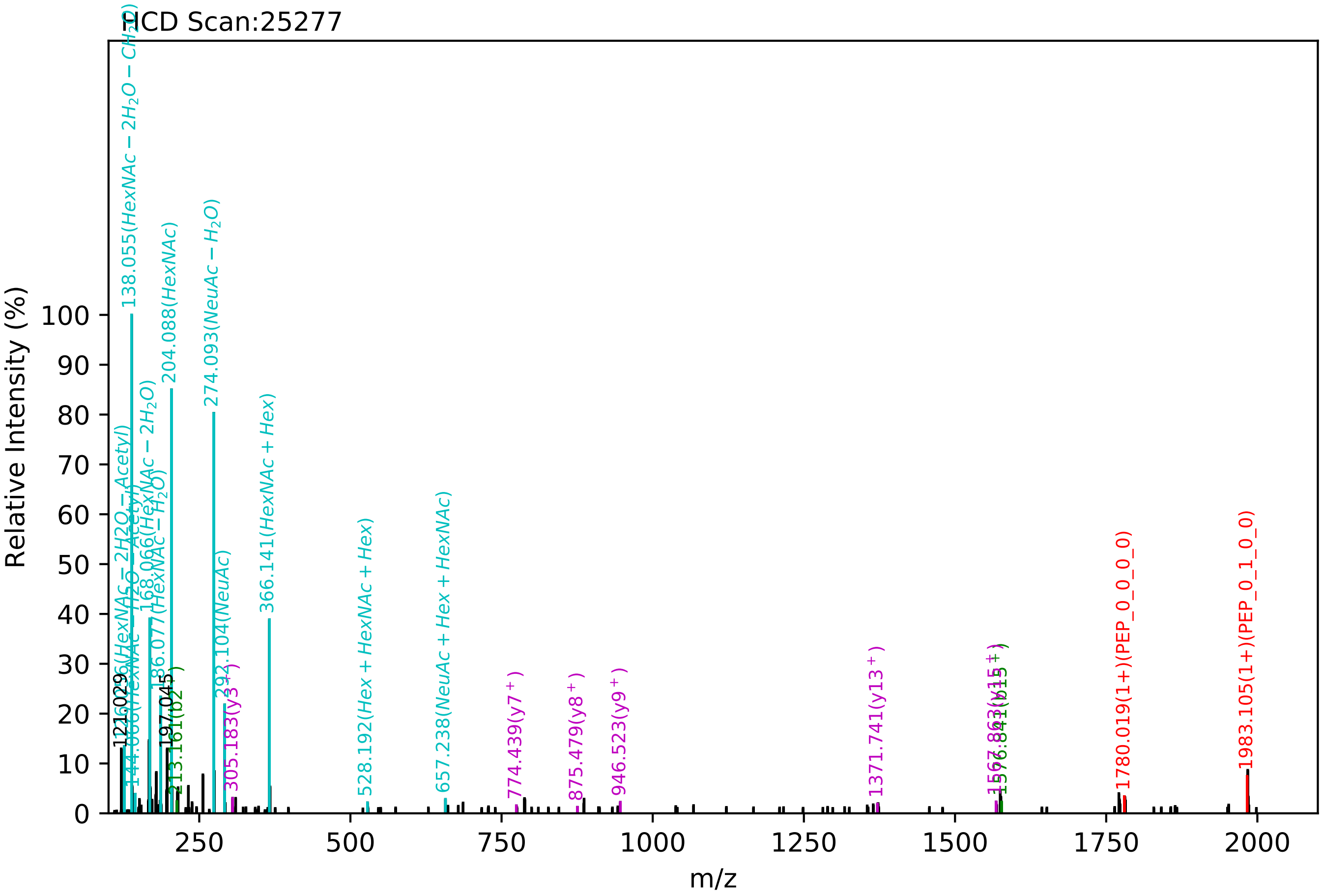

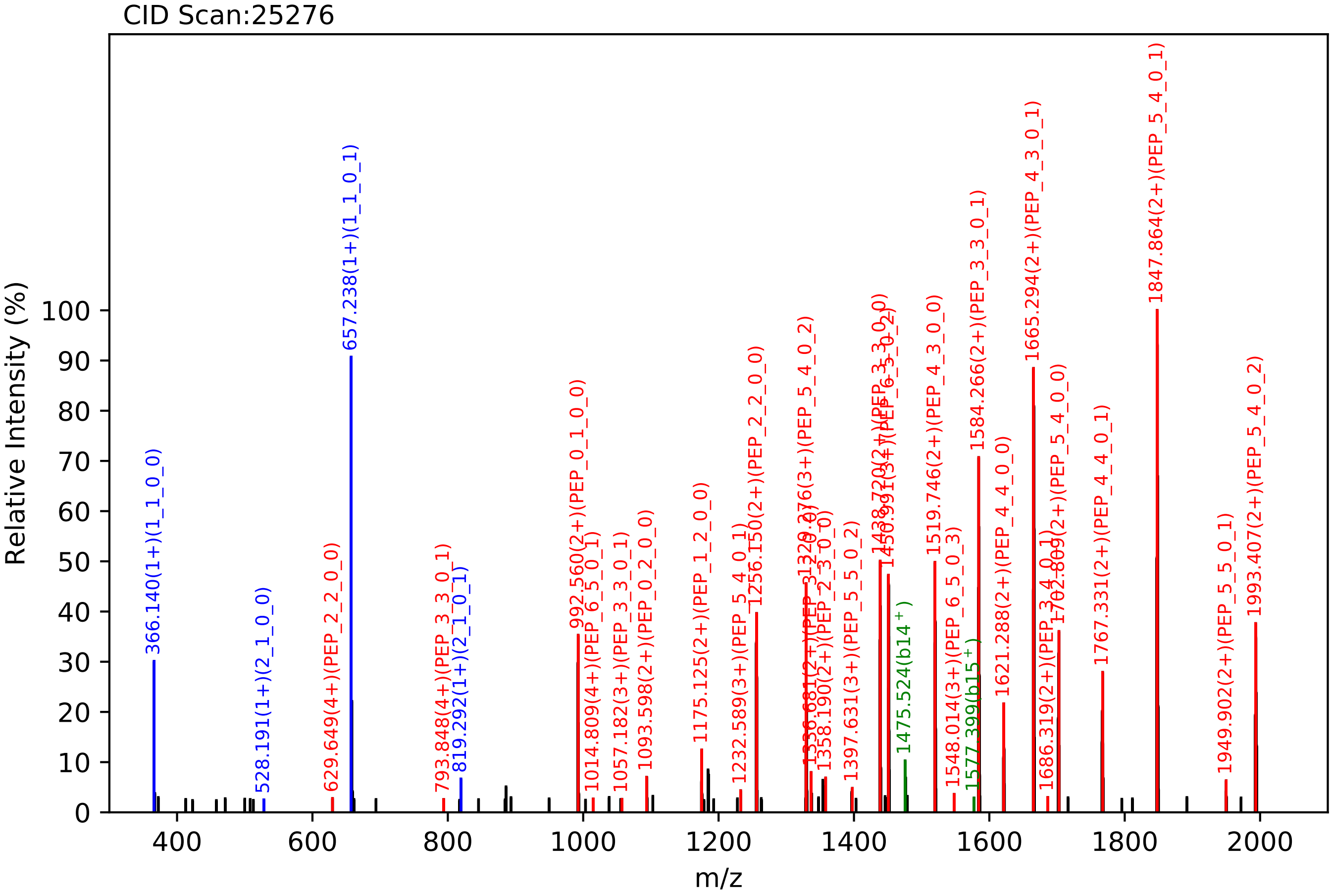

Test set no. 79, Experiment: AGP exp_4

LVPVPITNATLDQITGK(=PEP)_6_5_0_3, m/z:1161.01(4+), RT:62.85, Y-score:81.71

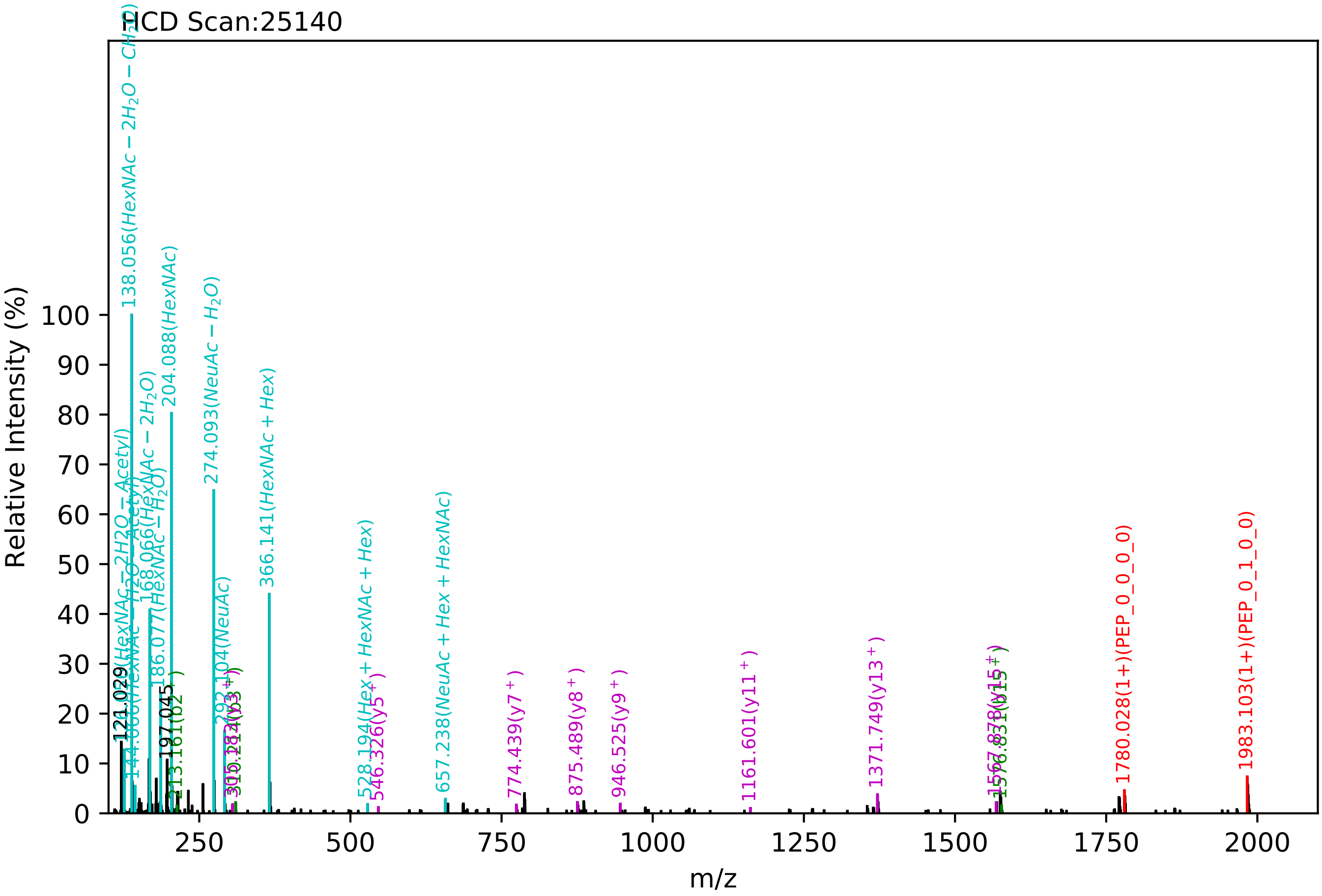

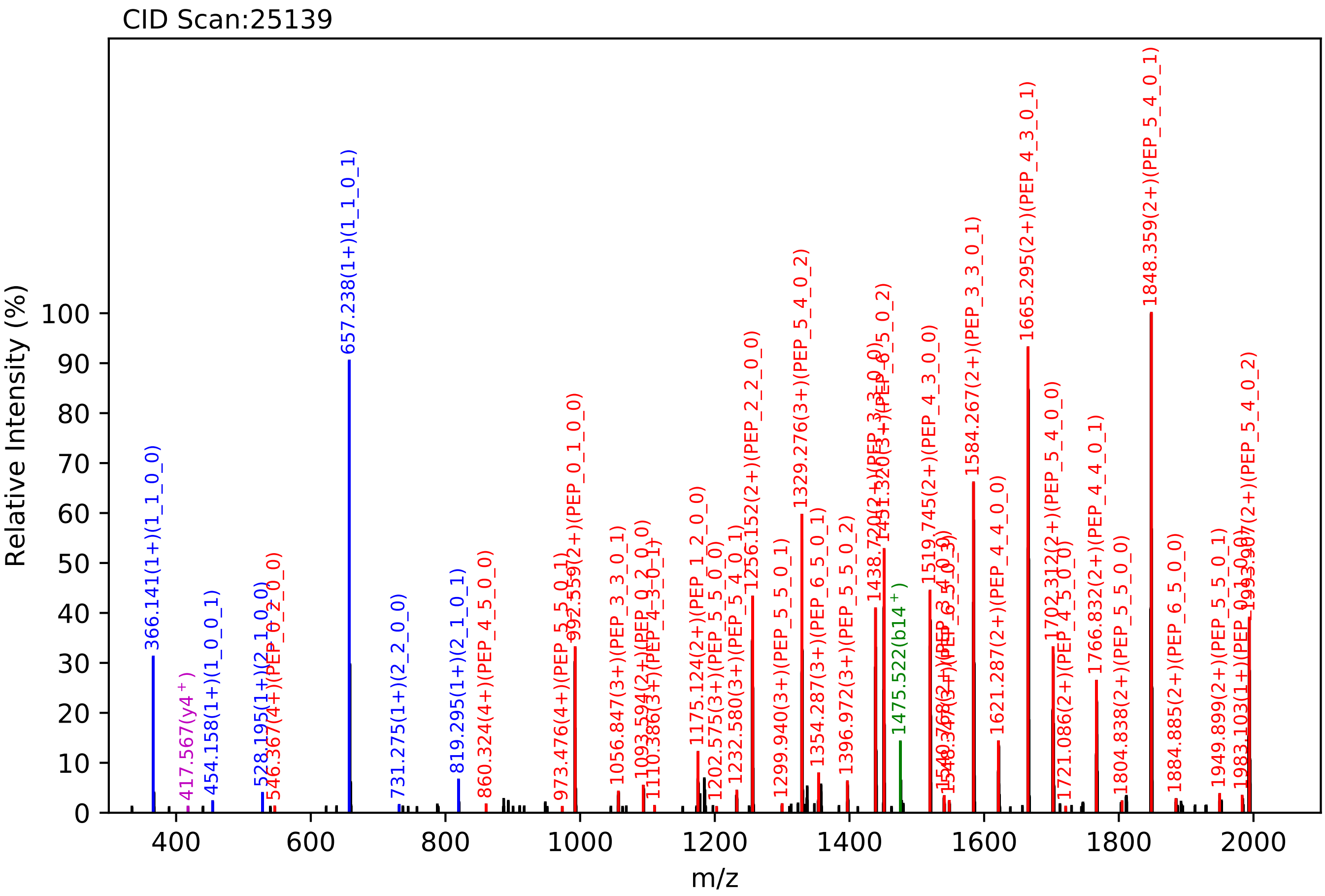

Test set no. 80, Experiment: AGP exp_3

LVPVPITNATLDQITGK(=PEP)_6_5_0_3, m/z:1160.76(4+), RT:63.38, Y-score:81.71

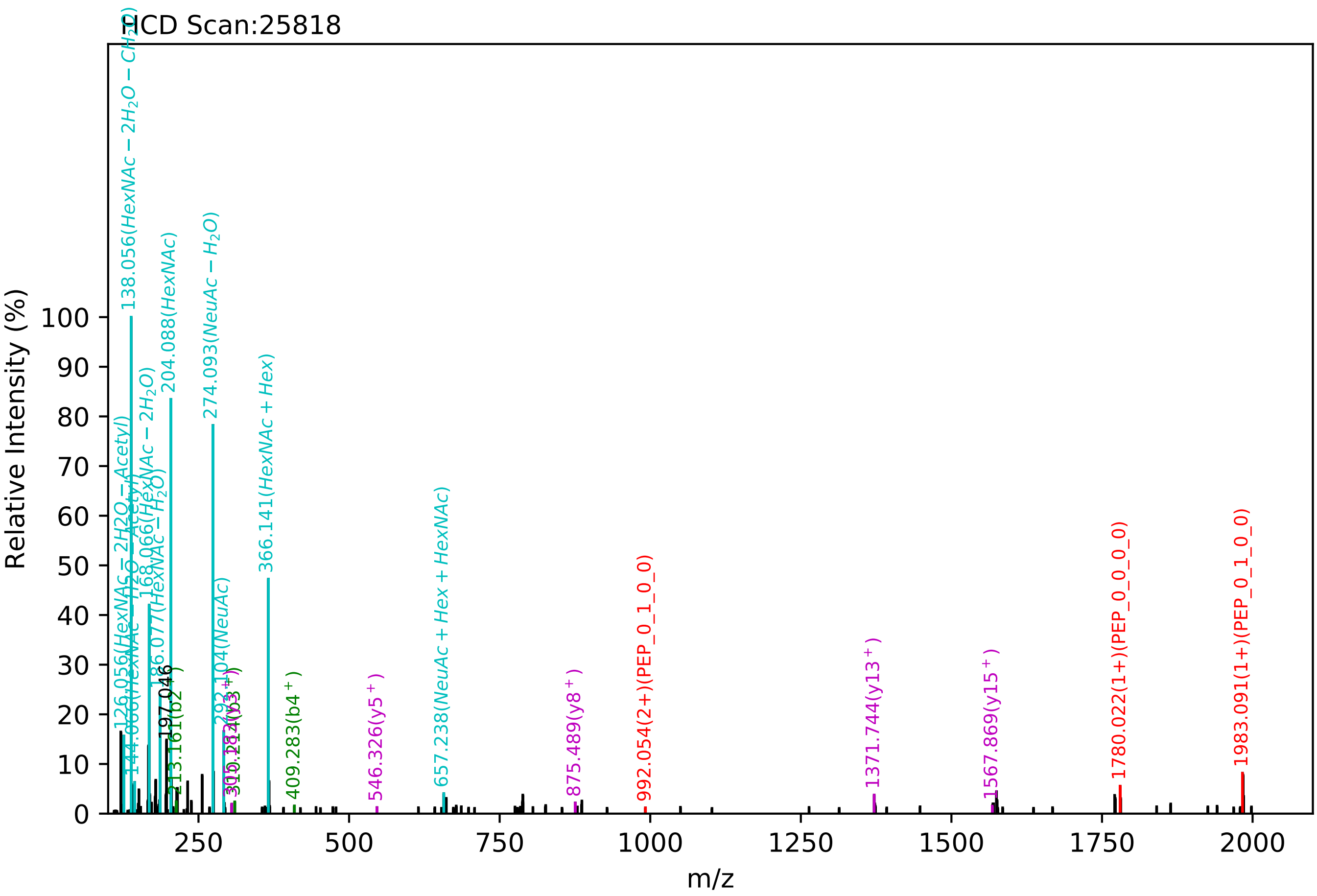

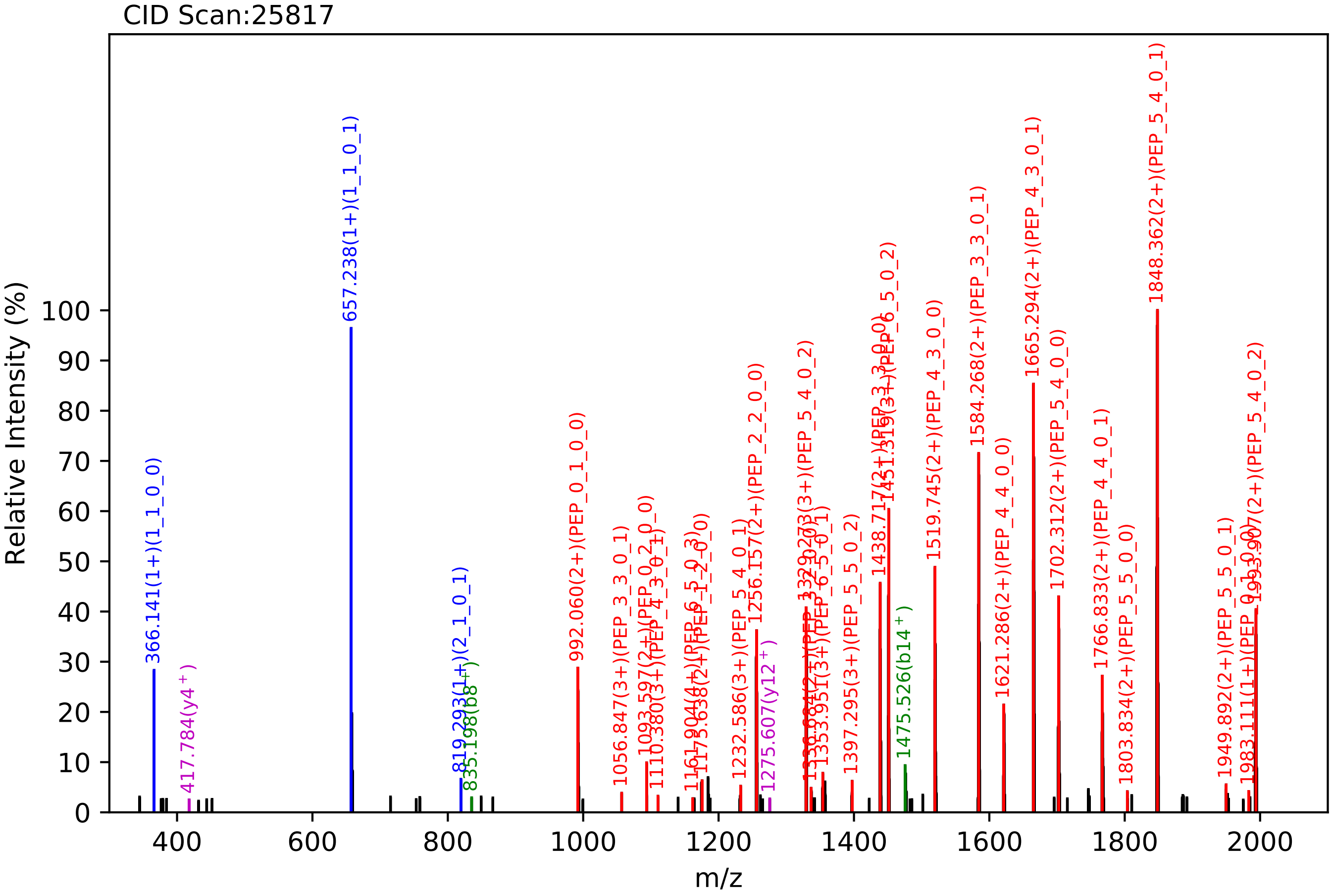

Test set no. 81, Experiment: AGP exp_4

LVPVPITNATLDQITGK(=PEP)_6_5_1_1, m/z:1402.30(3+), RT:48.44, Y-score:89.15

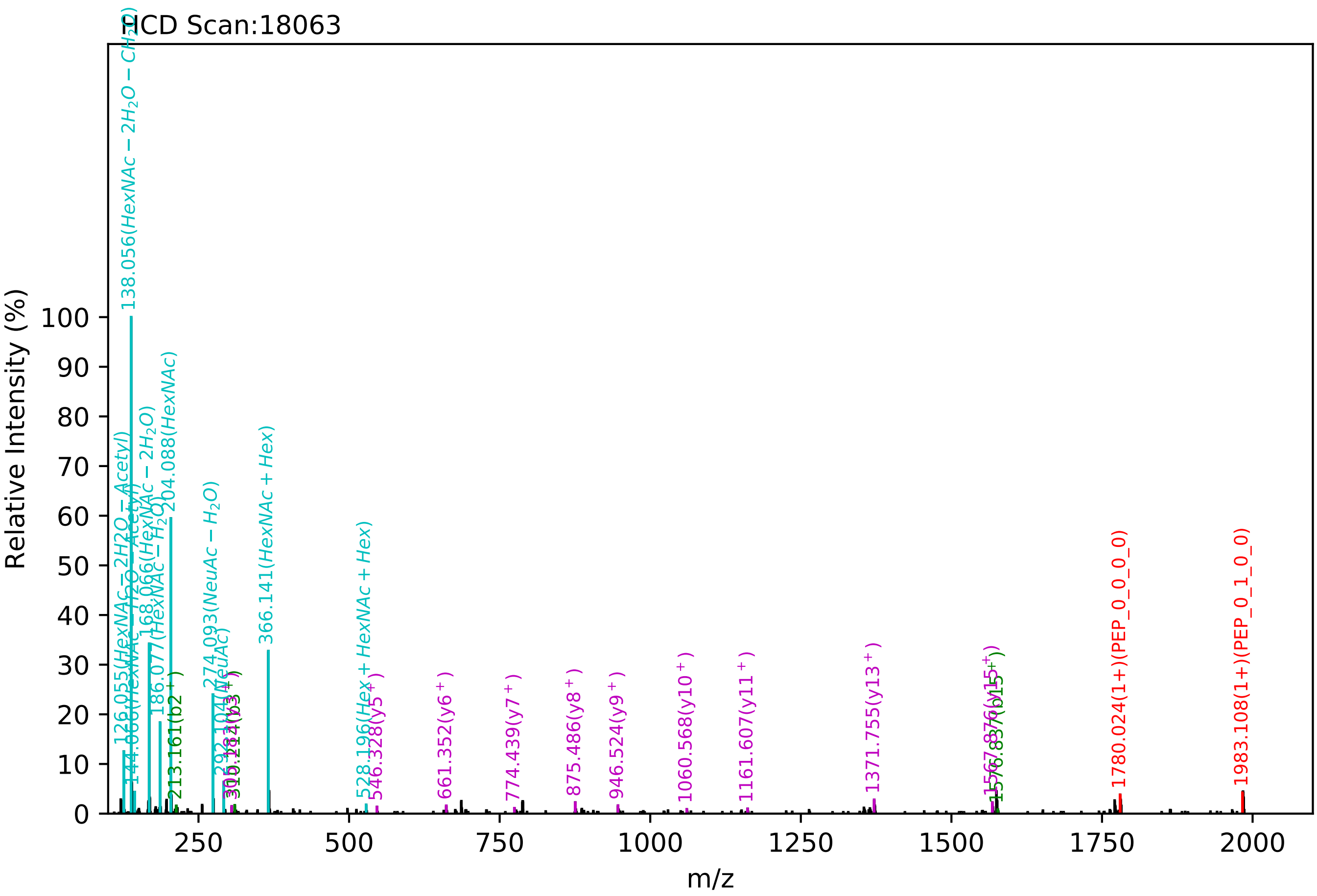

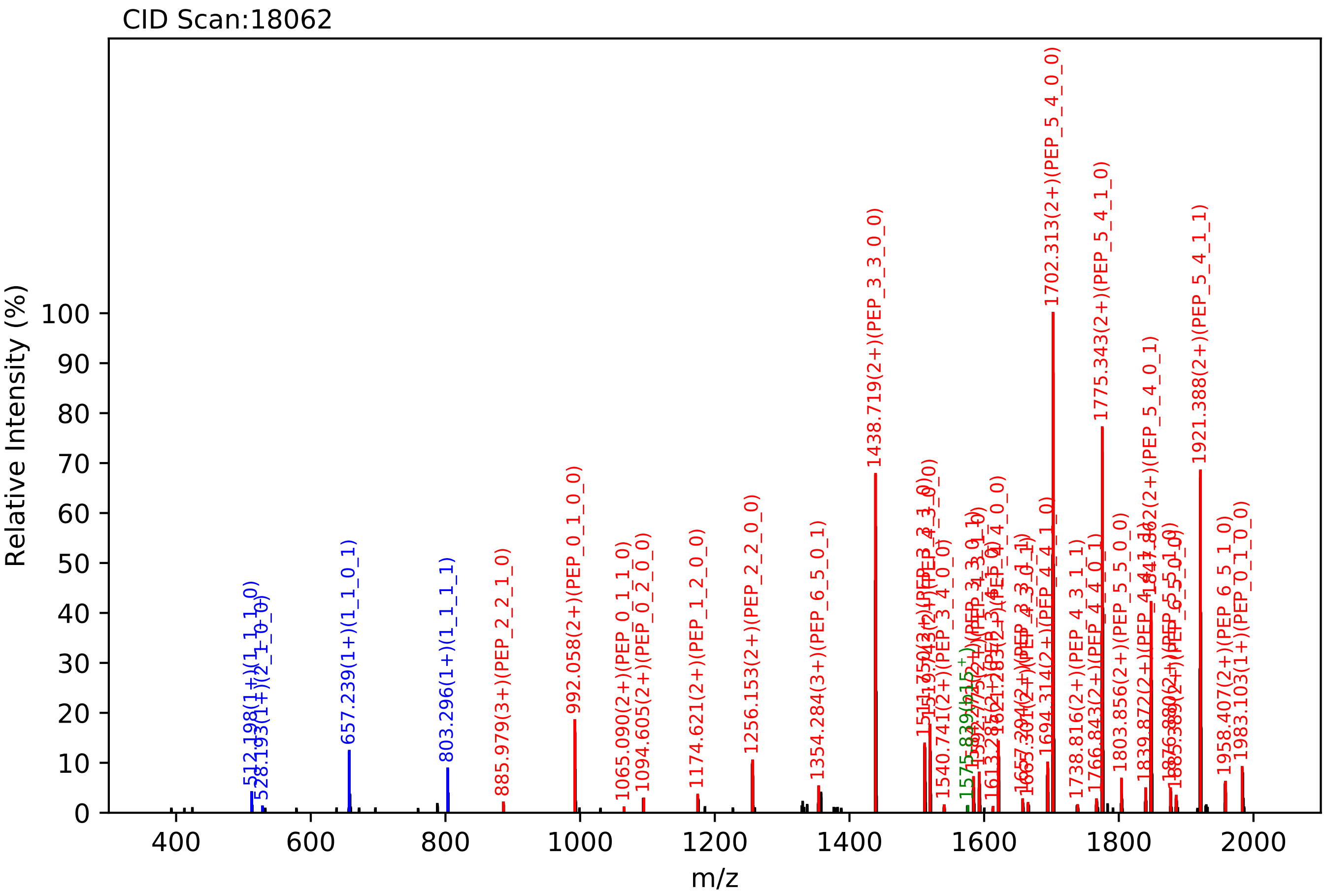

Test set no. 82, Experiment: AGP exp_3

LVPVPITNATLDQITGK(=PEP)_6_5_1_1, m/z:1402.30(3+), RT:48.57, Y-score:88.92

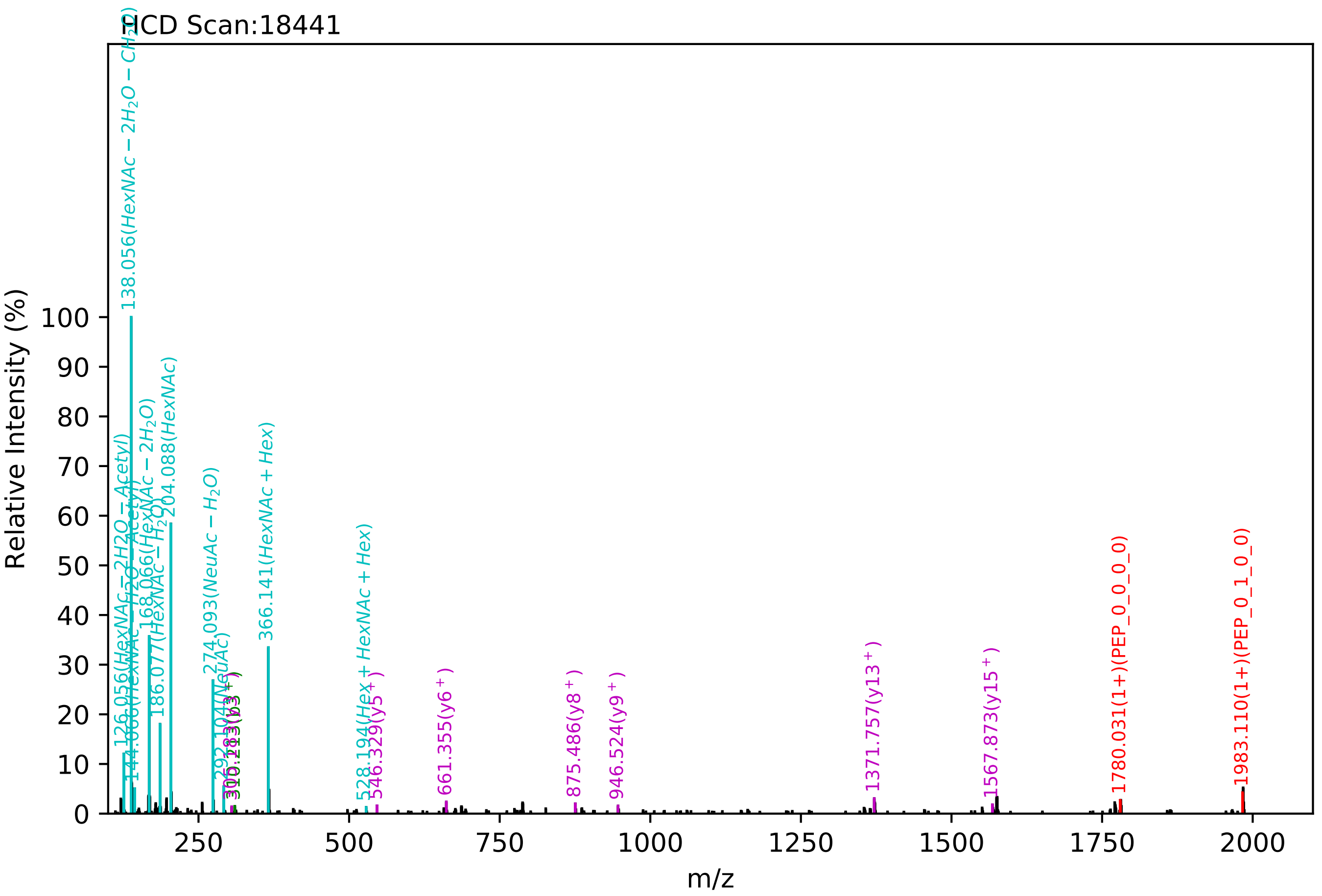

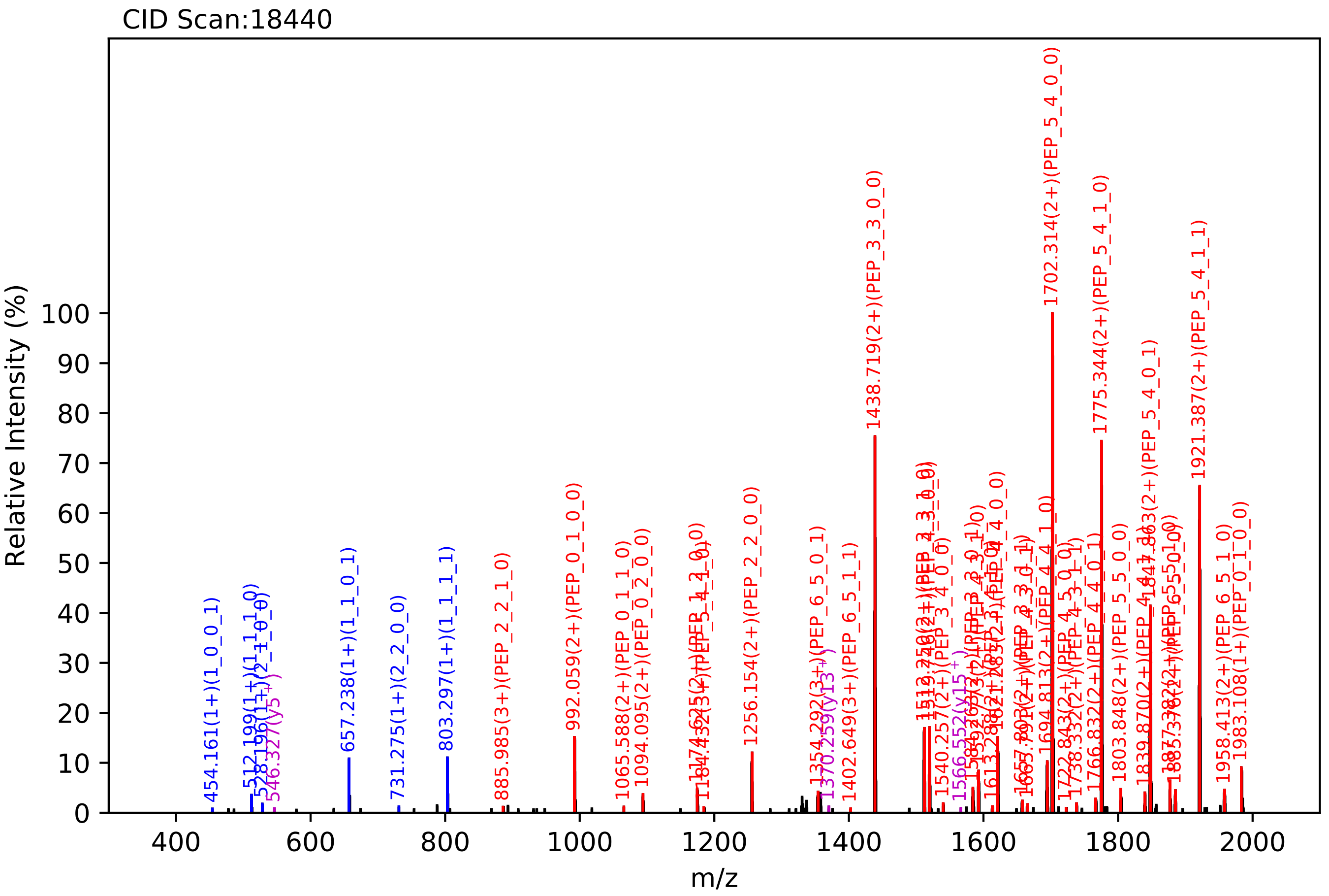

Test set no. 83, Experiment: AGP exp_4

LVPVPITNATLDQITGK(=PEP)_6_5_1_1, m/z:1051.98(4+), RT:48.56, Y-score:82.99

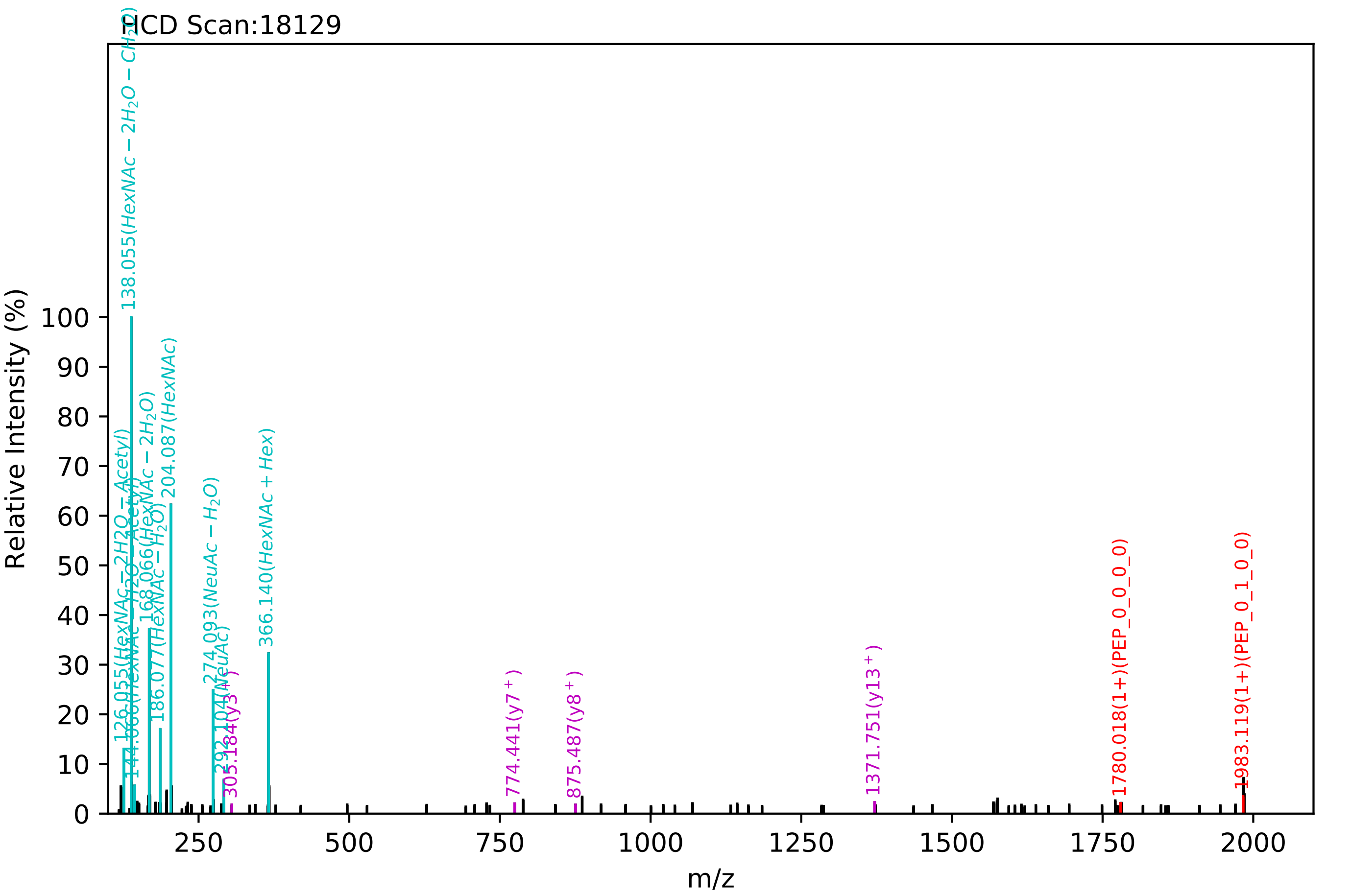

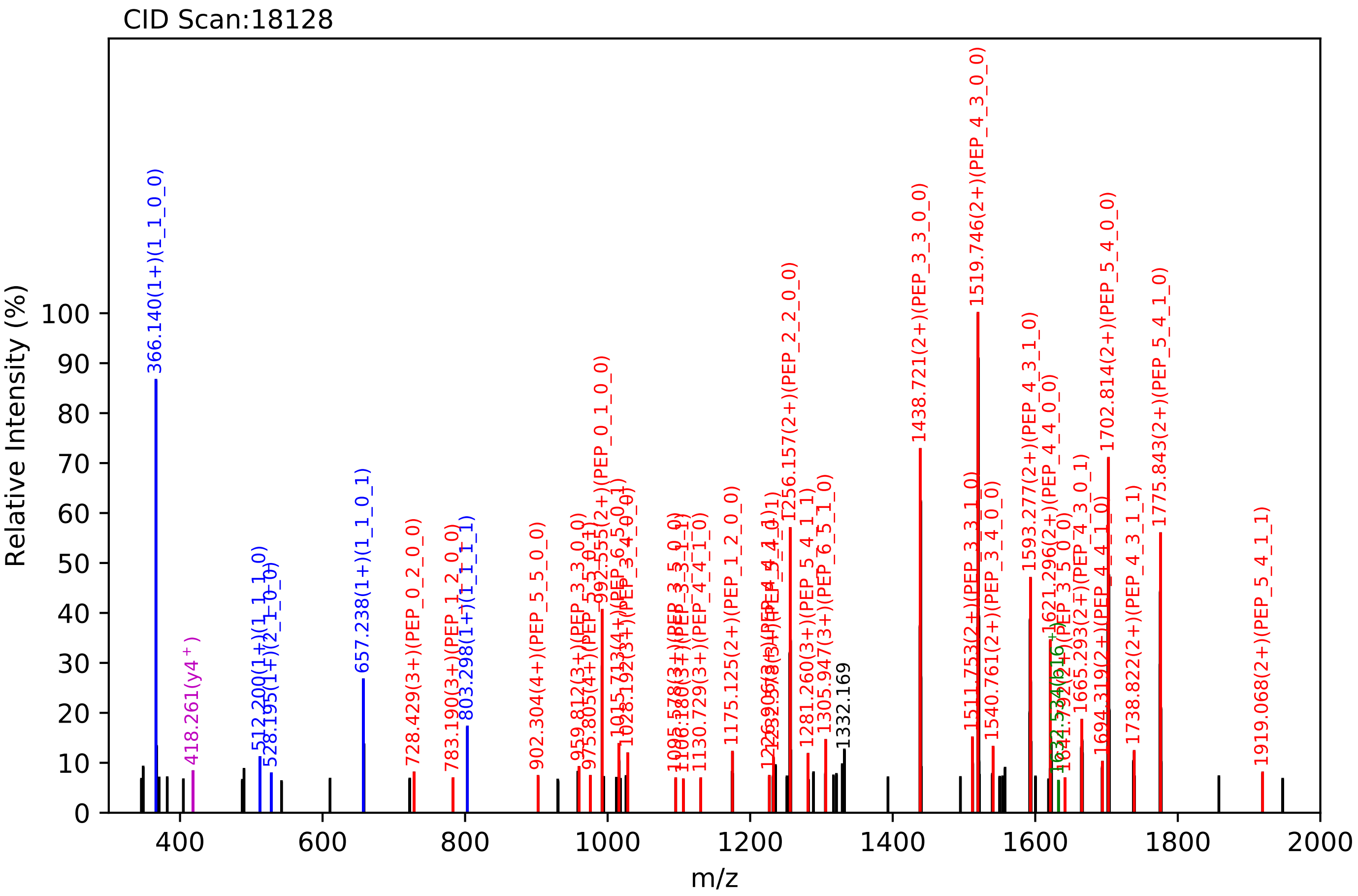

Test set no. 84, Experiment: AGP exp_4

LVPVPITNATLDQITGK(=PEP)_6_5_1_2, m/z:1499.34(3+), RT:54.84, Y-score:89.97

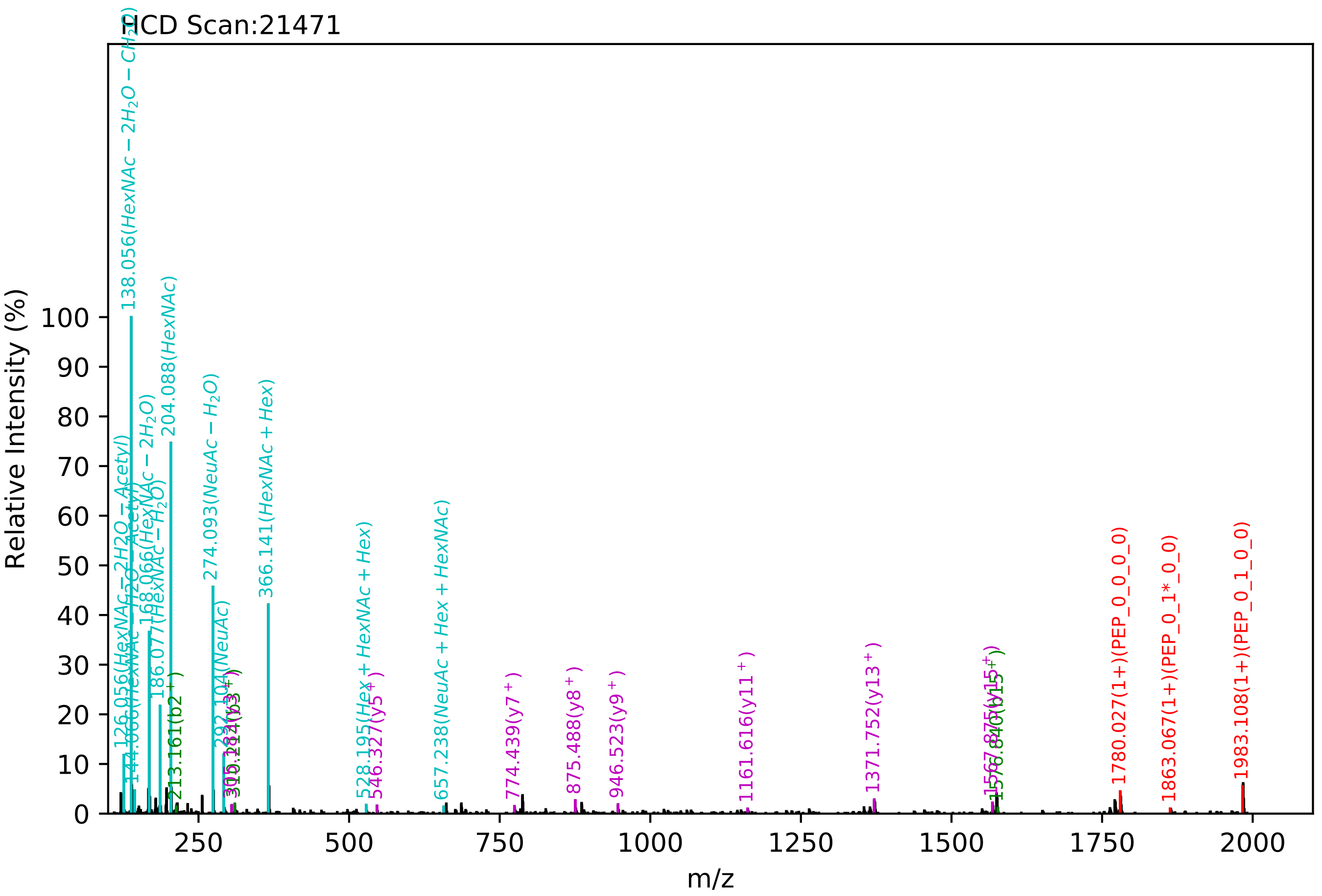

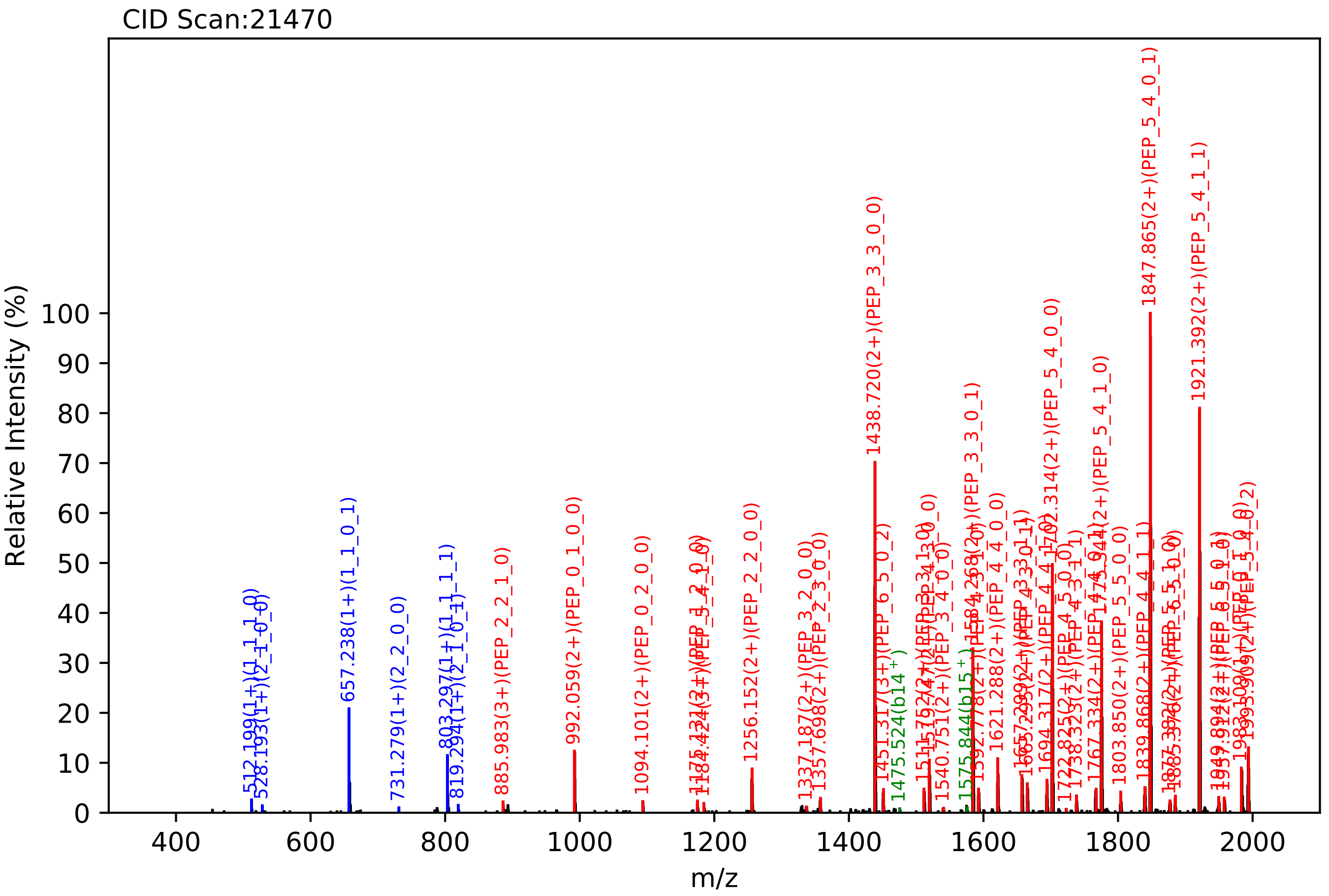

Test set no. 85, Experiment: AGP exp_3

LVPVPITNATLDQITGK(=PEP)_6_5_1_2, m/z:1499.34(3+), RT:54.80, Y-score:87.16

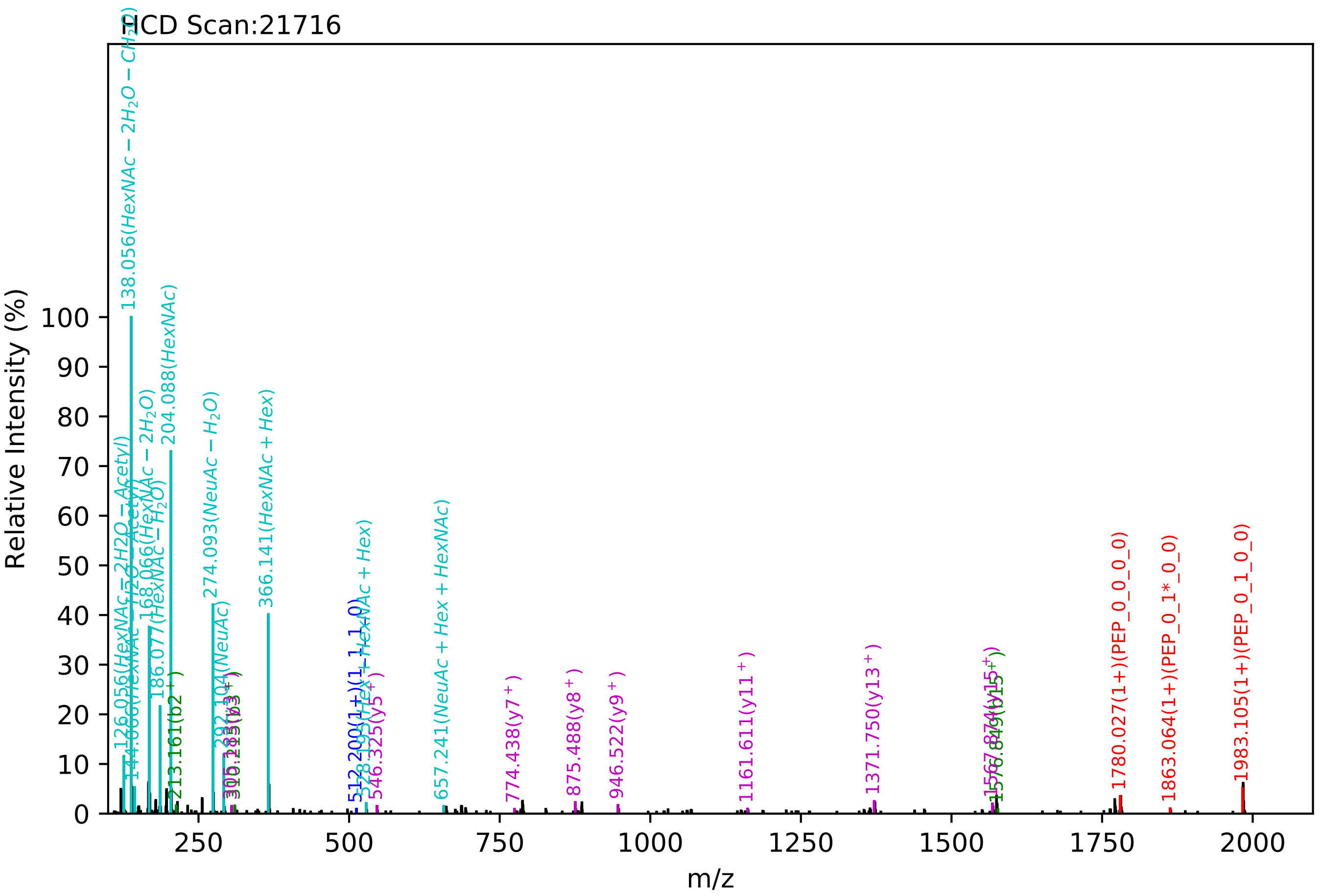

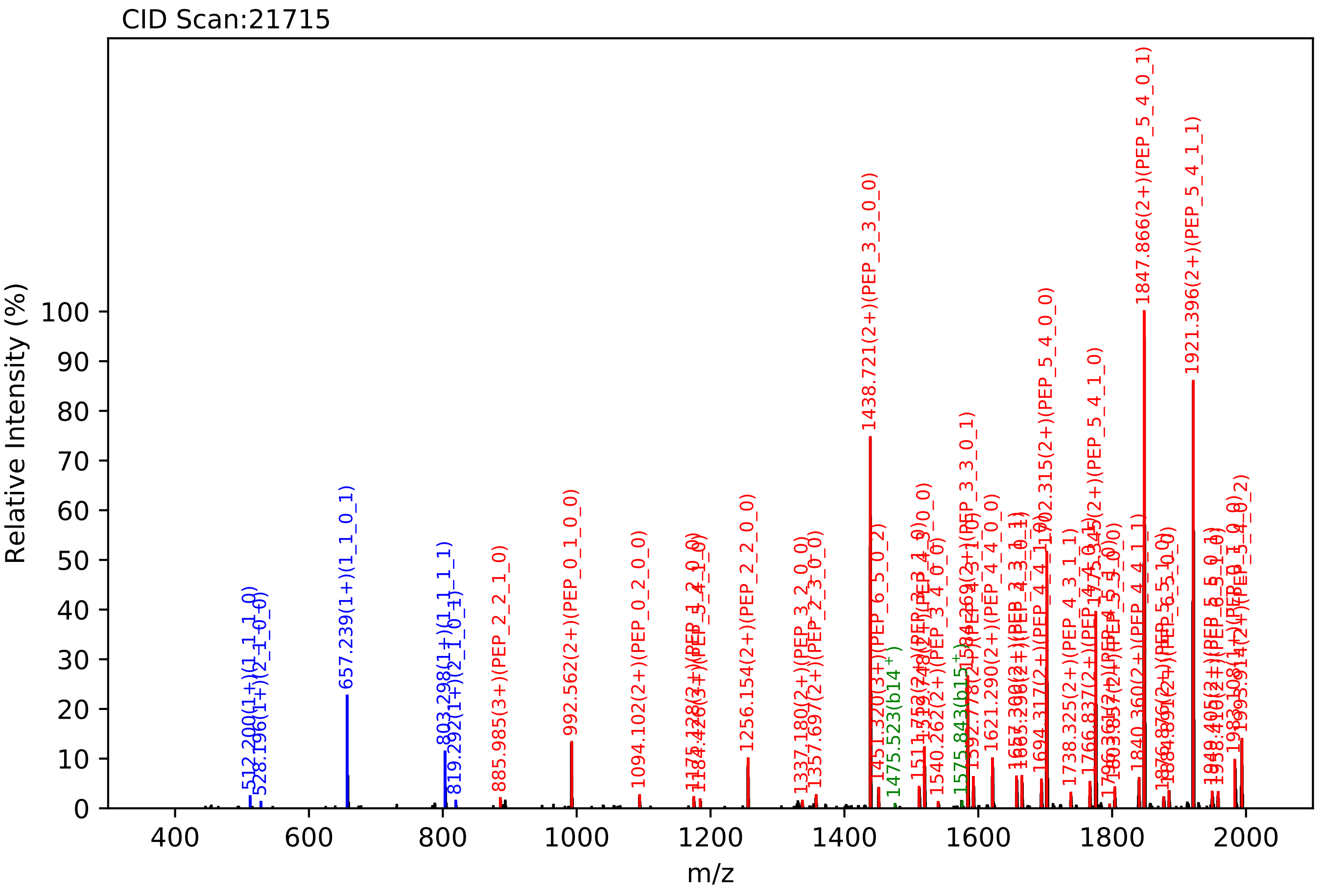

Test set no. 86, Experiment: AGP exp_3

LVPVPITNATLDQITGK(=PEP)_6_5_1_2, m/z:1499.34(3+), RT:55.37, Y-score:85.68

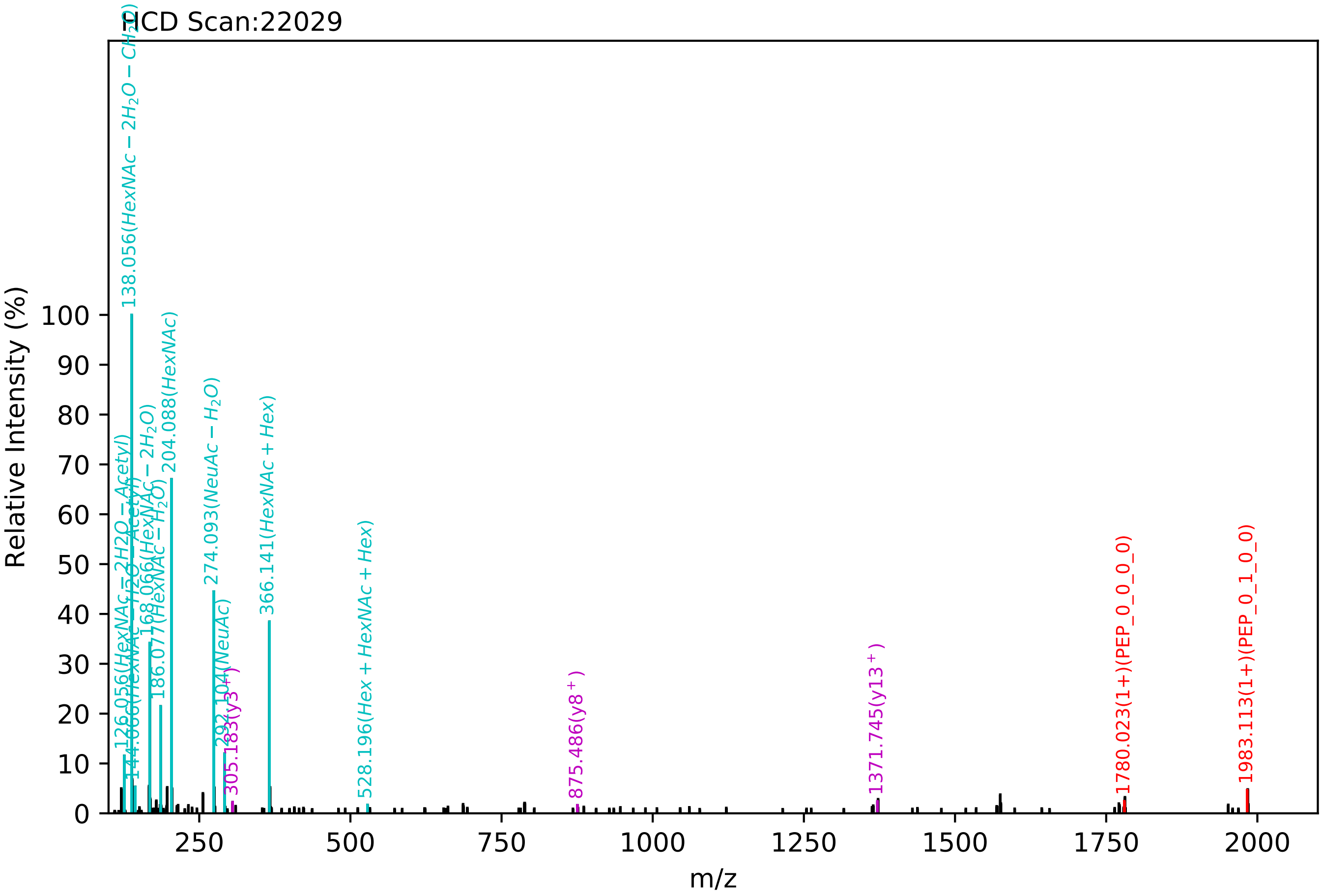

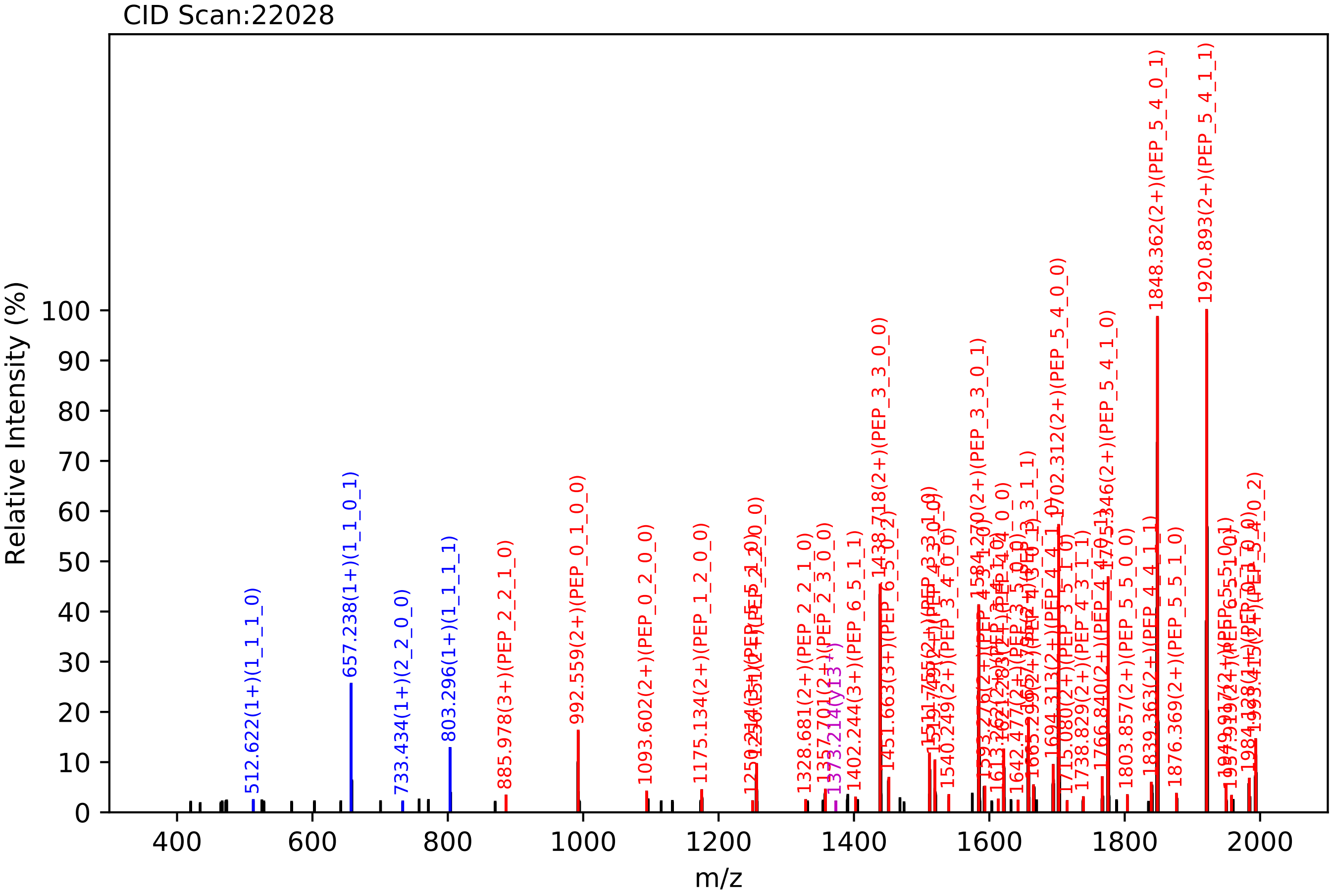

Test set no. 87, Experiment: AGP exp_4

LVPVPITNATLDQITGK(=PEP)_6_5_1_2, m/z:1124.75(4+), RT:54.85, Y-score:83.90

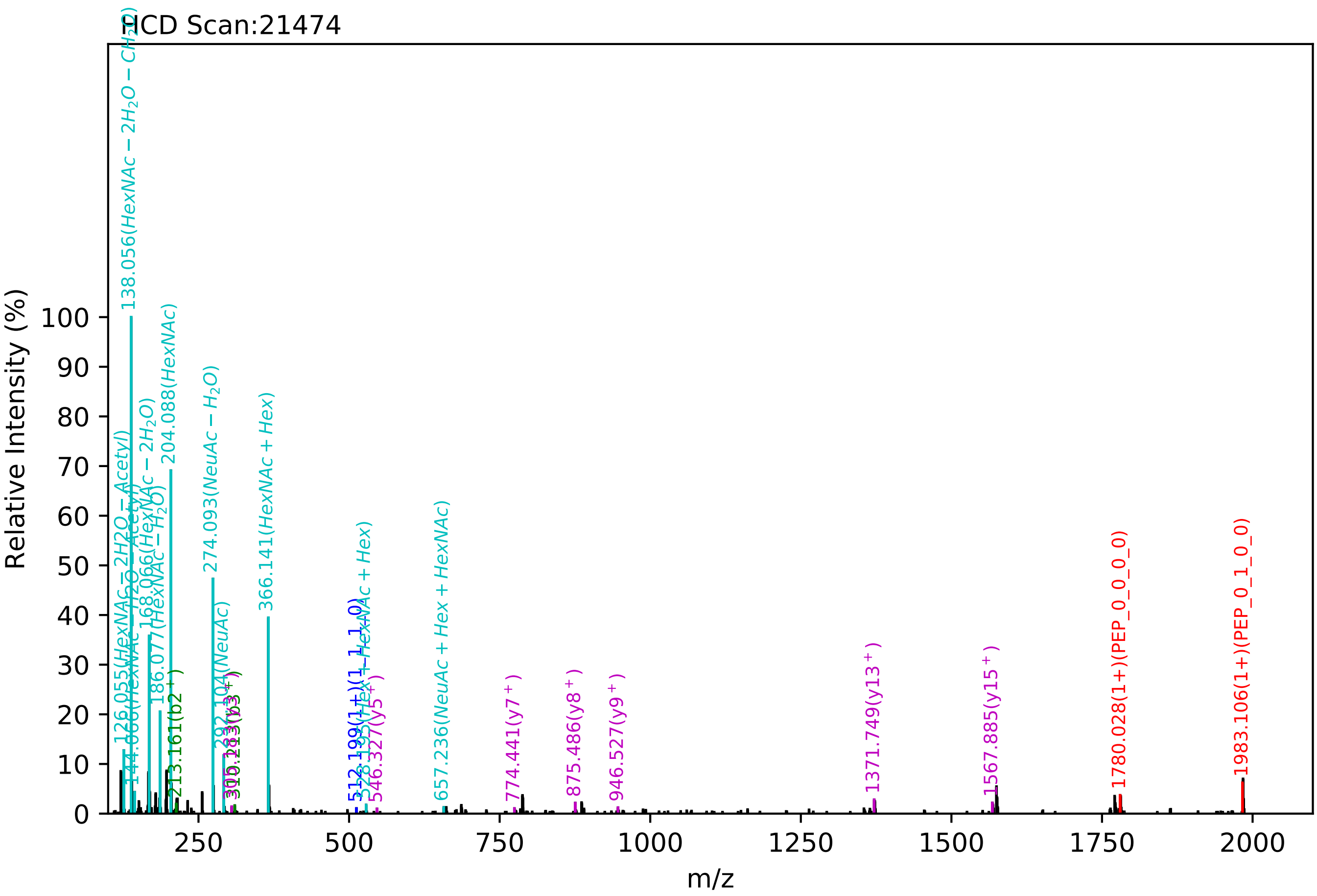

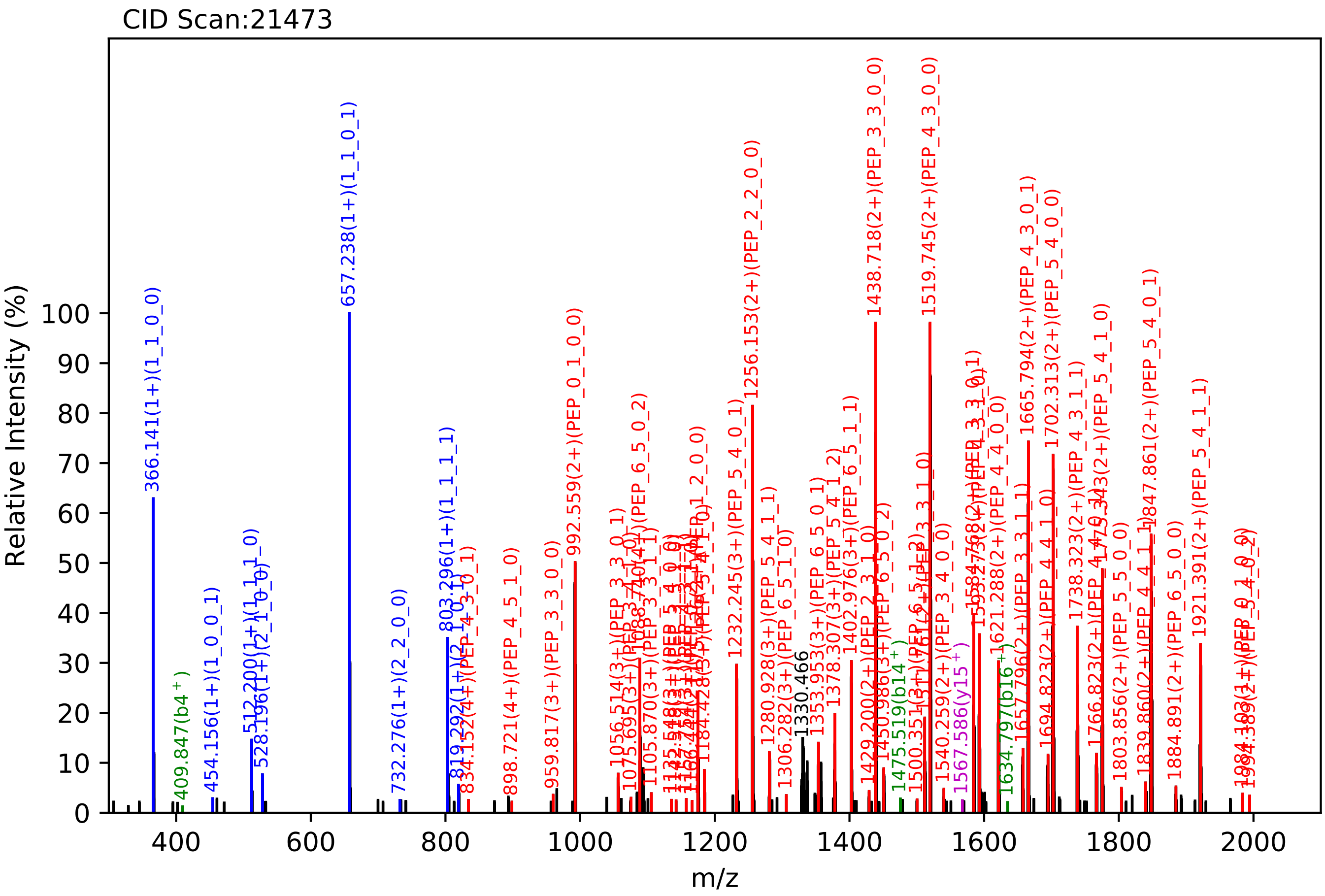

Test set no. 88, Experiment: AGP exp_3

LVPVPITNATLDQITGK(=PEP)_6_5_1_2, m/z:1124.75(4+), RT:54.93, Y-score:81.10

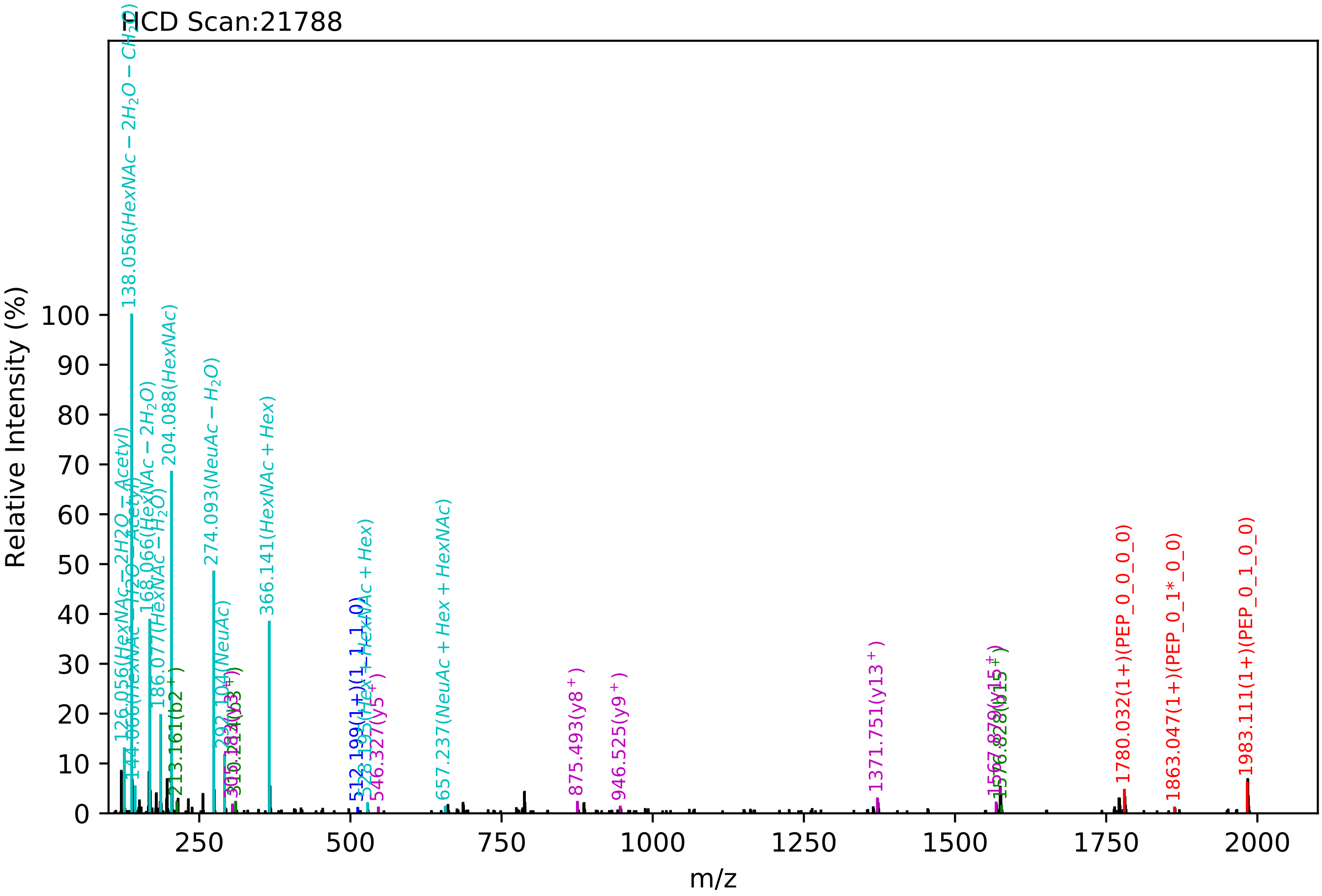

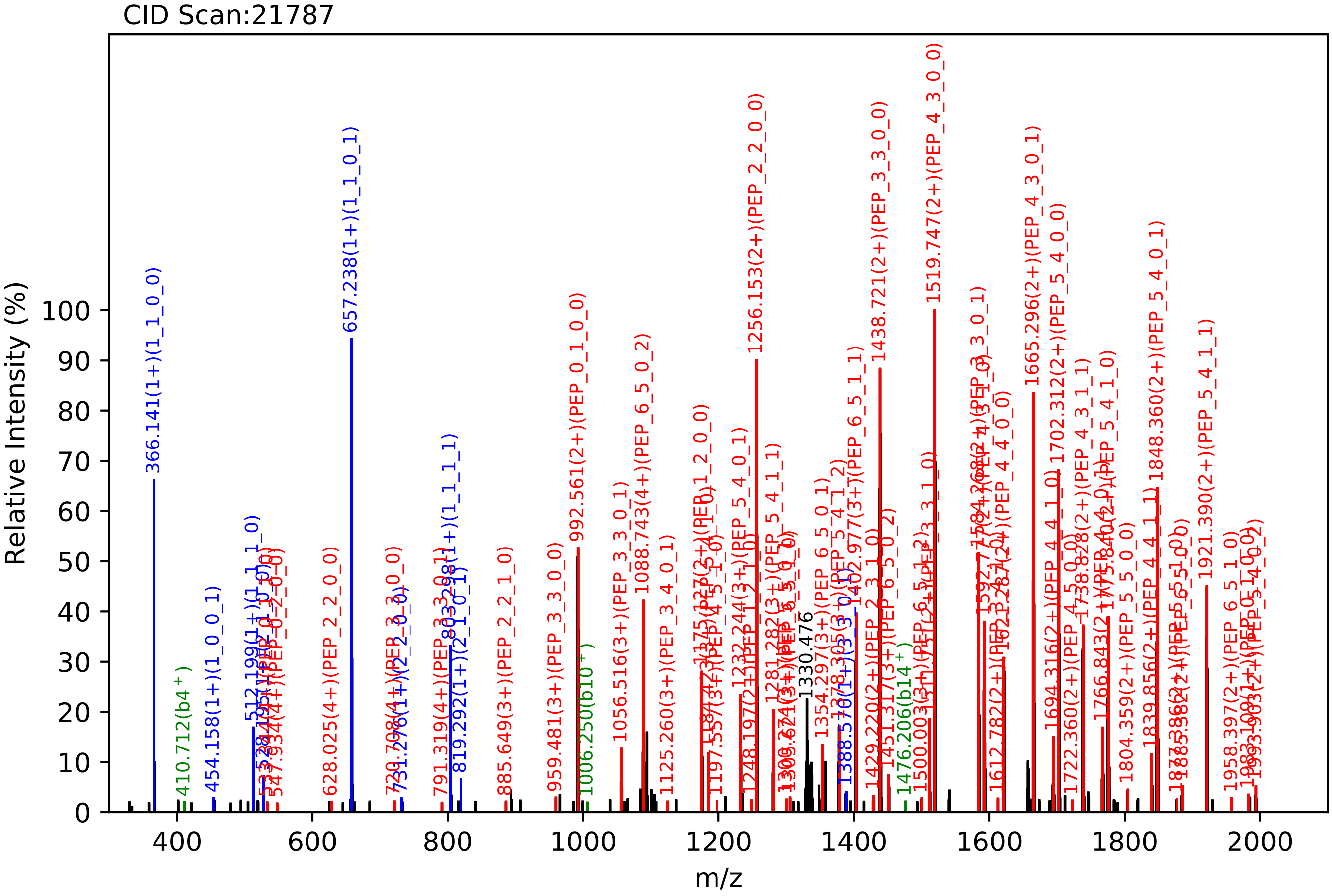

Test set no. 89, Experiment: AGP exp_4

LVPVPITNATLDQITGK(=PEP)_6_5_1_3, m/z:1596.36(3+), RT:62.44, Y-score:88.44

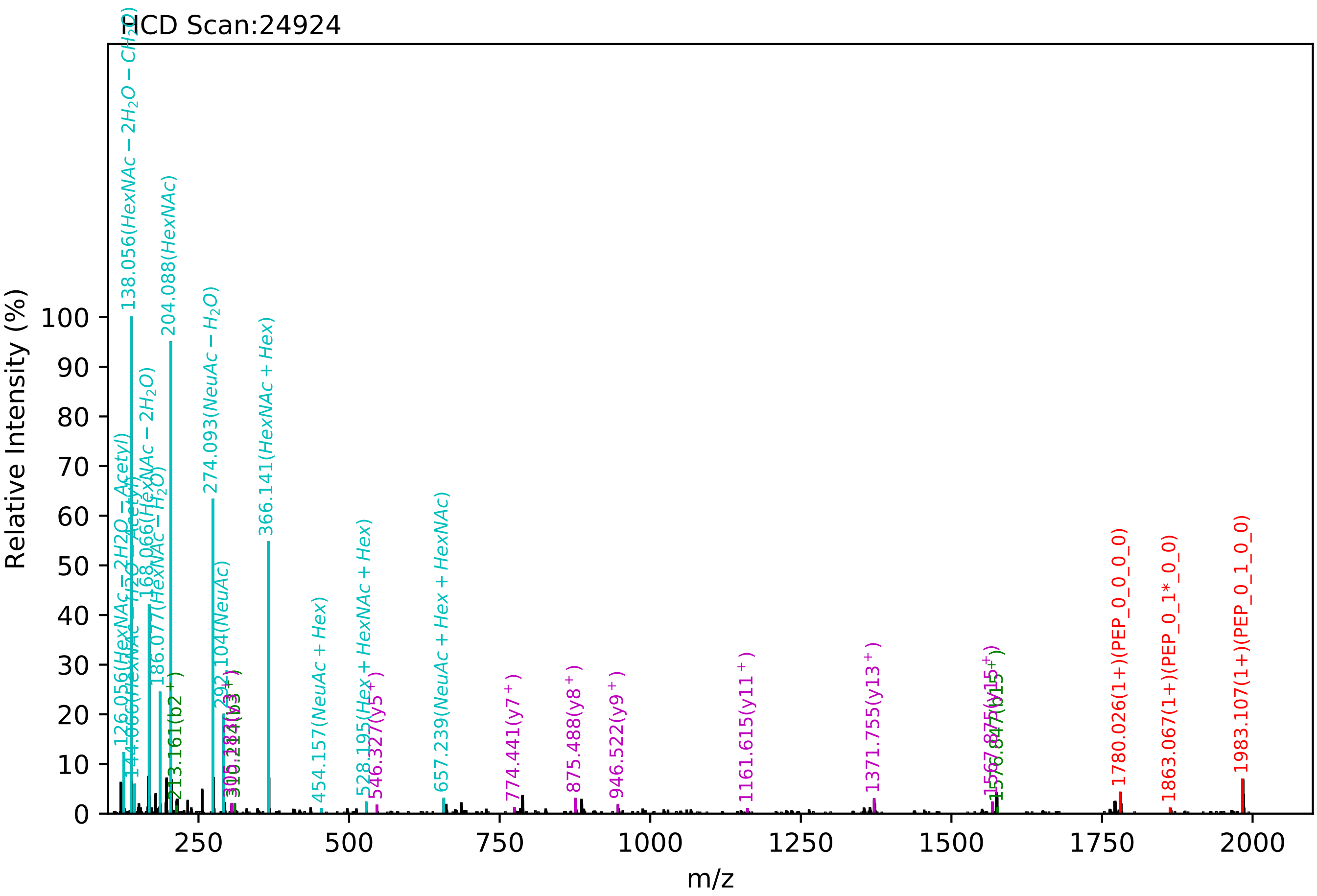

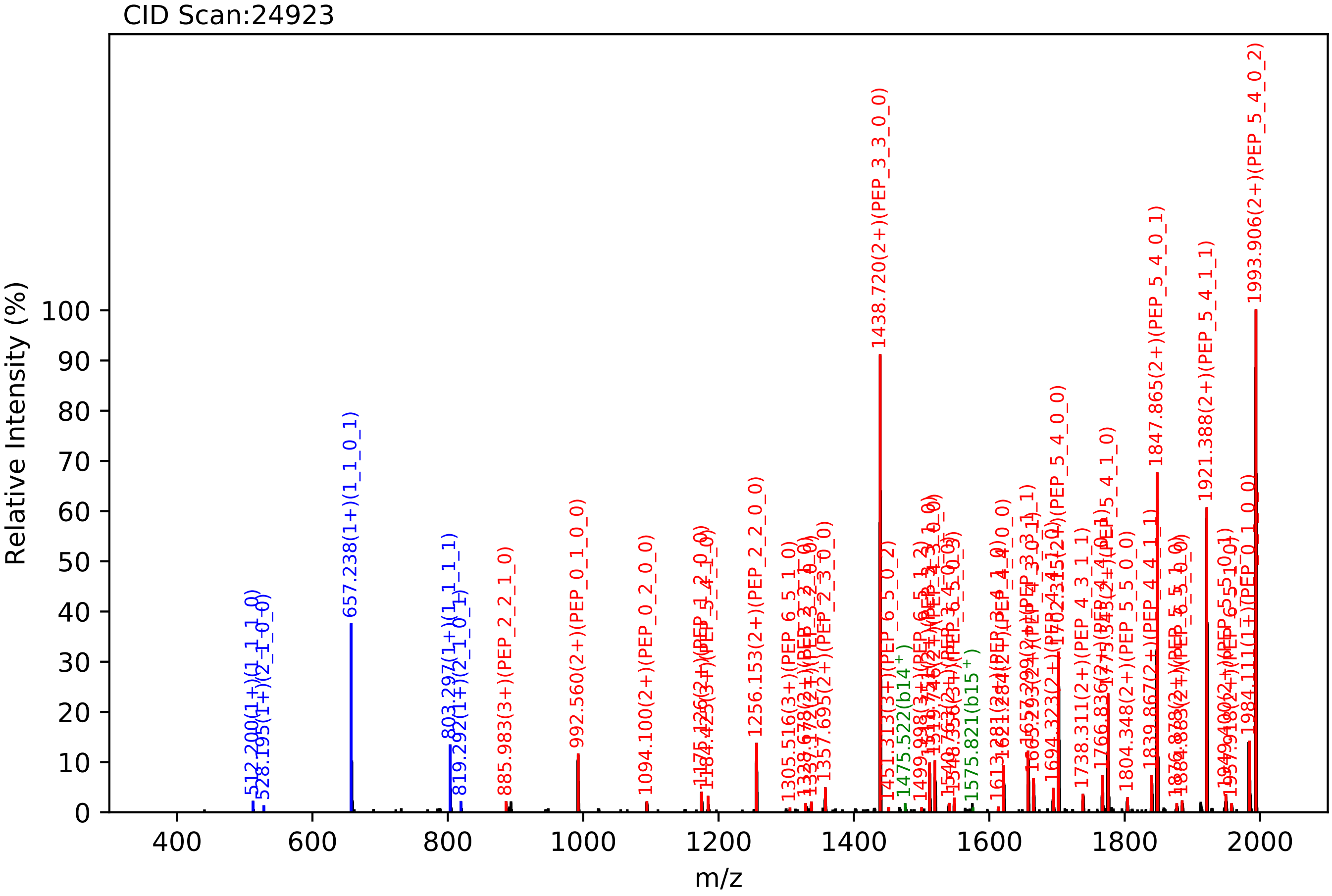

Test set no. 90, Experiment: AGP exp_4

LVPVPITNATLDQITGK(=PEP)_6_5_1_3, m/z:1596.37(3+), RT:62.97, Y-score:87.65

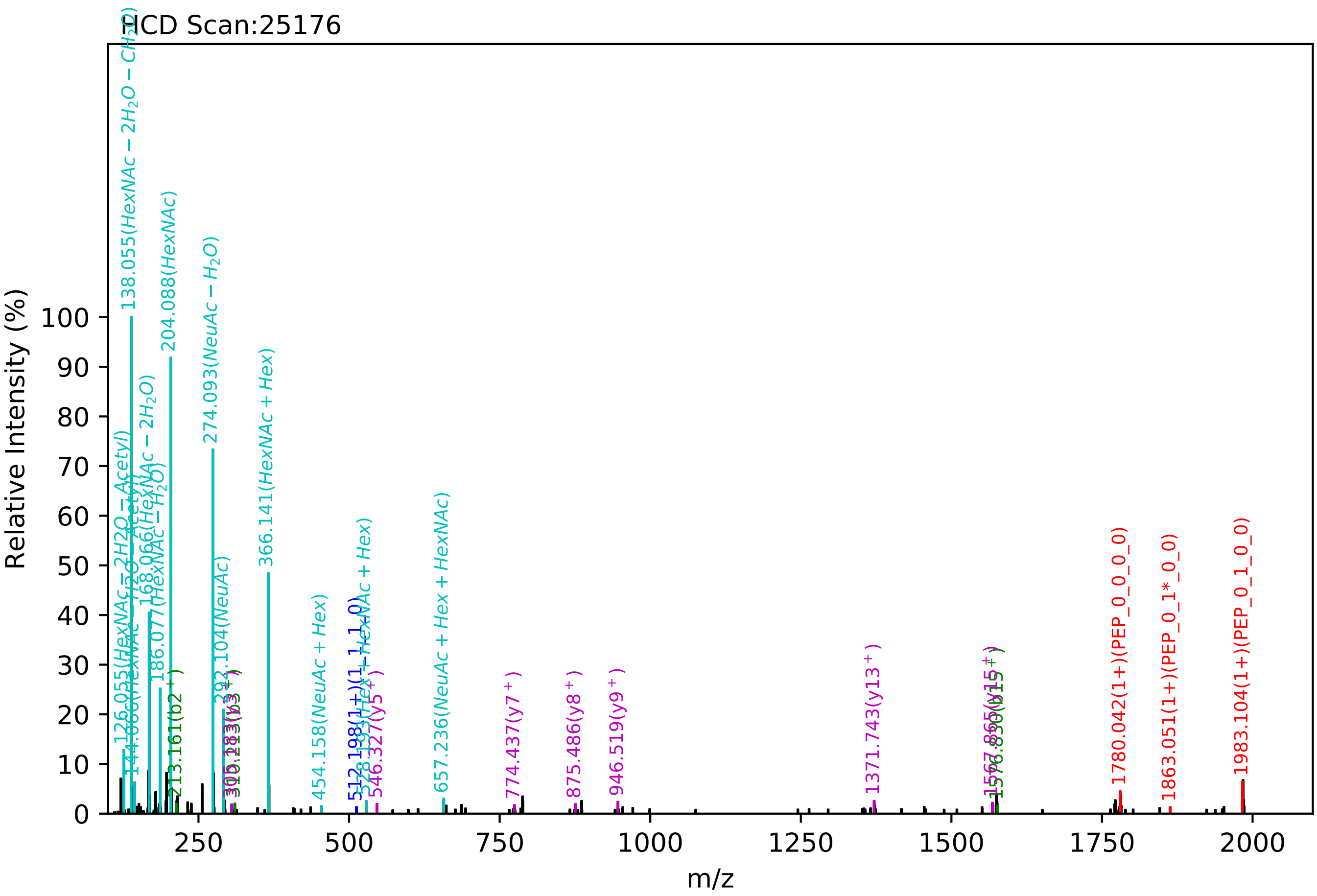

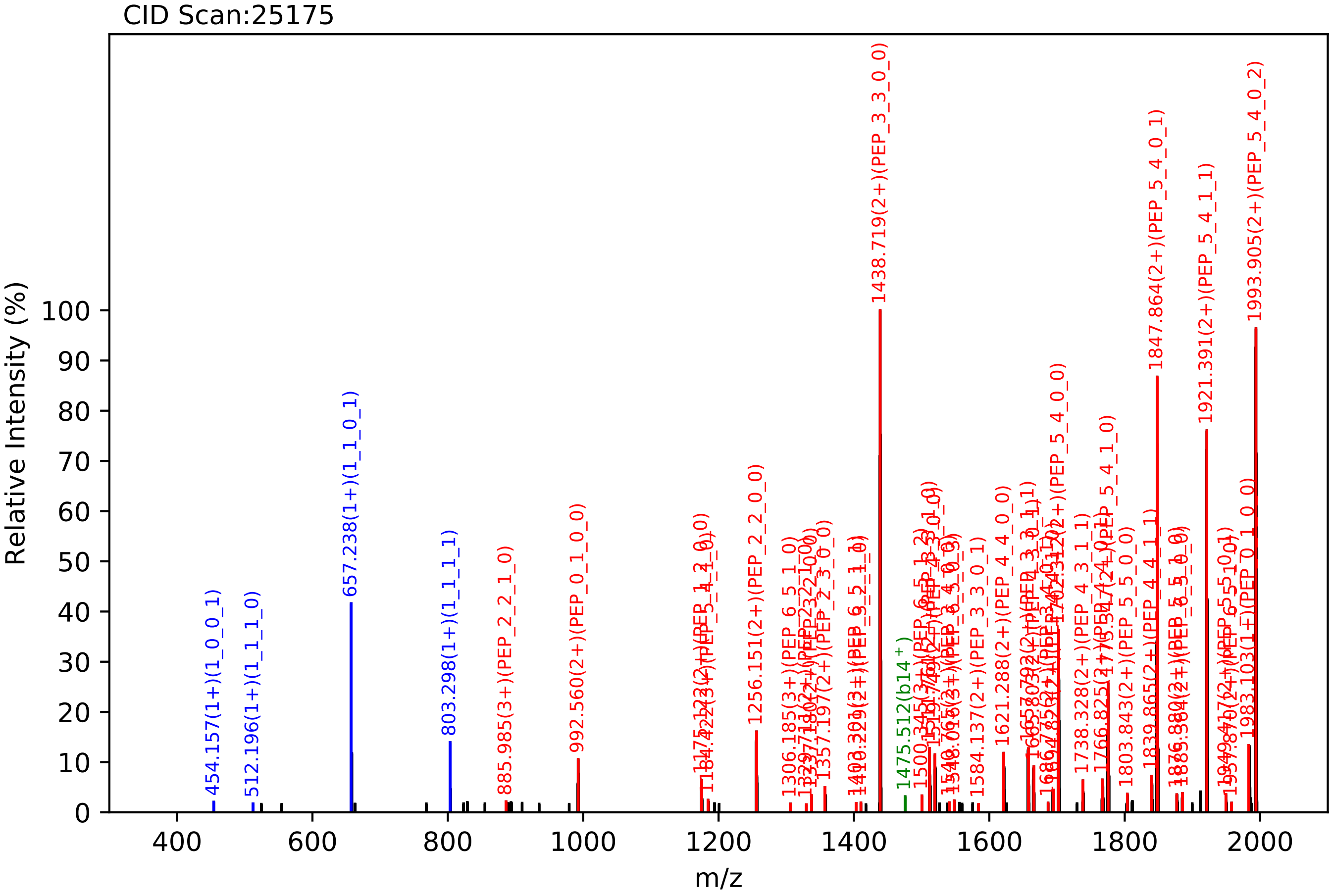

Test set no. 91, Experiment: AGP exp_3

LVPVPITNATLDQITGK(=PEP)_6_5_1_3, m/z:1596.38(3+), RT:62.44, Y-score:87.59

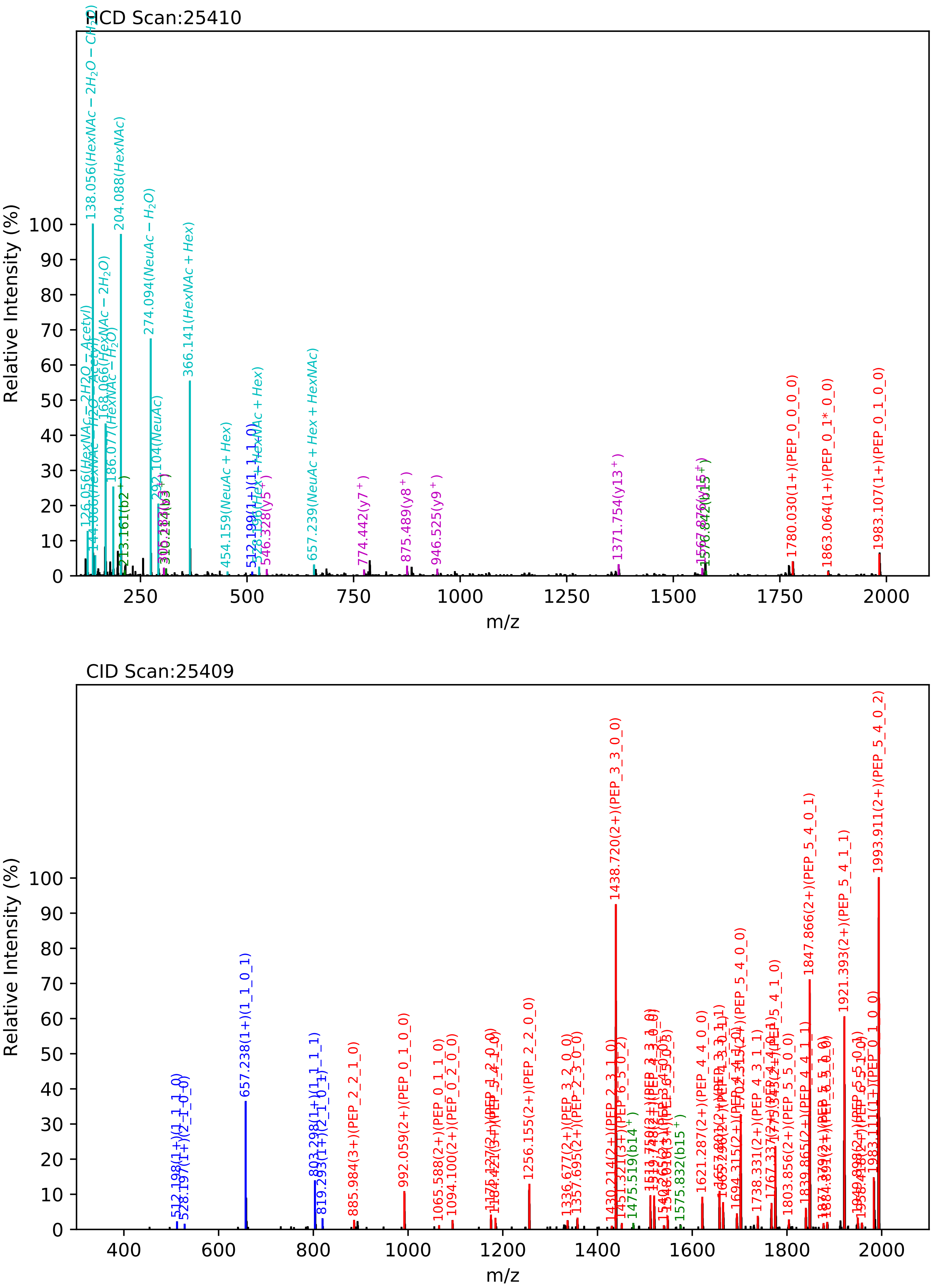

Test set no. 92, Experiment: AGP exp_3

LVPVPITNATLDQITGK(=PEP)_6_5_1_3, m/z:1197.53(4+), RT:62.49, Y-score:87.29

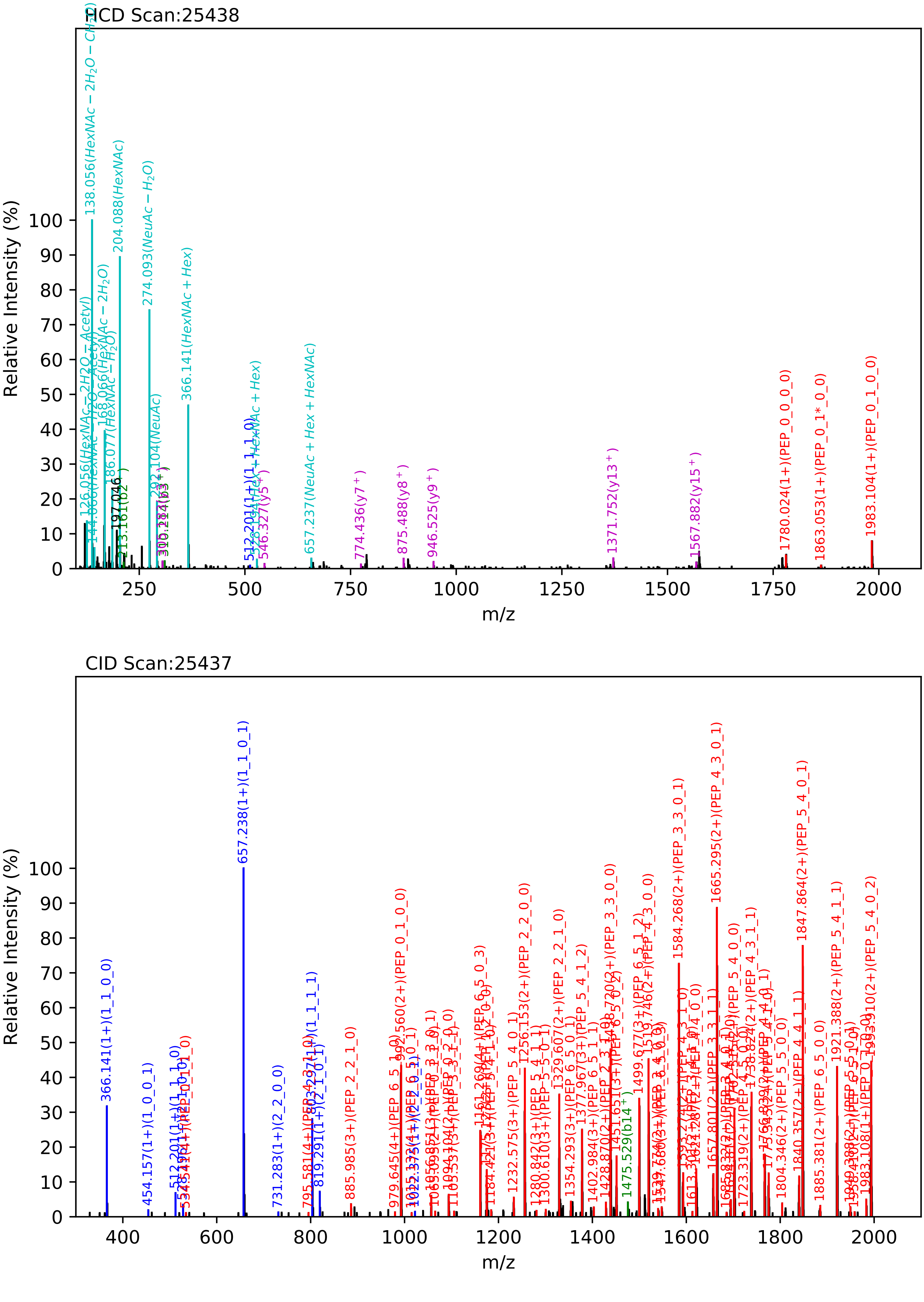

Test set no. 93, Experiment: AGP exp_3

LVPVPITNATLDQITGK(=PEP)_6_5_1_3, m/z:1596.38(3+), RT:62.46, Y-score:85.62

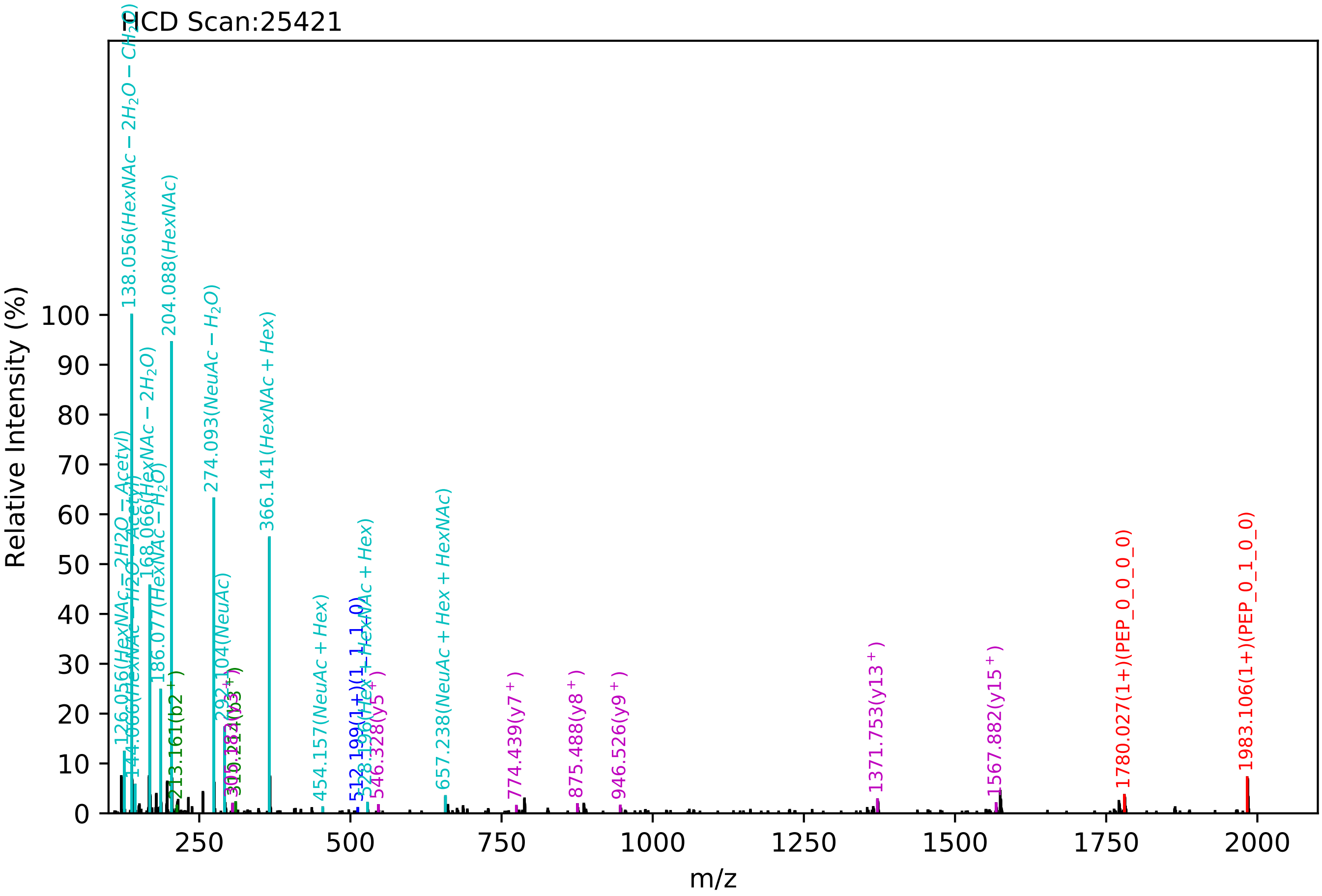

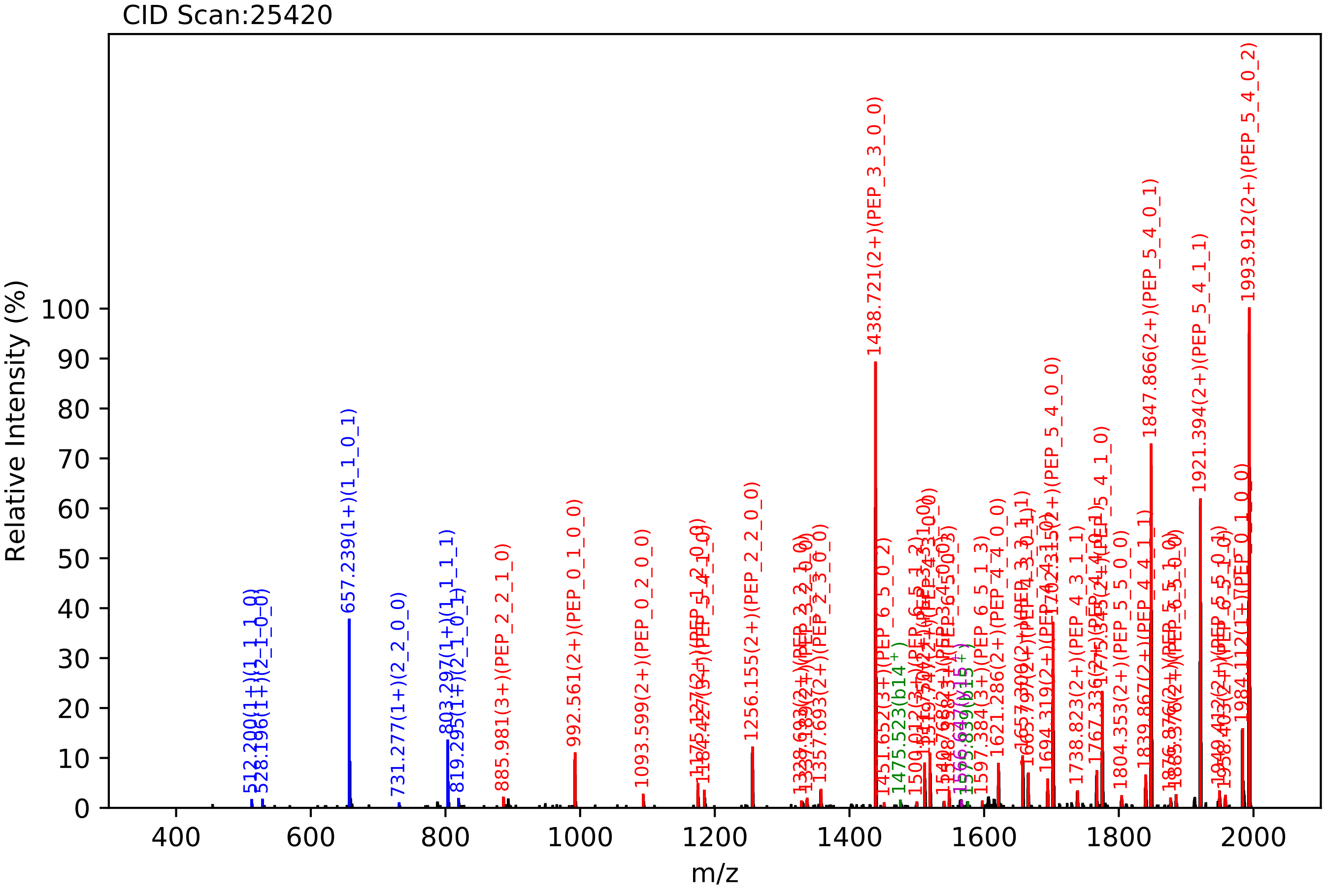

Test set no. 94, Experiment: AGP exp_3

LVPVPITNATLDQITGK(=PEP)_6_5_1_3, m/z:1596.37(3+), RT:62.99, Y-score:85.57

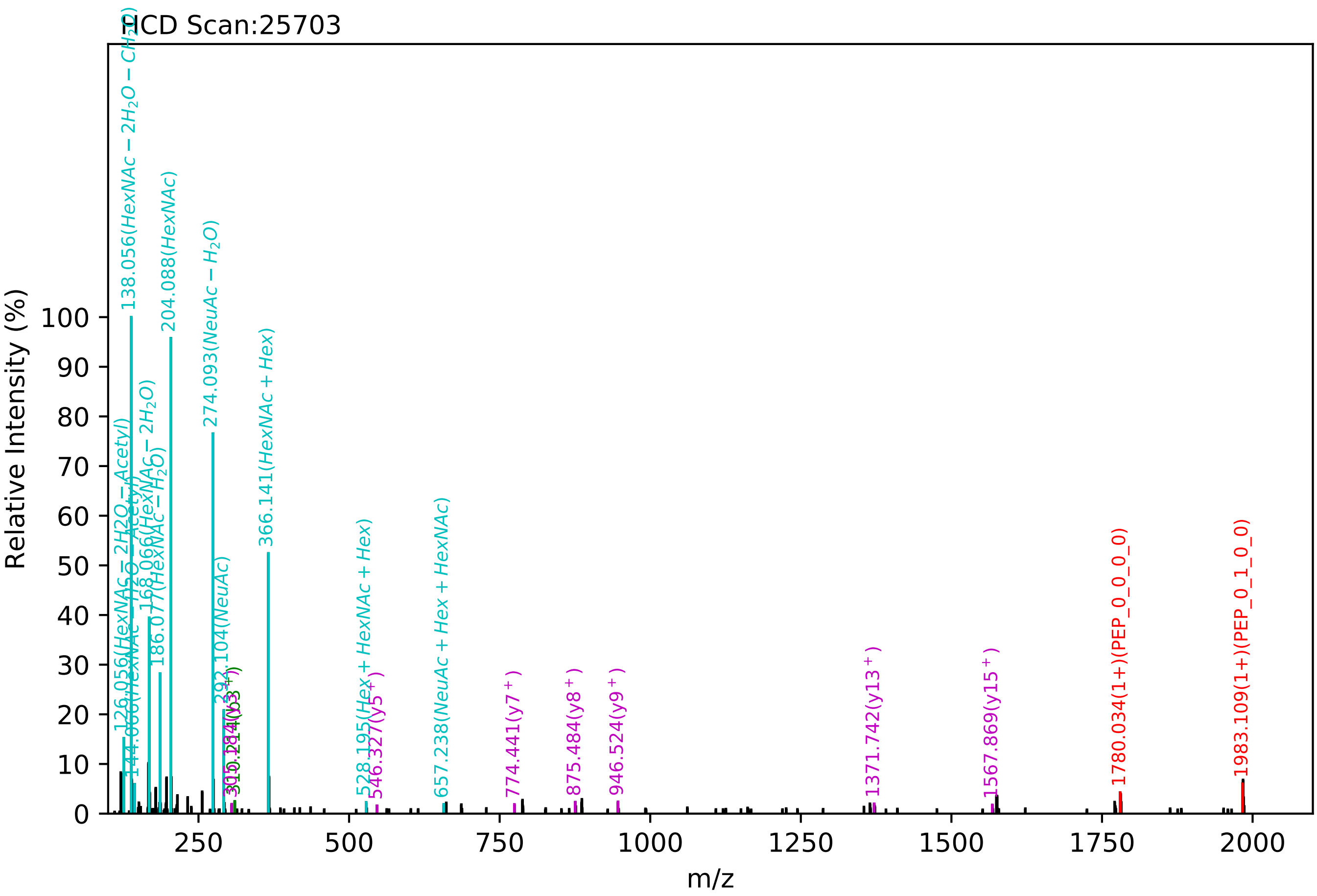

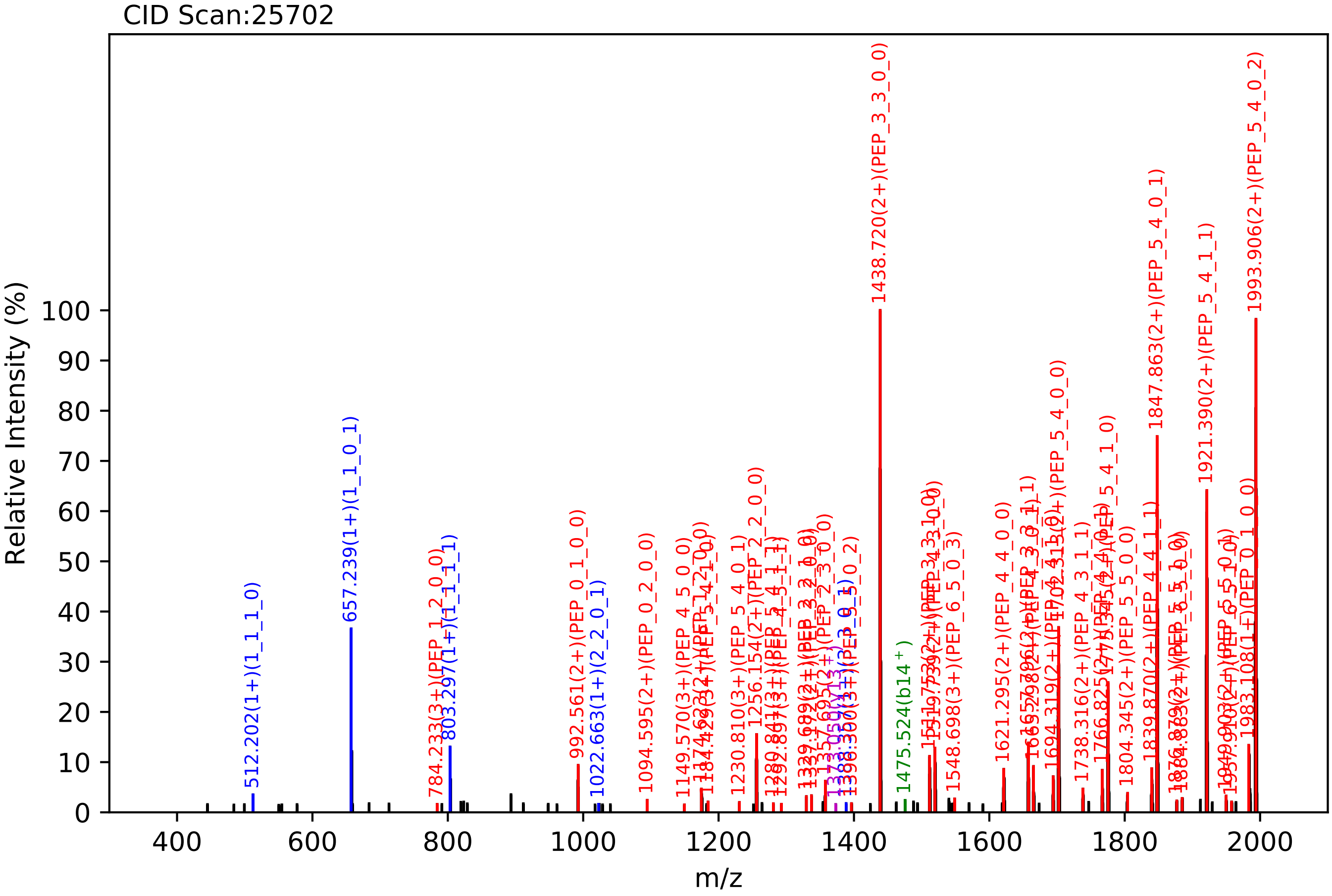

Test set no. 95, Experiment: AGP exp_3

LVPVPITNATLDQITGK(=PEP)_6_5_1_3, m/z:1197.53(4+), RT:62.35, Y-score:84.46

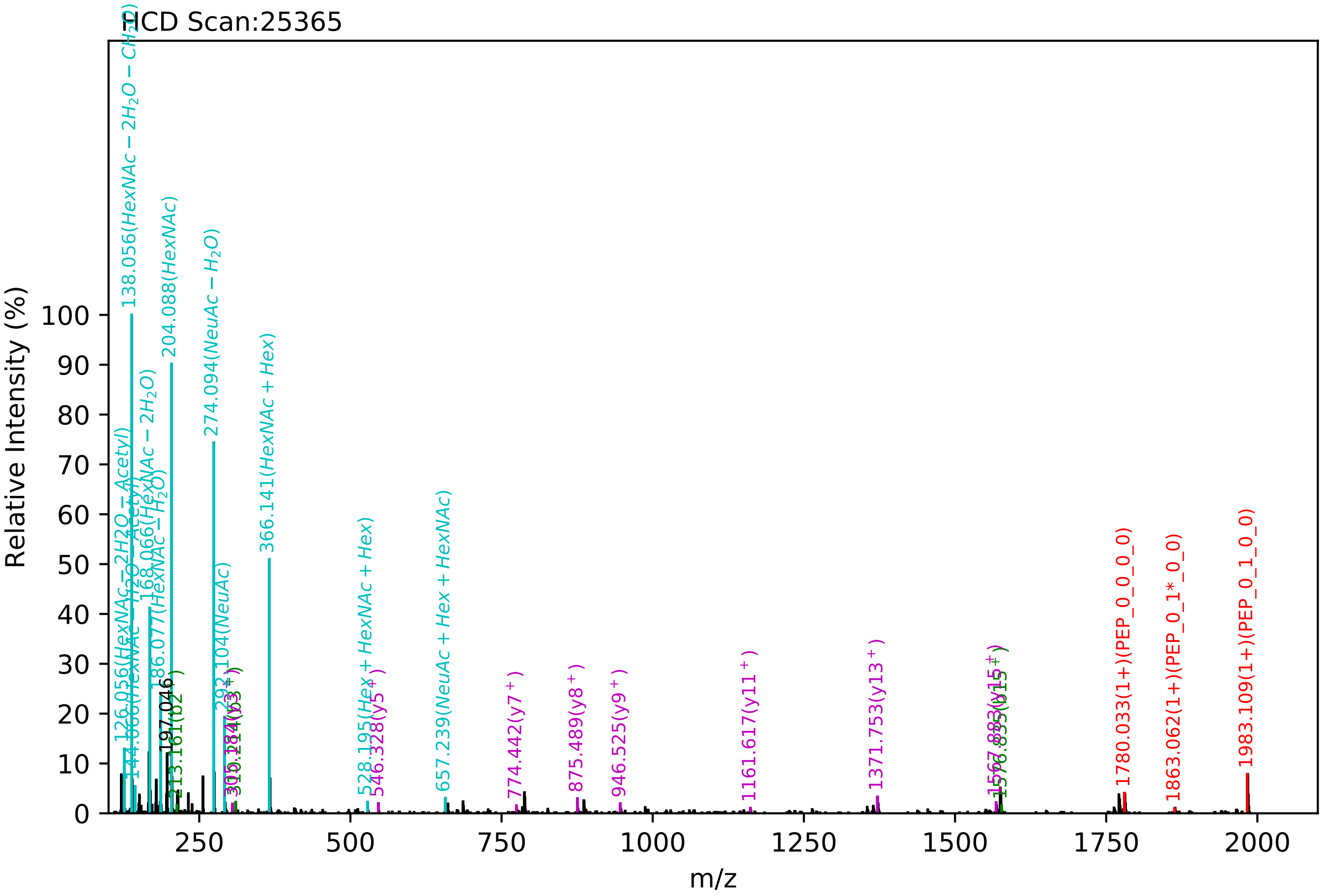

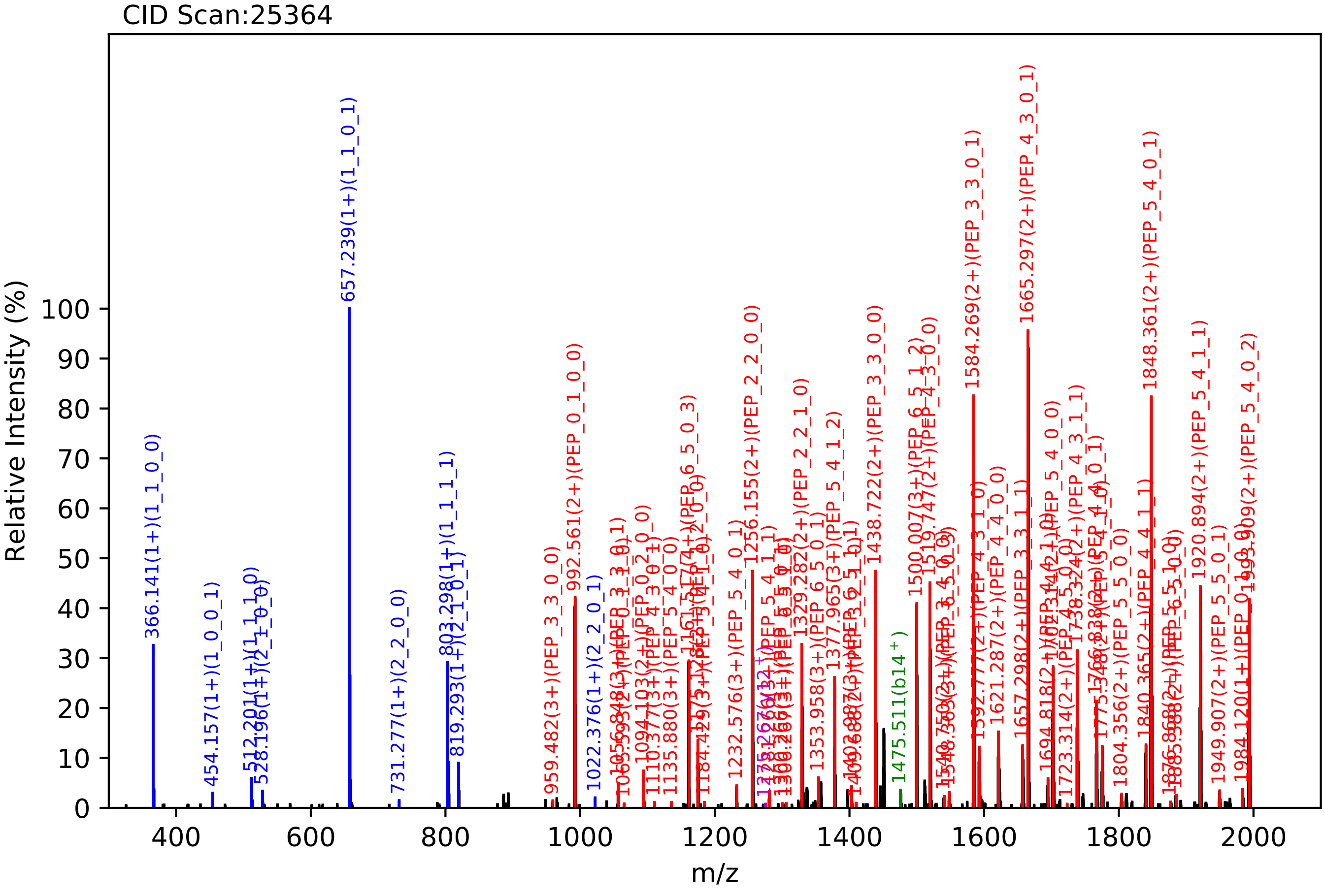

Test set no. 96, Experiment: AGP exp_3

LVPVPITNATLDQITGK(=PEP)_6_5_1_3, m/z:1197.53(4+), RT:63.01, Y-score:83.38

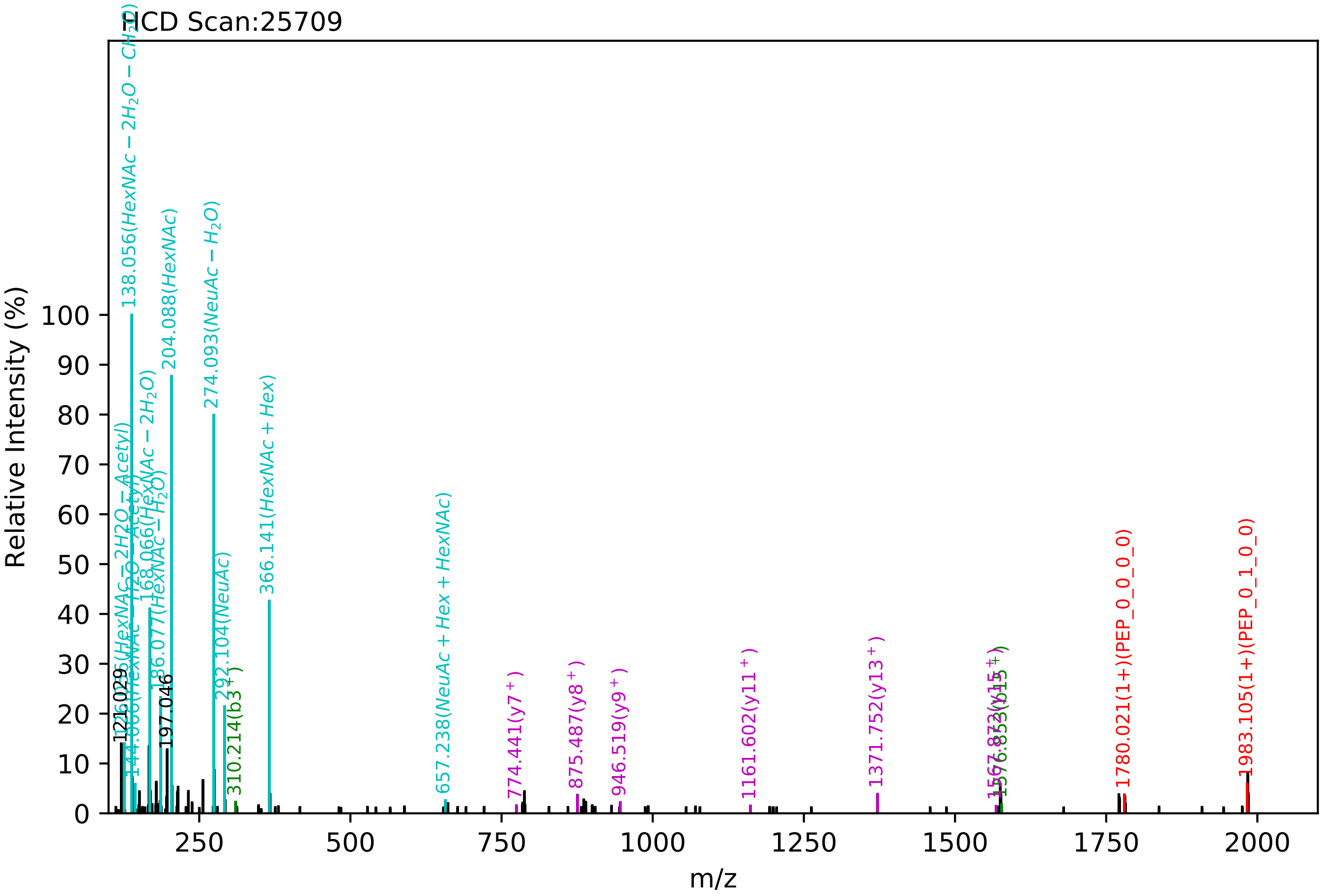

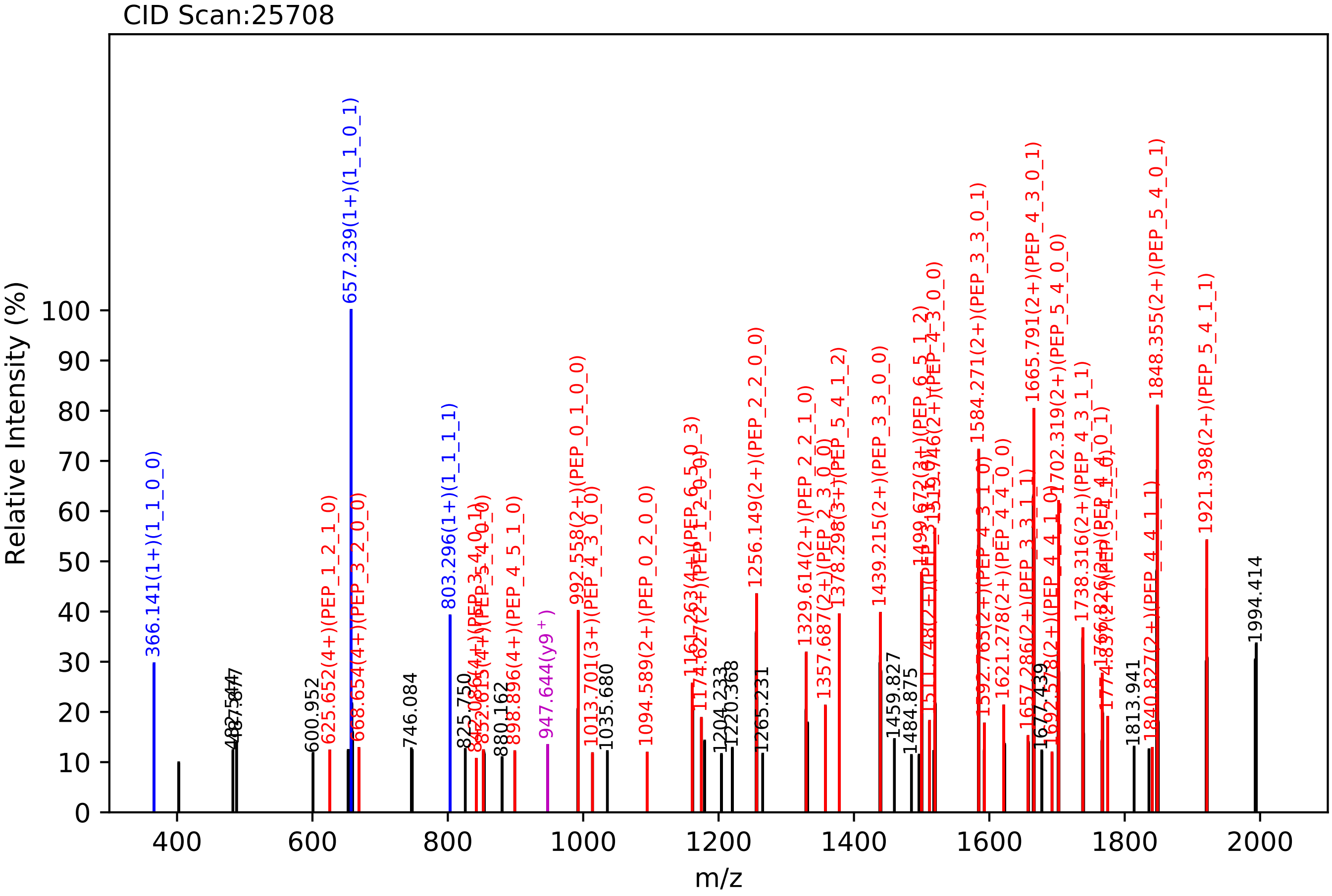

Test set no. 97, Experiment: AGP exp_4

LVPVPITNATLDQITGK(=PEP)_6_5_1_3, m/z:1197.53(4+), RT:62.45, Y-score:83.32

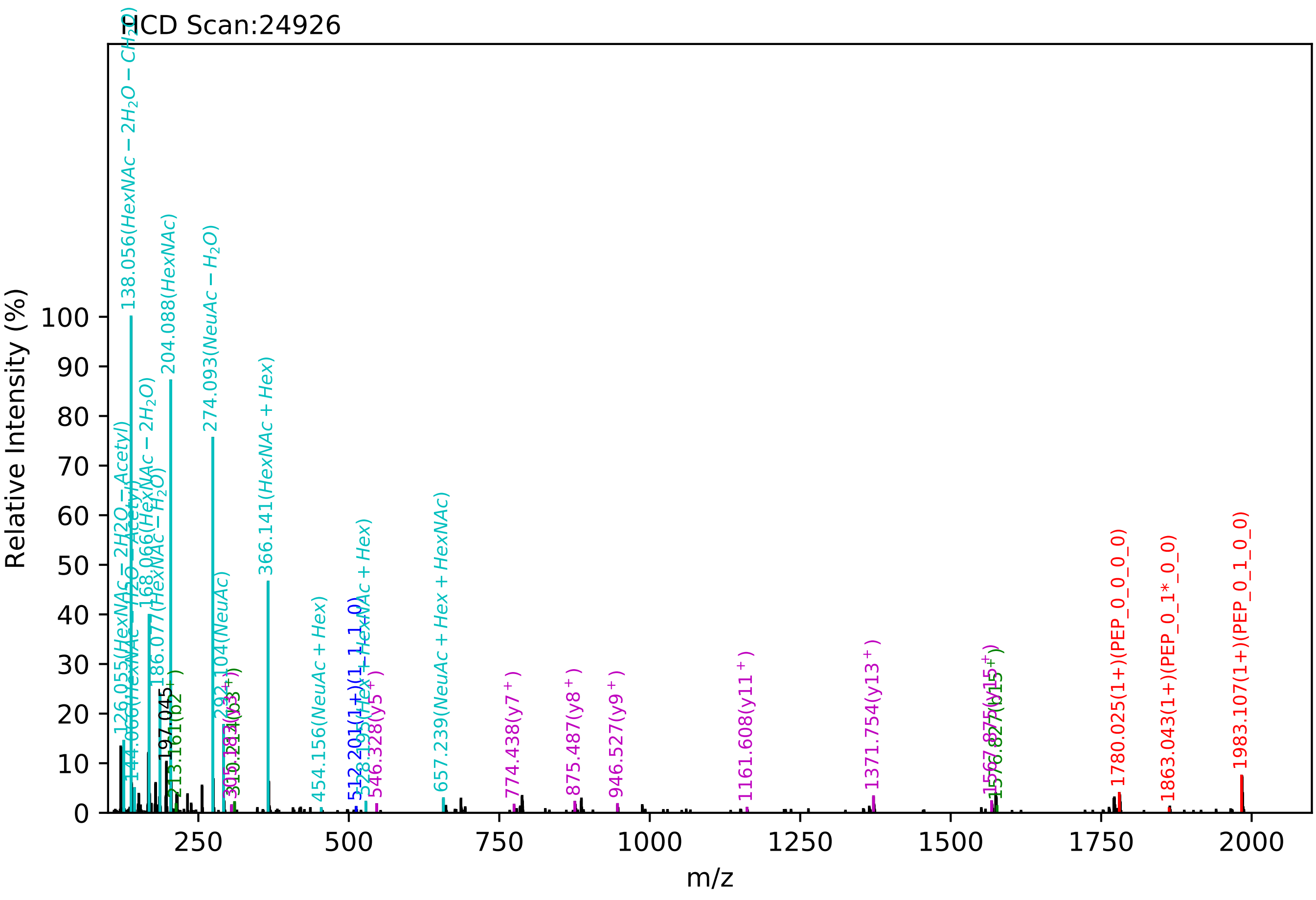

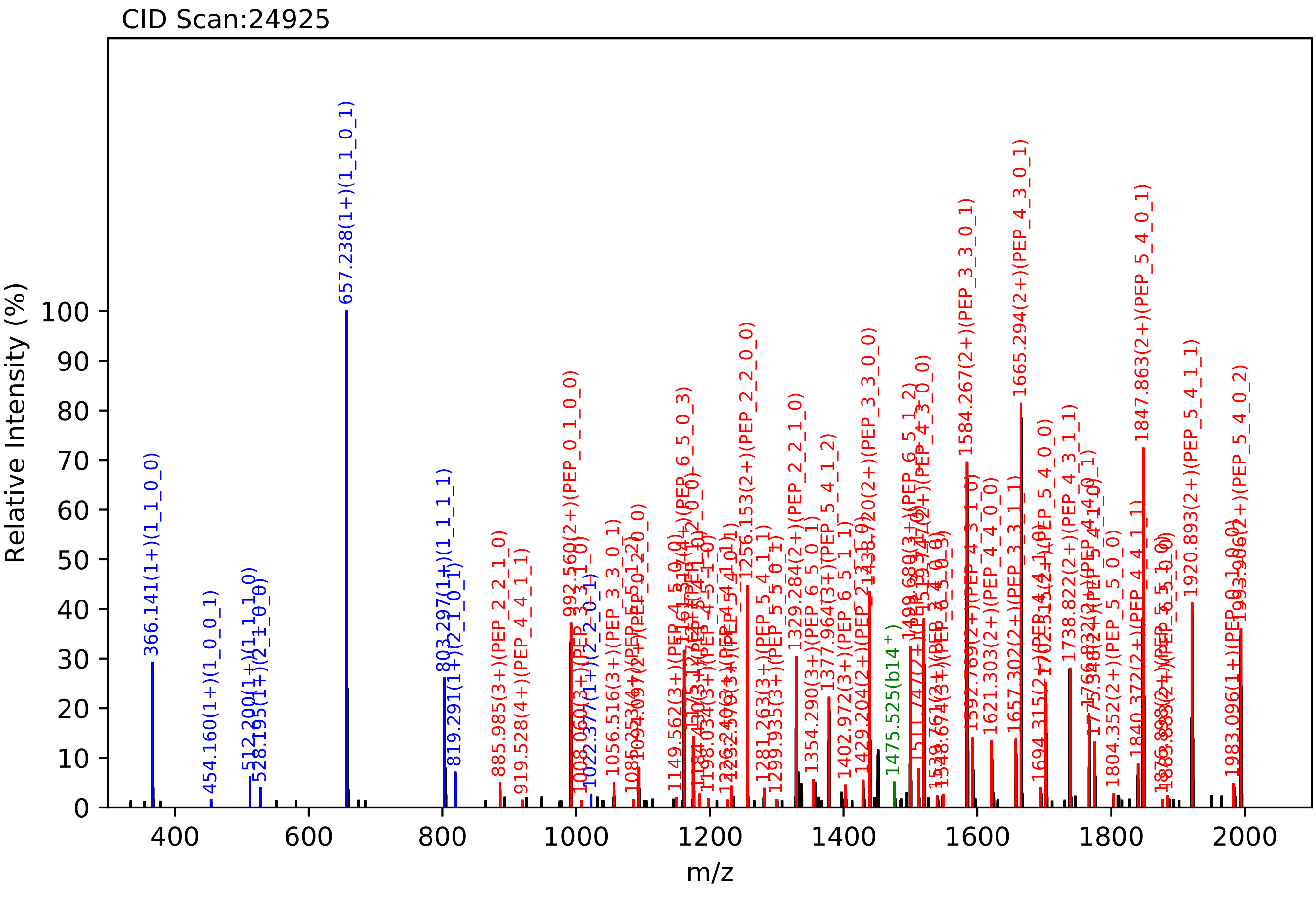

Test set no. 98, Experiment: AGP exp_4

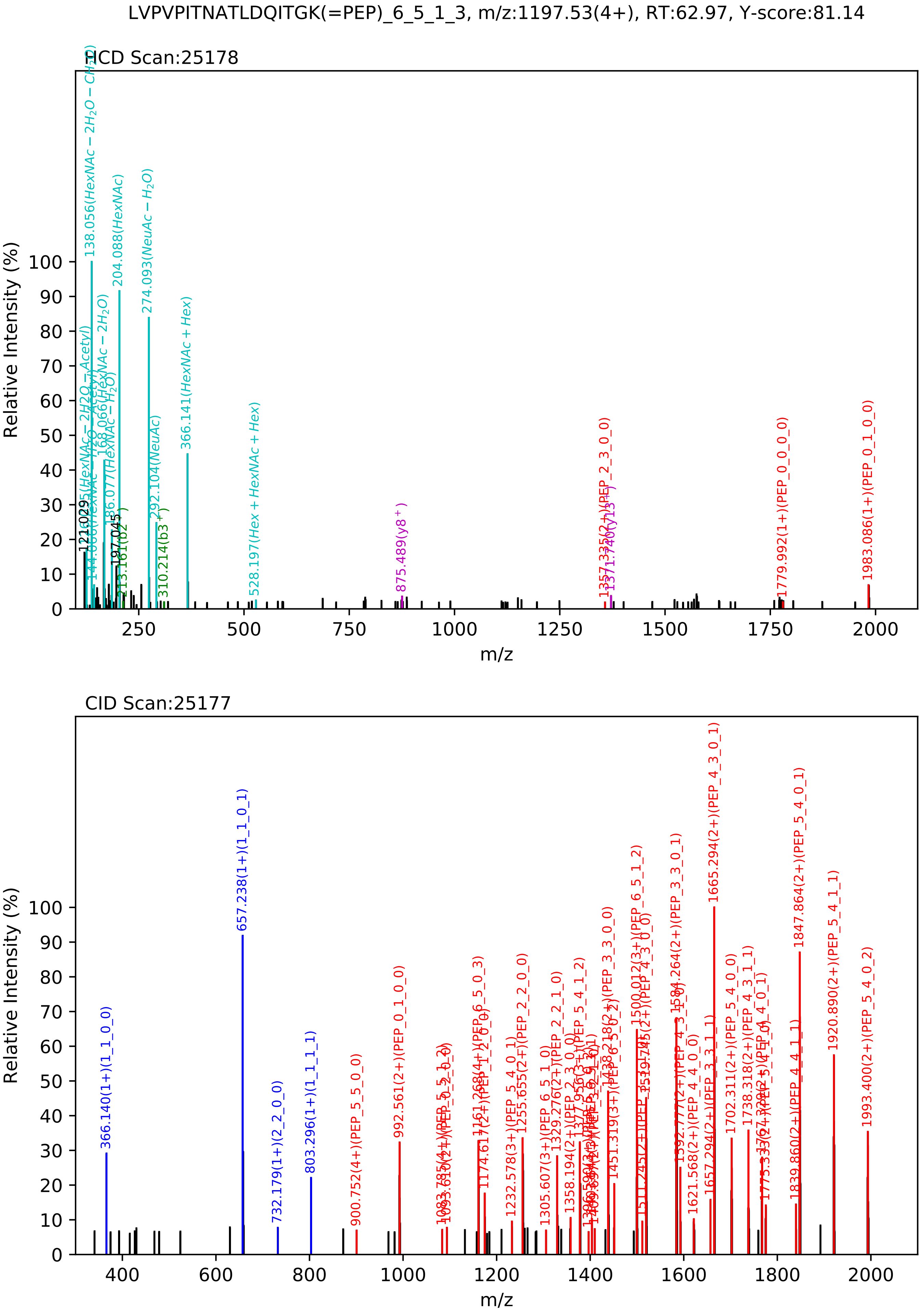

Test set no. 99, Experiment: AGP exp_18

LVPVPITNATLDQITGK(=PEP)_6_5_2_2, m/z:1548.02(3+), RT:96.58, Y-score:87.09

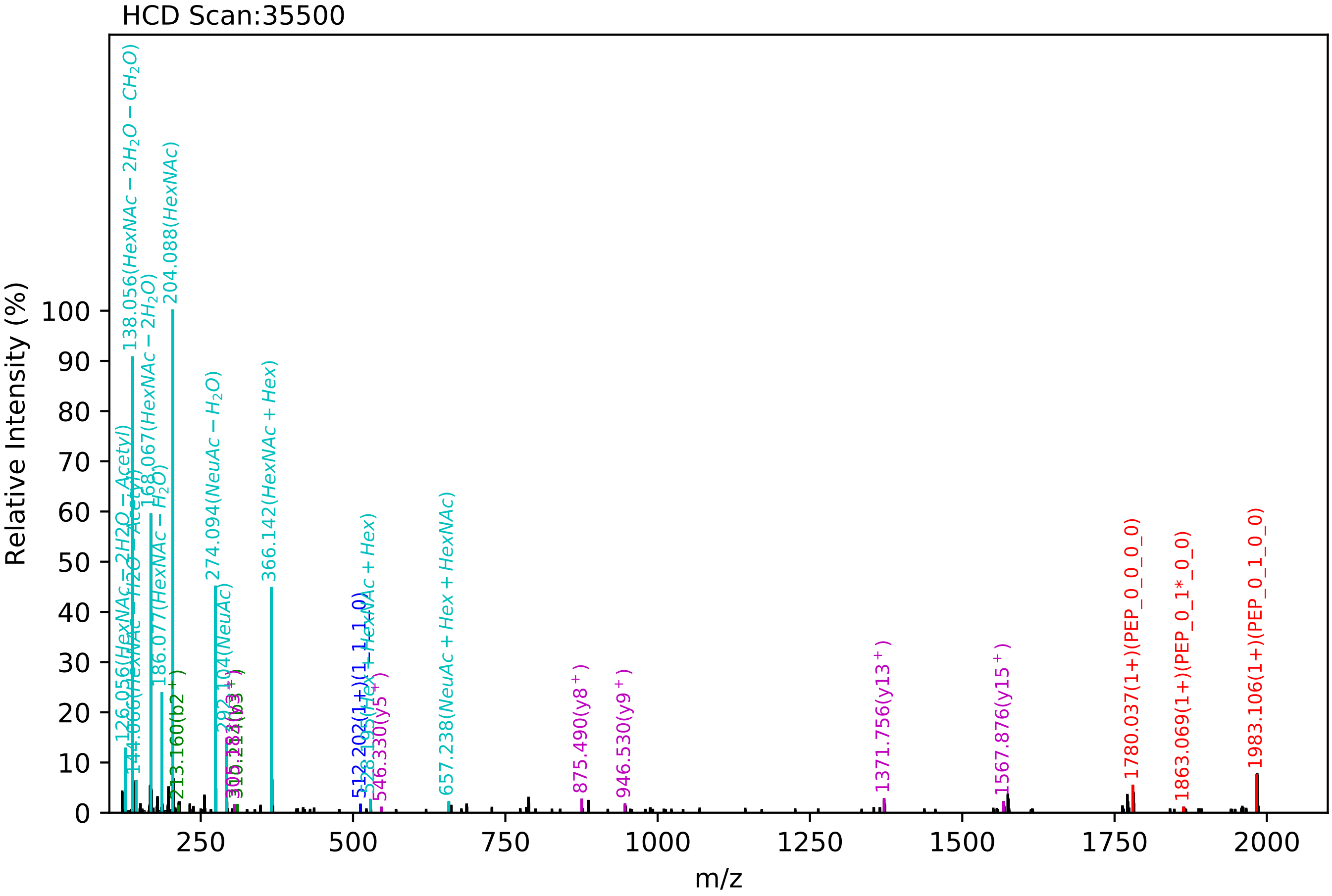

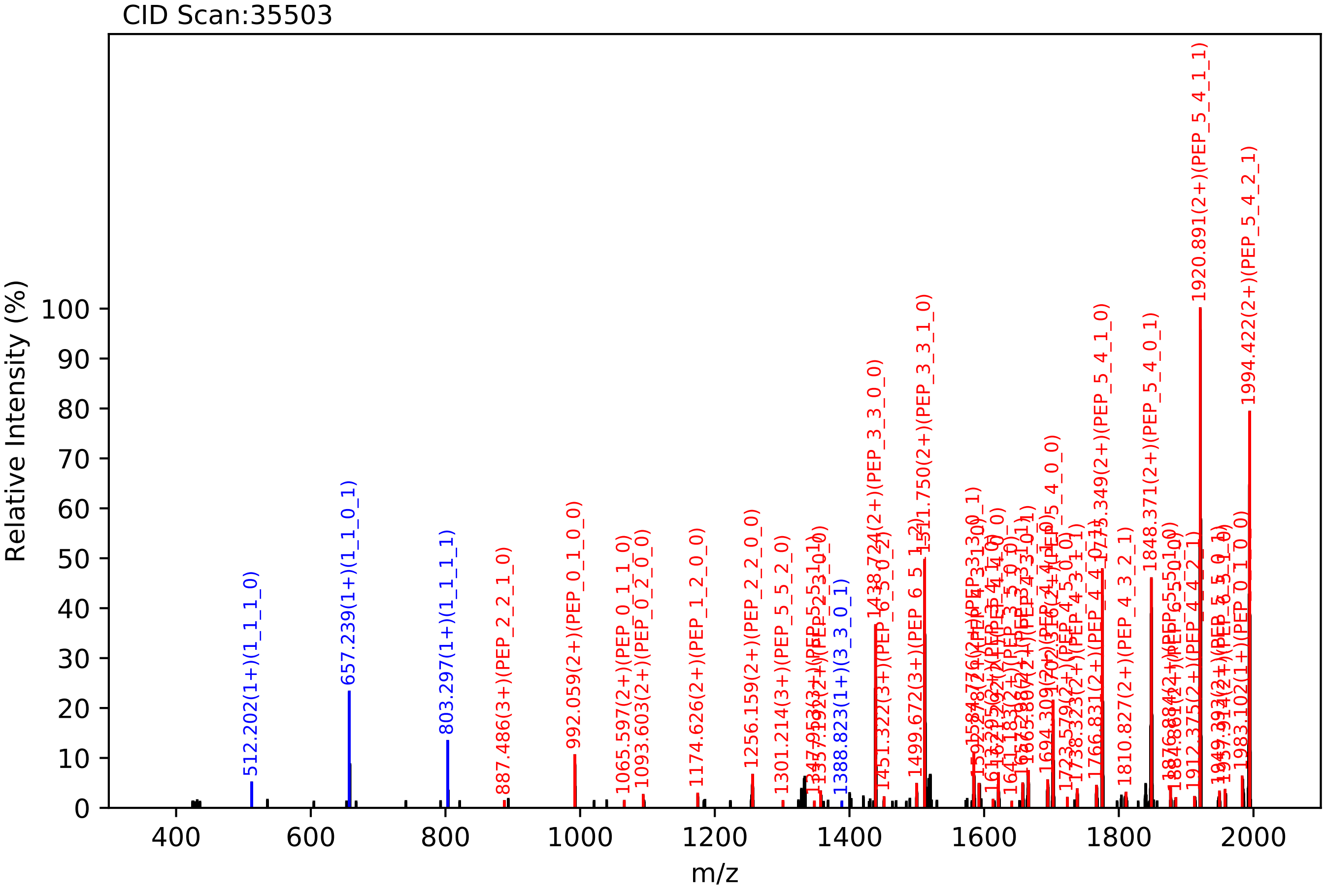

Test set no. 100, Experiment: AGP exp_7

LVPVPITNATLDQITGK(=PEP)_6_5_2_3, m/z:1234.04(4+), RT:83.42, Y-score:84.50

HCD Scan:29852

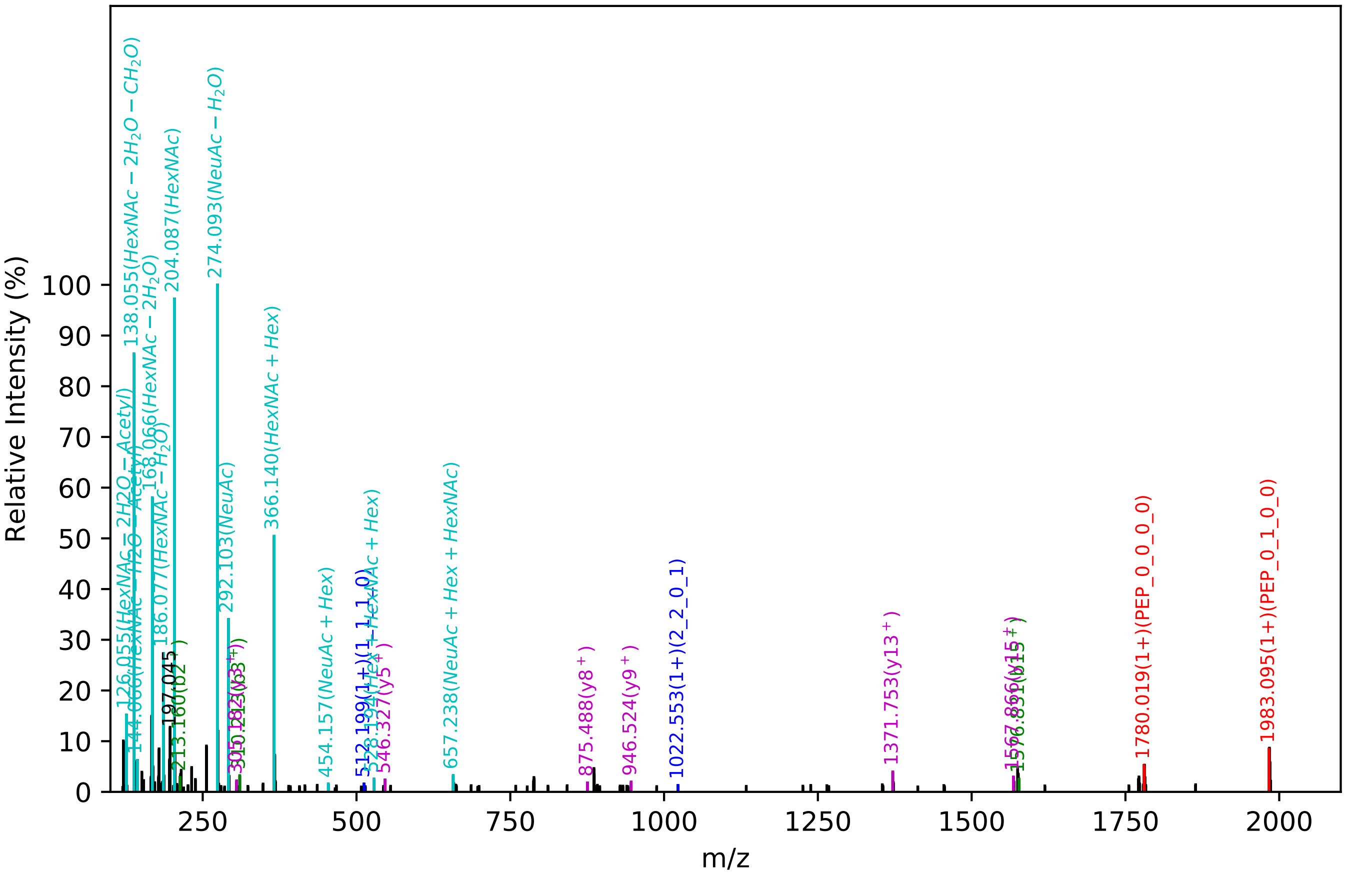

CID Scan:29857

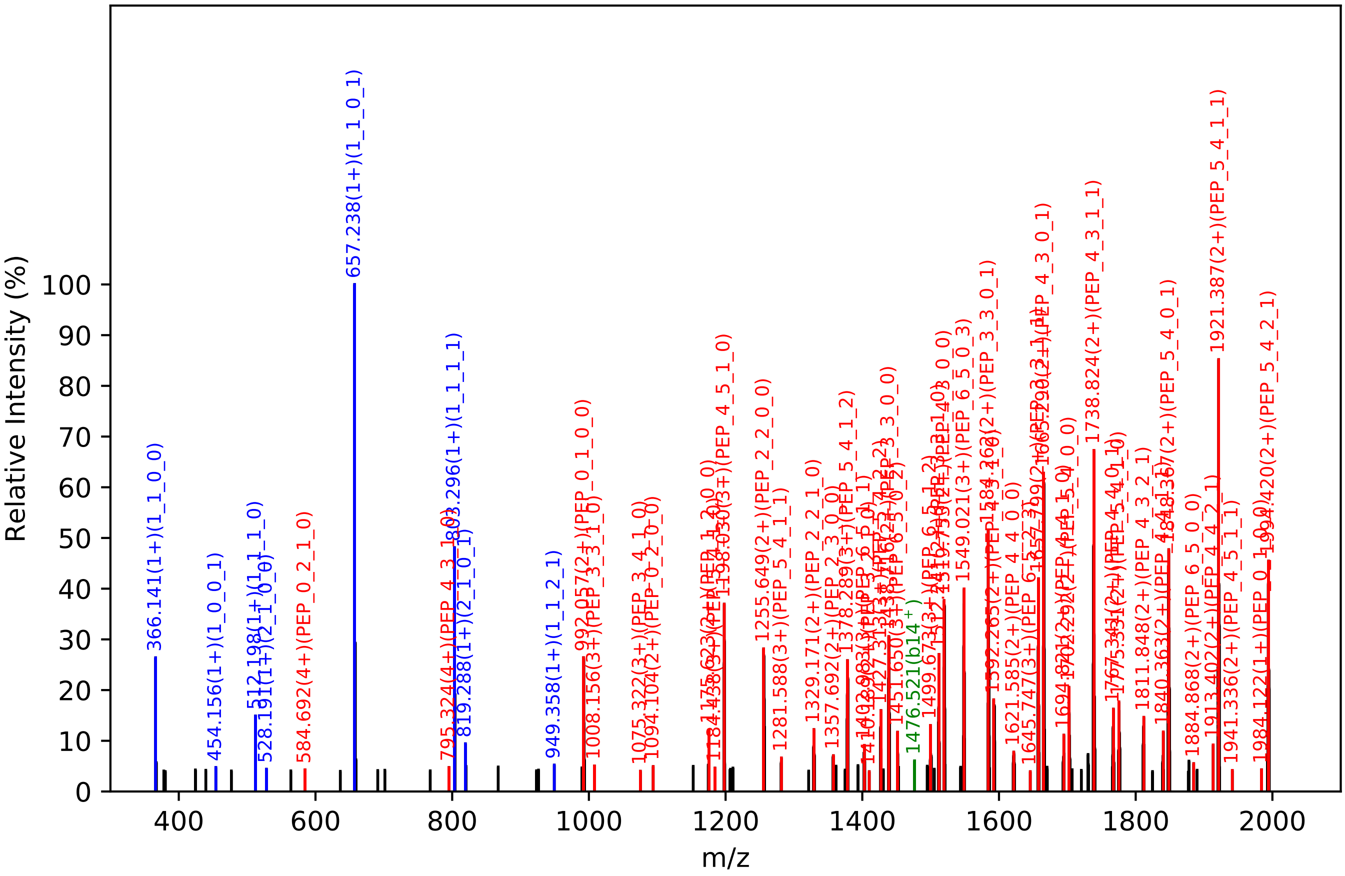

Test set no. 101, Experiment: AGP exp_38

LVPVPITNATLDQITGK(=PEP)_6_5_2_3, m/z:1234.04(4+), RT:83.98, Y-score:80.43

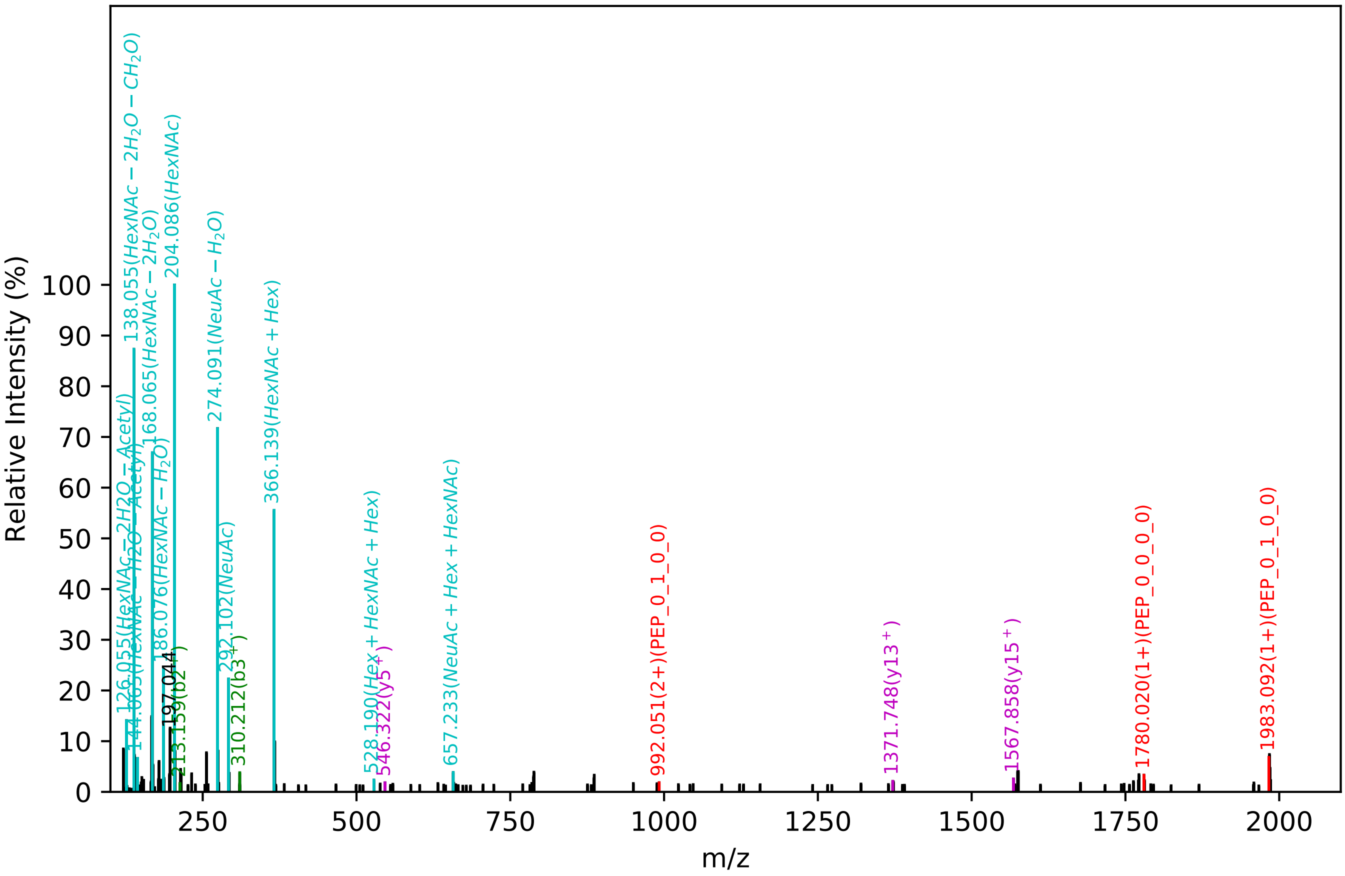

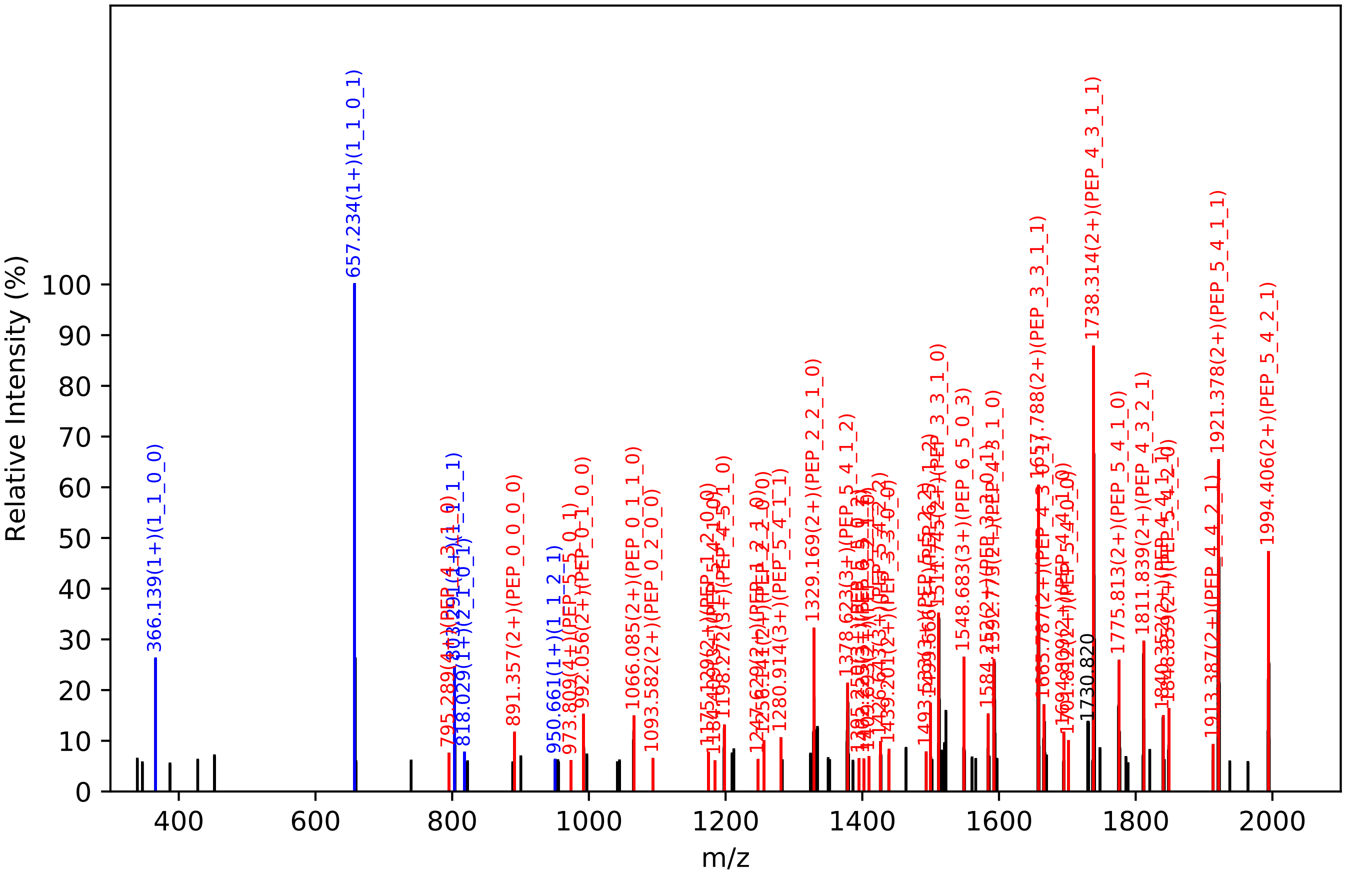

Test set no. 102, Experiment: AGP exp_32

LVPVPITNATLDQITGK(=PEP)_6_5_2_3, m/z:1234.04(4+), RT:82.70, Y-score:79.77

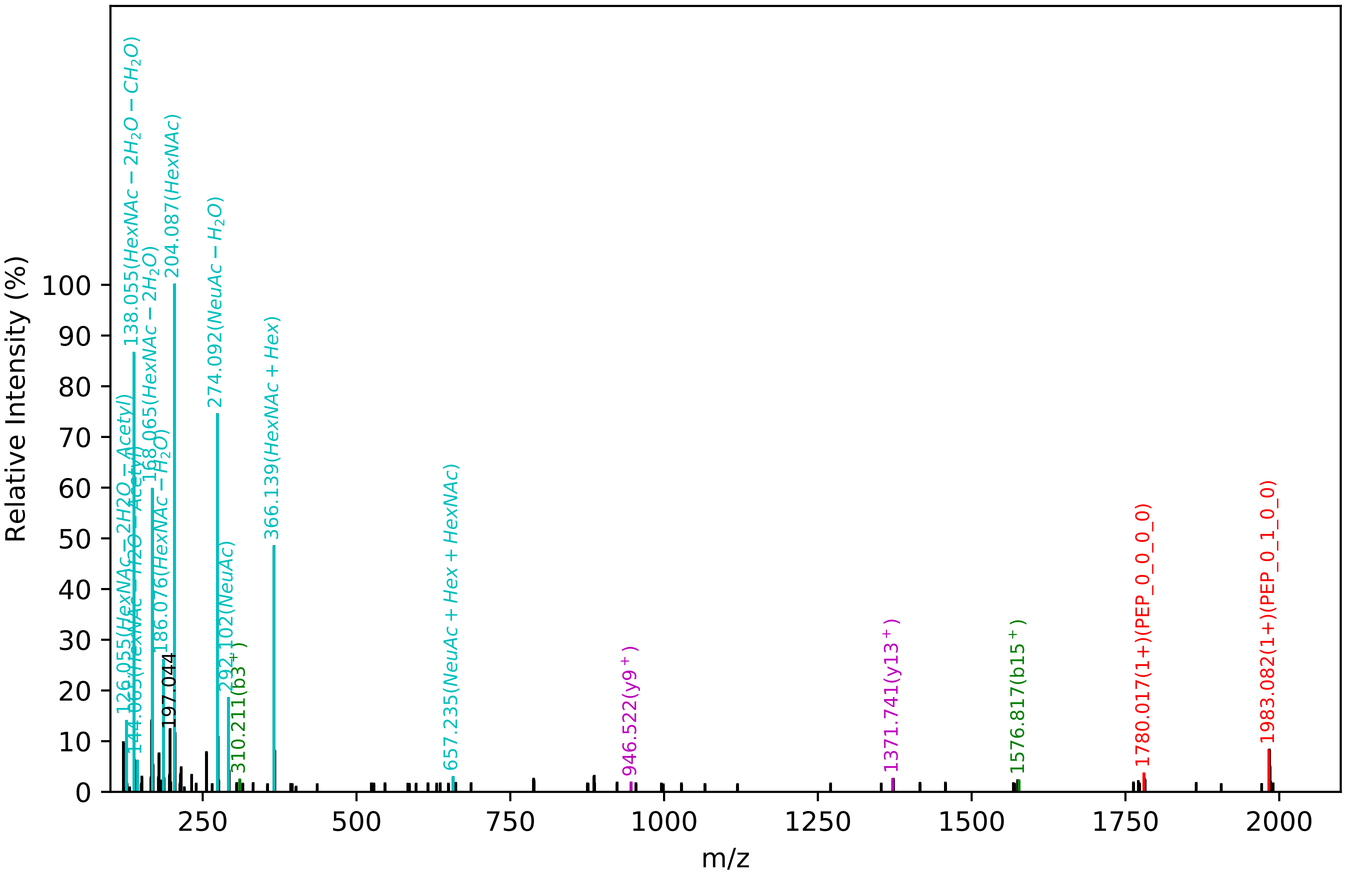

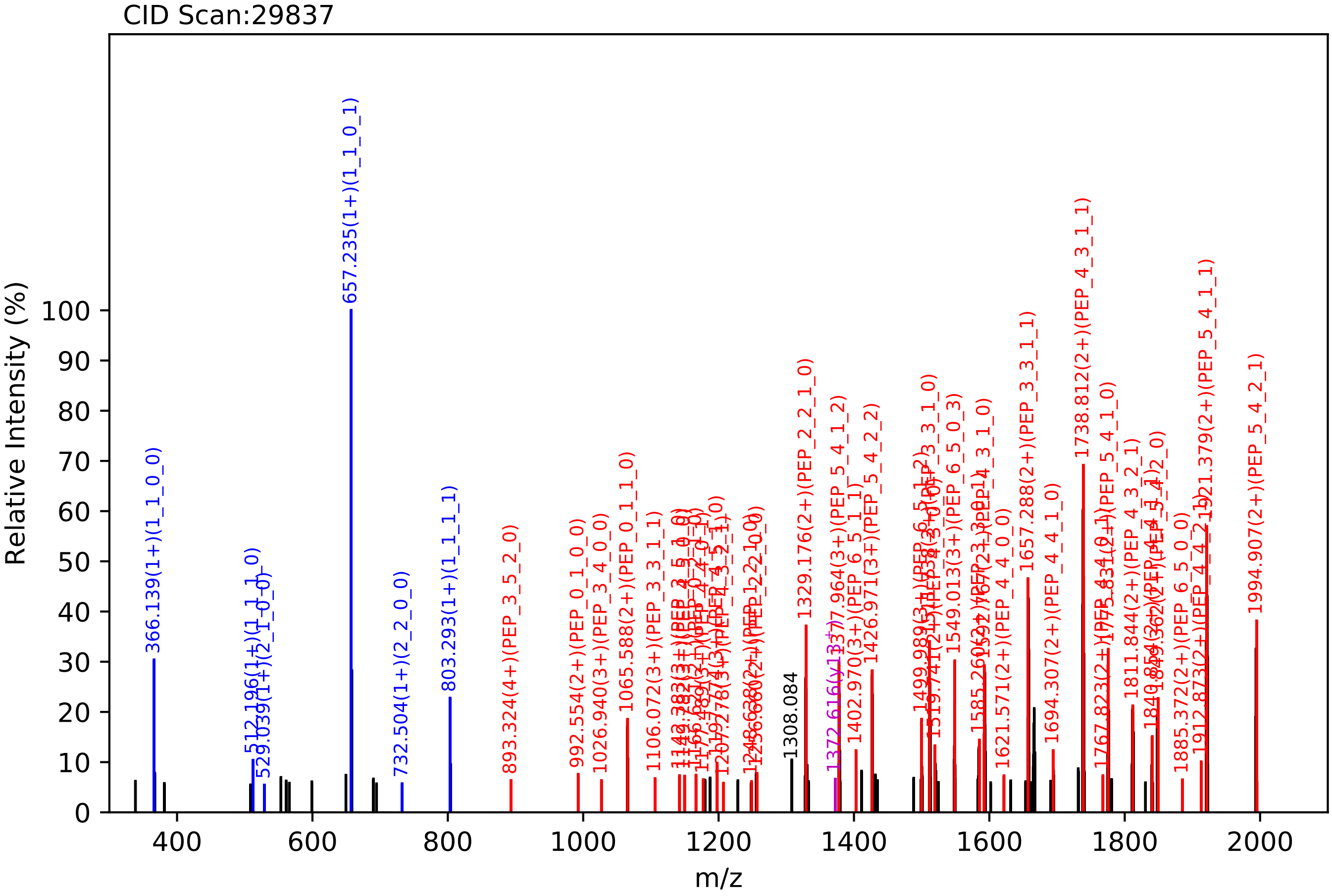

Test set no. 103, Experiment: AGP exp_30

LVPVPITNATLDQITGK(=PEP)_6_5_2_3, m/z:1234.04(4+), RT:83.14, Y-score:77.05

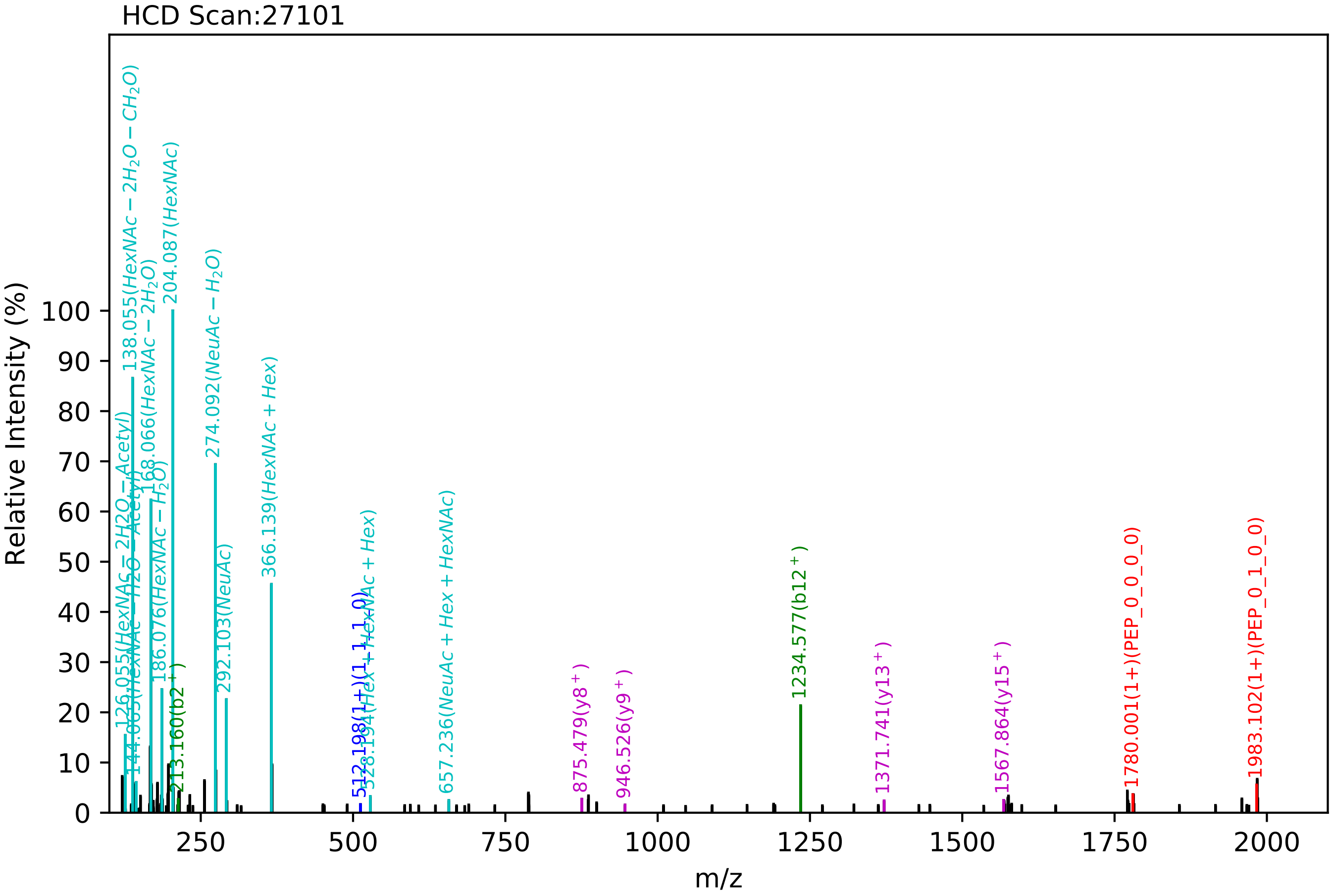

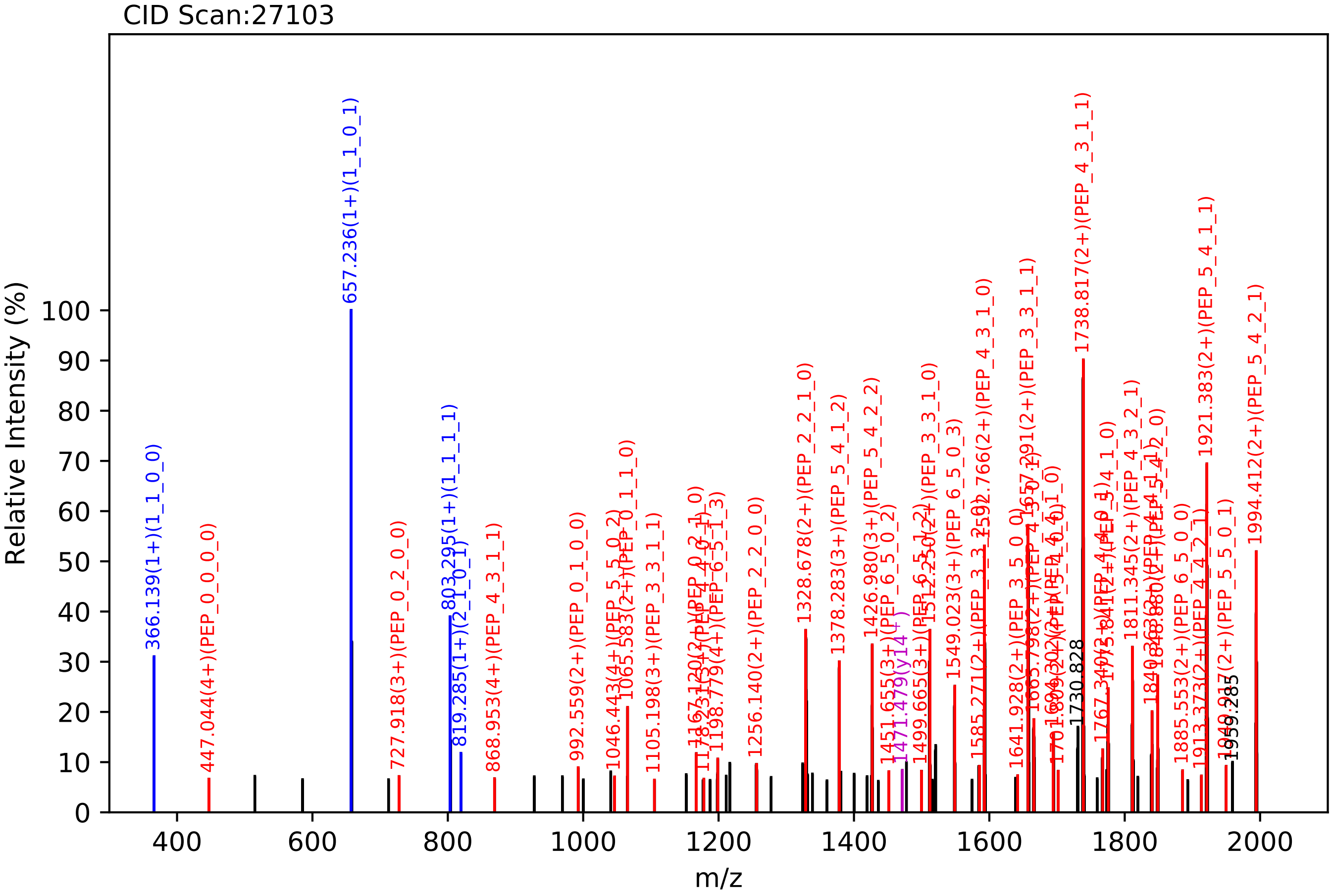

Test set no. 104, Experiment: AGP exp_4

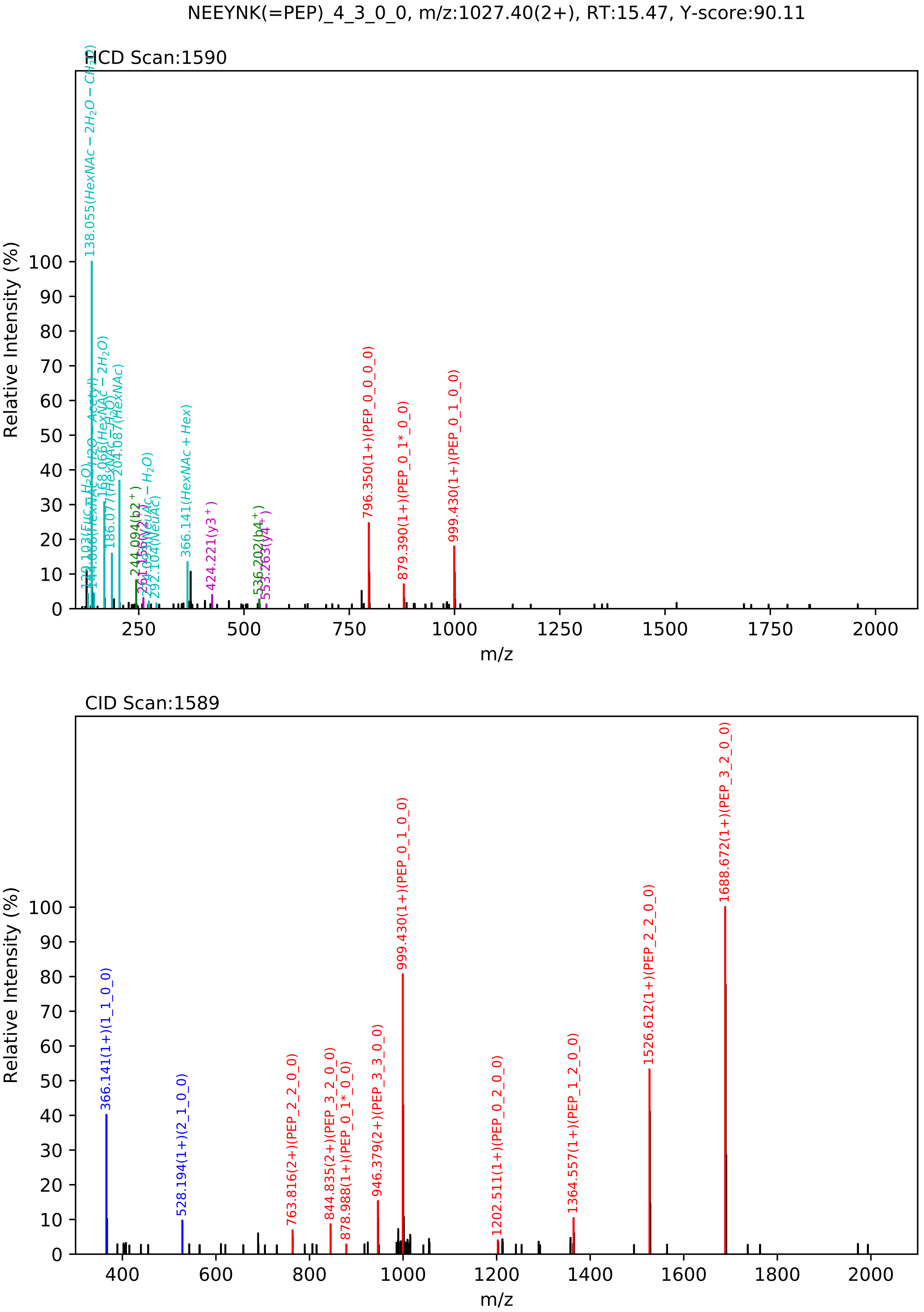

Test set no. 105, Experiment: AGP exp_3

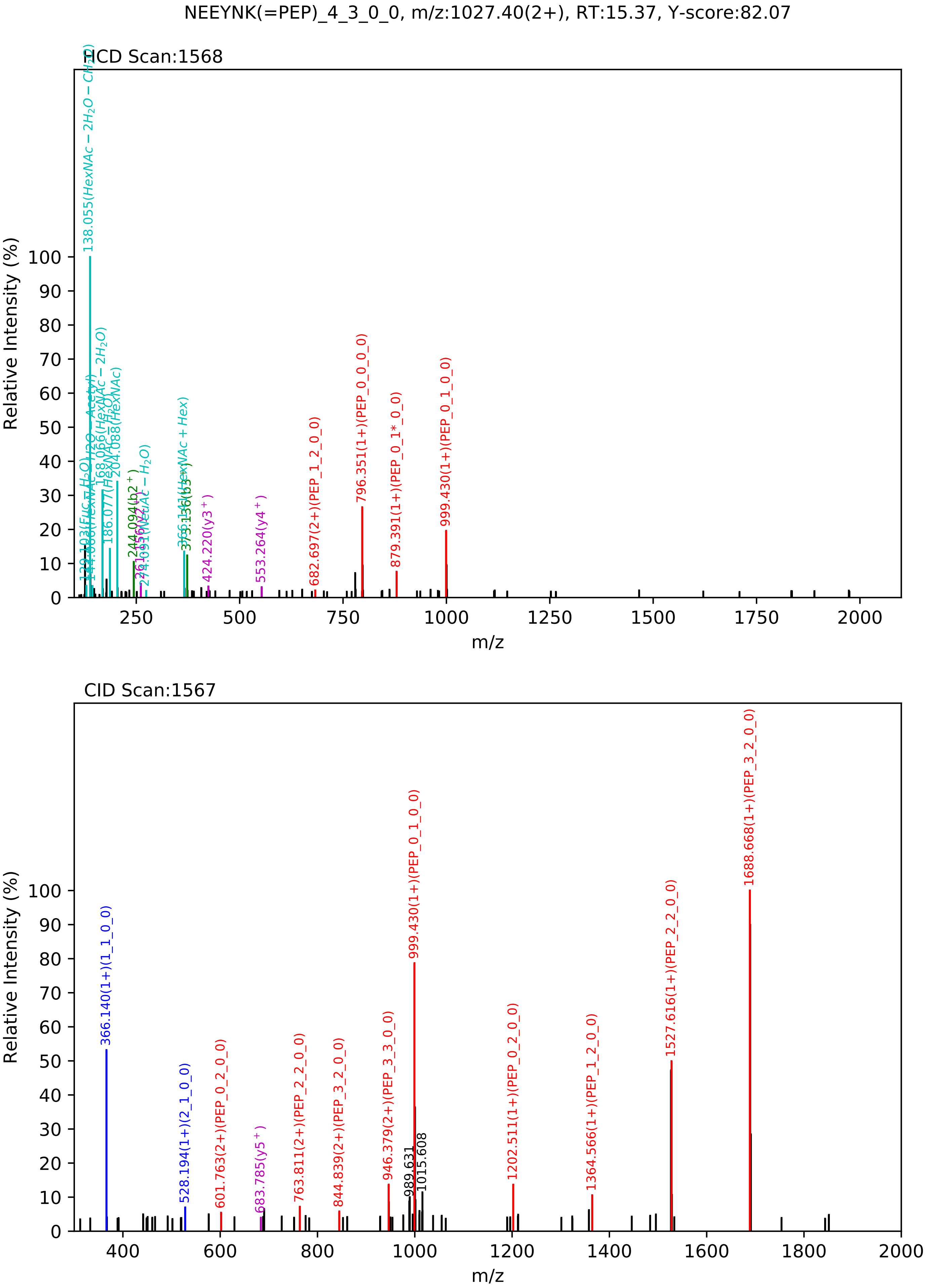

Test set no. 106, Experiment: AGP exp_4

NEEYNK(=PEP)_4_3_0_1, m/z:1172.95(2+), RT:16.18, Y-score:94.93

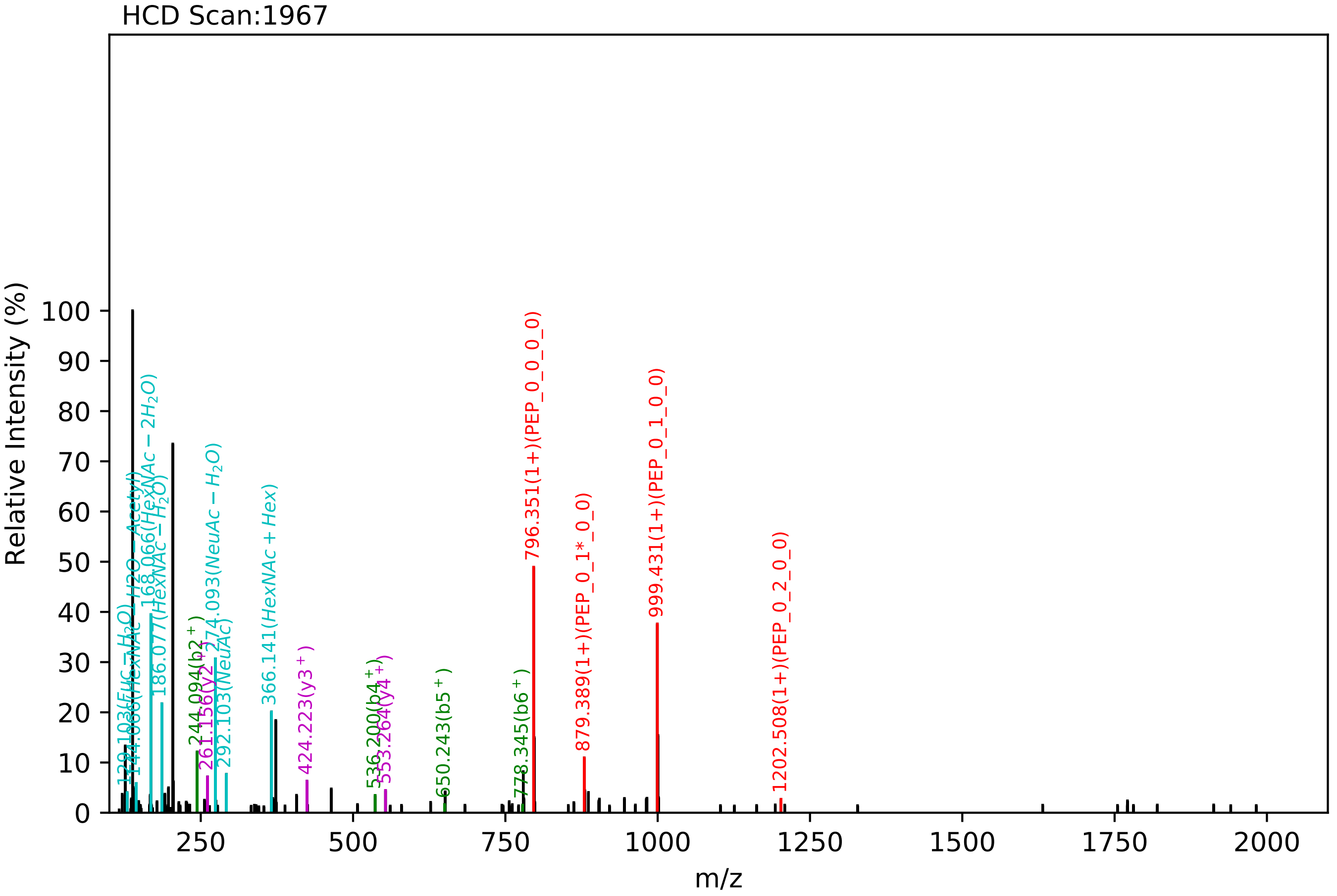

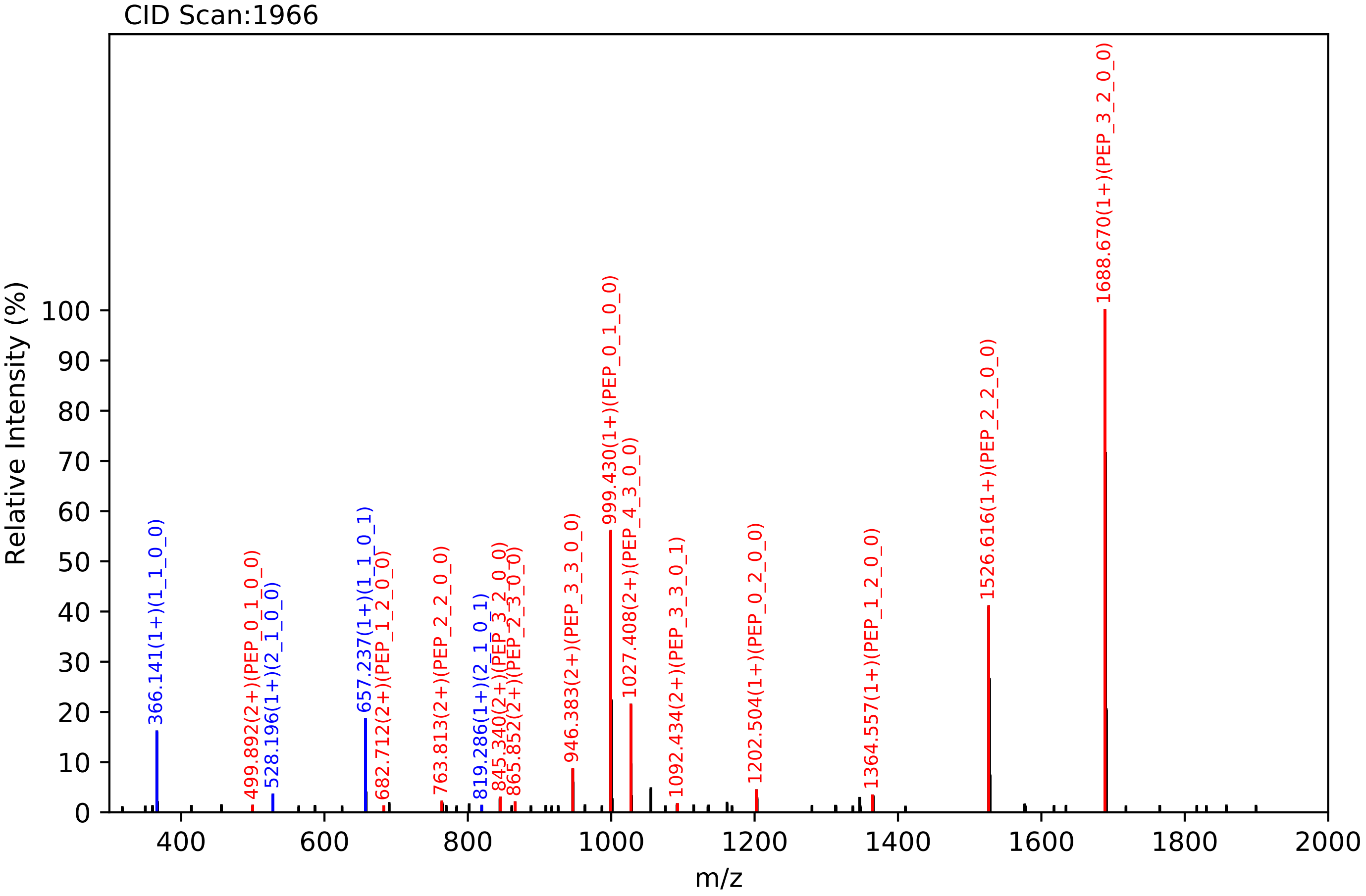

Test set no. 107, Experiment: AGP exp_3

NEEYNK(=PEP)_4_3_0_1, m/z:1172.95(2+), RT:14.57, Y-score:92.52

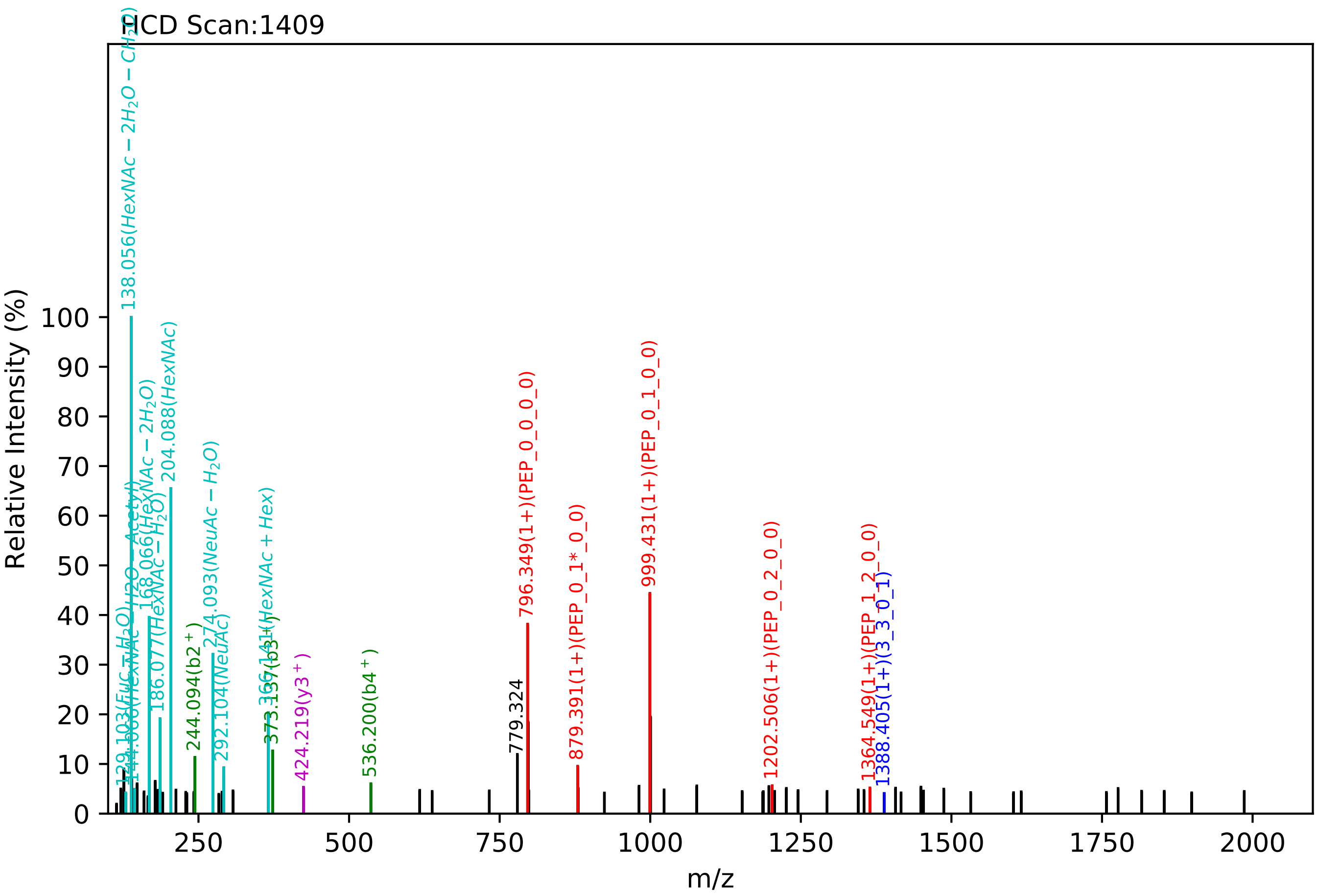

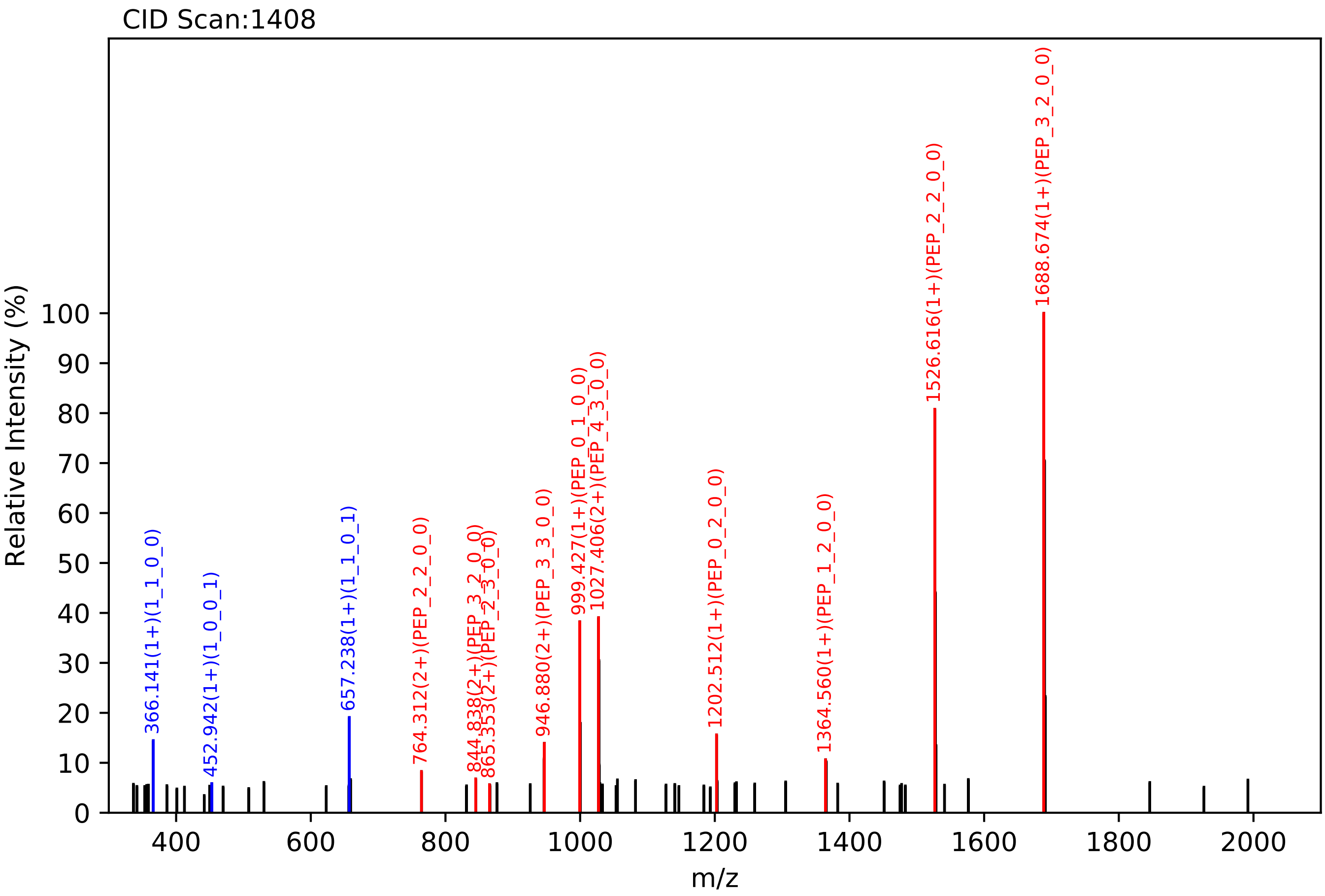

Test set no. 108, Experiment: AGP exp_3

NEEYNK(=PEP)_4_3_0_1, m/z:1172.95(2+), RT:16.07, Y-score:92.34

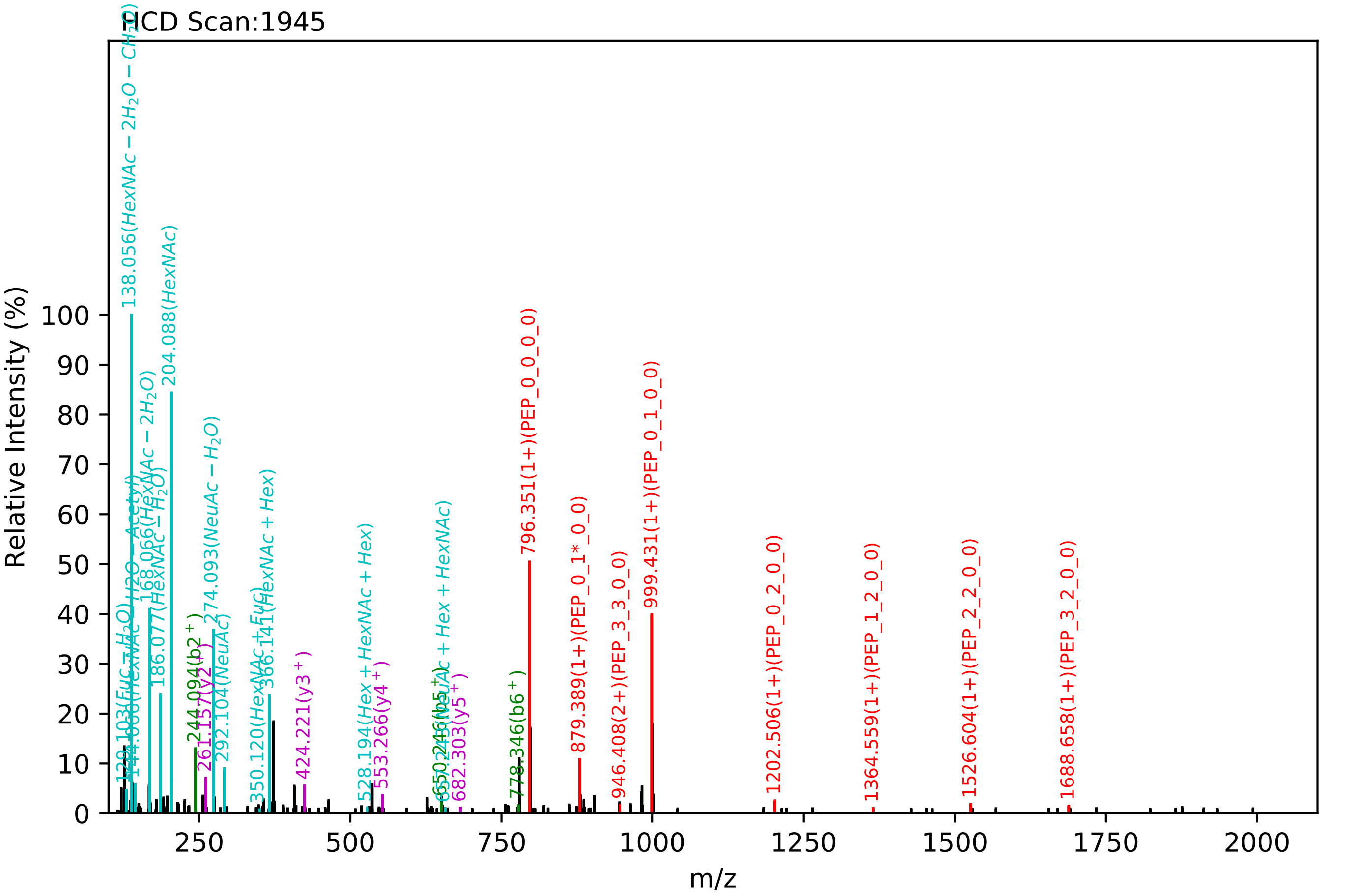

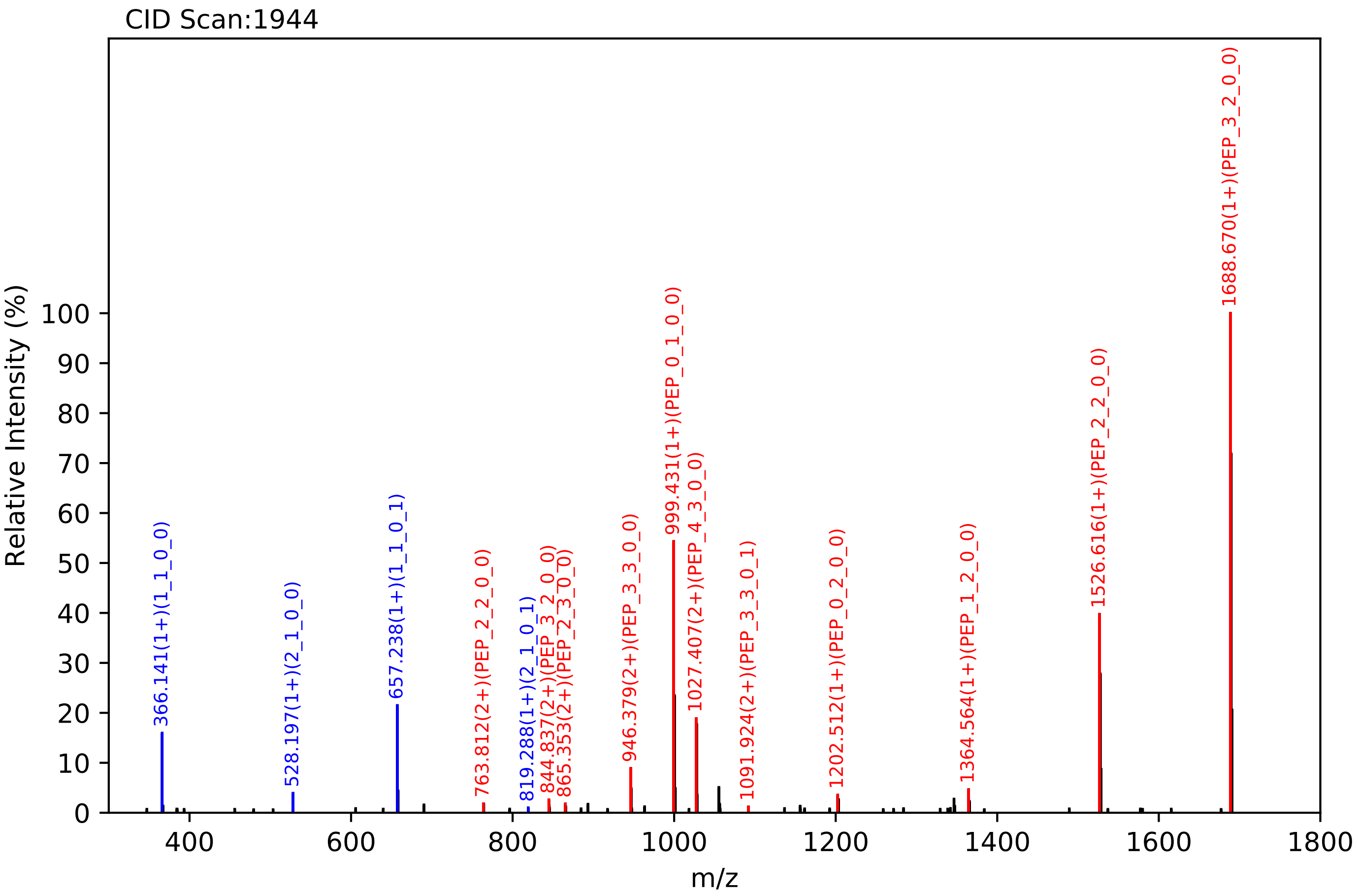

Test set no. 109, Experiment: AGP exp_4

NEEYNK(=PEP)_4_3_0_1, m/z:1172.95(2+), RT:14.69, Y-score:81.86

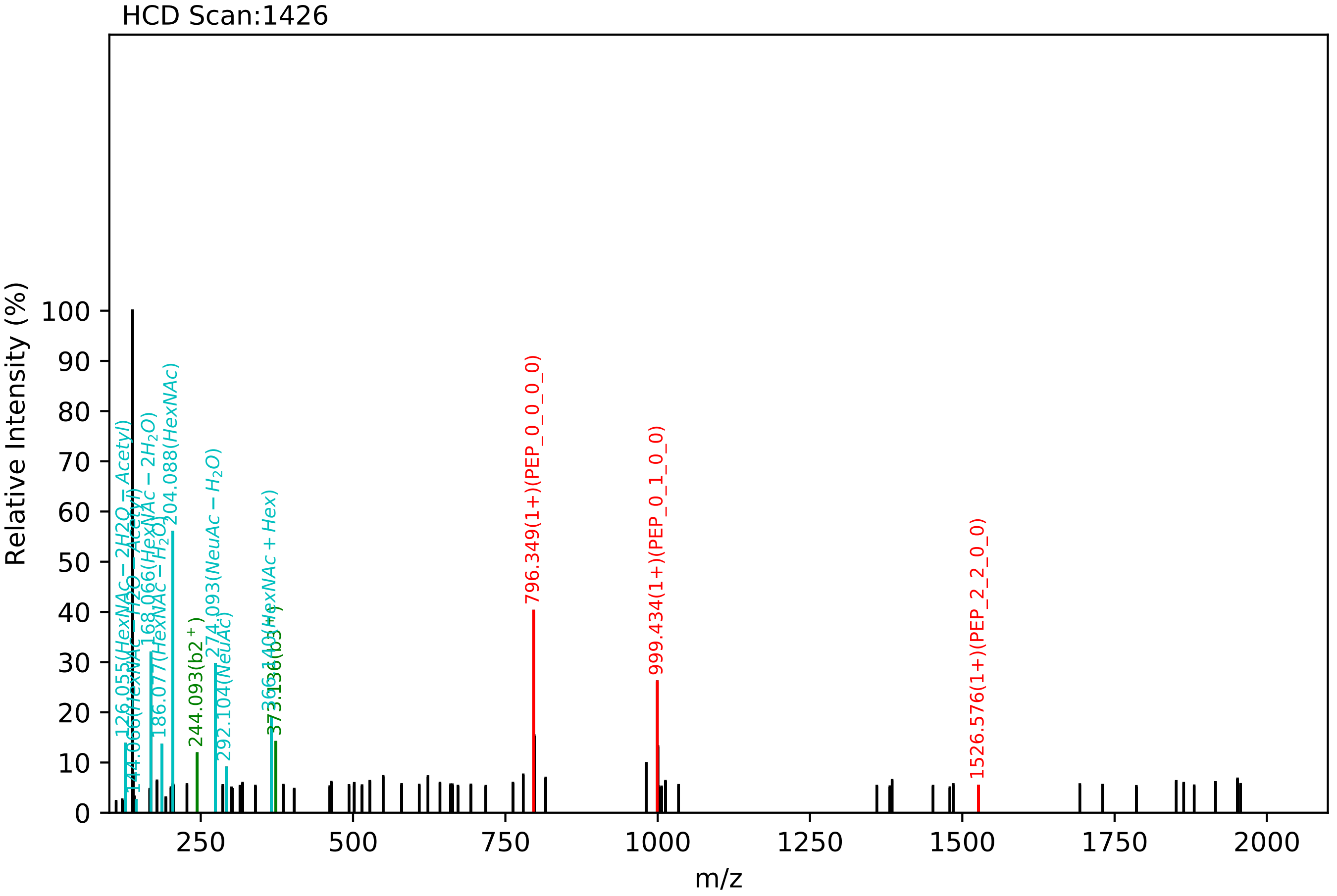

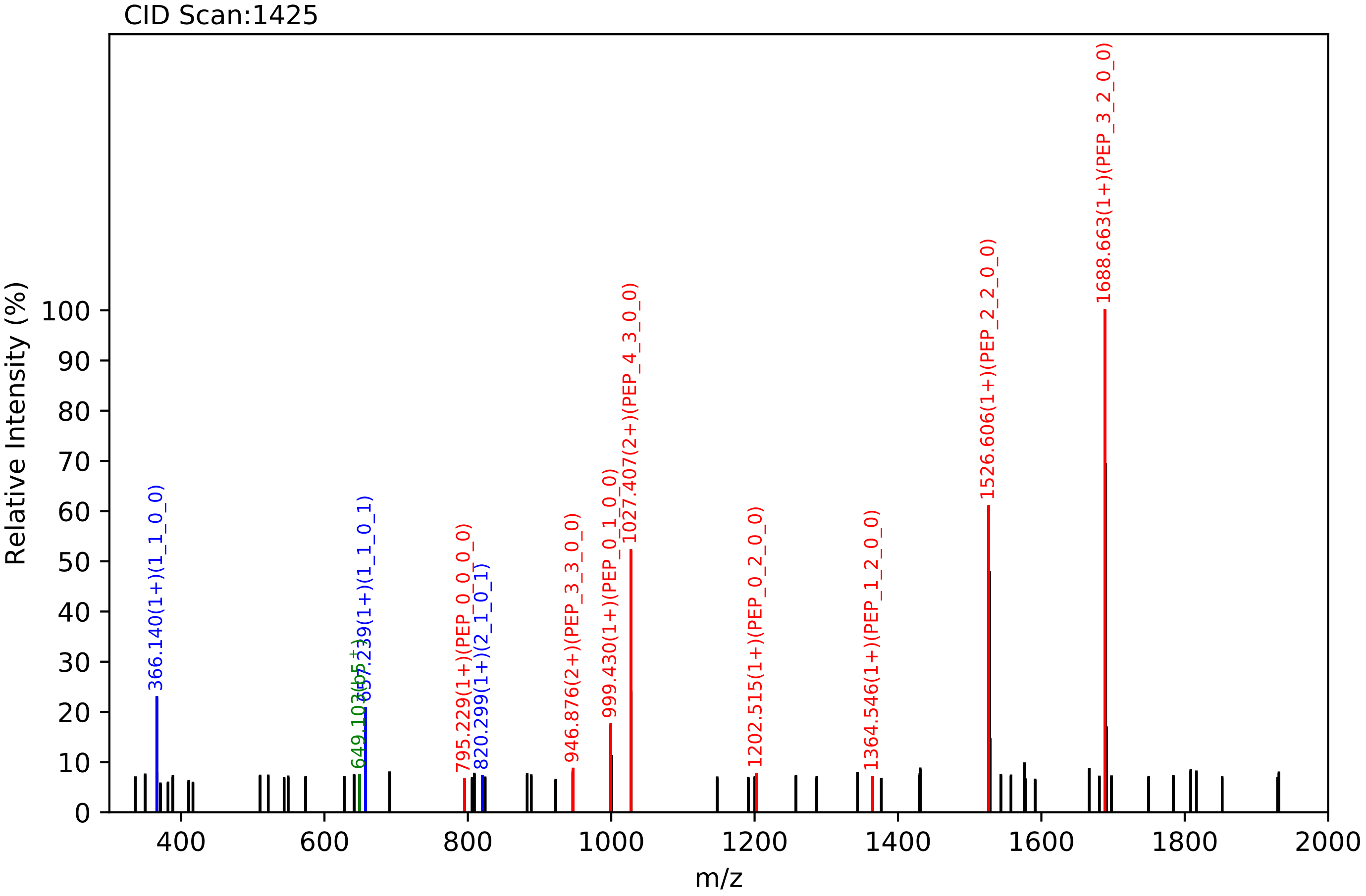

Test set no. 110, Experiment: AGP exp_4

NEEYNK(=PEP)_4_4_0_1, m/z:1274.49(2+), RT:15.36, Y-score:93.63

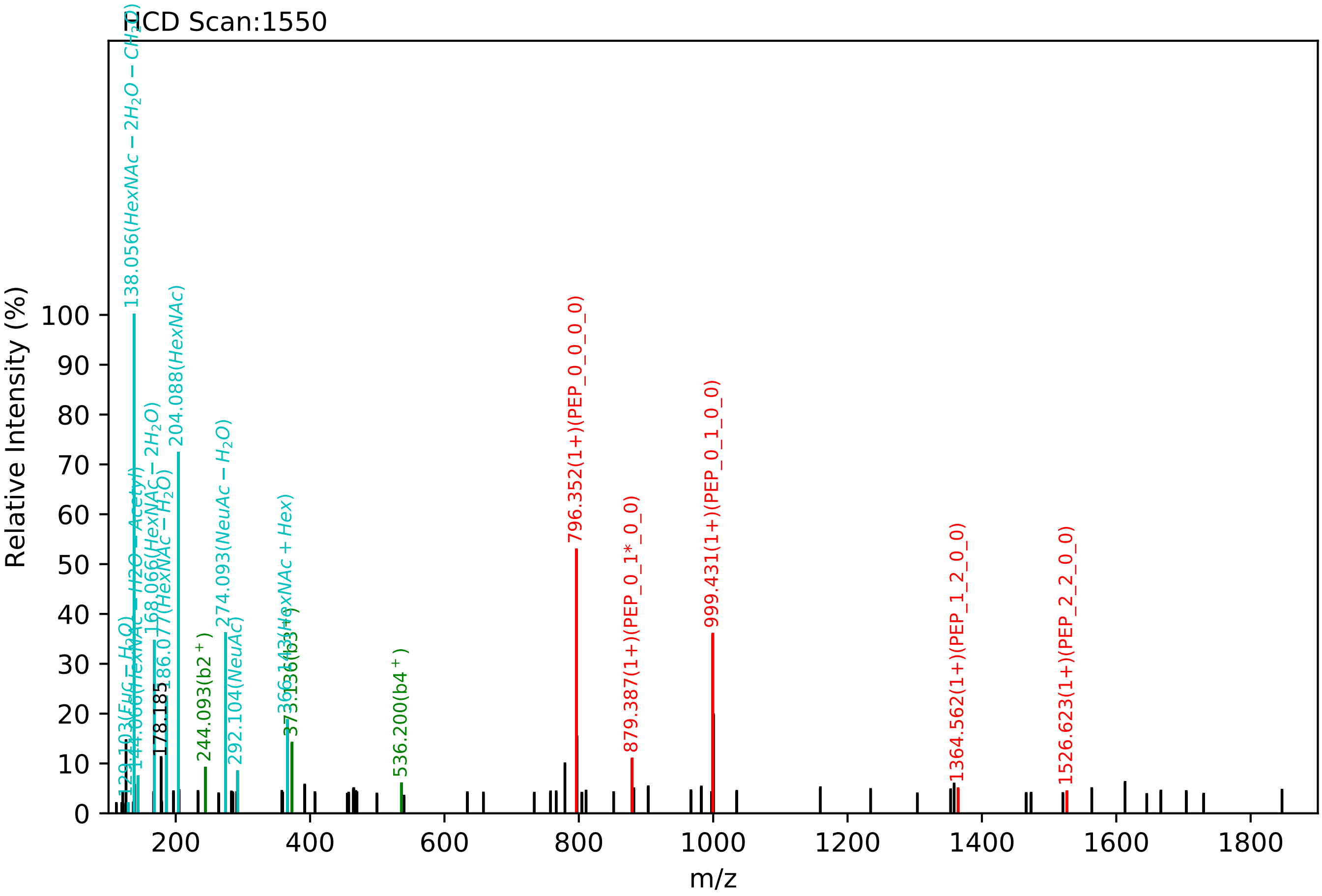

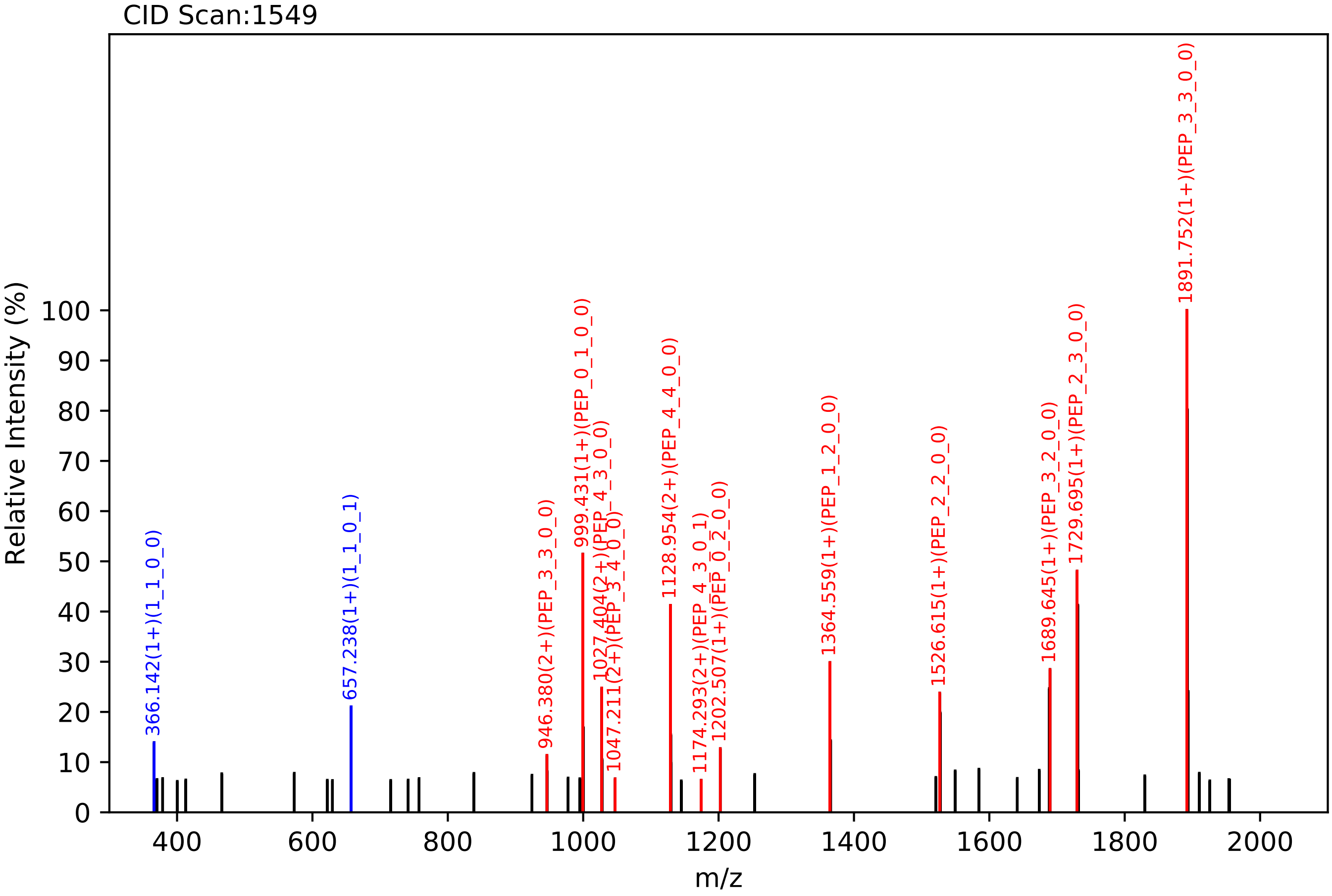

Test set no. 111, Experiment: AGP exp_4

NEEYNK(=PEP)_4_4_0_1, m/z:1274.49(2+), RT:15.86, Y-score:92.80

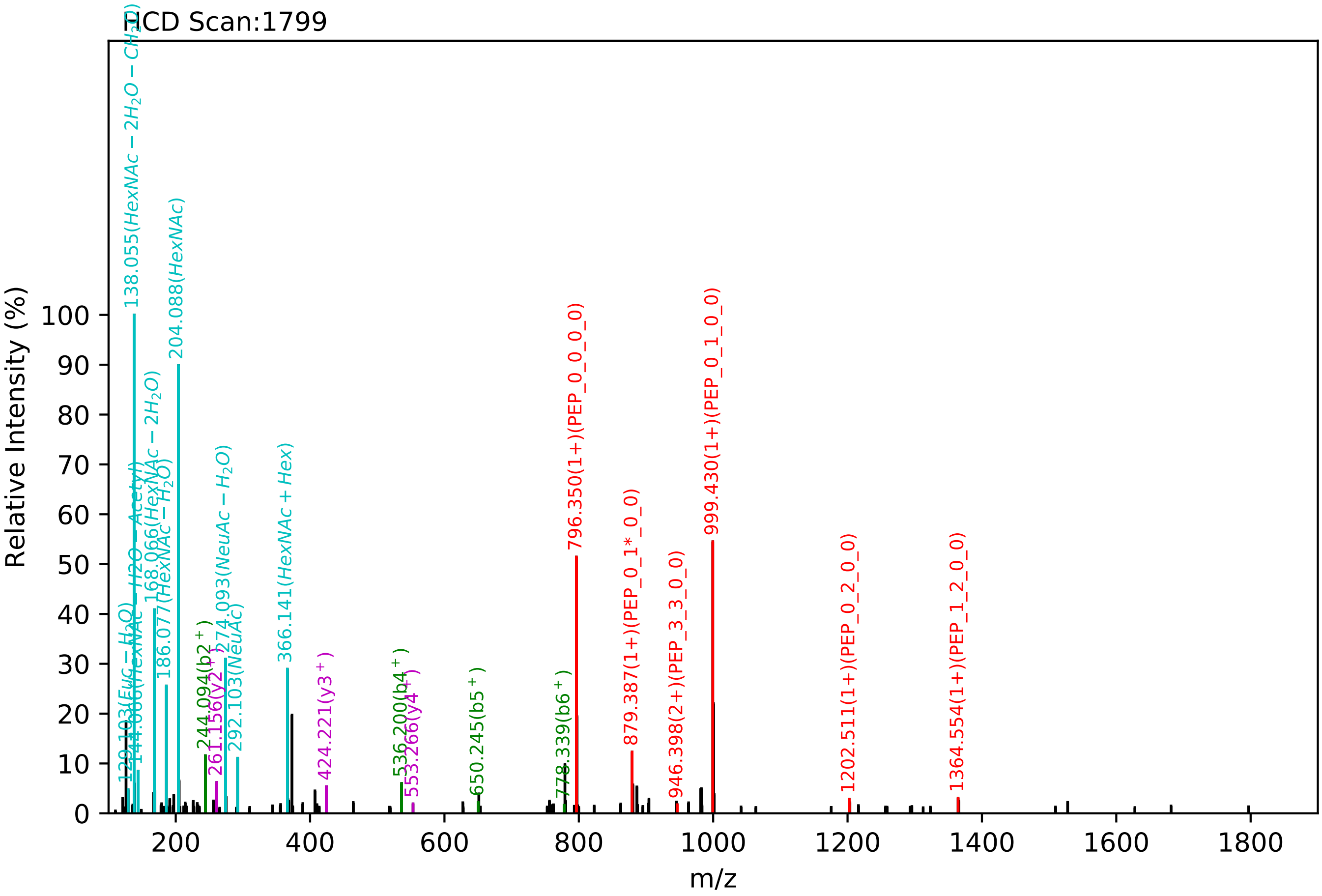

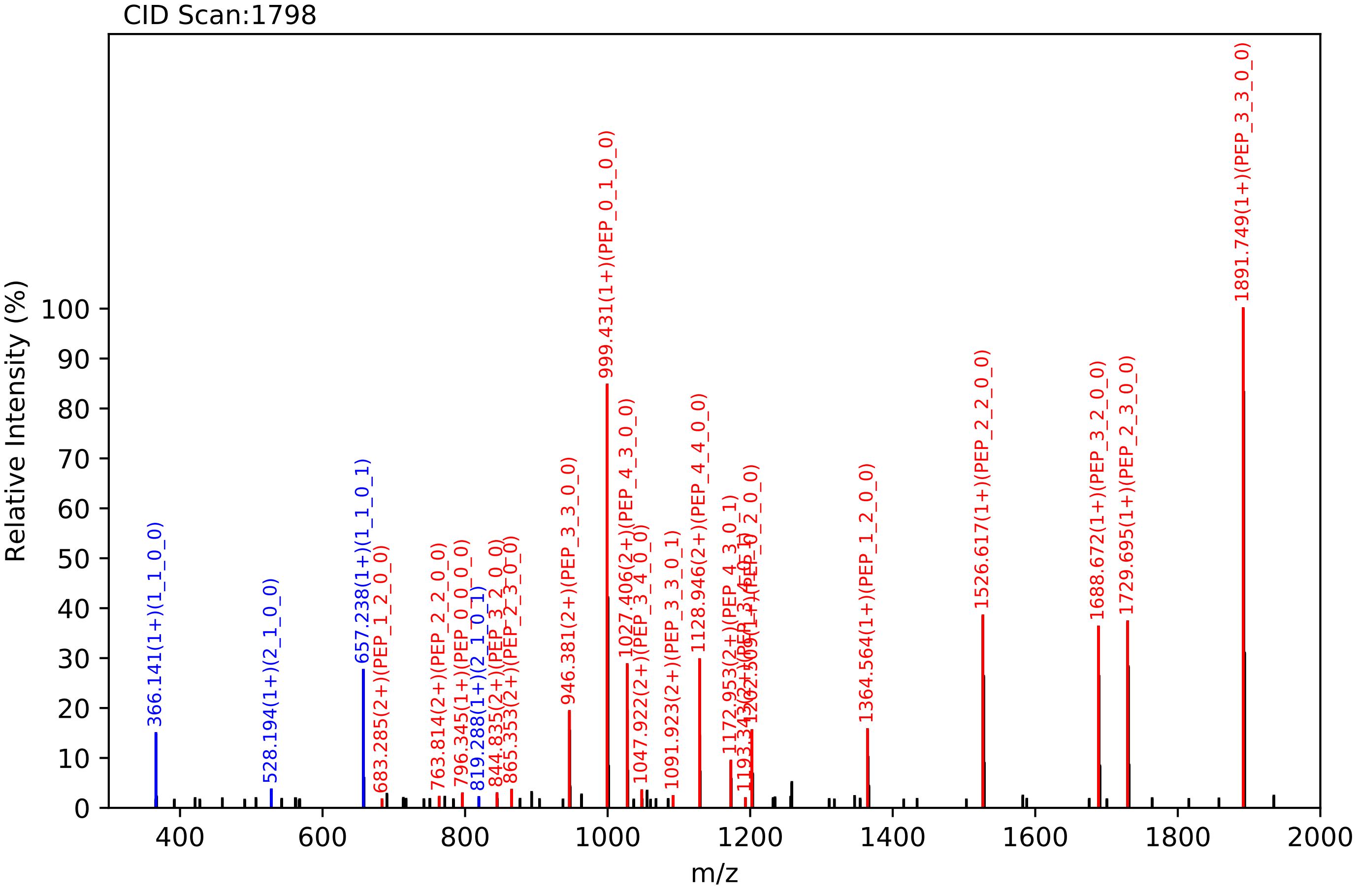

Test set no. 112, Experiment: AGP exp_3

NEEYNK(=PEP)_4_4_0_1, m/z:1274.49(2+), RT:15.78, Y-score:92.41

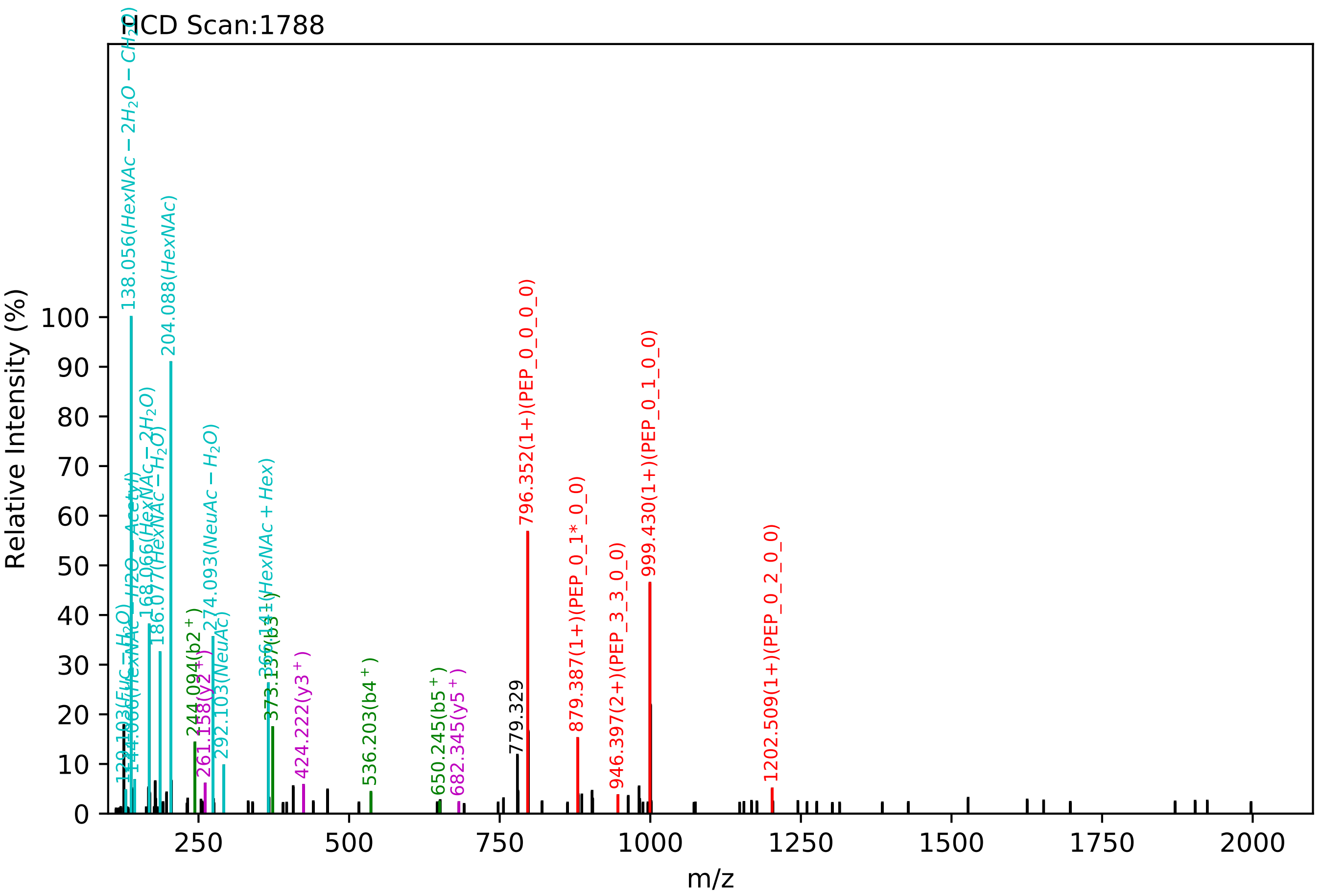

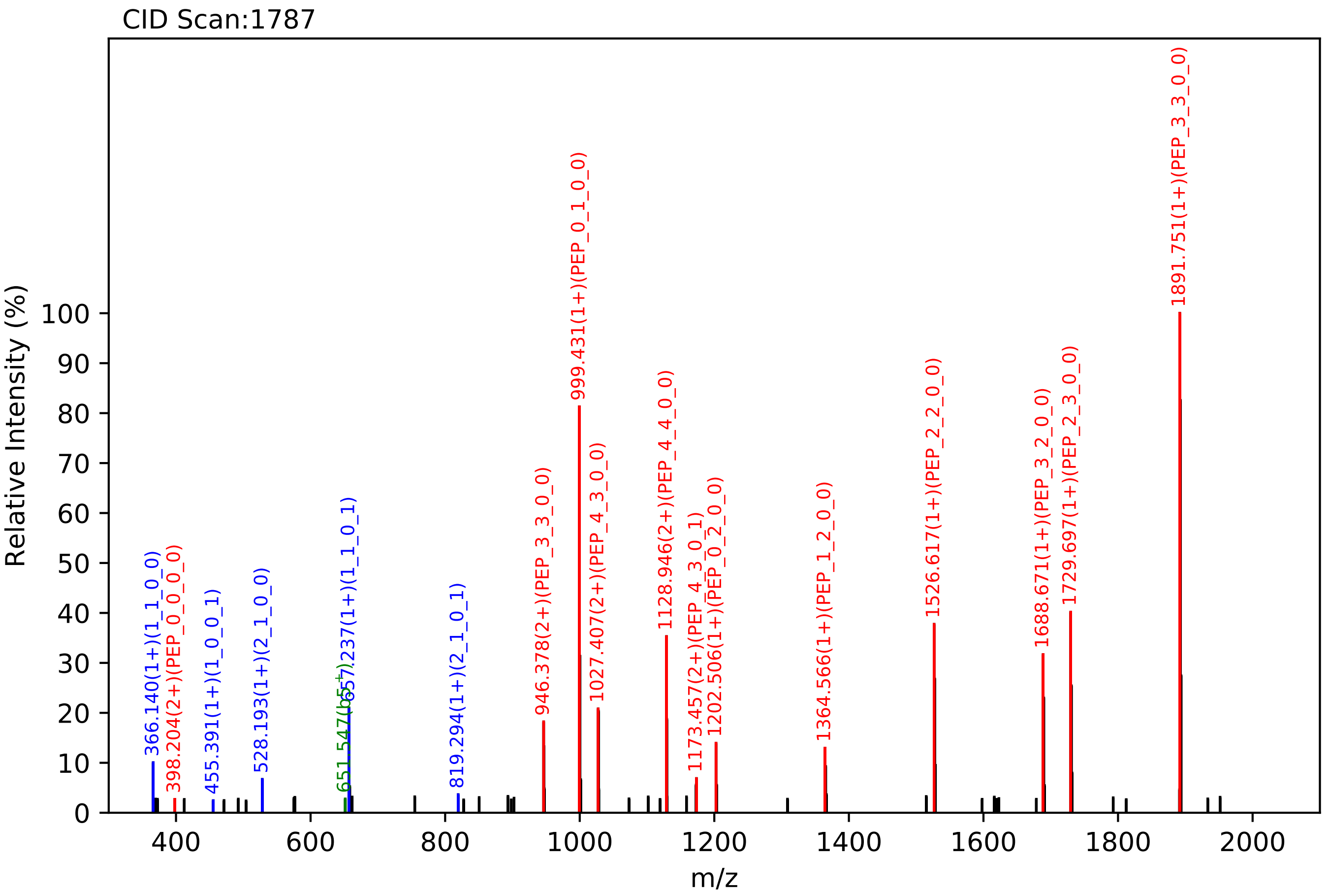

Test set no. 113, Experiment: AGP exp_3

NEEYNK(=PEP)_4_4_0_1, m/z:1274.49(2+), RT:15.26, Y-score:91.55

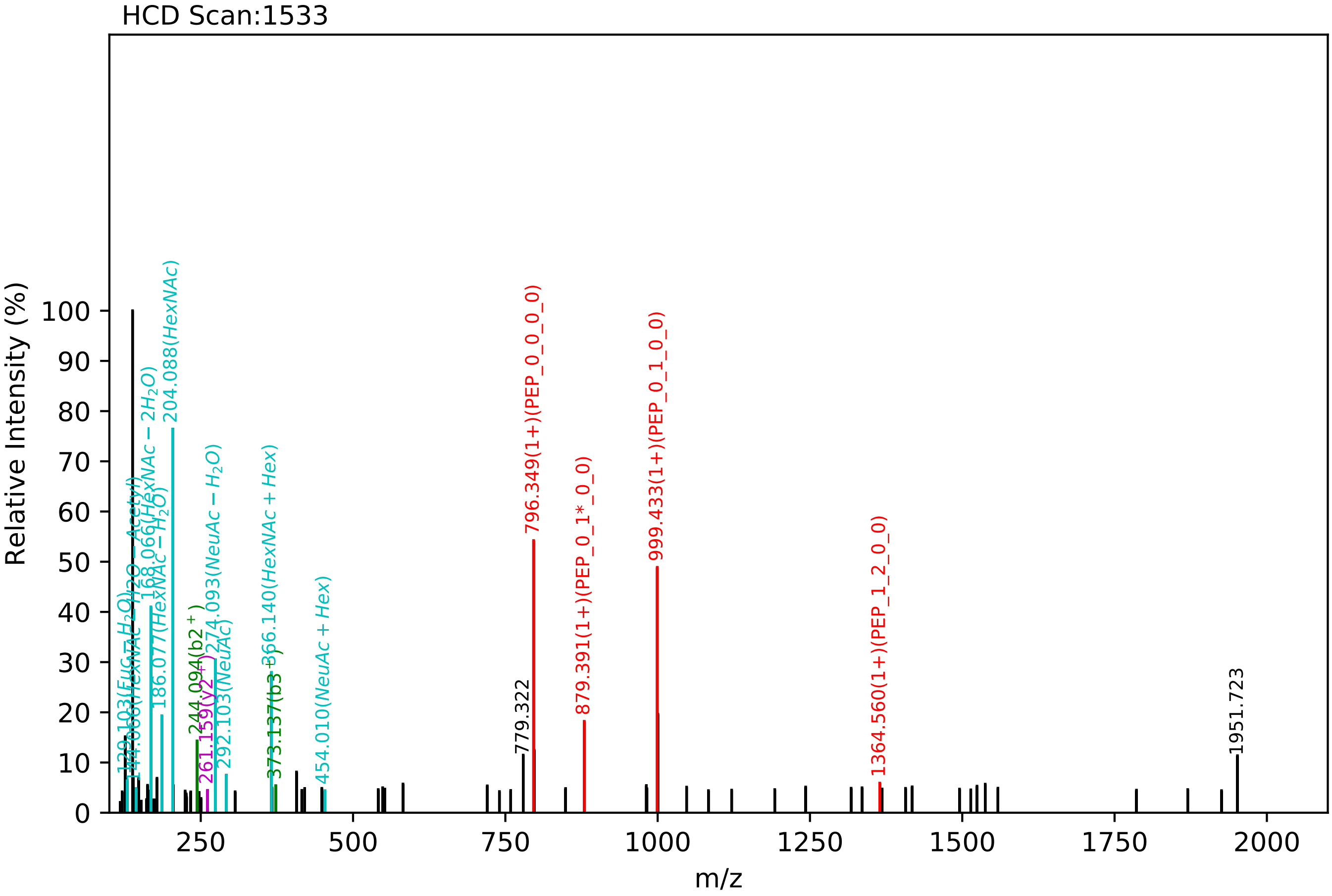

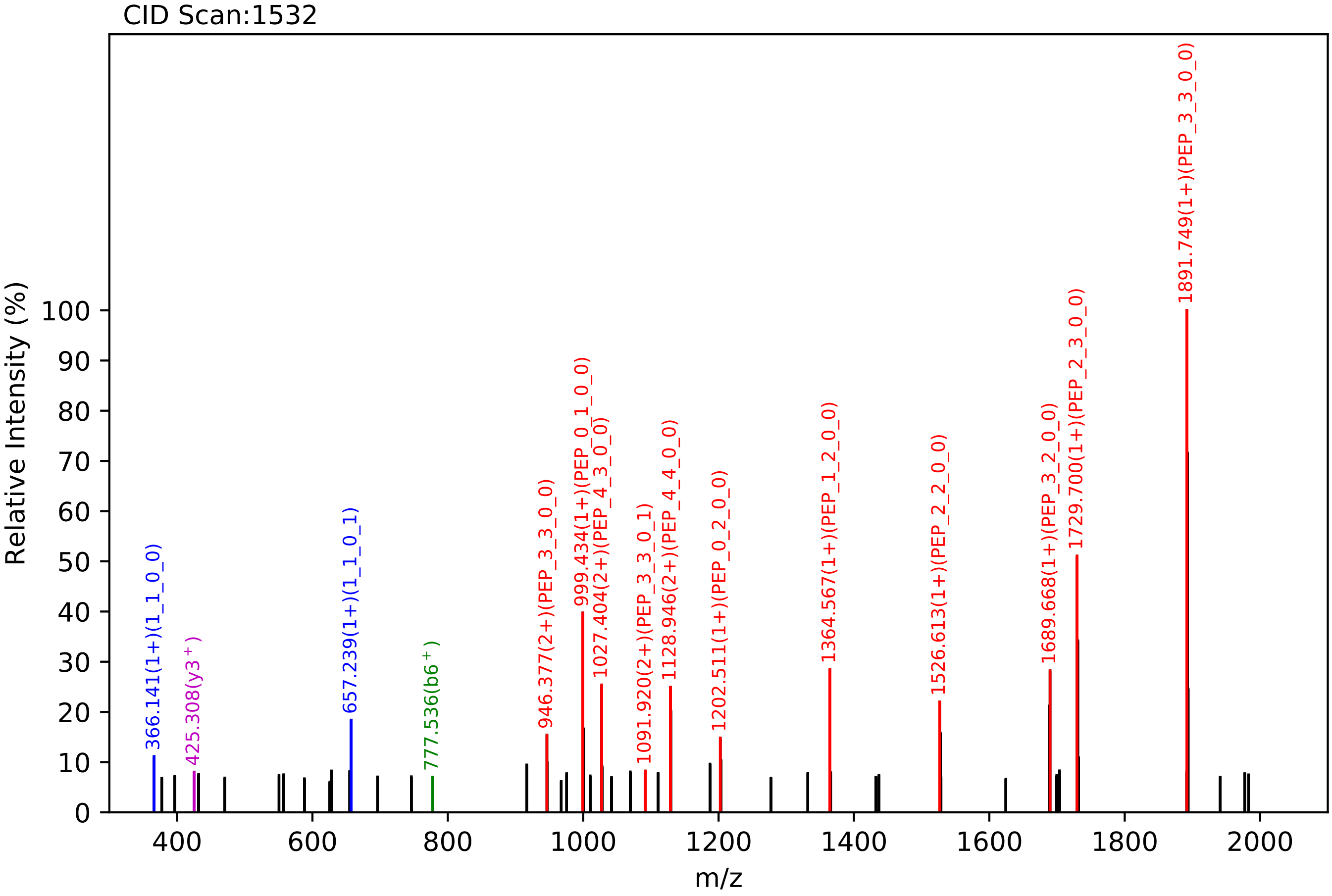

Test set no. 114, Experiment: AGP exp_3

NEEYNK(=PEP)_4_4_0_1, m/z:1274.49(2+), RT:16.30, Y-score:78.61

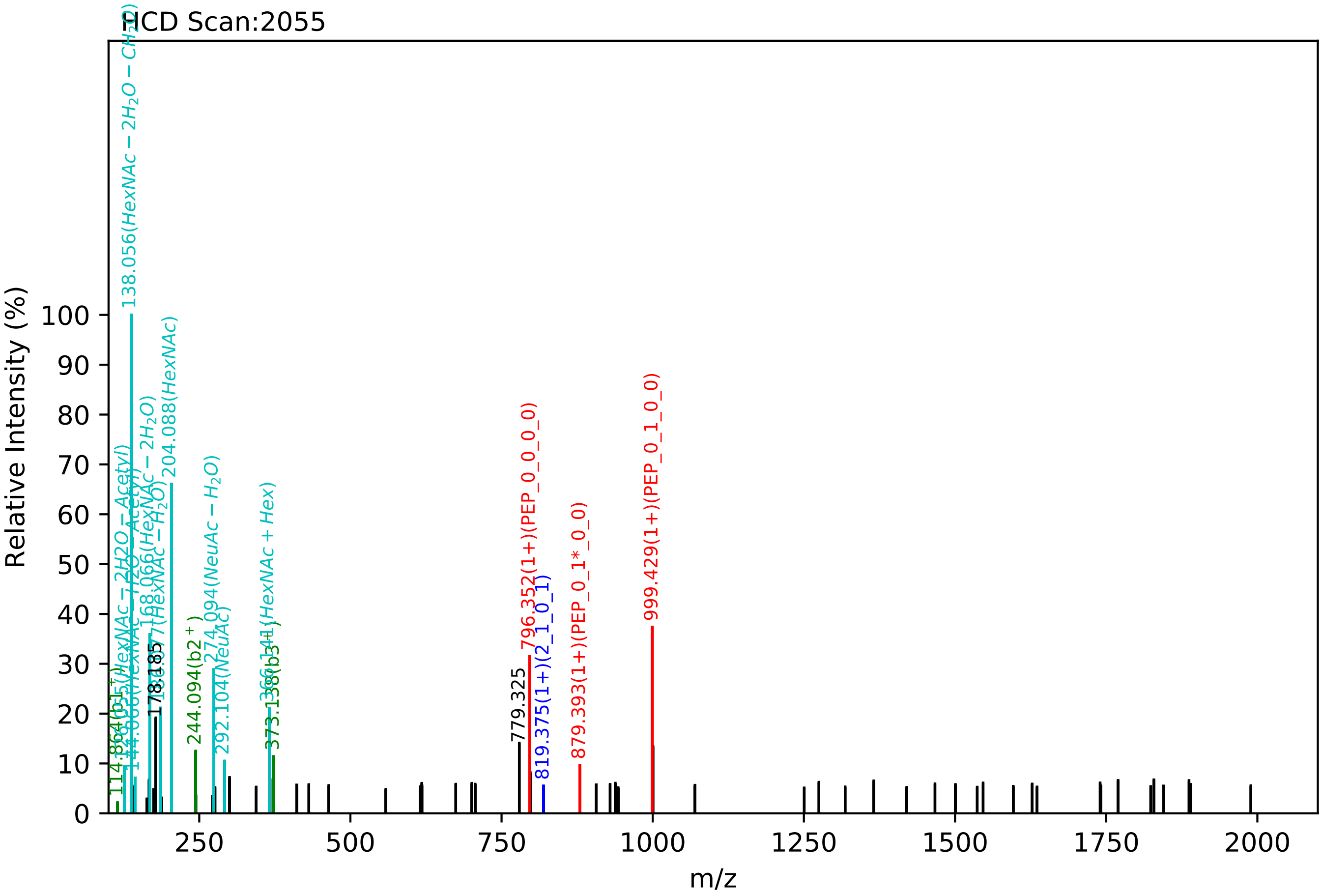

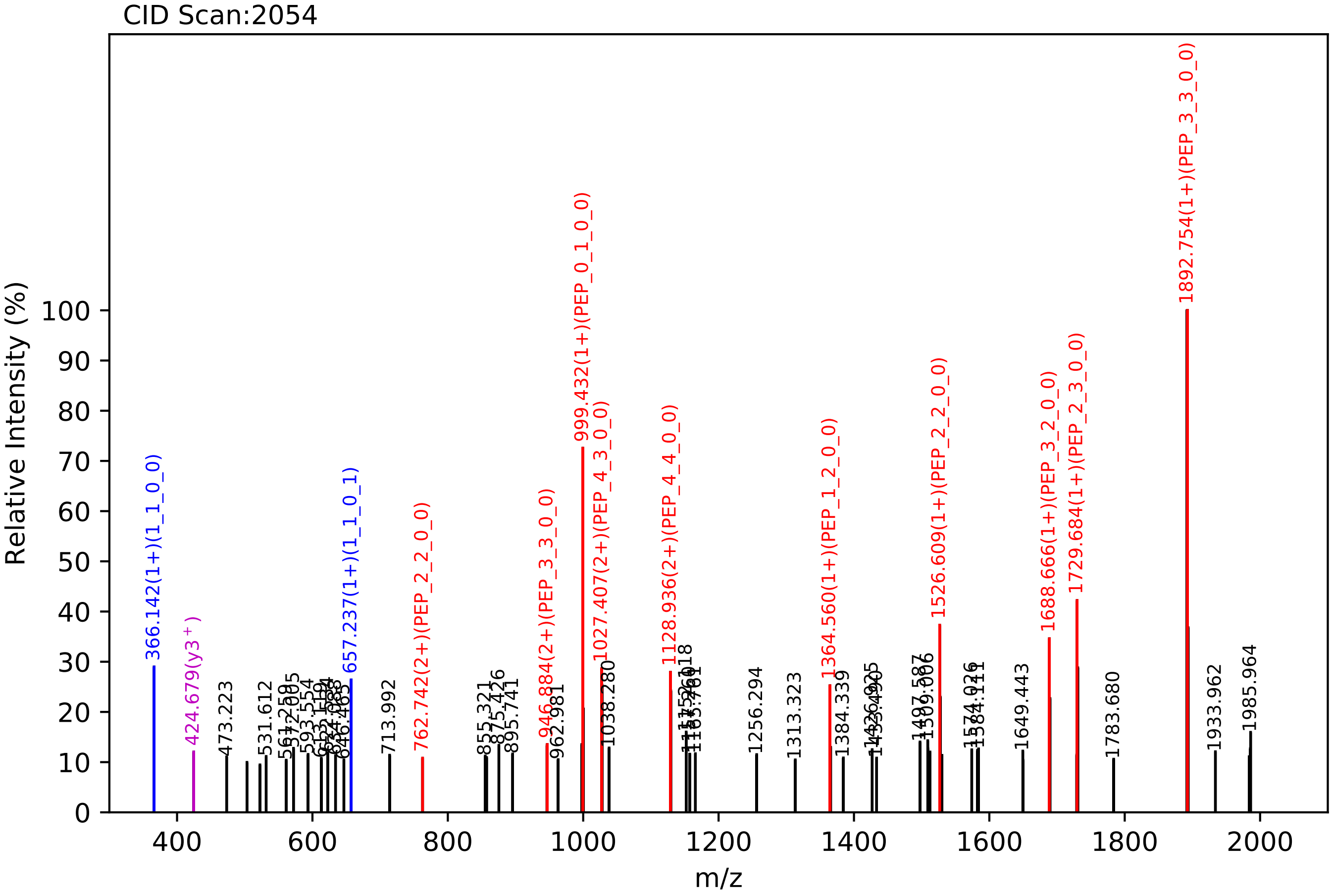

Test set no. 115, Experiment: AGP exp_3

NEEYNK(=PEP)_5_4_0_0, m/z:1209.97(2+), RT:14.28, Y-score:95.20

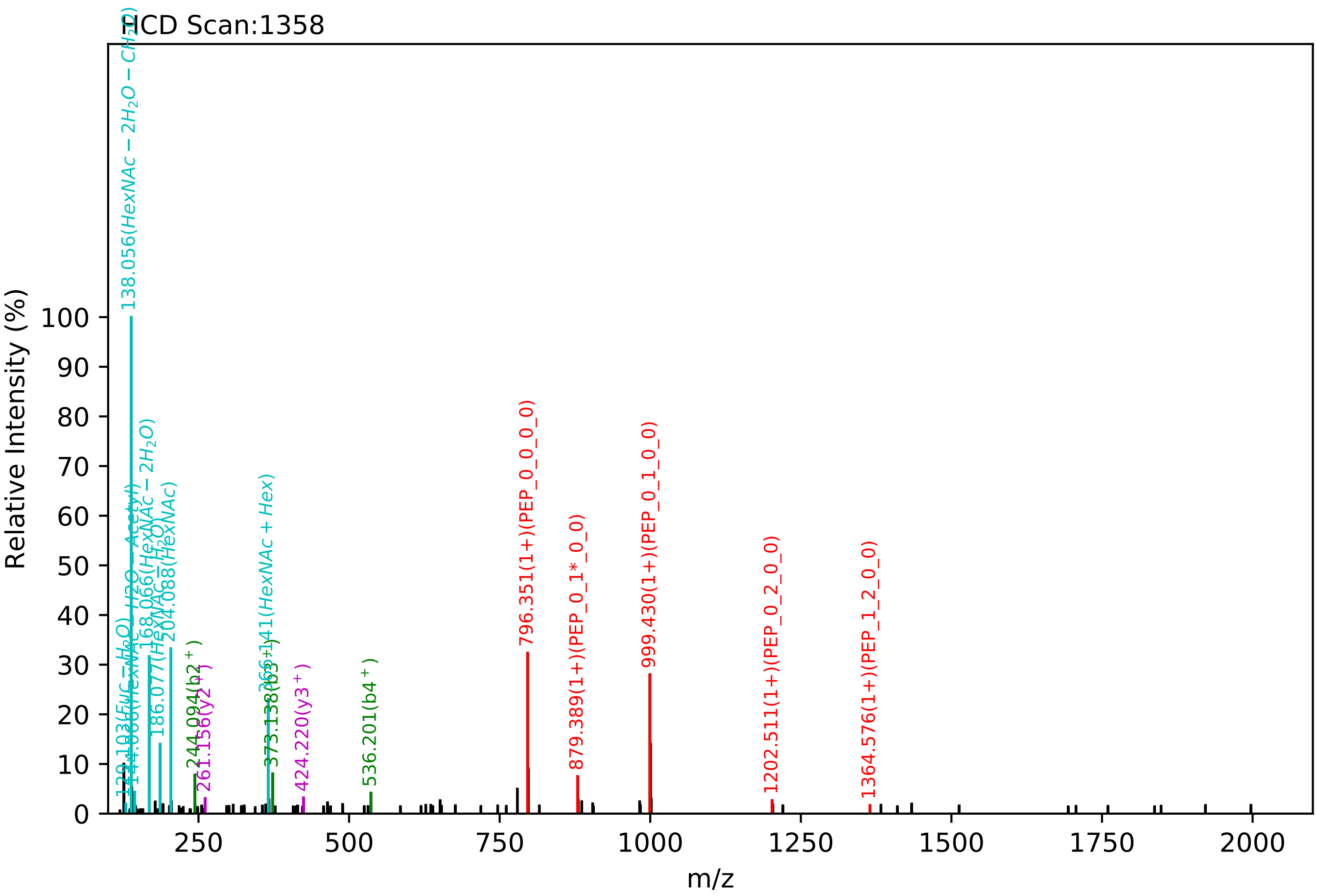

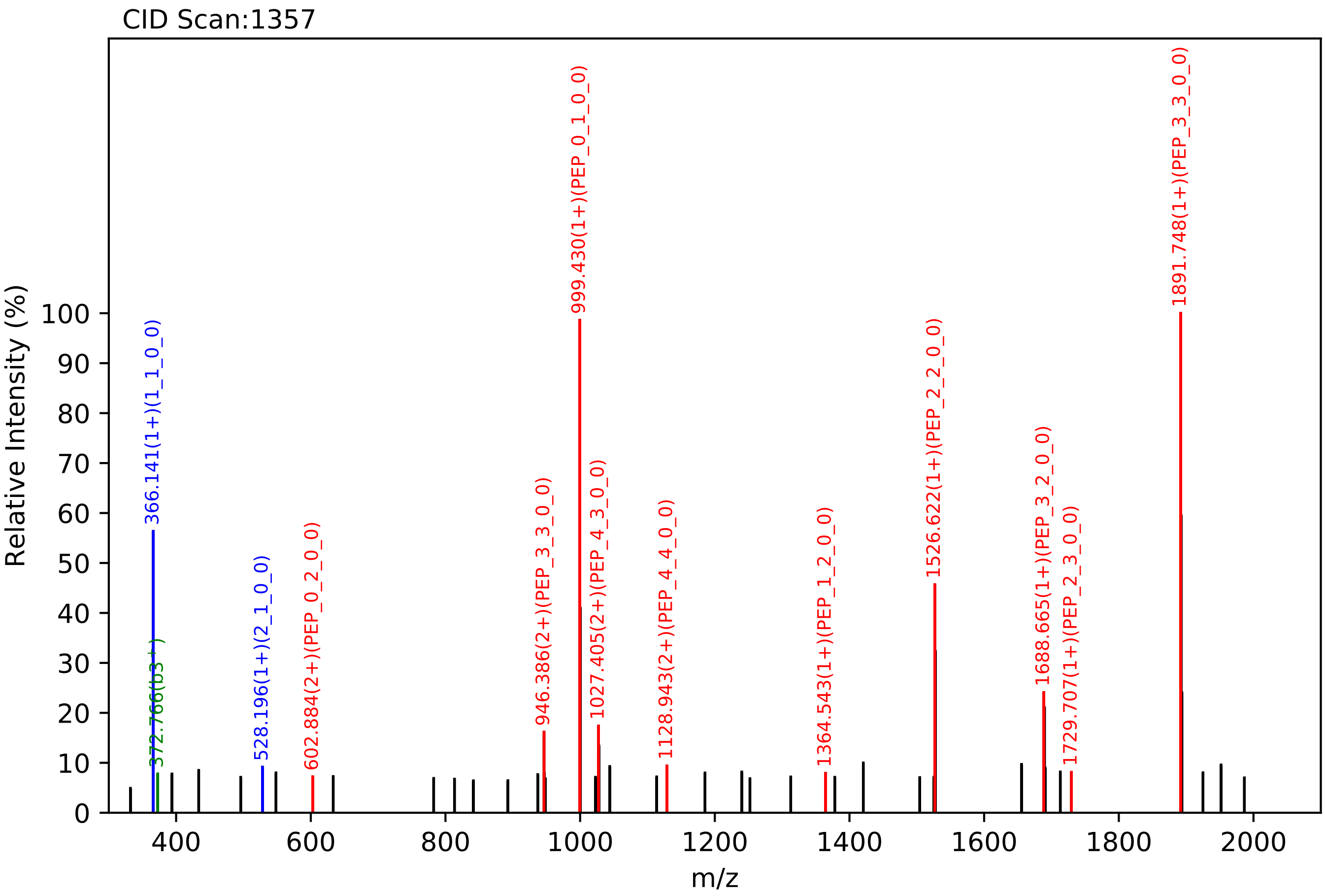

Test set no. 116, Experiment: AGP exp_4

NEEYNK(=PEP)_5_4_0_0, m/z:1209.97(2+), RT:14.28, Y-score:91.28

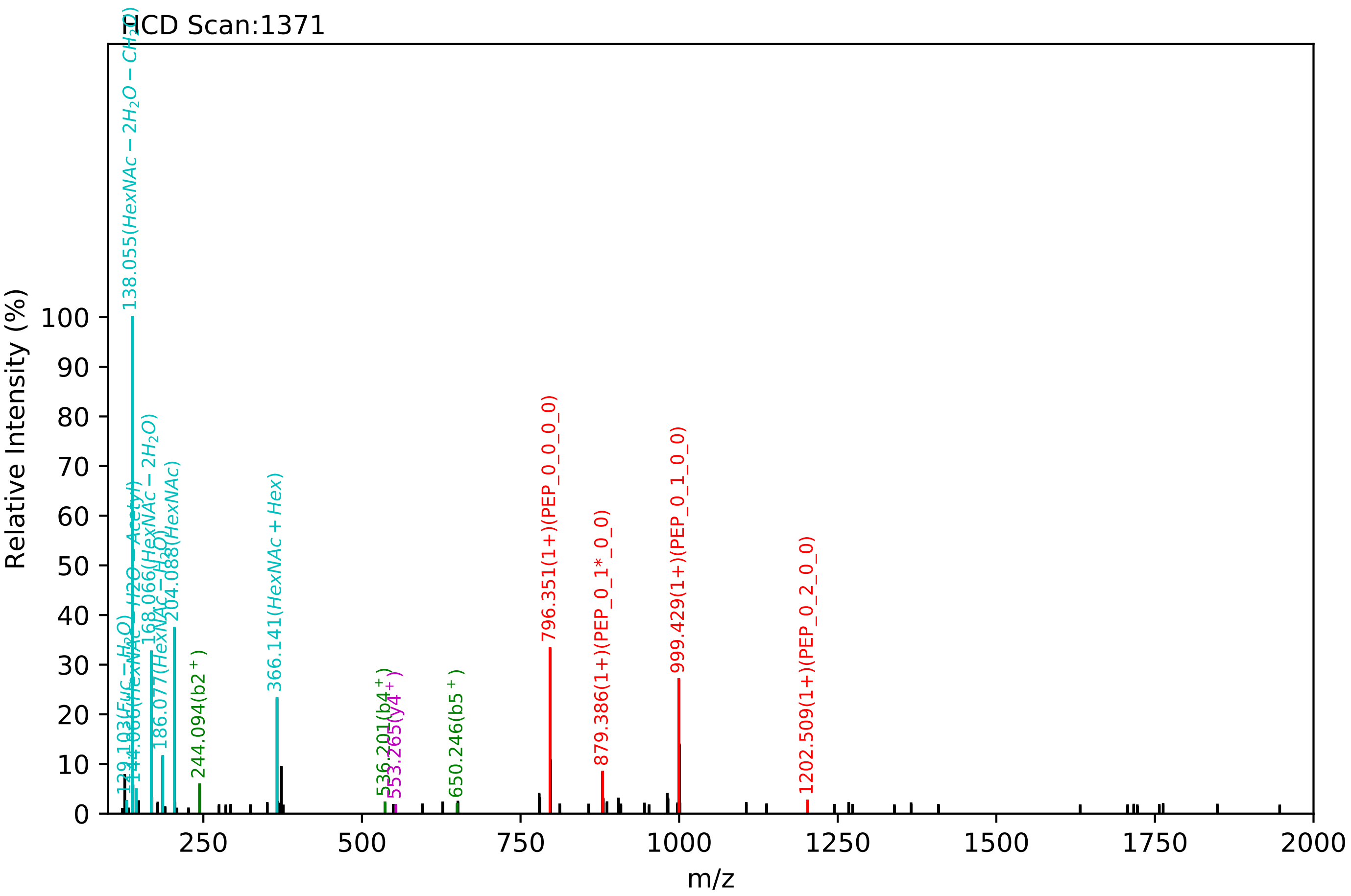

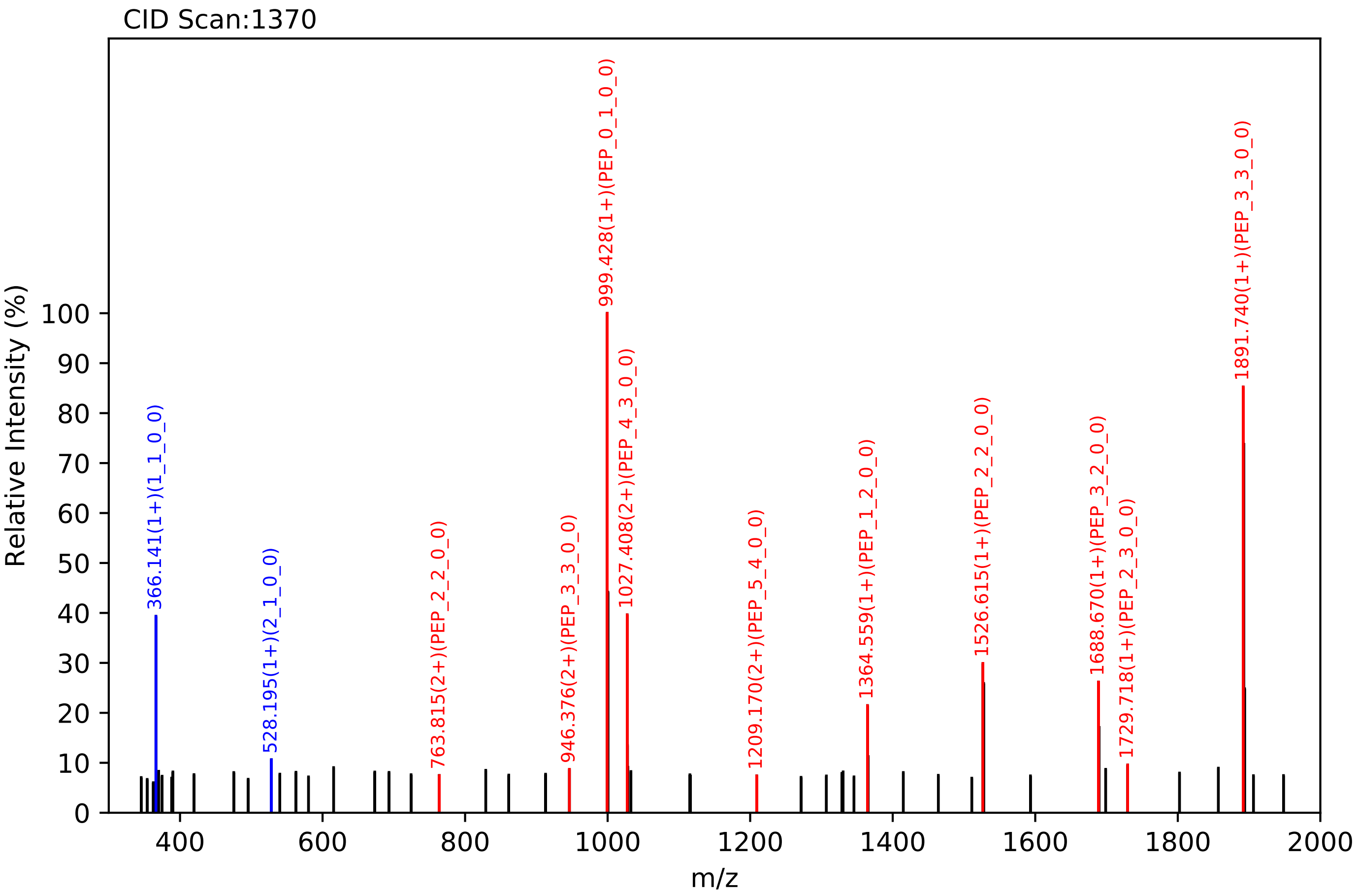

Test set no. 117, Experiment: AGP exp_3

NEEYNK(=PEP)_5_4_0_1, m/z:904.02(3+), RT:15.77, Y-score:94.05

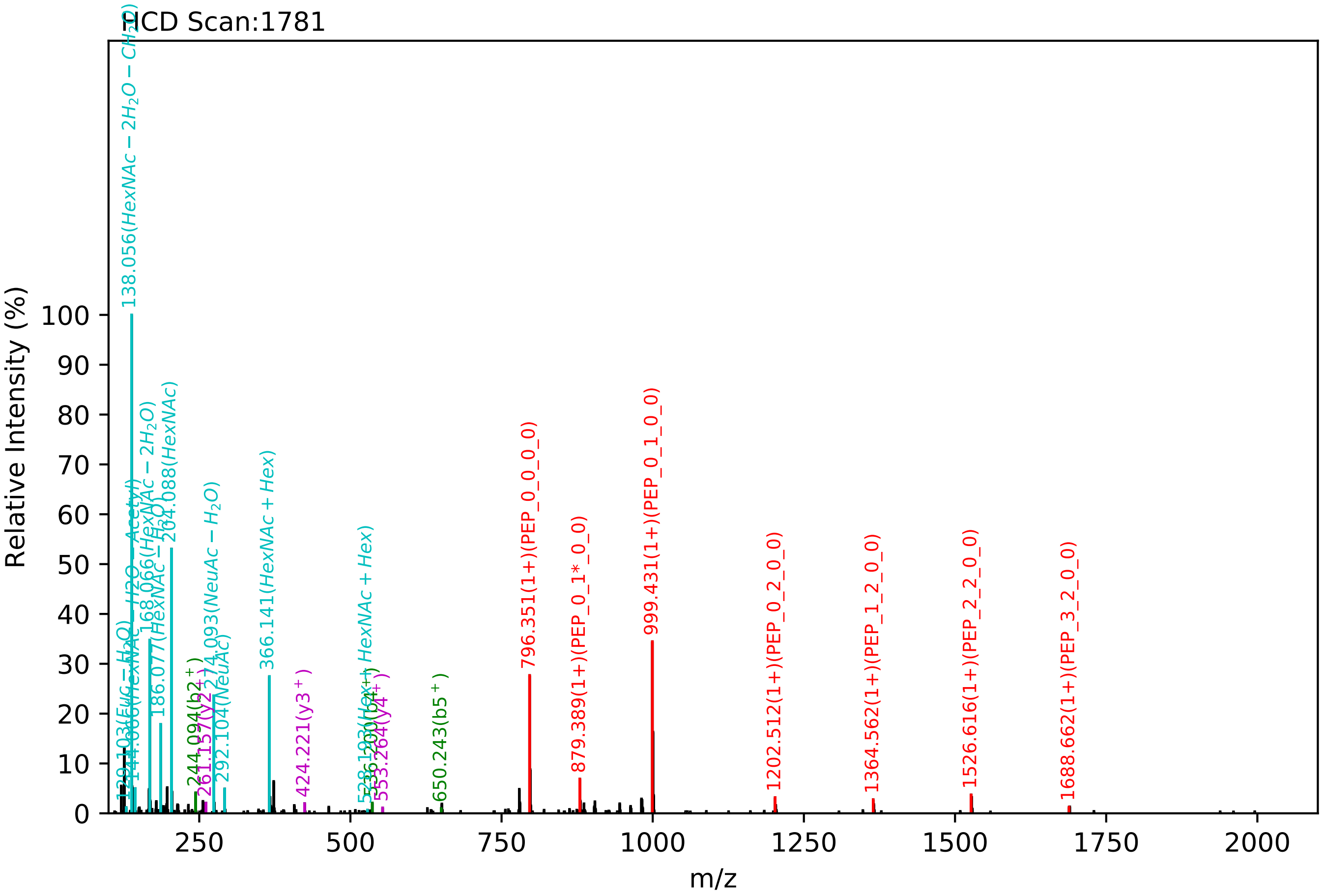

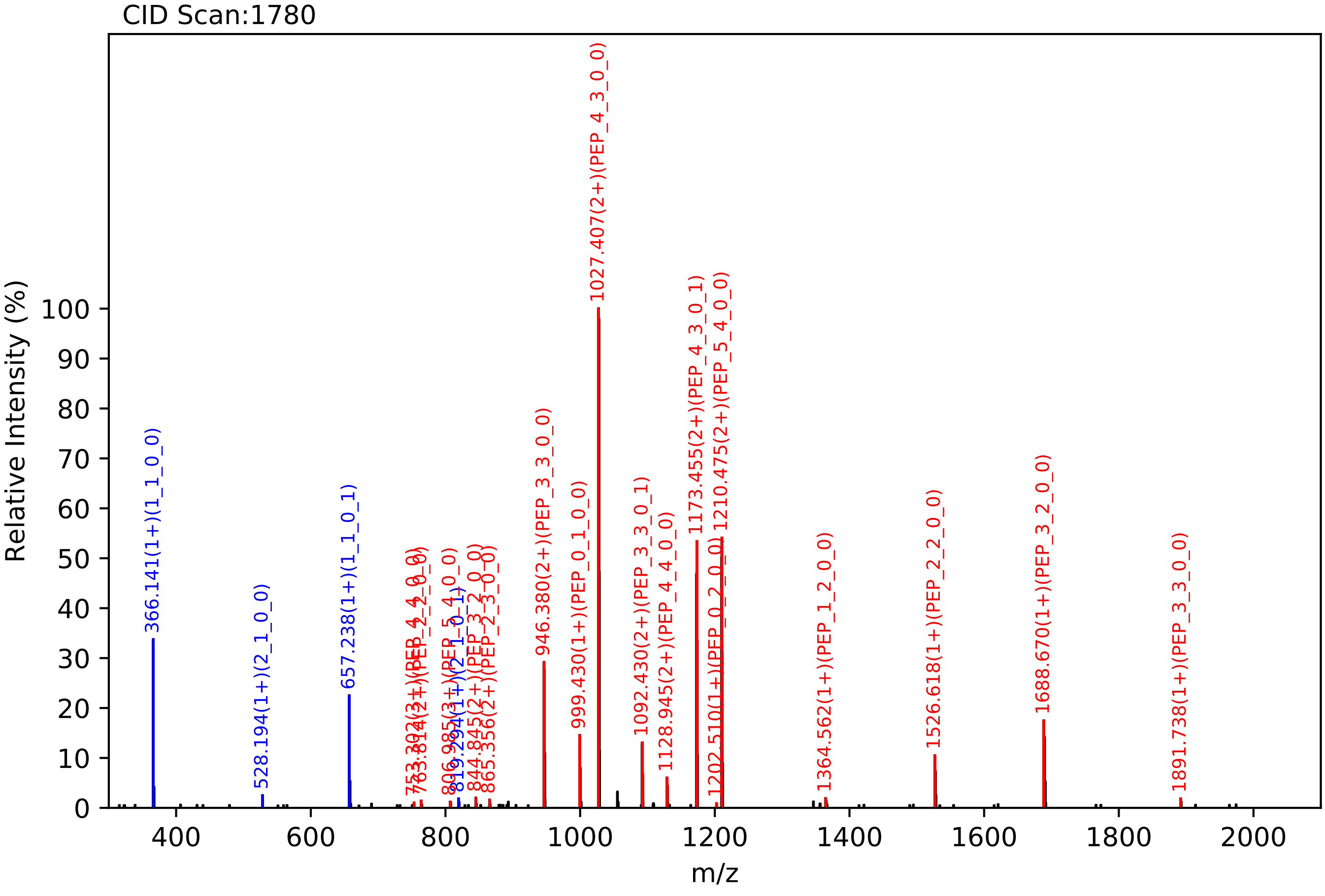

Test set no. 118, Experiment: AGP exp_3

NEEYNK(=PEP)_5_4_0_1, m/z:904.02(3+), RT:18.59, Y-score:93.75

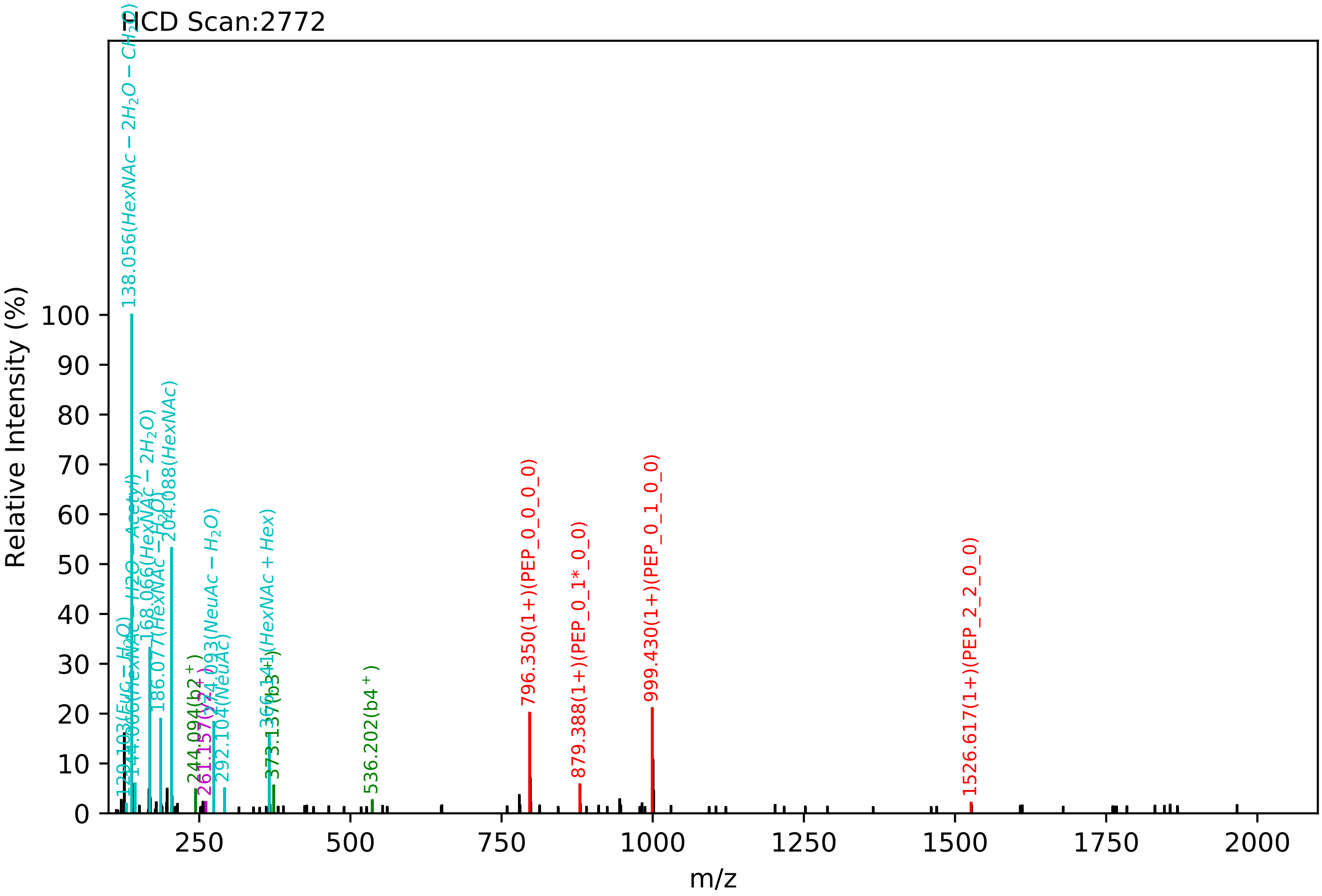

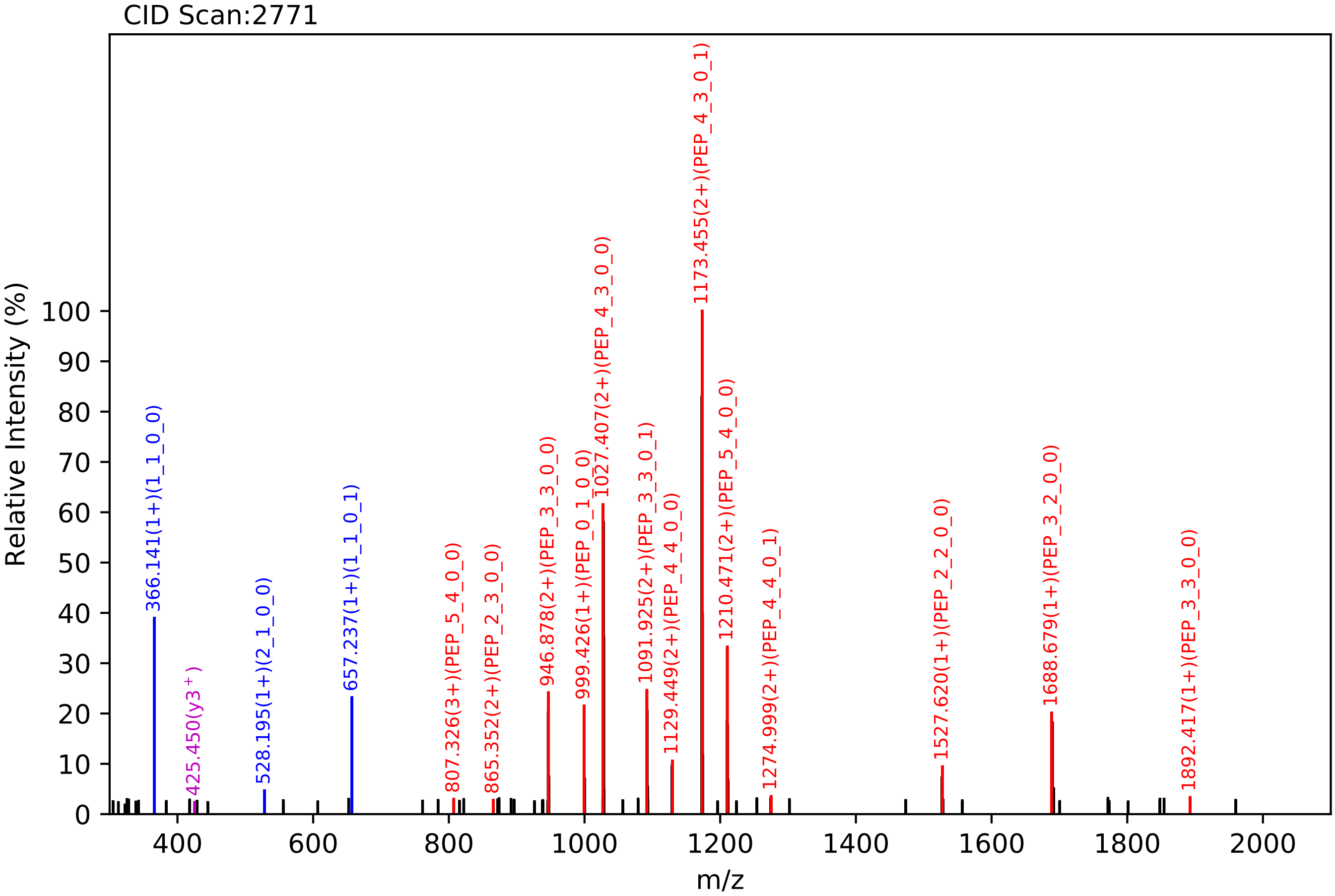

Test set no. 119, Experiment: AGP exp_4

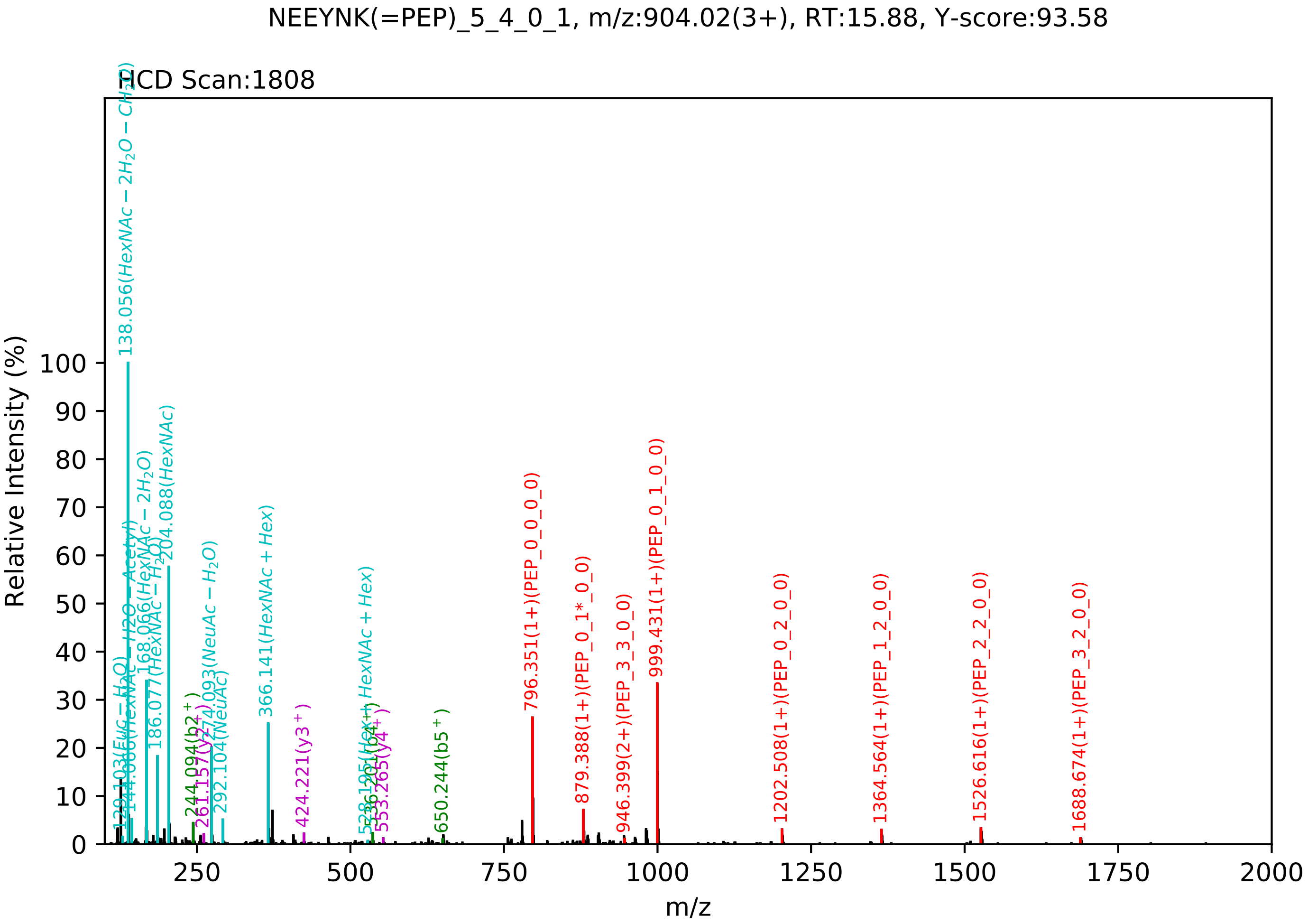

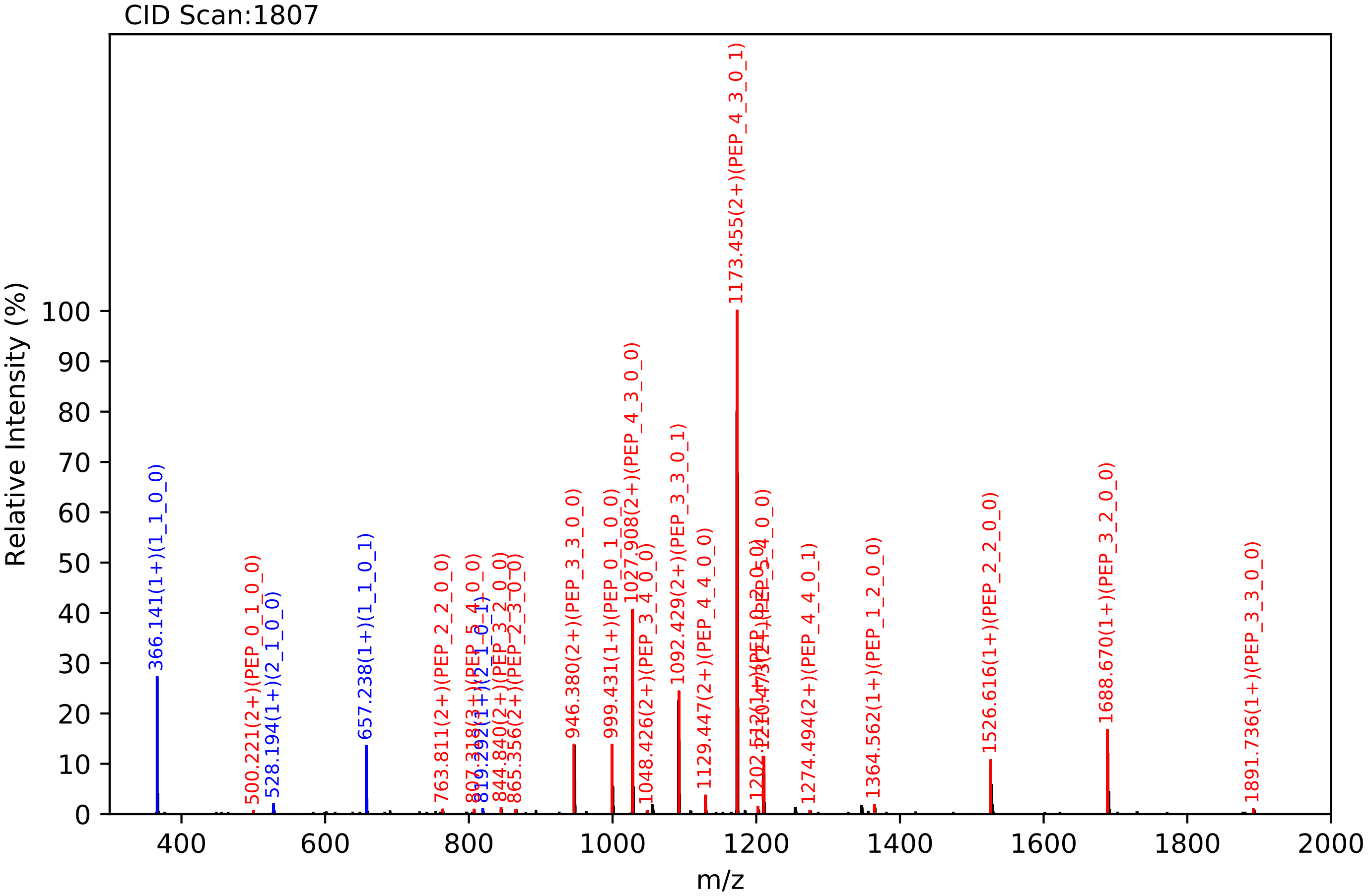

Test set no. 120, Experiment: AGP exp_3

NEEYNK(=PEP)_5_4_0_1, m/z:1356.02(2+), RT:16.28, Y-score:92.85

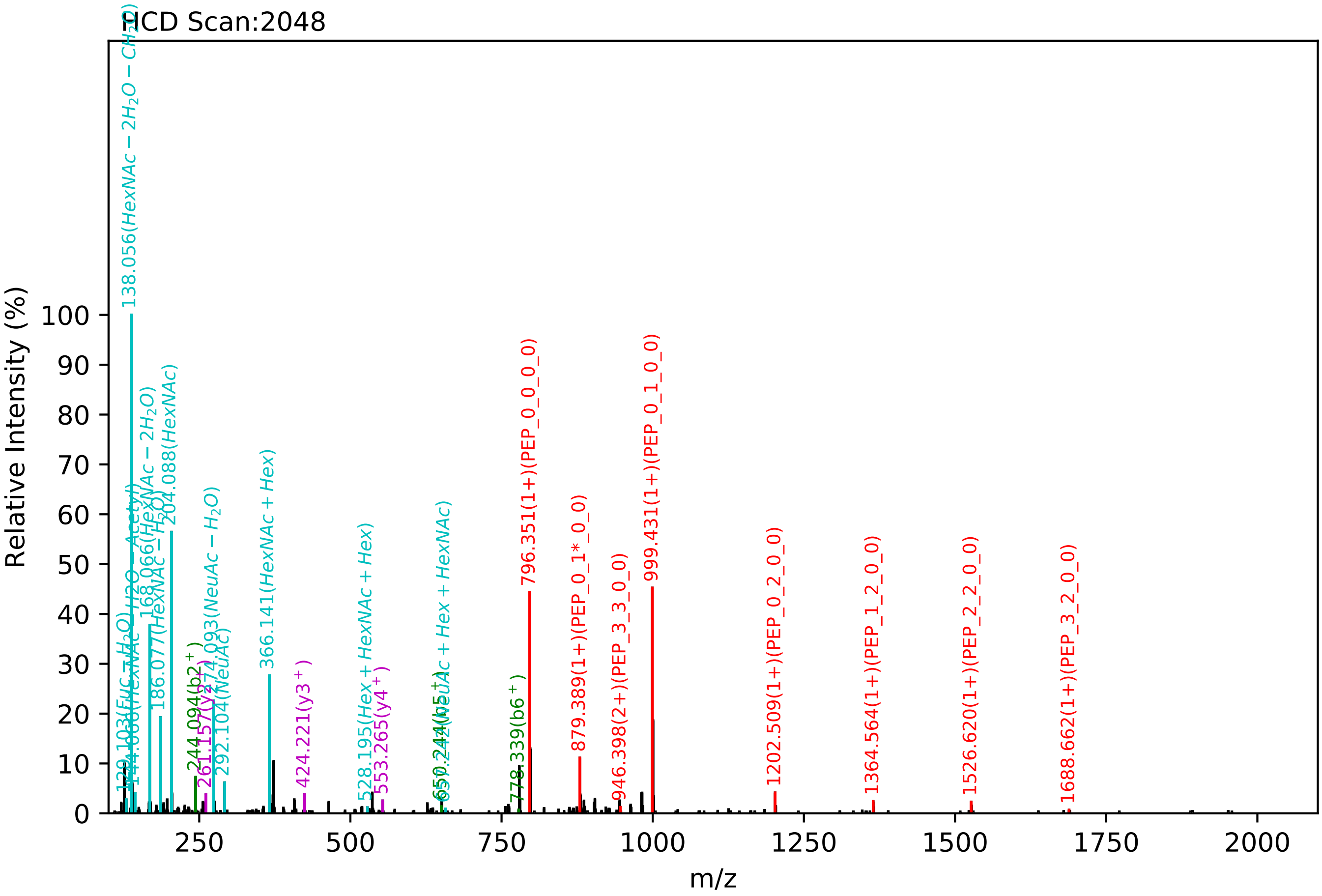

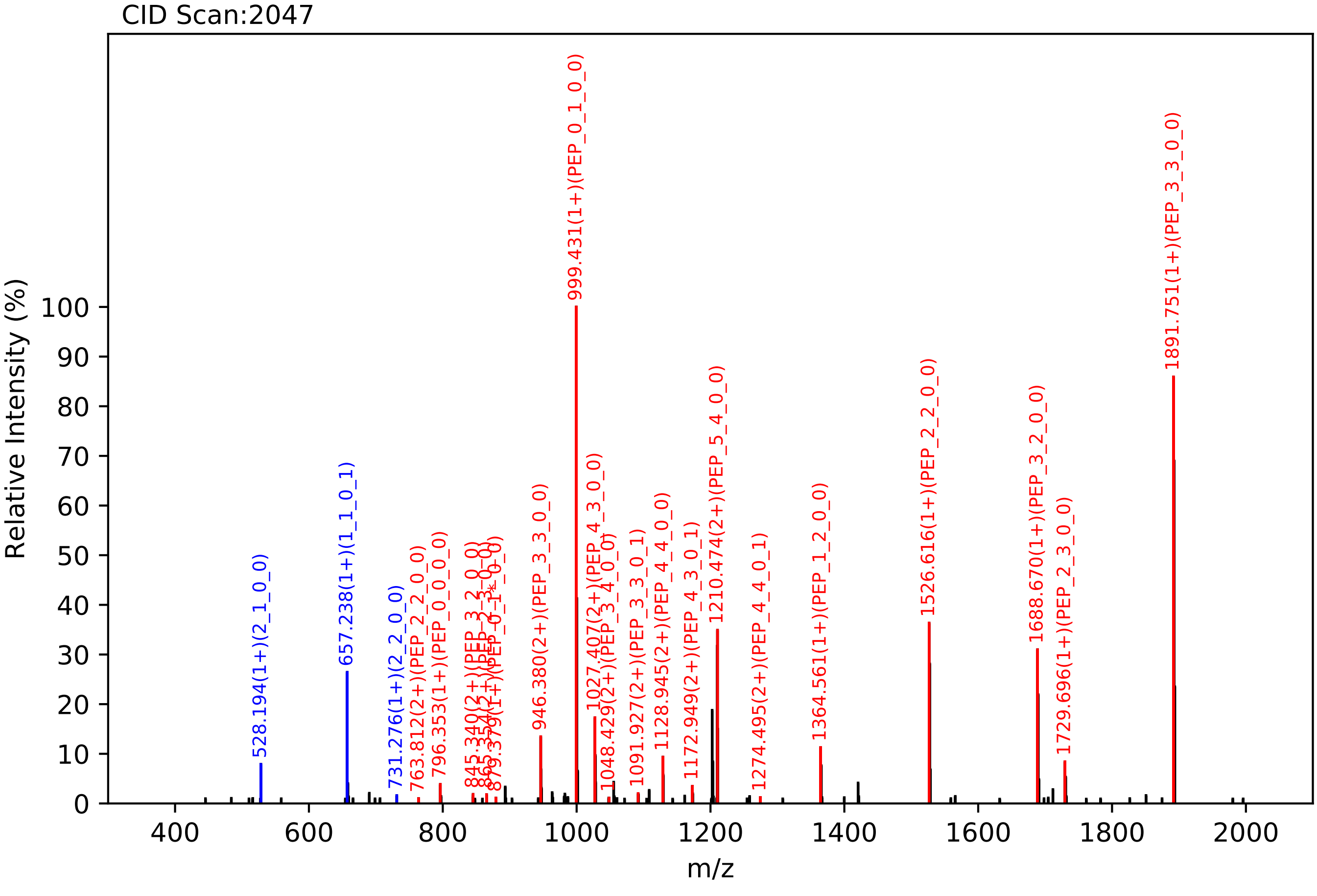

Test set no. 121, Experiment: AGP exp_4

NEEYNK(=PEP)_5_4_0_1, m/z:1356.02(2+), RT:15.86, Y-score:92.81

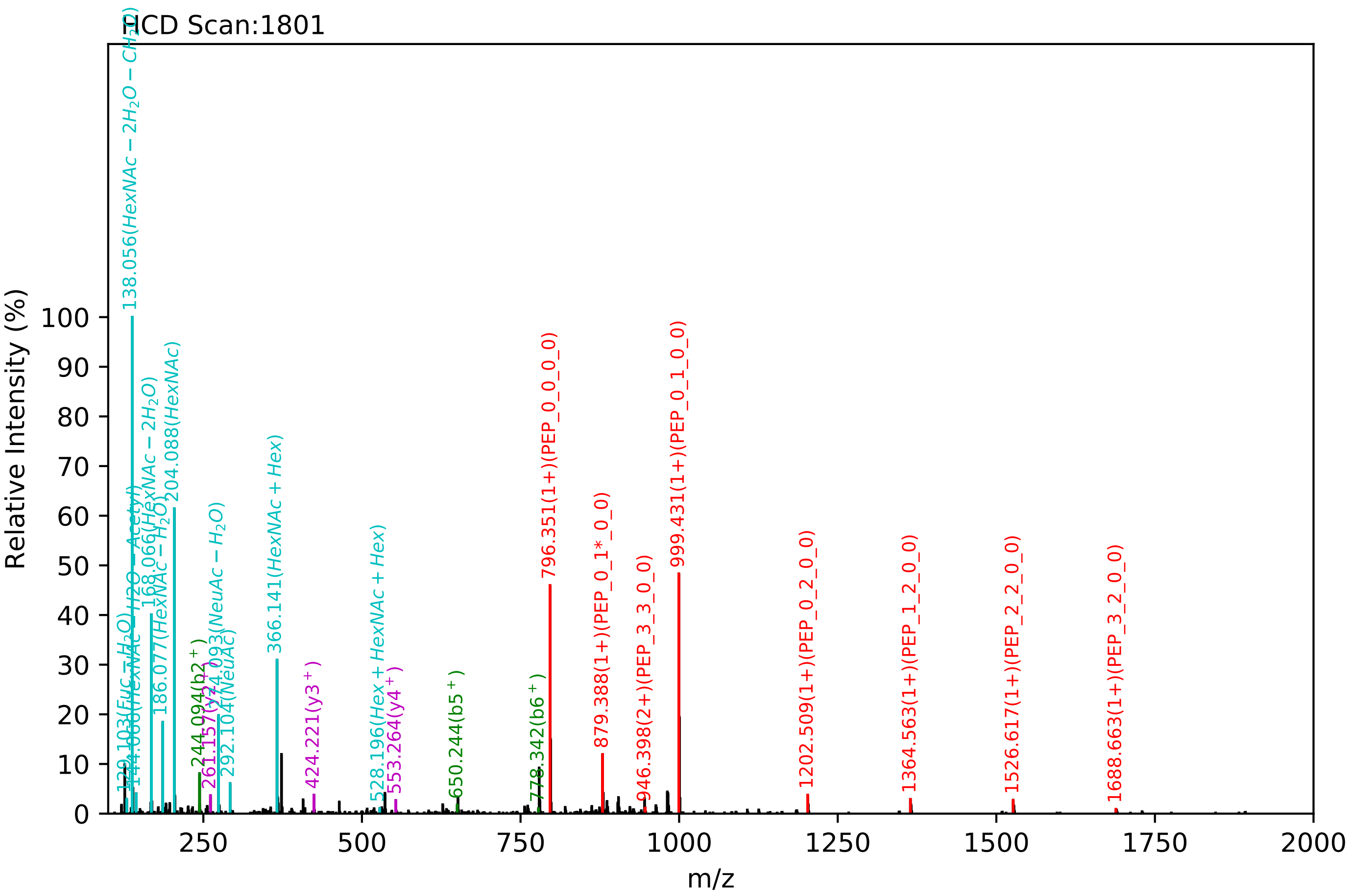

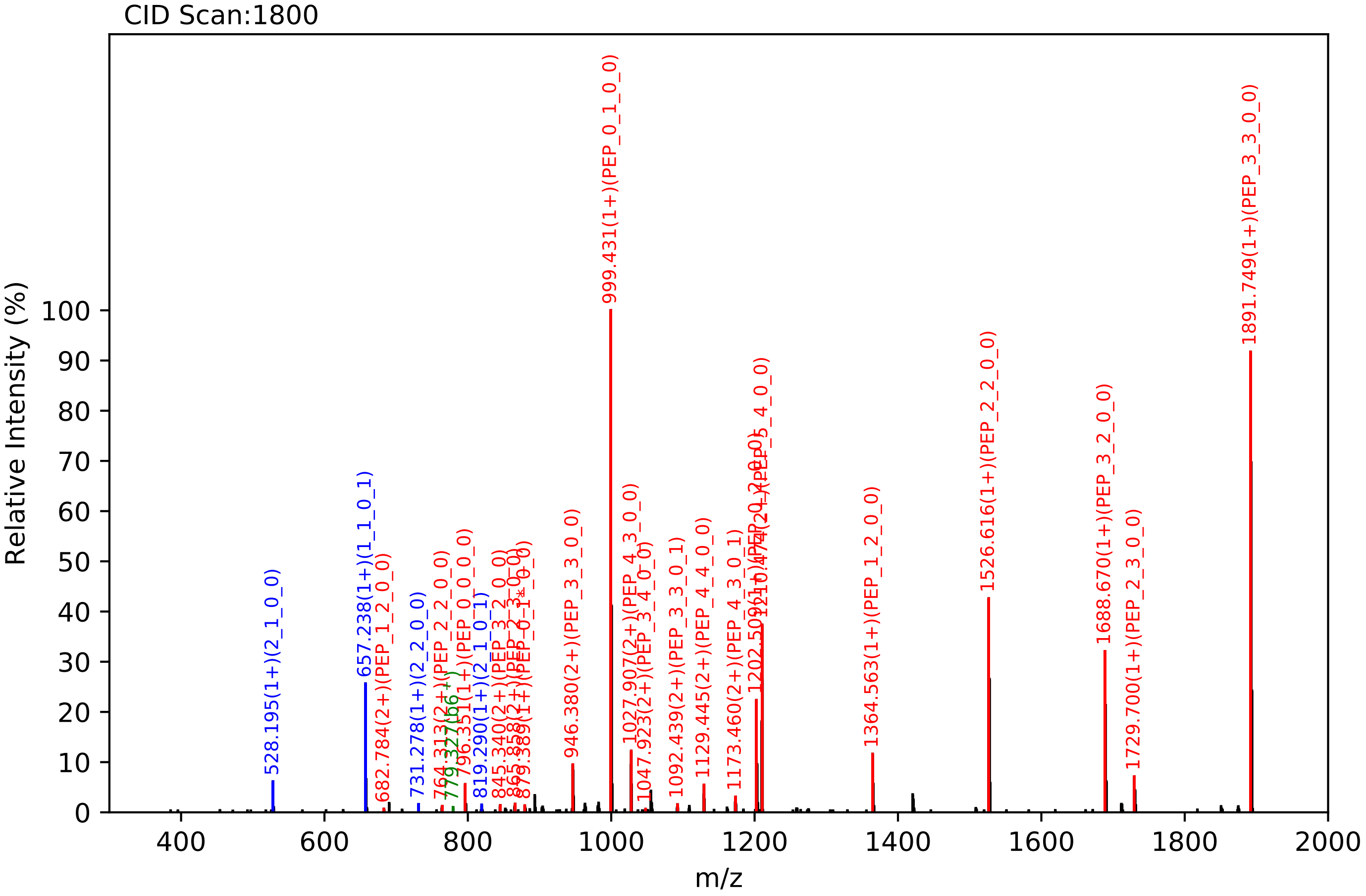

Test set no. 122, Experiment: AGP exp_4

NEEYNK(=PEP)_5_4_0_1, m/z:904.02(3+), RT:15.36, Y-score:91.96

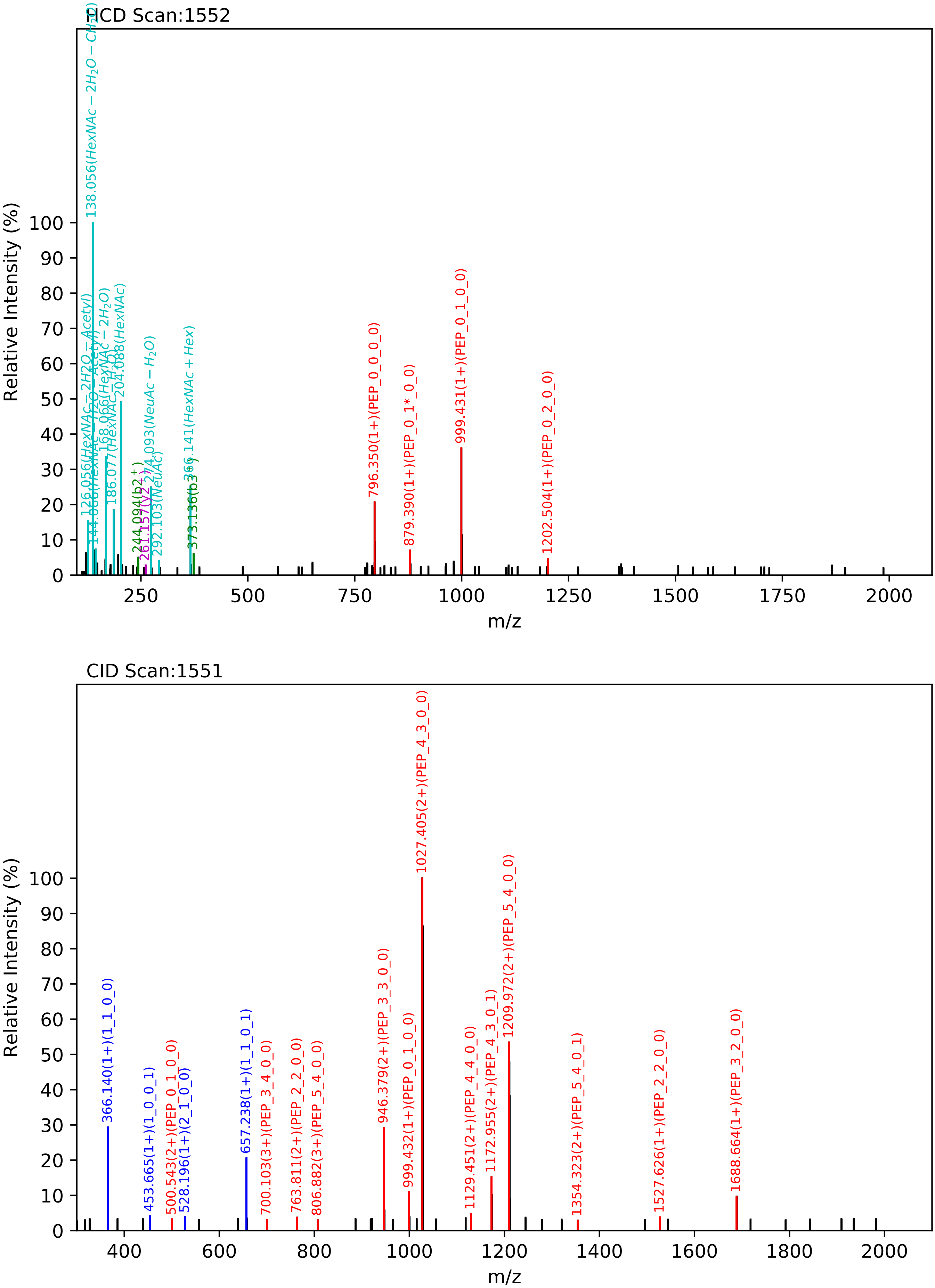

Test set no. 123, Experiment: AGP exp_3

NEEYNK(=PEP)_5_4_0_1, m/z:1356.02(2+), RT:15.76, Y-score:91.42

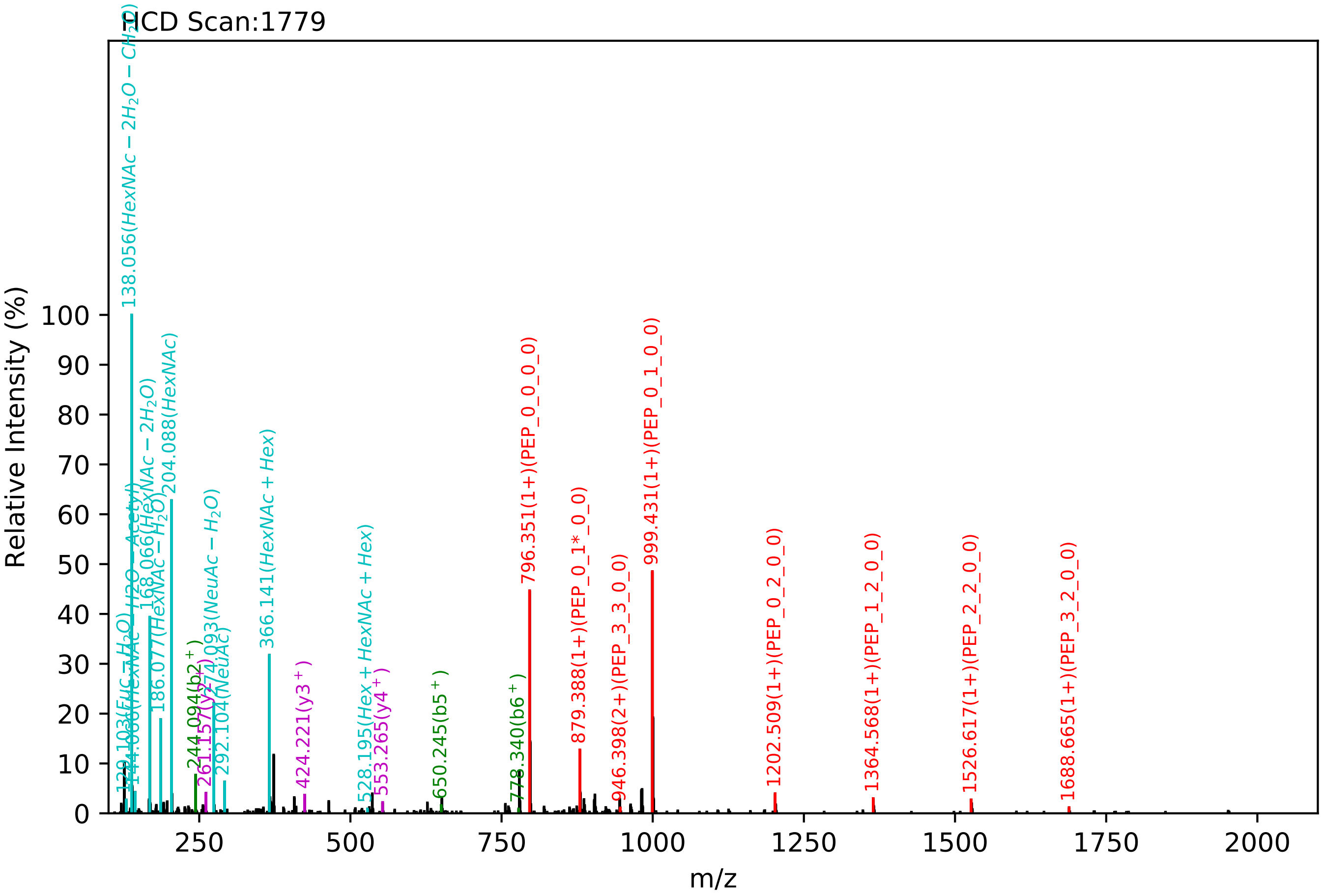

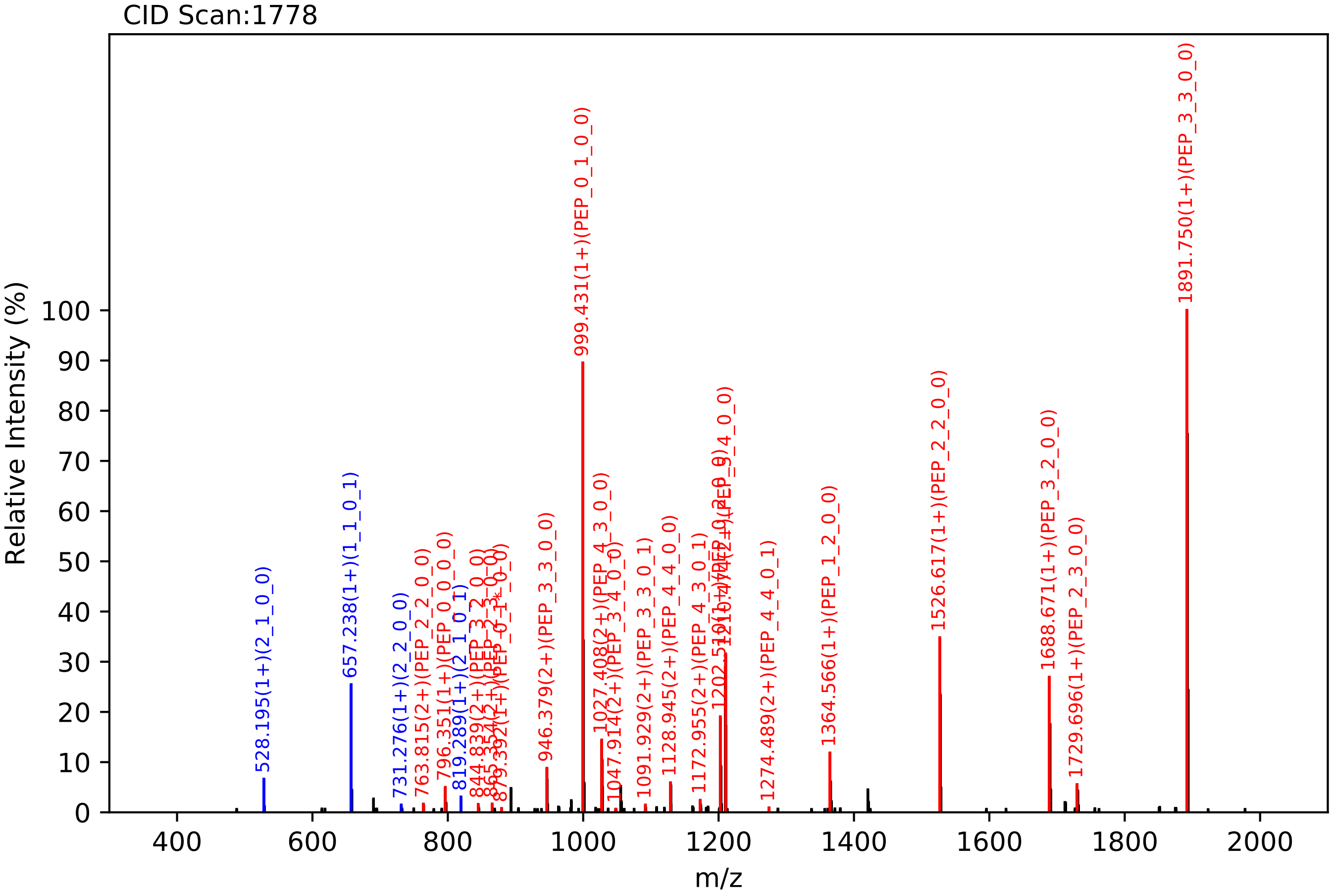

Test set no. 124, Experiment: AGP exp_4

NEEYNK(=PEP)_5_4_0_1, m/z:1355.52(2+), RT:15.35, Y-score:90.82

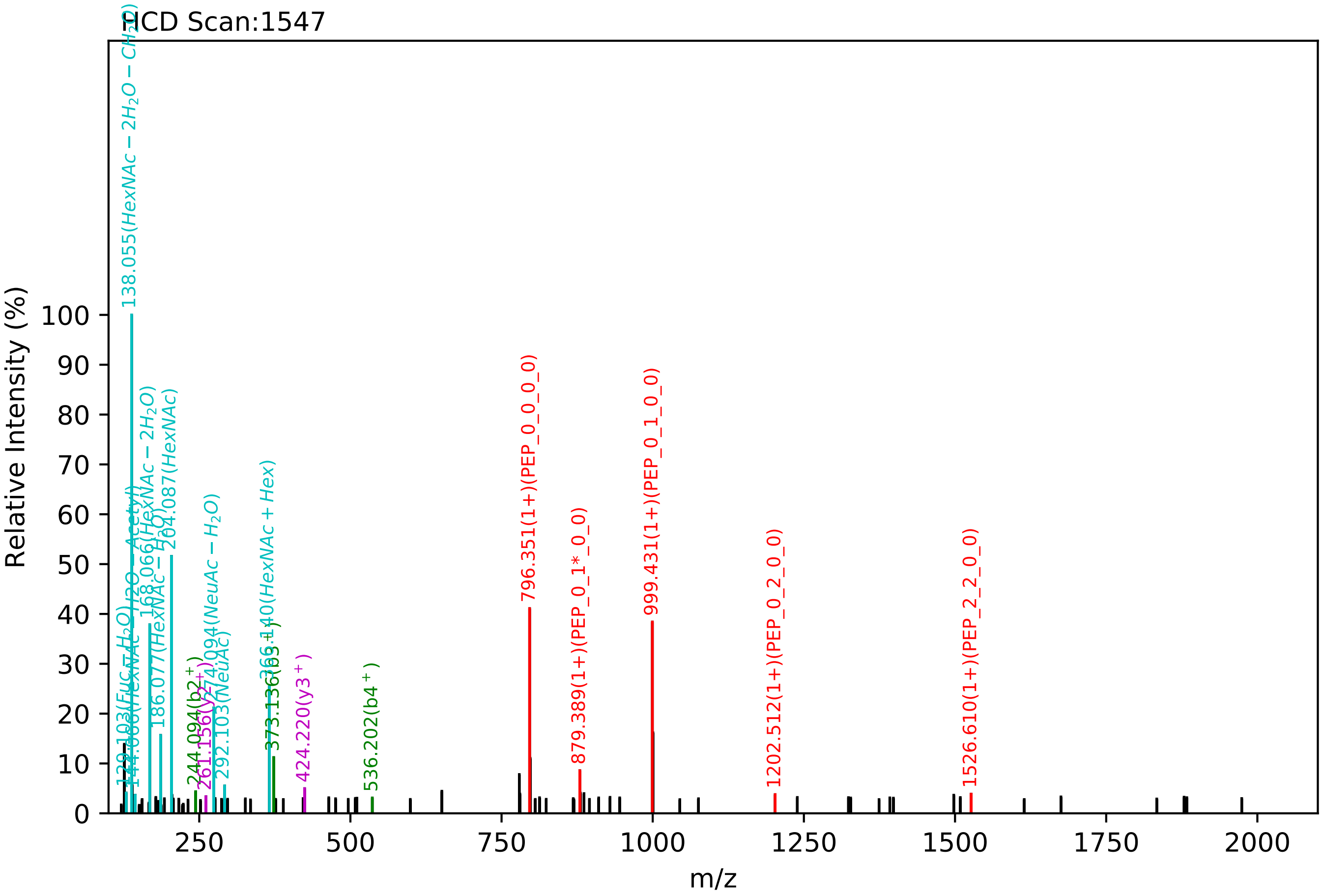

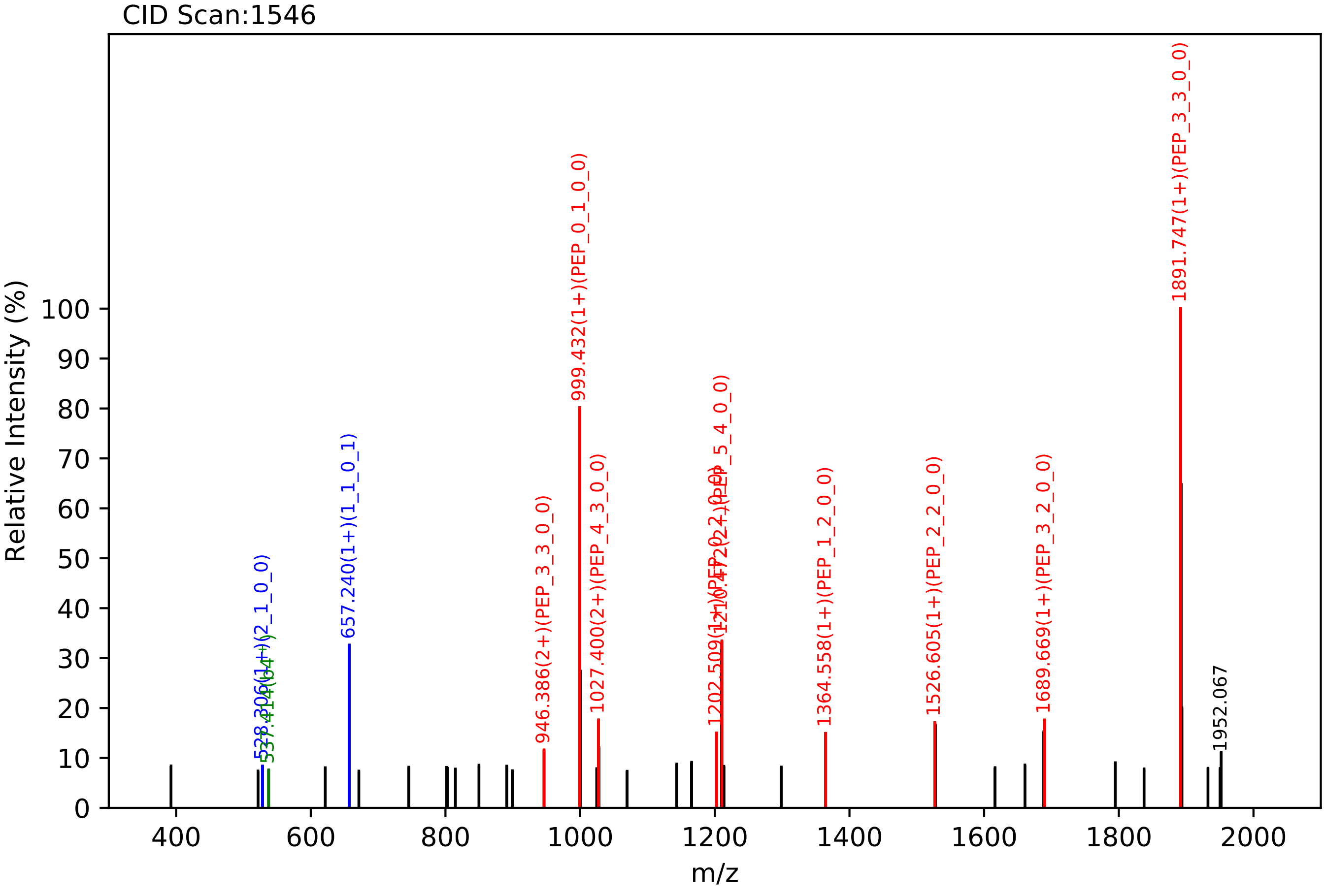

Test set no. 125, Experiment: AGP exp_3

NEEYNK(=PEP)_5_4_0_1, m/z:1355.52(2+), RT:18.88, Y-score:90.56

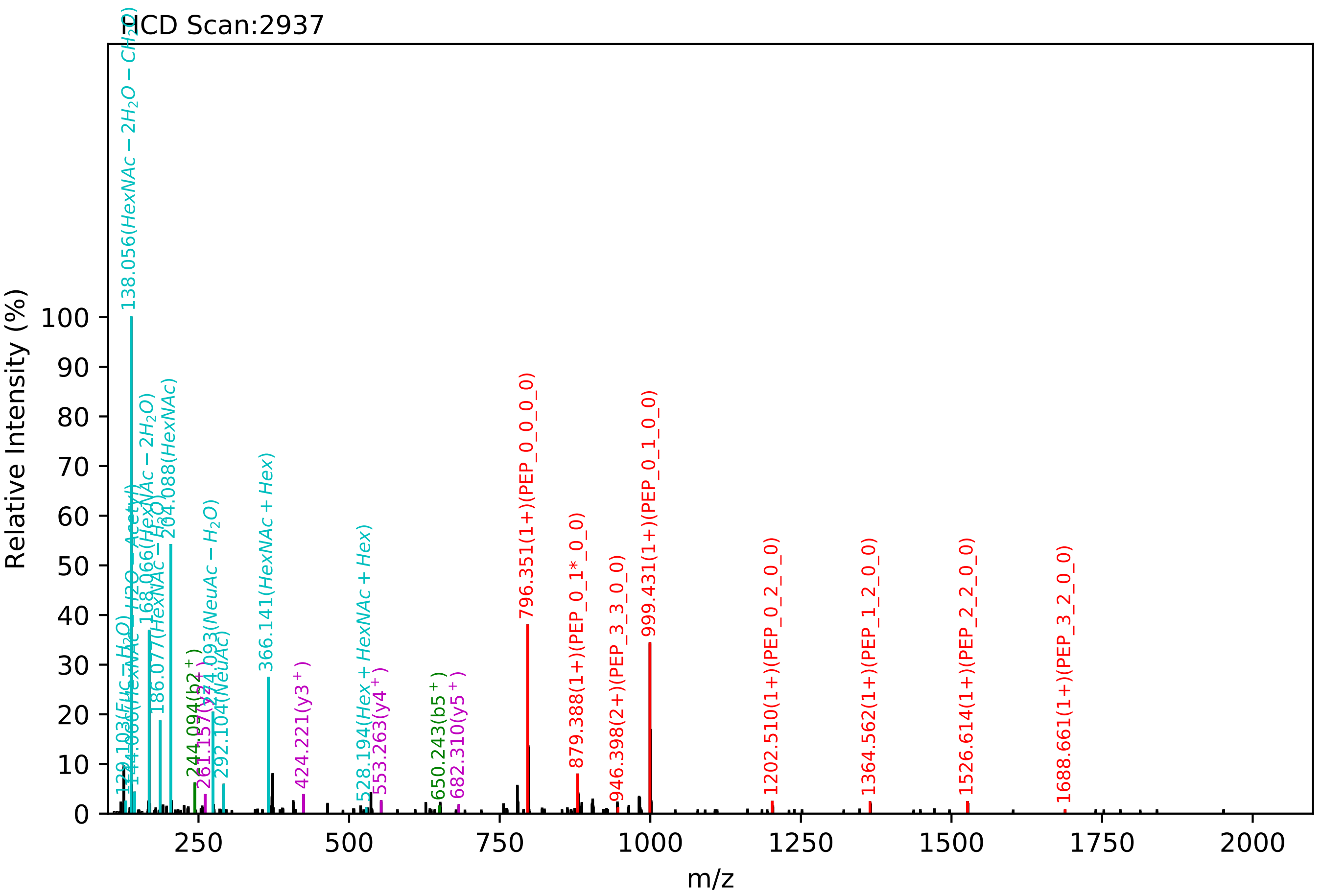

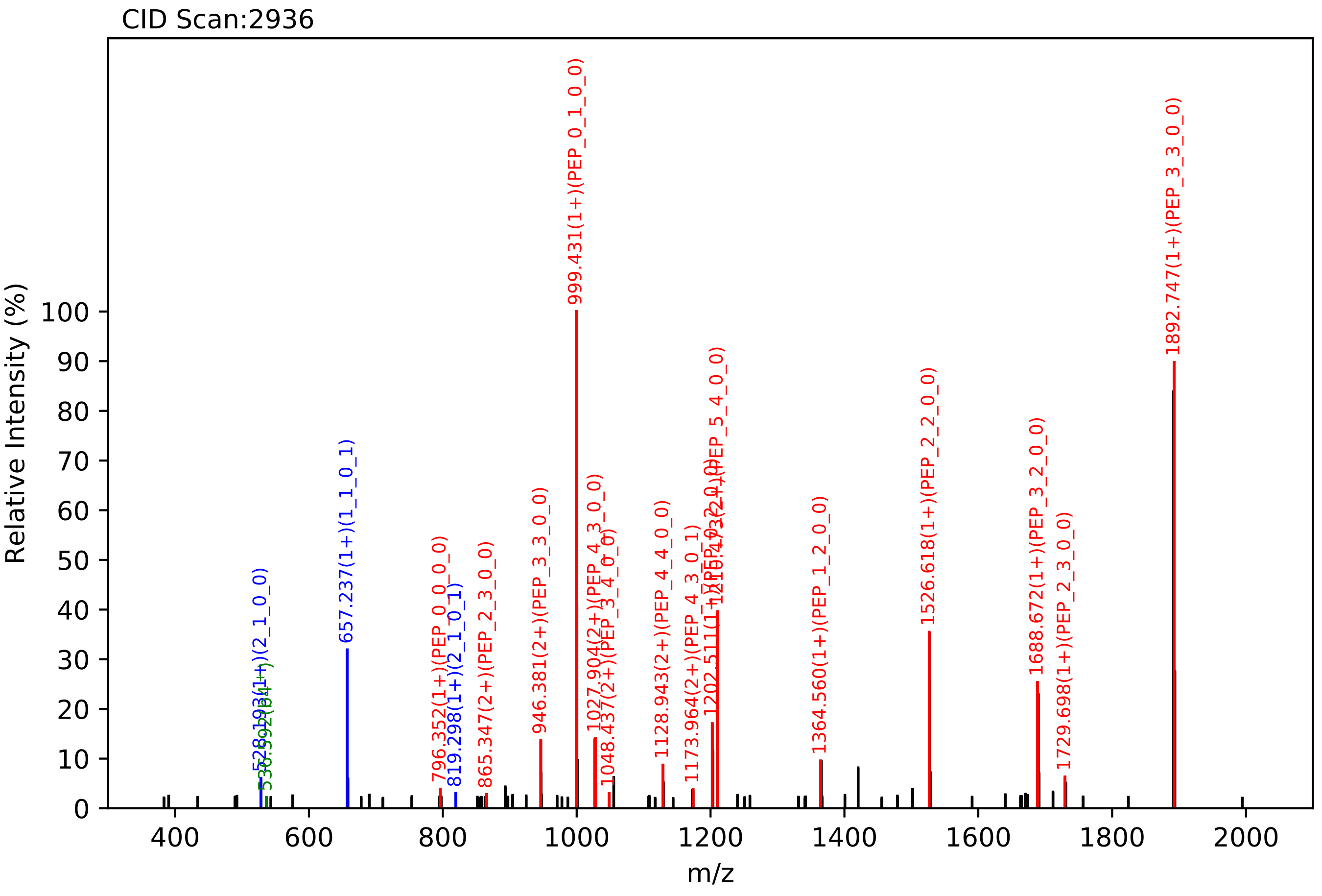

Test set no. 126, Experiment: AGP exp_4

NEEYNK(=PEP)_5_4_0_1, m/z:1355.52(2+), RT:16.37, Y-score:89.85

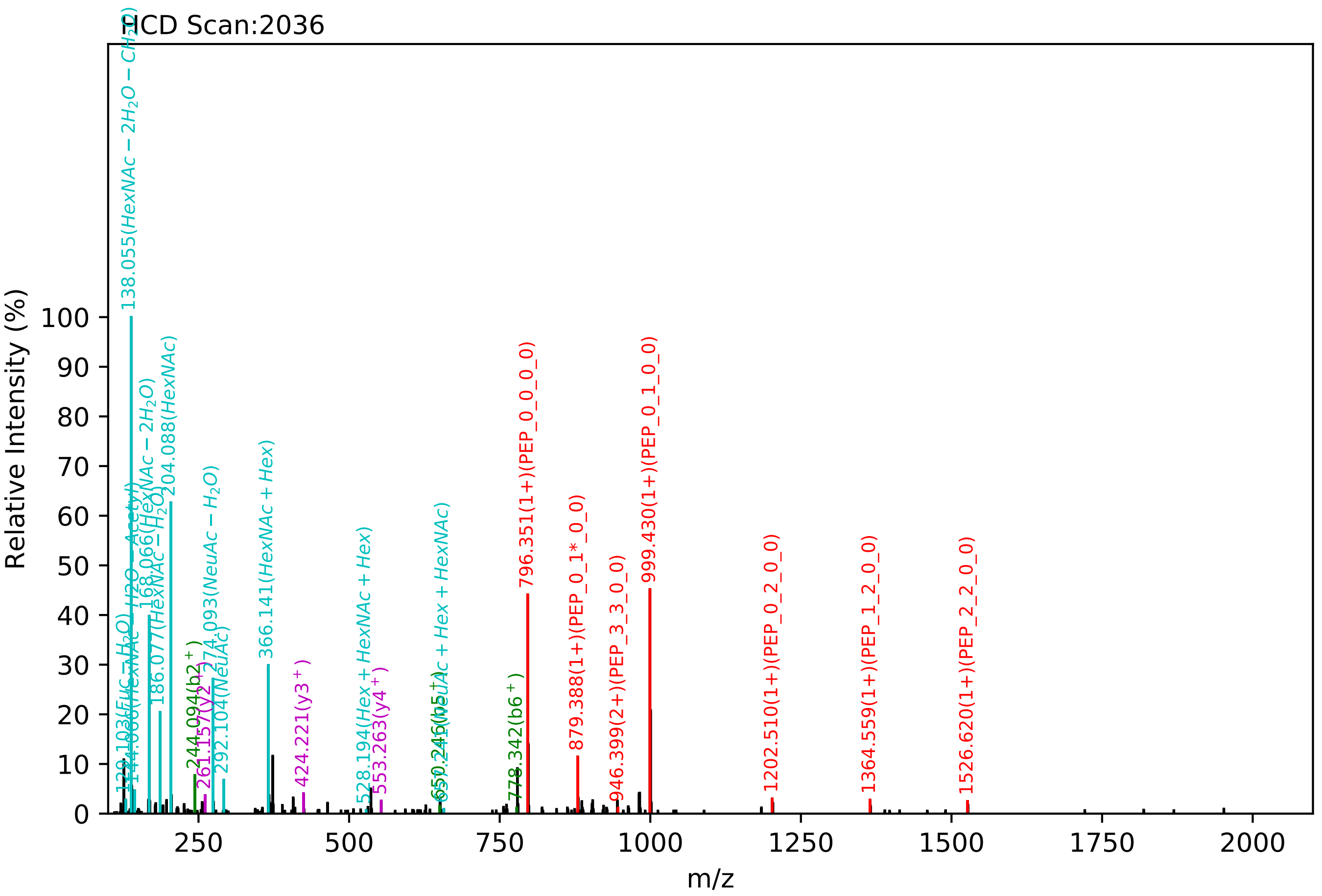

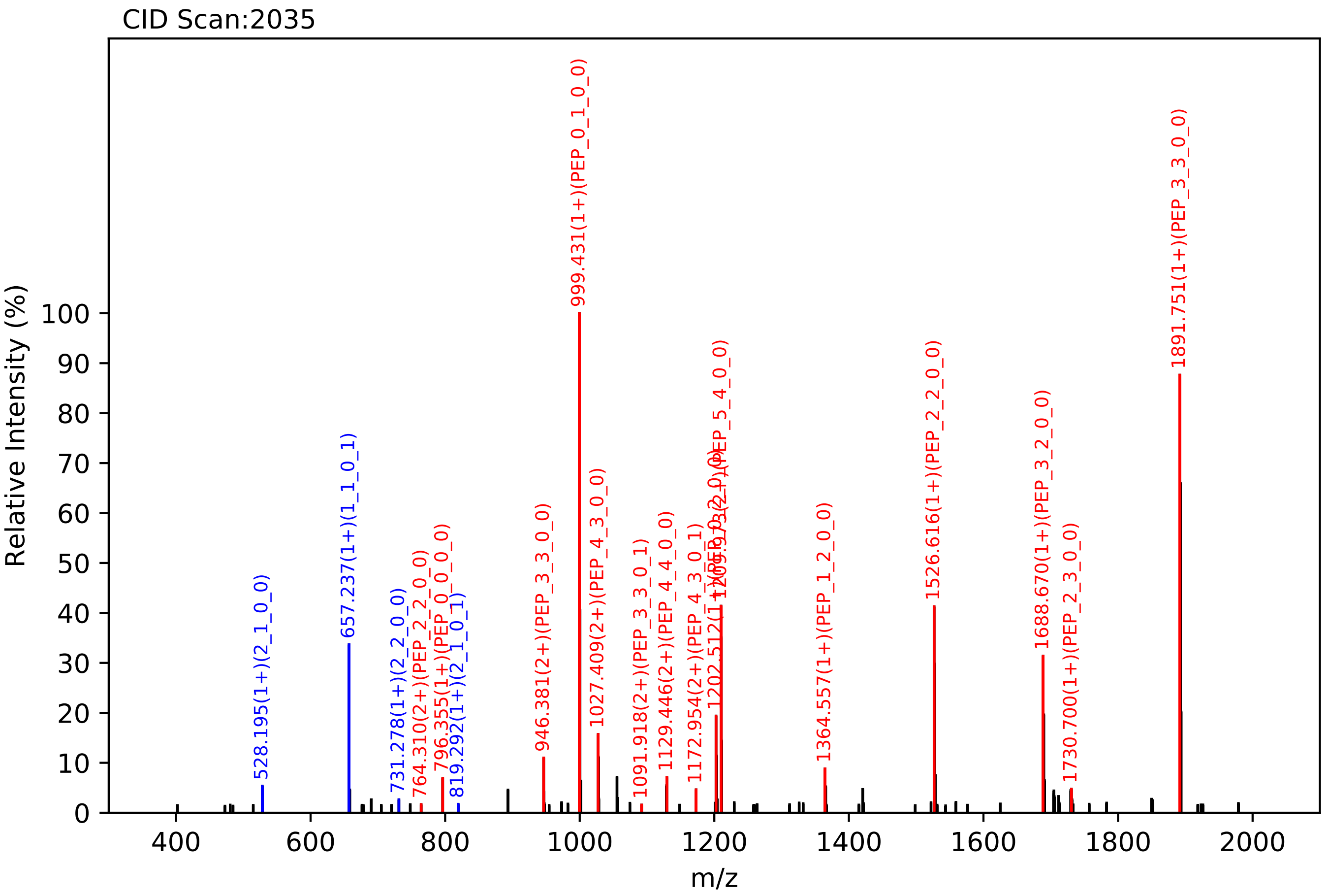

Test set no. 127, Experiment: AGP exp_3

NEEYNK(=PEP)_5_4_0_1, m/z:1356.02(2+), RT:19.46, Y-score:85.23

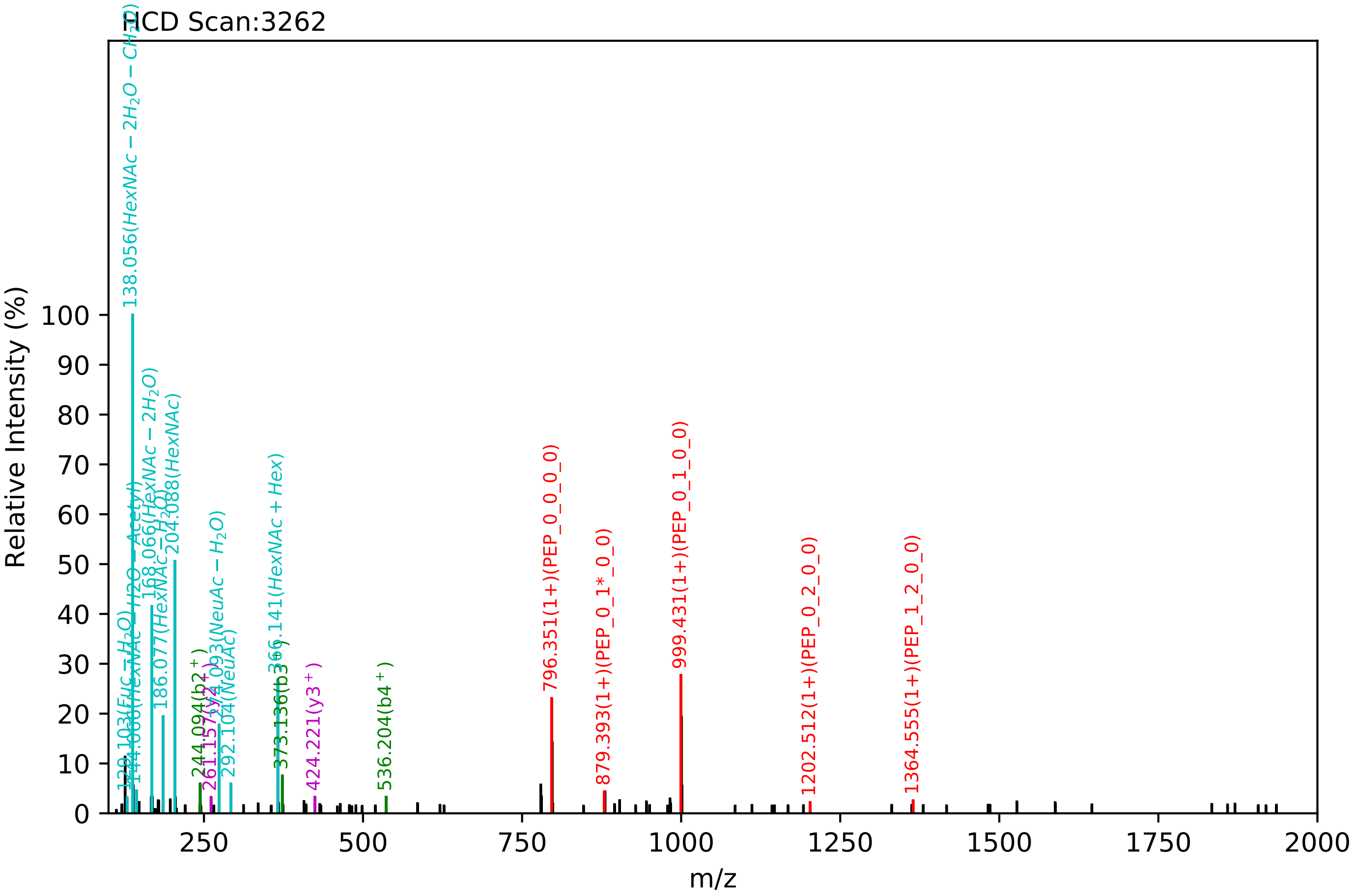

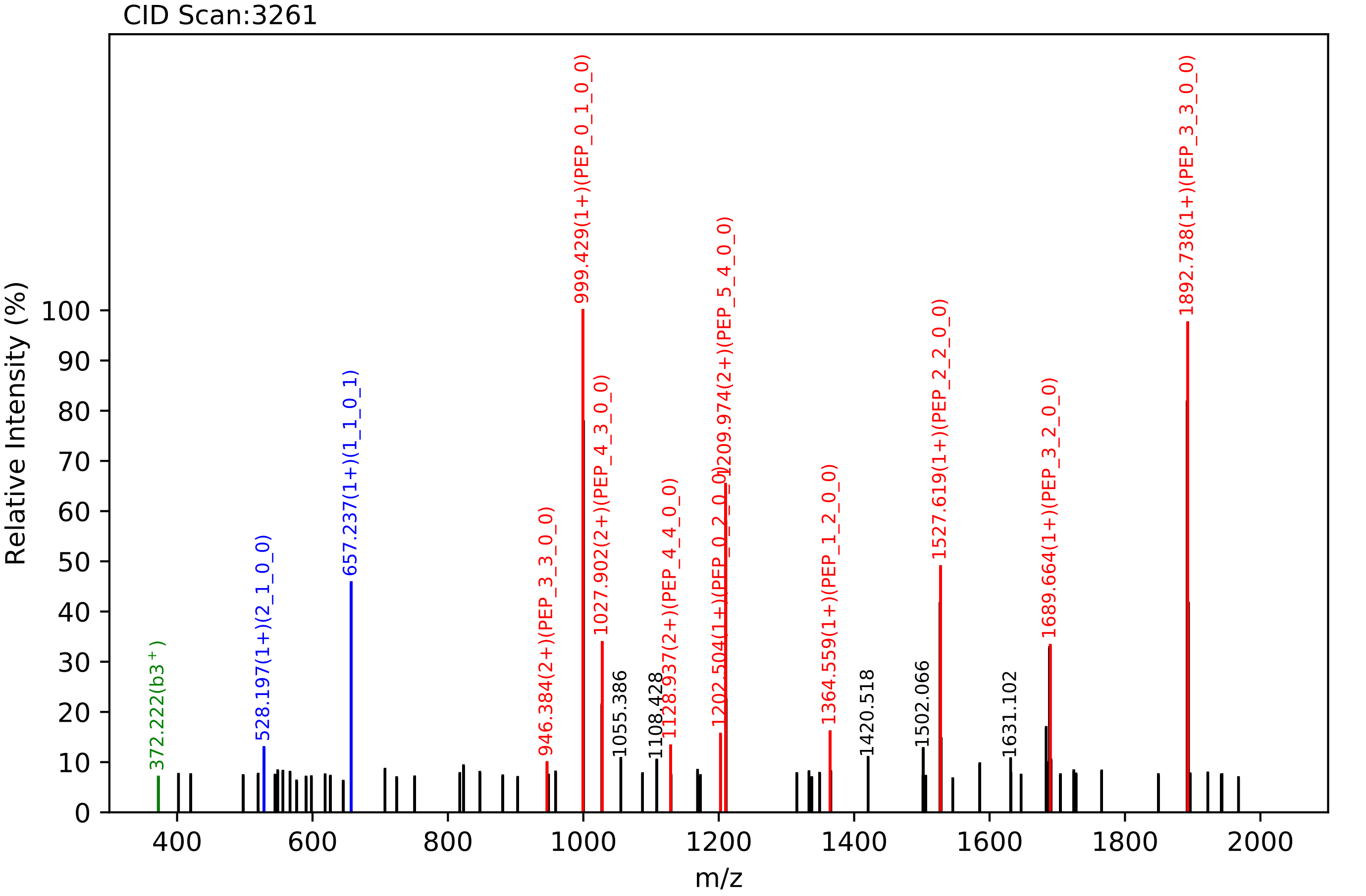

Test set no. 128, Experiment: AGP exp_3

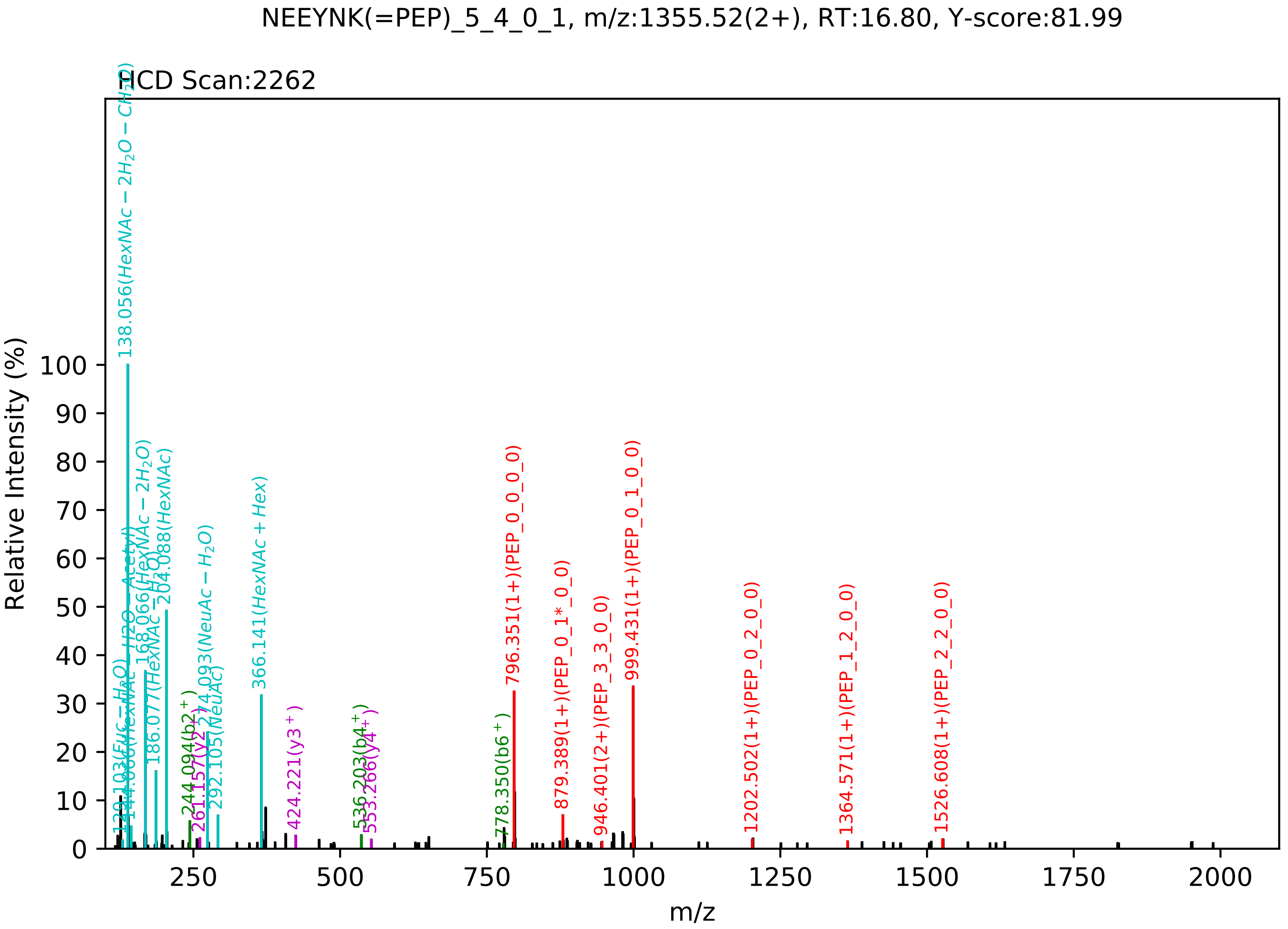

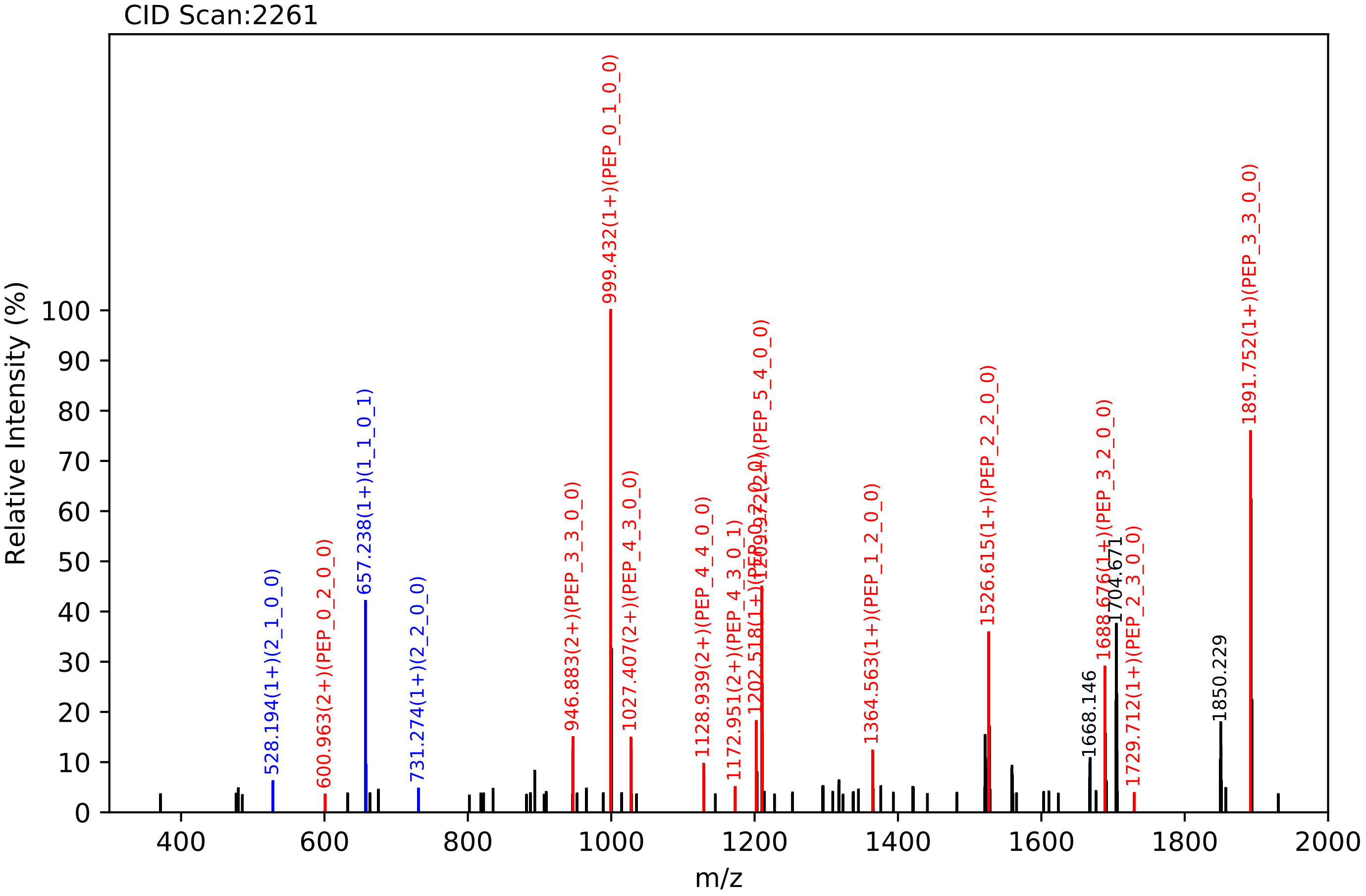

Test set no. 129, Experiment: AGP exp_4

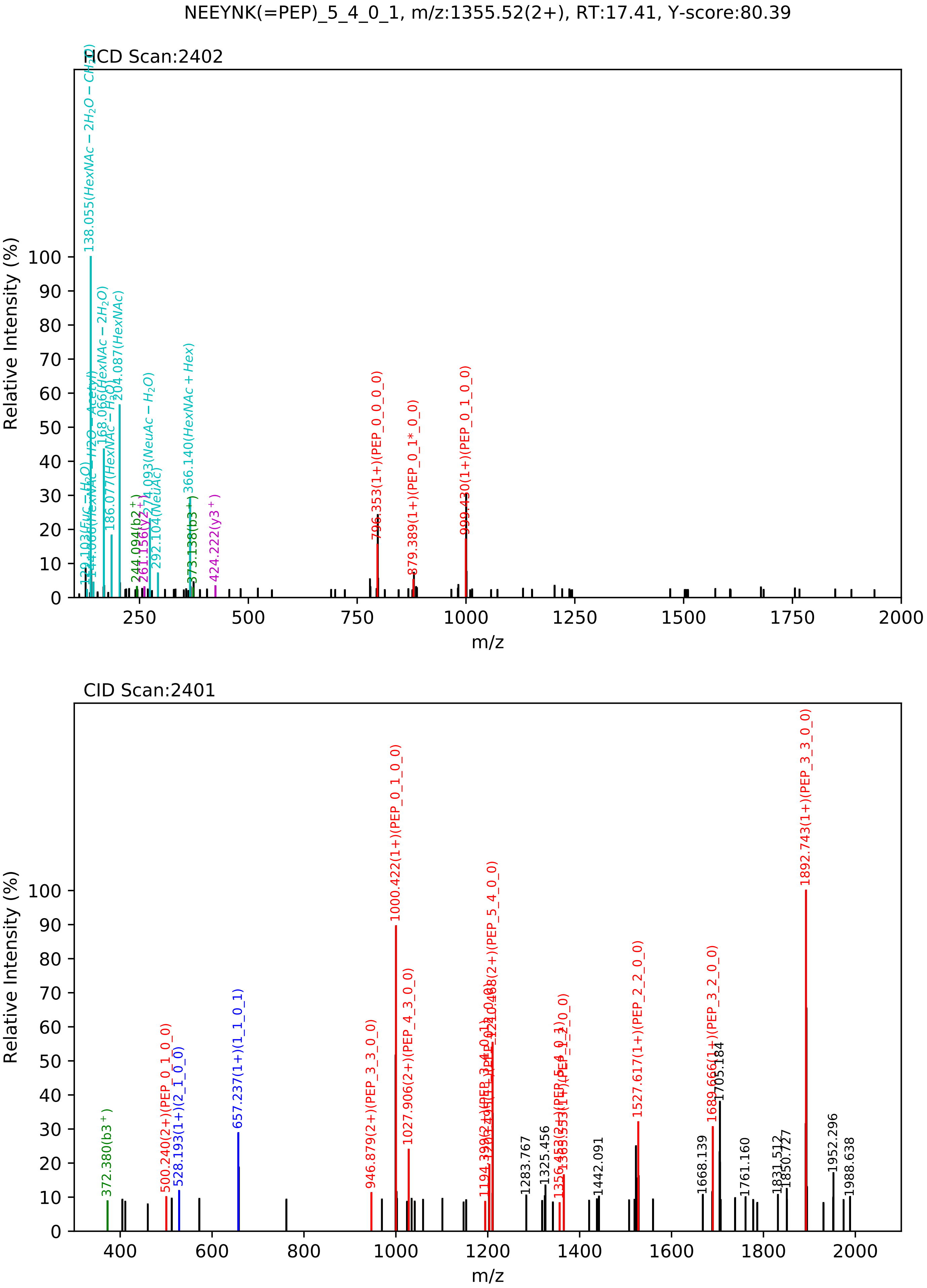

Test set no. 130, Experiment: AGP exp_4

NEEYNK(=PEP)_5_4_0_1, m/z:1355.52(2+), RT:16.90, Y-score:79.62

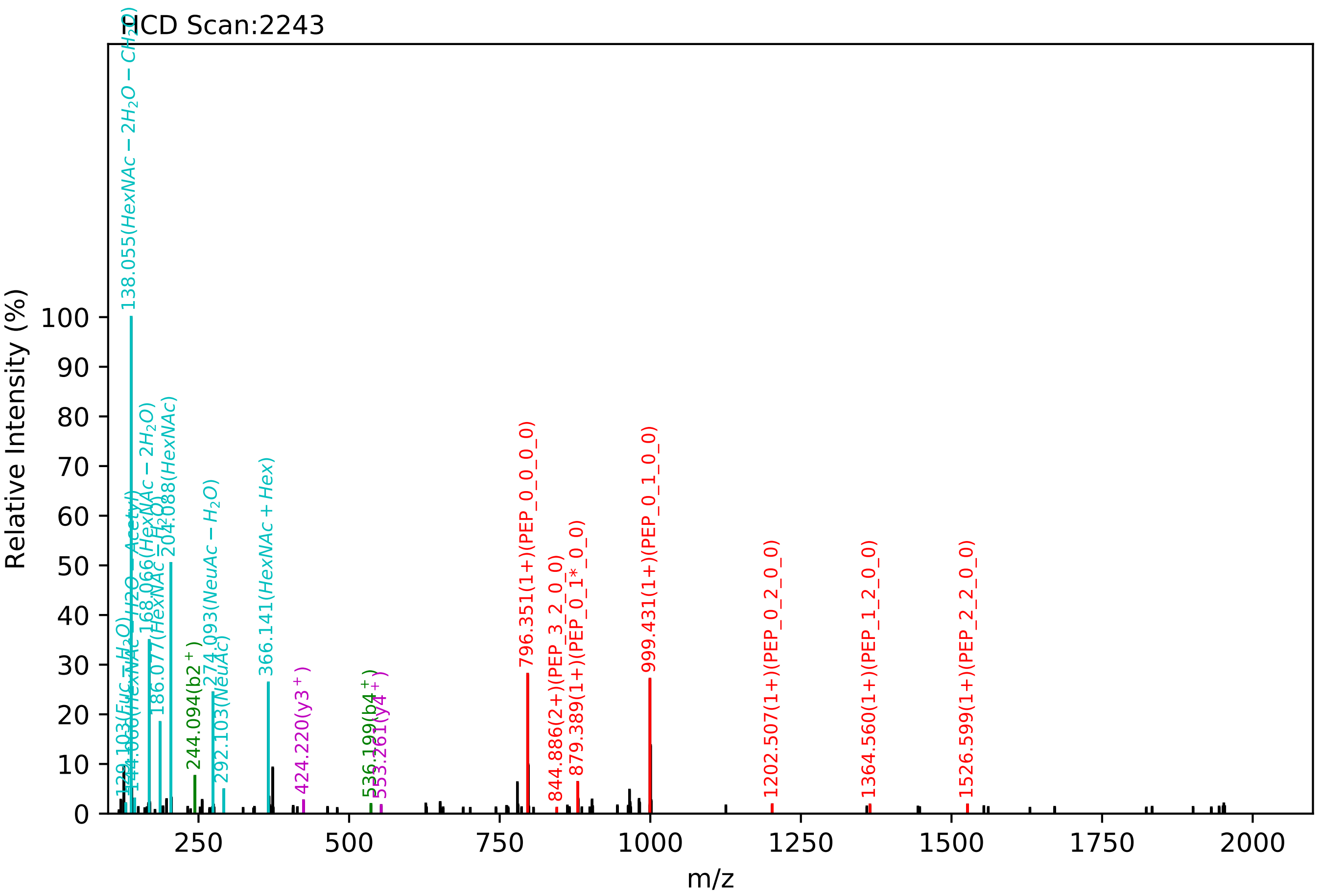

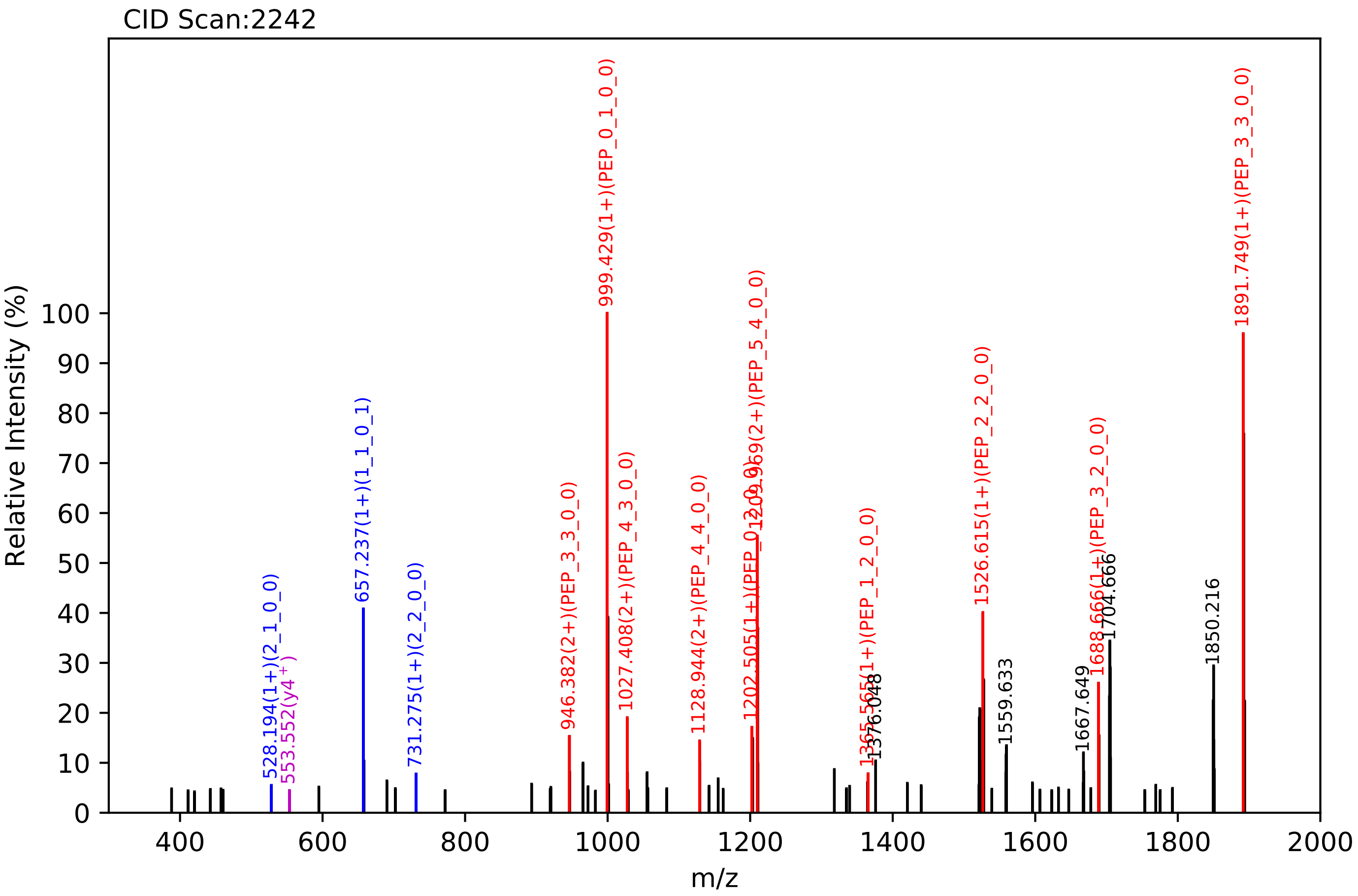

Test set no. 131, Experiment: AGP exp_3

NEEYNK(=PEP)_5_4_0_2, m/z:1002.05(3+), RT:18.65, Y-score:91.21

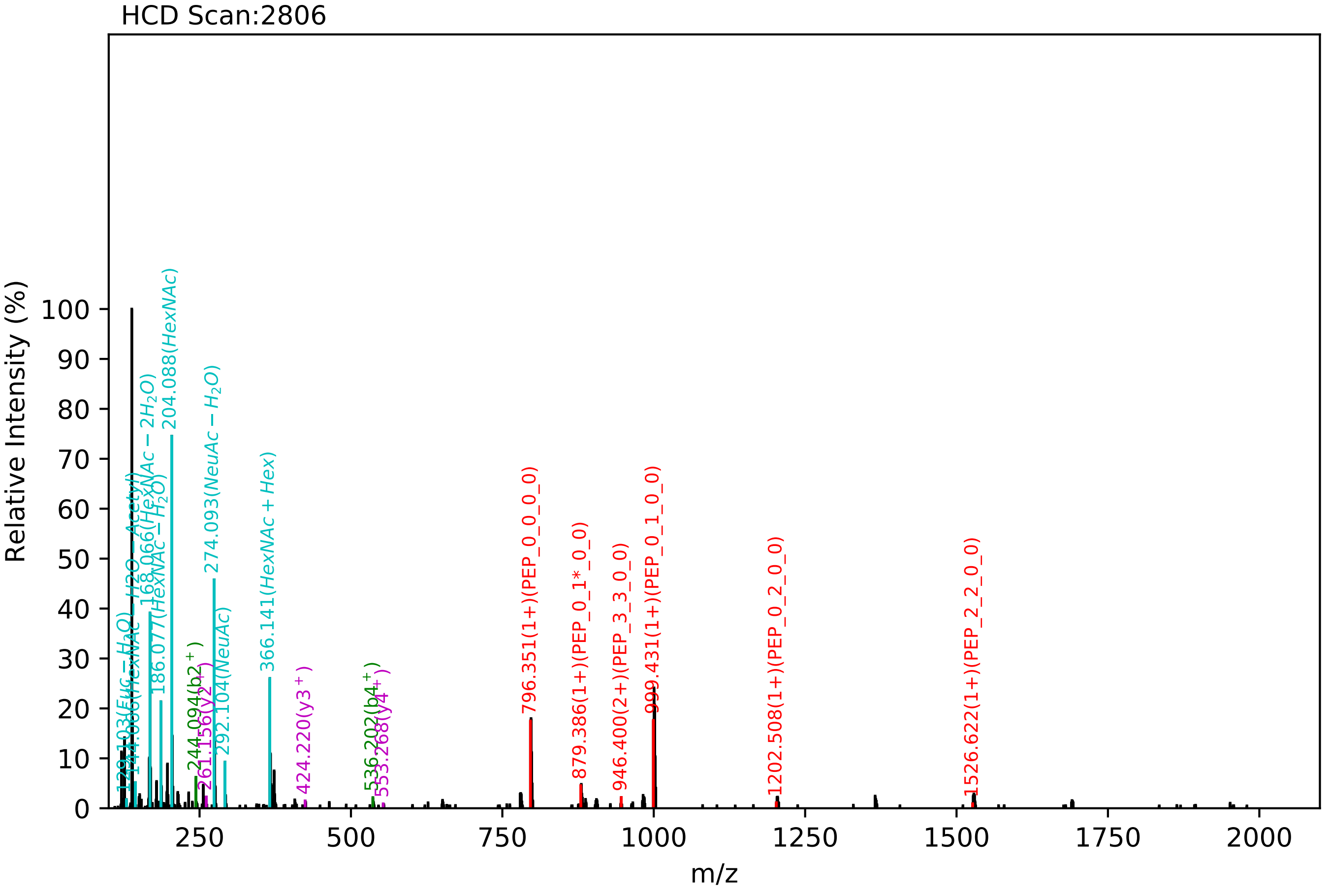

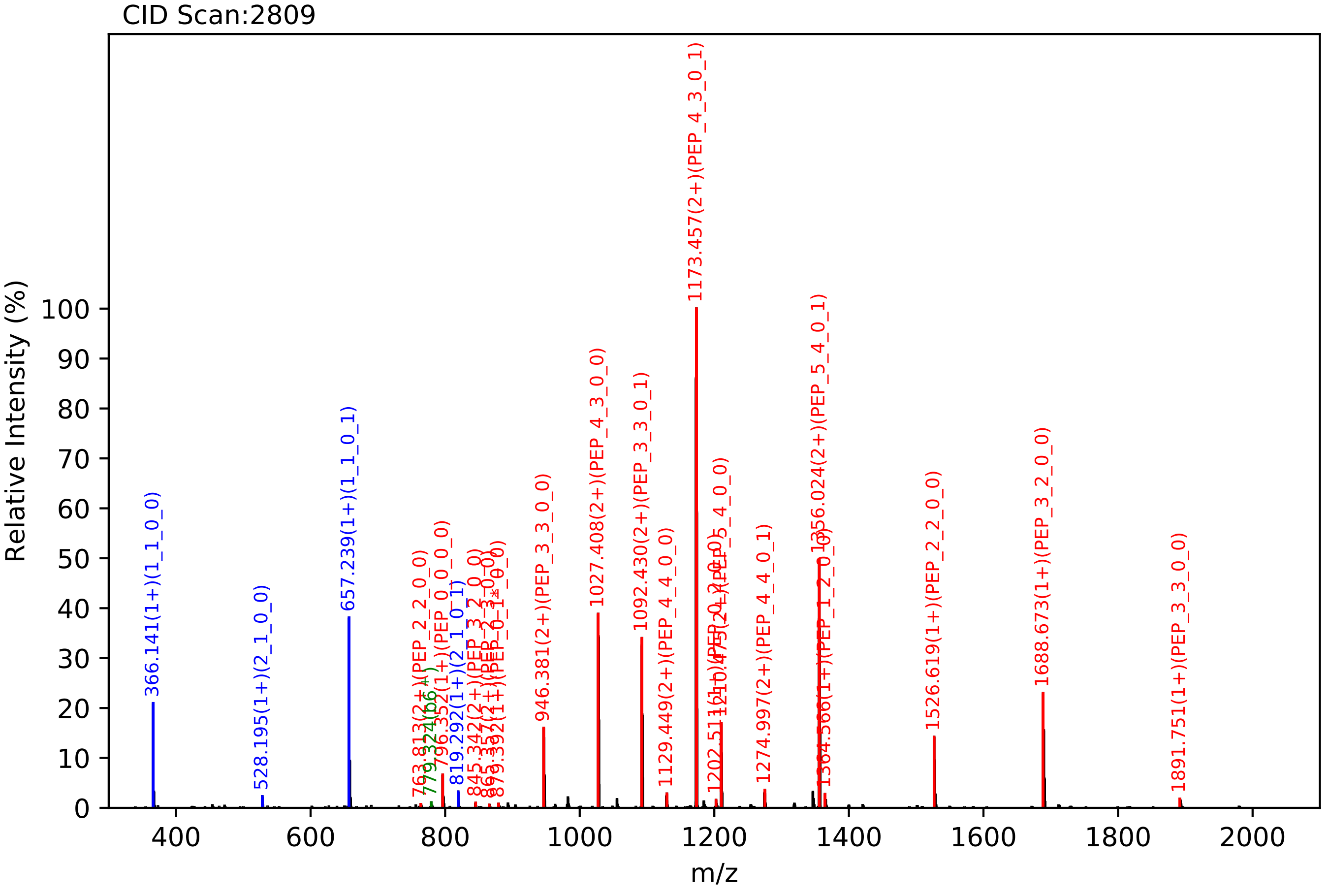

Test set no. 132, Experiment: AGP exp_3

NEEYNK(=PEP)_5_4_0_2, m/z:1001.05(3+), RT:19.92, Y-score:93.75

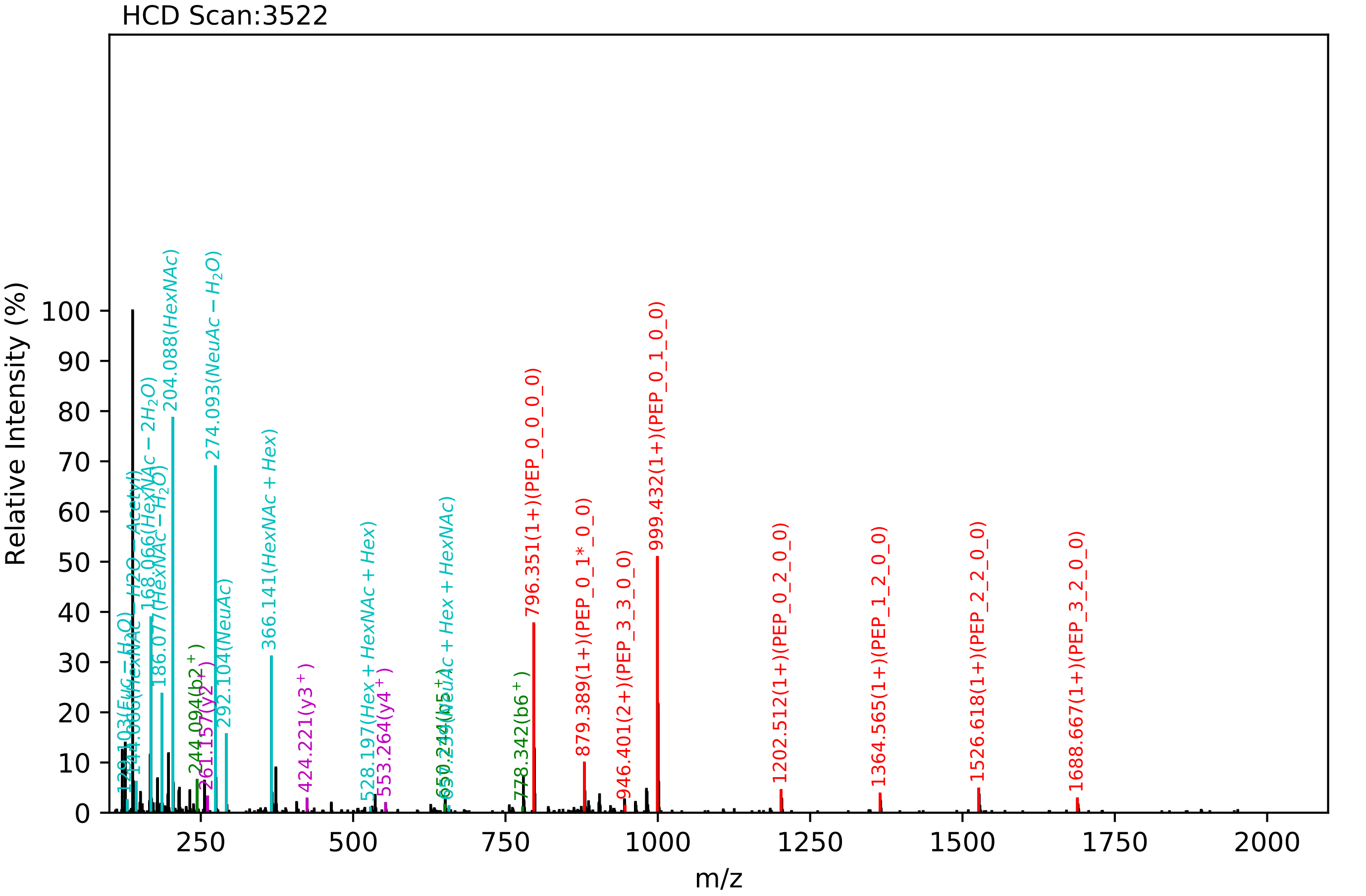

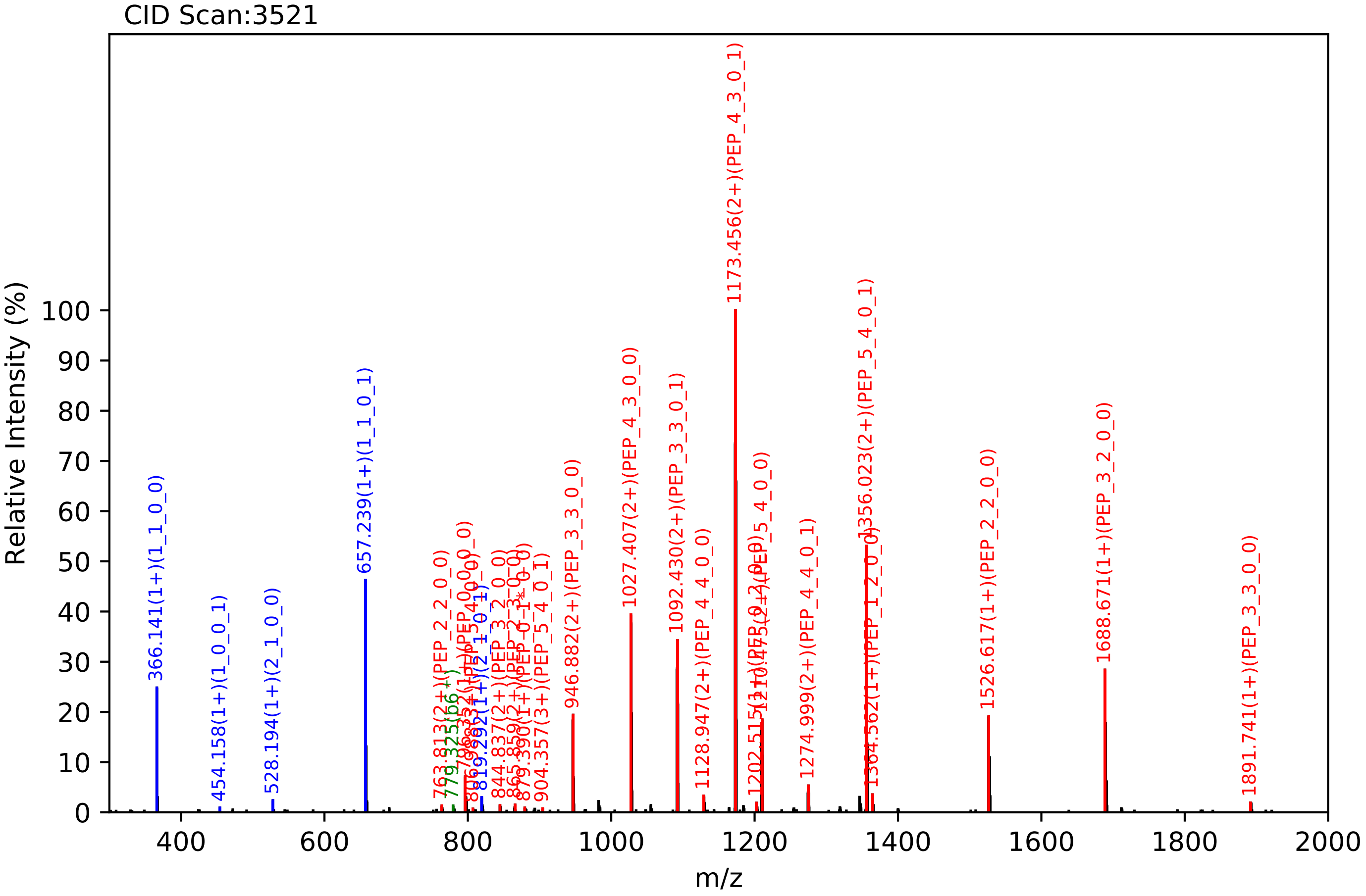

Test set no. 133, Experiment: AGP exp_3

NEEYNK(=PEP)_5_4_0_2, m/z:1001.05(3+), RT:19.29, Y-score:93.55

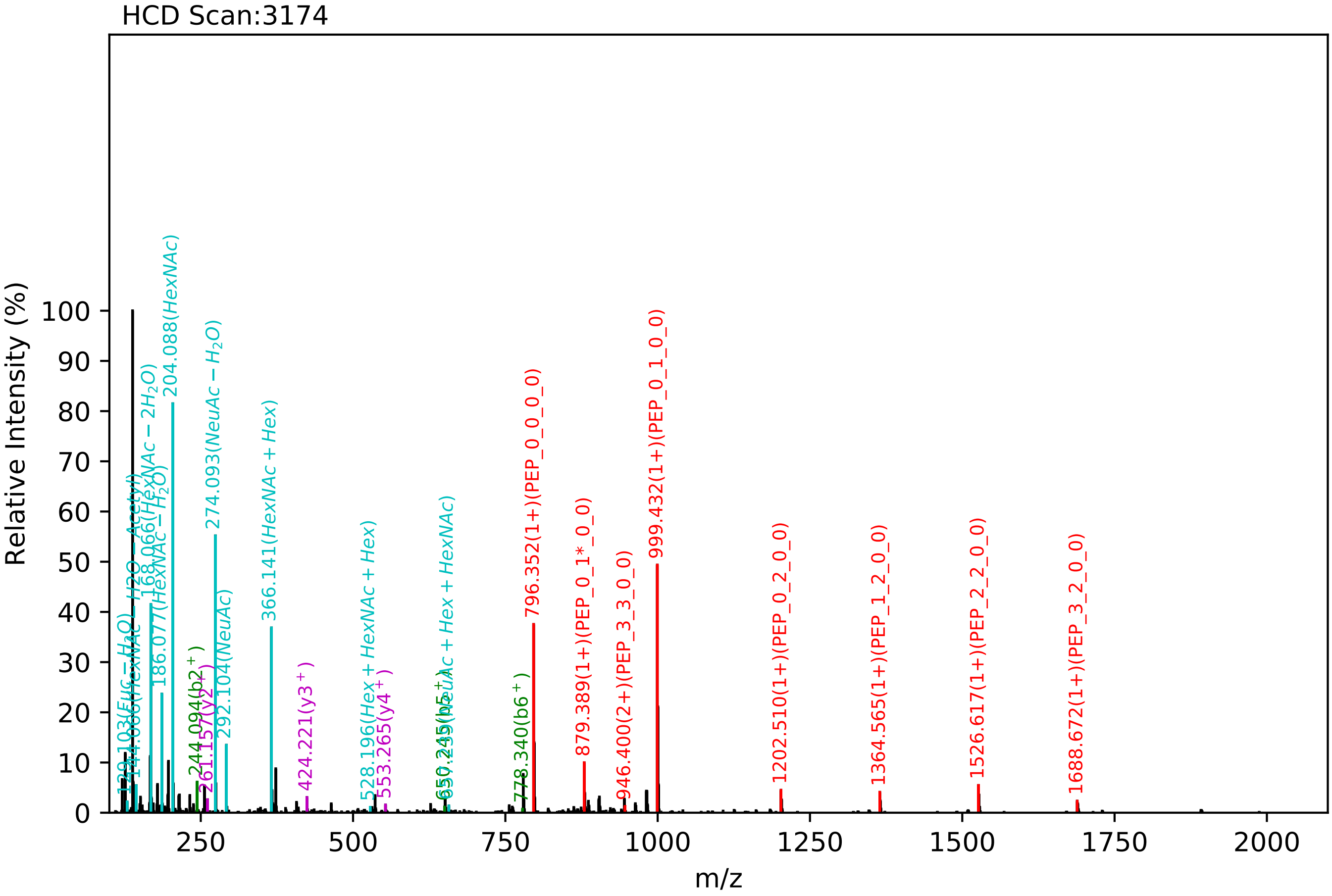

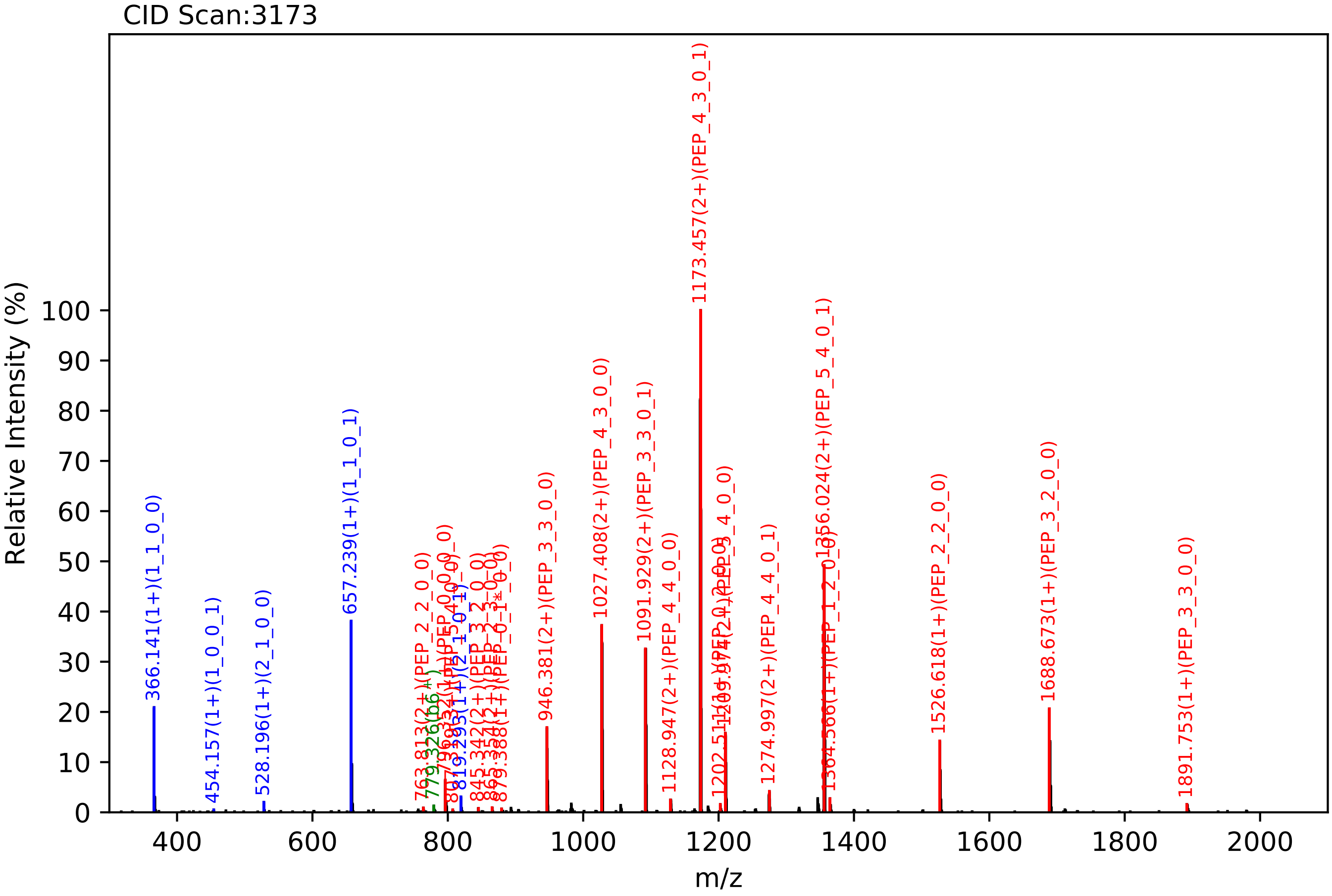

Test set no. 134, Experiment: AGP exp_4

NEEYNK(=PEP)_5_4_0_2, m/z:1002.05(3+), RT:18.70, Y-score:90.39

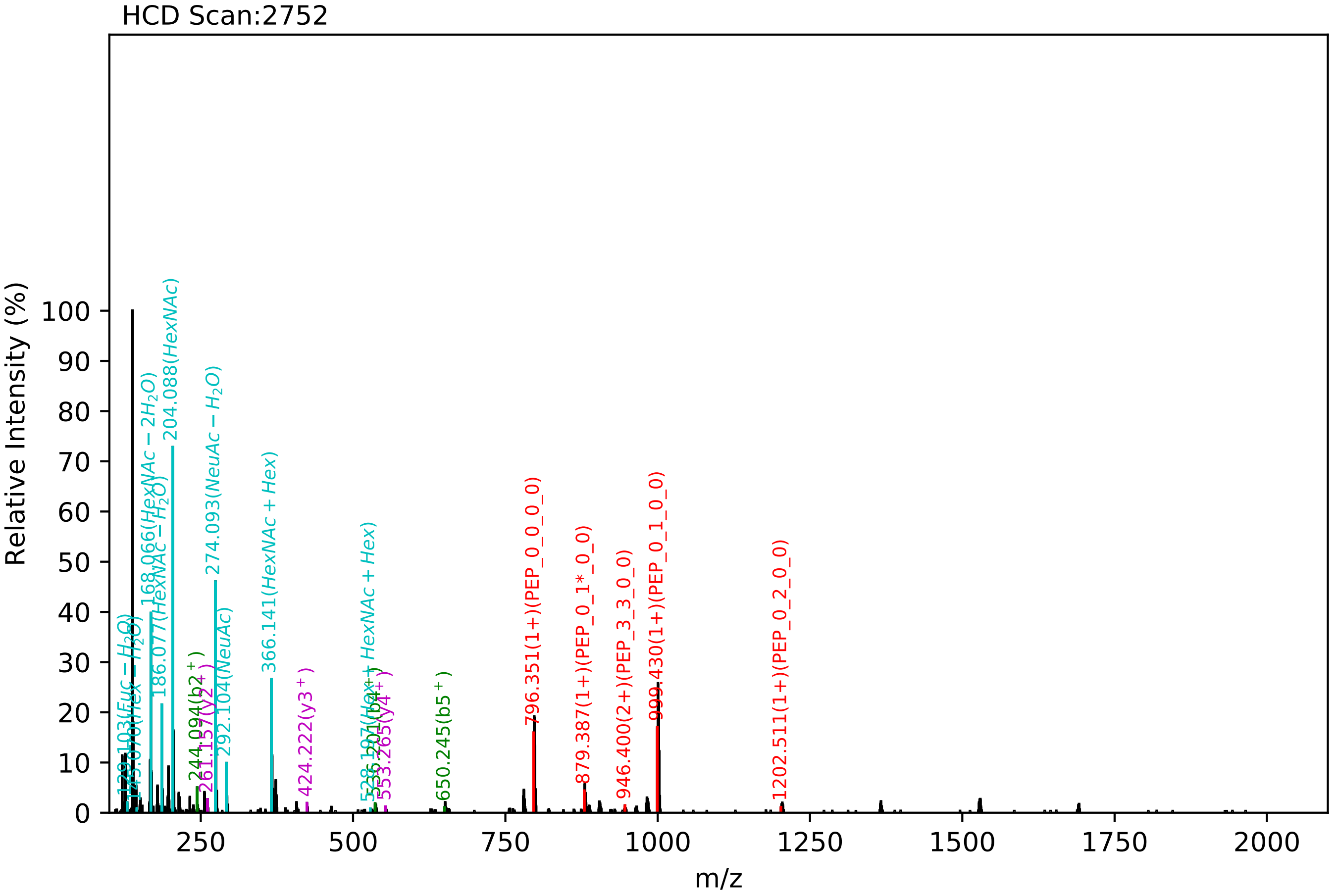

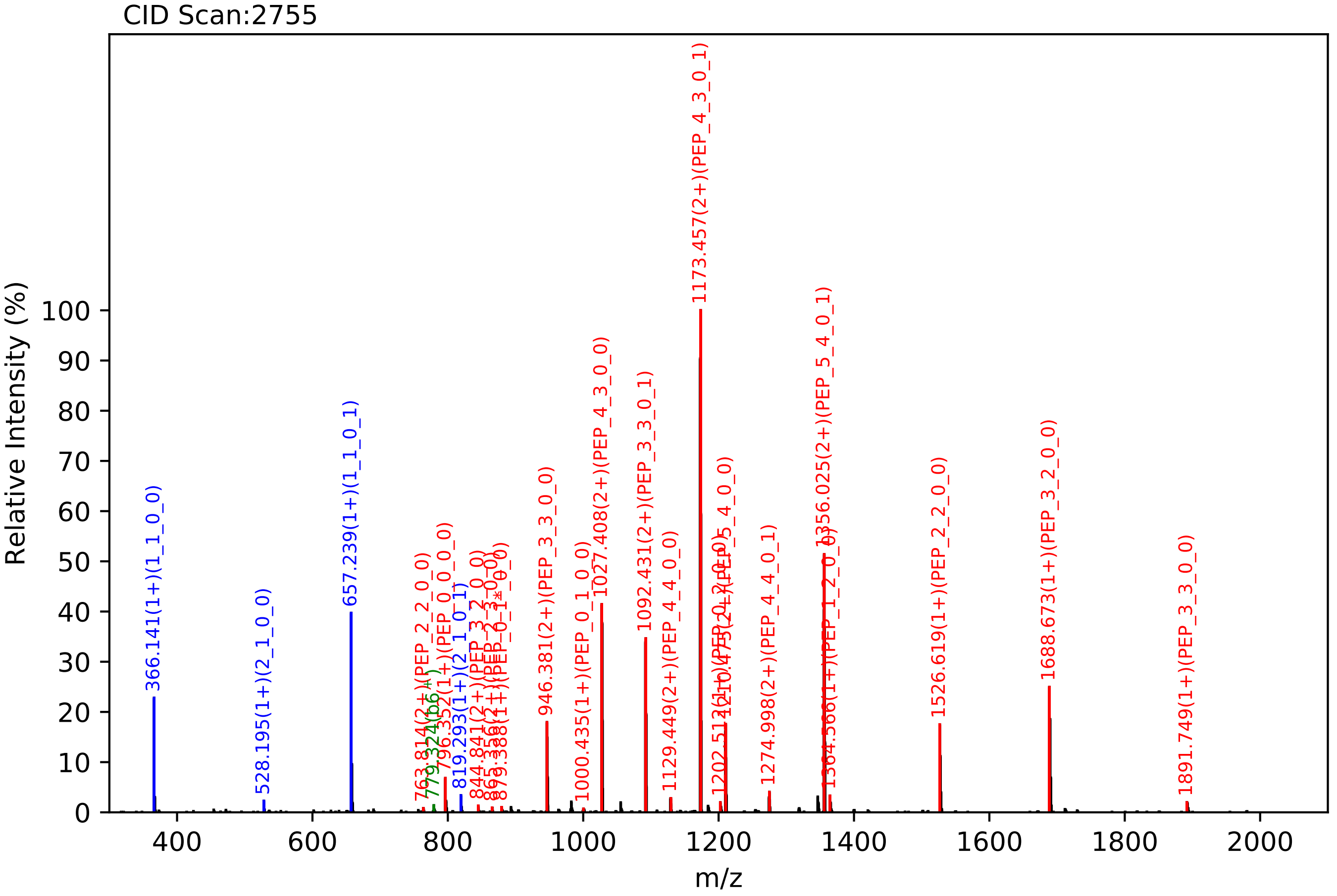

Test set no. 135, Experiment: AGP exp_4

NEEYNK(=PEP)_5_4_0_2, m/z:1501.07(2+), RT:19.51, Y-score:93.05

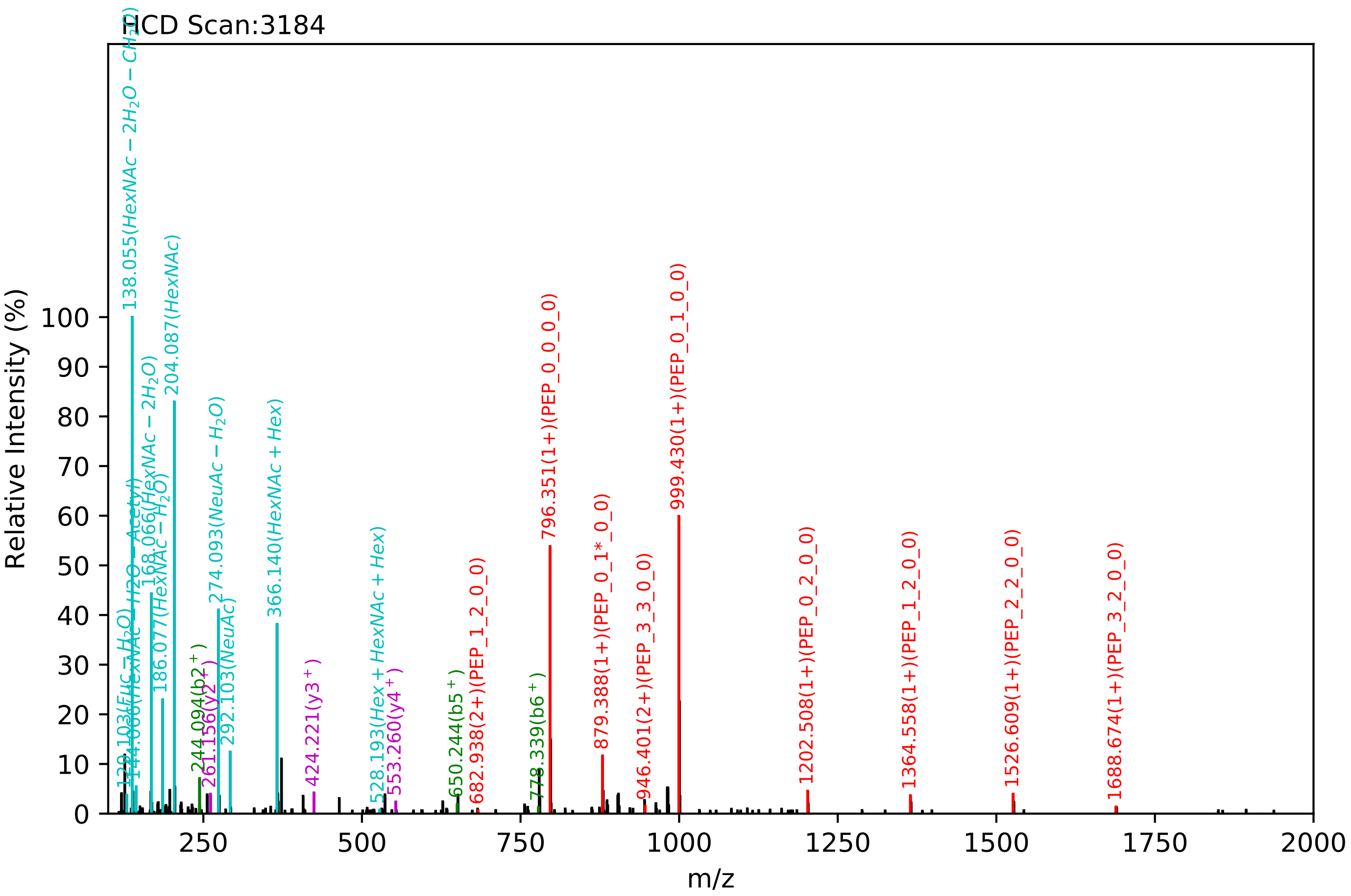

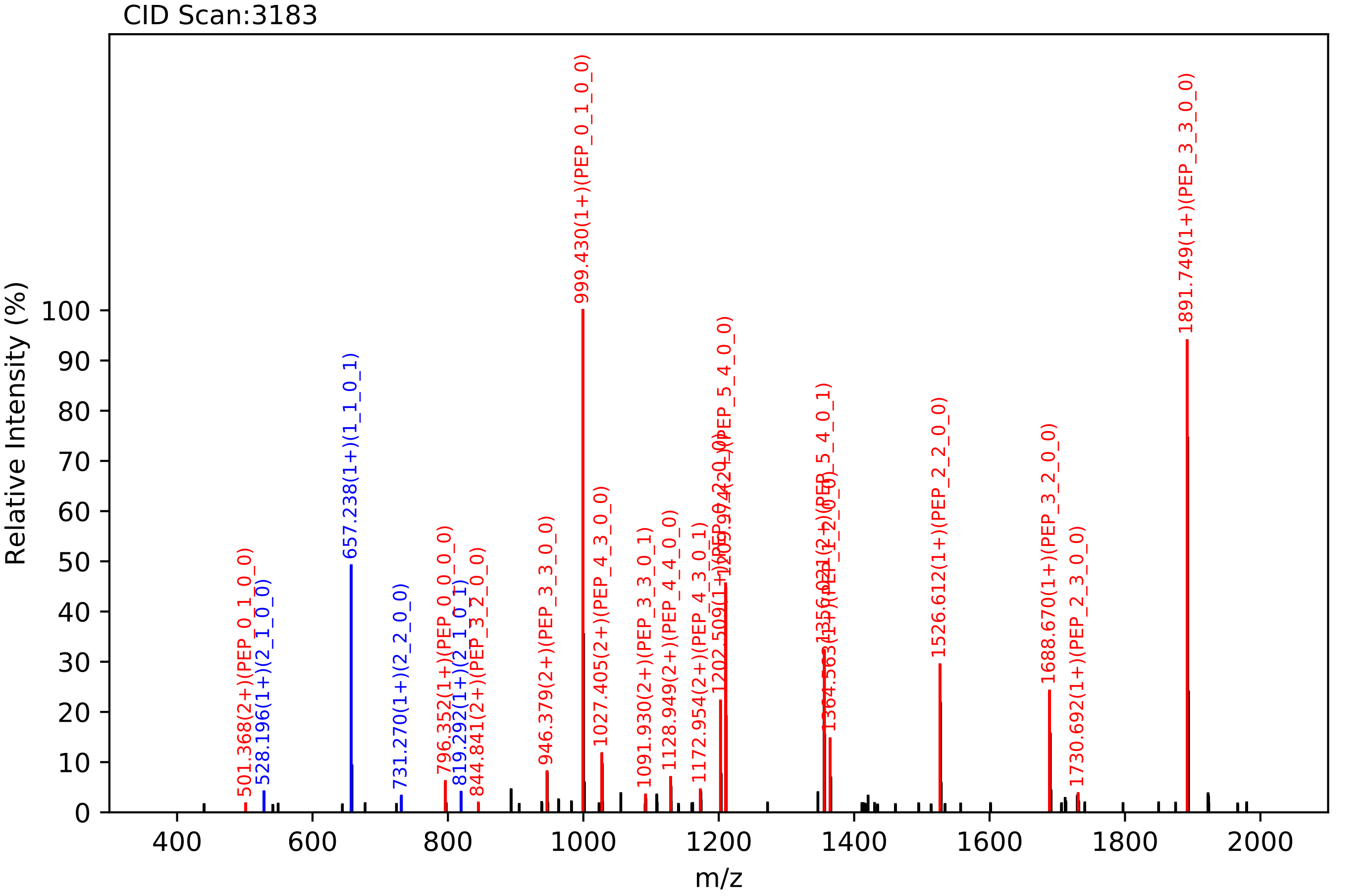

Test set no. 136, Experiment: AGP exp_4

NEEYNK(=PEP)_5_4_0_2, m/z:1001.05(3+), RT:20.27, Y-score:92.51

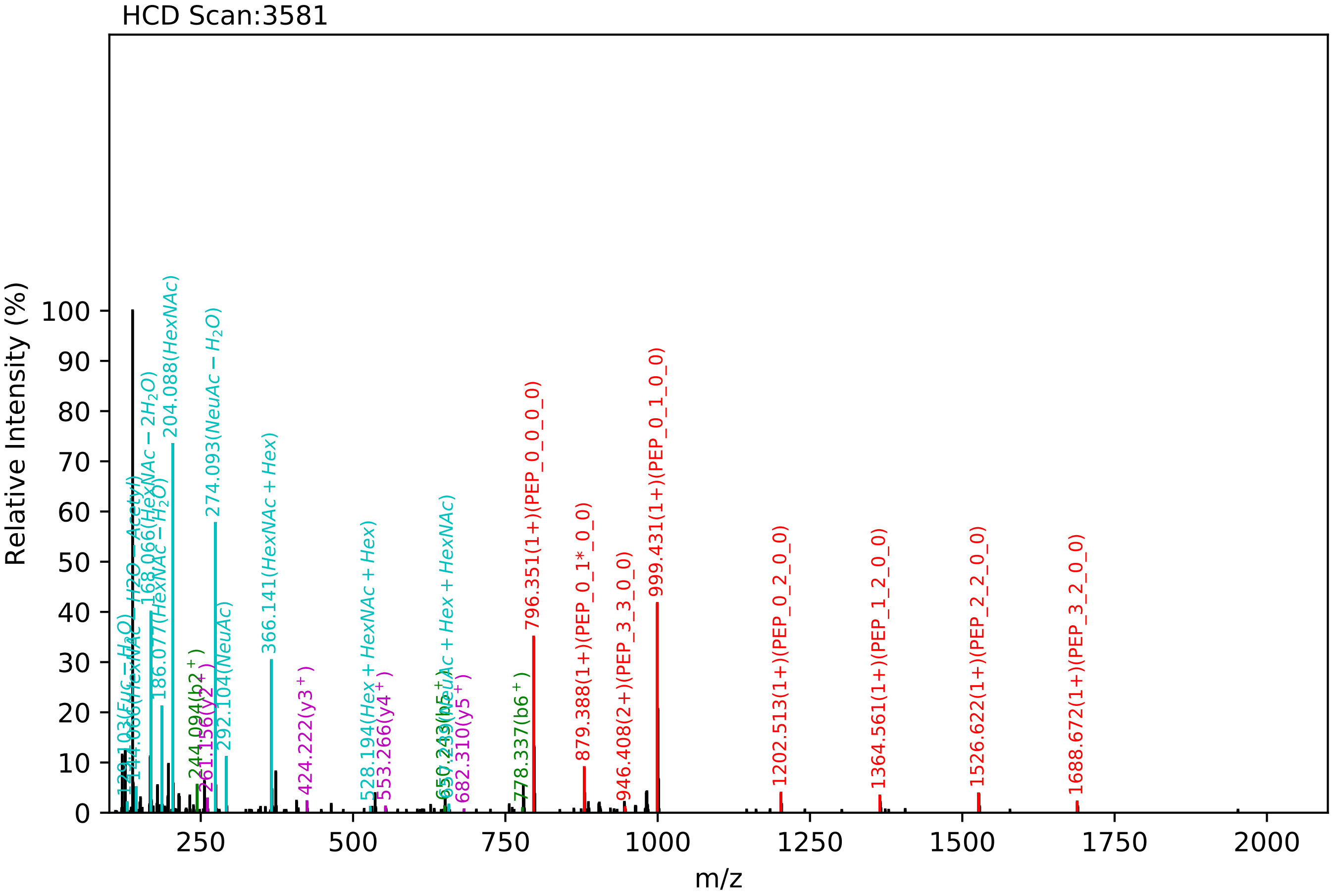

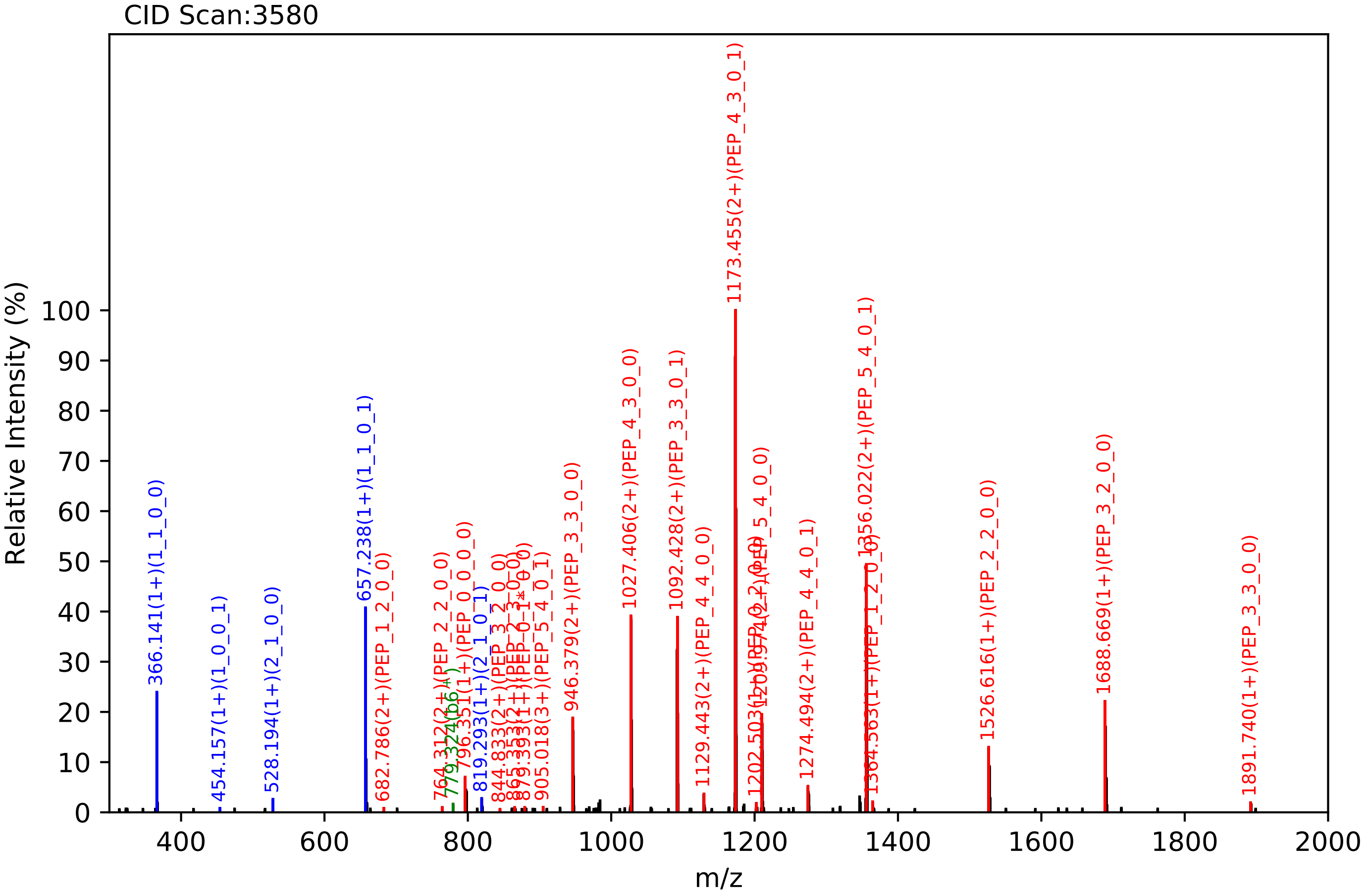

Test set no. 137, Experiment: AGP exp_3

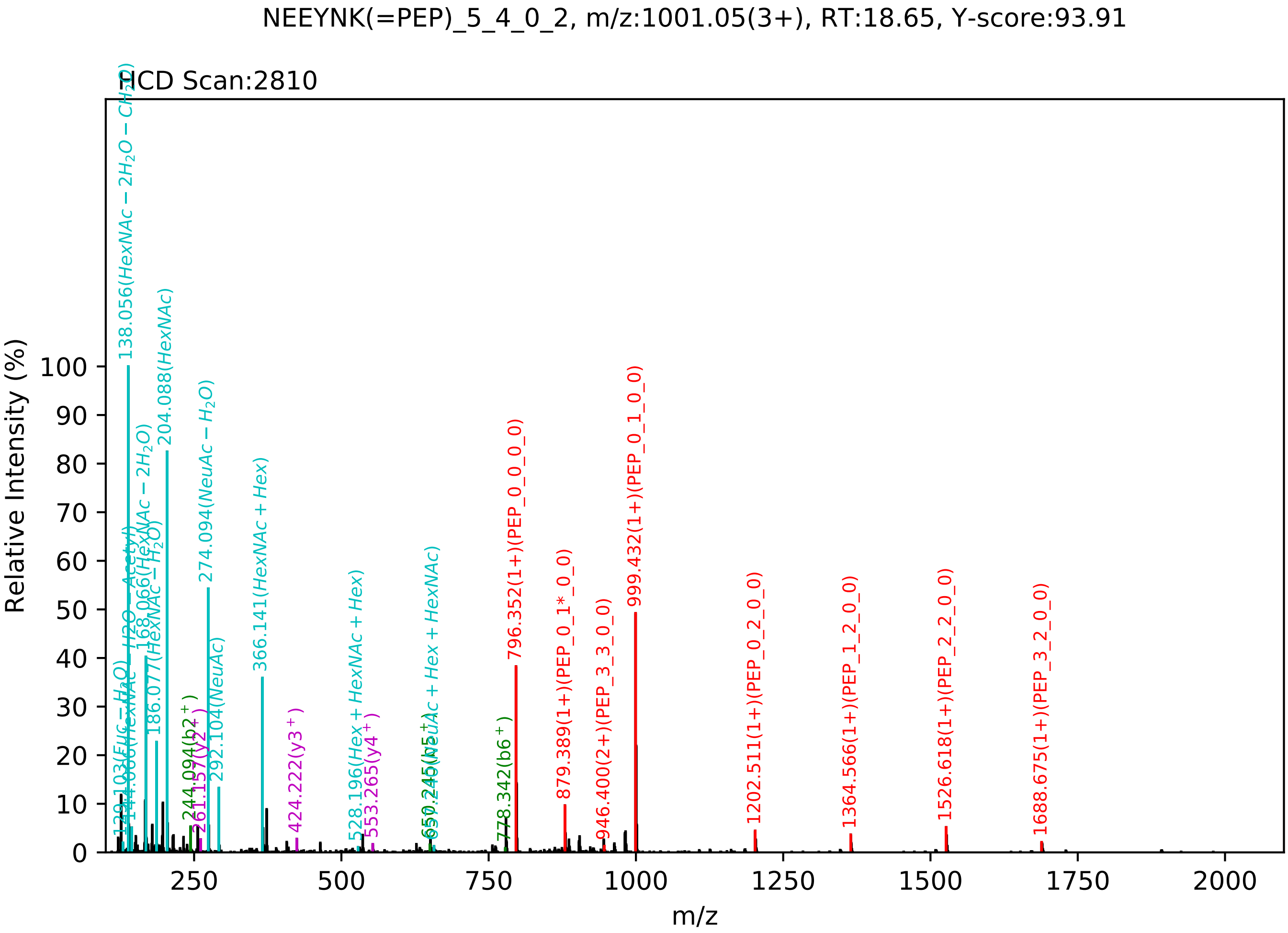

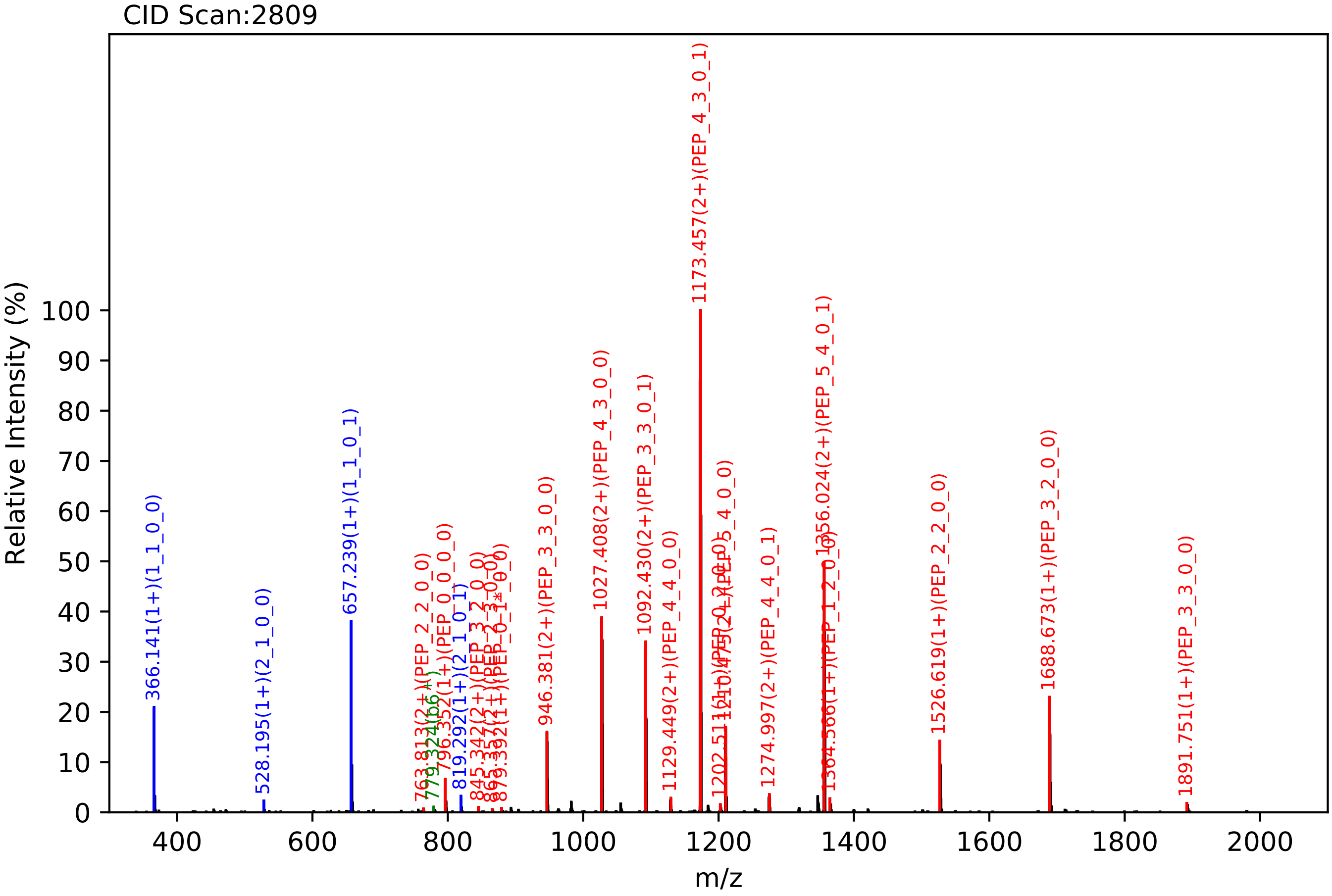

Test set no. 138, Experiment: AGP exp_4

NEEYNK(=PEP)_5_4_0_2, m/z:1001.05(3+), RT:19.73, Y-score:90.99

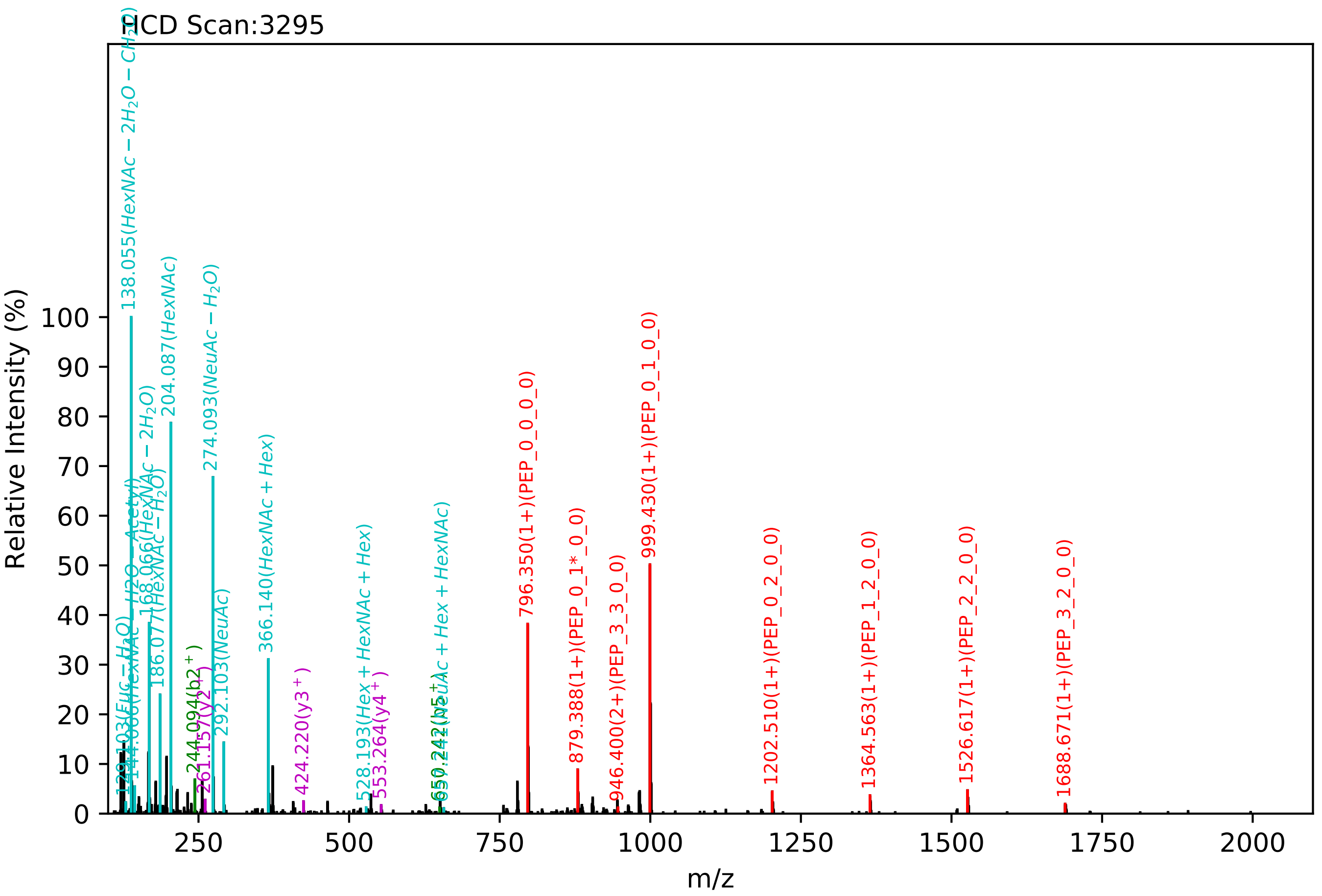

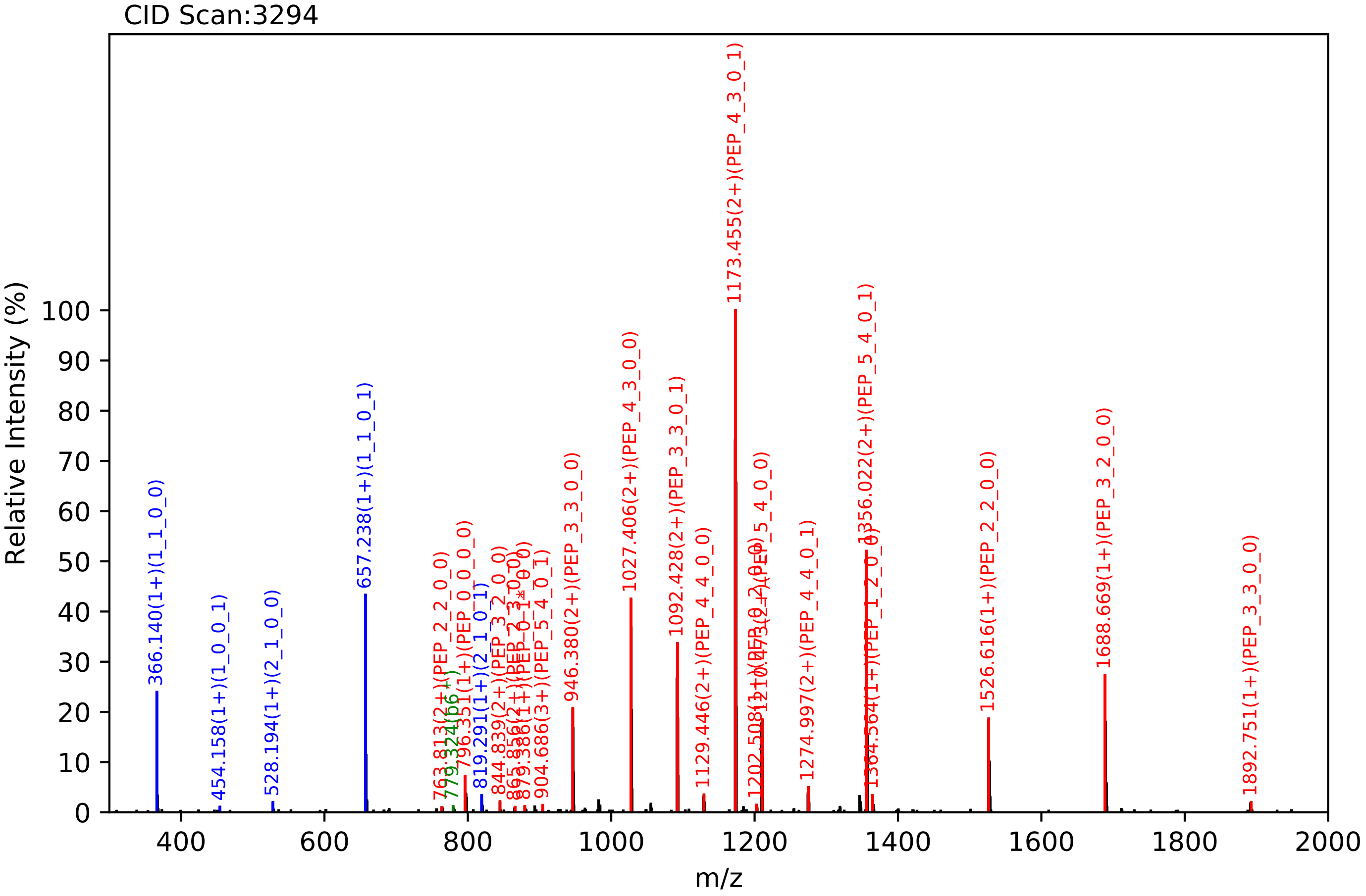

Test set no. 139, Experiment: AGP exp_3

NEEYNK(=PEP)_5_4_0_2, m/z:1501.06(2+), RT:19.64, Y-score:90.93

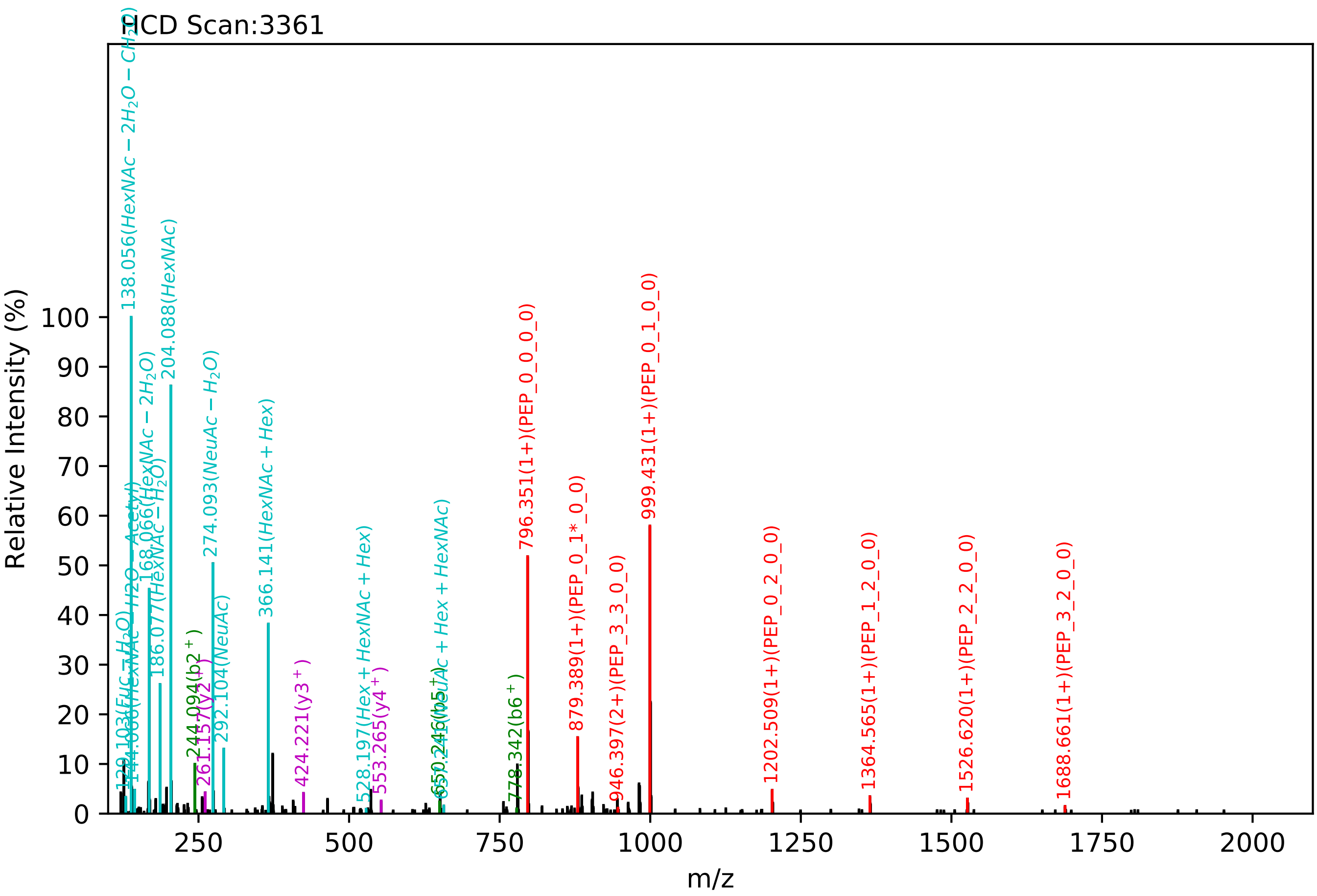

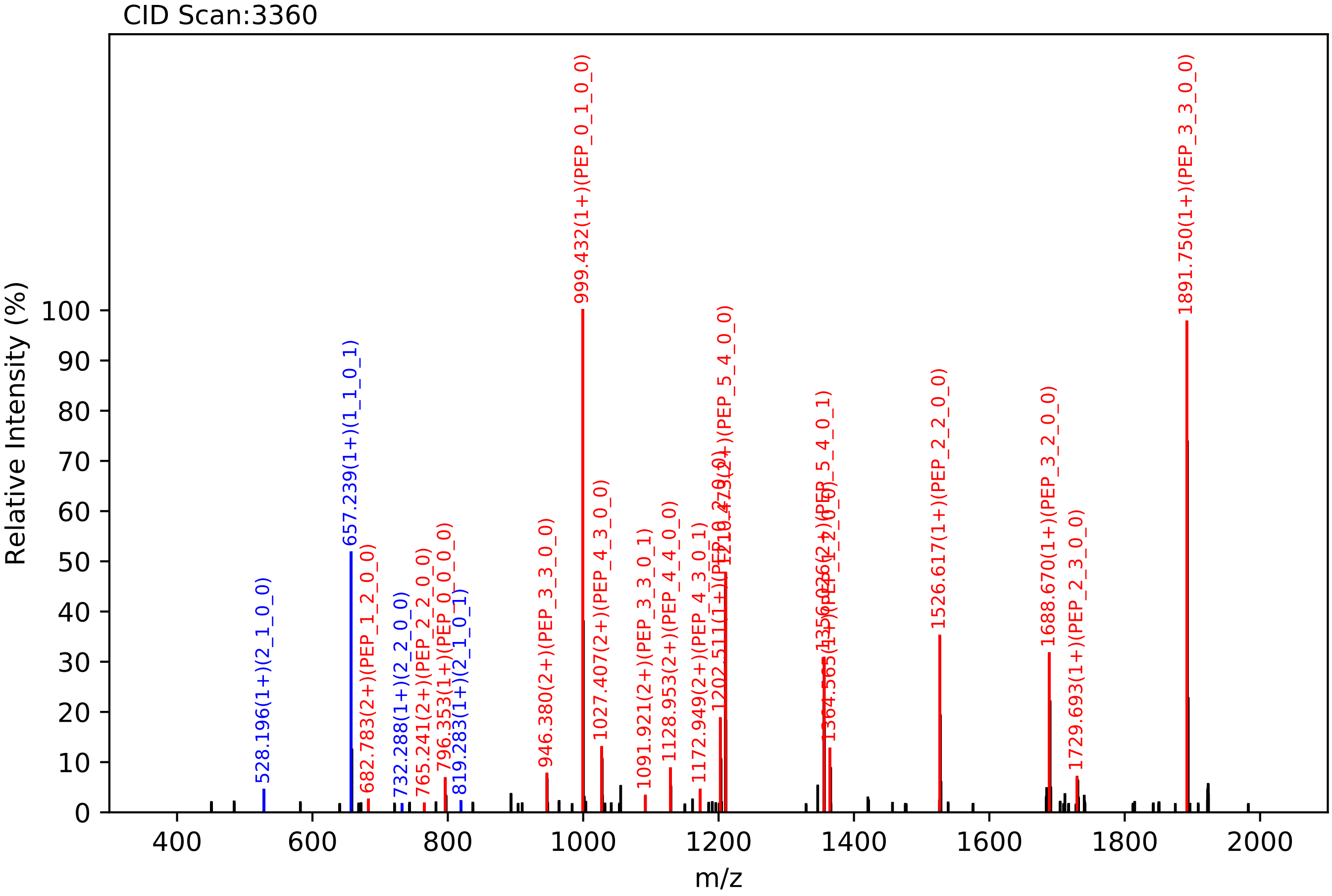

Test set no. 140, Experiment: AGP exp_3

NEEYNK(=PEP)_5_4_0_2, m/z:1001.05(3+), RT:20.50, Y-score:90.81

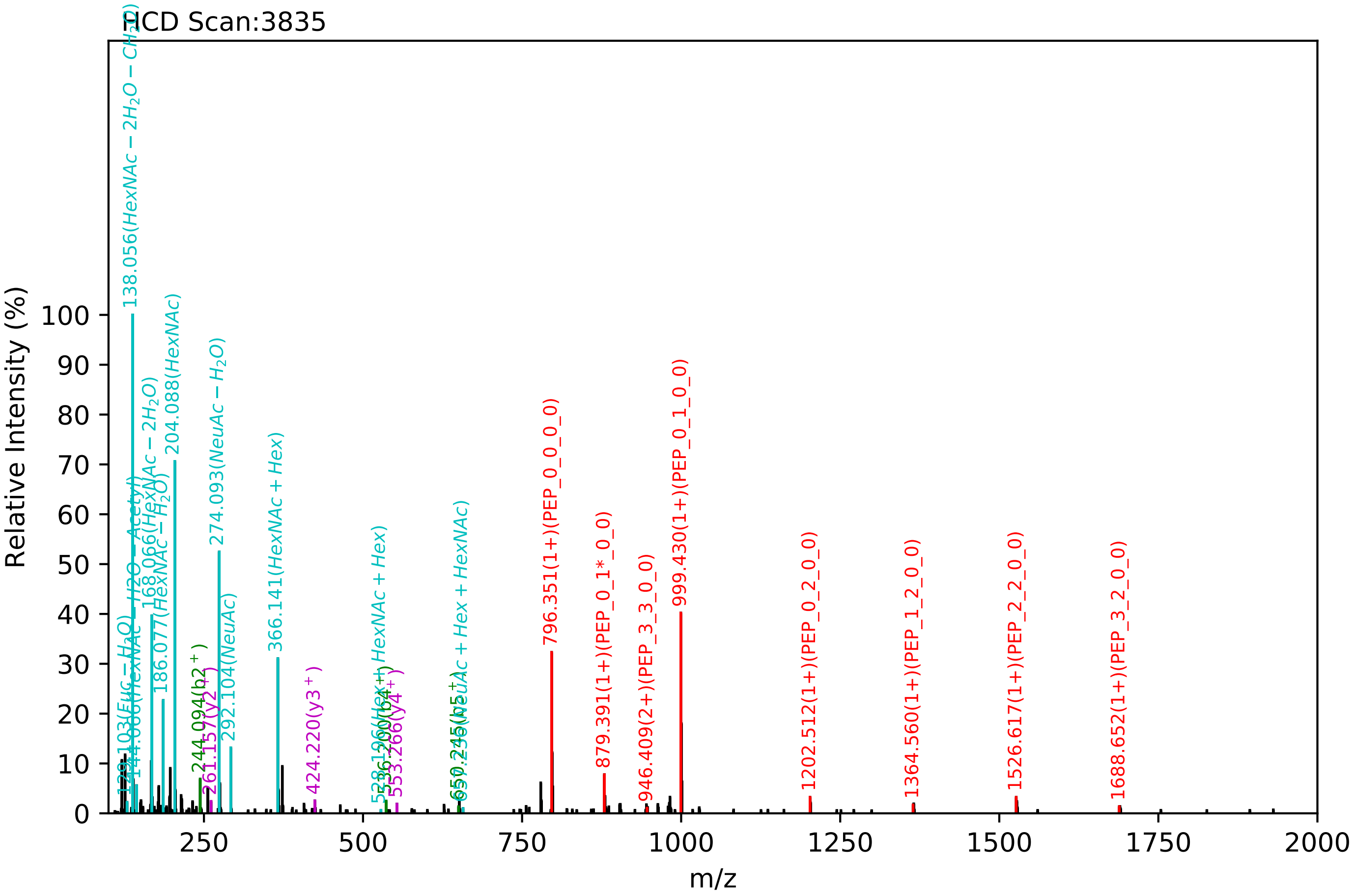

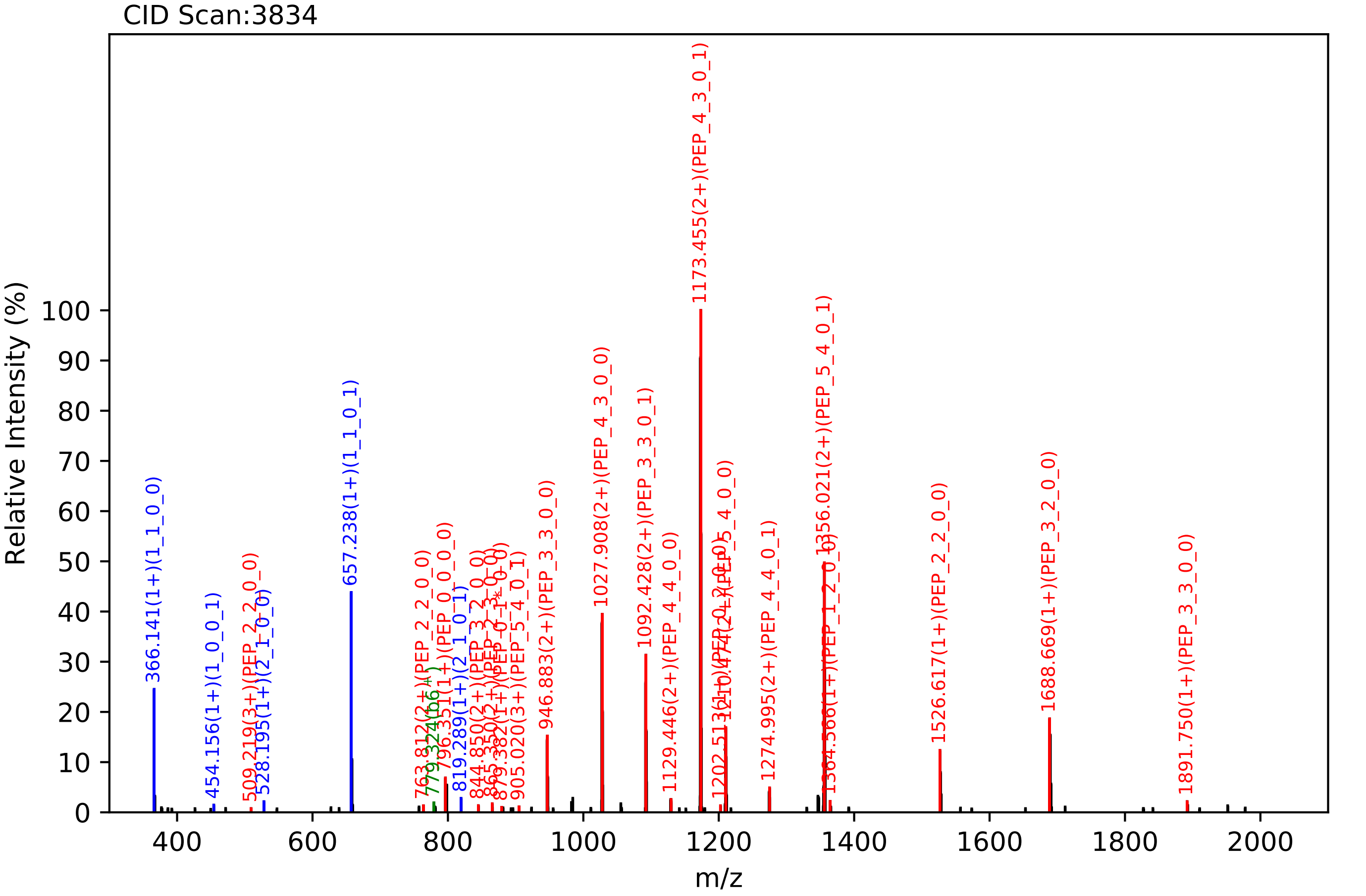

Test set no. 141, Experiment: AGP exp_4

NEEYNK(=PEP)_5_4_0_2, m/z:1001.05(3+), RT:18.71, Y-score:93.40

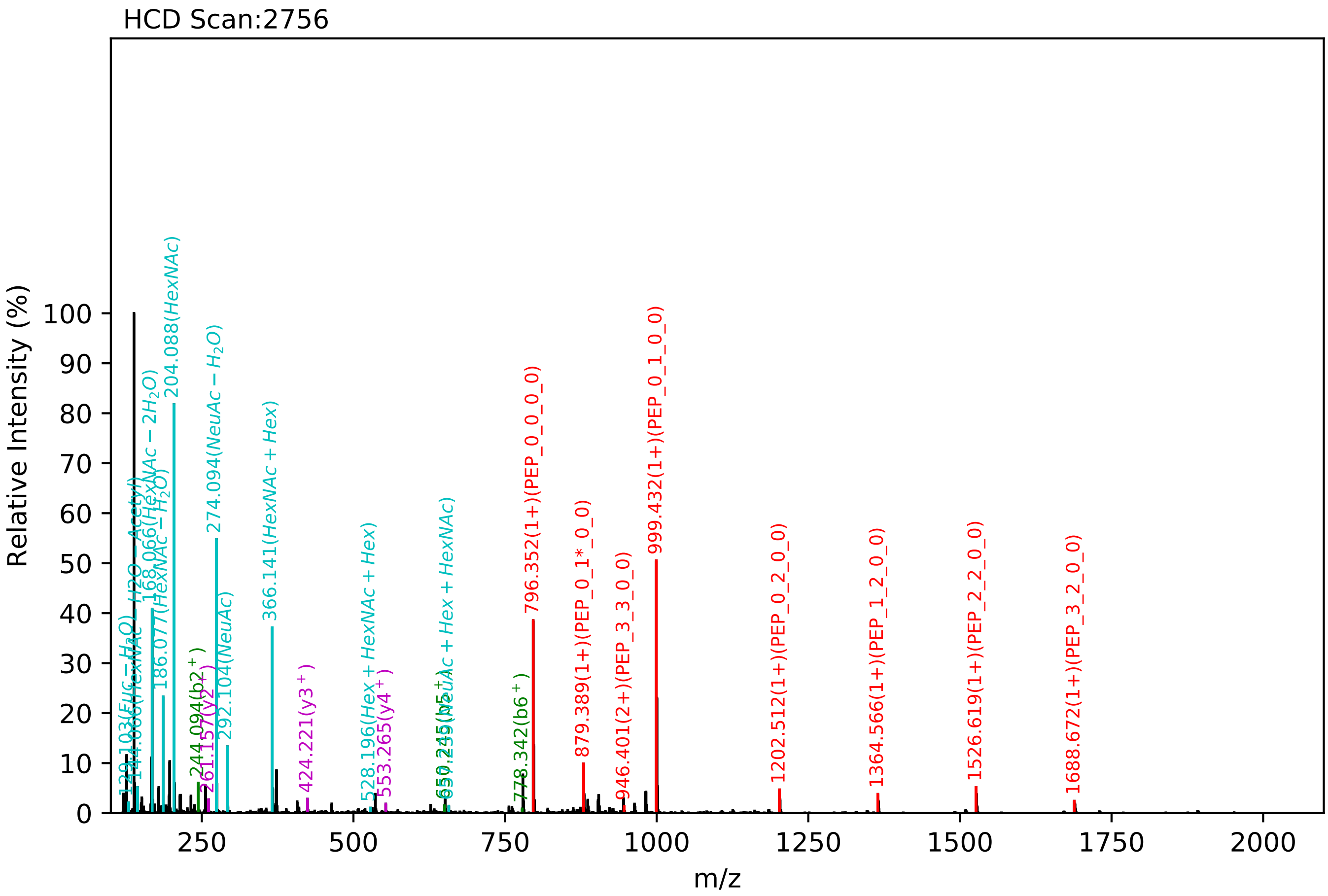

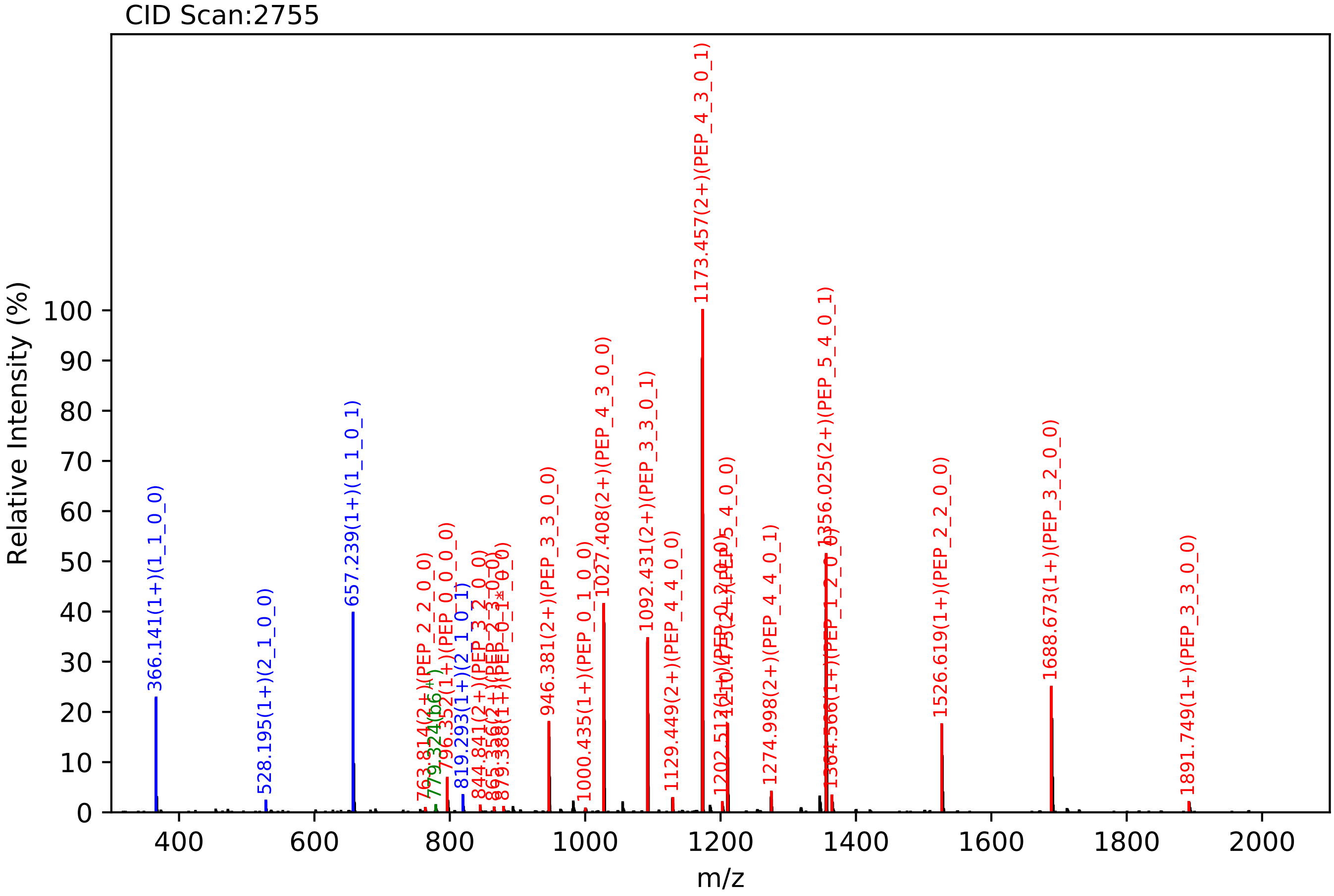

Test set no. 142, Experiment: AGP exp_3

NEEYNK(=PEP)_5_4_0_2, m/z:1501.57(2+), RT:18.45, Y-score:87.43

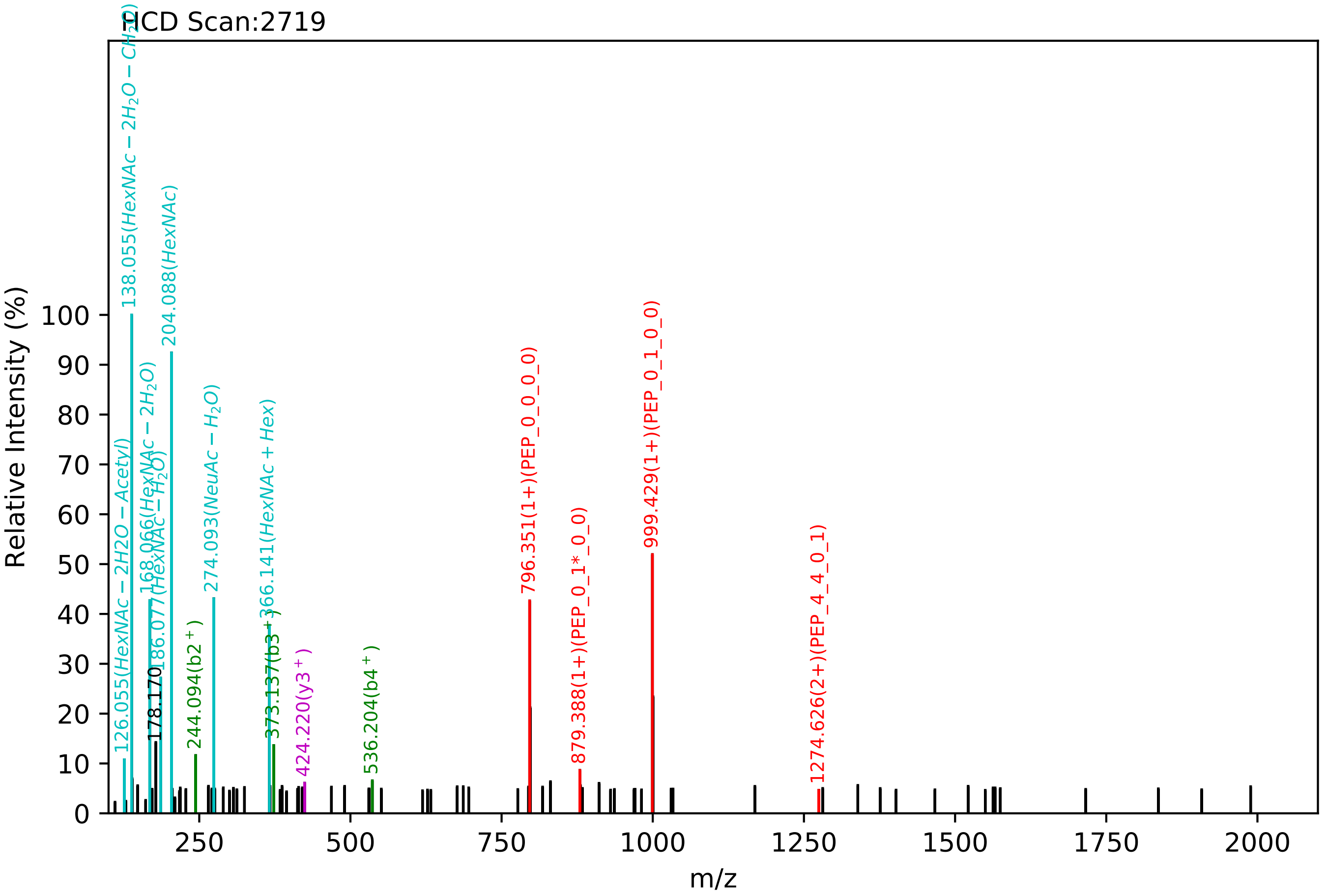

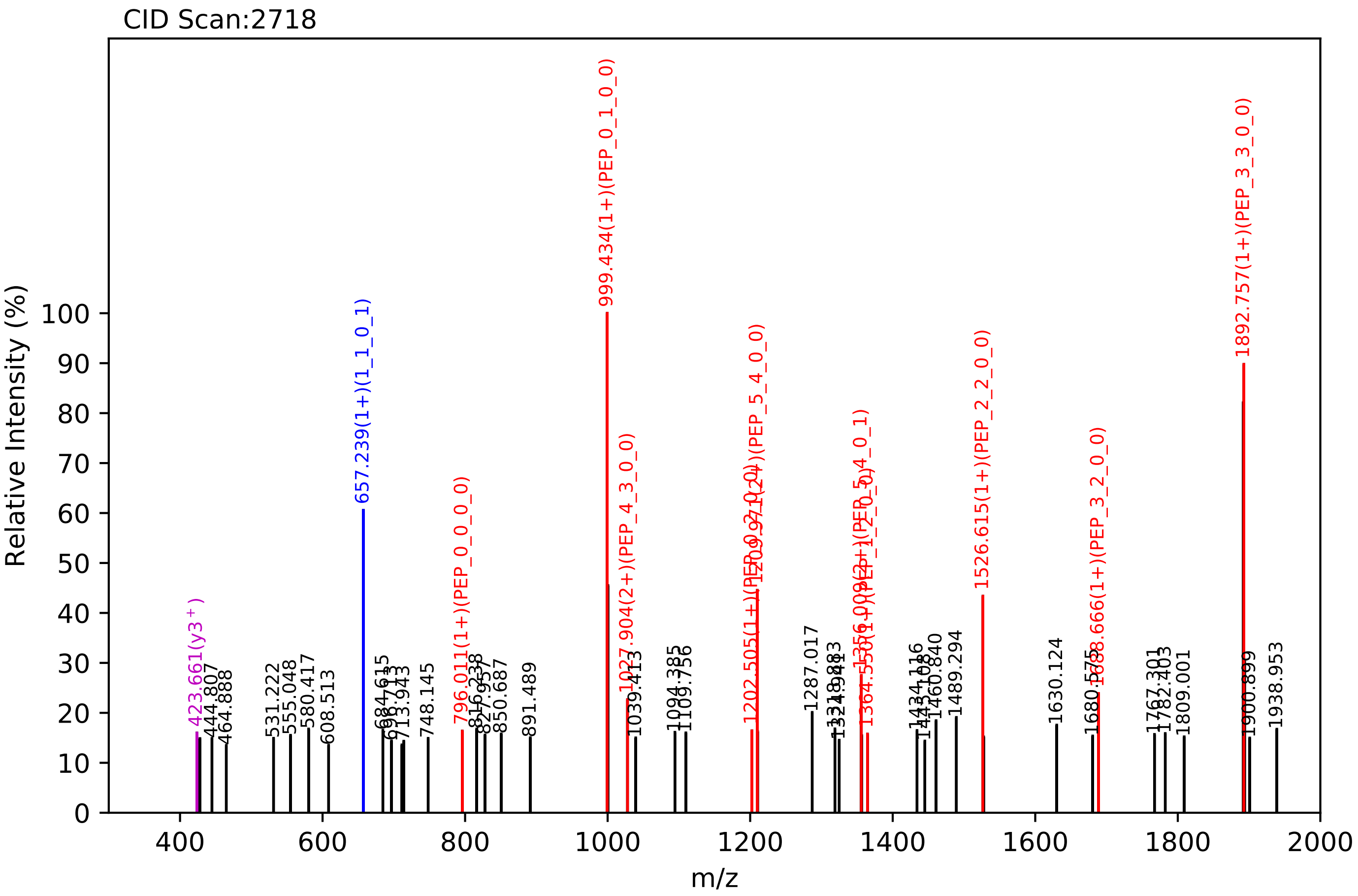

Test set no. 143, Experiment: AGP exp_3

NEEYNK(=PEP)_5_4_0_2, m/z:1501.57(2+), RT:22.12, Y-score:87.17

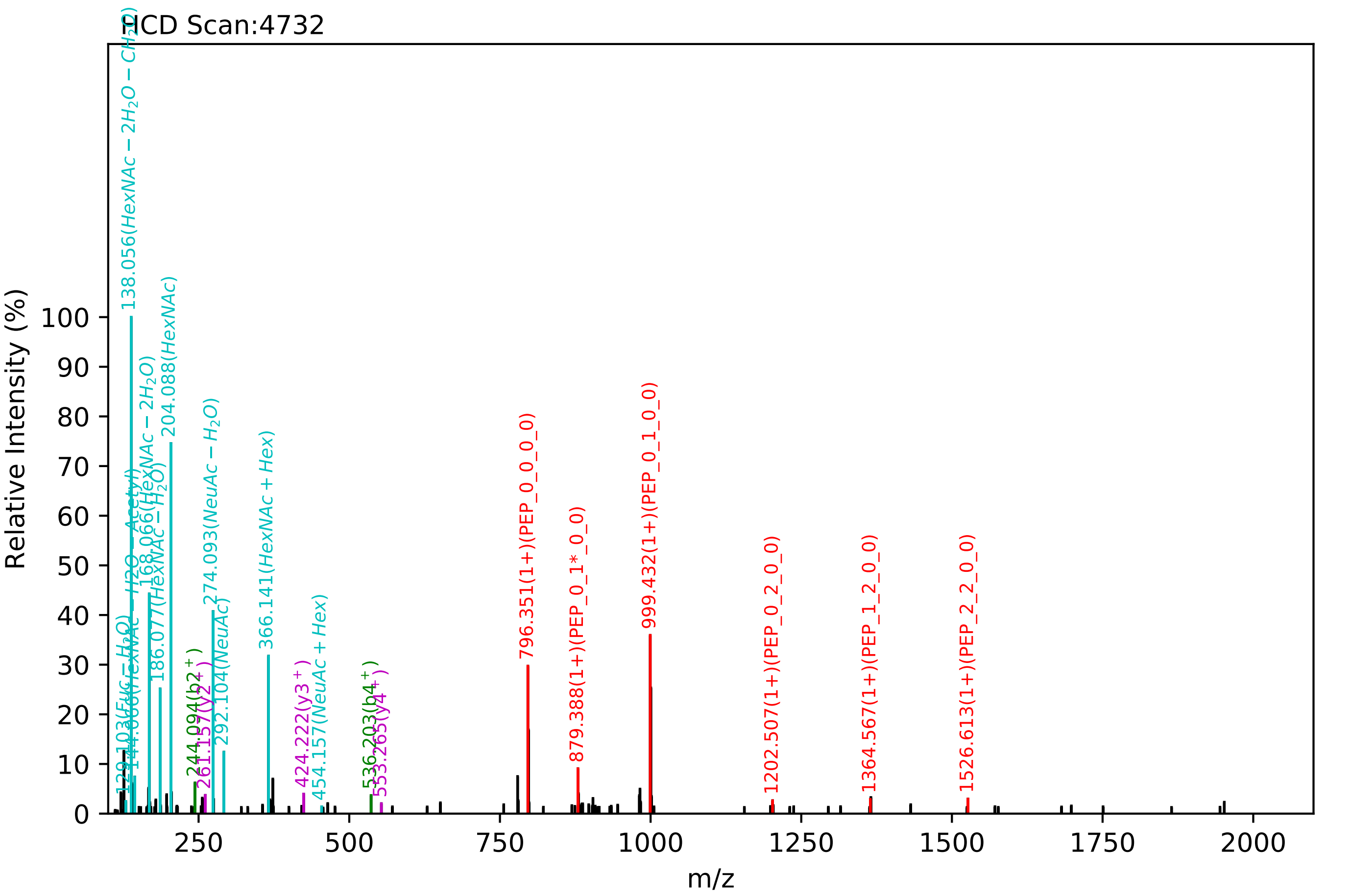

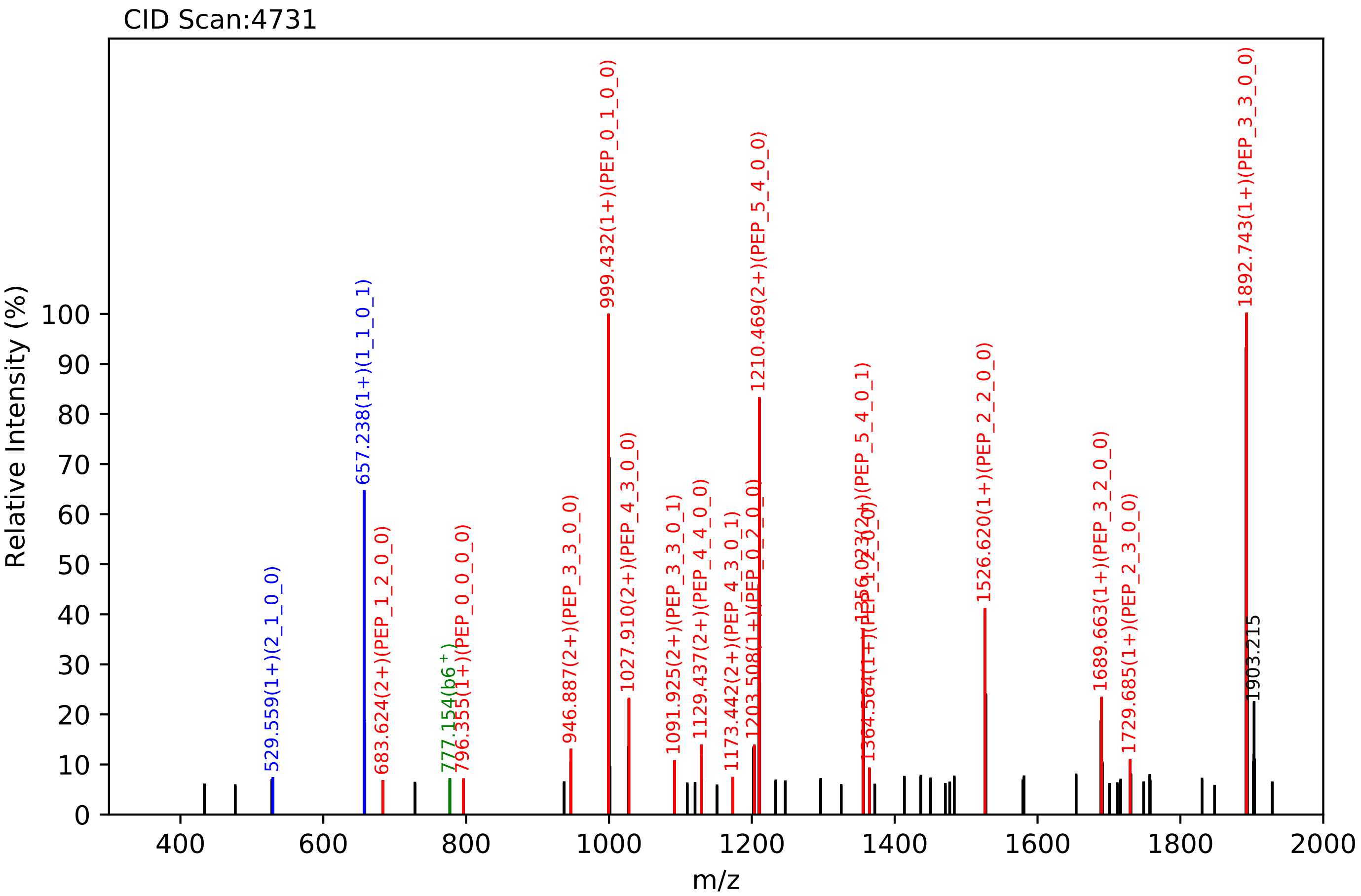

Test set no. 144, Experiment: AGP exp_4

NEEYNK(=PEP)_5_4_0_2, m/z:1500.56(2+), RT:20.08, Y-score:84.35

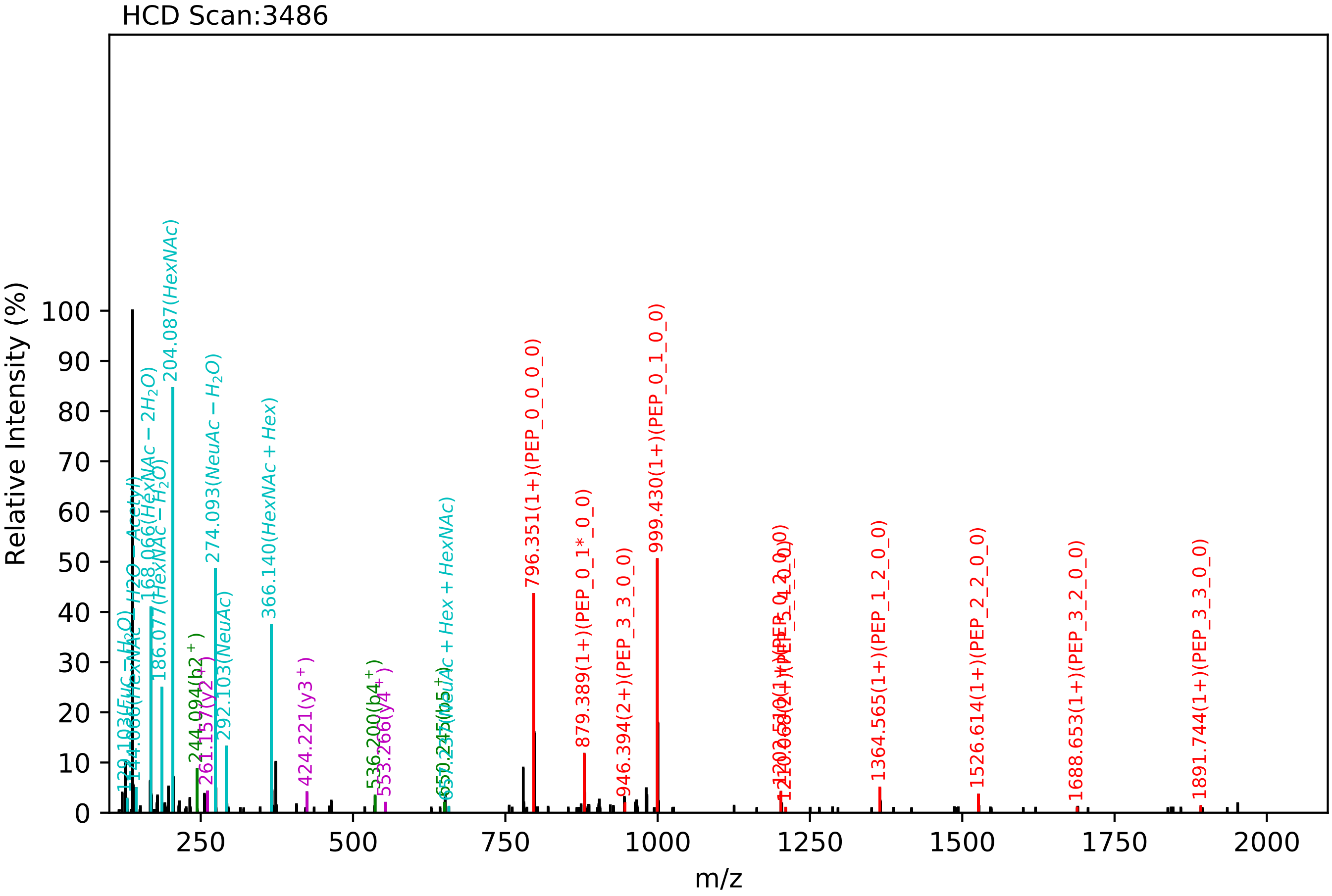

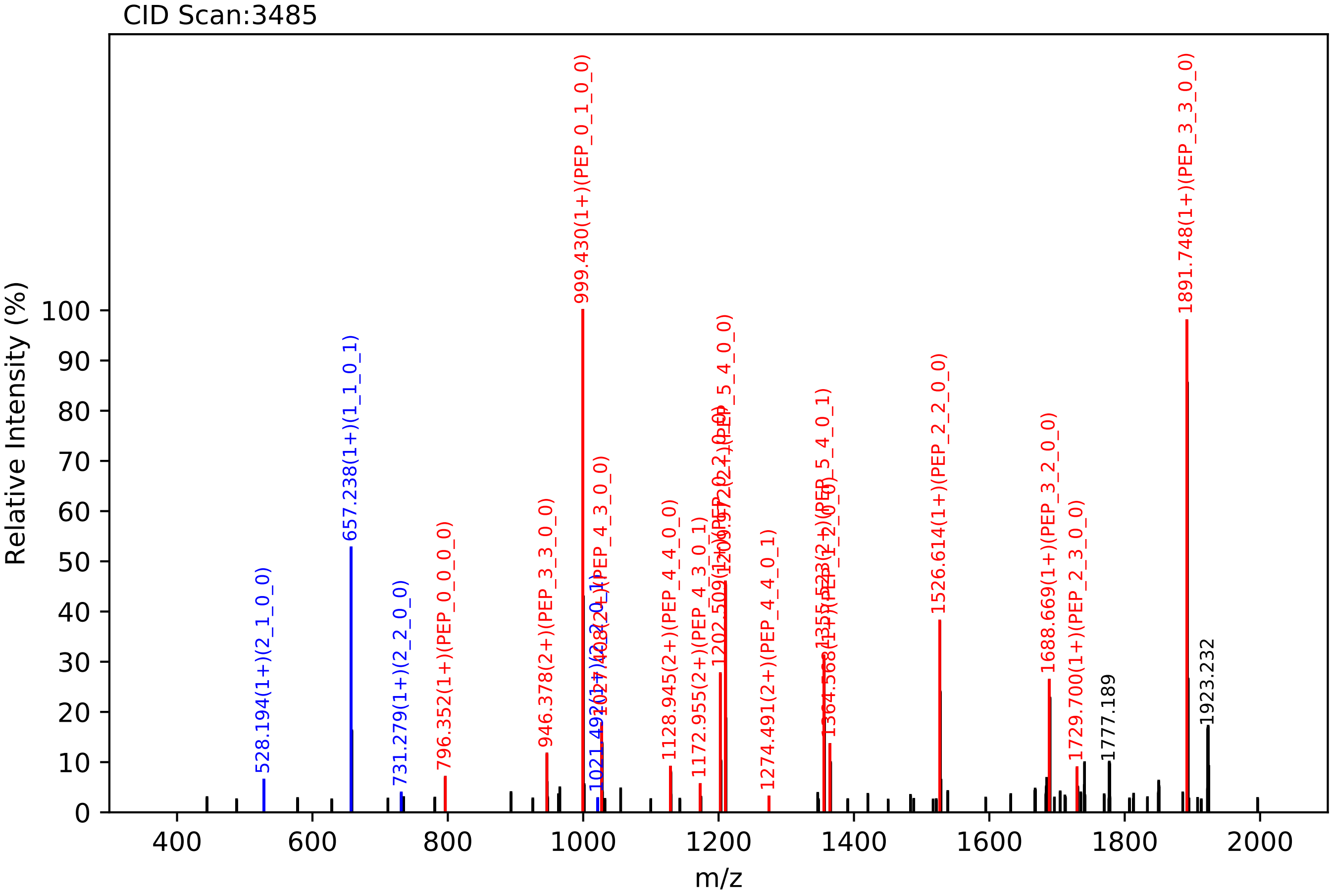

Test set no. 145, Experiment: AGP exp_3

NEEYNK(=PEP)_5_4_0_2, m/z:1501.07(2+), RT:20.23, Y-score:81.72

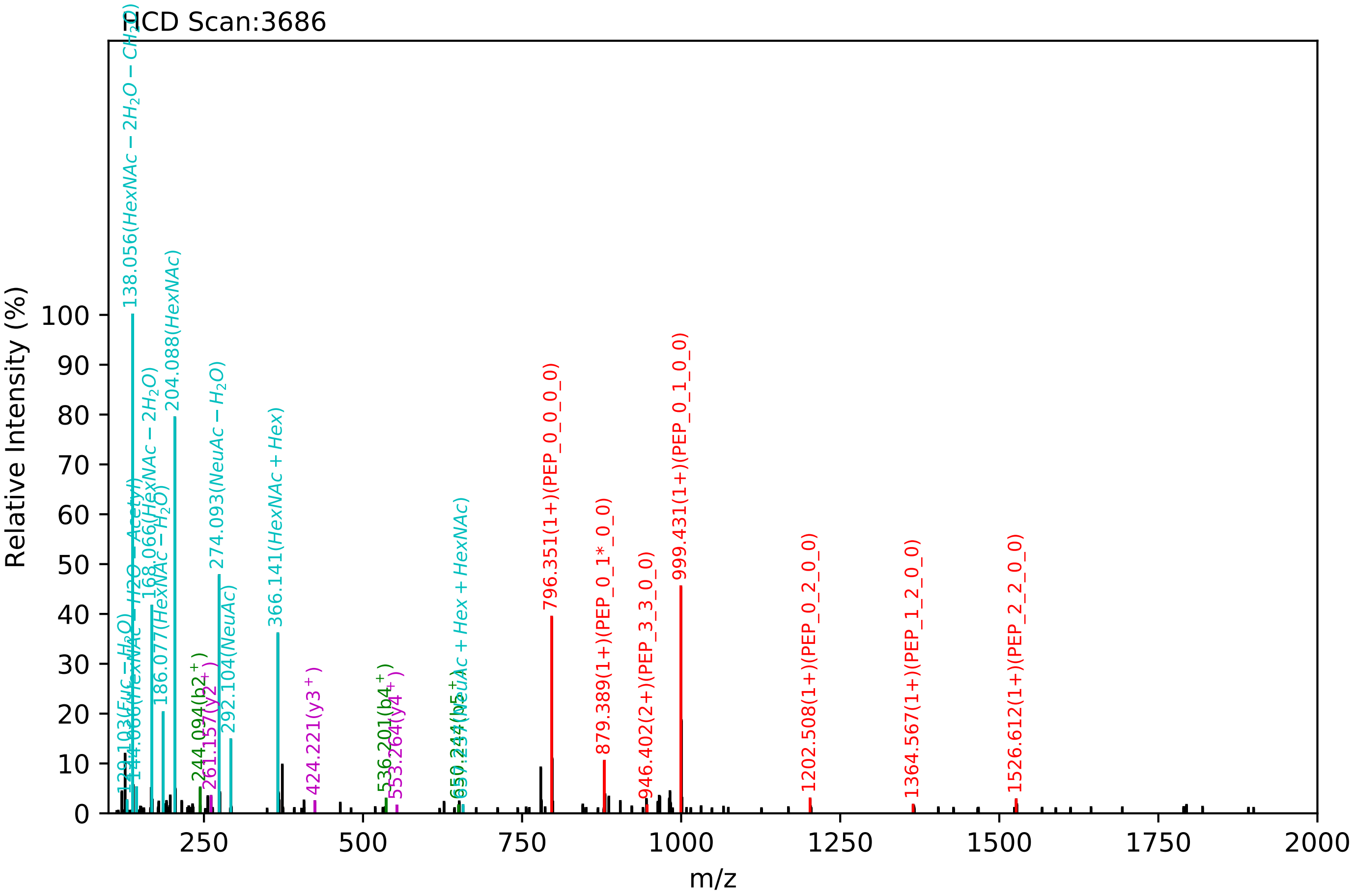

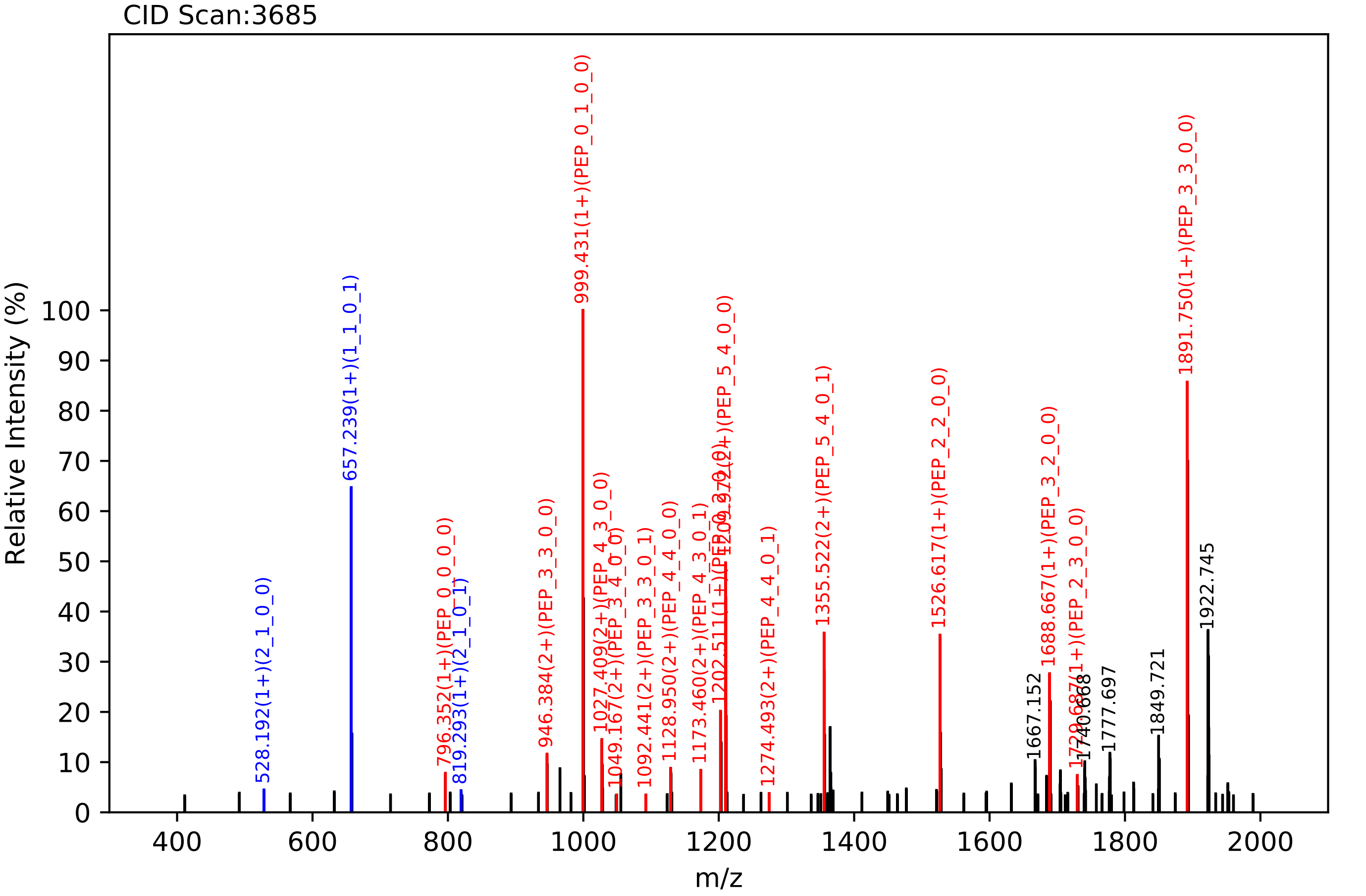

Test set no. 146, Experiment: AGP exp_4

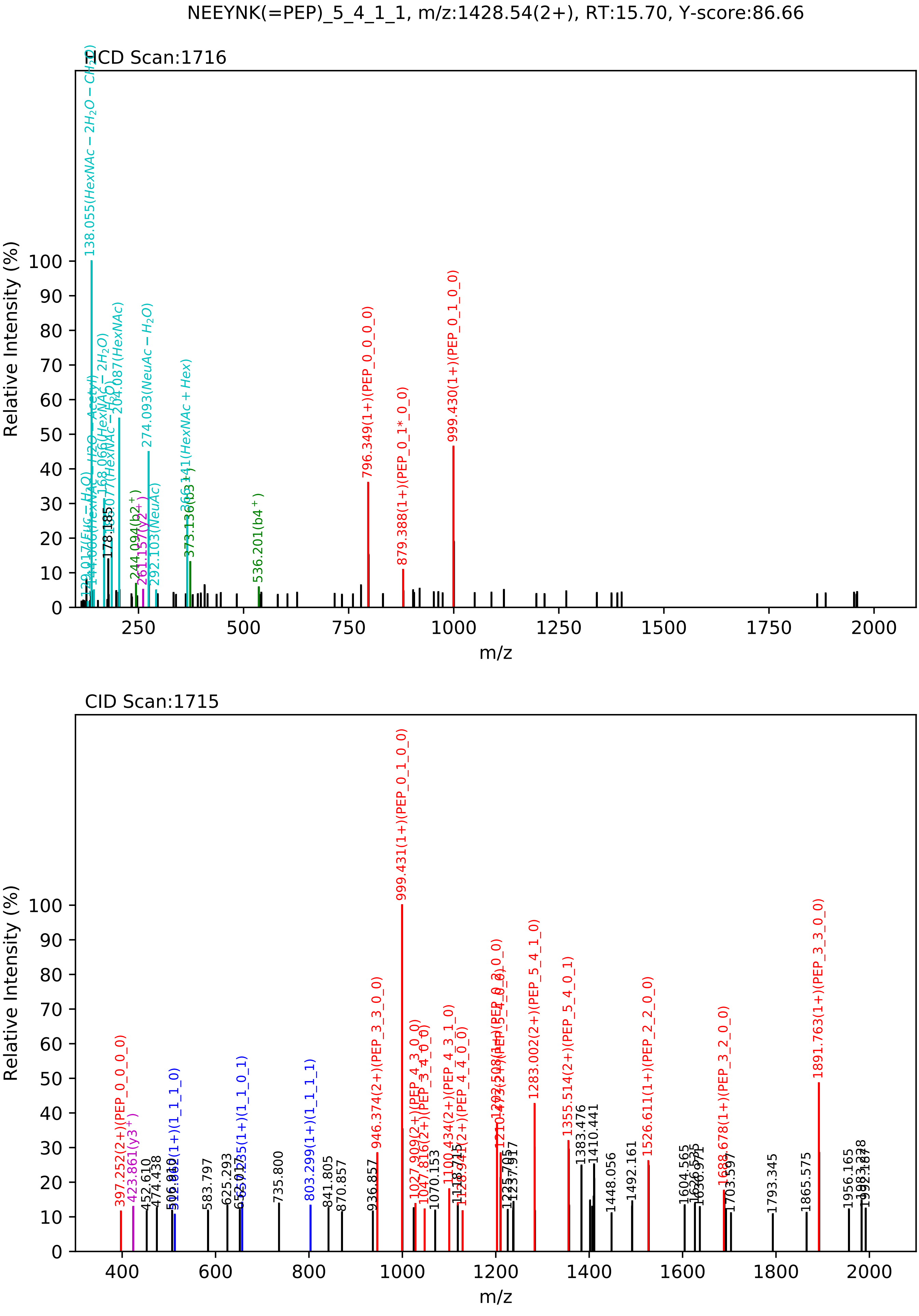

Test set no. 147, Experiment: AGP exp_4

NEEYNK(=PEP)_5_4_1_2, m/z:1049.73(3+), RT:19.38, Y-score:89.96

HCD Scan:3138

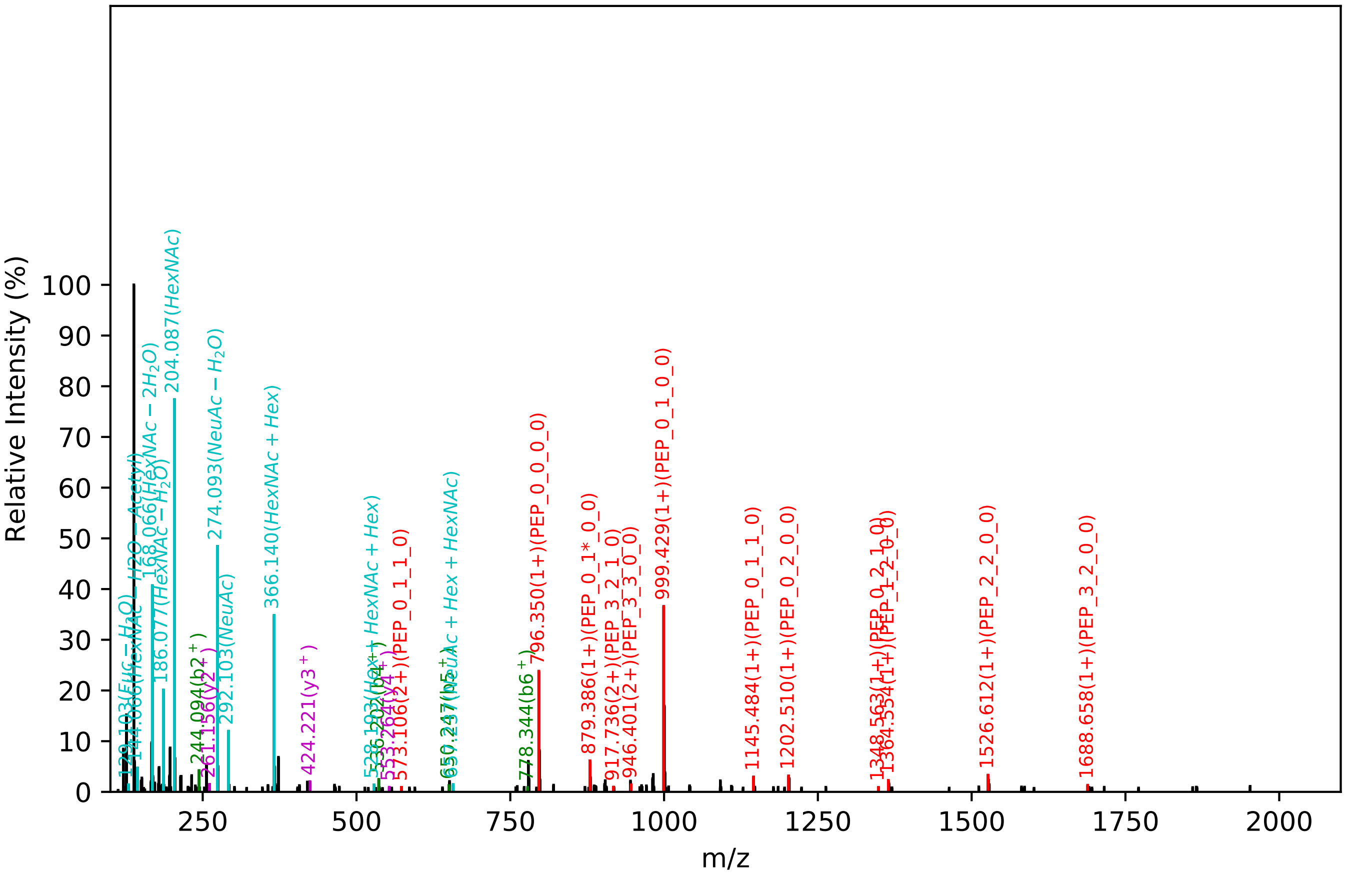

CID Scan:3137

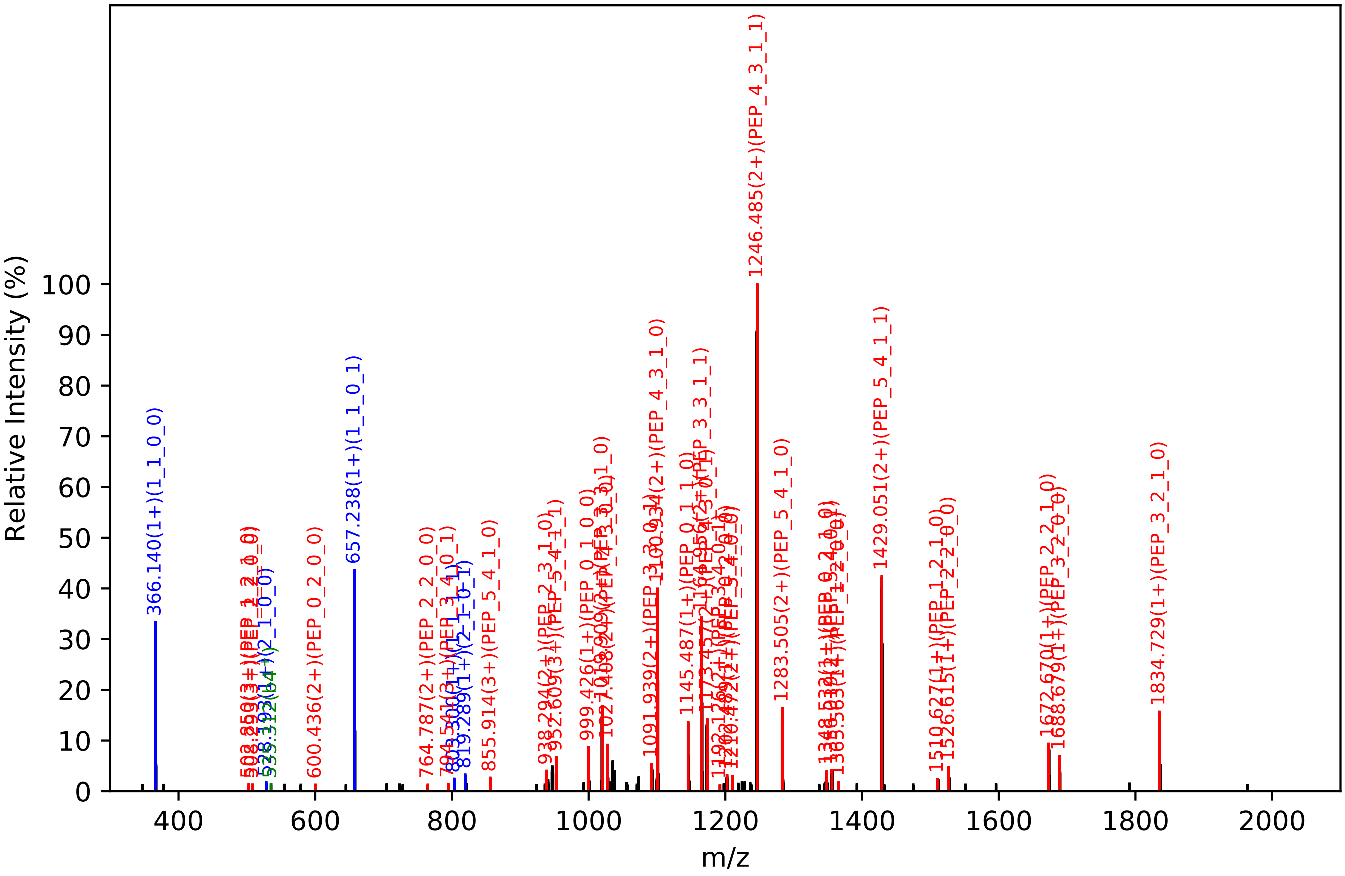

Test set no. 148, Experiment: AGP exp_4

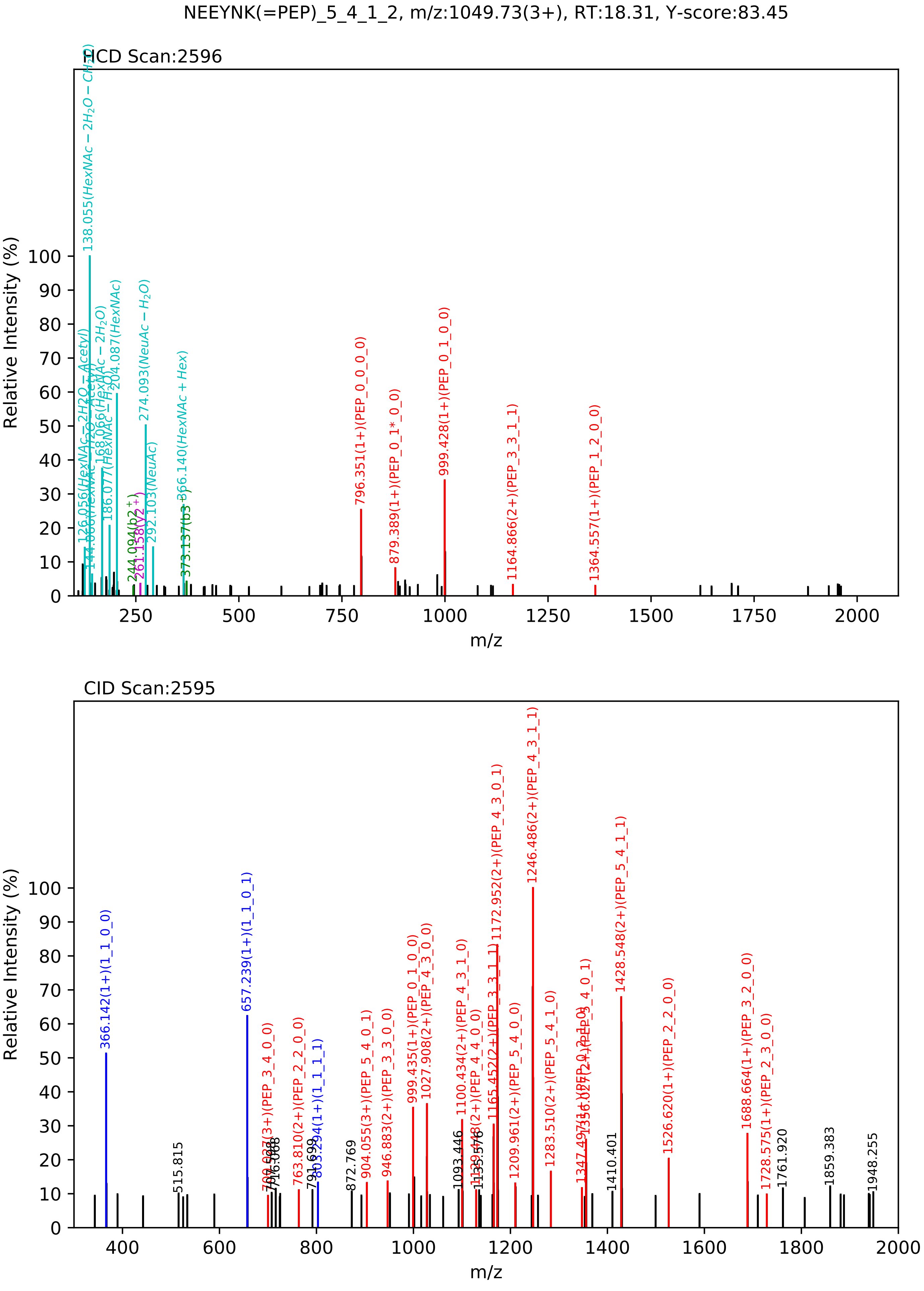

Test set no. 149, Experiment: AGP exp_3

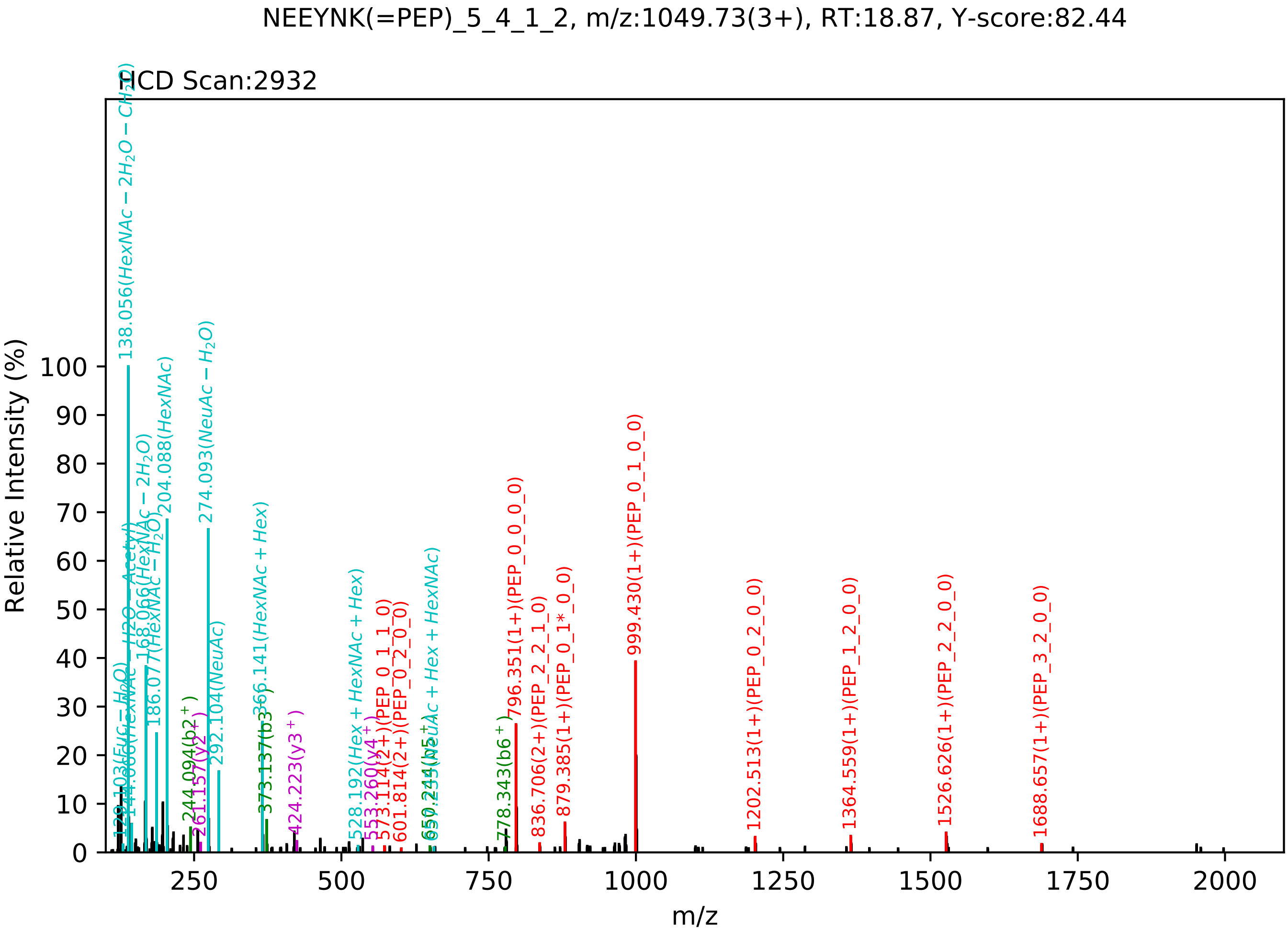

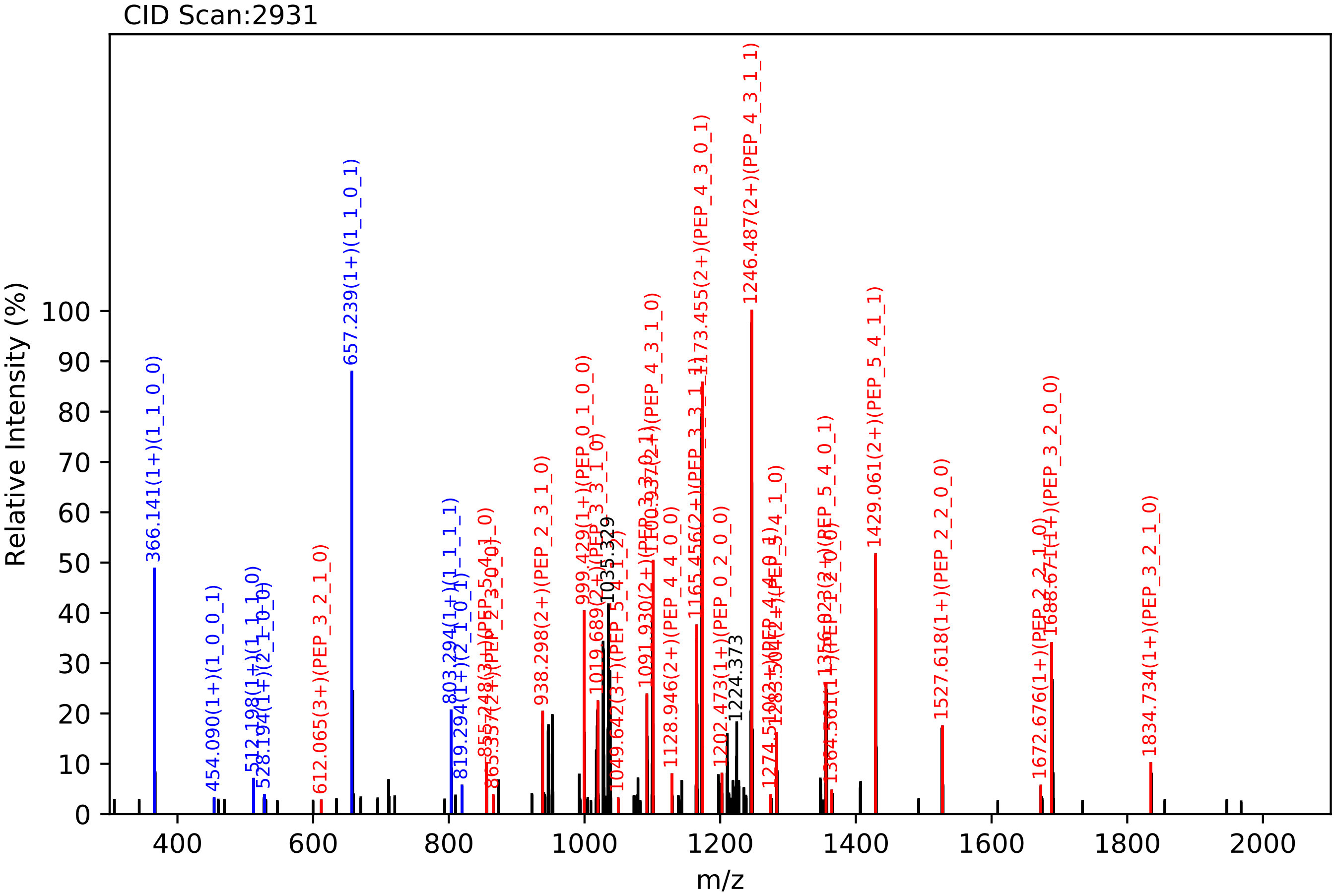

Test set no. 150, Experiment: AGP exp_3

NEEYNK(=PEP)_5_4_1_2, m/z:1049.73(3+), RT:18.30, Y-score:82.09

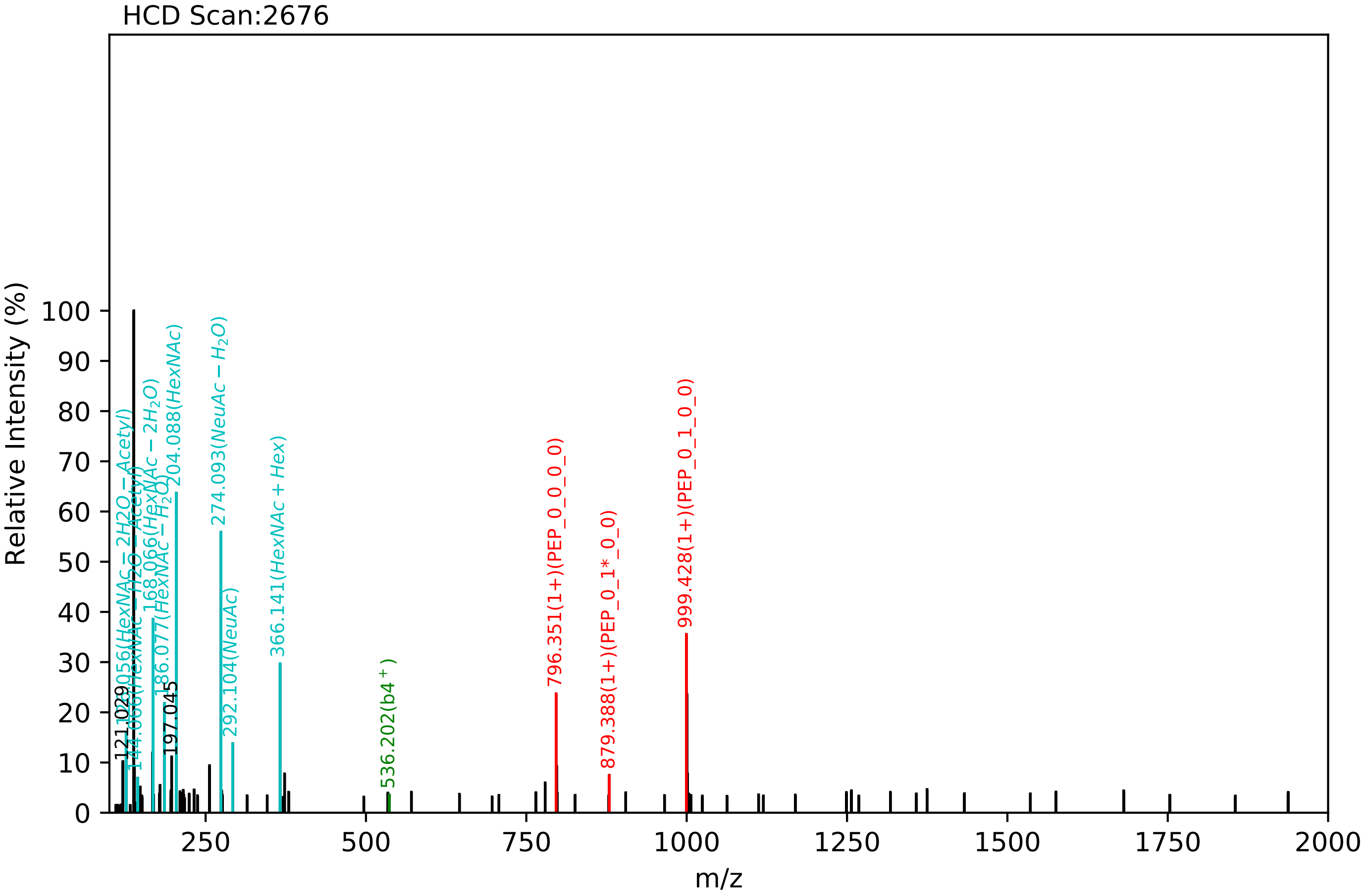

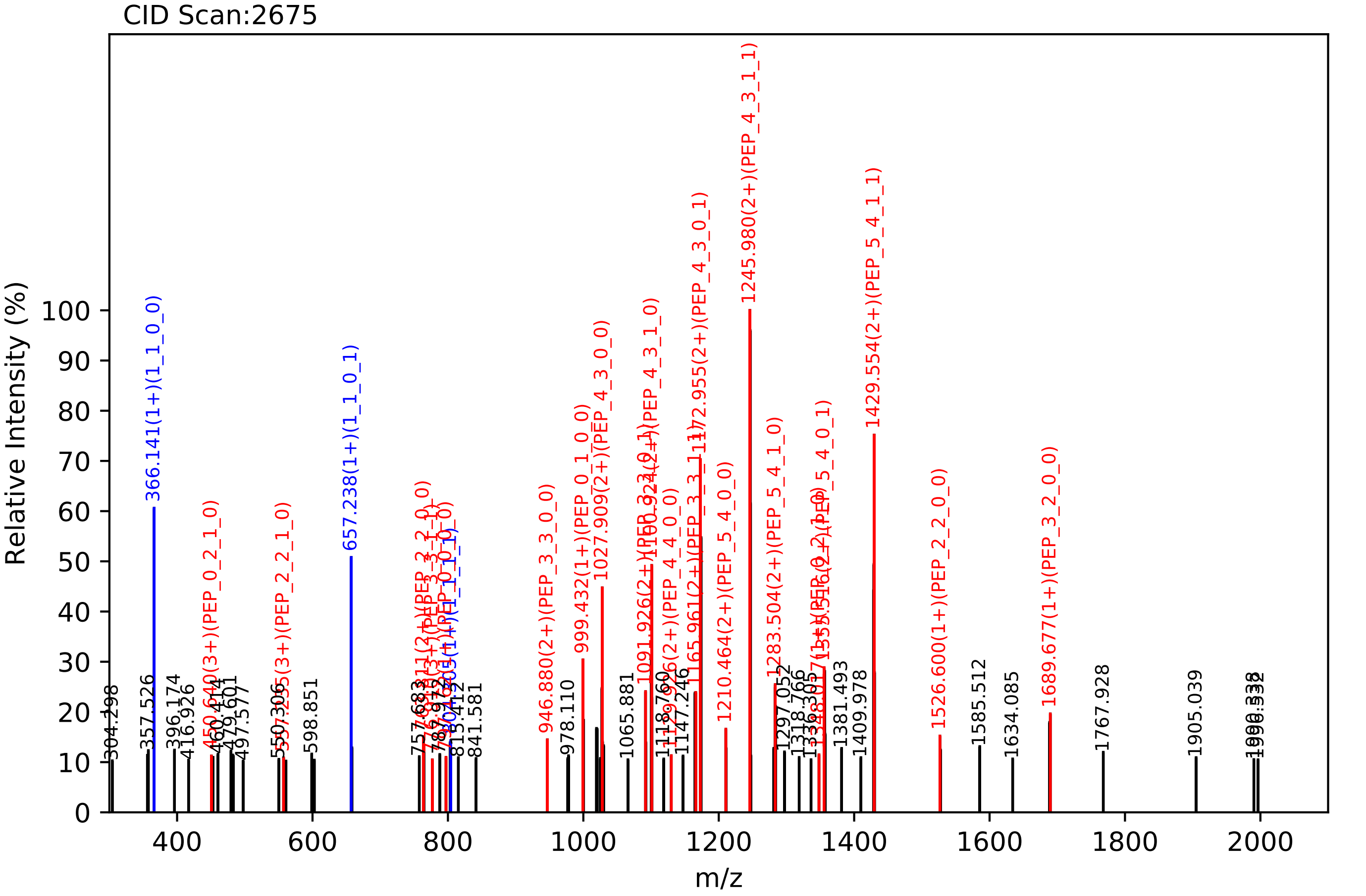

Test set no. 151, Experiment: AGP exp_4

NEEYNK(=PEP)_5_4_1_2, m/z:1049.73(3+), RT:18.87, Y-score:72.79

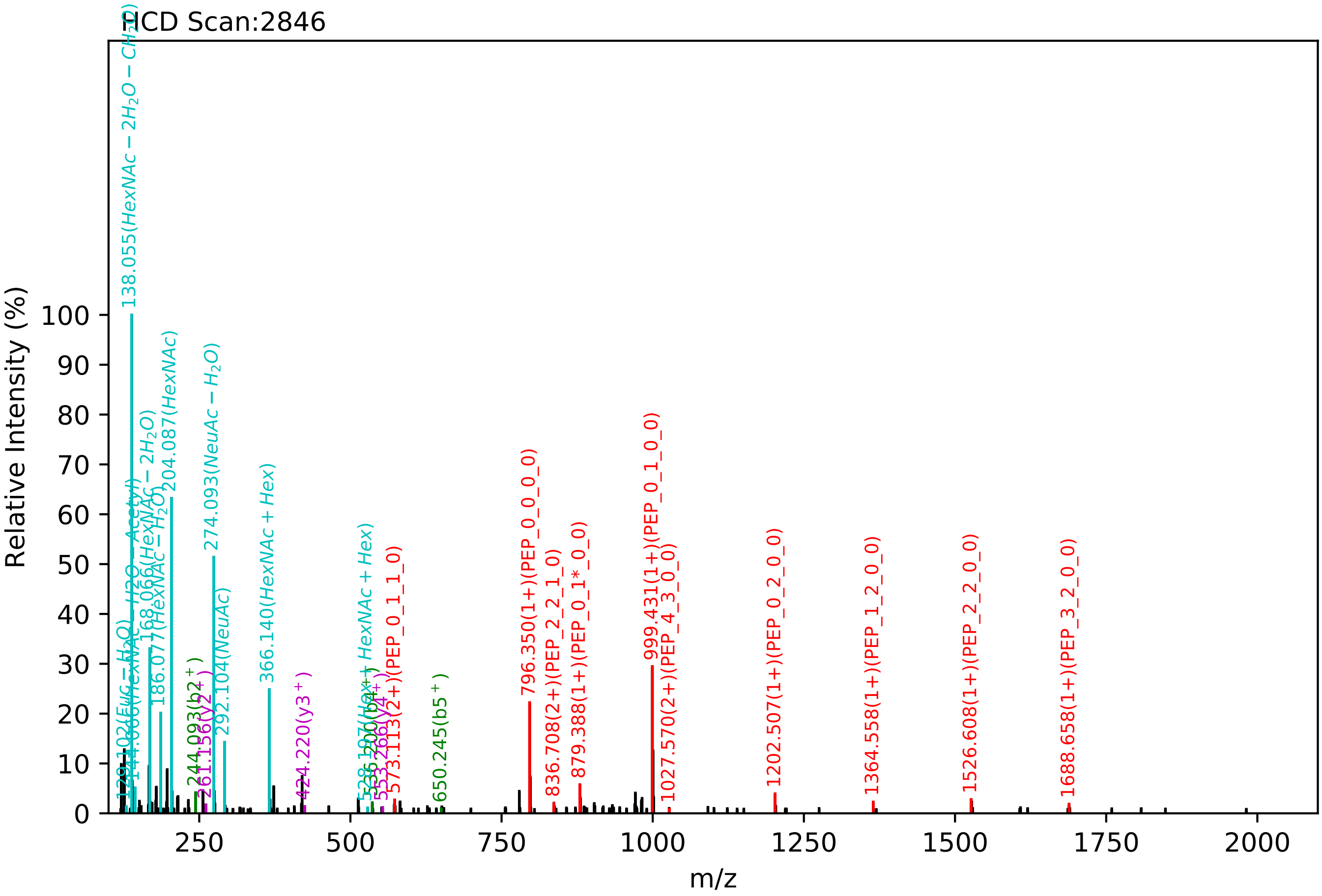

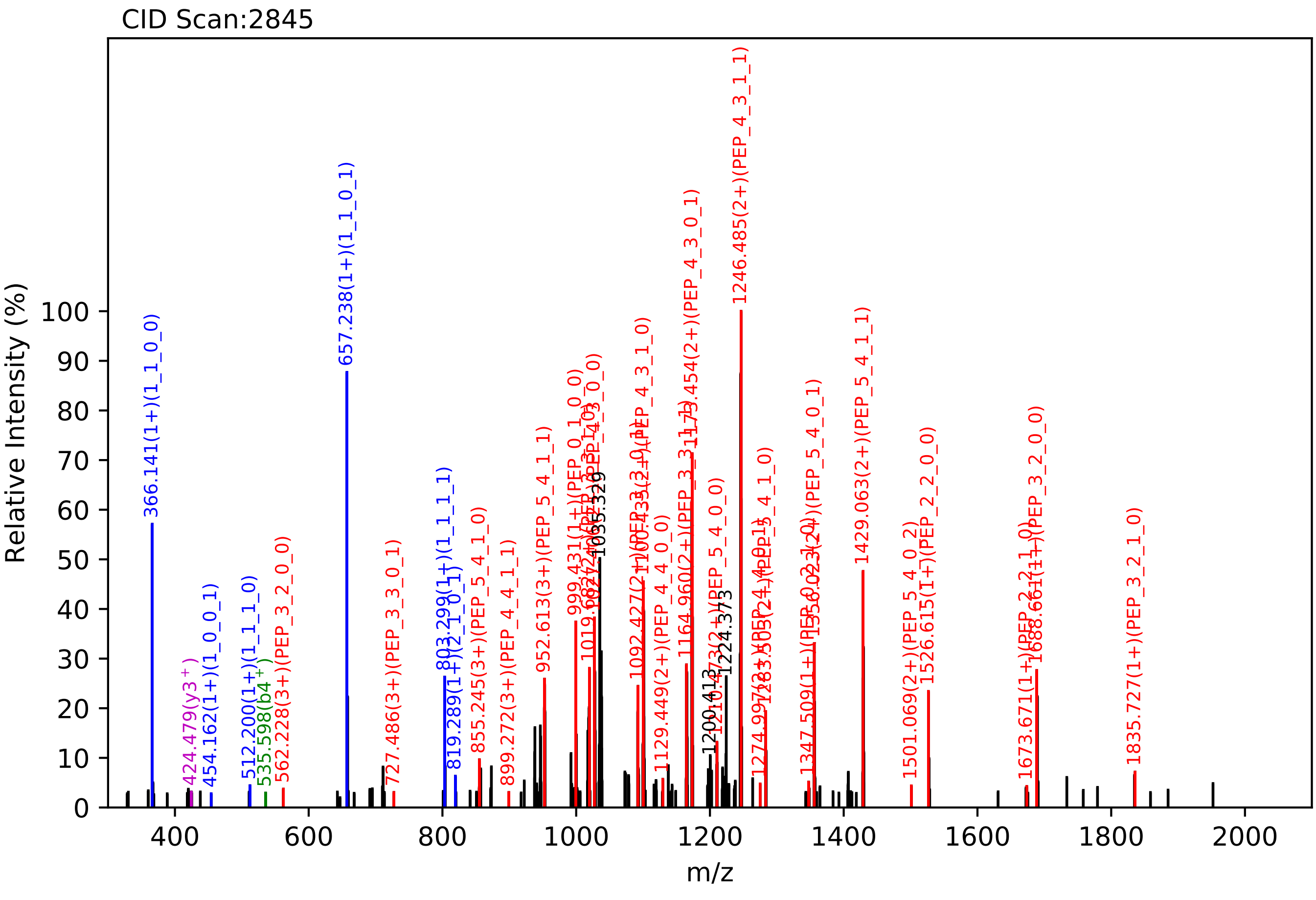

Test set no. 152, Experiment: AGP exp_27

NEEYNK(=PEP)_5_4_2_2, m/z:1098.42(3+), RT:21.48, Y-score:88.07

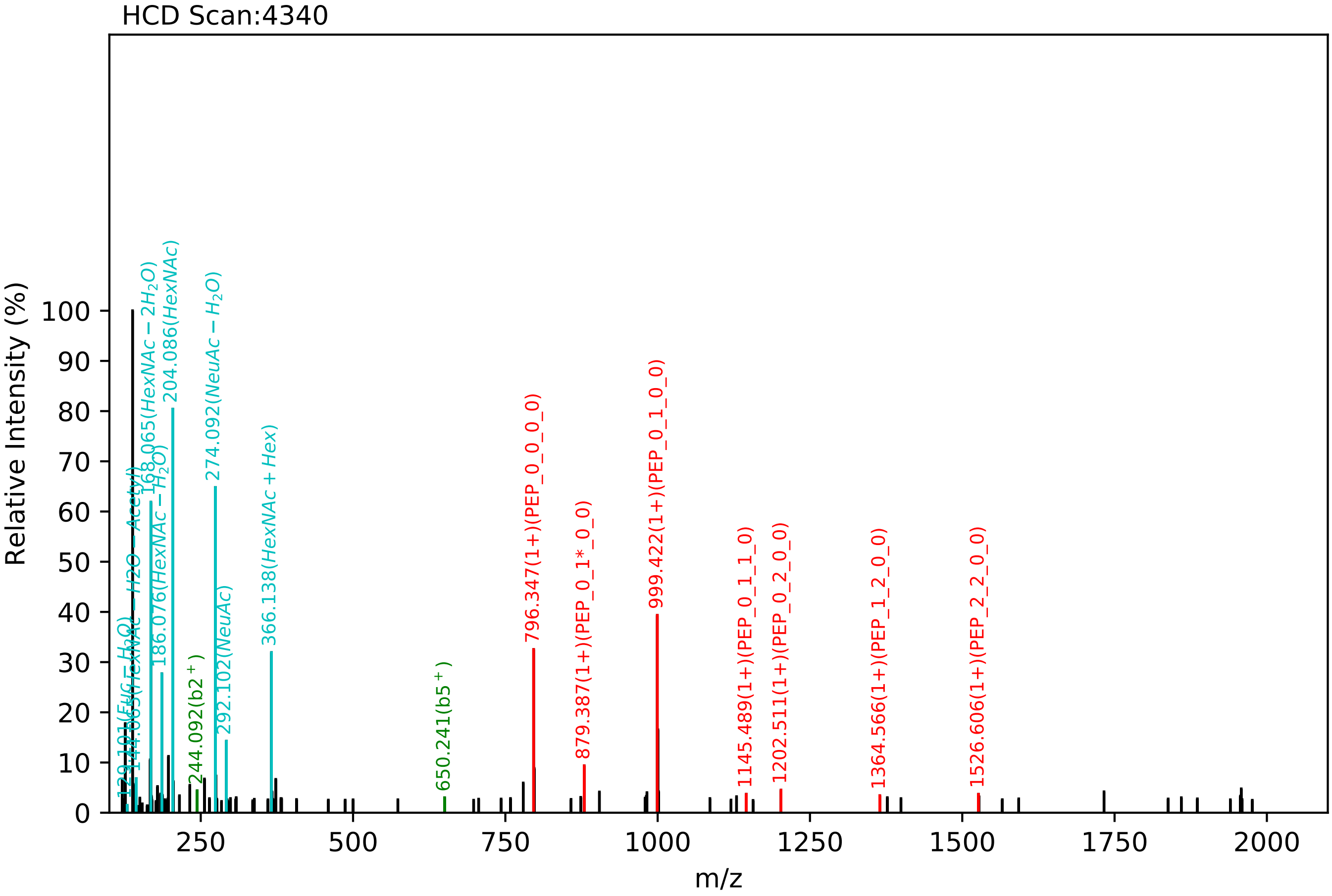

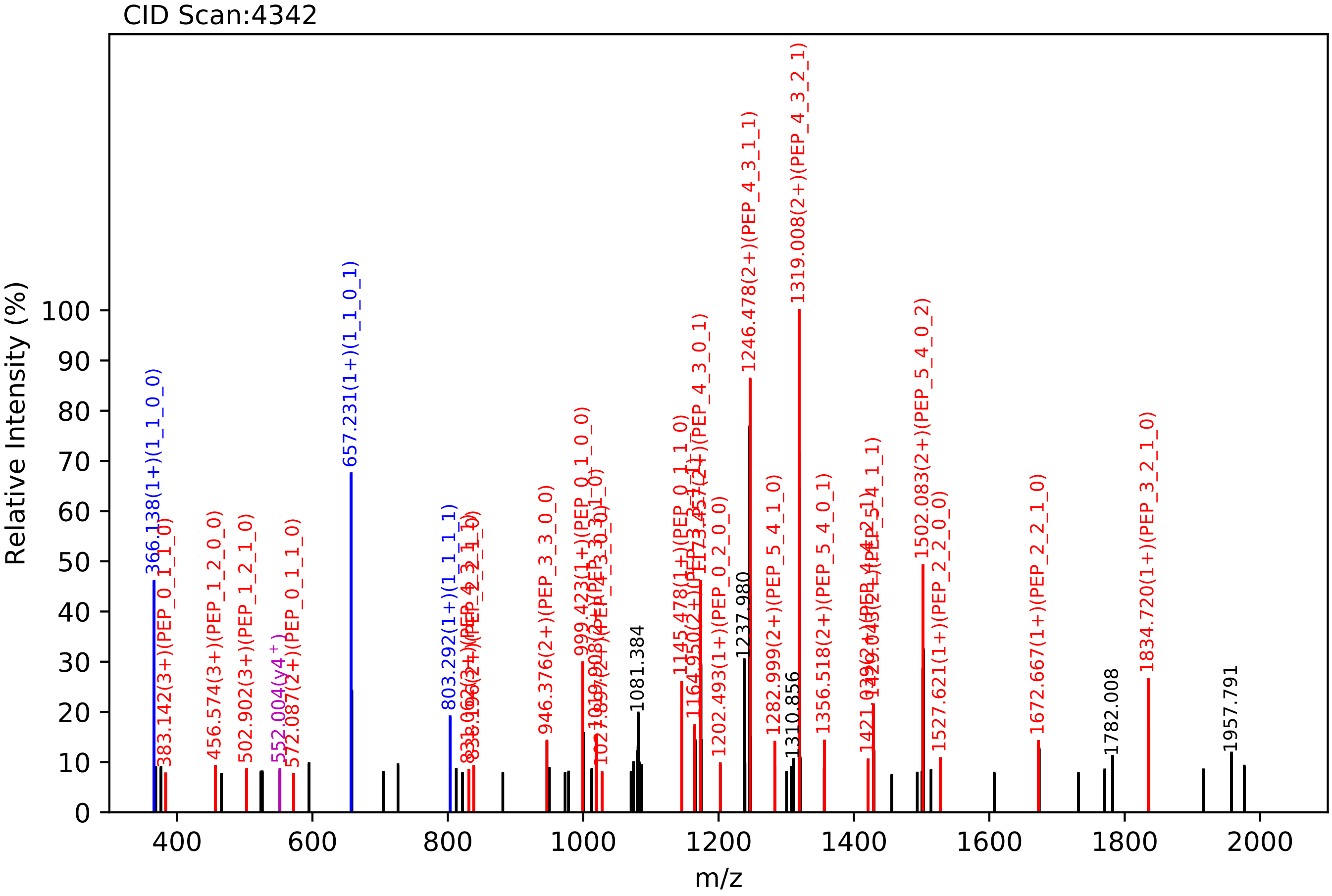

Test set no. 153, Experiment: AGP exp_18

NEEYNK(=PEP)_5_4_2_2, m/z:1098.42(3+), RT:21.13, Y-score:87.57

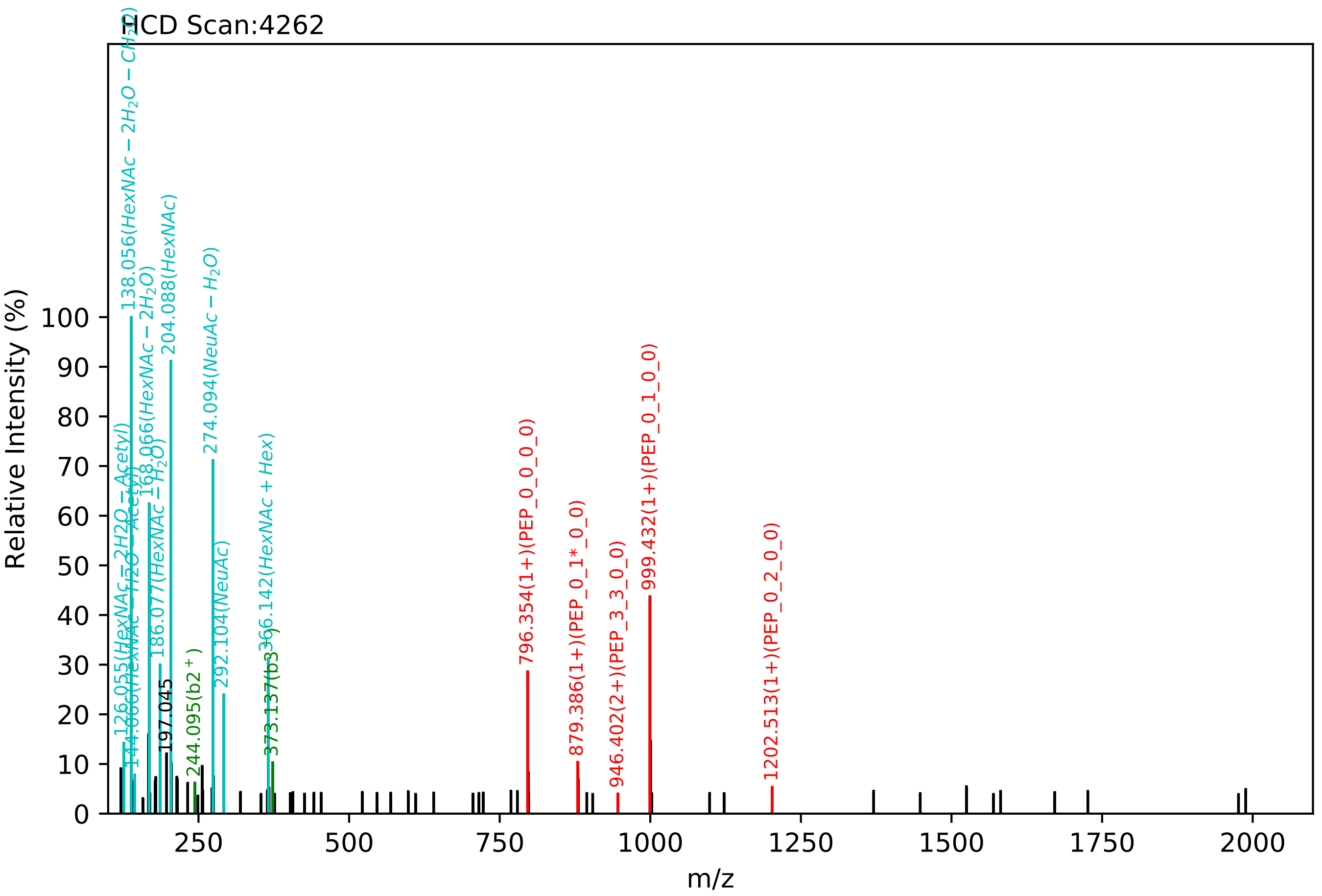

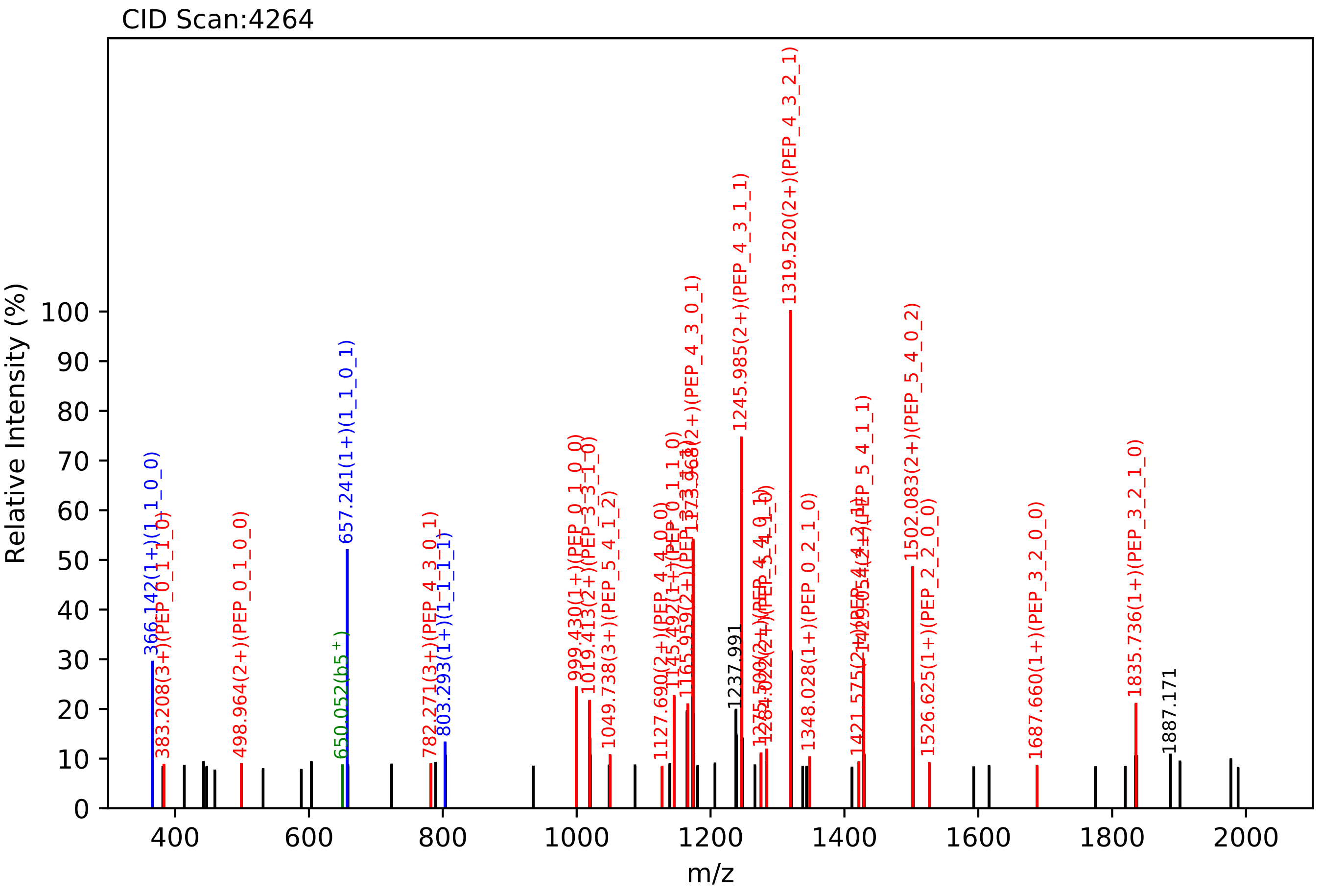

Test set no. 154, Experiment: AGP exp_14

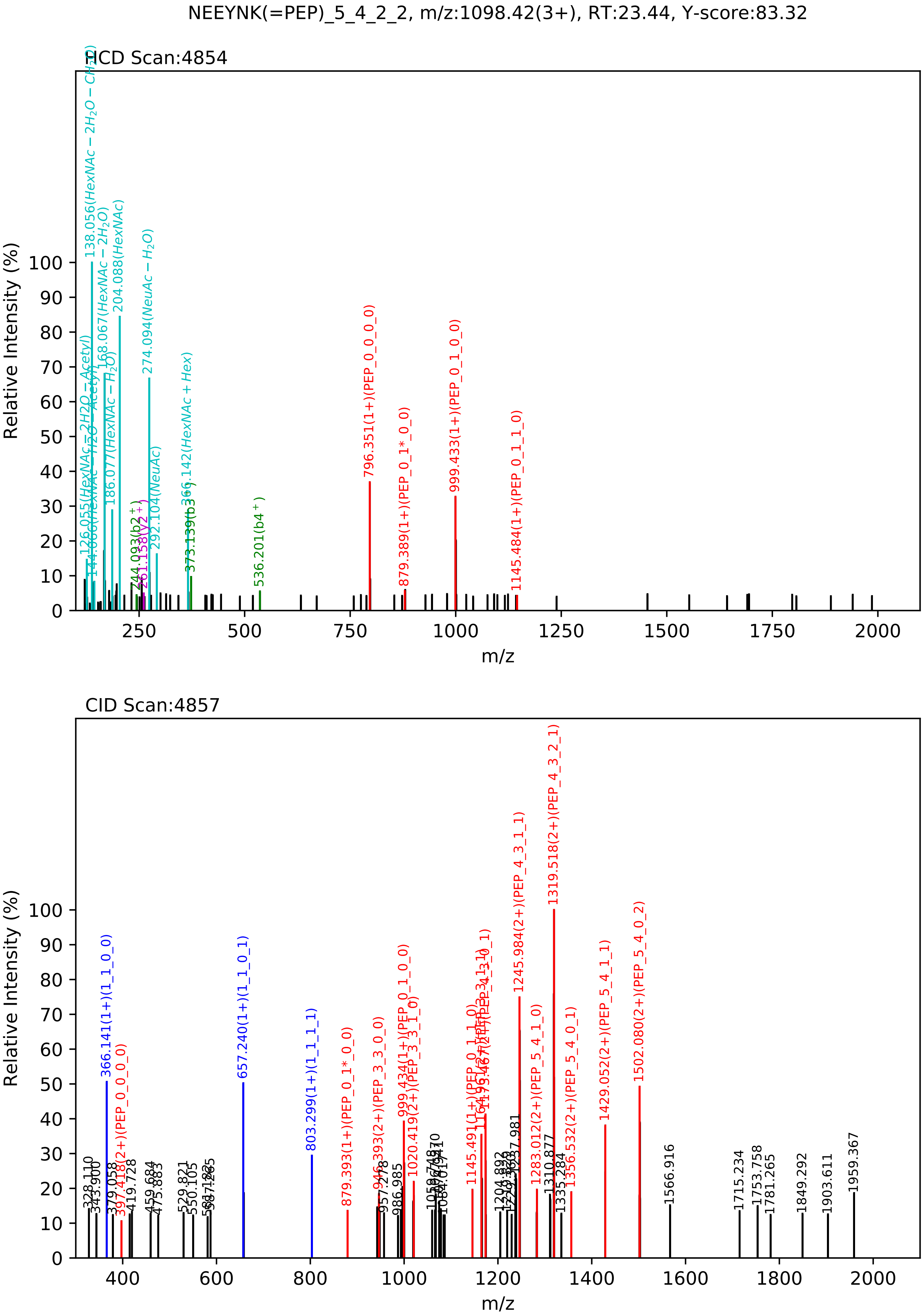

Test set no. 155, Experiment: AGP exp_10

NEEYNK(=PEP)_5_4_2_2, m/z:1098.42(3+), RT:21.04, Y-score:81.81

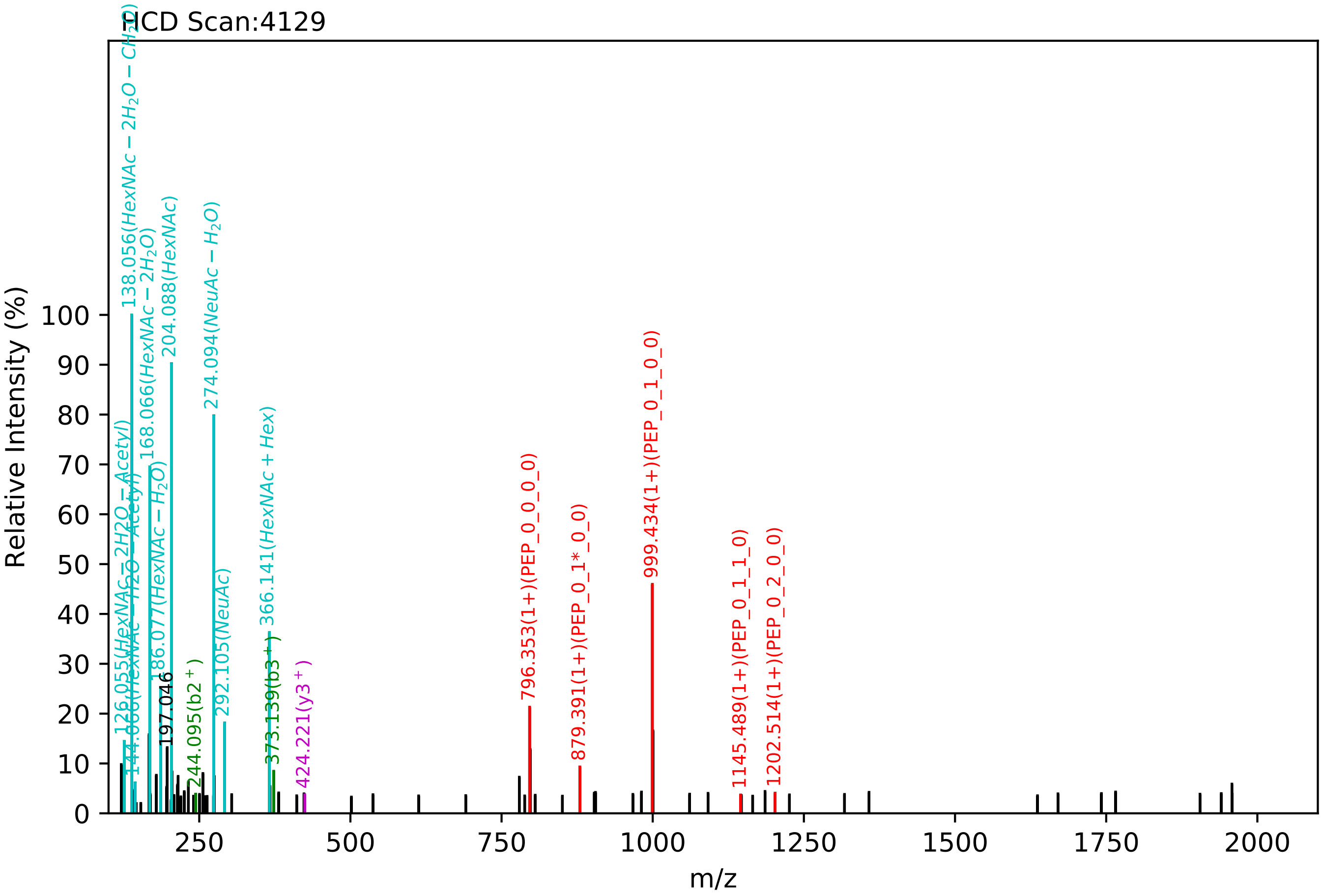

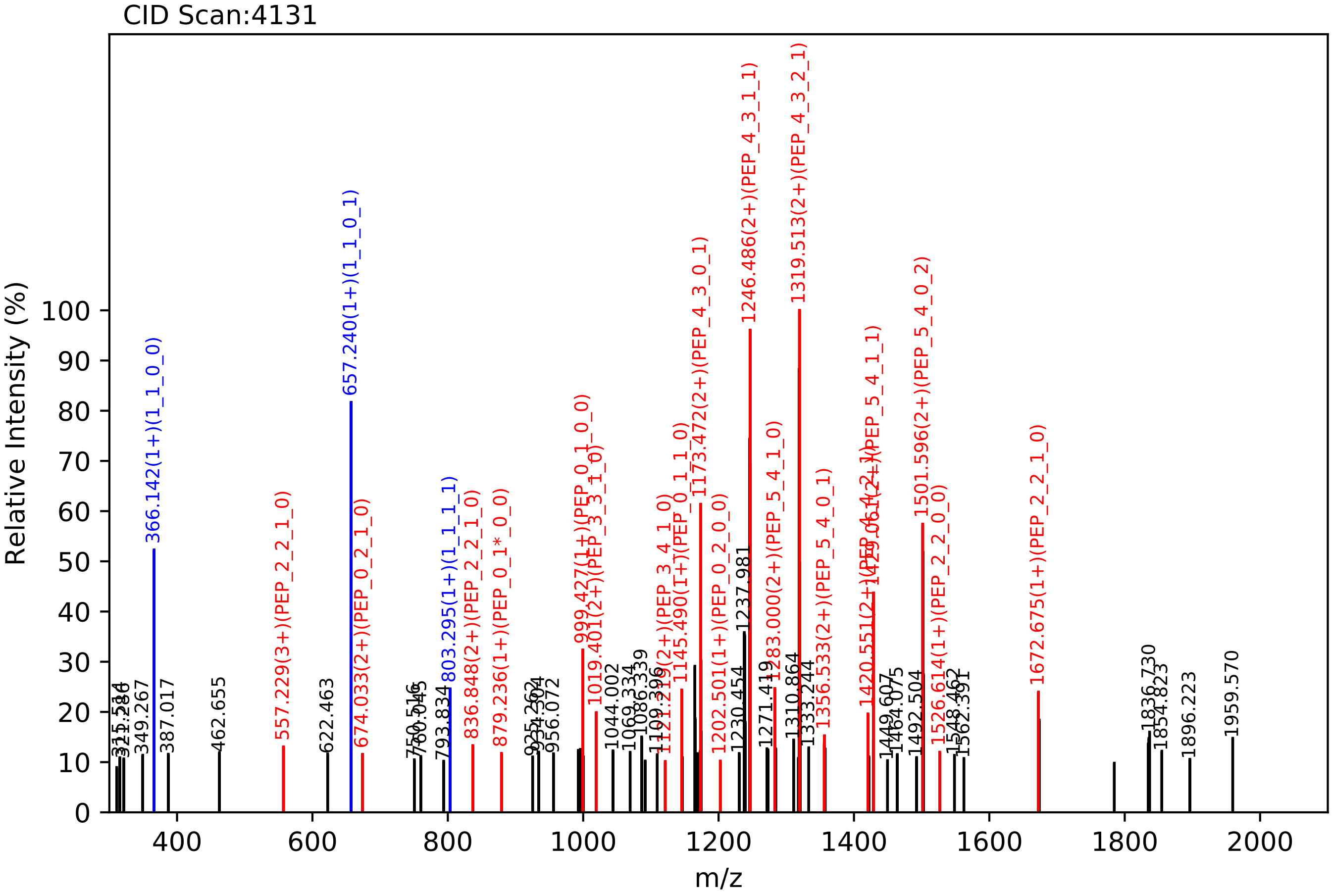

Test set no. 156, Experiment: AGP exp_16

NEEYNK(=PEP)_5_4_2_2, m/z:1098.42(3+), RT:23.20, Y-score:75.62

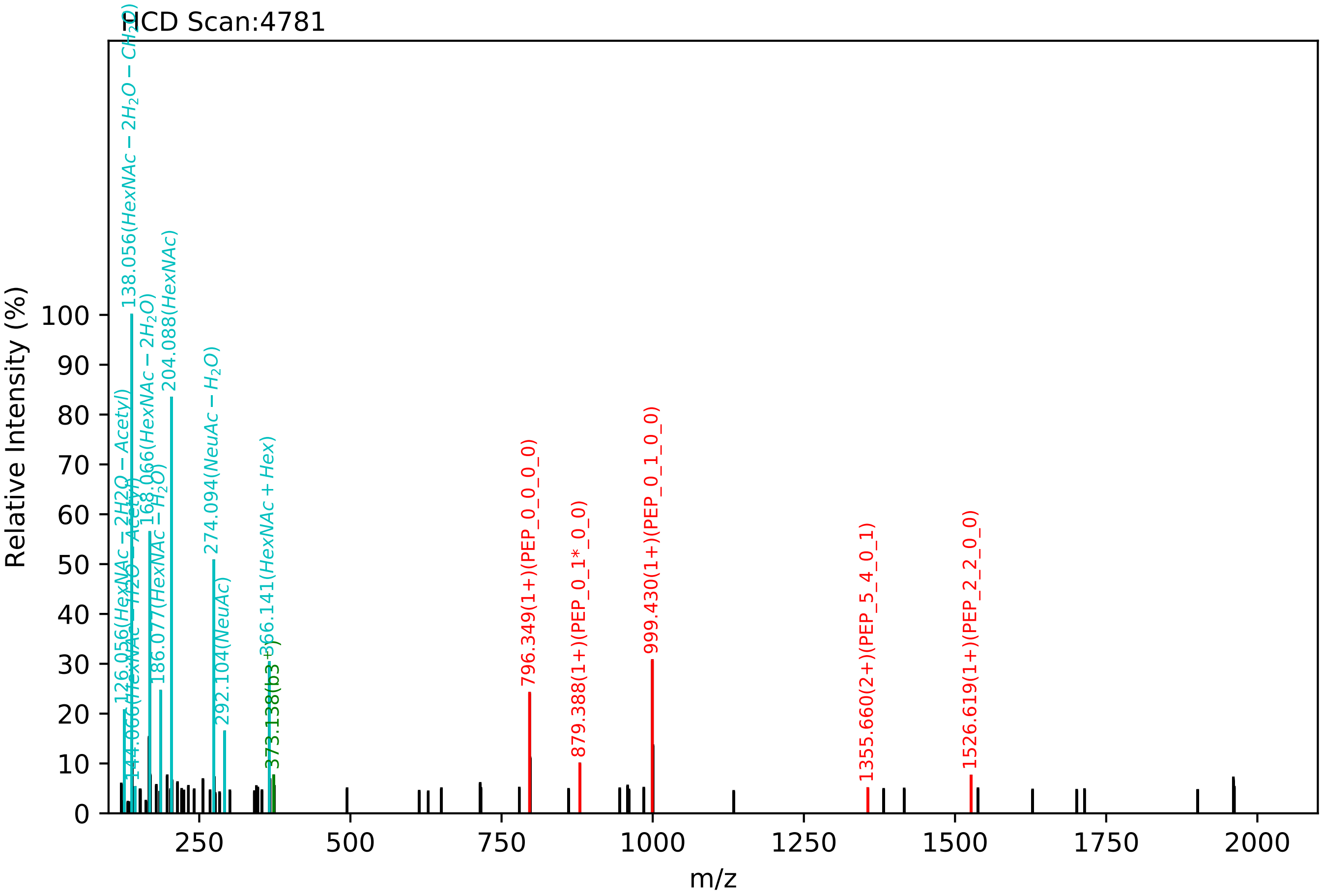

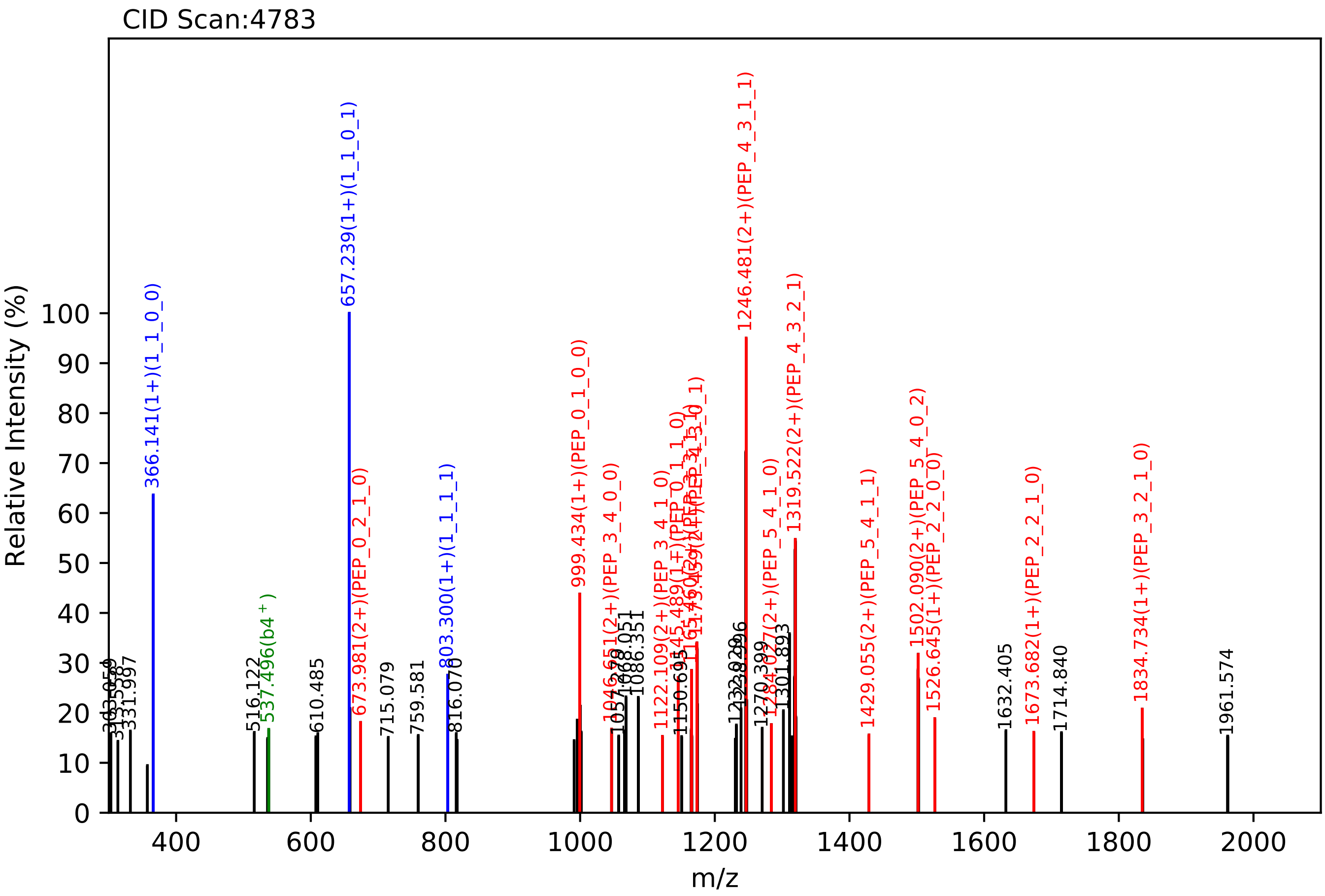

Test set no. 157, Experiment: AGP exp_3

NEEYNK(=PEP)_5_5_0_2, m/z:1068.74(3+), RT:20.78, Y-score:78.50

HCD Scan:3998

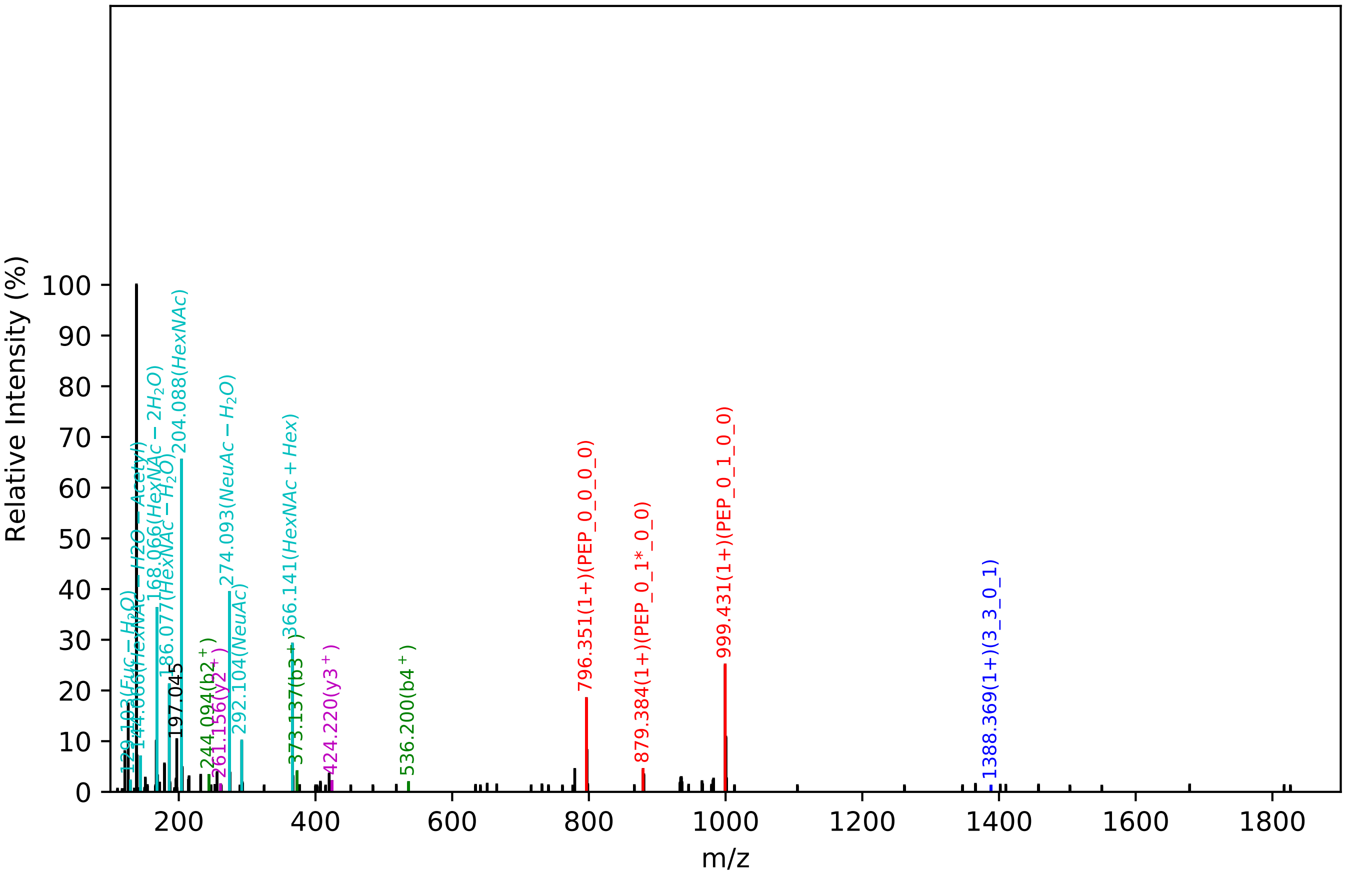

CID Scan:3997

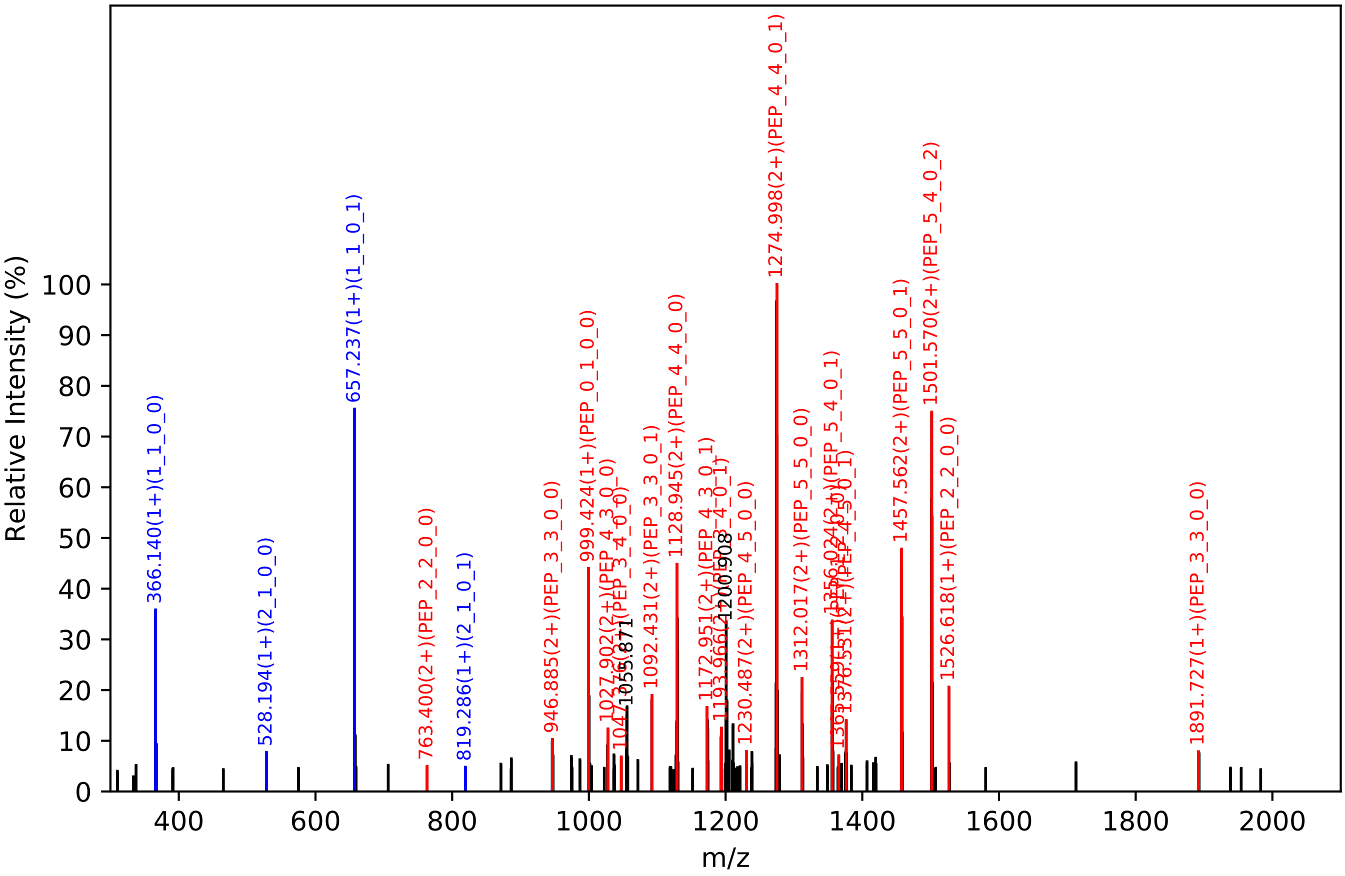

Test set no. 158, Experiment: AGP exp_3

NEEYNK(=PEP)_6_5_0_1, m/z:1025.73(3+), RT:16.84, Y-score:91.85

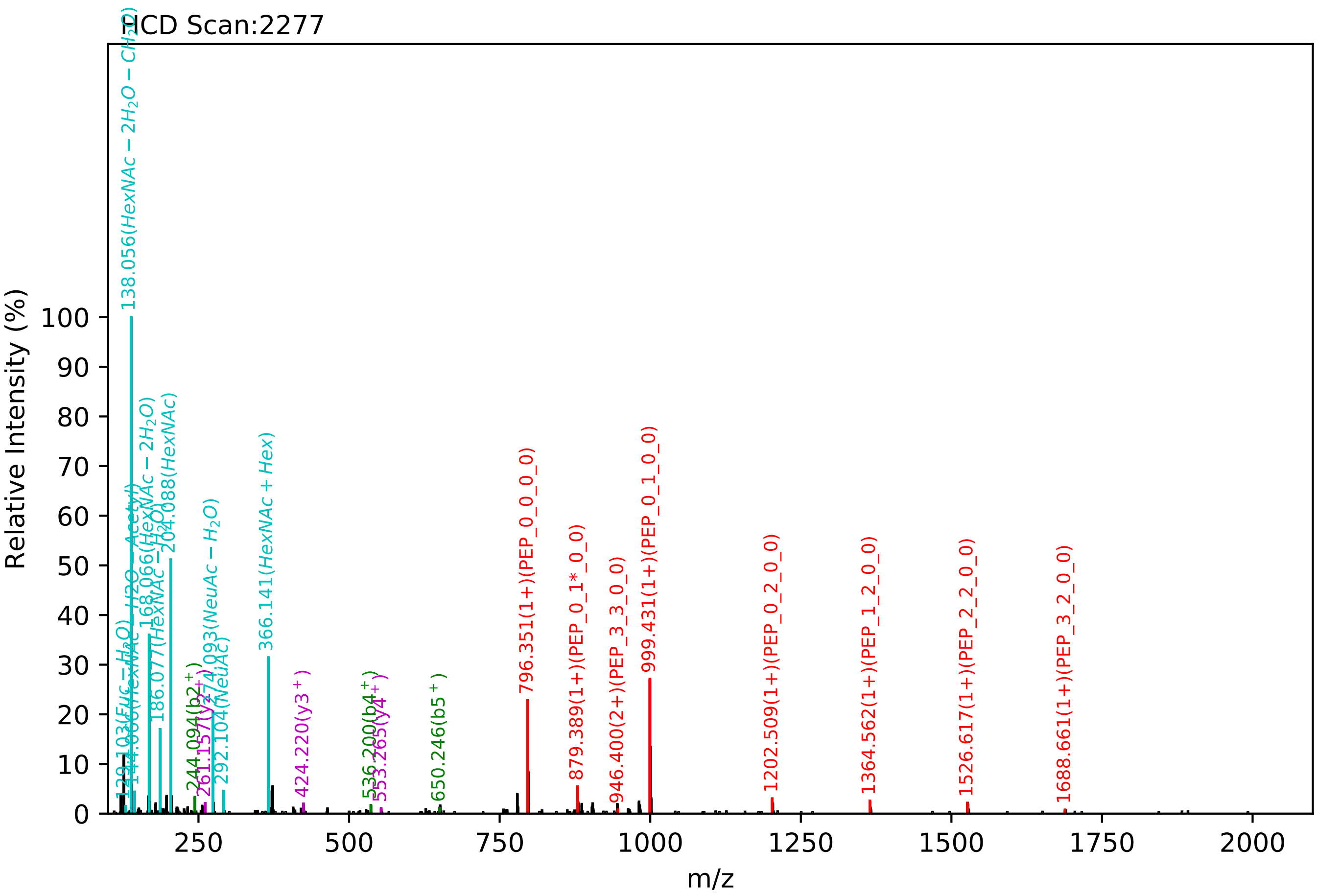

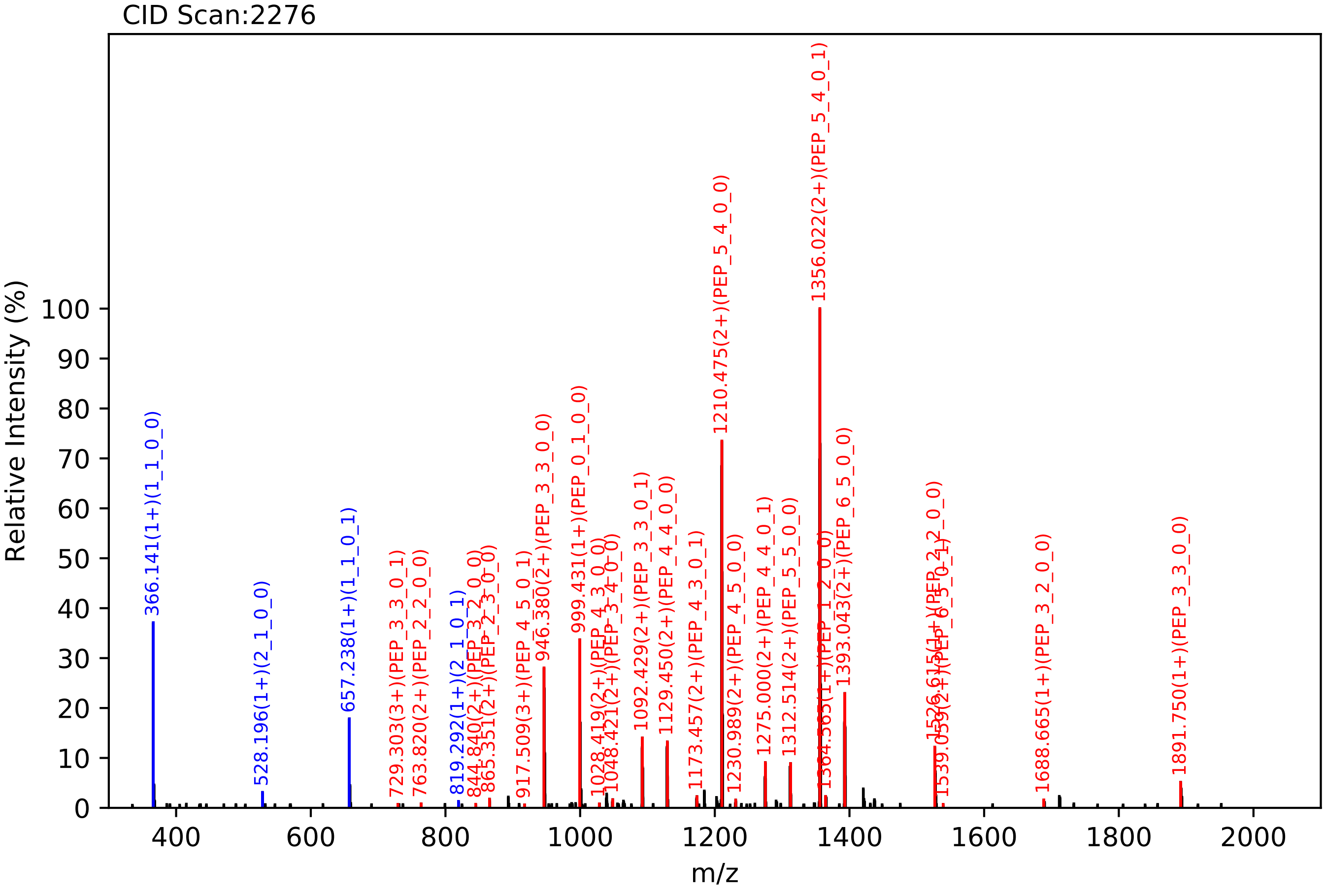

Test set no. 159, Experiment: AGP exp_3

NEEYNK(=PEP)_6_5_0_1, m/z:1025.73(3+), RT:17.37, Y-score:91.26

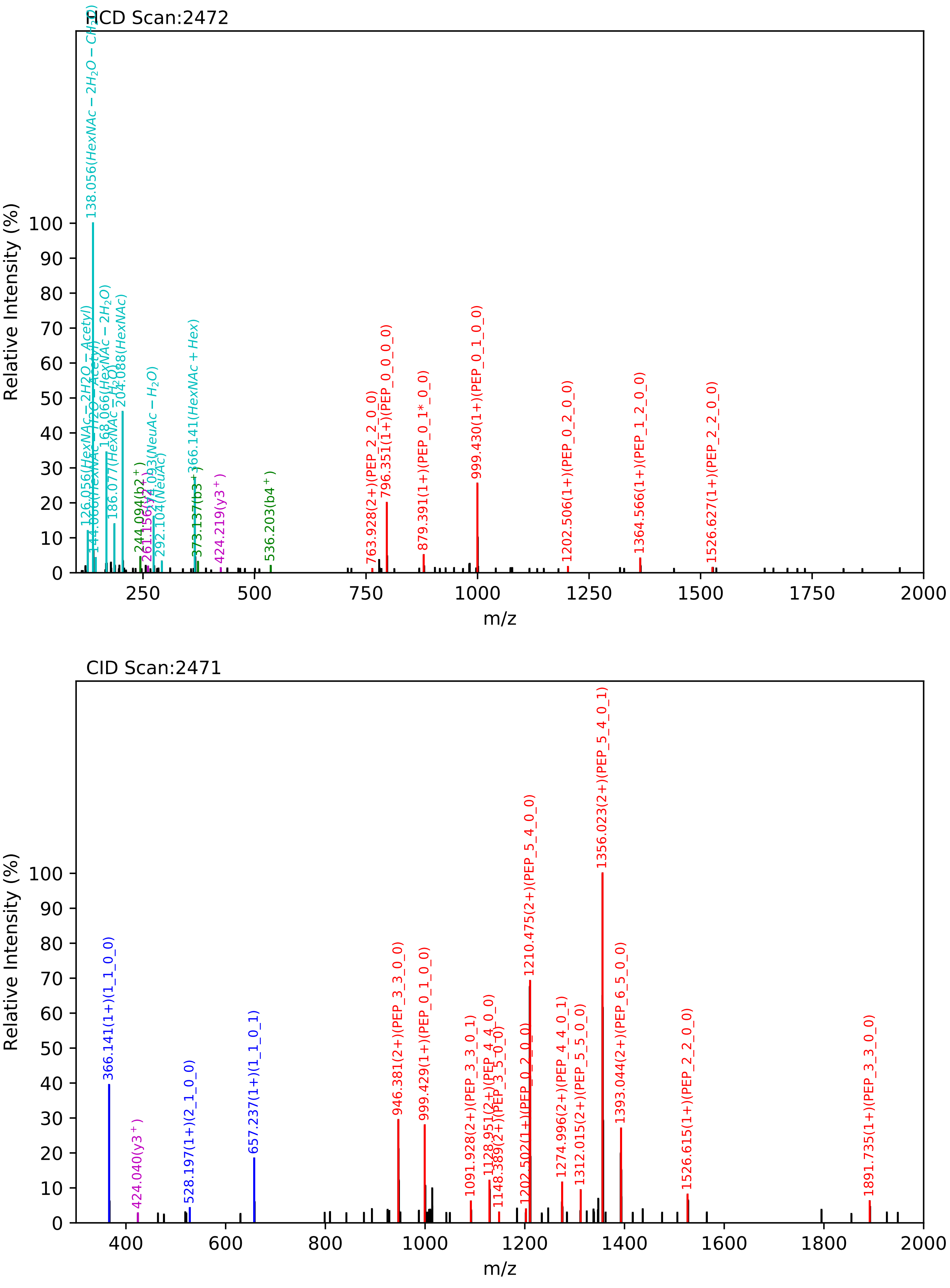

Test set no. 160, Experiment: AGP exp_4

NEEYNK(=PEP)_6_5_0_1, m/z:1538.08(2+), RT:16.98, Y-score:91.02

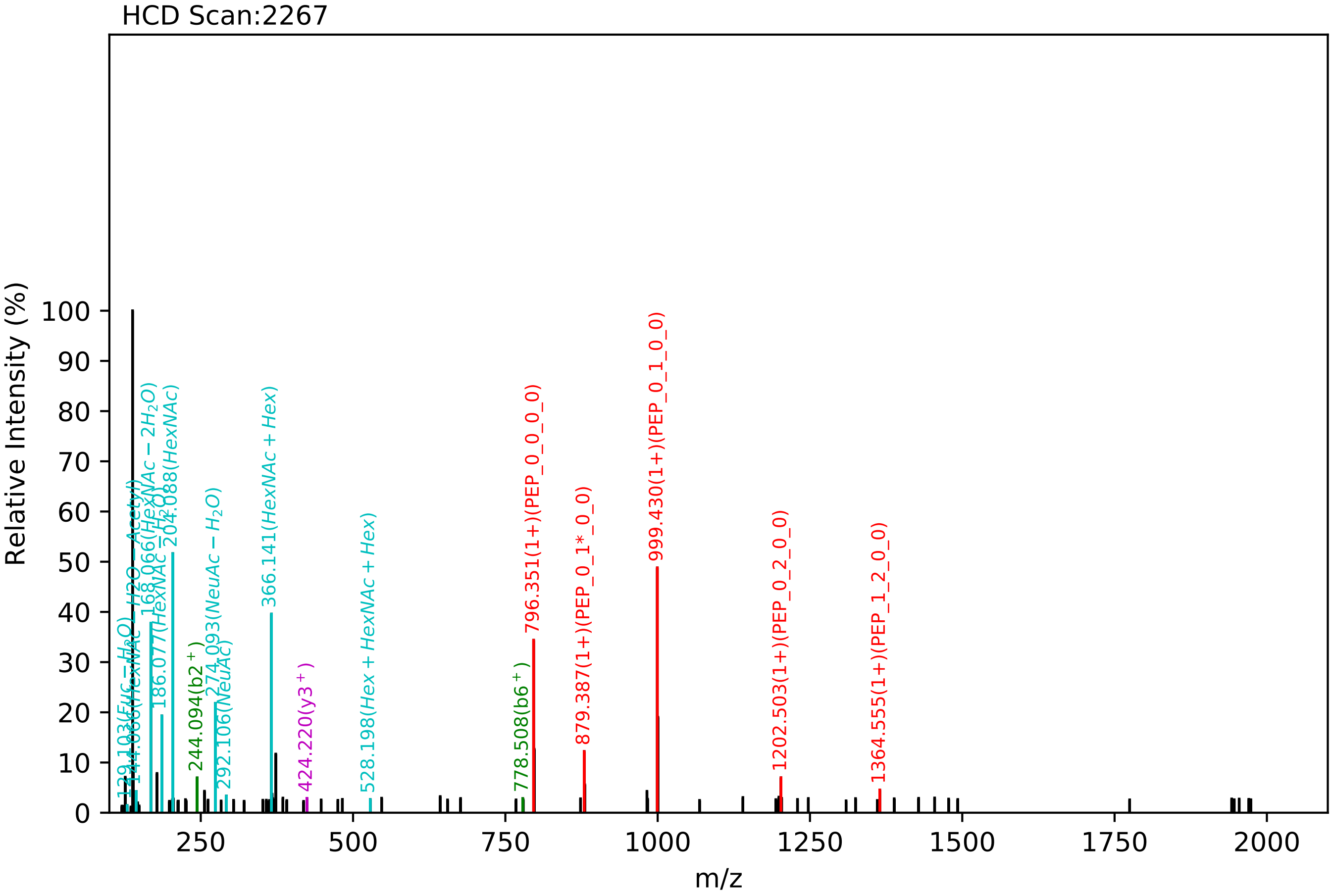

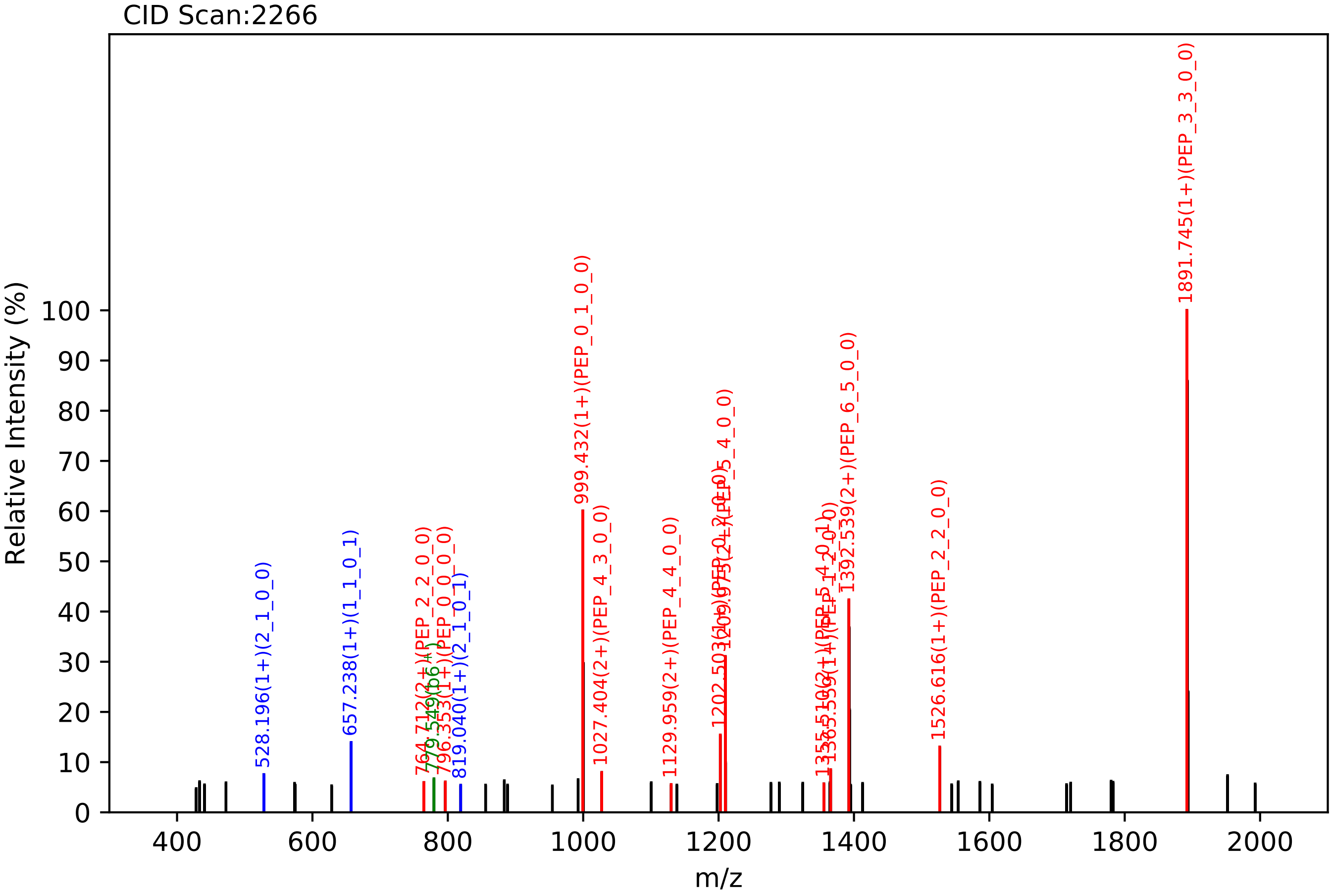

Test set no. 161, Experiment: AGP exp_4

NEEYNK(=PEP)_6_5_0_1, m/z:1025.73(3+), RT:17.37, Y-score:90.67

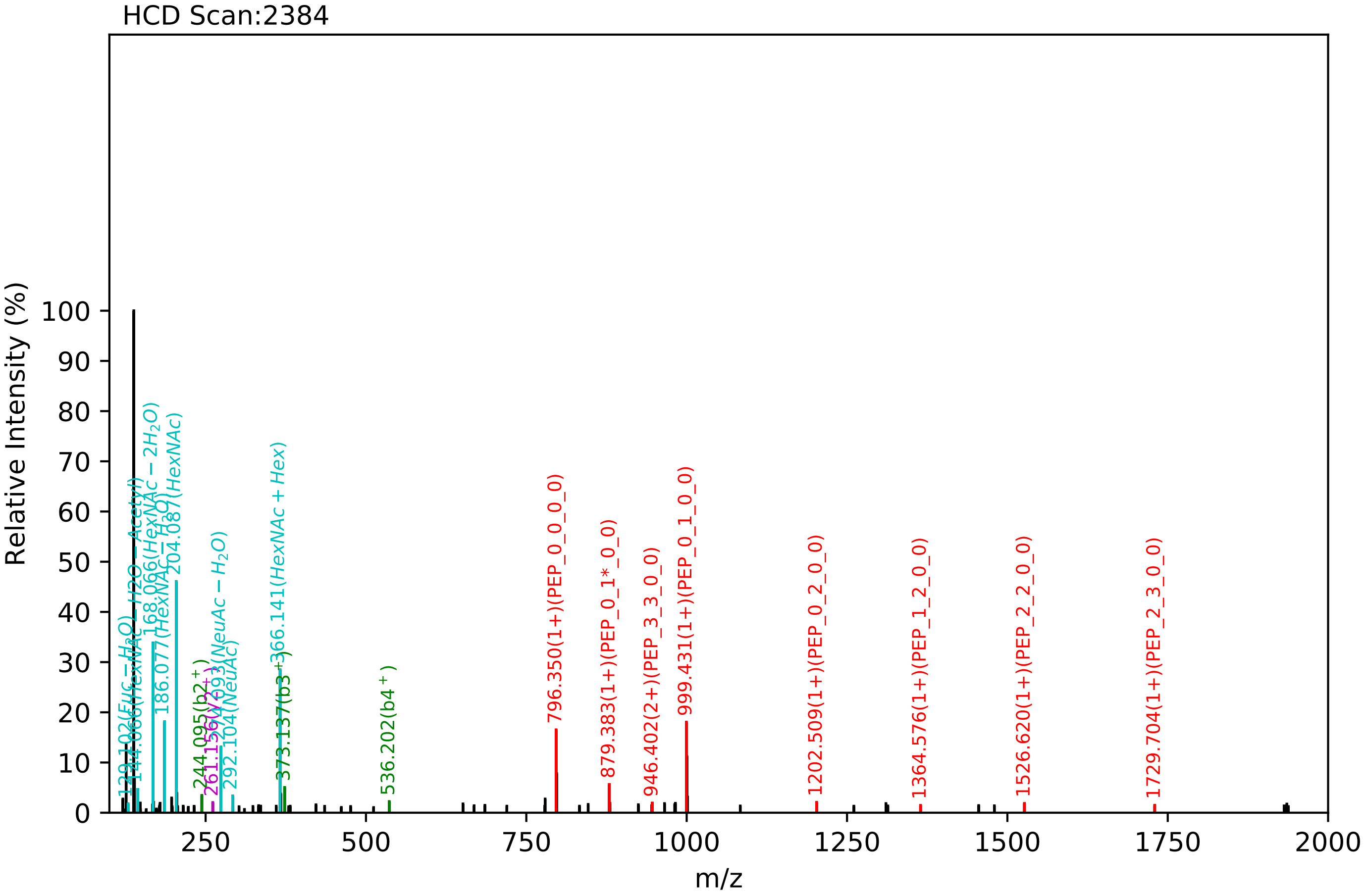

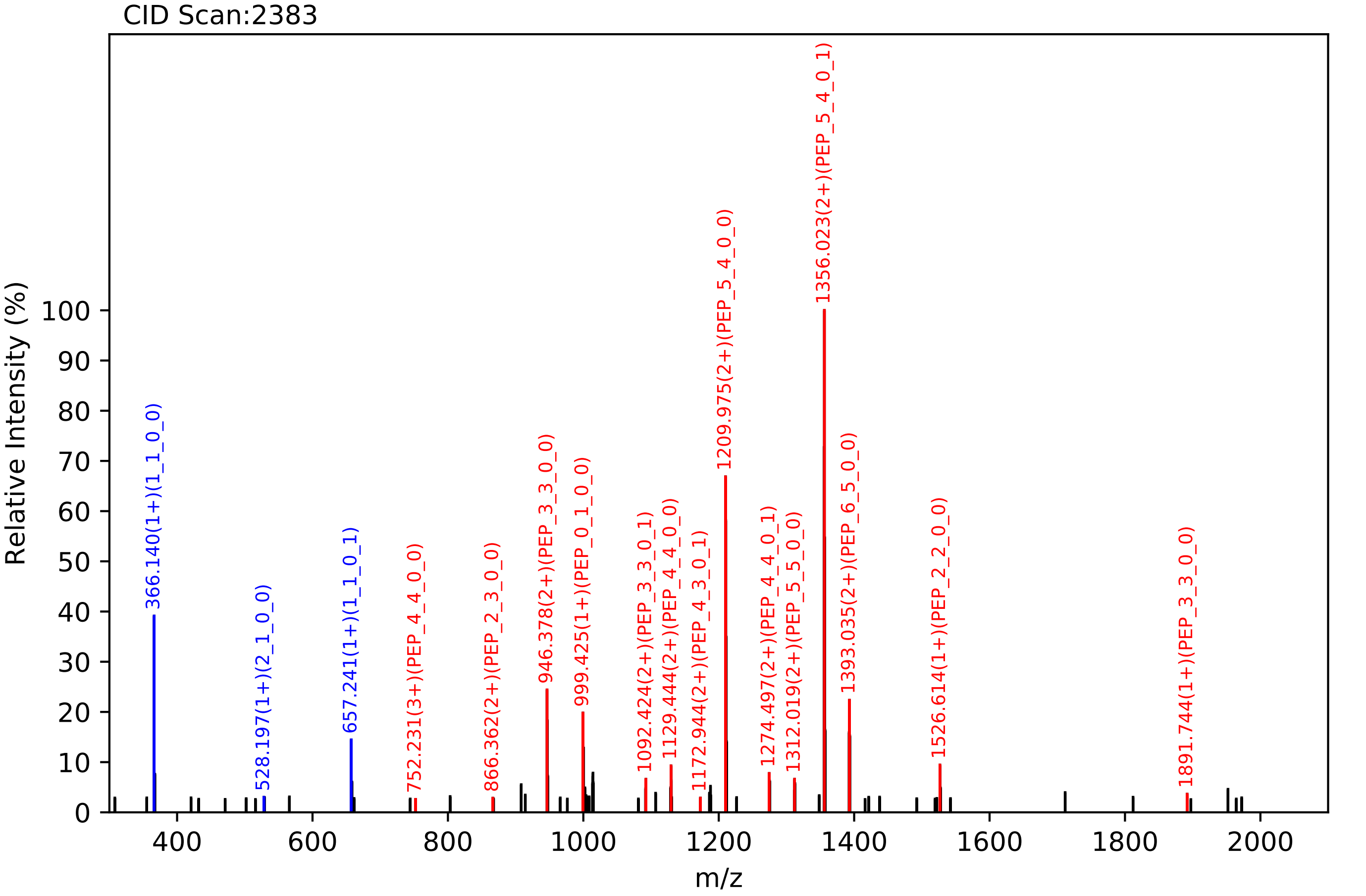

Test set no. 162, Experiment: AGP exp_4

NEEYNK(=PEP)_6_5_0_1, m/z:1025.73(3+), RT:15.81, Y-score:89.50

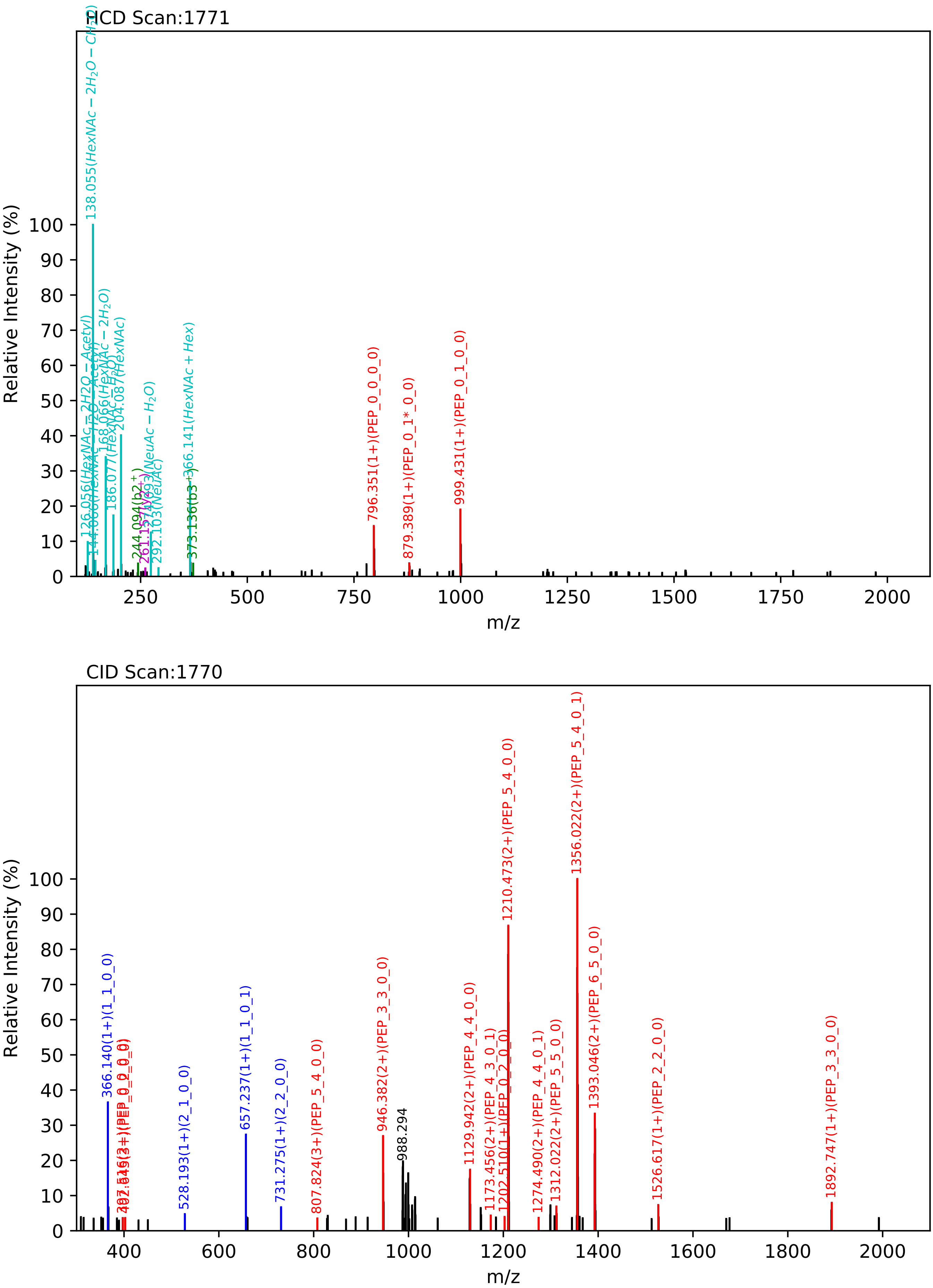

Test set no. 163, Experiment: AGP exp_4

NEEYNK(=PEP)_6_5_0_1, m/z:1025.73(3+), RT:16.86, Y-score:89.28

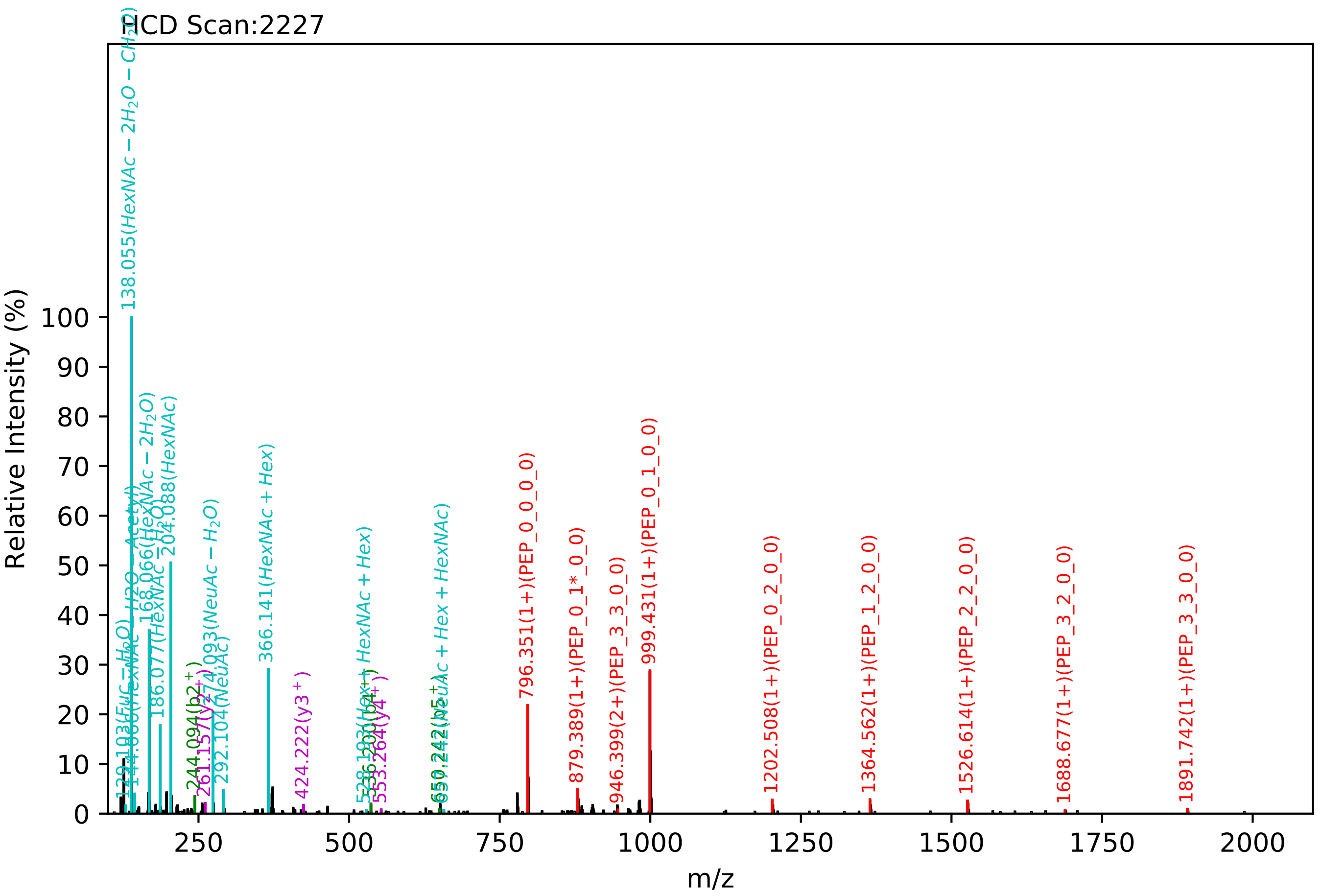

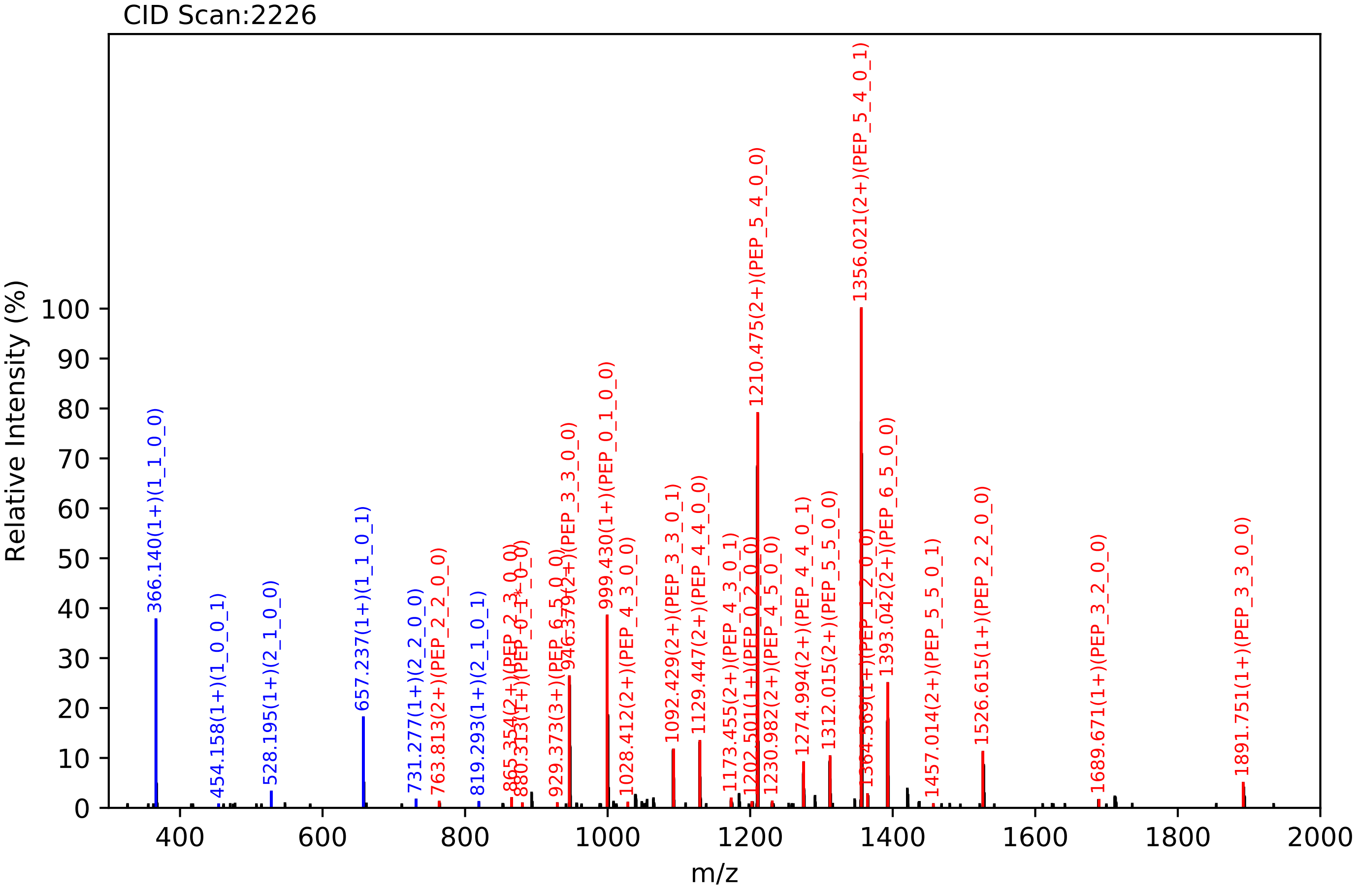

Test set no. 164, Experiment: AGP exp_3

NEEYNK(=PEP)_6_5_0_1, m/z:1025.73(3+), RT:16.33, Y-score:89.15

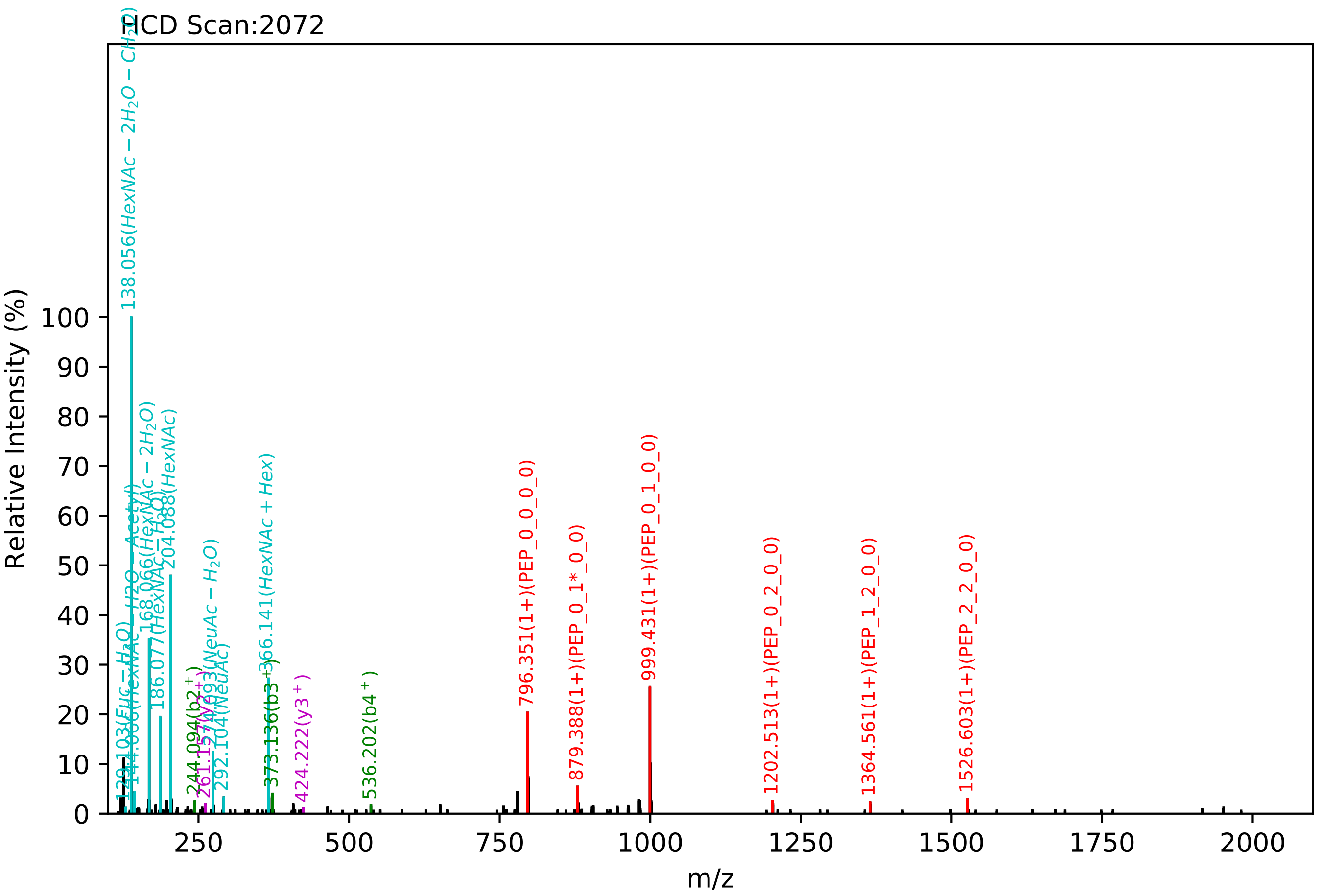

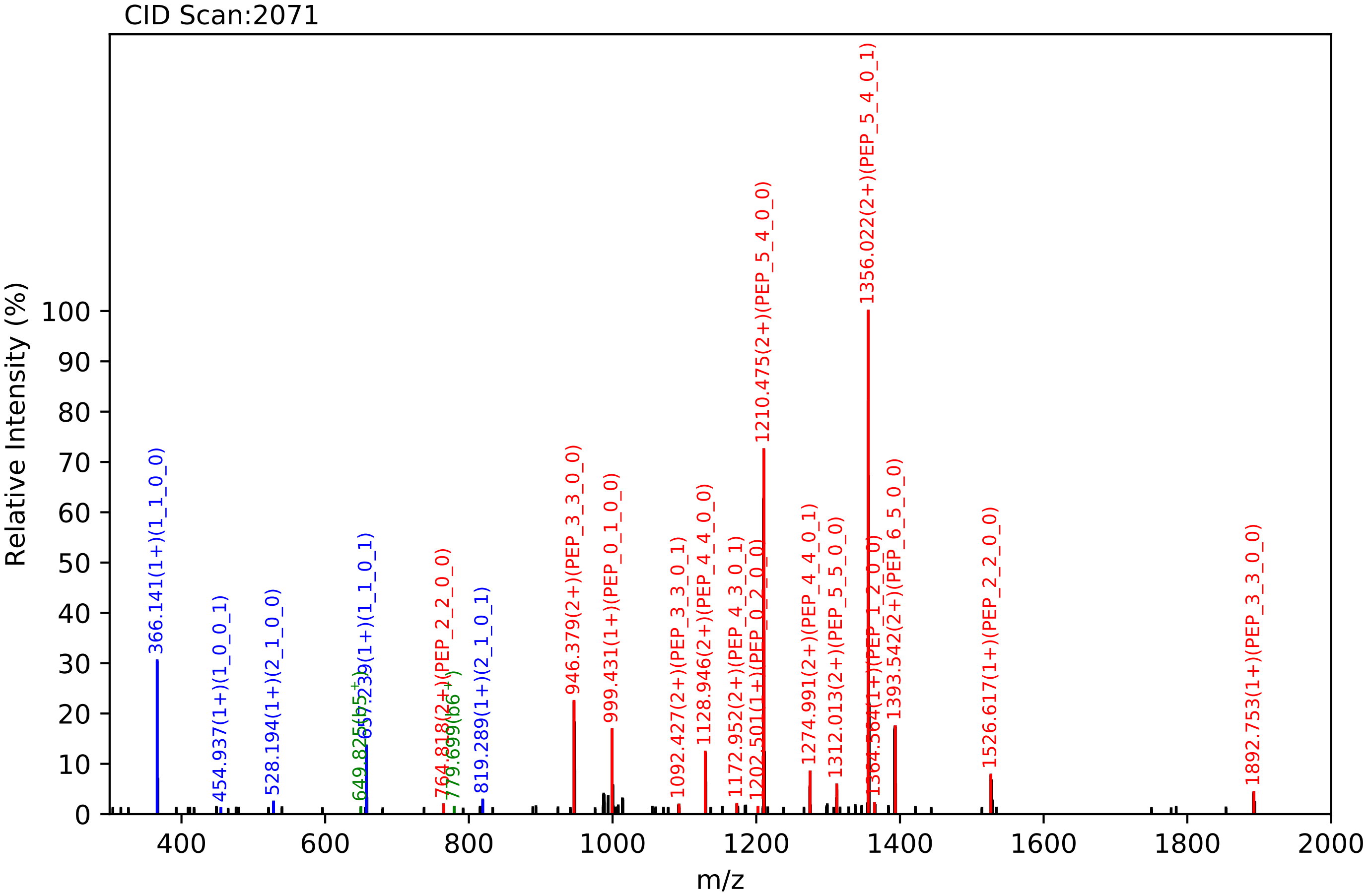

Test set no. 165, Experiment: AGP exp_4

NEEYNK(=PEP)_6_5_0_1, m/z:1538.09(2+), RT:15.95, Y-score:77.68

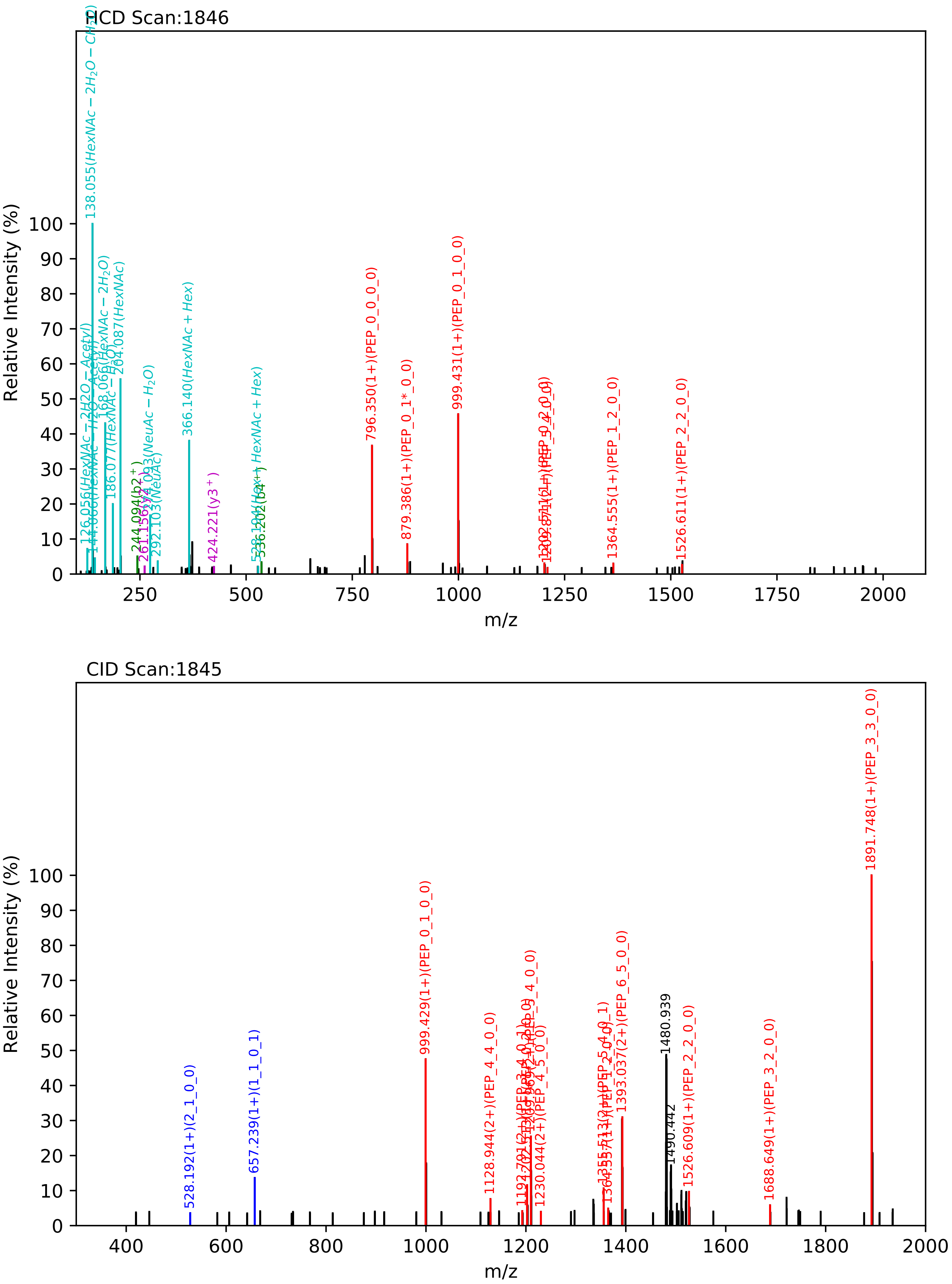

Test set no. 166, Experiment: AGP exp_3

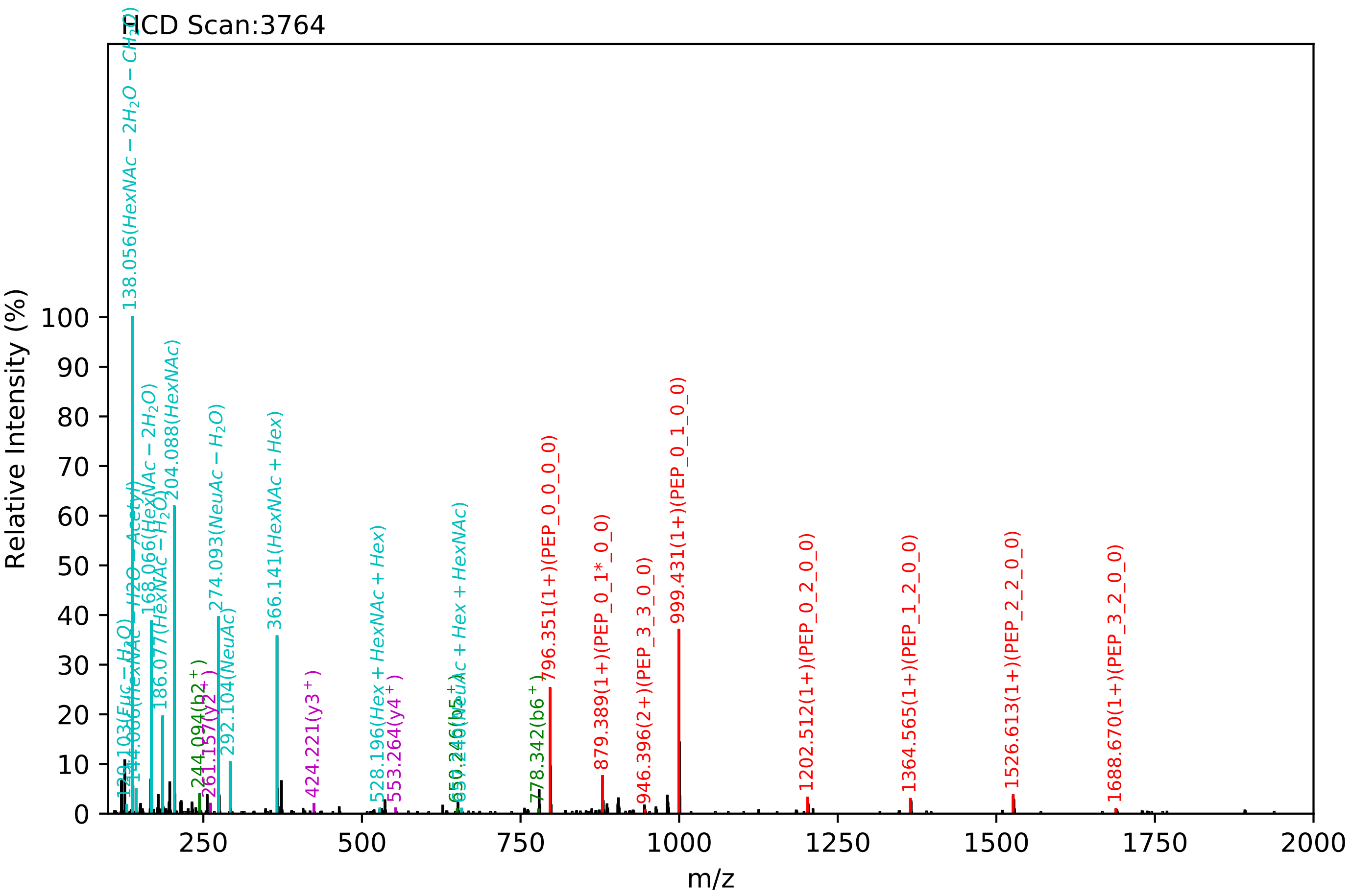

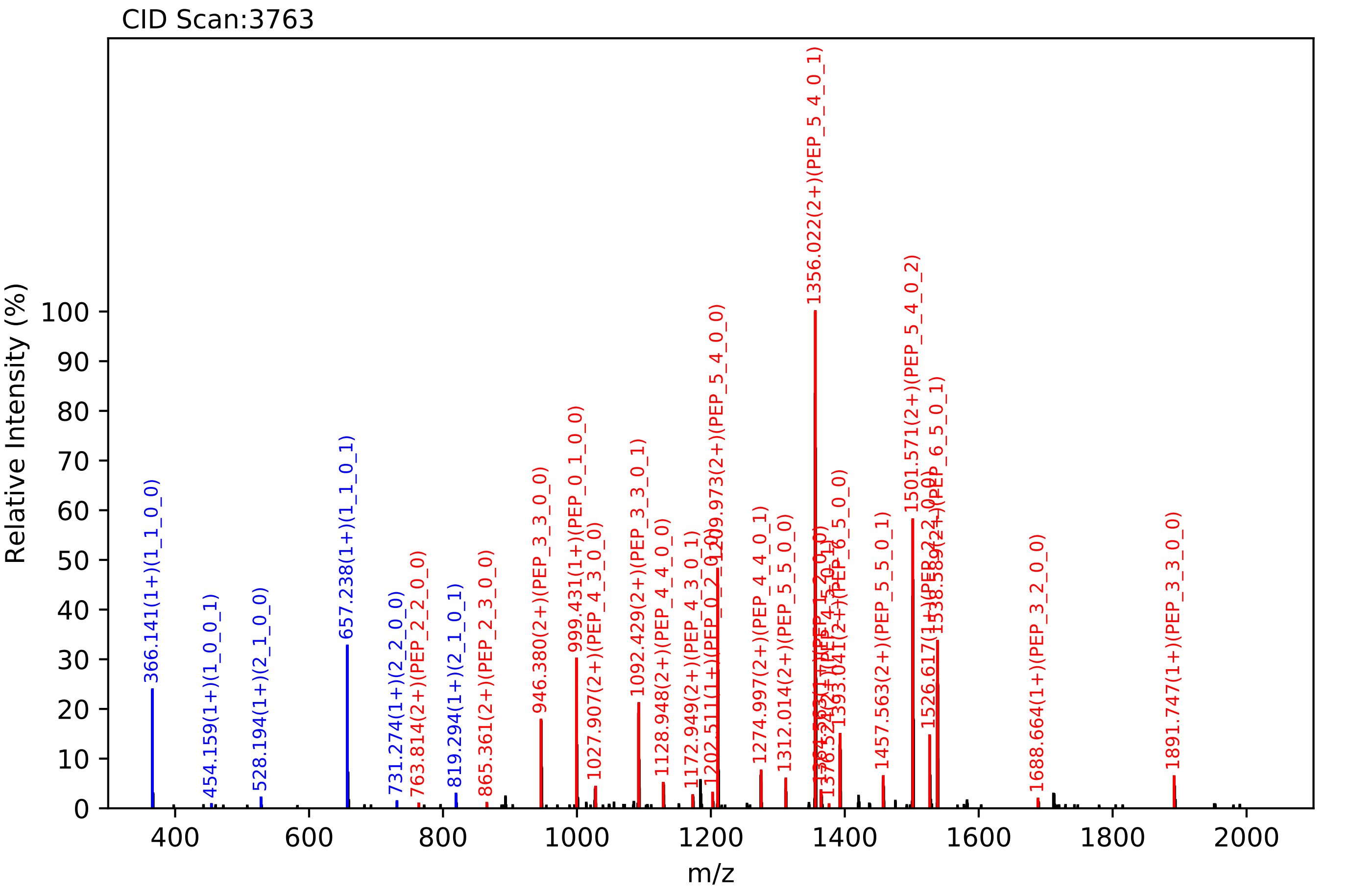

Test set no. 167, Experiment: AGP exp_4

NEEYNK(=PEP)_6_5_0_2, m/z:1122.76(3+), RT:20.17, Y-score:92.54

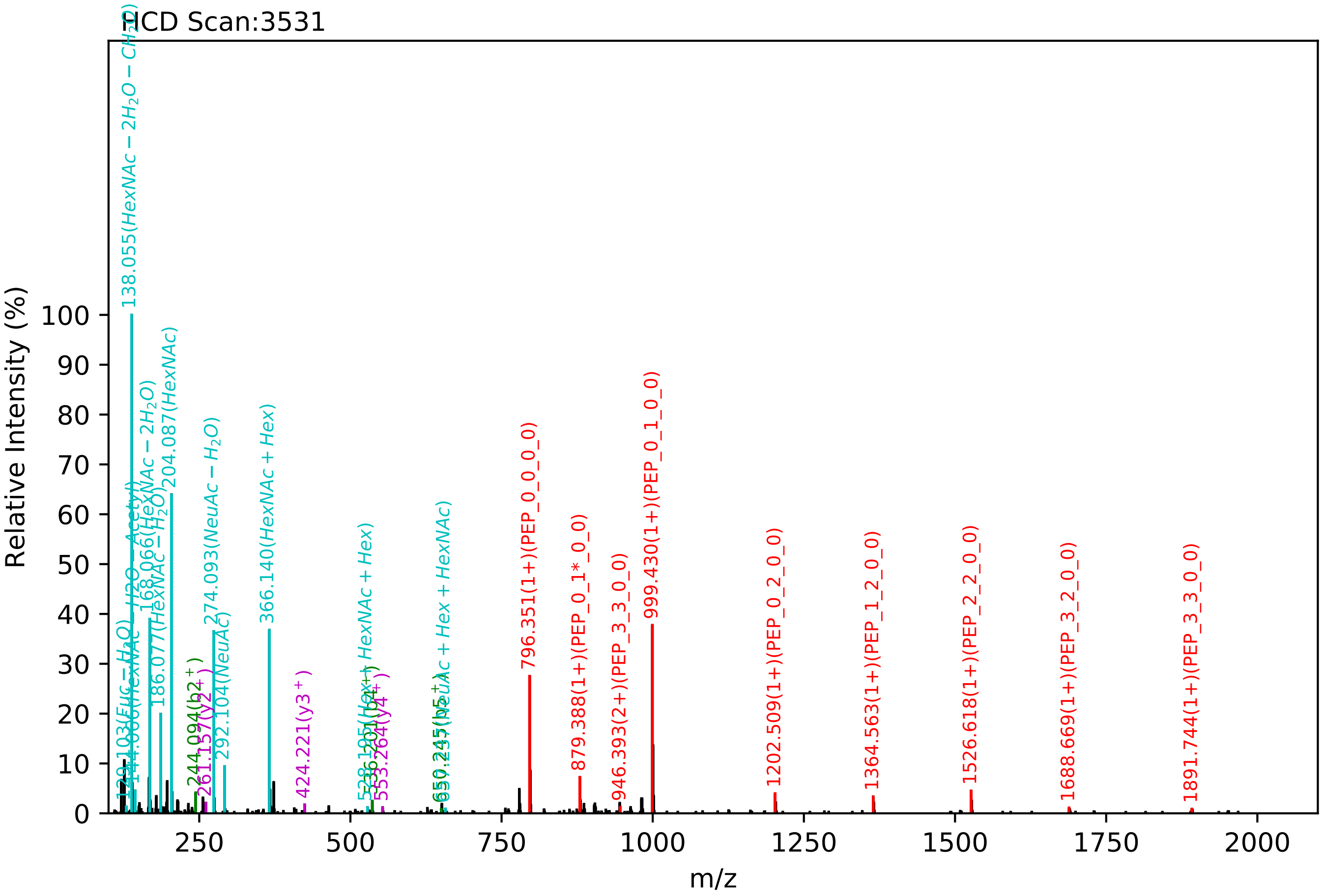

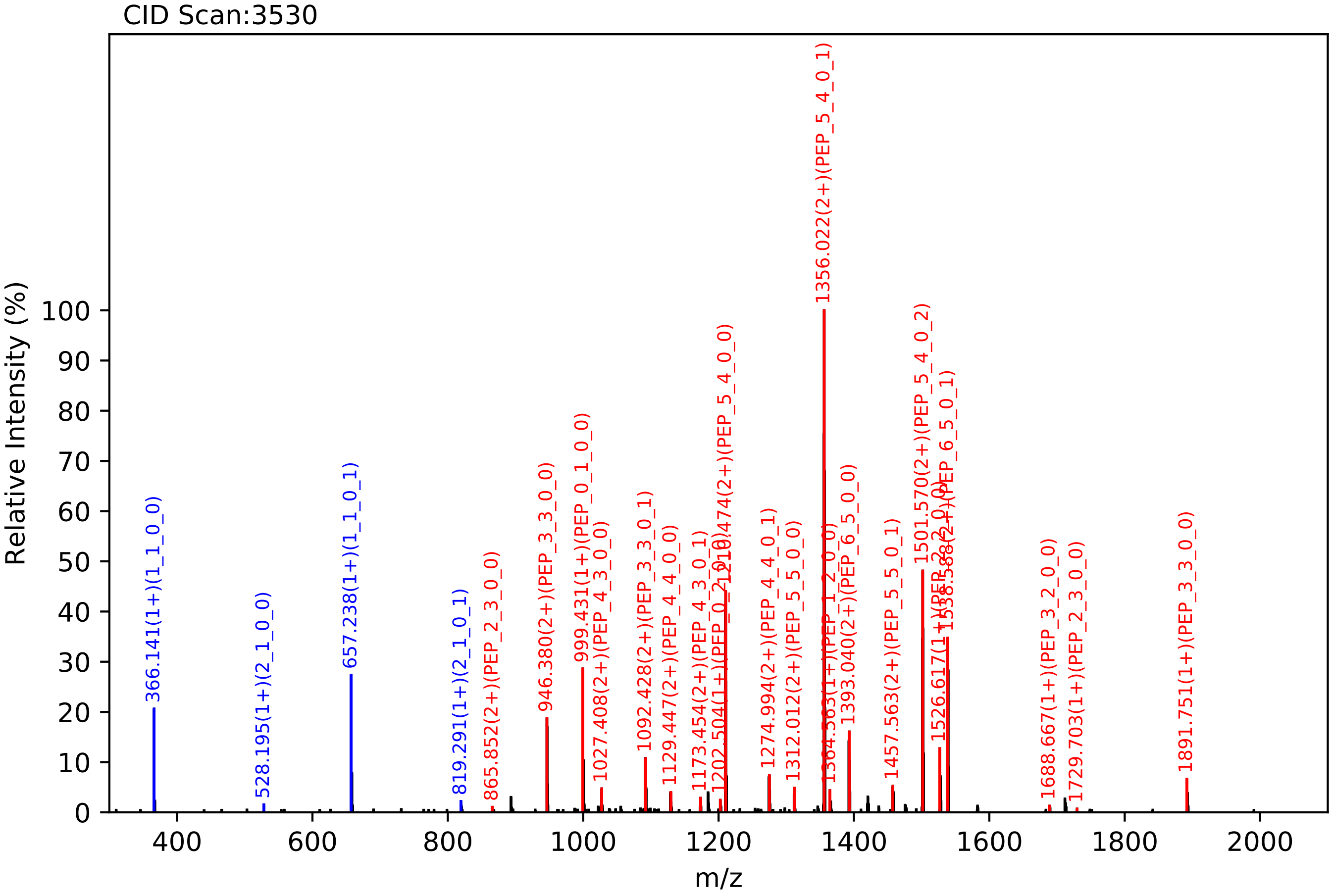

Test set no. 168, Experiment: AGP exp_4

NEEYNK(=PEP)_6_5_0_2, m/z:1122.76(3+), RT:19.61, Y-score:92.37

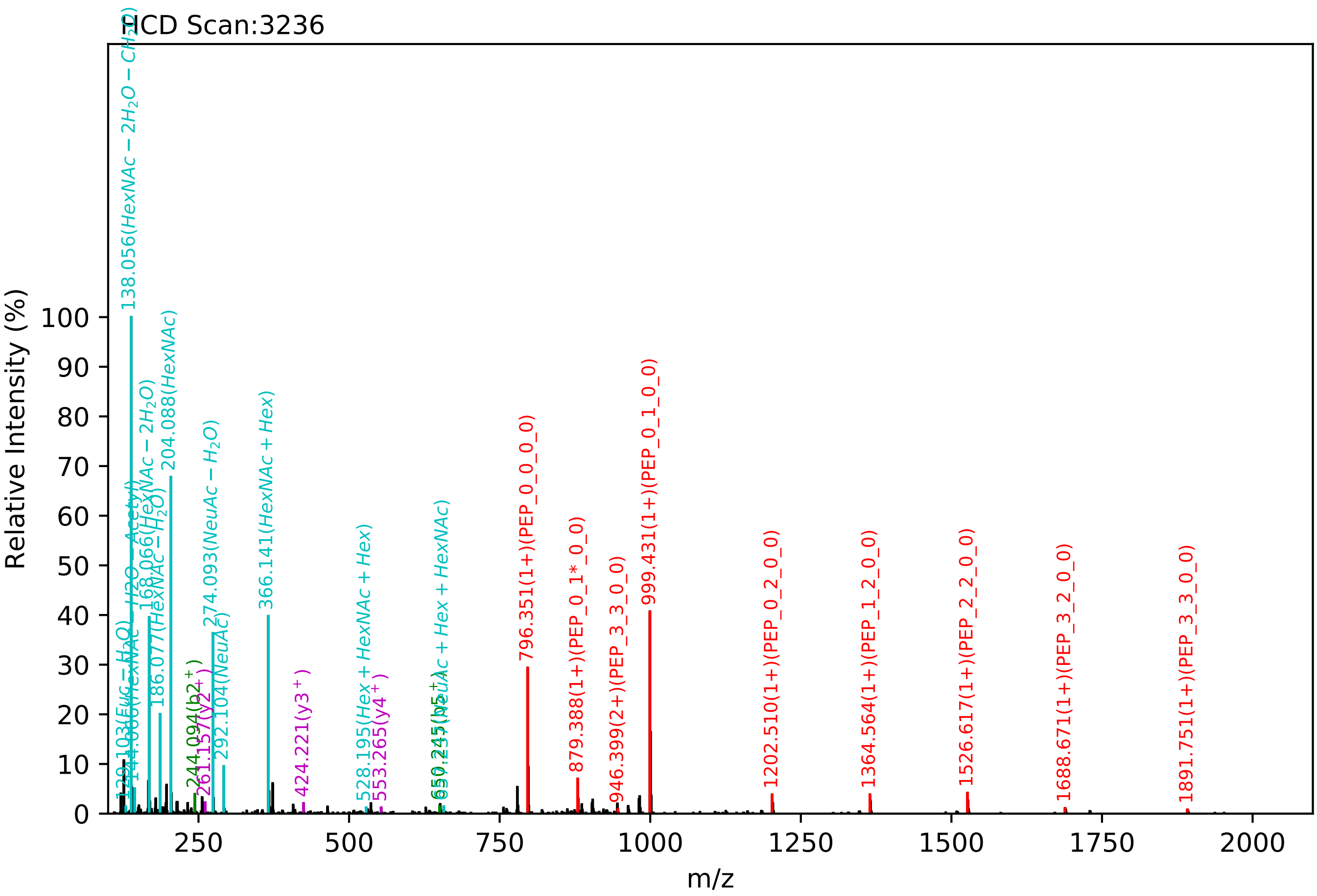

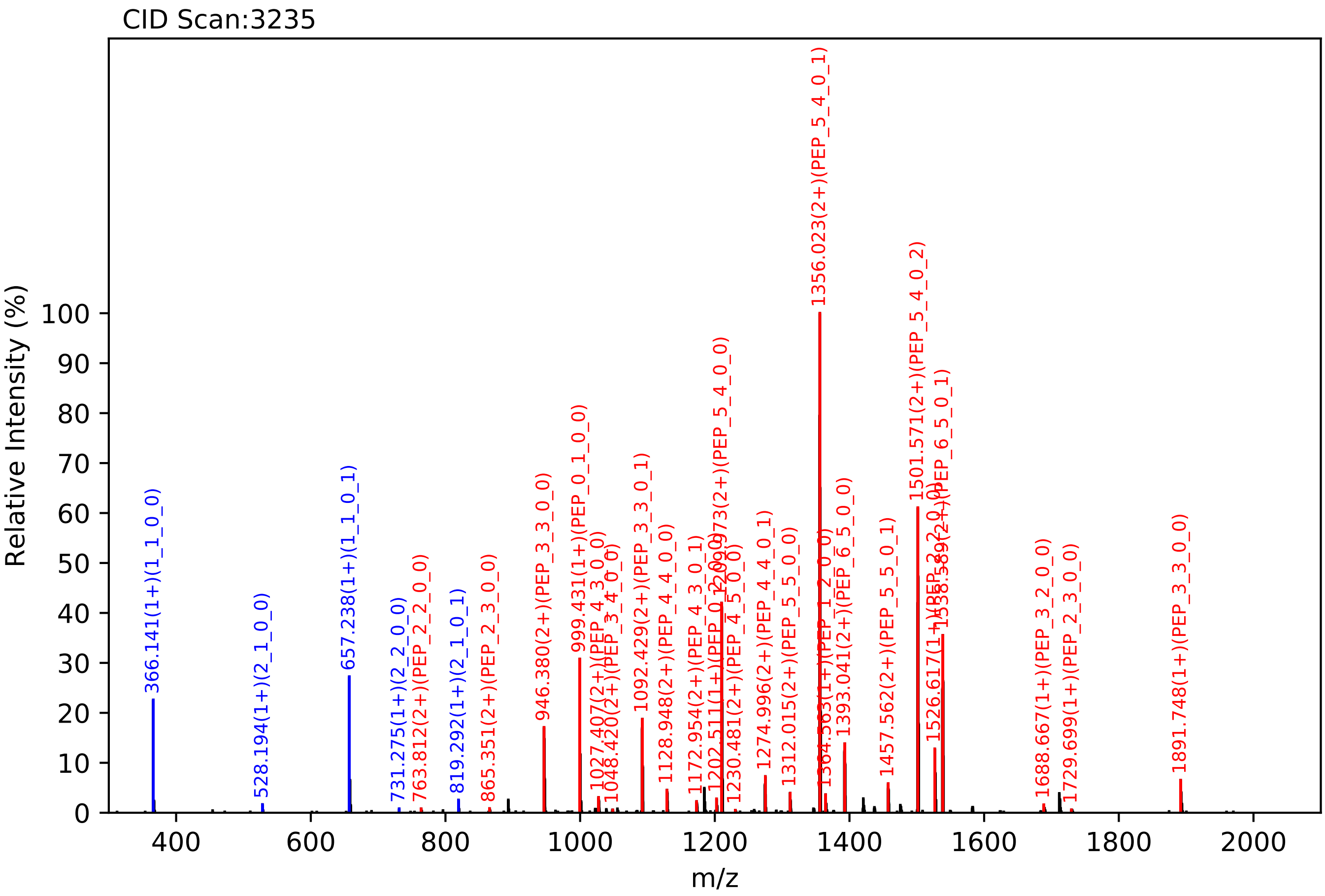

Test set no. 169, Experiment: AGP exp_4

NEEYNK(=PEP)_6_5_0_2, m/z:1122.76(3+), RT:20.78, Y-score:91.69

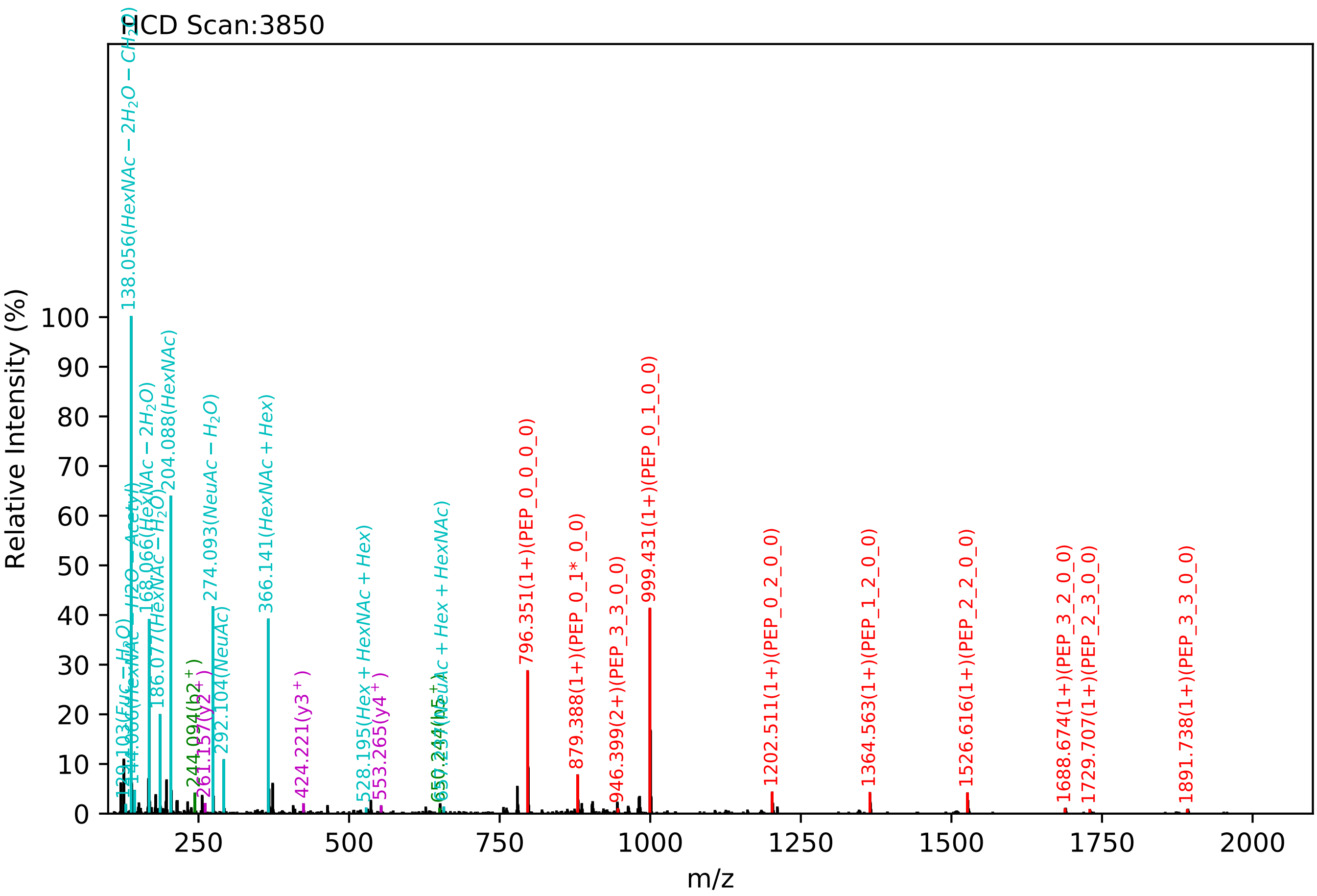

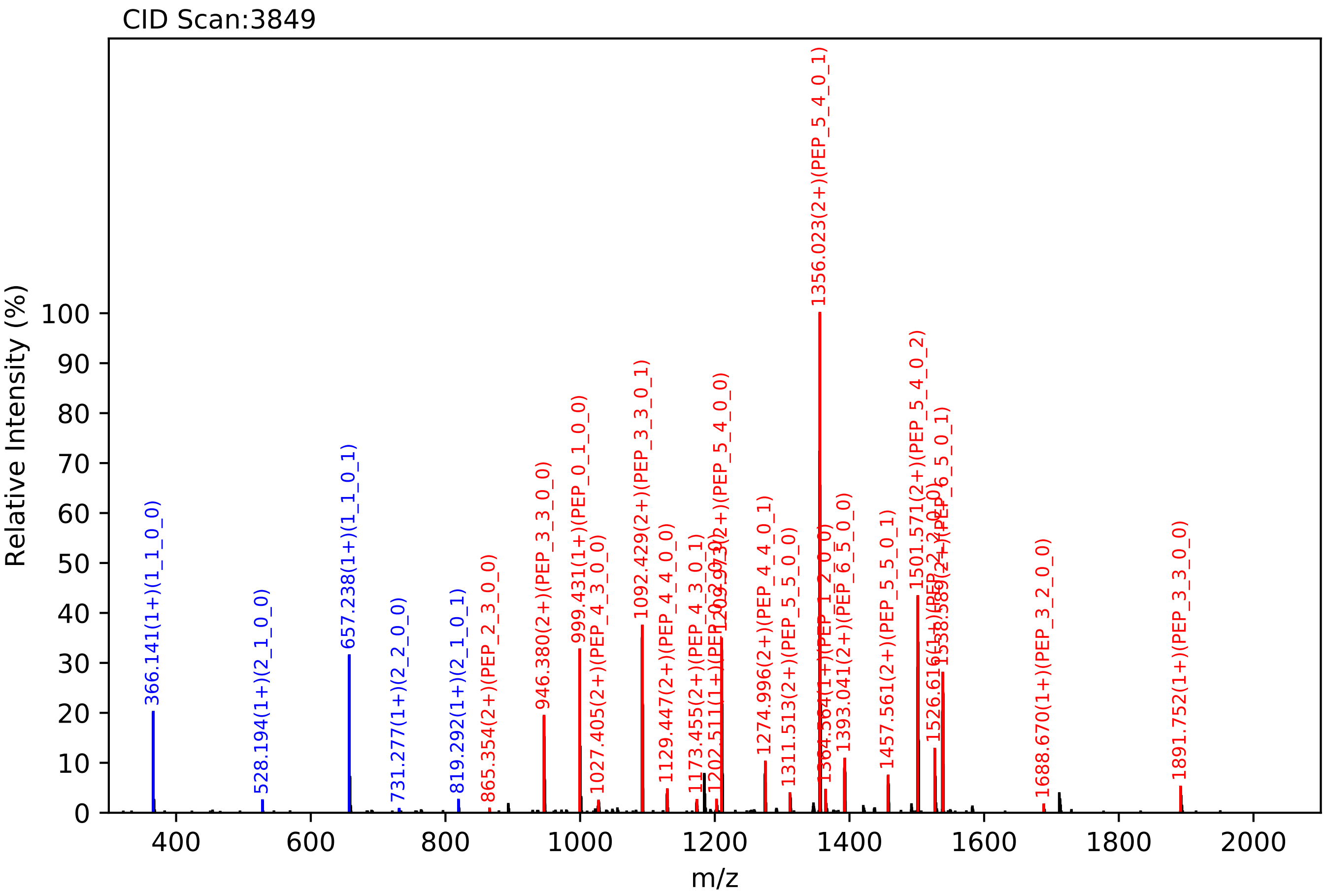

Test set no. 170, Experiment: AGP exp_3

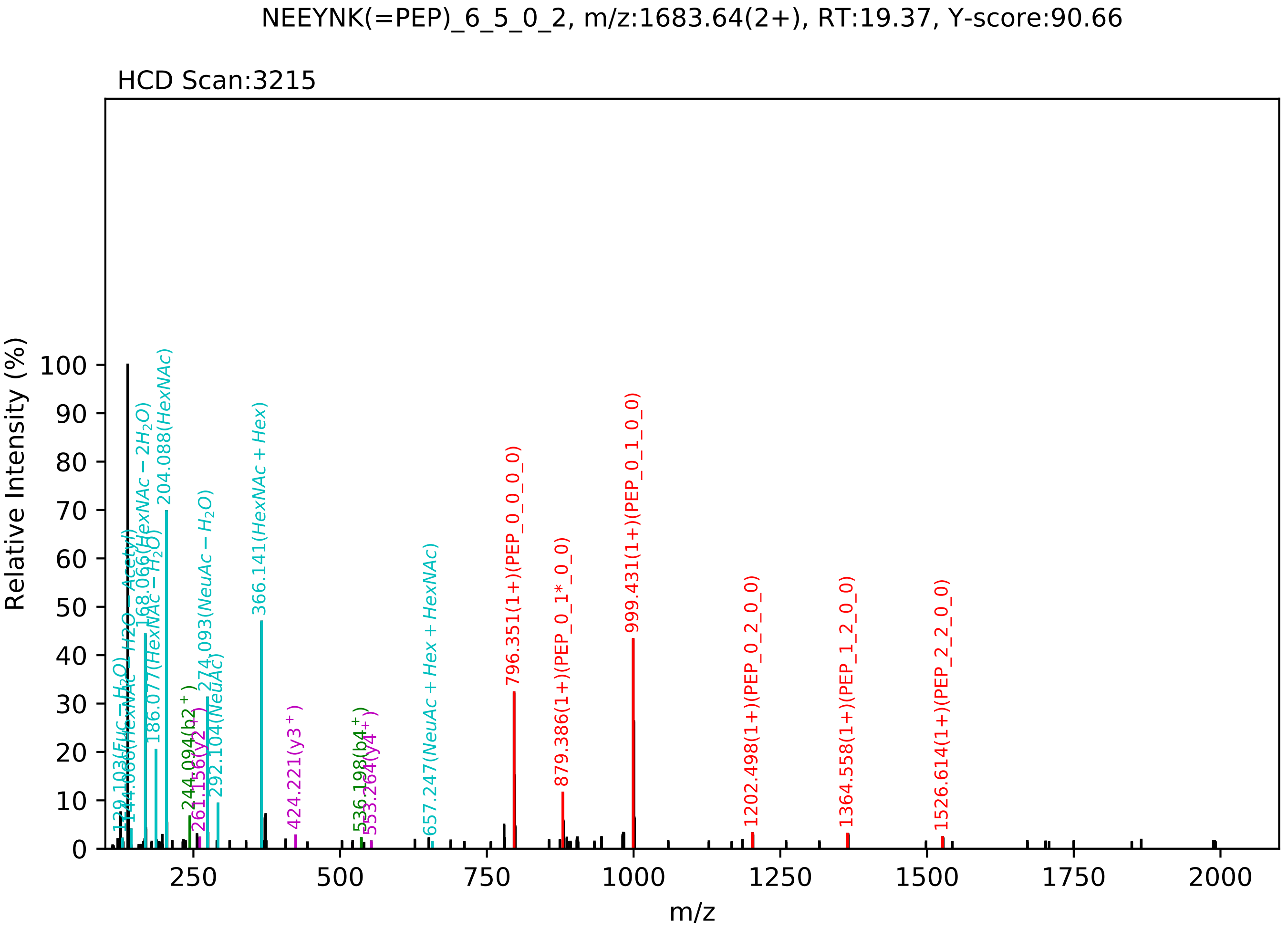

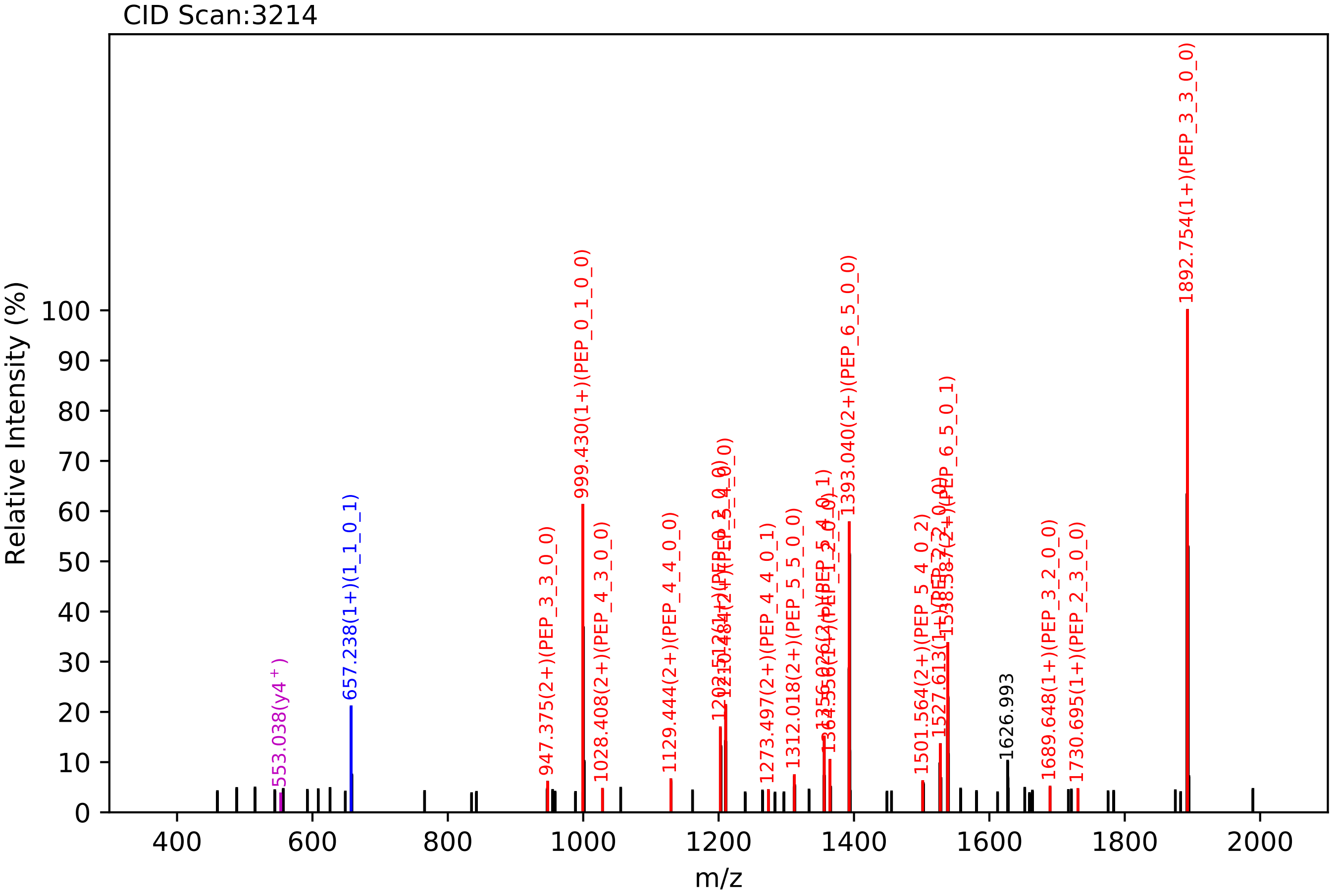

Test set no. 171, Experiment: AGP exp_4

NEEYNK(=PEP)_6_5_0_2, m/z:1683.63(2+), RT:20.57, Y-score:90.65

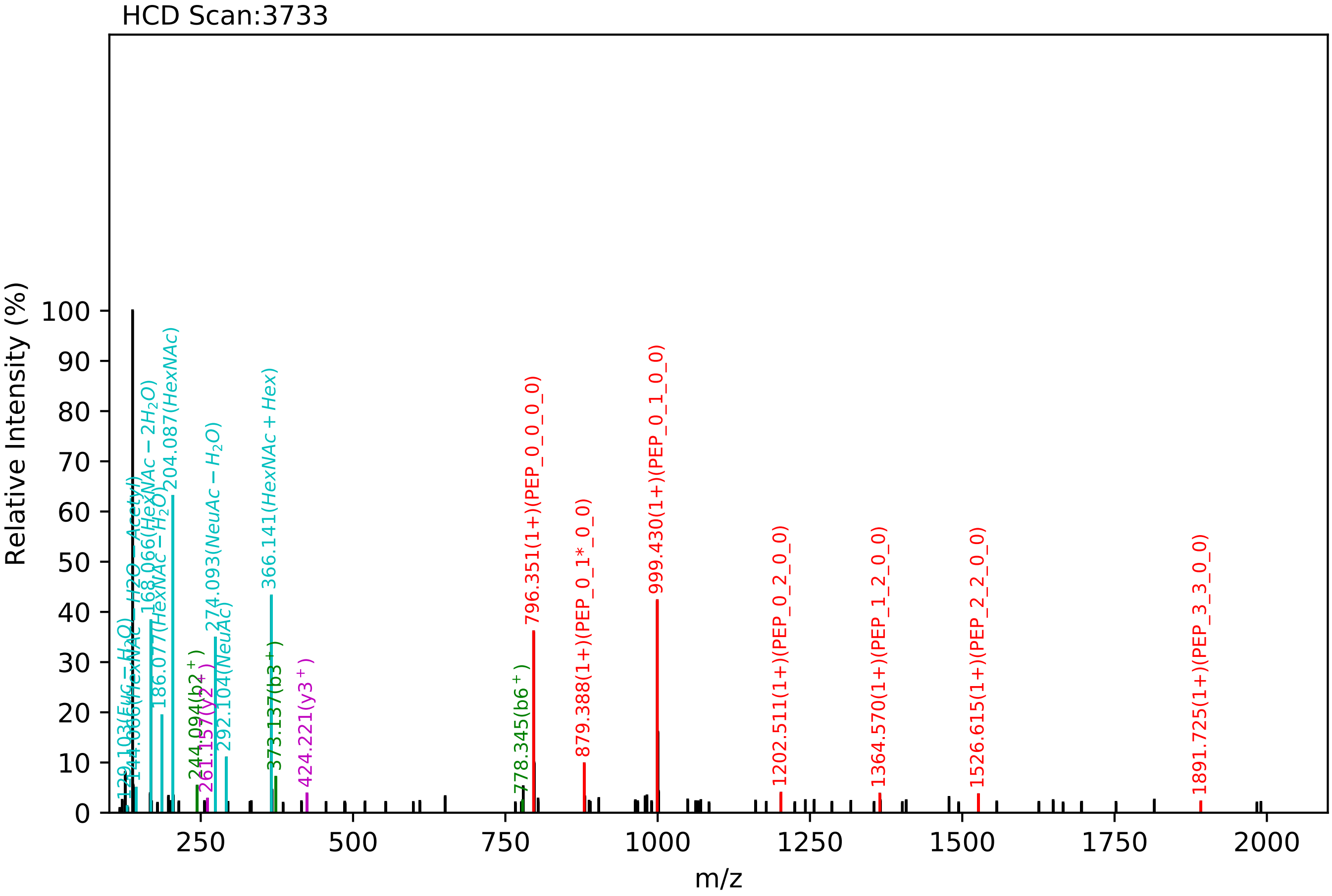

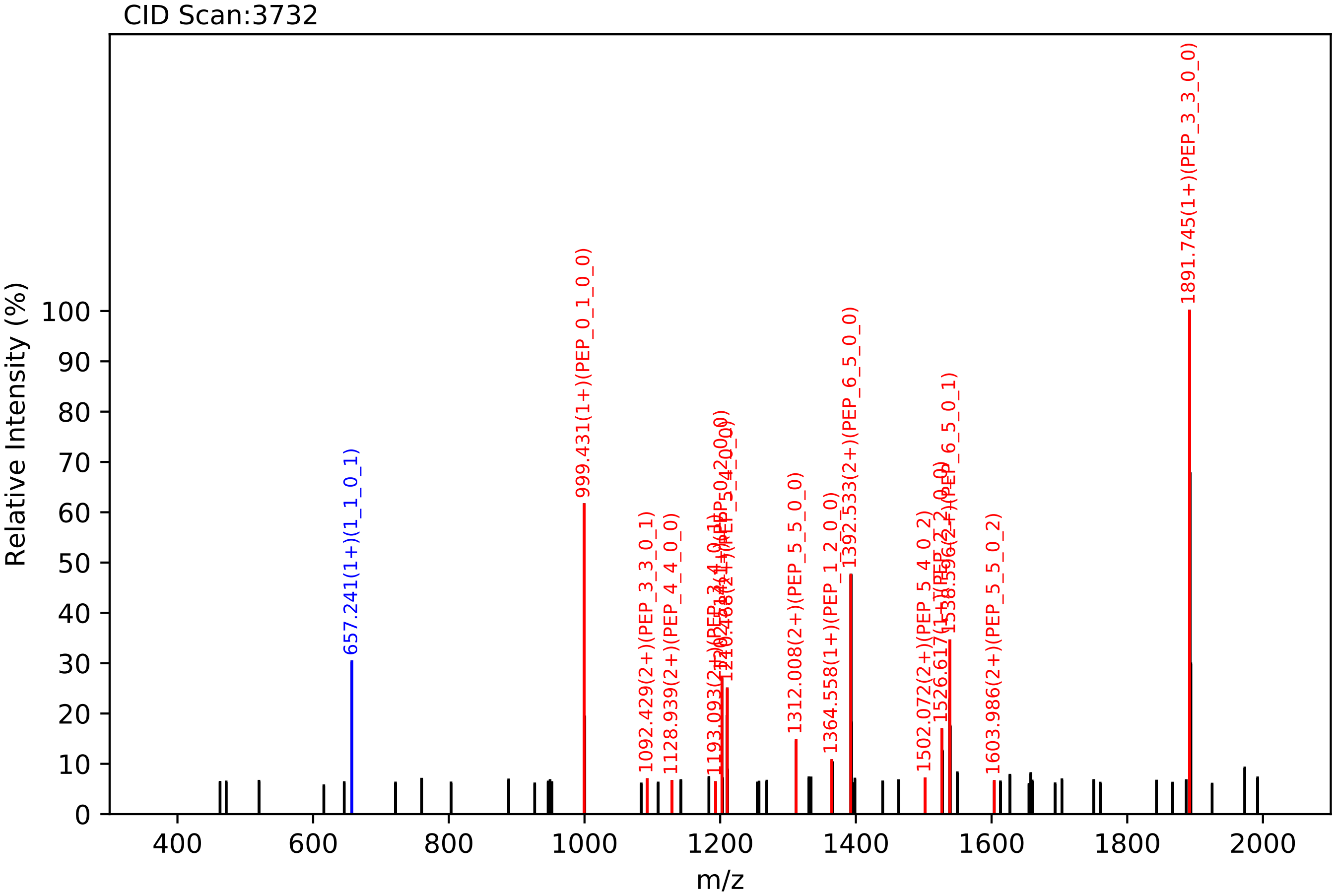

Test set no. 172, Experiment: AGP exp_3

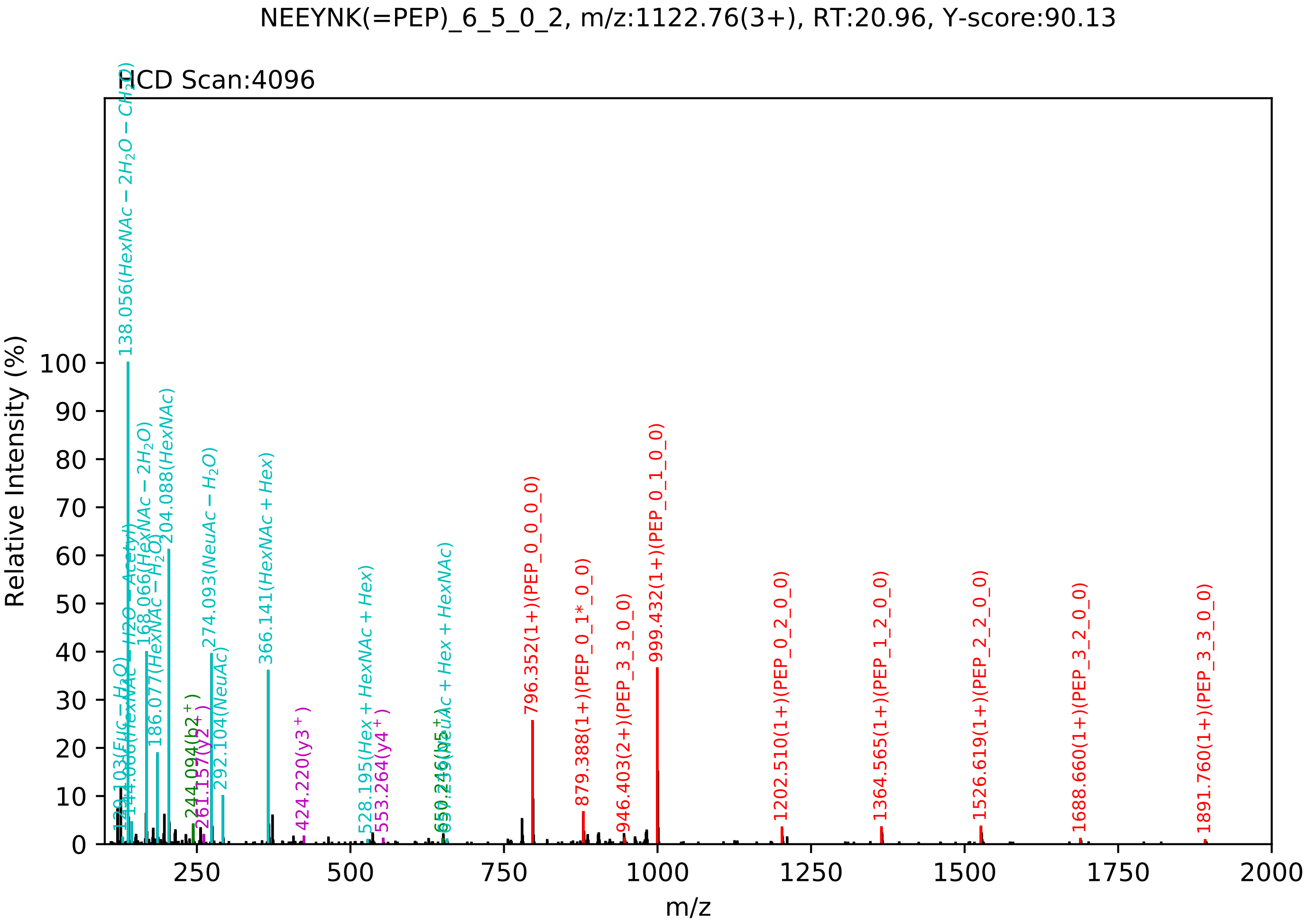

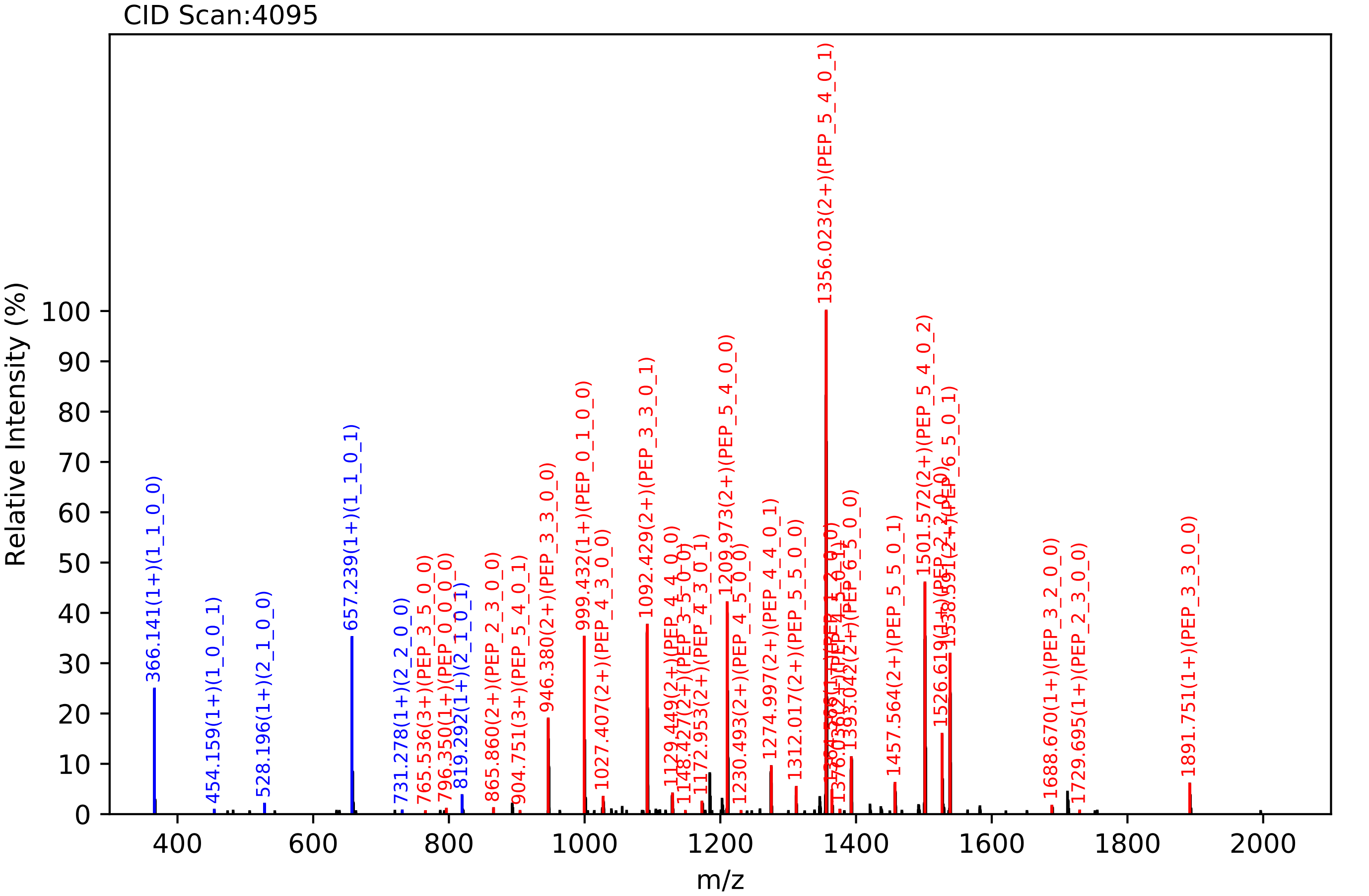

Test set no. 173, Experiment: AGP exp_3

NEEYNK(=PEP)_6_5_0_2, m/z:1122.76(3+), RT:19.22, Y-score:88.31

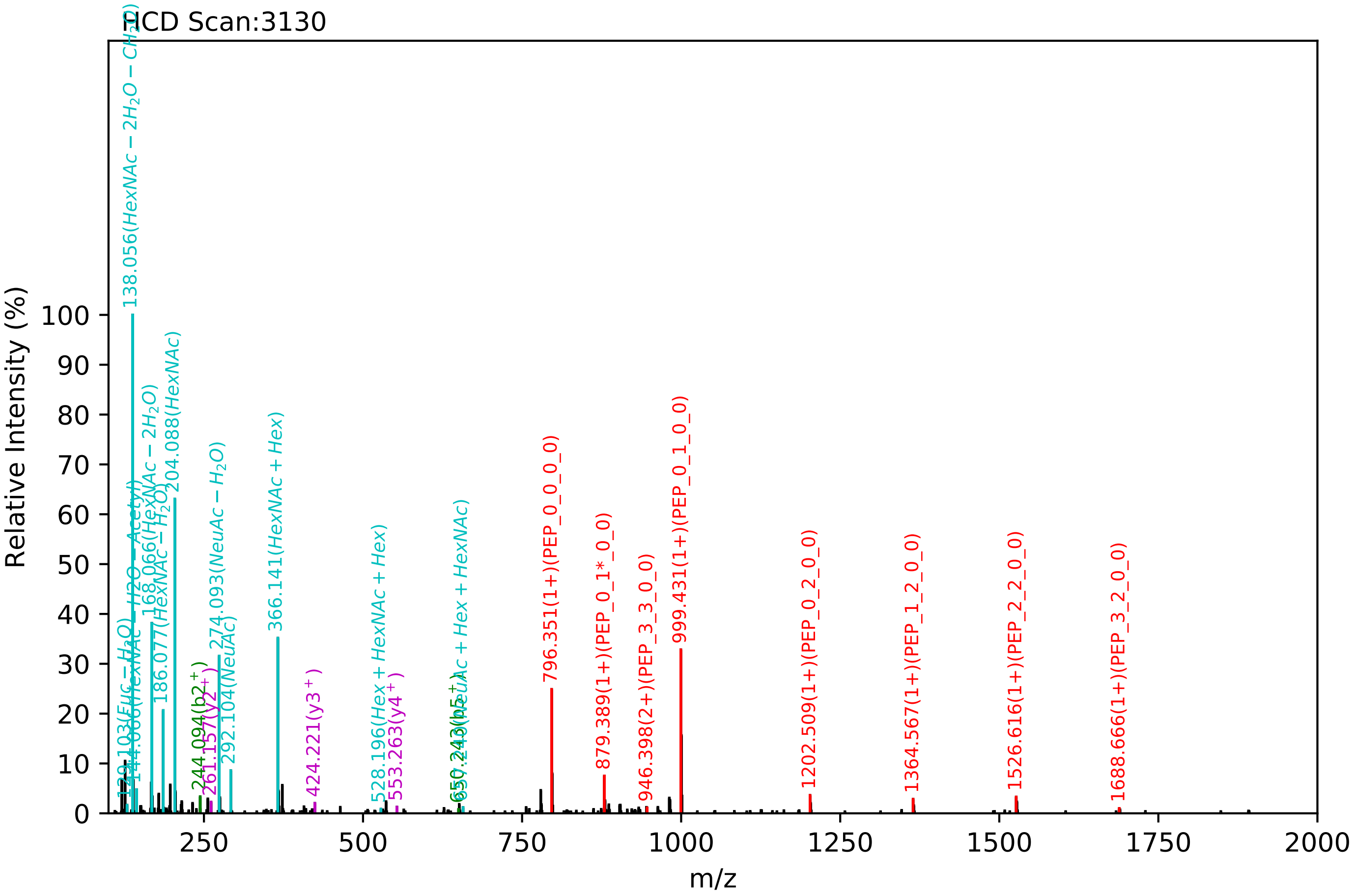

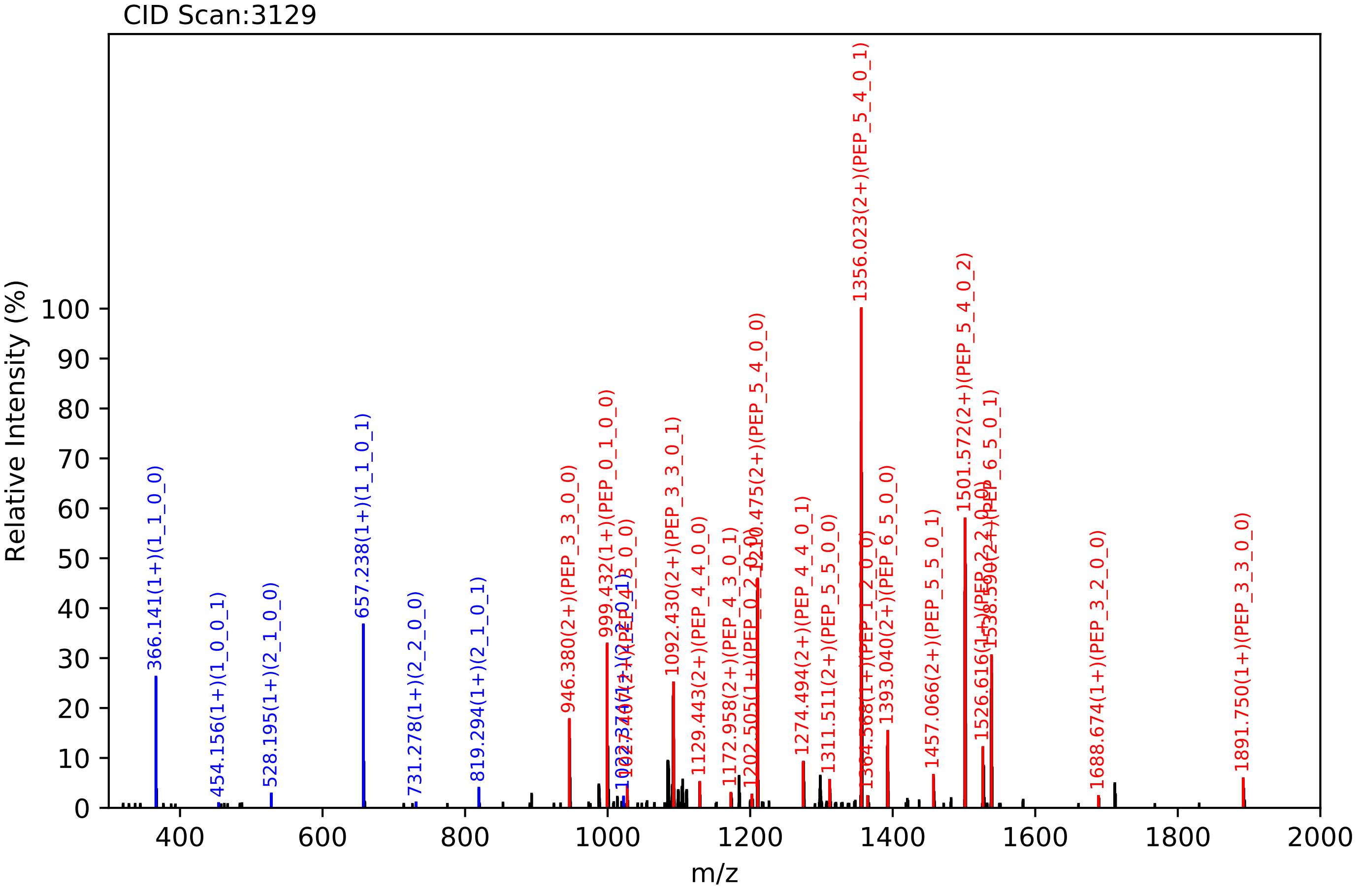

Test set no. 174, Experiment: AGP exp_4

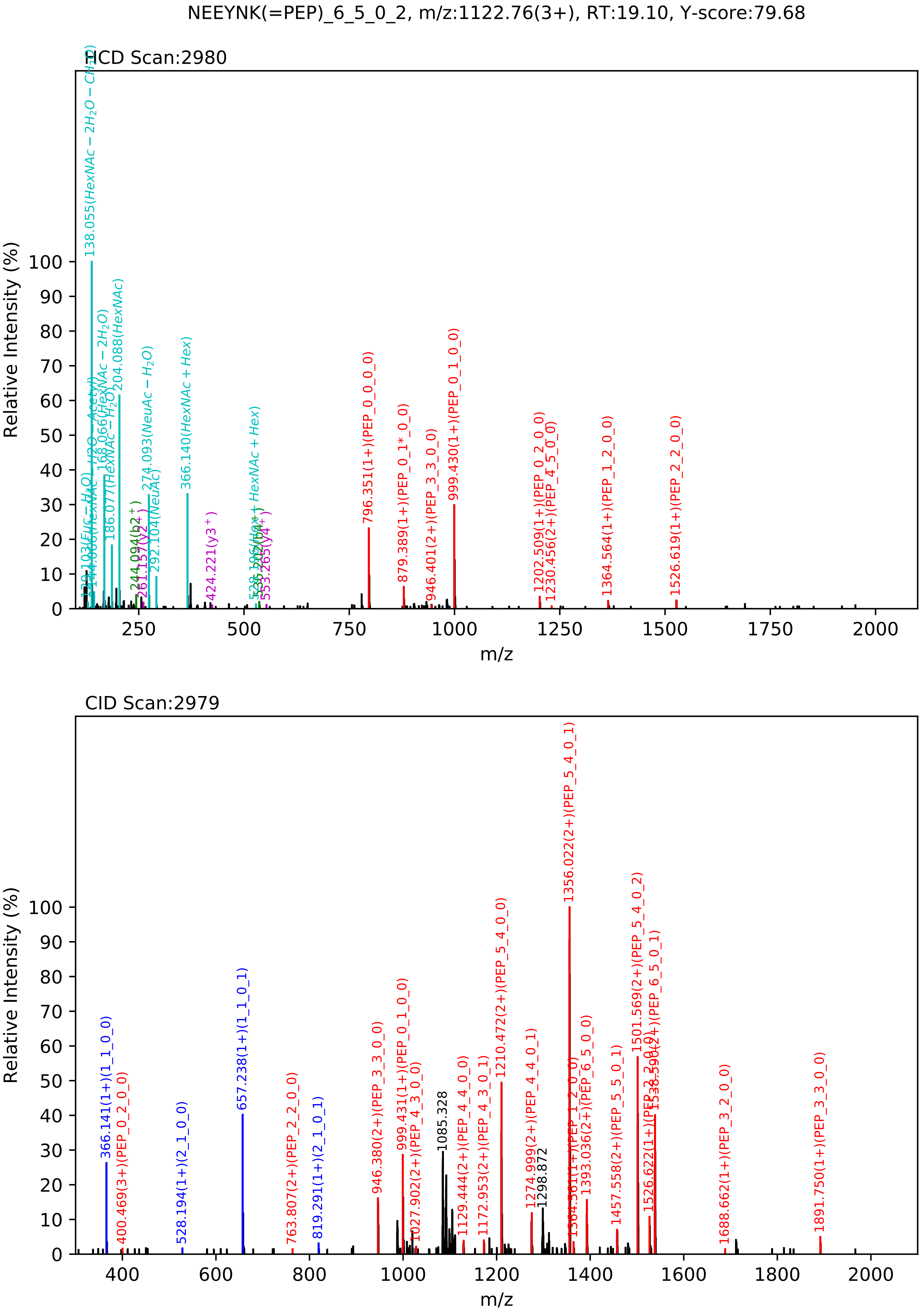

Test set no. 175, Experiment: AGP exp_3

NEEYNK(=PEP)_6_5_0_3, m/z:1219.79(3+), RT:22.20, Y-score:90.67

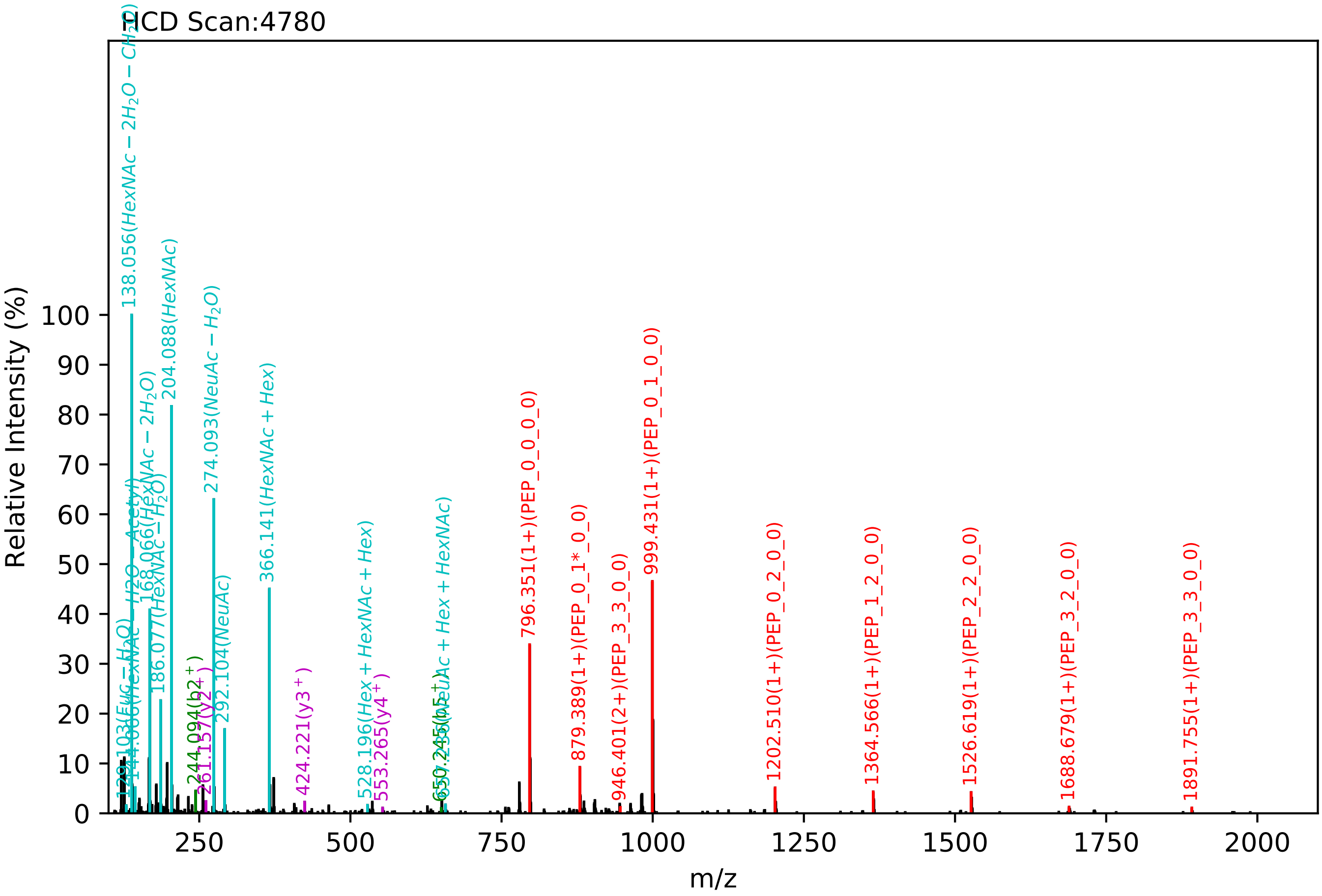

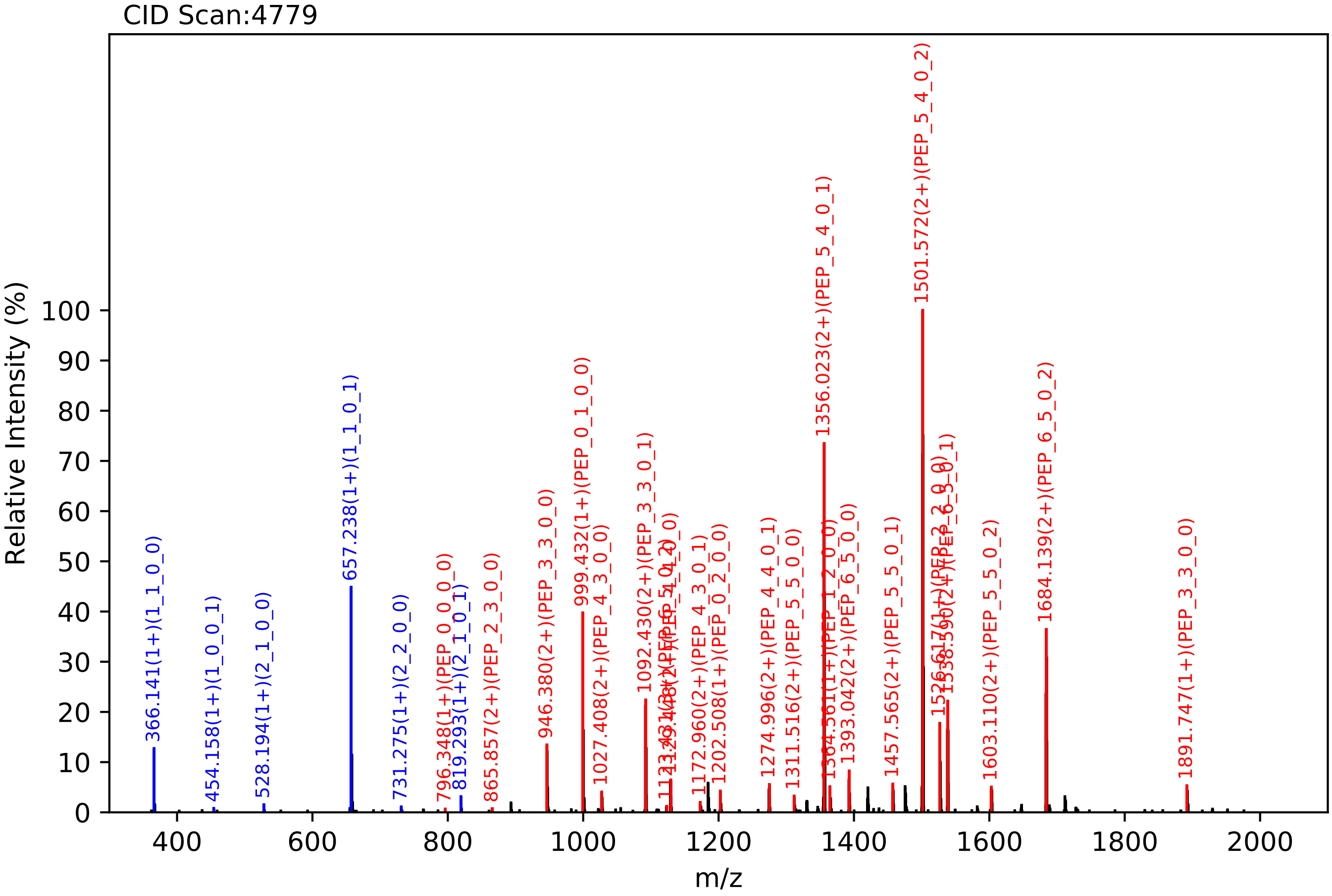

Test set no. 176, Experiment: AGP exp_4

NEEYNK(=PEP)_6_5_0_3, m/z:1219.45(3+), RT:22.15, Y-score:89.54

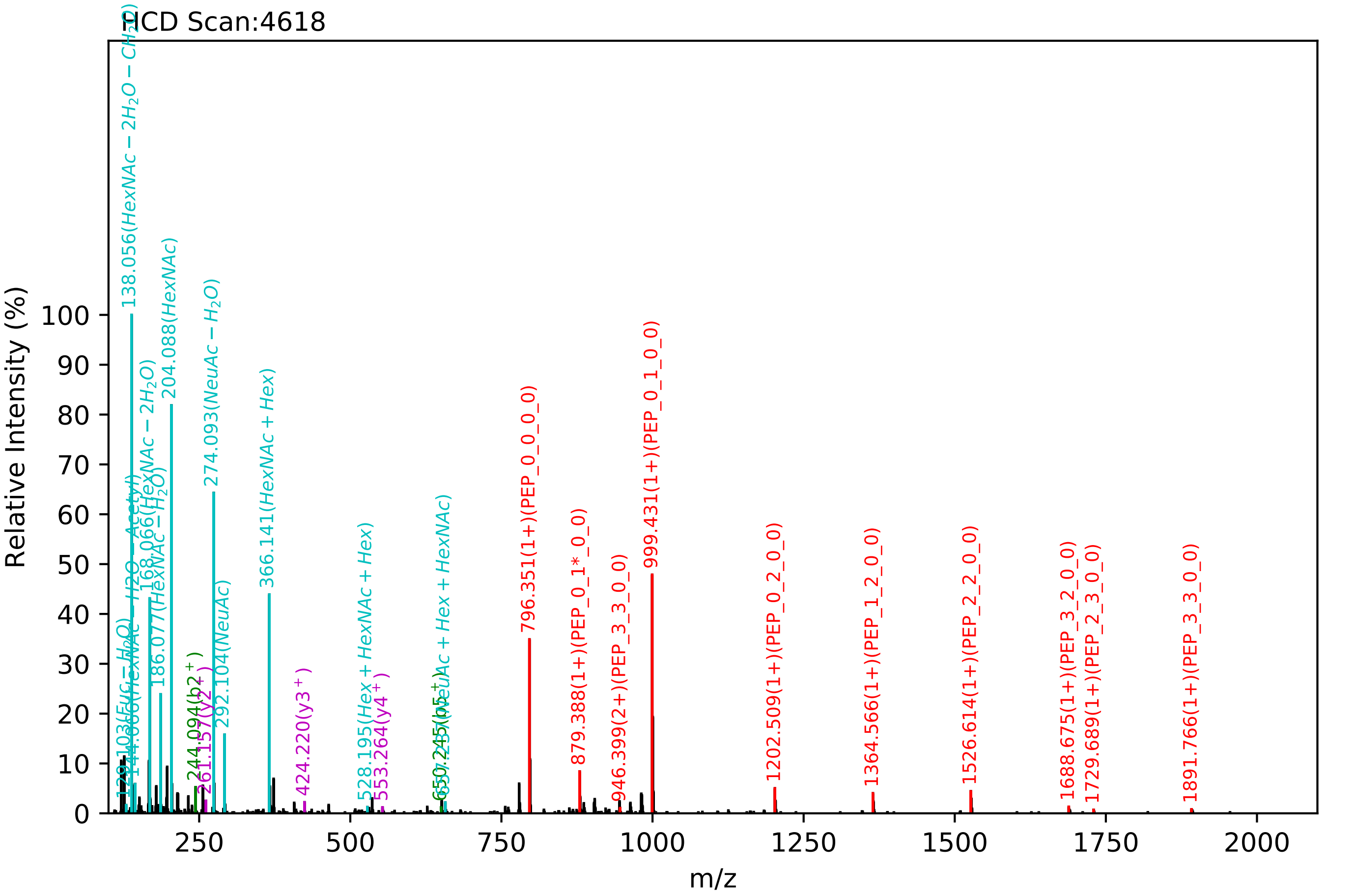

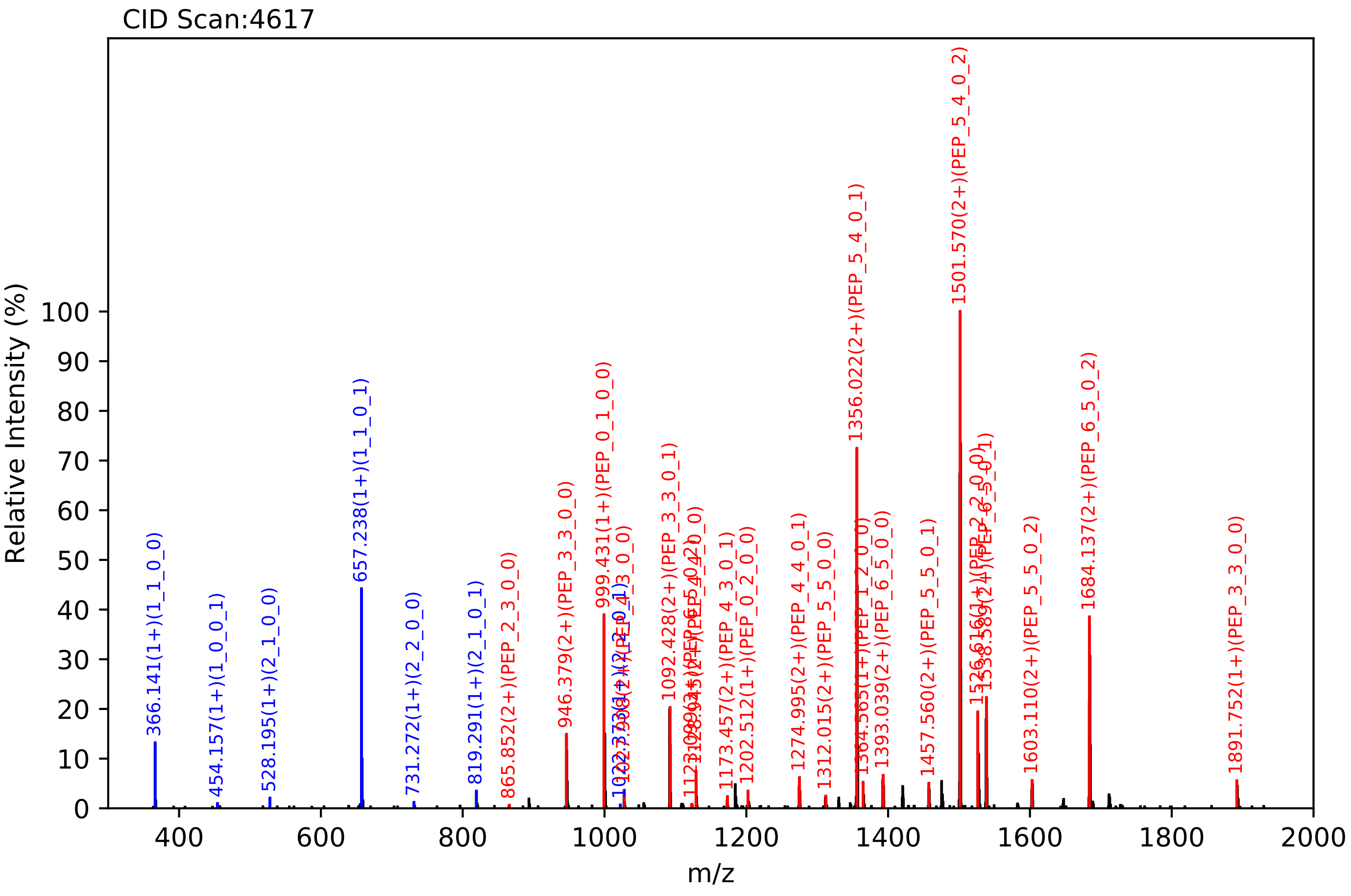

Test set no. 177, Experiment: AGP exp_4

NEEYNK(=PEP)_6_5_1_1, m/z:1074.41(3+), RT:15.70, Y-score:95.87

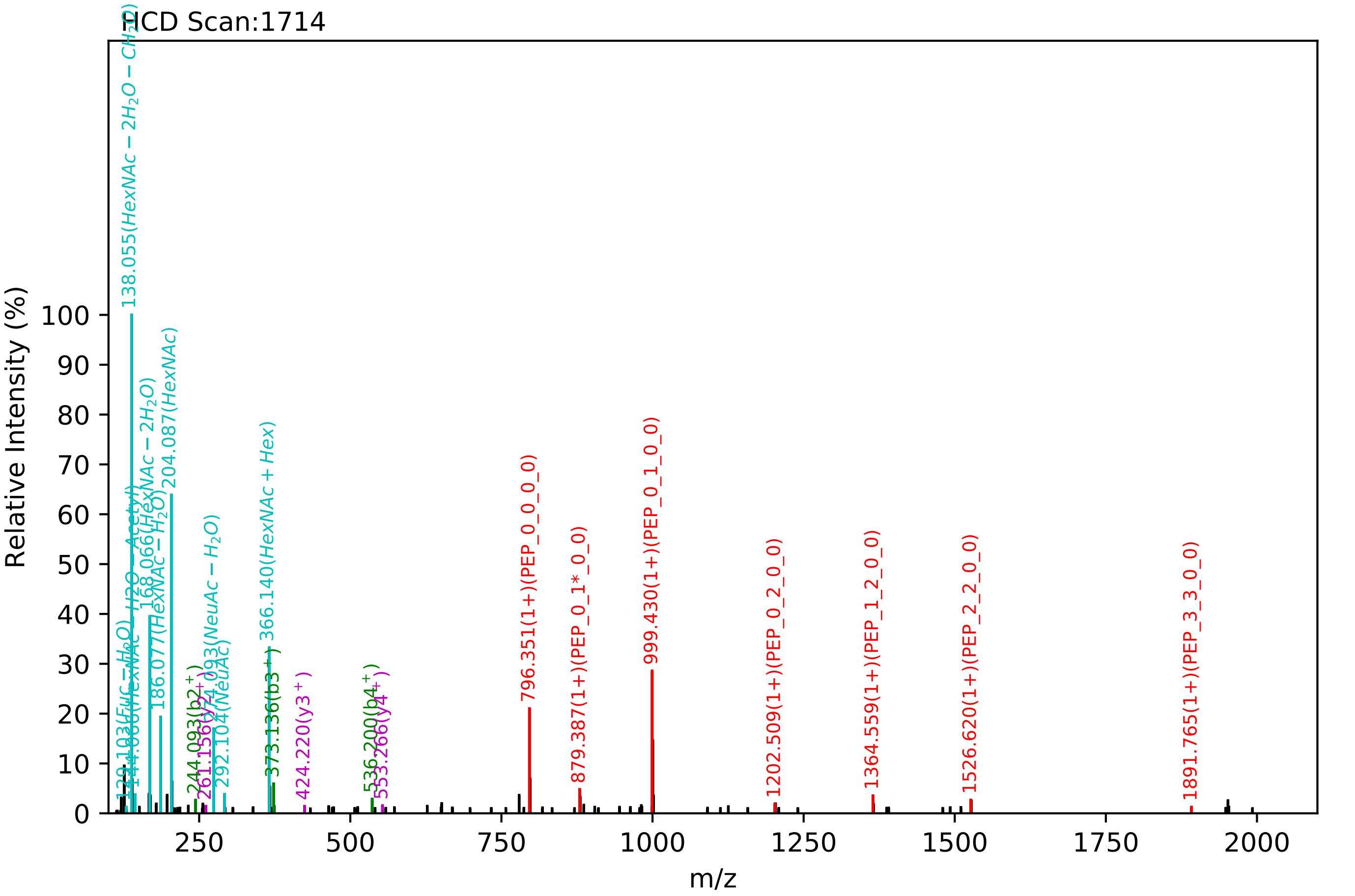

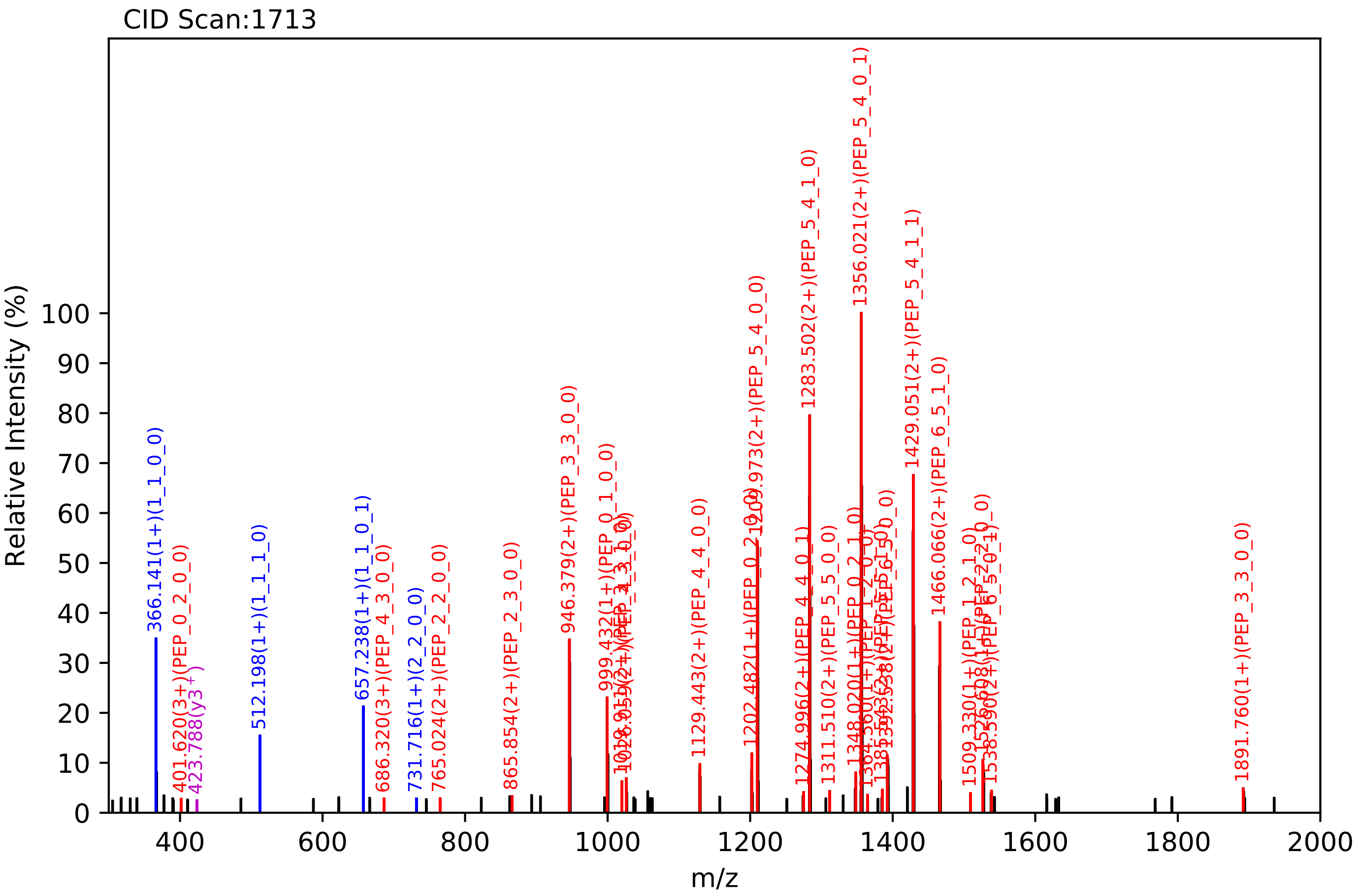

Test set no. 178, Experiment: AGP exp_4

NEEYNK(=PEP)_6_5_1_1, m/z:1074.41(3+), RT:16.24, Y-score:91.34

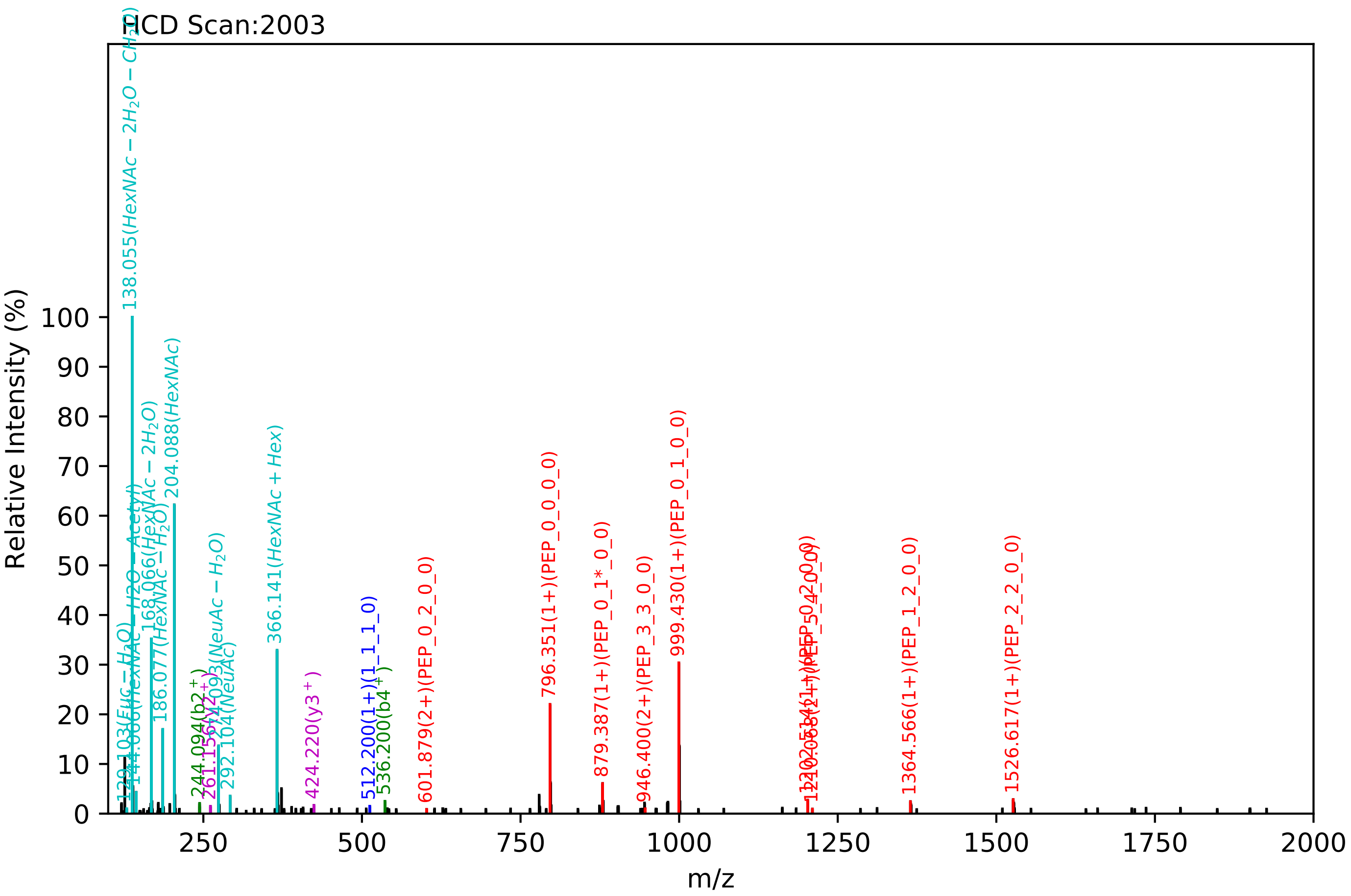

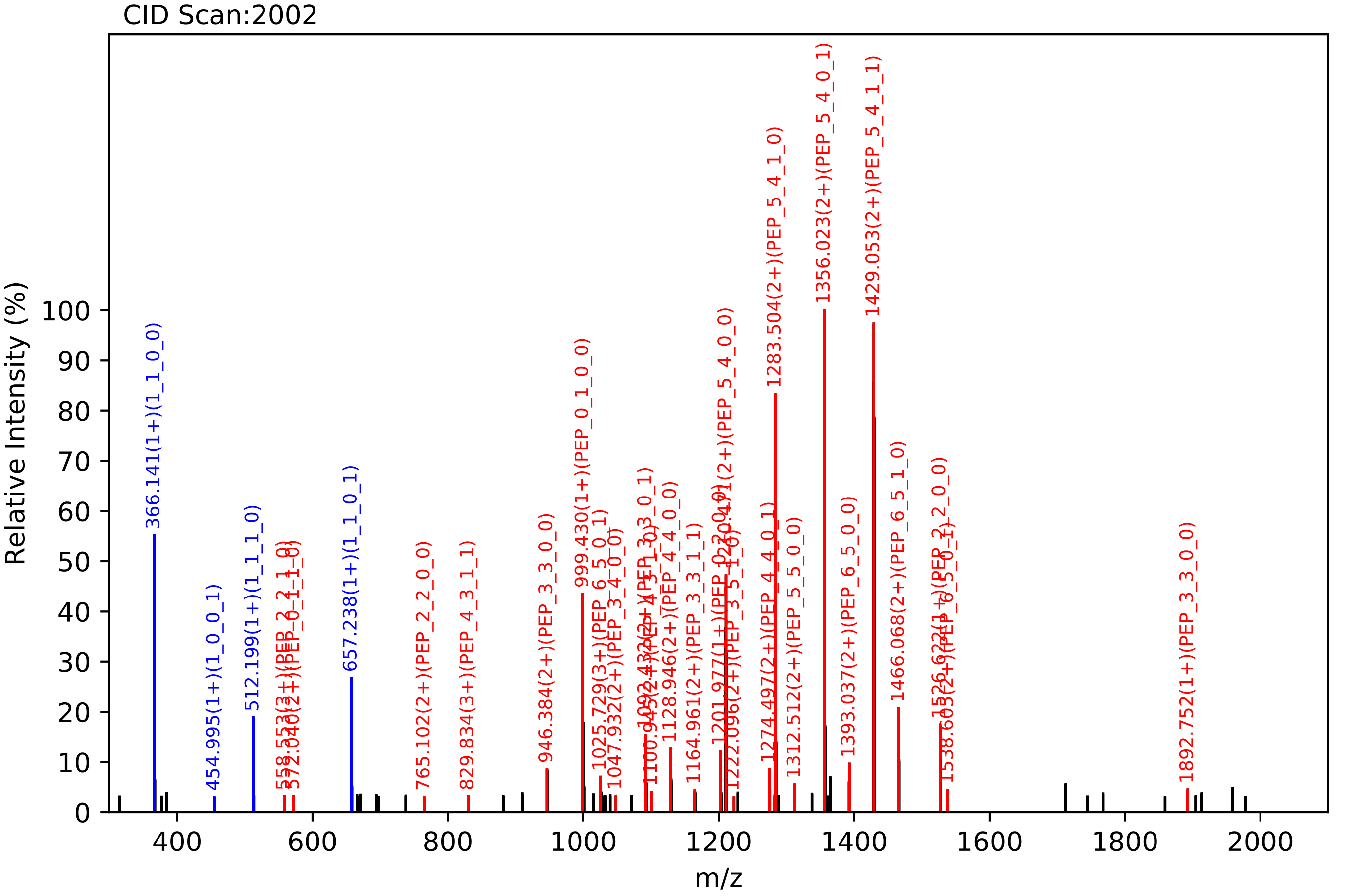

Test set no. 179, Experiment: AGP exp_4

NEEYNK(=PEP)_6_5_1_1, m/z:1074.41(3+), RT:16.76, Y-score:91.27

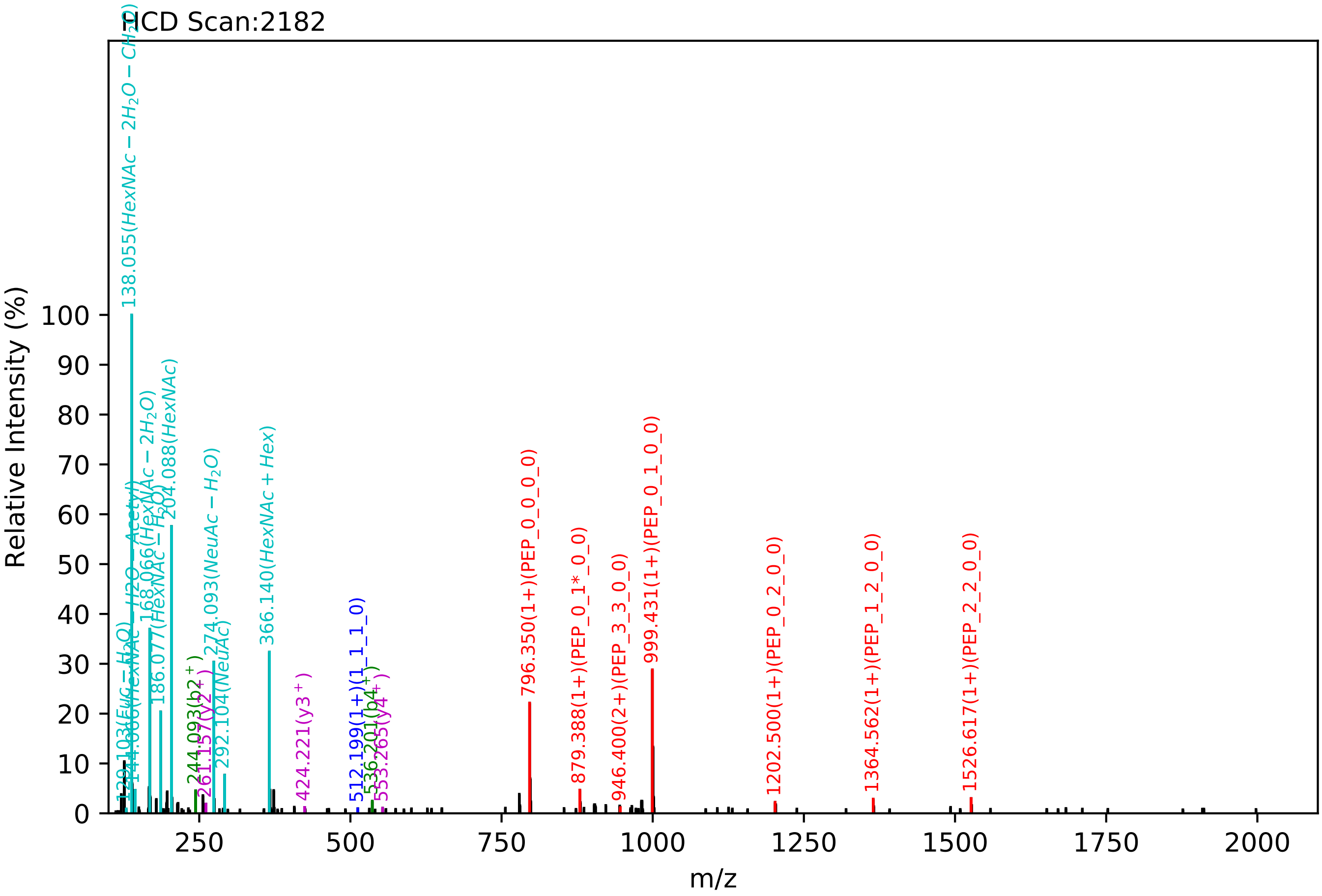

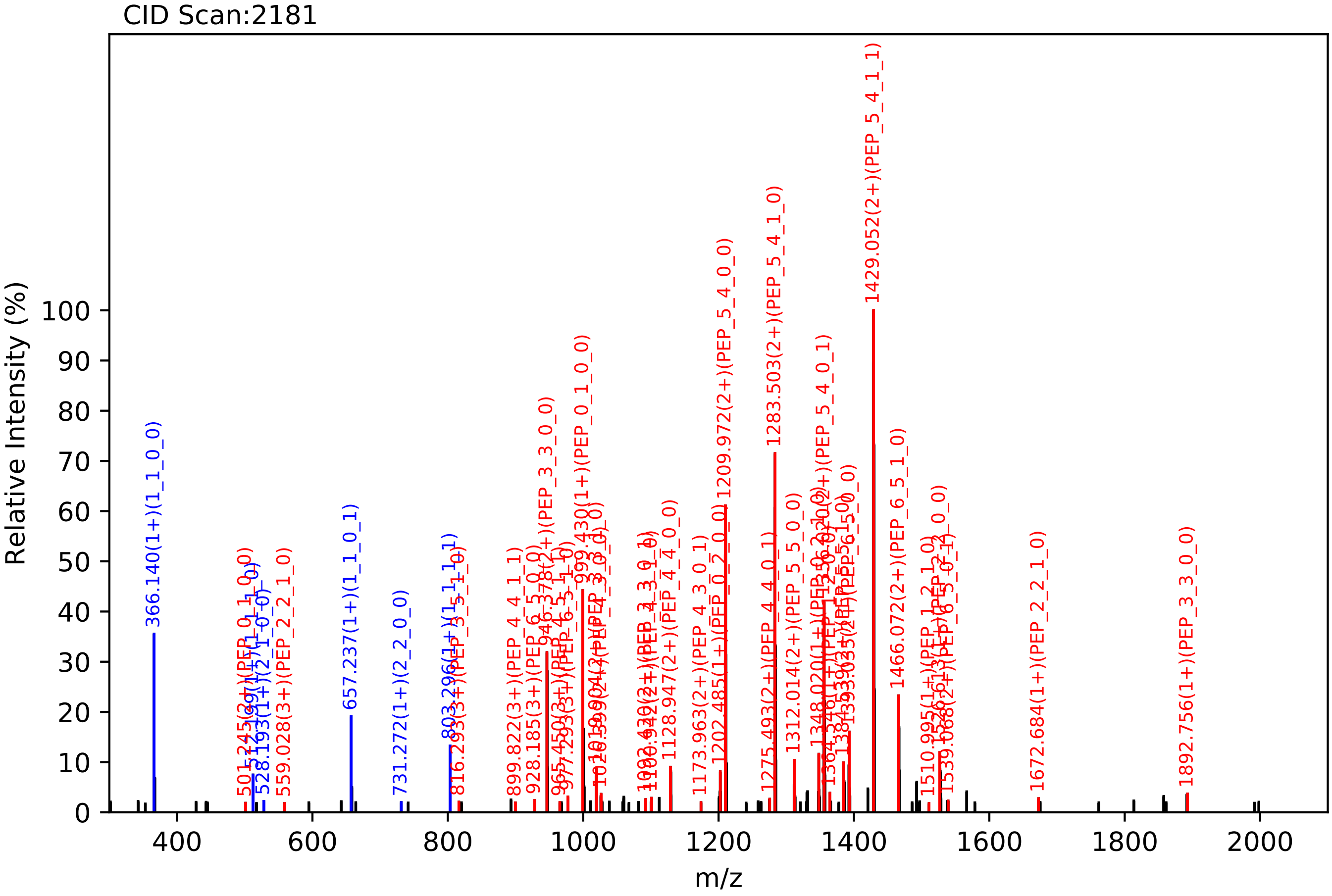

Test set no. 180, Experiment: AGP exp_3

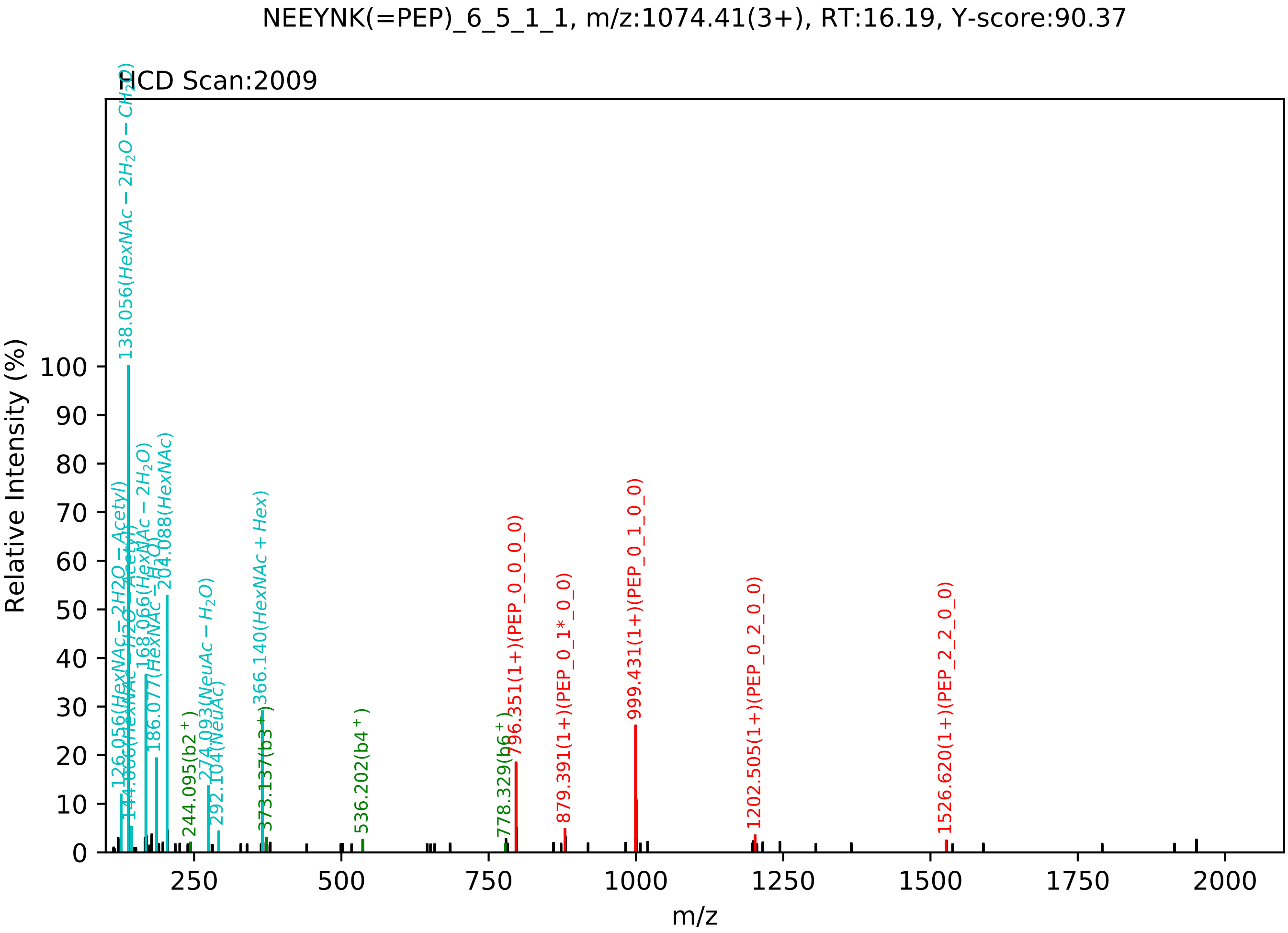

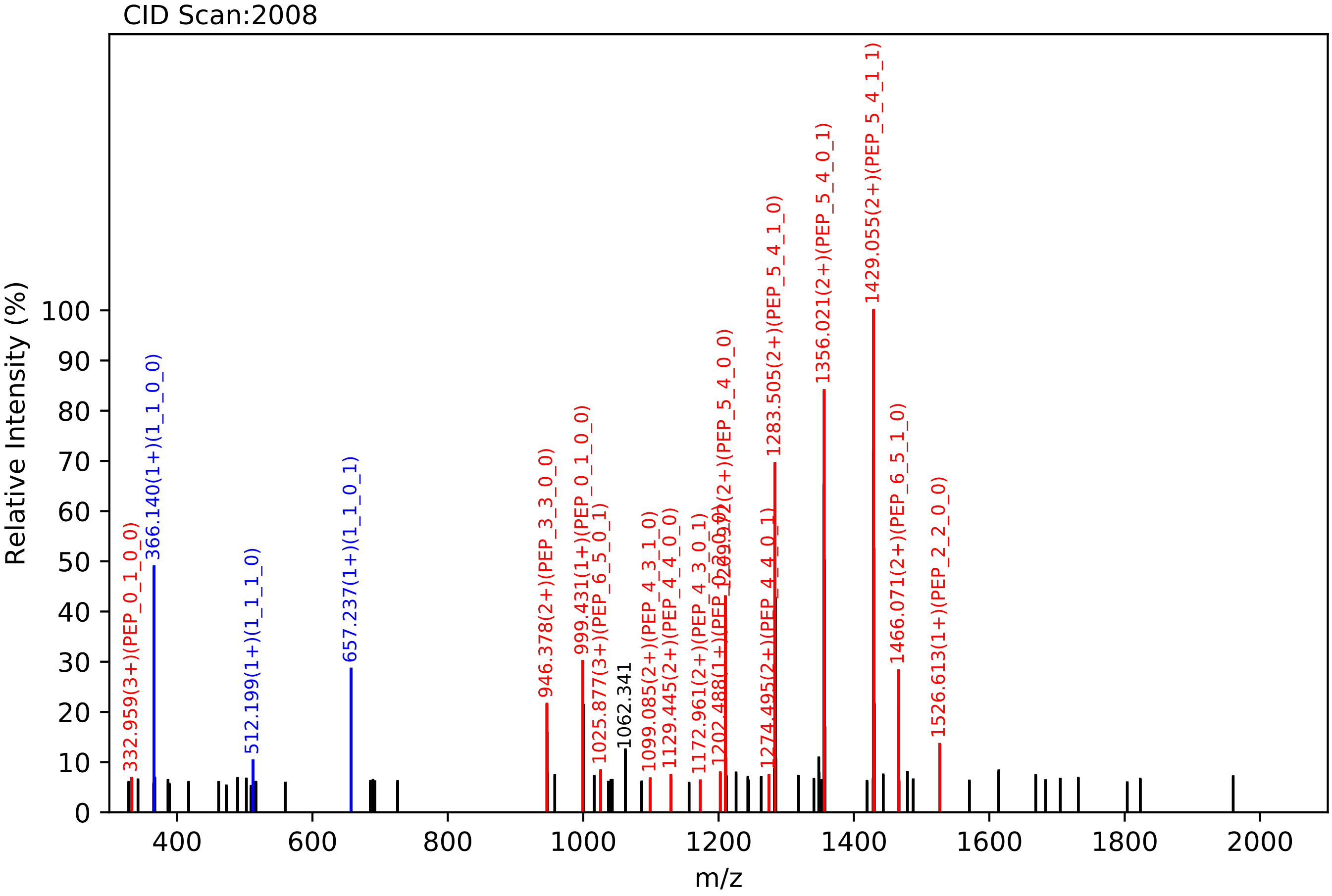

Test set no. 181, Experiment: AGP exp_3

NEEYNK(=PEP)_6_5_1_1, m/z:1074.41(3+), RT:16.70, Y-score:90.11

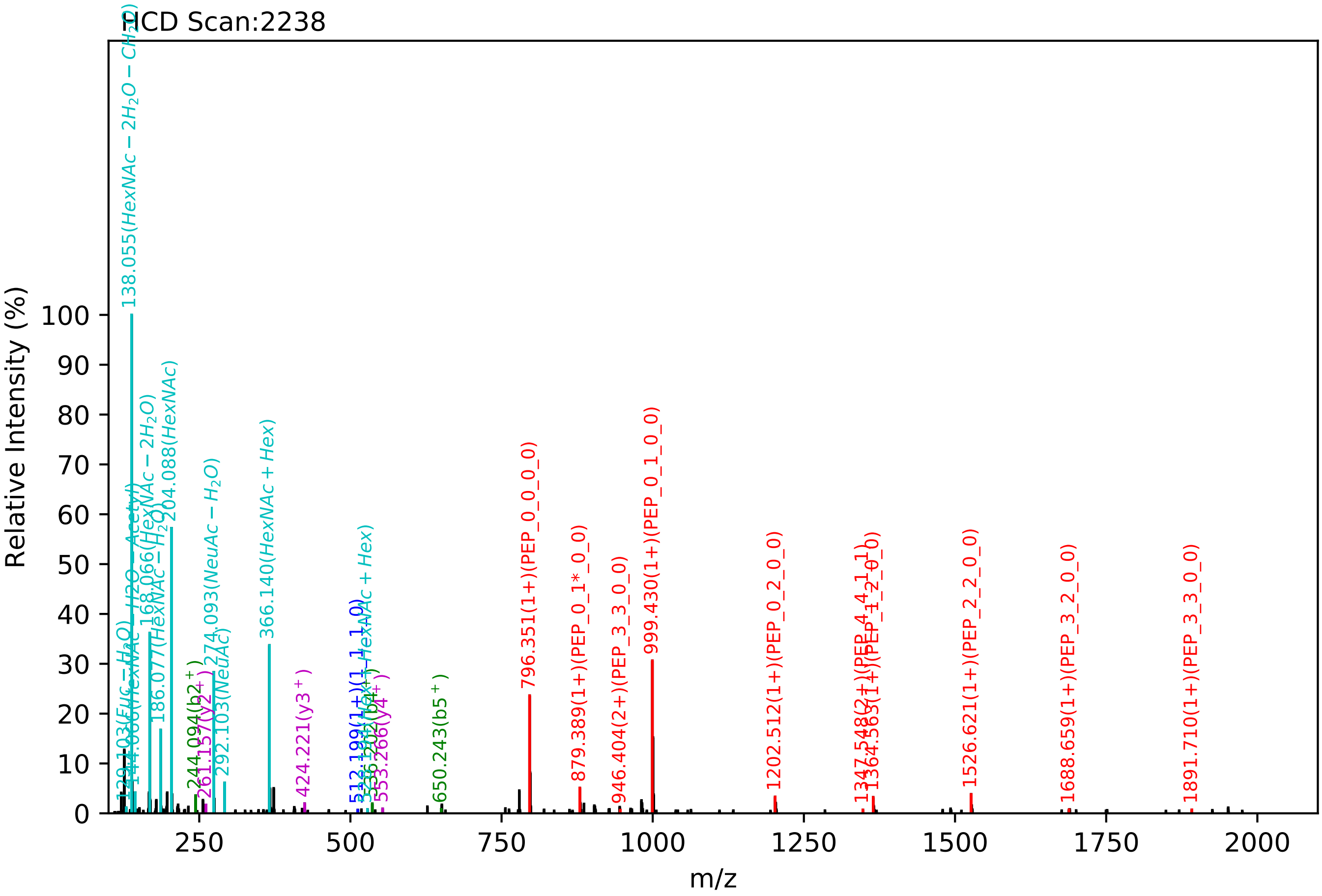

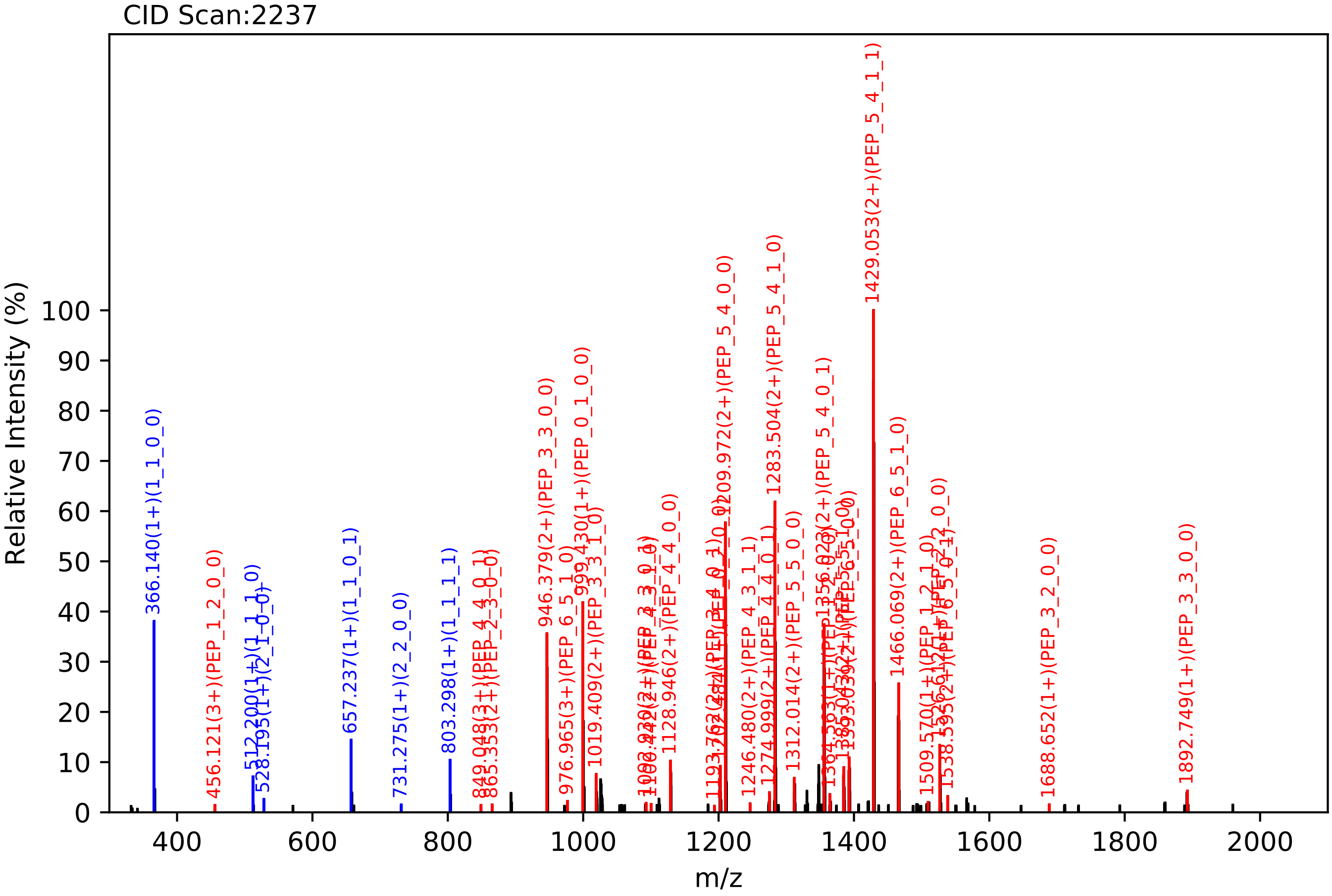

Test set no. 182, Experiment: AGP exp_3

NEEYNK(=PEP)_6_5_1_2, m/z:1171.45(3+), RT:20.22, Y-score:92.00

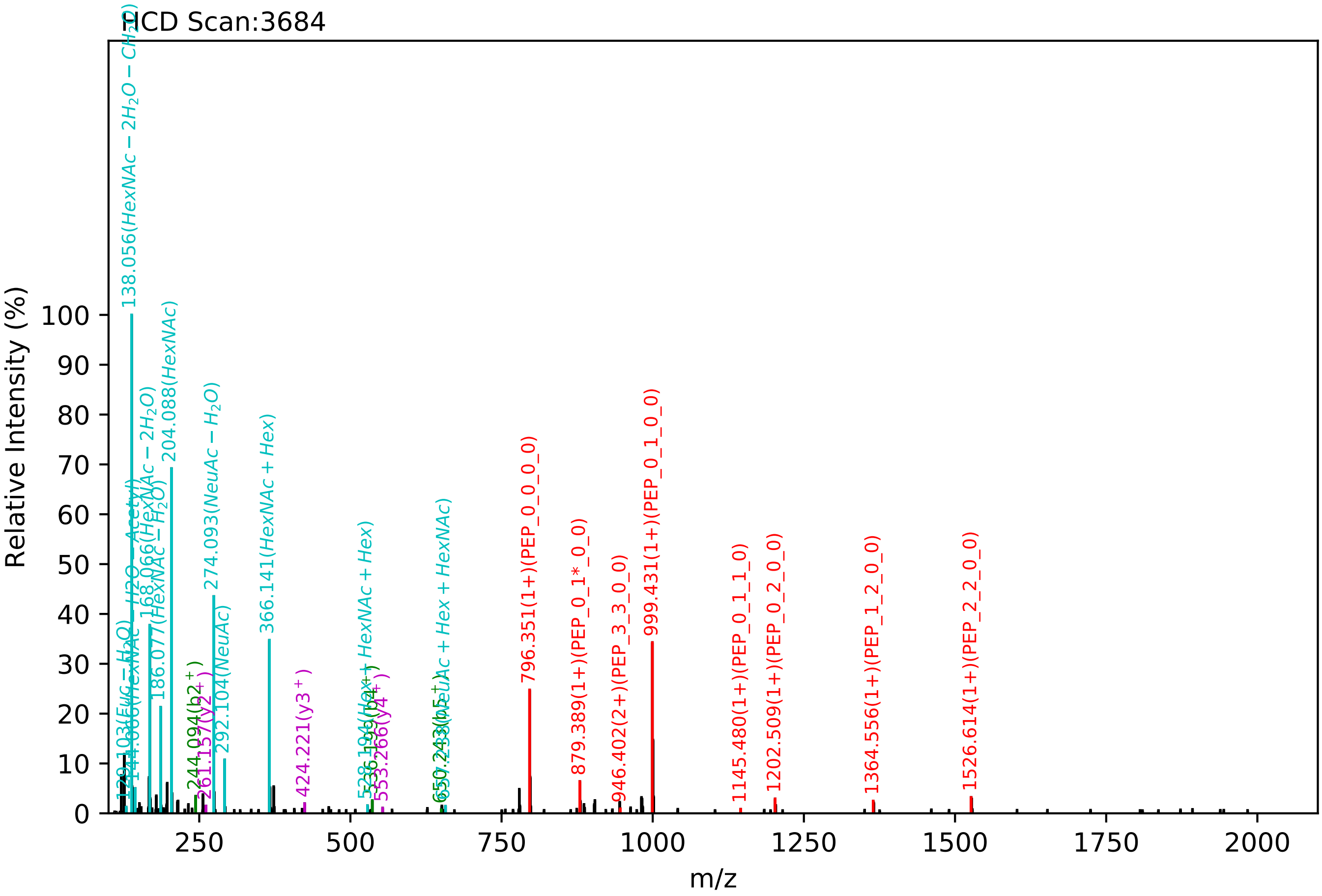

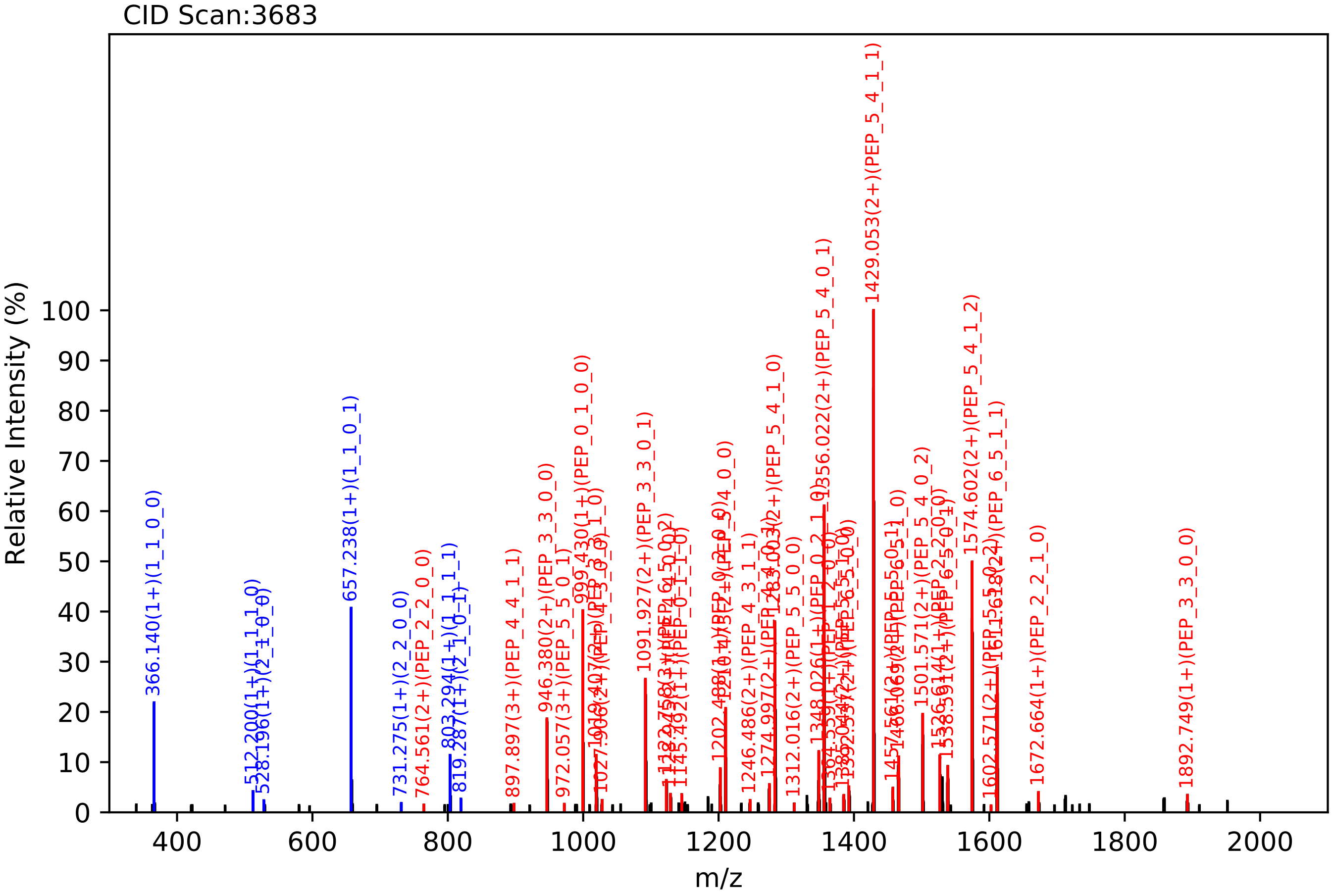

Test set no. 183, Experiment: AGP exp_3

NEEYNK(=PEP)_6_5_1_2, m/z:1171.44(3+), RT:19.06, Y-score:90.72

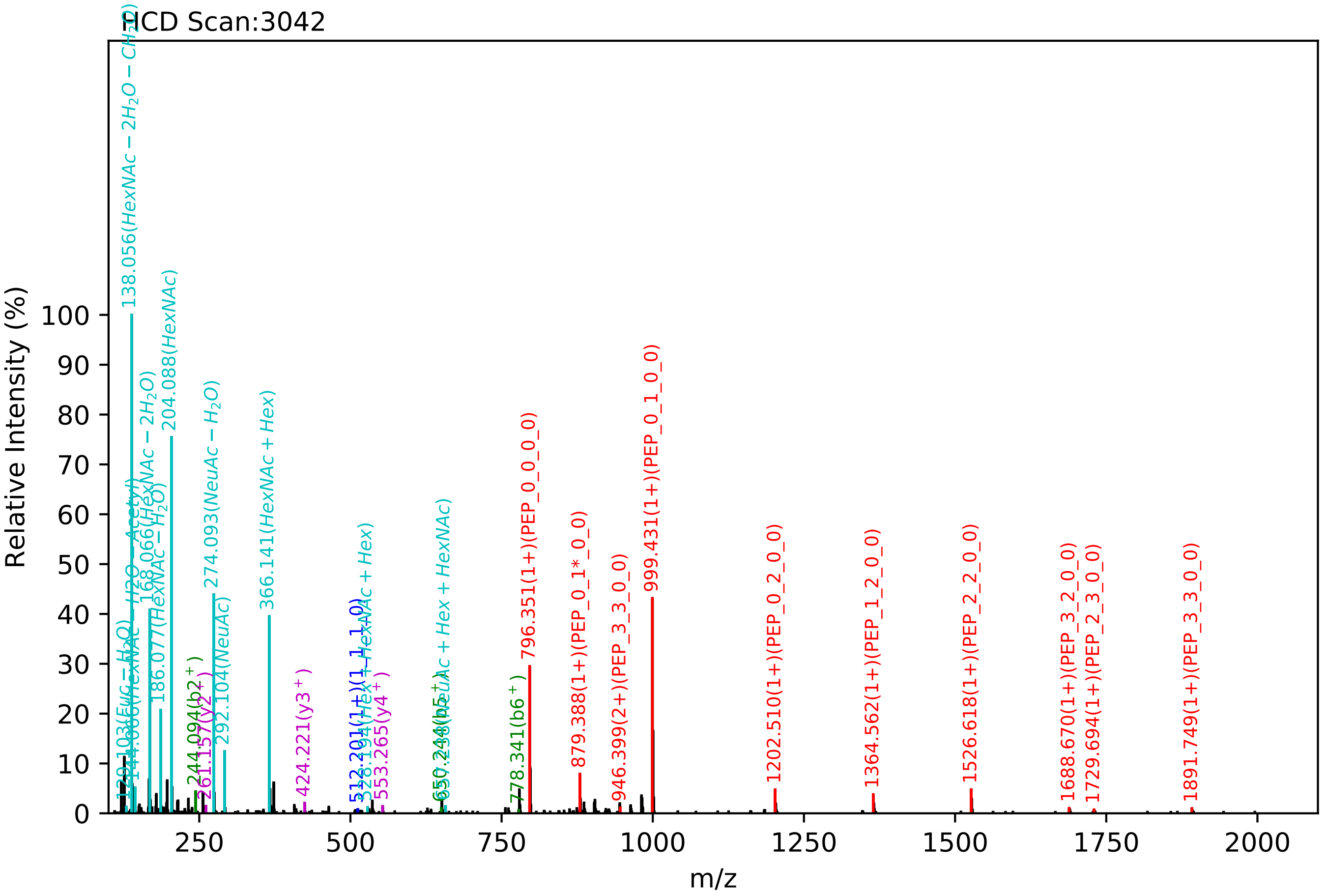

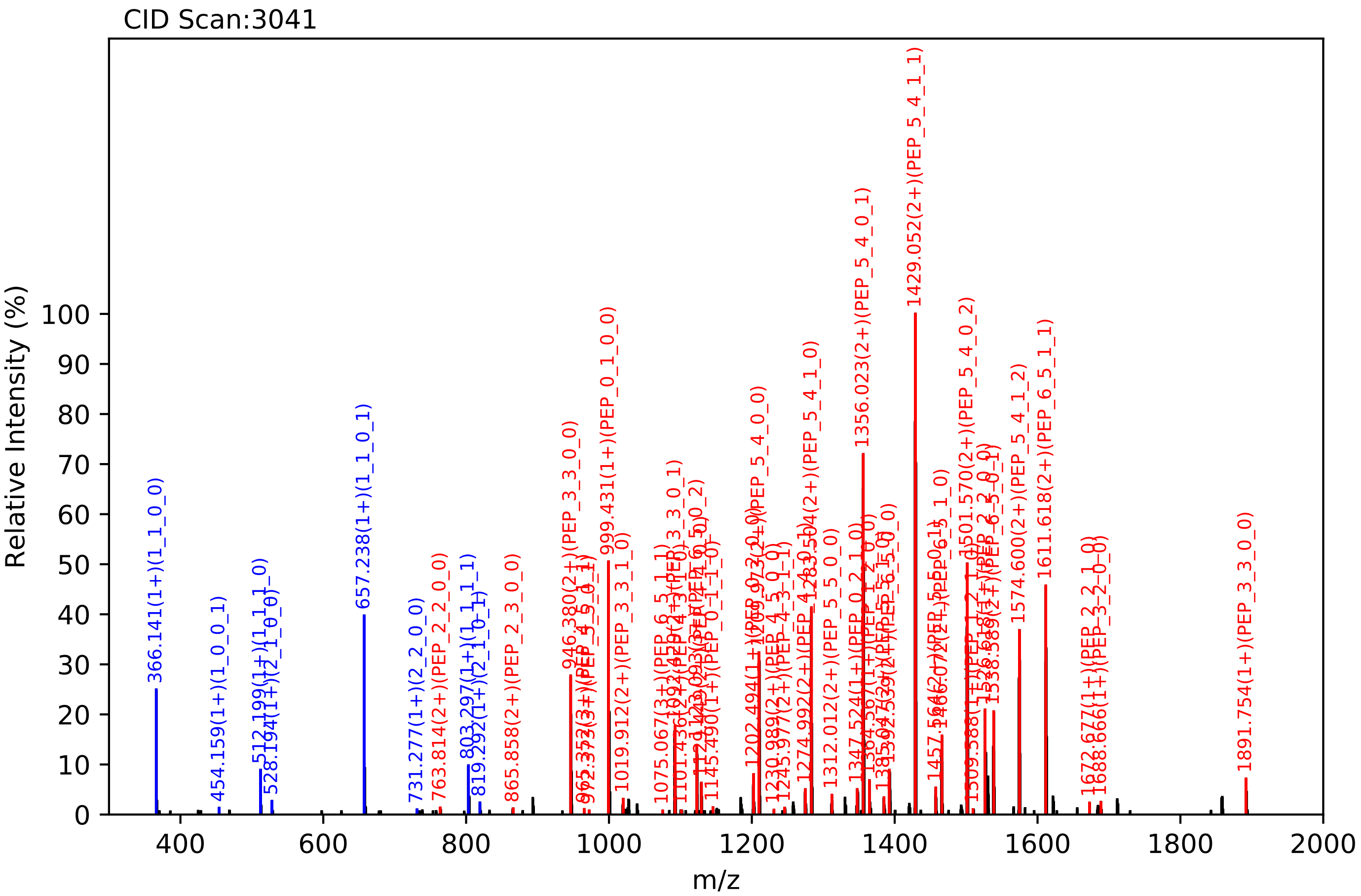

Test set no. 184, Experiment: AGP exp_4

NEEYNK(=PEP)_6_5_1_2, m/z:1173.05(3+), RT:19.55, Y-score:85.71

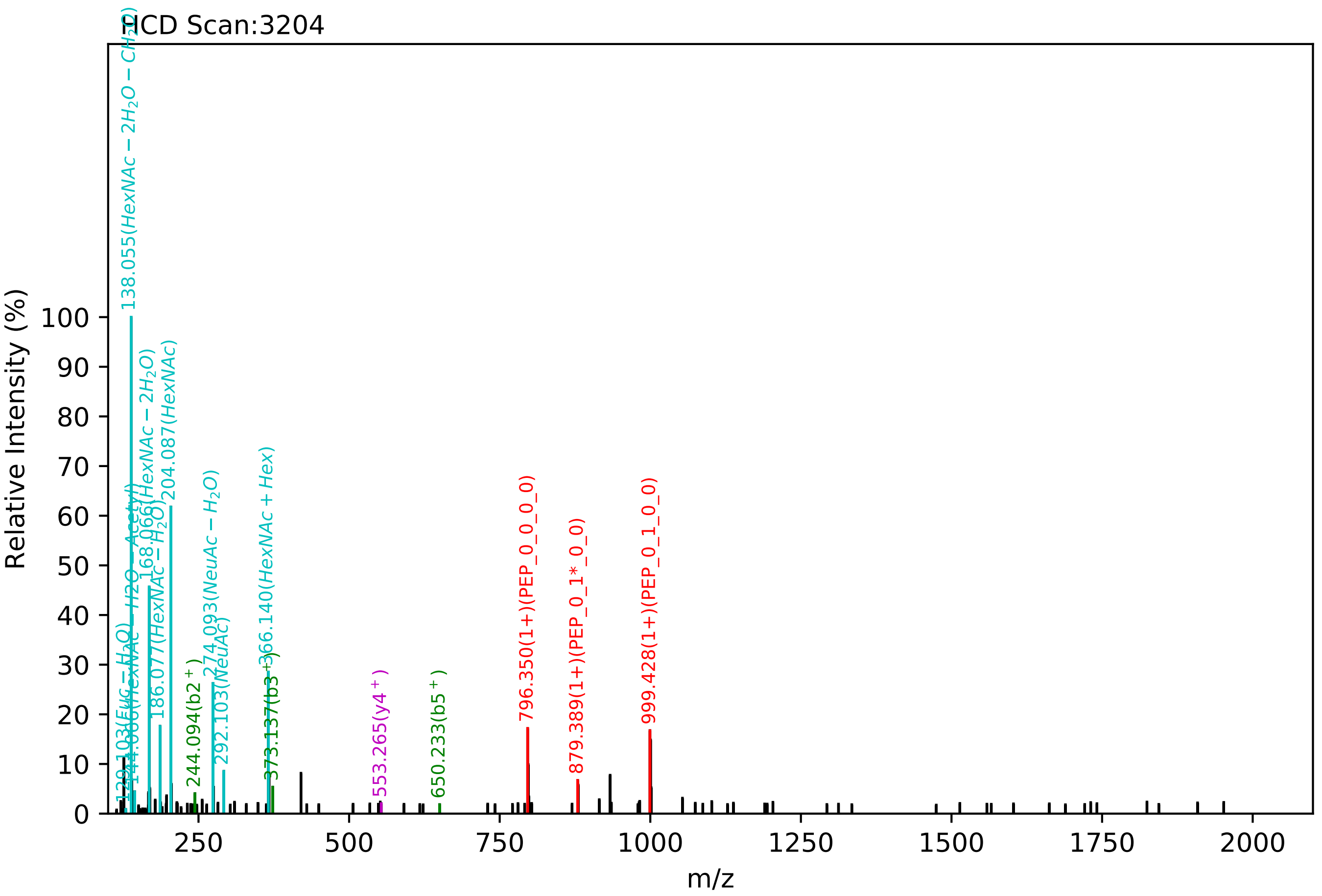

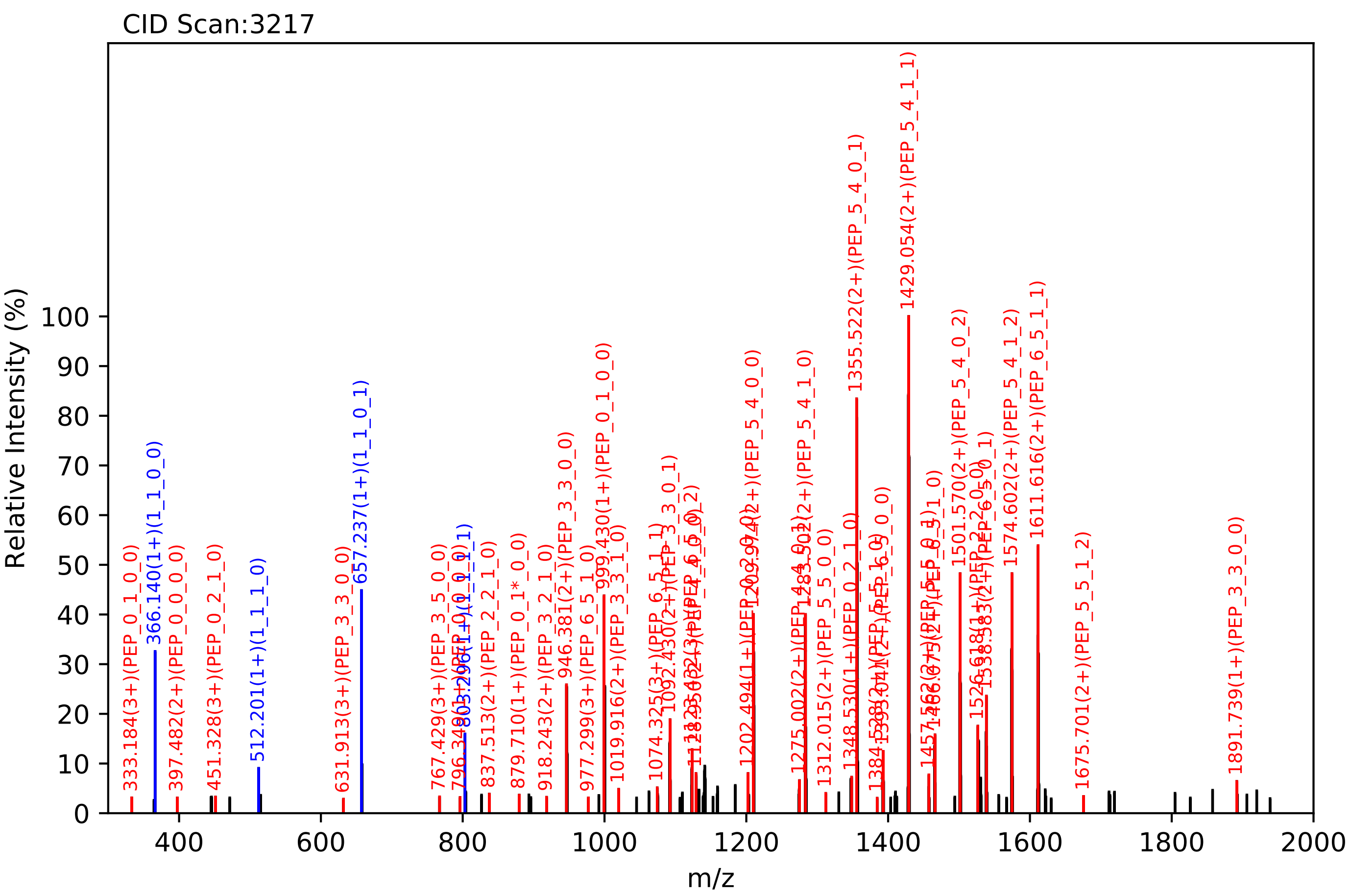

Test set no. 185, Experiment: AGP exp_4

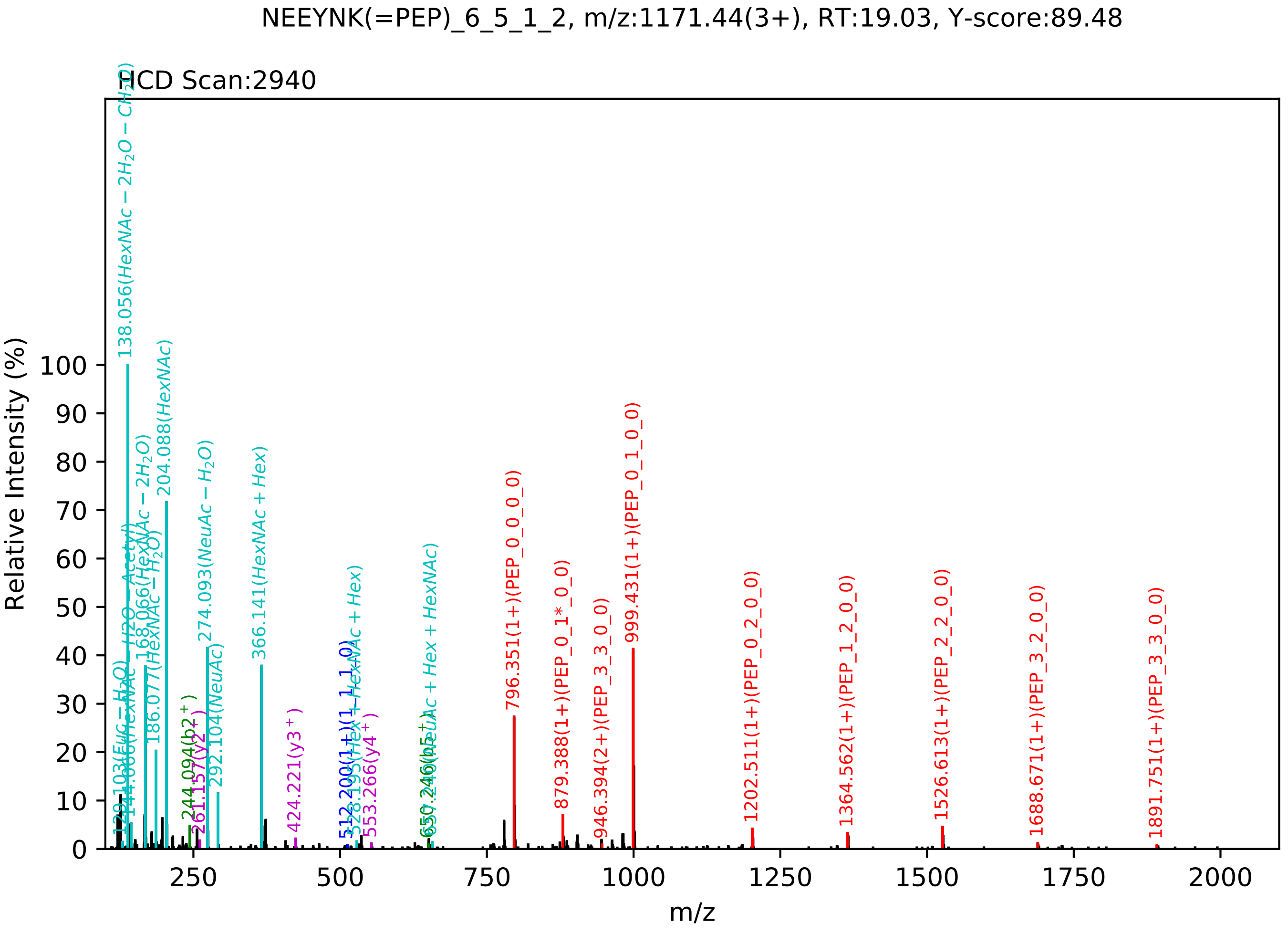

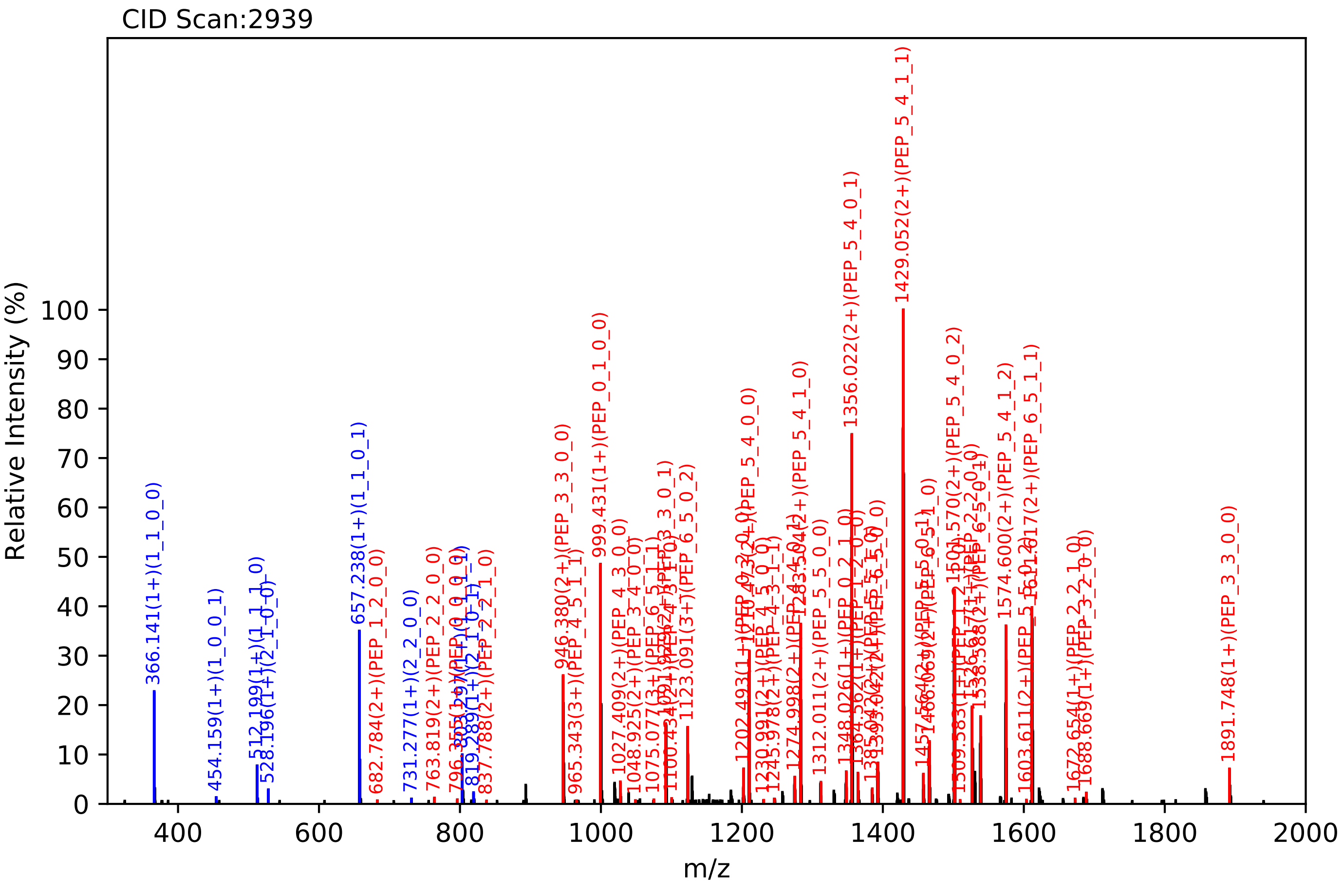

Test set no. 186, Experiment: AGP exp_3

NEEYNK(=PEP)_6_5_1_2, m/z:1171.44(3+), RT:19.64, Y-score:88.72

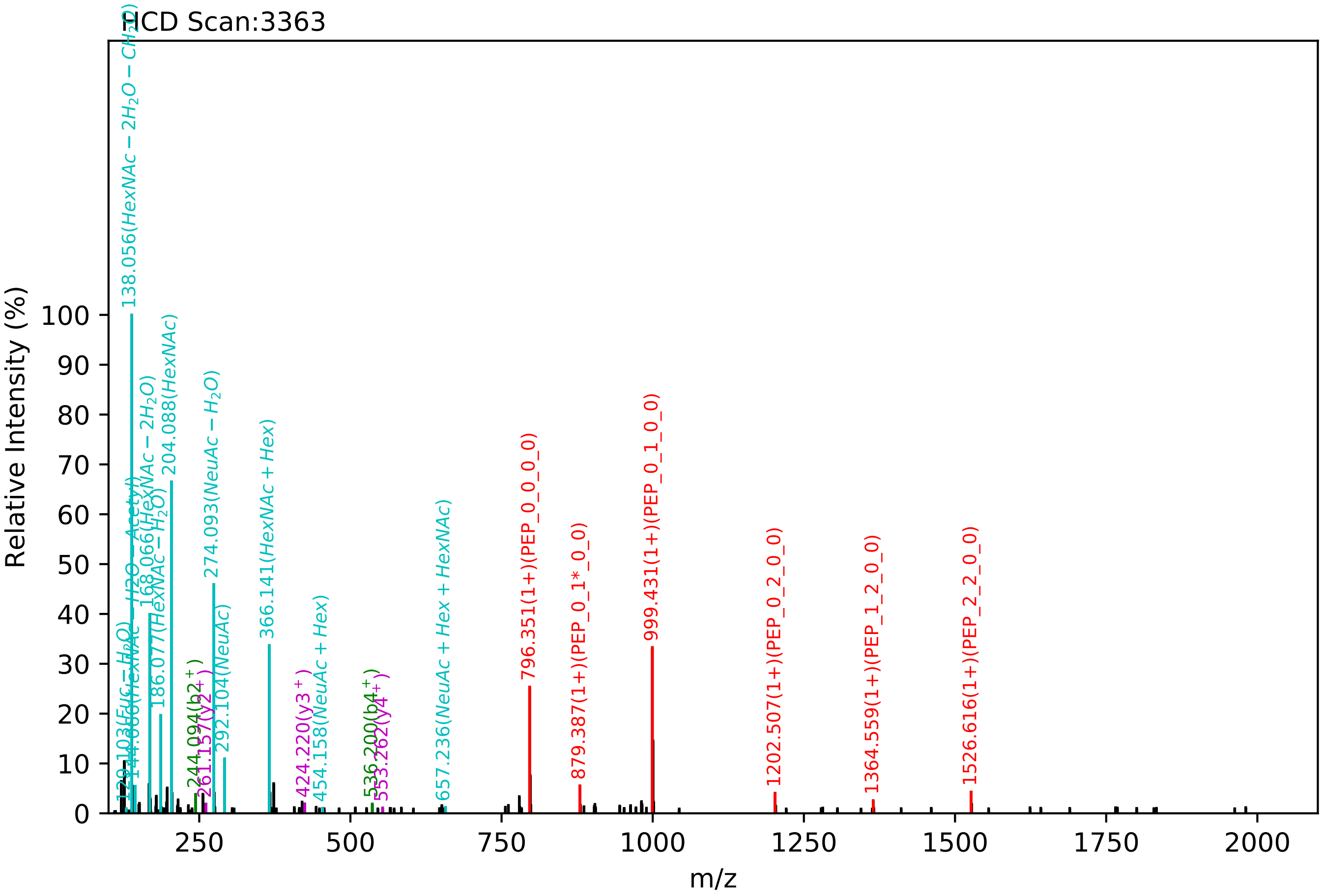

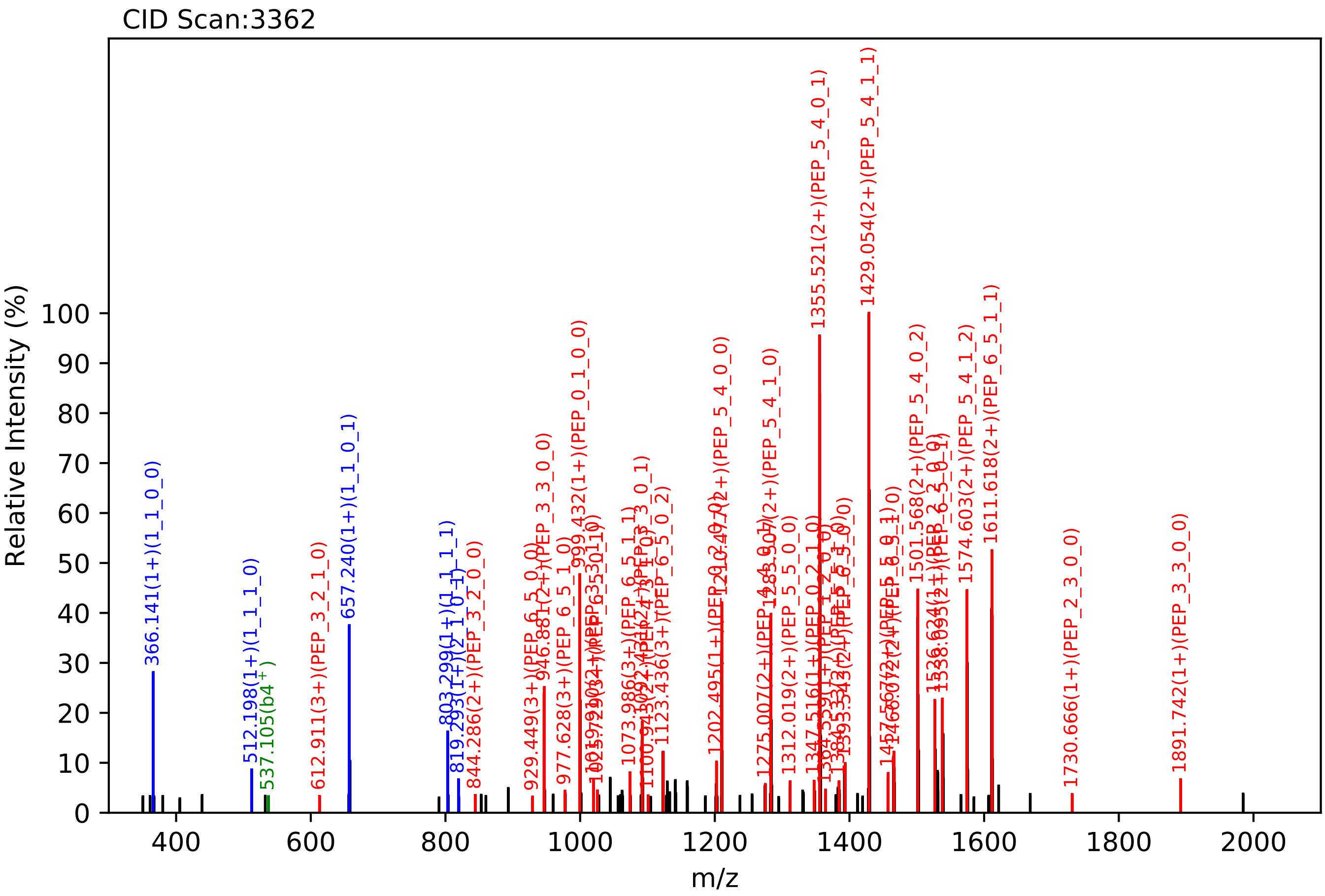

Test set no. 187, Experiment: AGP exp_4

NEEYNK(=PEP)_6_5_1_2, m/z:1171.44(3+), RT:18.46, Y-score:87.16

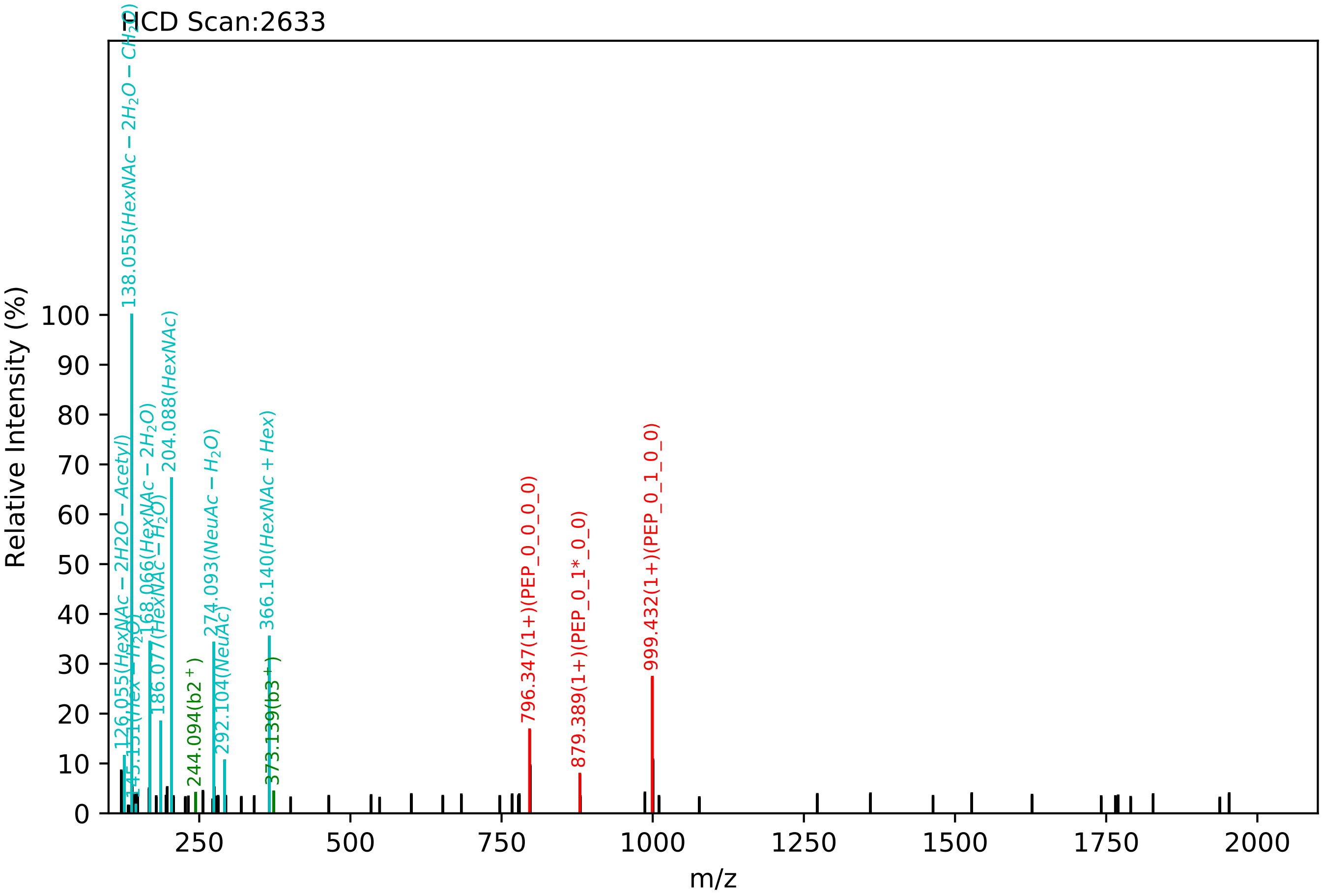

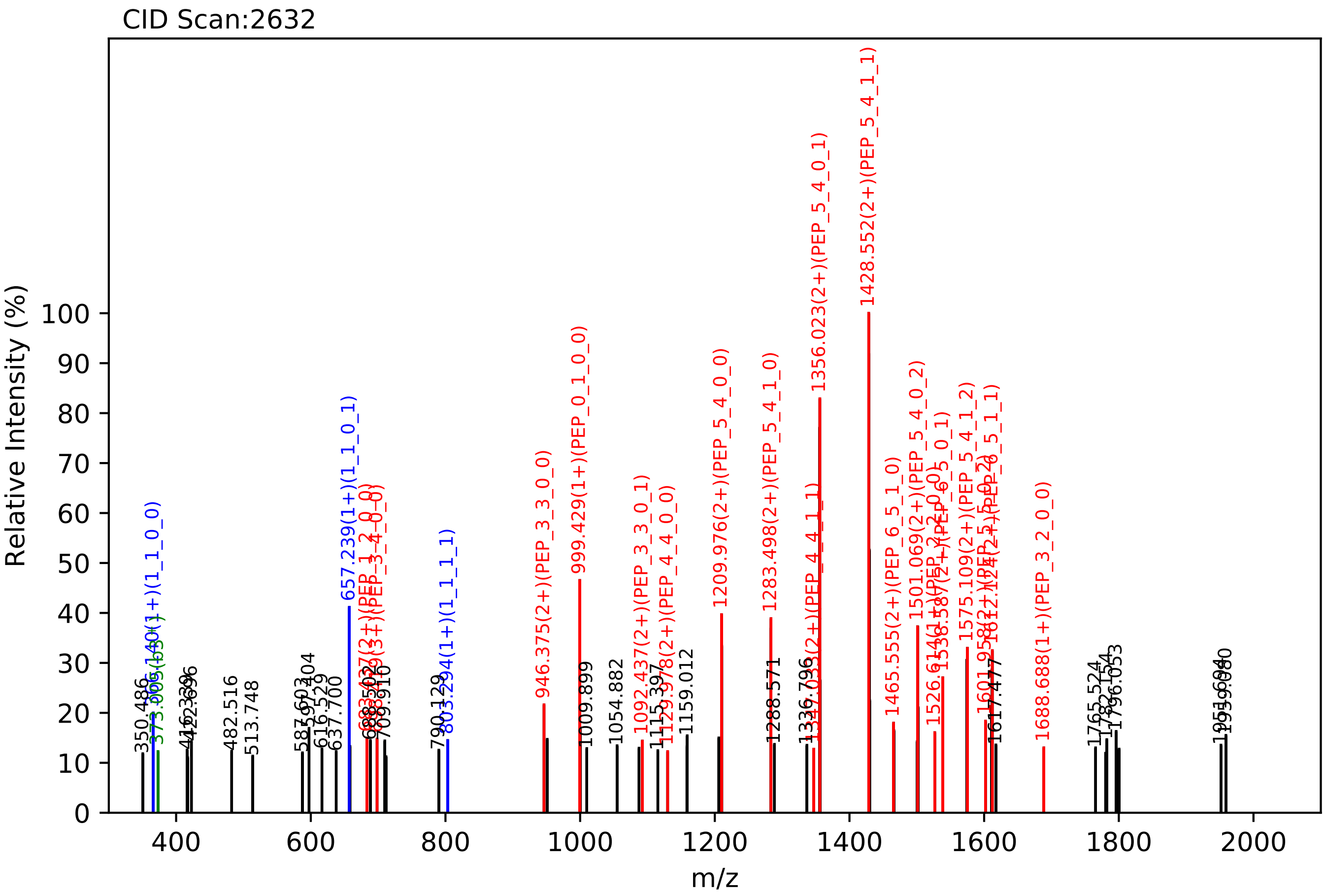

Test set no. 188, Experiment: AGP exp_4

NEEYNK(=PEP)_6_5_1_2, m/z:1171.44(3+), RT:20.69, Y-score:87.14

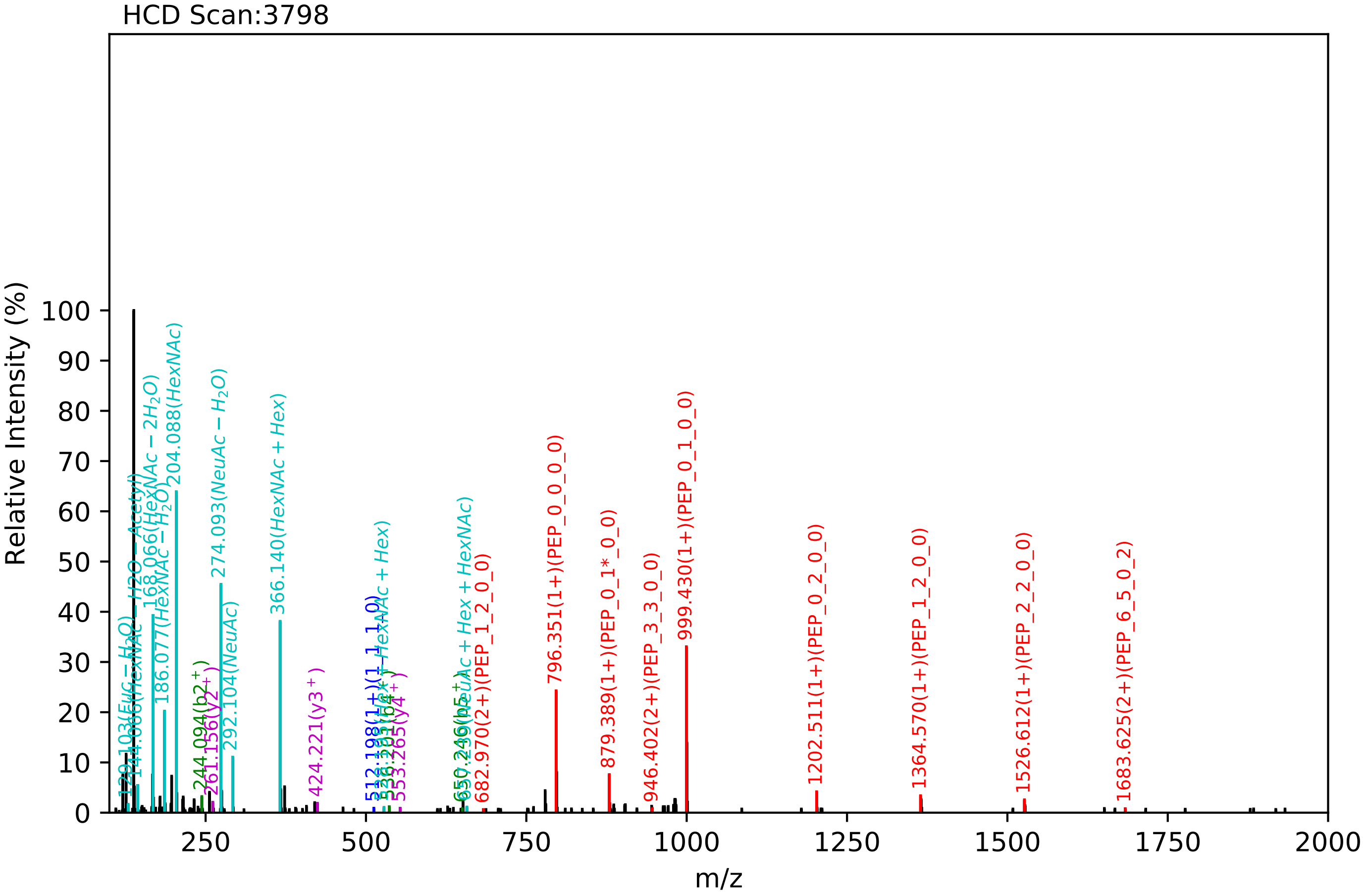

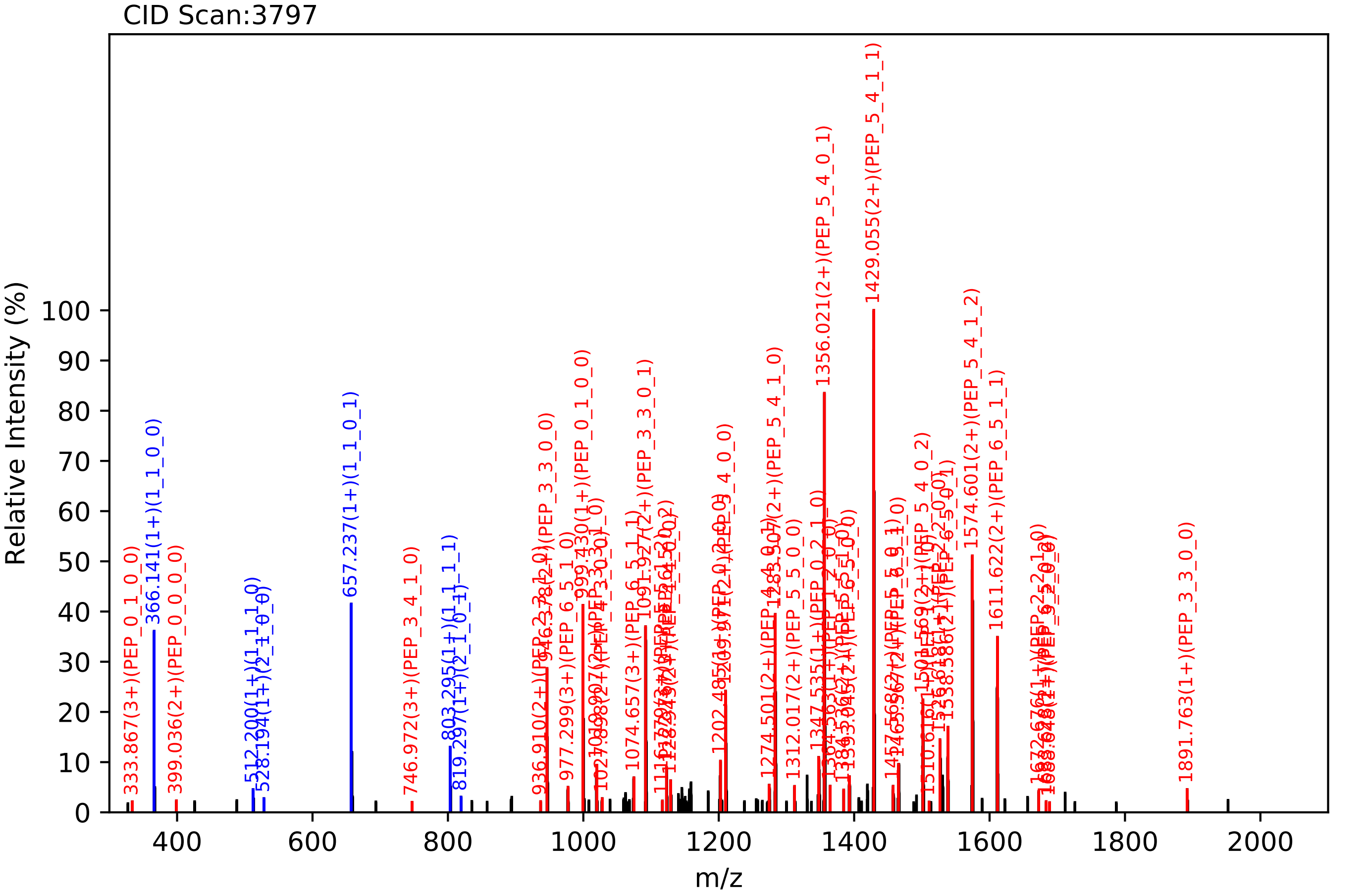

Test set no. 189, Experiment: AGP exp_3

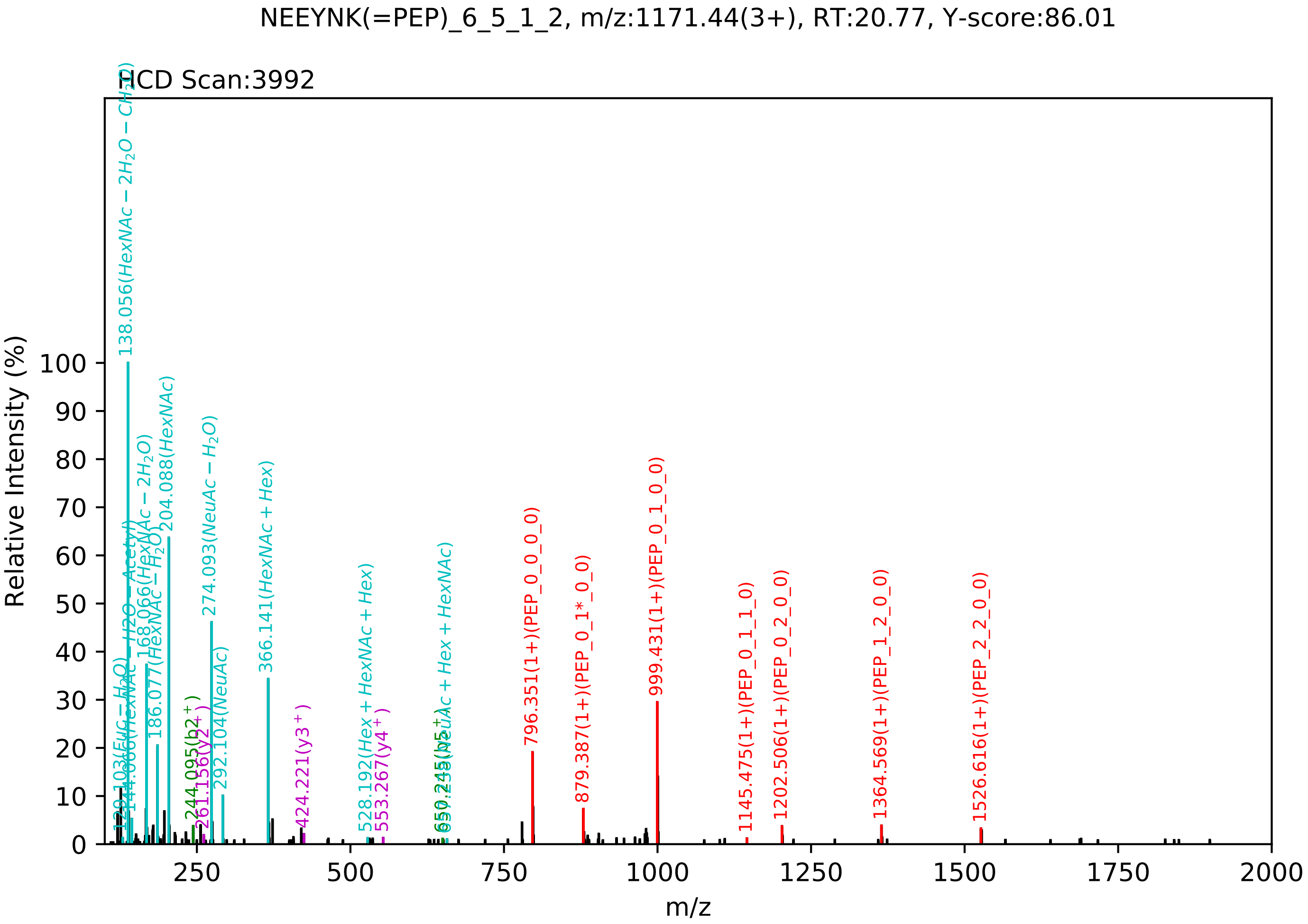

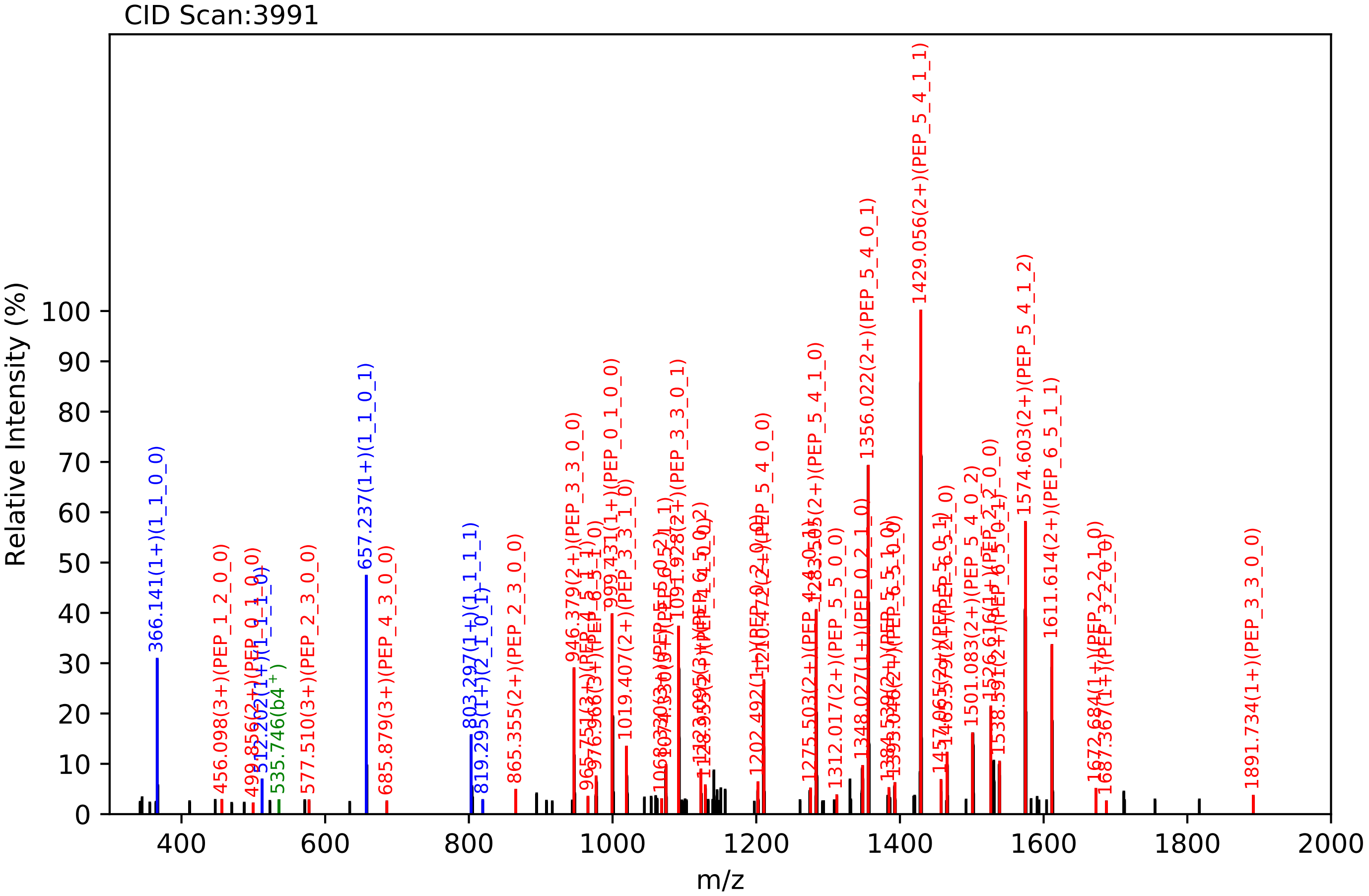

Test set no. 190, Experiment: AGP exp_4

NEEYNK(=PEP)_6_5_1_2, m/z:1171.44(3+), RT:19.58, Y-score:90.29

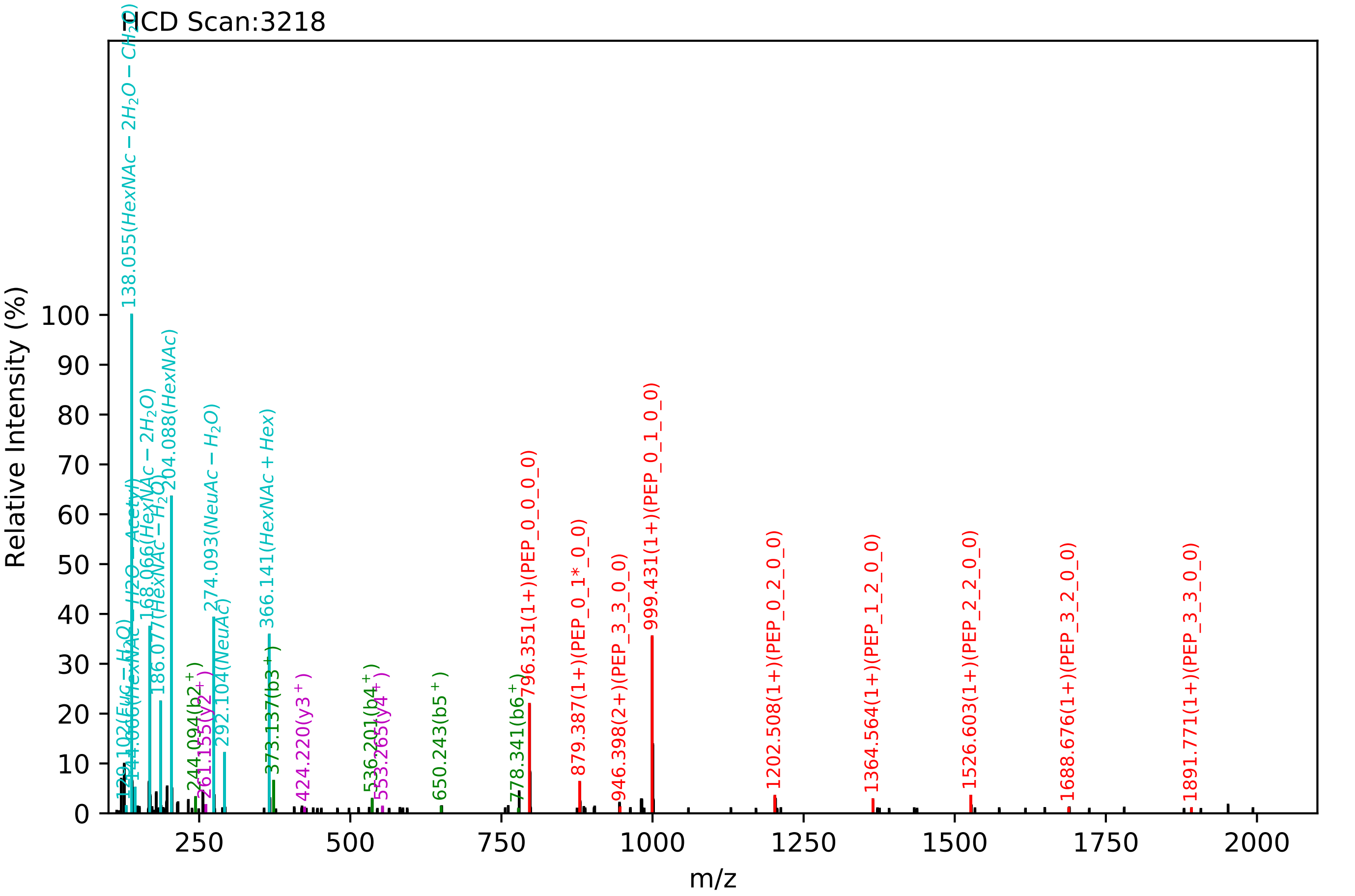

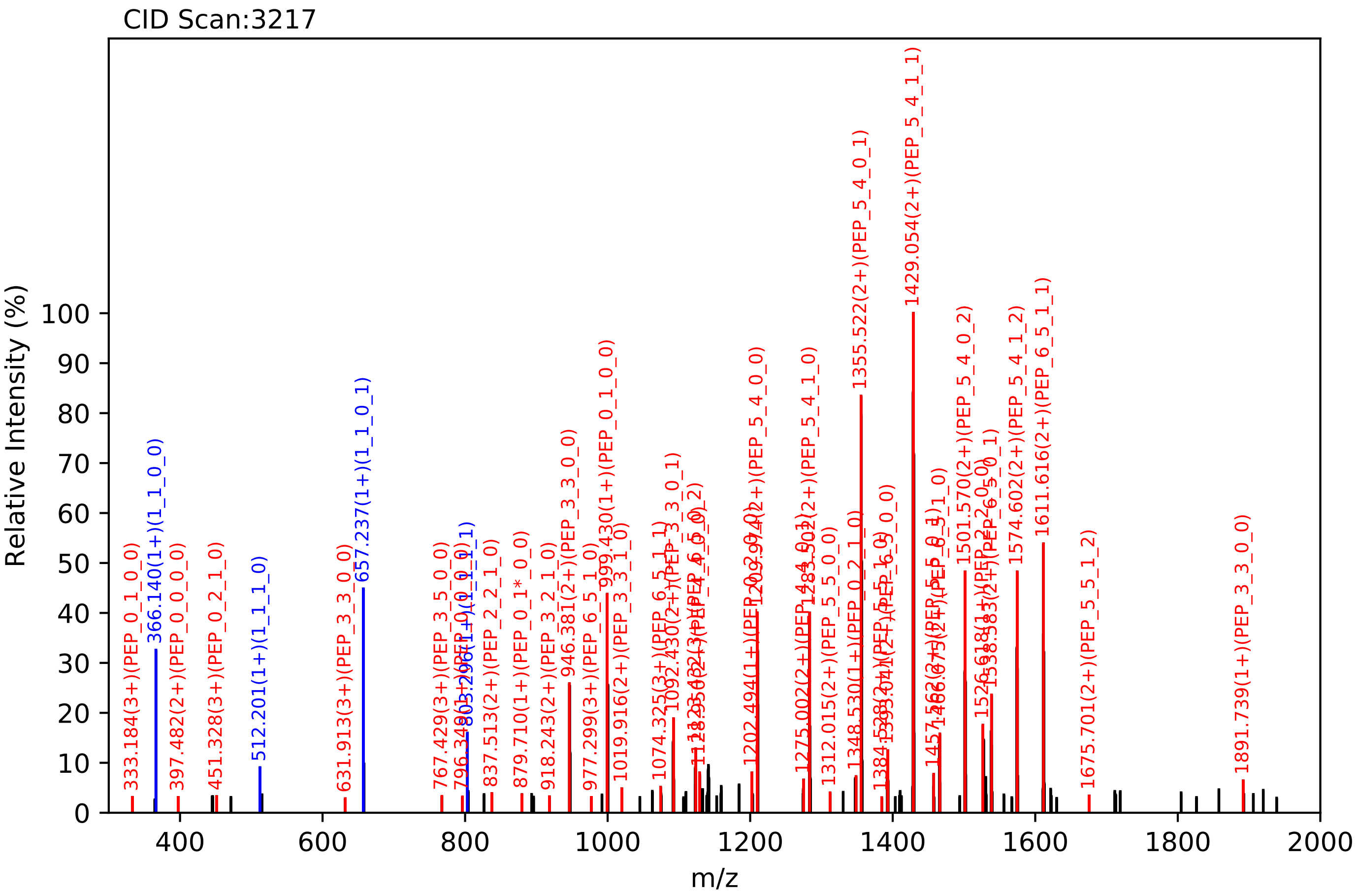

Test set no. 191, Experiment: AGP exp_3

NEEYNK(=PEP)_6_5_1_3, m/z:1269.15(3+), RT:22.15, Y-score:90.39

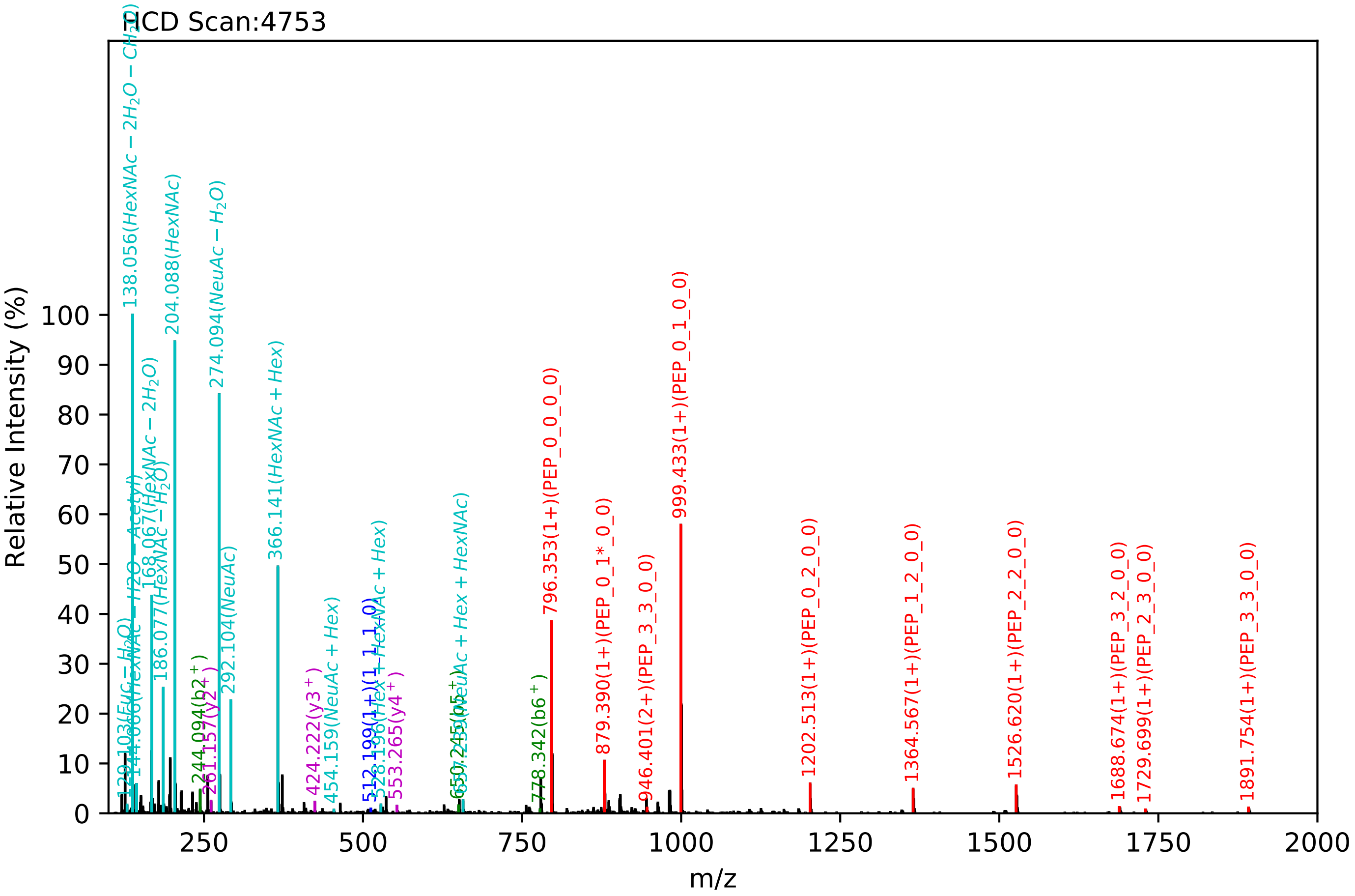

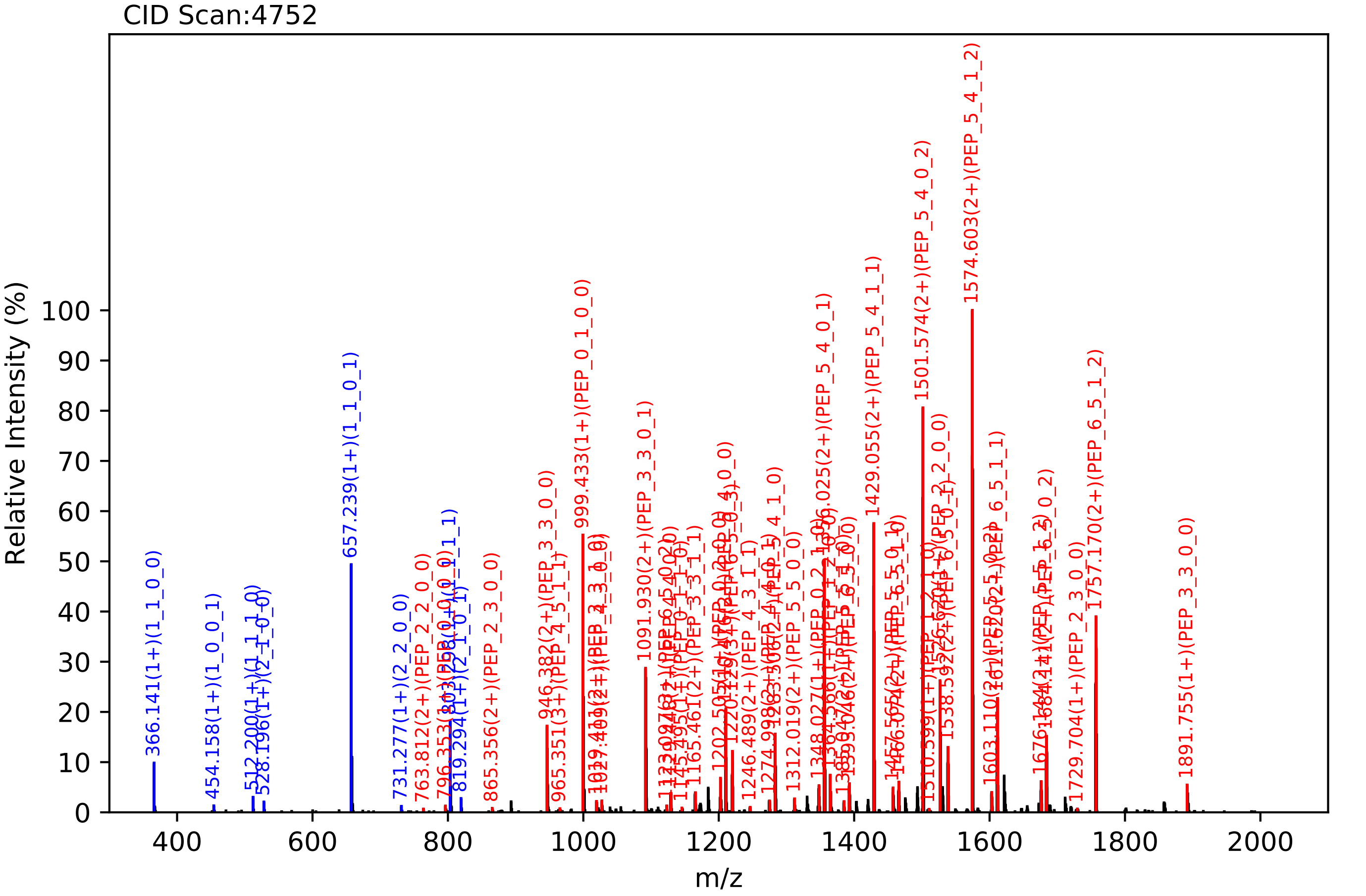

Test set no. 192, Experiment: AGP exp_4

NEEYNK(=PEP)_6_5_1_3, m/z:1268.14(3+), RT:21.98, Y-score:89.74

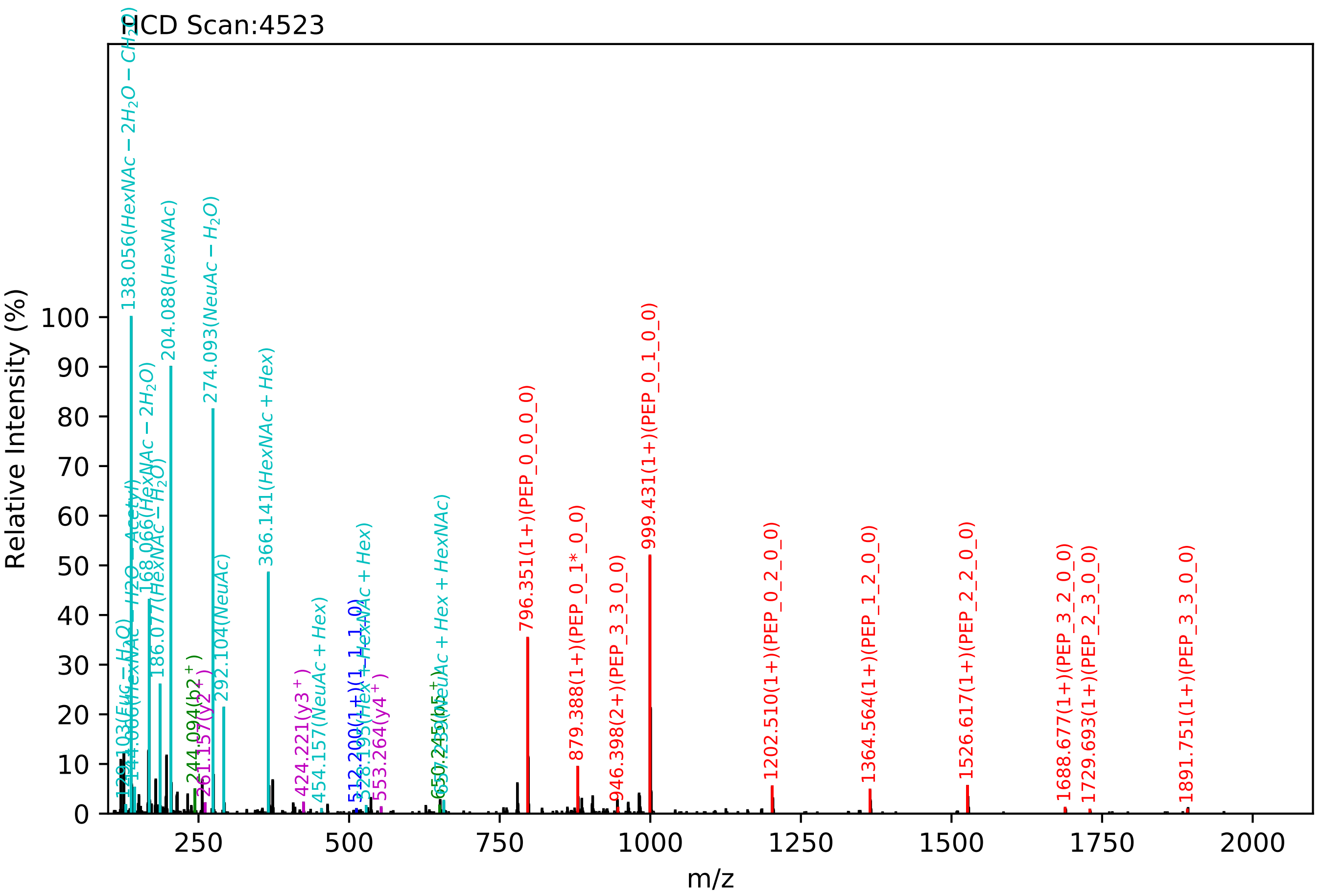

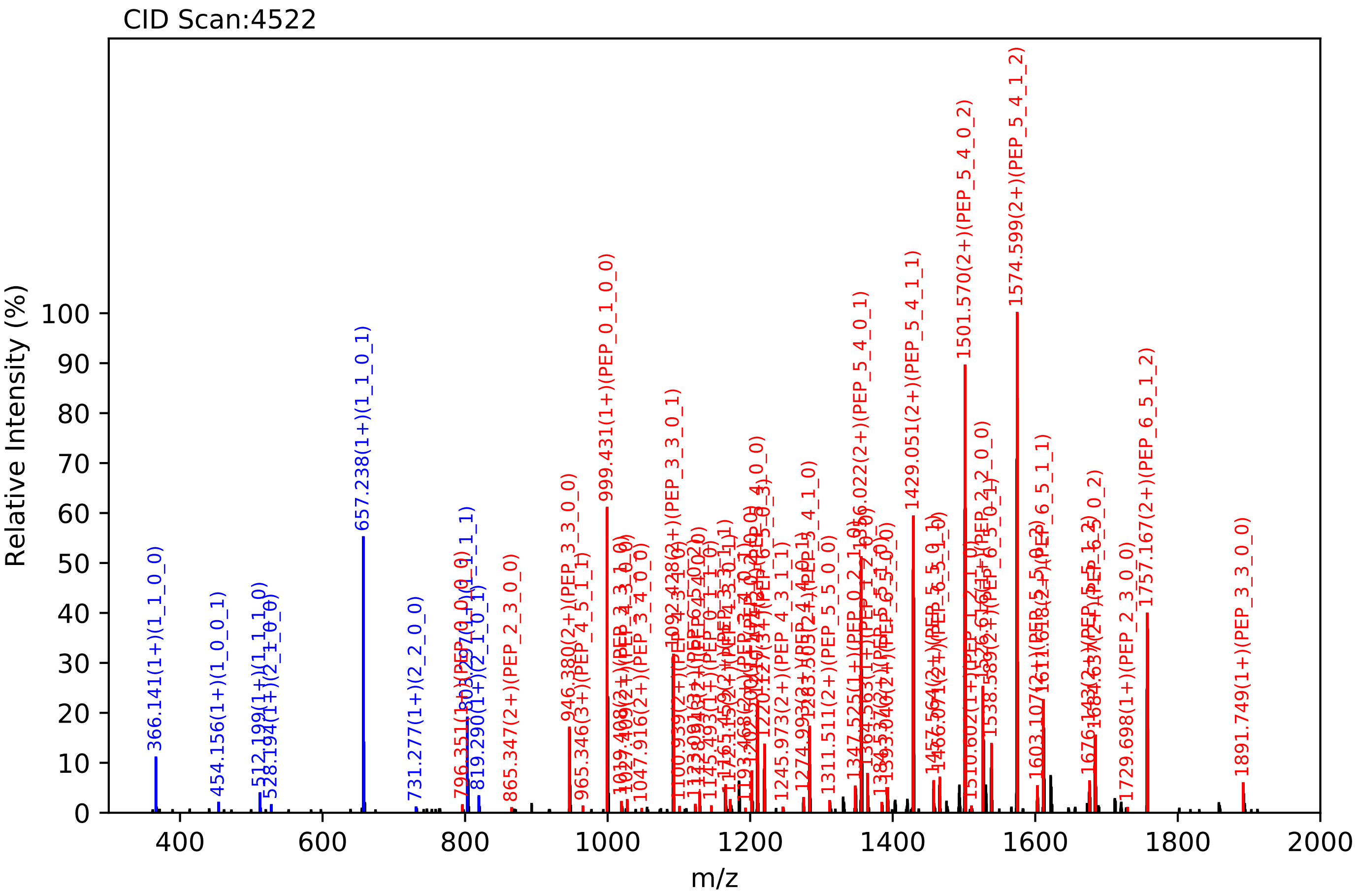

Test set no. 193, Experiment: AGP exp_32

NEEYNK(=PEP)_6_5_2_2, m/z:1220.13(3+), RT:24.16, Y-score:93.20

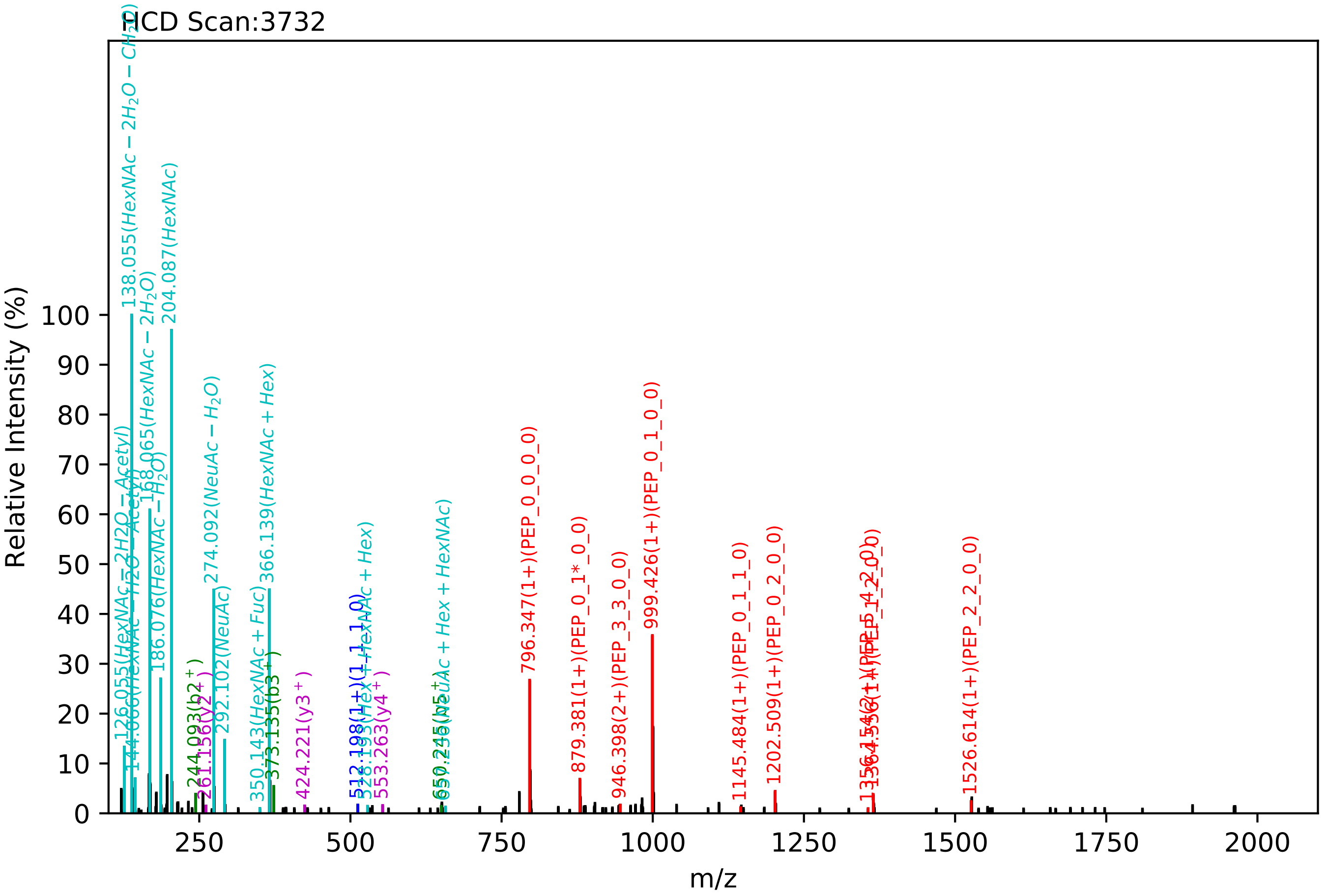

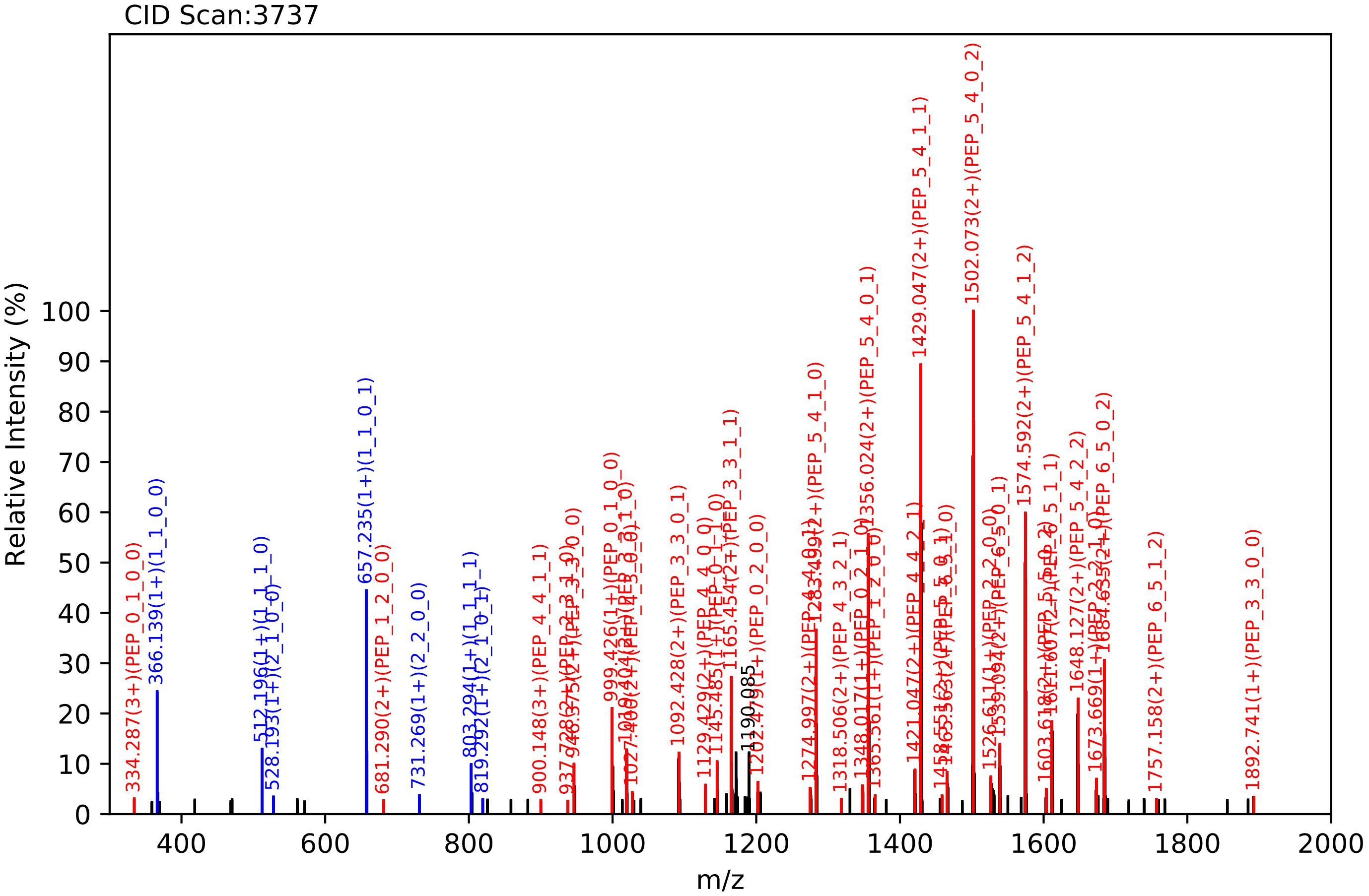

Test set no. 194, Experiment: AGP exp_27

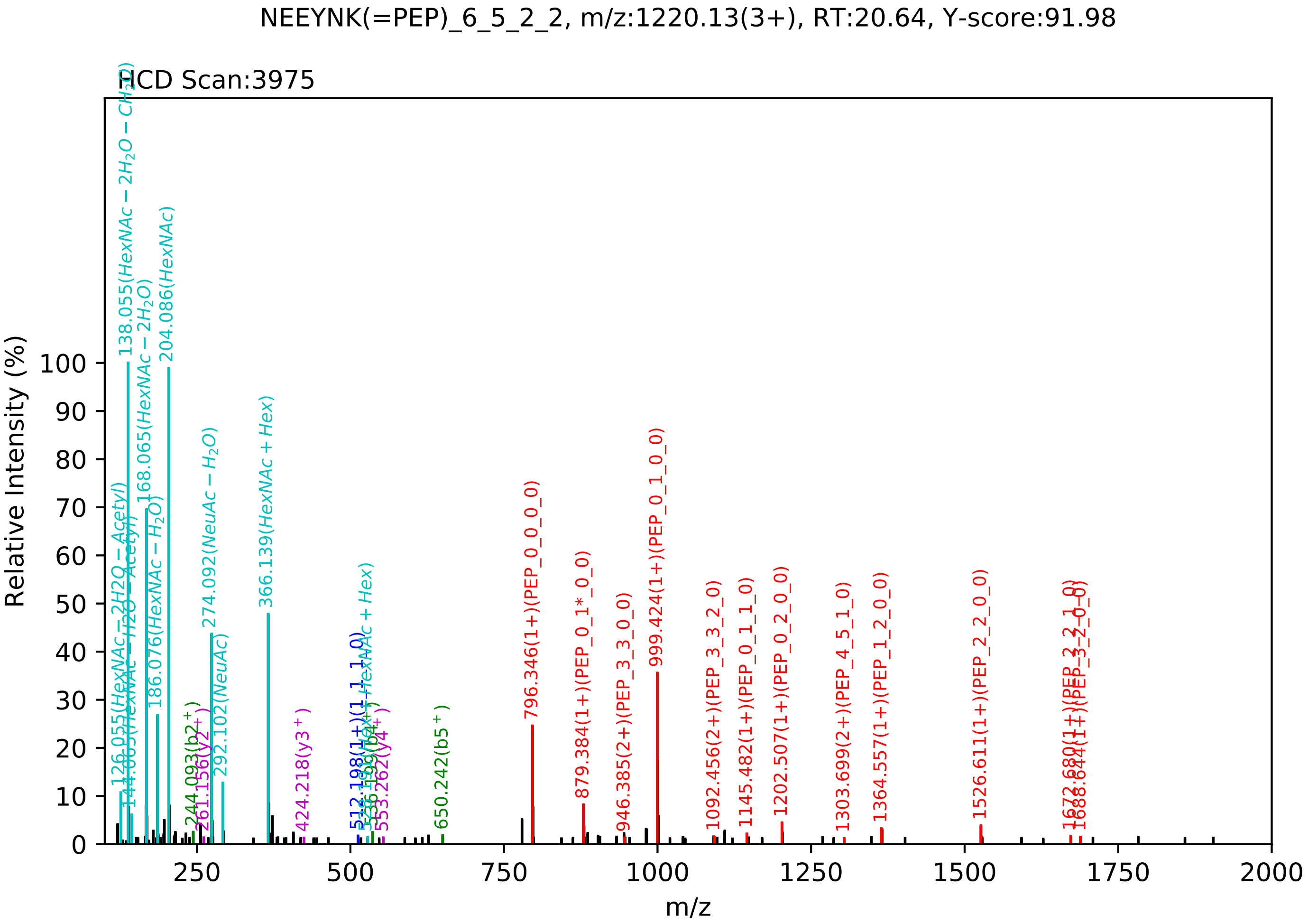

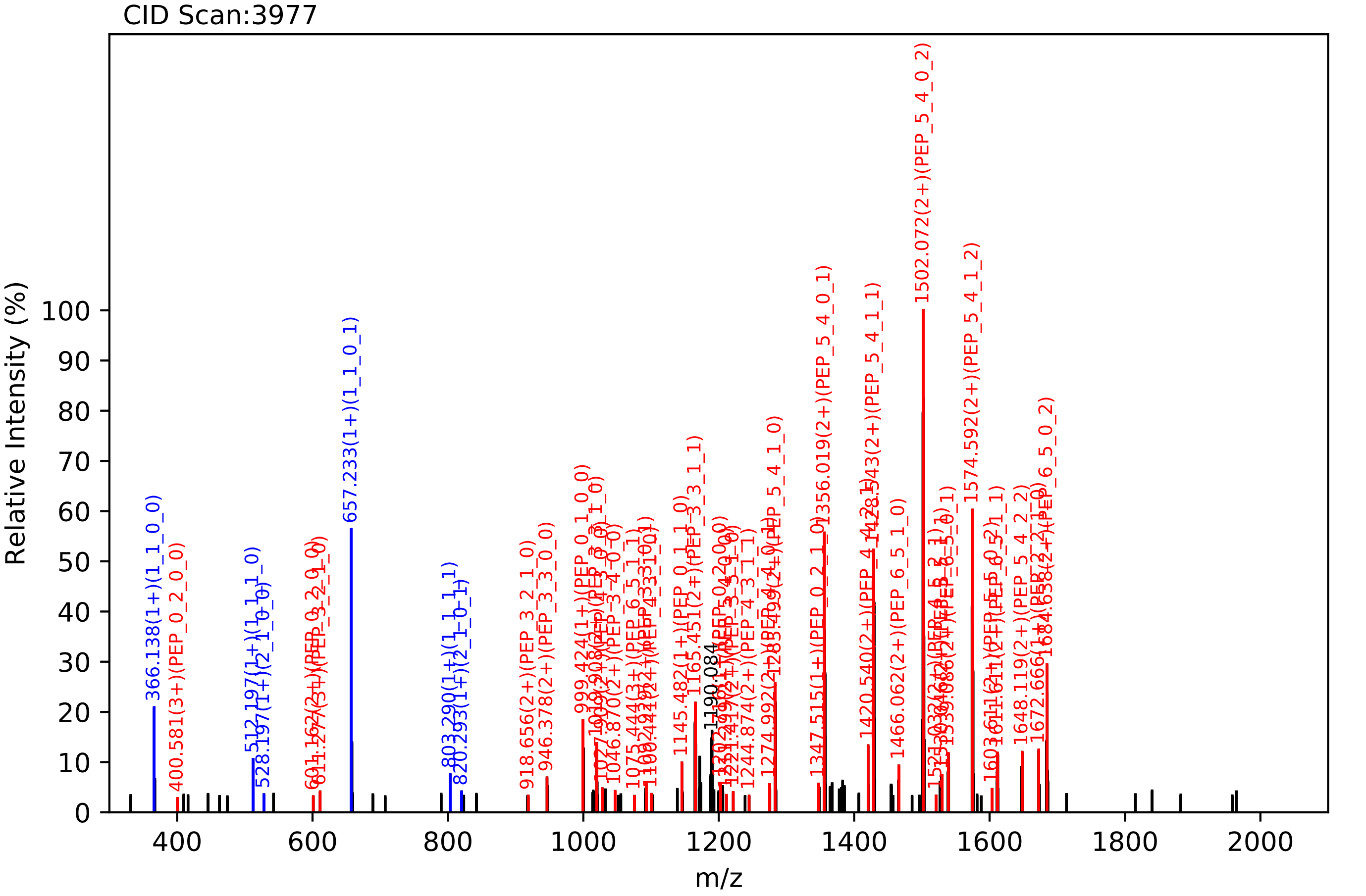

Test set no. 195, Experiment: AGP exp_35

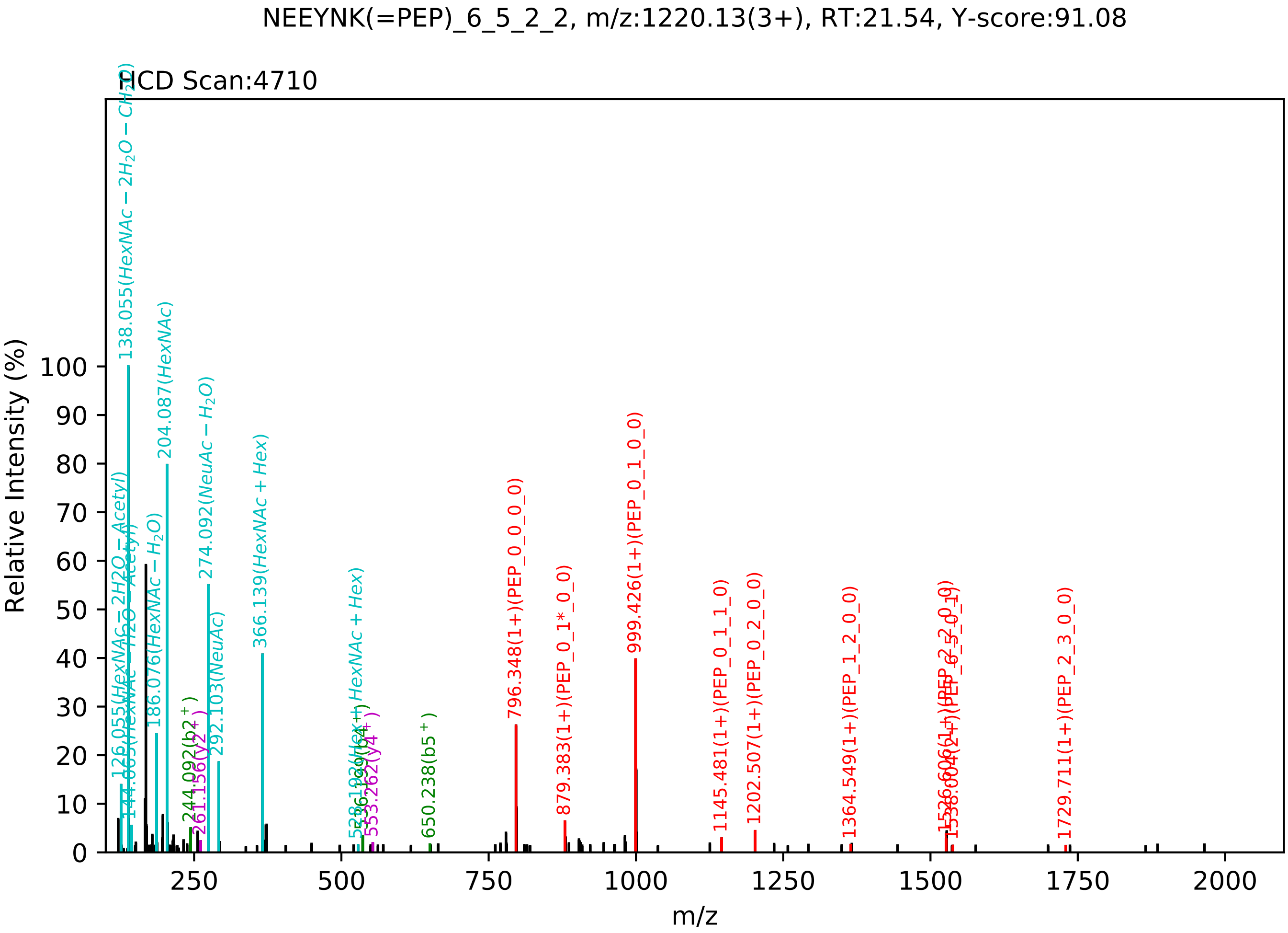

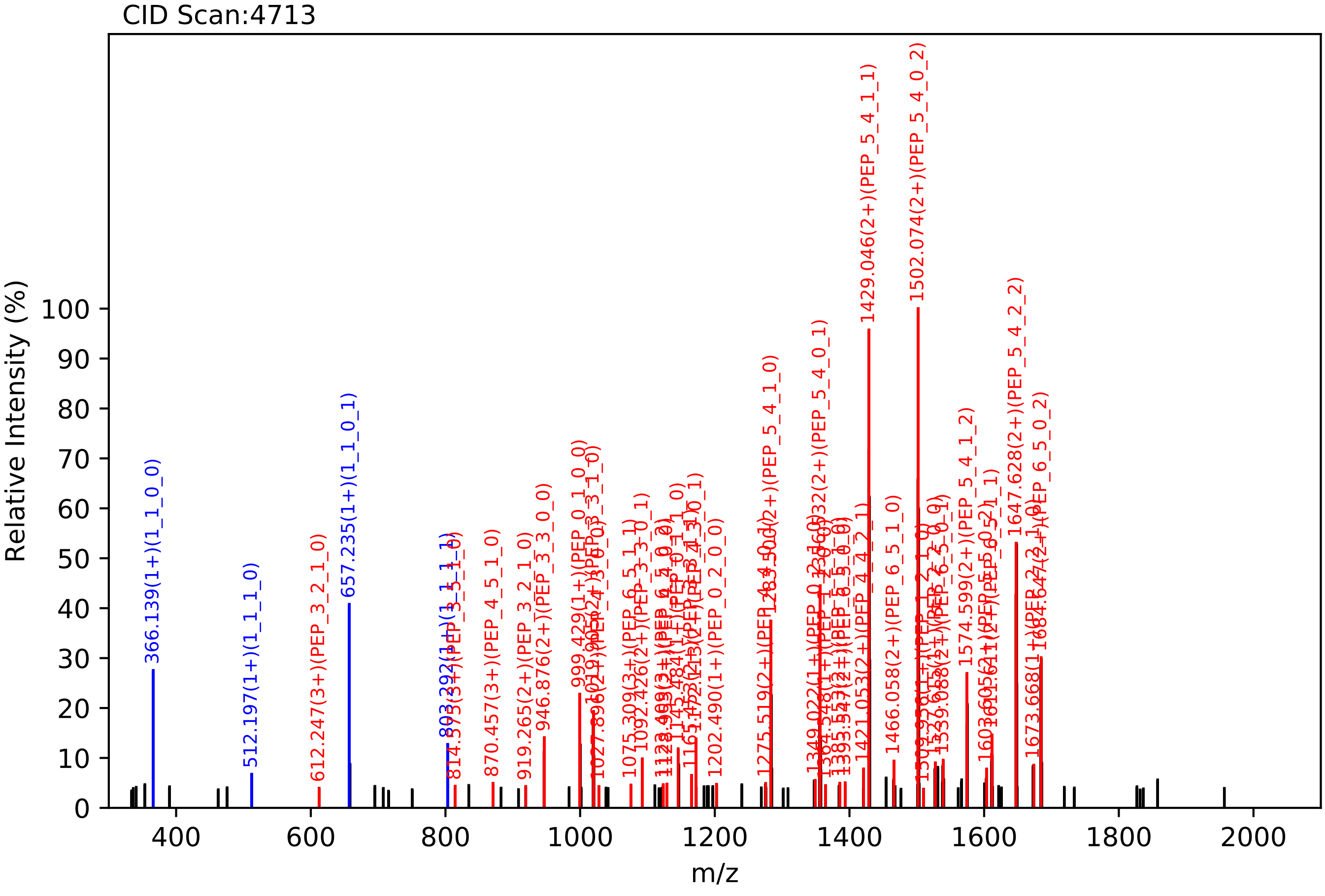

Test set no. 196, Experiment: AGP exp_33

NEEYNK(=PEP)_6_5_2_2, m/z:1219.79(3+), RT:24.19, Y-score:89.35

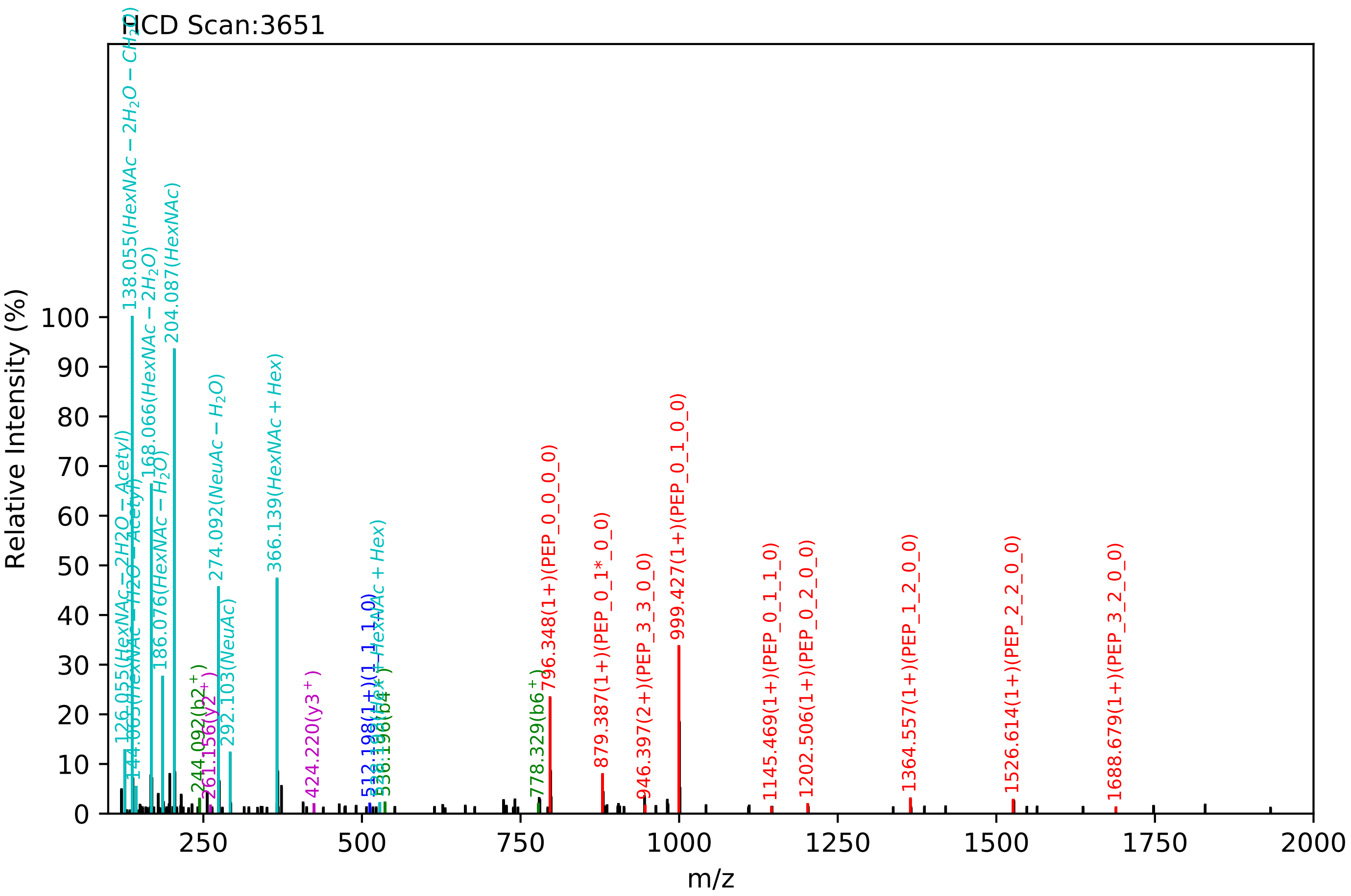

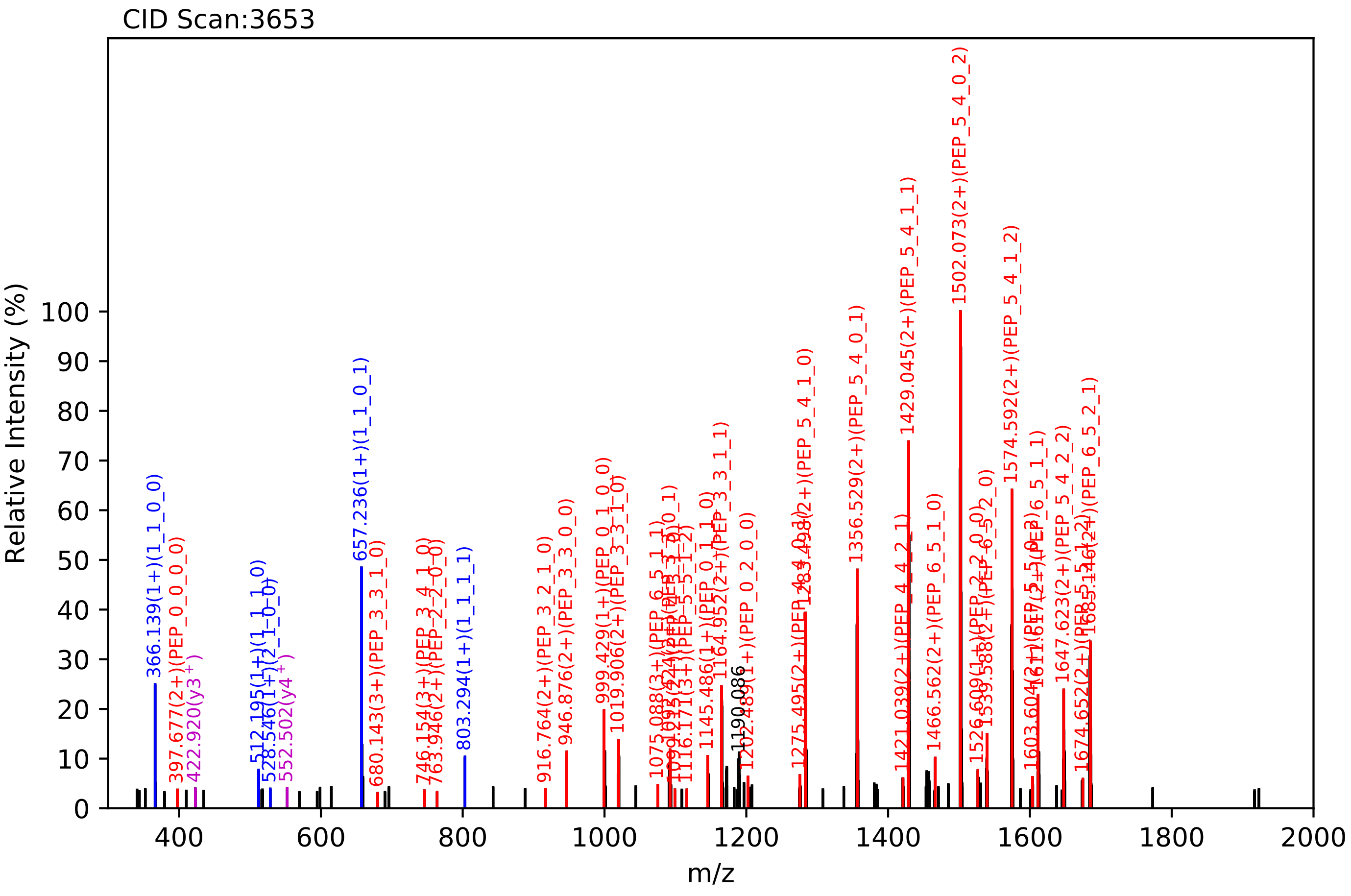

Test set no. 197, Experiment: AGP exp_39

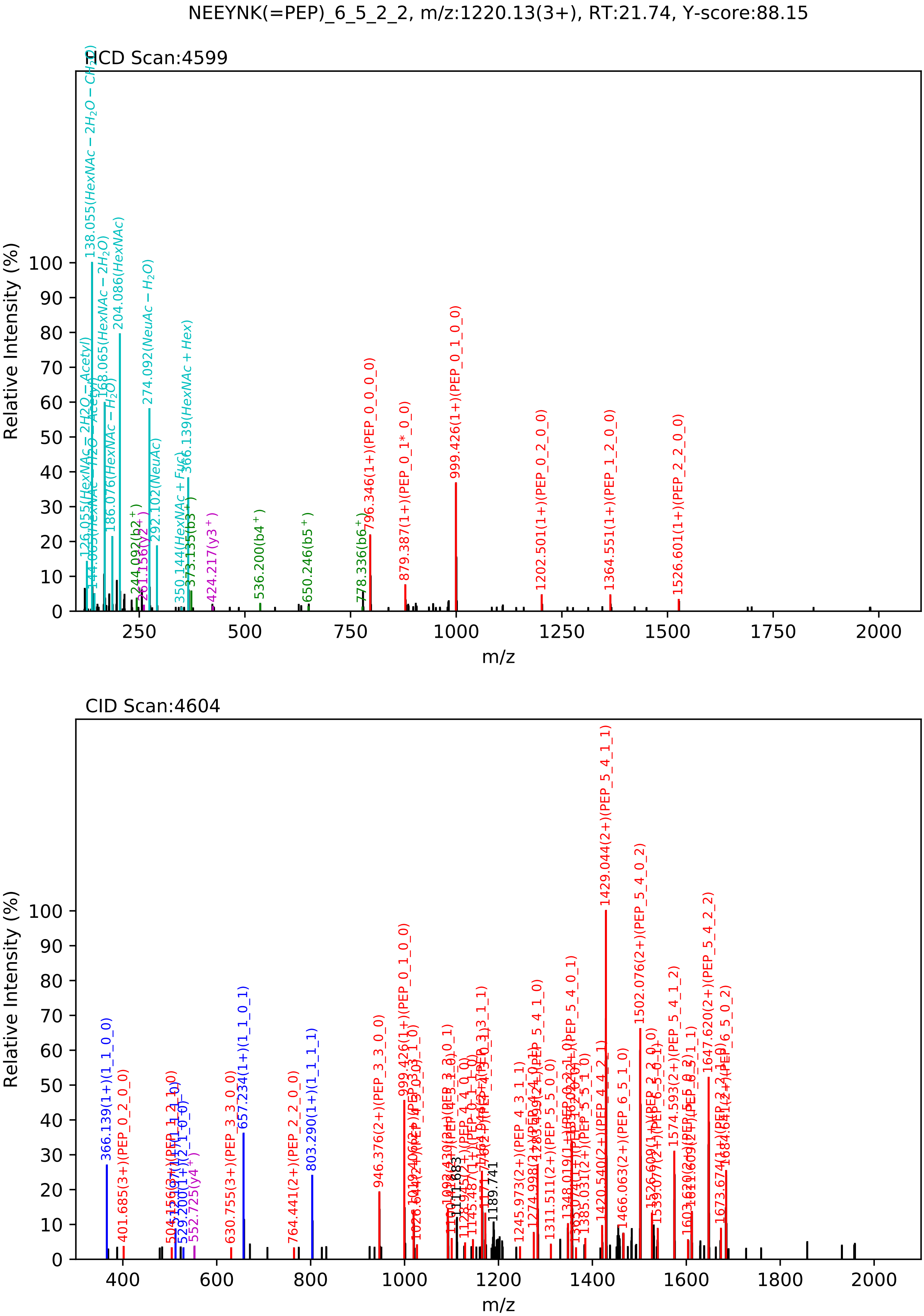

Test set no. 198, Experiment: AGP exp_10

NEEYNK(=PEP)_6_5_2_2, m/z:1220.13(3+), RT:20.24, Y-score:87.75

HCD Scan:3847

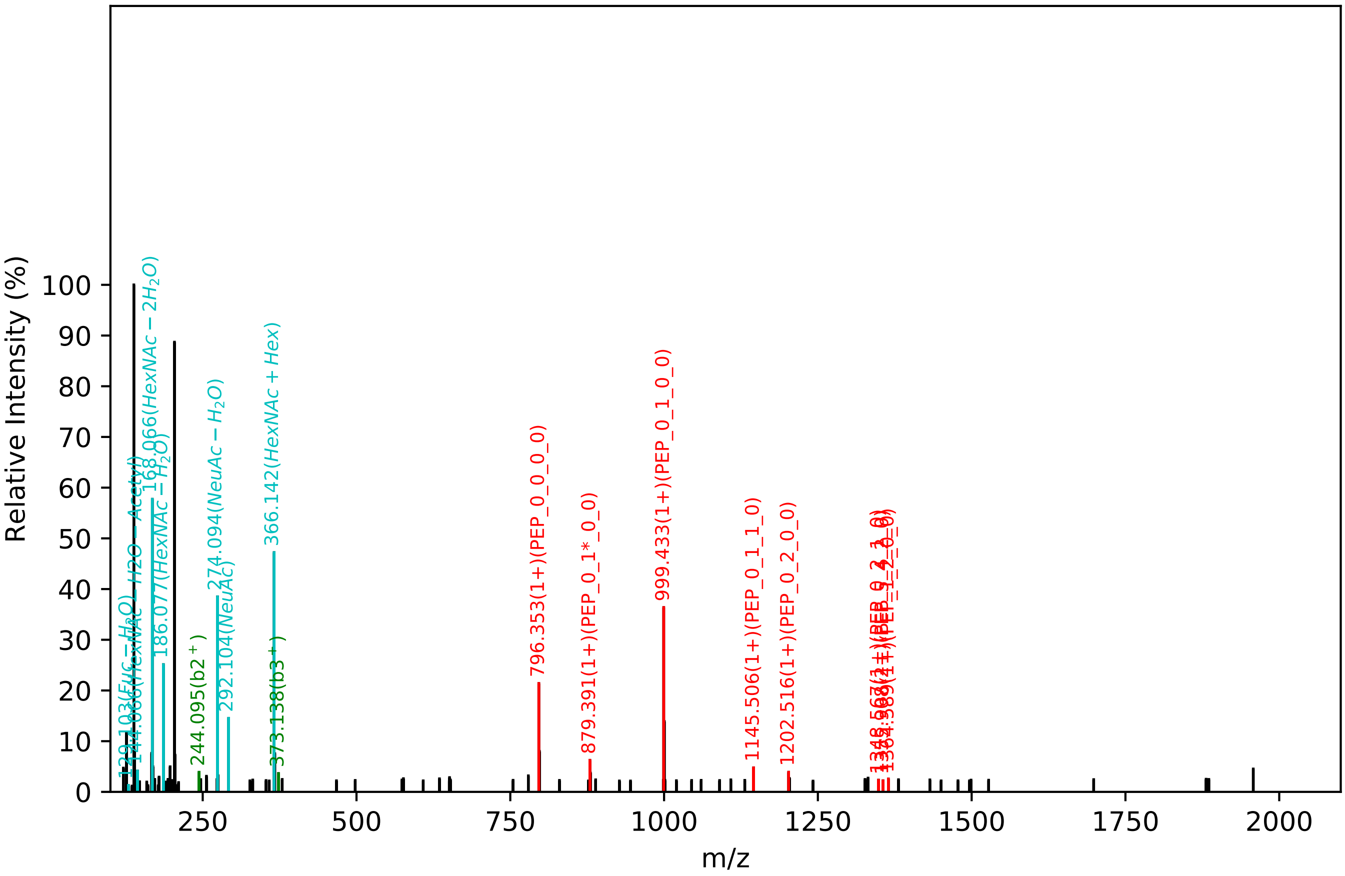

CID Scan:3850

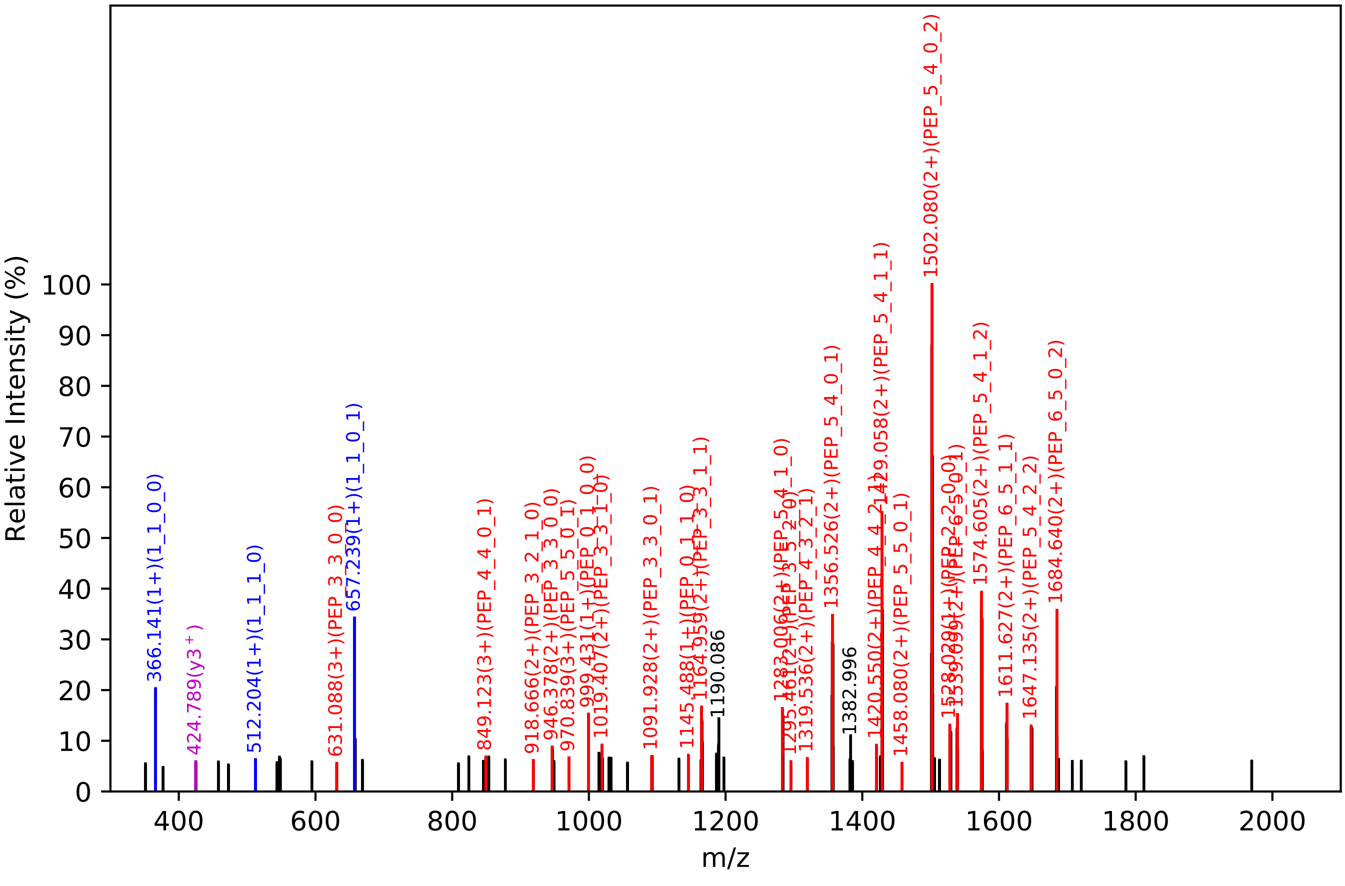

Test set no. 199, Experiment: AGP exp_33

NEEYNK(=PEP)_6_5_2_2, m/z:1220.13(3+), RT:25.05, Y-score:87.27

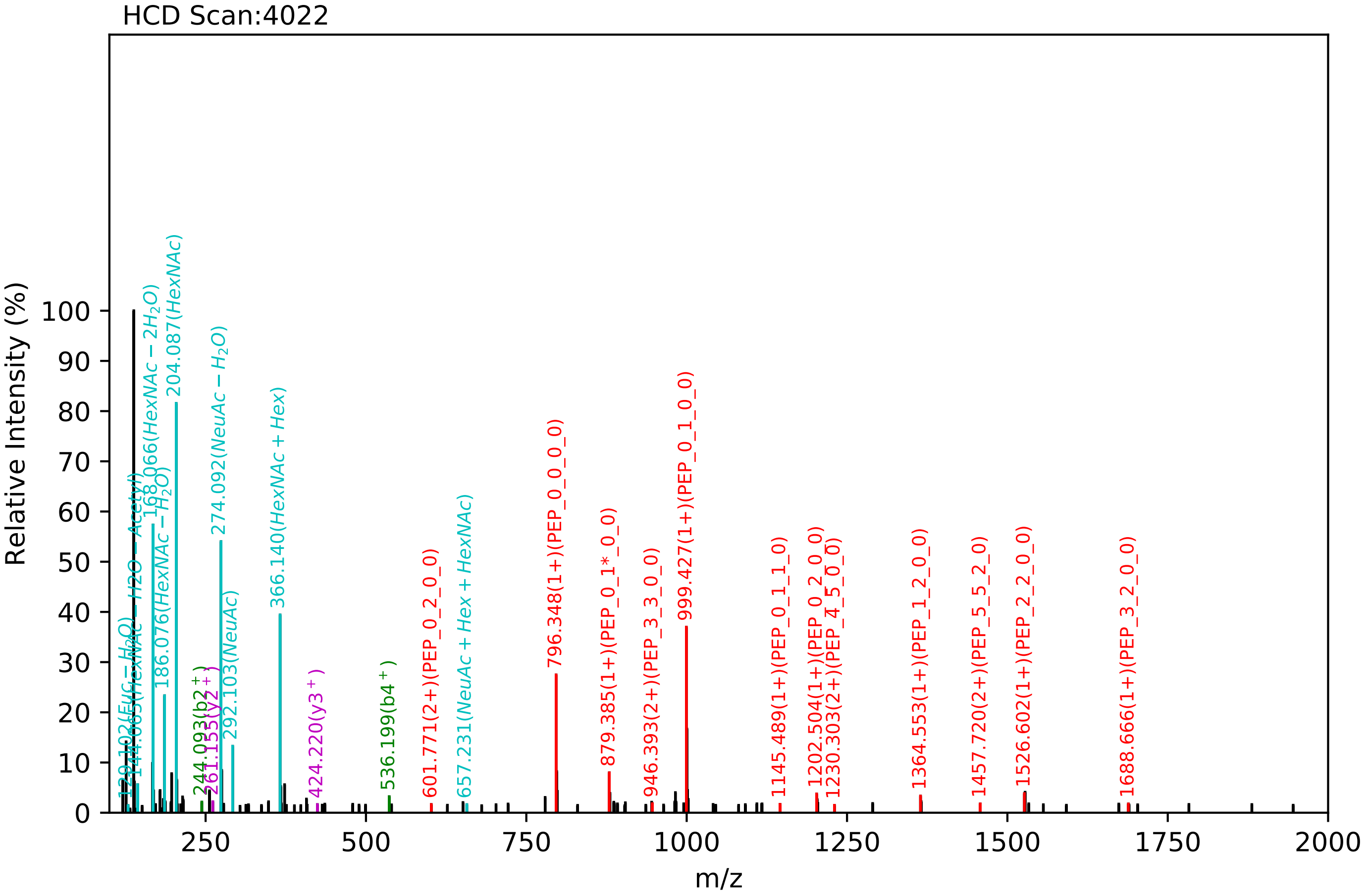

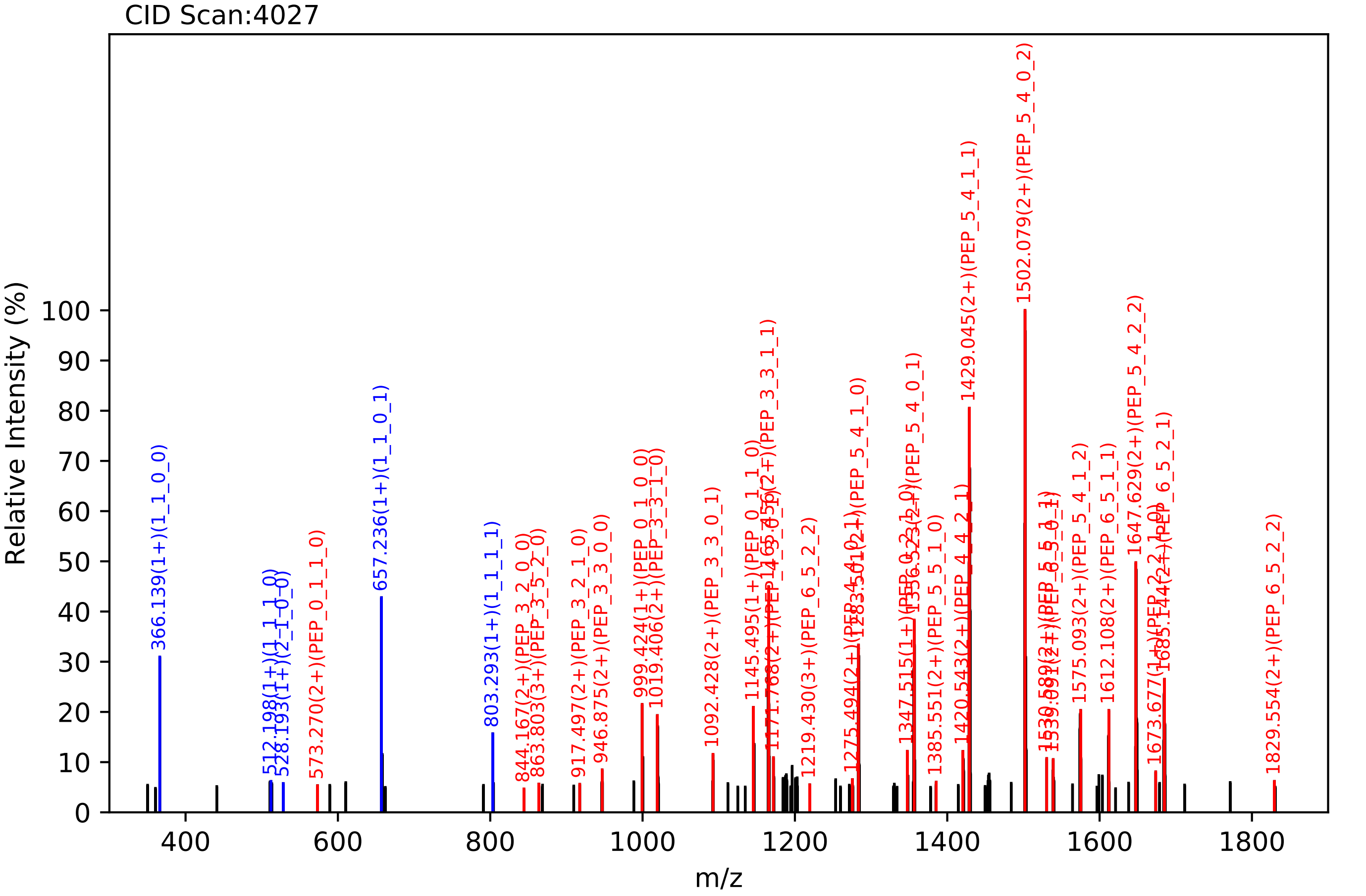

Test set no. 200, Experiment: AGP exp_43

NEEYNK(=PEP)_6_5_2_2, m/z:1220.13(3+), RT:21.56, Y-score:86.87

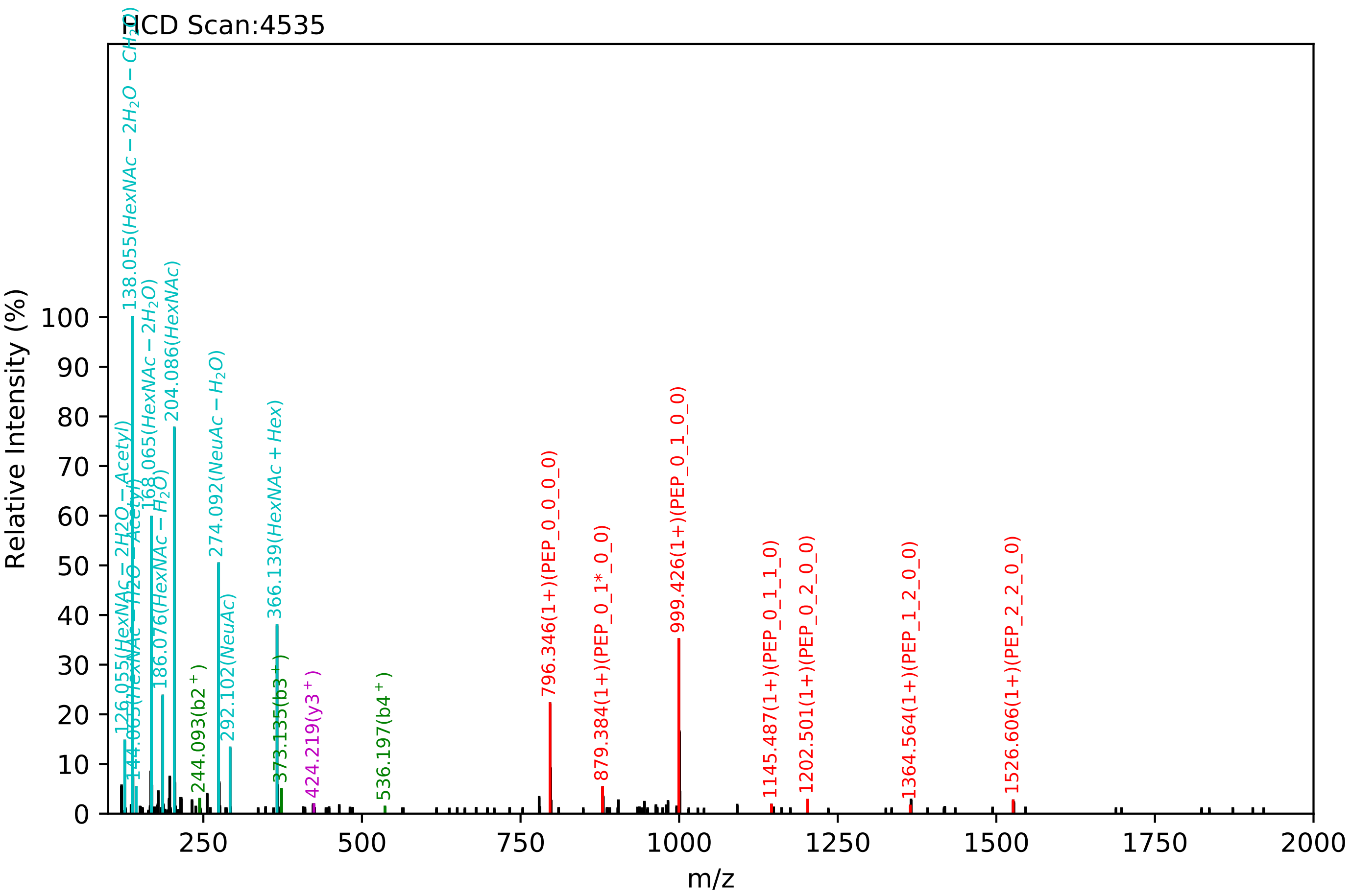

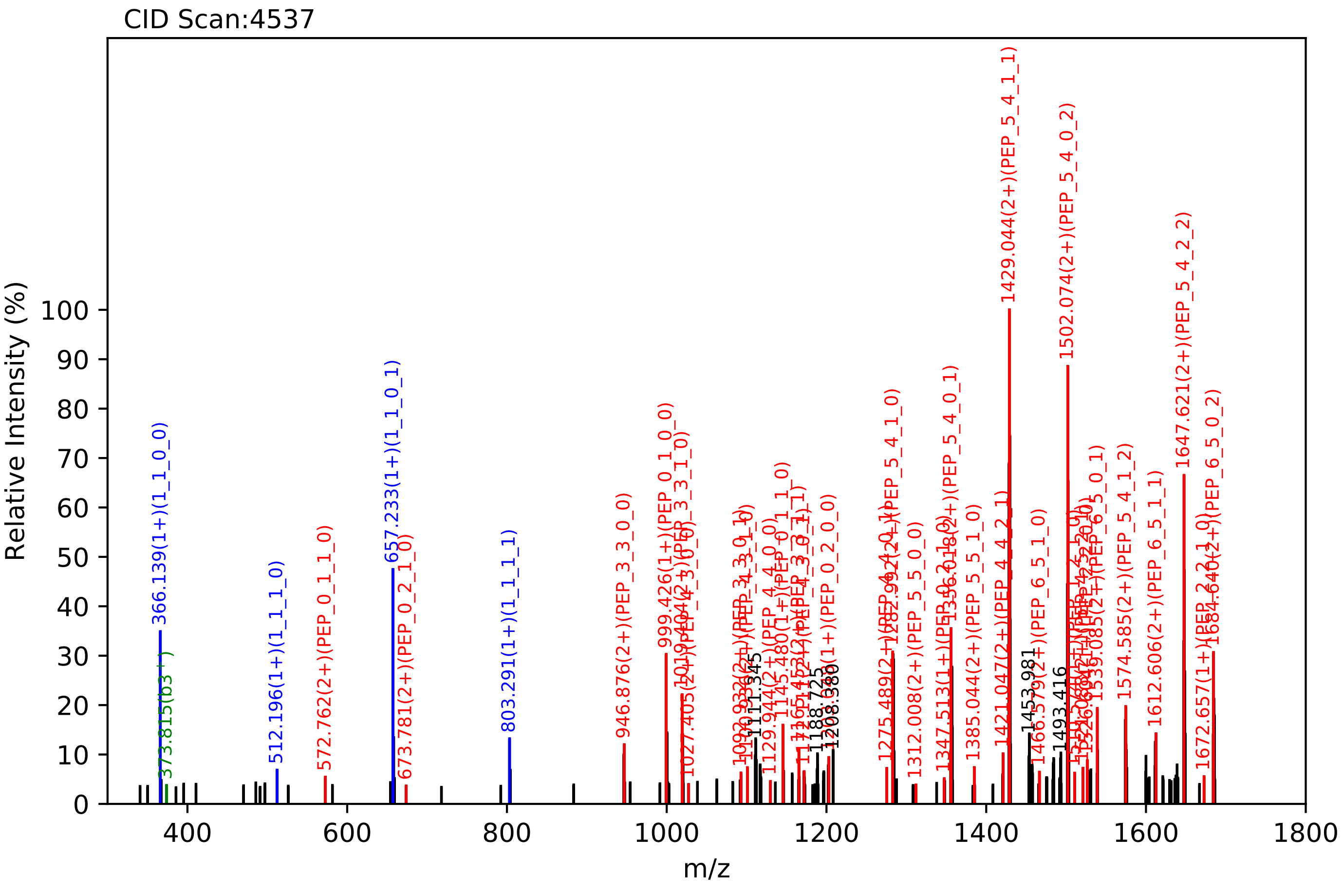

Test set no. 201, Experiment: AGP exp_10

NEEYNK(=PEP)_6_5_2_2, m/z:1220.13(3+), RT:20.88, Y-score:86.27

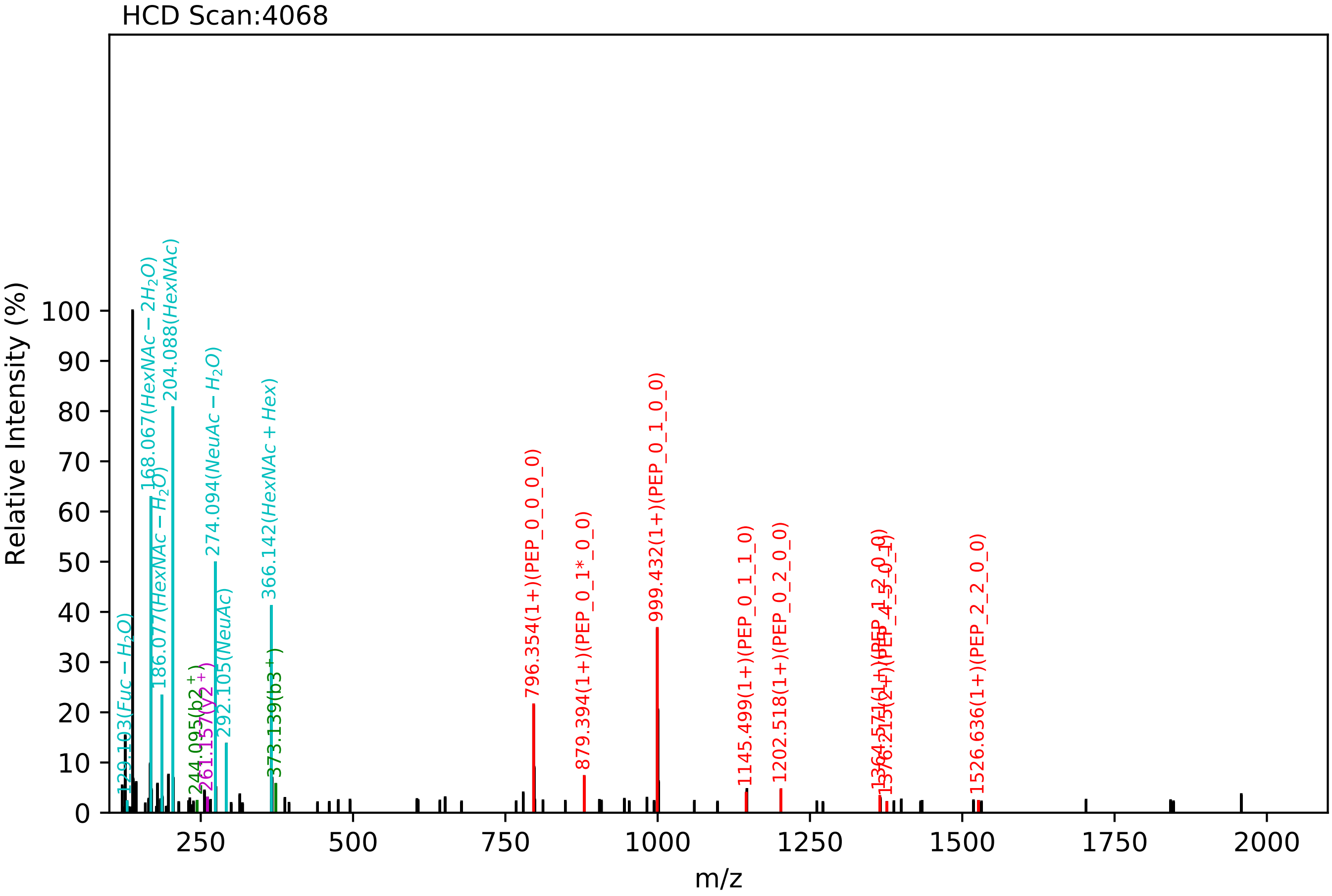

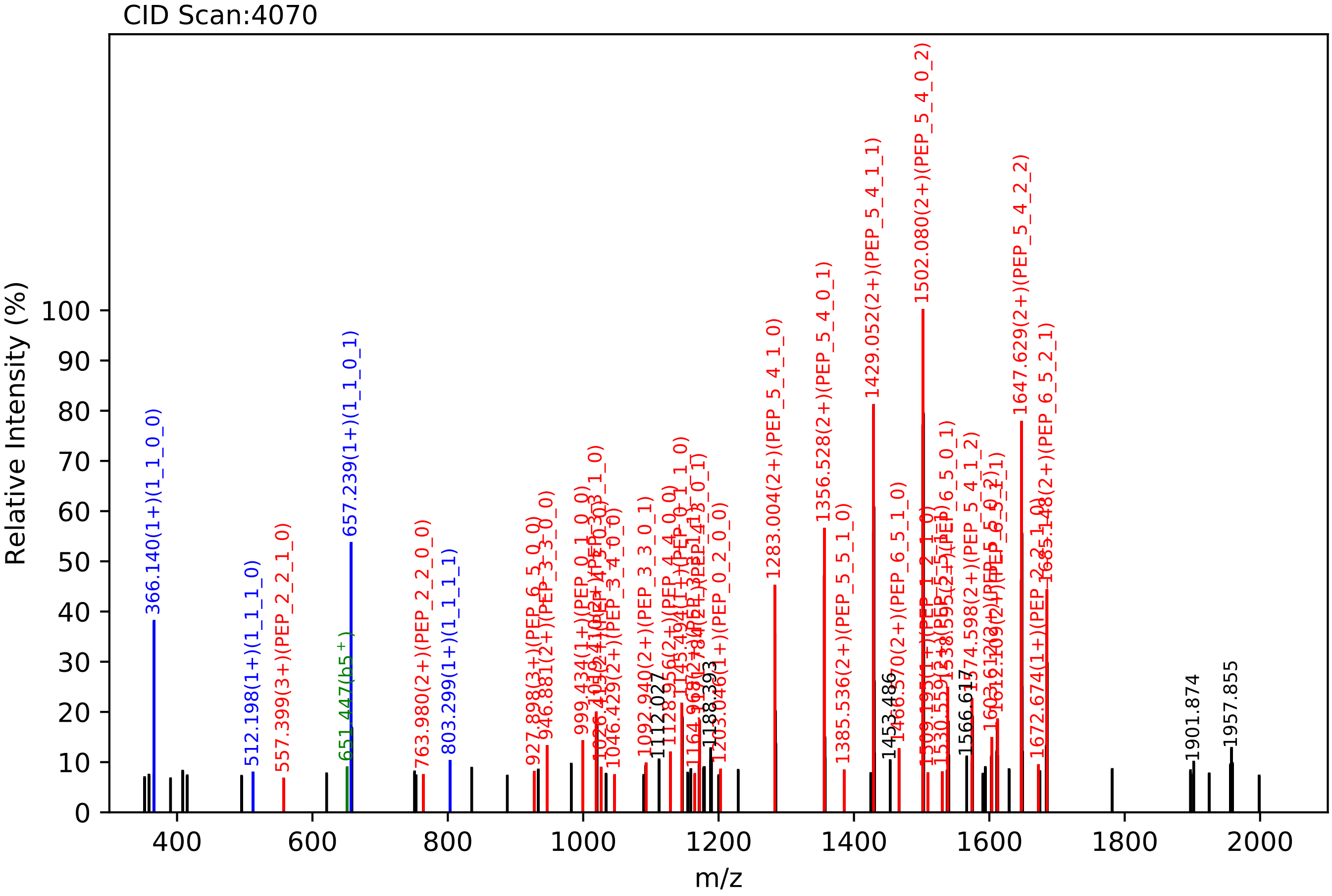

Test set no. 202, Experiment: AGP exp_3

NEEYNK(=PEP)_6_5_2_2, m/z:1220.13(3+), RT:18.54, Y-score:85.95

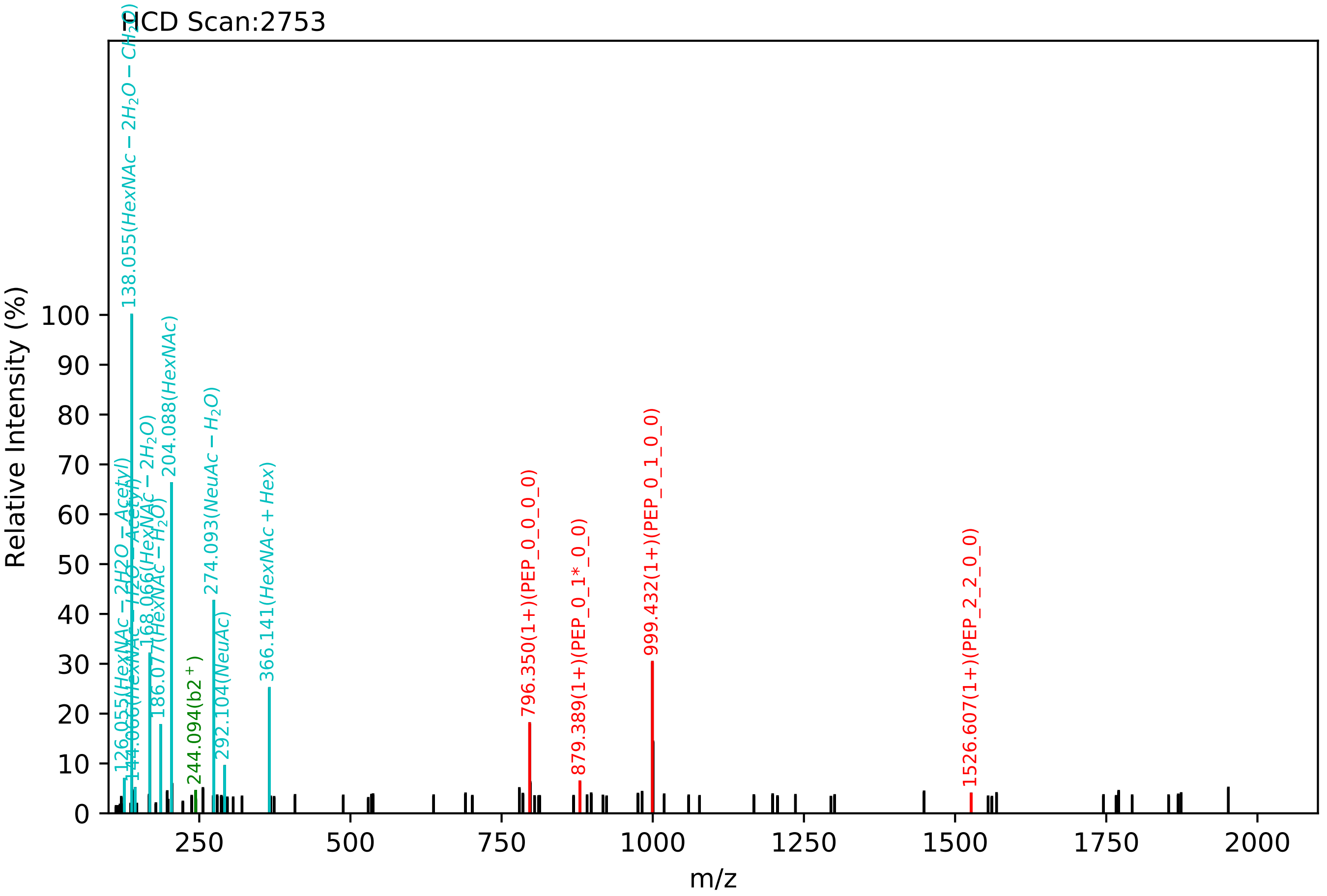

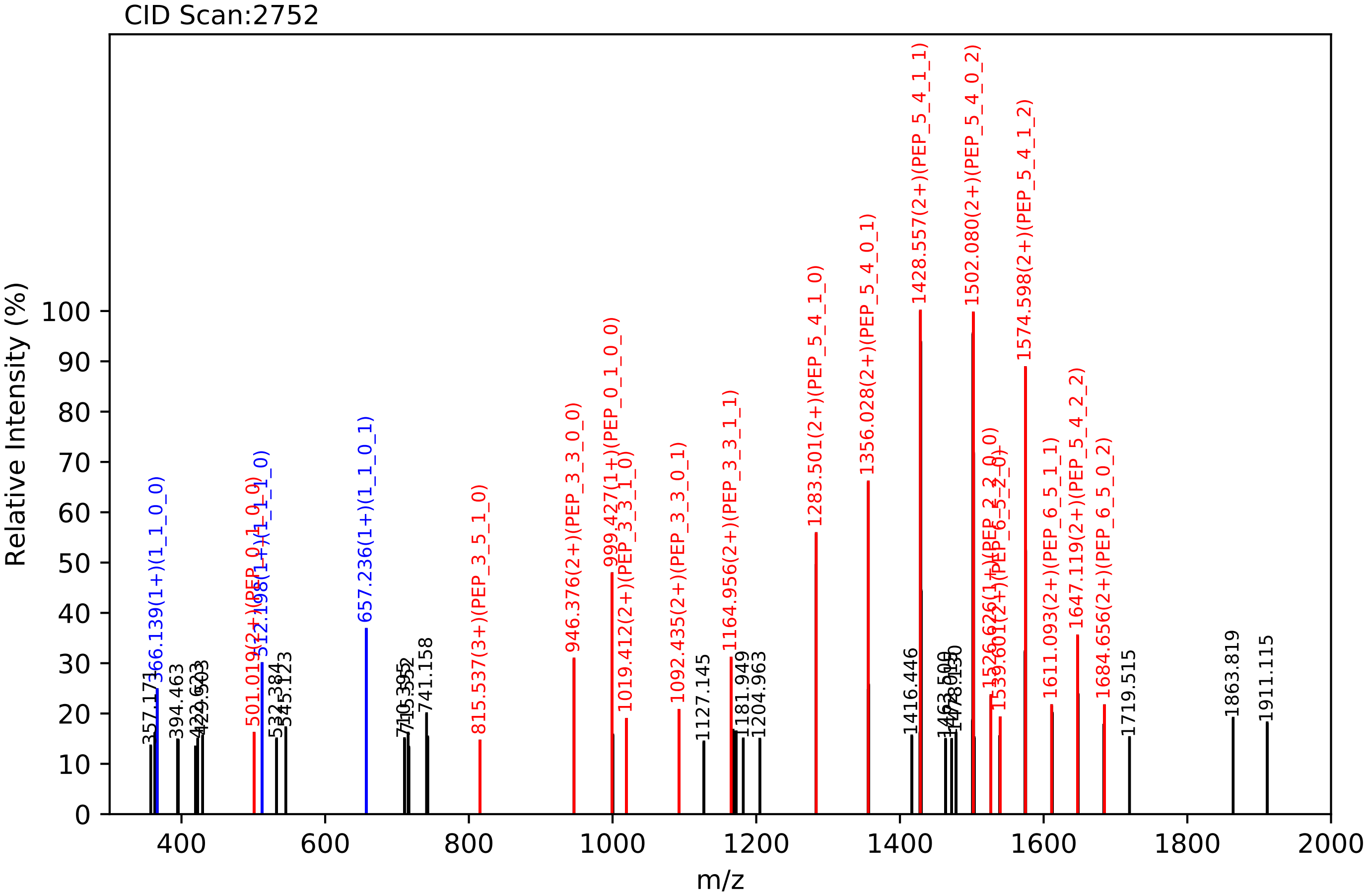

Test set no. 203, Experiment: AGP exp_37

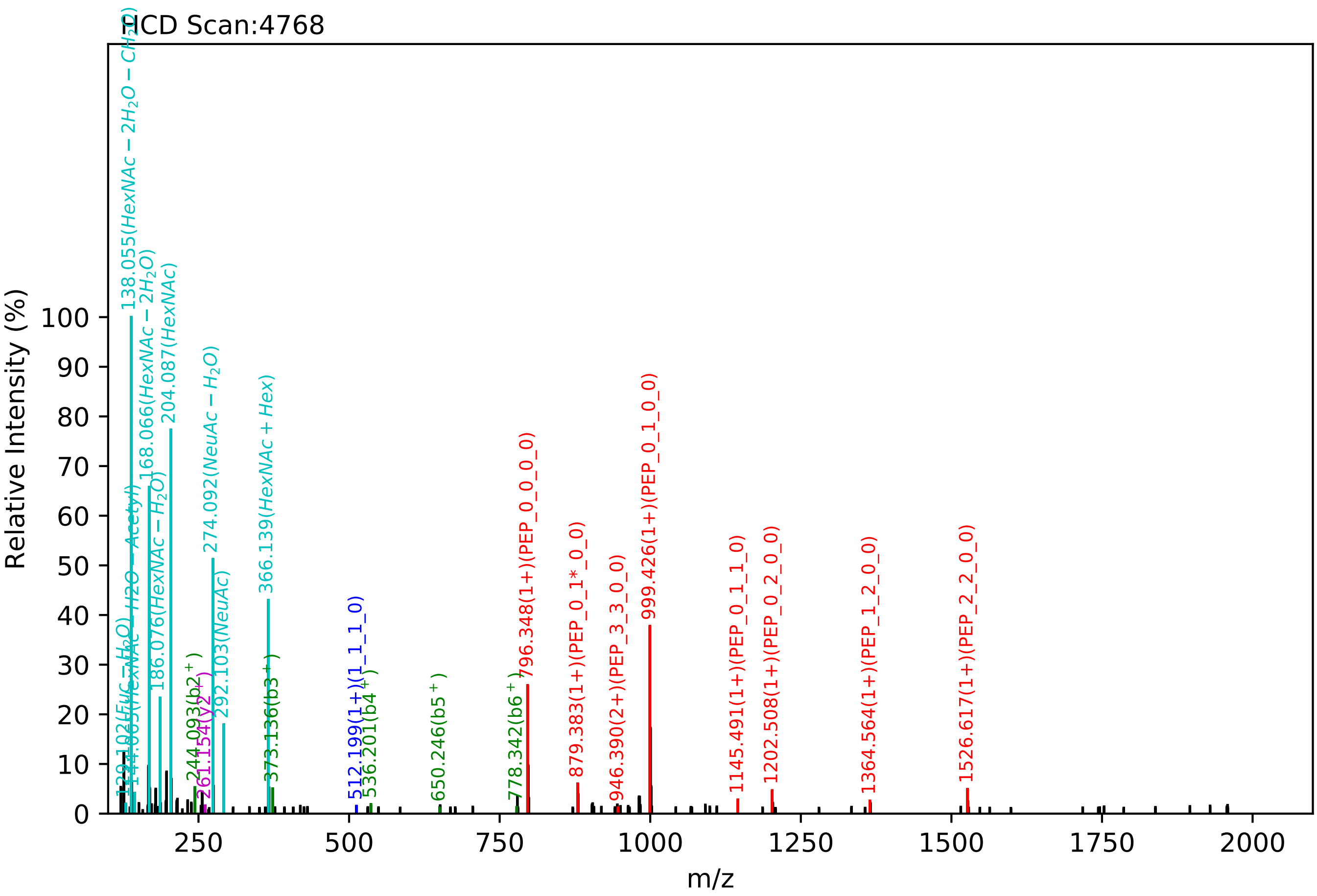

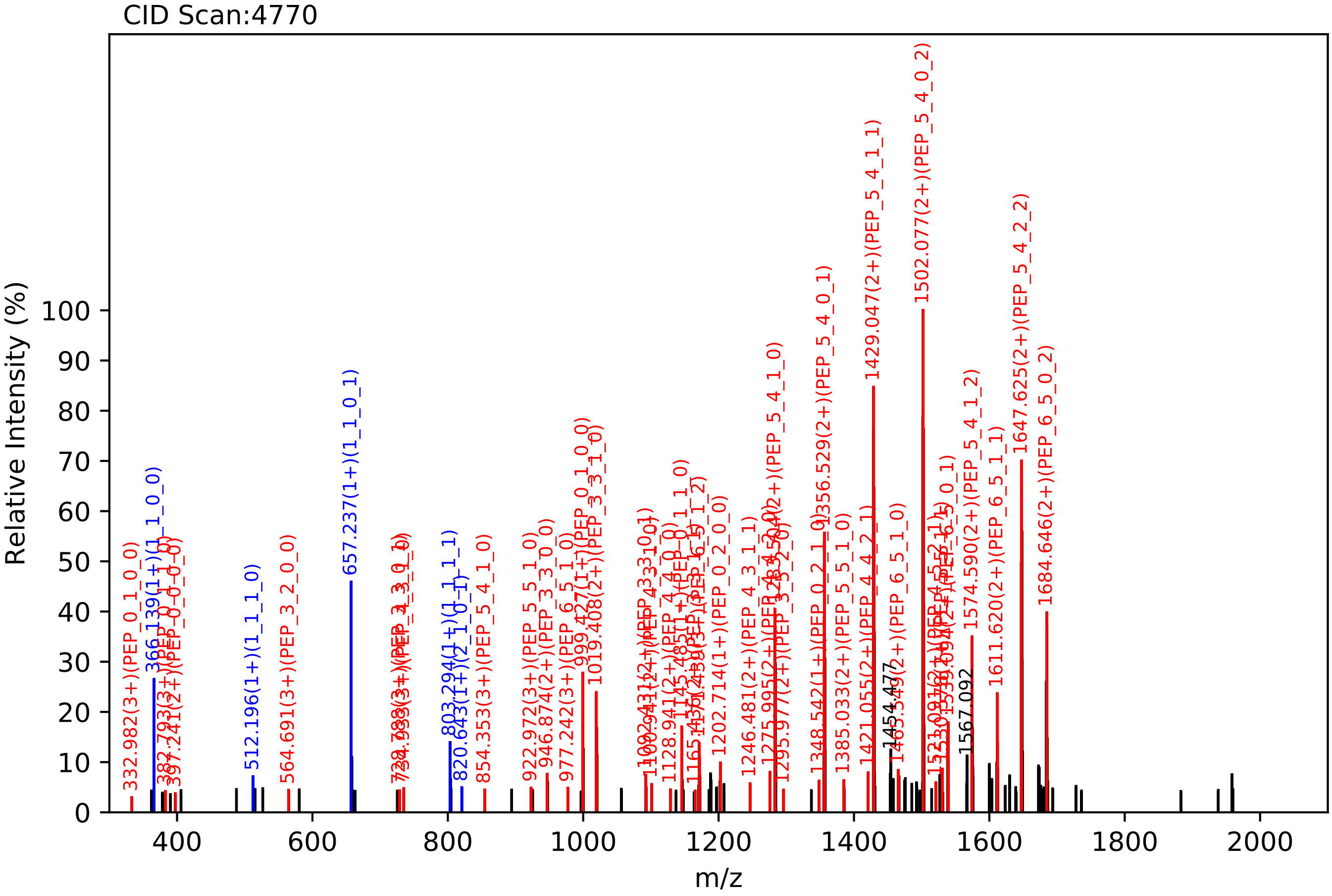

Test set no. 204, Experiment: AGP exp_42

NEEYNK(=PEP)_6_5_2_2, m/z:1220.13(3+), RT:21.86, Y-score:85.11

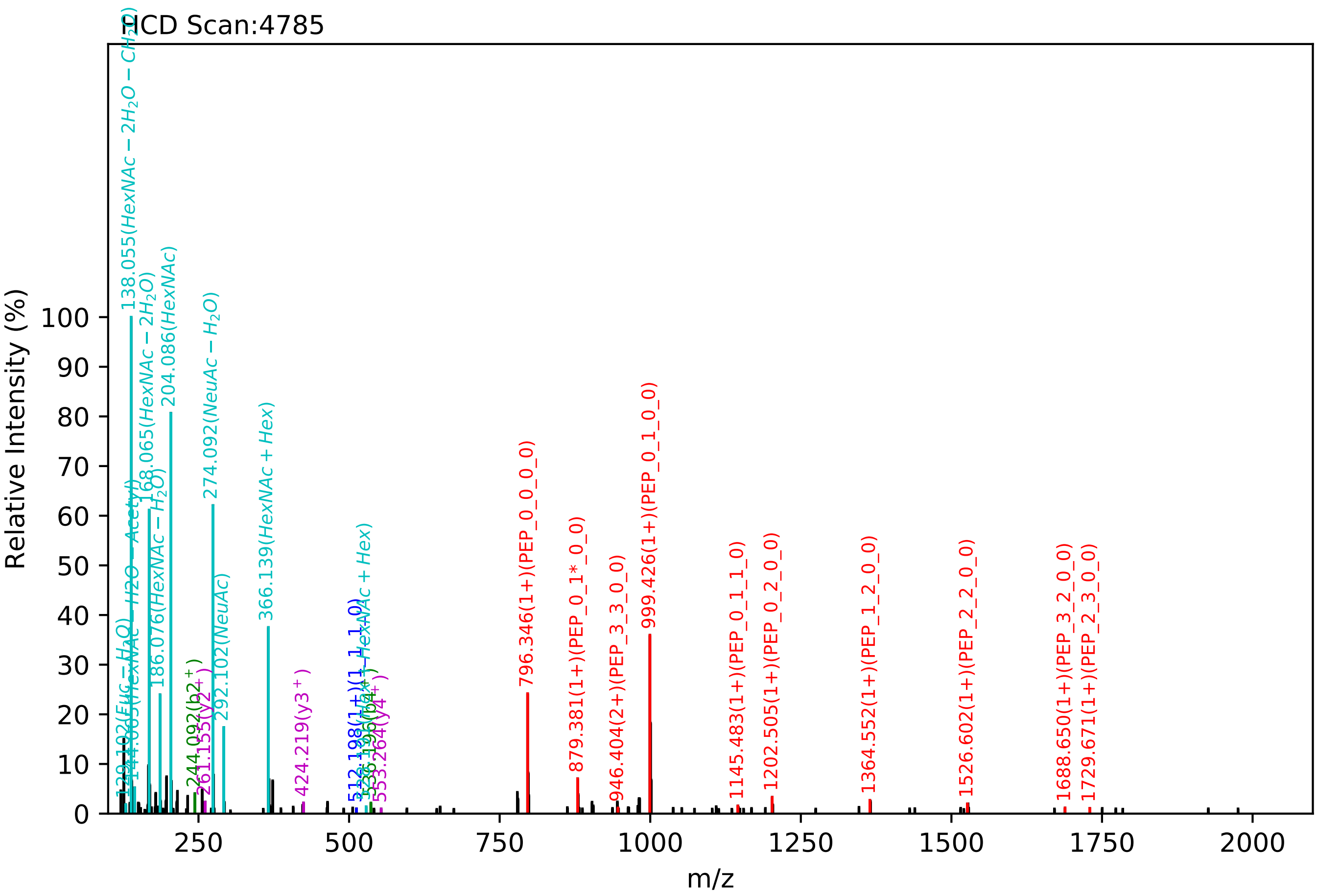

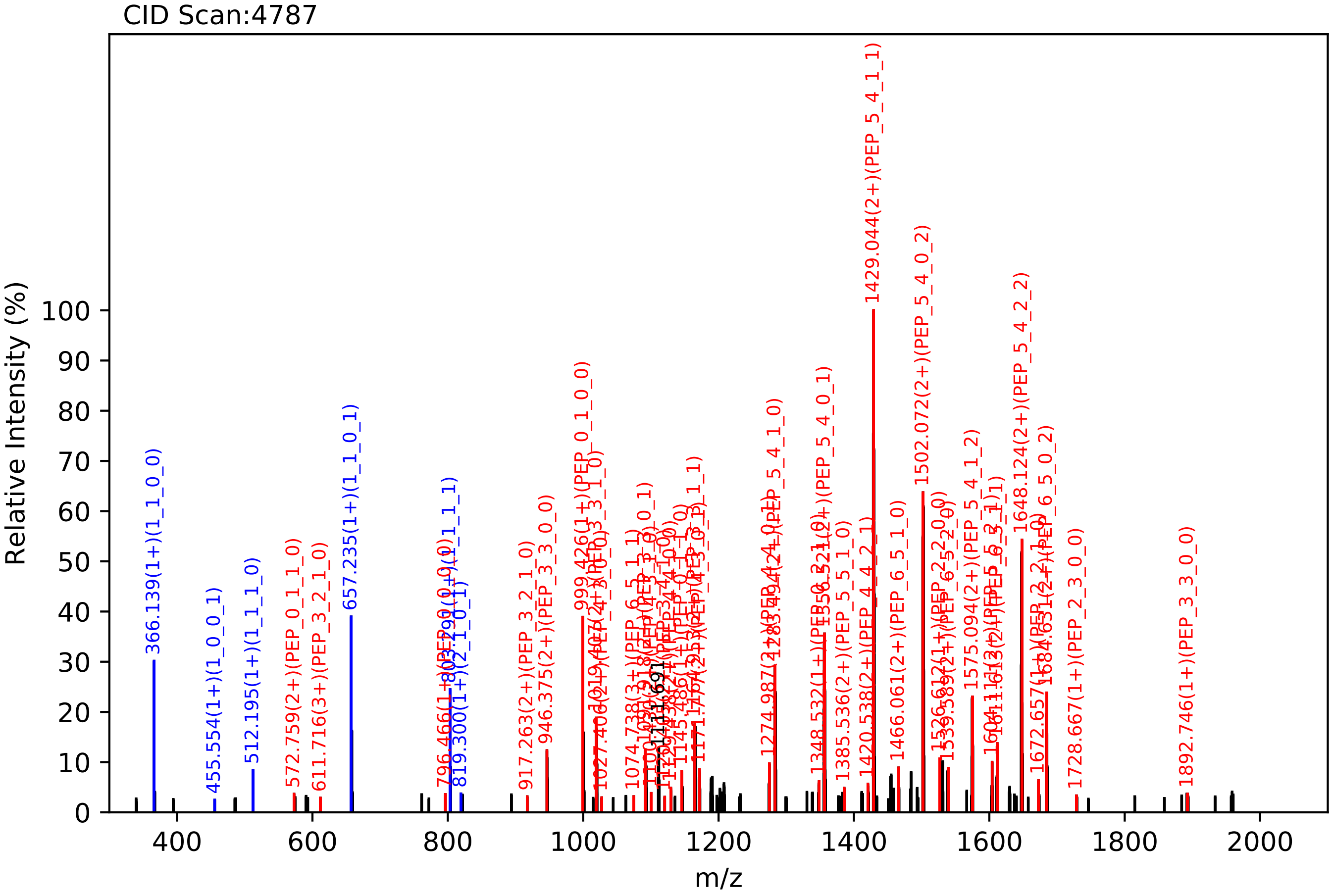

Test set no. 205, Experiment: AGP exp_38

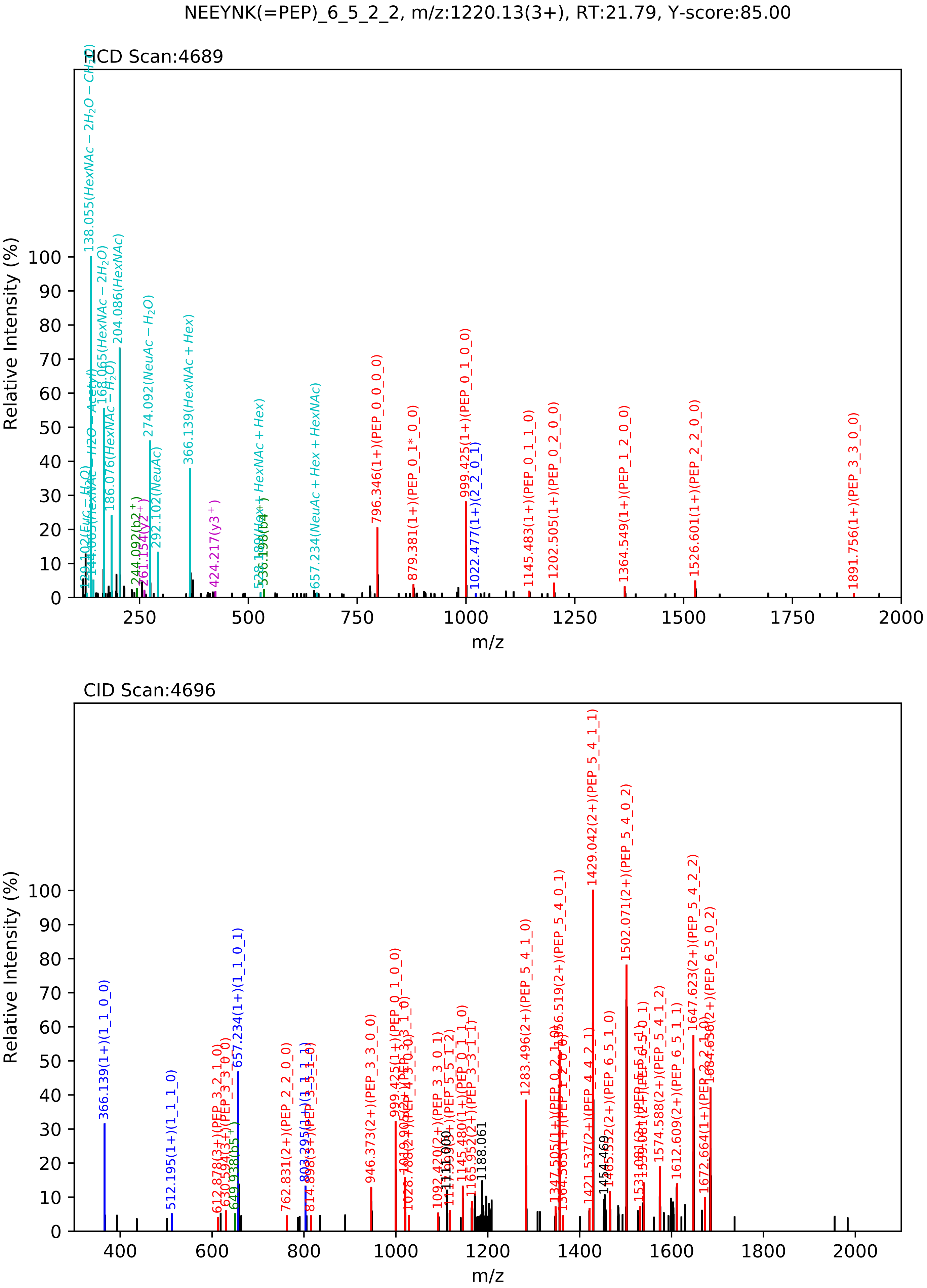

Test set no. 206, Experiment: AGP exp_28

NEEYNK(=PEP)_6_5_2_2, m/z:1220.13(3+), RT:22.26, Y-score:82.85

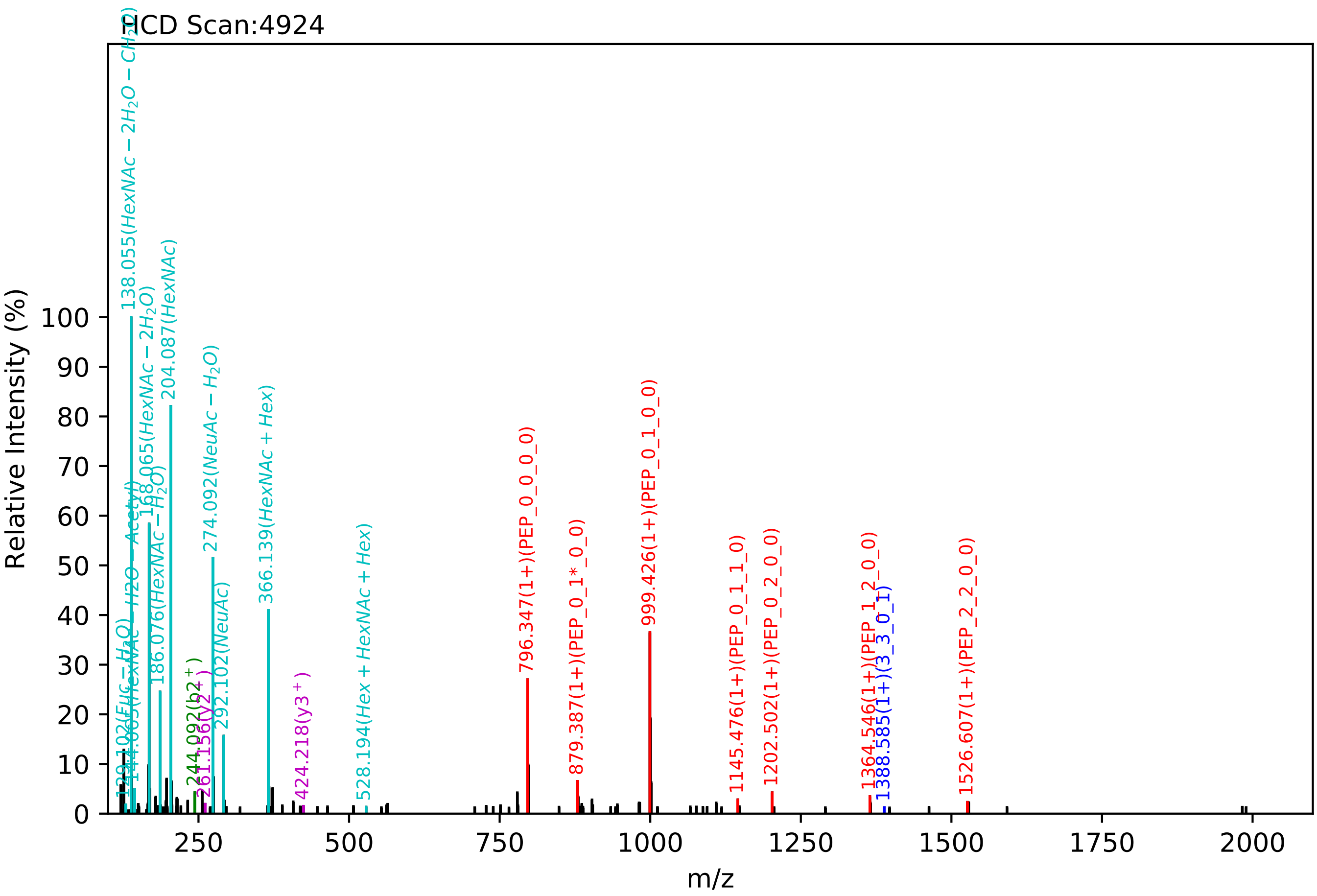

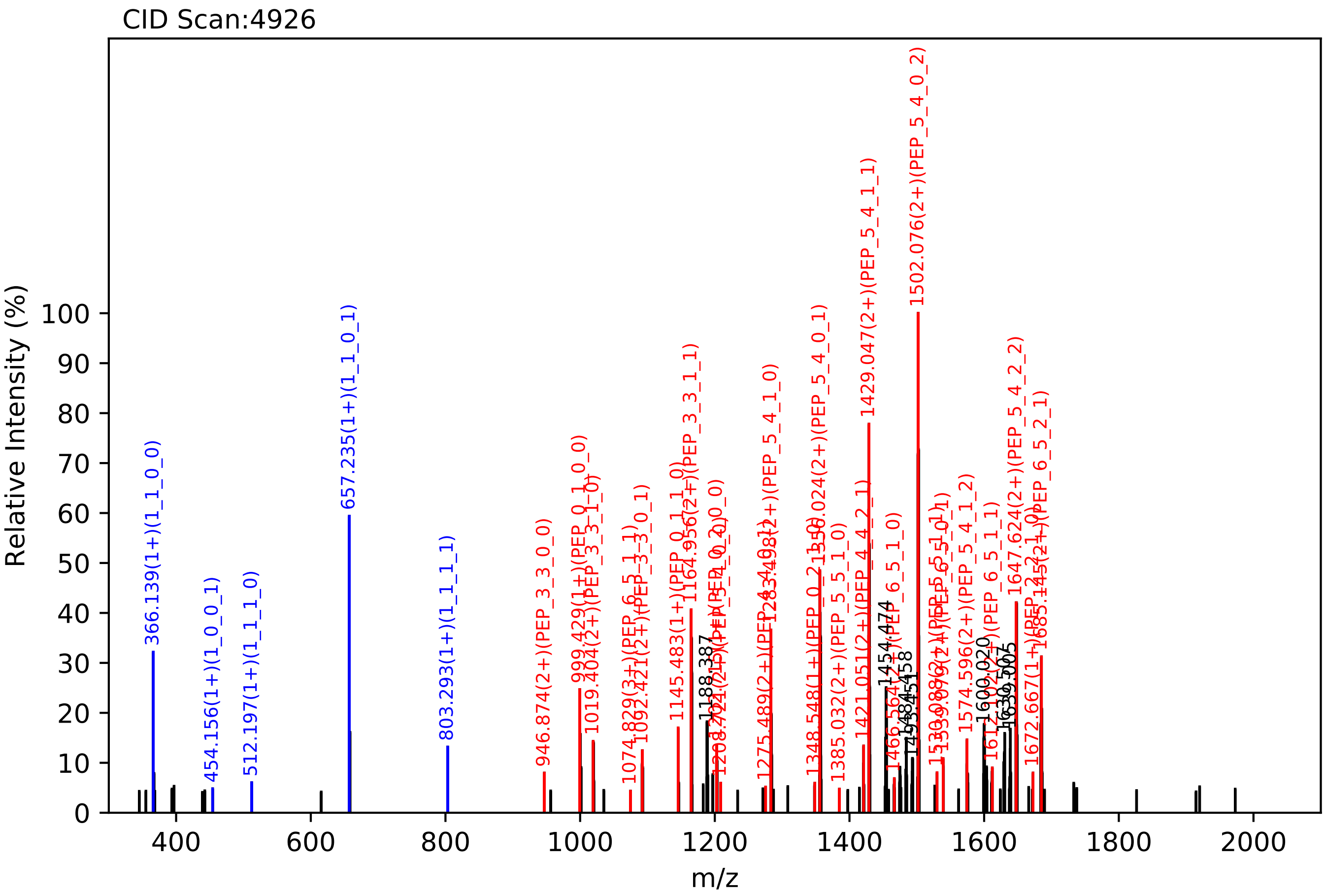

Test set no. 207, Experiment: AGP exp_16

NEEYNK(=PEP)_5_4_2_2, m/z:1220.13(3+), RT:24.66, Y-score:82.53

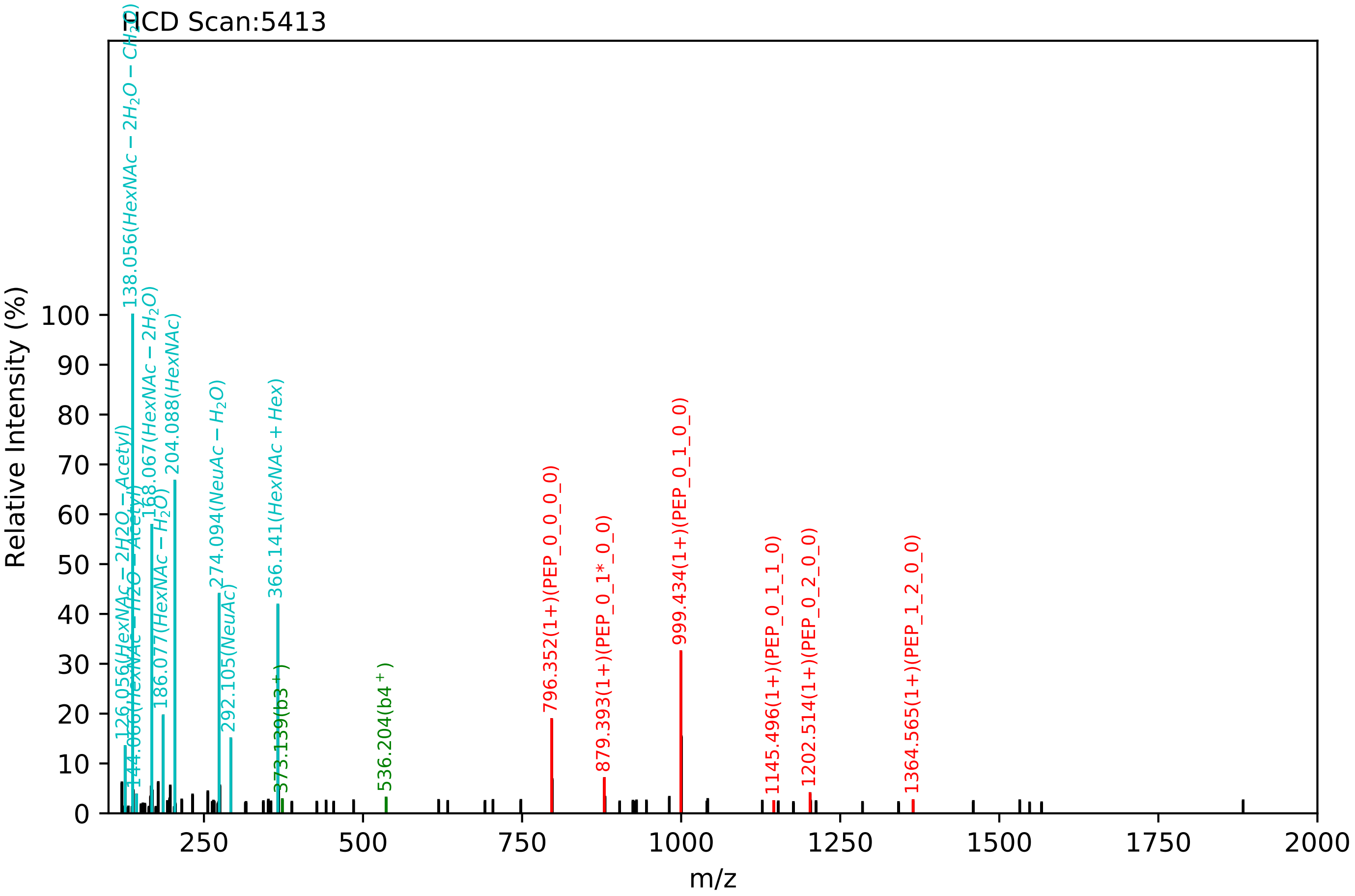

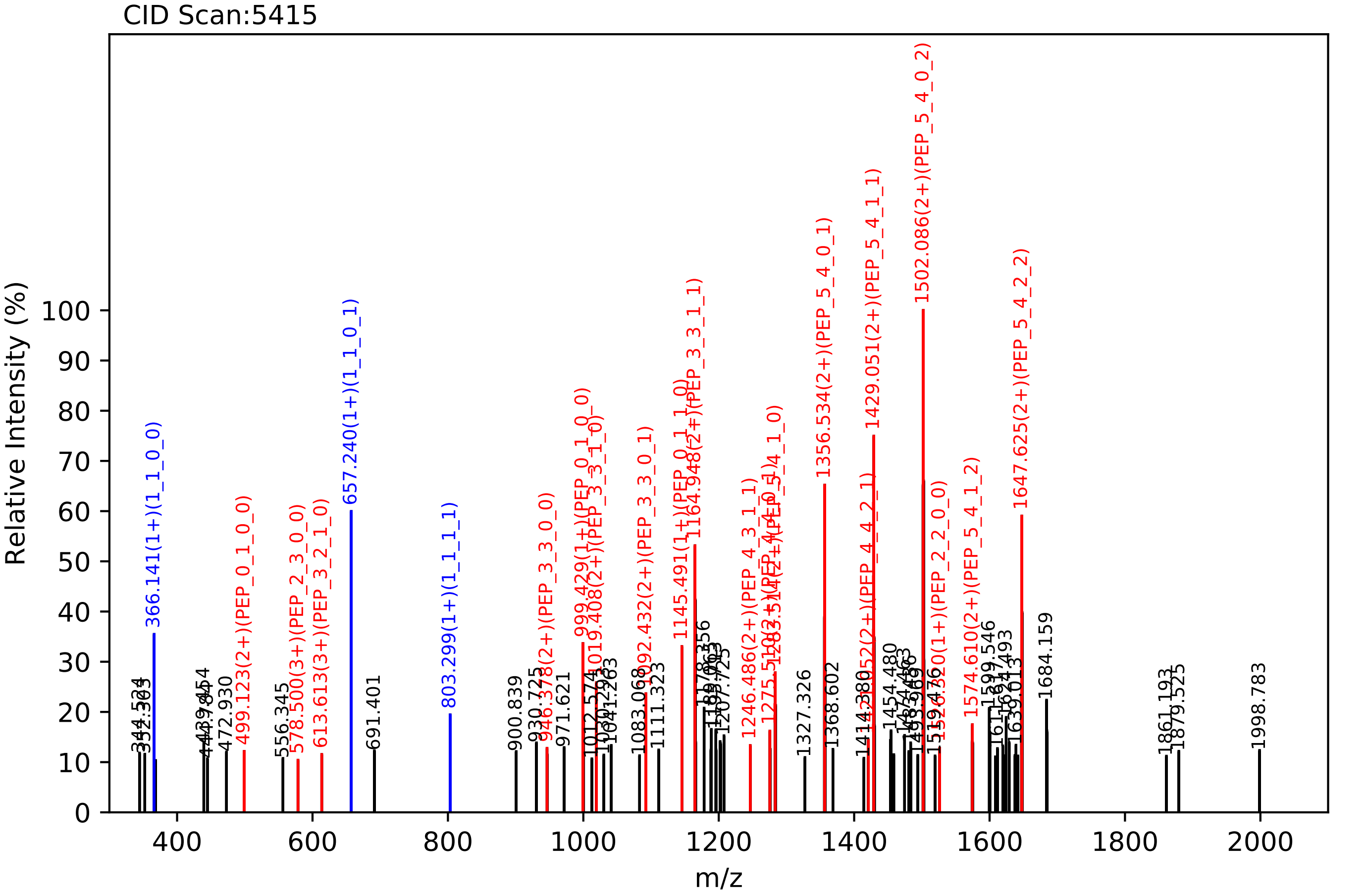

Test set no. 208, Experiment: AGP exp_40

NEEYNK(=PEP)_6_5_2_2, m/z:1220.13(3+), RT:21.58, Y-score:81.75

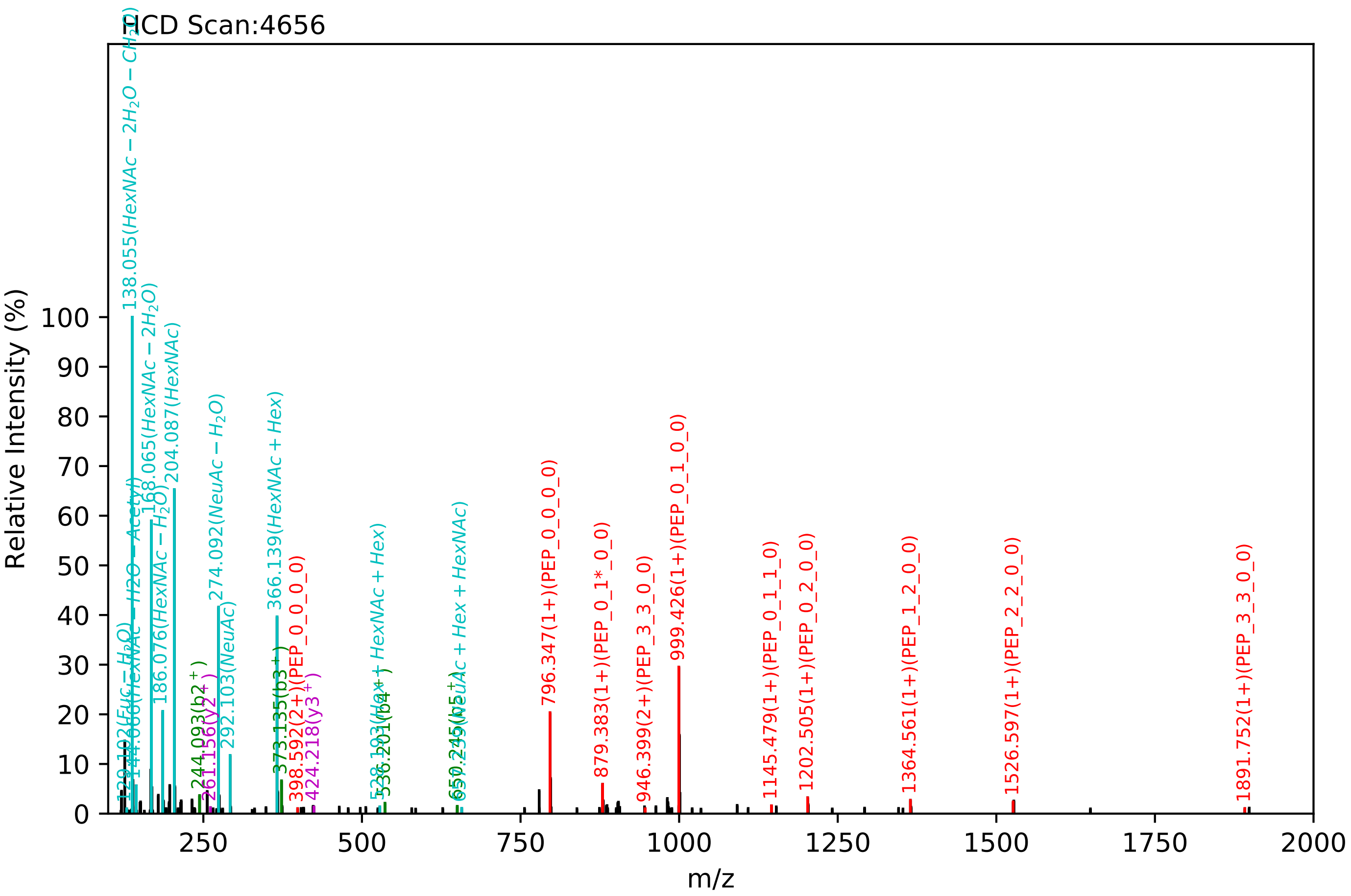

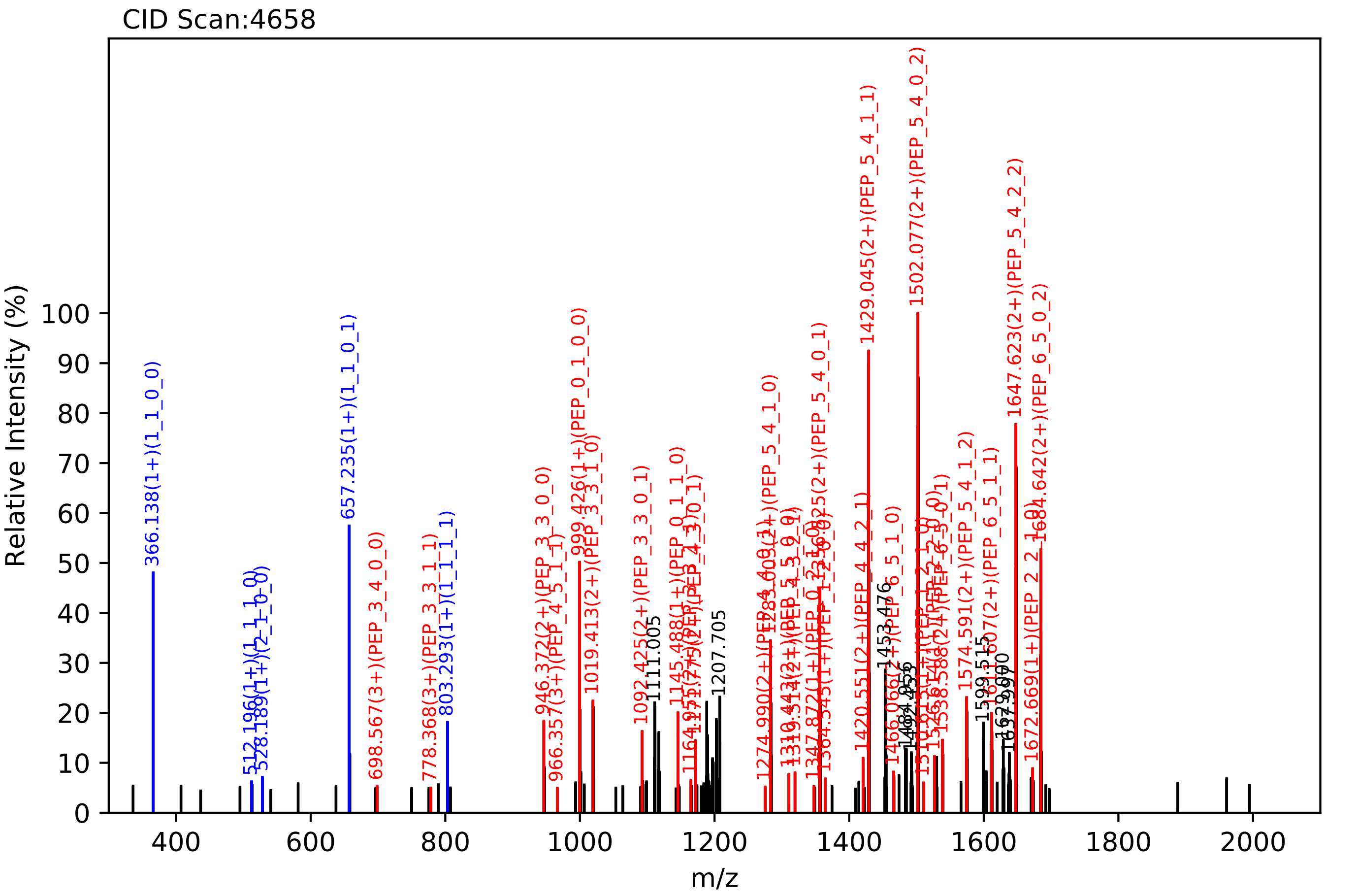

Test set no. 209, Experiment: AGP exp_13

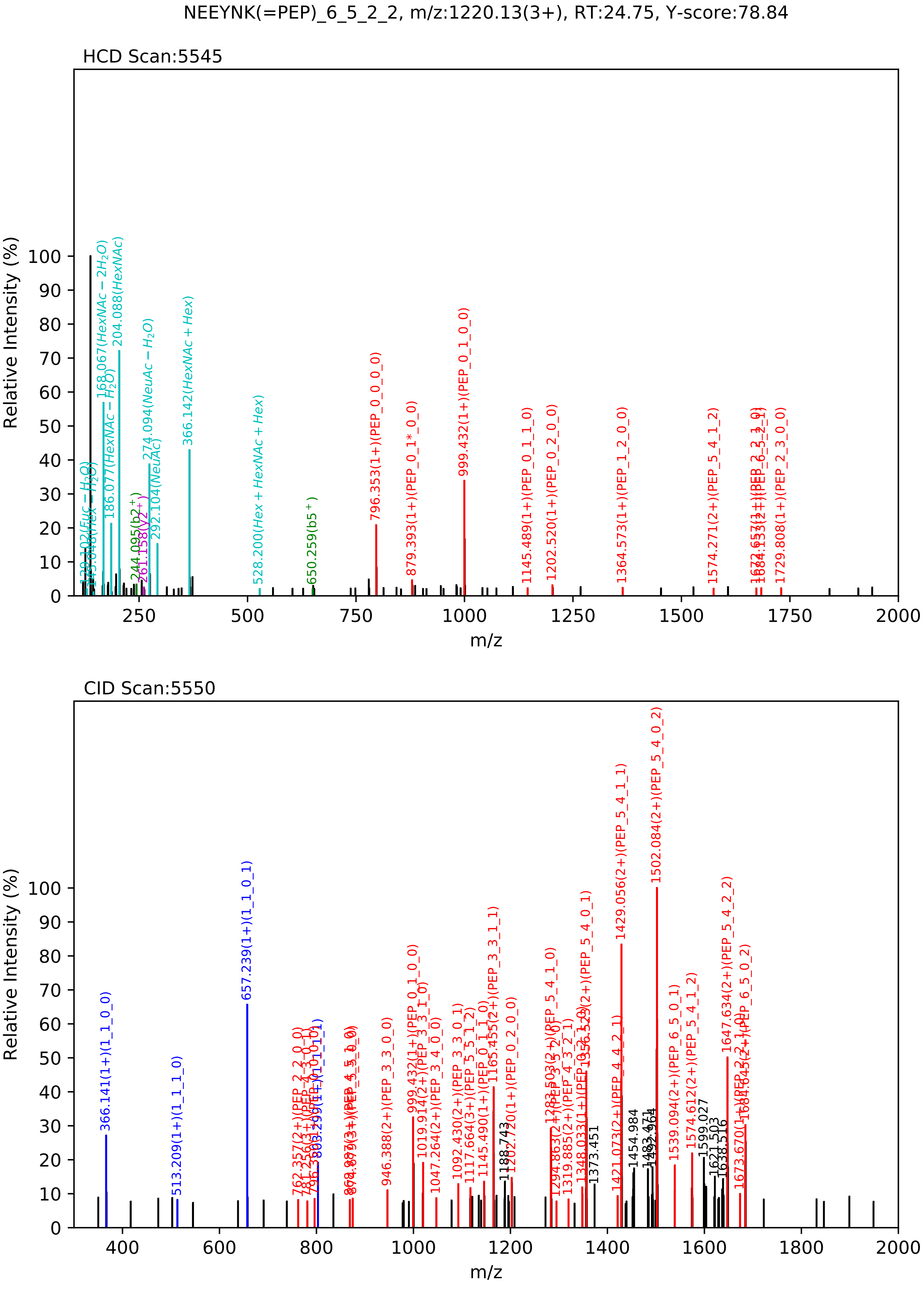

Test set no. 210, Experiment: AGP exp_26

NEEYNK(=PEP)_6_5_2_2, m/z:1220.13(3+), RT:22.08, Y-score:77.31

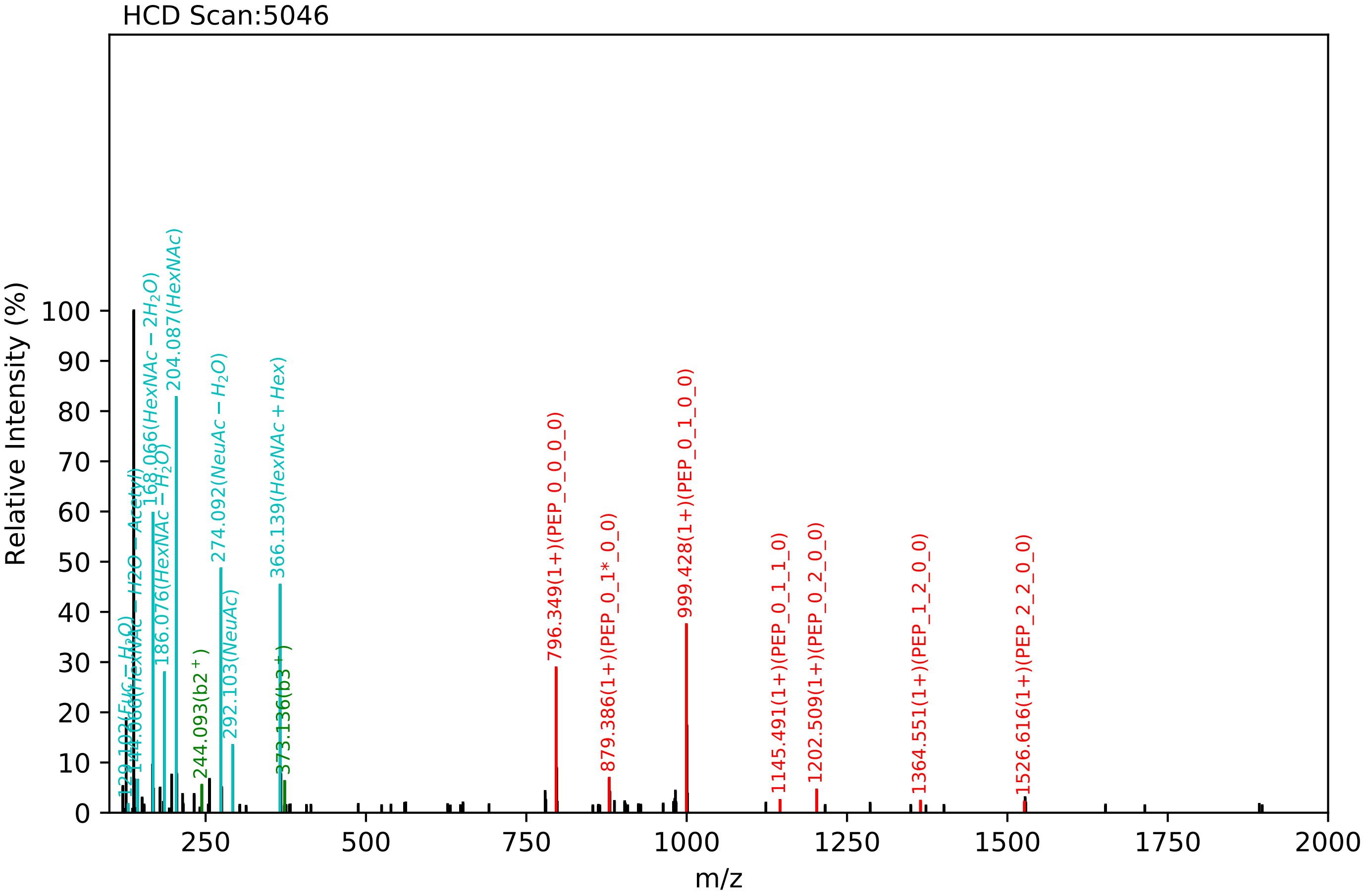

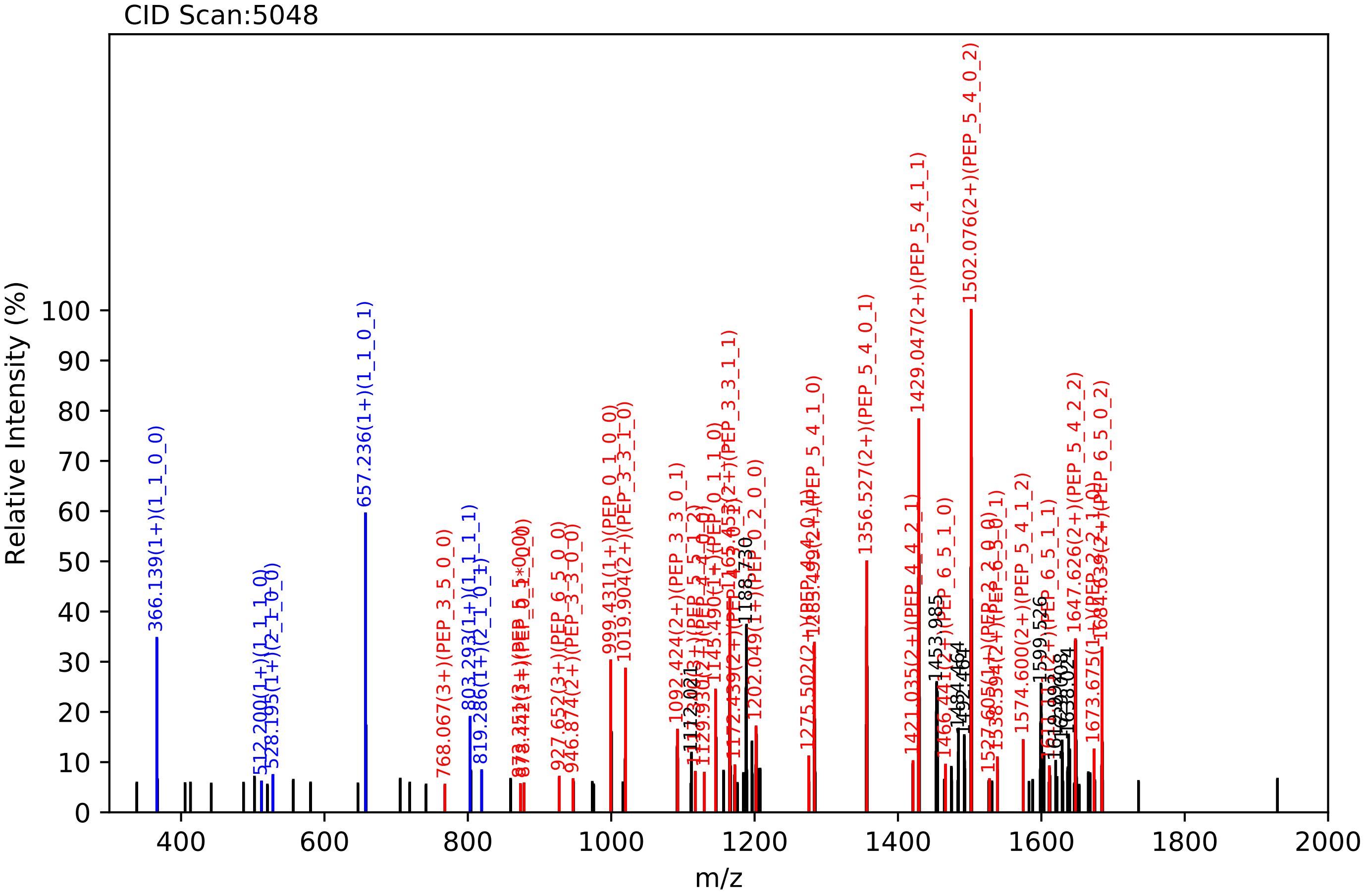

Test set no. 211, Experiment: AGP exp_40

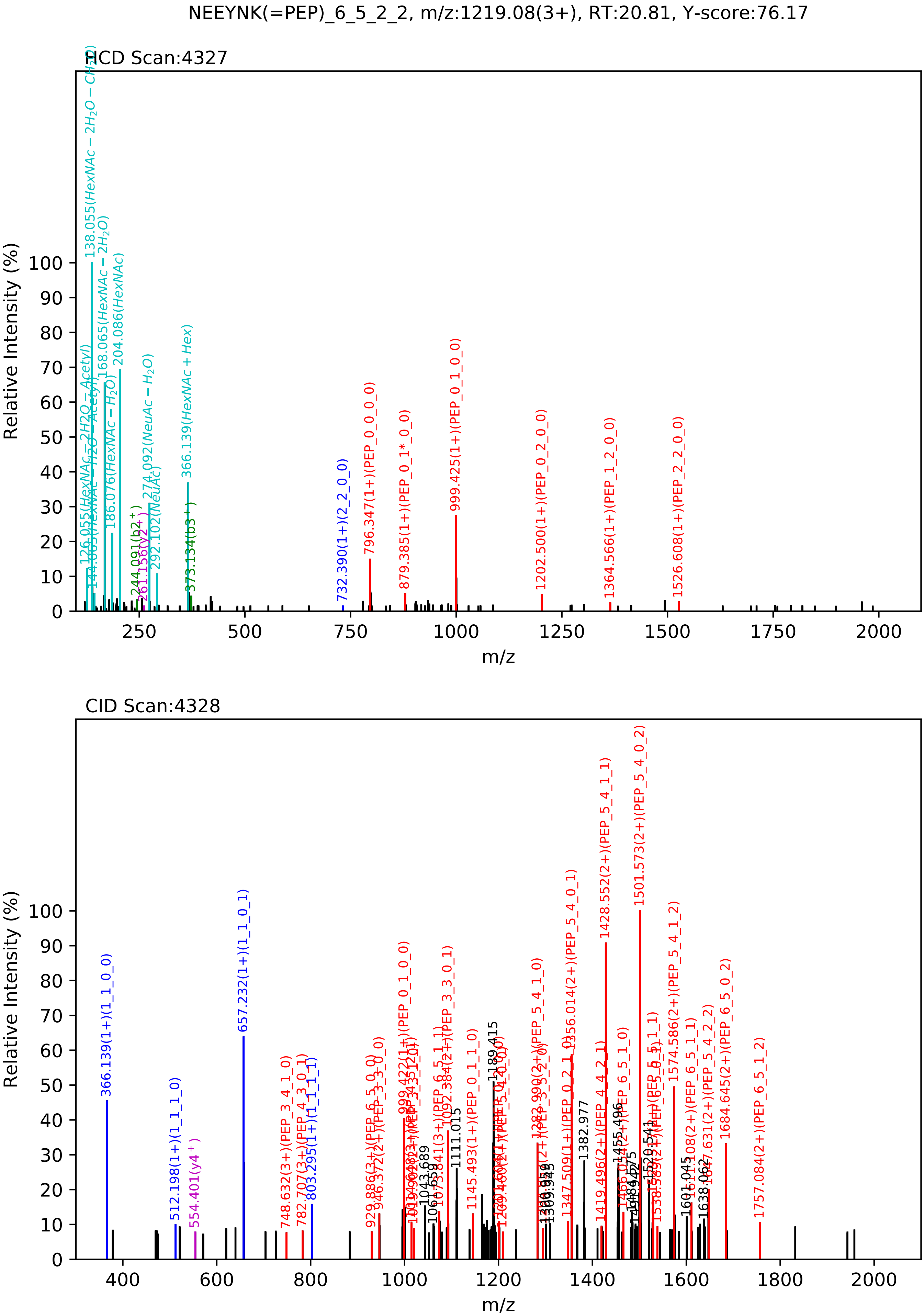

Test set no. 212, Experiment: AGP exp_43

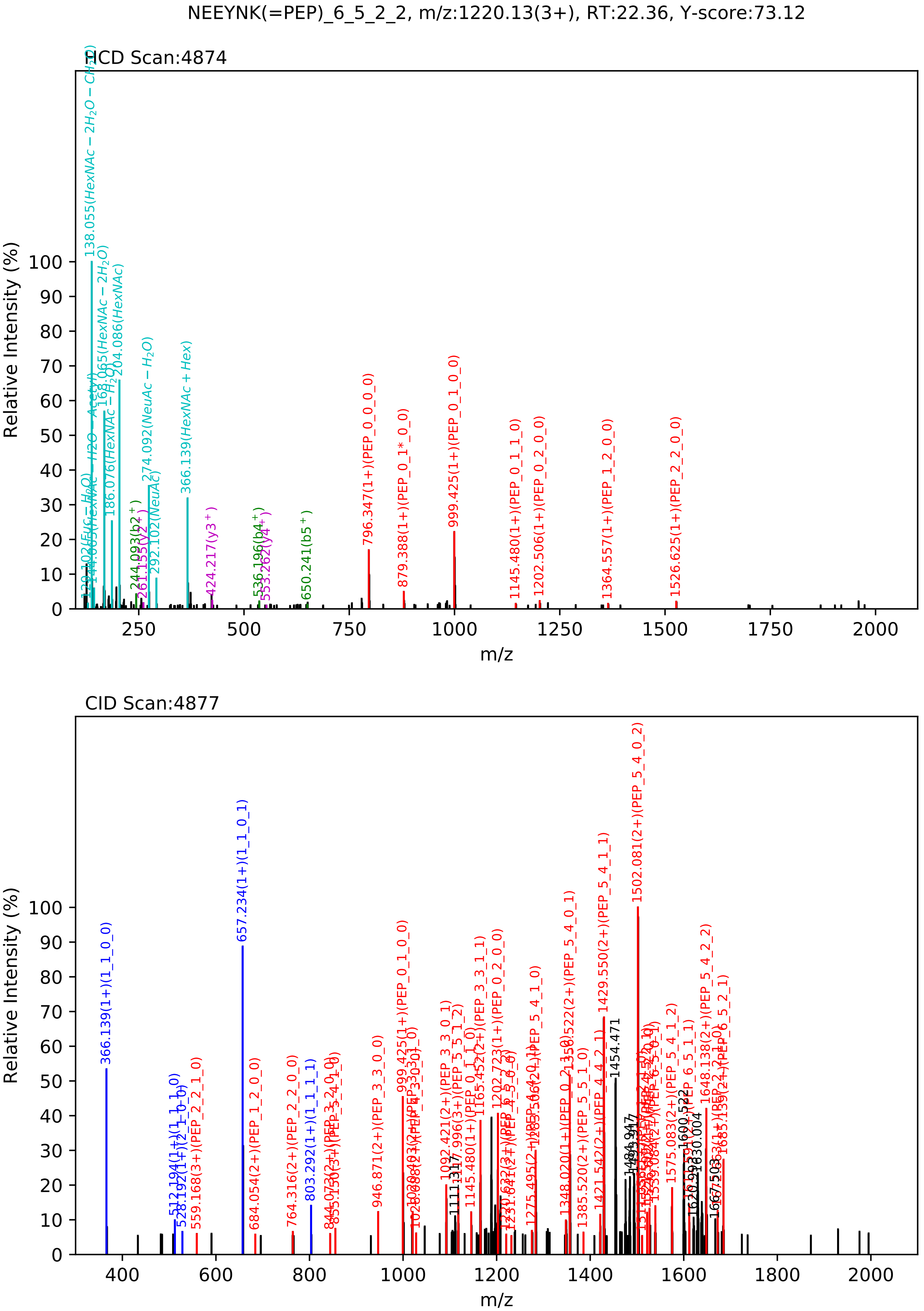

Test set no. 213, Experiment: AGP exp_16

NEEYNK(=PEP)_6_5_2_3, m/z:1317.16(3+), RT:36.71, Y-score:90.81

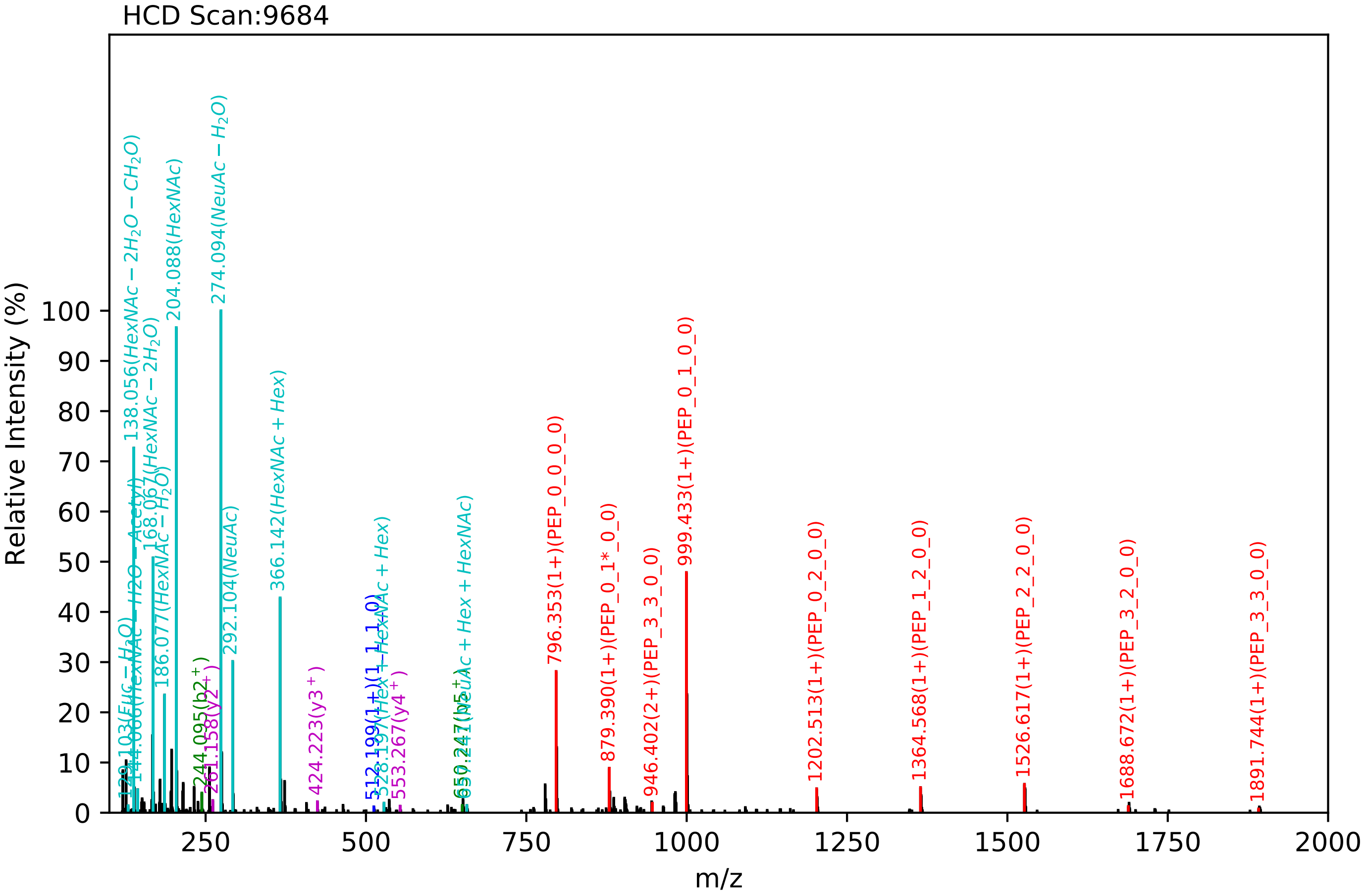

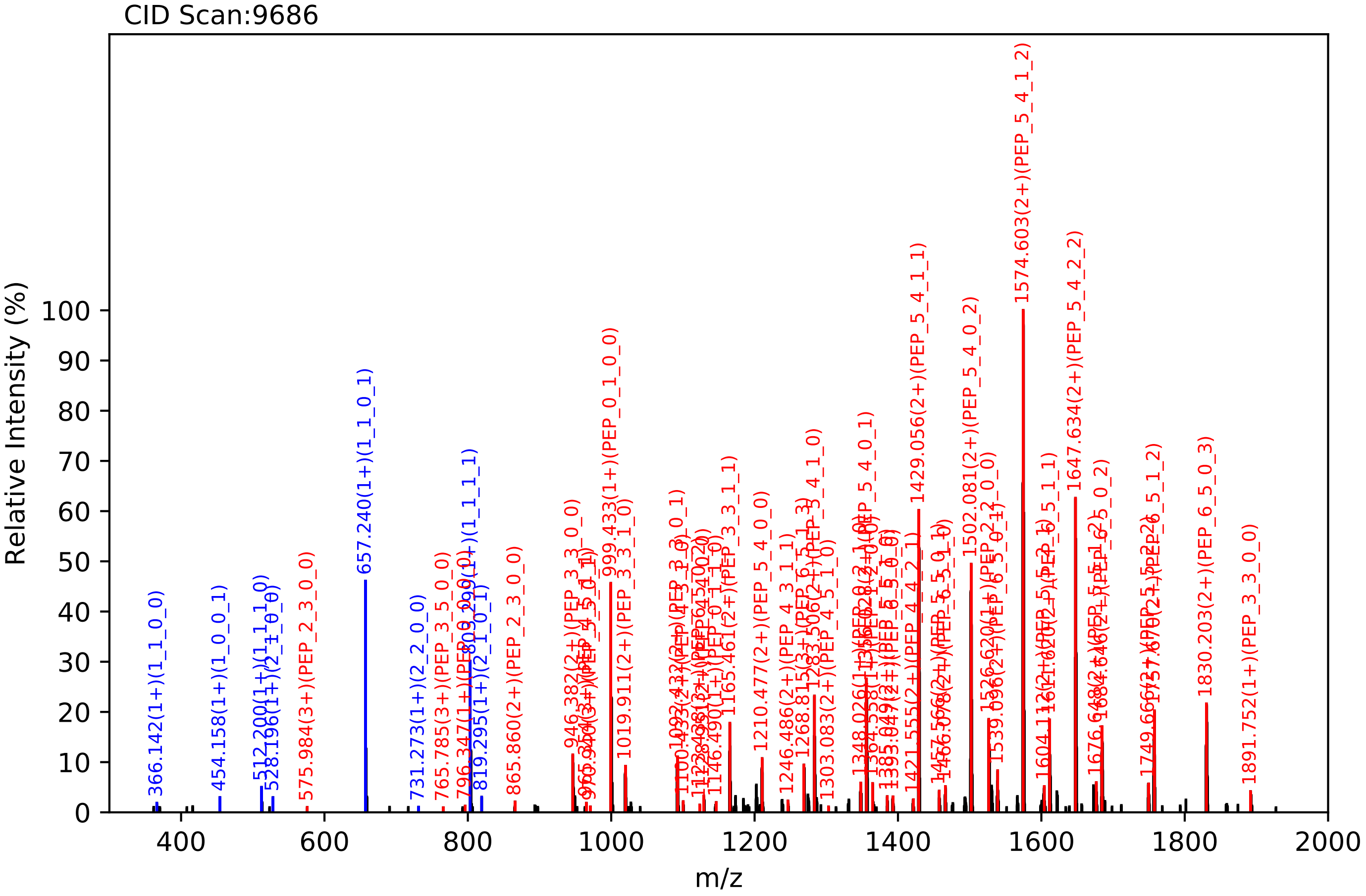

Test set no. 214, Experiment: AGP exp_10

NEEYNK(=PEP)_6_5_2_3, m/z:1317.16(3+), RT:30.49, Y-score:90.67

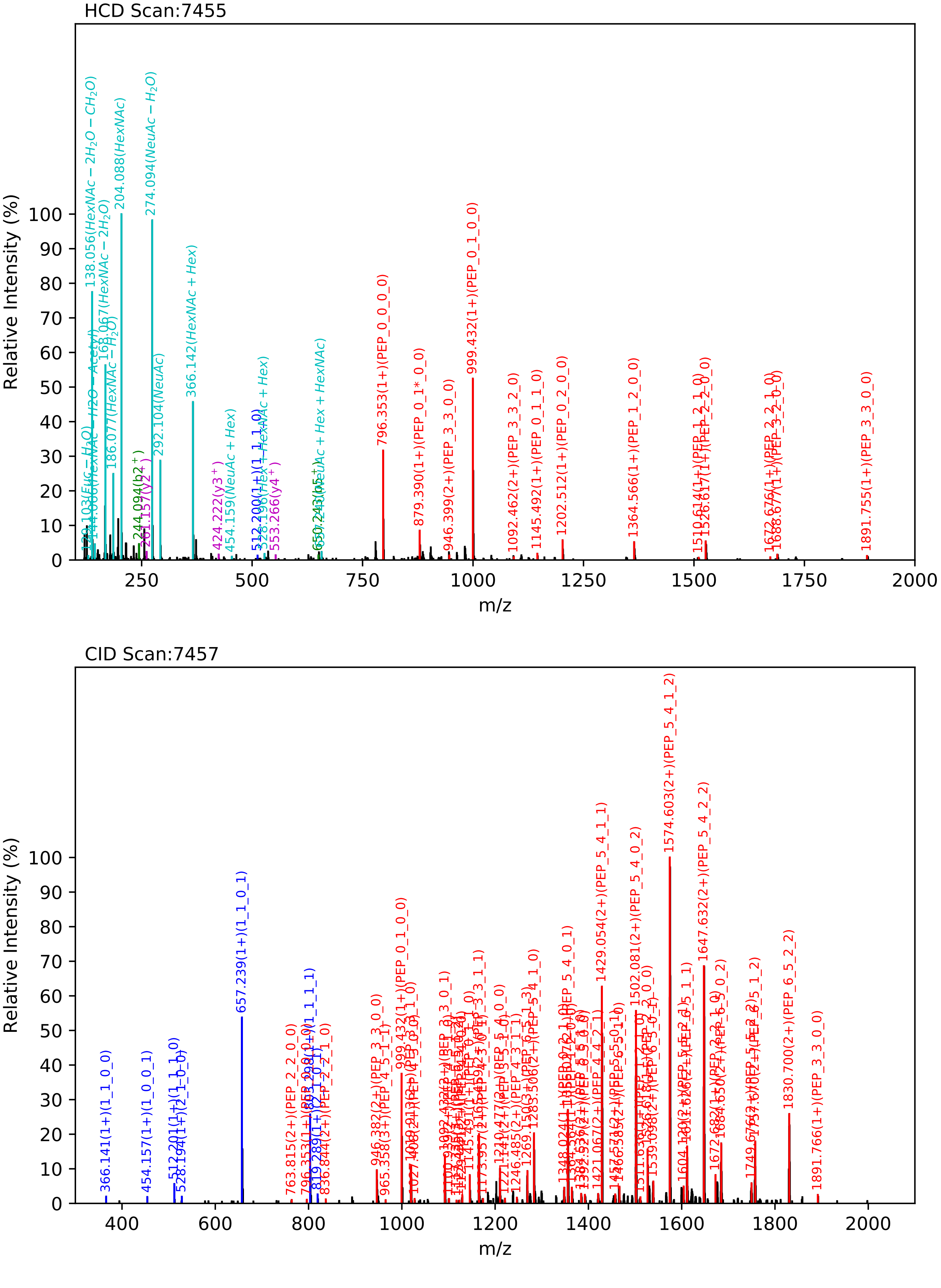

Test set no. 215, Experiment: AGP exp_15

NEEYNK(=PEP)_6_5_2_3, m/z:1317.16(3+), RT:36.71, Y-score:90.49

HCD Scan:9512

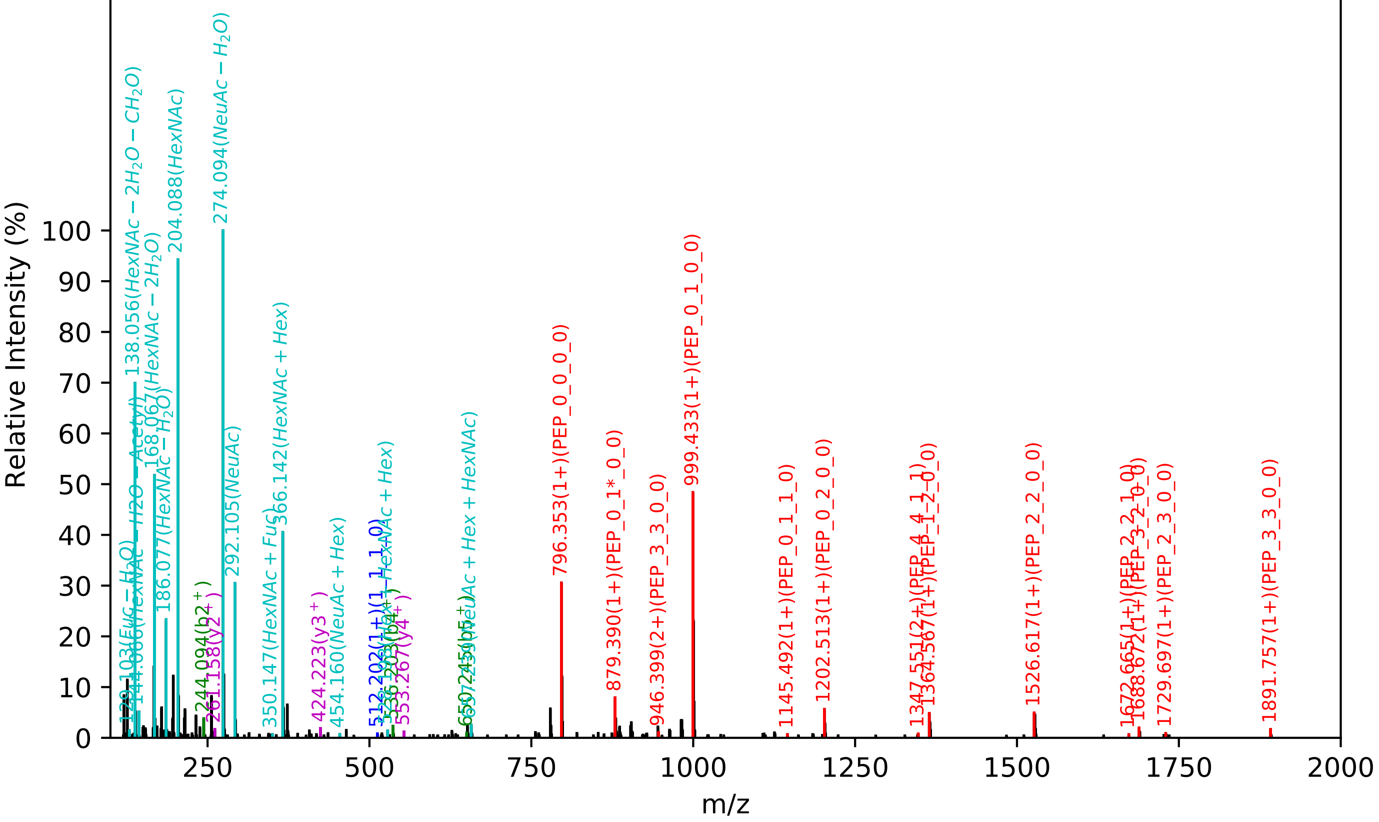

CID Scan:9515

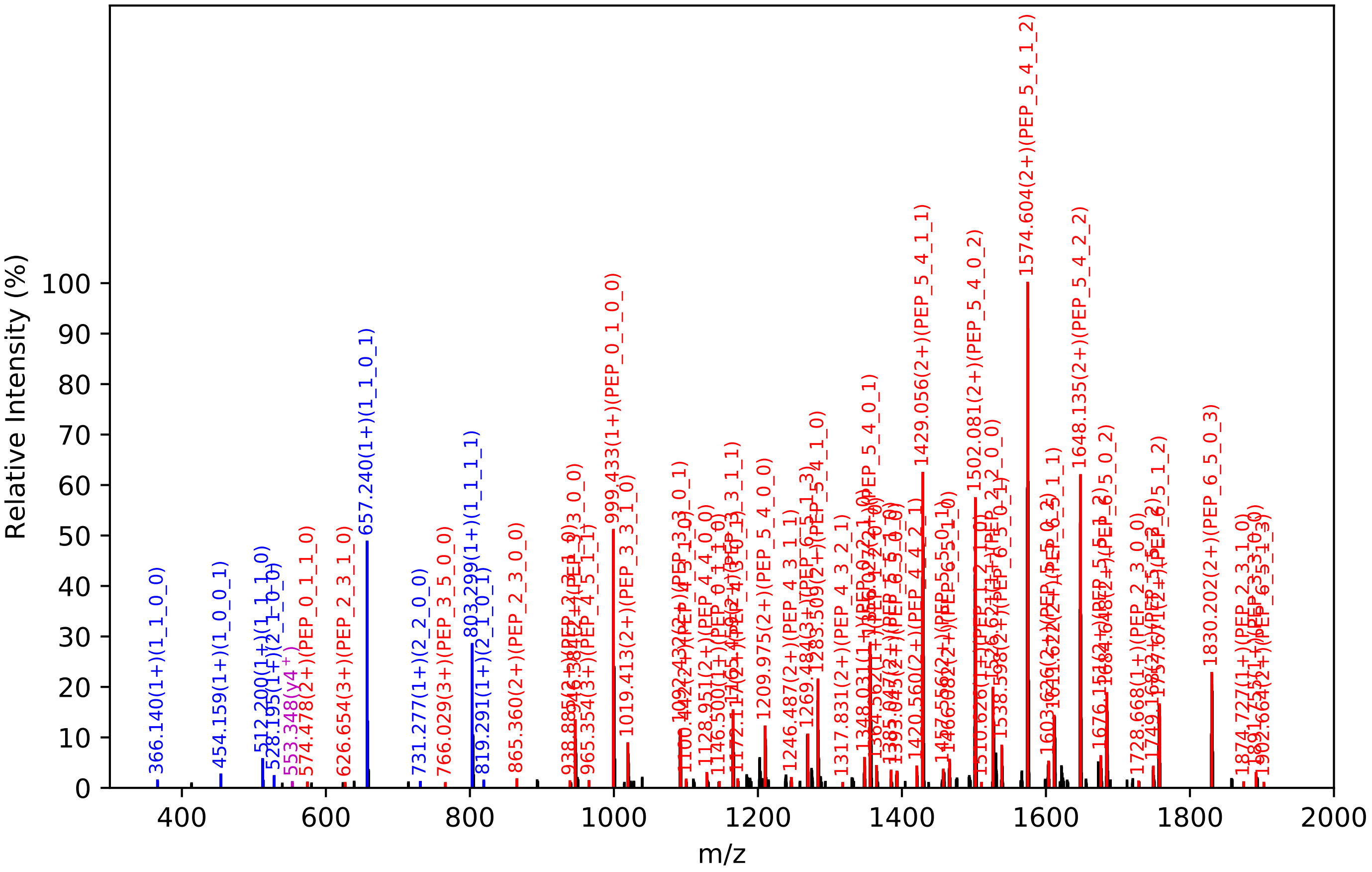

Test set no. 216, Experiment: AGP exp_43

NEEYNK(=PEP)_6_5_2_3, m/z:1317.16(3+), RT:31.35, Y-score:89.42

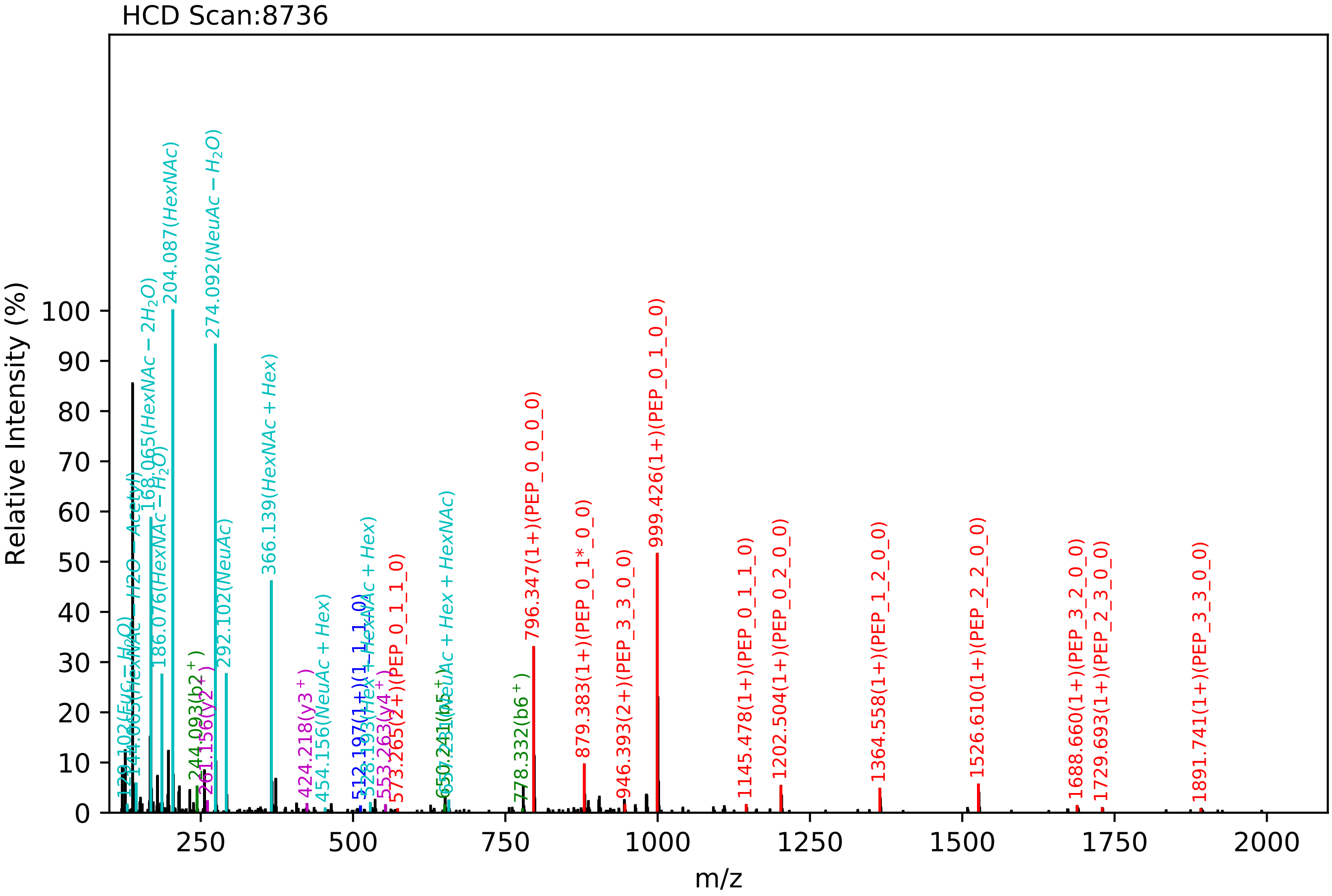

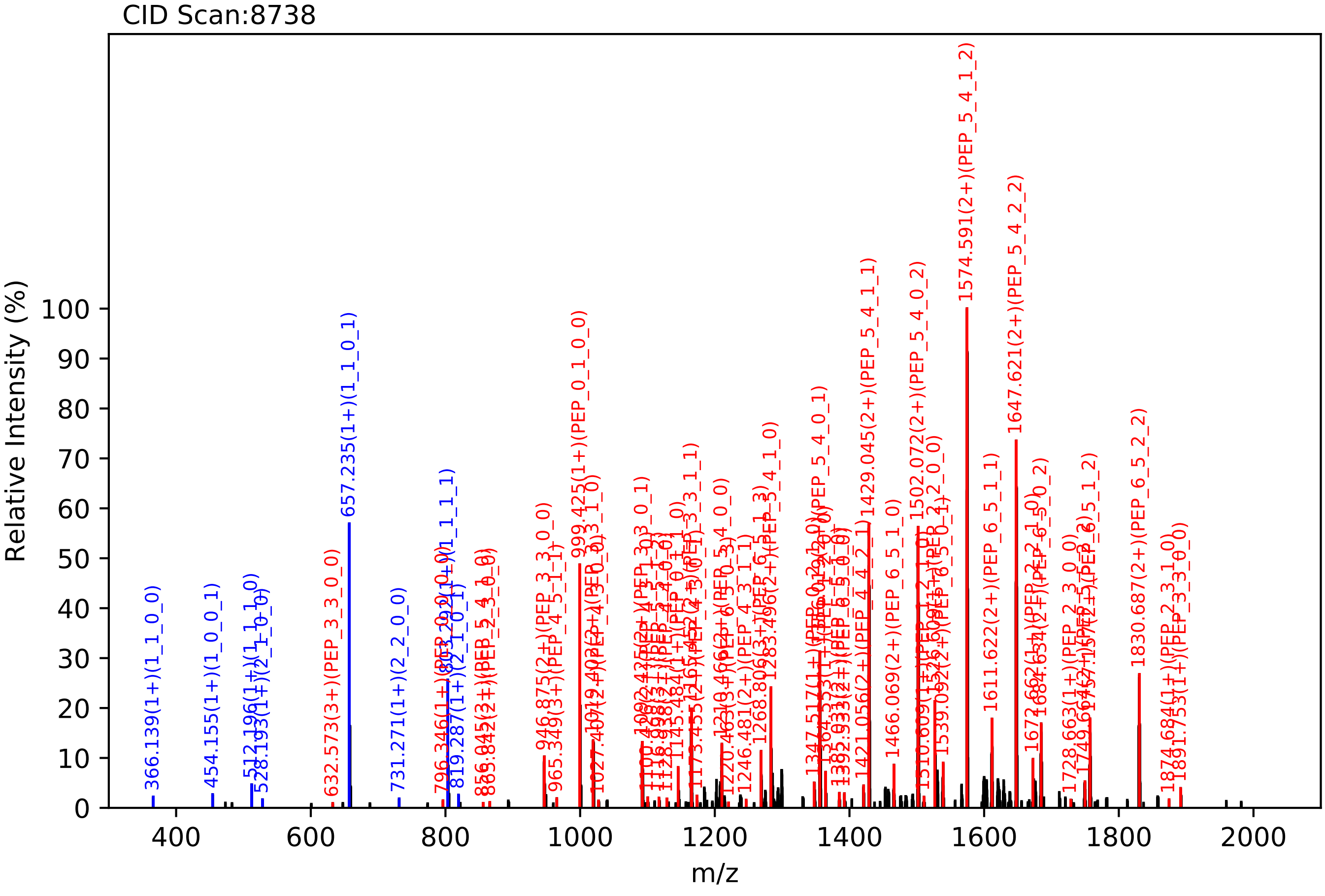

Test set no. 217, Experiment: AGP exp_39

NEEYNK(=PEP)_6_5_2_3, m/z:1317.16(3+), RT:31.22, Y-score:89.24

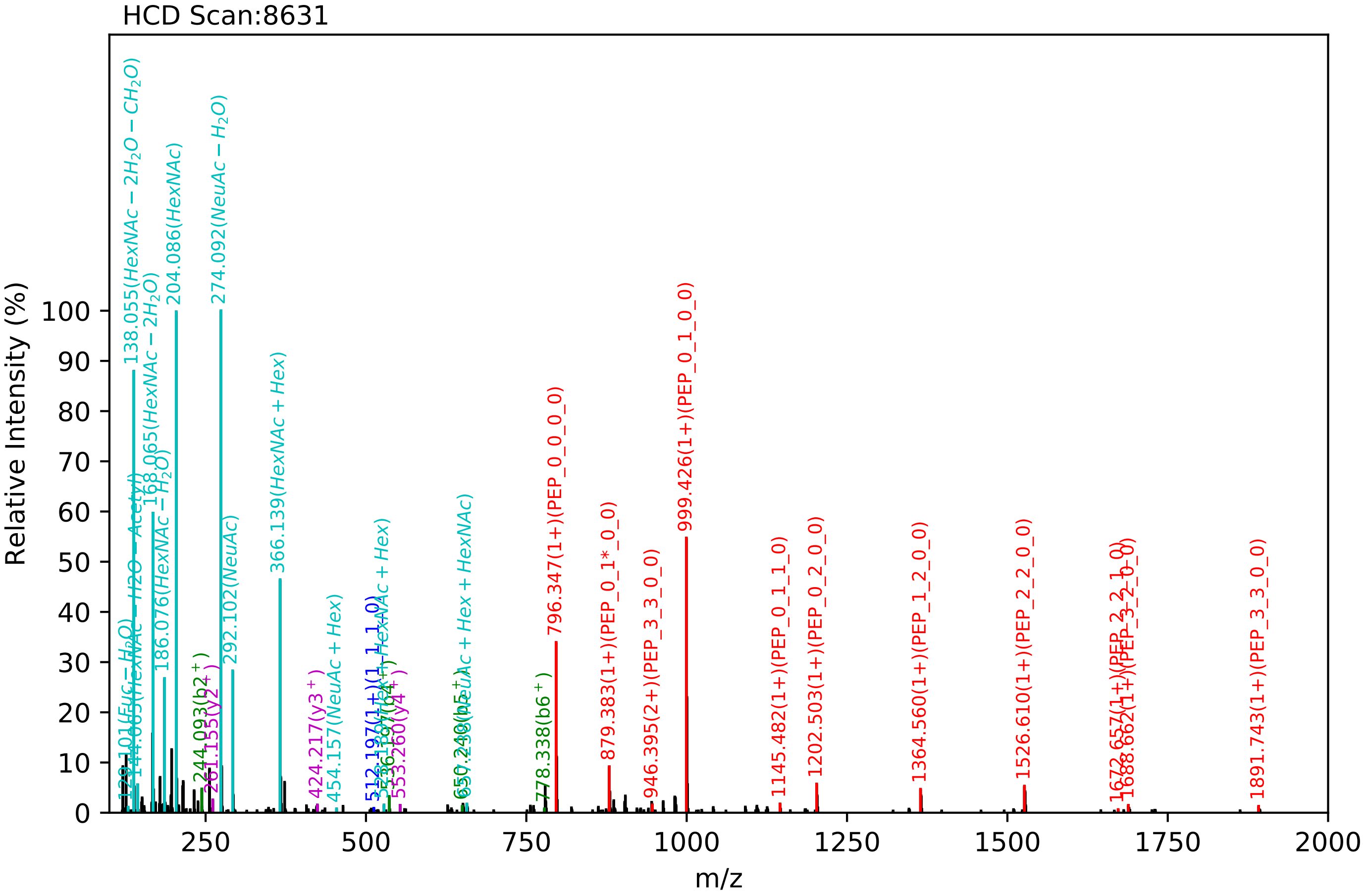

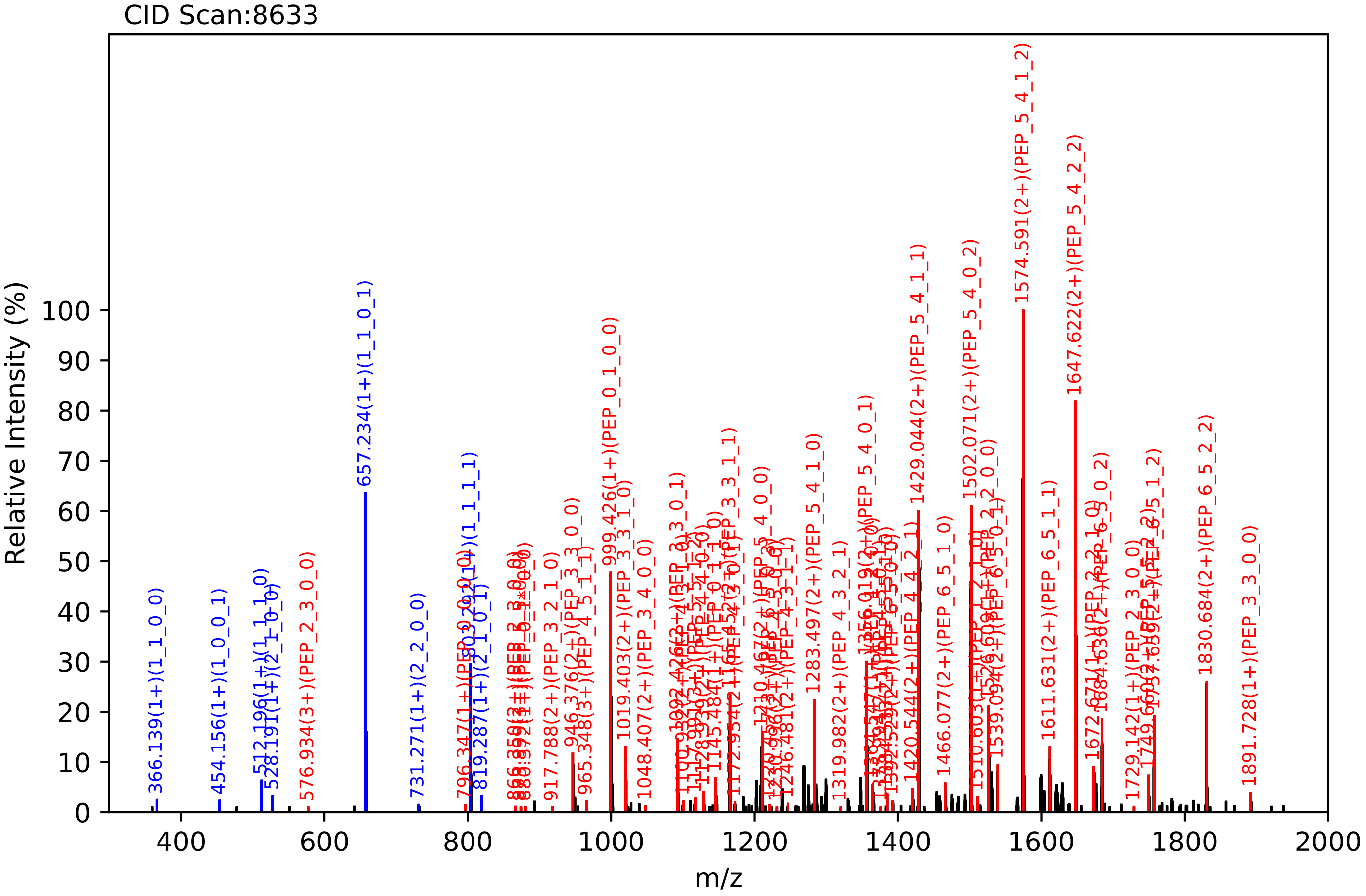

Test set no. 218, Experiment: AGP exp_30

NEEYNK(=PEP)_6_5_2_3, m/z:1317.16(3+), RT:30.94, Y-score:89.11

HCD Scan:3879

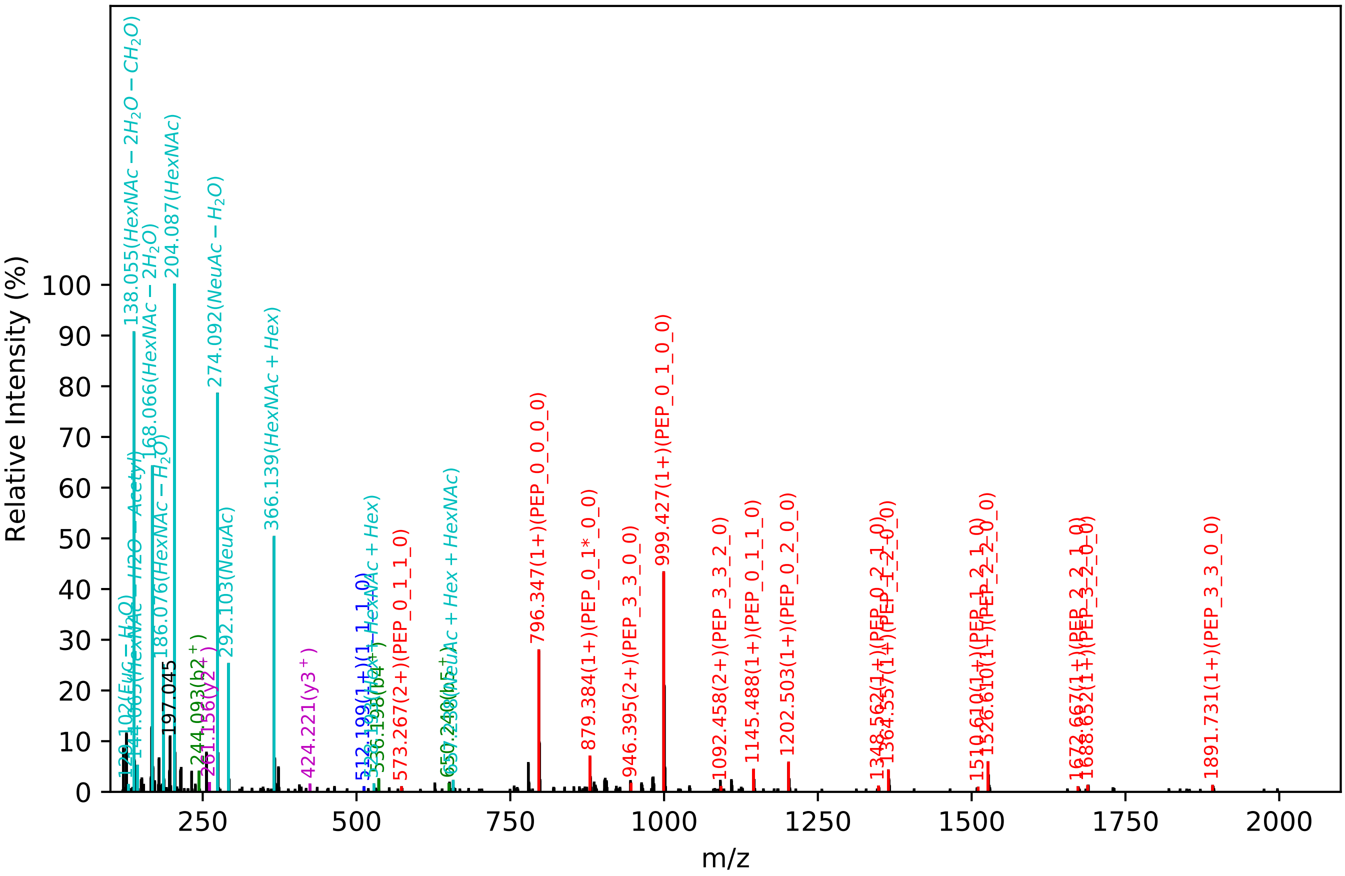

CID Scan:3882

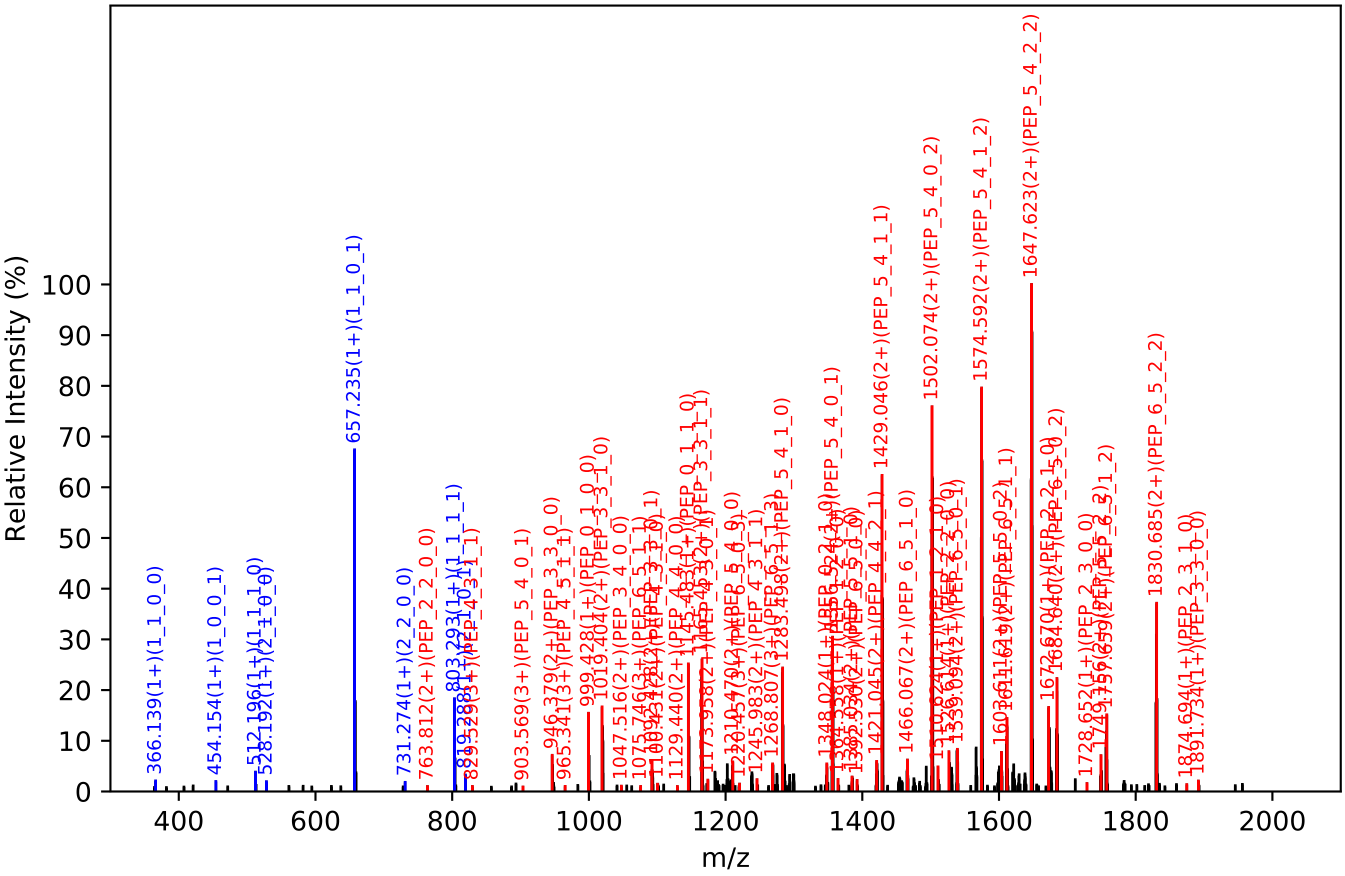

Test set no. 219, Experiment: AGP exp_27

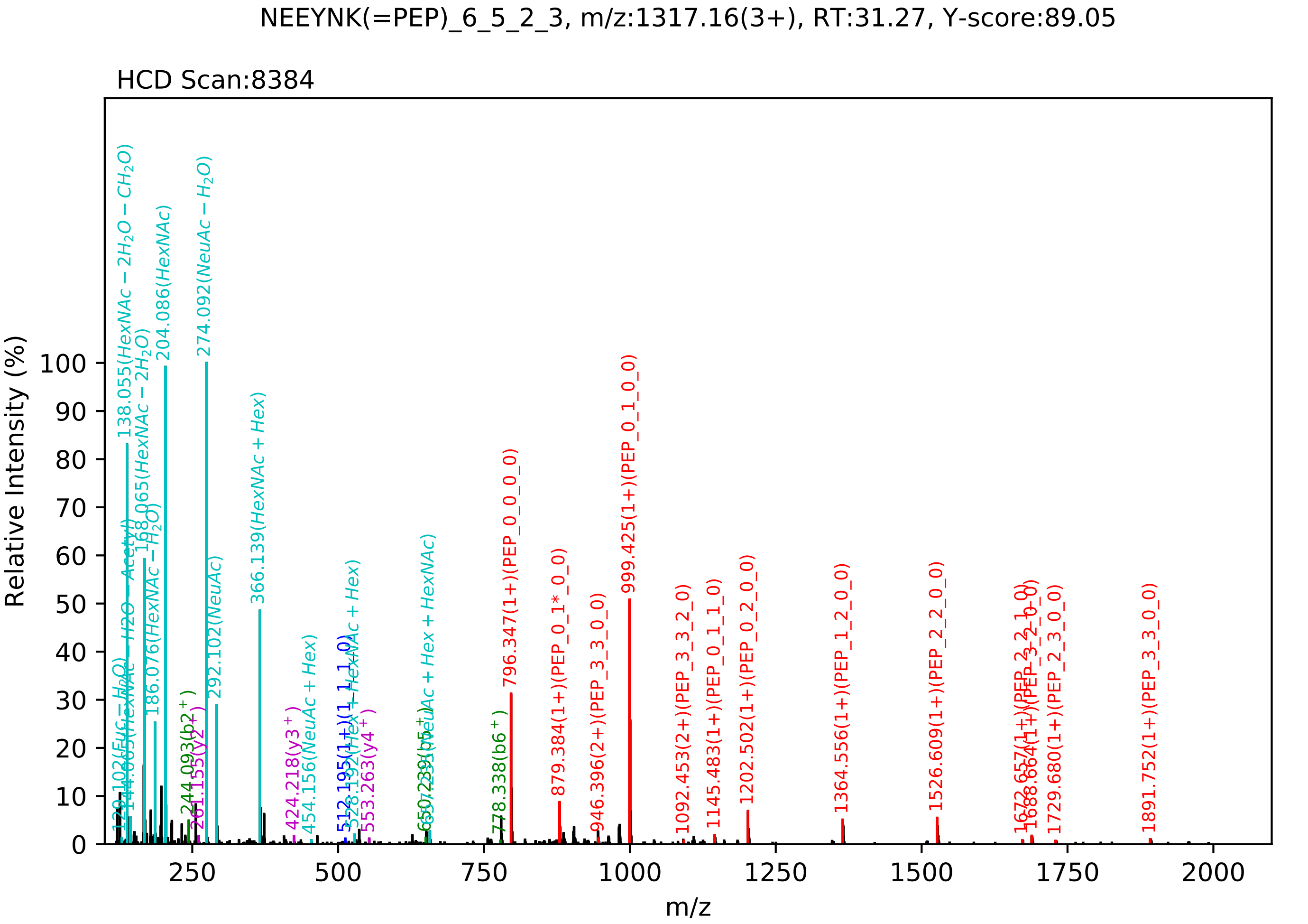

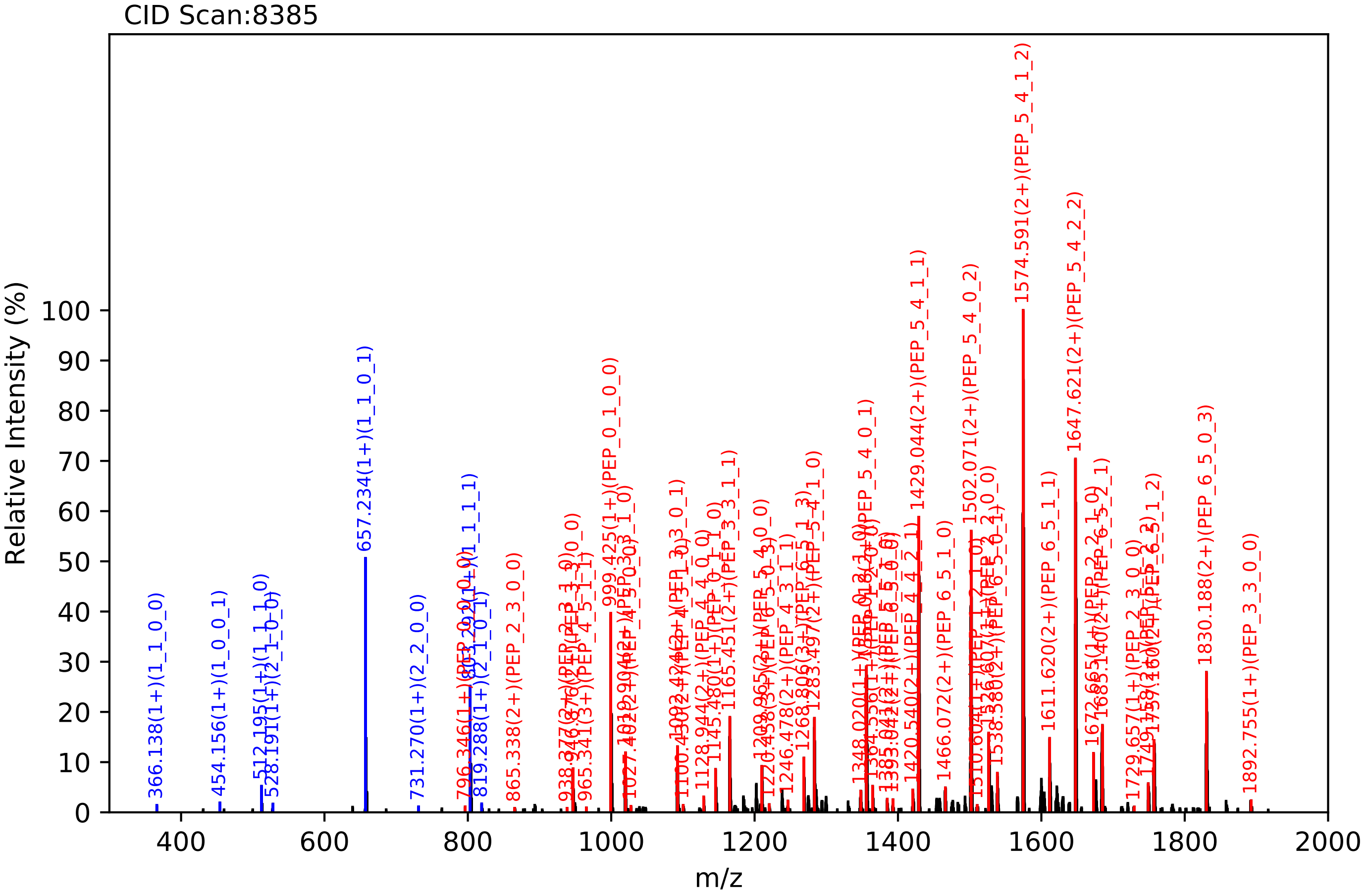

Test set no. 220, Experiment: AGP exp_22

NEEYNK(=PEP)_6_5_2_3, m/z:1317.49(3+), RT:30.64, Y-score:88.89

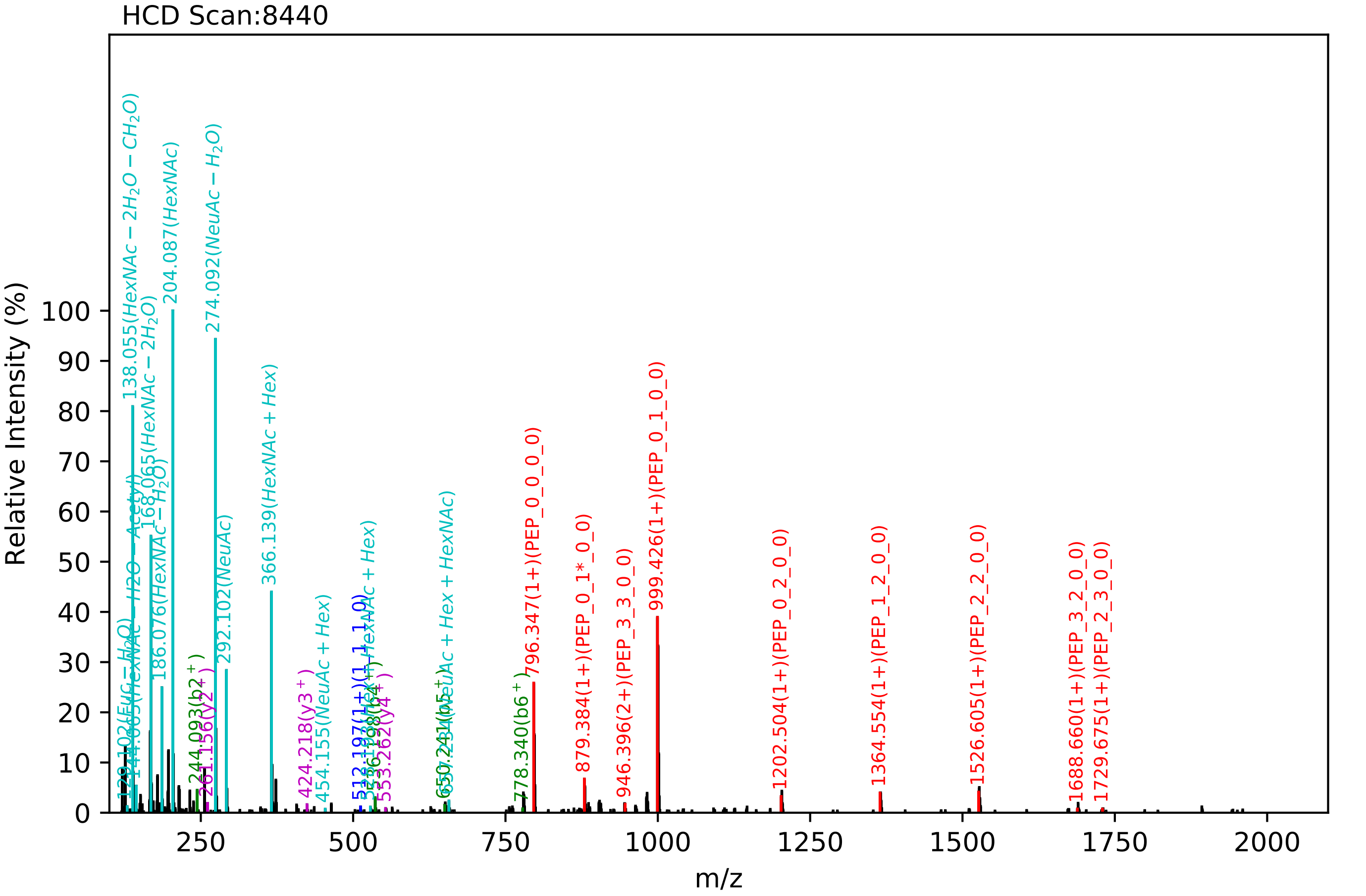

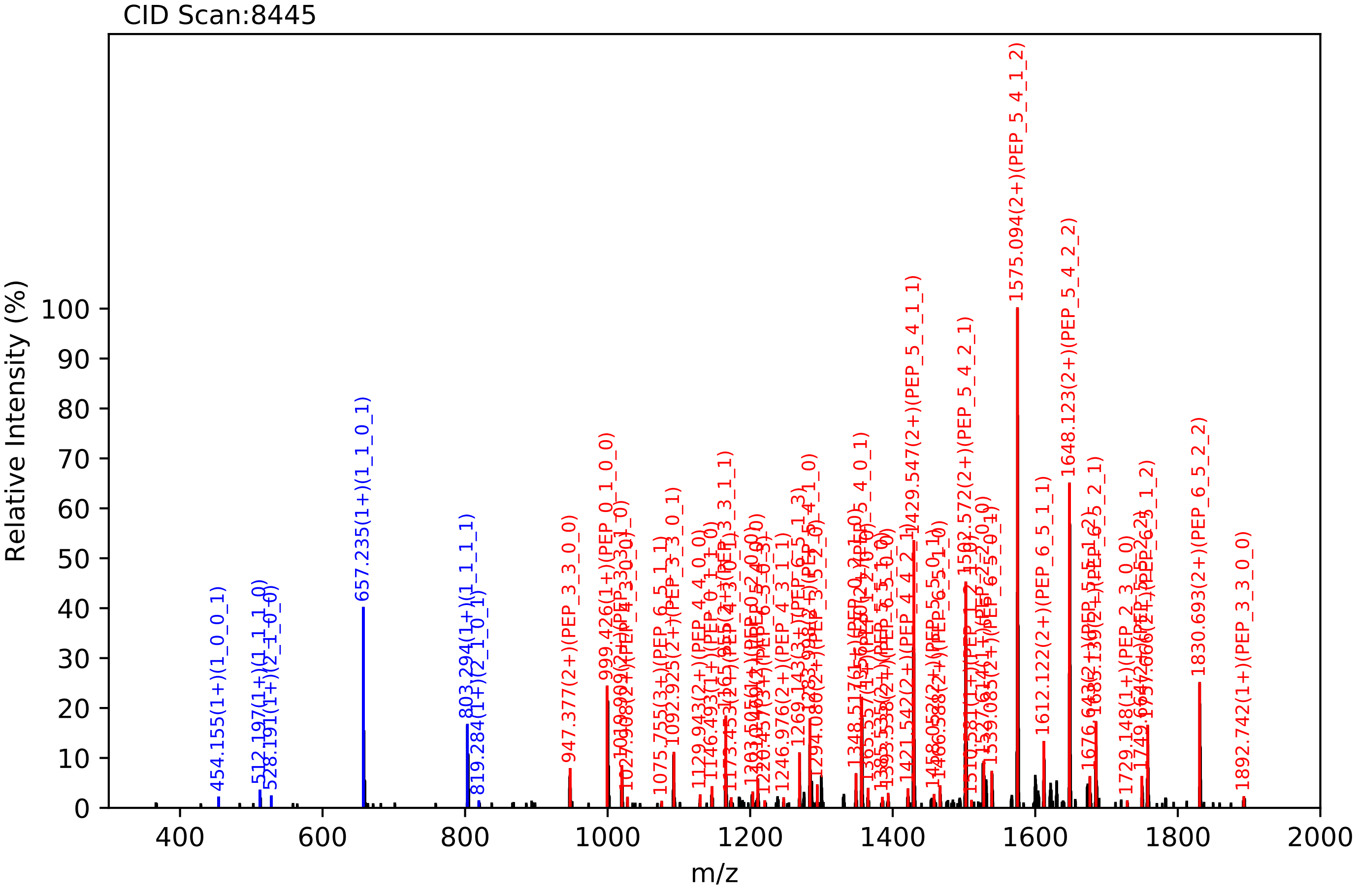

Test set no. 221, Experiment: AGP exp_35

NEEYNK(=PEP)_6_5_2_3, m/z:1317.16(3+), RT:30.96, Y-score:88.80

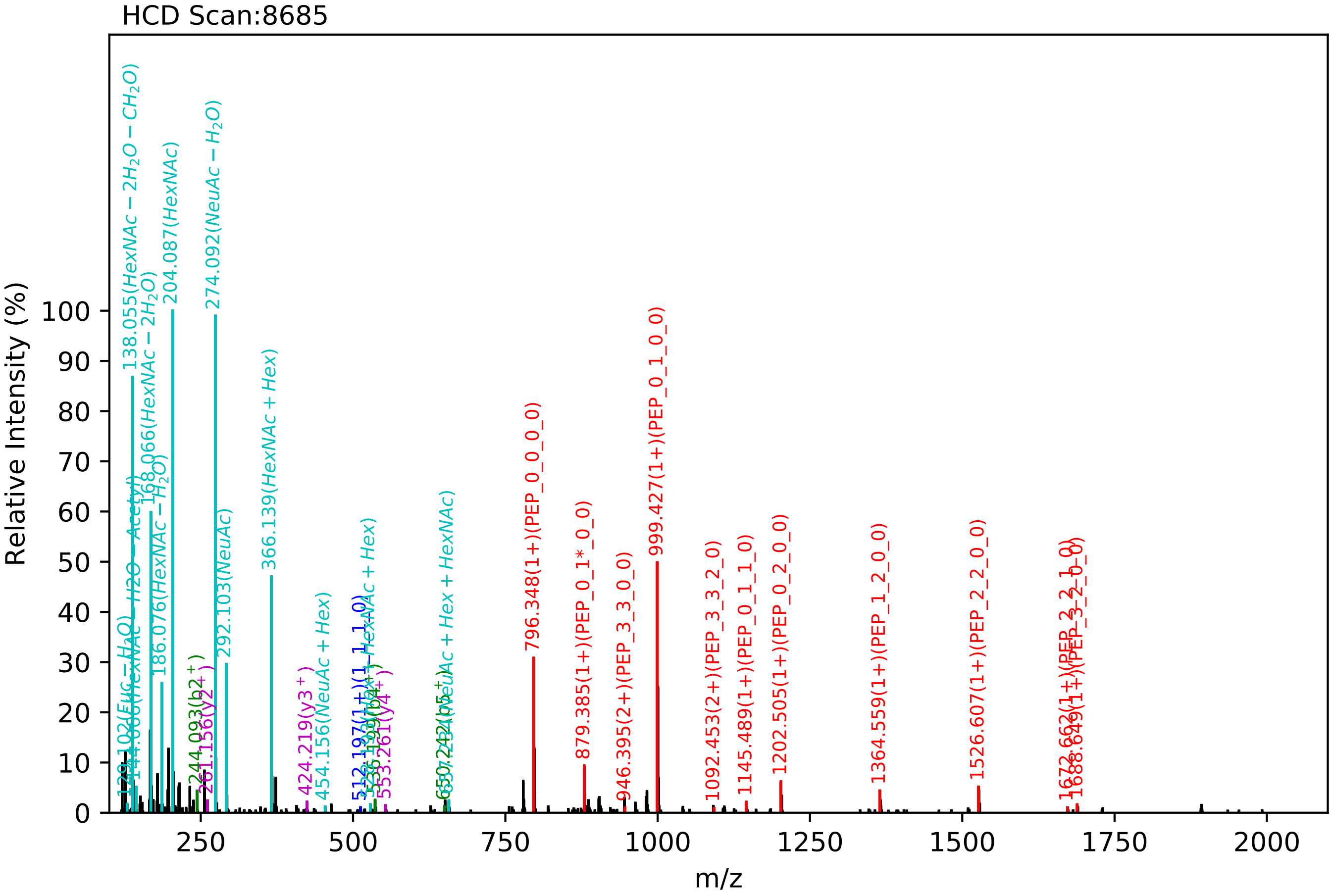

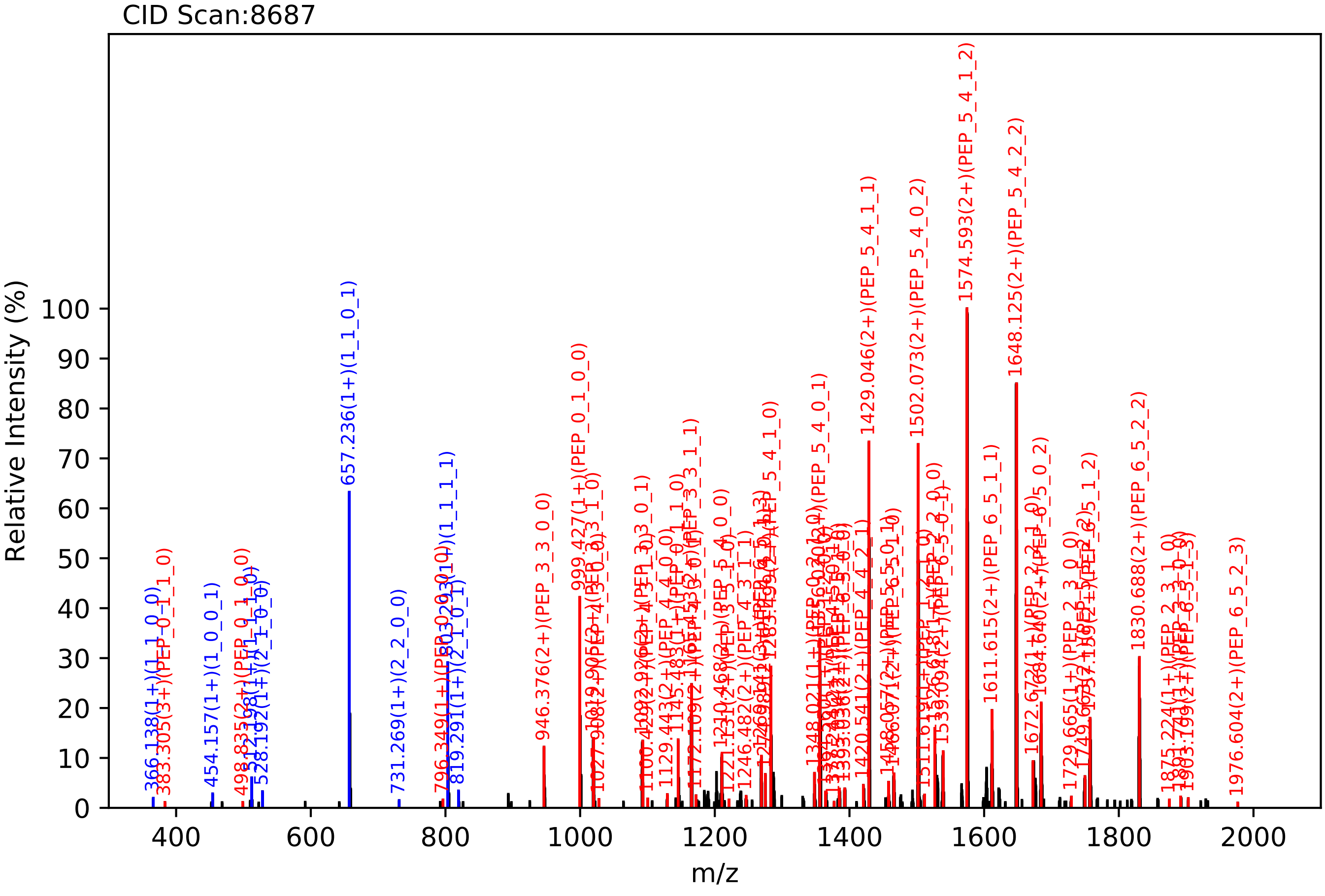

Test set no. 222, Experiment: AGP exp_18

NEEYNK(=PEP)_6_5_2_3, m/z:1317.16(3+), RT:30.66, Y-score:88.43

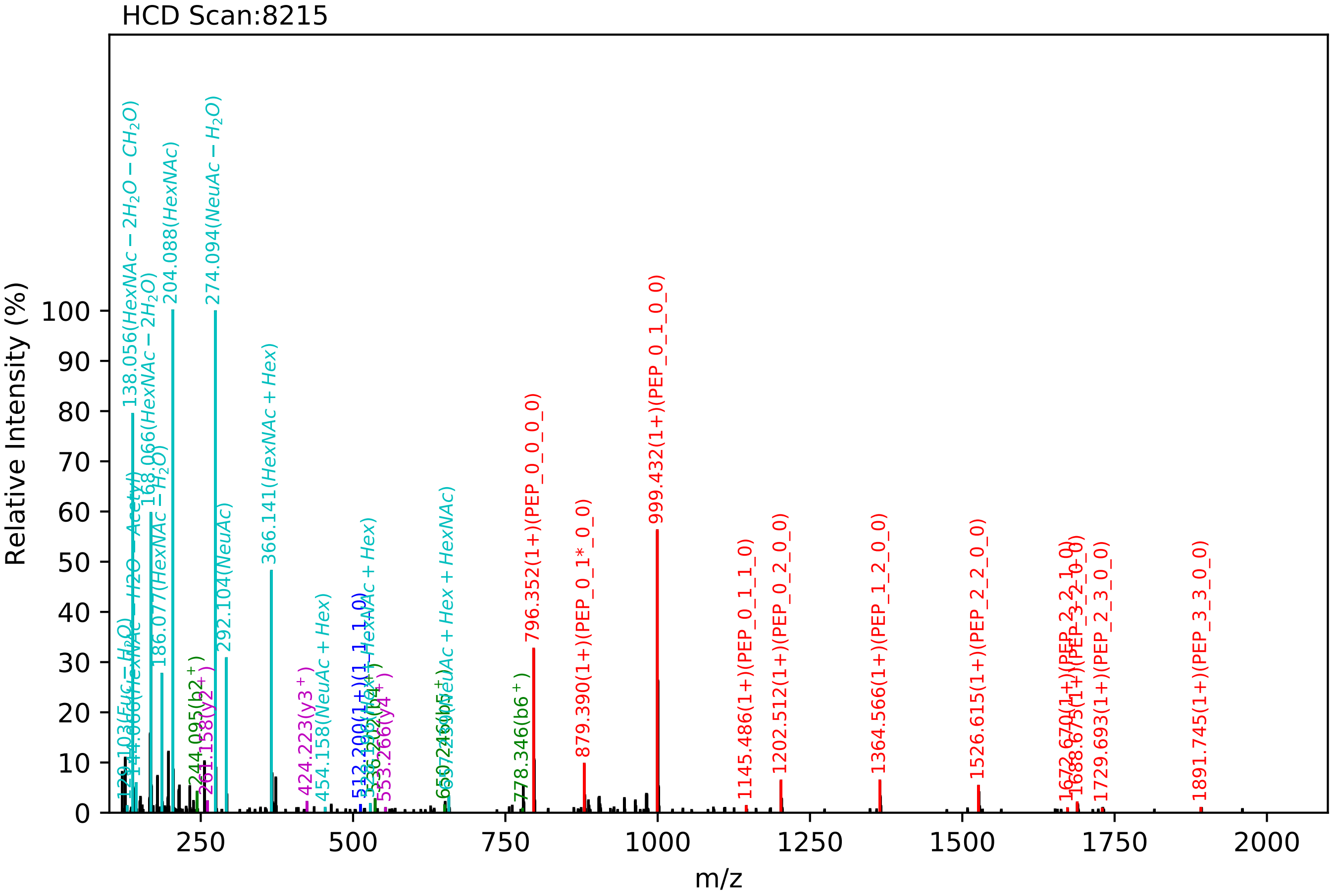

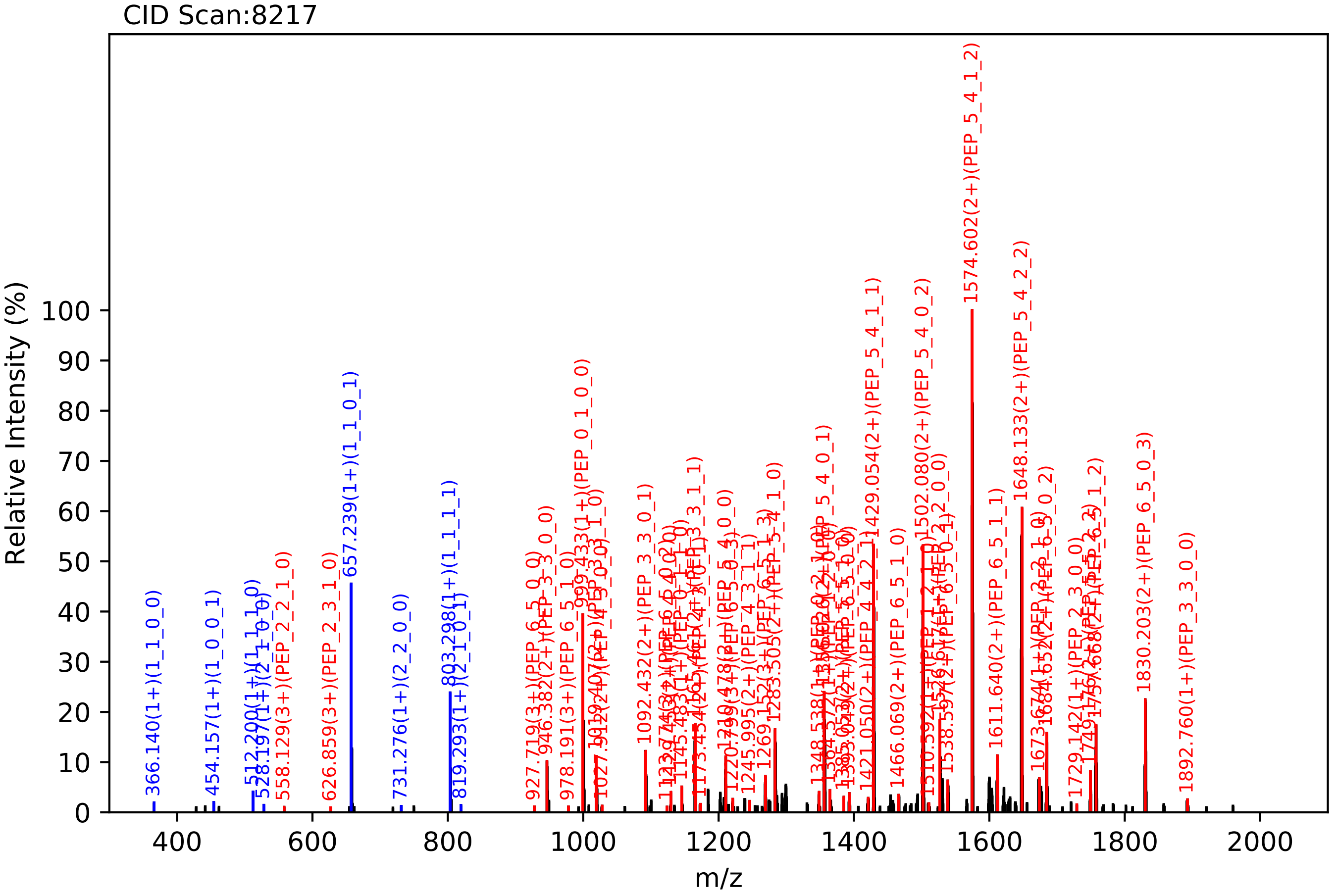

Test set no. 223, Experiment: AGP exp_24

NEEYNK(=PEP)_6_5_2_3, m/z:1317.16(3+), RT:31.31, Y-score:87.79

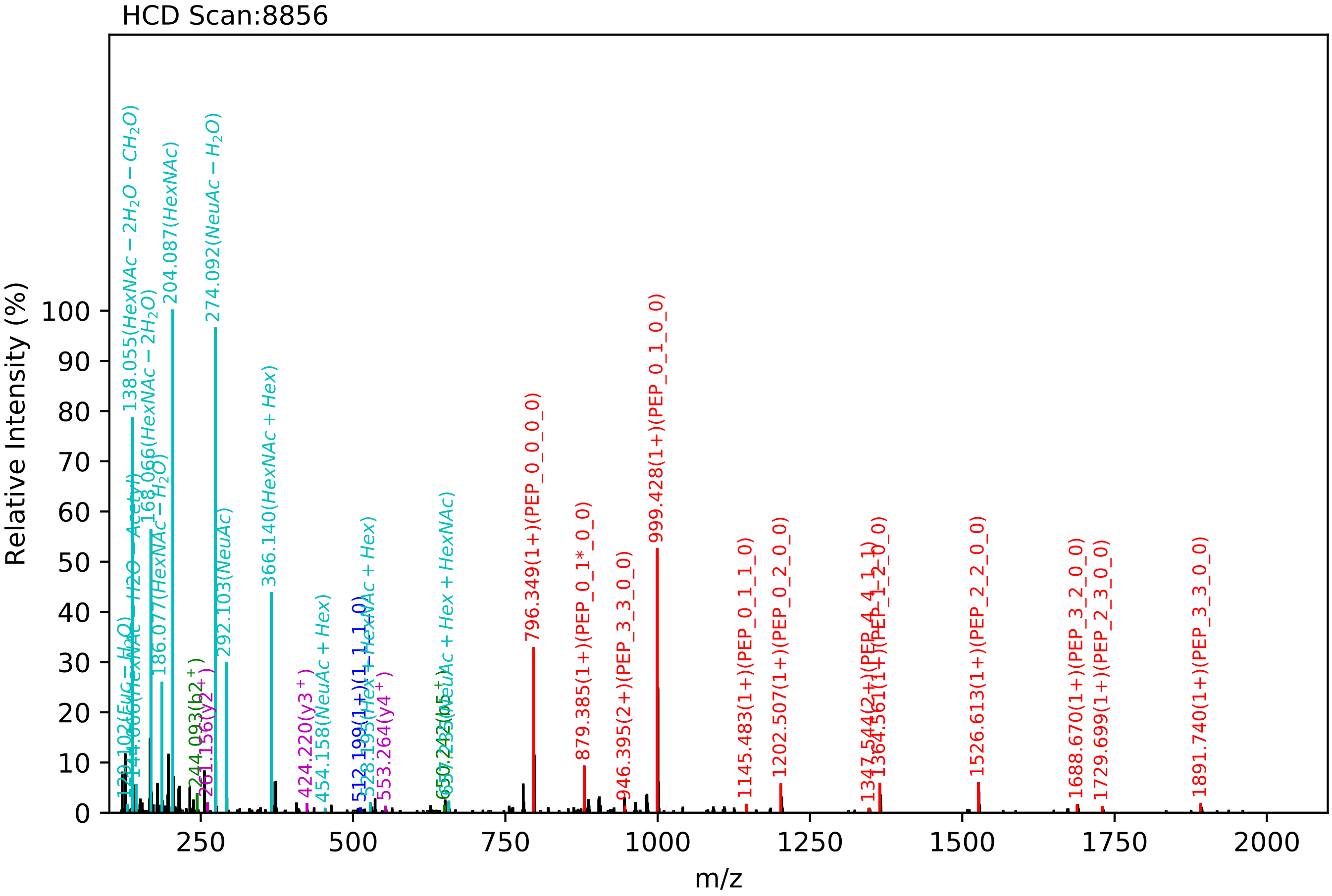

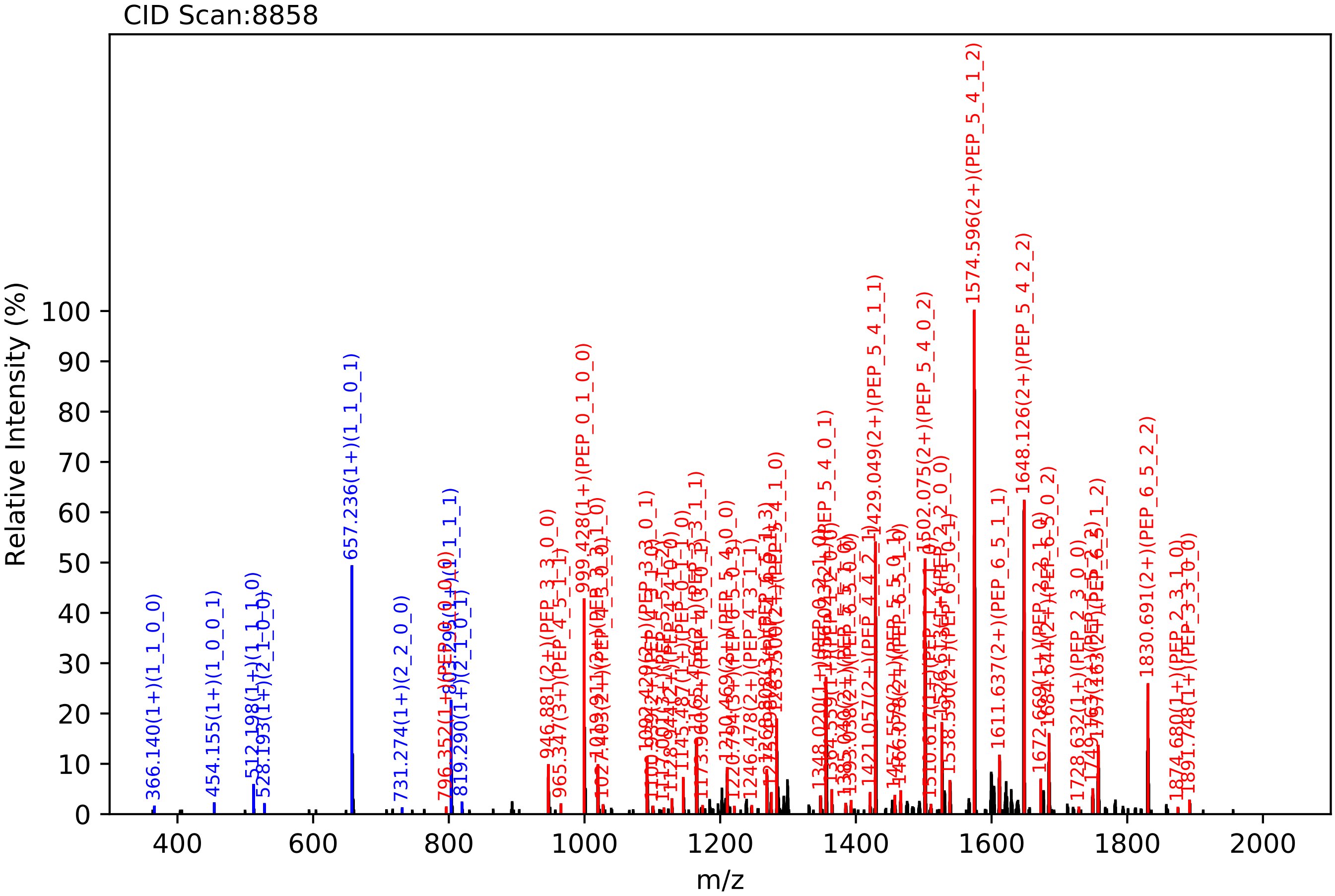

Test set no. 224, Experiment: AGP exp_29

NEEYNK(=PEP)_6_5_2_3, m/z:1317.16(3+), RT:31.03, Y-score:84.28

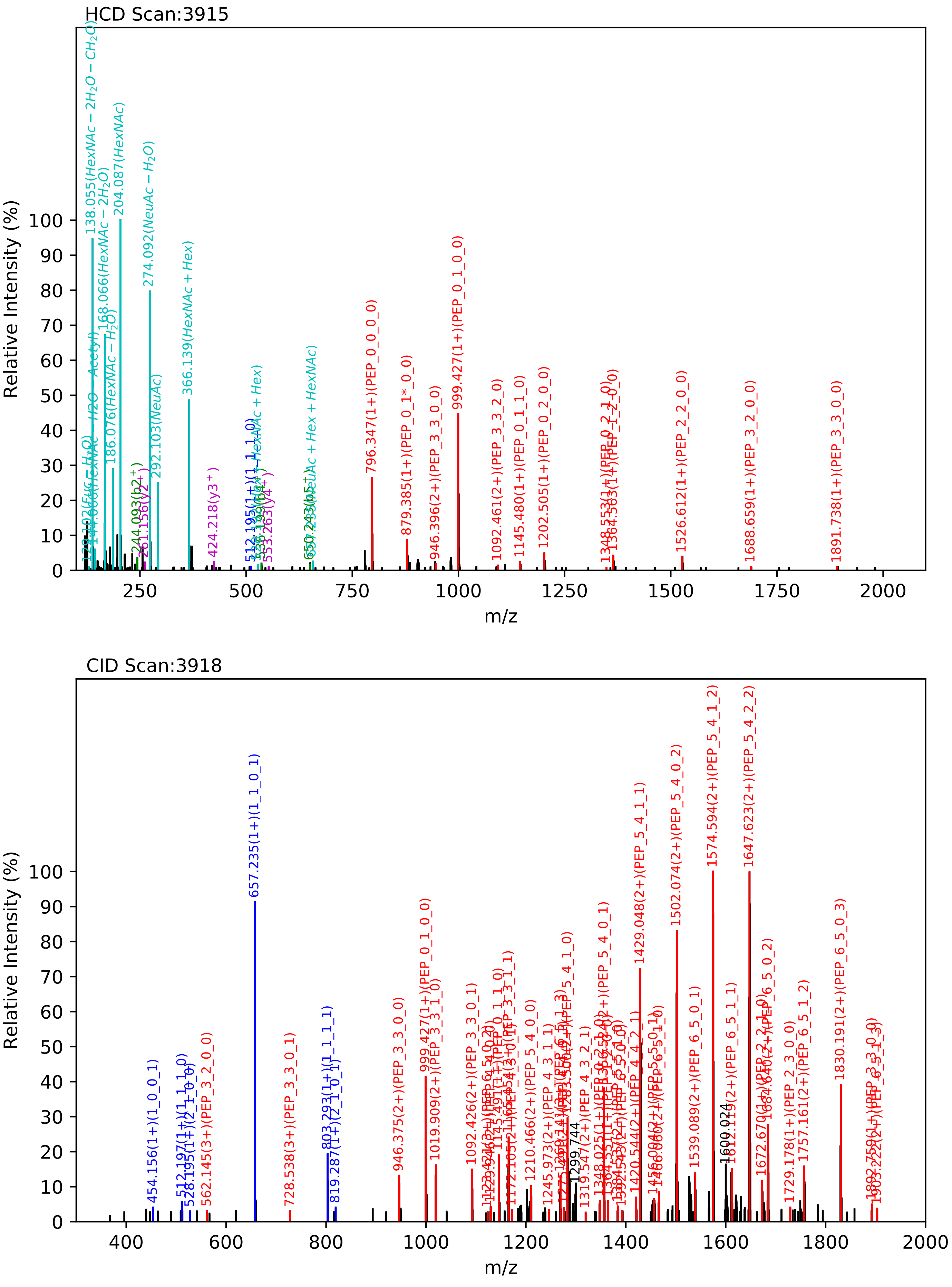

Test set no. 225, Experiment: AGP exp_4

NEEYNK(=PEP)_6_5_2_3, m/z:1317.16(3+), RT:22.15, Y-score:83.07

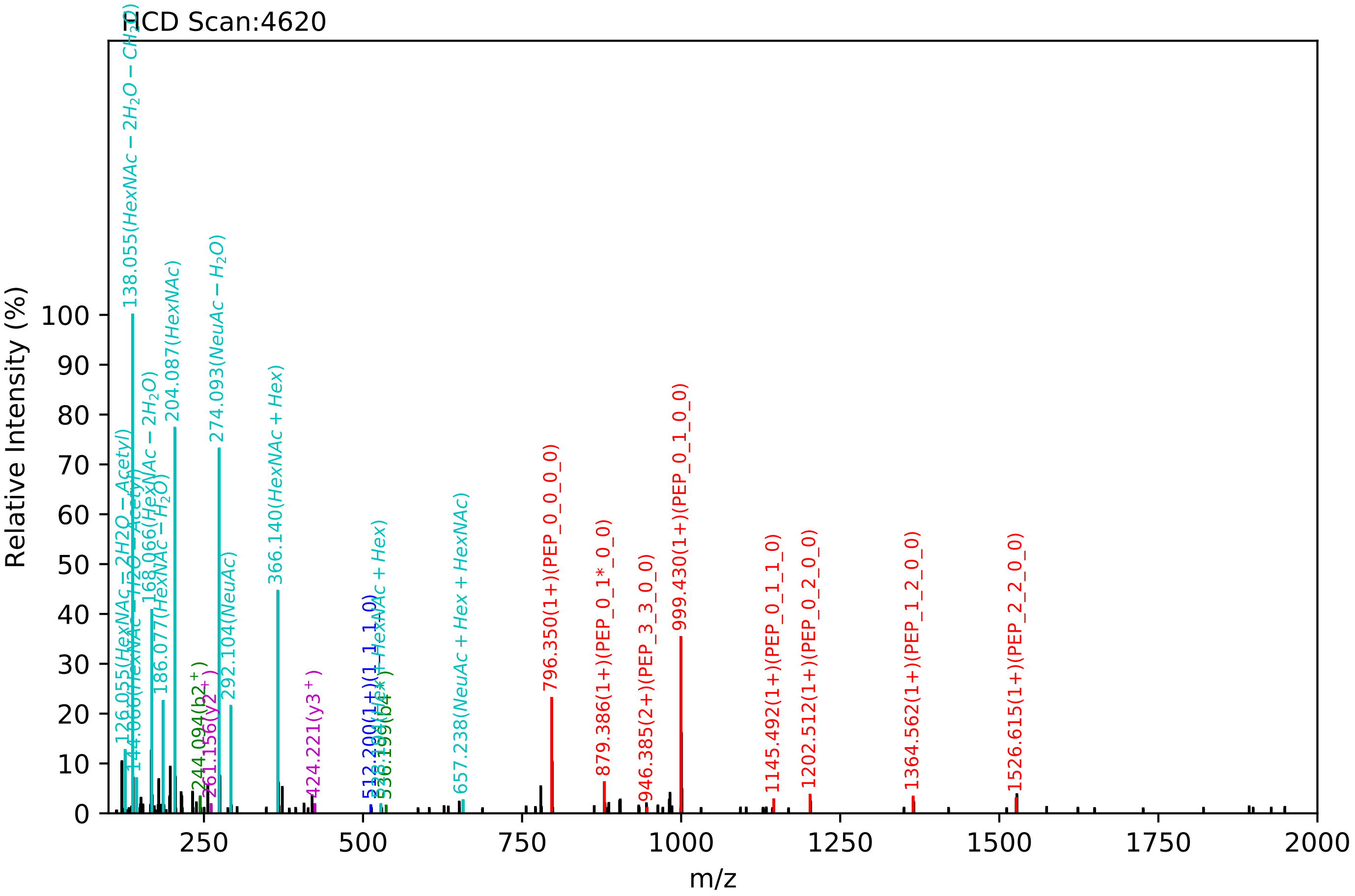

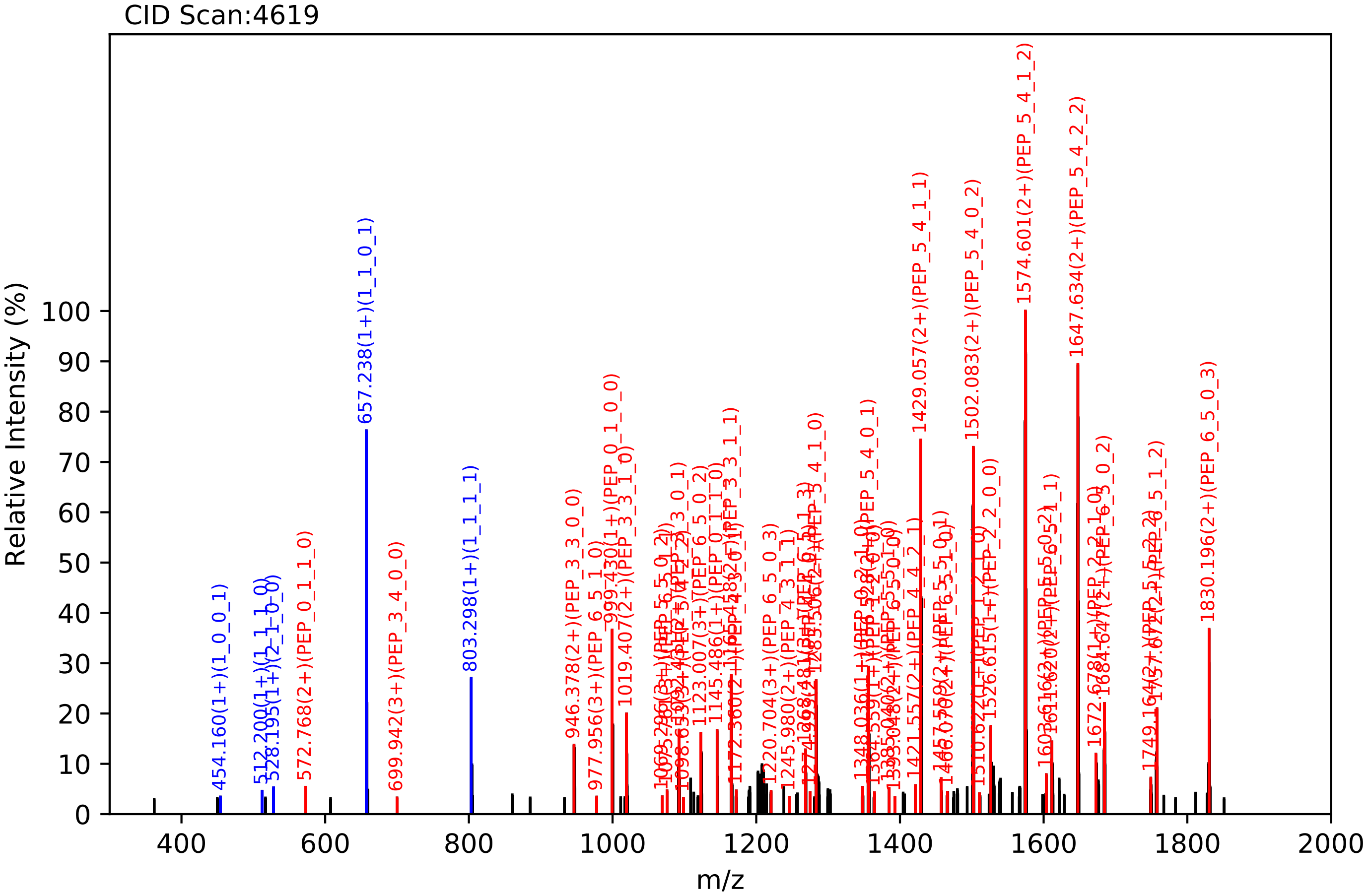

Test set no. 226, Experiment: AGP exp_13

NEEYNK(=PEP)_6_5_2_3, m/z:1317.16(3+), RT:37.27, Y-score:81.14

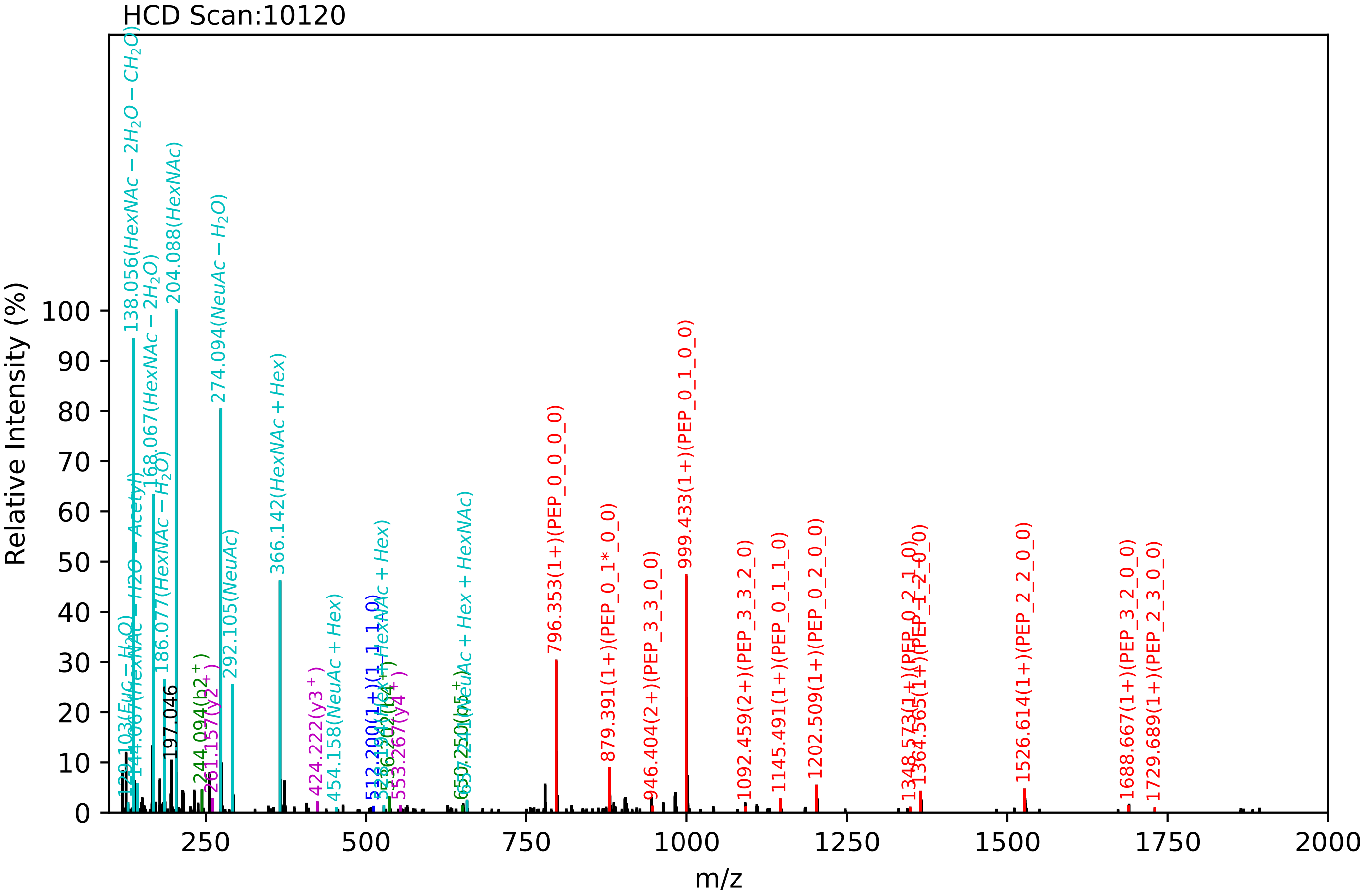

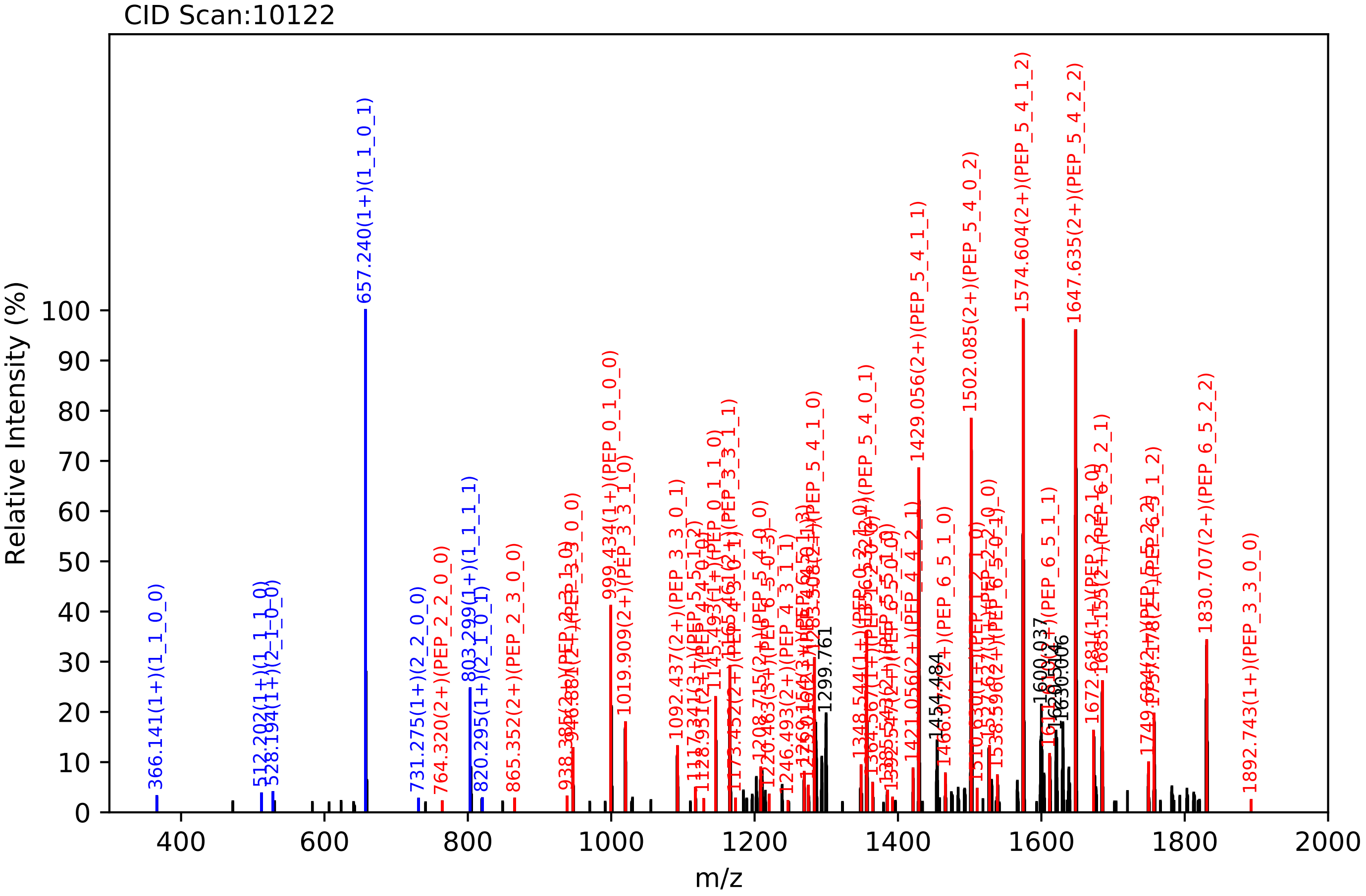

Test set no. 227, Experiment: AGP exp_6

NEEYNK(=PEP)_6_5_2_3, m/z:1317.16(3+), RT:29.70, Y-score:79.57

HCD Scan:5902

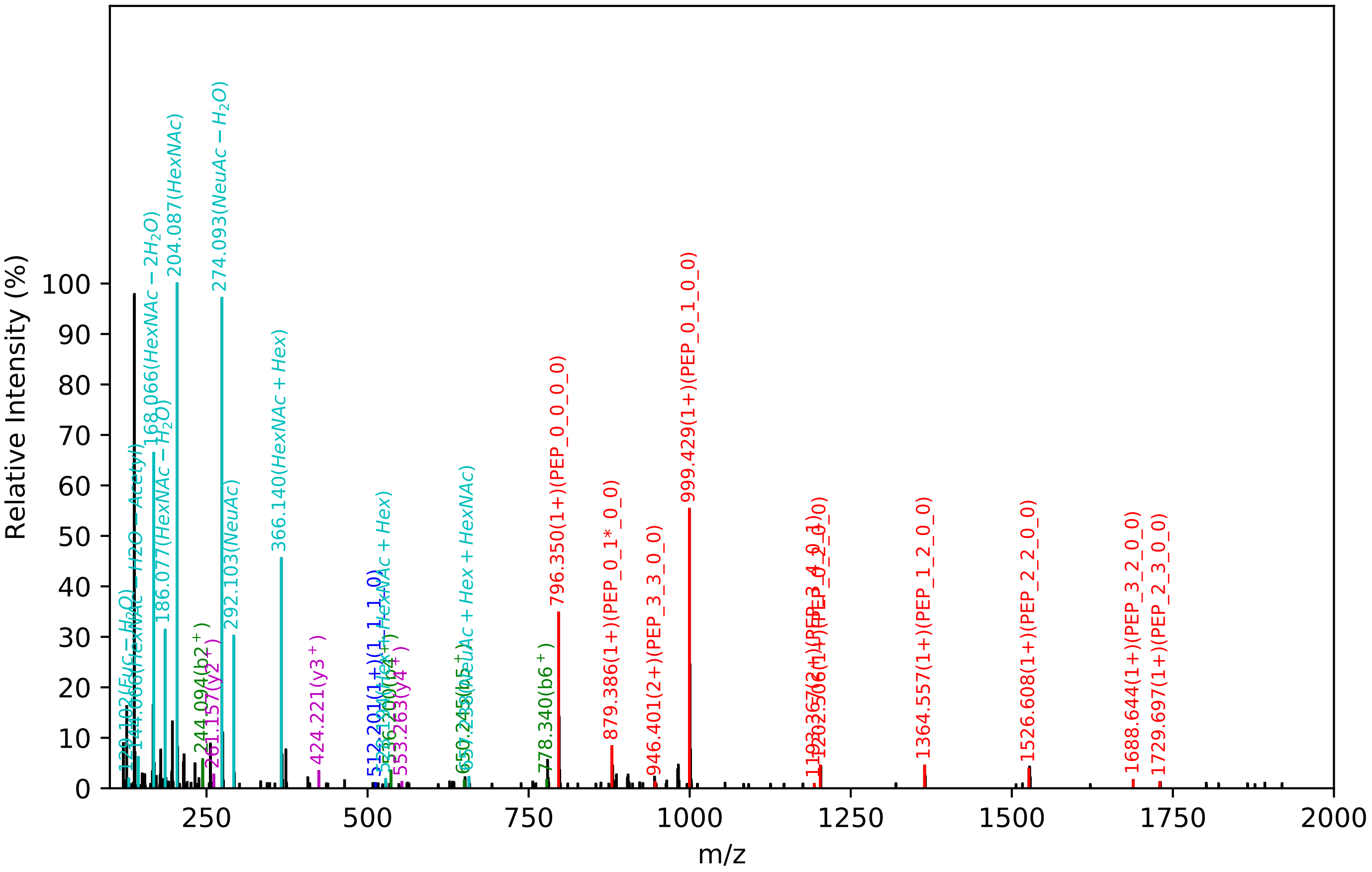

CID Scan:5905

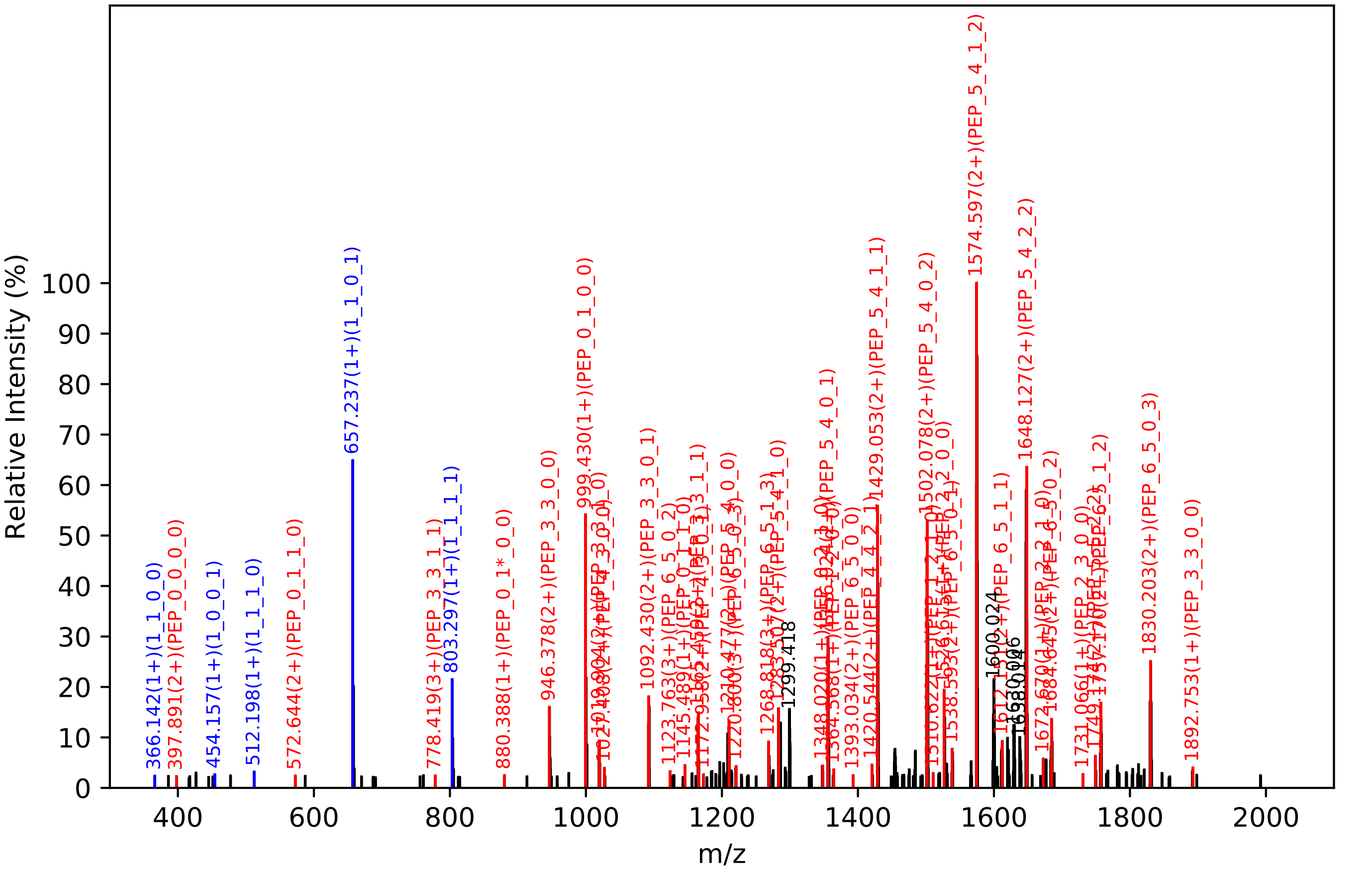

Test set no. 228, Experiment: AGP exp_3

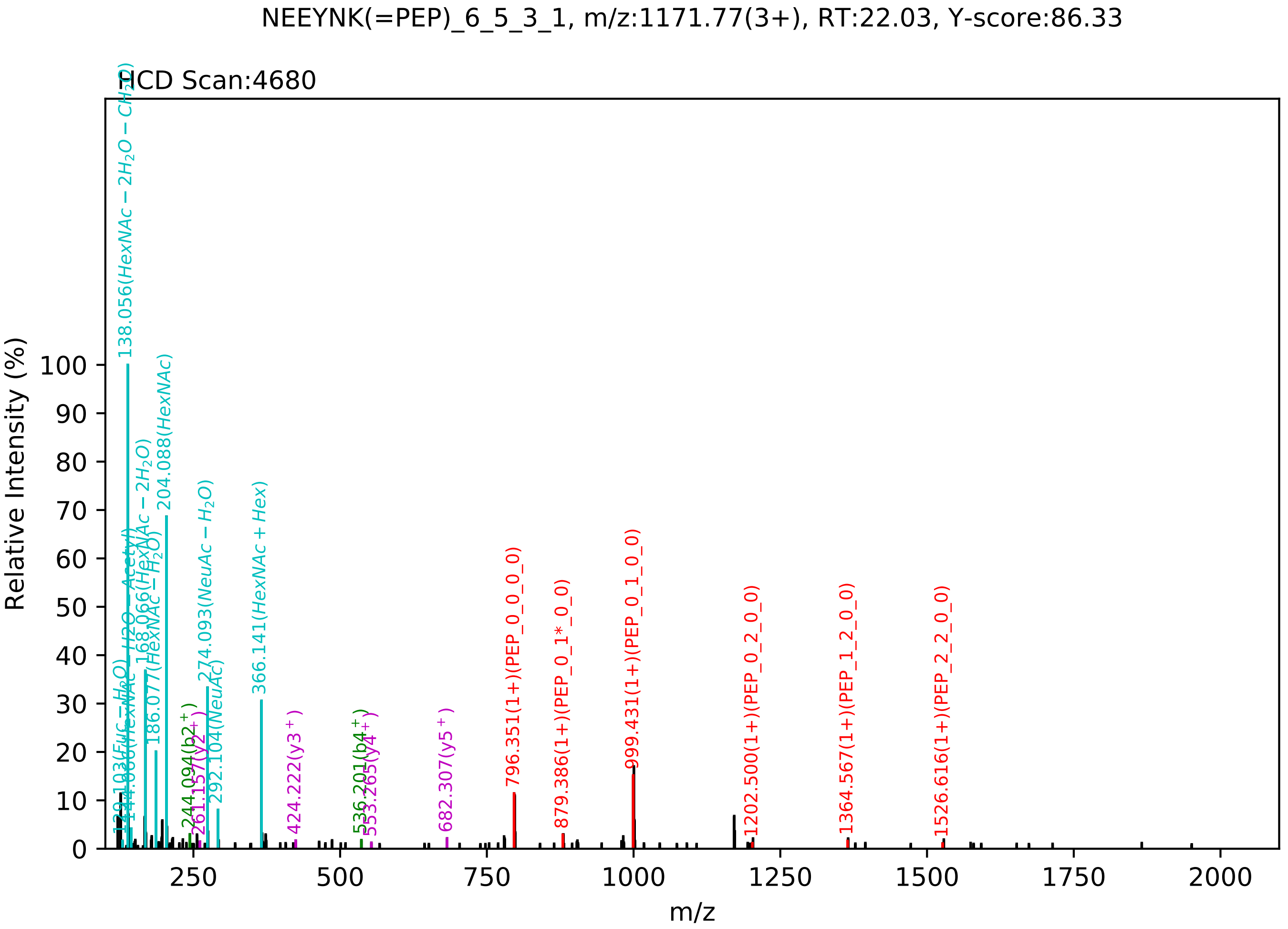

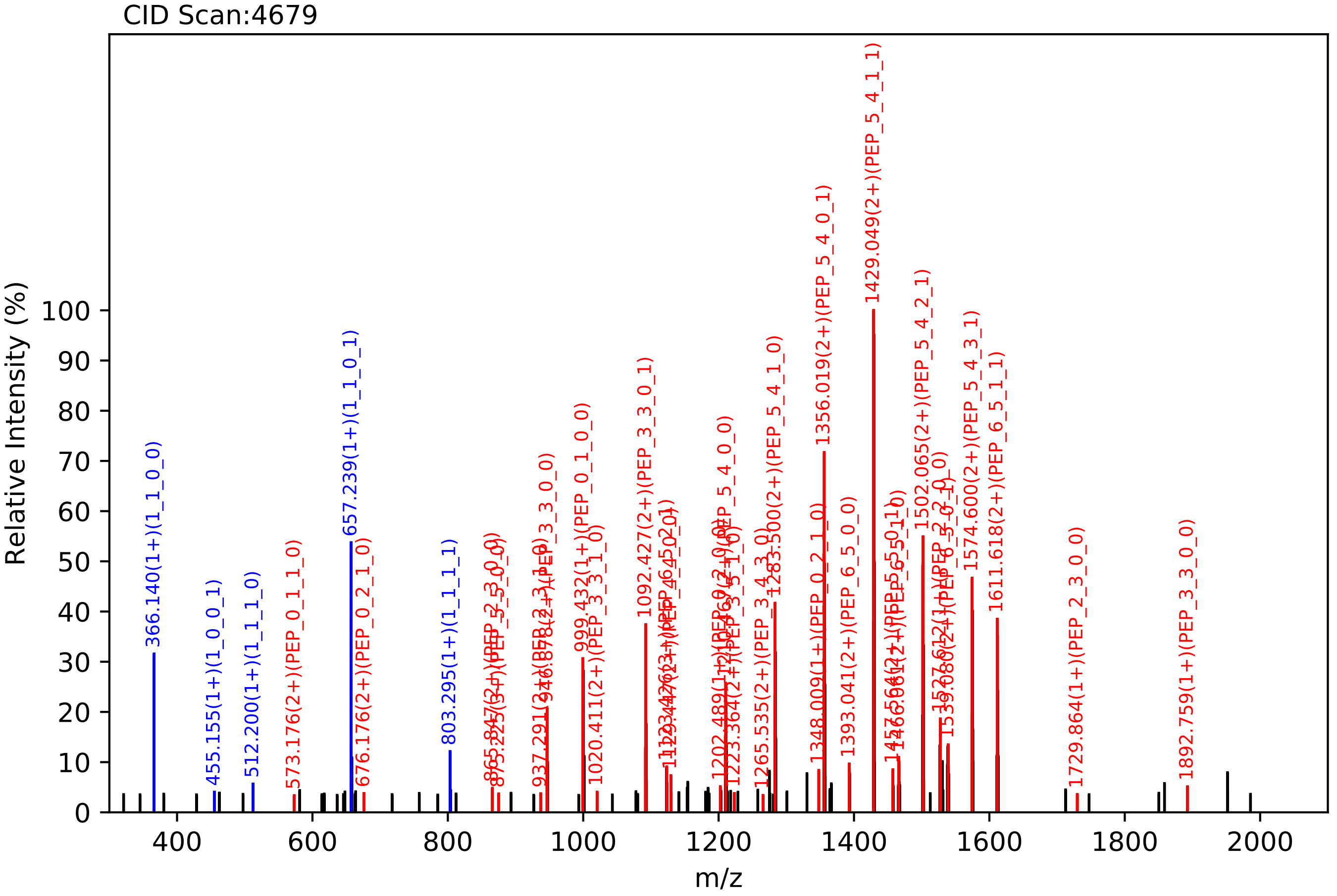

Test set no. 229, Experiment: AGP exp_3

NEEYNK(=PEP)_6_5_3_2, m/z:1268.80(3+), RT:24.25, Y-score:75.95

HCD Scan:5954

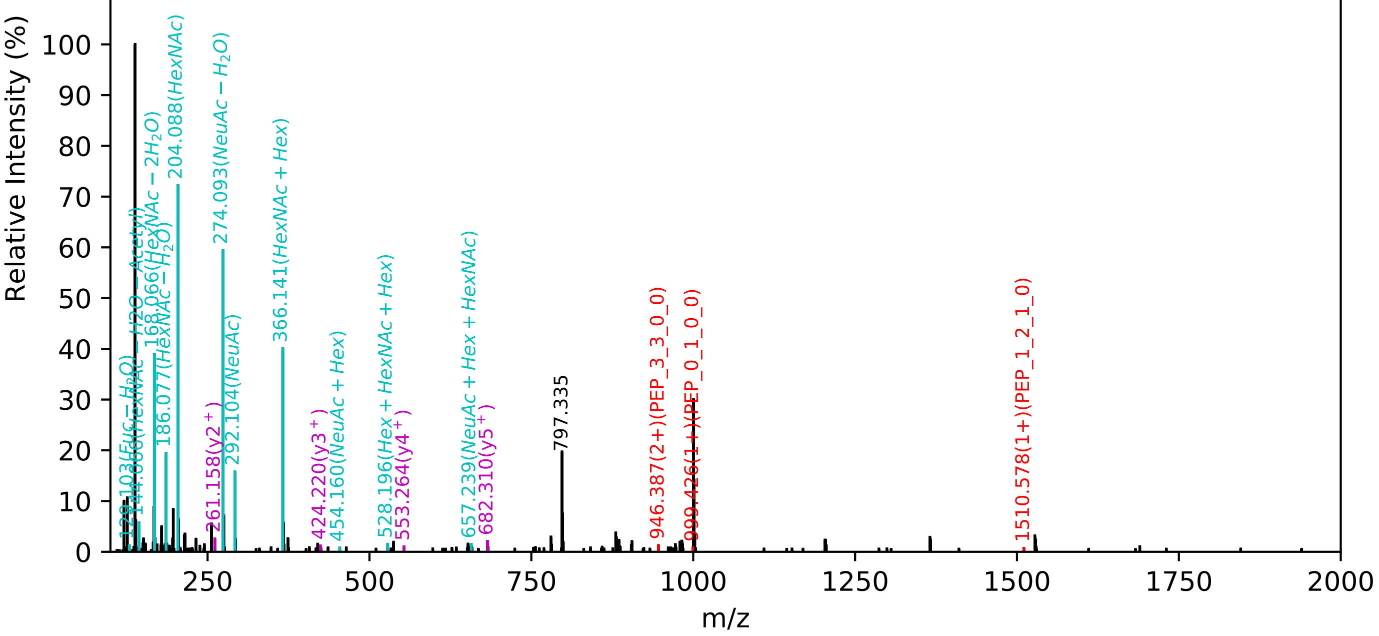

CID Scan:5953

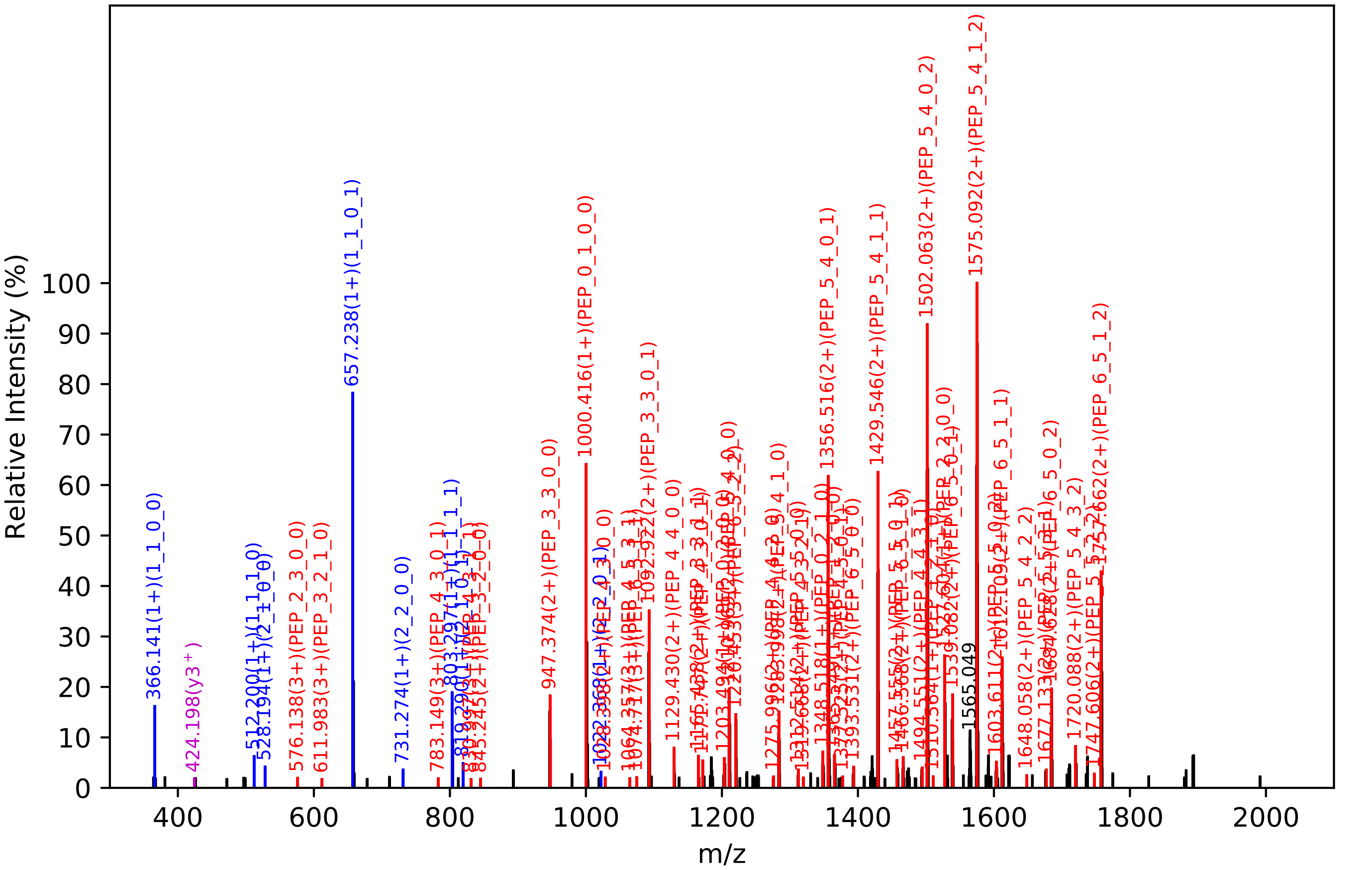

Test set no. 230, Experiment: AGP exp_4

SVQEIQATFFYFTPNK(=PEP)_6_5_0_3, m/z:1196.00(4+), RT:70.33, Y-score:75.03

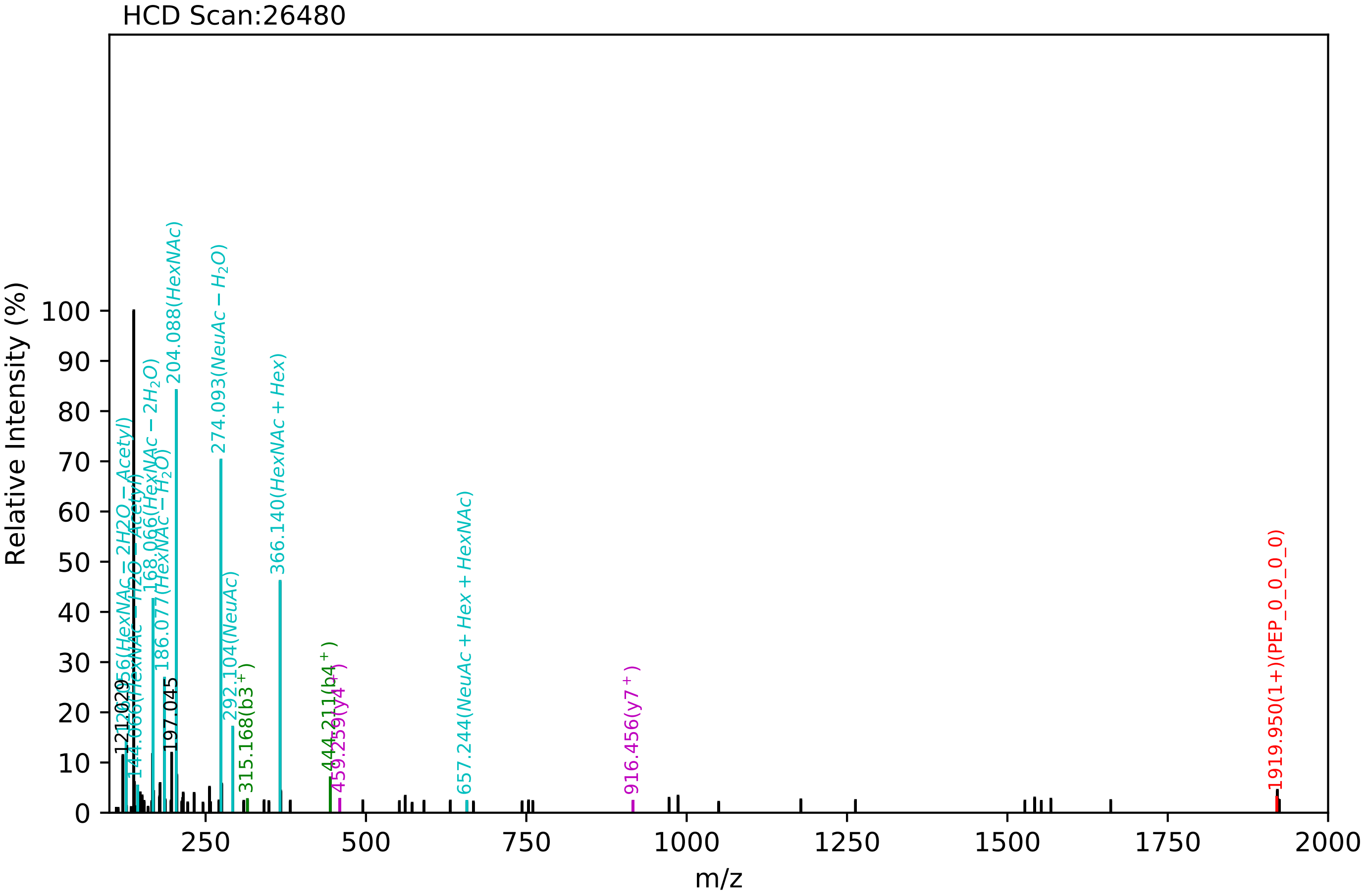

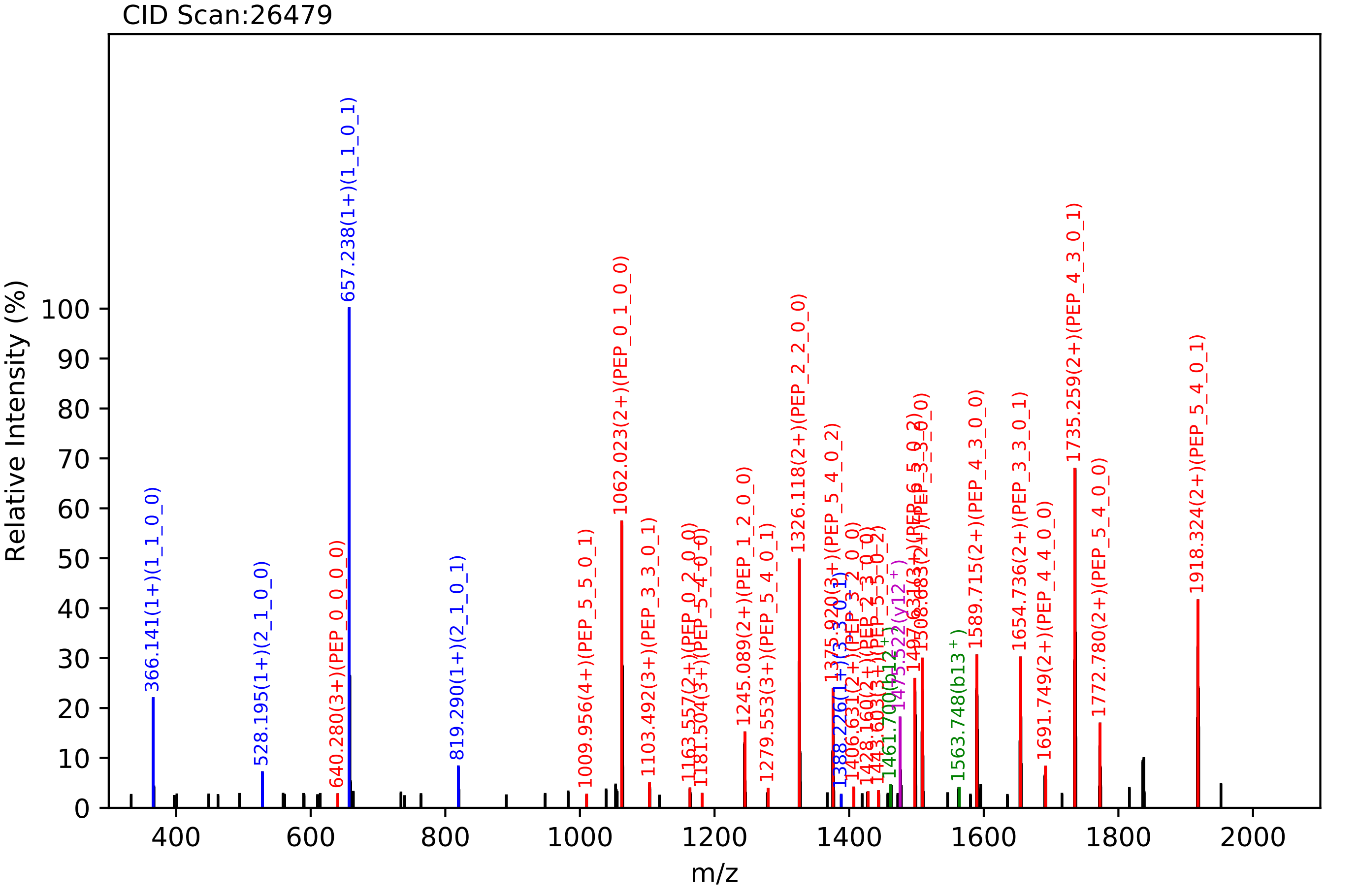

Test set no. 231, Experiment: AGP exp_4

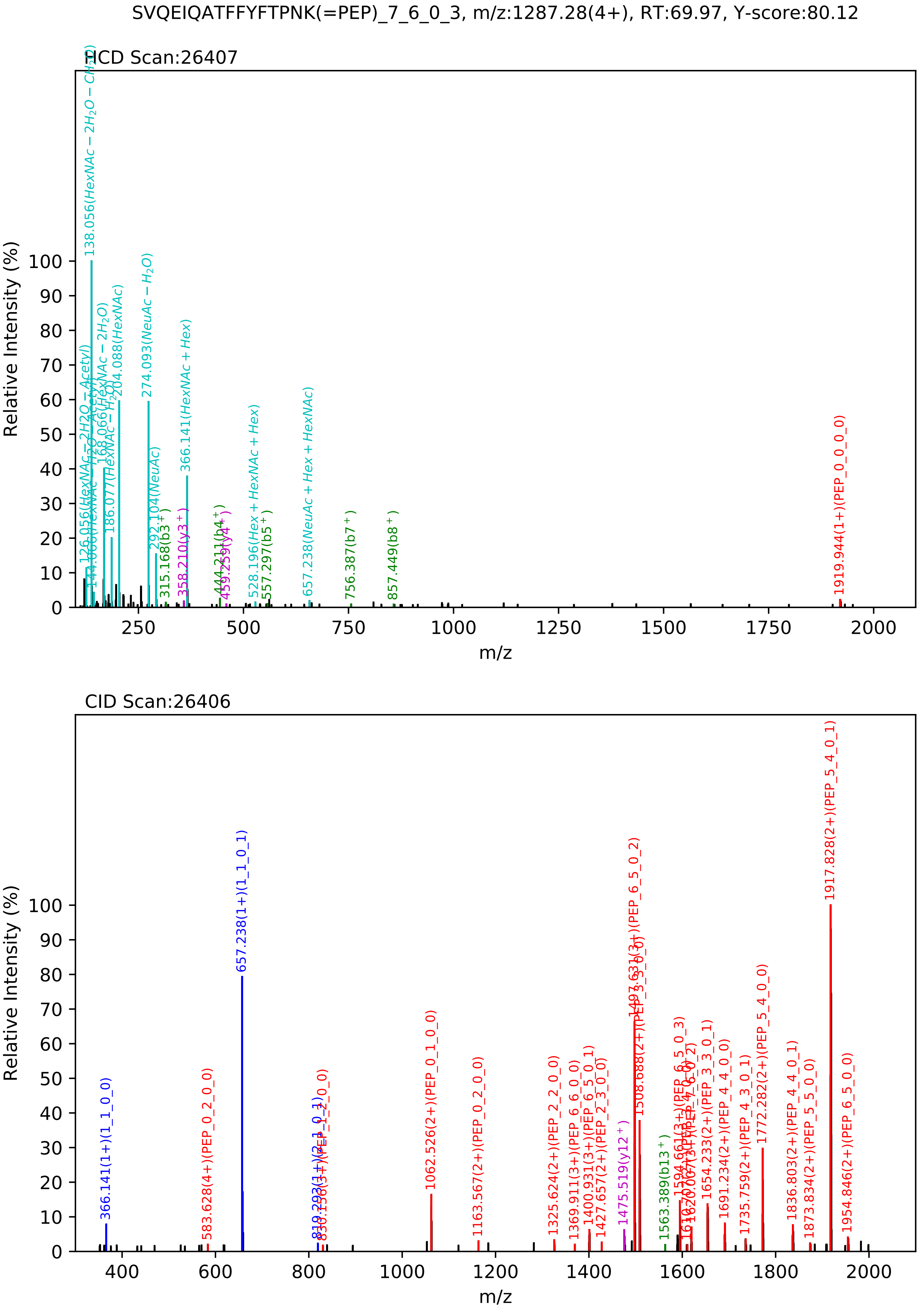

Test set no. 232, Experiment: AGP exp_4

ENGTVSR(=PEP)_6_5_0_1, m/z:1014.40(3+), RT:15.54, Y-score:83.38

HCD Scan:1623

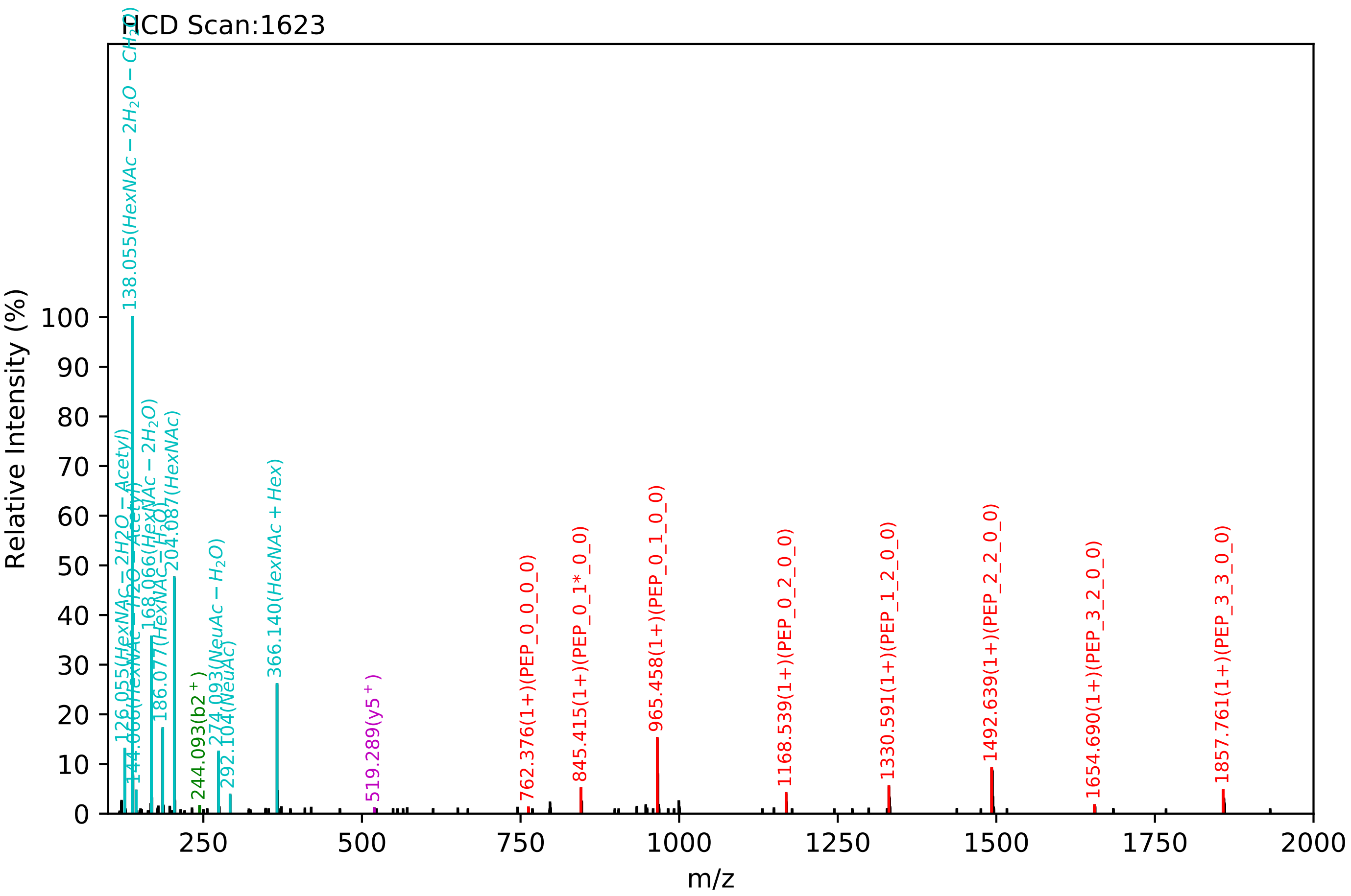

CID Scan:1622

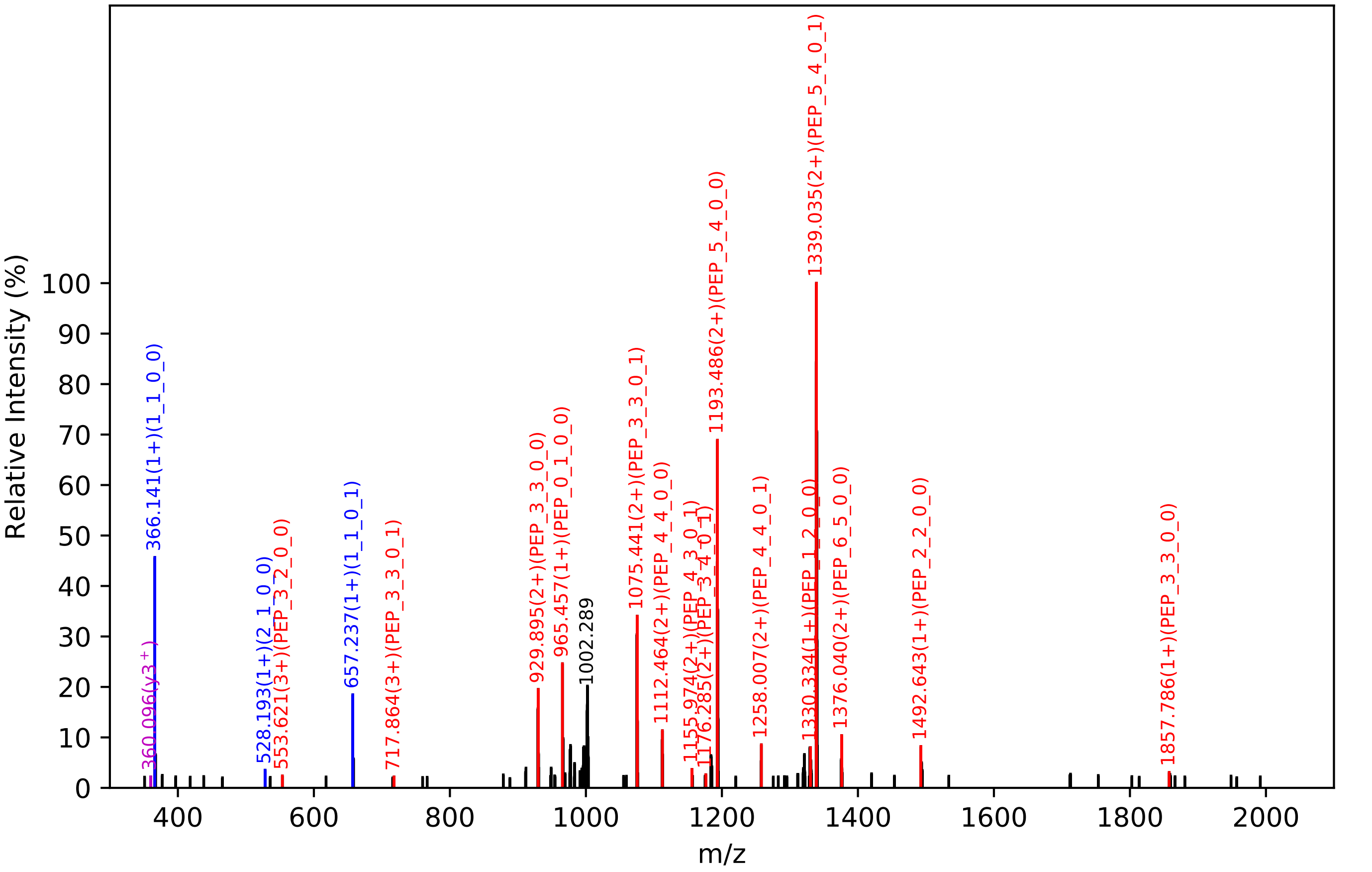

Test set no. 233, Experiment: AGP exp_4

ENGTVSR(=PEP)_6_5_0_2, m/z:1111.43(3+), RT:17.10, Y-score:97.82

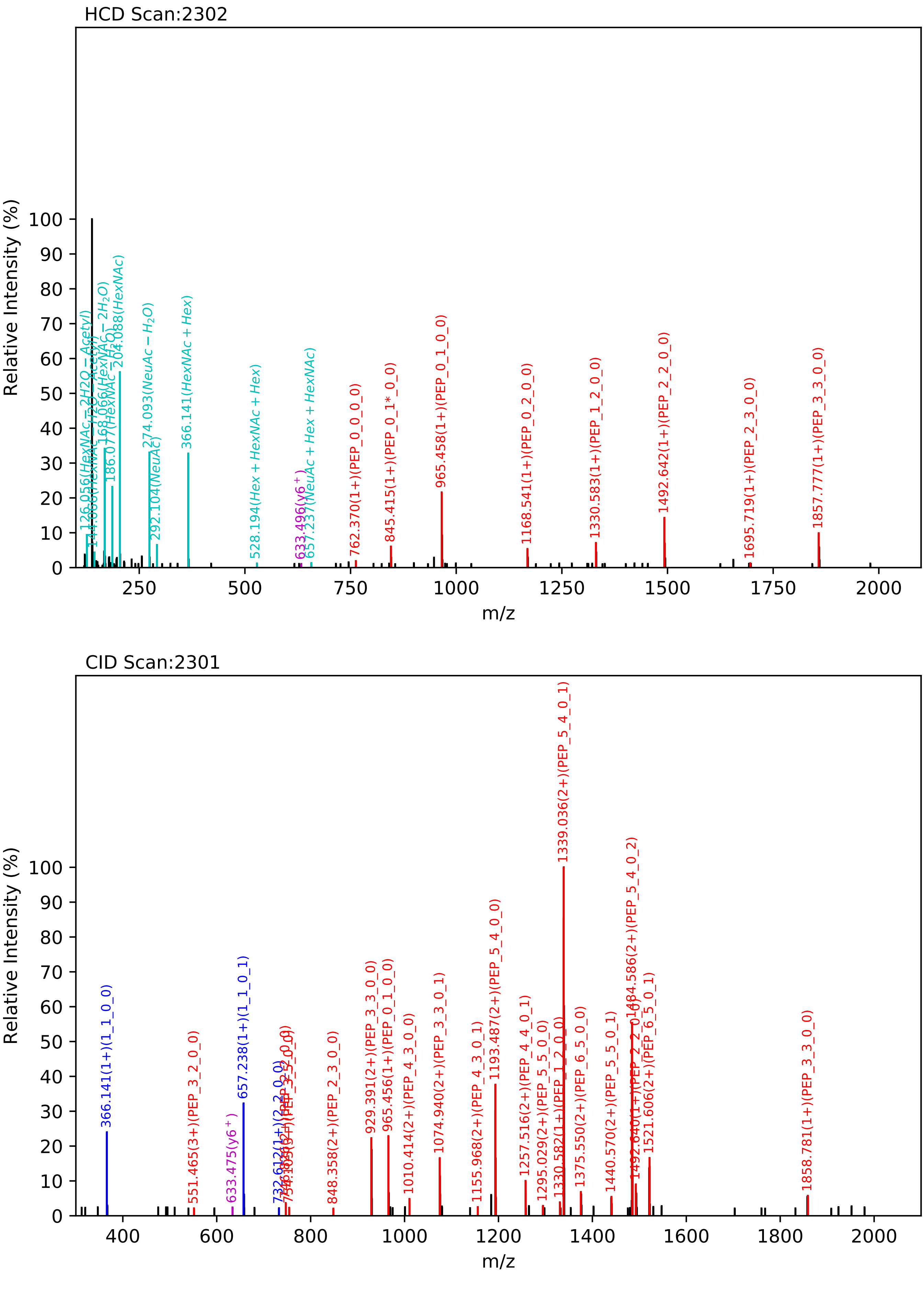

Test set no. 234, Experiment: AGP exp_3

ENGTVSR(=PEP)_6_5_0_2, m/z:1111.43(3+), RT:17.61, Y-score:93.82

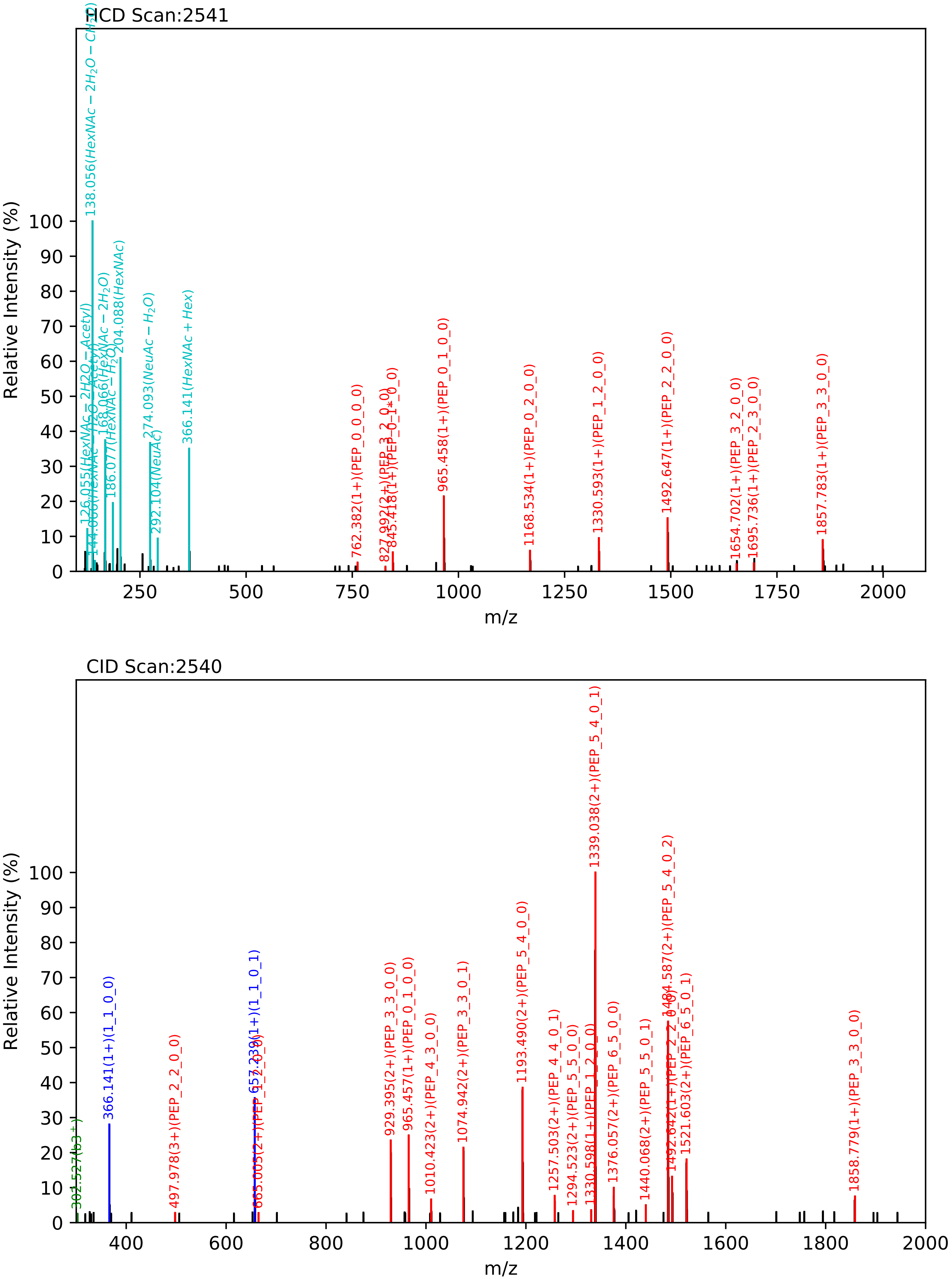

Test set no. 235, Experiment: AGP exp_4

ENGTVSR(=PEP)_6_5_0_2, m/z:1111.43(3+), RT:17.61, Y-score:93.77

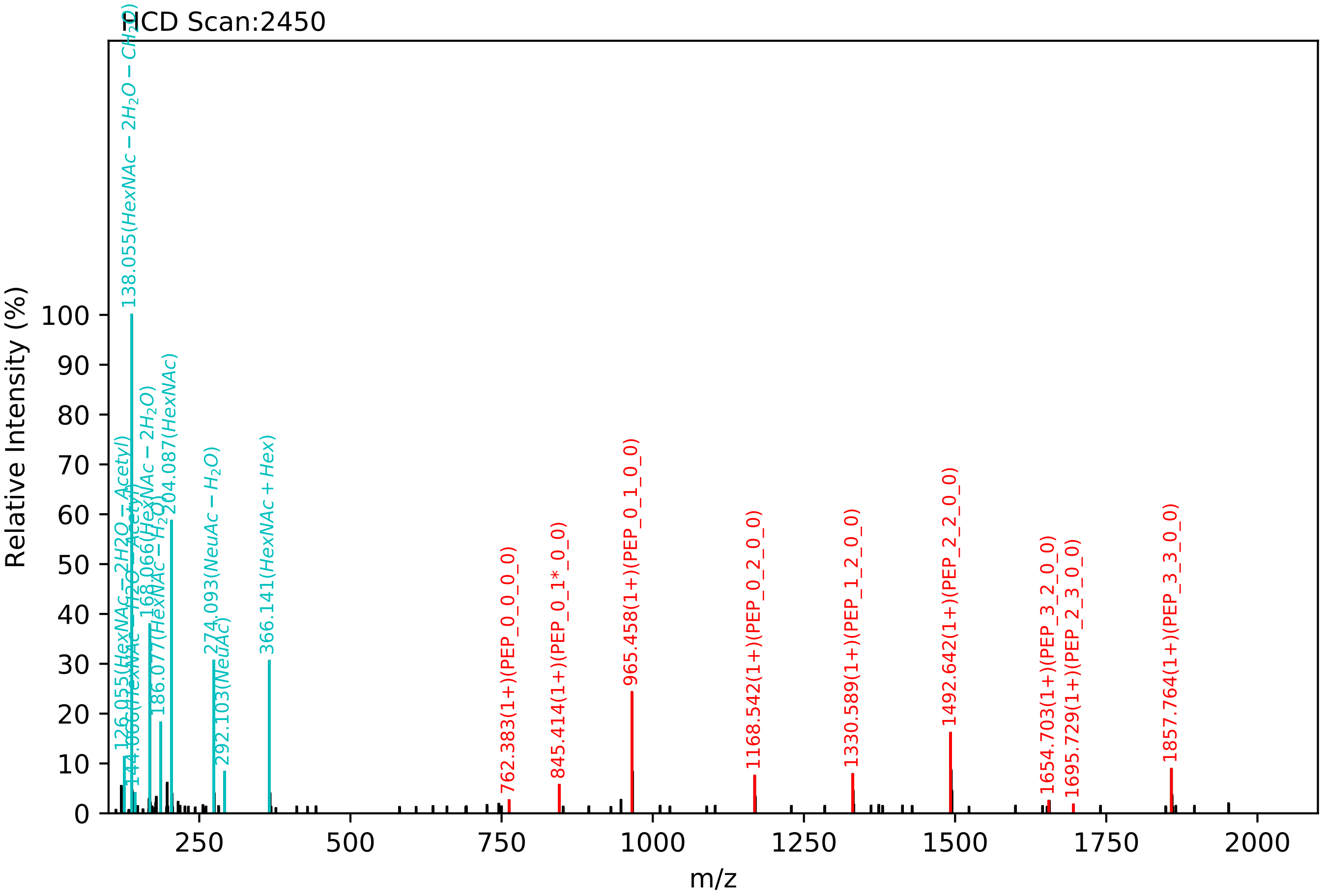

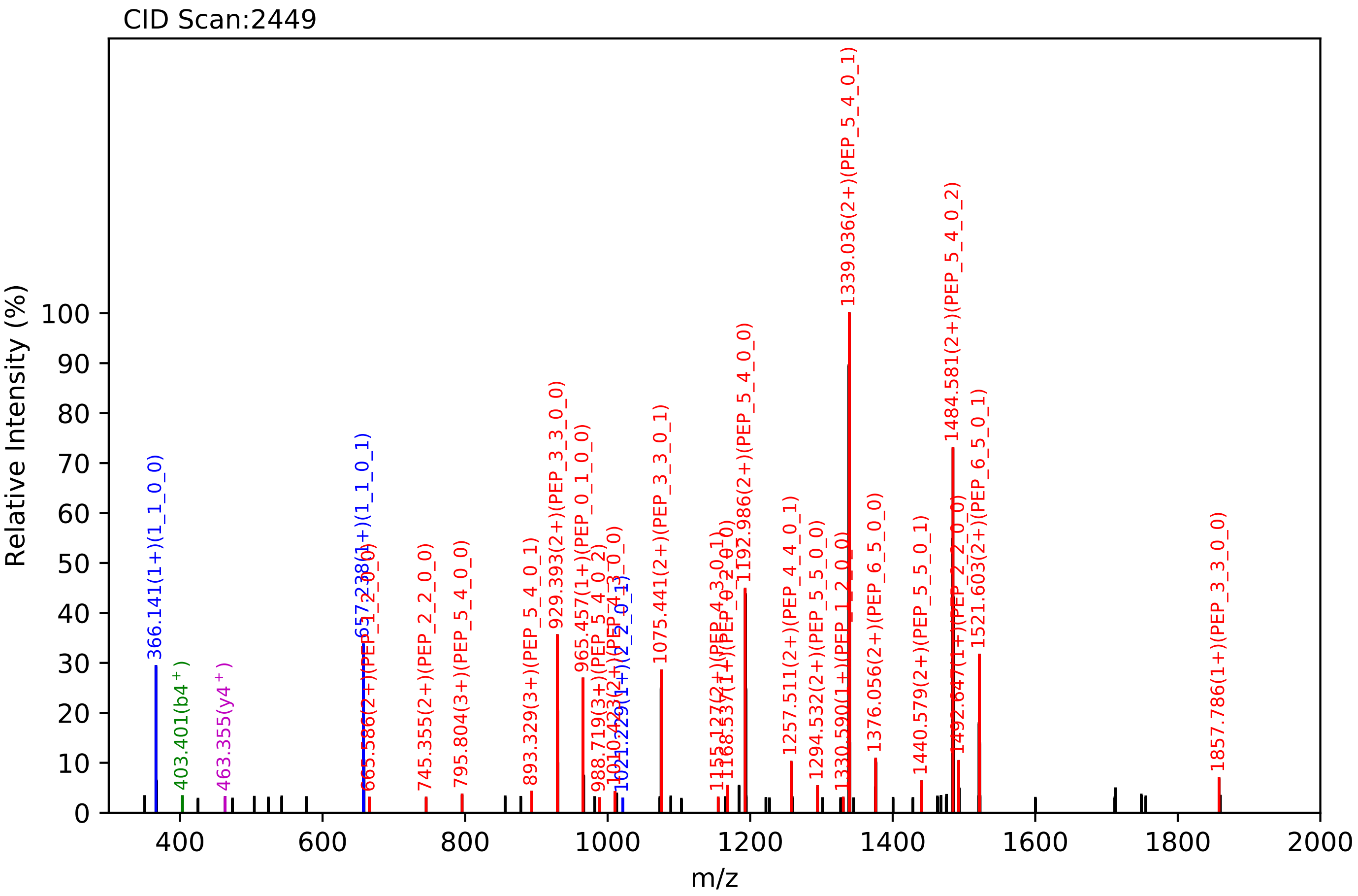

Test set no. 236, Experiment: AGP exp_3

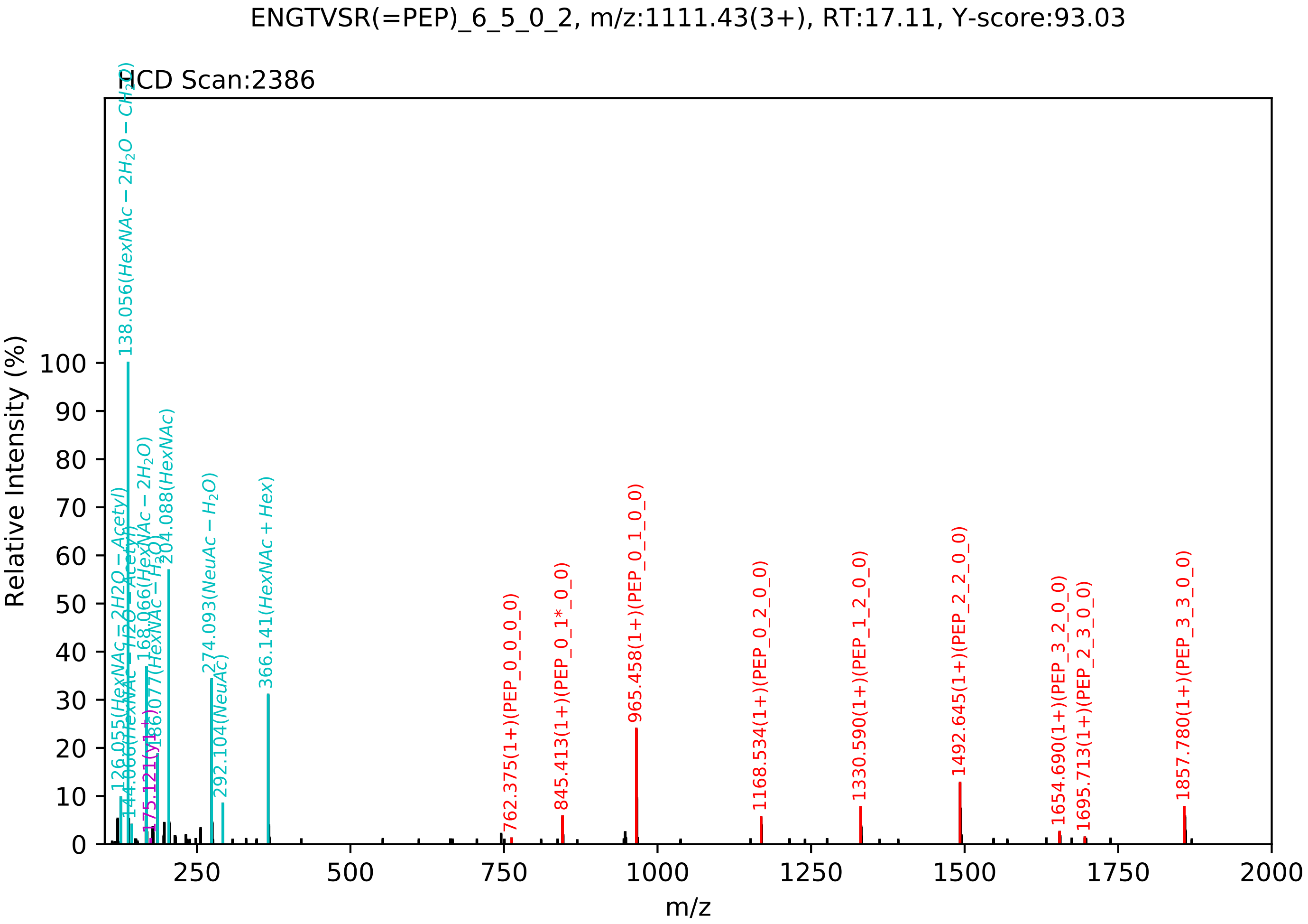

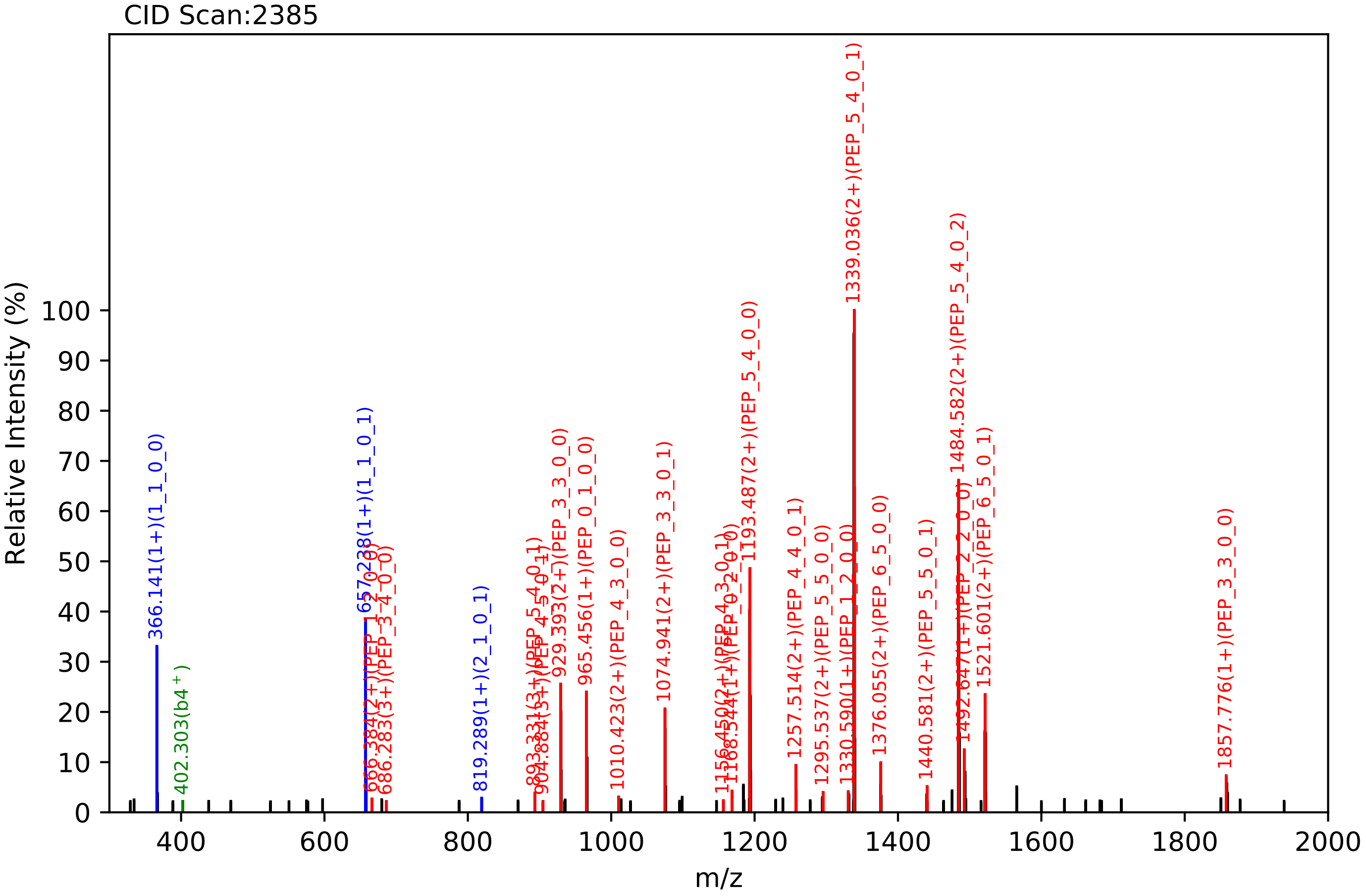

Test set no. 237, Experiment: AGP exp_4

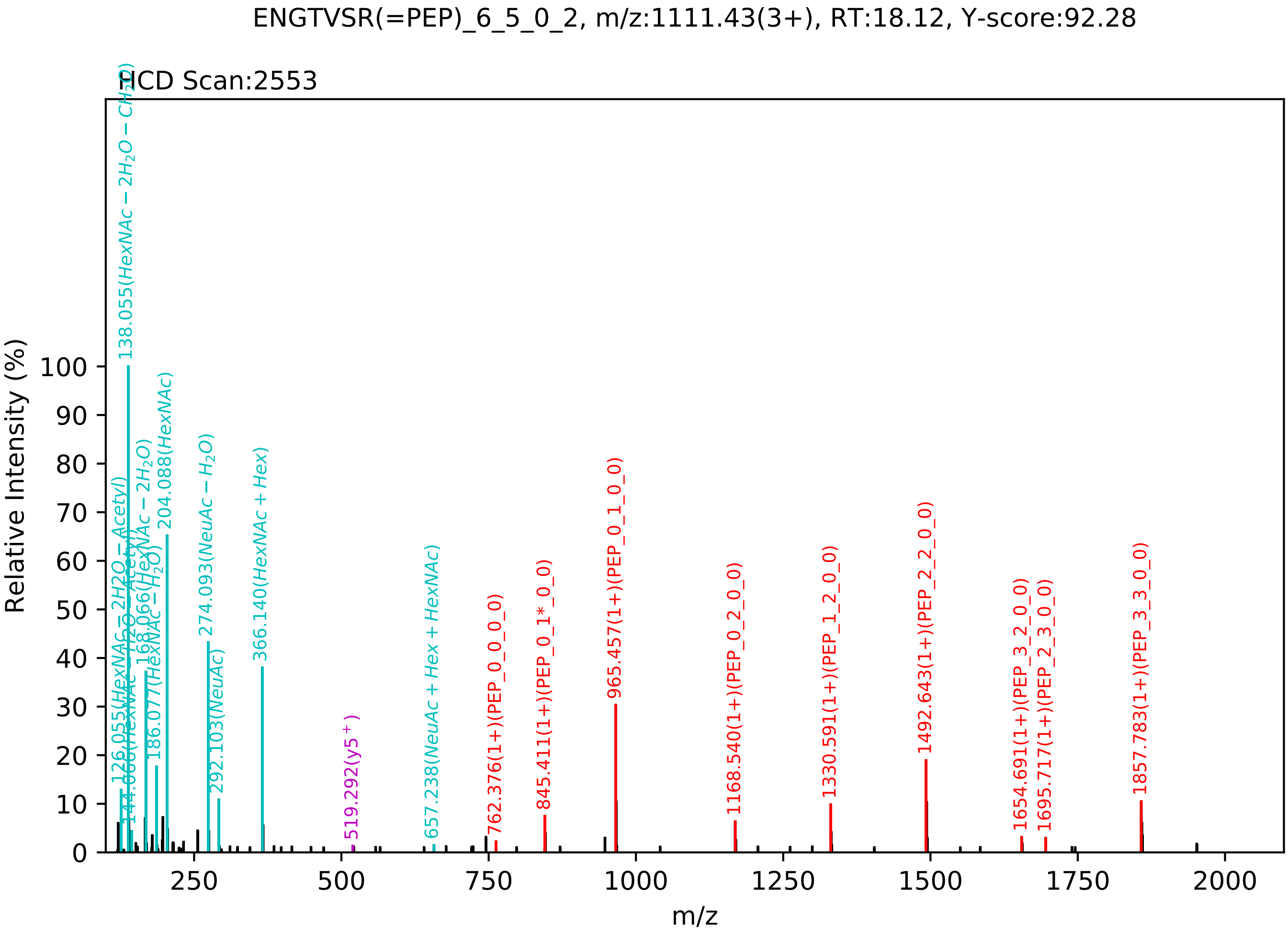

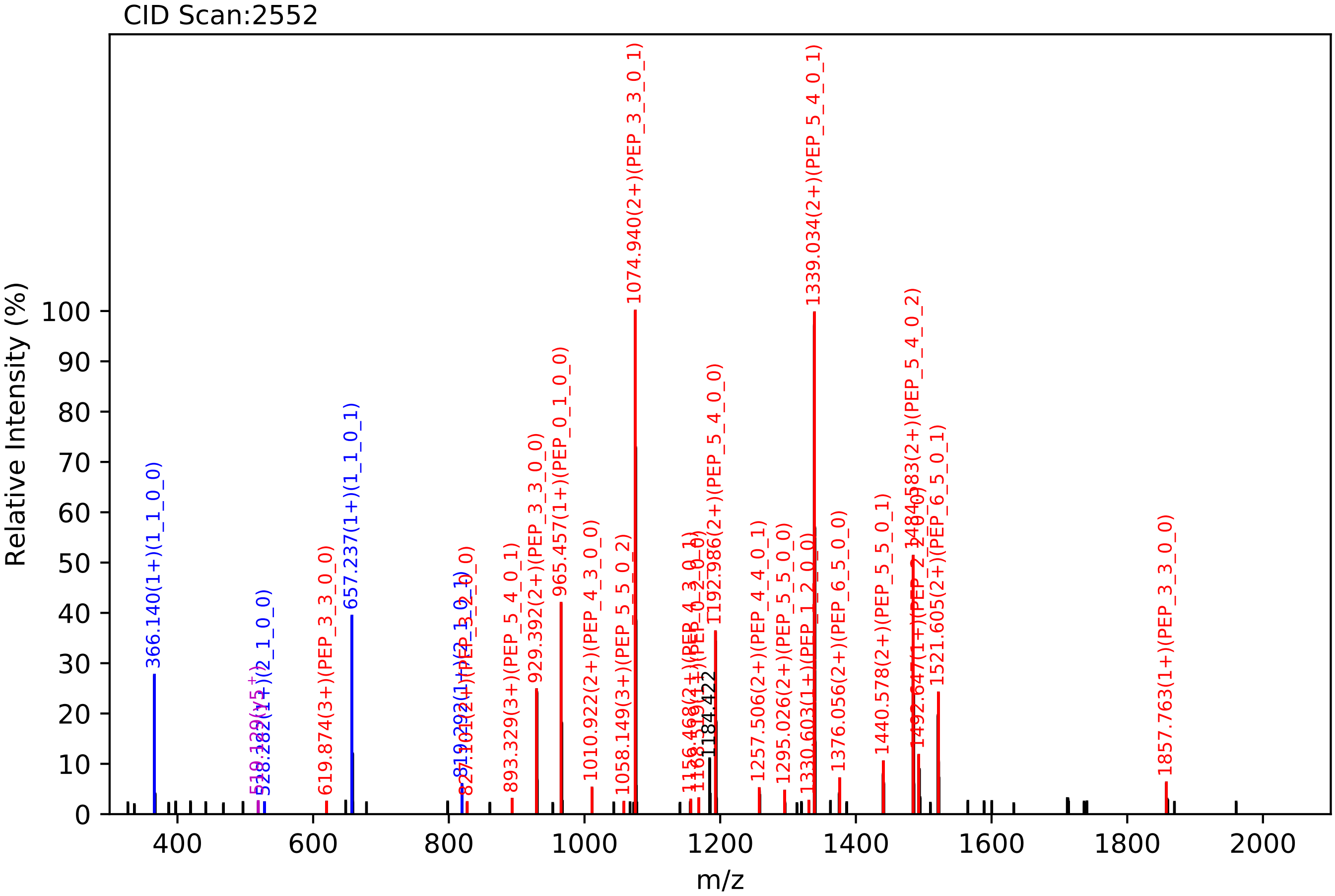

Test set no. 238, Experiment: AGP exp_3

ENGTVSR(=PEP)_6_5_0_2, m/z:1111.10(3+), RT:18.12, Y-score:89.95

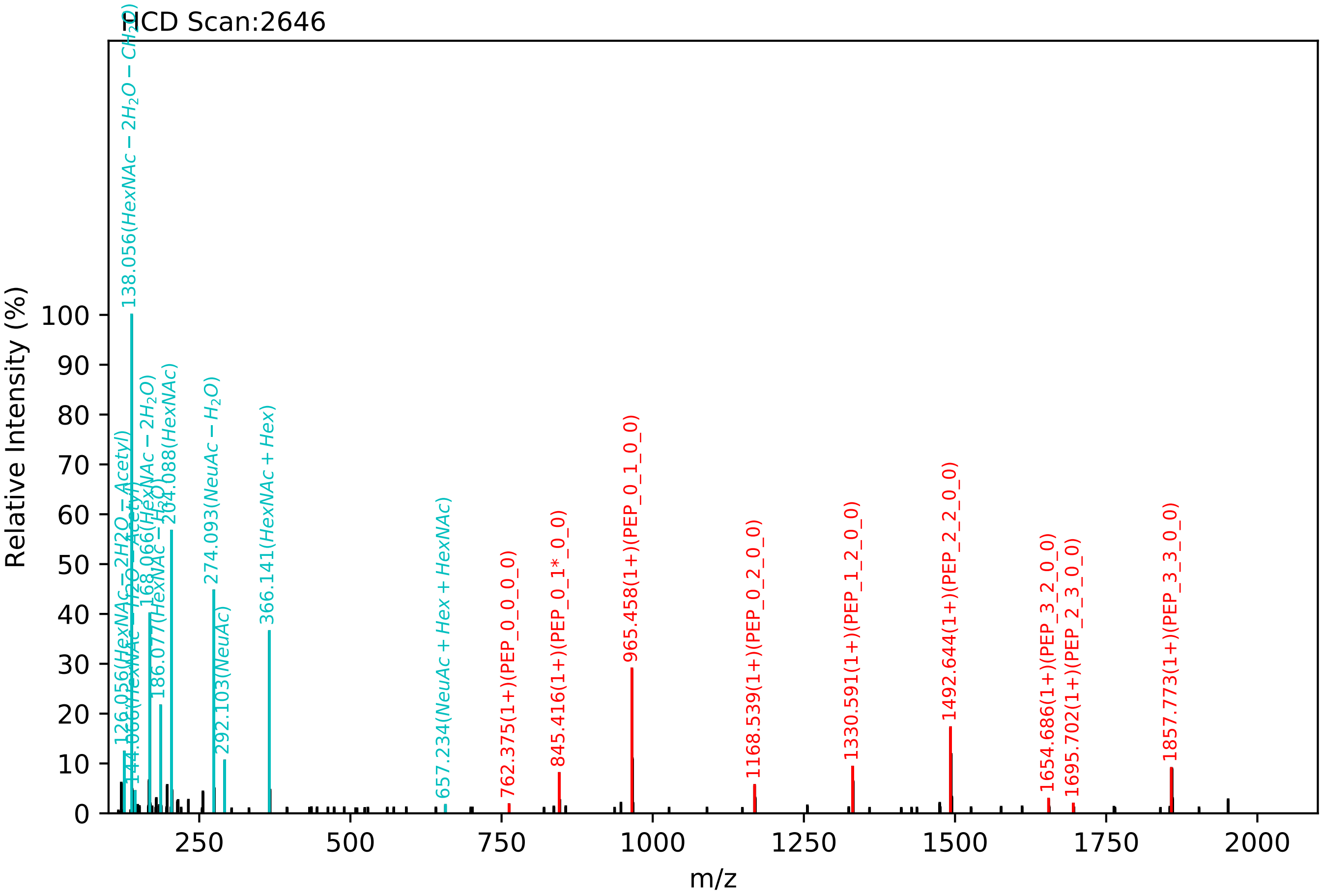

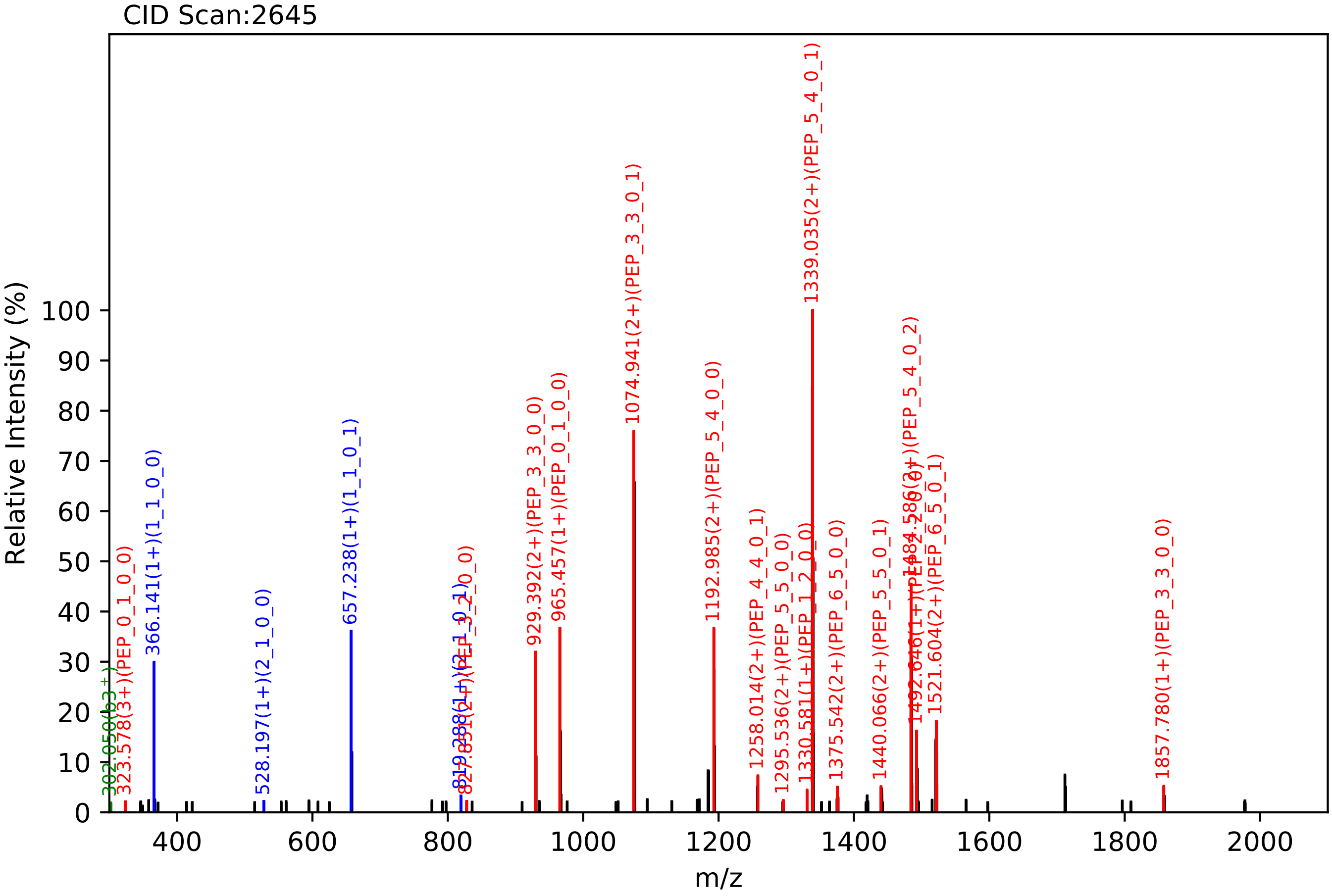

Test set no. 239, Experiment: AGP exp_4

ENGTVSR(=PEP)_6_5_0_3, m/z:1208.47(3+), RT:21.16, Y-score:90.79

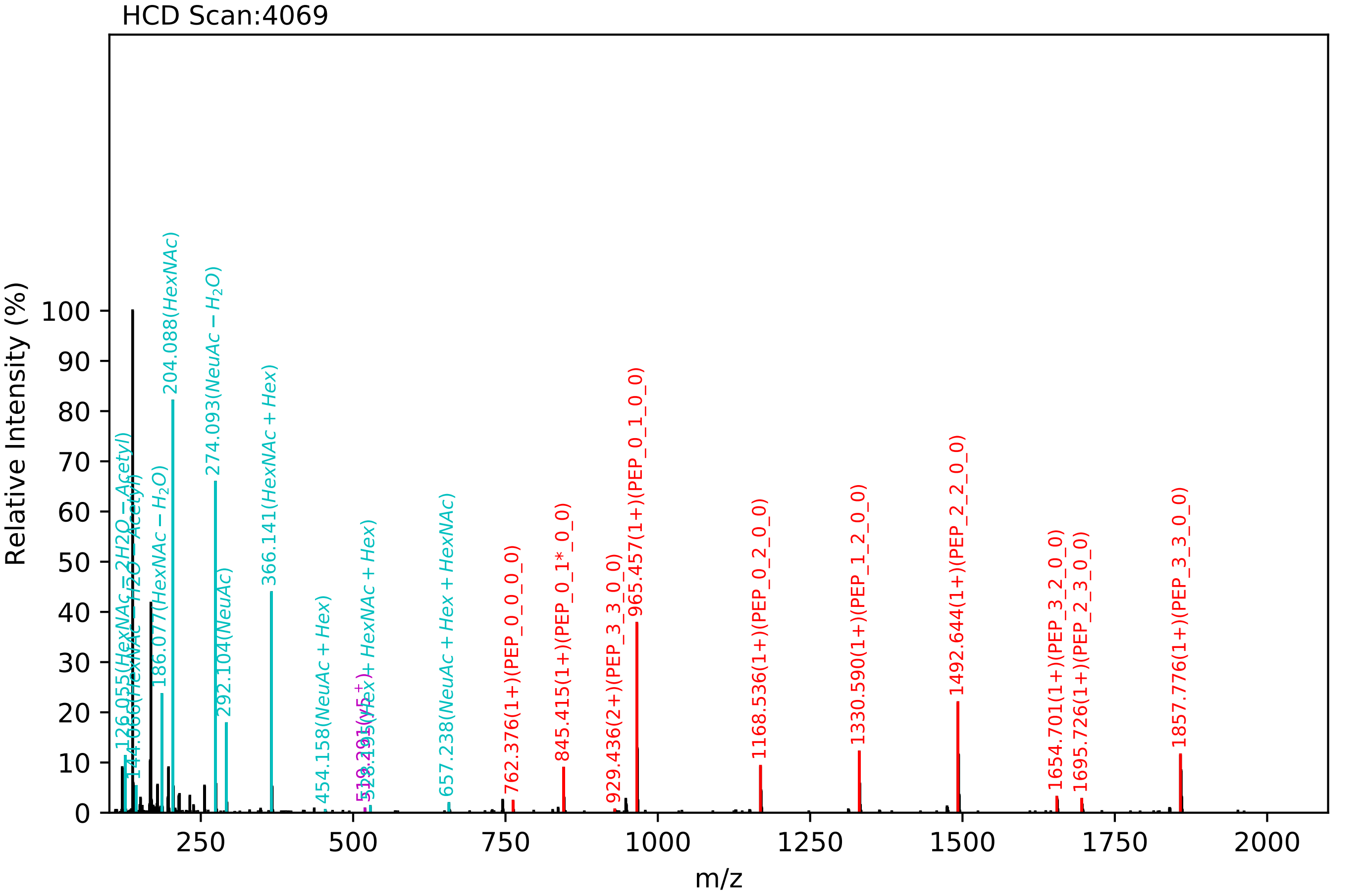

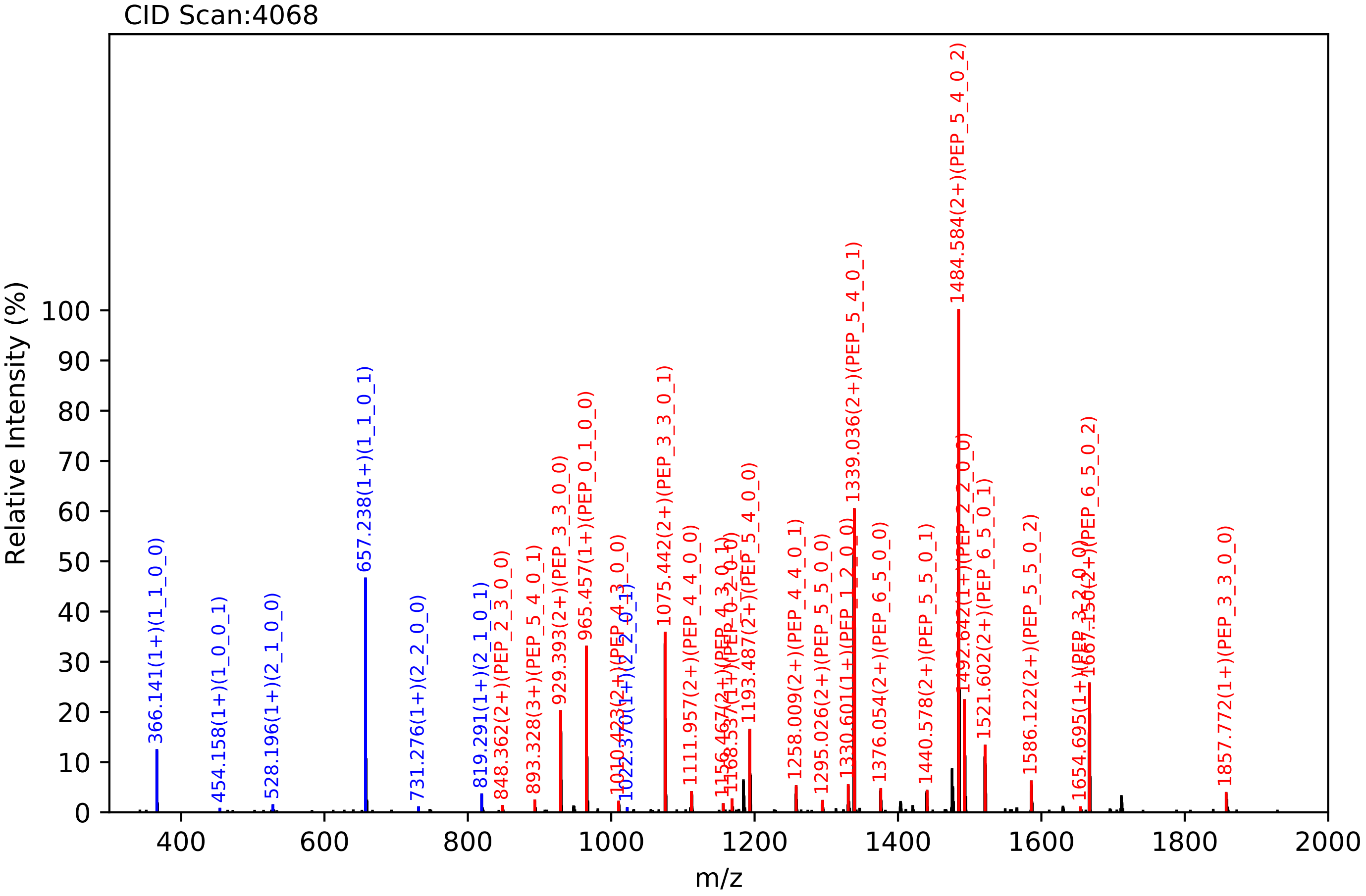

Test set no. 240, Experiment: AGP exp_3

ENGTVSR(=PEP)_6_5_0_3, m/z:1208.46(3+), RT:21.12, Y-score:90.31

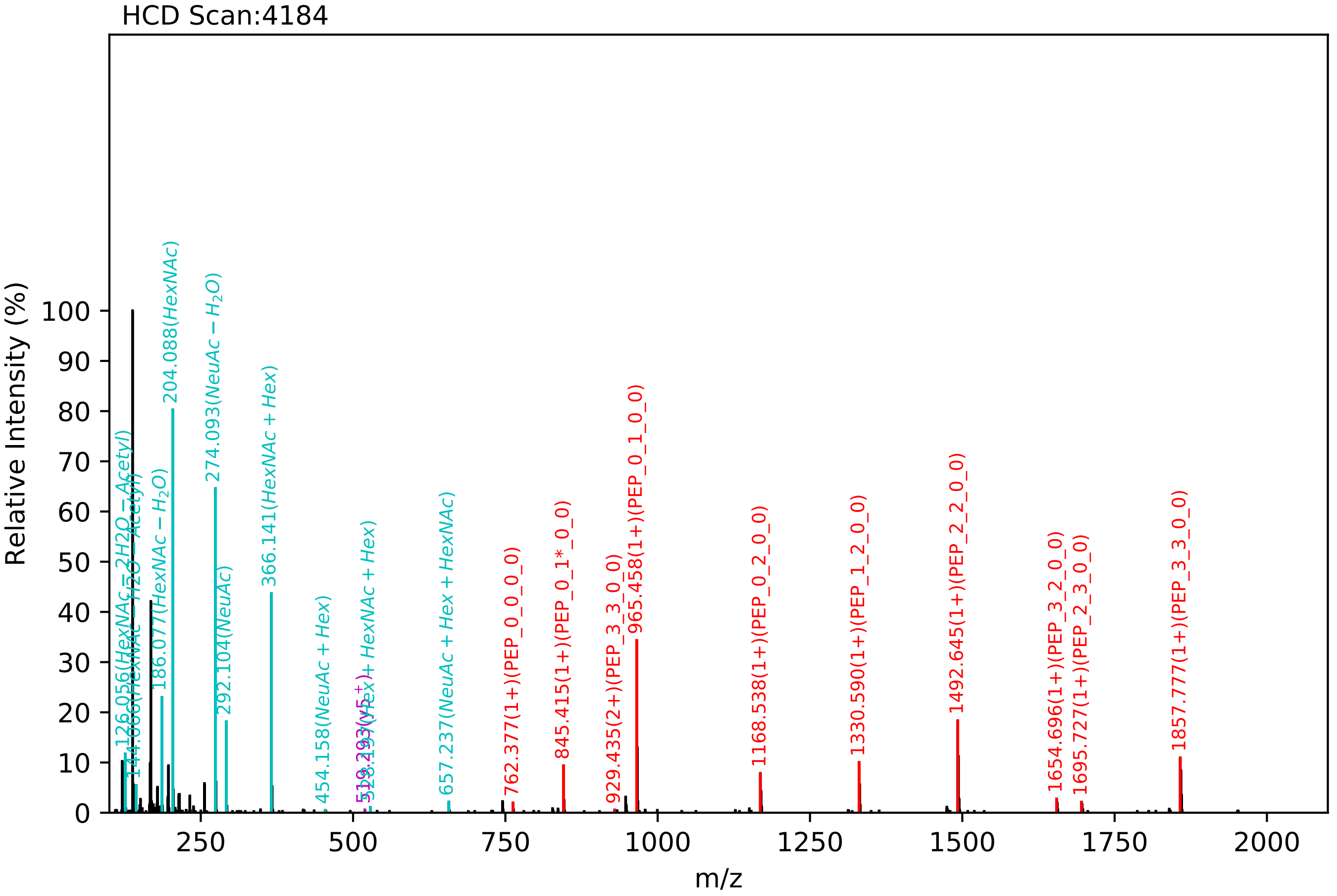

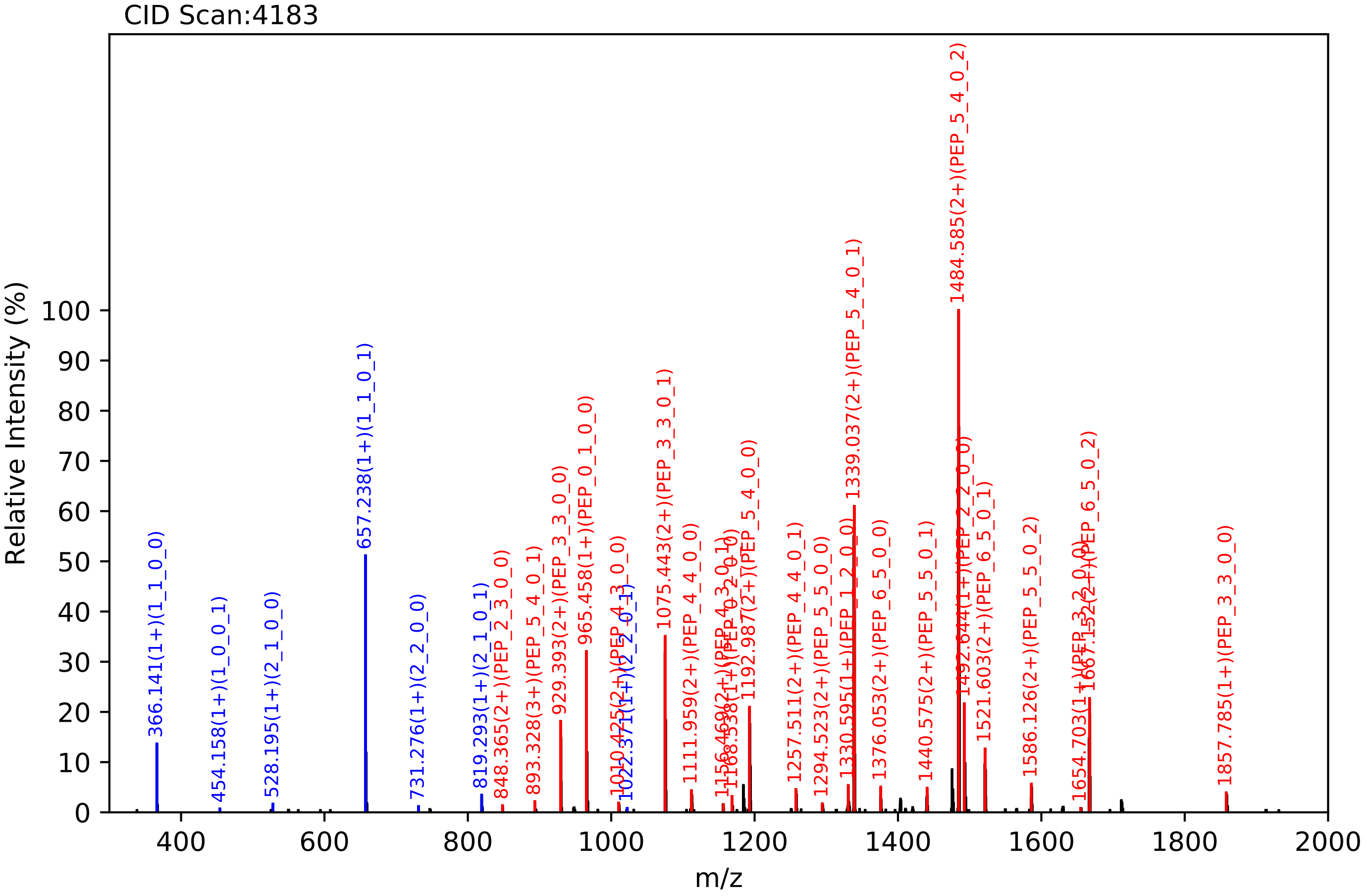

Test set no. 241, Experiment: AGP exp_3

ENGTVSR(=PEP)_6_5_1_1, m/z:1063.09(3+), RT:14.71, Y-score:89.50

HCD Scan:1432

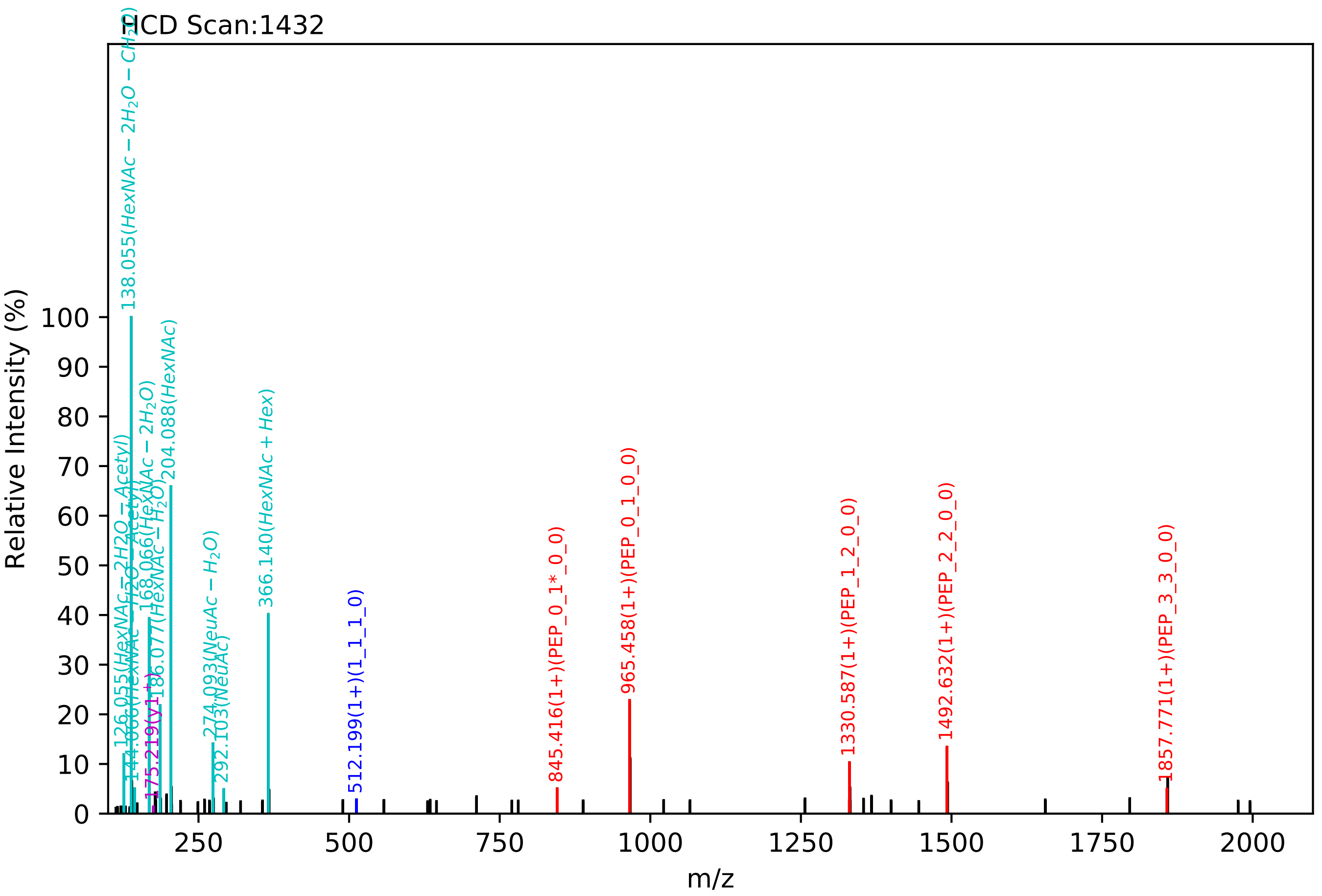

CID Scan:1431

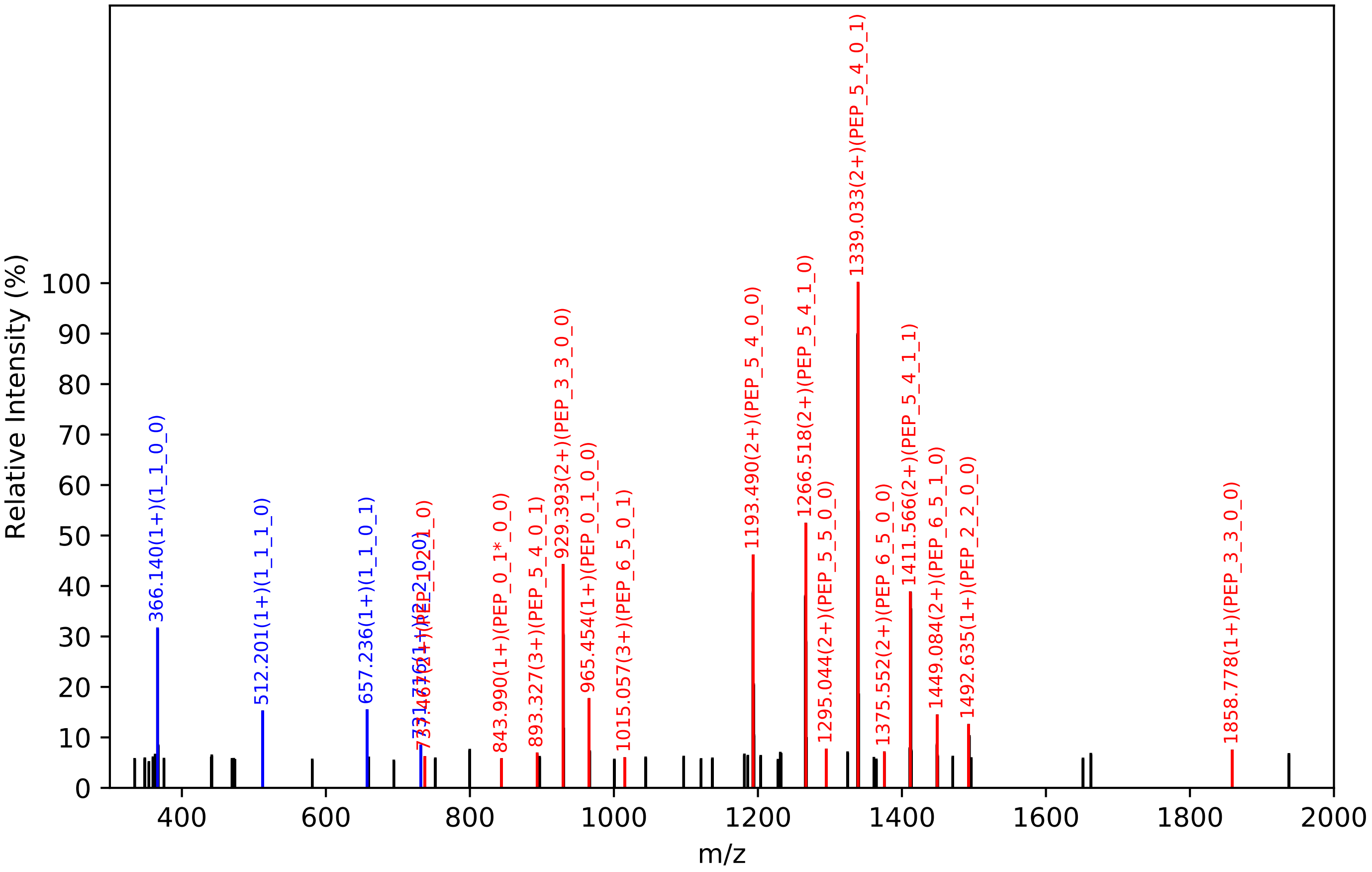

Test set no. 242, Experiment: AGP exp_4

ENGTVSR(=PEP)_6_5_1_1, m/z:1063.09(3+), RT:14.90, Y-score:87.34

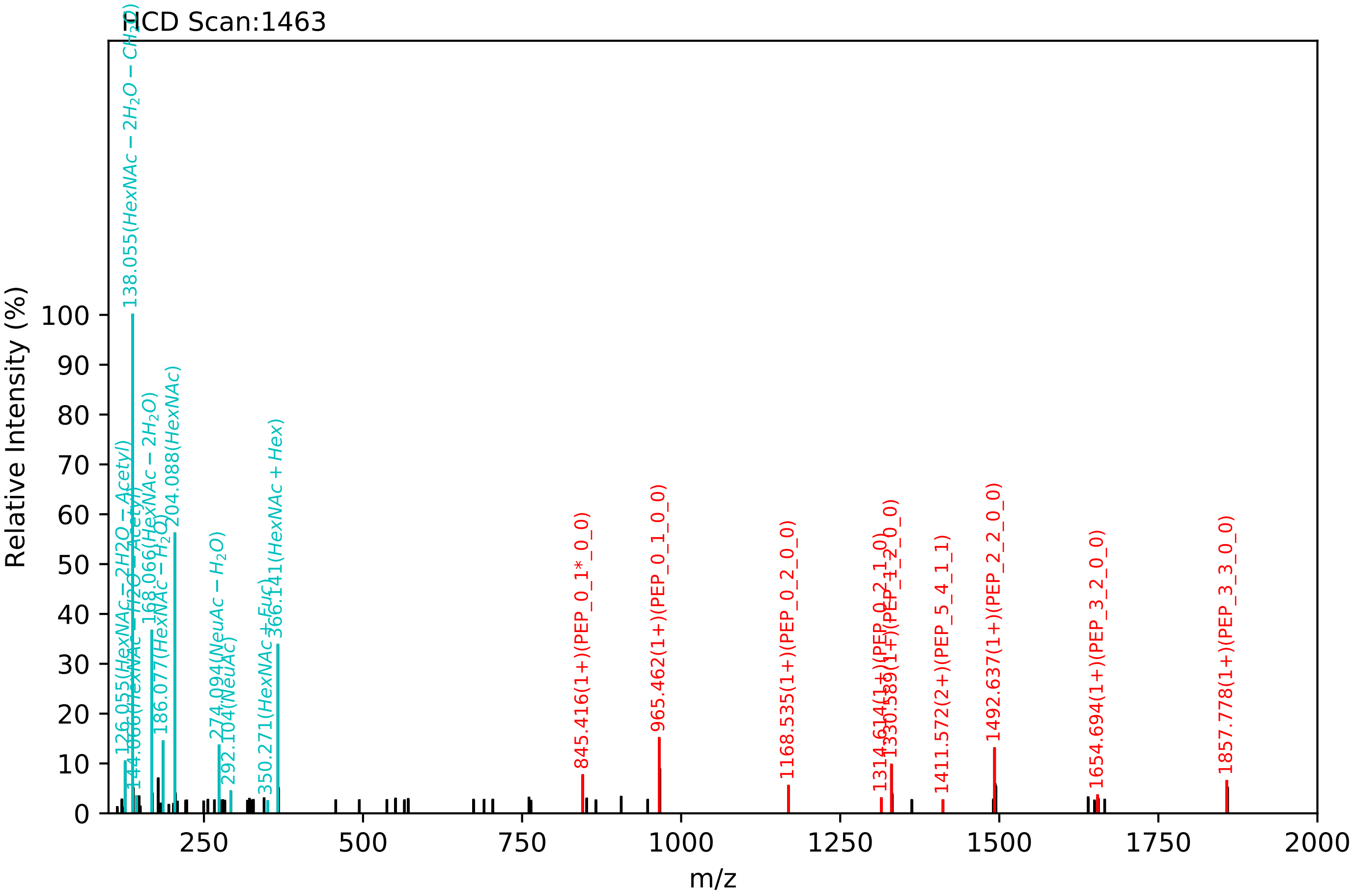

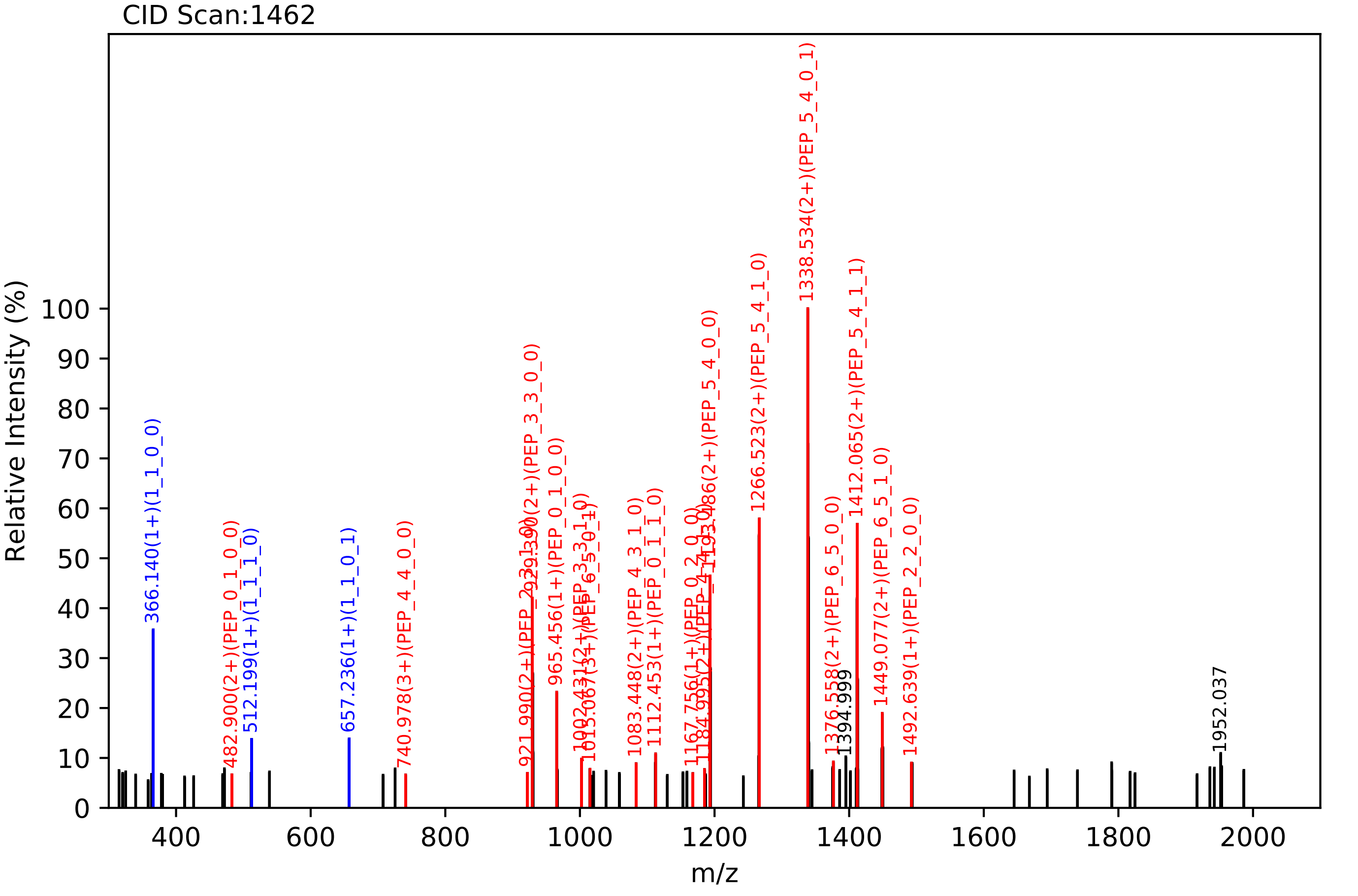

Test set no. 243, Experiment: AGP exp_4

ENGTVSR(=PEP)_6_5_1_1, m/z:1063.09(3+), RT:15.44, Y-score:87.27

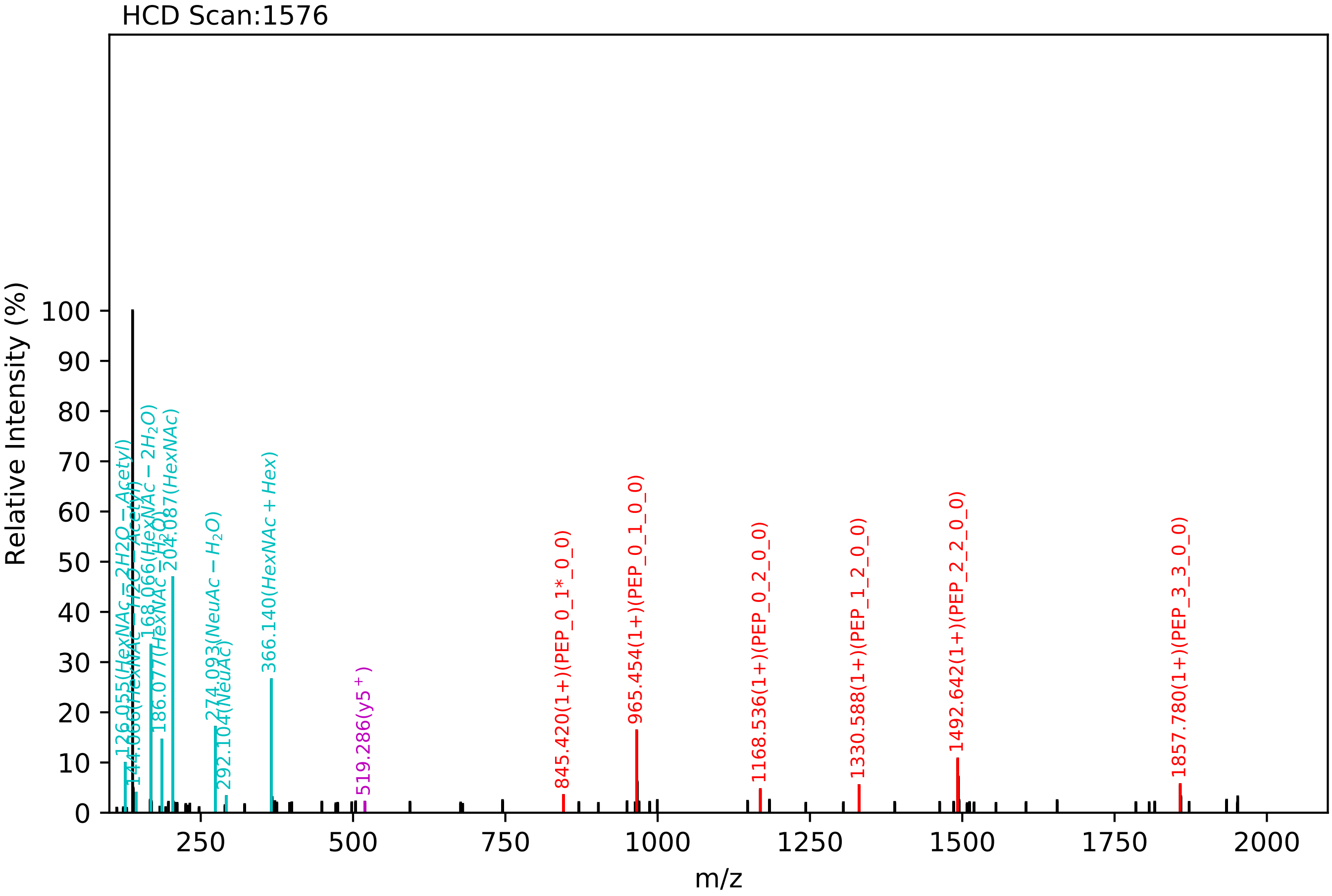

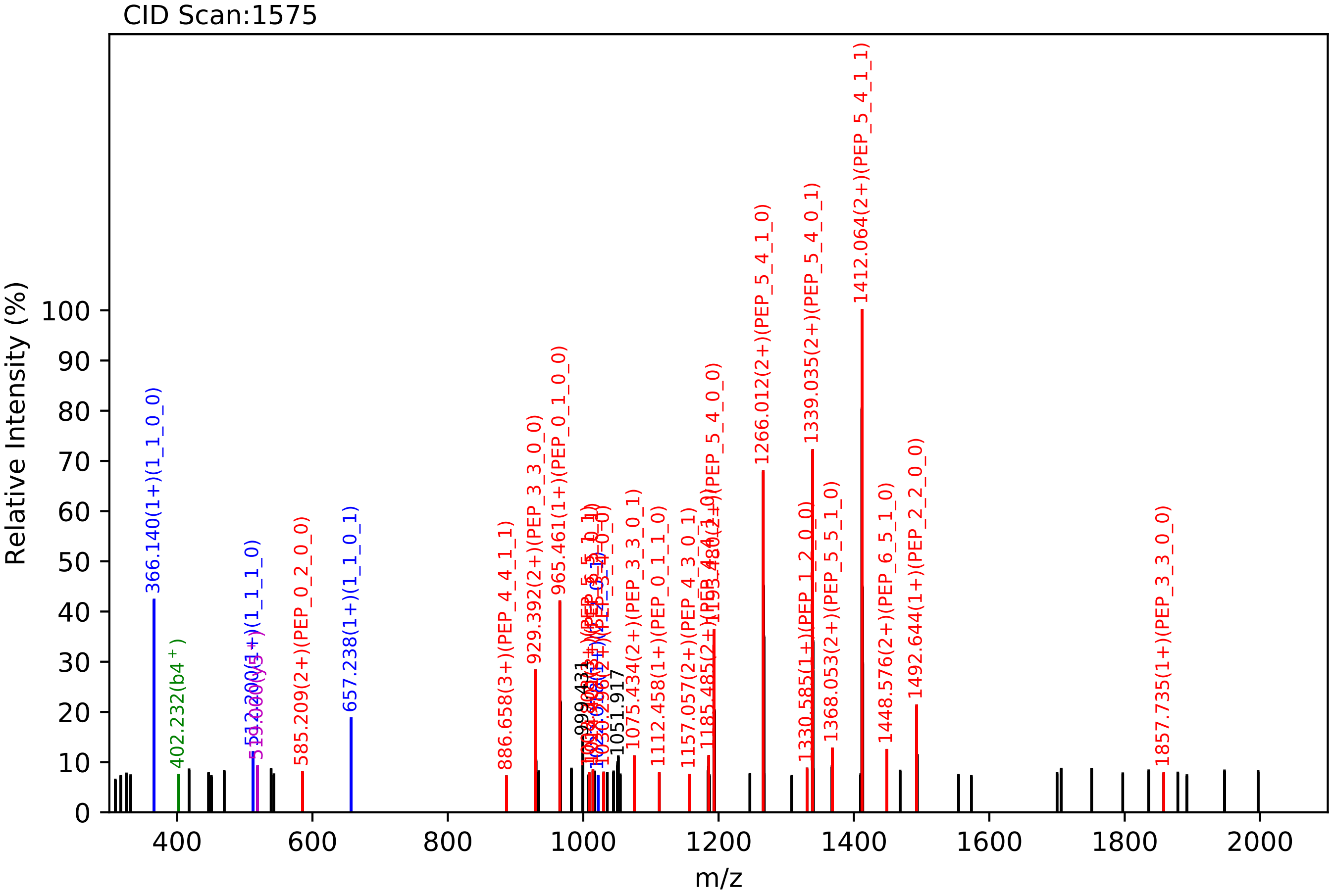

Test set no. 244, Experiment: AGP exp_3

ENGTVSR(=PEP)_6_5_1_2, m/z:1160.12(3+), RT:17.90, Y-score:96.69

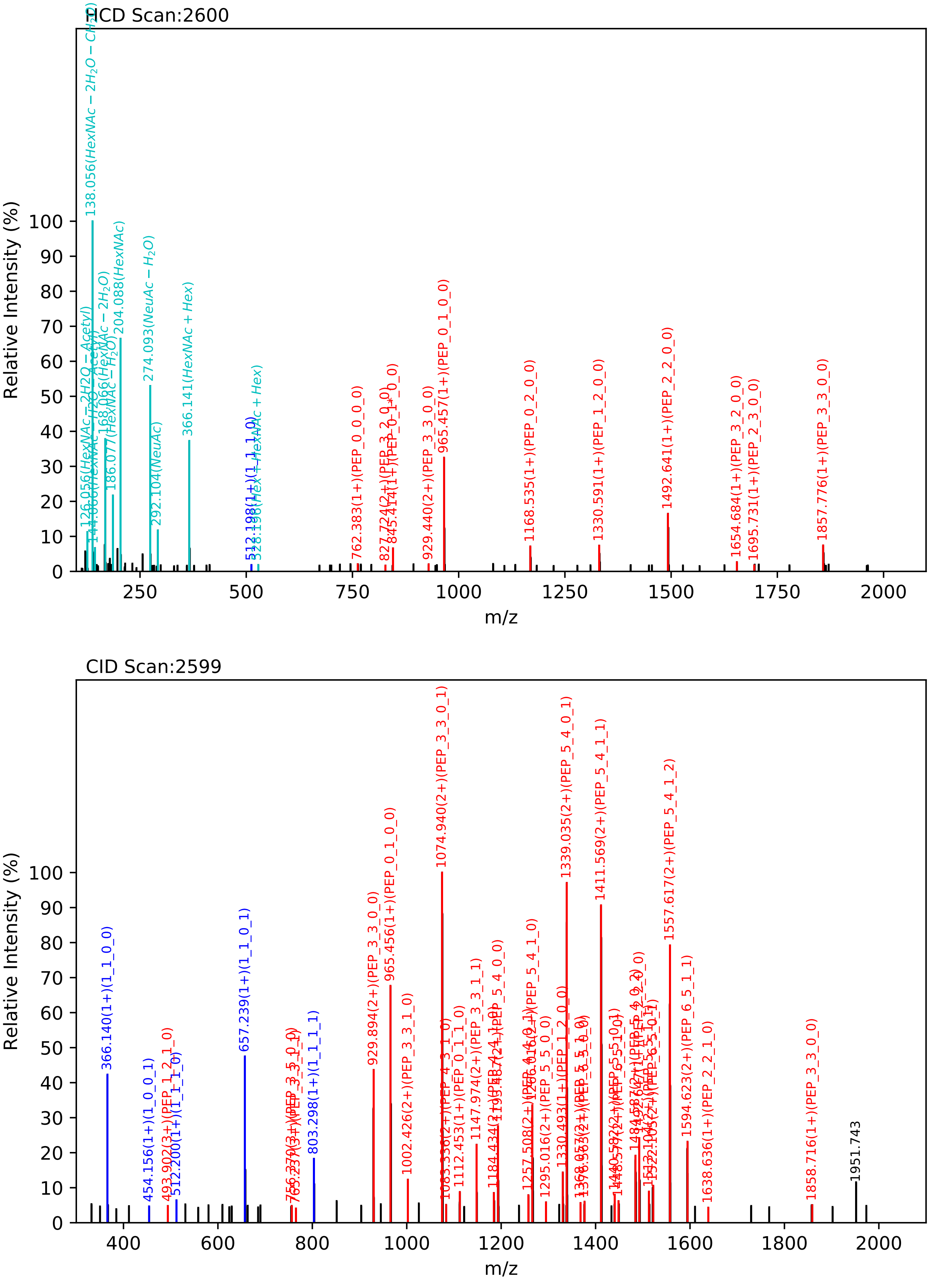

Test set no. 245, Experiment: AGP exp_4

ENGTVSR(=PEP)_6_5_1_2, m/z:1160.12(3+), RT:17.11, Y-score:94.38

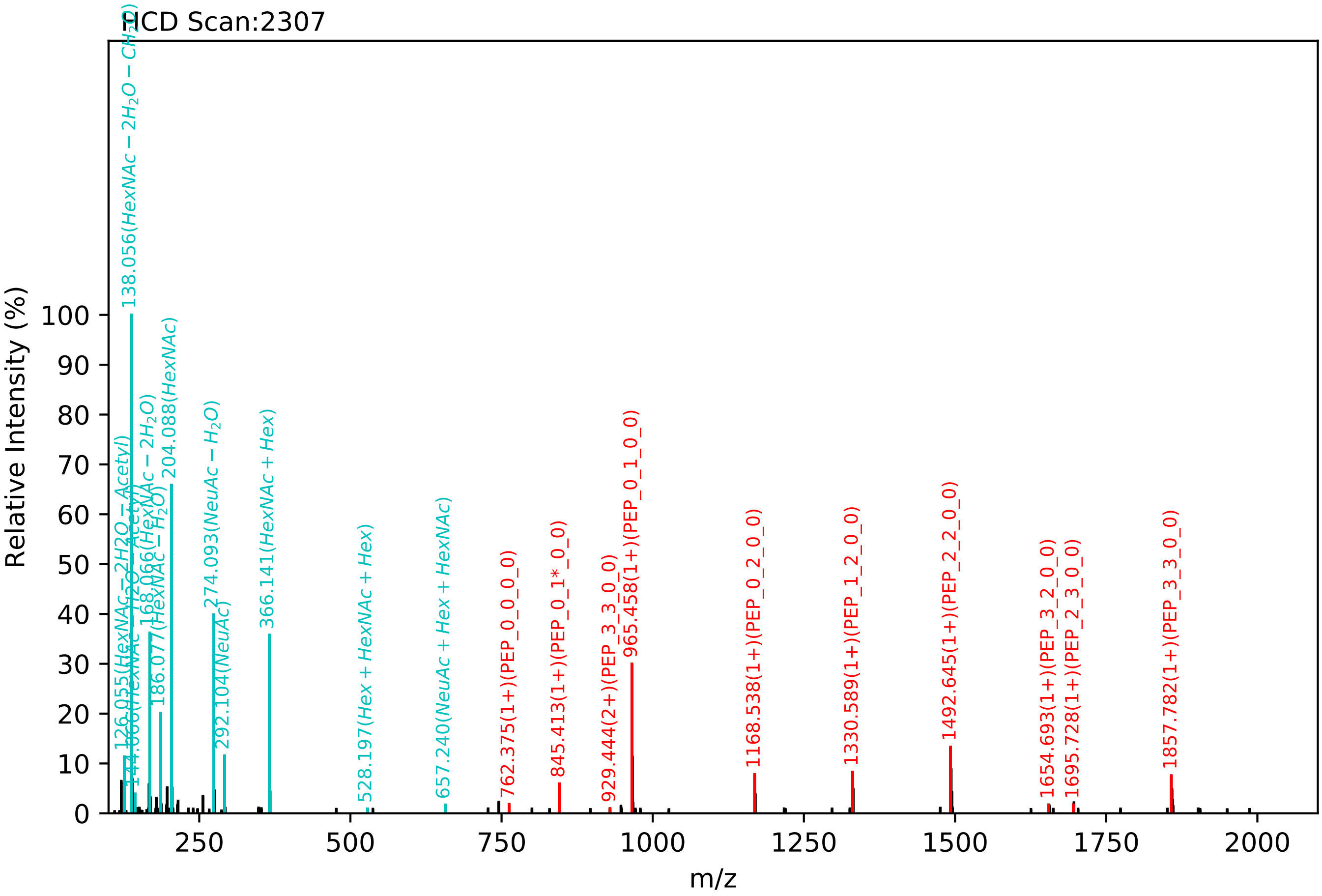

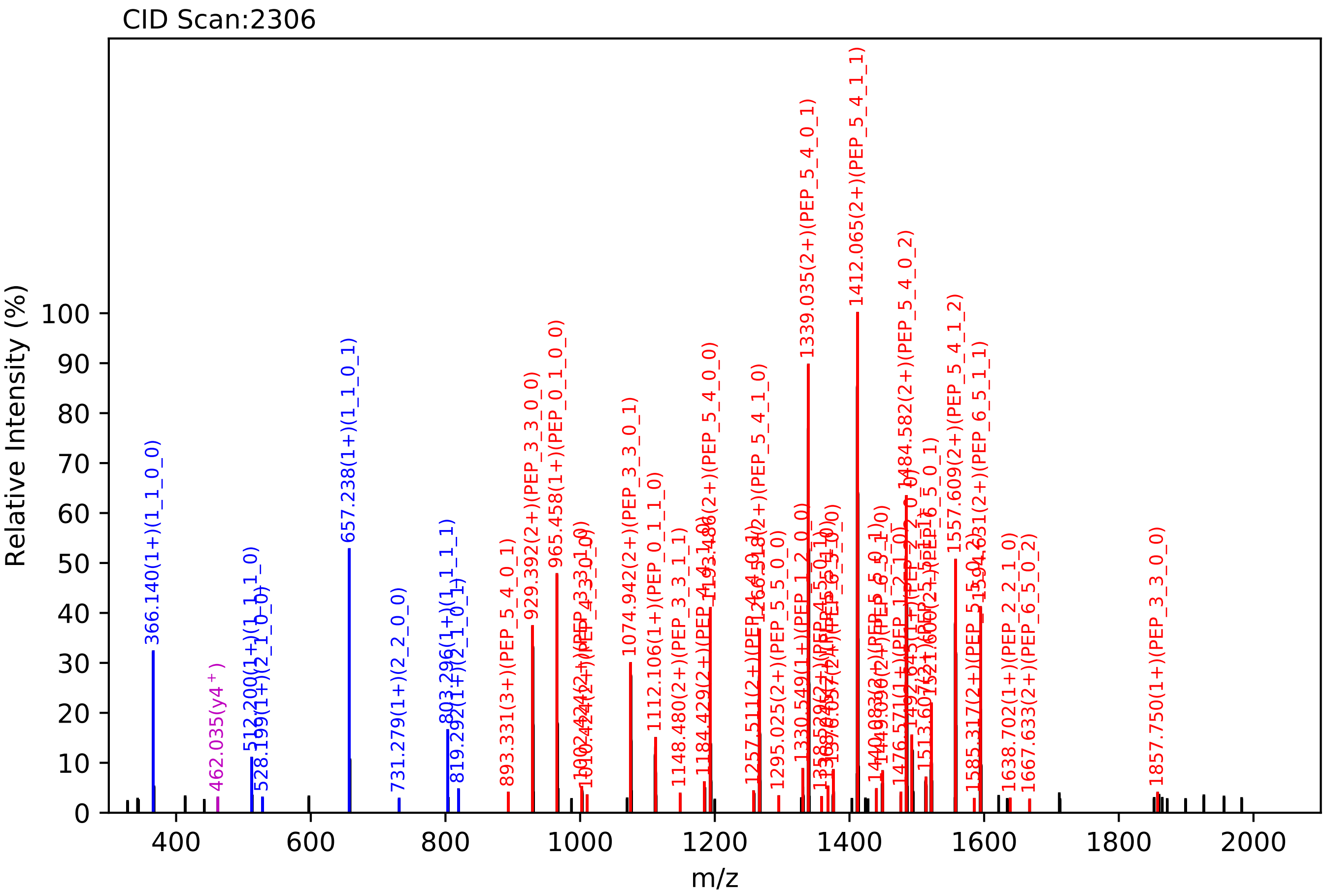

Test set no. 246, Experiment: AGP exp_4

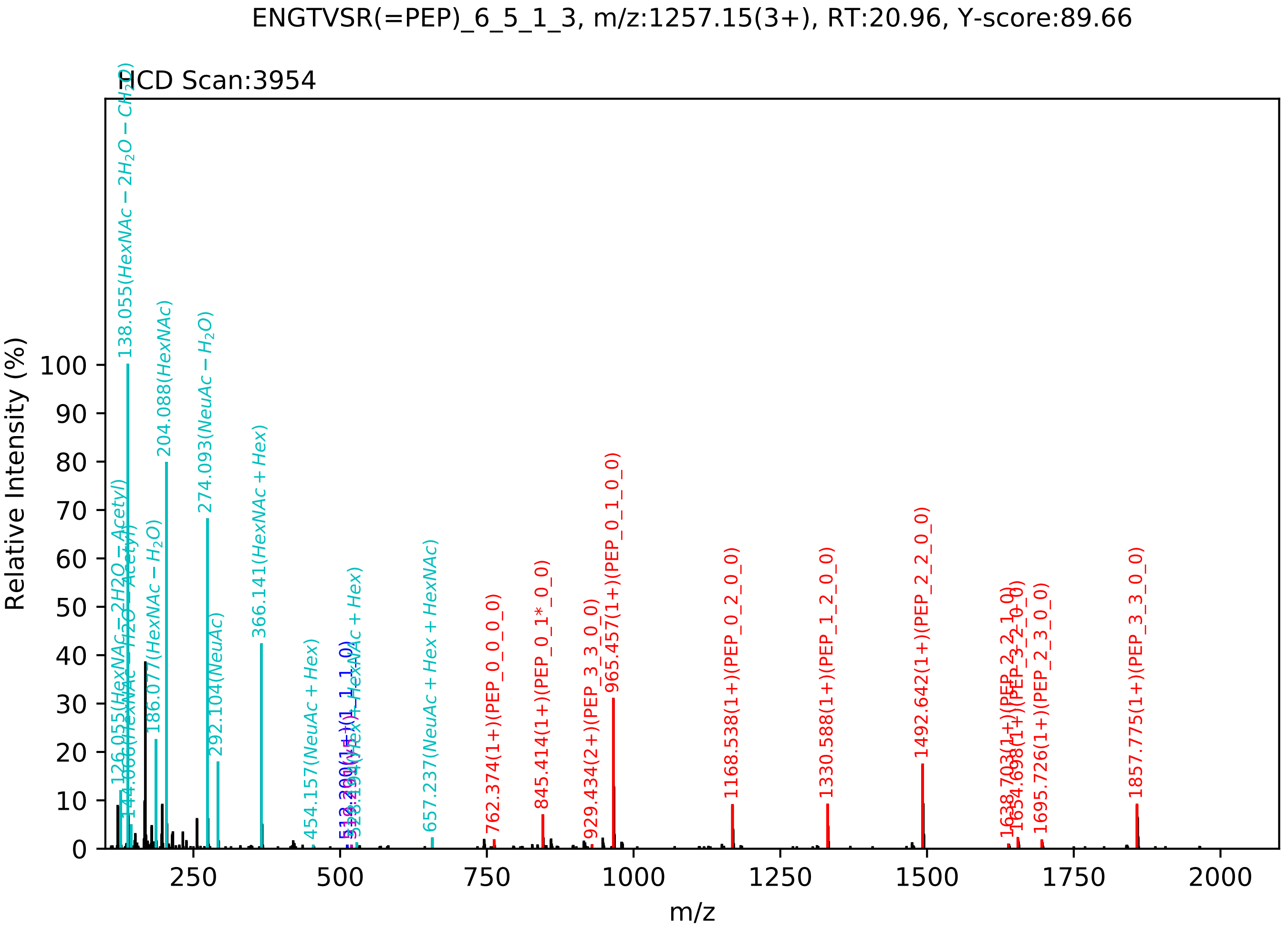

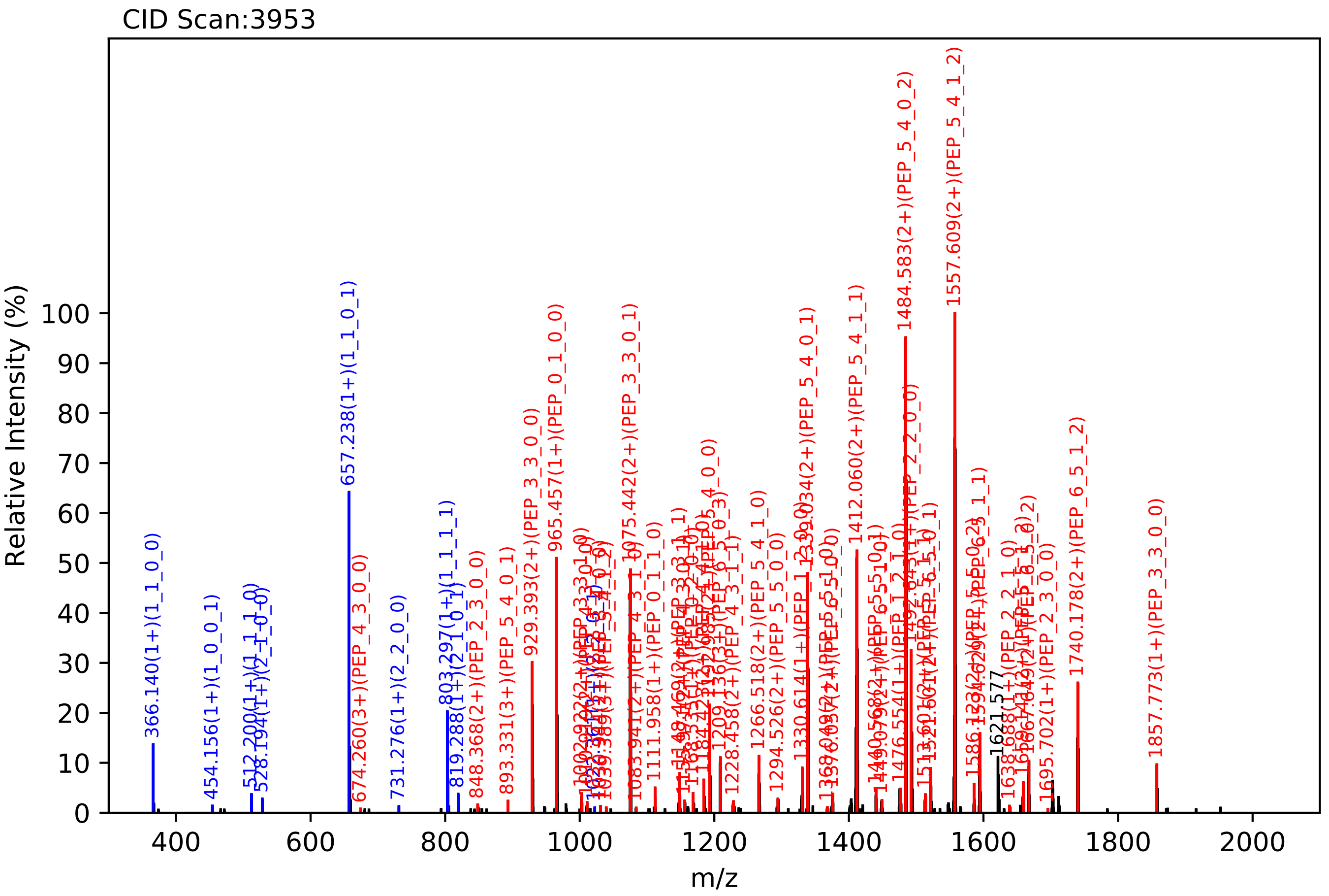

Test set no. 247, Experiment: AGP exp_3

ENGTVSR(=PEP)_6_5_1_3, m/z:1257.15(3+), RT:20.91, Y-score:87.38

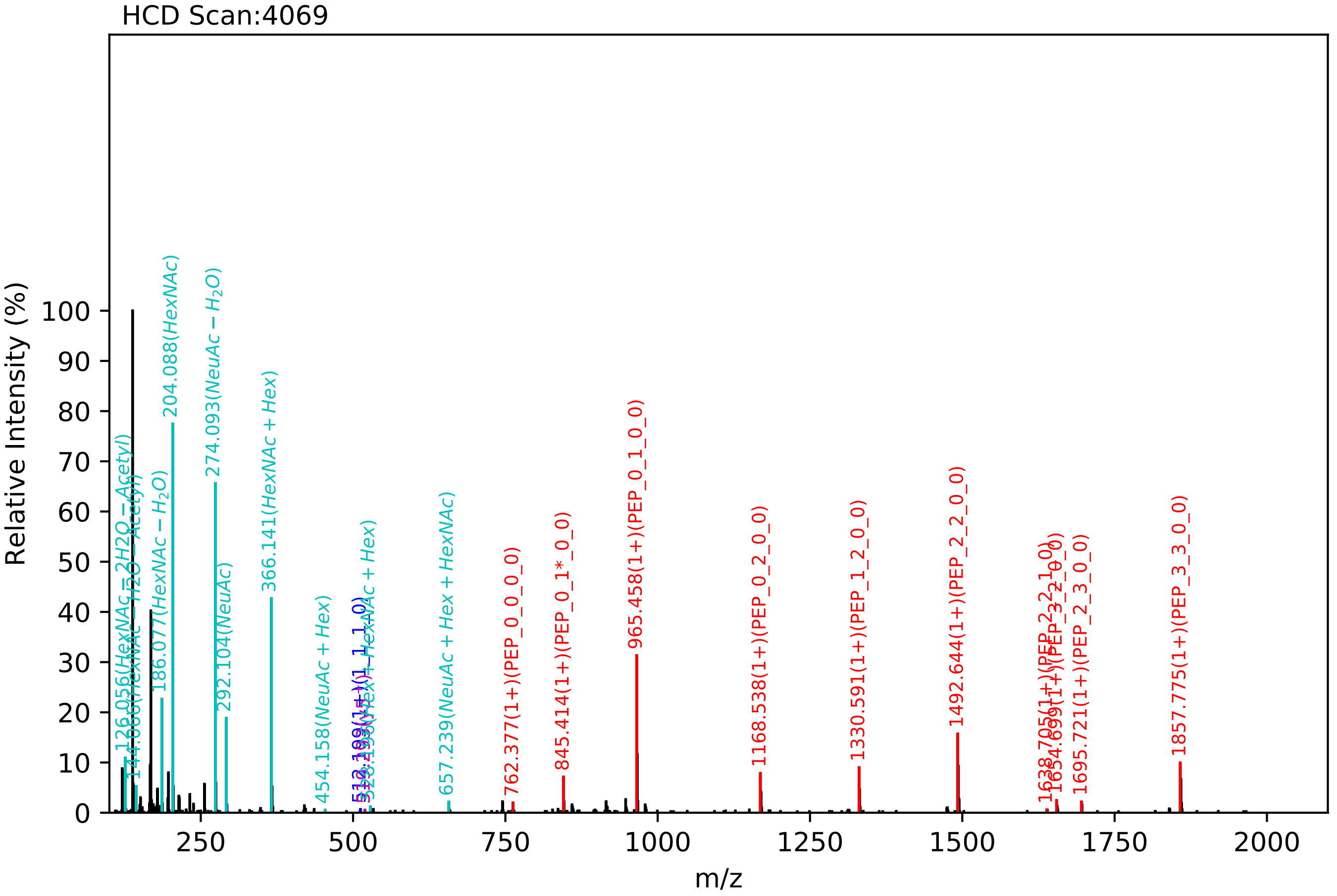

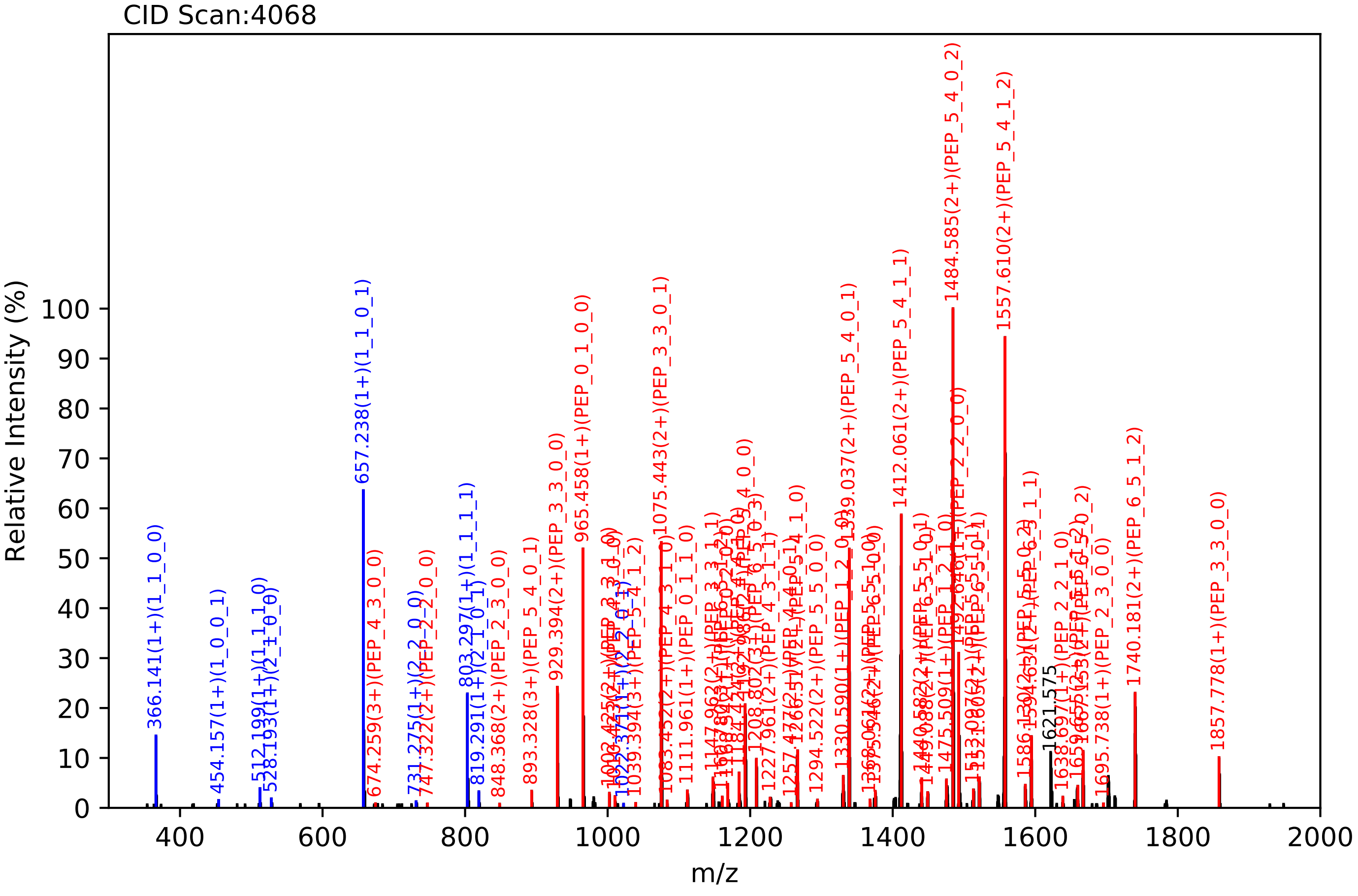

Test set no. 248, Experiment: AGP exp_23

ENGTVSR(=PEP)_6_5_2_3, m/z:1305.84(3+), RT:26.25, Y-score:92.73

HCD Scan:6763

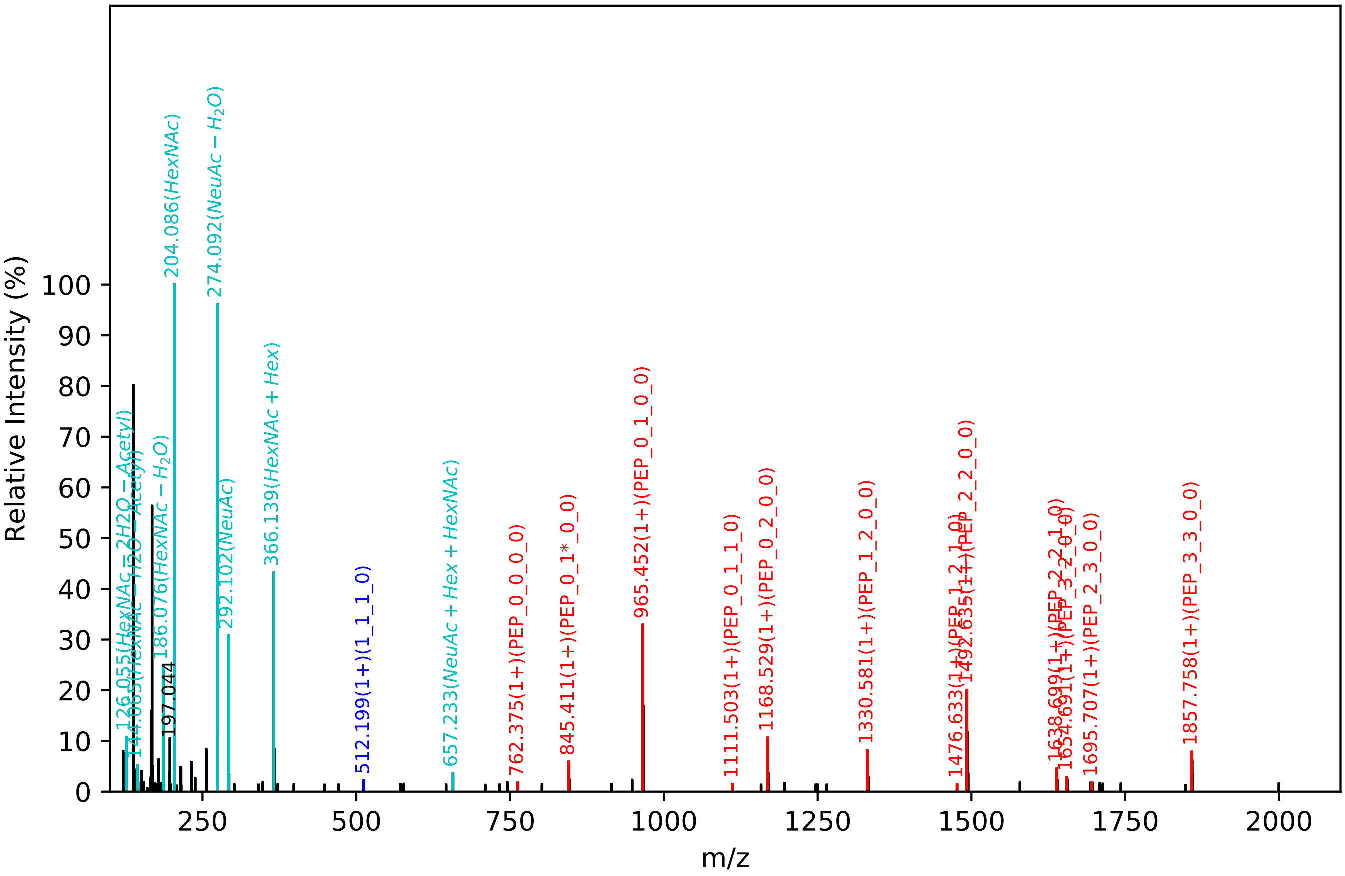

CID Scan:6765

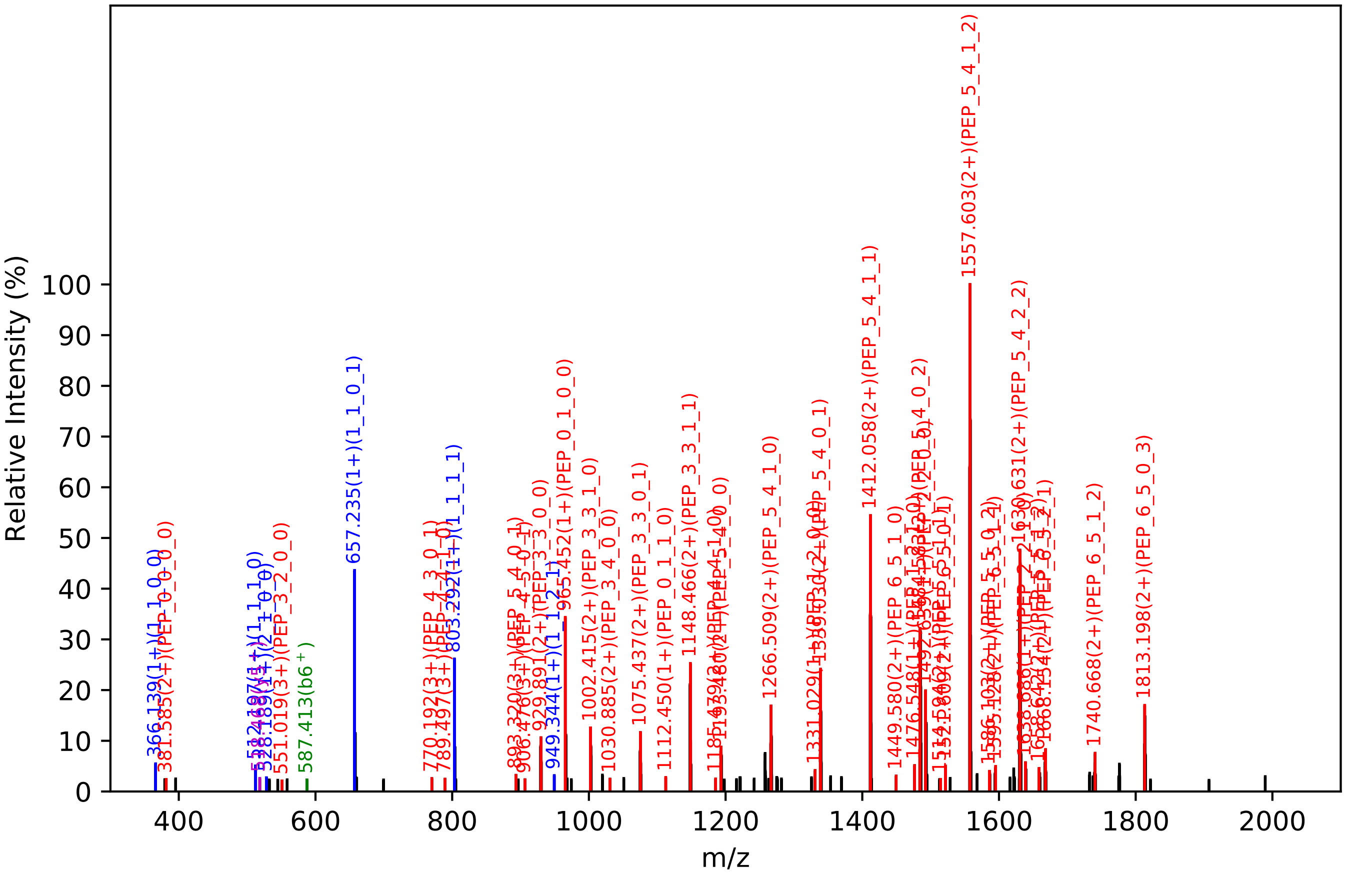

Test set no. 249, Experiment: AGP exp_9

ENGTVSR(=PEP)_6_5_2_3, m/z:1305.84(3+), RT:26.05, Y-score:91.34

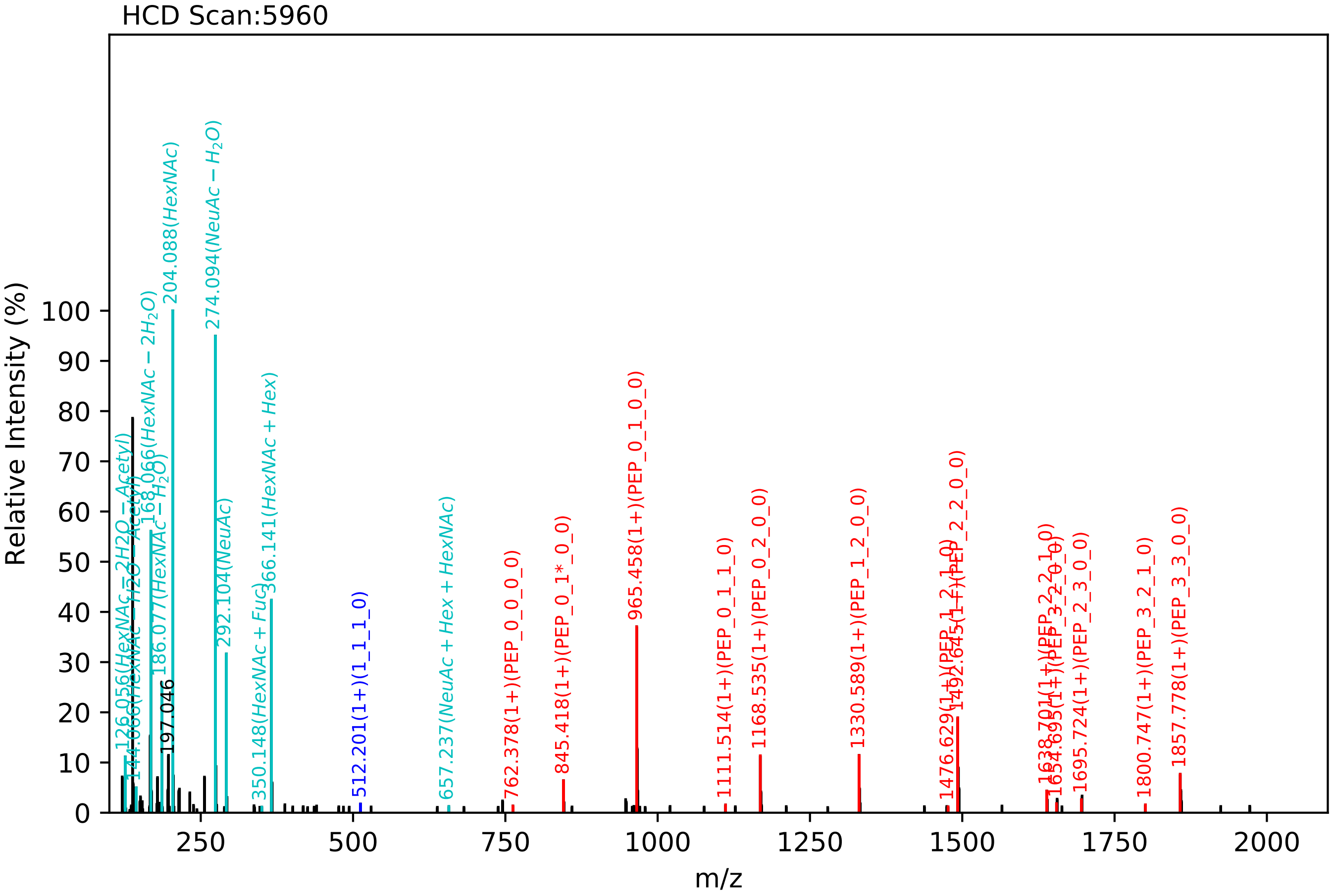

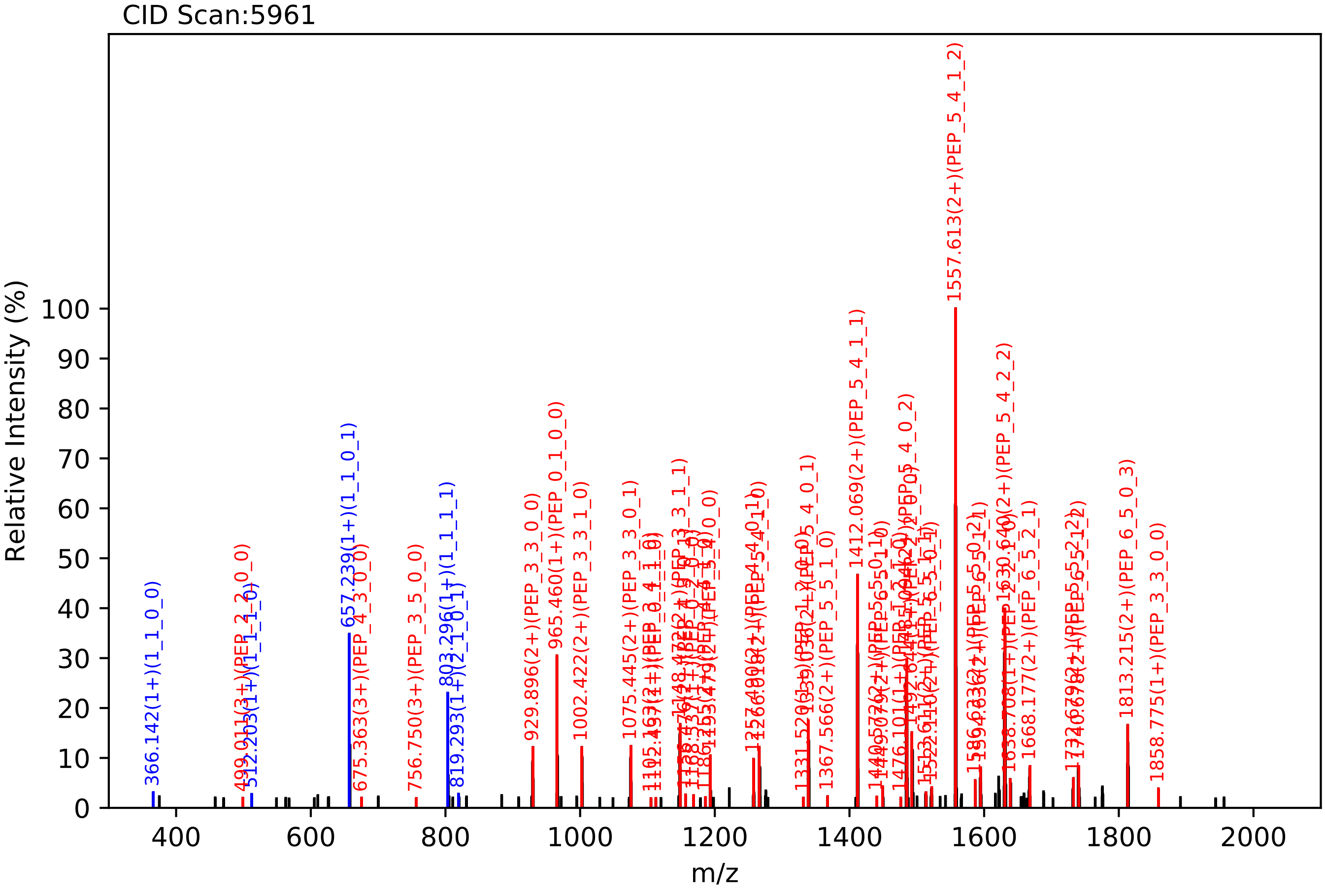

Test set no. 250, Experiment: AGP exp_40

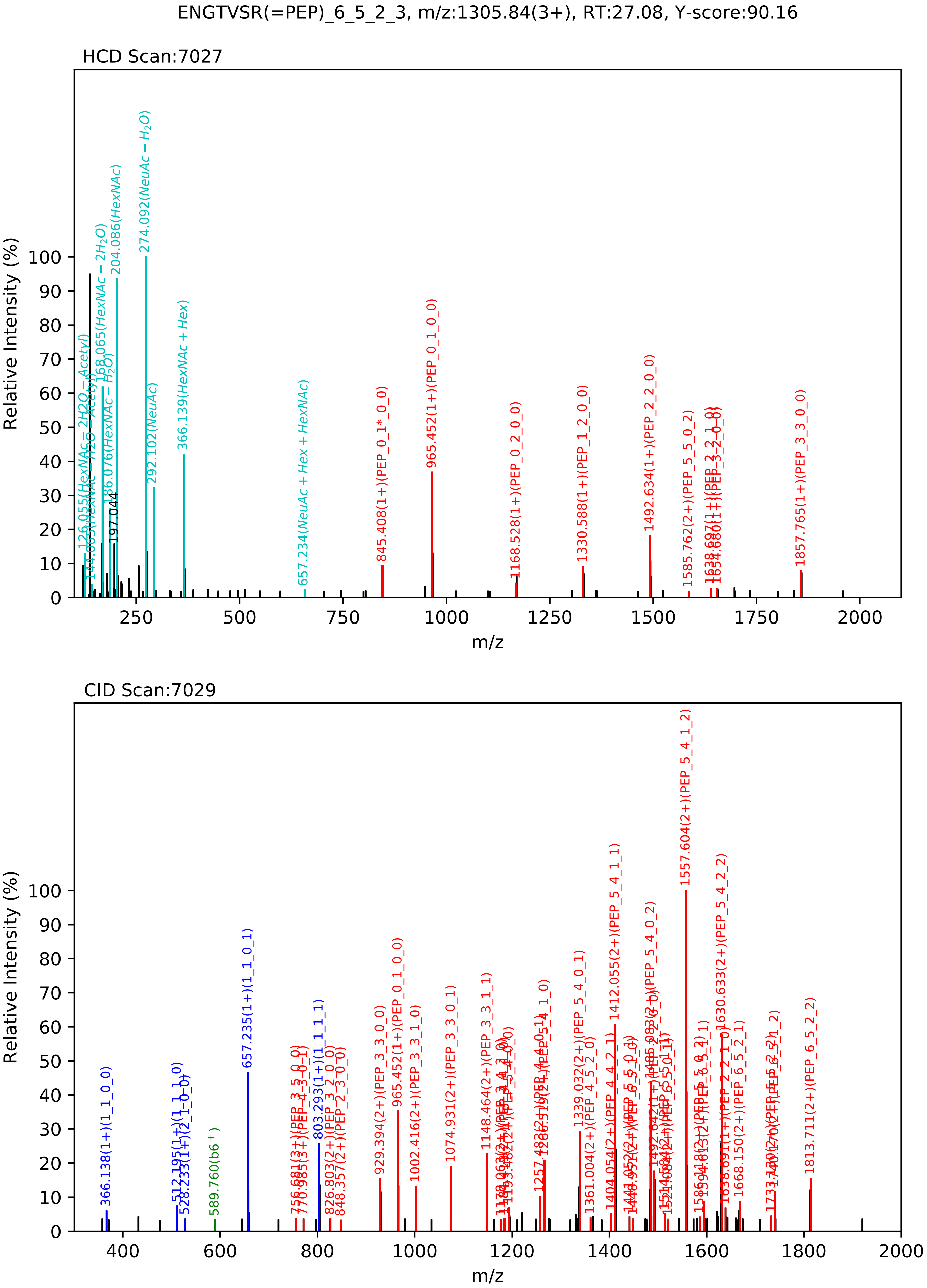

Test set no. 251, Experiment: AGP exp_41

ENGTVSR(=PEP)_6_5_2_3, m/z:1305.84(3+), RT:27.39, Y-score:90.06

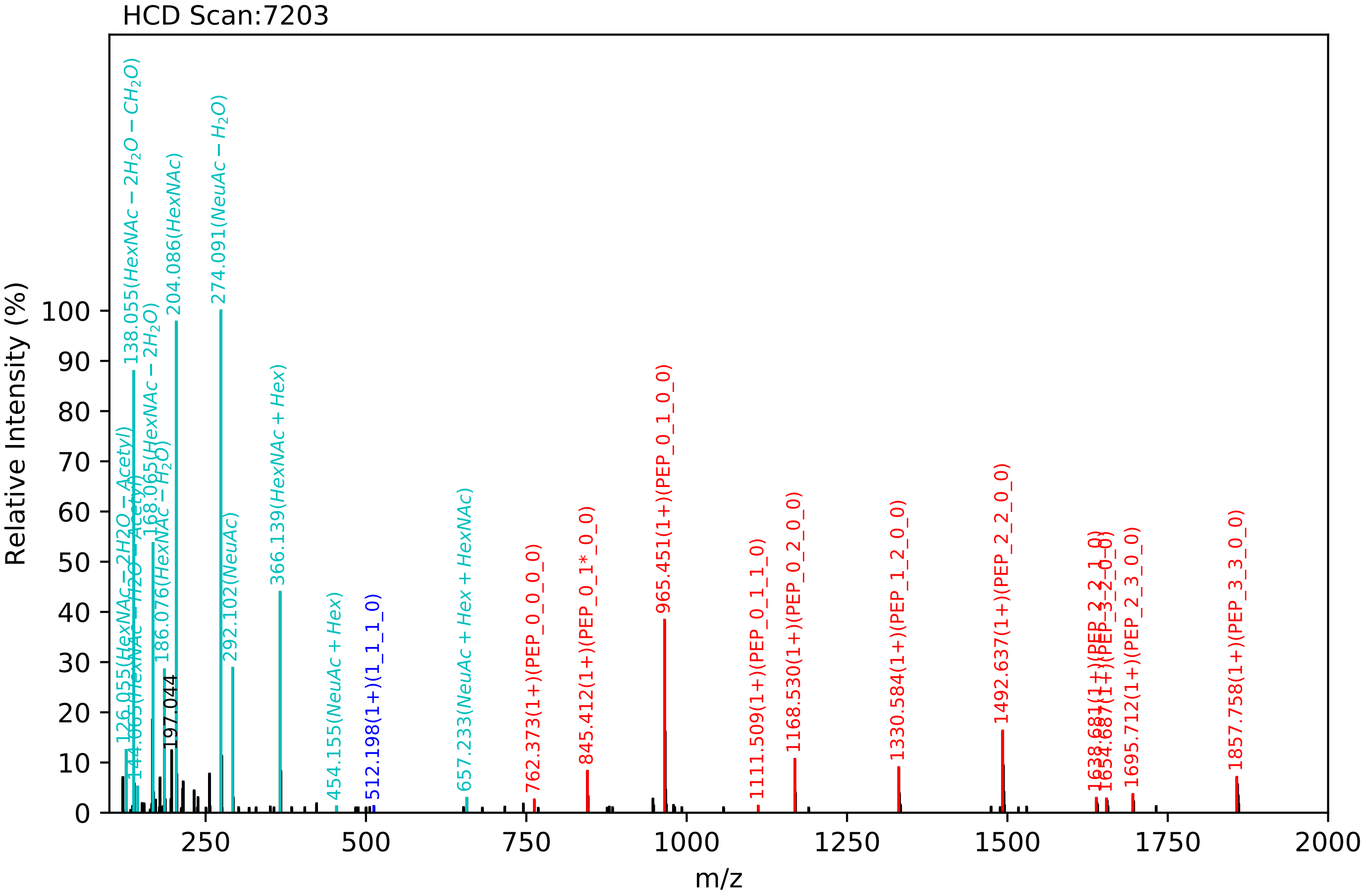

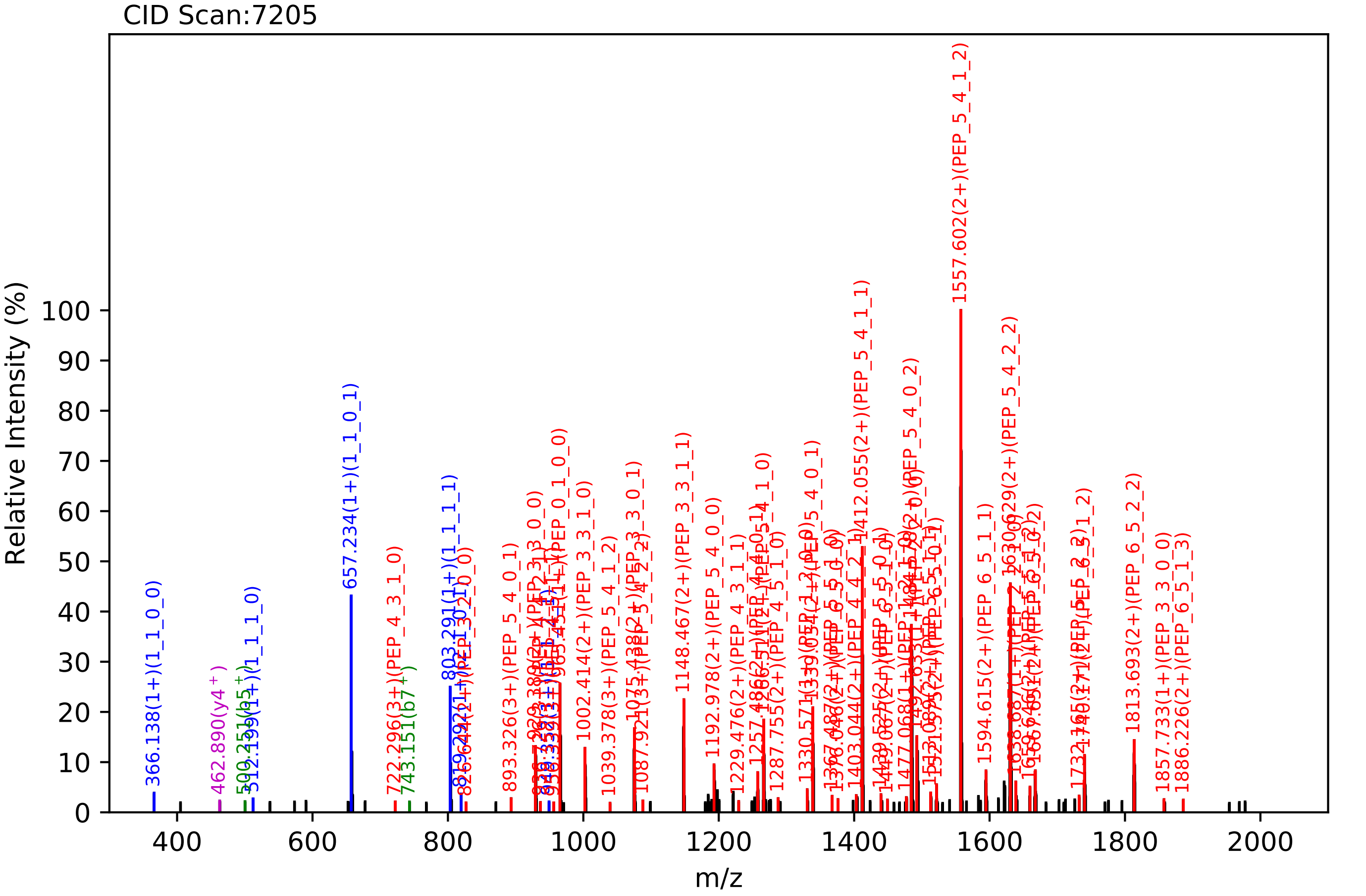

Test set no. 252, Experiment: AGP exp_38

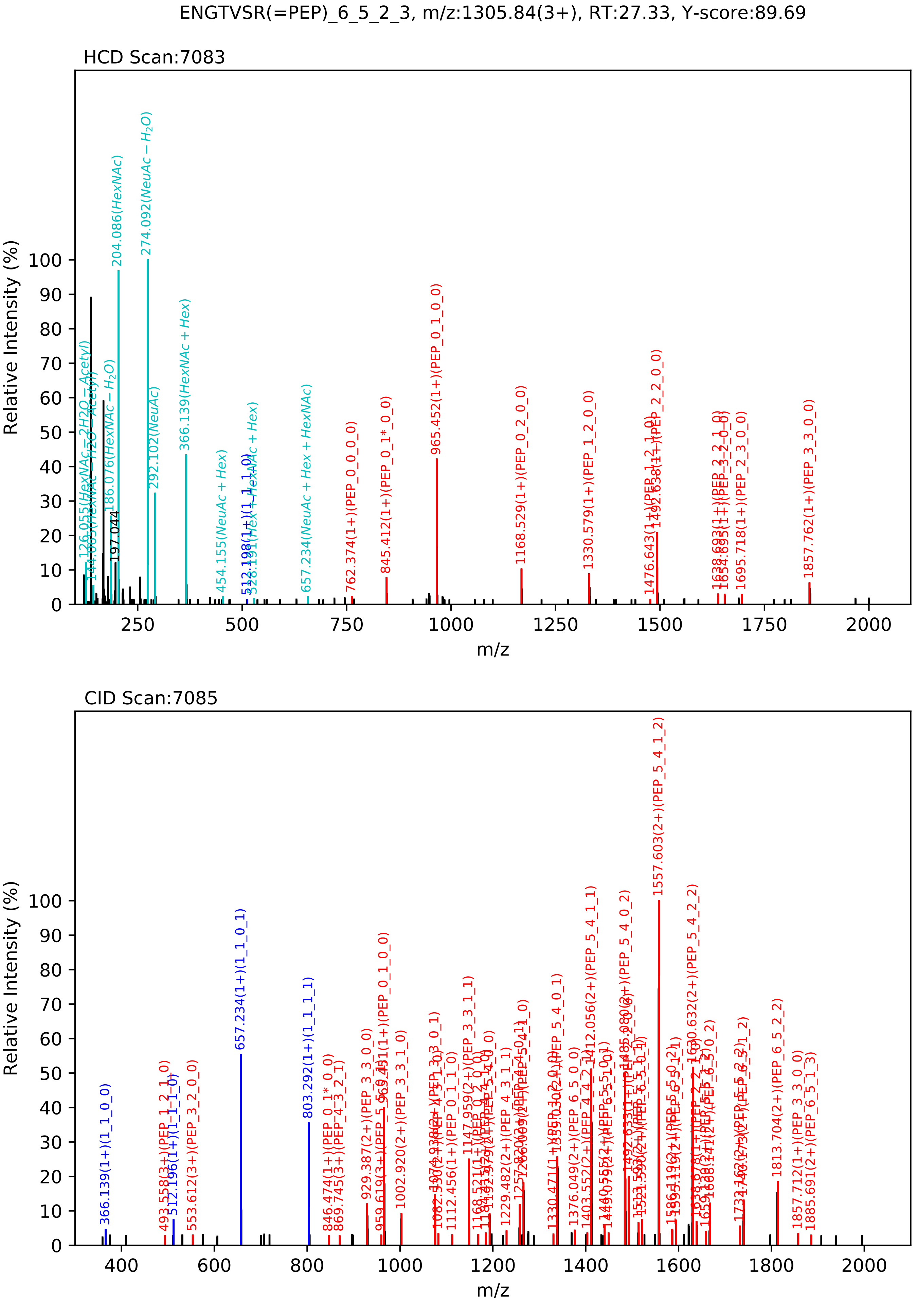

Test set no. 253, Experiment: AGP exp_34

ENGTVSR(=PEP)_6_5_2_3, m/z:1305.84(3+), RT:29.30, Y-score:89.04

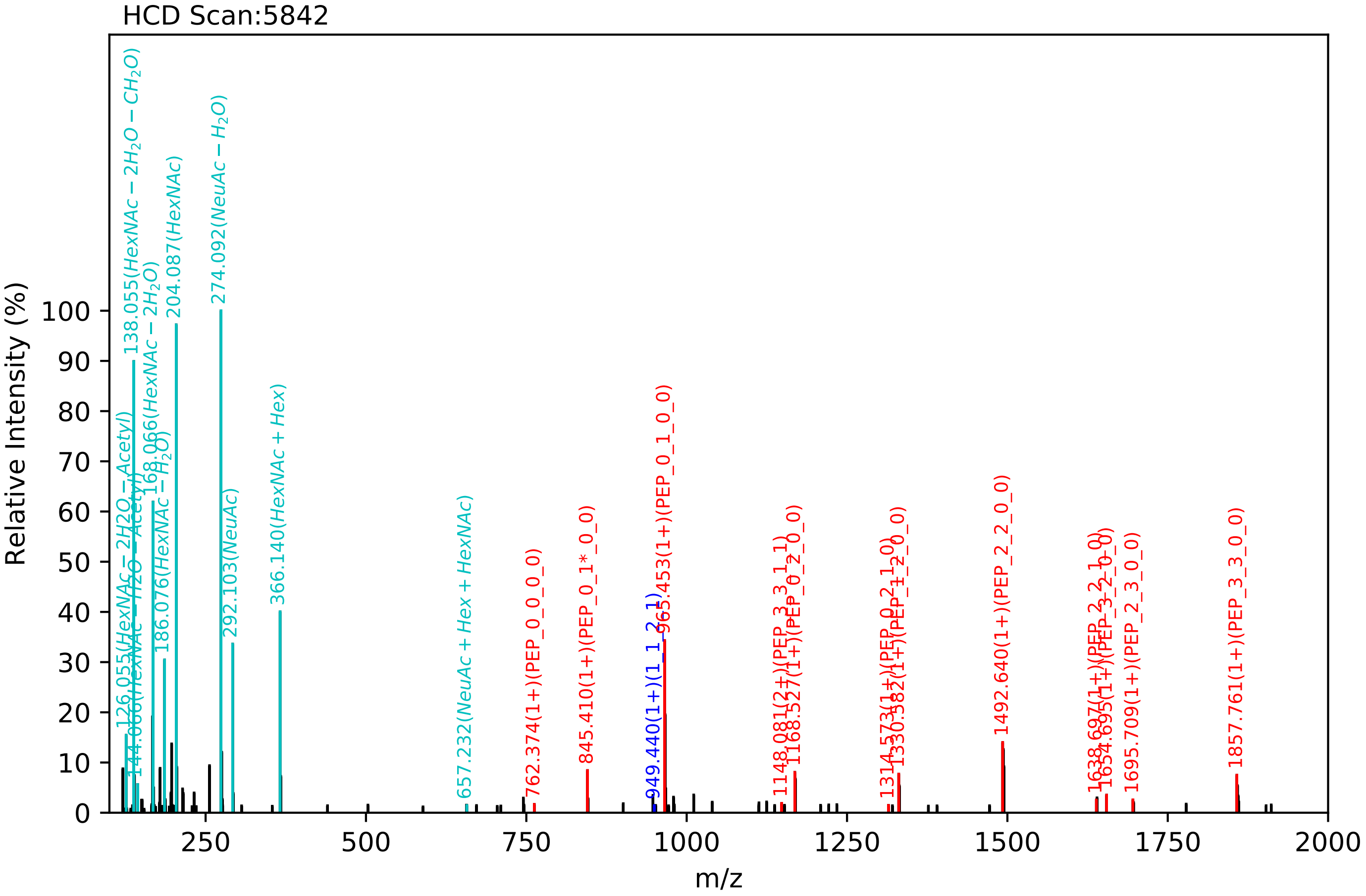

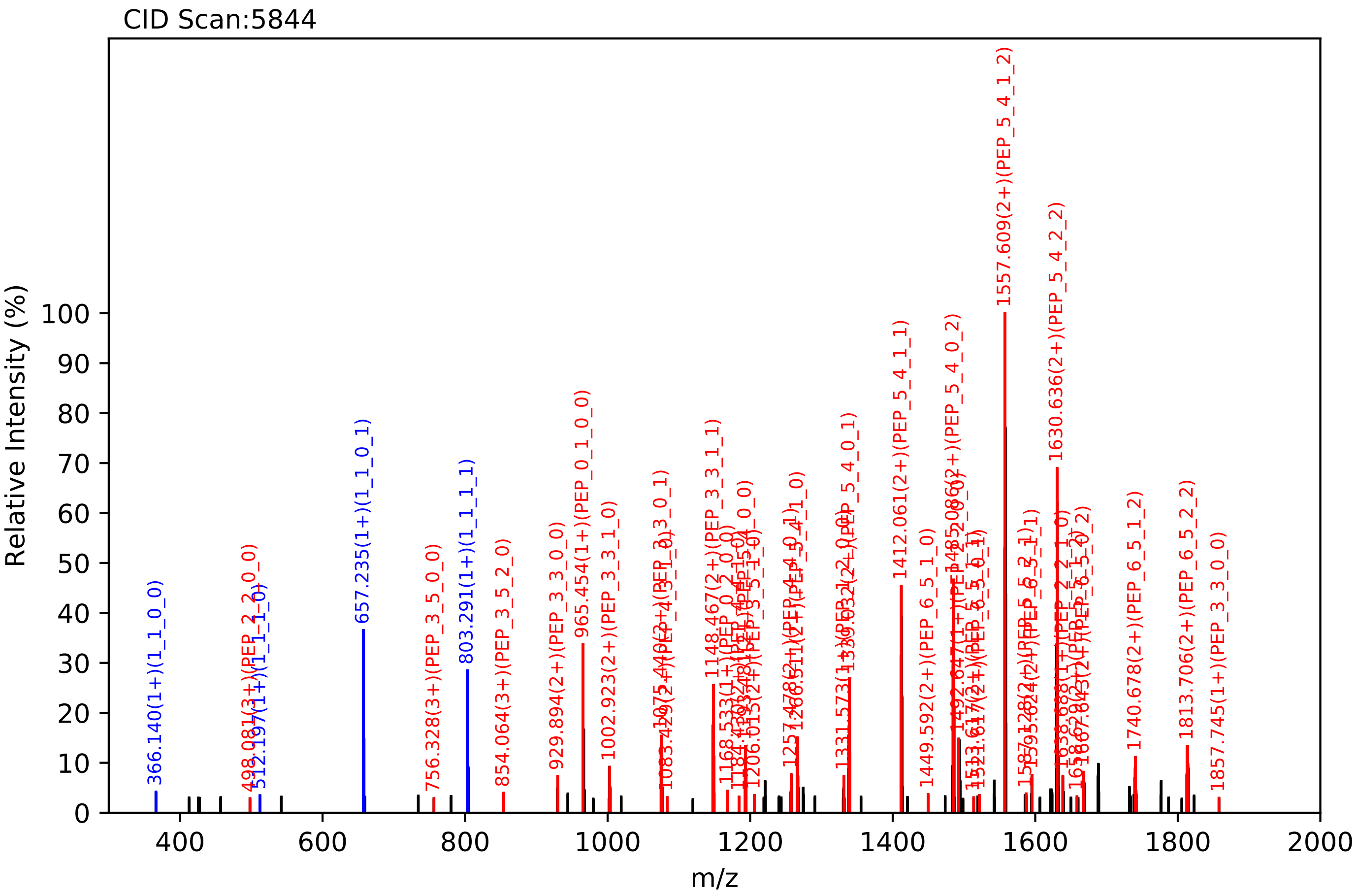

Test set no. 254, Experiment: AGP exp_42

ENGTVSR(=PEP)_6_5_2_3, m/z:1305.84(3+), RT:27.42, Y-score:86.50

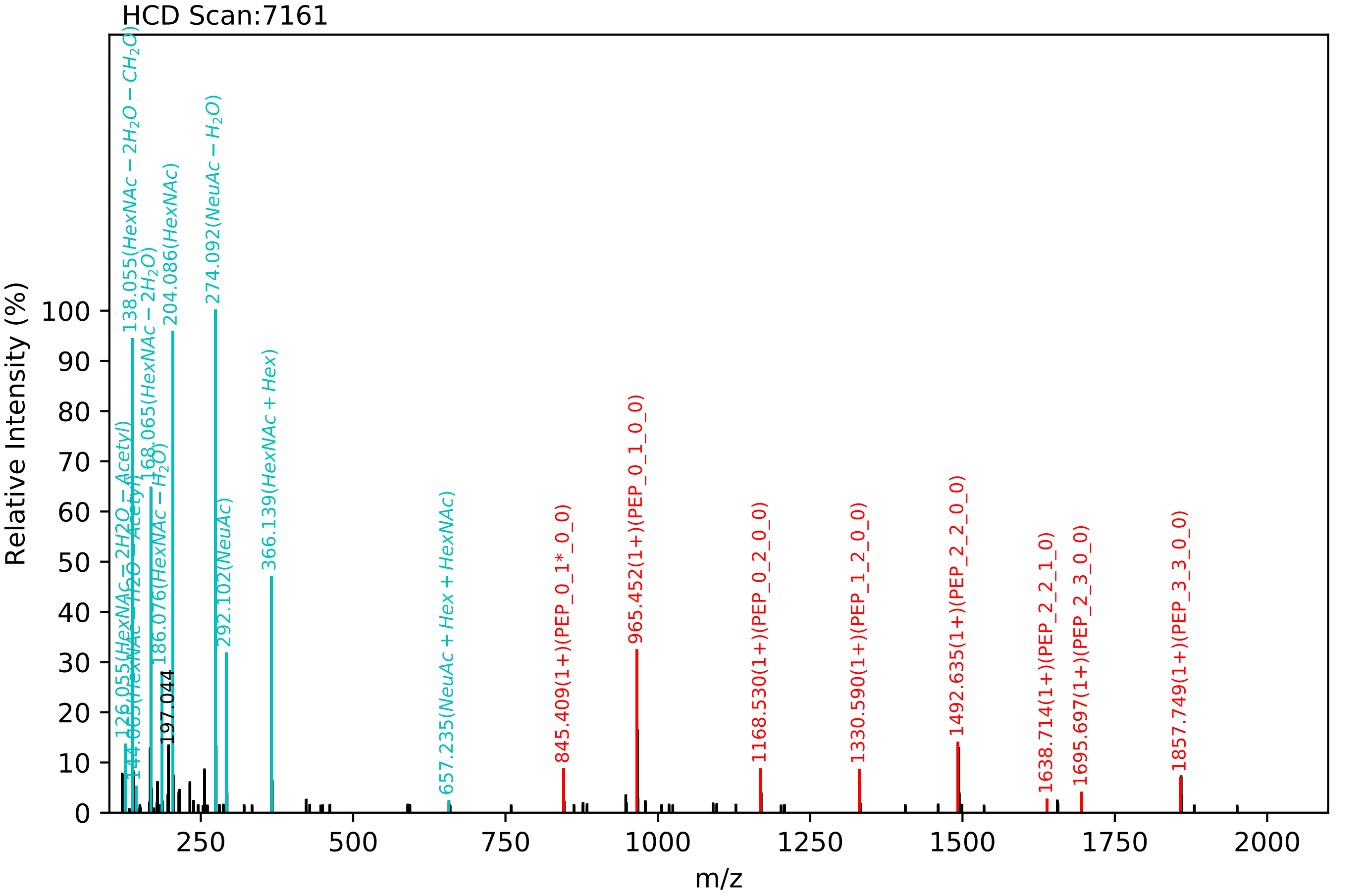

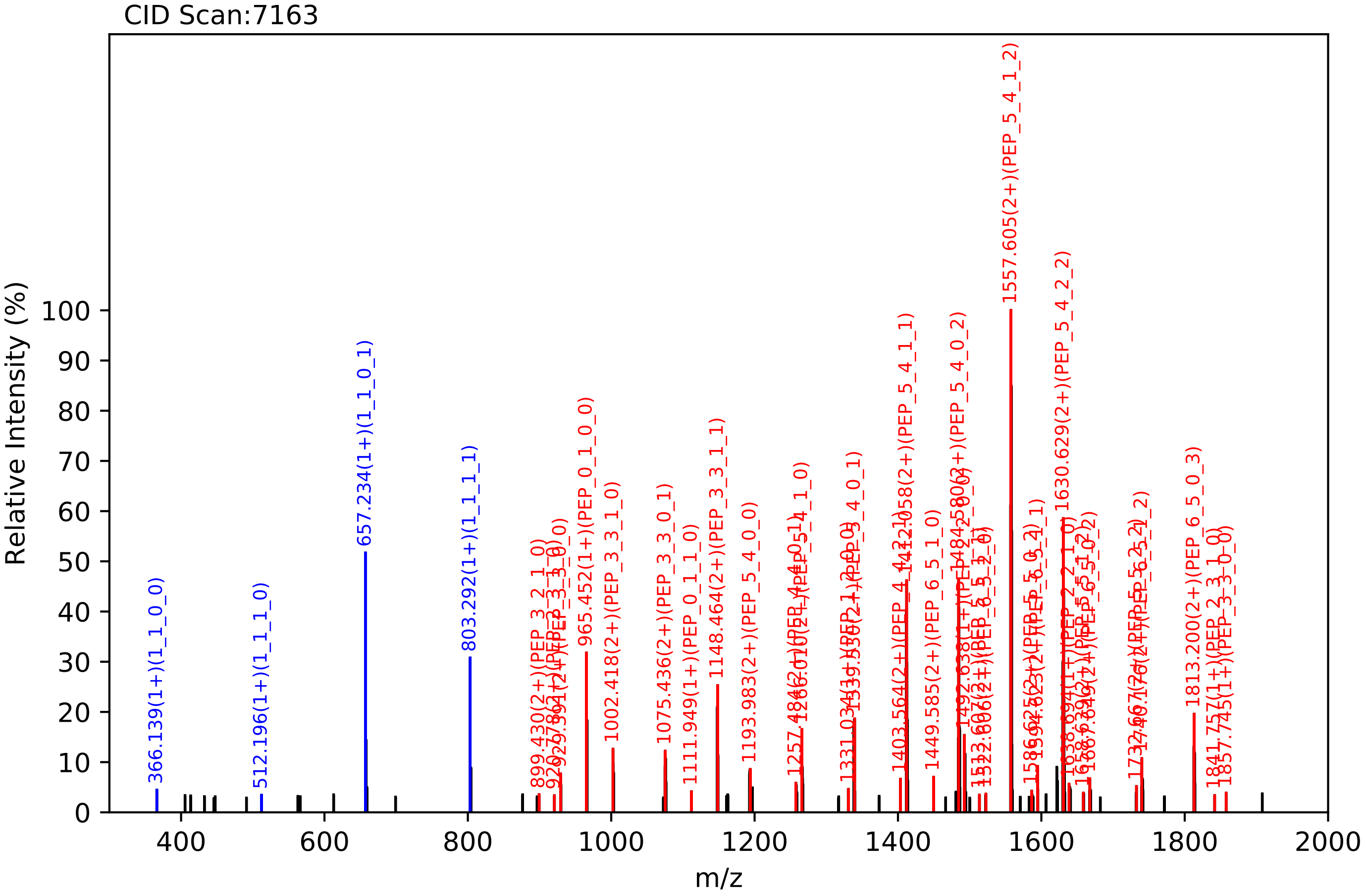

Test set no. 255, Experiment: AGP exp_32

ENGTVSR(=PEP)_6_5_2_3, m/z:1305.84(3+), RT:29.43, Y-score:84.01

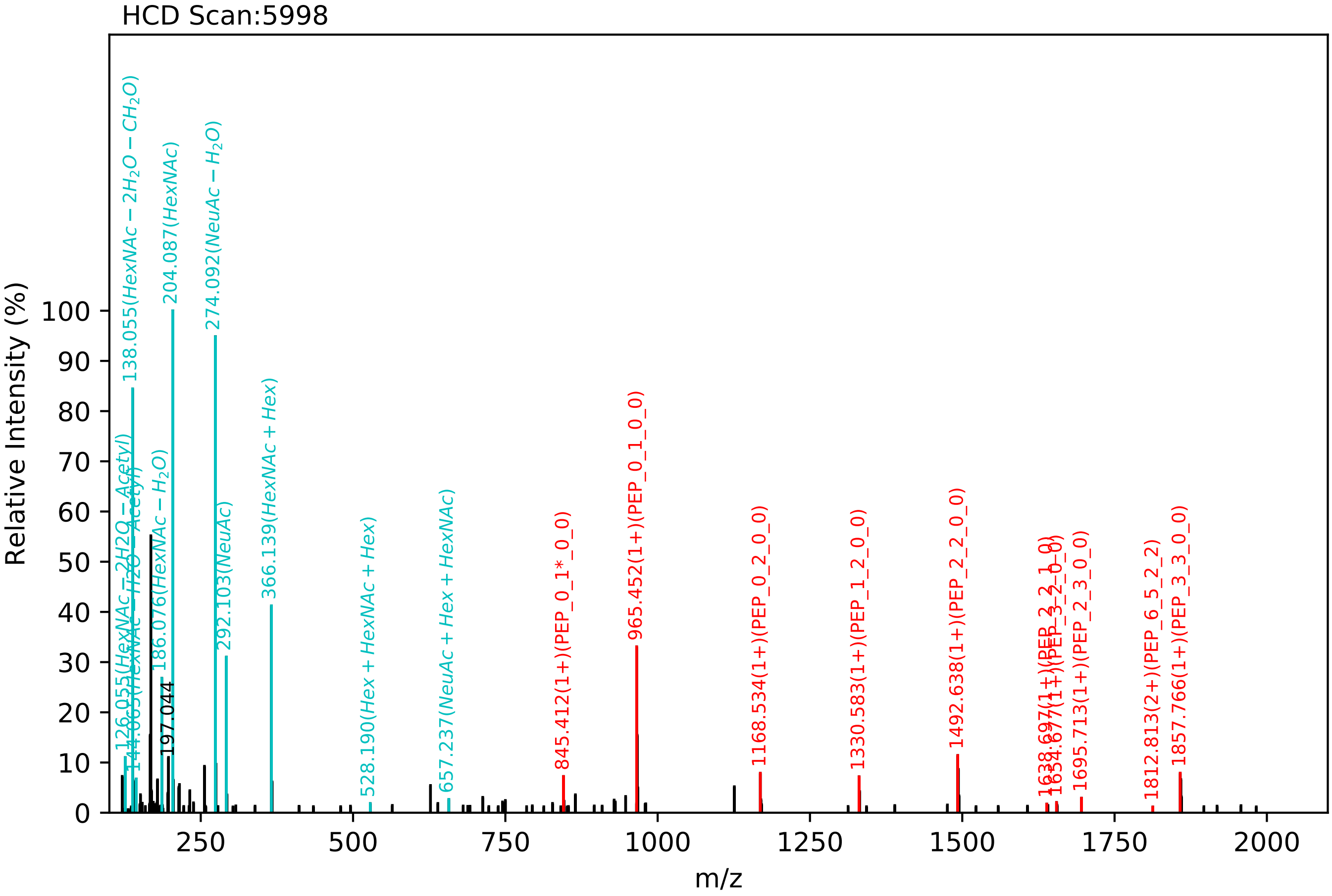

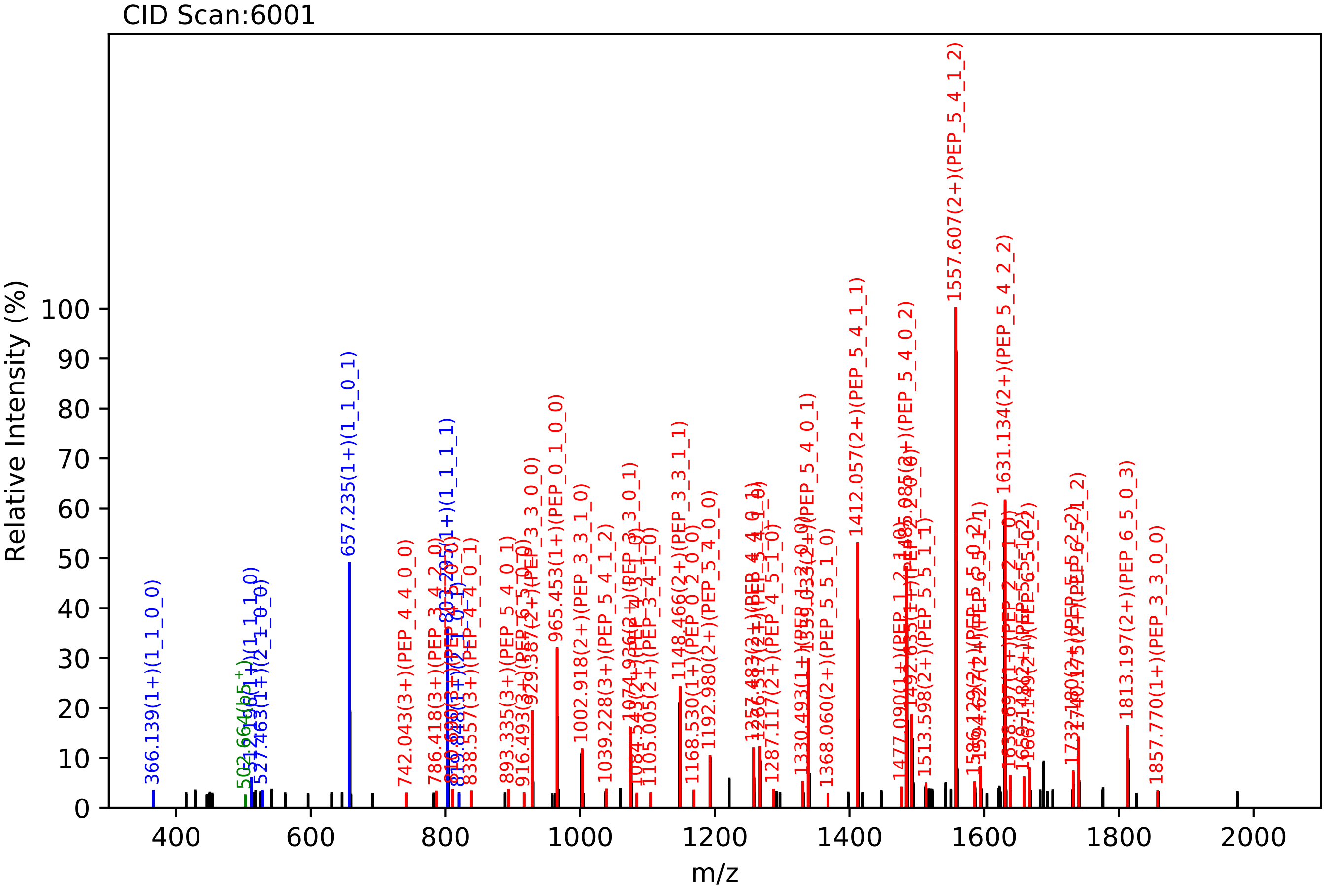

Test set no. 256, Experiment: AGP exp_4

ENGTVSR(=PEP)_7_6_1_2, m/z:1281.83(3+), RT:16.14, Y-score:93.60

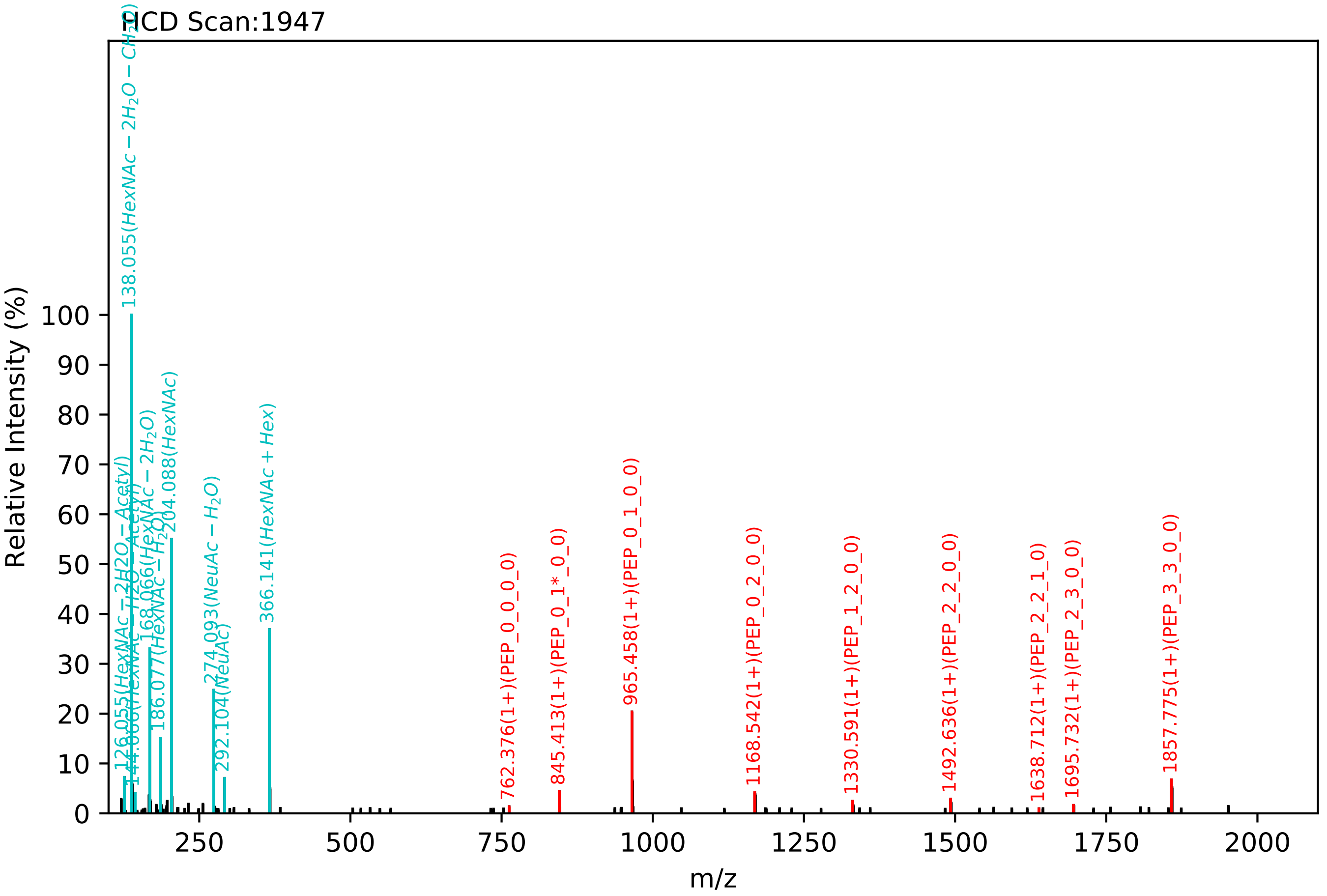

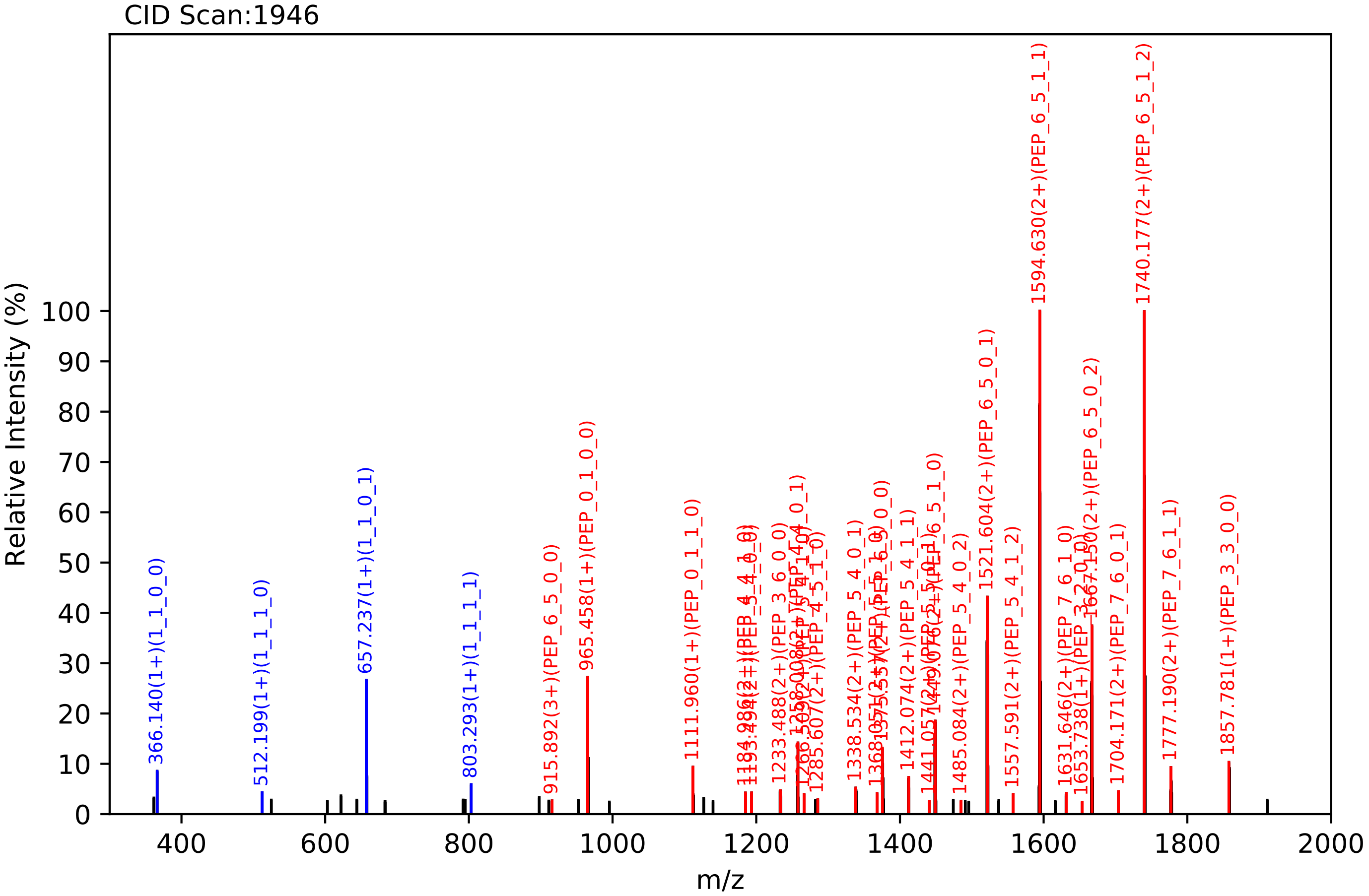

Test set no. 257, Experiment: AGP exp_3

ENGTVSR(=PEP)_7_6_1_2, m/z:1281.83(3+), RT:17.12, Y-score:93.26

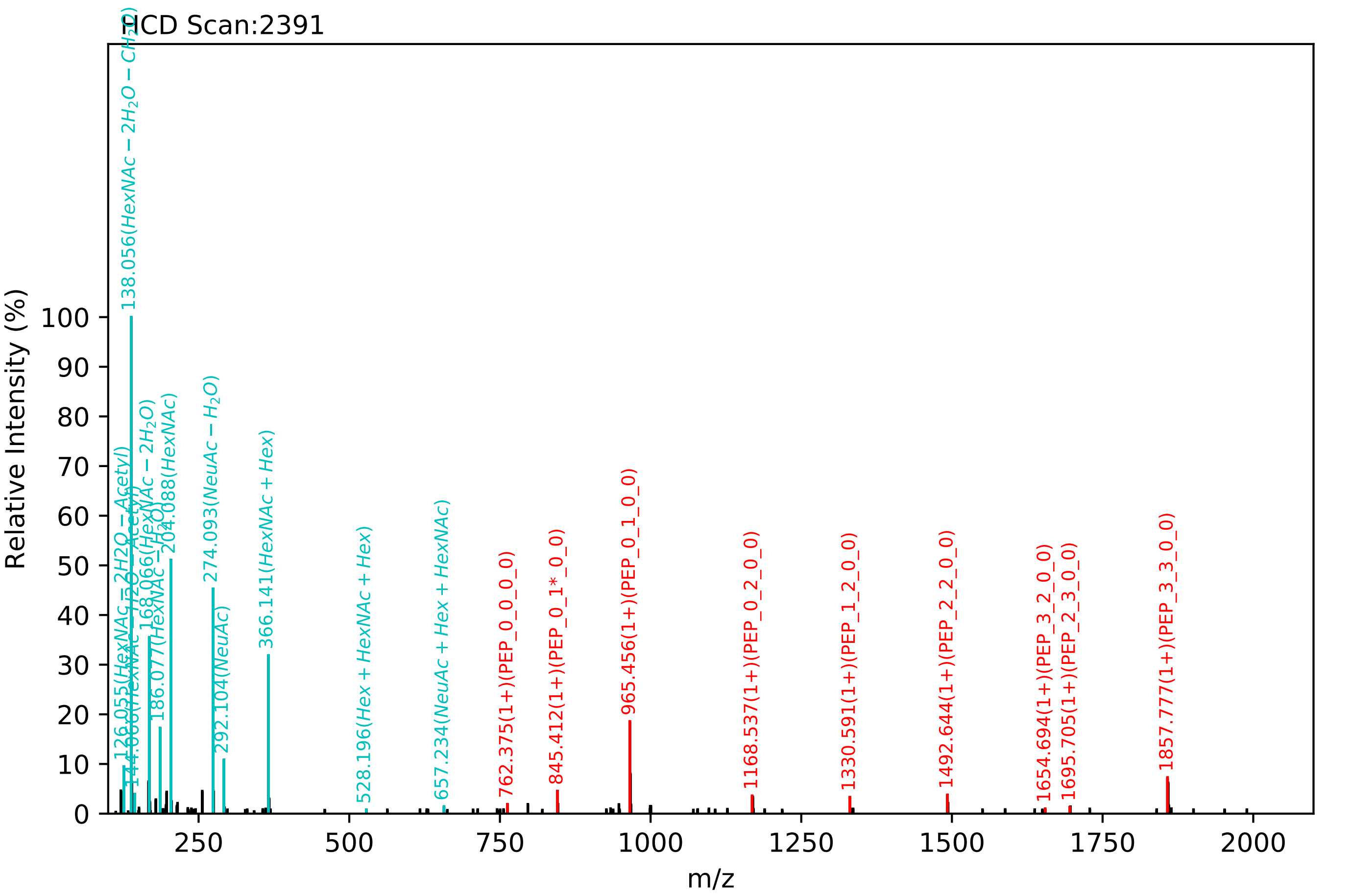

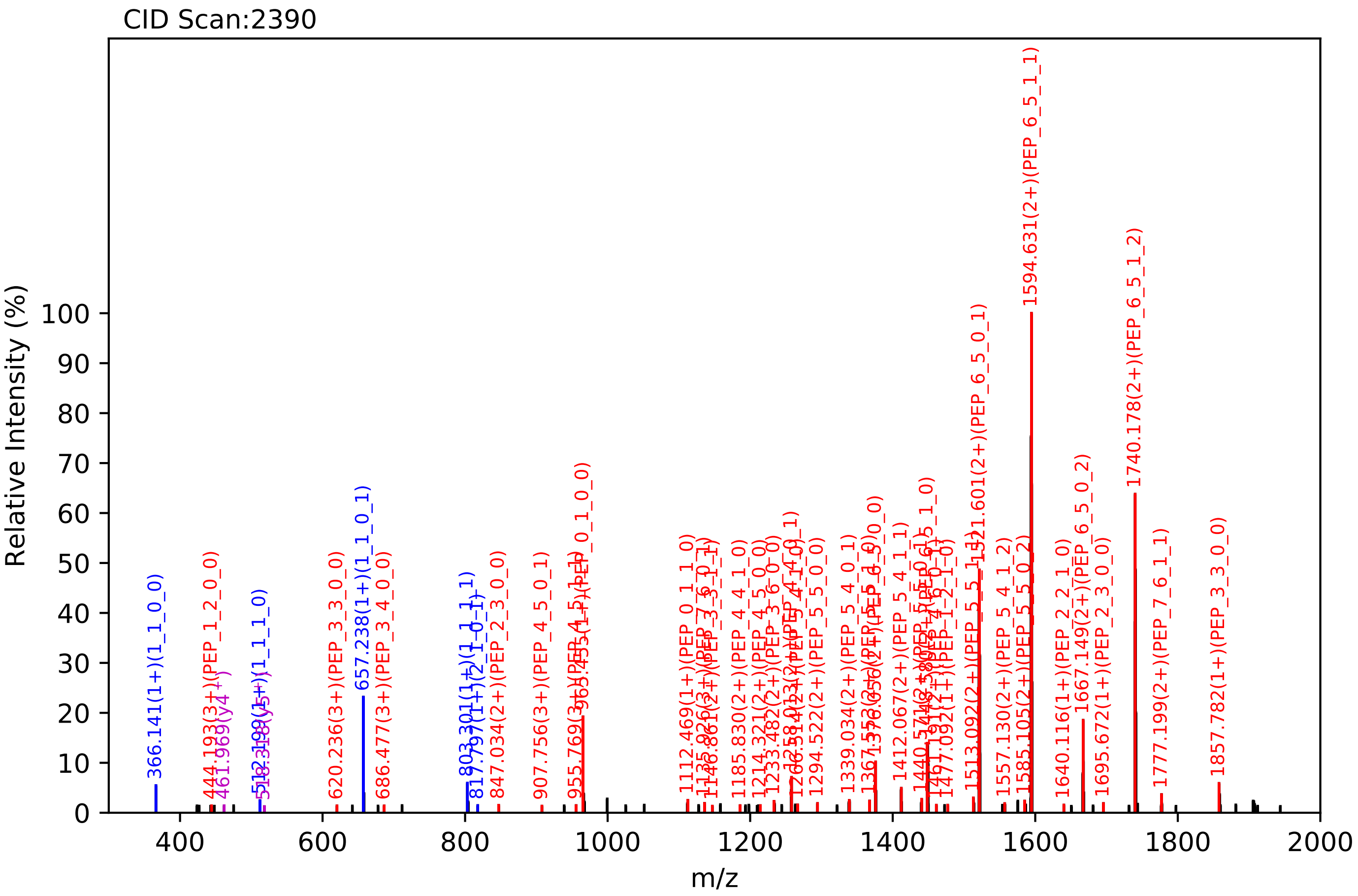

Test set no. 258, Experiment: AGP exp_3

ENGNGTVSR(=PEP)_7_6_1_2, m/z:1281.83(3+), RT:16.61, Y-score:92.72

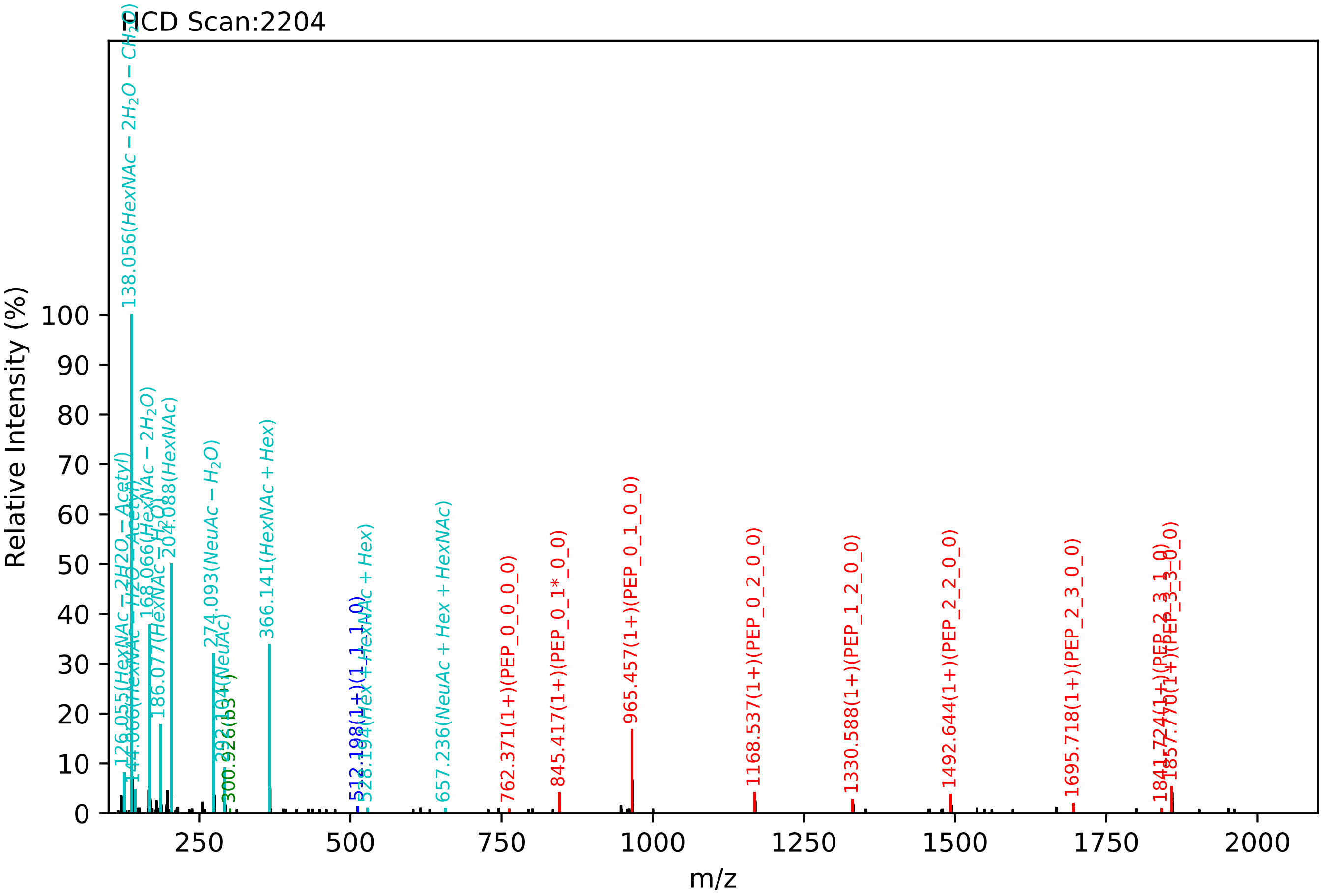

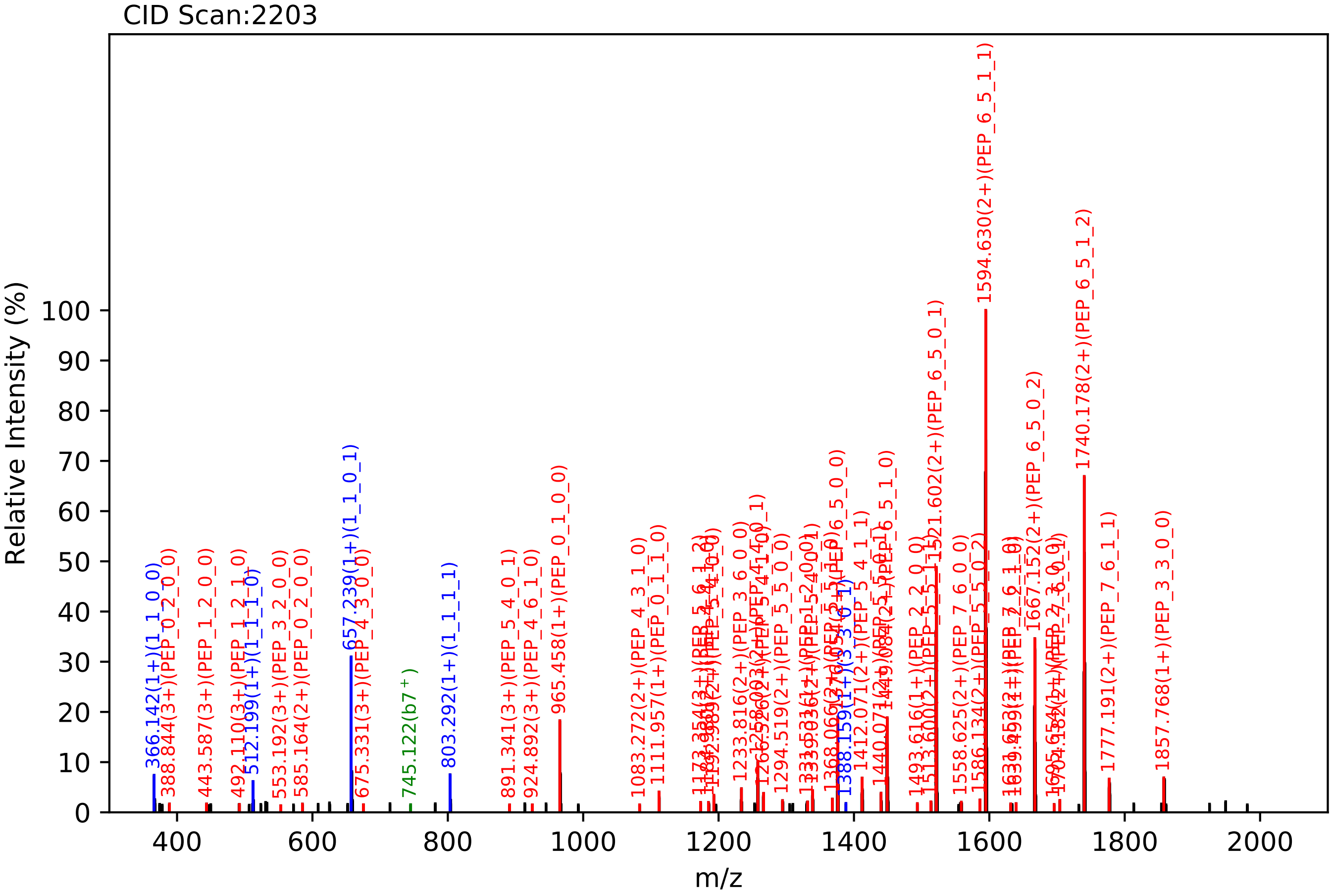

Test set no. 259, Experiment: AGP exp_3

ENGTVSR(=PEP)_7_6_1_2, m/z:1281.83(3+), RT:16.09, Y-score:90.66

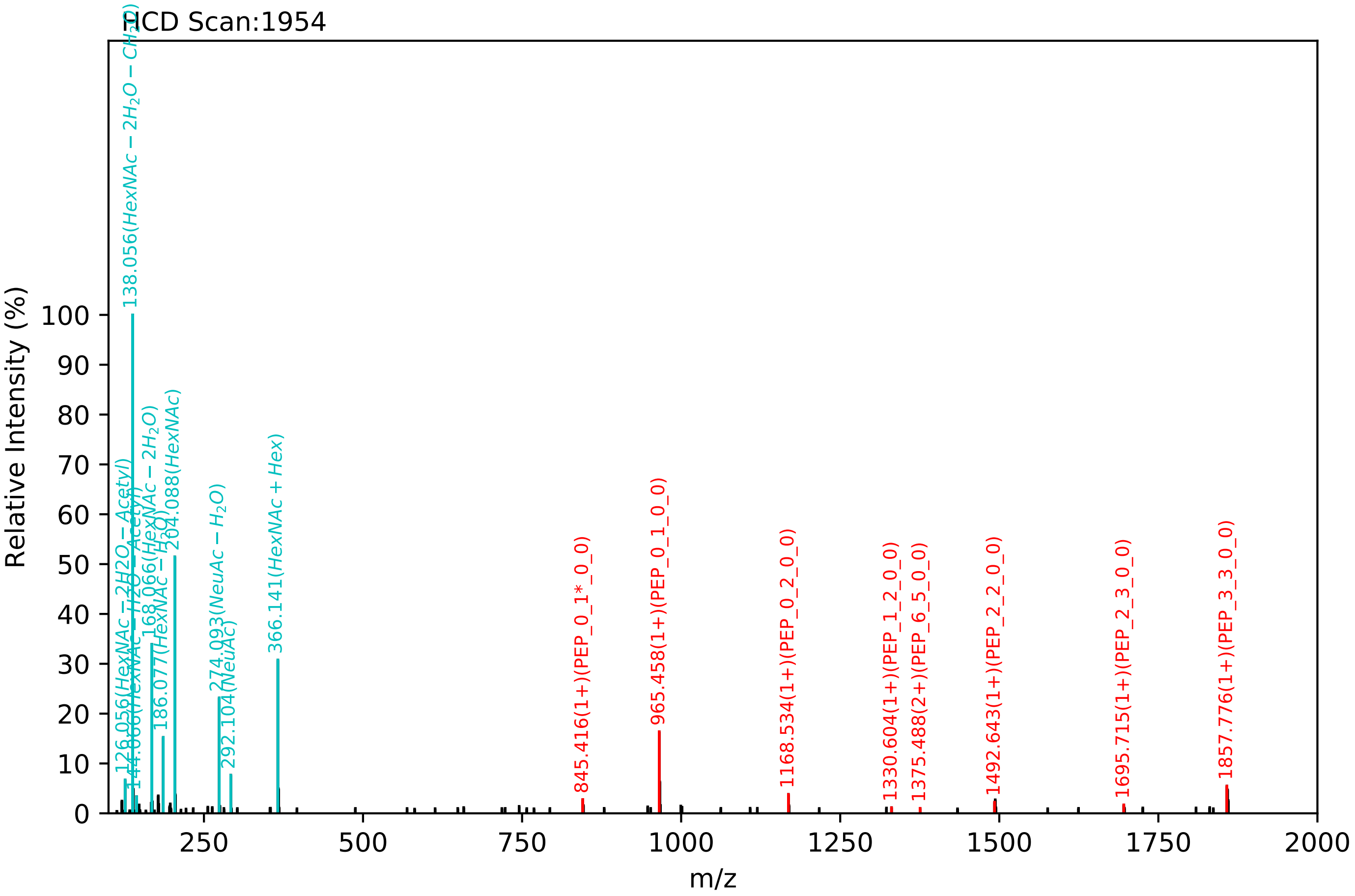

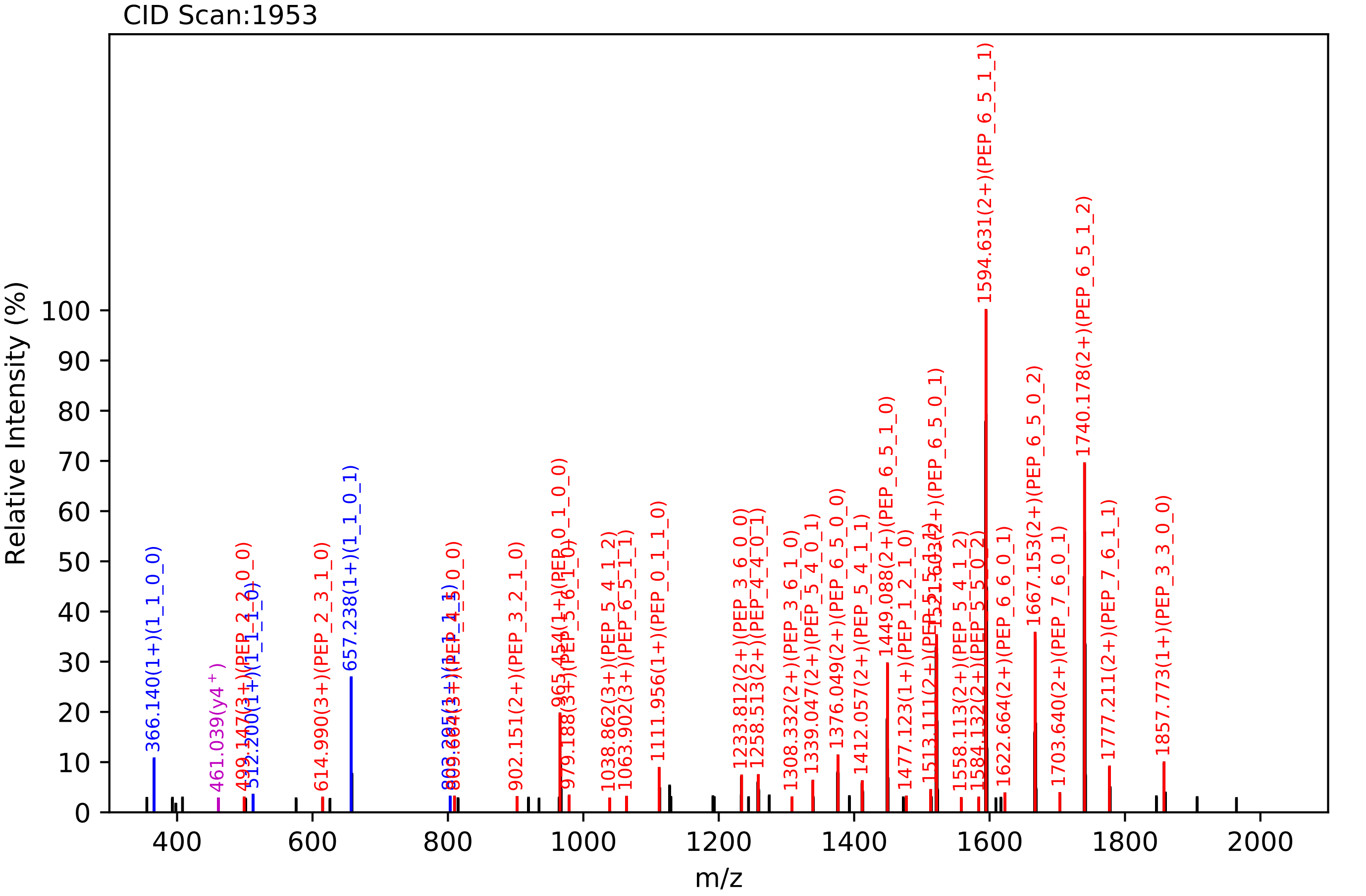

Test set no. 260, Experiment: AGP exp_4

ENGTVSR(=PEP)_7_6_1_2, m/z:1281.83(3+), RT:16.67, Y-score:90.25

HCD Scan:2137

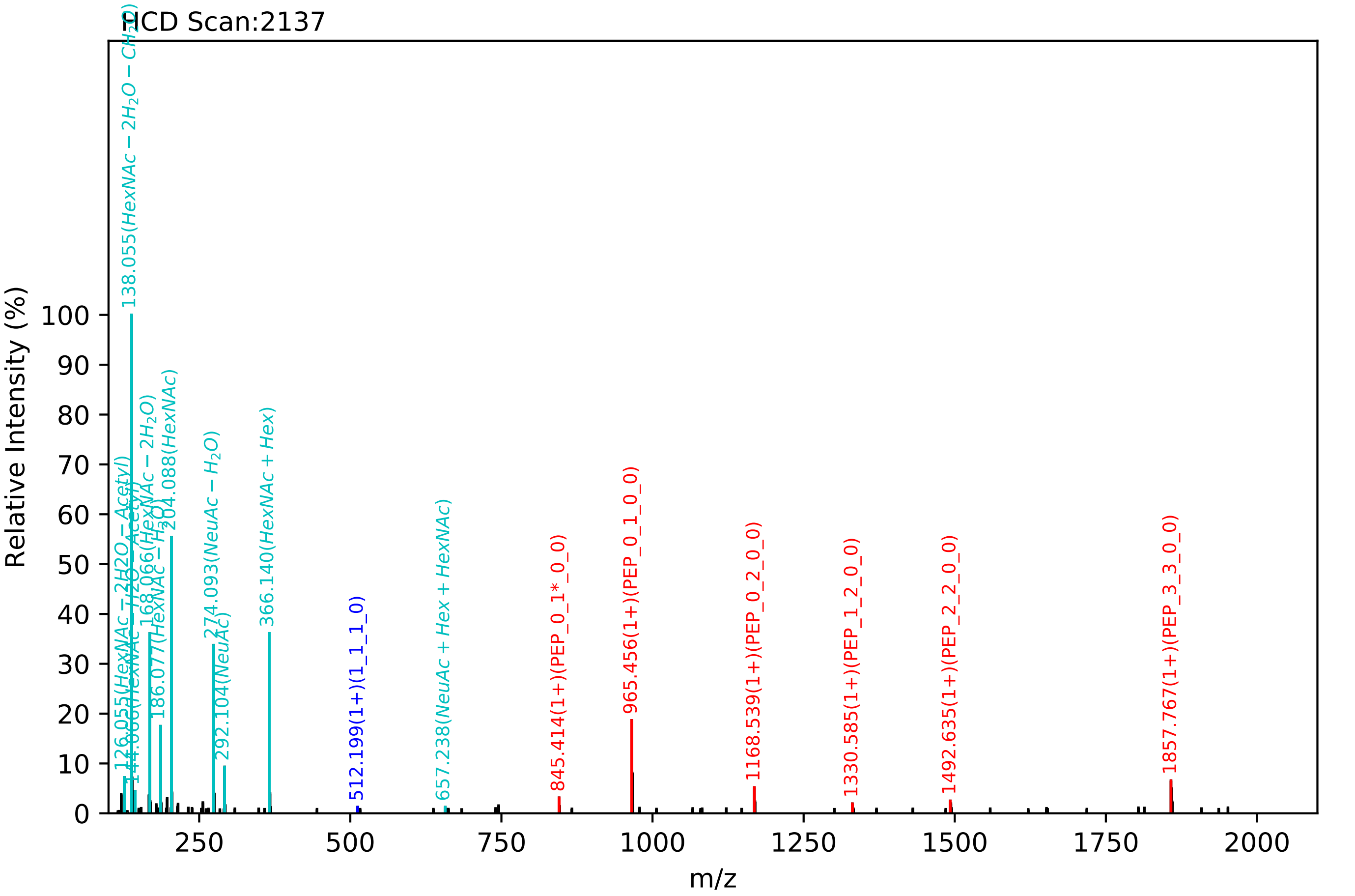

CID Scan:2136

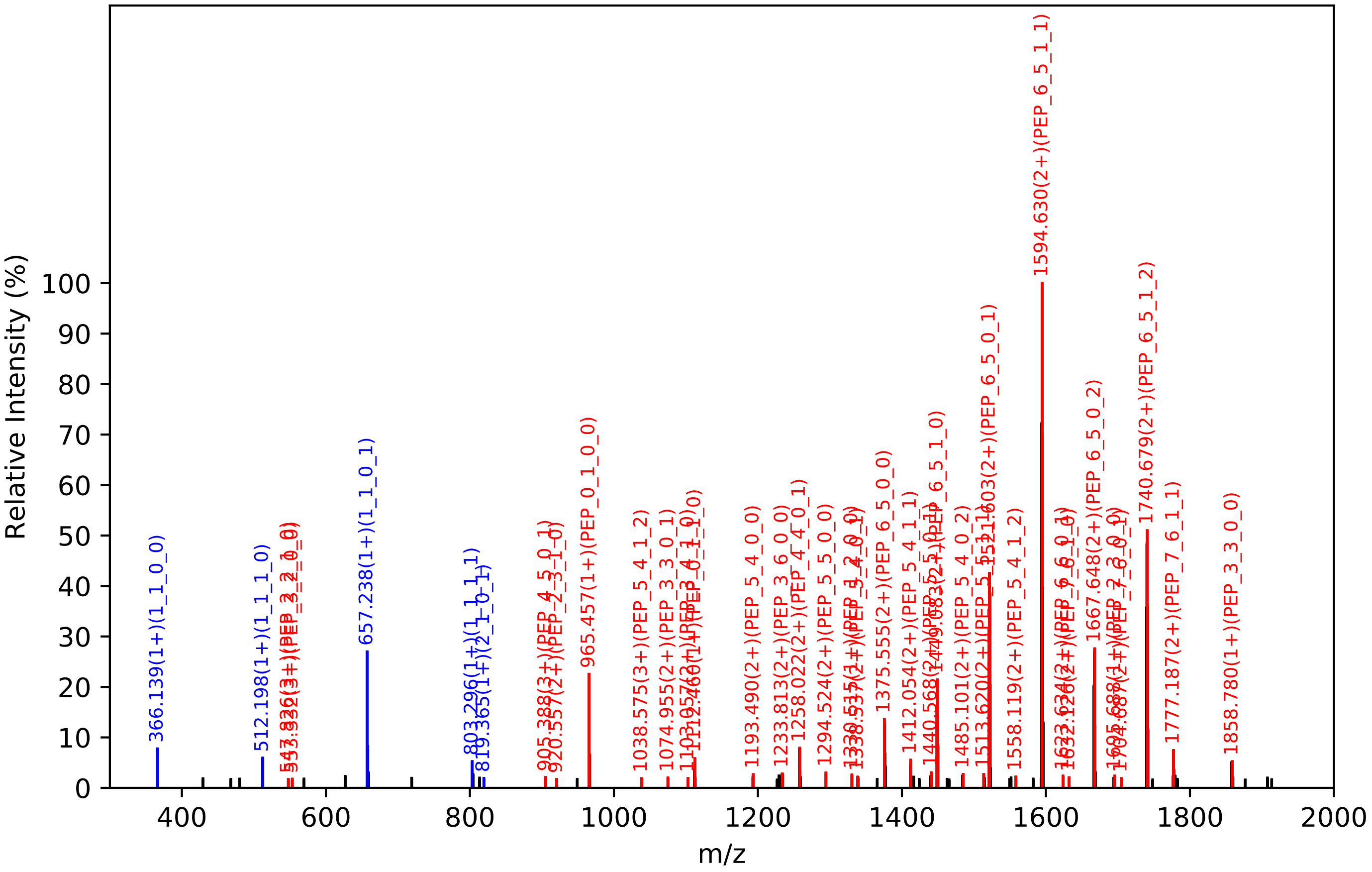

Test set no. 261, Experiment: AGP exp_4

ENGTVSR(=PEP)_7_6_1_2, m/z:1281.83(3+), RT:16.70, Y-score:90.13

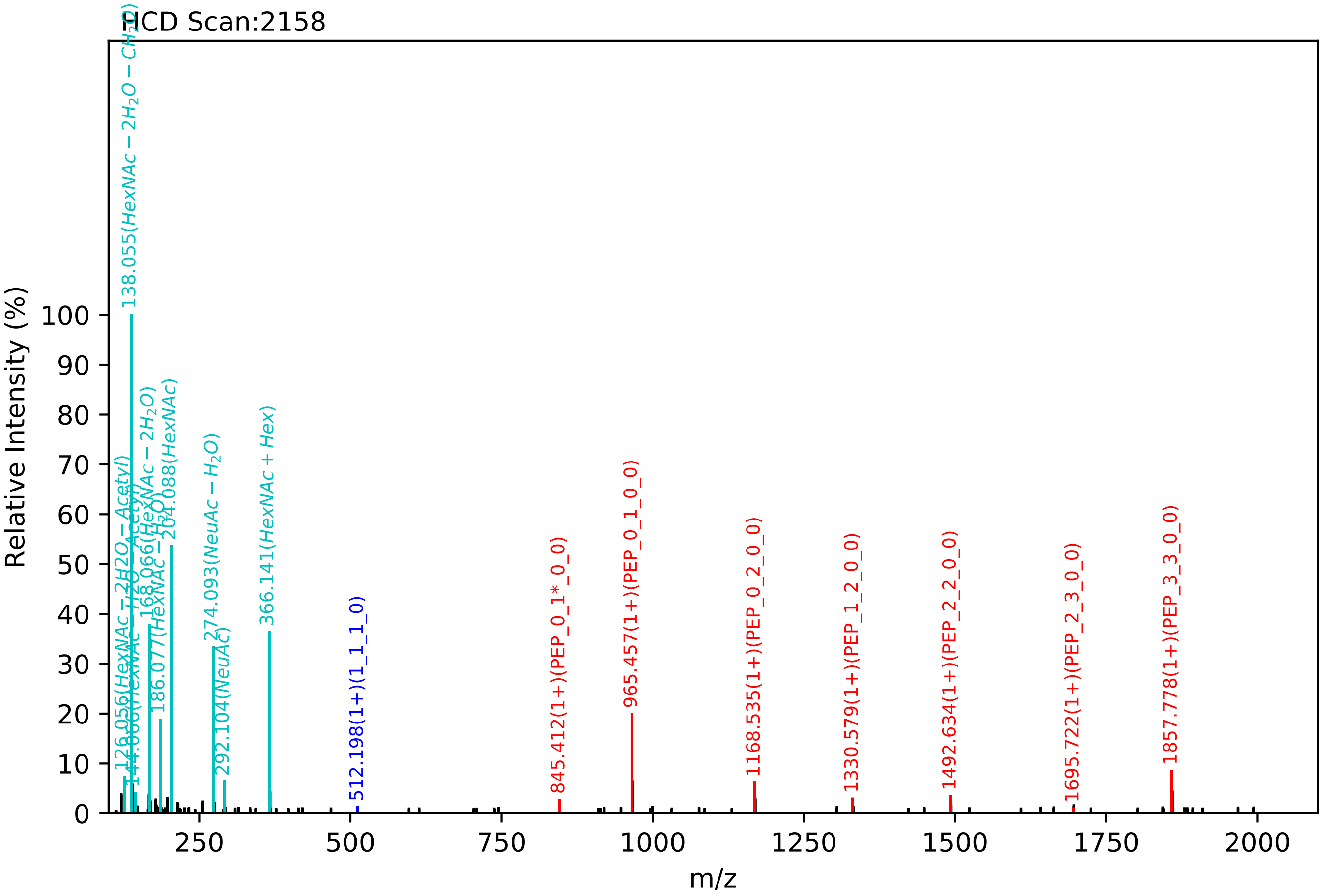

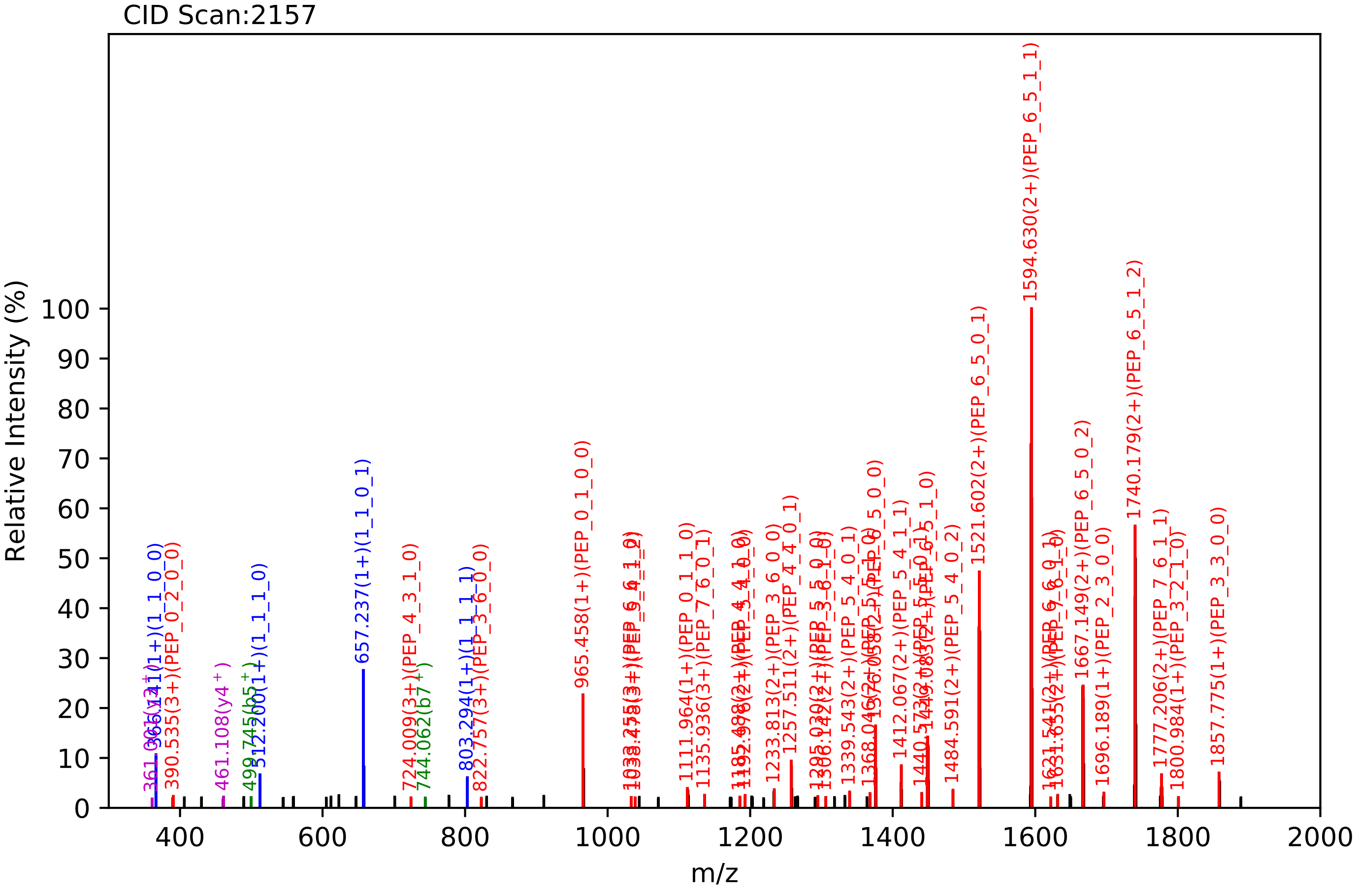

Test set no. 262, Experiment: AGP exp_3

ENGTVSR(=PEP)_7_6_1_4, m/z:1107.17(4+), RT:22.96, Y-score:85.23

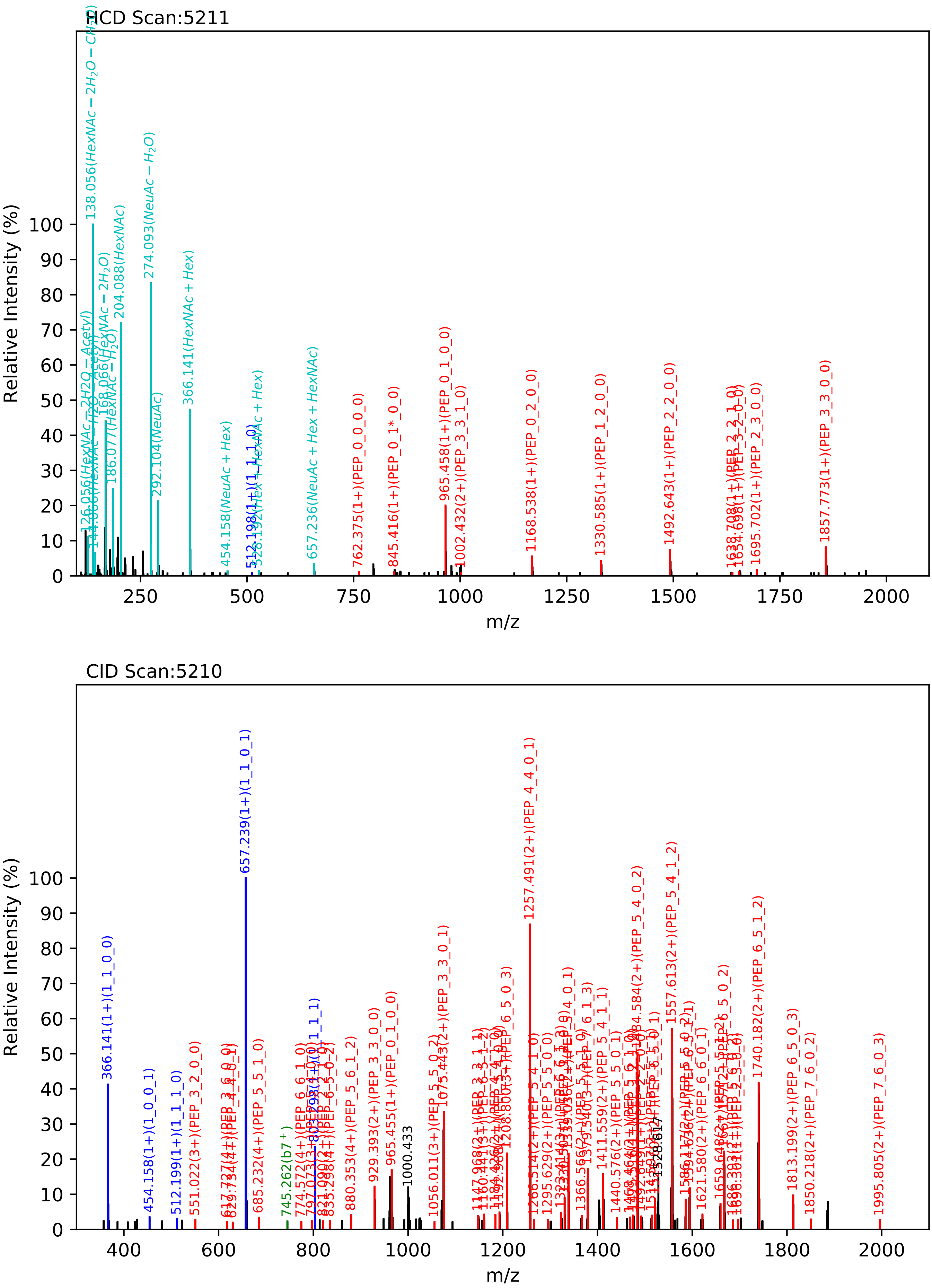

Test set no. 263, Experiment: AGP exp_4

ENGTVSR(=PEP)_7_6_1_4, m/z:1107.17(4+), RT:22.97, Y-score:84.56

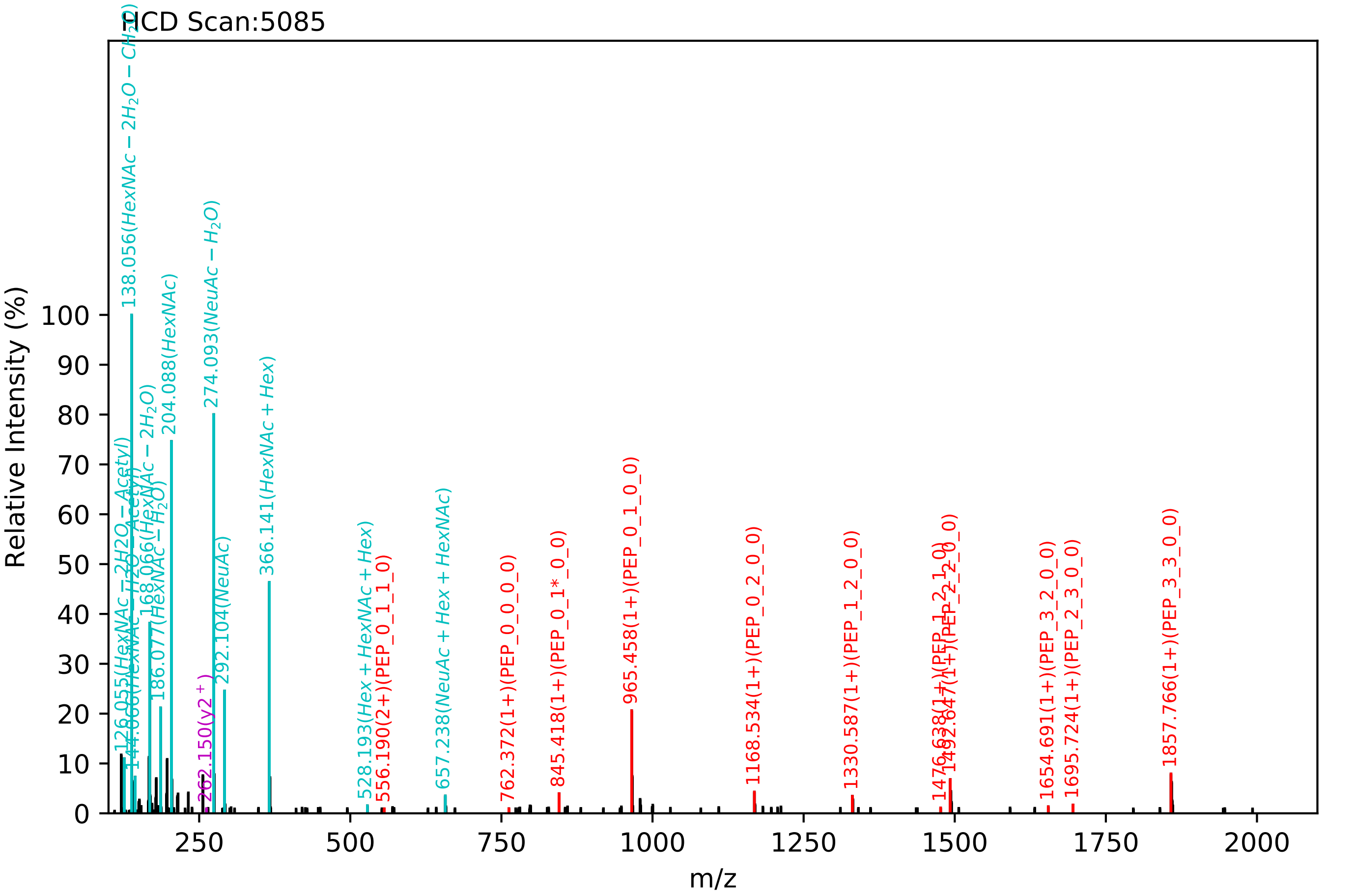

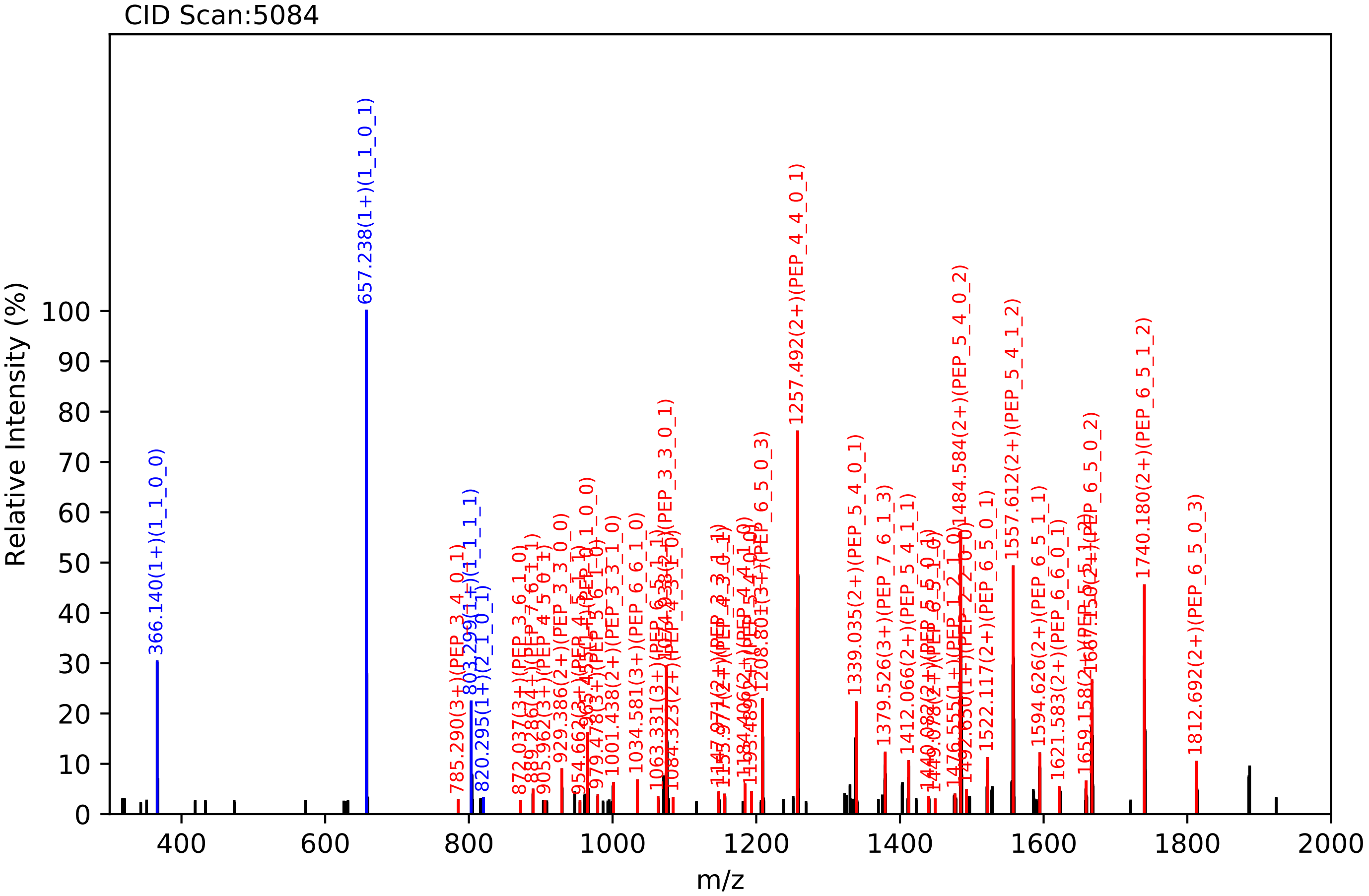

Test set no. 264, Experiment: AGP exp_4

LVPVPITNATLDR(=PEP)_3_3_0_0, m/z:1252.61(2+), RT:42.59, Y-score:77.72

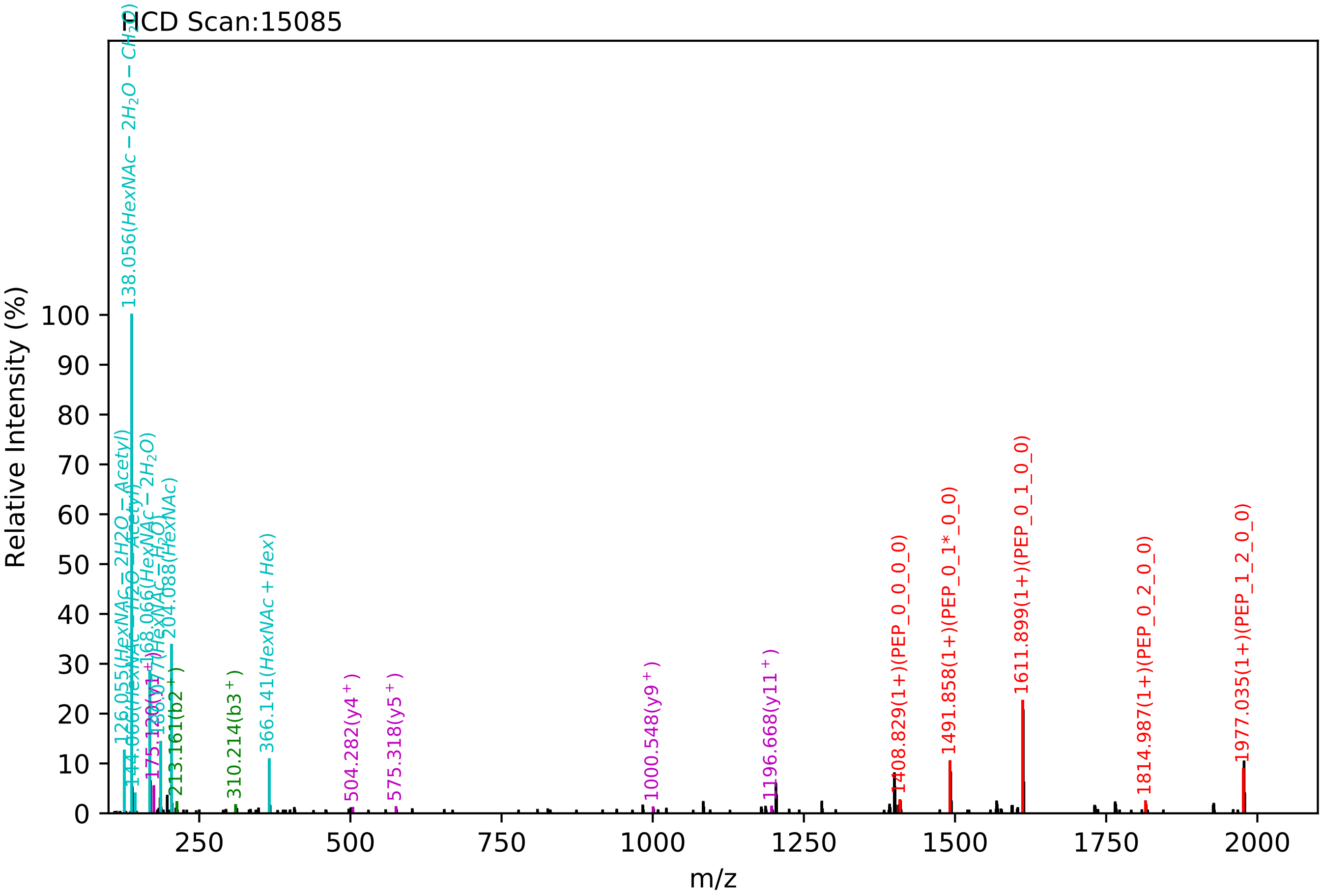

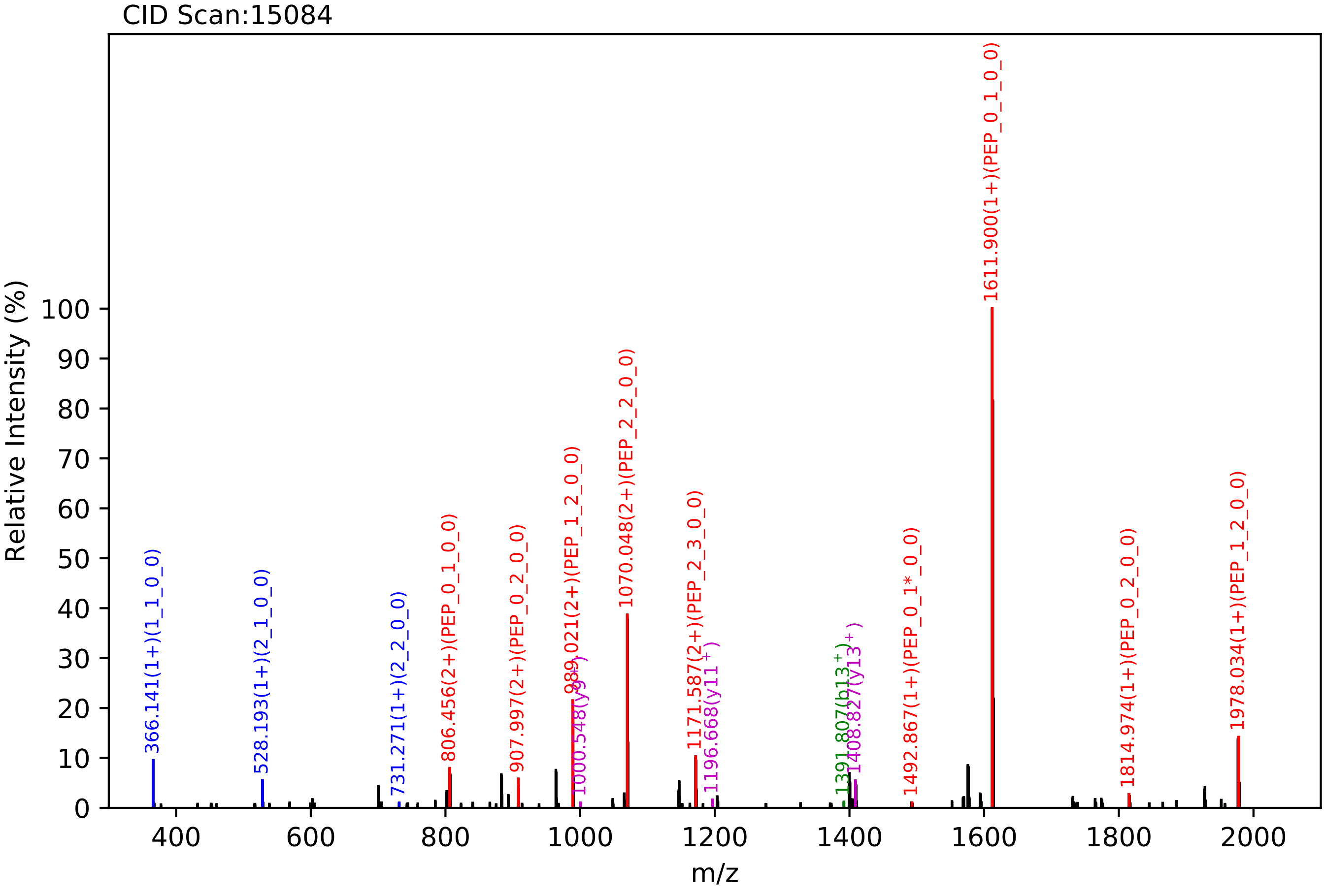

Test set no. 265, Experiment: AGP exp_3

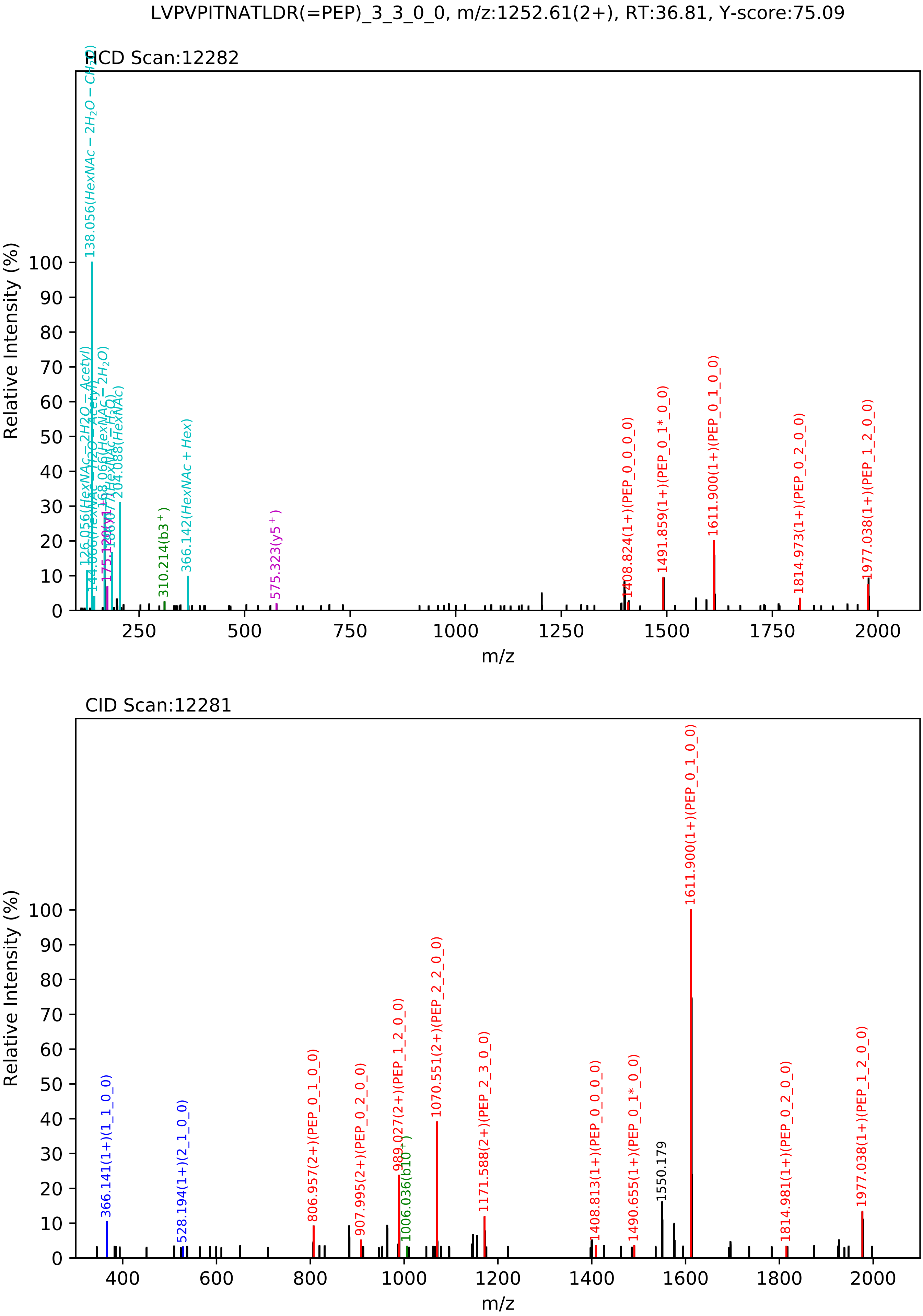

Test set no. 266, Experiment: AGP exp_3

LVPVPITNATLDR(=PEP)_4_4_0_1, m/z:1054.15(3+), RT:37.68, Y-score:86.66

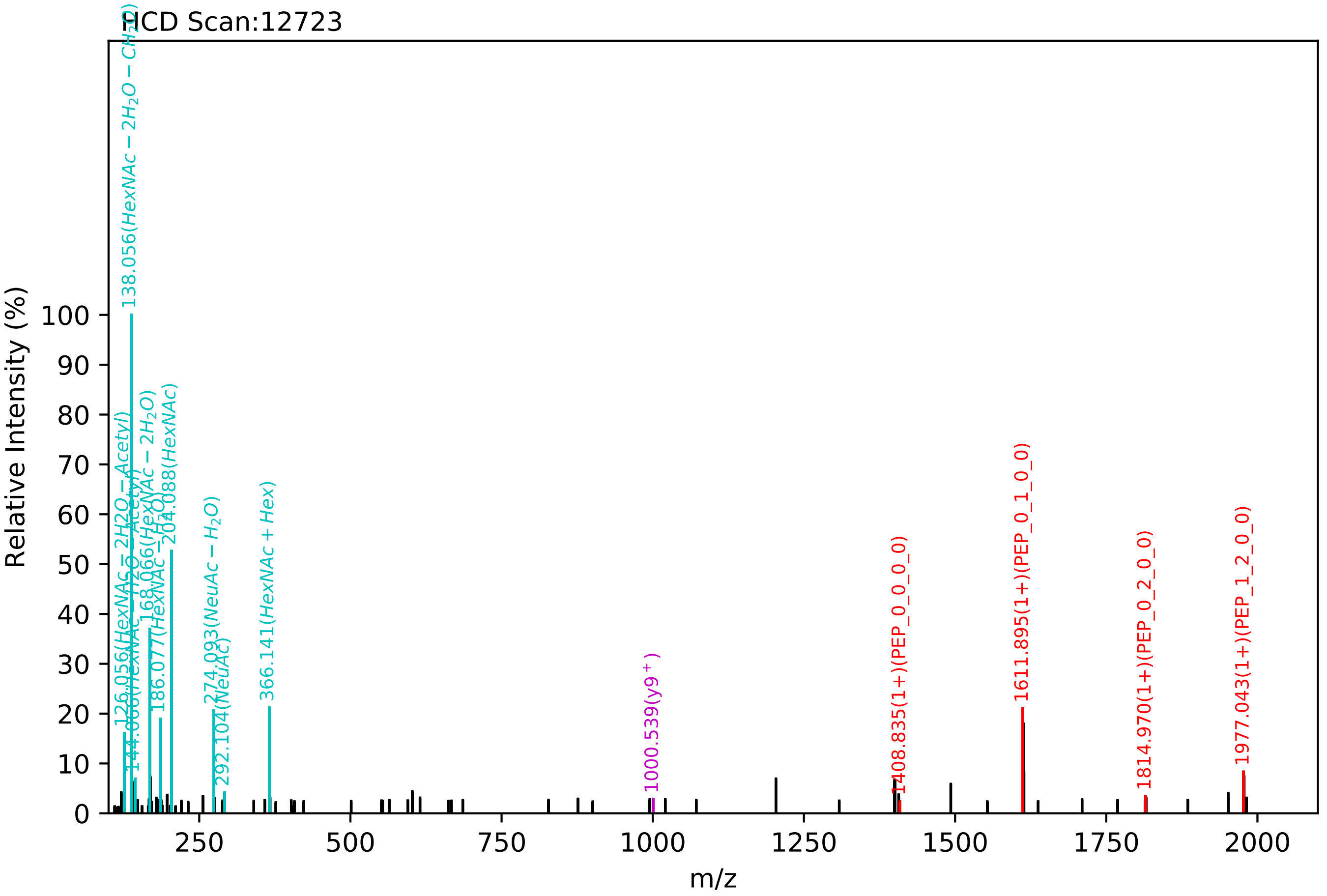

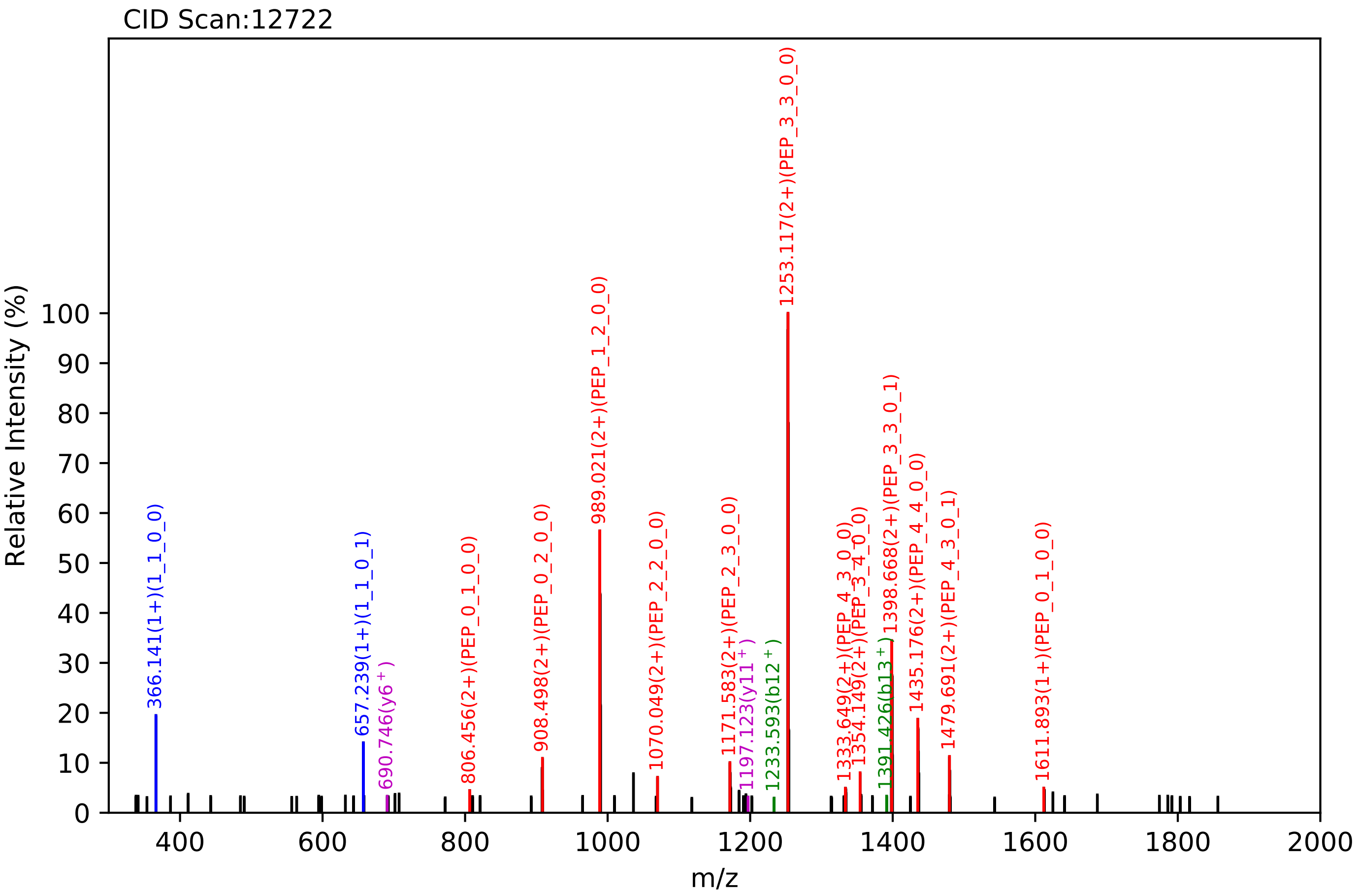

Test set no. 267, Experiment: AGP exp_3

LVPVPITNATLDR(=PEP)_4_4_0_1, m/z:1054.15(3+), RT:37.69, Y-score:82.65

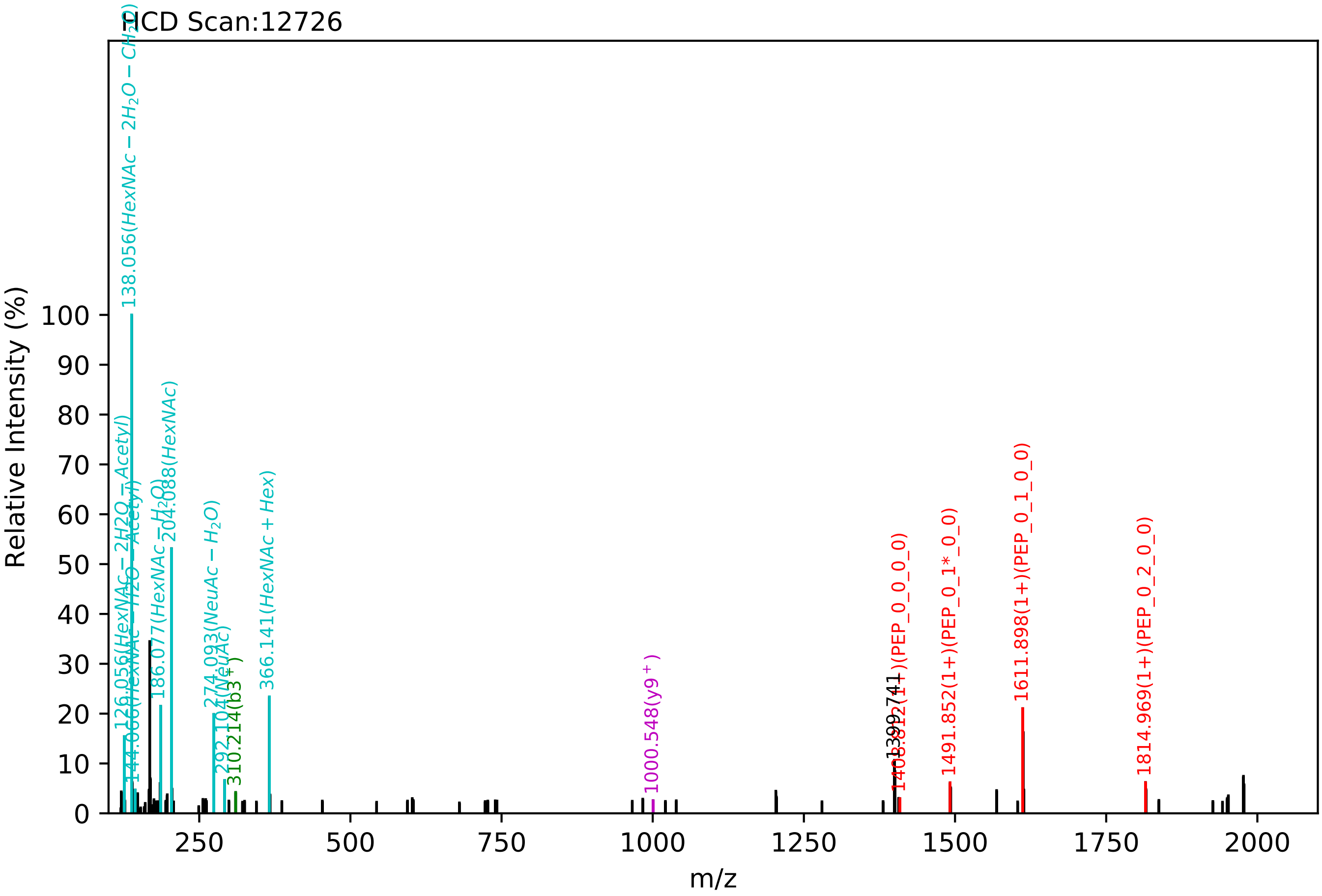

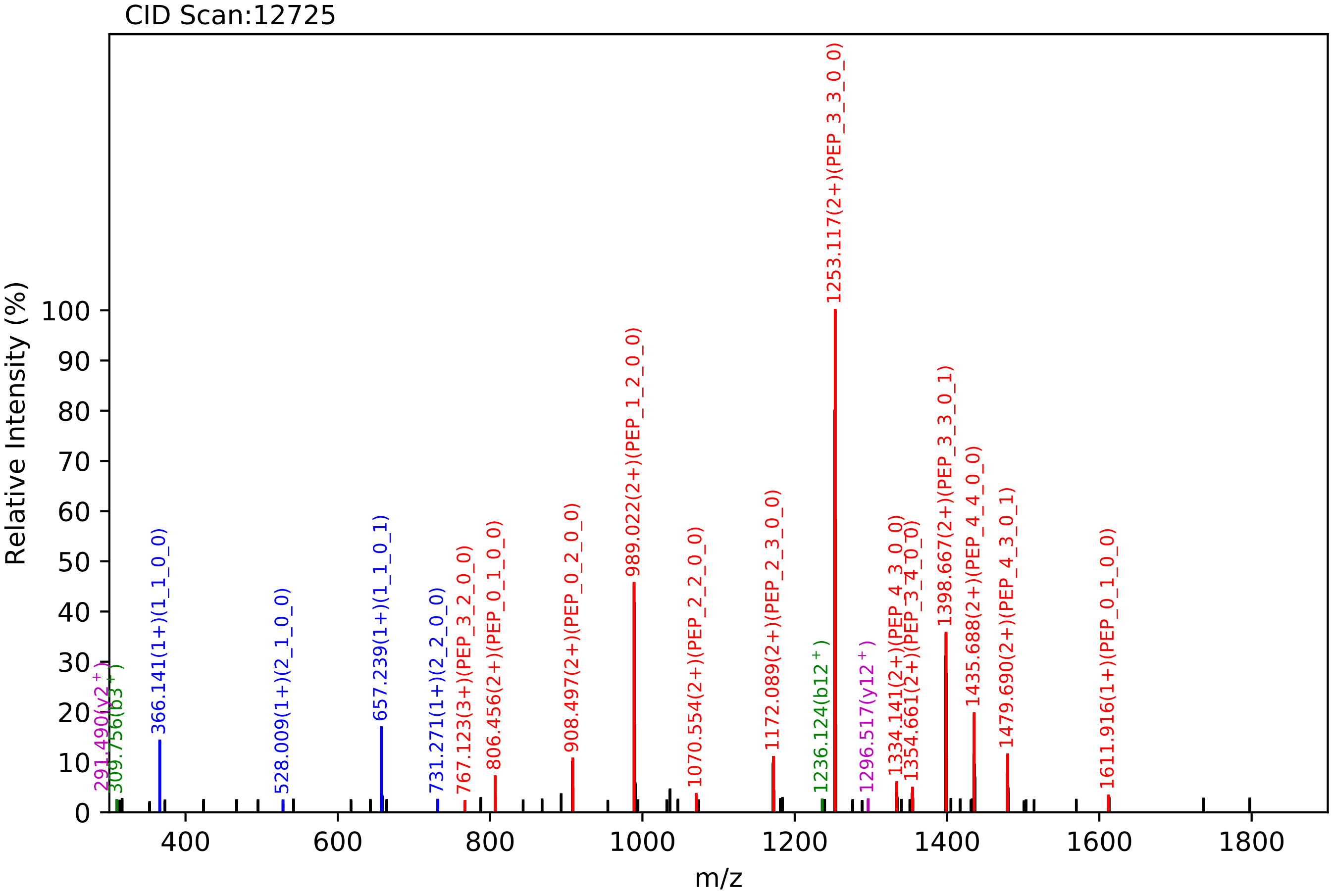

Test set no. 268, Experiment: AGP exp_3

LVPVPITNATLDR(=PEP)_5_4_0_1, m/z:1661.76(2+), RT:42.55, Y-score:90.50

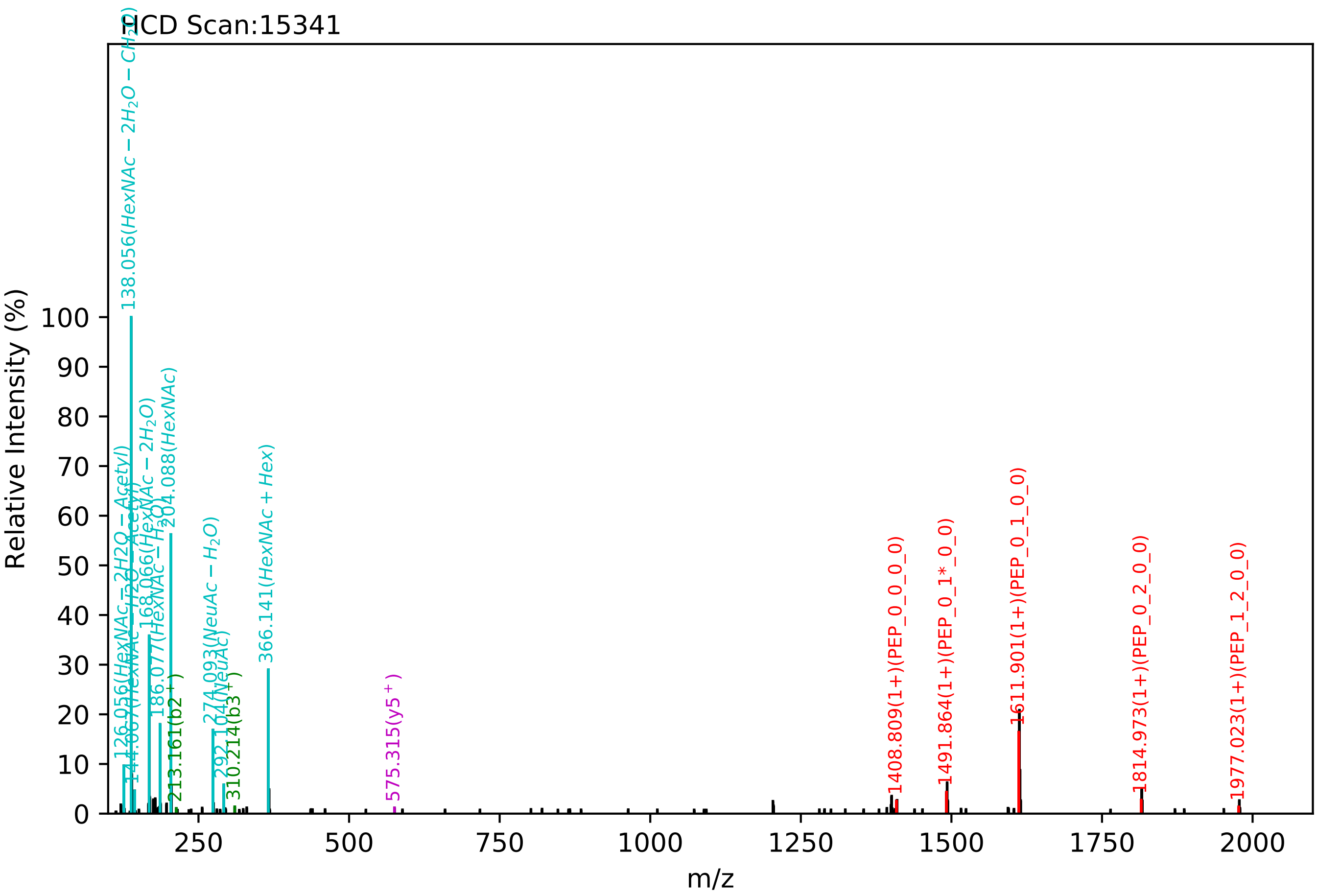

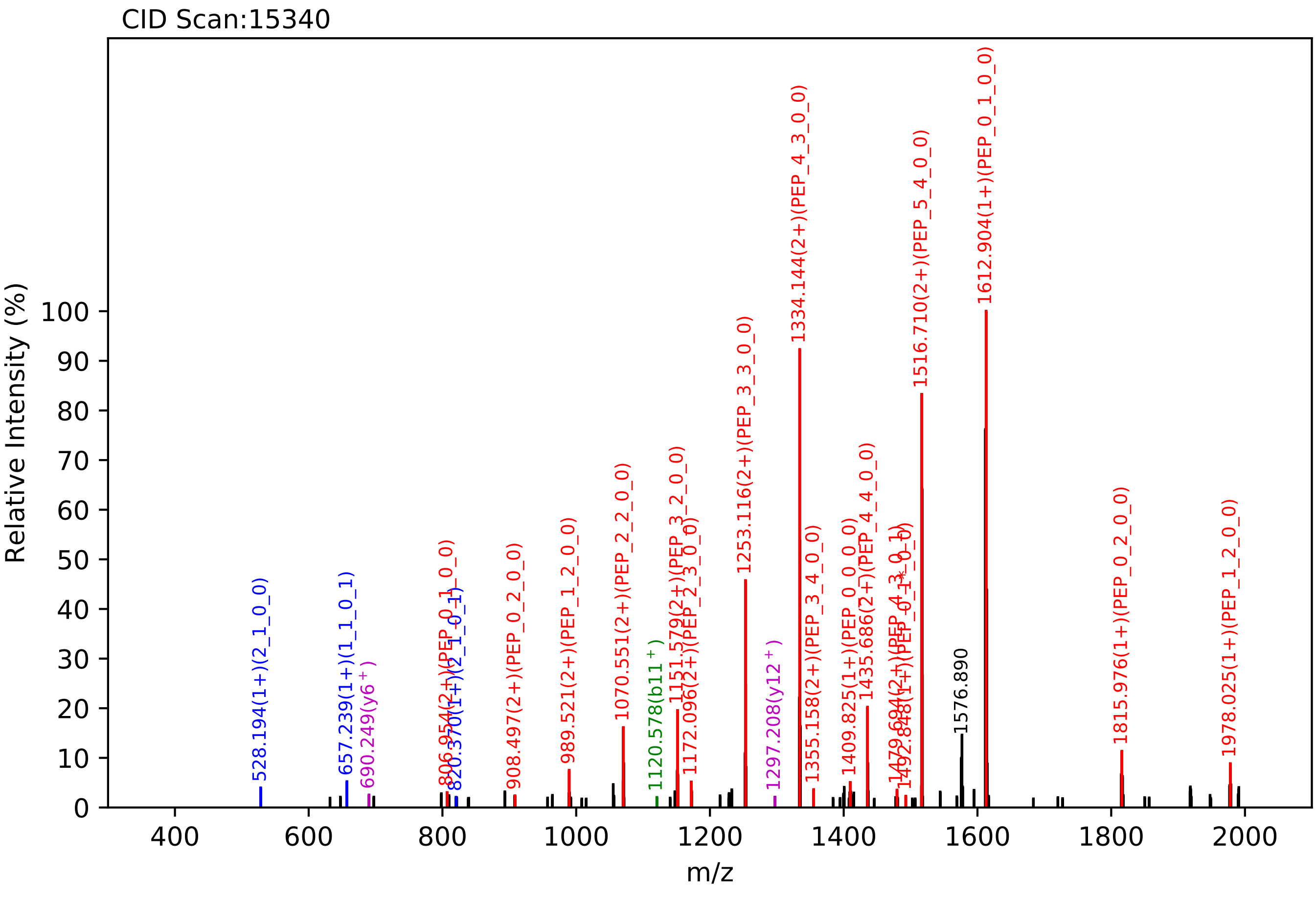

Test set no. 269, Experiment: AGP exp_3

LVPVPITNATLDR(=PEP)_5_4_0_1, m/z:1108.17(3+), RT:37.45, Y-score:89.92

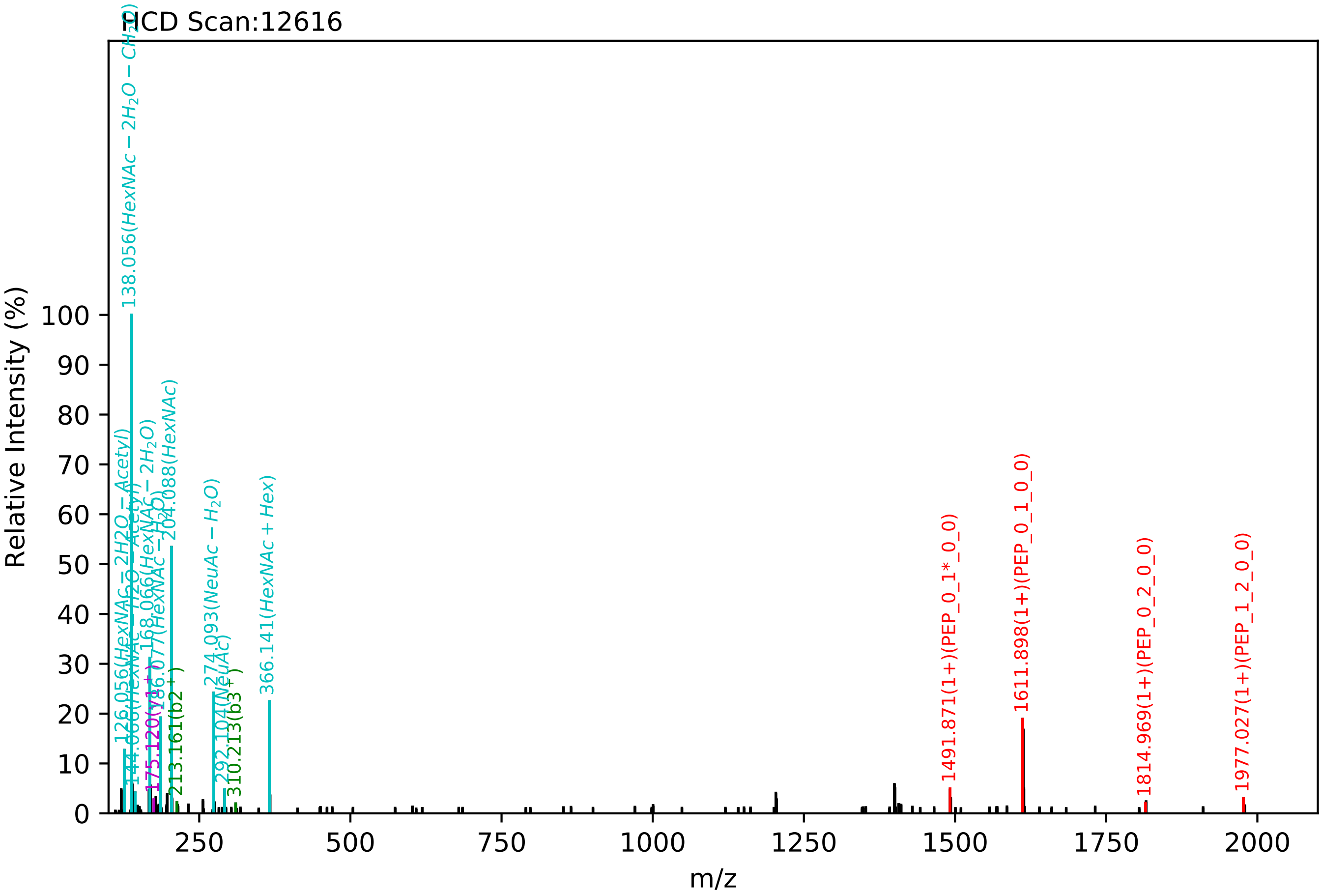

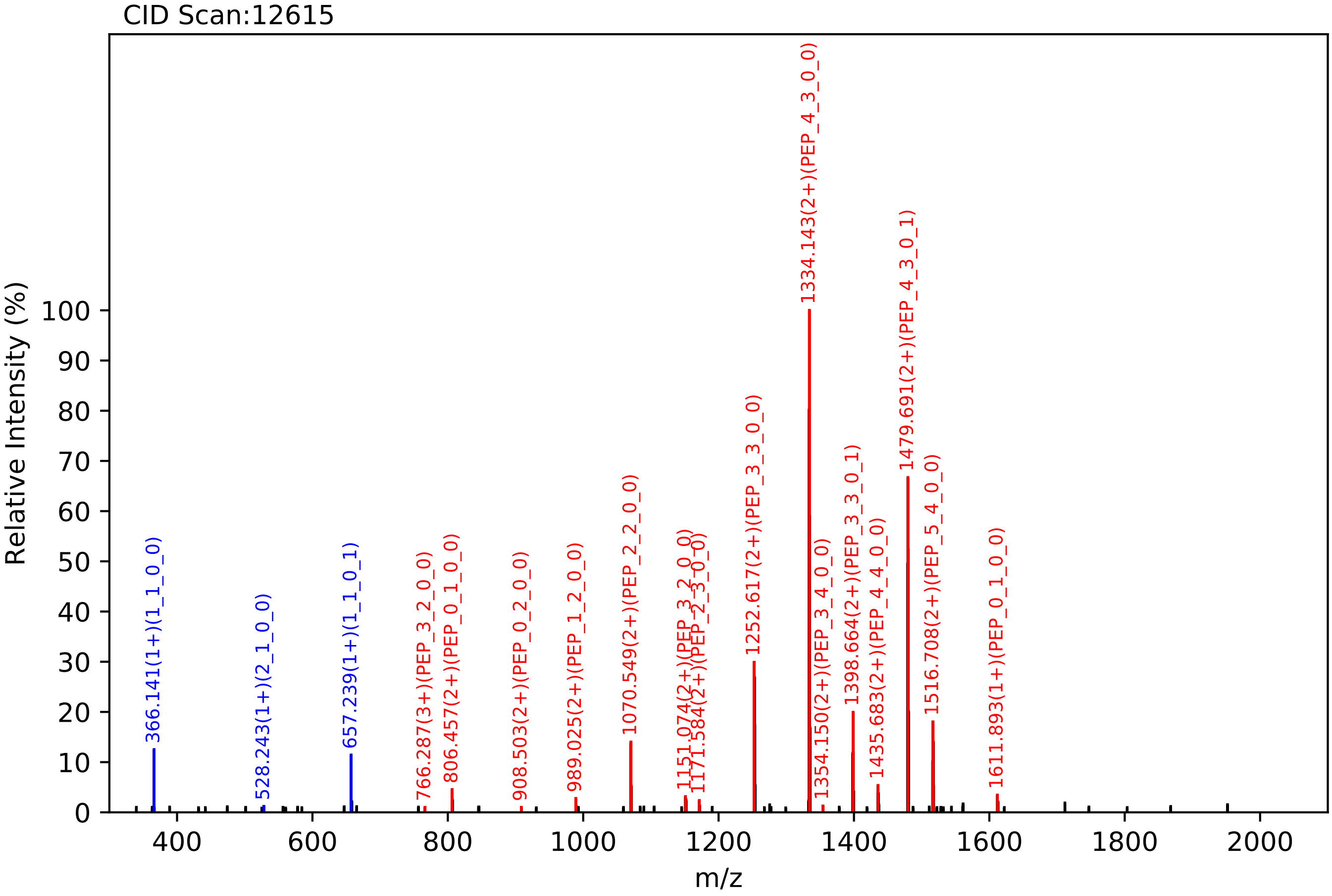

Test set no. 270, Experiment: AGP exp_4

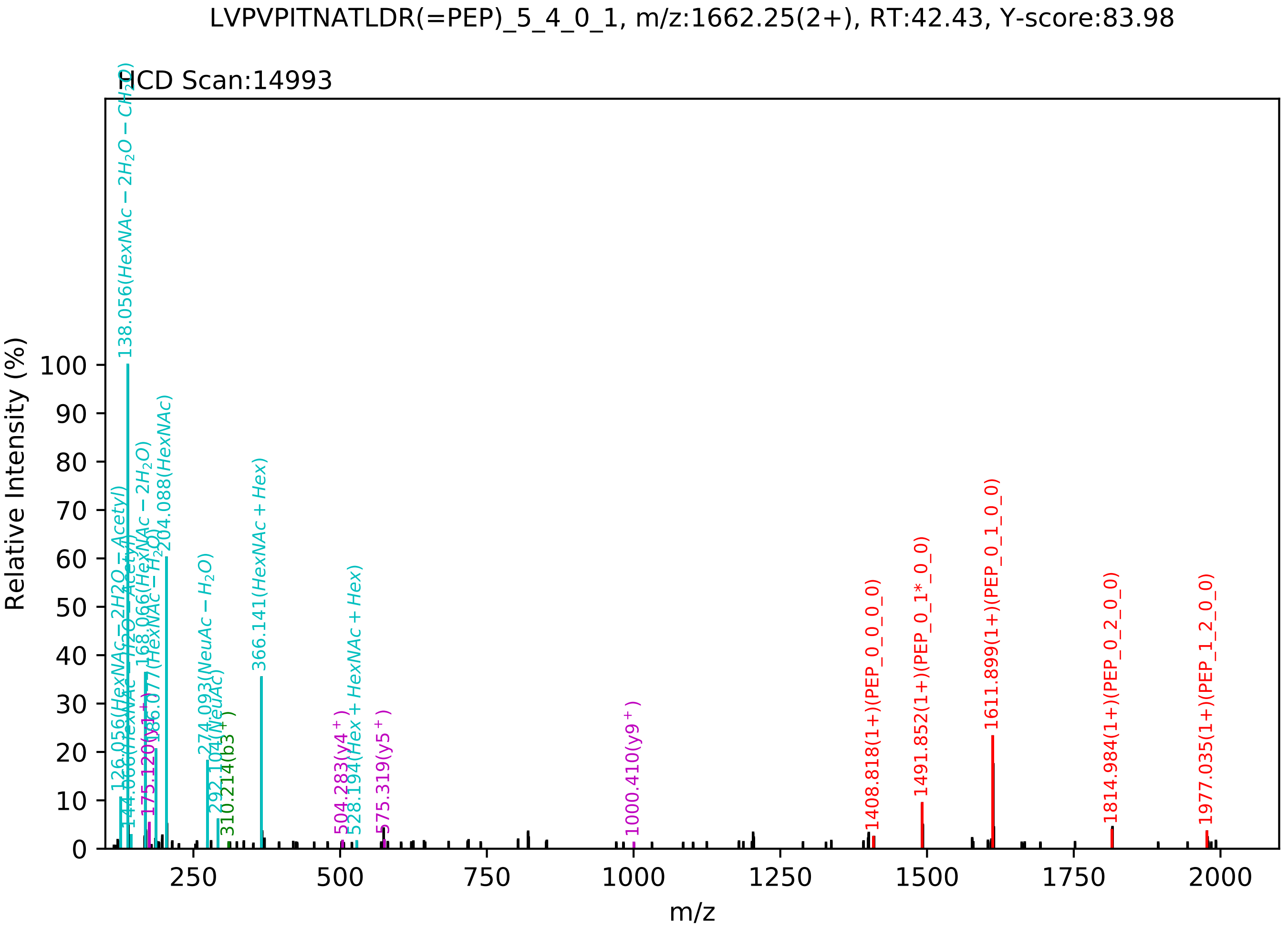

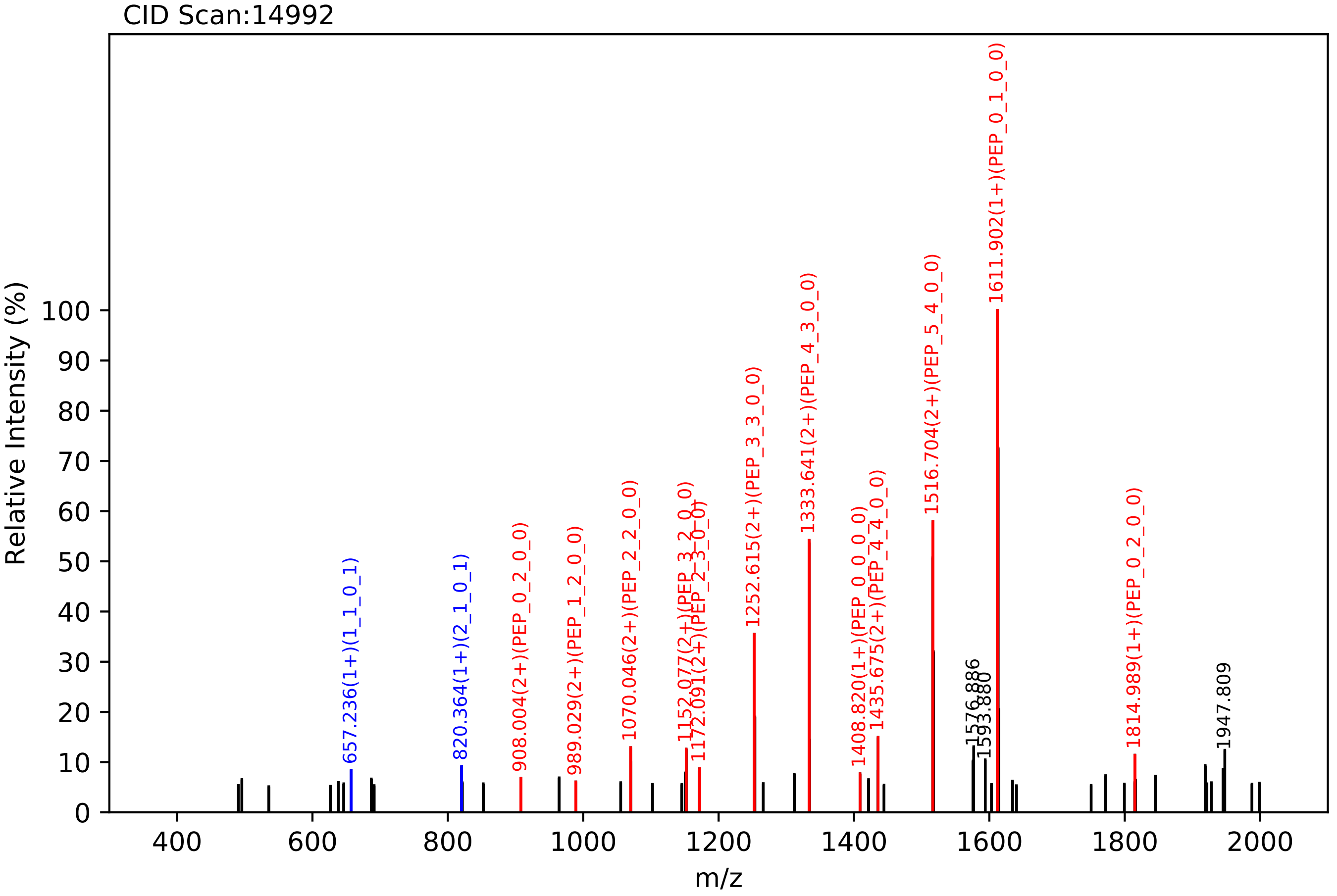

Test set no. 271, Experiment: AGP exp_3

LVPVPITNATLDR(=PEP)_5_4_0_1, m/z:831.38(4+), RT:36.85, Y-score:73.42

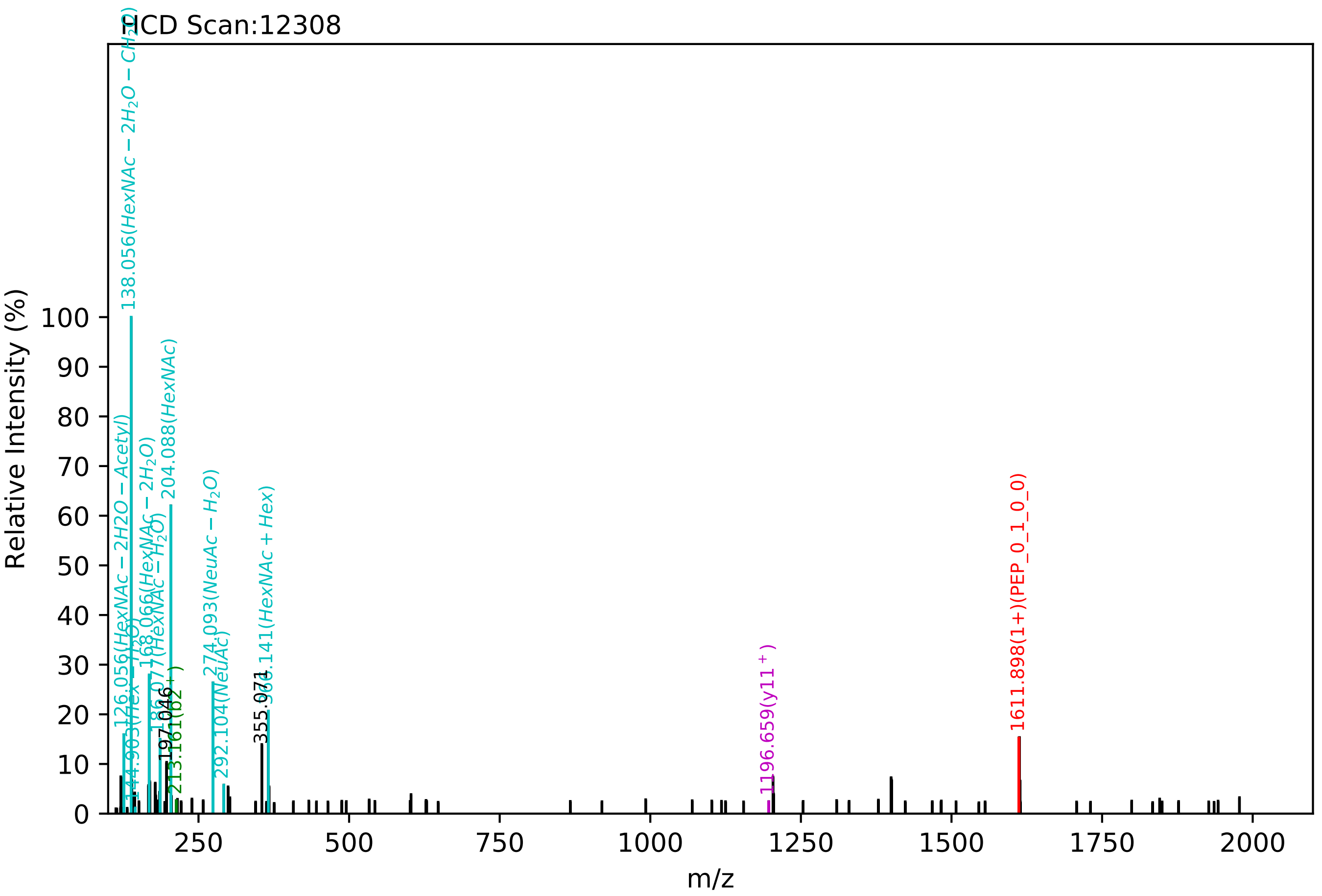

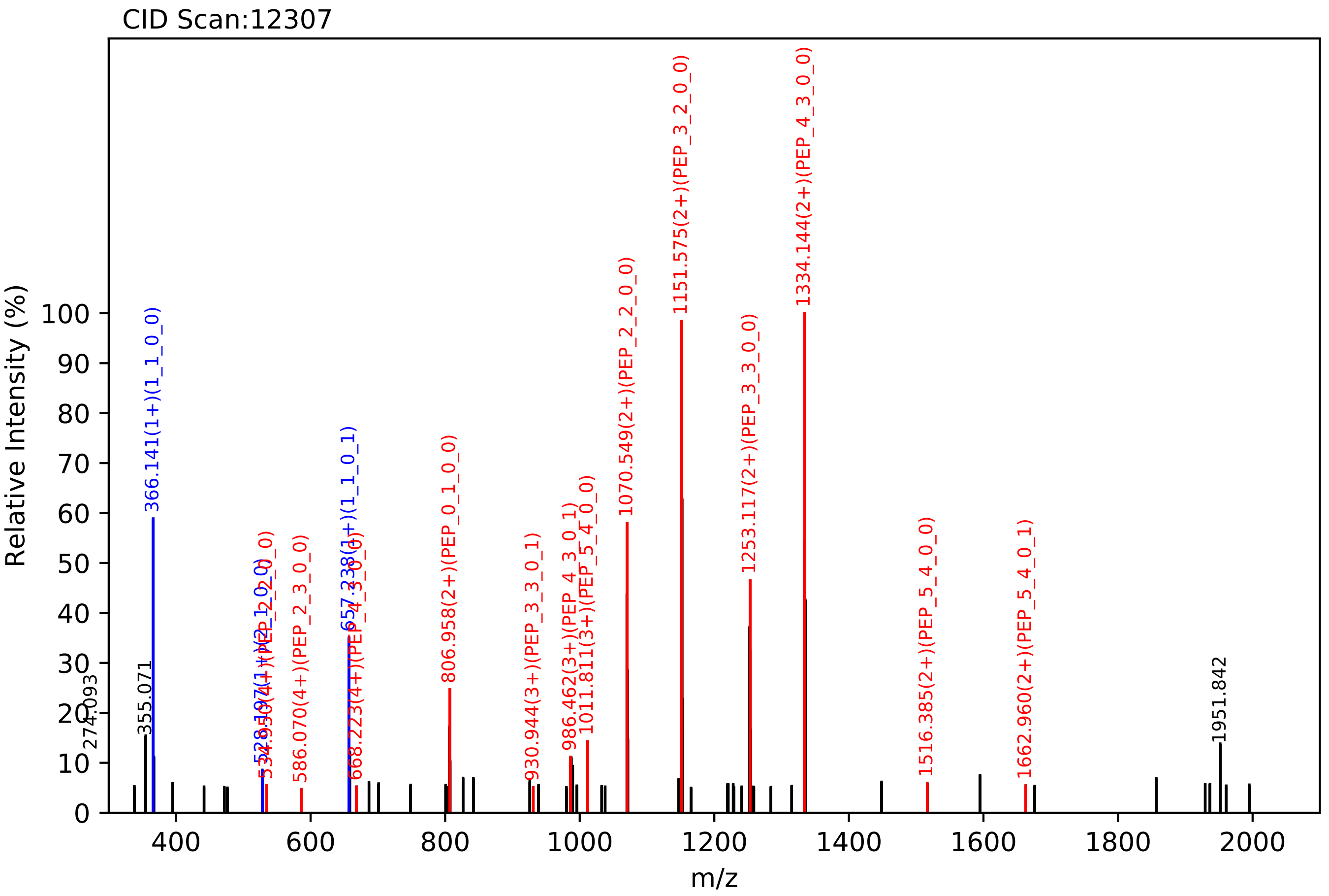

Test set no. 272, Experiment: AGP exp_4

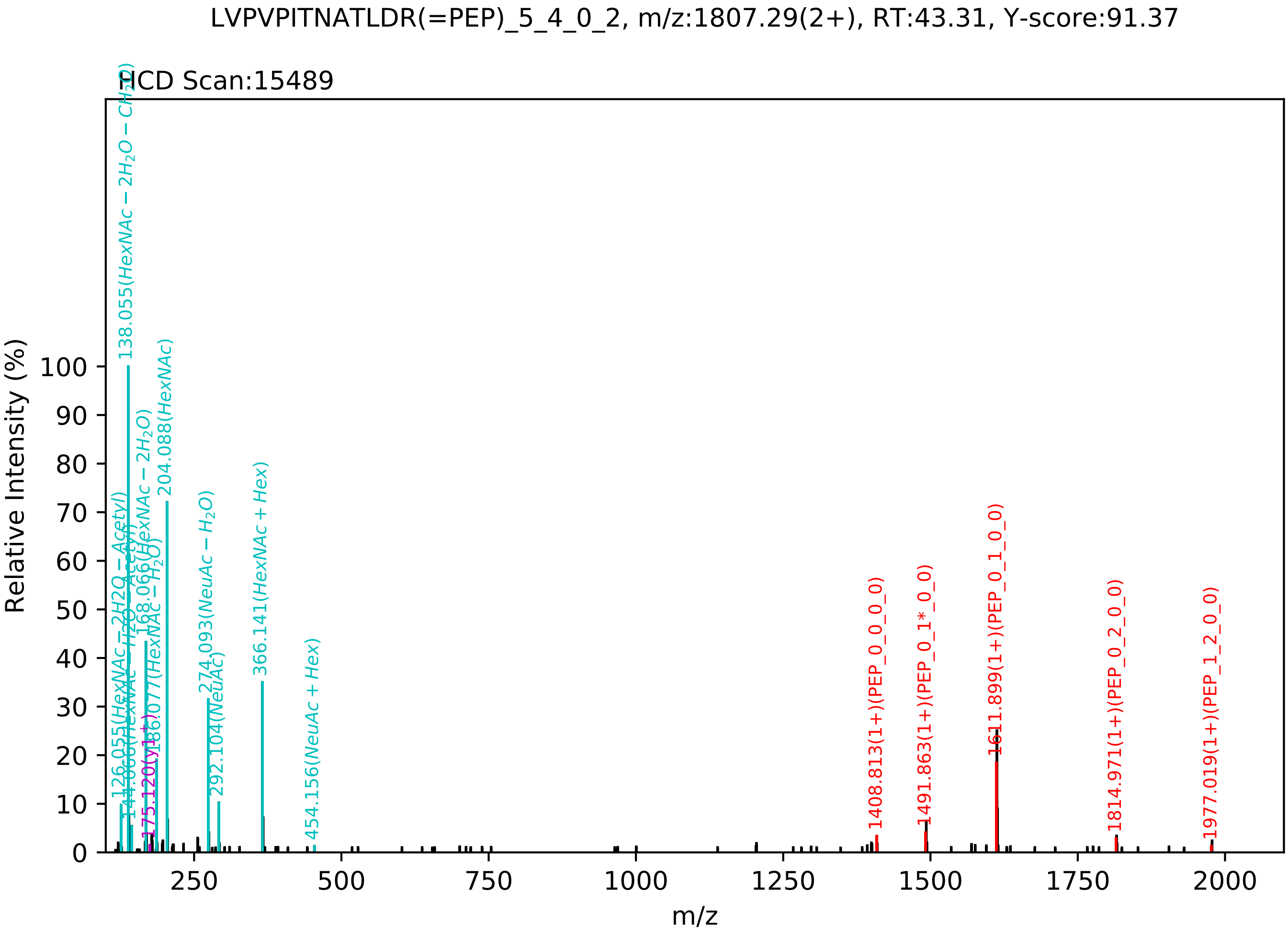

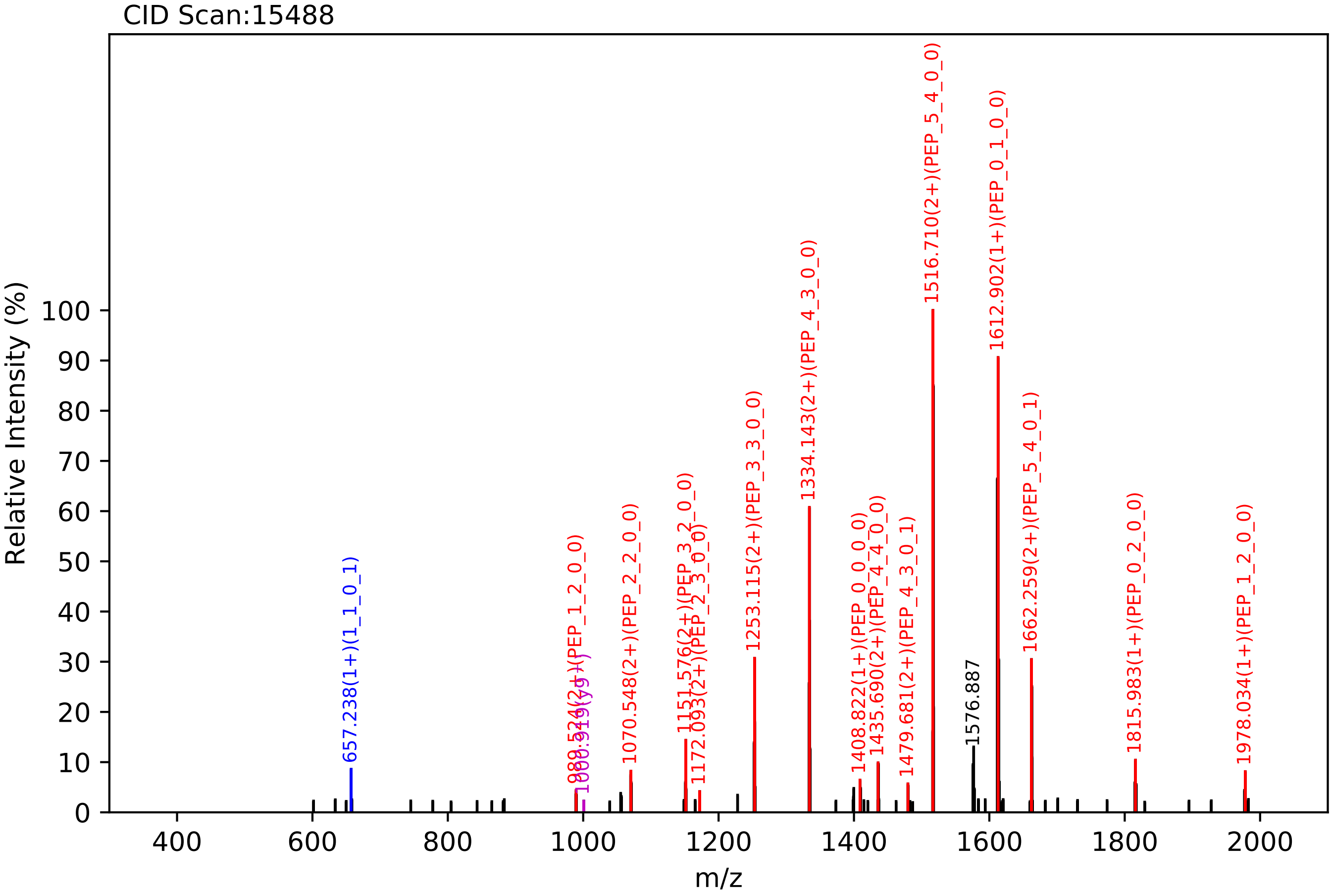

Test set no. 273, Experiment: AGP exp_3

LVPVPITNATLDR(=PEP)_5_4_0_2, m/z:1205.21(3+), RT:43.84, Y-score:90.91

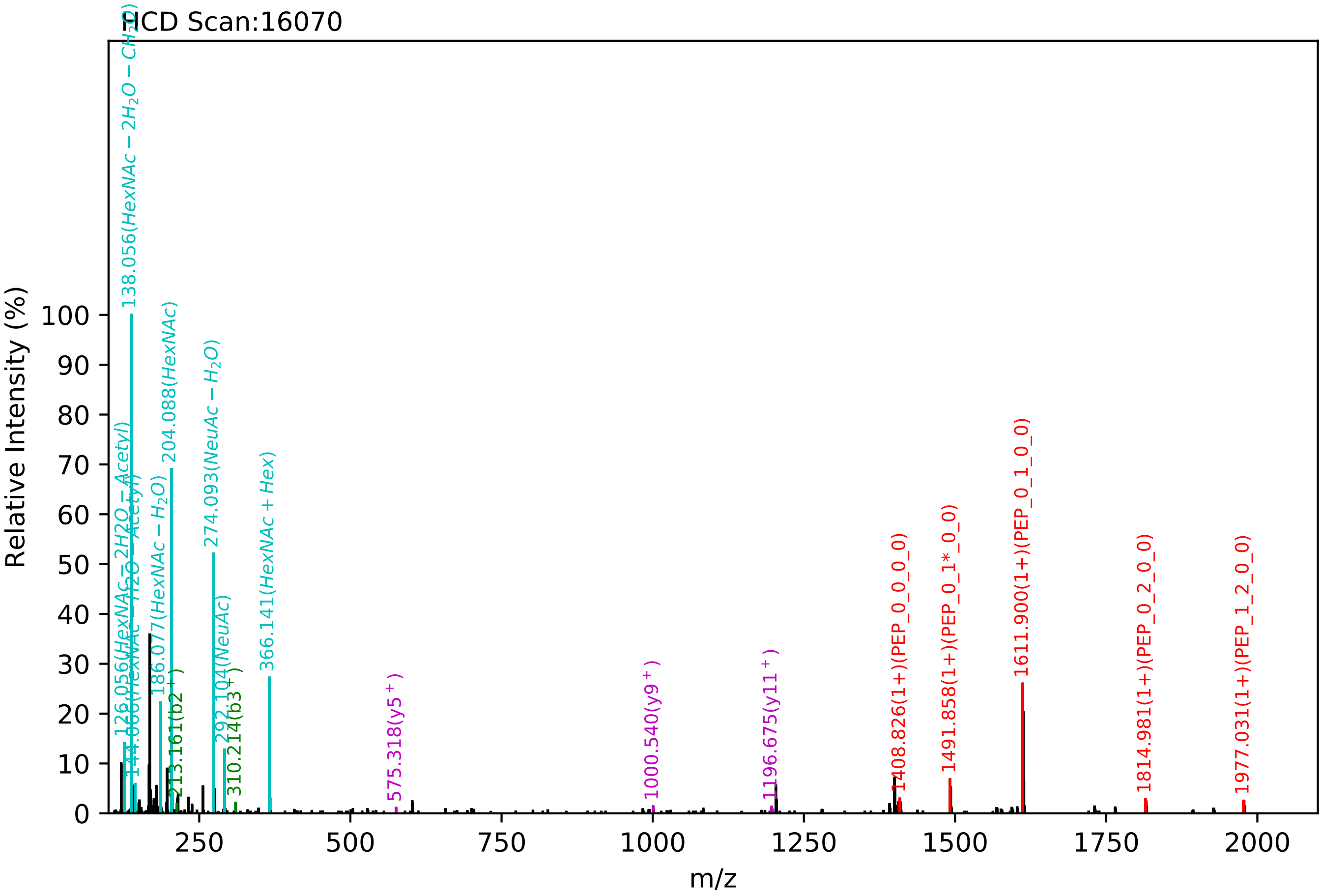

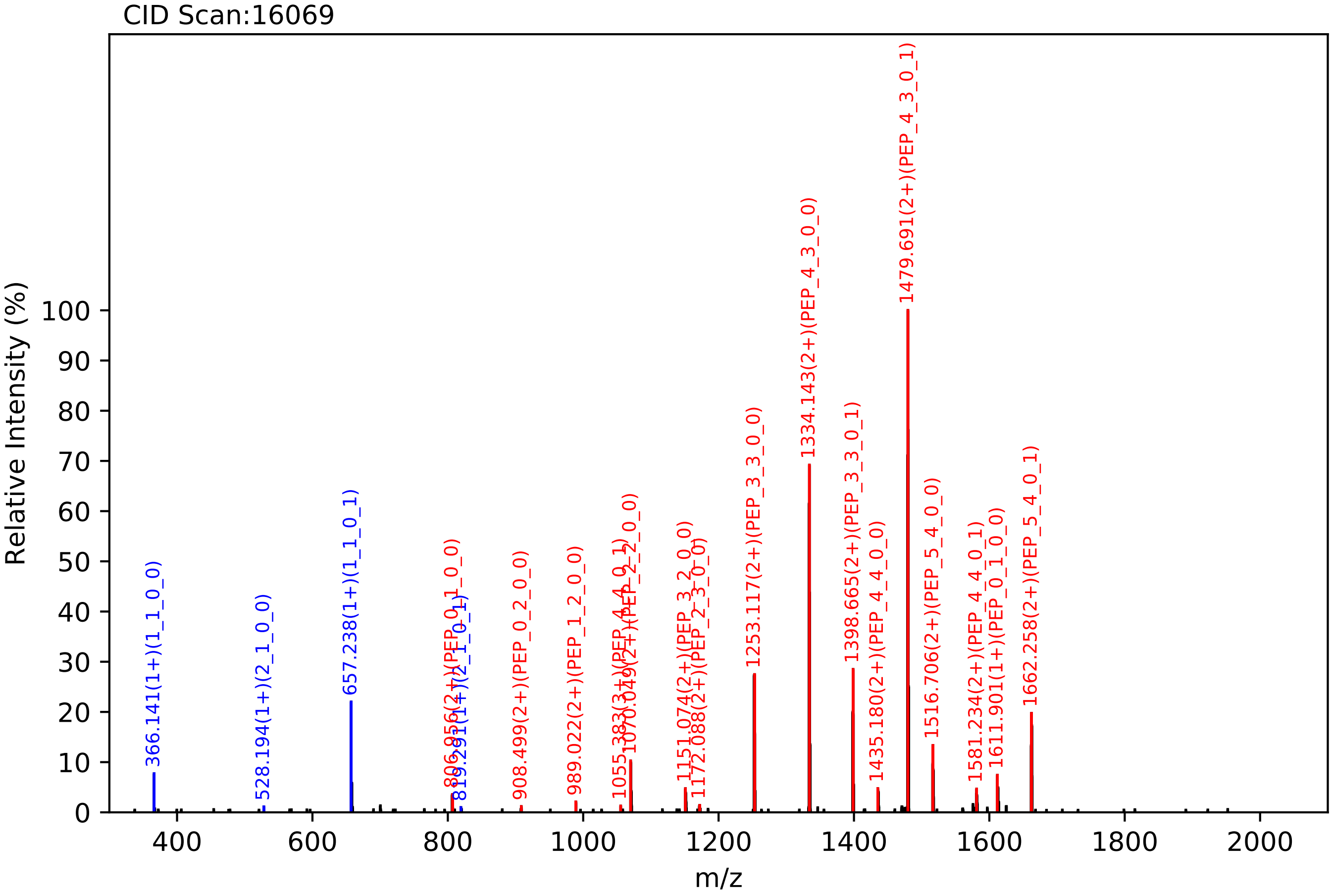

Test set no. 274, Experiment: AGP exp_3

LVPVPITNATLDR(=PEP)_5_4_0_2, m/z:904.15(4+), RT:43.35, Y-score:82.53

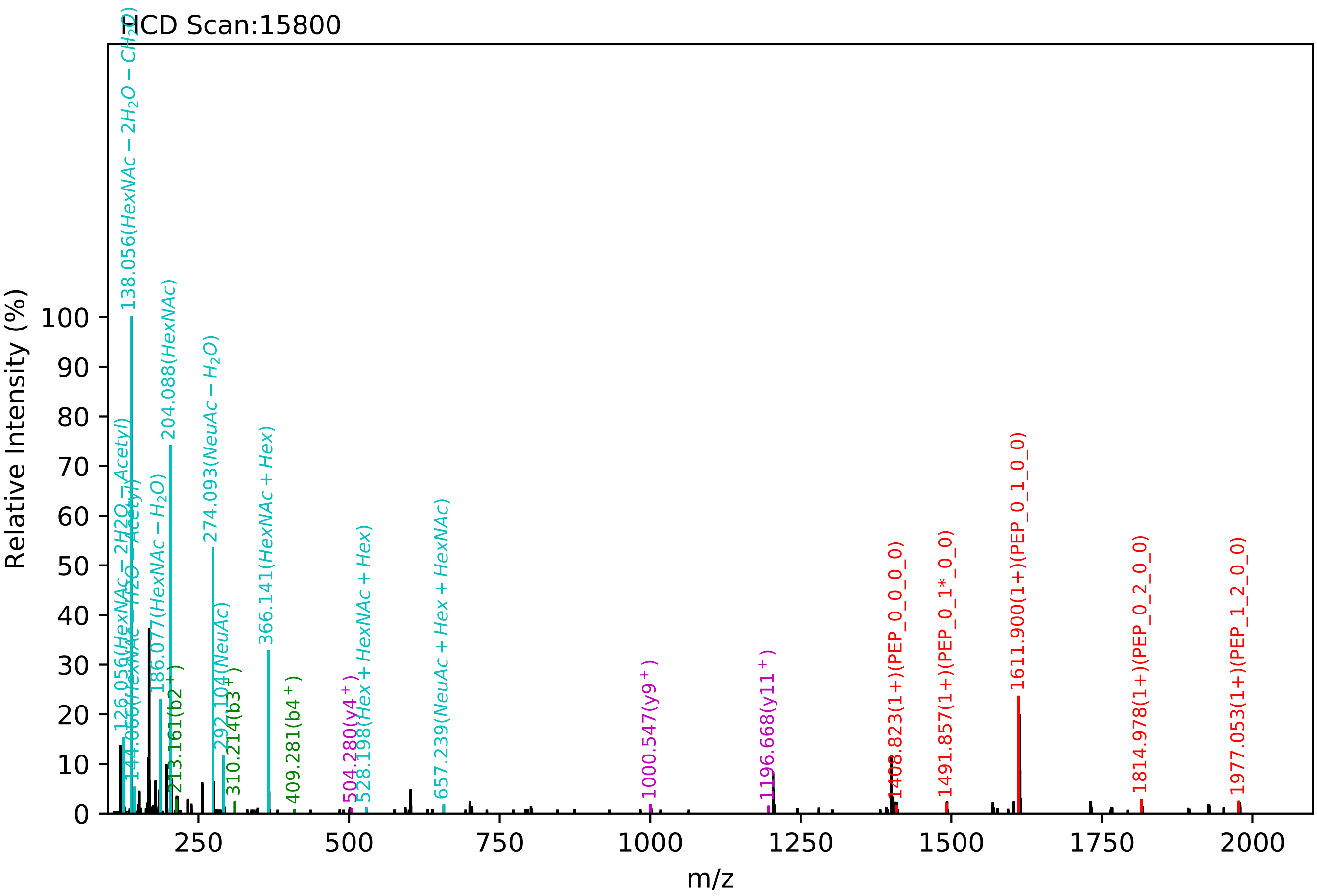

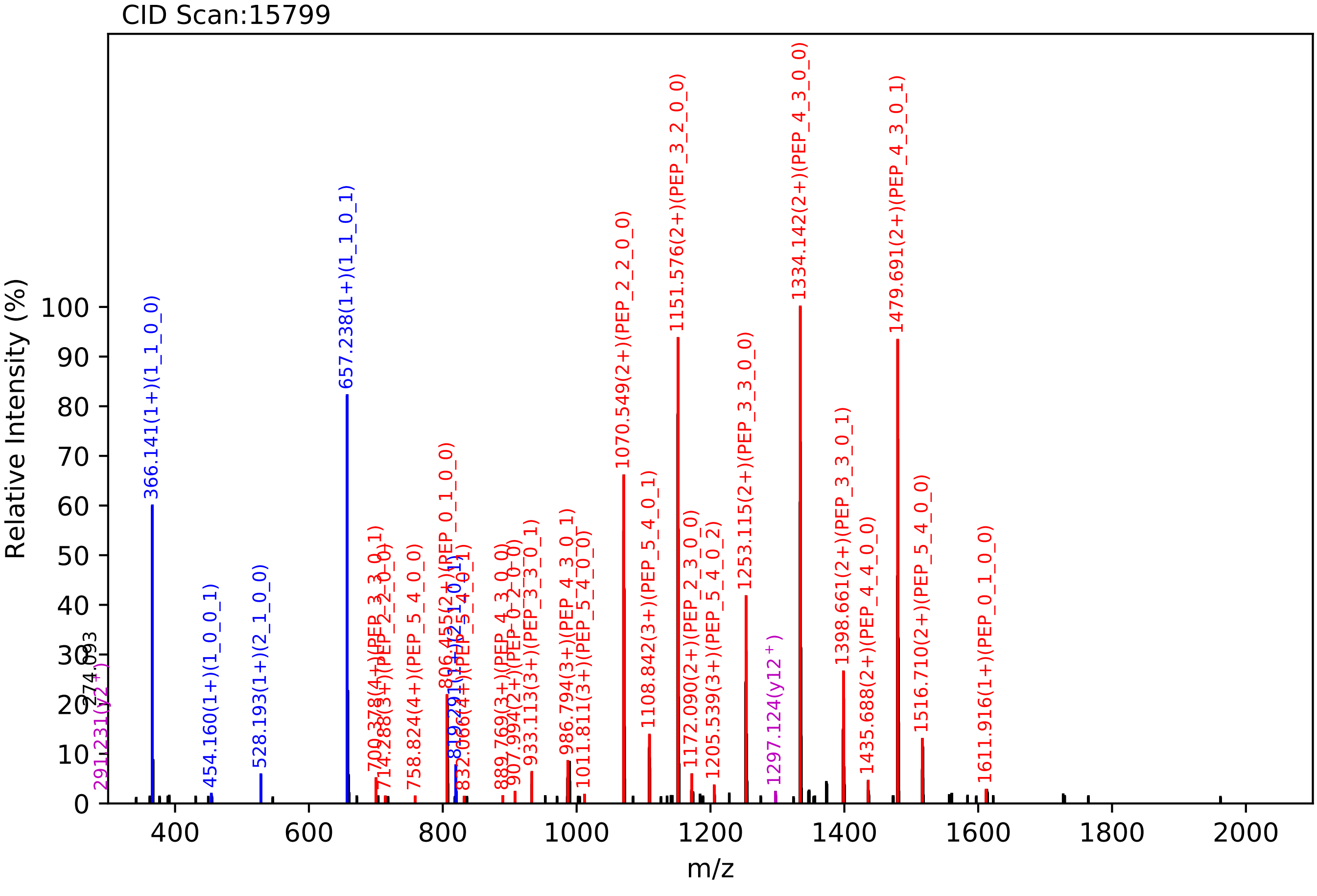

Test set no. 275, Experiment: AGP exp_3

LVPVPITNATLDR(=PEP)_5_4_0_2, m/z:1205.20(3+), RT:50.47, Y-score:81.27

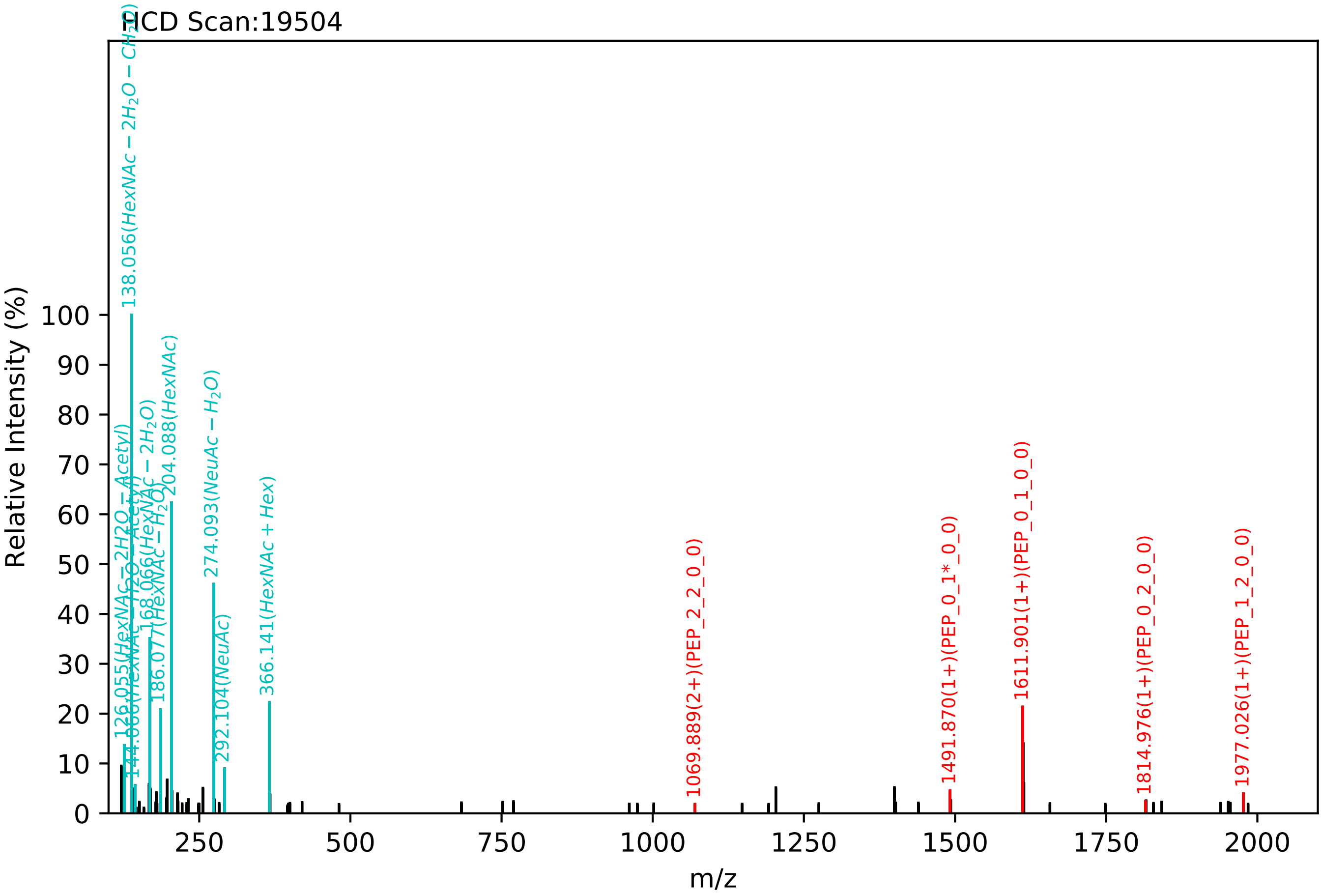

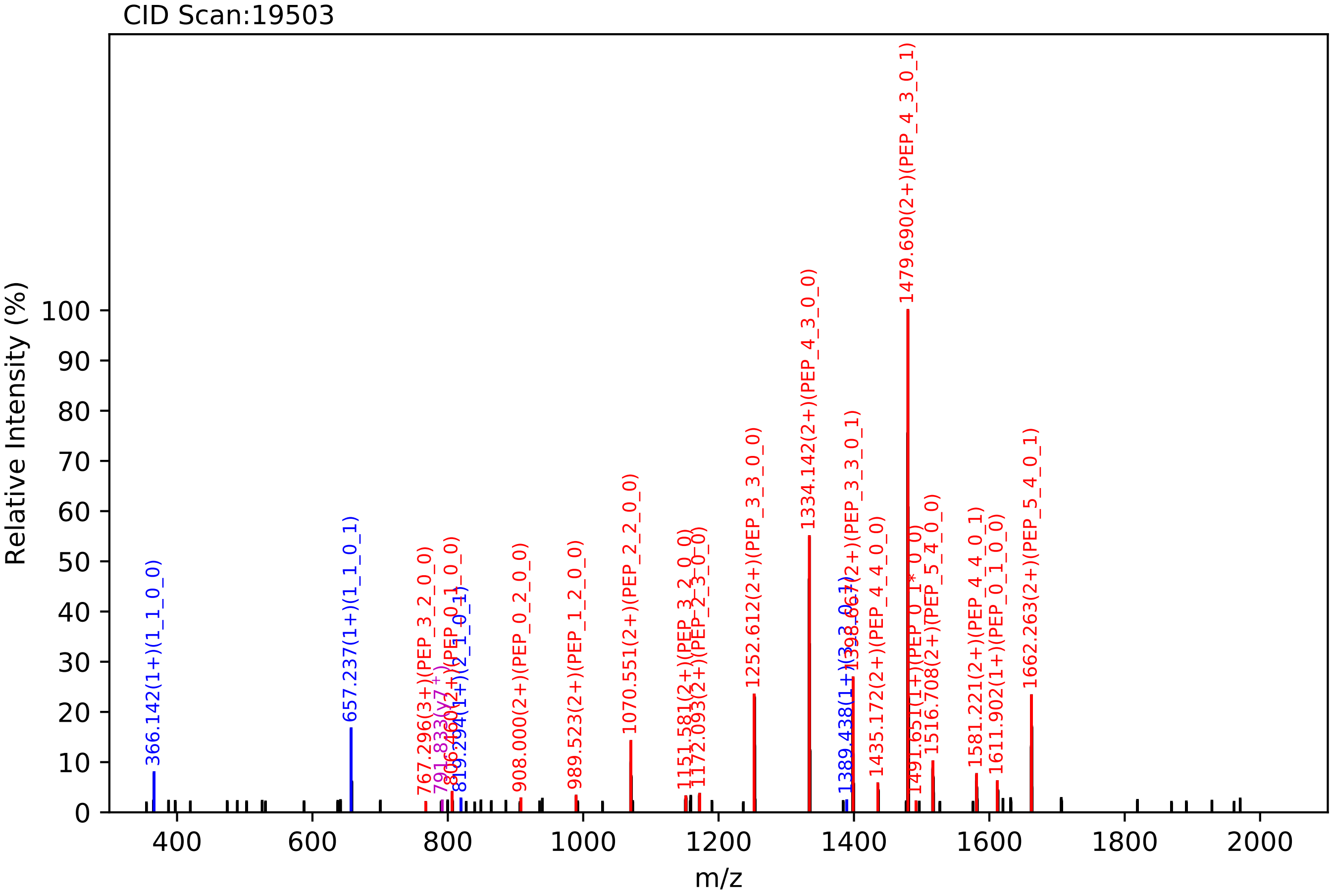

Test set no. 276, Experiment: AGP exp_3

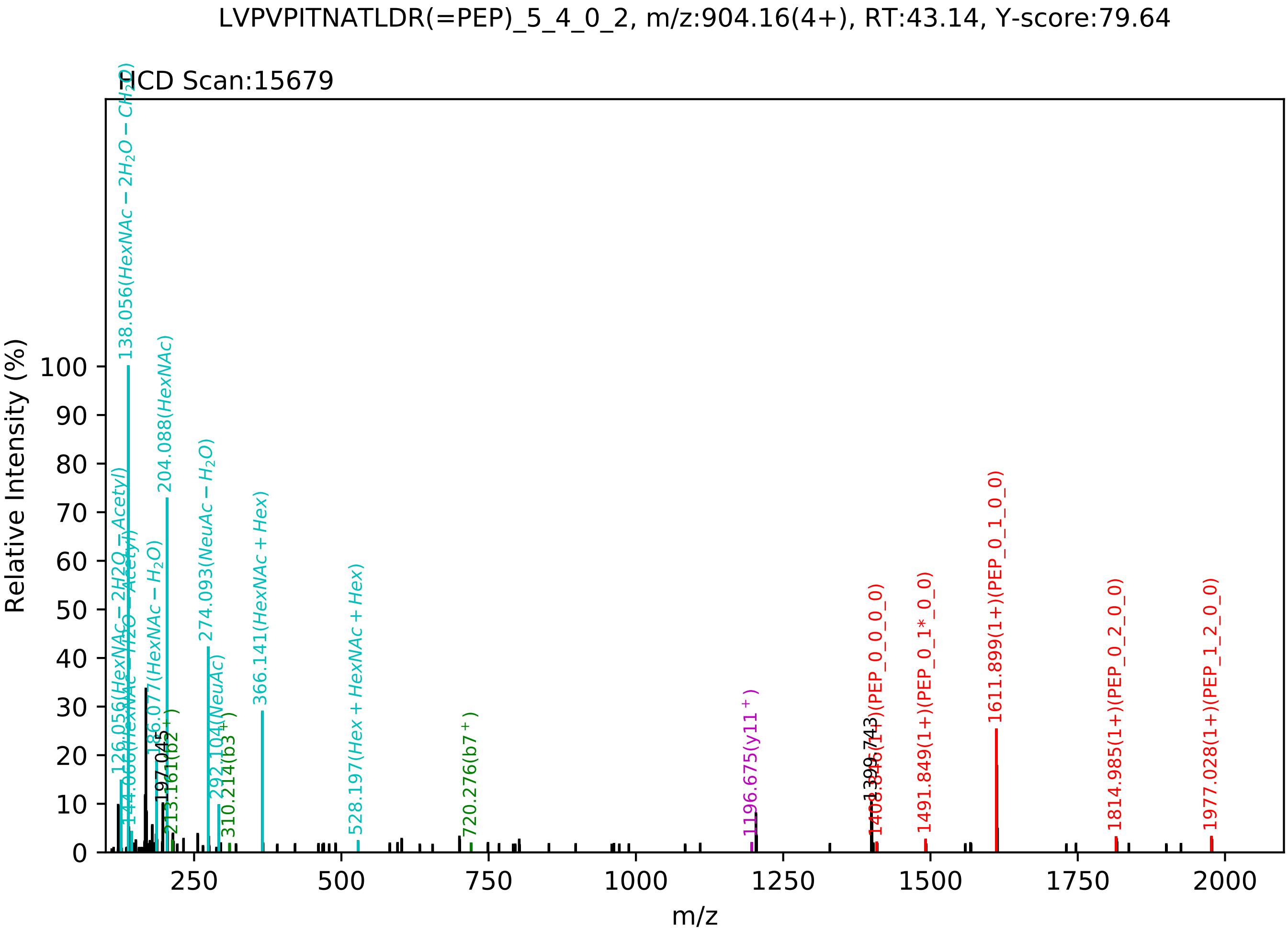

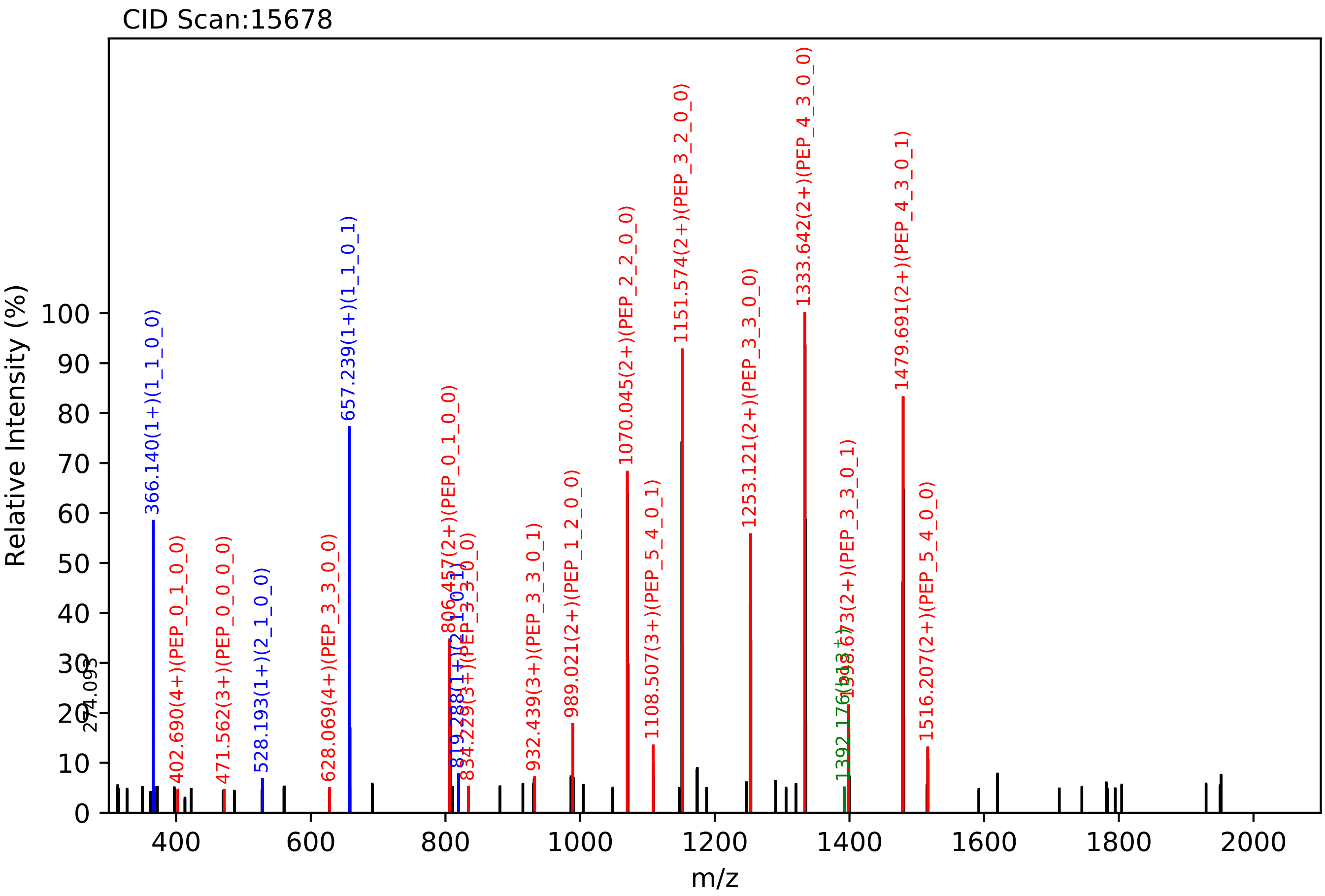

Test set no. 277, Experiment: AGP exp_4

LVPVPITNATLDR(=PEP)_5_4_0_2, m/z:904.16(4+), RT:43.11, Y-score:76.80

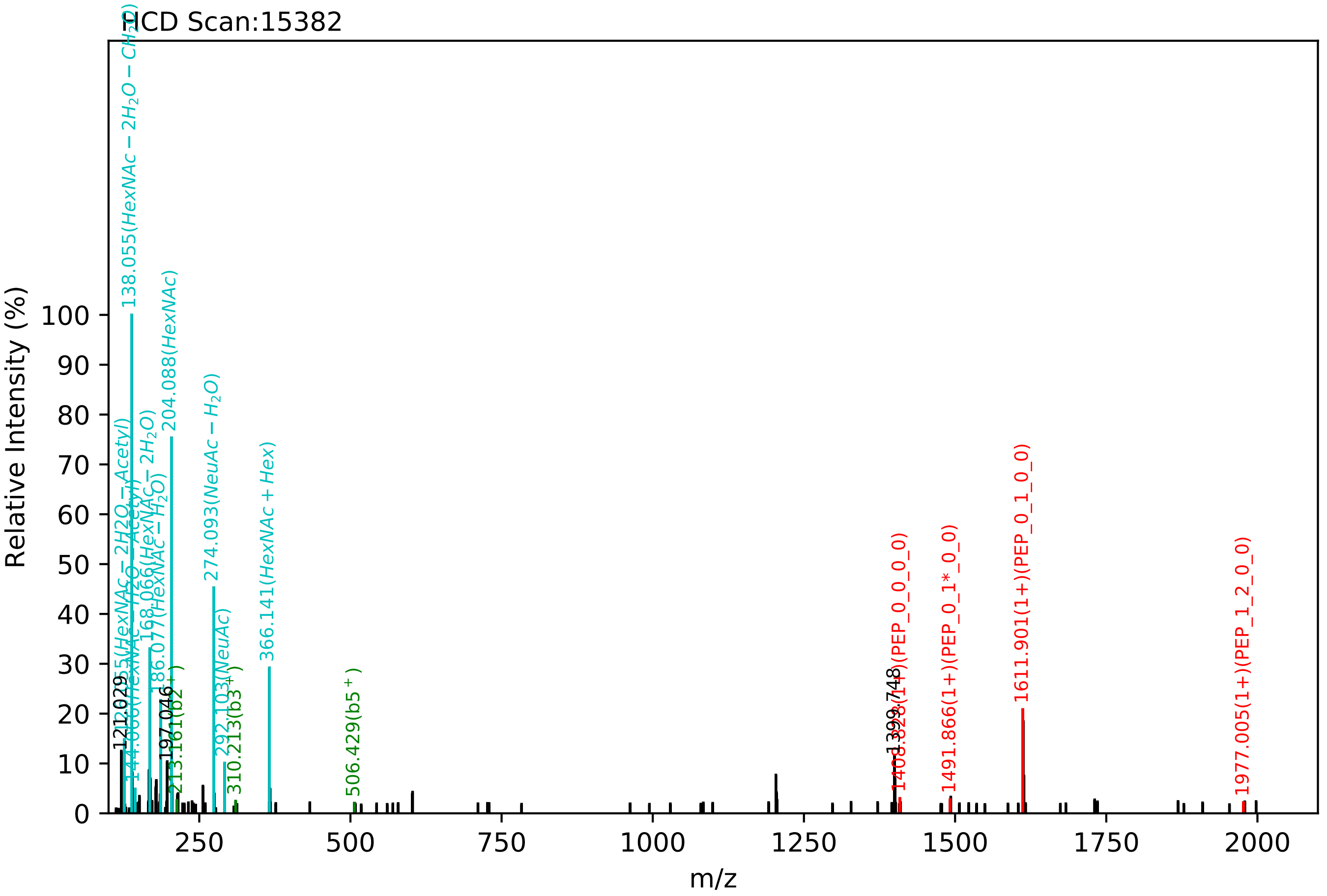

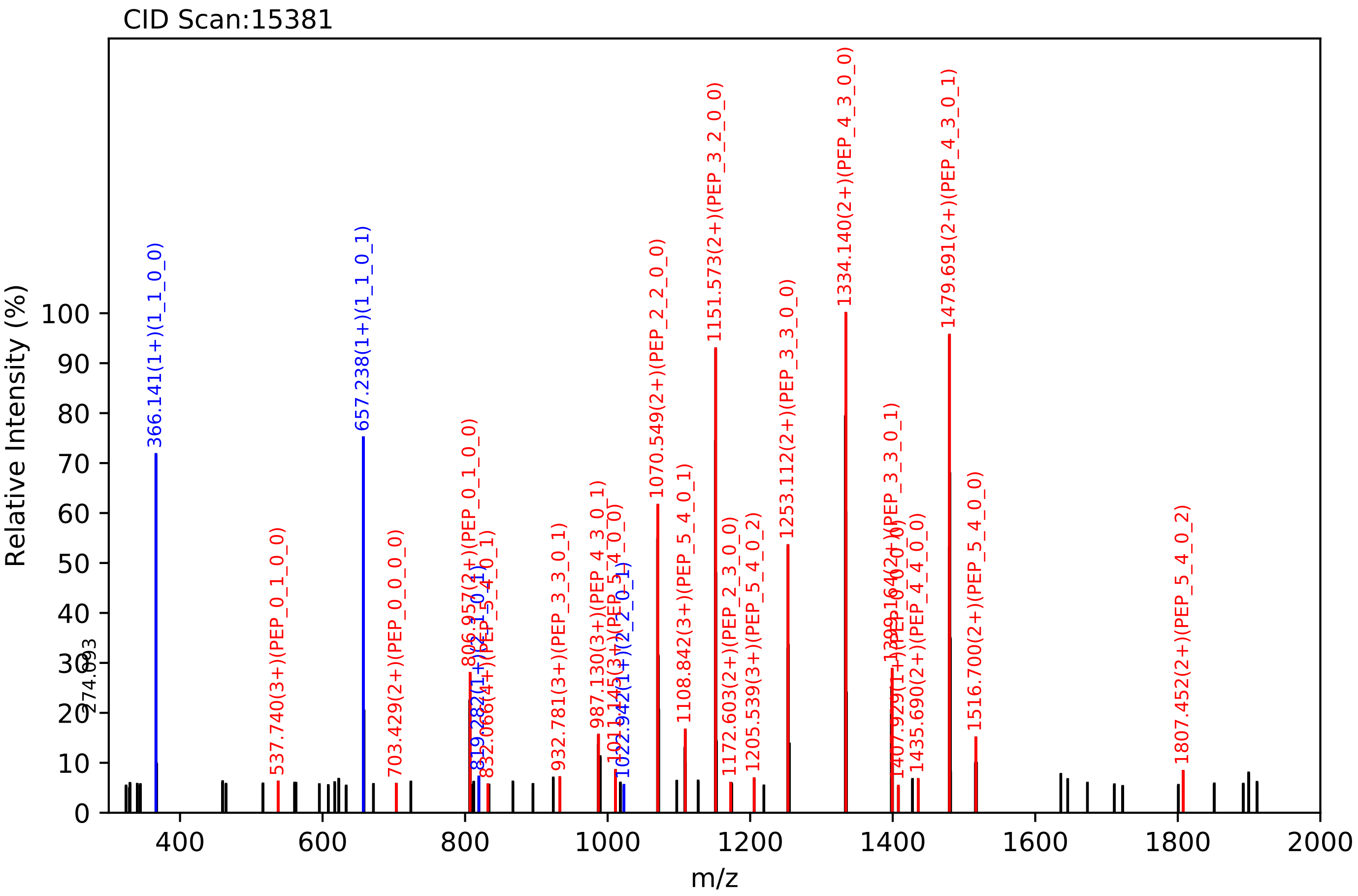

Test set no. 278, Experiment: AGP exp_3

LVPVPITNATLDR(=PEP)_5_4_1_2, m/z:1253.89(3+), RT:43.29, Y-score:77.75

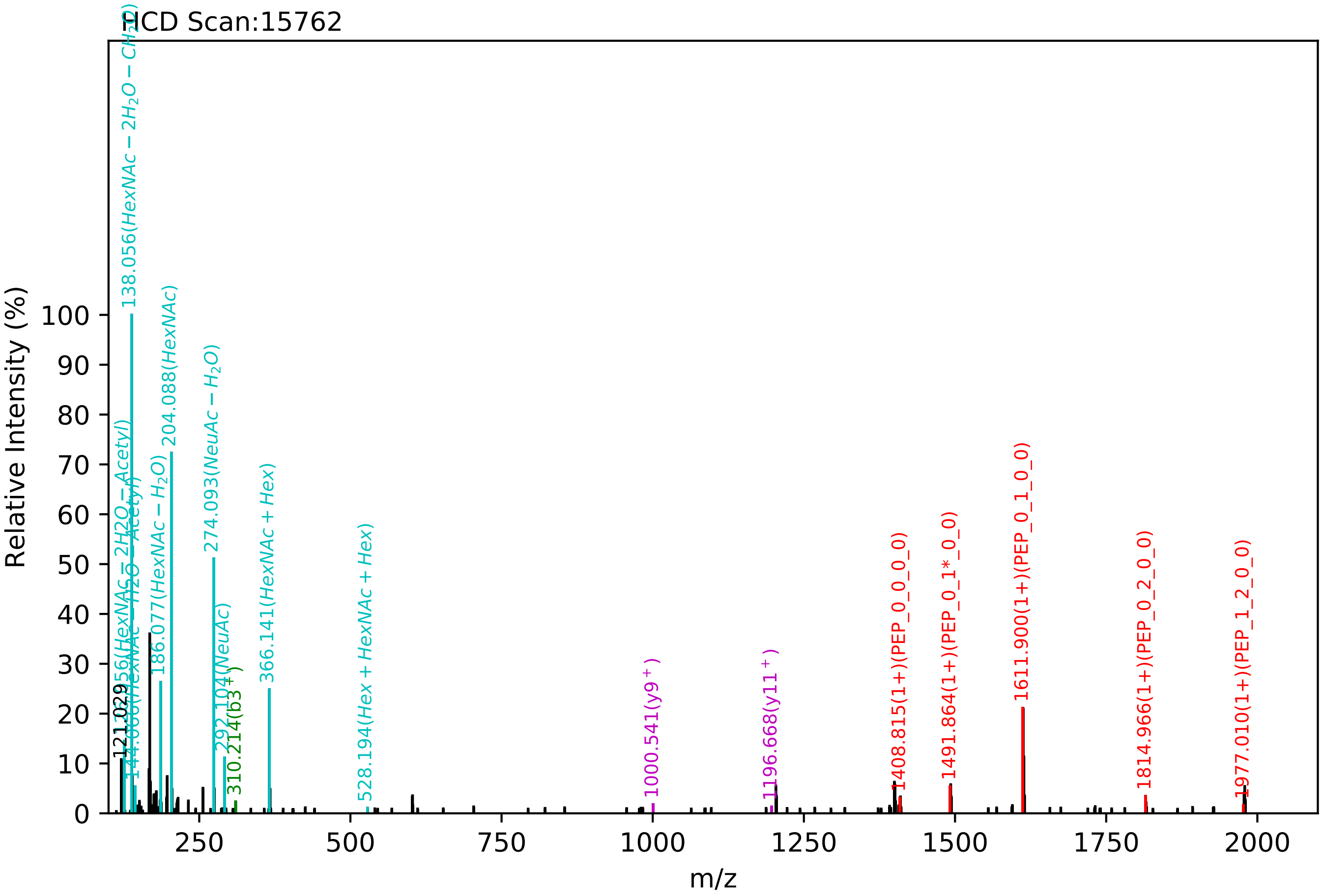

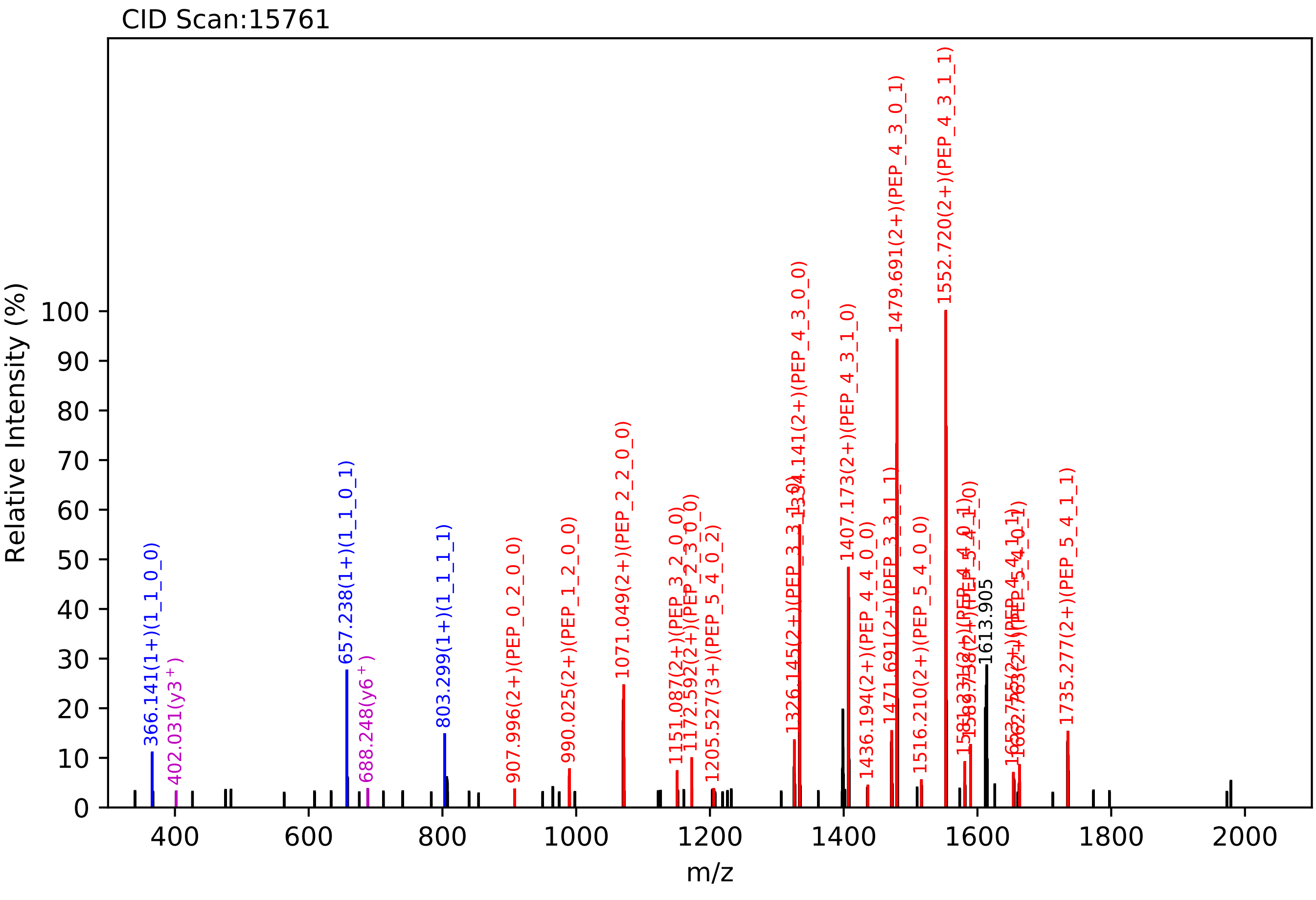

Test set no. 279, Experiment: AGP exp_4

LVPVPITNATLDR(=PEP)_5_4_1_2, m/z:1253.89(3+), RT:43.09, Y-score:69.07

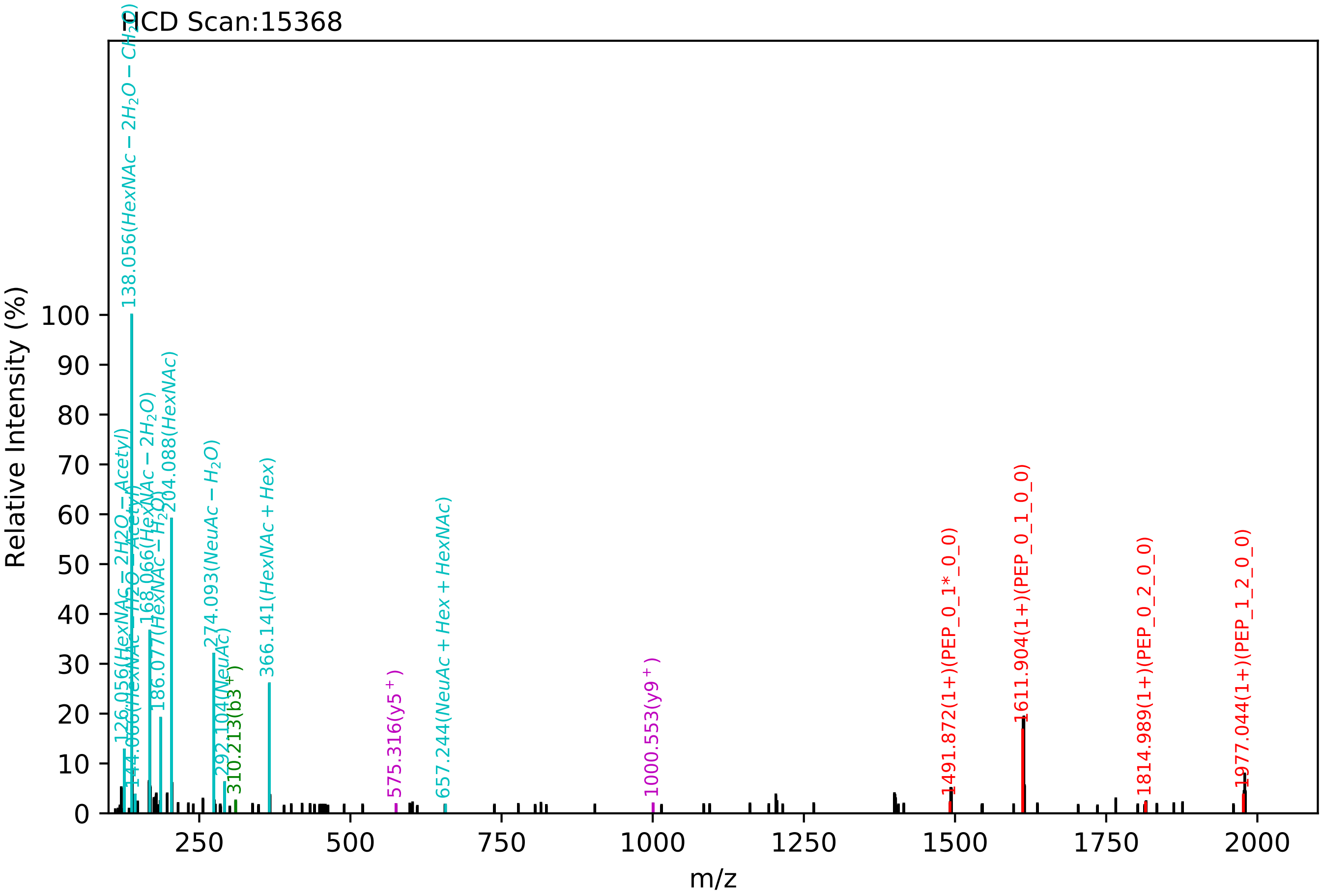

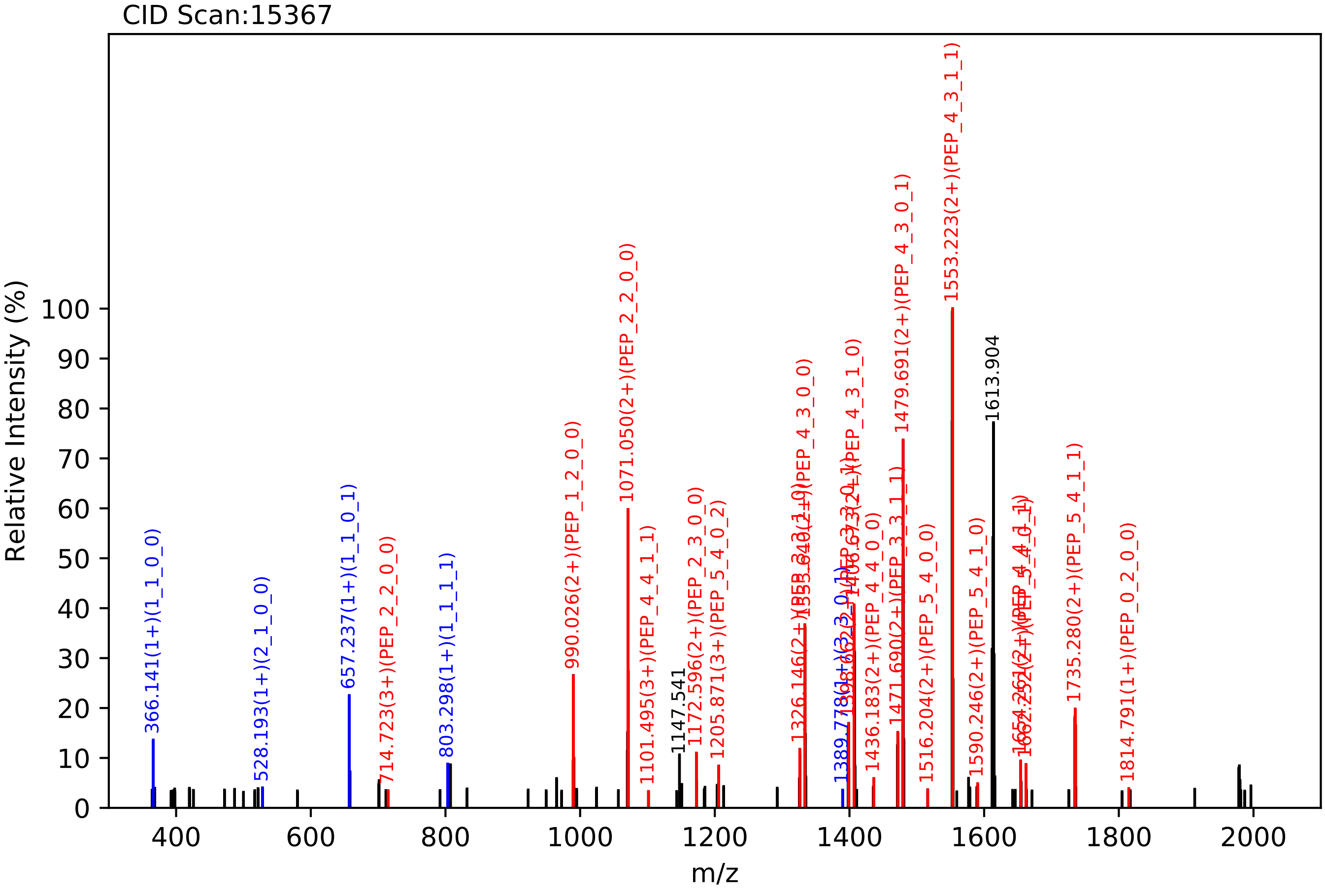

Test set no. 280, Experiment: AGP exp_3

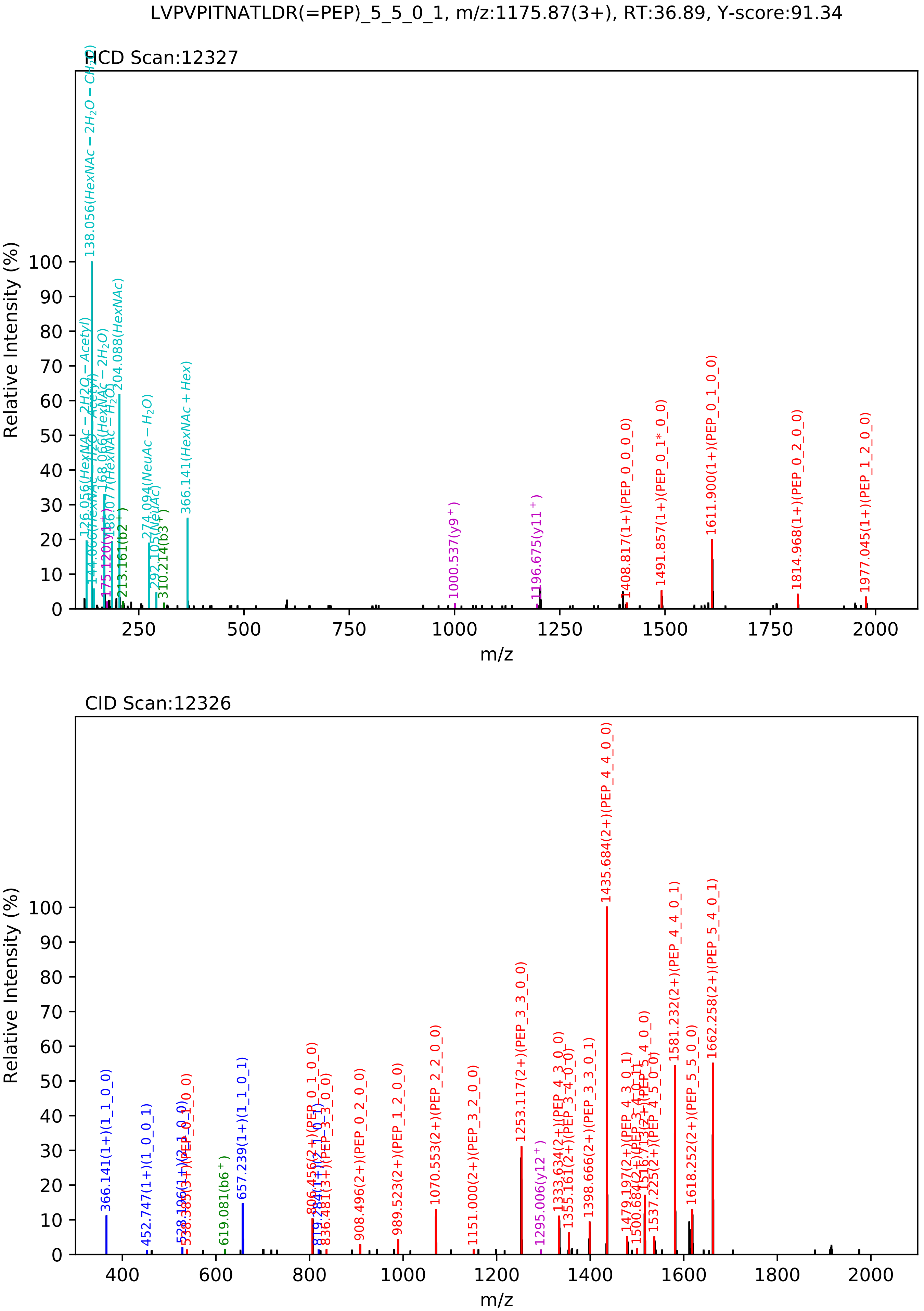

Test set no. 281, Experiment: AGP exp_4

LVPVPITNATLDR(=PEP)_5_5_0_1, m/z:1175.87(3+), RT:36.93, Y-score:88.20

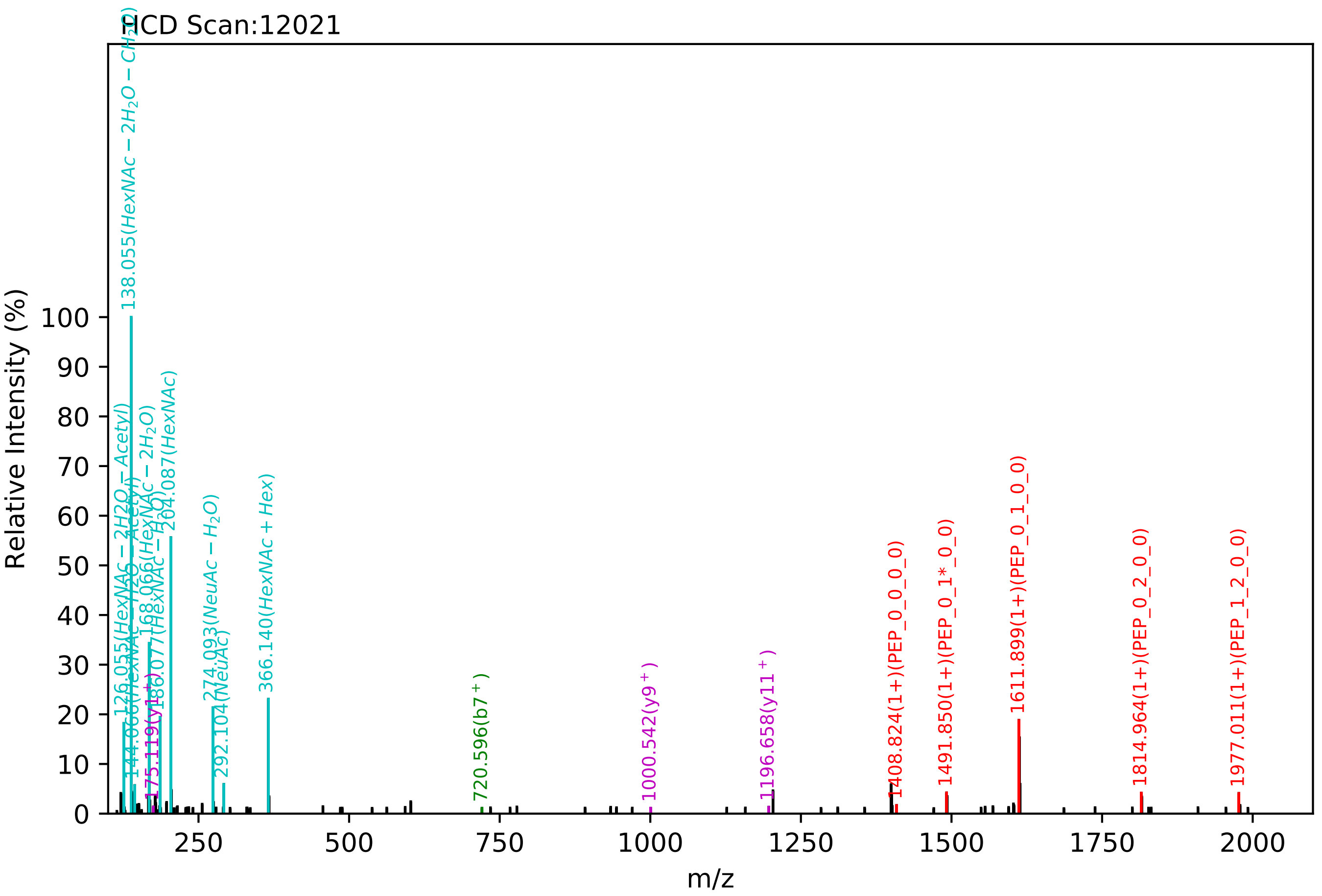

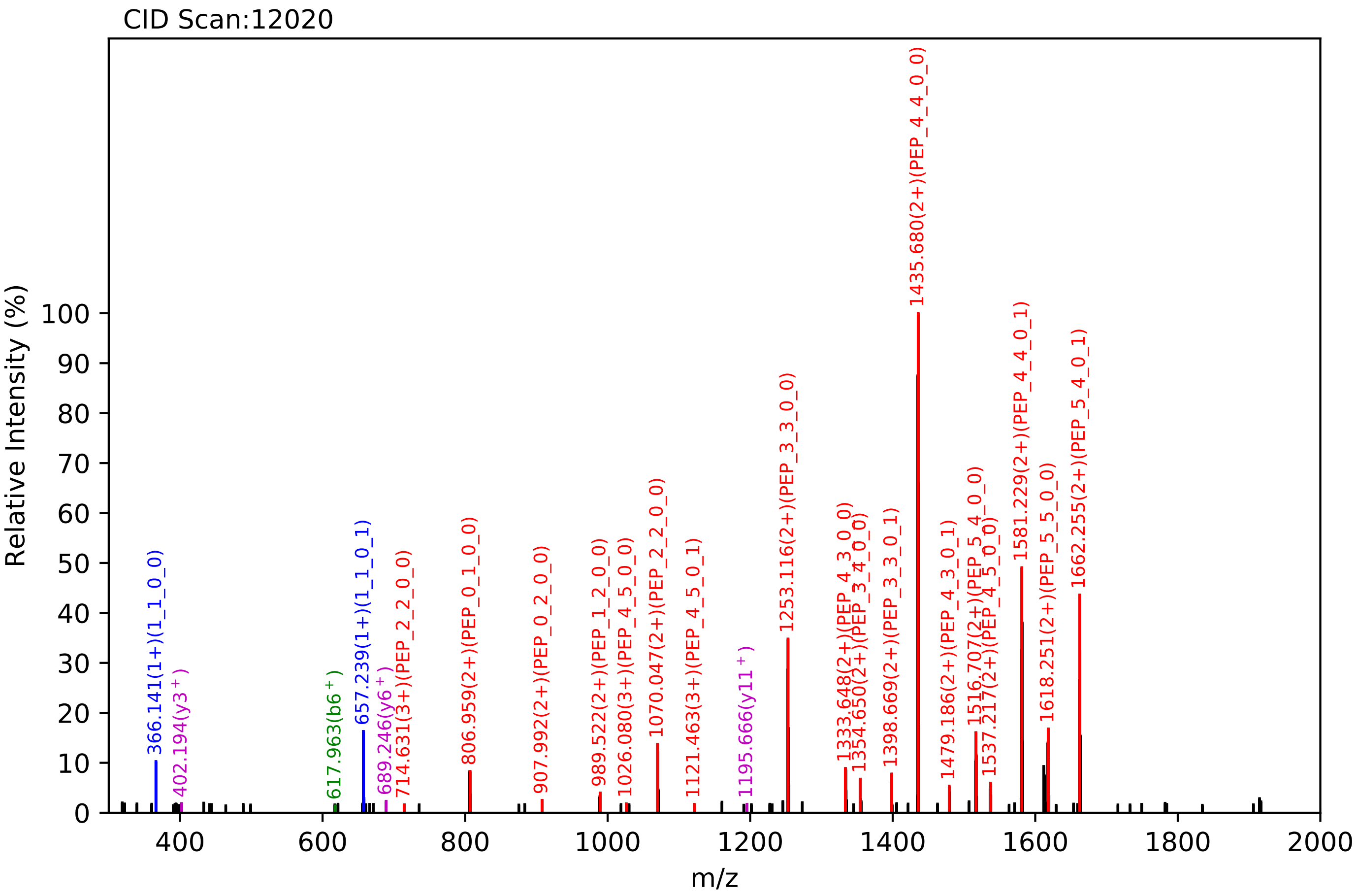

Test set no. 282, Experiment: AGP exp_3

LVPVPITNATLDR(=PEP)_5_5_1_2, m/z:1321.59(3+), RT:42.89, Y-score:80.77

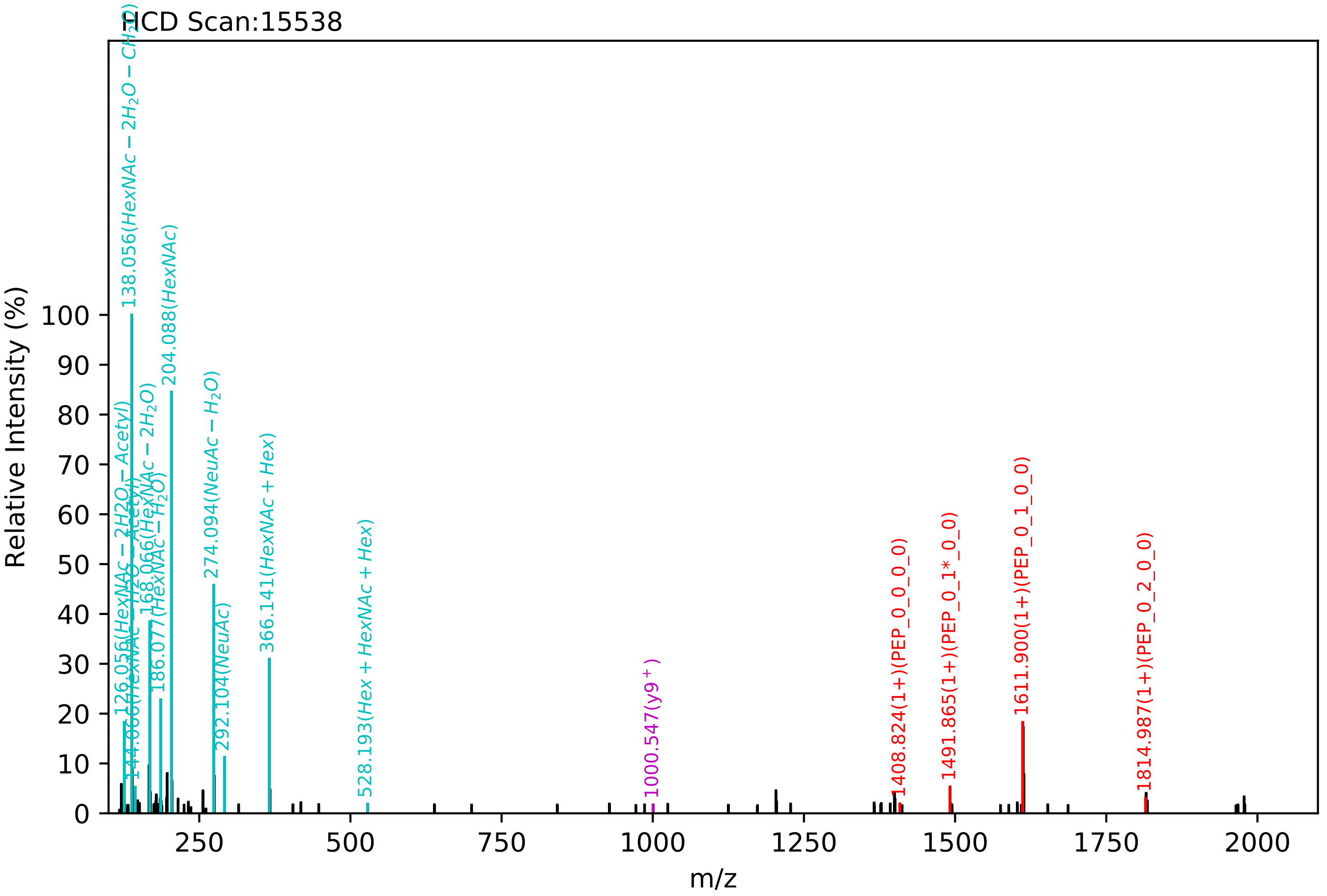

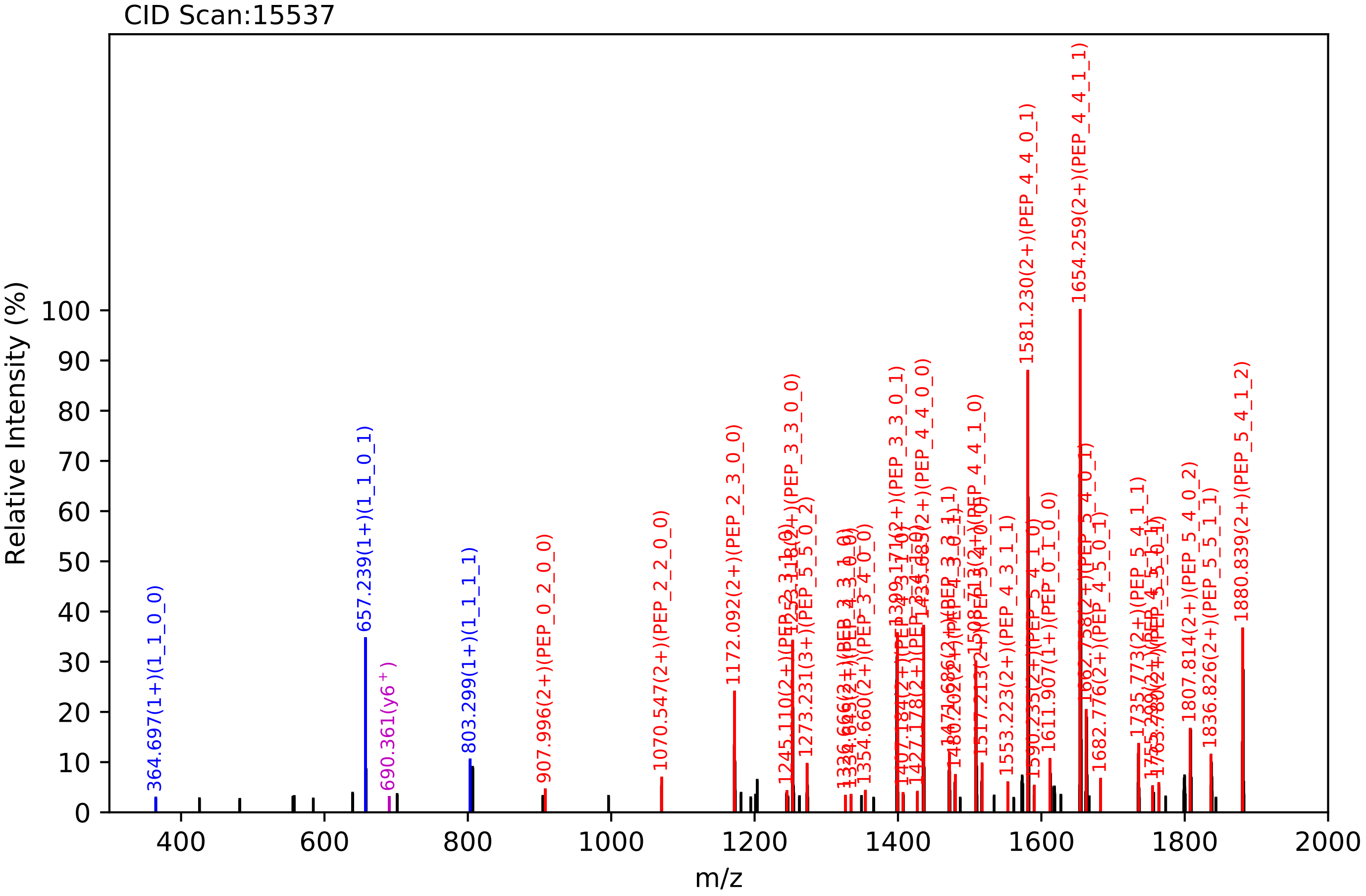

Test set no. 283, Experiment: AGP exp_4

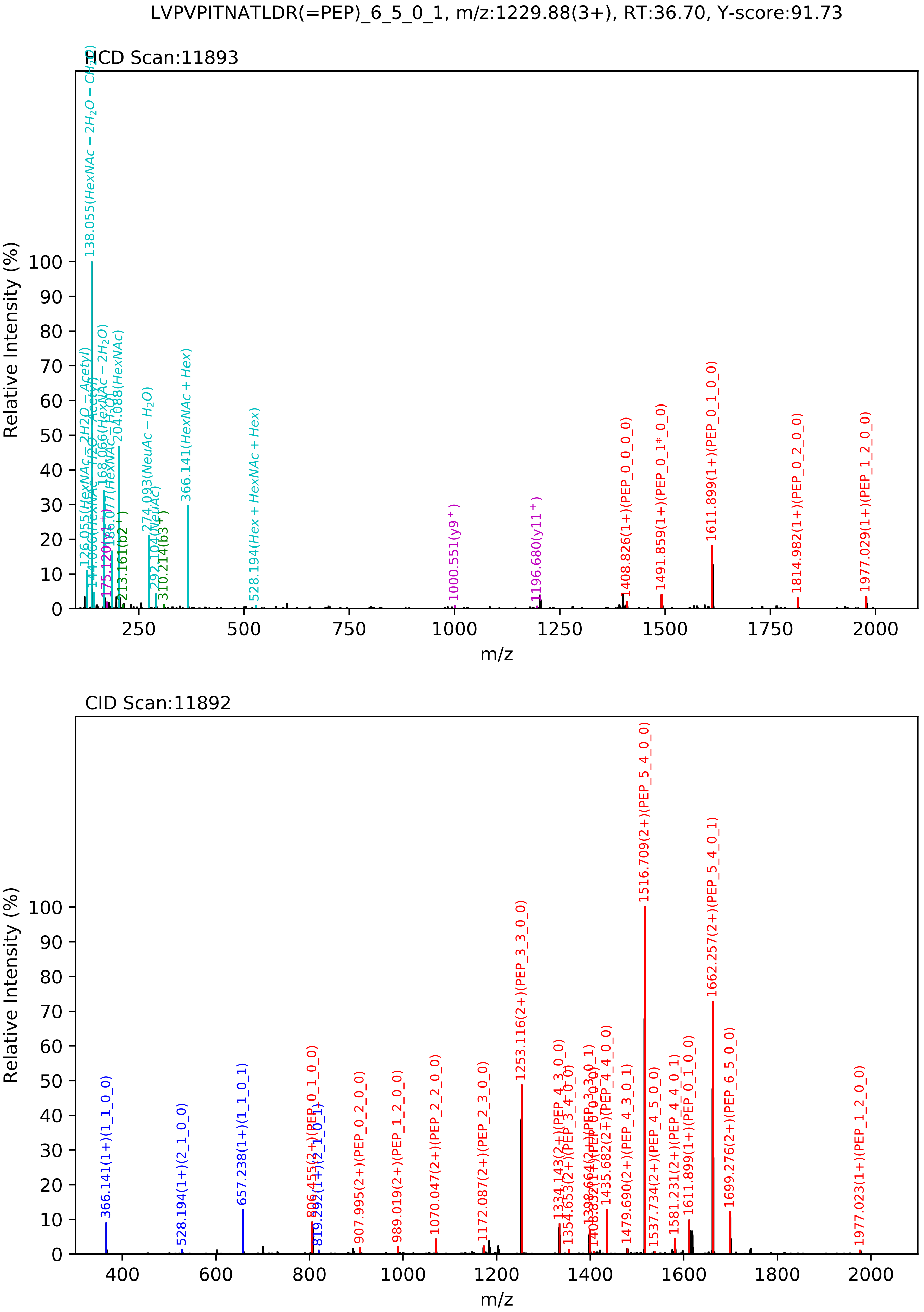

Test set no. 284, Experiment: AGP exp_3

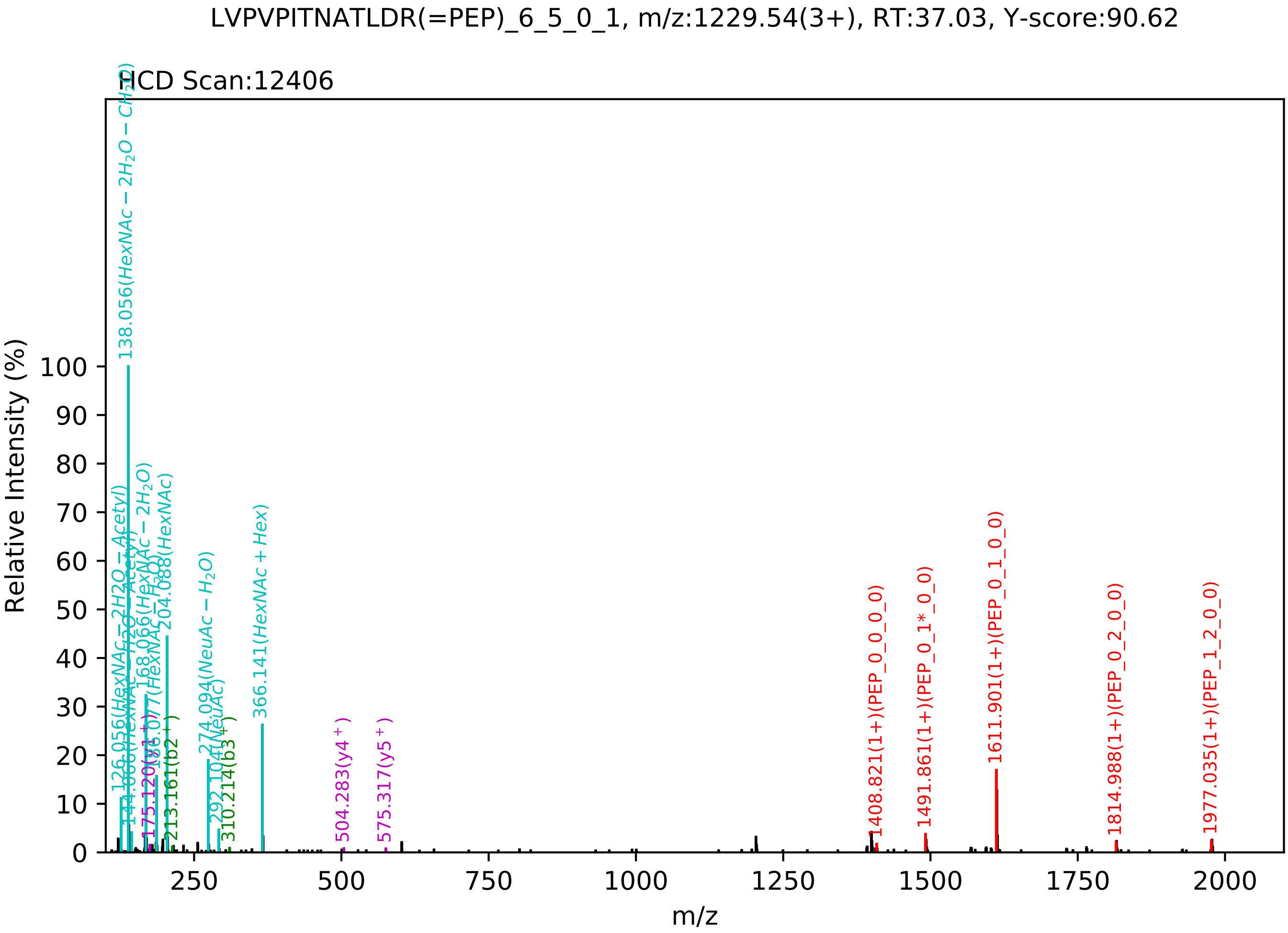

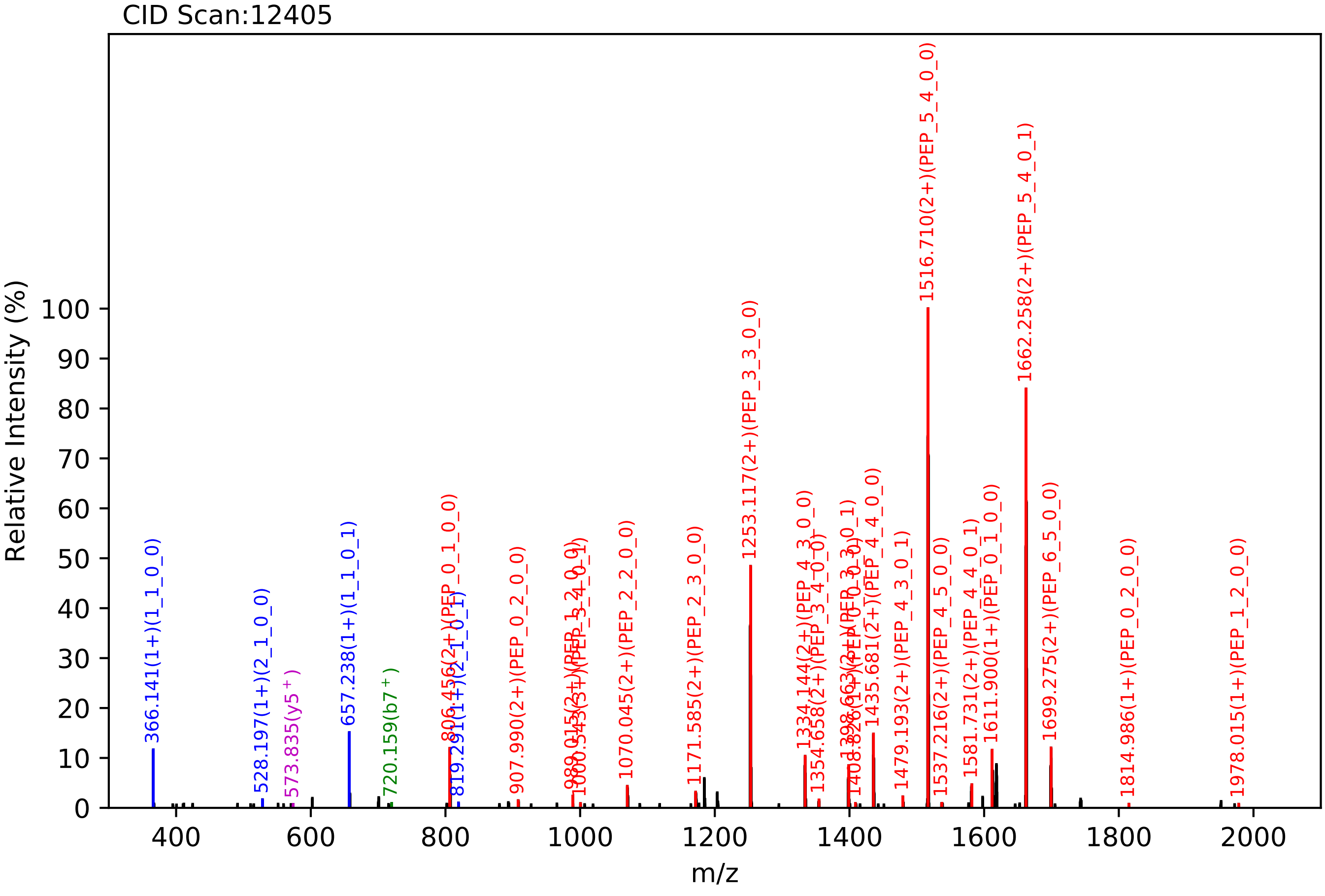

Test set no. 285, Experiment: AGP exp_4

LVPVPITNATLDR(=PEP)_6_5_0_1, m/z:922.67(4+), RT:36.62, Y-score:87.86

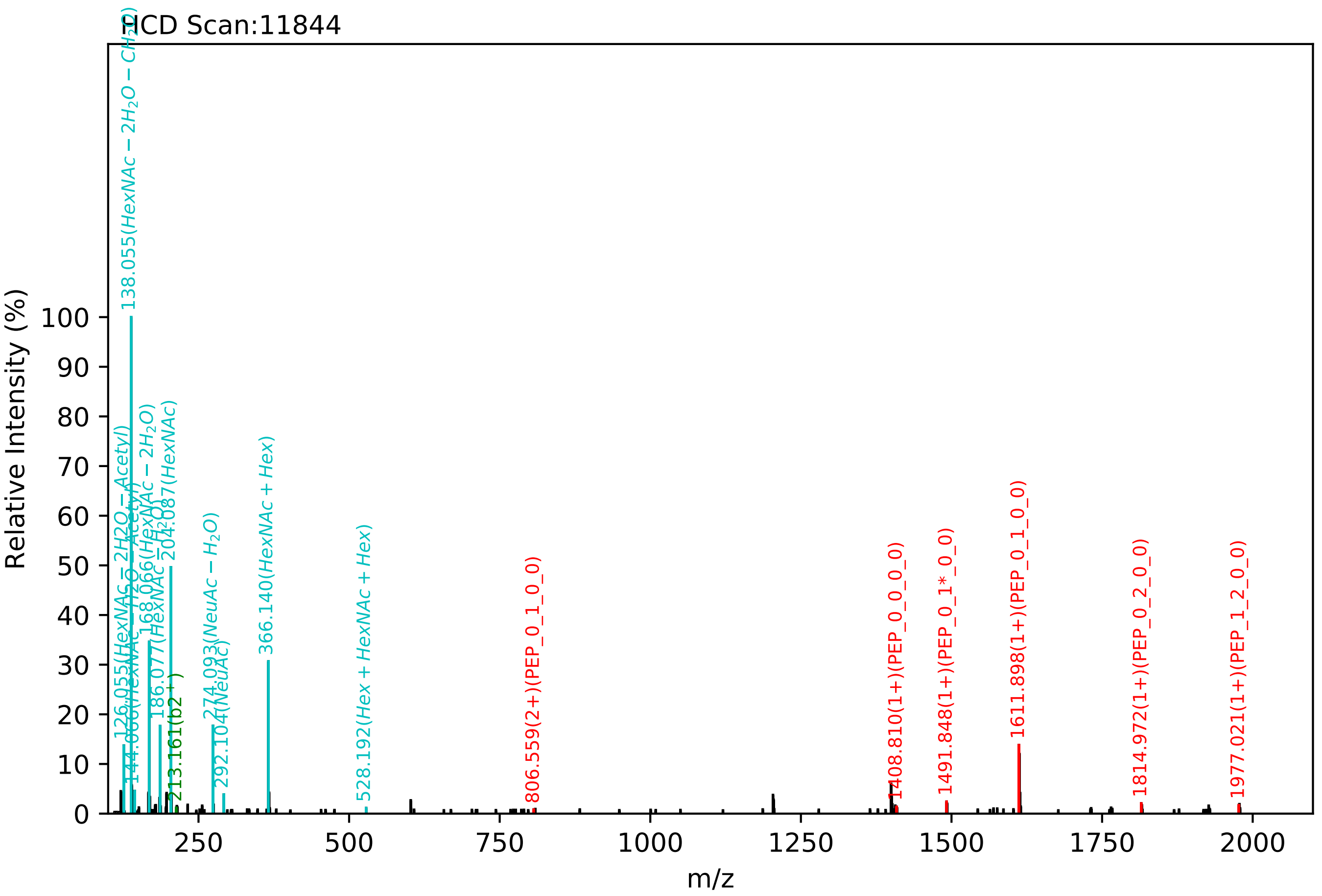

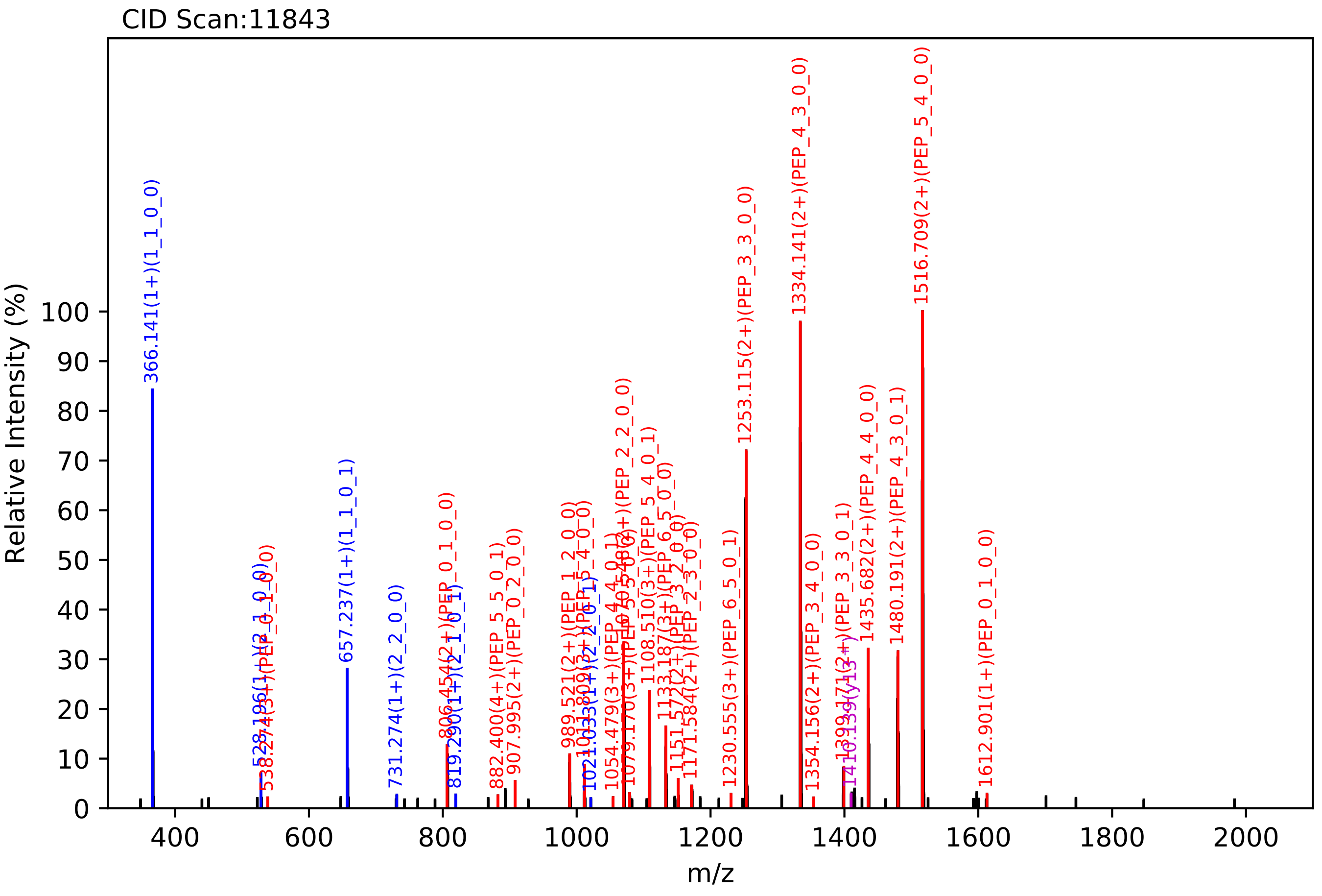

Test set no. 286, Experiment: AGP exp_3

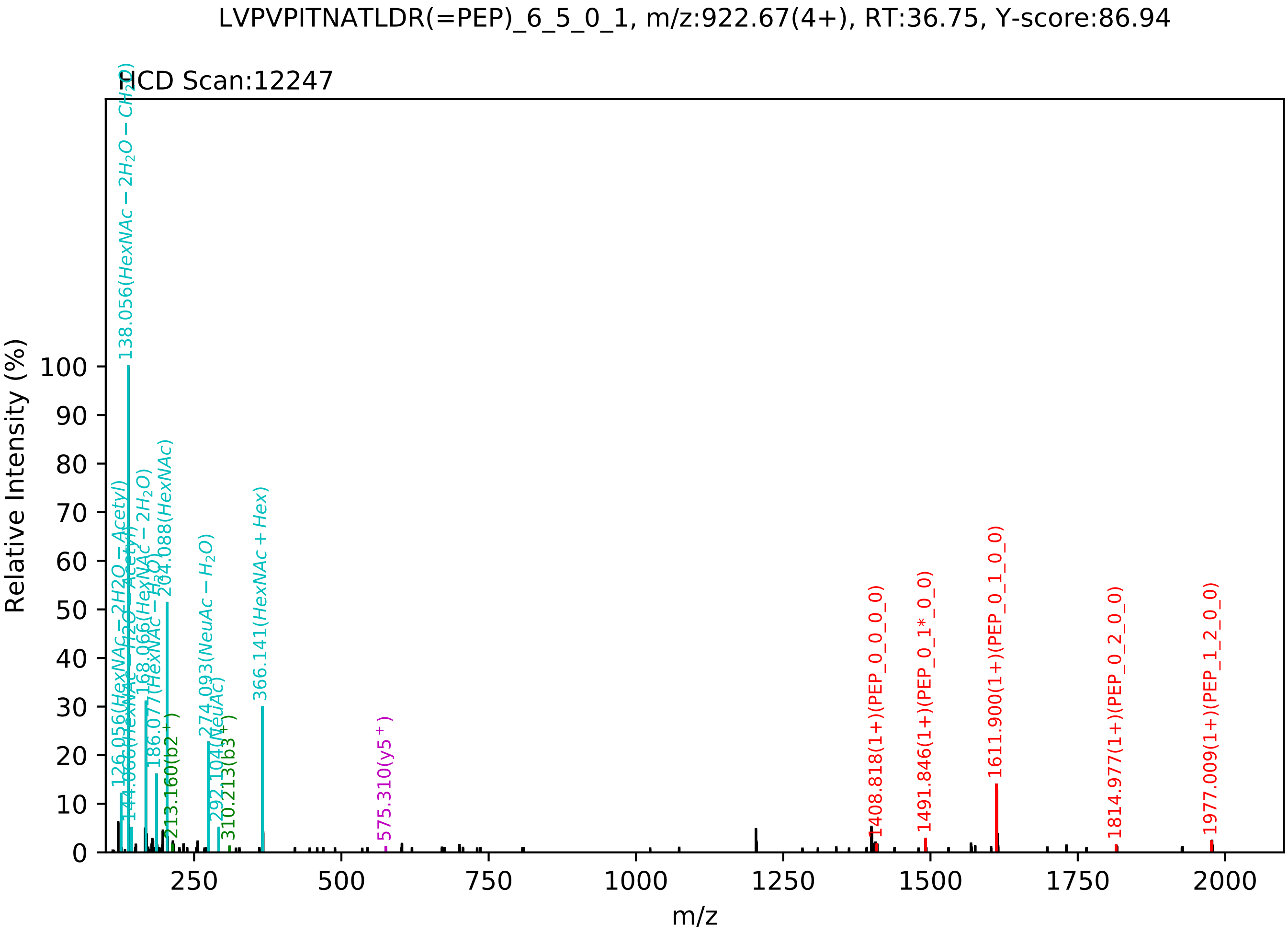

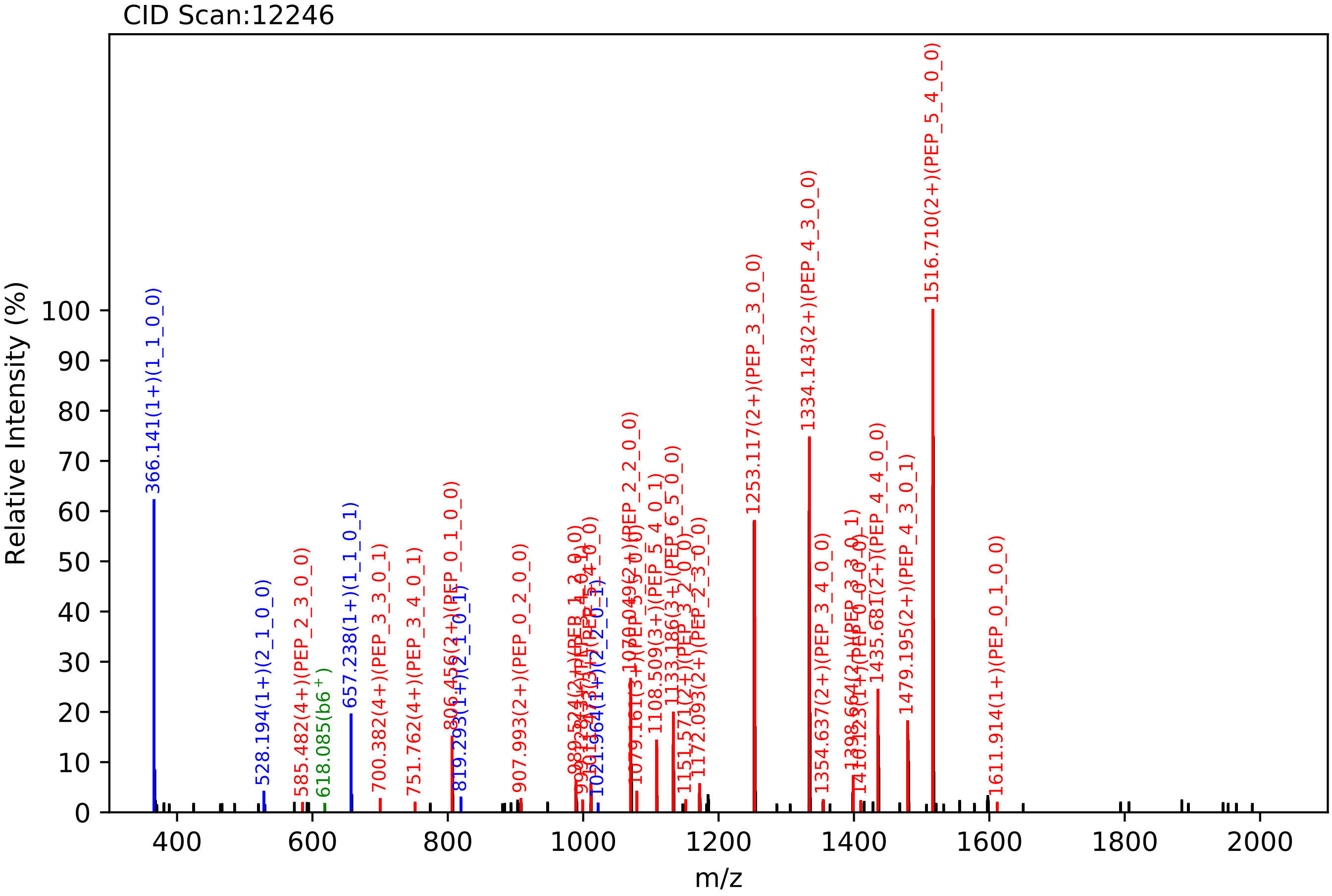

Test set no. 287, Experiment: AGP exp_3

LVPVPITNATLDR(=PEP)_6_5_0_1, m/z:922.67(4+), RT:36.19, Y-score:78.76

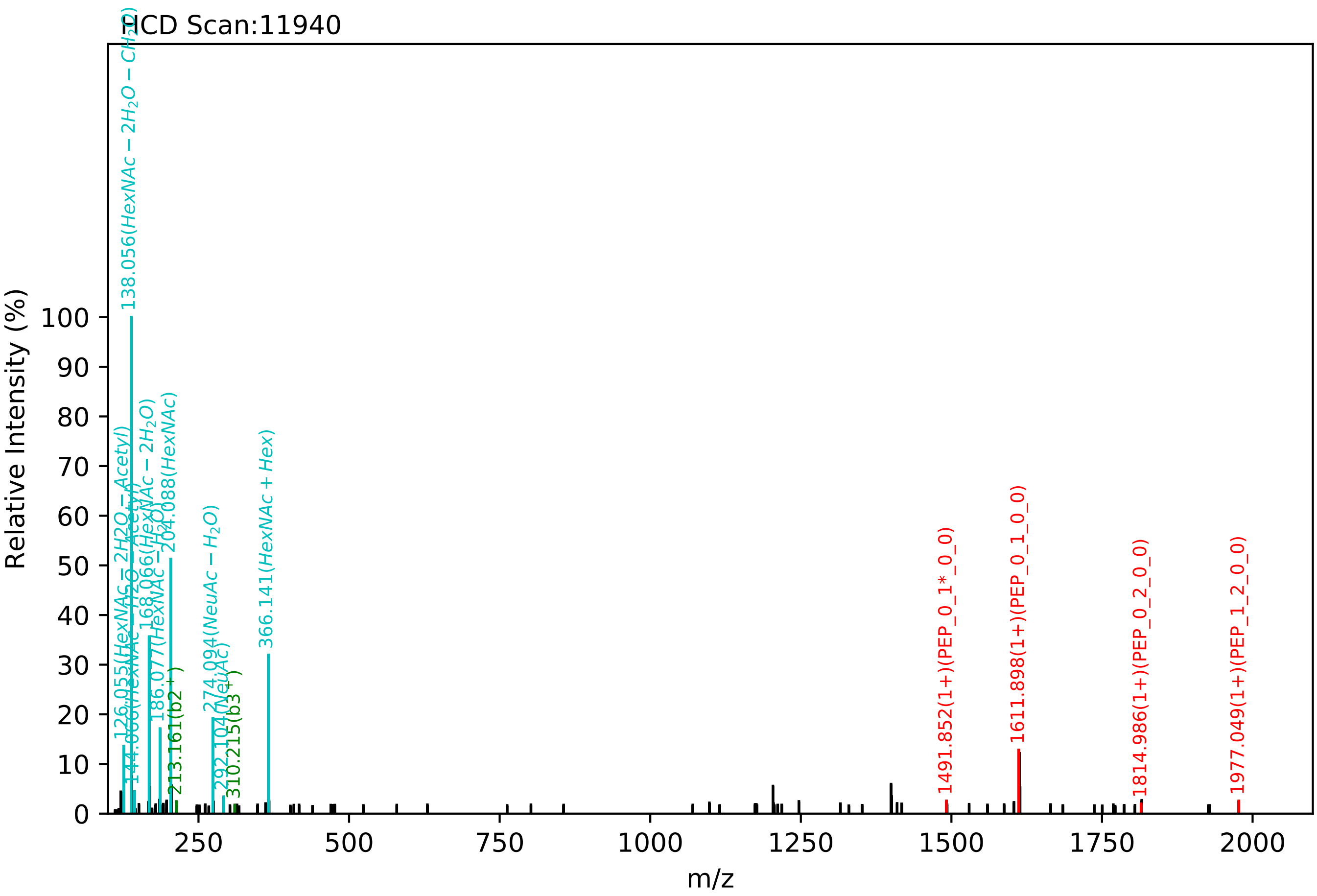

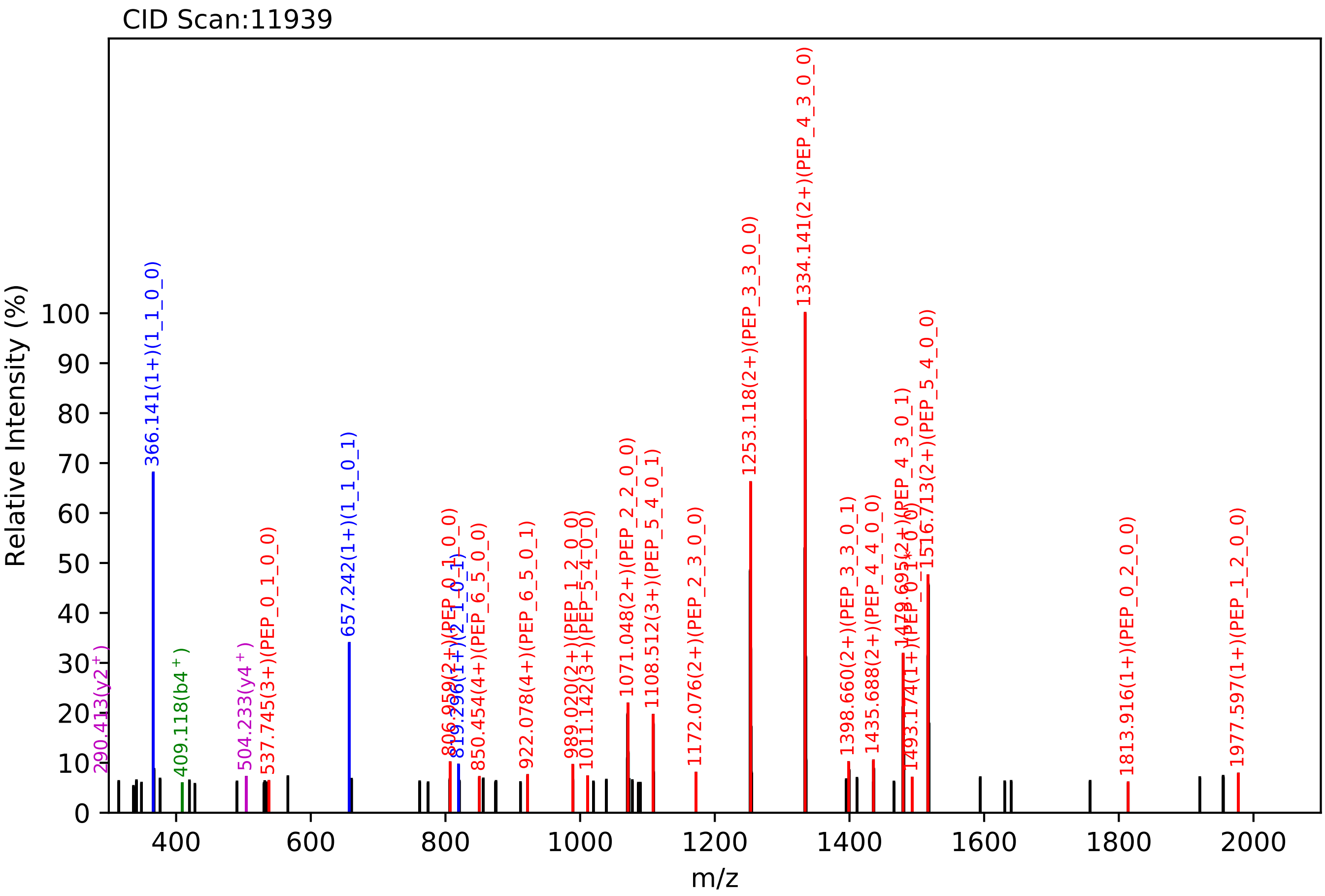

Test set no. 288, Experiment: AGP exp_3

LVPVPITNATLDR(=PEP)_6_5_0_2, m/z:995.45(4+), RT:43.06, Y-score:87.48

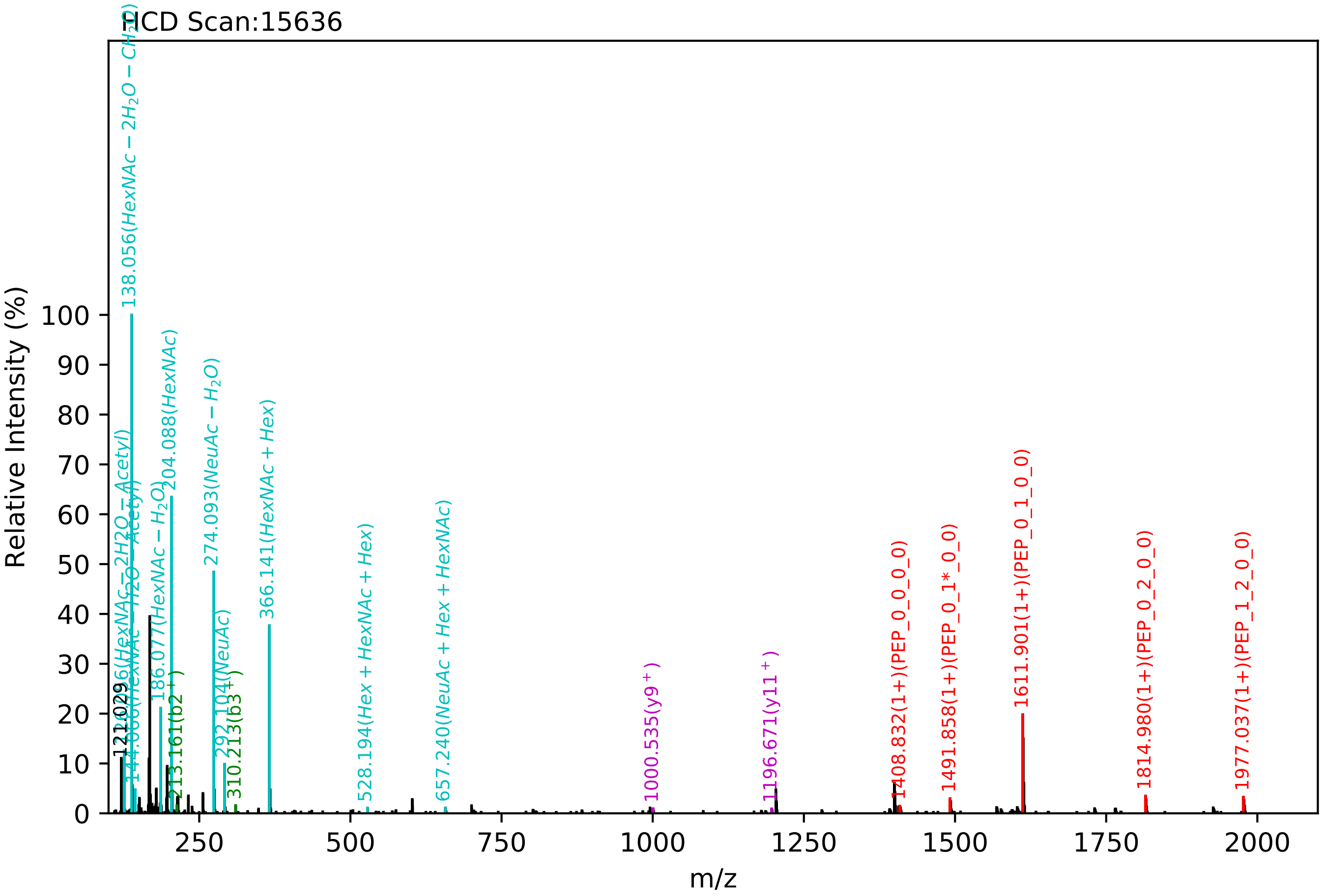

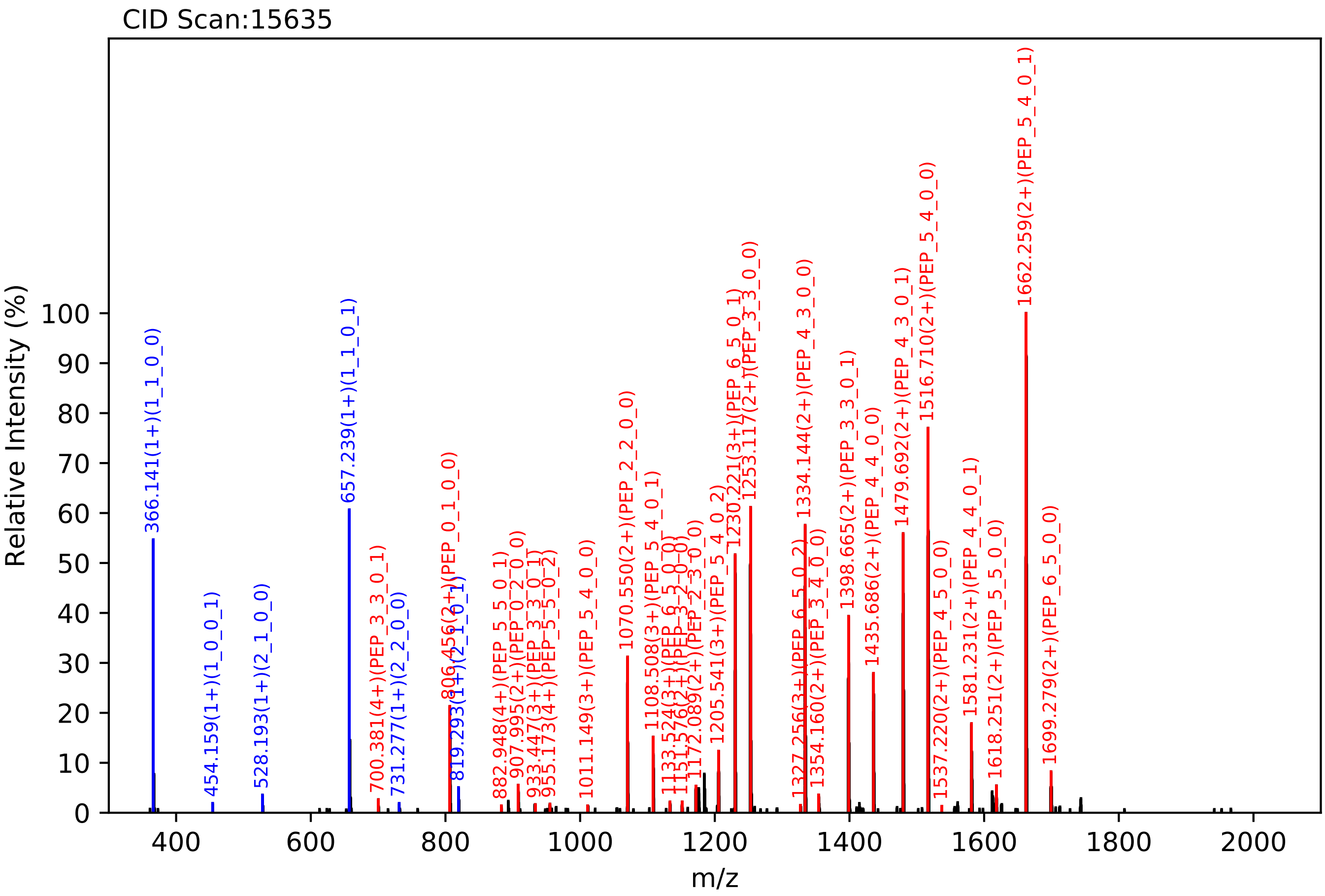

Test set no. 289, Experiment: AGP exp_3

LVPVPITNATLDR(=PEP)_6_5_0_2, m/z:995.44(4+), RT:42.88, Y-score:86.99

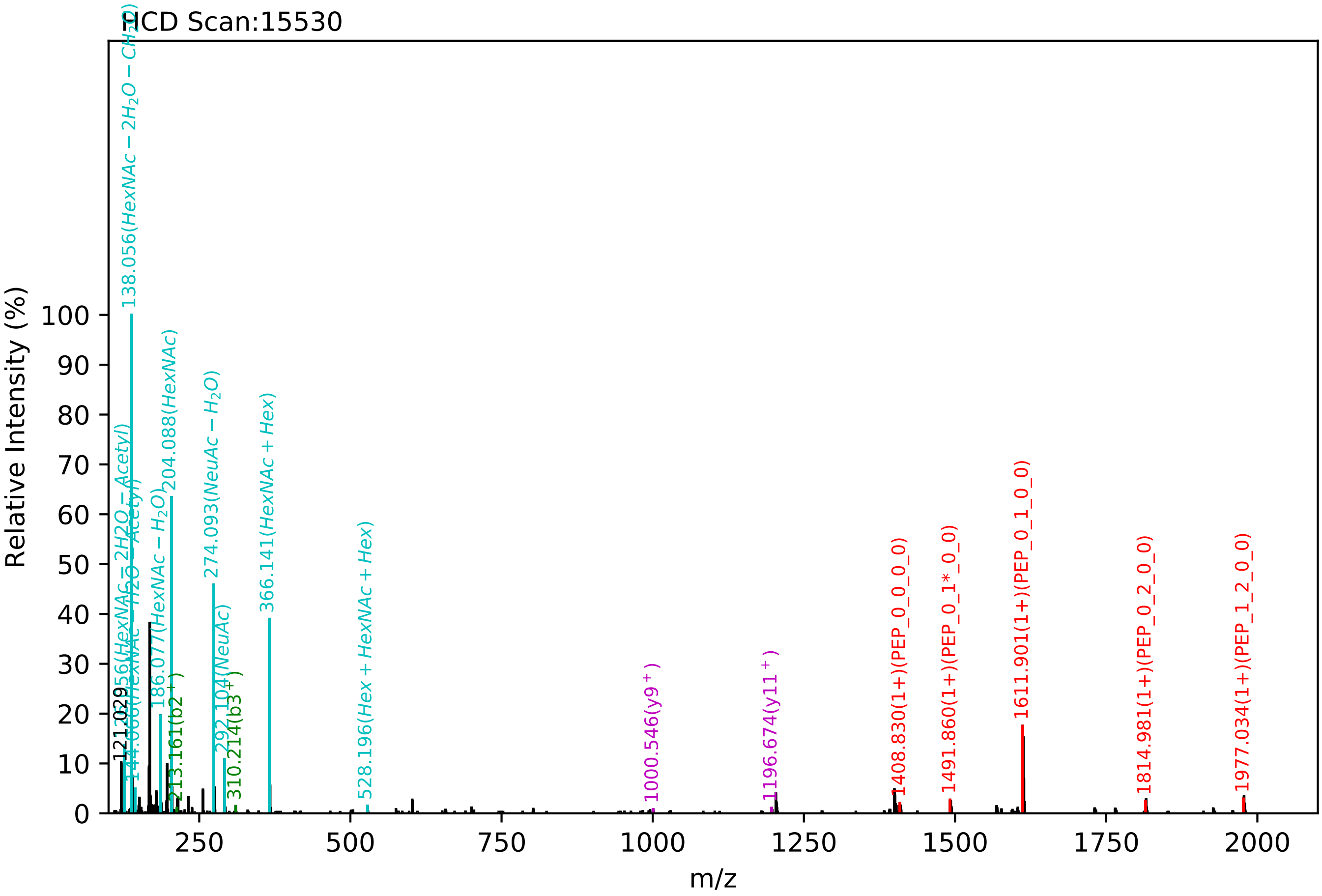

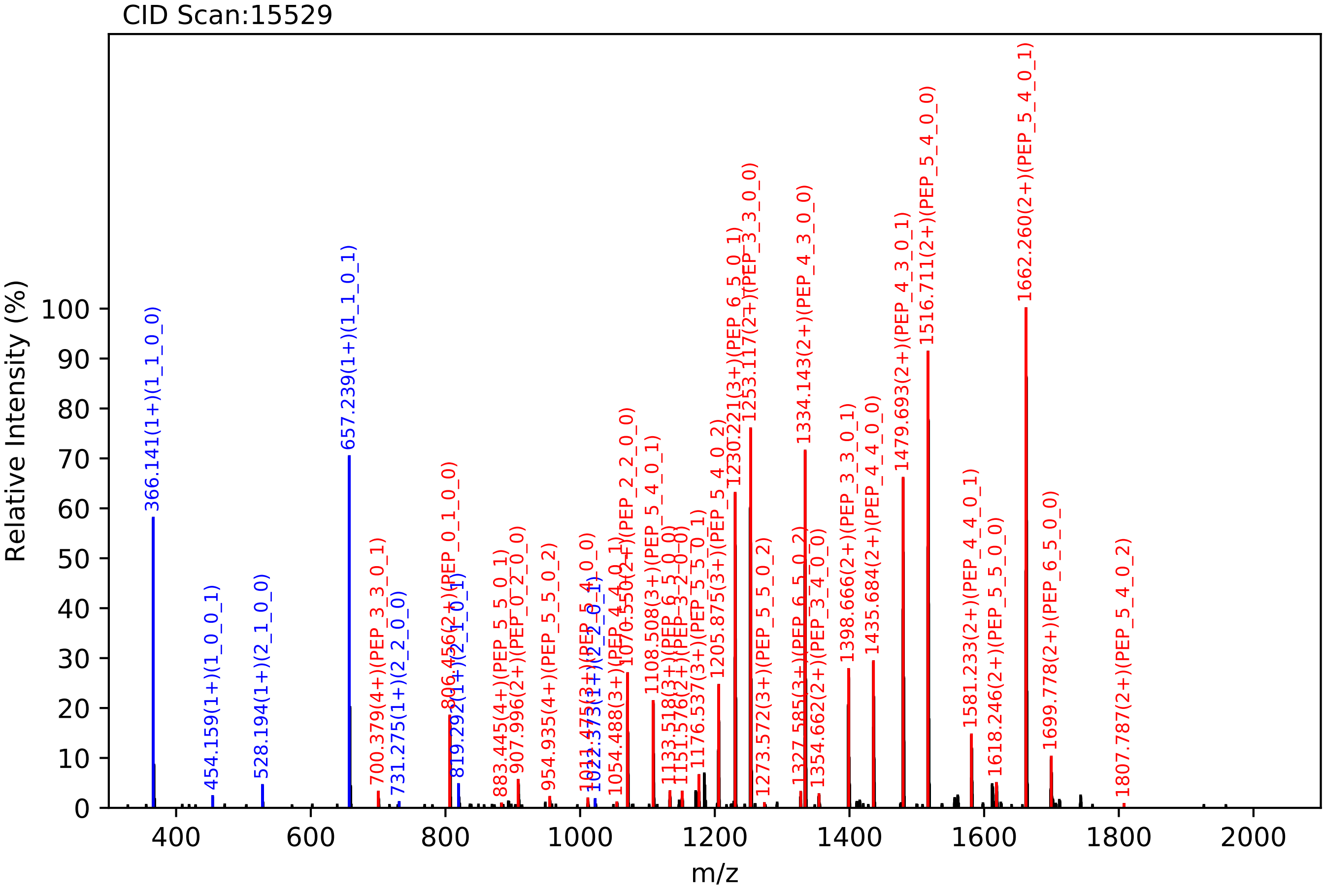

Test set no. 290, Experiment: AGP exp_4

LVPVPITNATLDR(=PEP)_6_5_0_2, m/z:995.45(4+), RT:42.94, Y-score:84.35

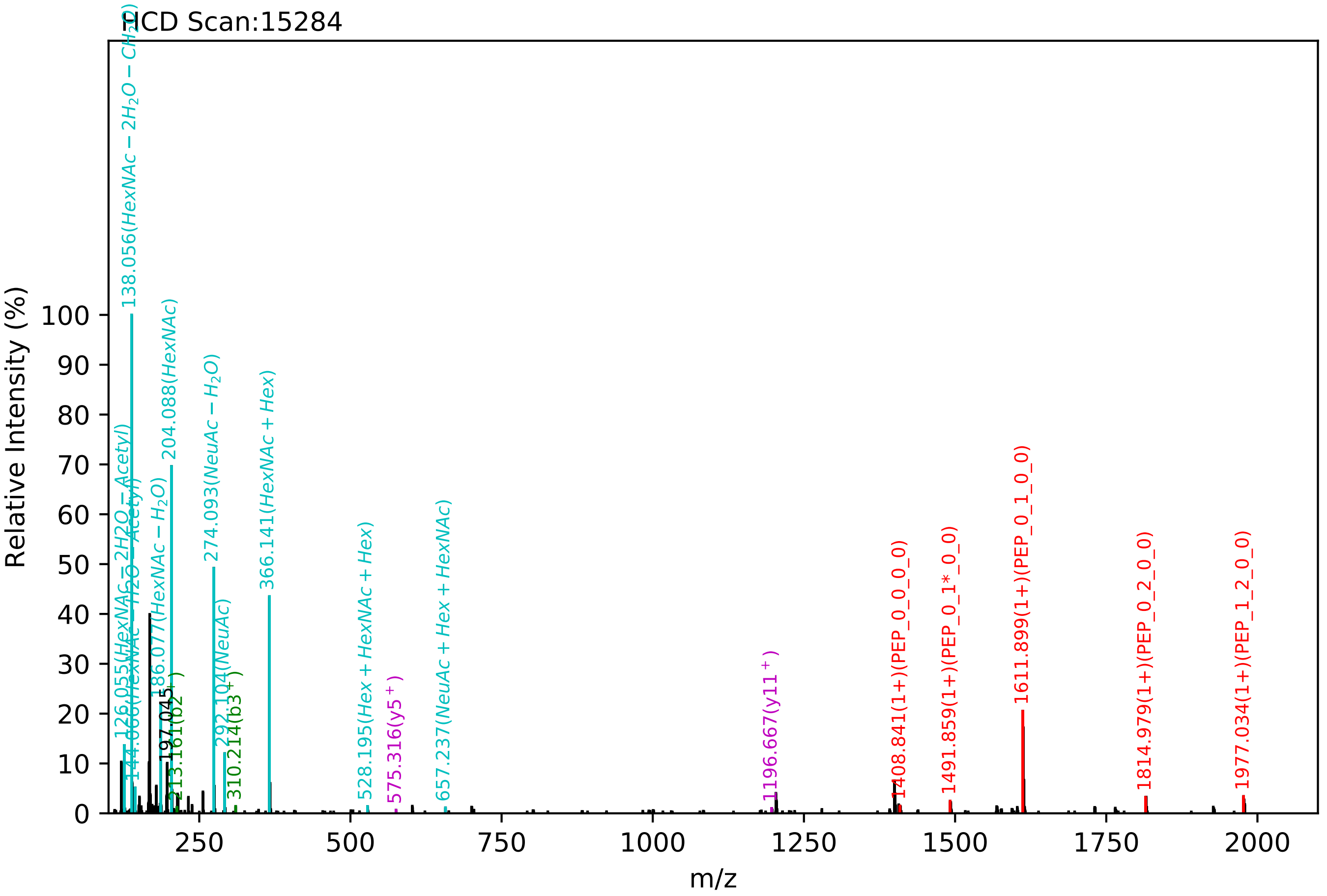

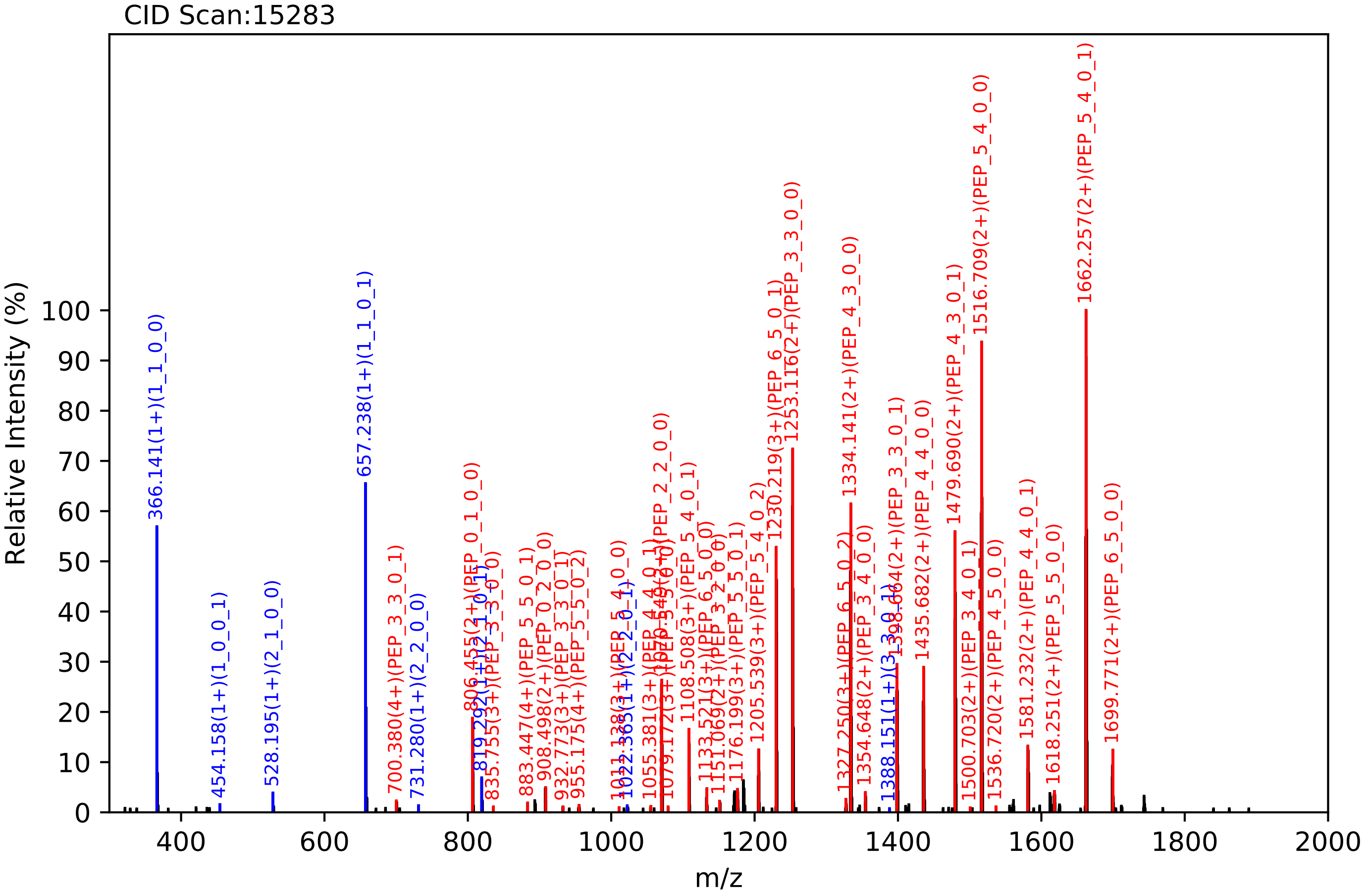

Test set no. 291, Experiment: AGP exp_4

LVPVPITNATLDR(=PEP)_6_5_0_2, m/z:995.44(4+), RT:42.32, Y-score:84.20

HCD Scan:14930

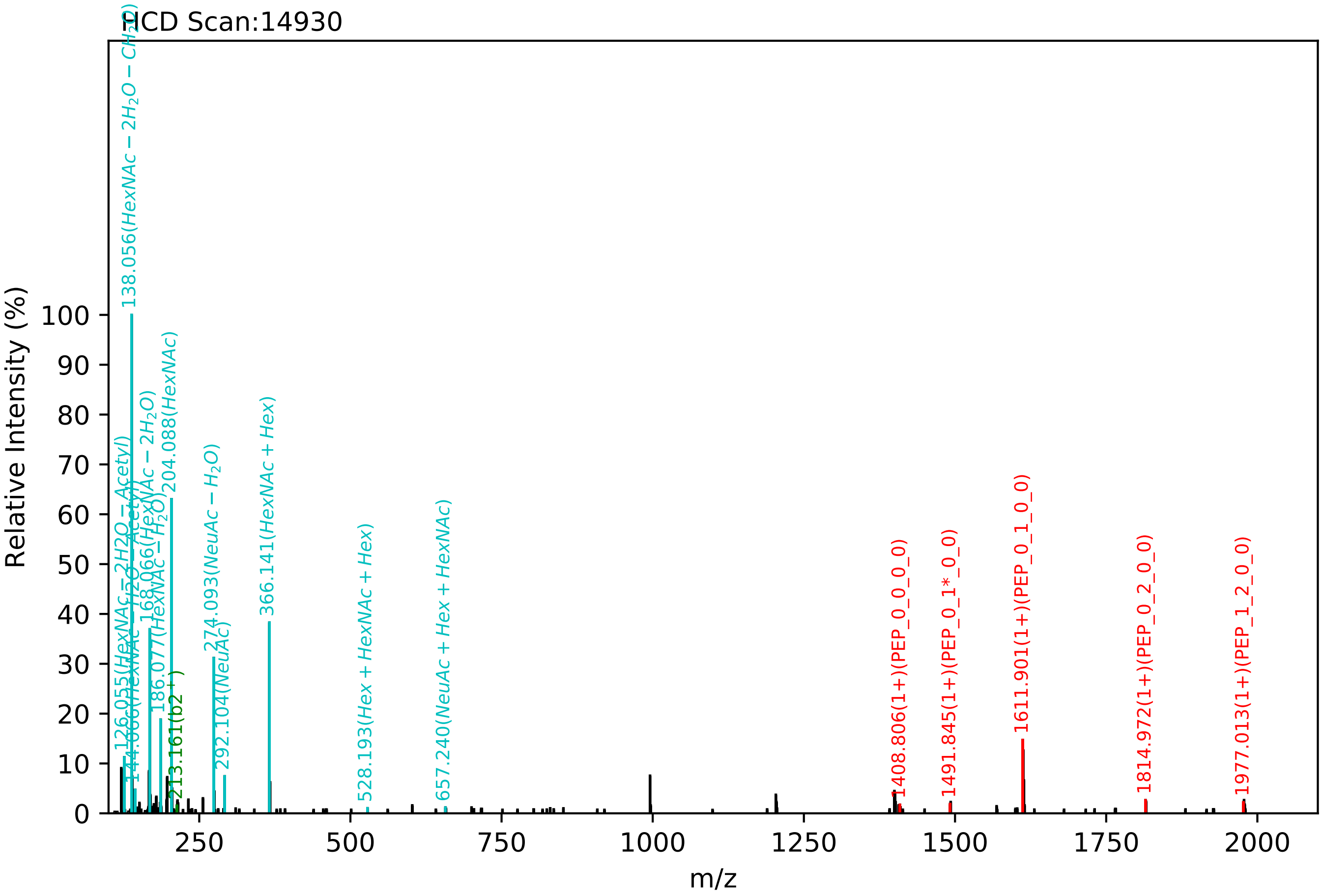

CID Scan:14929

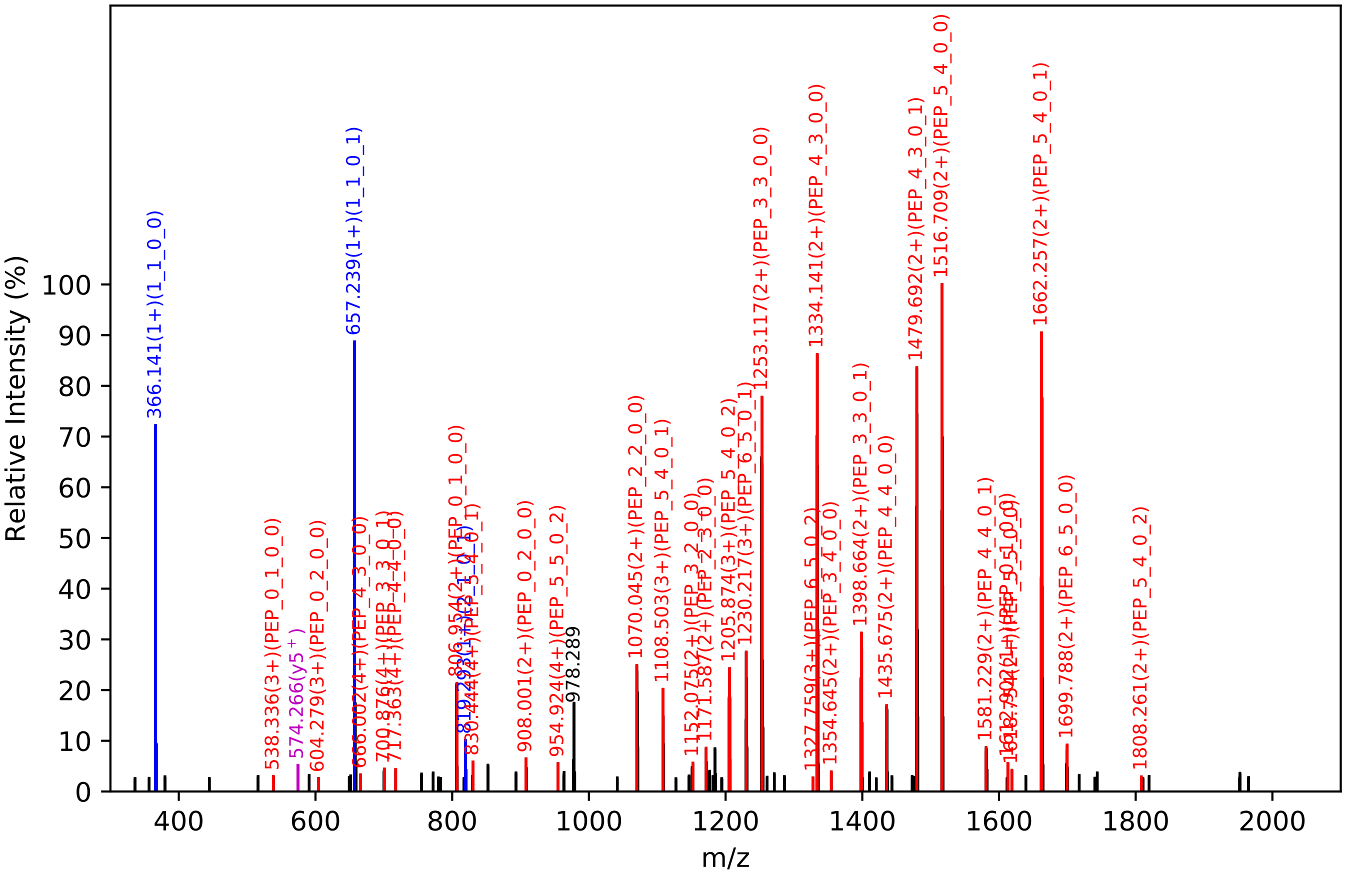

Test set no. 292, Experiment: AGP exp_4

LVPVPITNATLDR(=PEP)_6_5_0_2, m/z:995.44(4+), RT:43.49, Y-score:79.17

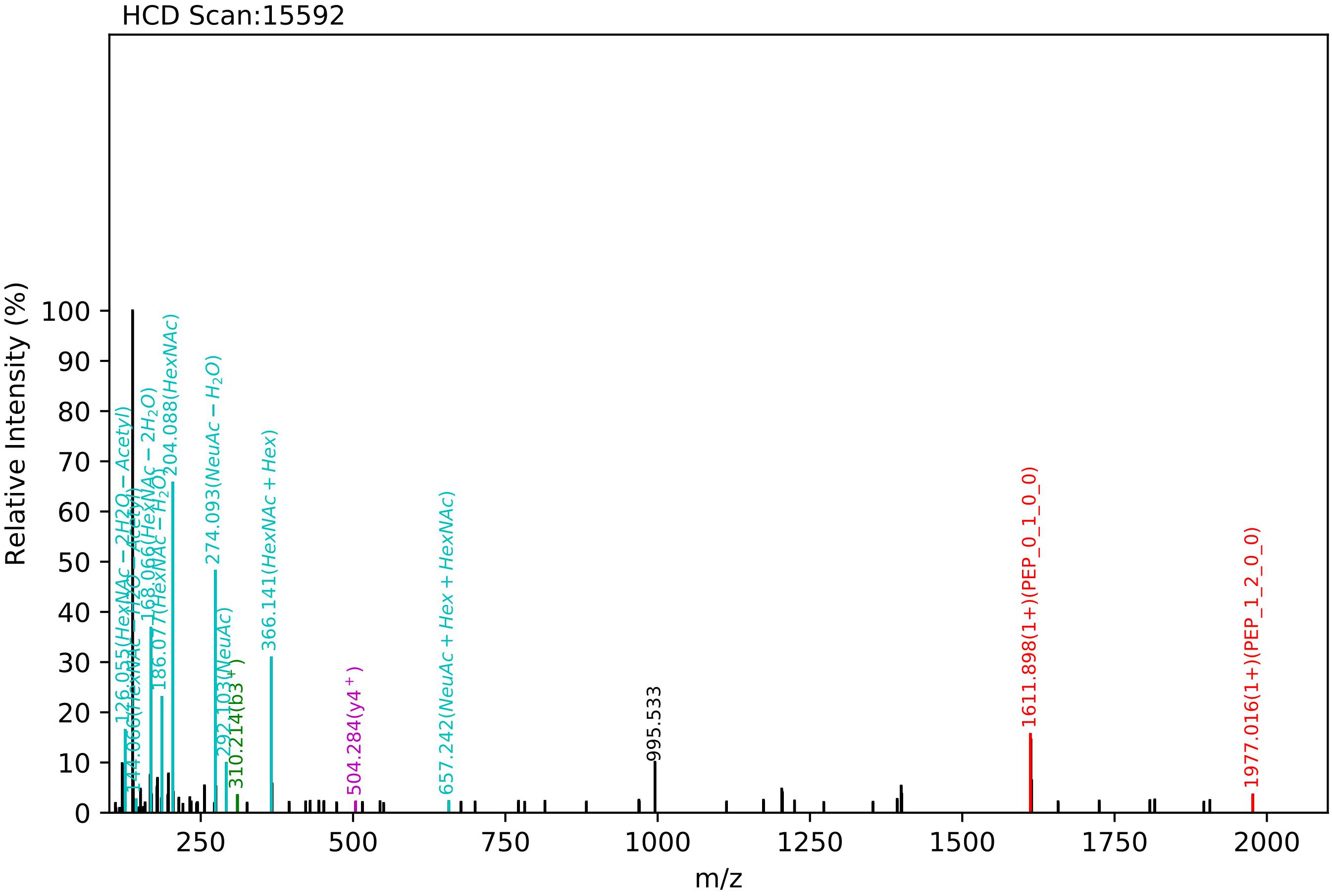

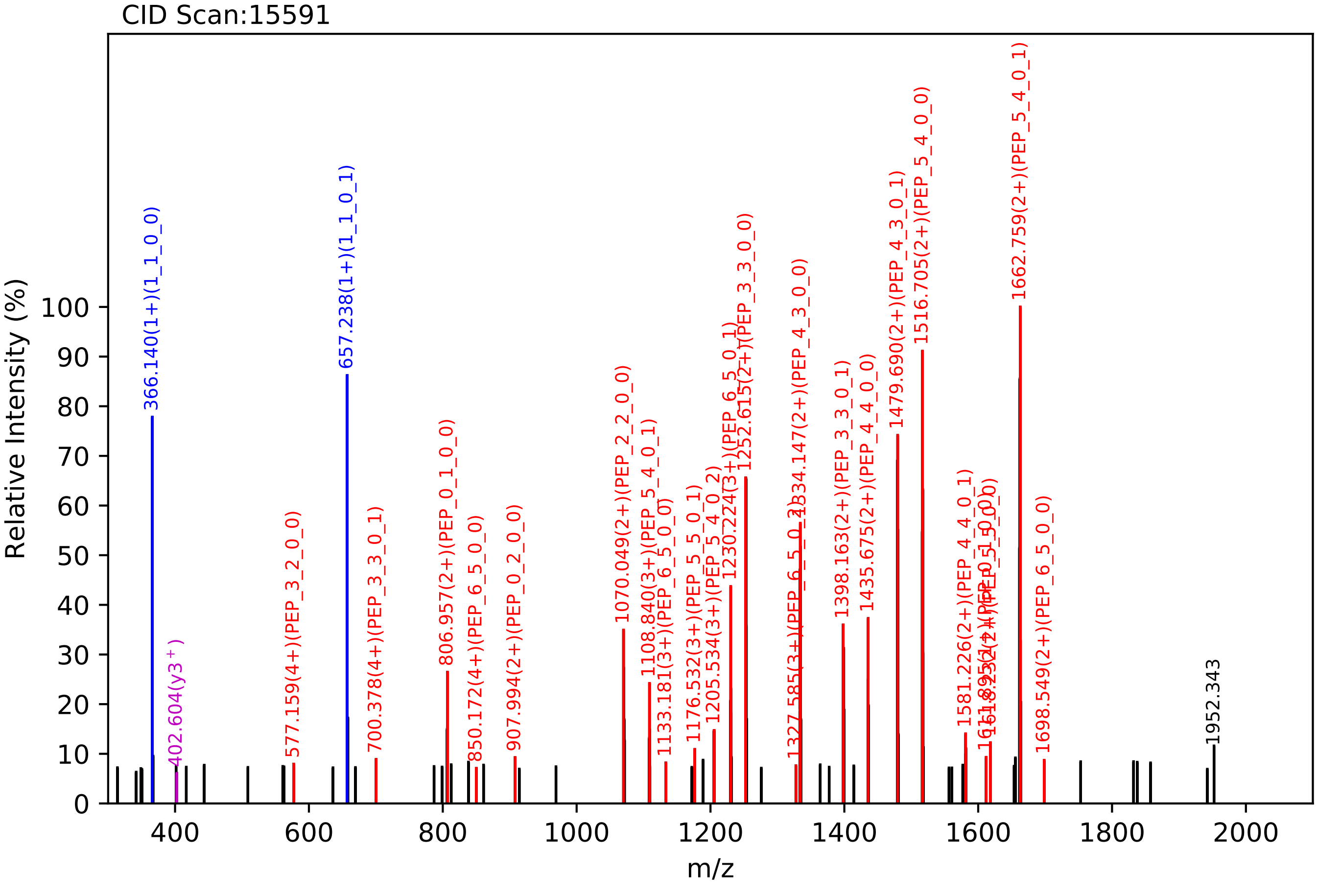

Test set no. 293, Experiment: AGP exp_4

LVPVPITNATLDR(=PEP)_6_5_0_3, m/z:1068.21(4+), RT:50.62, Y-score:83.08

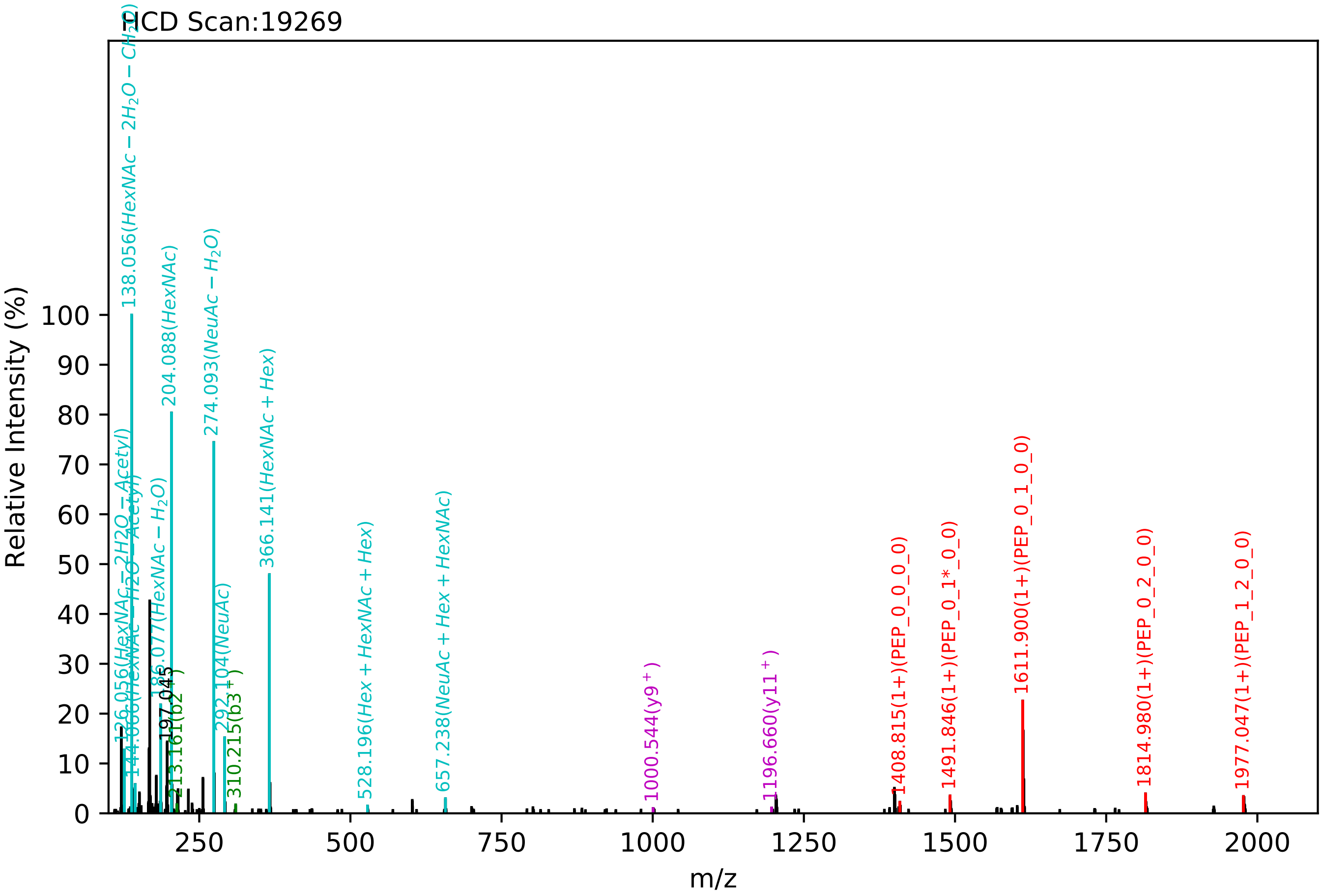

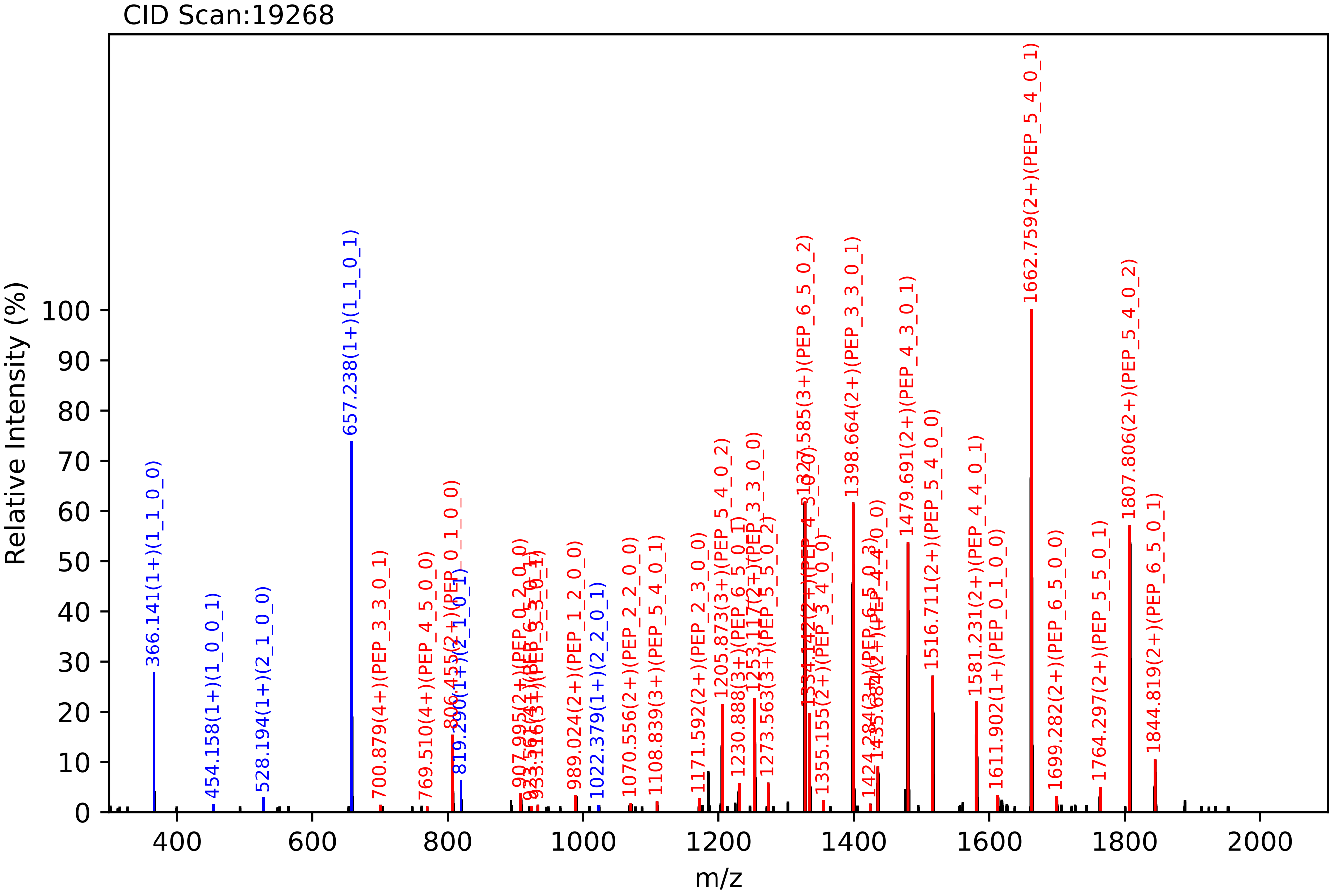

Test set no. 294, Experiment: AGP exp_4

LVPVPITNATLDR(=PEP)_6_5_0_3, m/z:1068.21(4+), RT:50.42, Y-score:82.02

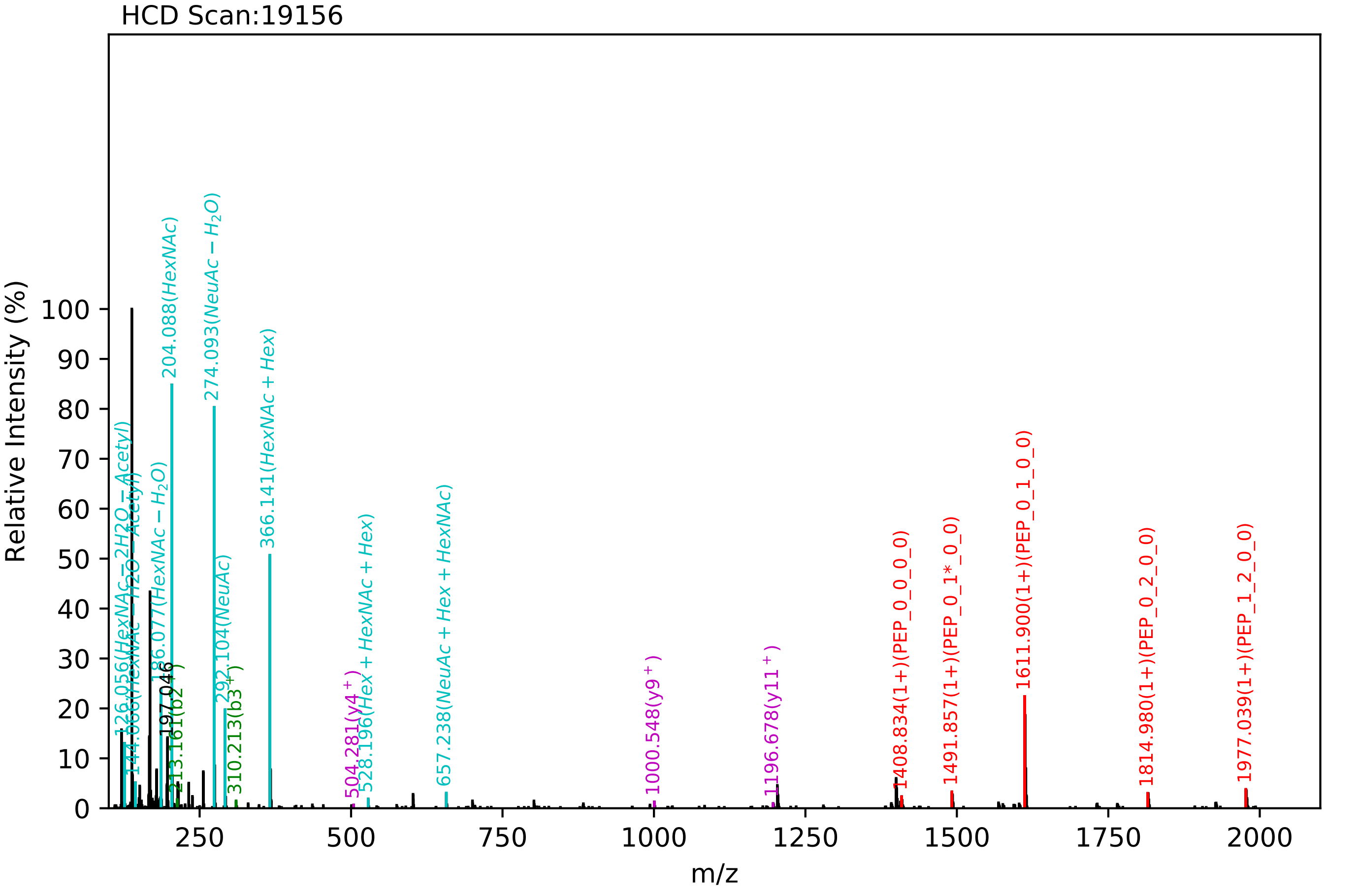

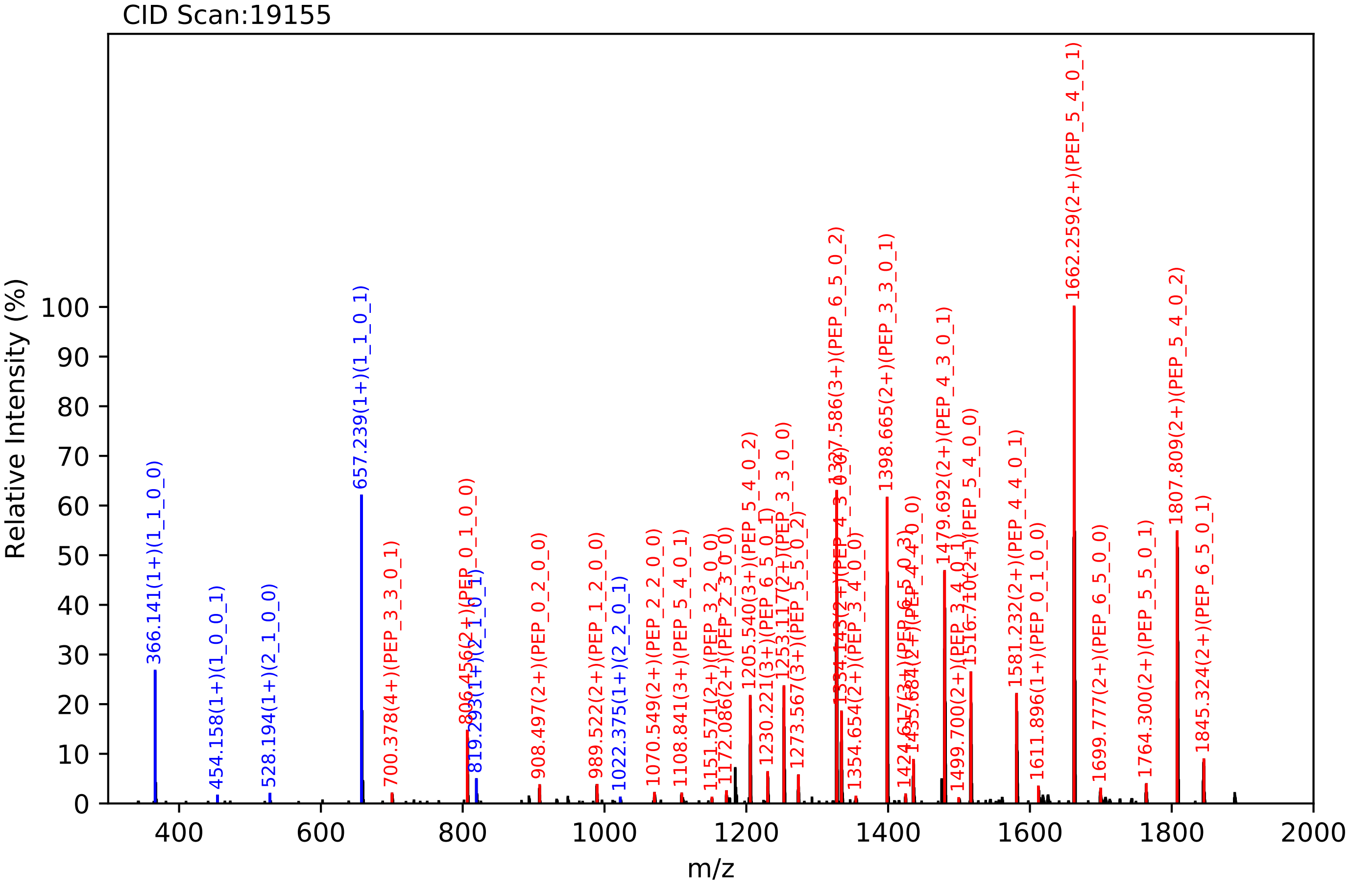

Test set no. 295, Experiment: AGP exp_3

LVPVPITNATLDR(=PEP)_6_5_0_3, m/z:1067.96(4+), RT:50.14, Y-score:81.90

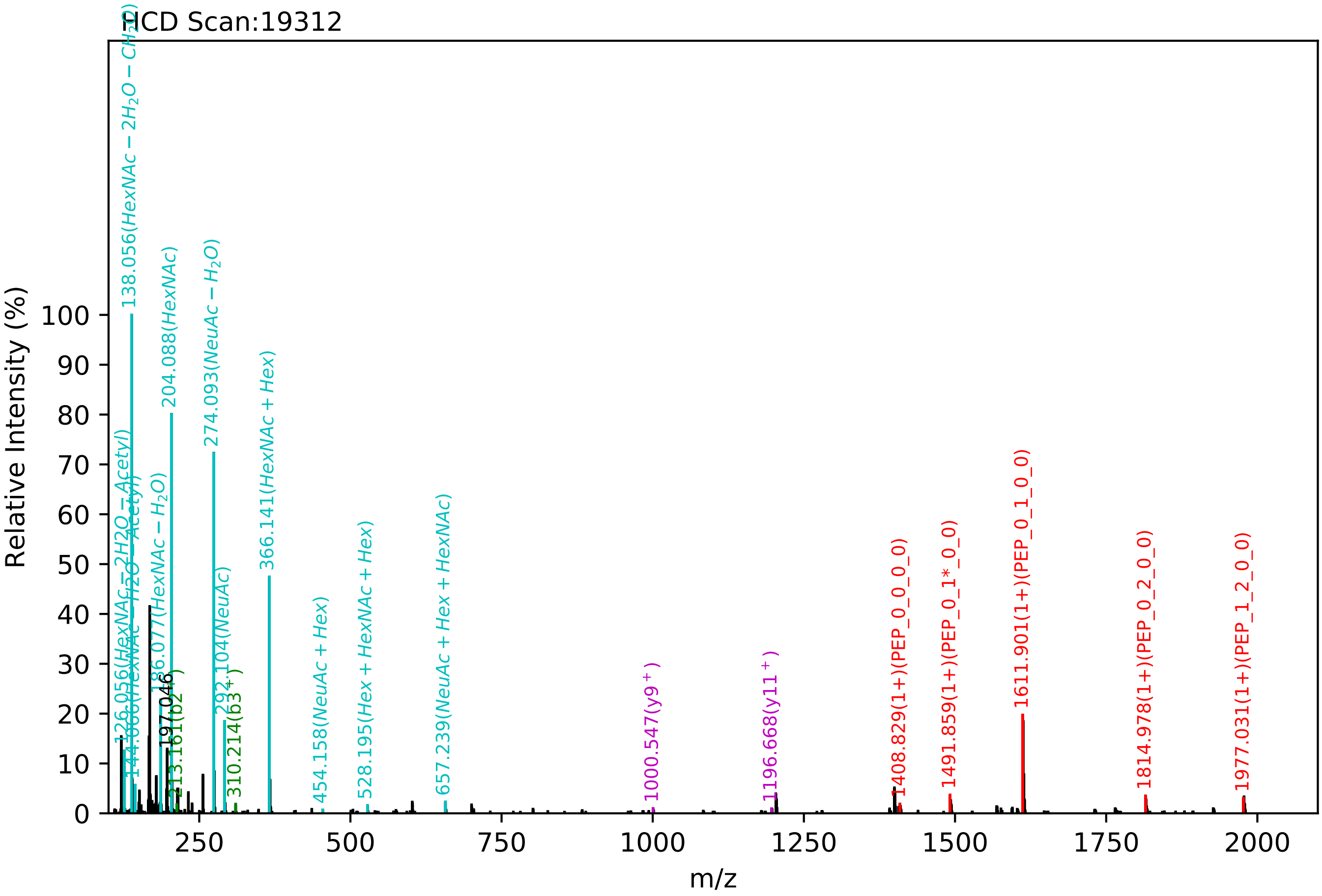

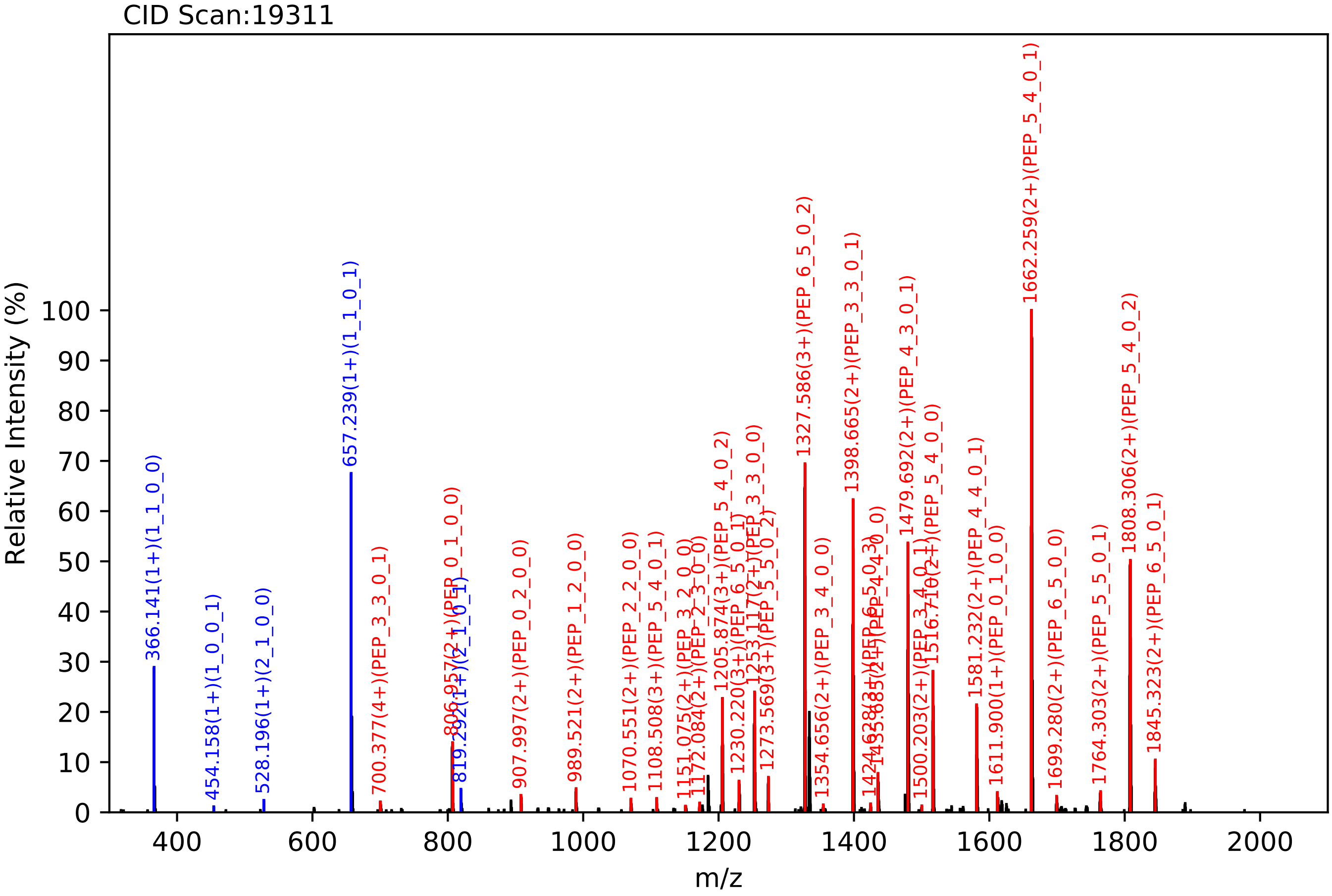

Test set no. 296, Experiment: AGP exp_4

LVPVPITNATLDR(=PEP)_6_5_0_3, m/z:1068.21(4+), RT:49.85, Y-score:80.98

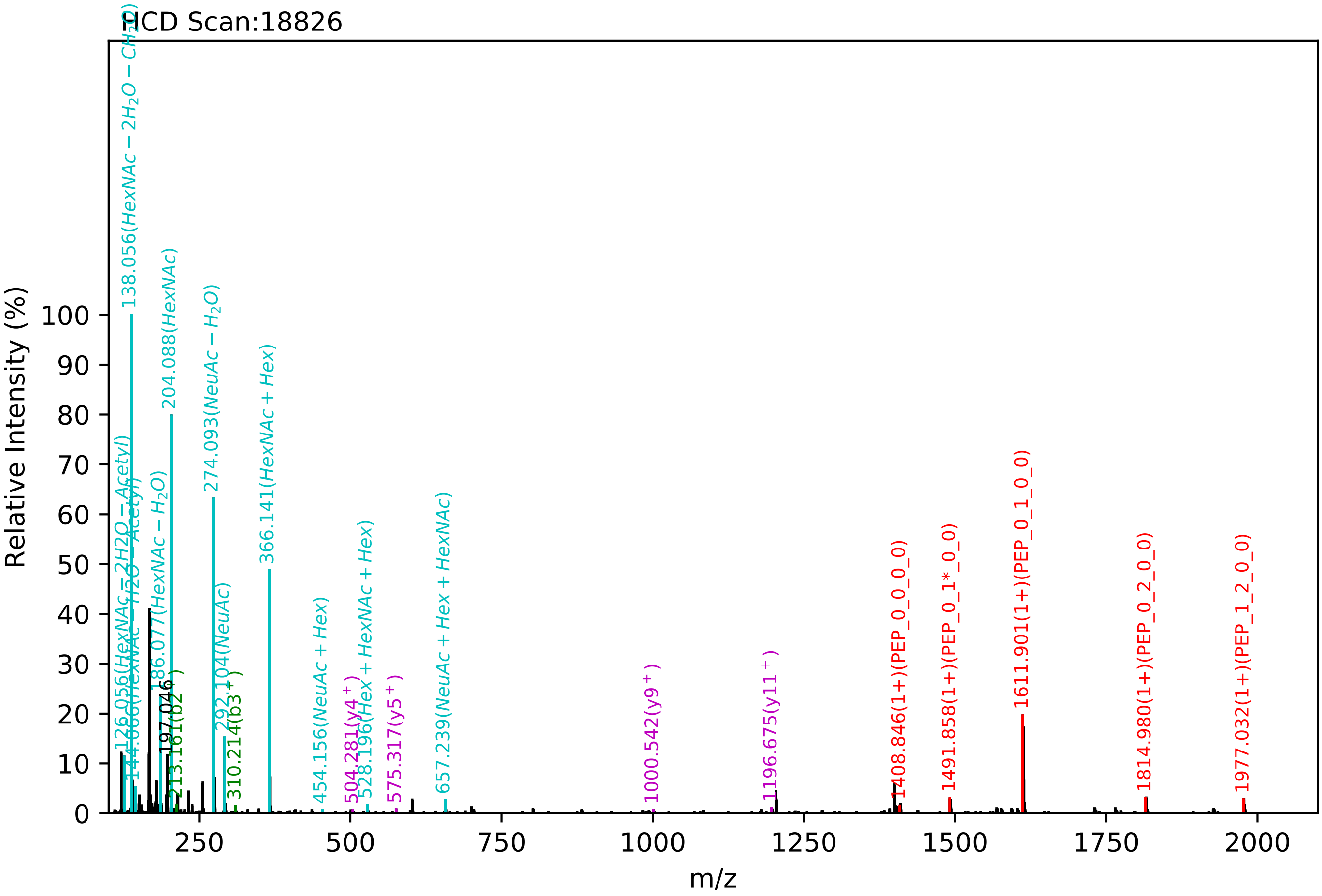

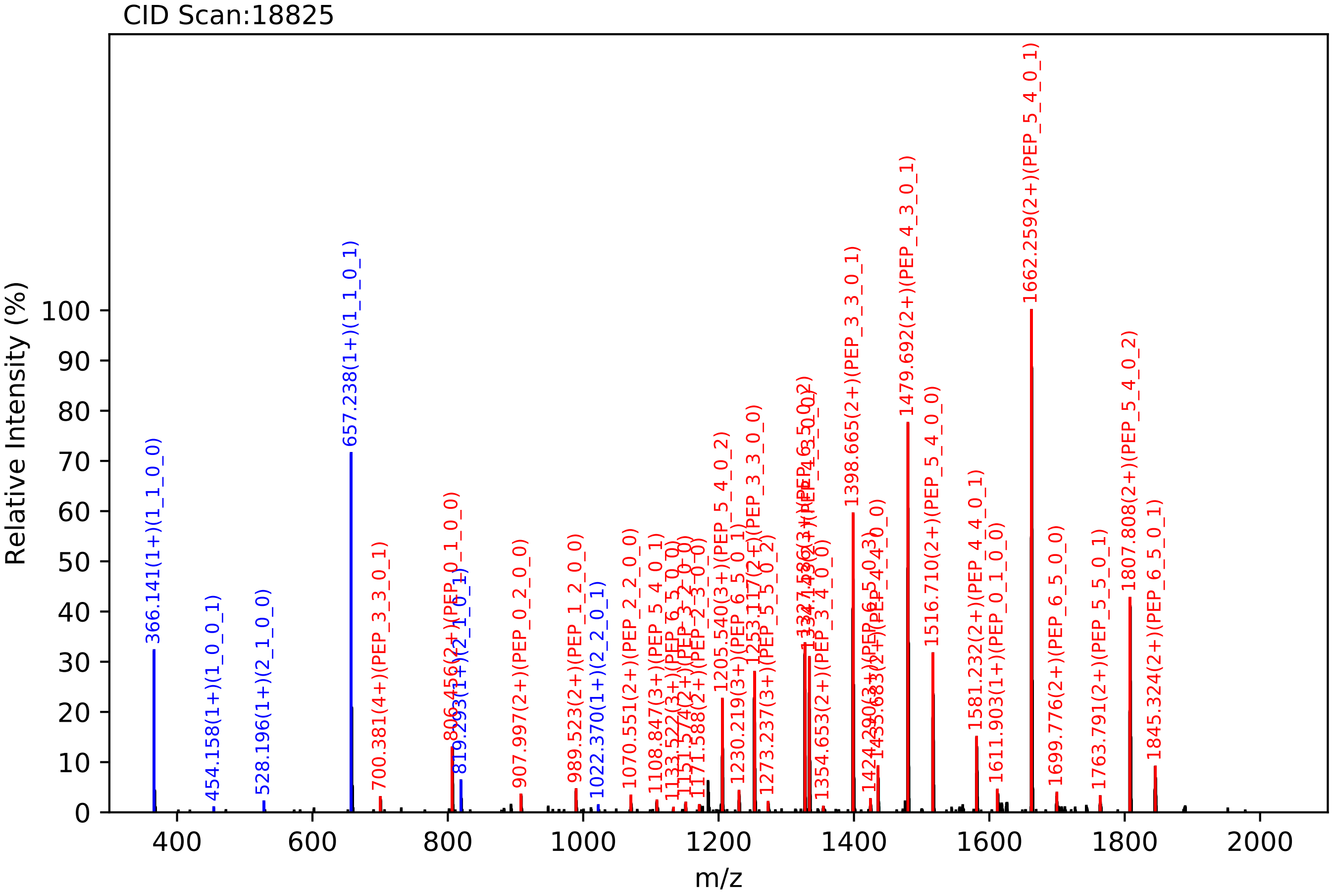

Test set no. 297, Experiment: AGP exp_3

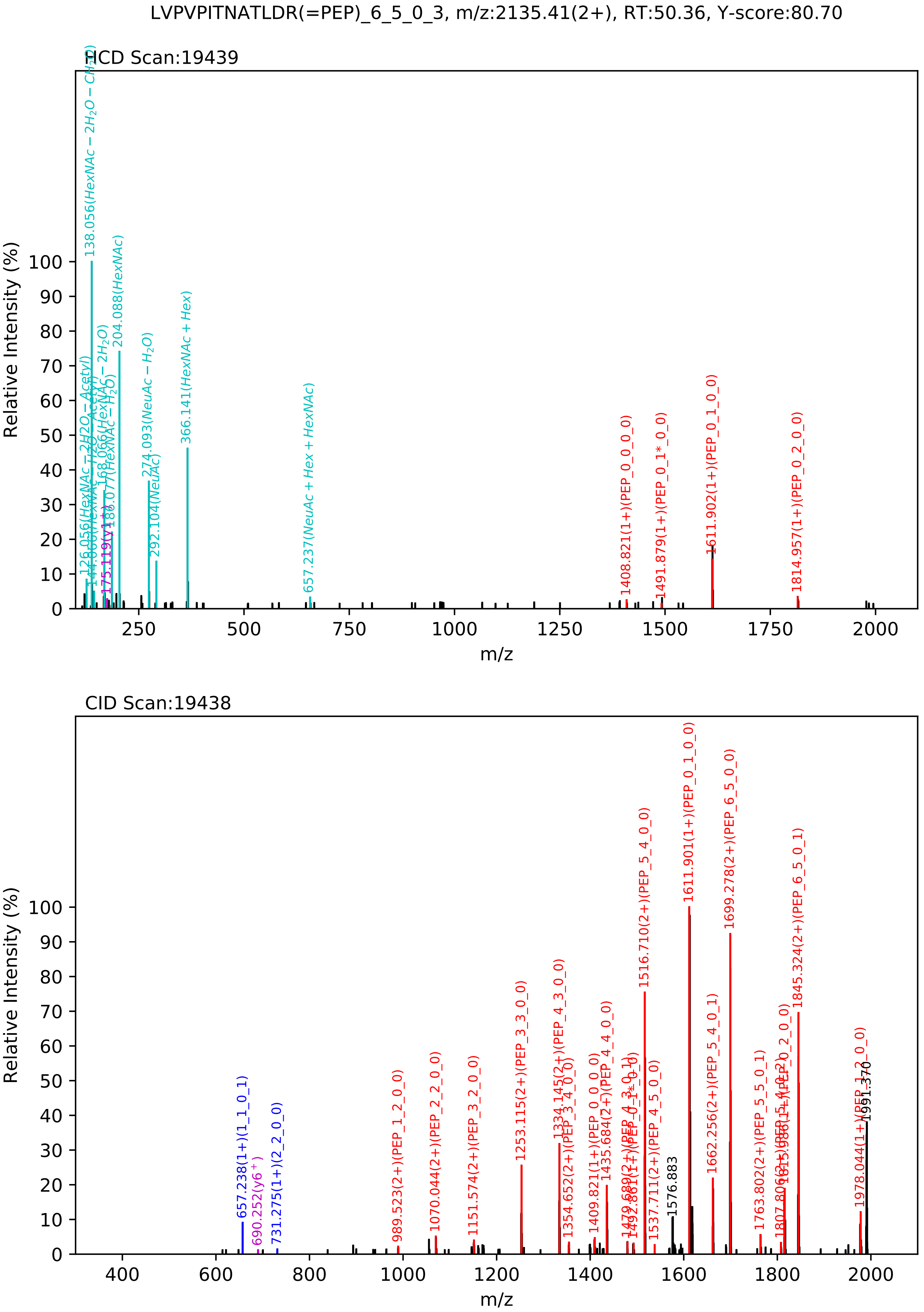

Test set no. 298, Experiment: AGP exp_4

LVPVPITNATLDR(=PEP)_6_5_1_1, m/z:1278.57(3+), RT:36.31, Y-score:89.64

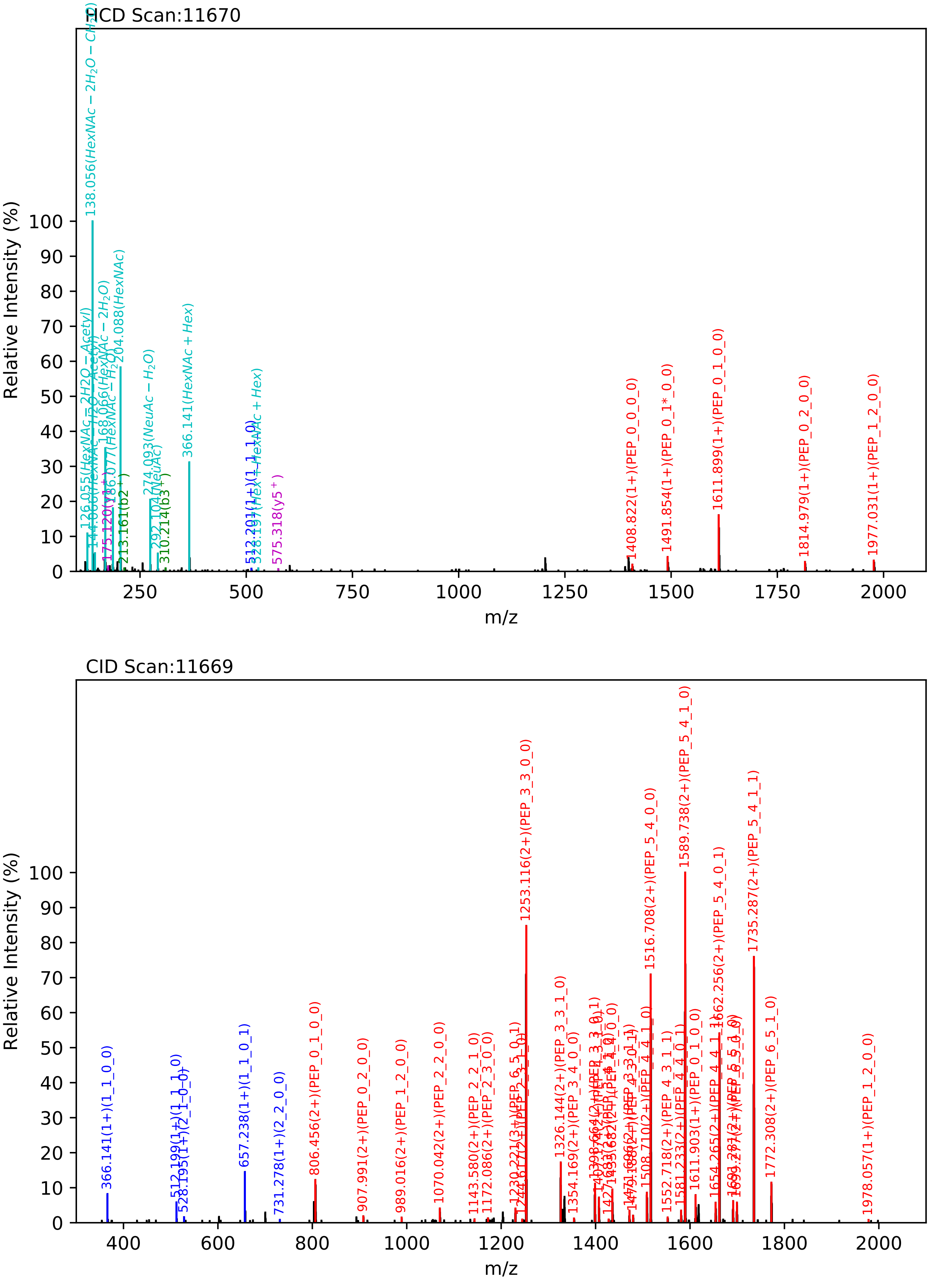

Test set no. 299, Experiment: AGP exp_4

LVPVPITNATLDR(=PEP)_6_5_1_1, m/z:1278.57(3+), RT:42.59, Y-score:87.55

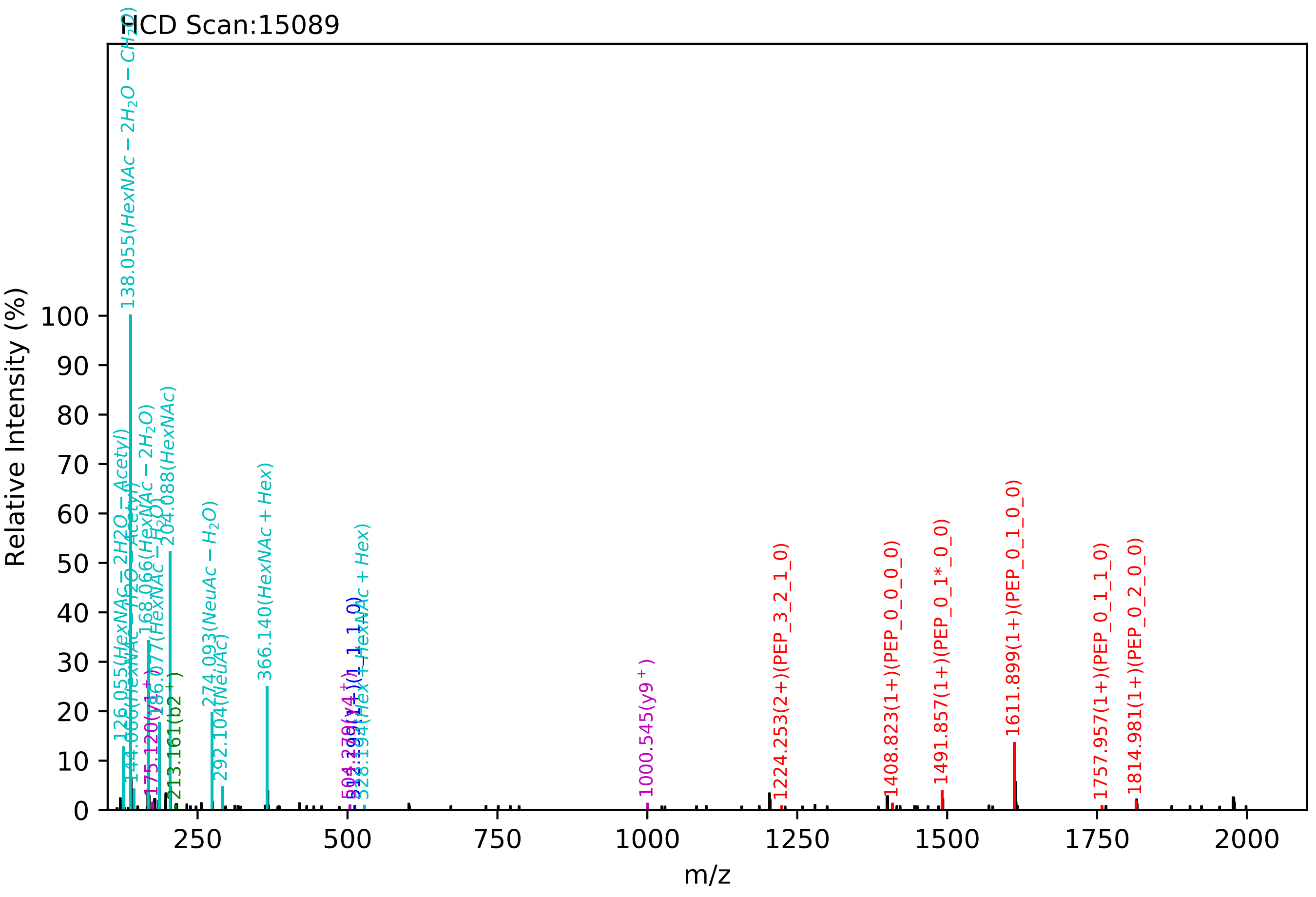

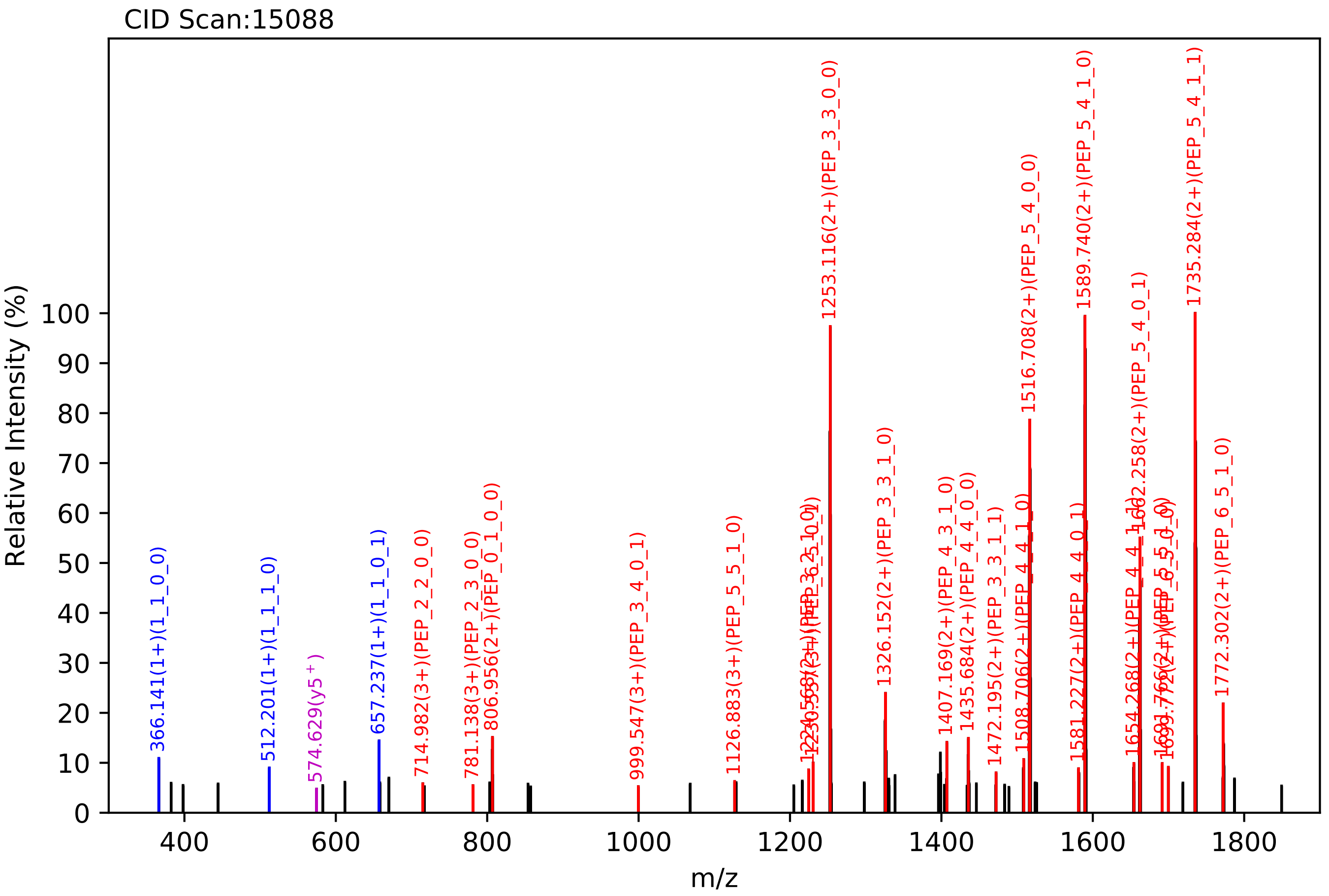

Test set no. 300, Experiment: AGP exp_3

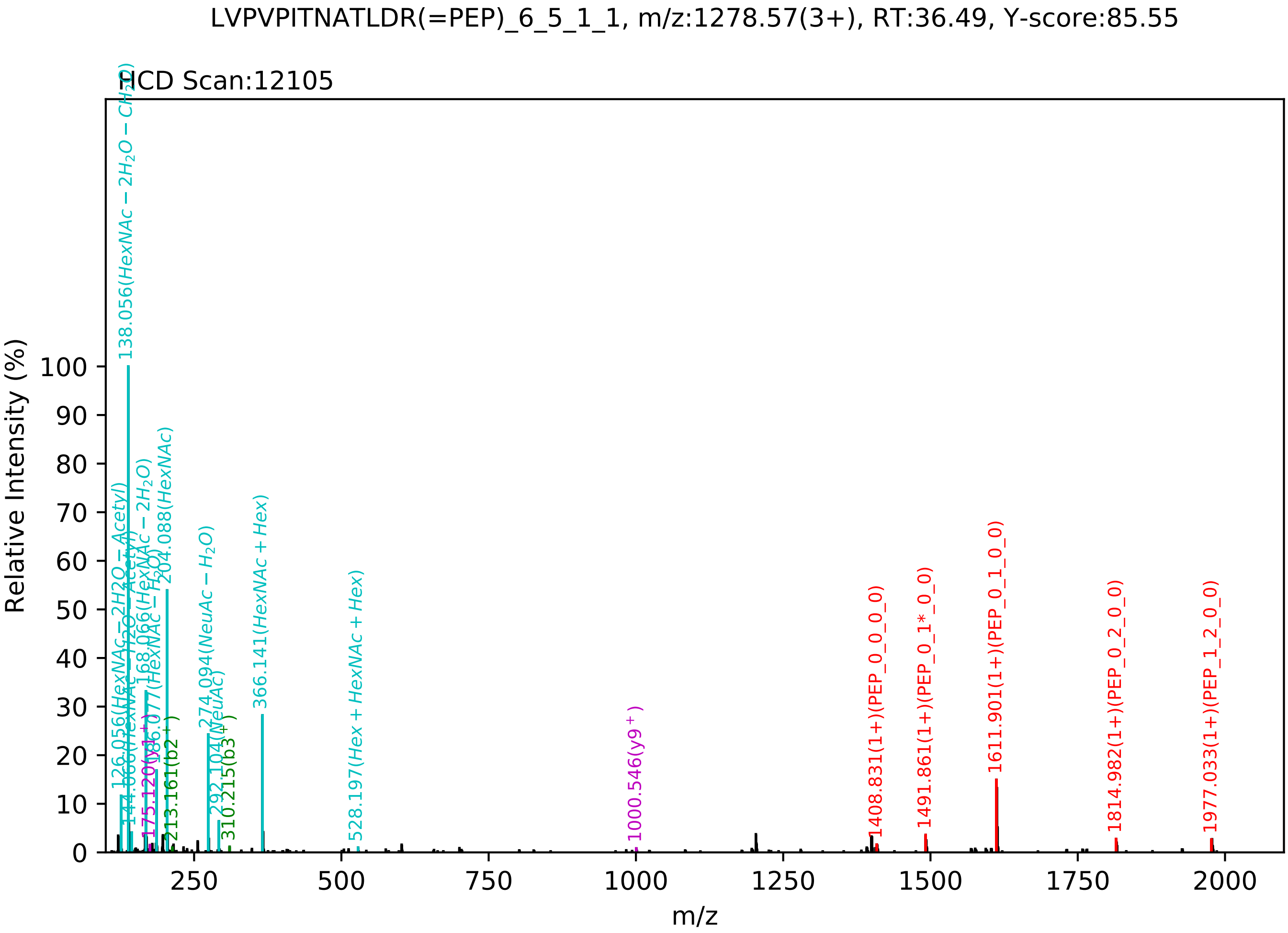

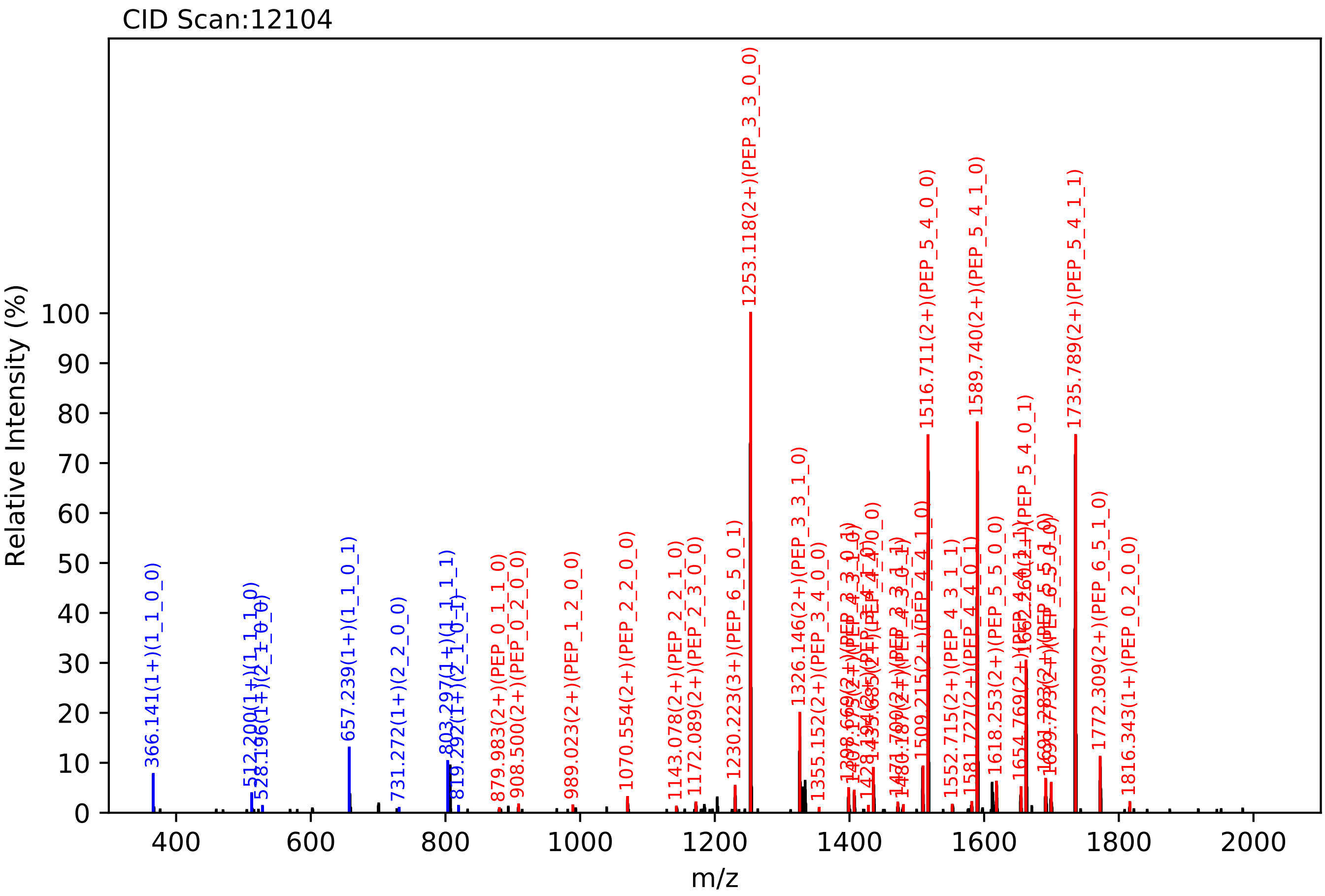

Test set no. 301, Experiment: AGP exp_4

LVPVPITNATLDR(=PEP)_6_5_1_1, m/z:1278.57(3+), RT:36.88, Y-score:80.40

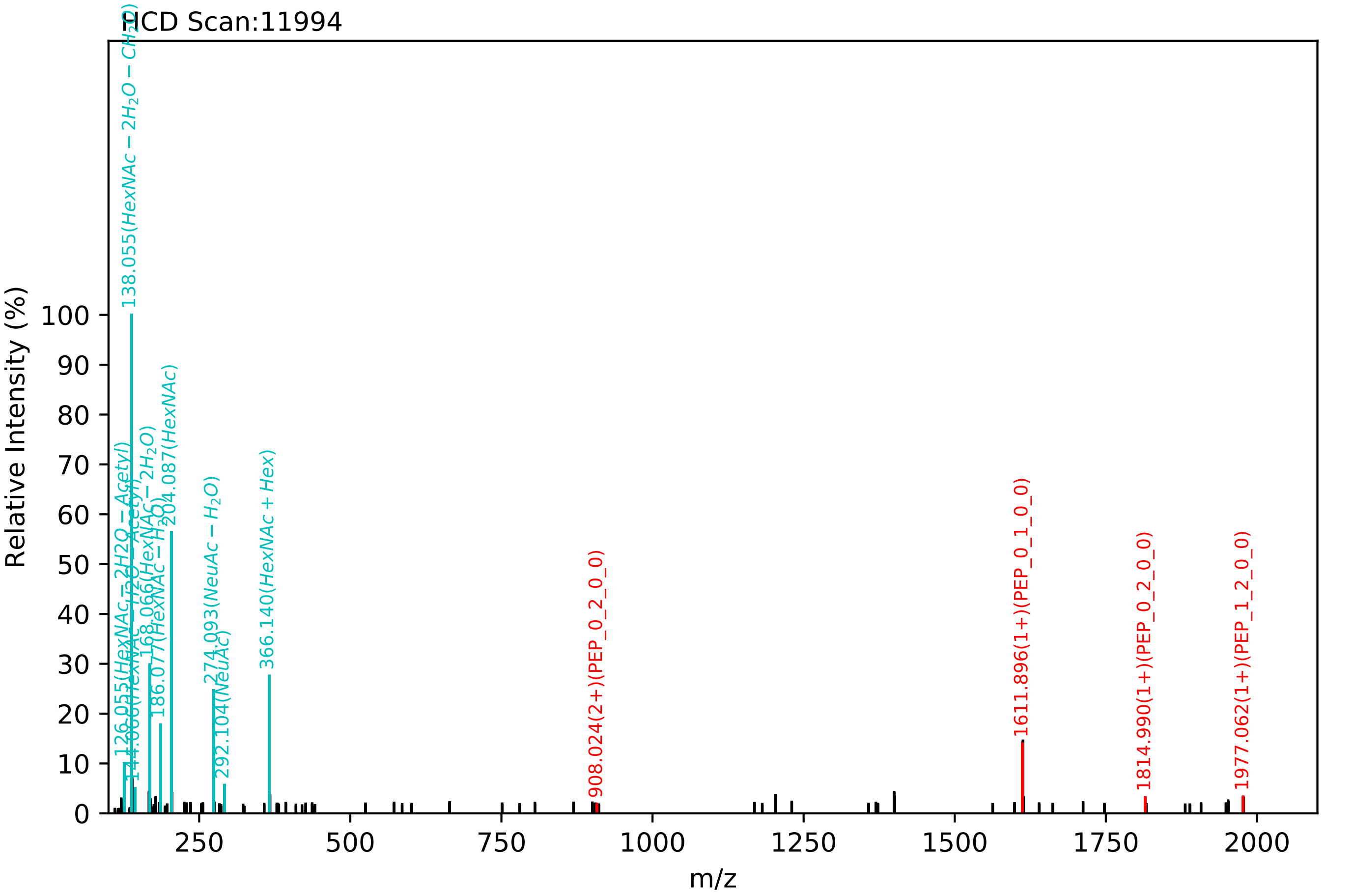

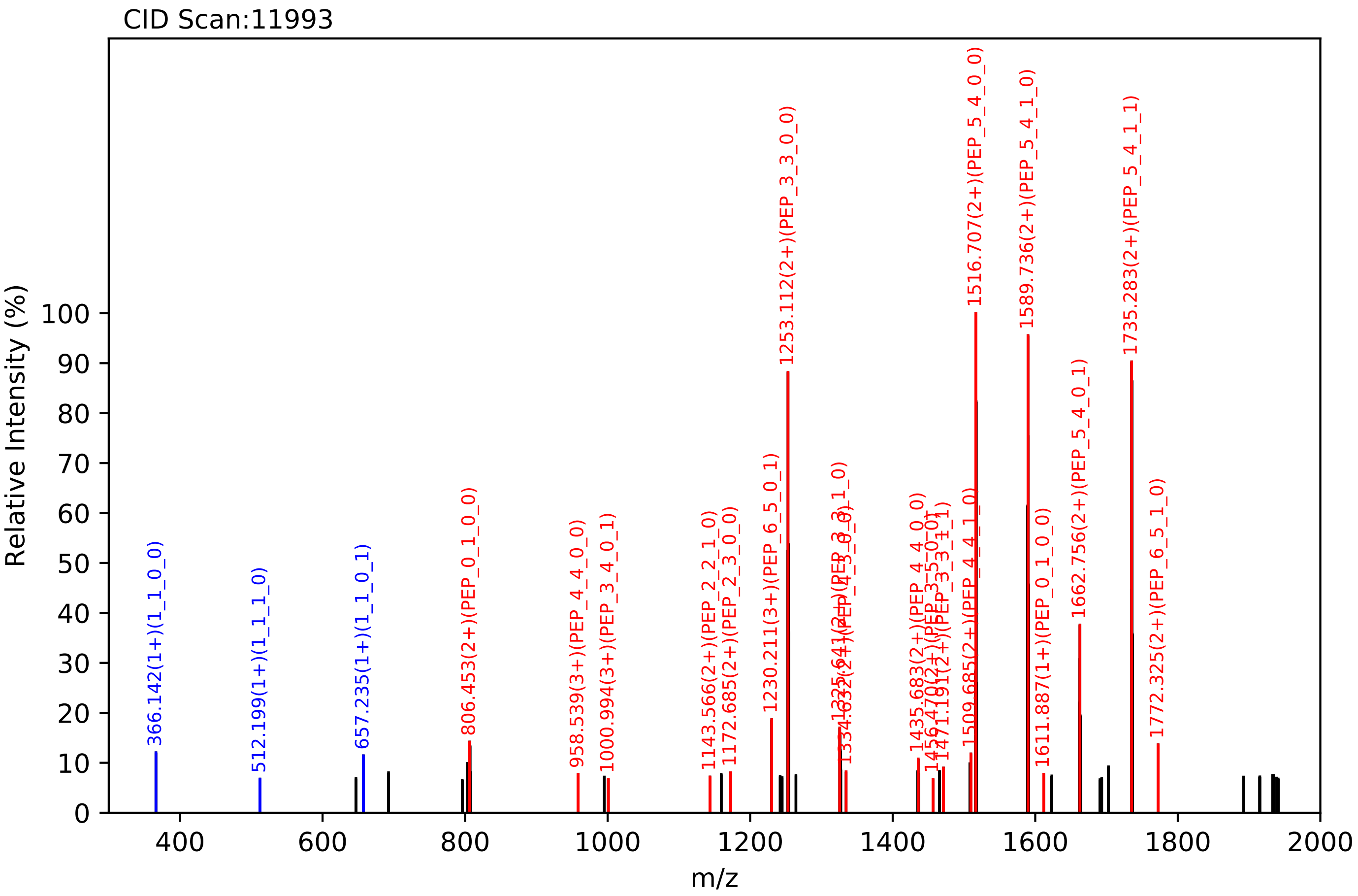

Test set no. 302, Experiment: AGP exp_3

LVPVPITNATLDR(=PEP)_6_5_1_2, m/z:1375.60(3+), RT:43.07, Y-score:92.55

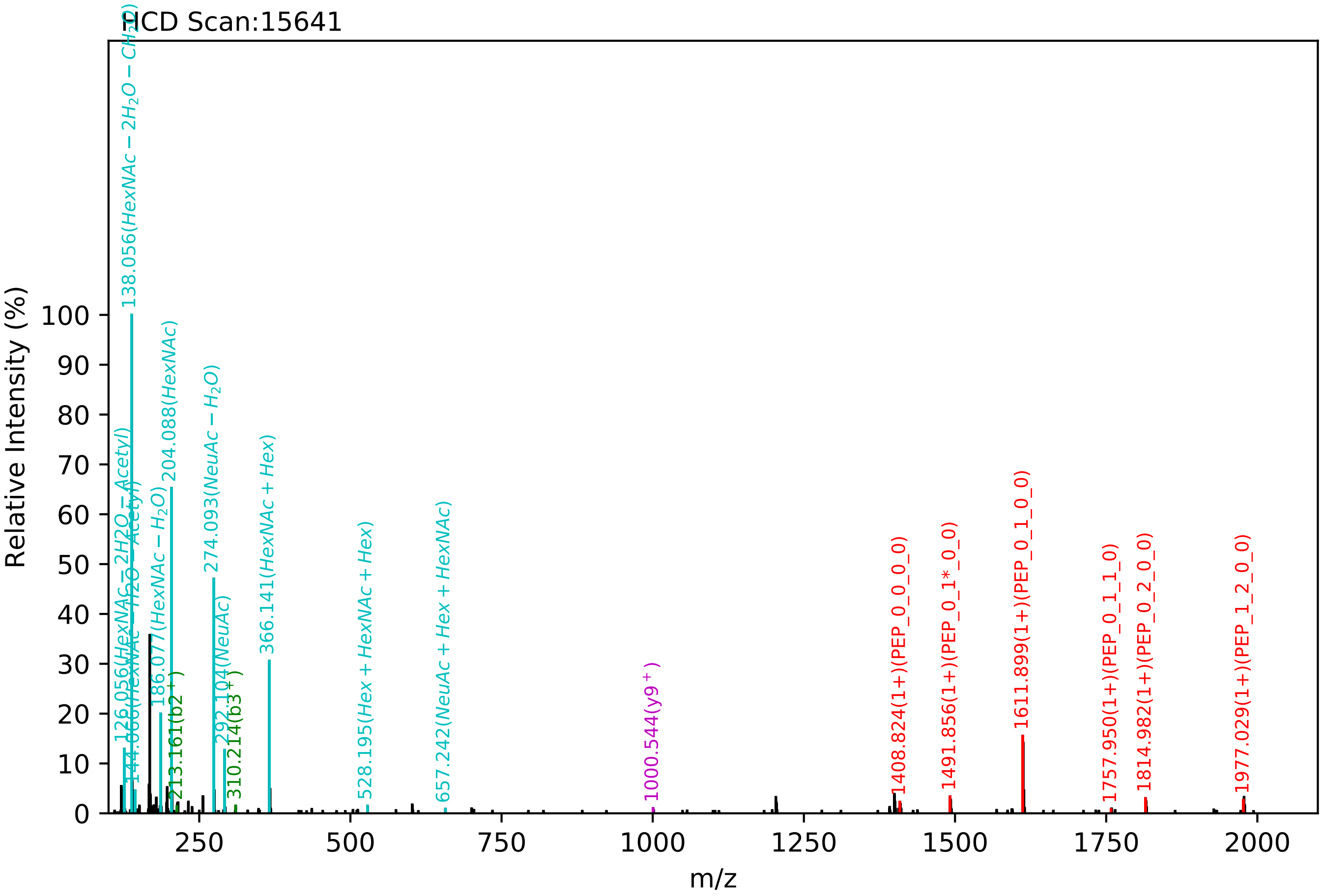

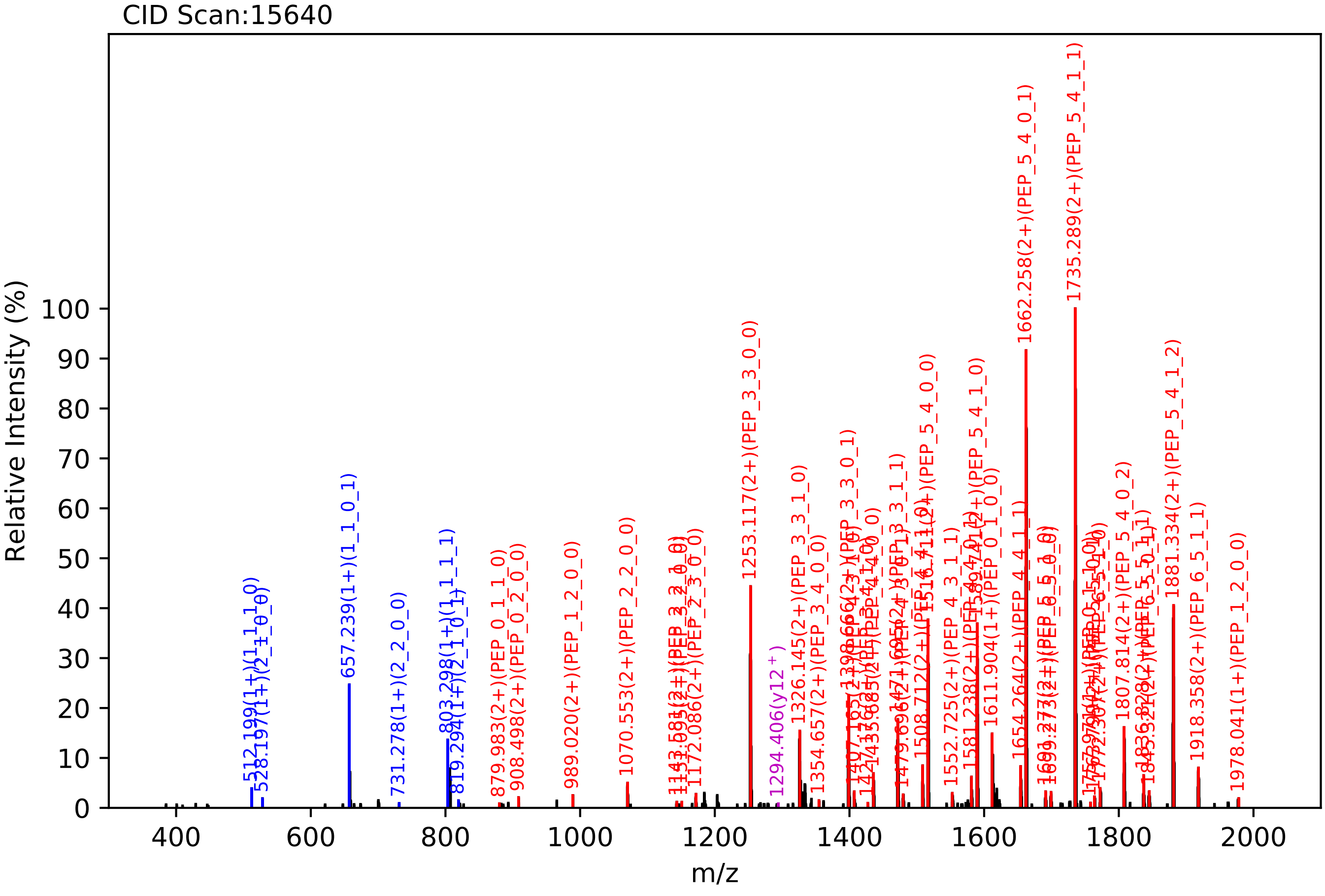

Test set no. 303, Experiment: AGP exp_4

LVPVPITNATLDR(=PEP)_6_5_1_2, m/z:1375.60(3+), RT:42.67, Y-score:90.15

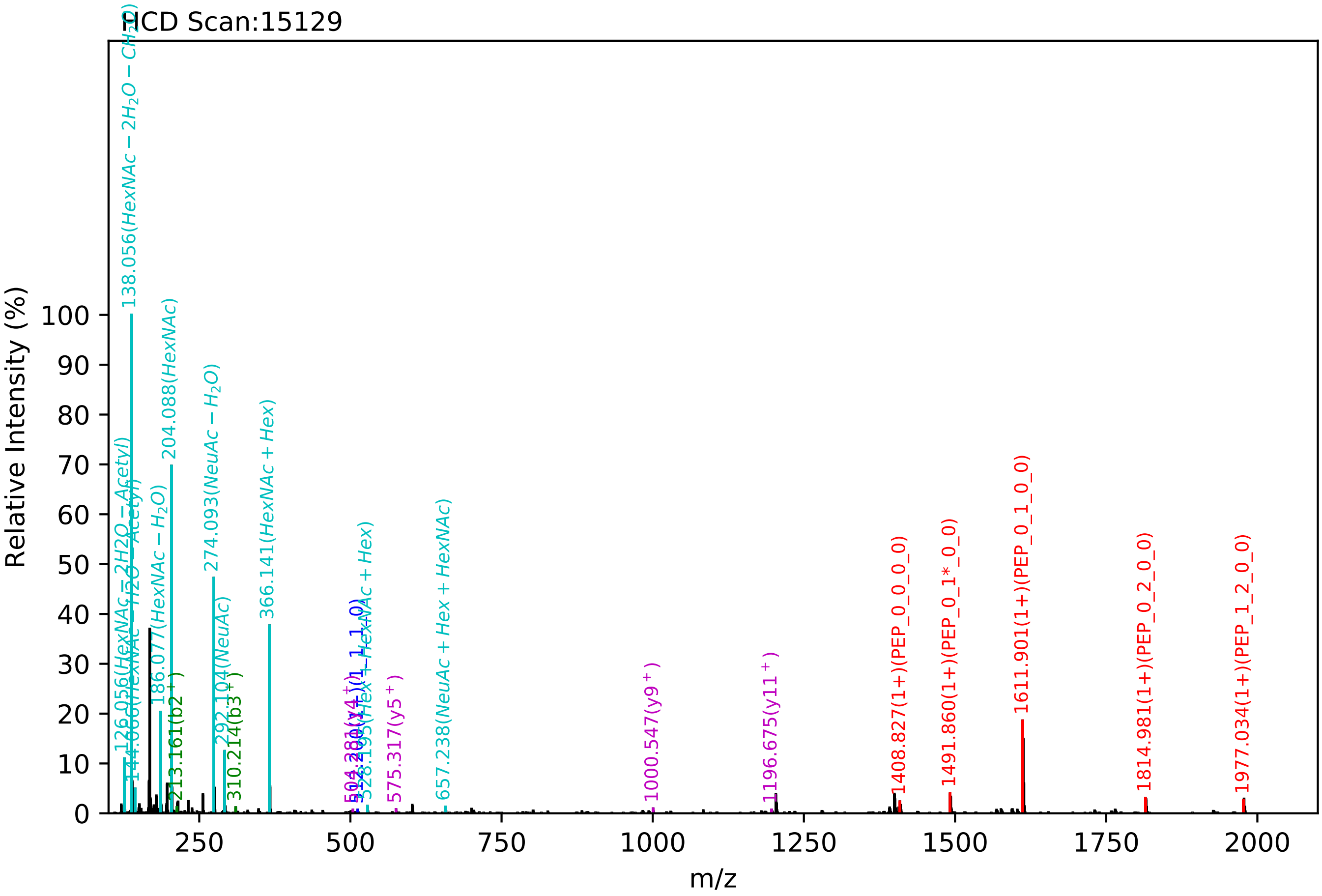

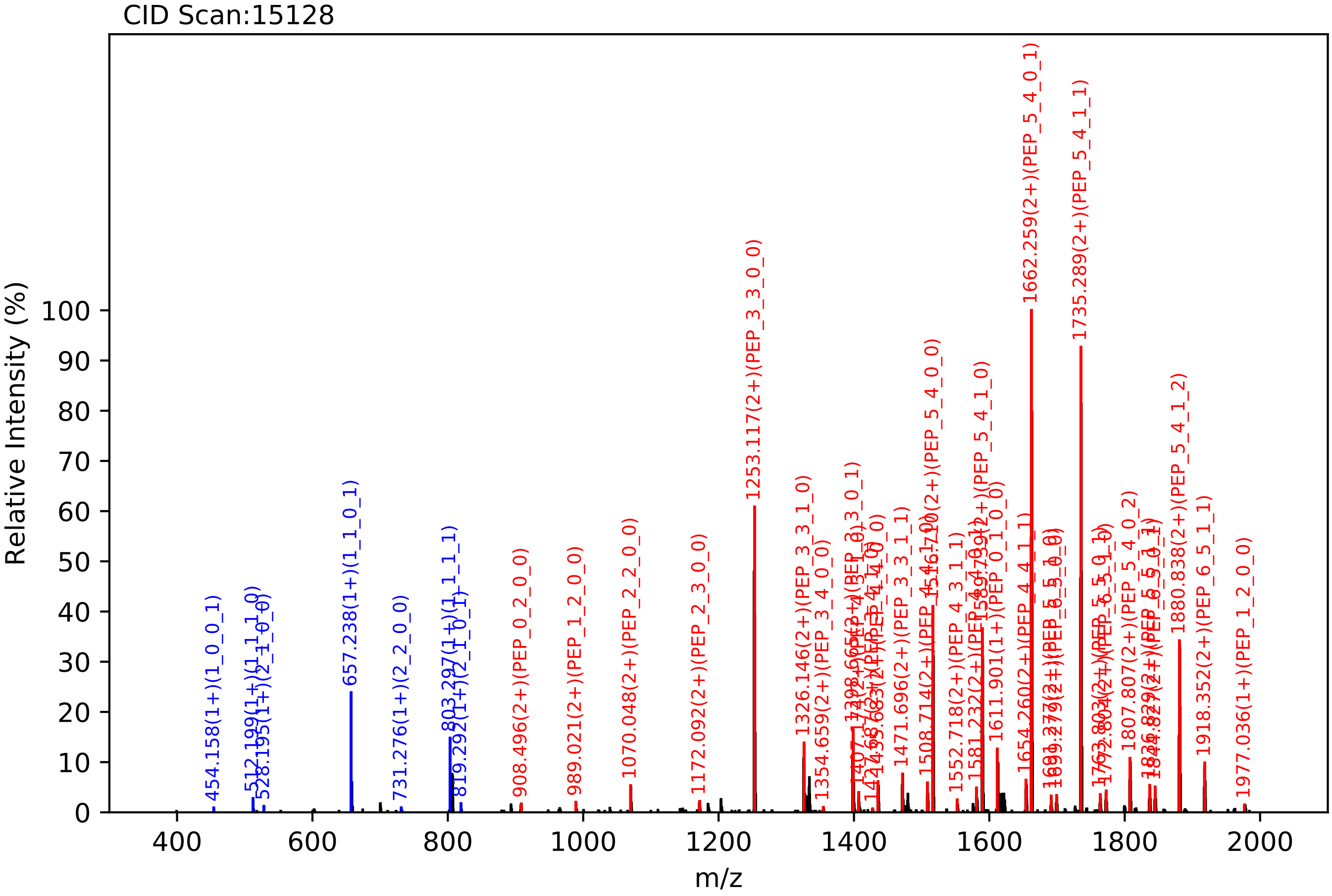

Test set no. 304, Experiment: AGP exp_4

LVPVPITNATLDR(=PEP)_6_5_1_2, m/z:1375.60(3+), RT:43.23, Y-score:90.01

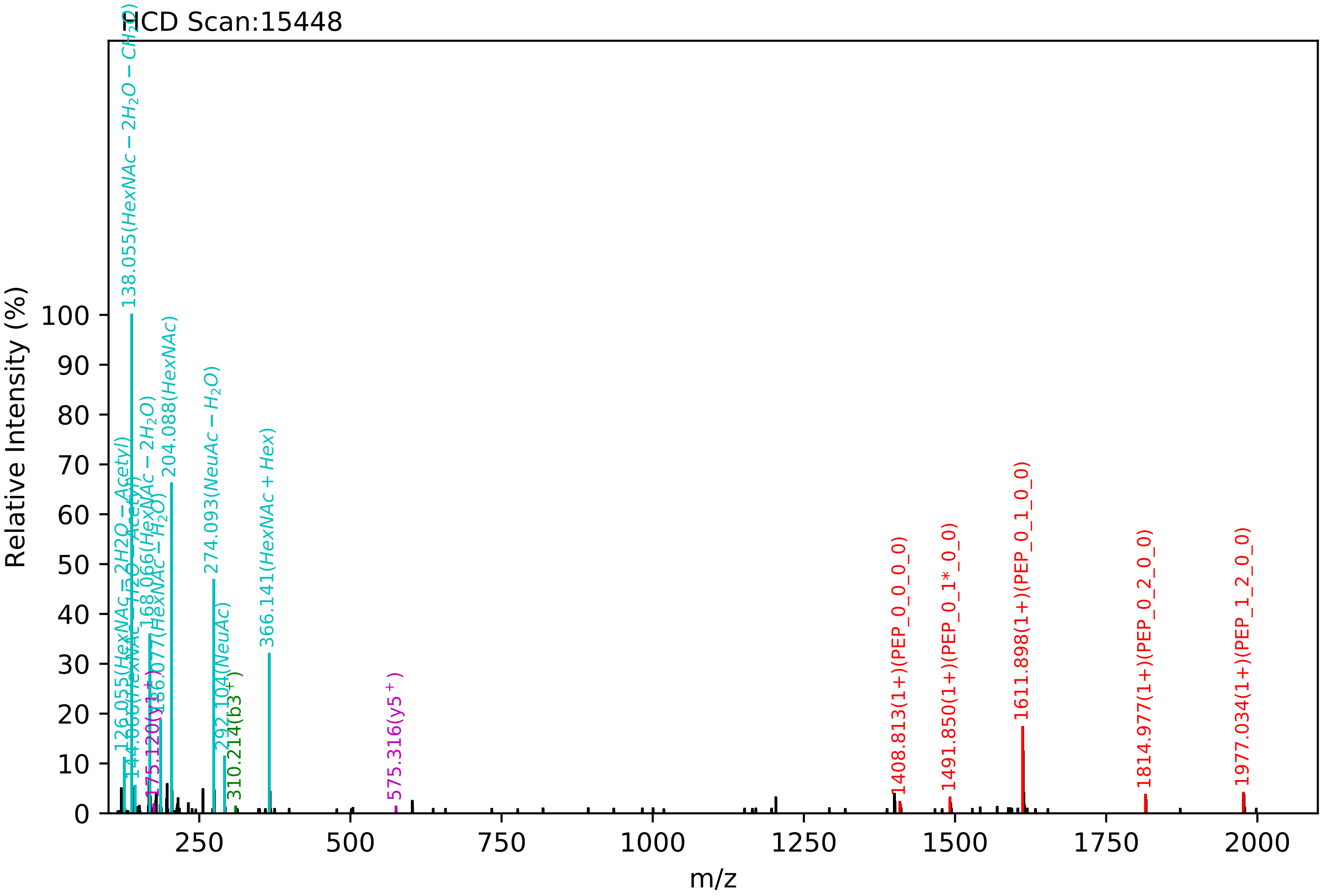

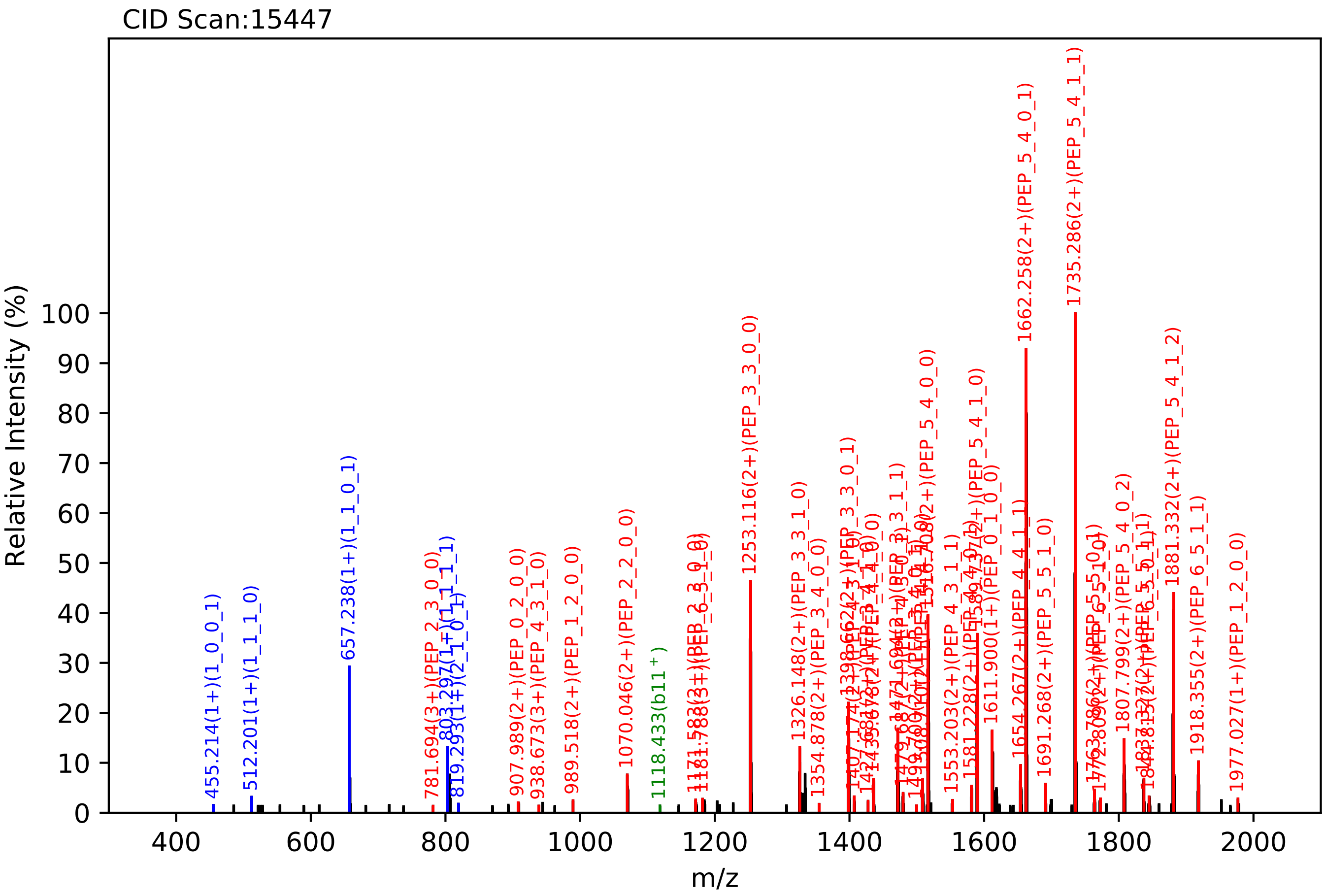

Test set no. 305, Experiment: AGP exp_4

LVPVPITNATLDR(=PEP)_6_5_1_2, m/z:1375.60(3+), RT:42.10, Y-score:89.19

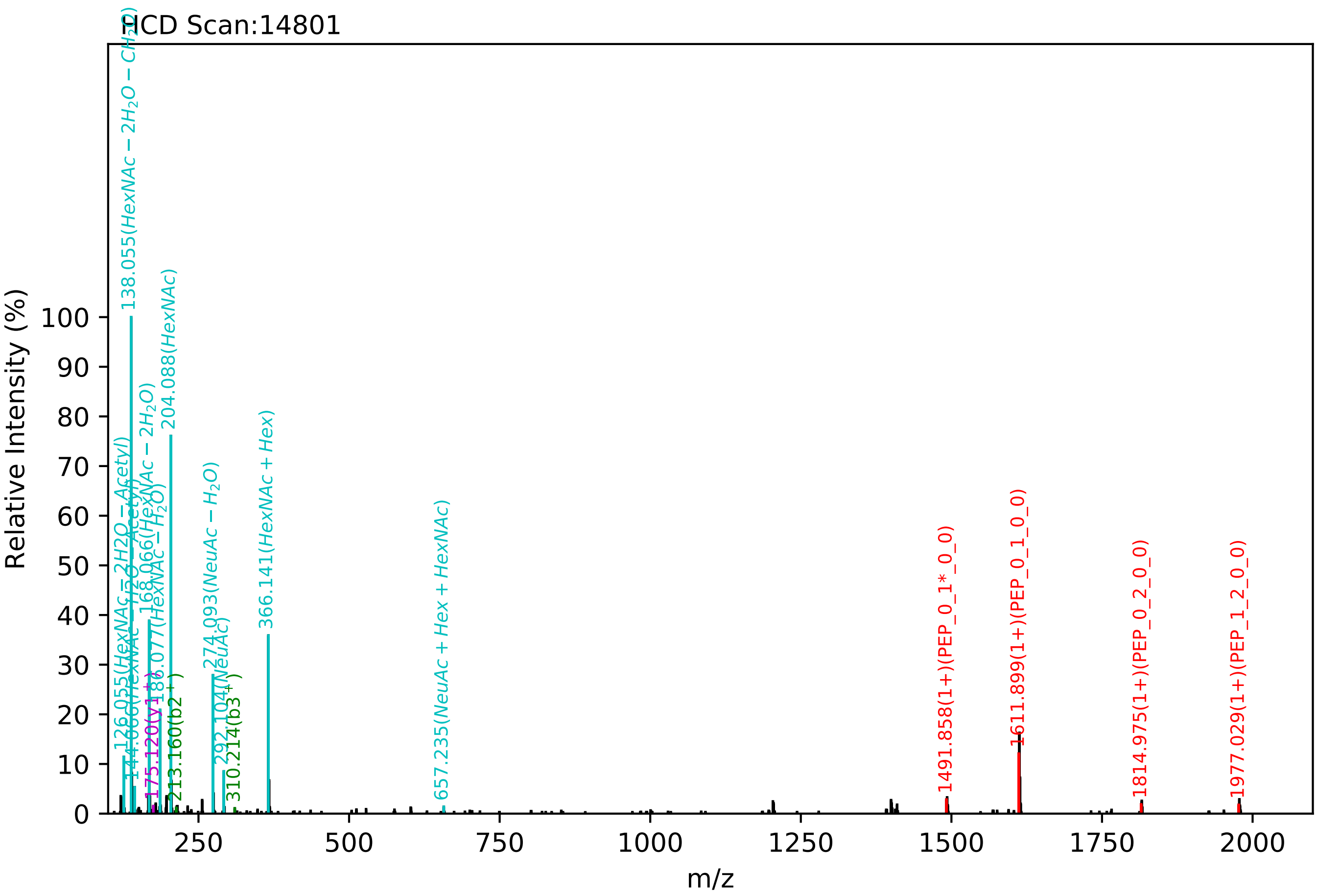

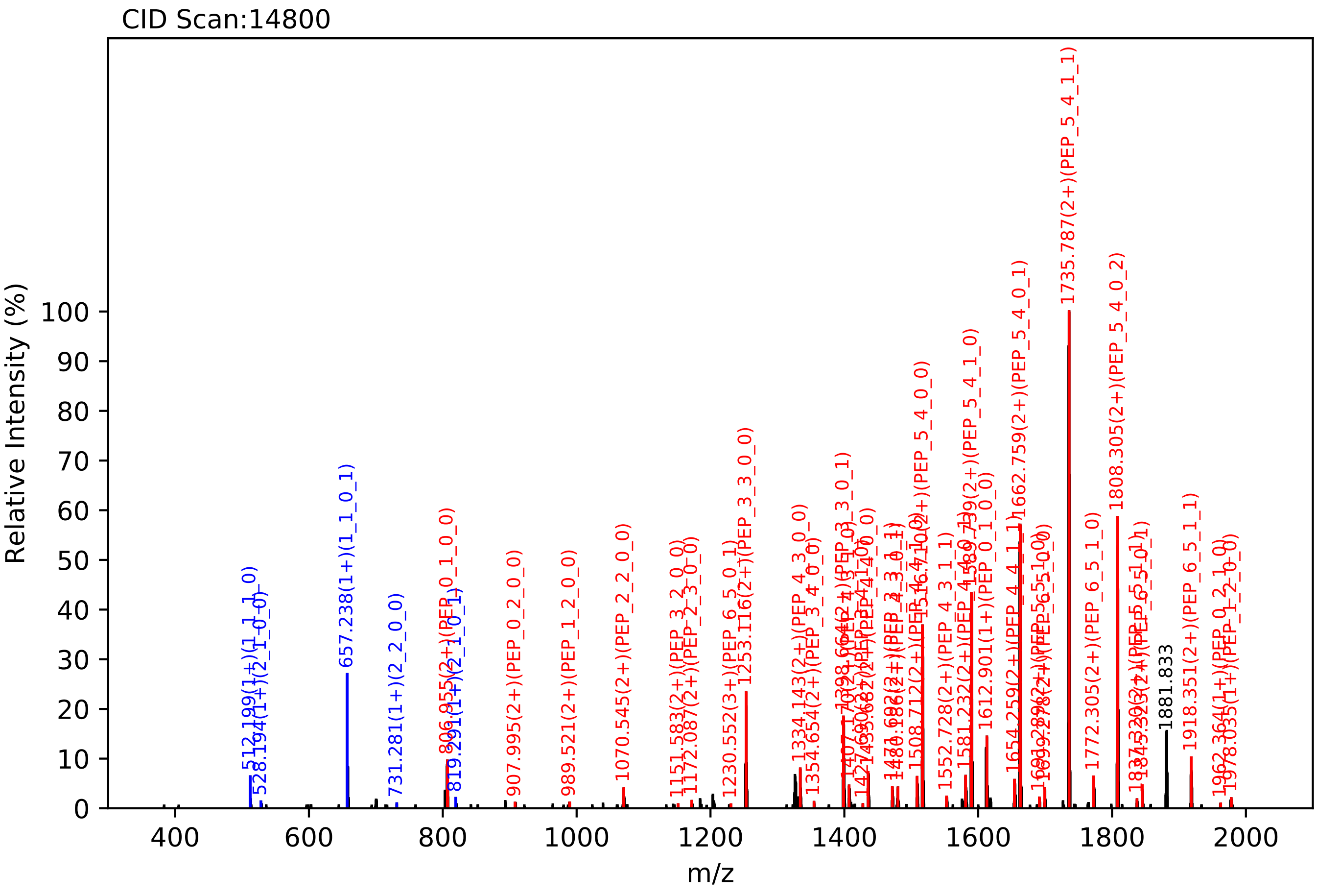

Test set no. 306, Experiment: AGP exp_3

LVPVPITNATLDR(=PEP)_6_5_1_2, m/z:1031.95(4+), RT:42.45, Y-score:85.43

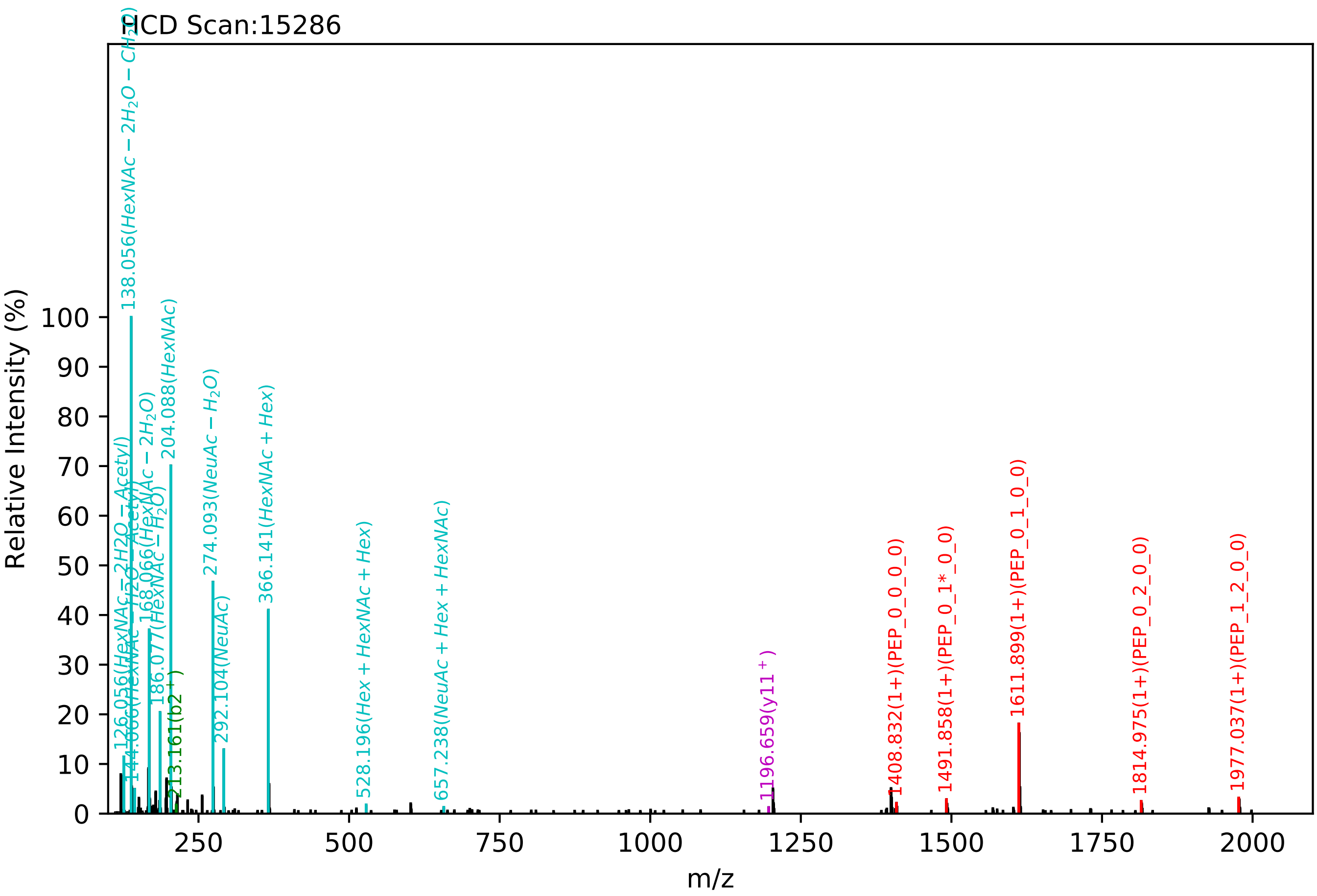

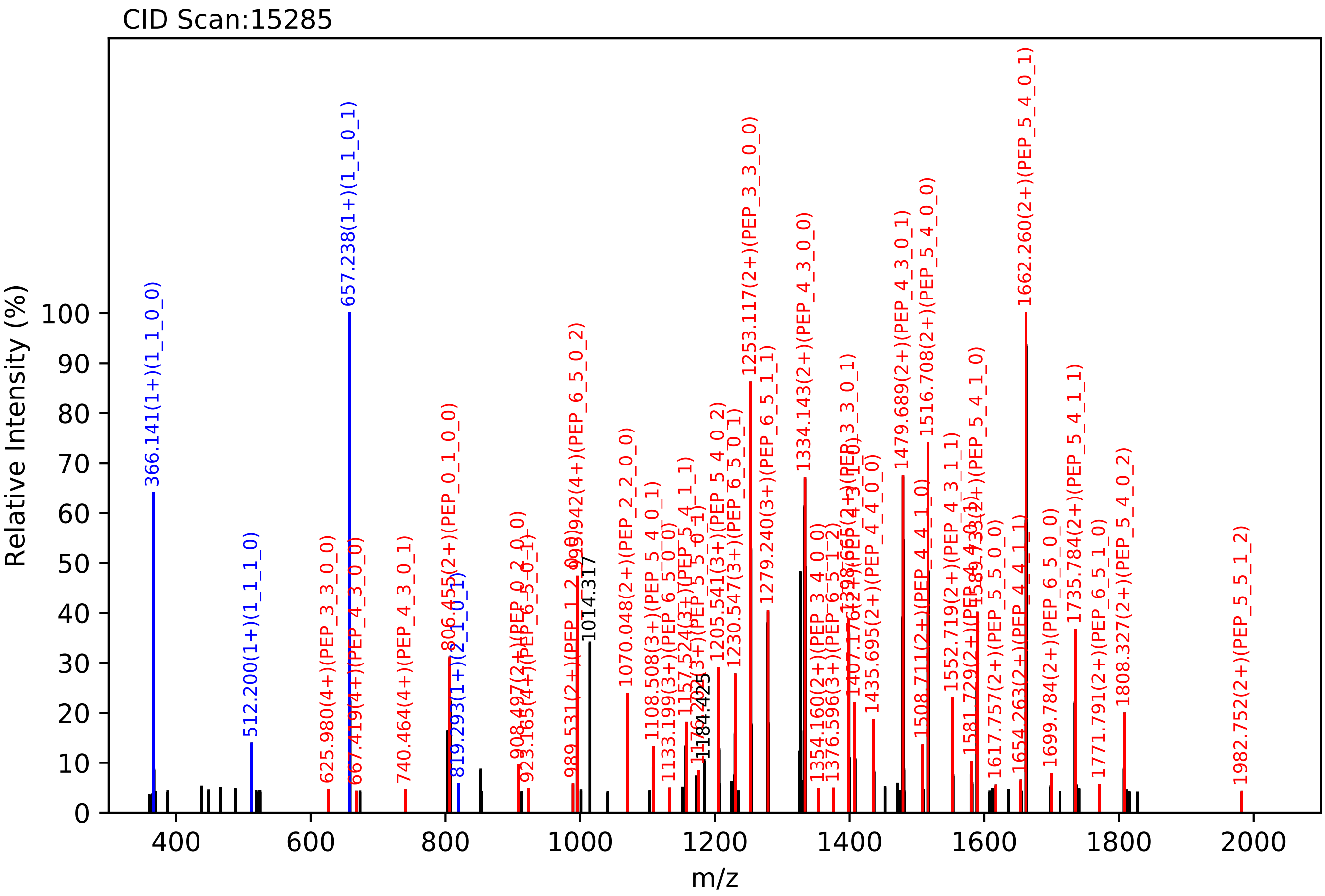

Test set no. 307, Experiment: AGP exp_4

LVPVPITNATLDR(=PEP)_6_5_1_2, m/z:1031.95(4+), RT:42.74, Y-score:80.44

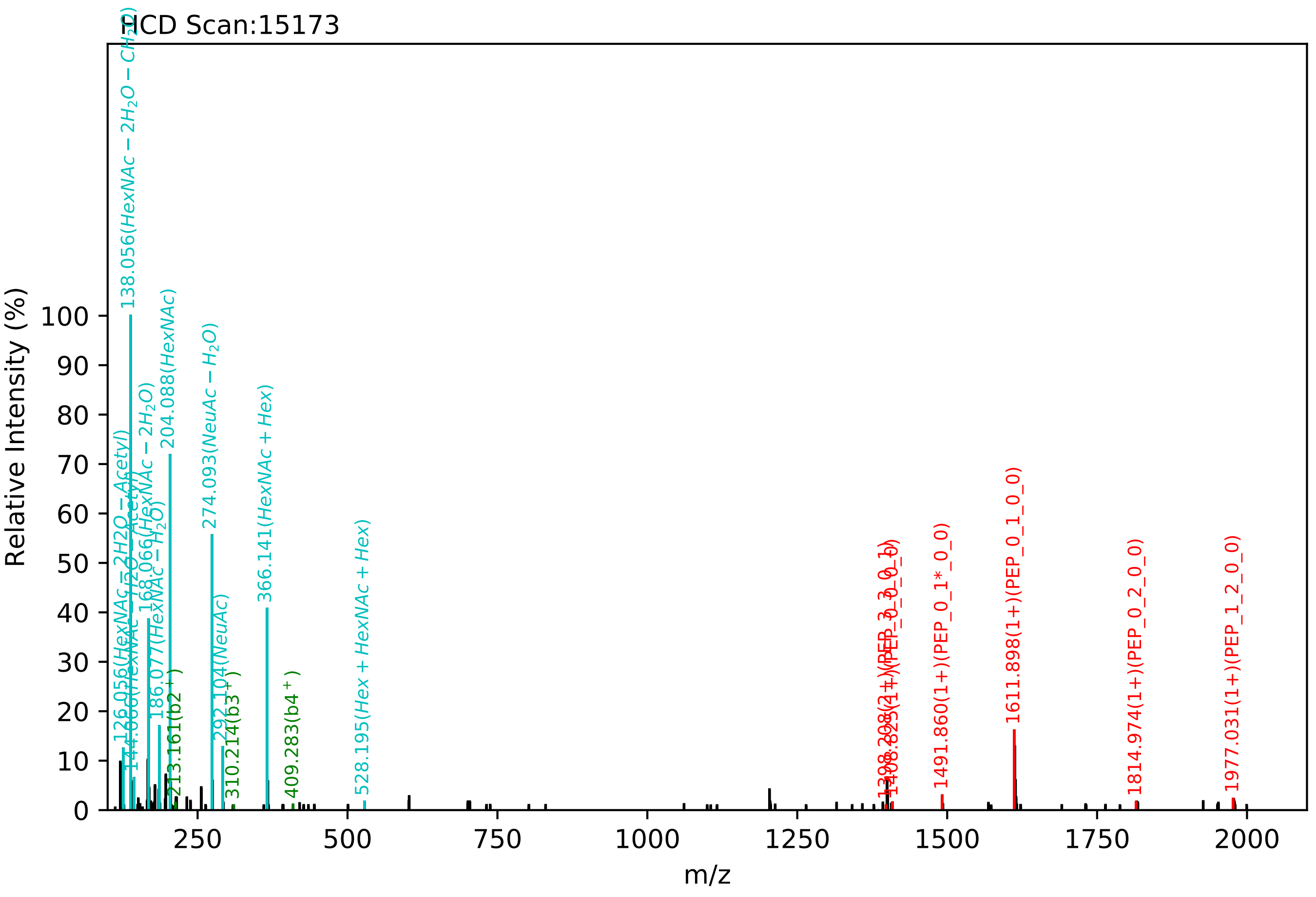

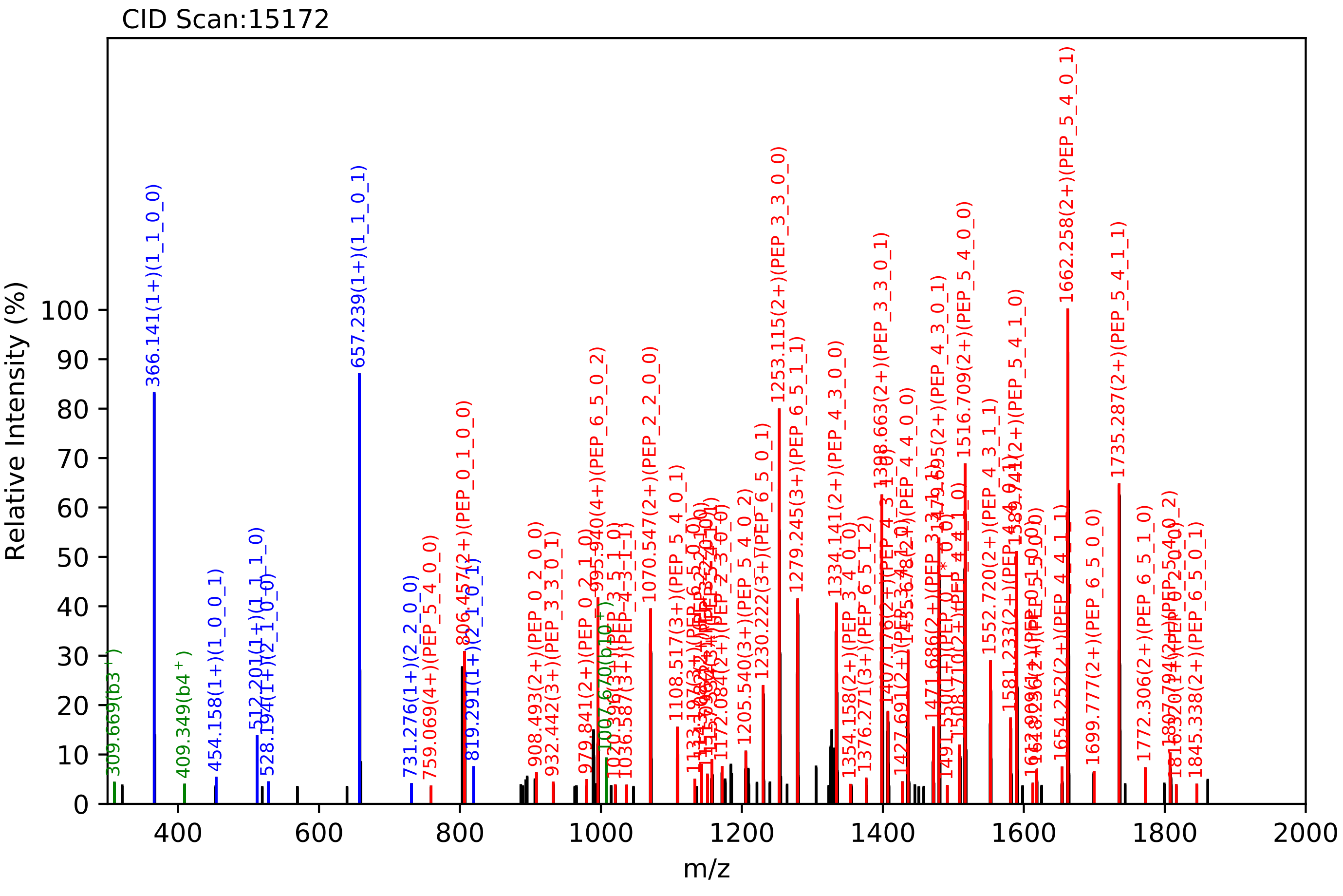

Test set no. 308, Experiment: AGP exp_4

LVPVPITNATLDR(=PEP)_6_5_1_2, m/z:1031.95(4+), RT:42.16, Y-score:75.73

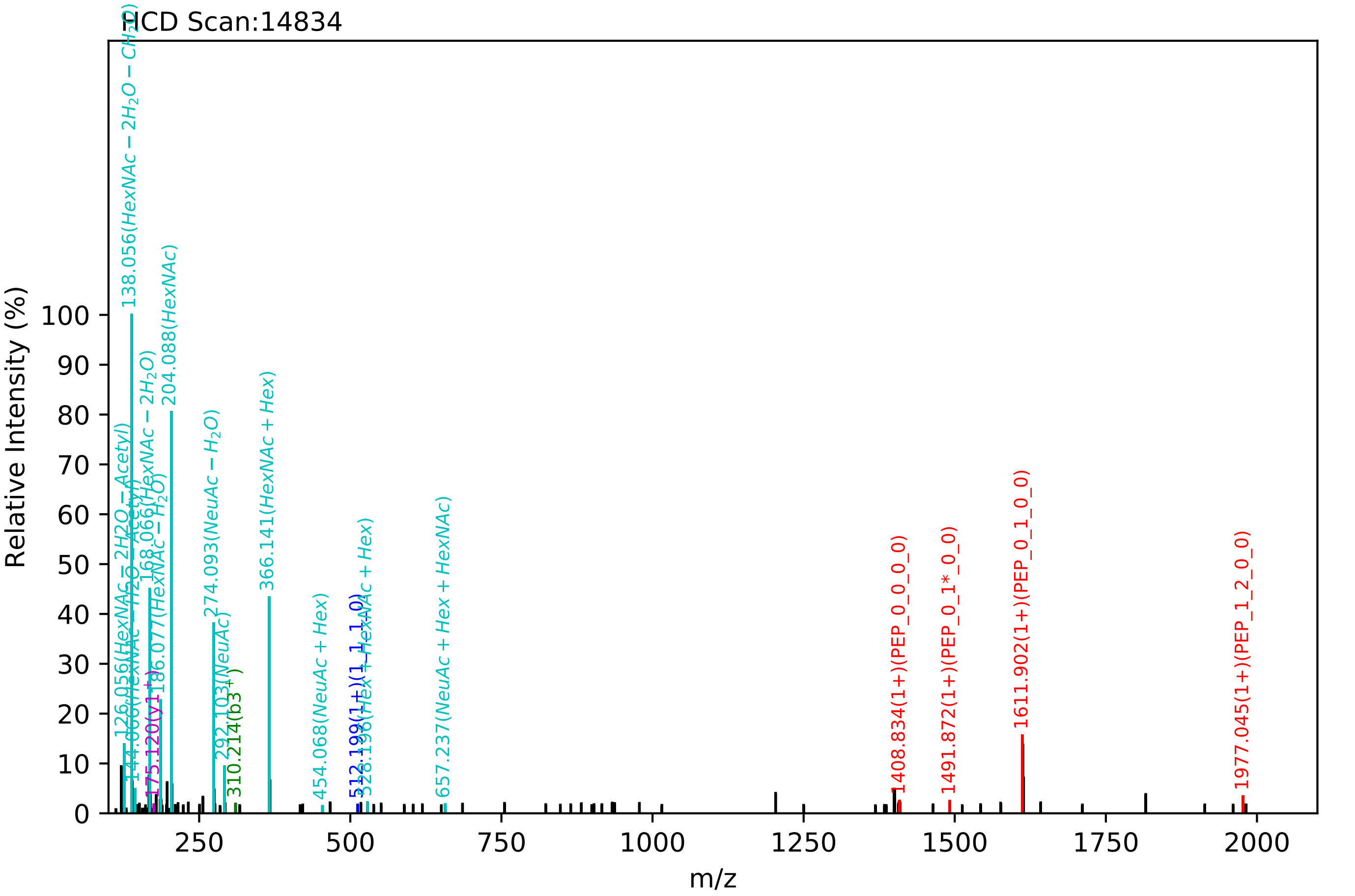

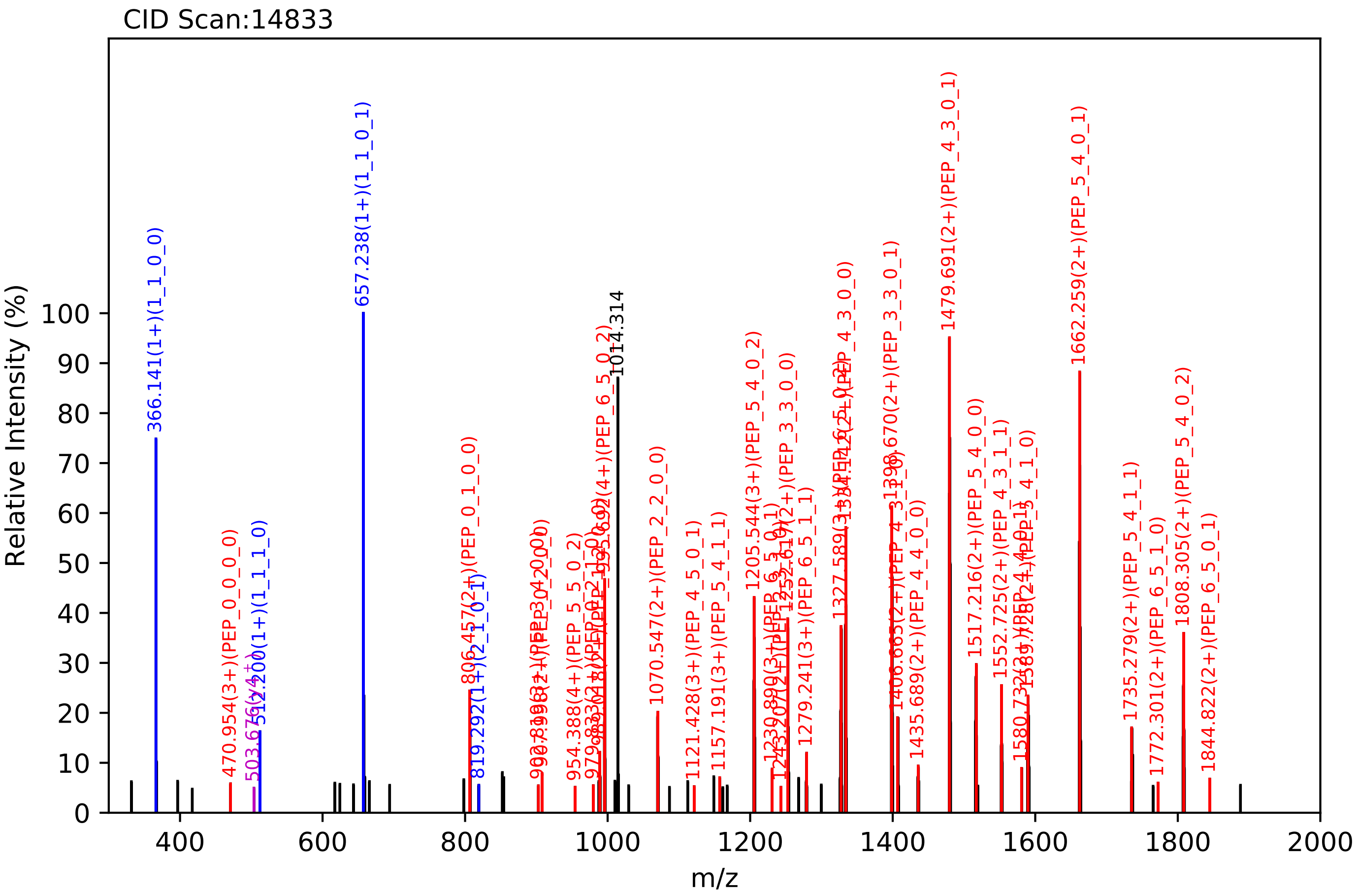

Test set no. 309, Experiment: AGP exp_4

LVPVPITNATLDR(=PEP)_6_5_1_3, m/z:1472.63(3+), RT:50.64, Y-score:86.92

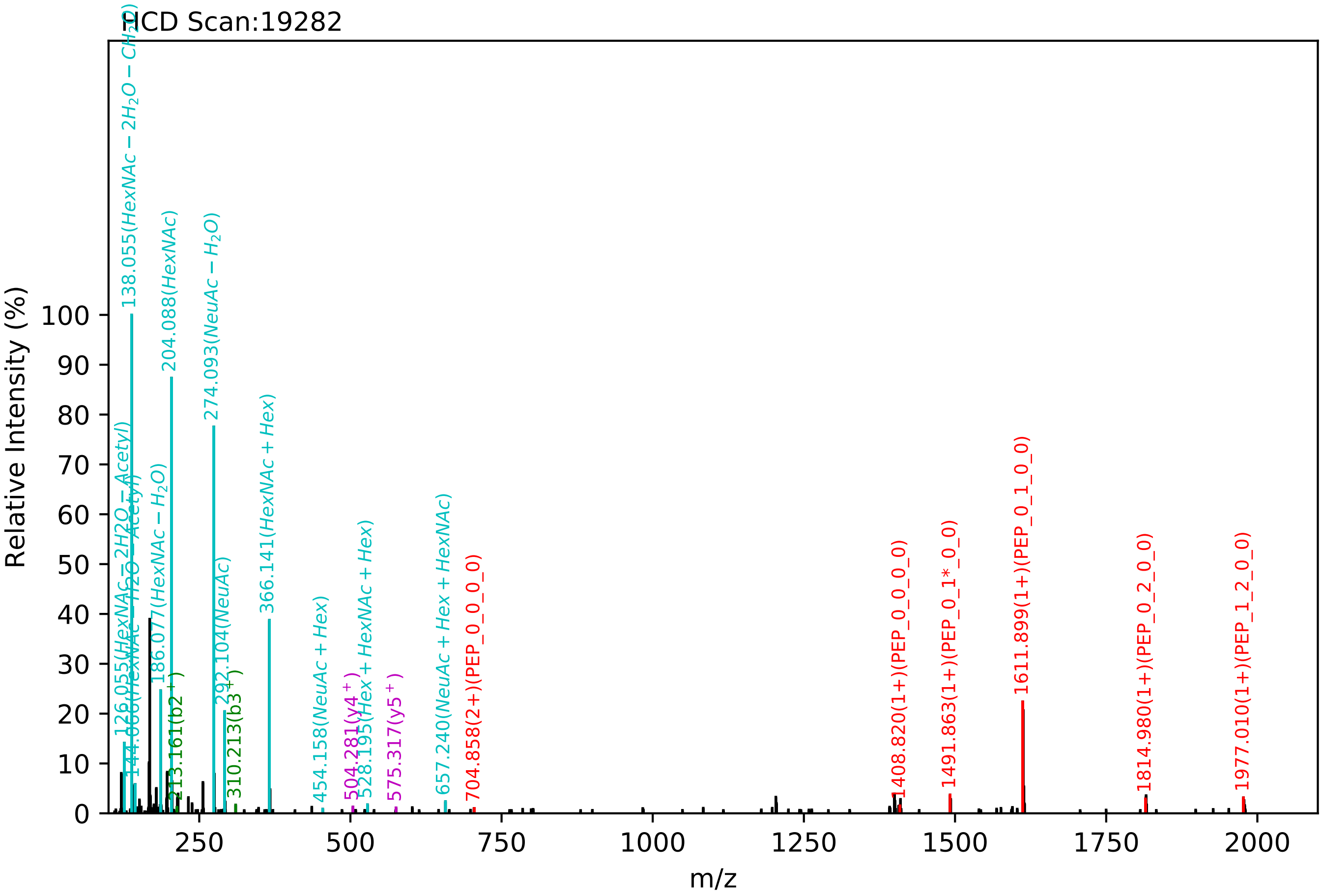

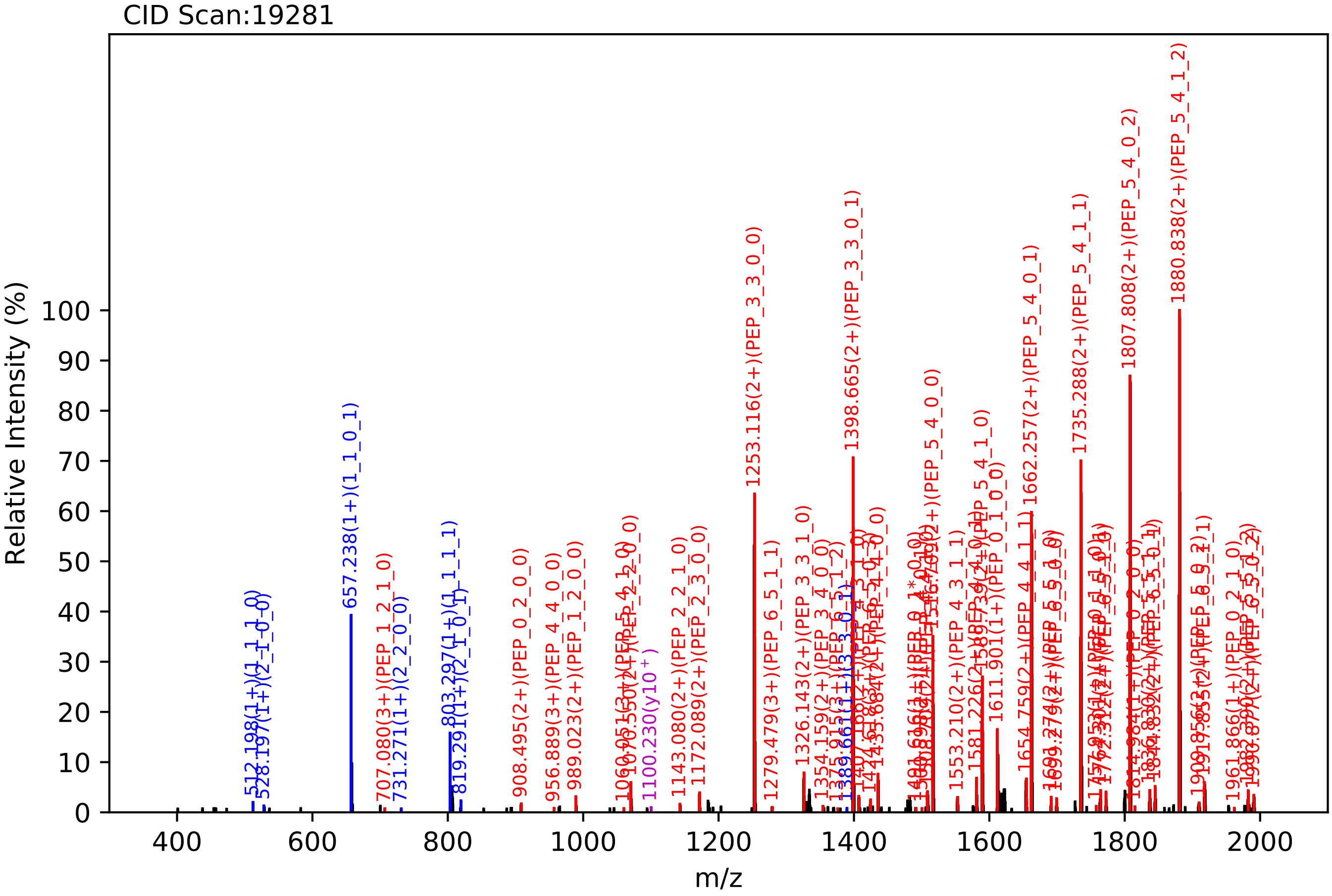

Test set no. 310, Experiment: AGP exp_3

LVPVPITNATLDR(=PEP)_6_5_1_3, m/z:1472.63(3+), RT:50.74, Y-score:85.82

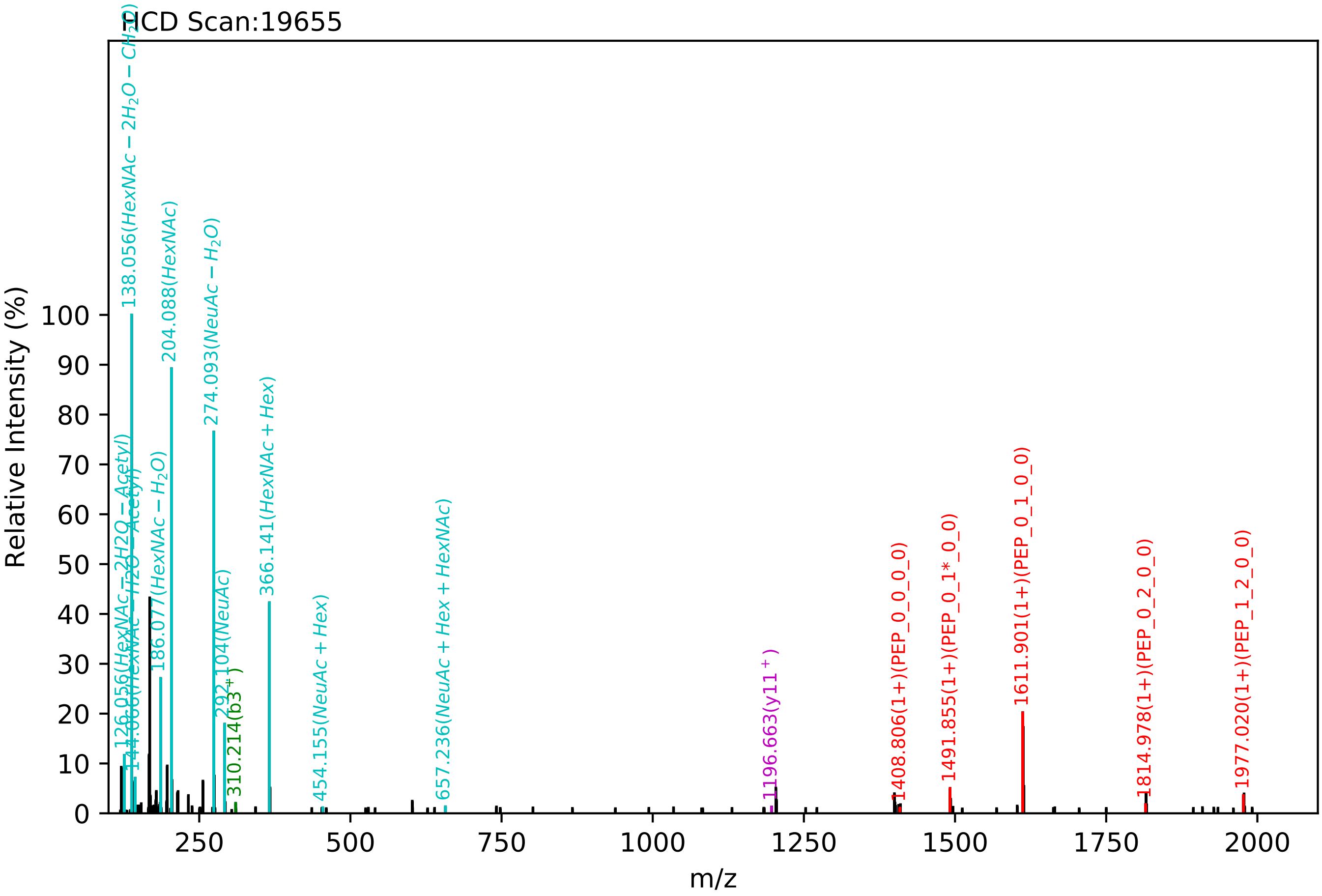

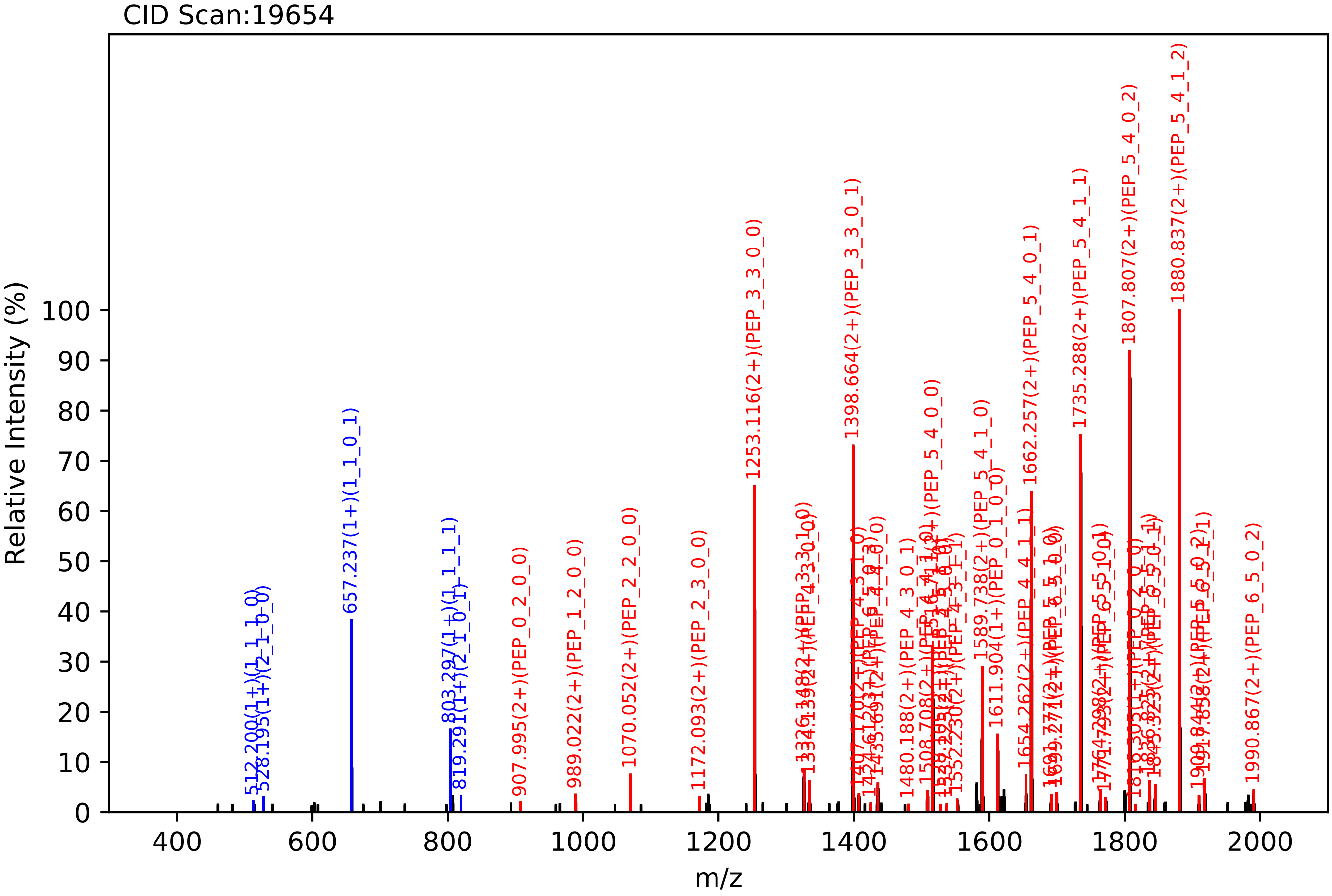

Test set no. 311, Experiment: AGP exp_3

LVPVPITNATLDR(=PEP)_6_5_1_3, m/z:1104.73(4+), RT:49.74, Y-score:77.70

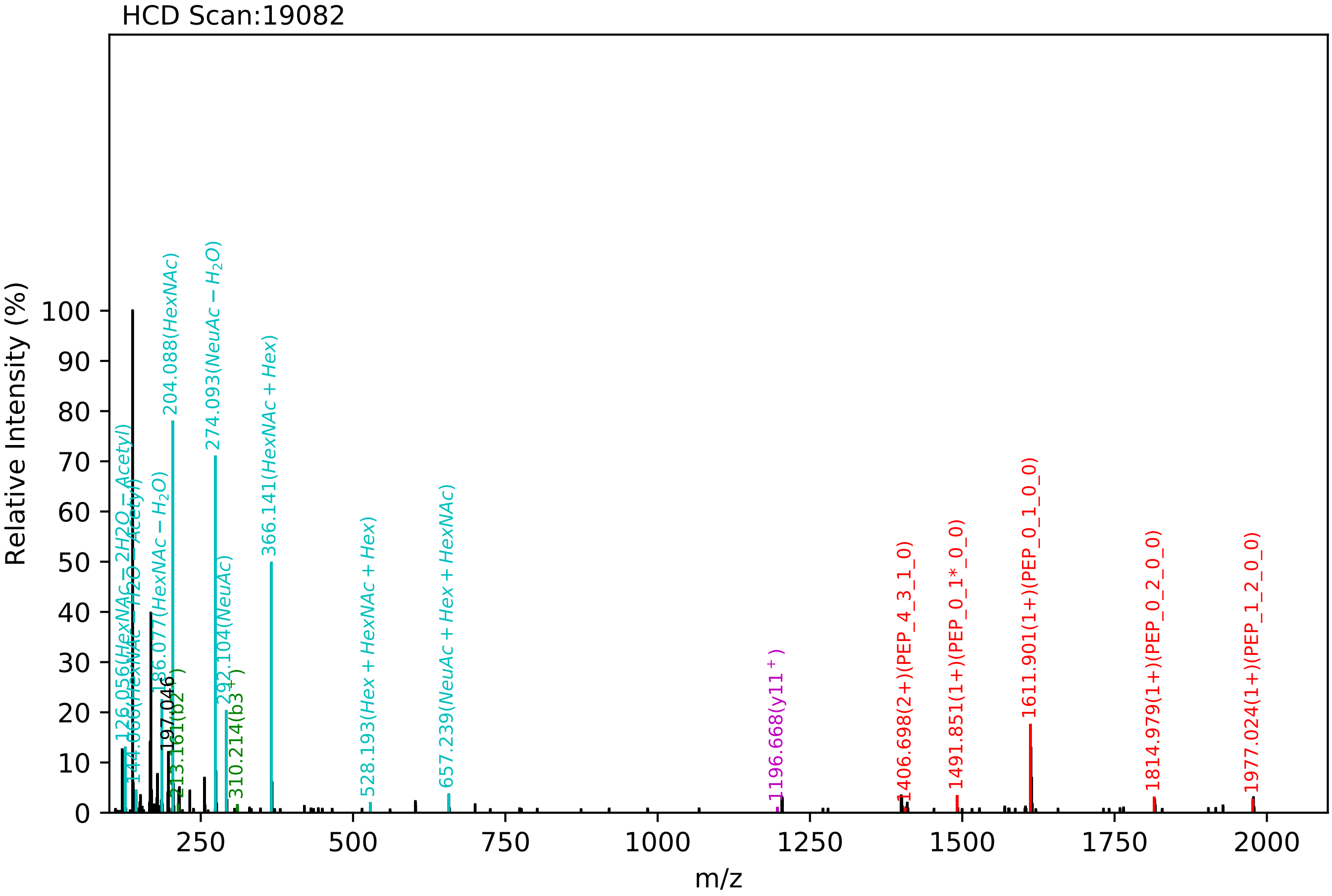

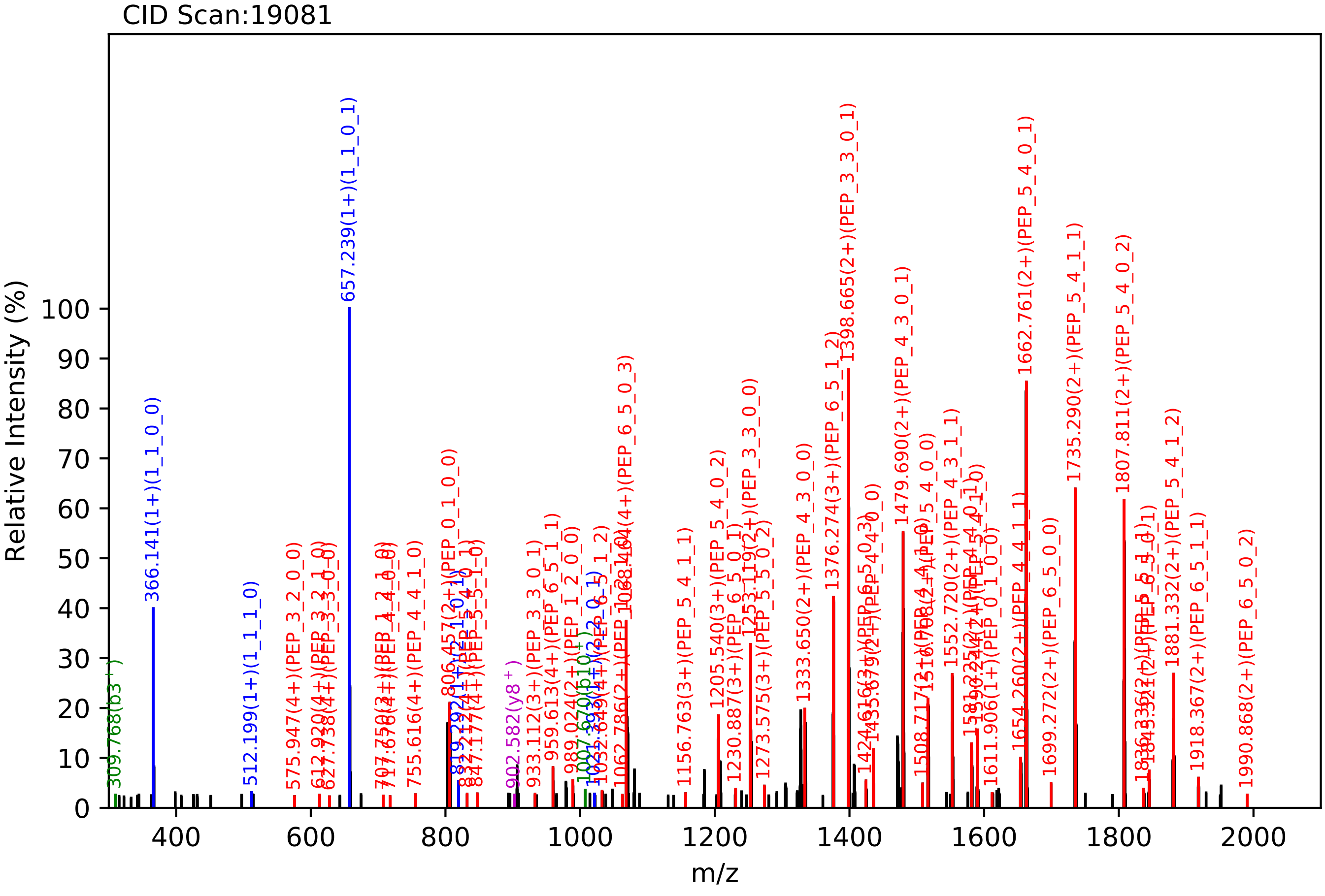

Test set no. 312, Experiment: IgG exp_7

EEQYNSTYR(=PEP)_3_3_0_0, m/z:1142.96(2+), RT:15.65, Y-score:85.81

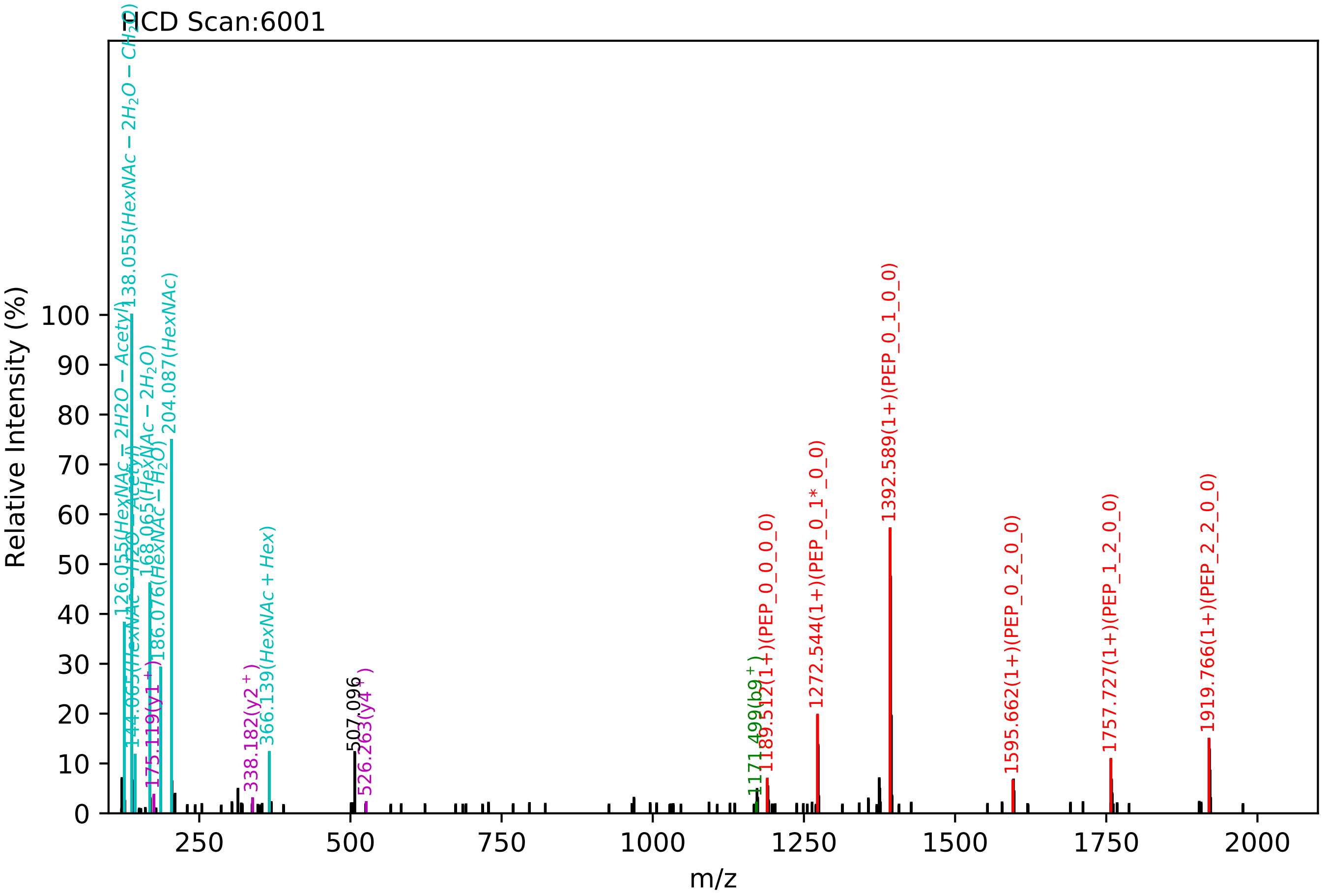

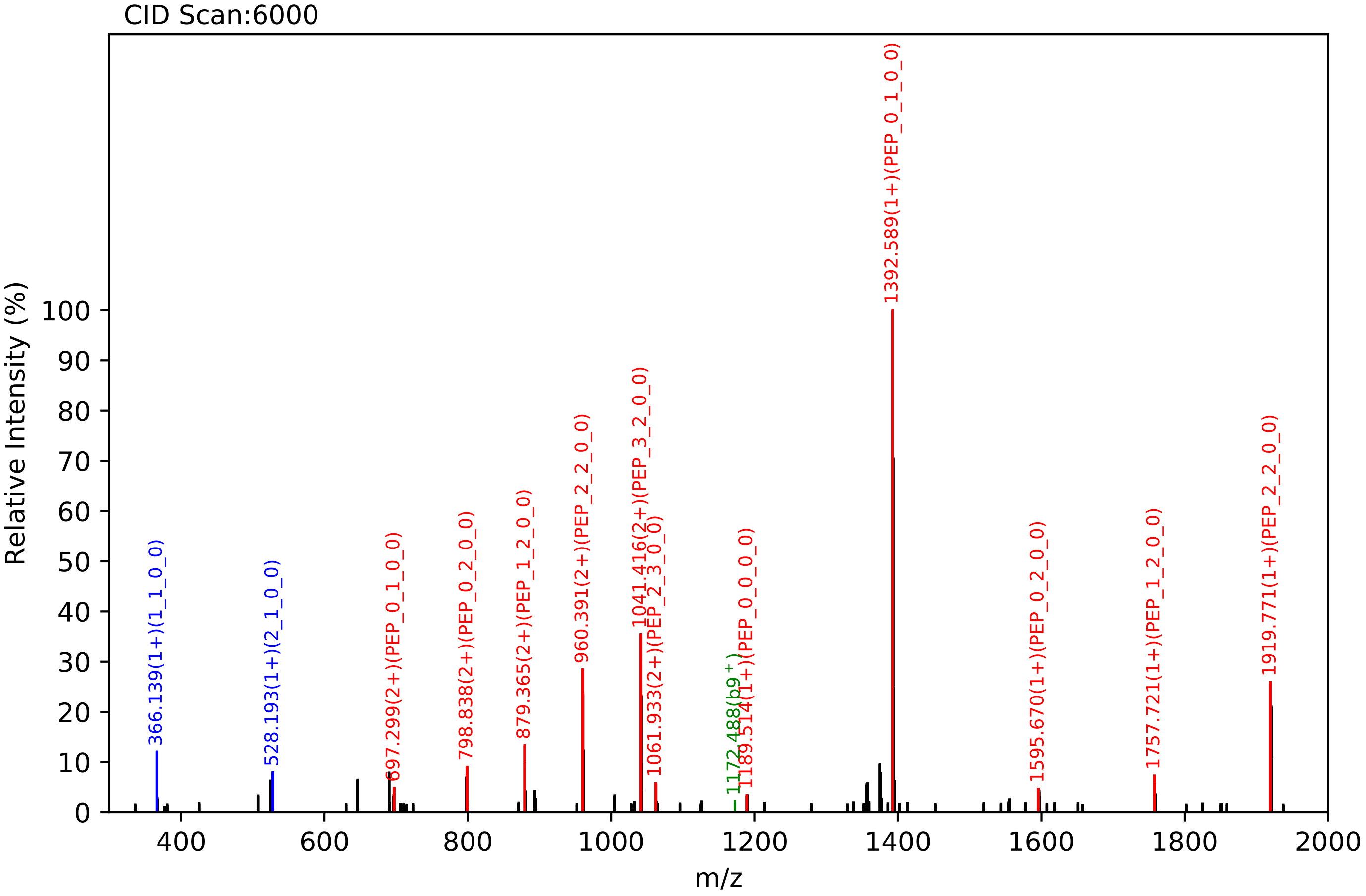

Test set no. 313, Experiment: IgG exp_7

EEQYNSTYR(=PEP)_3_3_1_0, m/z:1215.99(2+), RT:15.75, Y-score:89.01

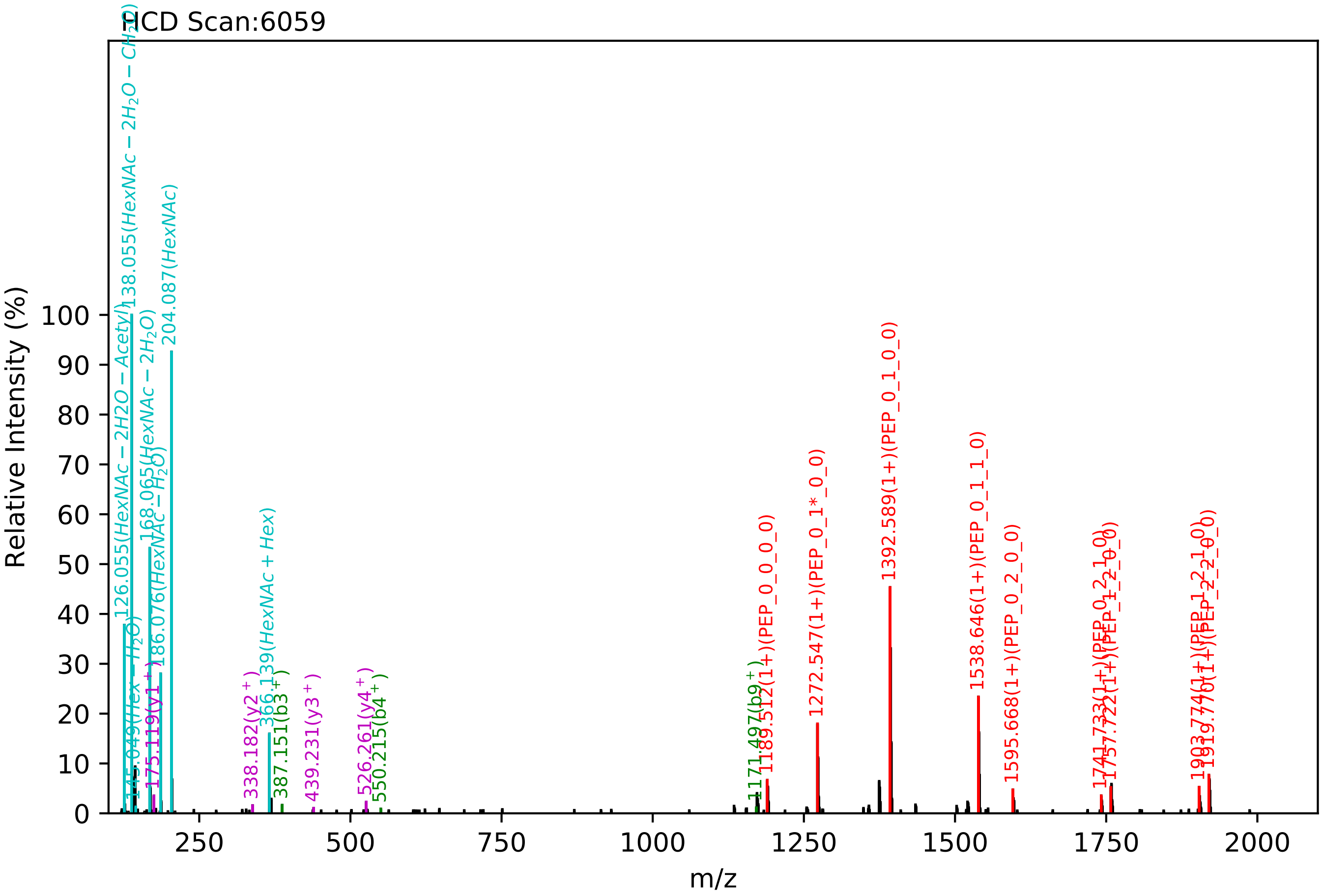

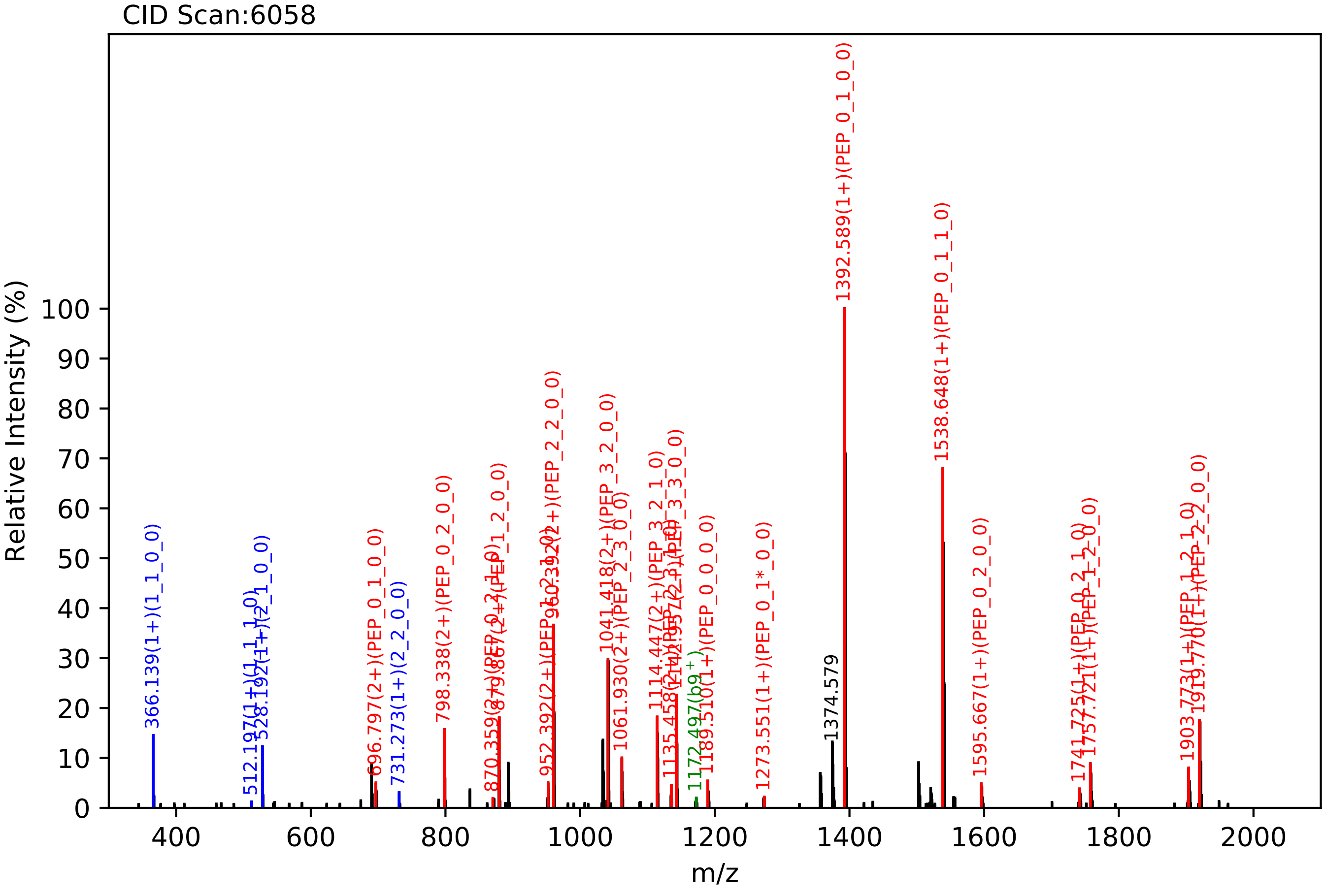

Test set no. 314, Experiment: IgG exp_7

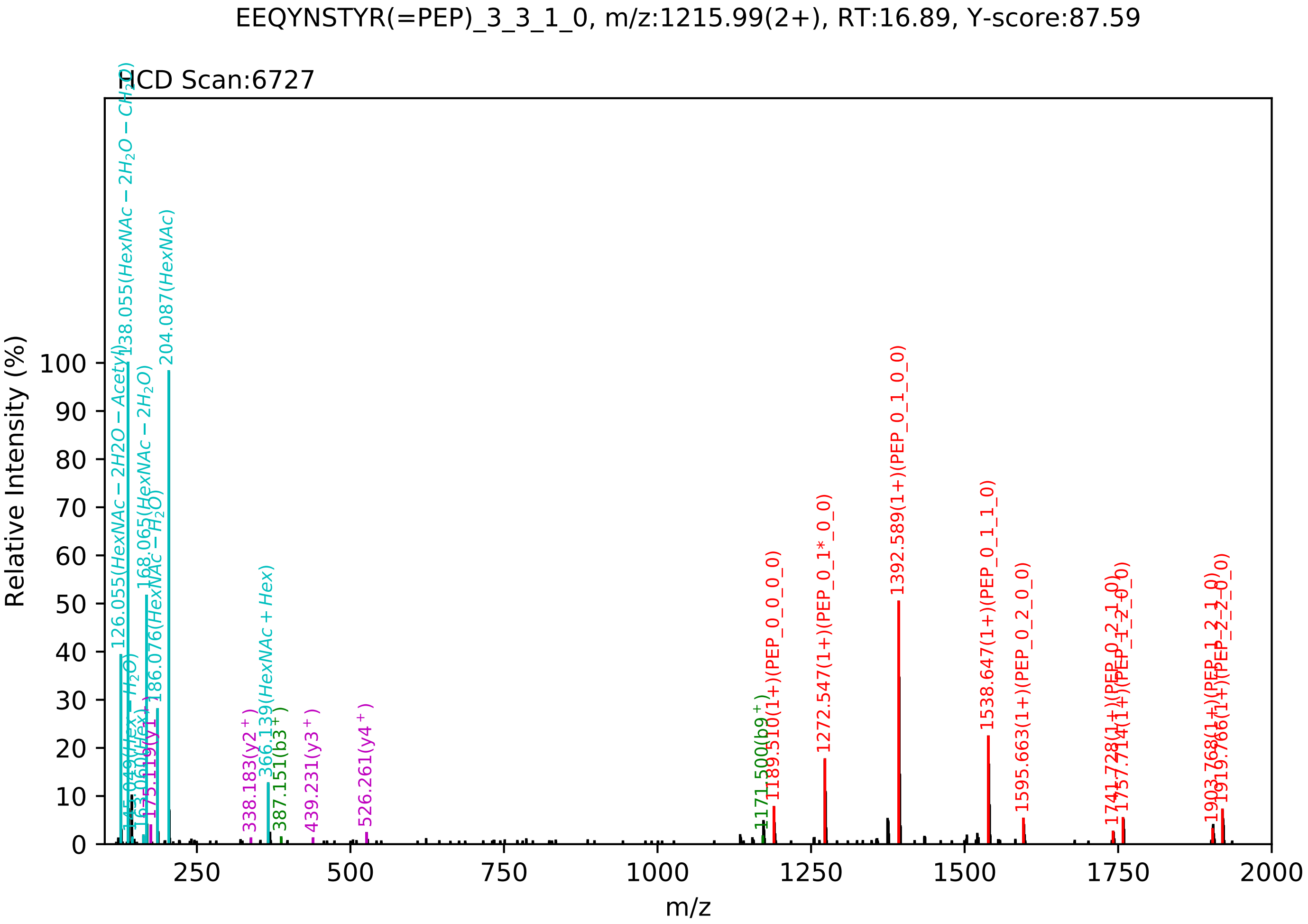

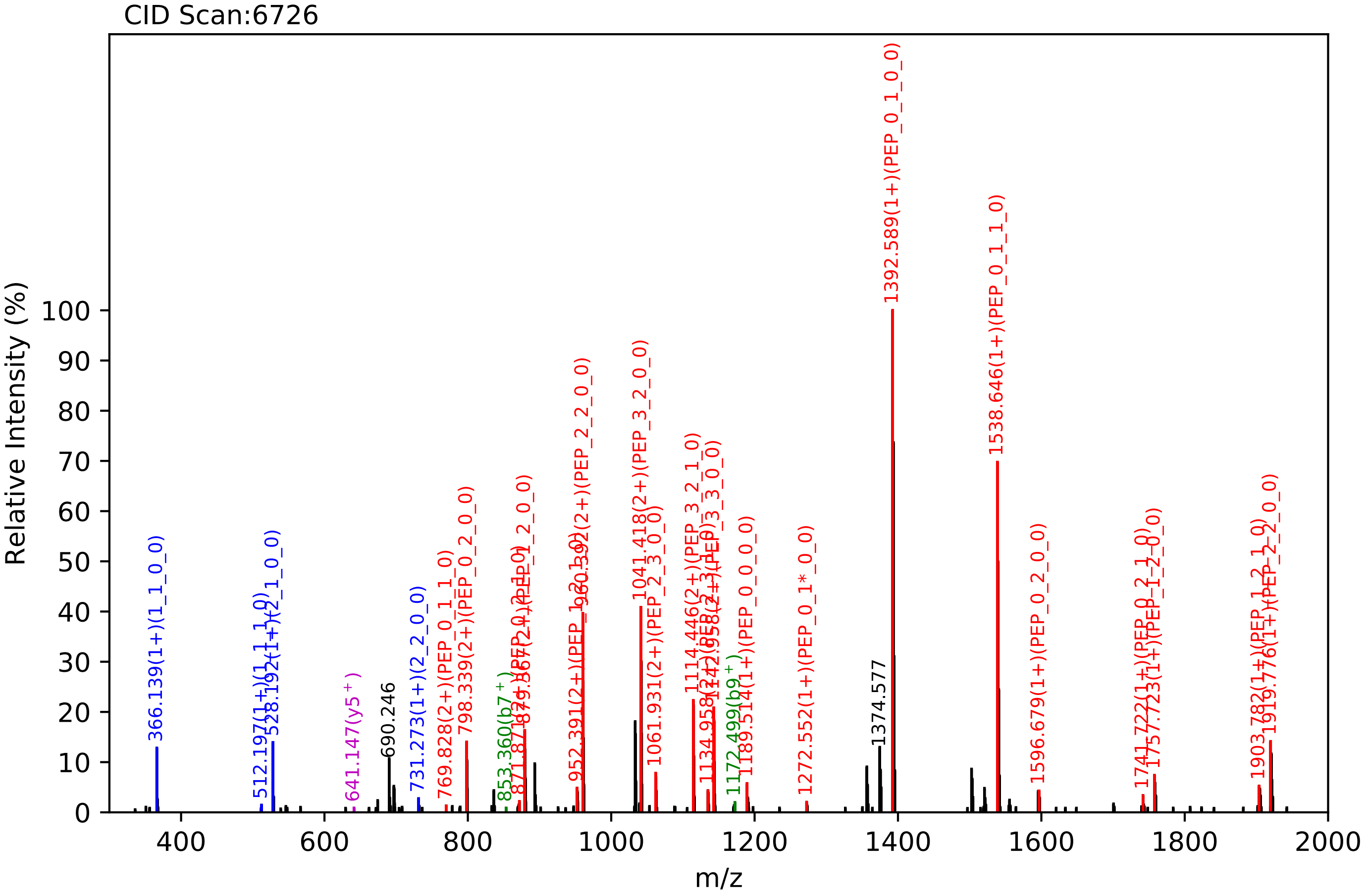

Test set no. 315, Experiment: IgG exp_7

EEQYNSTYR(=PEP)_3_3_1_0, m/z:1215.99(2+), RT:16.32, Y-score:87.51

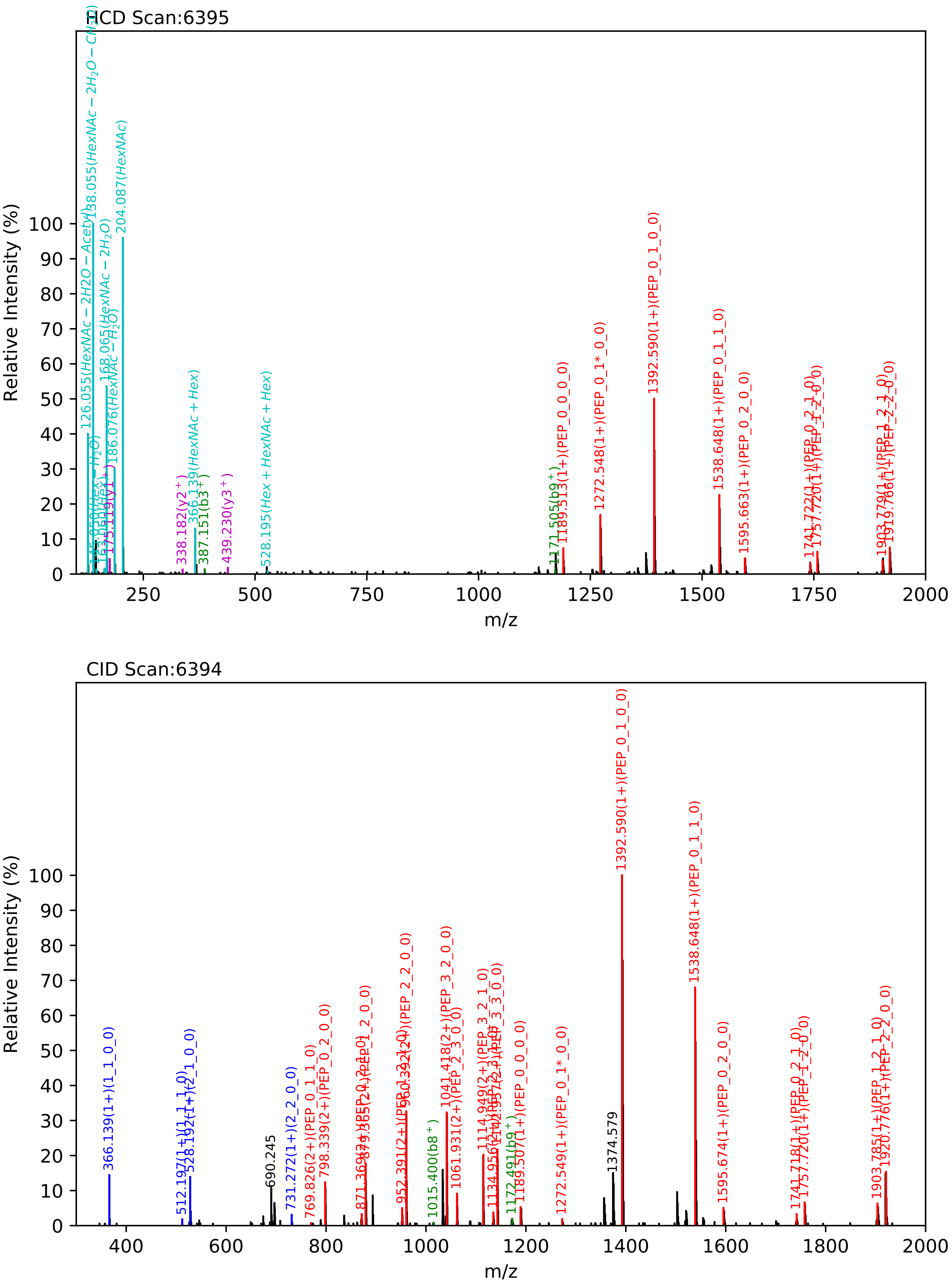

Test set no. 316, Experiment: IgG exp_7

EEQYNSTYR(=PEP)_3_3_1_0, m/z:1215.99(2+), RT:17.40, Y-score:85.23

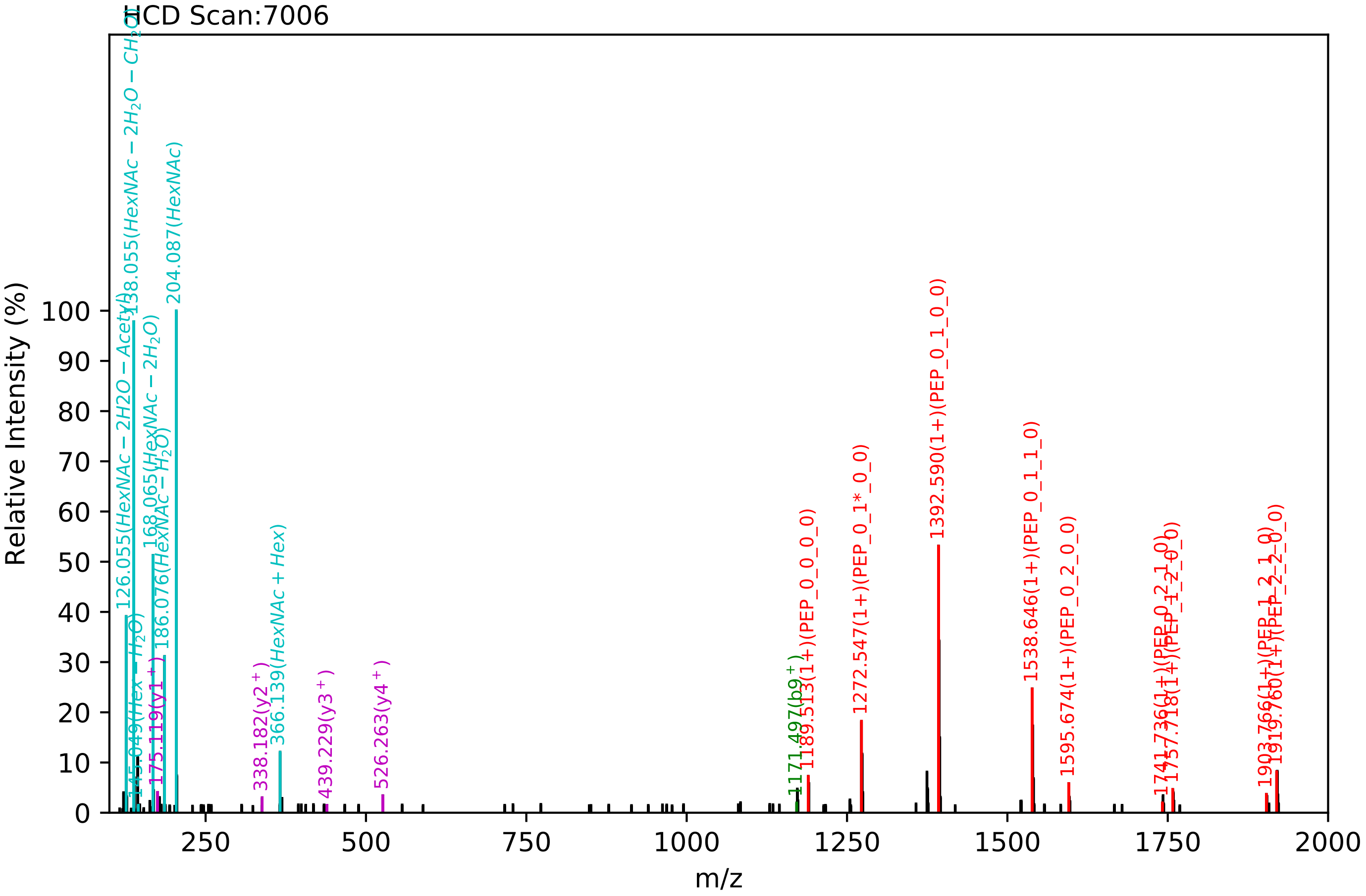

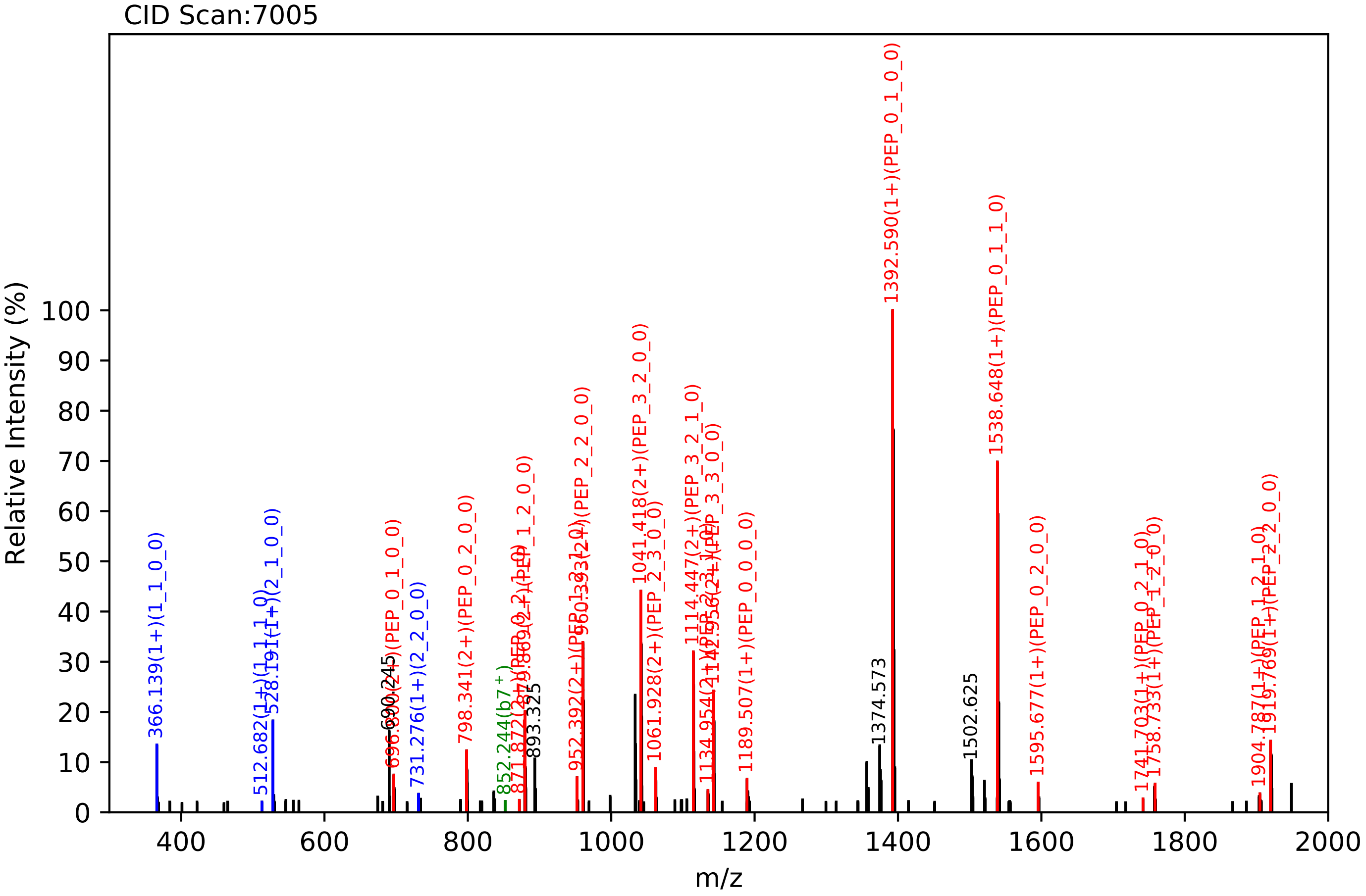

Test set no. 317, Experiment: IgG exp_6

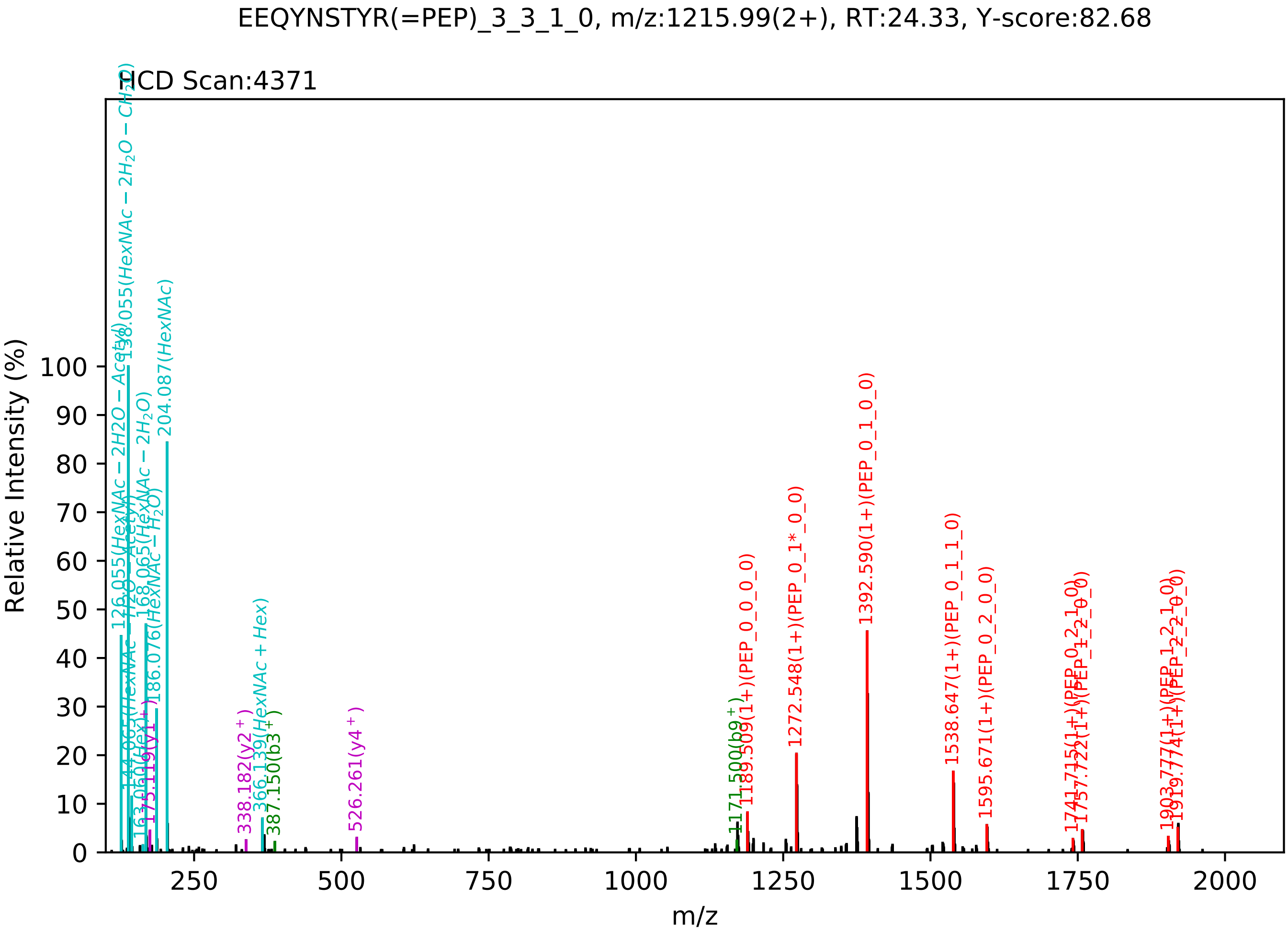

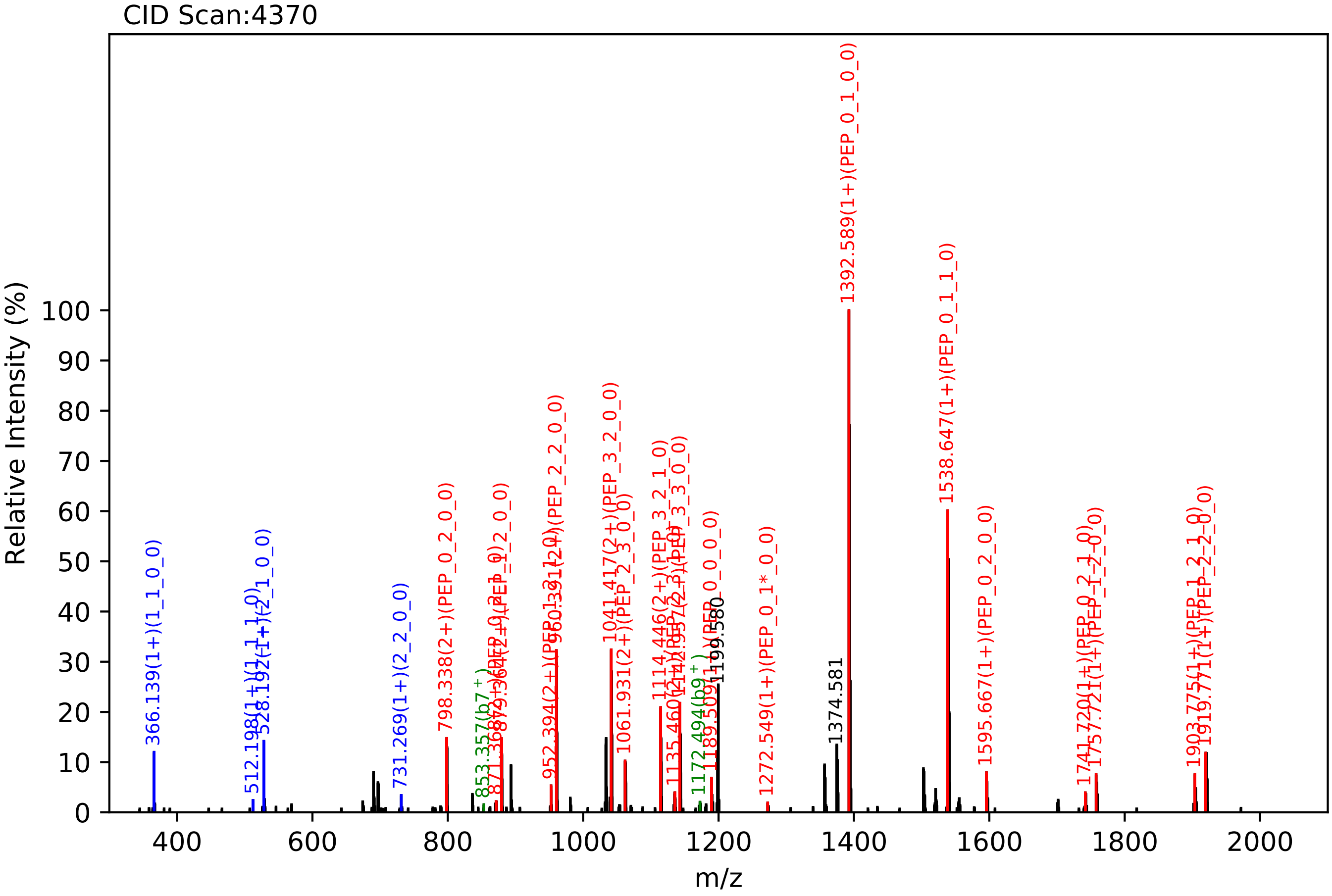

Test set no. 318, Experiment: IgG exp_7

EEQYNSTYR(=PEP)_3_4_0_0, m/z:1244.50(2+), RT:16.18, Y-score:87.19

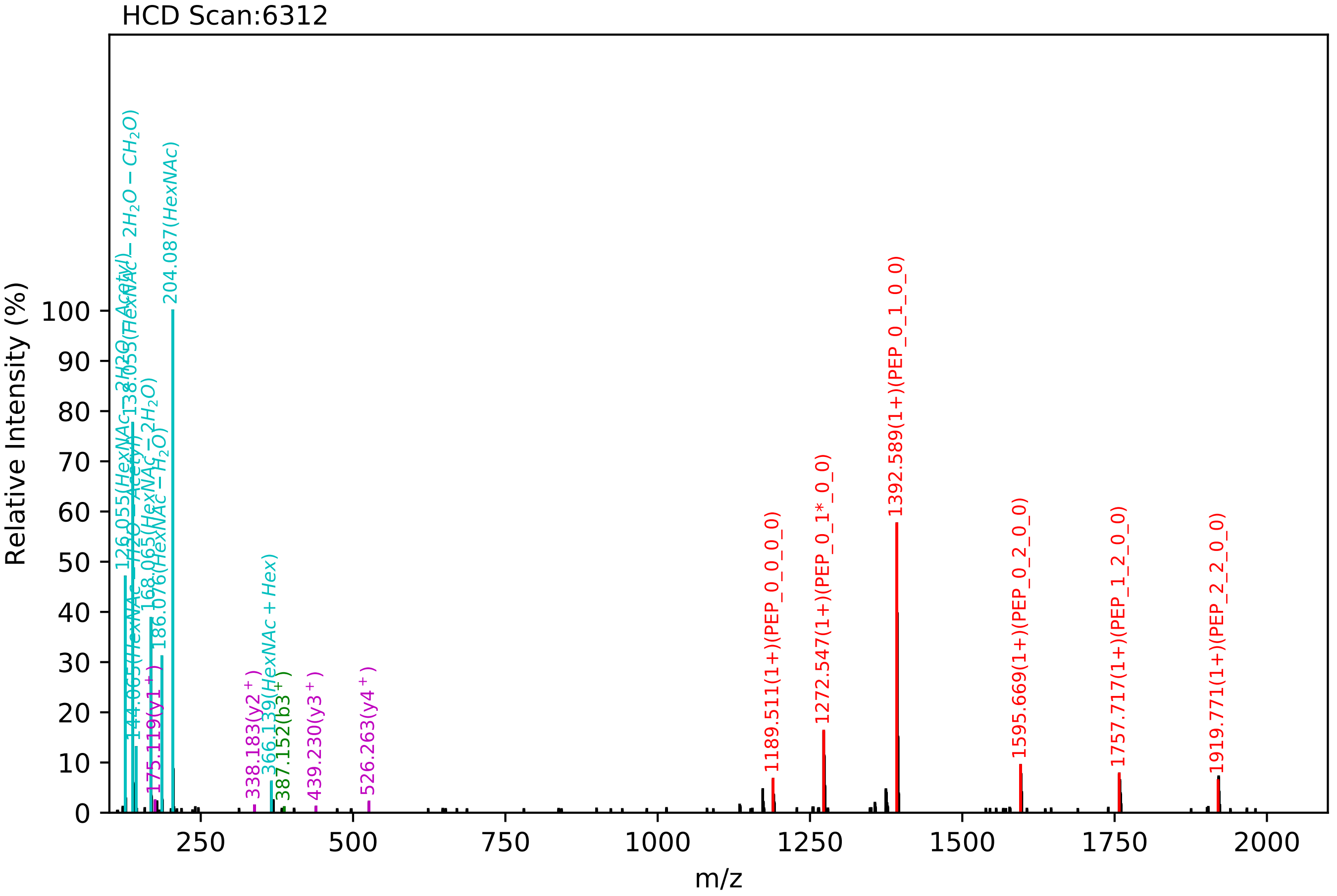

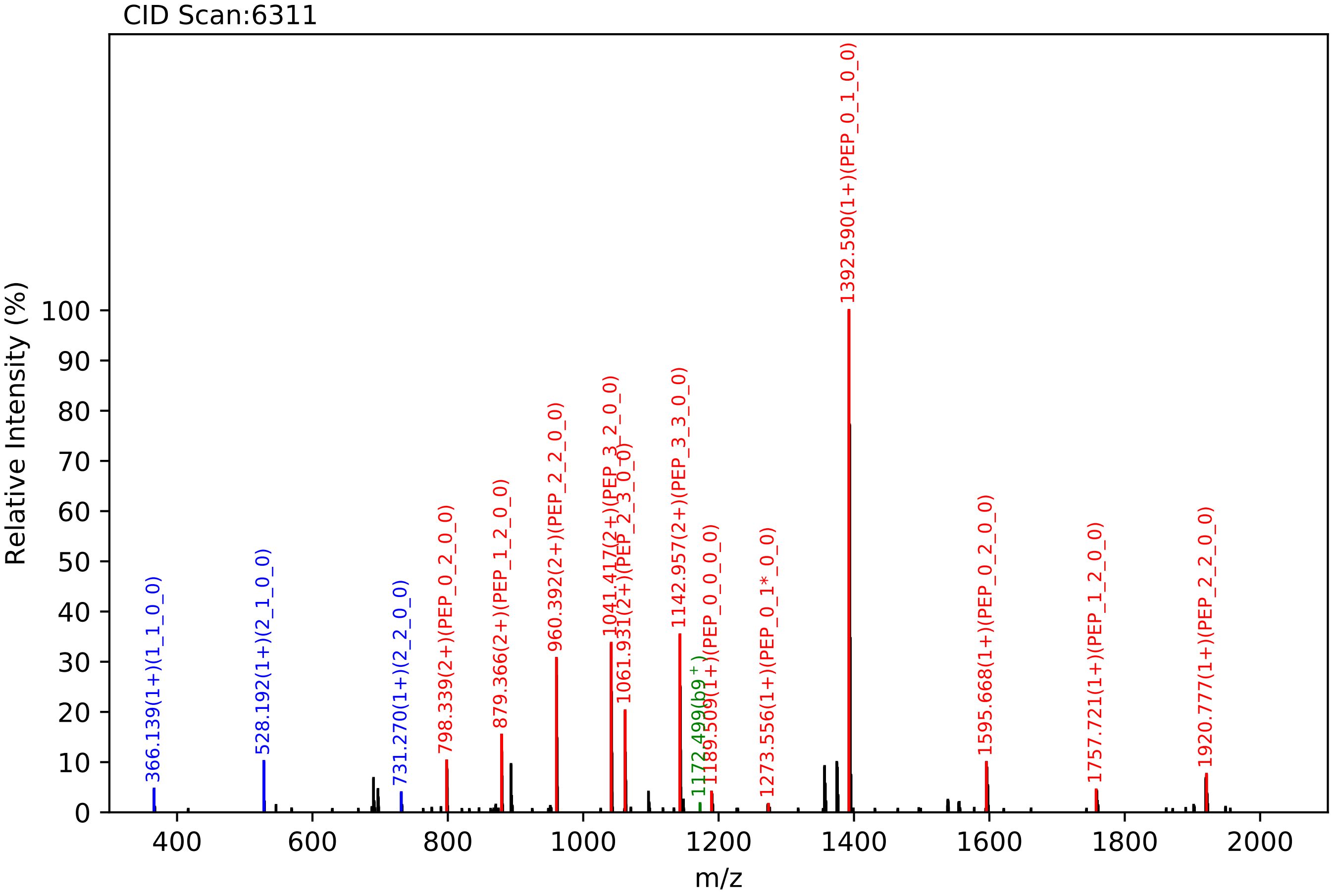

Test set no. 319, Experiment: IgG exp_7

EEQYNSTYR(=PEP)_3_4_0_0, m/z:1244.50(2+), RT:16.72, Y-score:86.96

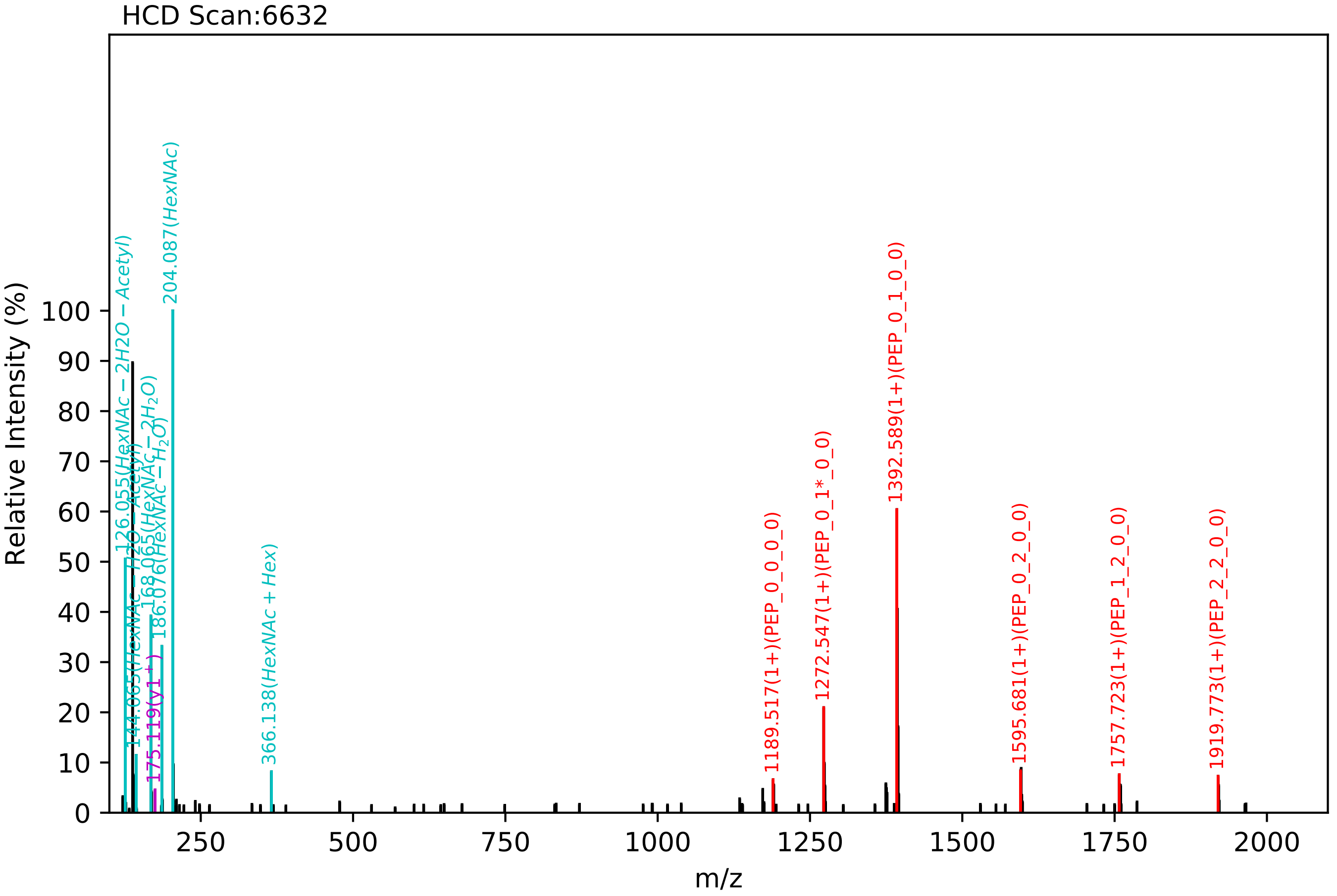

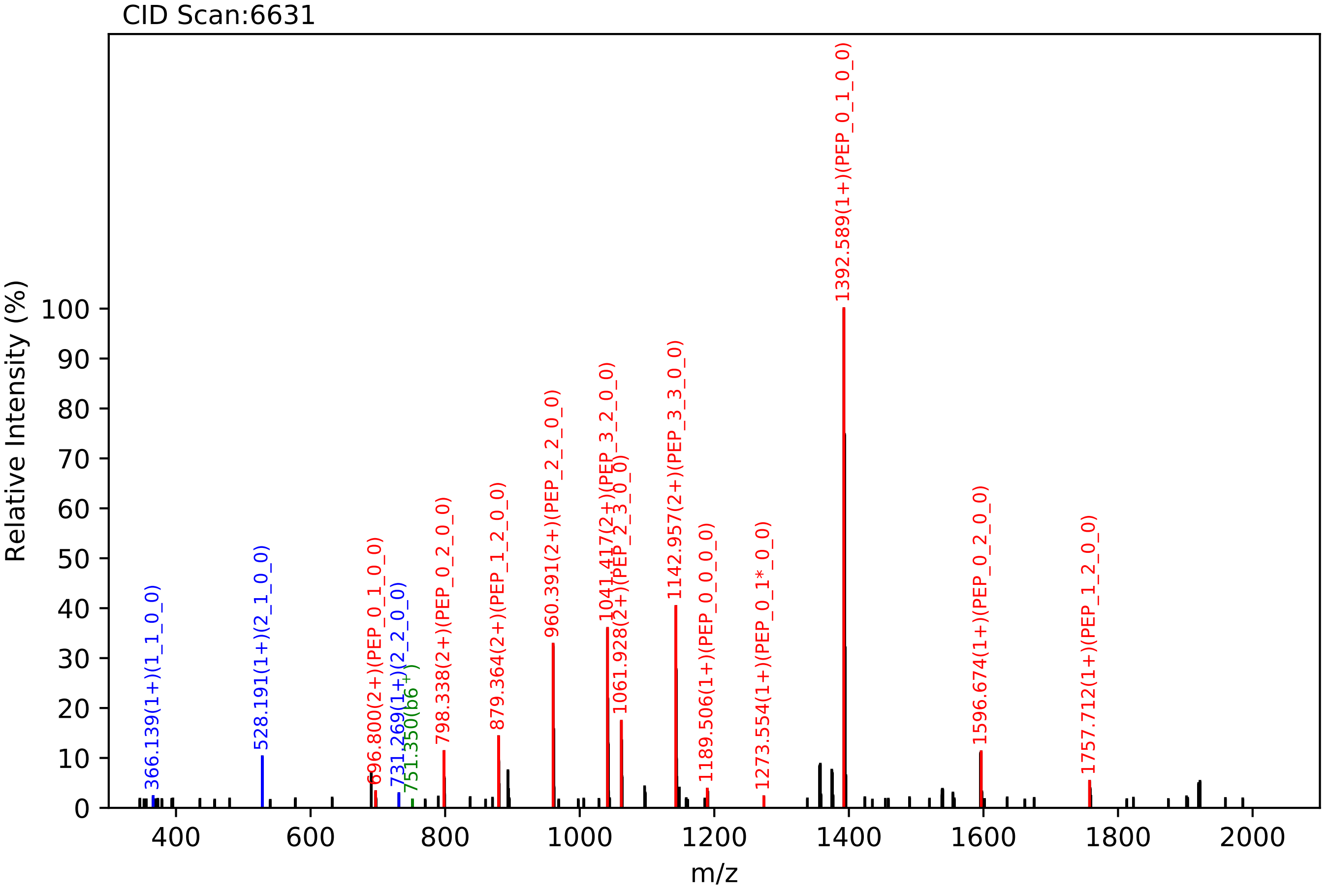

Test set no. 320, Experiment: IgG exp_7

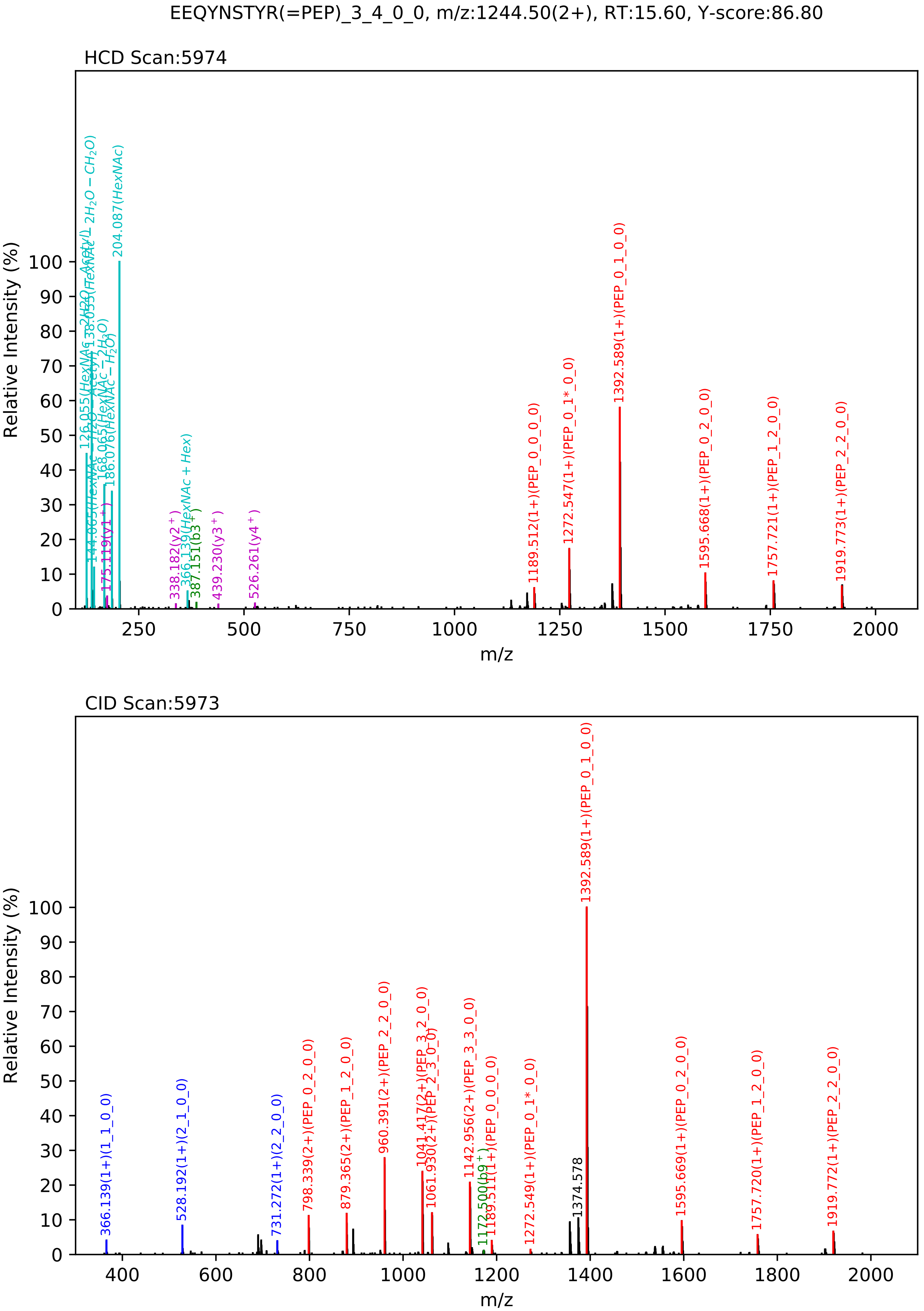

Test set no. 321, Experiment: IgG exp_6

EEQYNSTYR(=PEP)_3_4_0_0, m/z:1244.50(2+), RT:24.37, Y-score:84.47

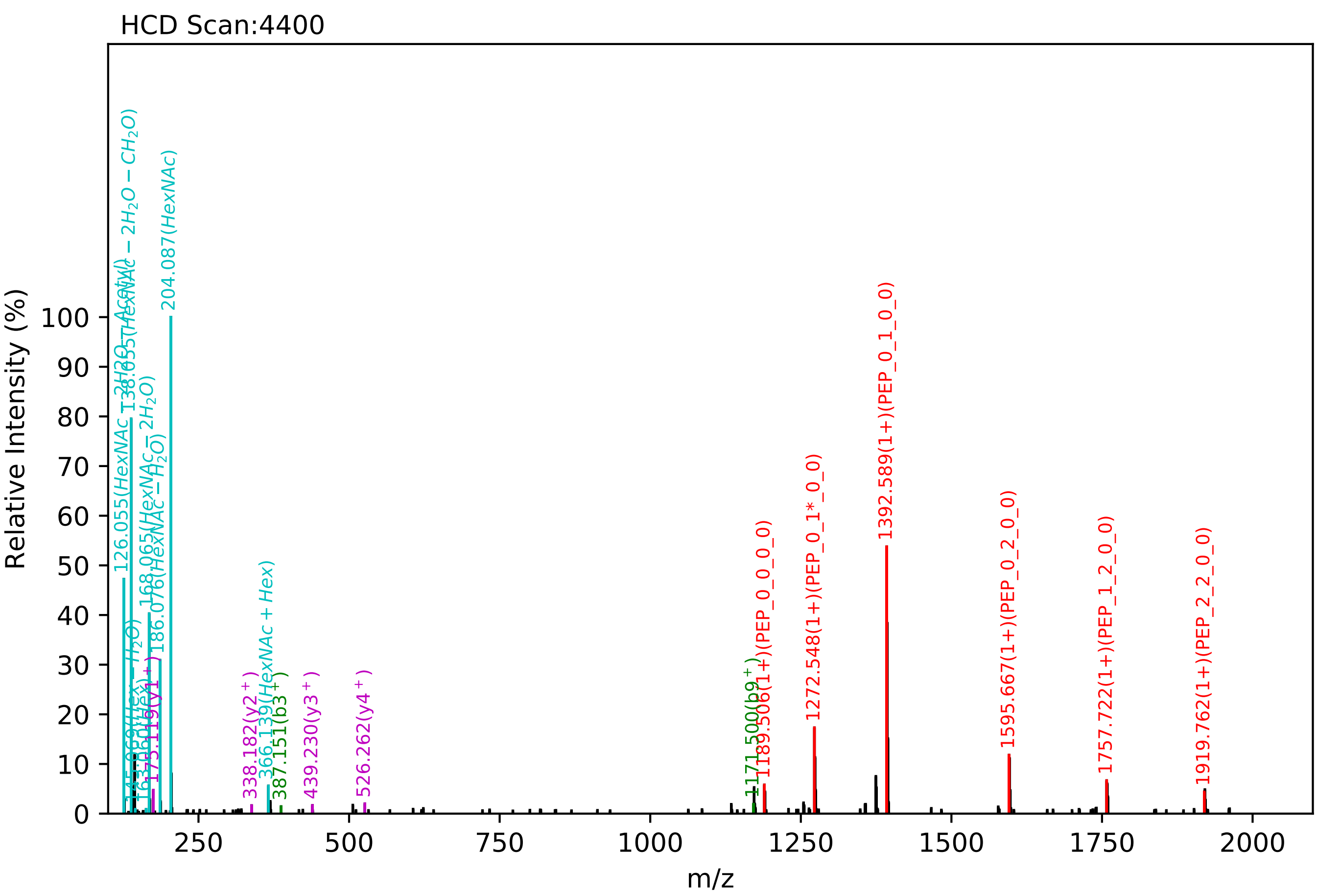

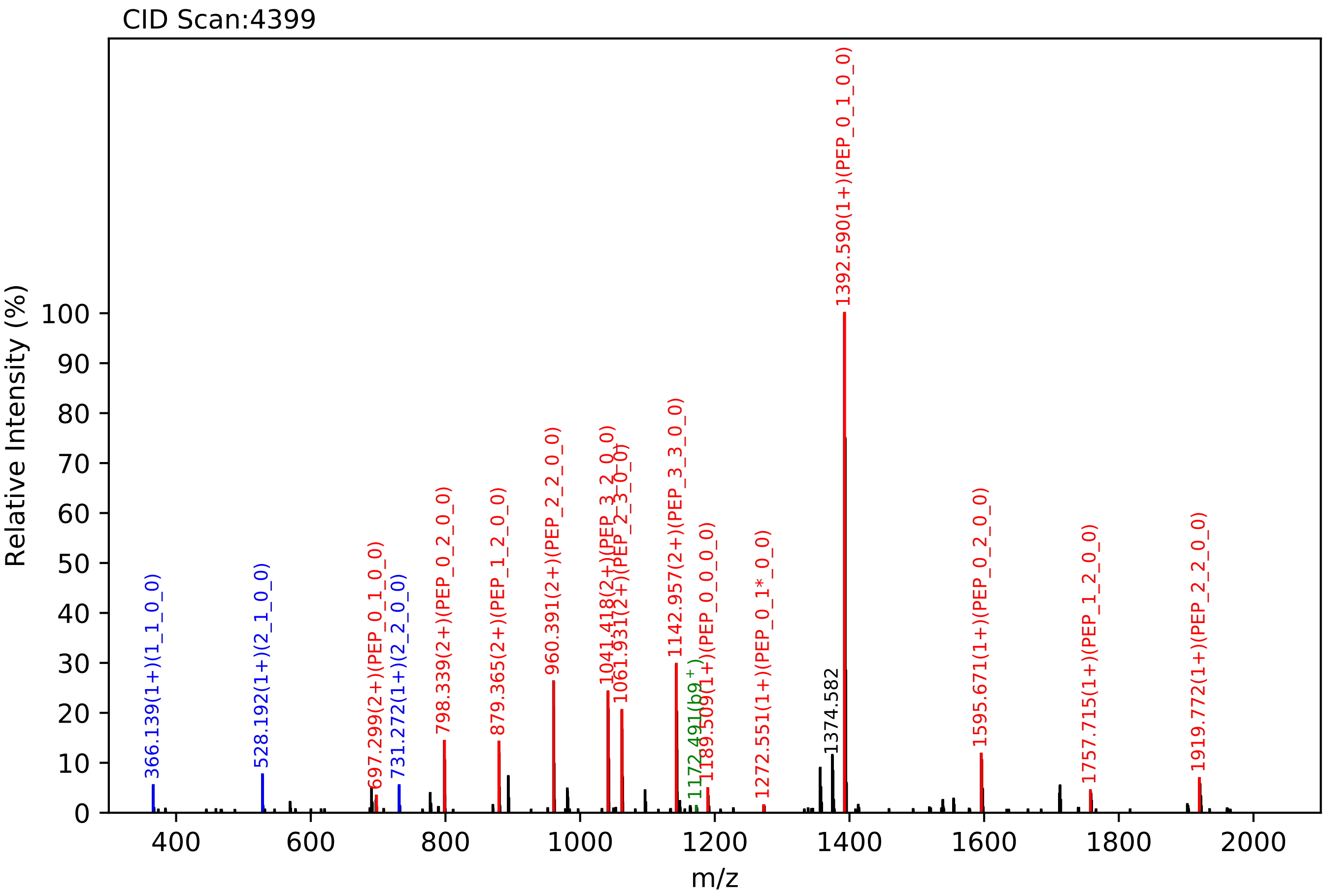

Test set no. 322, Experiment: IgG exp_7

EEQYNSTYR(=PEP)_3_4_1_0, m/z:1317.53(2+), RT:19.49, Y-score:90.24

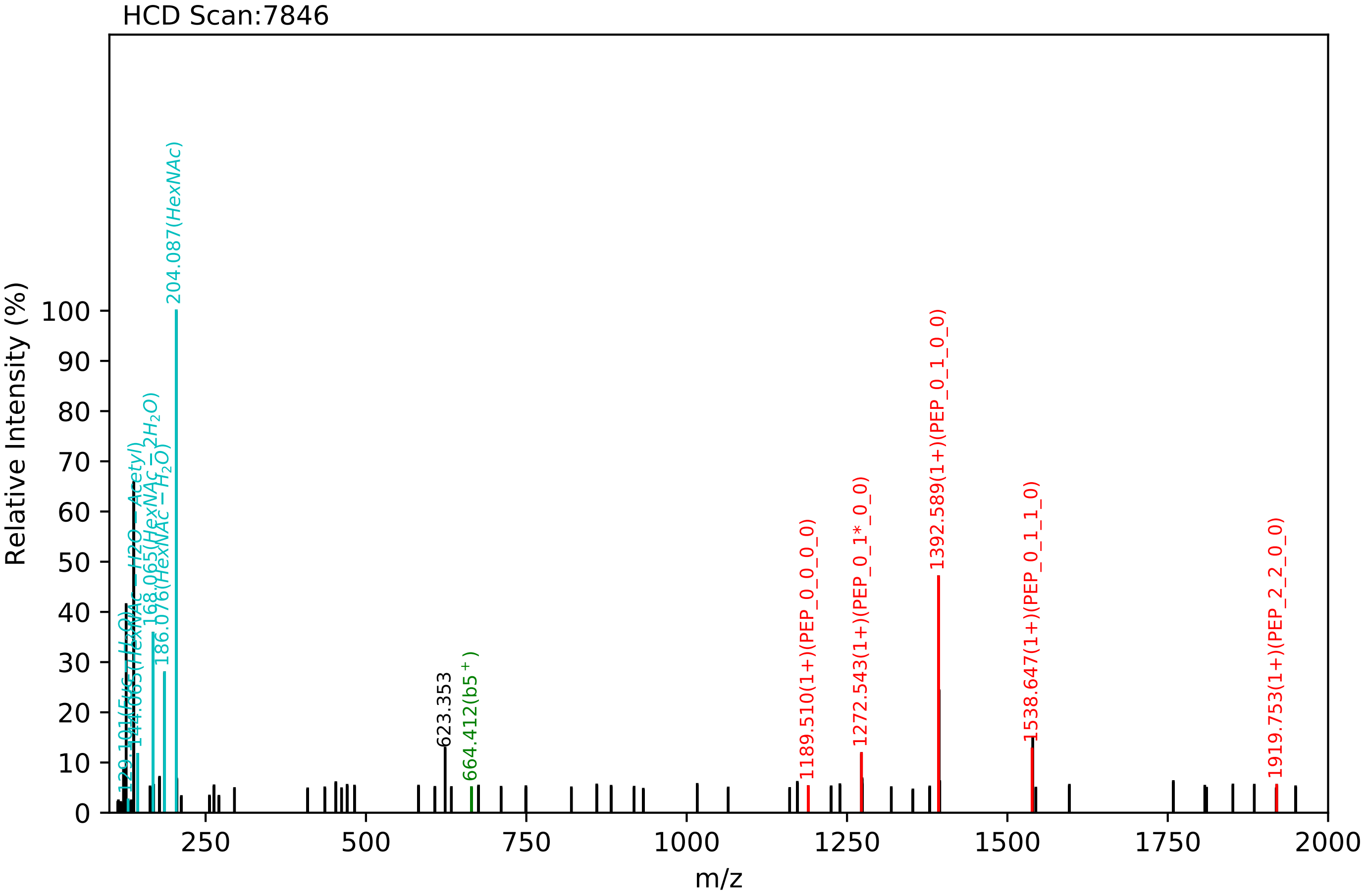

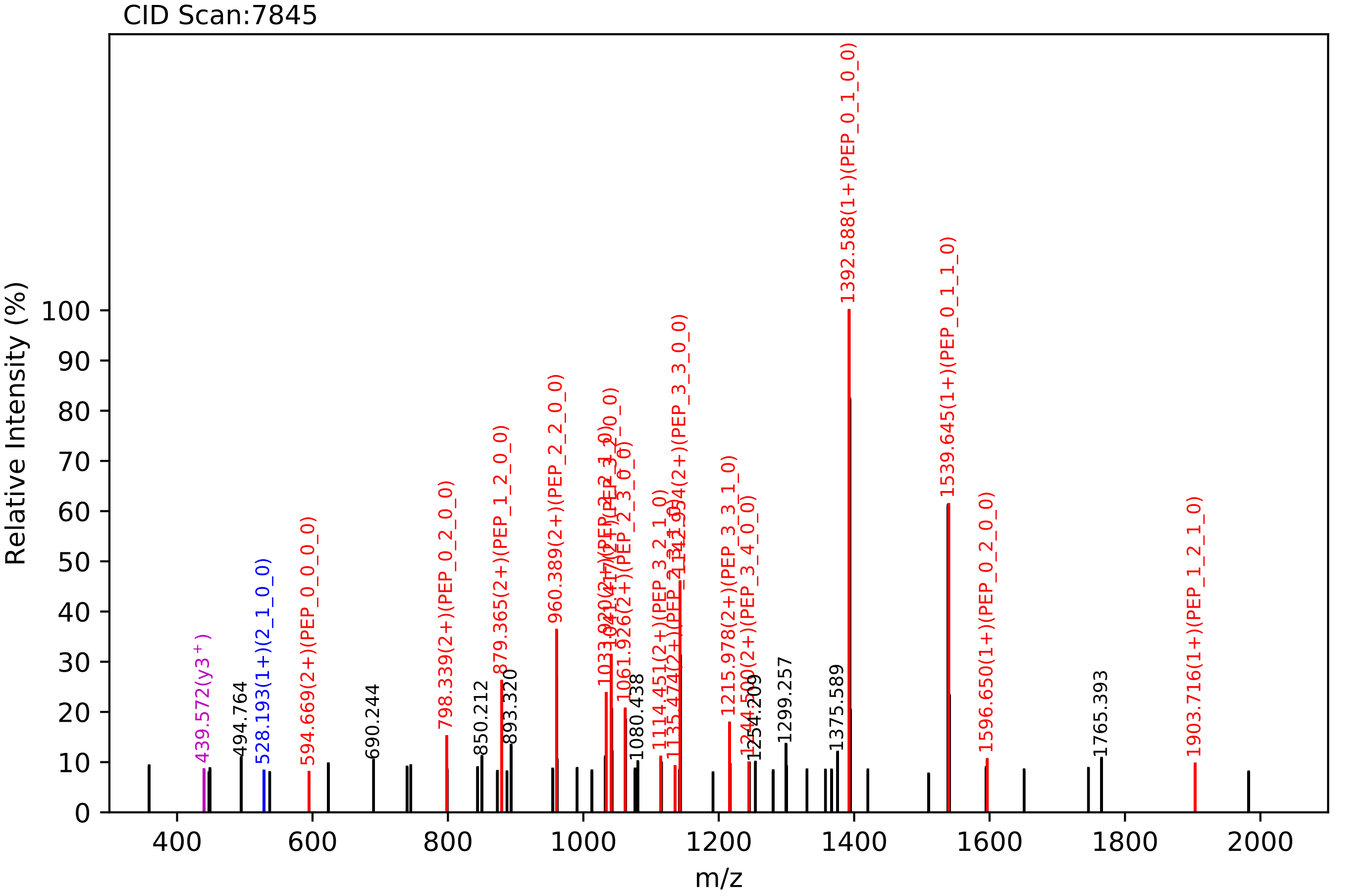

Test set no. 323, Experiment: IgG exp_7

EEQYNSTYR(=PEP)_3_4_1_0, m/z:1317.53(2+), RT:14.20, Y-score:89.94

HCD Scan:5296

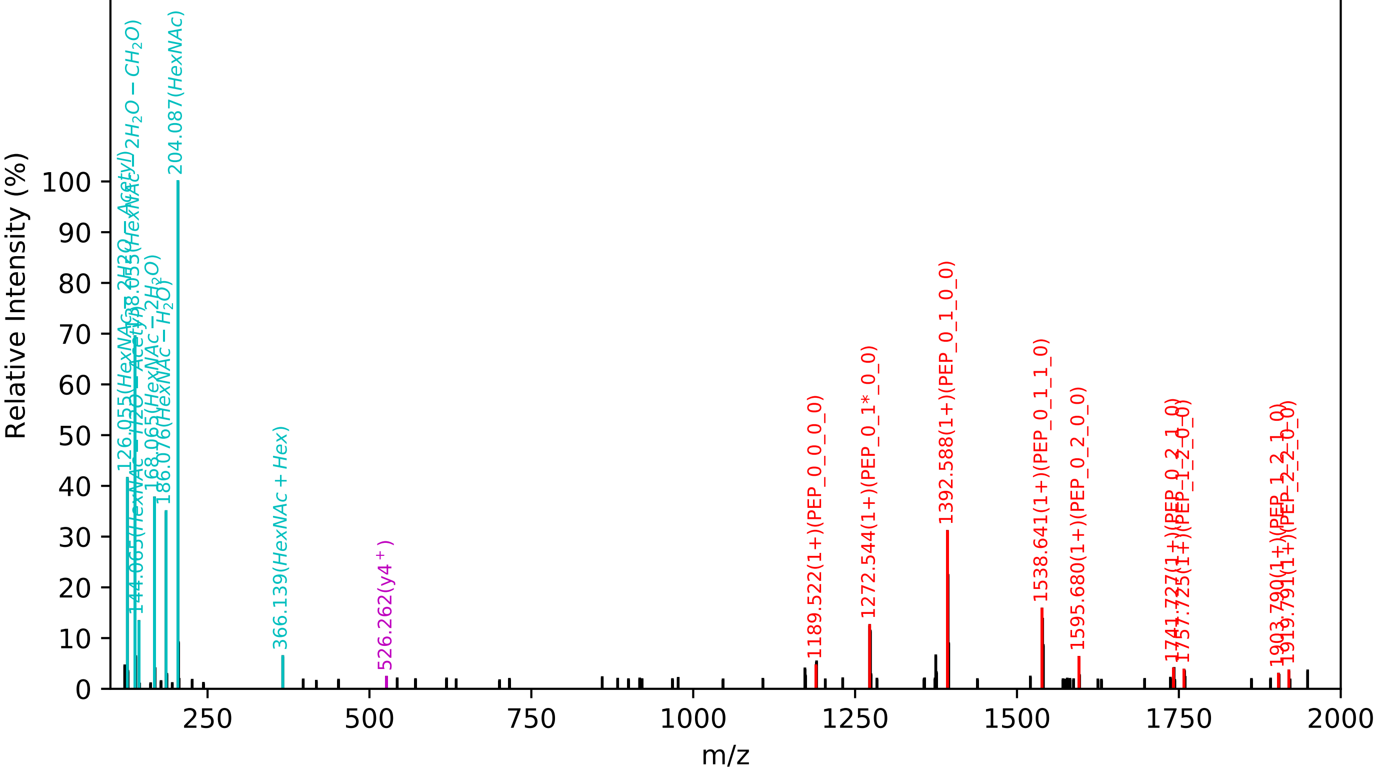

CID Scan:5295

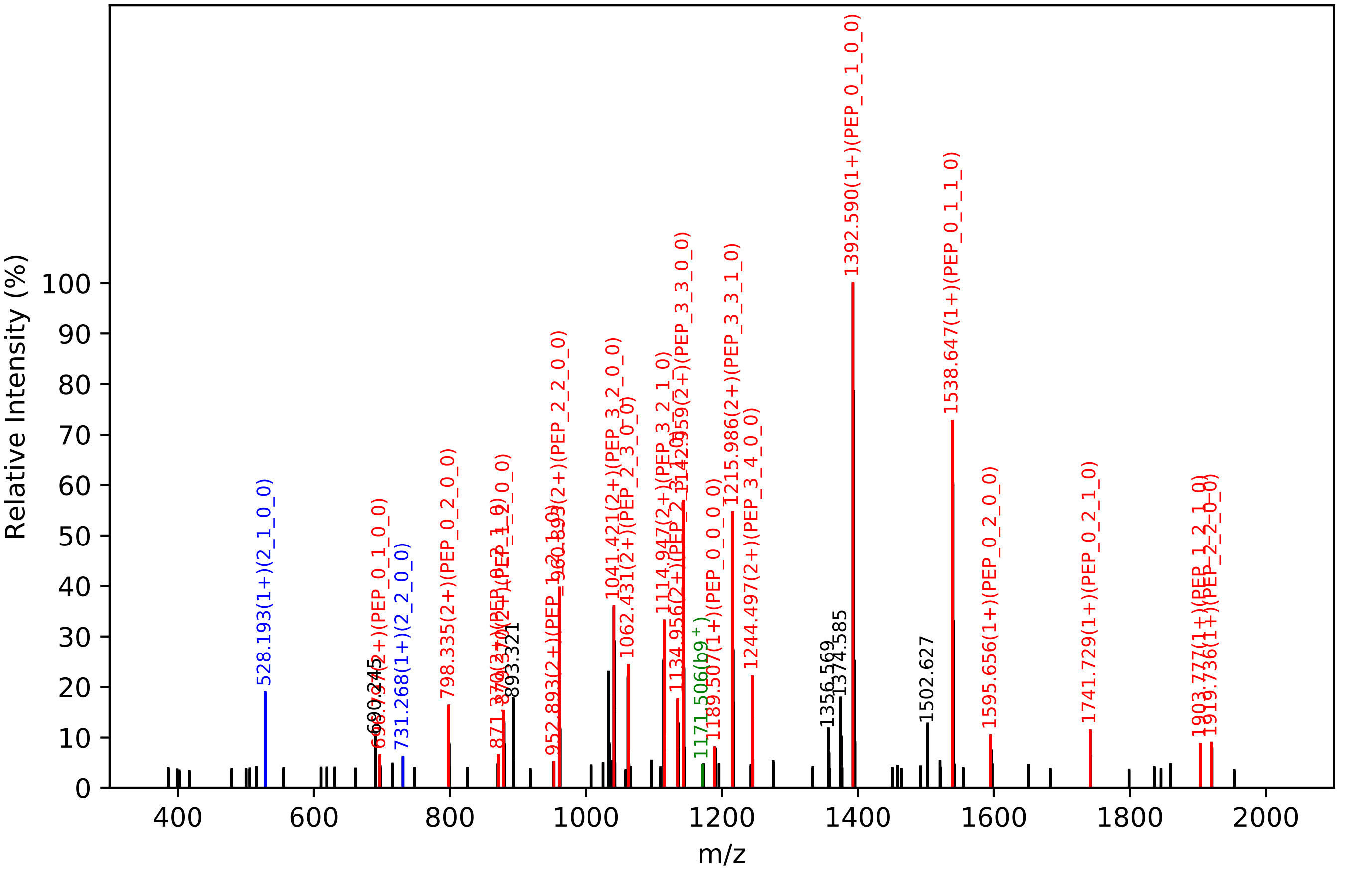

Test set no. 324, Experiment: IgG exp_7

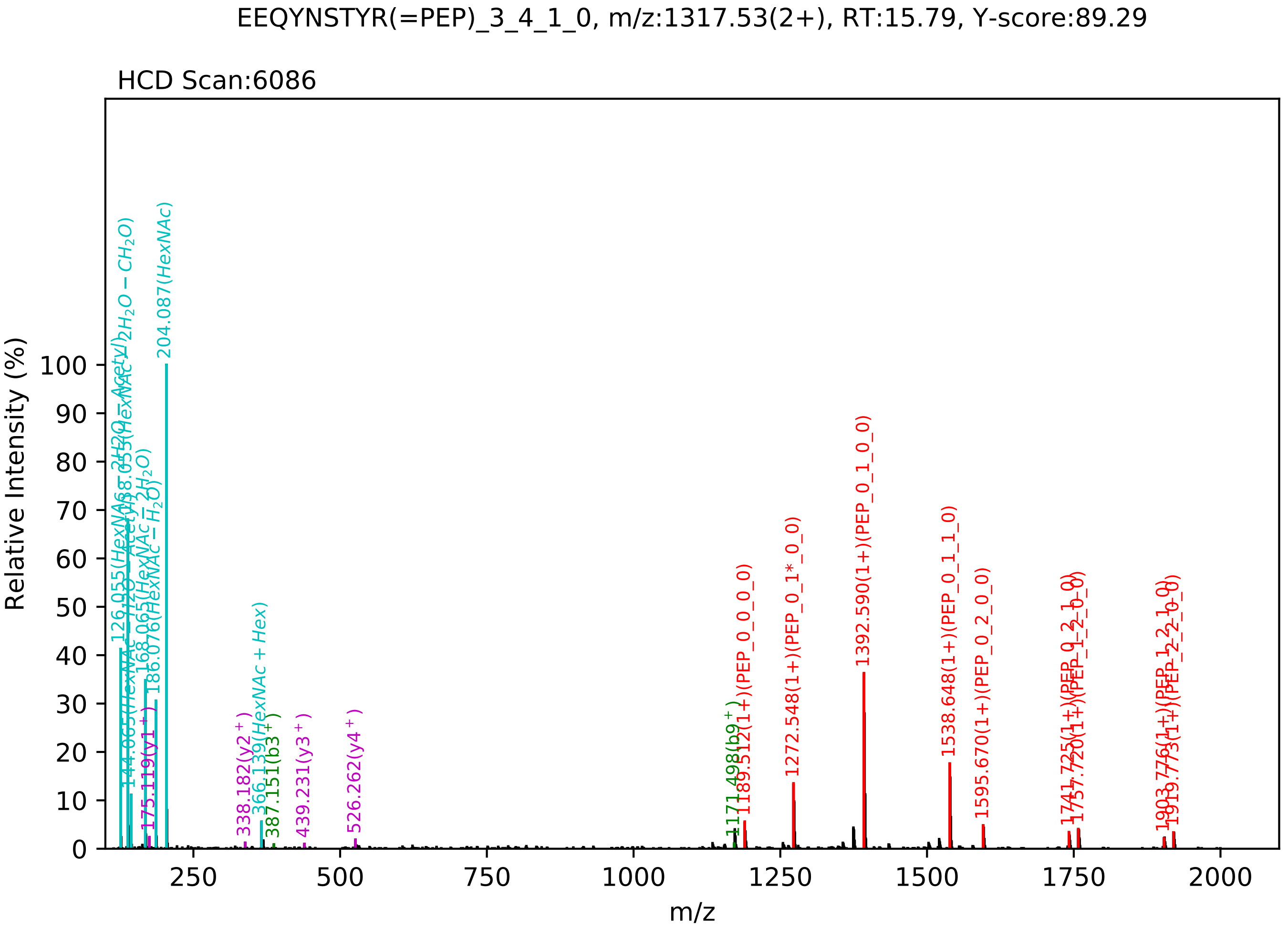

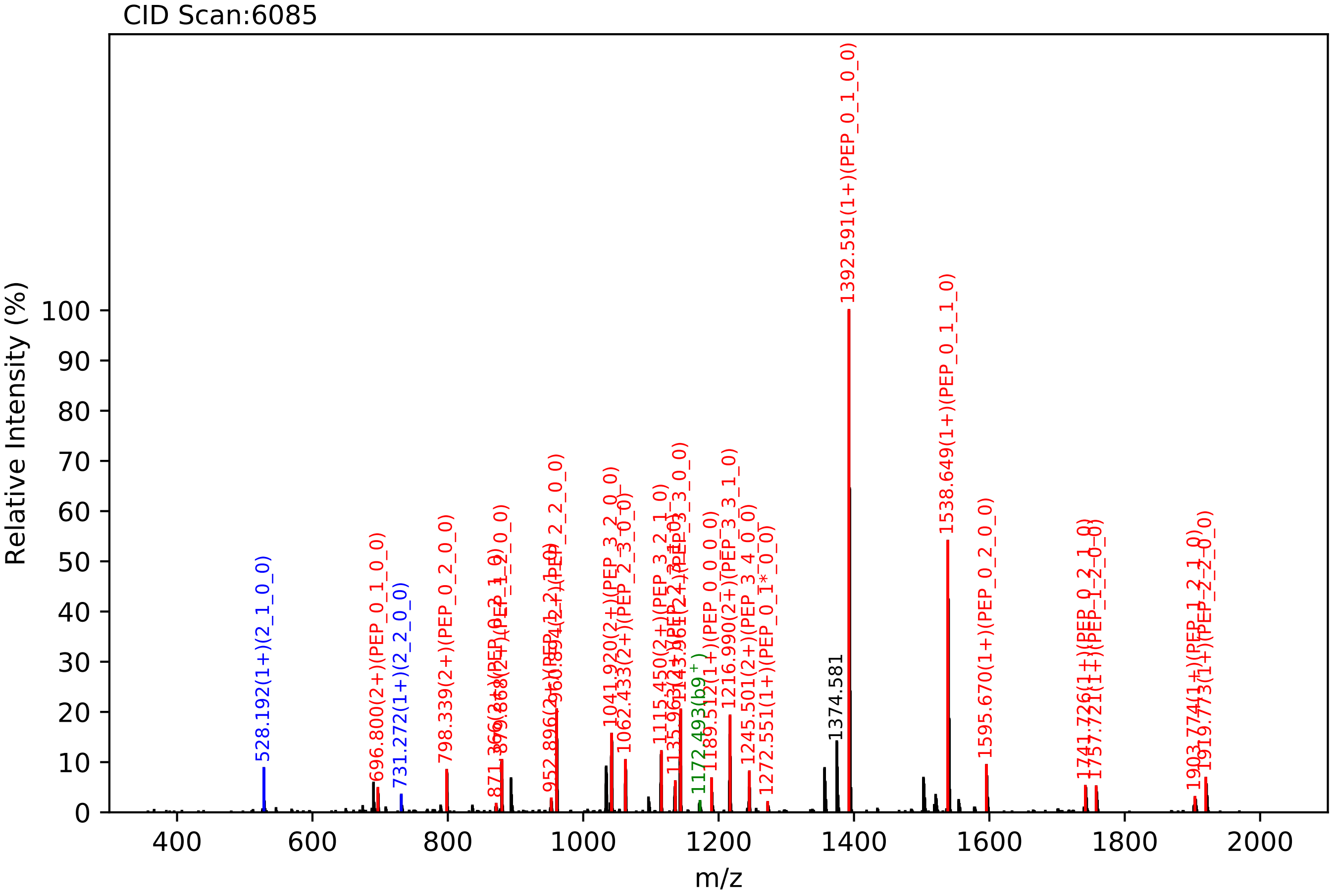

Test set no. 325, Experiment: IgG exp_7

EEQYNSTYR(=PEP)_3_4_1_0, m/z:1317.53(2+), RT:16.36, Y-score:89.19

HCD Scan:6422

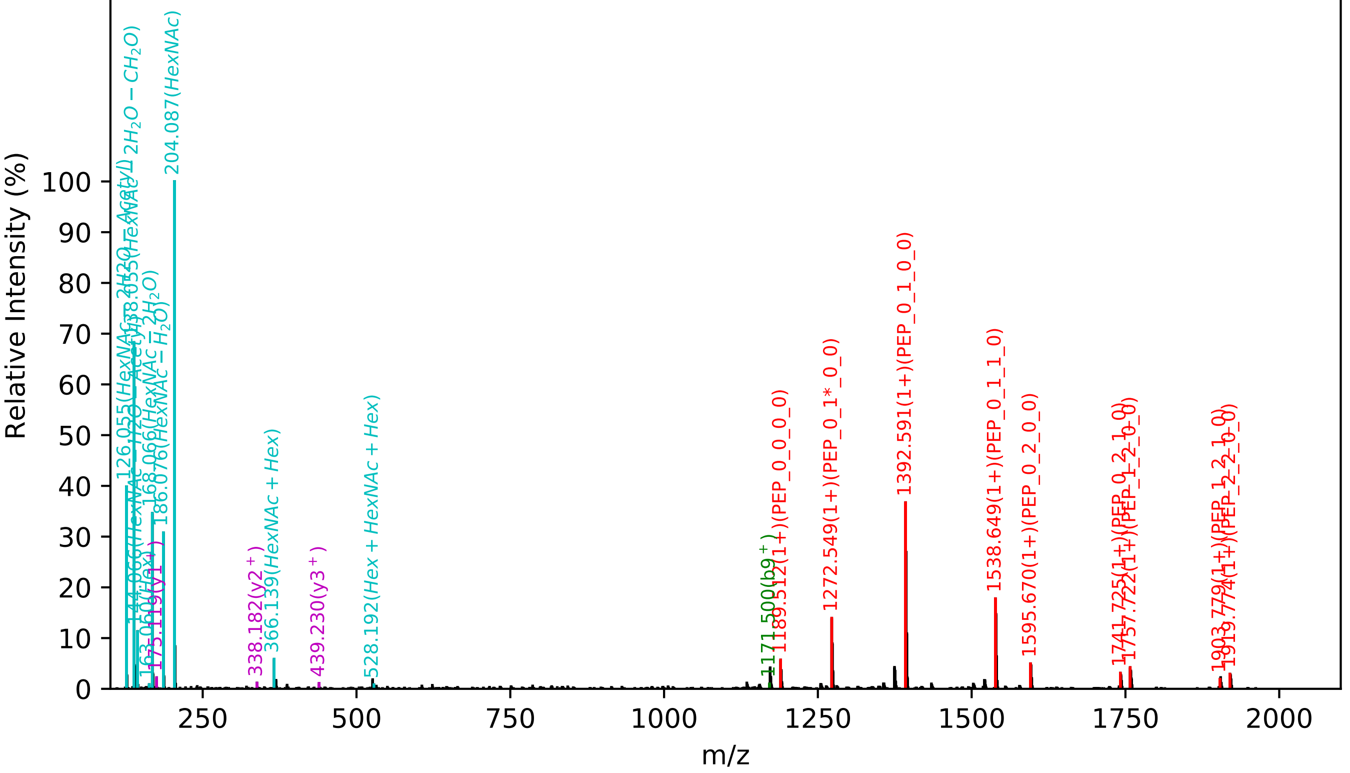

CID Scan:6421

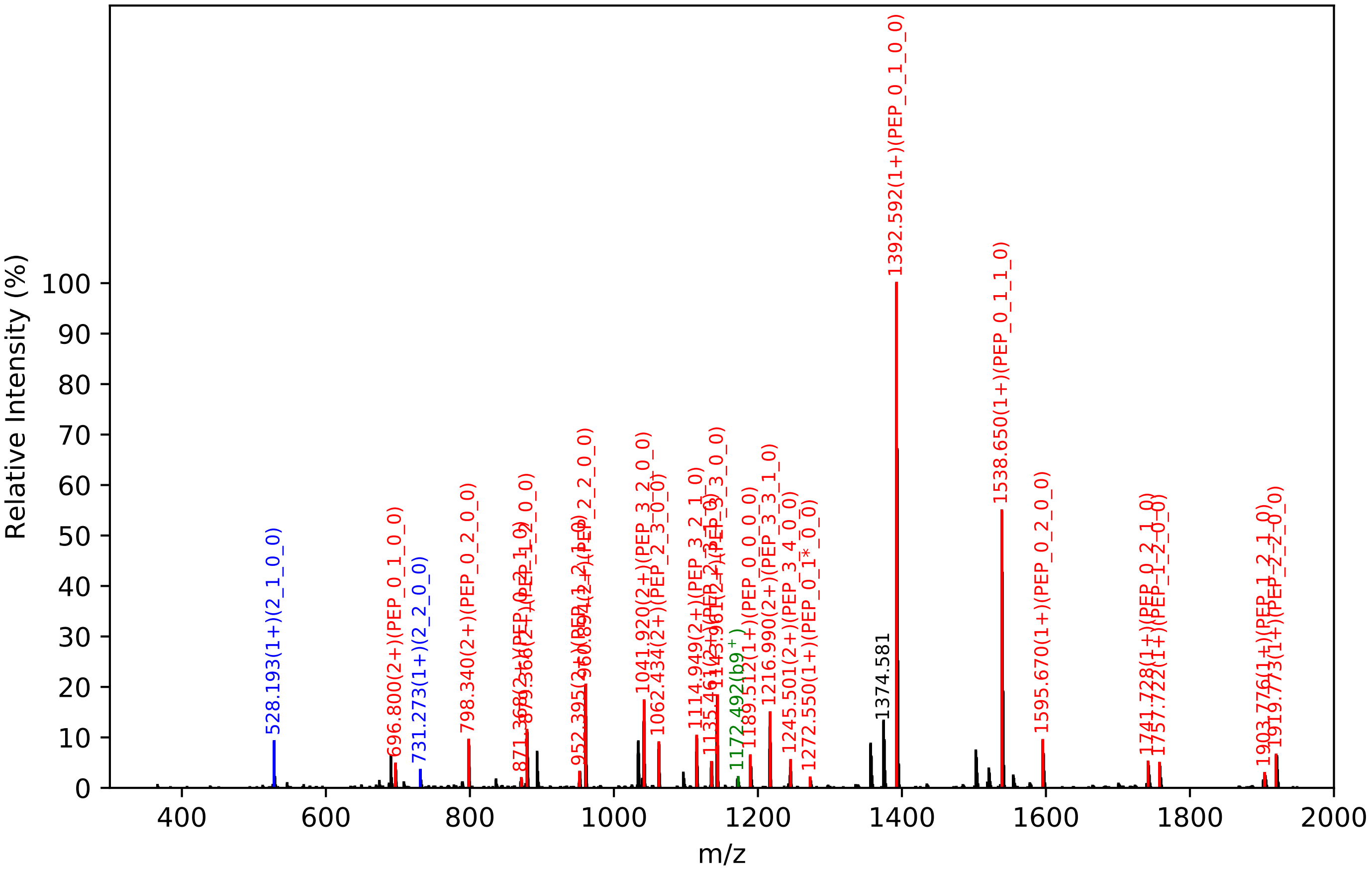

Test set no. 326, Experiment: IgG exp_7

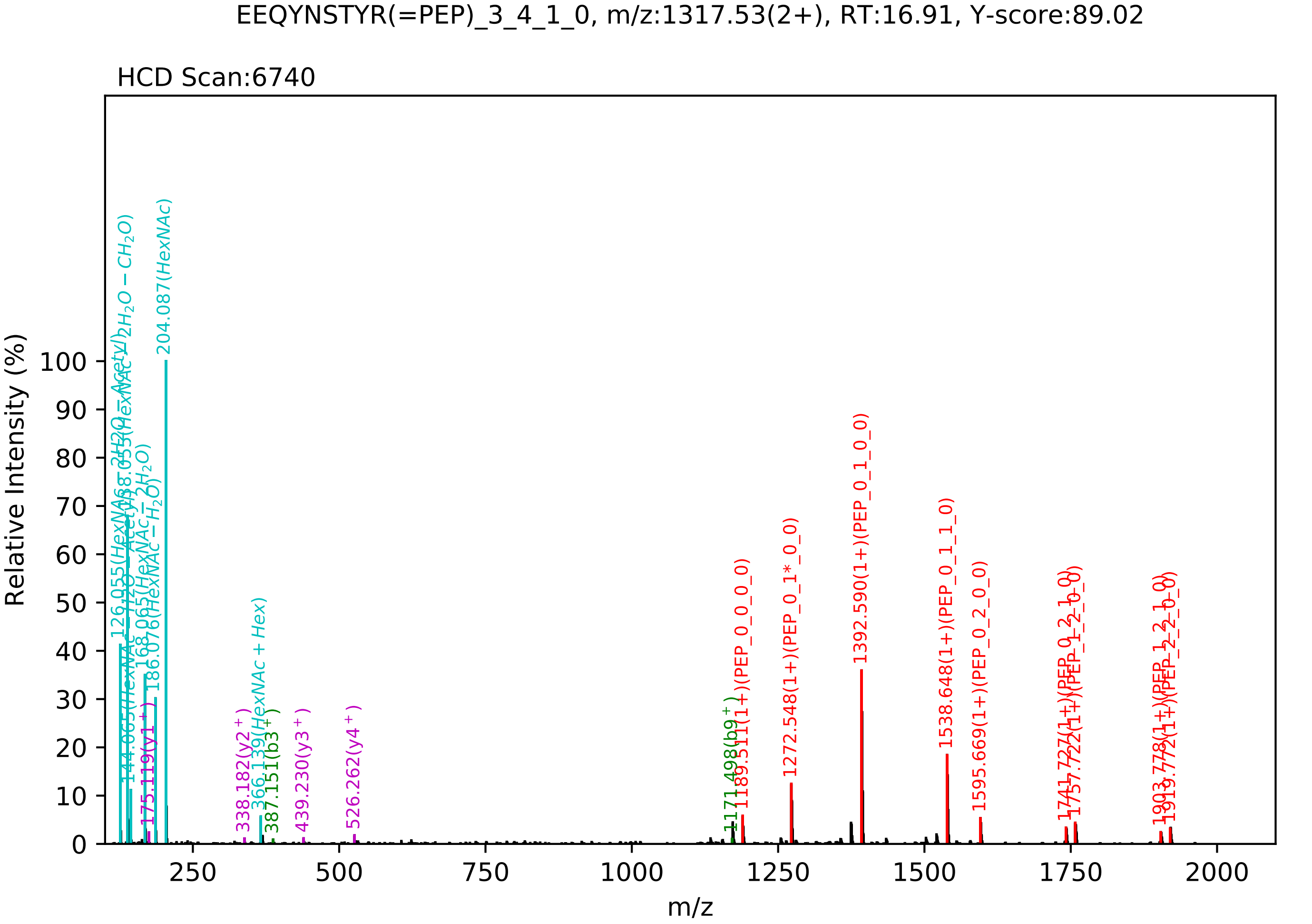

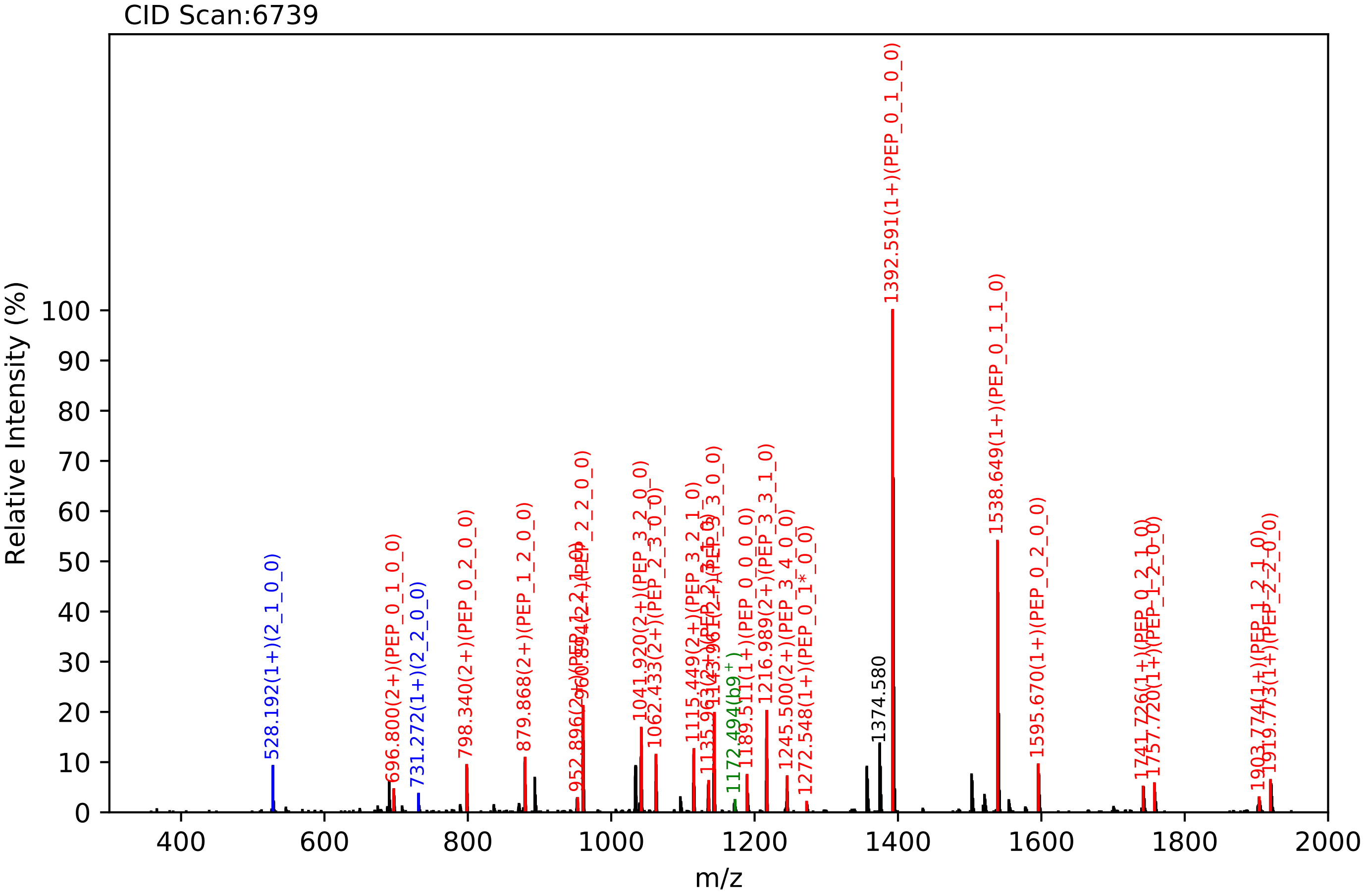

Test set no. 327, Experiment: IgG exp_7

EEQYNSTYR(=PEP)_3_4_1_0, m/z:1317.53(2+), RT:18.47, Y-score:88.41

HCD Scan:7491

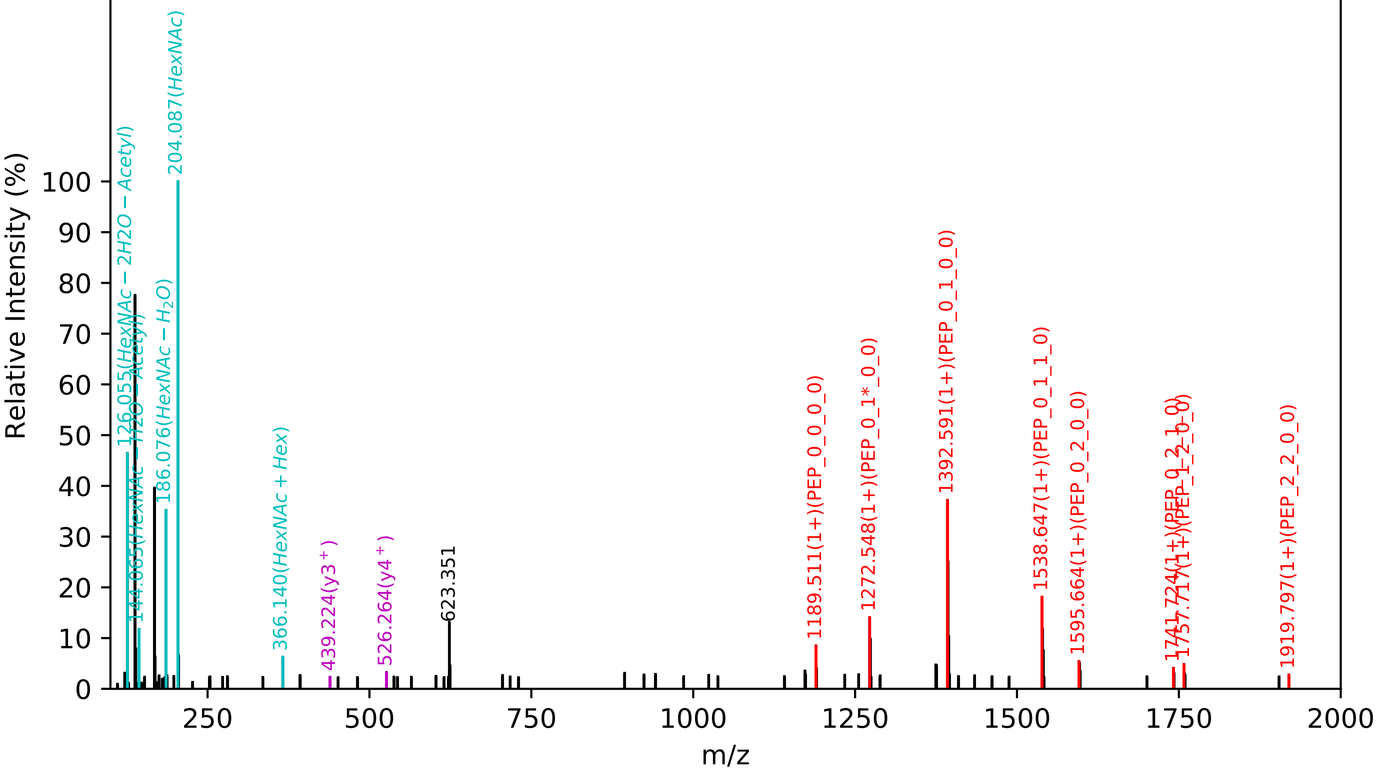

CID Scan:7490

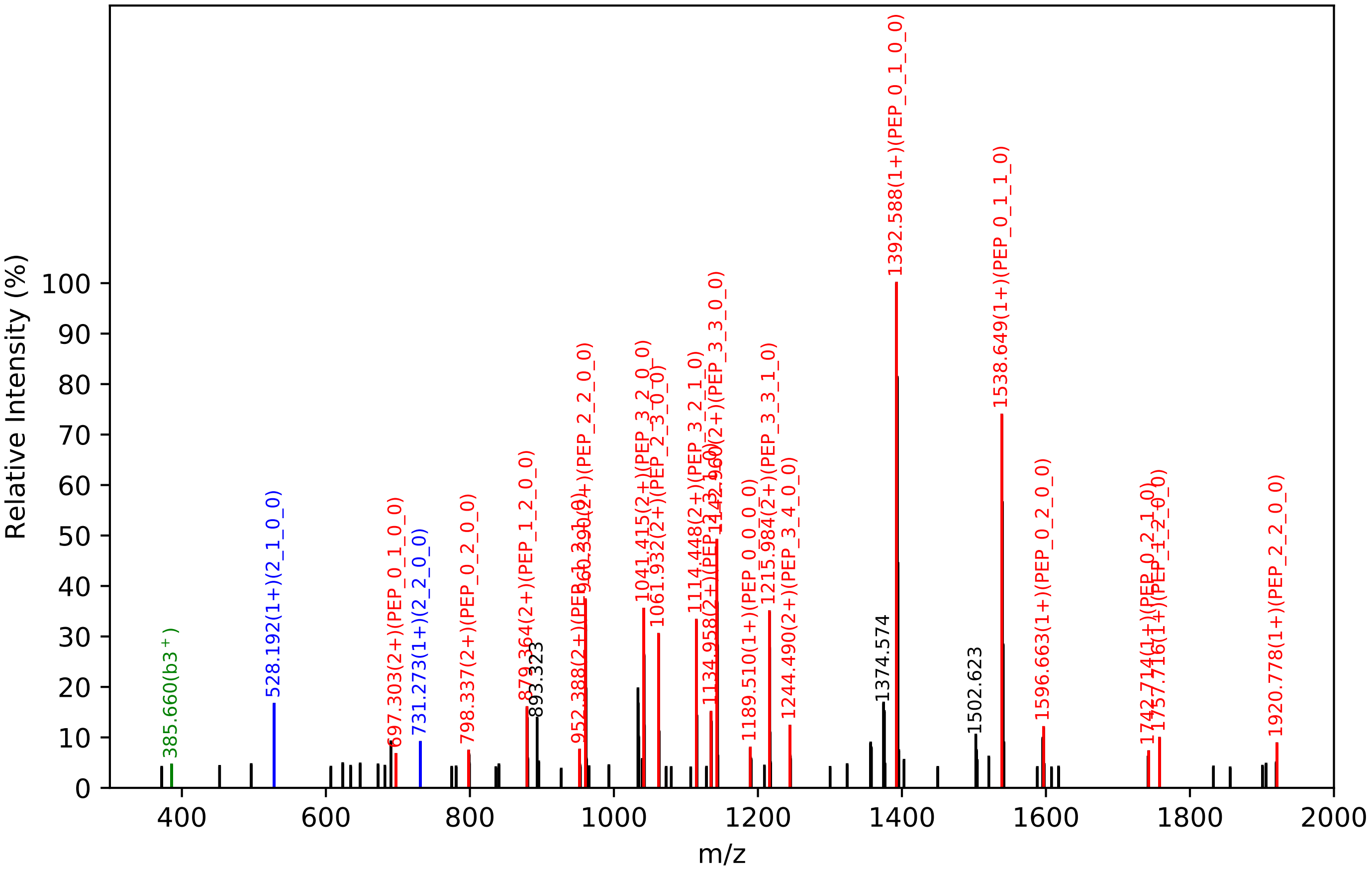

Test set no. 328, Experiment: IgG exp_7

EEQYNSTYR(=PEP)_3_4_1_0, m/z:1317.53(2+), RT:17.44, Y-score:88.36

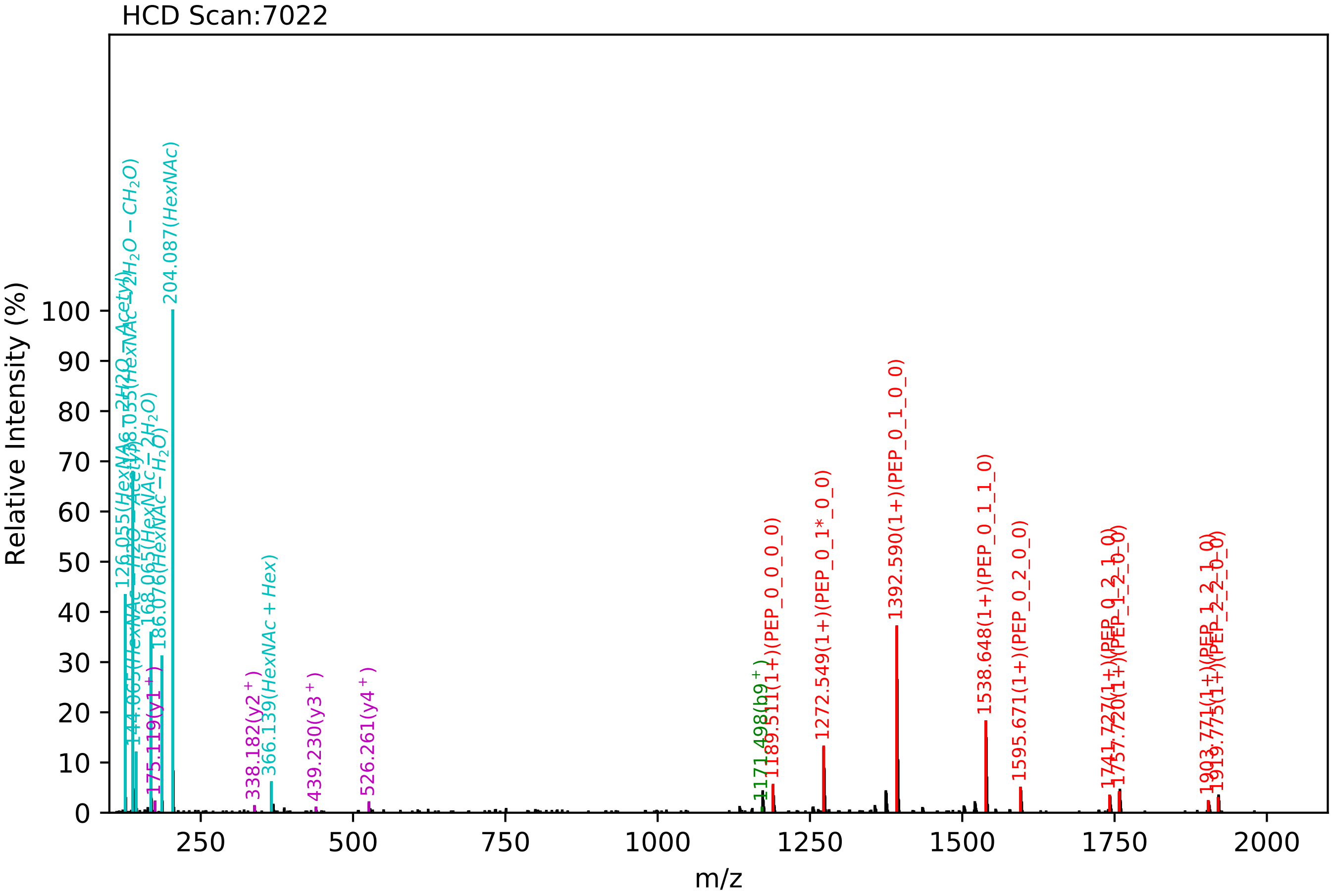

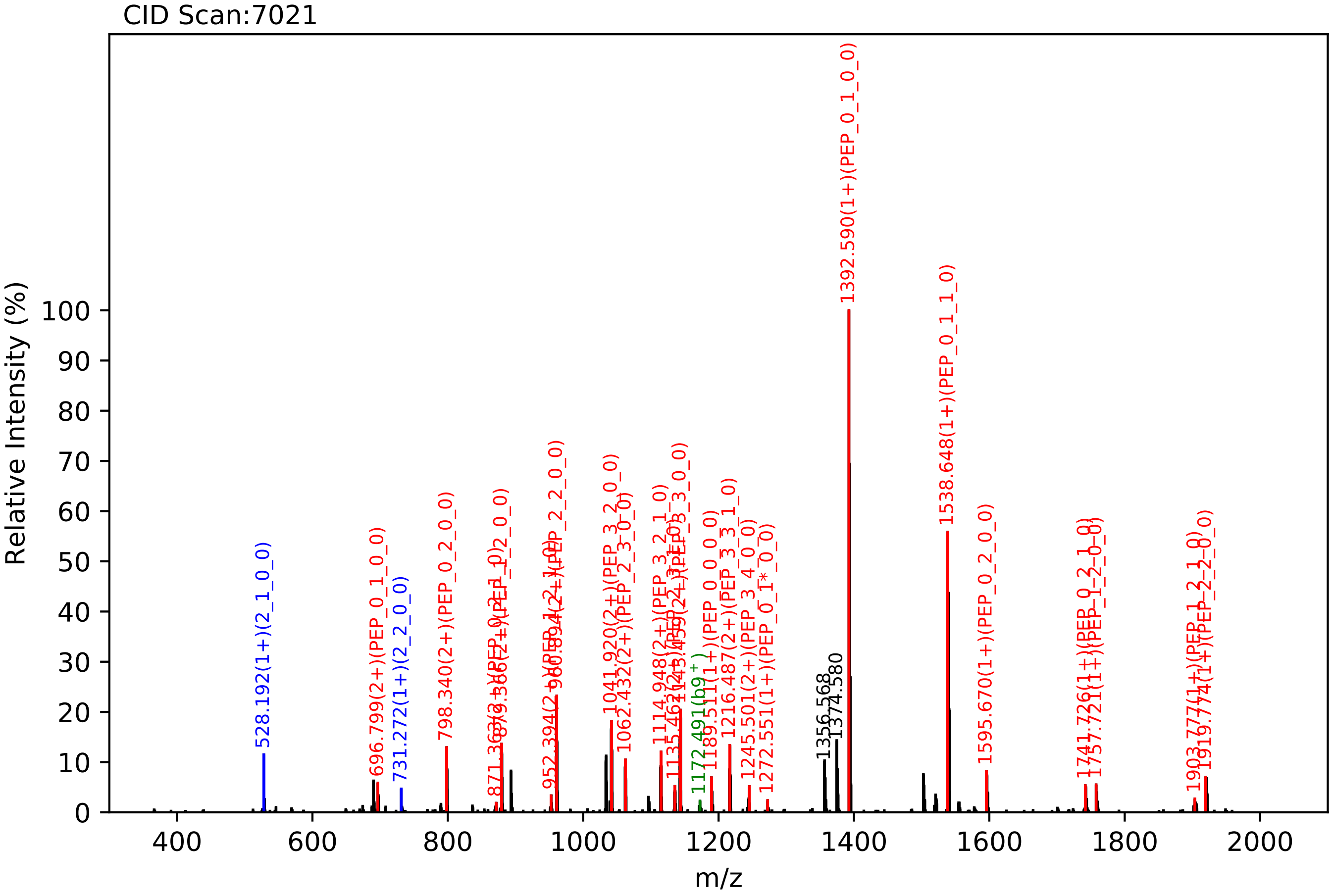

Test set no. 329, Experiment: IgG exp_5

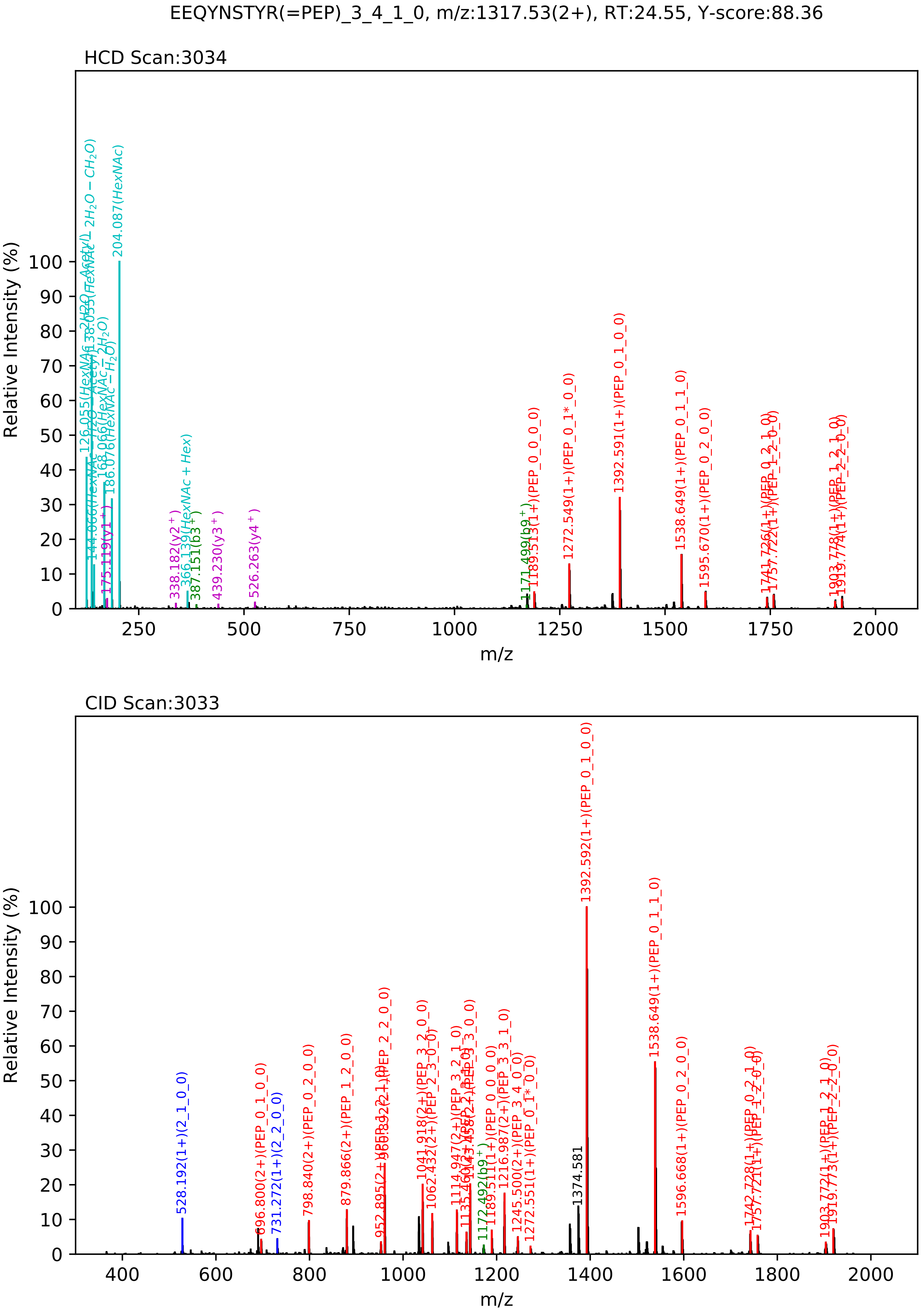

Test set no. 330, Experiment: IgG exp_7

EEQYNSTYR(=PEP)_3_4_1_0, m/z:1317.53(2+), RT:15.24, Y-score:86.83

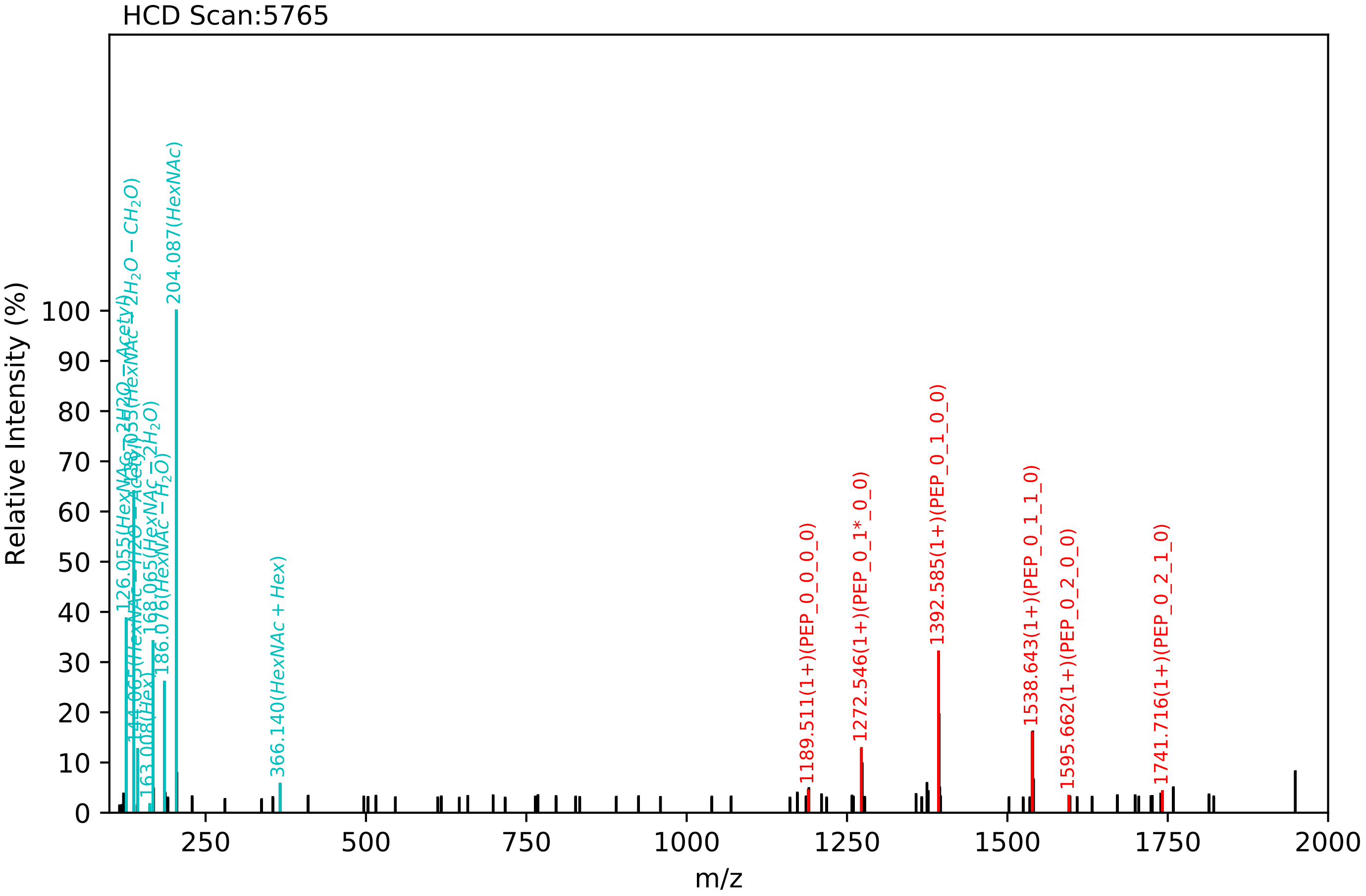

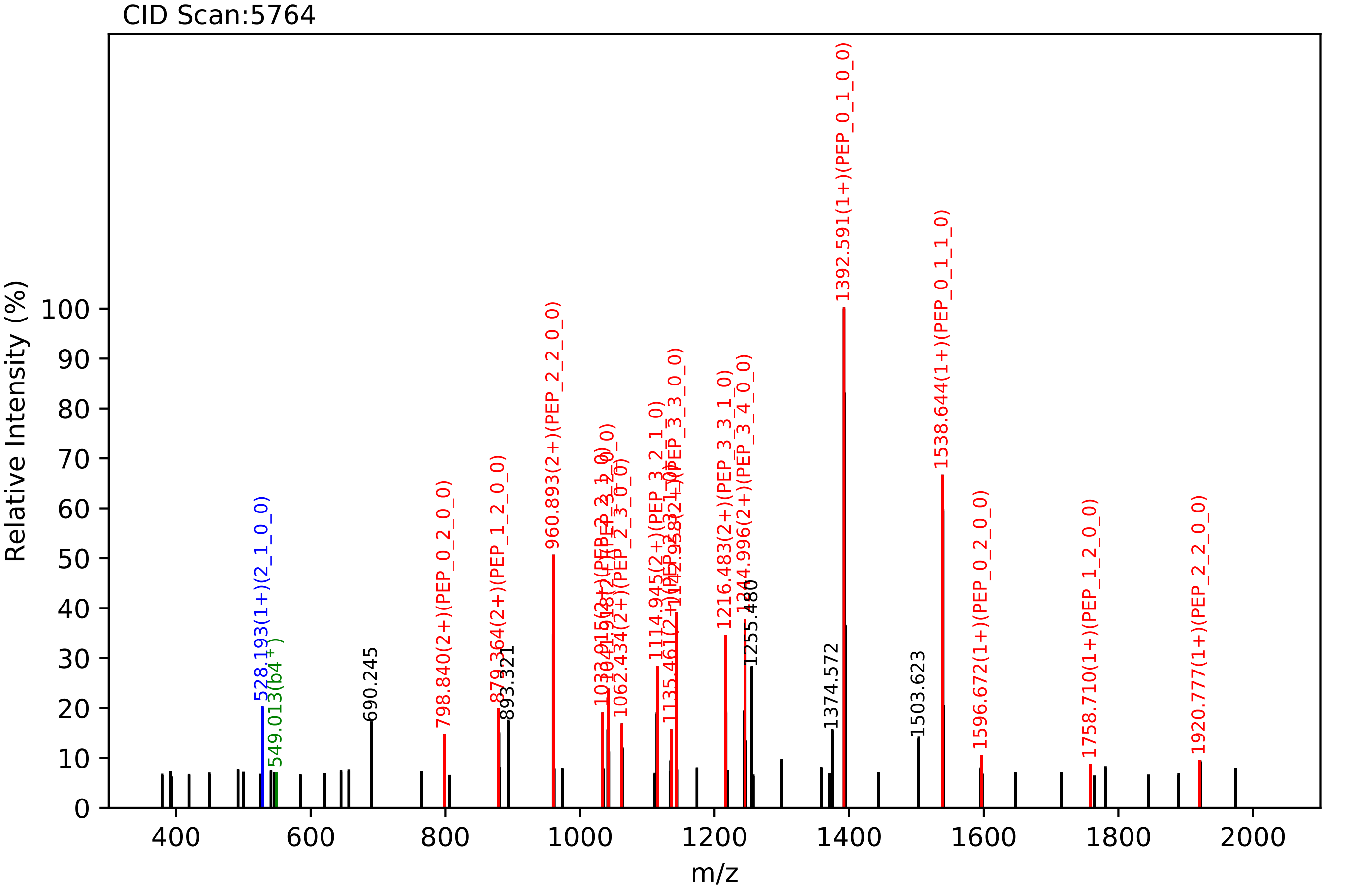

Test set no. 331, Experiment: IgG exp_7

EEQYNSTYR(=PEP)_3_4_1_0, m/z:1317.53(2+), RT:18.98, Y-score:85.46

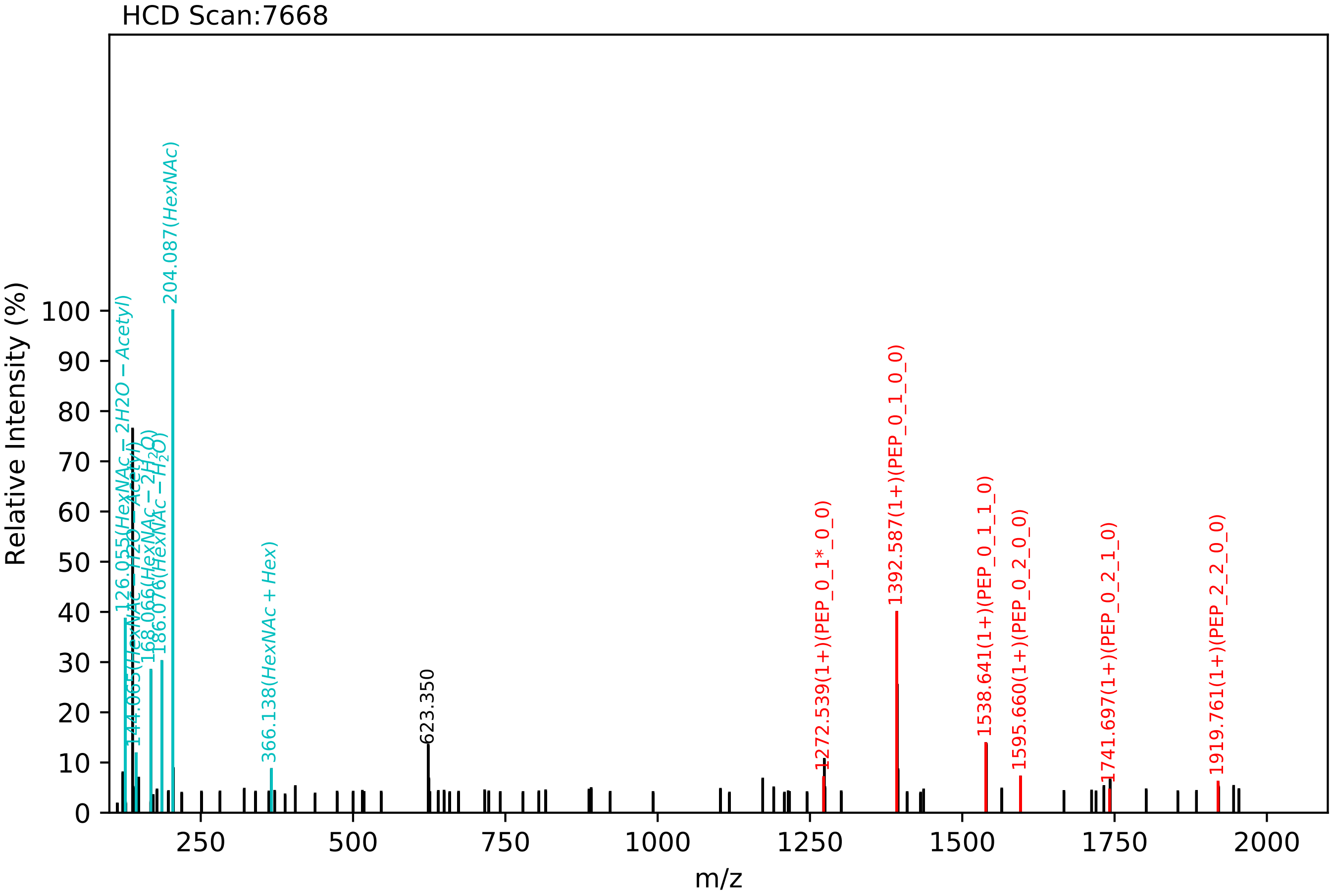

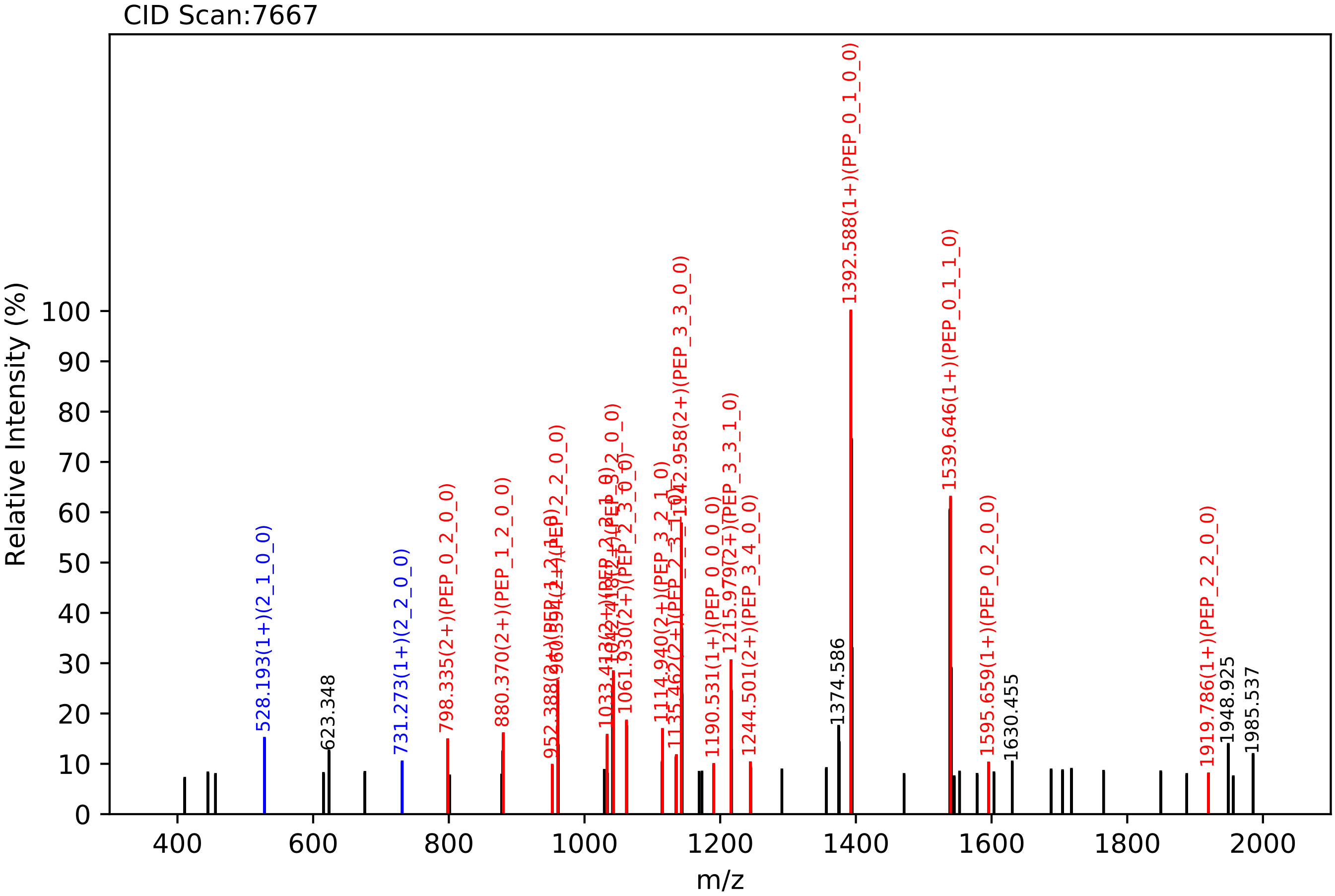

Test set no. 332, Experiment: IgG exp_7

EEQYNSTYR(=PEP)_3_4_1_0, m/z:1317.53(2+), RT:17.96, Y-score:85.21

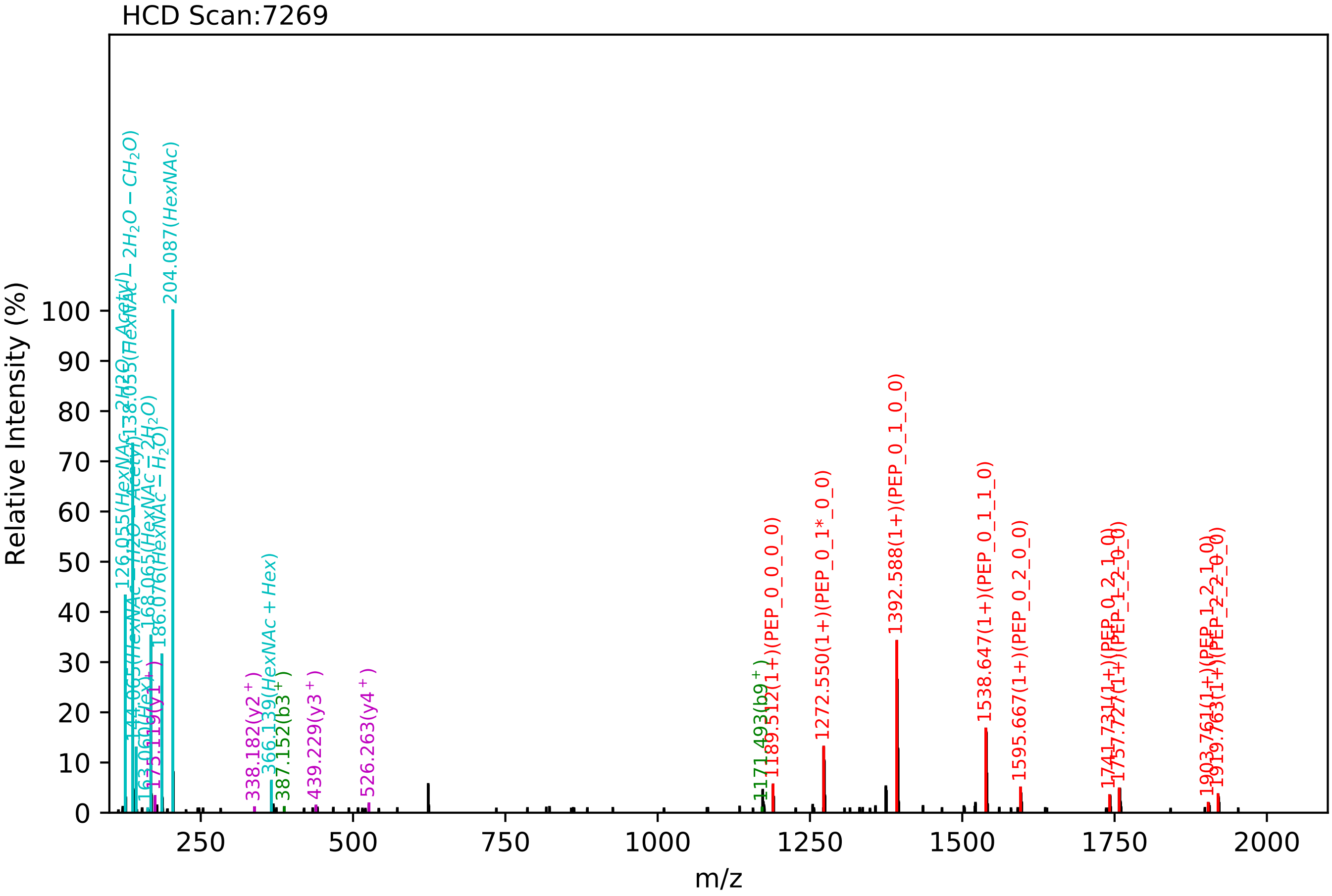

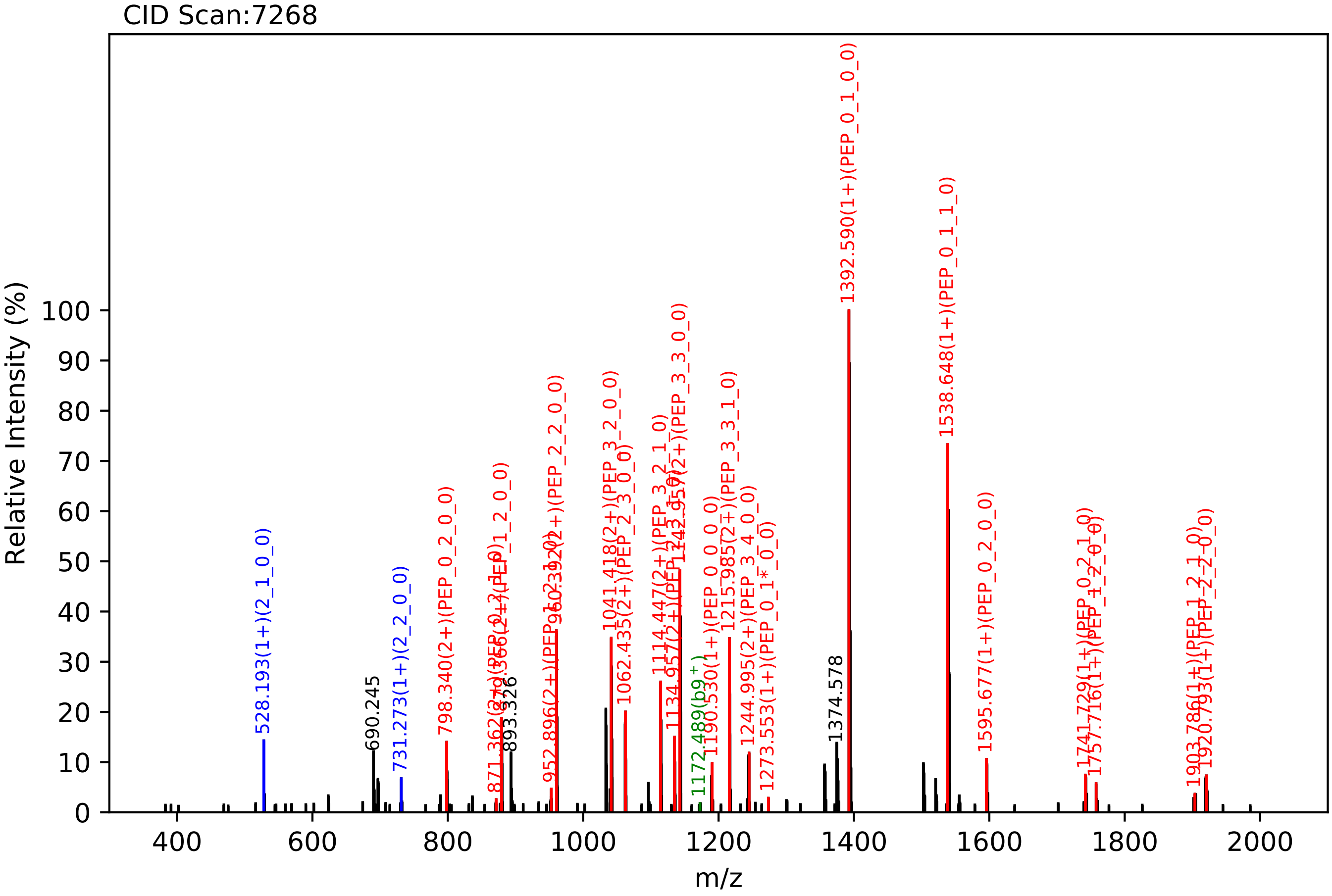

Test set no. 333, Experiment: IgG exp_7

EEQYNSTYR(=PEP)_3_4_1_0, m/z:1318.03(2+), RT:20.56, Y-score:79.75

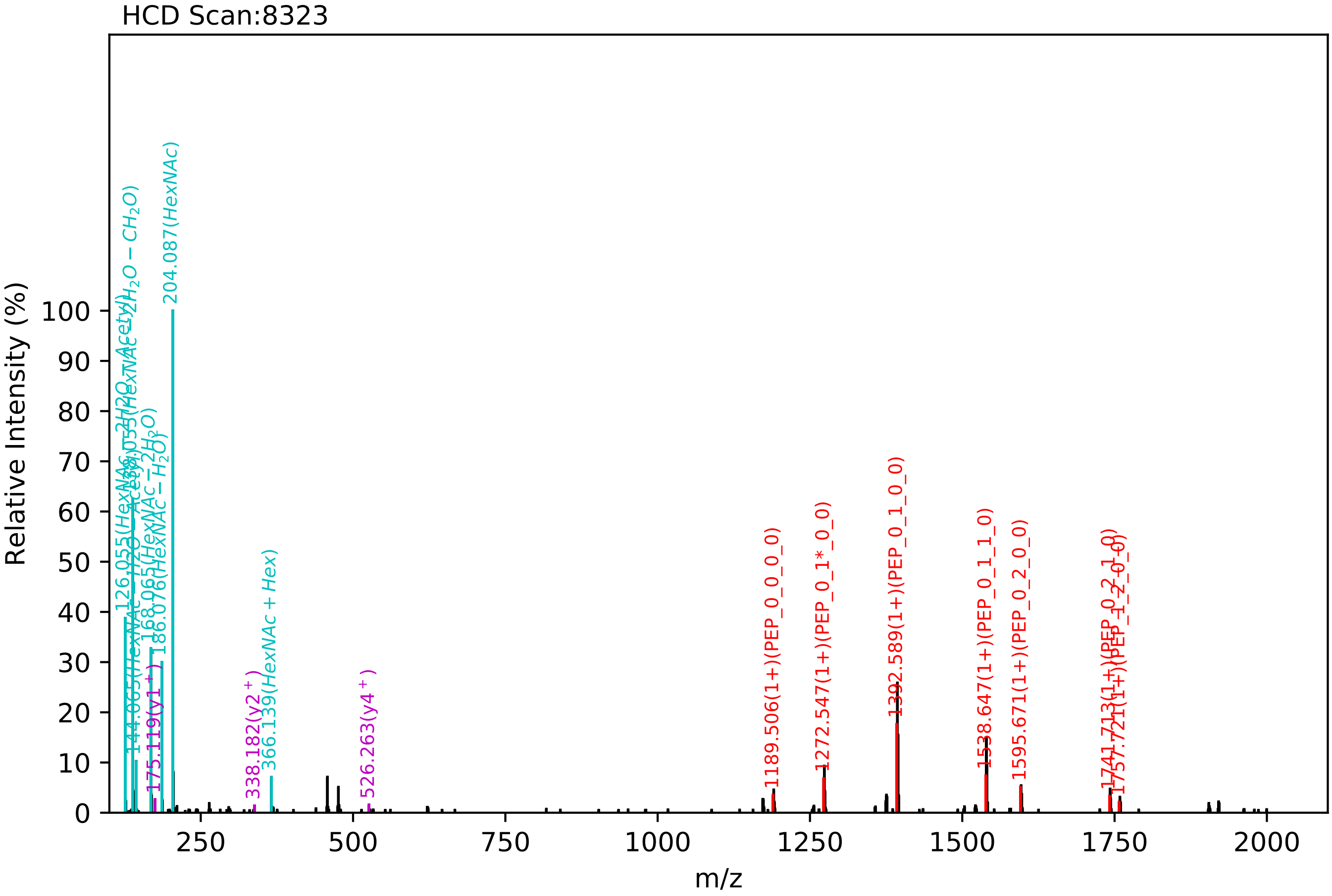

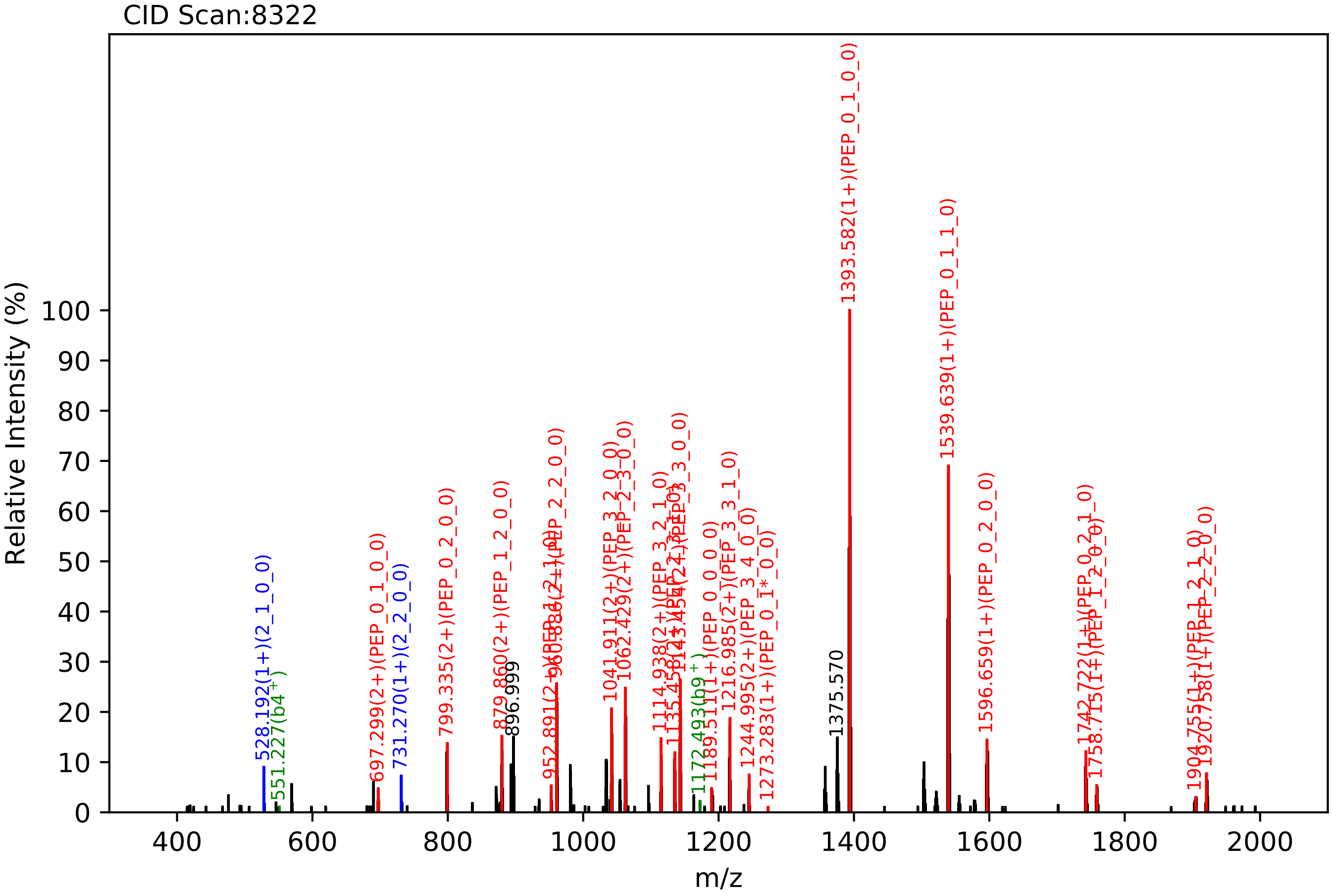

Test set no. 334, Experiment: IgG exp_7

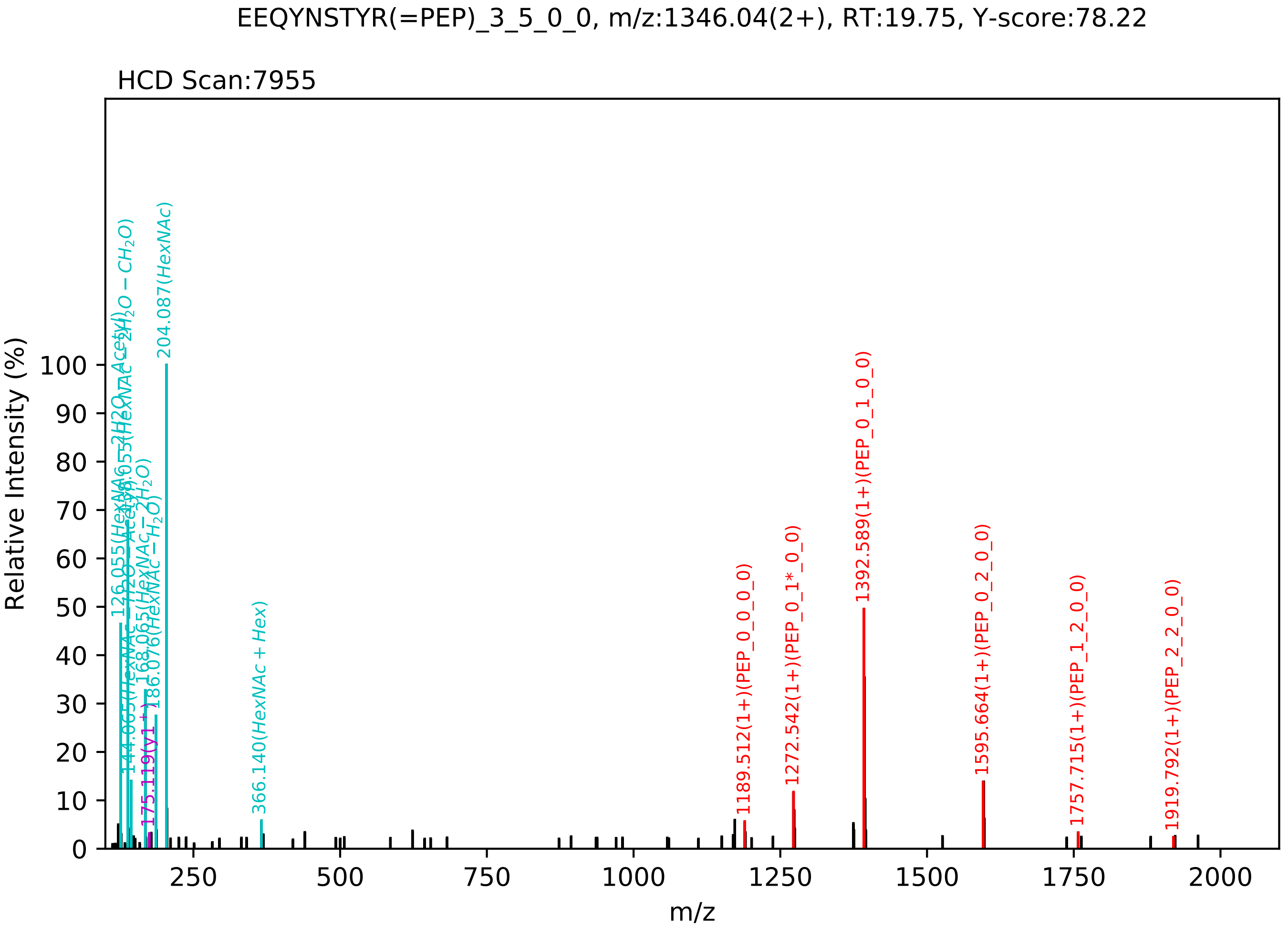

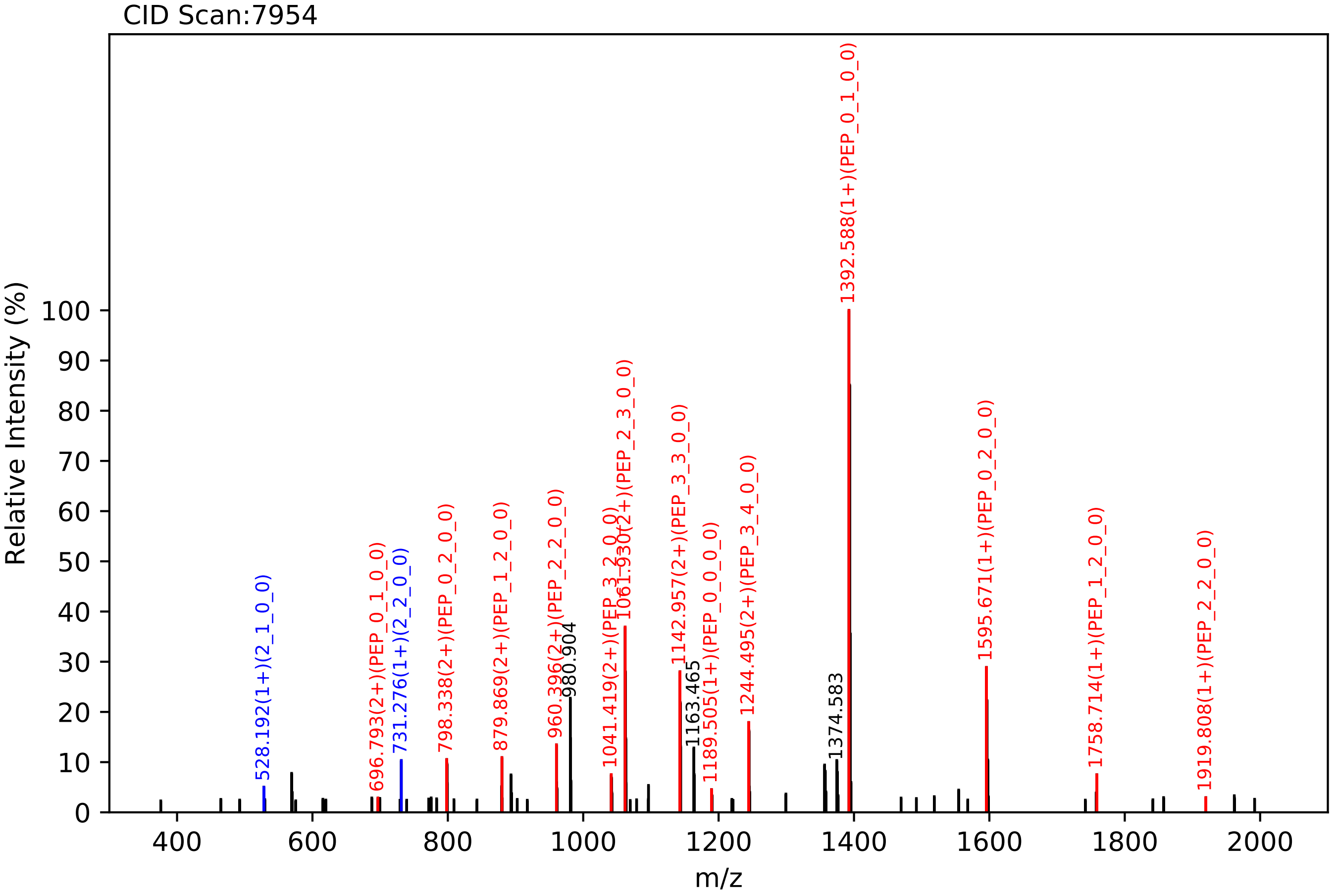

Test set no. 335, Experiment: IgG exp_7

EEQYNSTYR(=PEP)_3_5_1_0, m/z:1419.07(2+), RT:21.14, Y-score:83.72

HCD Scan:8667

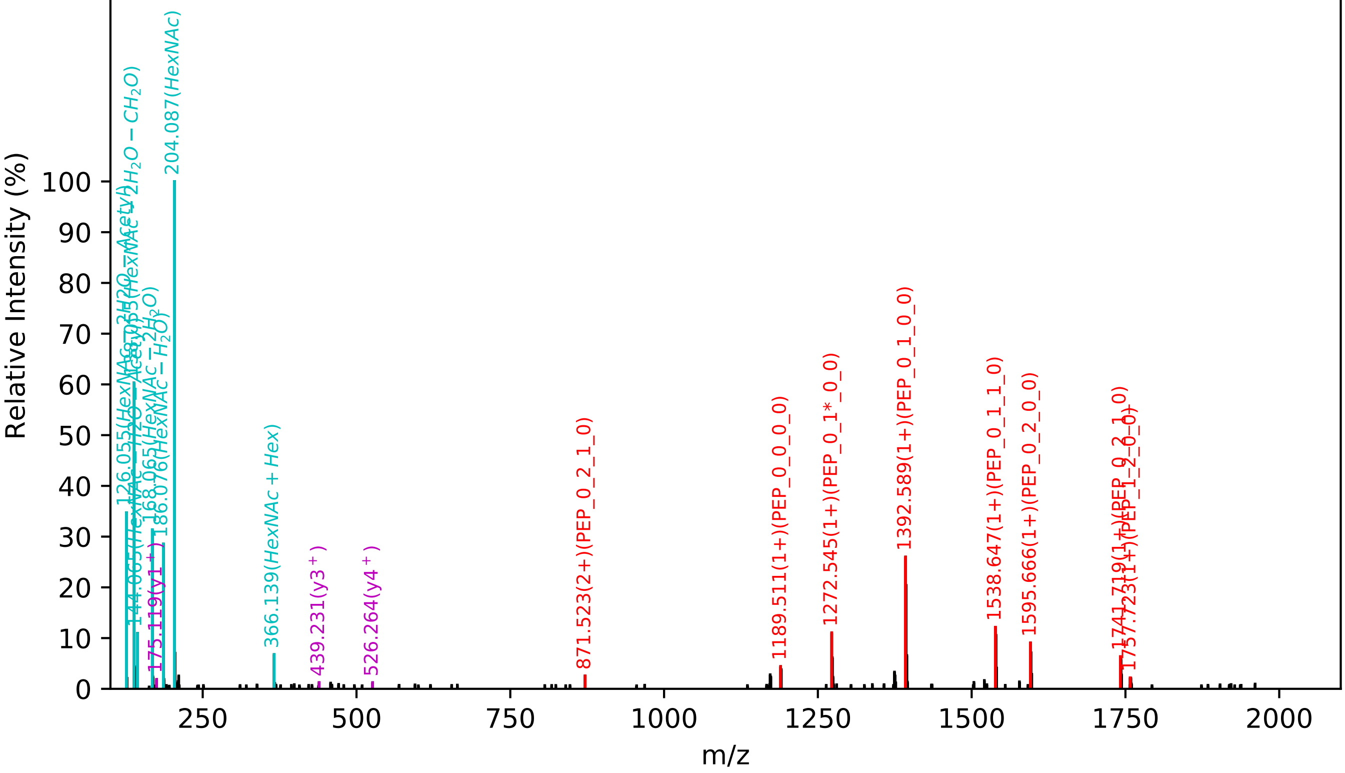

CID Scan:8666

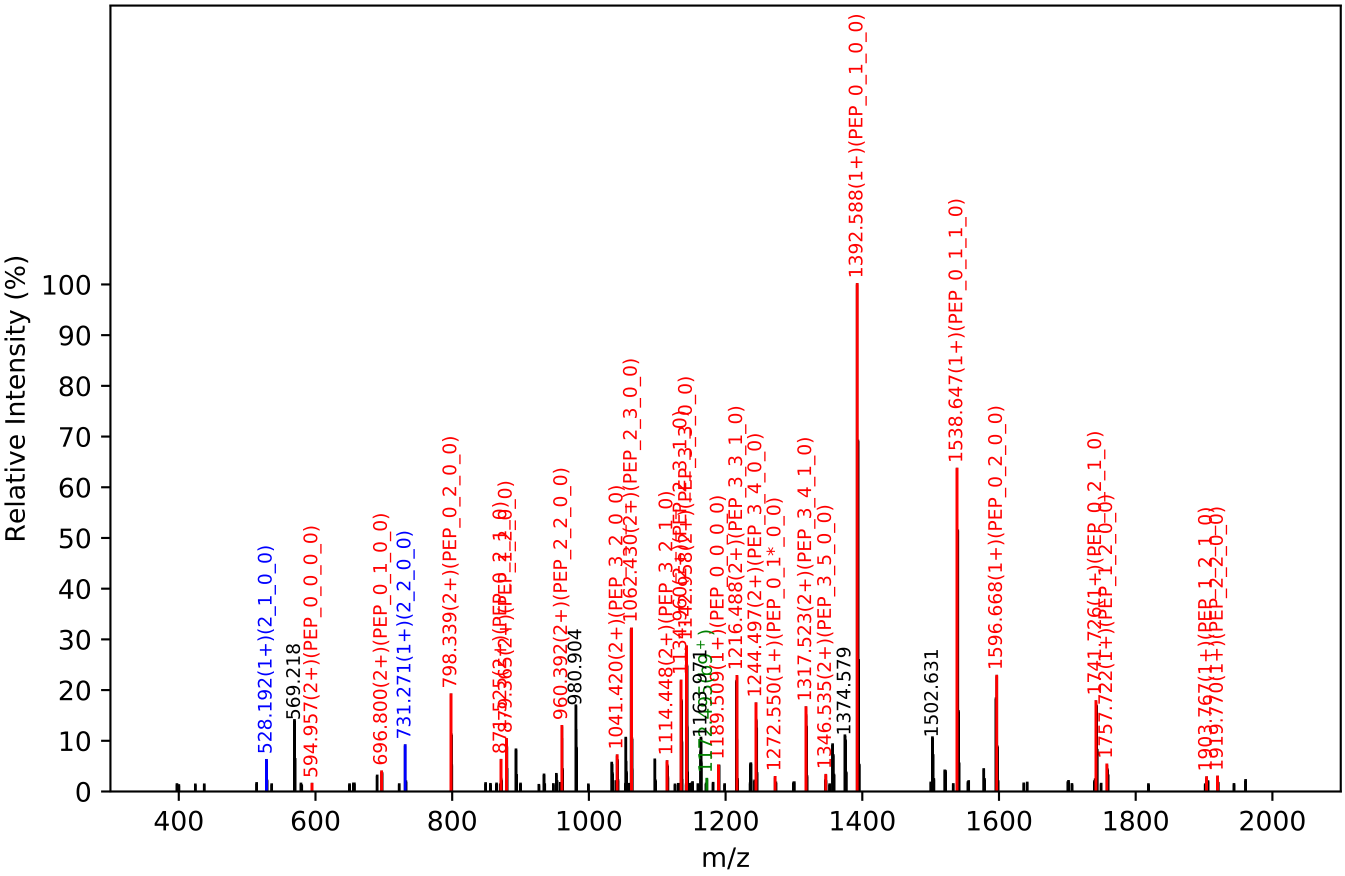

Test set no. 336, Experiment: IgG exp_6

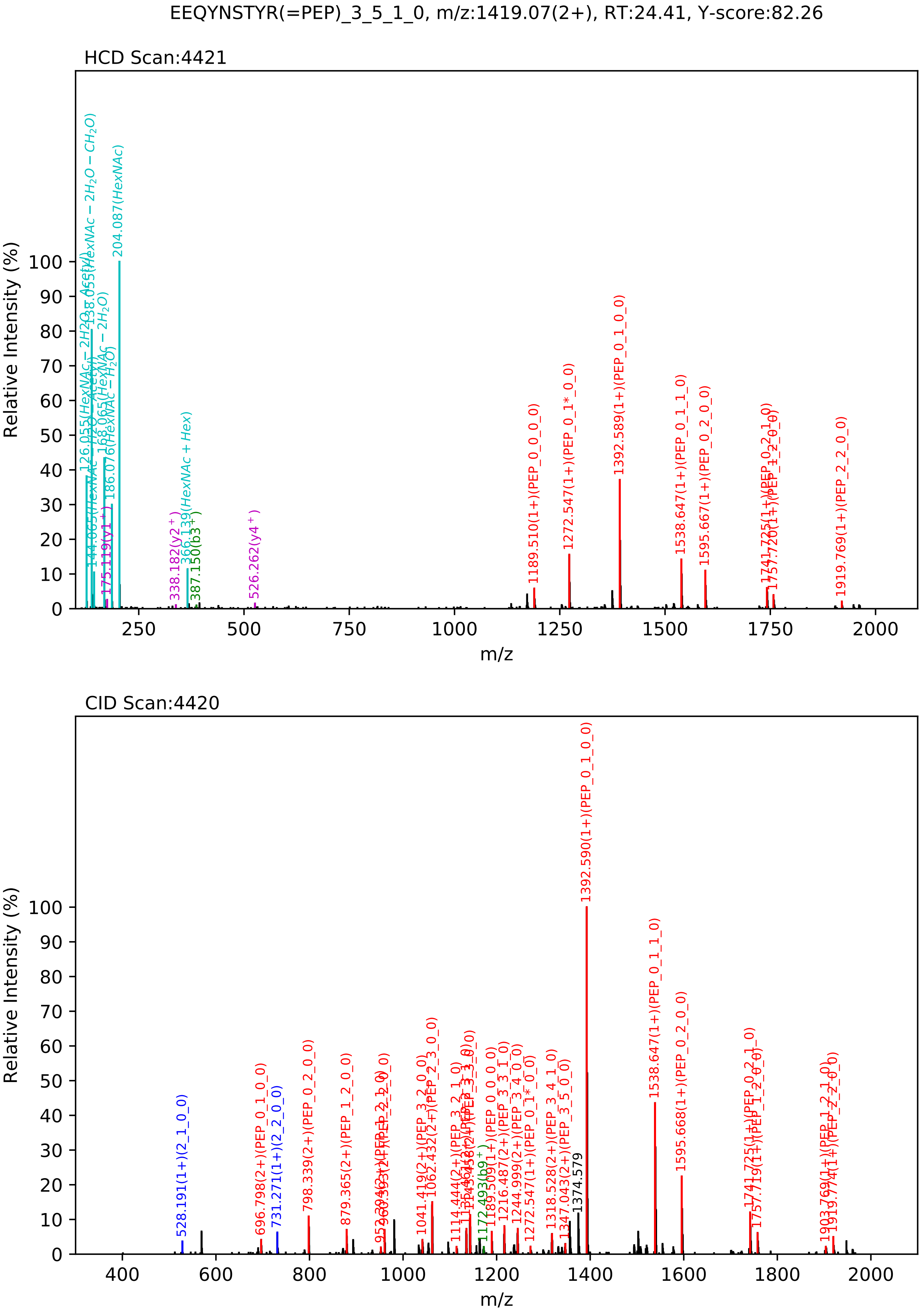

Test set no. 337, Experiment: IgG exp_7

EEQYNSTYR(=PEP)_3_5_1_0, m/z:1419.07(2+), RT:20.54, Y-score:82.13

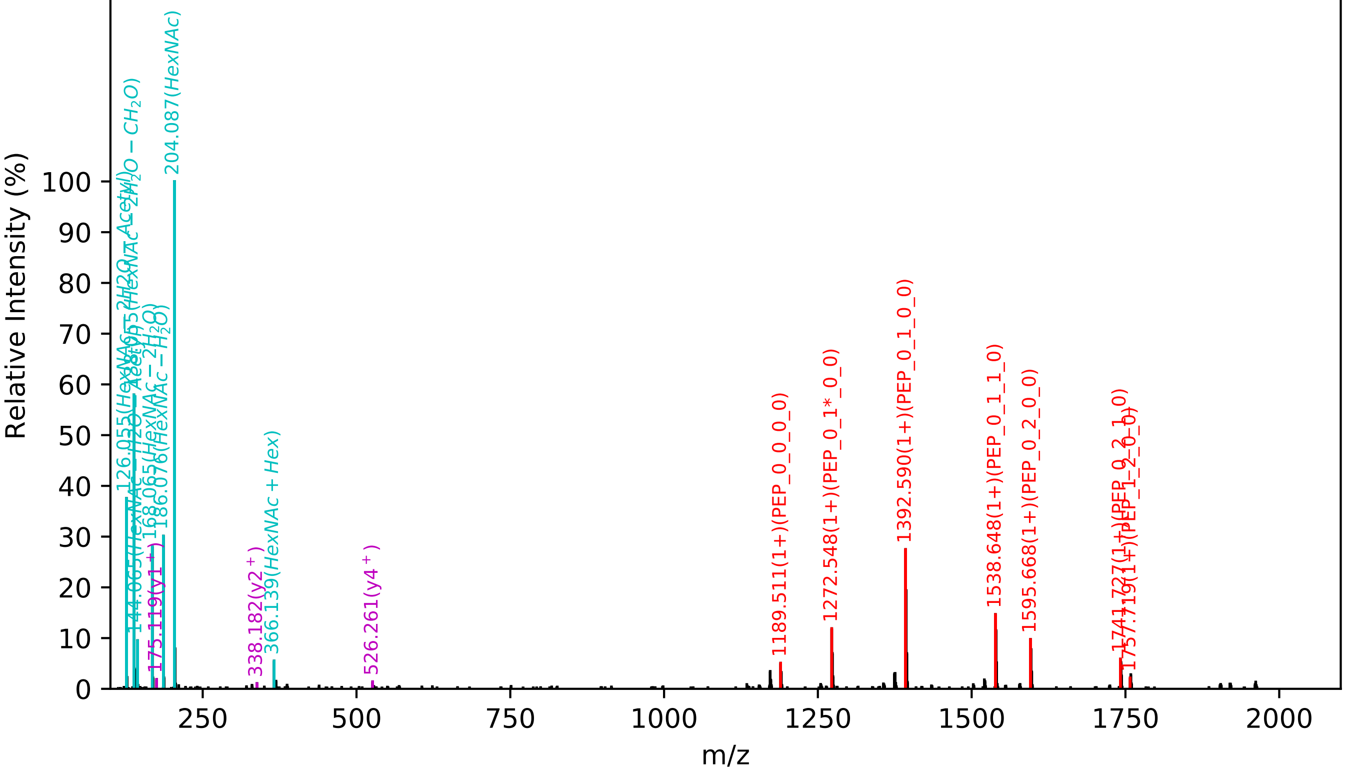

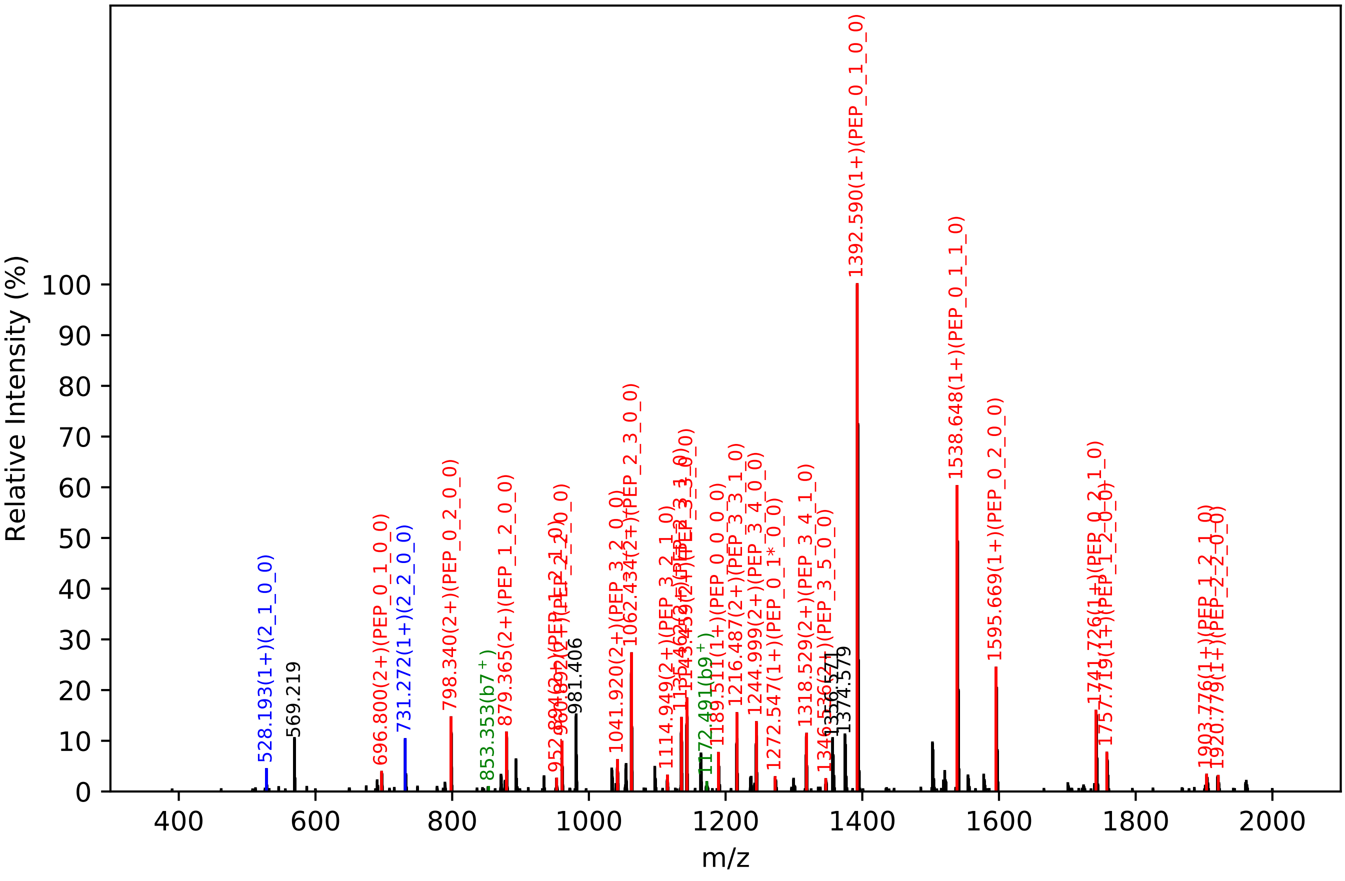

Test set no. 338, Experiment: IgG exp_7

EEQYNSTYR(=PEP)_4_3_1_0, m/z:1297.02(2+), RT:15.99, Y-score:88.45

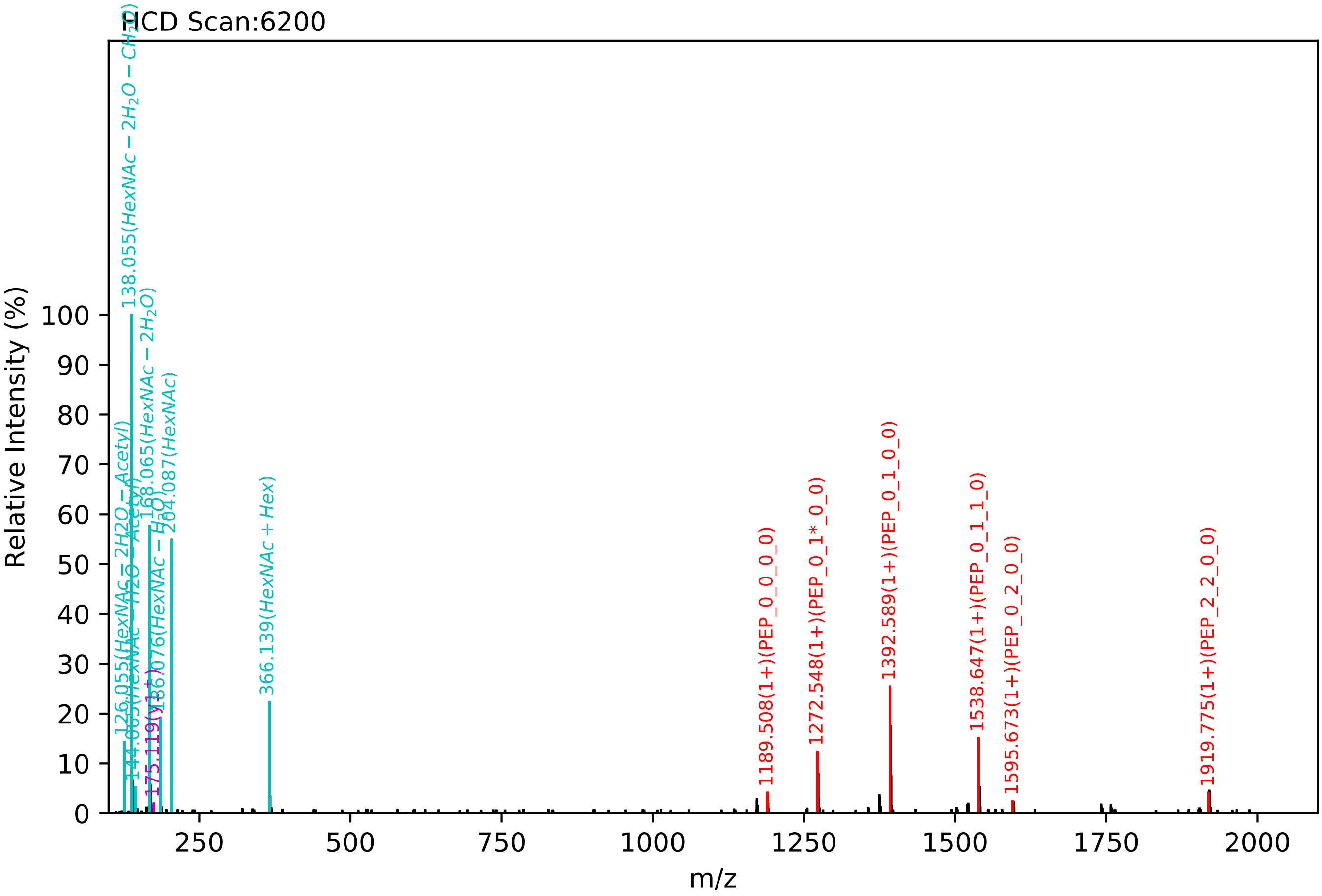

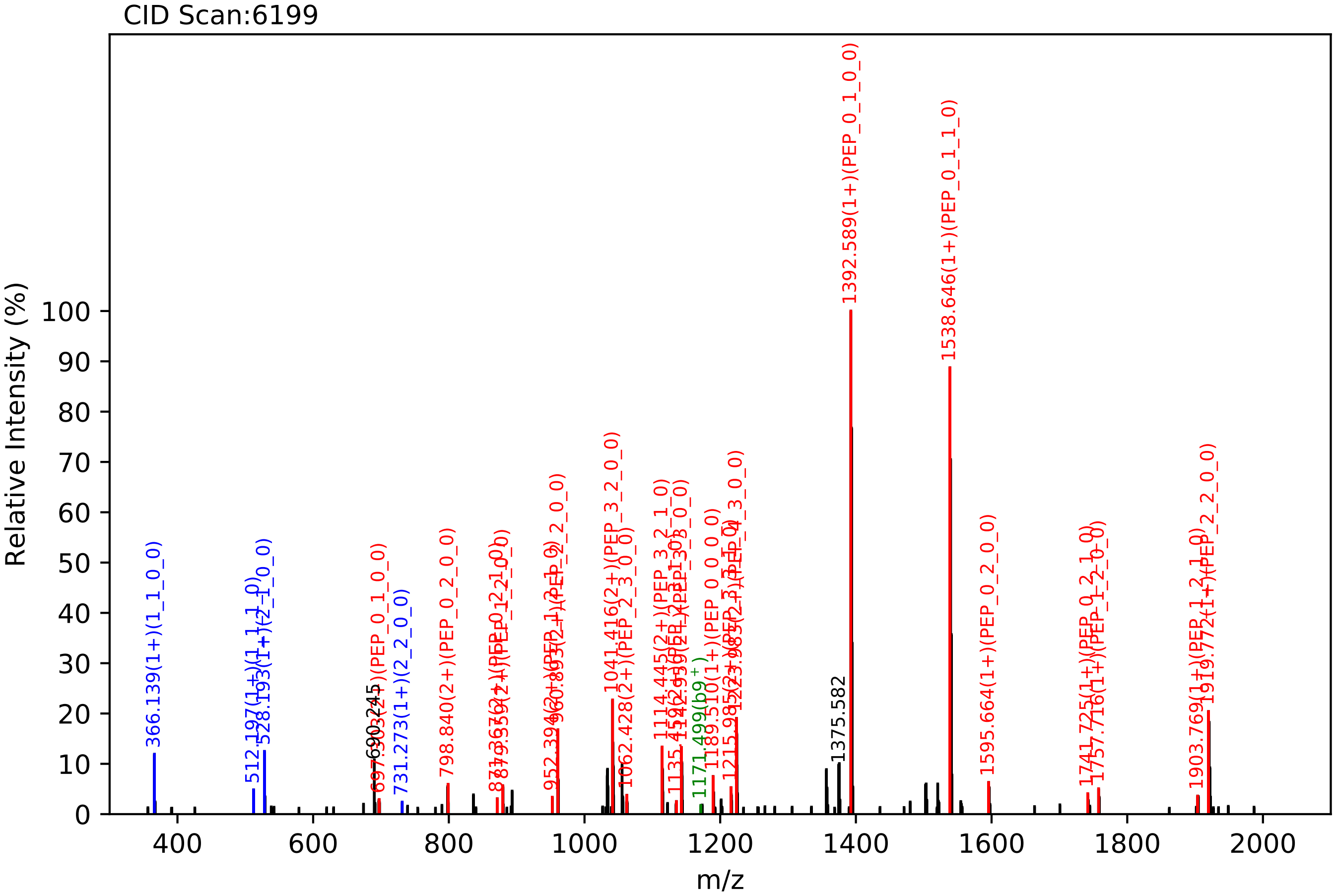

Test set no. 339, Experiment: IgG exp_7

EEQYNSTYR(=PEP)_4_3_1_0, m/z:1297.02(2+), RT:16.56, Y-score:88.43

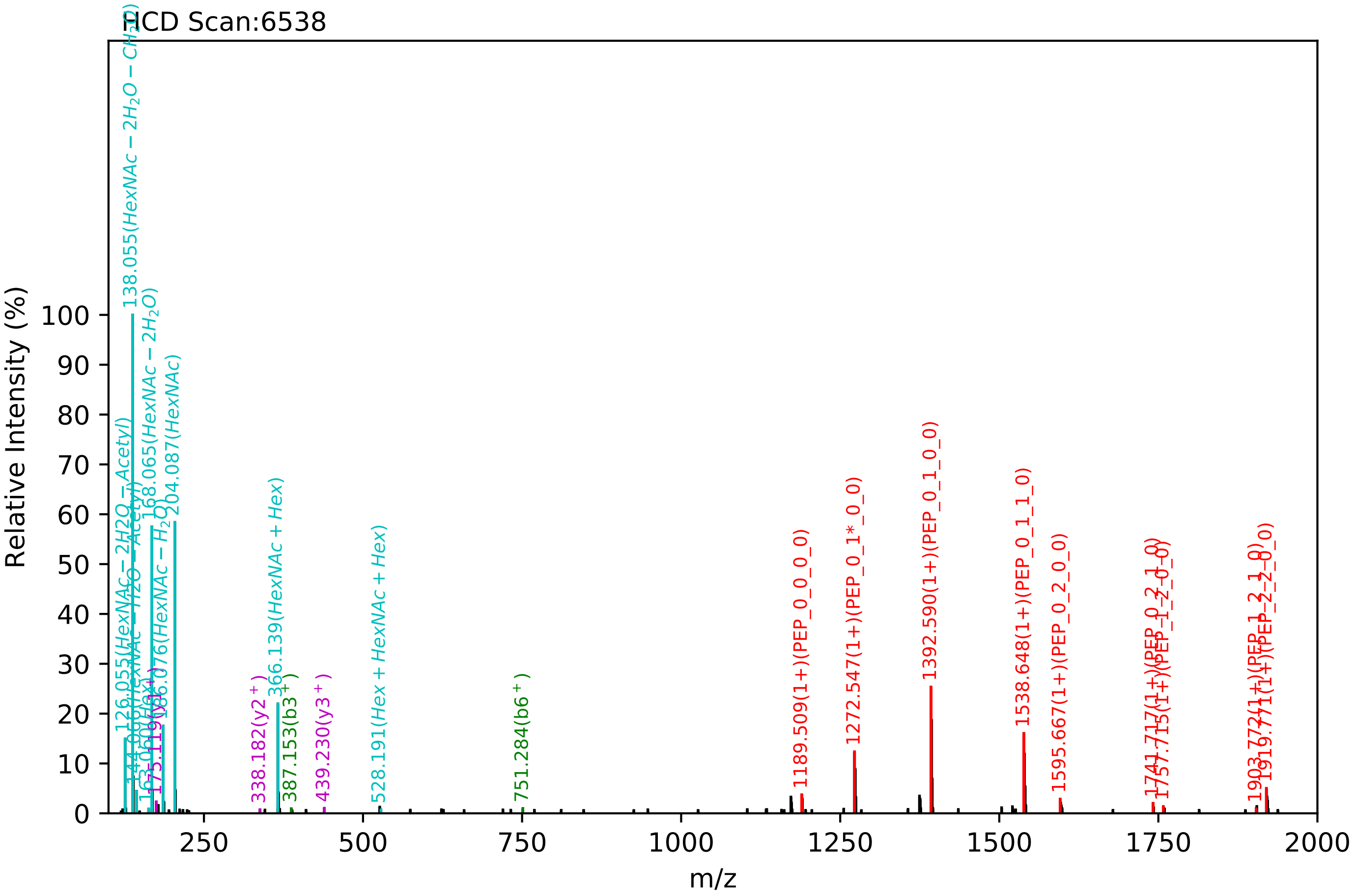

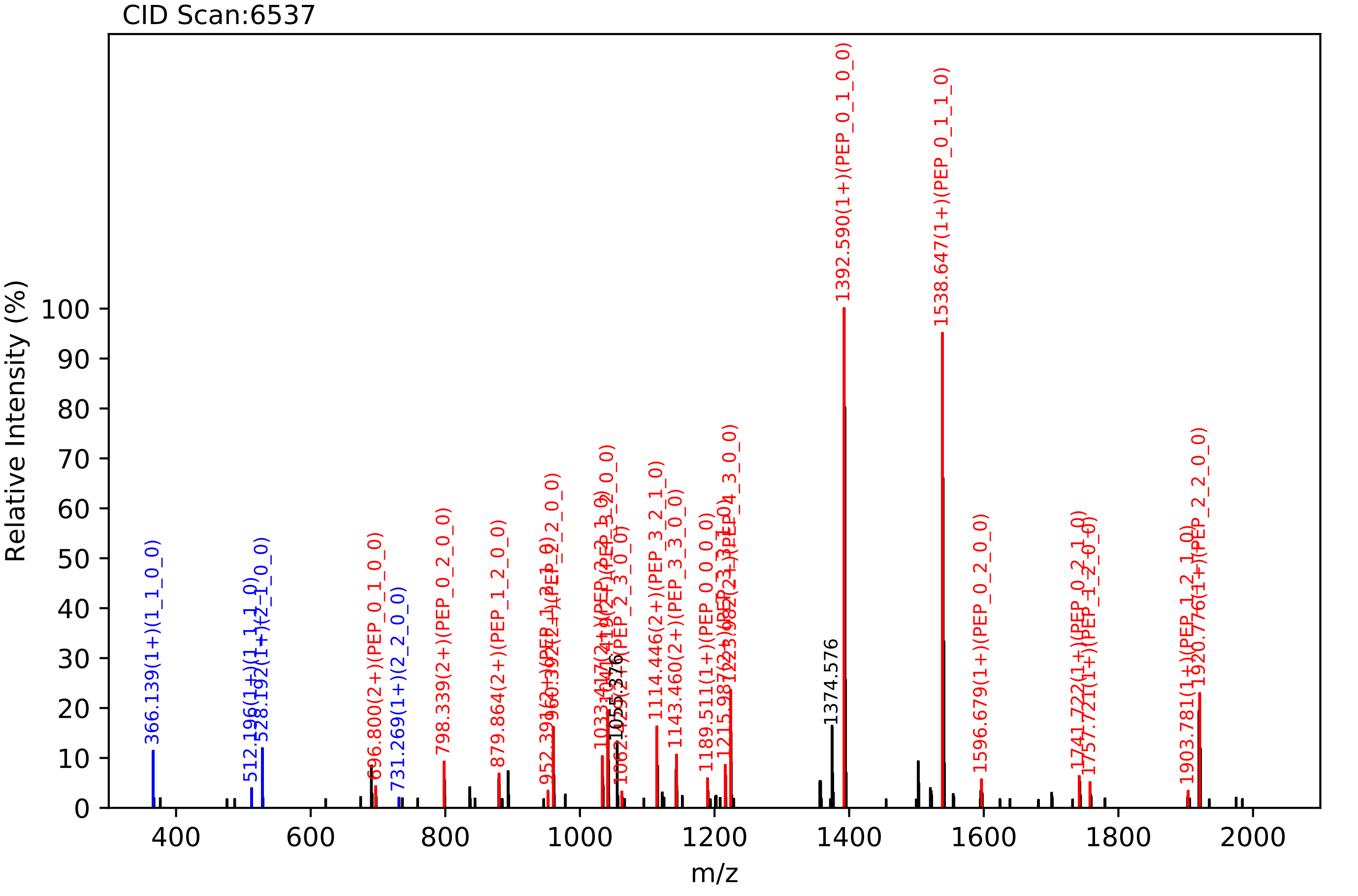

Test set no. 340, Experiment: IgG exp_6

EEQYNSTYR(=PEP)_4_4_0_0, m/z:1325.52(2+), RT:24.31, Y-score:88.10

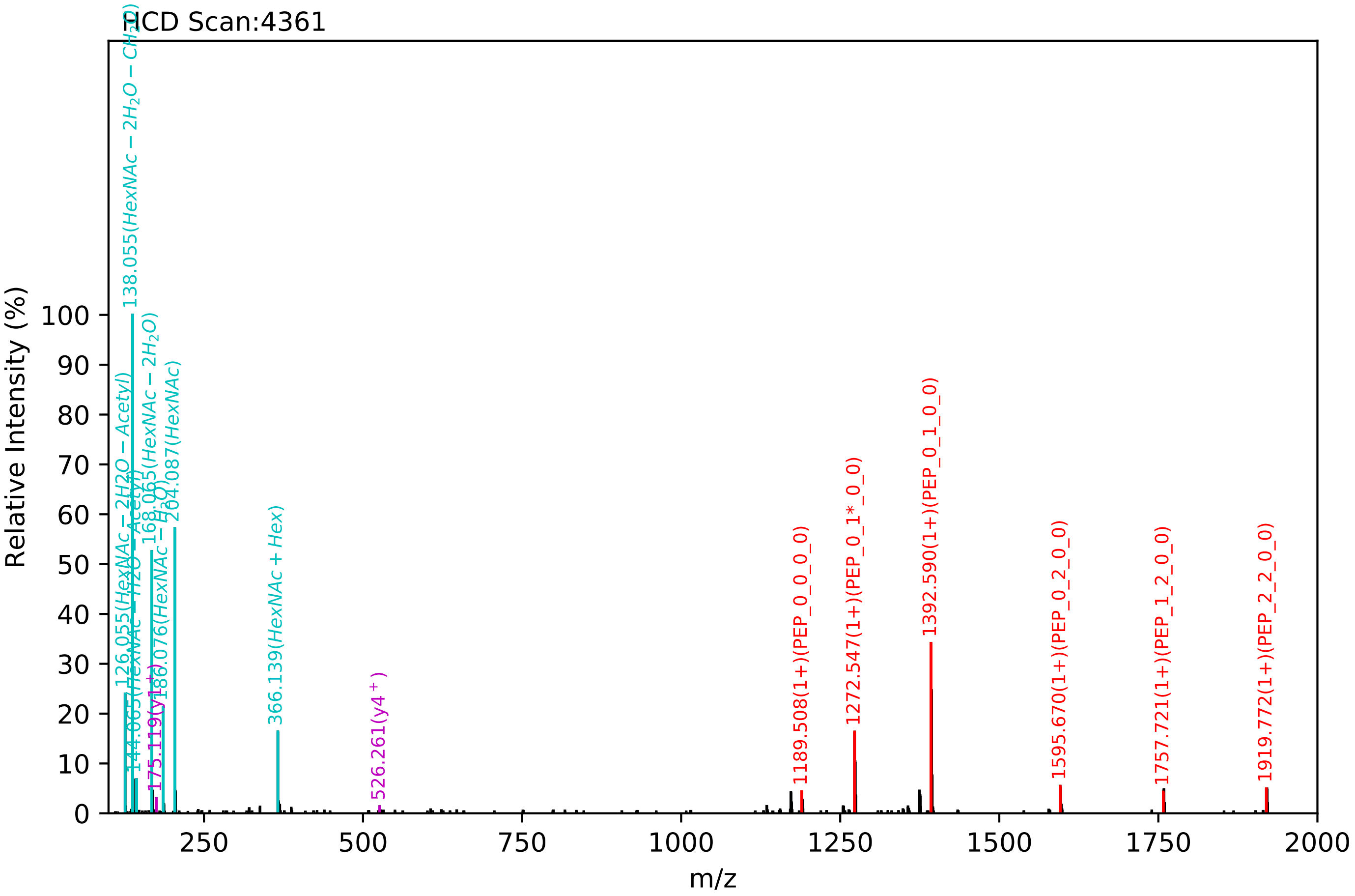

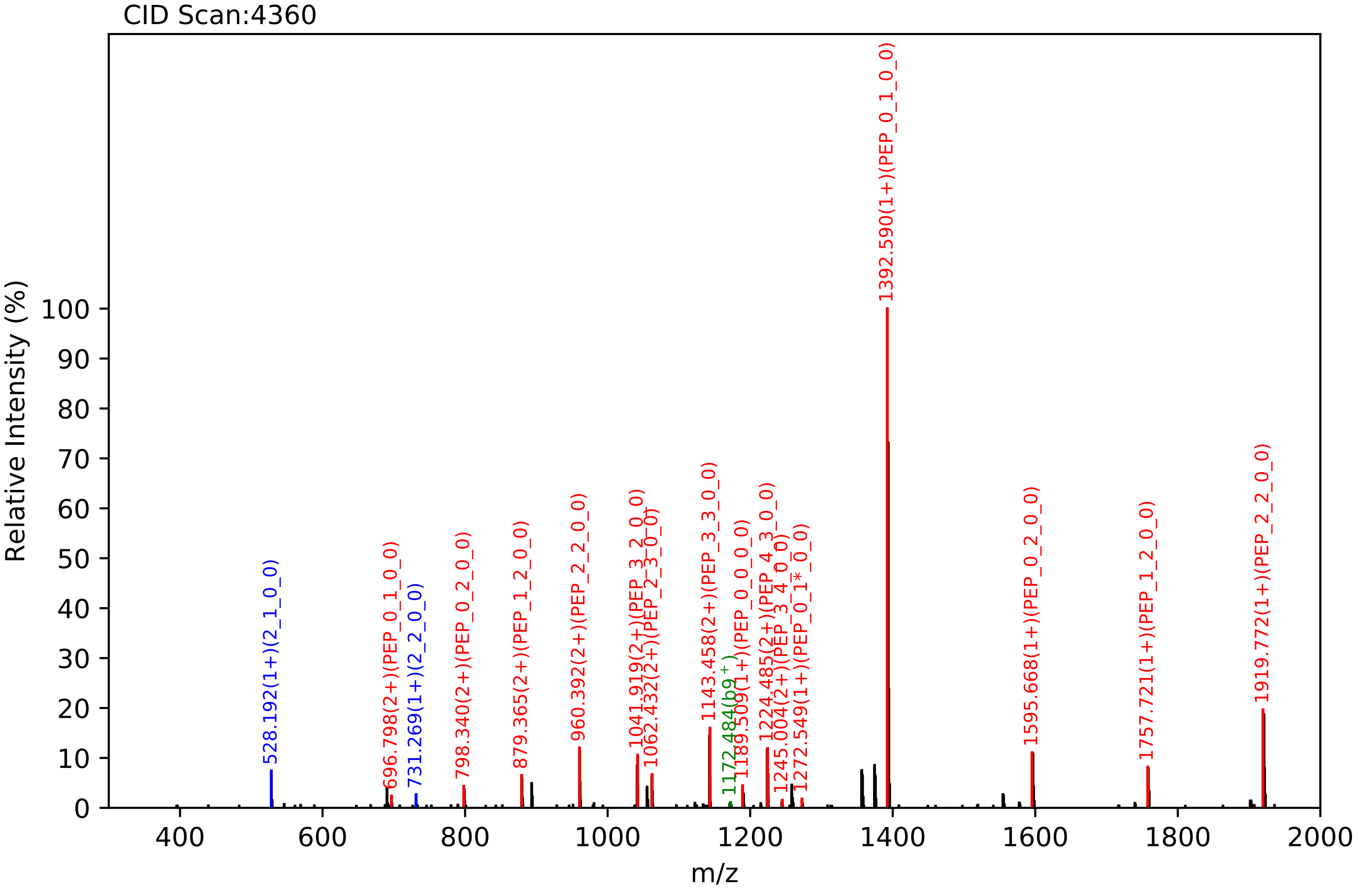

Test set no. 341, Experiment: IgG exp_7

EEQYNSTYR(=PEP)_4_4_0_0, m/z:1325.52(2+), RT:15.23, Y-score:87.35

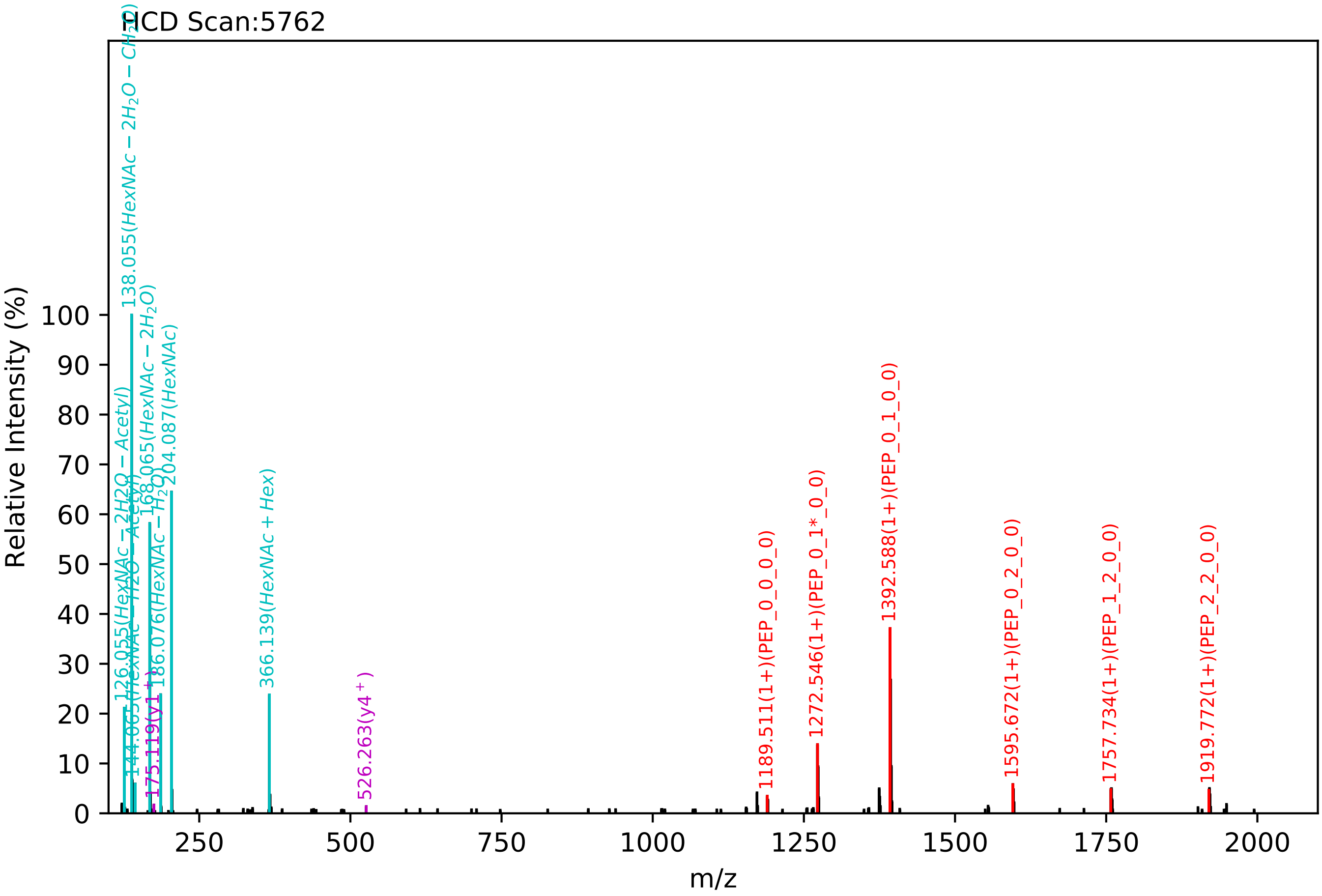

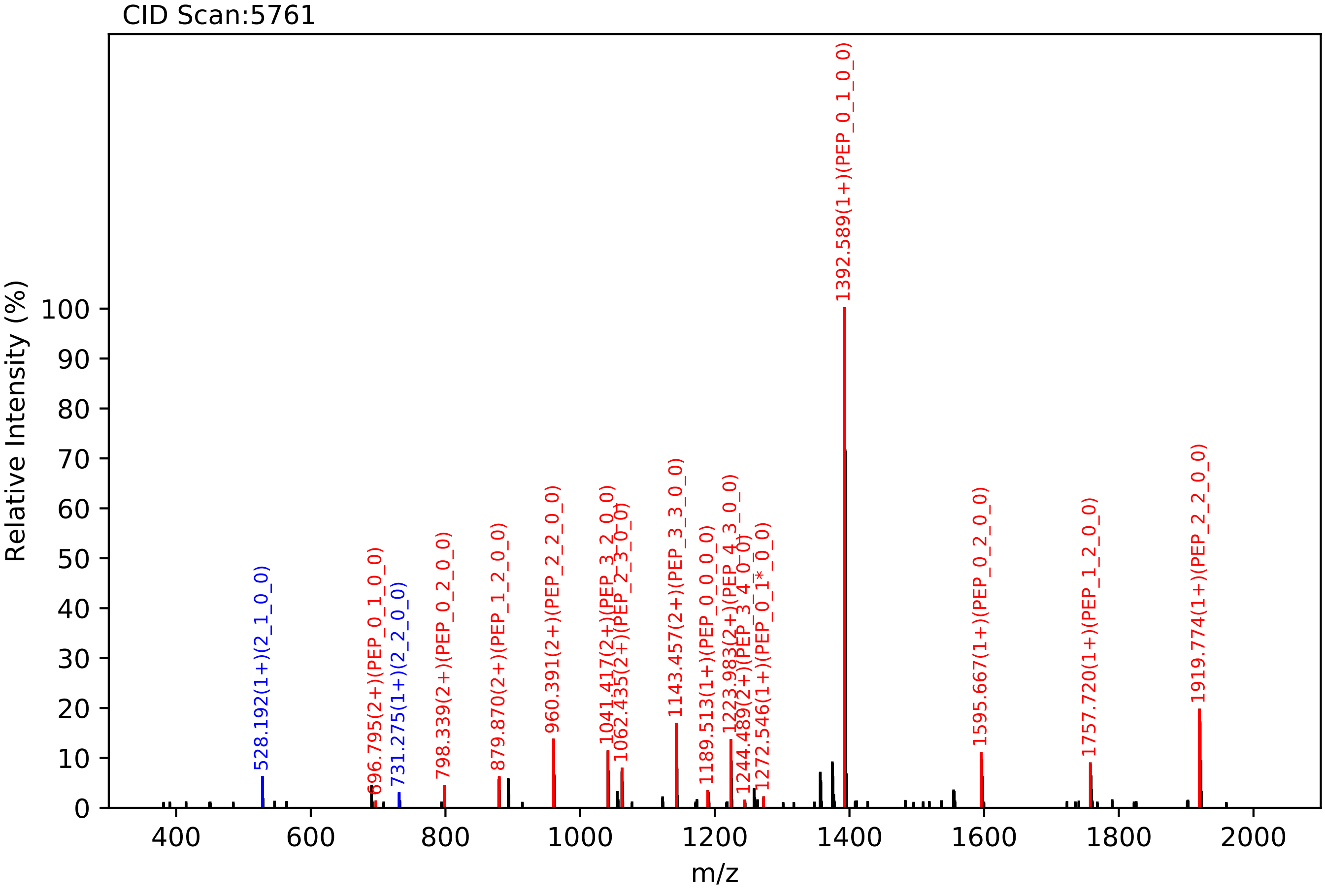

Test set no. 342, Experiment: IgG exp_7

EEQYNSTYR(=PEP)_4_4_0_0, m/z:1325.53(2+), RT:15.80, Y-score:86.55

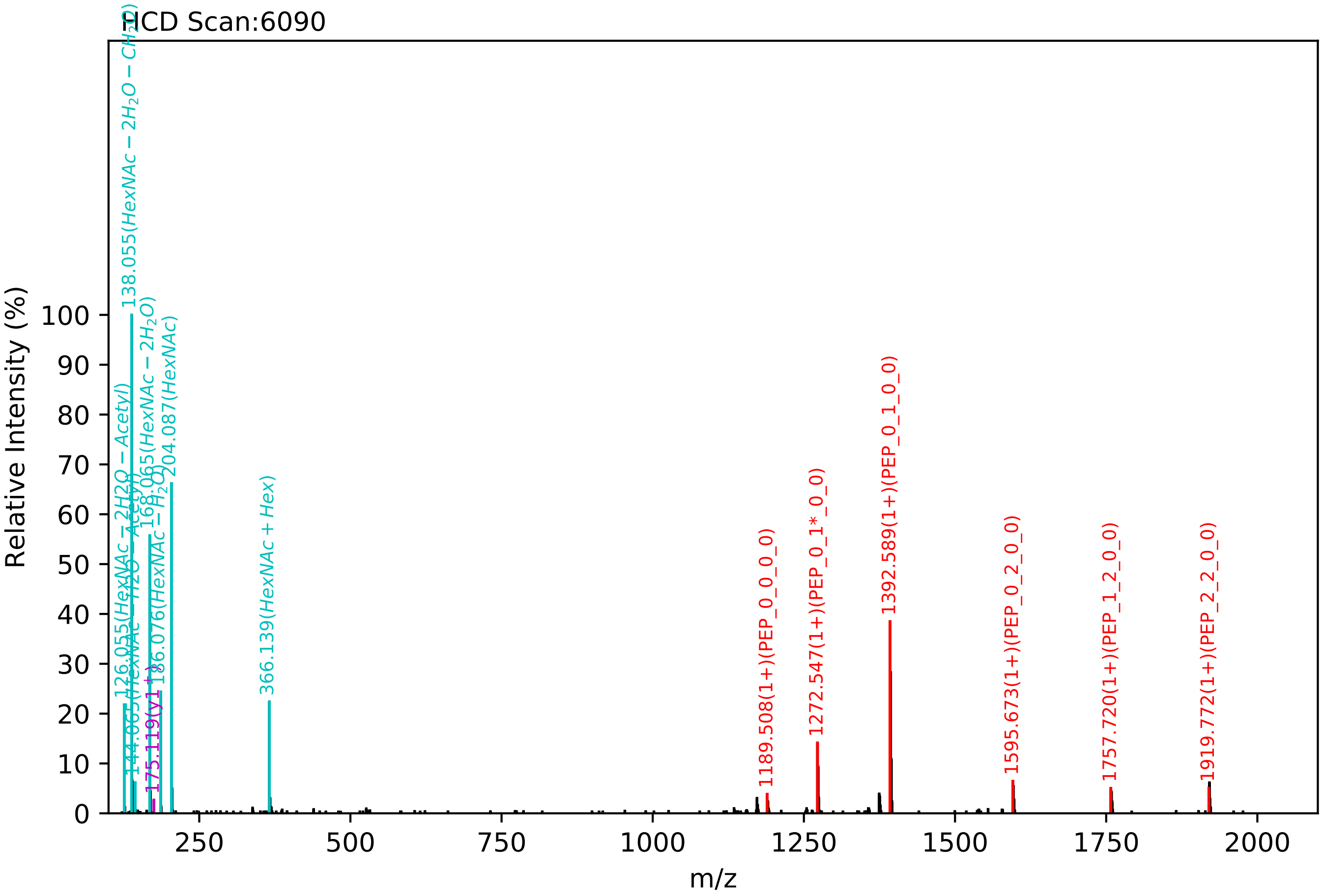

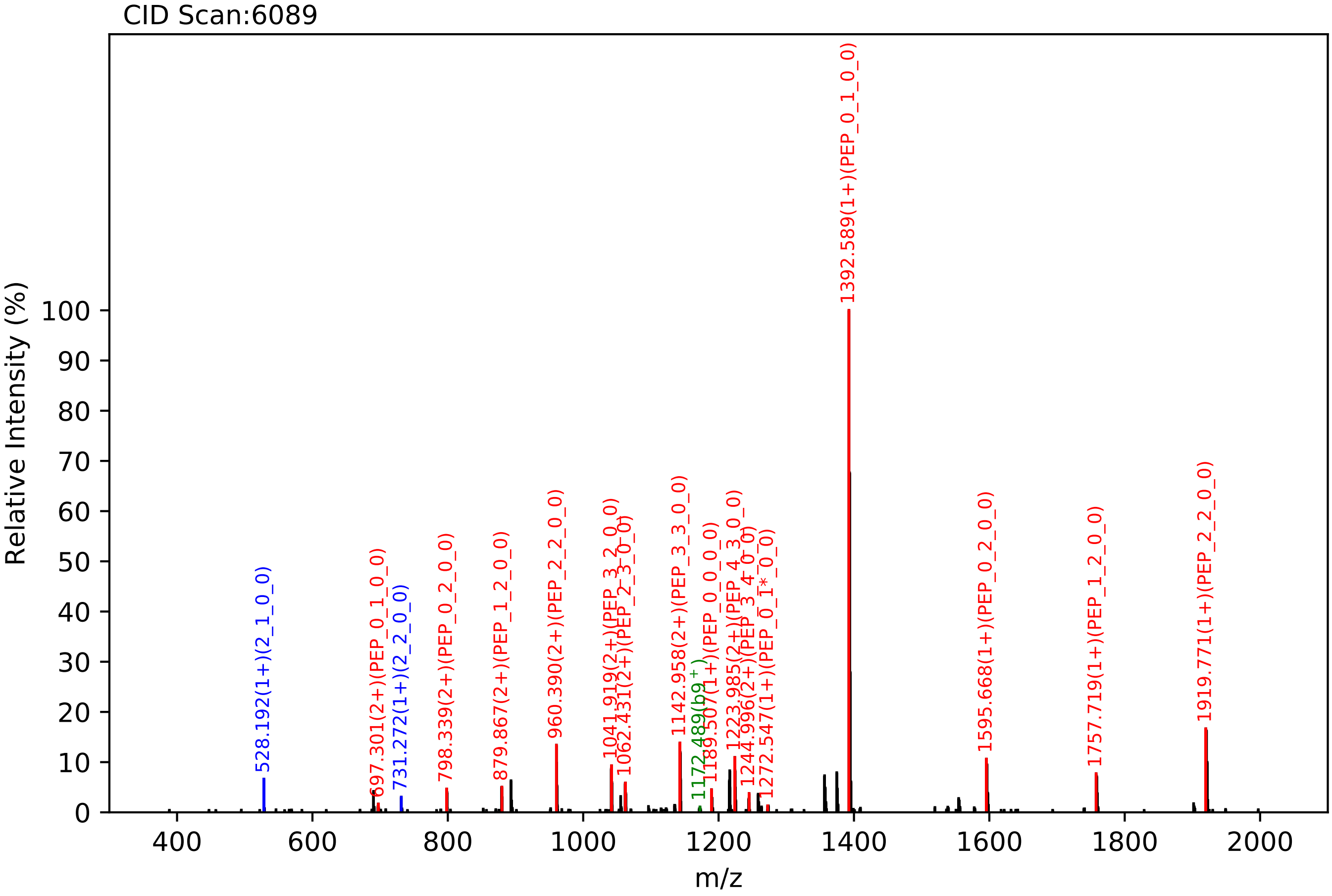

Test set no. 343, Experiment: IgG exp_7

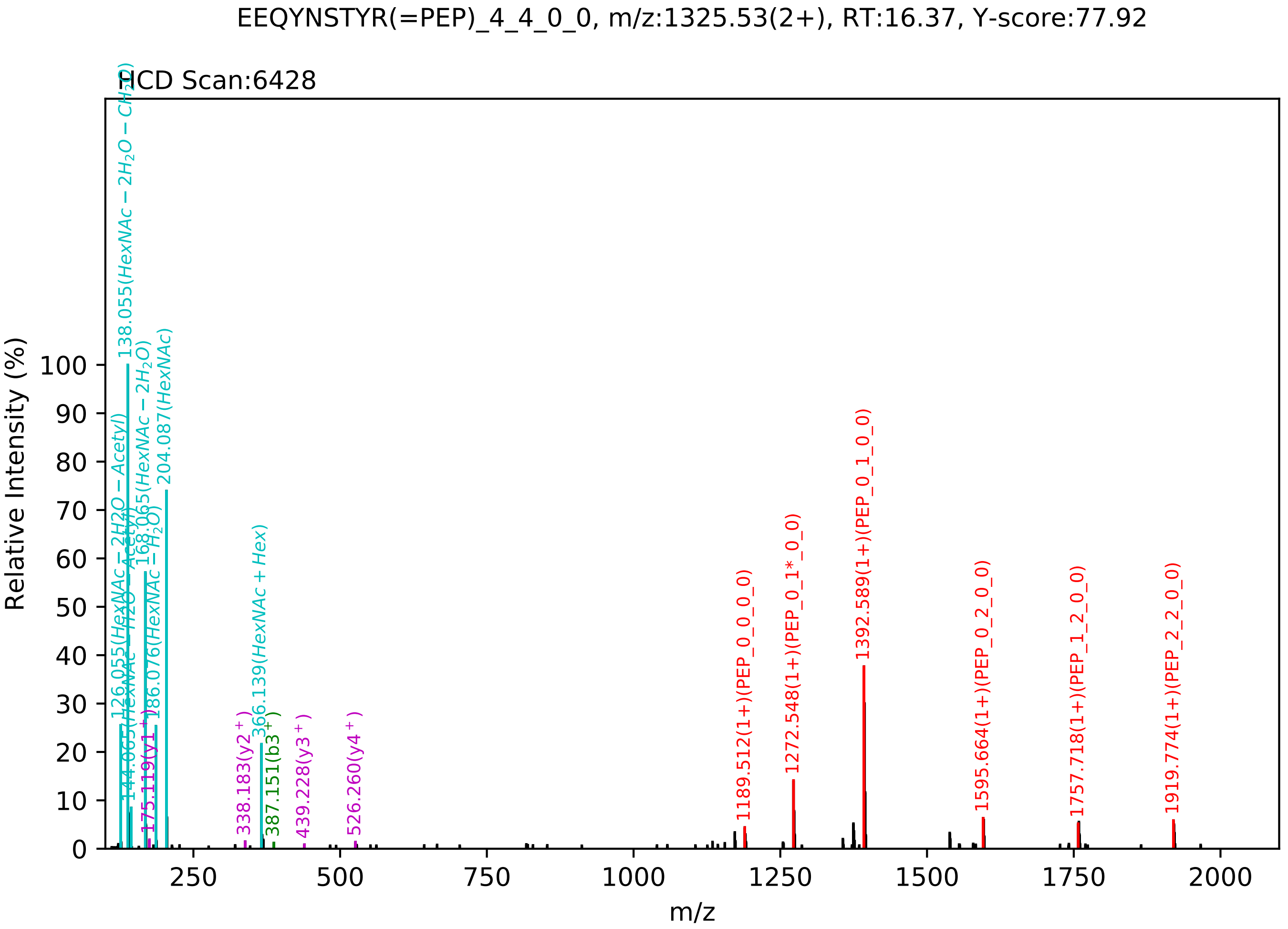

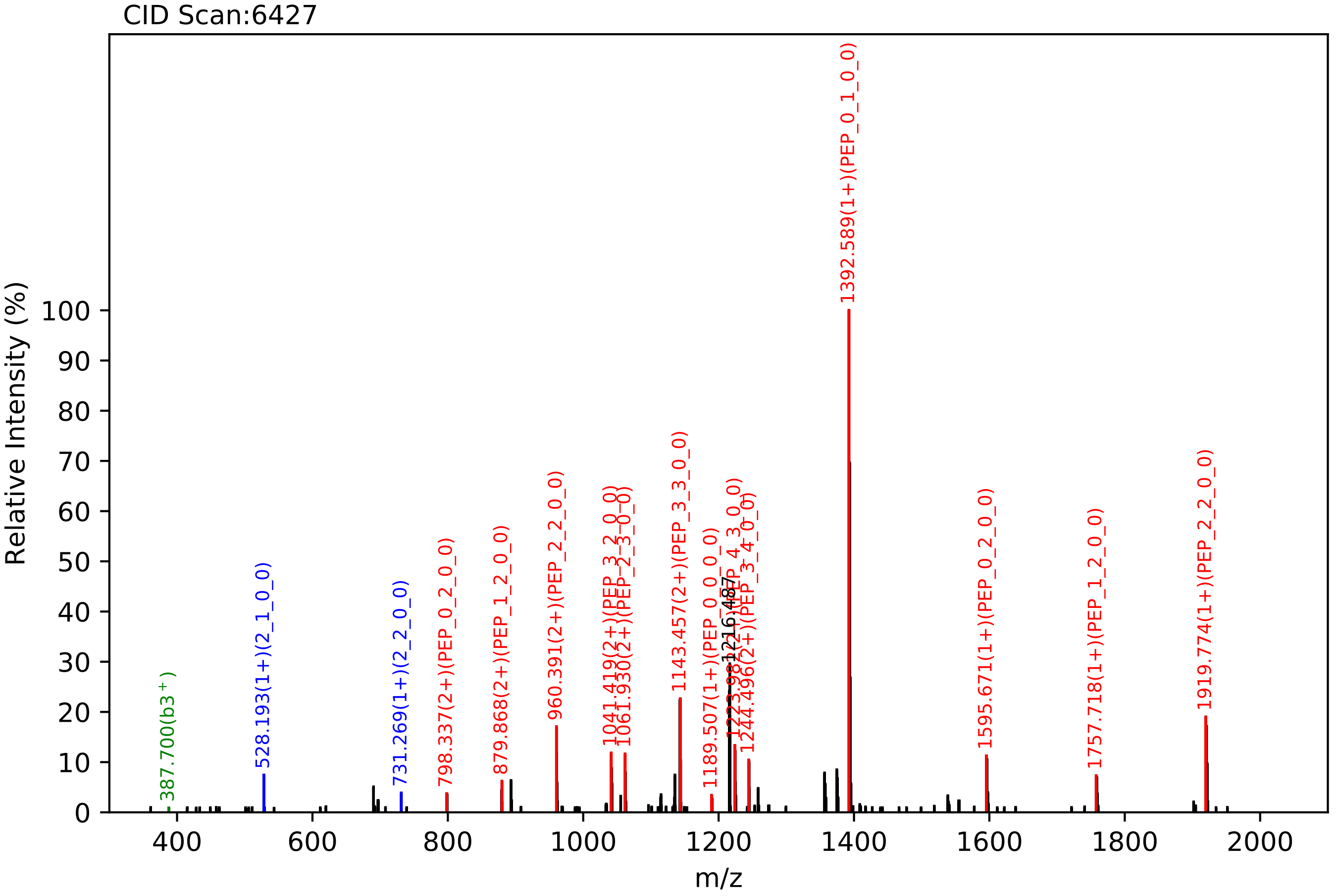

Test set no. 344, Experiment: IgG exp_6

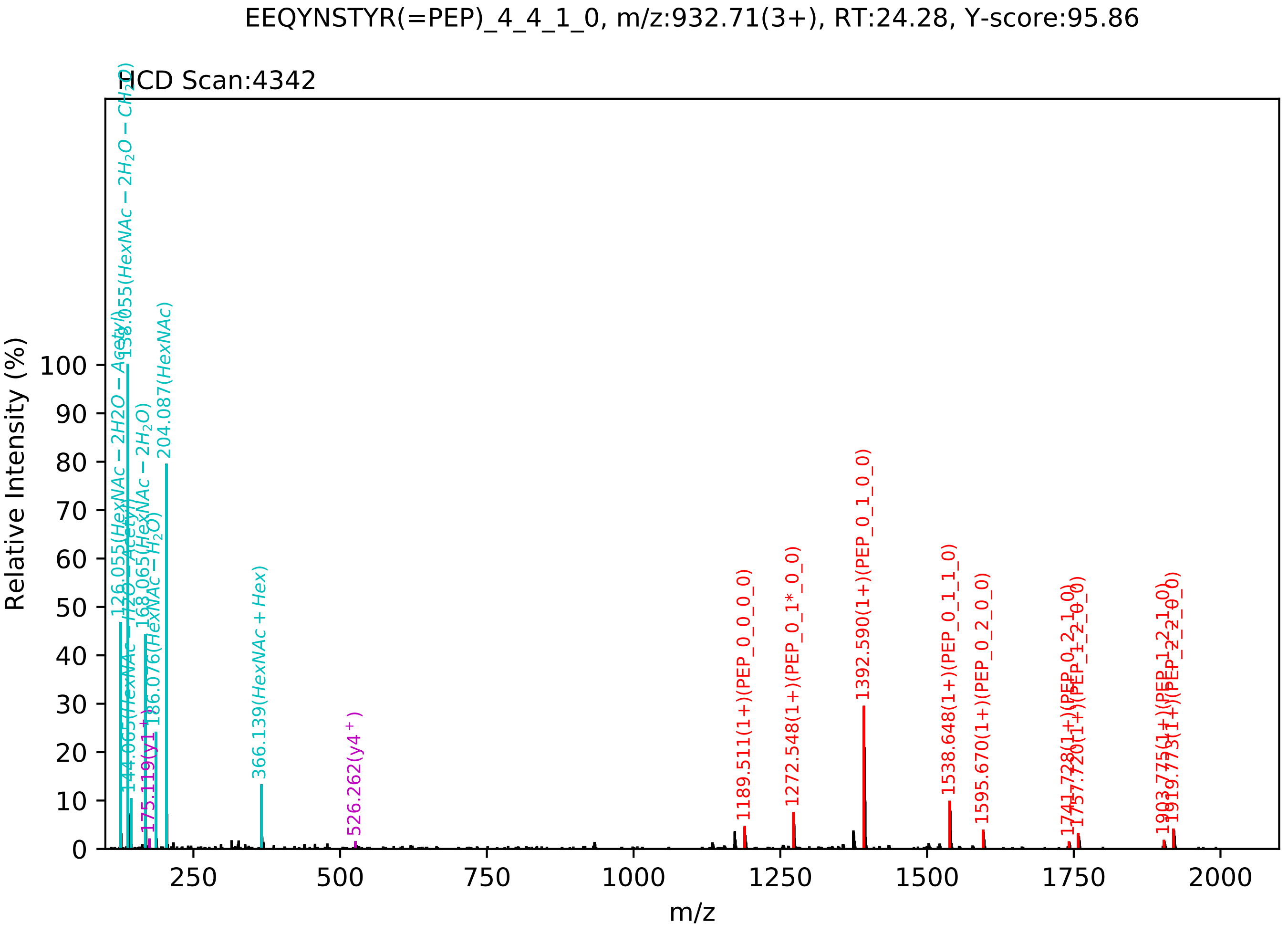

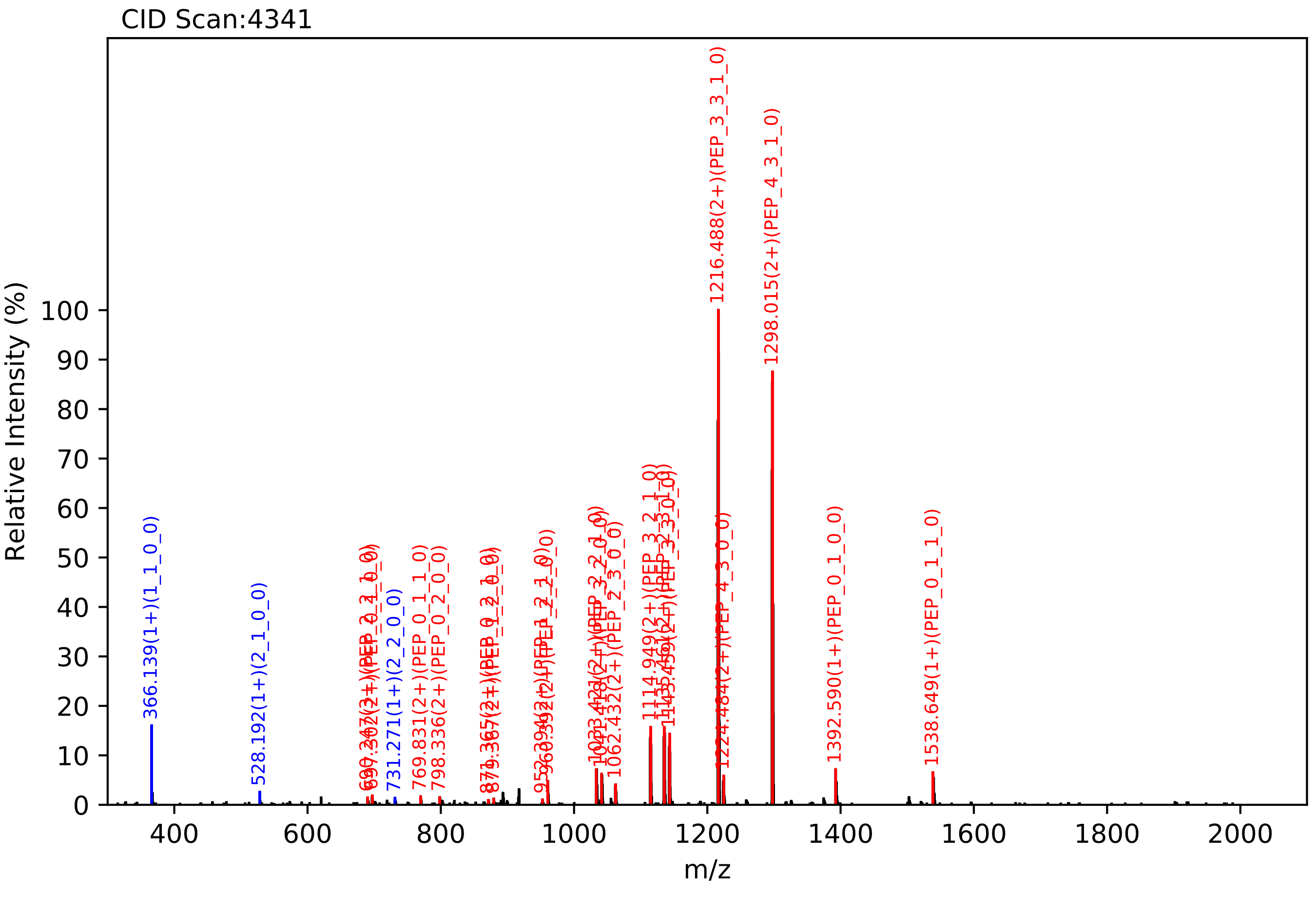

Test set no. 345, Experiment: IgG exp_7

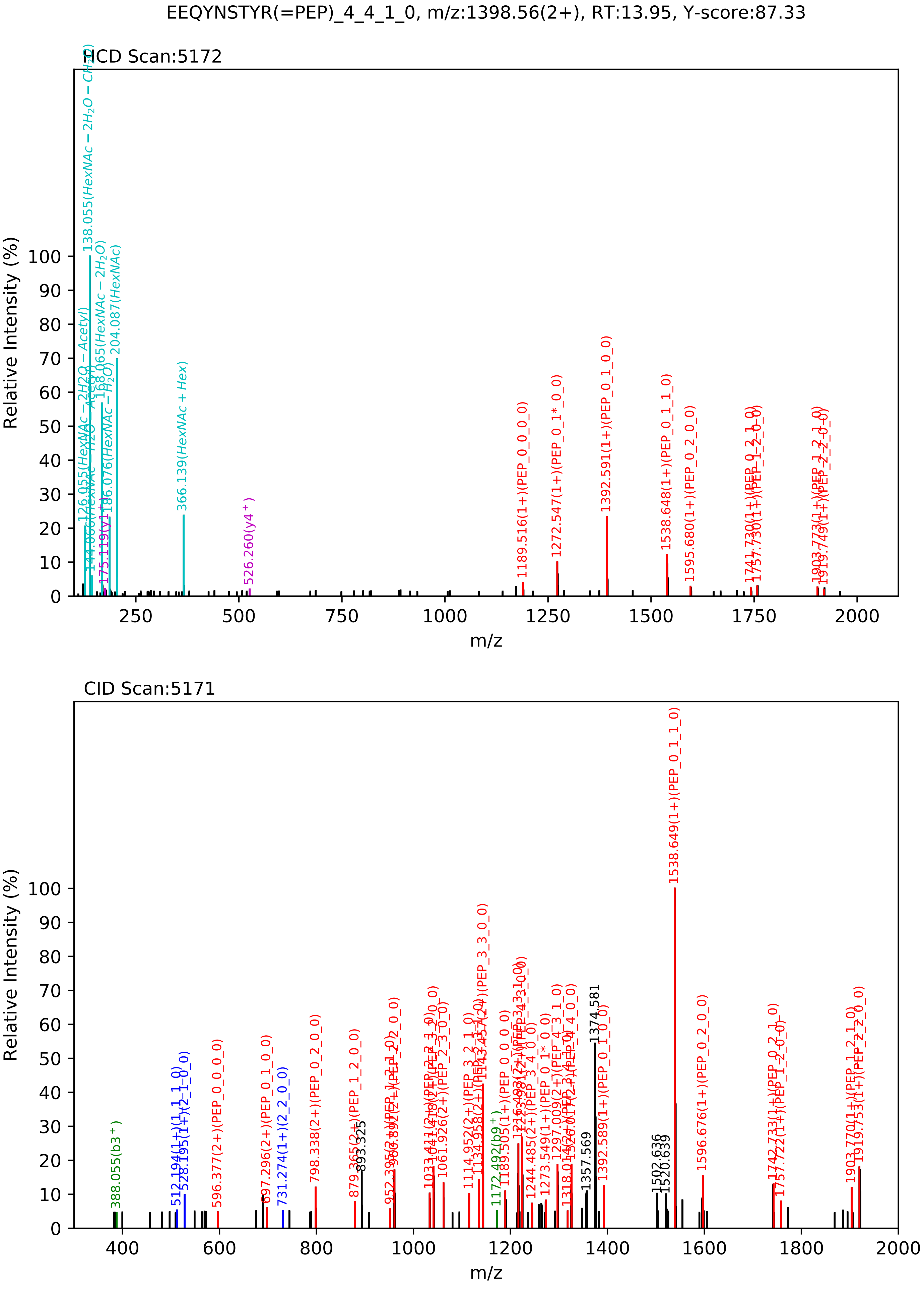

Test set no. 346, Experiment: IgG exp_7

EEQYNSTYR(=PEP)_4_4_1_0, m/z:1398.55(2+), RT:15.00, Y-score:85.04

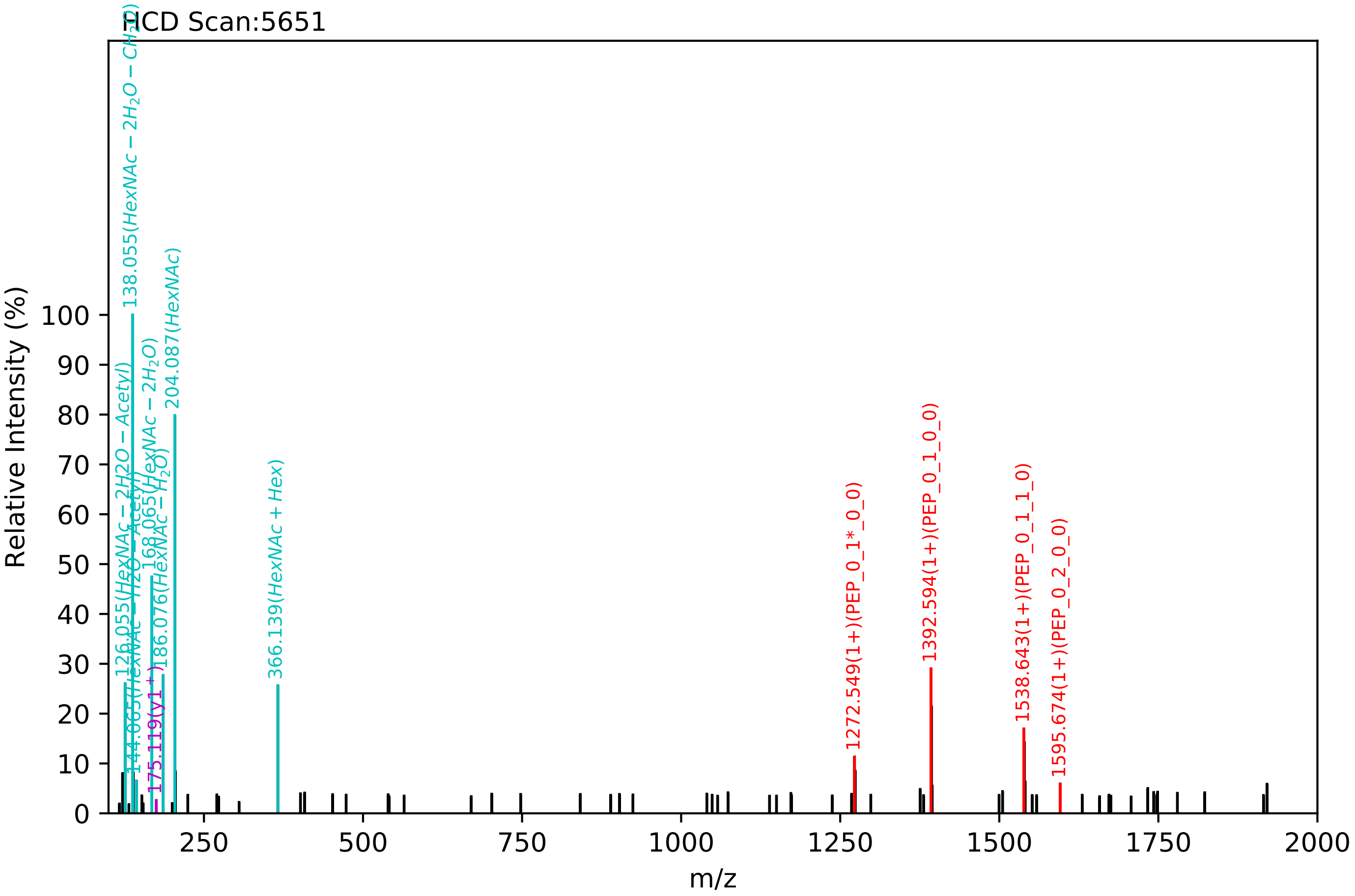

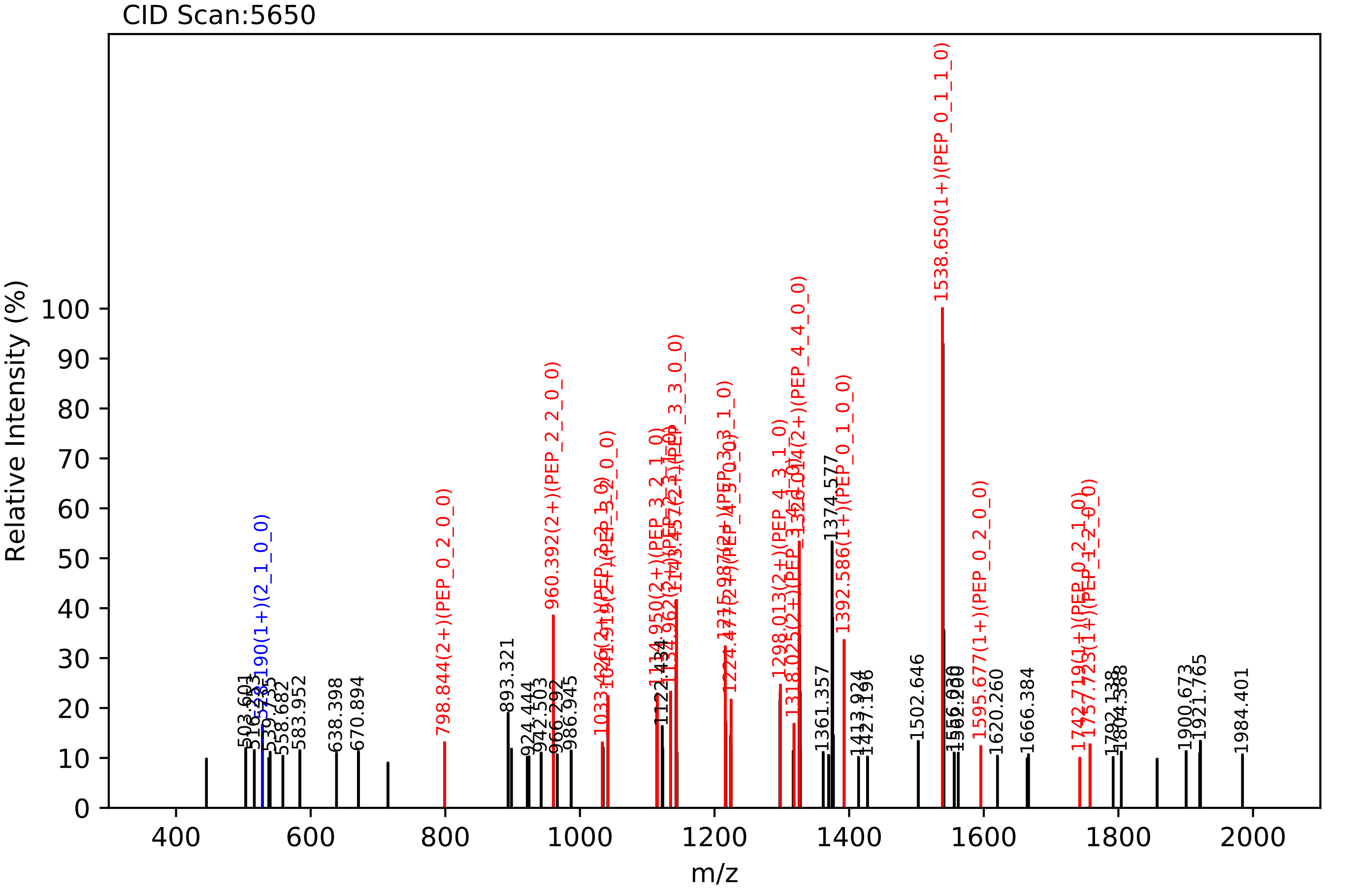

Test set no. 347, Experiment: IgG exp_7

EEQYNSTYR(=PEP)_4_4_1_0, m/z:1398.55(2+), RT:17.23, Y-score:84.62

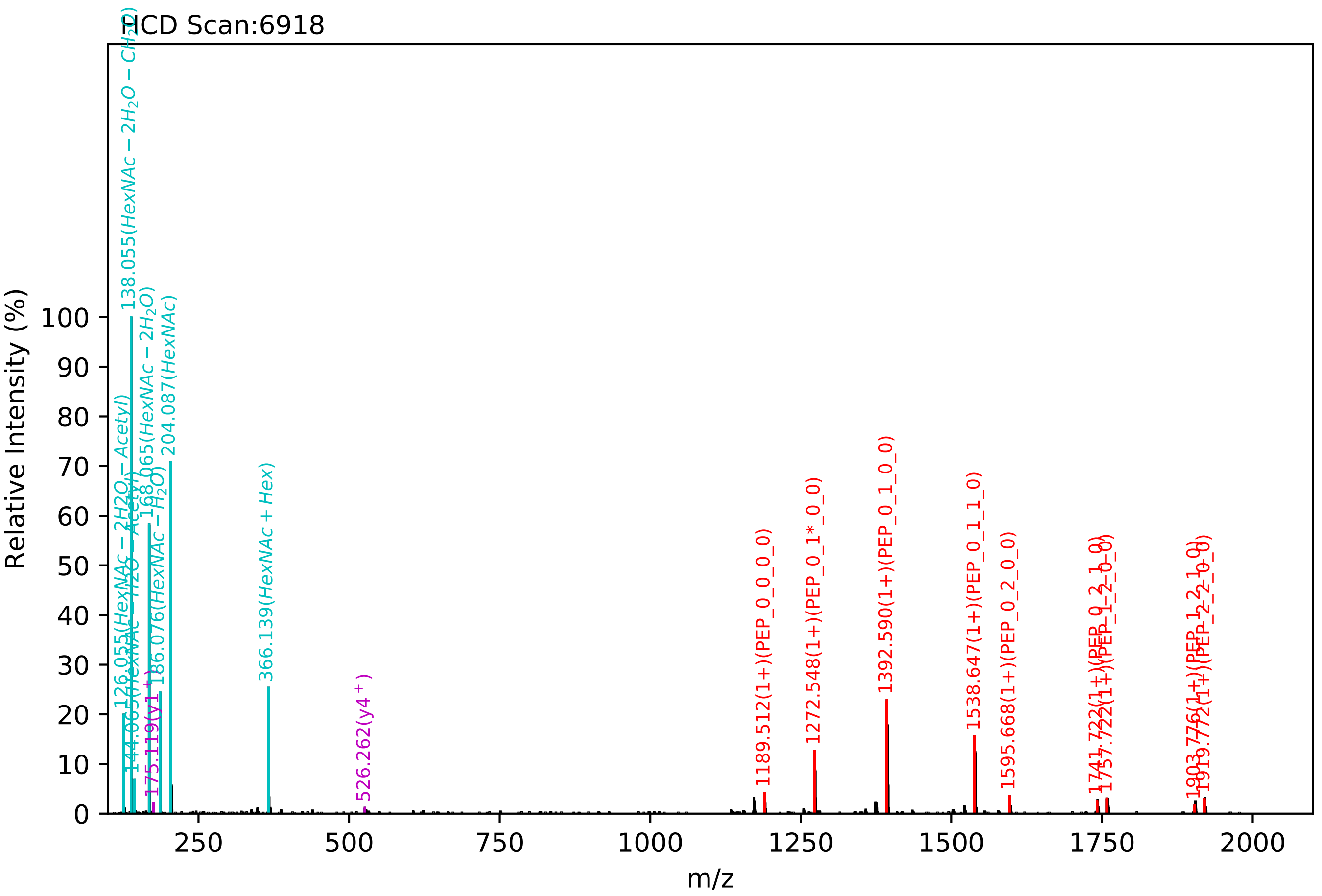

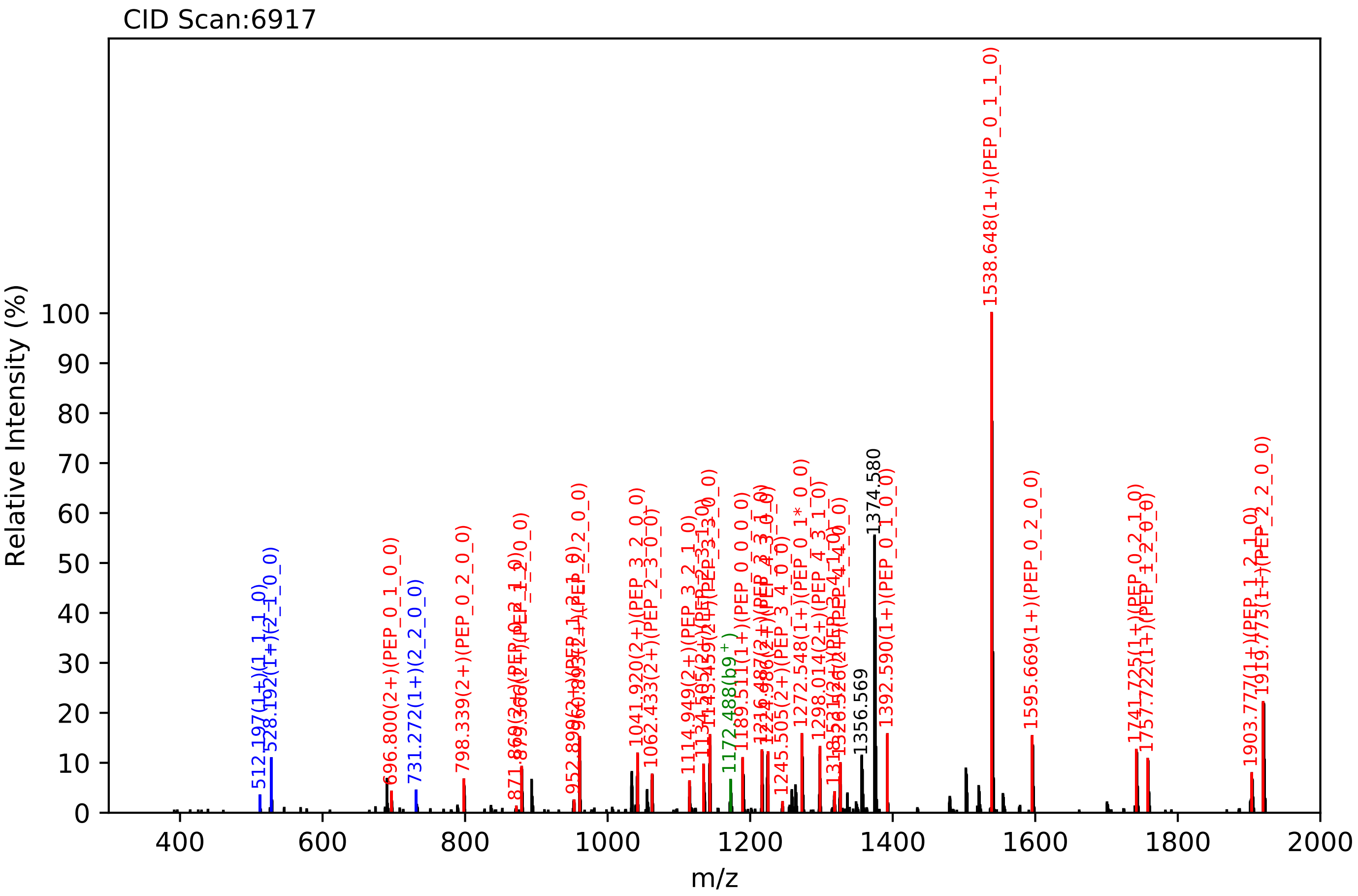

Test set no. 348, Experiment: IgG exp_7

EEQYNSTYR(=PEP)_4_4_1_0, m/z:1398.55(2+), RT:15.55, Y-score:84.19

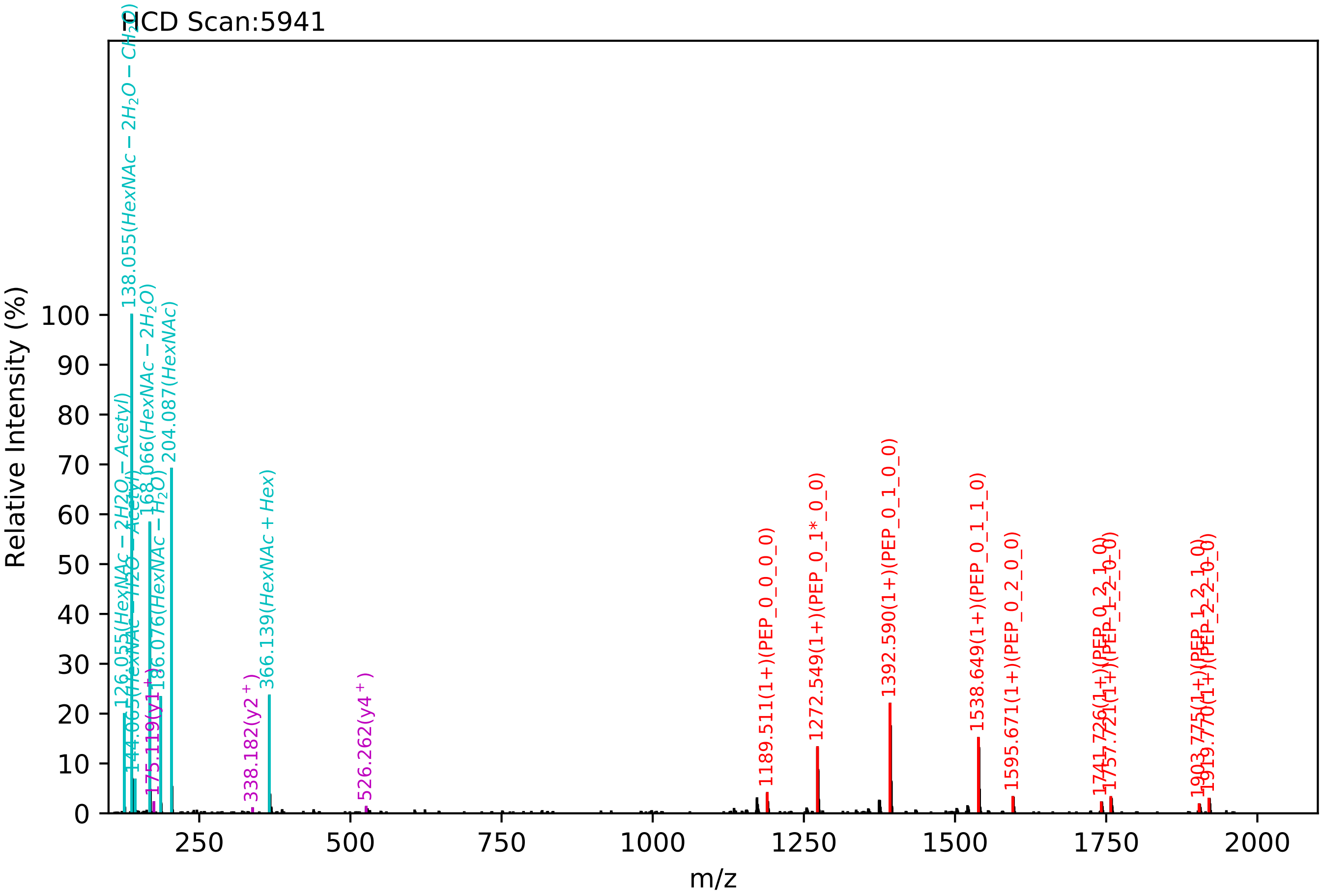

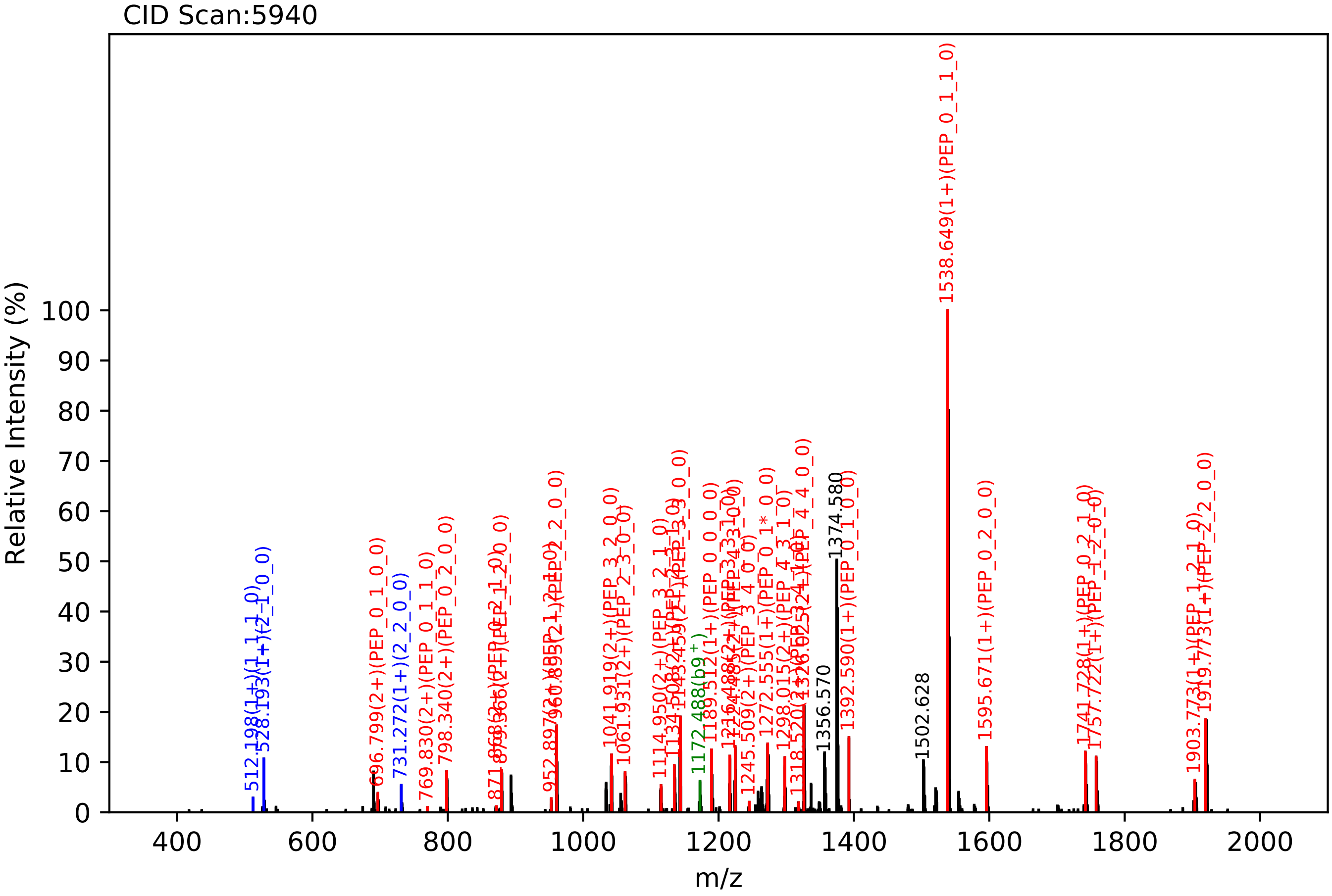

Test set no. 349, Experiment: IgG exp_7

EEQYNSTYR(=PEP)_4_4_1_0, m/z:1398.56(2+), RT:20.90, Y-score:83.78

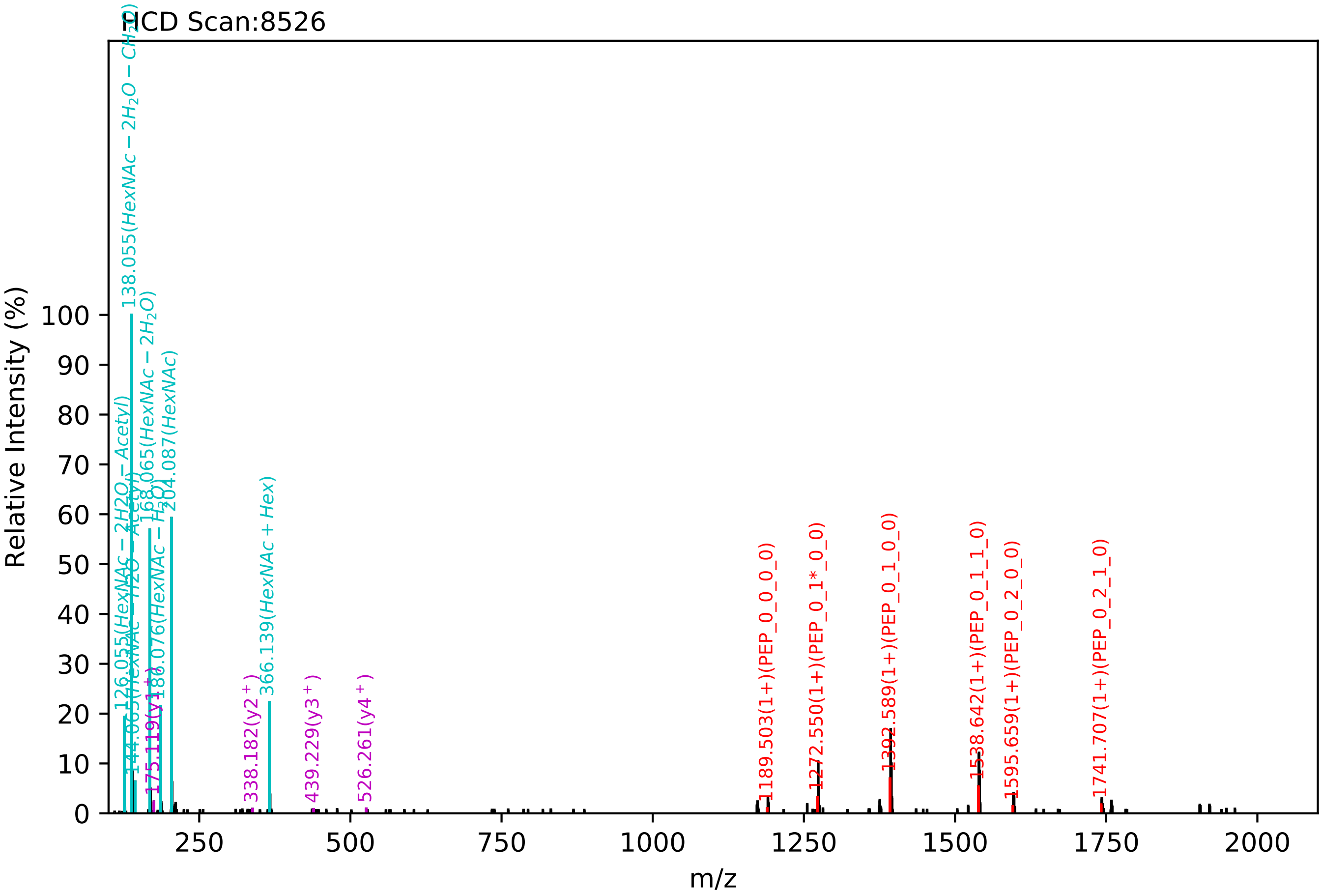

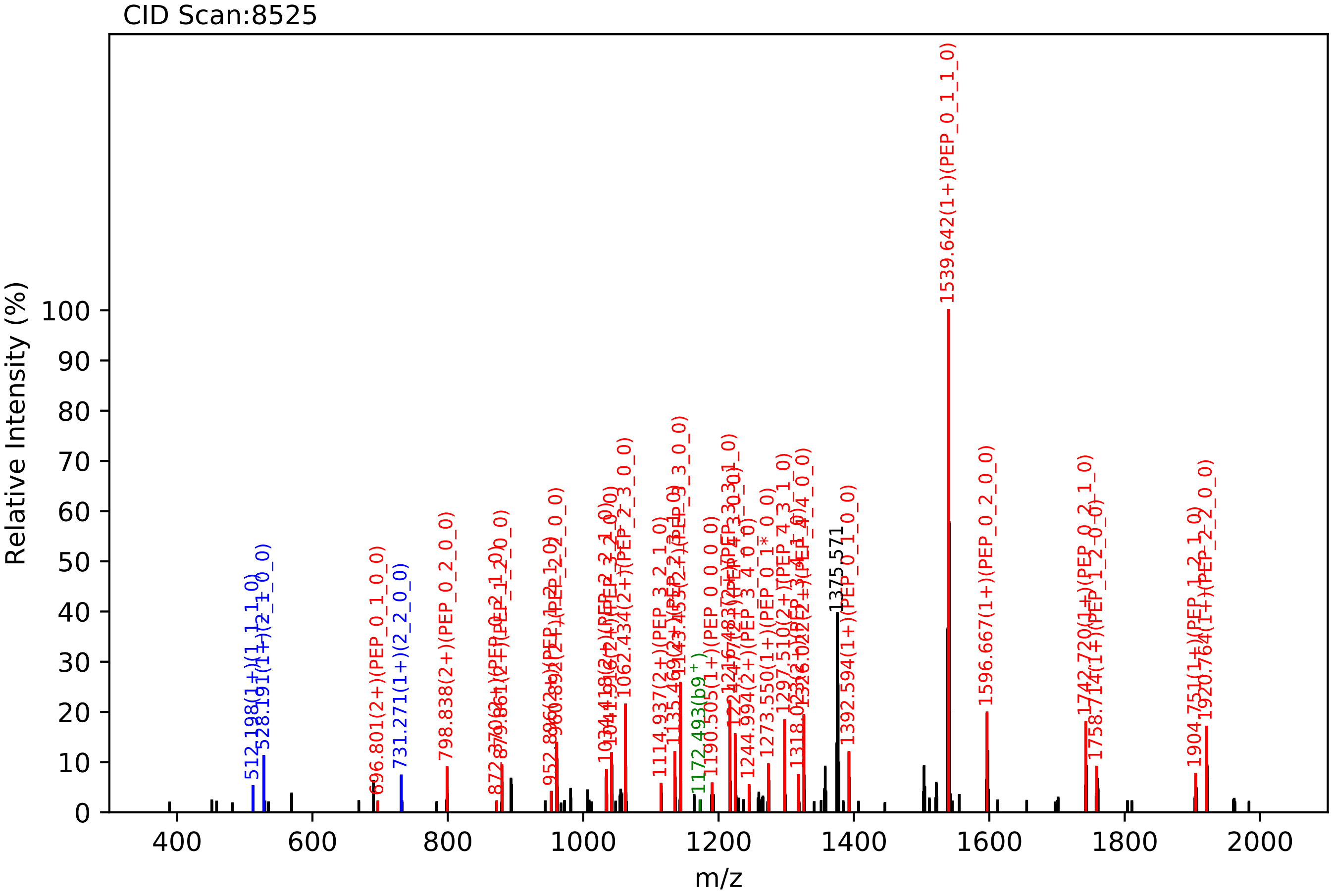

Test set no. 350, Experiment: IgG exp_7

EEQYNSTYR(=PEP)_4_4_1_0, m/z:1398.56(2+), RT:16.69, Y-score:83.16

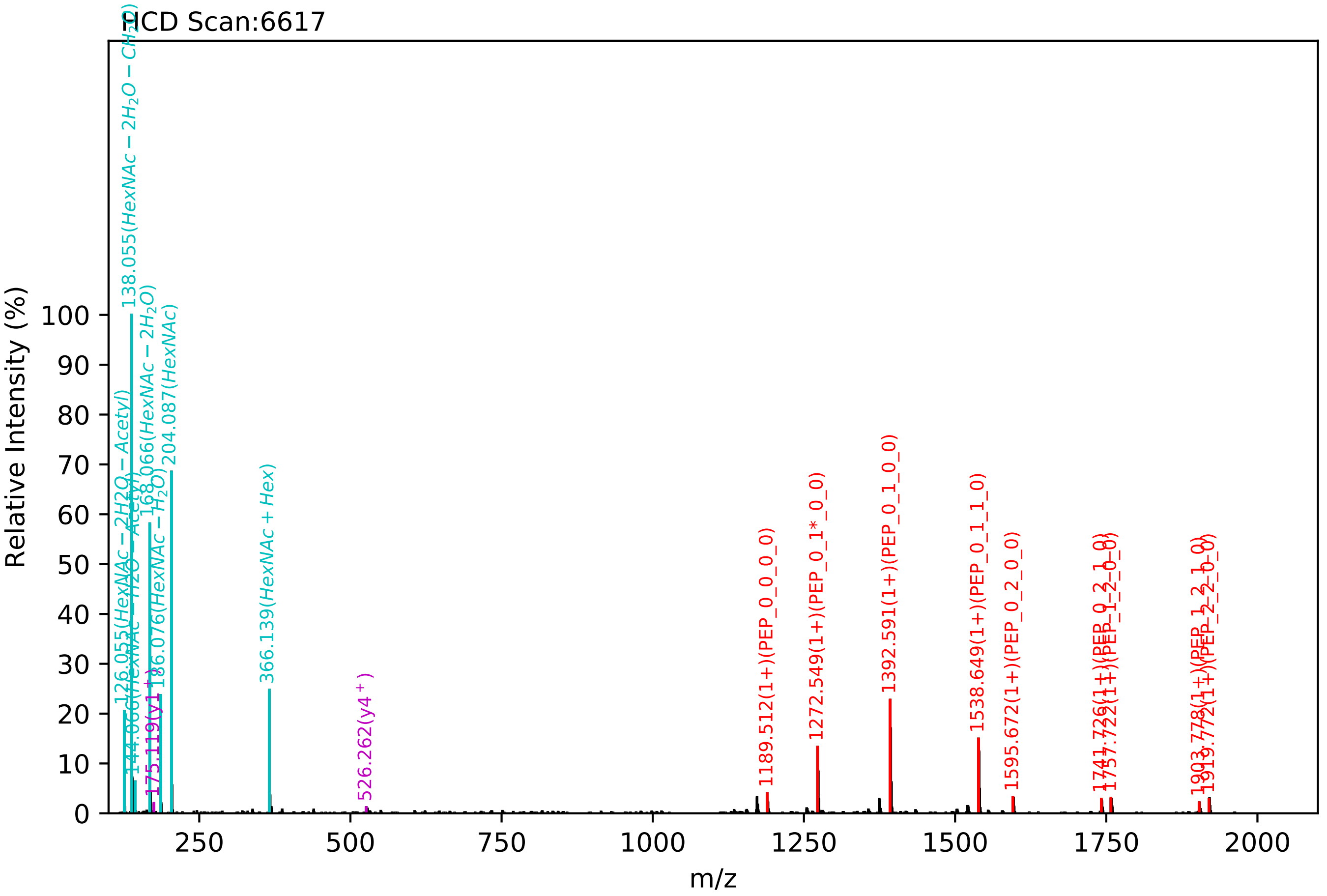

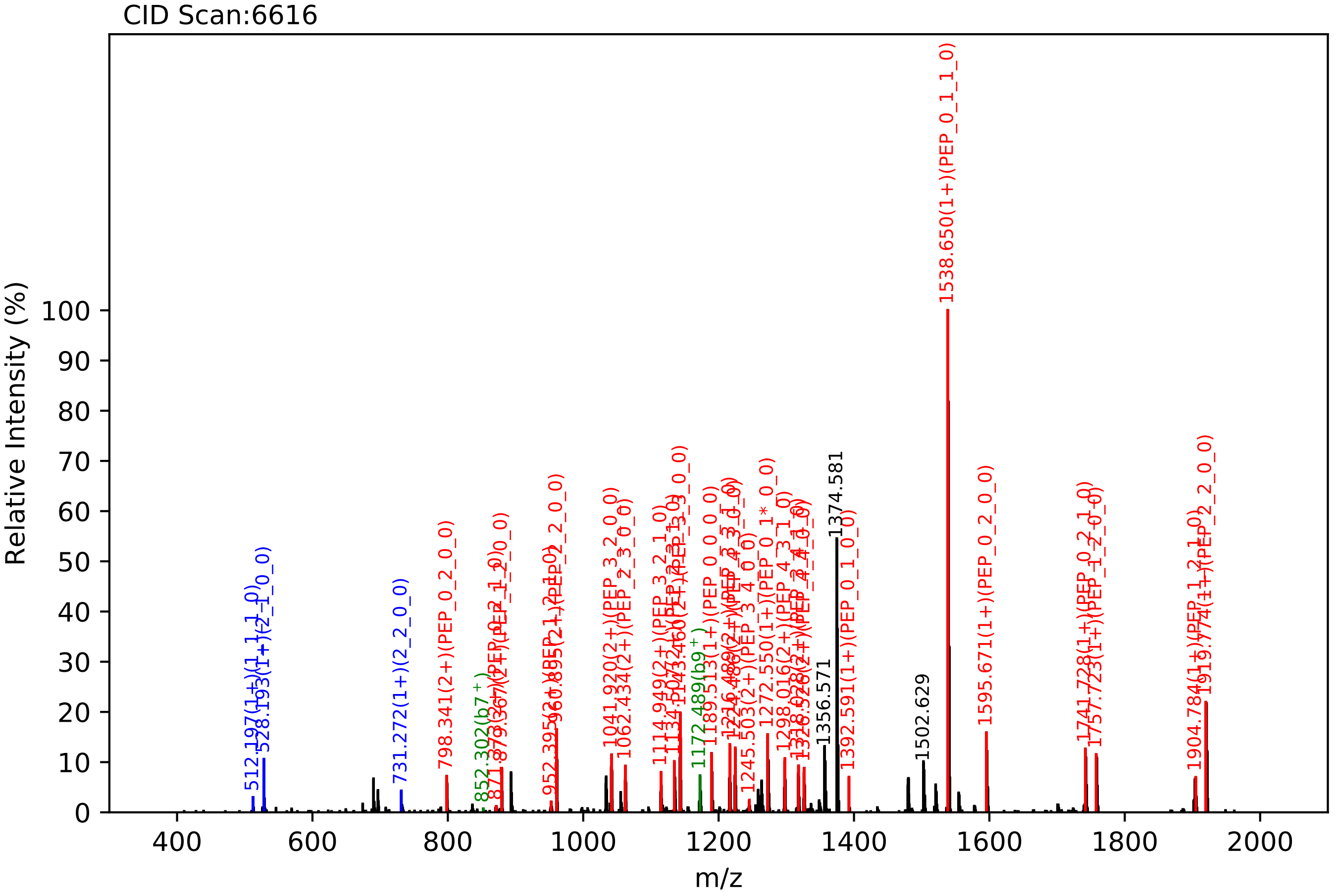

Test set no. 351, Experiment: IgG exp_7

EEQYNSTYR(=PEP)_4_4_1_0, m/z:1398.55(2+), RT:17.75, Y-score:83.13

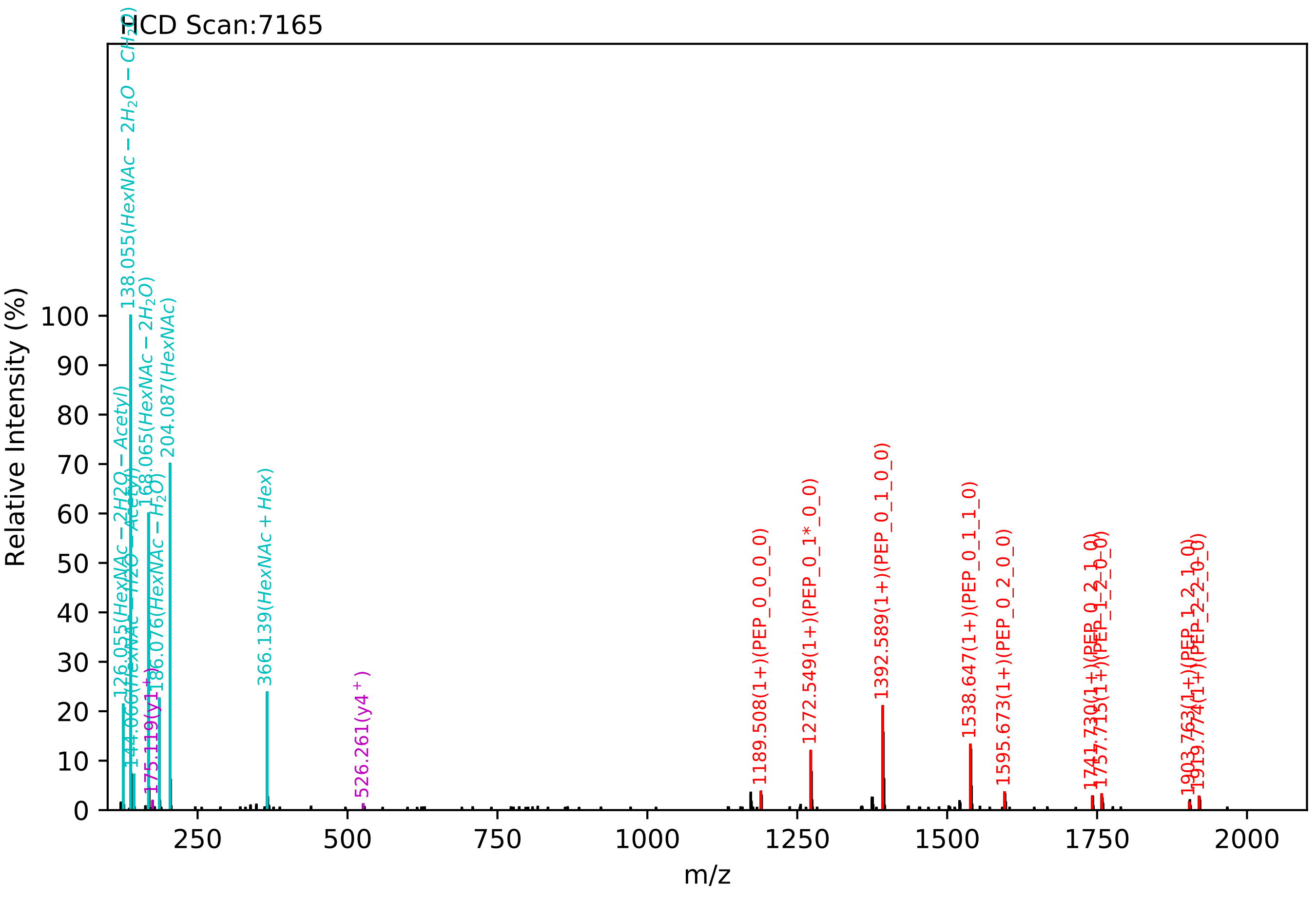

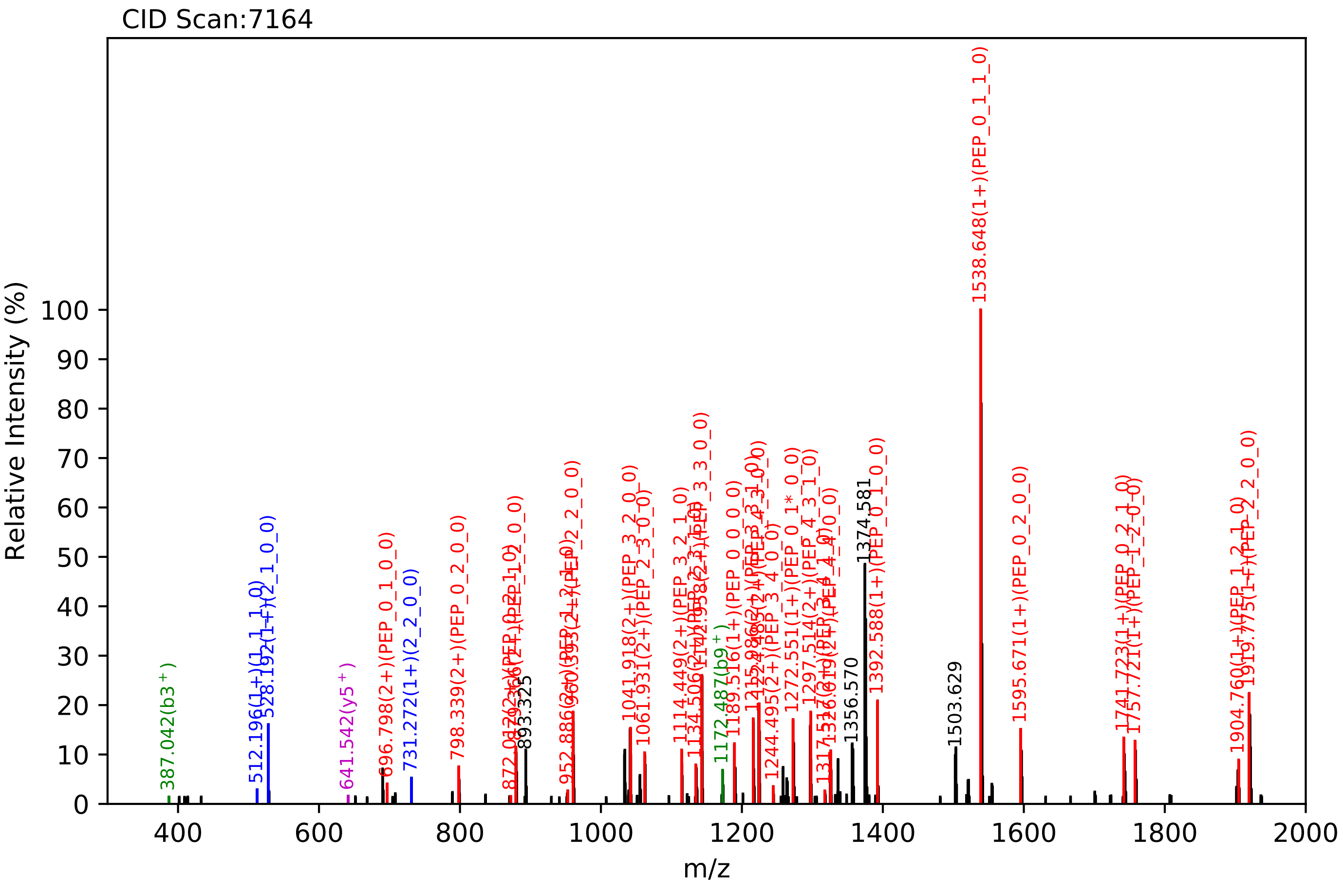

Test set no. 352, Experiment: IgG exp_7

EEQYNSTYR(=PEP)_4_4_1_0, m/z:1398.55(2+), RT:16.12, Y-score:83.11

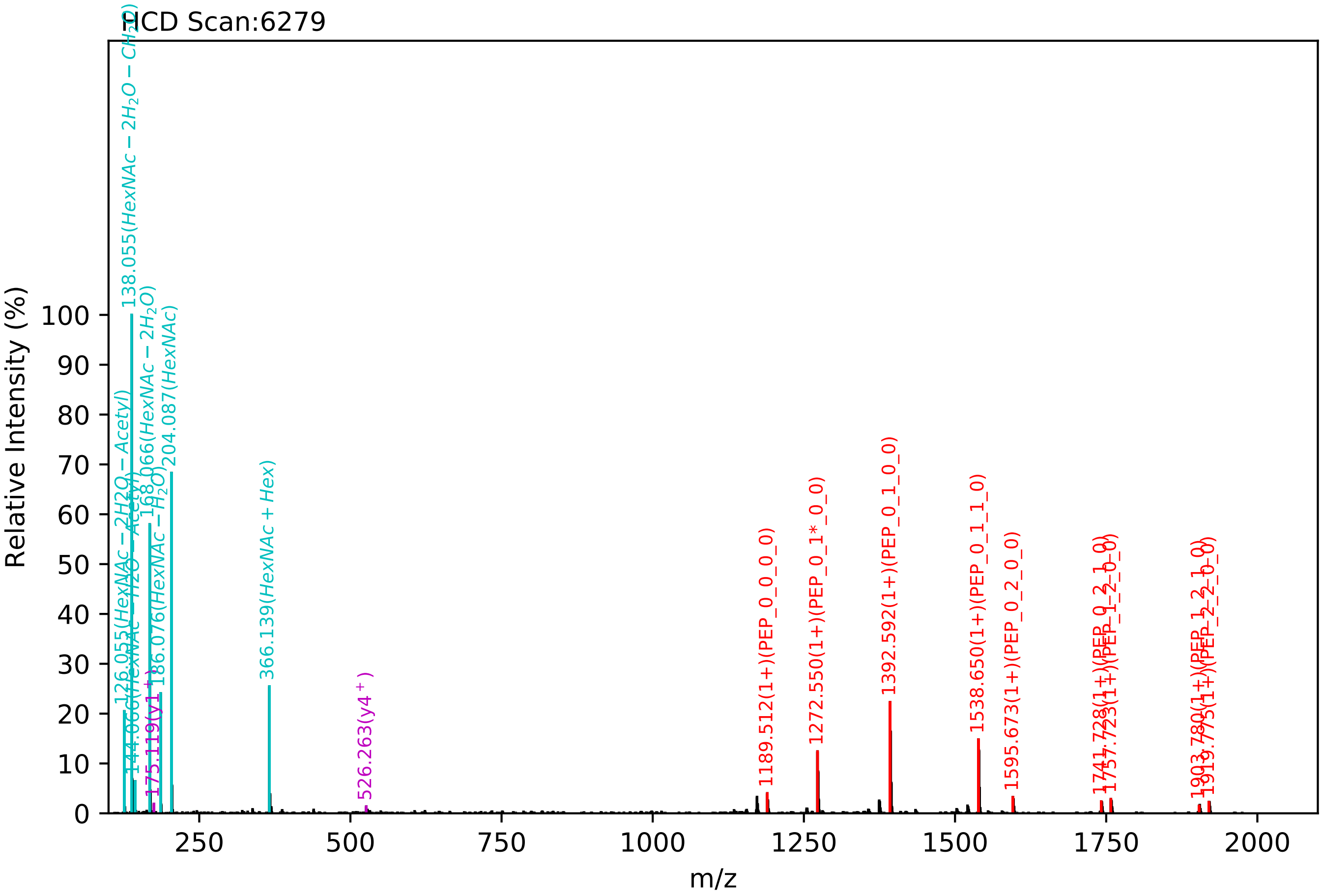

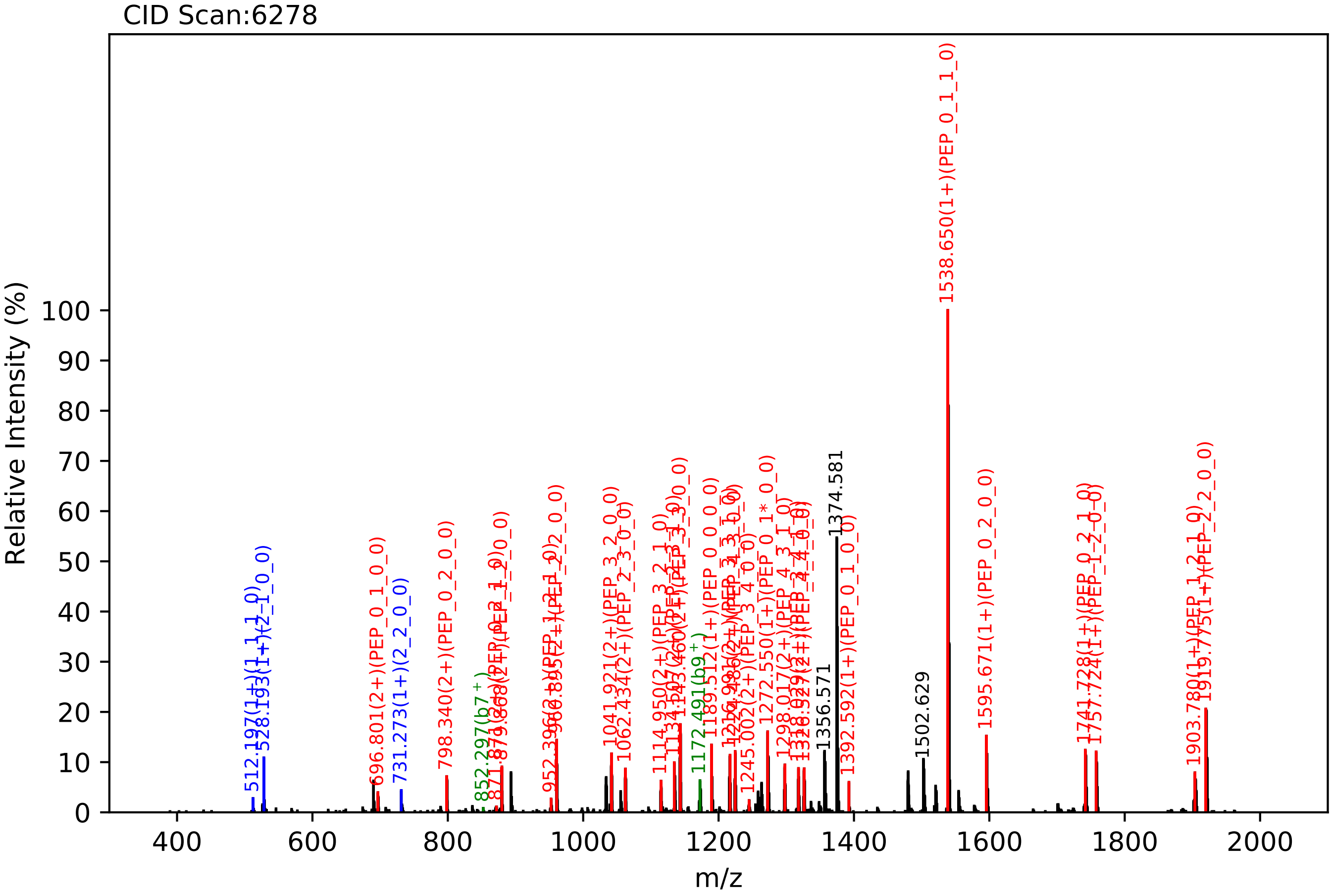

Test set no. 353, Experiment: IgG exp_5

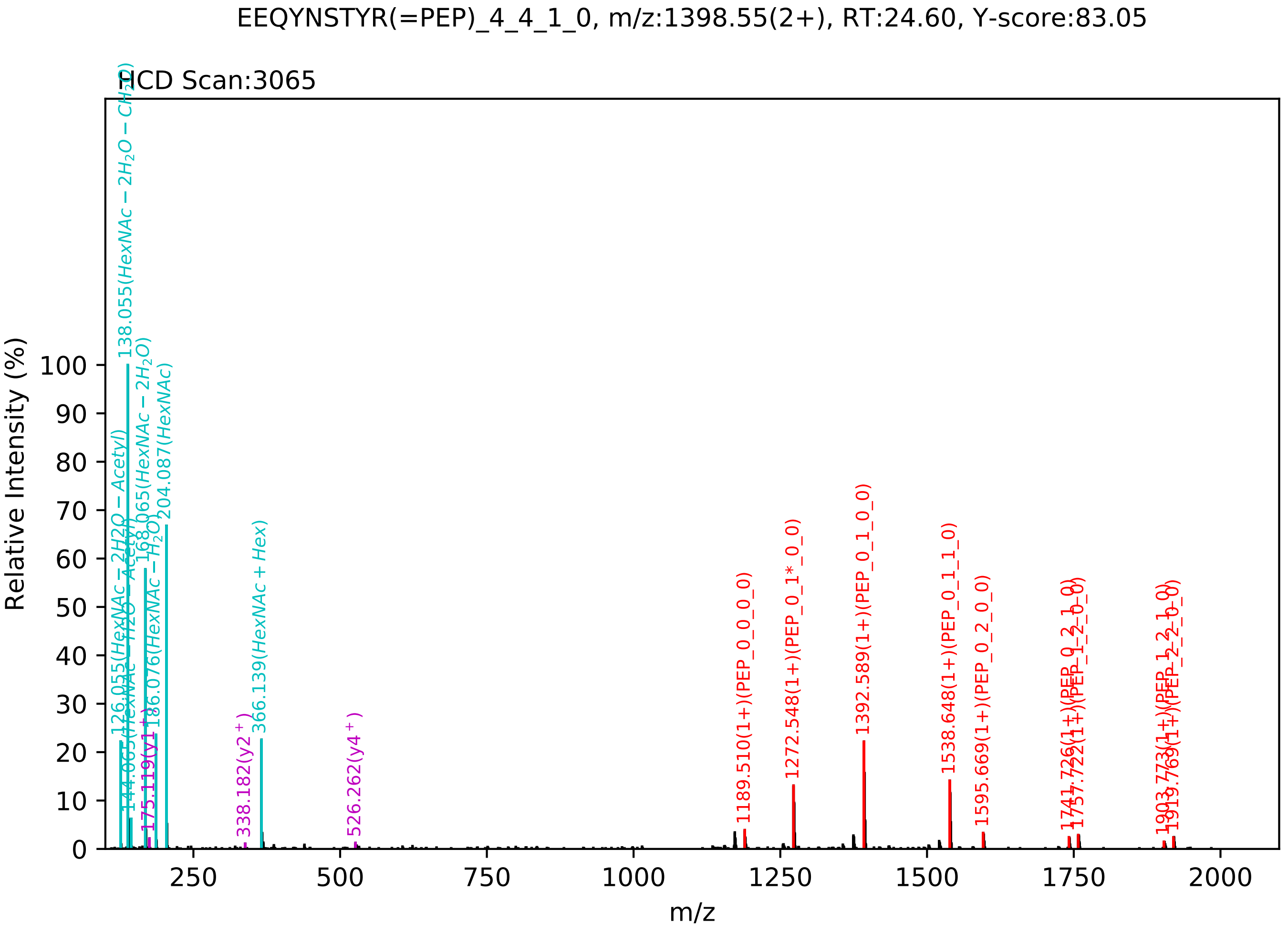

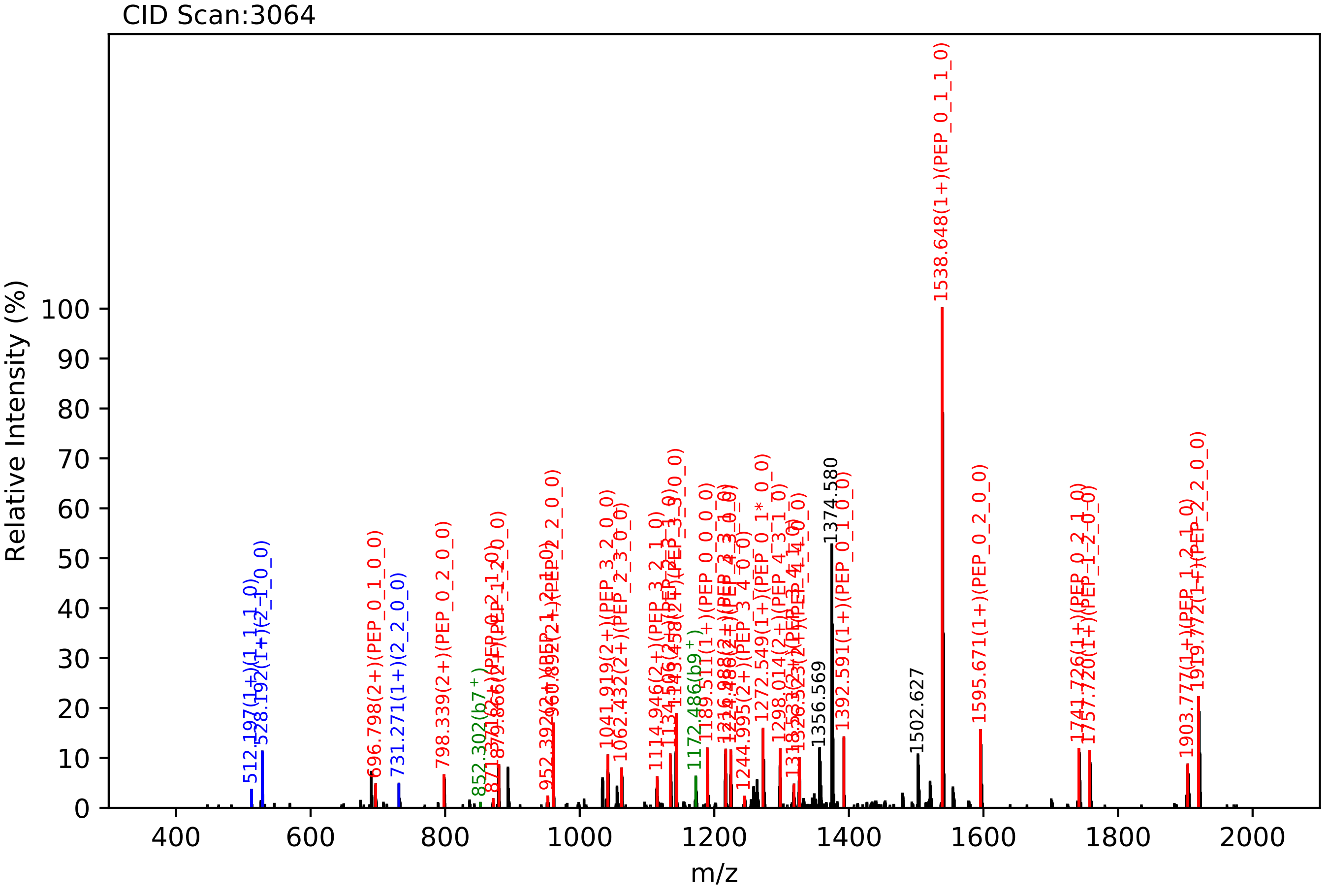

Test set no. 354, Experiment: IgG exp_7

EEQYNSTYR(=PEP)_4_4_1_0, m/z:1398.55(2+), RT:18.26, Y-score:82.34

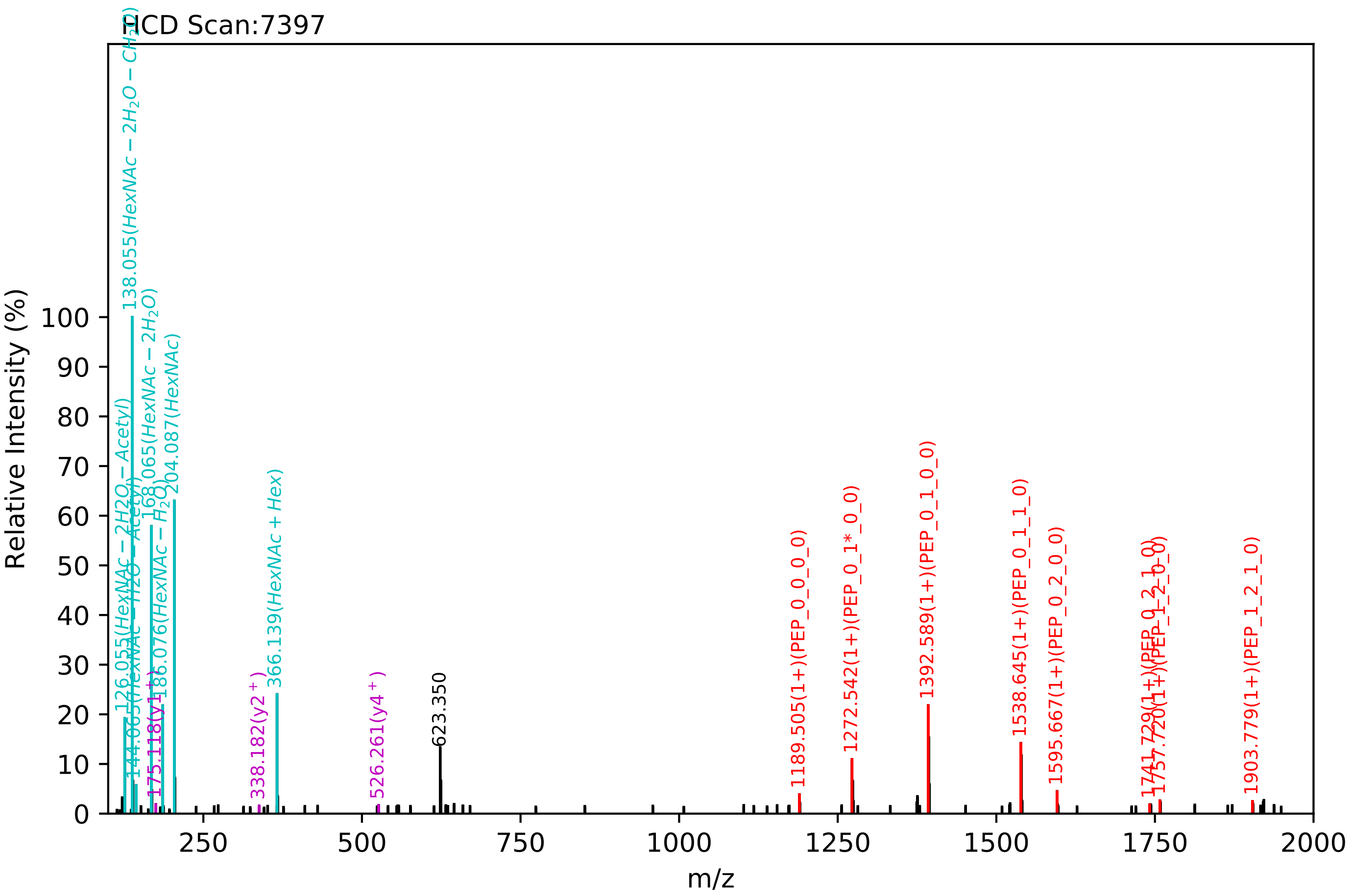

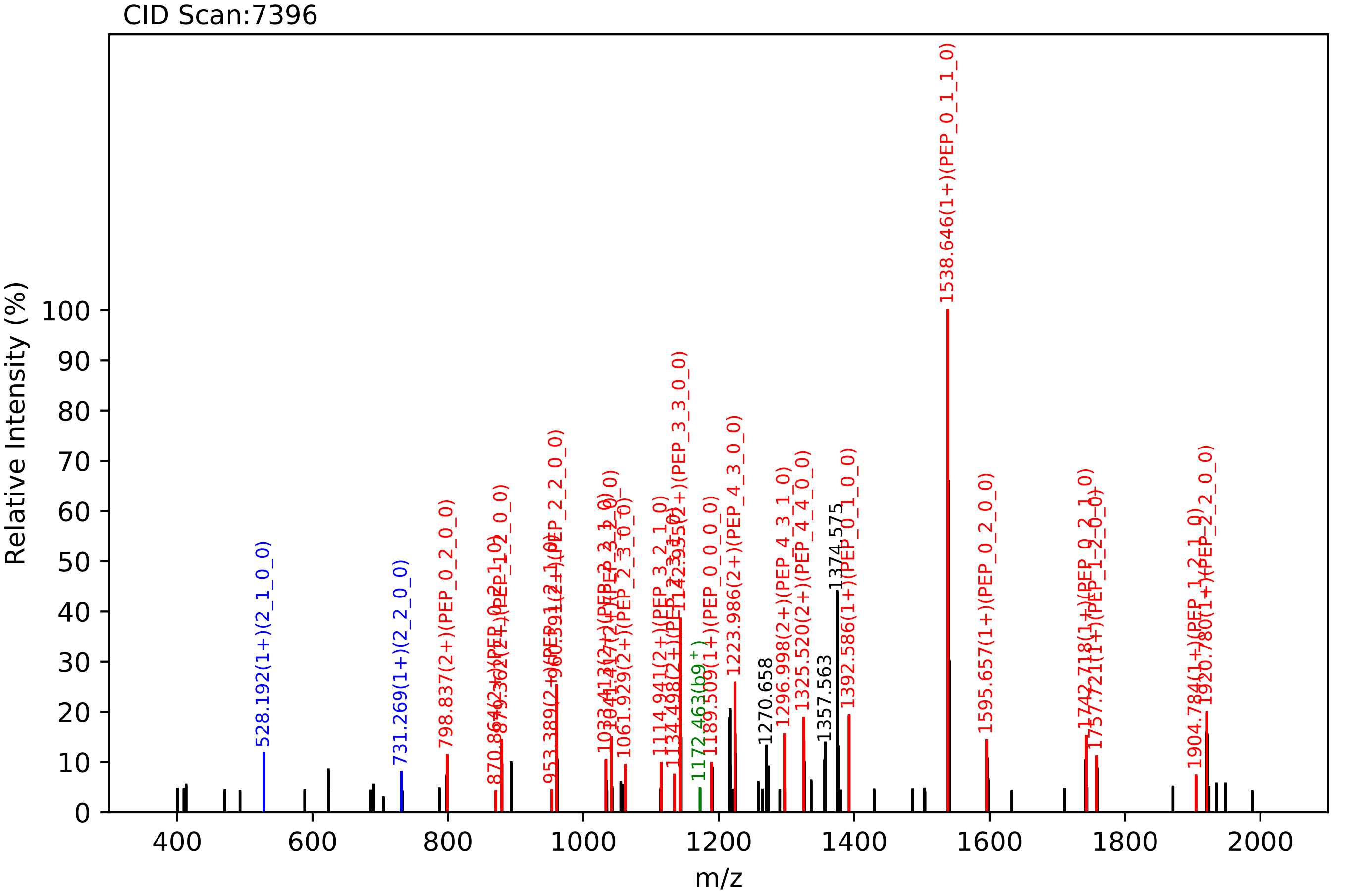

Test set no. 355, Experiment: IgG exp_6

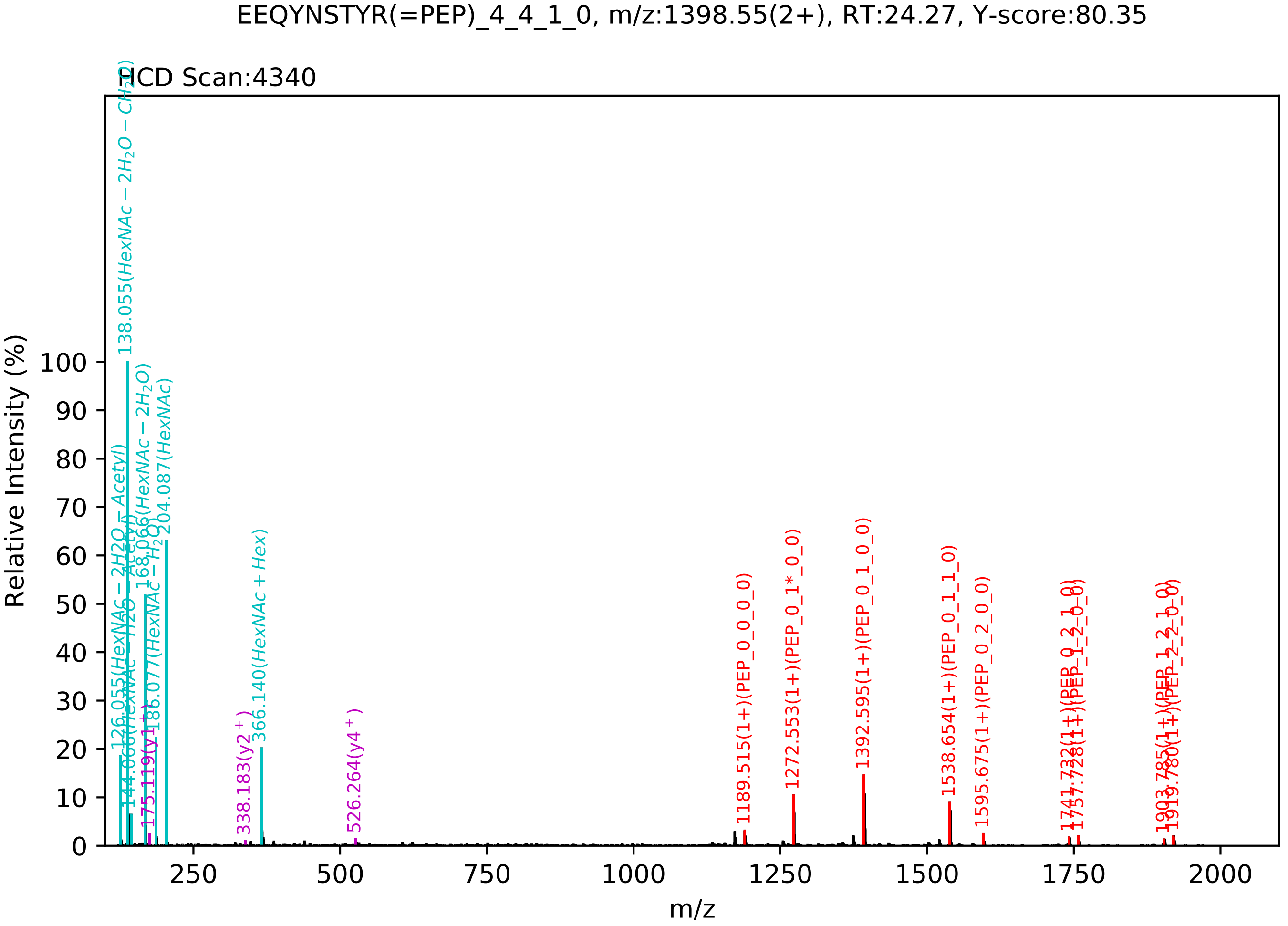

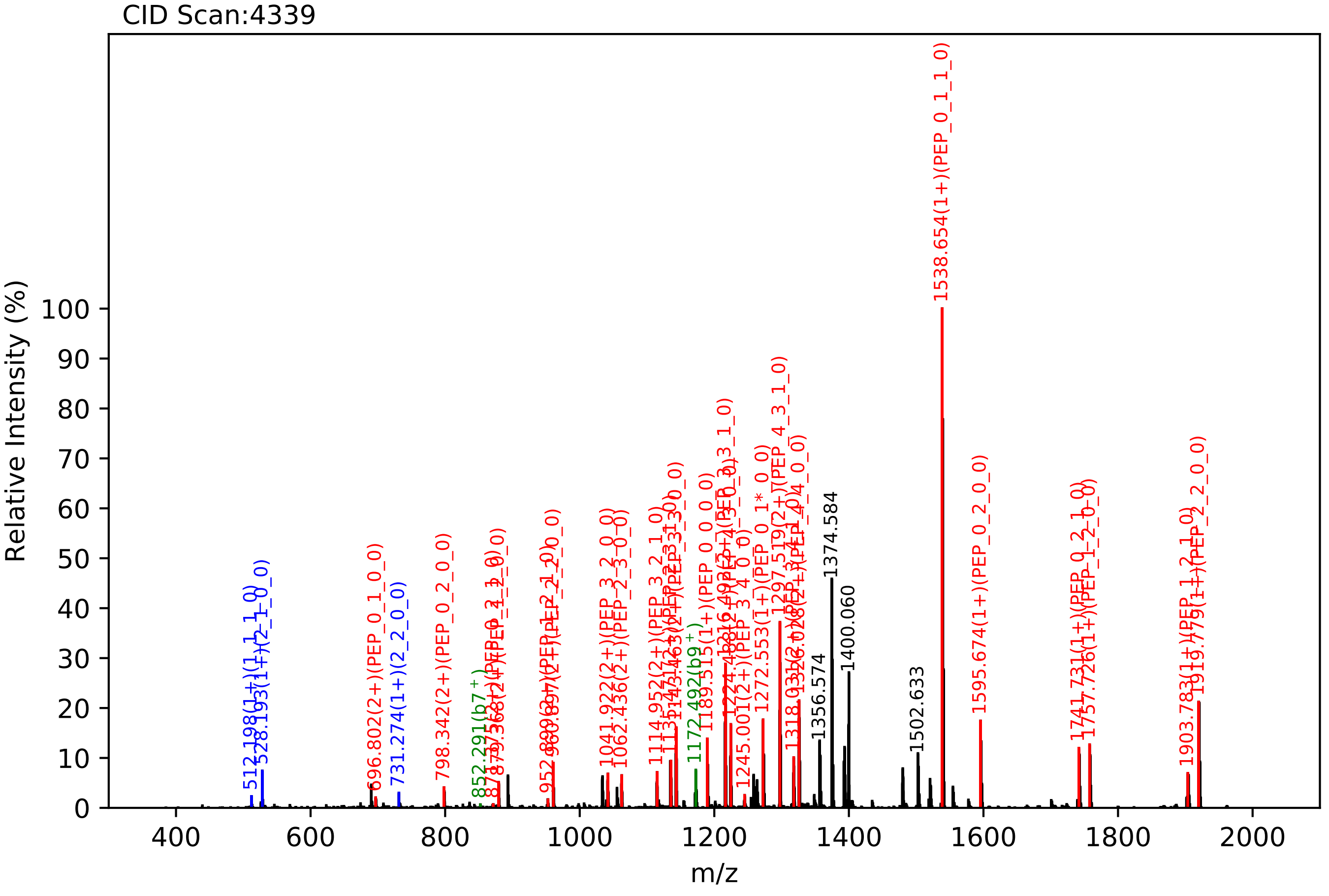

Test set no. 356, Experiment: IgG exp_7

EEQYNSTYR(=PEP)_4_4_1_1, m/z:1029.74(3+), RT:23.08, Y-score:94.22

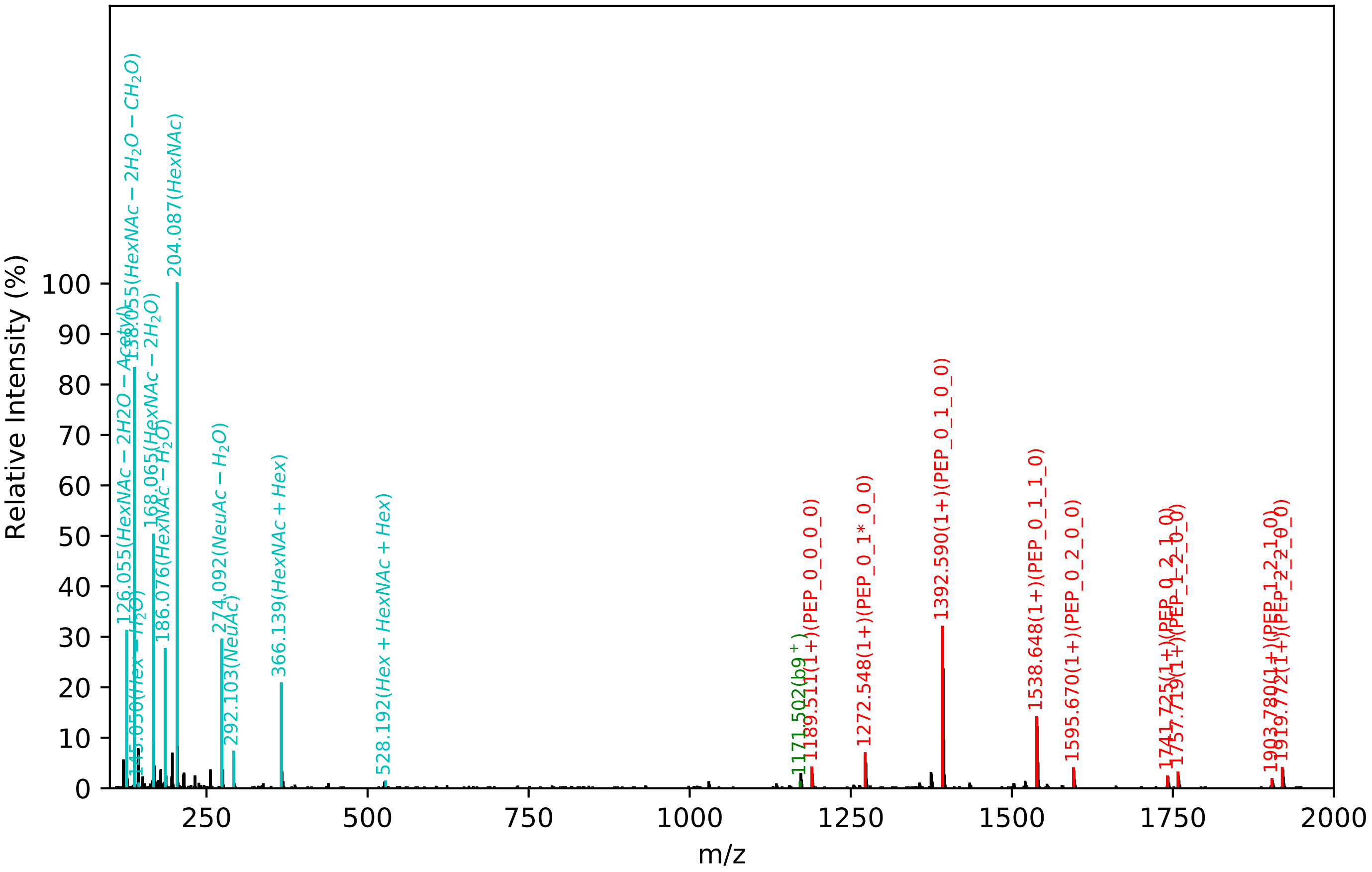

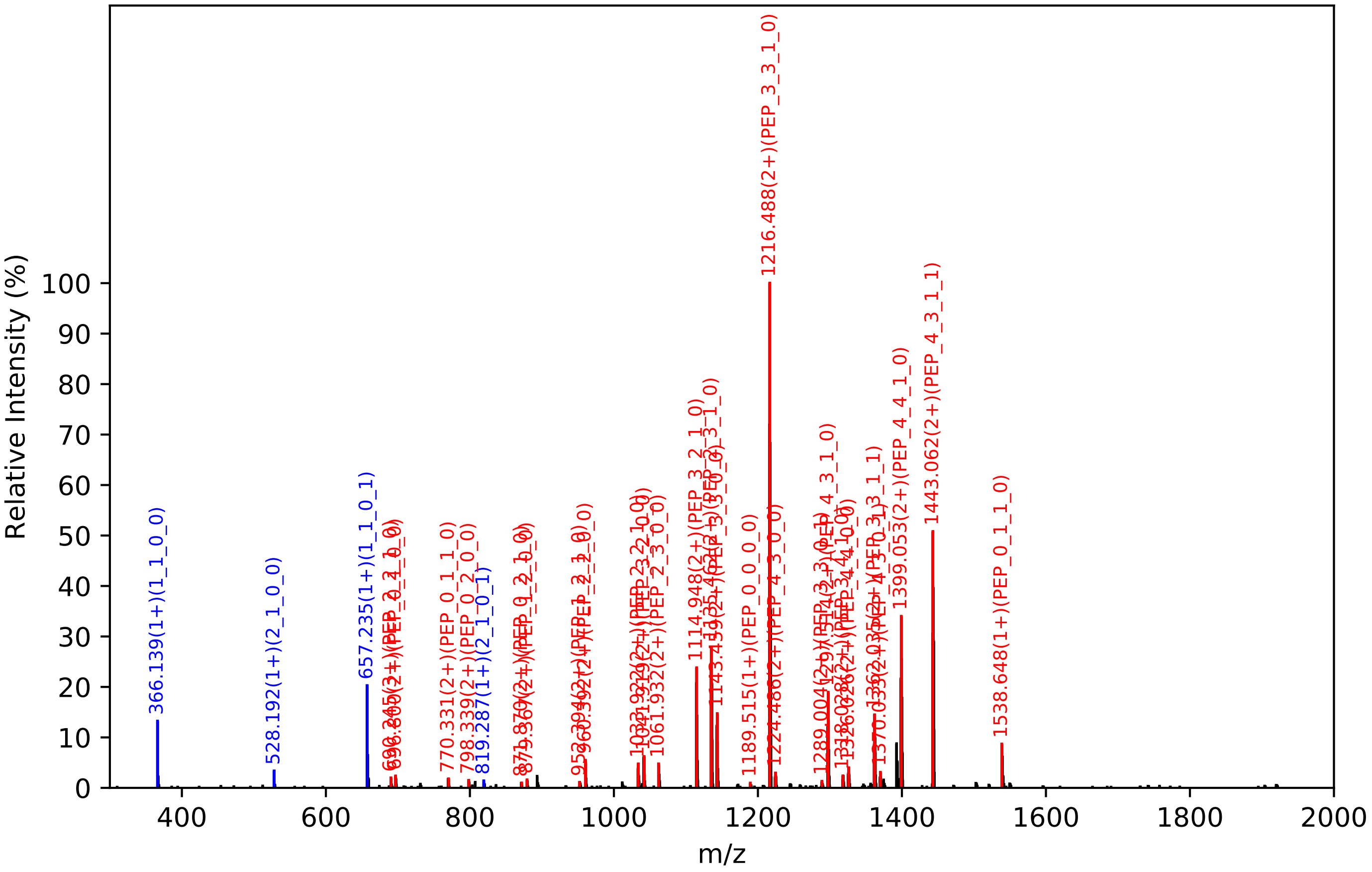

Test set no. 357, Experiment: IgG exp_7

EEQYNSTYR(=PEP)_4_4_1_1, m/z:1544.10(2+), RT:23.10, Y-score:81.13

HCD Scan:9813

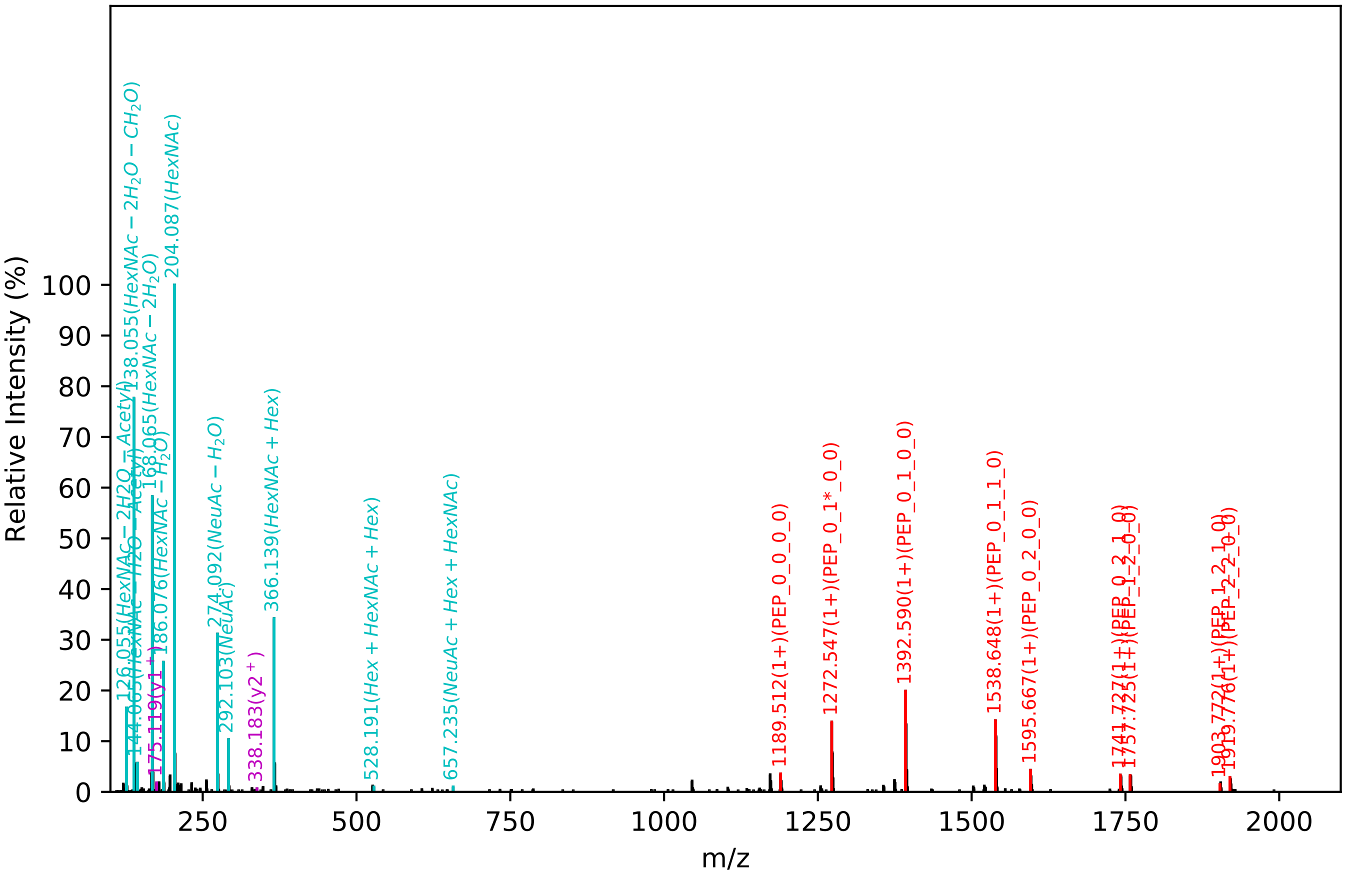

CID Scan:9812

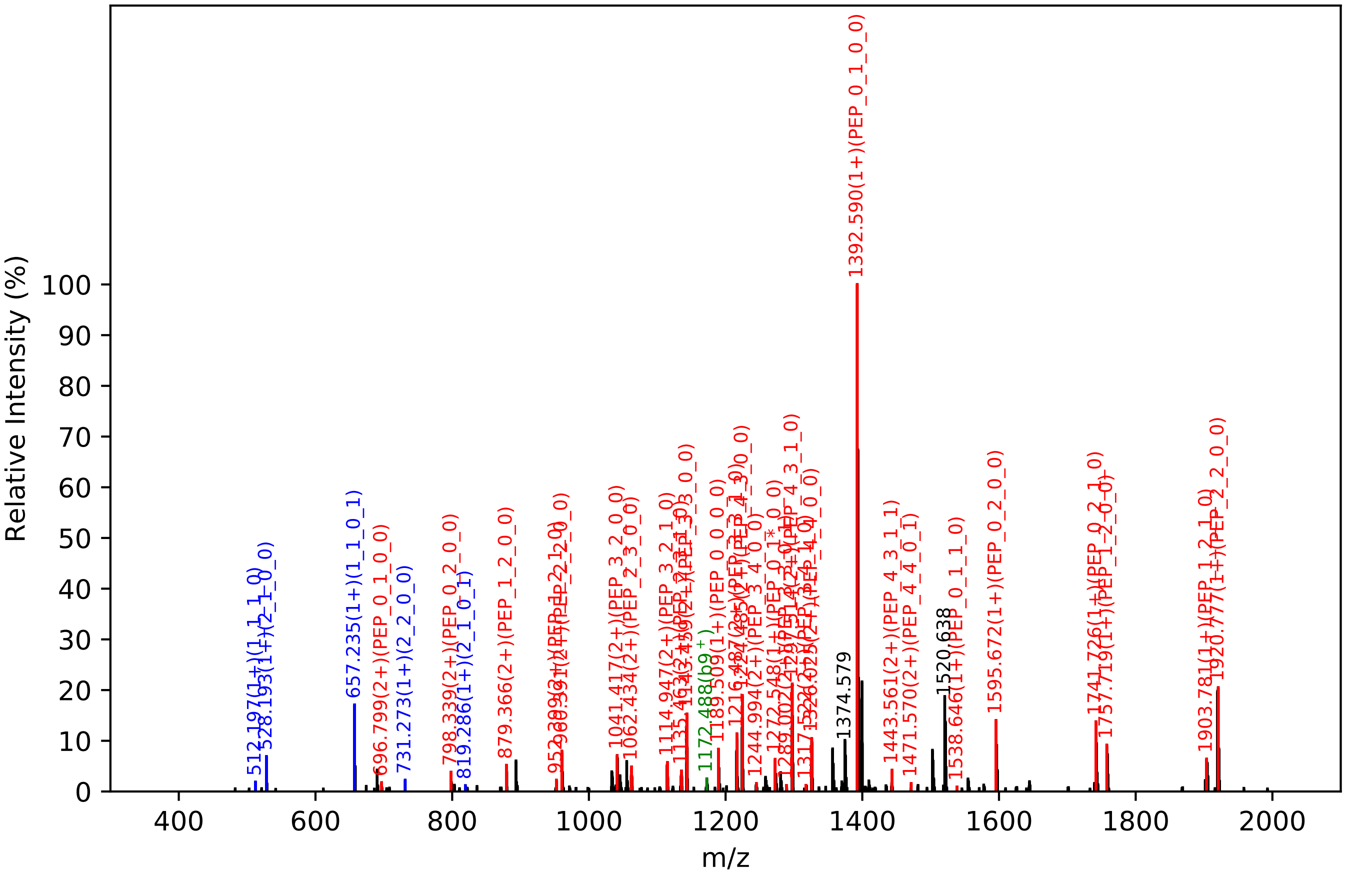

Test set no. 358, Experiment: IgG exp_6

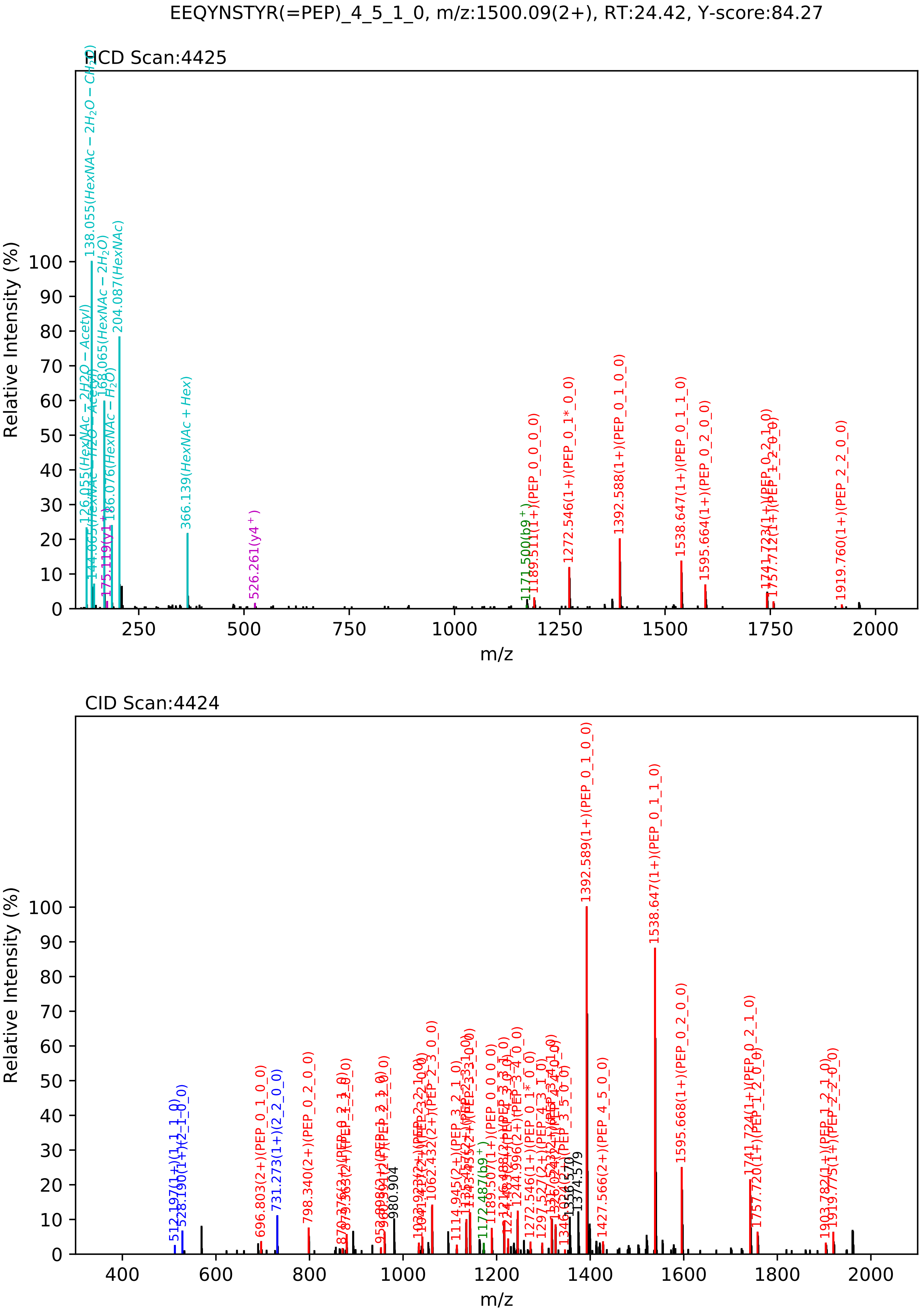

Test set no. 359, Experiment: IgG exp_7

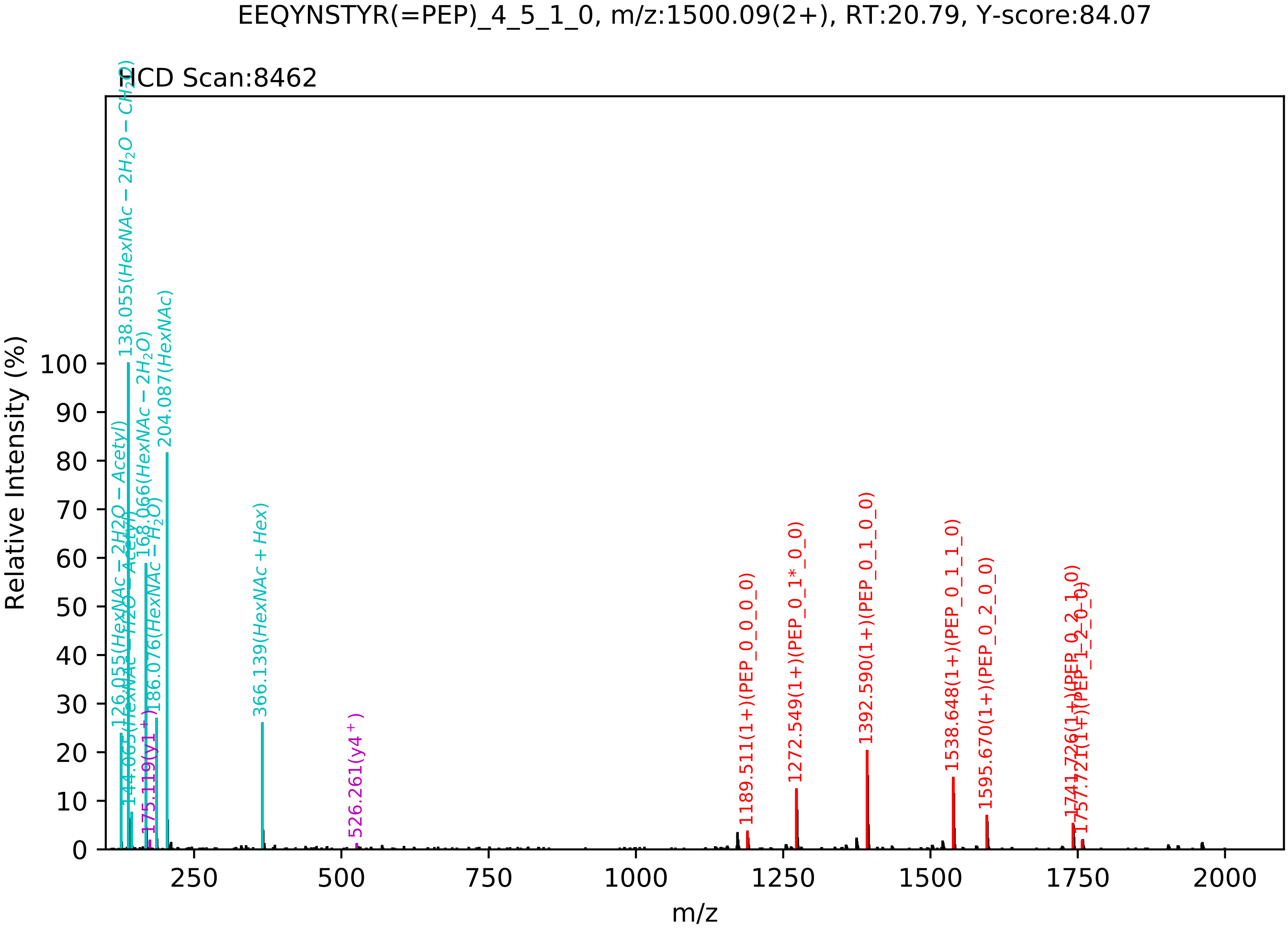

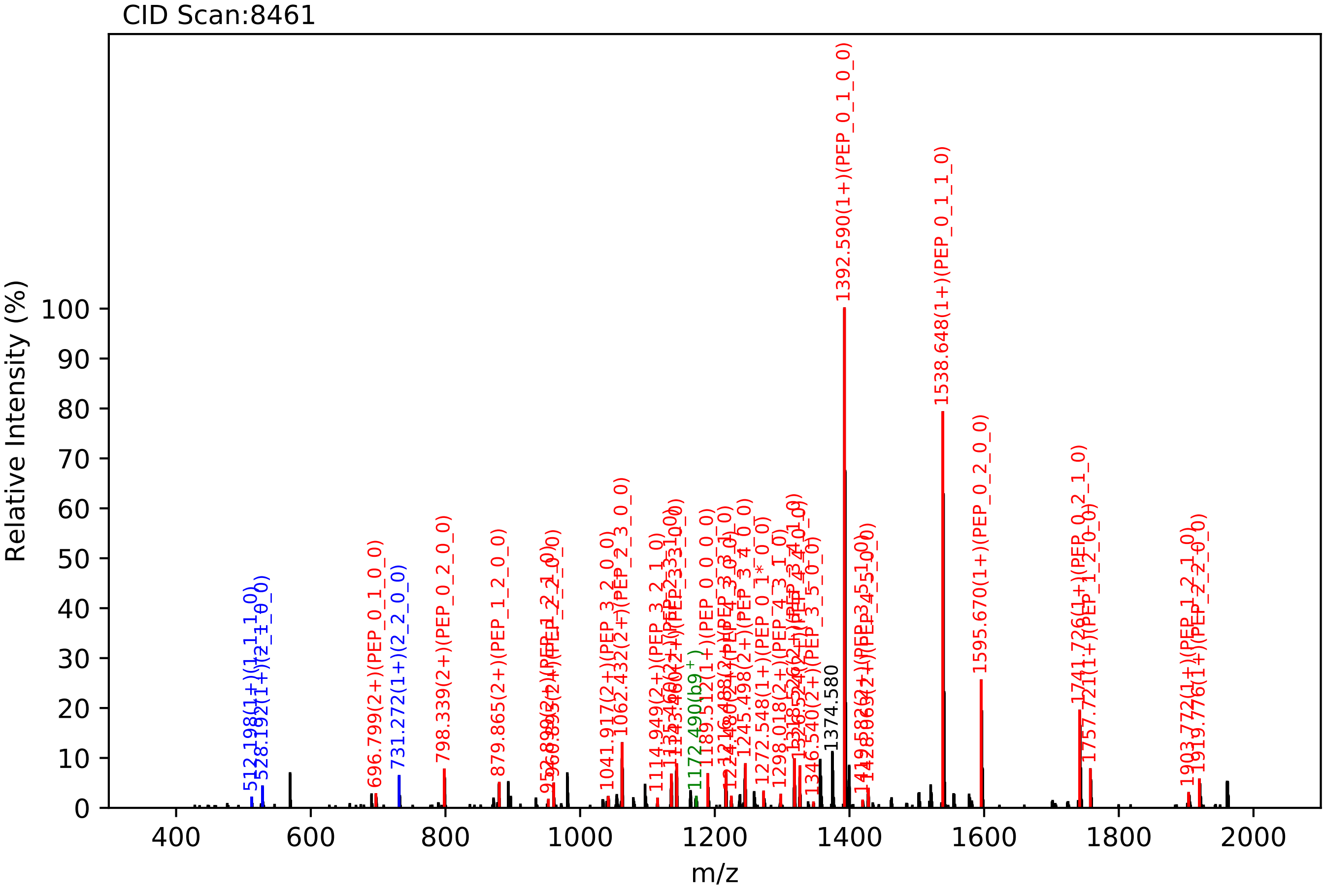

Test set no. 360, Experiment: IgG exp_7

EEQYNSTYR(=PEP)_5_4_0_0, m/z:1406.55(2+), RT:15.61, Y-score:70.72

HCD Scan:5976

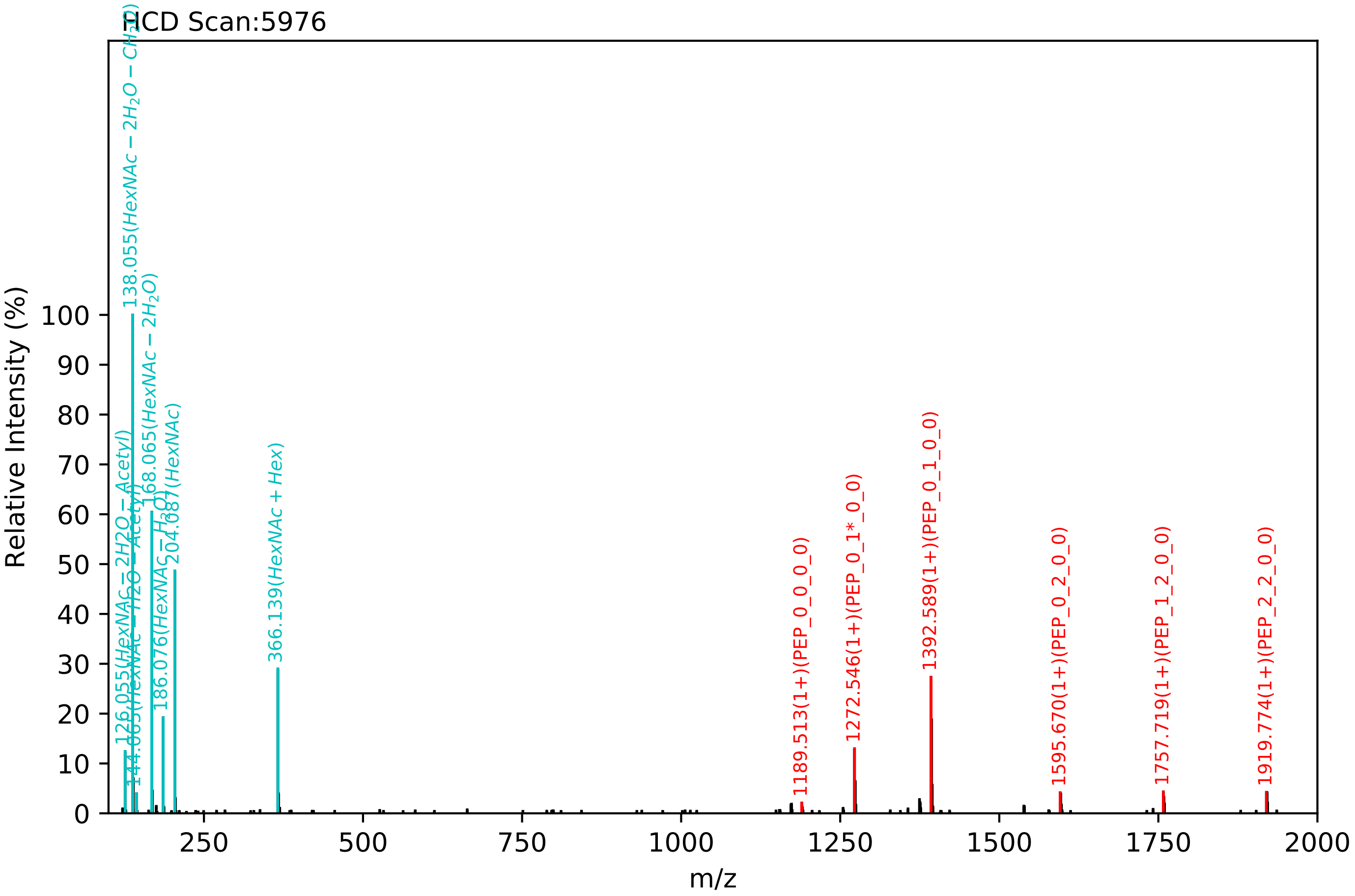

CID Scan:5975

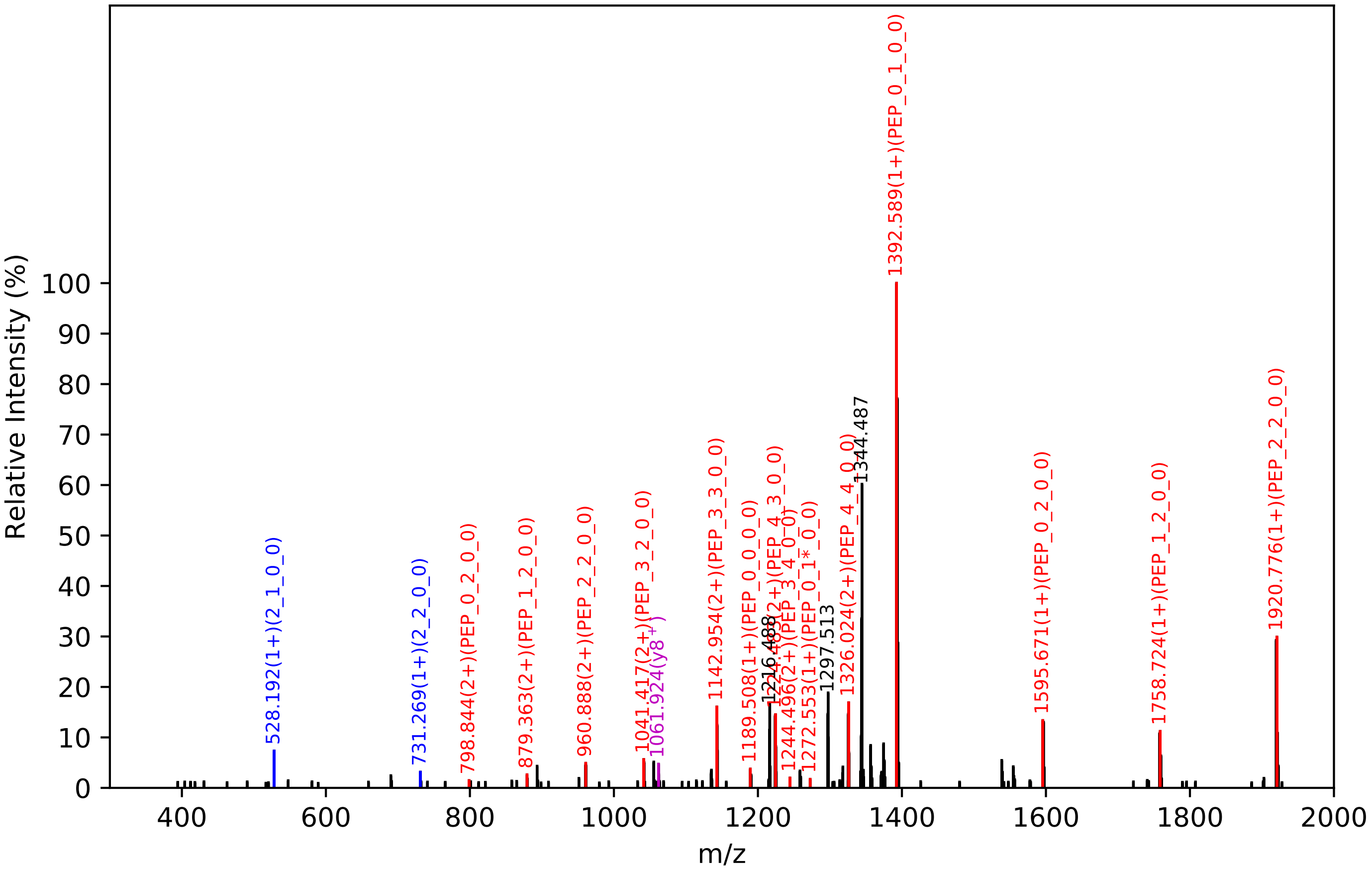

Test set no. 361, Experiment: IgG exp_5

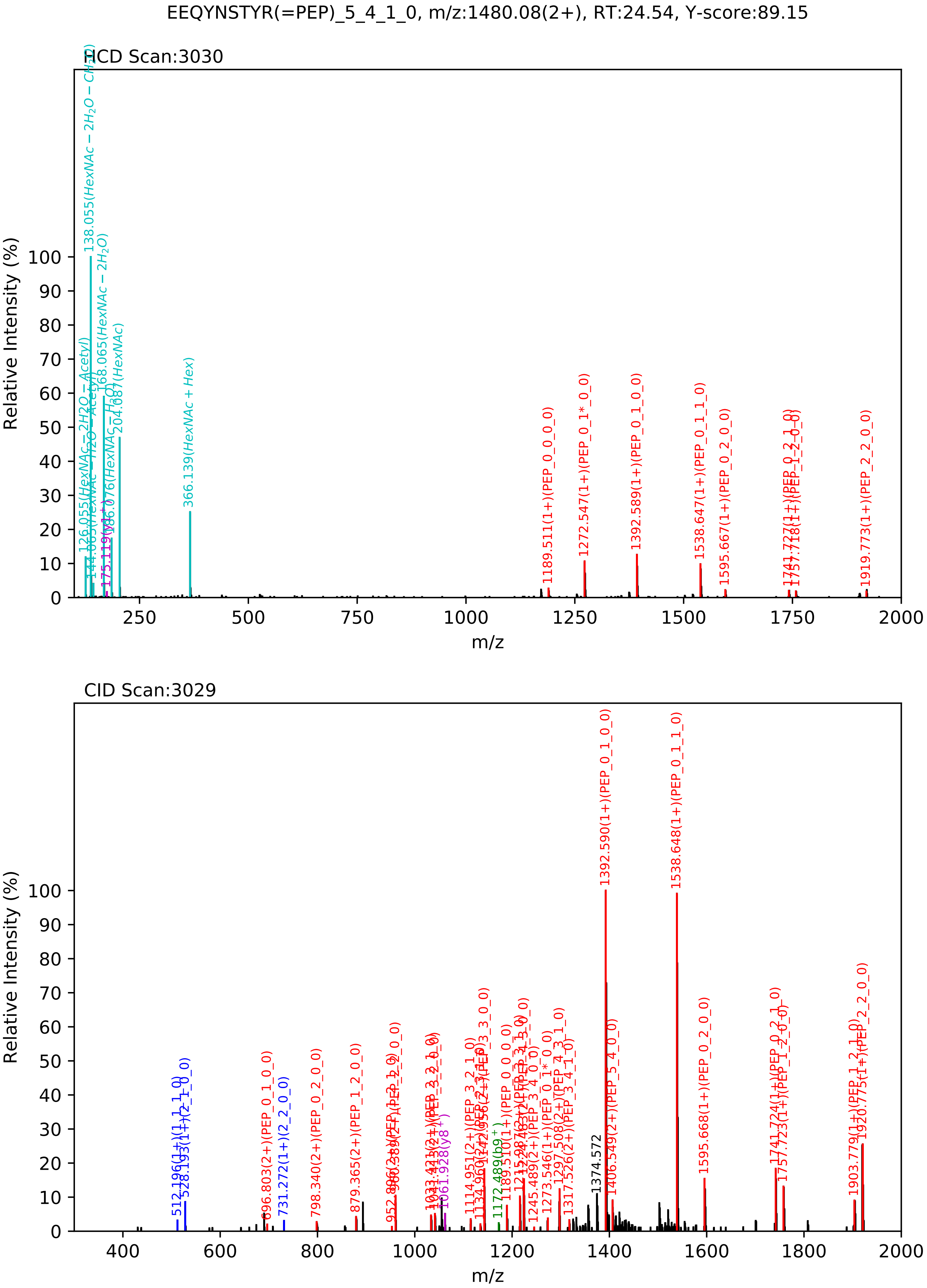

Test set no. 362, Experiment: IgG exp_7

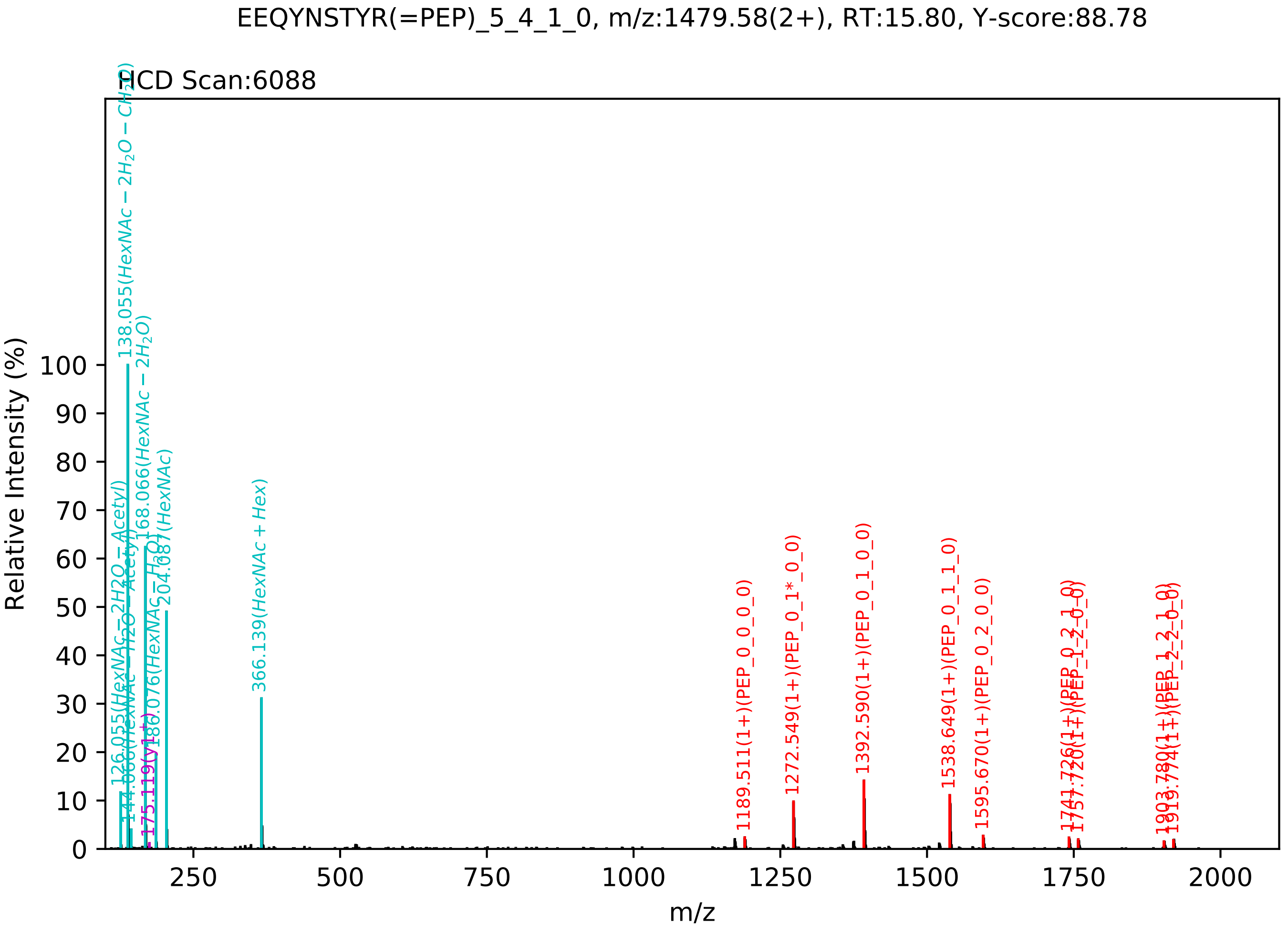

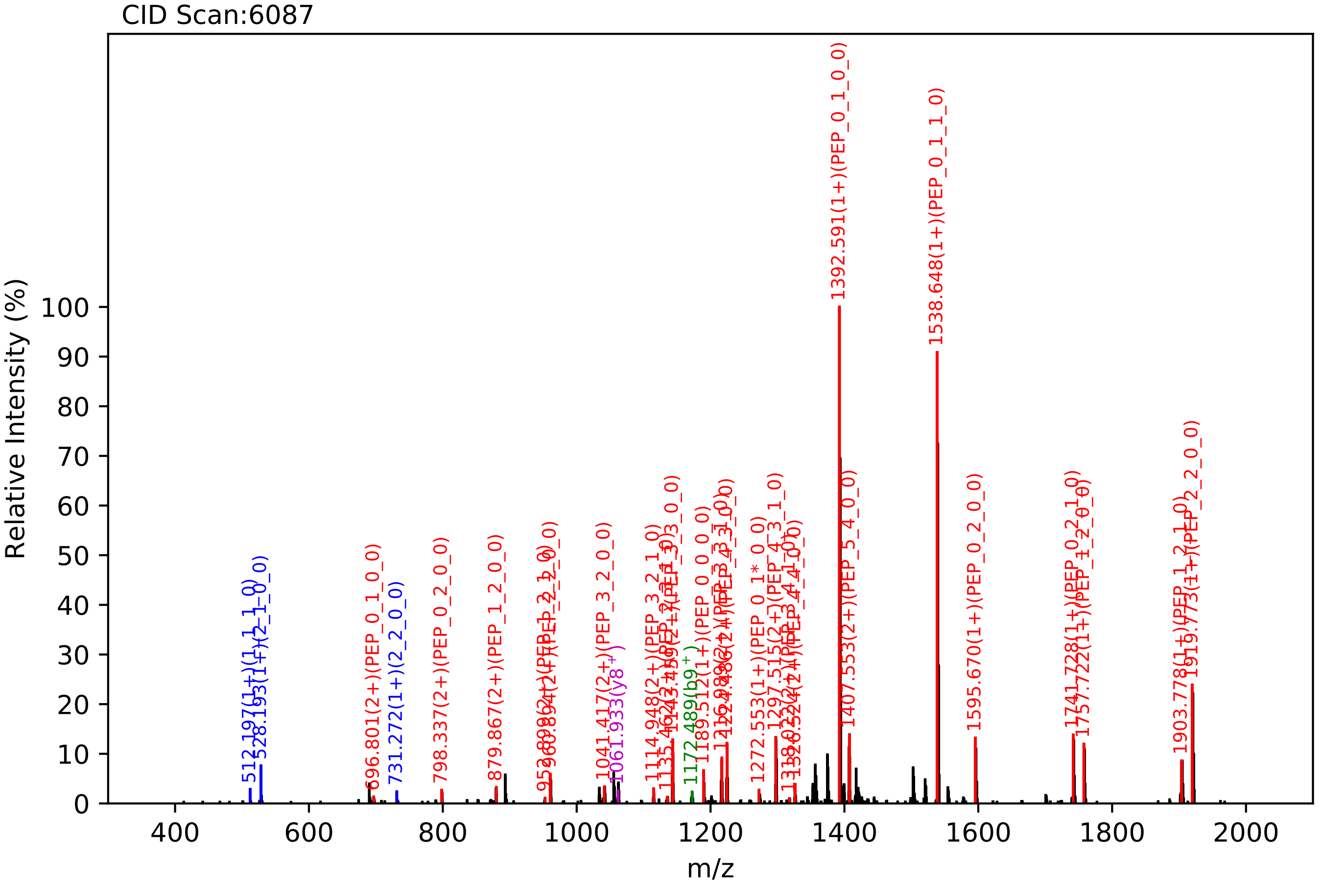

Test set no. 363, Experiment: IgG exp_7

EEQYNSTYR(=PEP)_5_4_1_0, m/z:1479.58(2+), RT:16.37, Y-score:88.32

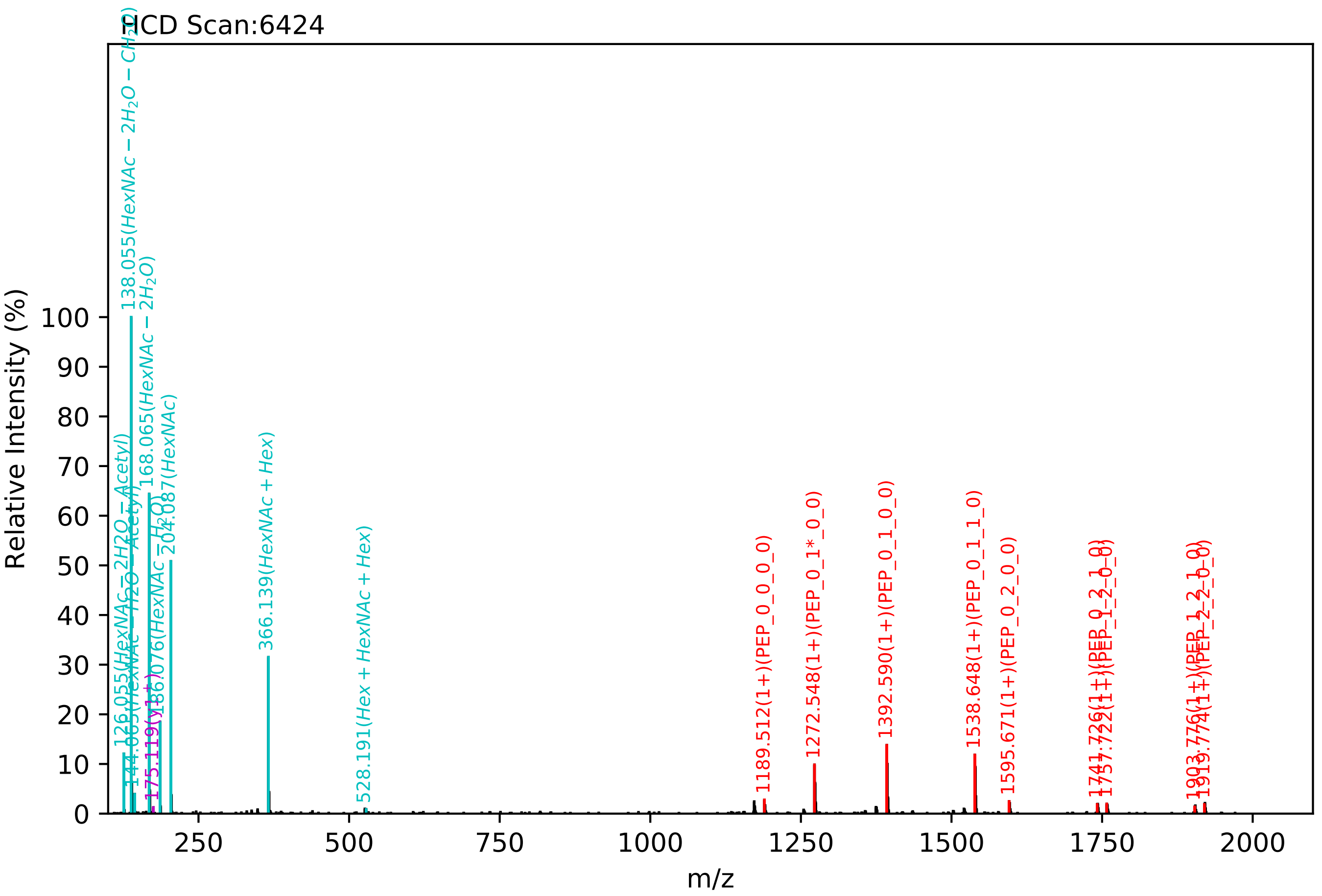

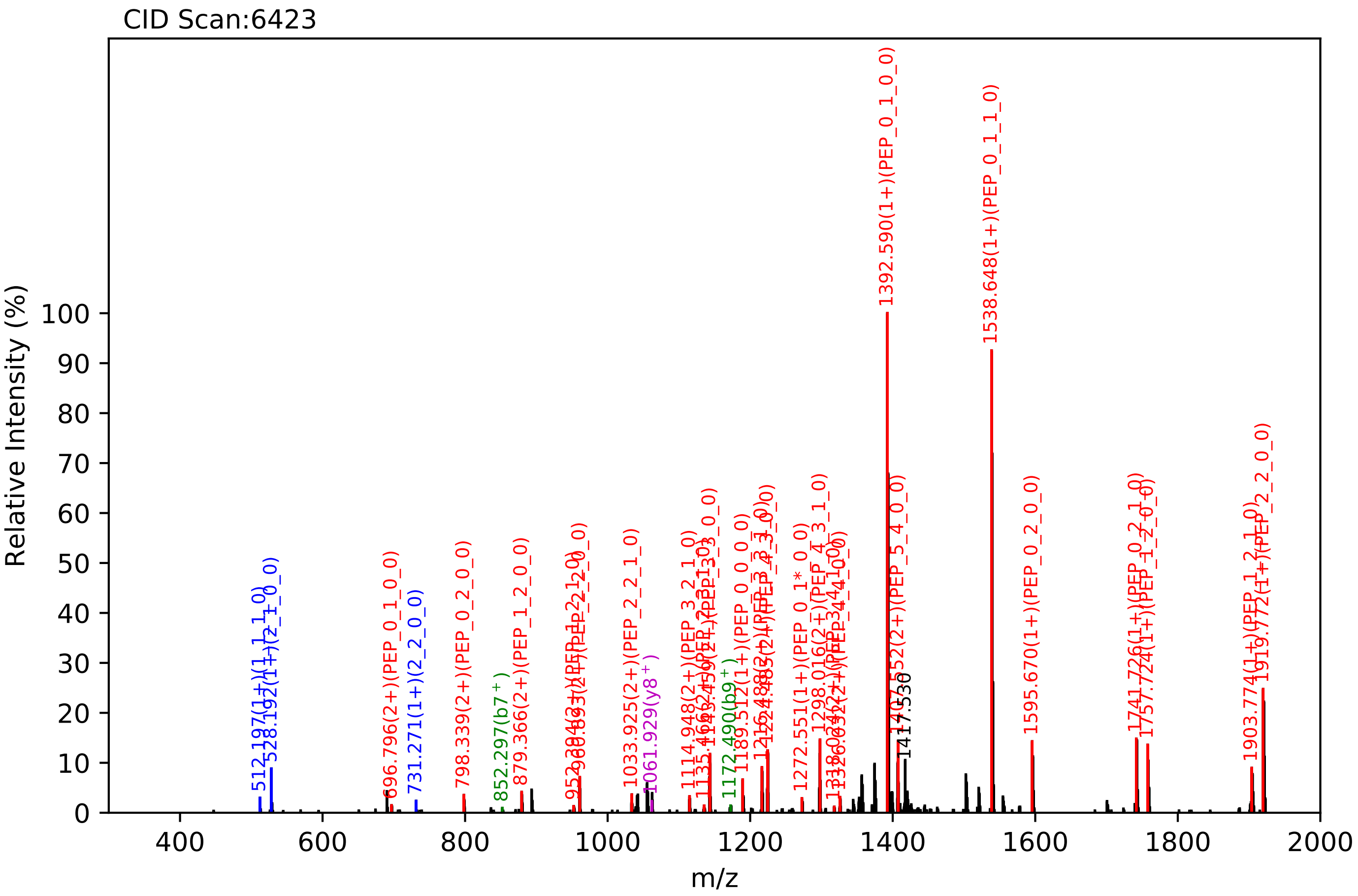

Test set no. 364, Experiment: IgG exp_7

EEQYNSTYR(=PEP)_5_4_1_0, m/z:1479.58(2+), RT:16.92, Y-score:85.98

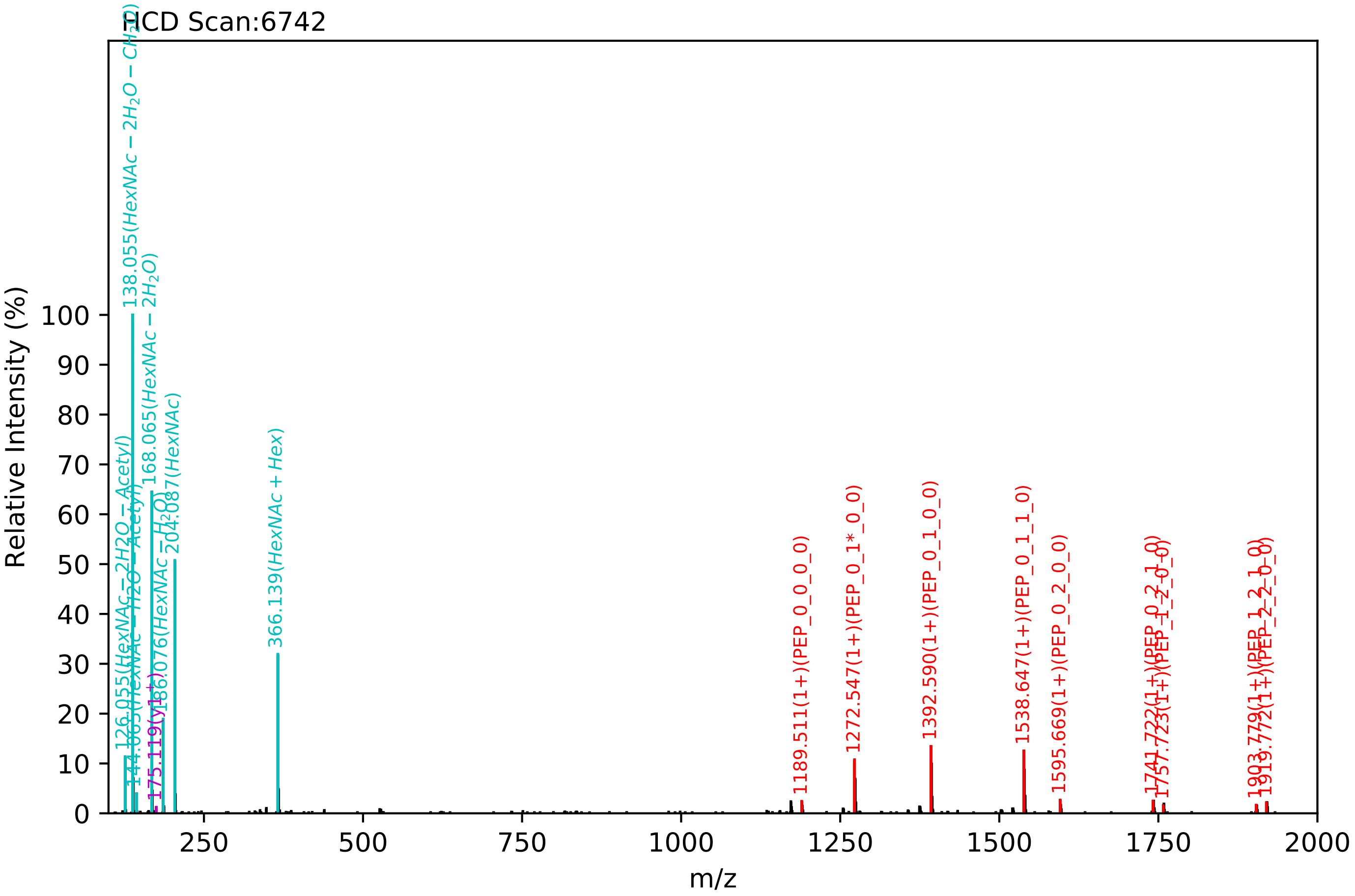

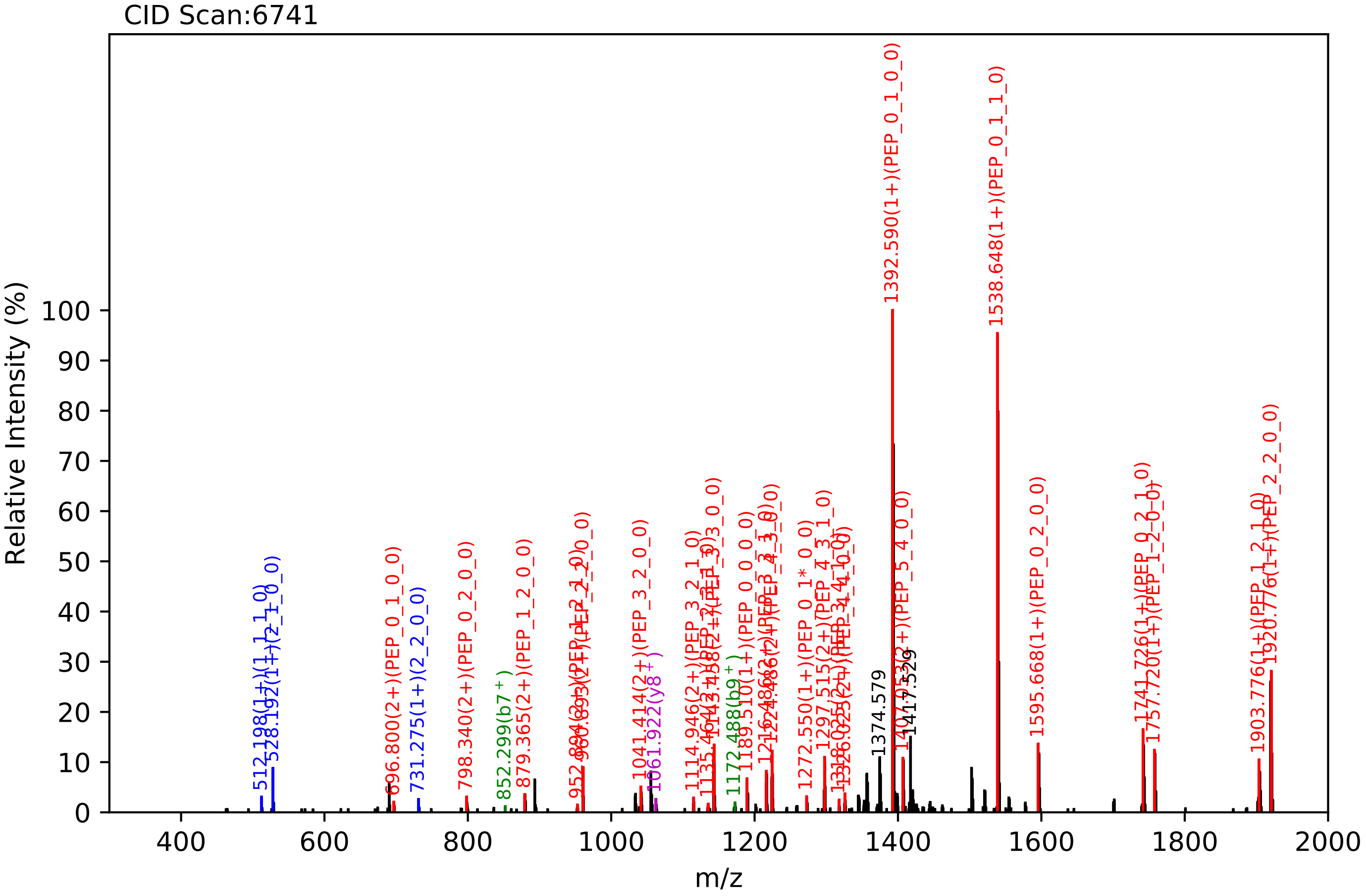

Test set no. 365, Experiment: IgG exp_7

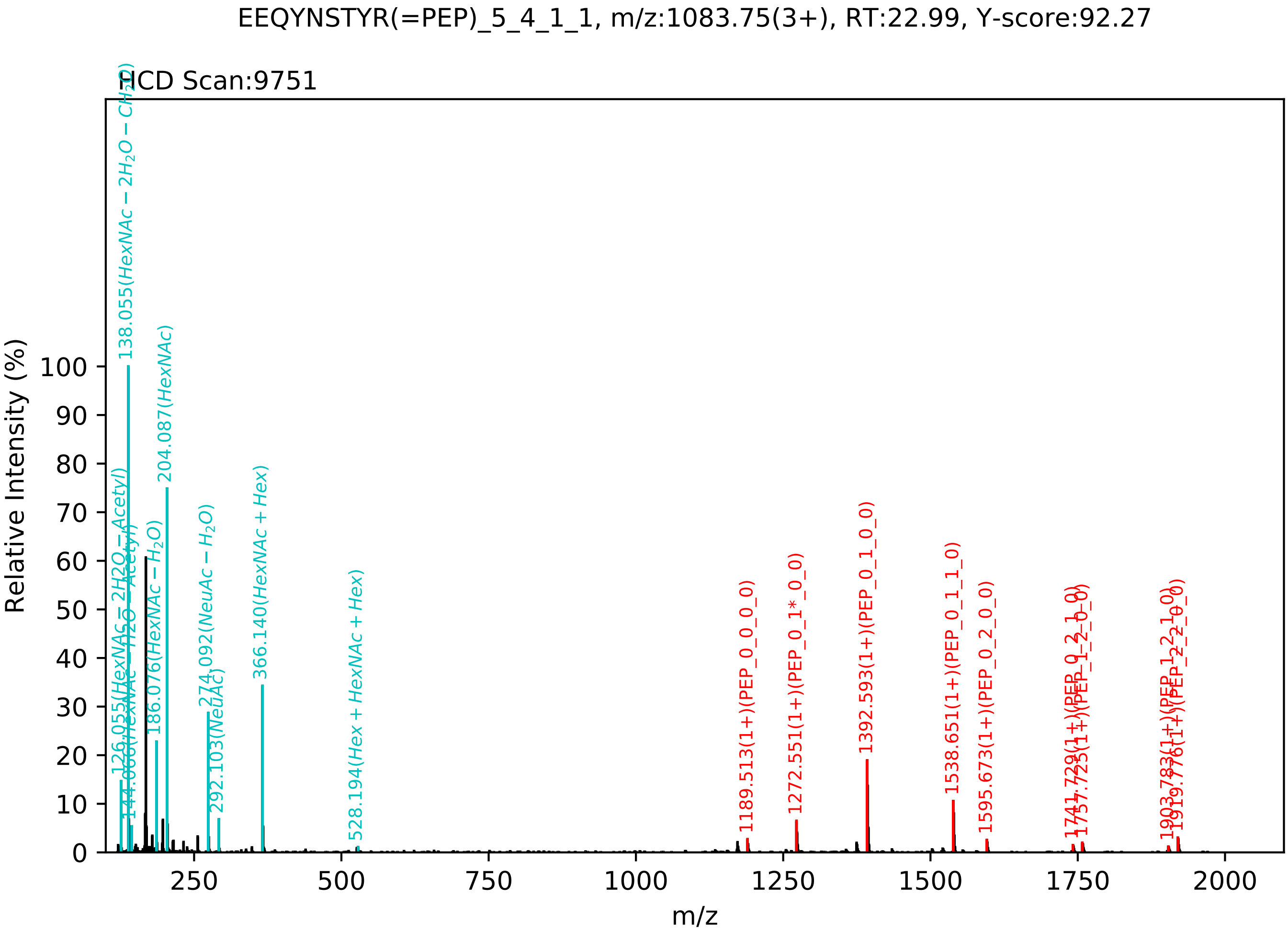

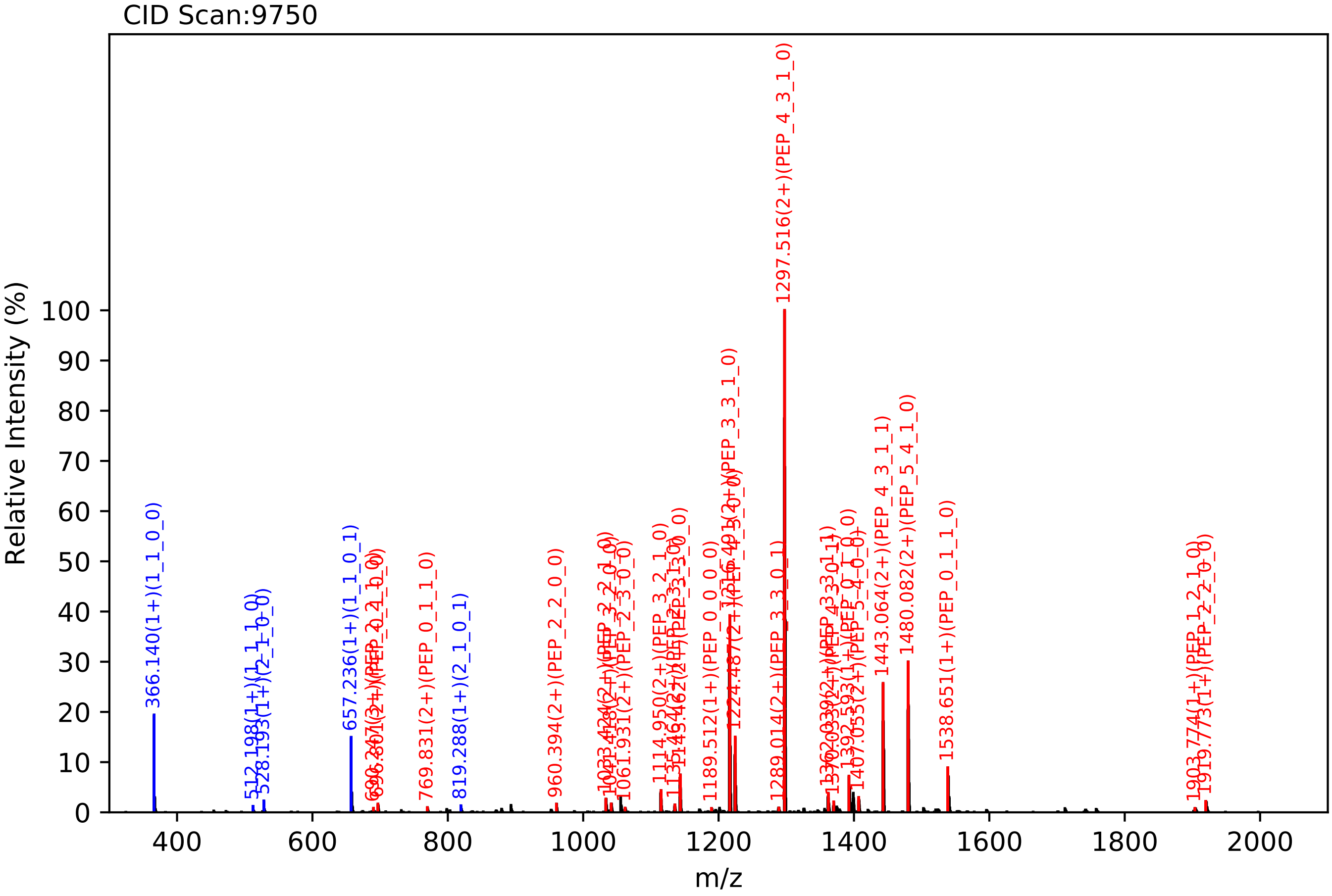

Test set no. 366, Experiment: IgG exp_6

EEQYNSTYR(=PEP)_5_4_1_1, m/z:1625.13(2+), RT:25.16, Y-score:90.11

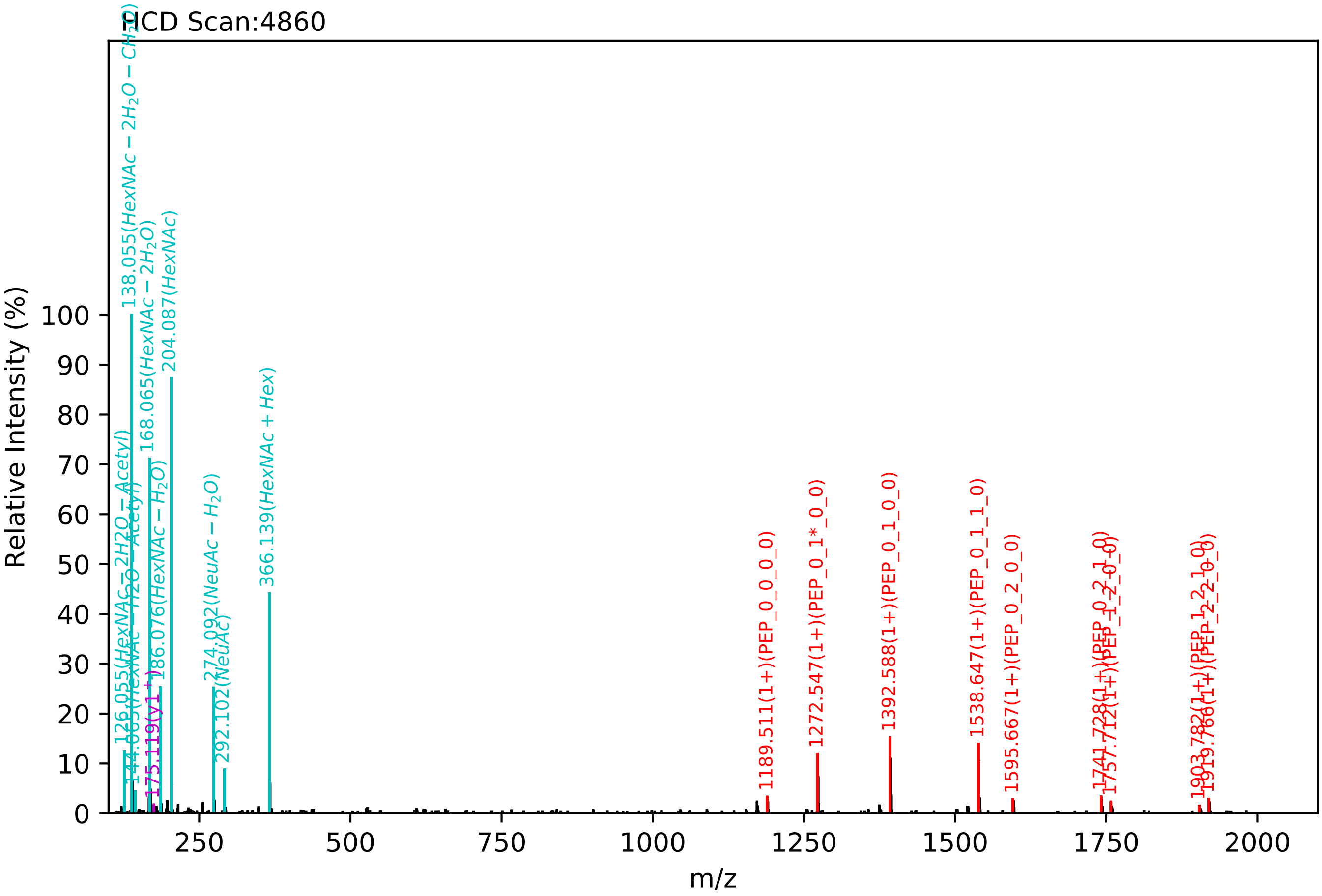

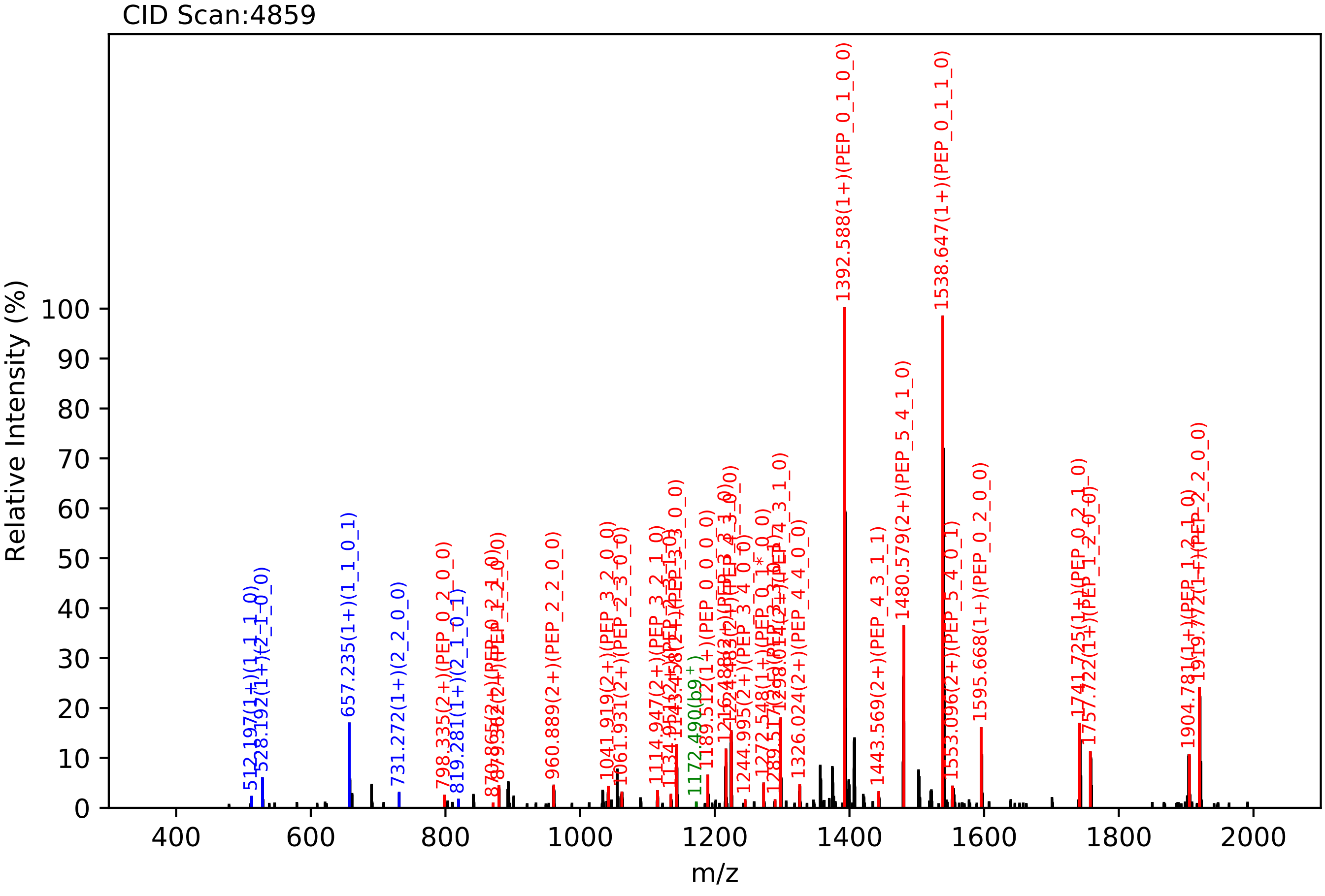

Test set no. 367, Experiment: IgG exp_7

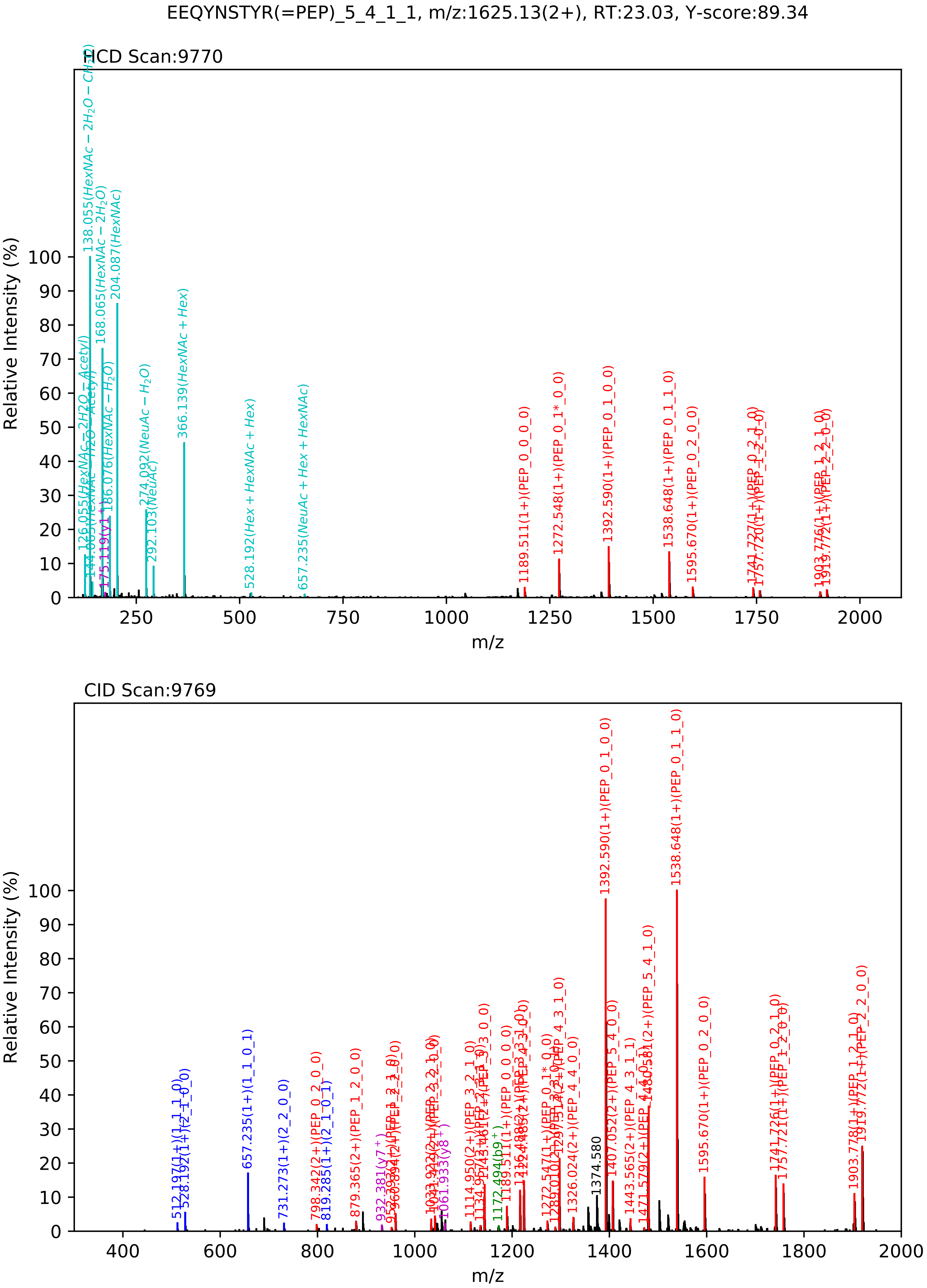

Test set no. 368, Experiment: IgG exp_7

EEQFNSTFR(=PEP)_3_3_1_0, m/z:1199.99(2+), RT:24.42, Y-score:88.61

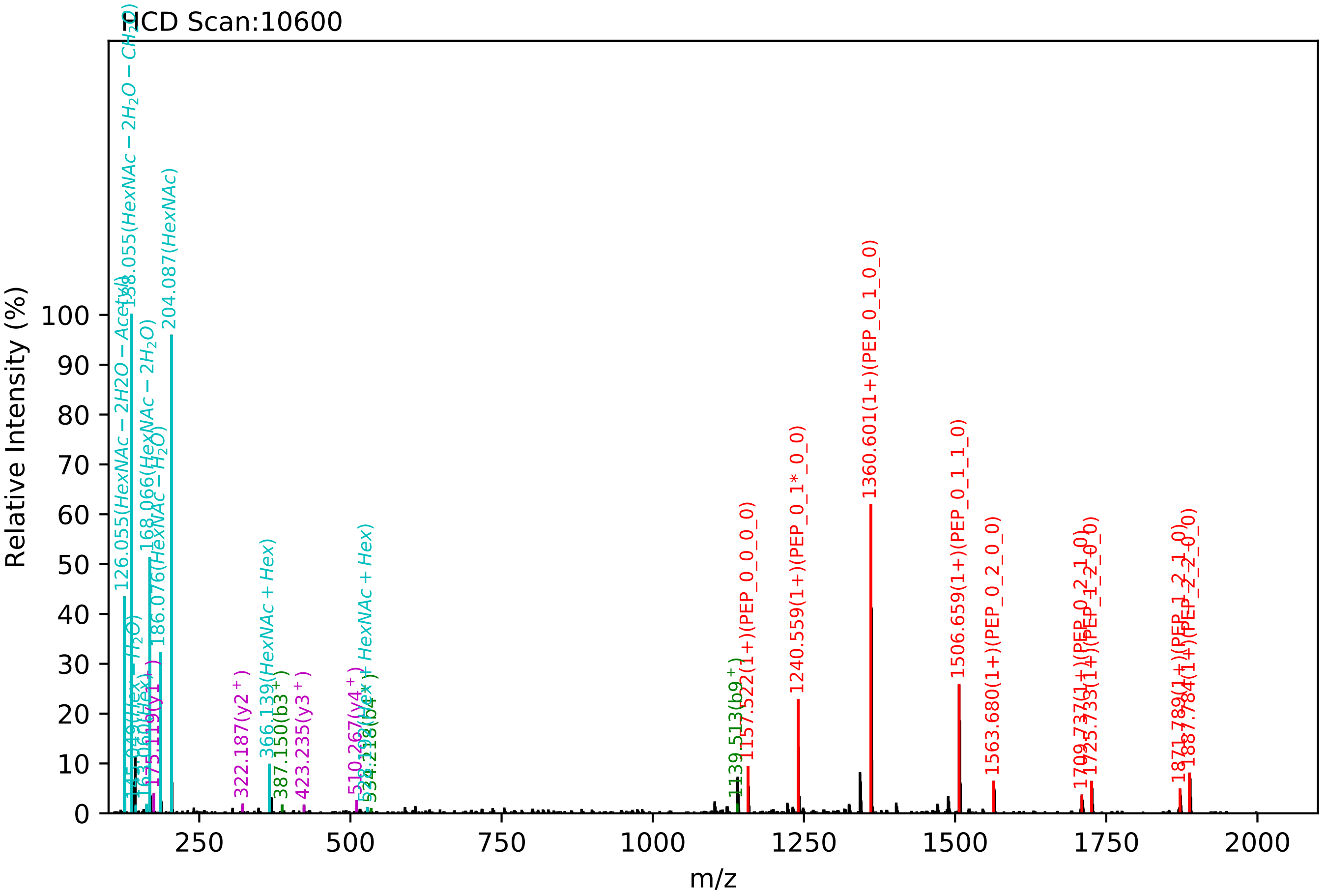

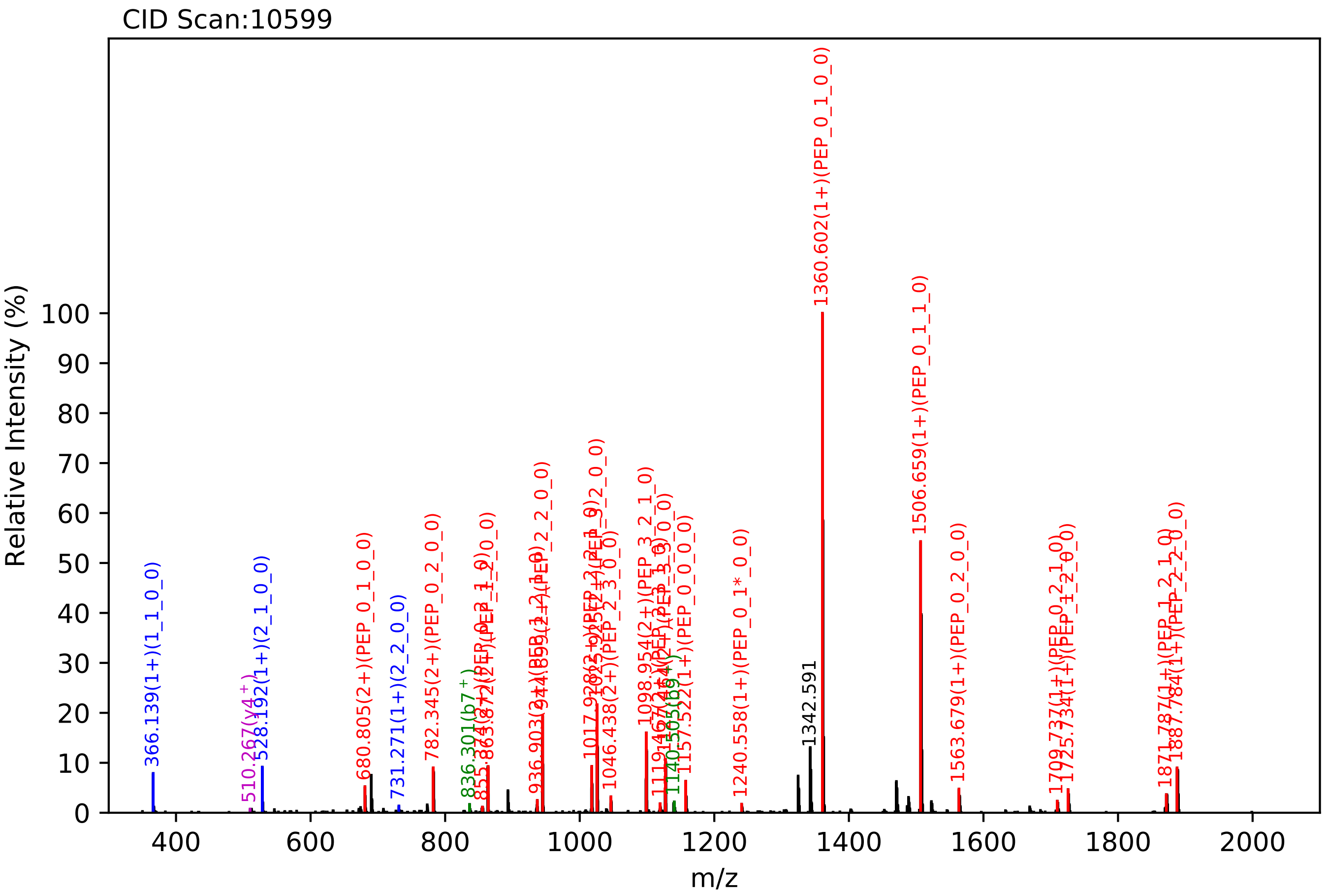

Test set no. 369, Experiment: IgG exp_7

EEQFNSTFR(=PEP)_3_4_0_0, m/z:1228.50(2+), RT:24.57, Y-score:88.48

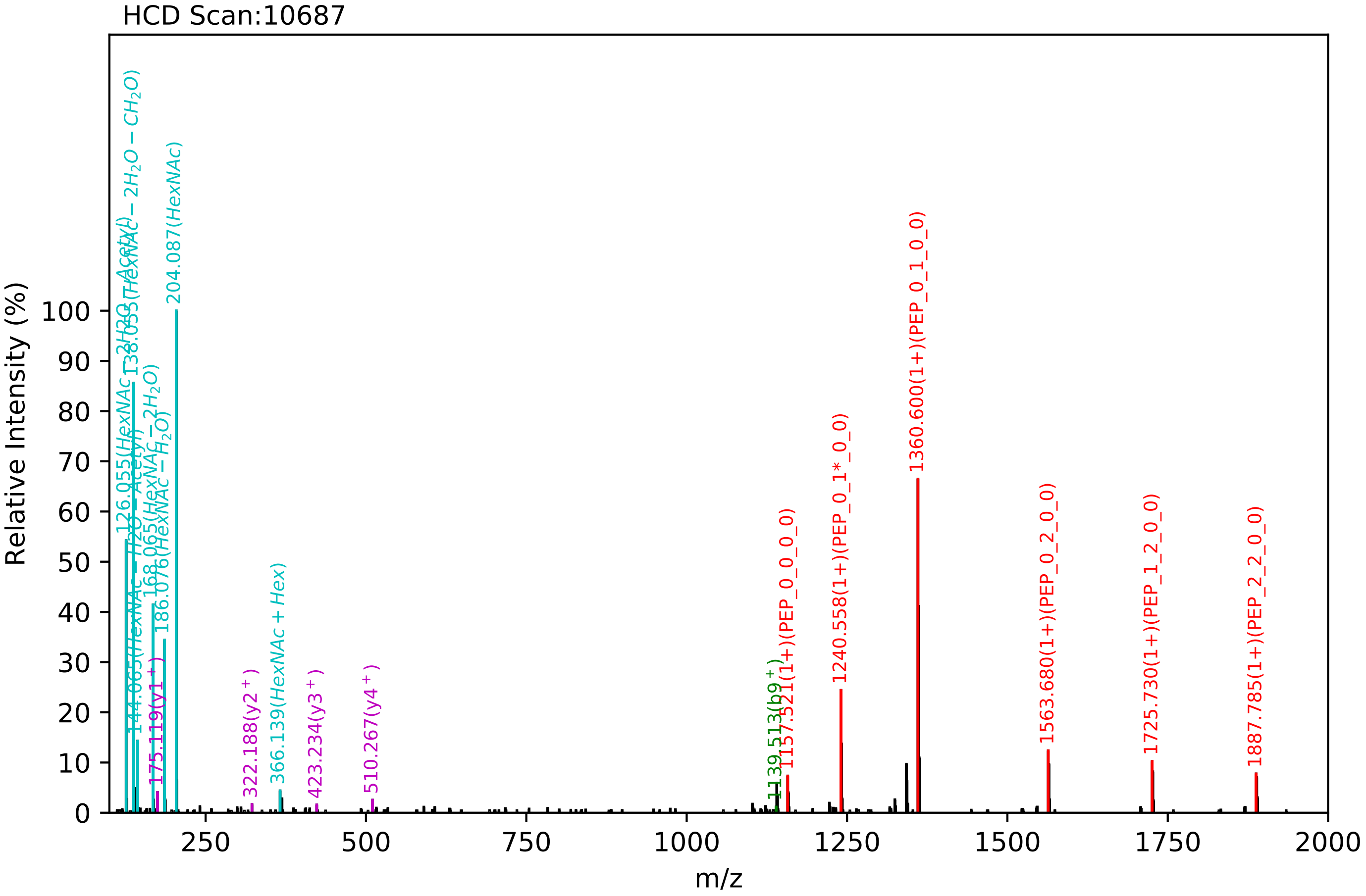

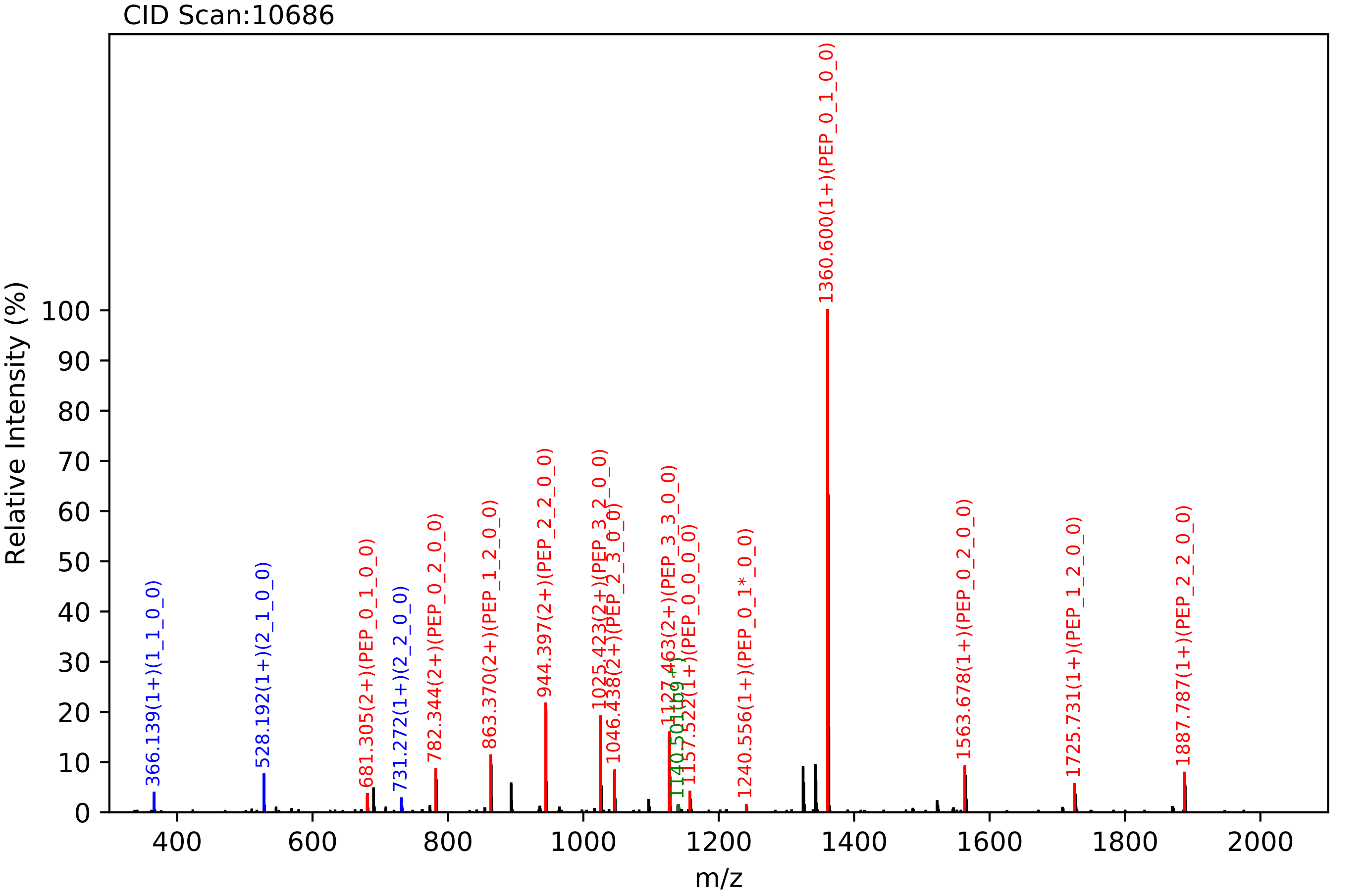

Test set no. 370, Experiment: IgG exp_5

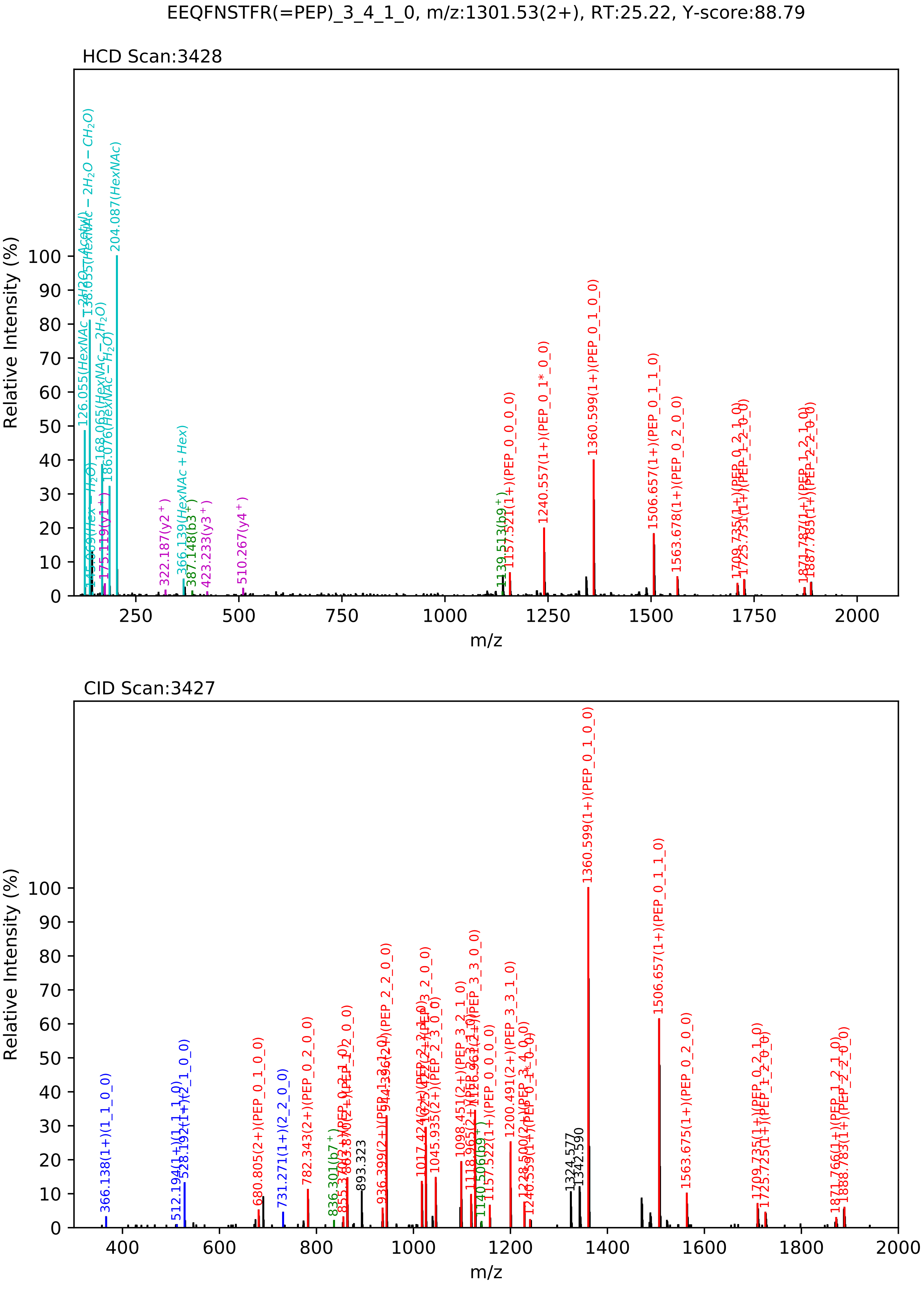

Test set no. 371, Experiment: IgG exp_7

EEQFNSTFR(=PEP)_3_4_1_0, m/z:1301.53(2+), RT:24.20, Y-score:87.41

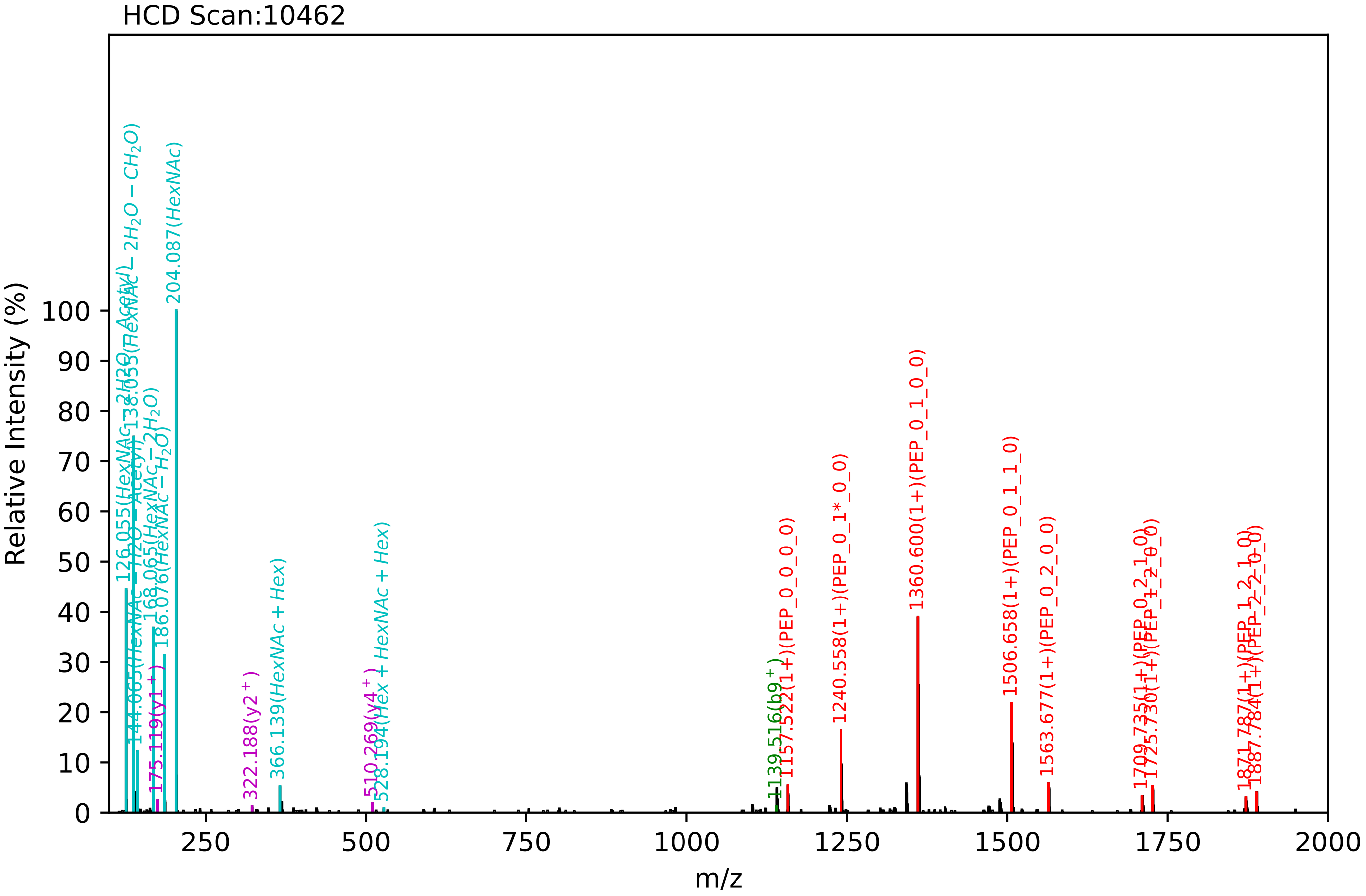

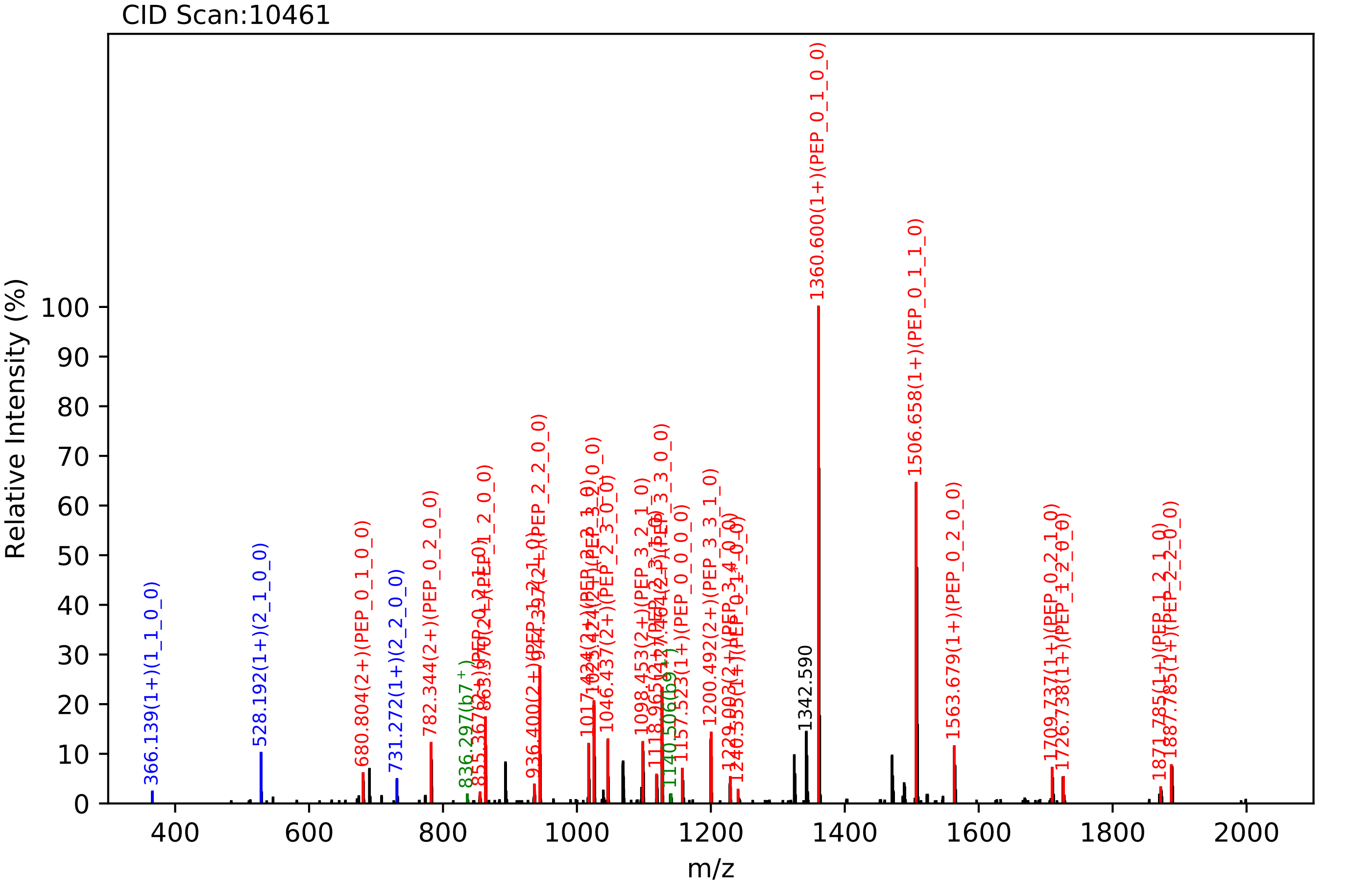

Test set no. 372, Experiment: IgG exp_7

EEQFNSTFR(=PEP)_3_4_1_0, m/z:1301.53(2+), RT:24.78, Y-score:86.66

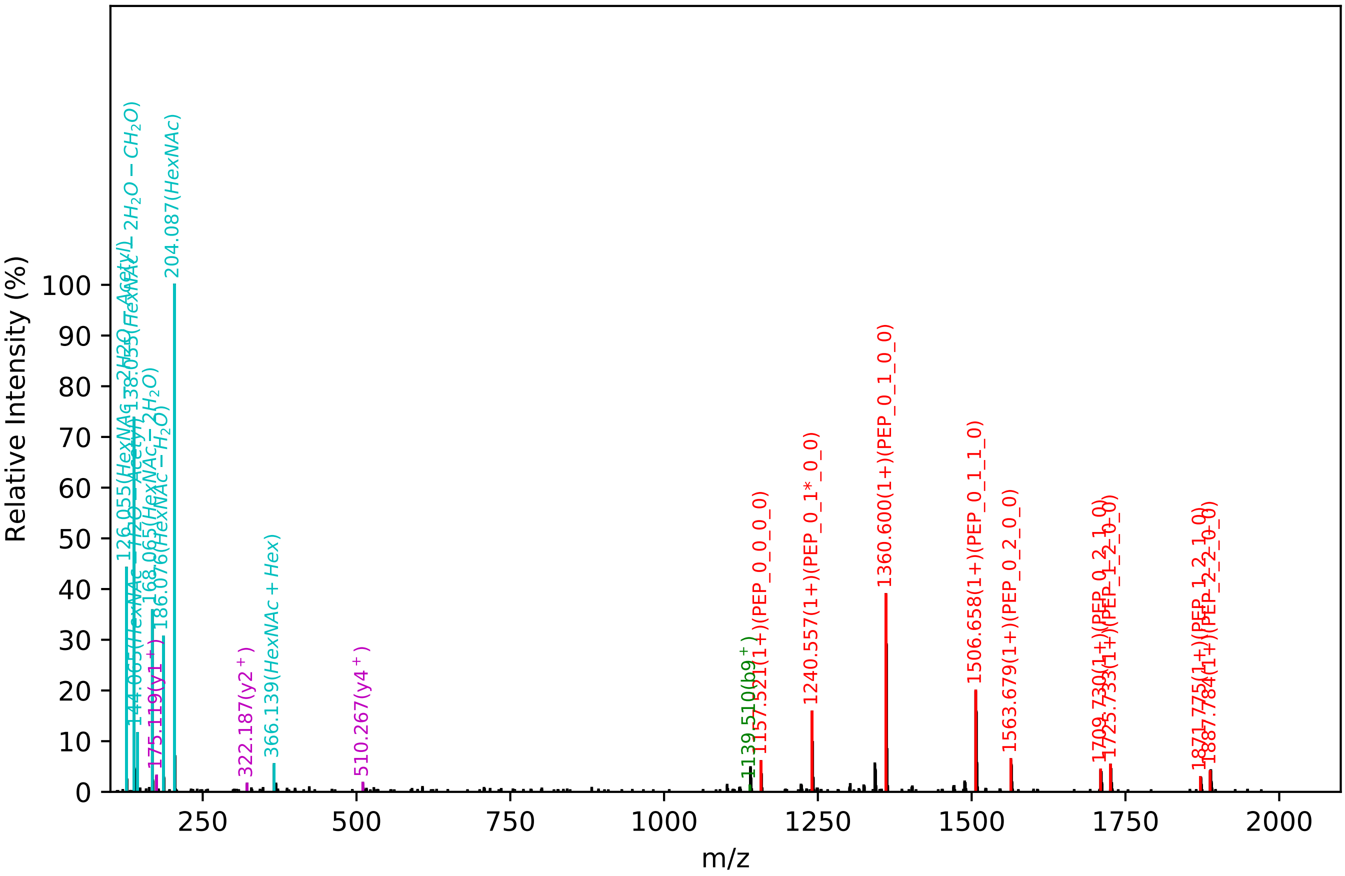

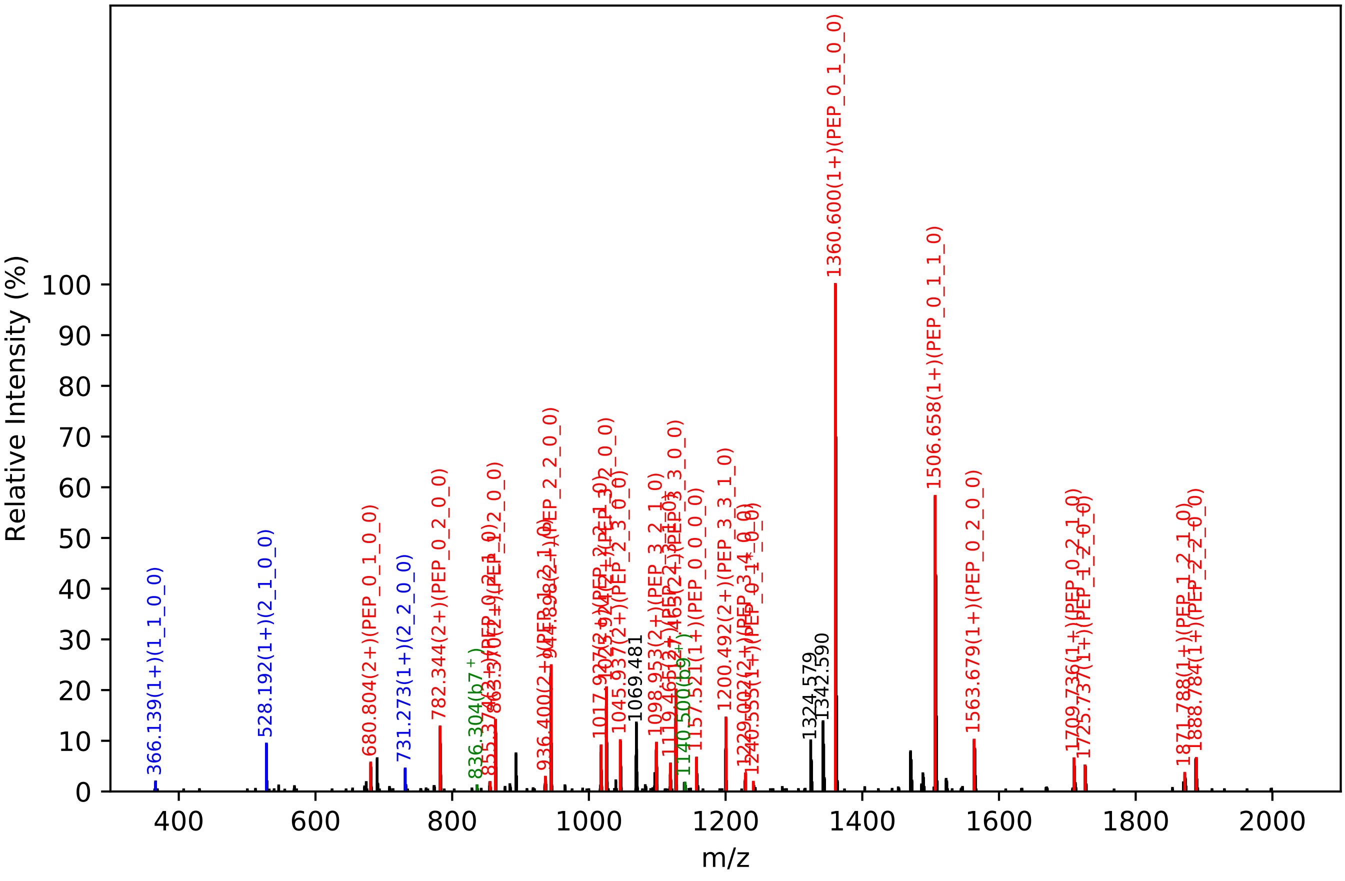

Test set no. 373, Experiment: IgG exp_7

EEQFNSTFR(=PEP)_3_5_1_0, m/z:935.72(3+), RT:24.58, Y-score:88.96

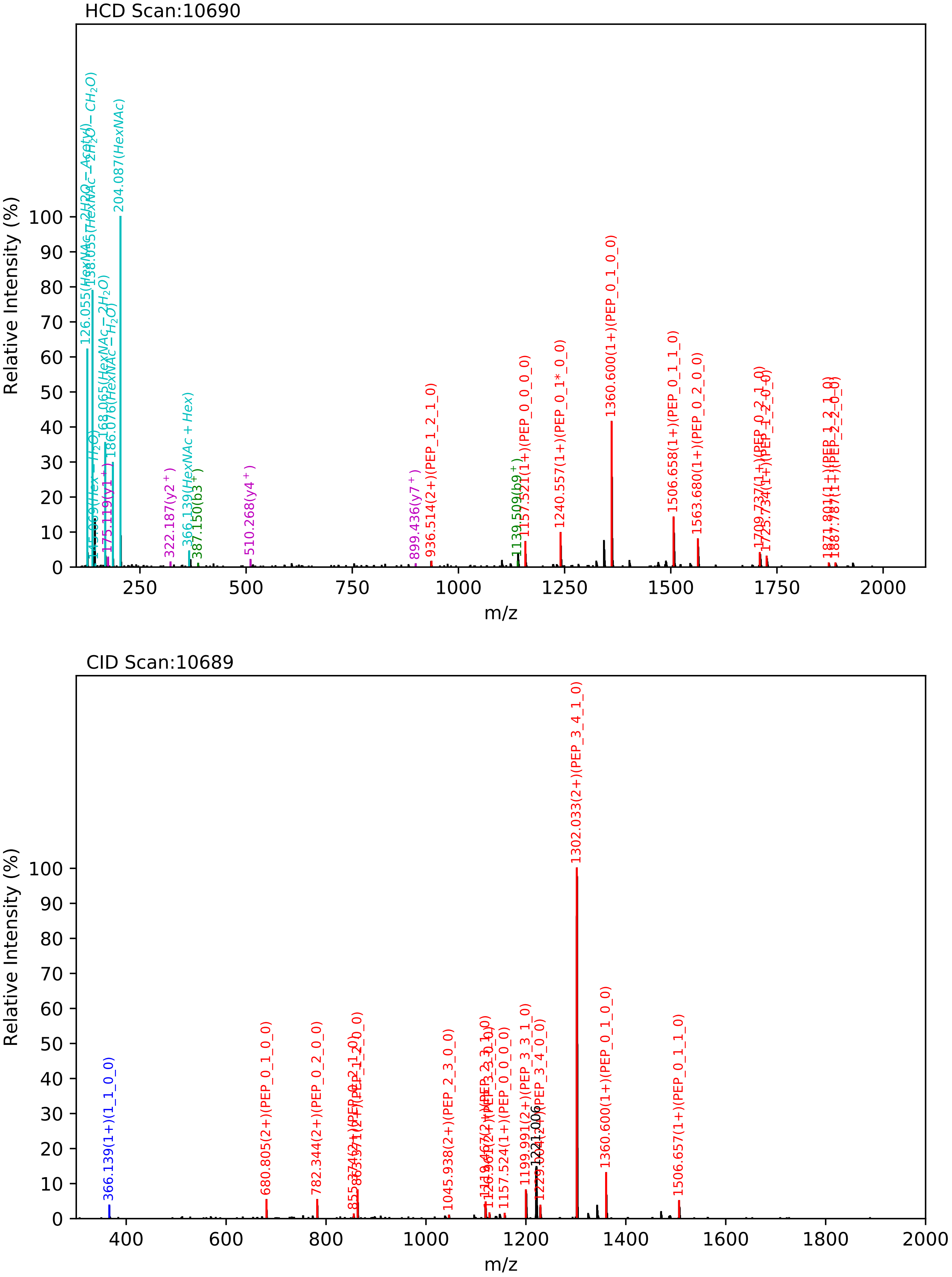

Test set no. 374, Experiment: IgG exp_7

EEQFNSTFR(=PEP)_3_5_1_0, m/z:1403.07(2+), RT:24.58, Y-score:82.74

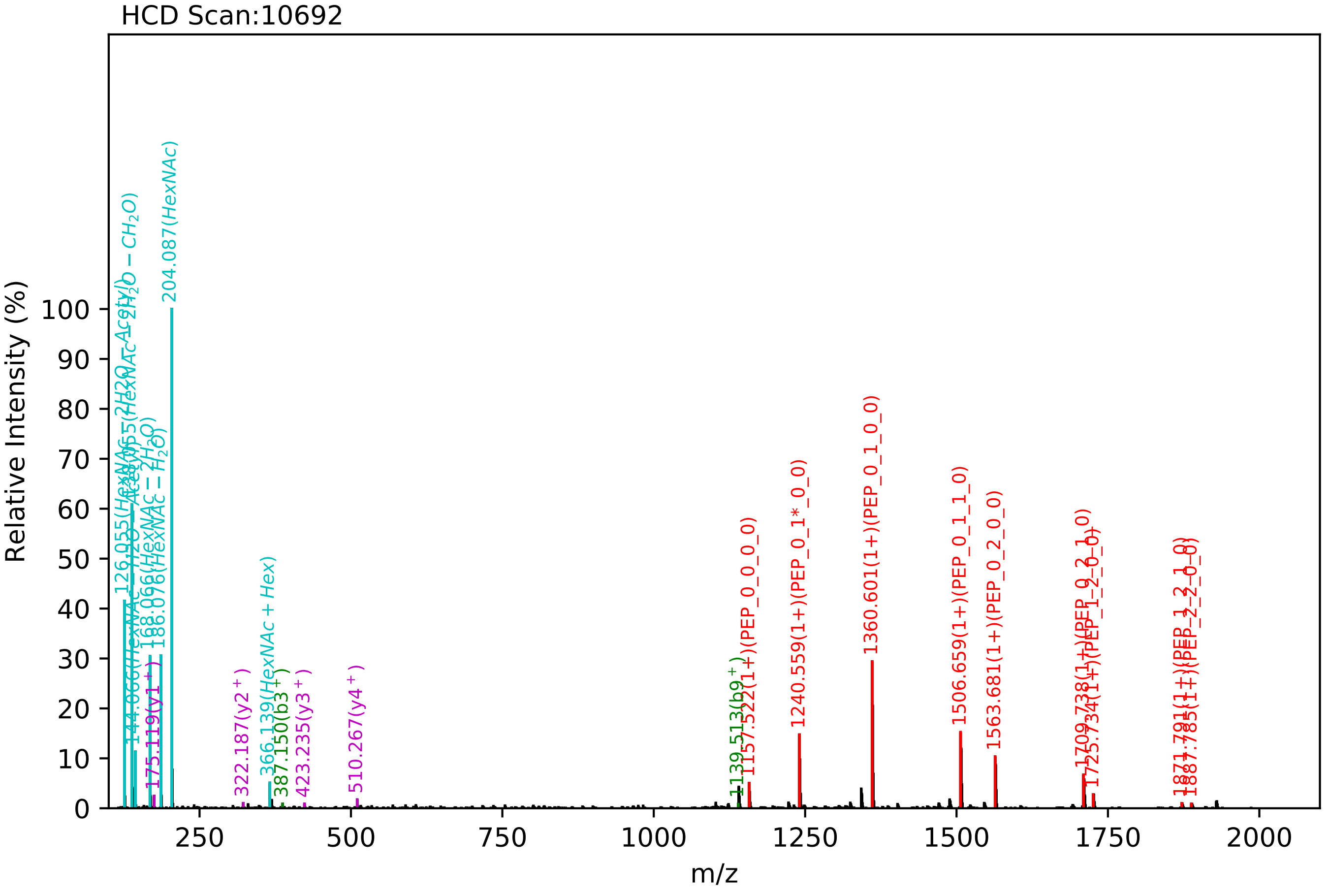

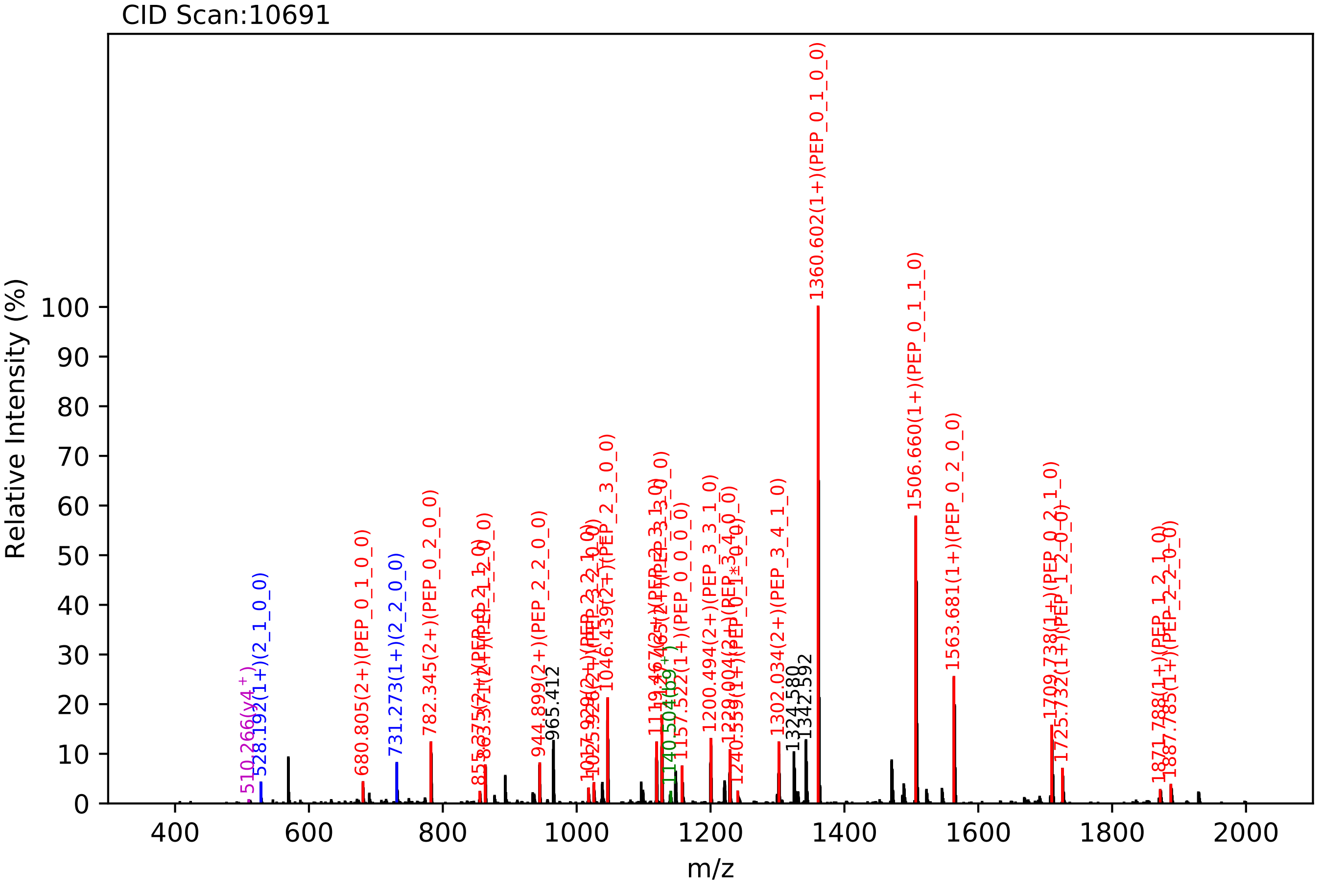

Test set no. 375, Experiment: IgG exp_5

EEQFNSTFR(=PEP)_3_5_1_0, m/z:1403.07(2+), RT:25.26, Y-score:78.99

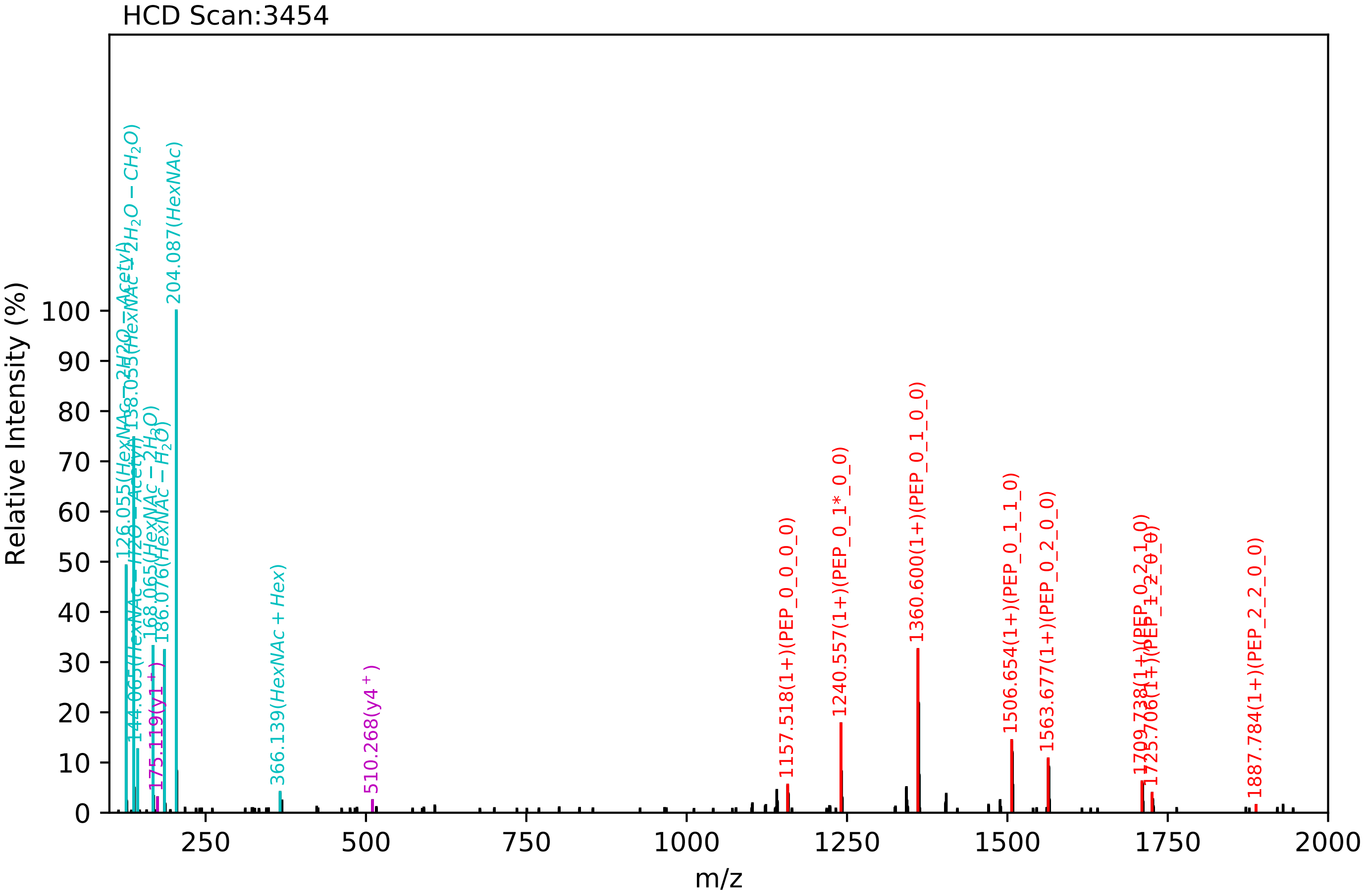

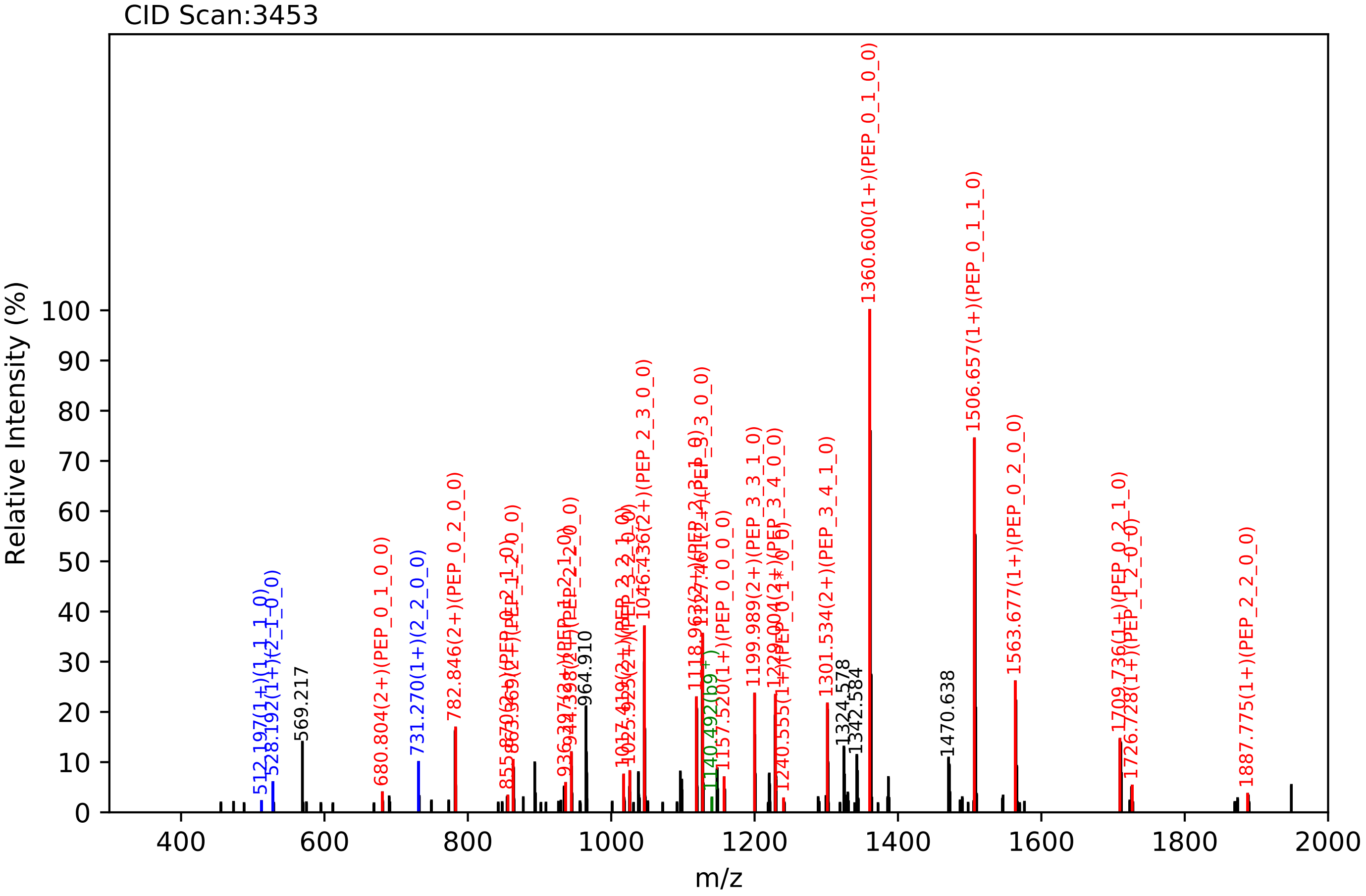

Test set no. 376, Experiment: IgG exp_7

EEQFNSTFR(=PEP)_4_3_1_0, m/z:1281.02(2+), RT:24.40, Y-score:86.44

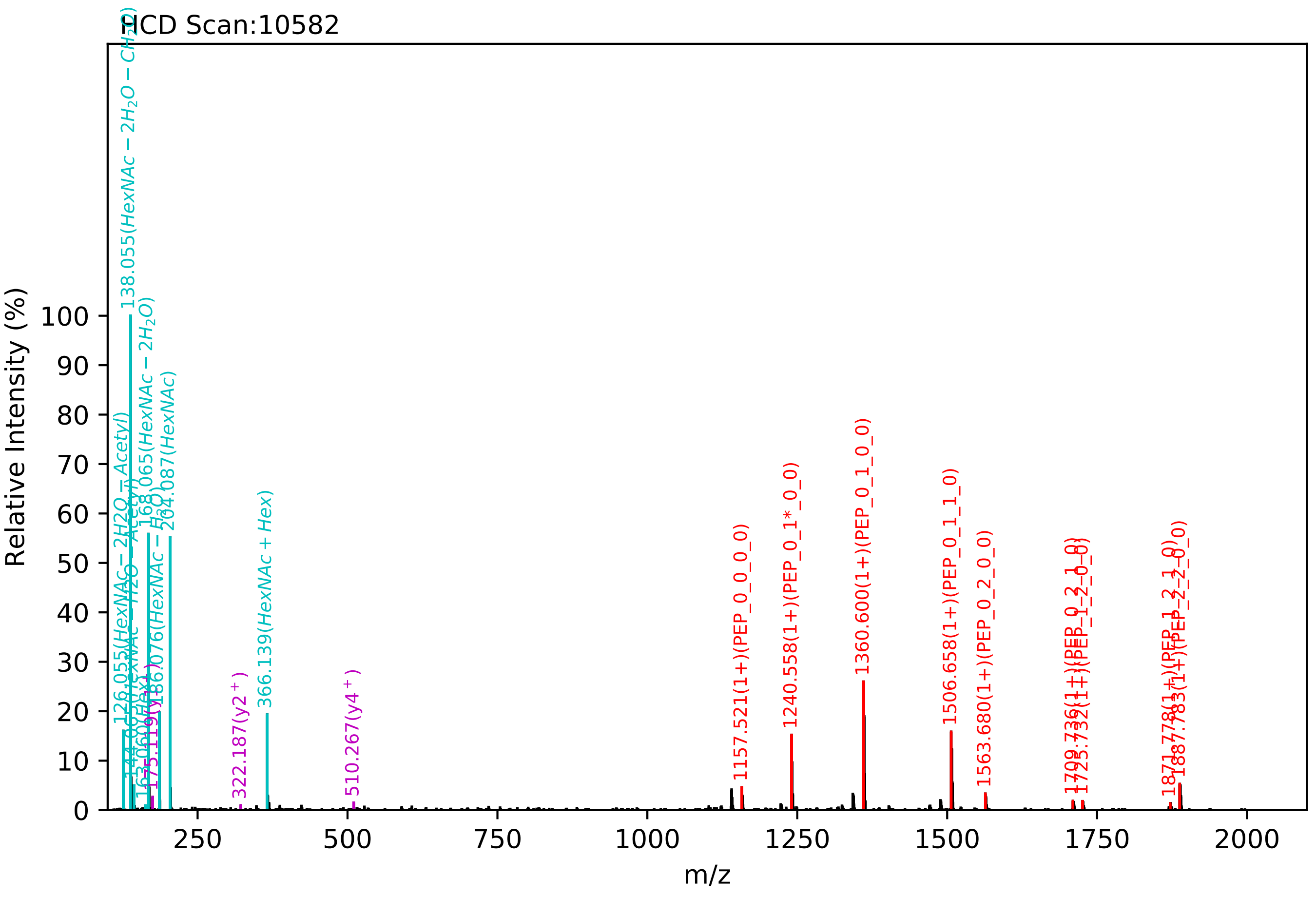

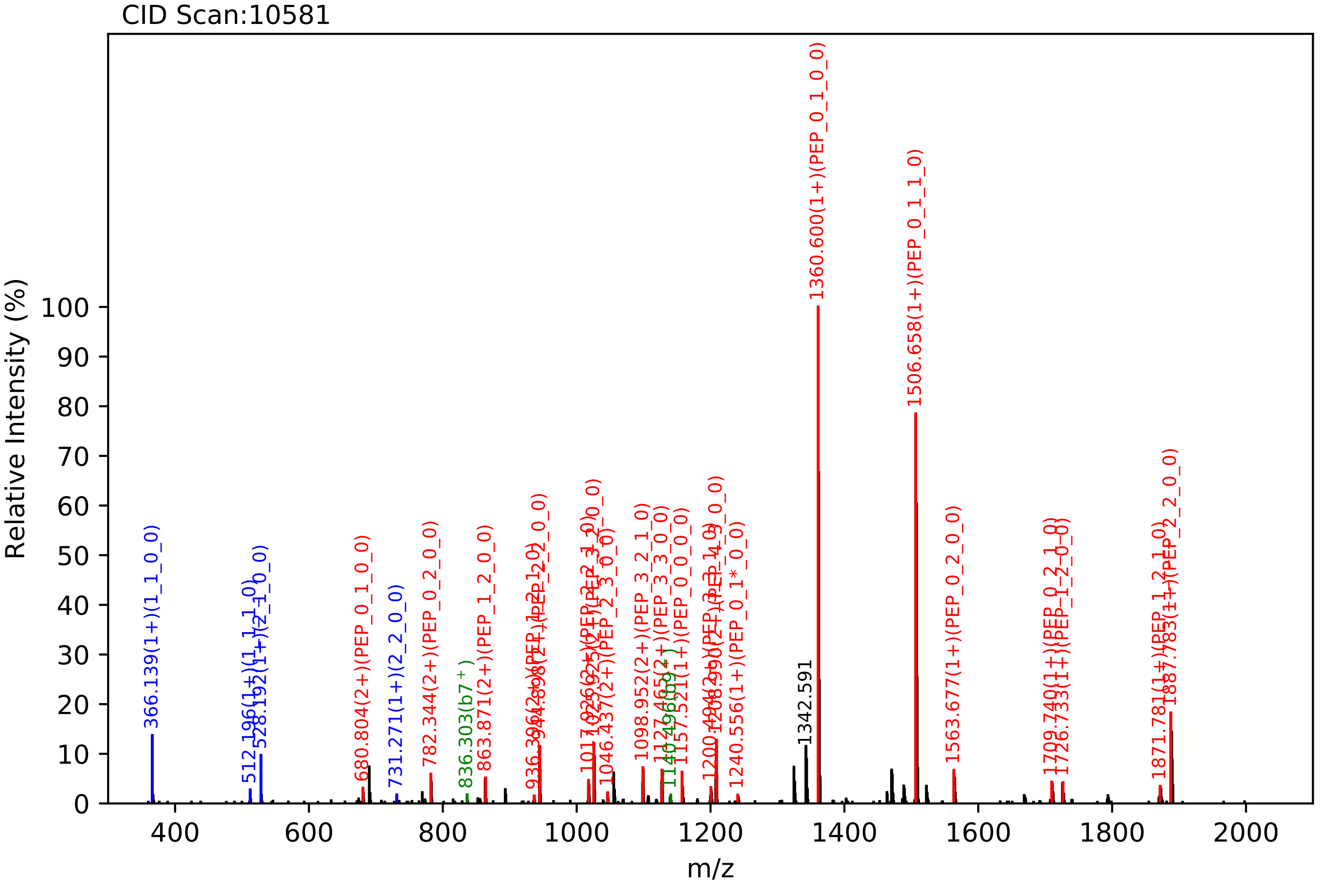

Test set no. 377, Experiment: IgG exp_7

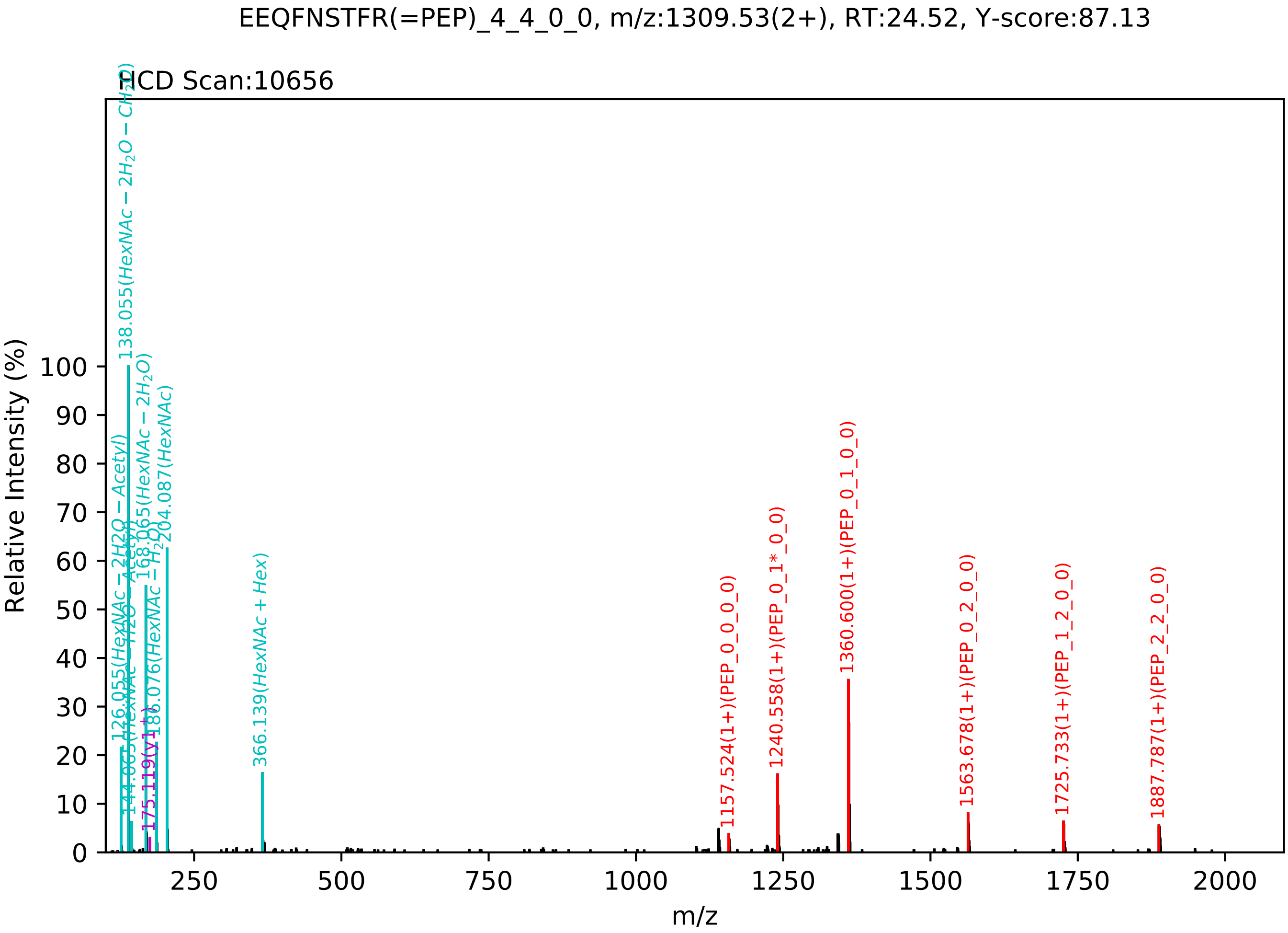

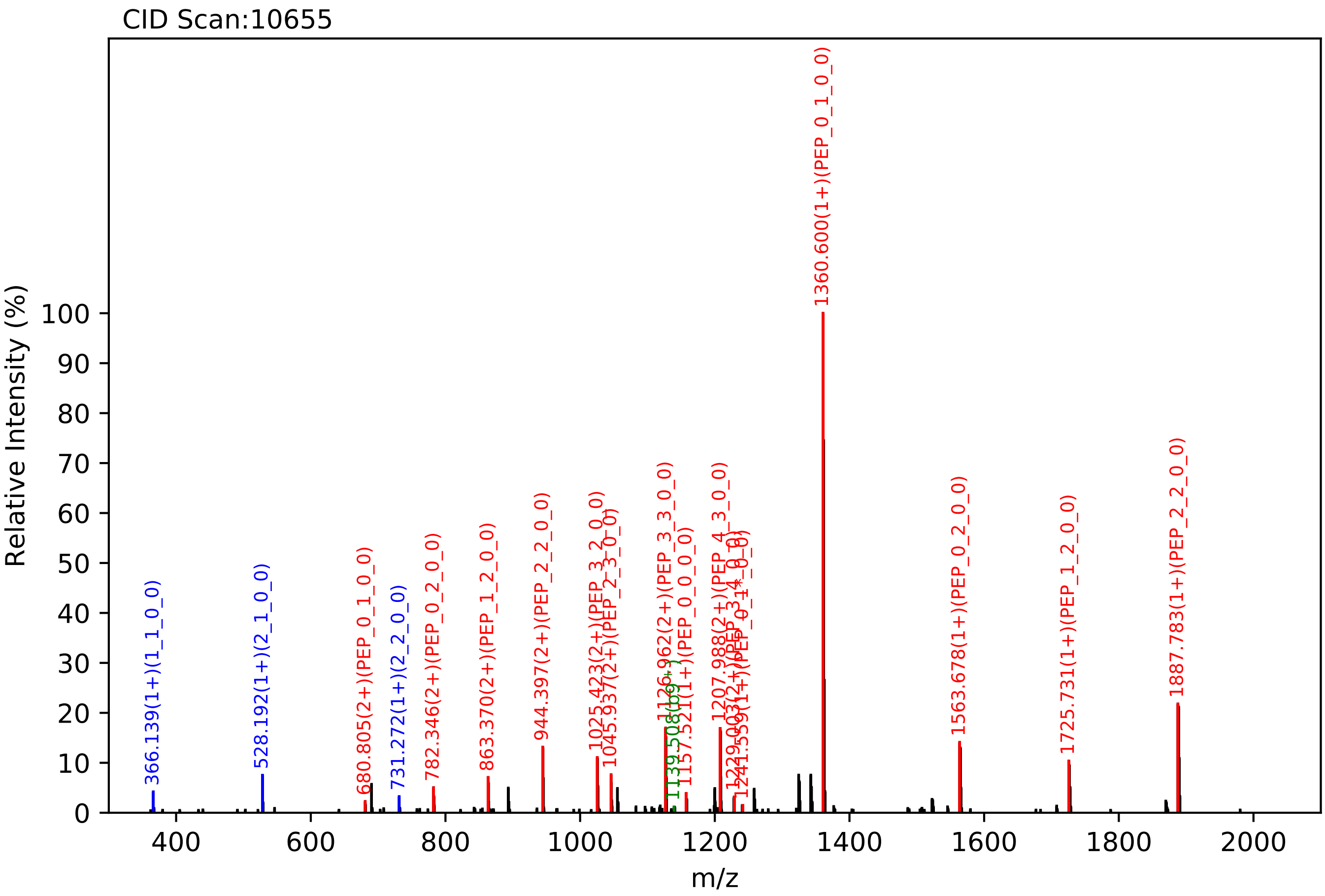

Test set no. 378, Experiment: IgG exp_5

EEQFNSTFR(=PEP)_4_4_1_0, m/z:1382.56(2+), RT:25.18, Y-score:88.34

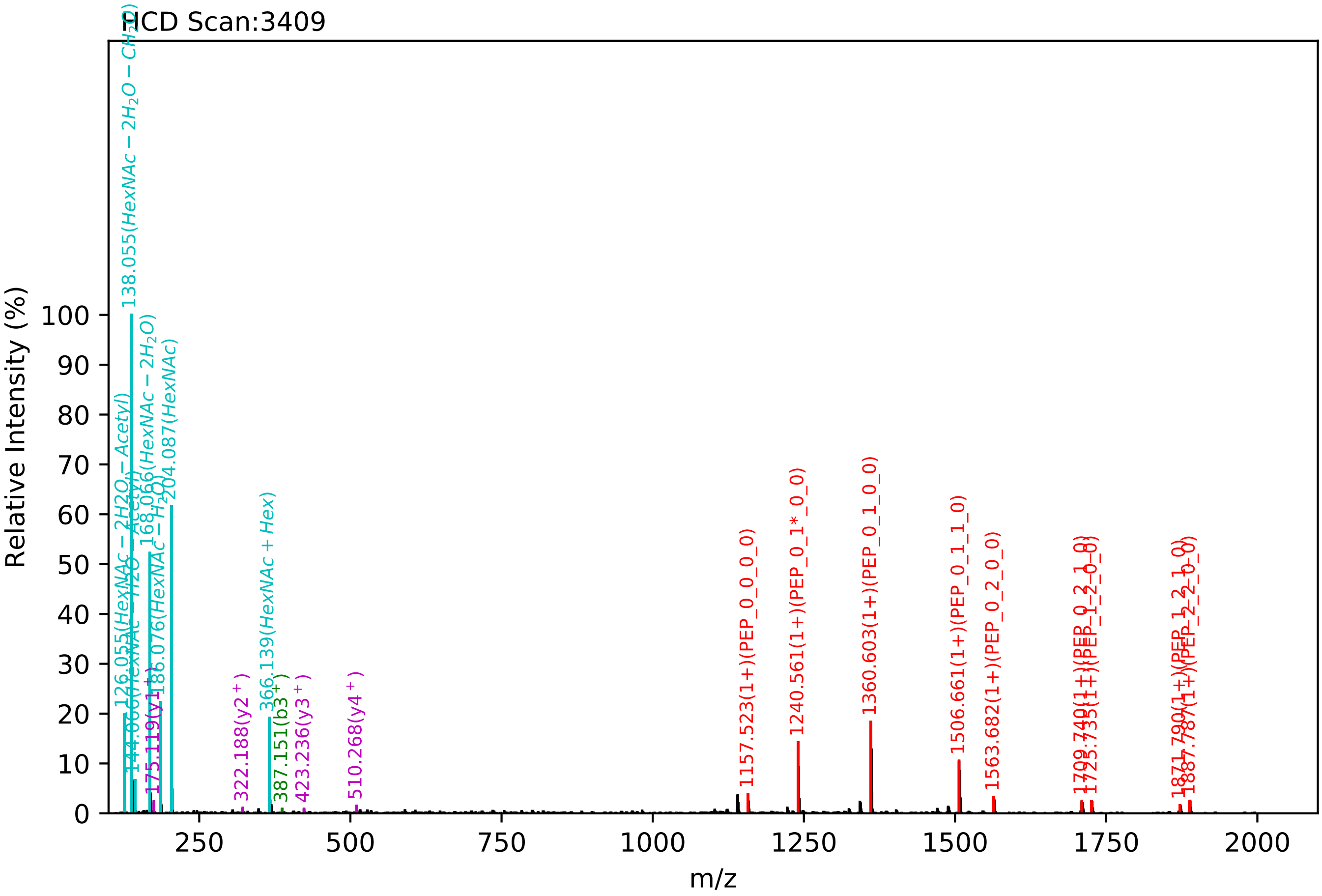

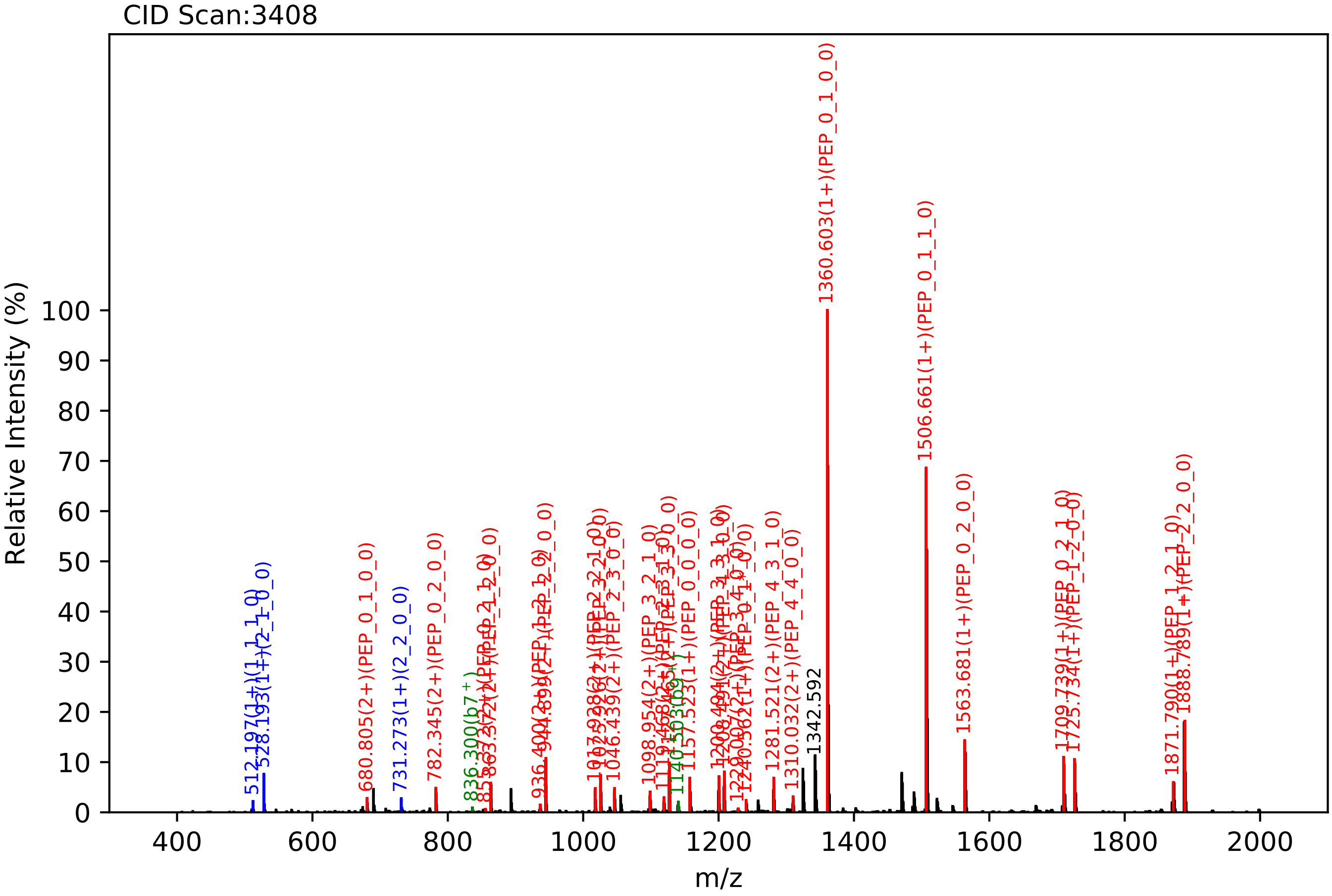

Test set no. 379, Experiment: IgG exp_7

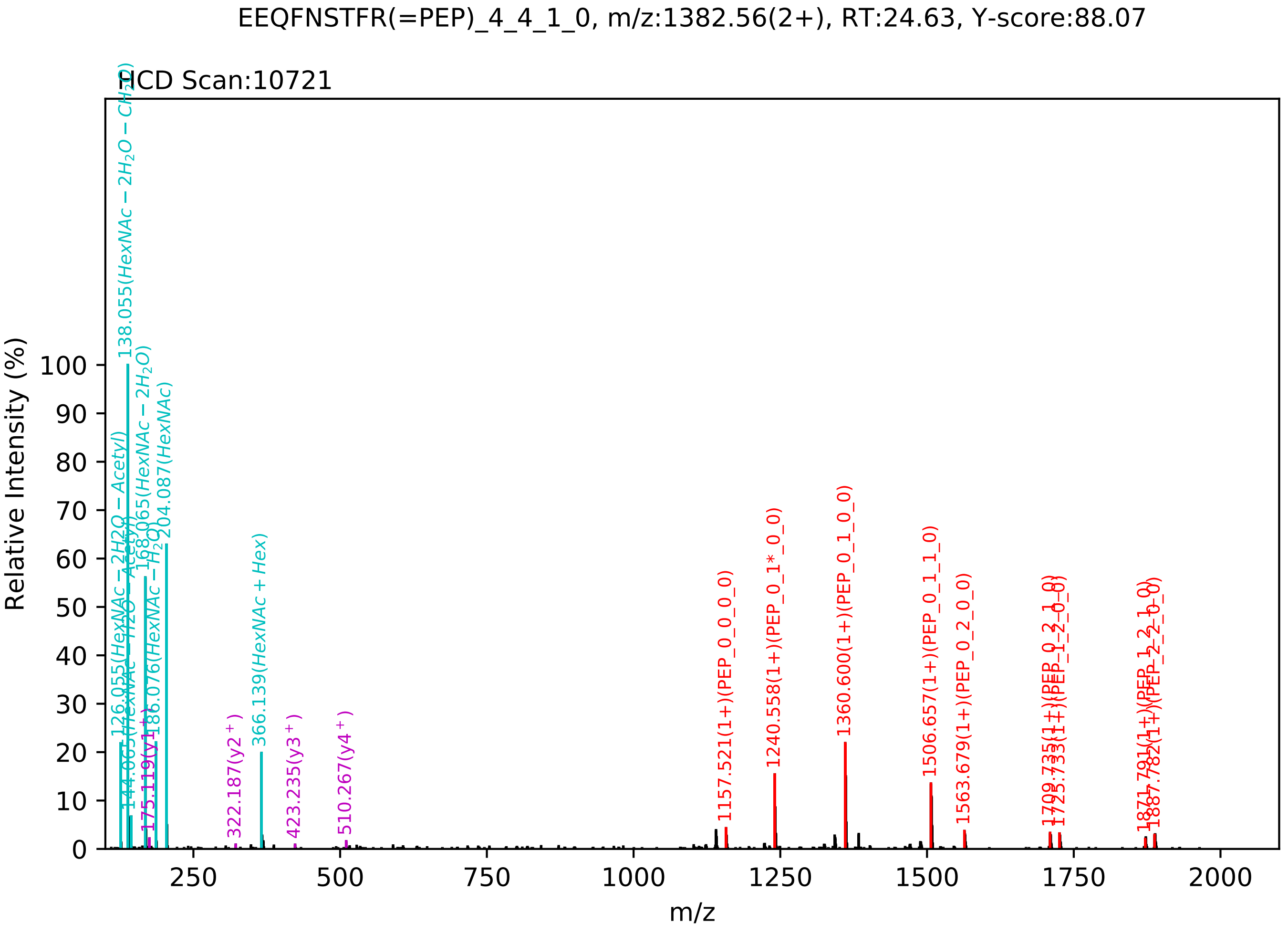

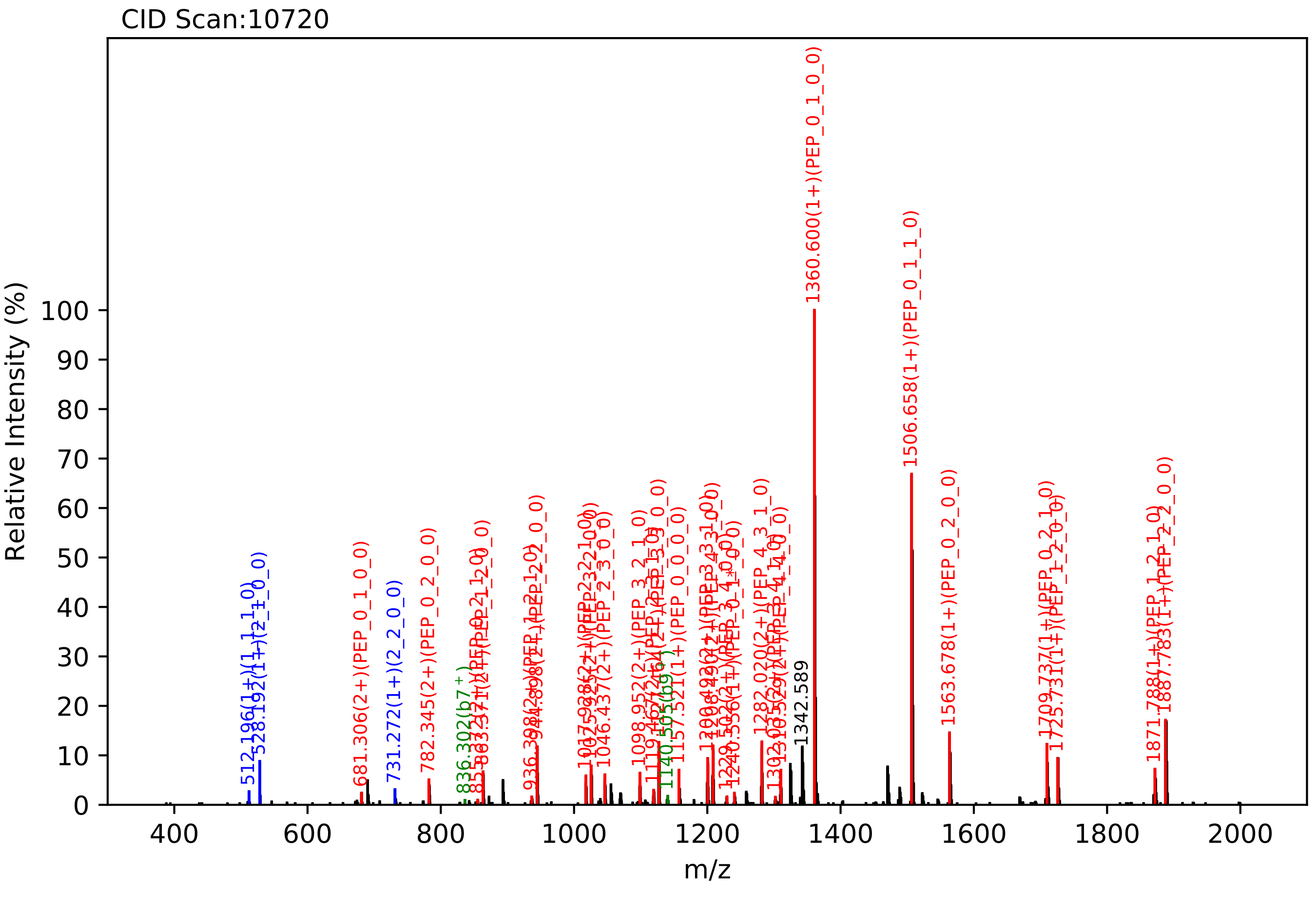

Test set no. 380, Experiment: IgG exp_6

EEQFNSTFR(=PEP)_4_4_1_0, m/z:1382.56(2+), RT:27.22, Y-score:86.18

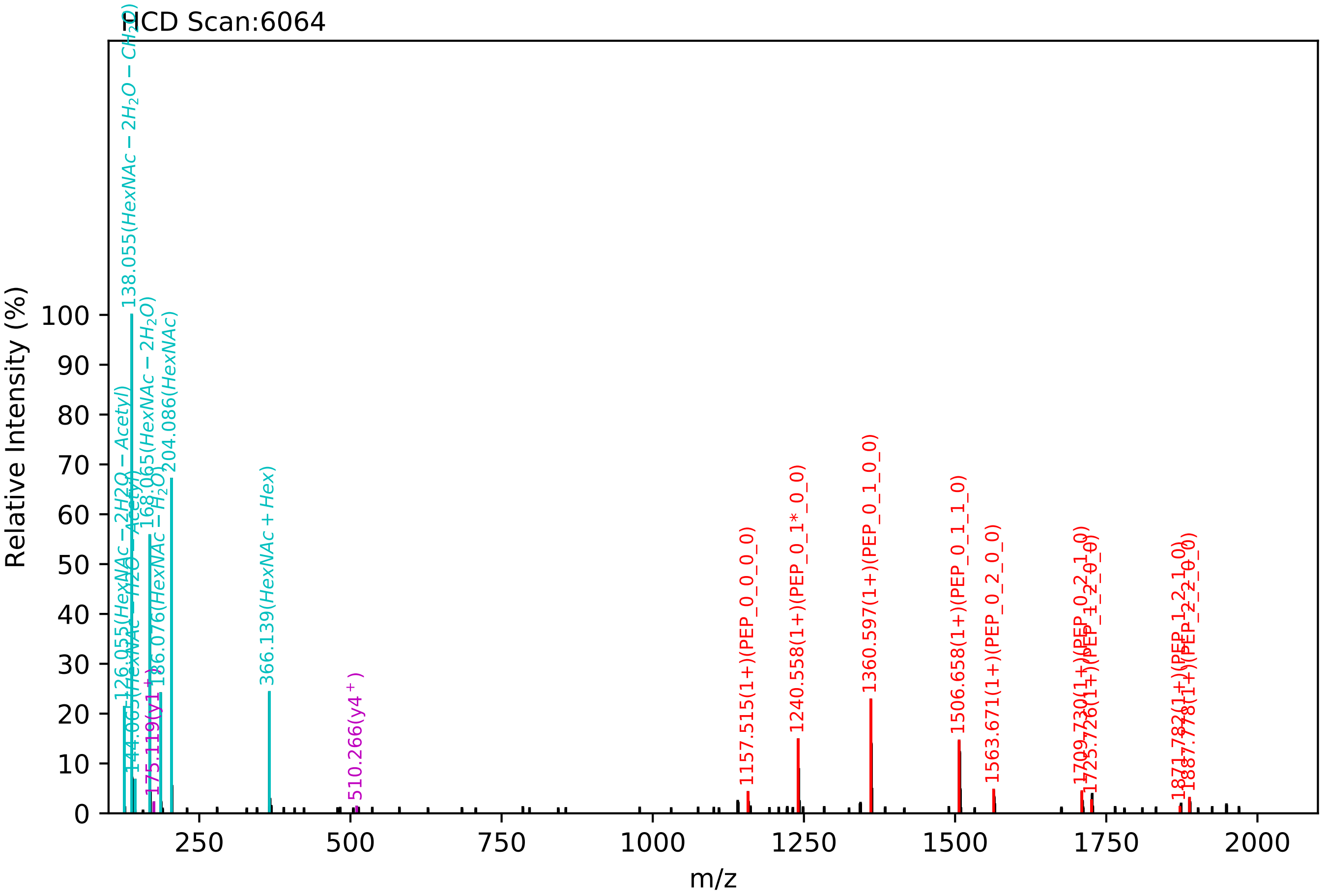

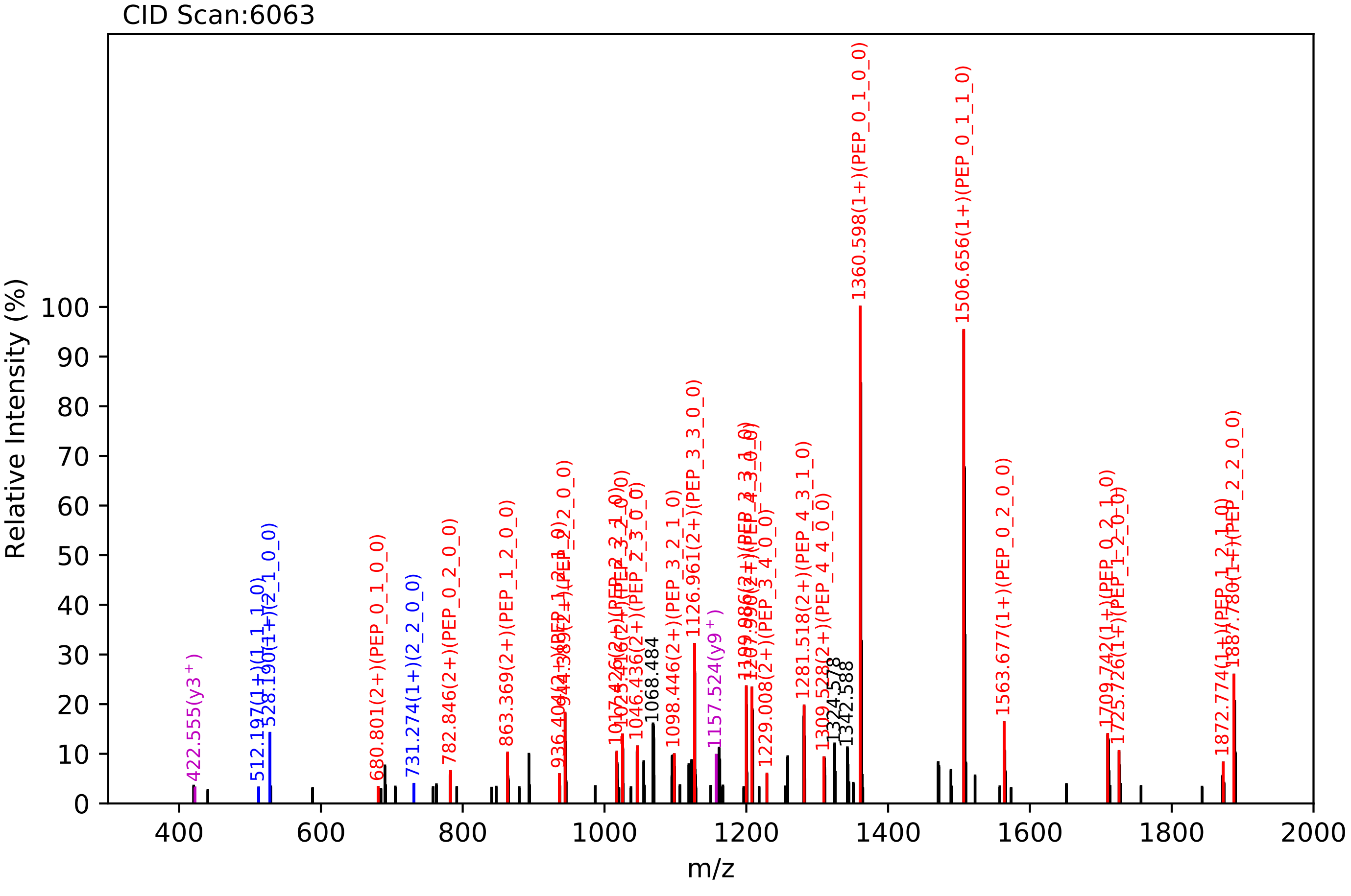

Test set no. 381, Experiment: IgG exp_7

EEQFNSTFR(=PEP)_4_4_1_0, m/z:1382.56(2+), RT:24.06, Y-score:82.80

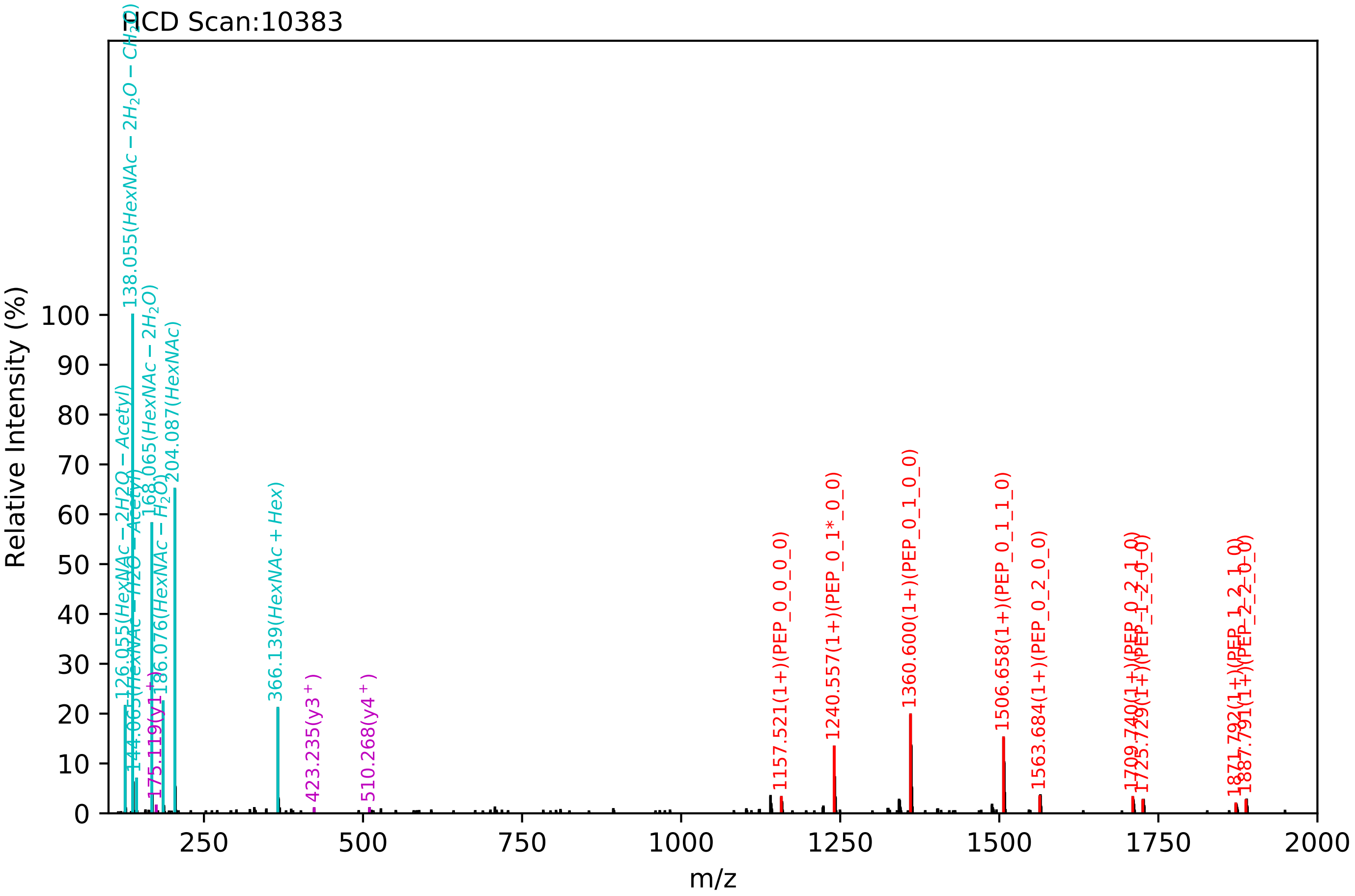

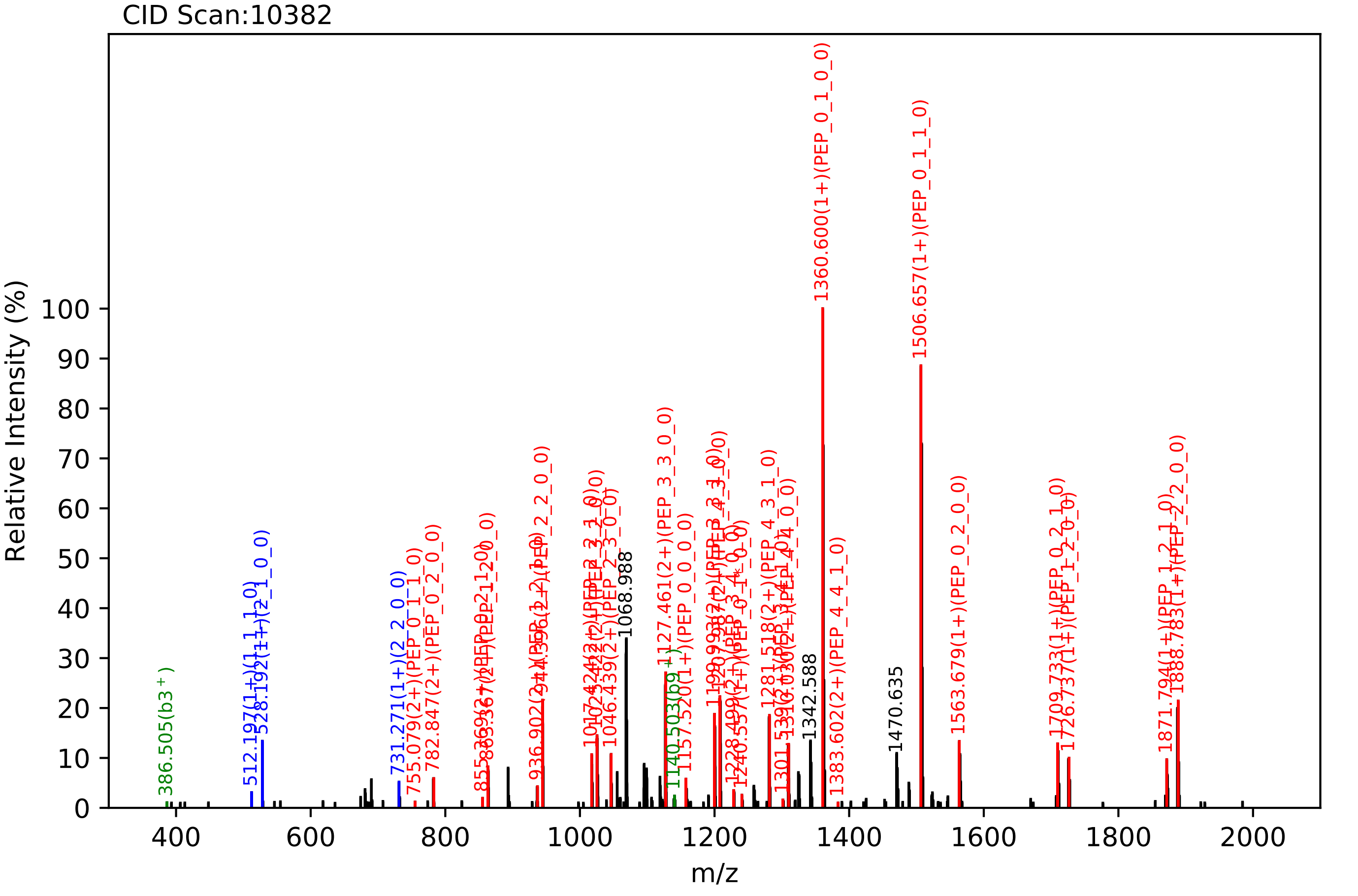

Test set no. 382, Experiment: IgG exp_6

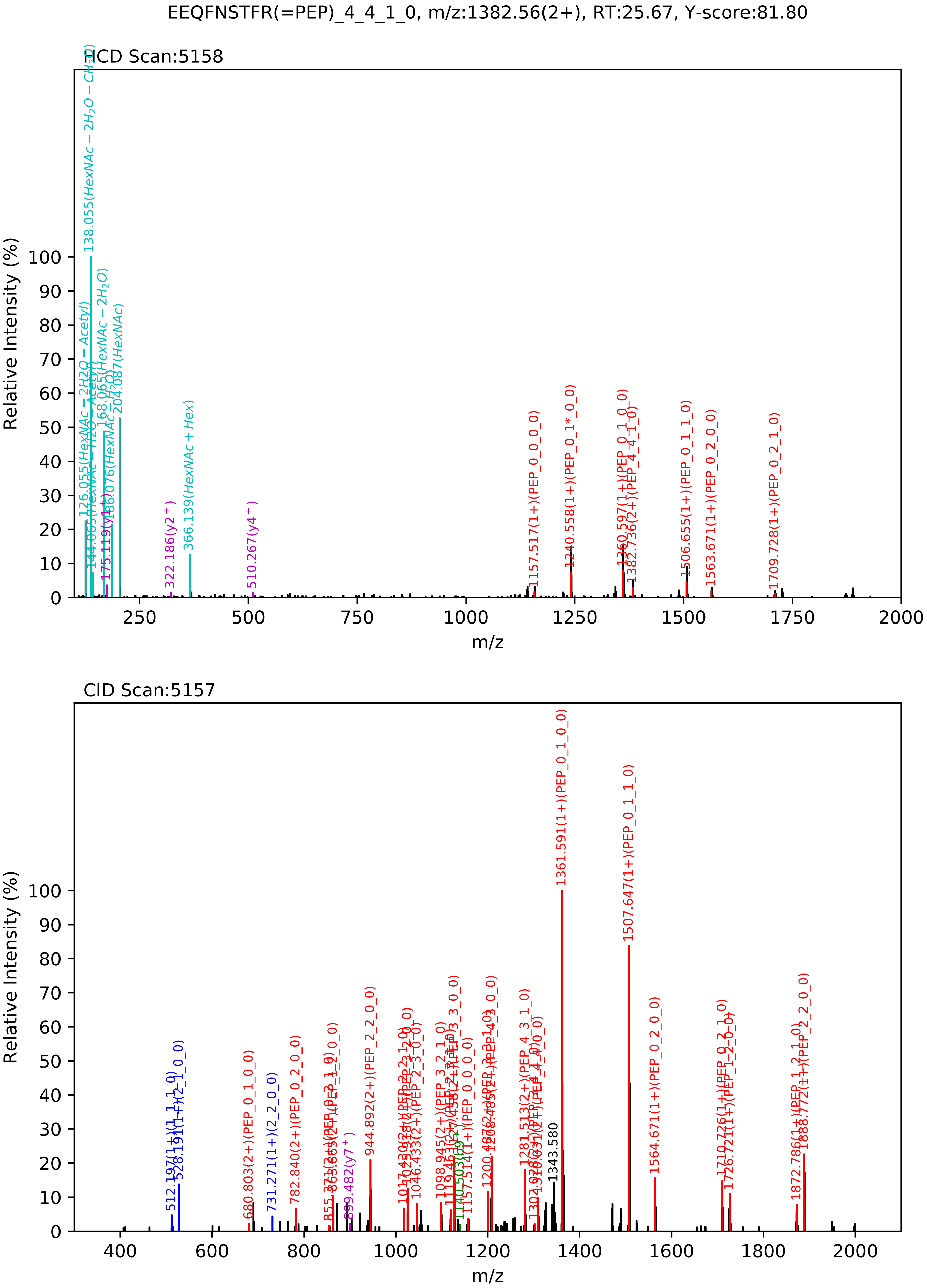

Test set no. 383, Experiment: IgG exp_7

EEQFNSTFR(=PEP)_4_4_1_0, m/z:922.04(3+), RT:24.63, Y-score:78.50

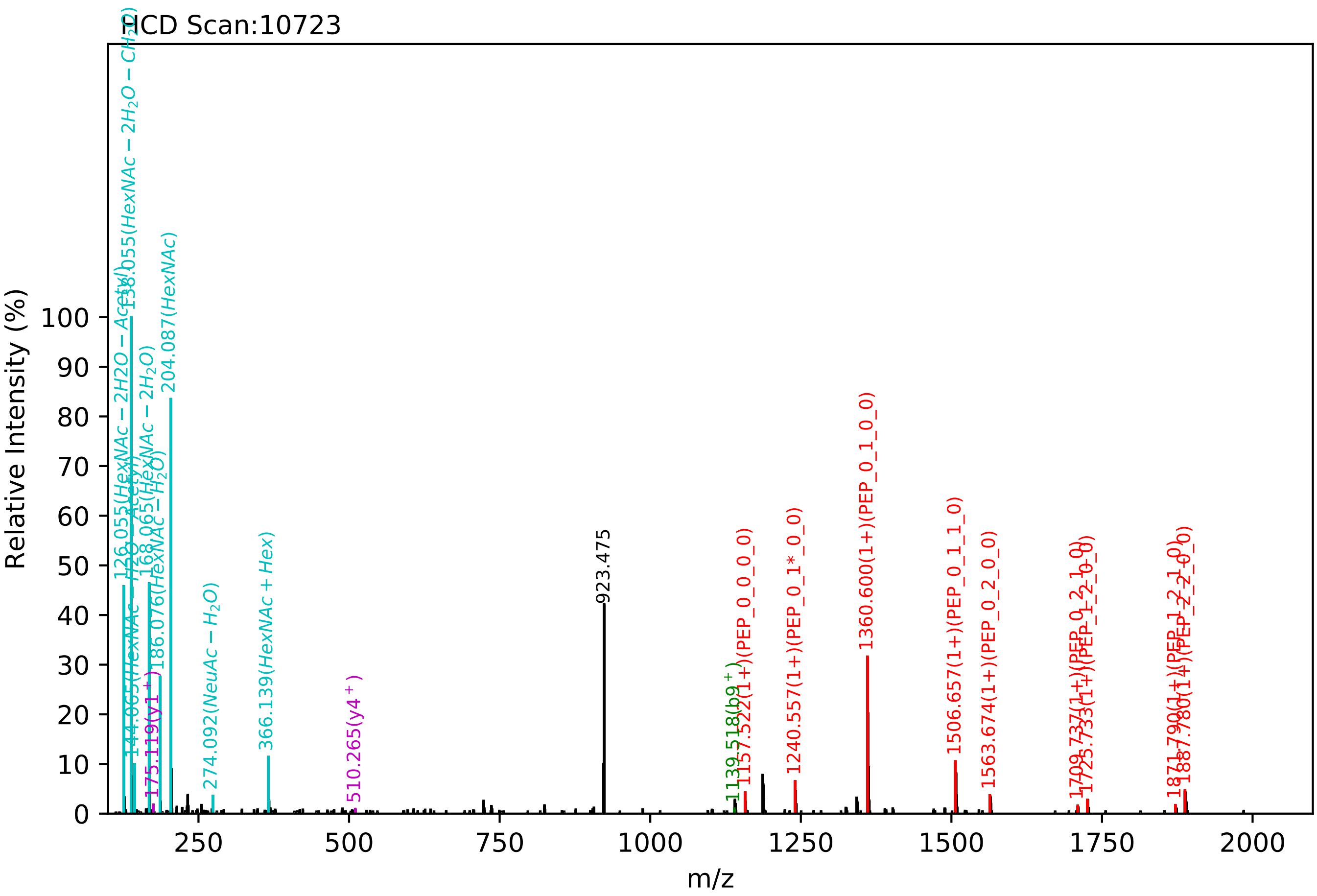

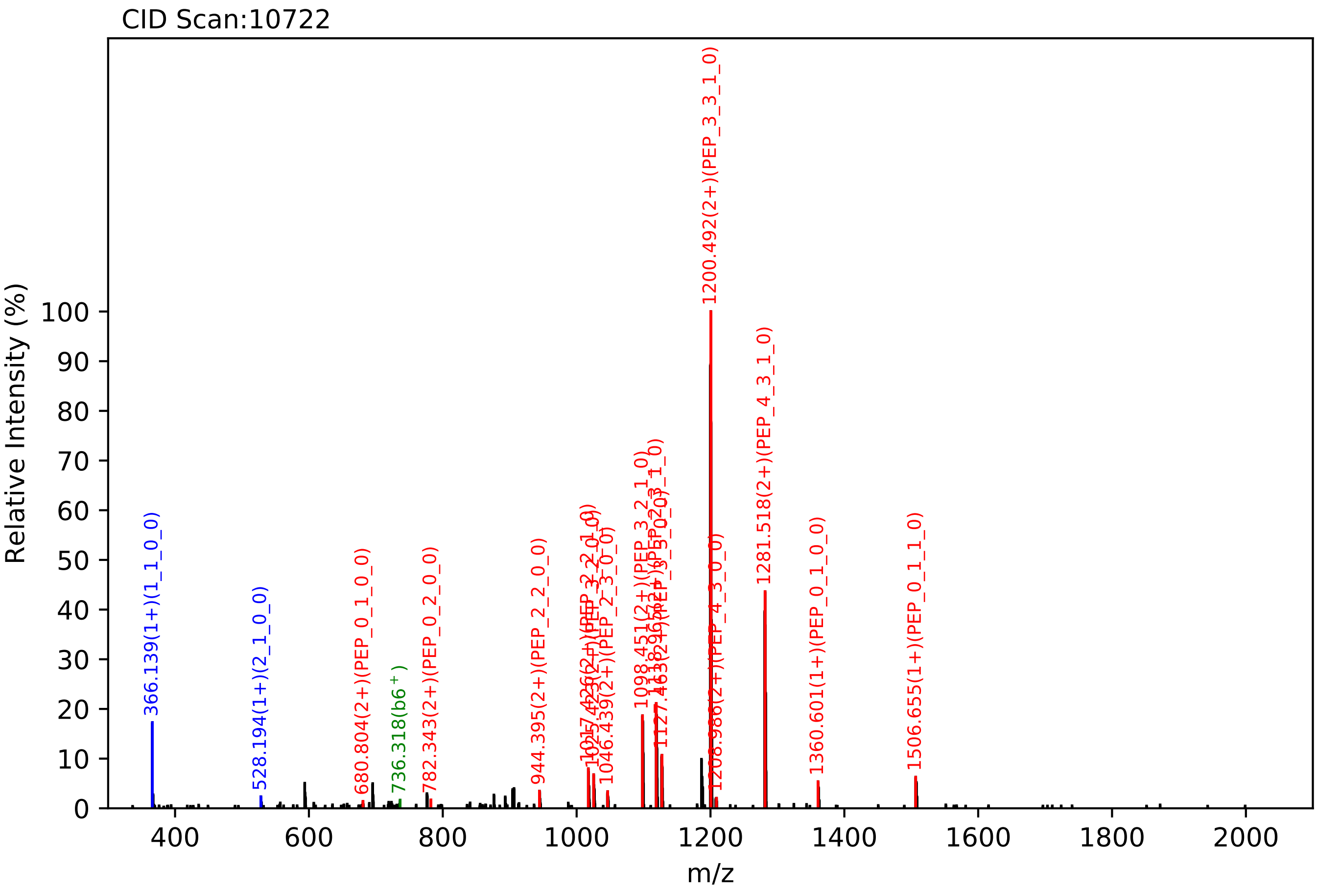

Test set no. 384, Experiment: IgG exp_7

EEQFNSTFR(=PEP)_4_4_1_1, m/z:1019.07(3+), RT:27.01, Y-score:93.54

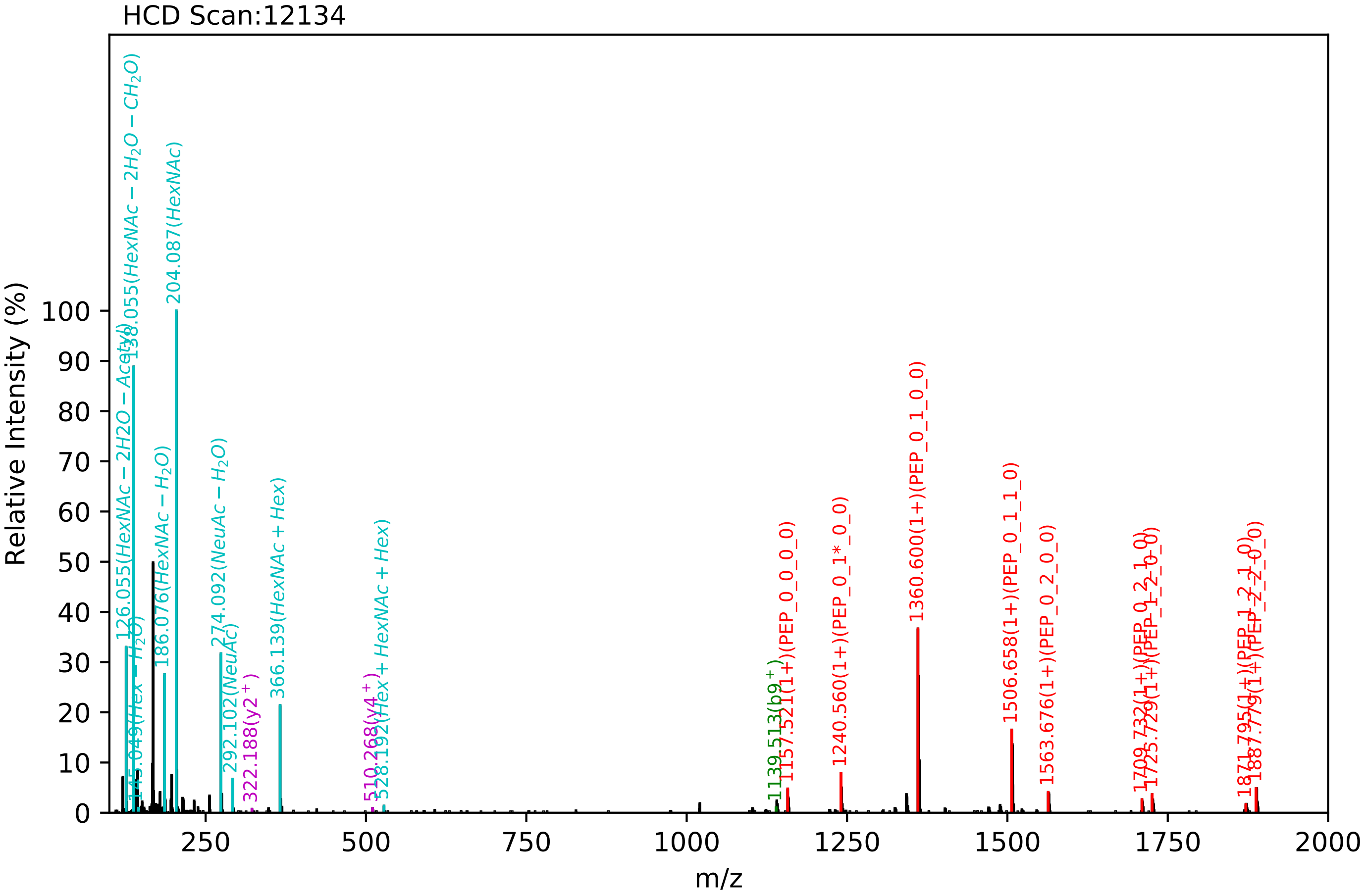

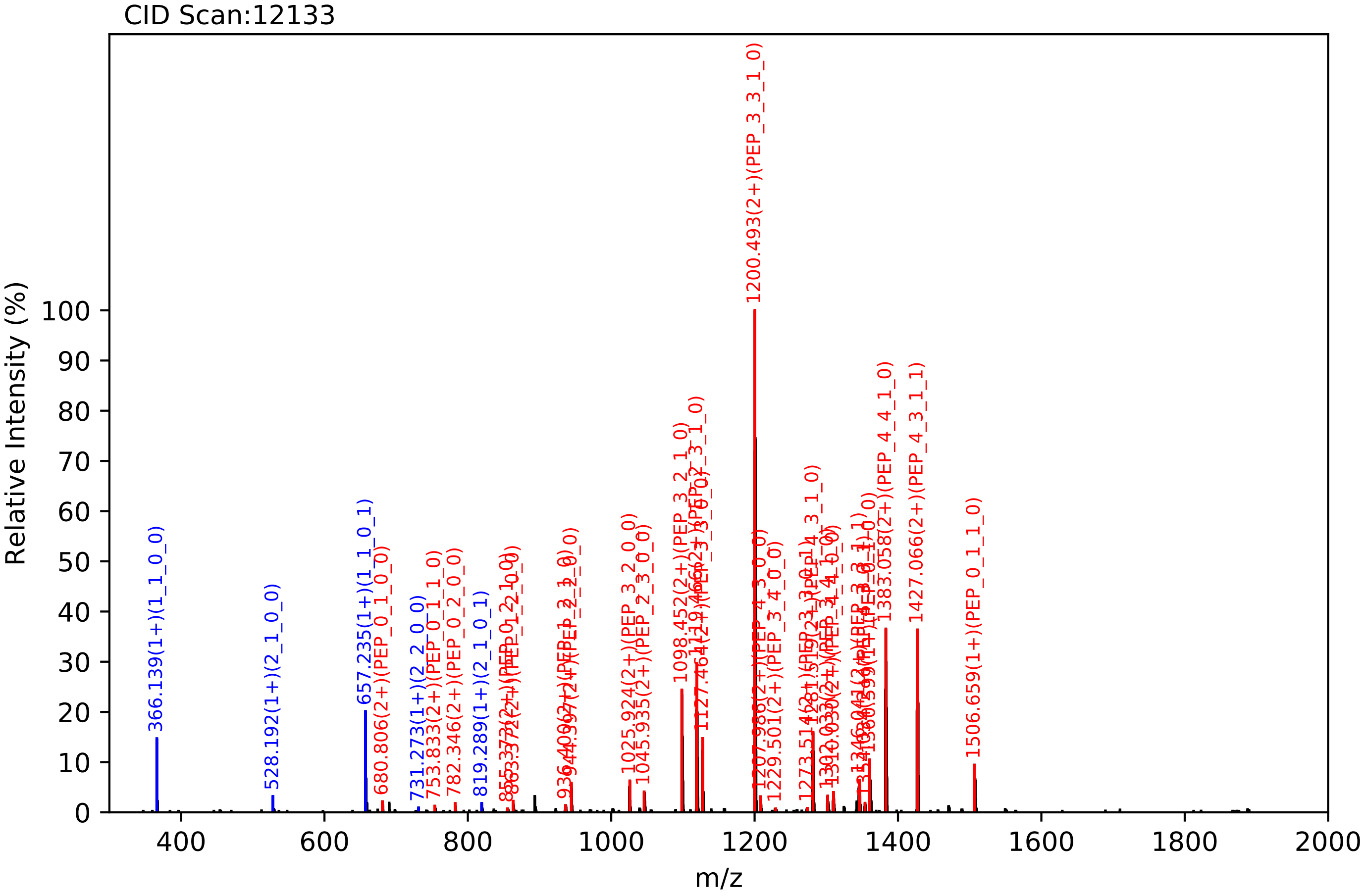

Test set no. 385, Experiment: IgG exp_5

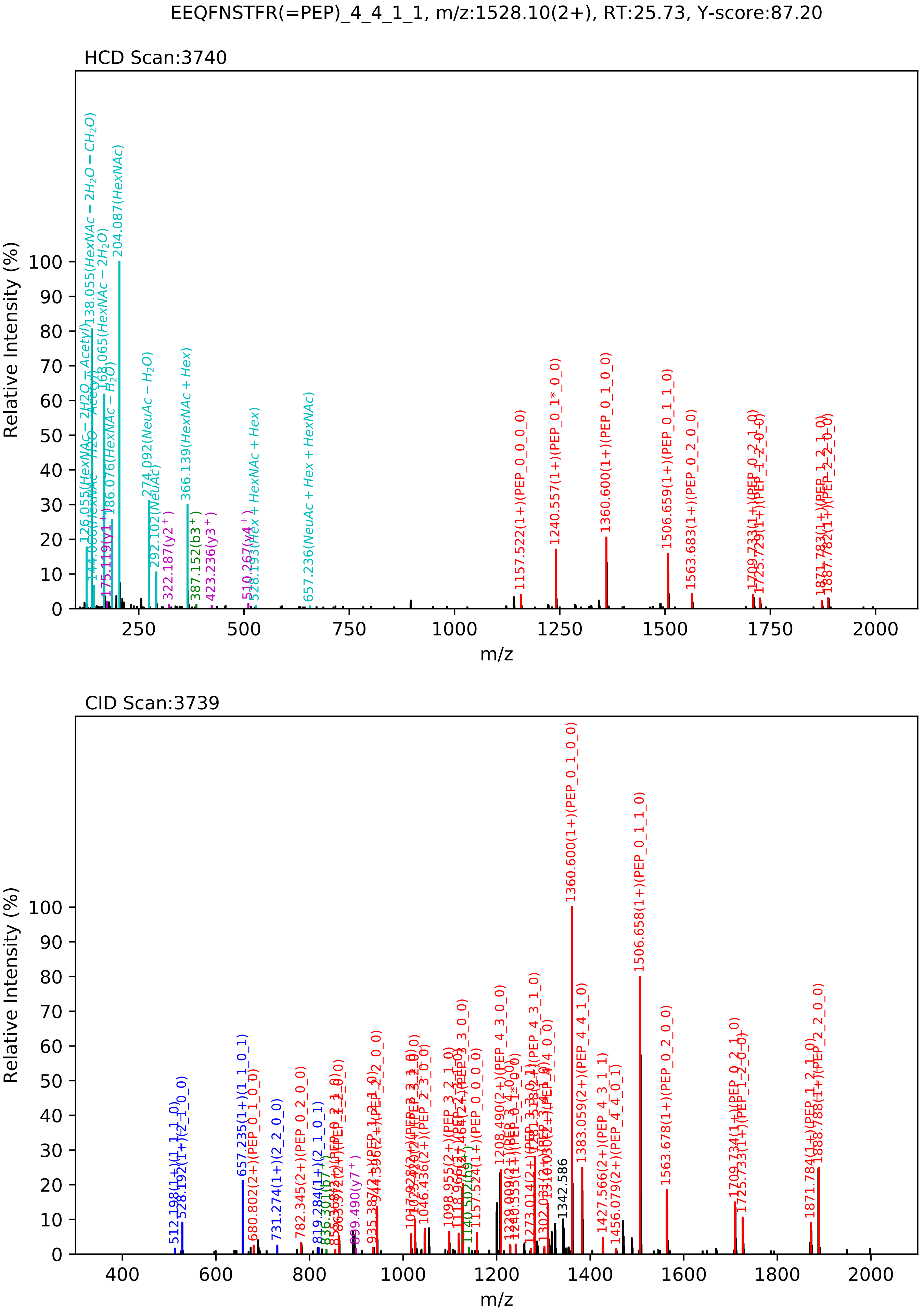

Test set no. 386, Experiment: IgG exp_7

EEQFNSTFR(=PEP)_4_4_1_1, m/z:1528.11(2+), RT:27.05, Y-score:86.45

HCD Scan:12155

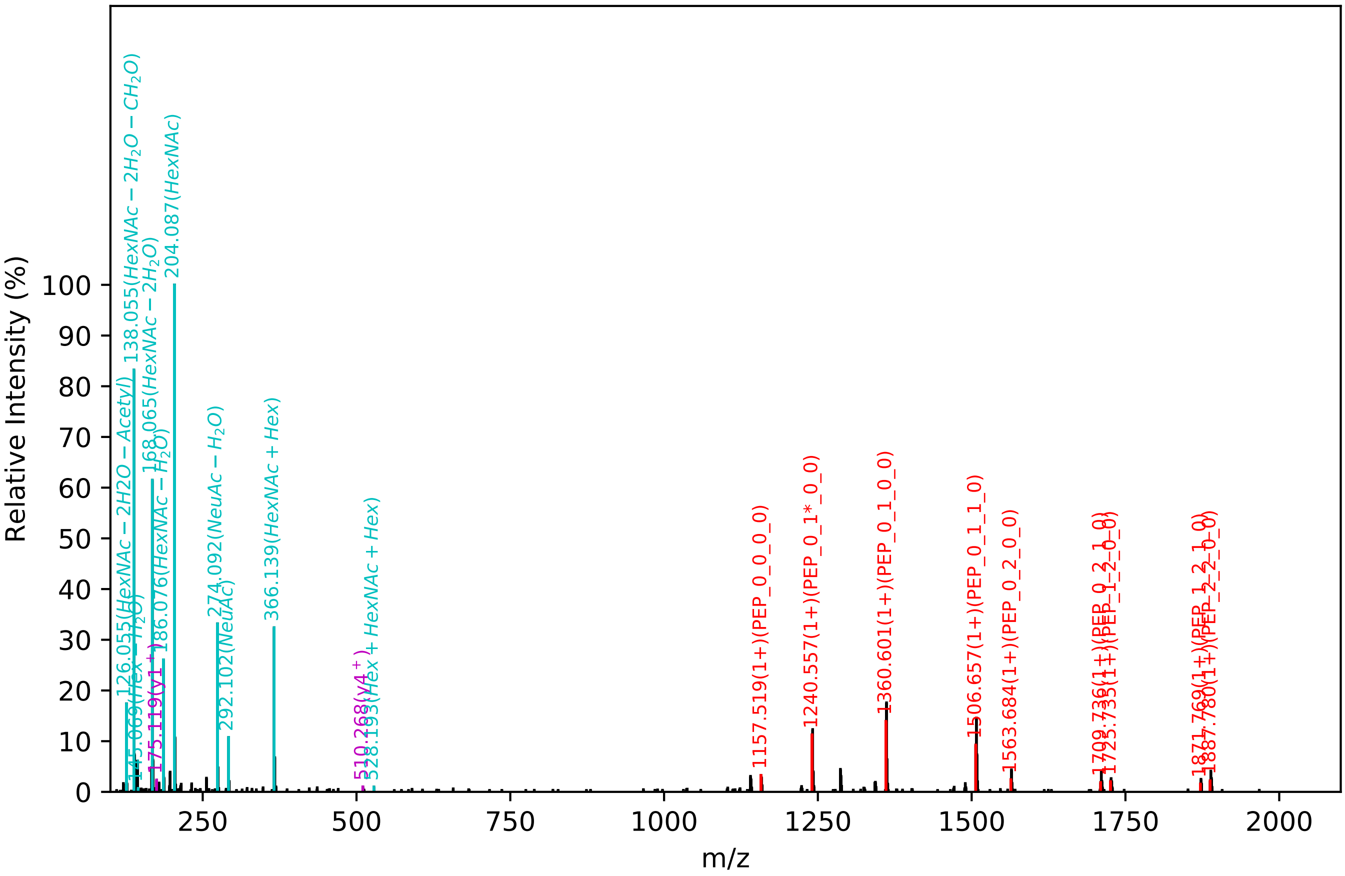

CID Scan:12154

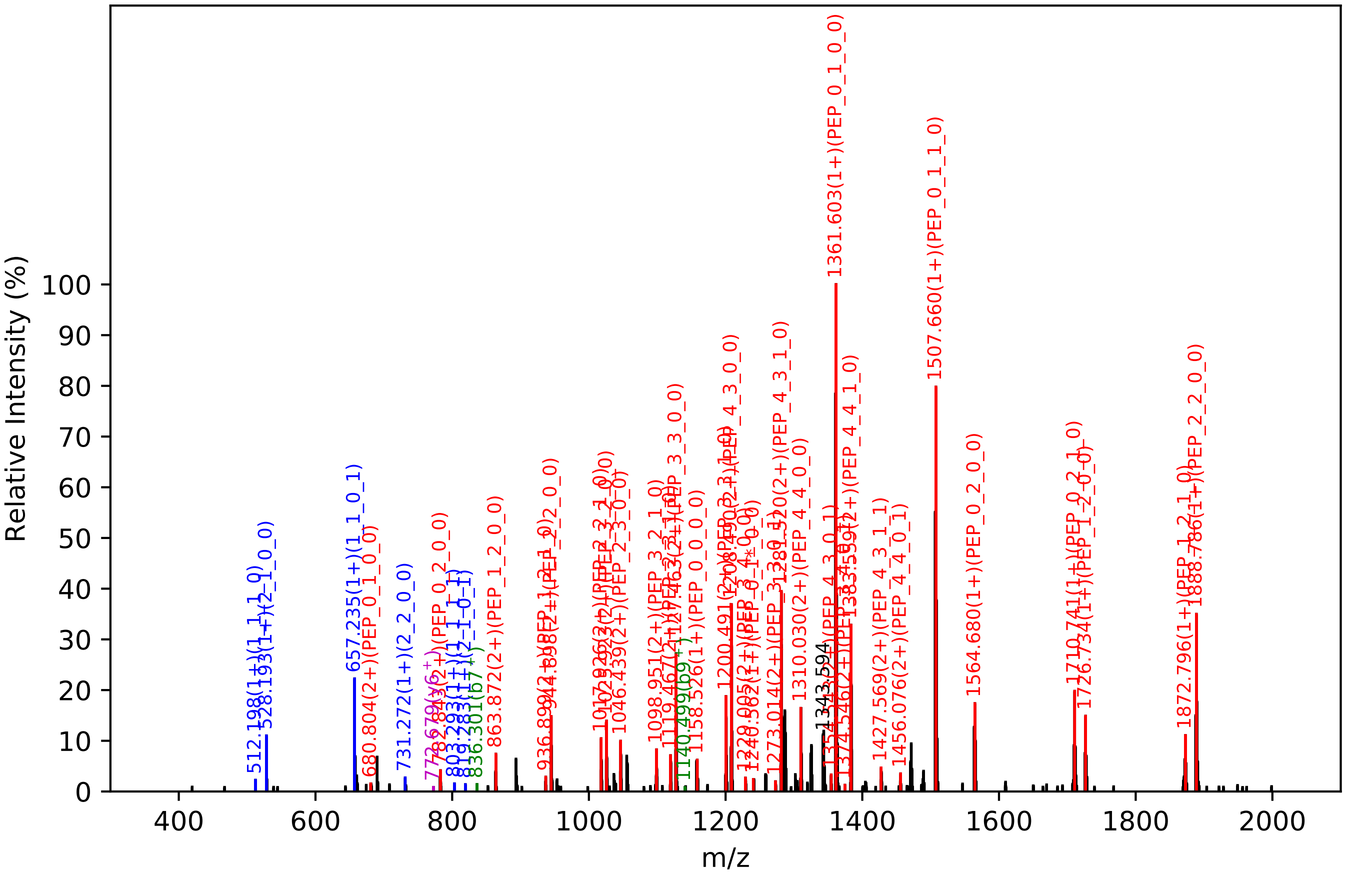

Test set no. 387, Experiment: IgG exp_5

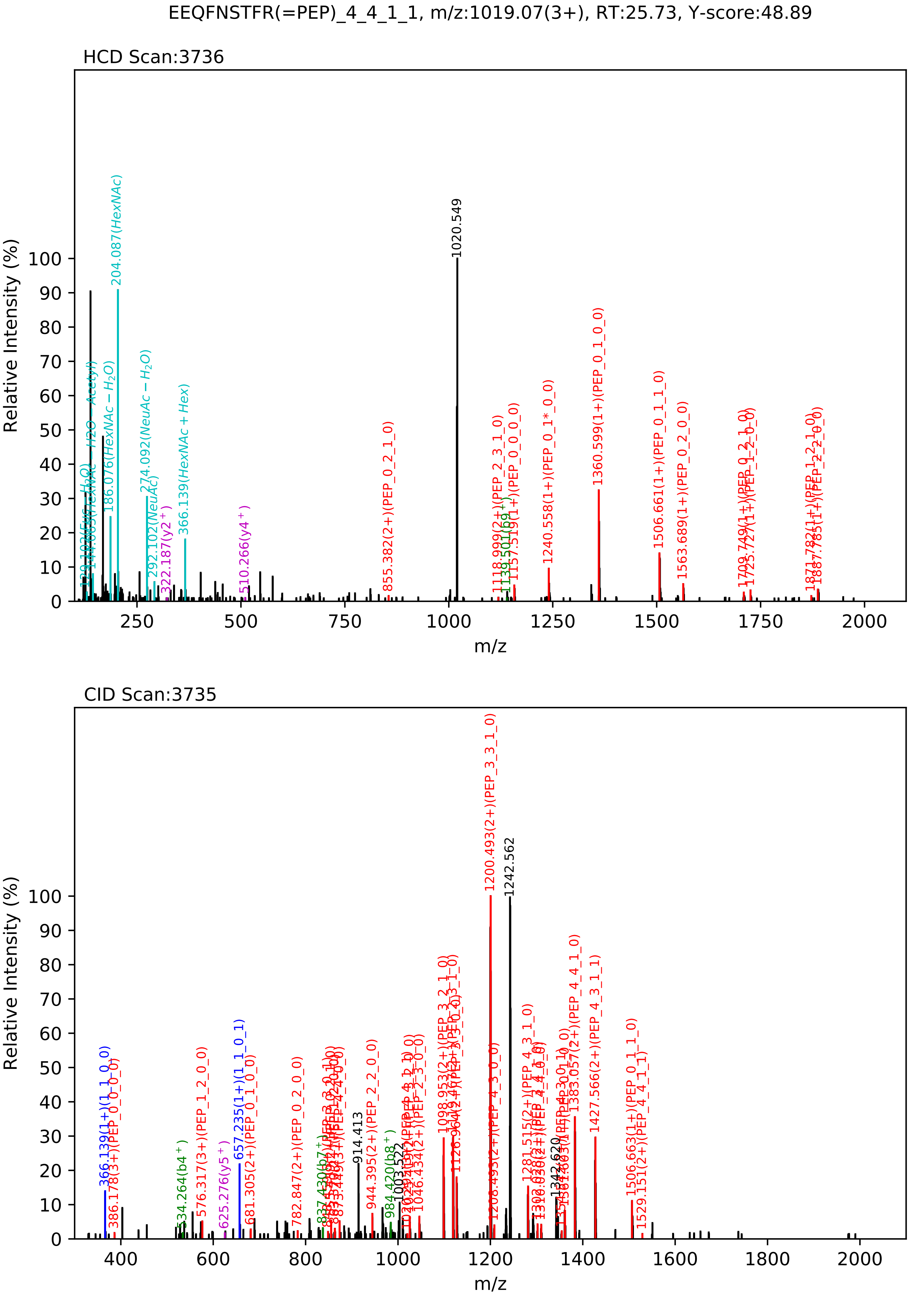

Test set no. 388, Experiment: IgG exp_7

EEQFNSTFR(=PEP)_4_5_1_0, m/z:1484.10(2+), RT:24.52, Y-score:84.03

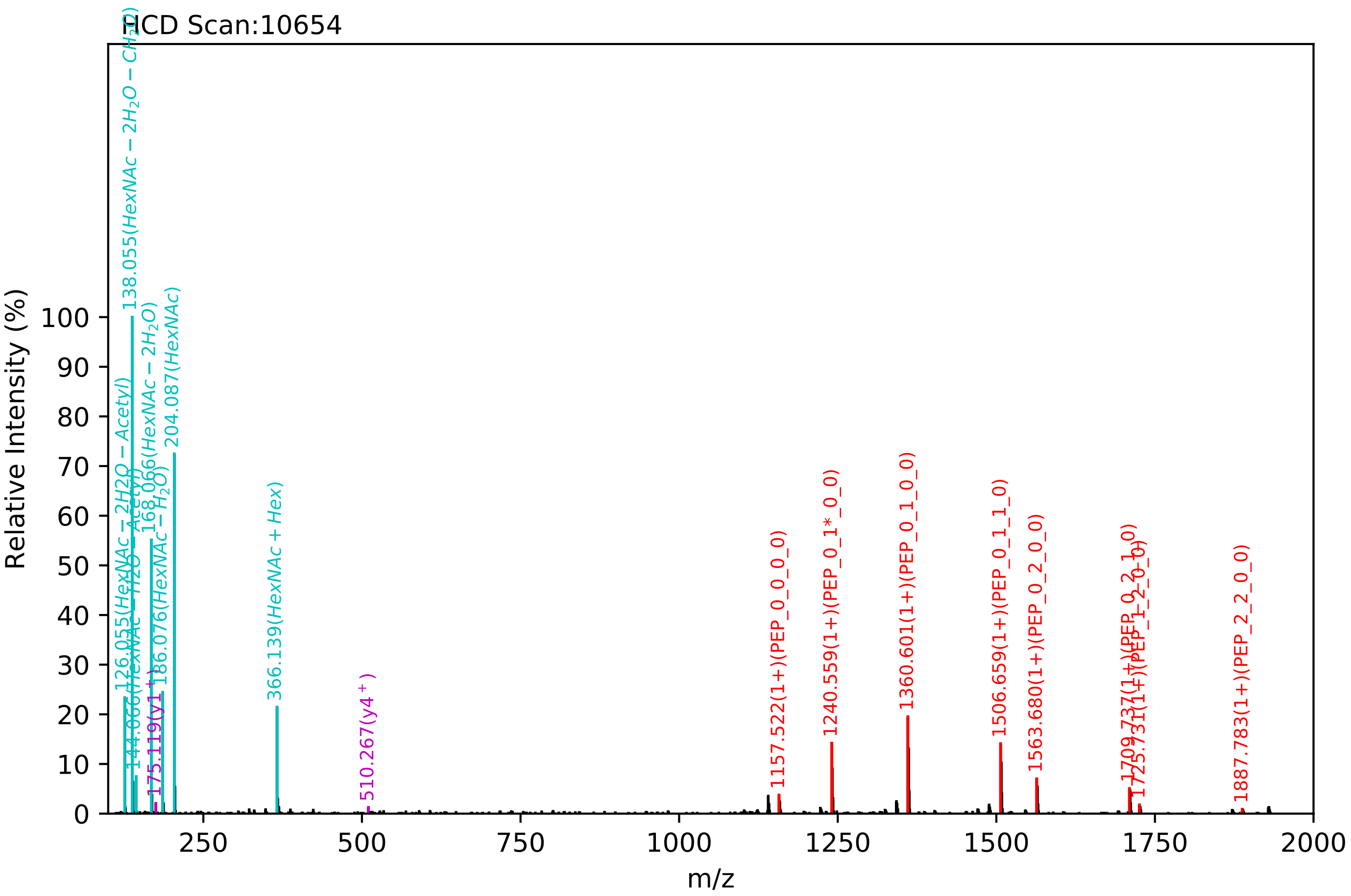

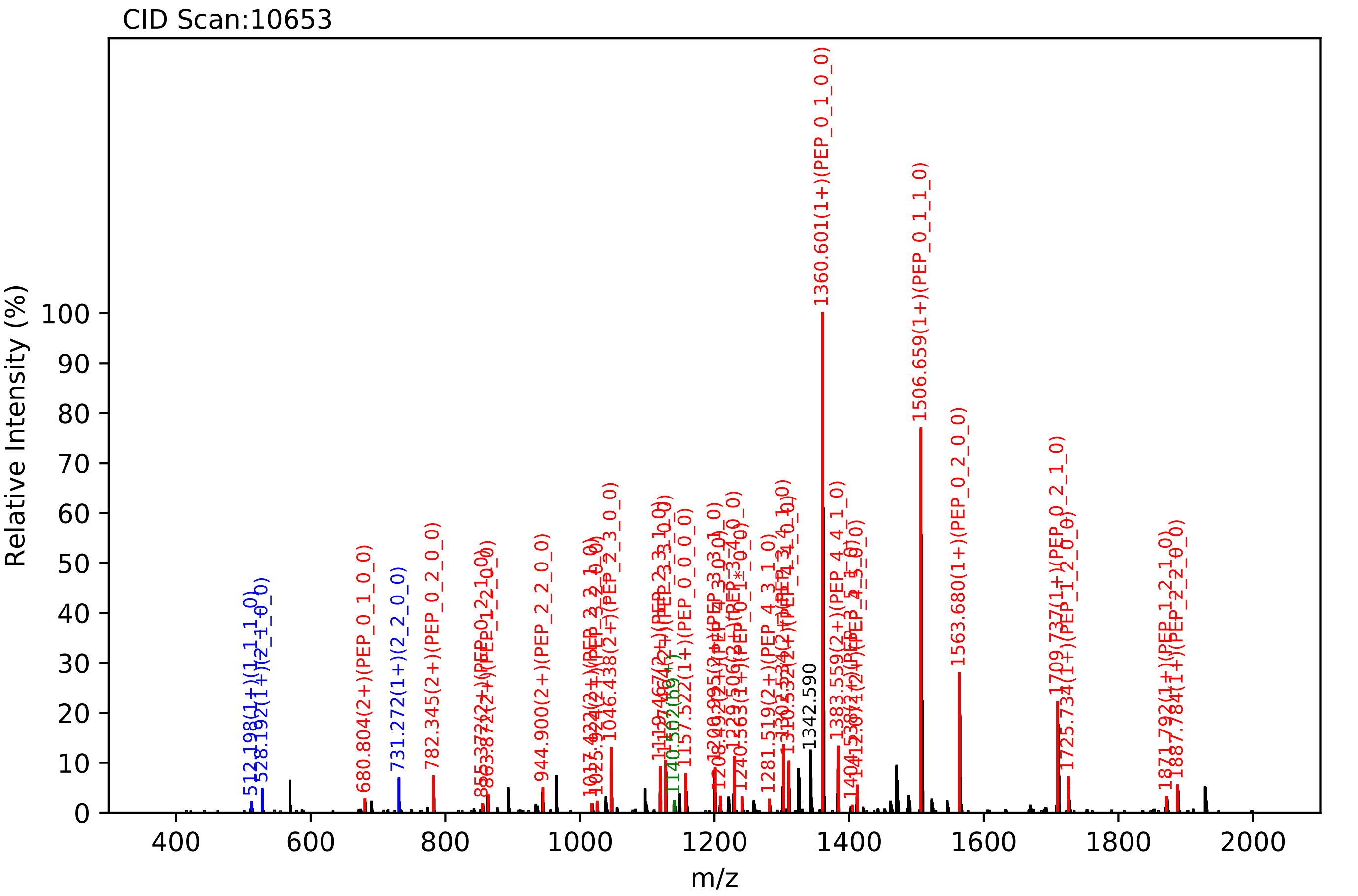

Test set no. 389, Experiment: IgG exp_5

EEQFNSTFR(=PEP)_5_4_1_0, m/z:1463.58(2+), RT:25.17, Y-score:88.75

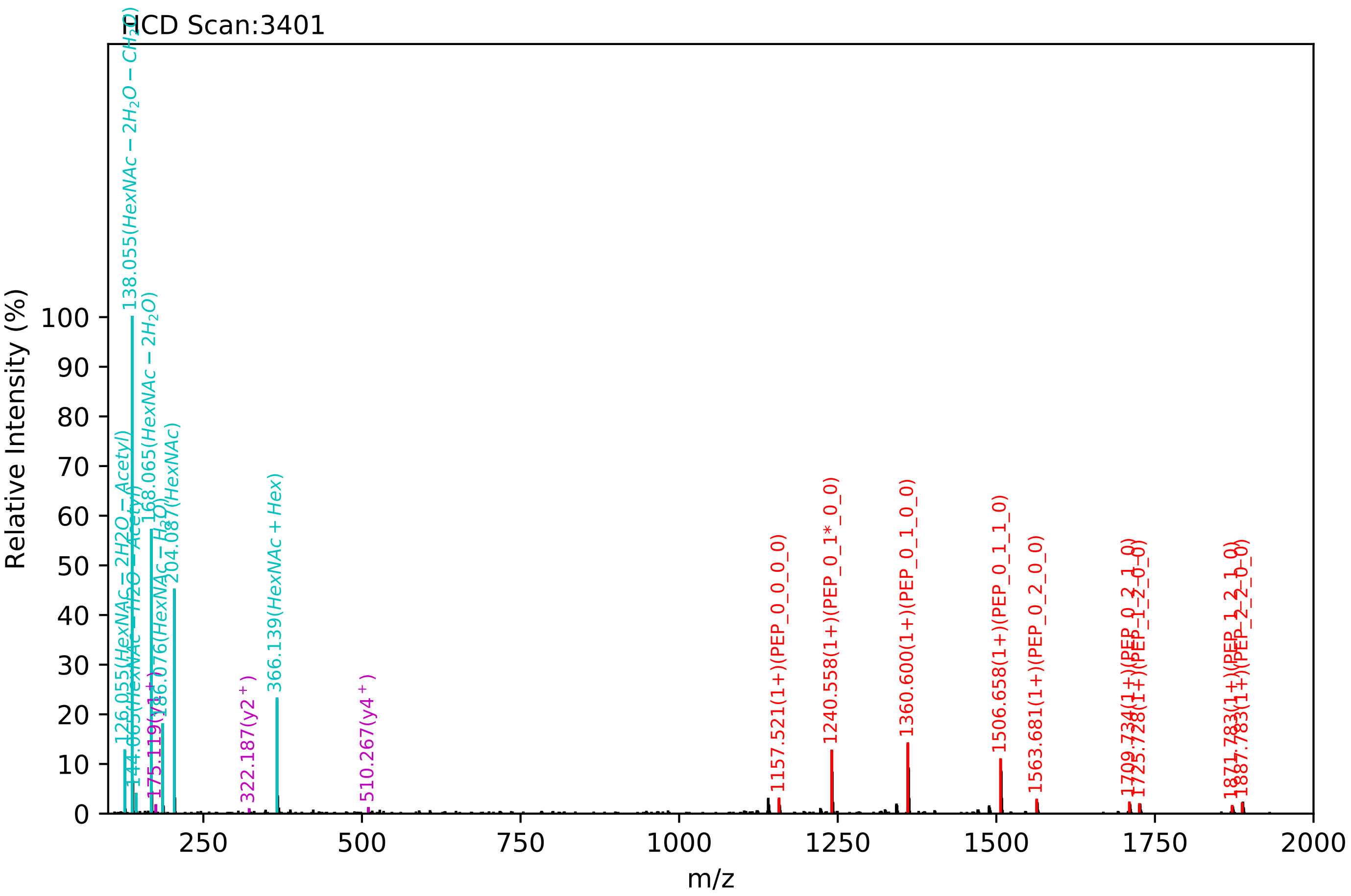

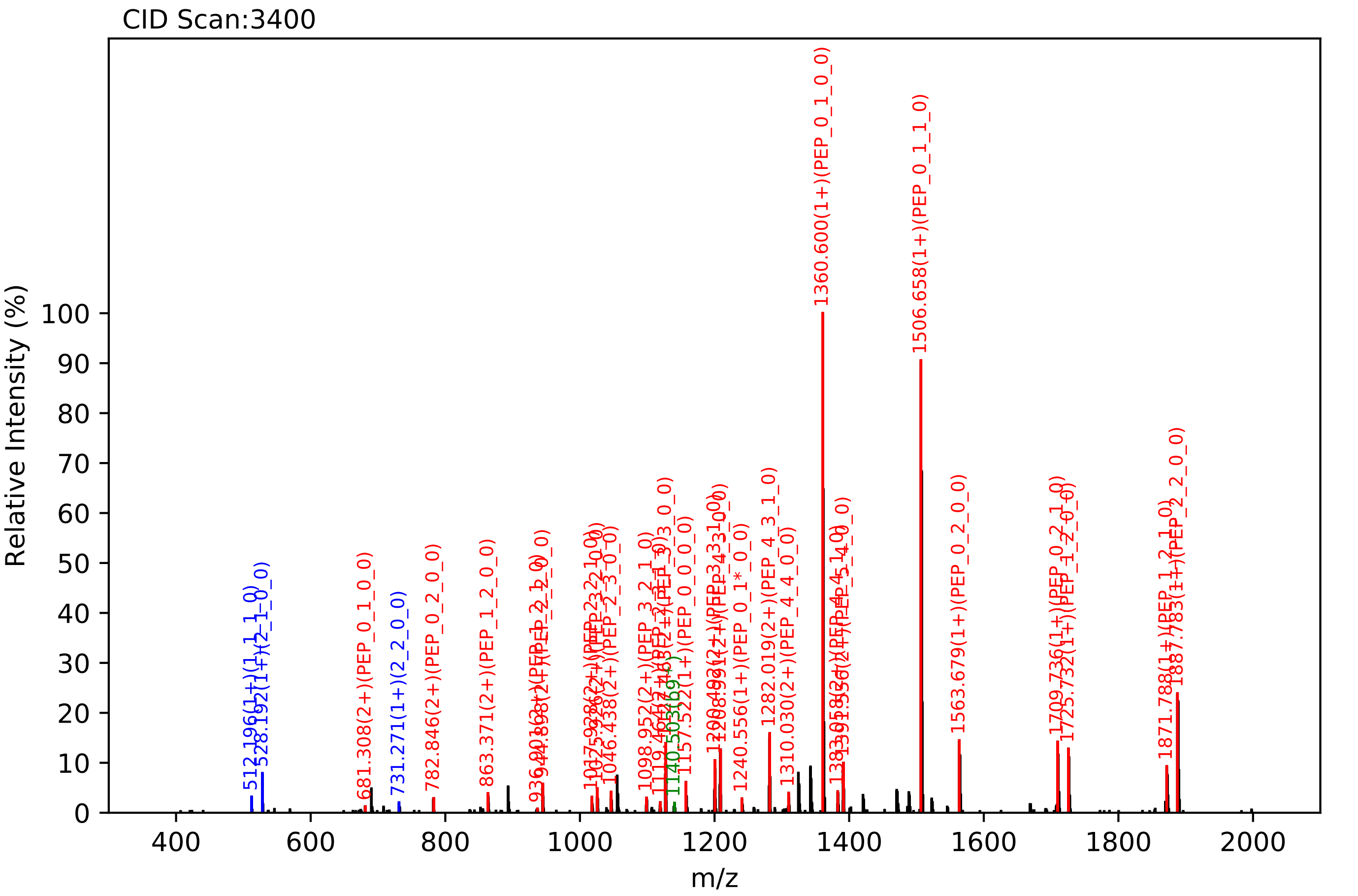

Test set no. 390, Experiment: IgG exp_7

EEQFNSTFR(=PEP)_5_4_1_0, m/z:1463.59(2+), RT:24.34, Y-score:88.58

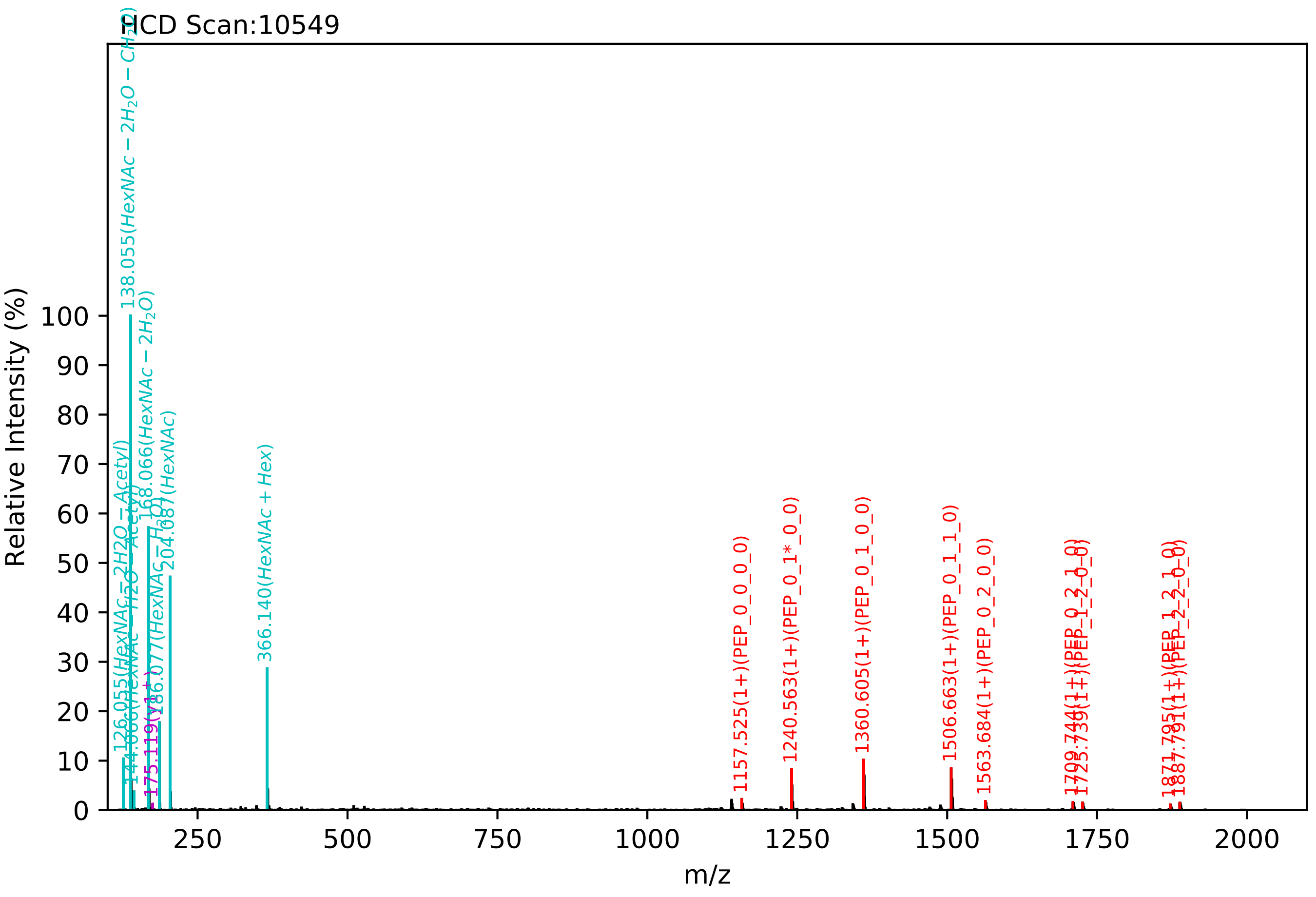

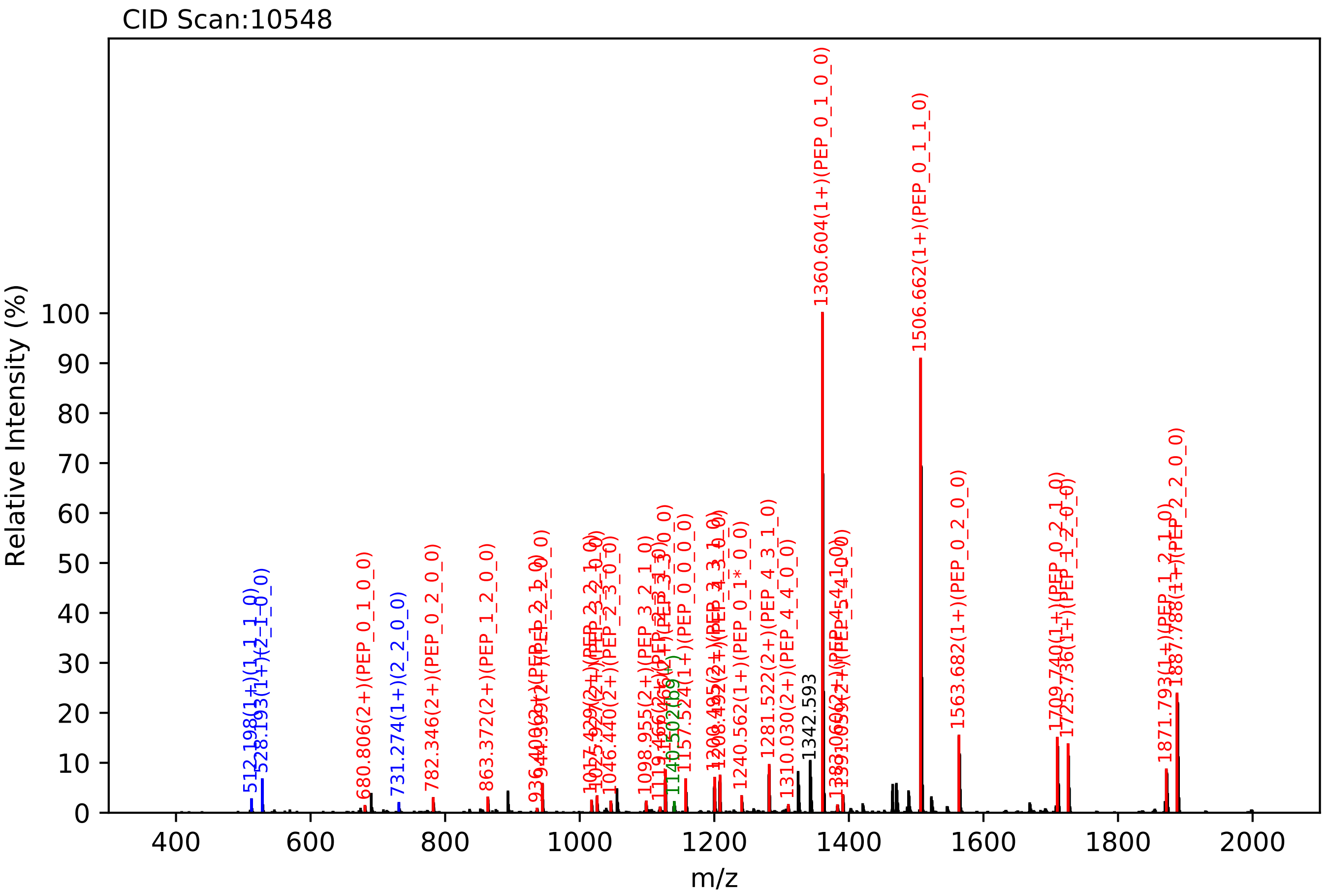

Test set no. 391, Experiment: IgG exp_7

EEQFNSTFR(=PEP)_5_4_1_1, m/z:1609.13(2+), RT:26.94, Y-score:87.82

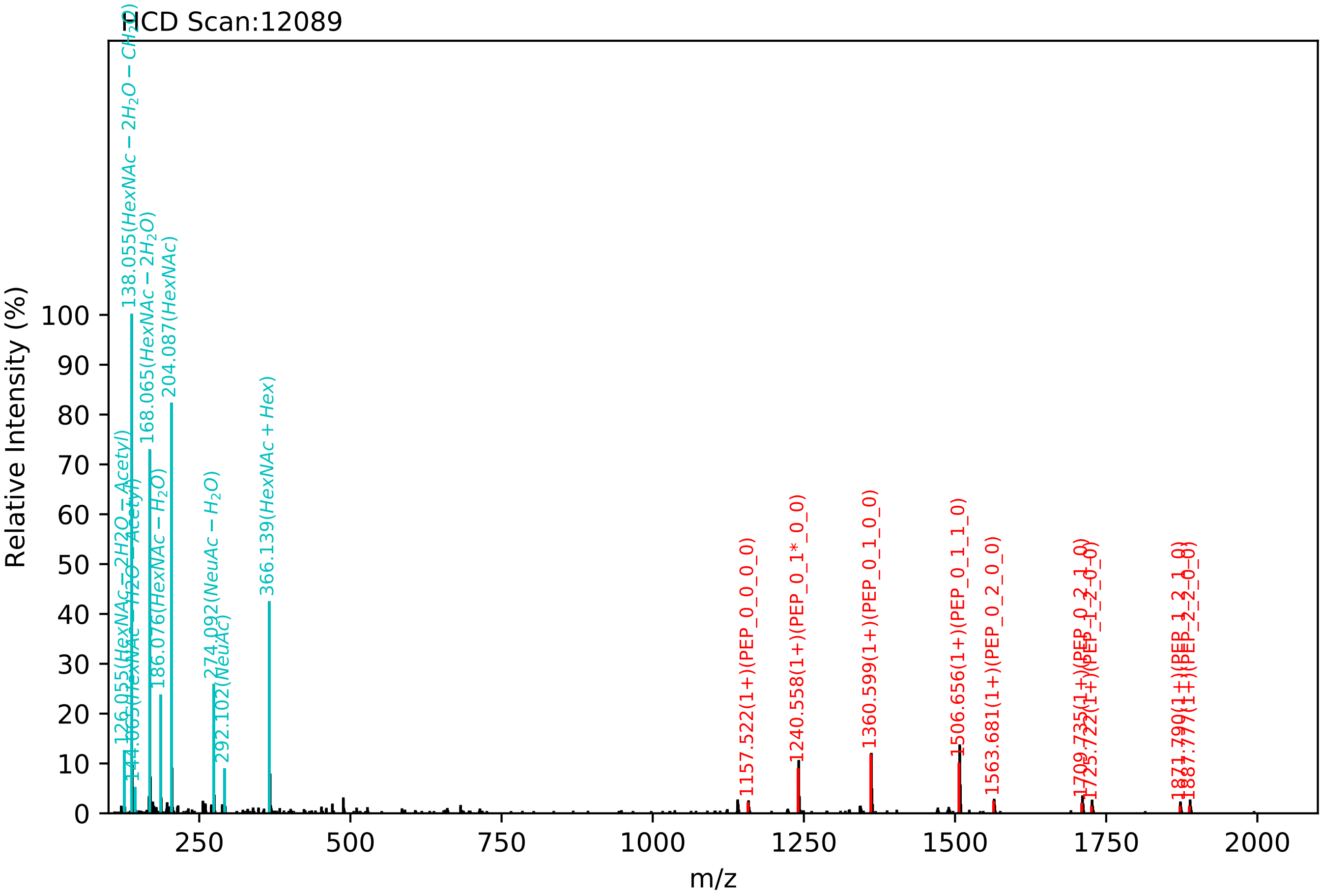

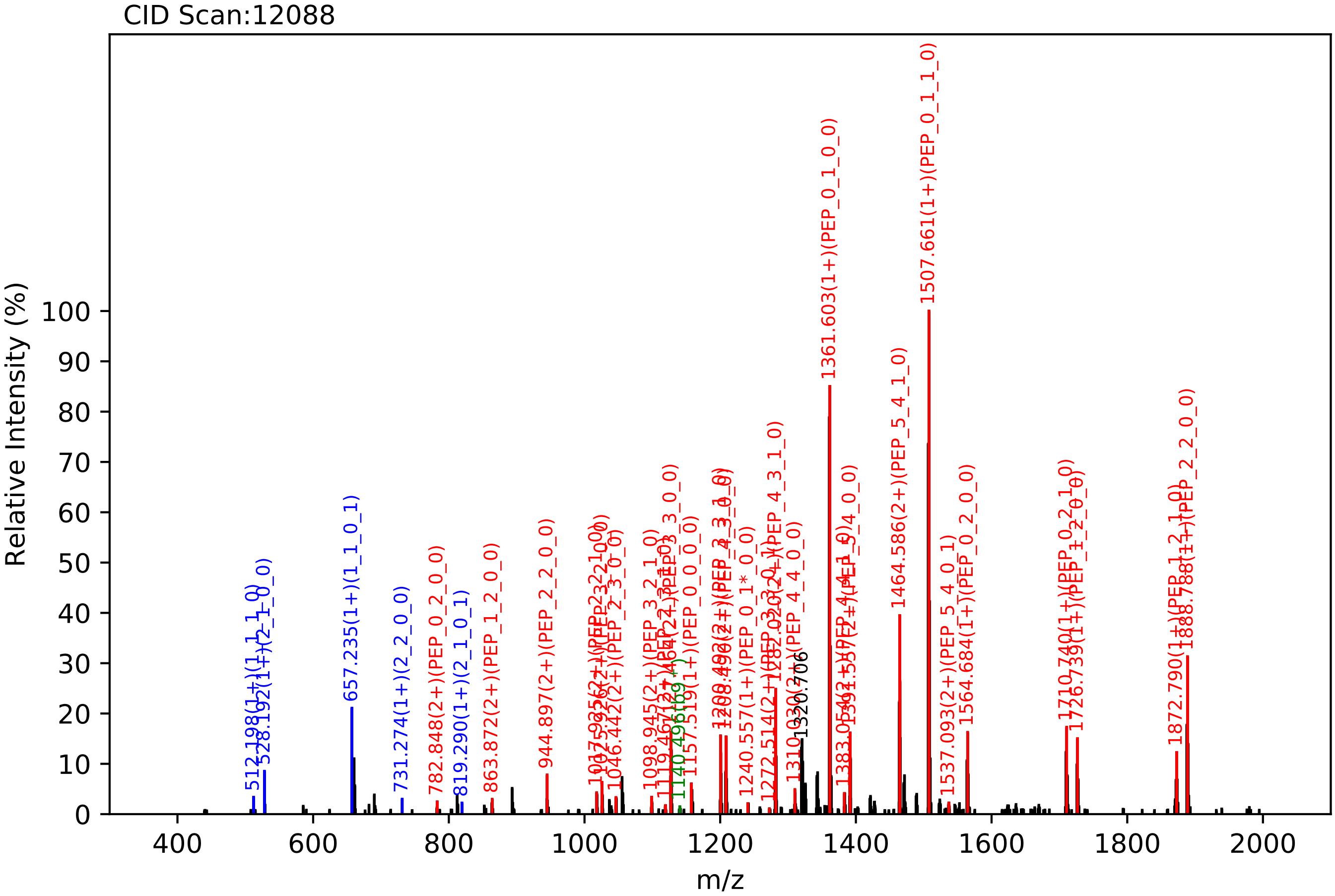

Test set no. 392, Experiment: IgG exp_7

EEQFNSTYR(=PEP)_3_4_1_0, m/z:1309.53(2+), RT:22.93, Y-score:88.63

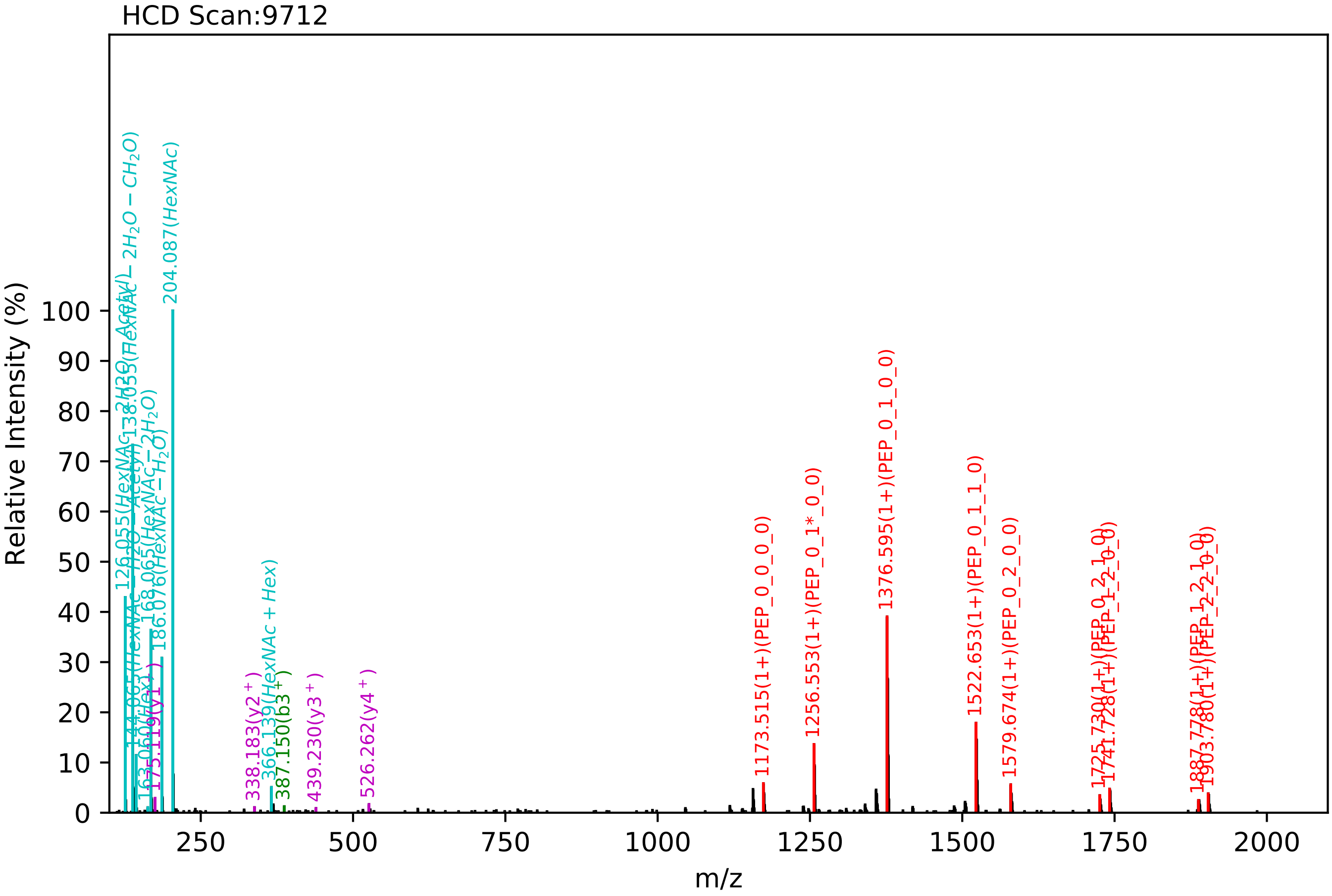

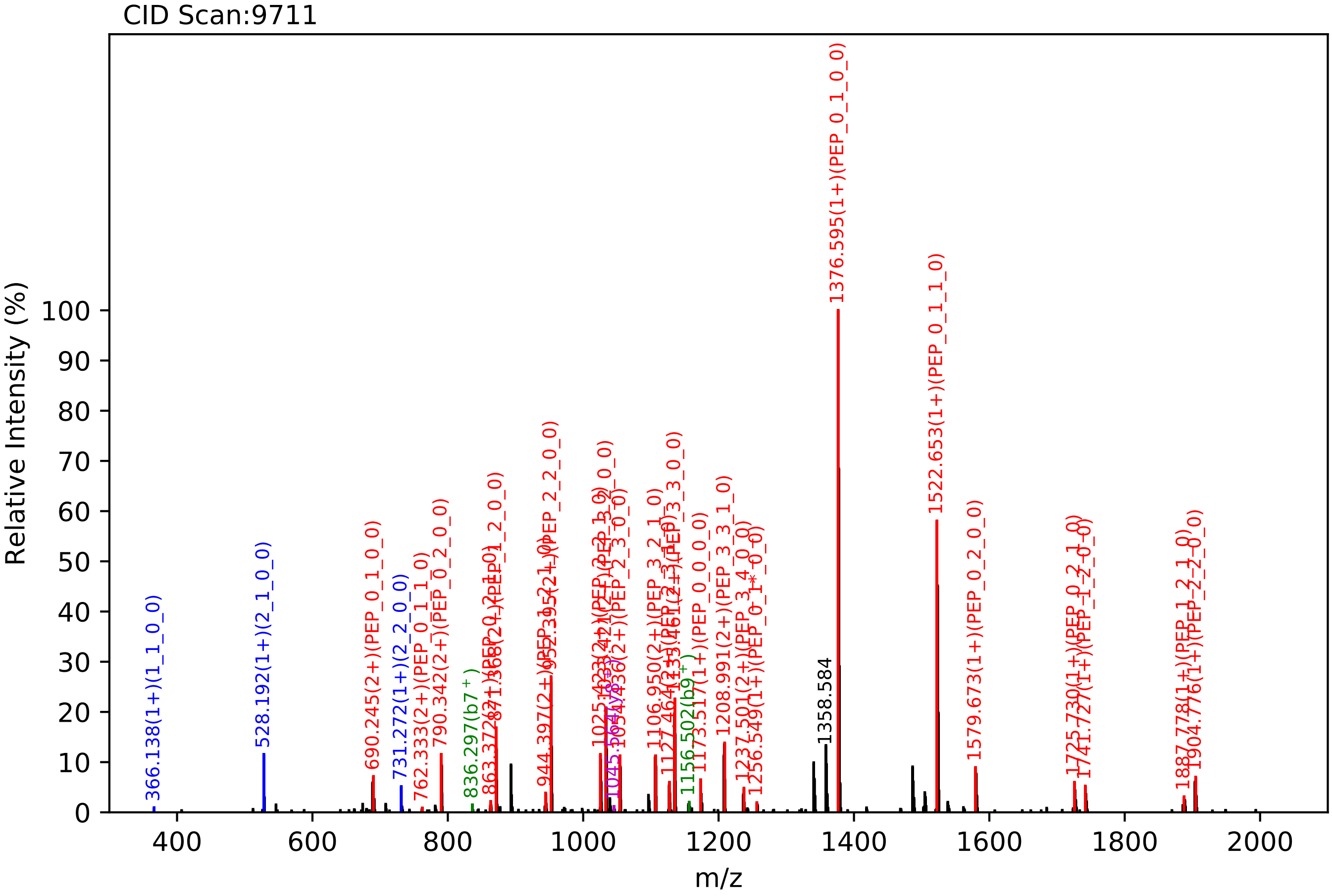

Test set no. 393, Experiment: IgG exp_7

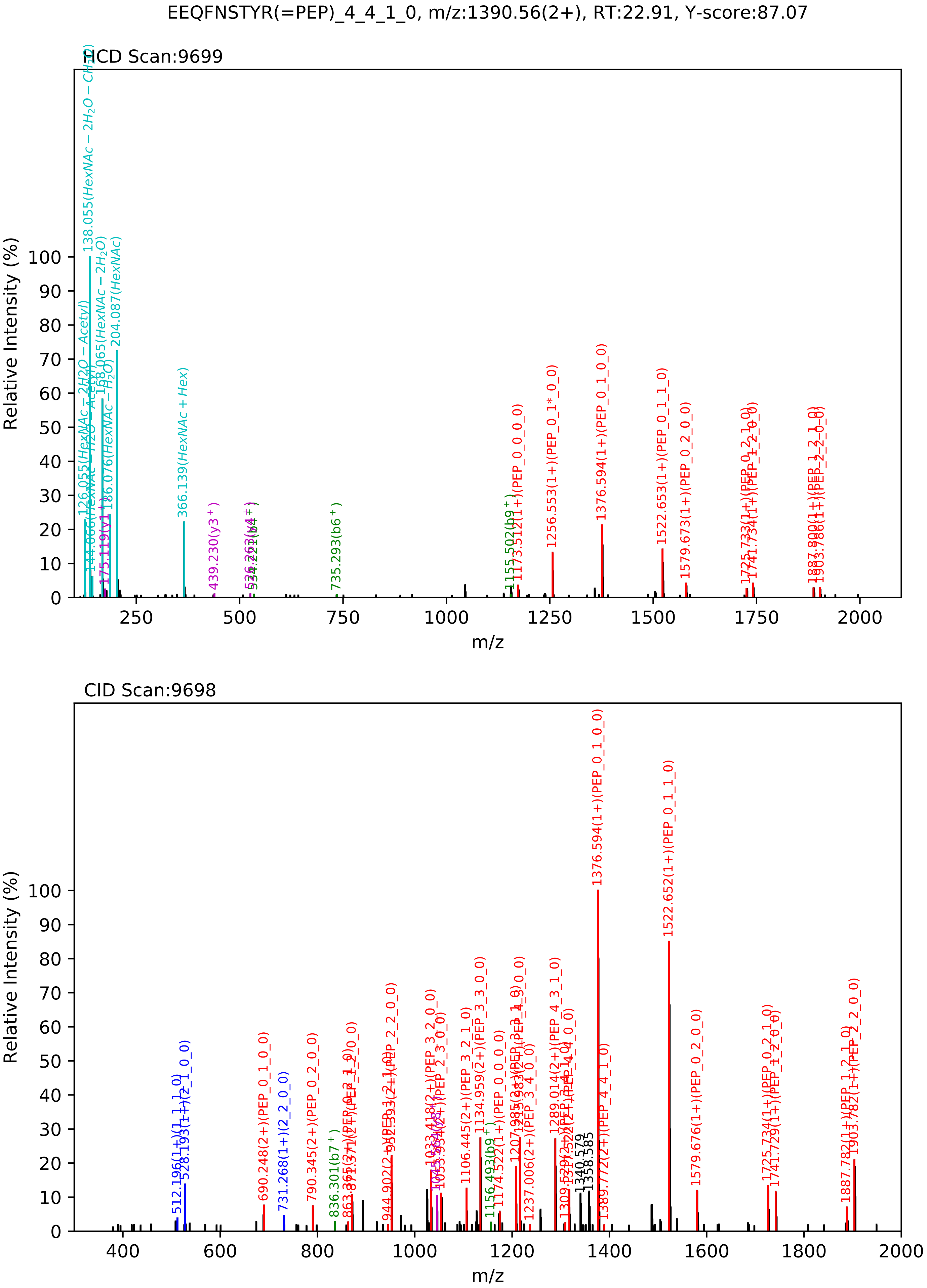

Supplementary Figure 4. MS/MS spectra of N-glycopeptides identified from human plasma for unknown set.

Unknown set no. 1, Experiment: Human Plasma exp_1

DIENFNSTQK(=PEP)_5_4_0_2, m/z:850.84(4+), RT:65.95, Y-score:90.02

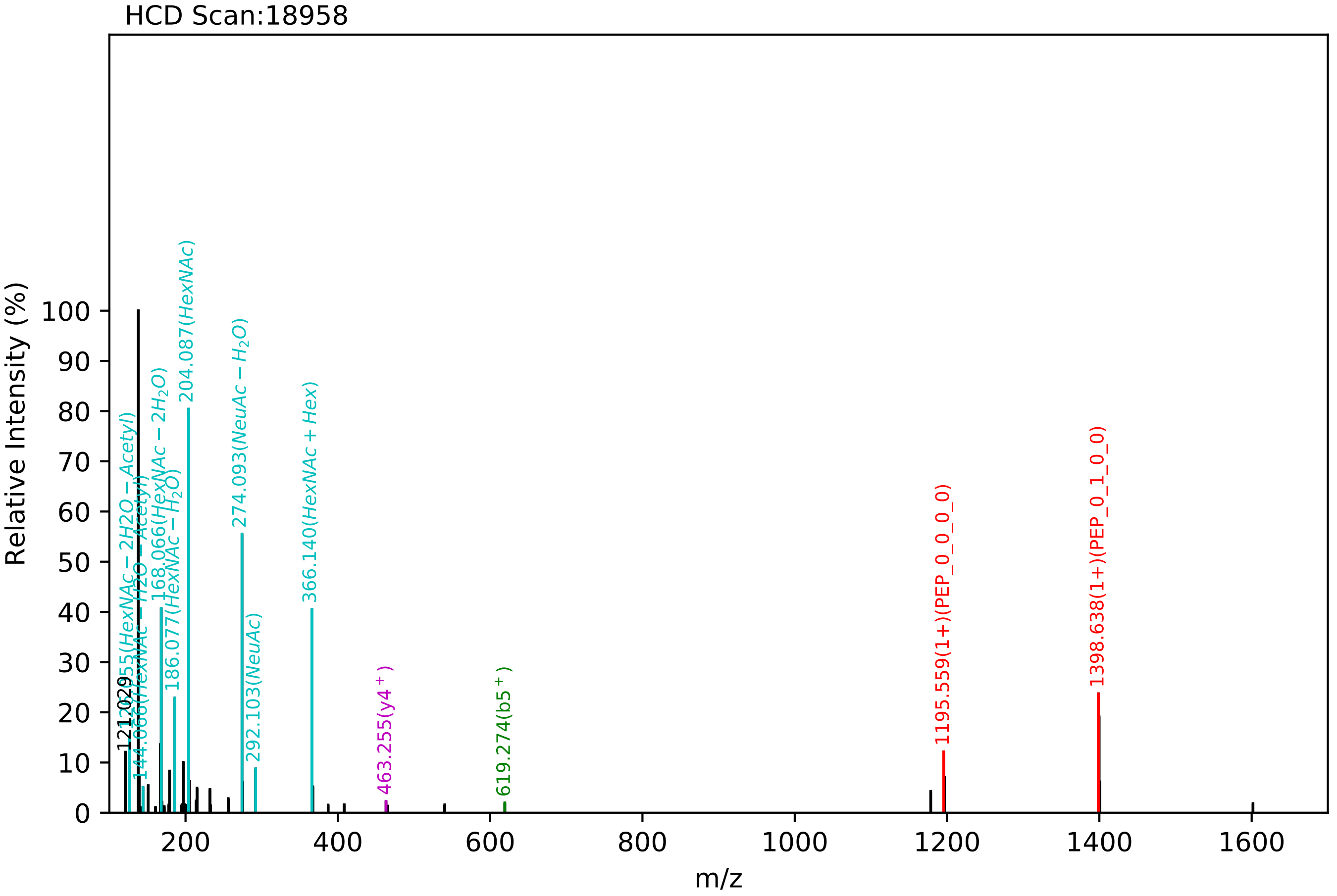

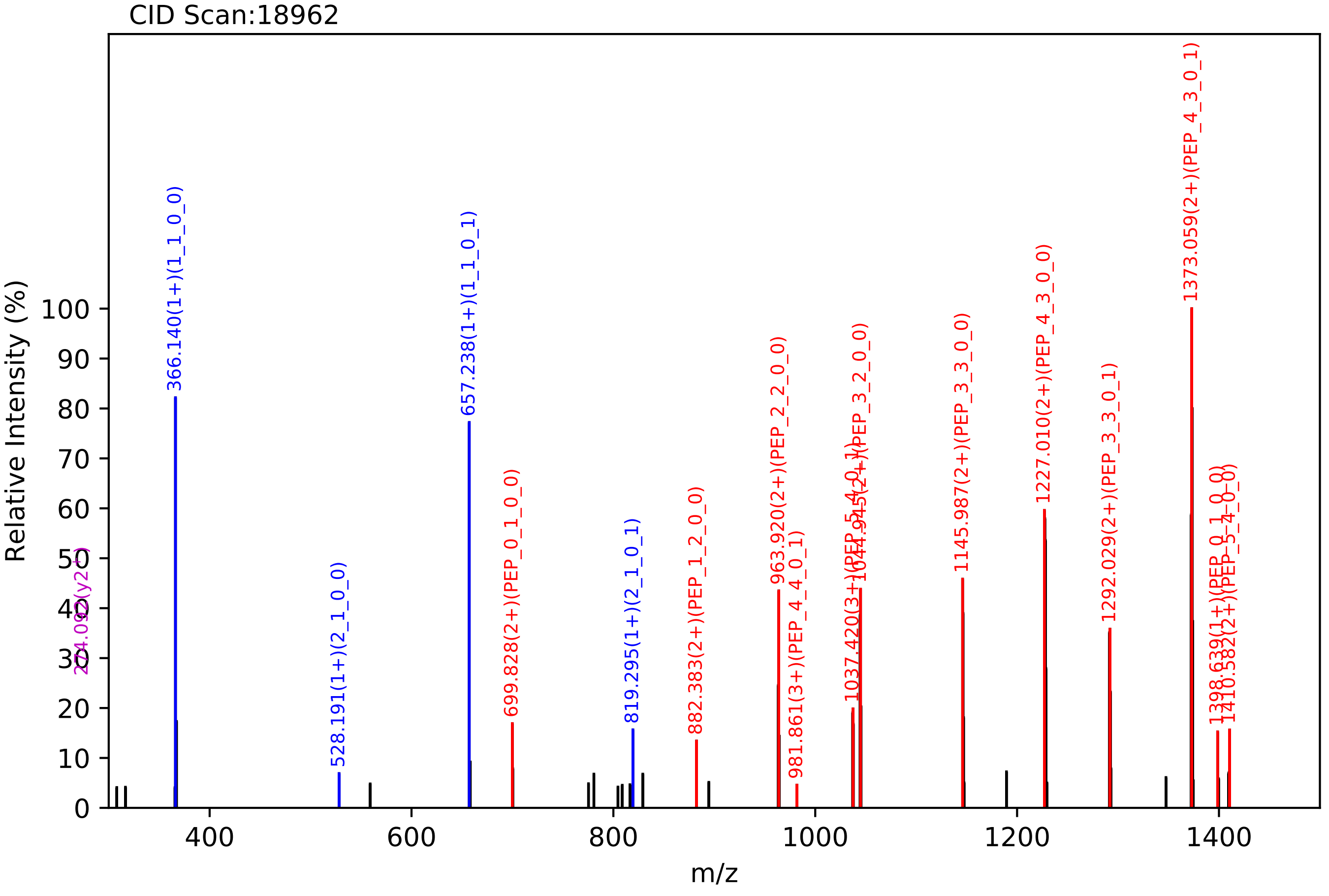

Unknown set no. 2, Experiment: Human Plasma exp_1

DIENFNSTQK(=PEP)_5_4_0_2, m/z:1134.12(3+), RT:65.95, Y-score:93.70

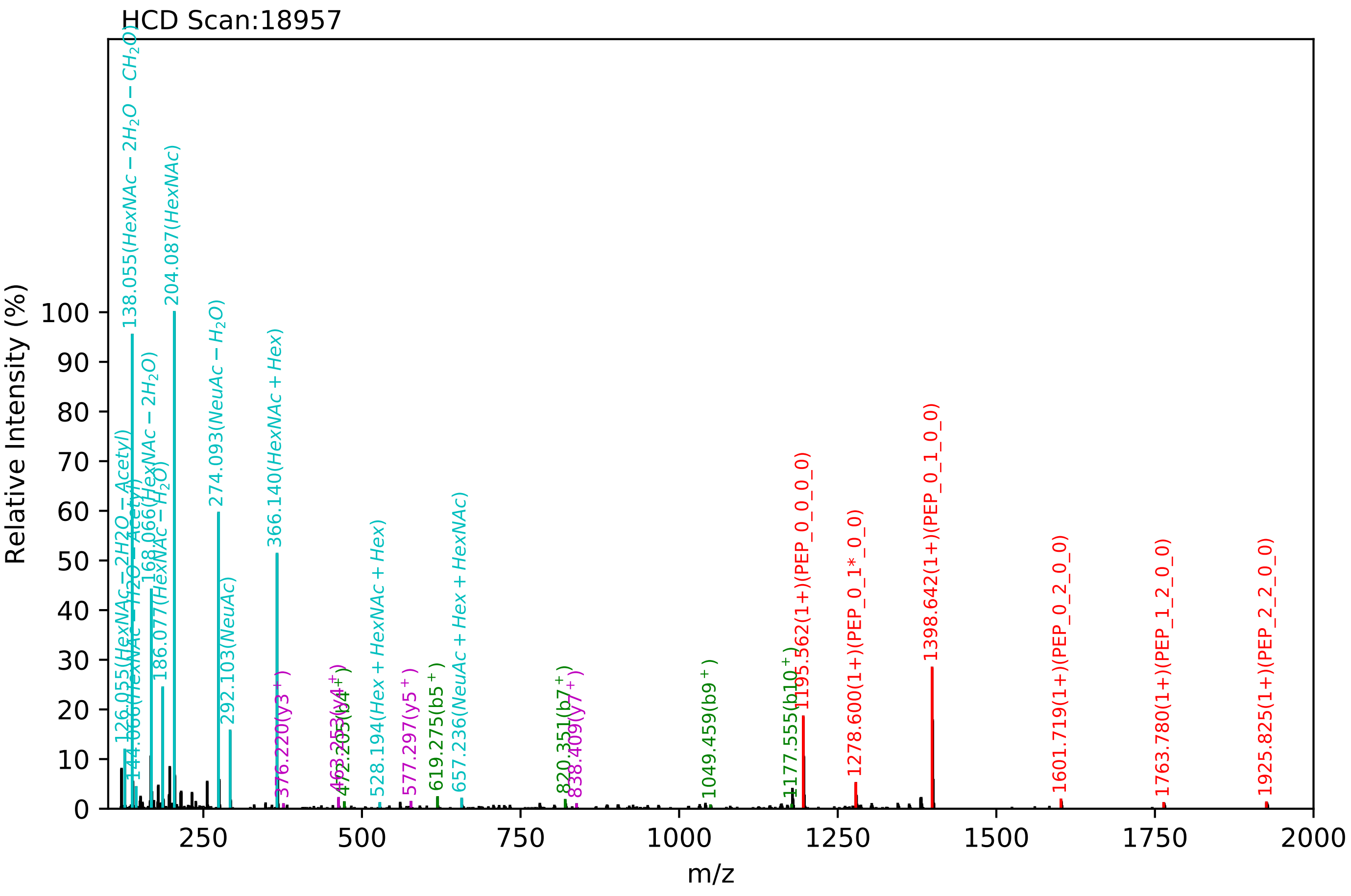

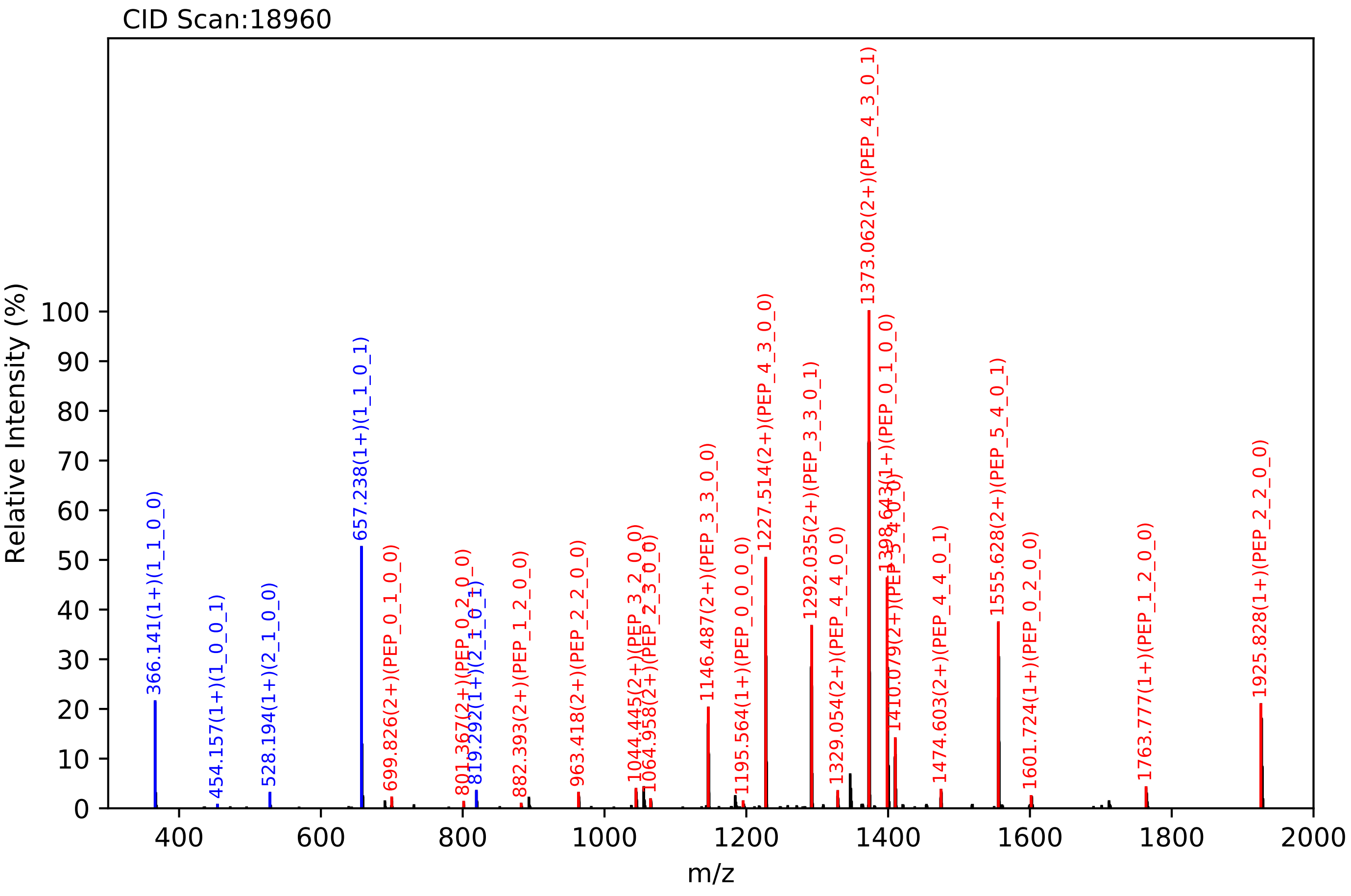

Unknown set no. 3, Experiment: Human Plasma exp_1

DIENFNSTQK(=PEP)_5_4_0_2, m/z:1700.67(2+), RT:65.84, Y-score:100.00

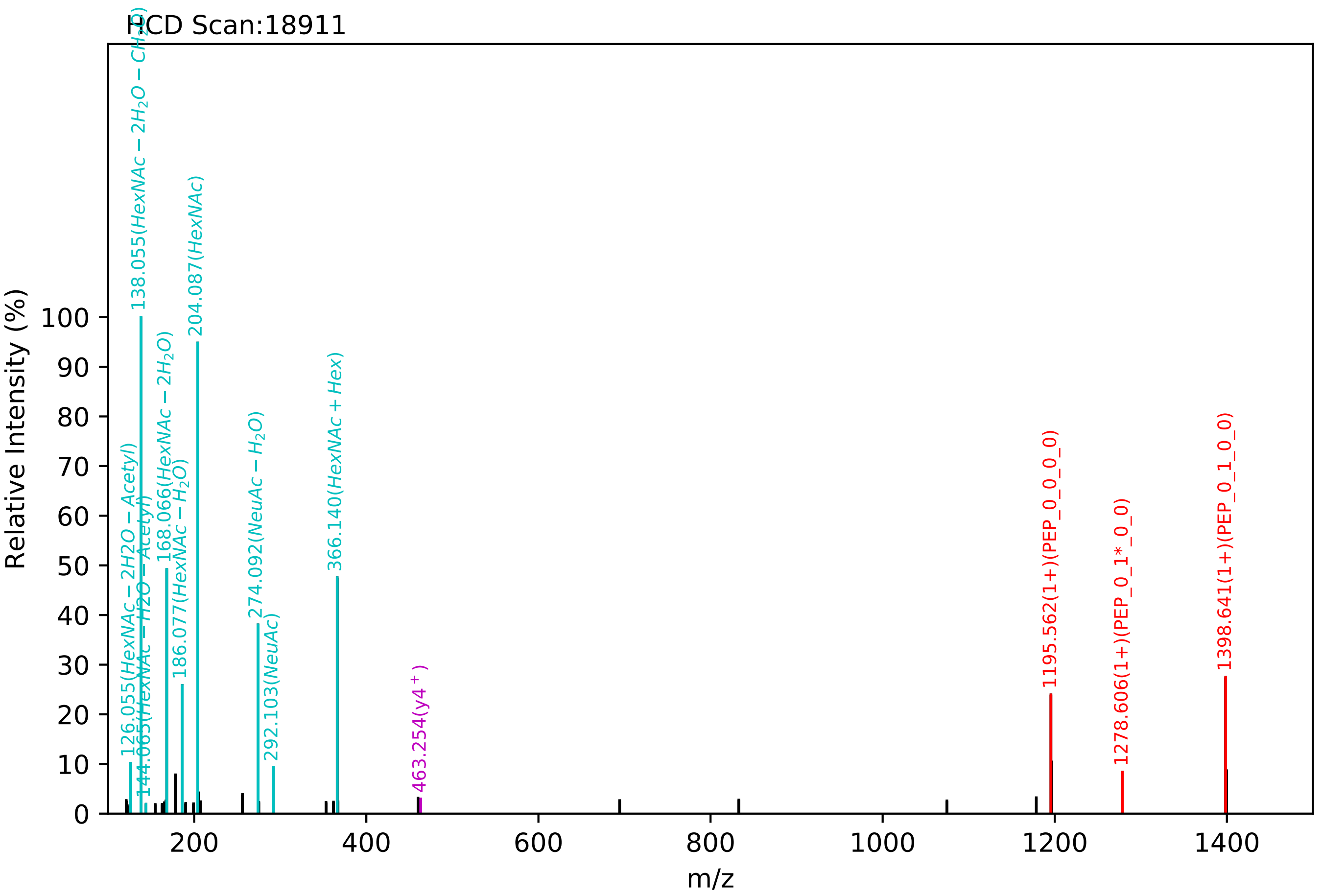

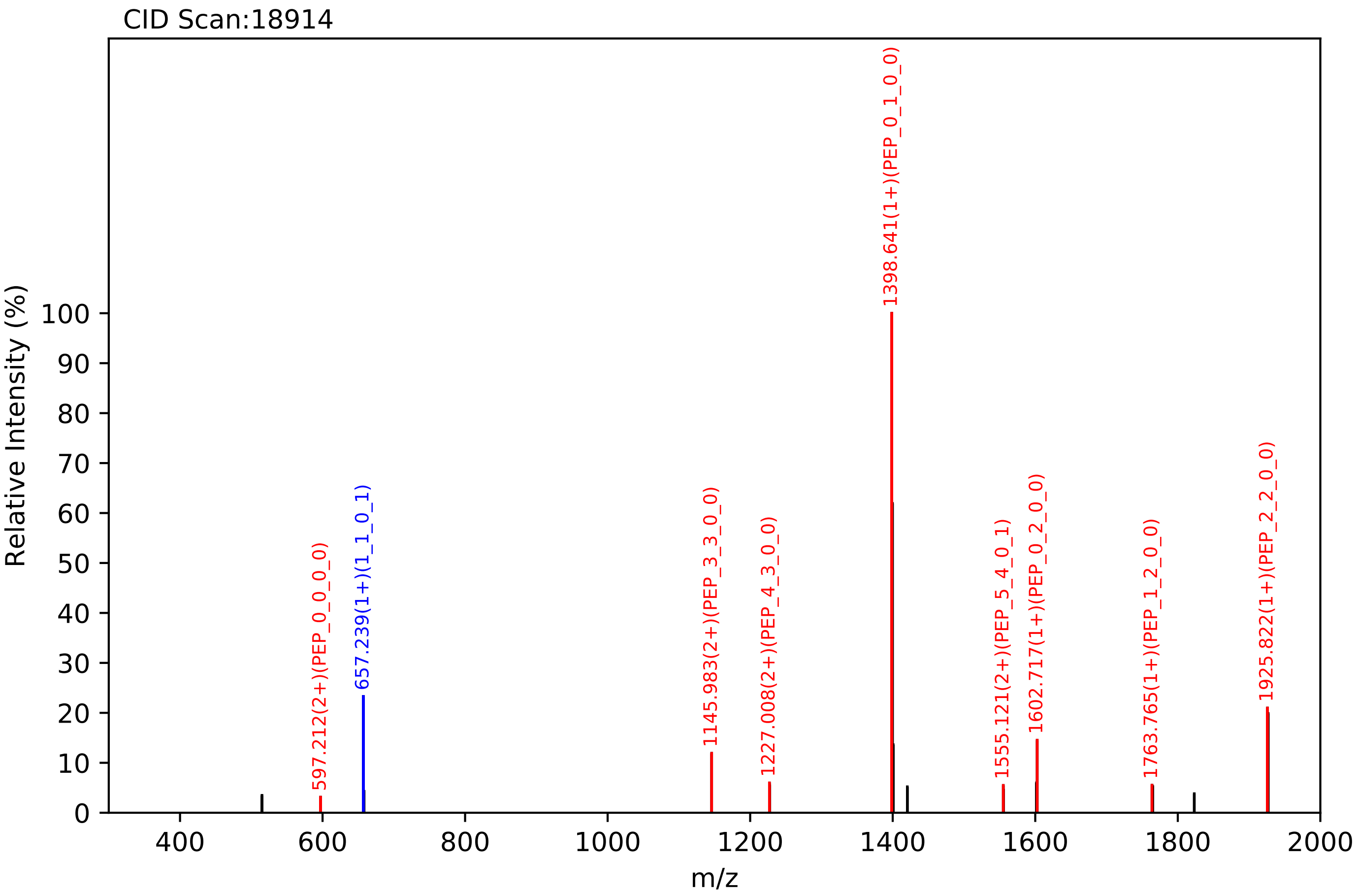

Unknown set no. 4, Experiment: Human Plasma exp_2

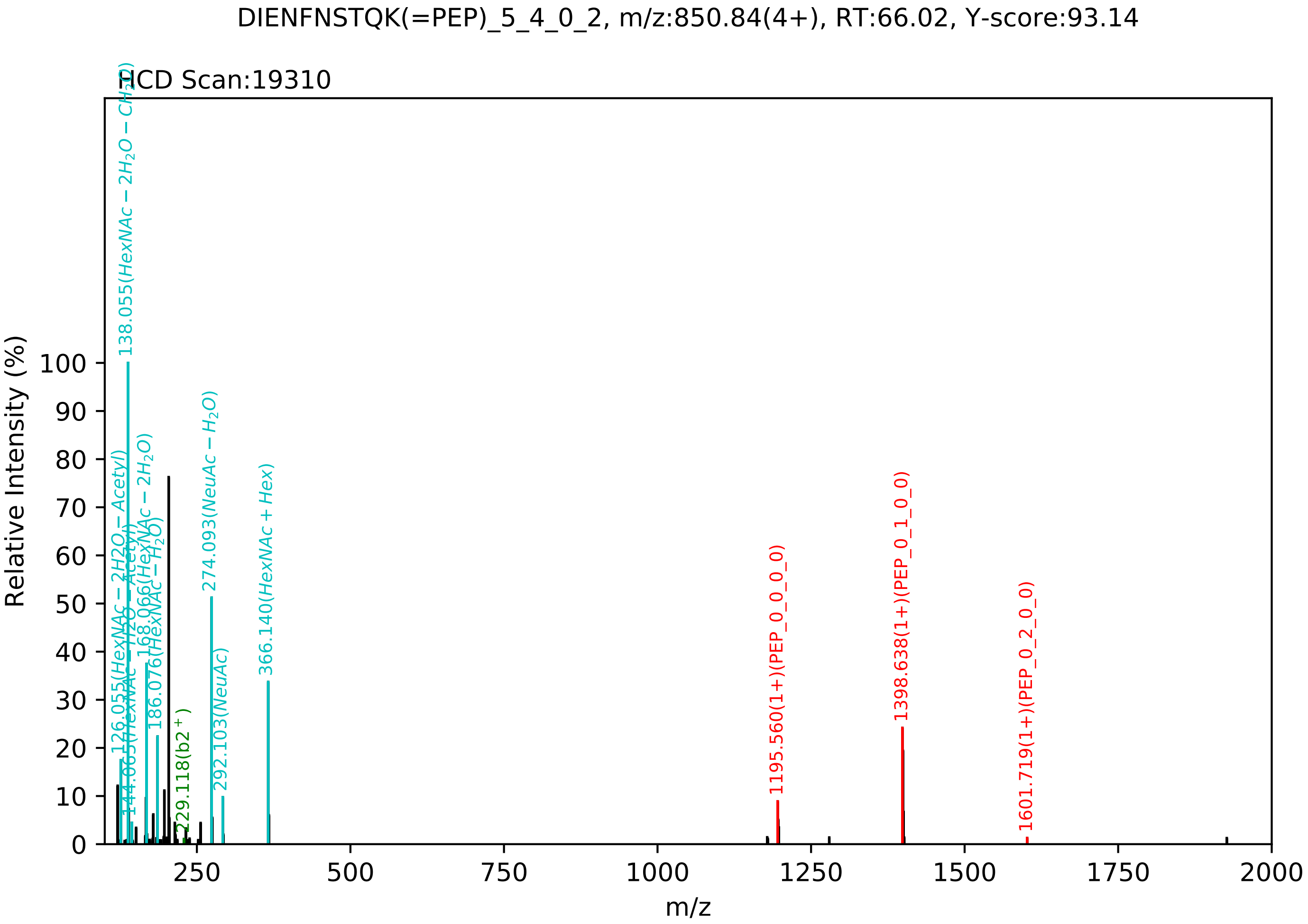

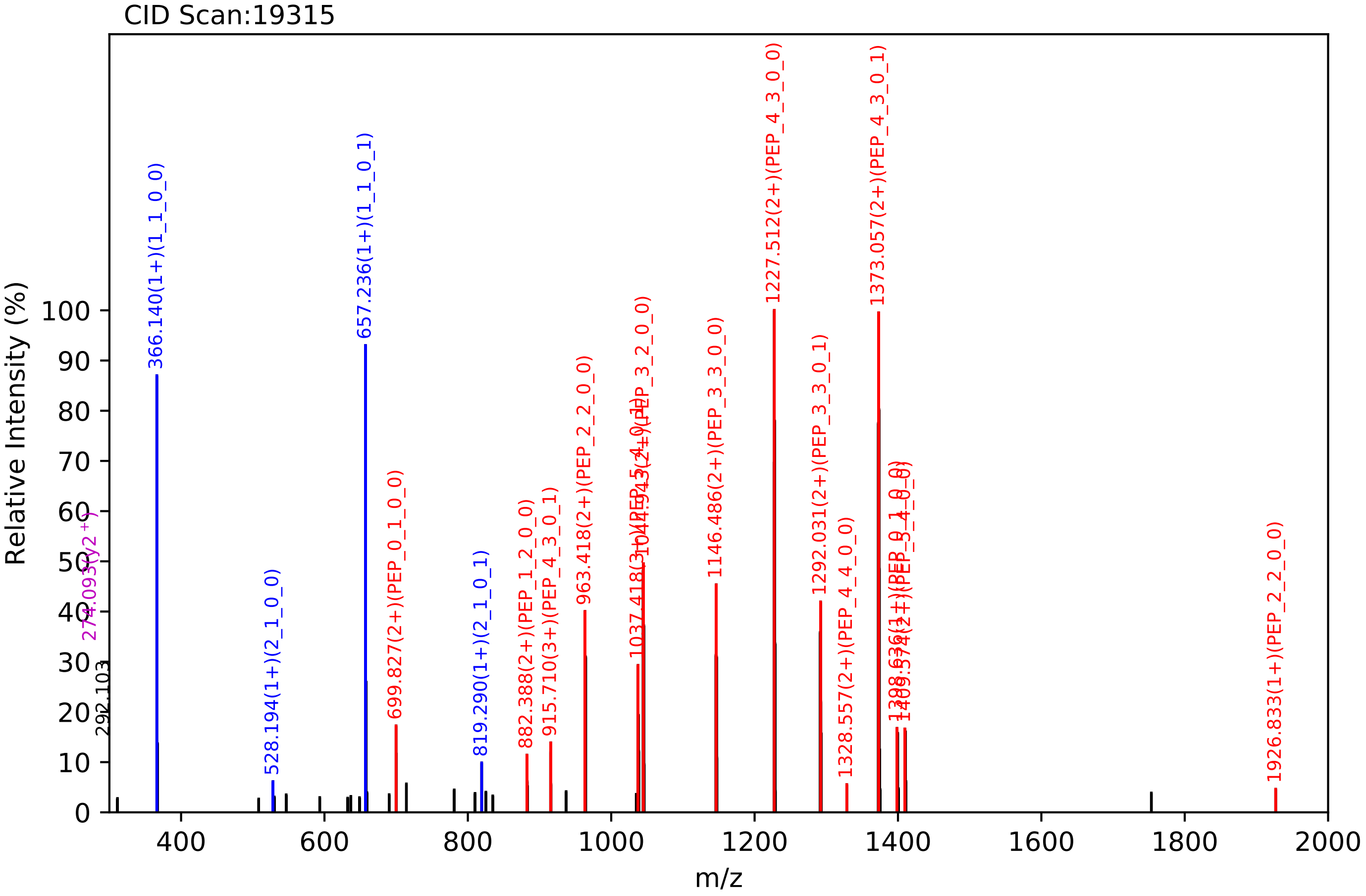

Unknown set no. 5, Experiment: Human Plasma exp_2

DIENFNSTQK(=PEP)_5_4_0_2, m/z:1134.12(3+), RT:66.14, Y-score:93.04

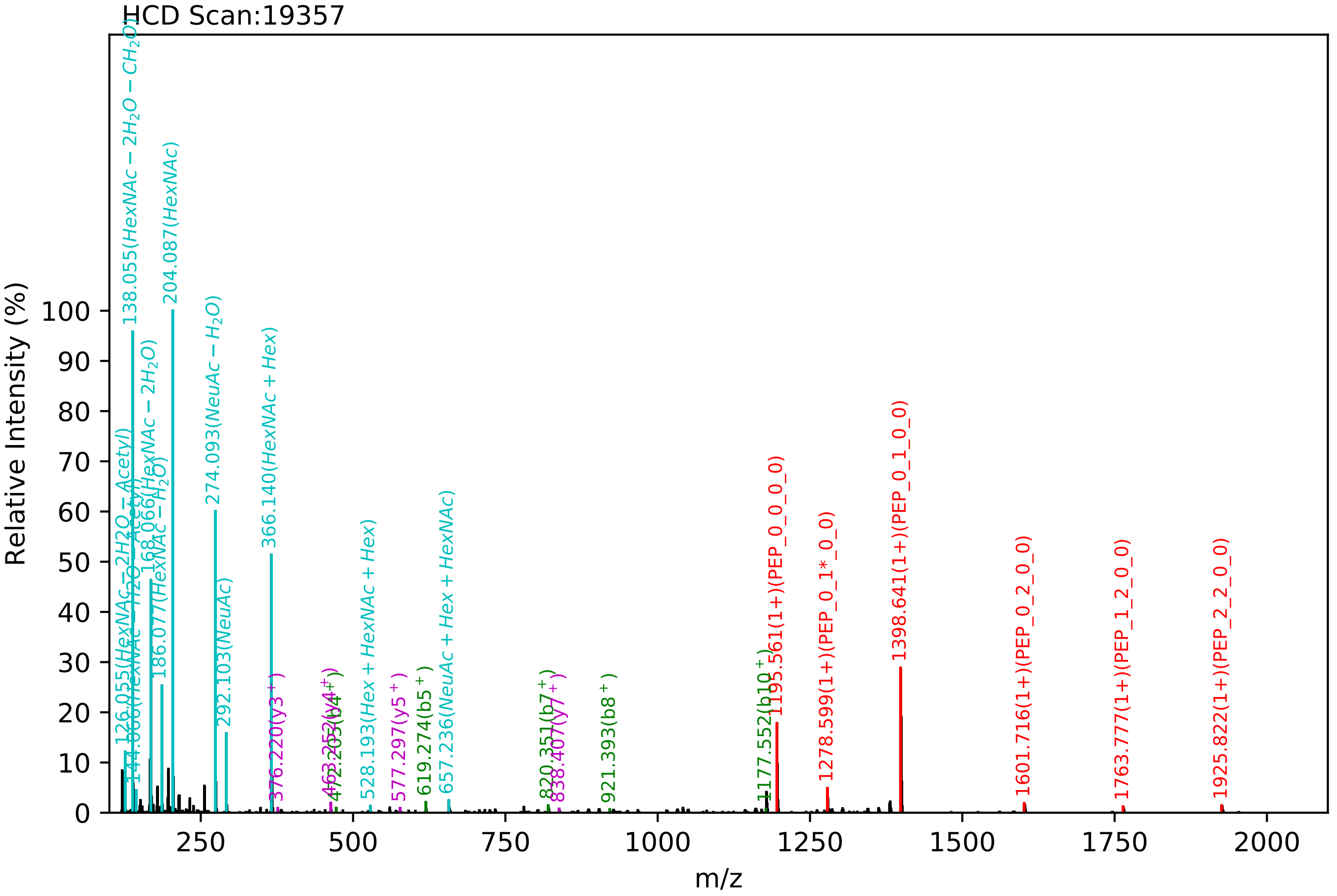

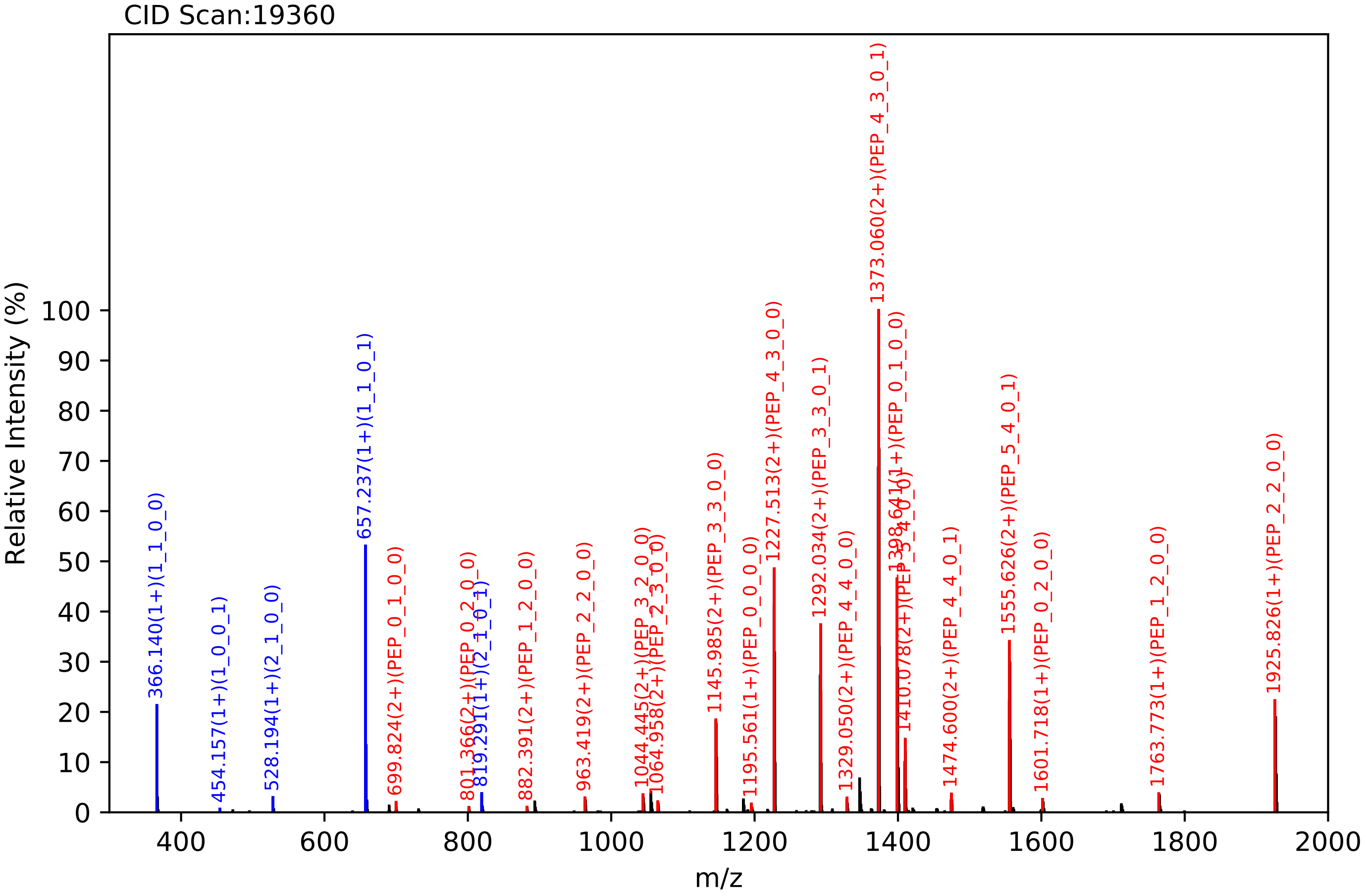

Unknown set no. 6, Experiment: Human Plasma exp_2

DIENFNSTQK(=PEP)_5_4_0_2, m/z:1700.67(2+), RT:66.00, Y-score:83.01

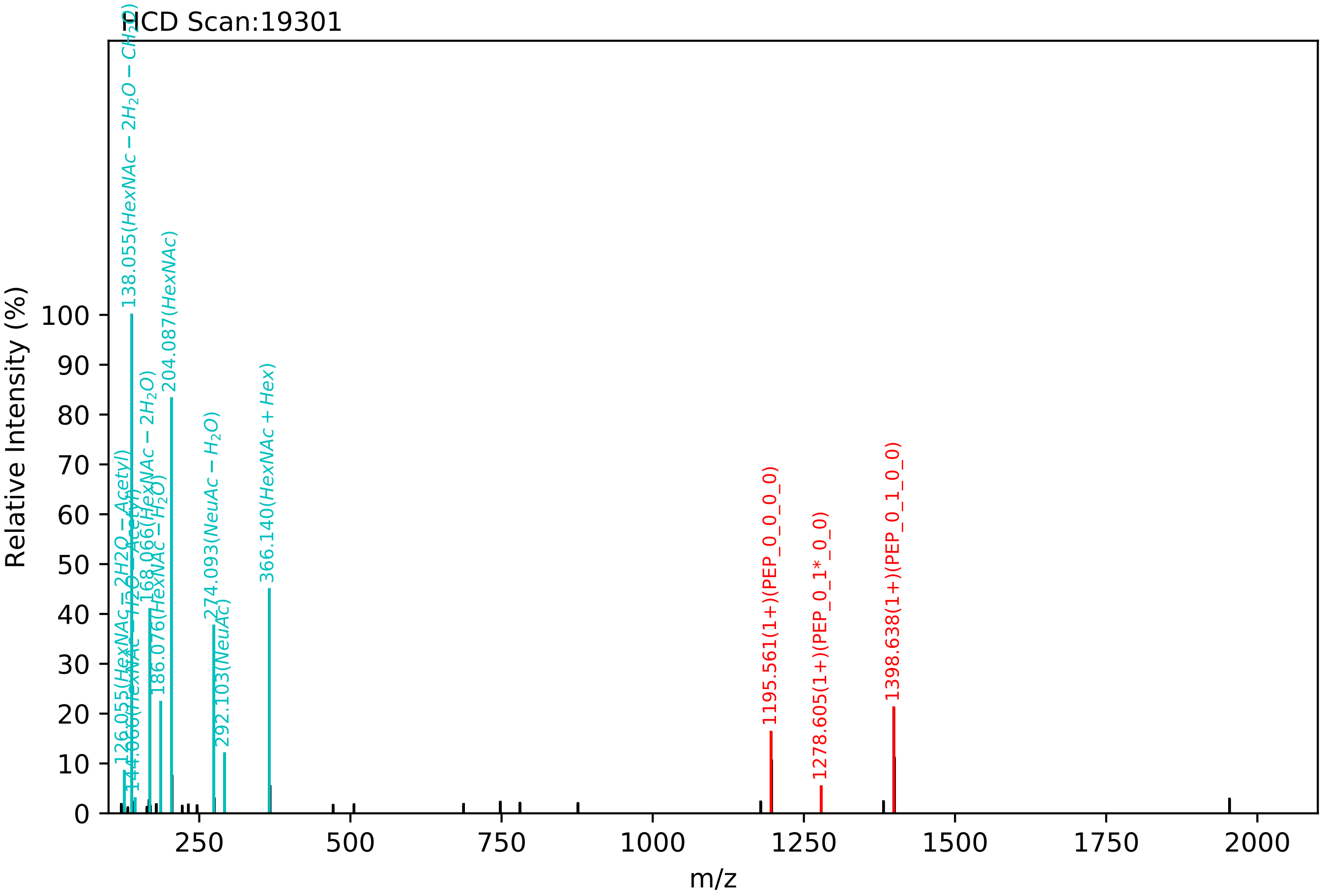

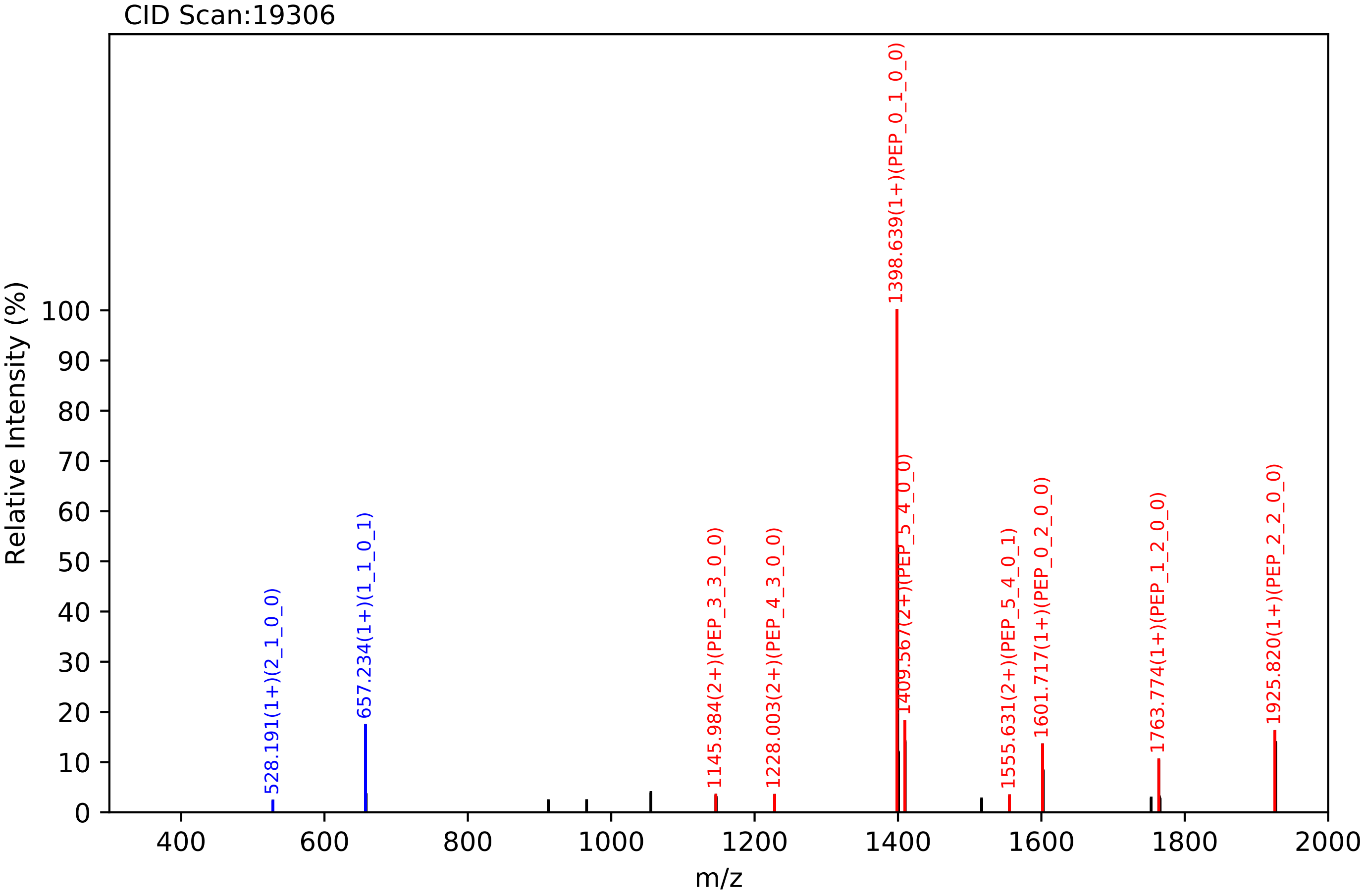

Unknown set no. 7, Experiment: Human Plasma exp_3

DIENFNSTQK(=PEP)_5_4_0_2, m/z:850.84(4+), RT:66.06, Y-score:91.82

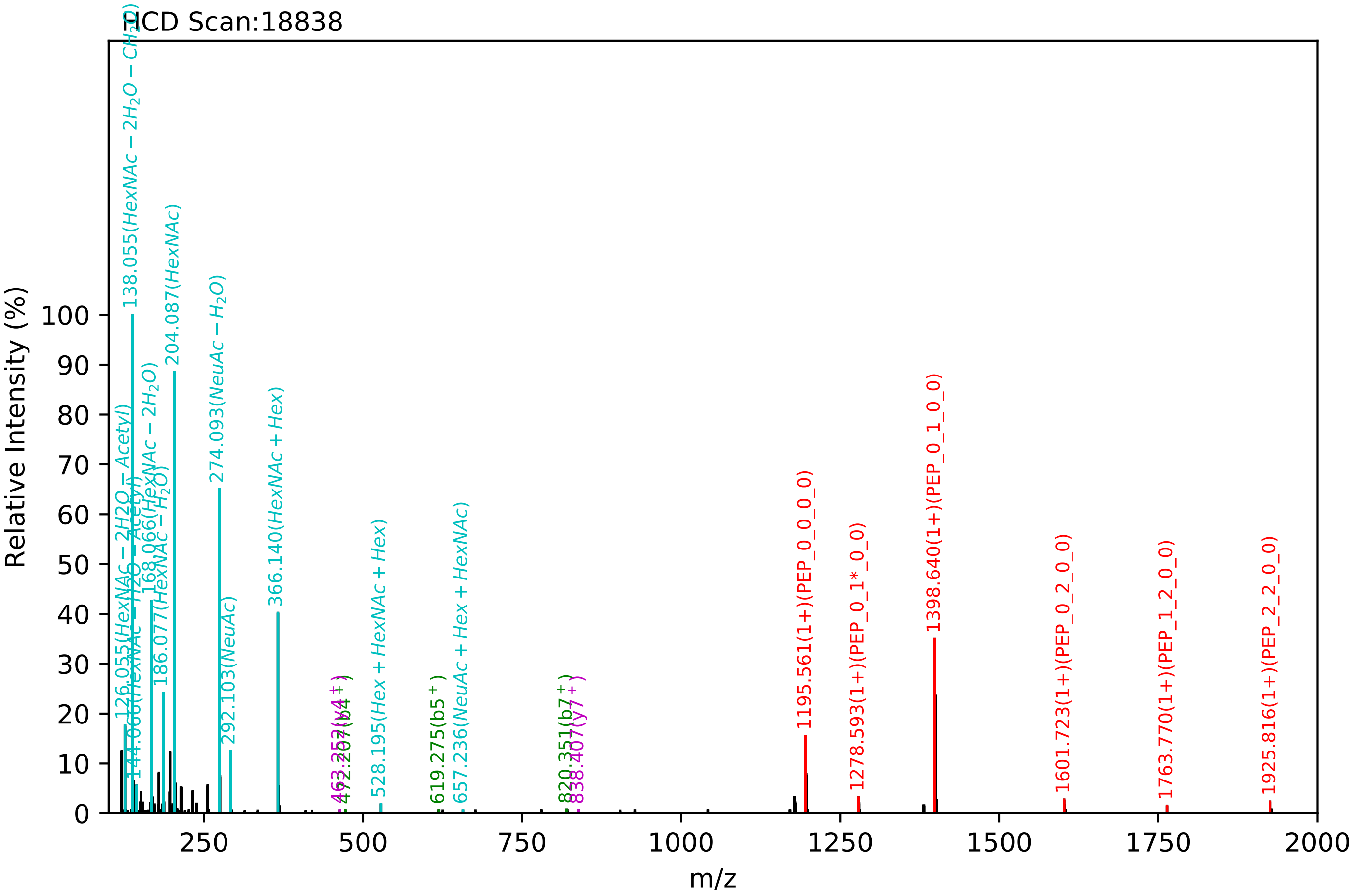

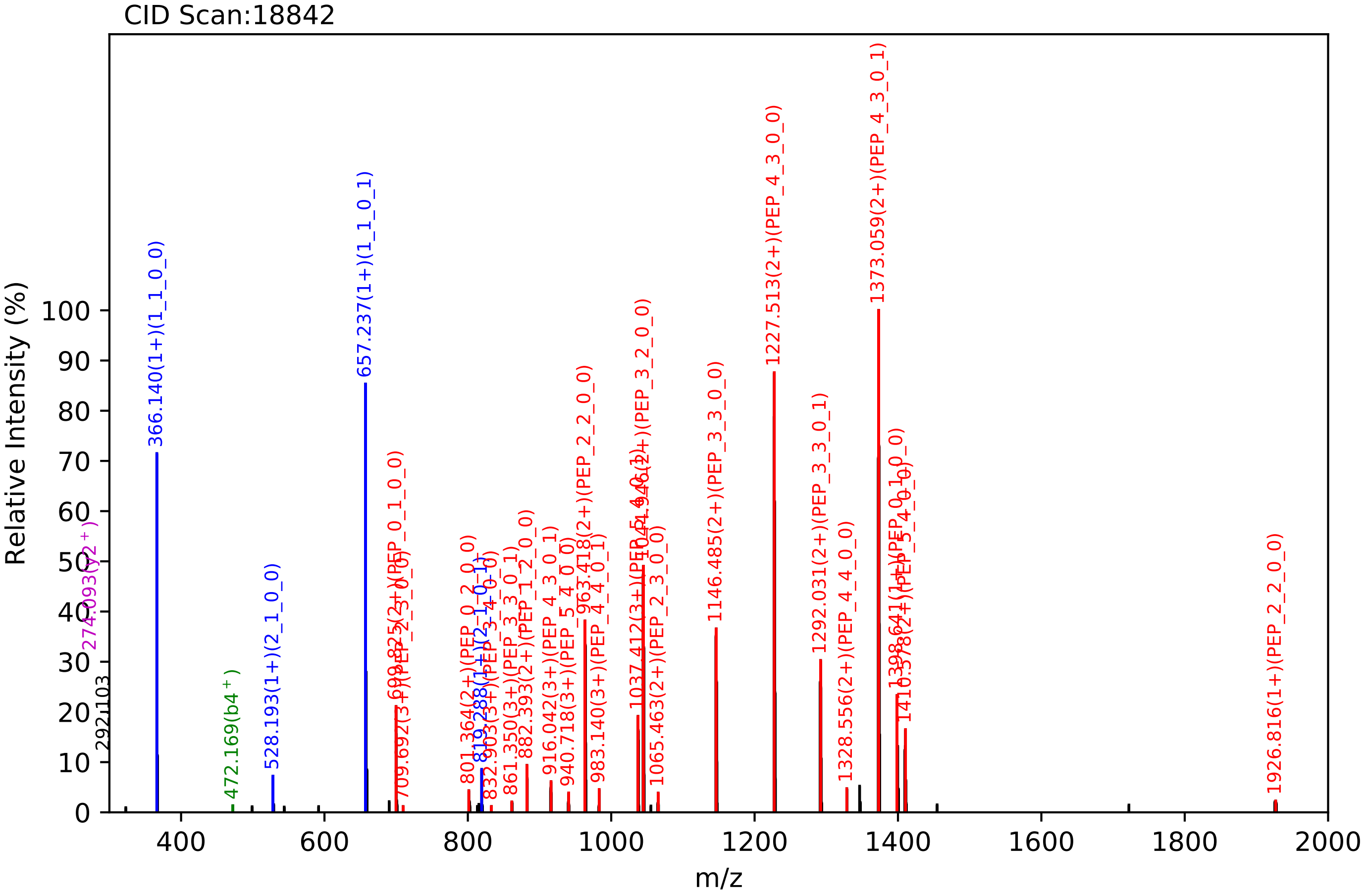

Unknown set no. 8, Experiment: Human Plasma exp_4

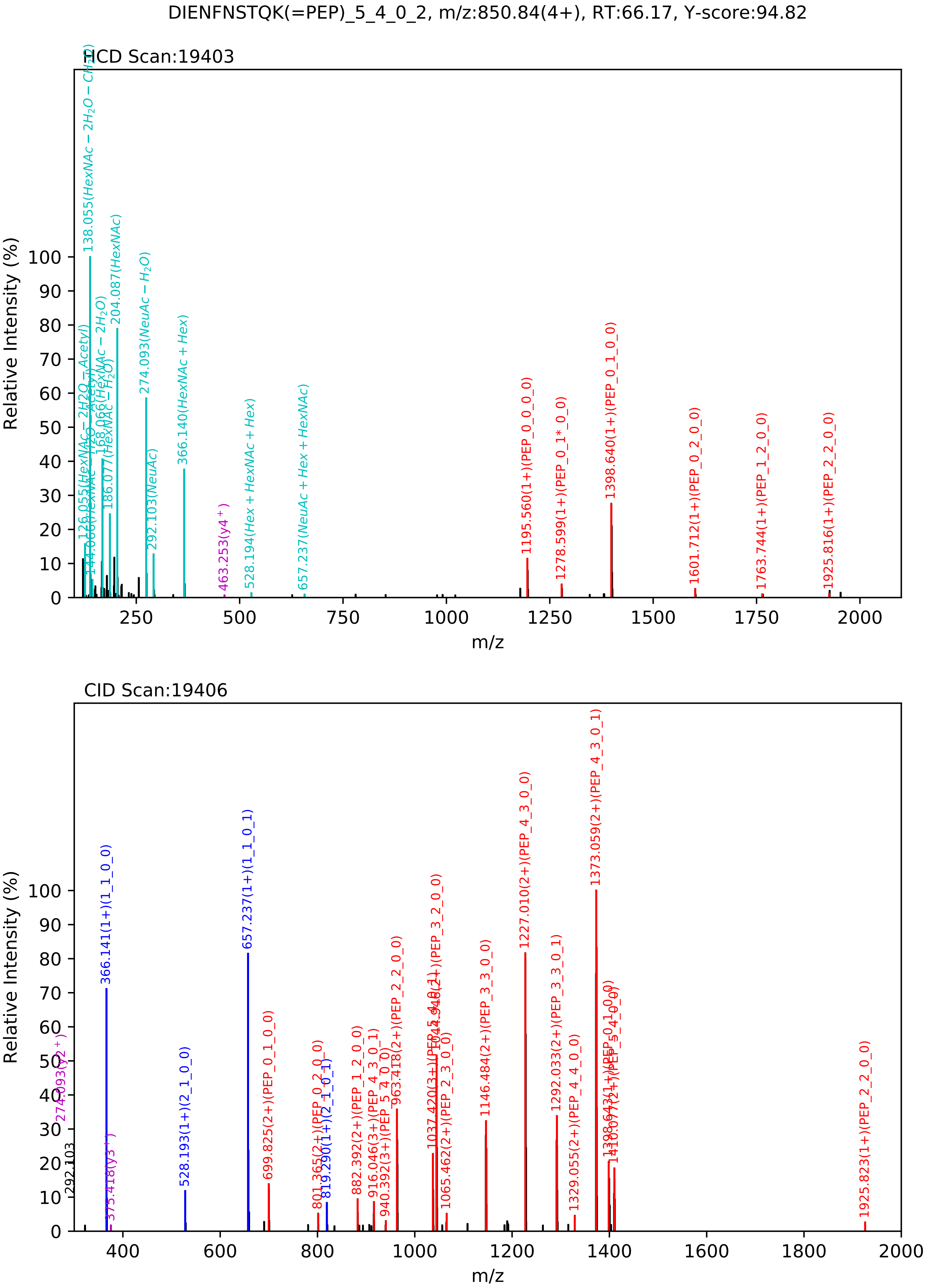

Unknown set no. 9, Experiment: Human Plasma exp_4

DIENFNSTQK(=PEP)_5_4_0_2, m/z:1134.12(3+), RT:66.27, Y-score:91.86

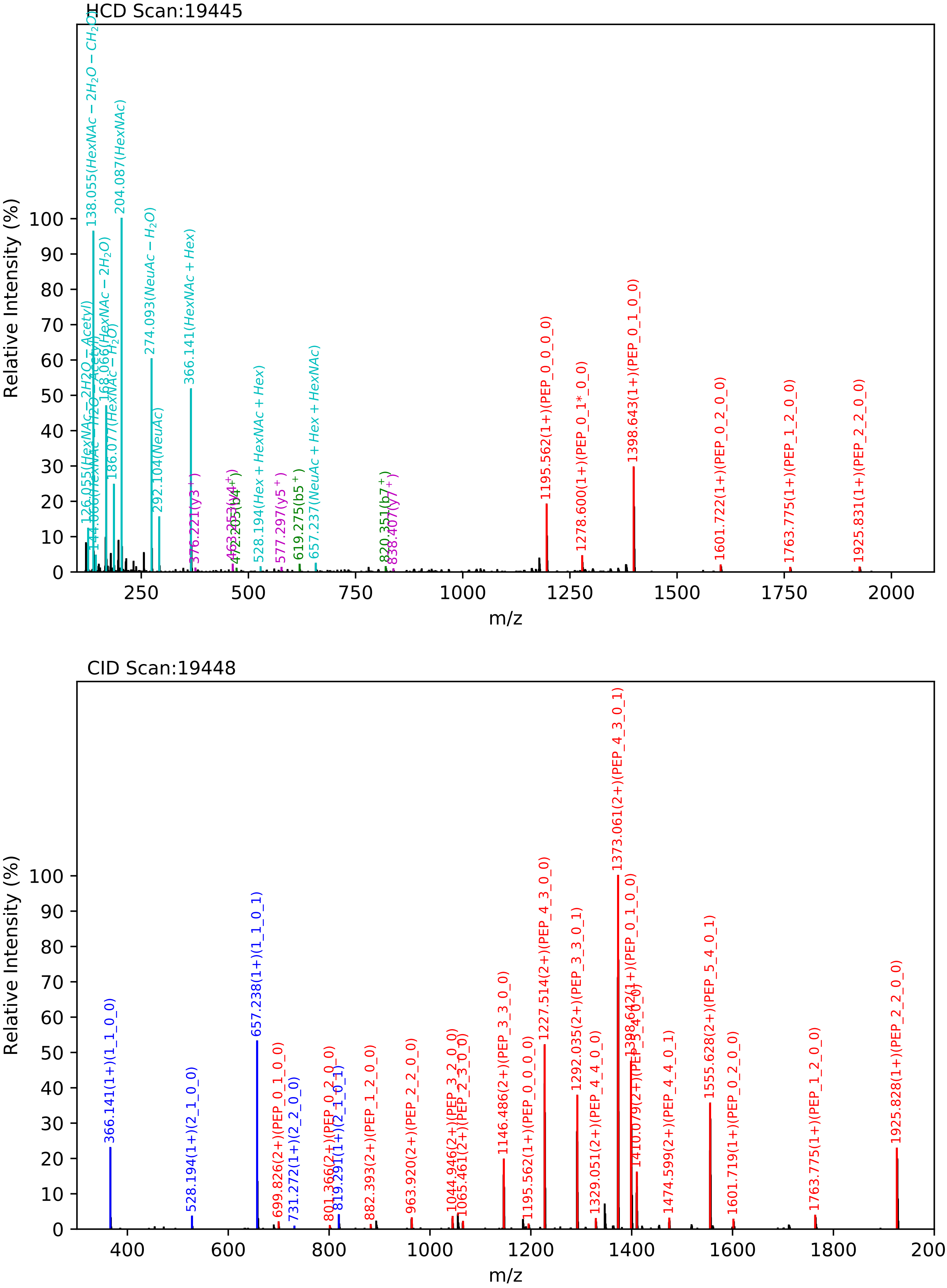

Unknown set no. 10, Experiment: Human Plasma exp_5

DIENFNSTQK(=PEP)_5_4_0_2, m/z:850.84(4+), RT:65.98, Y-score:91.44

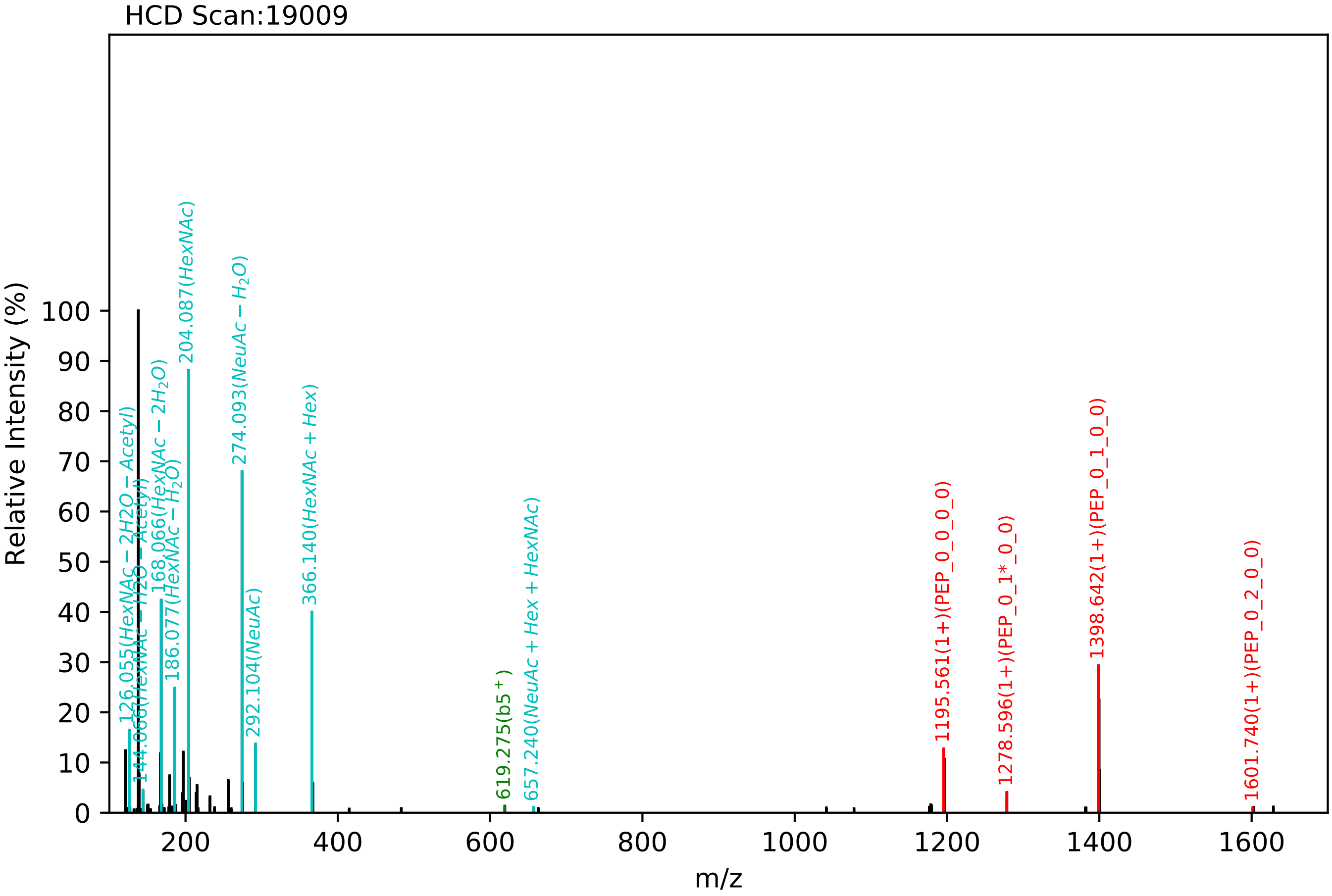

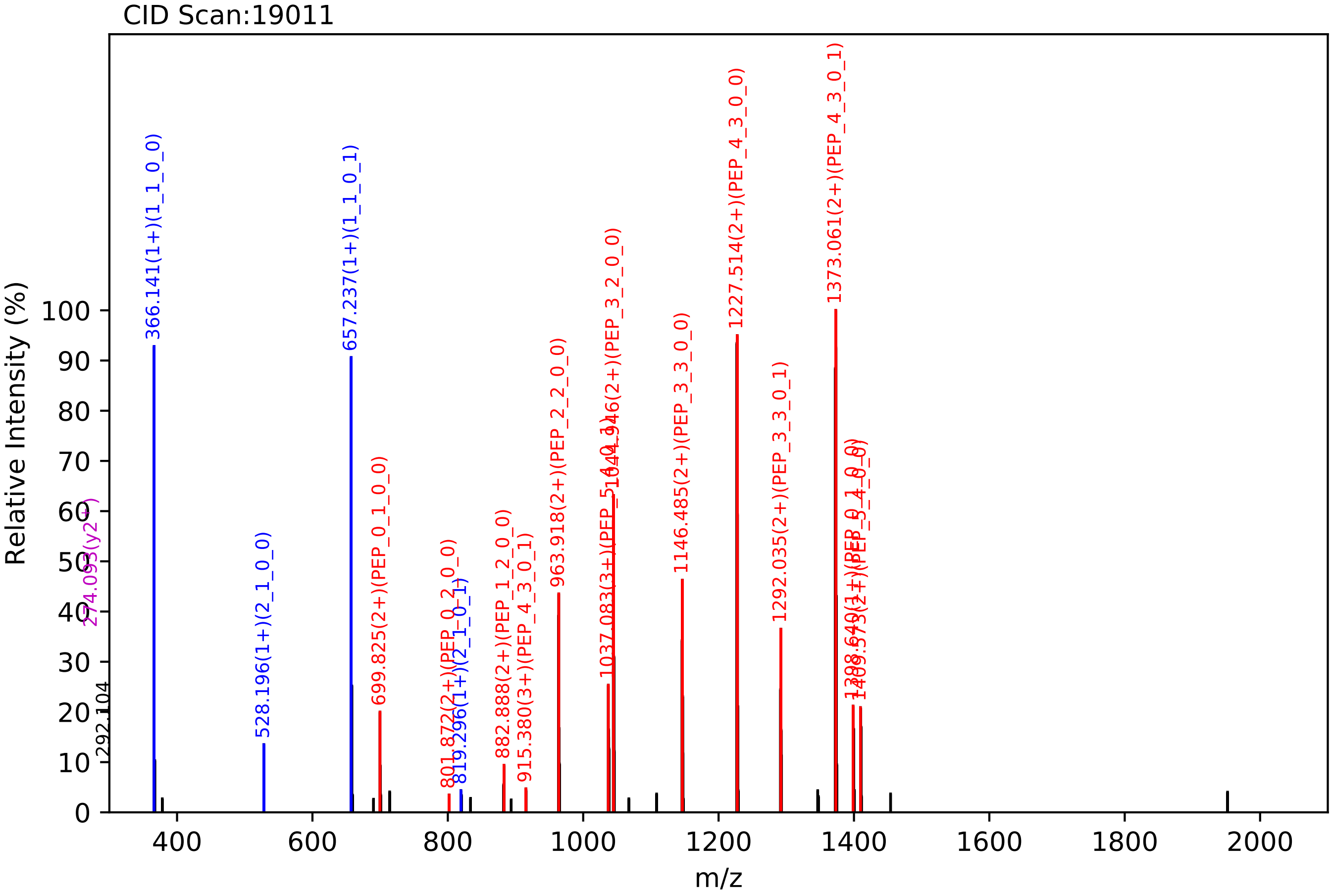

Unknown set no. 11, Experiment: Human Plasma exp_5

DIENFNSTQK(=PEP)_5_4_0_2, m/z:1134.12(3+), RT:66.30, Y-score:95.82

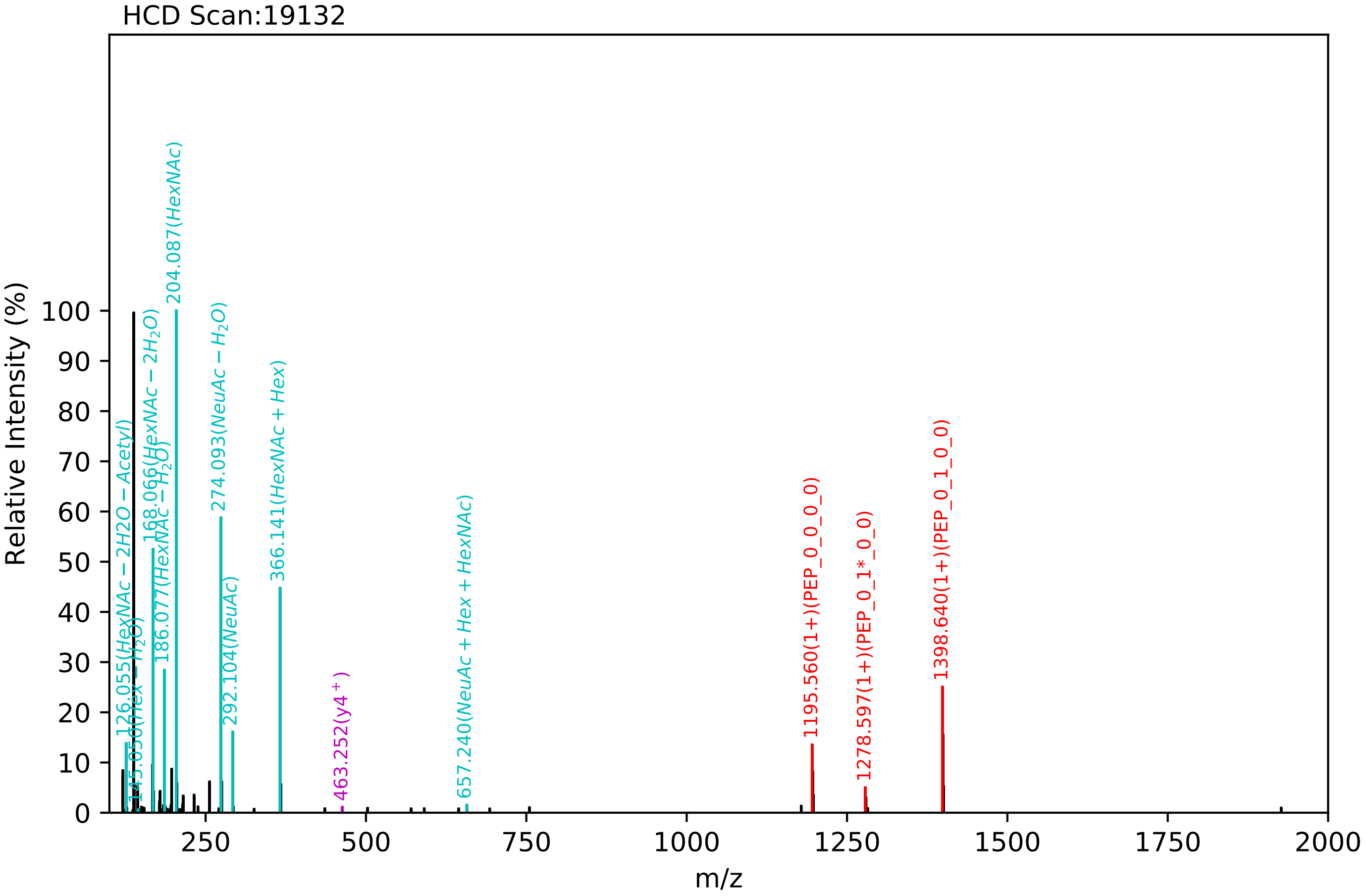

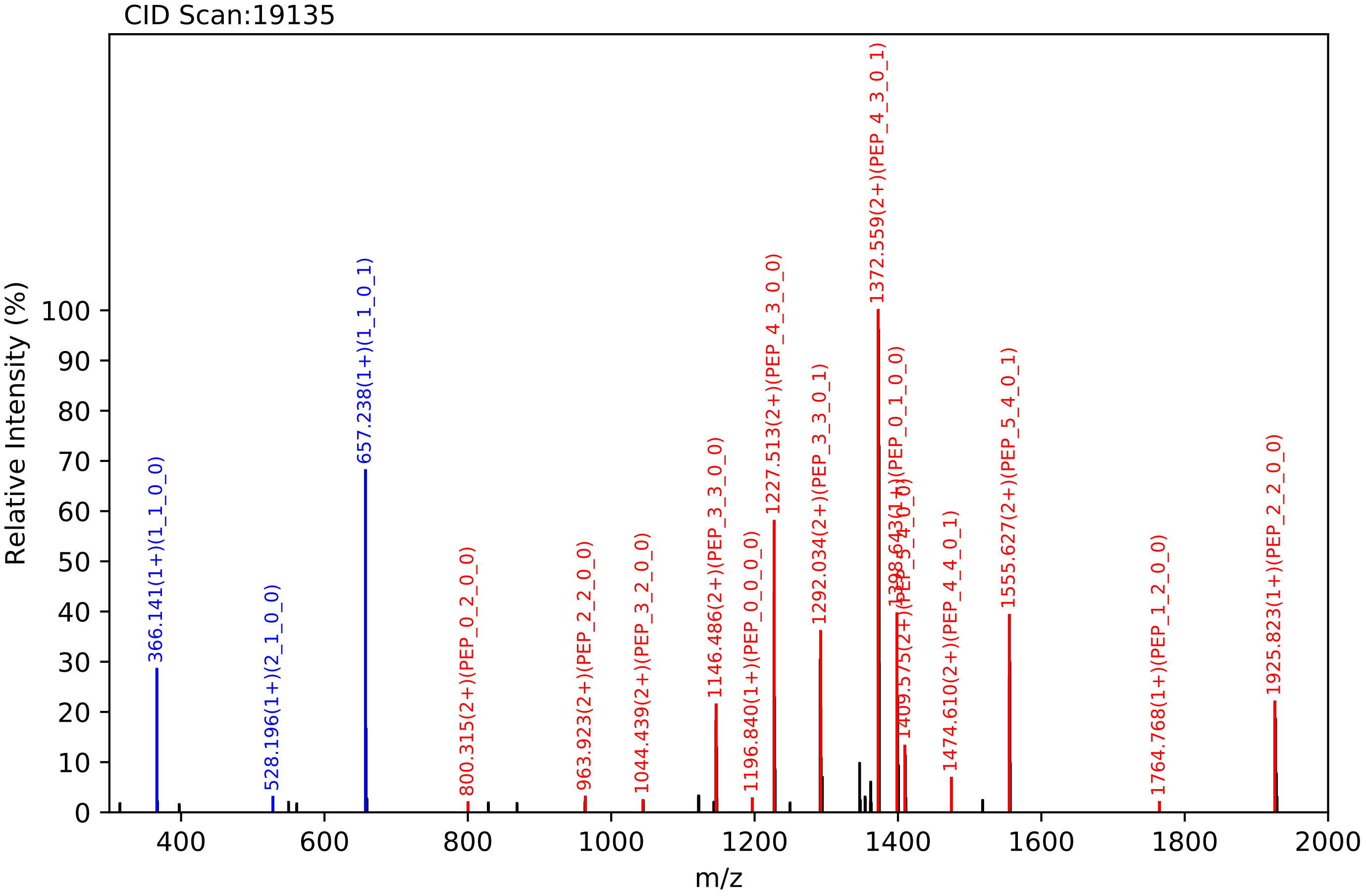

Unknown set no. 12, Experiment: Human Plasma exp_5

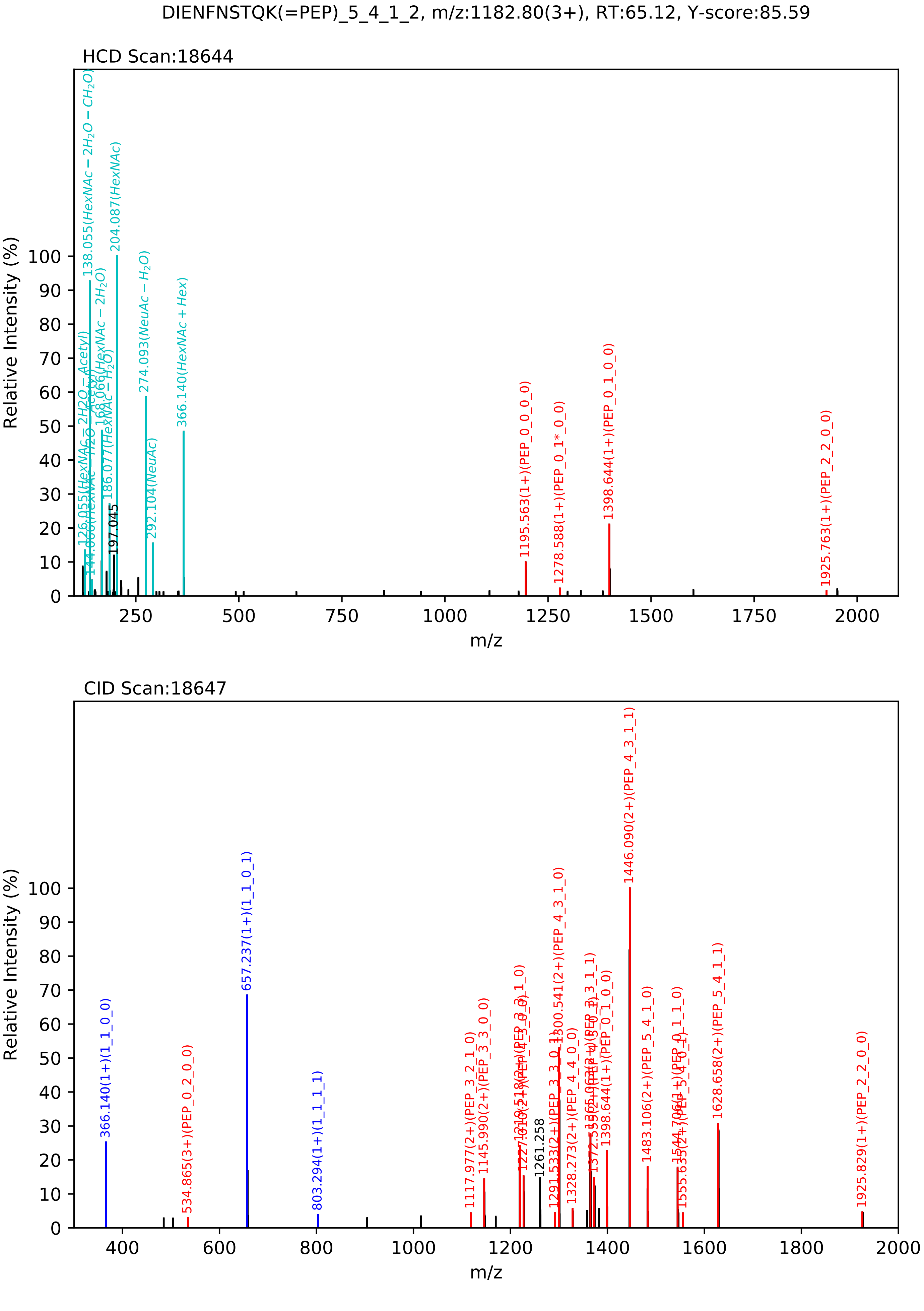

Unknown set no. 13, Experiment: Human Plasma exp_3

FNETTEK(=PEP)_5_4_0_2, m/z:1025.07(3+), RT:34.33, Y-score:90.92

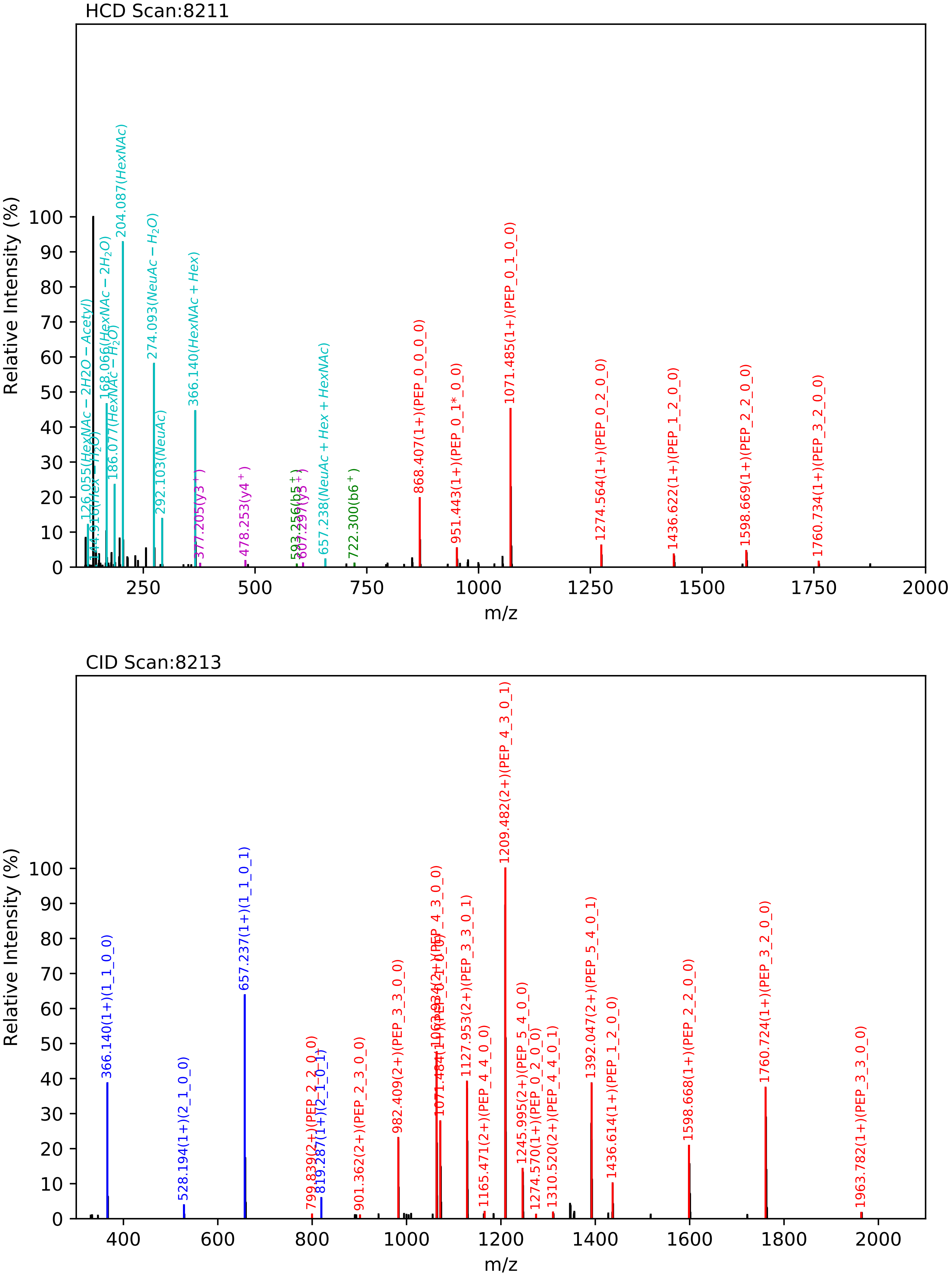

Unknown set no. 14, Experiment: Human Plasma exp_5

FNETTEK(=PEP)_5_4_0_2, m/z:1025.07(3+), RT:33.86, Y-score:94.91

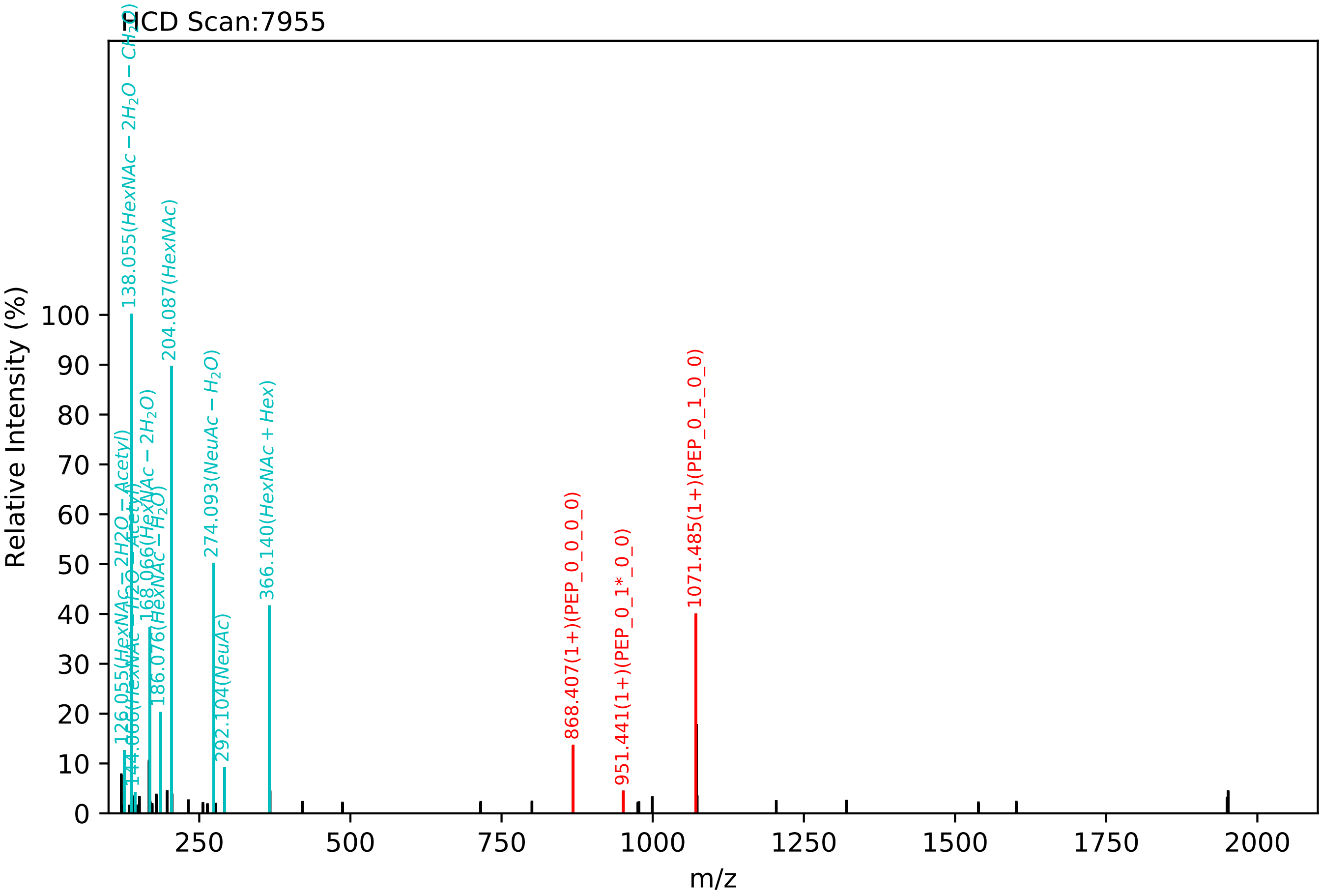

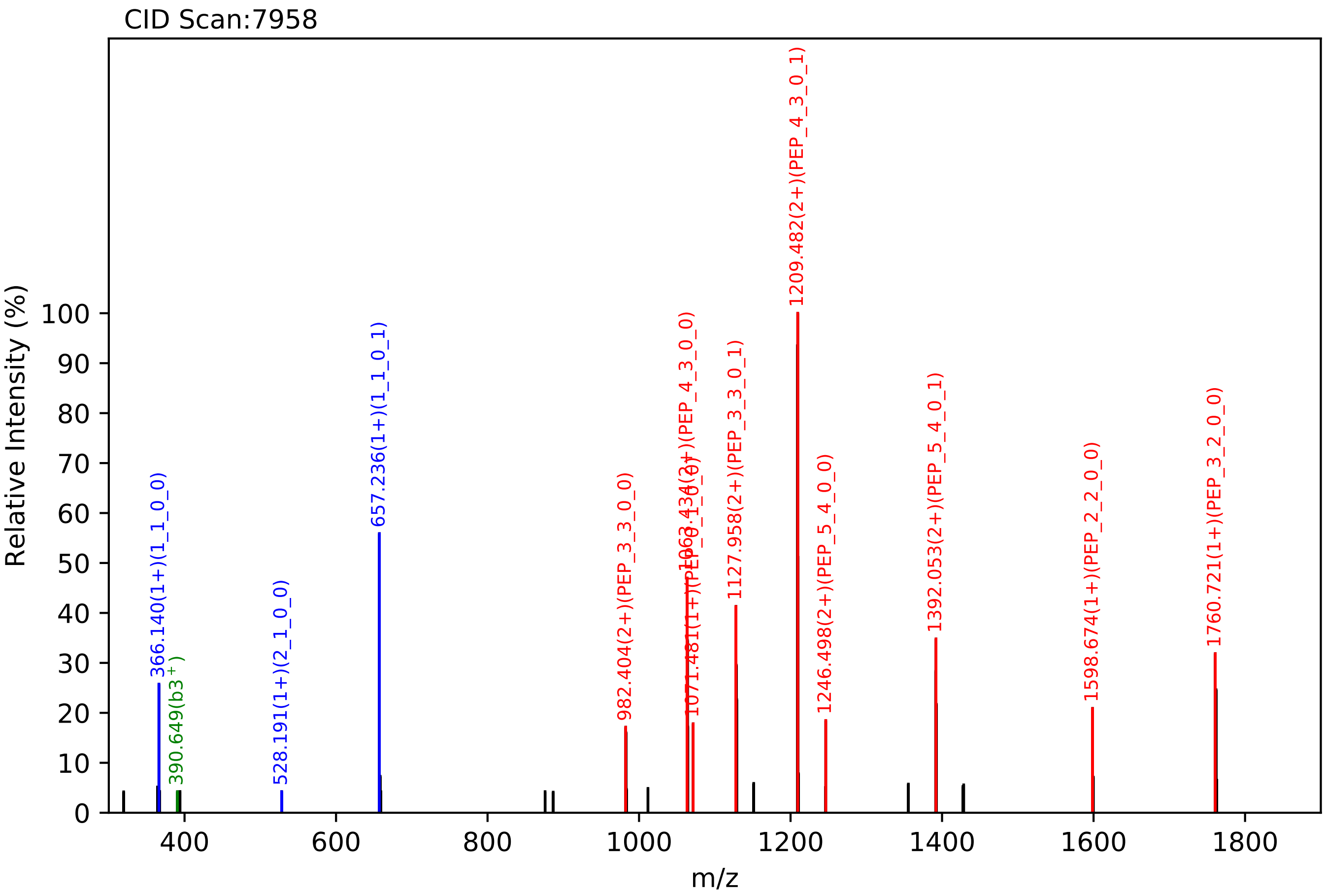

Unknown set no. 15, Experiment: Human Plasma exp_5

FNETTEK(=PEP)_6_5_0_3, m/z:1243.81(3+), RT:50.10, Y-score:91.68

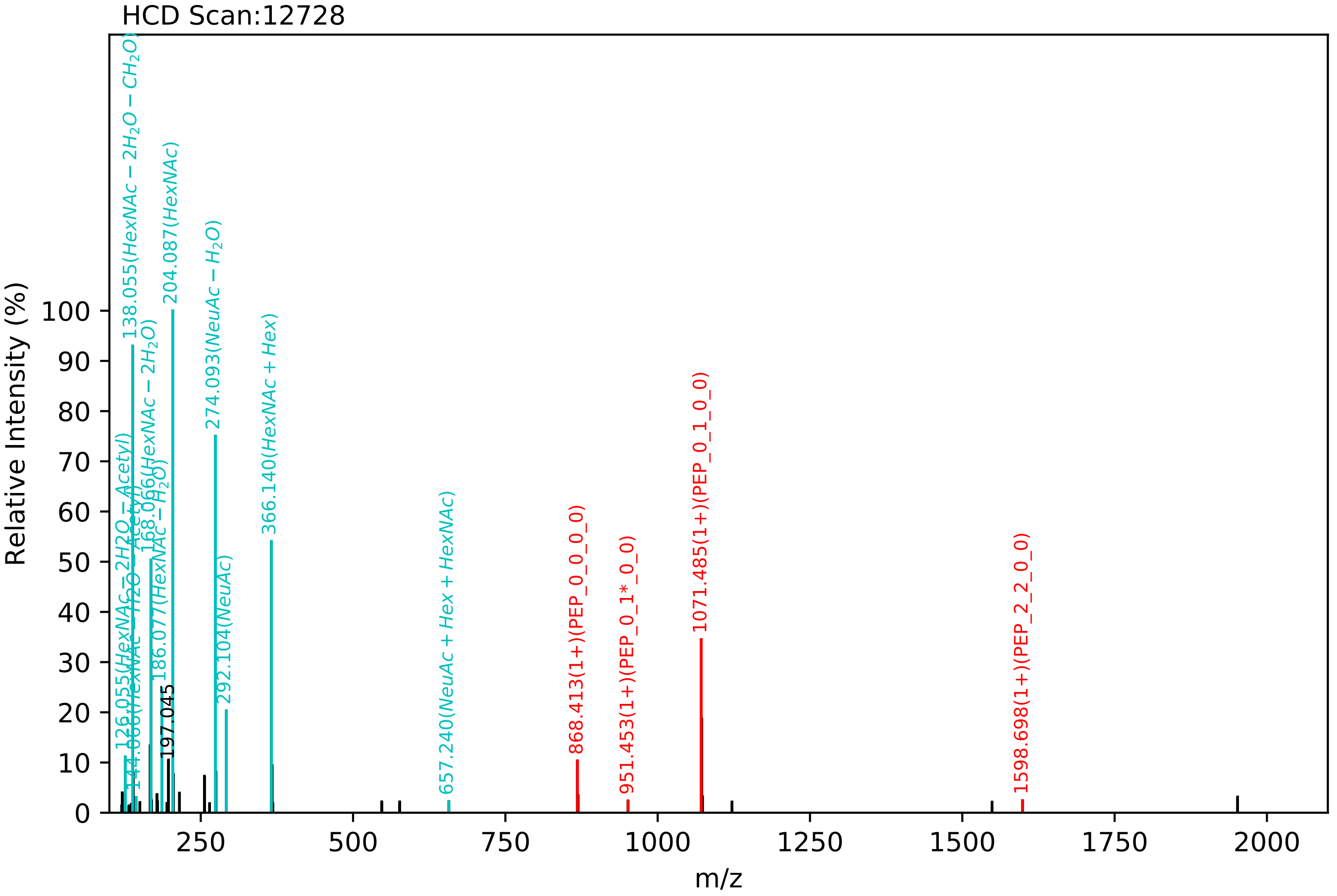

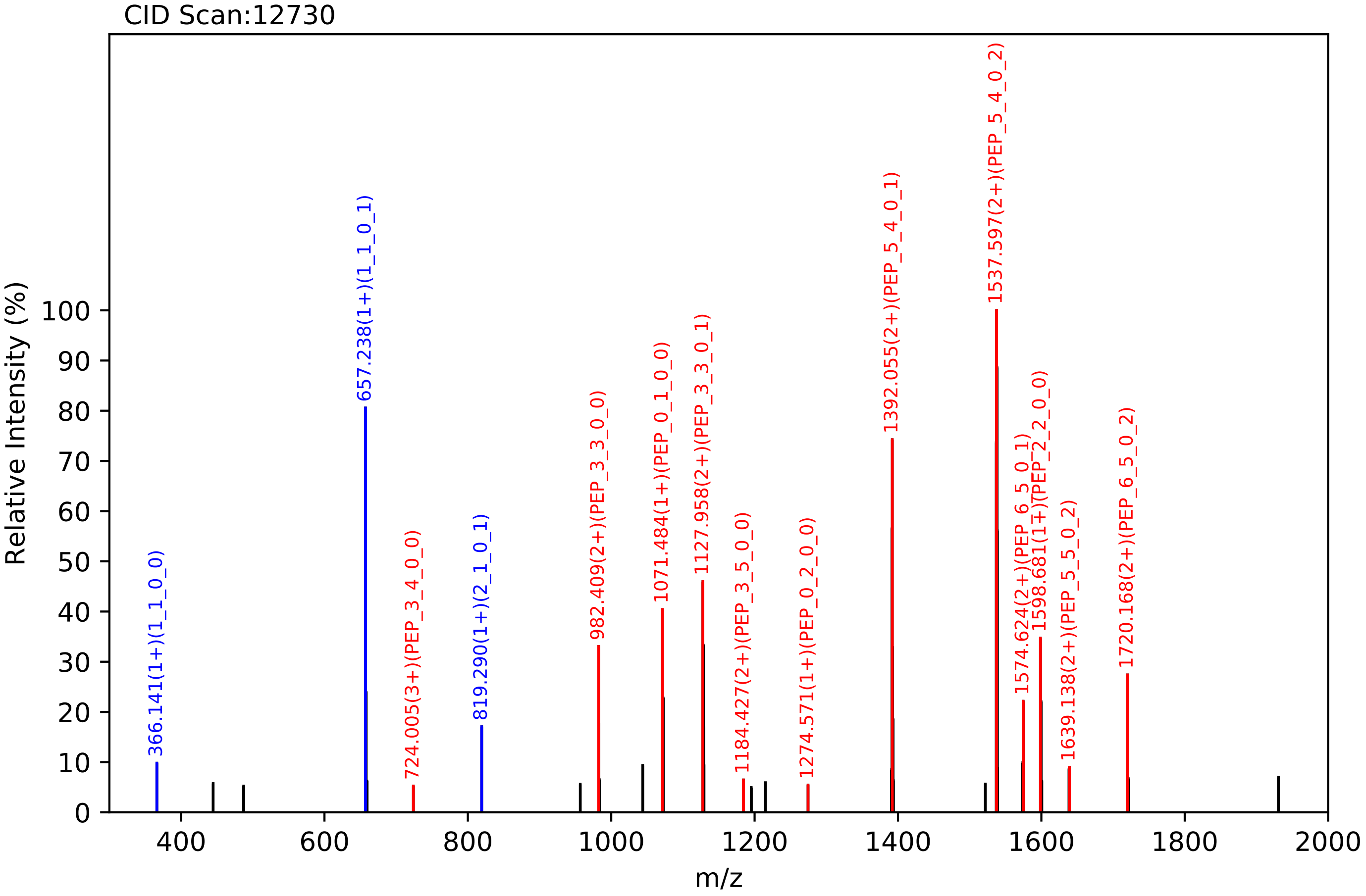

Unknown set no. 16, Experiment: Human Plasma exp_1

HNFSHCCSK(=PEP)_5_4_0_2, m/z:846.07(4+), RT:11.77, Y-score:86.79

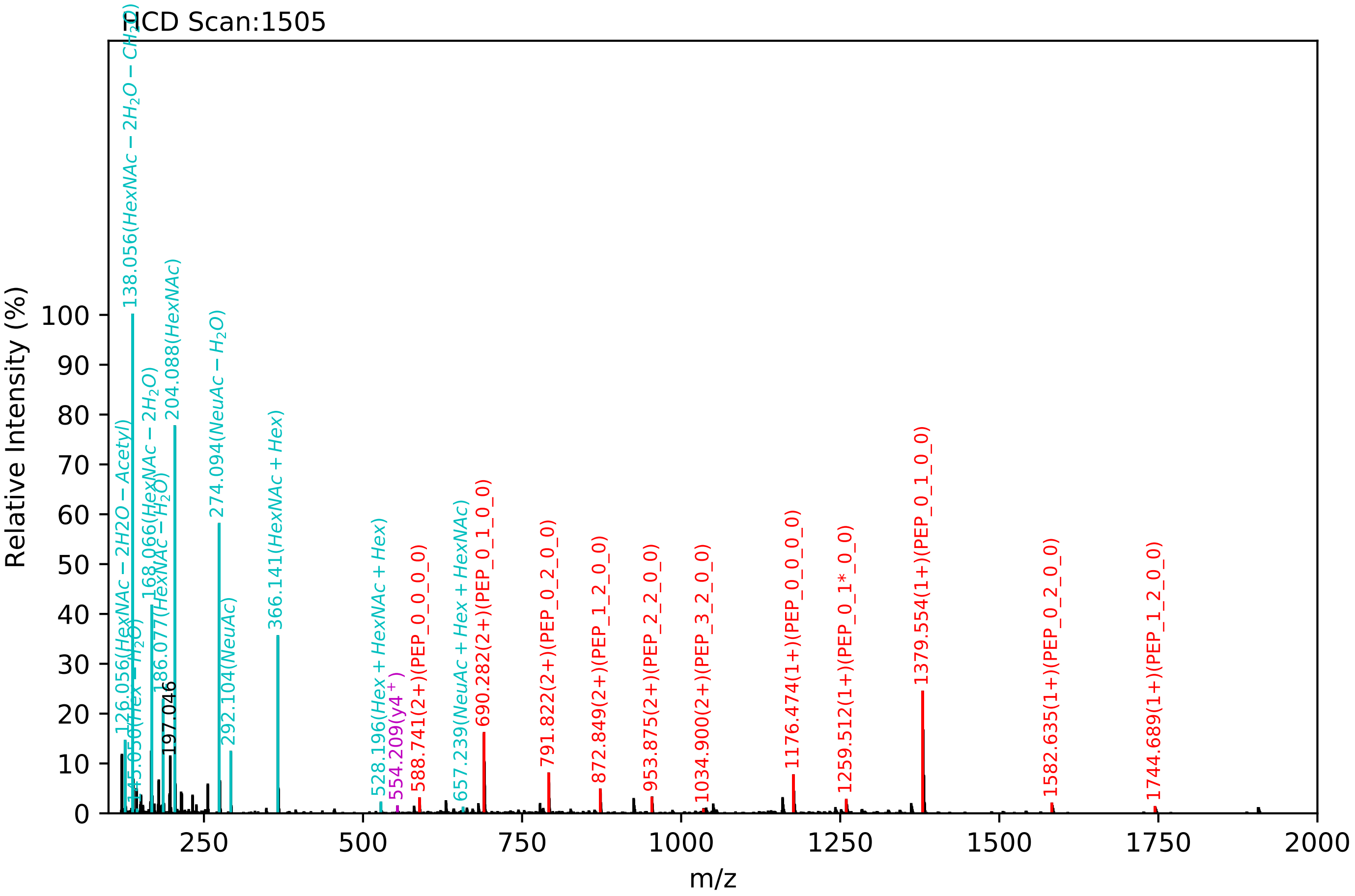

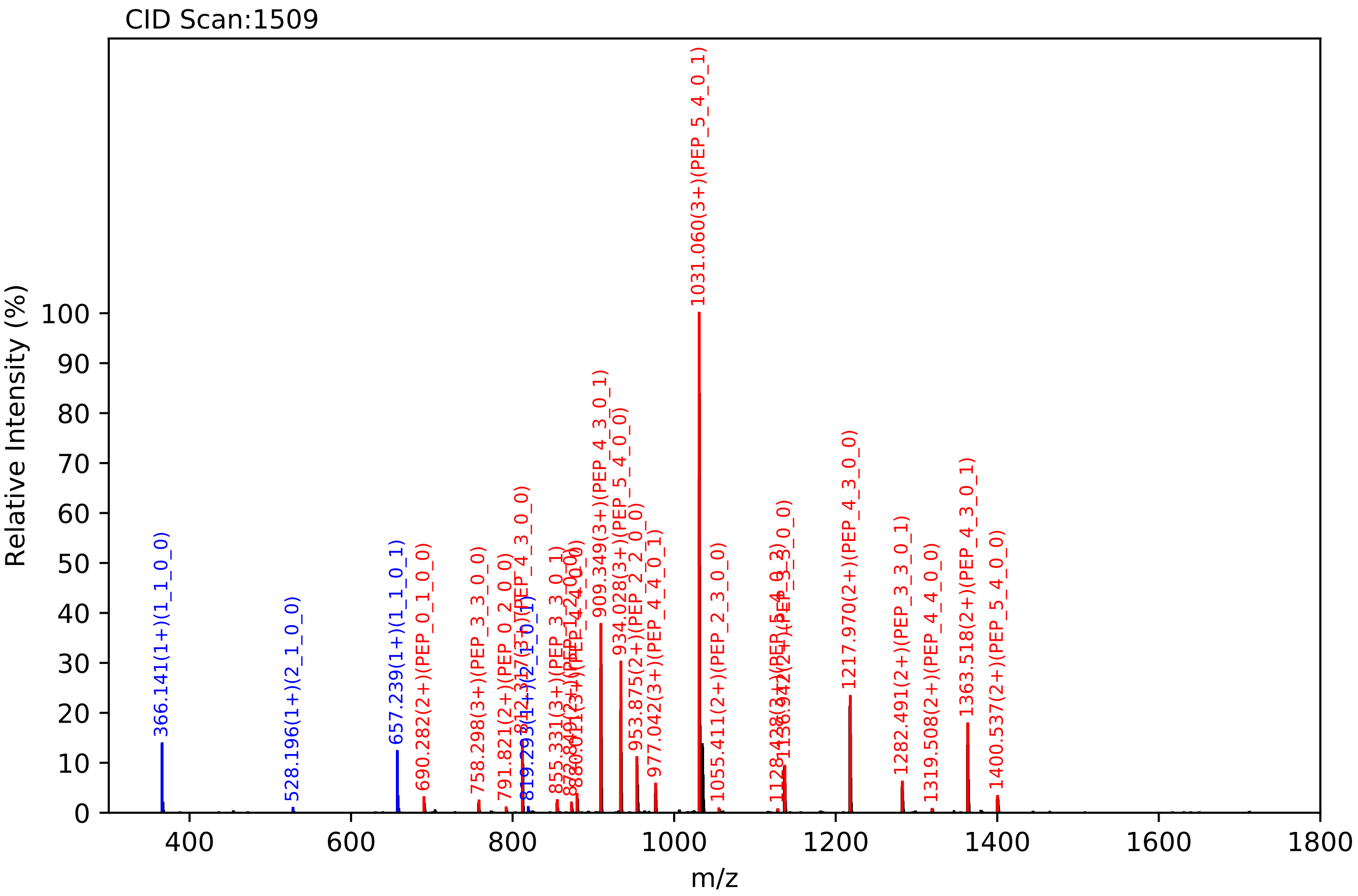

Unknown set no. 17, Experiment: Human Plasma exp_2

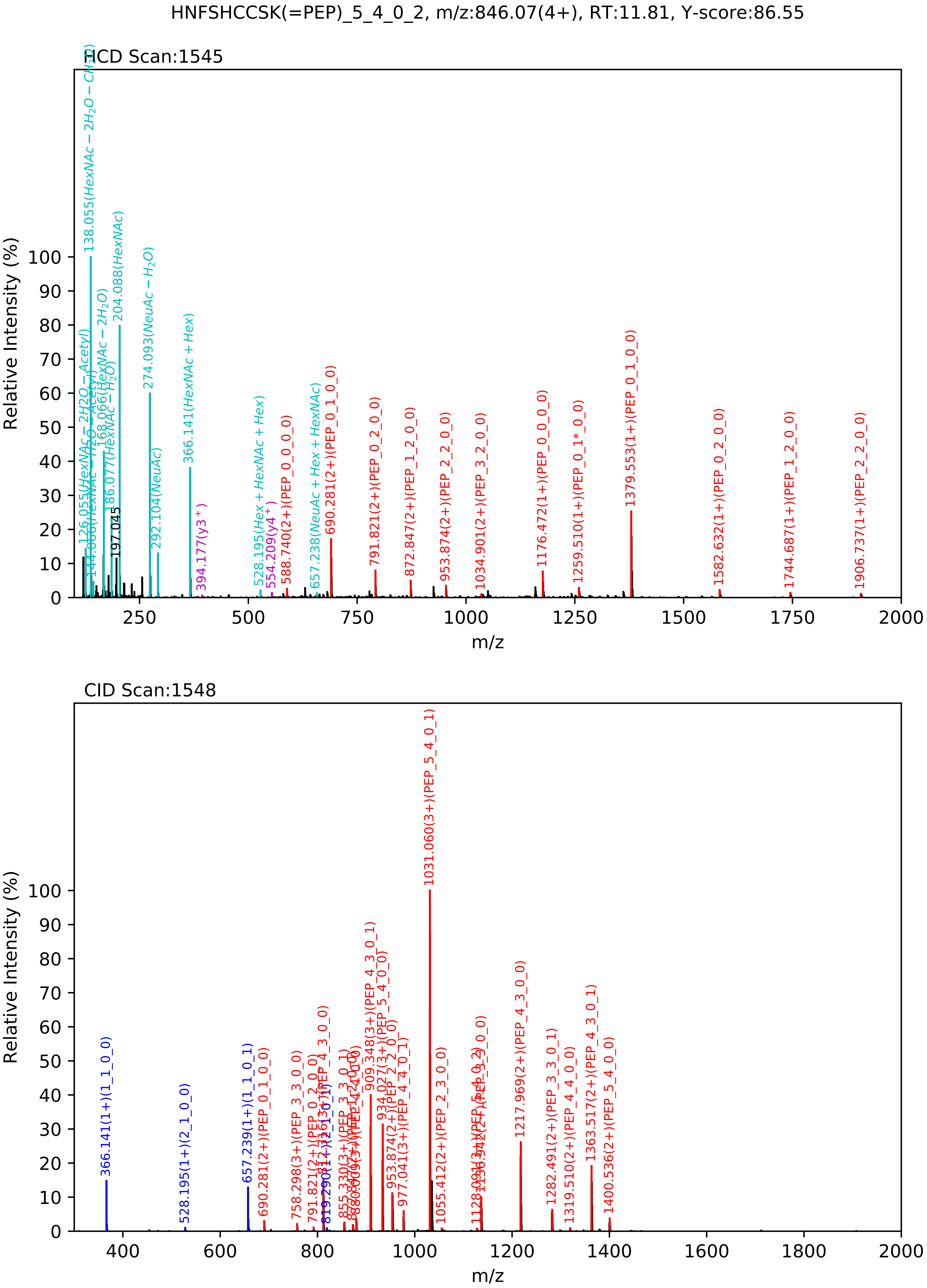

Unknown set no. 18, Experiment: Human Plasma exp_4

HNFSHCCSK(=PEP)_5_4_0_2, m/z:1127.75(3+), RT:11.95, Y-score:83.63

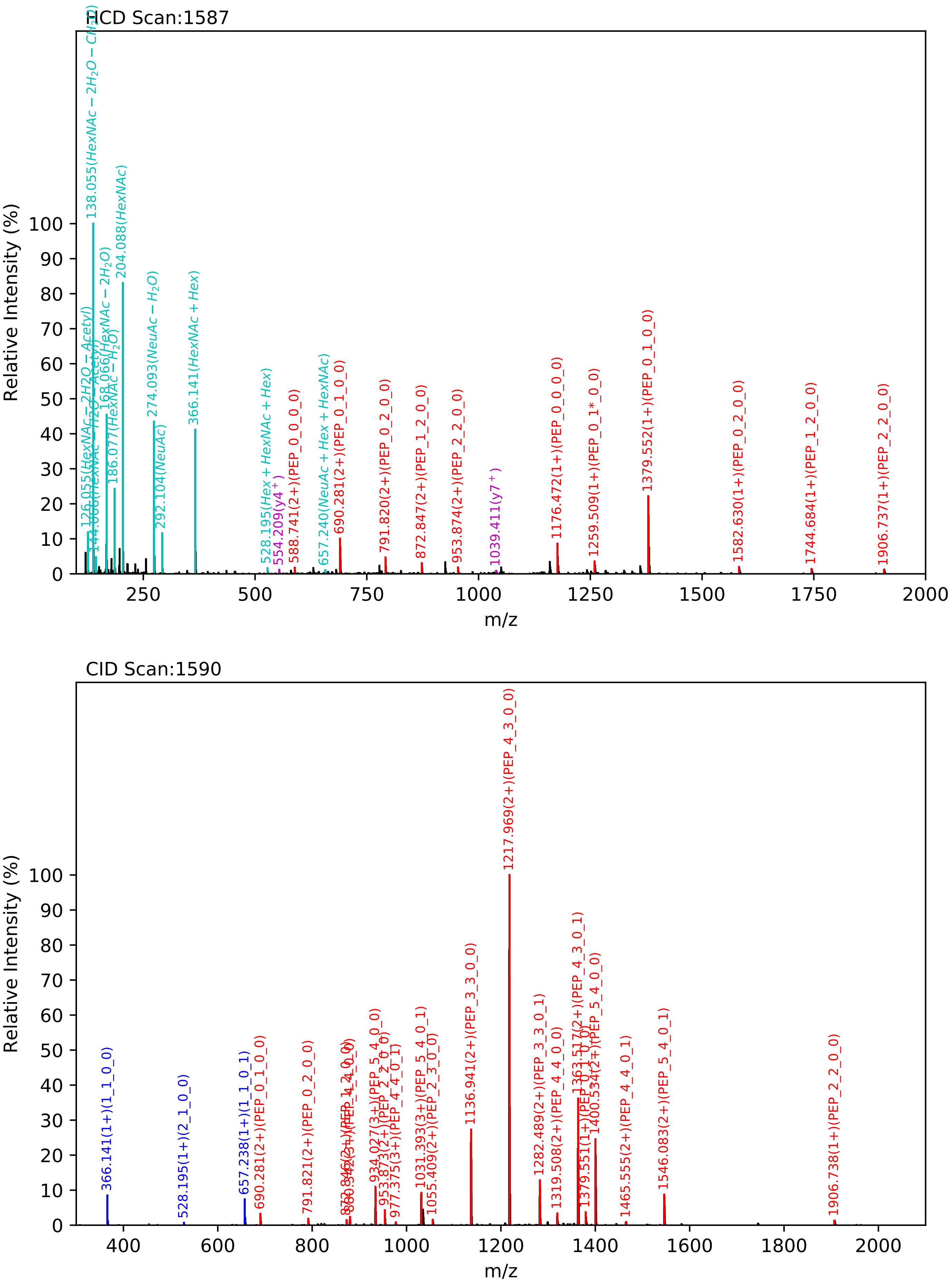

Unknown set no. 19, Experiment: Human Plasma exp_5

HNFSHCCSK(=PEP)_5_4_0_2, m/z:846.07(4+), RT:11.75, Y-score:86.94

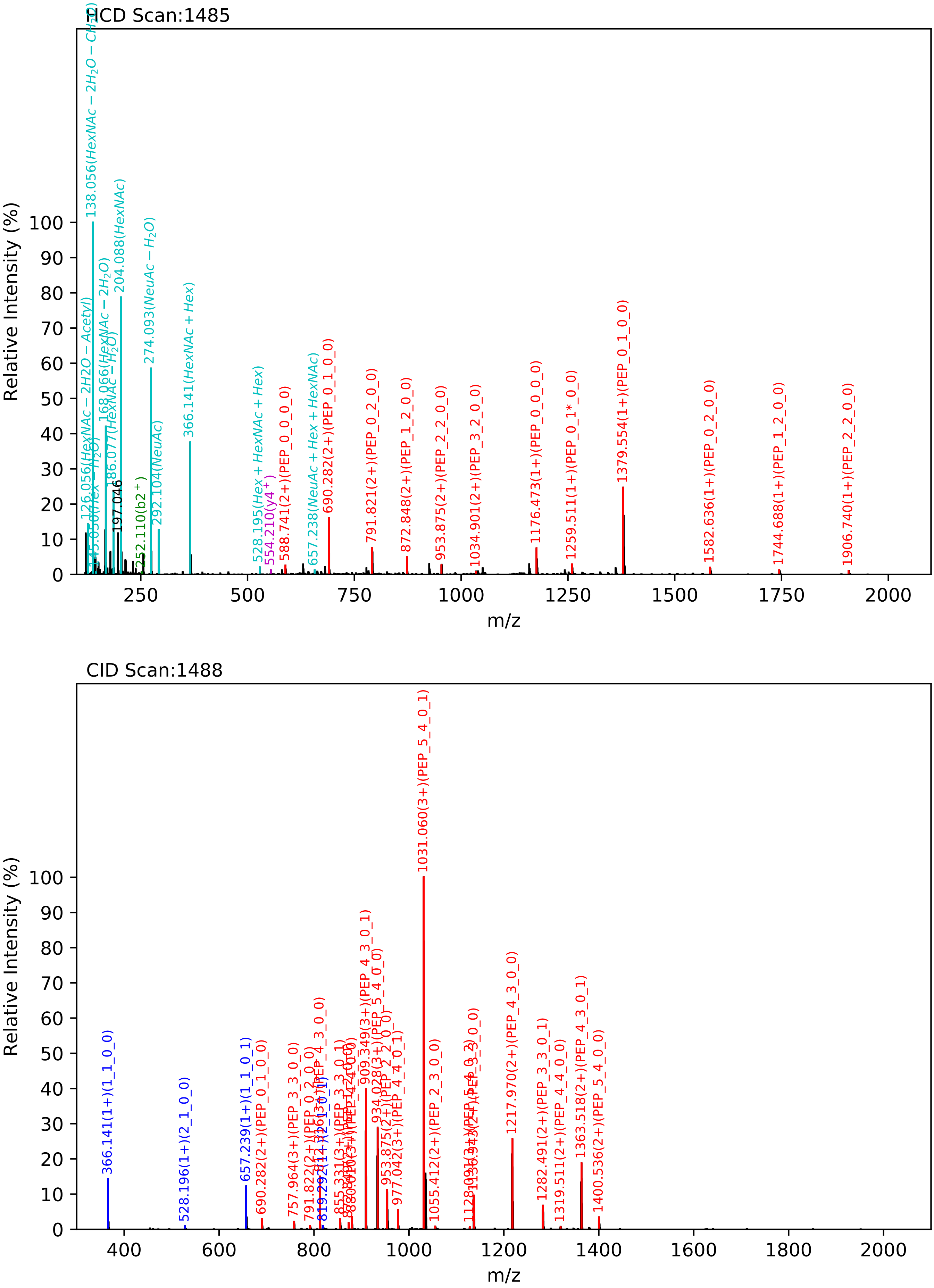

Unknown set no. 20, Experiment: Human Plasma exp_5

HNFSHCCSK(=PEP)_5_4_0_2, m/z:1127.75(3+), RT:11.76, Y-score:83.41

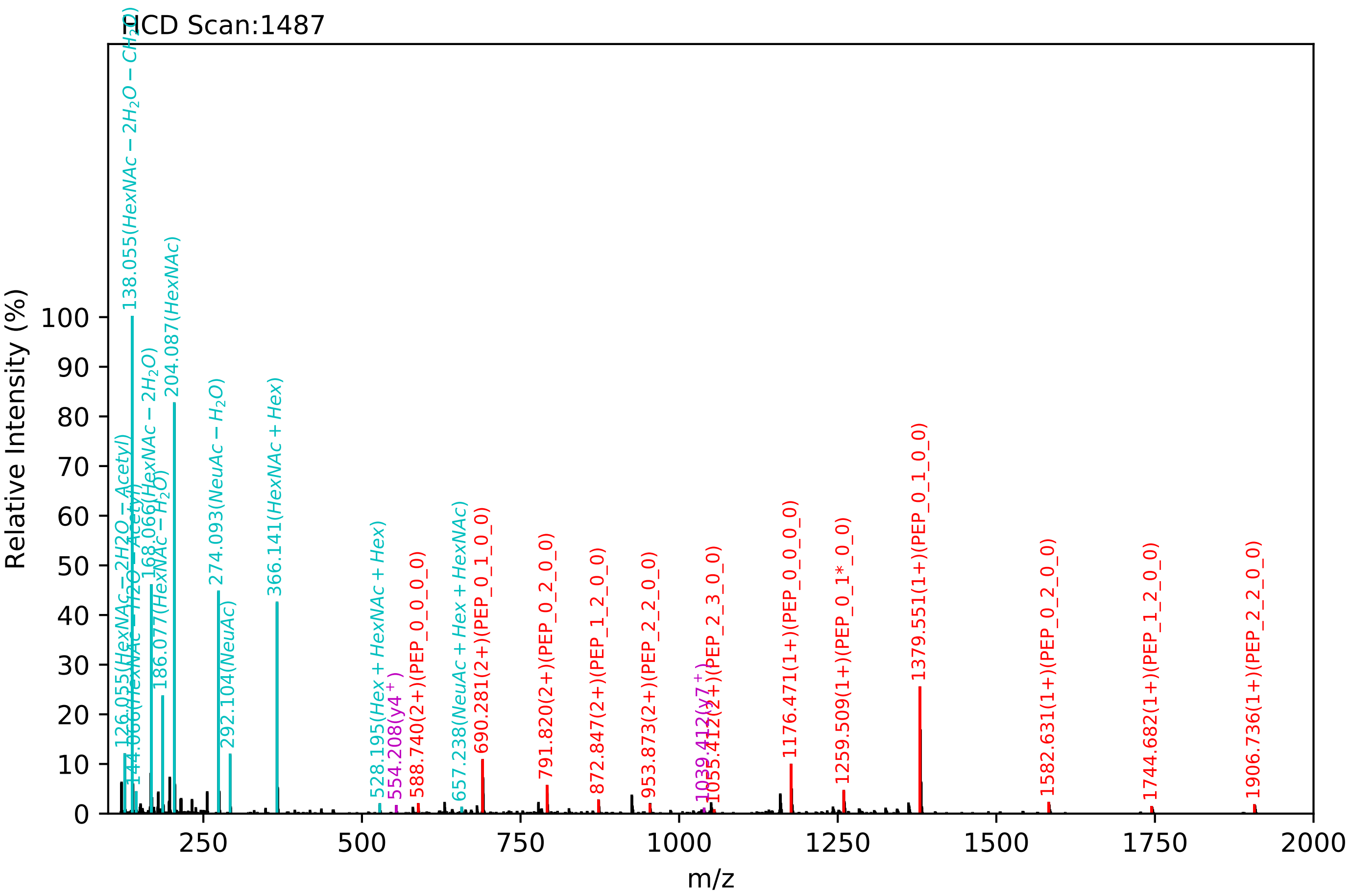

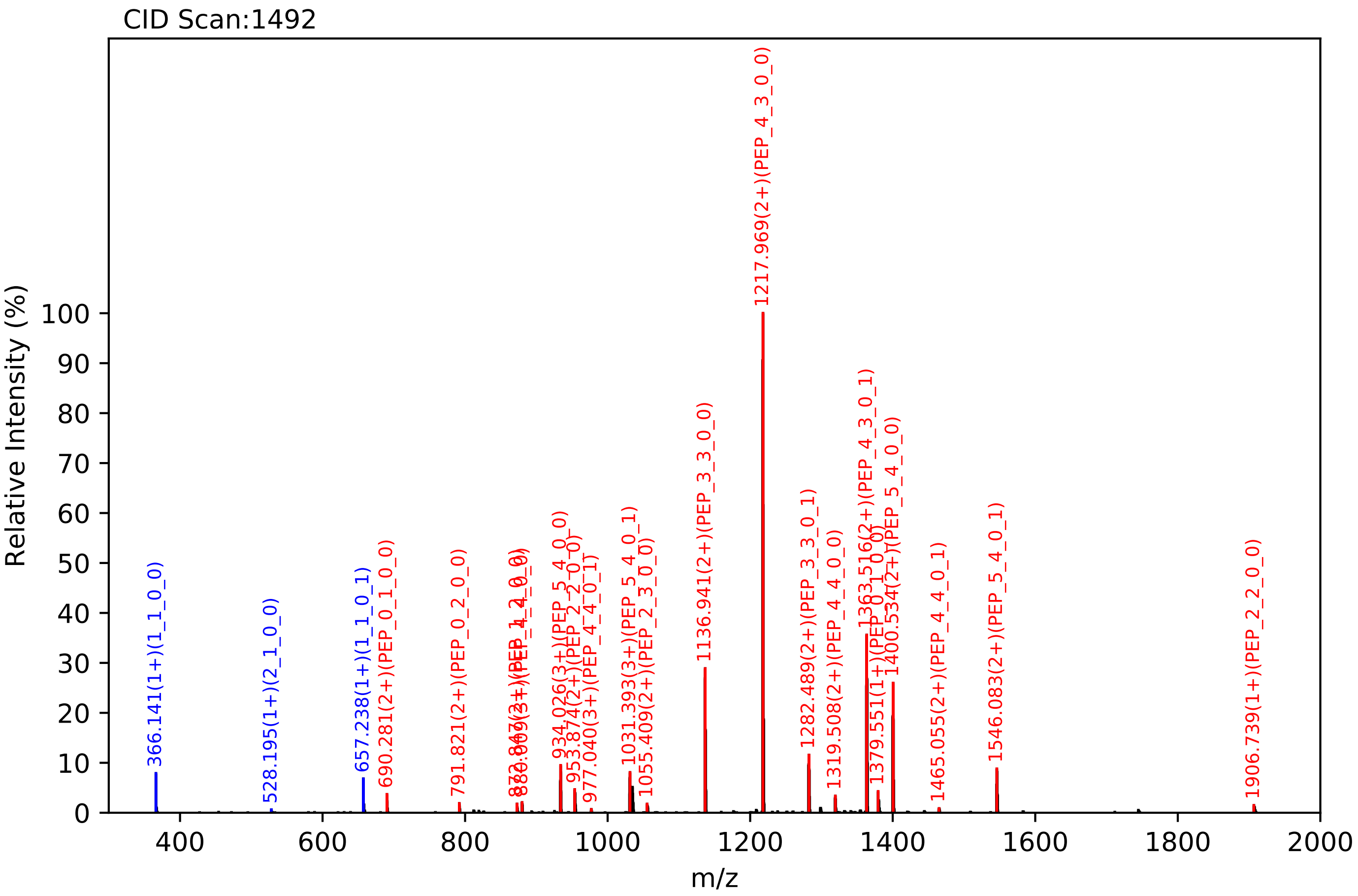

Unknown set no. 21, Experiment: Human Plasma exp_1

NCCNTENPPGCYR(=PEP)_5_4_0_2, m/z:962.36(4+), RT:52.44, Y-score:87.99

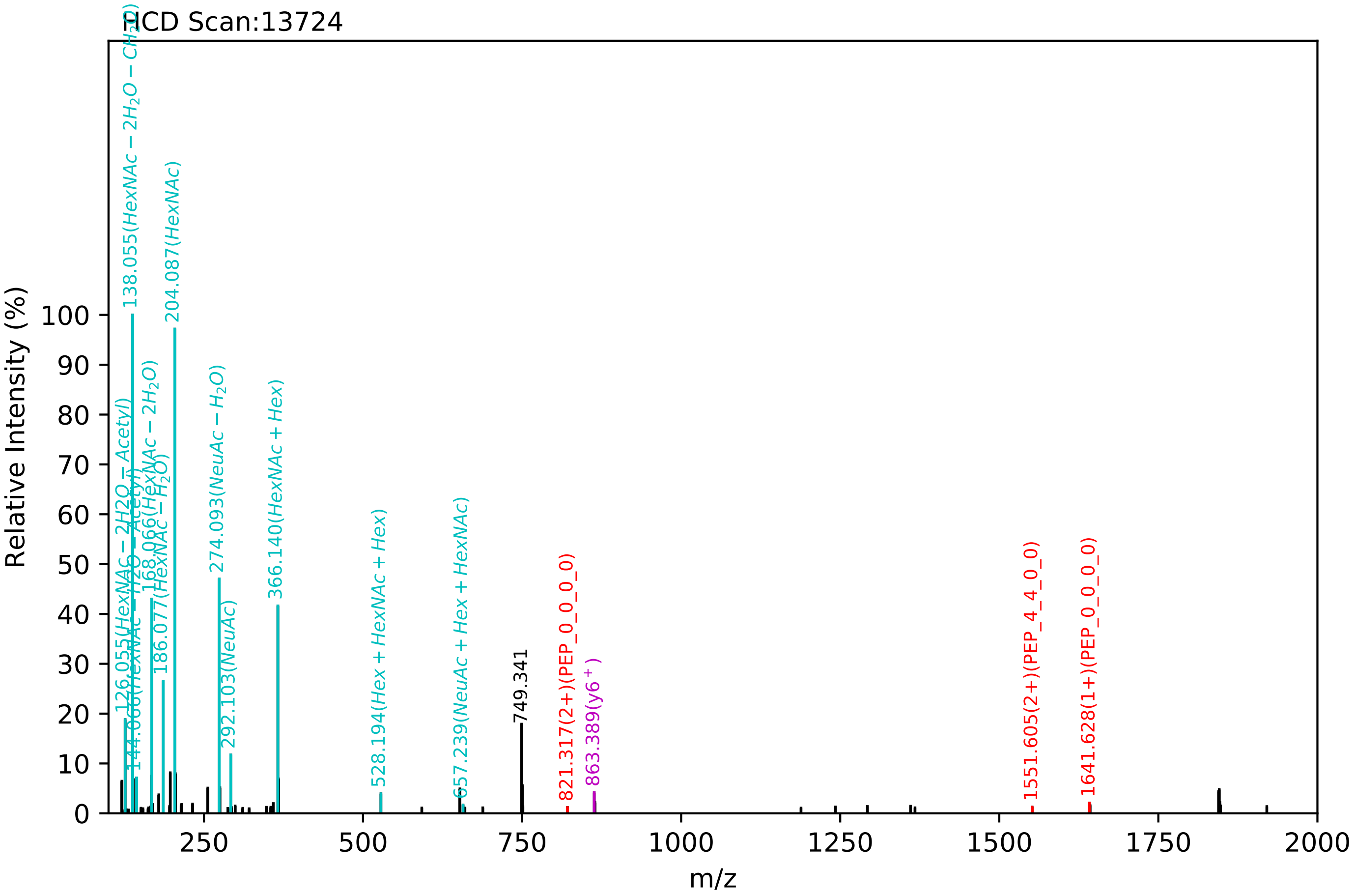

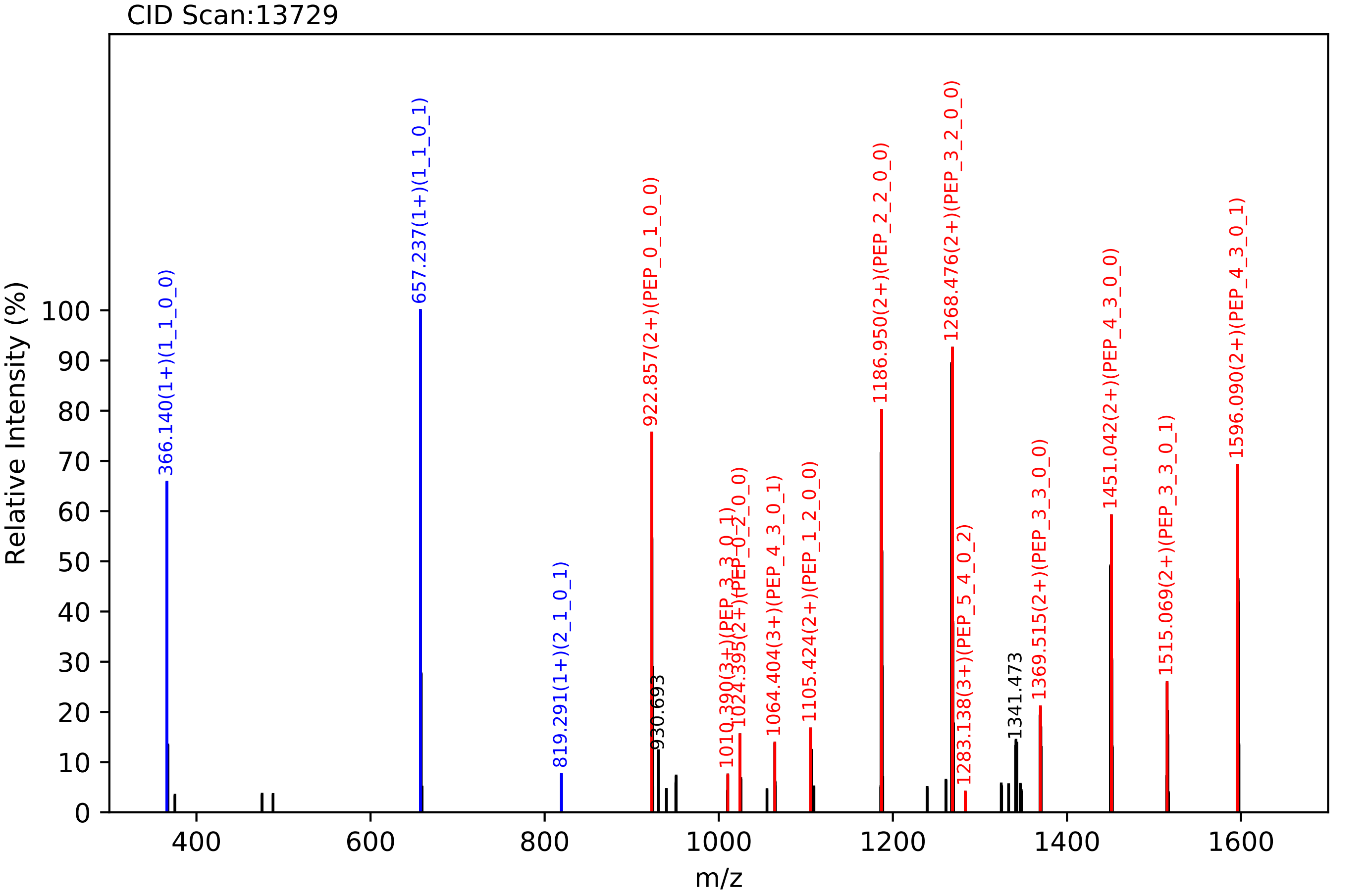

Unknown set no. 22, Experiment: Human Plasma exp_1

NCCNTENPPGCYR(=PEP)_5_4_0_2, m/z:1282.80(3+), RT:52.43, Y-score:86.86

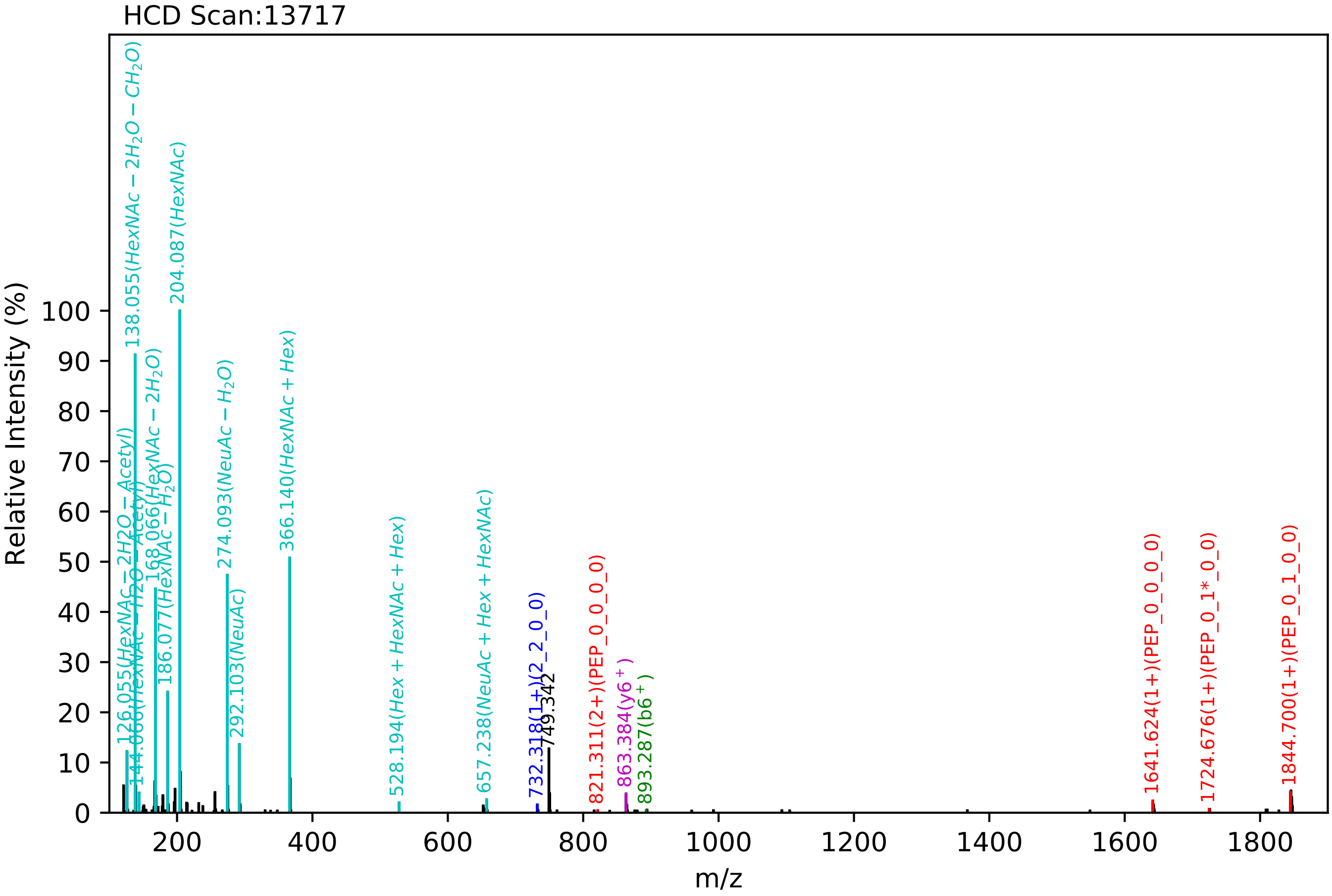

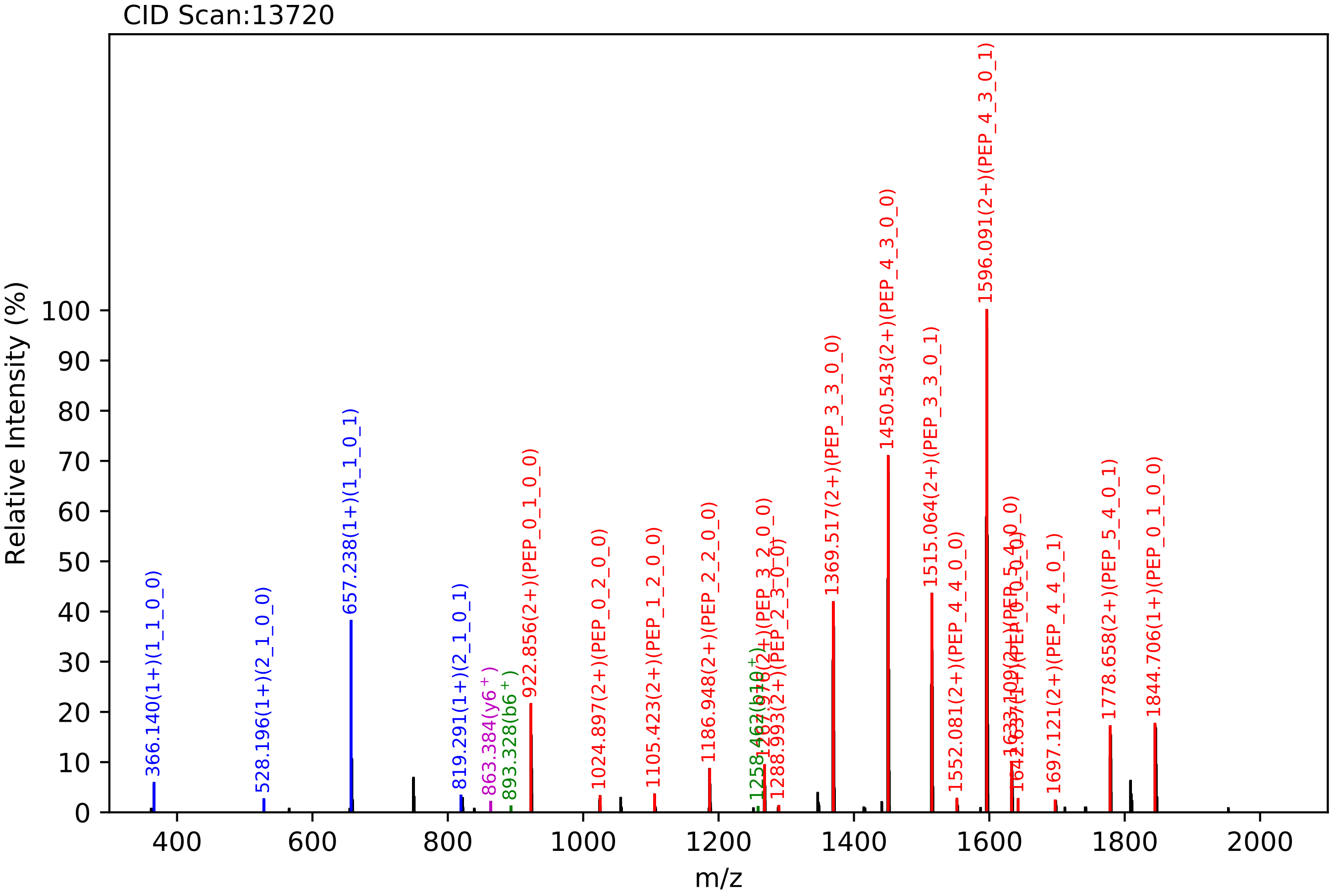

Unknown set no. 23, Experiment: Human Plasma exp_4

NCCNTENPPGCYR(=PEP)_5_4_0_2, m/z:962.36(4+), RT:52.56, Y-score:89.45

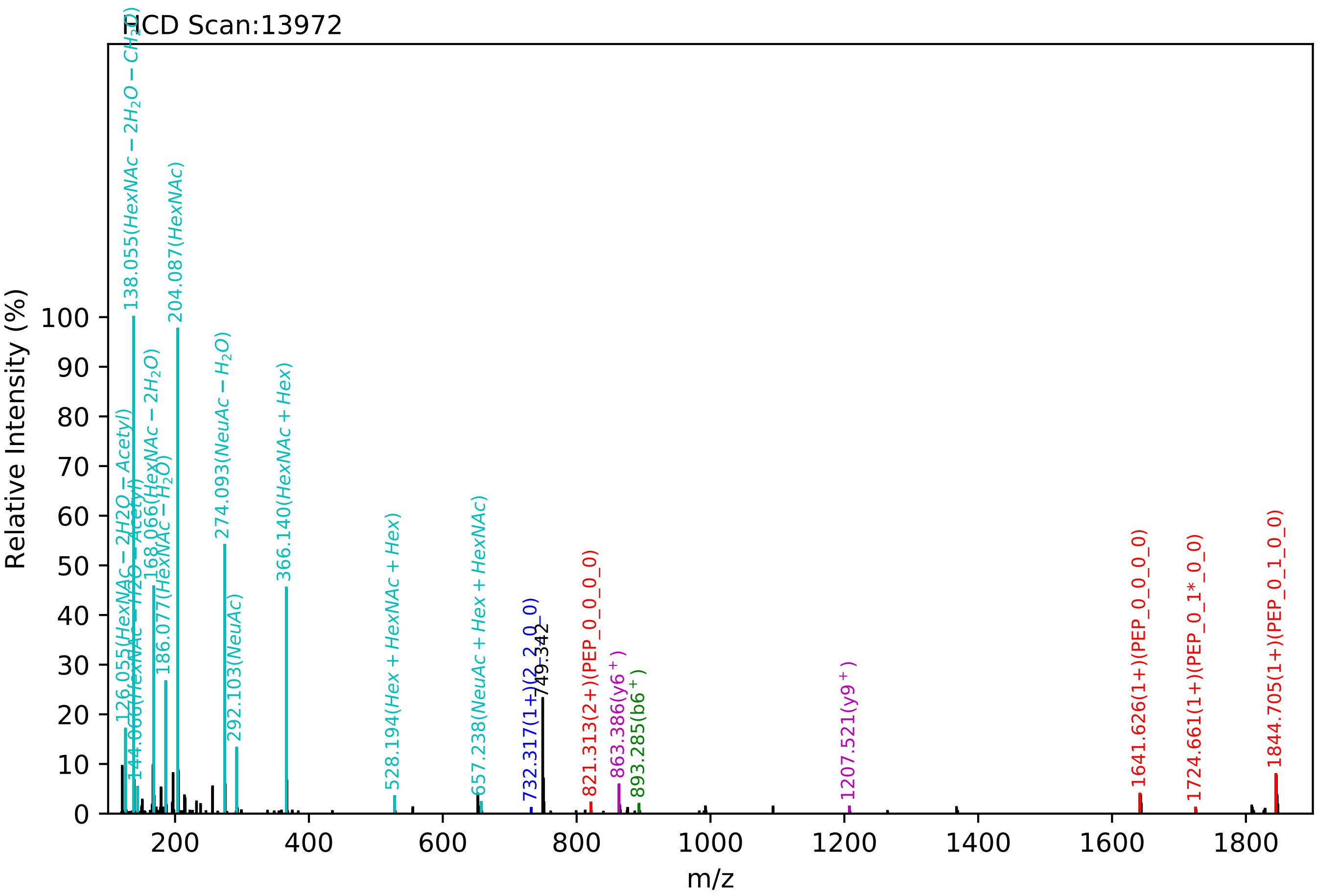

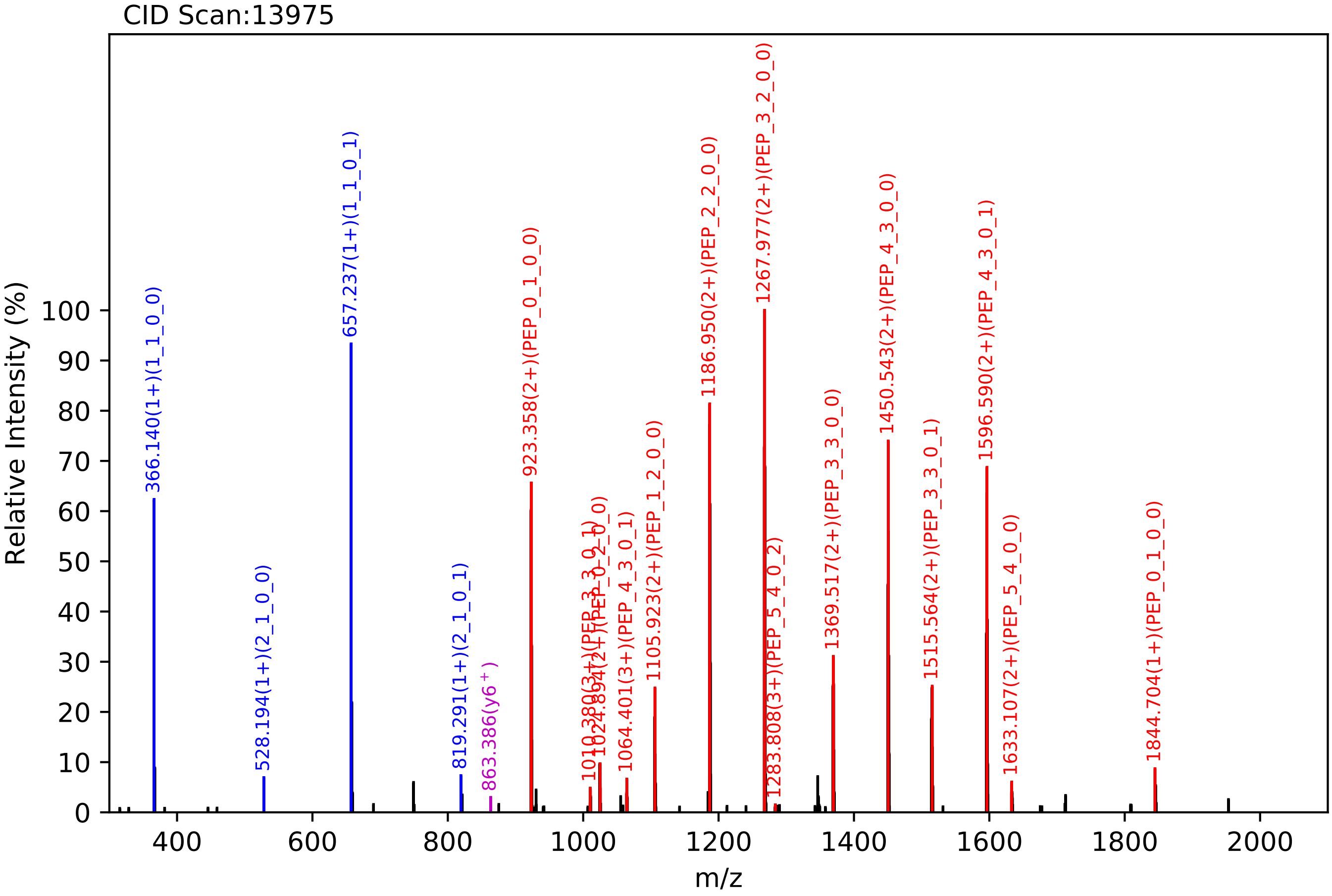

Unknown set no. 24, Experiment: Human Plasma exp_4

NCCNTENPPGCYR(=PEP)_5_4_0_2, m/z:1282.80(3+), RT:52.17, Y-score:87.52

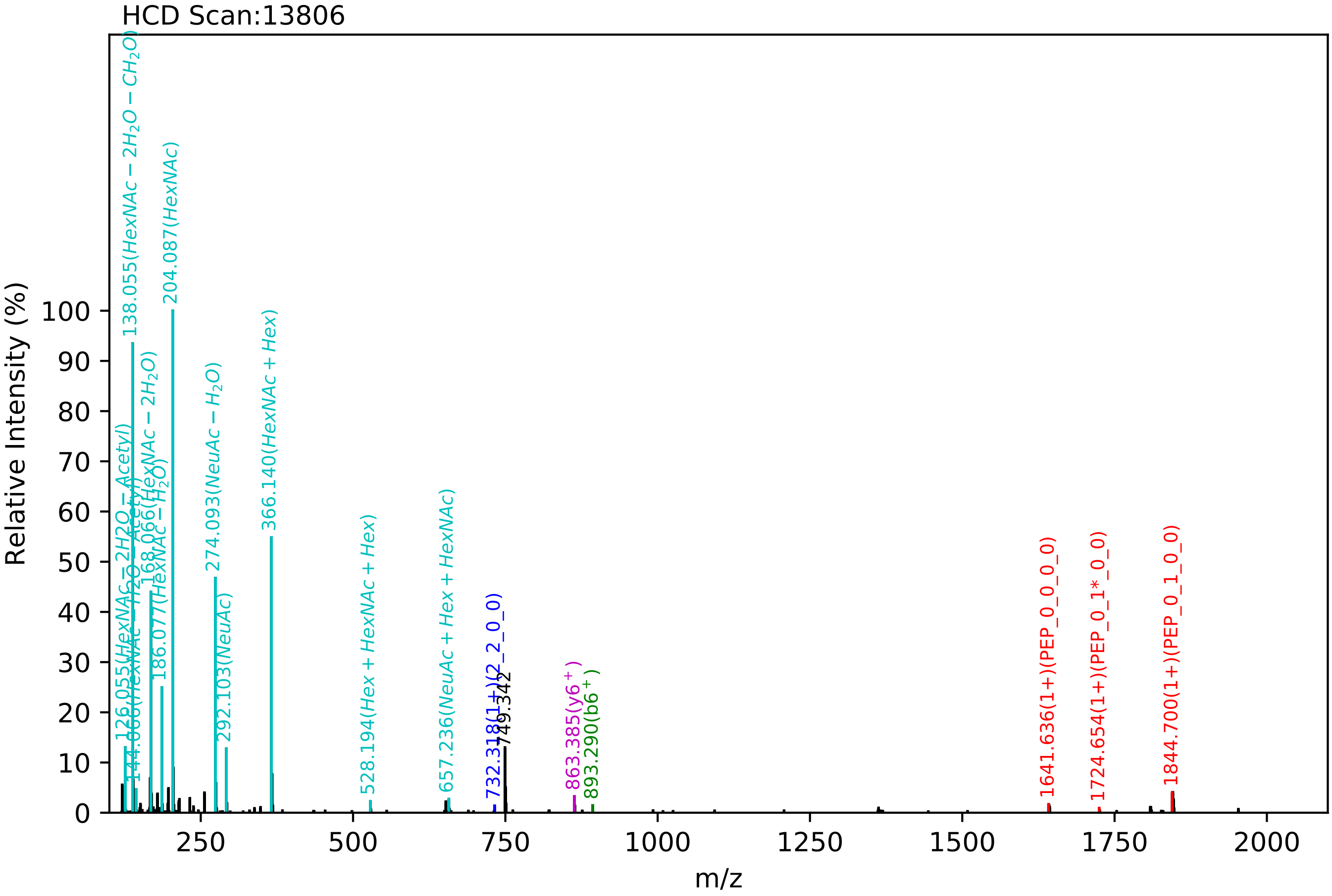

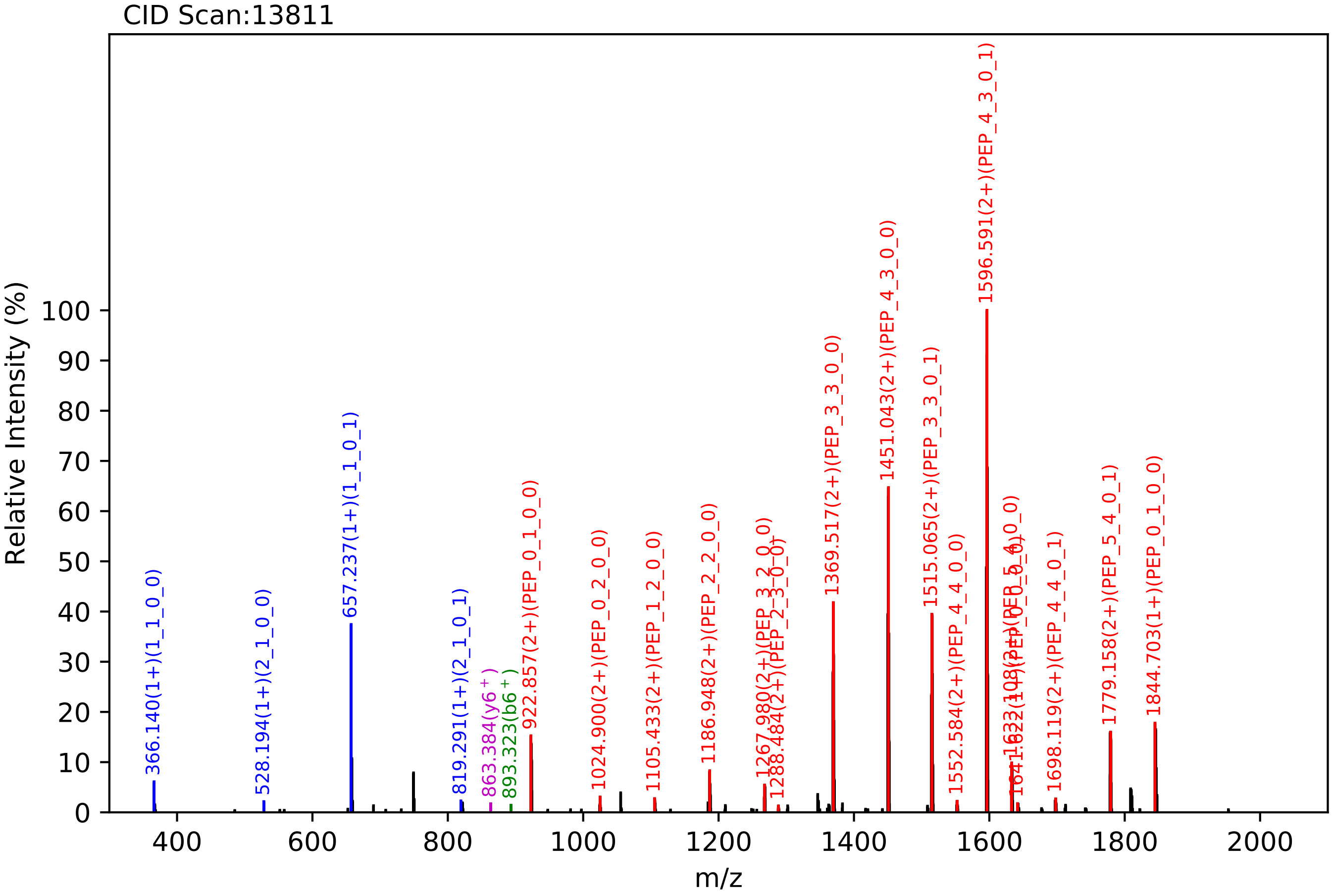

Unknown set no. 25, Experiment: Human Plasma exp_1

ENGTISR(=PEP)_5_4_0_2, m/z:994.40(3+), RT:23.98, Y-score:92.32

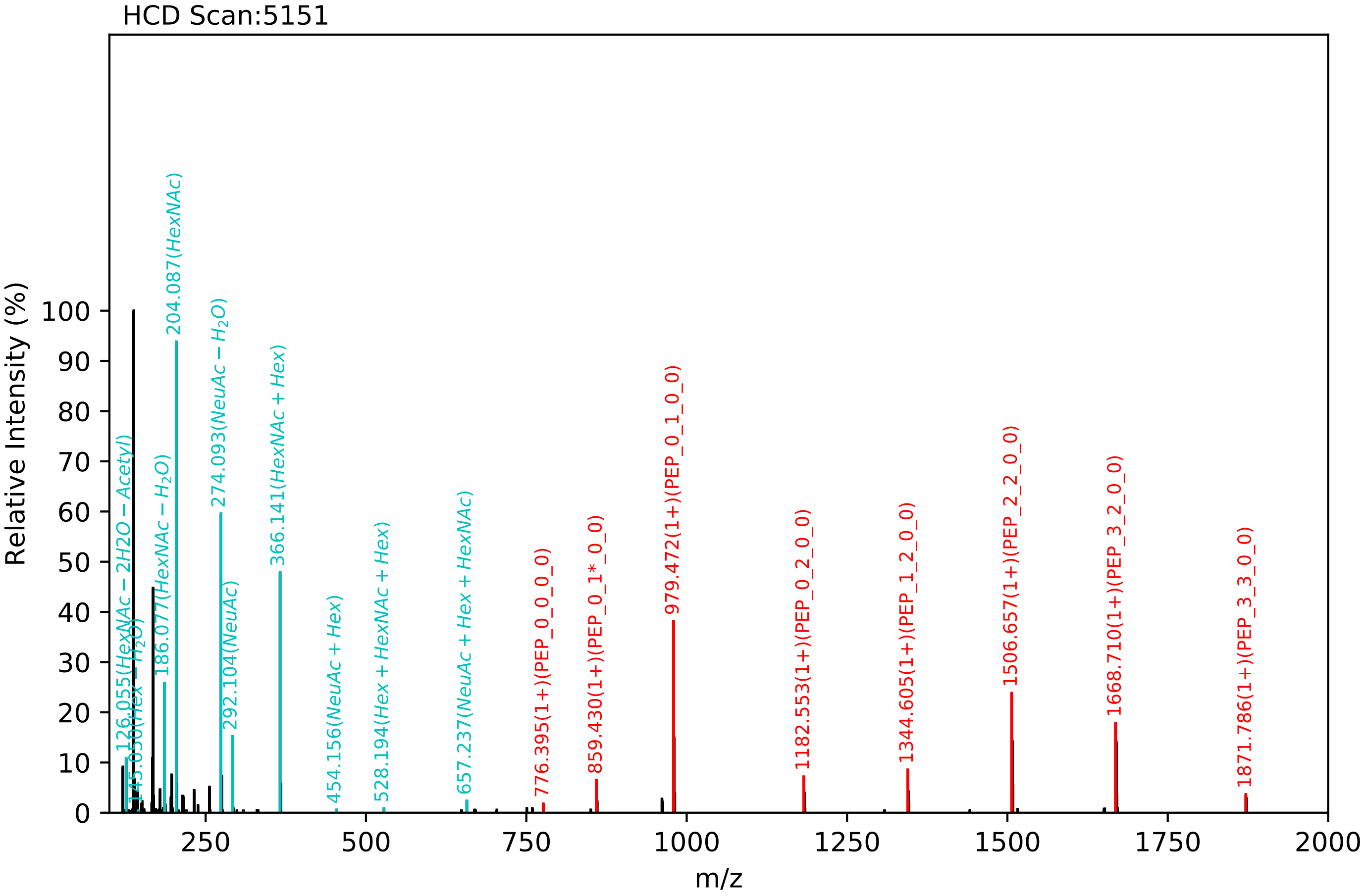

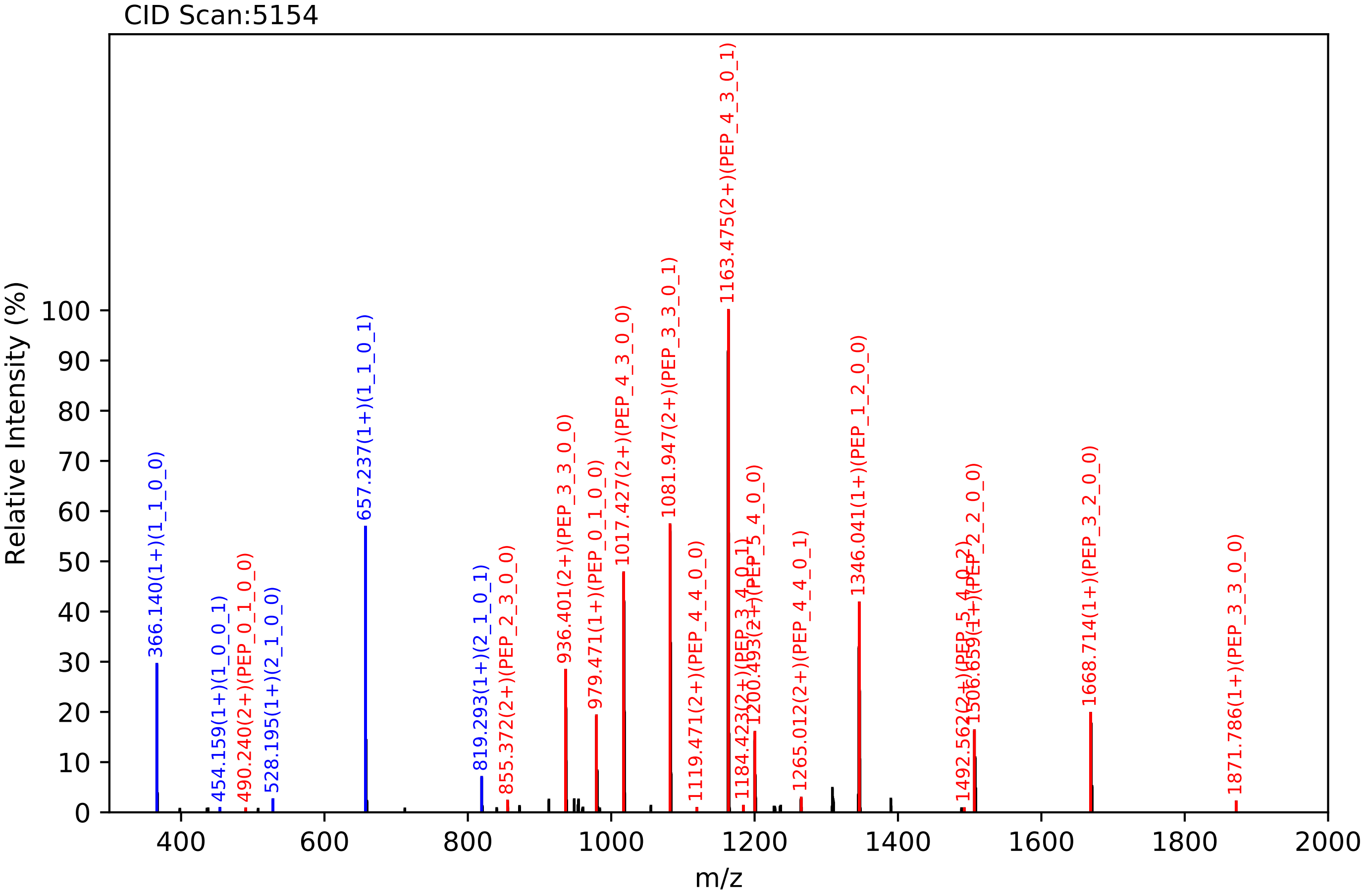

Unknown set no. 26, Experiment: Human Plasma exp_2

ENGTISR(=PEP)_5_4_0_2, m/z:994.40(3+), RT:24.14, Y-score:94.32

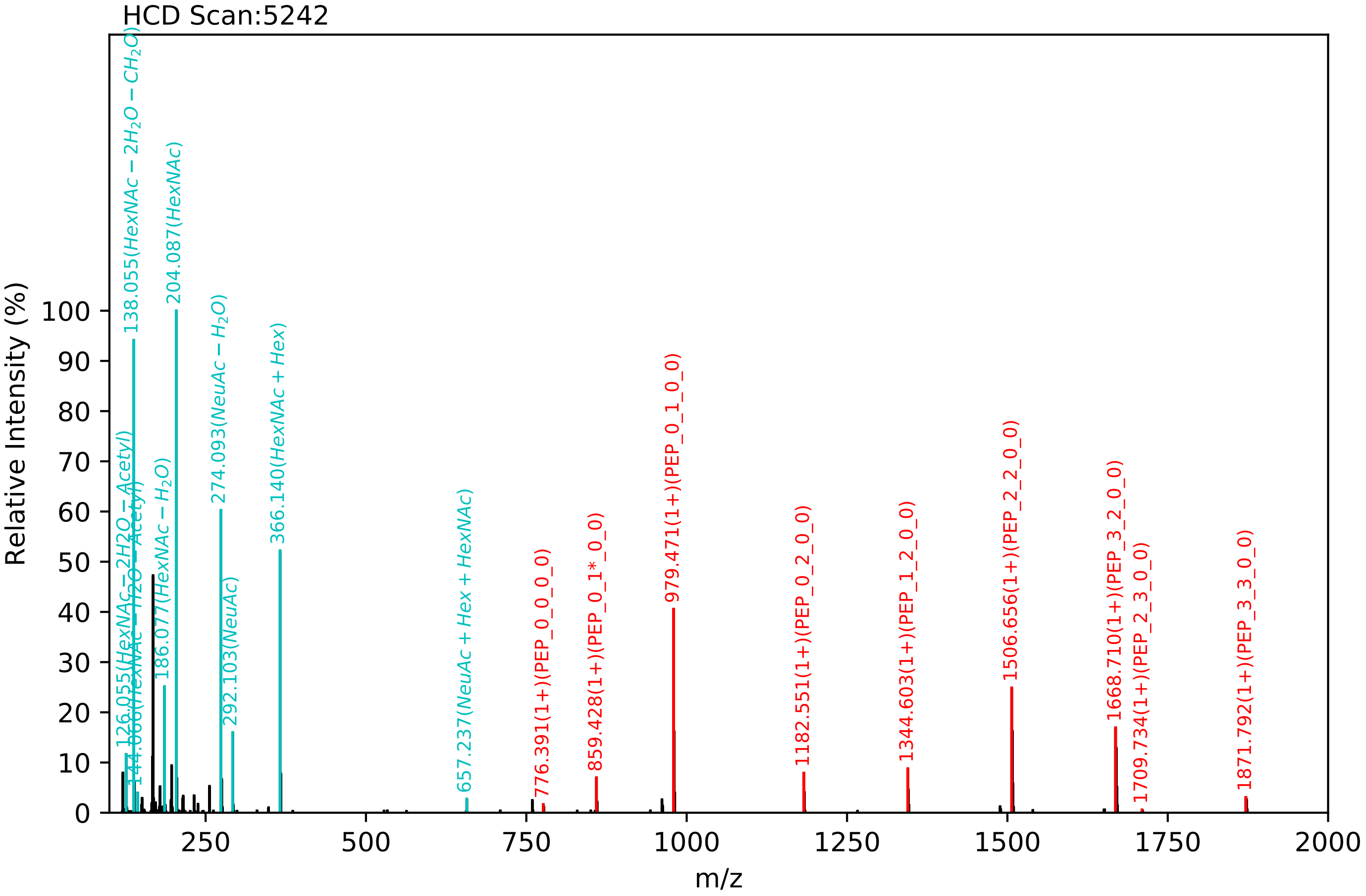

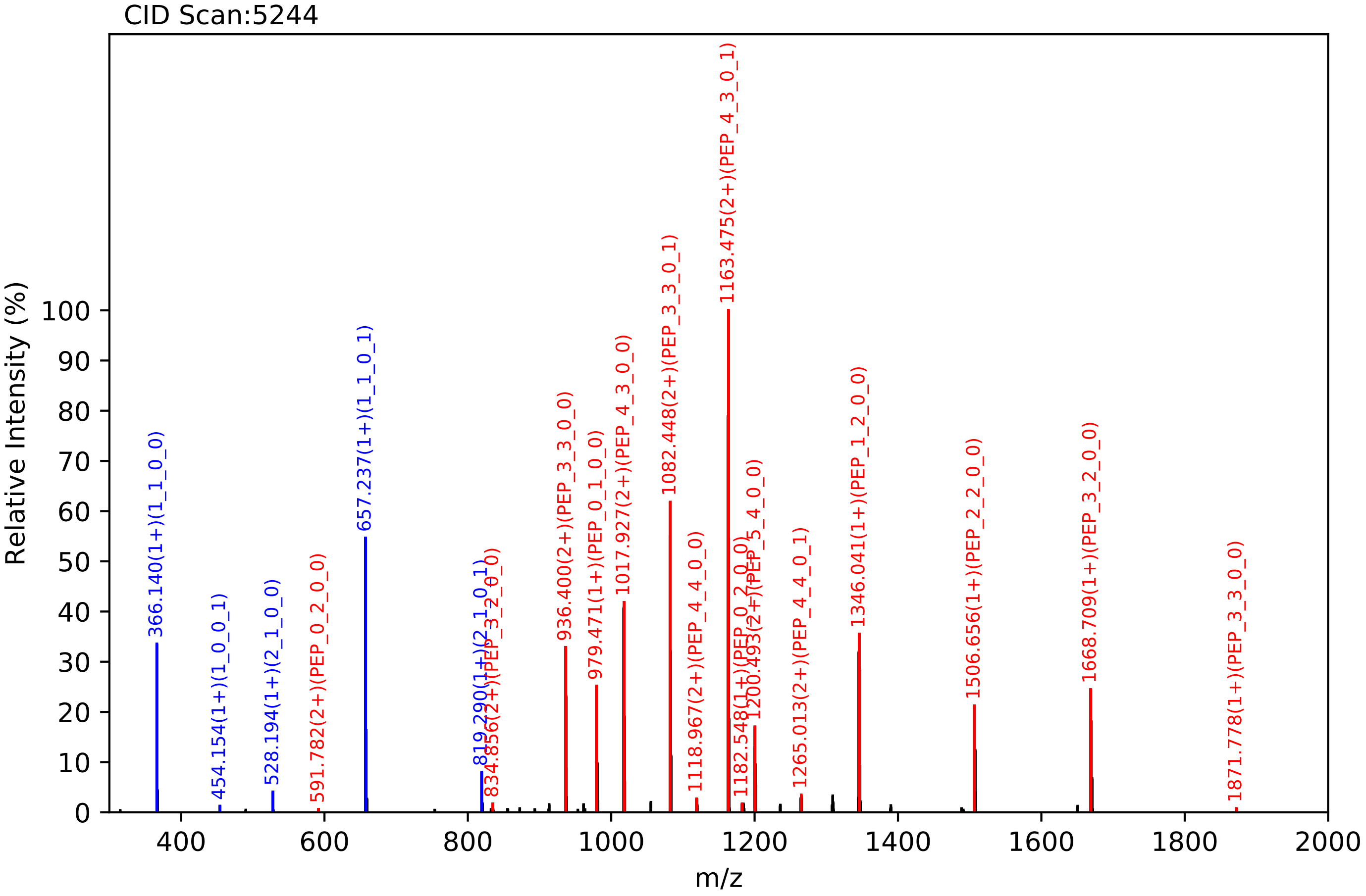

Unknown set no. 27, Experiment: Human Plasma exp_3

ENGTISR(=PEP)_5_4_0_2, m/z:994.40(3+), RT:24.34, Y-score:91.46

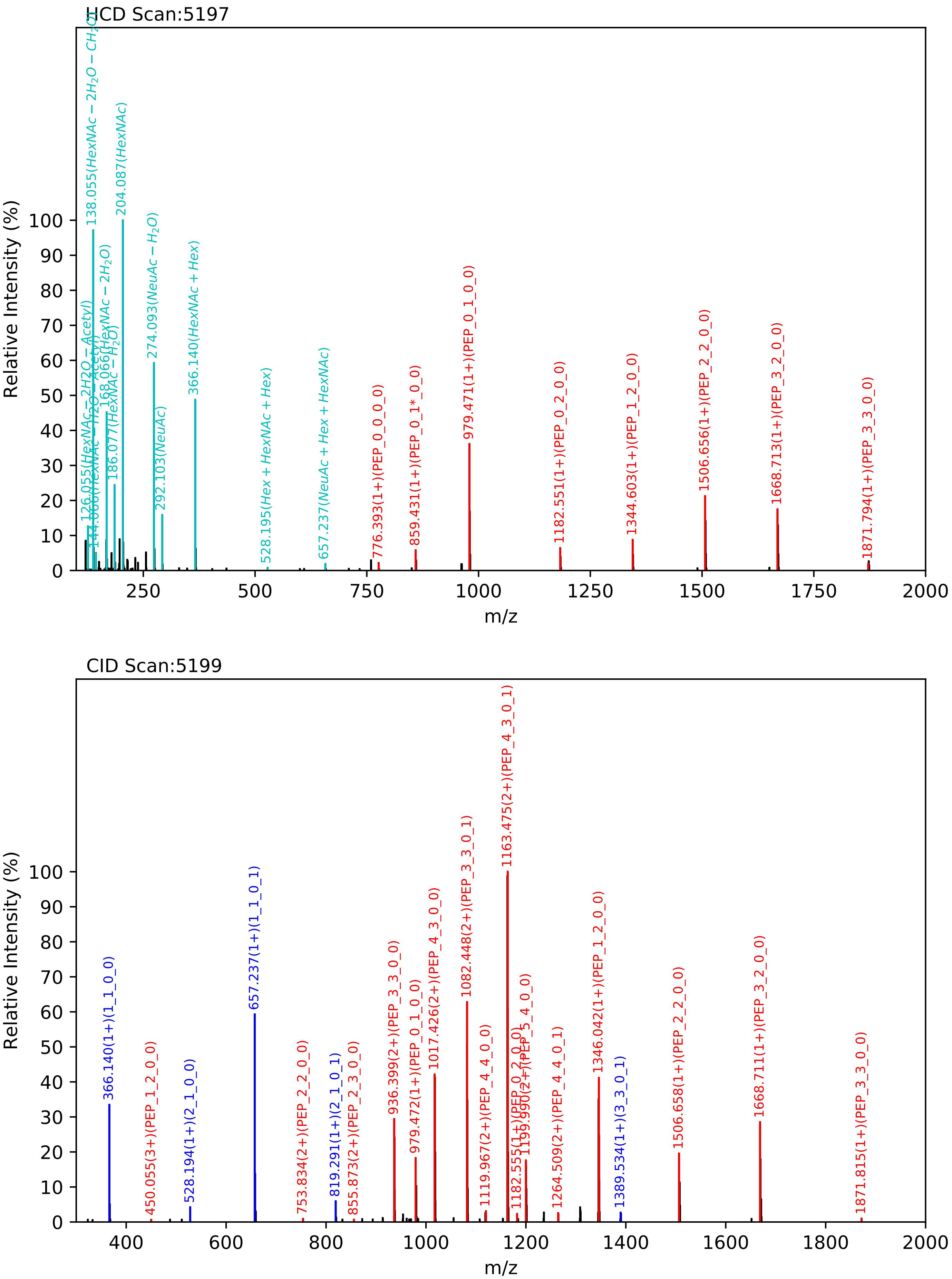

Unknown set no. 28, Experiment: Human Plasma exp_4

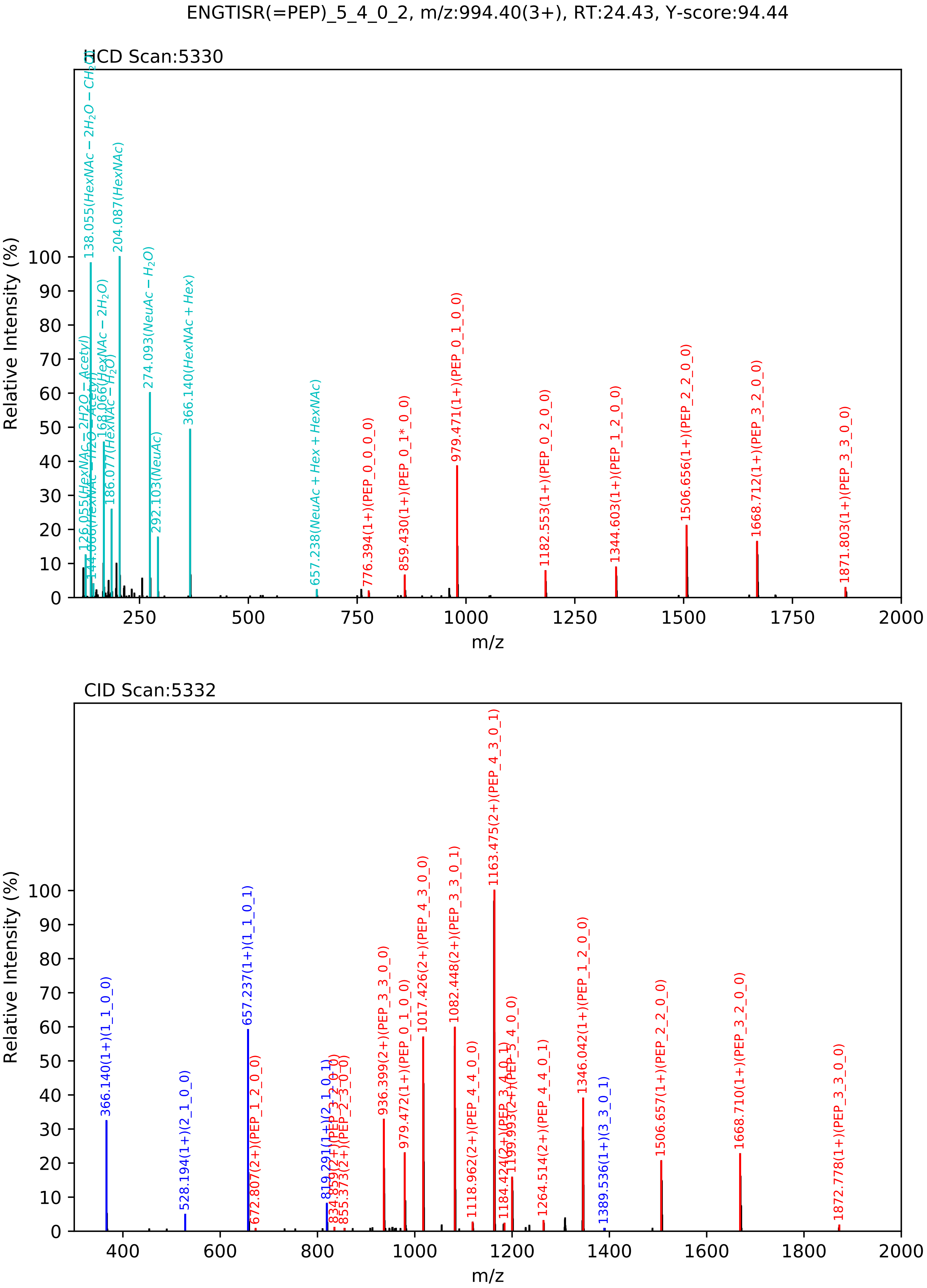

Unknown set no. 29, Experiment: Human Plasma exp_5

ENGTISR(=PEP)_5_4_0_2, m/z:994.40(3+), RT:24.41, Y-score:91.08

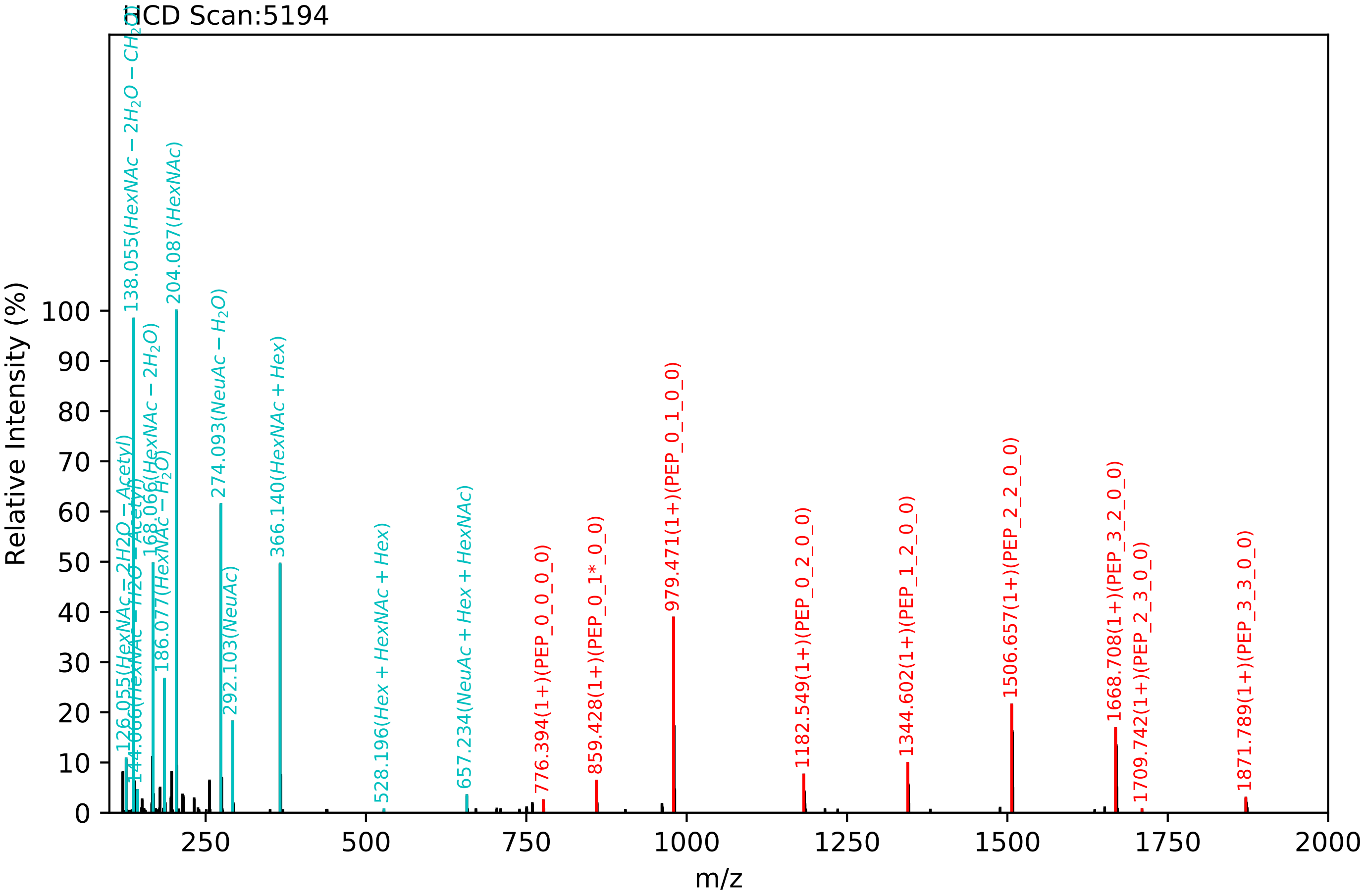

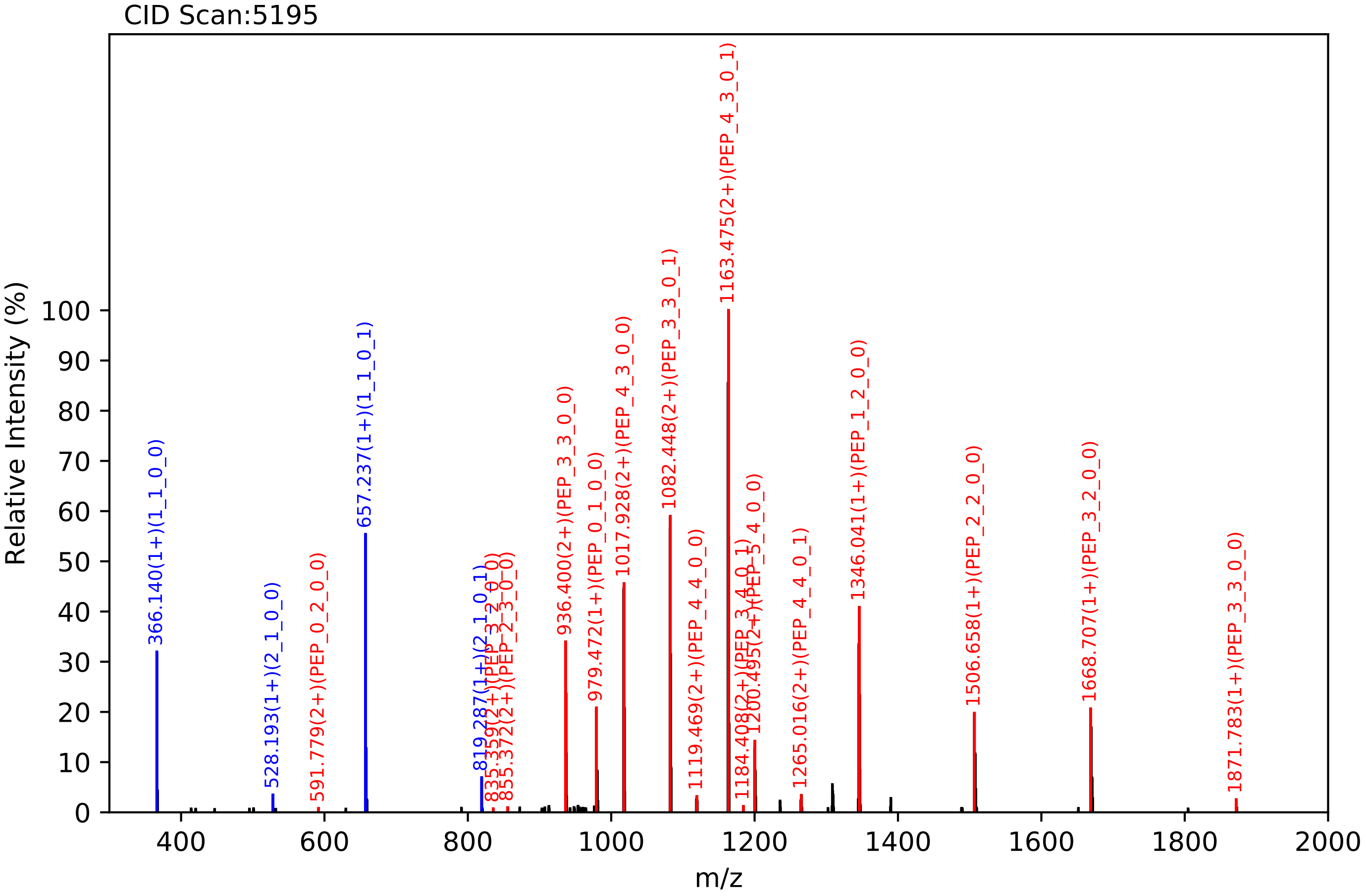

Unknown set no. 30, Experiment: Human Plasma exp_1

ENGTISR(=PEP)_5_4_1_2, m/z:1043.08(3+), RT:23.77, Y-score:80.88

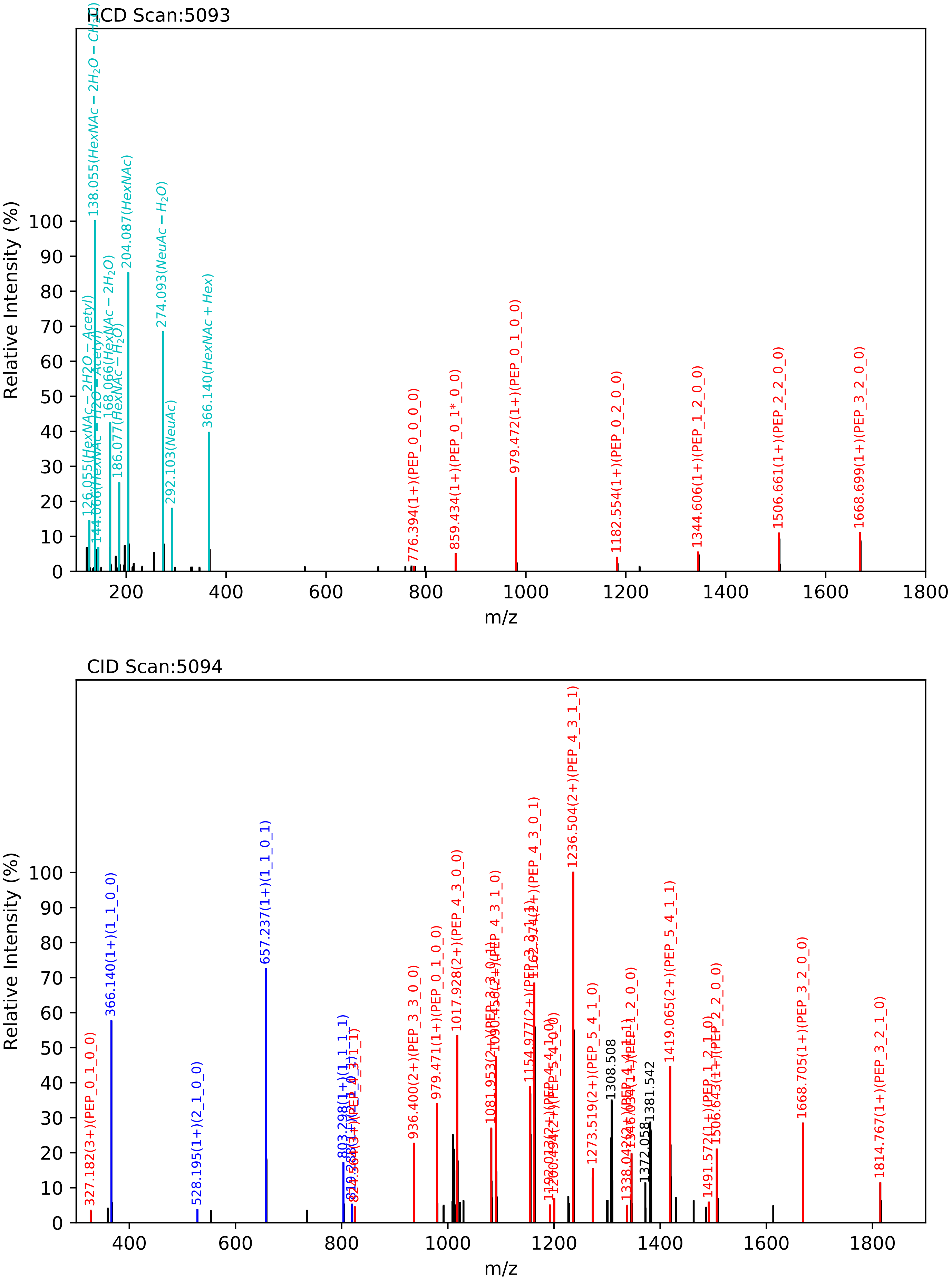

Unknown set no. 31, Experiment: Human Plasma exp_2

ENGTISR(=PEP)_5_4_1_2, m/z:1043.08(3+), RT:24.01, Y-score:79.50

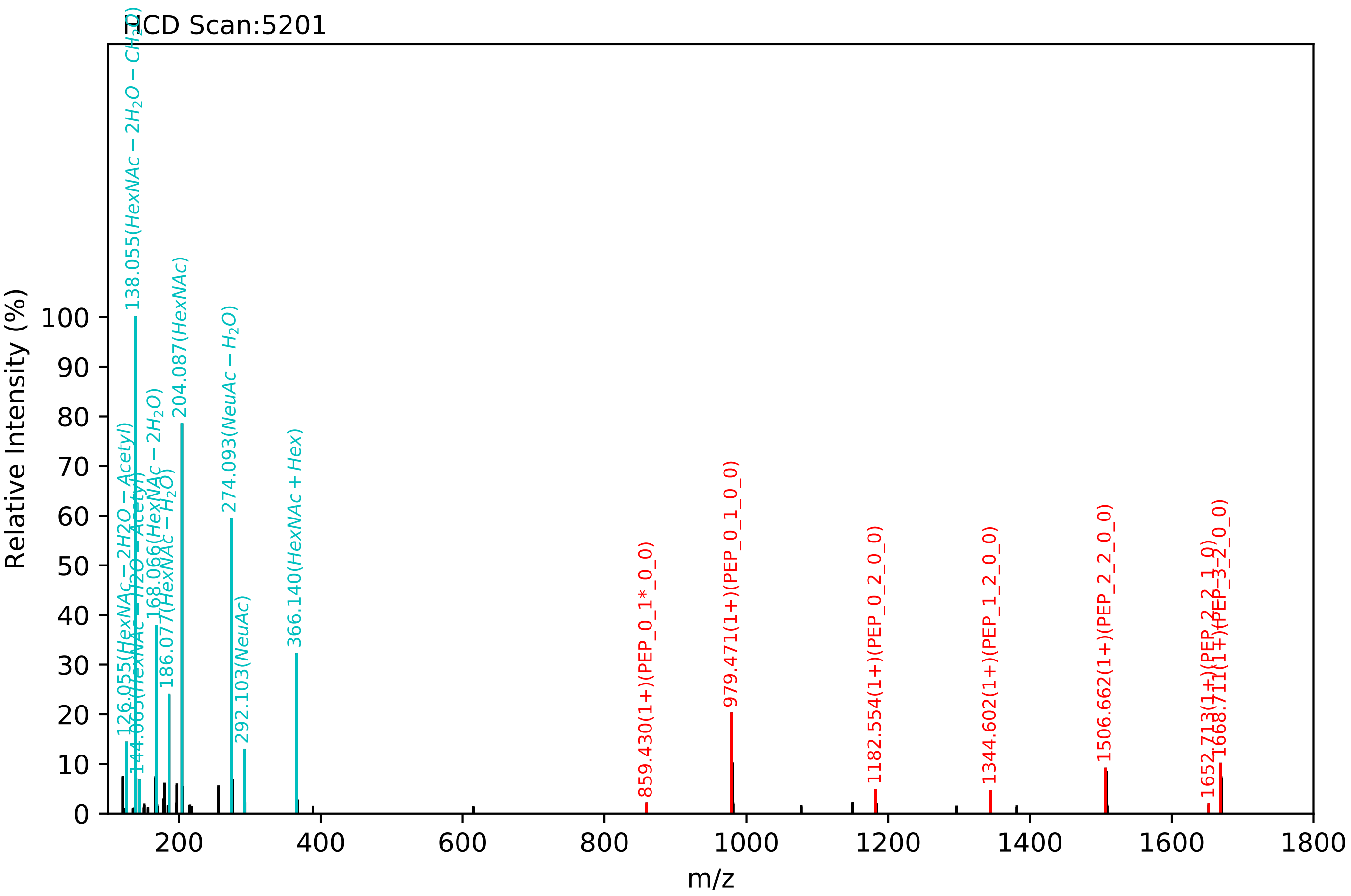

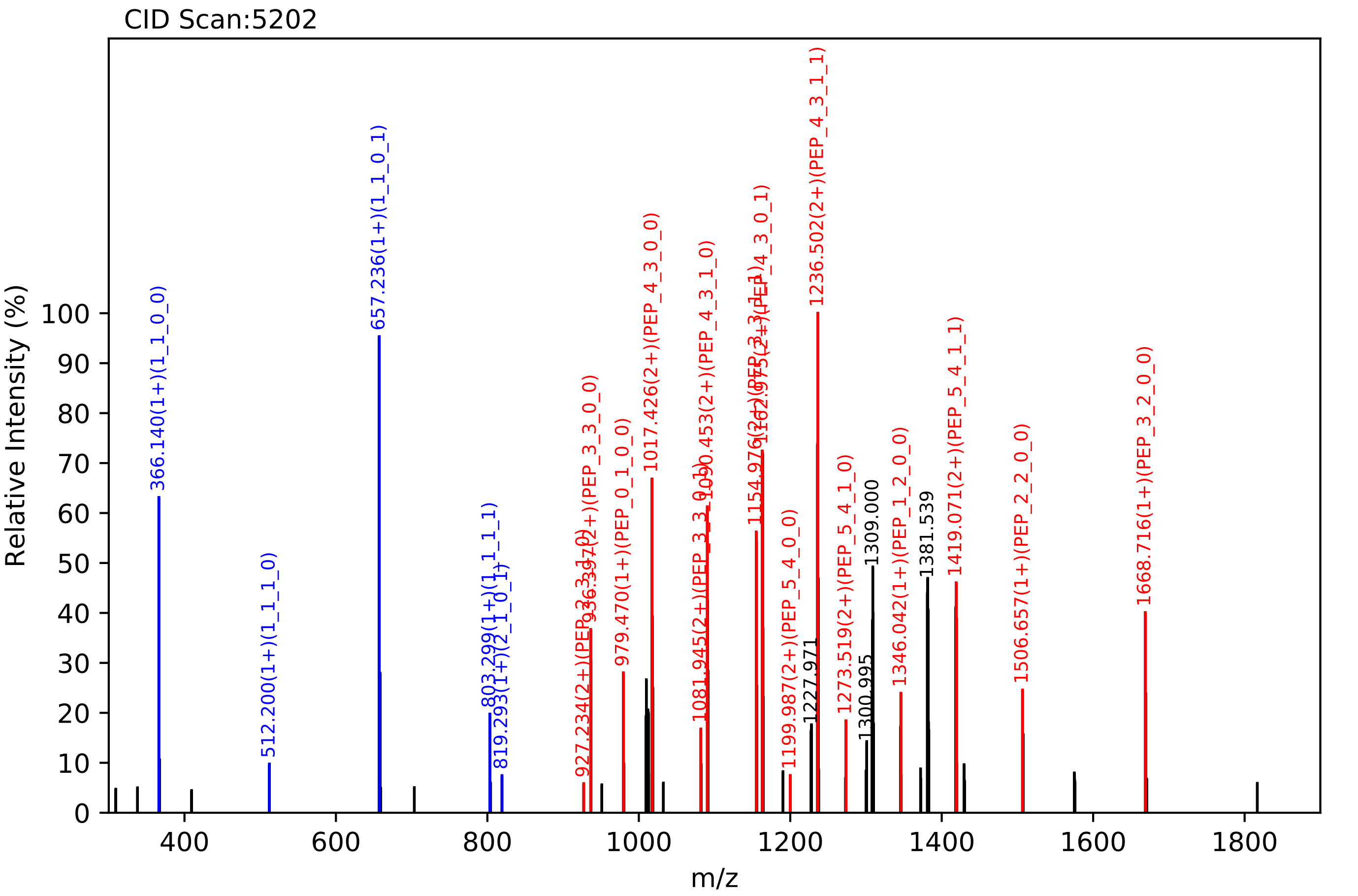

Unknown set no. 32, Experiment: Human Plasma exp_5

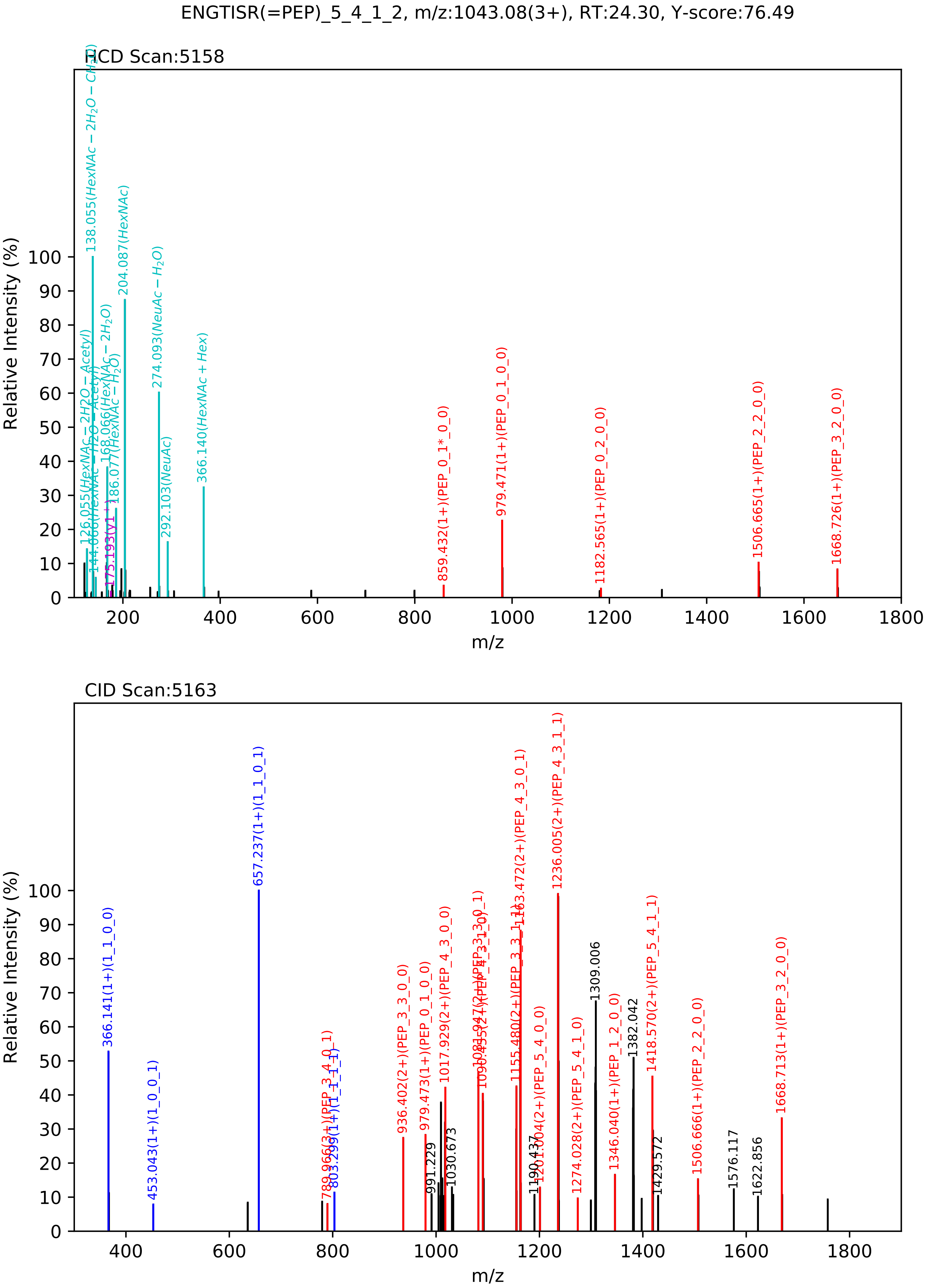

Unknown set no. 33, Experiment: Human Plasma exp_2

ENGTISR(=PEP)_6_5_0_3, m/z:910.10(4+), RT:38.17, Y-score:89.18

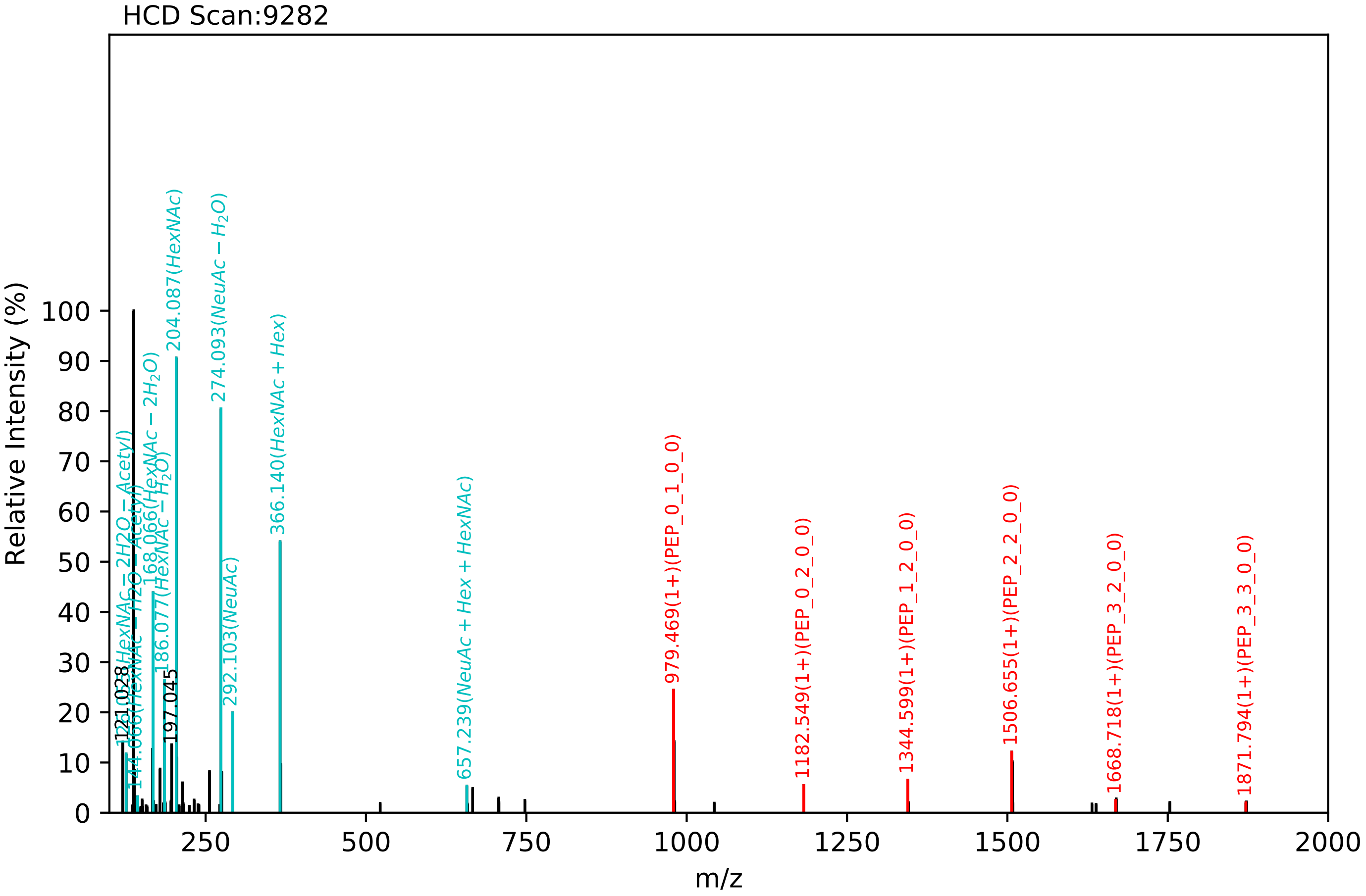

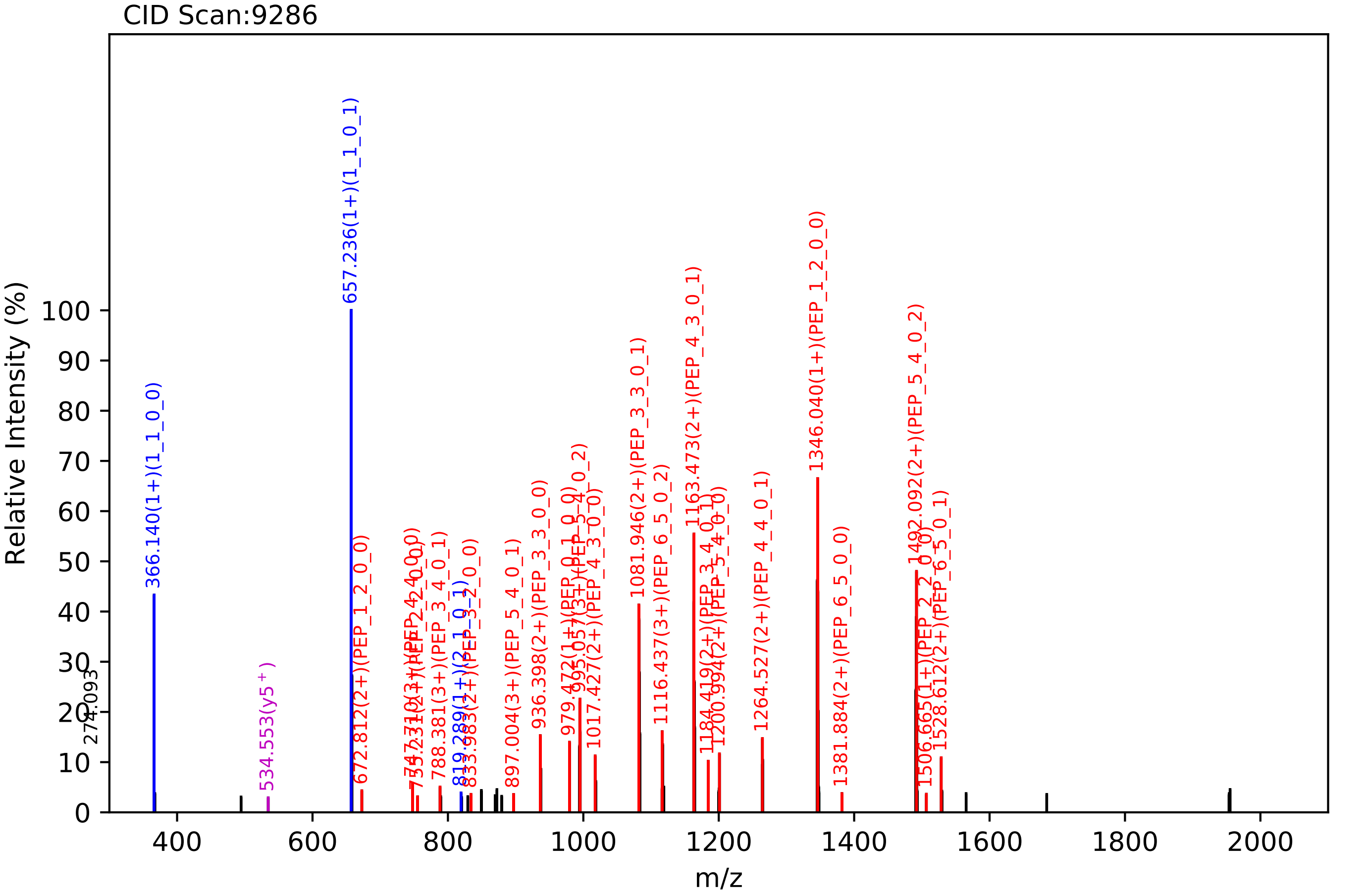

Unknown set no. 34, Experiment: Human Plasma exp_3

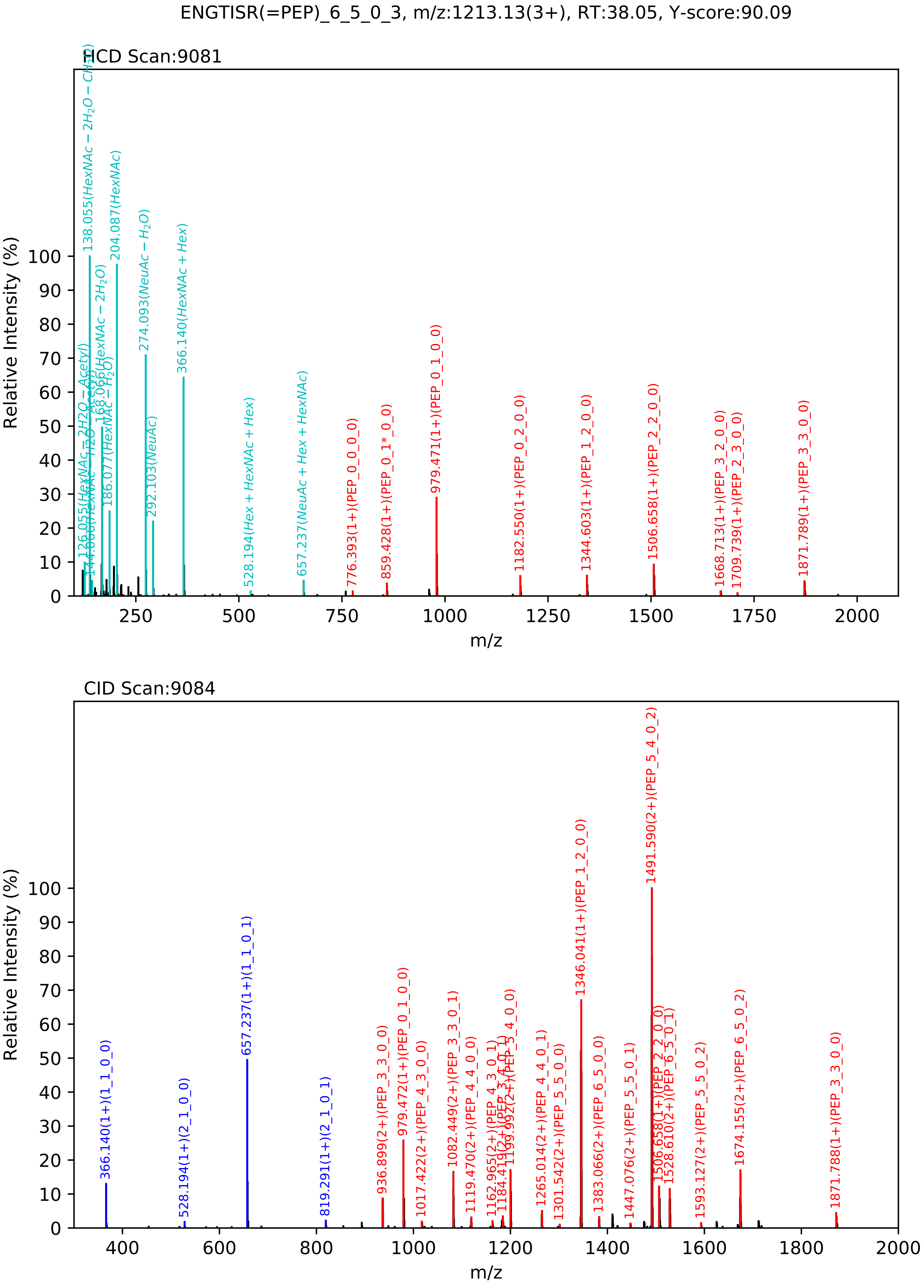

Unknown set no. 35, Experiment: Human Plasma exp_4

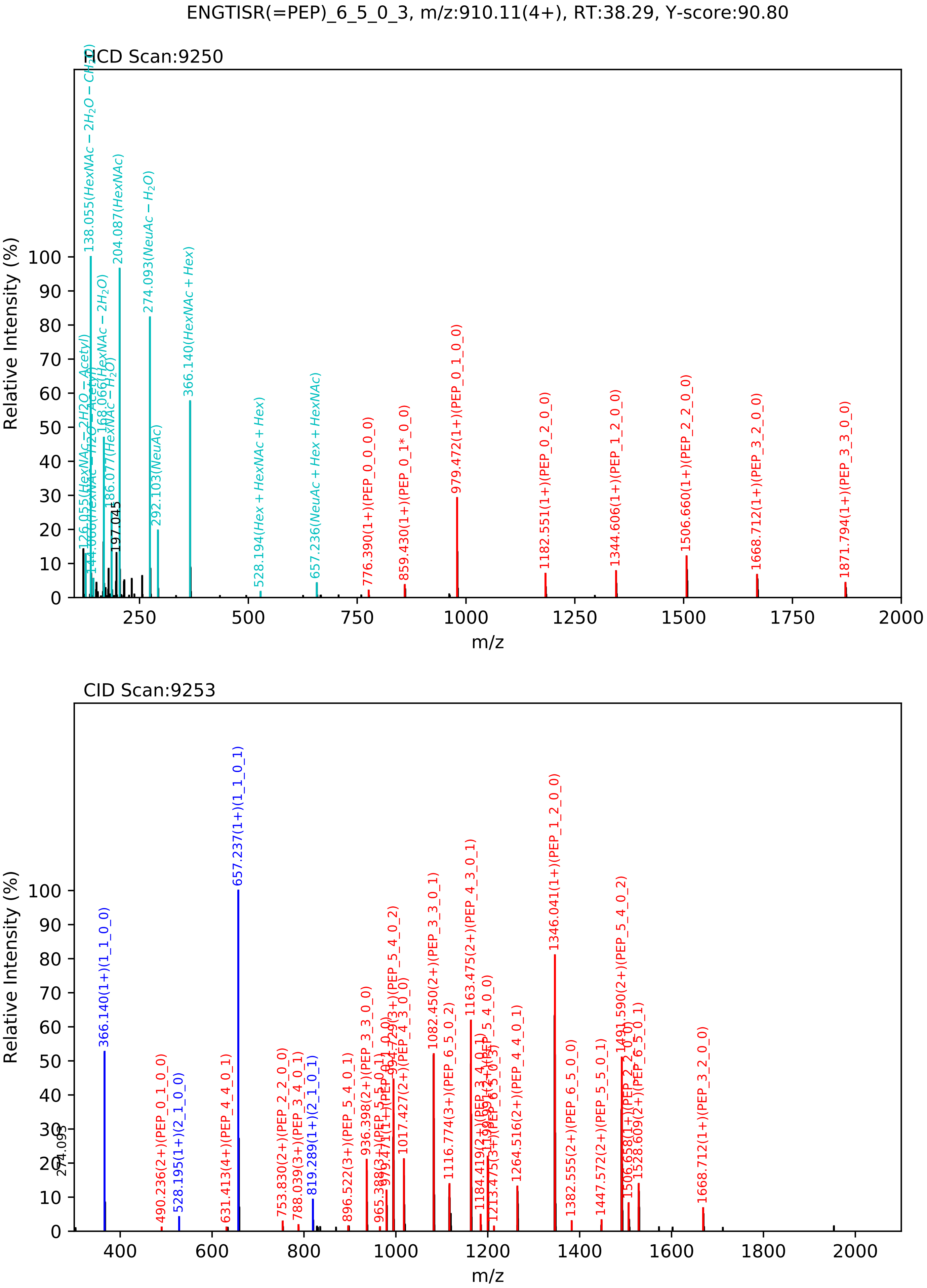

Unknown set no. 36, Experiment: Human Plasma exp_1

ENGTISR(=PEP)_6_5_1_3, m/z:1261.82(3+), RT:39.41, Y-score:93.76

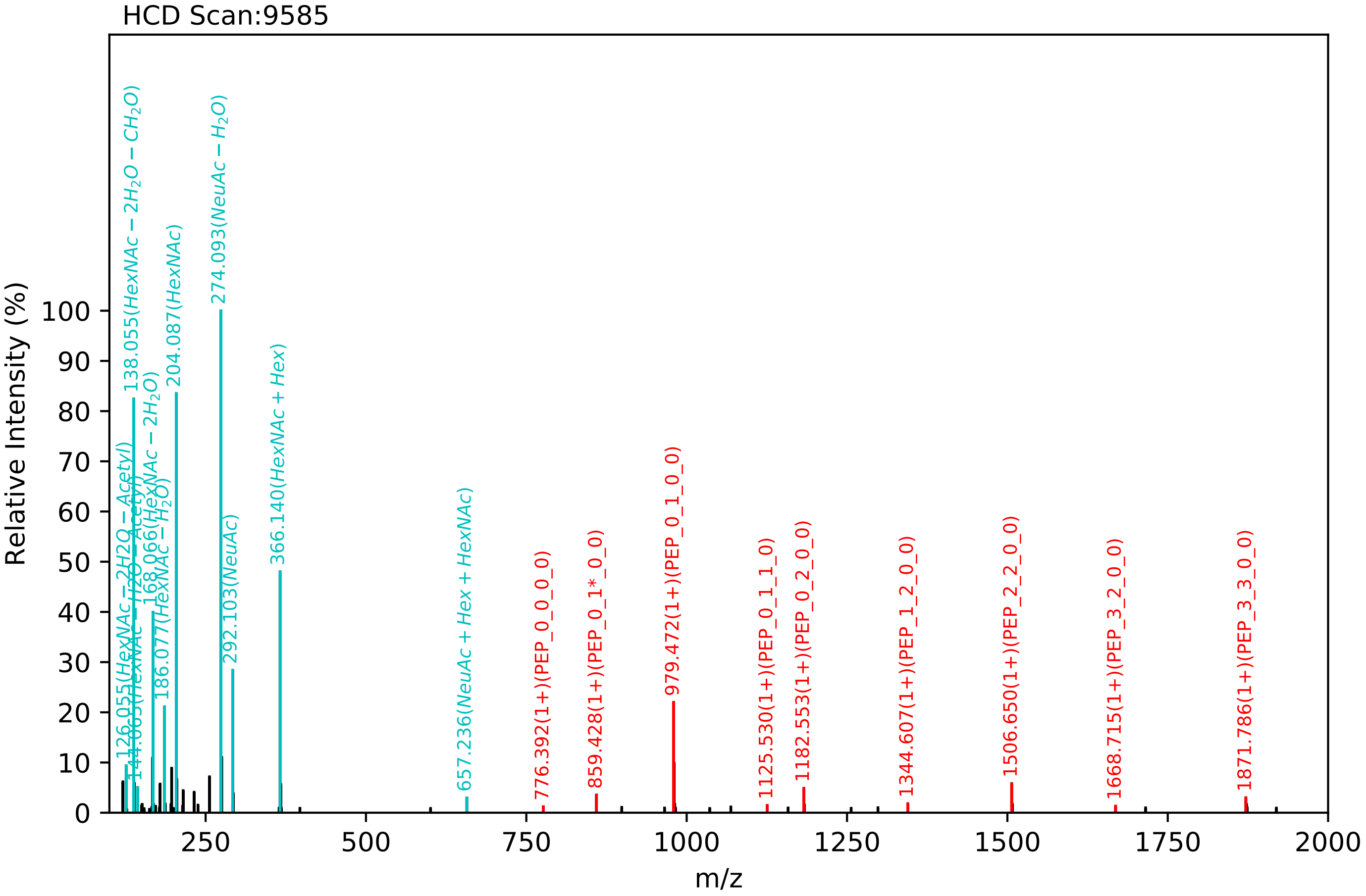

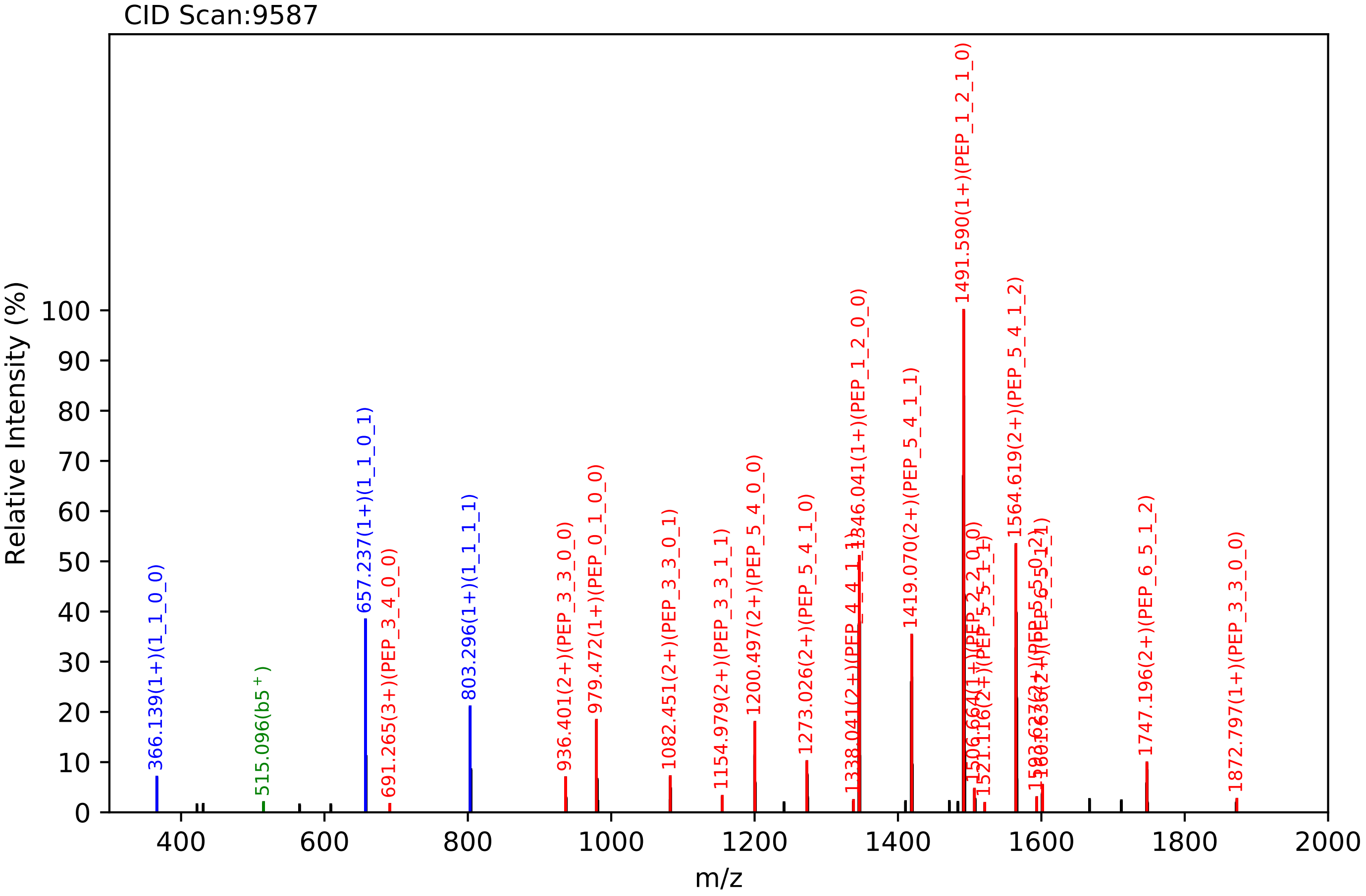

Unknown set no. 37, Experiment: Human Plasma exp_2

ENGTISR(=PEP)_6_5_1_3, m/z:946.62(4+), RT:37.31, Y-score:91.74

HCD Scan:8915

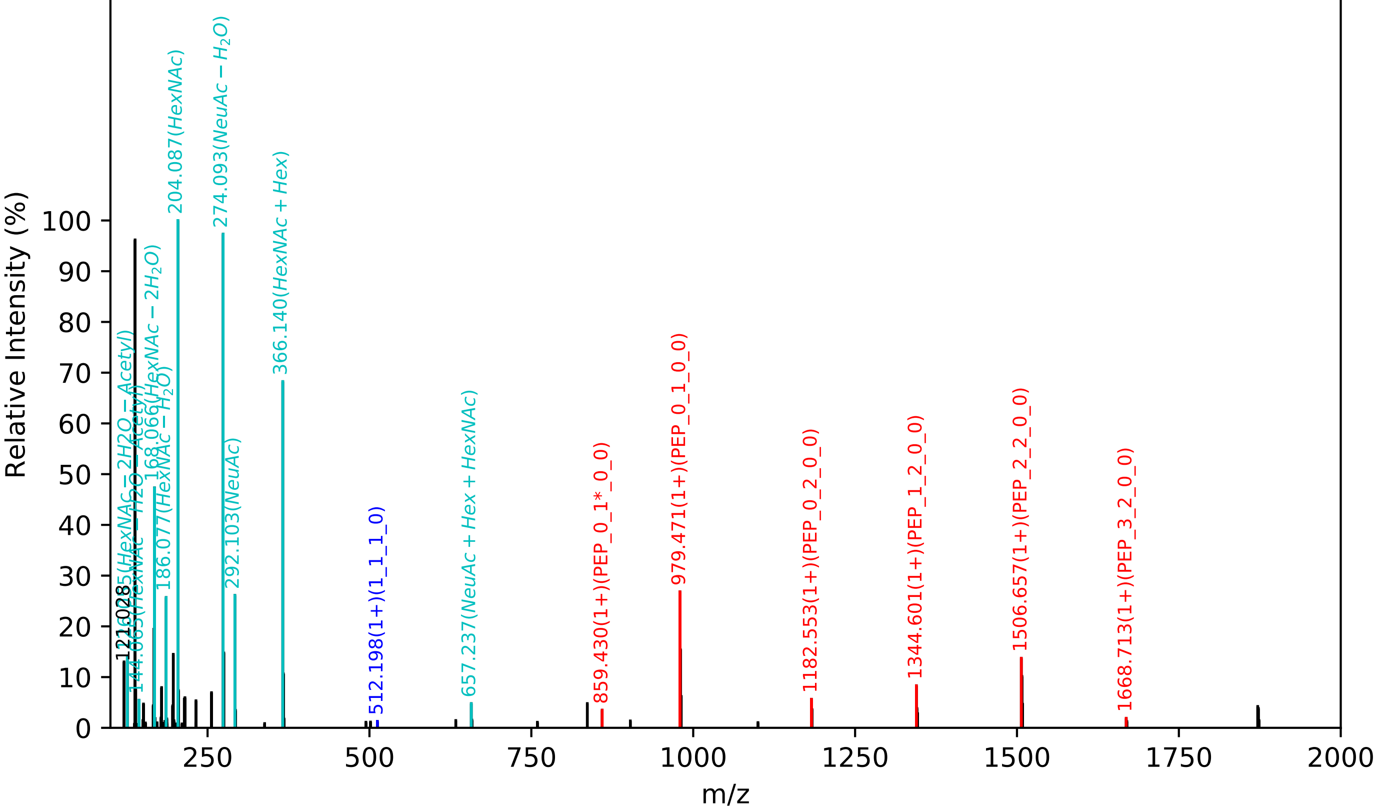

CID Scan:8919

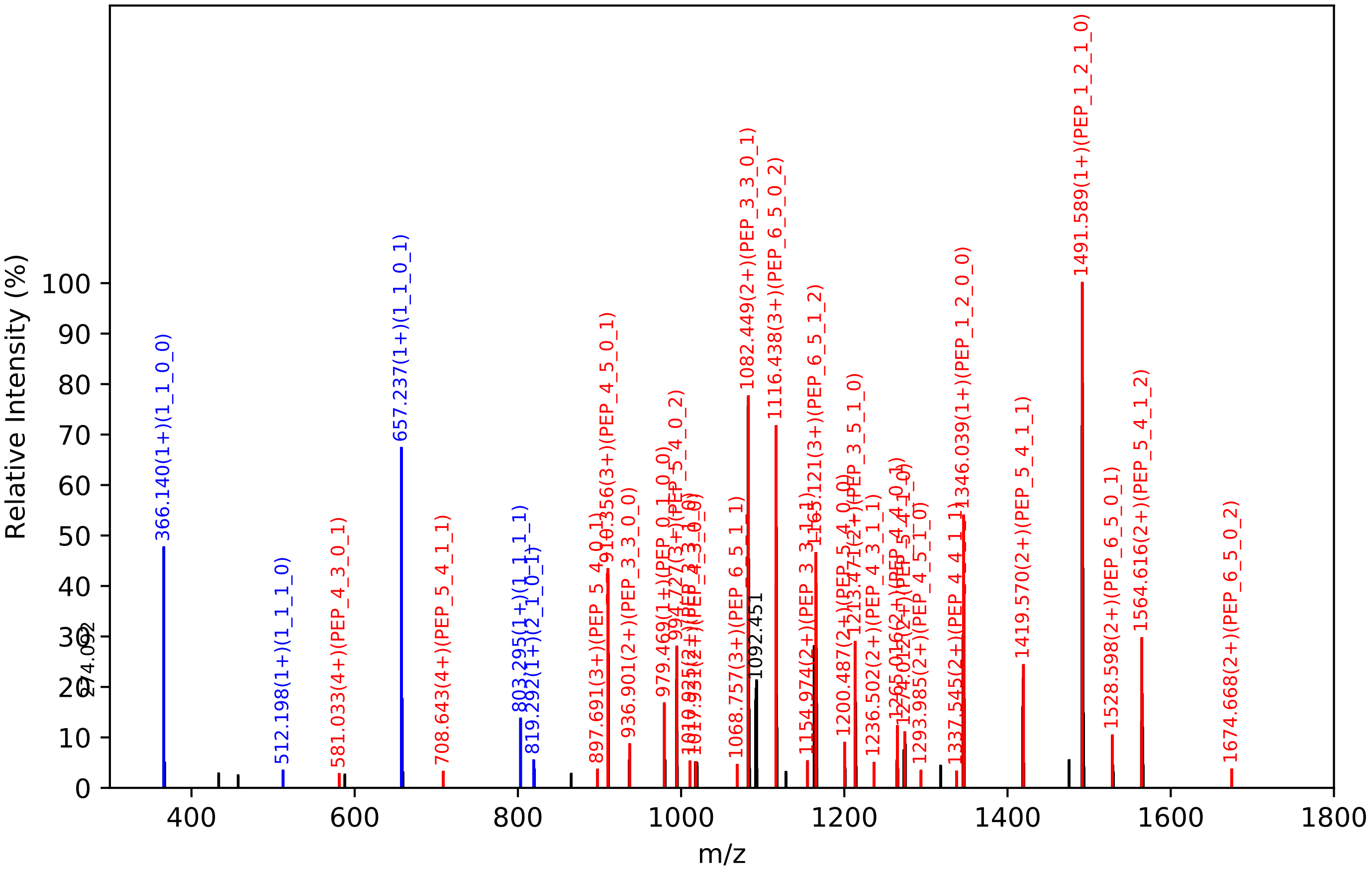

Unknown set no. 38, Experiment: Human Plasma exp_3

ENGTISR(=PEP)_6_5_1_3, m/z:946.62(4+), RT:37.60, Y-score:87.50

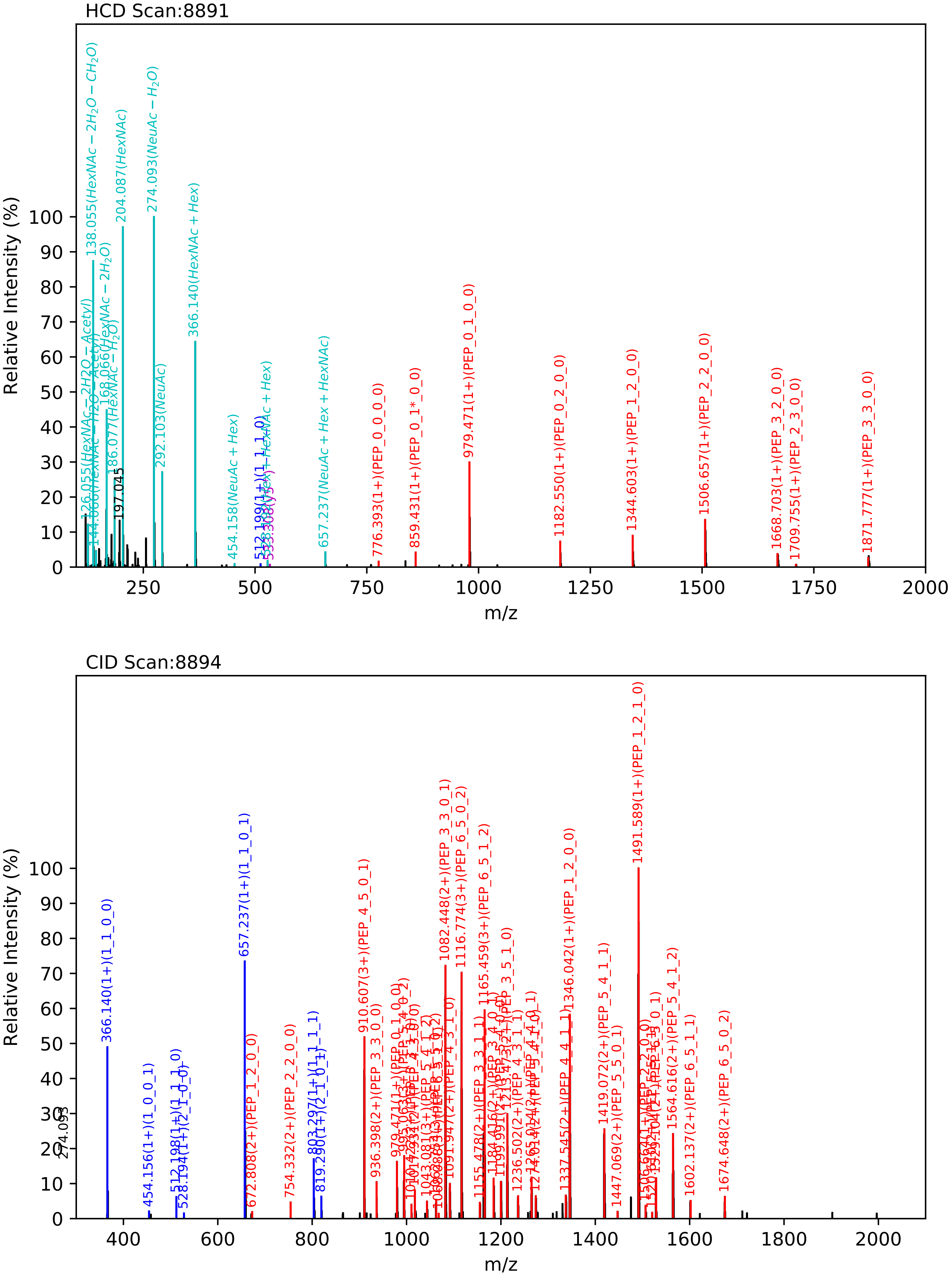

Unknown set no. 39, Experiment: Human Plasma exp_3

ENGTISR(=PEP)_6_5_1_3, m/z:1261.82(3+), RT:40.36, Y-score:95.92

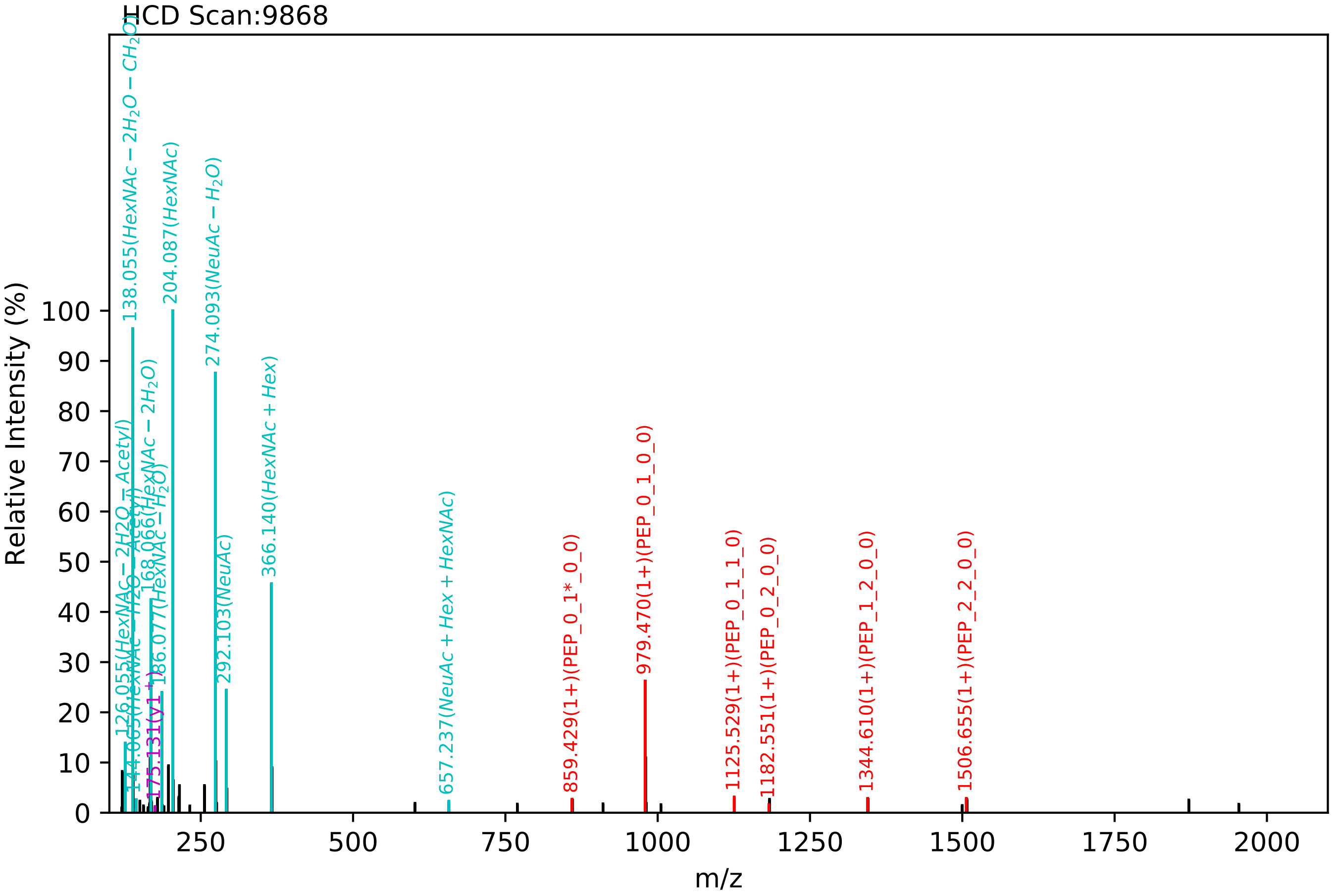

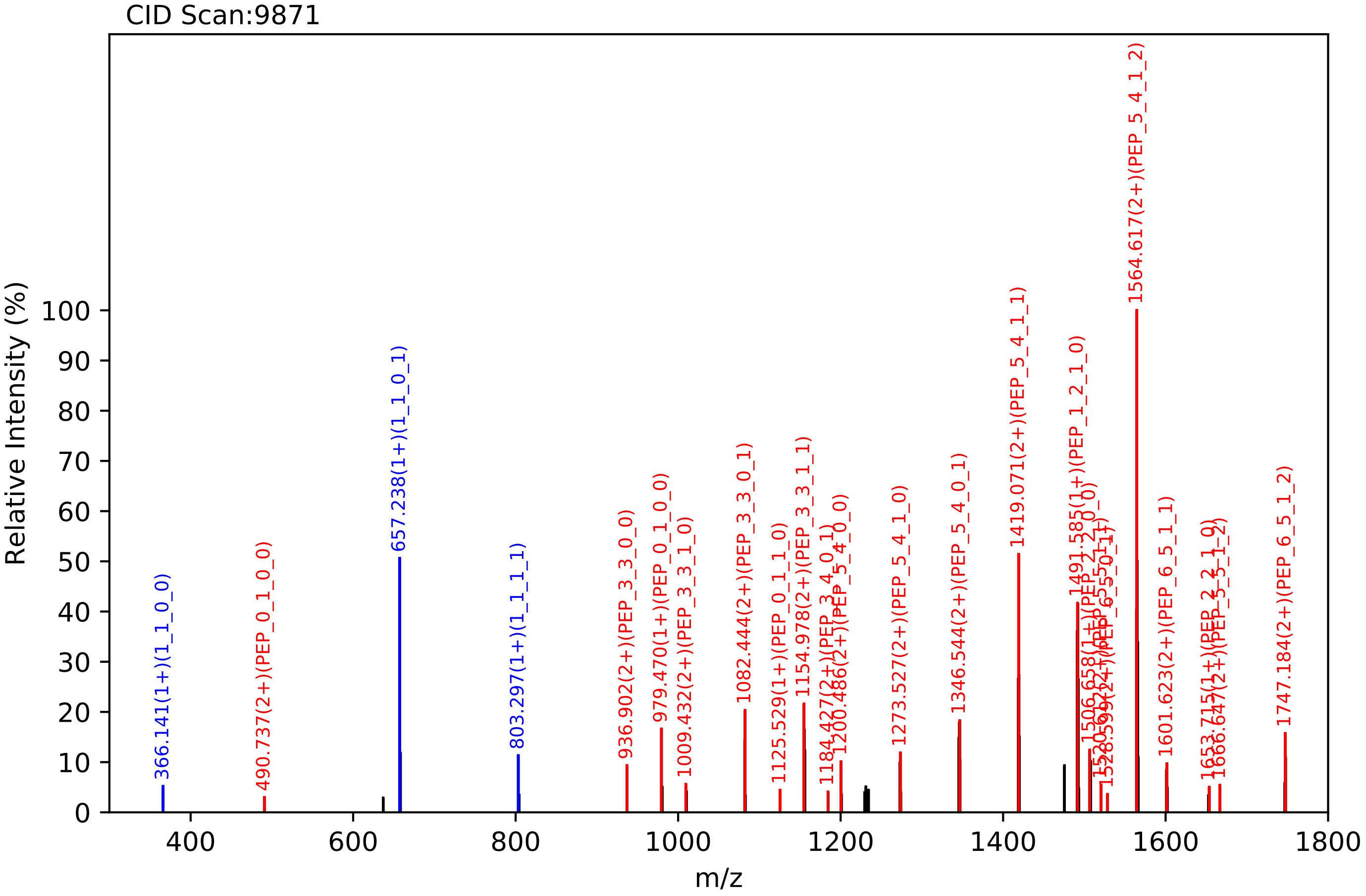

Unknown set no. 40, Experiment: Human Plasma exp_4

ENGTISR(=PEP)_6_5_1_3, m/z:946.62(4+), RT:37.65, Y-score:87.88

HCD Scan:8973

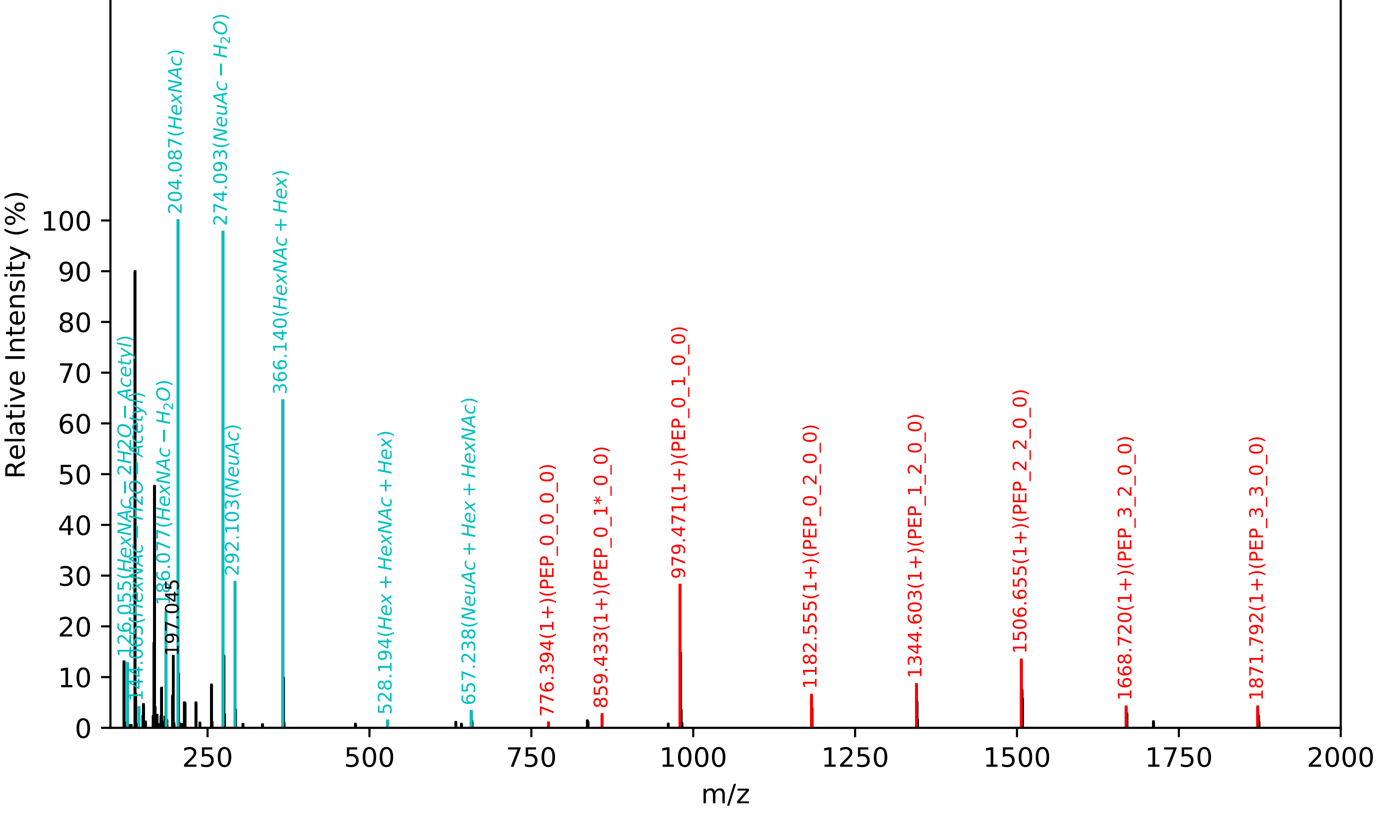

CID Scan:8976

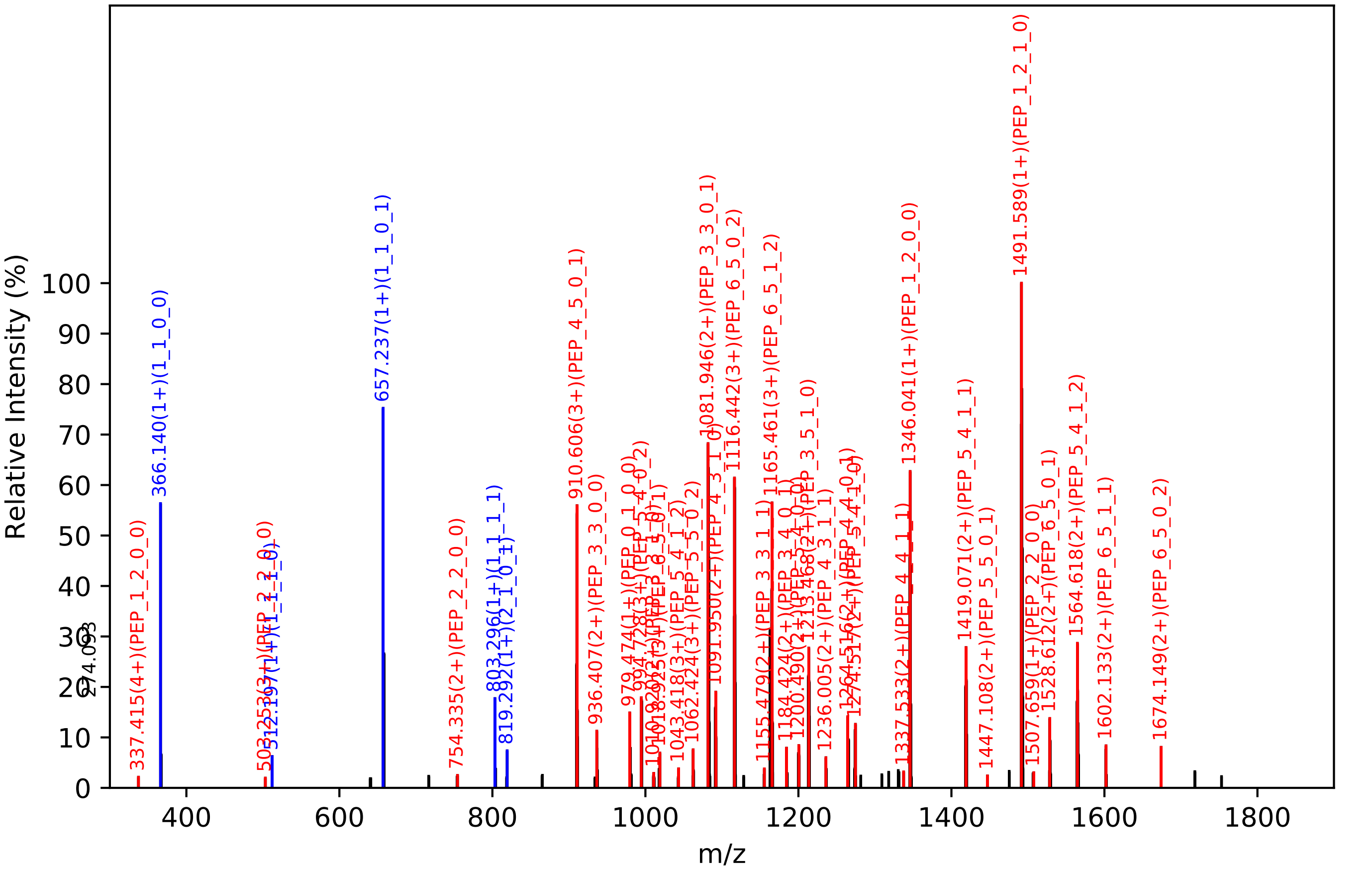

Unknown set no. 41, Experiment: Human Plasma exp_4

ENGTISR(=PEP)_6_5_1_3, m/z:1261.82(3+), RT:38.03, Y-score:91.02

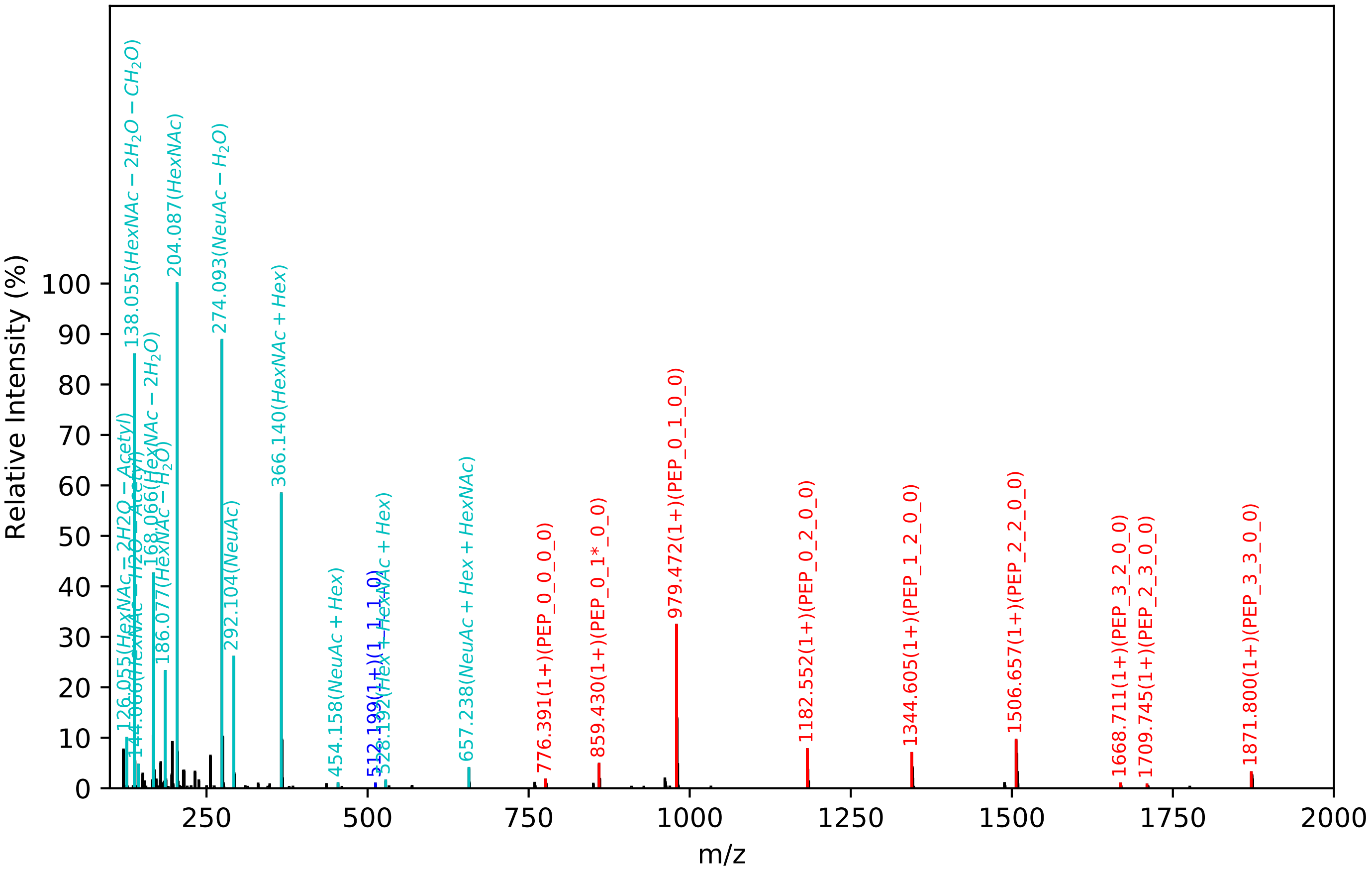

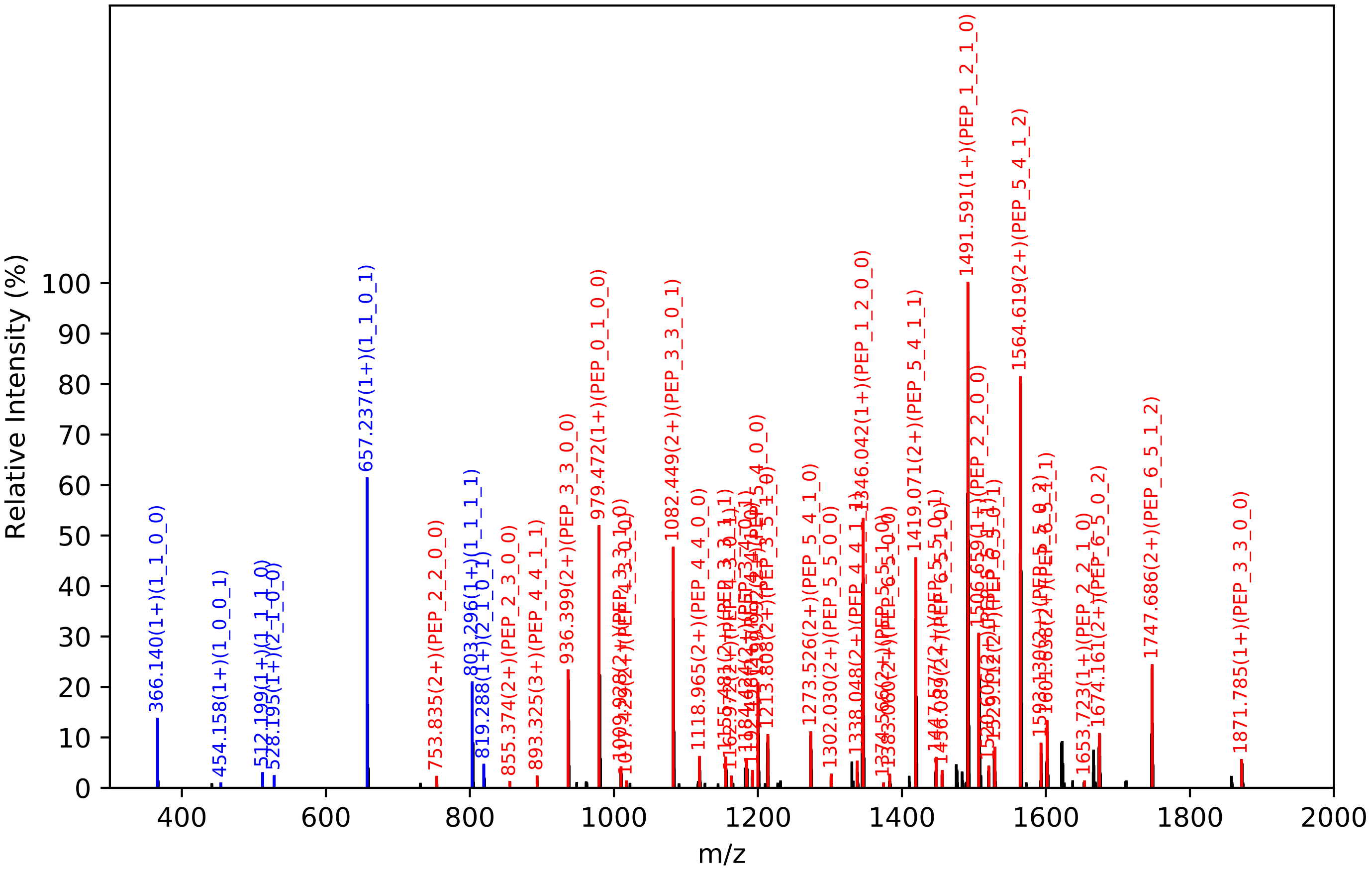

Unknown set no. 42, Experiment: Human Plasma exp_5

ENGTISR(=PEP)_6_5_1_3, m/z:946.62(4+), RT:37.69, Y-score:87.98

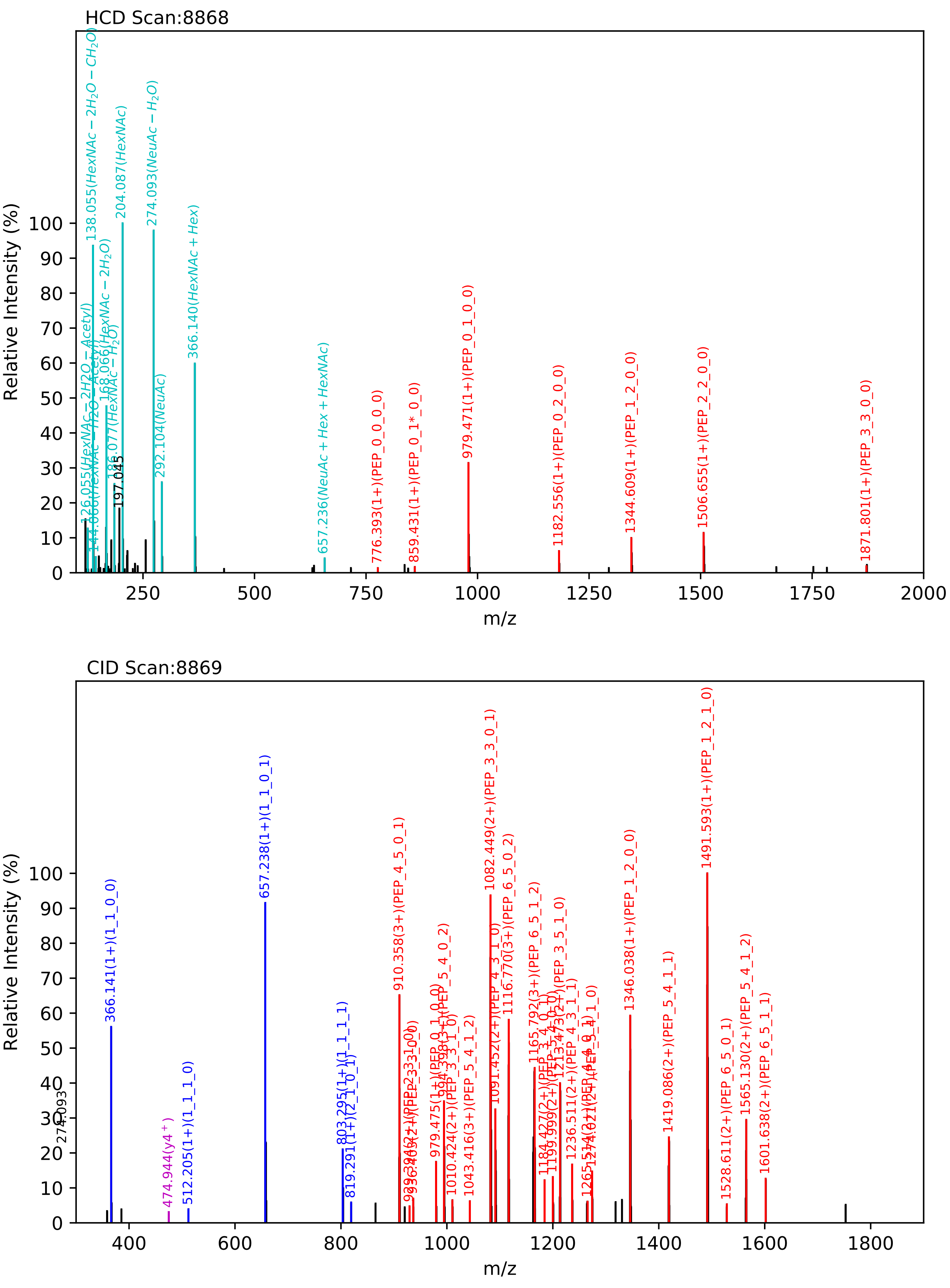

Unknown set no. 43, Experiment: Human Plasma exp_5

ENGTISR(=PEP)_6_5_1_3, m/z:1261.82(3+), RT:38.51, Y-score:94.37

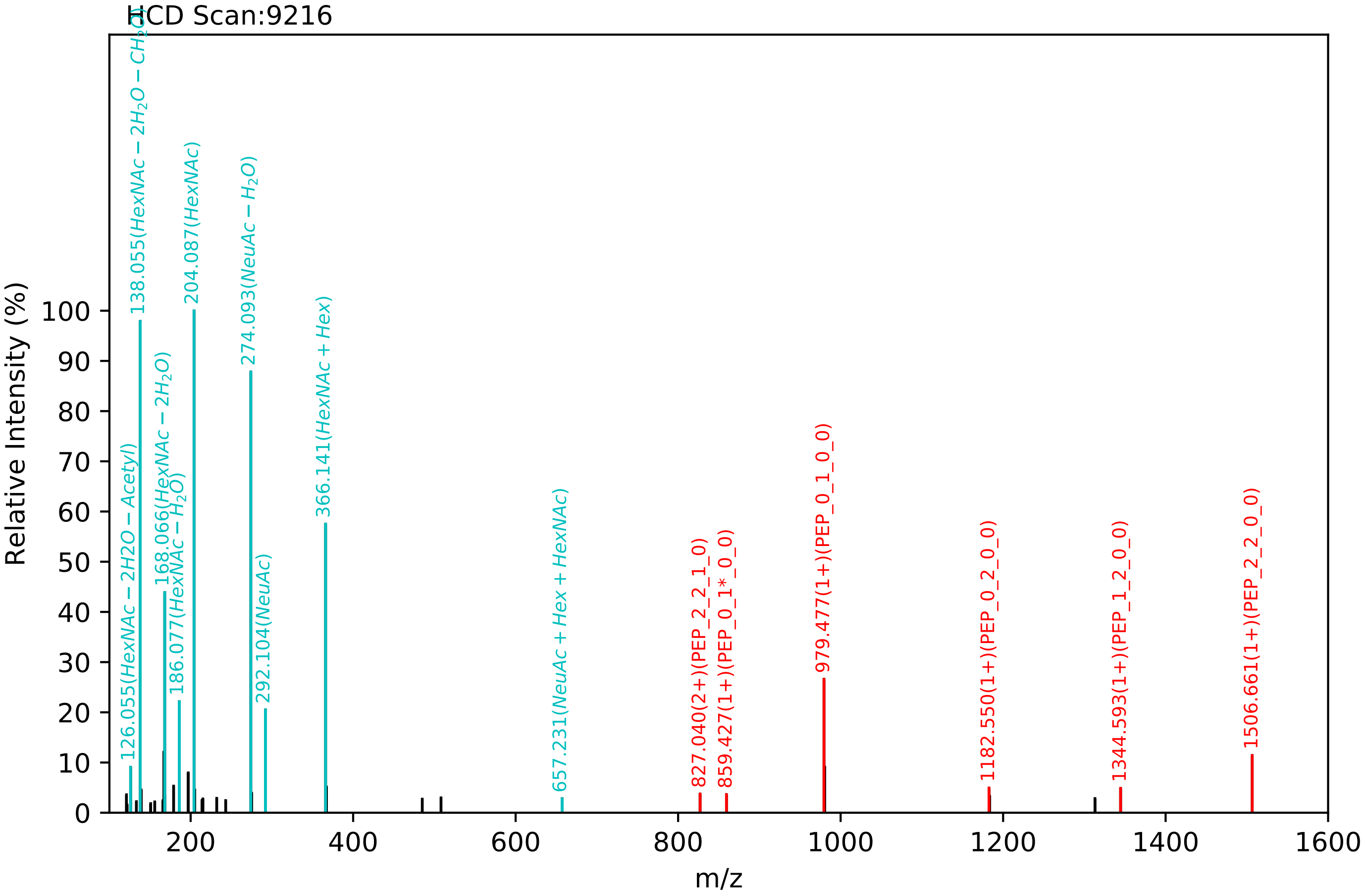

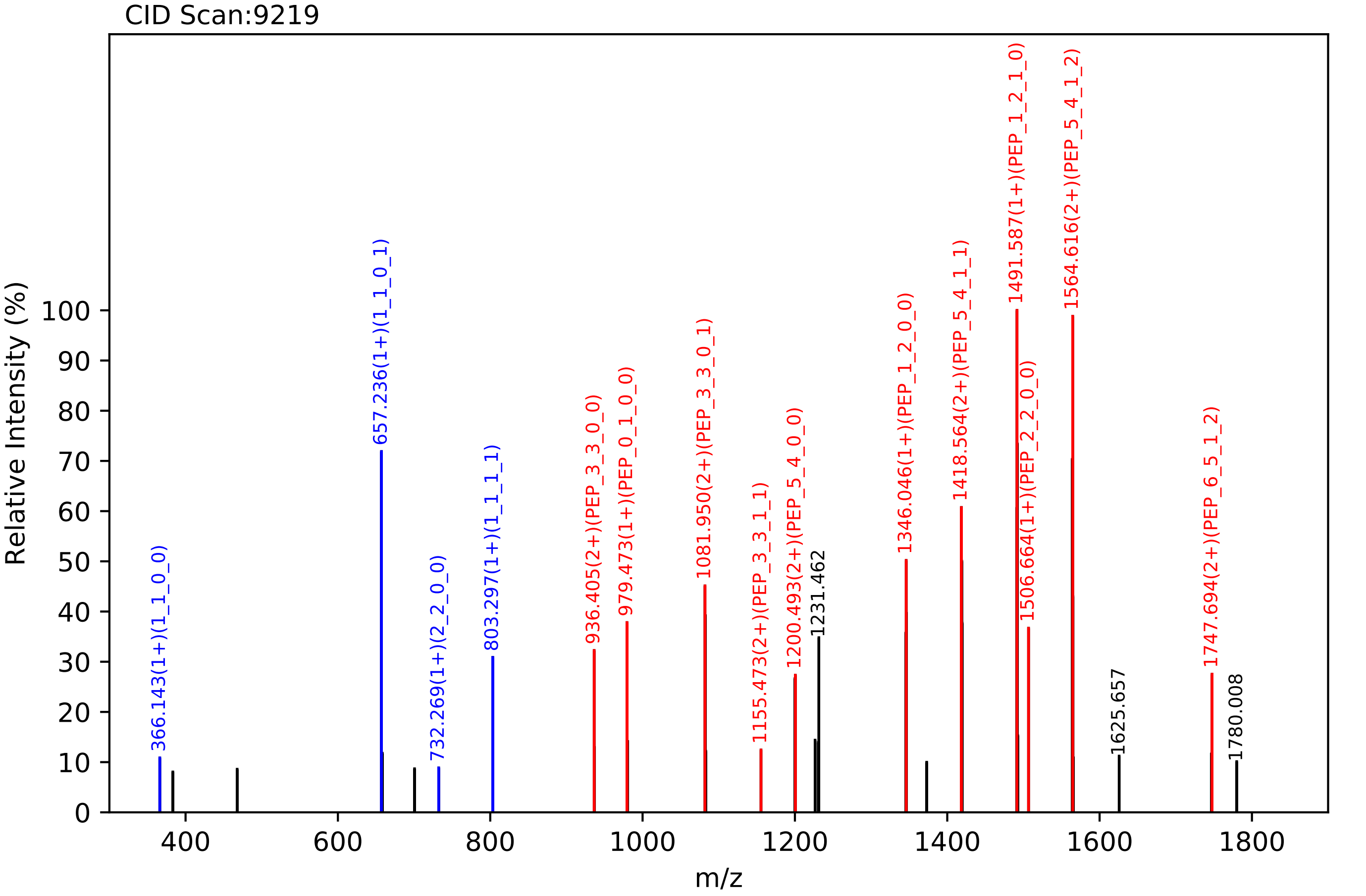

Unknown set no. 44, Experiment: Human Plasma exp_3

ENGTISR(=PEP)_7_6_1_4, m/z:1110.67(4+), RT:54.11, Y-score:90.77

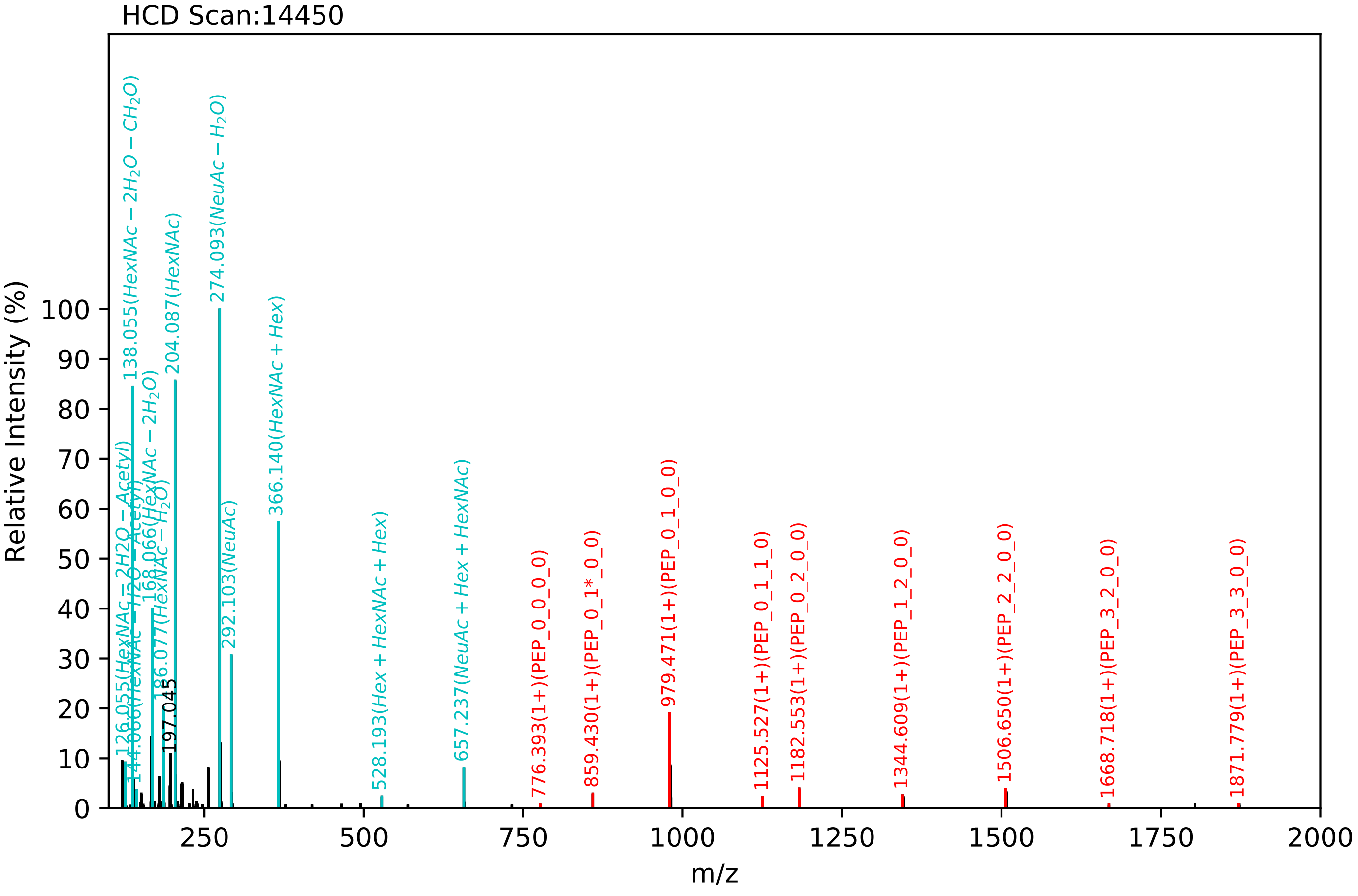

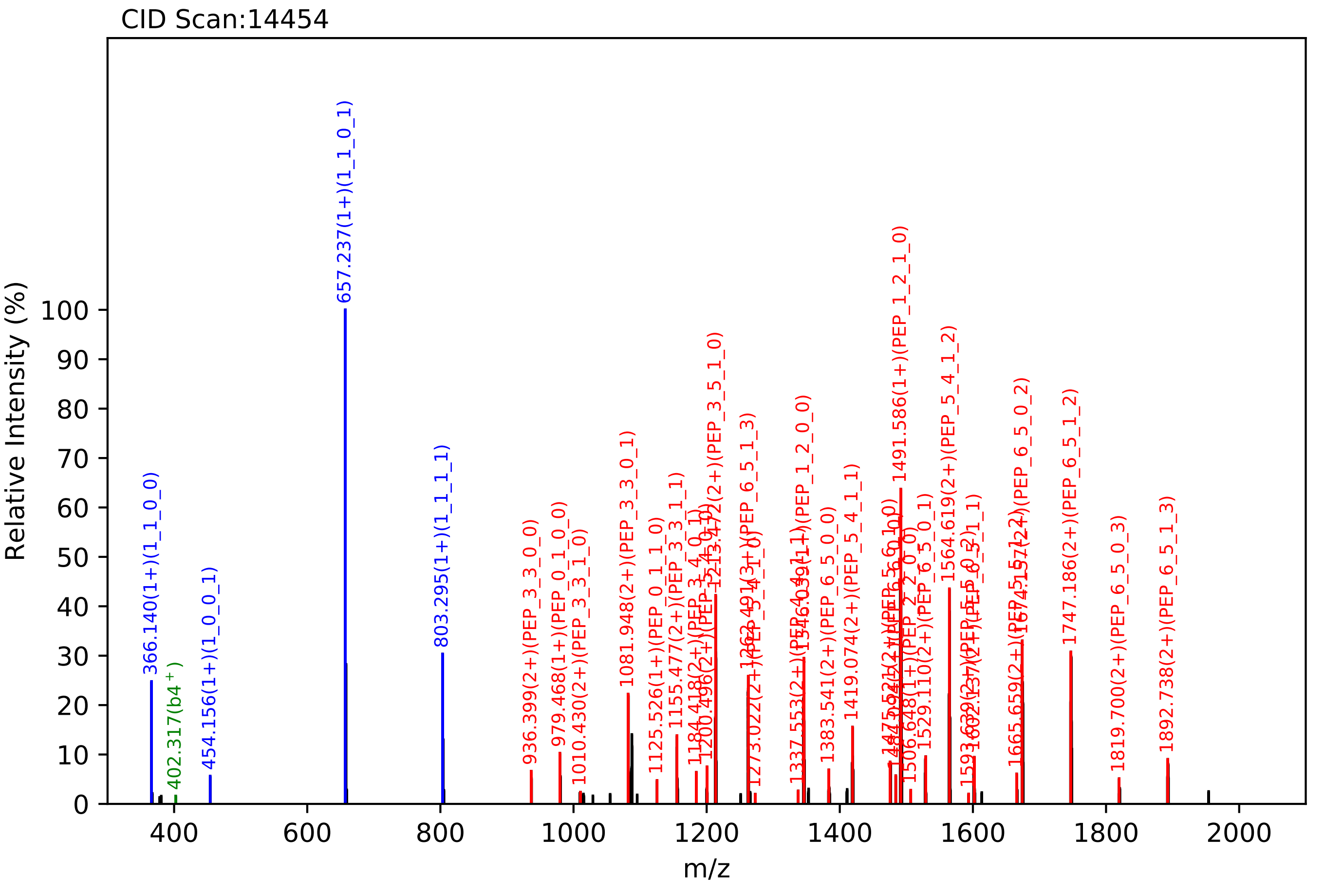

Unknown set no. 45, Experiment: Human Plasma exp_4

ENGTISR(=PEP)_7_6_1_4, m/z:1110.67(4+), RT:52.35, Y-score:88.44

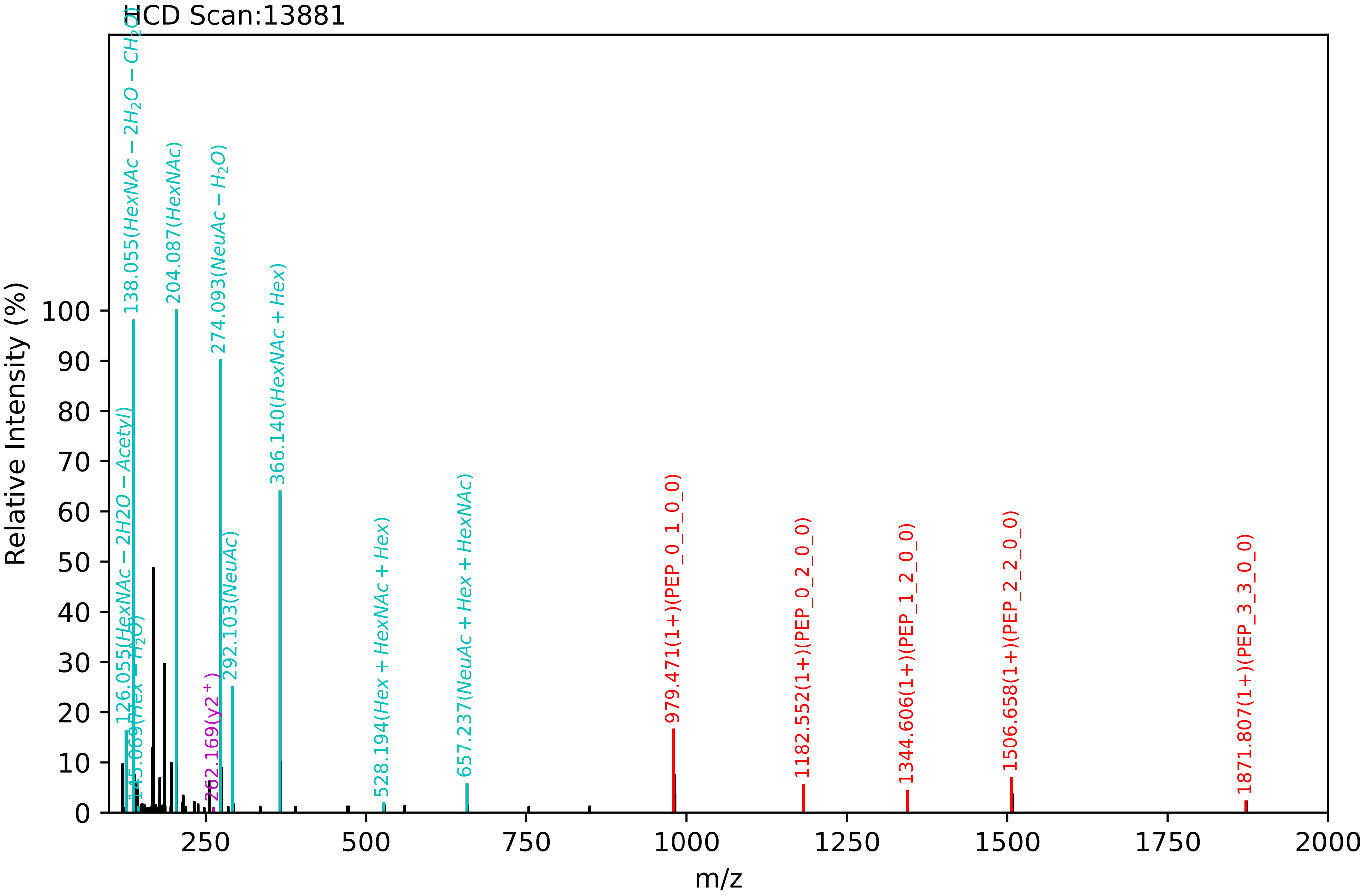

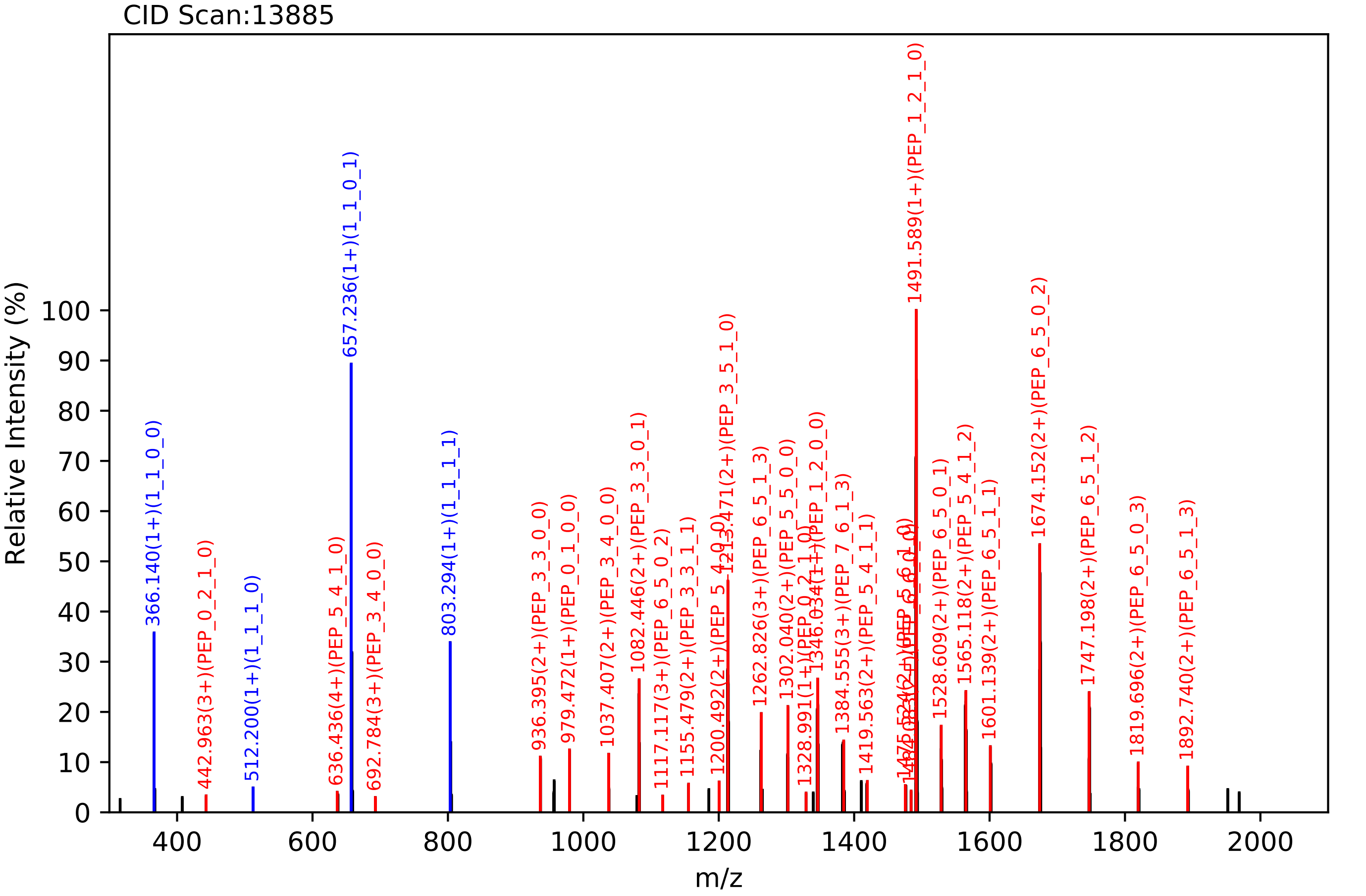

Unknown set no. 46, Experiment: Human Plasma exp_5

ENGTISR(=PEP)_7_6_1_4, m/z:1110.68(4+), RT:55.45, Y-score:85.90

HCD Scan:14873

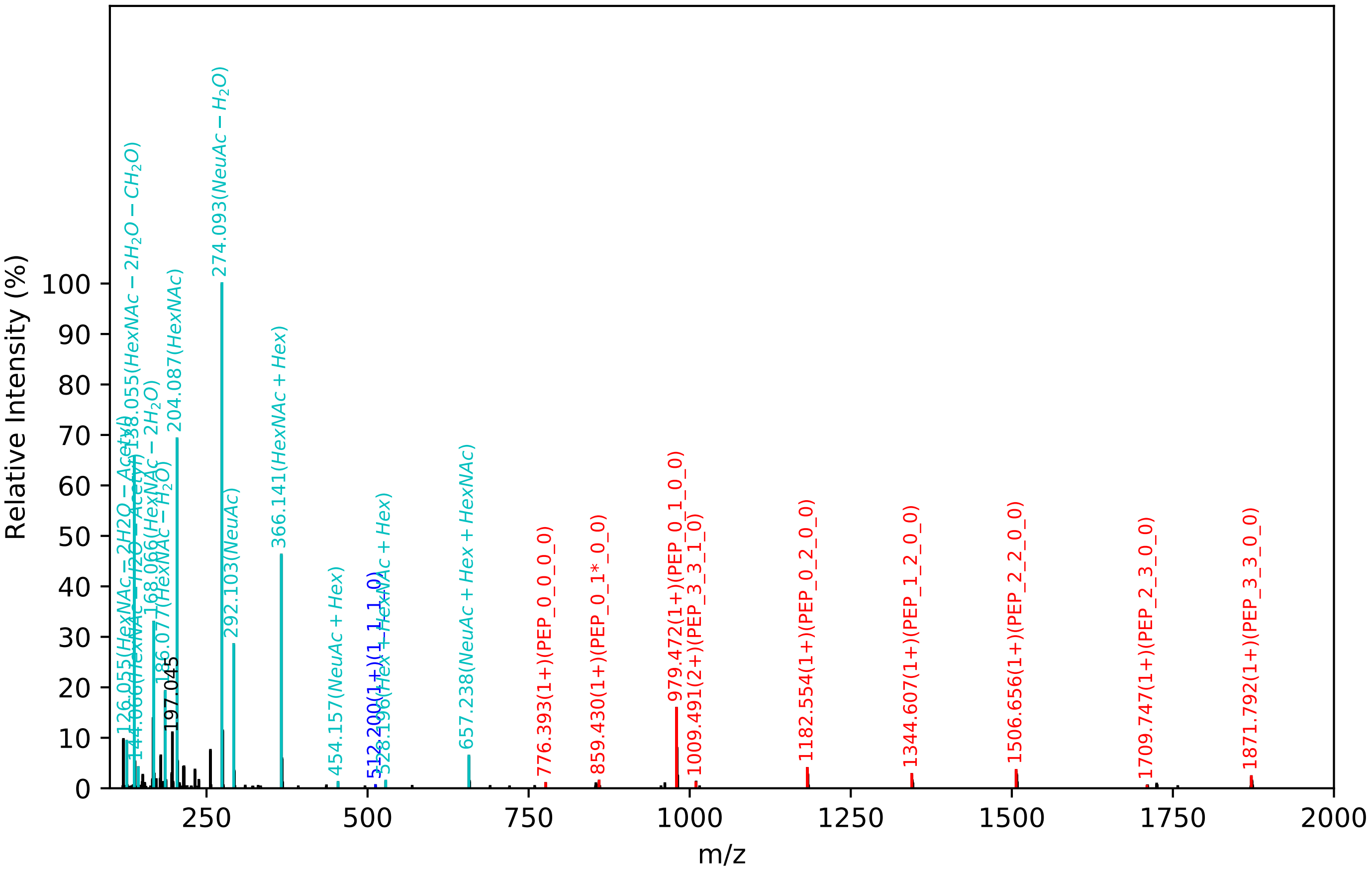

CID Scan:14875

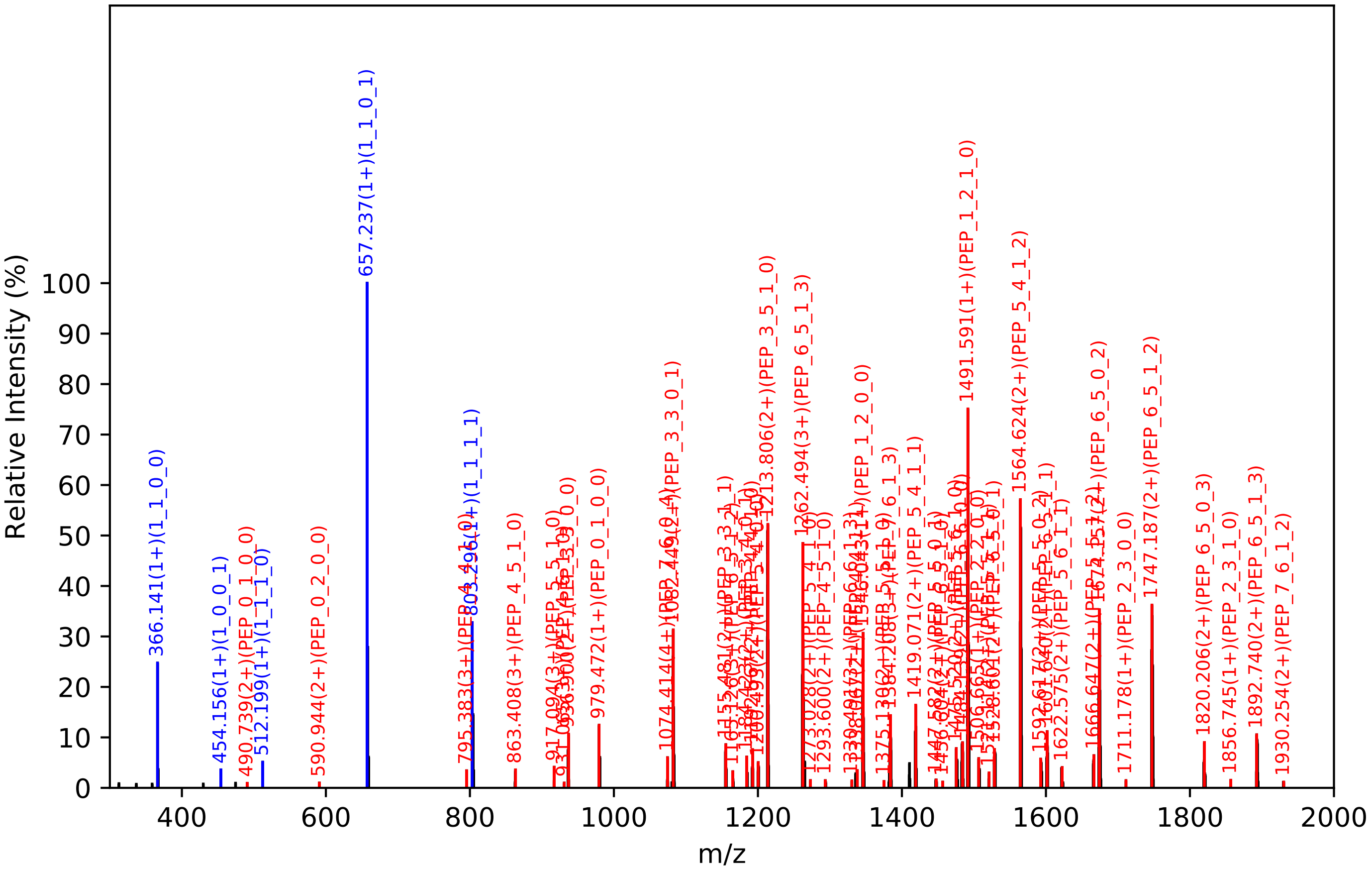

Unknown set no. 47, Experiment: Human Plasma exp_2

ENGTISR(=PEP)_7_6_2_4, m/z:1147.19(4+), RT:52.13, Y-score:83.94

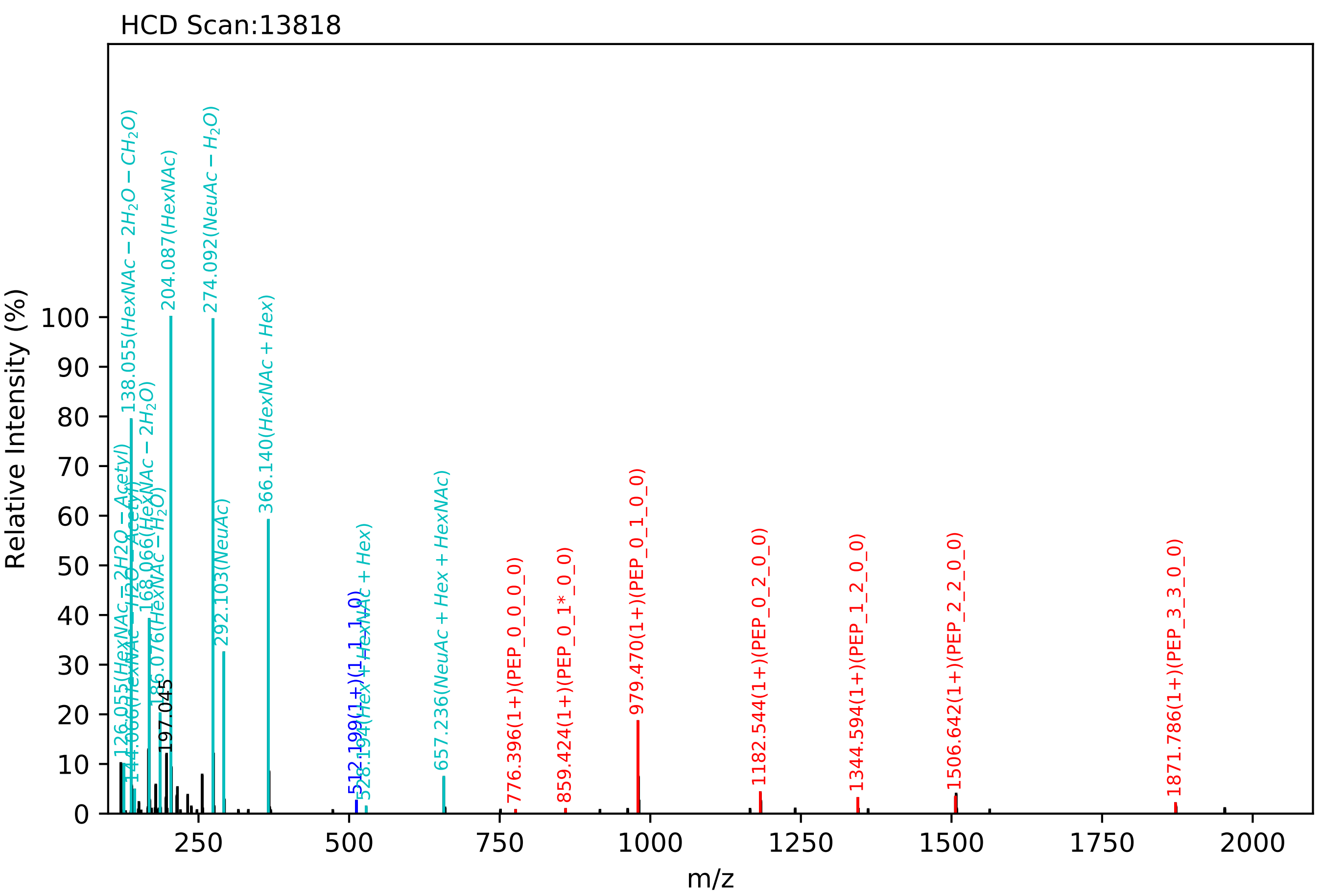

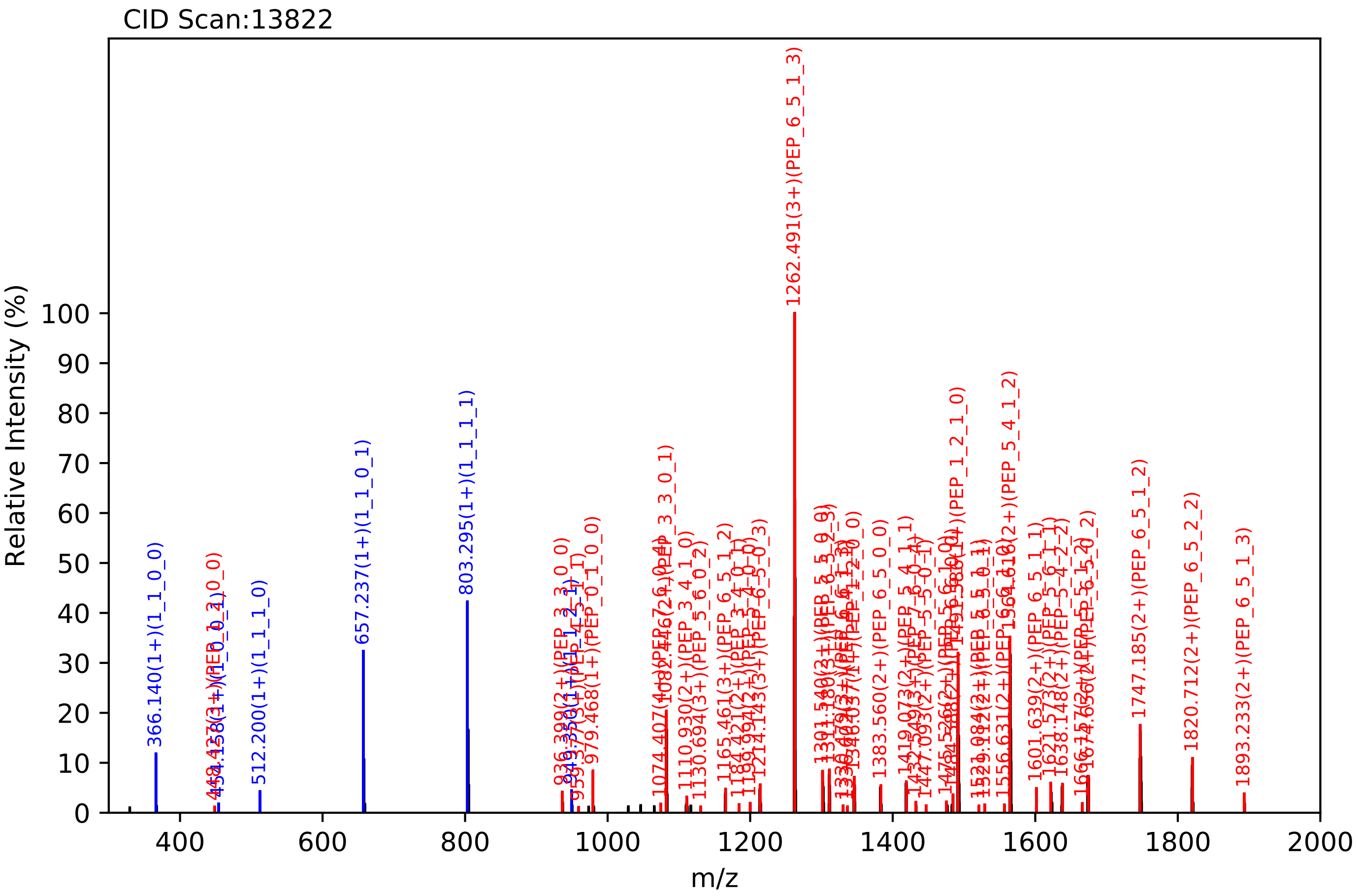

Unknown set no. 48, Experiment: Human Plasma exp_3

ENGTISR(=PEP)_7_6_2_4, m/z:1147.19(4+), RT:54.23, Y-score:84.92

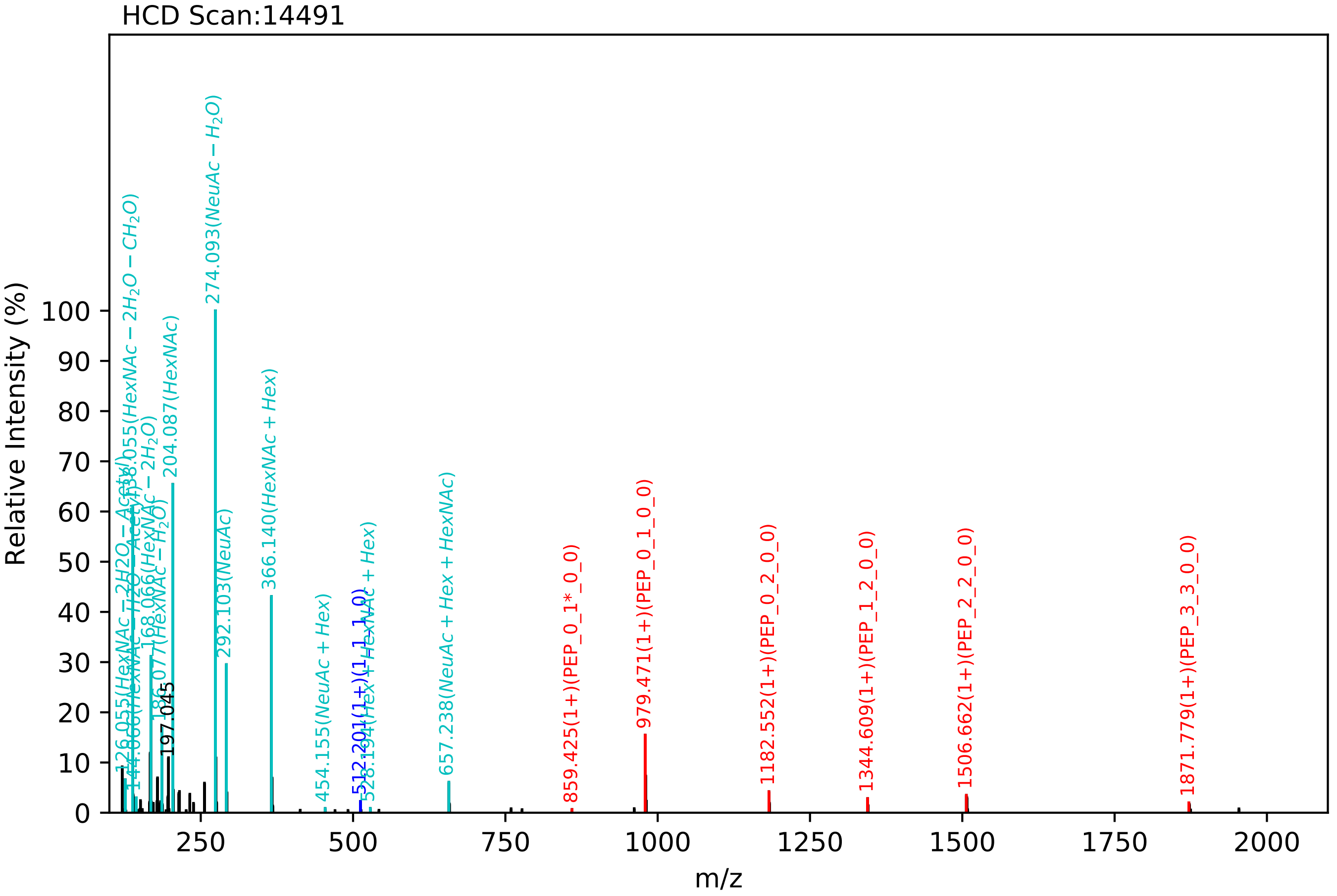

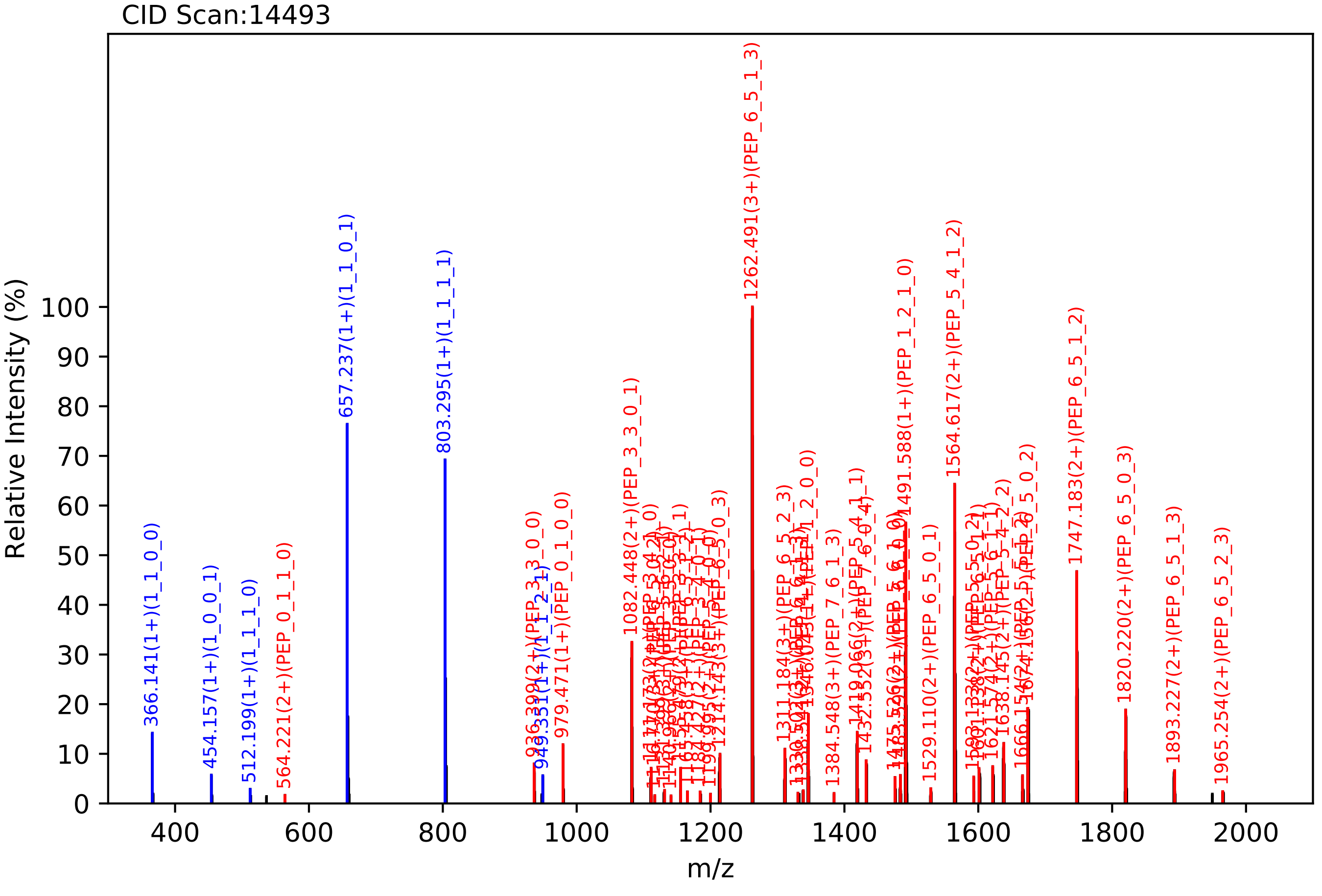

Unknown set no. 49, Experiment: Human Plasma exp_4

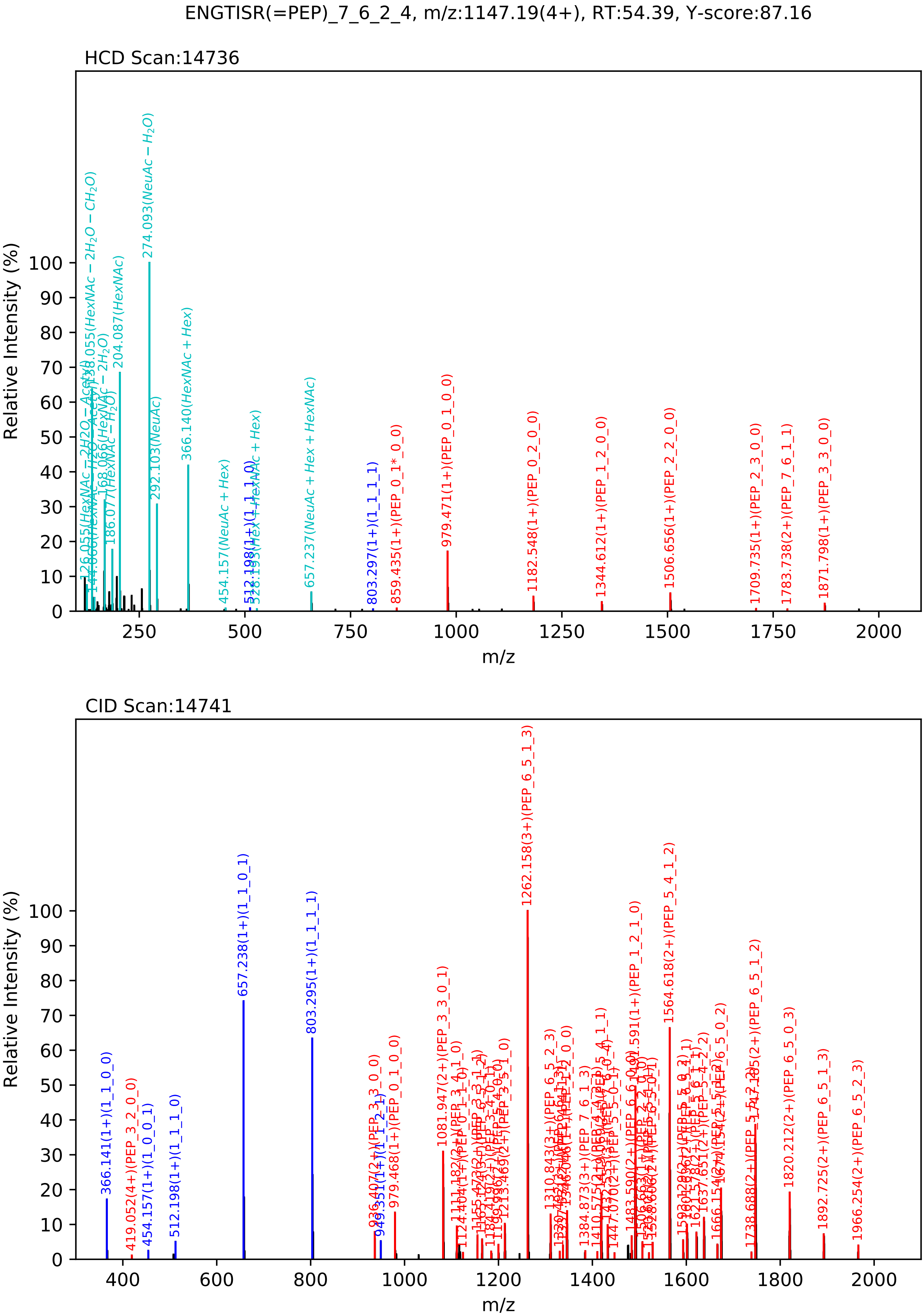

Unknown set no. 50, Experiment: Human Plasma exp_5

ENGTISR(=PEP)_7_6_2_4, m/z:1147.19(4+), RT:52.73, Y-score:88.21

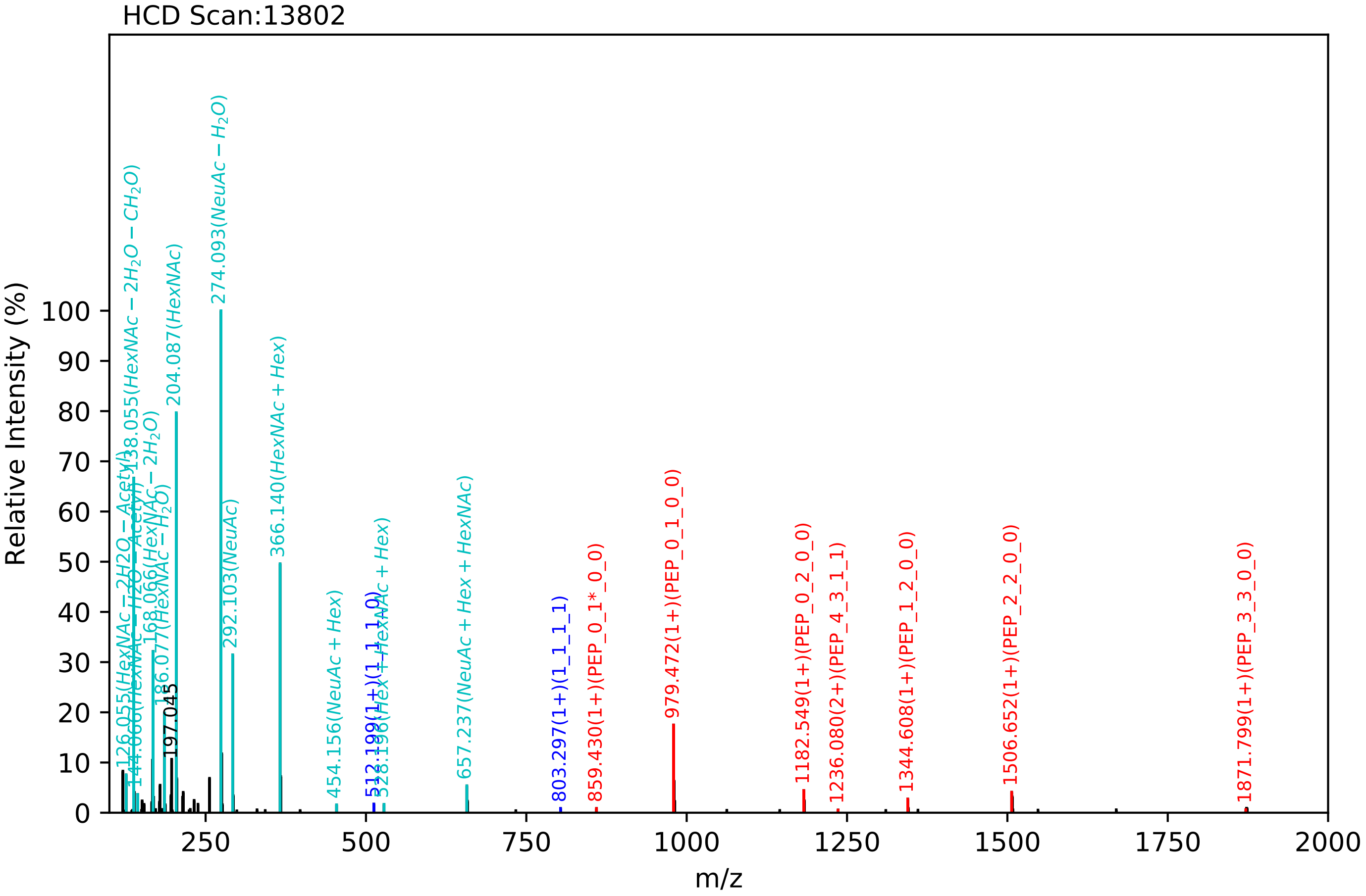

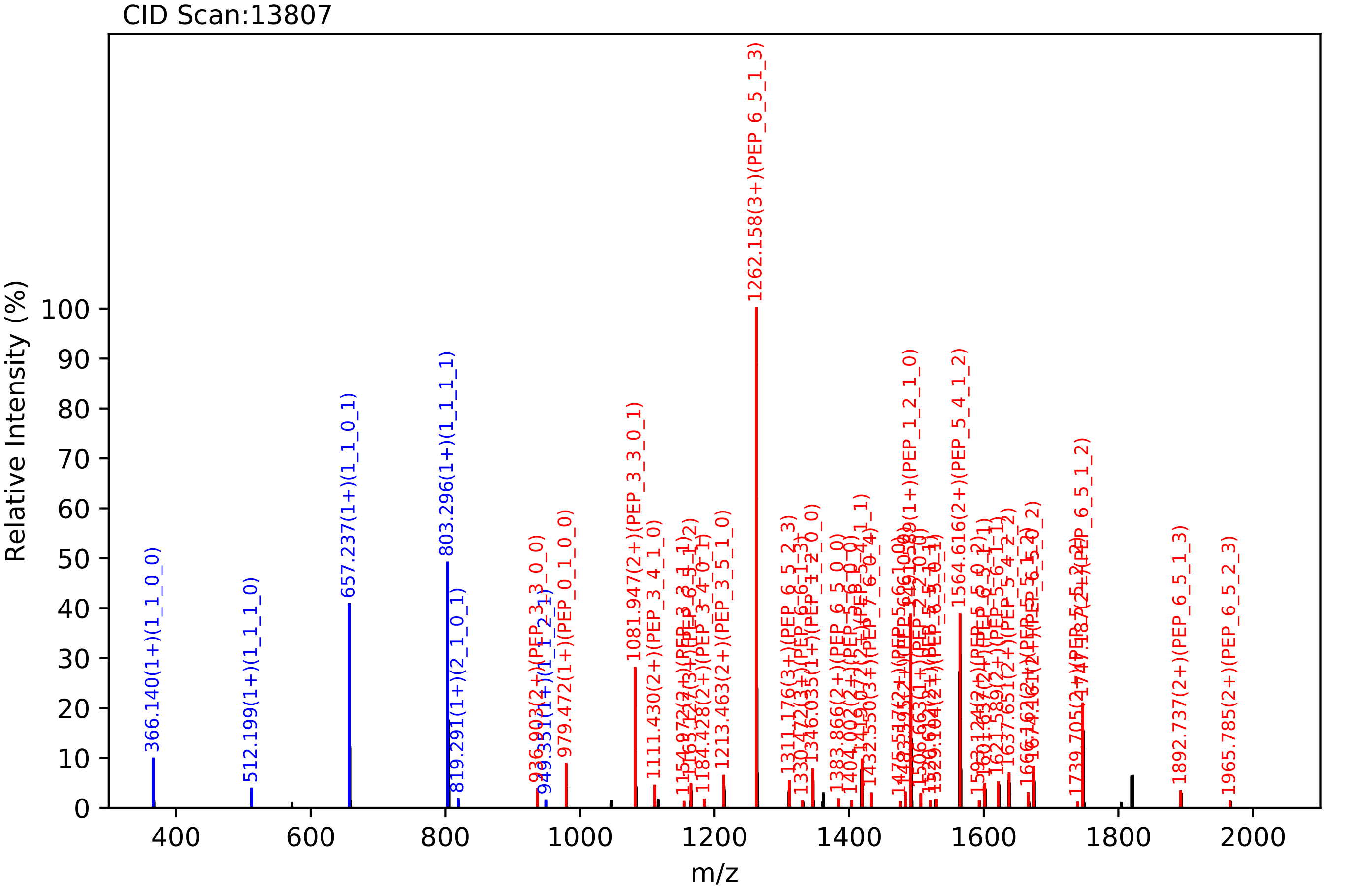

Unknown set no. 51, Experiment: Human Plasma exp_5

ENGTISR(=PEP)_7_6_3_4, m/z:1183.70(4+), RT:53.56, Y-score:83.95

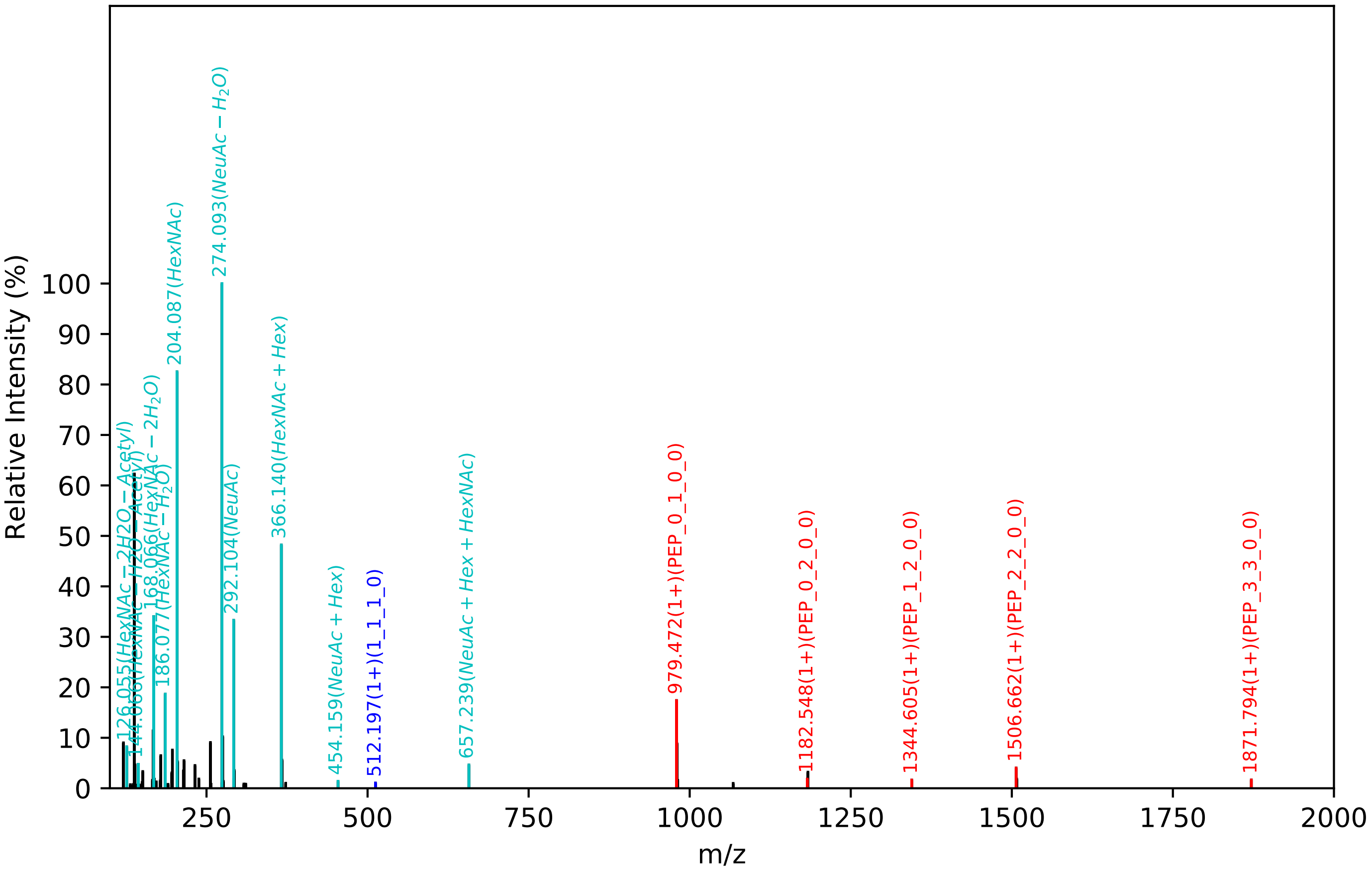

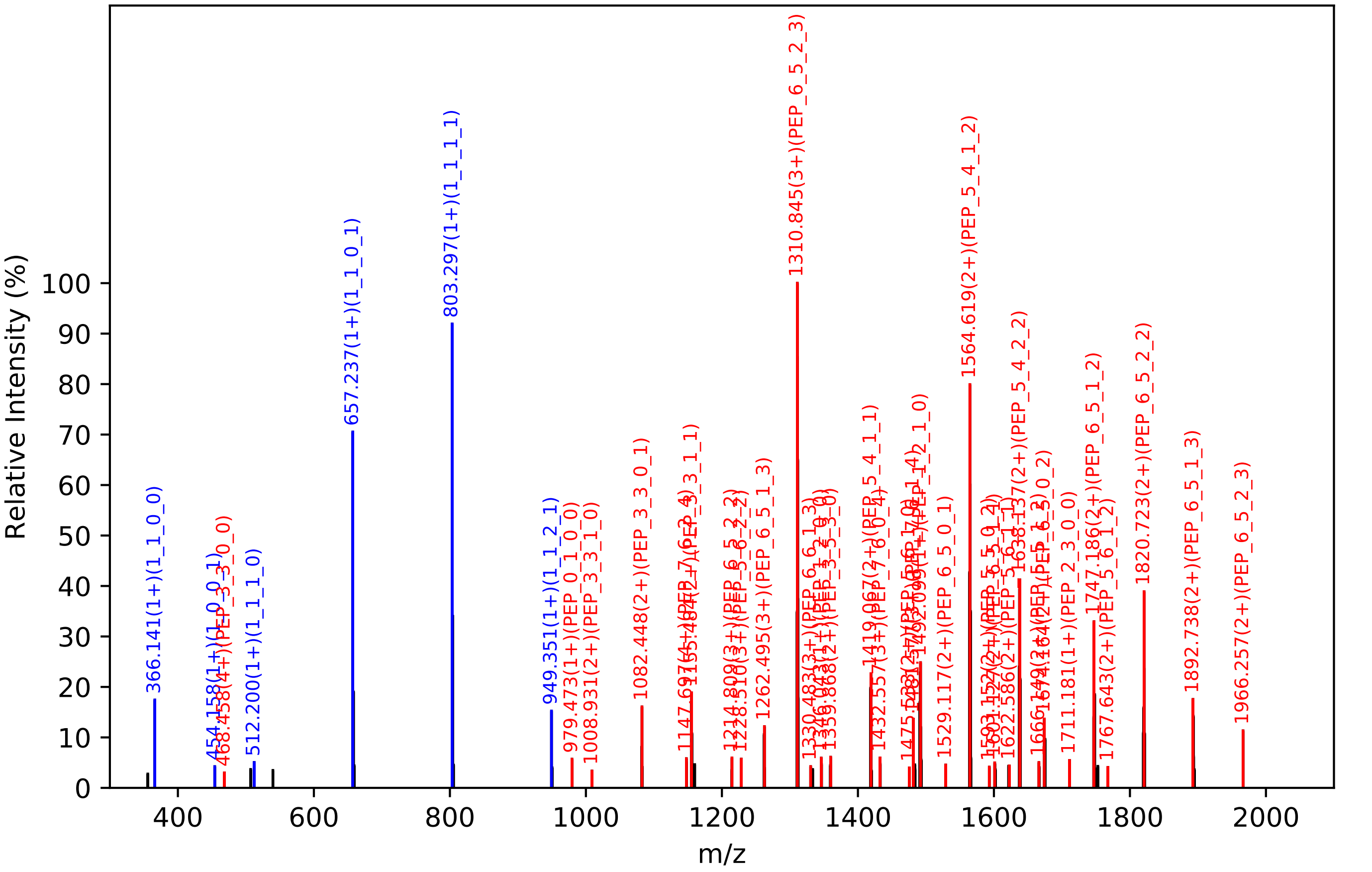

Unknown set no. 52, Experiment: Human Plasma exp_2

LVPVPITNATLDQITGK(=PEP)_5_4_0_1, m/z:924.18(4+), RT:104.20, Y-score:78.46

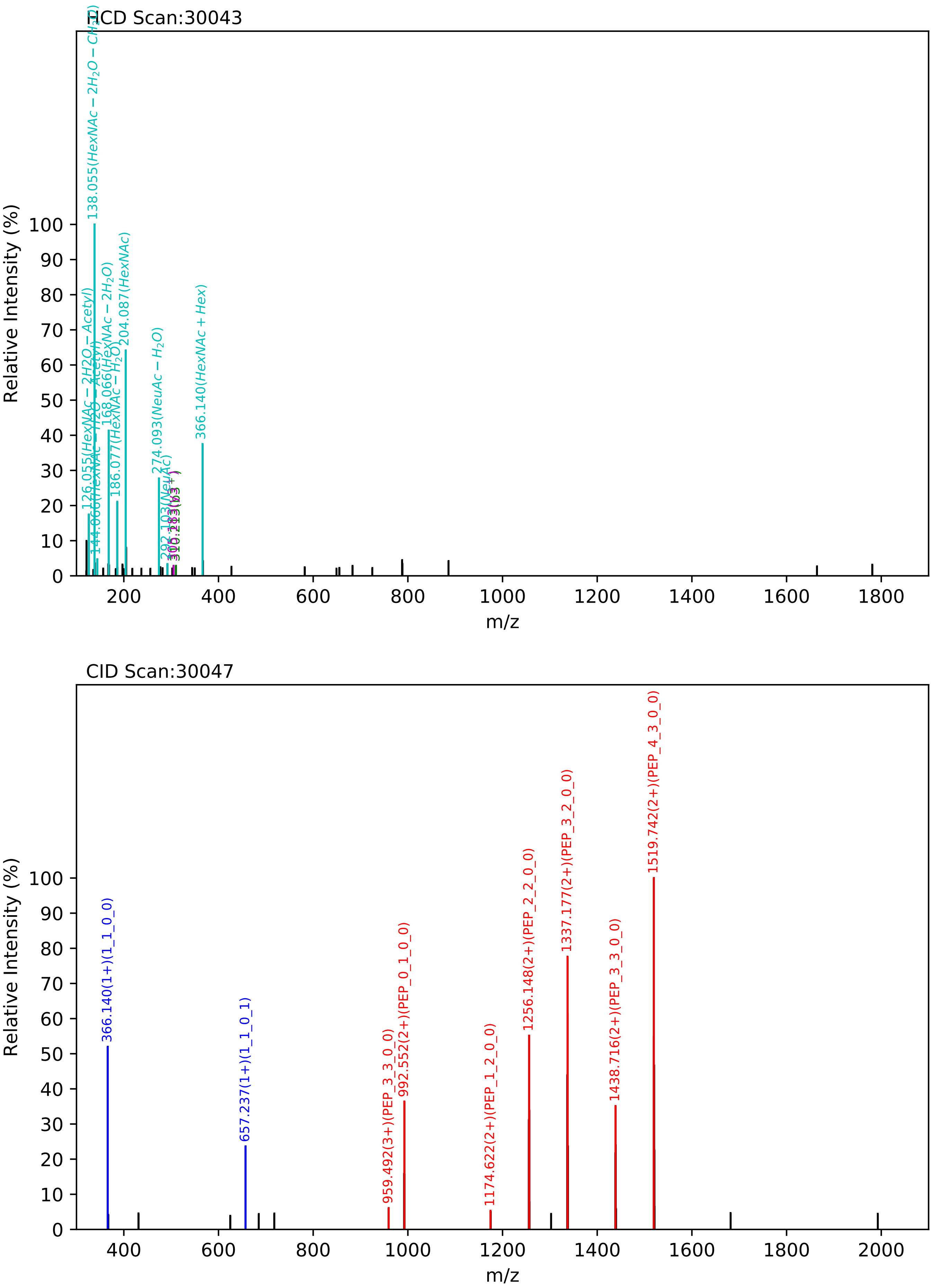

Unknown set no. 53, Experiment: Human Plasma exp_3

LVPVPITNATLDQITGK(=PEP)_5_4_0_1, m/z:924.18(4+), RT:104.16, Y-score:86.58

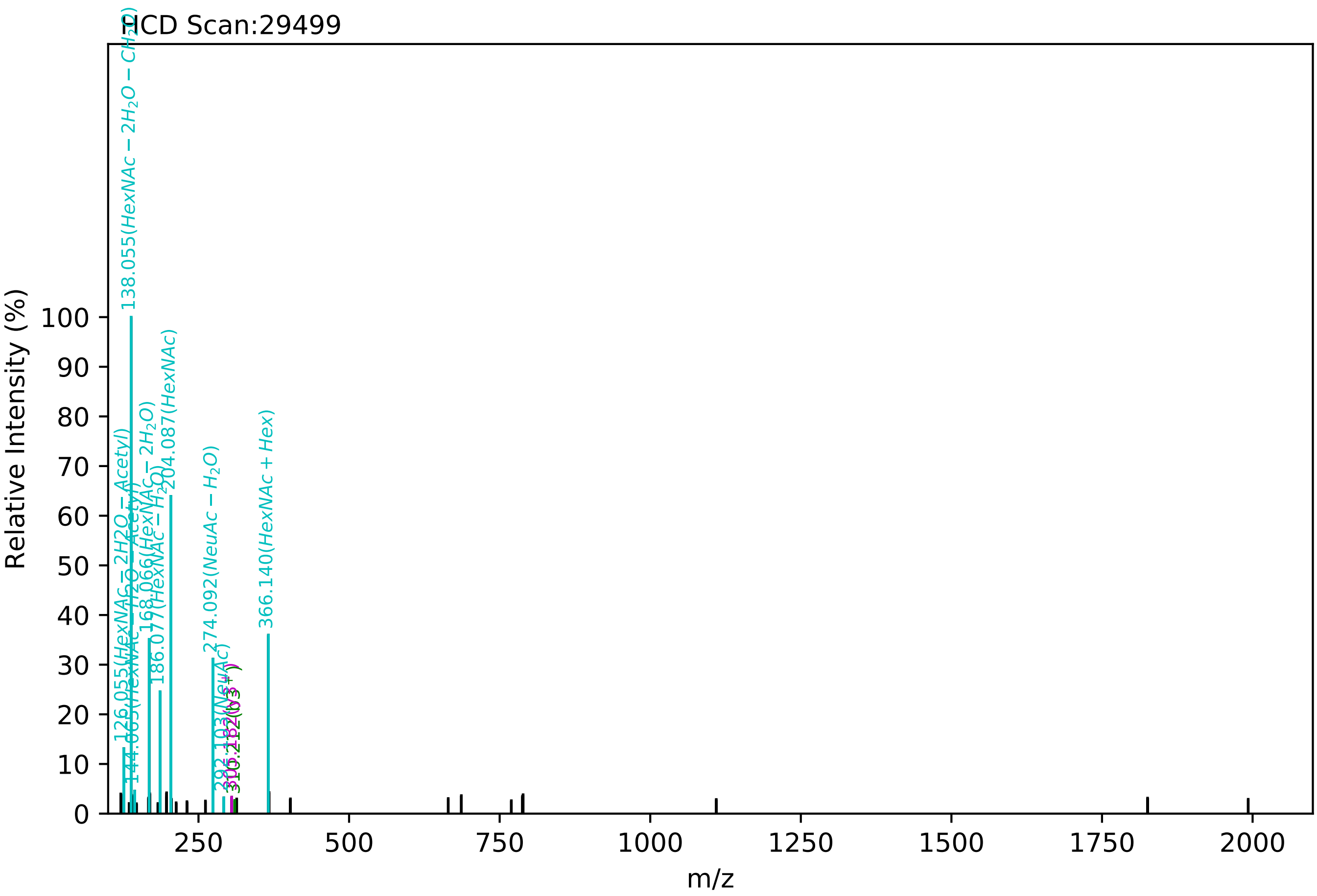

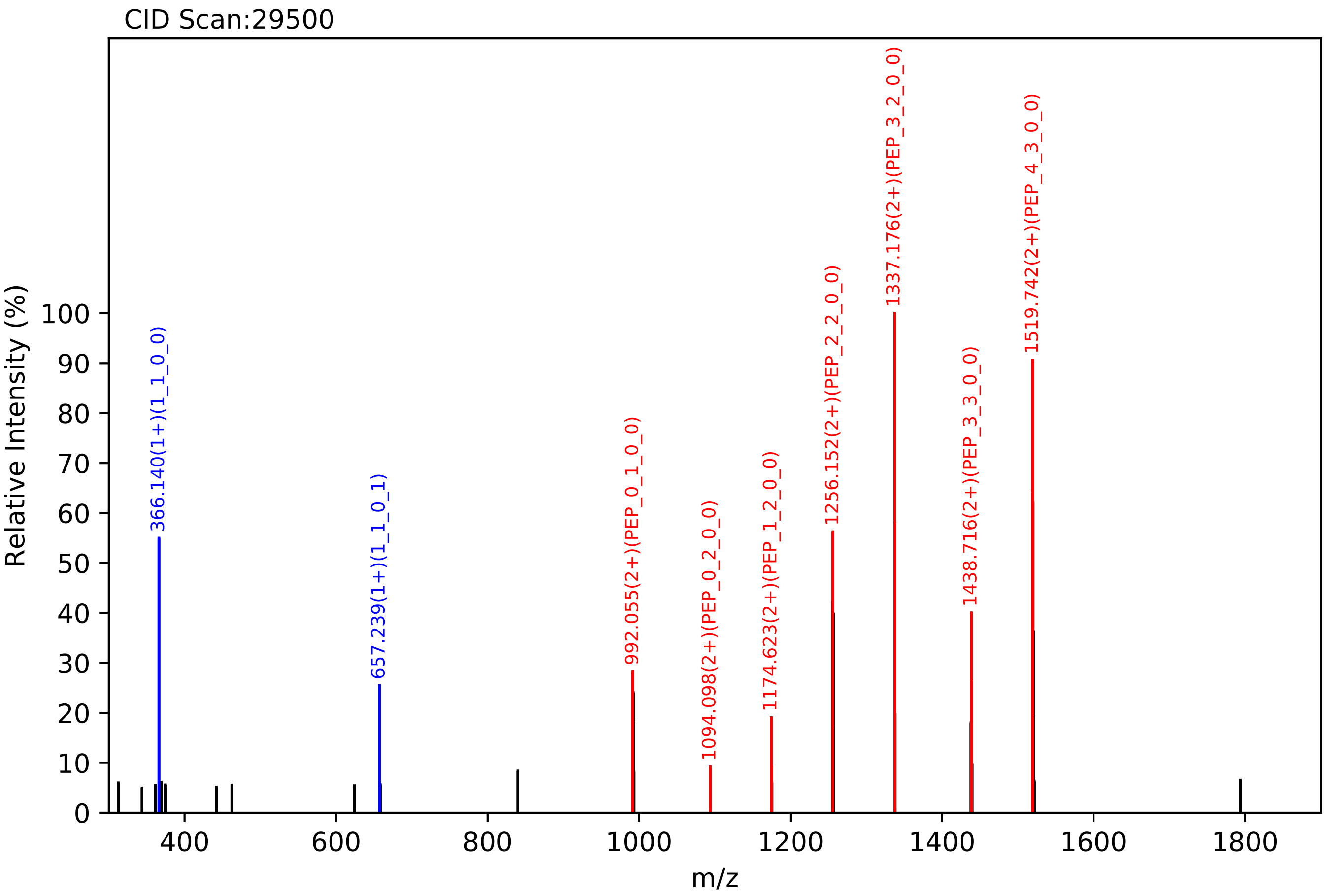

Unknown set no. 54, Experiment: Human Plasma exp_4

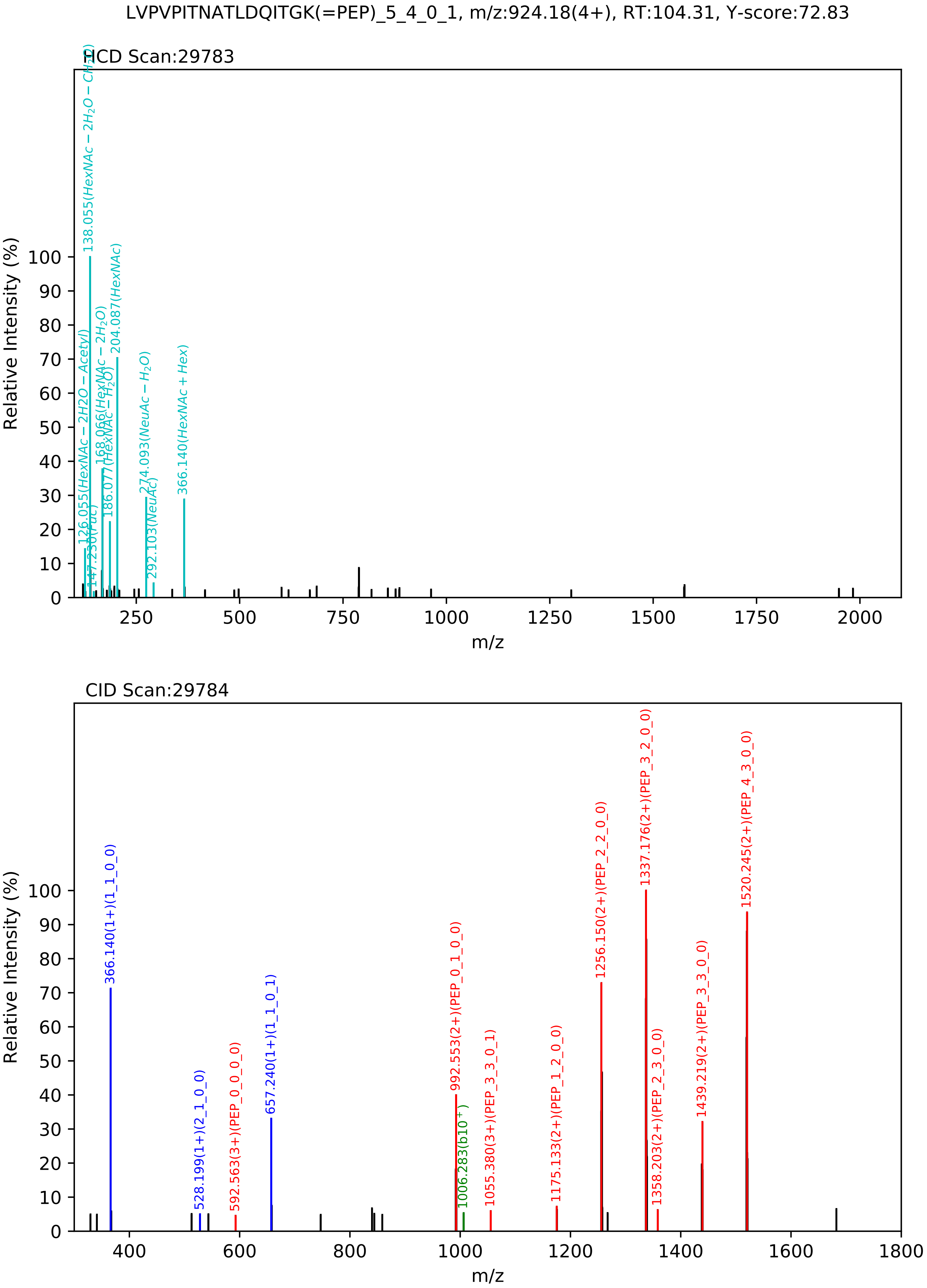

Unknown set no. 55, Experiment: Human Plasma exp_4

LVPVPITNATLDQITGK(=PEP)_5_4_0_2, m/z:996.96(4+), RT:115.23, Y-score:76.10

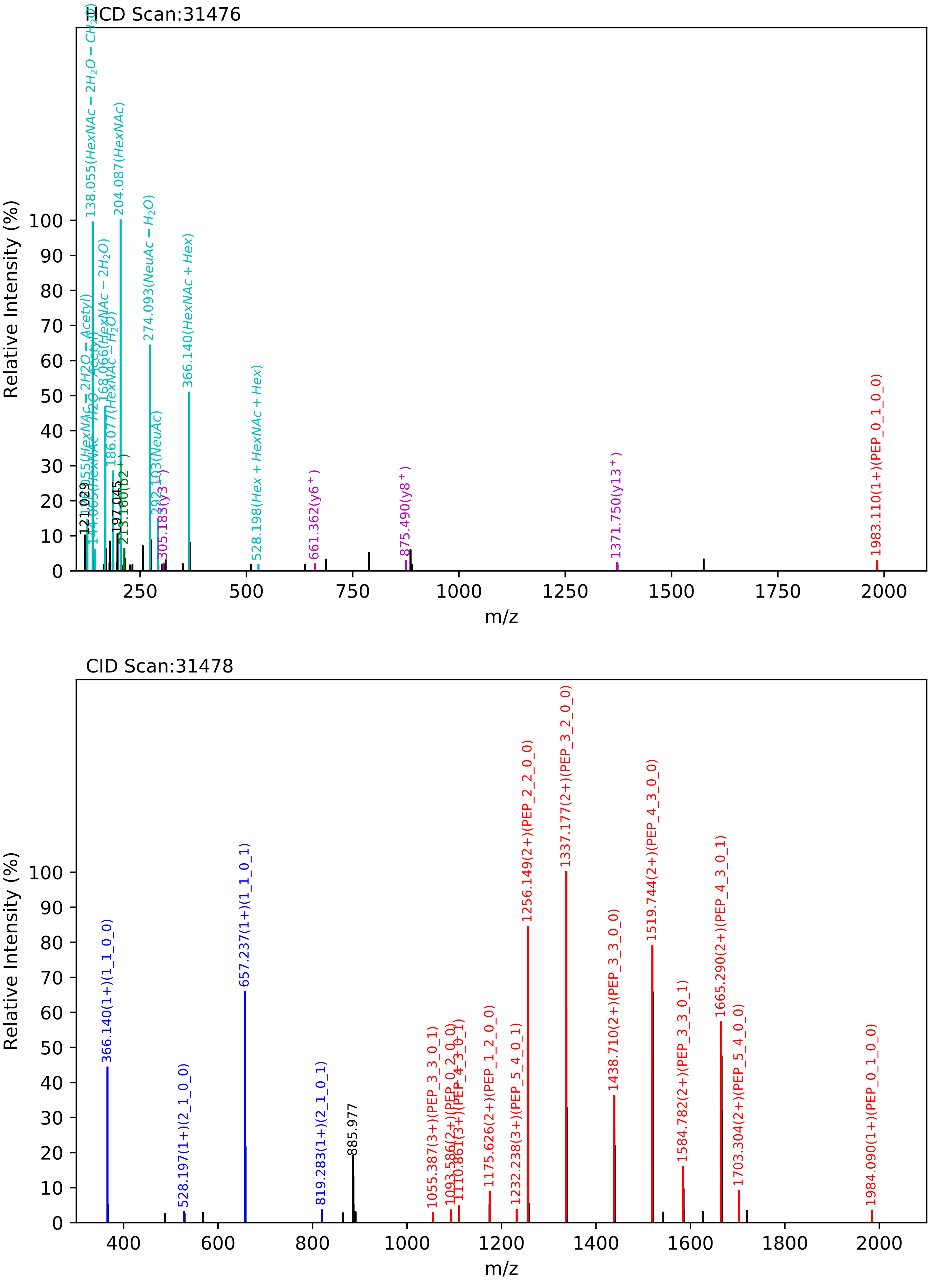

Unknown set no. 56 Experiment: Human Plasma exp_1

LVPVPITNATLDQITGK(=PEP)_6_5_0_3, m/z:1161.01(4+), RT:127.59, Y-score:79.92

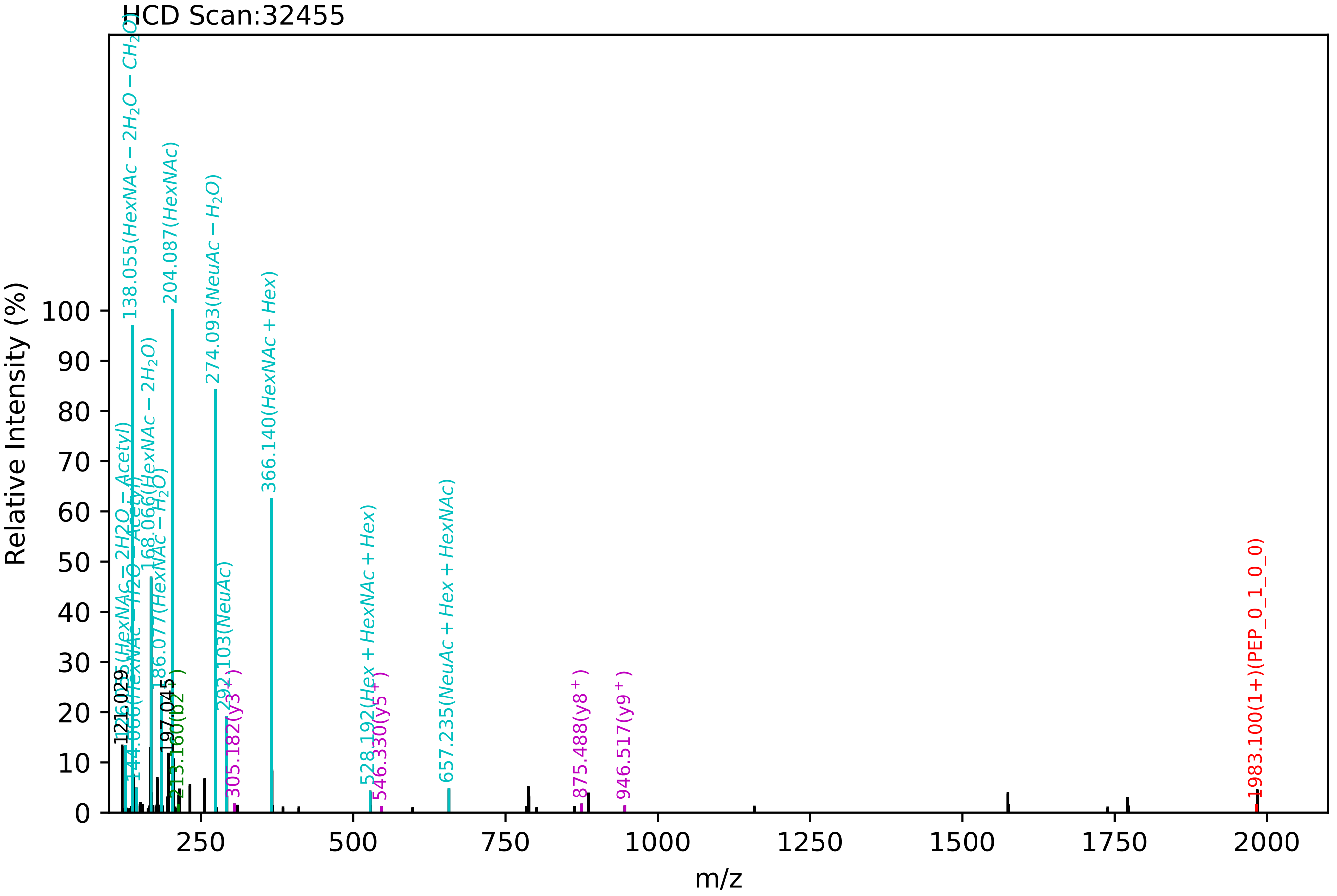

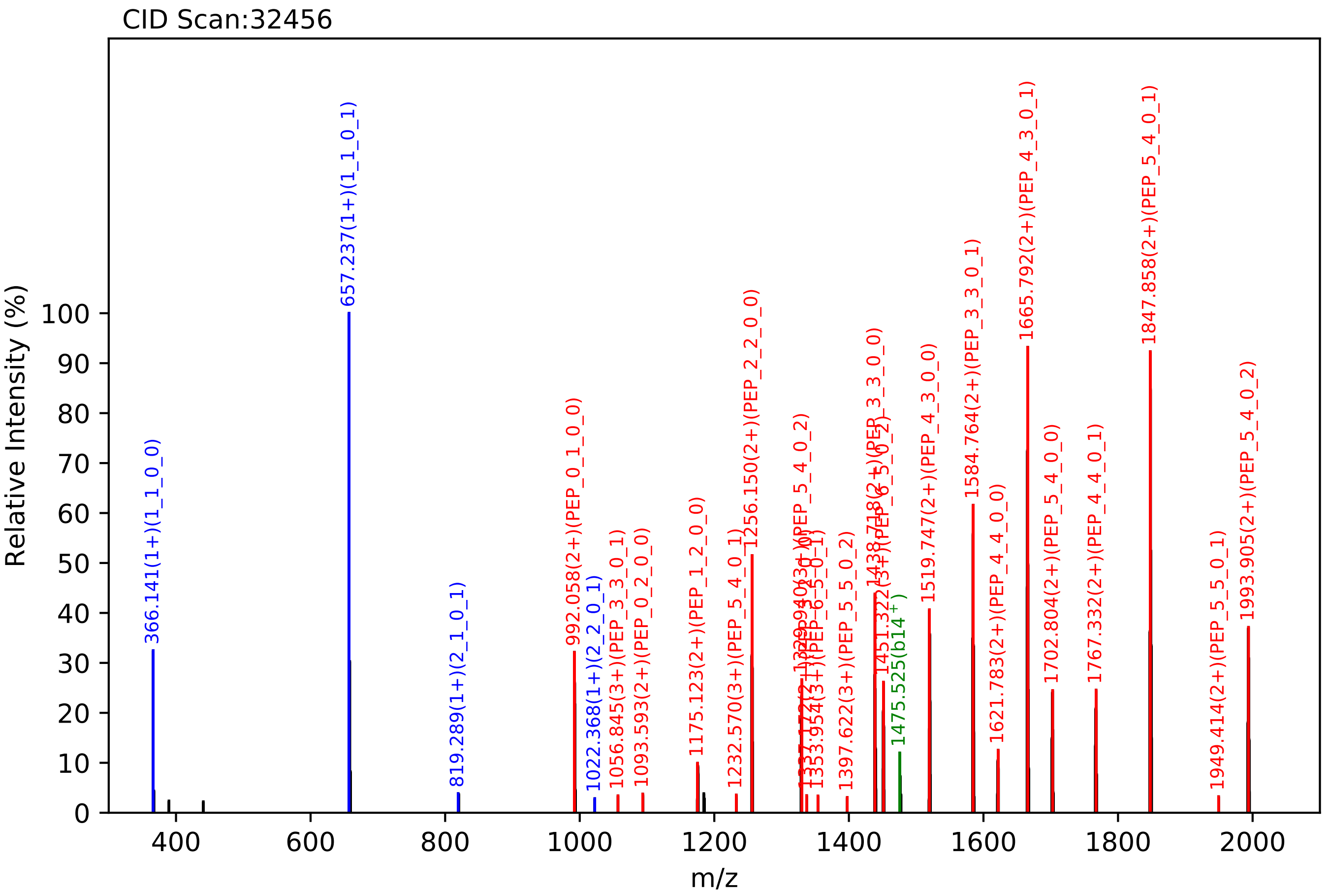

Unknown set no. 57, Experiment: Human Plasma exp_4

LVPVPITNATLDQITGK(=PEP)_6_5_0_3, m/z:1161.01(4+), RT:127.99, Y-score:84.12

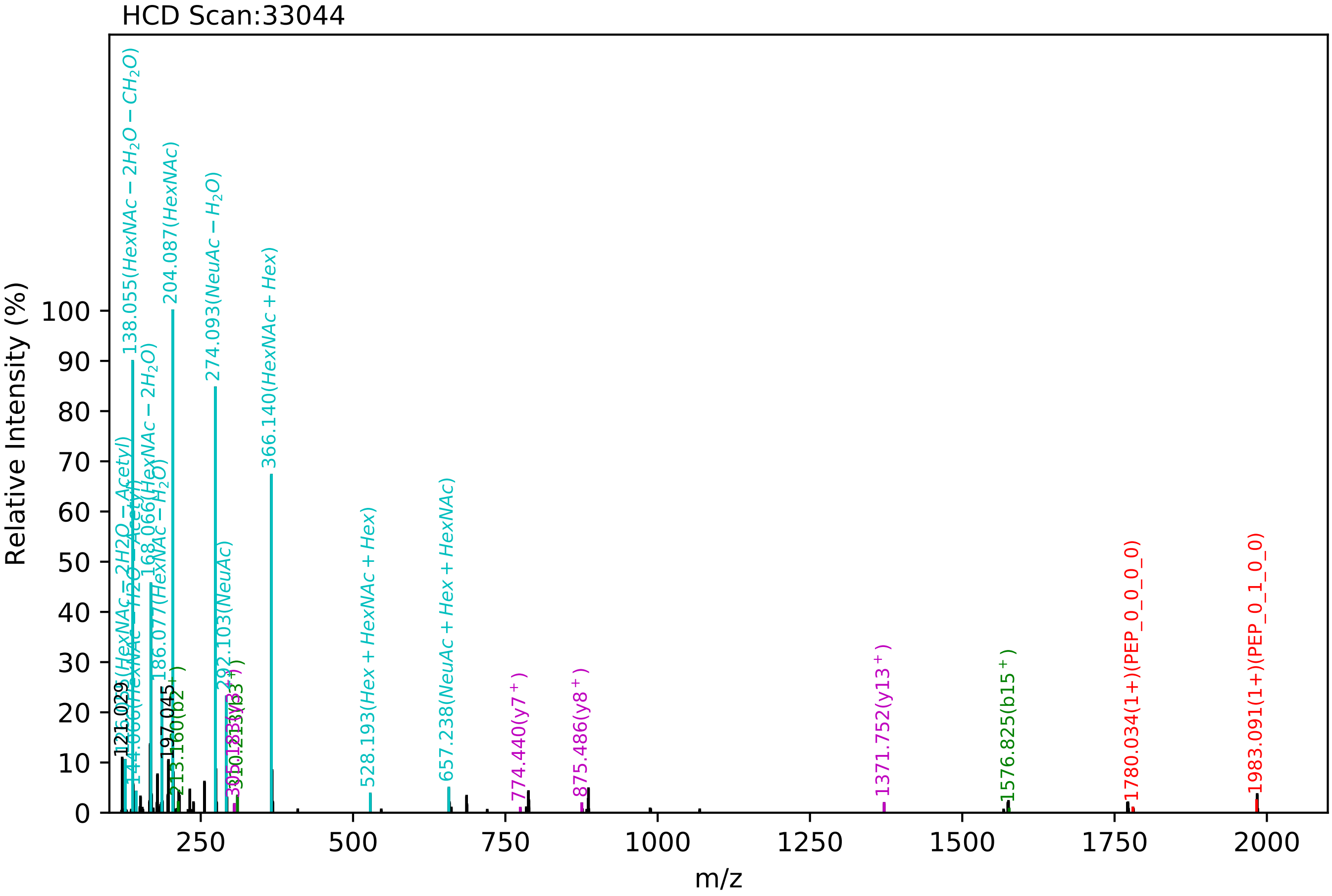

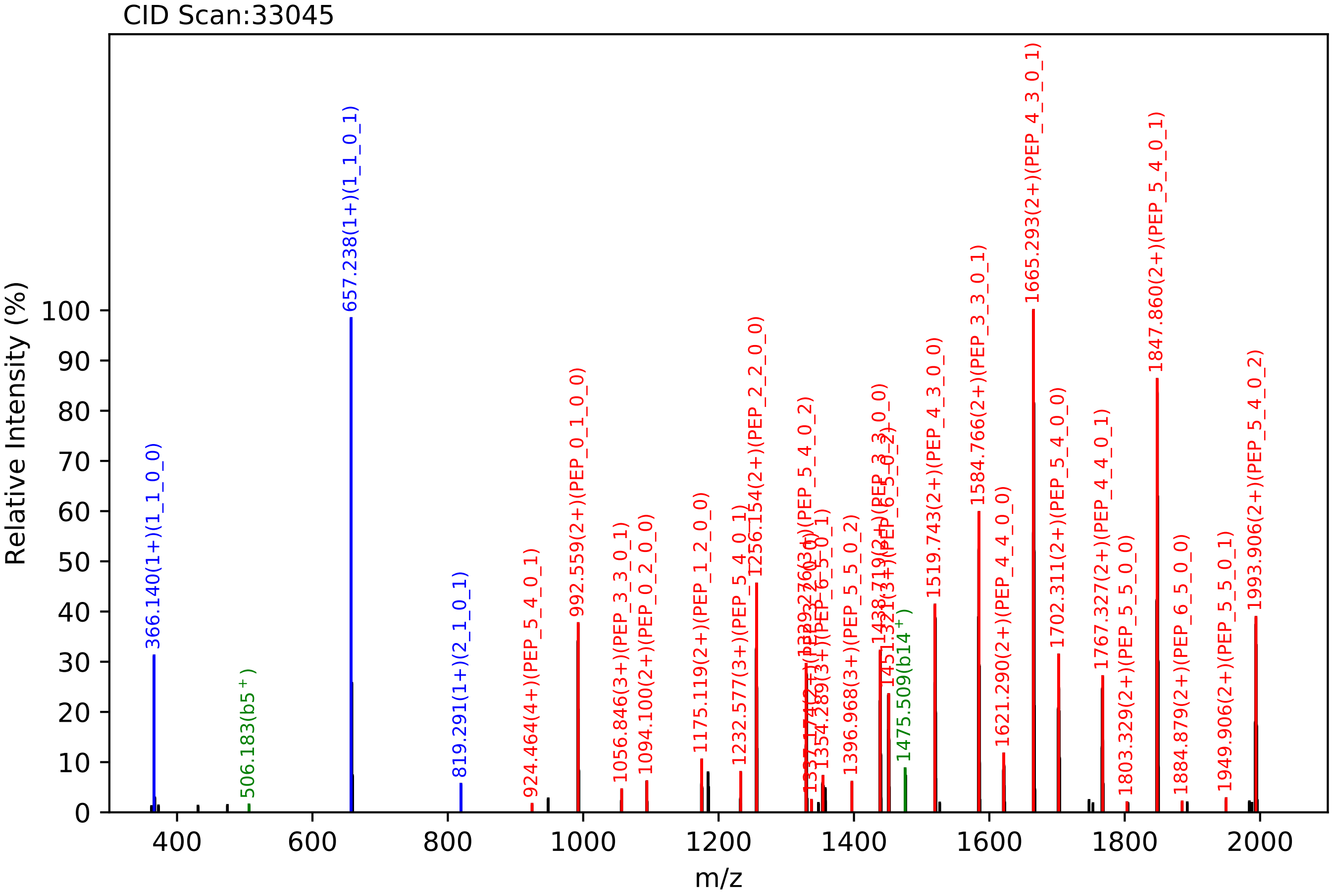

Unknown set no. 58, Experiment: Human Plasma exp_1

LVPVPITNATLDQITGK(=PEP)_6_5_1_2, m/z:1124.75(4+), RT:113.97, Y-score:77.81

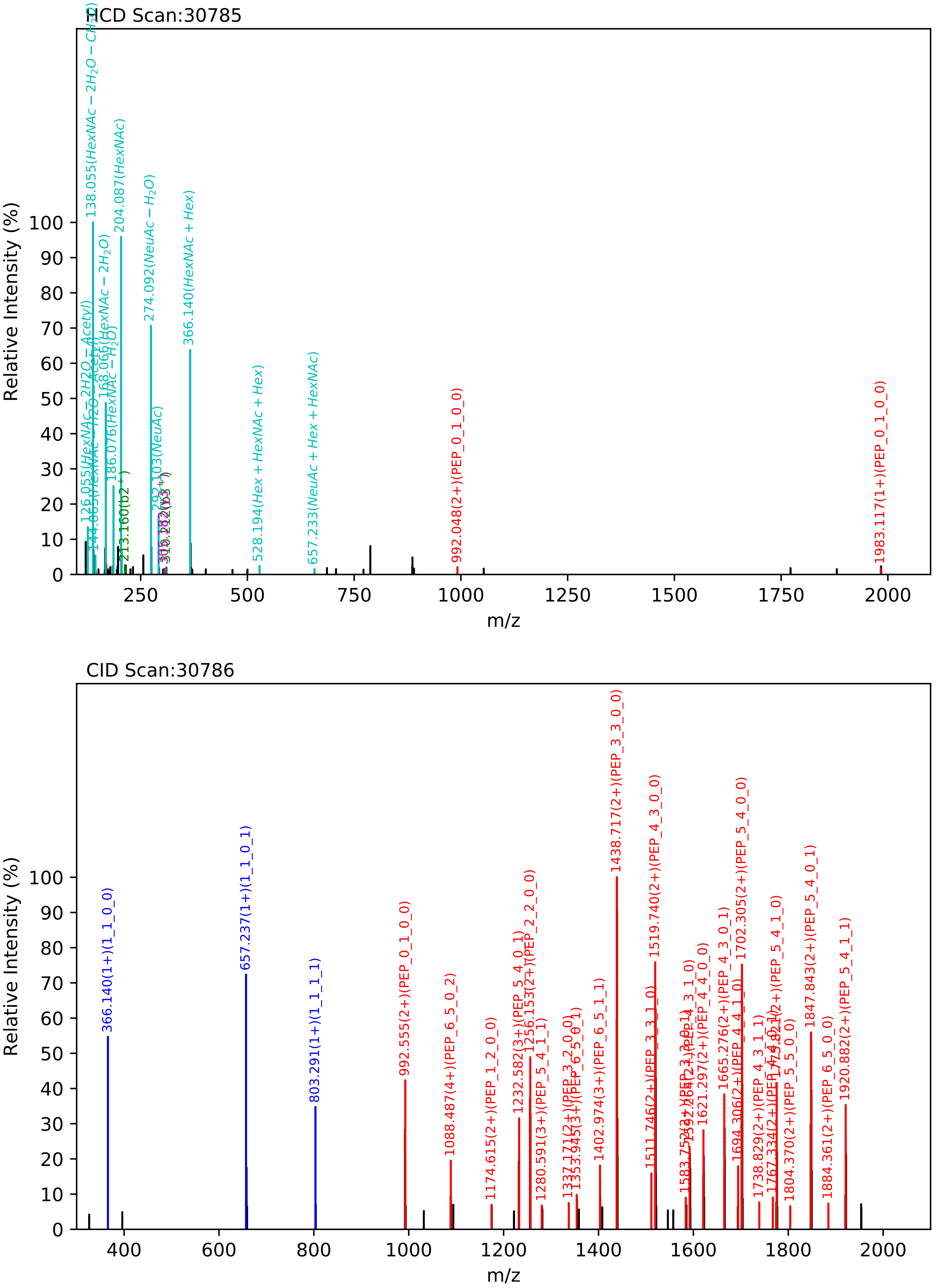

Unknown set no. 59, Experiment: Human Plasma exp_4

LVPVPITNATLDQITGK(=PEP)_6_5_1_2, m/z:1124.75(4+), RT:114.28, Y-score:82.03

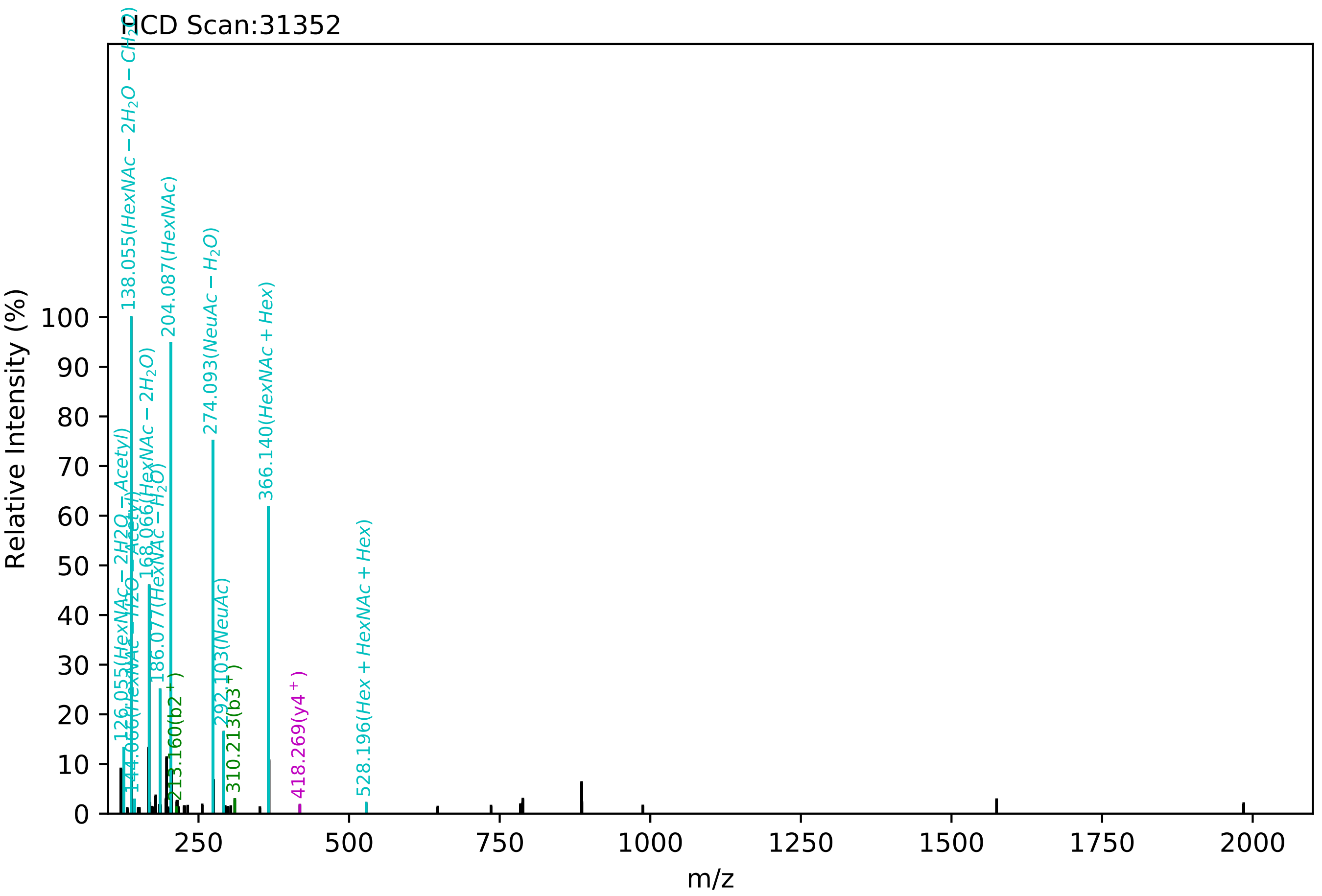

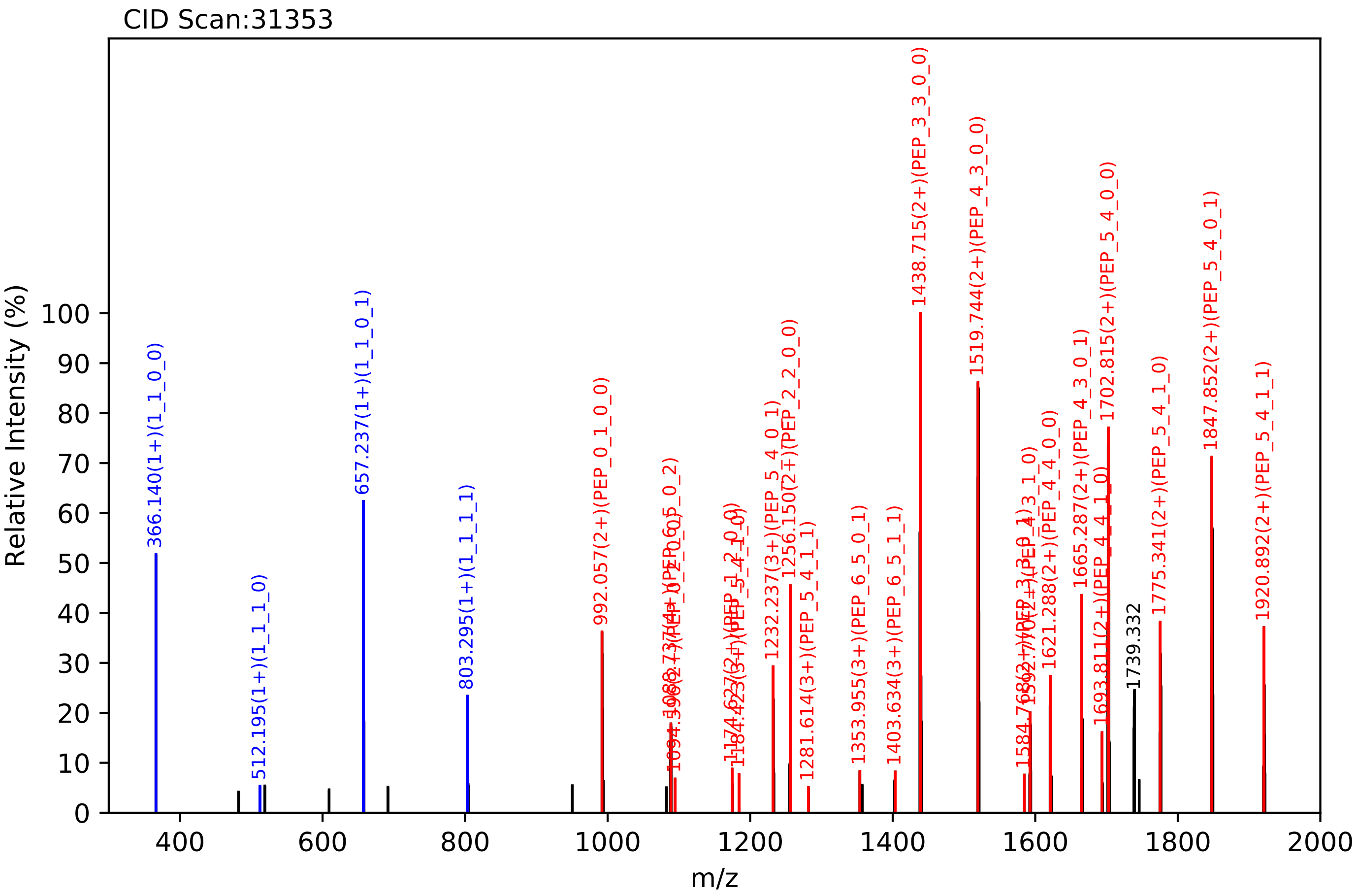

Unknown set no. 60, Experiment: Human Plasma exp_4

LVPVPITNATLDQITGK(=PEP)_6_5_1_3, m/z:1197.53(4+), RT:127.44, Y-score:74.27

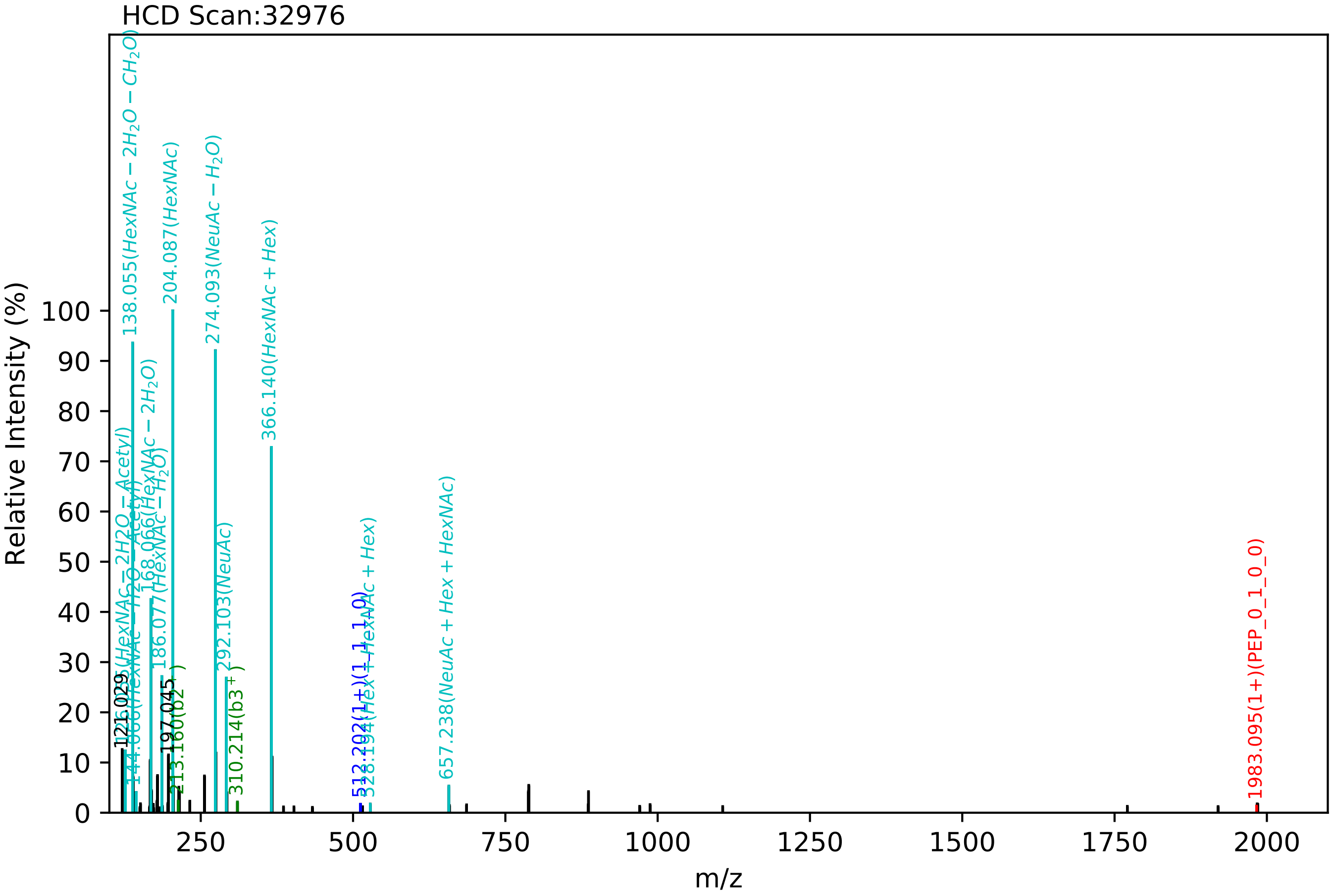

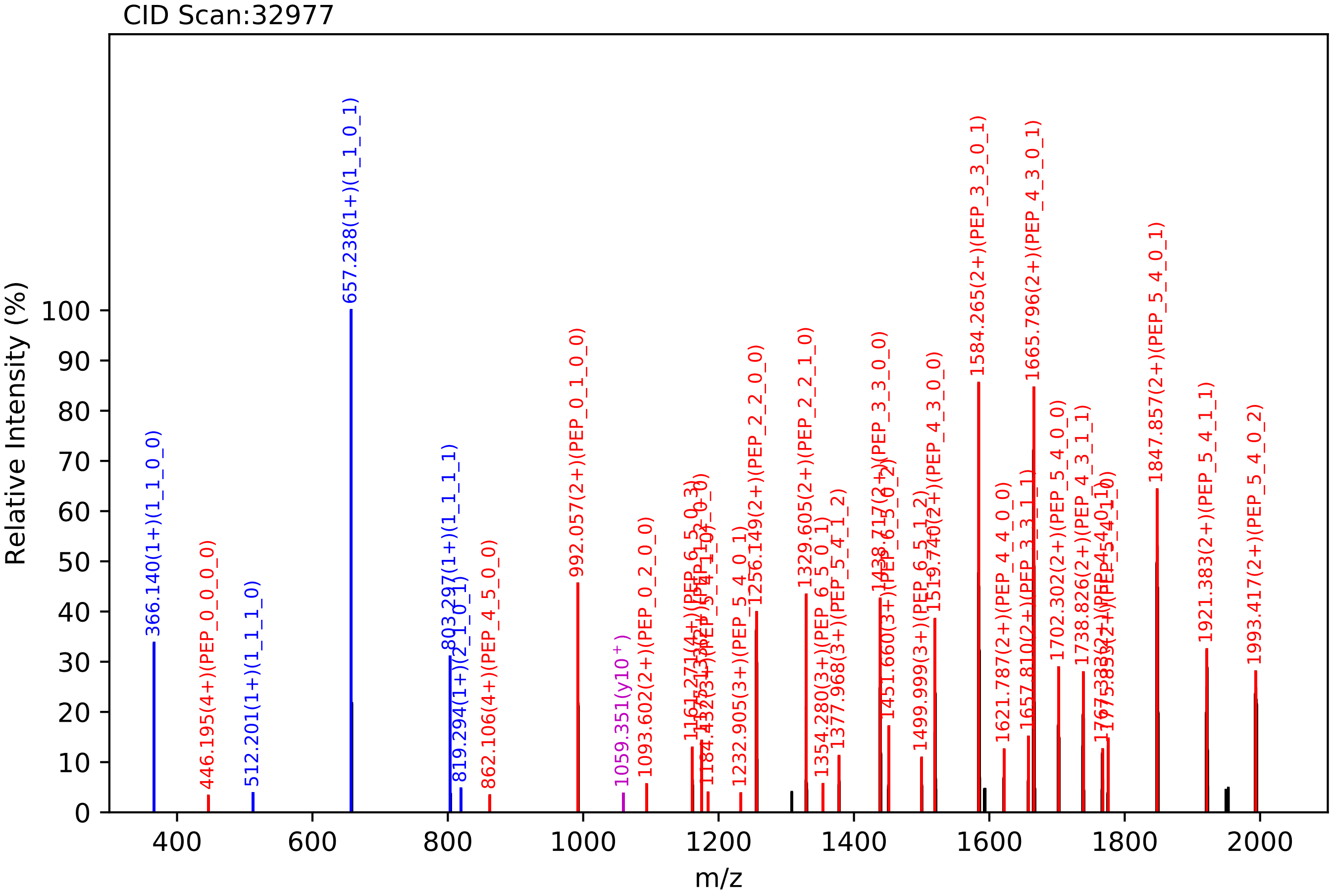

Unknown set no. 61, Experiment: Human Plasma exp_2

QDQCIYNTTYLNVQR(=PEP)_5_4_0_2, m/z:1030.92(4+), RT:92.35, Y-score:78.08

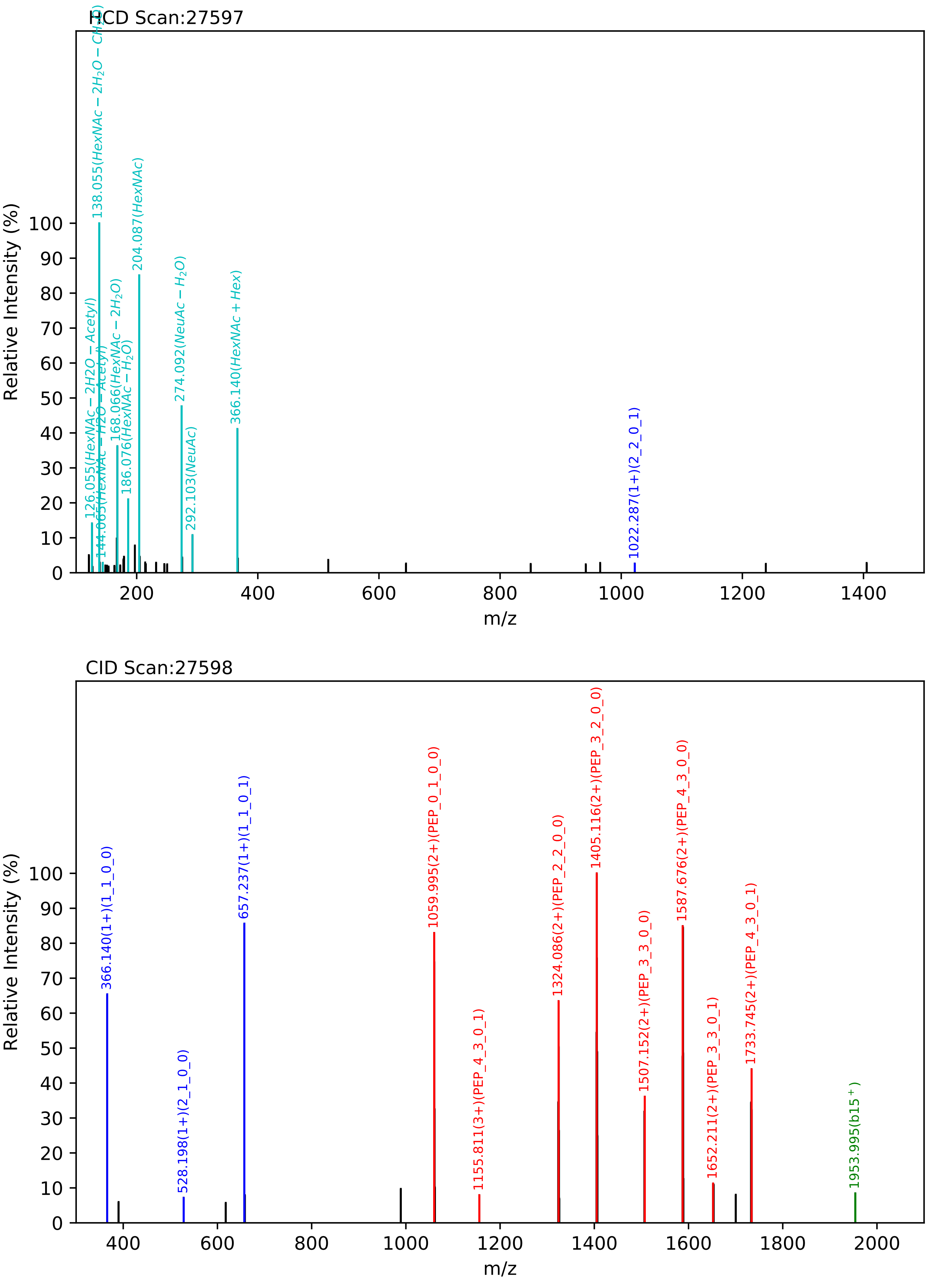

Unknown set no. 62, Experiment: Human Plasma exp_3

QDQCIYNTTYLNVQR(=PEP)_5_4_0_2, m/z:1030.92(4+), RT:92.41, Y-score:89.85

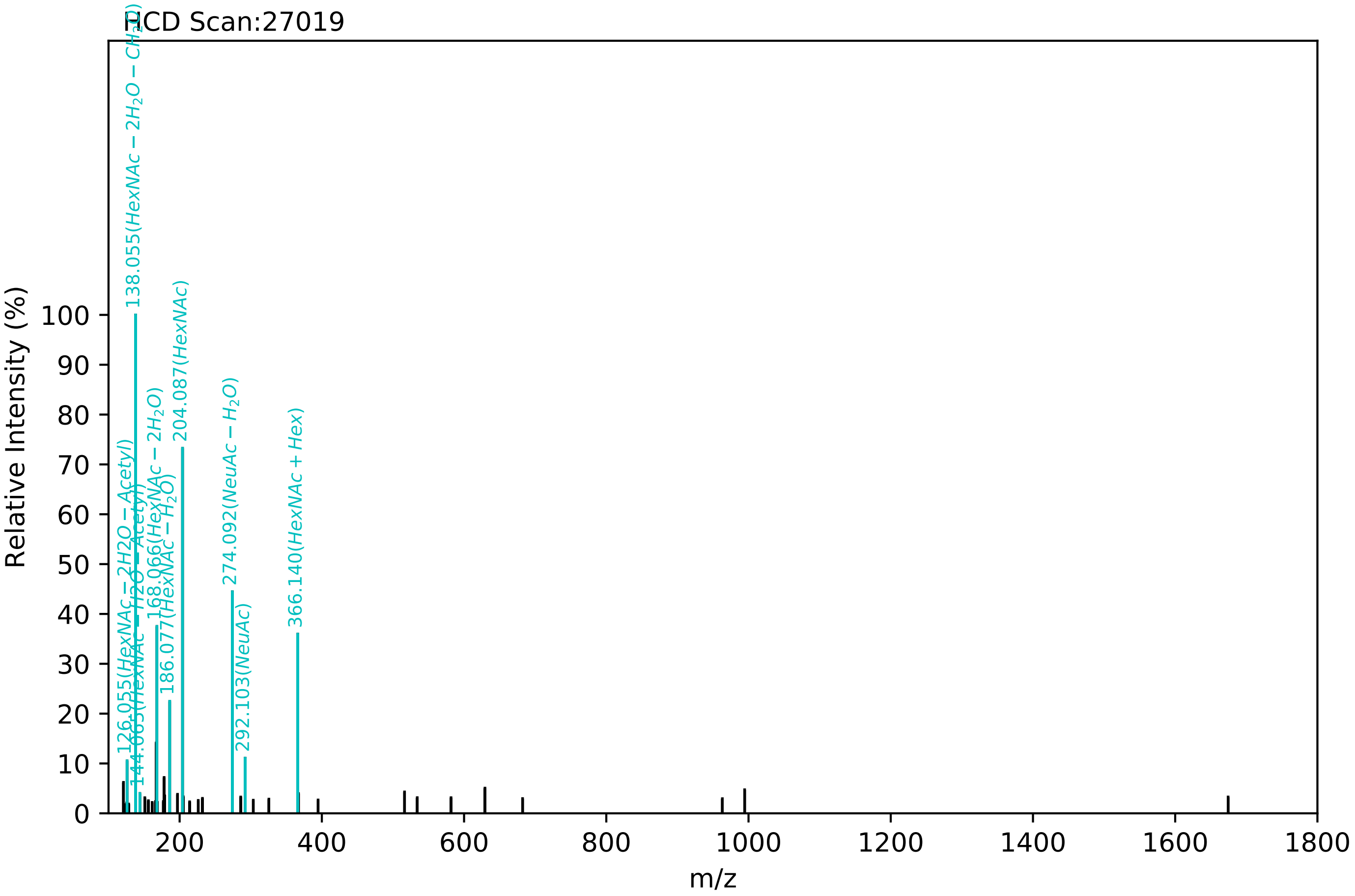

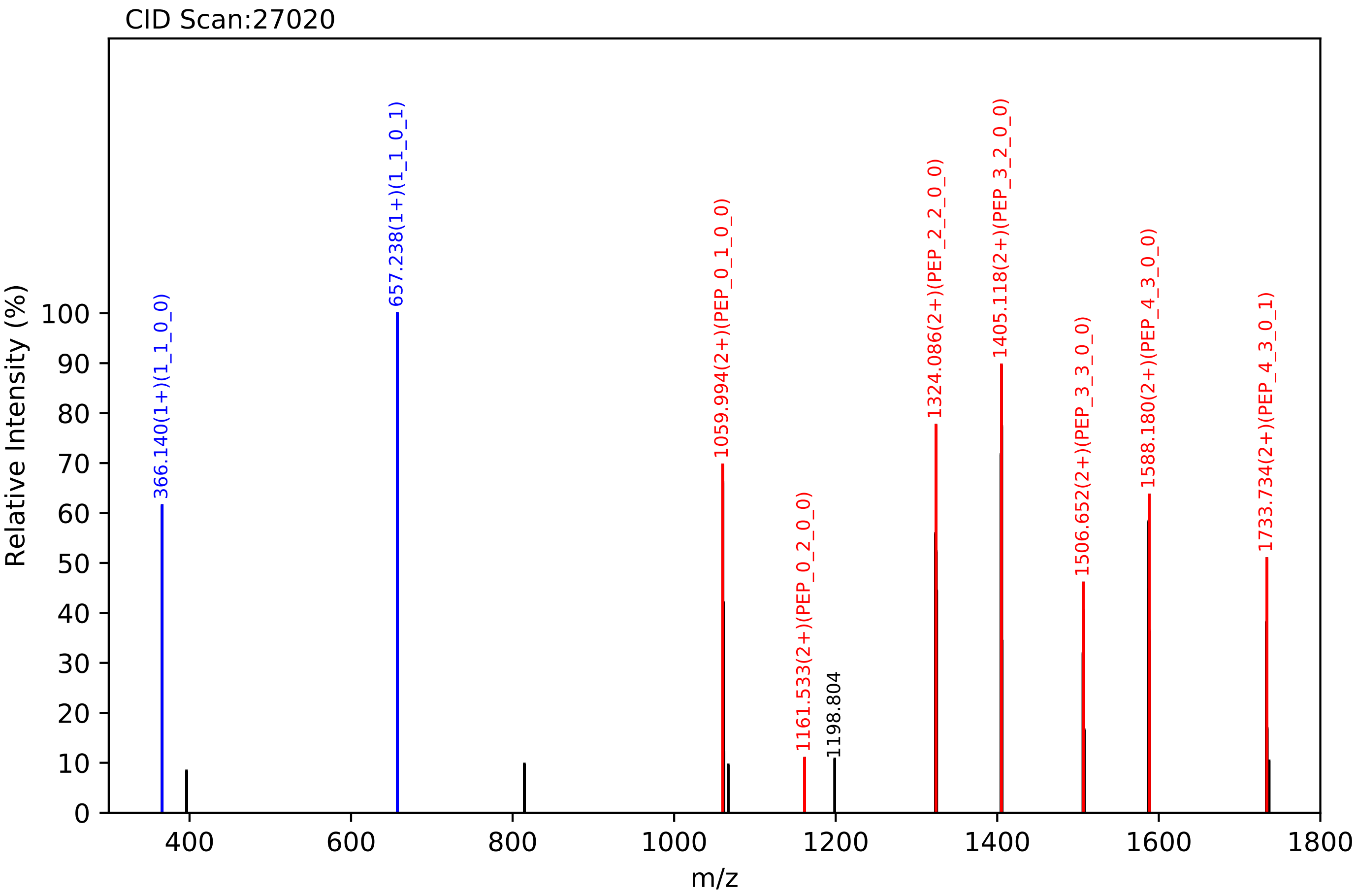

Unknown set no. 63, Experiment: Human Plasma exp_1

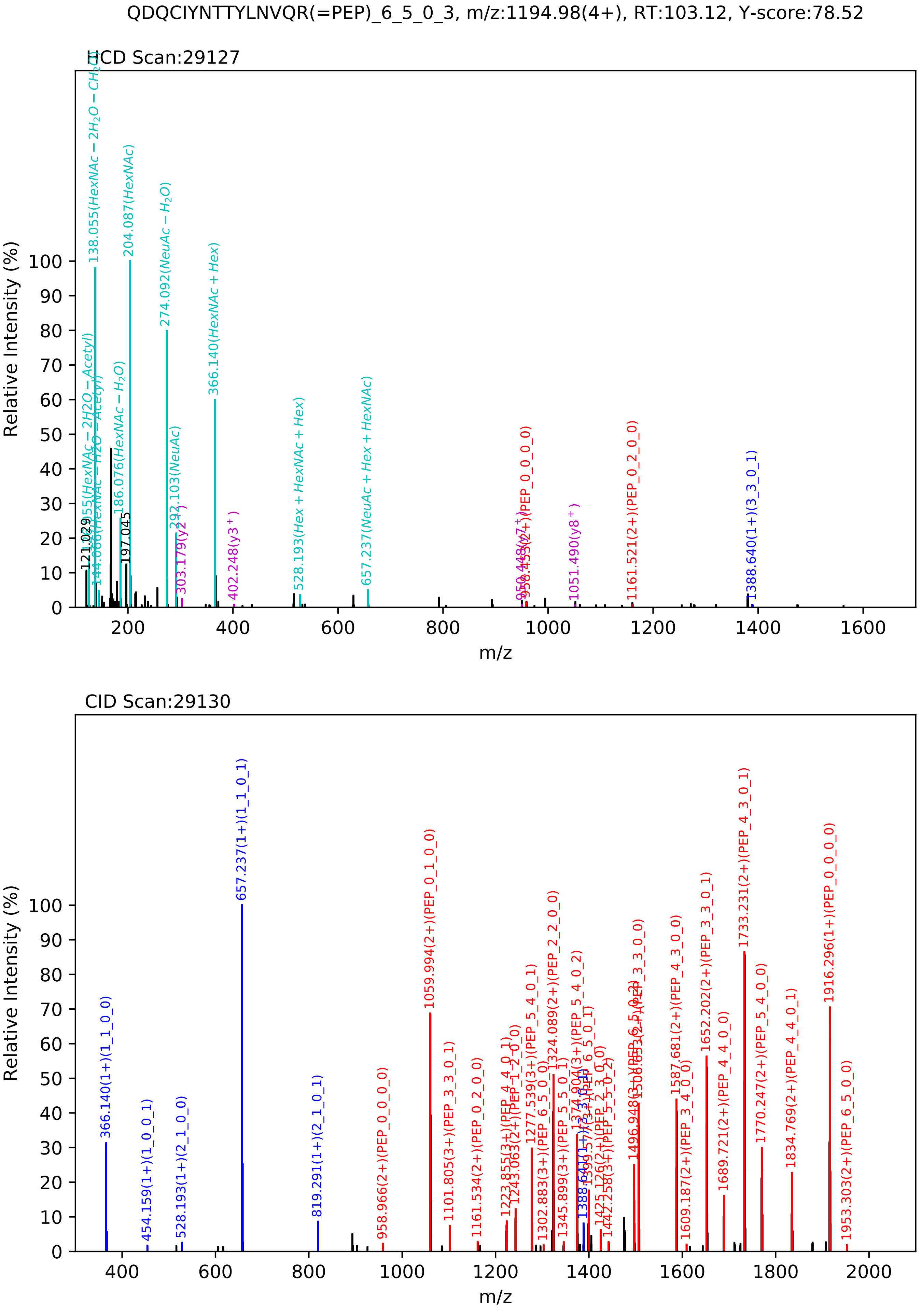

Unknown set no. 64, Experiment: Human Plasma exp_2

QDQCIYNTTYLNVQR(=PEP)_6_5_0_3, m/z:1194.98(4+), RT:103.23, Y-score:81.67

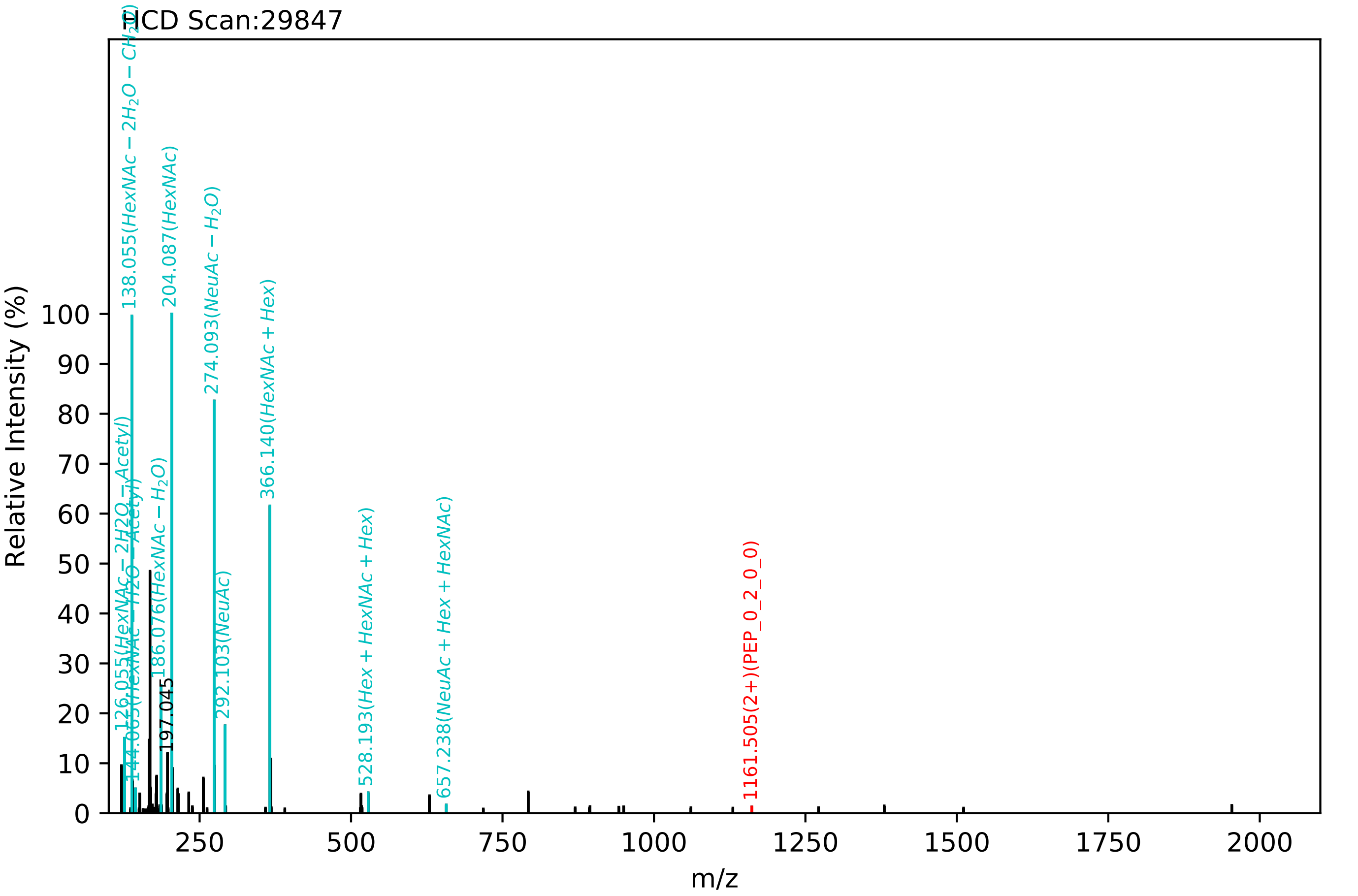

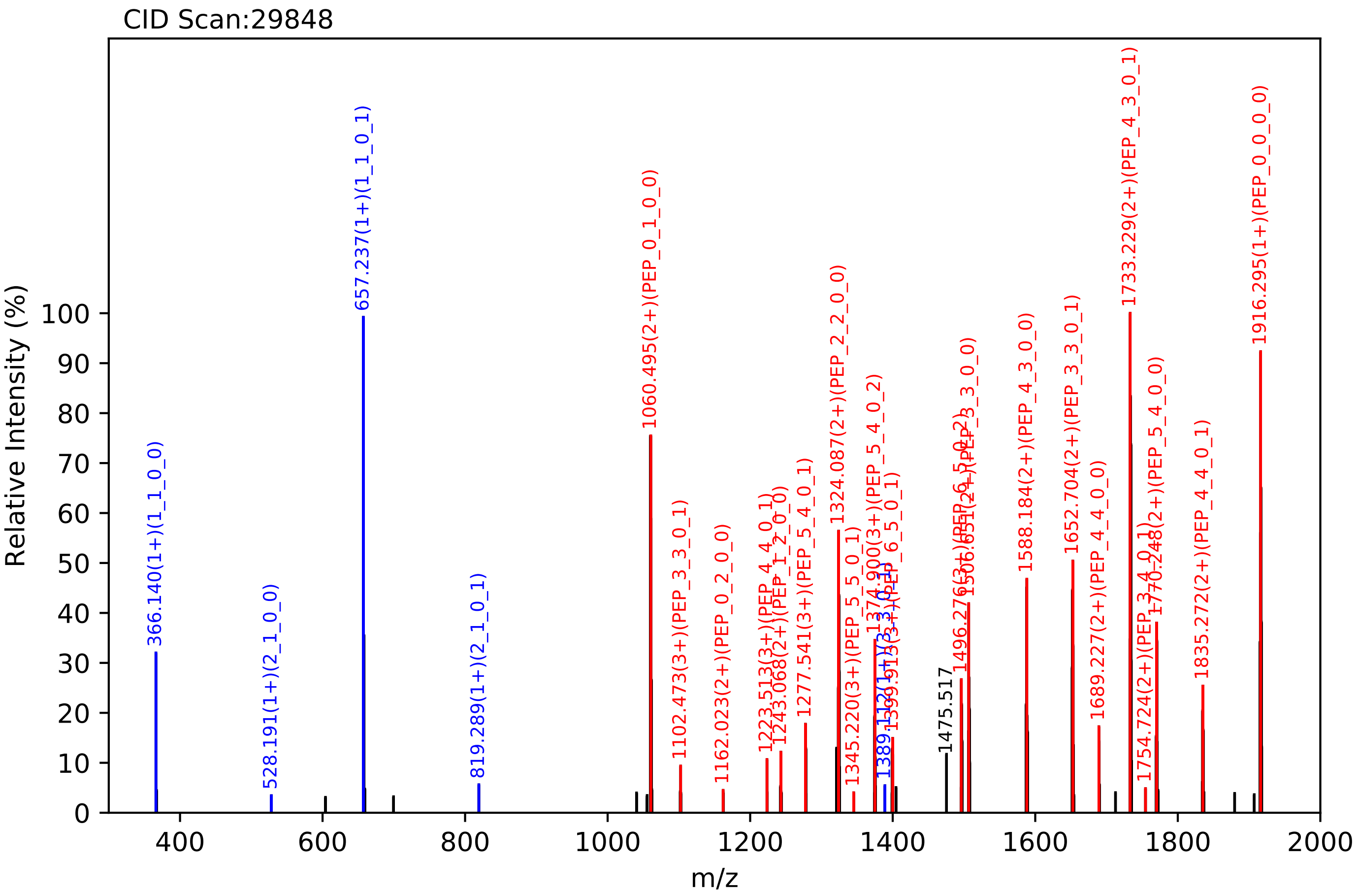

Unknown set no. 65, Experiment: Human Plasma exp_4

QDQCIYNTTYLNVQR(=PEP)_6_5_1_3, m/z:1231.50(4+), RT:103.03, Y-score:78.76

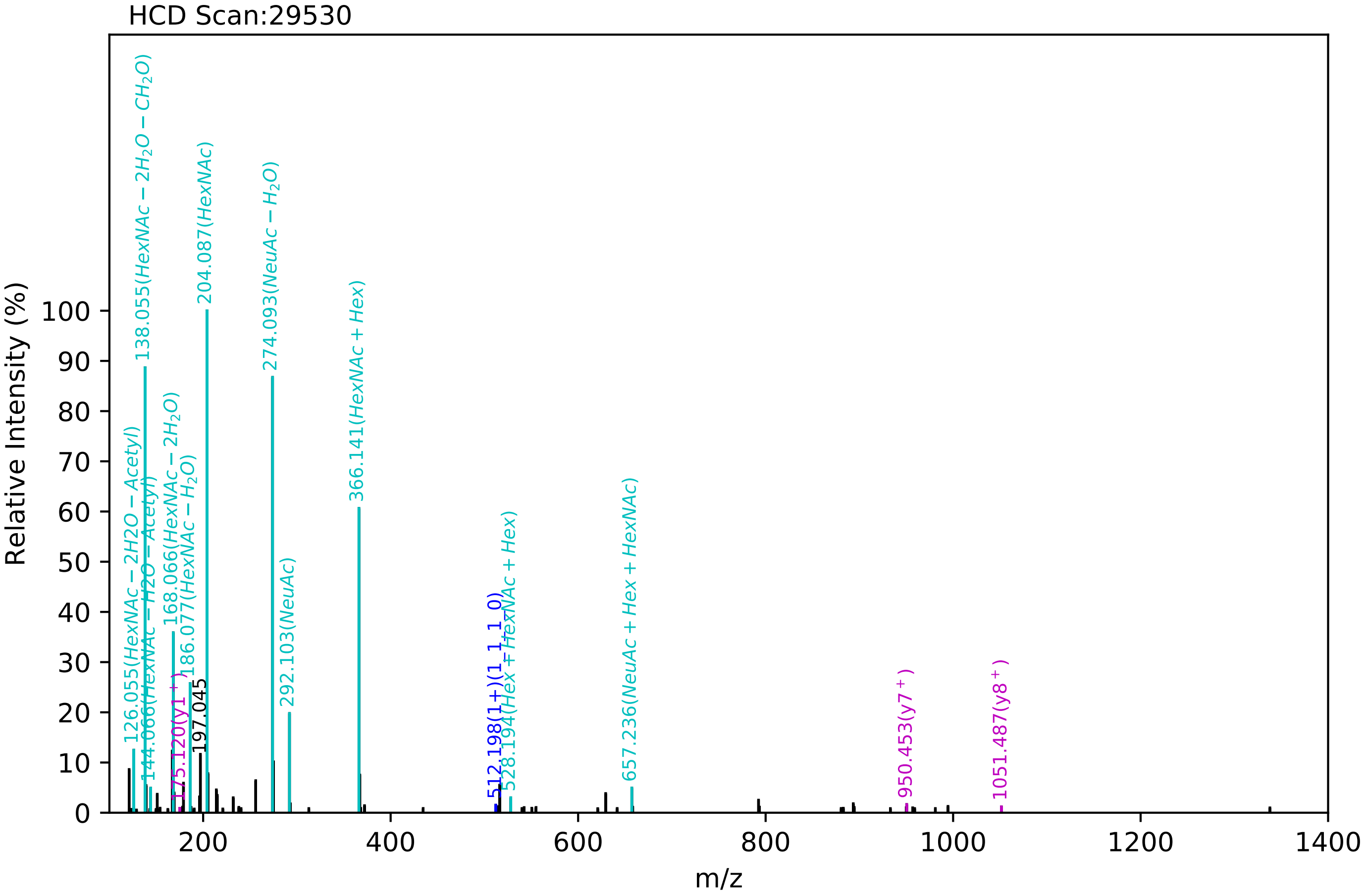

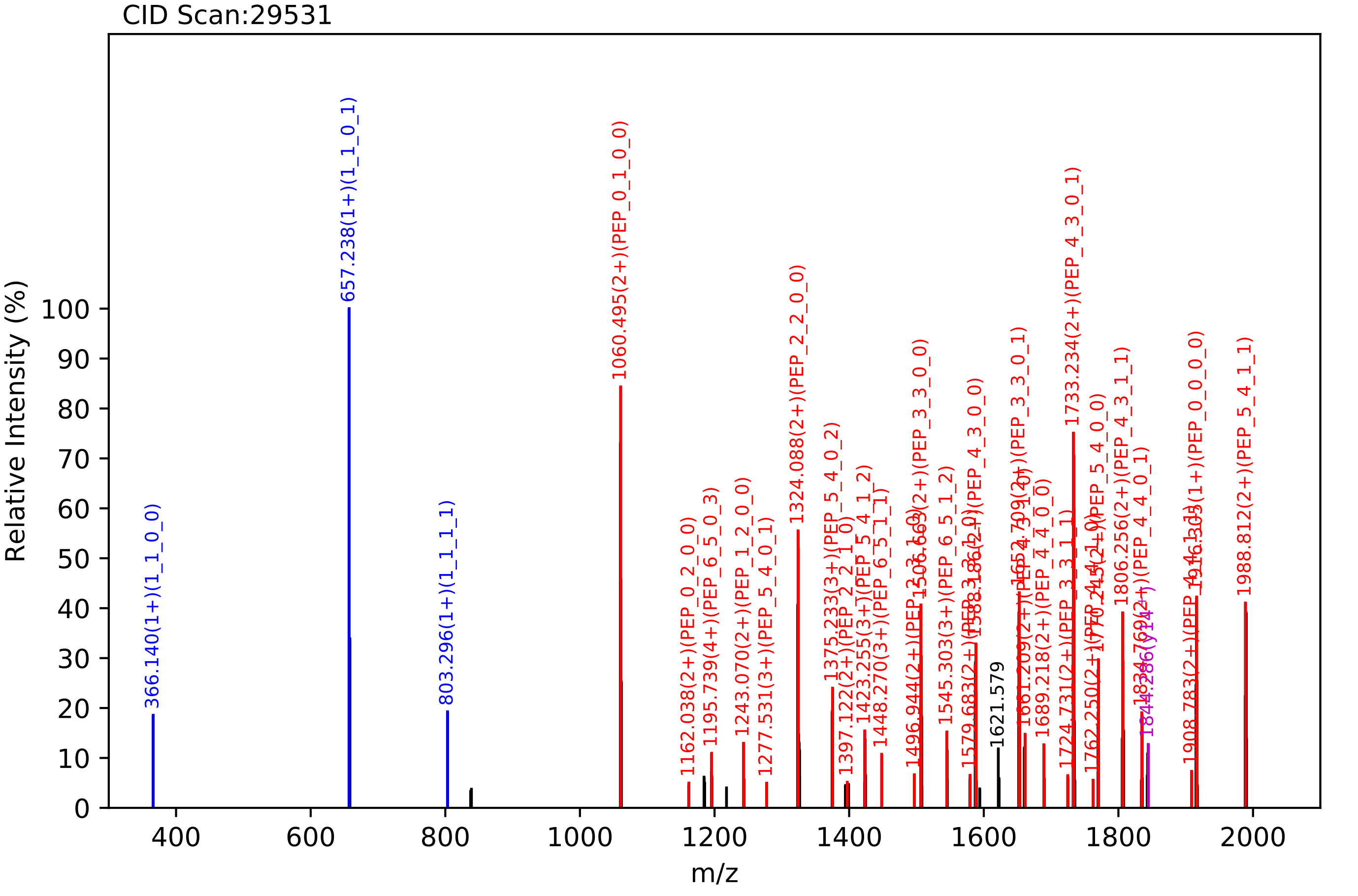

Unknown set no. 66, Experiment: Human Plasma exp_1

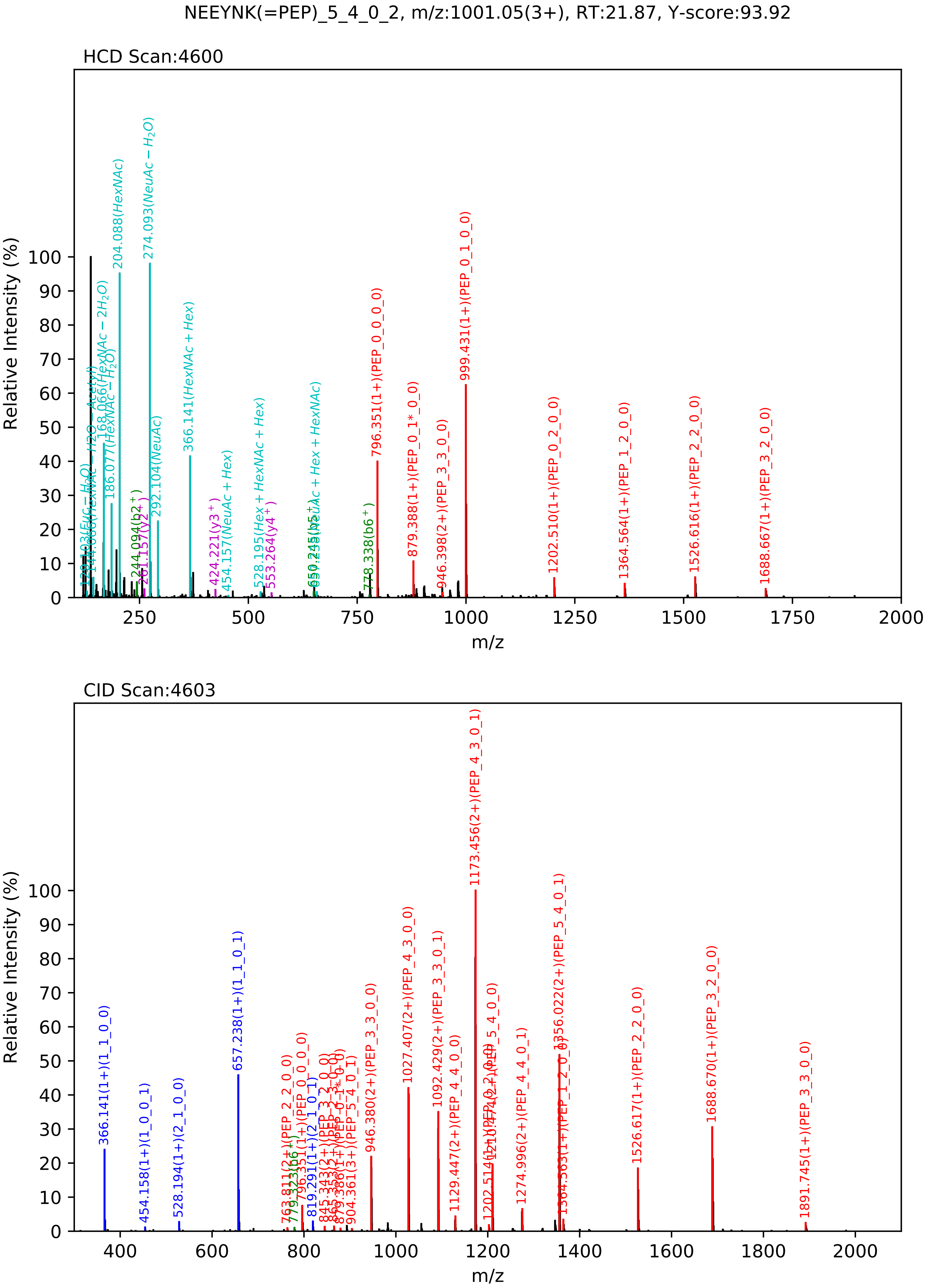

Unknown set no. 67, Experiment: Human Plasma exp_2

NEEYNK(=PEP)_5_4_0_2, m/z:1001.05(3+), RT:21.98, Y-score:94.15

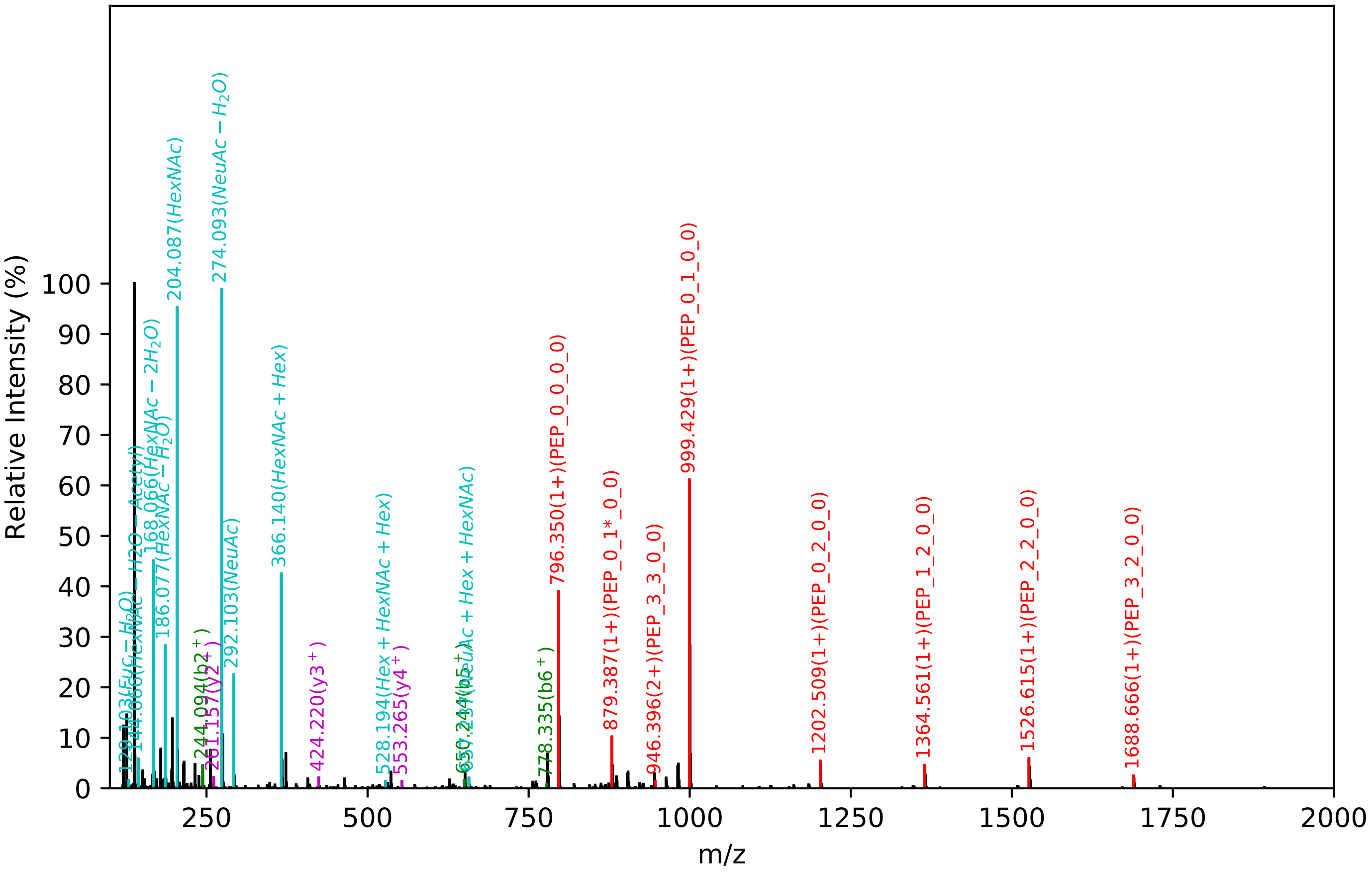

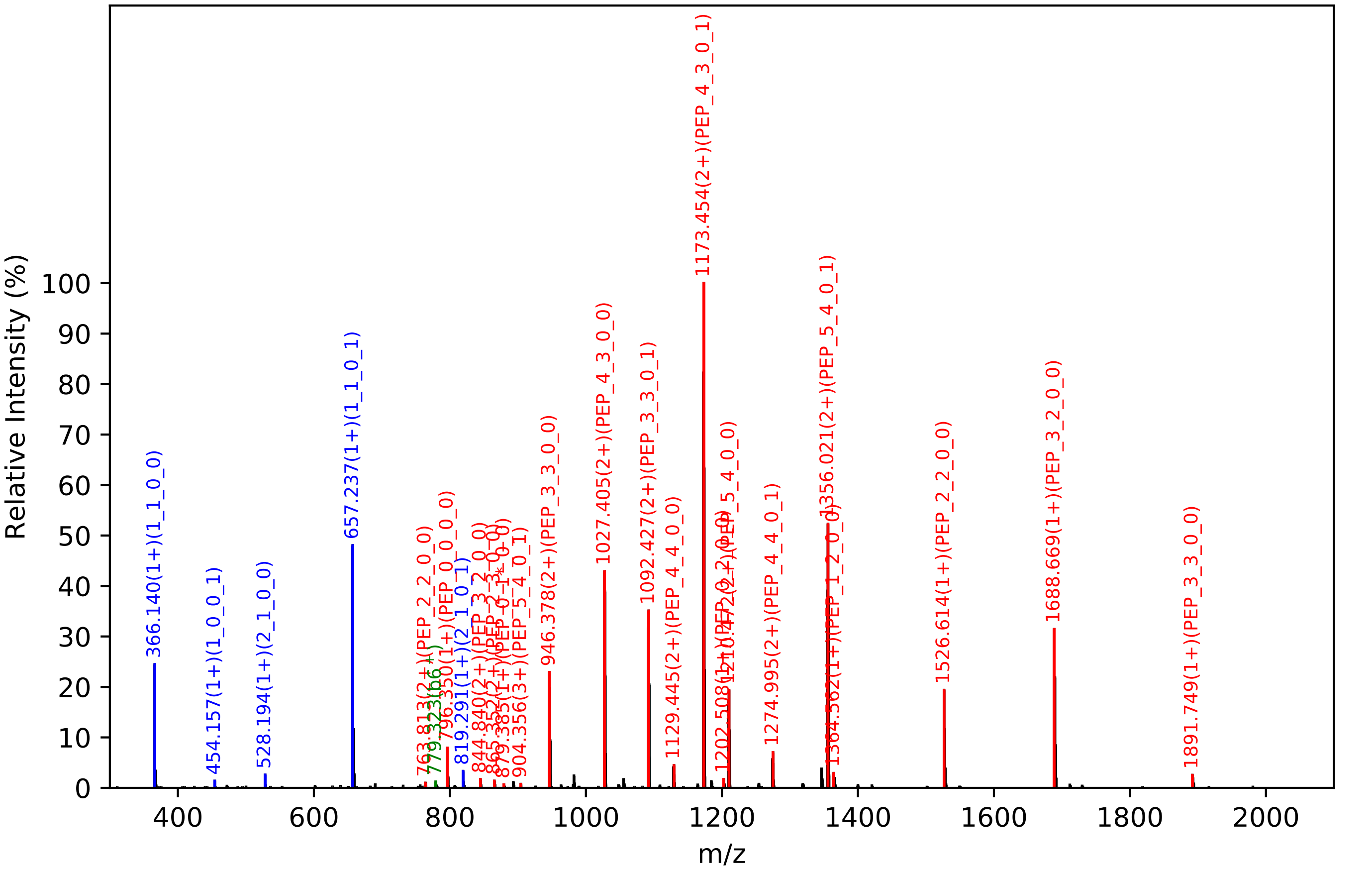

Unknown set no. 68, Experiment: Human Plasma exp_3

NEEYNK(=PEP)_5_4_0_2, m/z:1001.05(3+), RT:22.18, Y-score:94.41

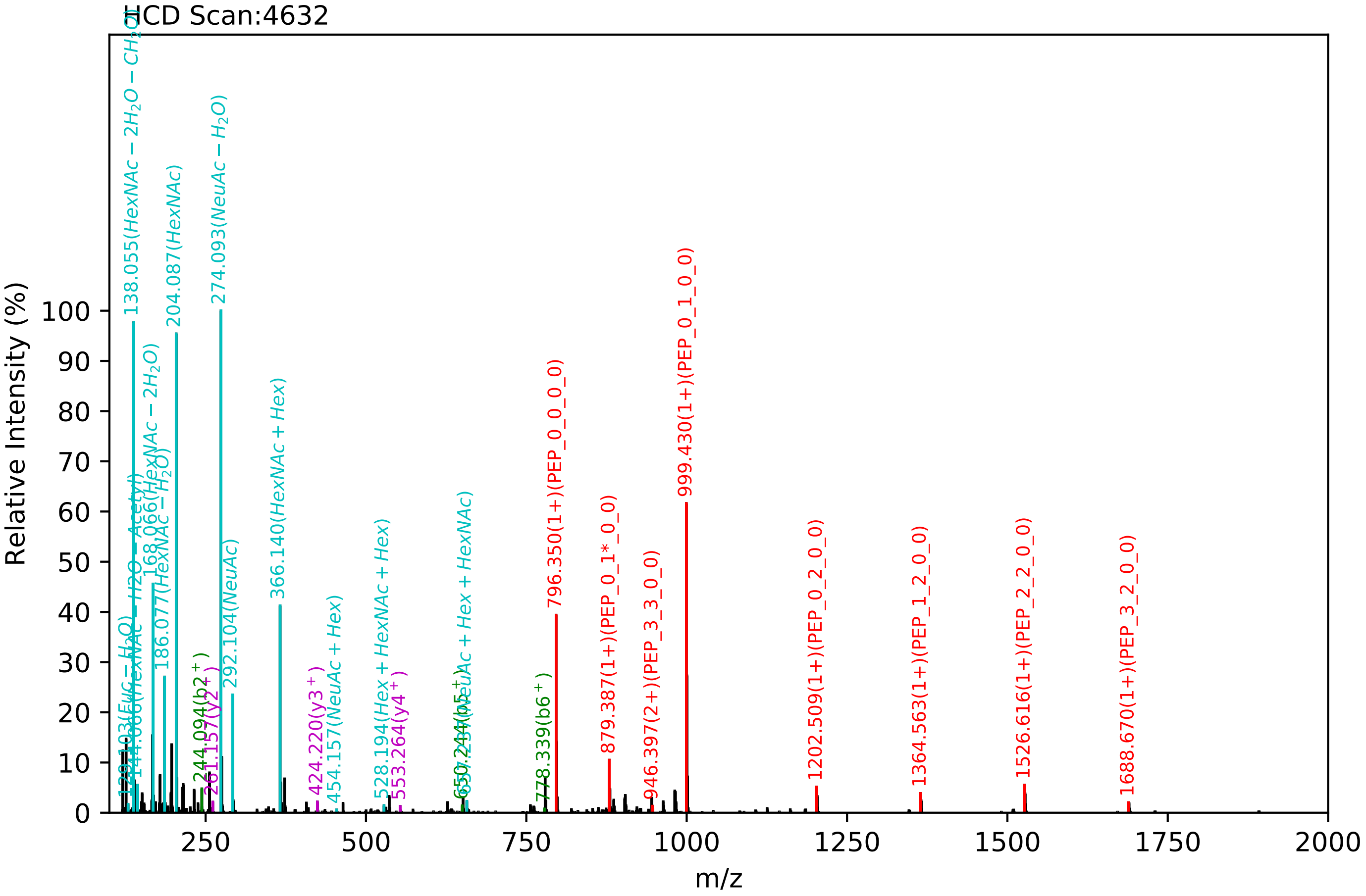

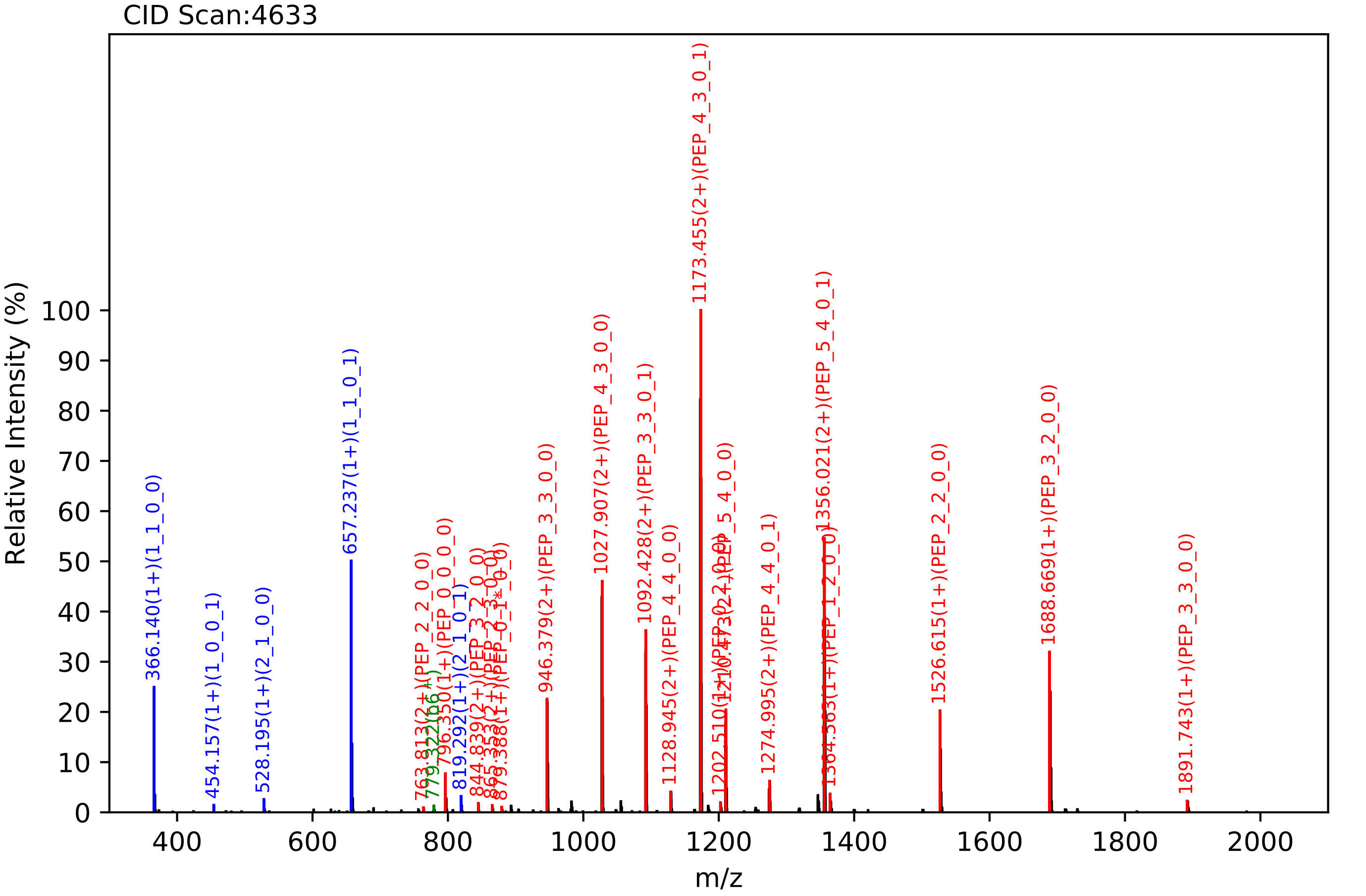

Unknown set no. 69, Experiment: Human Plasma exp_4

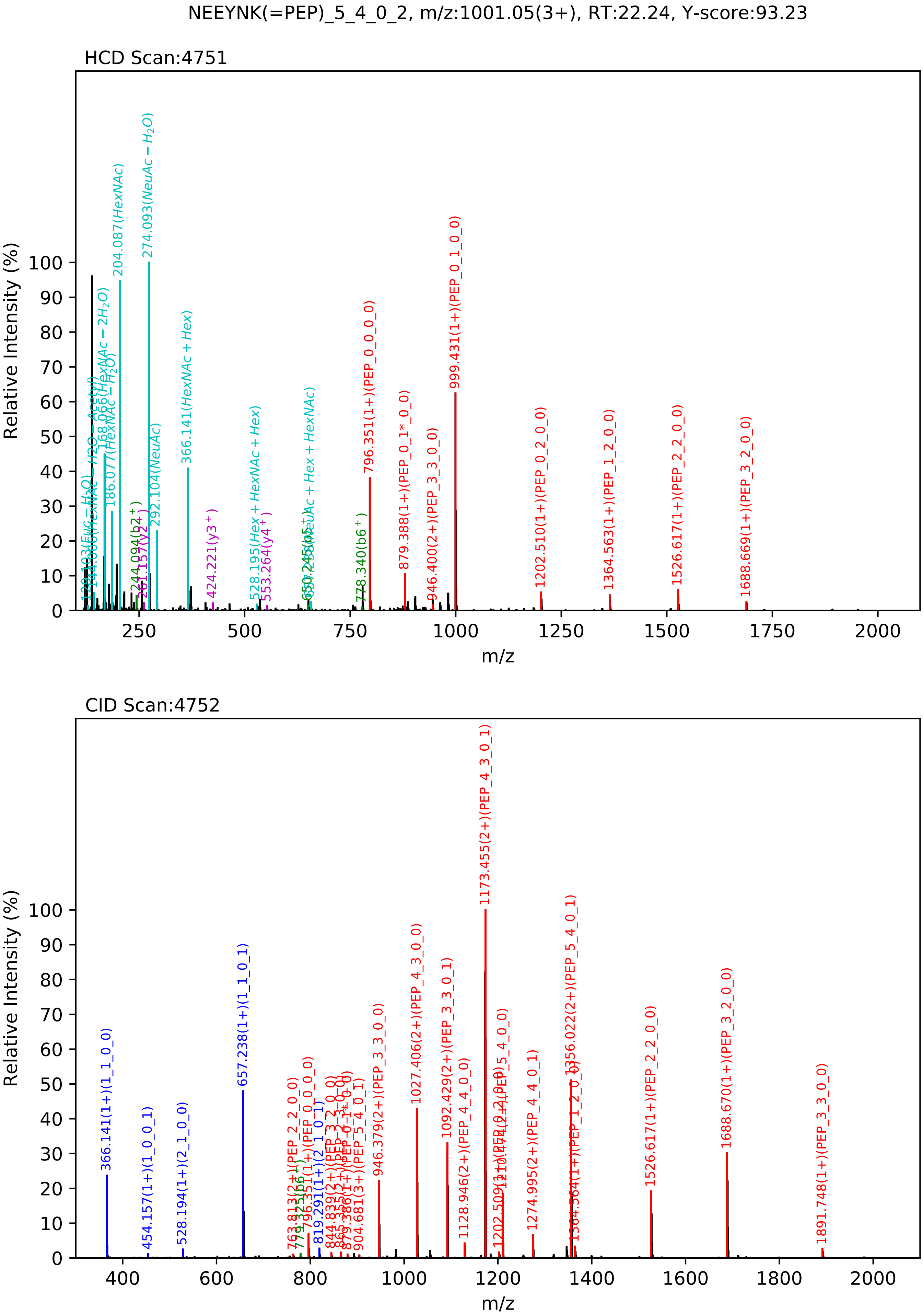

Unknown set no. 70, Experiment: Human Plasma exp_5

NEEYNK(=PEP)_5_4_0_2, m/z:1501.06(2+), RT:20.95, Y-score:79.37

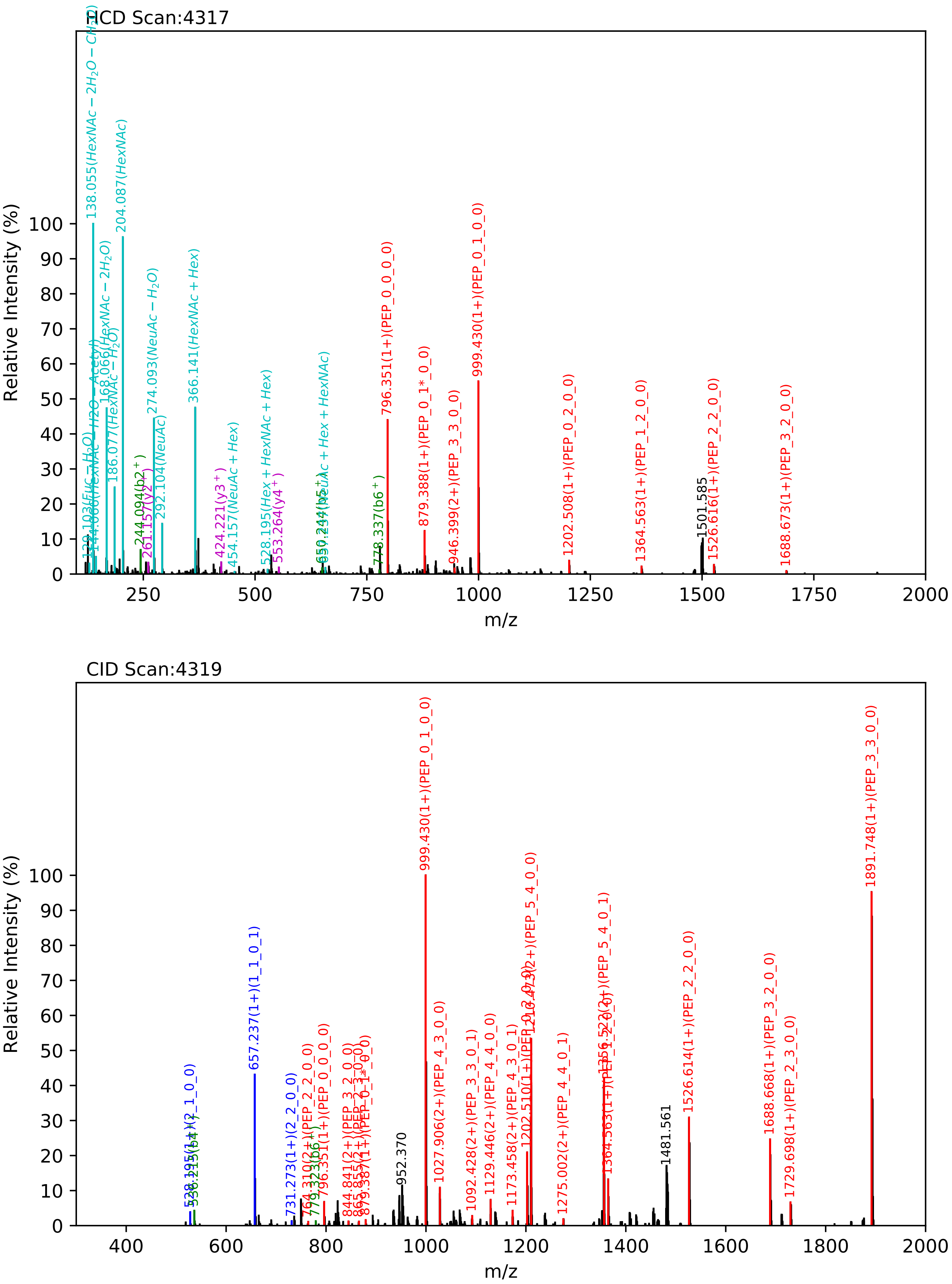

Unknown set no. 71, Experiment: Human Plasma exp_1

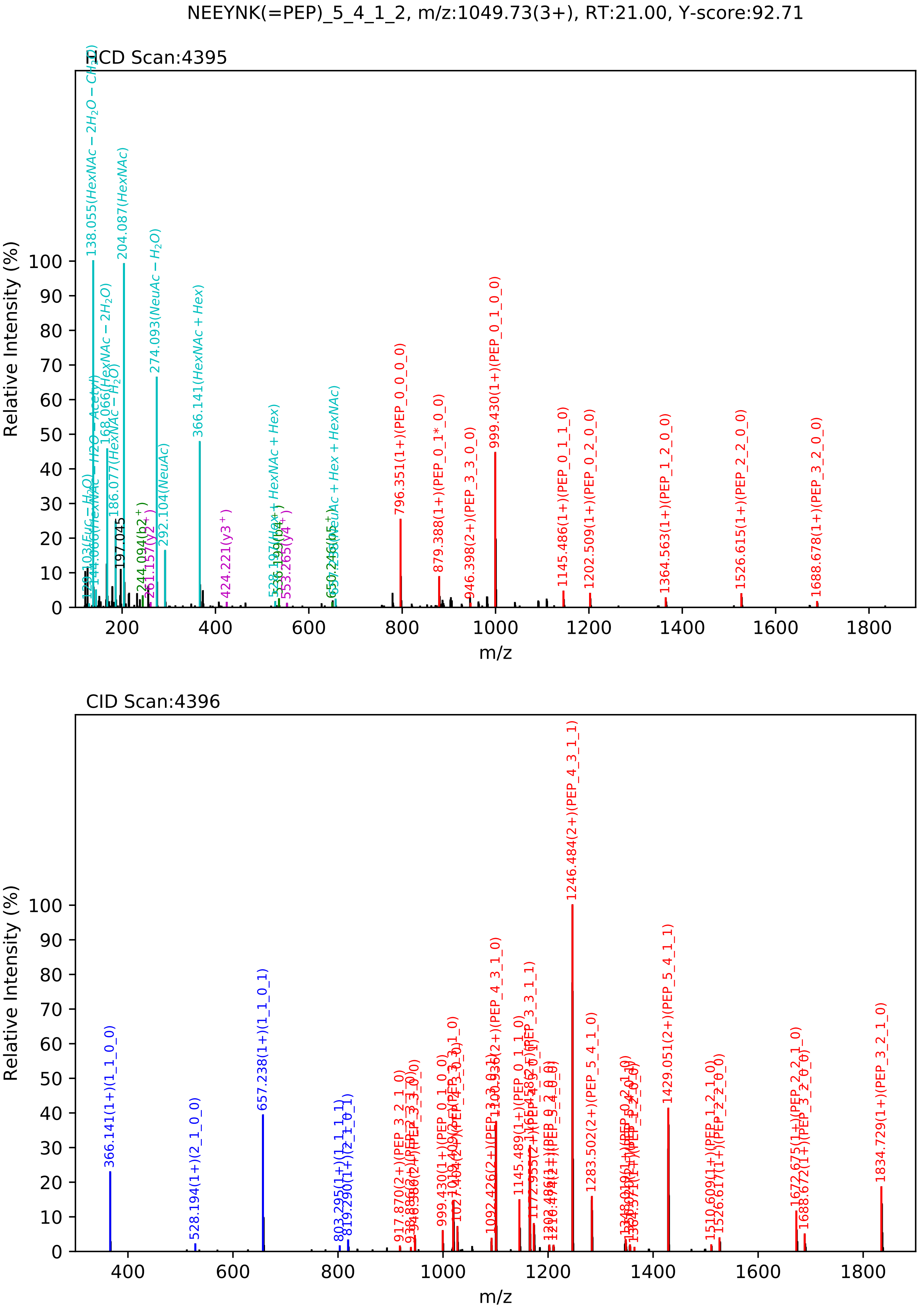

Unknown set no. 72, Experiment: Human Plasma exp_2

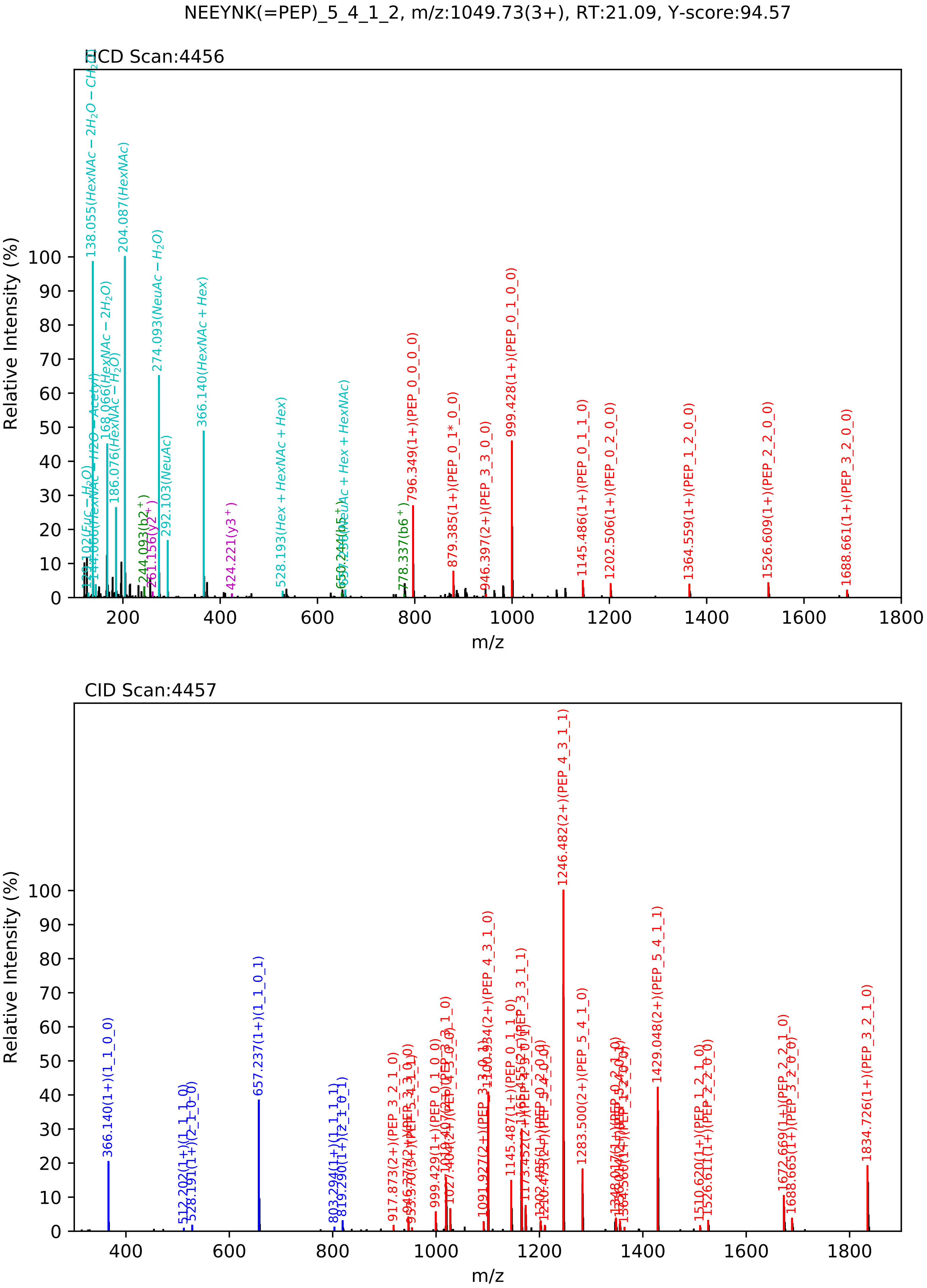

Unknown set no. 73, Experiment: Human Plasma exp_3

NEEYNK(=PEP)_5_4_1_2, m/z:1049.73(3+), RT:21.24, Y-score:94.14

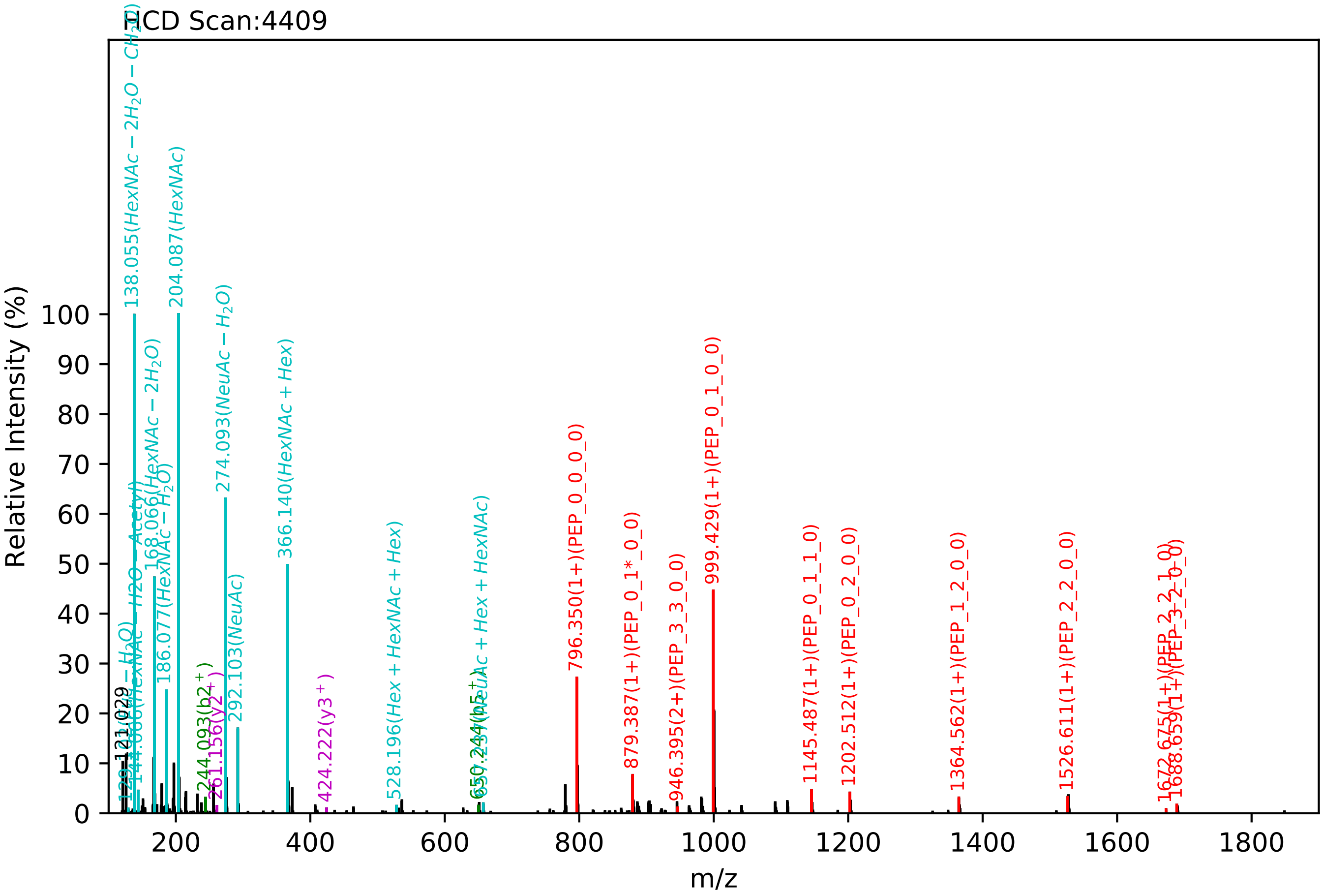

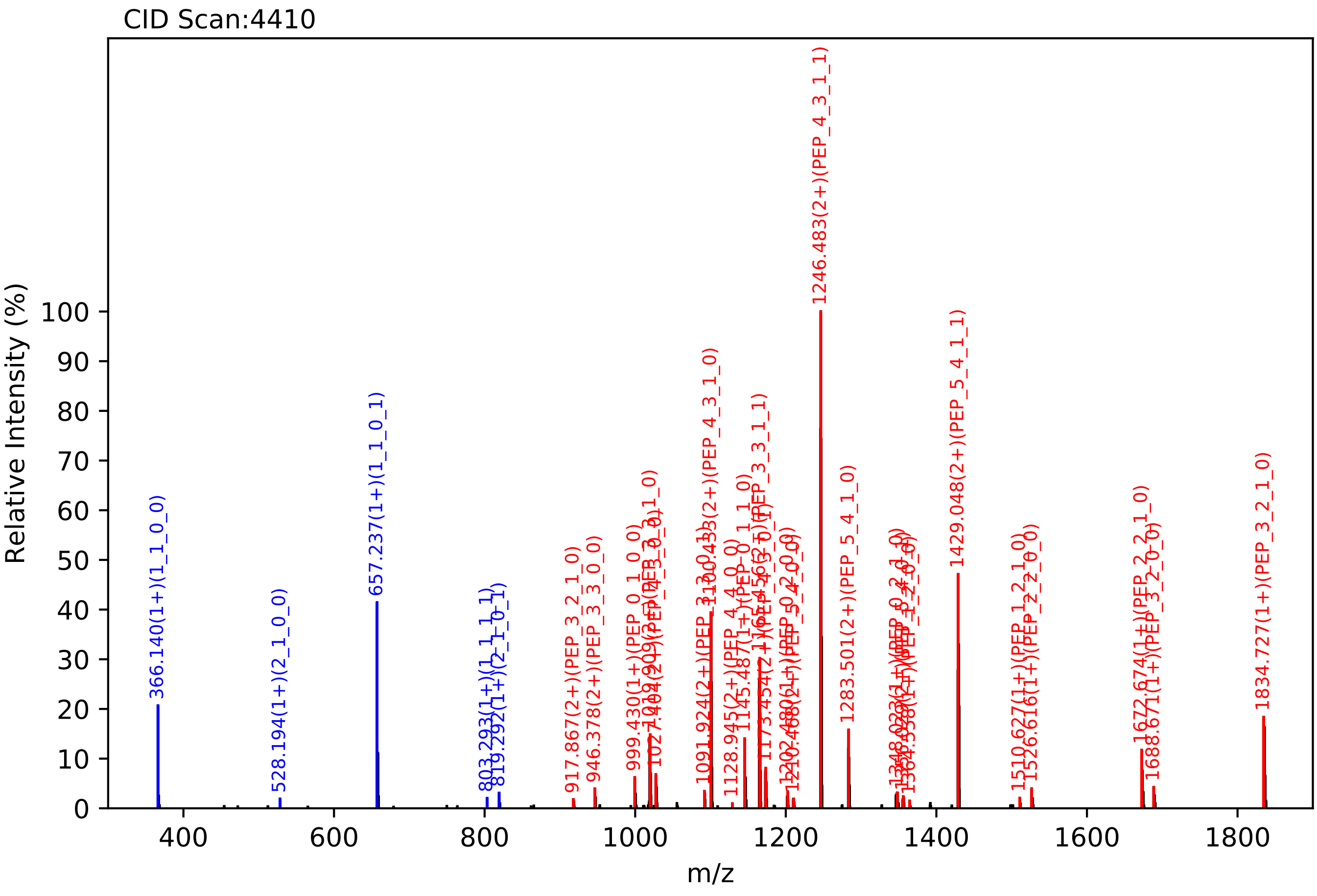

Unknown set no. 74, Experiment: Human Plasma exp_4

NEEYNK(=PEP)_5_4_1_2, m/z:1049.73(3+), RT:21.33, Y-score:93.63

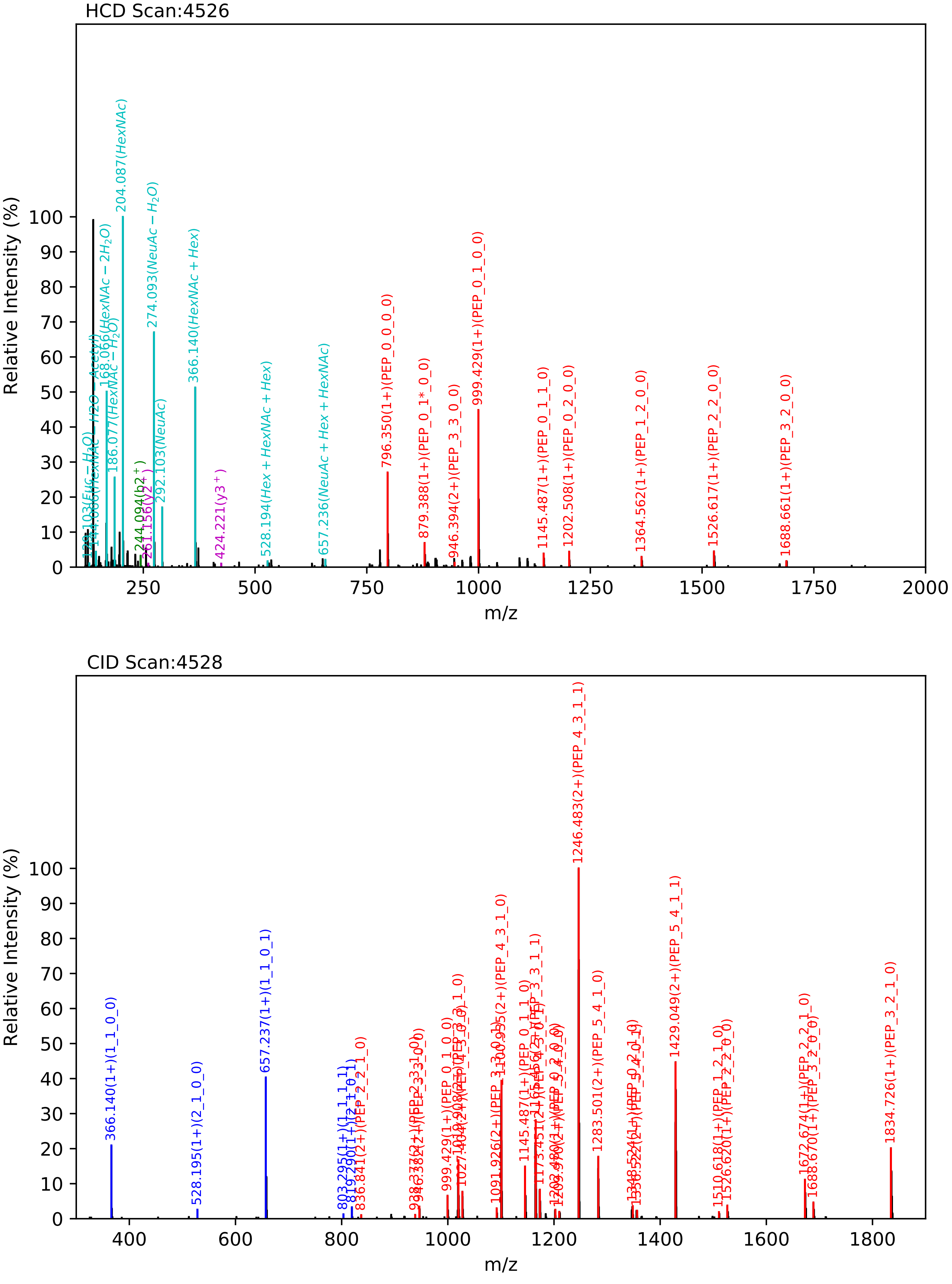

Unknown set no. 75, Experiment: Human Plasma exp_5

NEEYNK(=PEP)_5_4_1_2, m/z:1049.73(3+), RT:21.29, Y-score:94.45

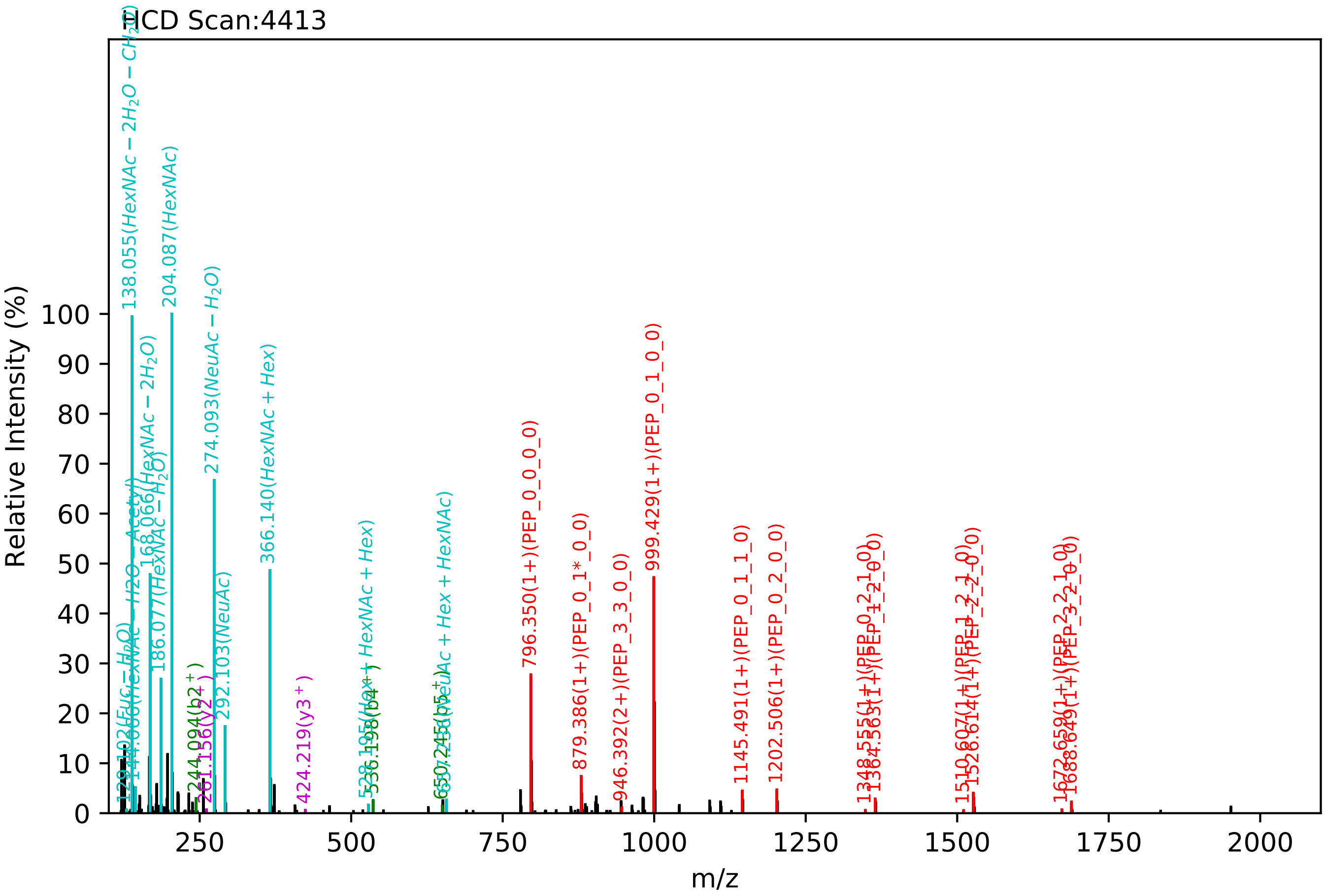

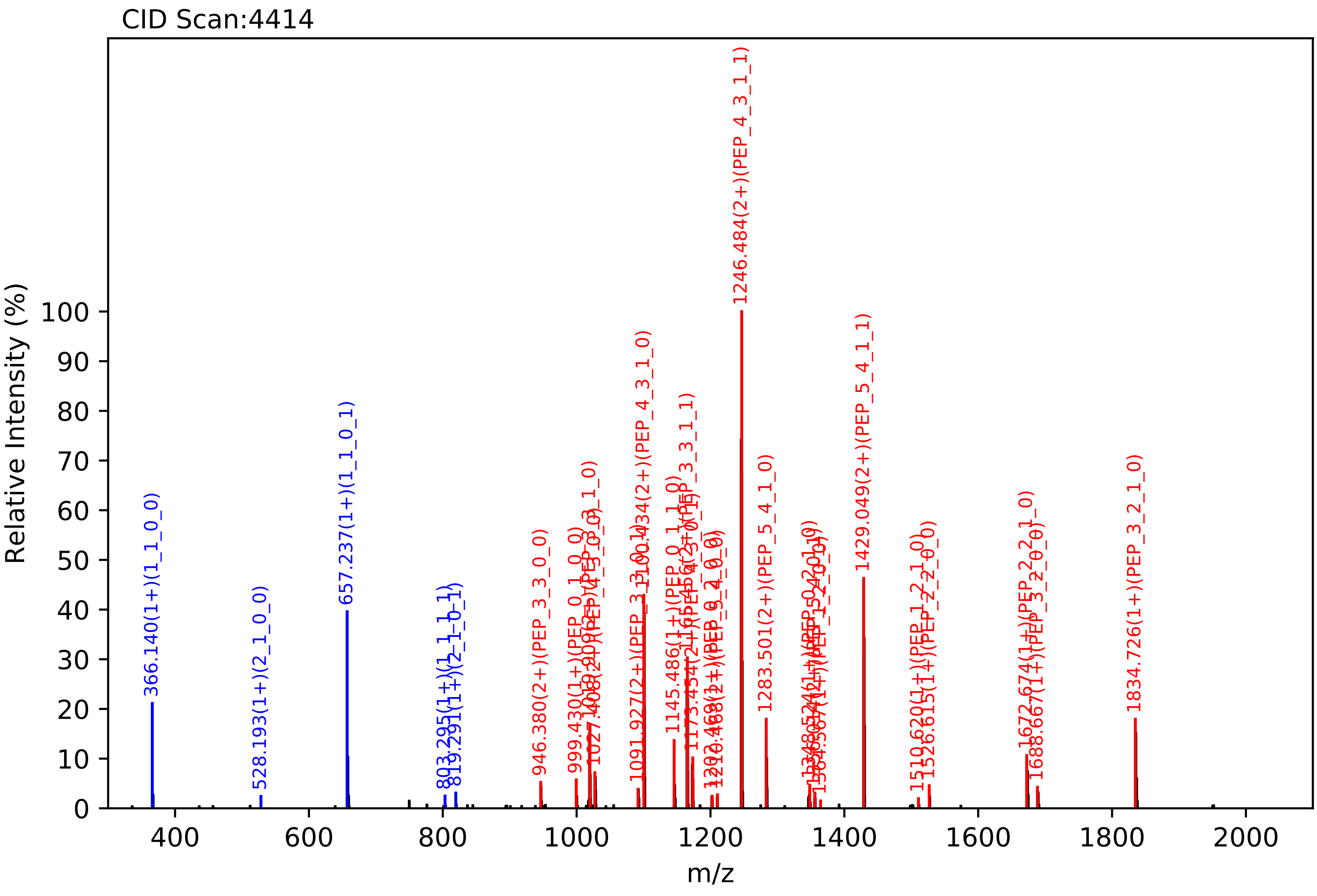

Unknown set no. 76, Experiment: Human Plasma exp_2

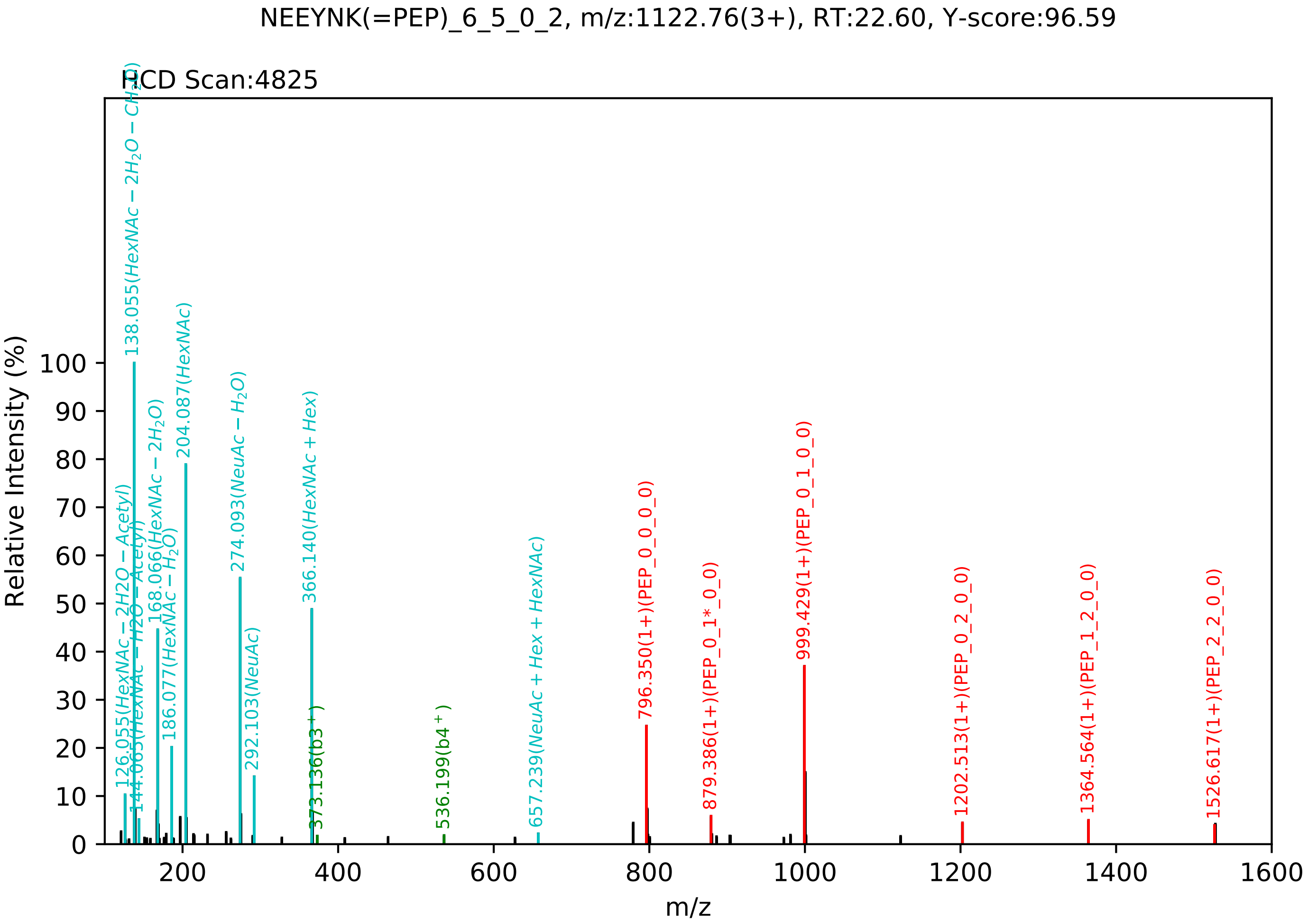

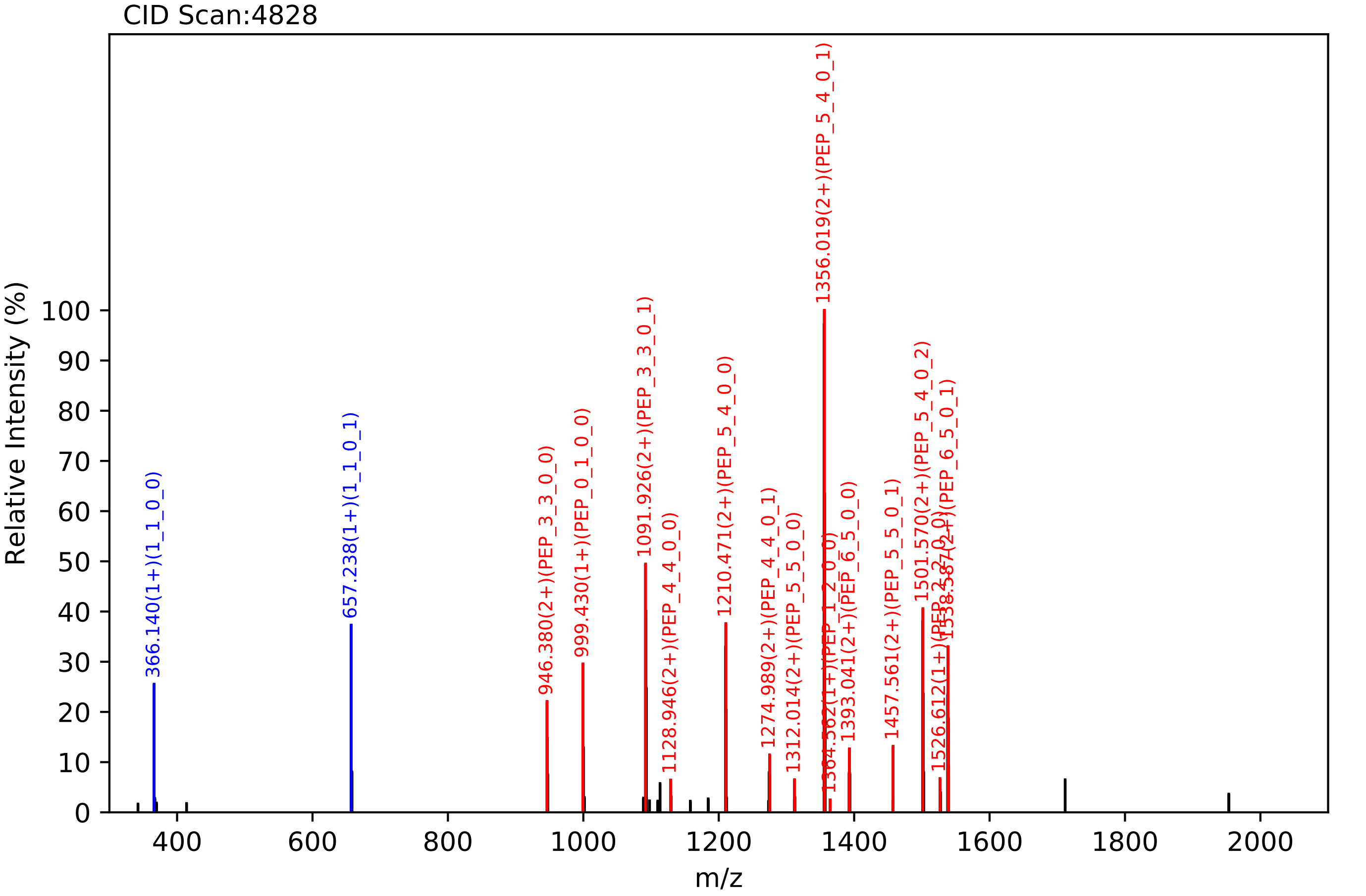

Unknown set no. 77, Experiment: Human Plasma exp_4

NEEYNK(=PEP)_6_5_0_2, m/z:1122.76(3+), RT:21.12, Y-score:93.31

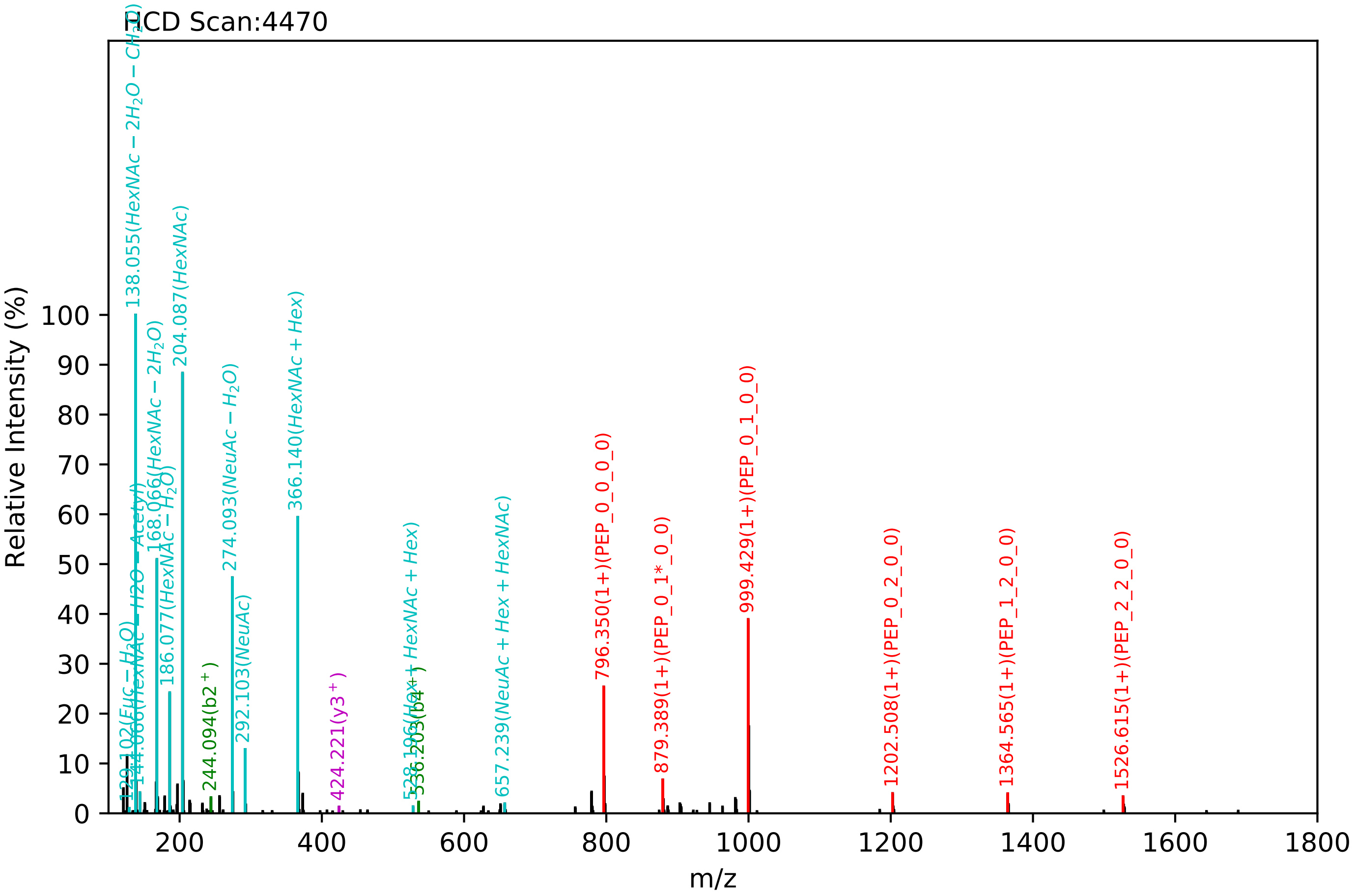

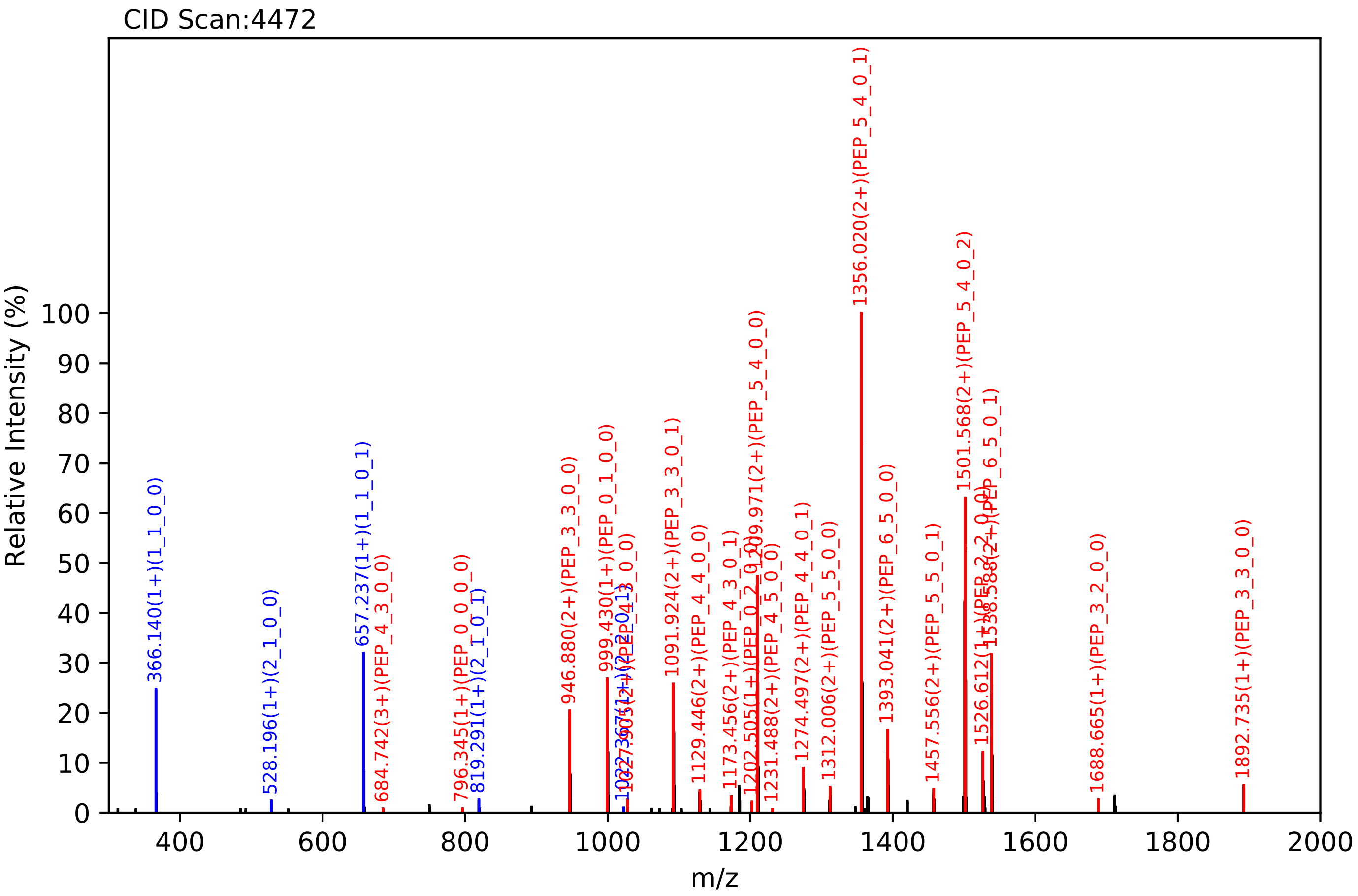

Unknown set no. 78, Experiment: Human Plasma exp_1

NEEYNK(=PEP)_6_5_0_3, m/z:1219.79(3+), RT:34.55, Y-score:95.39

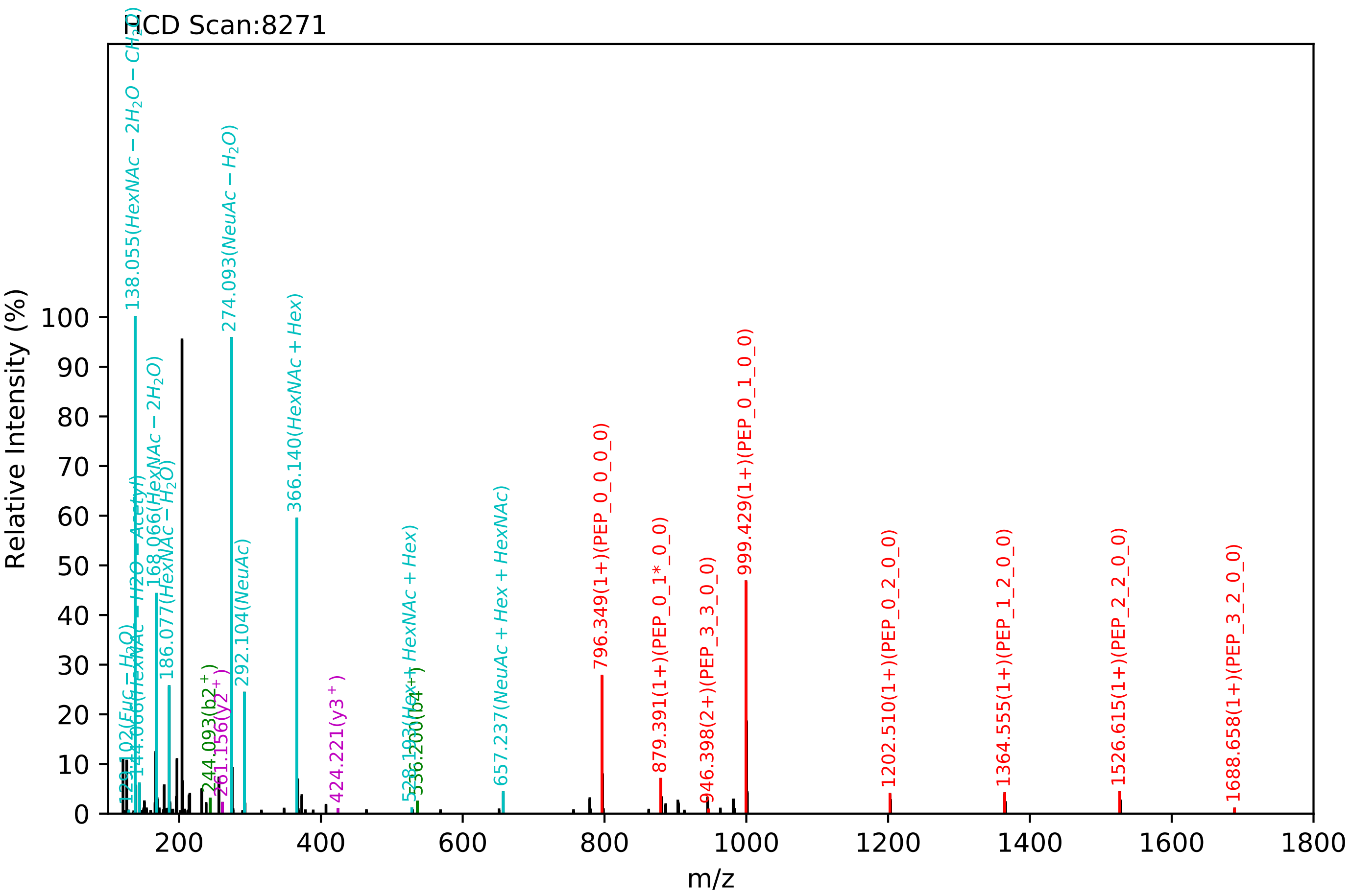

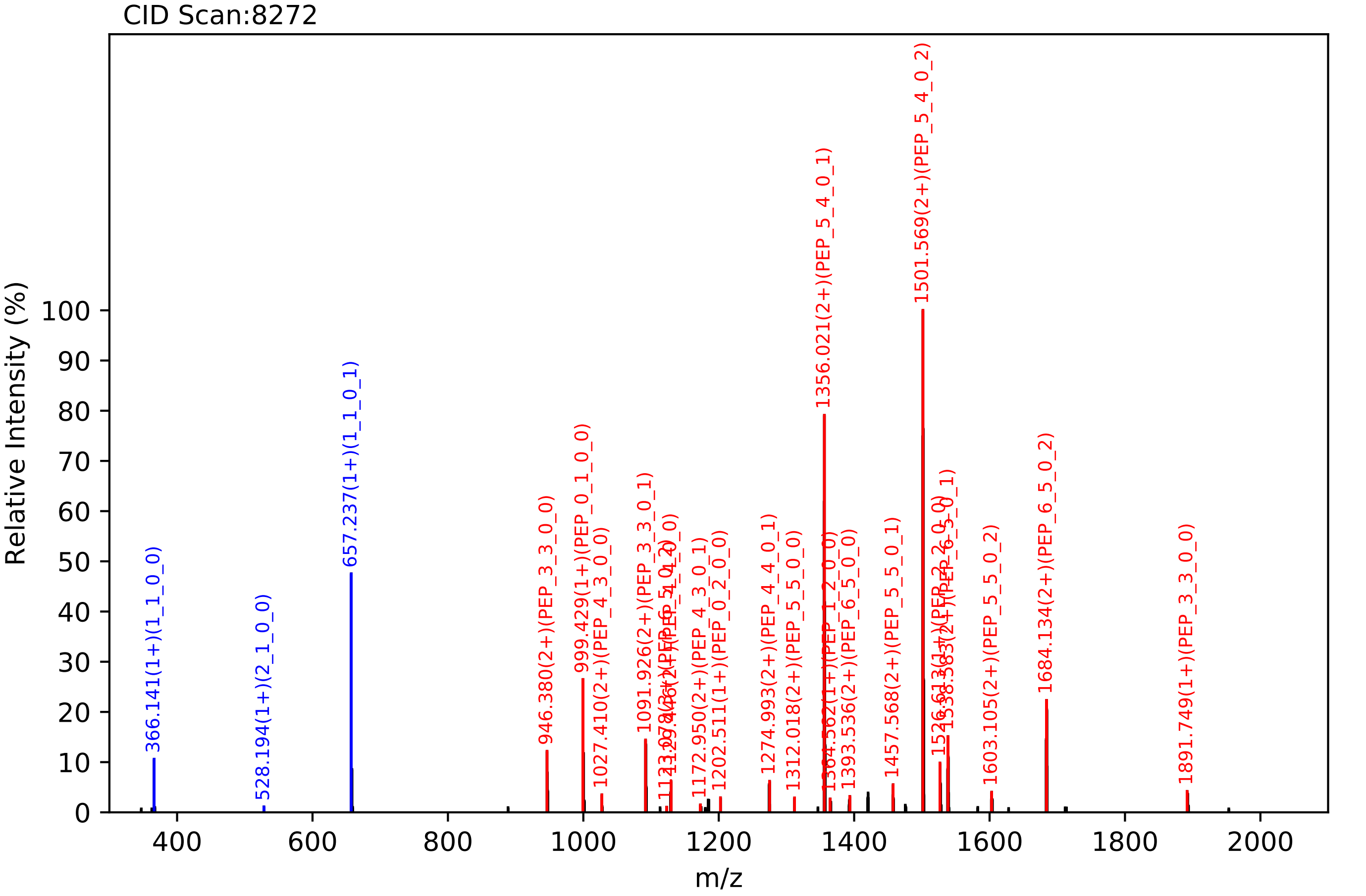

Unknown set no. 79, Experiment: Human Plasma exp_3

NEEYNK(=PEP)_6_5_0_3, m/z:1219.79(3+), RT:33.16, Y-score:95.89

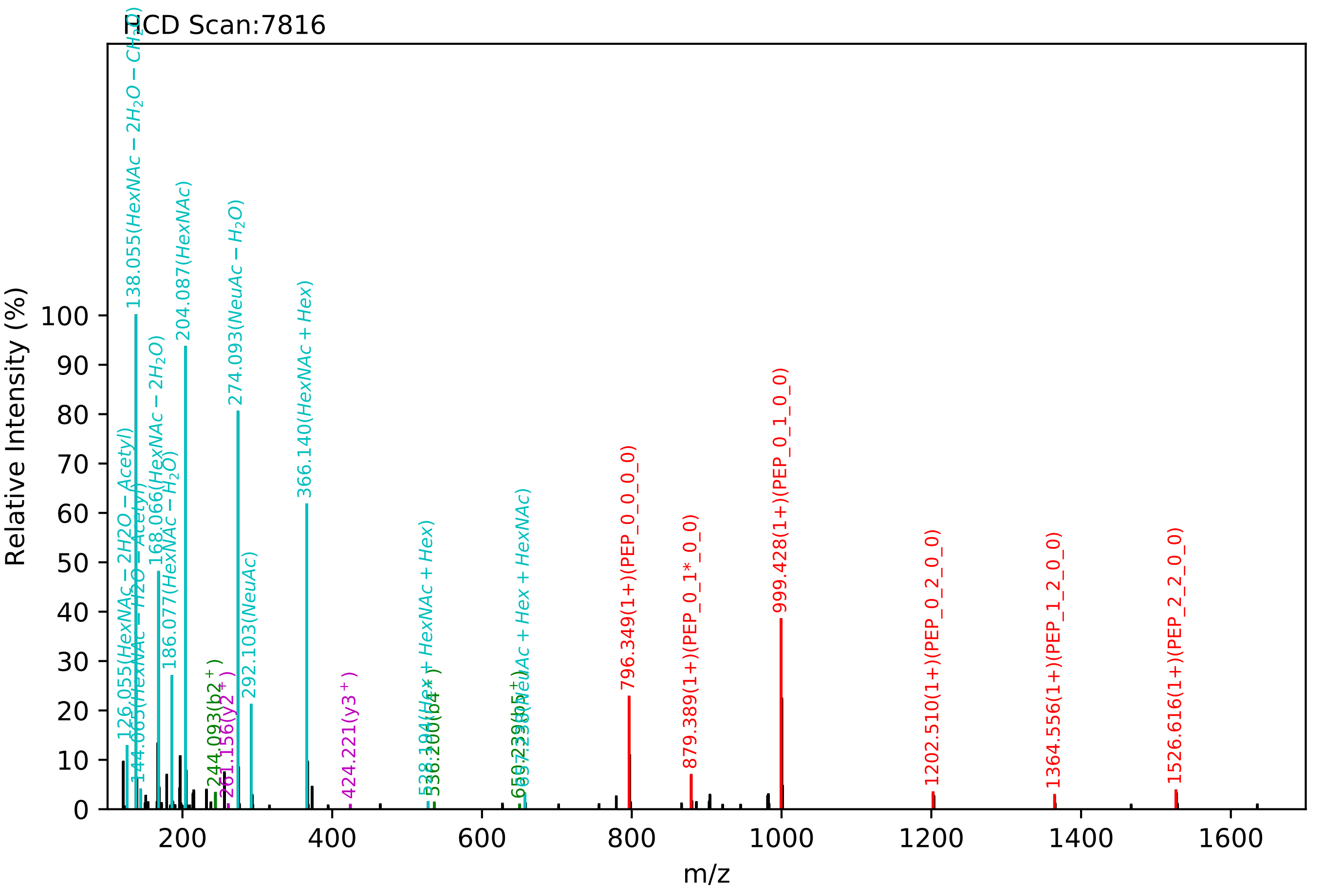

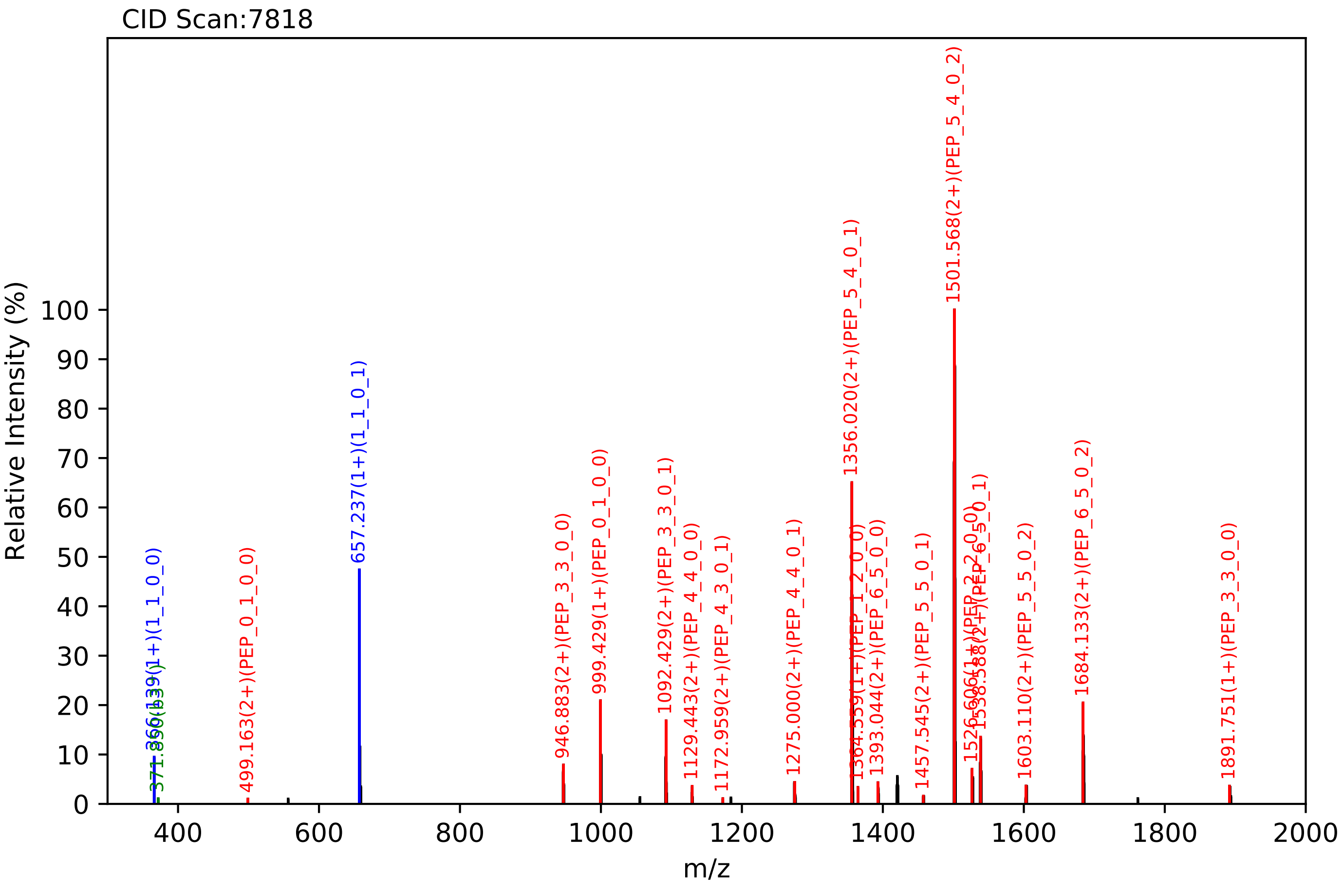

Unknown set no. 80, Experiment: Human Plasma exp_2

NEEYNK(=PEP)_6_5_1_2, m/z:1171.44(3+), RT:19.64, Y-score:94.69

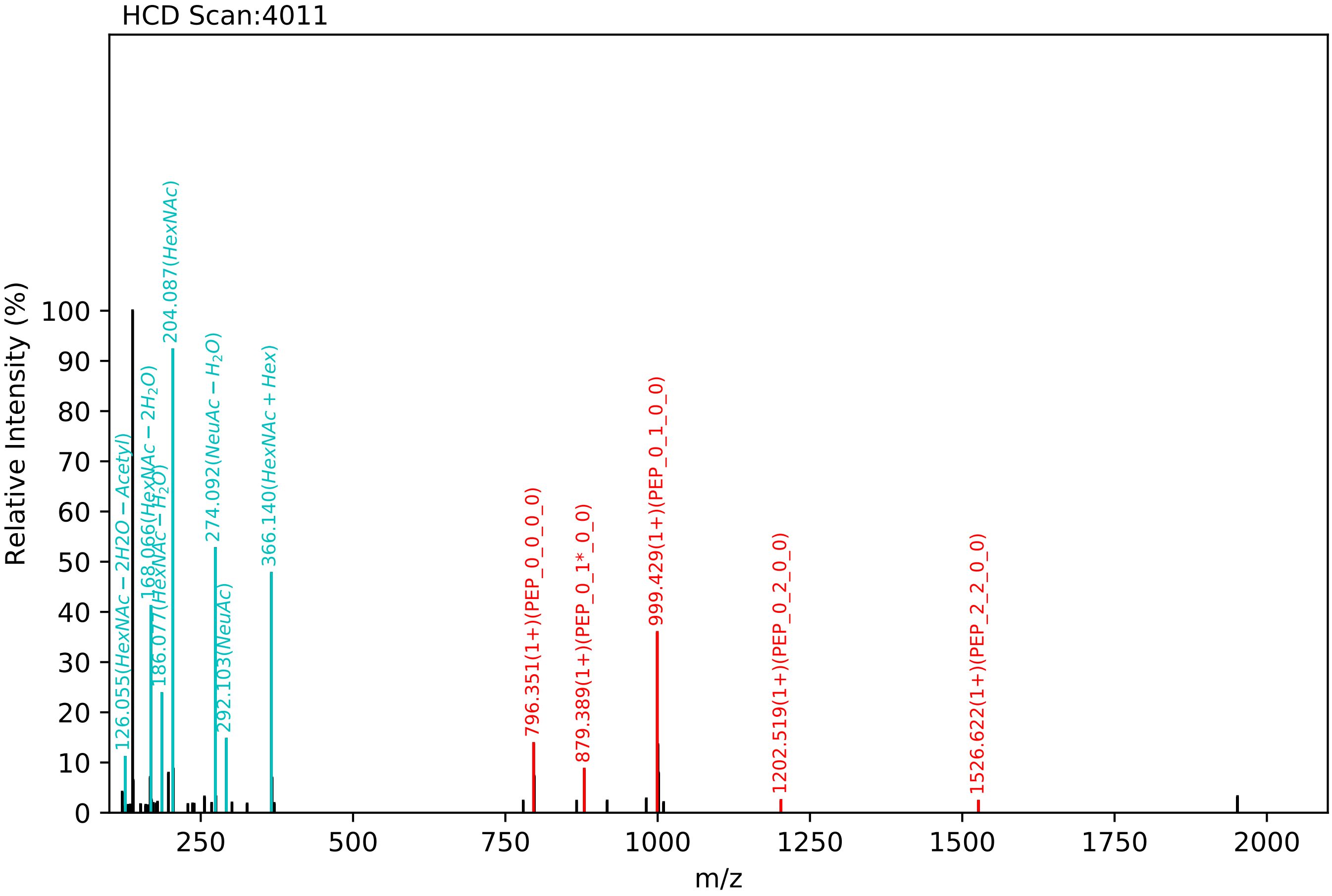

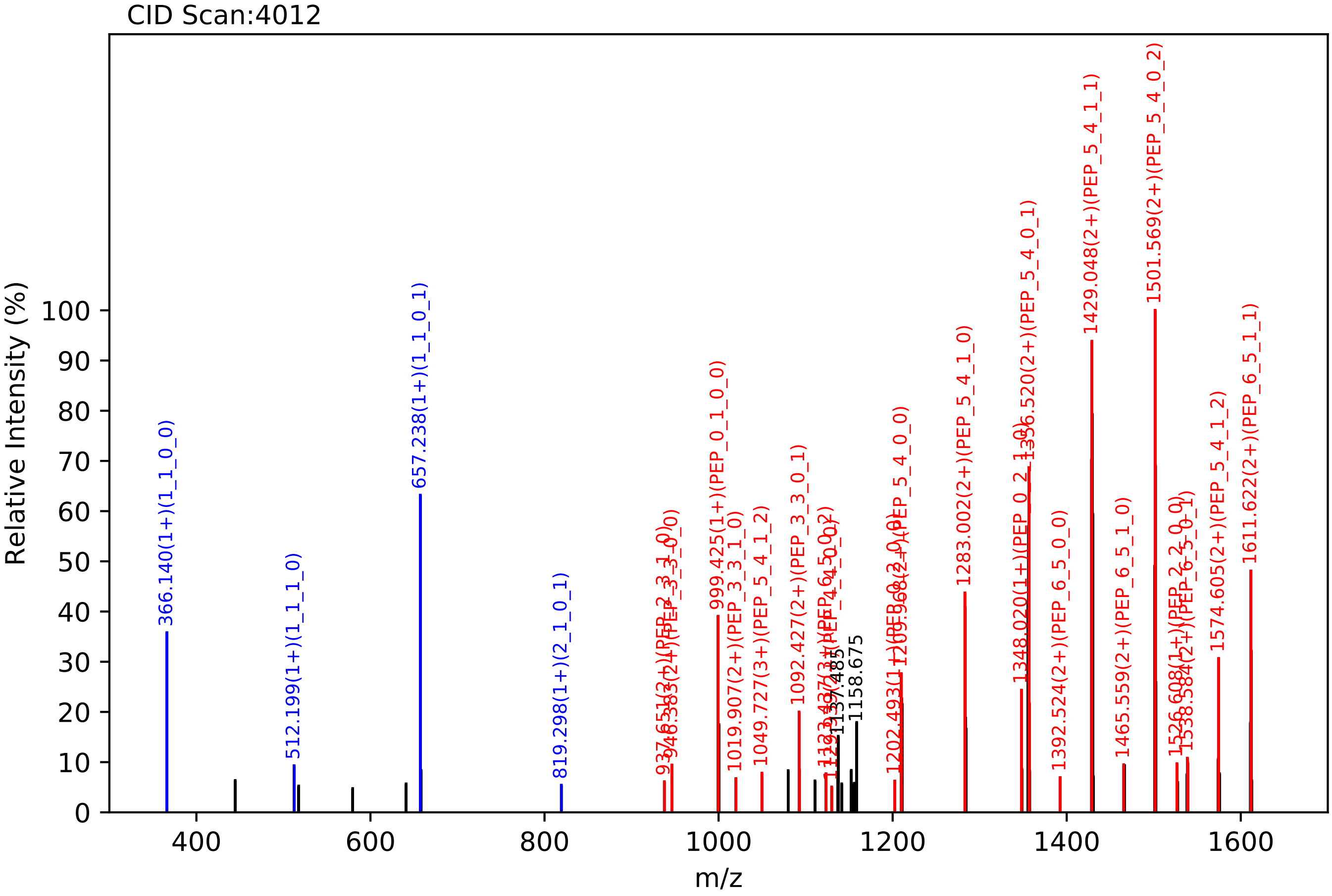

Unknown set no. 81, Experiment: Human Plasma exp_4

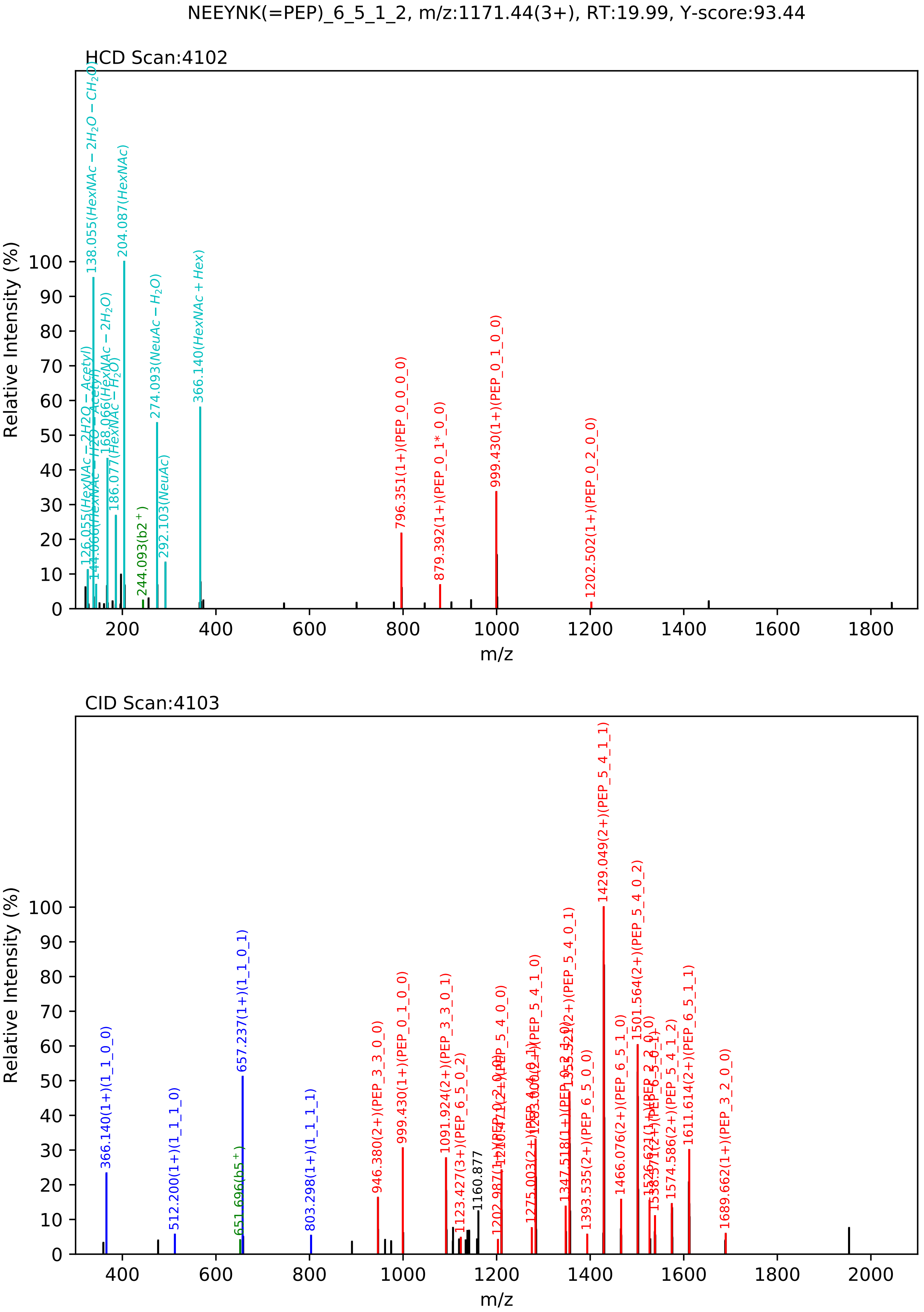

Unknown set no. 82, Experiment: Human Plasma exp_5

NEEYNK(=PEP)_6_5_1_2, m/z:1171.44(3+), RT:19.93, Y-score:89.95

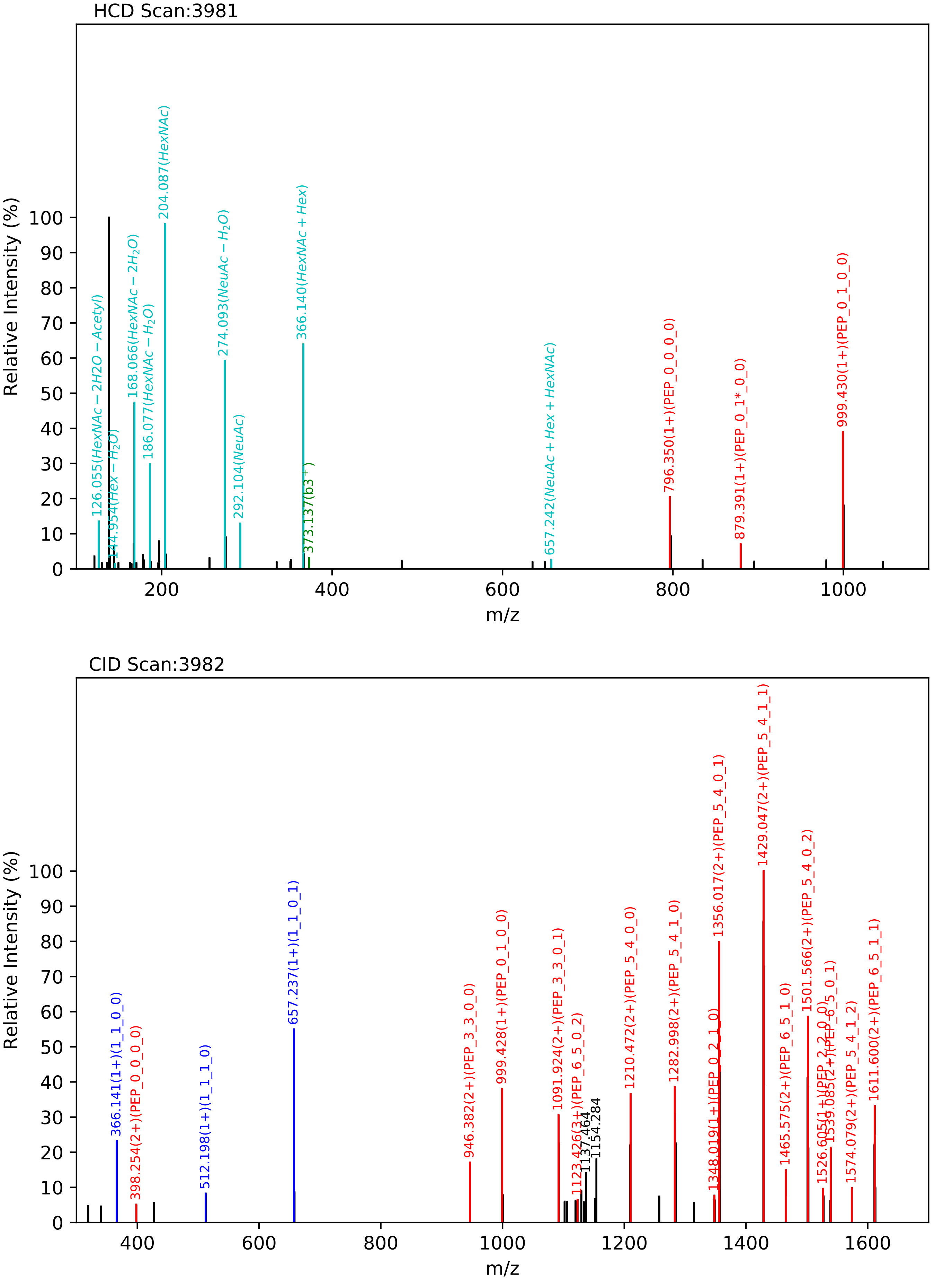

Unknown set no. 83, Experiment: Human Plasma exp_1

NEEYNK(=PEP)_6_5_1_3, m/z:1268.47(3+), RT:31.97, Y-score:91.75

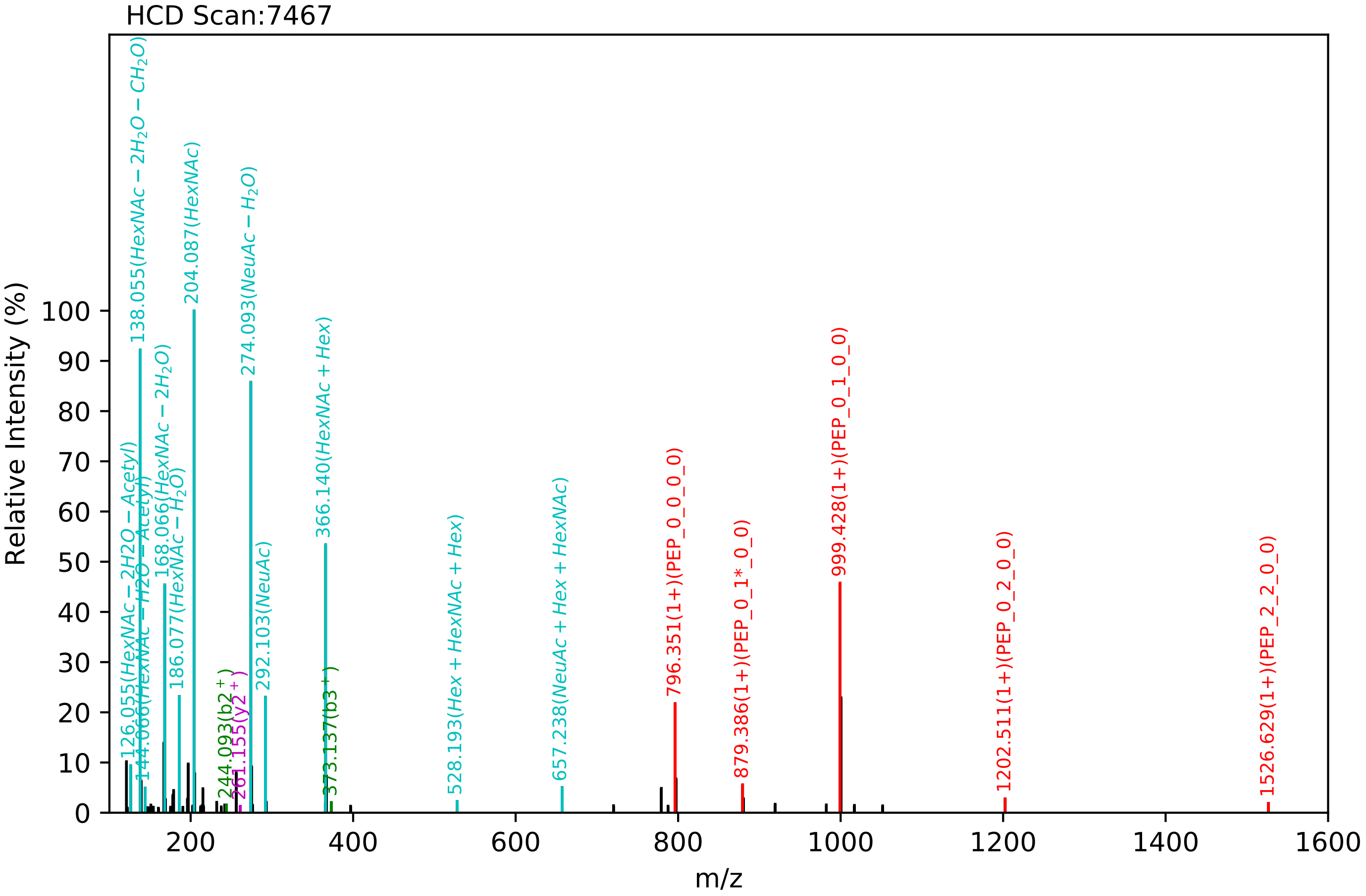

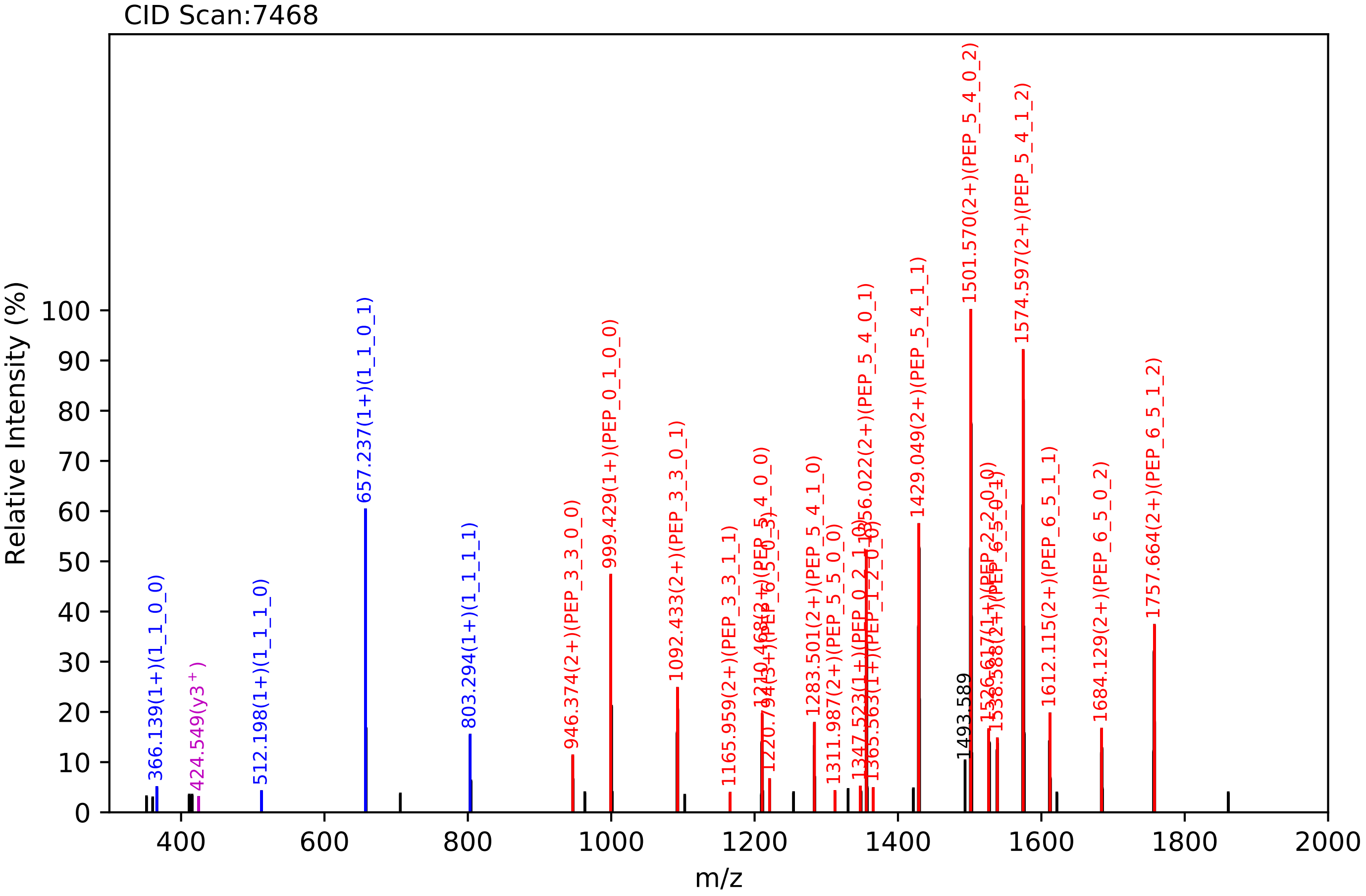

Unknown set no. 84, Experiment: Human Plasma exp_2

NEEYNK(=PEP)_6_5_1_3, m/z:951.61(4+), RT:32.93, Y-score:88.53

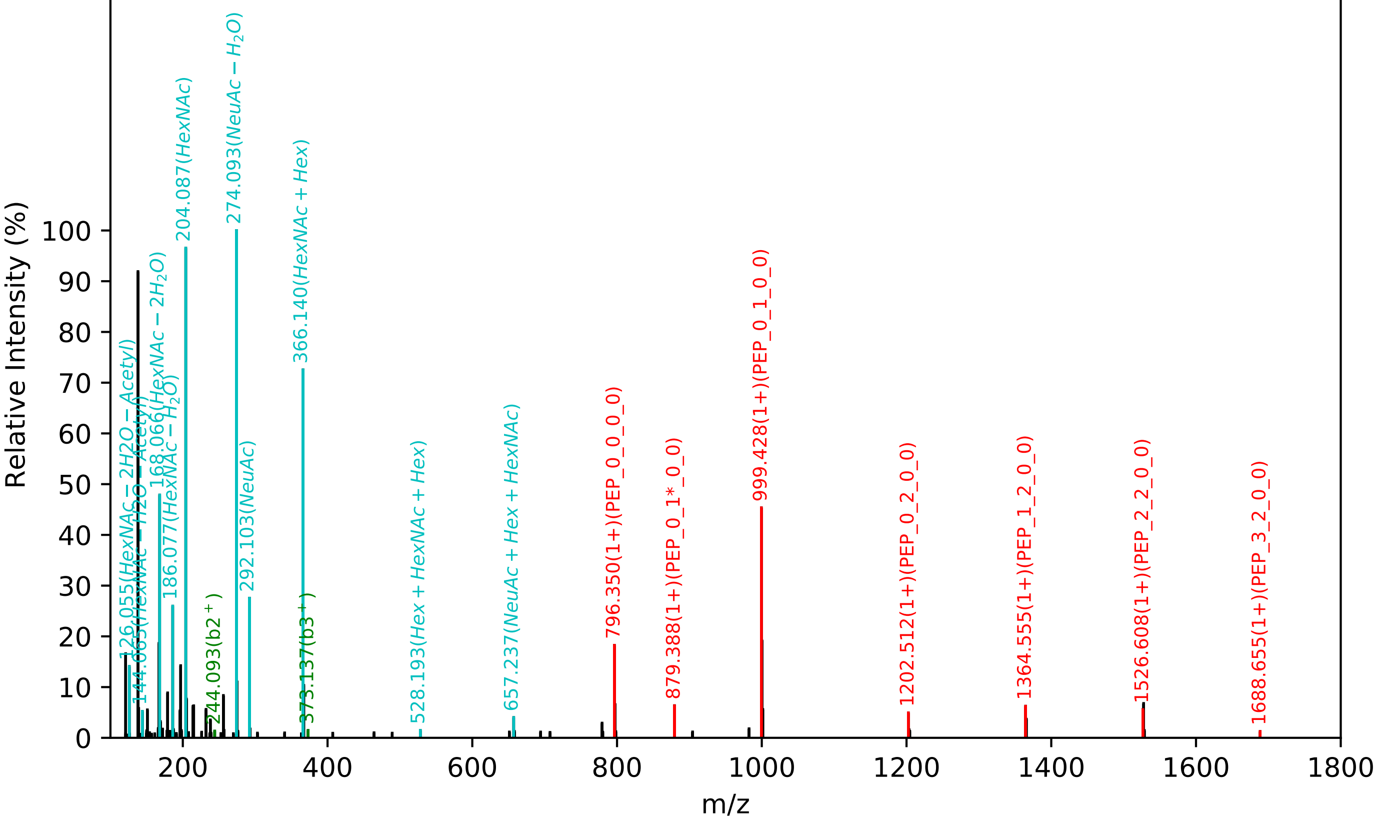

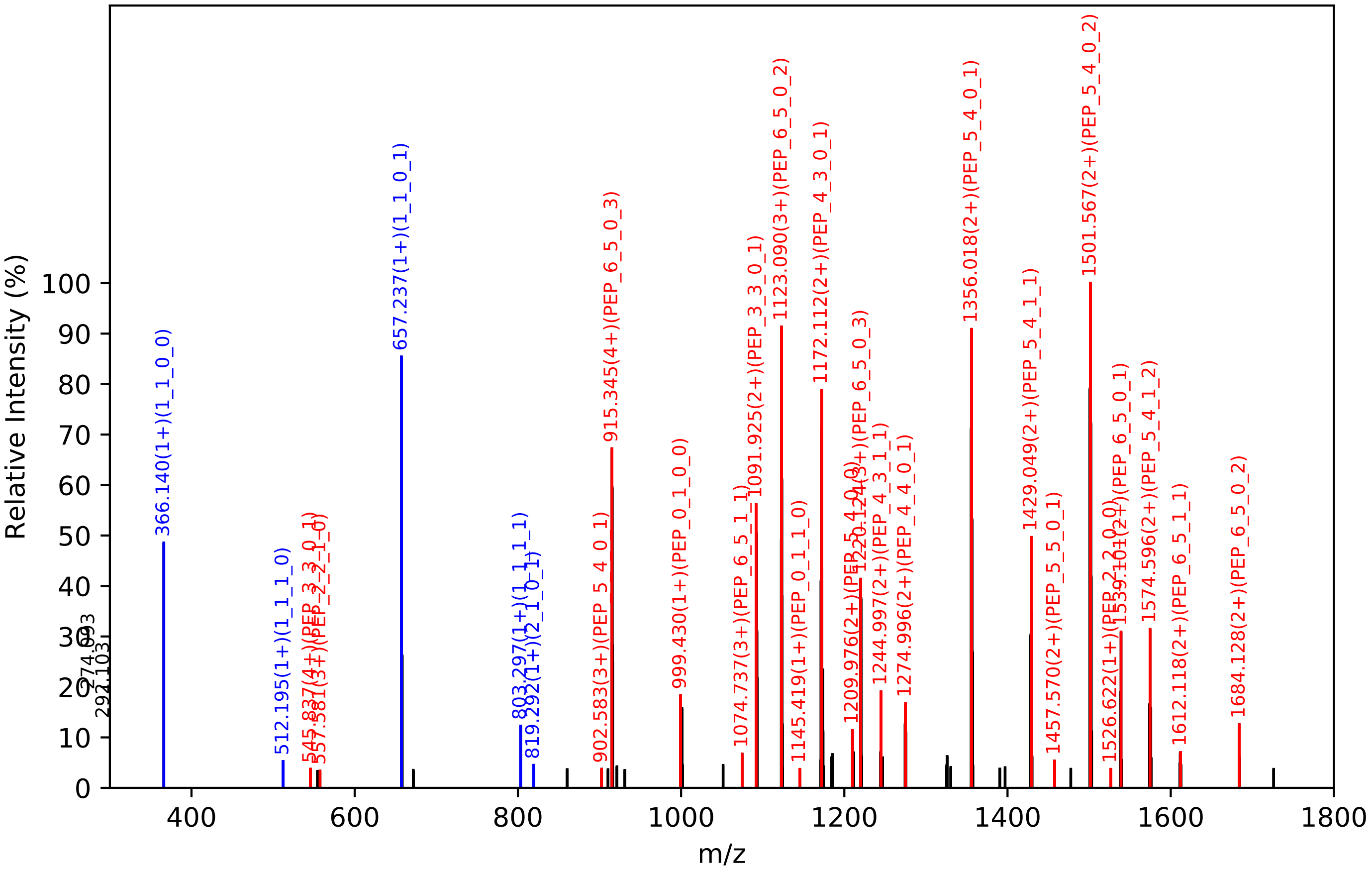

Unknown set no. 85, Experiment: Human Plasma exp_2

NEEYNK(=PEP)_6_5_1_3, m/z:1268.48(3+), RT:32.13, Y-score:95.70

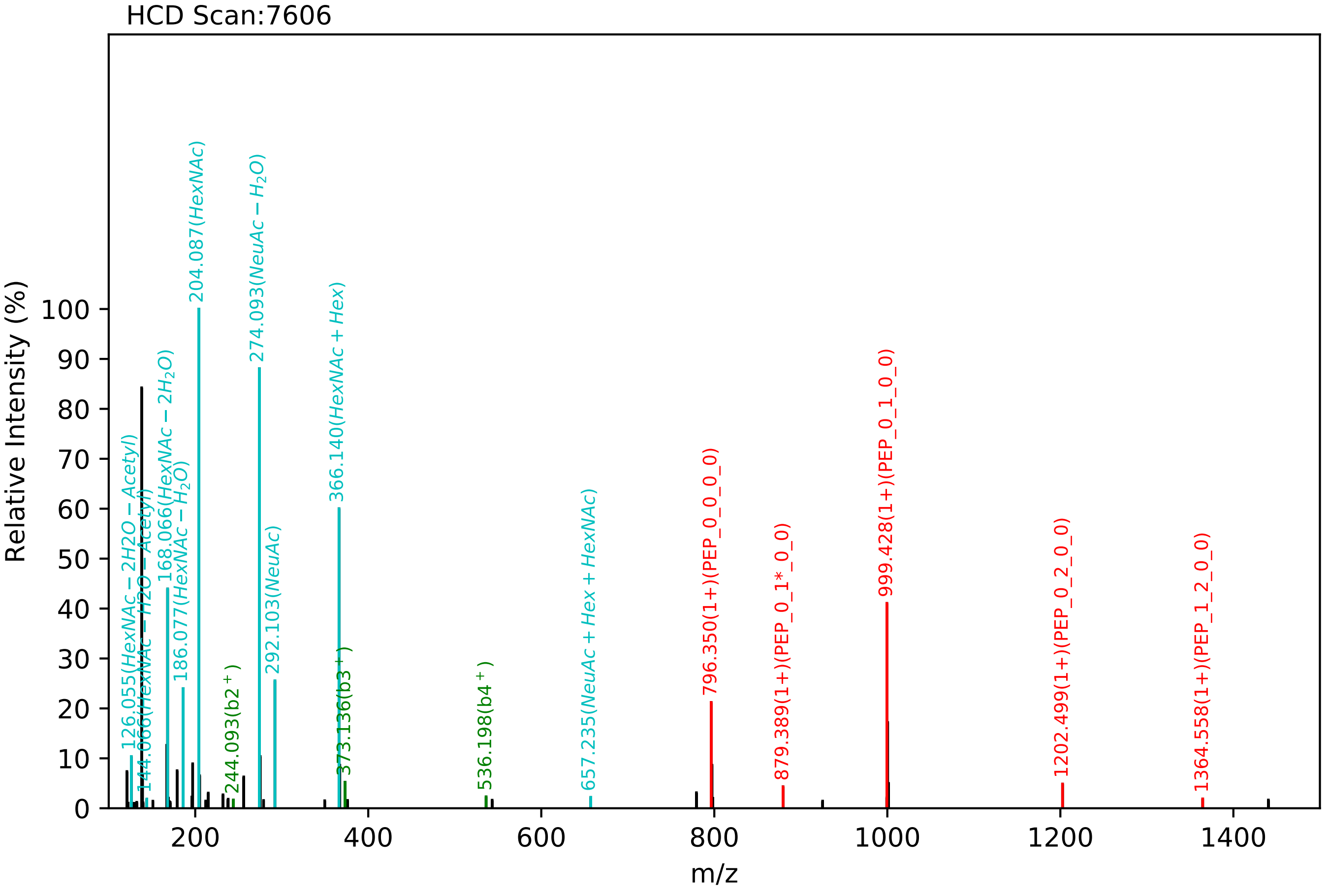

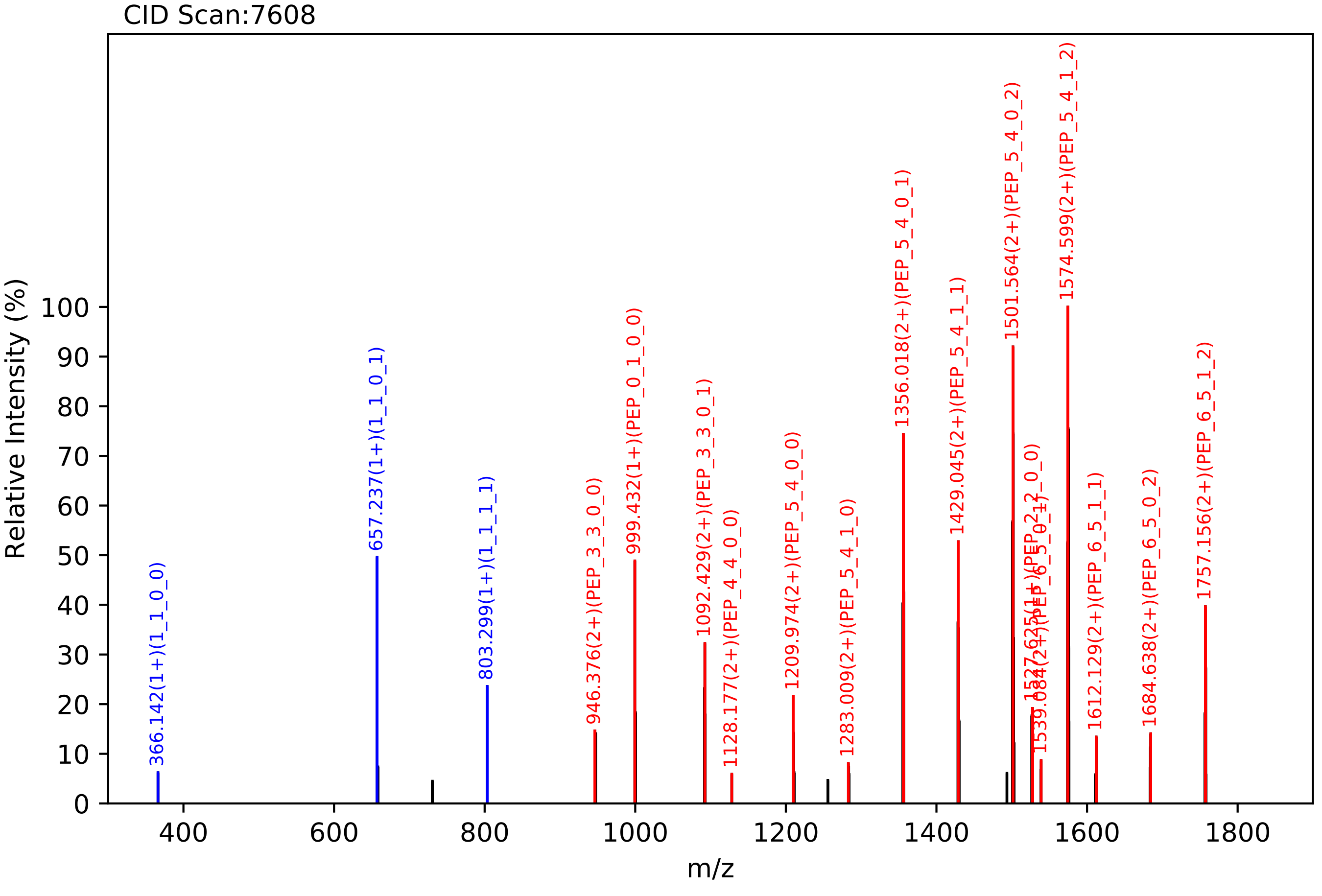

Unknown set no. 86, Experiment: Human Plasma exp_3

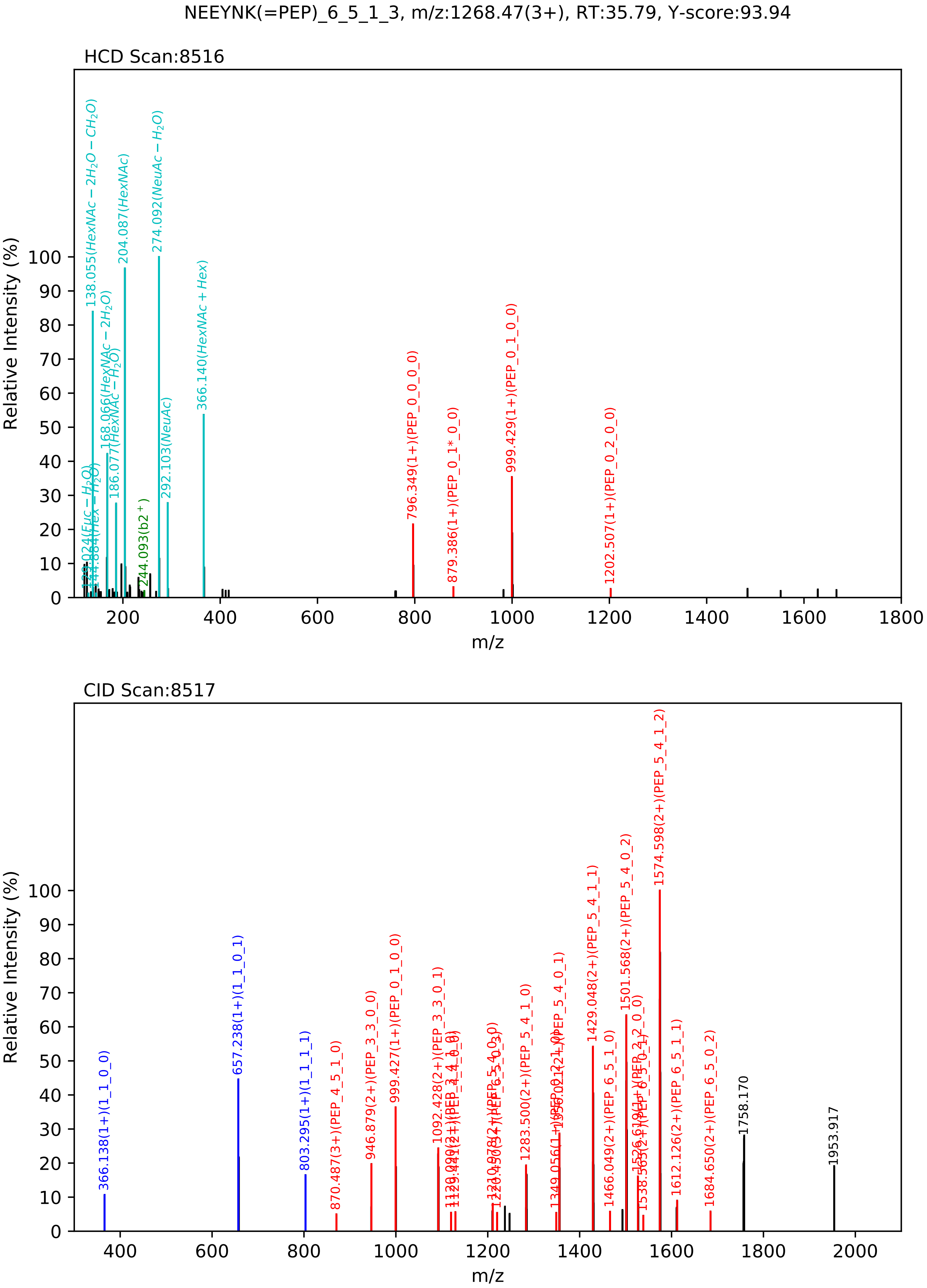

Unknown set no. 87, Experiment: Human Plasma exp_4

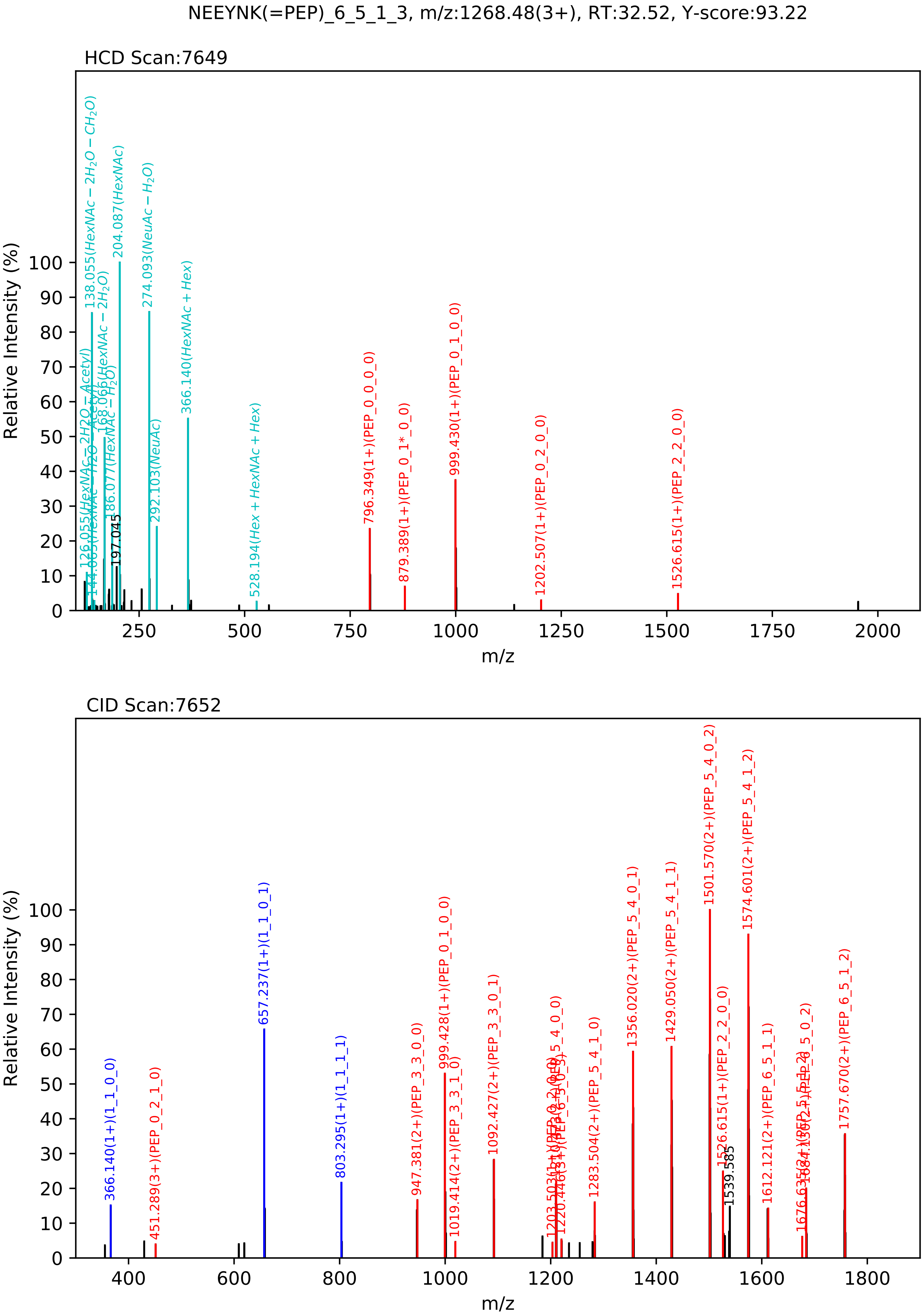

Unknown set no. 88, Experiment: Human Plasma exp_5

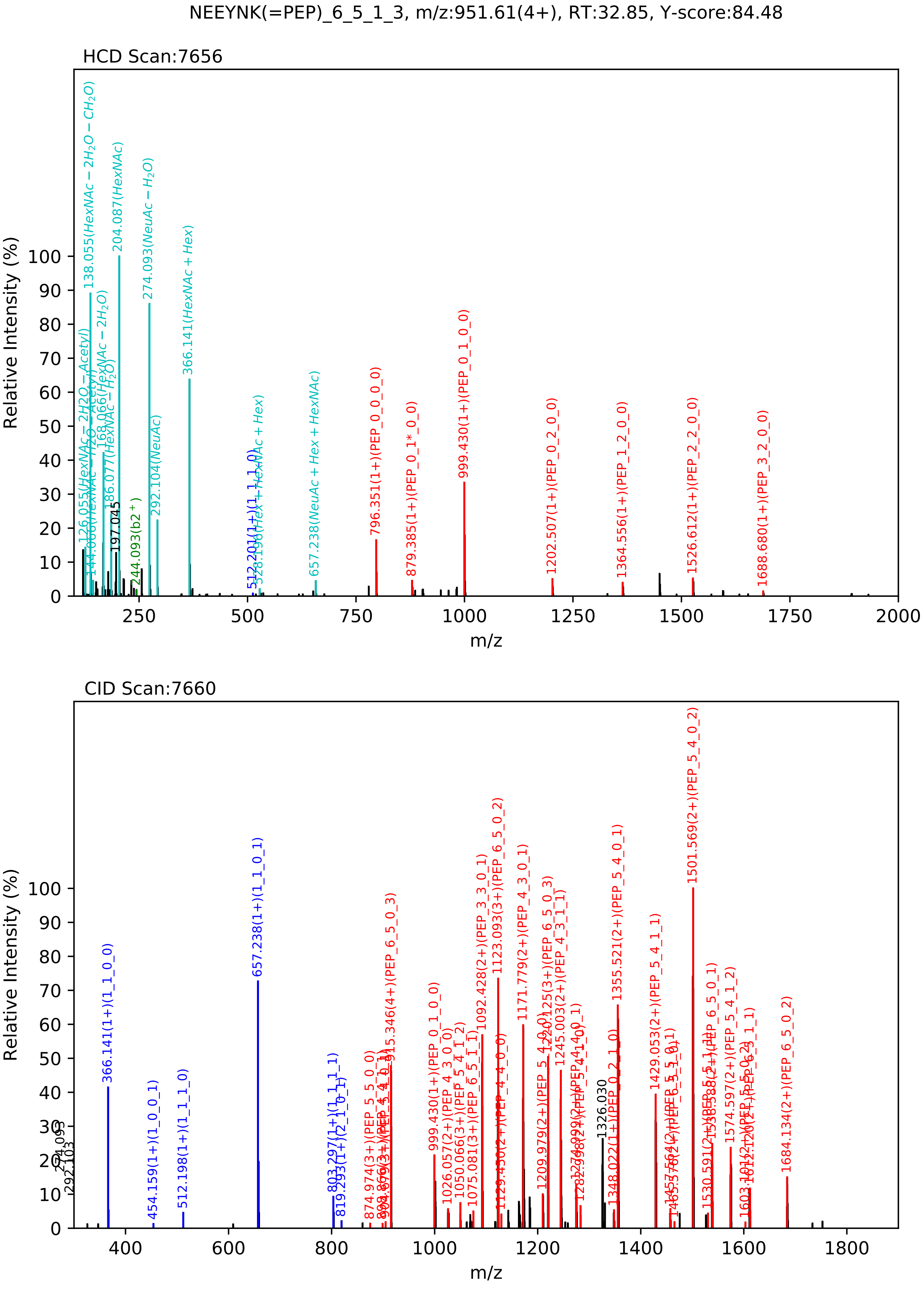

Unknown set no. 89, Experiment: Human Plasma exp_5

NEEYNK(=PEP)_6_5_1_3, m/z:1268.48(3+), RT:32.52, Y-score:97.35

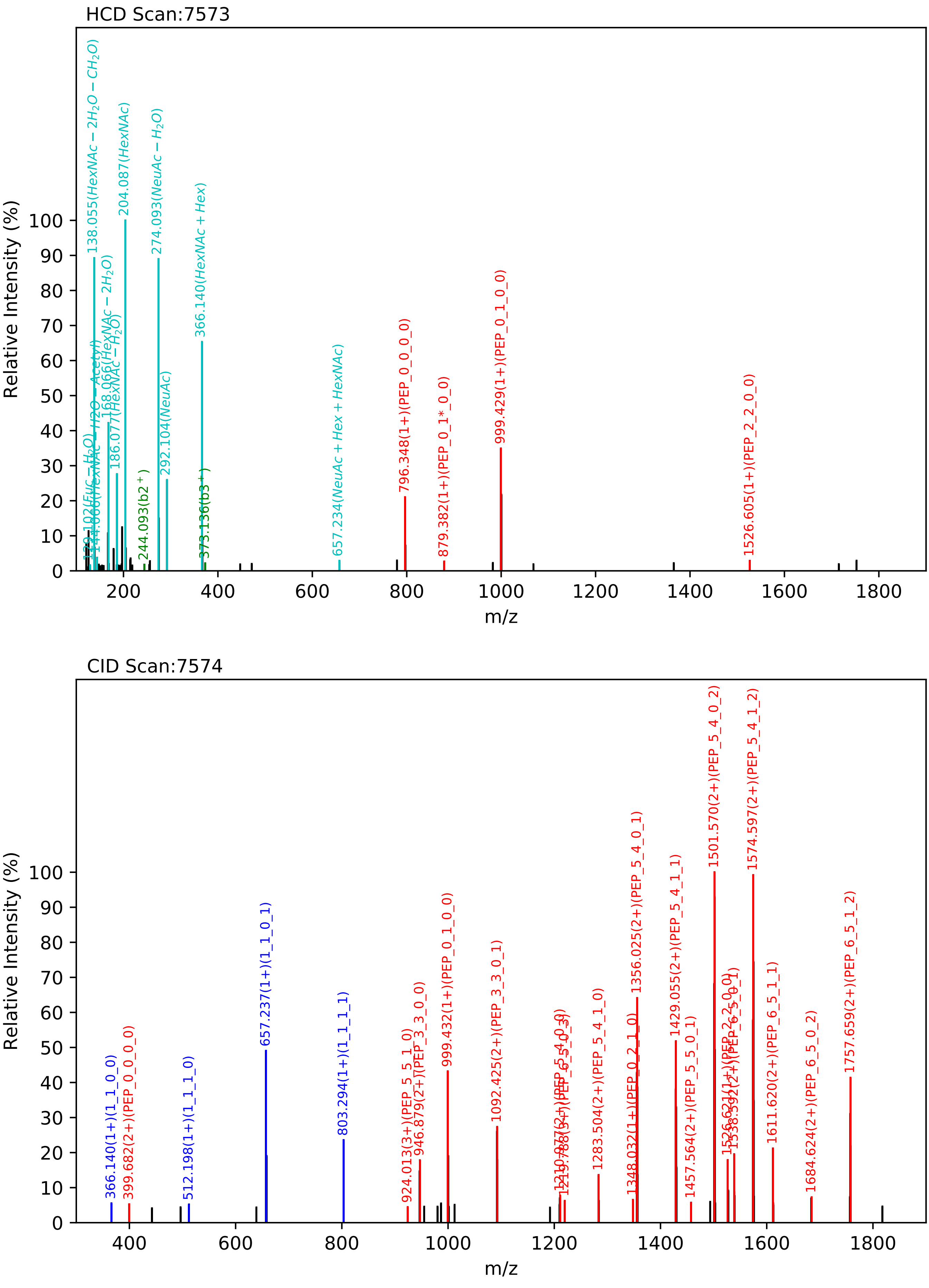

Unknown set no. 90, Experiment: Human Plasma exp_1

NEEYNK(=PEP)_6_5_2_3, m/z:1317.16(3+), RT:33.20, Y-score:92.38

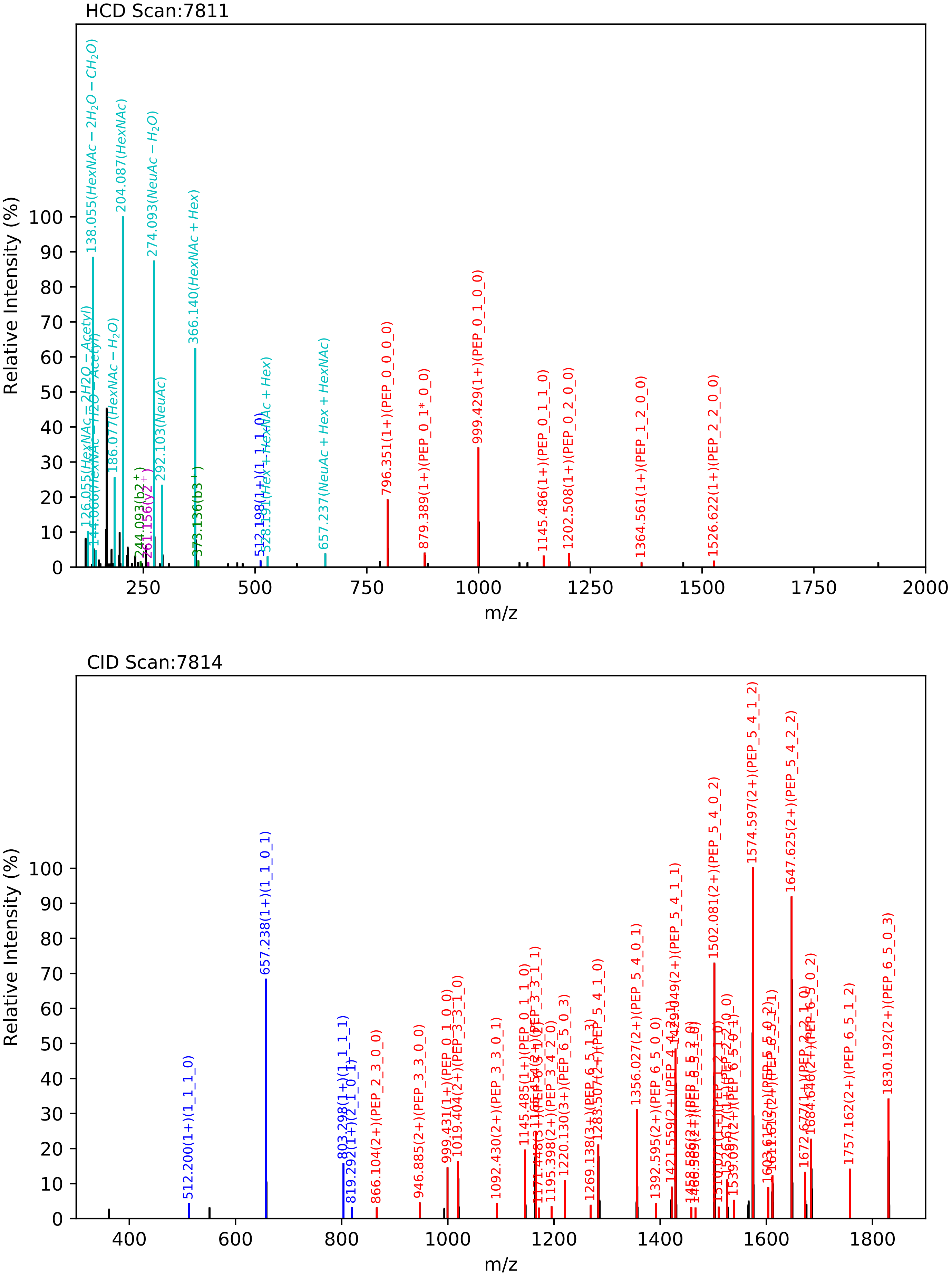

Unknown set no. 91, Experiment: Human Plasma exp_2

NEEYNK(=PEP)_6_5_2_3, m/z:1317.16(3+), RT:33.27, Y-score:92.83

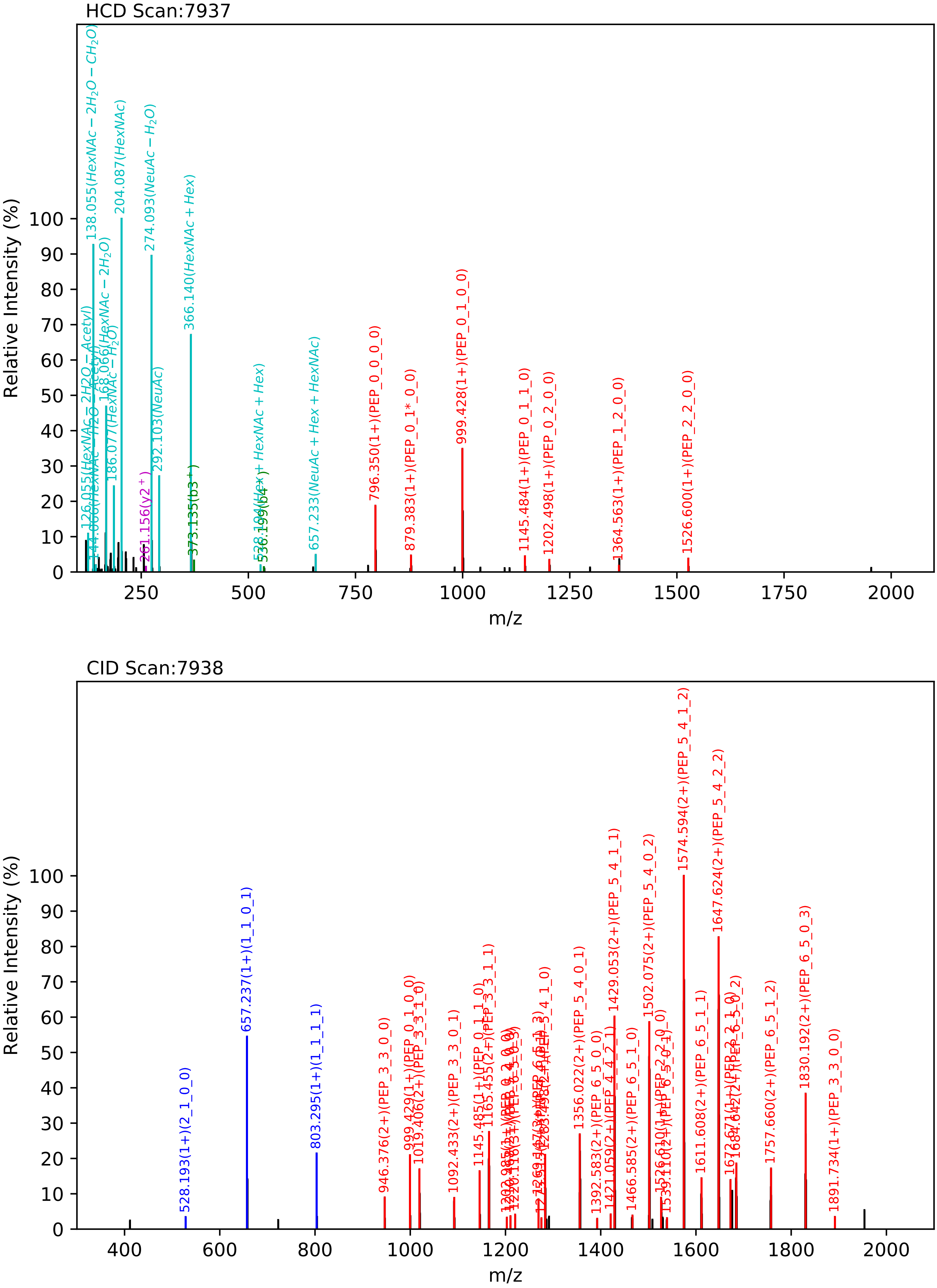

Unknown set no. 92, Experiment: Human Plasma exp_4

NEEYNK(=PEP)_6_5_2_3, m/z:1317.16(3+), RT:33.51, Y-score:91.80

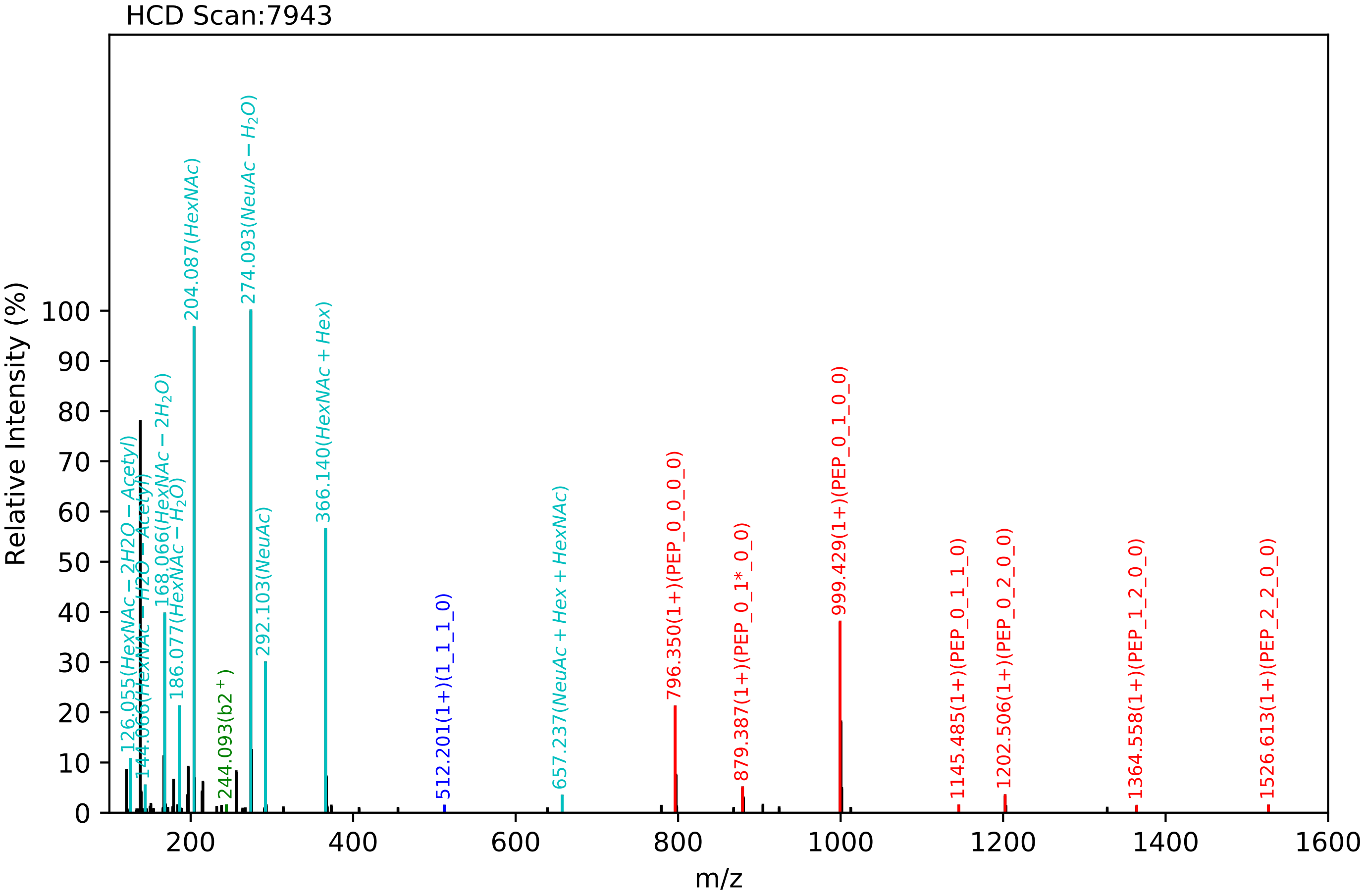

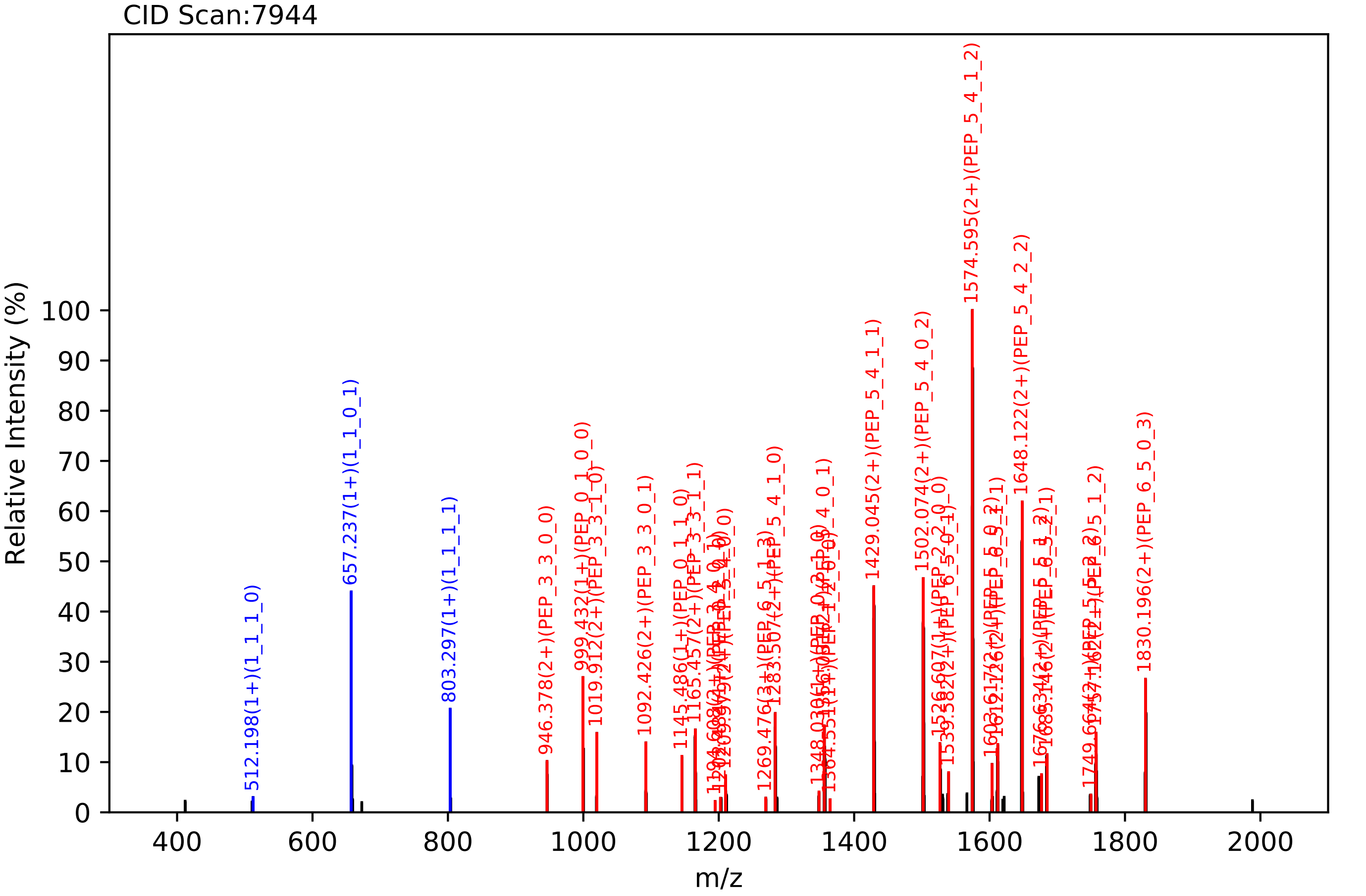

Unknown set no. 93, Experiment: Human Plasma exp_2

ENGTVSR(=PEP)_5_4_0_2, m/z:989.72(3+), RT:17.88, Y-score:97.26

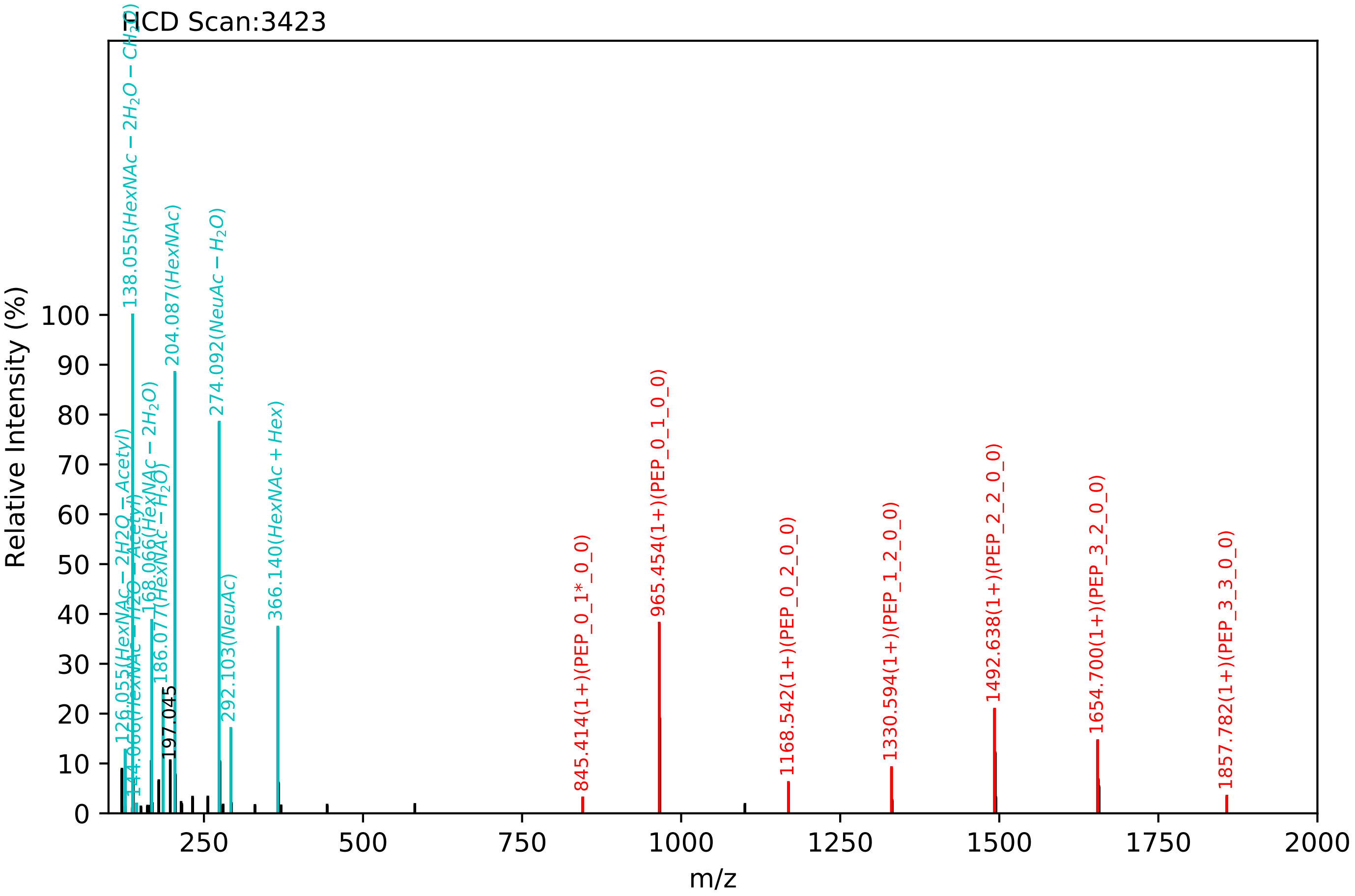

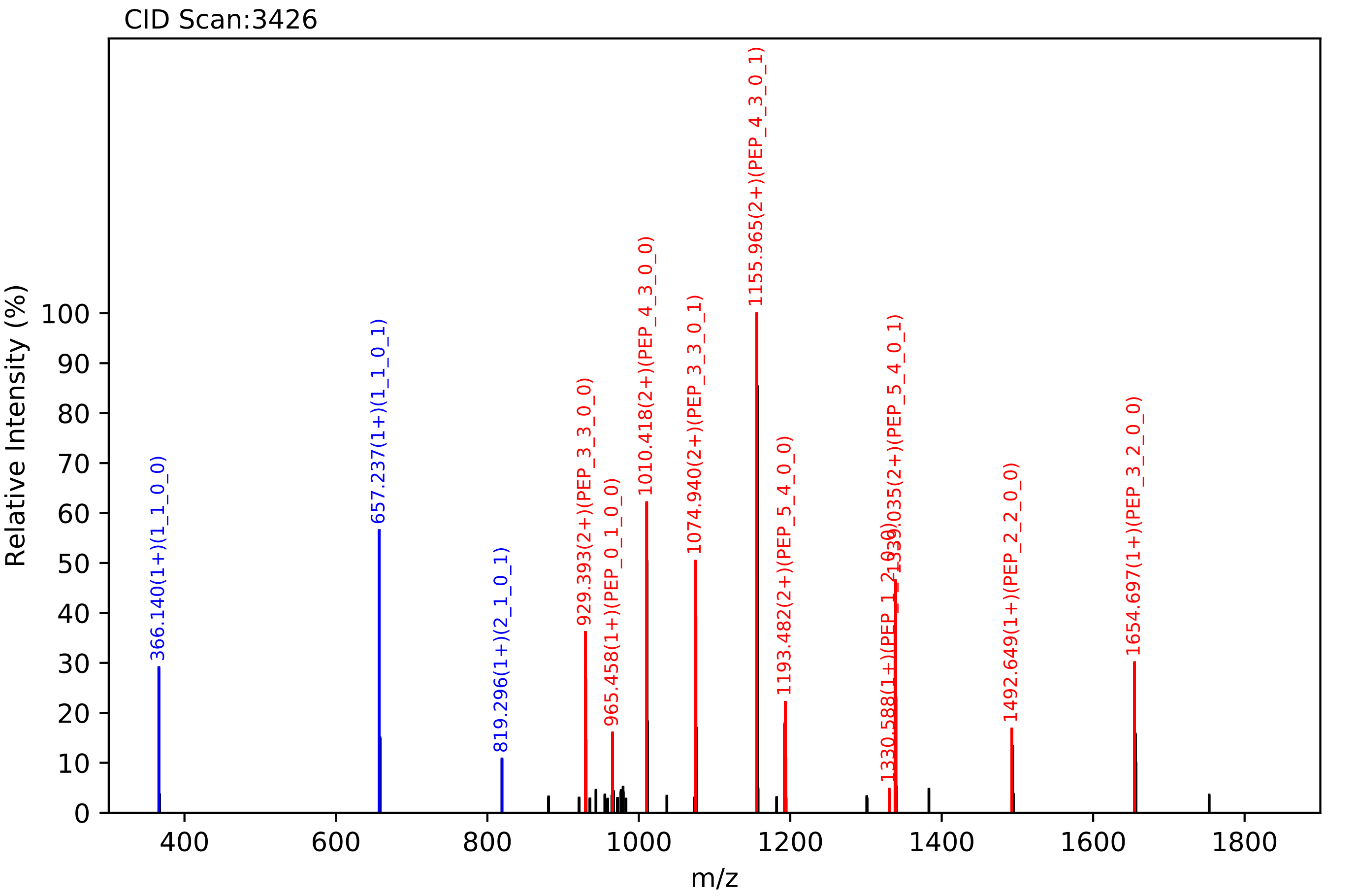

Unknown set no. 94, Experiment: Human Plasma exp_3

ENGTVSR(=PEP)_5_4_0_2, m/z:989.72(3+), RT:18.04, Y-score:95.10

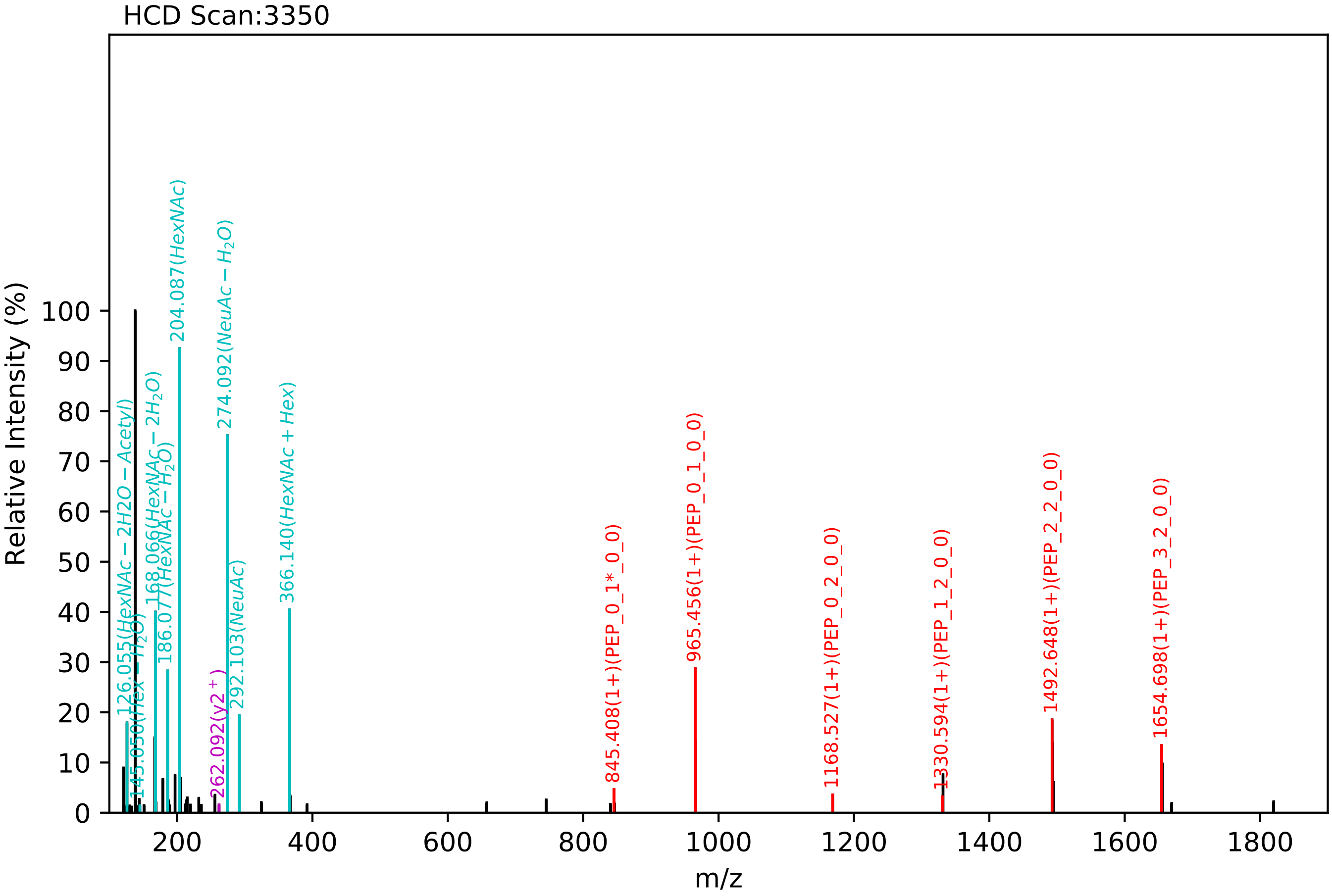

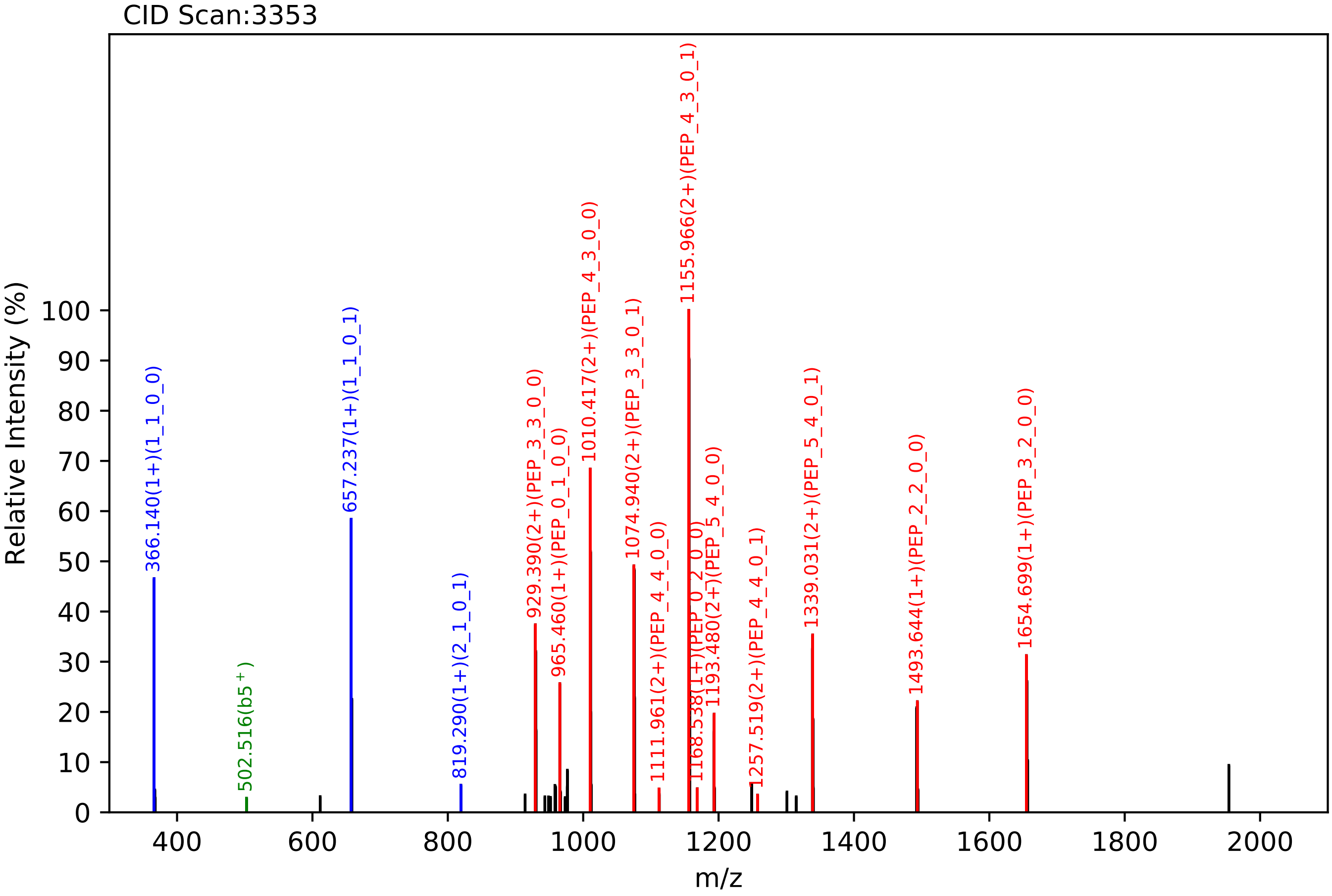

Unknown set no. 95, Experiment: Human Plasma exp_4

ENGTVSR(=PEP)_5_4_0_2, m/z:989.72(3+), RT:18.10, Y-score:93.53

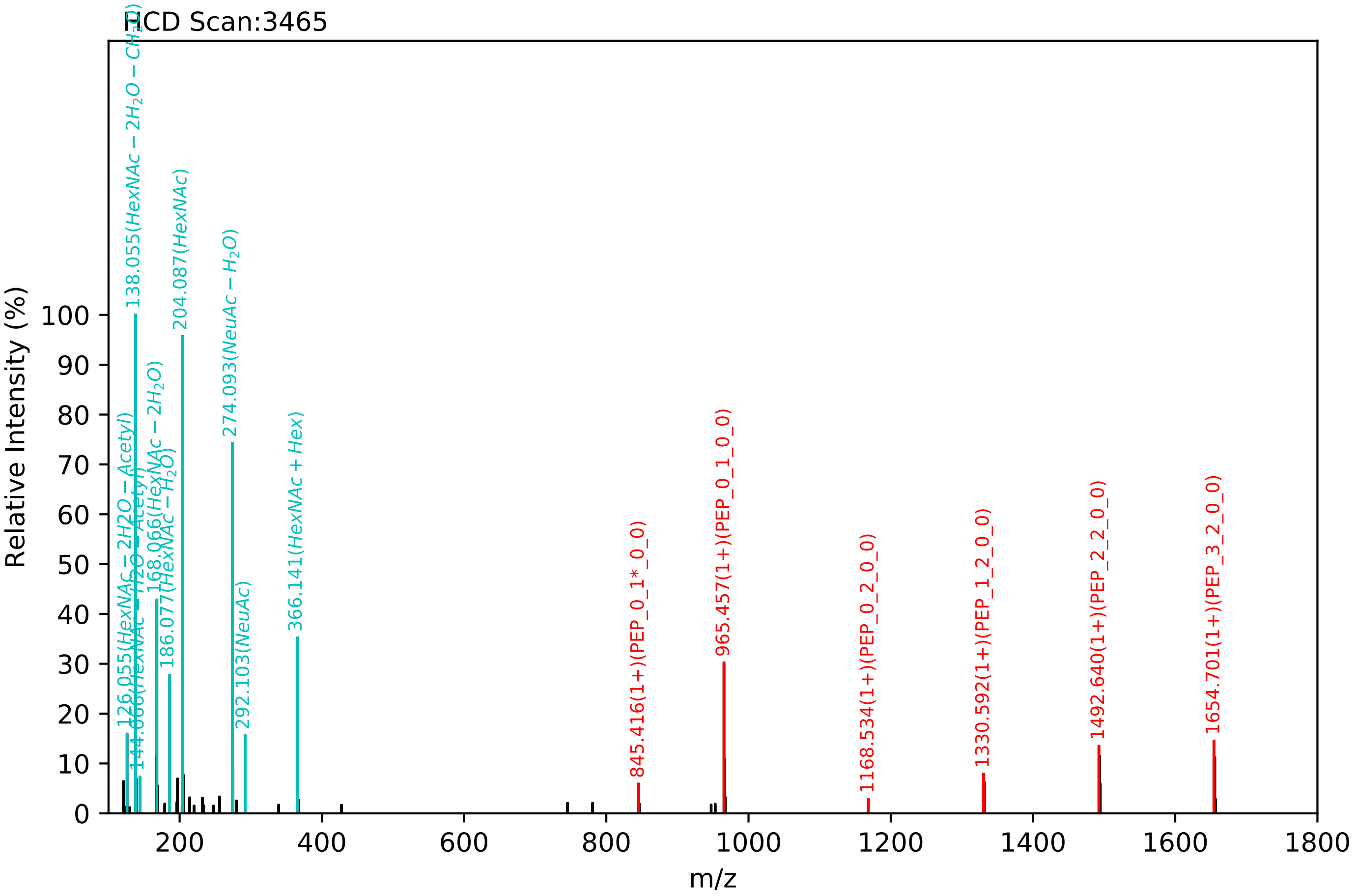

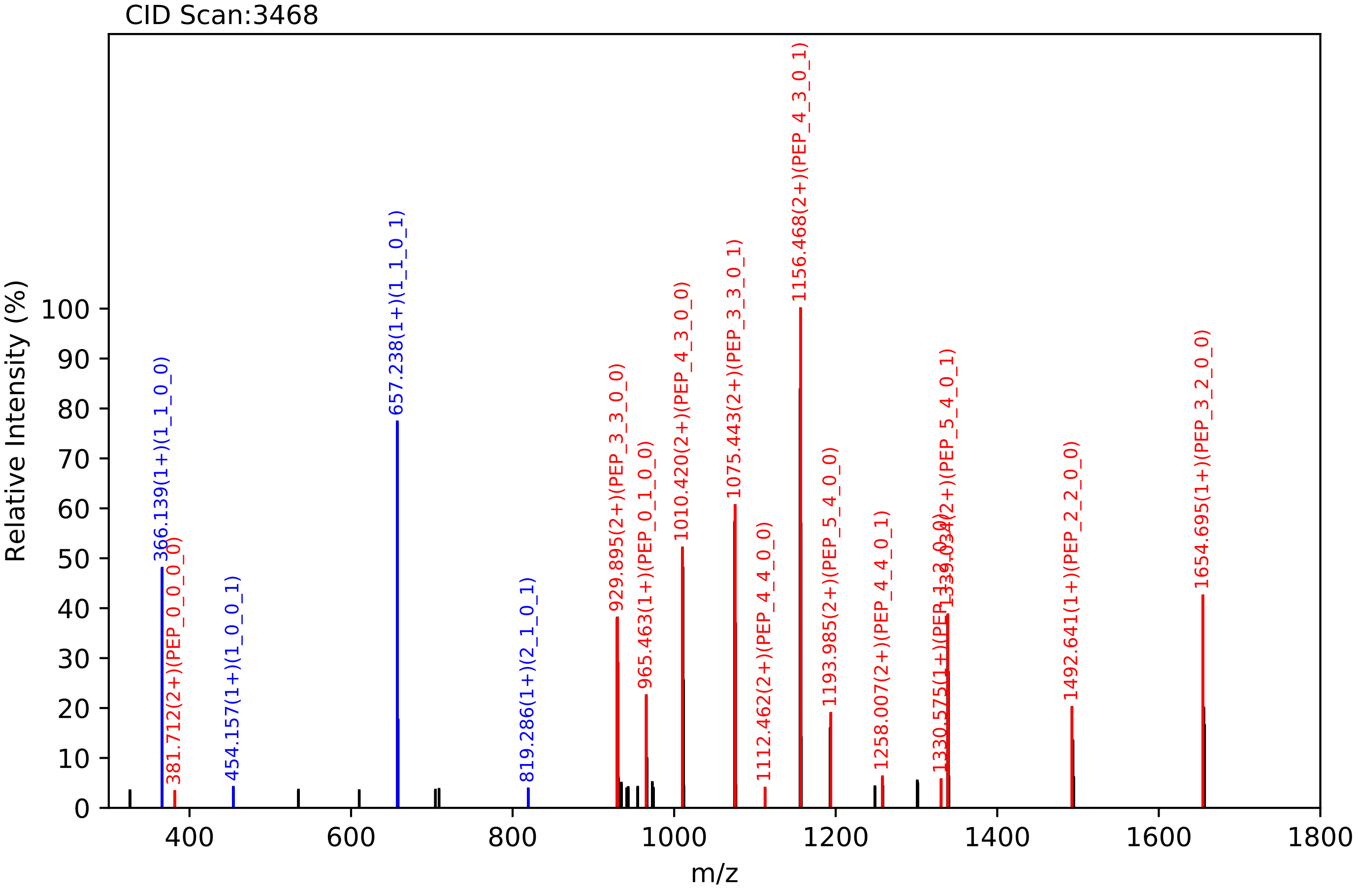

Unknown set no. 96, Experiment: Human Plasma exp_1

ENGTVSR(=PEP)_6_5_0_2, m/z:1111.43(3+), RT:16.97, Y-score:93.70

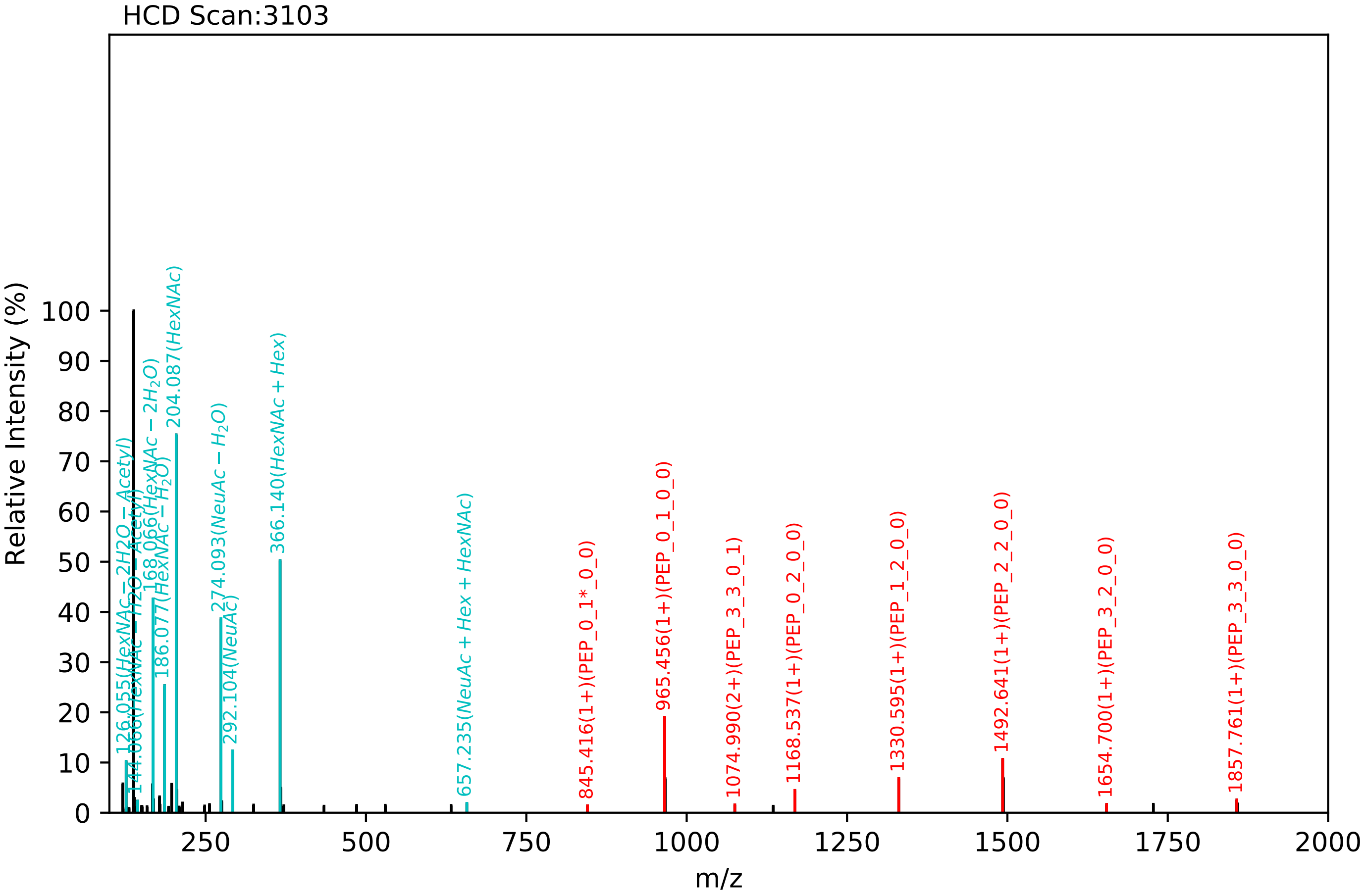

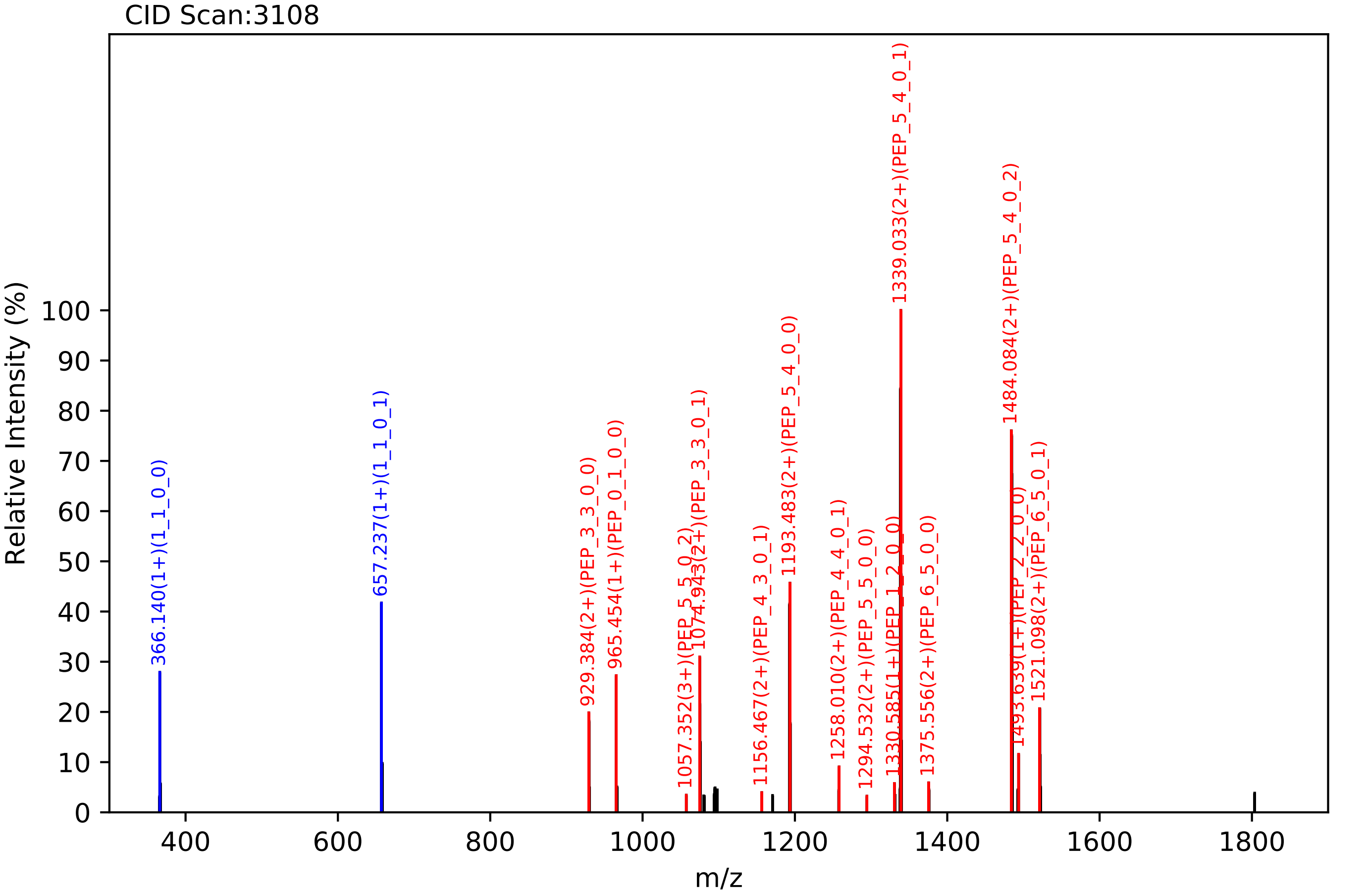

Unknown set no. 97, Experiment: Human Plasma exp_4

ENGTVSR(=PEP)_6_5_0_2, m/z:1111.43(3+), RT:17.40, Y-score:92.27

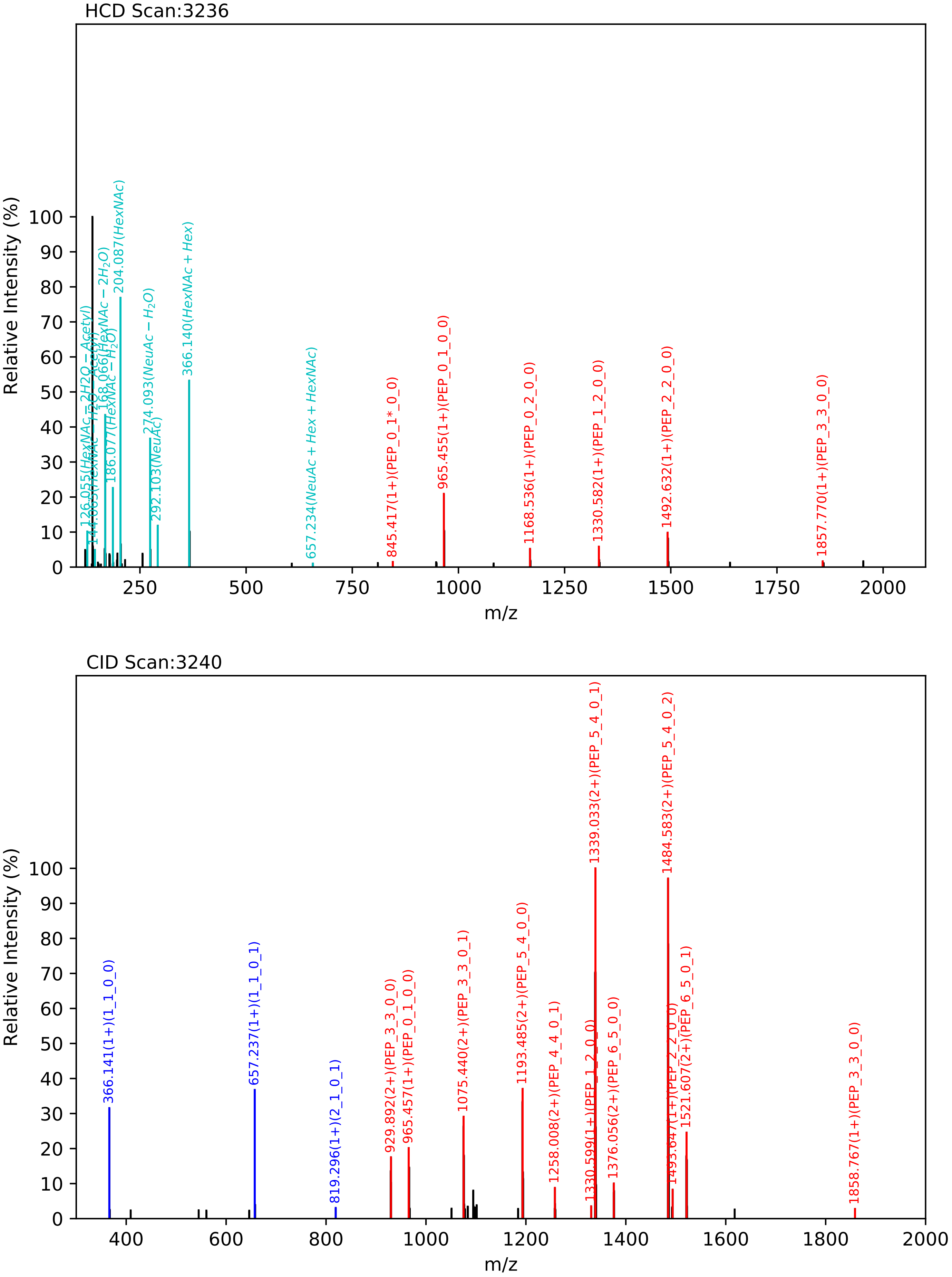

Unknown set no. 98, Experiment: Human Plasma exp_1

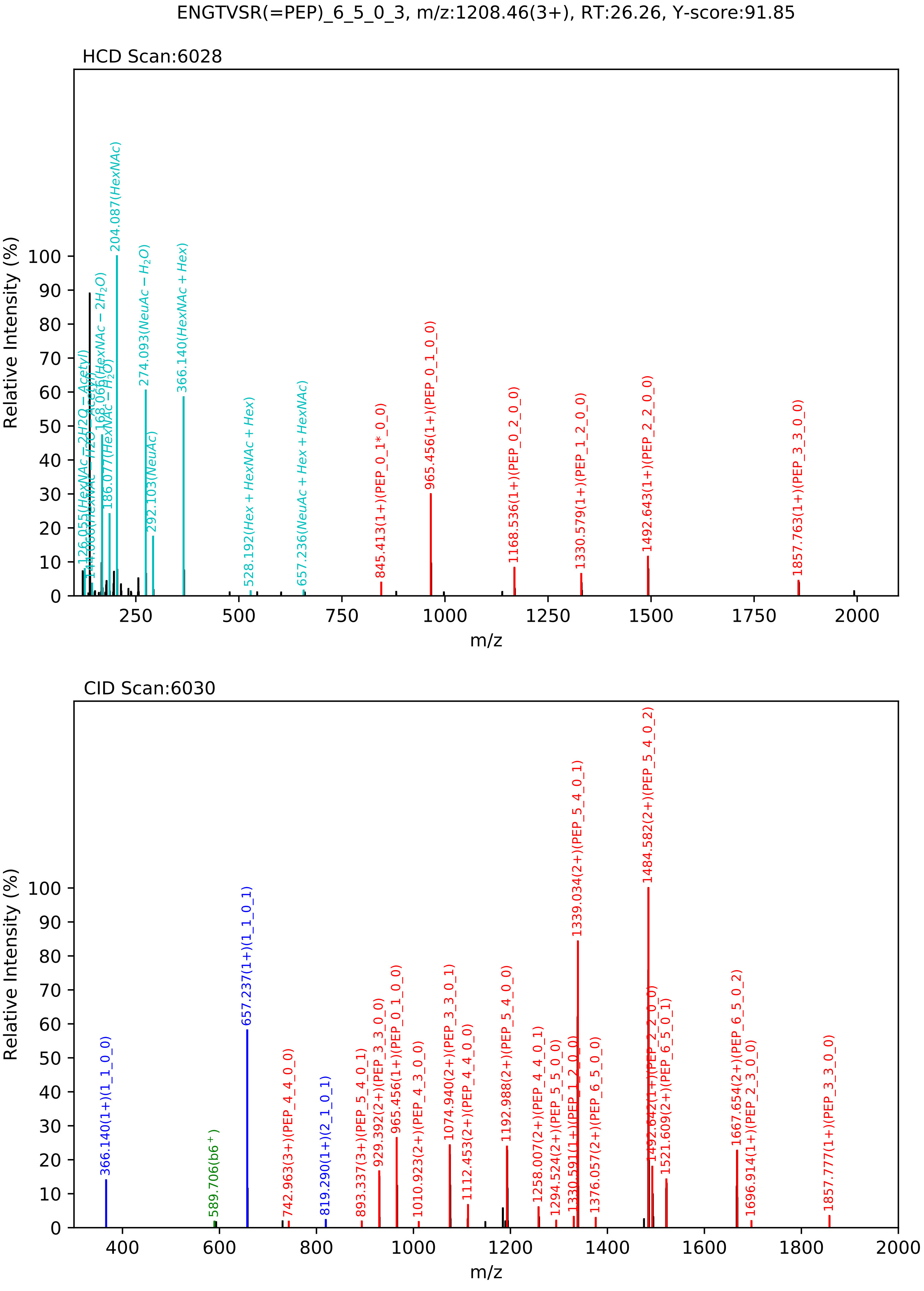

Unknown set no. 99, Experiment: Human Plasma exp_4

ENGTVSR(=PEP)_6_5_0_3, m/z:1208.47(3+), RT:26.57, Y-score:100.00

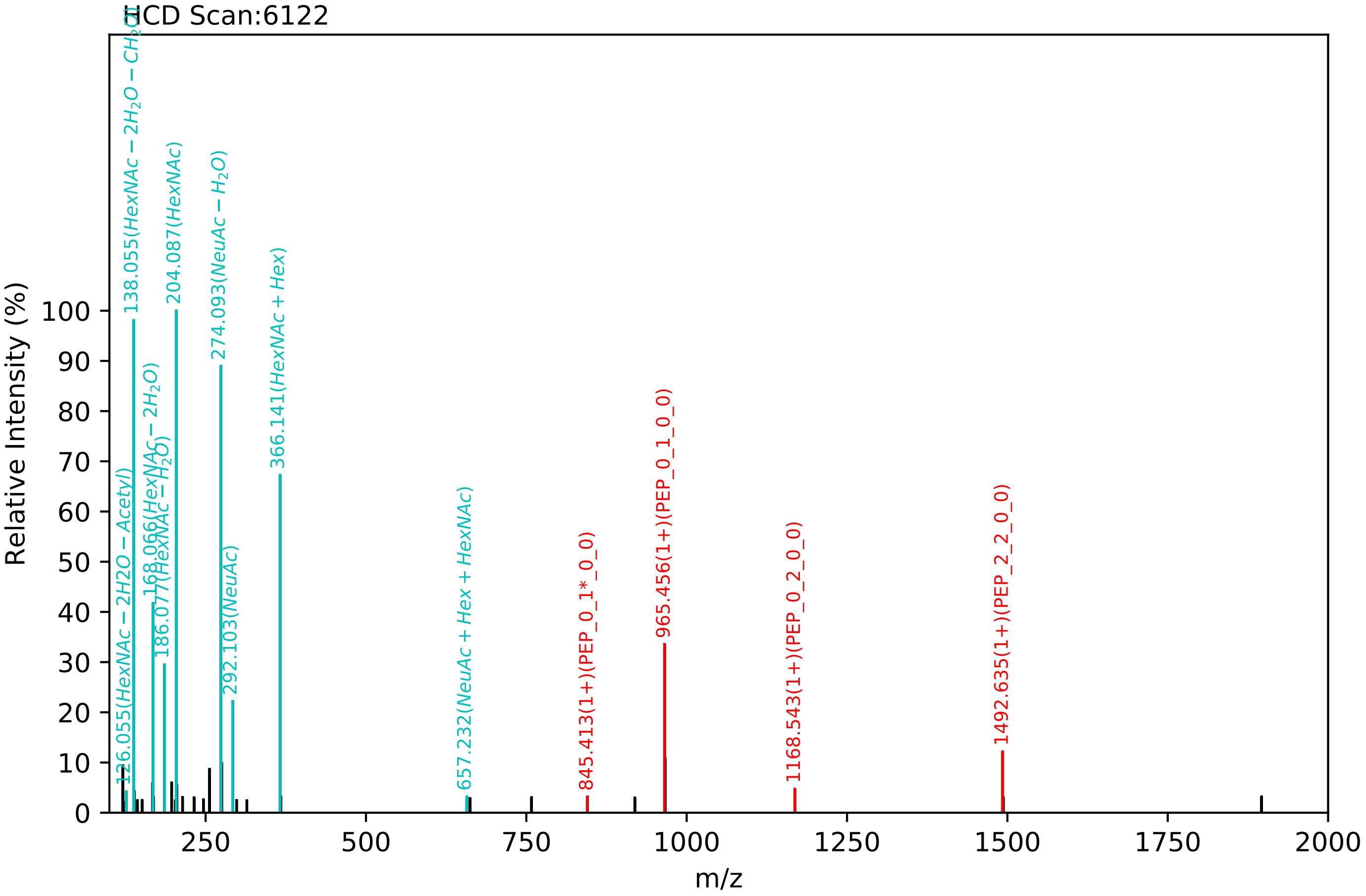

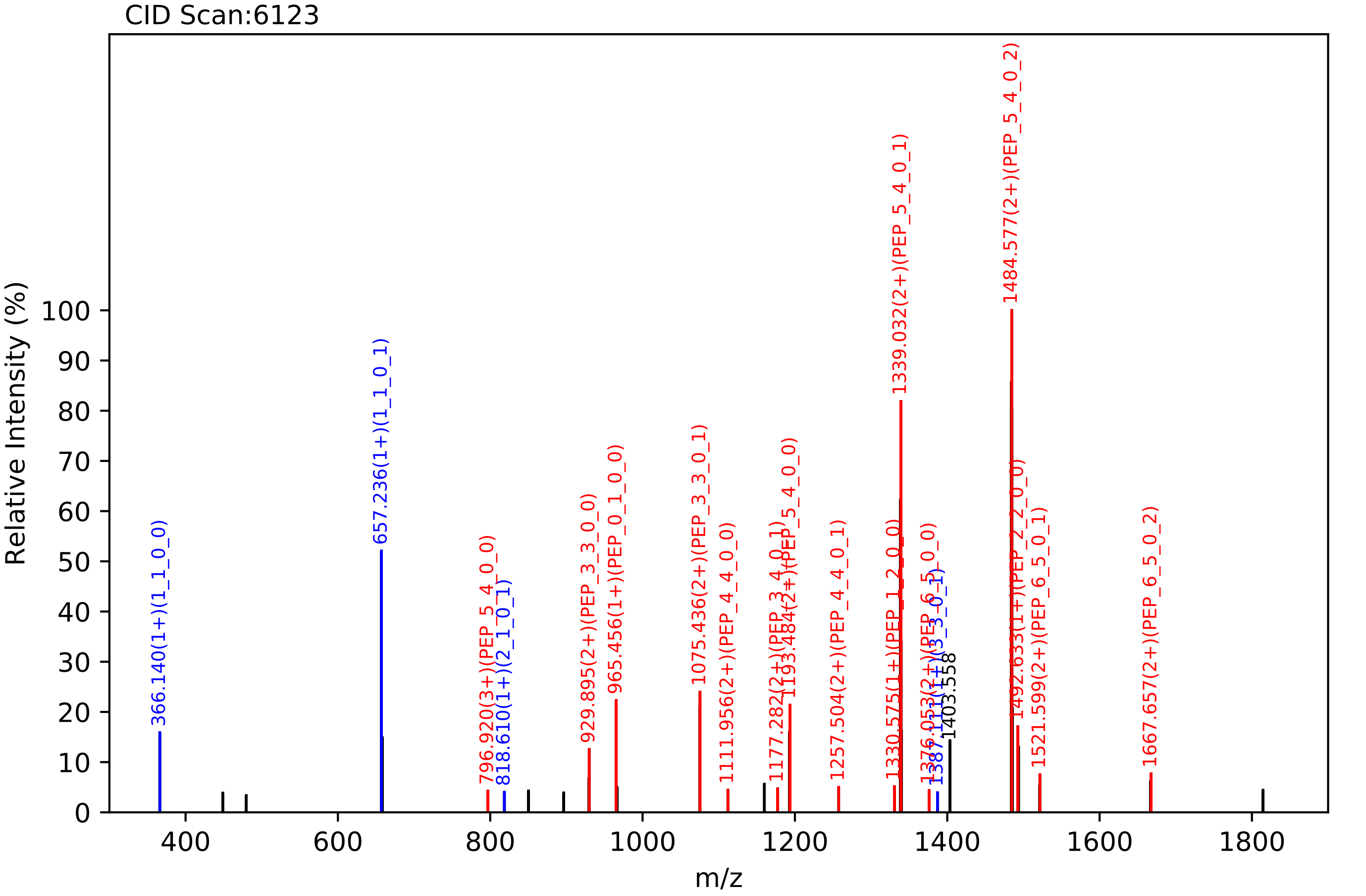

Unknown set no. 100, Experiment: Human Plasma exp_1

ENGTVSR(=PEP)_6_5_1_3, m/z:1257.15(3+), RT:26.16, Y-score:89.53

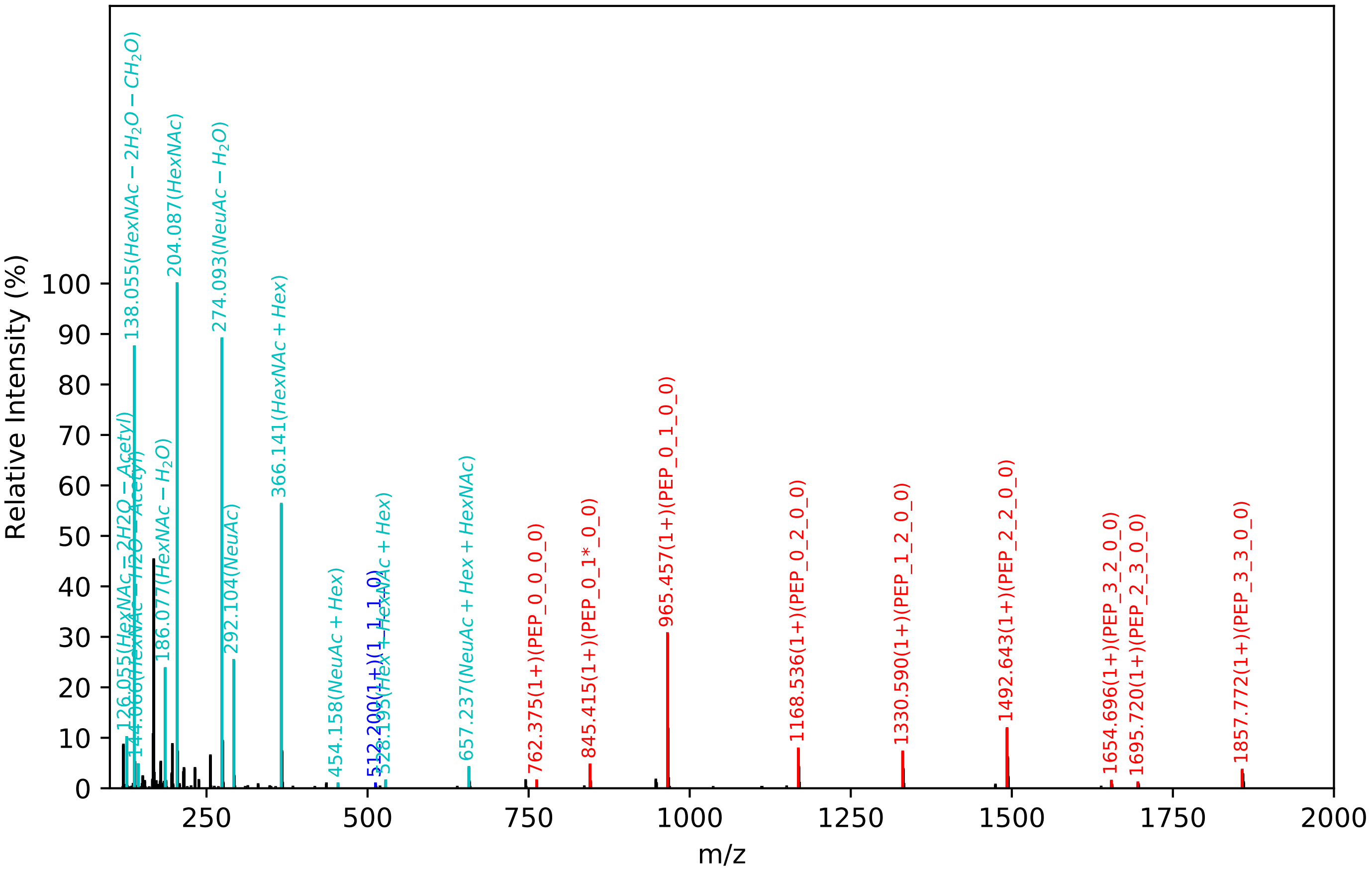

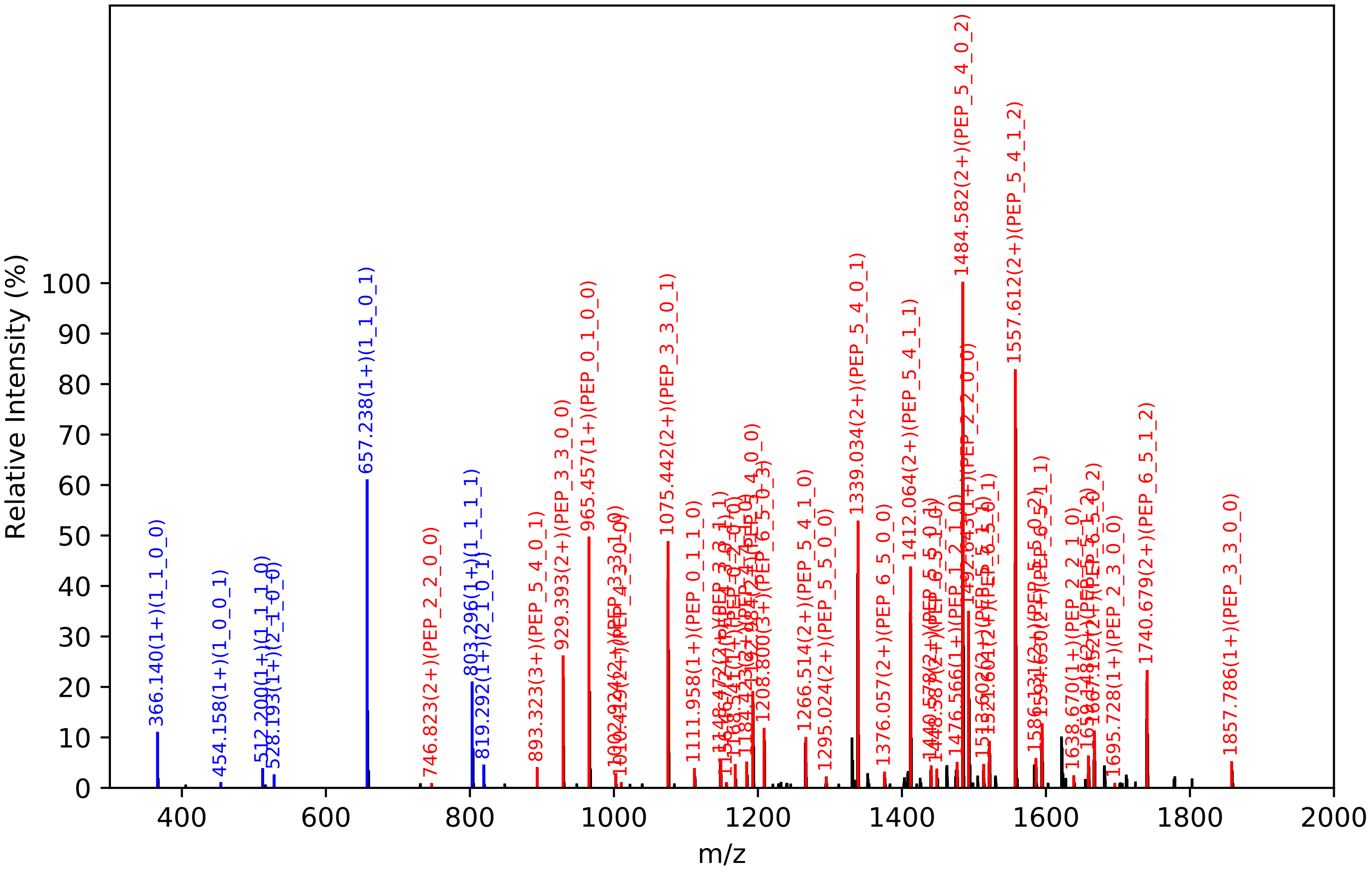

Unknown set no. 101, Experiment: Human Plasma exp_2

ENGTVSR(=PEP)_6_5_1_3, m/z:1257.15(3+), RT:26.34, Y-score:88.80

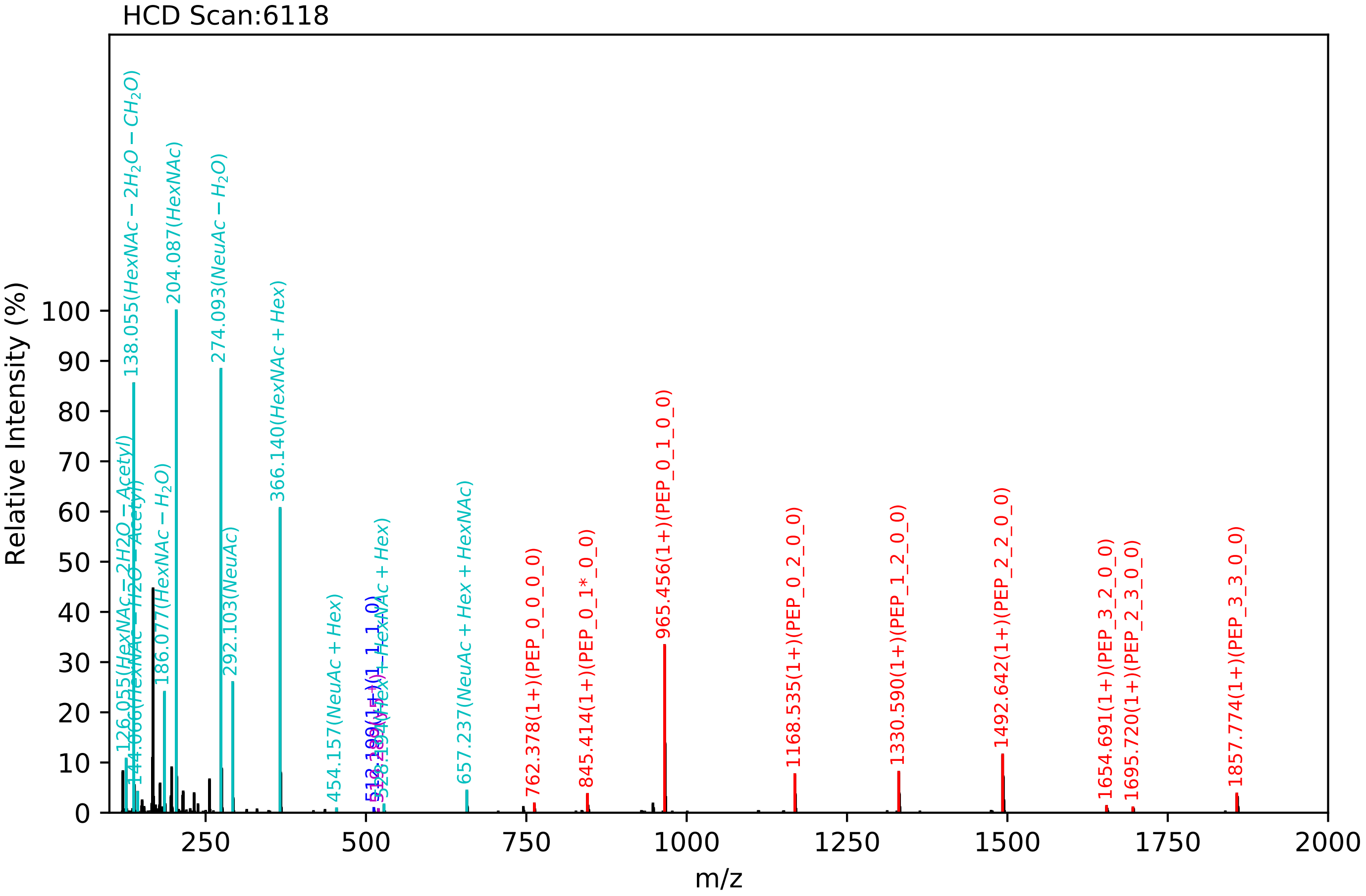

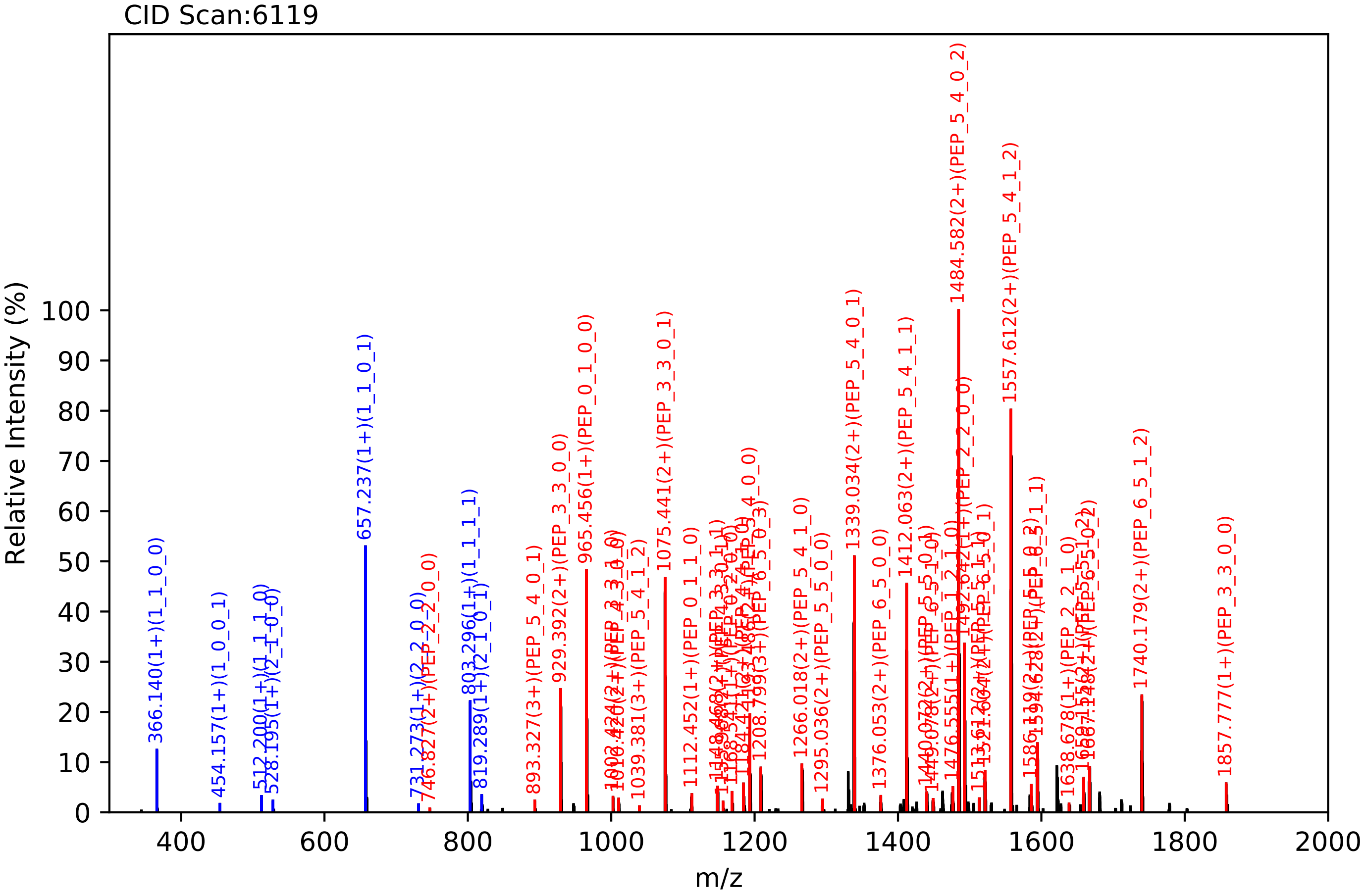

Unknown set no. 102, Experiment: Human Plasma exp_3

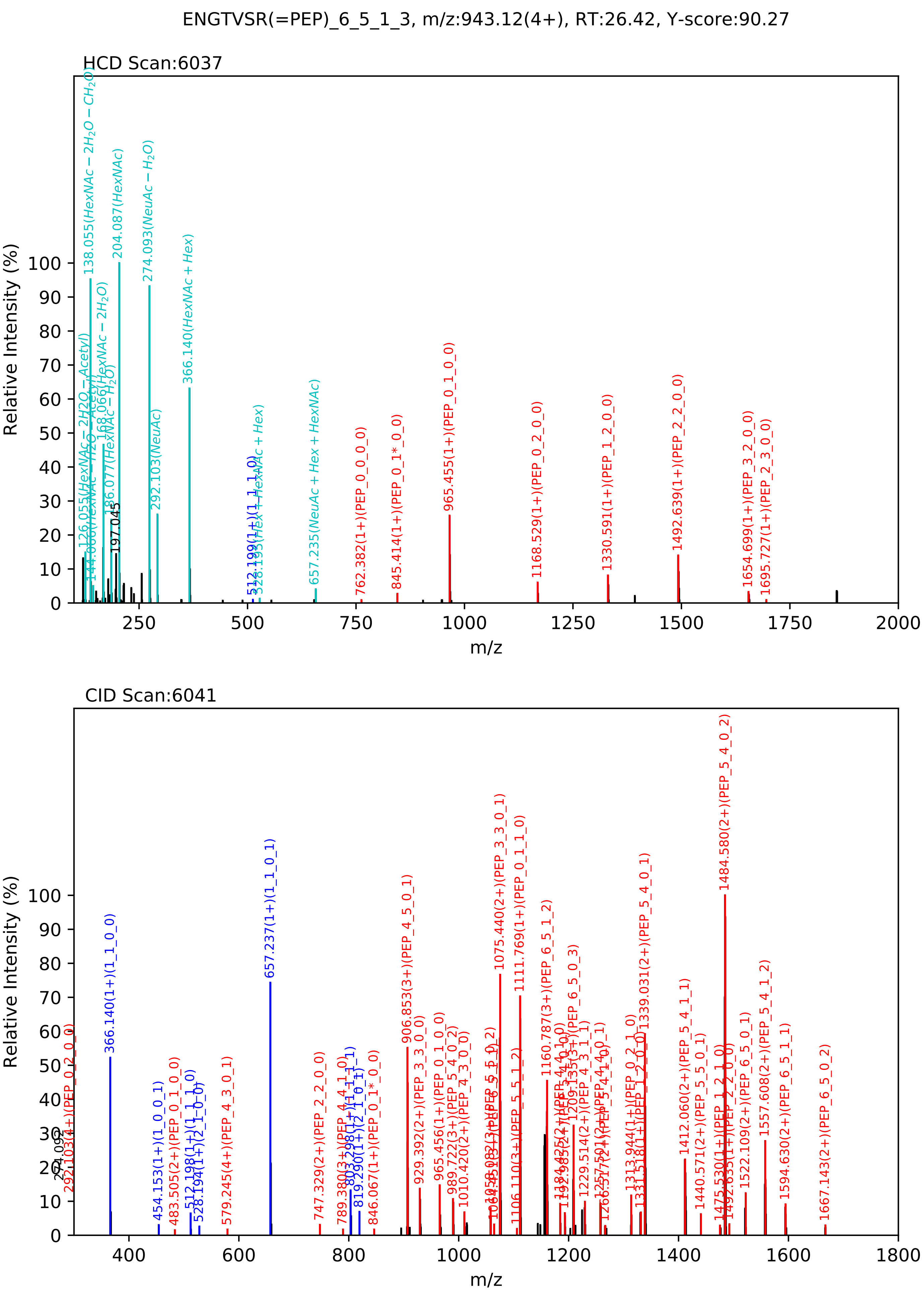

Unknown set no. 103, Experiment: Human Plasma exp_5

ENGTVSR(=PEP)_6_5_1_3, m/z:943.12(4+), RT:26.46, Y-score:90.76

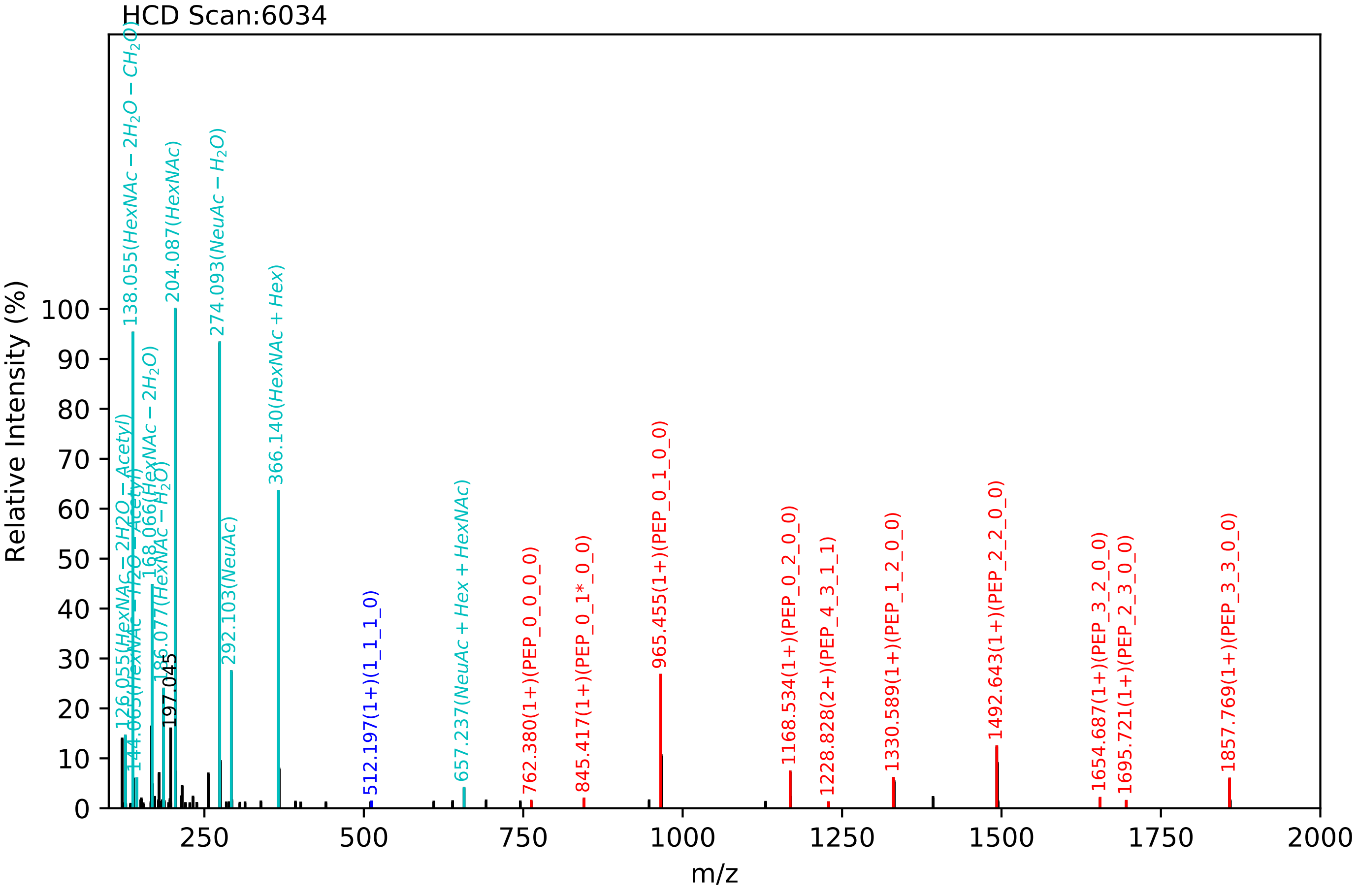

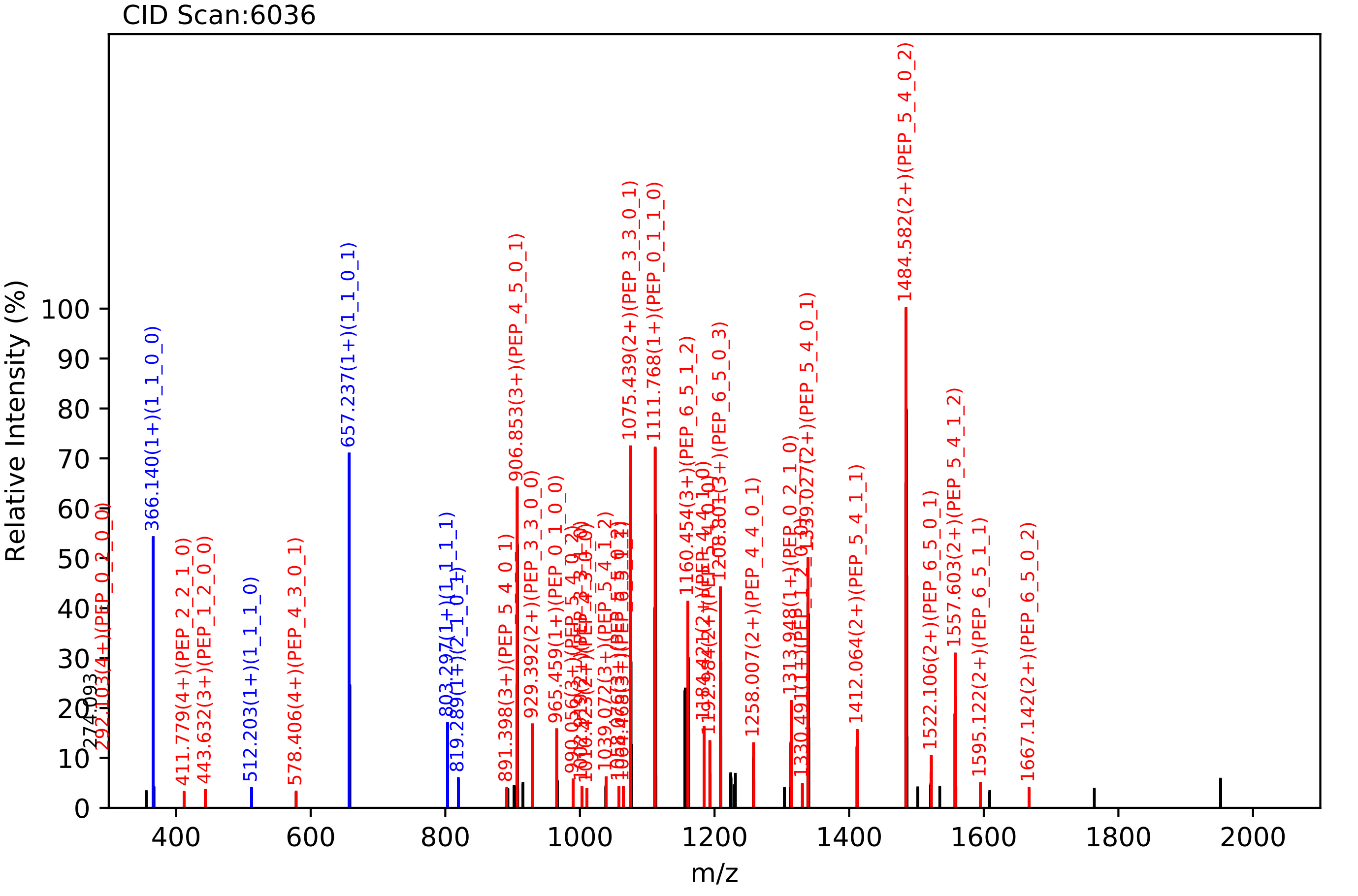

Unknown set no. 104, Experiment: Human Plasma exp_5

ENGTVSR(=PEP)_6_5_1_3, m/z:1257.15(3+), RT:26.51, Y-score:89.54

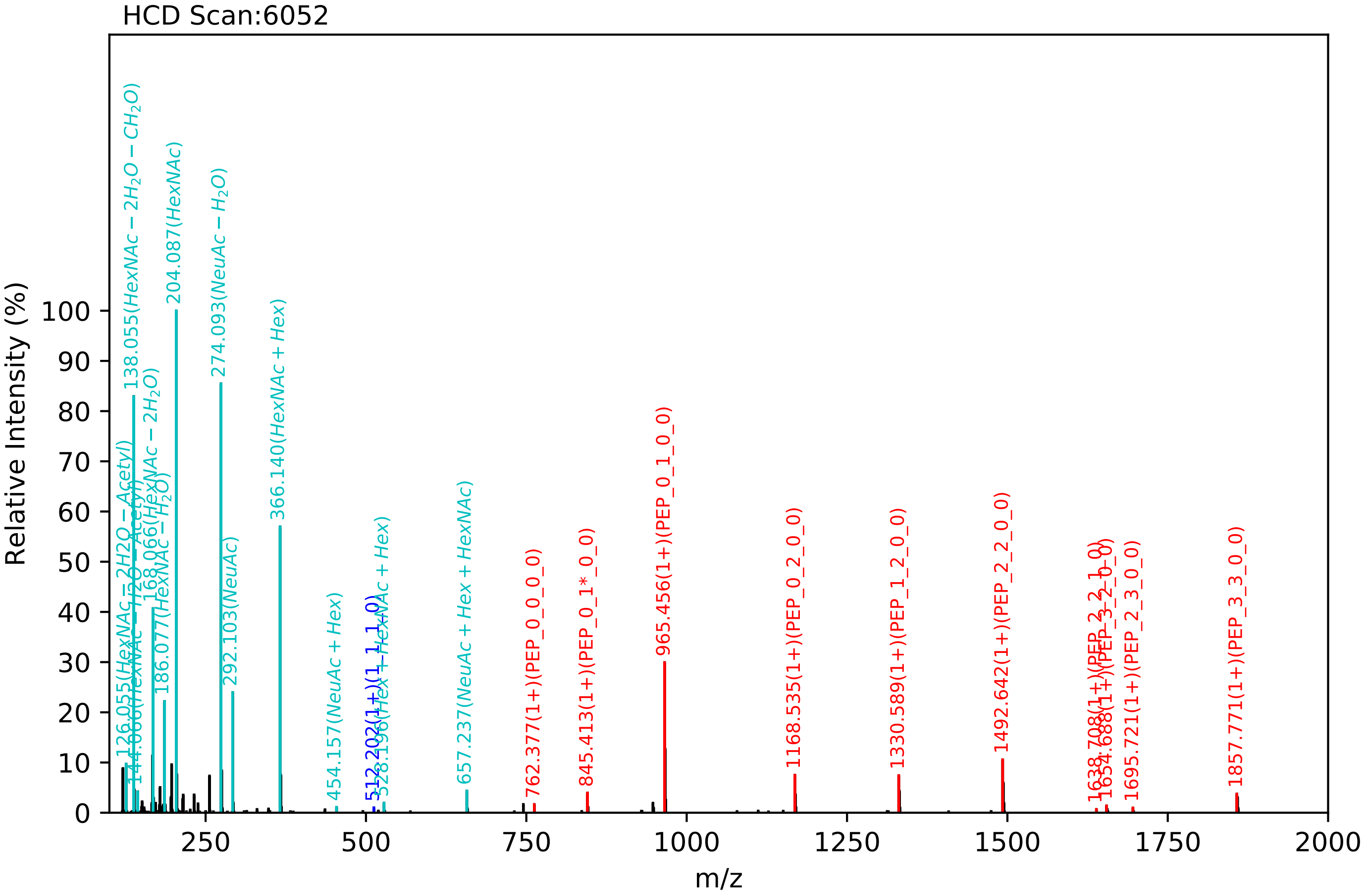

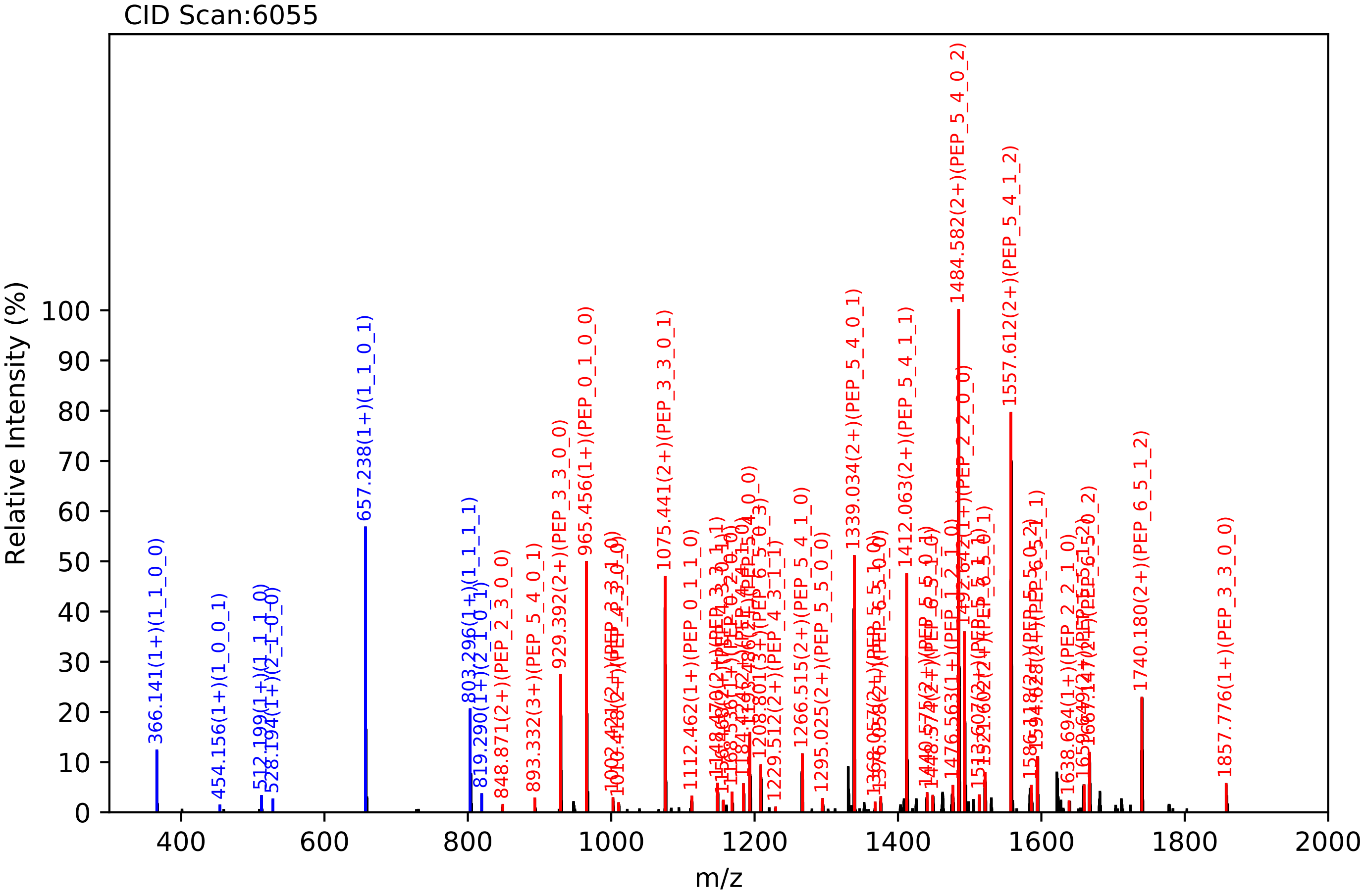

Unknown set no. 105, Experiment: Human Plasma exp_5

ENGTVSR(=PEP)_6_5_2_3, m/z:1305.84(3+), RT:26.28, Y-score:83.59

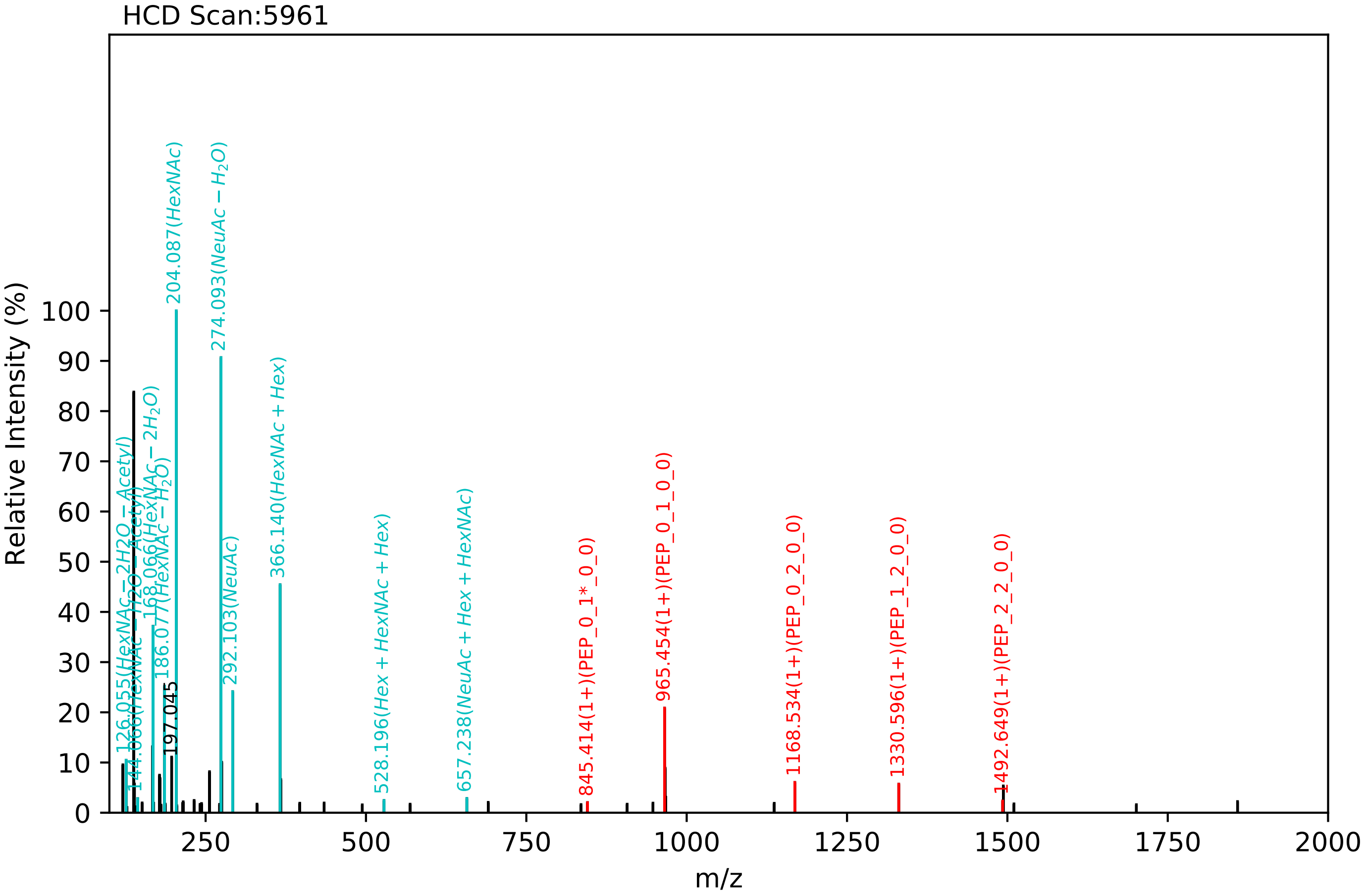

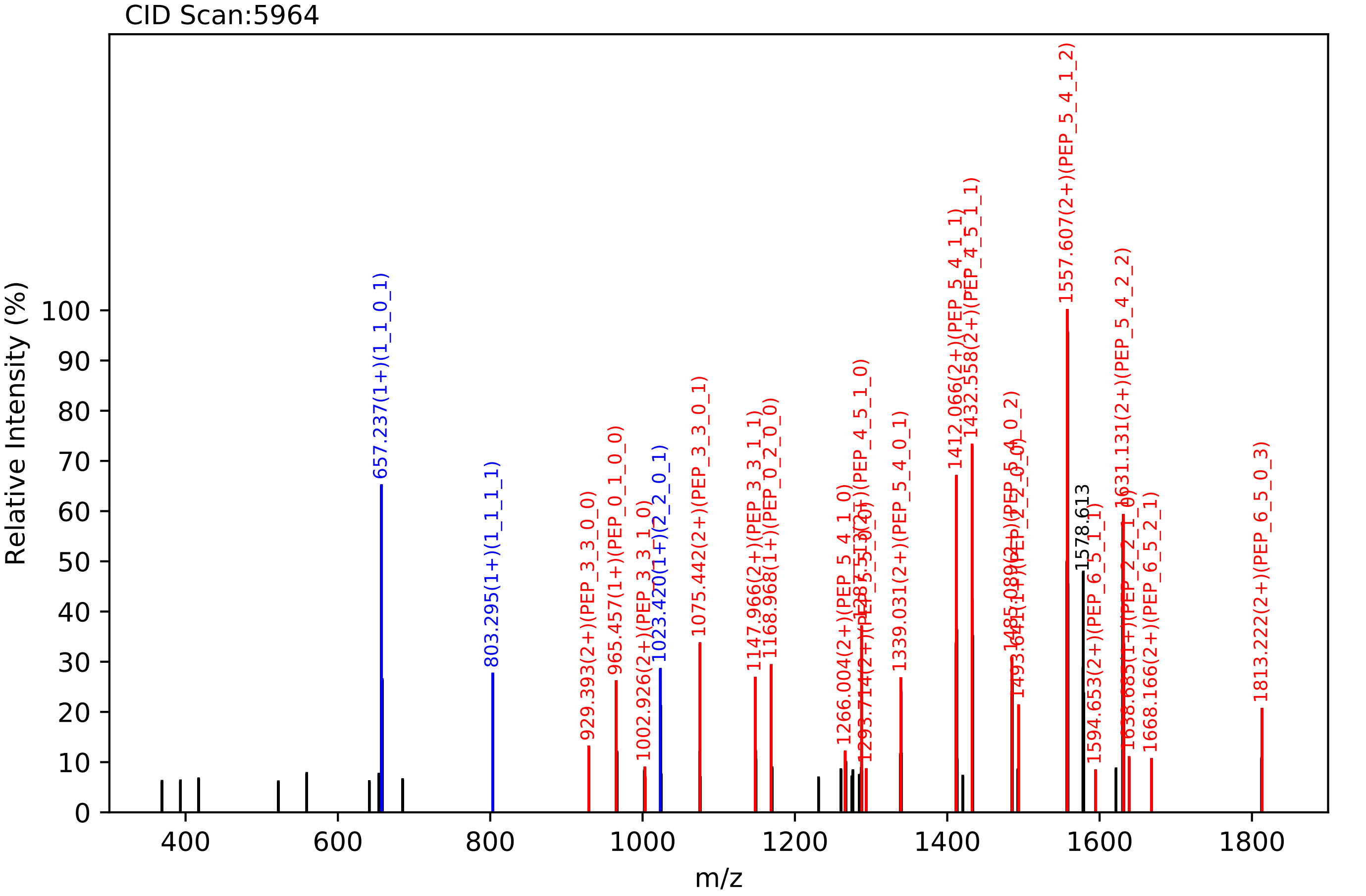

Unknown set no. 106, Experiment: Human Plasma exp_1

ENGTVSR(=PEP)_7_6_1_4, m/z:1107.17(4+), RT:40.97, Y-score:87.76

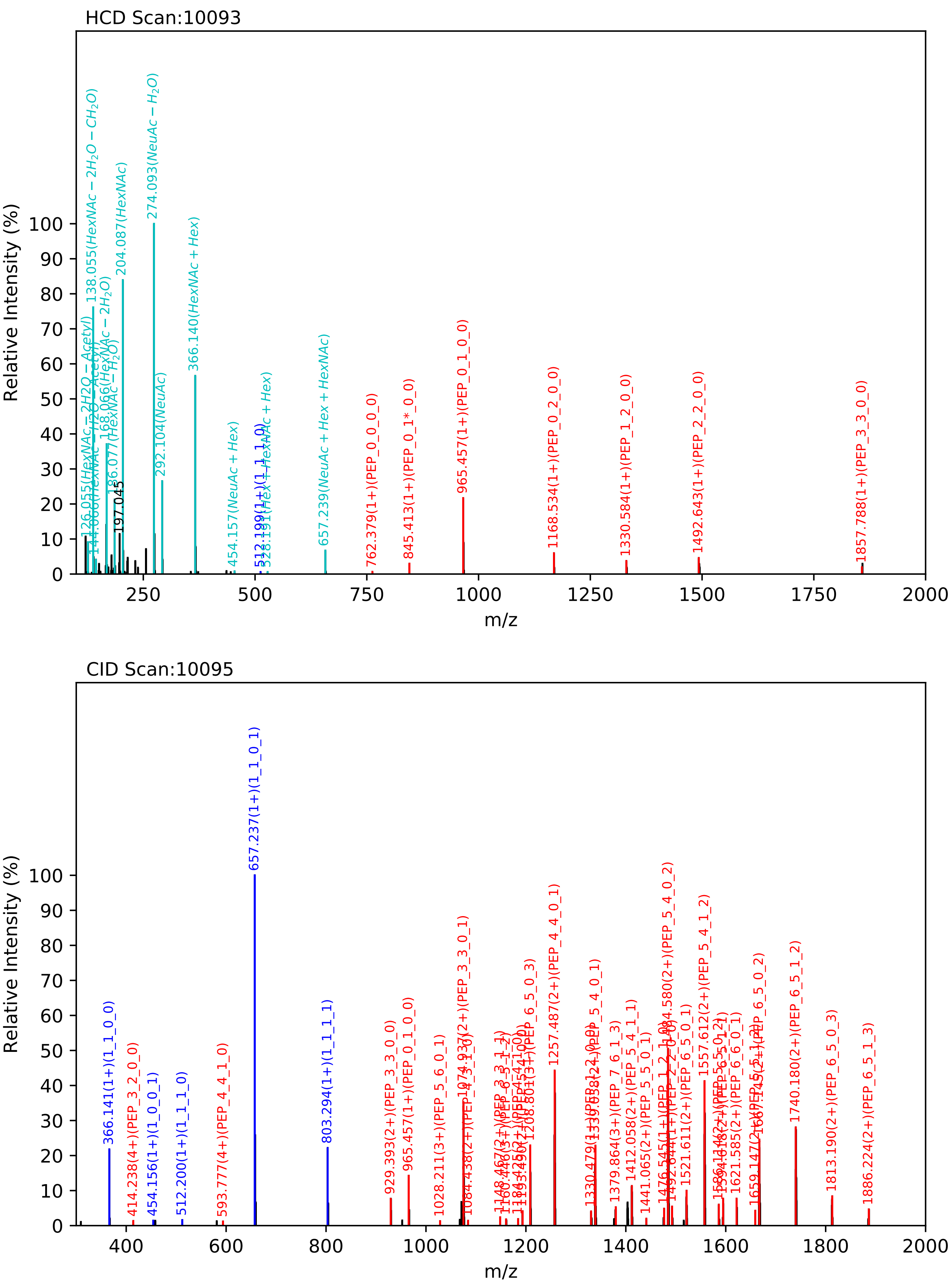

Unknown set no. 107, Experiment: Human Plasma exp_5

ENGTVSR(=PEP)_7_6_1_4, m/z:1107.17(4+), RT:41.89, Y-score:89.07

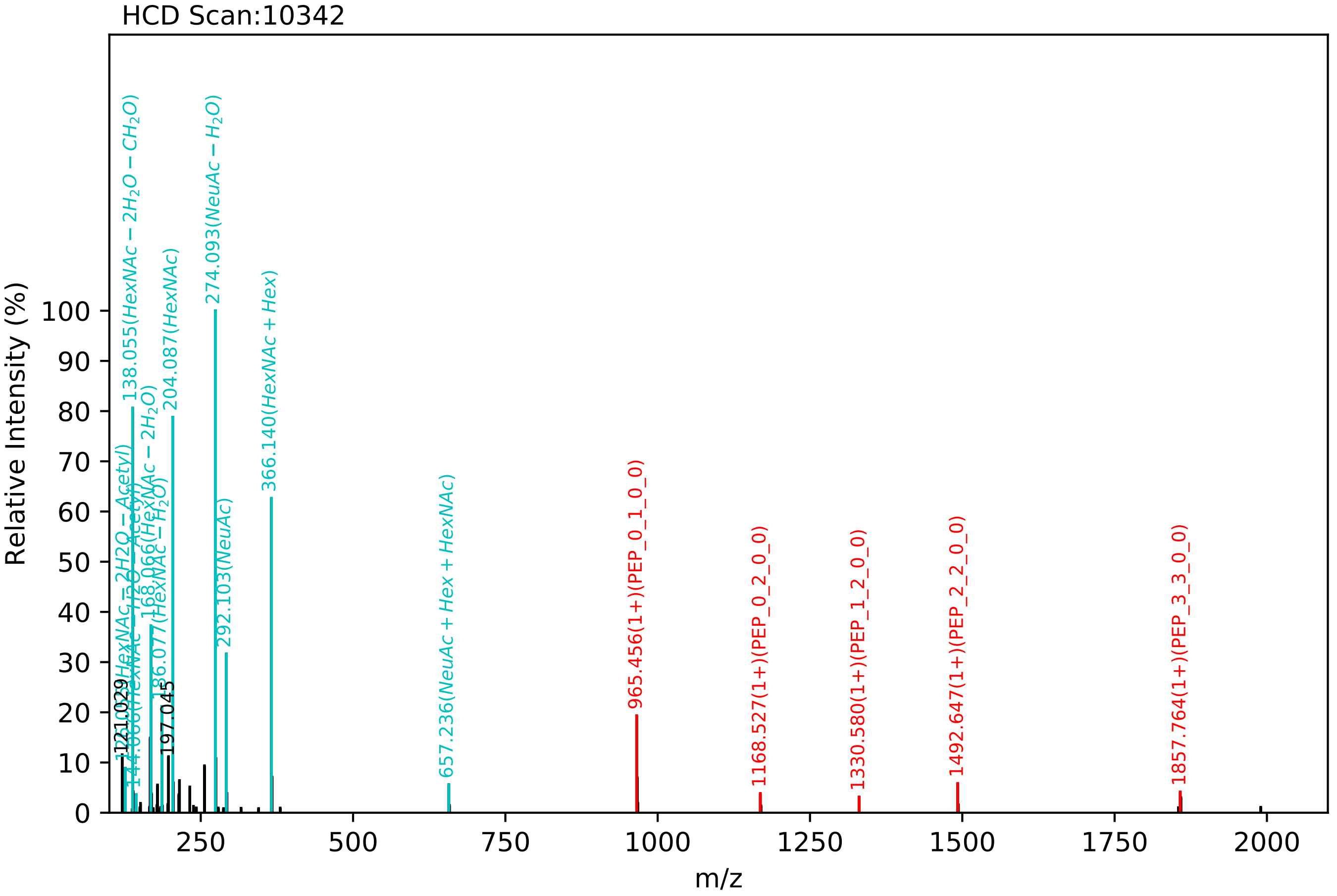

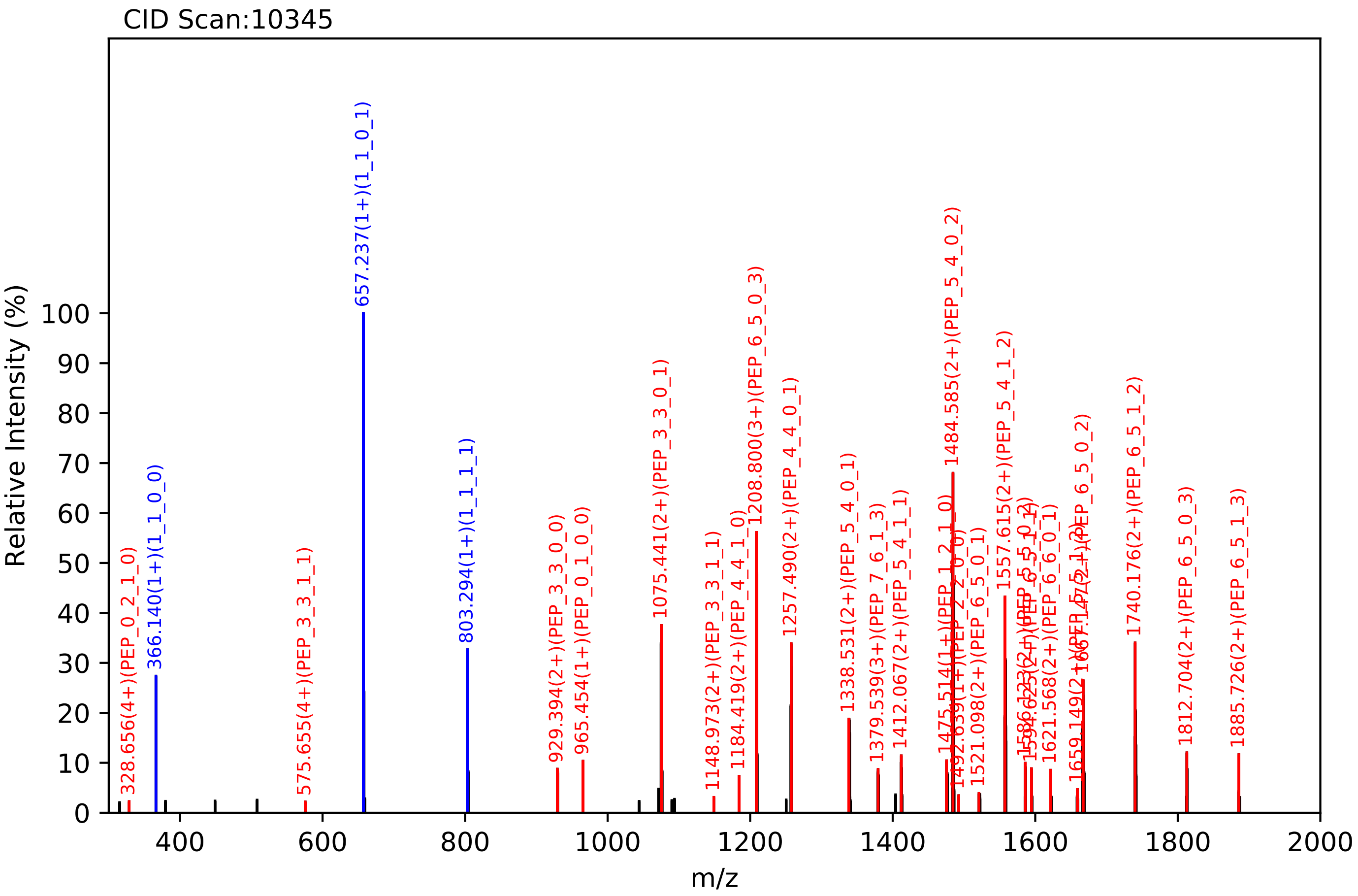

Unknown set no. 108, Experiment: Human Plasma exp_2

ENGTVSR(=PEP)_7_6_2_4, m/z:1143.69(4+), RT:42.95, Y-score:84.88

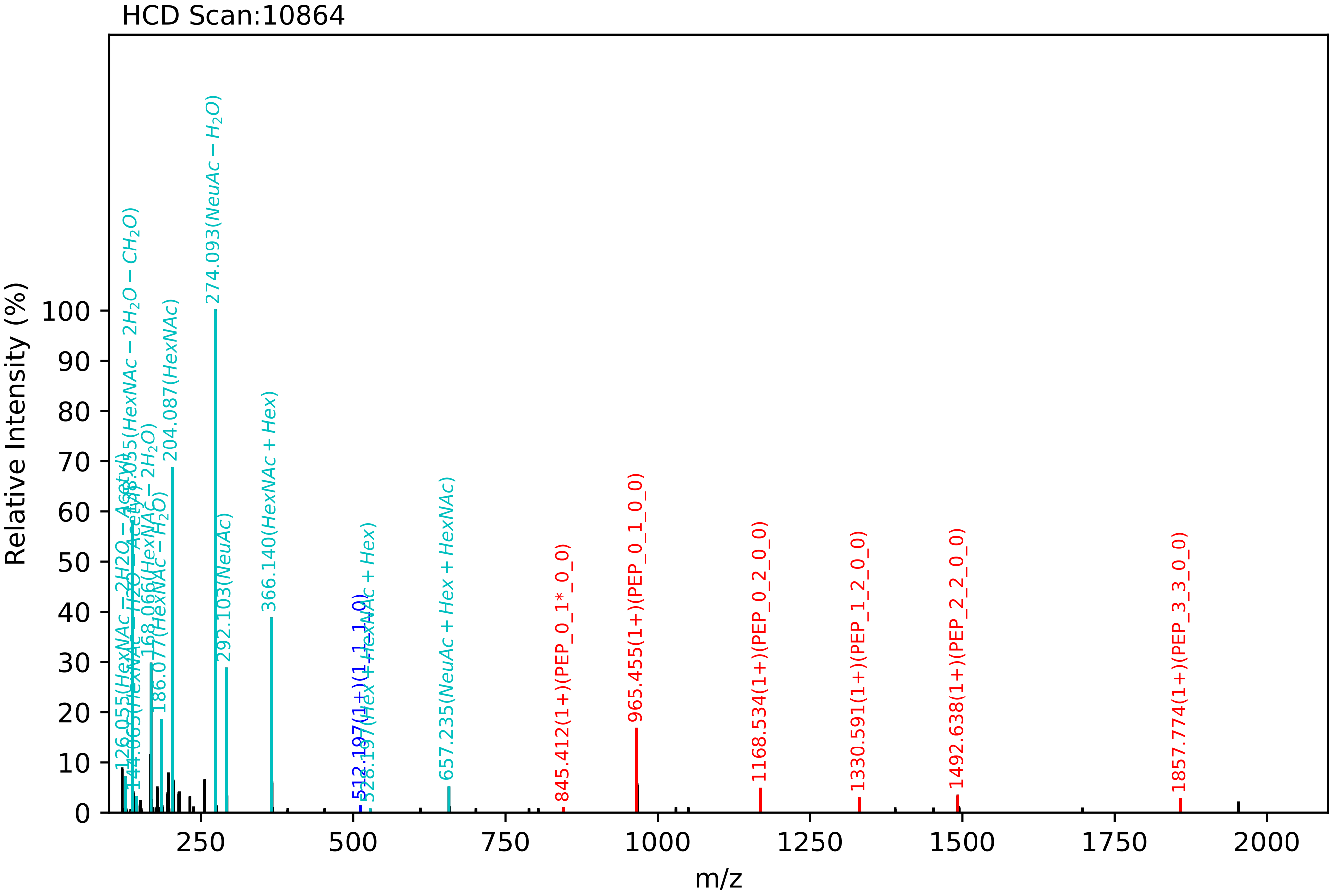

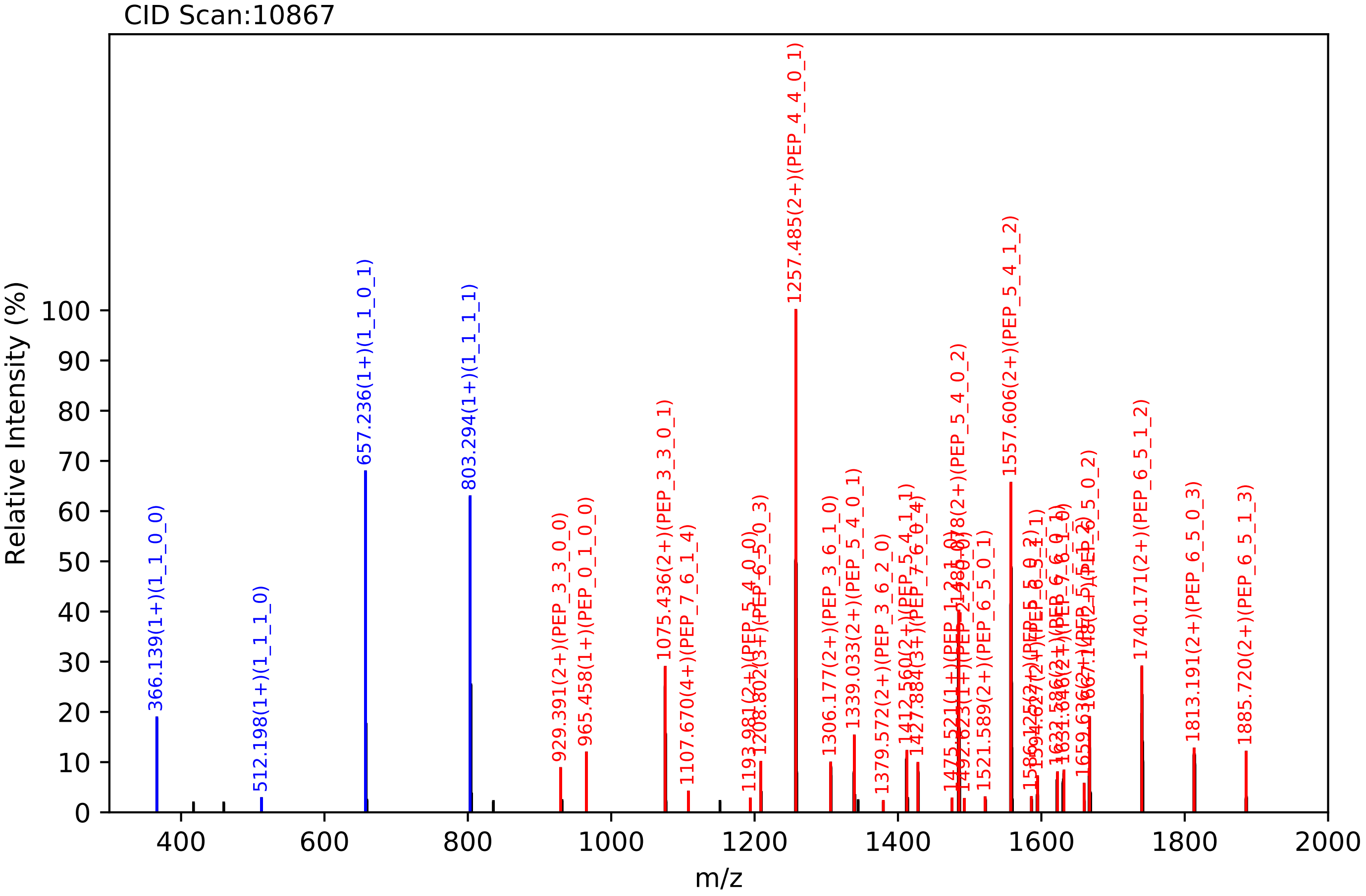

Unknown set no. 109, Experiment: Human Plasma exp_3

ENGTVSR(=PEP)_7_6_2_4, m/z:1143.68(4+), RT:41.91, Y-score:82.64

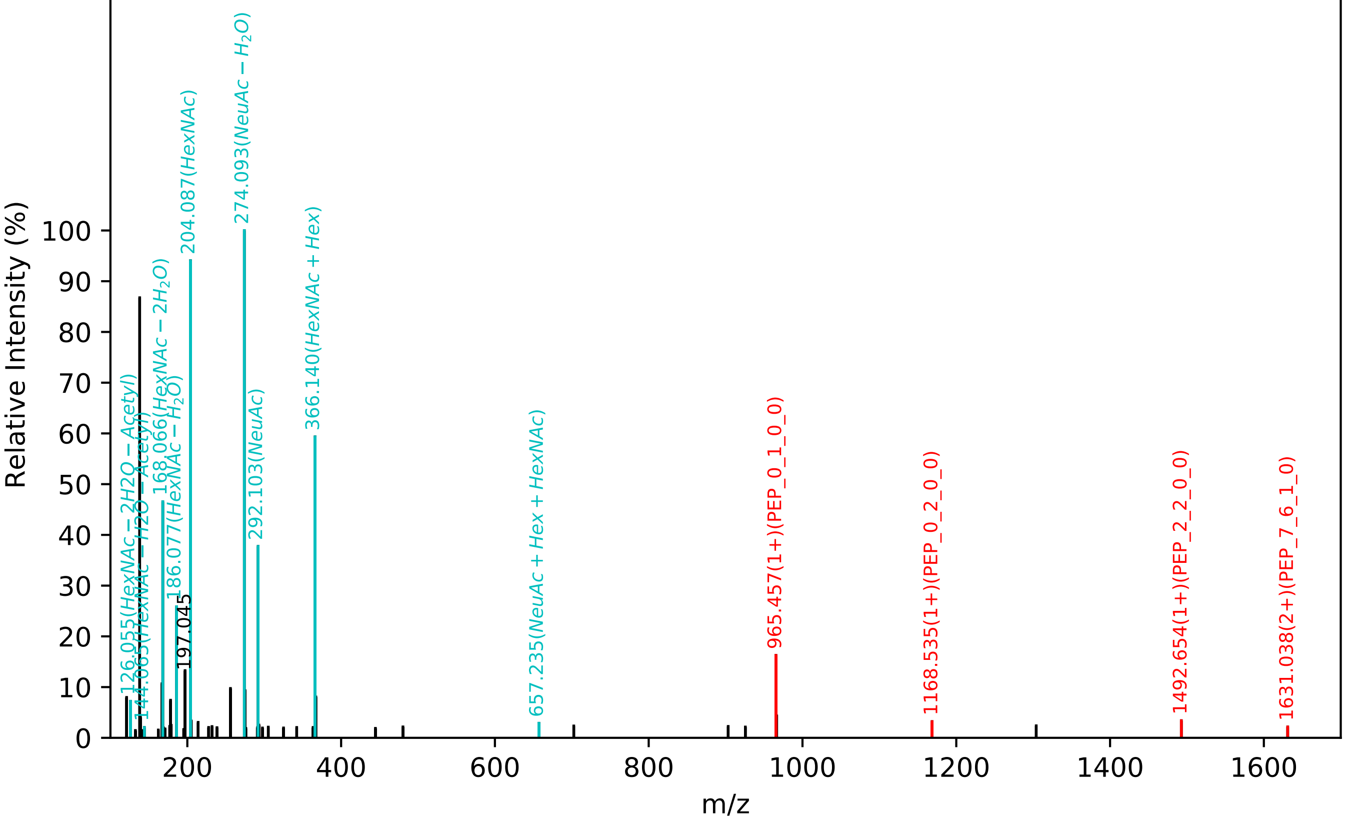

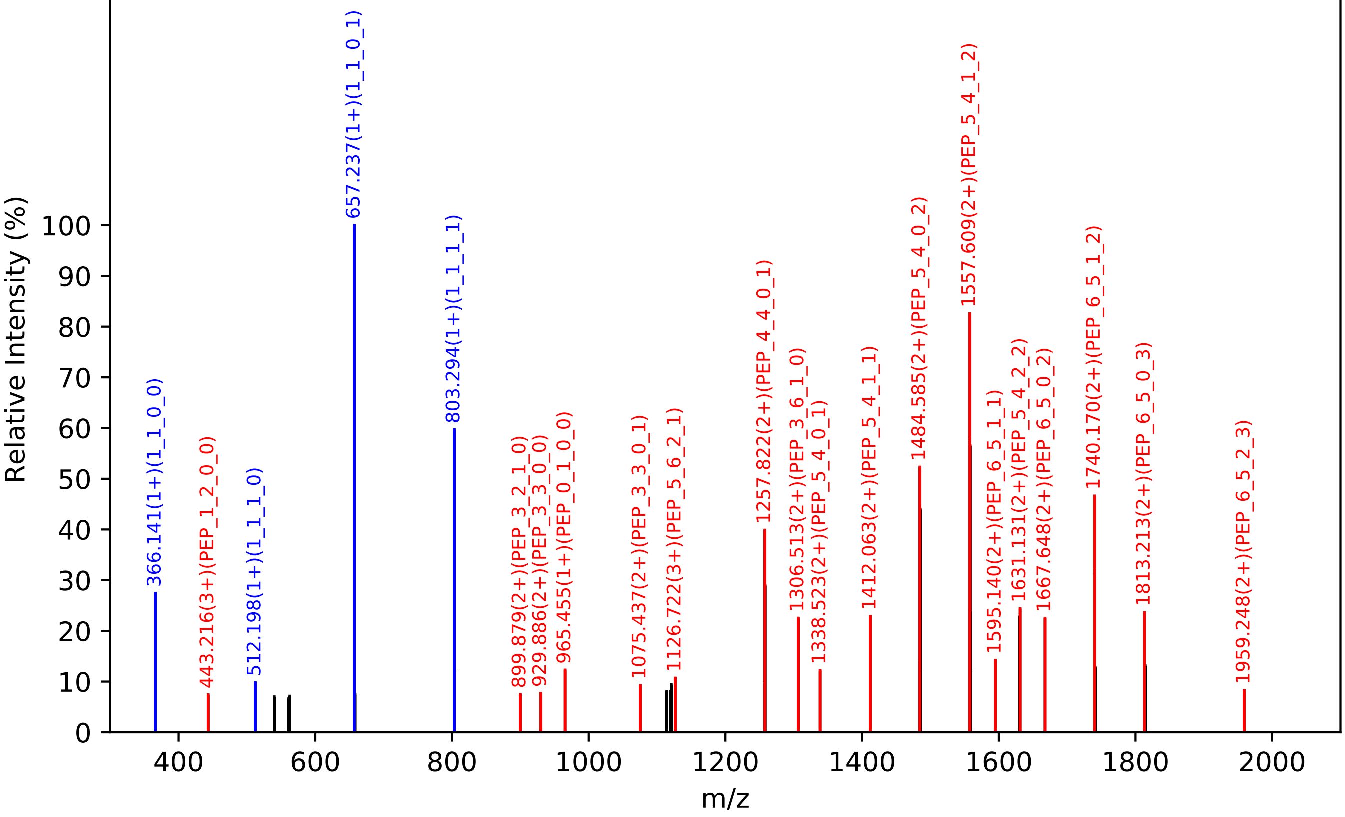

Unknown set no. 110, Experiment: Human Plasma exp_5

ENGTVSR(=PEP)_7_6_2_4, m/z:1143.69(4+), RT:43.10, Y-score:84.65

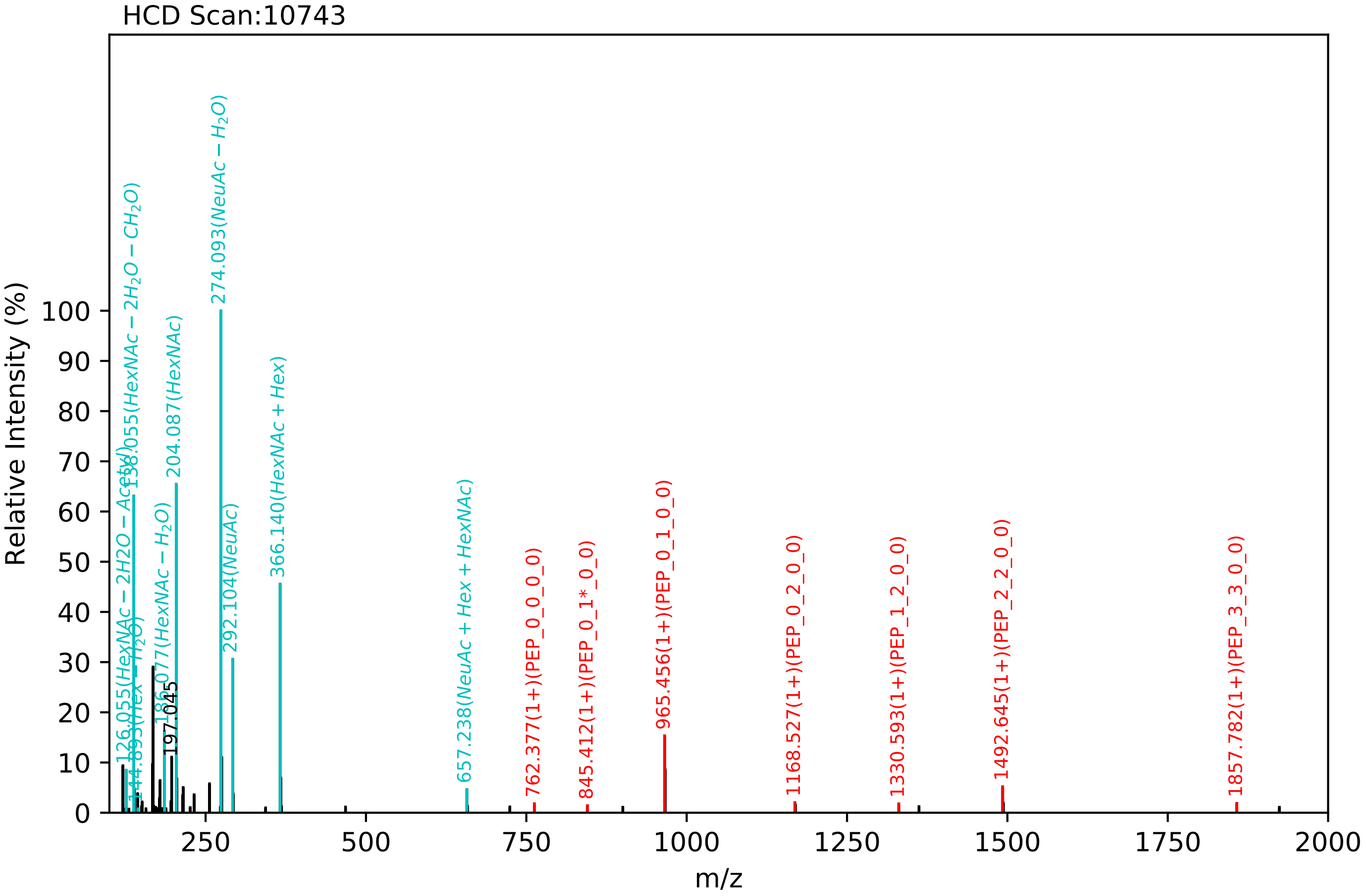

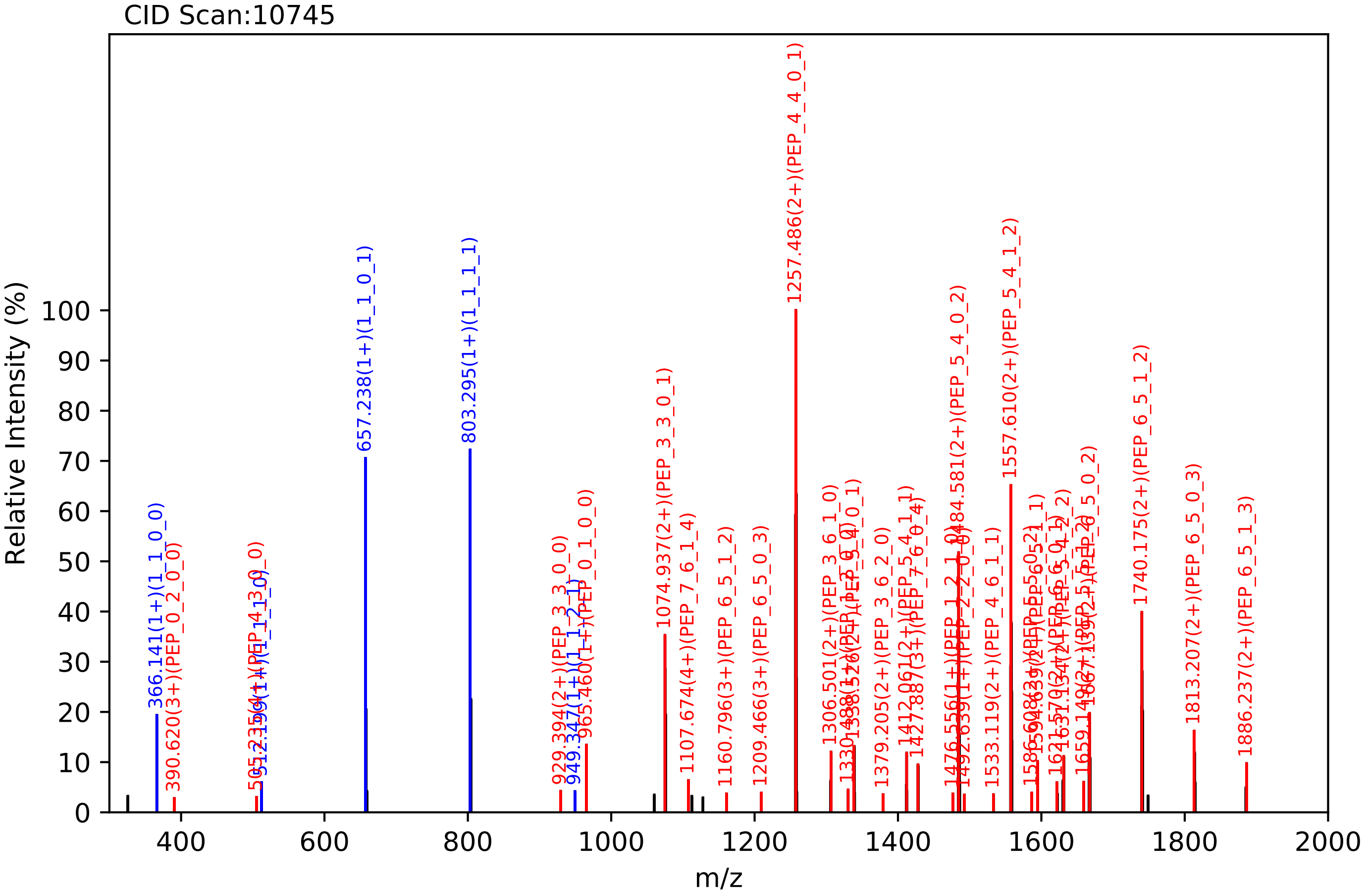

Unknown set no. 111, Experiment: Human Plasma exp_2

ENGTVSR(=PEP)_7_6_3_4, m/z:1180.20(4+), RT:41.40, Y-score:83.96

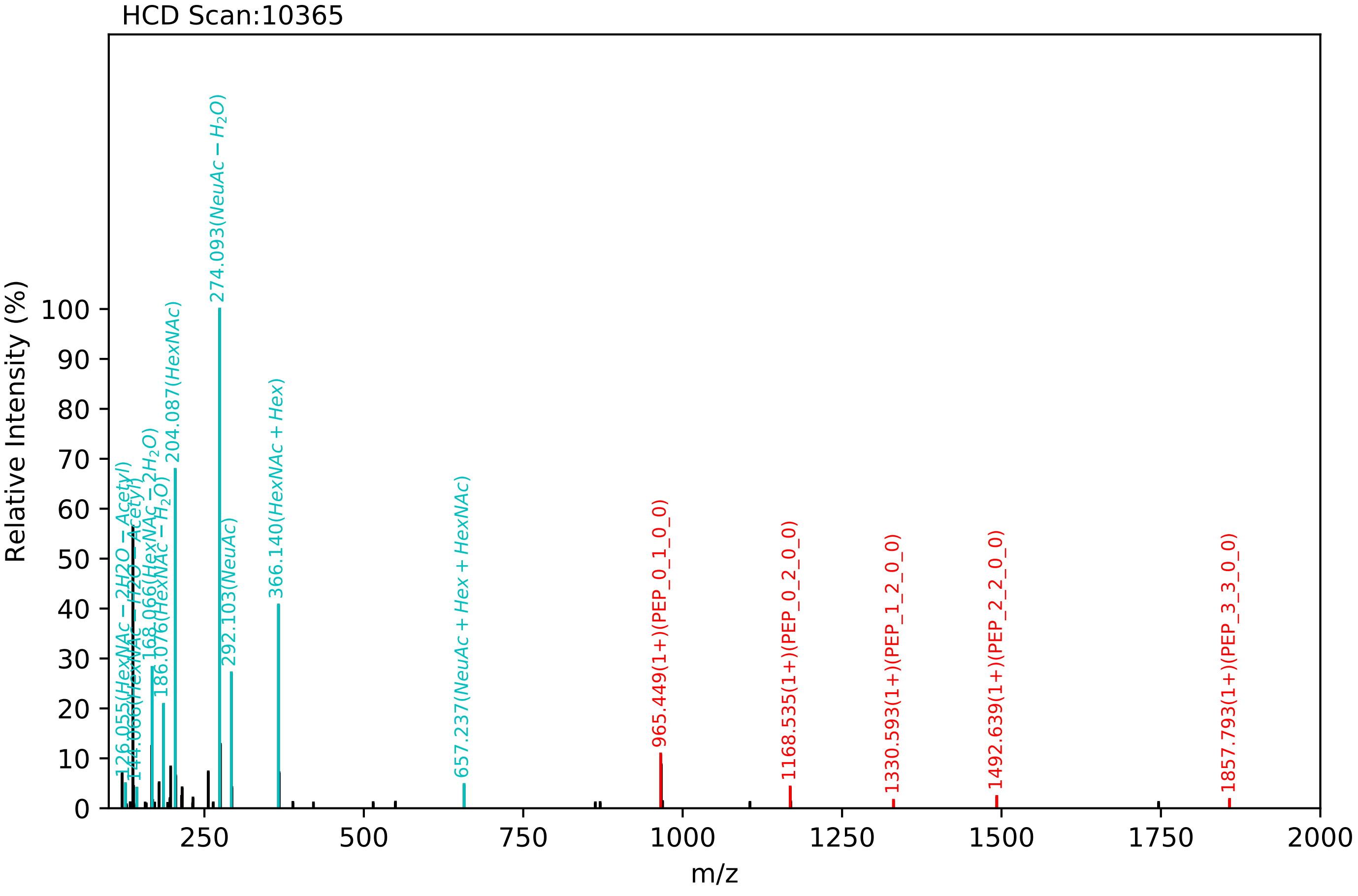

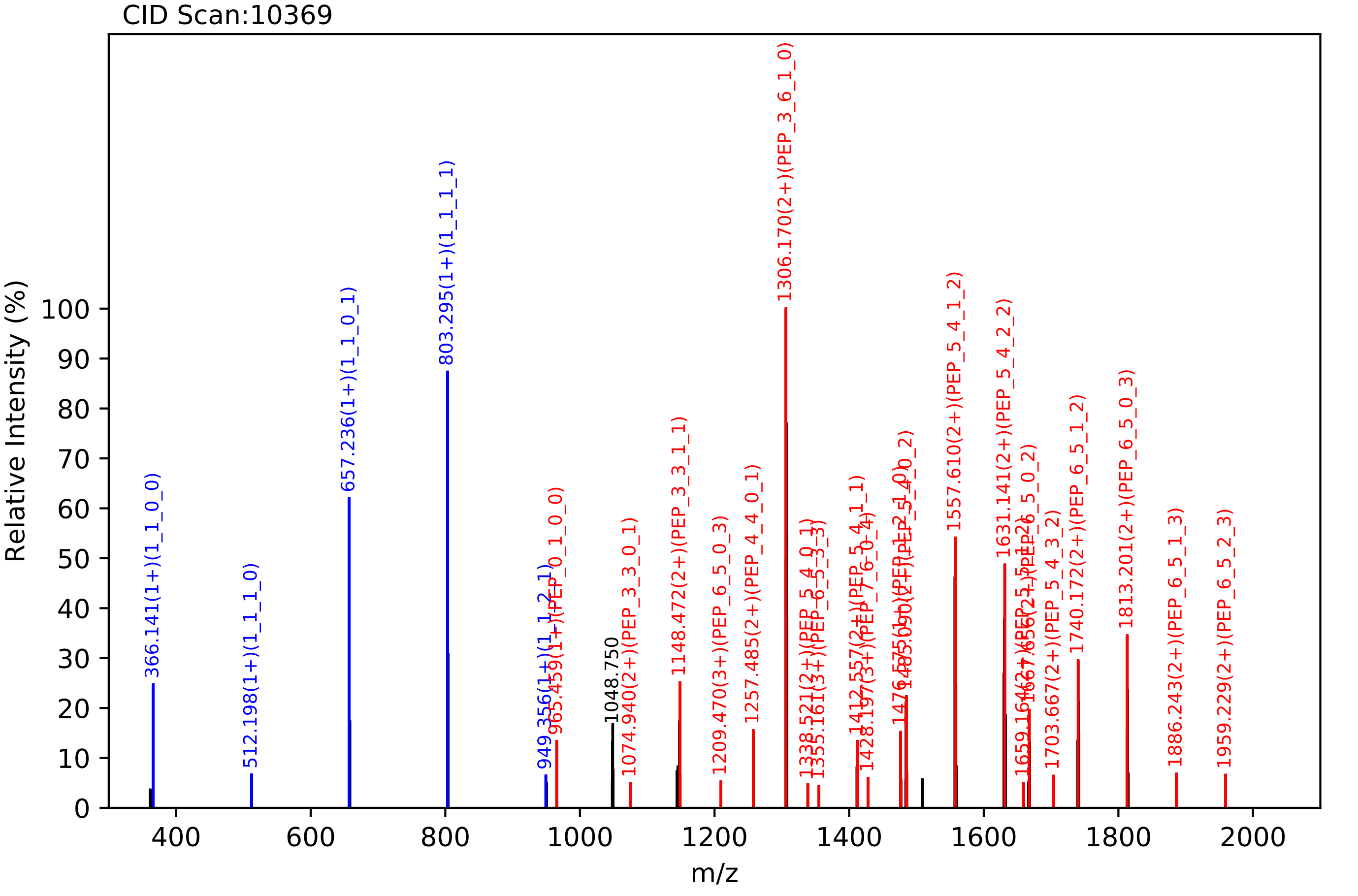

Unknown set no. 112, Experiment: Human Plasma exp_3

ENGTVSR(=PEP)_7_6_3_4, m/z:1180.20(4+), RT:41.80, Y-score:87.51

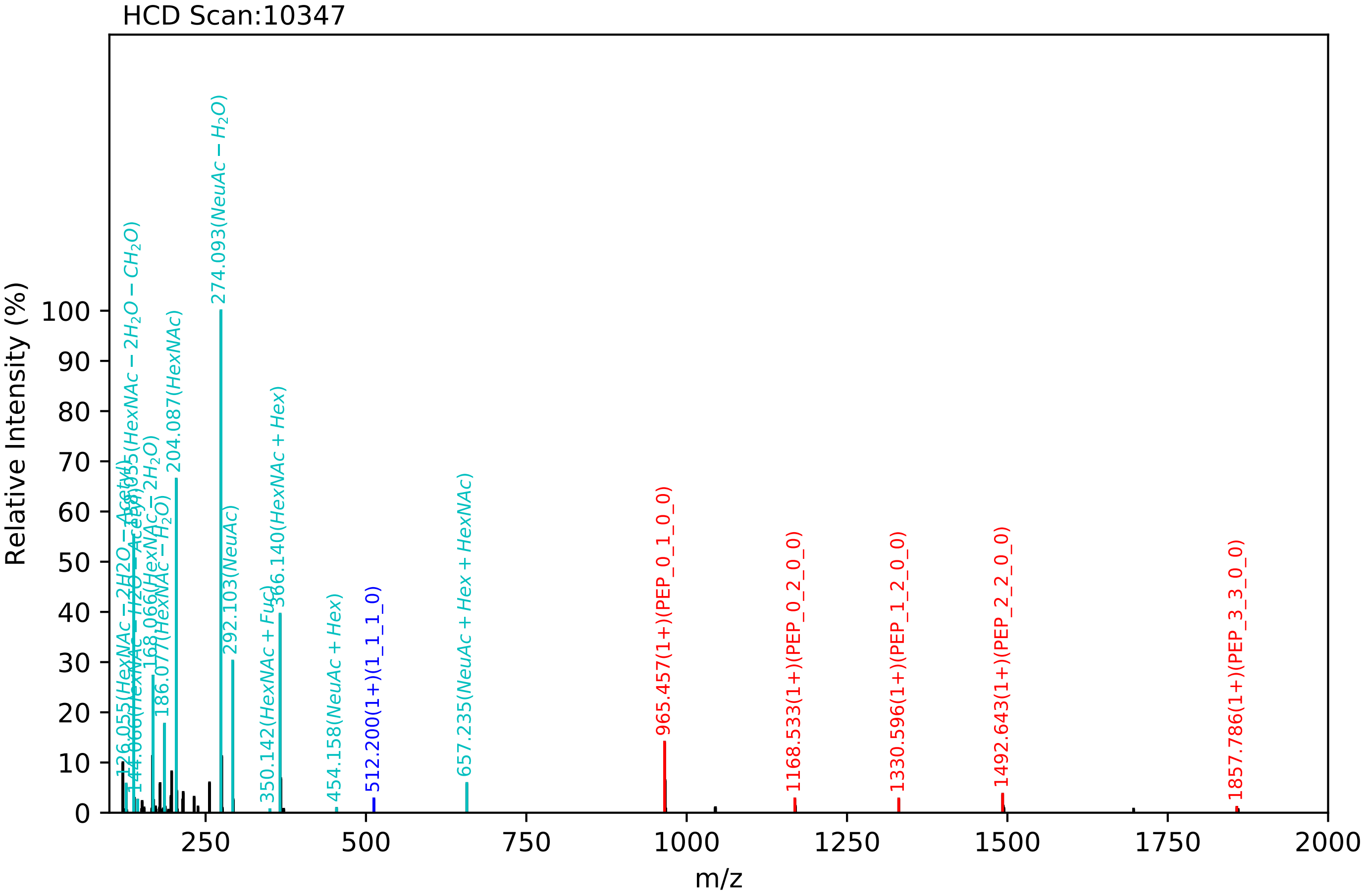

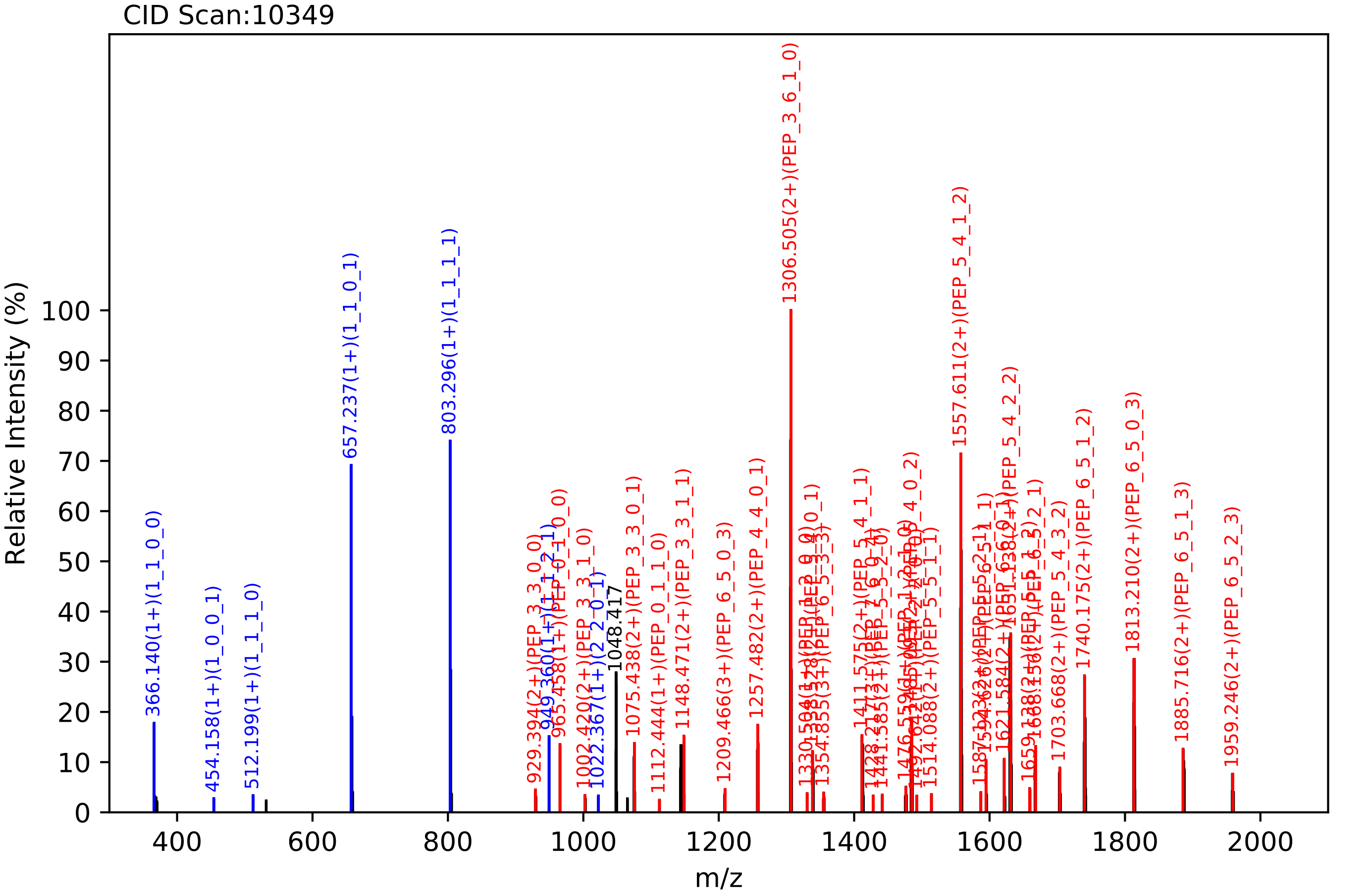

Unknown set no. 113, Experiment: Human Plasma exp_4

ENGTVSR(=PEP)_7_6_3_4, m/z:1180.20(4+), RT:41.75, Y-score:85.19

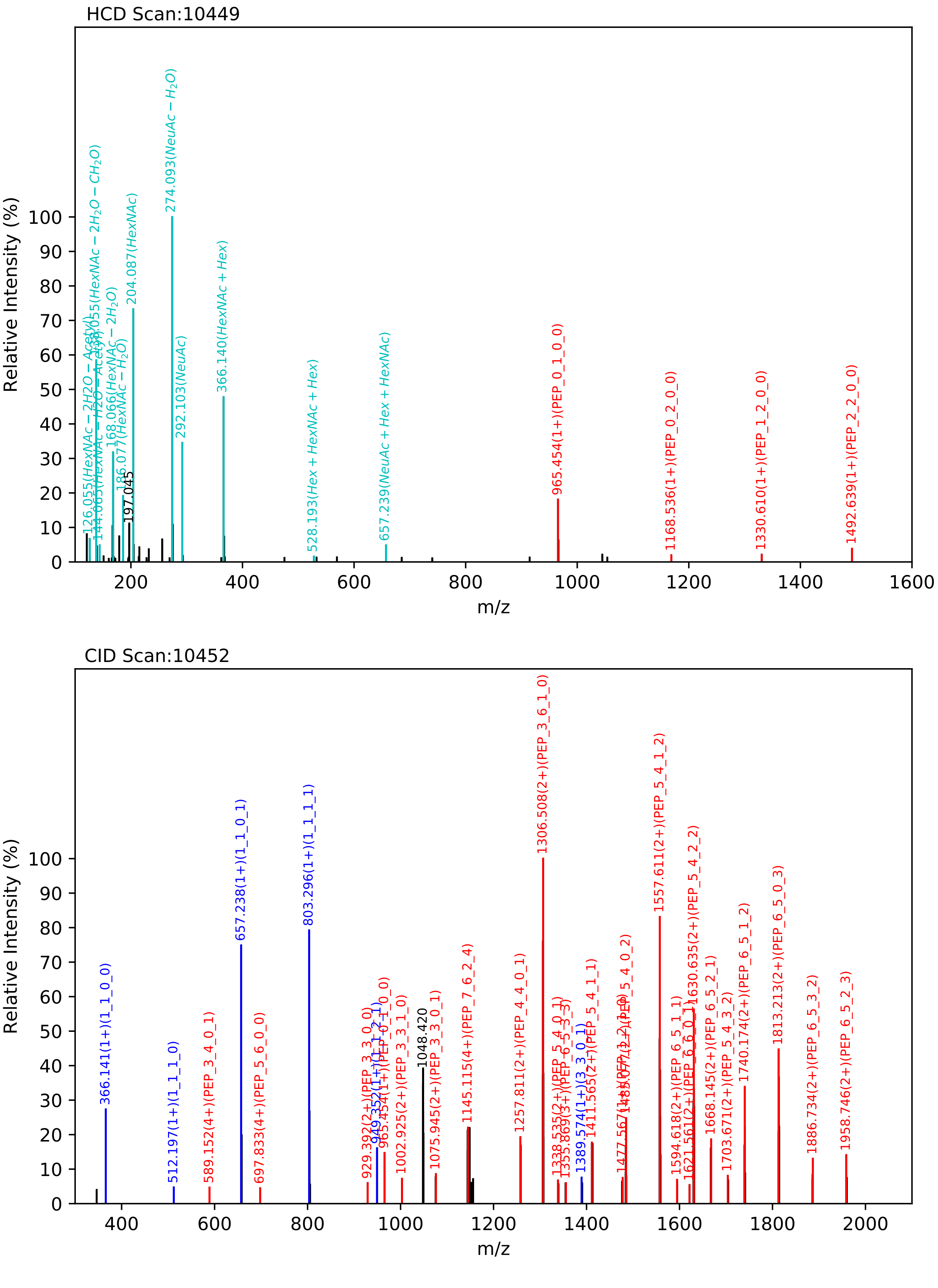

Unknown set no. 114, Experiment: Human Plasma exp_1

ENGTVSR(=PEP)_8_7_0_4, m/z:1161.94(4+), RT:41.40, Y-score:81.98

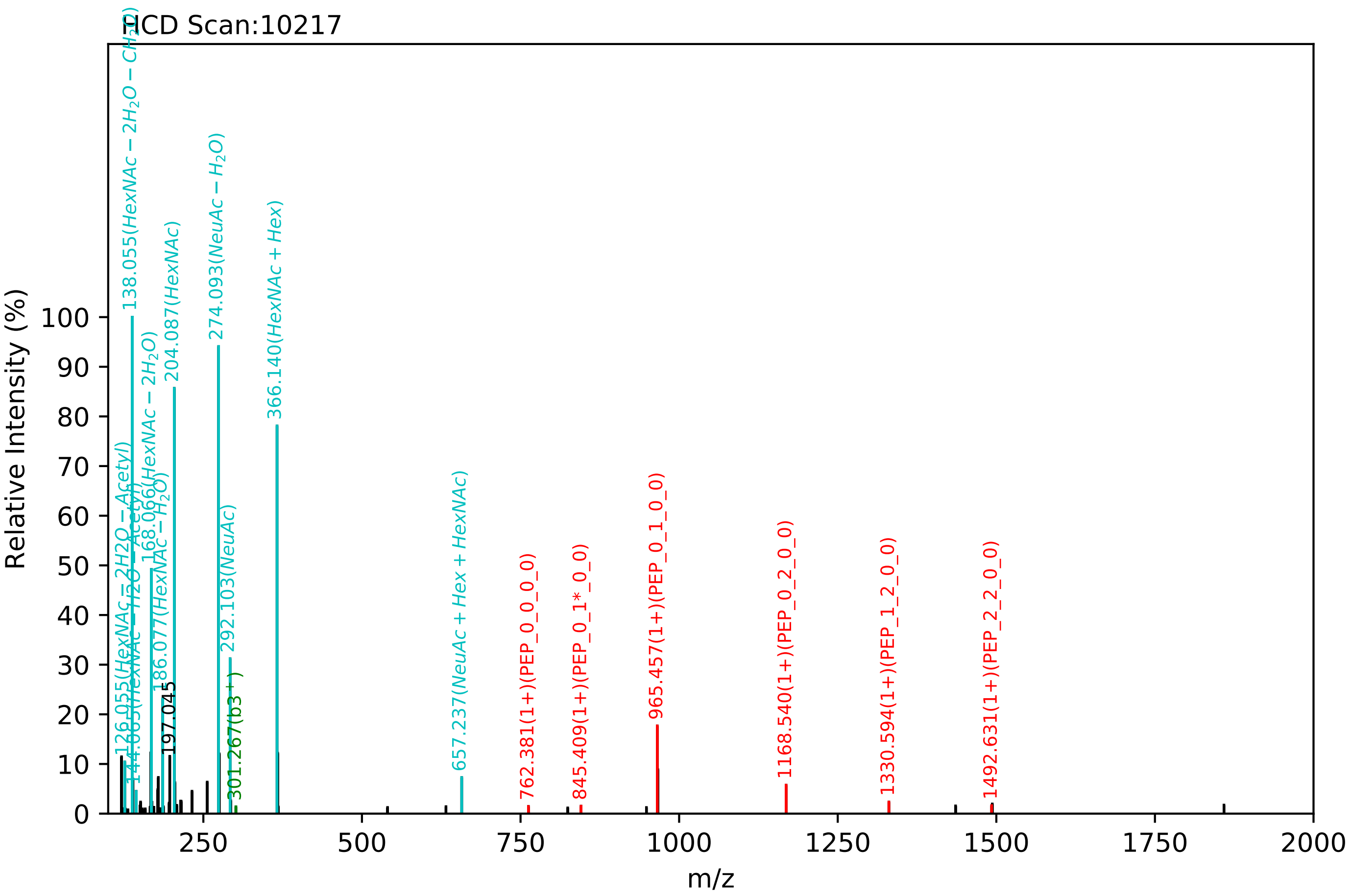

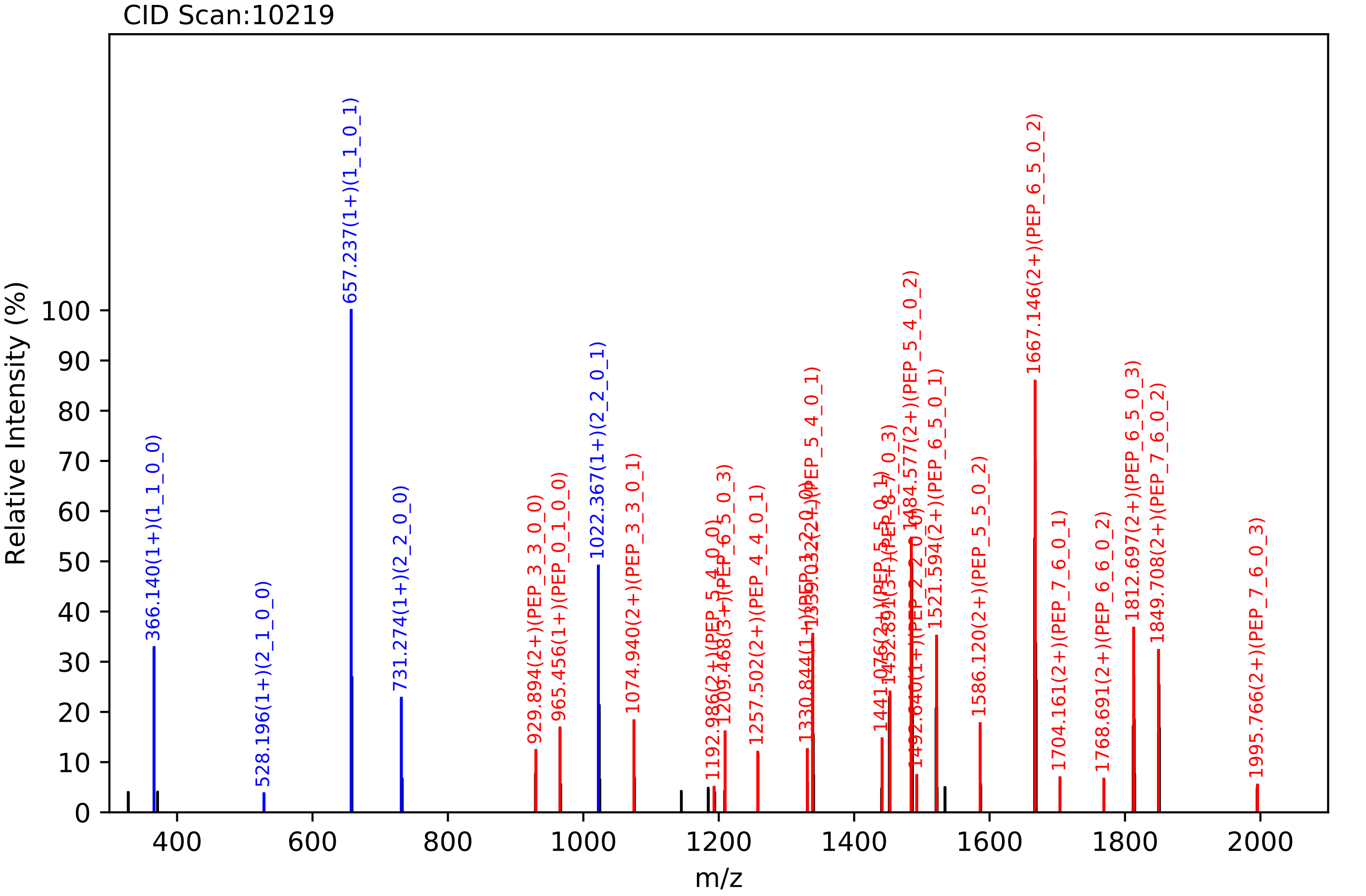

Unknown set no. 115, Experiment: Human Plasma exp_2

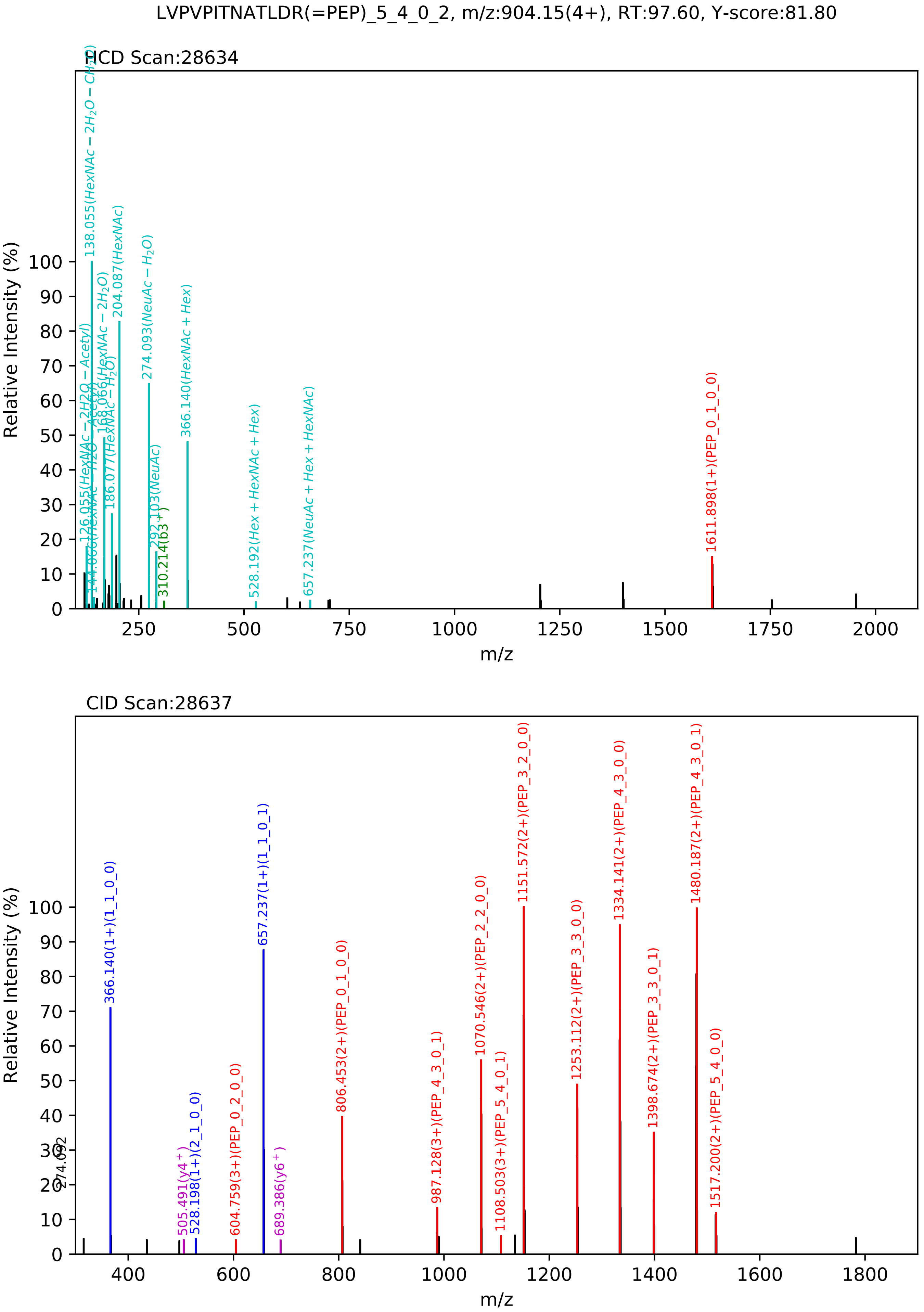

Unknown set no. 116, Experiment: Human Plasma exp_3

LVPVPITNATLDR(=PEP)_5_4_0_2, m/z:904.15(4+), RT:97.80, Y-score:81.08

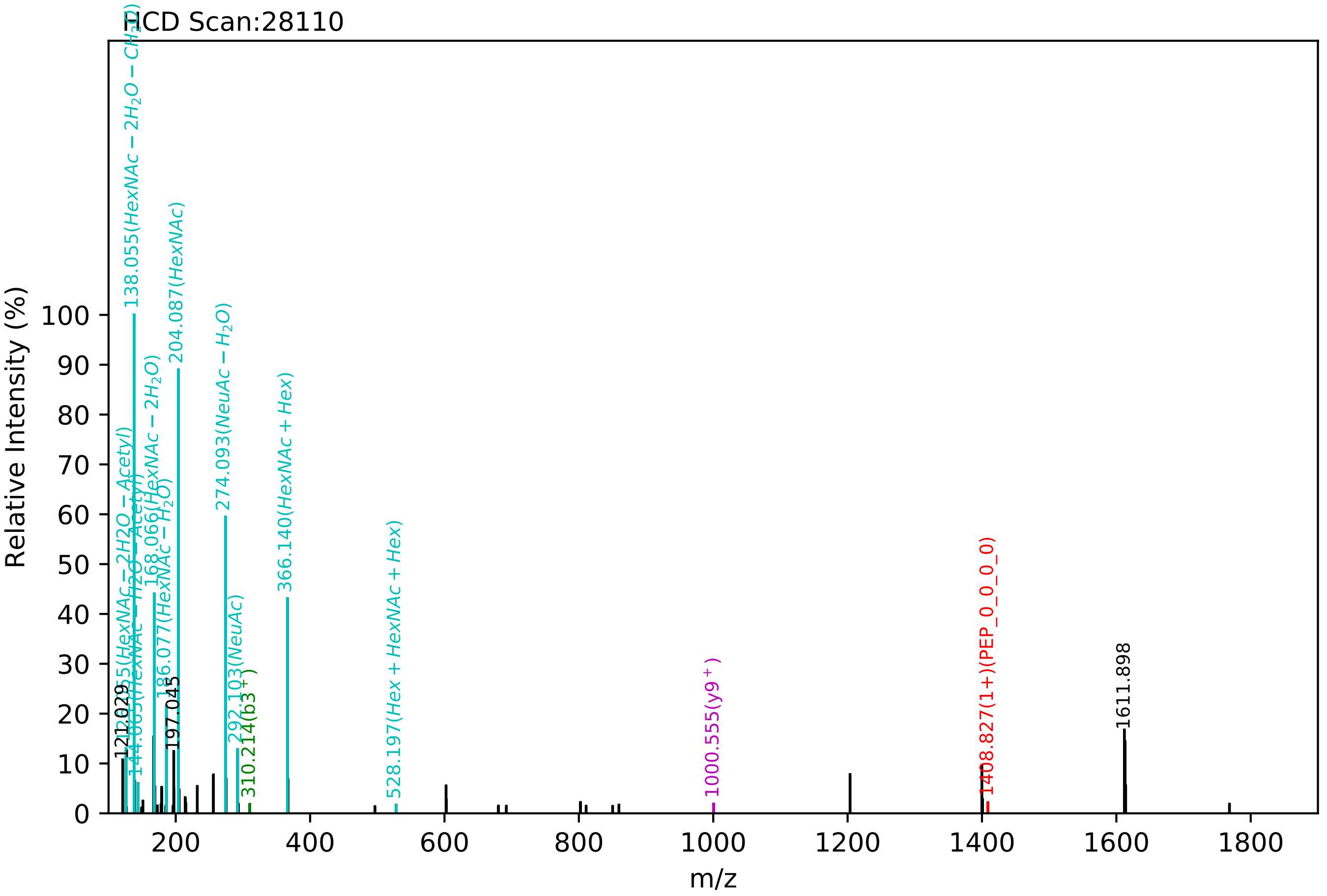

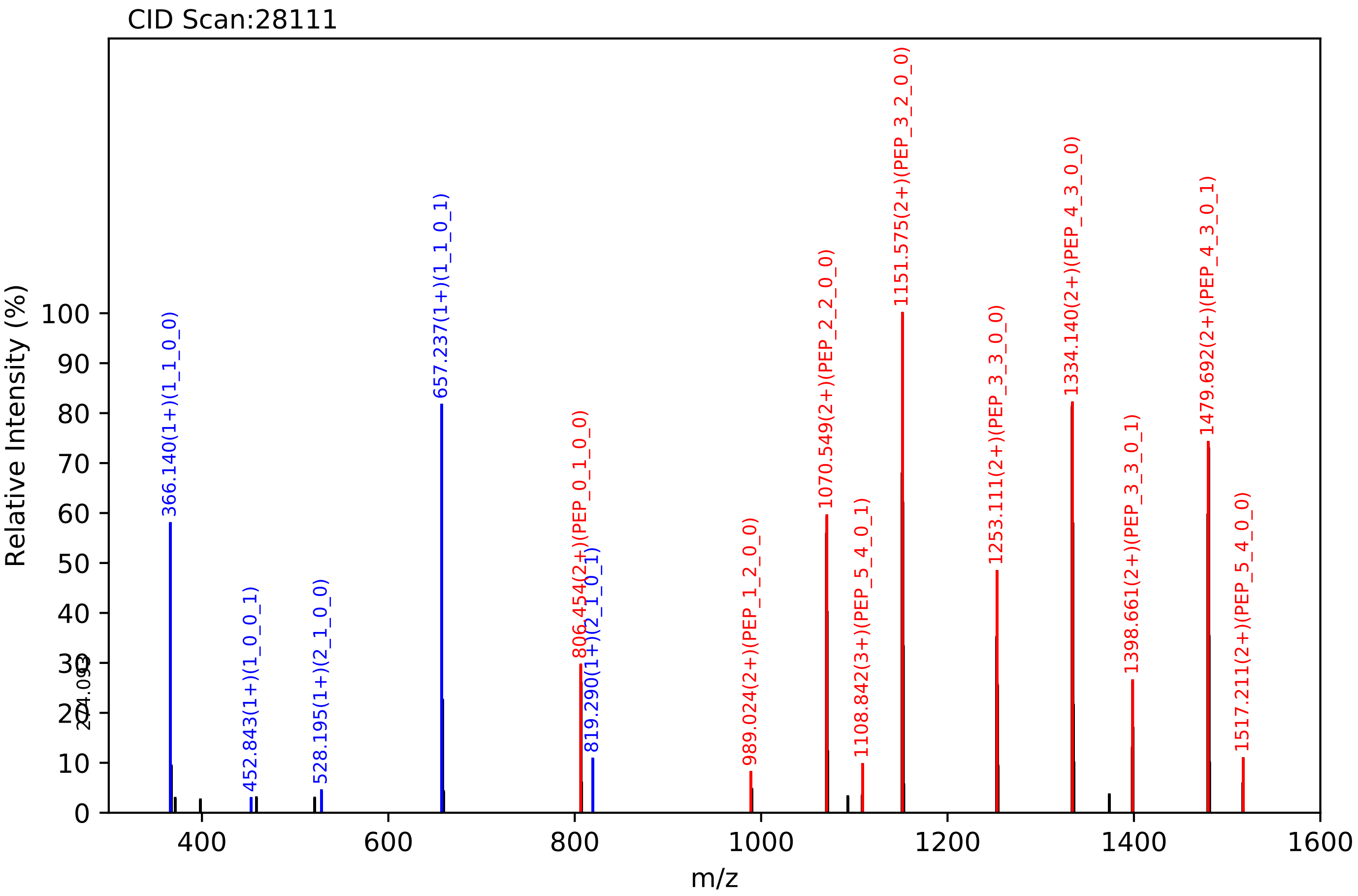

Unknown set no. 117, Experiment: Human Plasma exp_4

LVPVPITNATLDR(=PEP)_5_4_0_2, m/z:904.15(4+), RT:98.76, Y-score:79.68

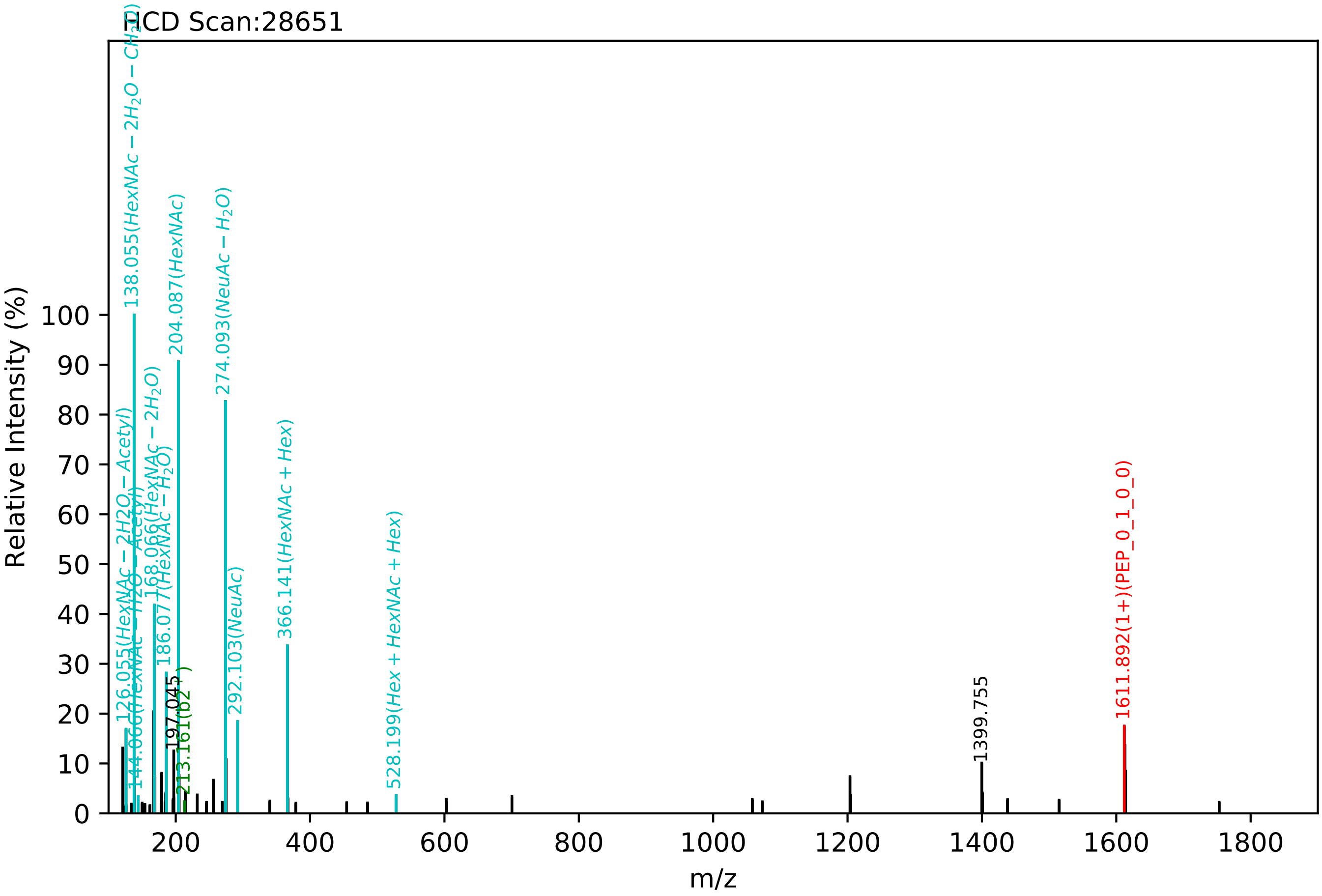

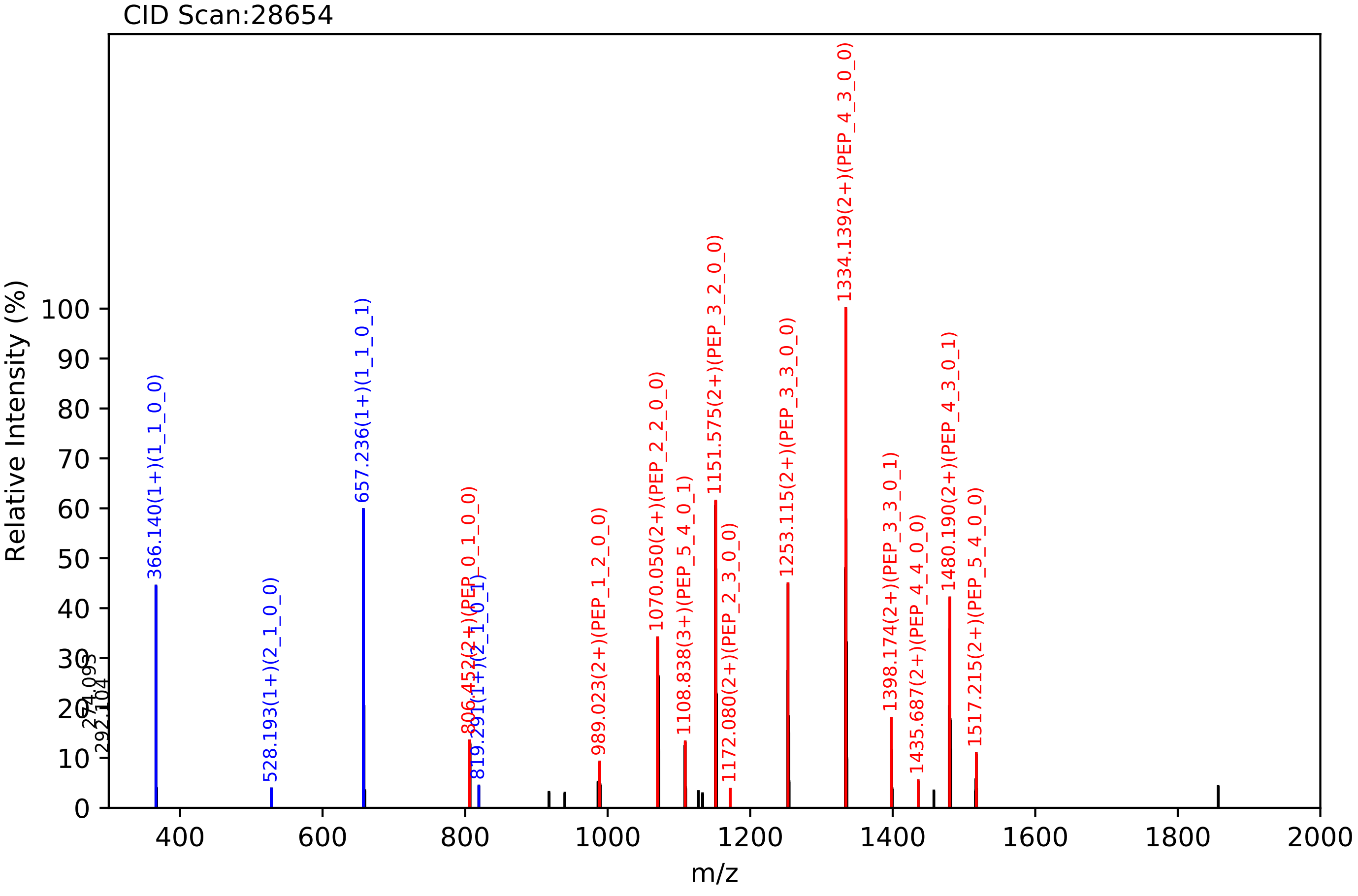

Unknown set no. 118, Experiment: Human Plasma exp_5

LVPVPITNATLDR(=PEP)_5_4_0_2, m/z:904.15(4+), RT:97.41, Y-score:80.30

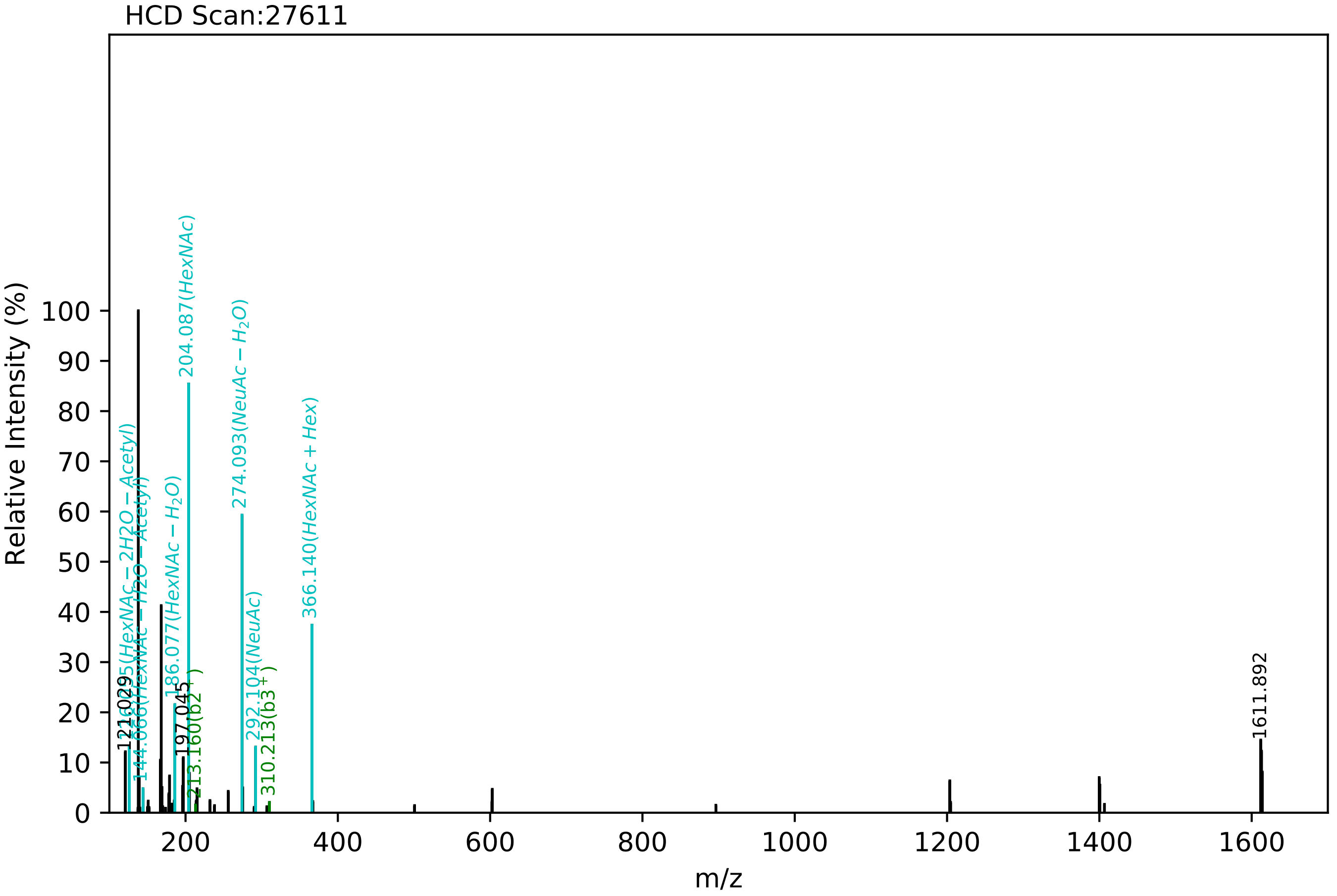

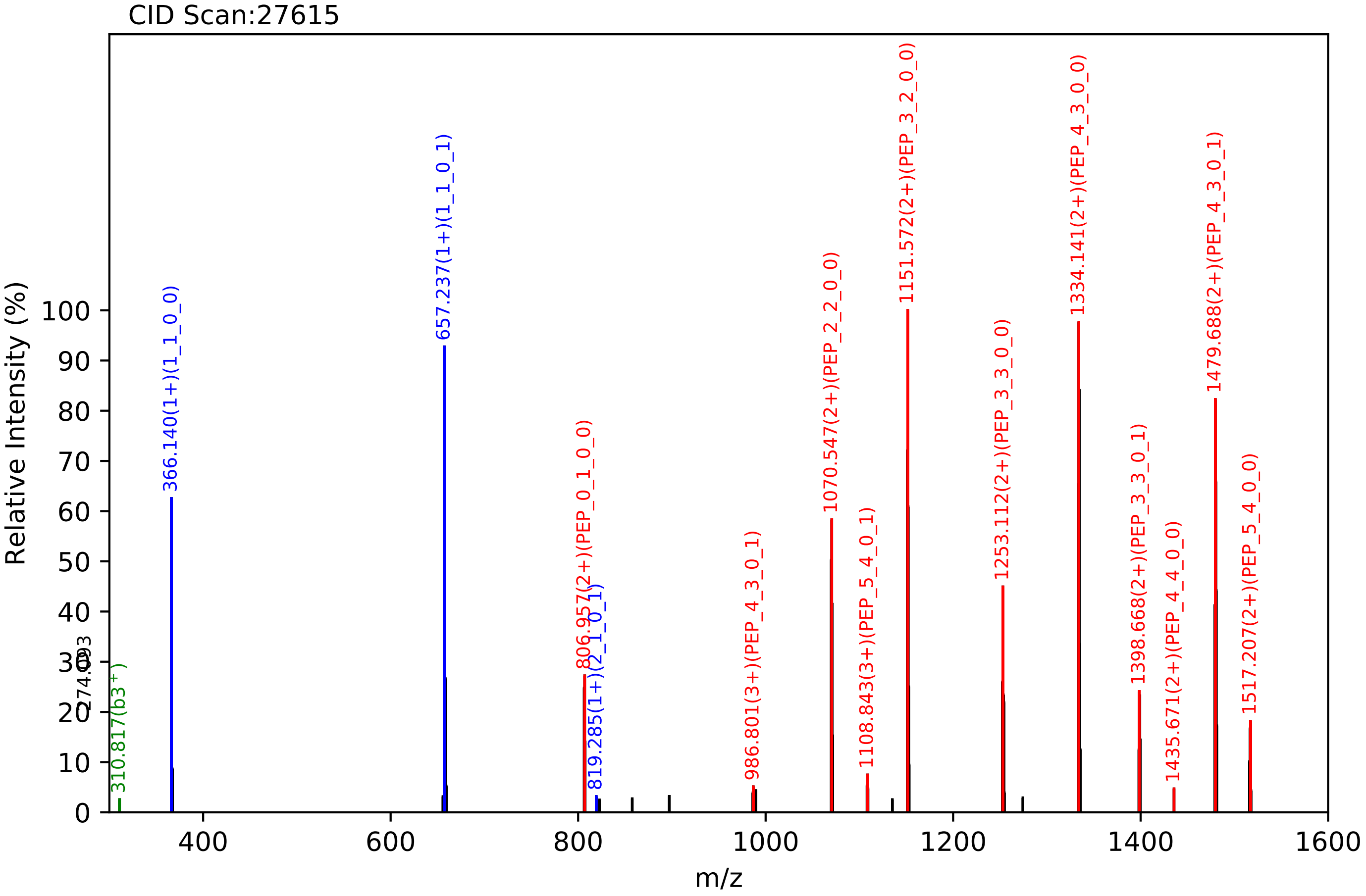

Unknown set no. 119, Experiment: Human Plasma exp_1

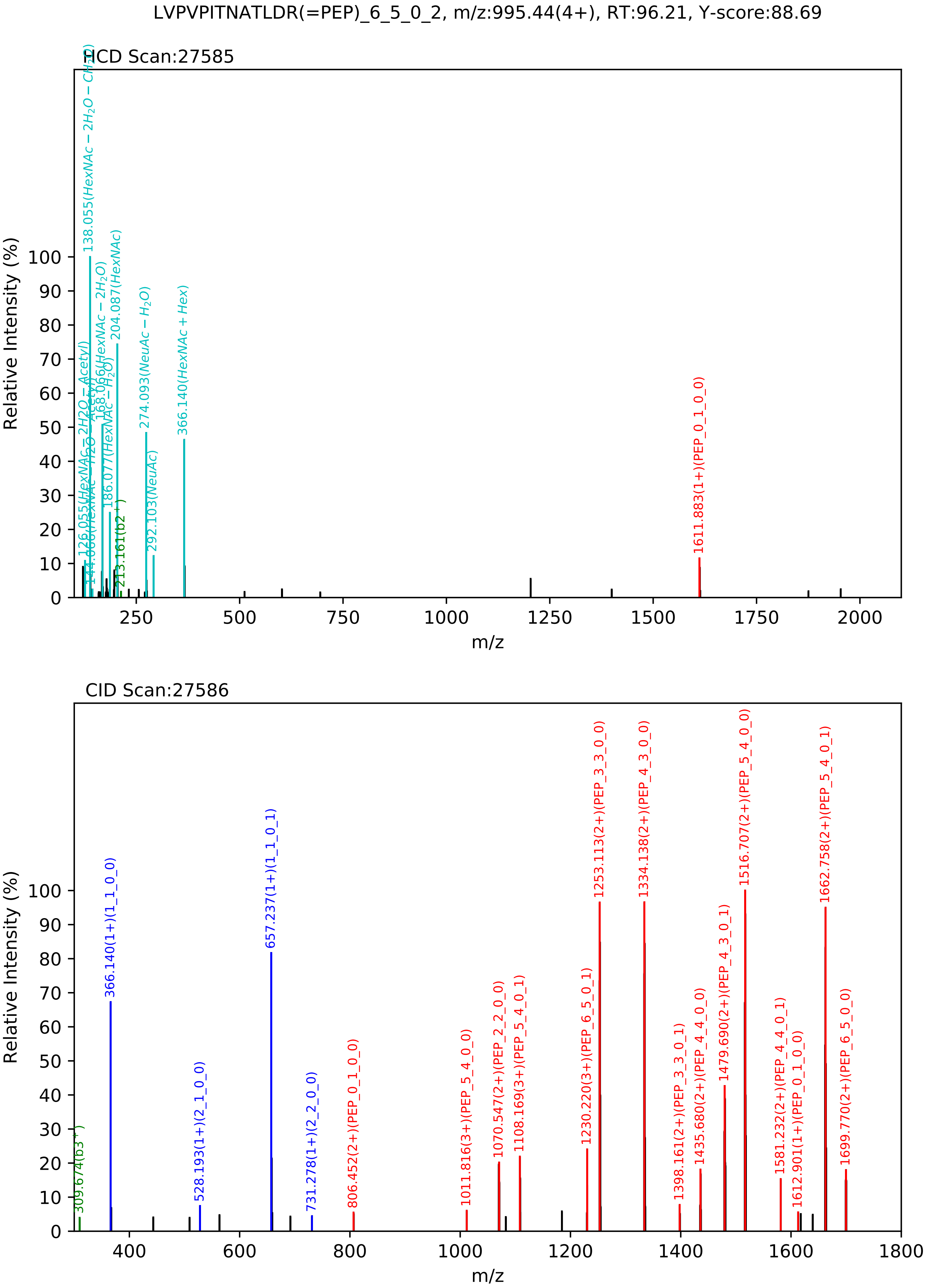

Unknown set no. 120, Experiment: Human Plasma exp_3

LVPVPITNATLDR(=PEP)_6_5_0_2, m/z:995.44(4+), RT:96.37, Y-score:87.37

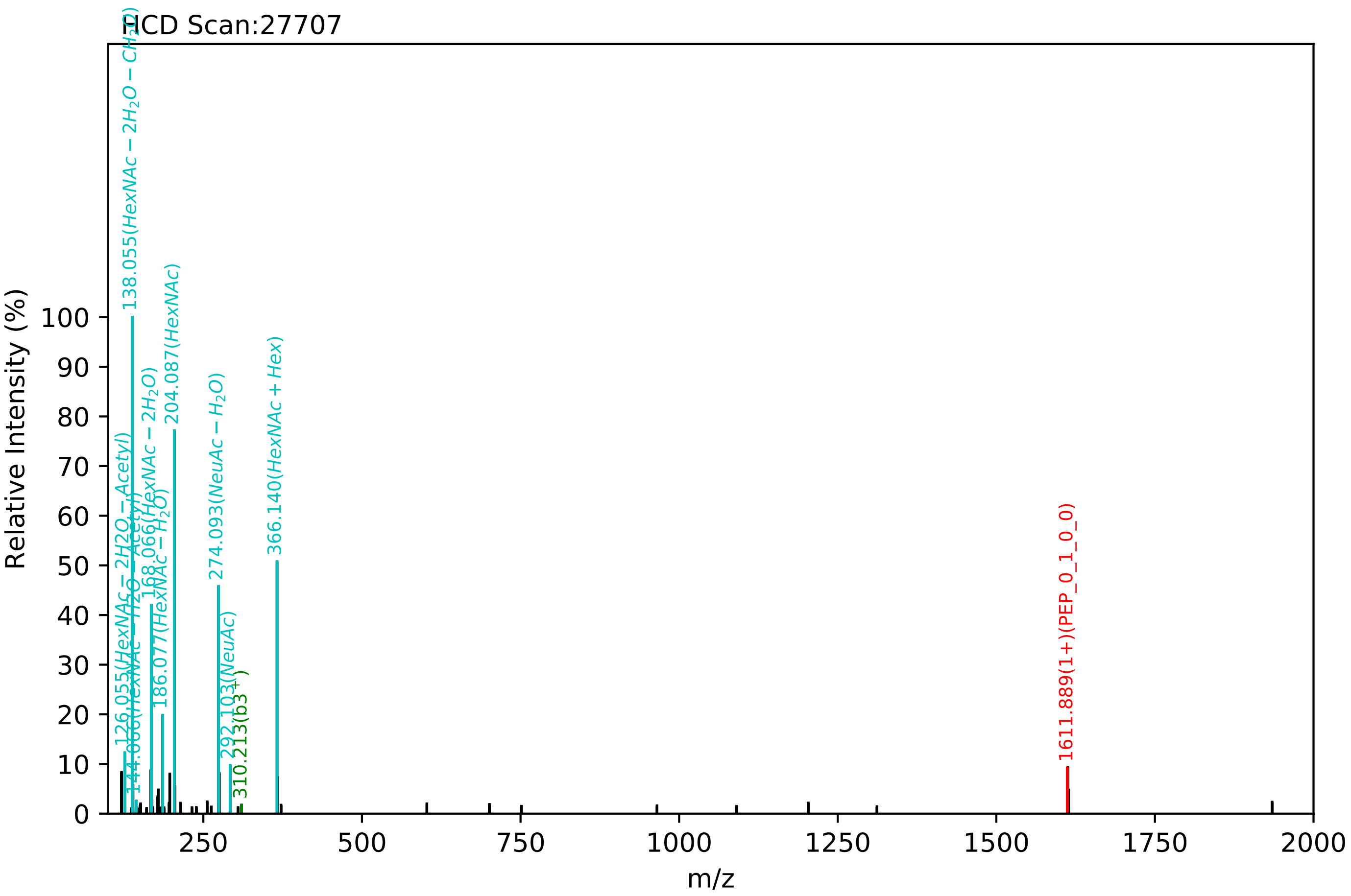

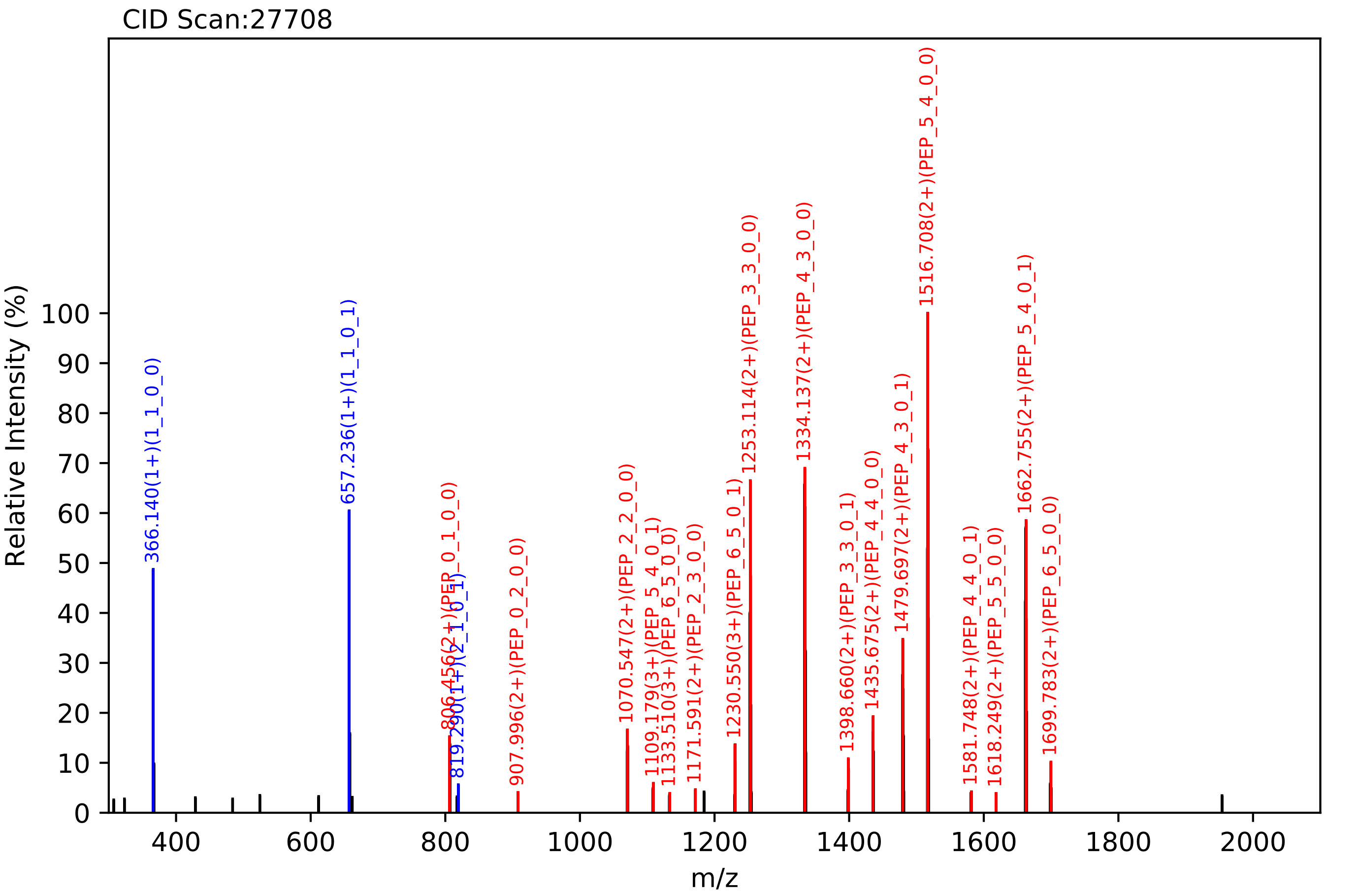

Unknown set no. 121, Experiment: Human Plasma exp_5

LVPVPITNATLDR(=PEP)_6_5_0_2, m/z:995.44(4+), RT:96.79, Y-score:84.74

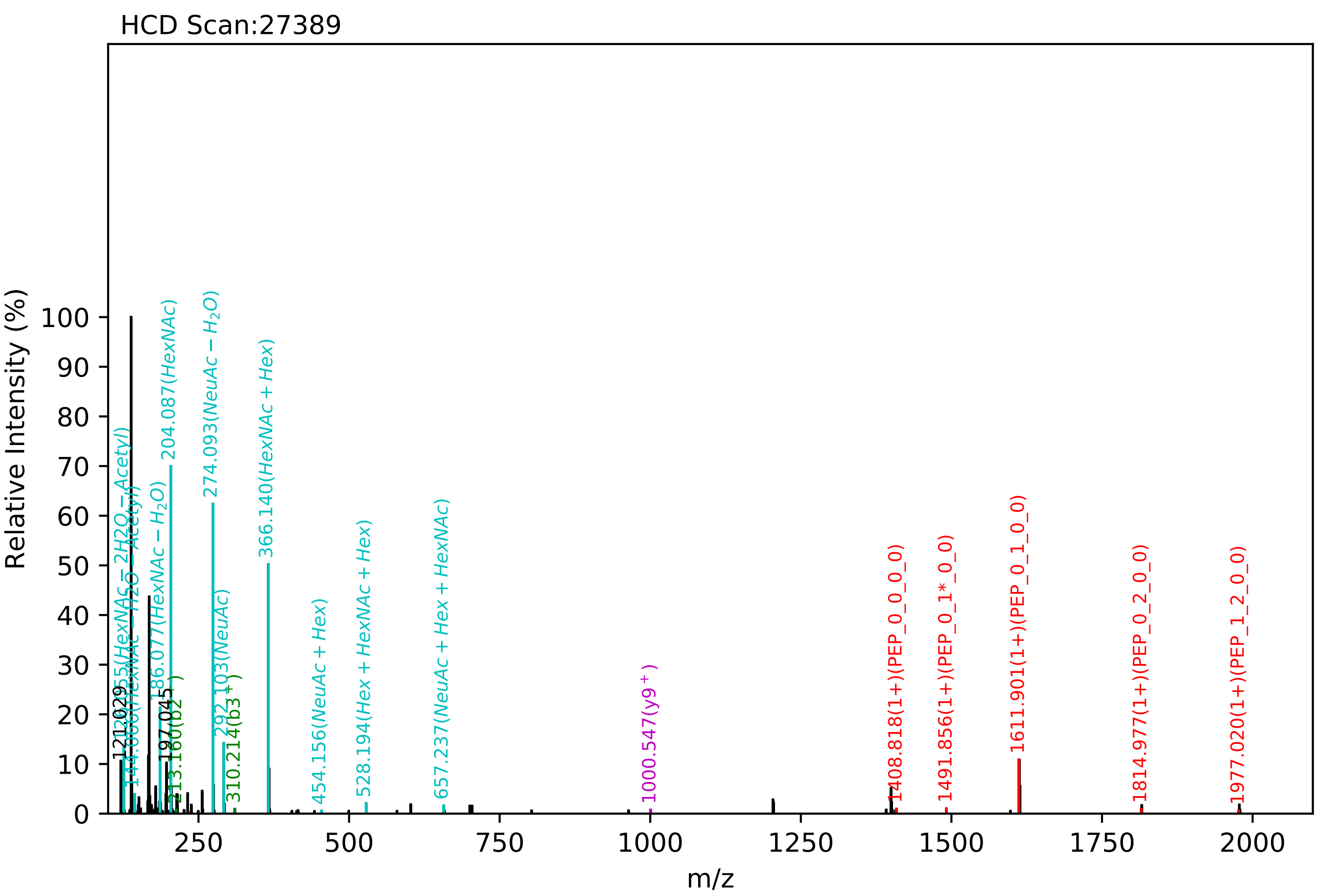

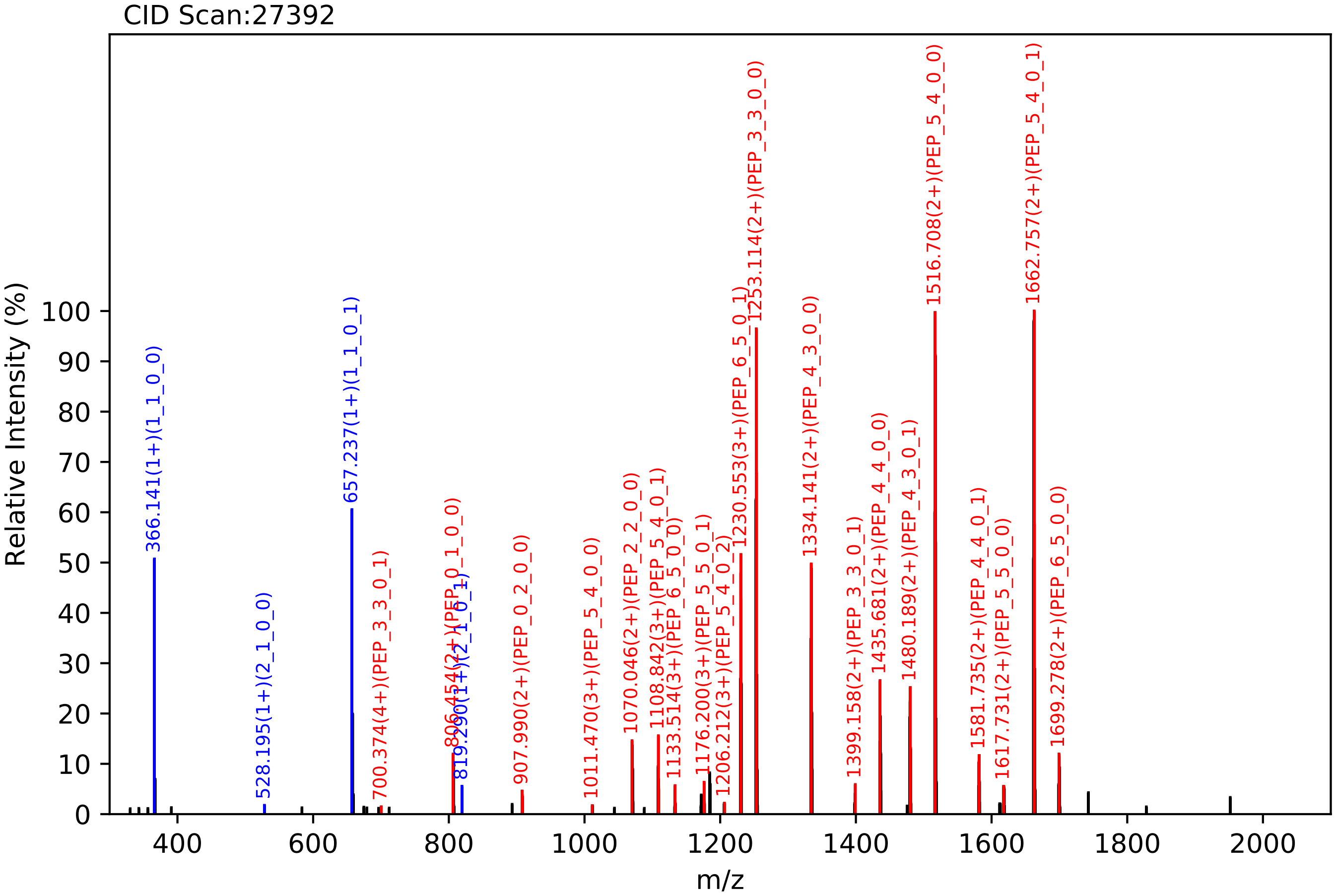

Unknown set no. 122, Experiment: Human Plasma exp_4

LVPVPITNATLDR(=PEP)_6_5_0_3, m/z:854.77(5+), RT:110.33, Y-score:80.35

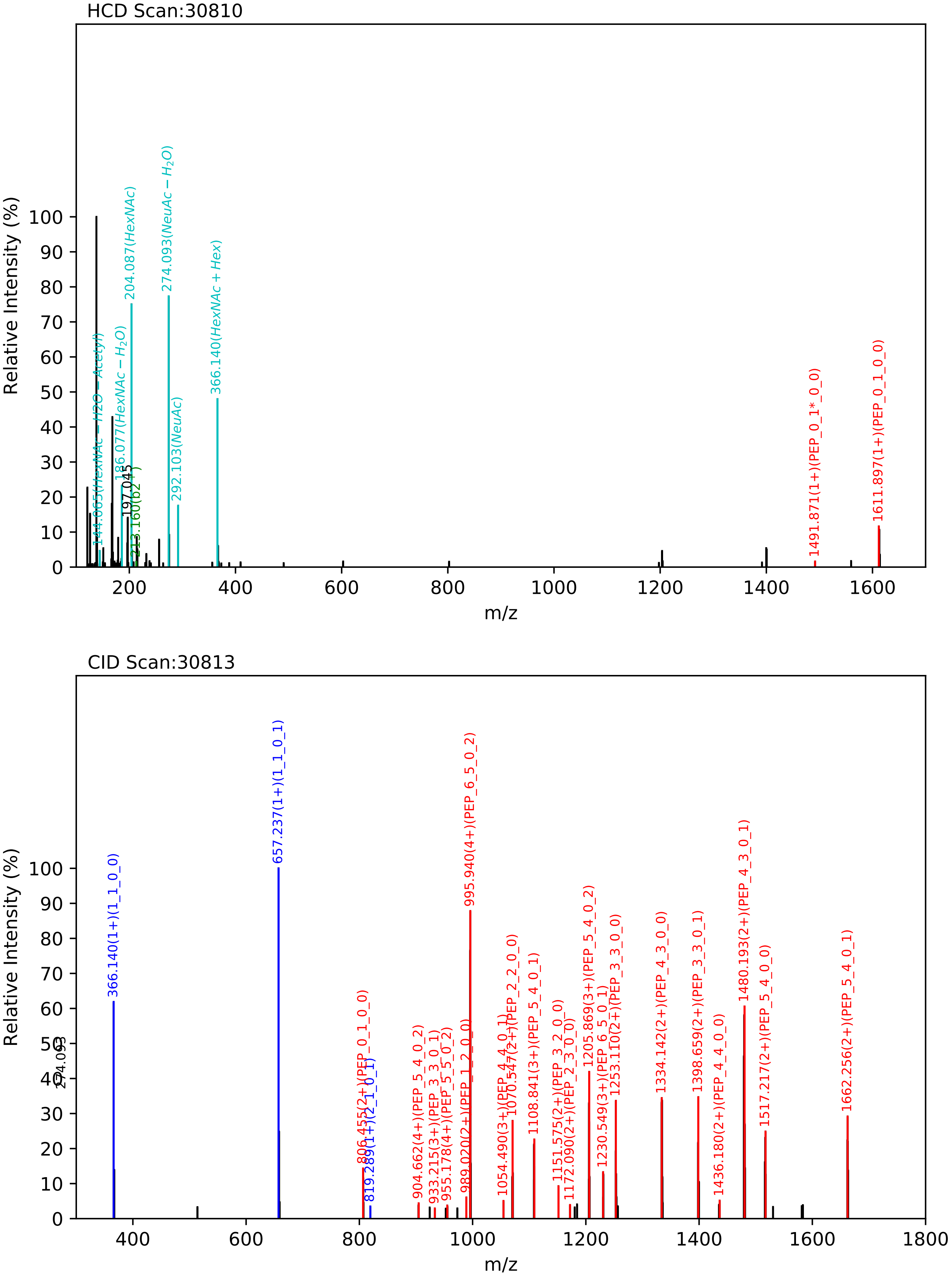

Unknown set no. 123, Experiment: Human Plasma exp_4

LVPVPITNATLDR(=PEP)_6_5_0_3, m/z:1068.21(4+), RT:109.13, Y-score:82.40

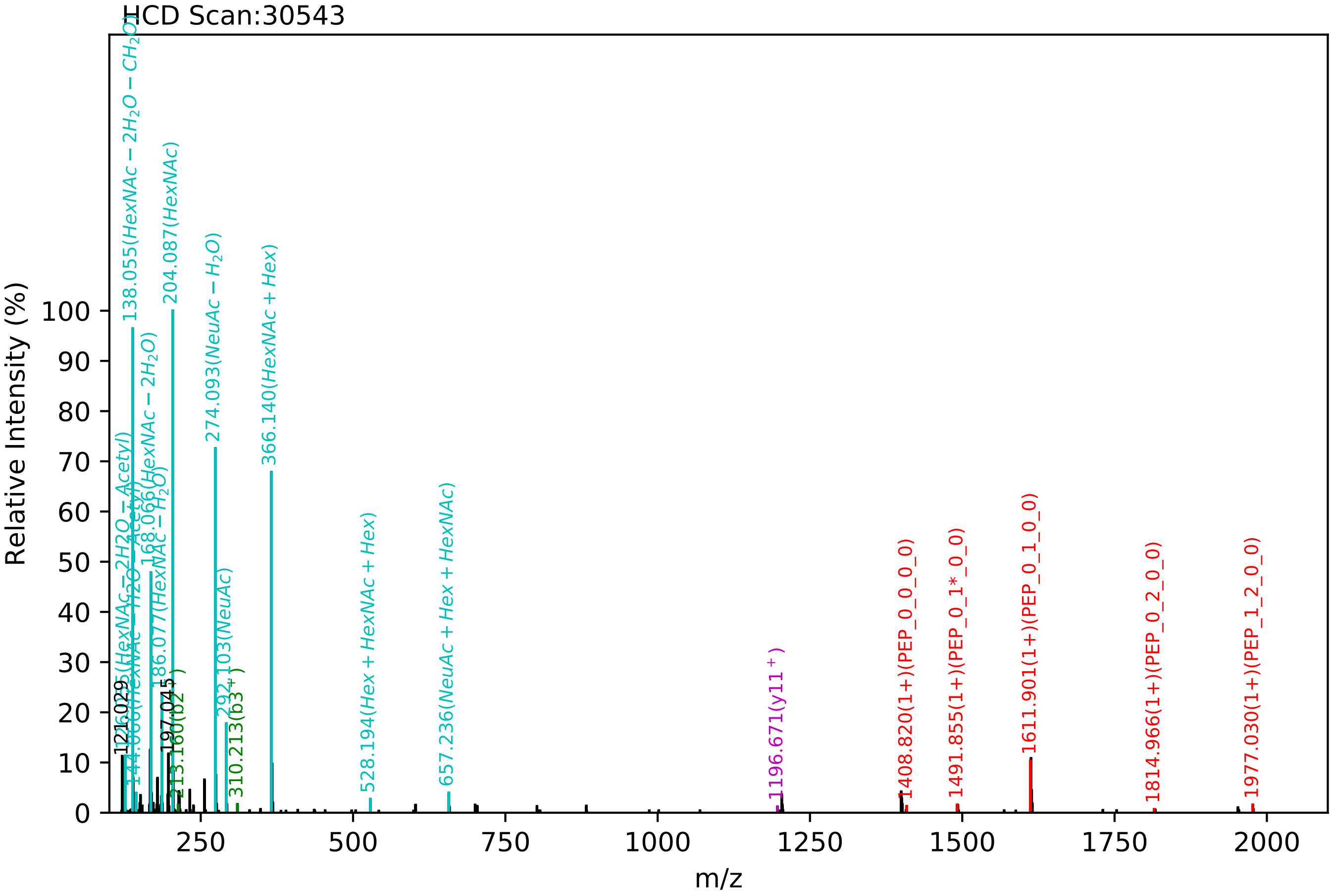

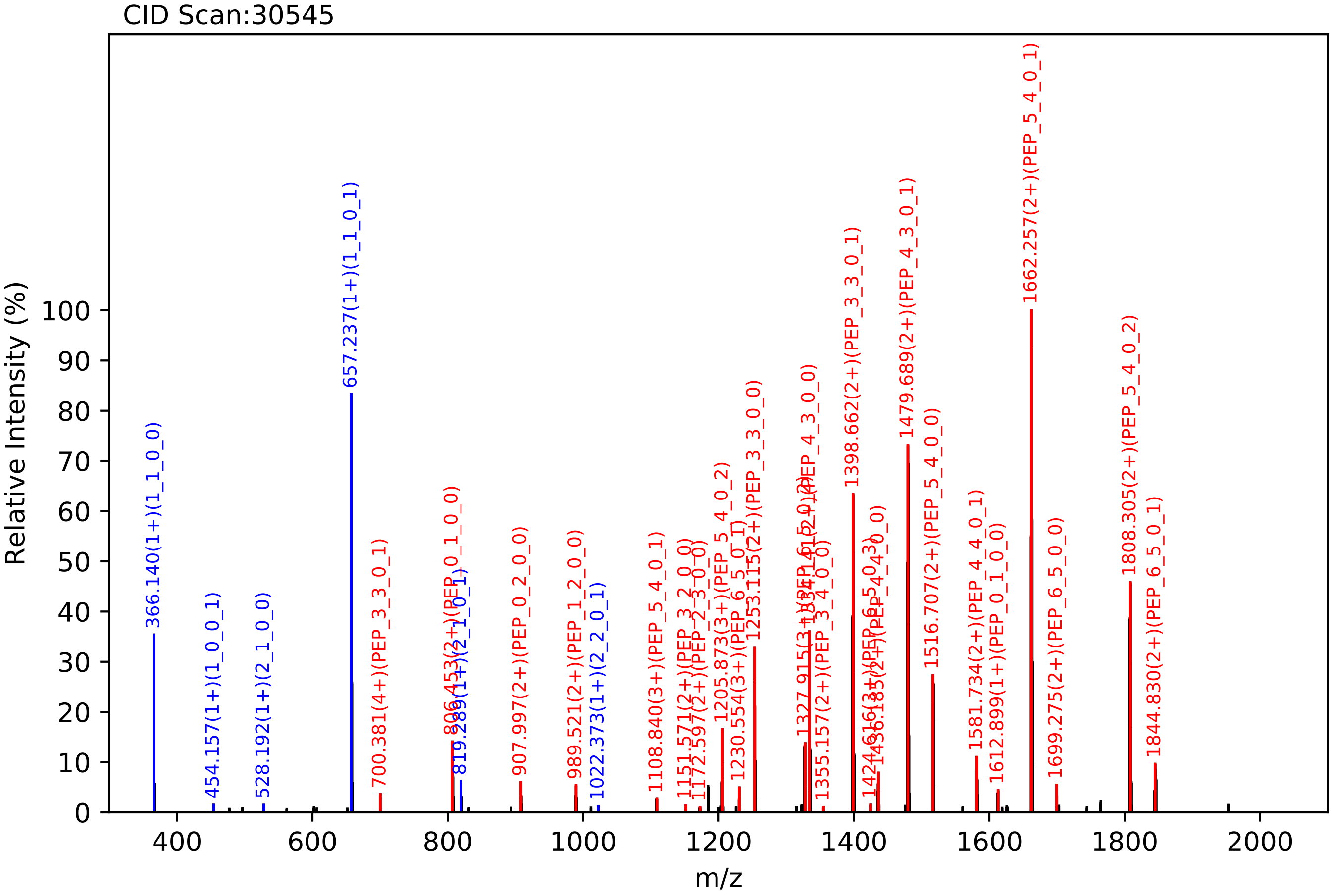

Unknown set no. 124, Experiment: Human Plasma exp_2

LVPVPITNATLDR(=PEP)_6_5_1_3, m/z:1104.73(4+), RT:109.33, Y-score:82.30

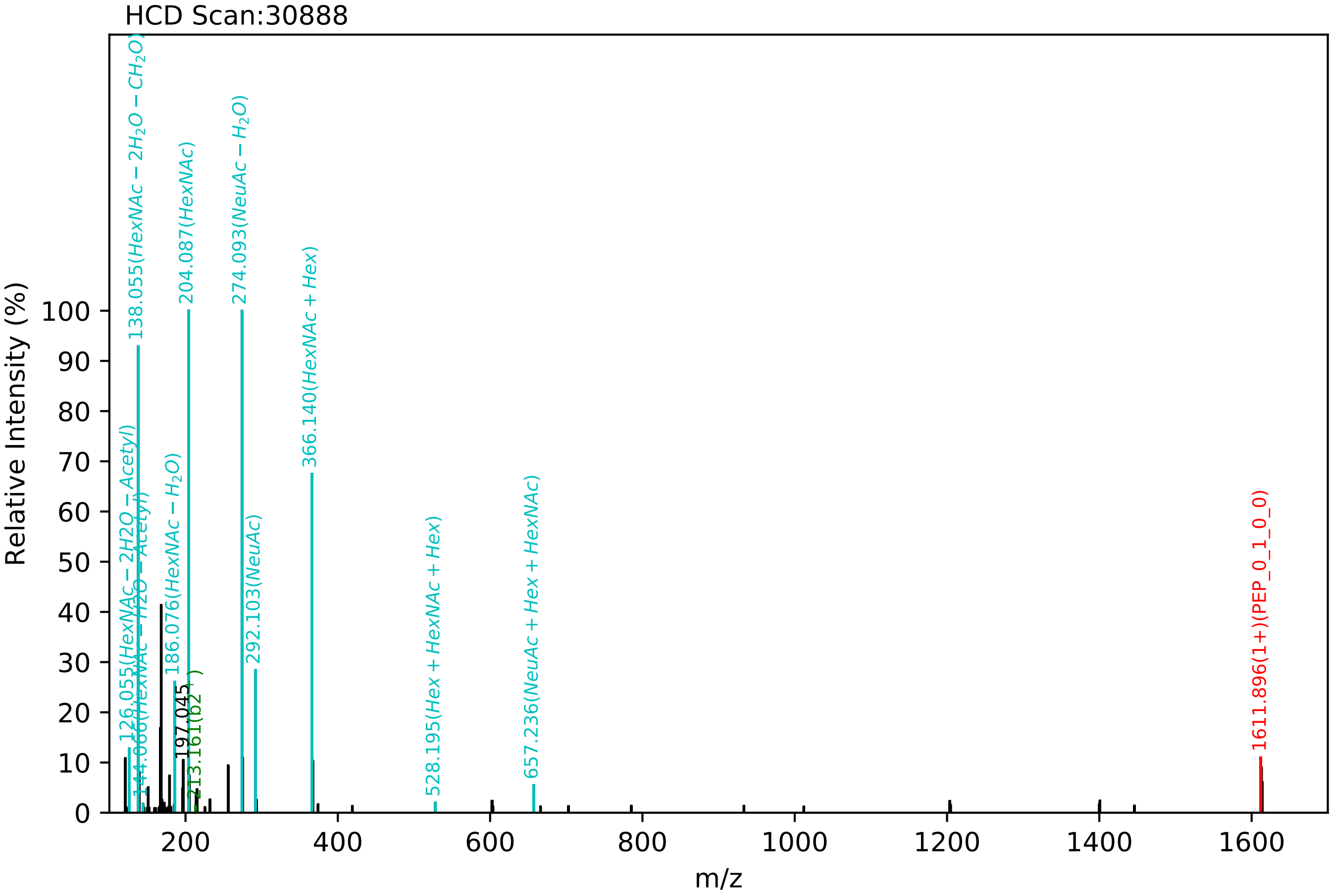

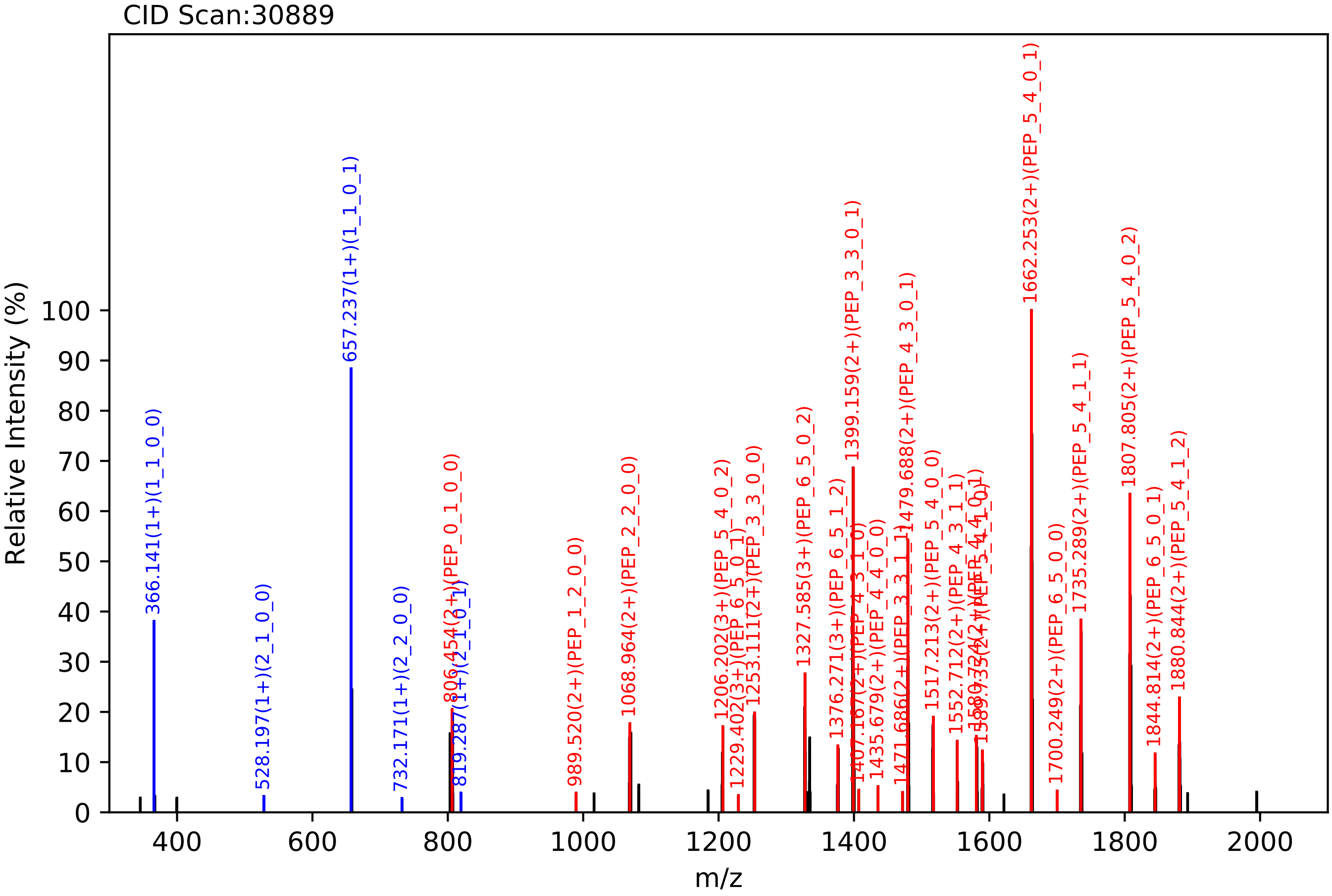

Unknown set no. 125, Experiment: Human Plasma exp_4

LVPVPITNATLDR(=PEP)_6_5_1_3, m/z:1104.73(4+), RT:109.44, Y-score:81.05

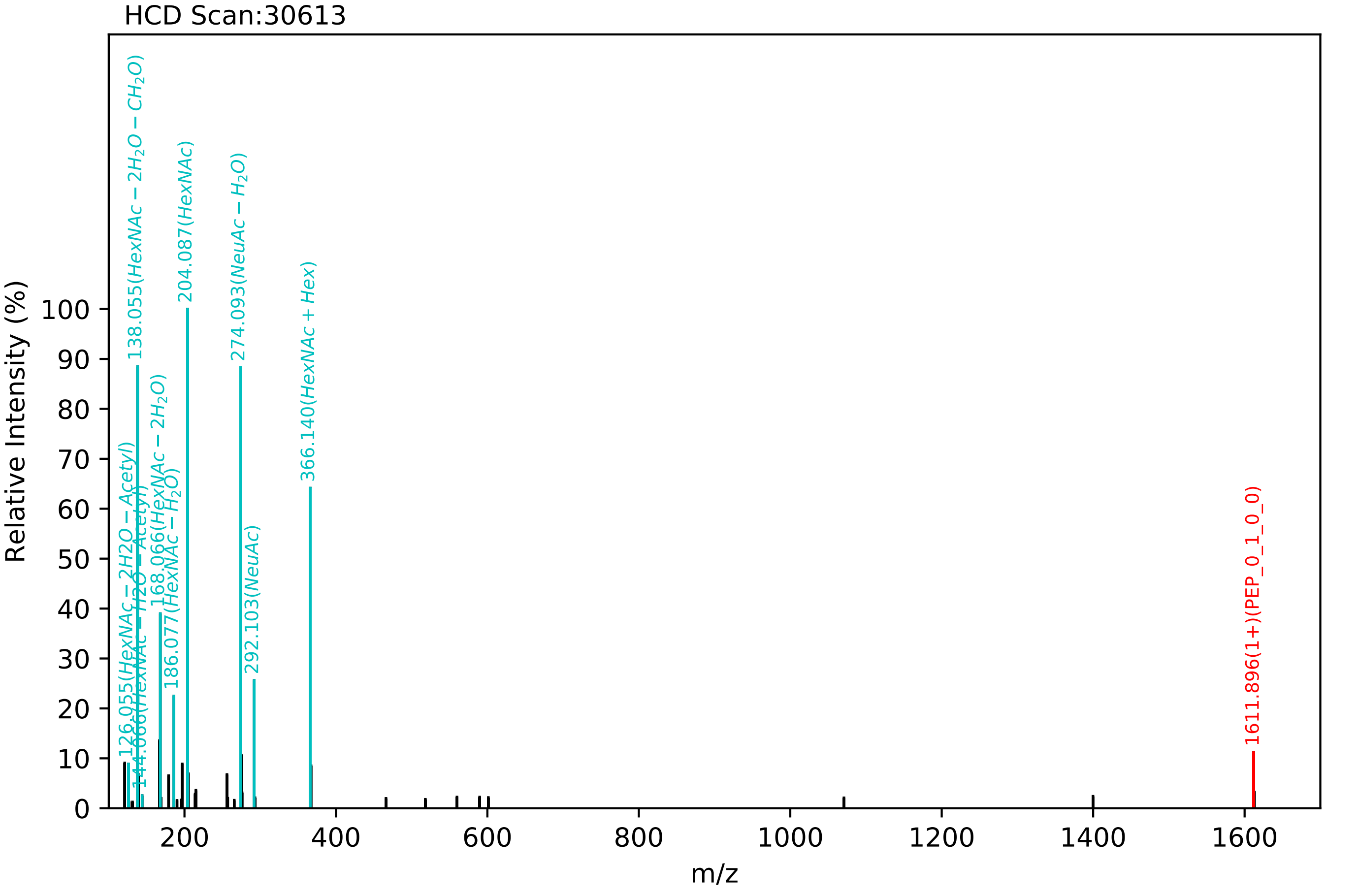

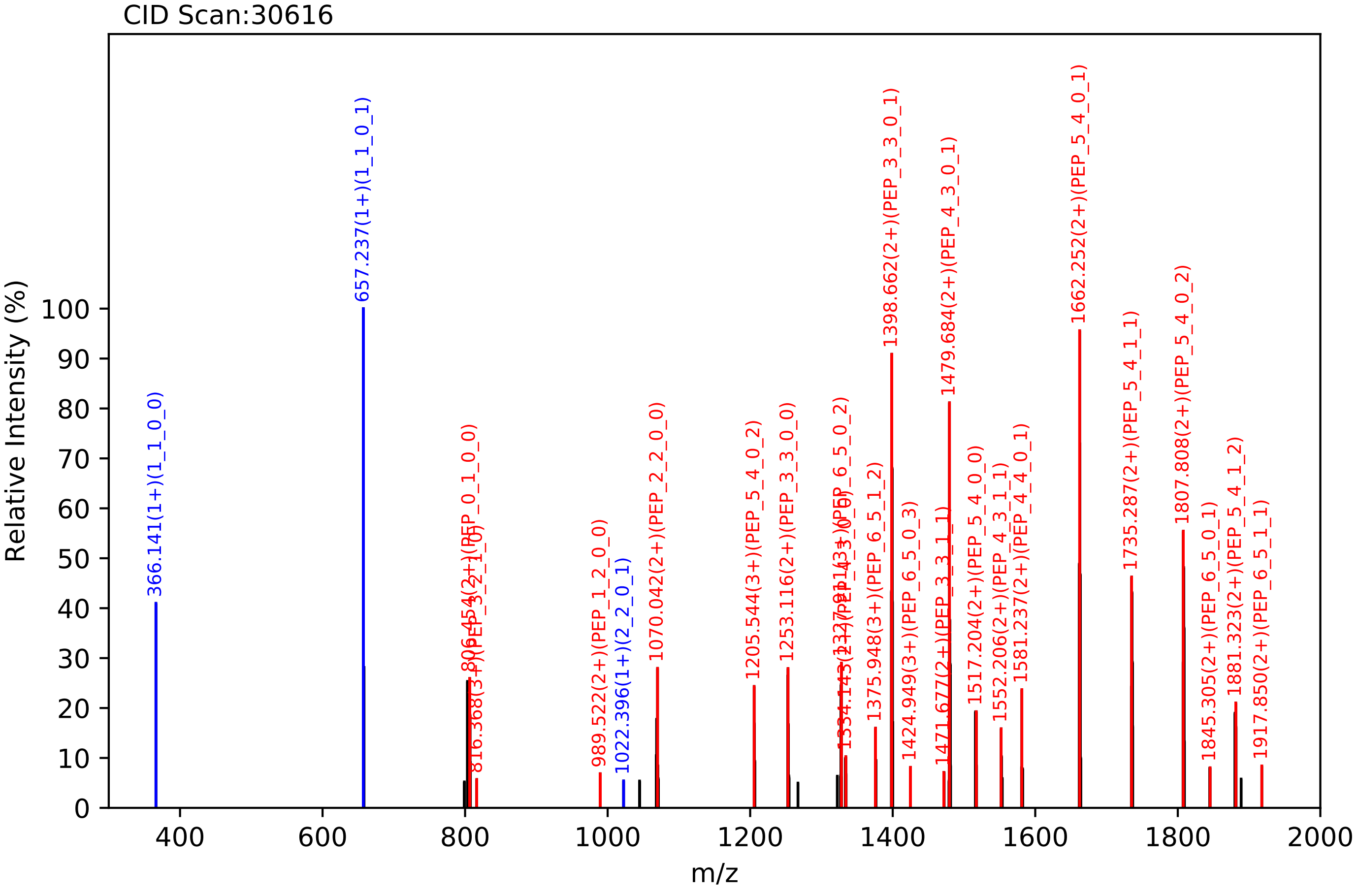

Unknown set no. 126, Experiment: Human Plasma exp_4

LVPVPITNATLDR(=PEP)_7_6_0_3, m/z:1159.49(4+), RT:107.65, Y-score:78.32

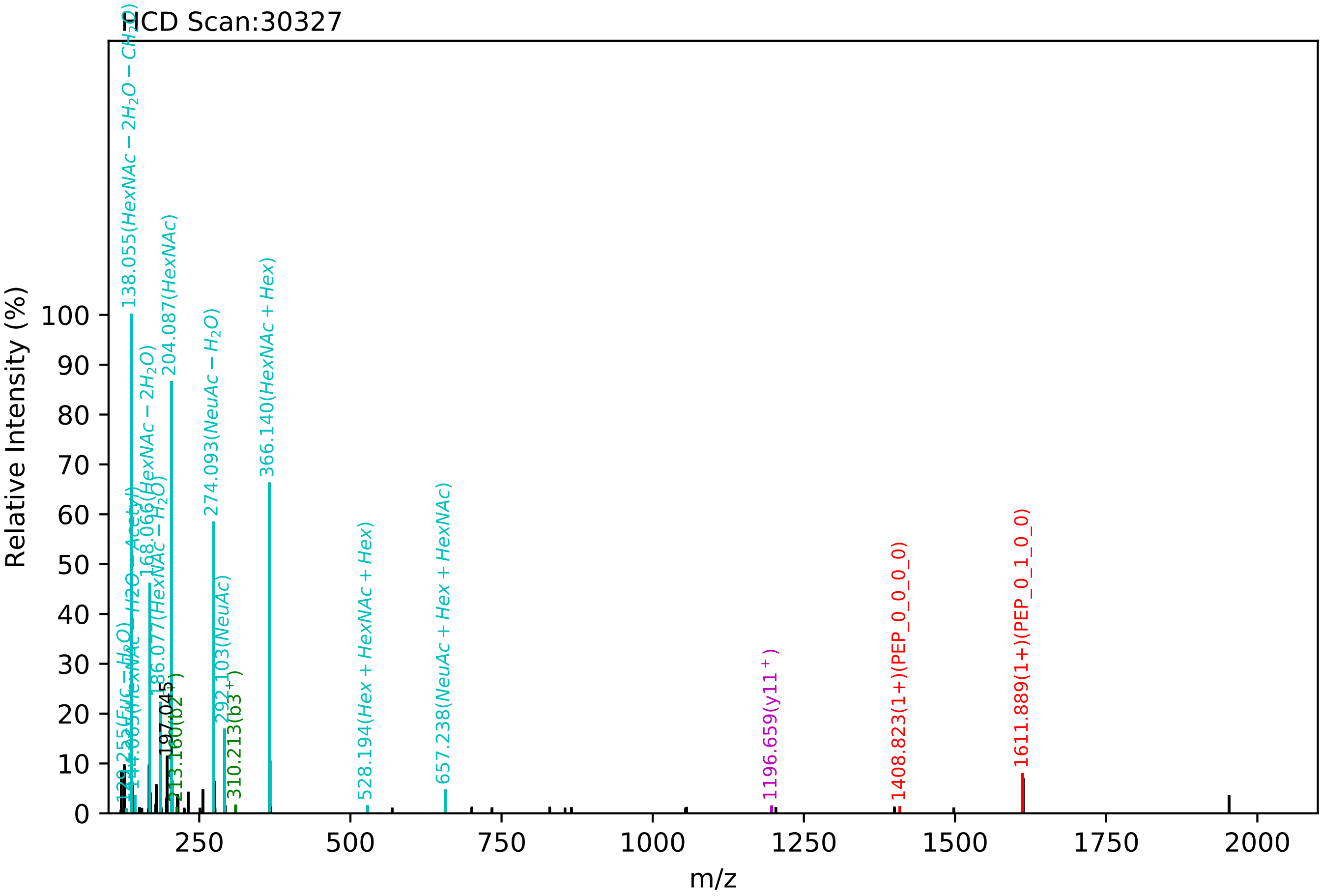

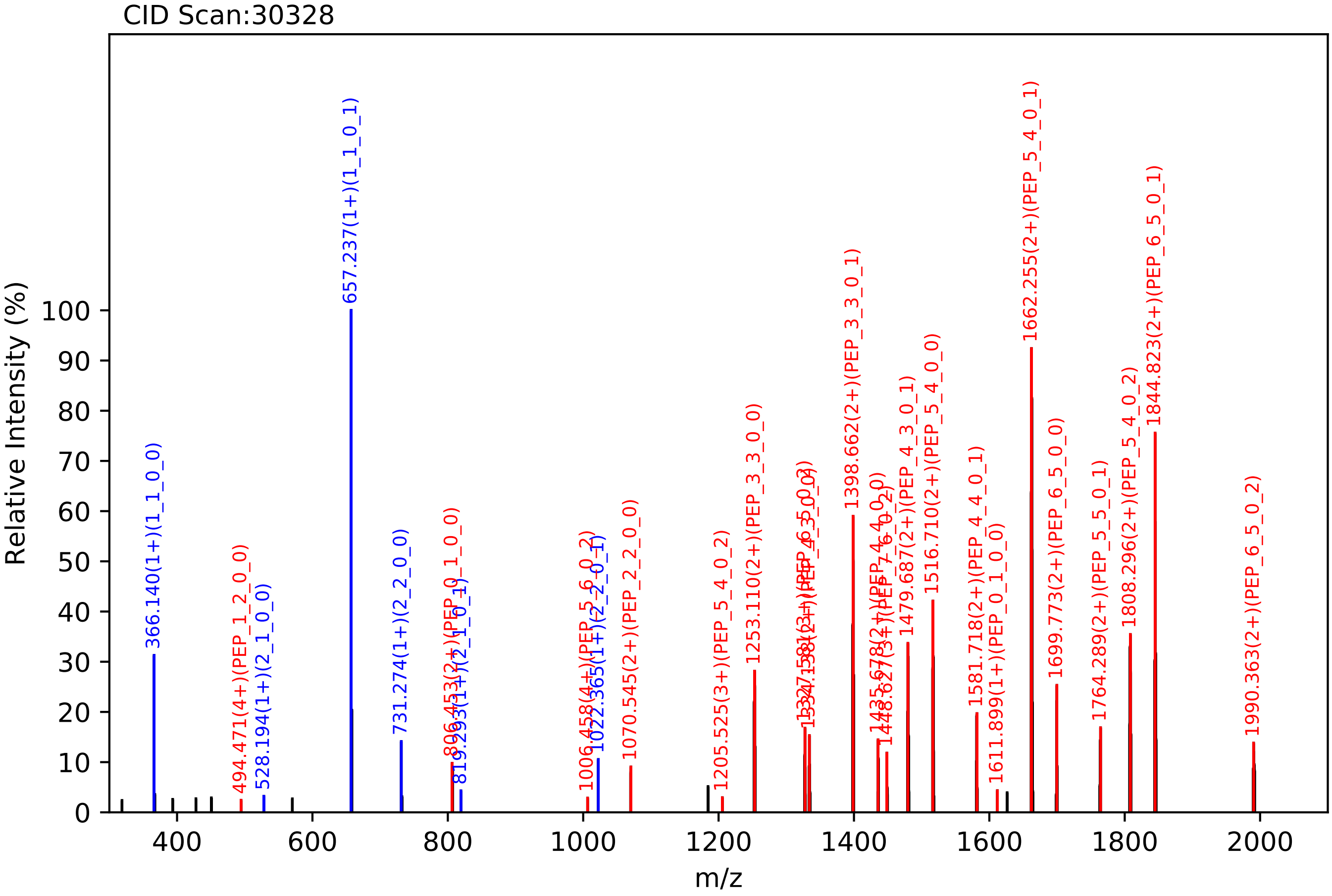

Unknown set no. 127, Experiment: Human Plasma exp_4

QNQCFYNSSYLNVQR(=PEP)_6_5_0_3, m/z:1196.22(4+), RT:101.65, Y-score:75.27

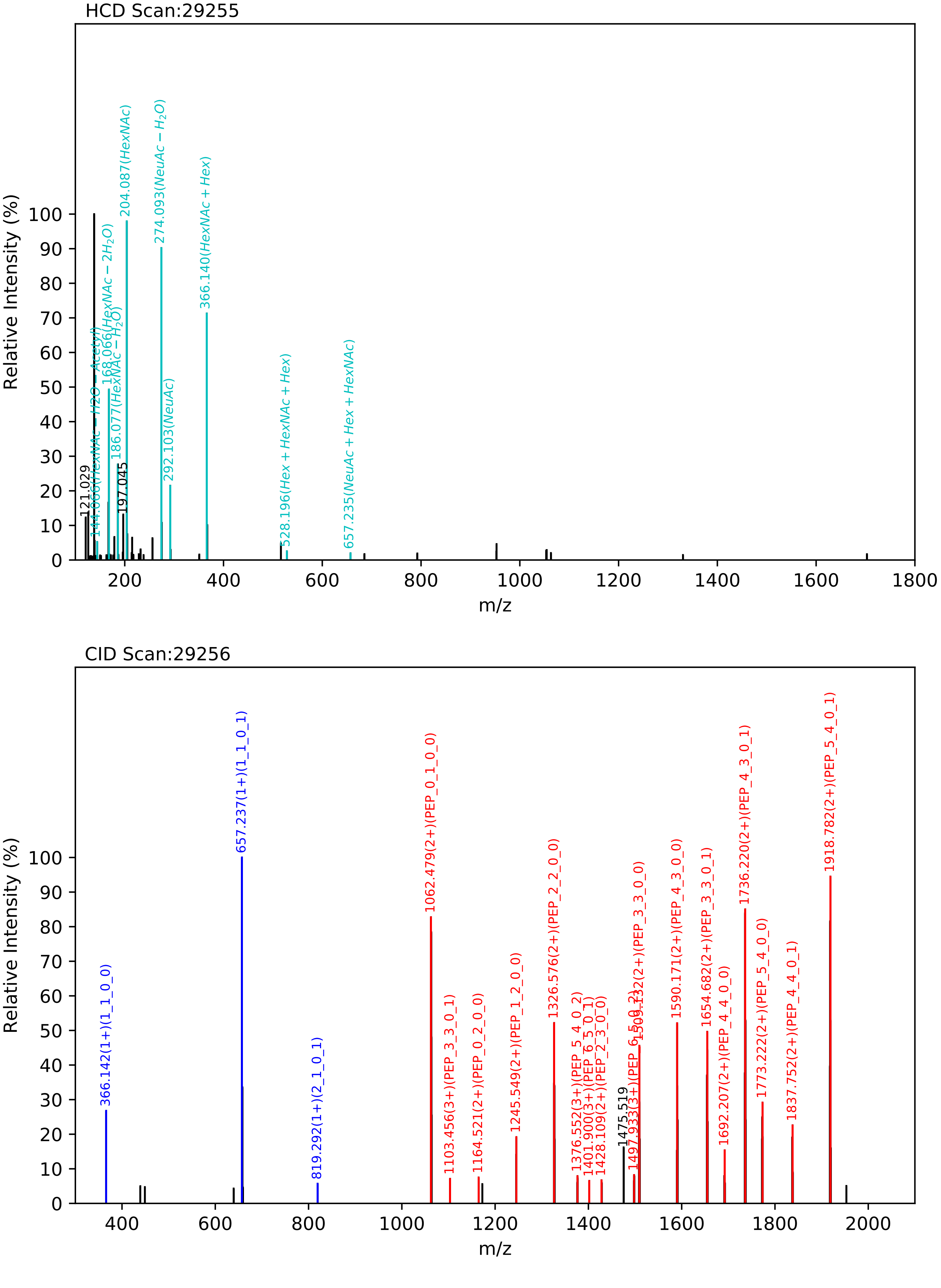

Unknown set no. 128, Experiment: Human Plasma exp_5

QNQCFYNSSYLNVQR(=PEP)_6_5_0_3, m/z:1196.22(4+), RT:101.38, Y-score:77.06

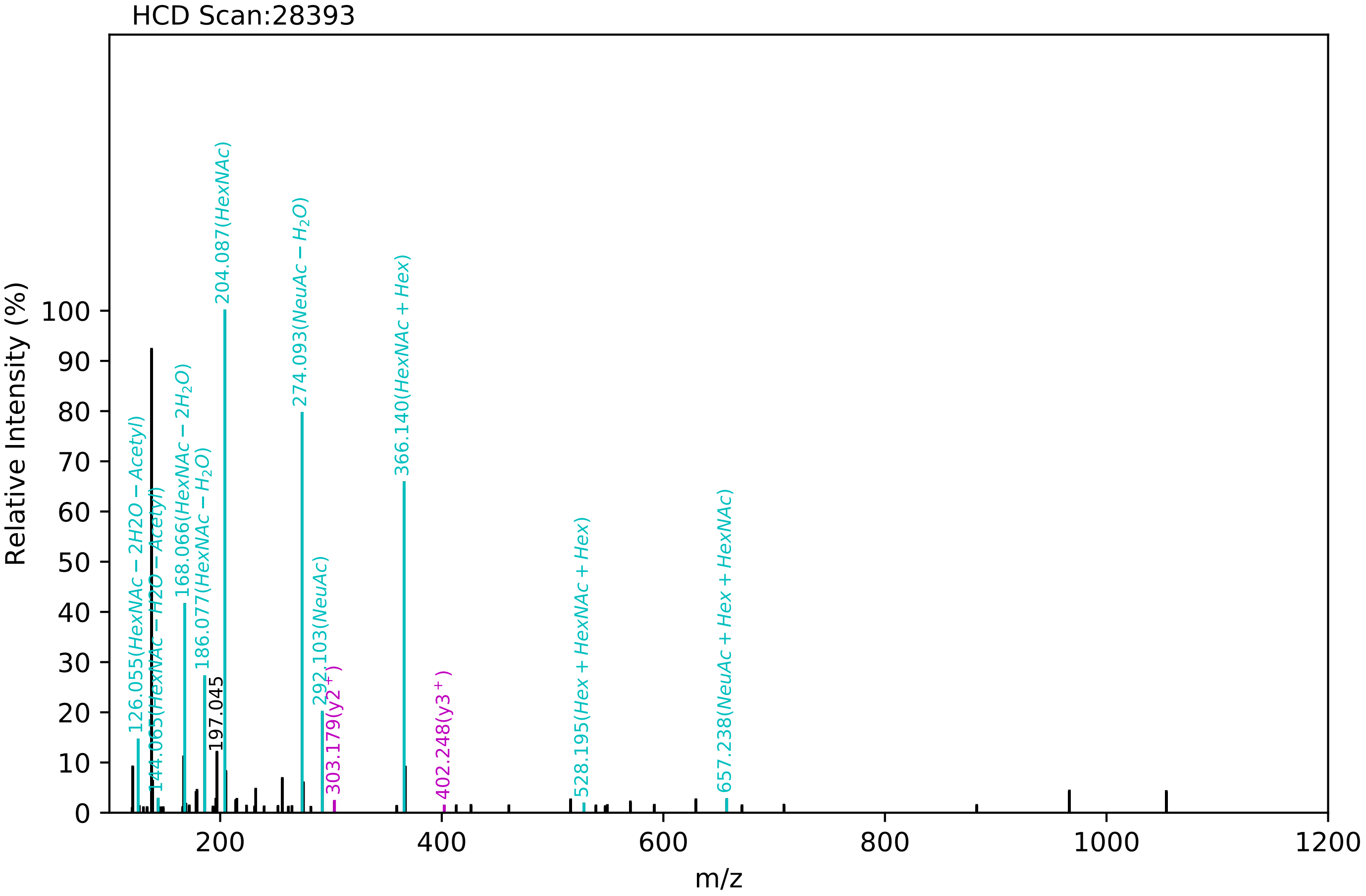

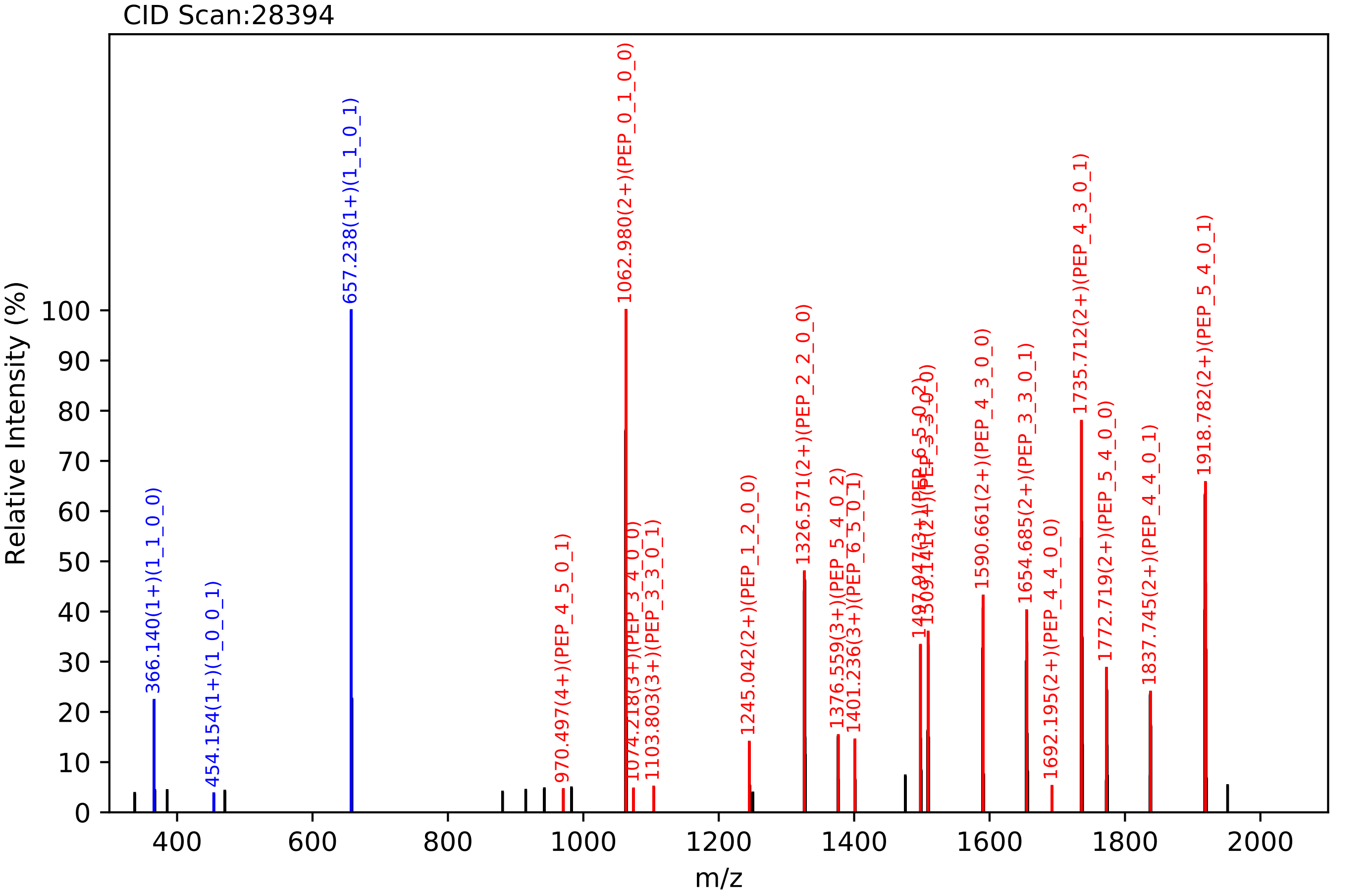

Unknown set no. 129, Experiment: Human Plasma exp_1

NGTR(=PEP)_6_5_0_3, m/z:1103.42(3+), RT:13.49, Y-score:84.67

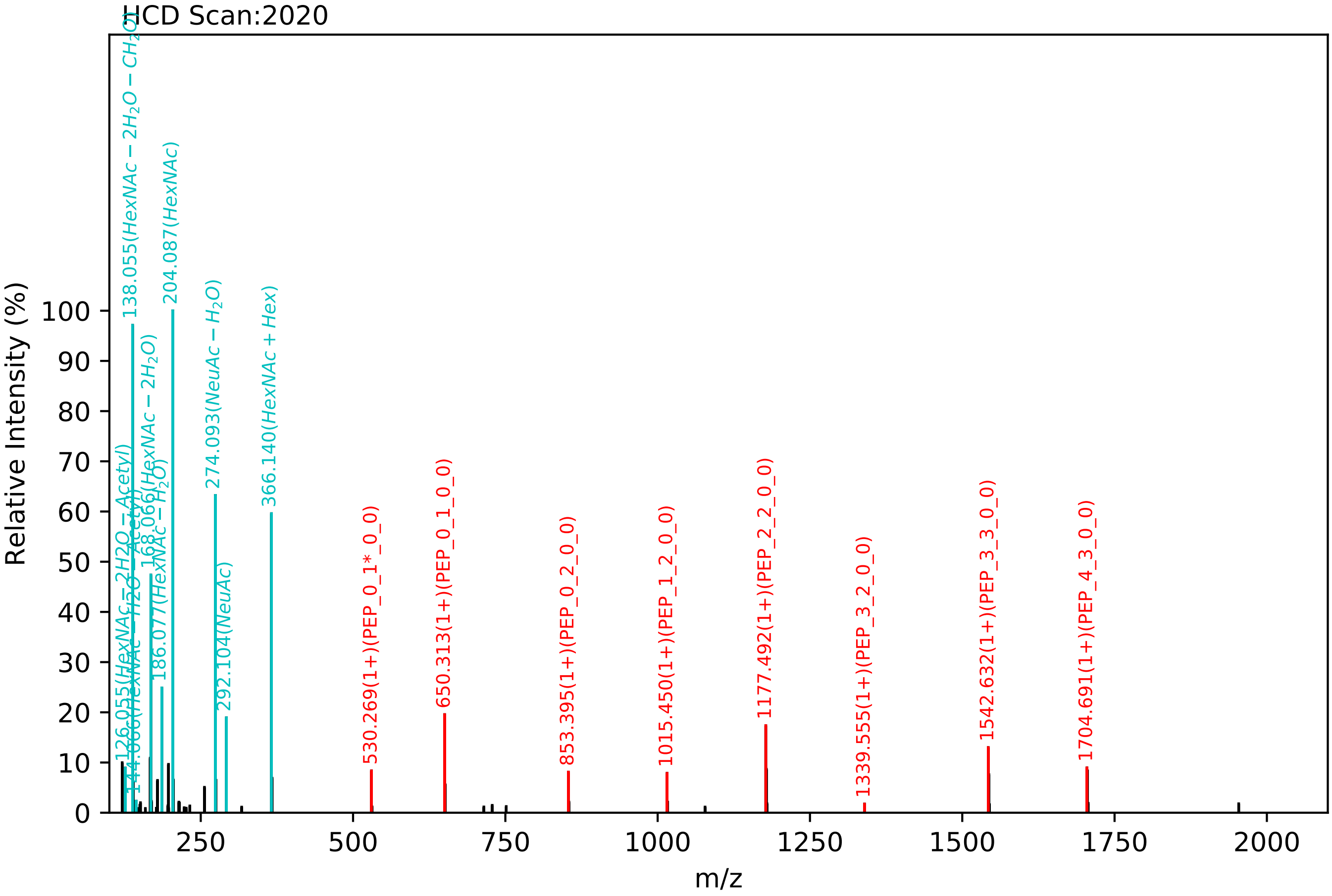

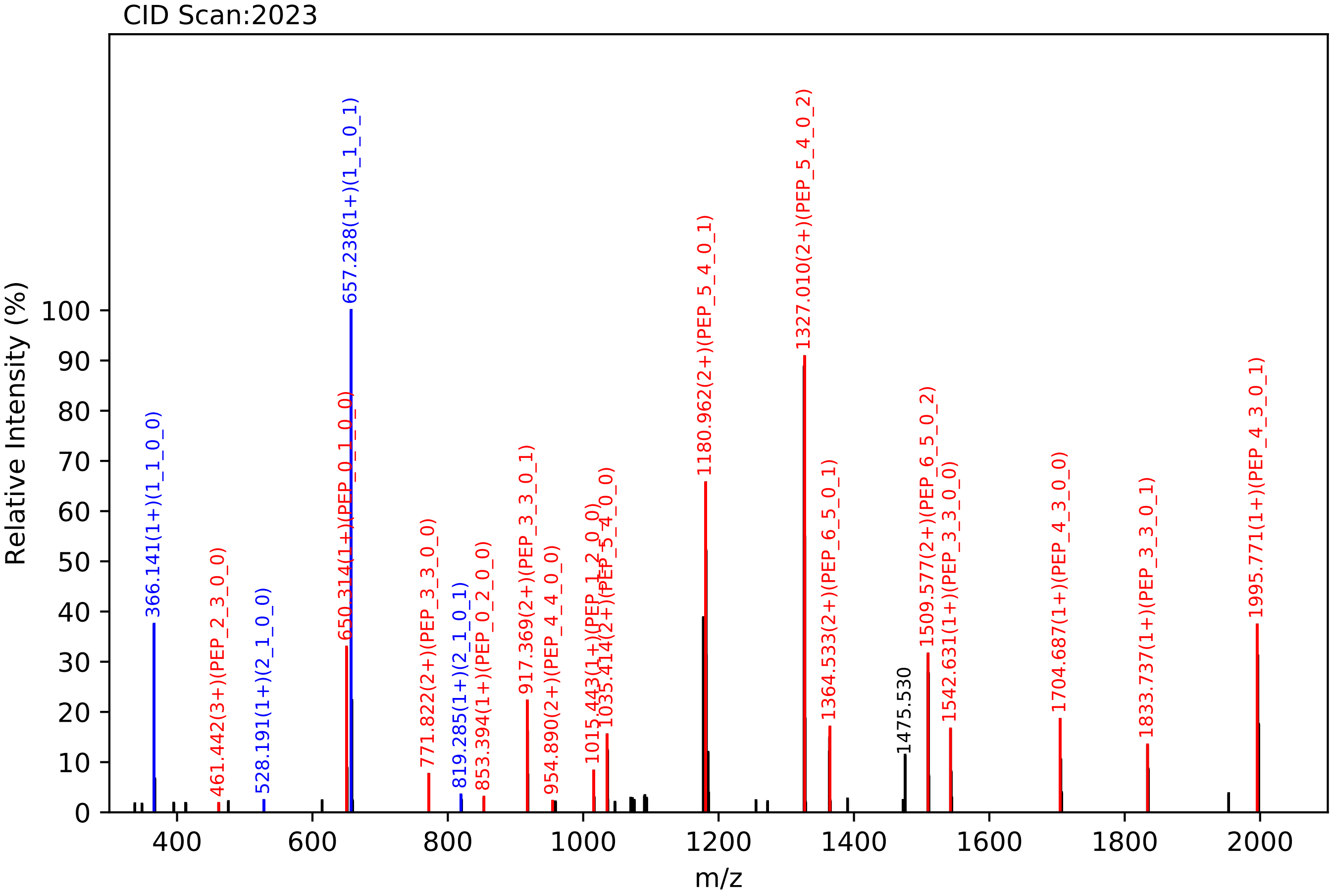

Unknown set no. 130, Experiment: Human Plasma exp_2

NGTR(=PEP)_6_5_0_3, m/z:1103.42(3+), RT:14.46, Y-score:80.01

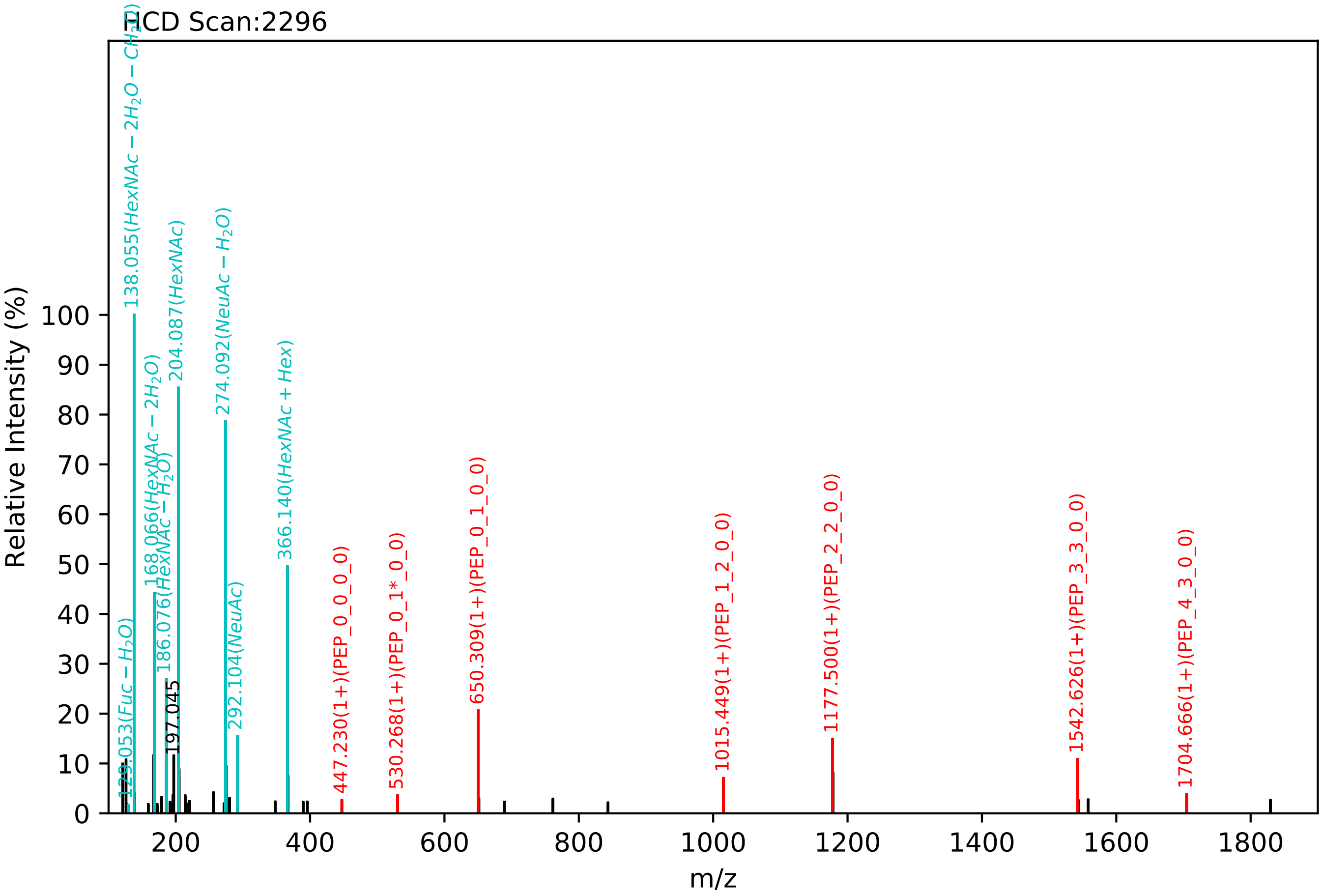

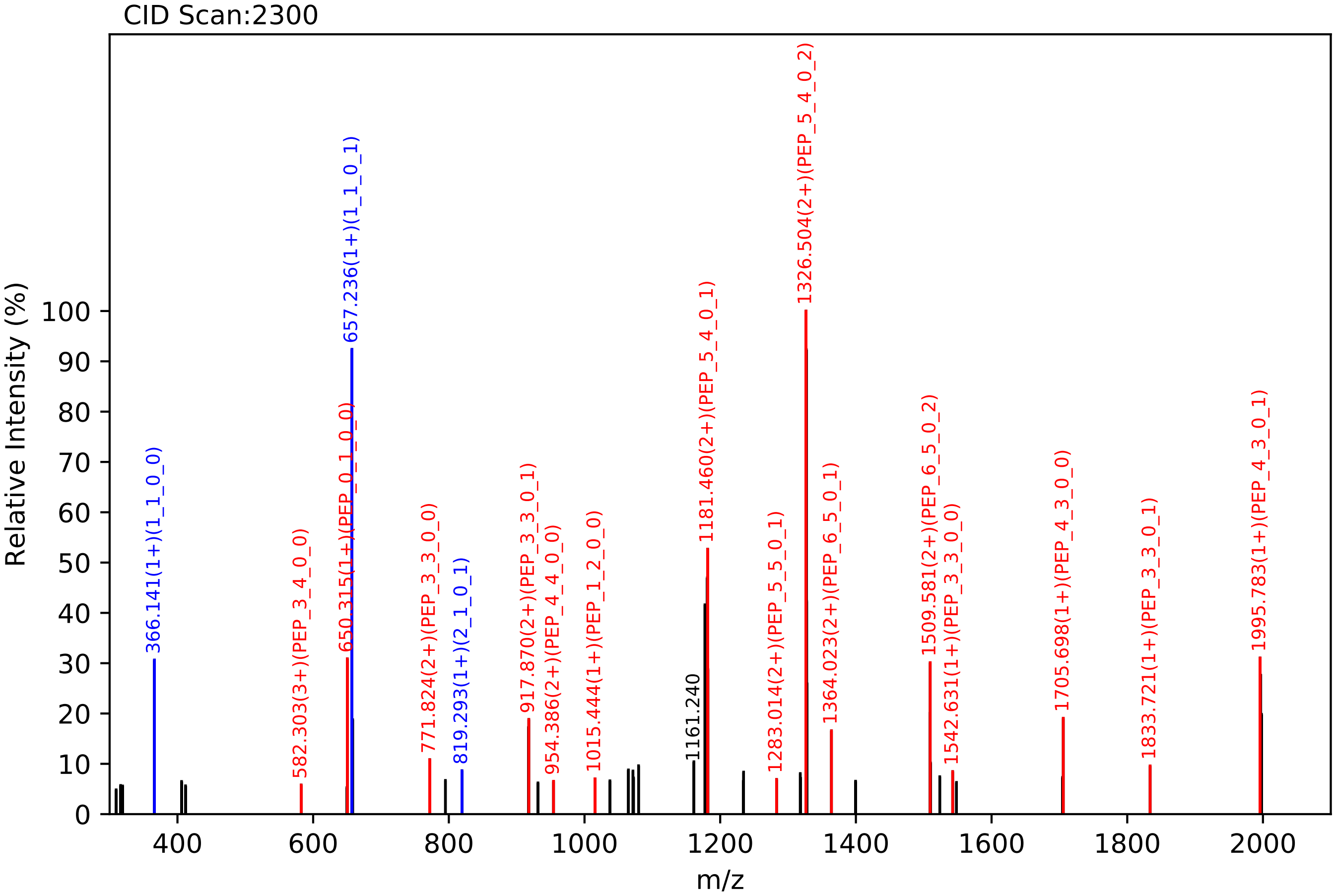

Unknown set no. 131, Experiment: Human Plasma exp_3

NGTR(=PEP)_6_5_0_3, m/z:1103.42(3+), RT:13.73, Y-score:85.64

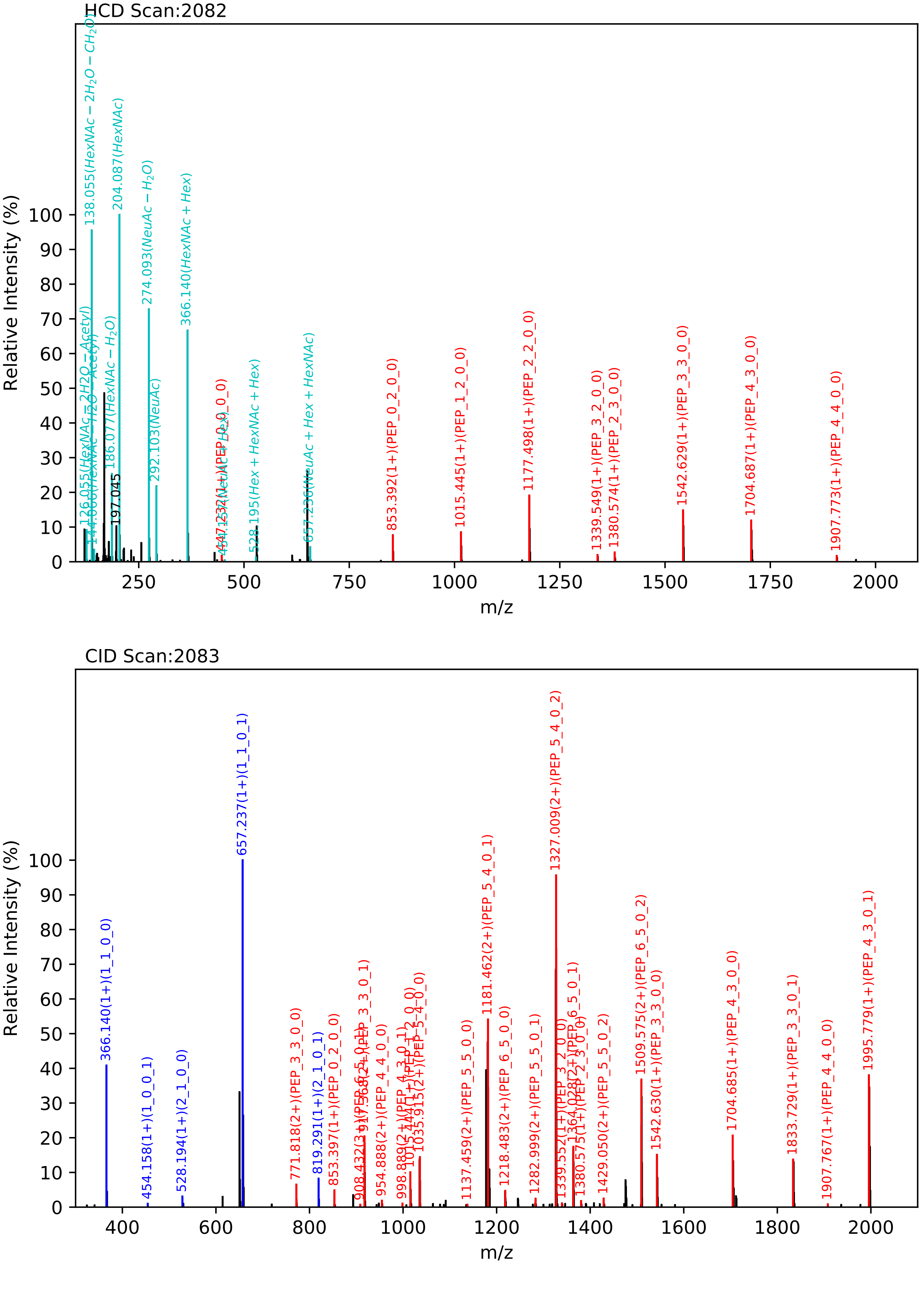

Unknown set no. 132, Experiment: Human Plasma exp_4

NGTR(=PEP)_6_5_0_3, m/z:1103.09(3+), RT:13.93, Y-score:84.43

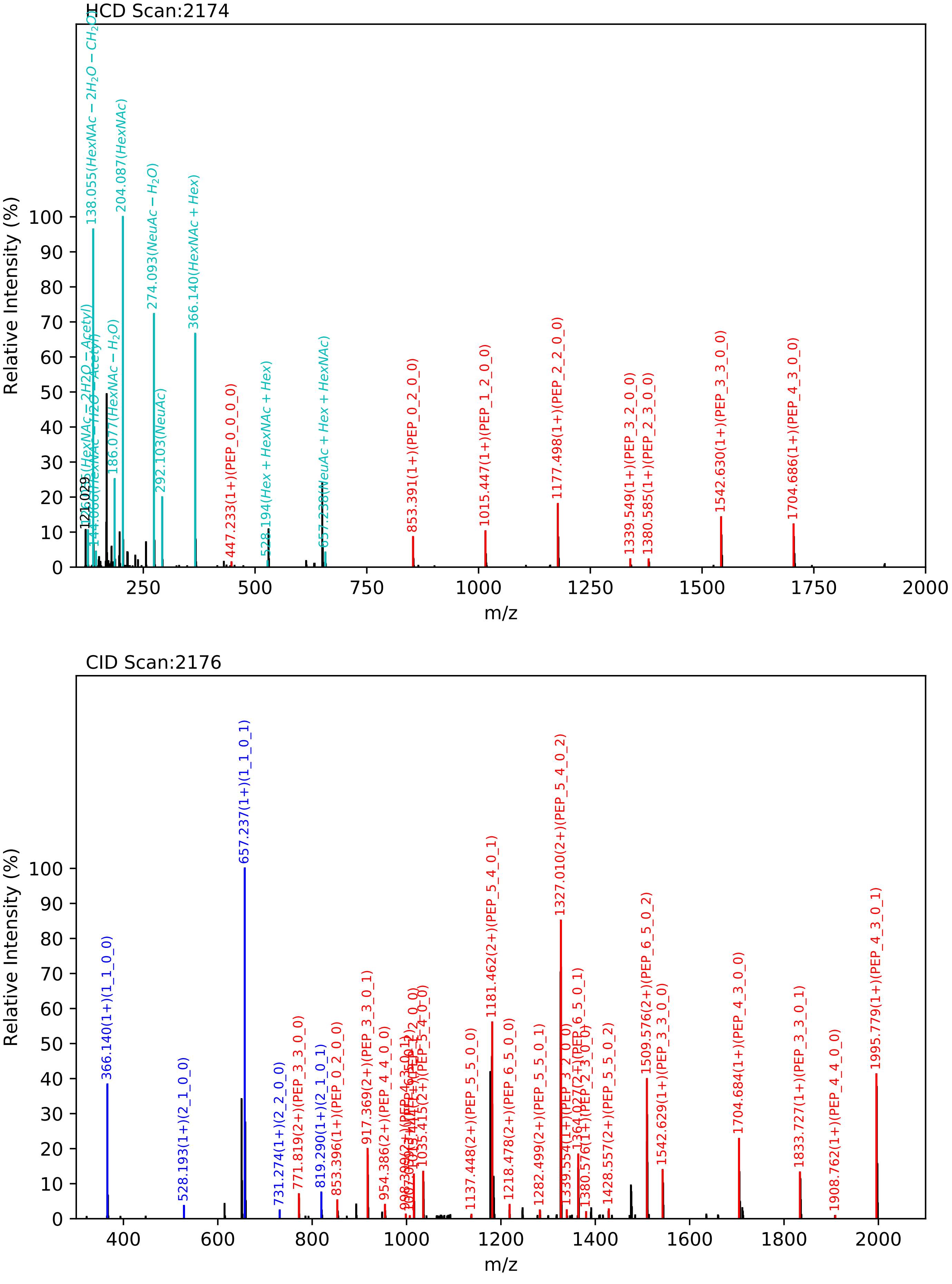

Unknown set no. 133, Experiment: Human Plasma exp_5

NGTR(=PEP)_6_5_0_3, m/z:1103.42(3+), RT:14.43, Y-score:85.37

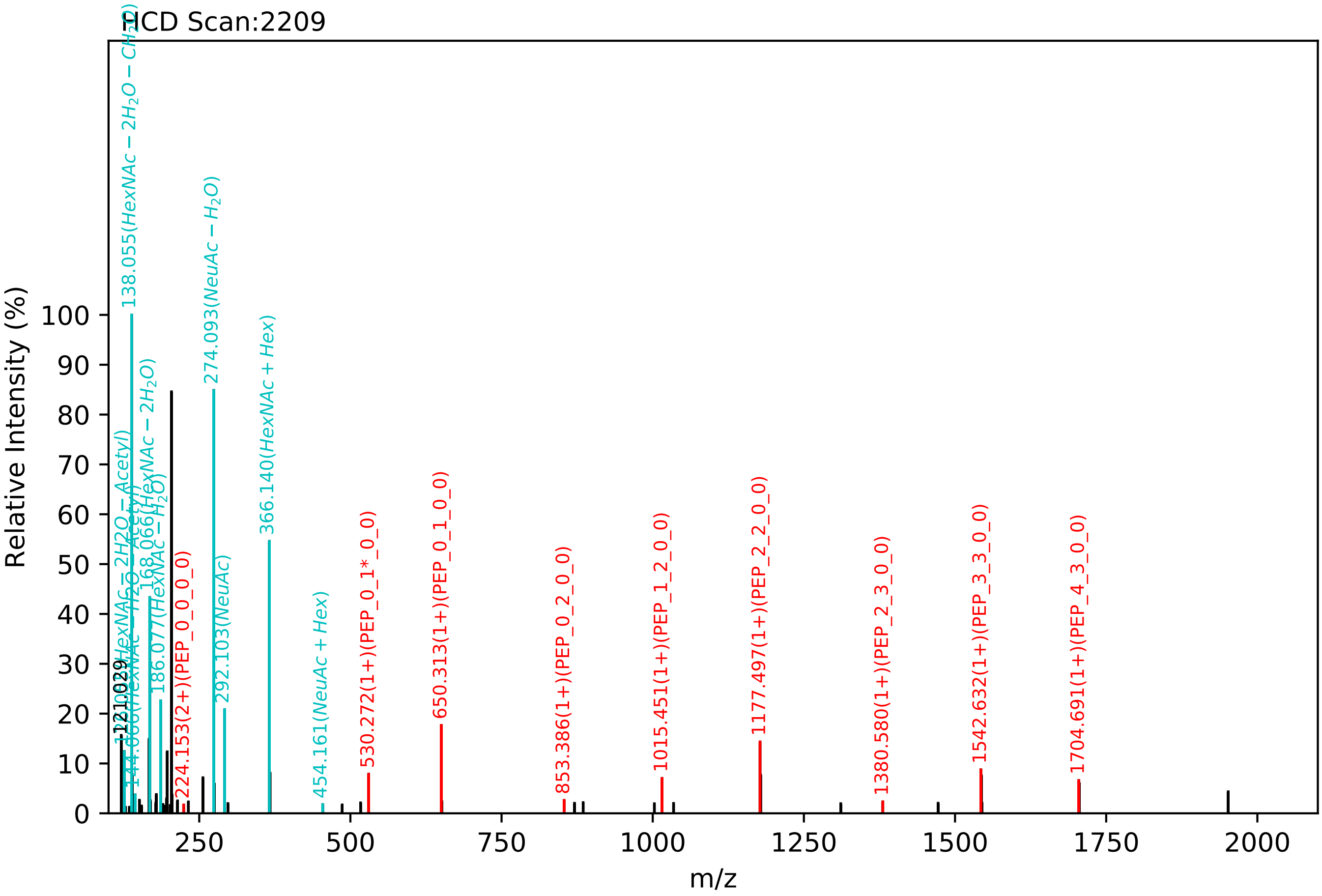

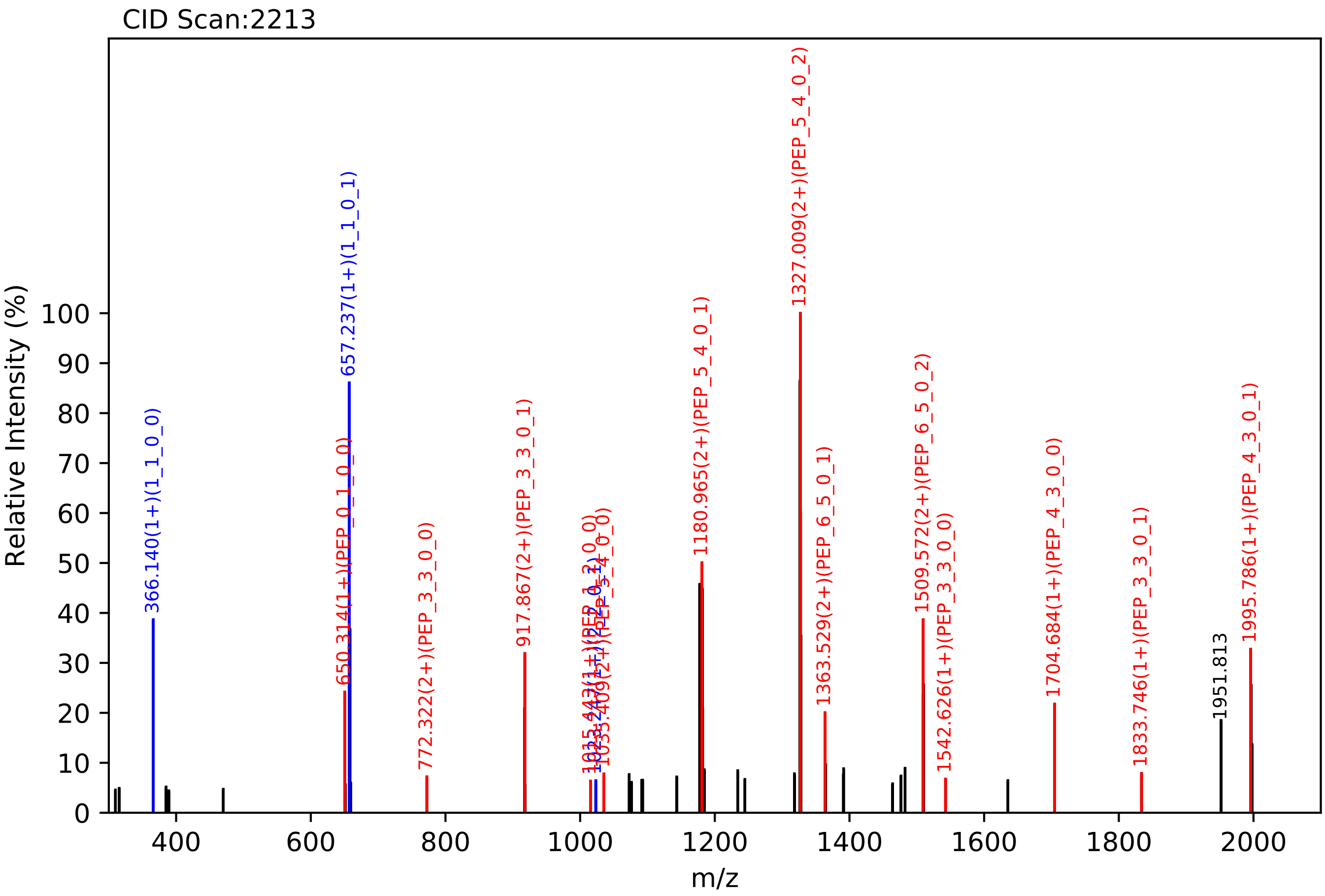

Unknown set no. 134, Experiment: Human Plasma exp_1

NGTR(=PEP)_6_5_1_3, m/z:1152.10(3+), RT:14.08, Y-score:80.17

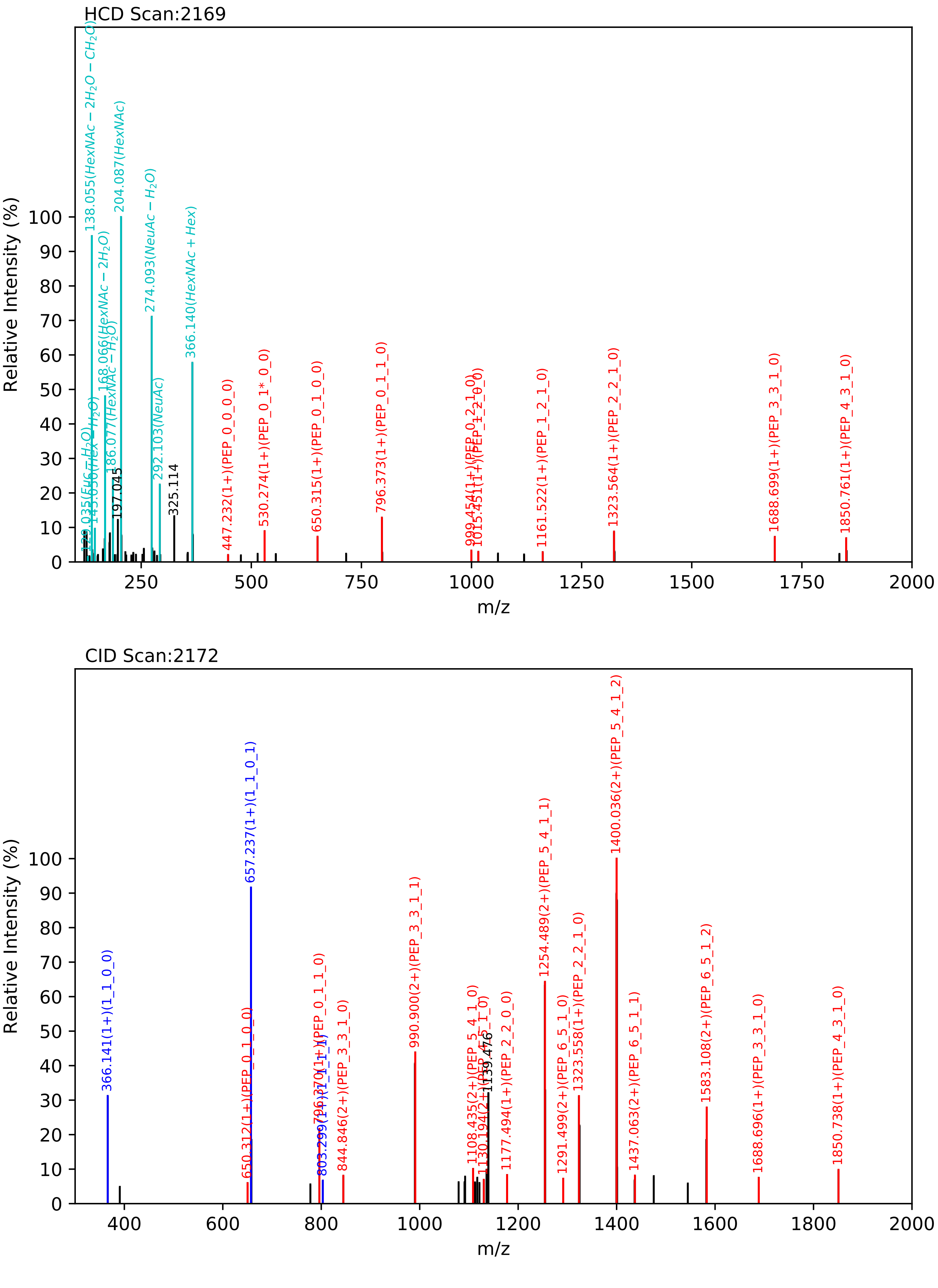

Unknown set no. 135, Experiment: Human Plasma exp_2

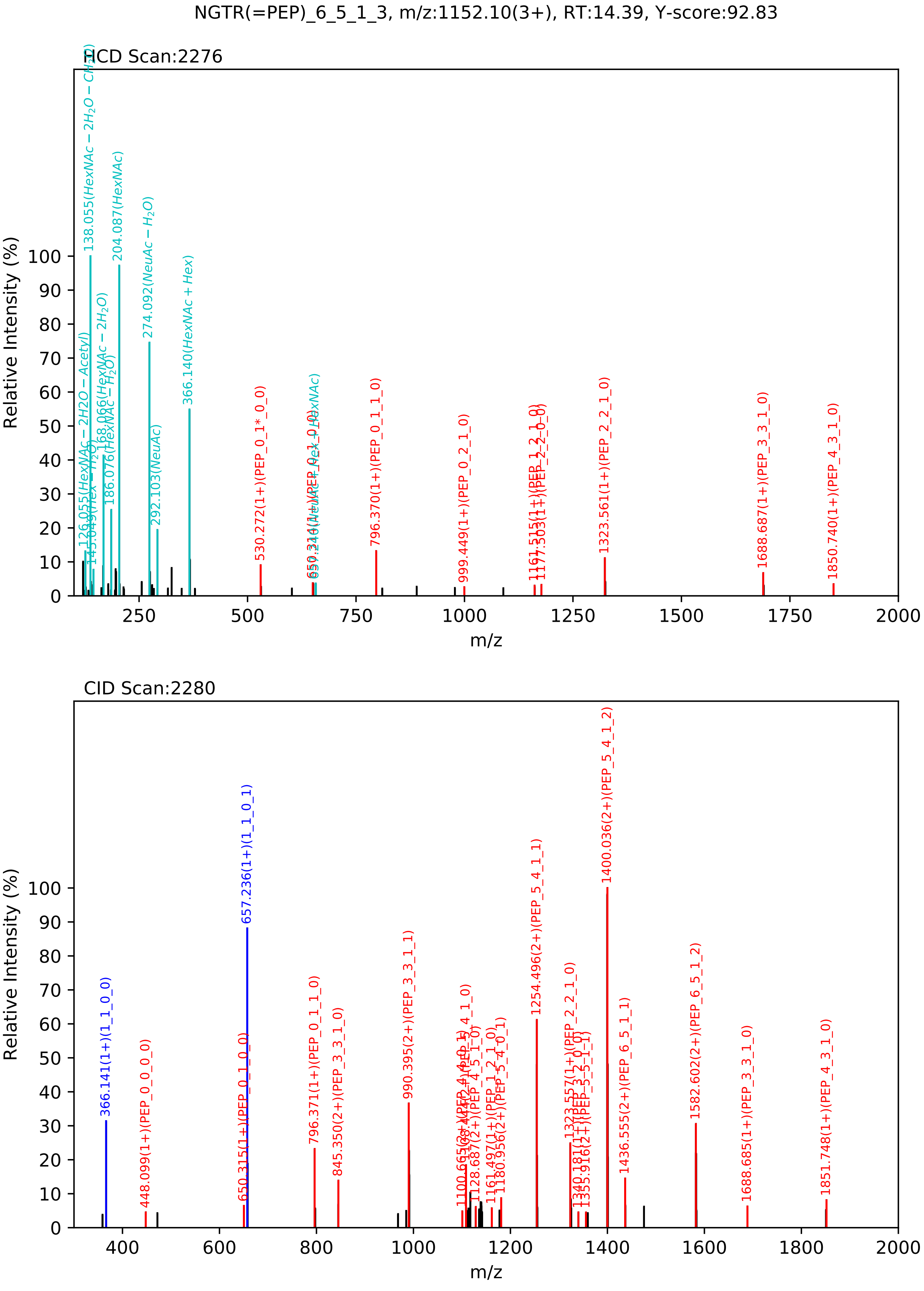

Unknown set no. 136, Experiment: Human Plasma exp_4

NGTR(=PEP)_6_5_1_3, m/z:1152.10(3+), RT:14.47, Y-score:78.47

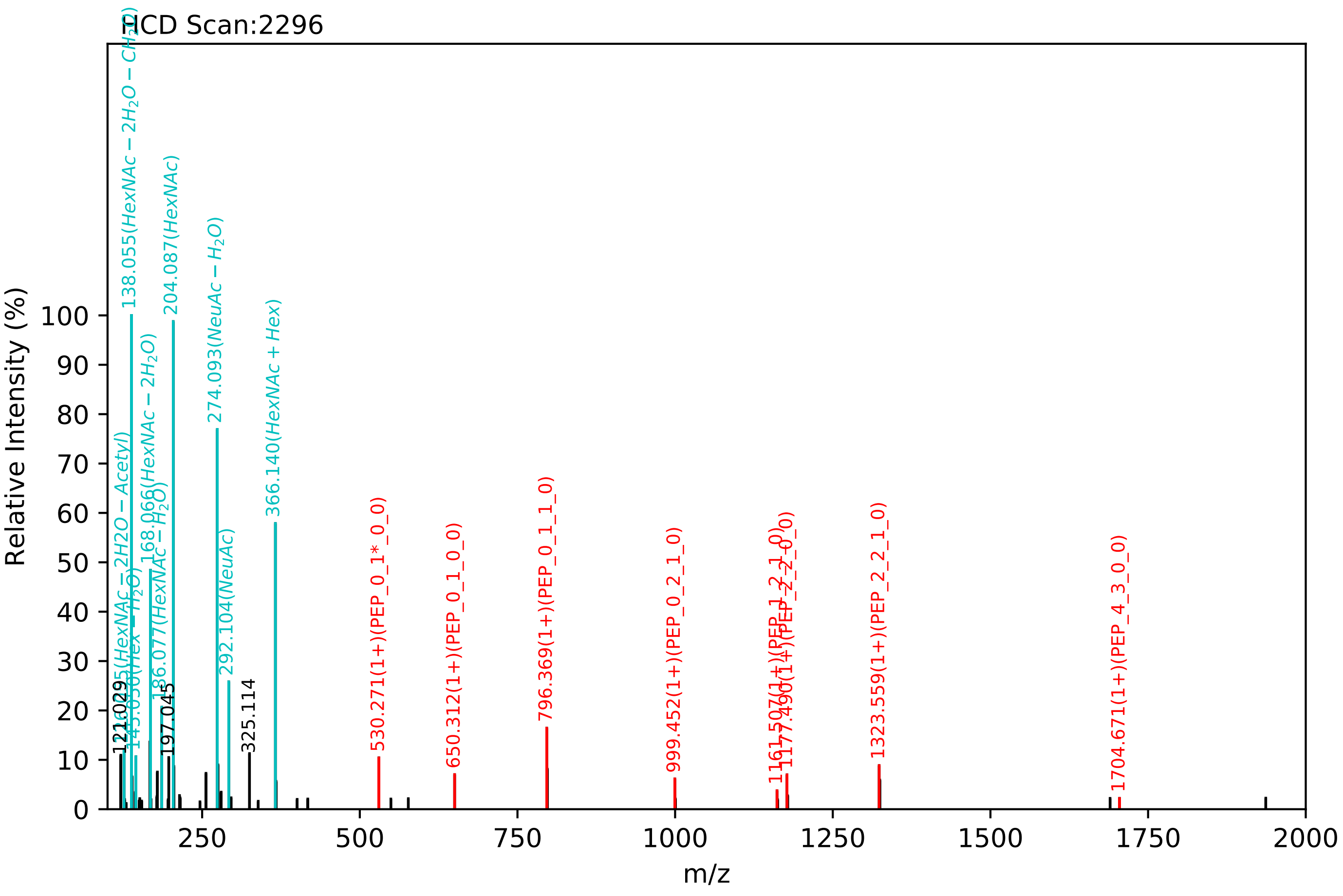

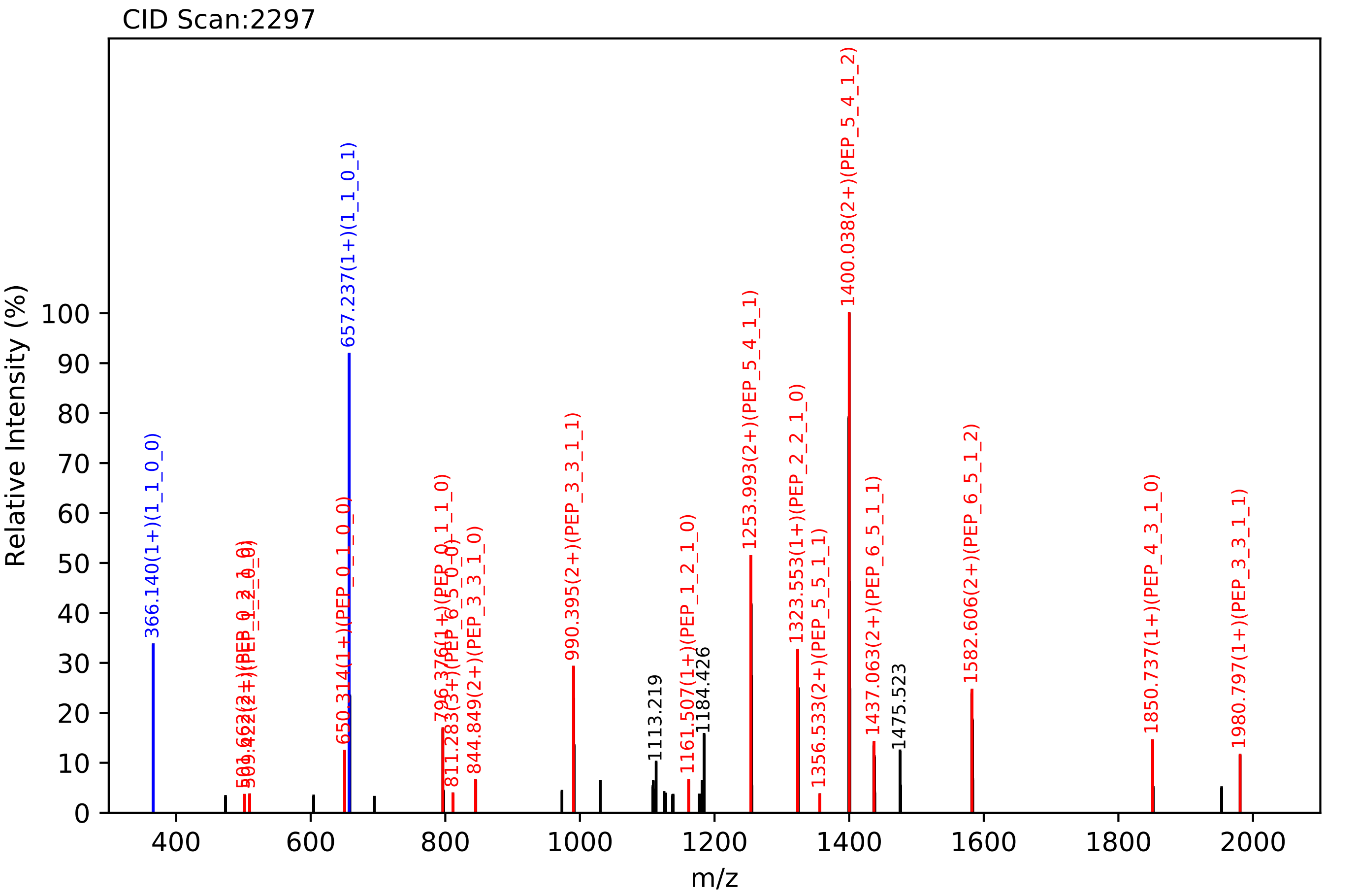

Unknown set no. 137, Experiment: Human Plasma exp_1

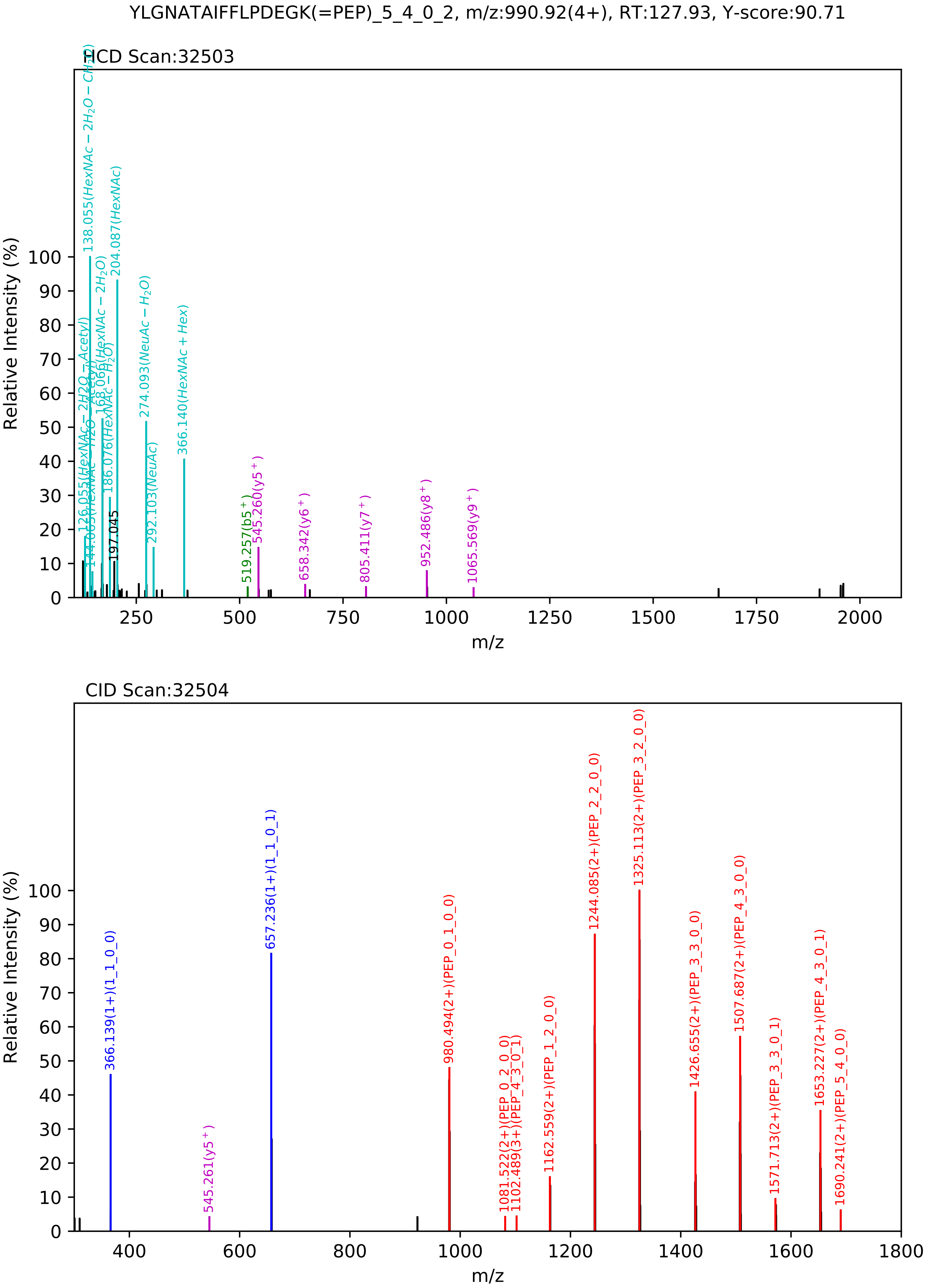

Unknown set no. 138, Experiment: Human Plasma exp_2

YLGNATAIFFLPDEGK(=PEP)_5_4_0_2, m/z:990.92(4+), RT:128.12, Y-score:87.83

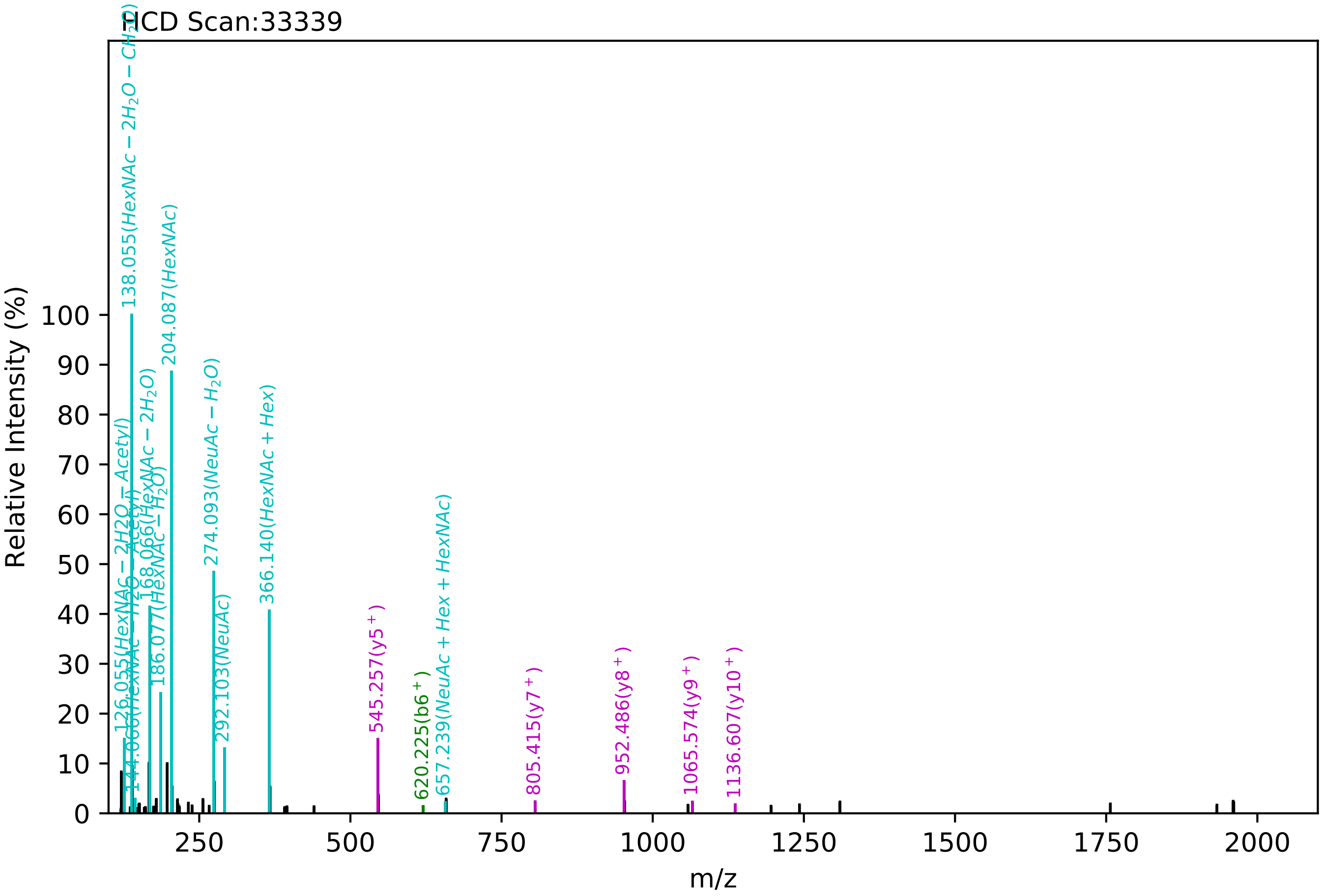

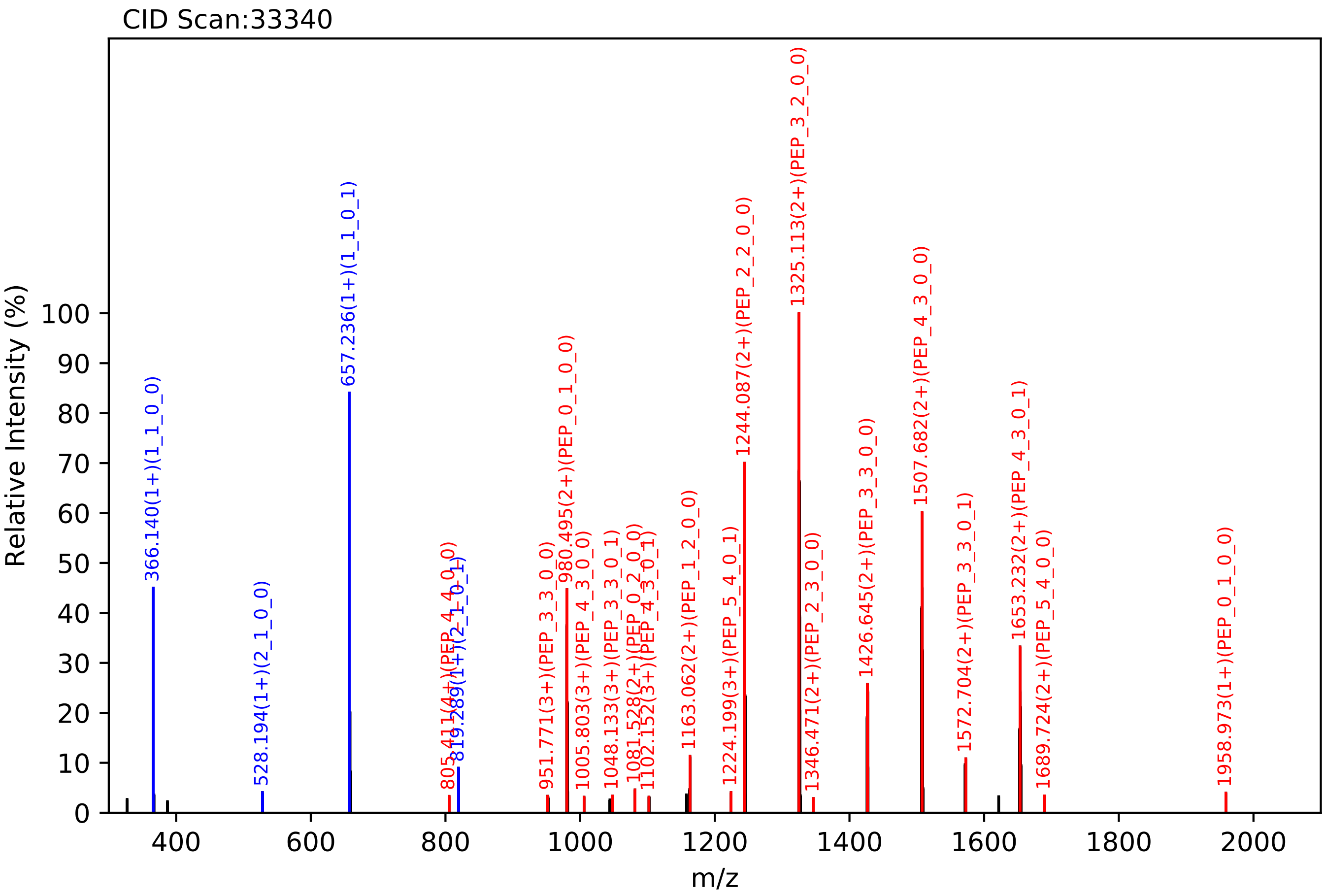

Unknown set no. 139, Experiment: Human Plasma exp_3

YLGNATAIFFLPDEGK(=PEP)_5_4_0_2, m/z:990.92(4+), RT:128.20, Y-score:92.21

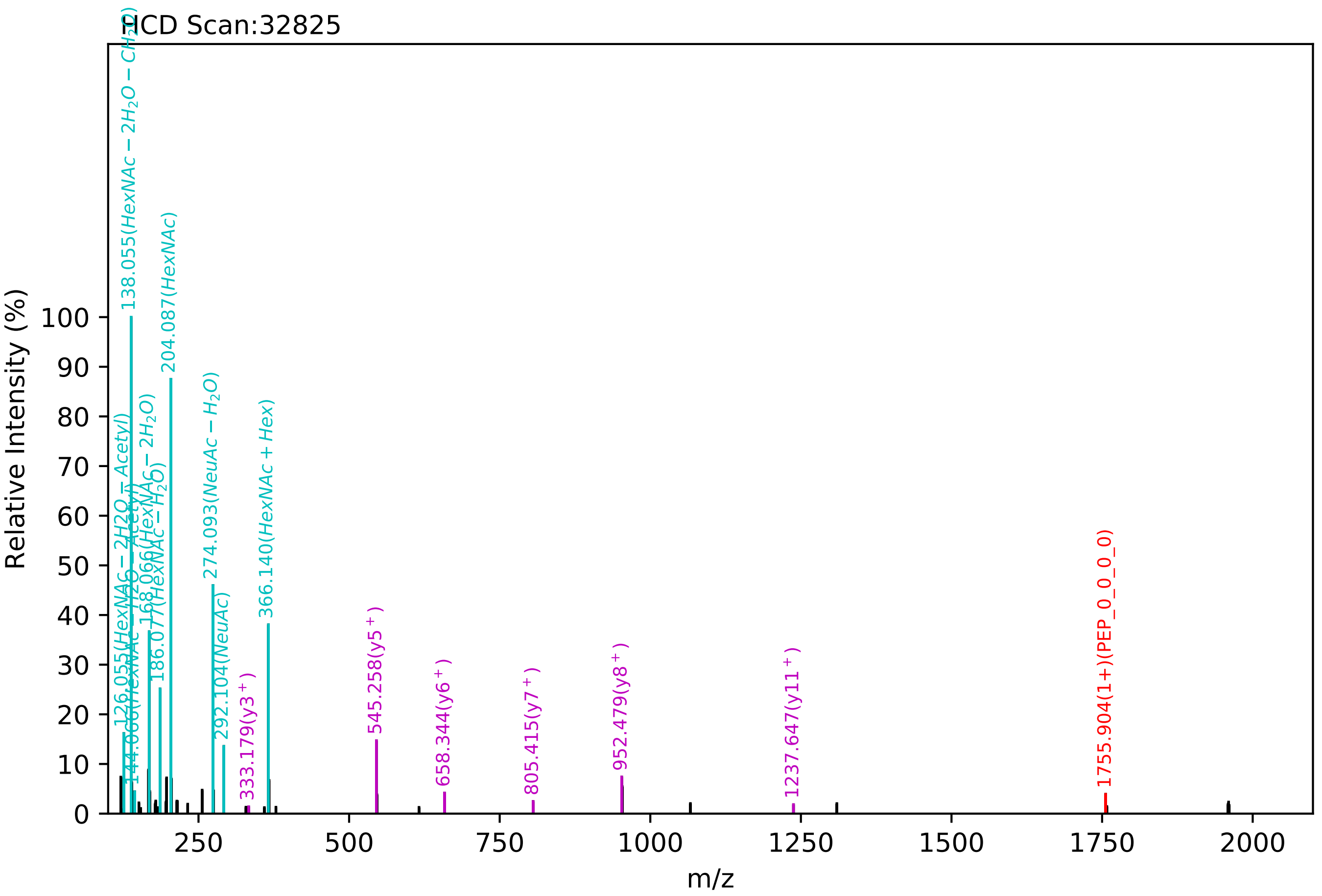

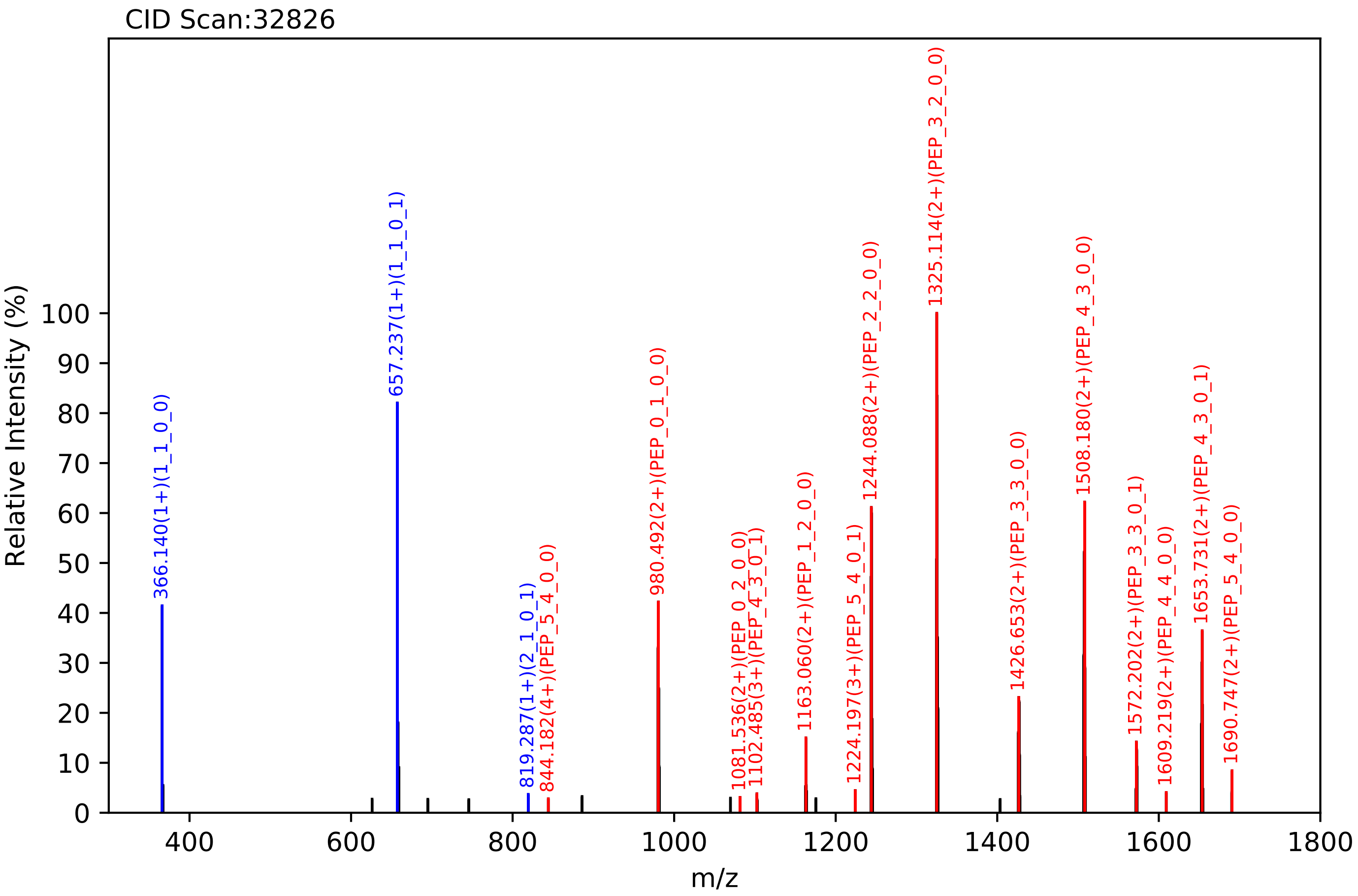

Unknown set no. 140, Experiment: Human Plasma exp_4

YLGNATAIFFLPDEGK(=PEP)_5_4_0_2, m/z:990.92(4+), RT:128.18, Y-score:90.85

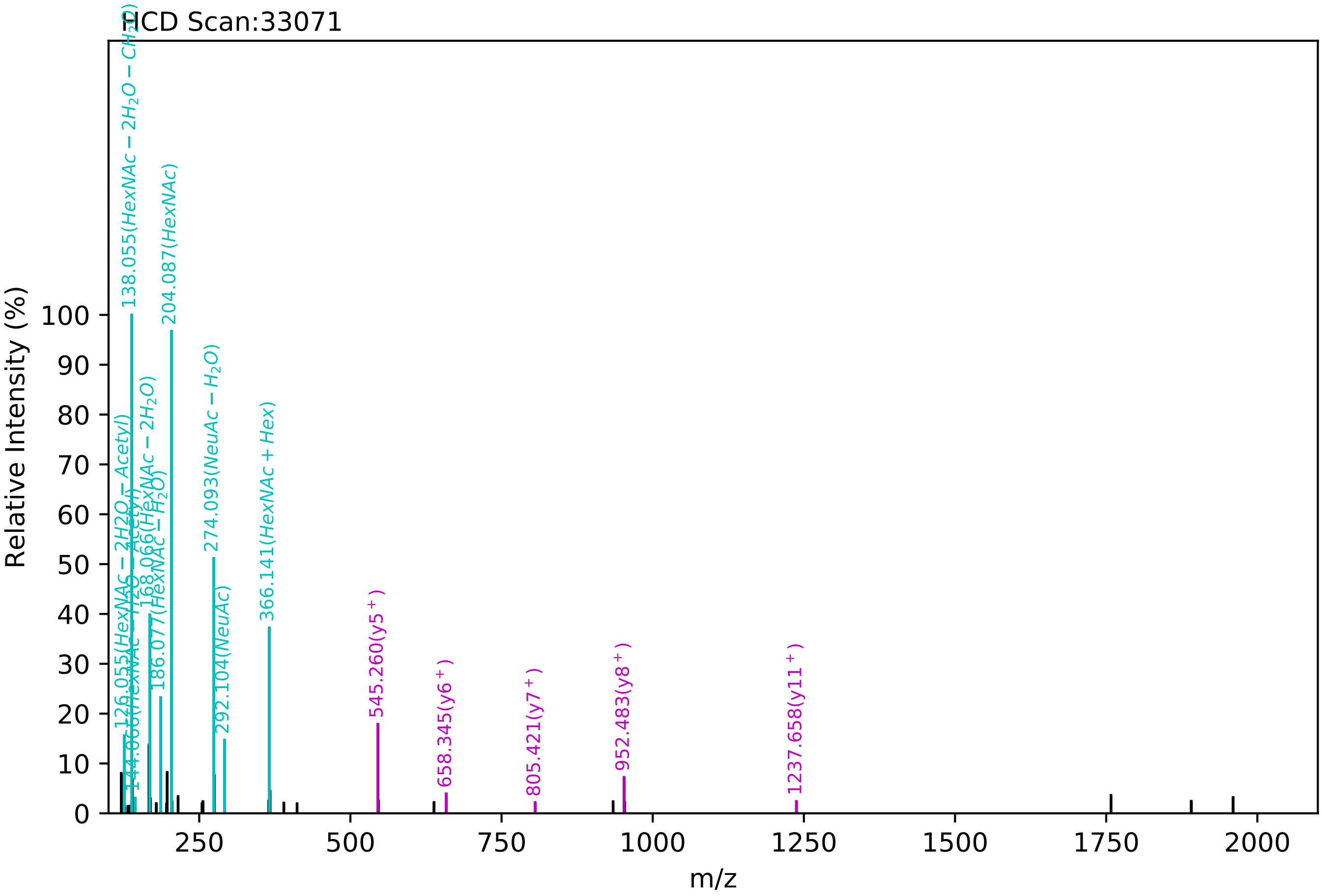

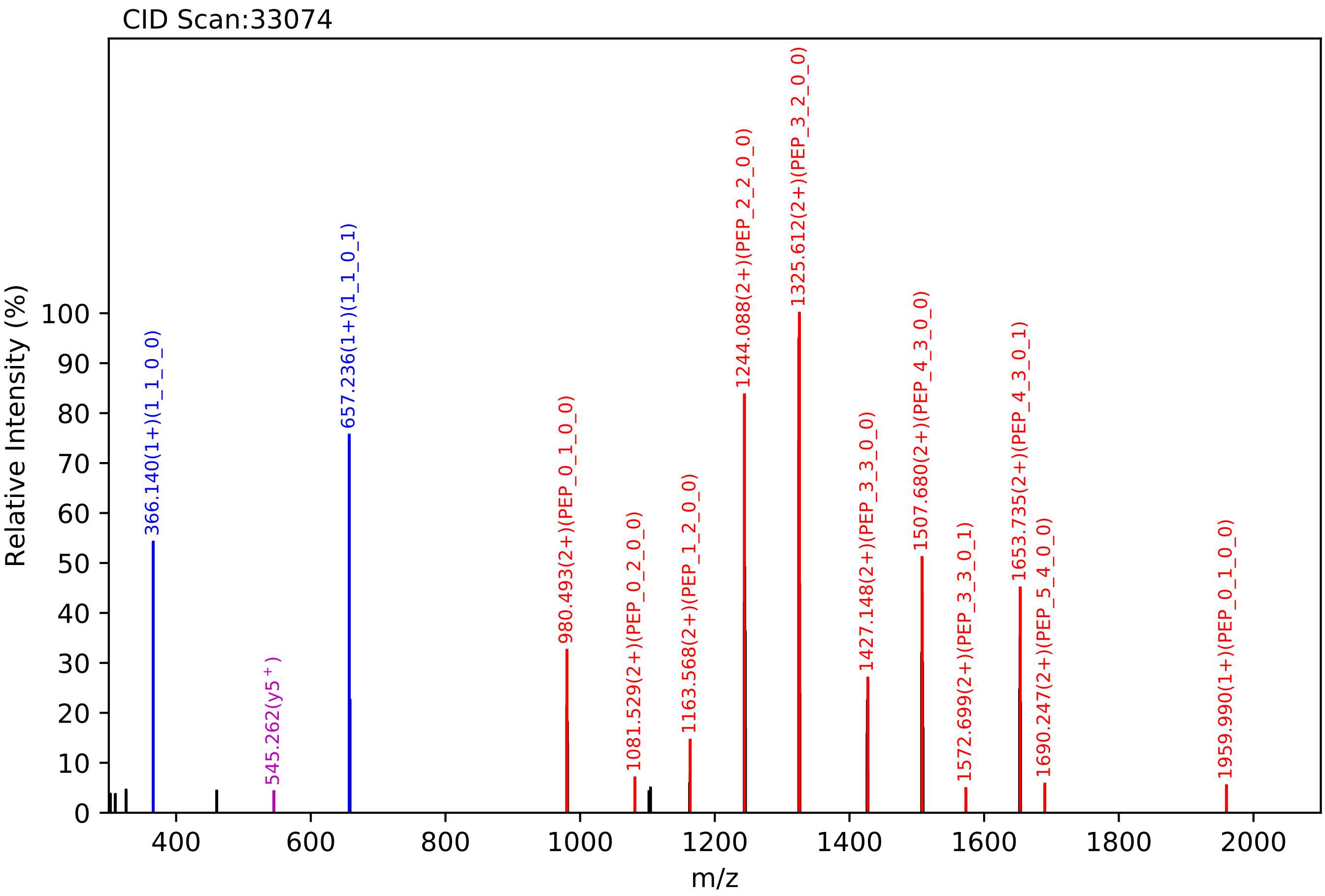

Unknown set no. 141, Experiment: Human Plasma exp_5

YLGNATAIFFLPDEGK(=PEP)_5_4_0_2, m/z:990.92(4+), RT:127.88, Y-score:87.01

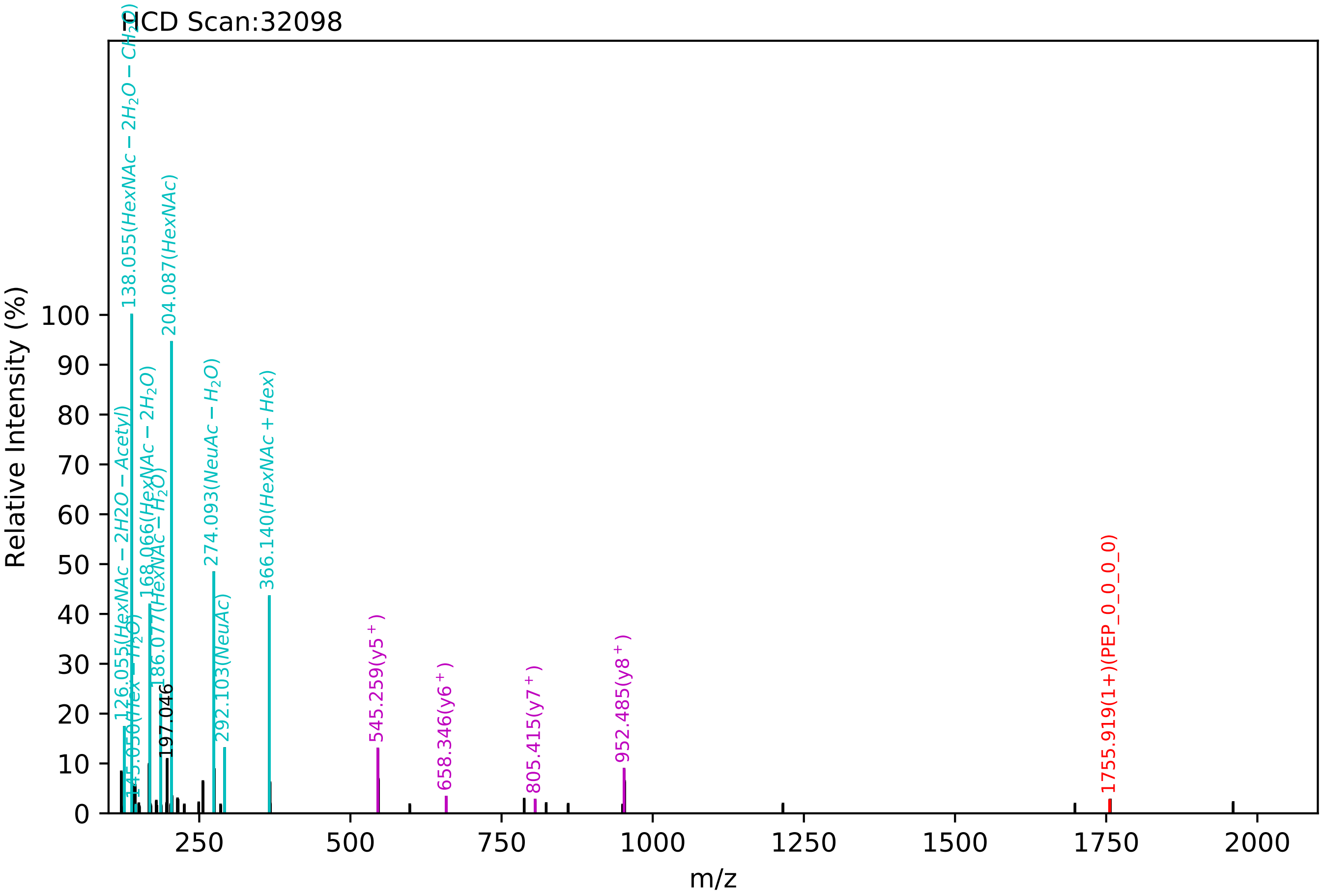

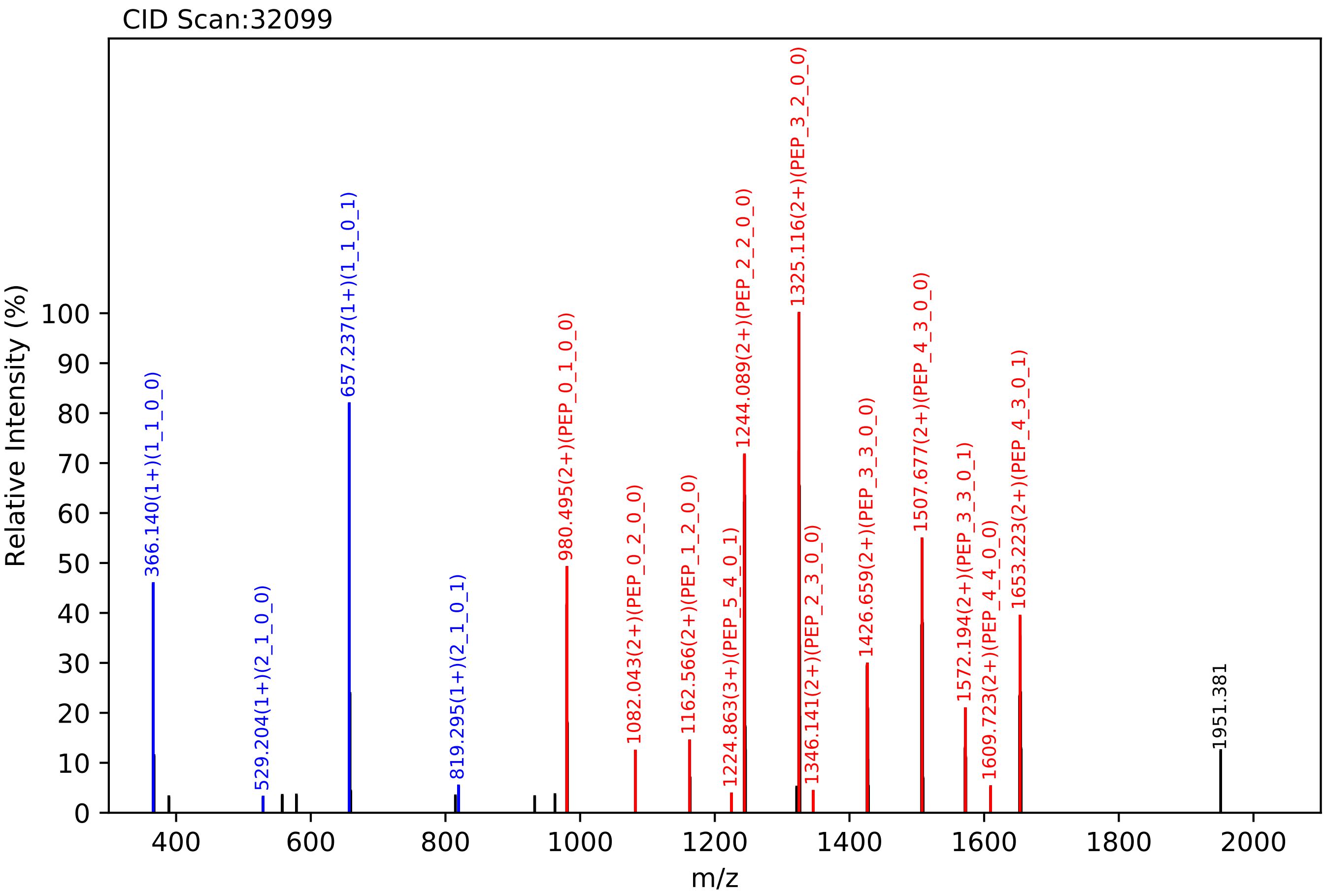

Unknown set no. 142, Experiment: Human Plasma exp_1

VCQDCPLLAPLNDTR(=PEP)_5_4_0_2, m/z:994.91(4+), RT:101.83, Y-score:81.63

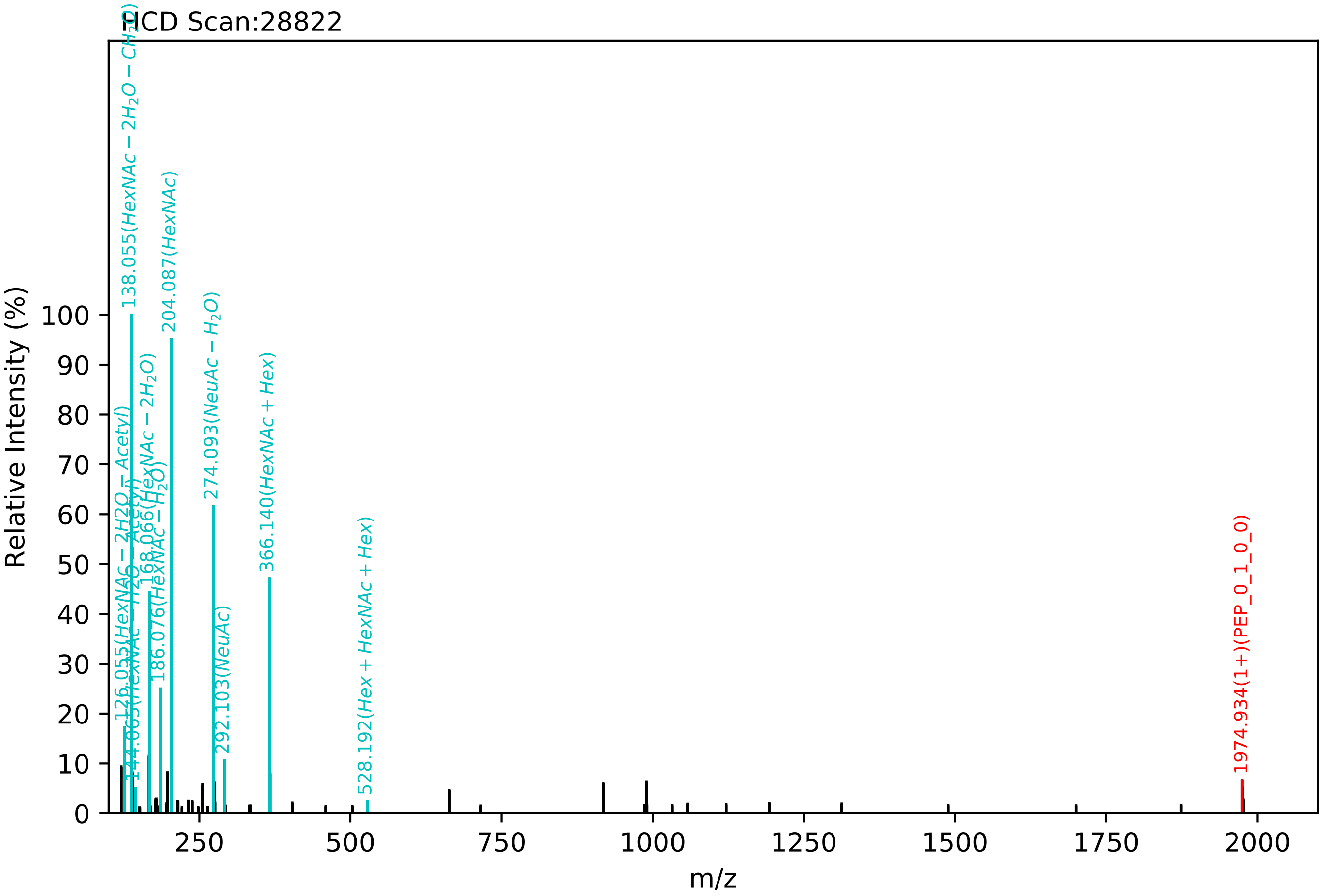

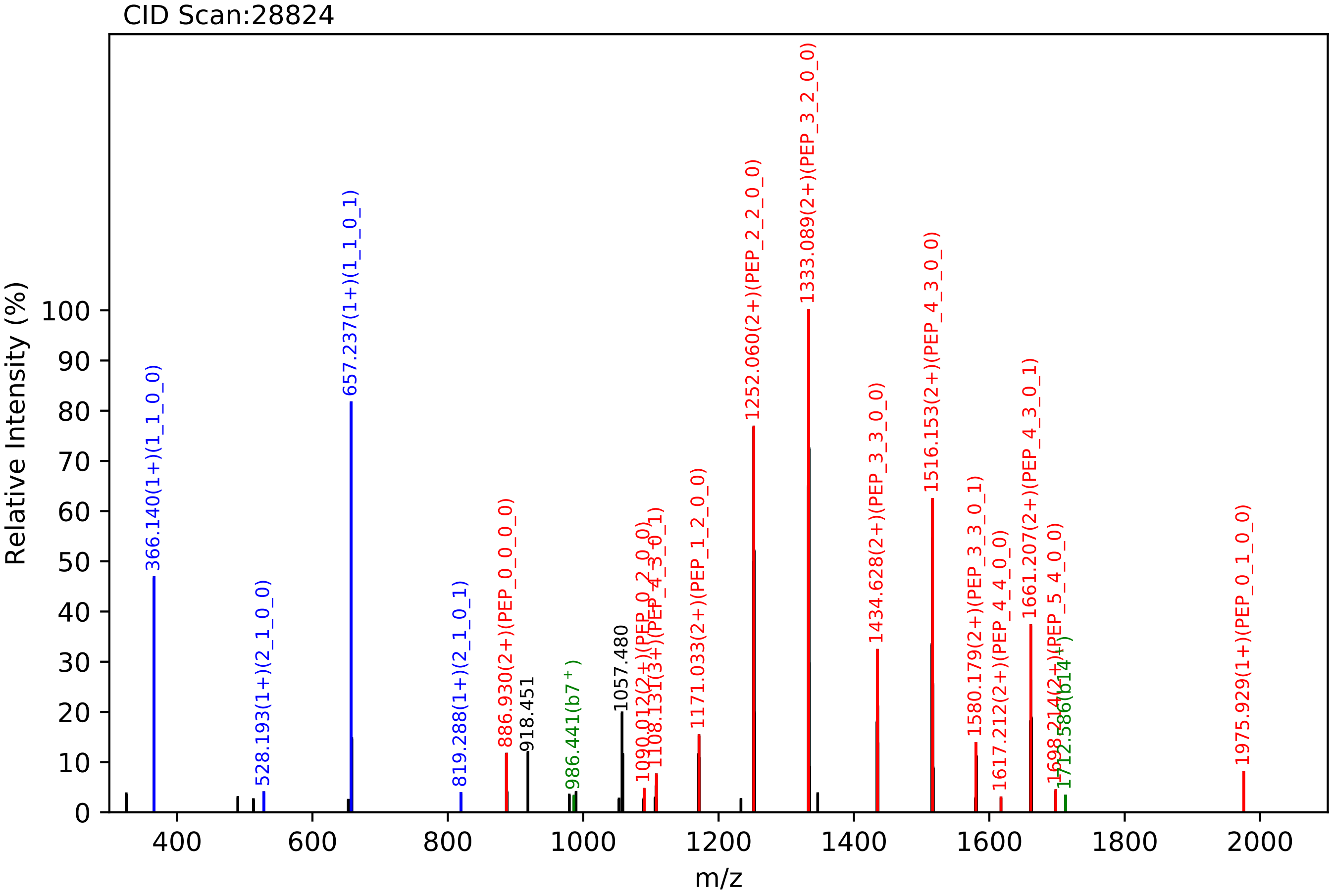

Unknown set no. 143, Experiment: Human Plasma exp_4

VCQDCPLLAPLNDTR(=PEP)_5_4_0_2, m/z:994.91(4+), RT:103.23, Y-score:80.62

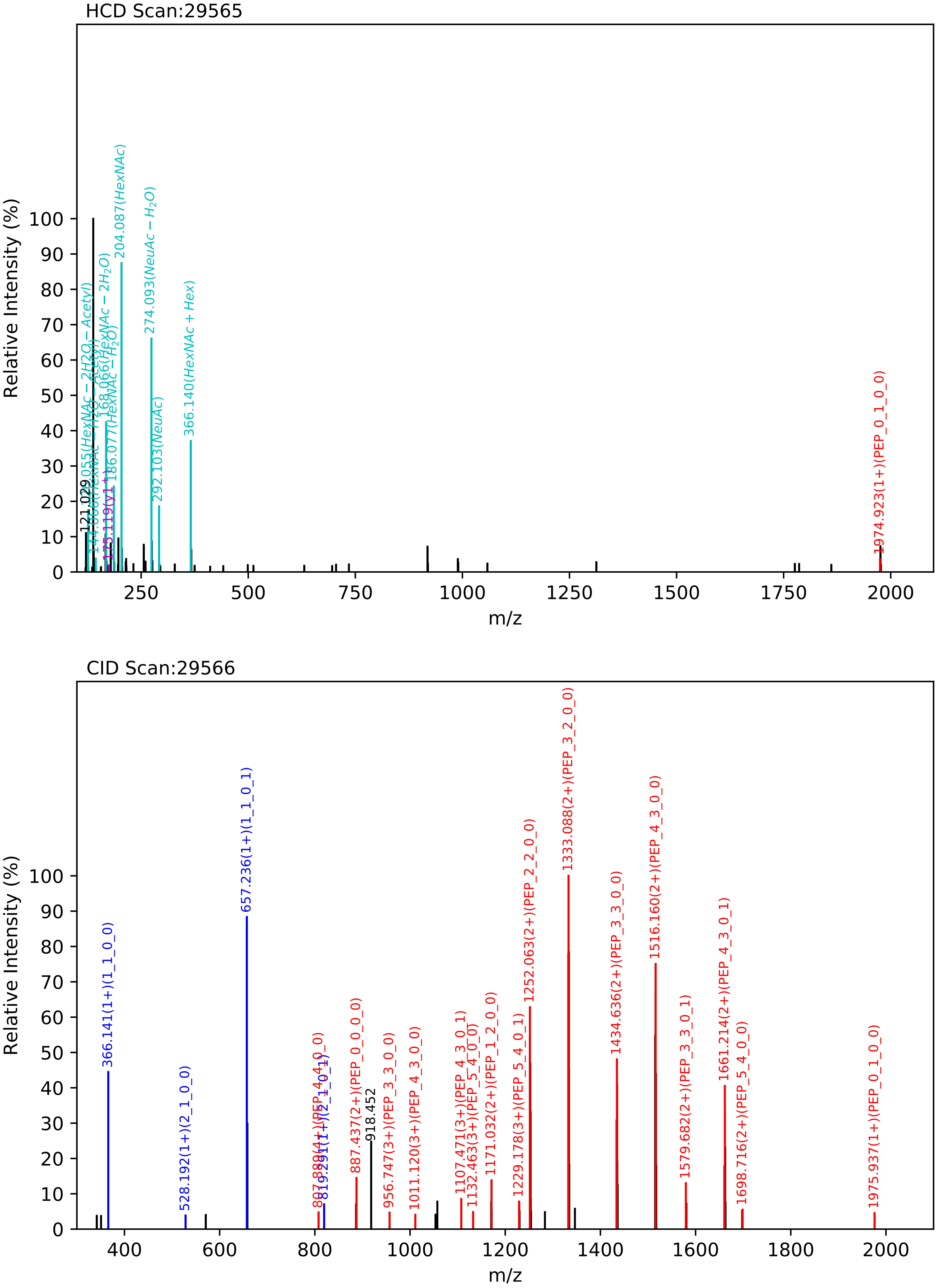

Unknown set no. 144, Experiment: Human Plasma exp_5

VCQDCPLLAPLNDTR(=PEP)_5_4_0_2, m/z:994.91(4+), RT:101.73, Y-score:76.40

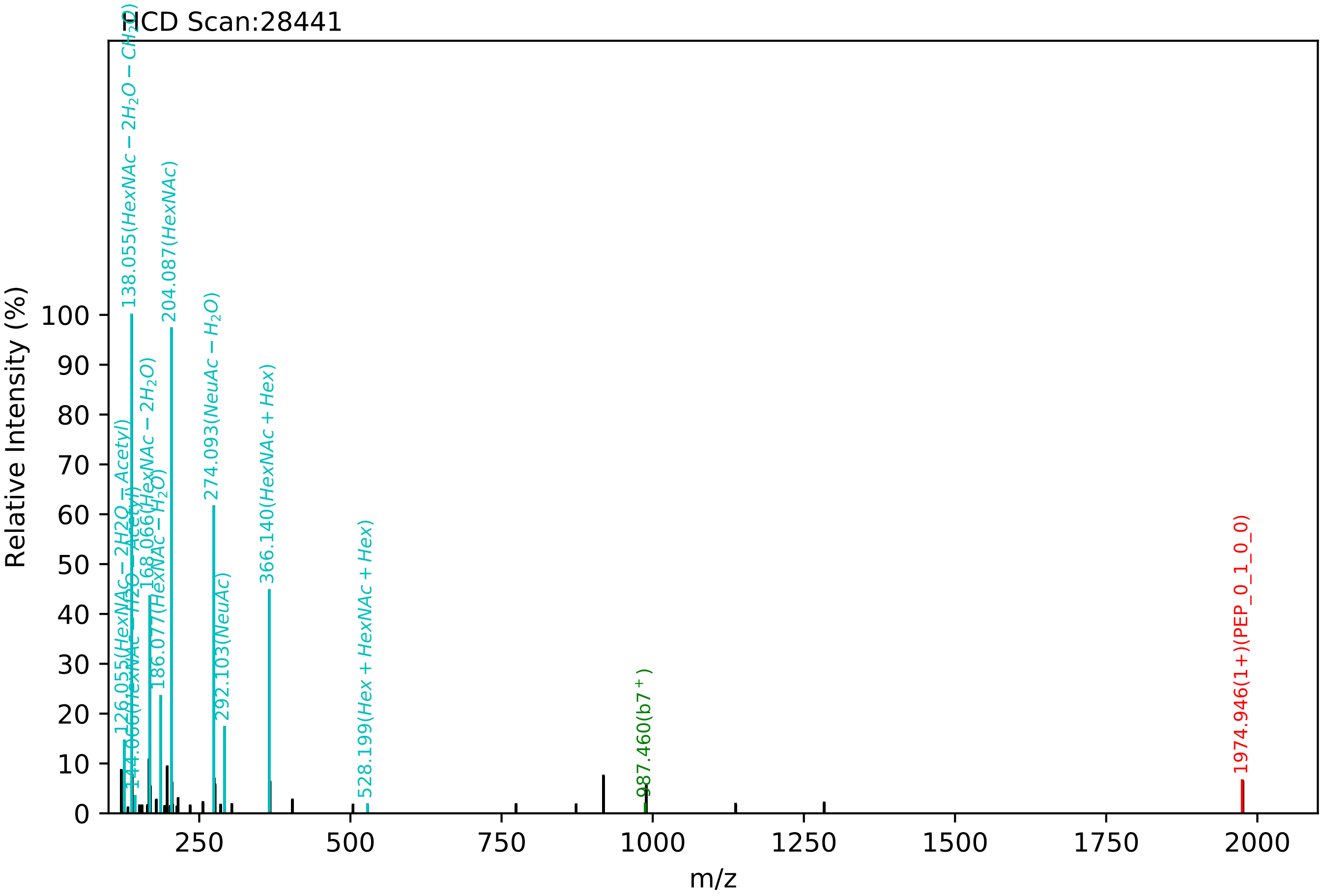

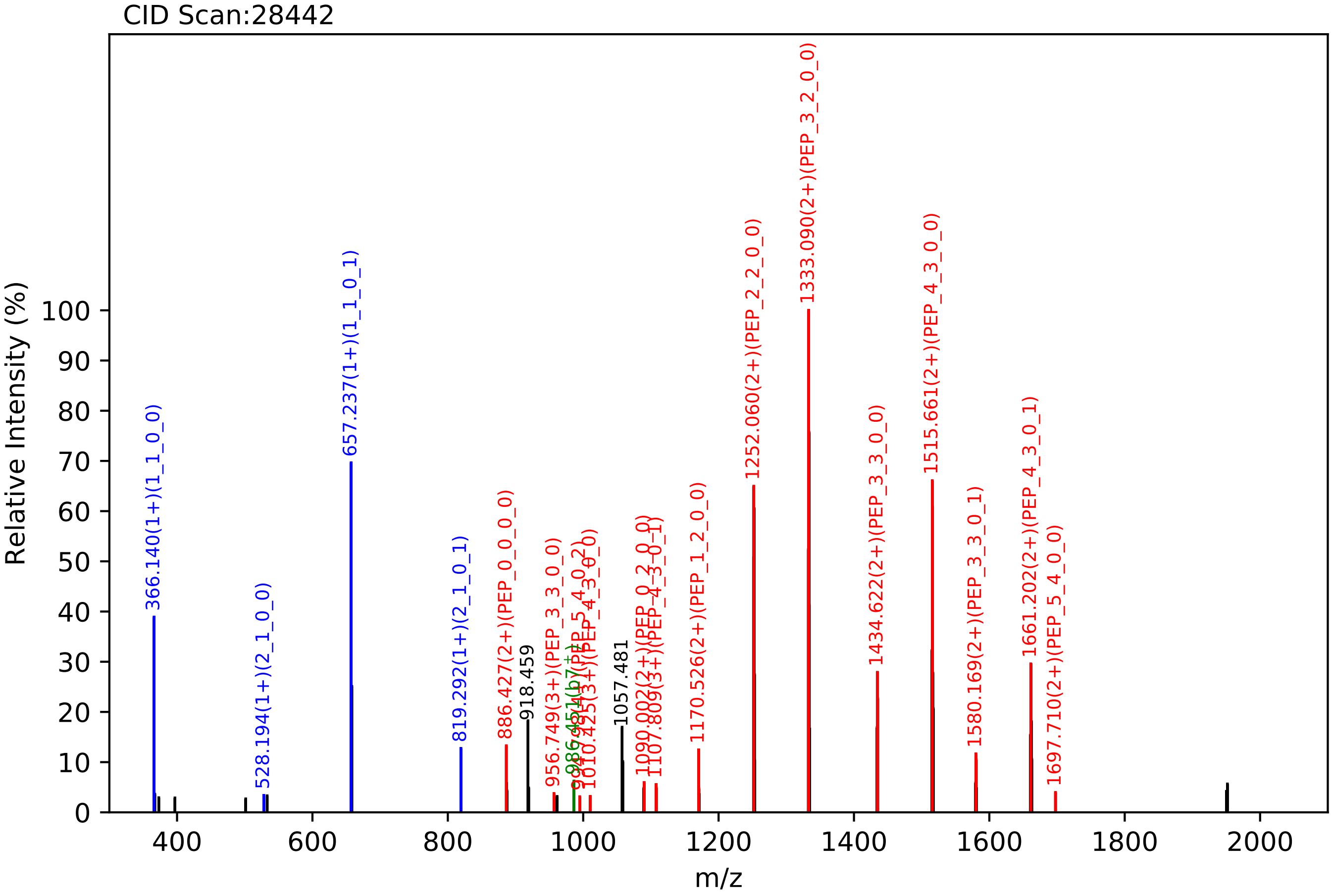

Unknown set no. 145, Experiment: Human Plasma exp_5

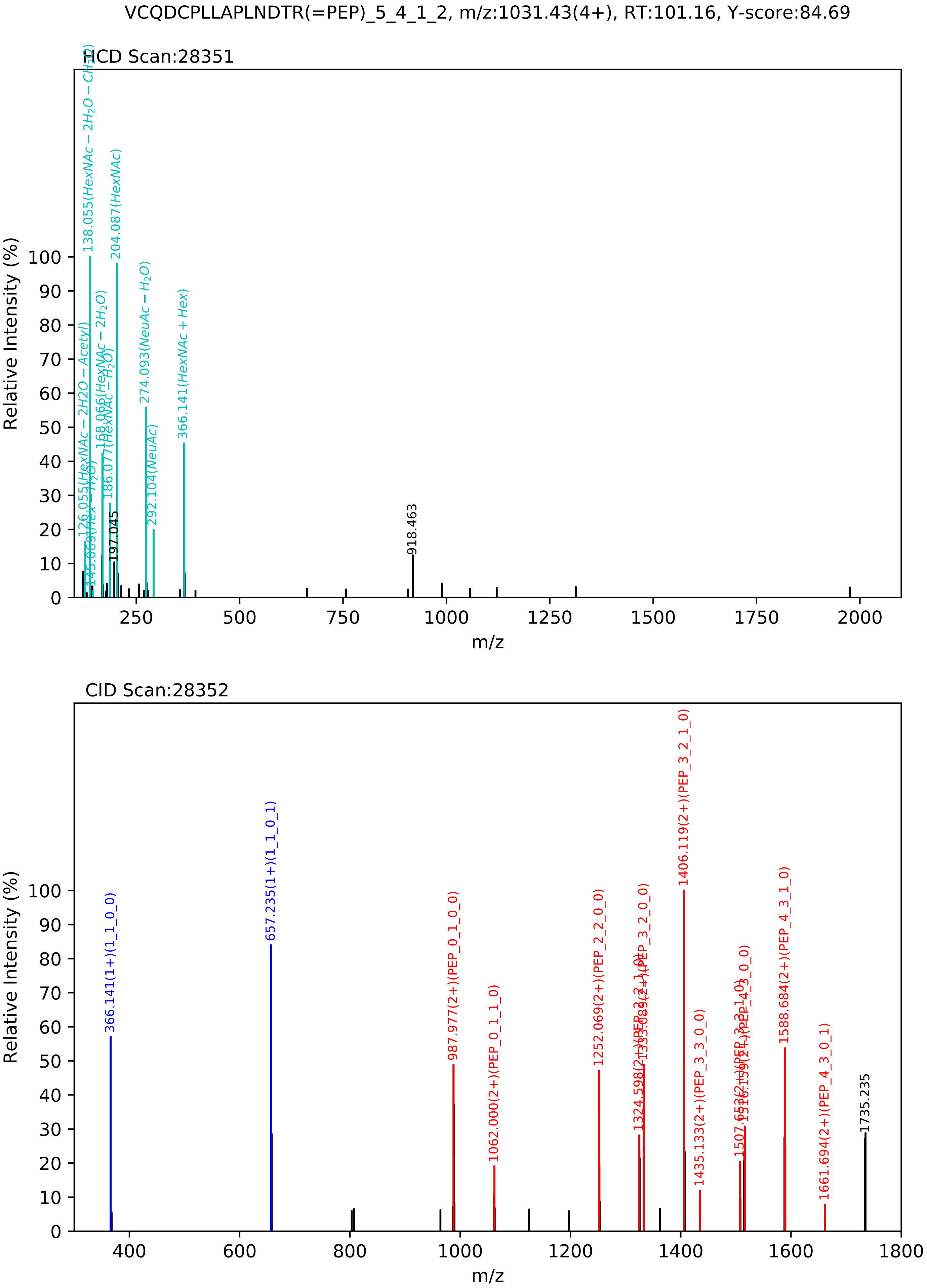

Unknown set no. 146, Experiment: Human Plasma exp_4

VCQDCPLLAPLNDTR(=PEP)_6_5_1_3, m/z:1195.48(4+), RT:115.61, Y-score:70.80

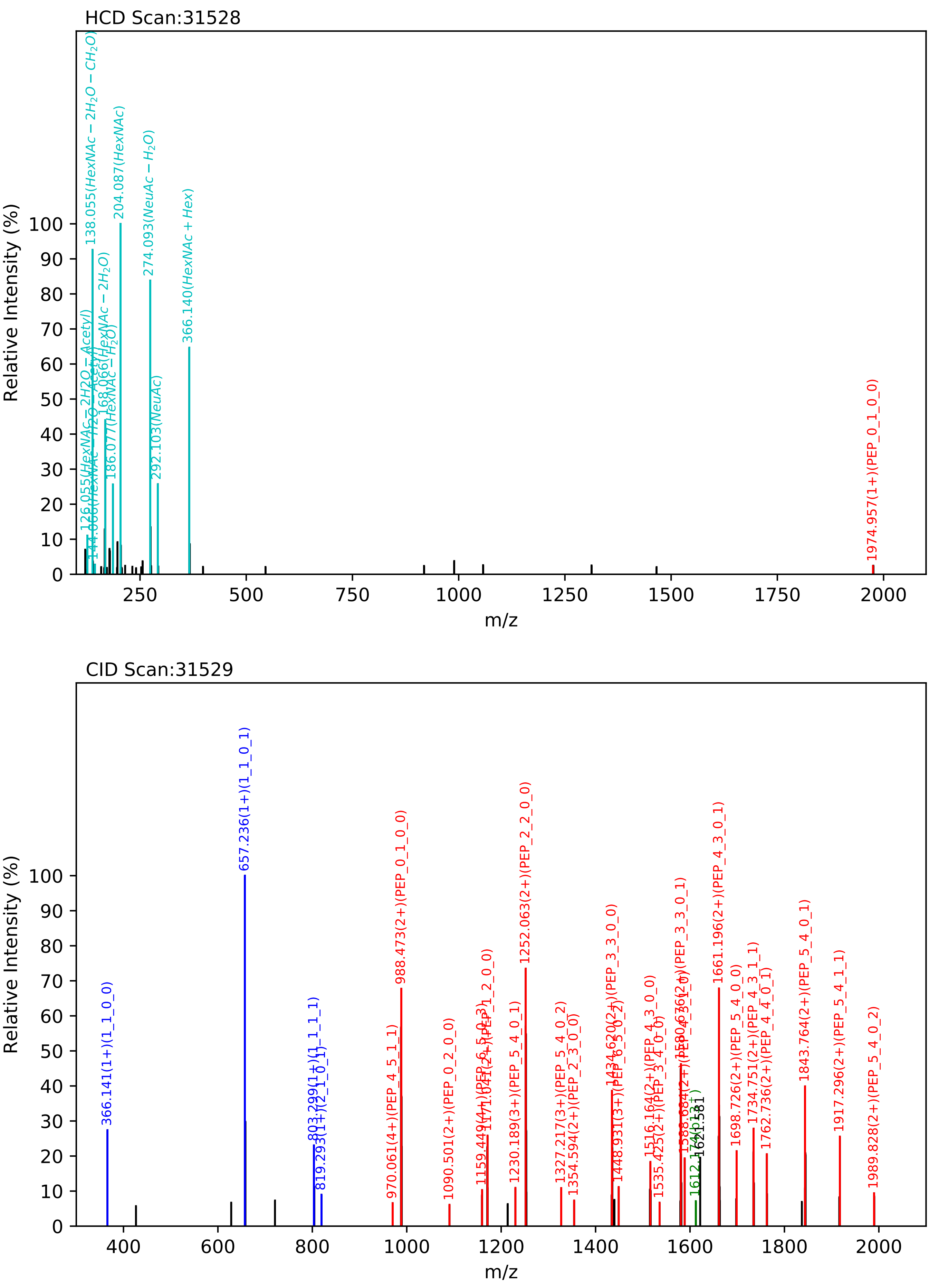

Unknown set no. 147, Experiment: Human Plasma exp_5

VCQDCPLLAPLNDTR(=PEP)_6_5_1_3, m/z:1195.48(4+), RT:115.58, Y-score:81.49

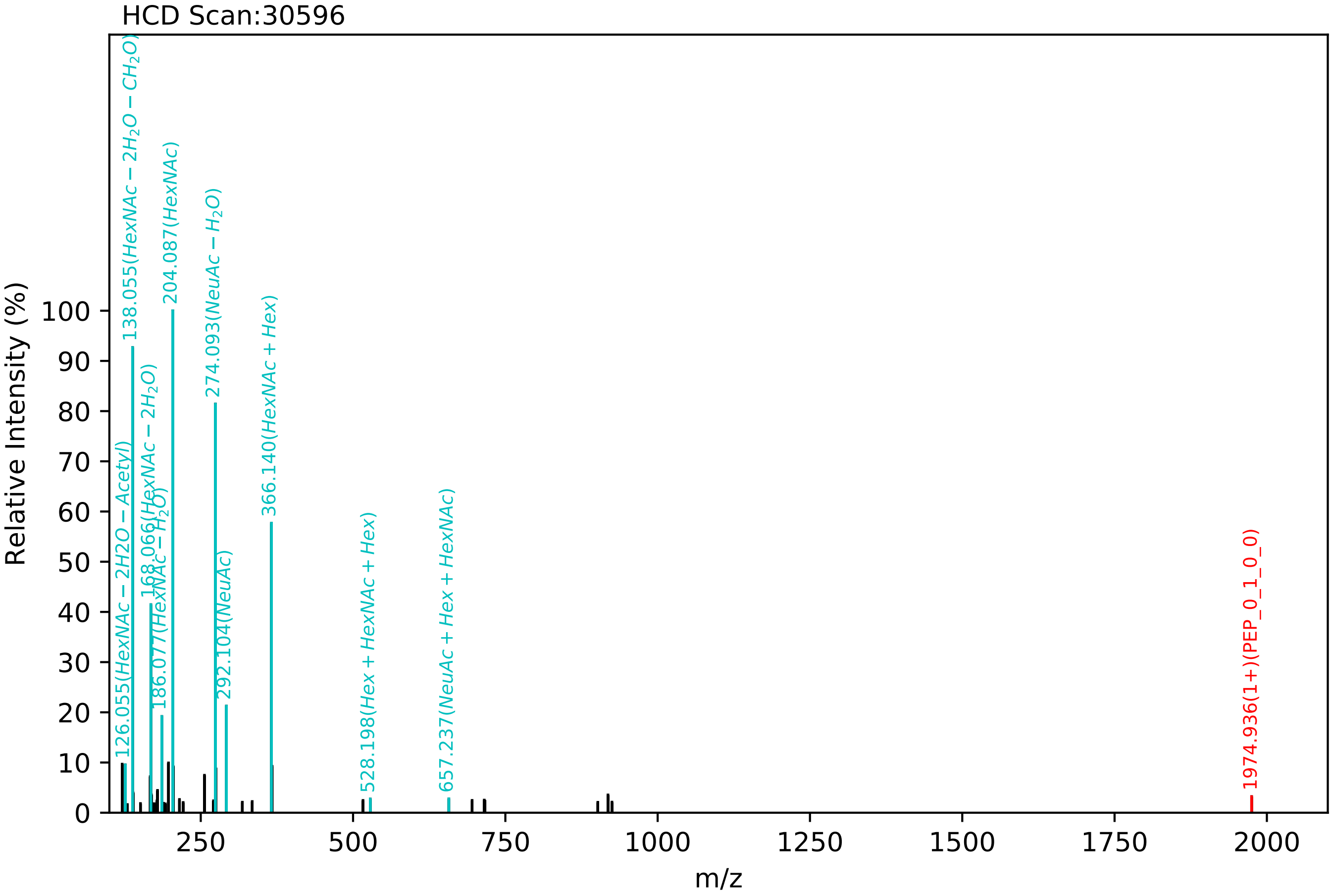

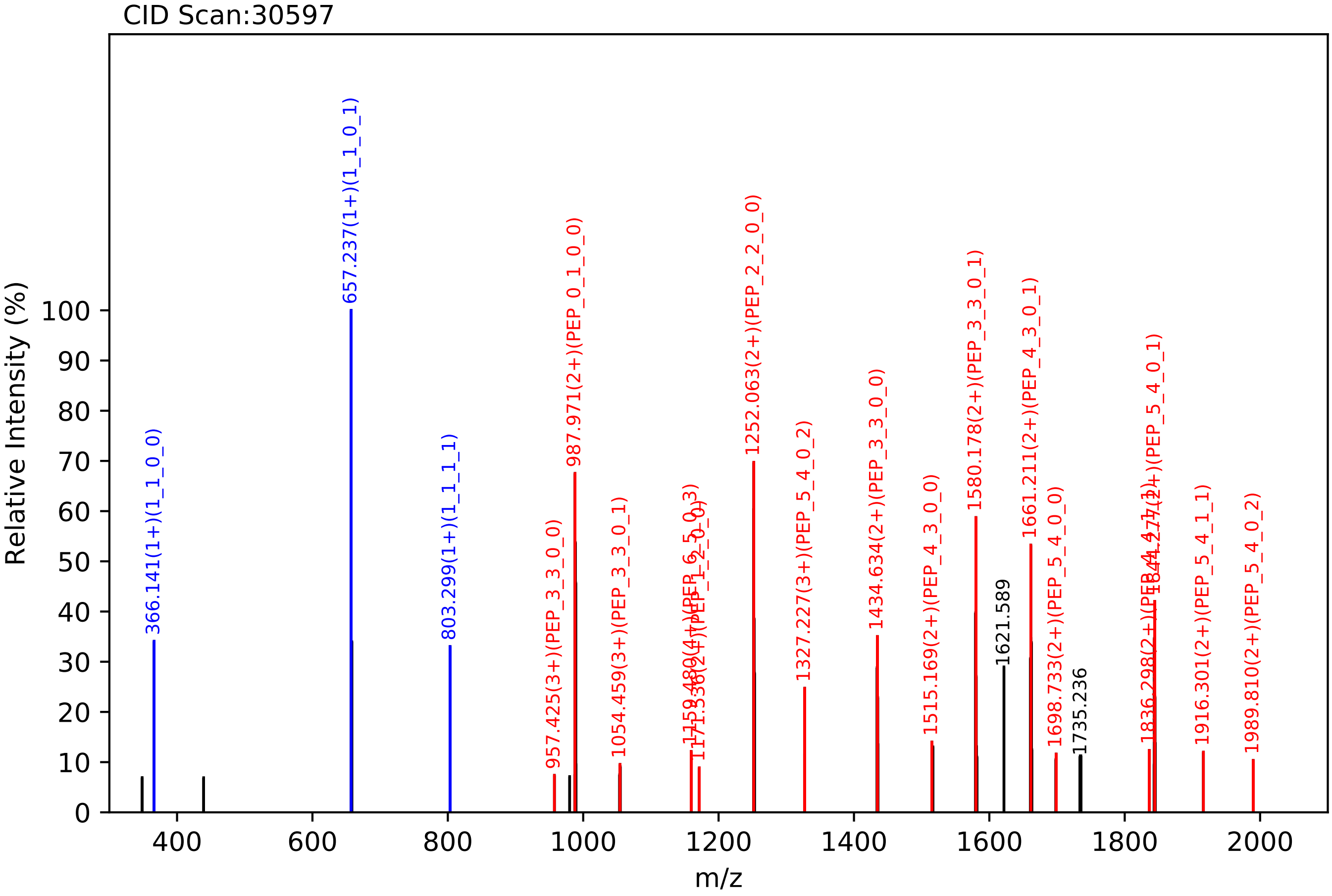

Unknown set no. 148, Experiment: Human Plasma exp_5

VPVTLALNNTLFLIEER(=PEP)_4_4_0_1, m/z:924.18(4+), RT:104.10, Y-score:77.10

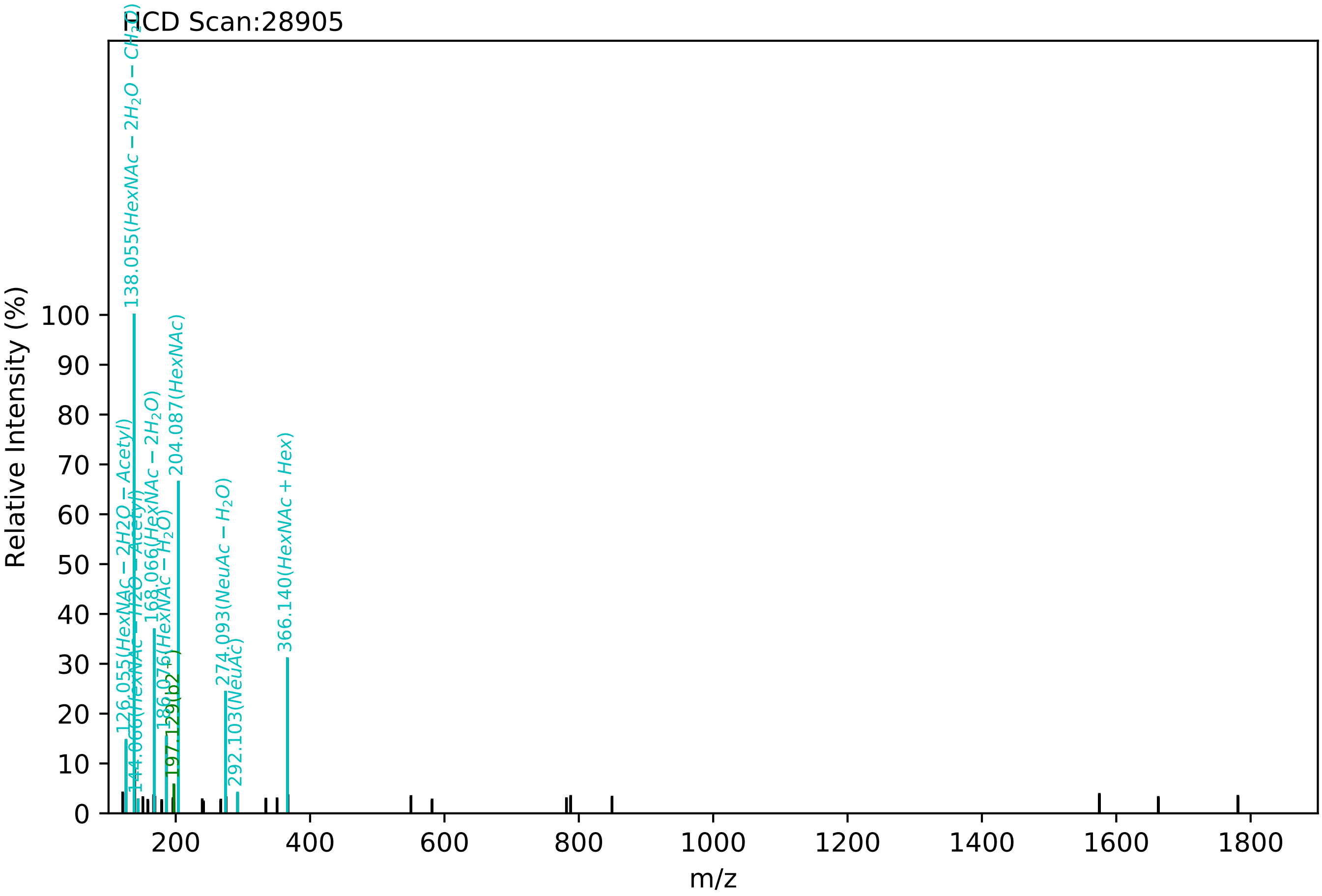

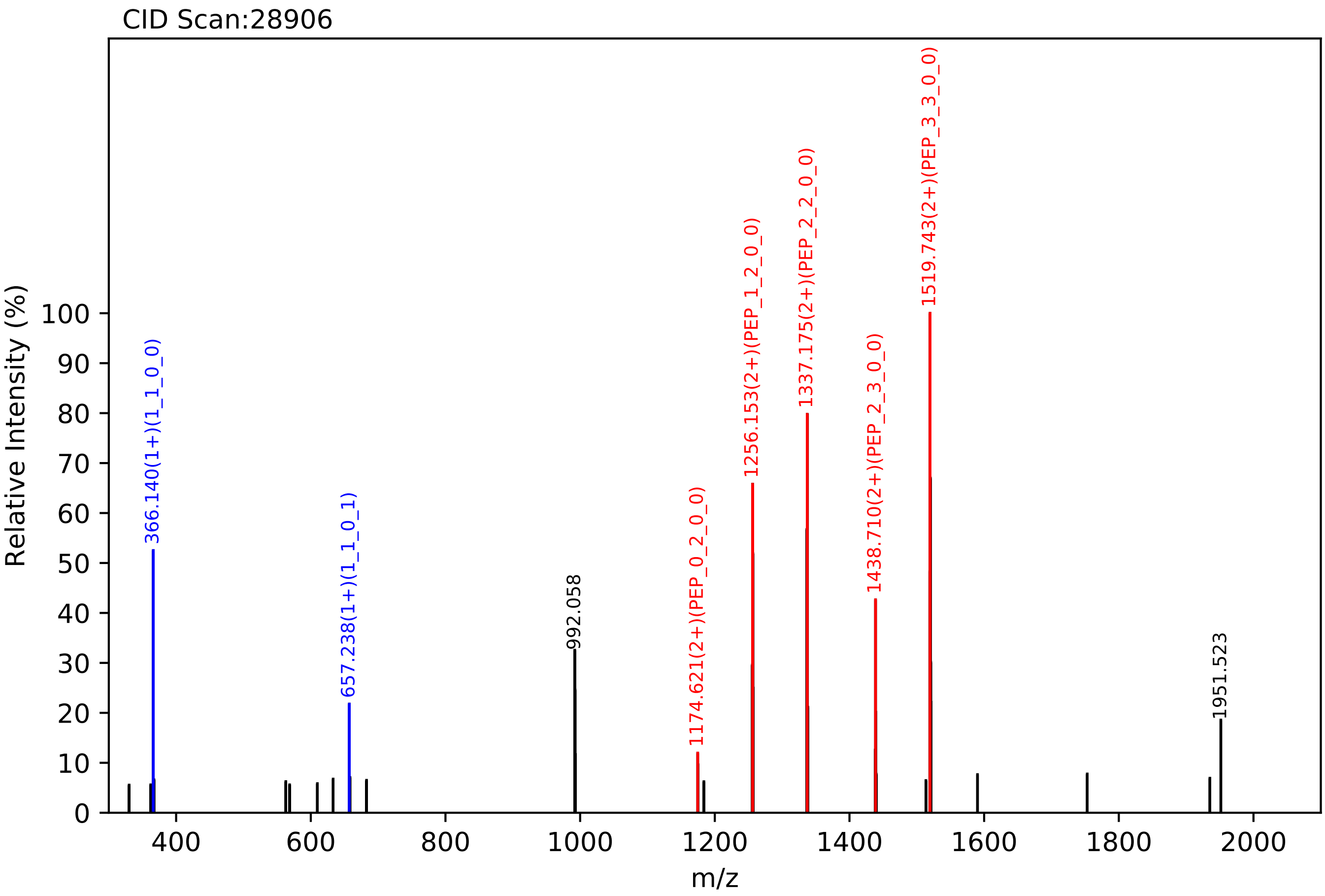

Unknown set no. 149, Experiment: Human Plasma exp_1

NCTSR(=PEP)_5_4_0_2, m/z:948.02(3+), RT:11.83, Y-score:83.90

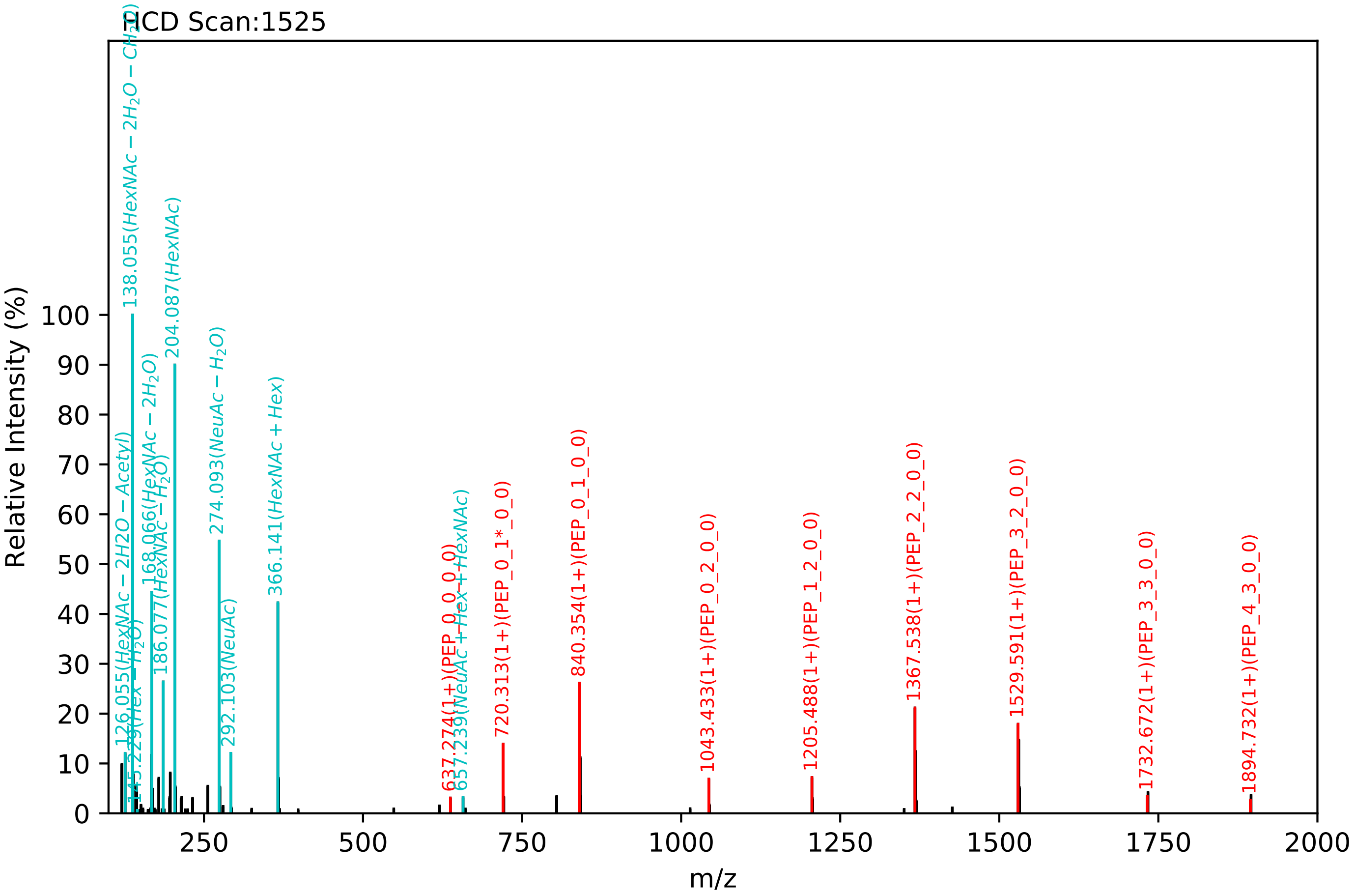

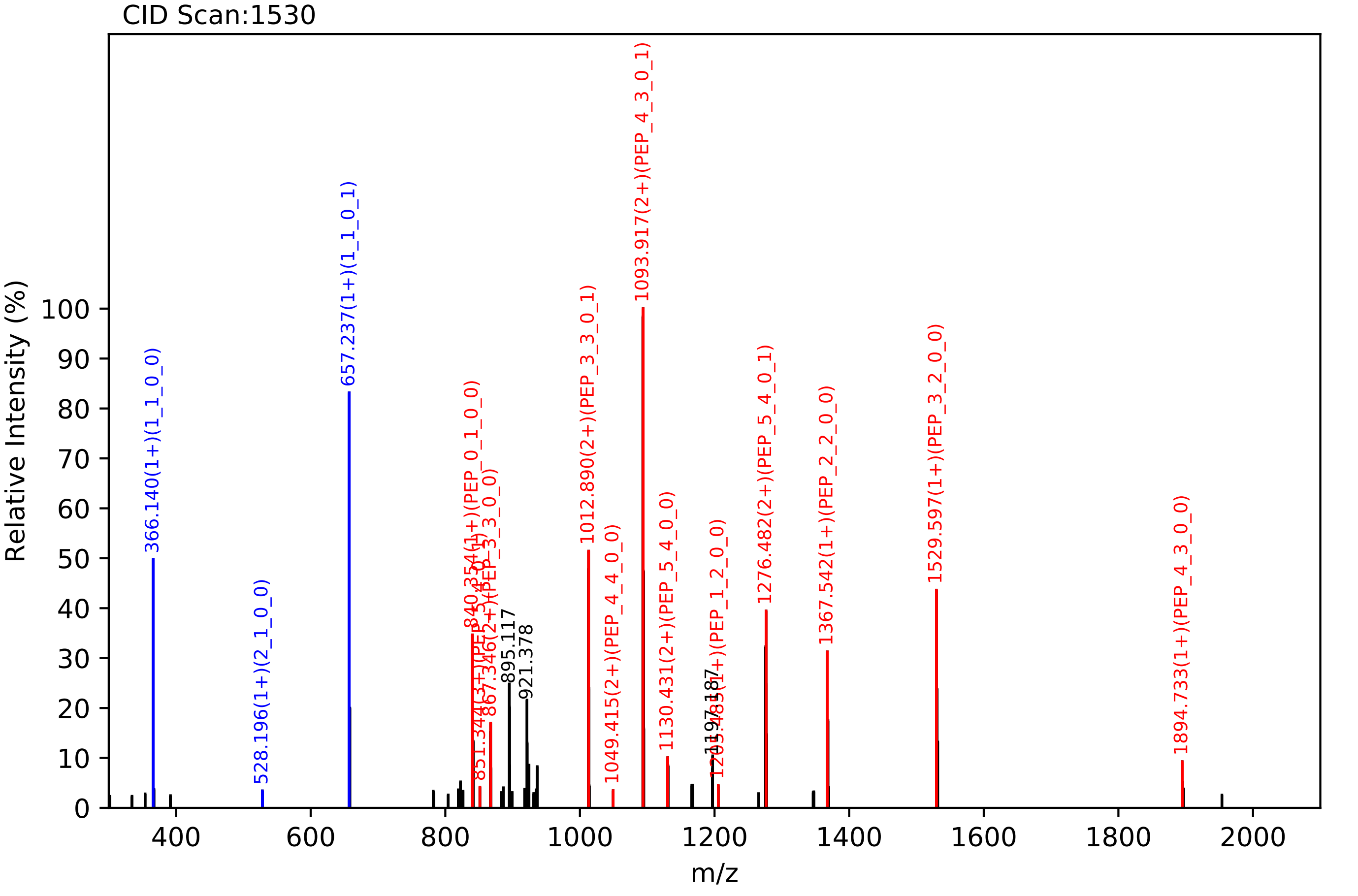

Unknown set no. 150, Experiment: Human Plasma exp_2

NCTSR(=PEP)_5_4_0_2, m/z:948.02(3+), RT:11.84, Y-score:91.12

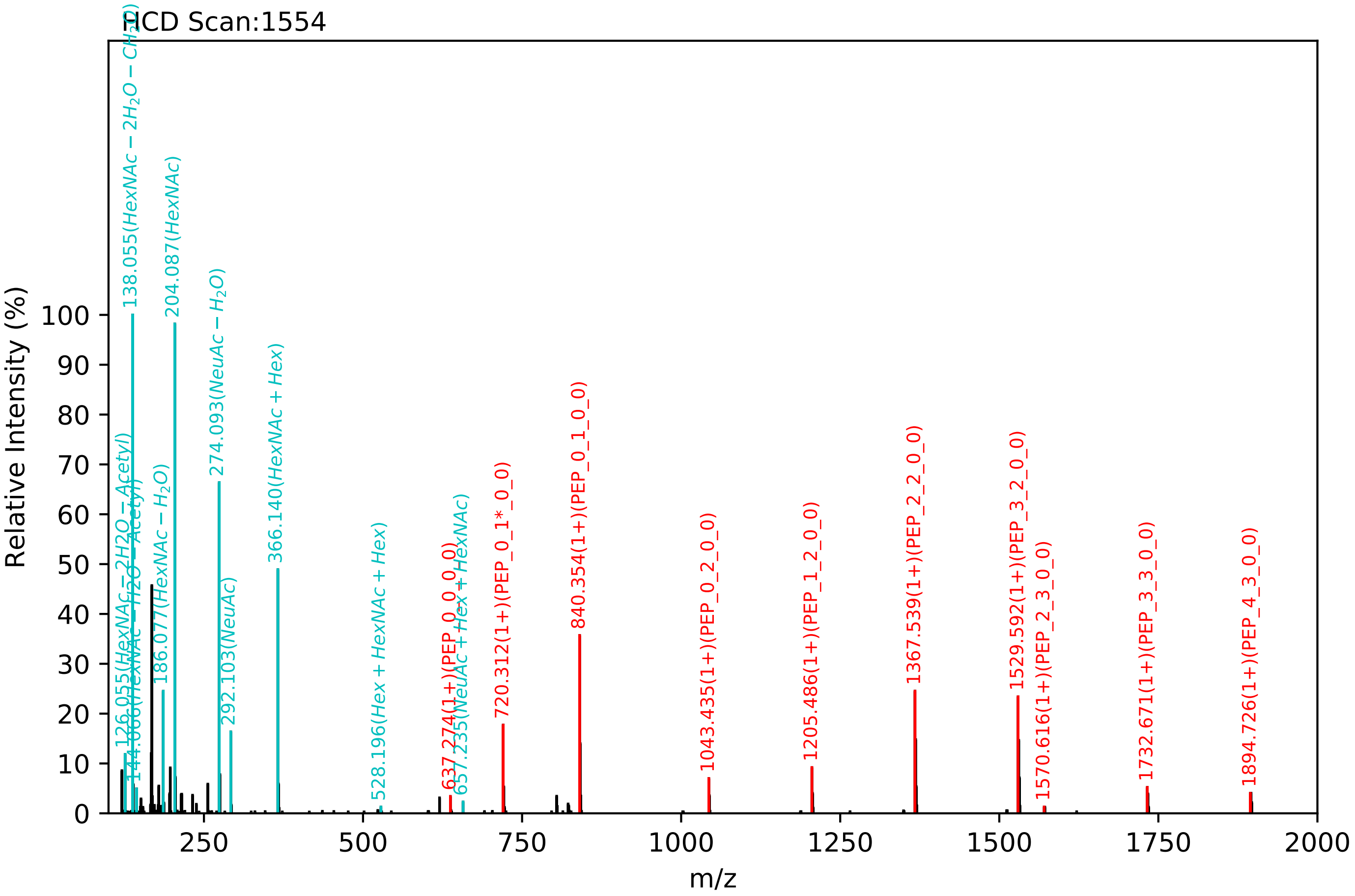

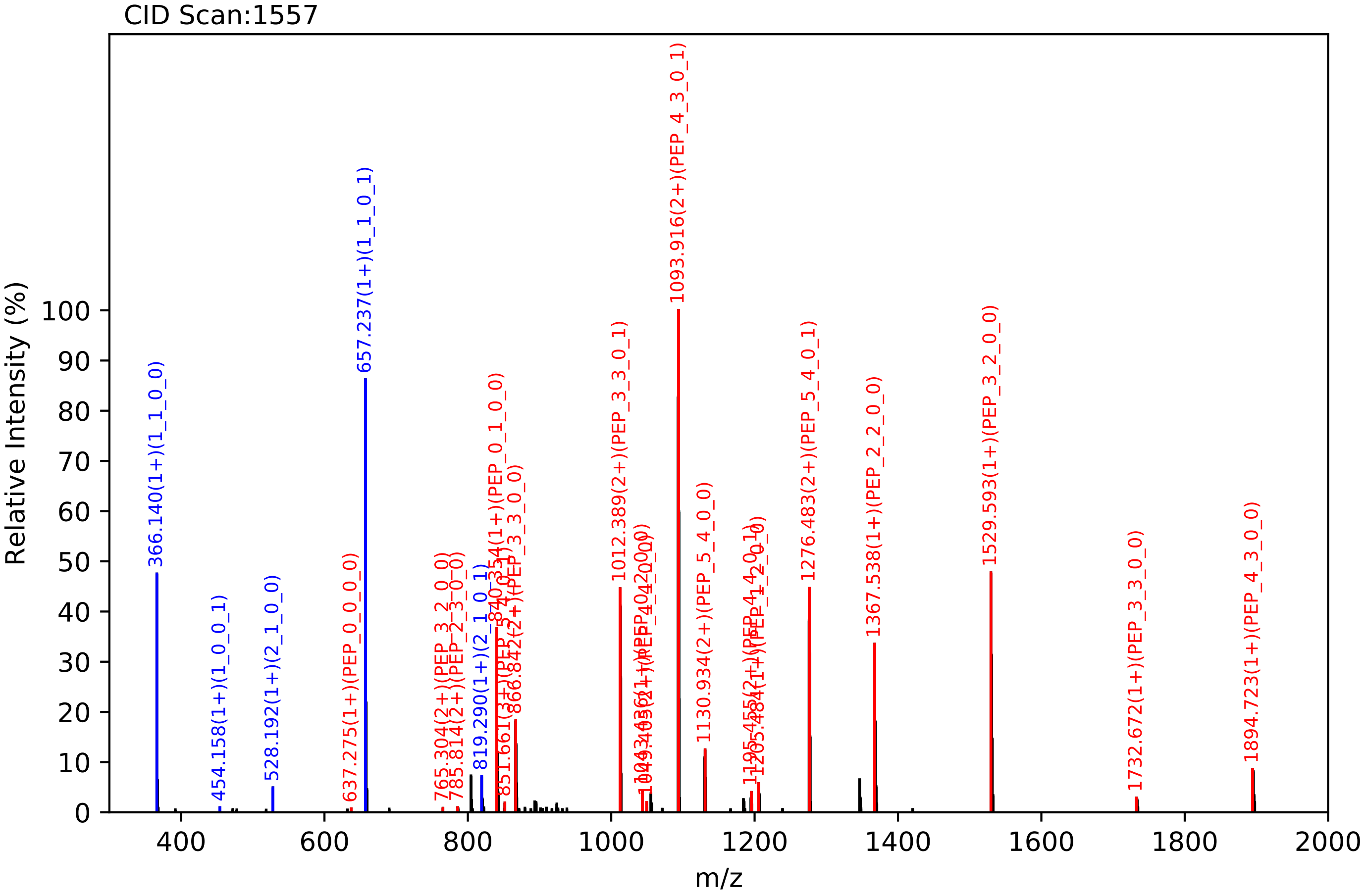

Unknown set no. 151, Experiment: Human Plasma exp_3

NCTSR(=PEP)_5_4_0_2, m/z:948.02(3+), RT:11.87, Y-score:90.66

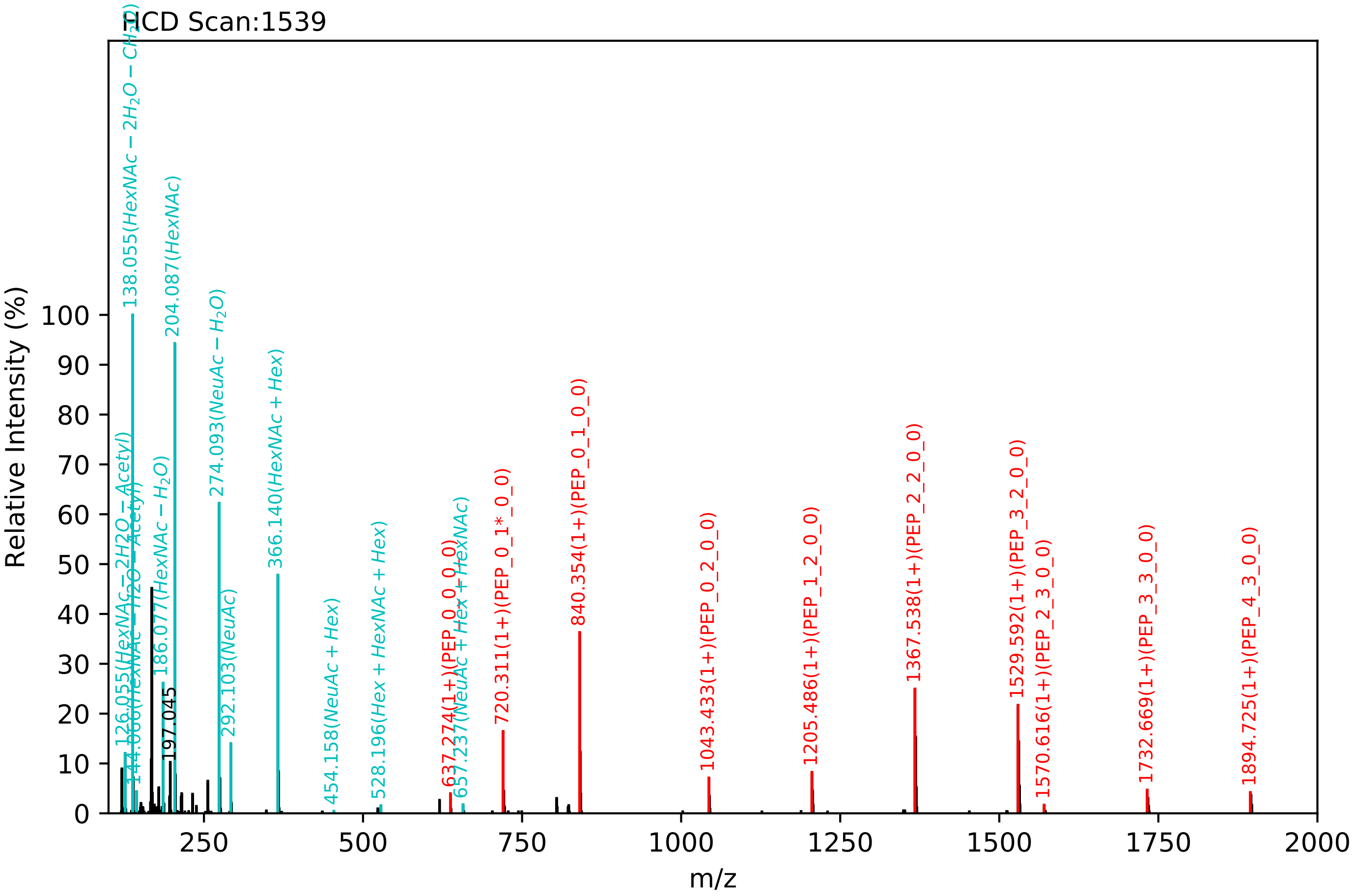

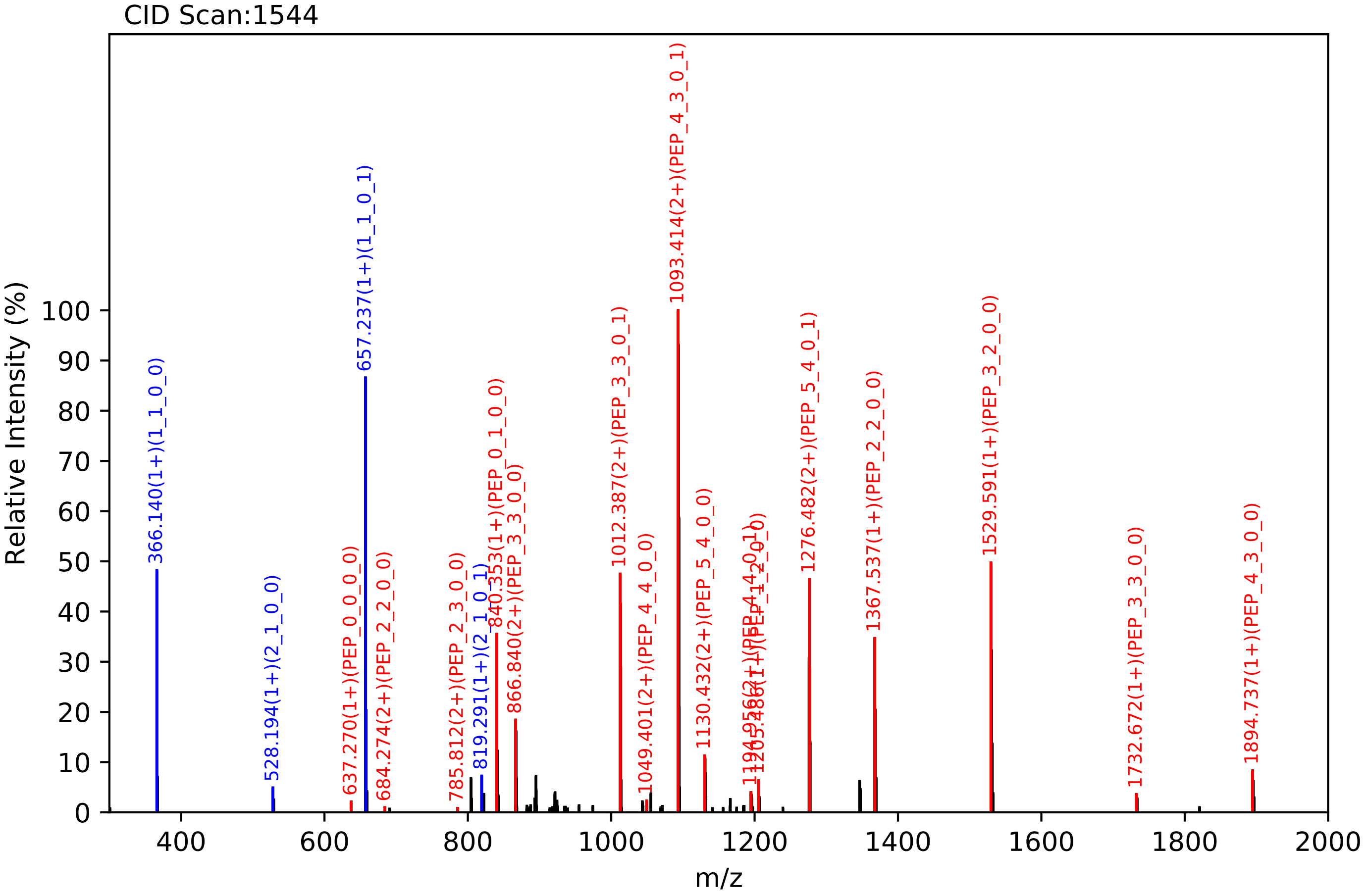

Unknown set no. 152, Experiment: Human Plasma exp_4

NCTSR(=PEP)_5_4_0_2, m/z:948.02(3+), RT:11.95, Y-score:91.49

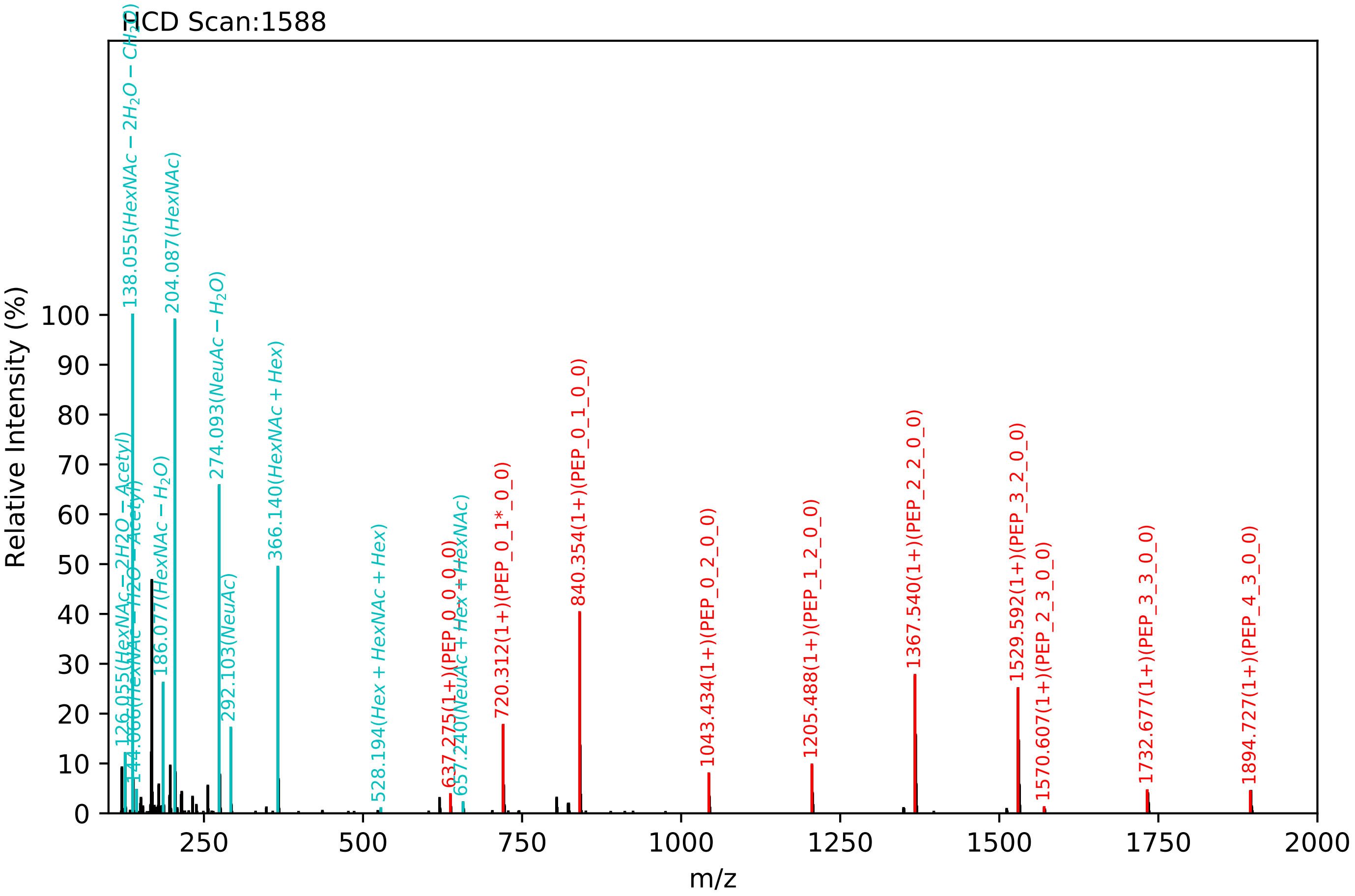

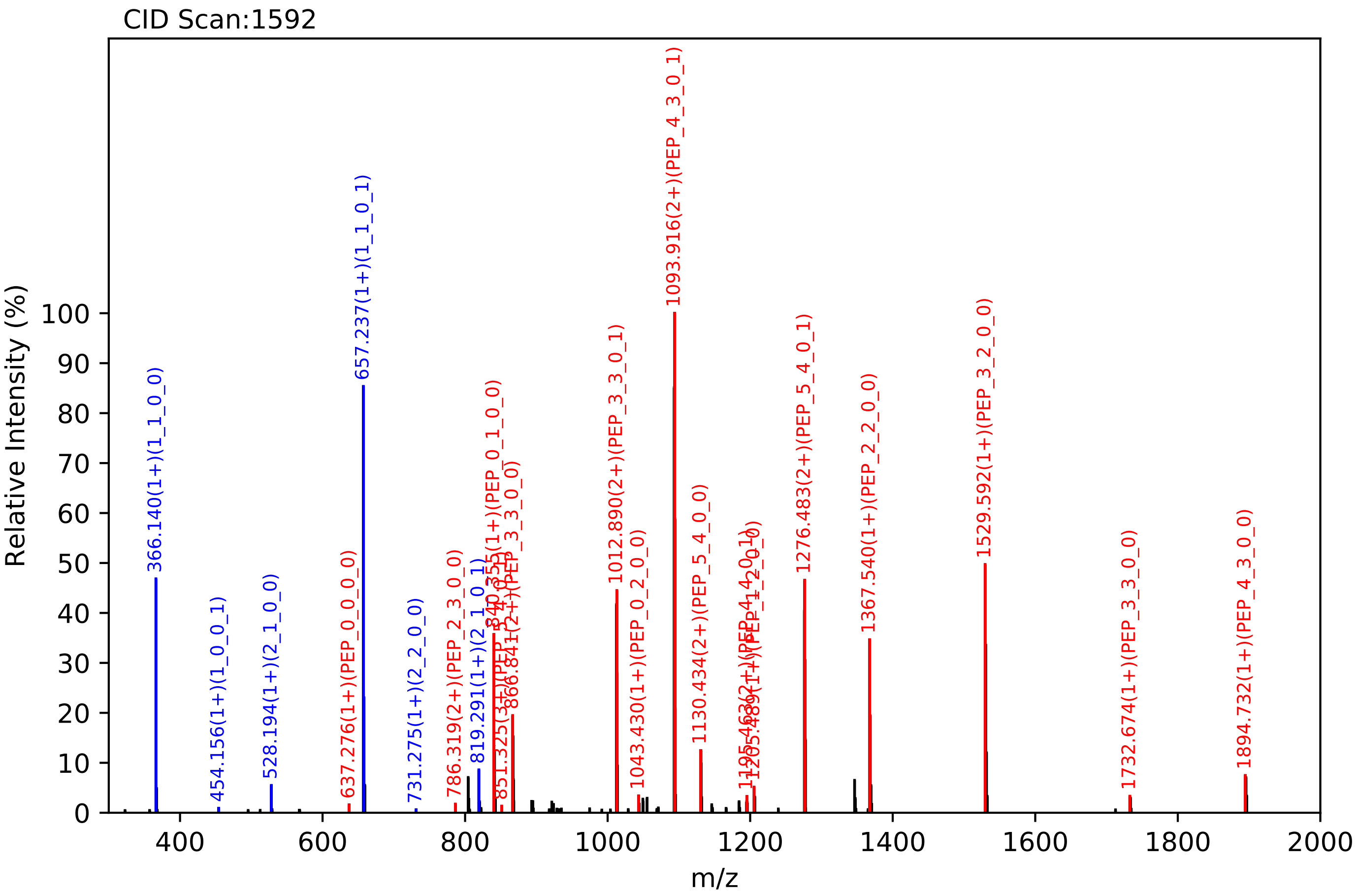

Unknown set no. 153, Experiment: Human Plasma exp_5

NCTSR(=PEP)_5_4_0_2, m/z:948.02(3+), RT:11.81, Y-score:89.49

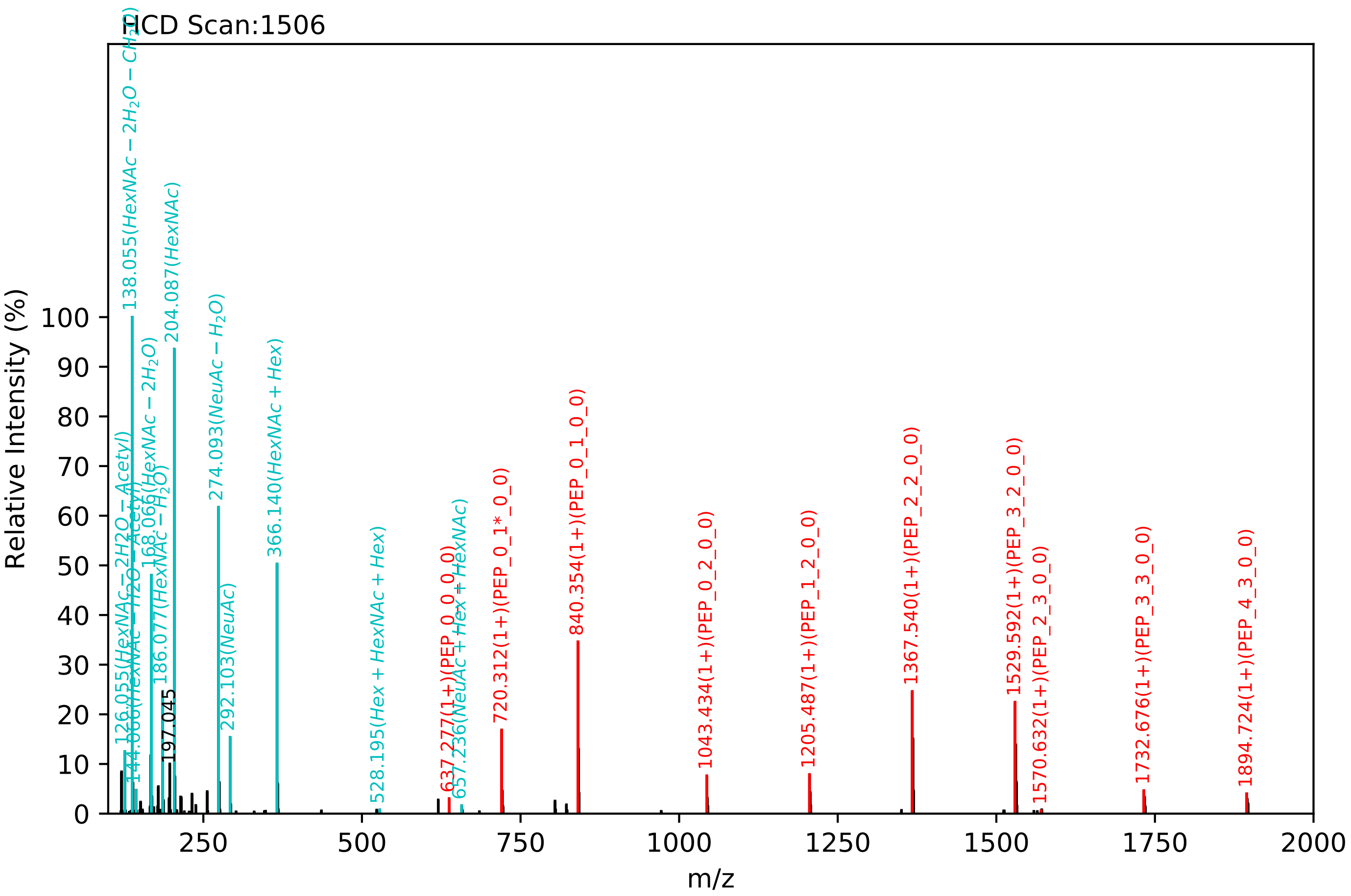

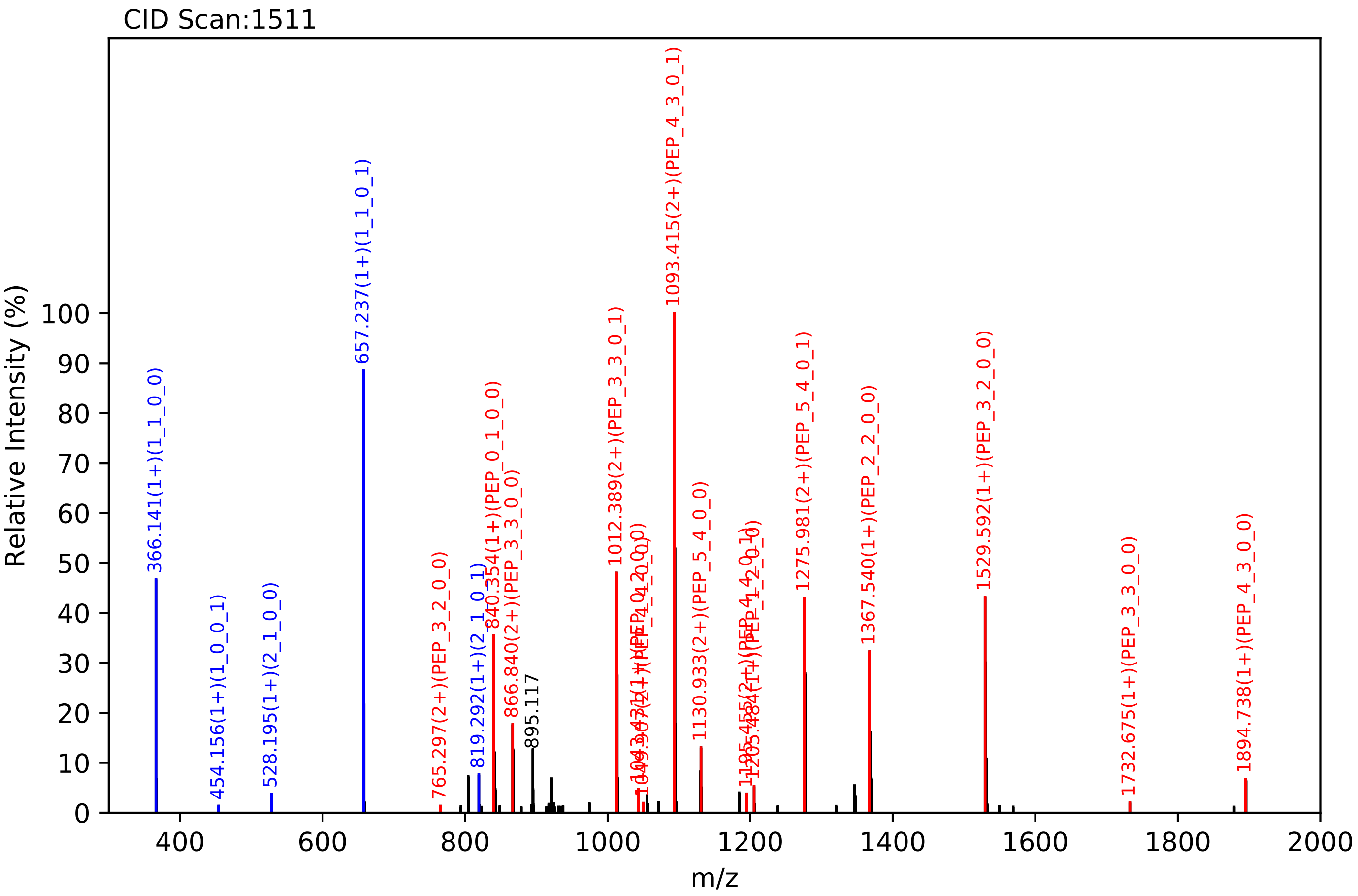

Unknown set no. 154, Experiment: Human Plasma exp_1

NCTSR(=PEP)_6_5_0_3, m/z:1166.76(3+), RT:17.25, Y-score:81.70

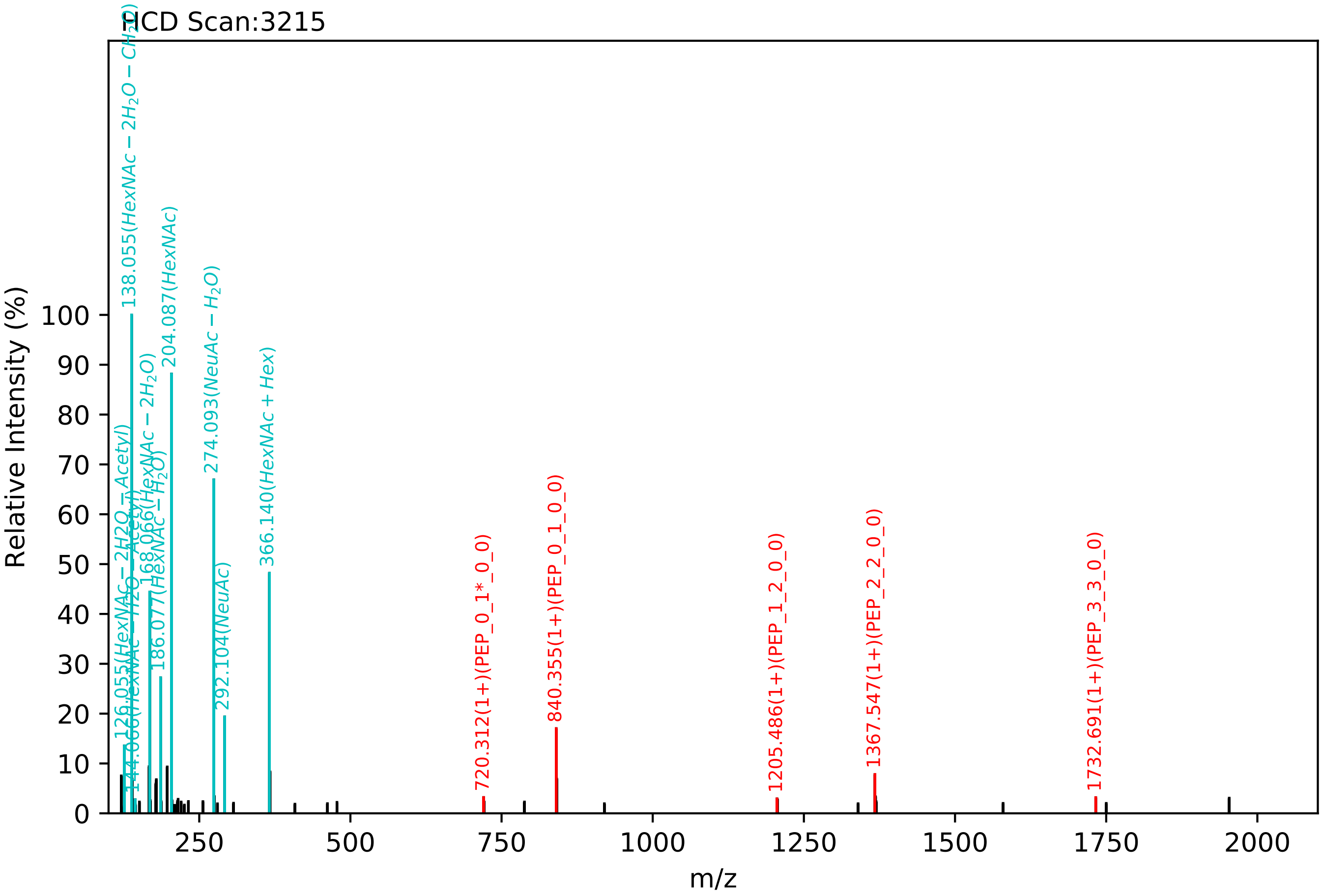

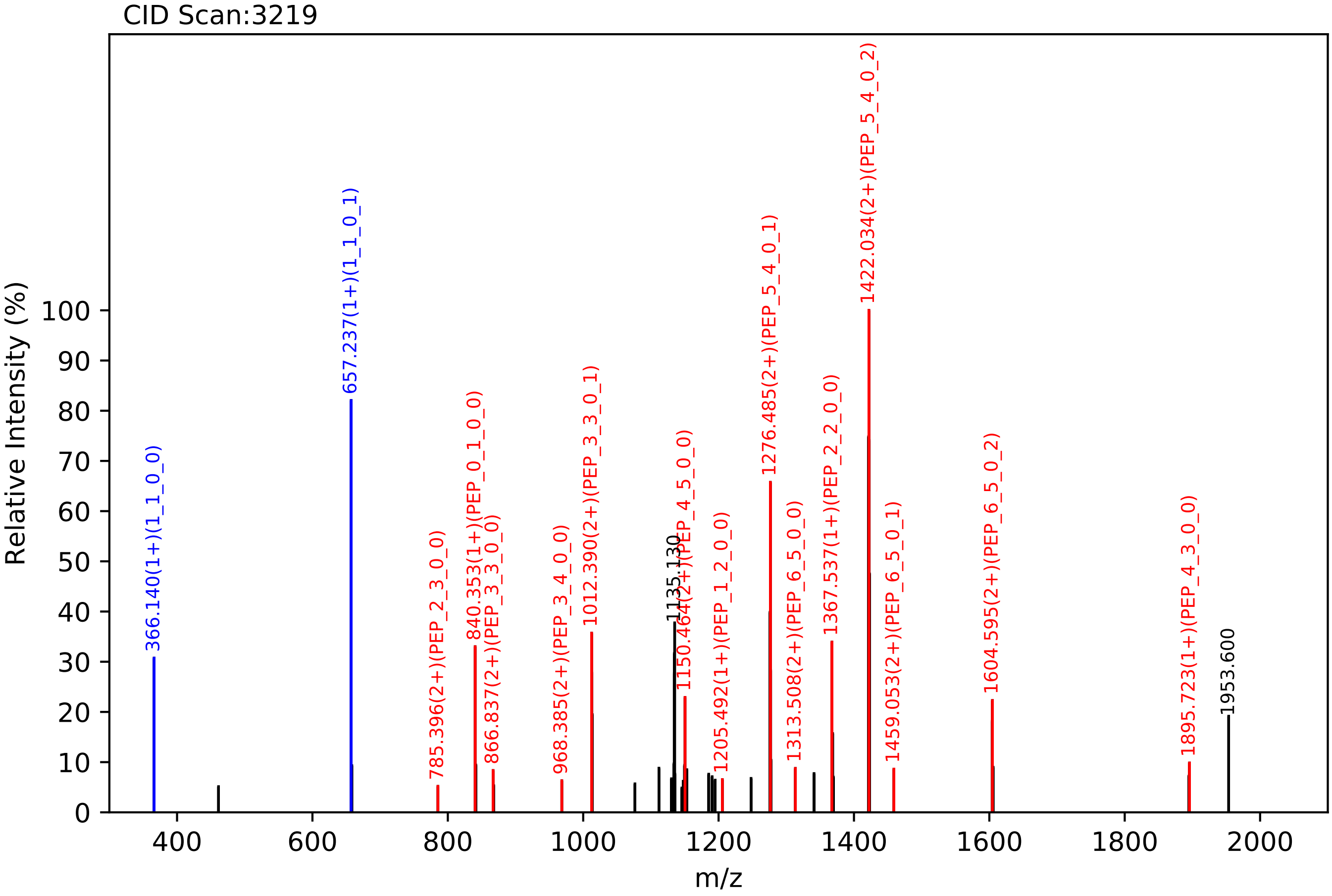

Unknown set no. 155, Experiment: Human Plasma exp_2

NCTSR(=PEP)_6_5_0_3, m/z:1166.76(3+), RT:17.39, Y-score:84.02

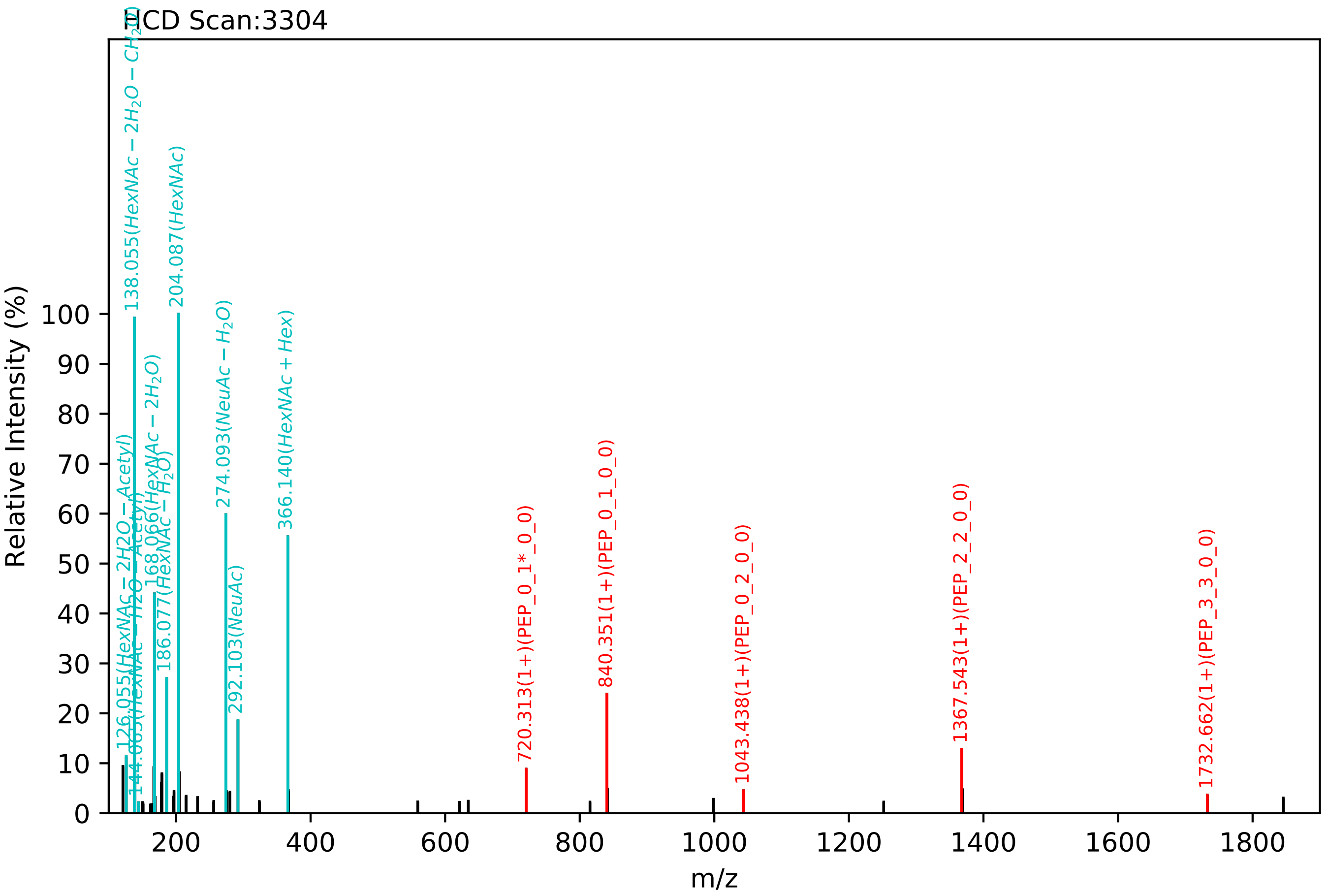

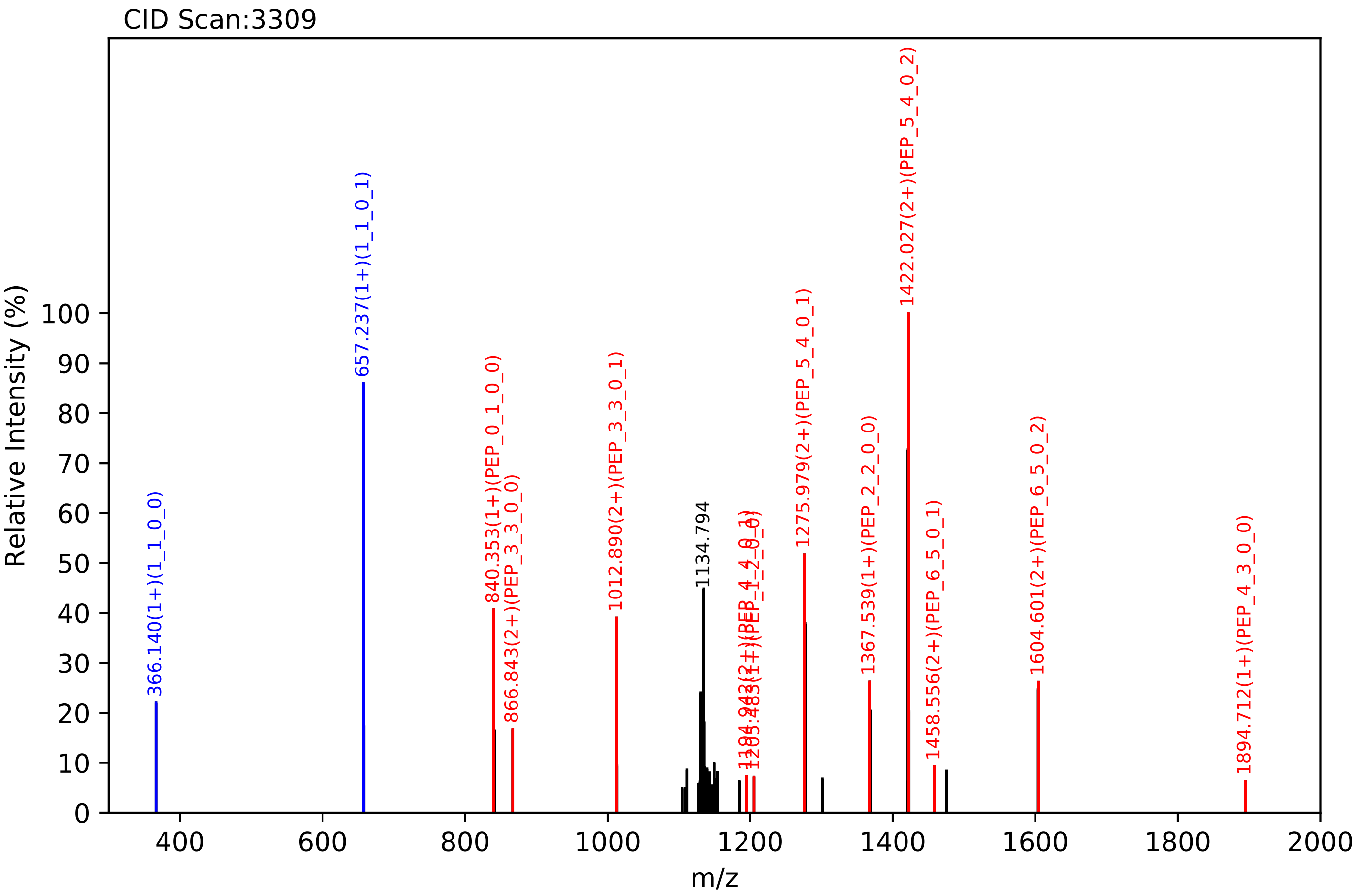

Unknown set no. 156, Experiment: Human Plasma exp_5

NCTSR(=PEP)_6_5_0_3, m/z:1166.76(3+), RT:17.56, Y-score:90.90

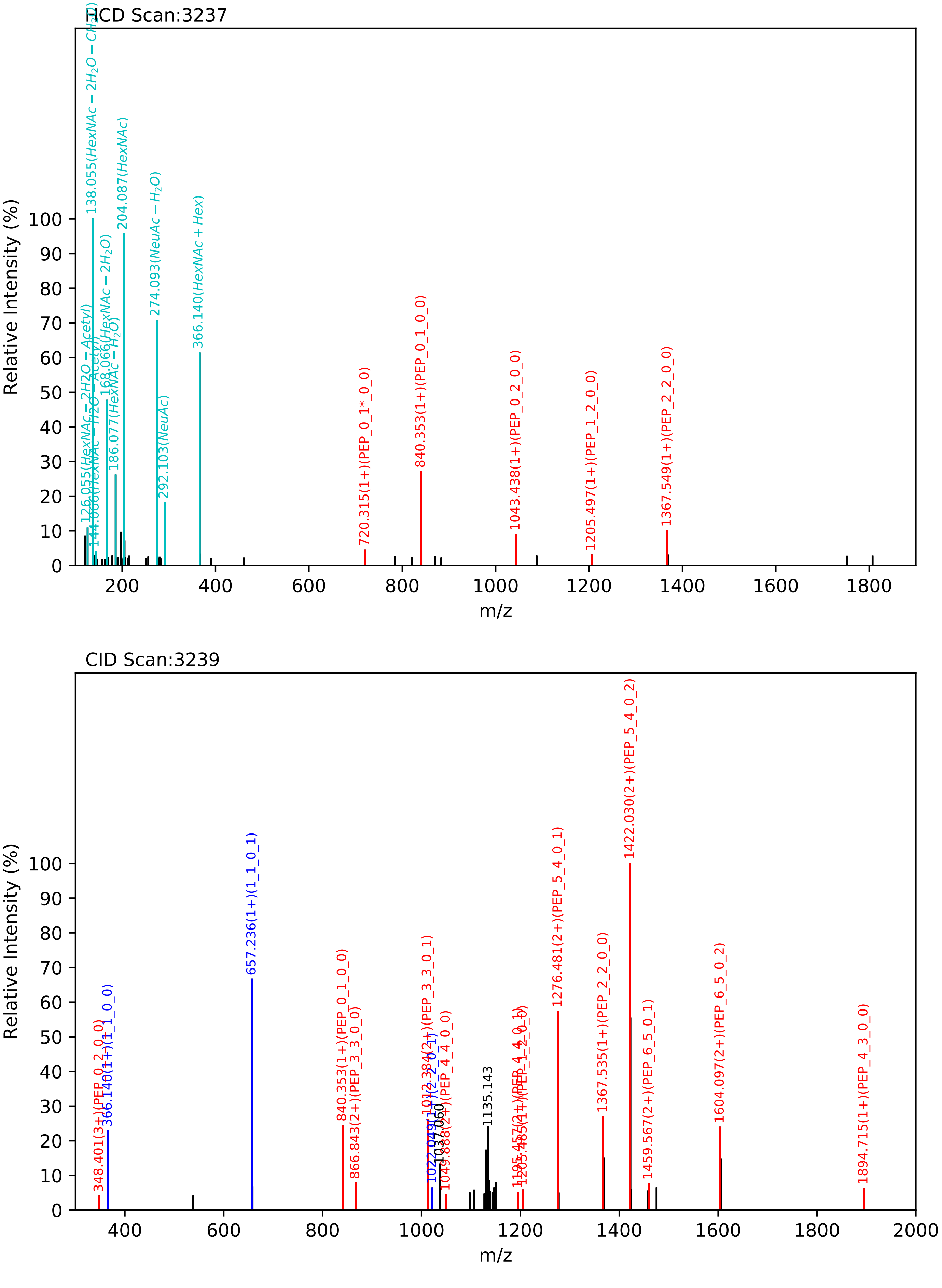

Unknown set no. 157, Experiment: Human Plasma exp_1

ELLETVVNR(=PEP)_5_4_0_2, m/z:1093.13(3+), RT:84.93, Y-score:79.67

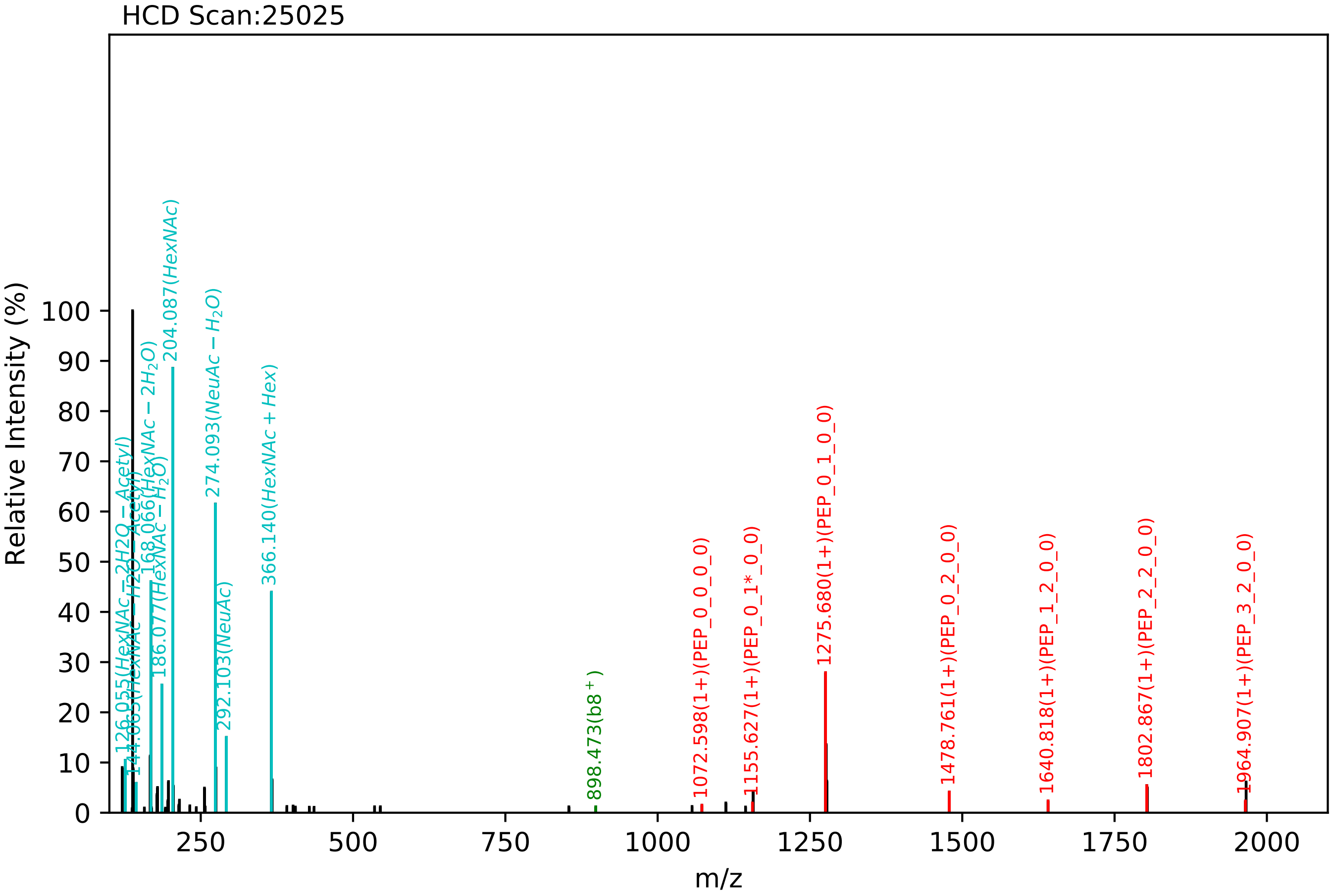

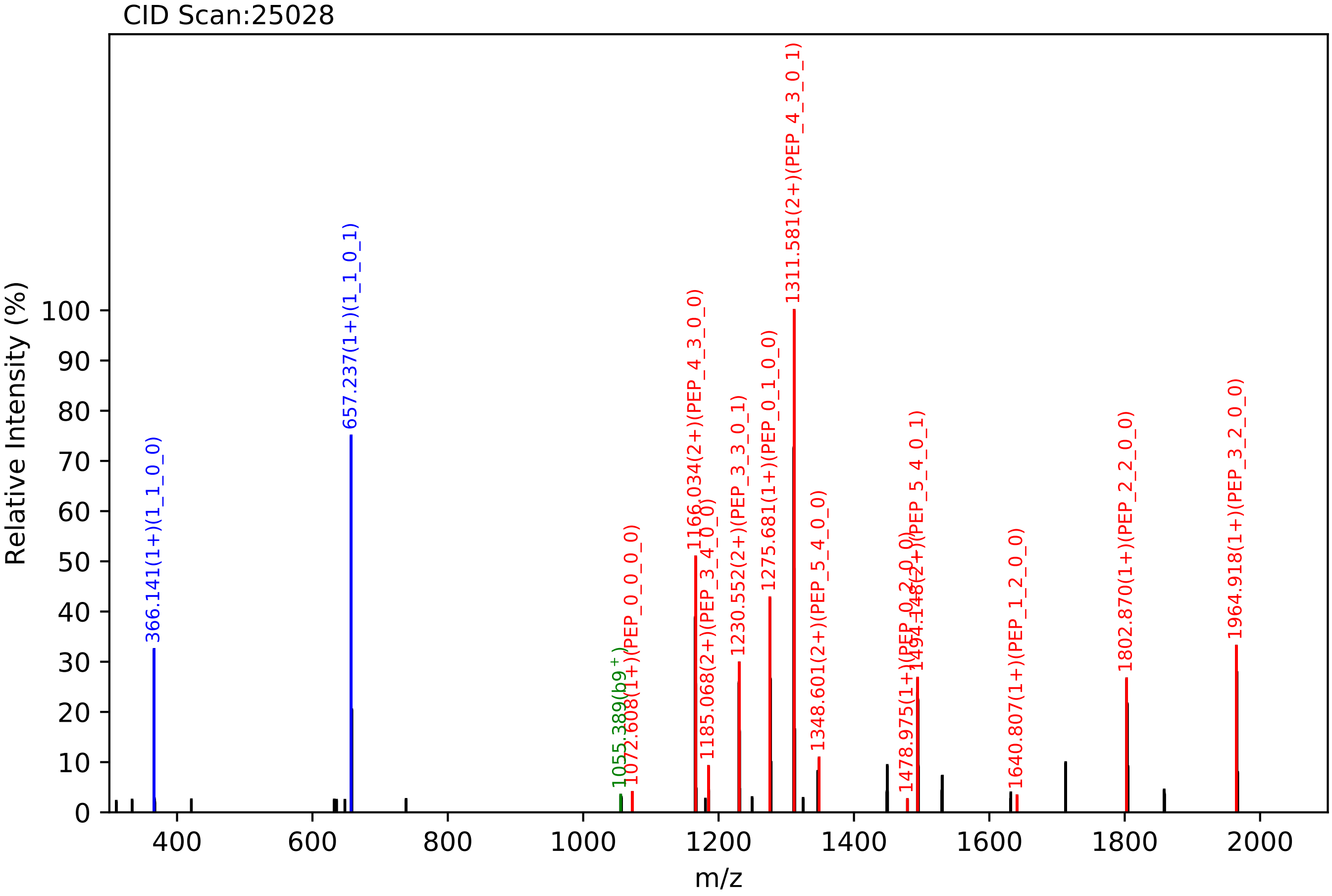

Unknown set no. 158, Experiment: Human Plasma exp_5

ELLETVVNR(=PEP)_5_4_0_2, m/z:1093.13(3+), RT:84.91, Y-score:80.63

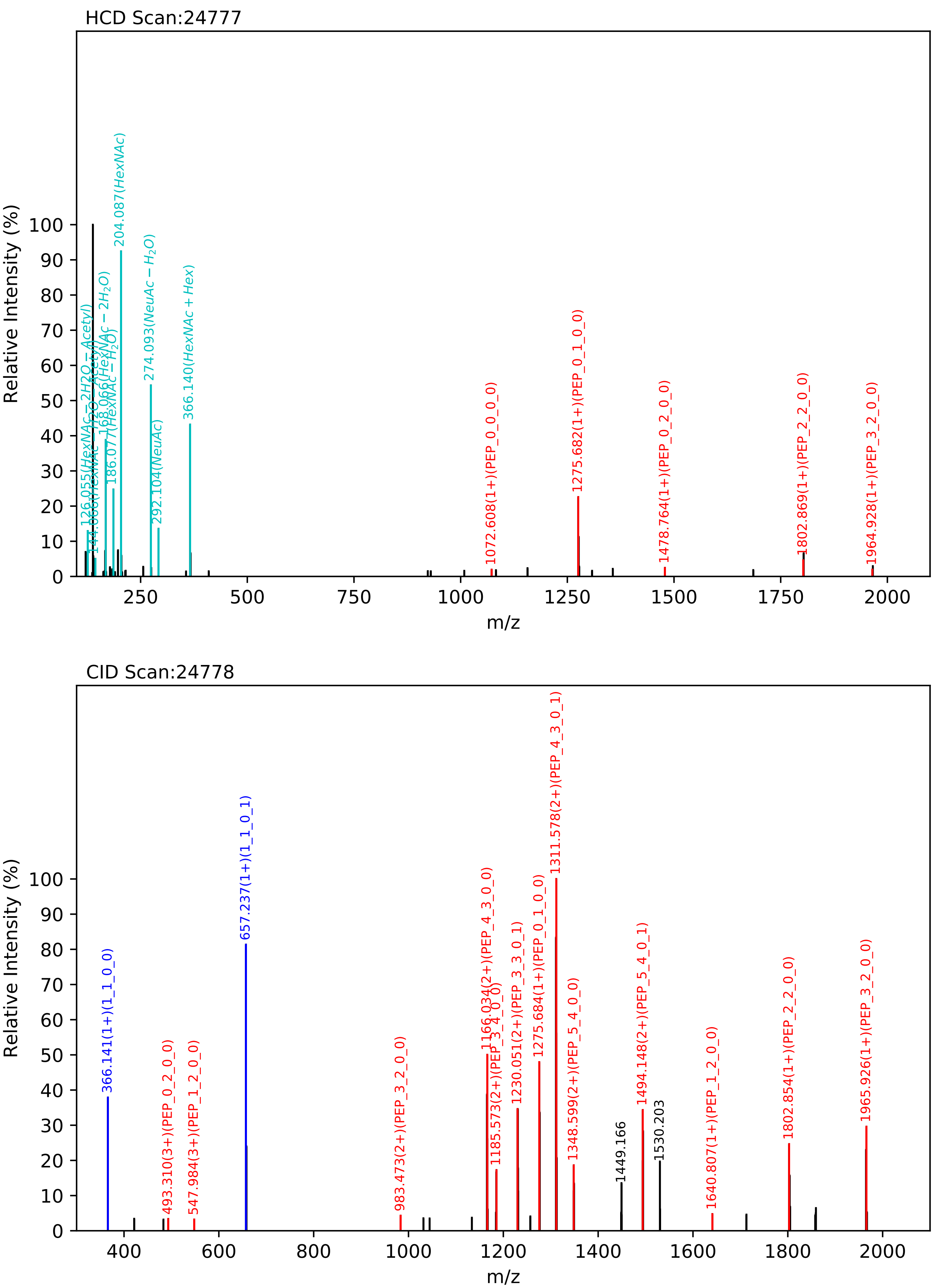

Unknown set no. 159, Experiment: Human Plasma exp_5

ADGTVNQIEGEATPVNLTEPAK(=PEP)_5_4_0_1, m/z:1042.71(4+), RT:84.73, Y-score:83.65

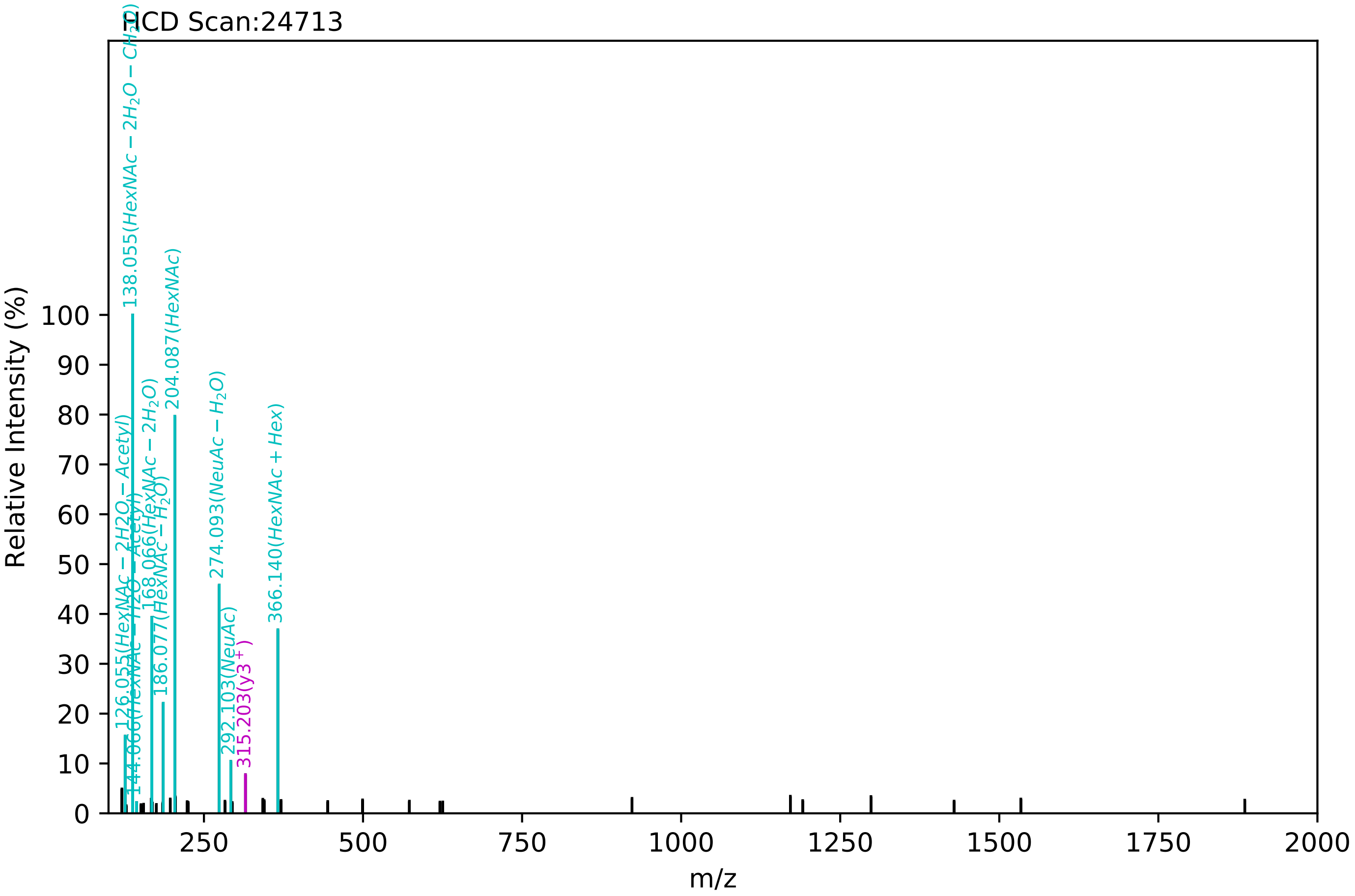

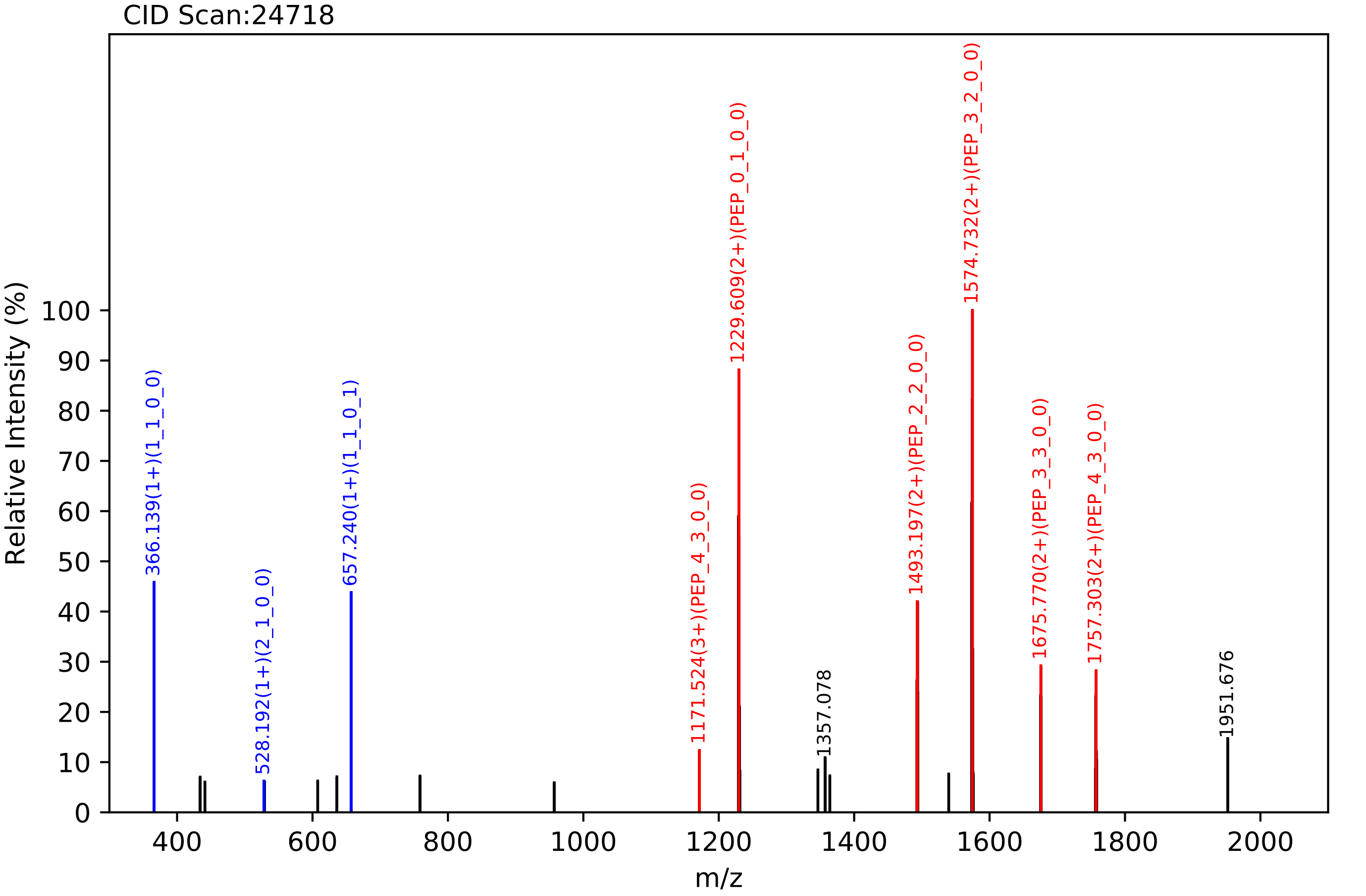

Unknown set no. 160, Experiment: Human Plasma exp_2

ADGTVNQIEGEATPVNLTEPAK(=PEP)_5_4_1_1, m/z:1079.22(4+), RT:84.31, Y-score:83.33

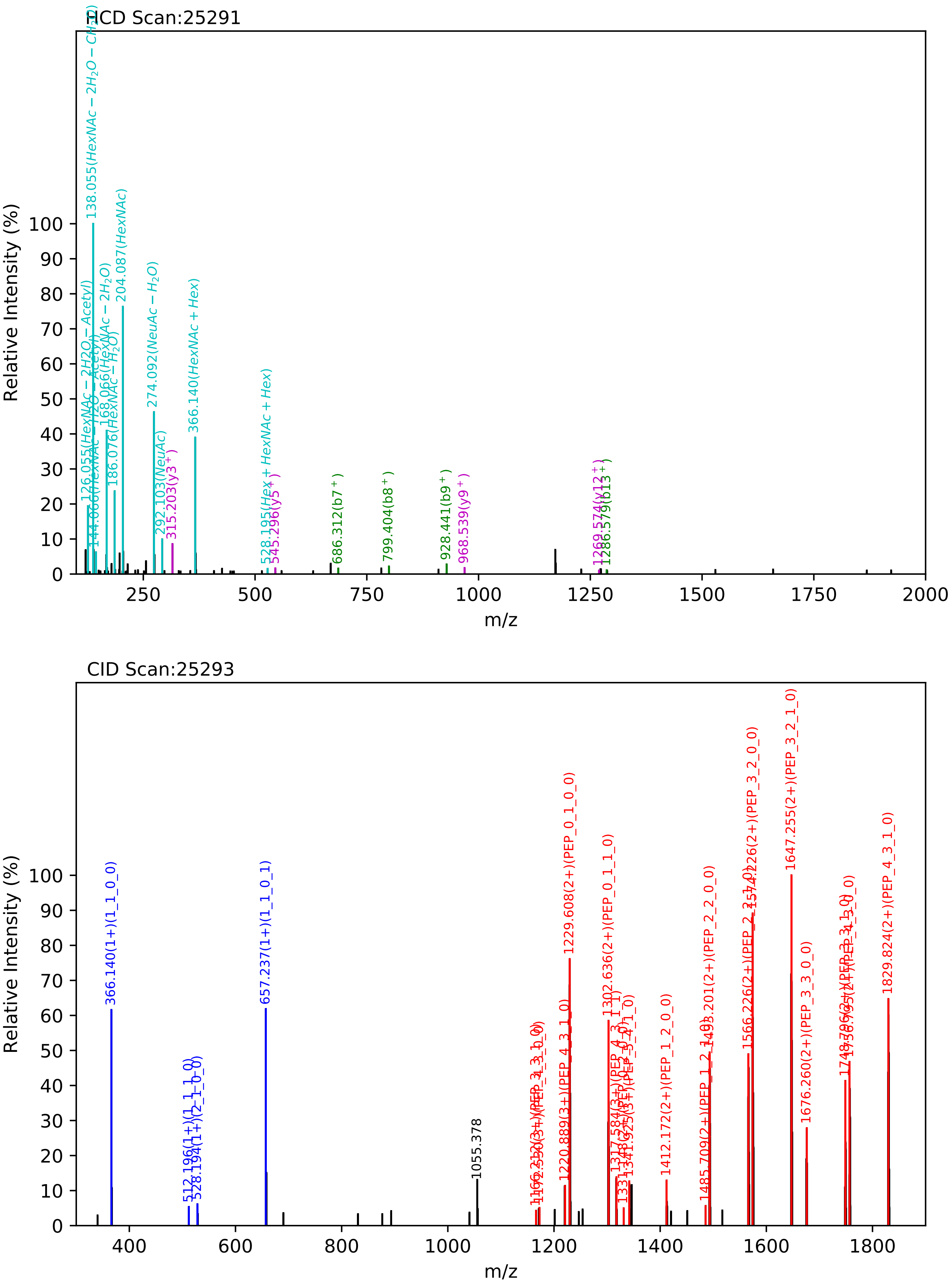

Unknown set no. 161, Experiment: Human Plasma exp_3

ADGTVNQIEGEATPVNLTEPAK(=PEP)_5_4_1_1, m/z:1079.22(4+), RT:84.48, Y-score:85.14

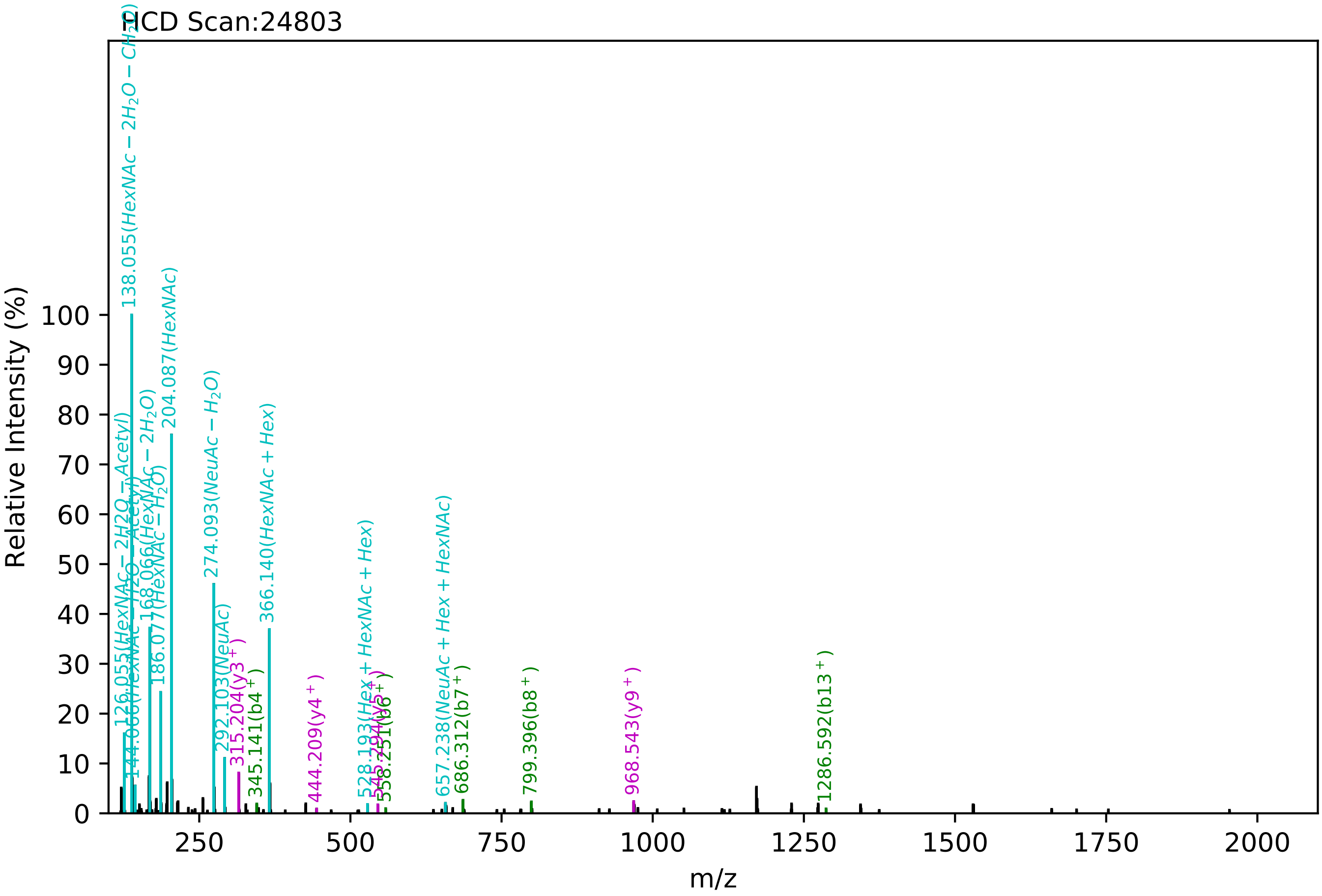

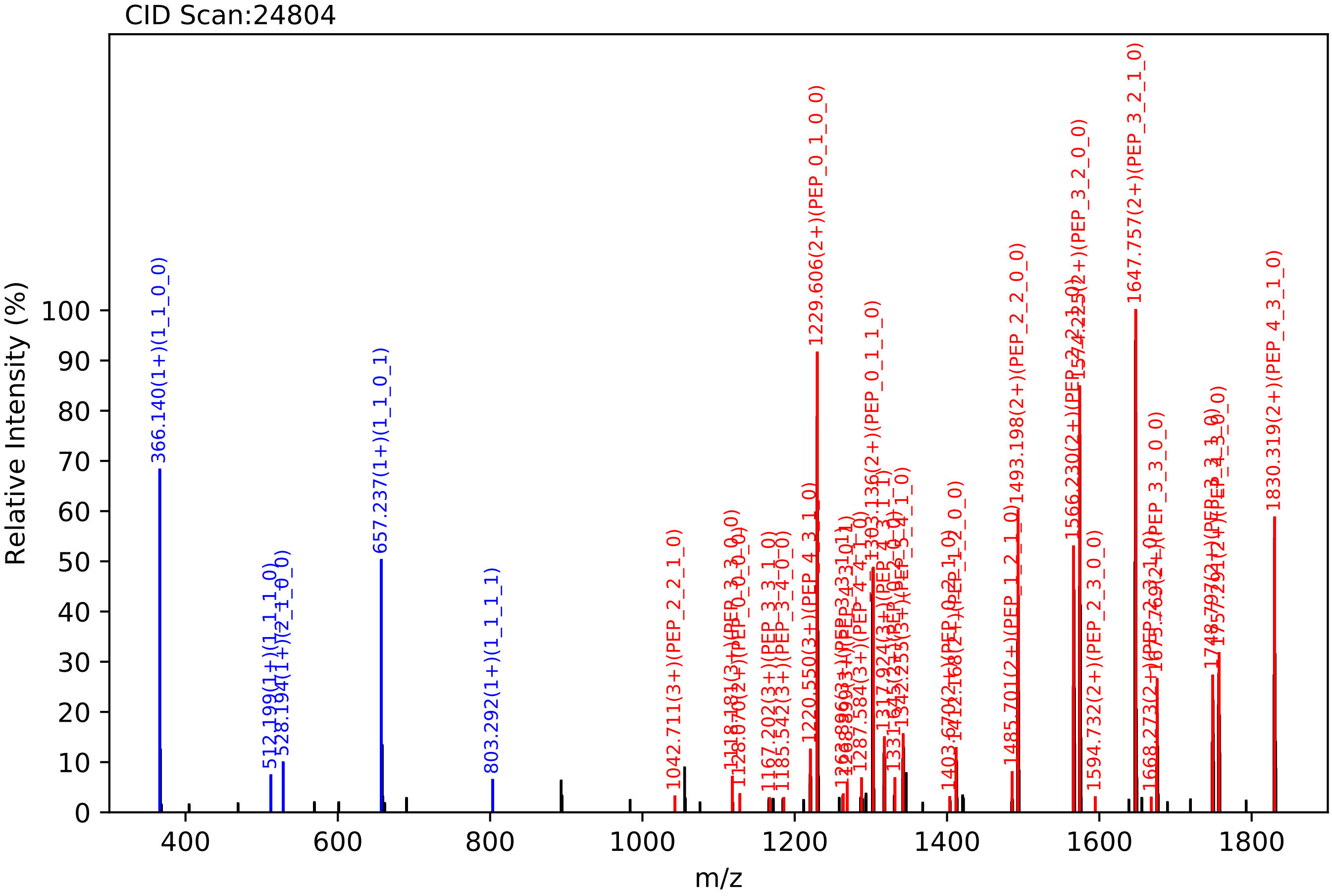

Unknown set no. 162, Experiment: Human Plasma exp_4

ADGTVNQIEGEATPVNLTEPAK(=PEP)_5_4_1_1, m/z:1079.22(4+), RT:84.52, Y-score:88.08

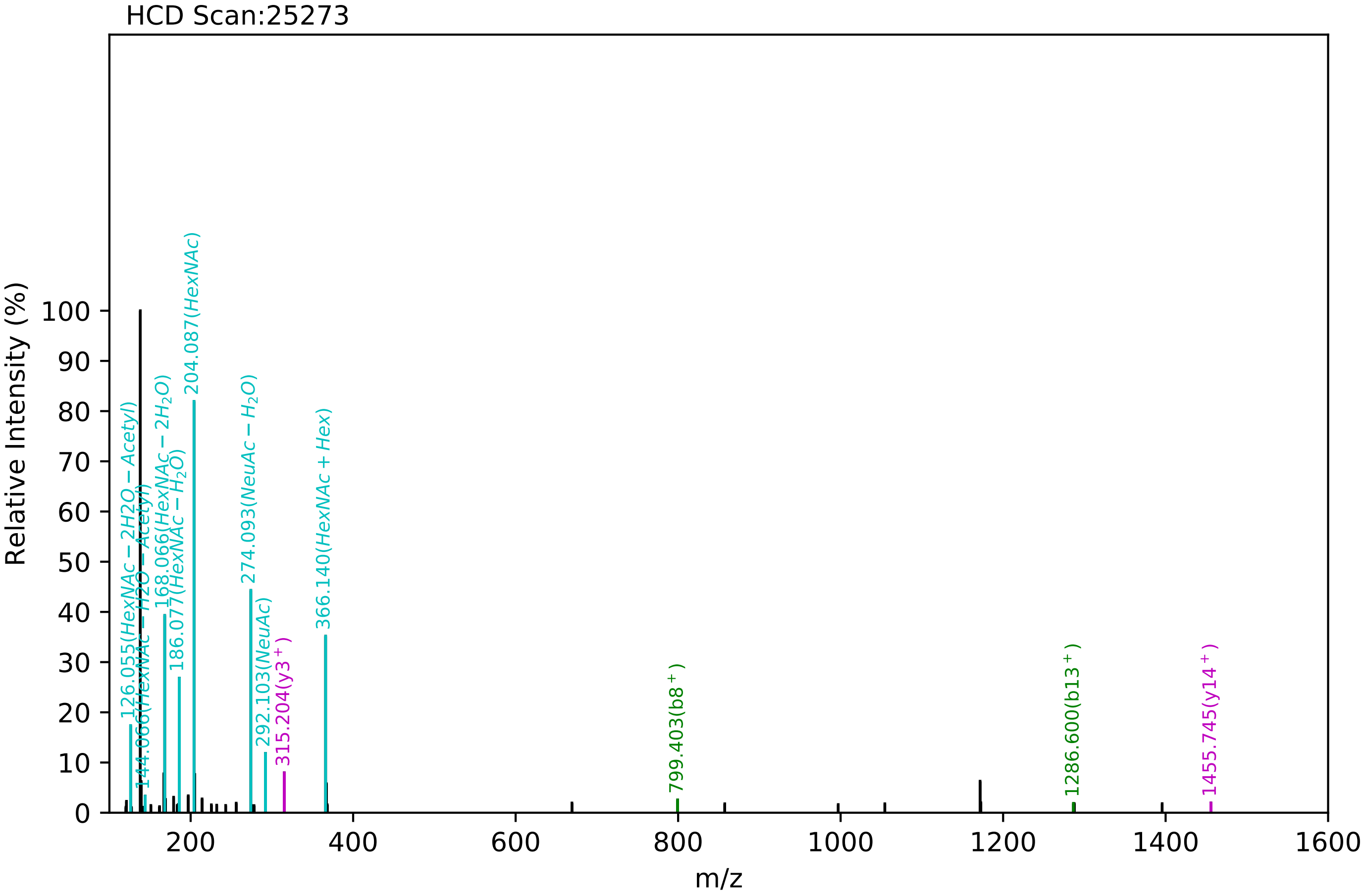

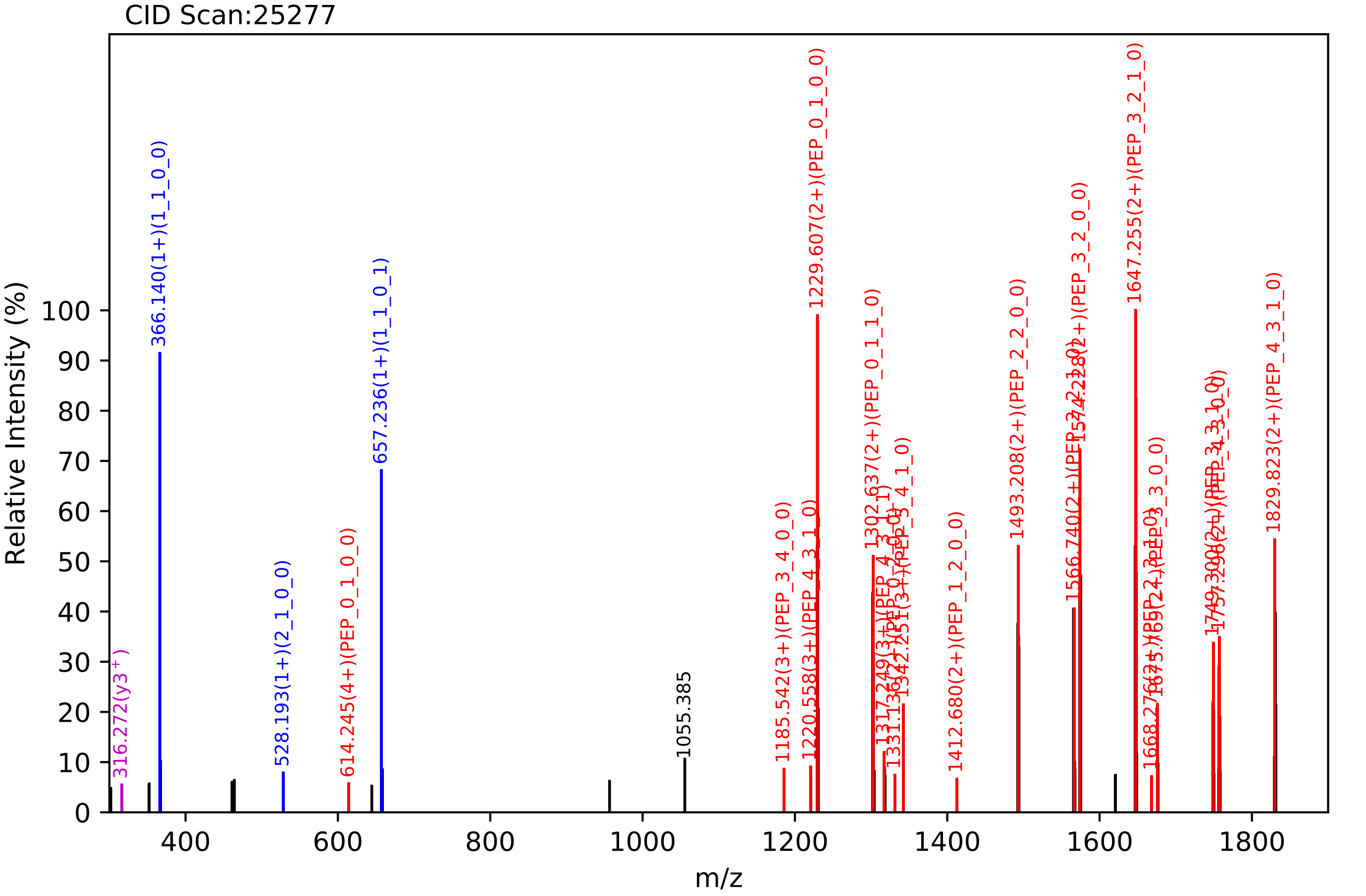

Unknown set no. 163, Experiment: Human Plasma exp_2

ADGTVNQIEGEATPVNLTEPAK(=PEP)_5_4_1_2, m/z:1151.99(4+), RT:93.59, Y-score:79.44

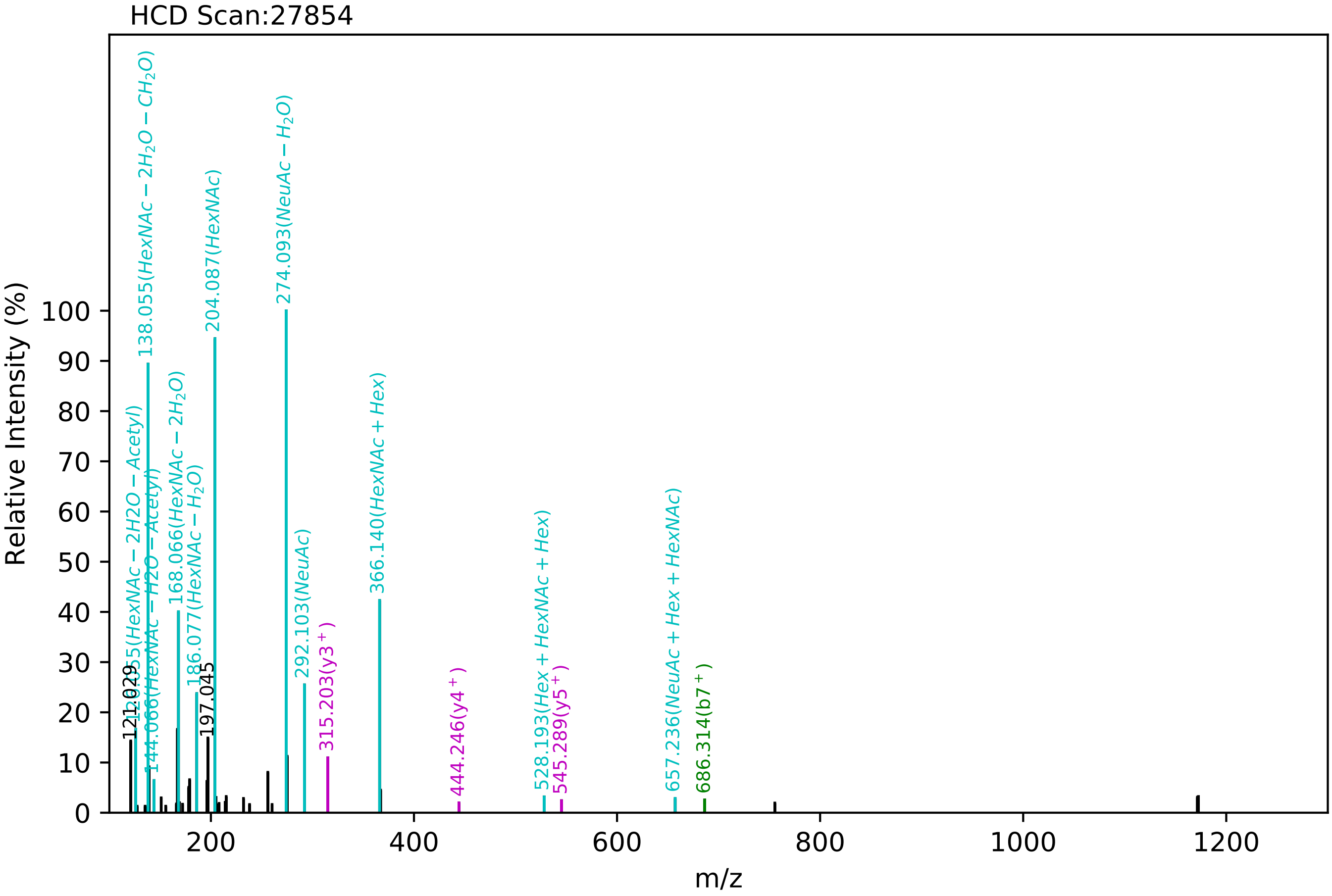

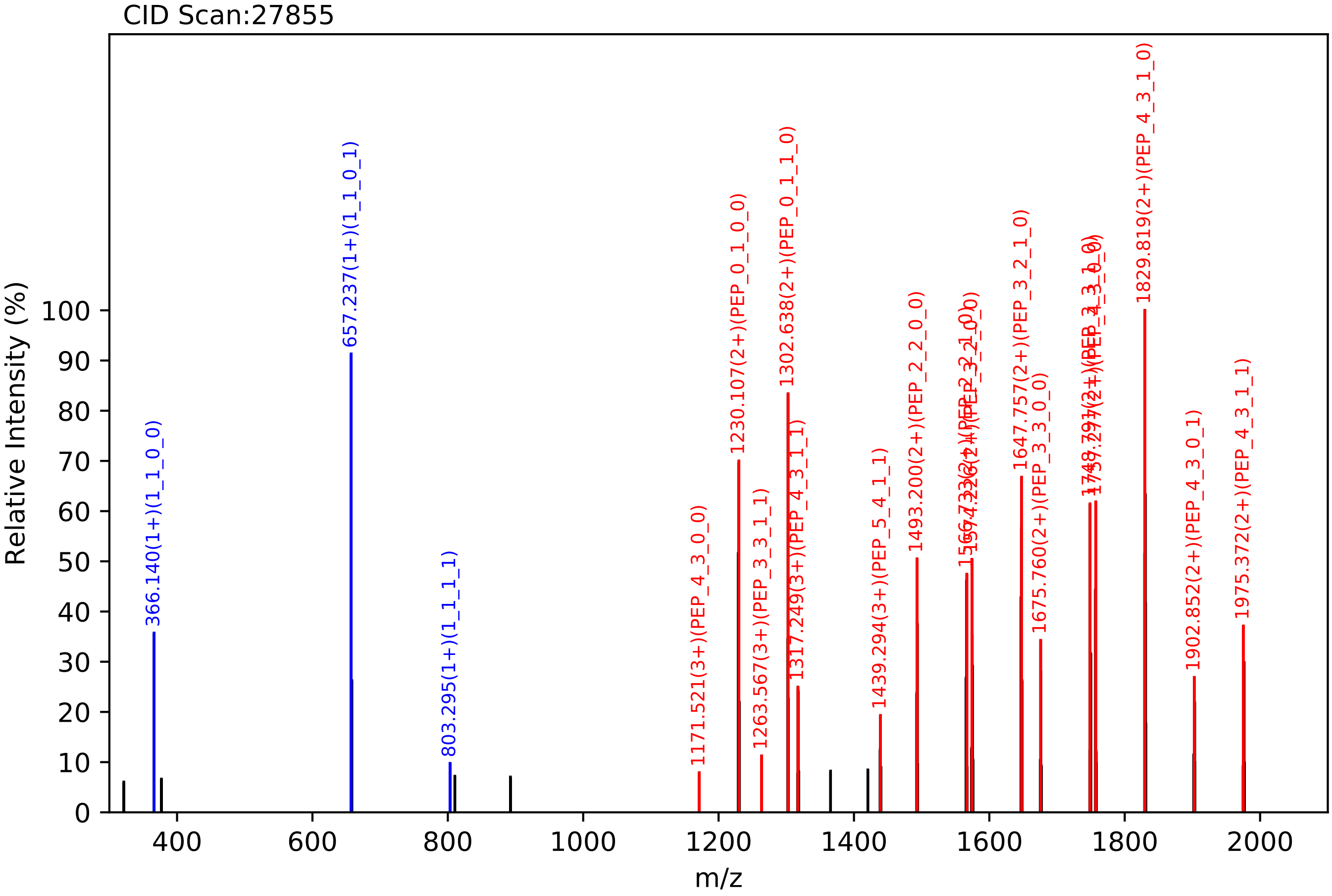

Unknown set no. 164, Experiment: Human Plasma exp_3

LGNWSAMPSCK(=PEP)_5_4_0_2, m/z:864.59(4+), RT:83.18, Y-score:93.04

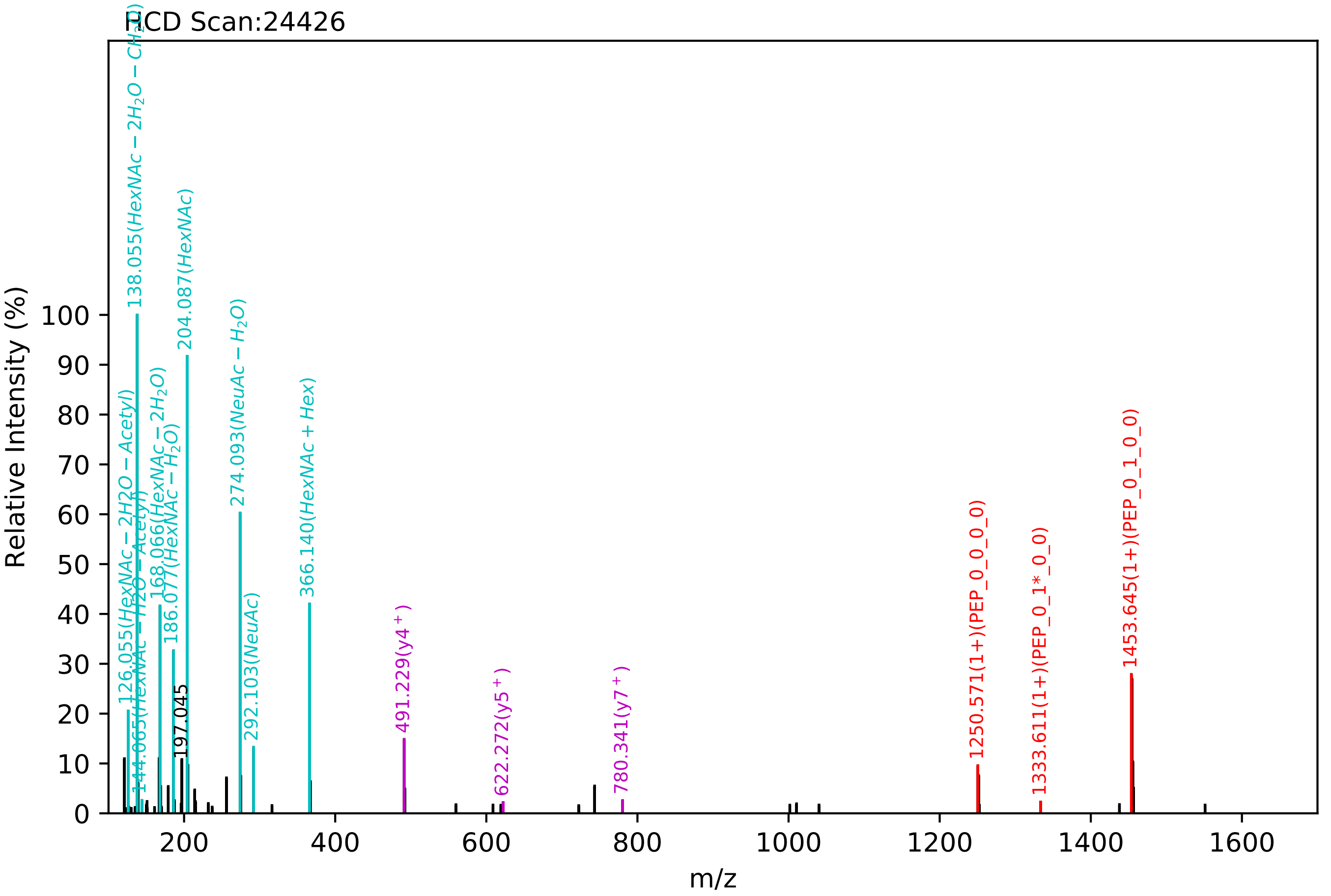

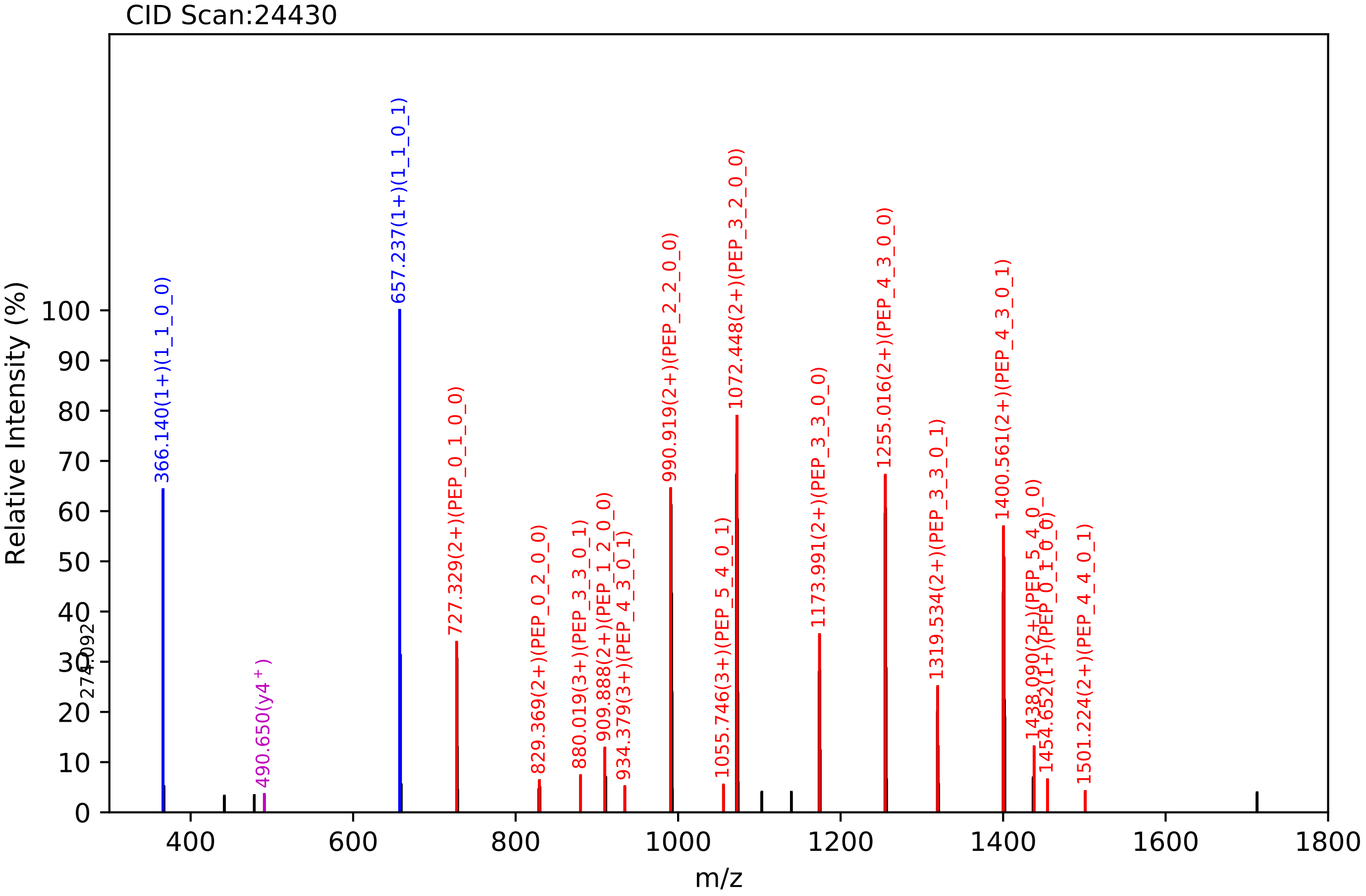

Unknown set no. 165, Experiment: Human Plasma exp_4

LGNWSAMPSCK(=PEP)_5_4_0_2, m/z:864.59(4+), RT:83.26, Y-score:98.11

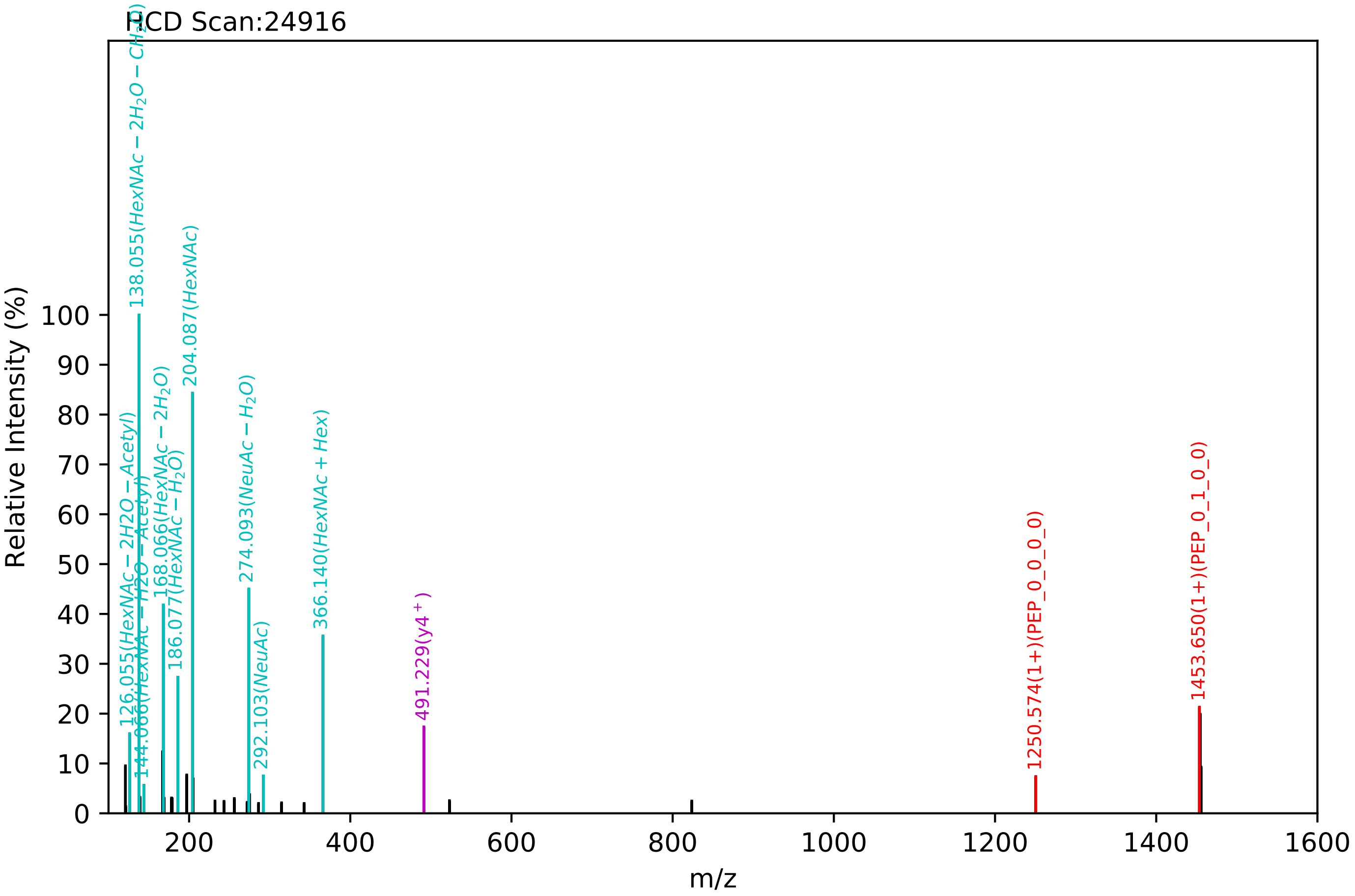

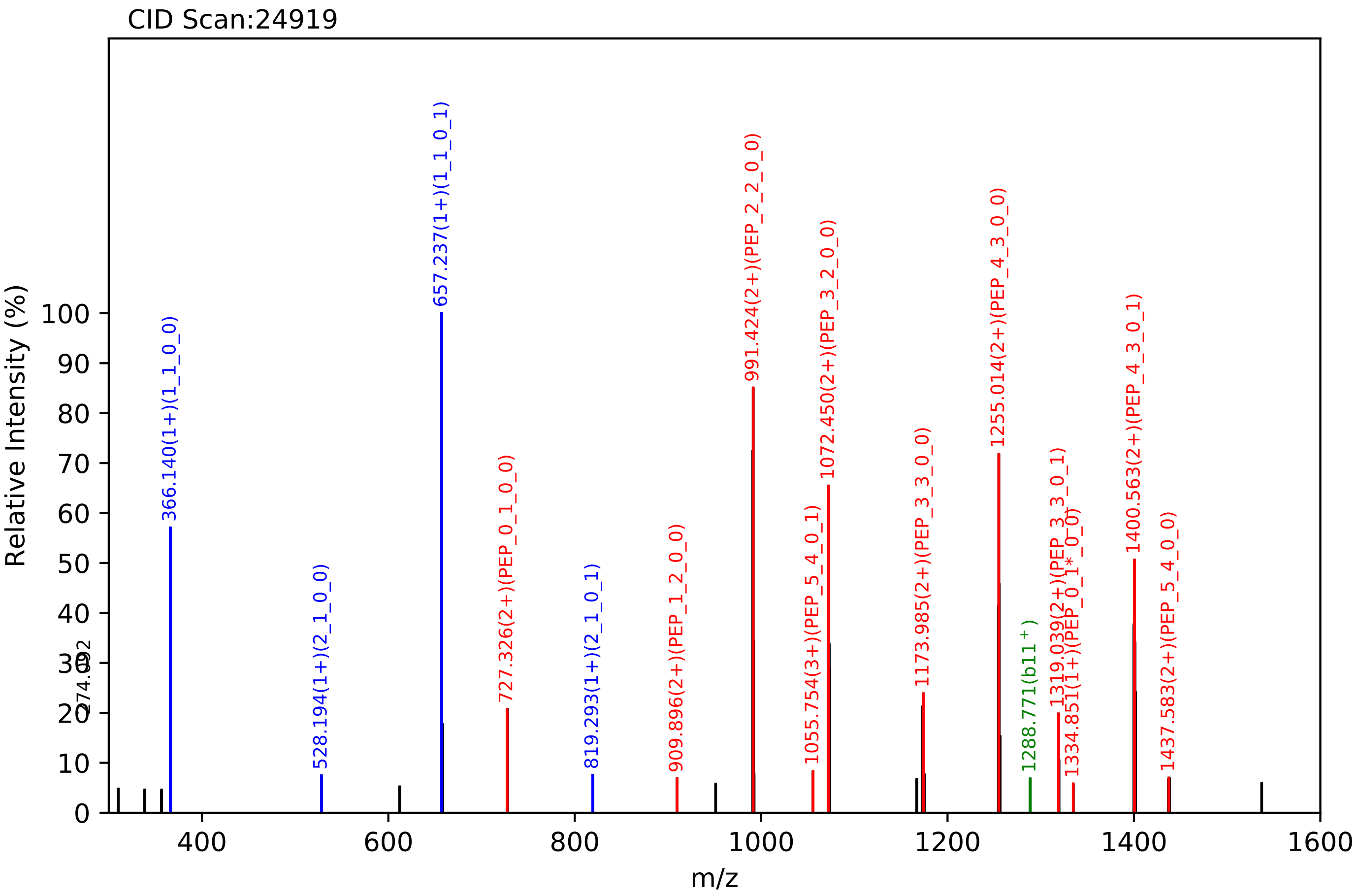

Unknown set no. 166, Experiment: Human Plasma exp_2

VYKPSAGNNSLYR(=PEP)_4_3_0_1, m/z:1006.44(3+), RT:56.72, Y-score:83.58

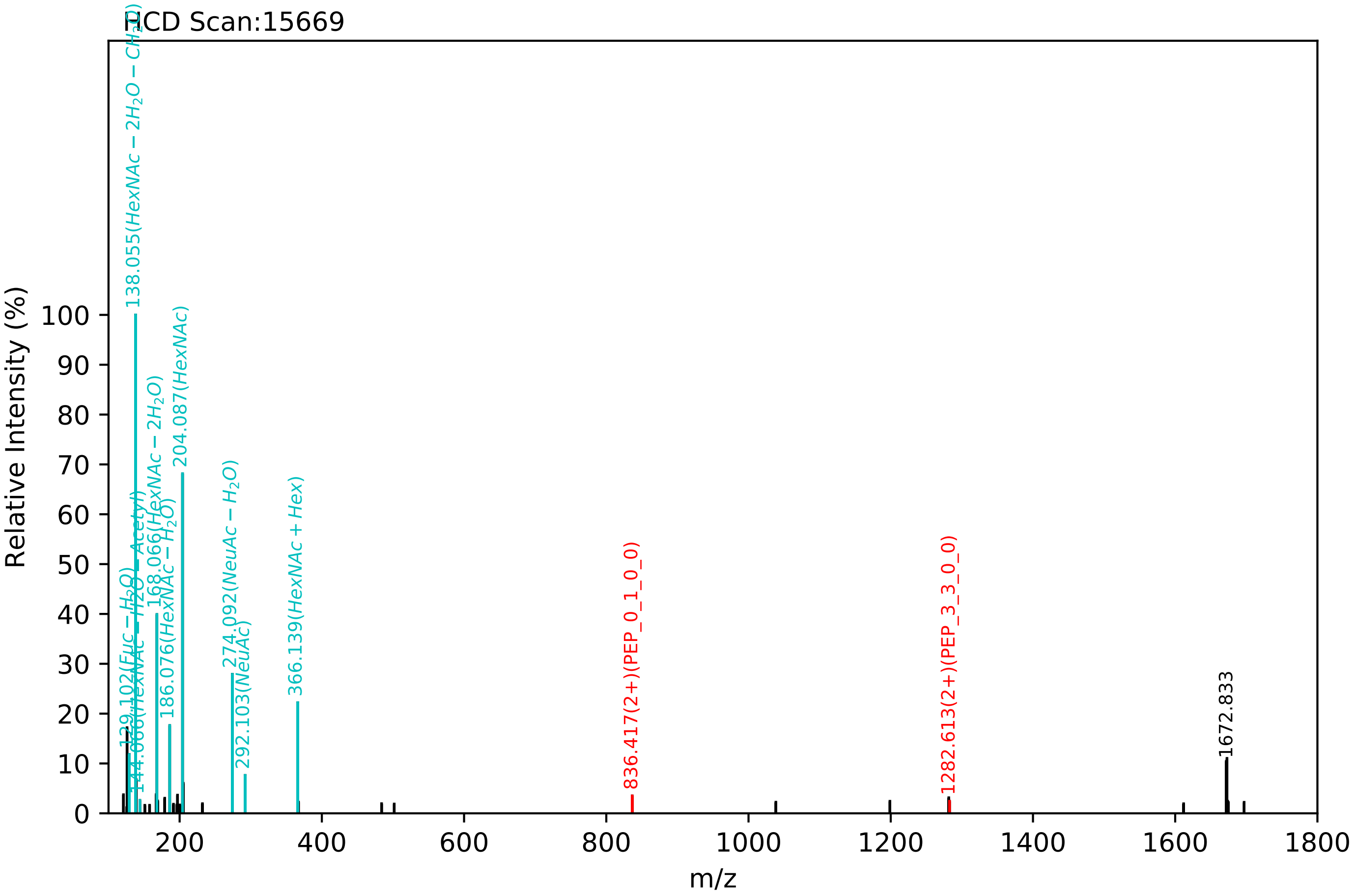

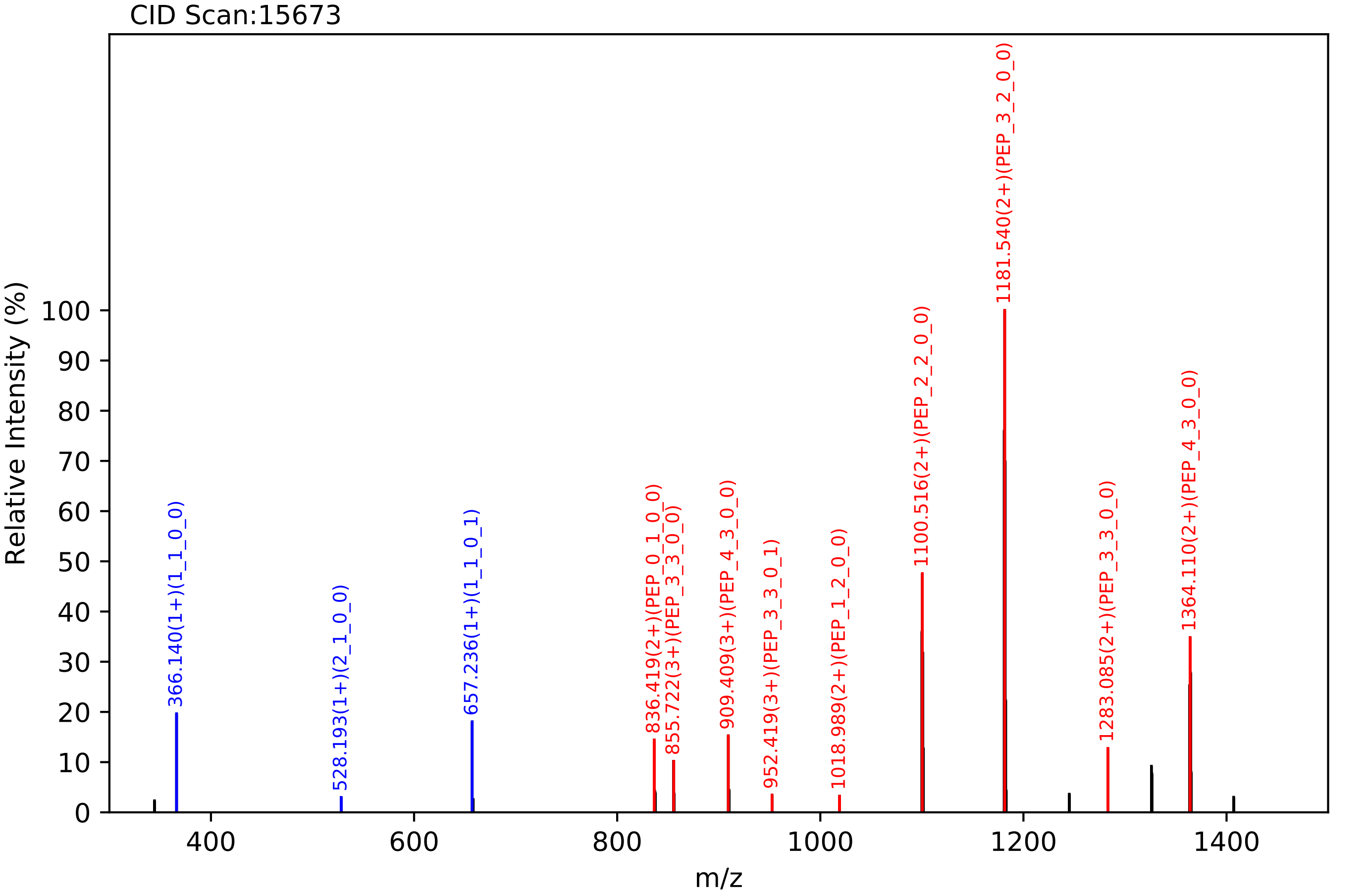

Unknown set no. 167, Experiment: Human Plasma exp_4

VYKPSAGNNSLYR(=PEP)_6_5_1_3, m/z:1119.71(4+), RT:63.99, Y-score:71.99

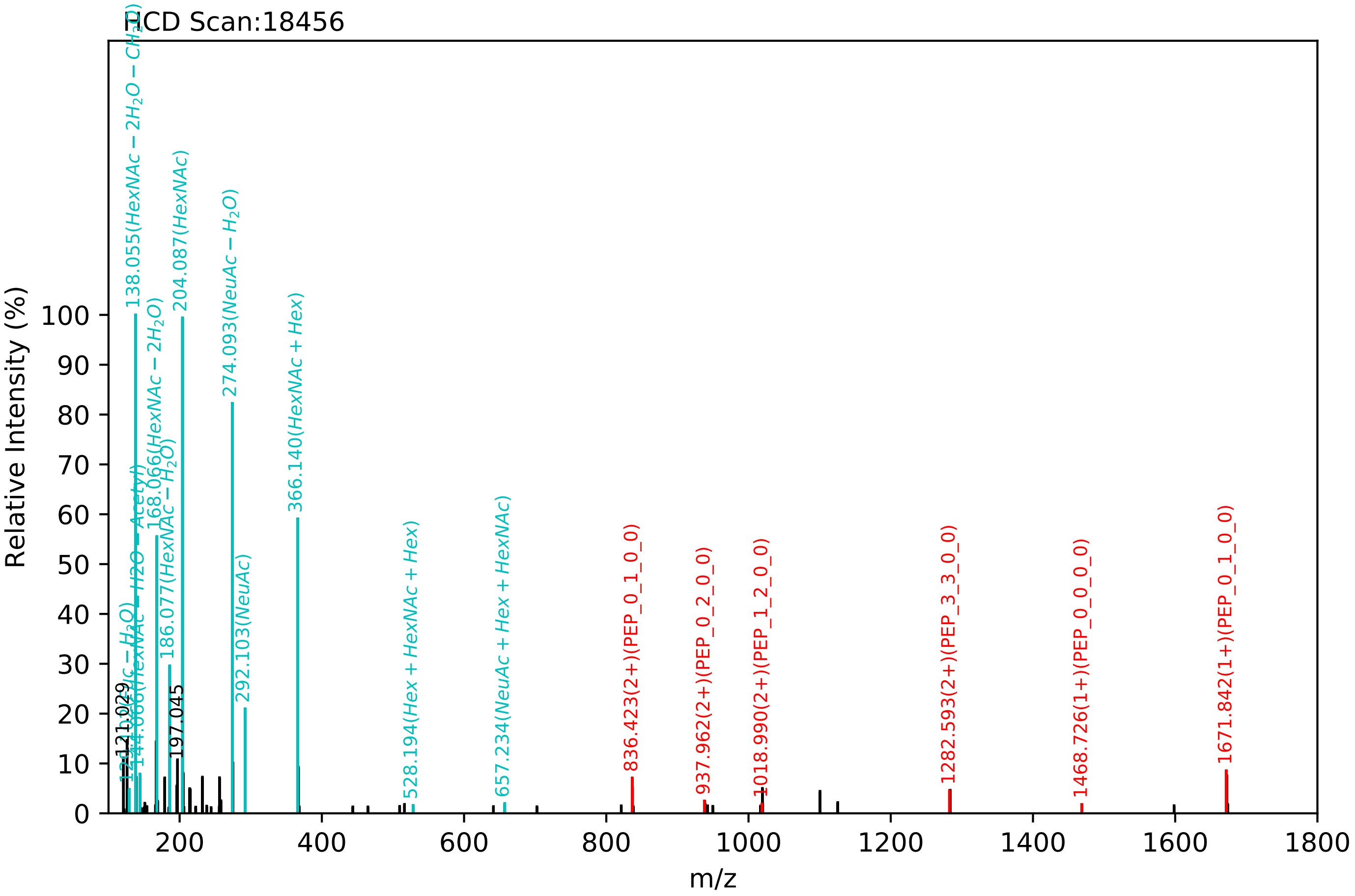

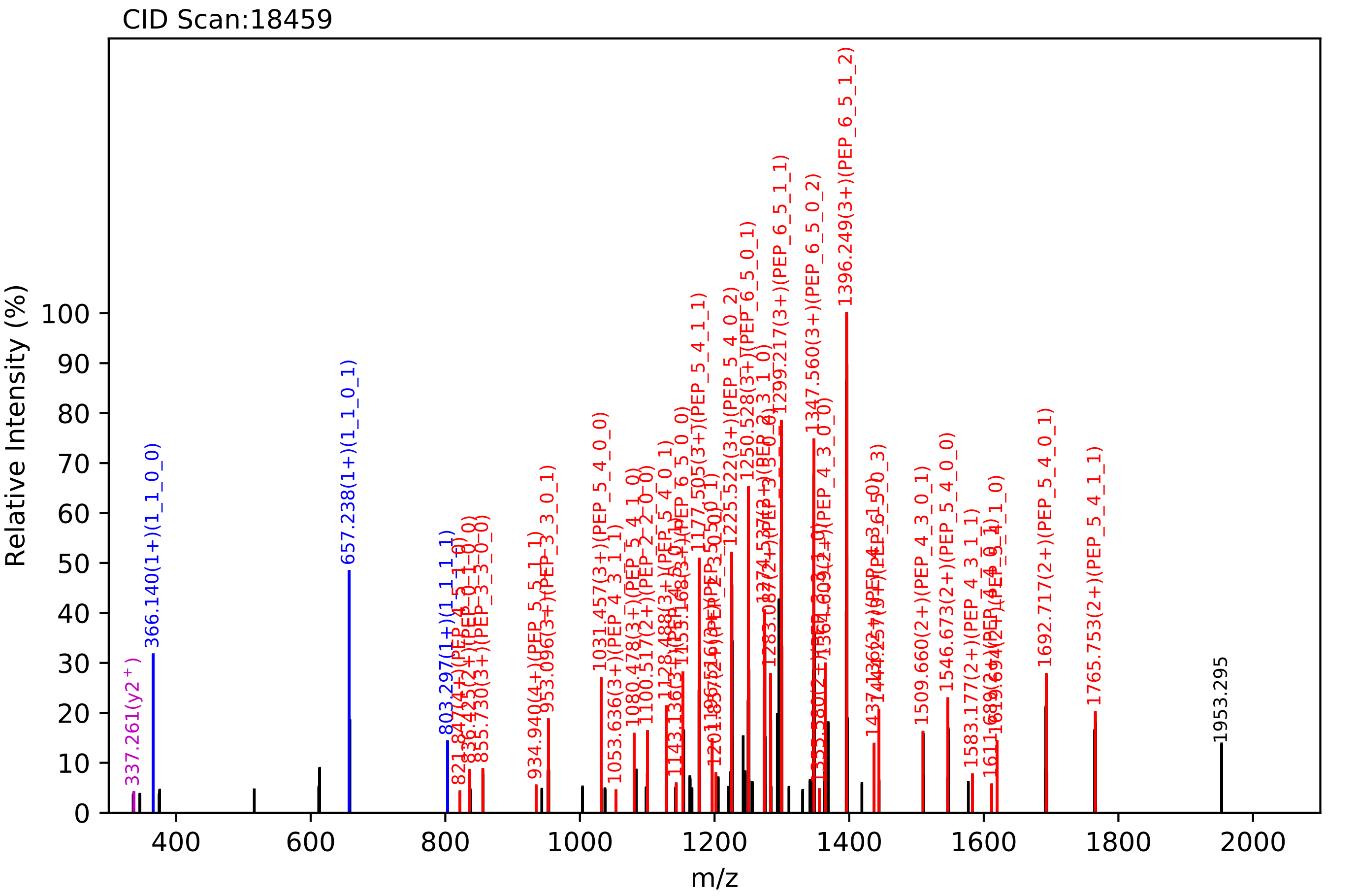

Unknown set no. 168, Experiment: Human Plasma exp_5

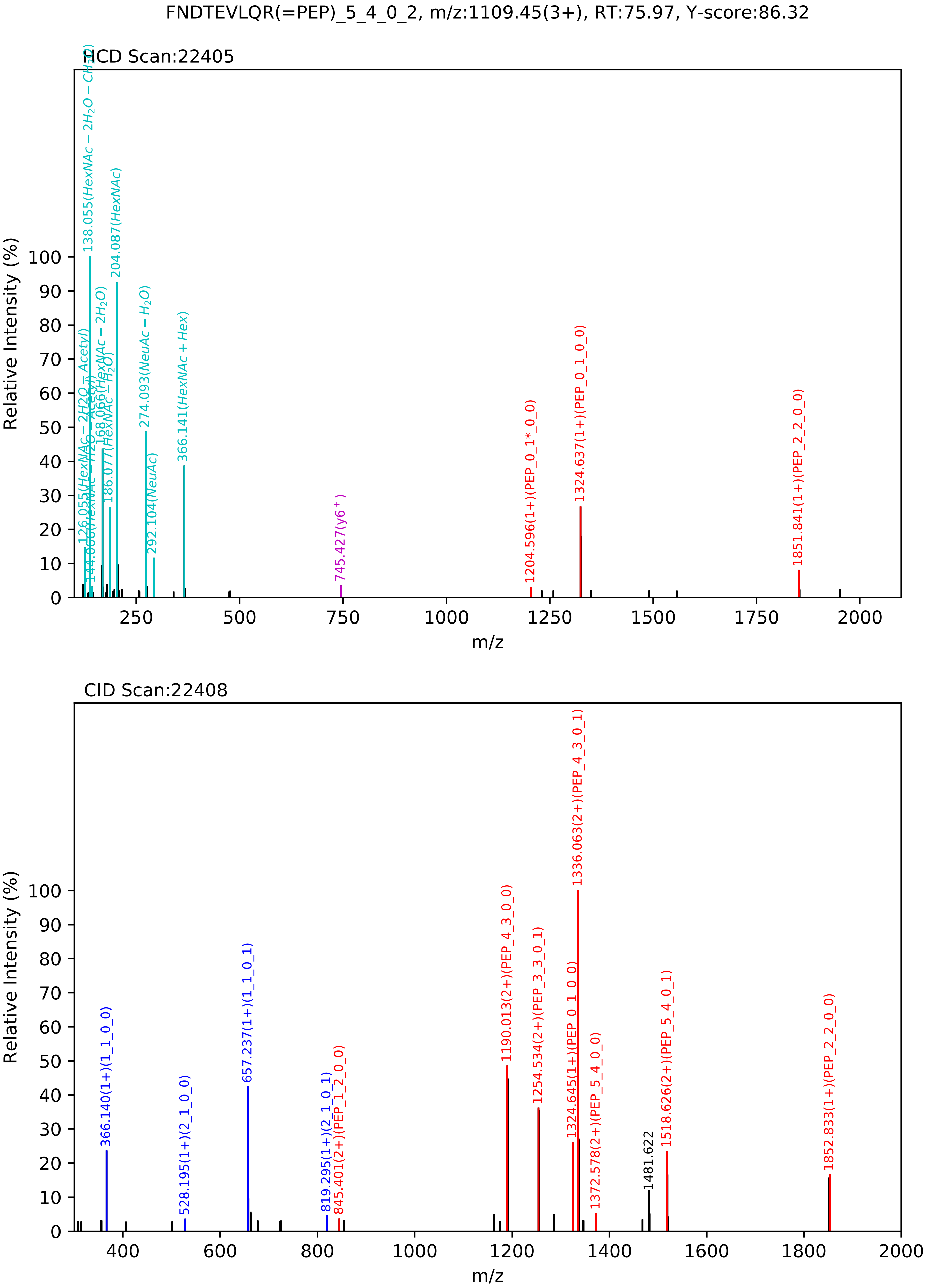

Unknown set no. 169, Experiment: Human Plasma exp_1

NPVGLIGAENATGETDPSHSK(=PEP)_6_5_0_3, m/z:1239.51(4+), RT:82.46, Y-score:78.38

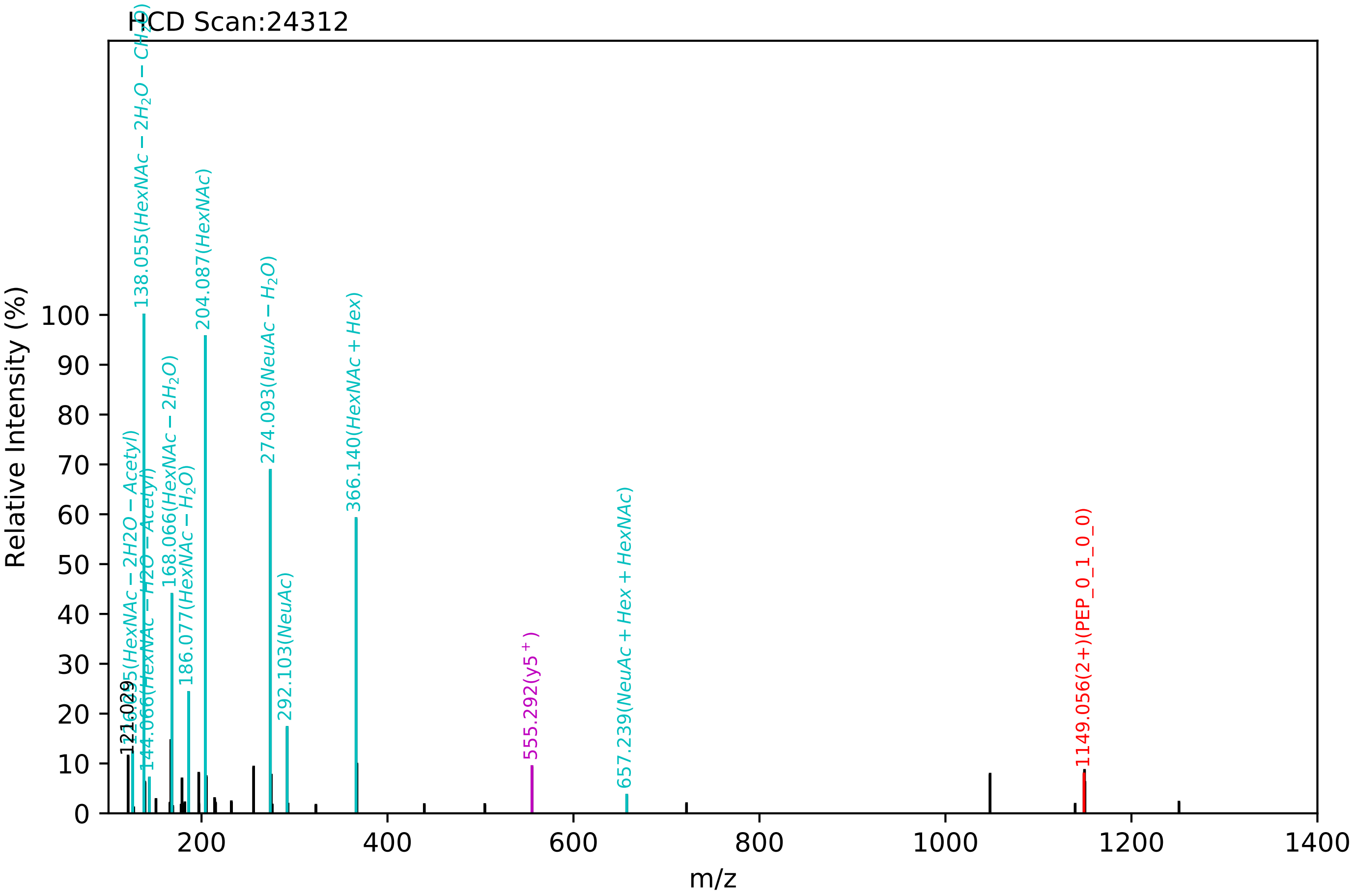

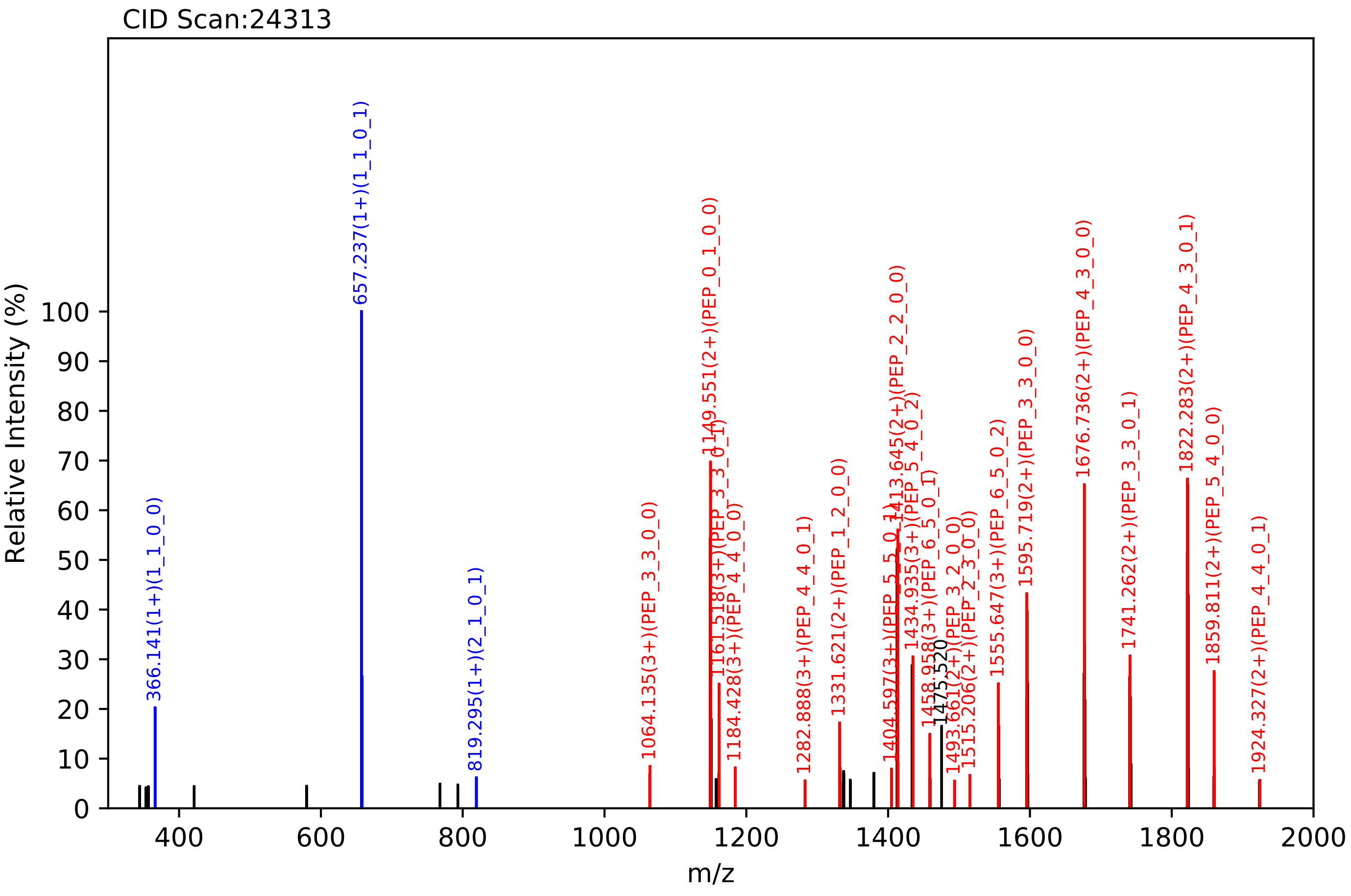

Unknown set no. 170, Experiment: Human Plasma exp_3

AGLQAFFQVQECNK(=PEP)_5_4_0_2, m/z:961.90(4+), RT:108.31, Y-score:96.32

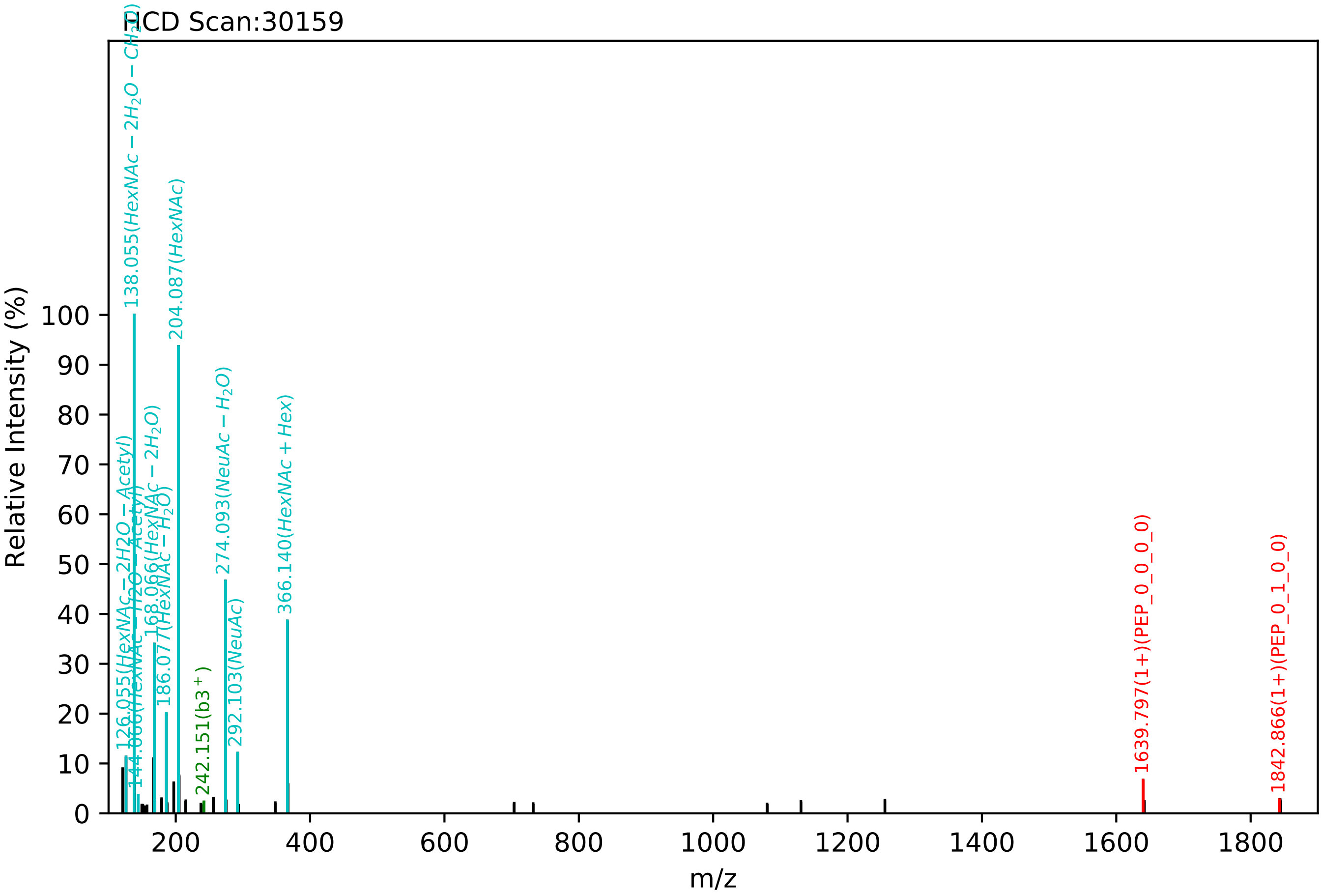

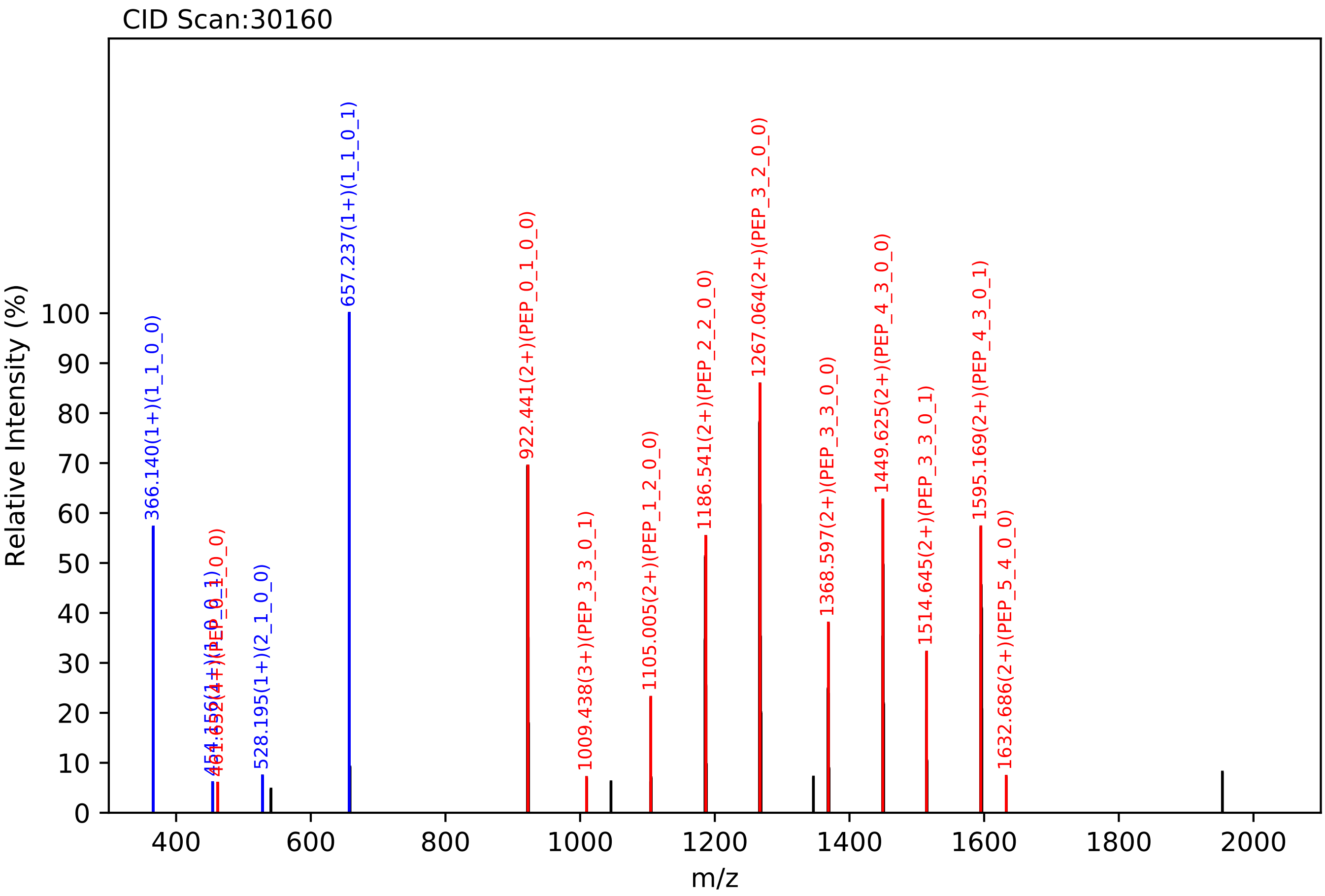

Unknown set no. 171, Experiment: Human Plasma exp_4

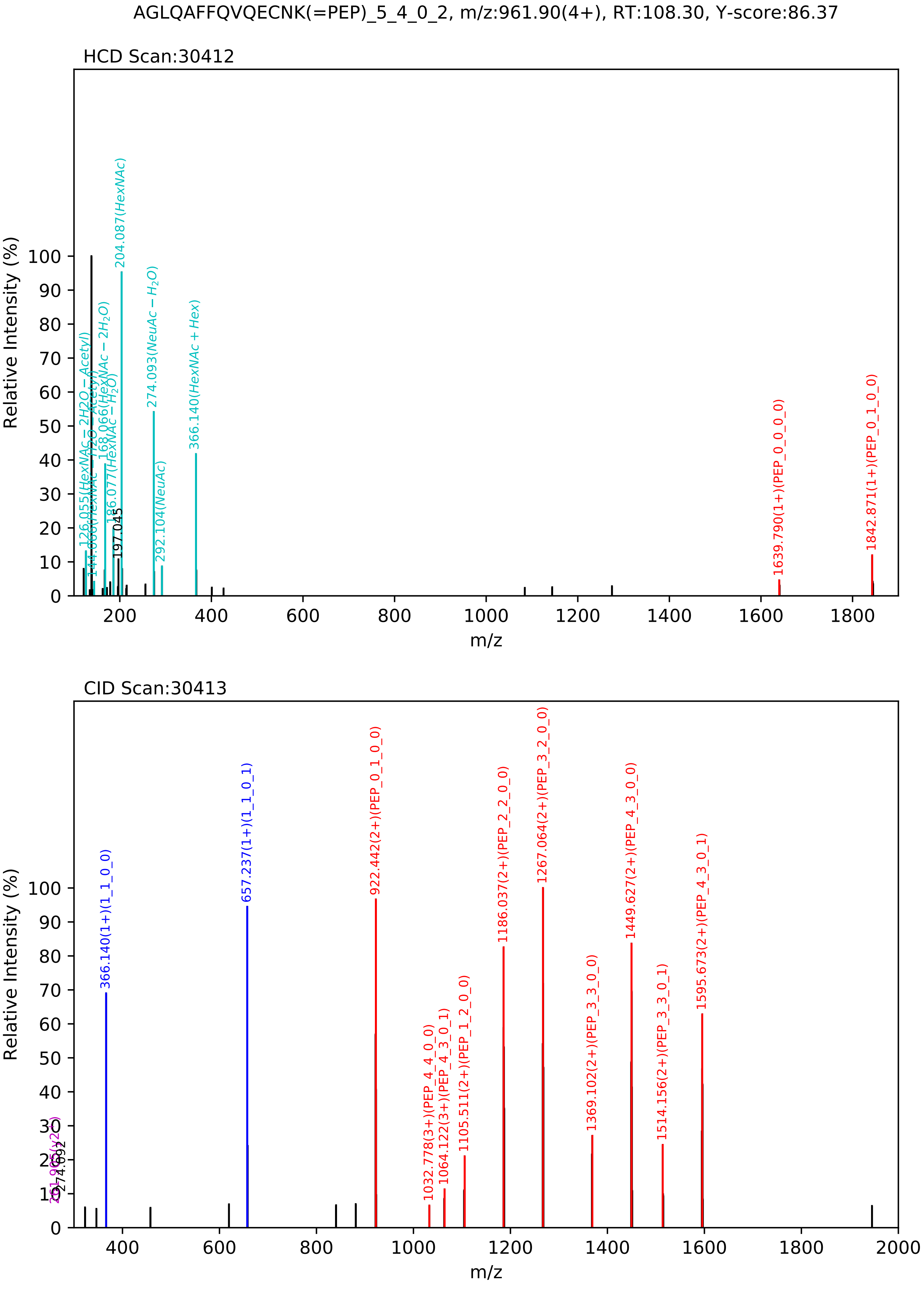

Unknown set no. 172, Experiment: Human Plasma exp_3

EHEGAIYPDNTTDFQR(=PEP)_4_3_0_1, m/z:1147.80(3+), RT:70.73, Y-score:84.13

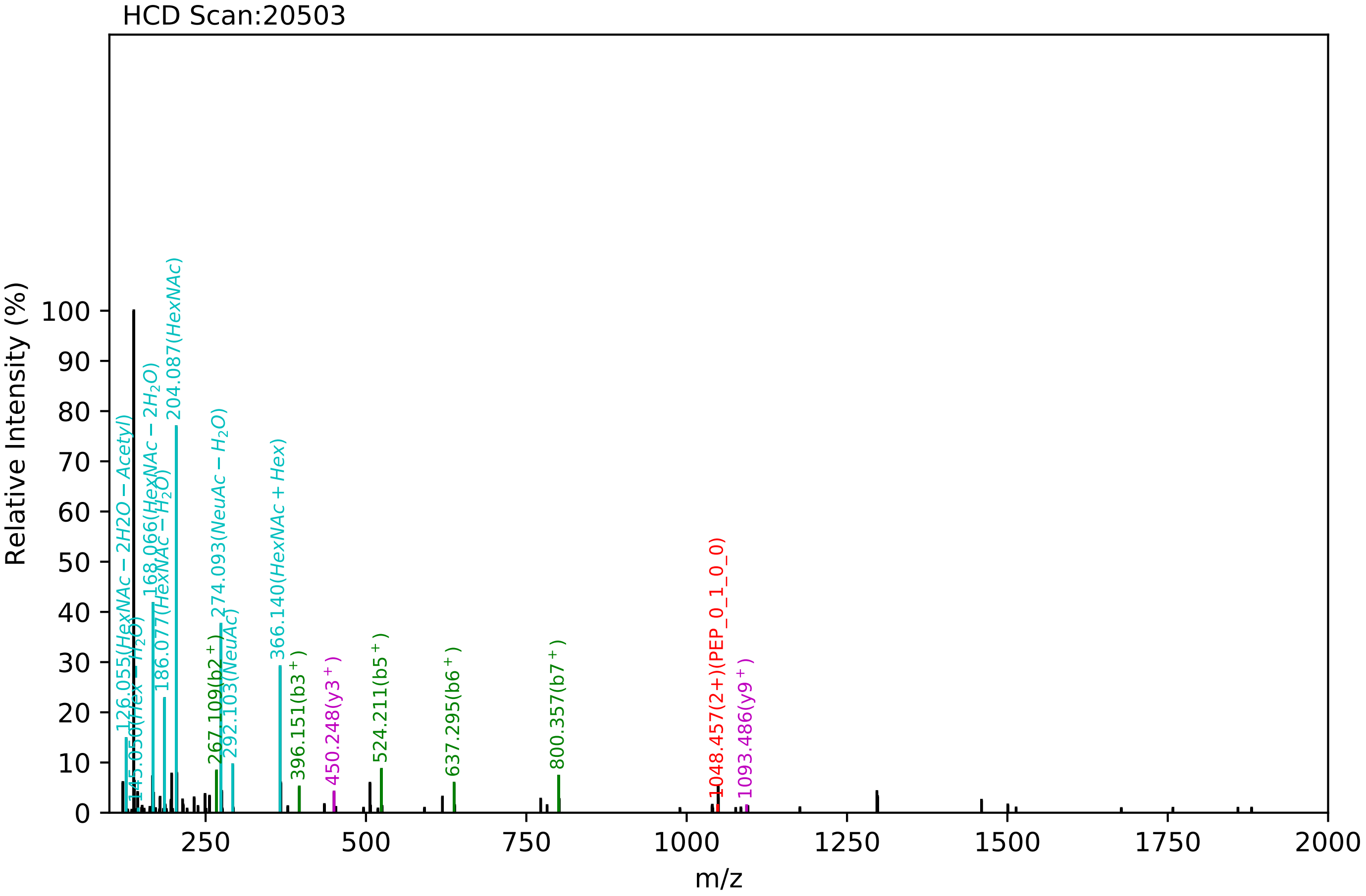

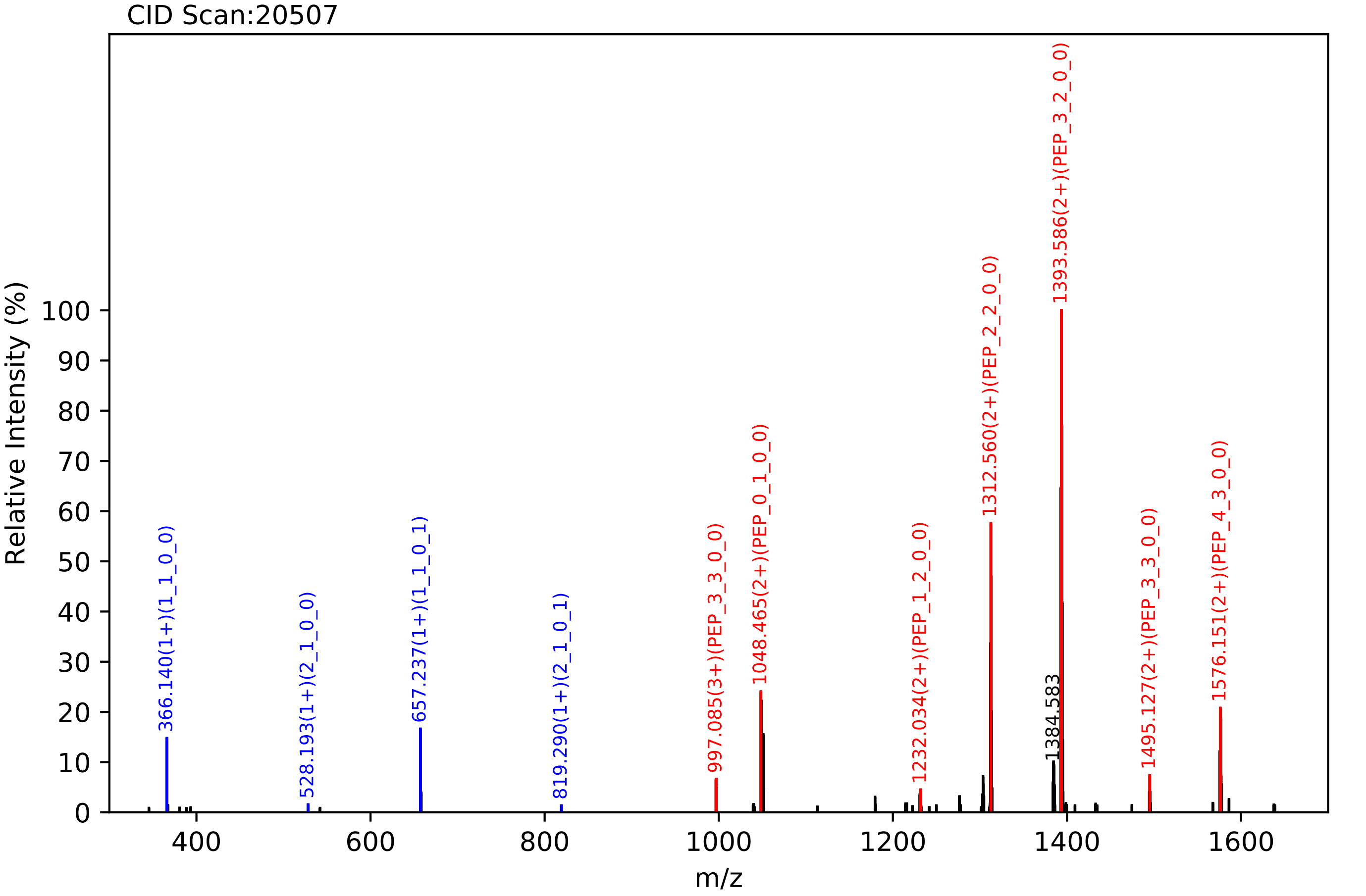

Unknown set no. 173, Experiment: Human Plasma exp_3

EHEGAIYPDNTTDFQR(=PEP)_4_3_1_1, m/z:1196.49(3+), RT:70.01, Y-score:86.46

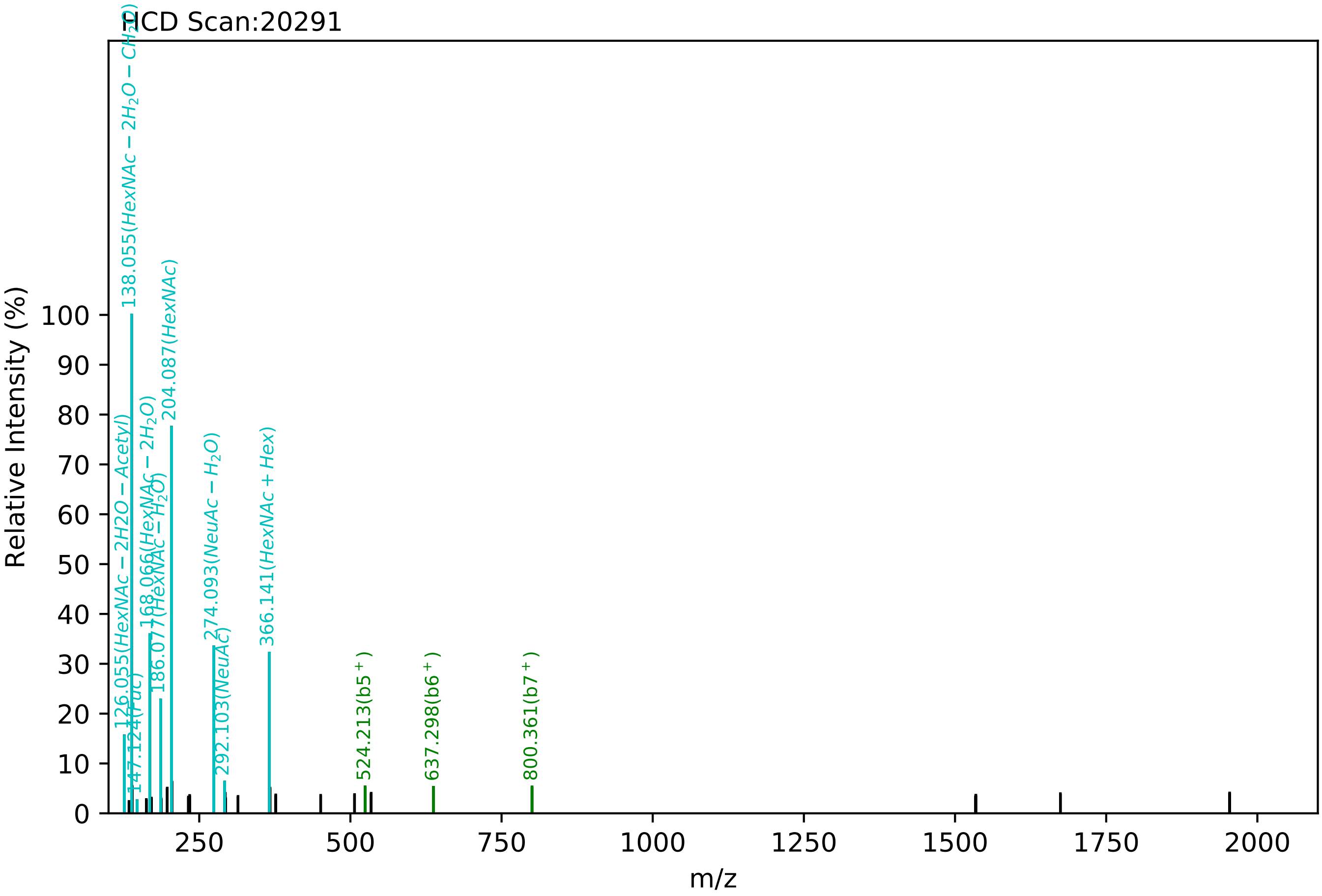

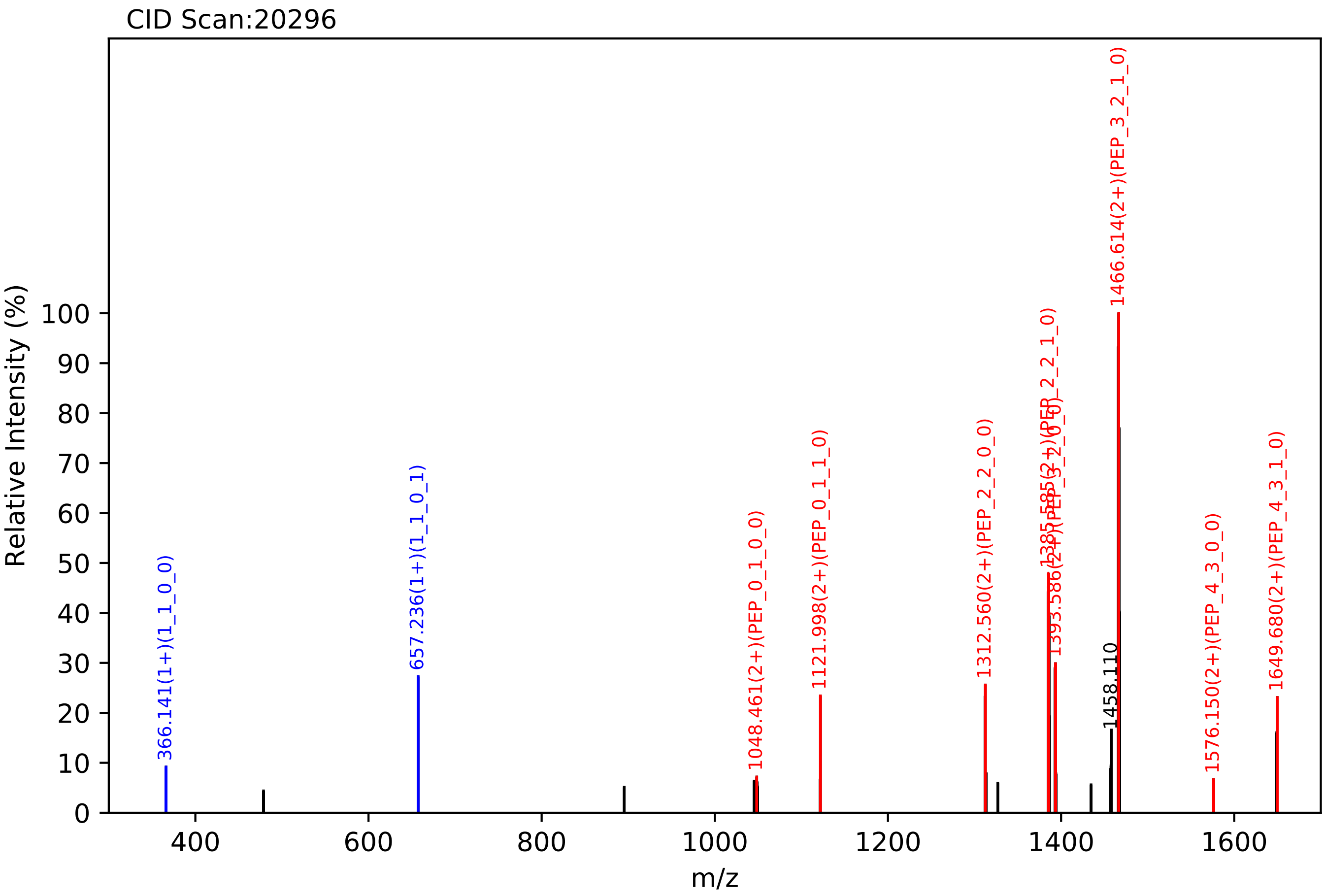

Unknown set no. 174, Experiment: Human Plasma exp_2

ELHHLQEQNVSNAFLDK(=PEP)_5_4_0_2, m/z:1057.45(4+), RT:71.98, Y-score:78.18

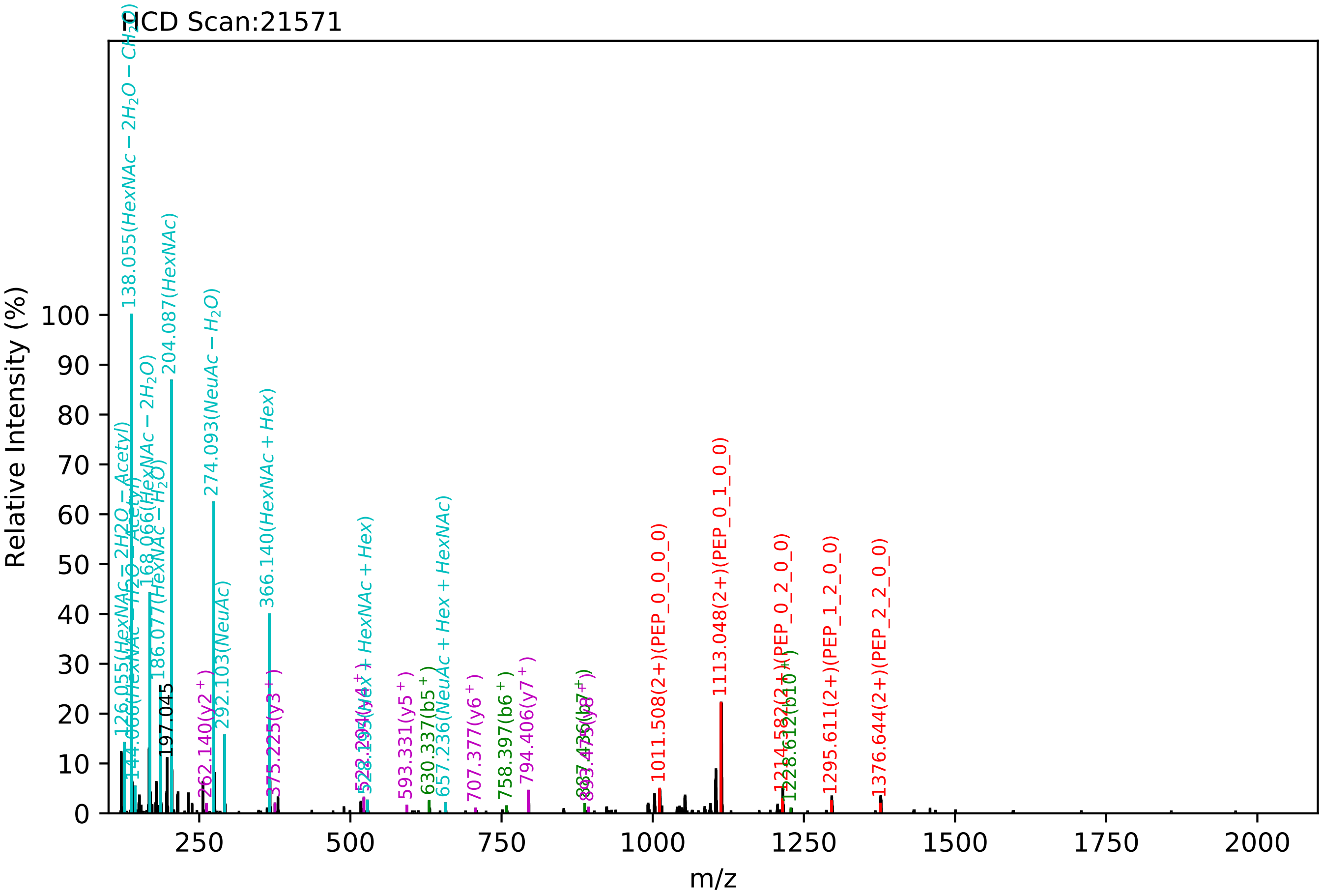

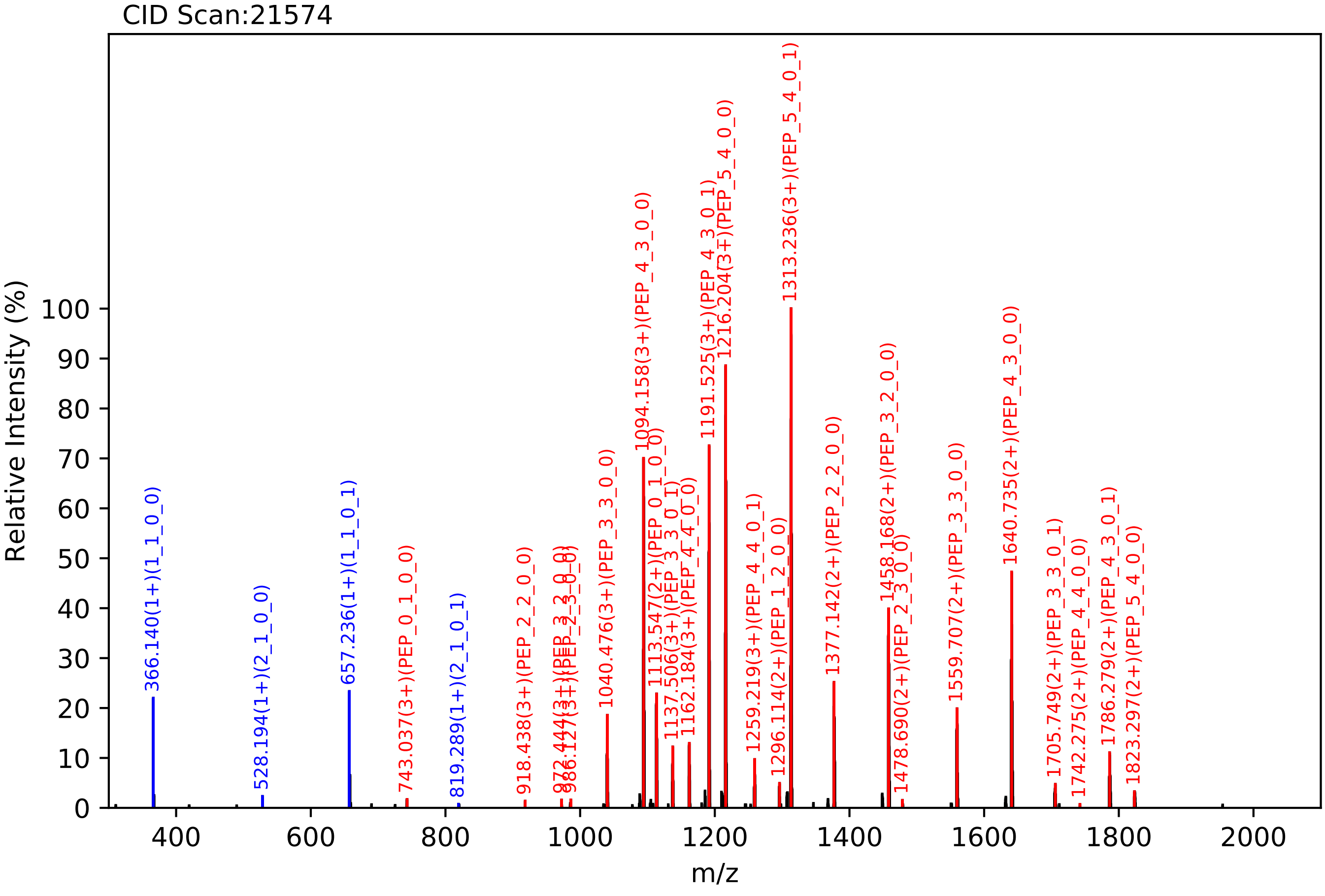

Unknown set no. 175, Experiment: Human Plasma exp_2

ELHHLQEQNVSNAFLDK(=PEP)_5_4_1_2, m/z:1093.97(4+), RT:71.04, Y-score:77.84

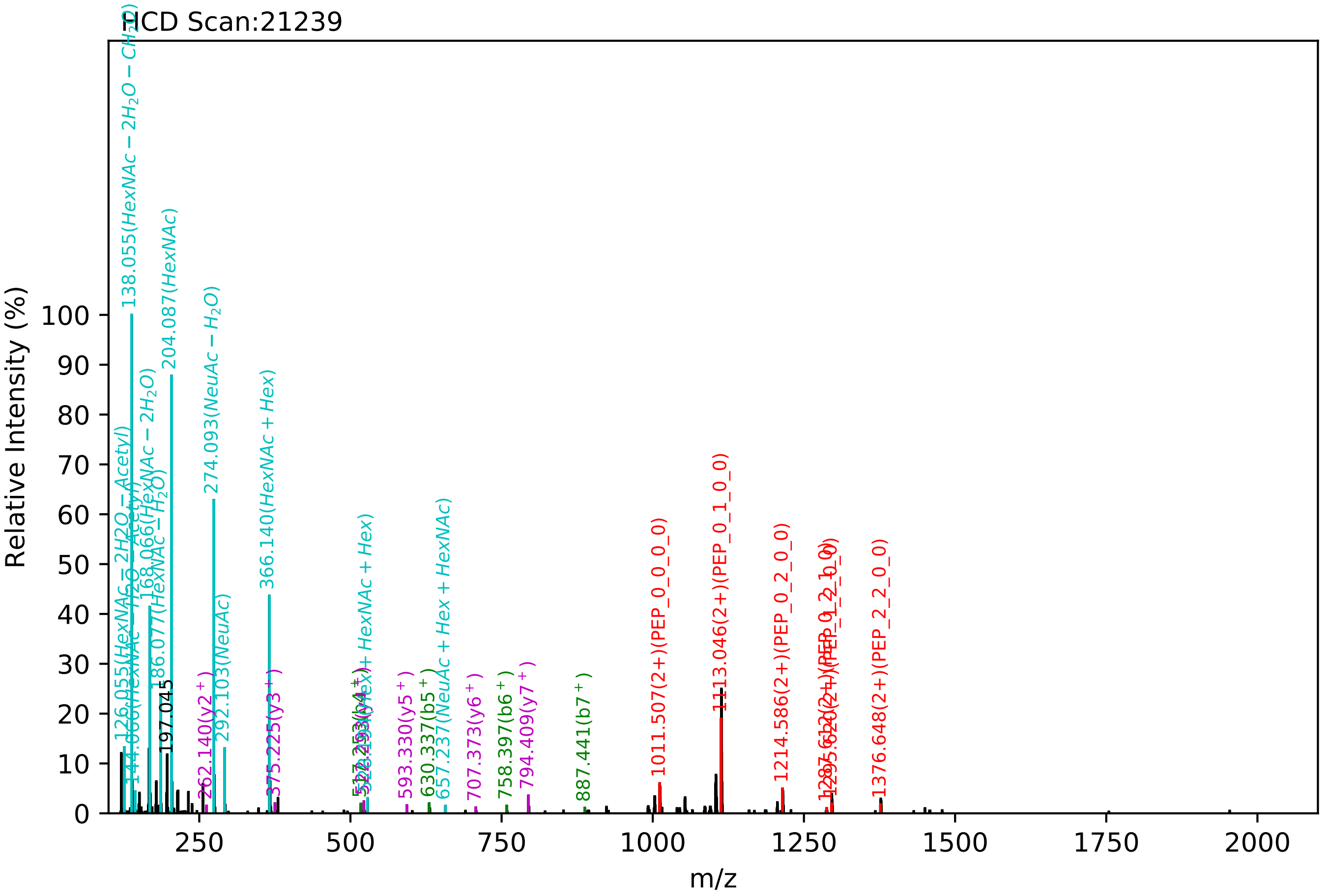

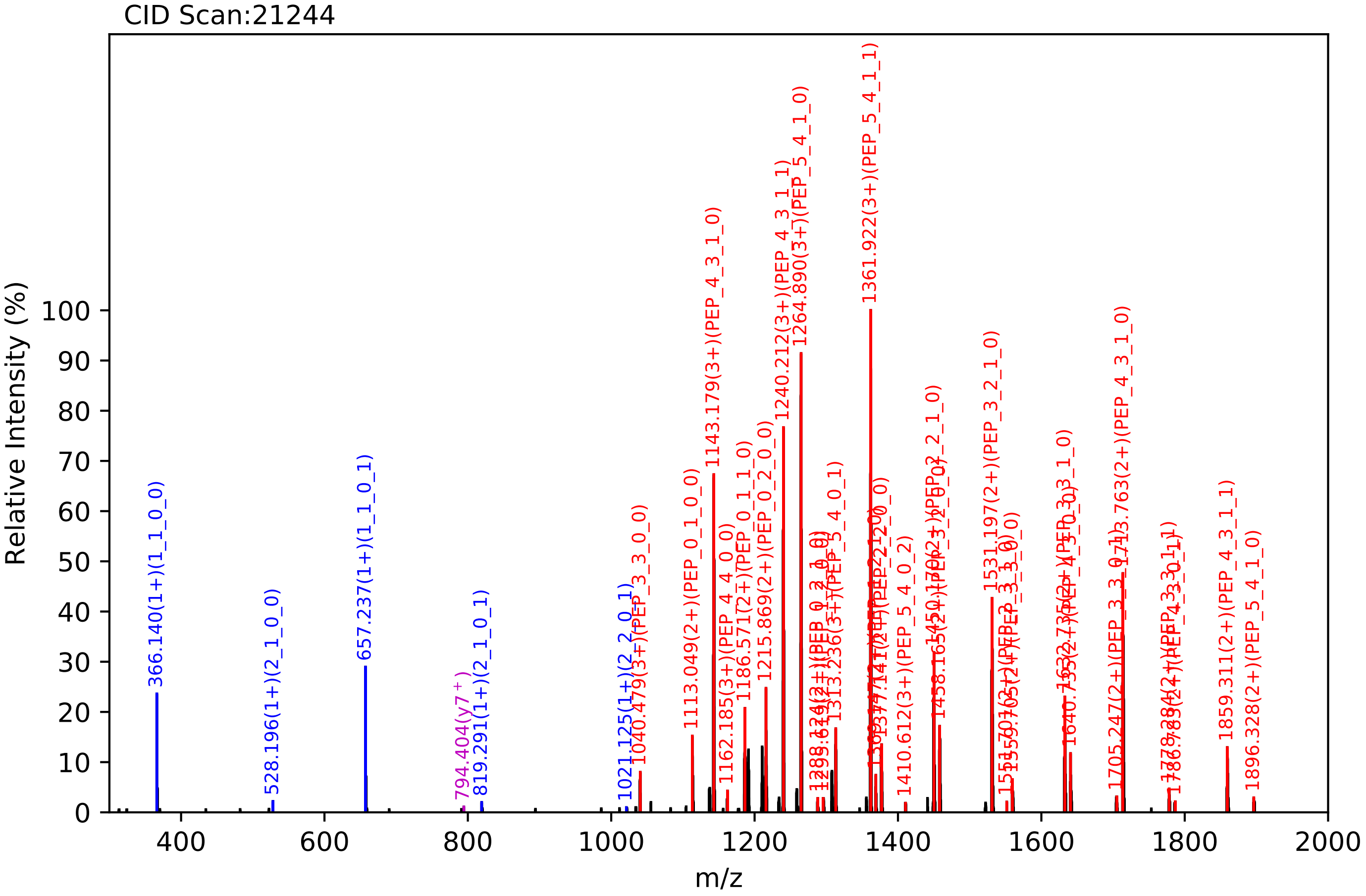

Unknown set no. 176, Experiment: Human Plasma exp_1

ENLTAPGSDSAVFFEQGTTR(=PEP)_5_4_0_2, m/z:1083.70(4+), RT:103.12, Y-score:95.07

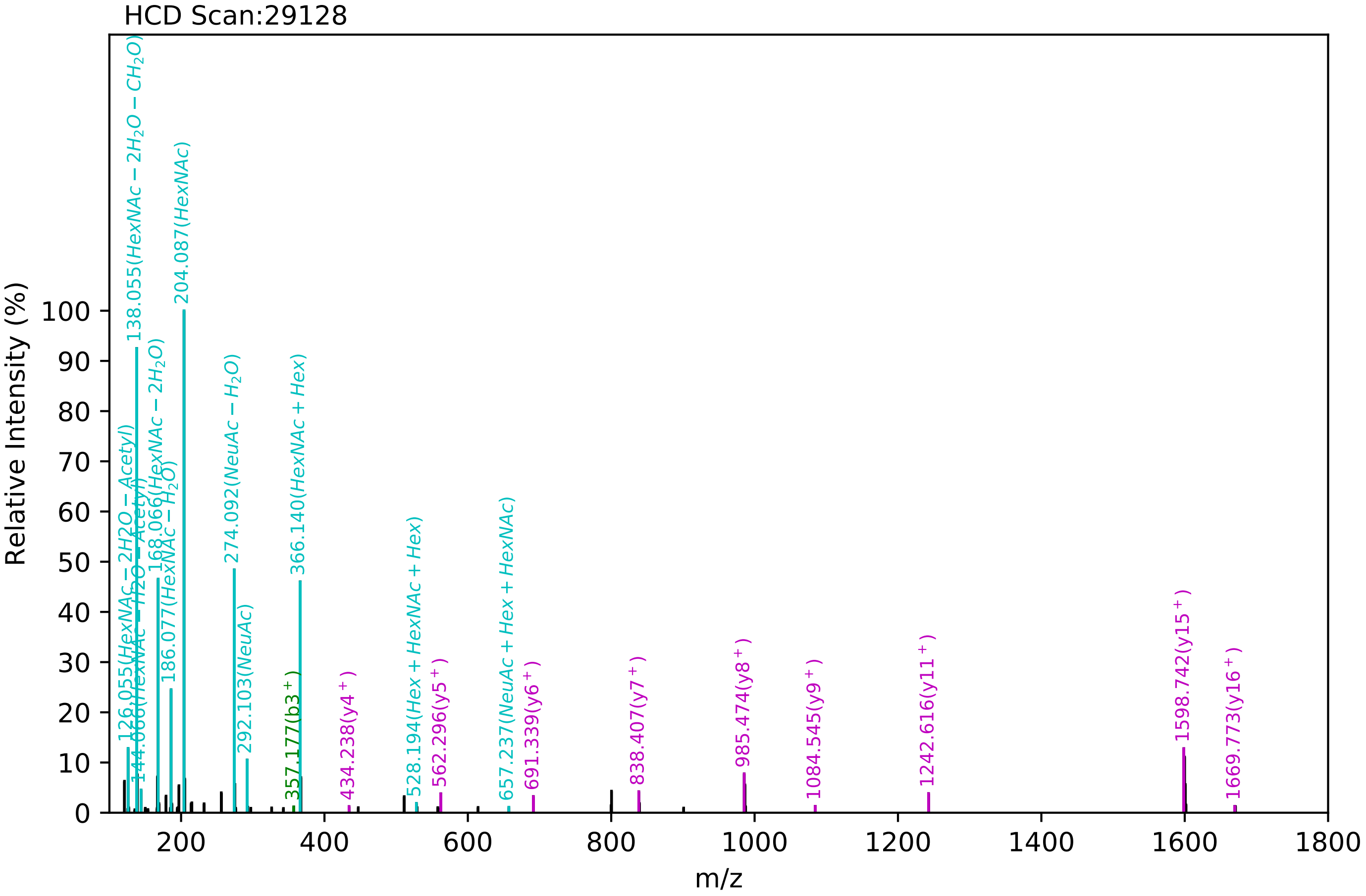

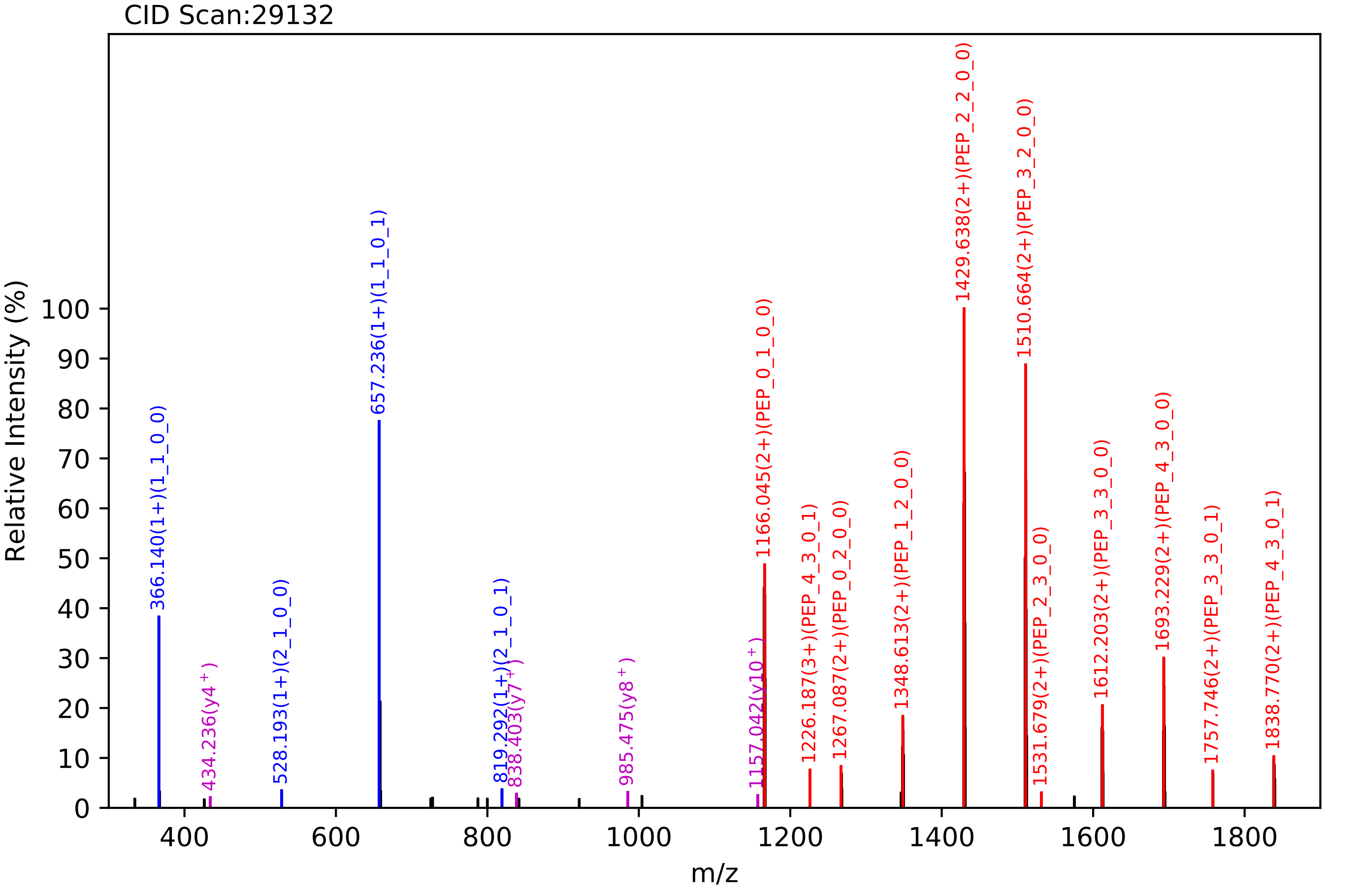

Unknown set no. 177, Experiment: Human Plasma exp_2

ENLTAPGSDSAVFFEQGTTR(=PEP)_5_4_0_2, m/z:1083.70(4+), RT:103.34, Y-score:94.64

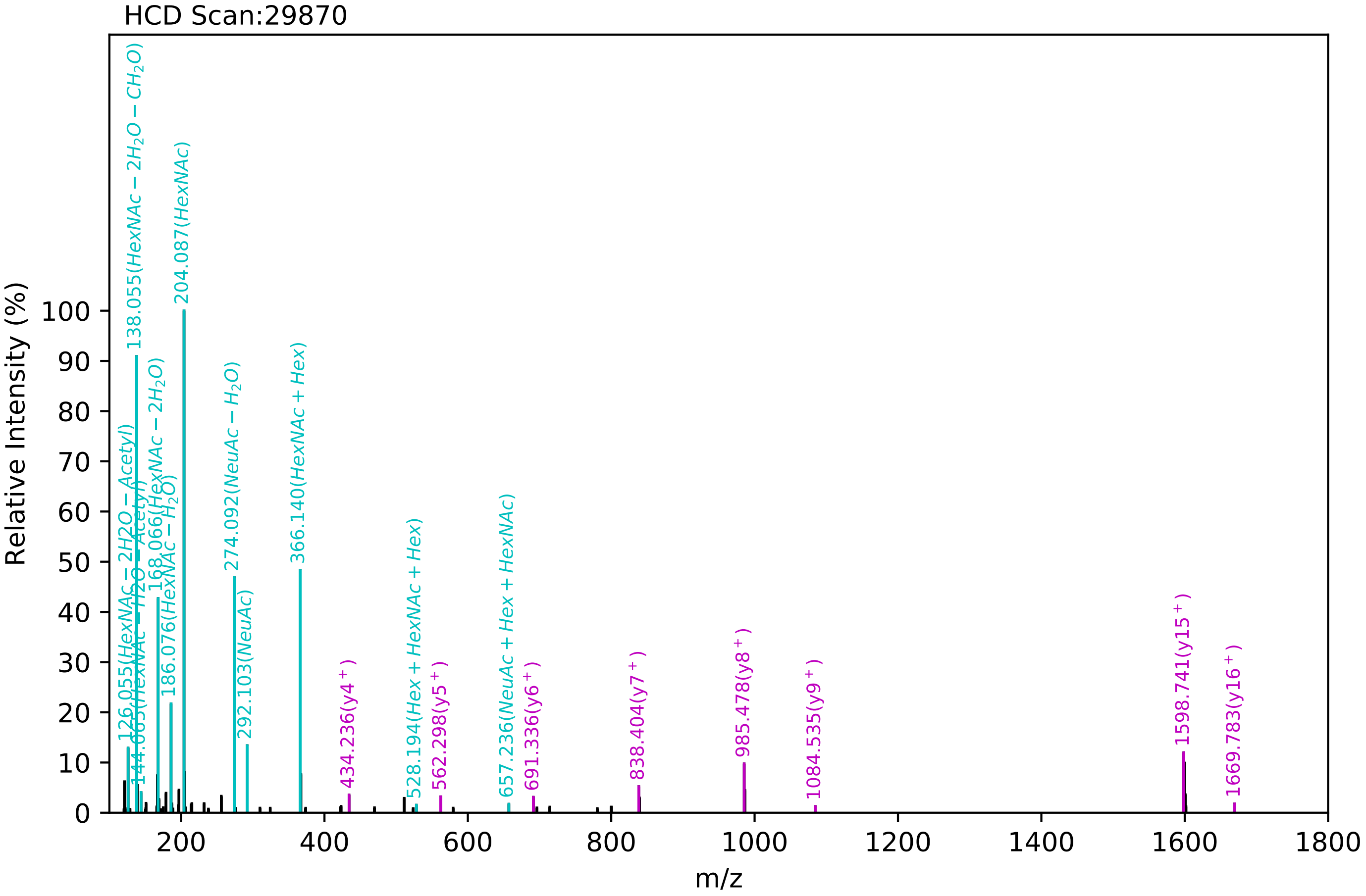

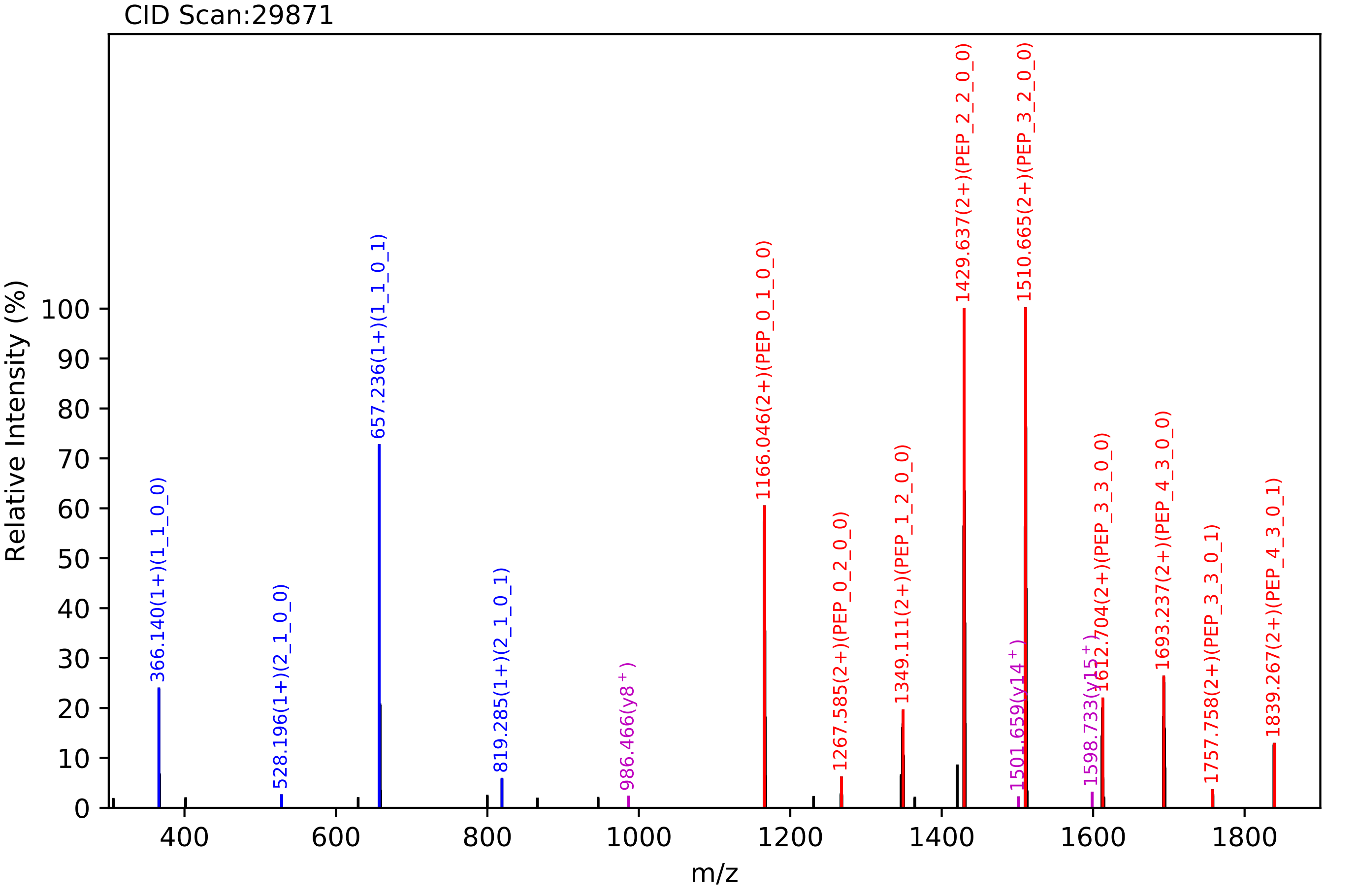

Unknown set no. 178, Experiment: Human Plasma exp_3

ENLTAPGSDSAVFFEQGTTR(=PEP)_5_4_0_2, m/z:1083.70(4+), RT:103.72, Y-score:93.43

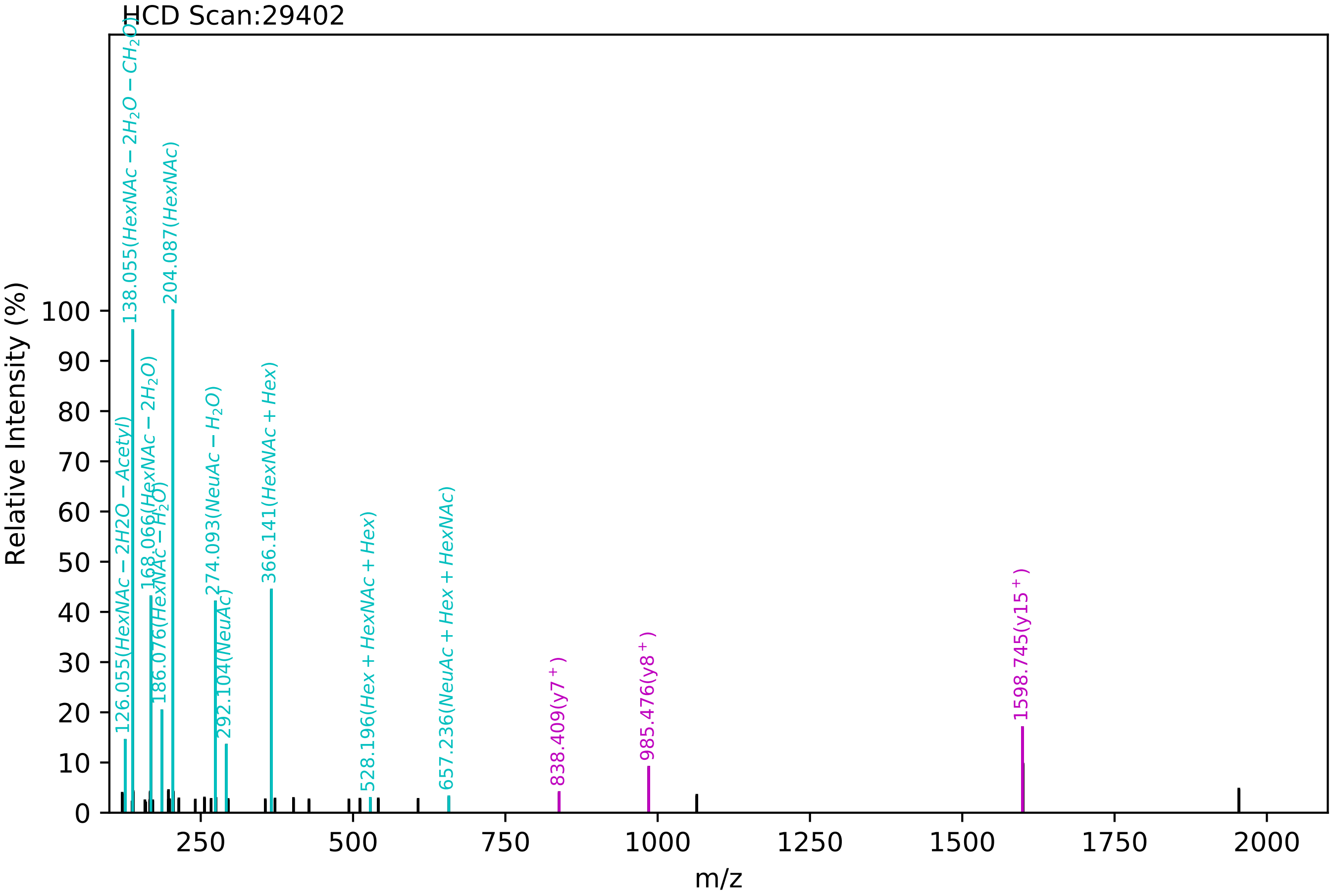

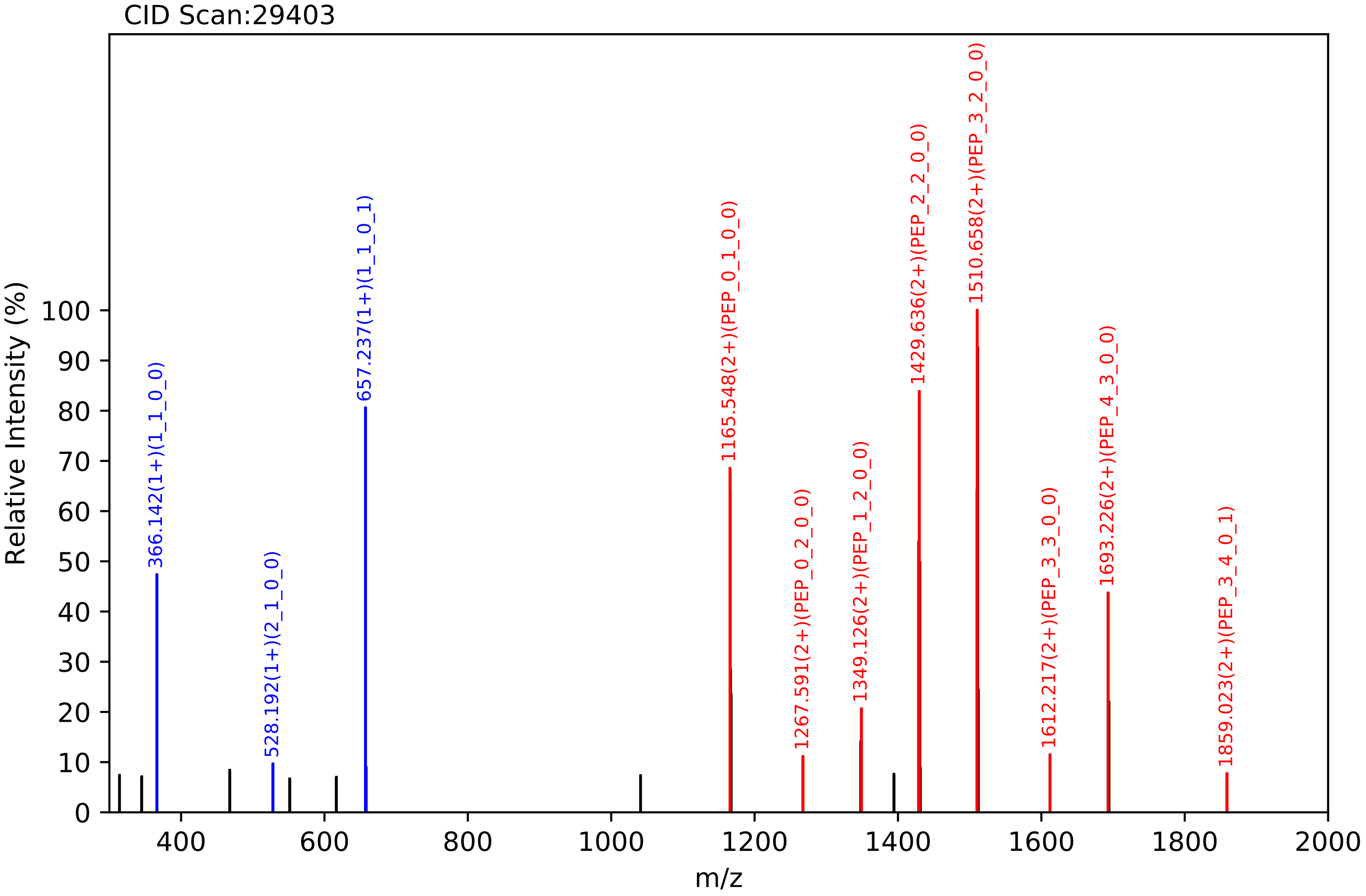

Unknown set no. 179, Experiment: Human Plasma exp_4

ENLTAPGSDSAVFFEQGTTR(=PEP)_5_4_0_2, m/z:1083.70(4+), RT:103.49, Y-score:91.26

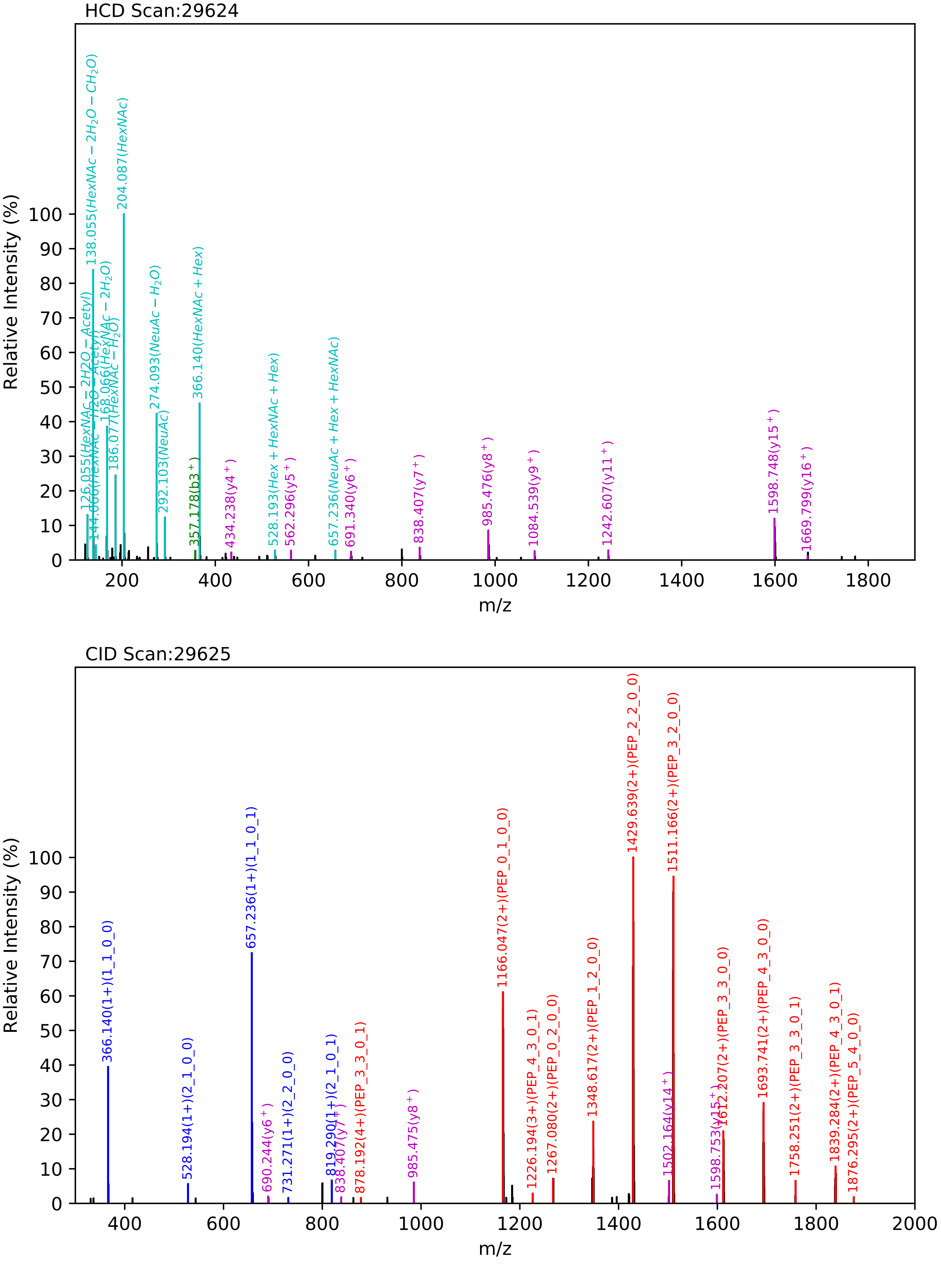

Unknown set no. 180, Experiment: Human Plasma exp_5

ENLTAPGSDSAVFFEQGTTR(=PEP)_5_4_0_2, m/z:1083.70(4+), RT:103.11, Y-score:98.45

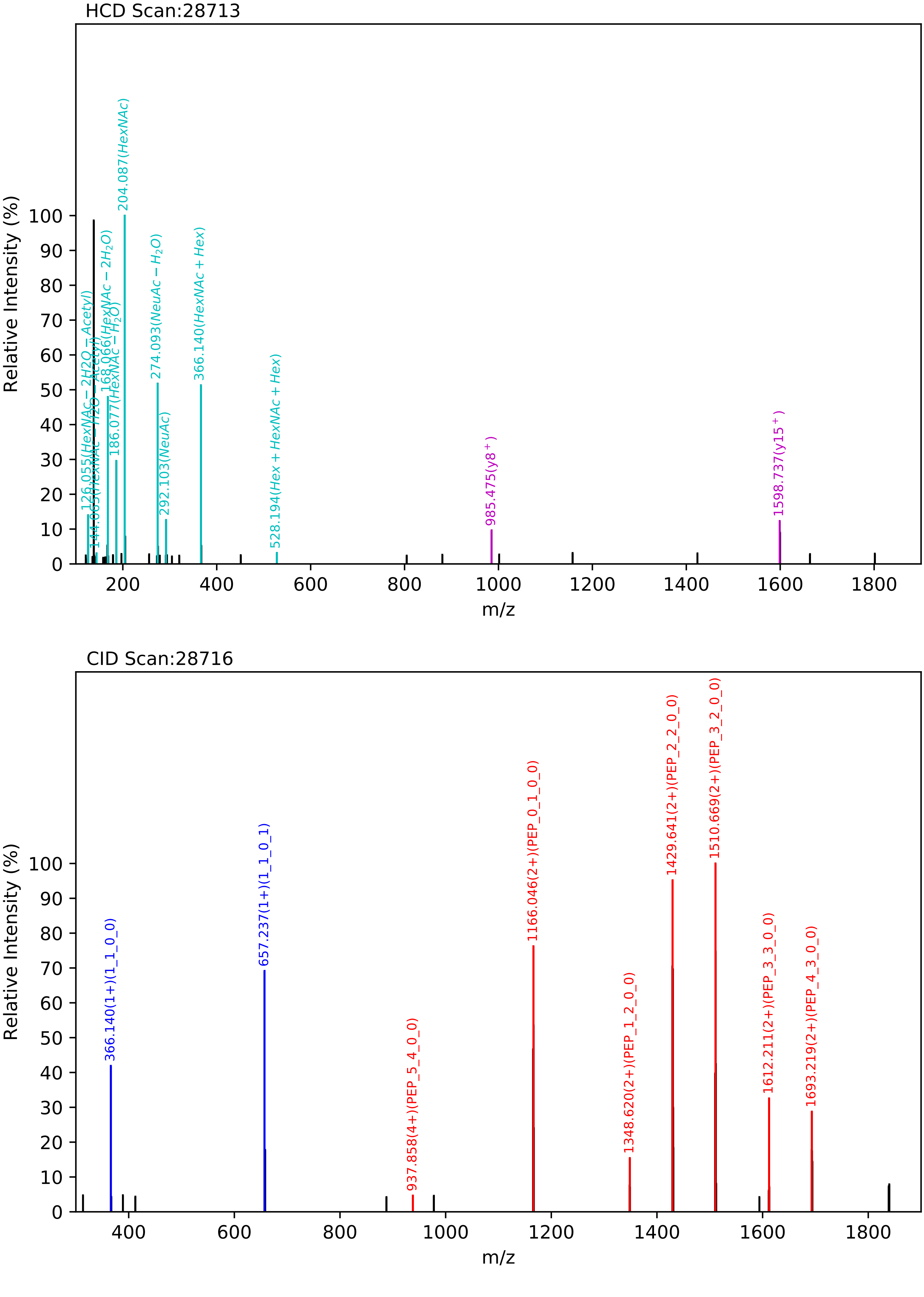

Unknown set no. 181, Experiment: Human Plasma exp_1

DNNSIITR(=PEP)_5_4_0_1, m/z:949.39(3+), RT:45.38, Y-score:94.78

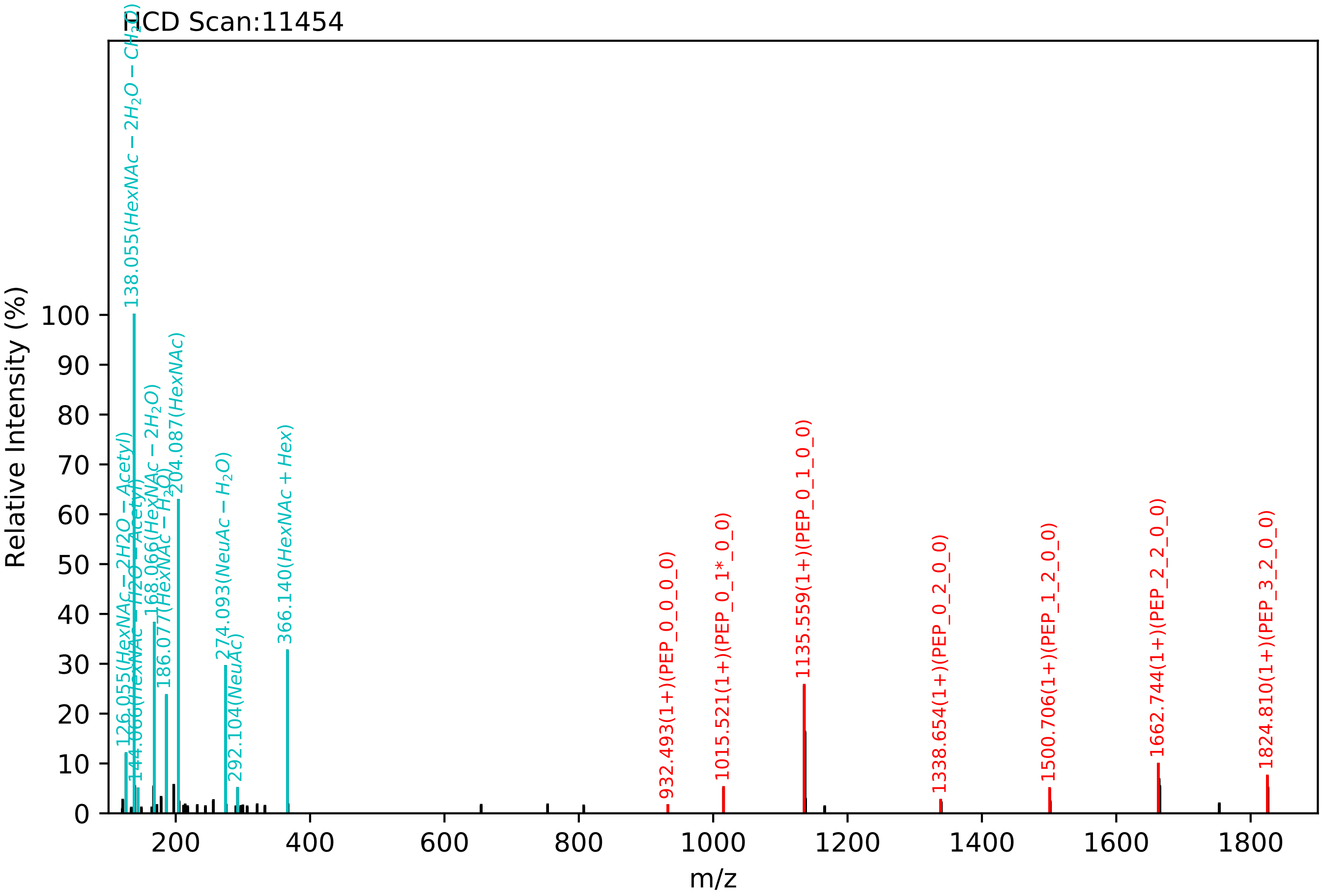

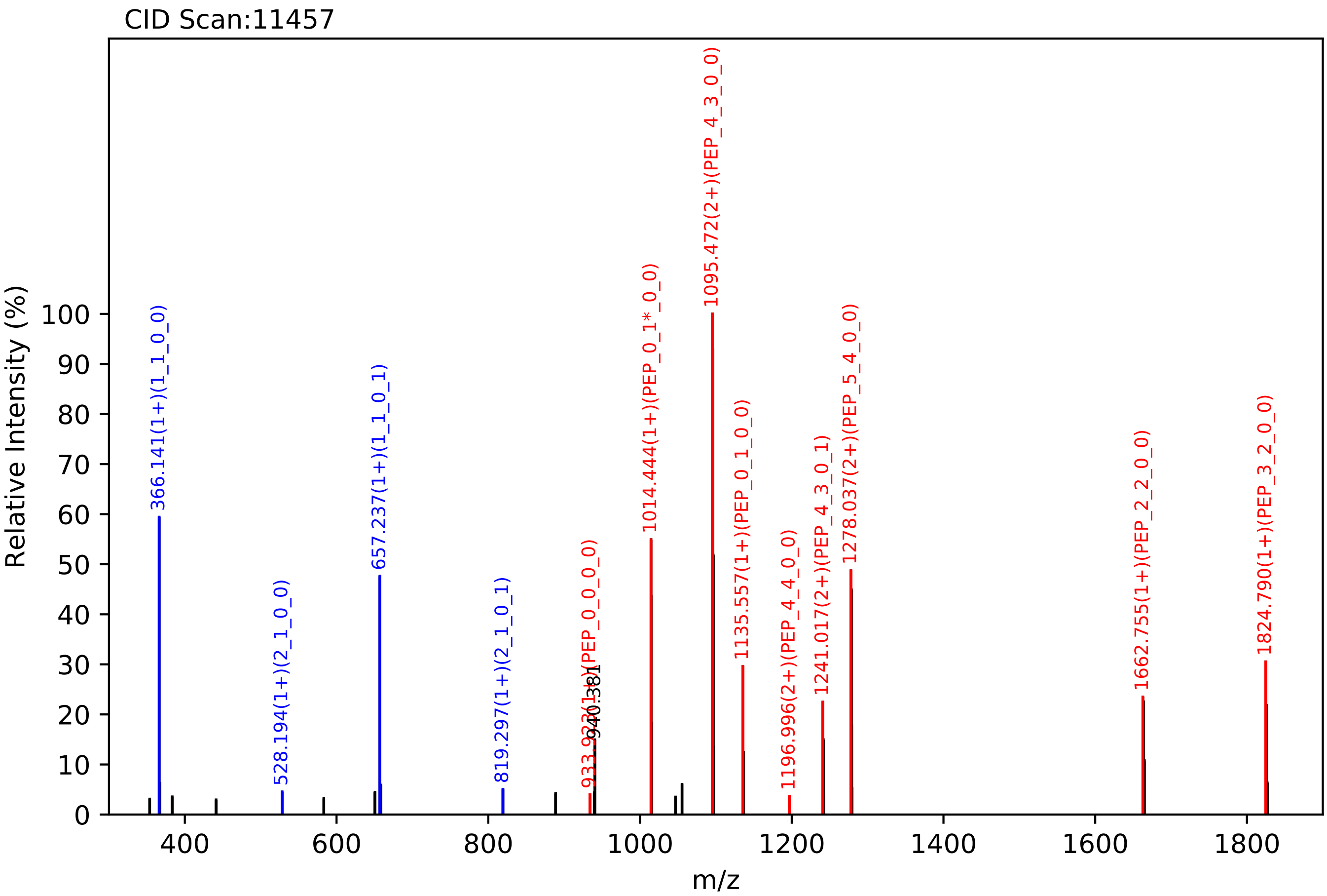

Unknown set no. 182, Experiment: Human Plasma exp_2

DNNSIITR(=PEP)_5_4_0_1, m/z:949.39(3+), RT:45.46, Y-score:93.55

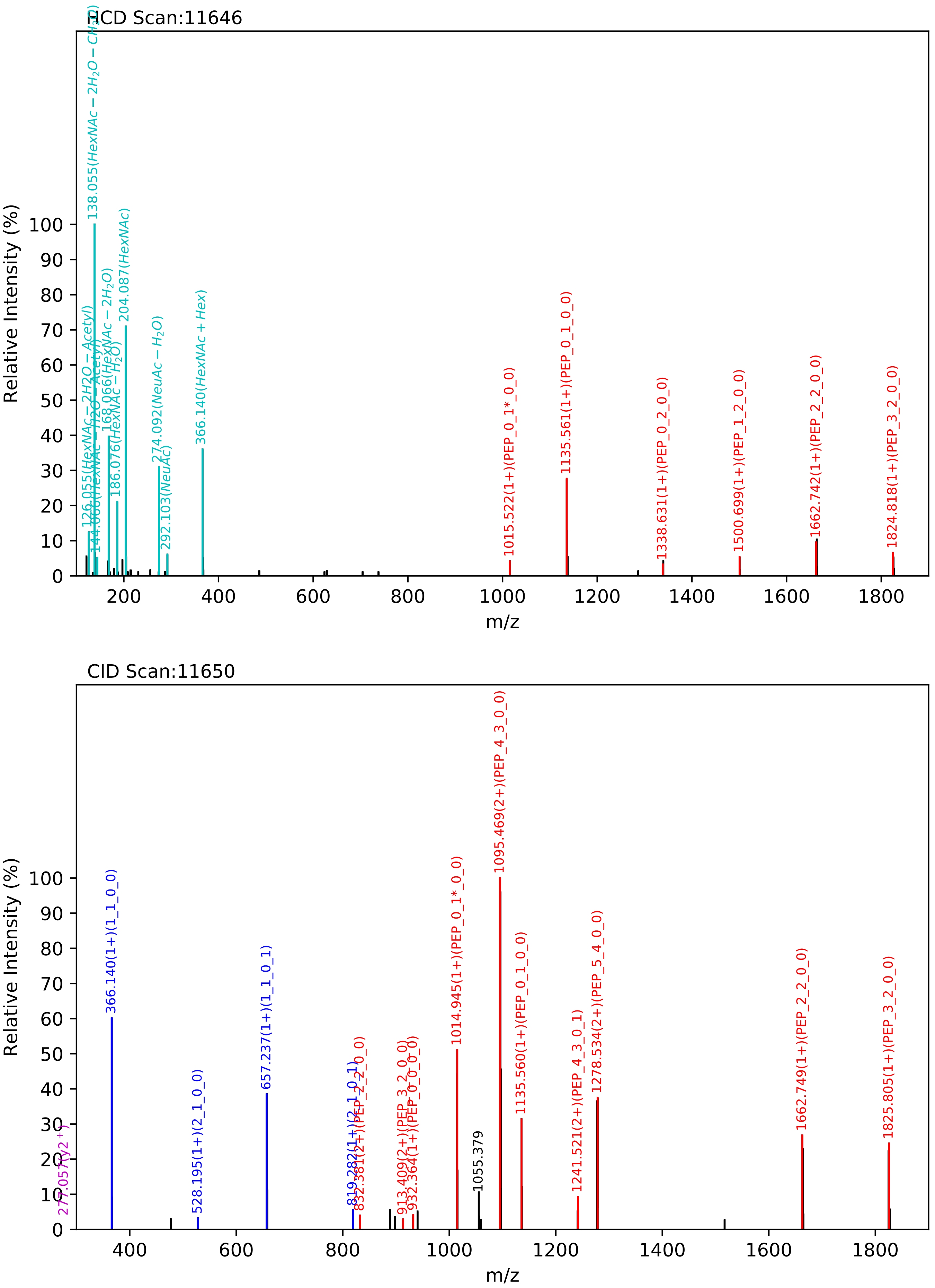

Unknown set no. 183, Experiment: Human Plasma exp_5

DNNSIITR(=PEP)_5_4_0_1, m/z:949.39(3+), RT:45.69, Y-score:93.77

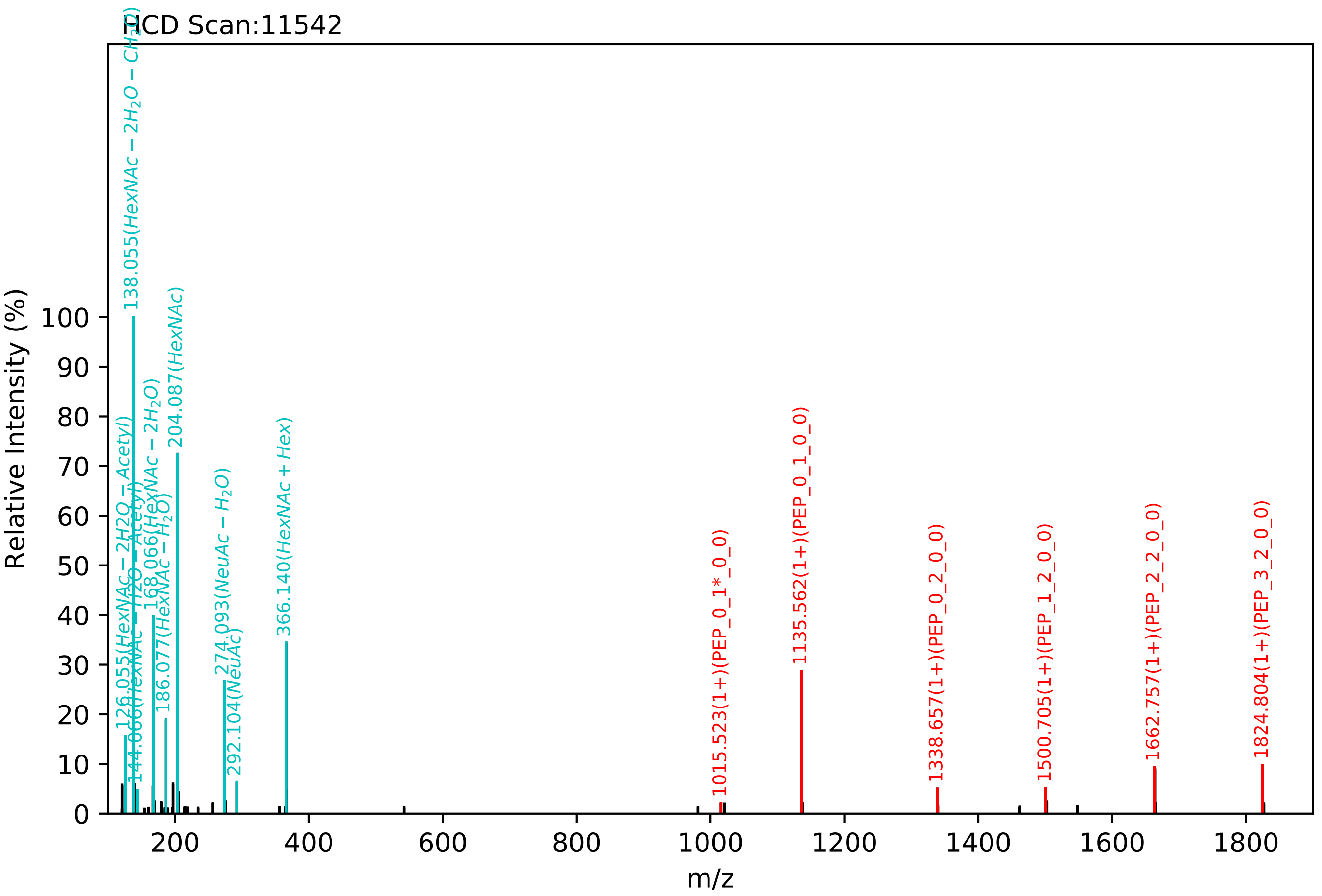

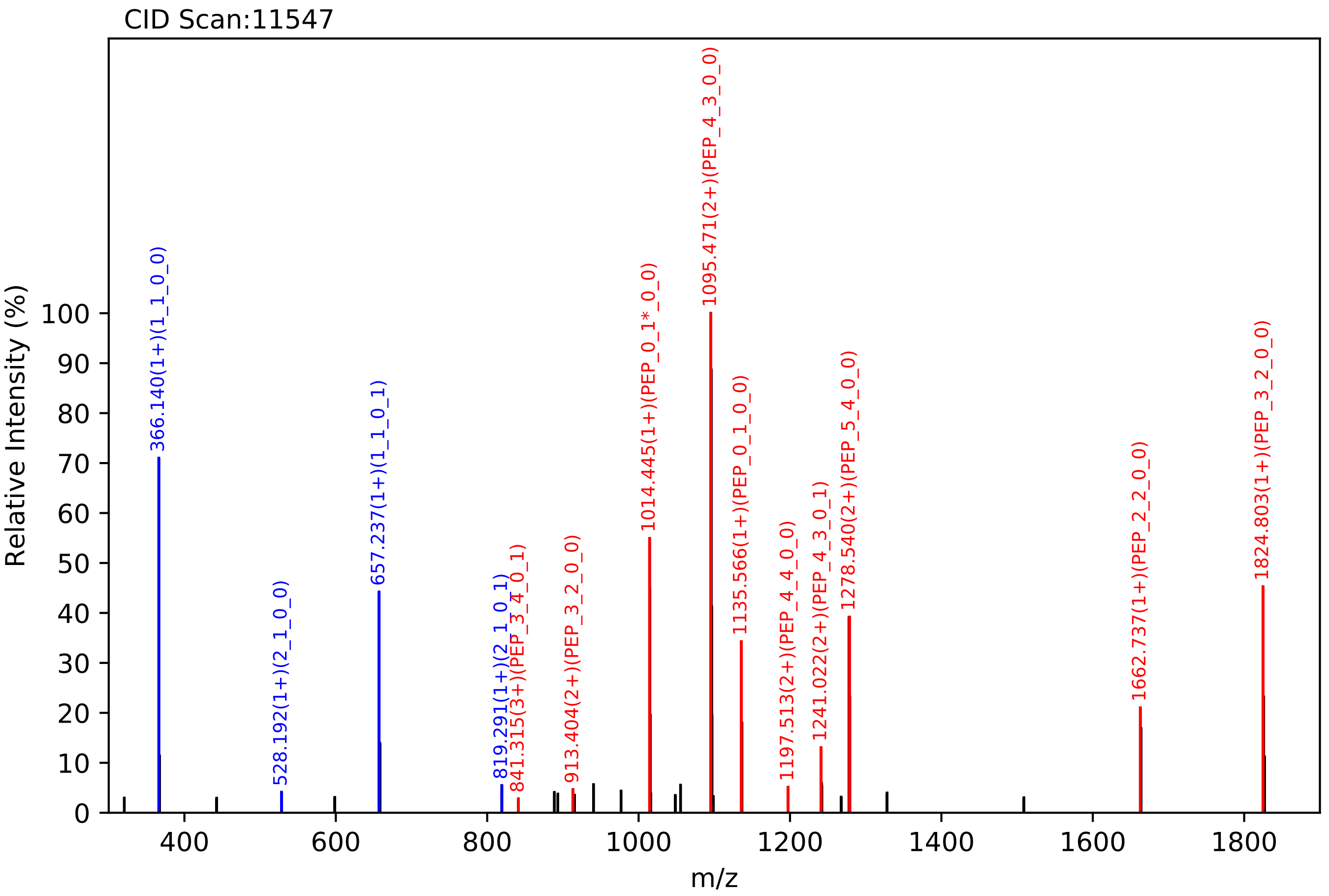

Unknown set no. 184, Experiment: Human Plasma exp_5

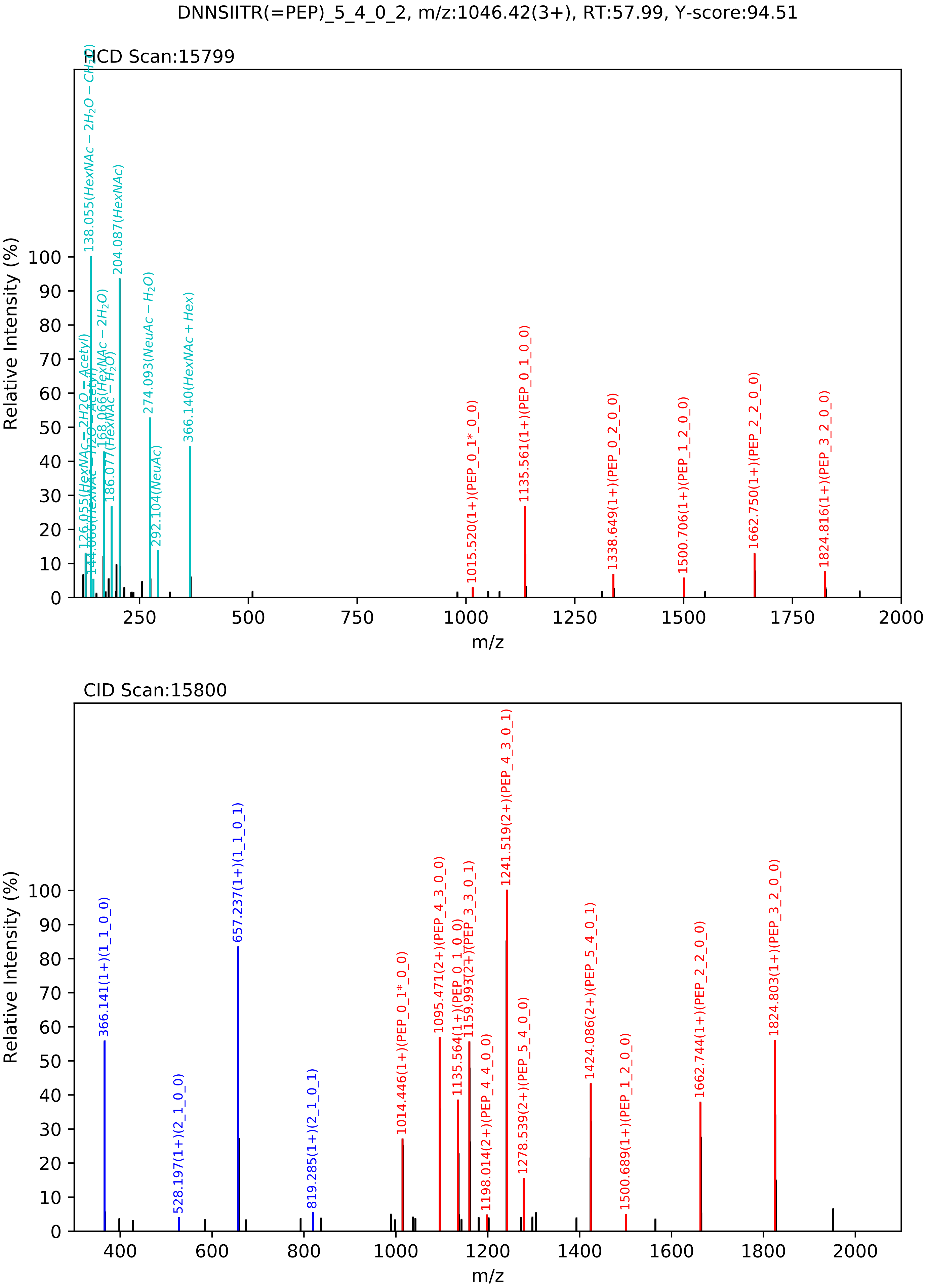

Unknown set no. 185, Experiment: Human Plasma exp_1

DNYTK(=PEP)_5_4_0_2, m/z:949.03(3+), RT:21.41, Y-score:92.28

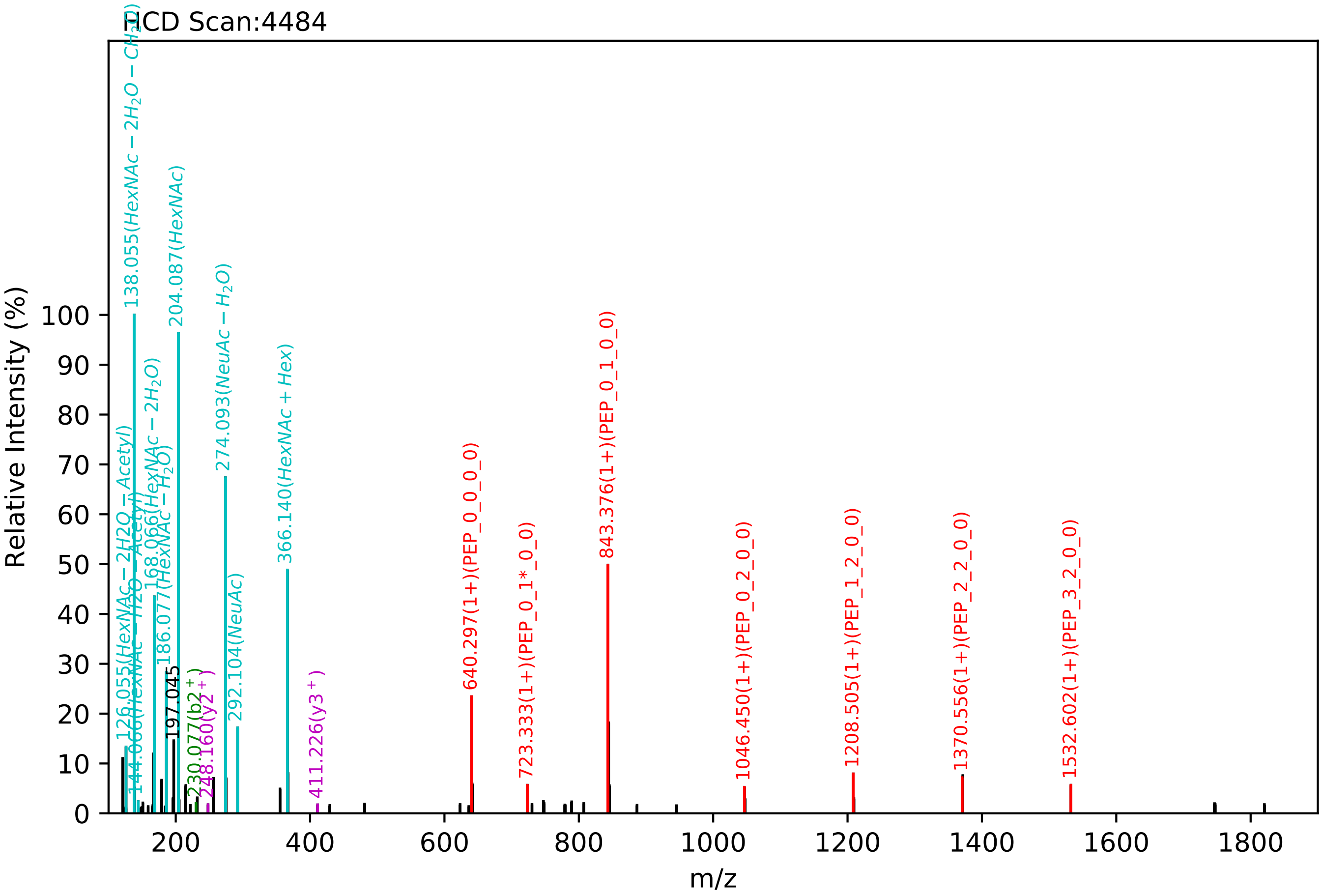

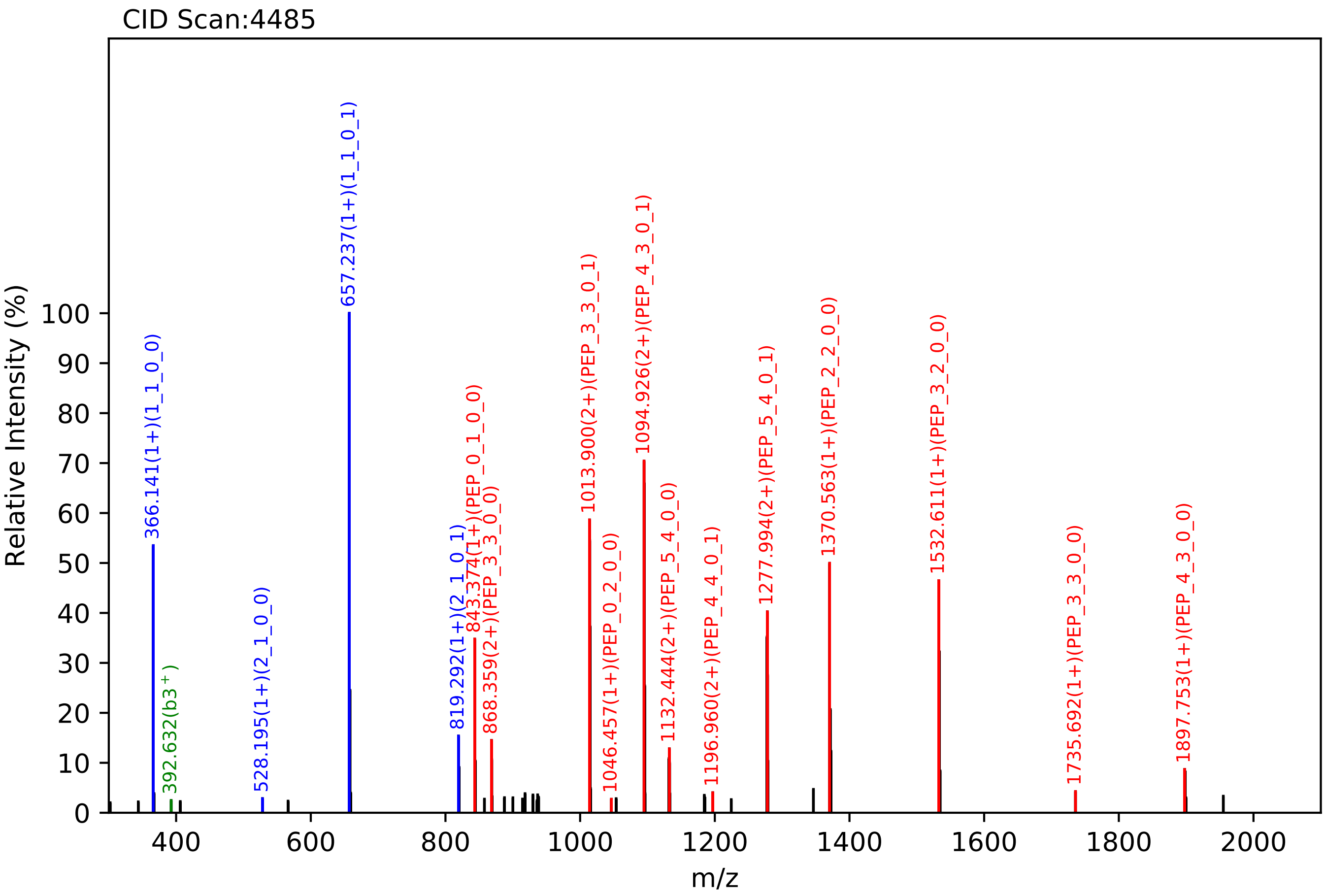

Unknown set no. 186, Experiment: Human Plasma exp_2

DNYTK(=PEP)_5_4_0_2, m/z:949.03(3+), RT:21.52, Y-score:94.98

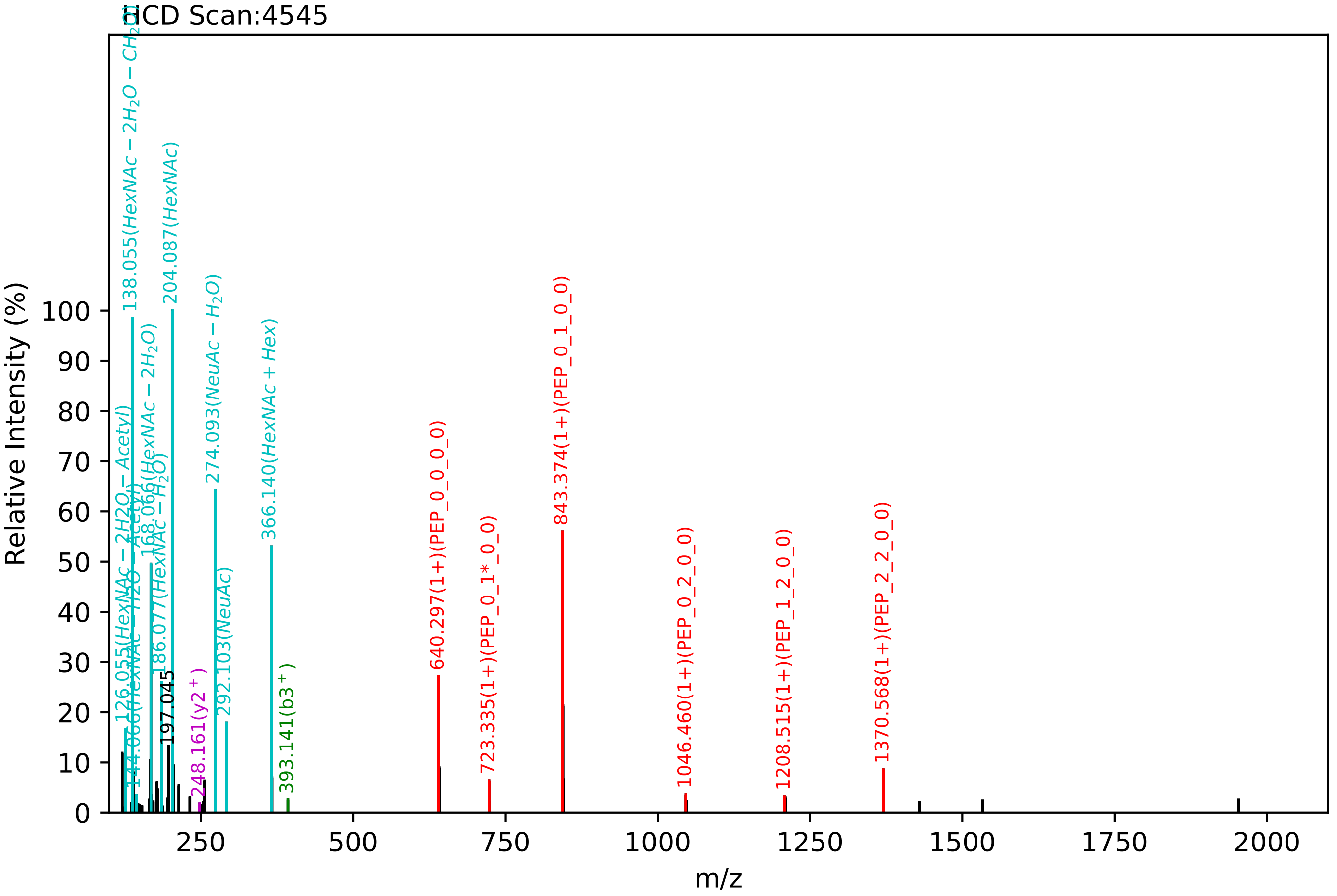

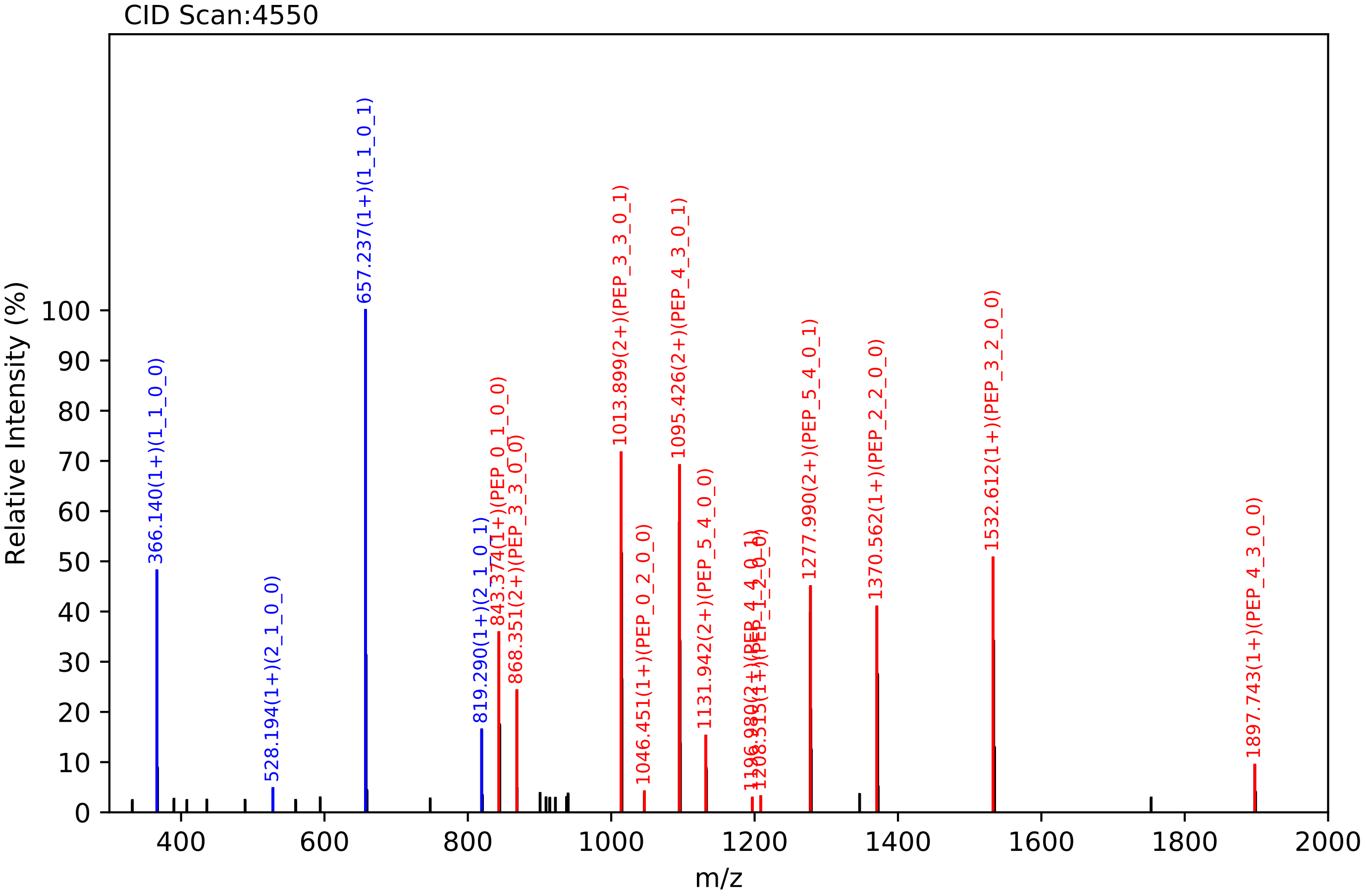

Unknown set no. 187, Experiment: Human Plasma exp_3

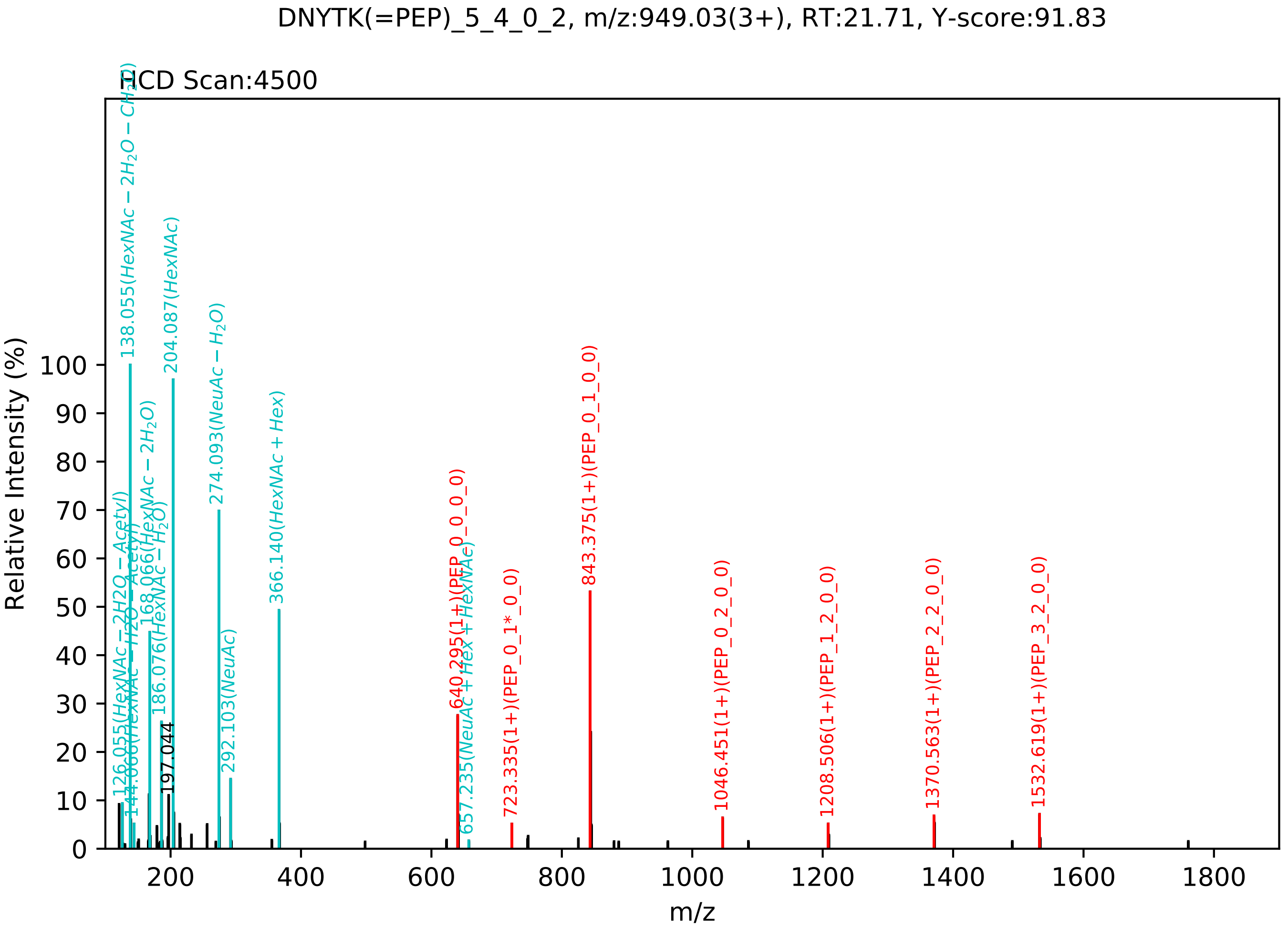

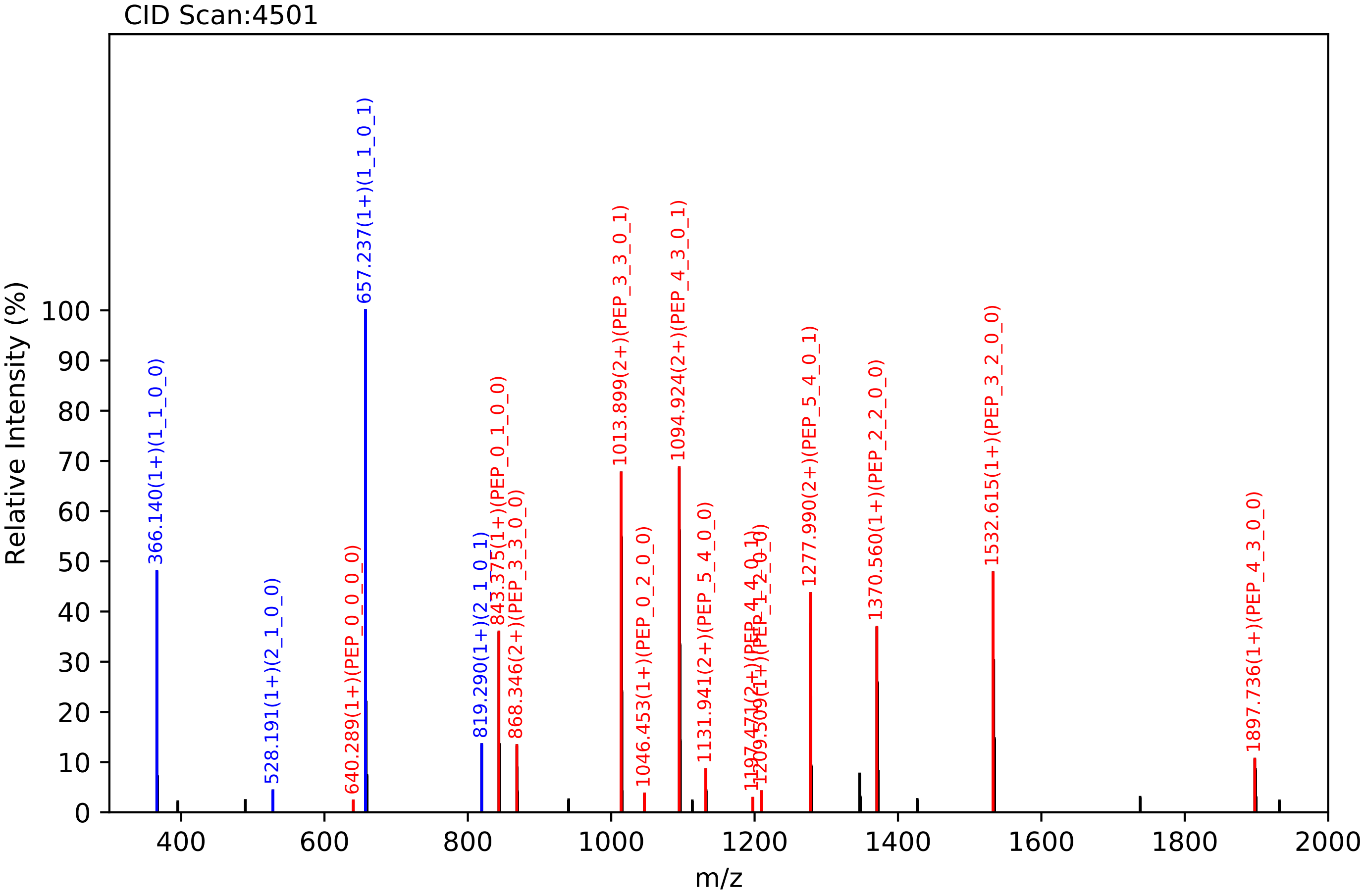

Unknown set no. 188, Experiment: Human Plasma exp_4

DNYTK(=PEP)_5_4_0_2, m/z:949.03(3+), RT:21.78, Y-score:94.32

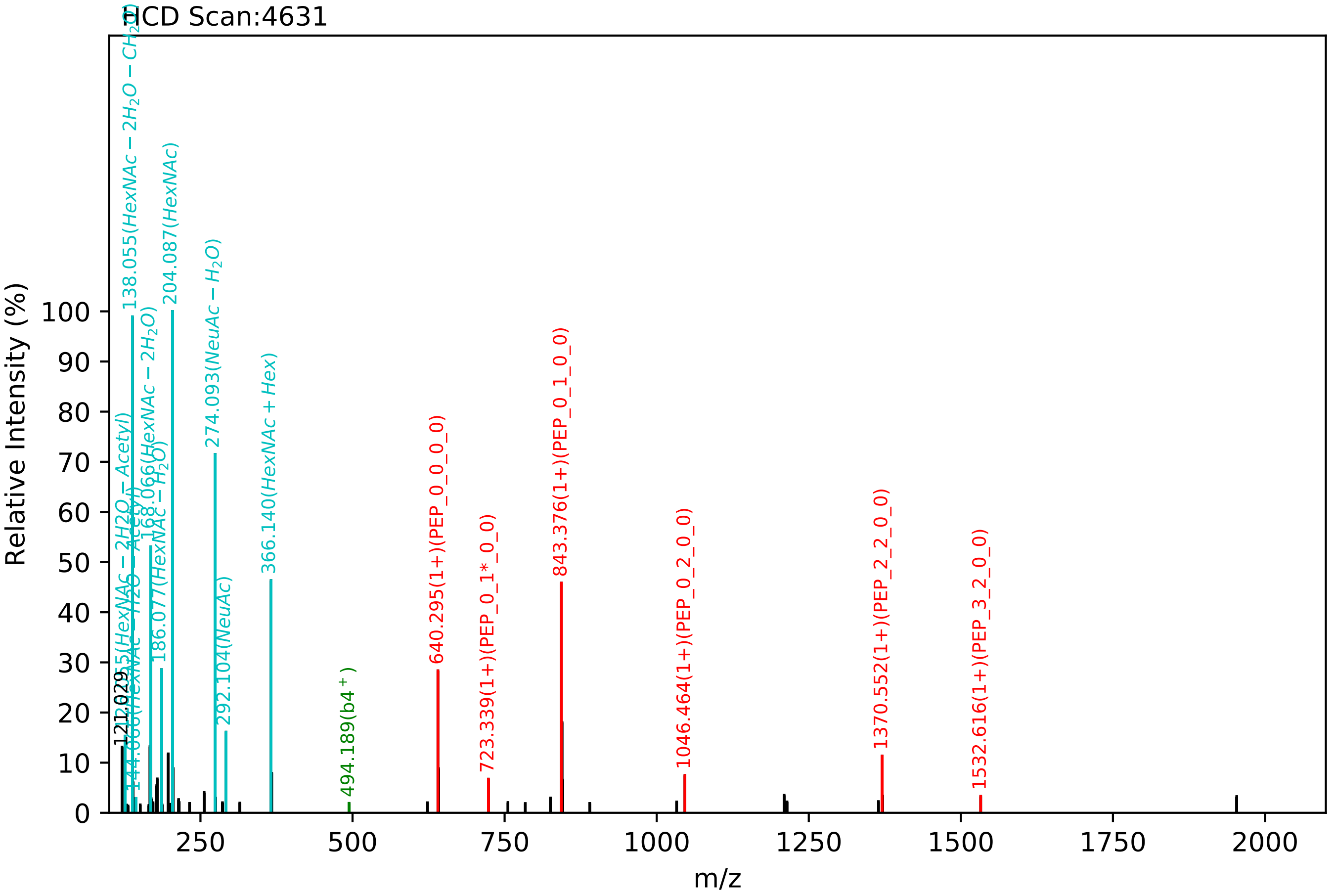

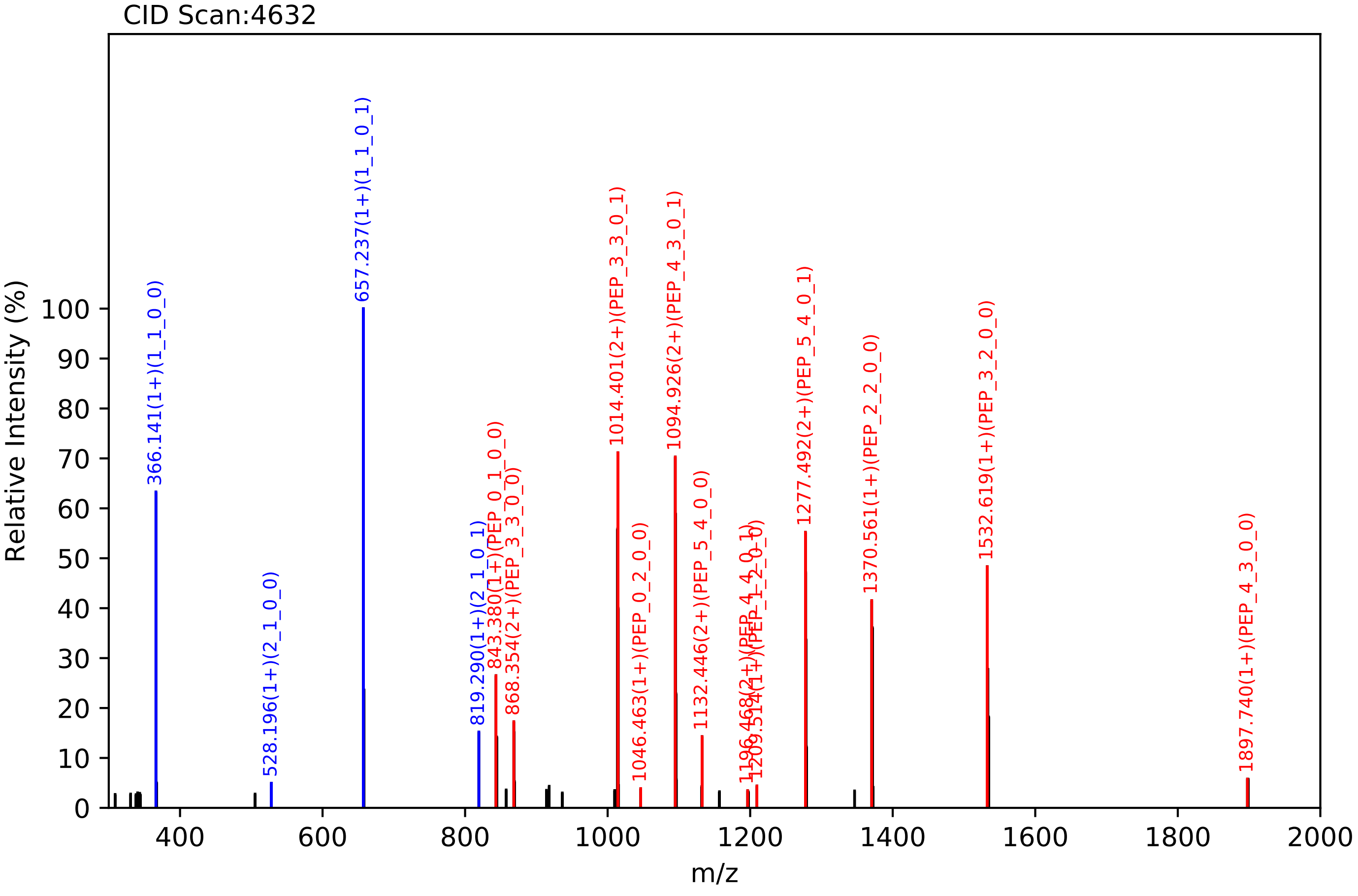

Unknown set no. 189, Experiment: Human Plasma exp_5

DNYTK(=PEP)_5_4_0_2, m/z:949.03(3+), RT:21.80, Y-score:94.79

HCD Scan:4513

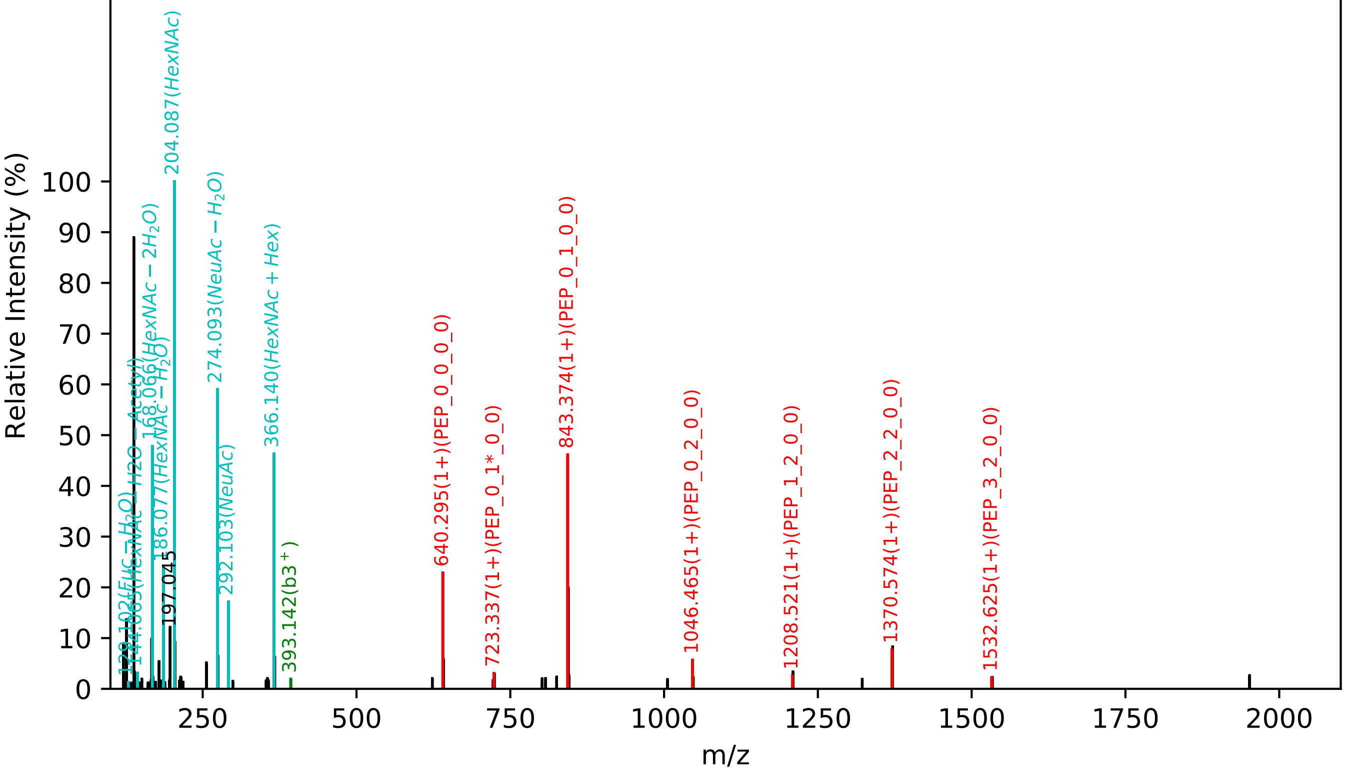

CID Scan:4514

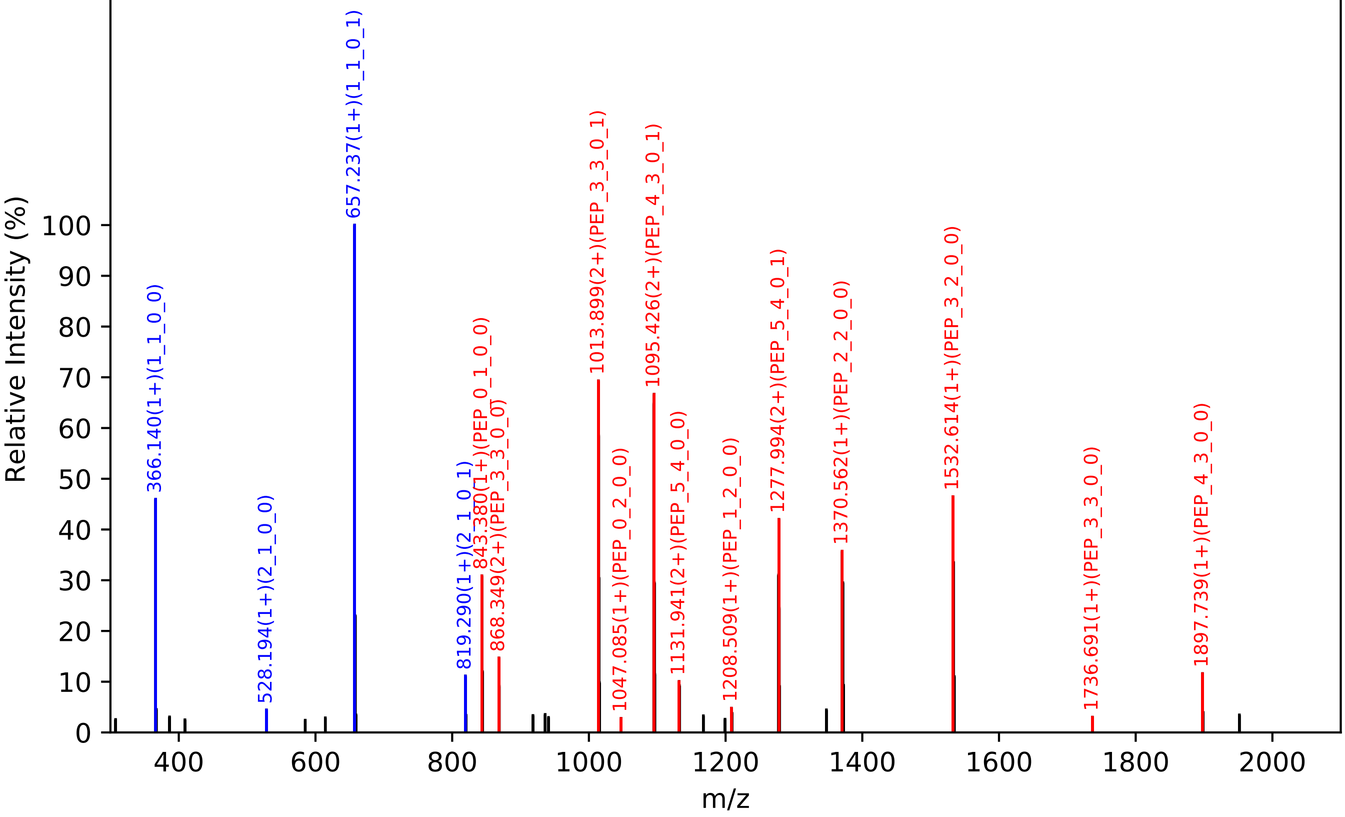

Unknown set no. 190, Experiment: Human Plasma exp_2

ENETEIIK(=PEP)_5_4_0_2, m/z:1060.77(3+), RT:60.50, Y-score:86.43

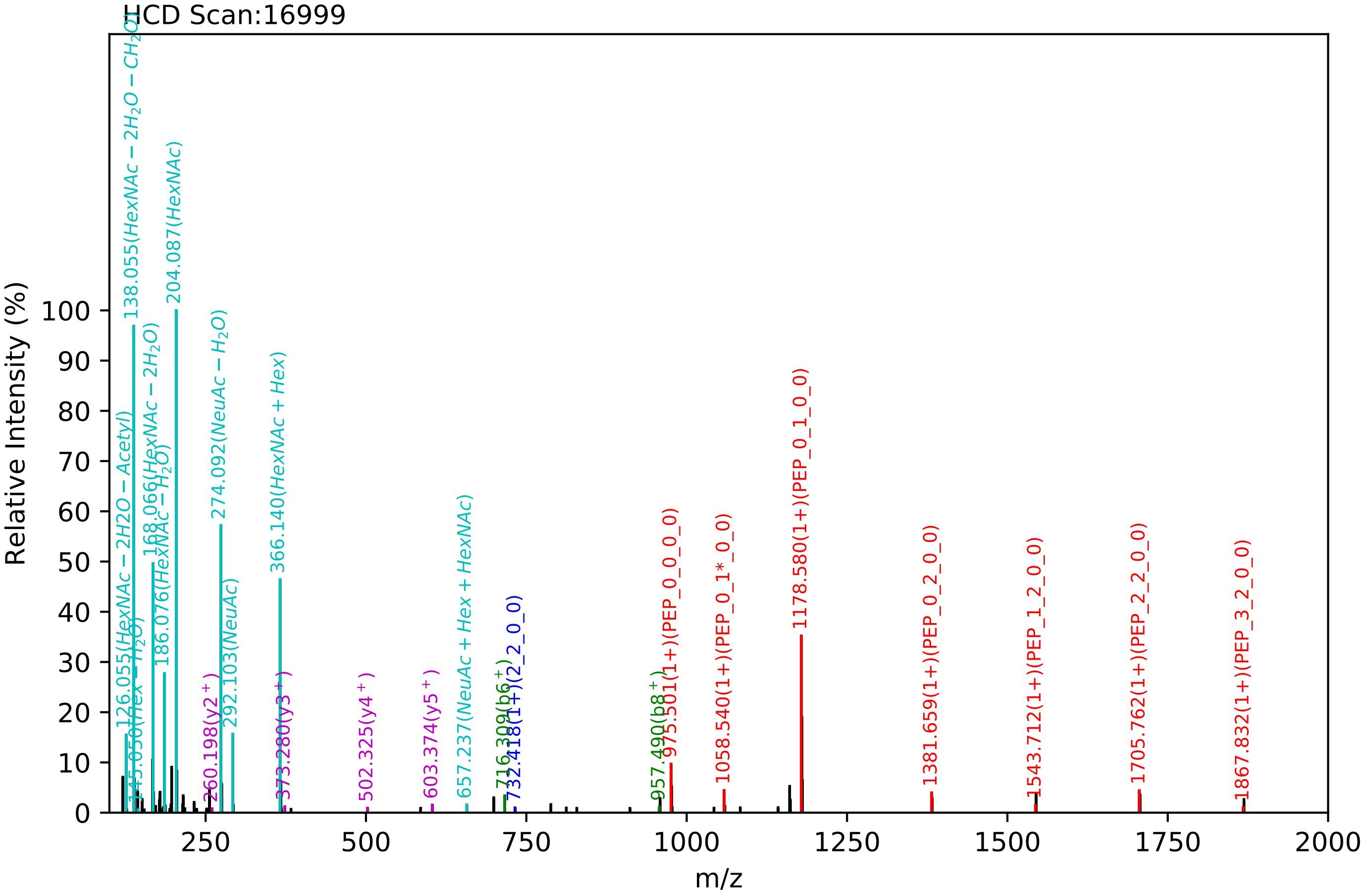

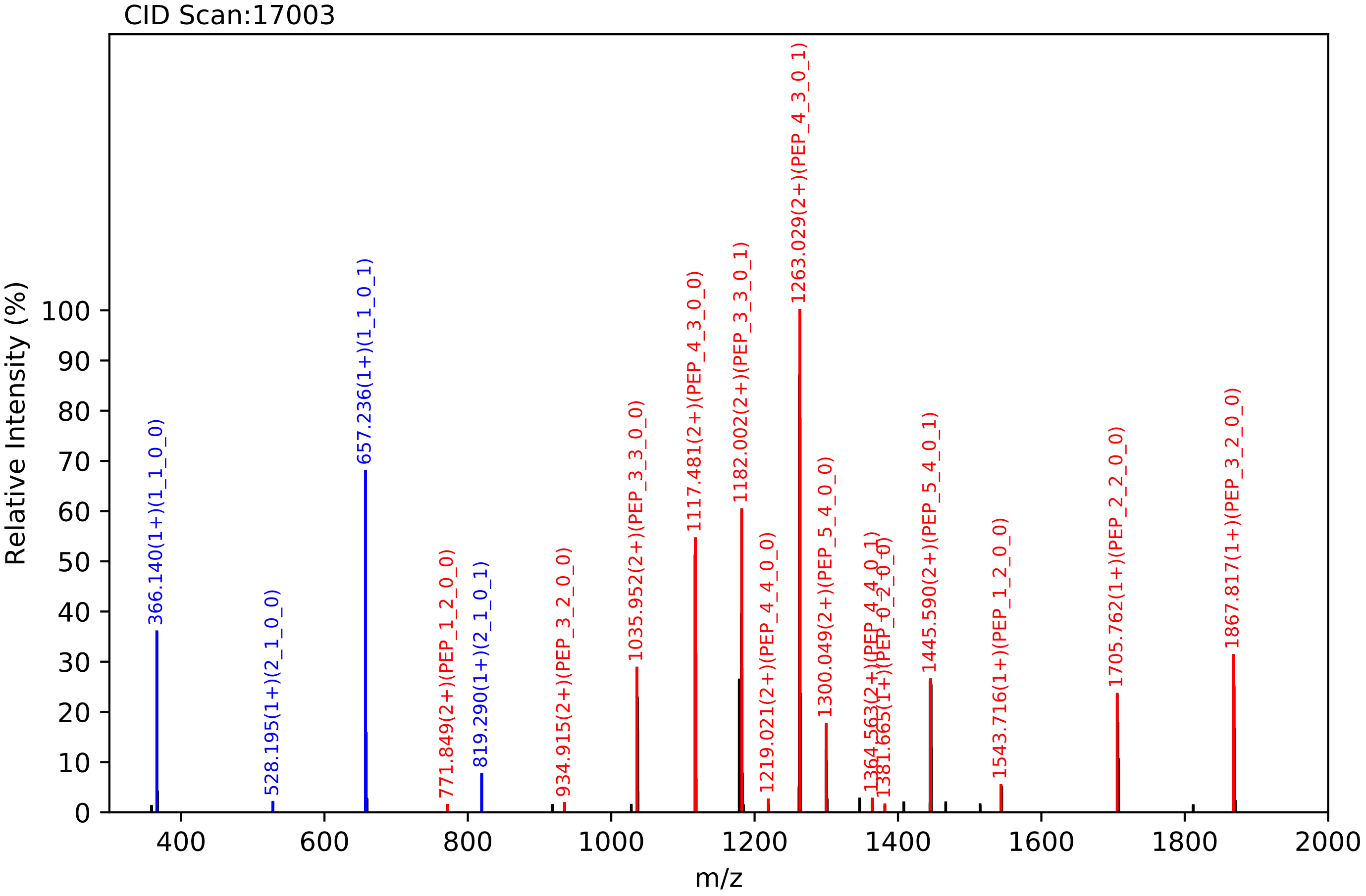

Unknown set no. 191, Experiment: Human Plasma exp_5

ENETEIIK(=PEP)_5_4_0_2, m/z:1060.77(3+), RT:60.56, Y-score:83.99

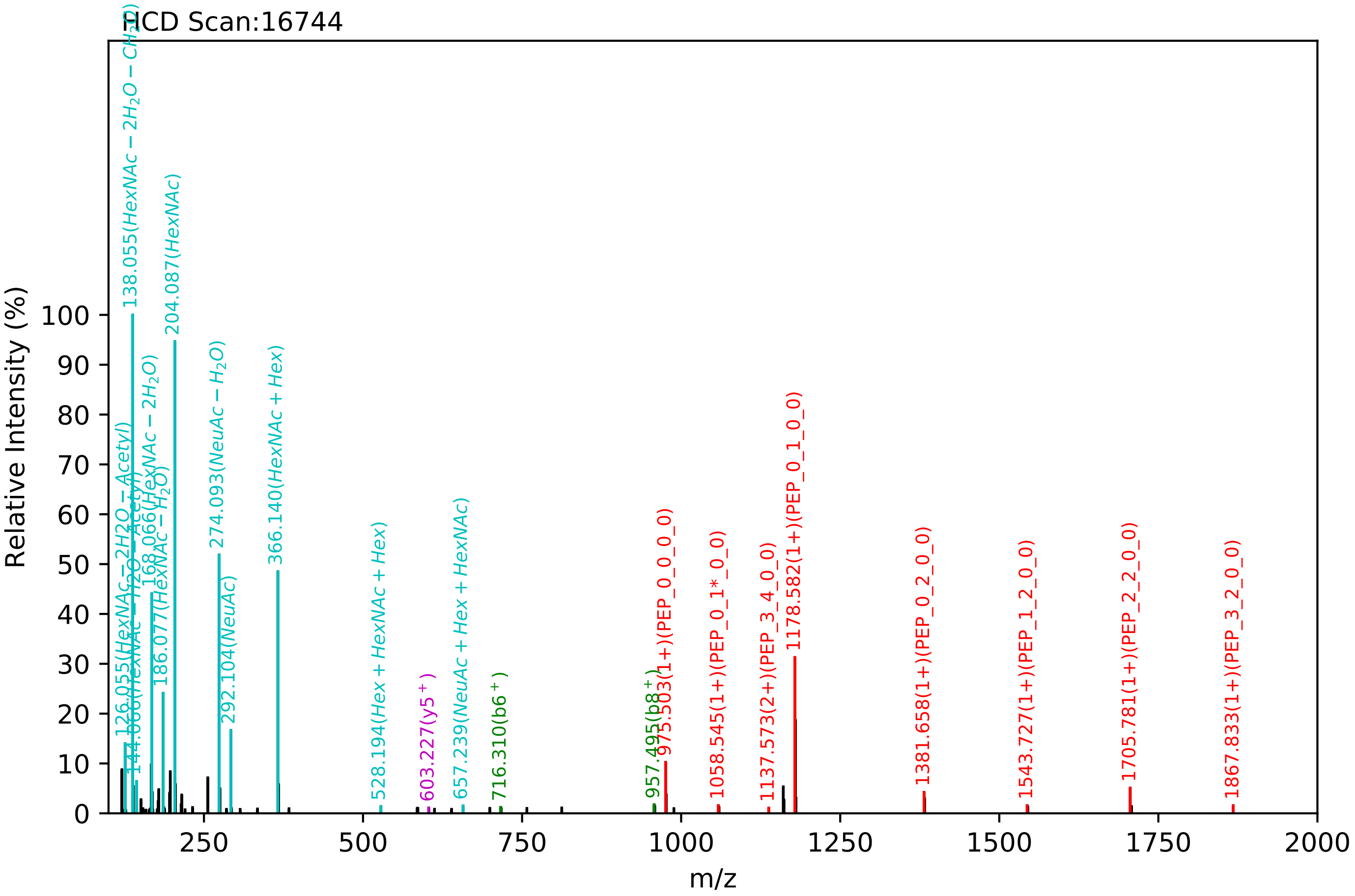

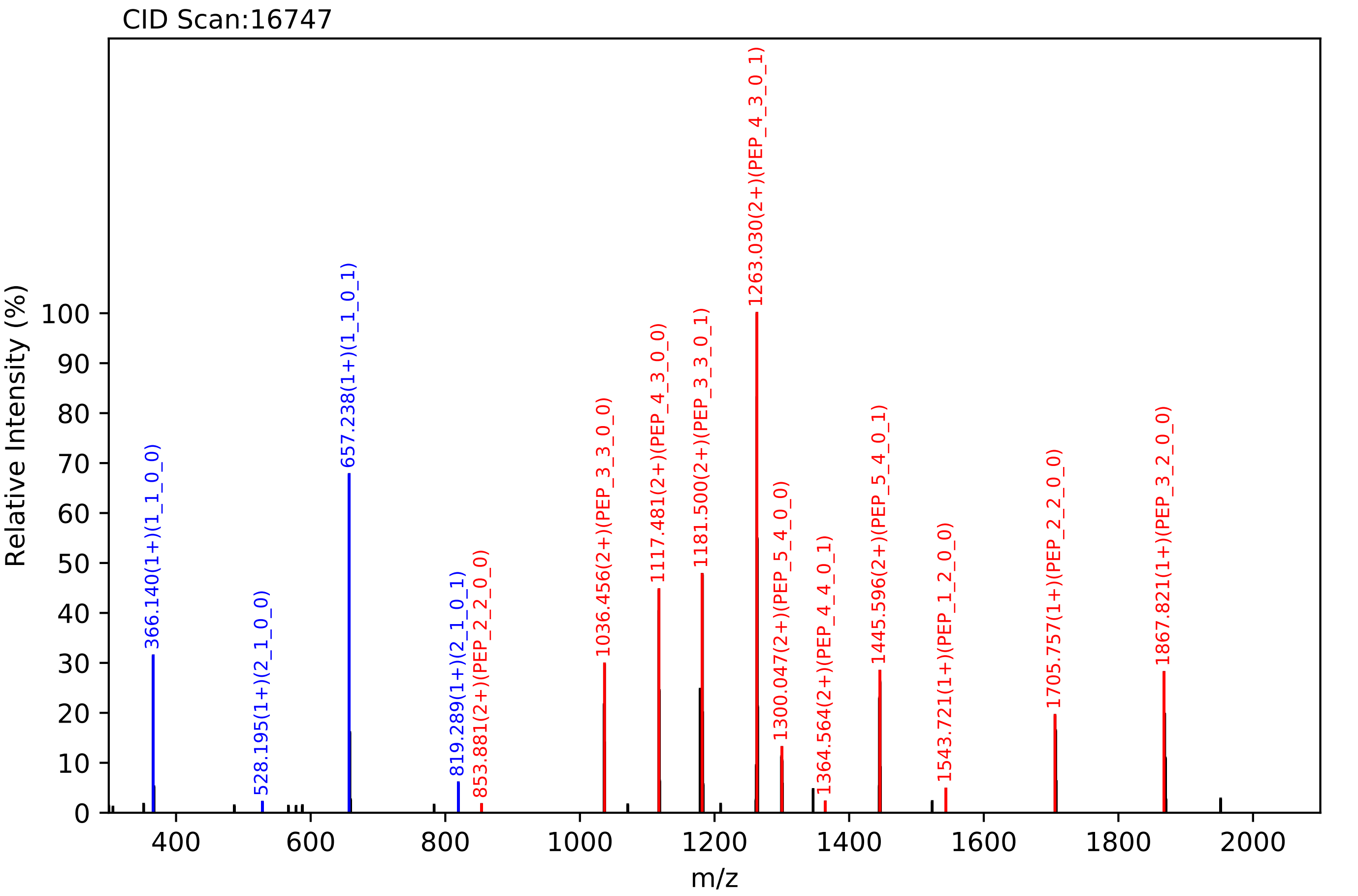

Unknown set no. 192, Experiment: Human Plasma exp_1

EDALNETR(=PEP)_5_4_0_2, m/z:1051.41(3+), RT:49.30, Y-score:78.63

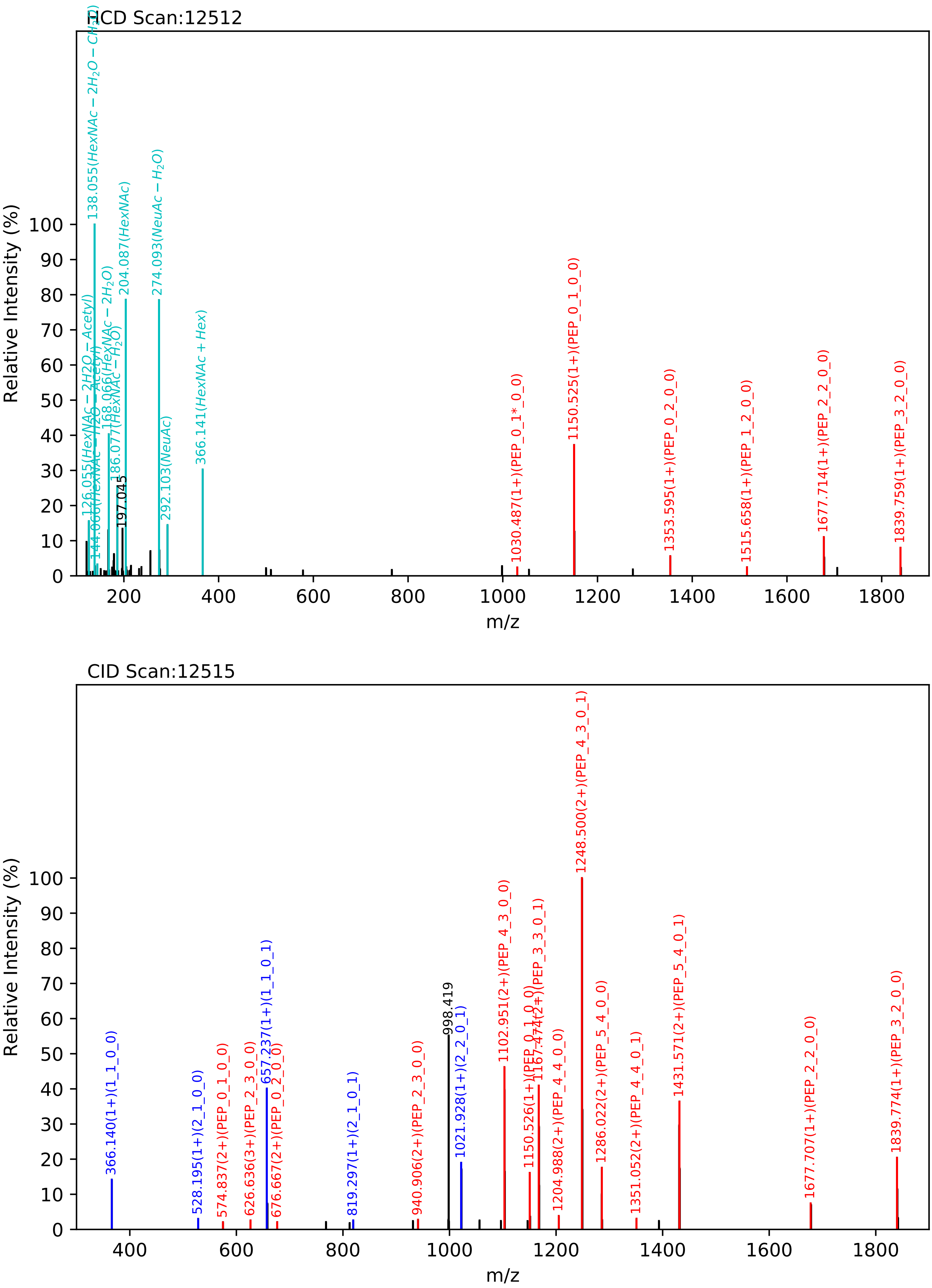

Unknown set no. 193, Experiment: Human Plasma exp_1

EDALNETR(=PEP)_6_5_0_3, m/z:1270.15(3+), RT:59.45, Y-score:91.56

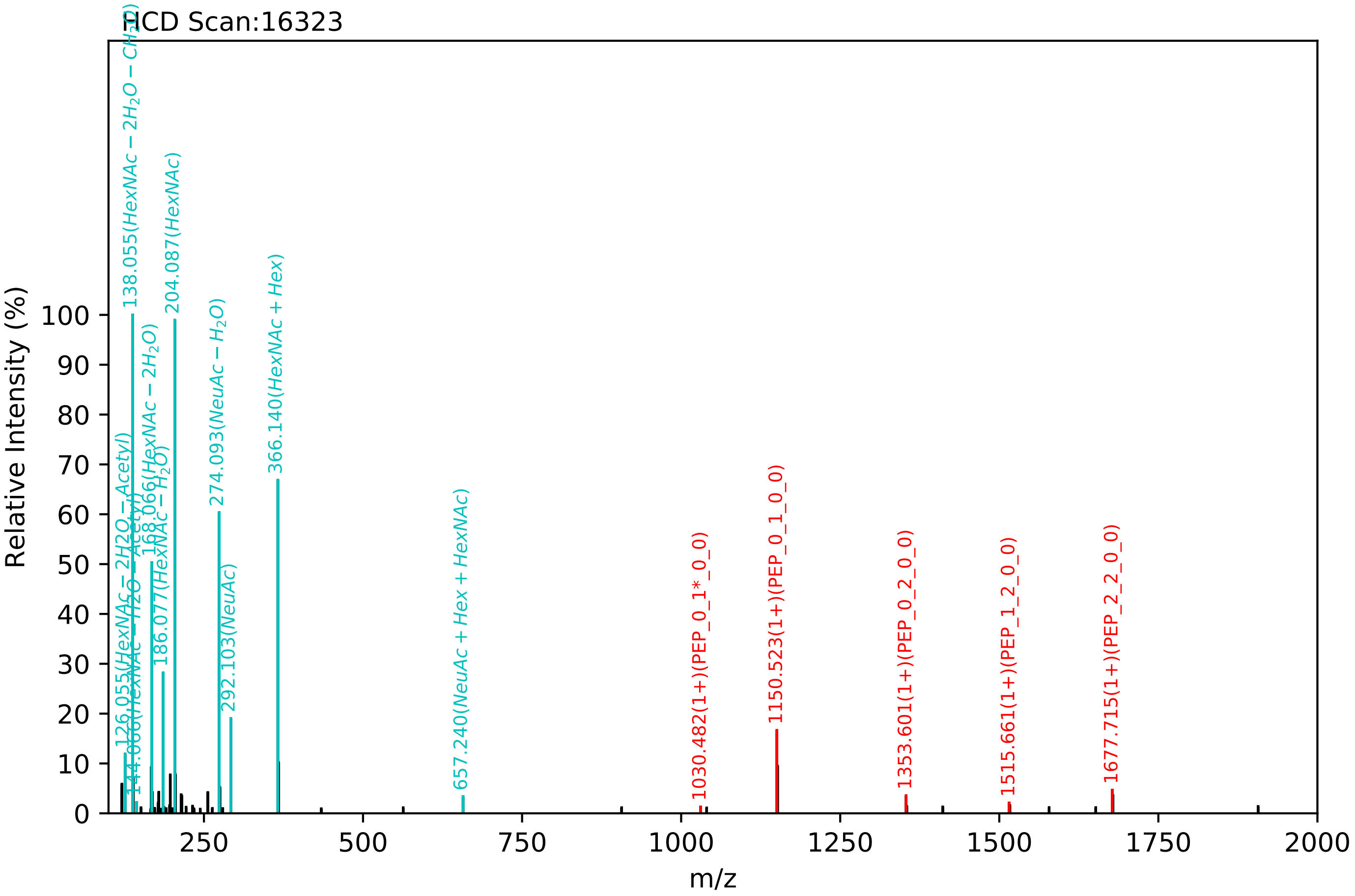

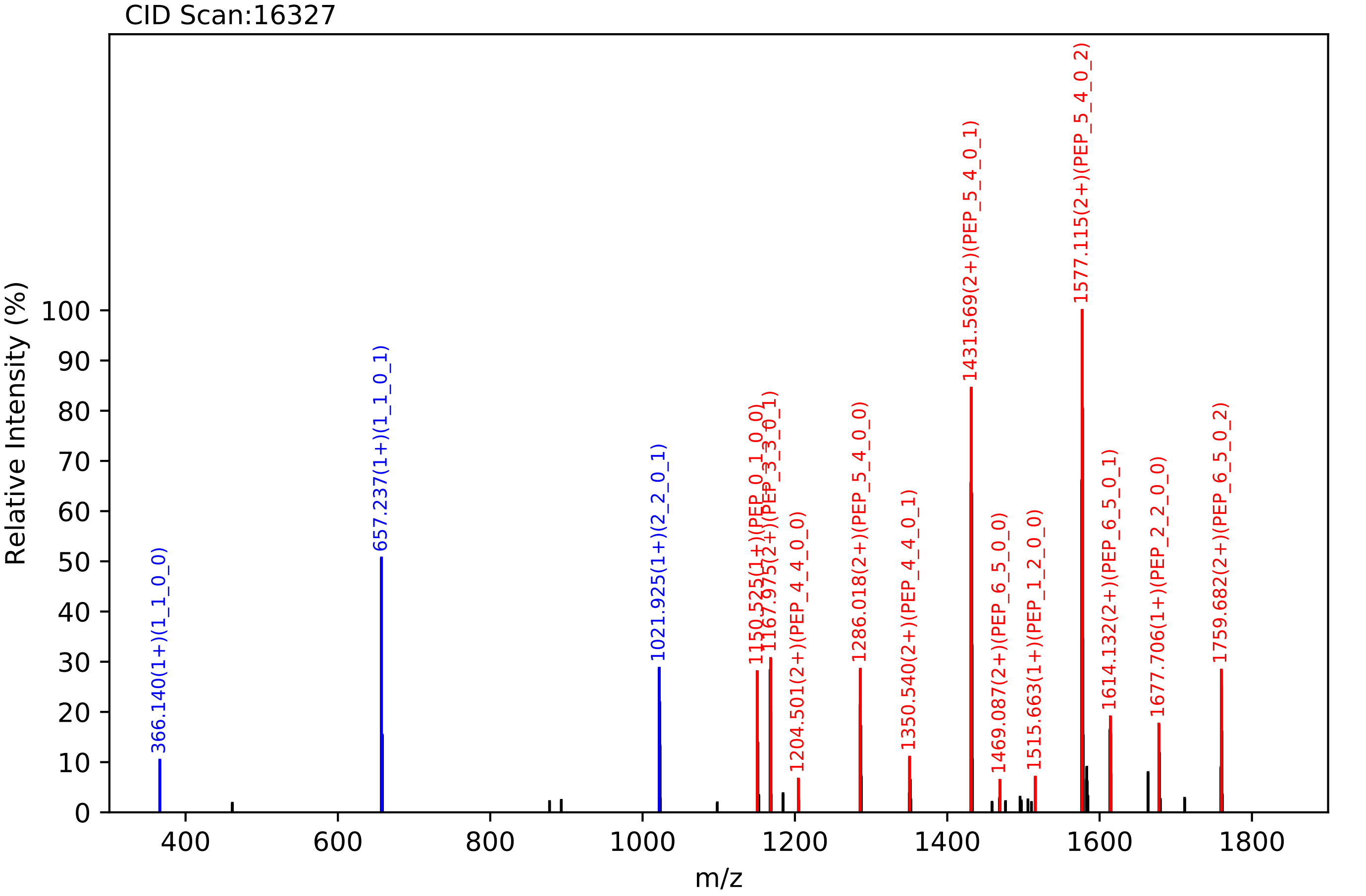

Unknown set no. 194, Experiment: Human Plasma exp_3

EDALNETR(=PEP)_6_5_1_3, m/z:989.38(4+), RT:59.64, Y-score:83.60

HCD Scan:16374

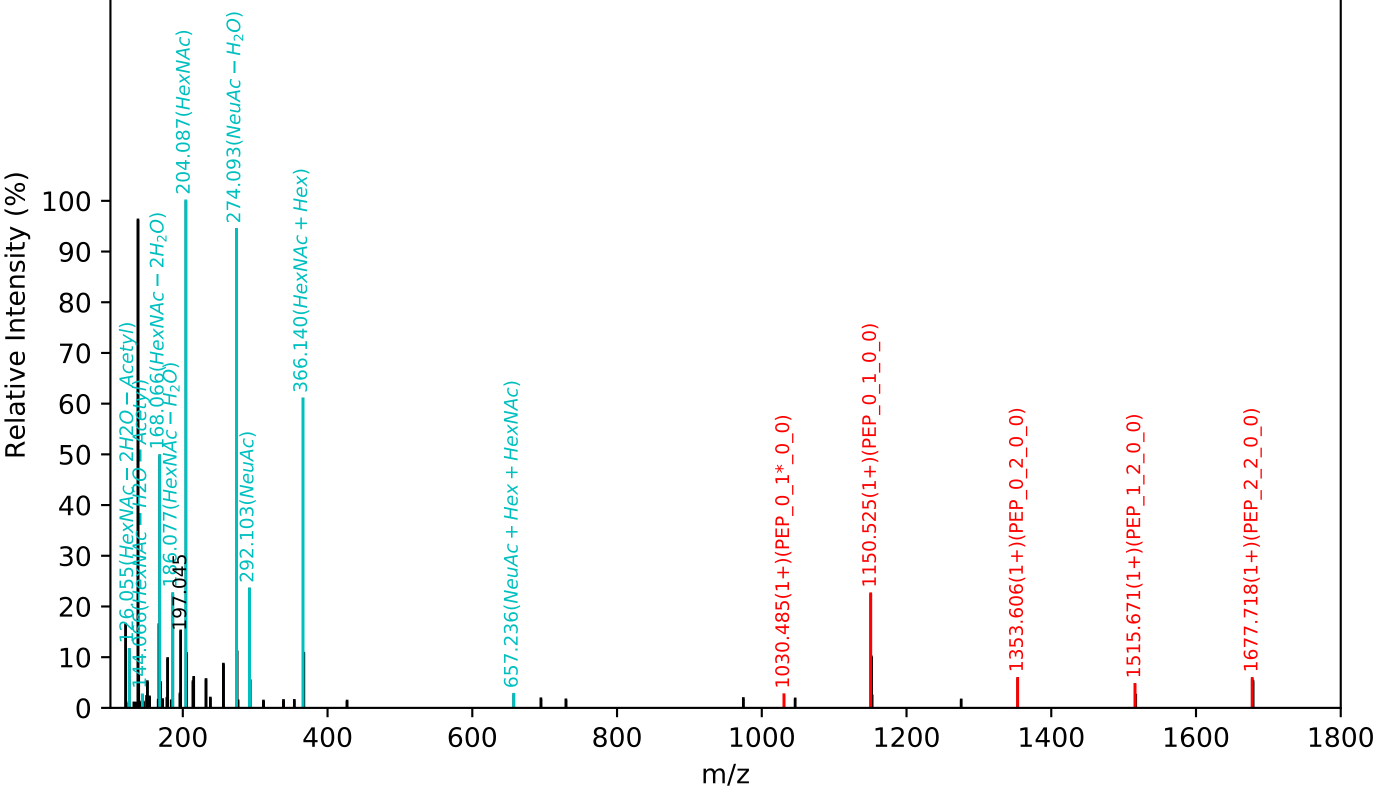

CID Scan:16379

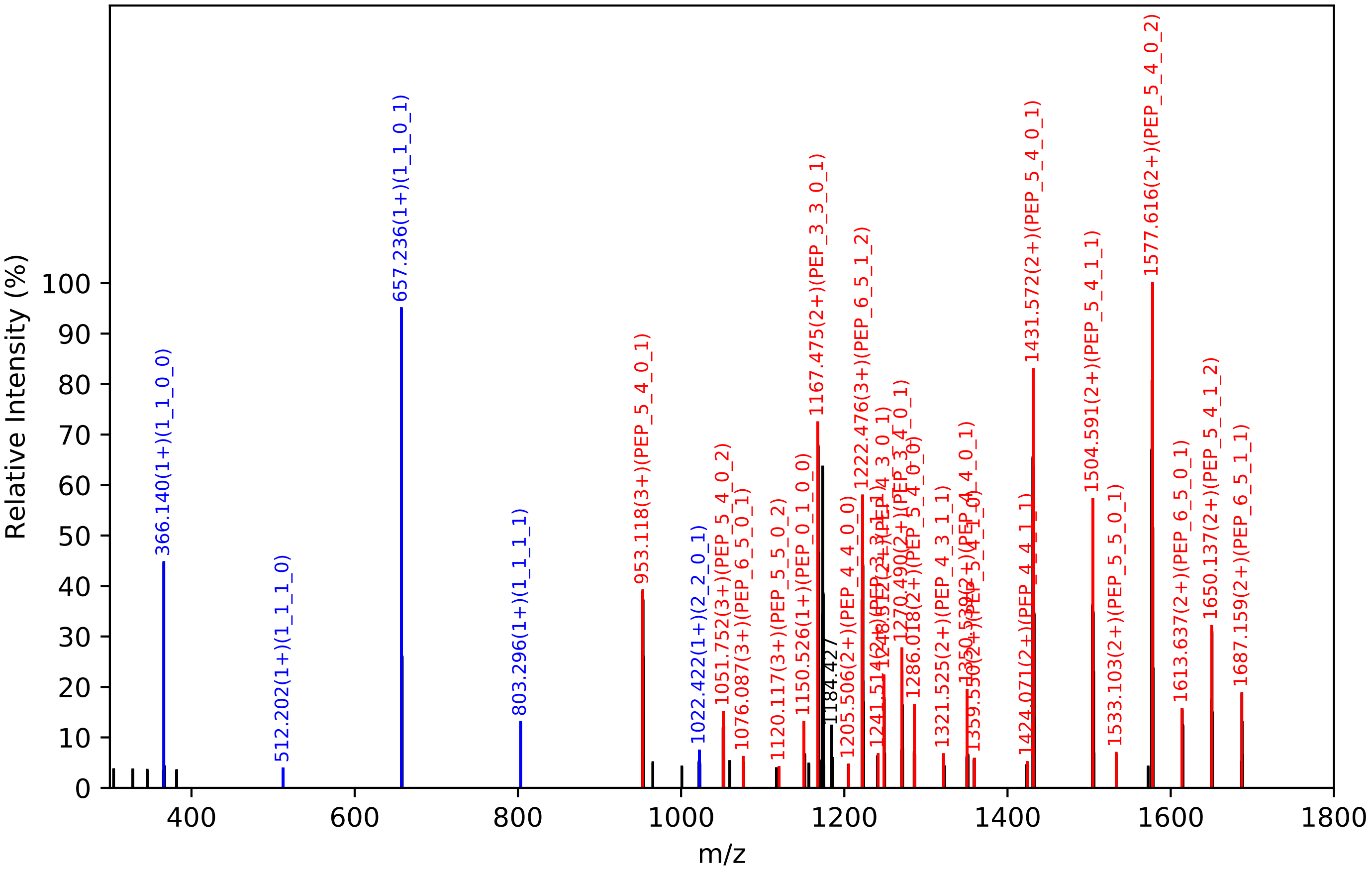

Unknown set no. 195, Experiment: Human Plasma exp_4

EDALNETR(=PEP)_6_5_1_3, m/z:1318.84(3+), RT:59.67, Y-score:87.67

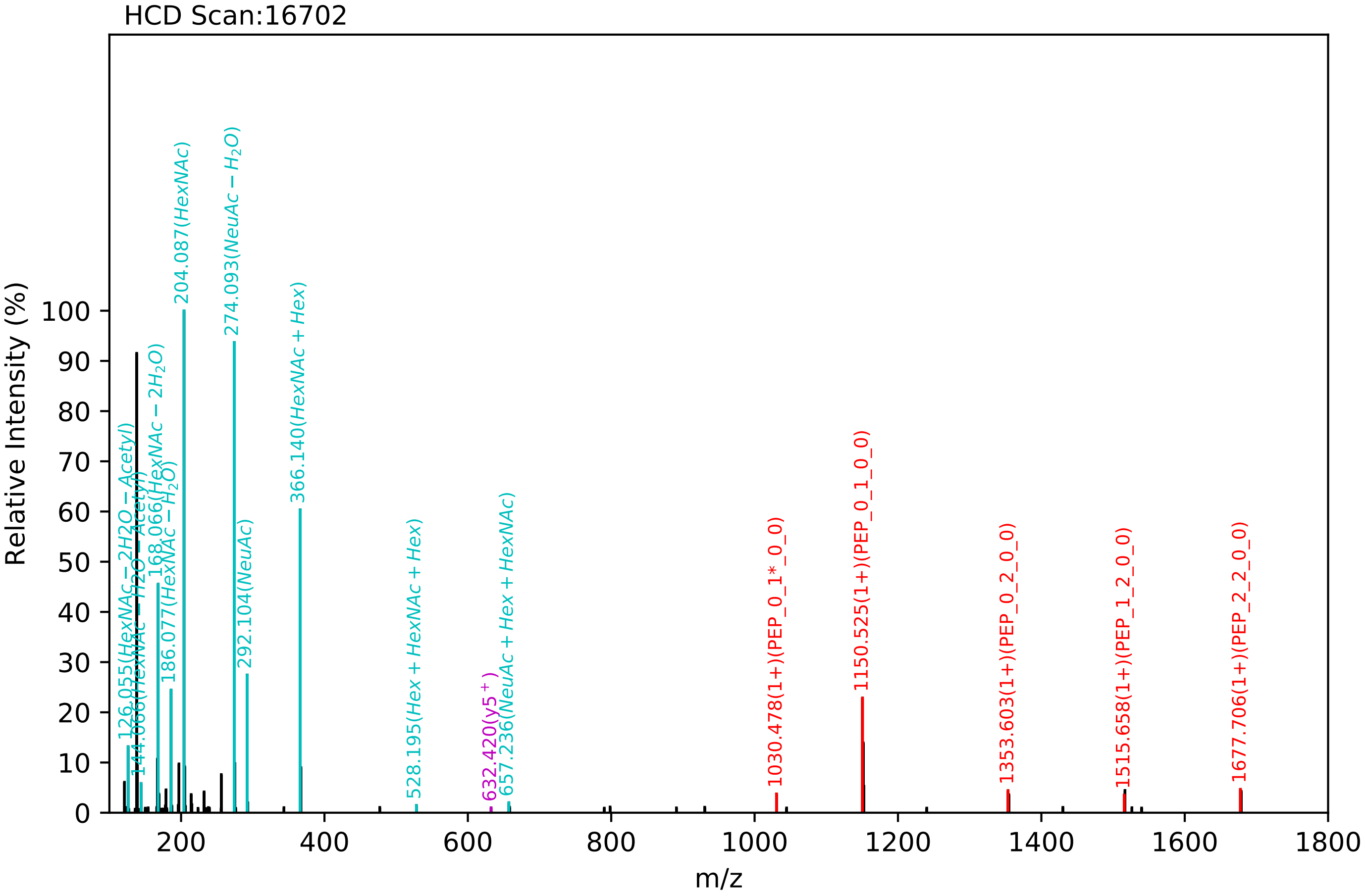

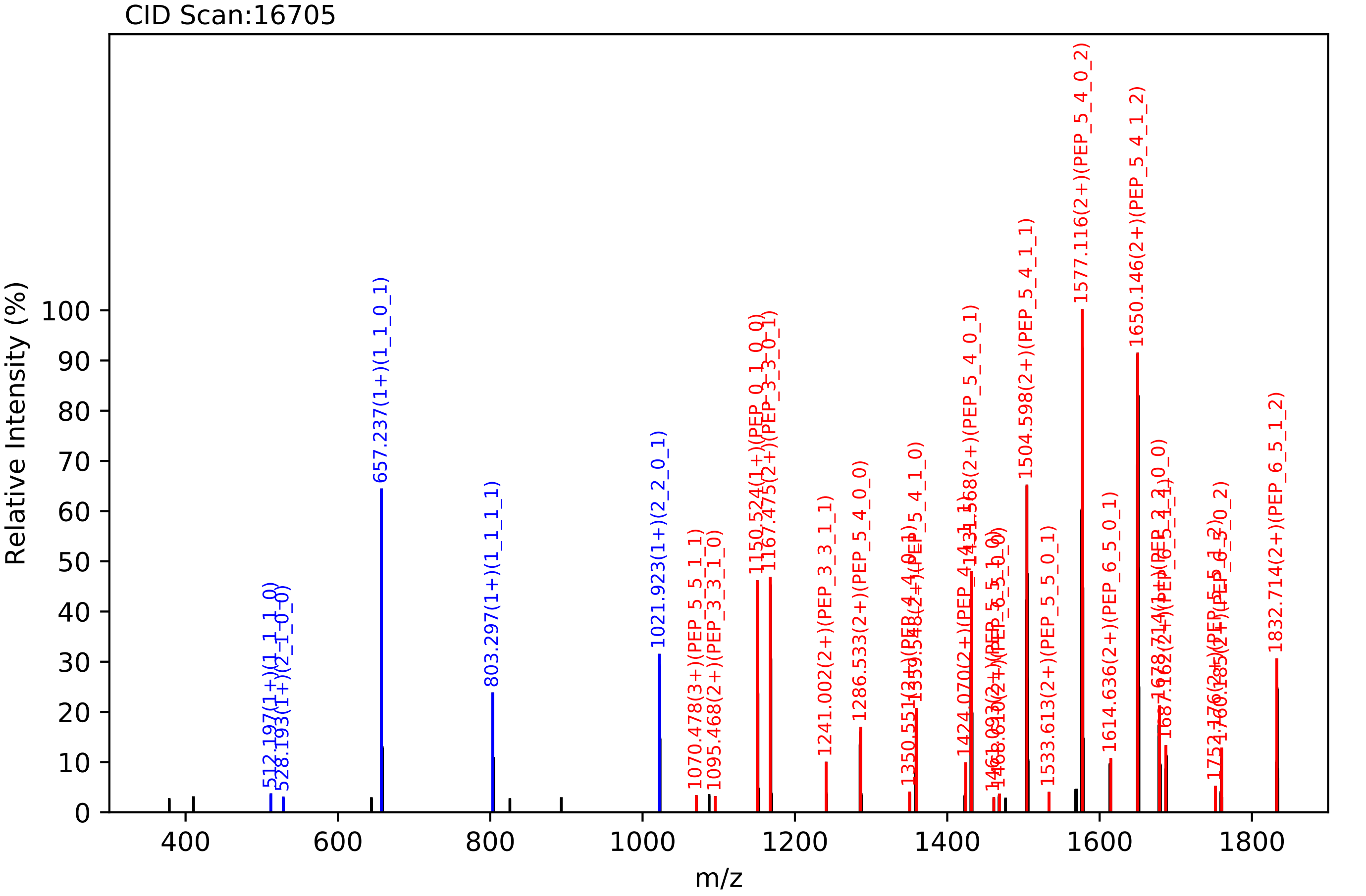

Unknown set no. 196, Experiment: Human Plasma exp_5

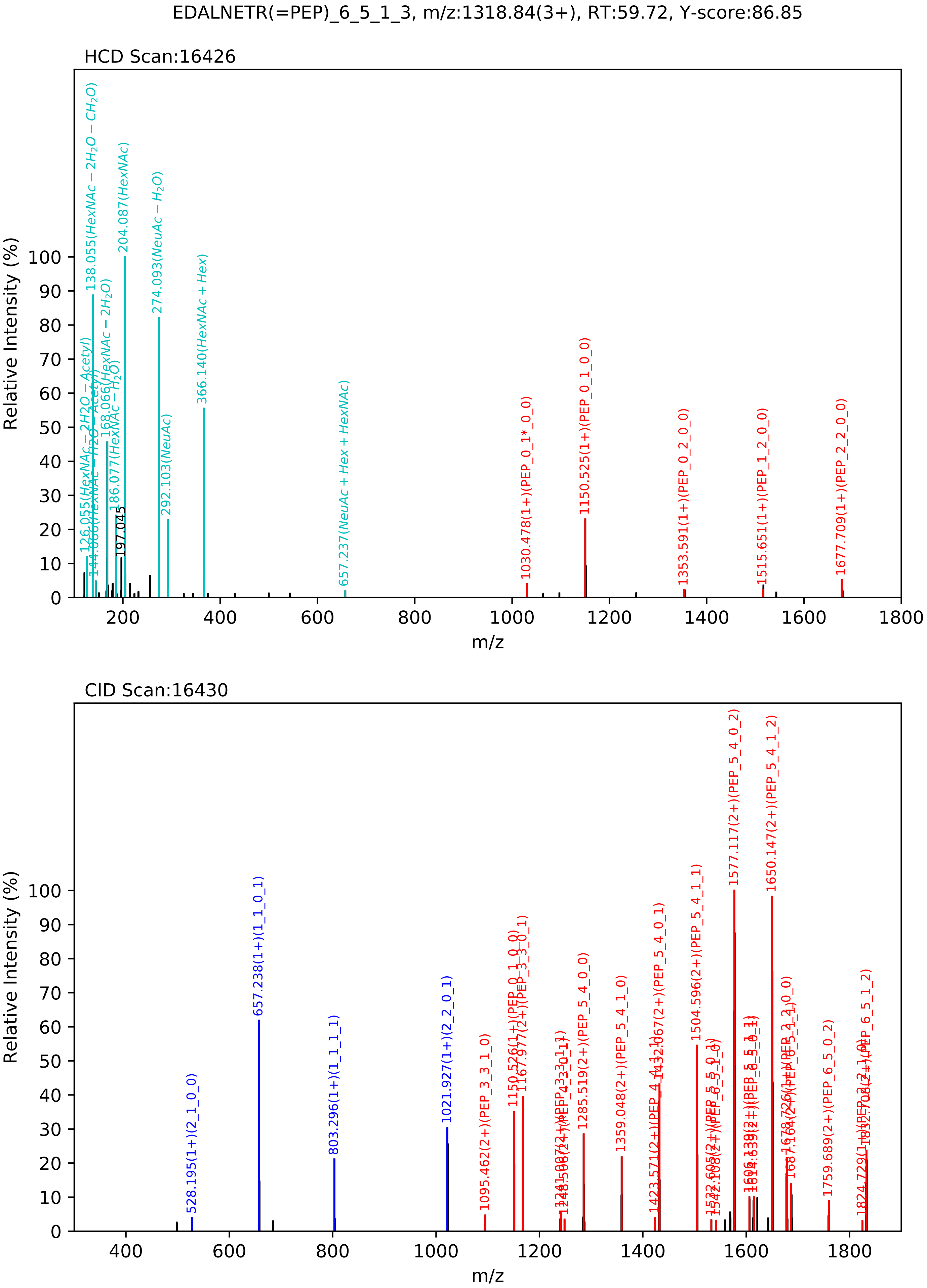

Unknown set no. 197, Experiment: Human Plasma exp_5

HNSTGCLR(=PEP)_6_5_0_3, m/z:952.12(4+), RT:24.32, Y-score:81.89

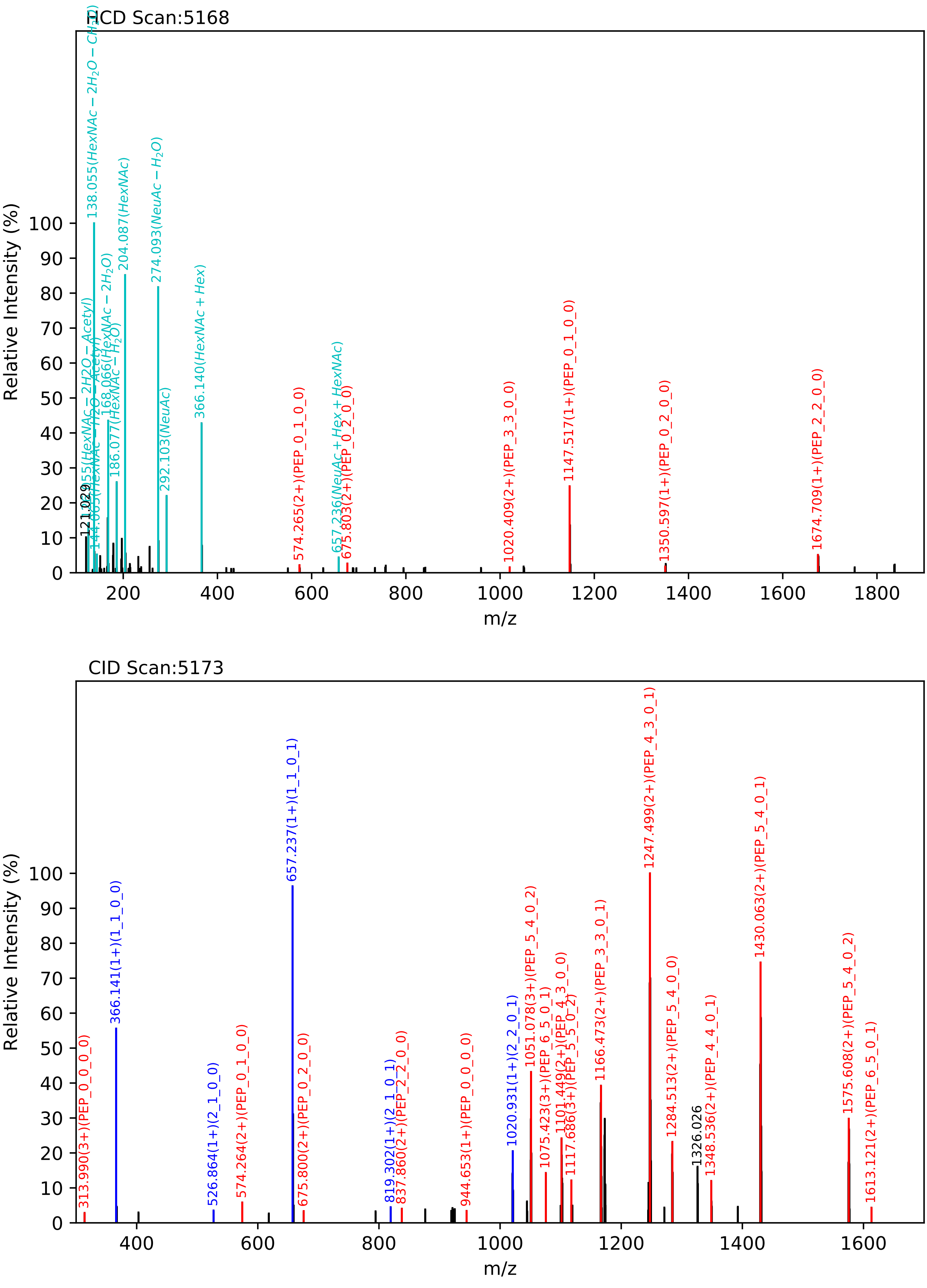

Unknown set no. 198, Experiment: Human Plasma exp_5

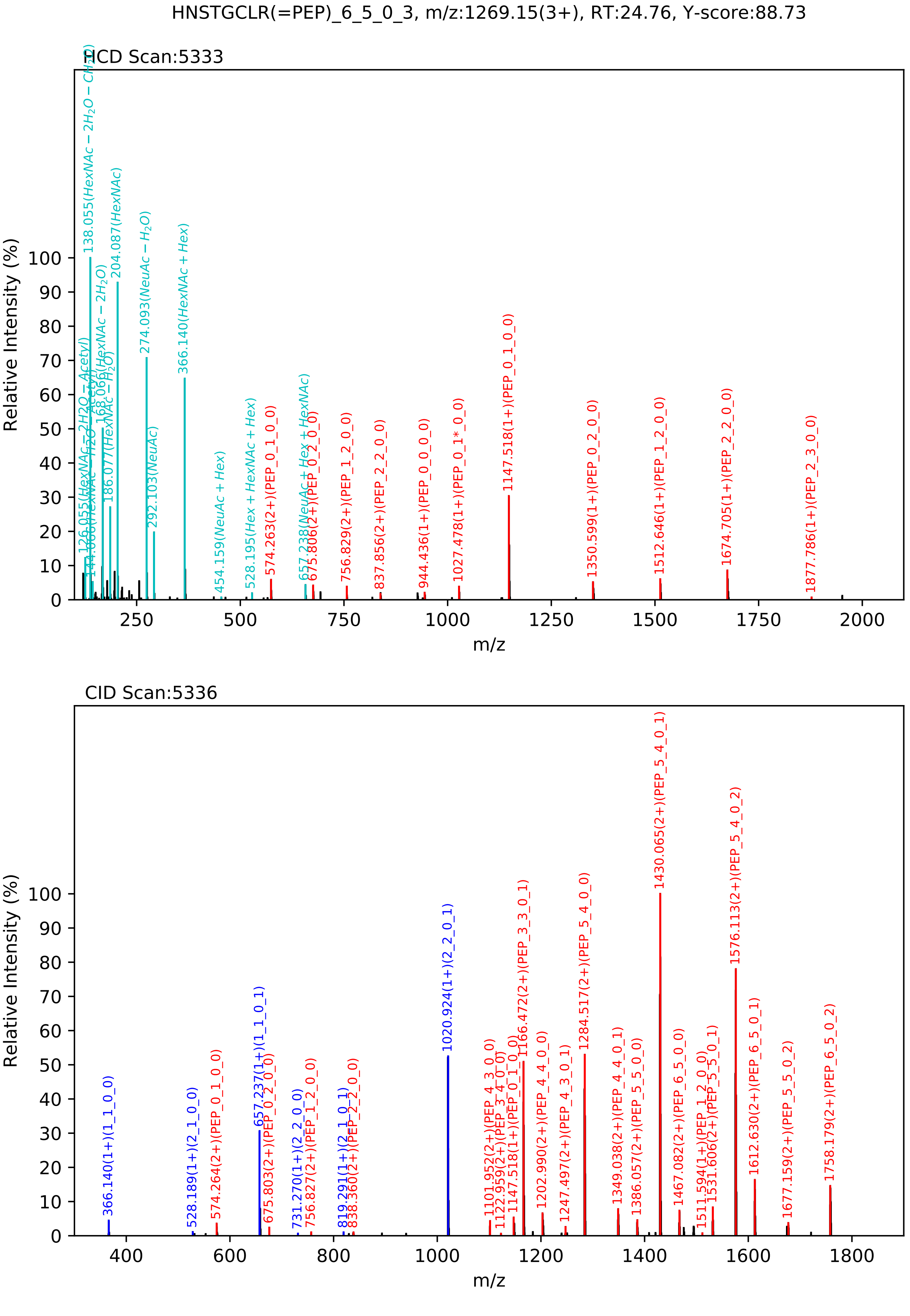

Unknown set no. 199, Experiment: Human Plasma exp_1

LANLTQGEDQYYLR(=PEP)_5_4_0_2, m/z:972.91(4+), RT:92.84, Y-score:86.40

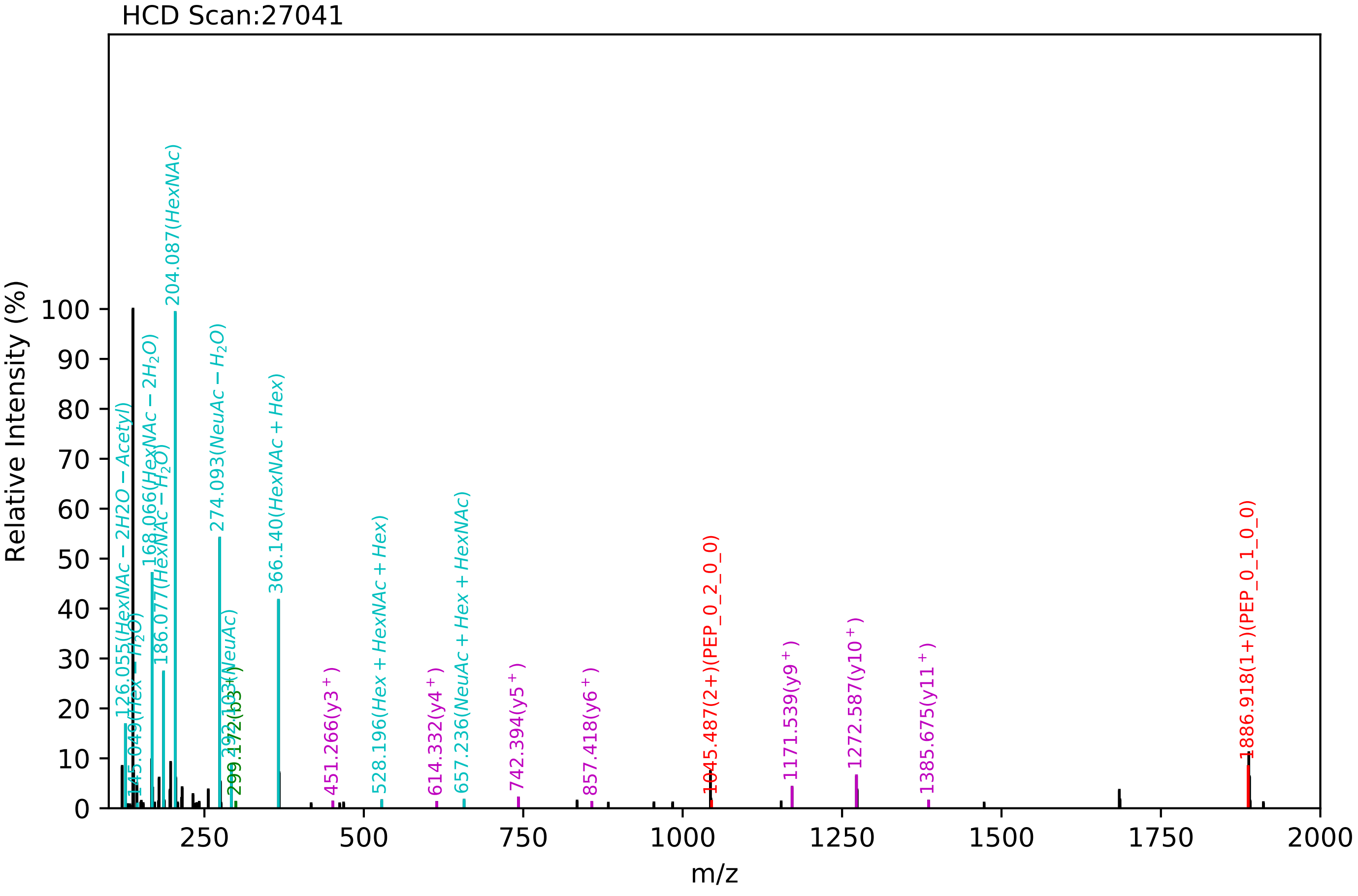

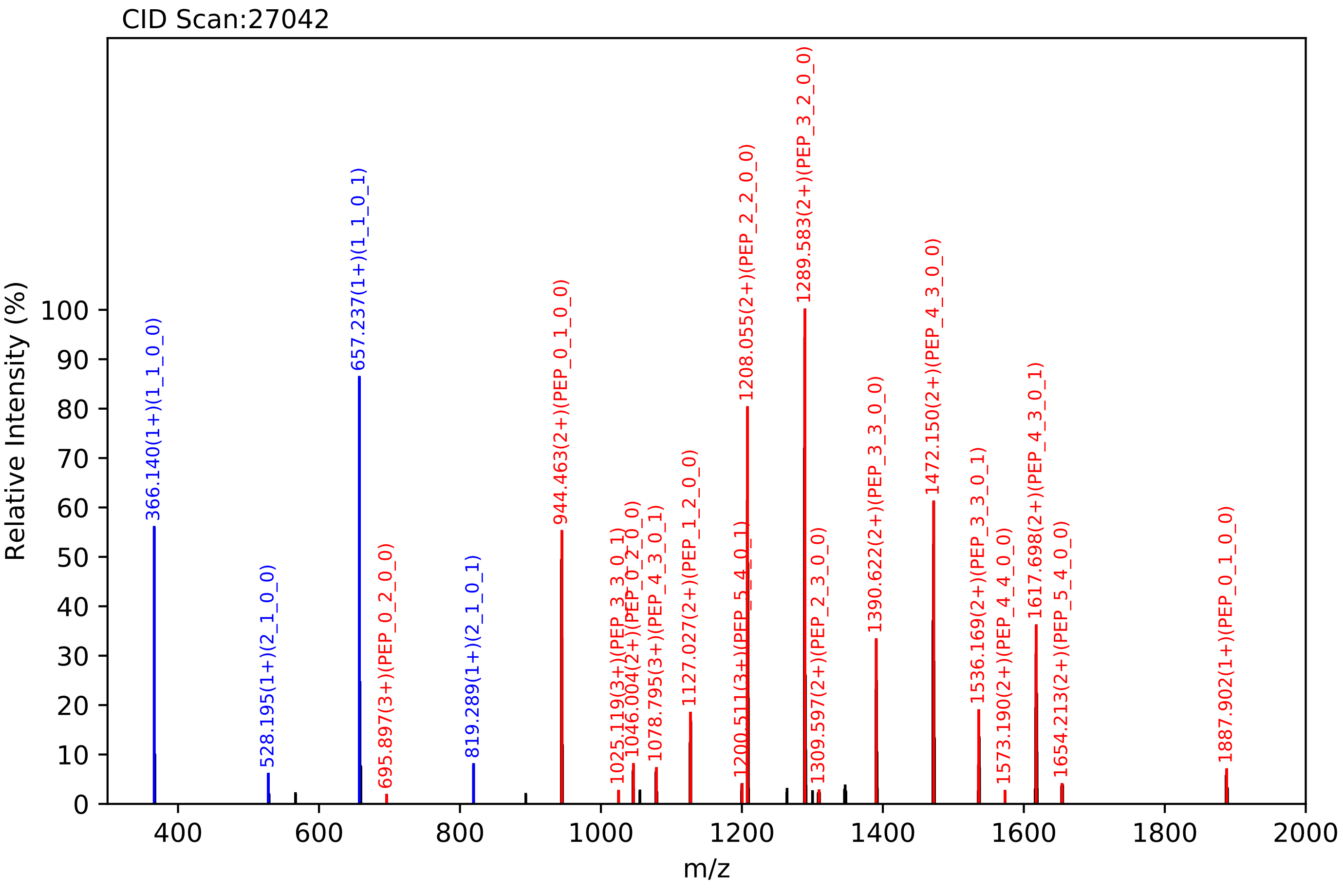

Unknown set no. 200, Experiment: Human Plasma exp_2

LANLTQGEDQYYLR(=PEP)_5_4_0_2, m/z:972.91(4+), RT:92.95, Y-score:87.69

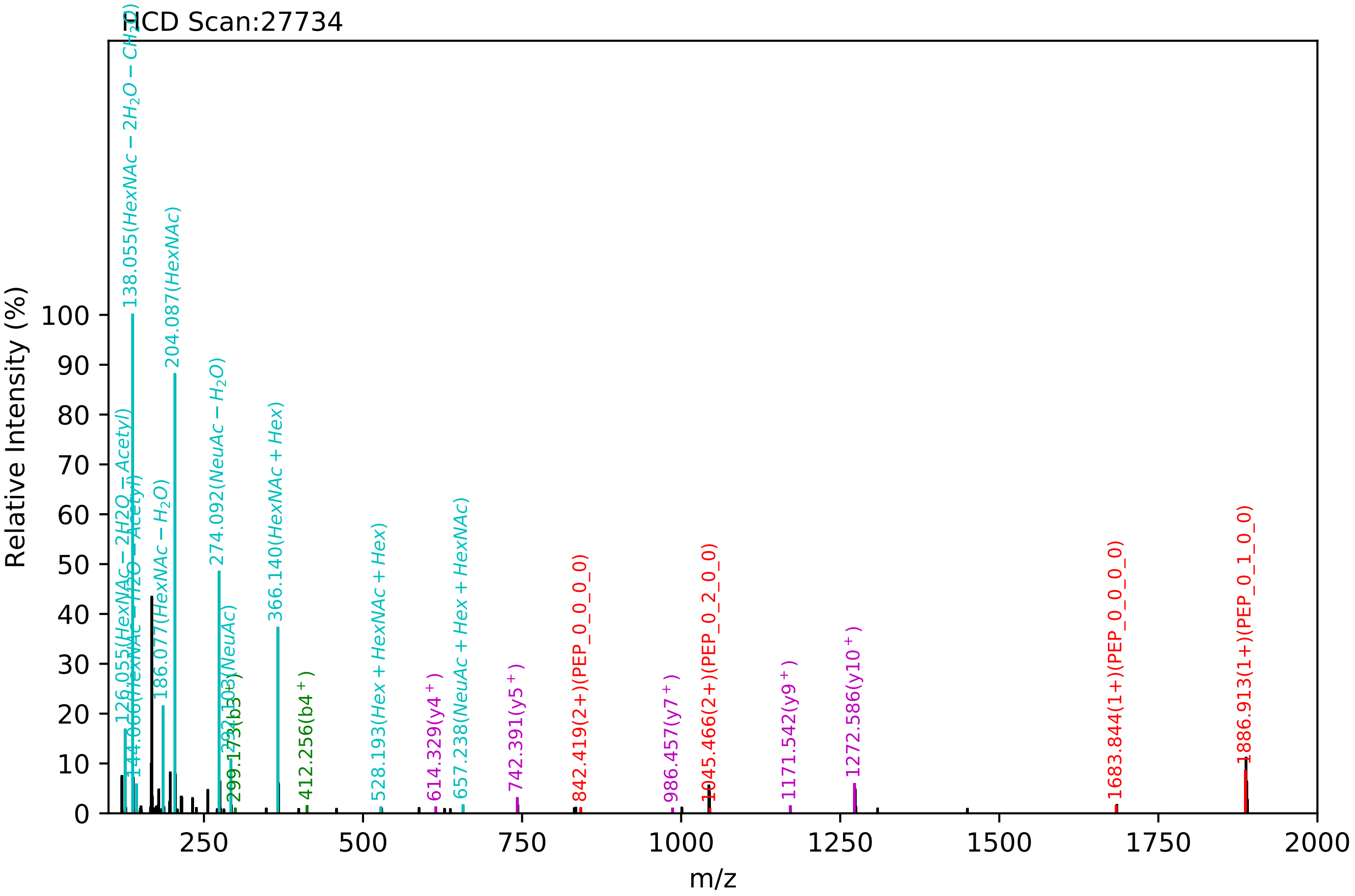

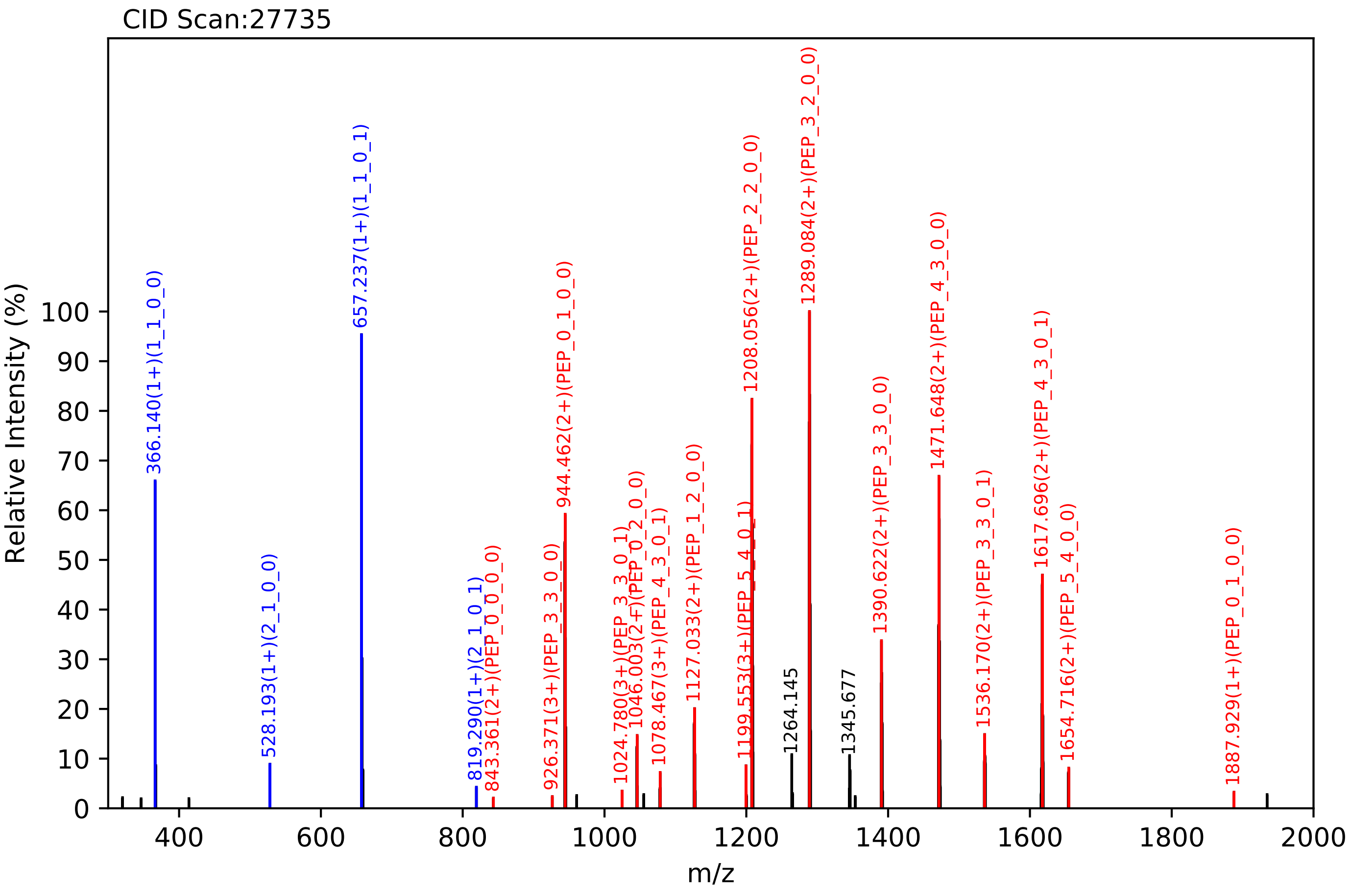

Unknown set no. 201, Experiment: Human Plasma exp_3

LANLTQGEDQYYLR(=PEP)_5_4_0_2, m/z:972.91(4+), RT:93.01, Y-score:83.06

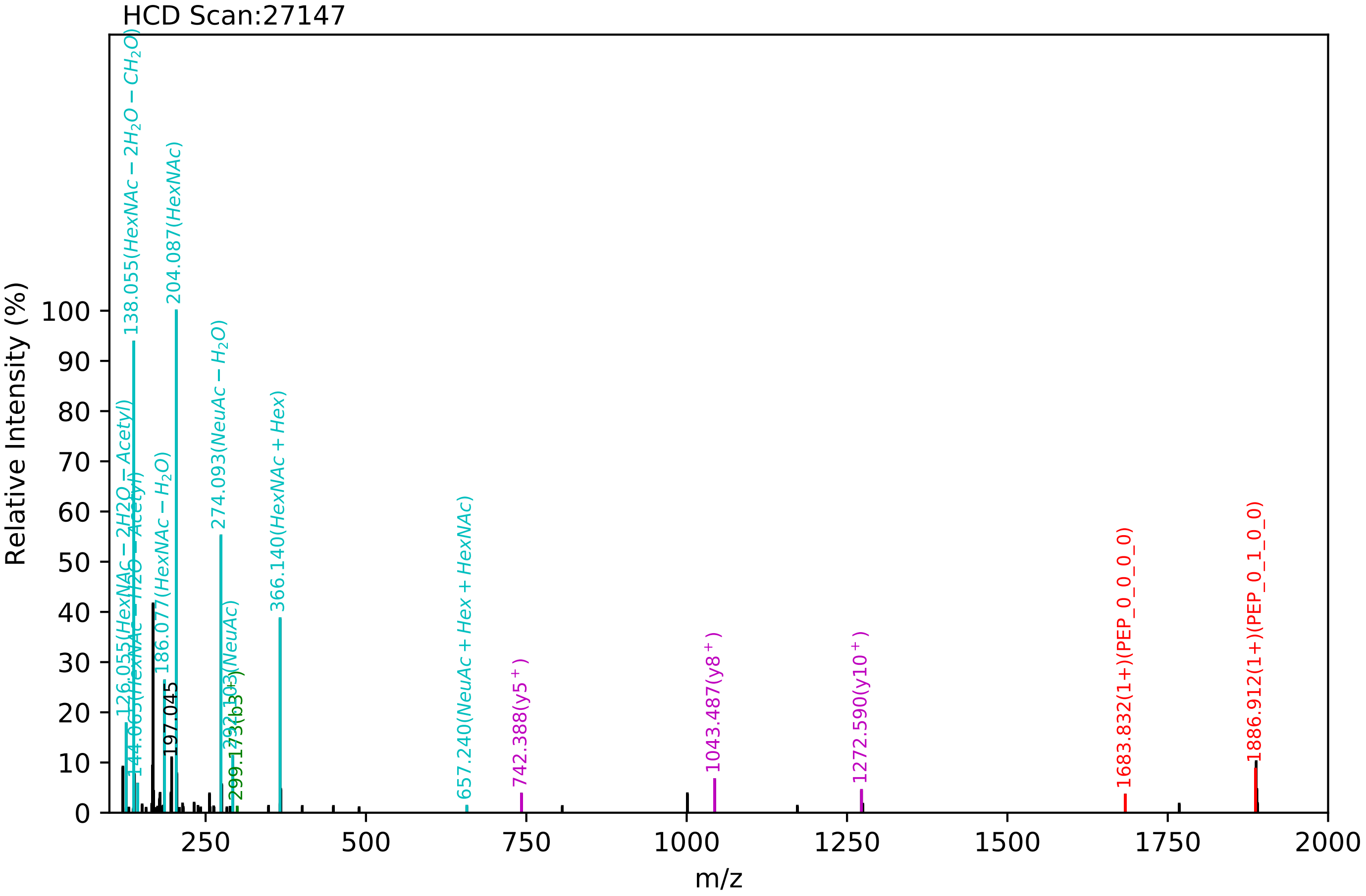

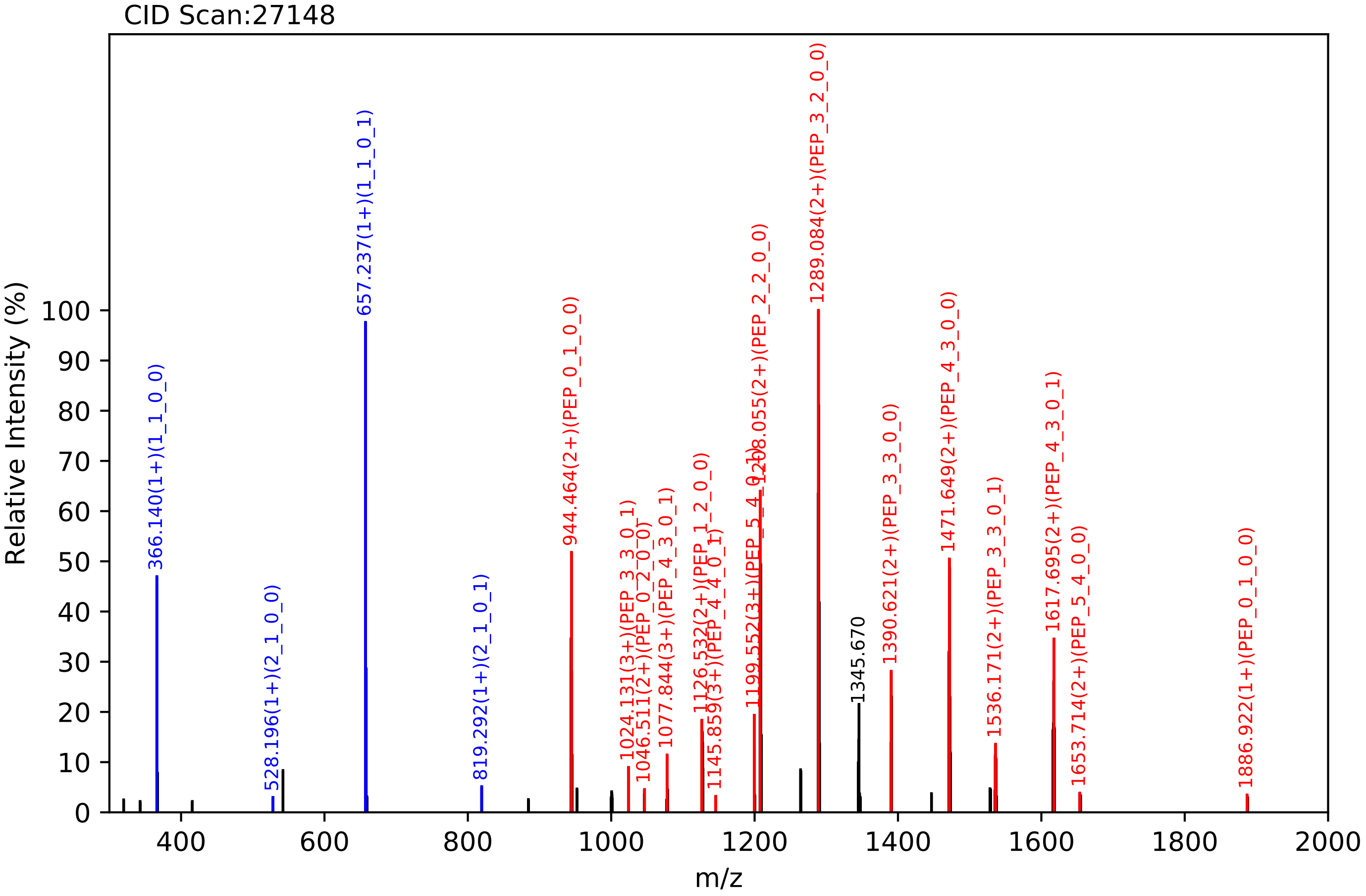

Unknown set no. 202, Experiment: Human Plasma exp_4

LANLTQGEDQYYLR(=PEP)_5_4_0_2, m/z:972.91(4+), RT:93.16, Y-score:91.88

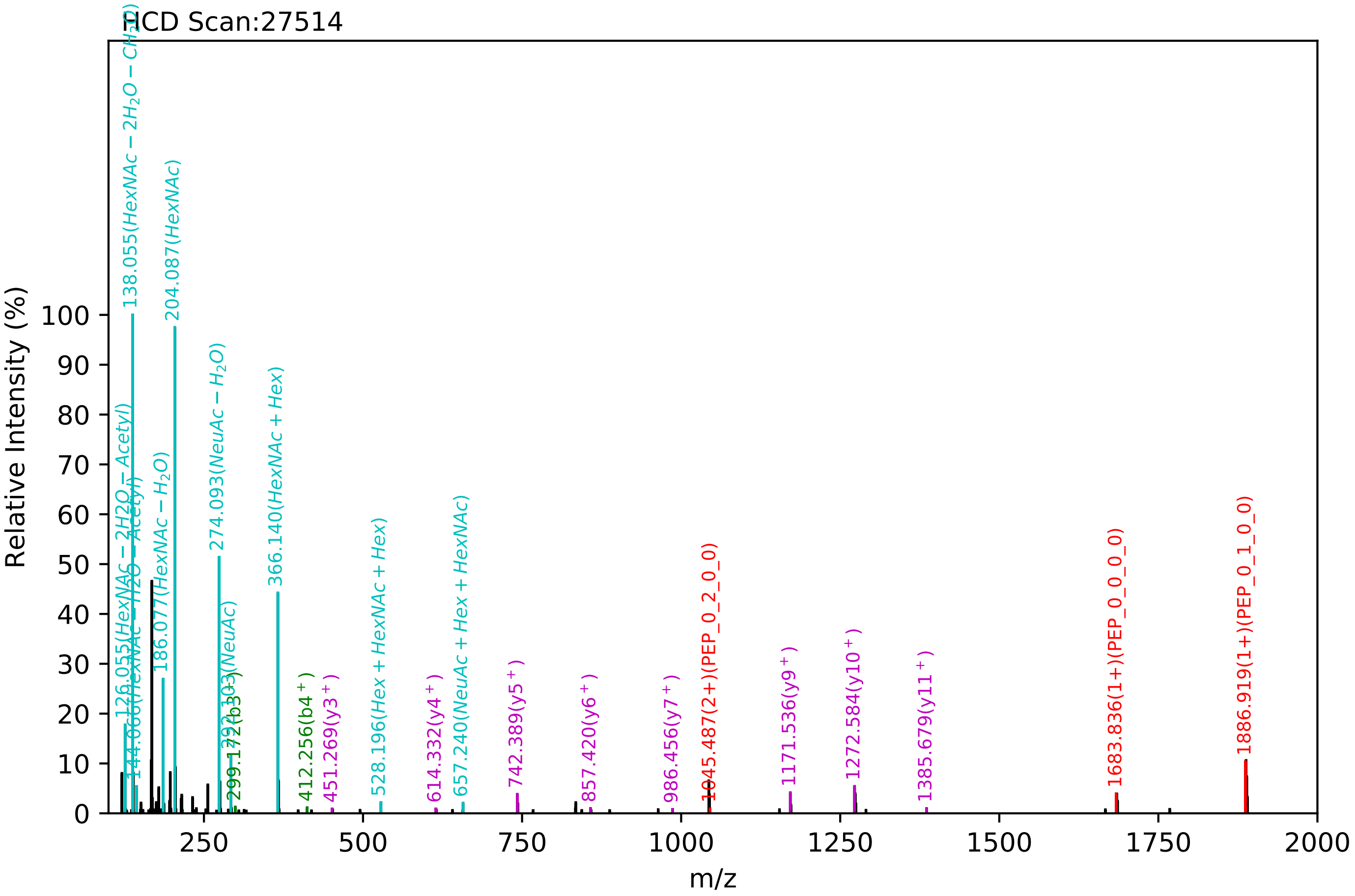

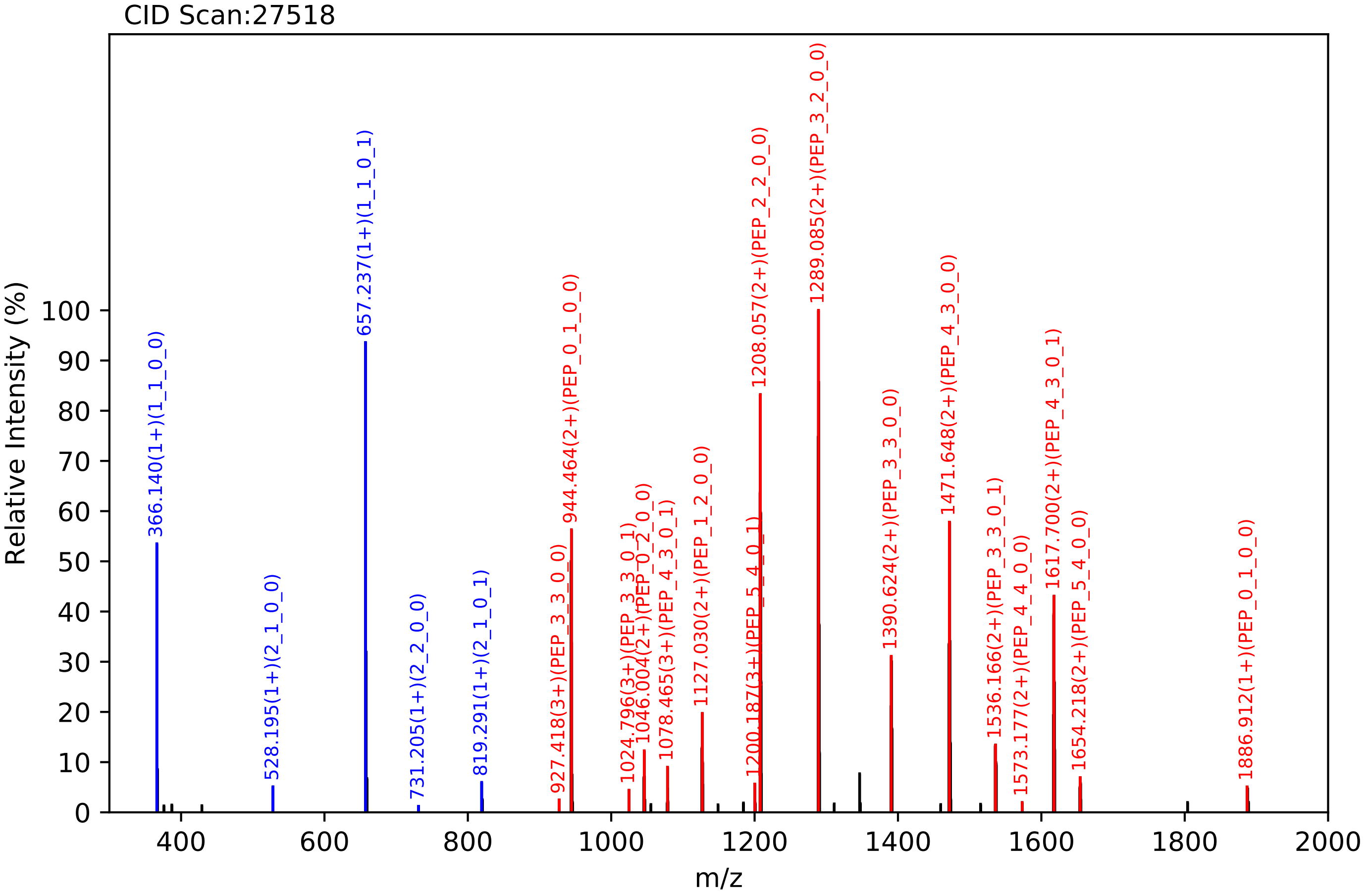

Unknown set no. 203, Experiment: Human Plasma exp_5

LANLTQGEDQYYLR(=PEP)_5_4_0_2, m/z:972.91(4+), RT:92.65, Y-score:87.90

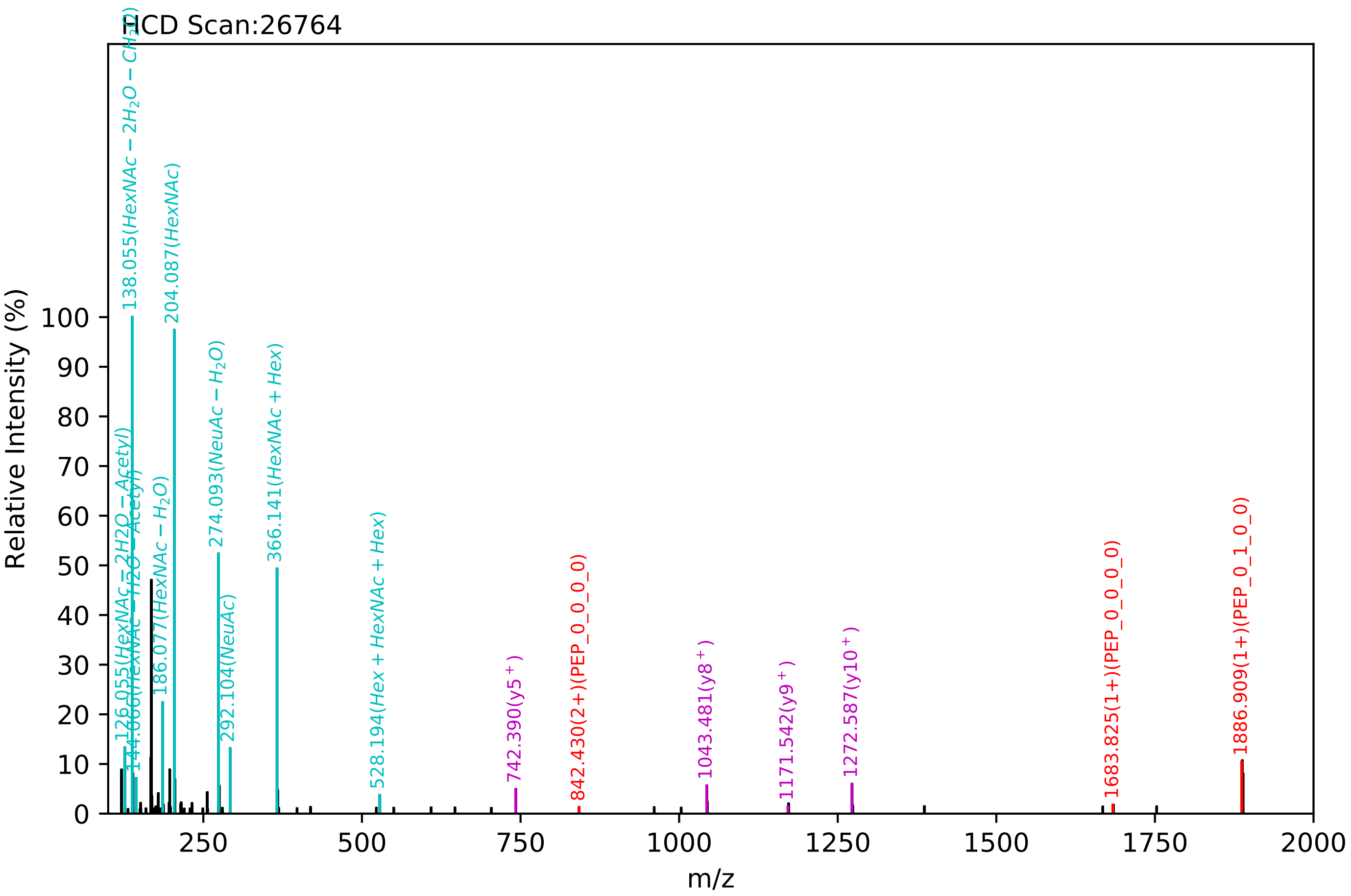

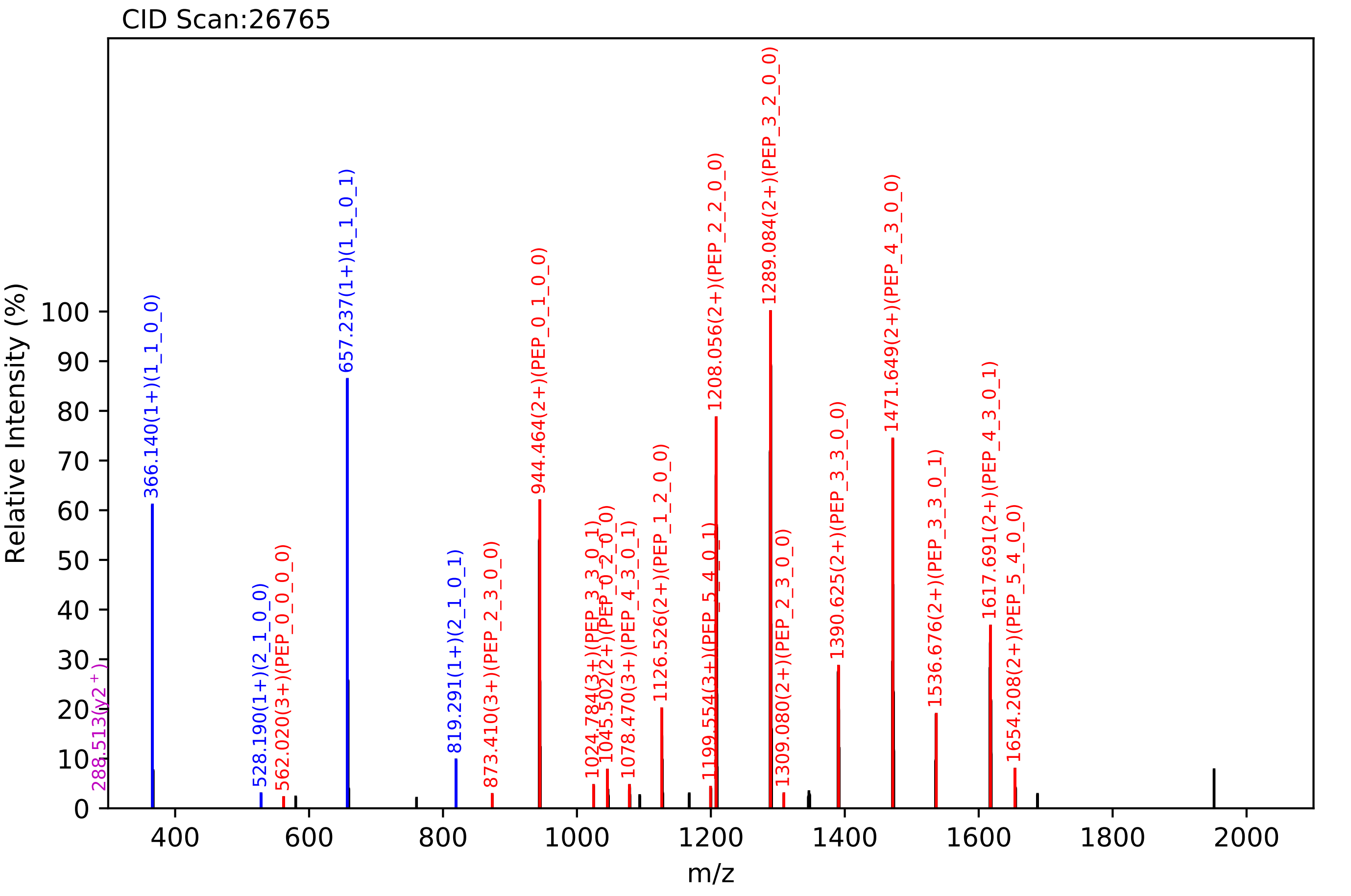

Unknown set no. 204, Experiment: Human Plasma exp_2

LANLTQGEDQYYLR(=PEP)_6_5_1_3, m/z:1173.48(4+), RT:104.03, Y-score:95.14

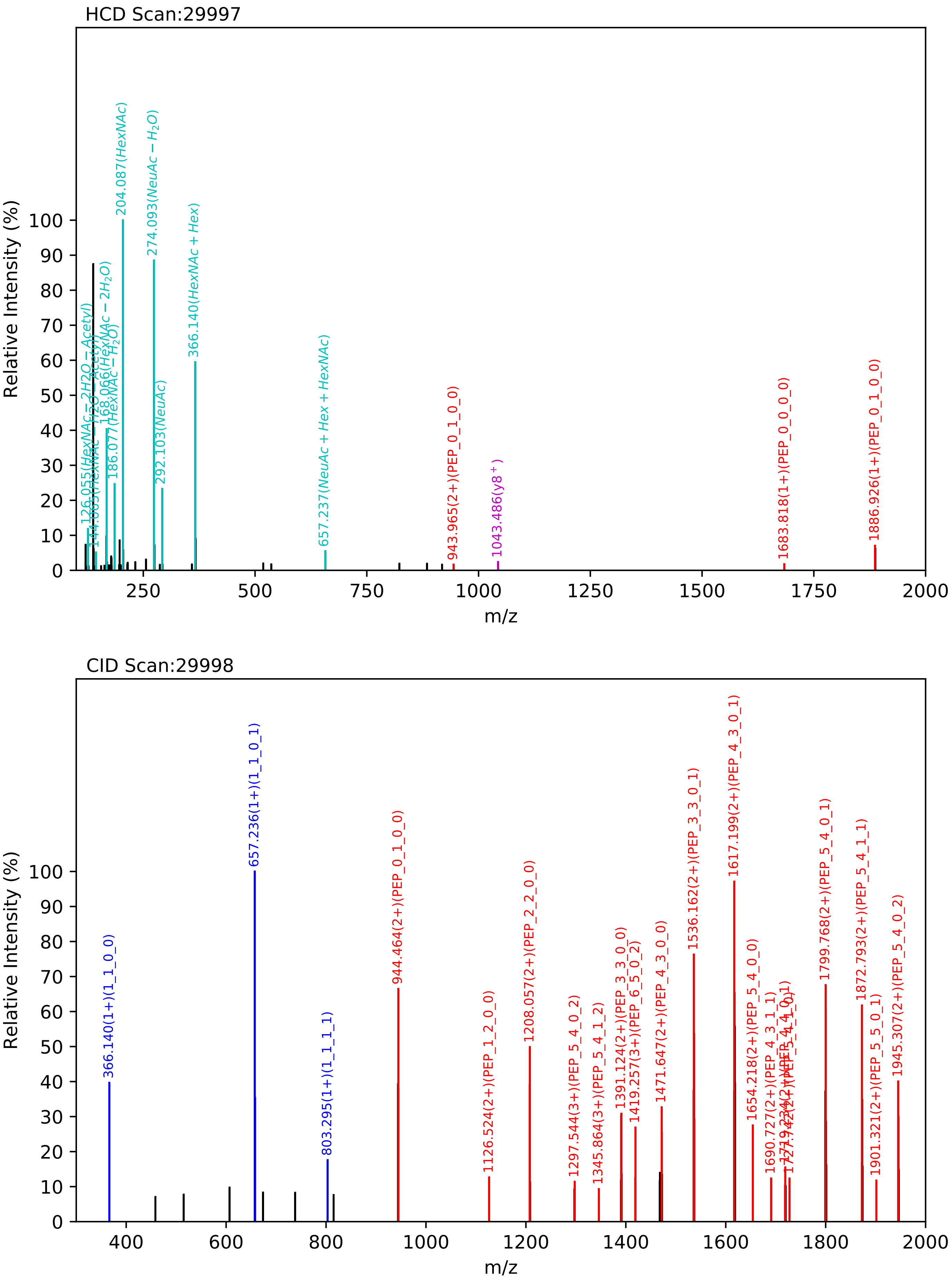

Unknown set no. 205, Experiment: Human Plasma exp_3

LANLTQGEDQYYLR(=PEP)_6_5_1_3, m/z:1173.48(4+), RT:104.08, Y-score:83.15

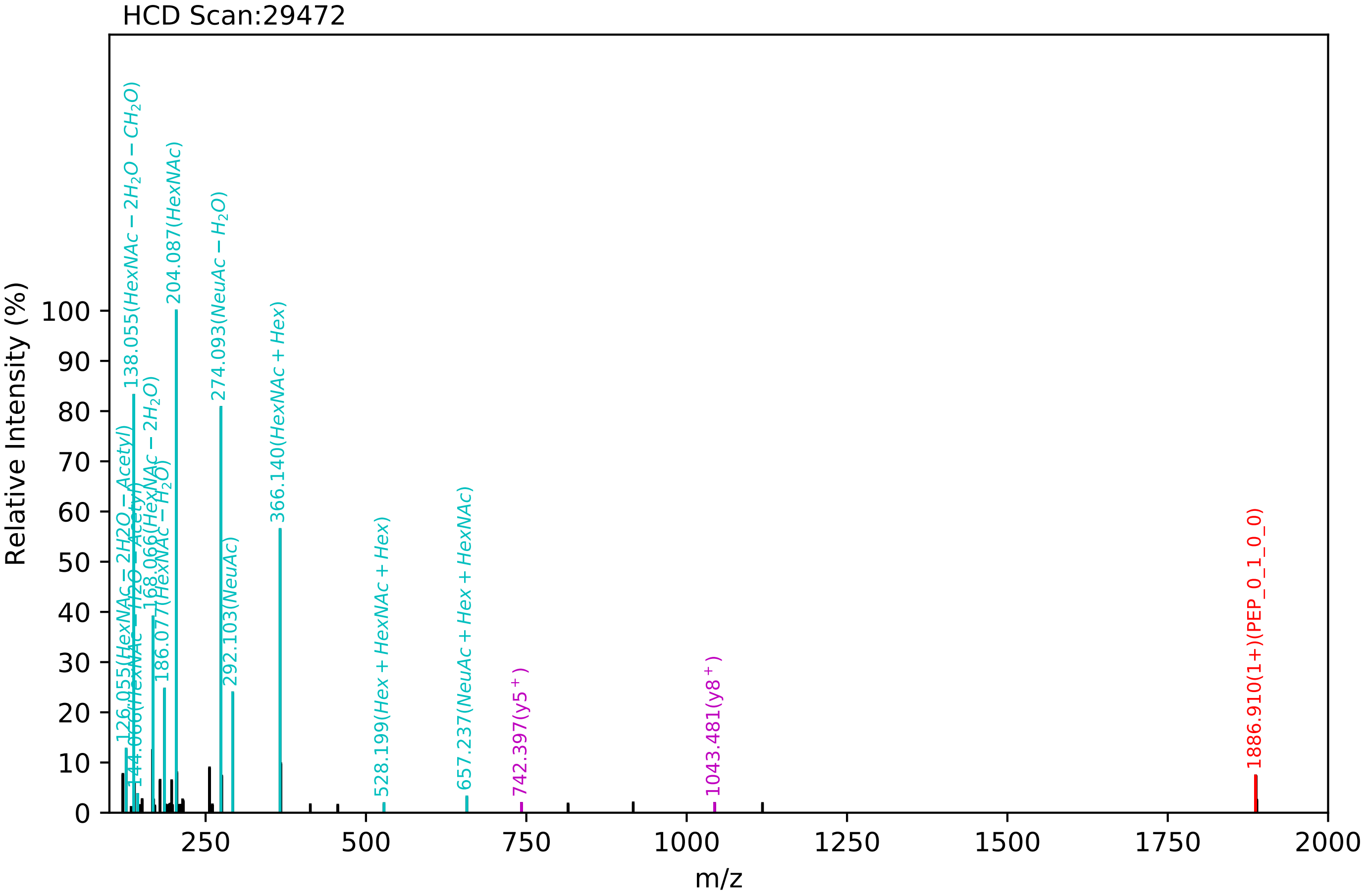

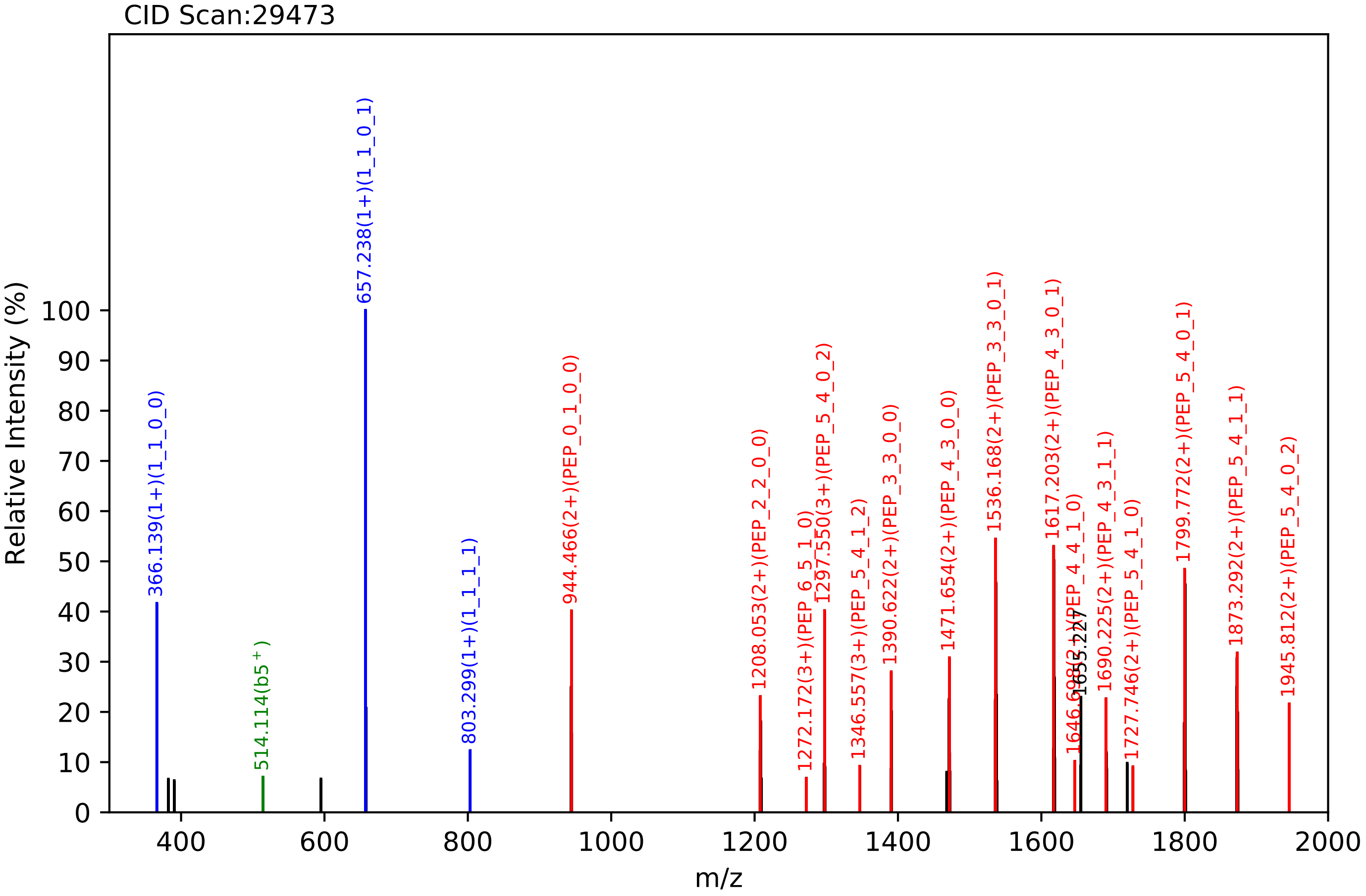

Unknown set no. 206, Experiment: Human Plasma exp_4

LANLTQGEDQYYLR(=PEP)_6_5_1_3, m/z:1173.48(4+), RT:104.25, Y-score:79.03

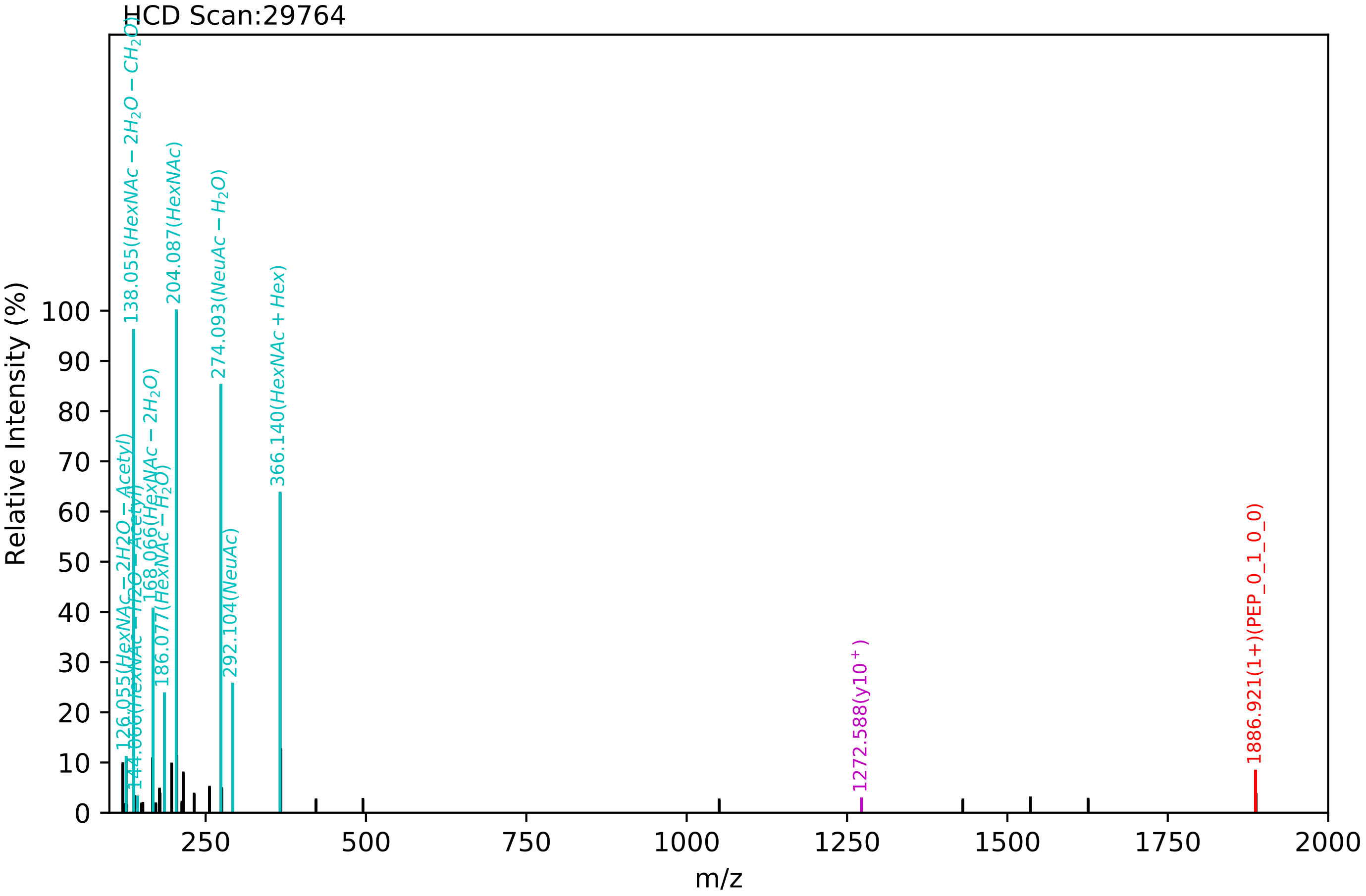

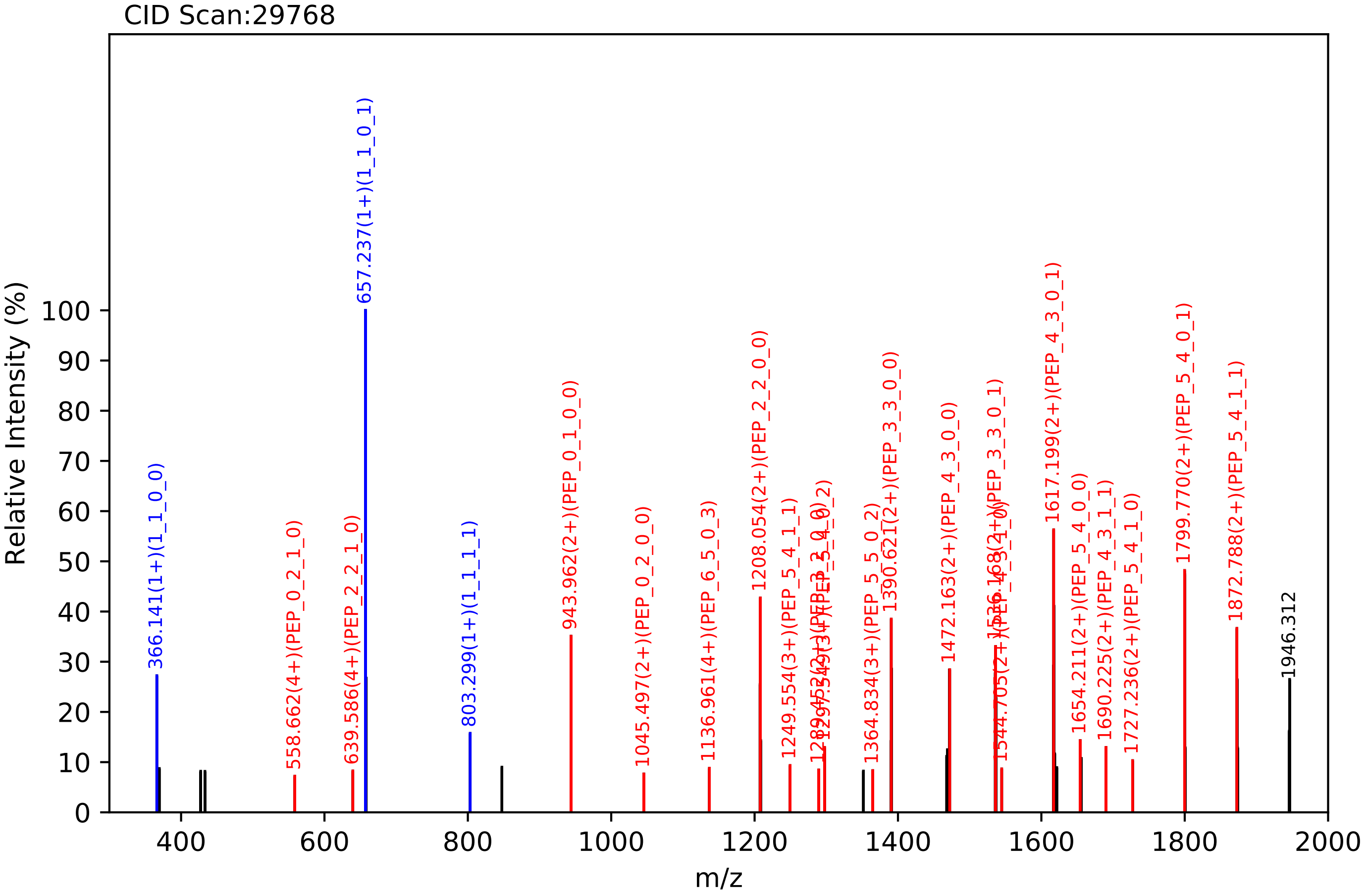

Unknown set no. 207, Experiment: Human Plasma exp_1

NHSCEPCQTLAVR(=PEP)_5_4_0_2, m/z:944.88(4+), RT:53.87, Y-score:79.80

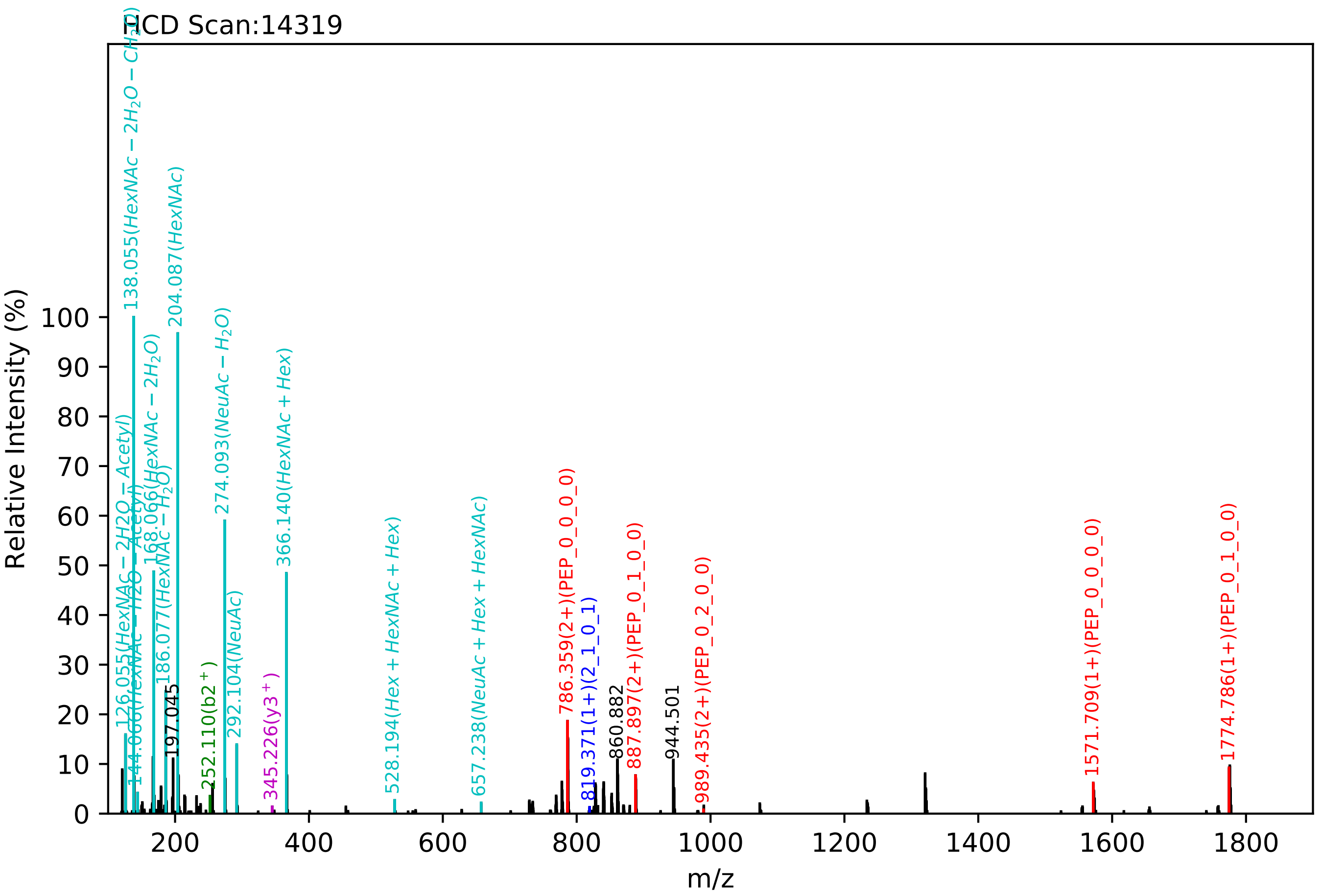

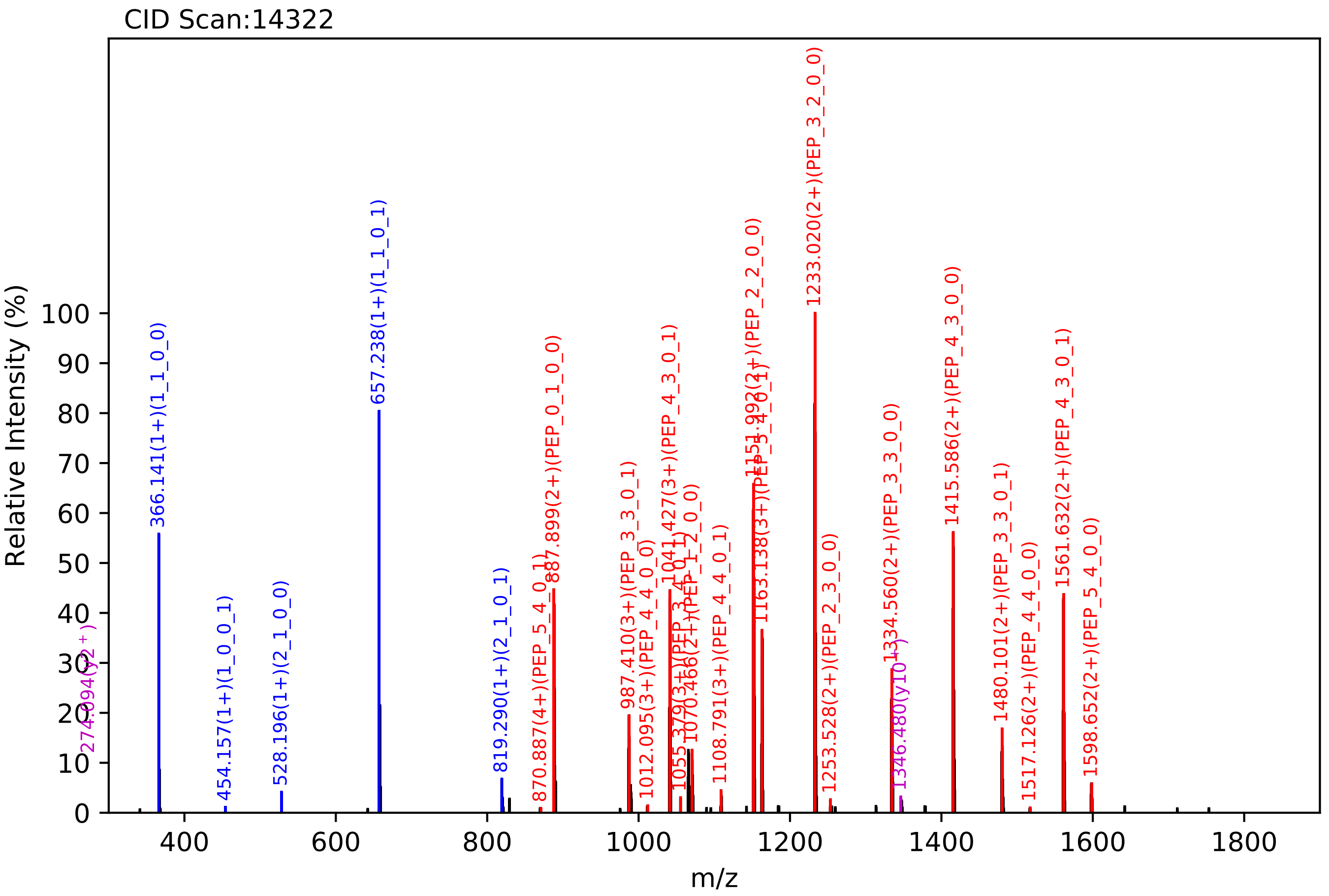

Unknown set no. 208, Experiment: Human Plasma exp_1

NHSCEPCQTLAVR(=PEP)_5_4_0_2, m/z:1259.50(3+), RT:53.91, Y-score:81.27

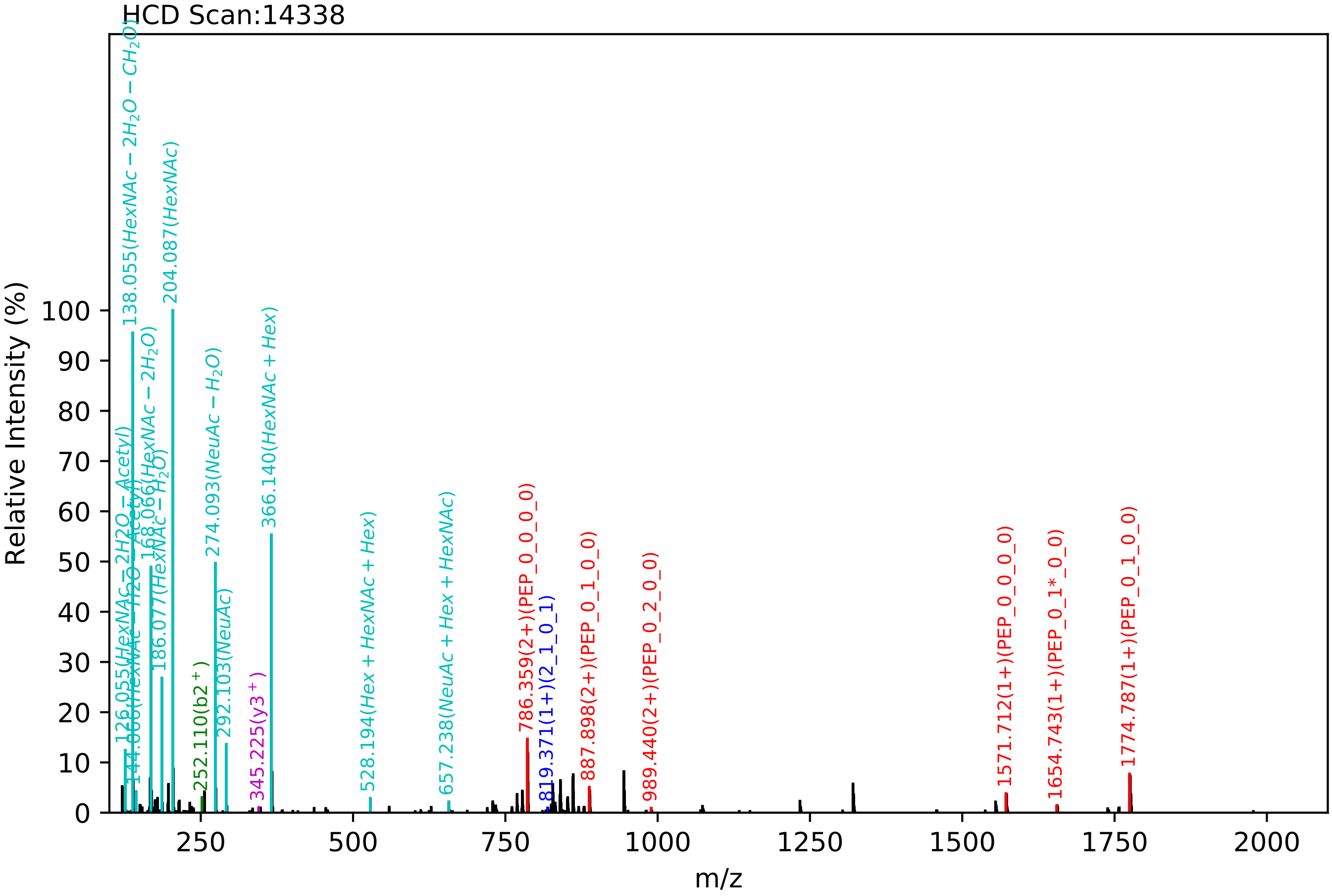

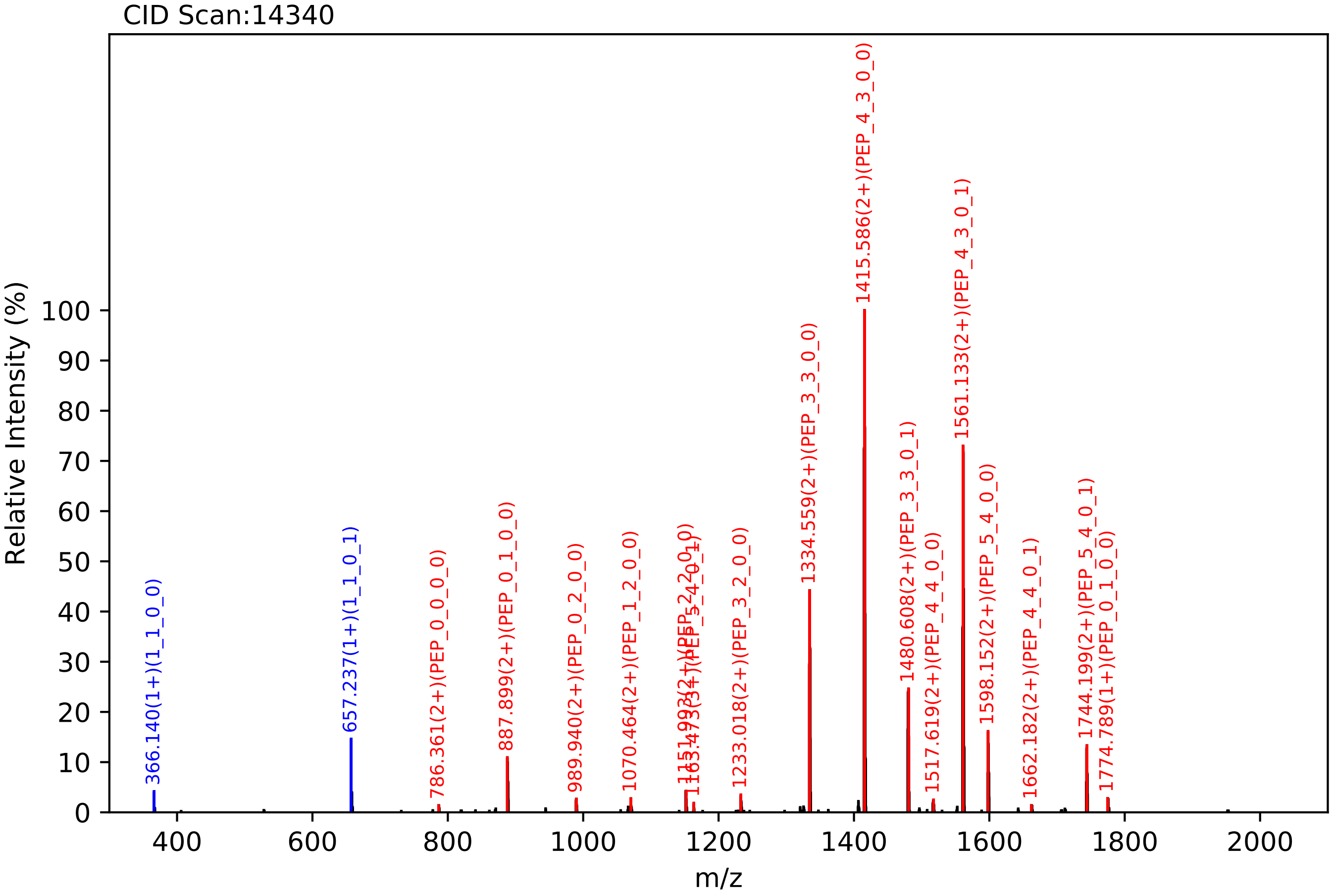

Unknown set no. 209, Experiment: Human Plasma exp_2

NHSCEPCQTLAVR(=PEP)_5_4_0_2, m/z:944.88(4+), RT:53.85, Y-score:78.22

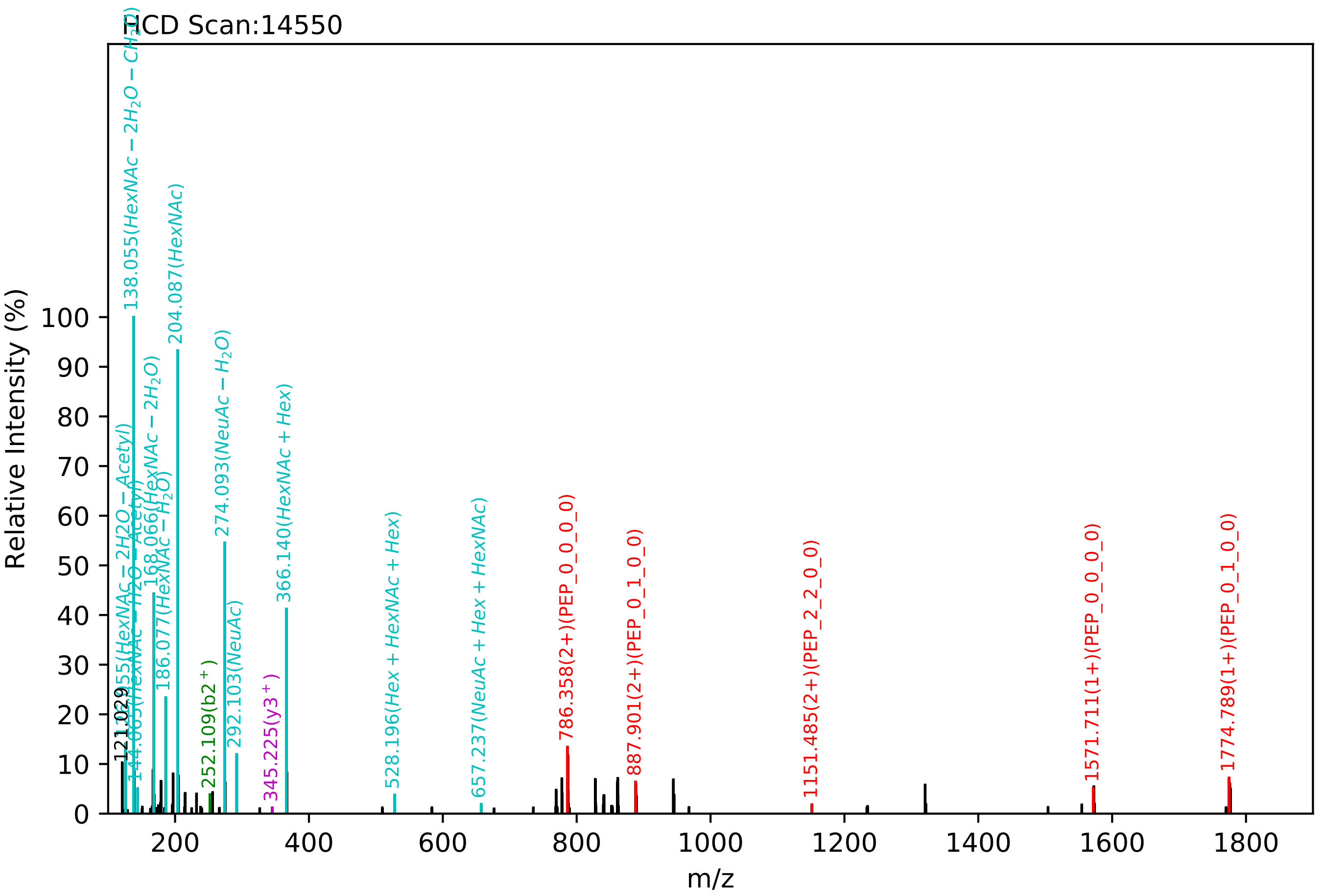

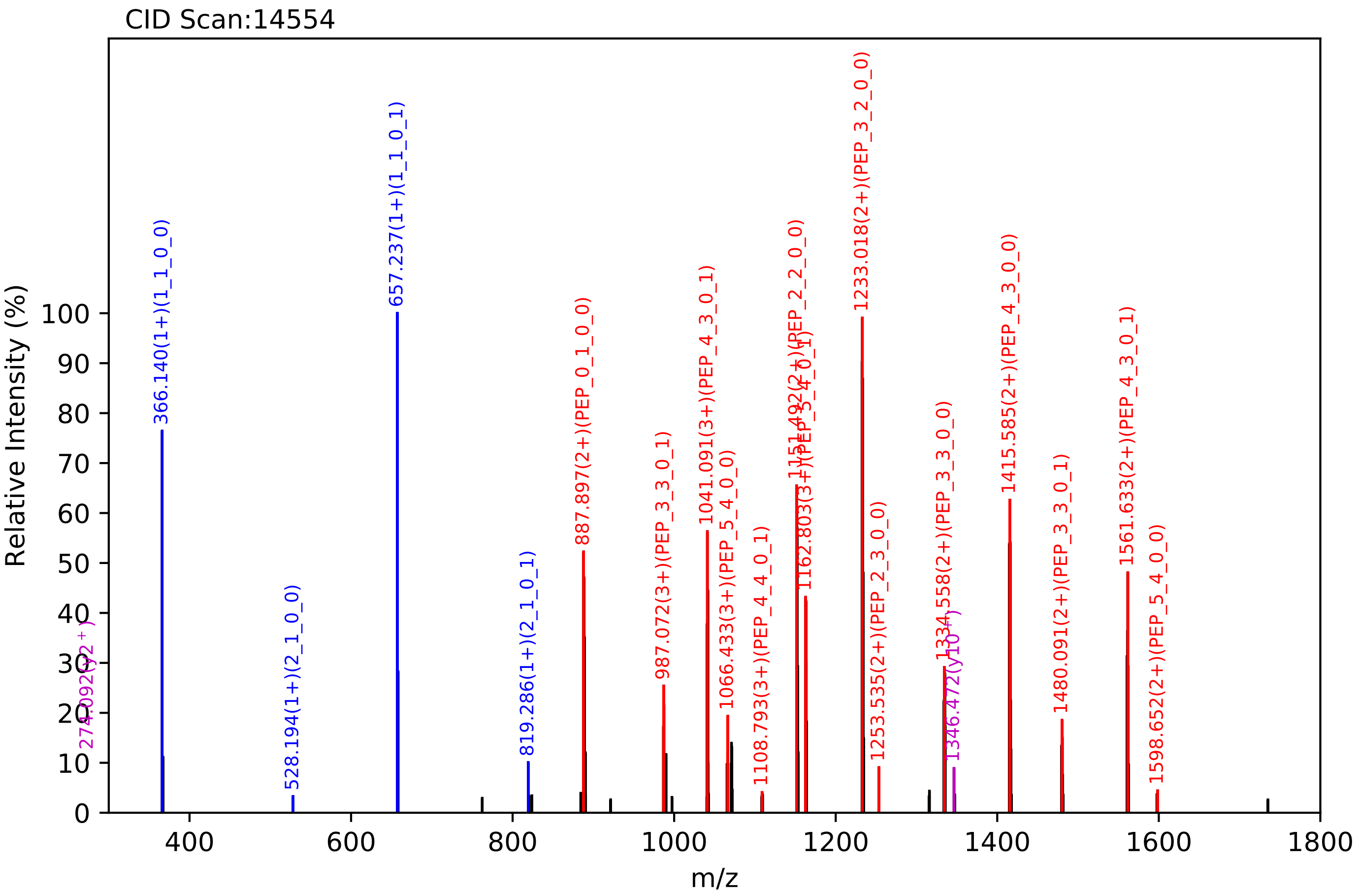

Unknown set no. 210, Experiment: Human Plasma exp_2

NHSCEPCQTLAVR(=PEP)_5_4_0_2, m/z:1259.50(3+), RT:54.01, Y-score:81.85

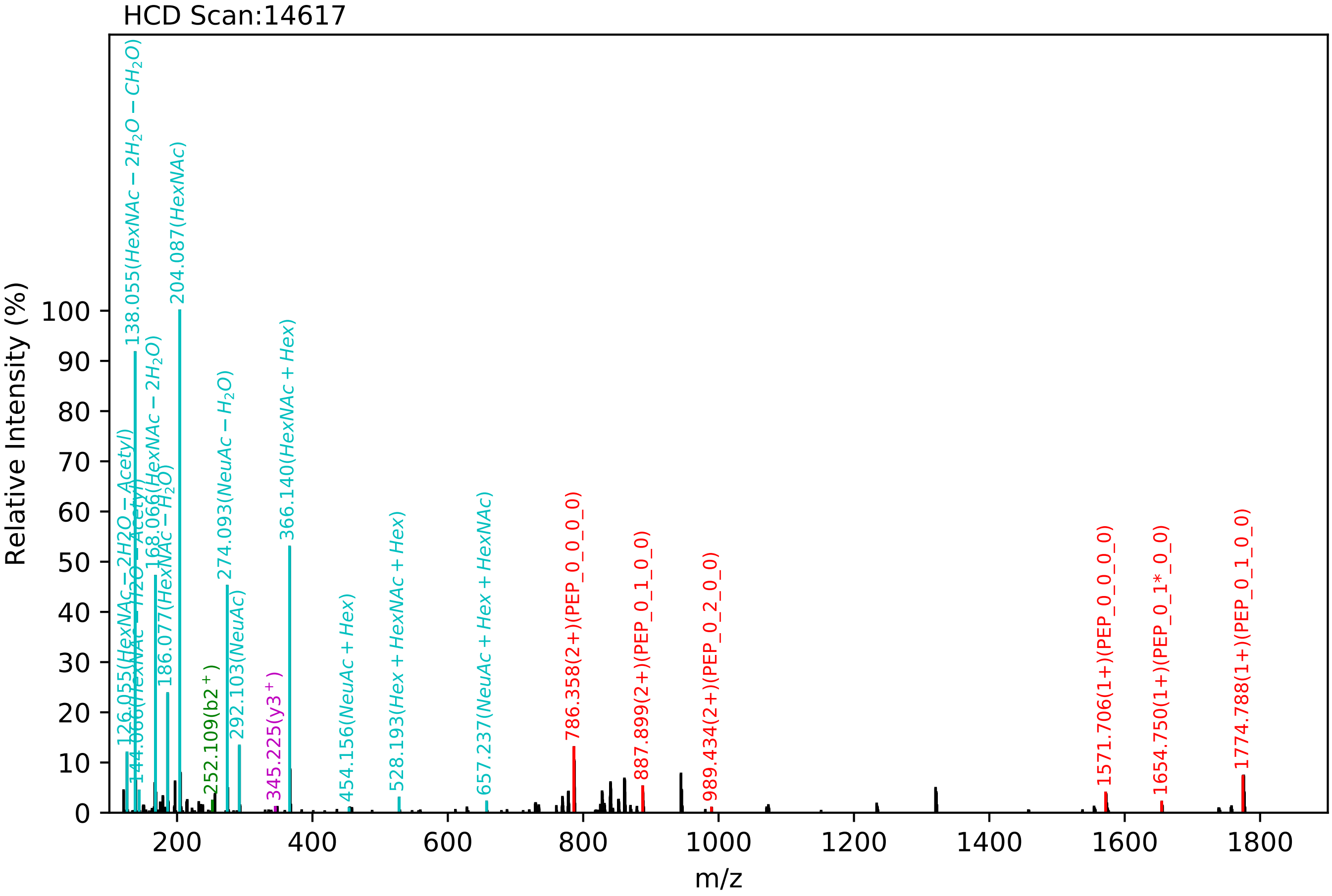

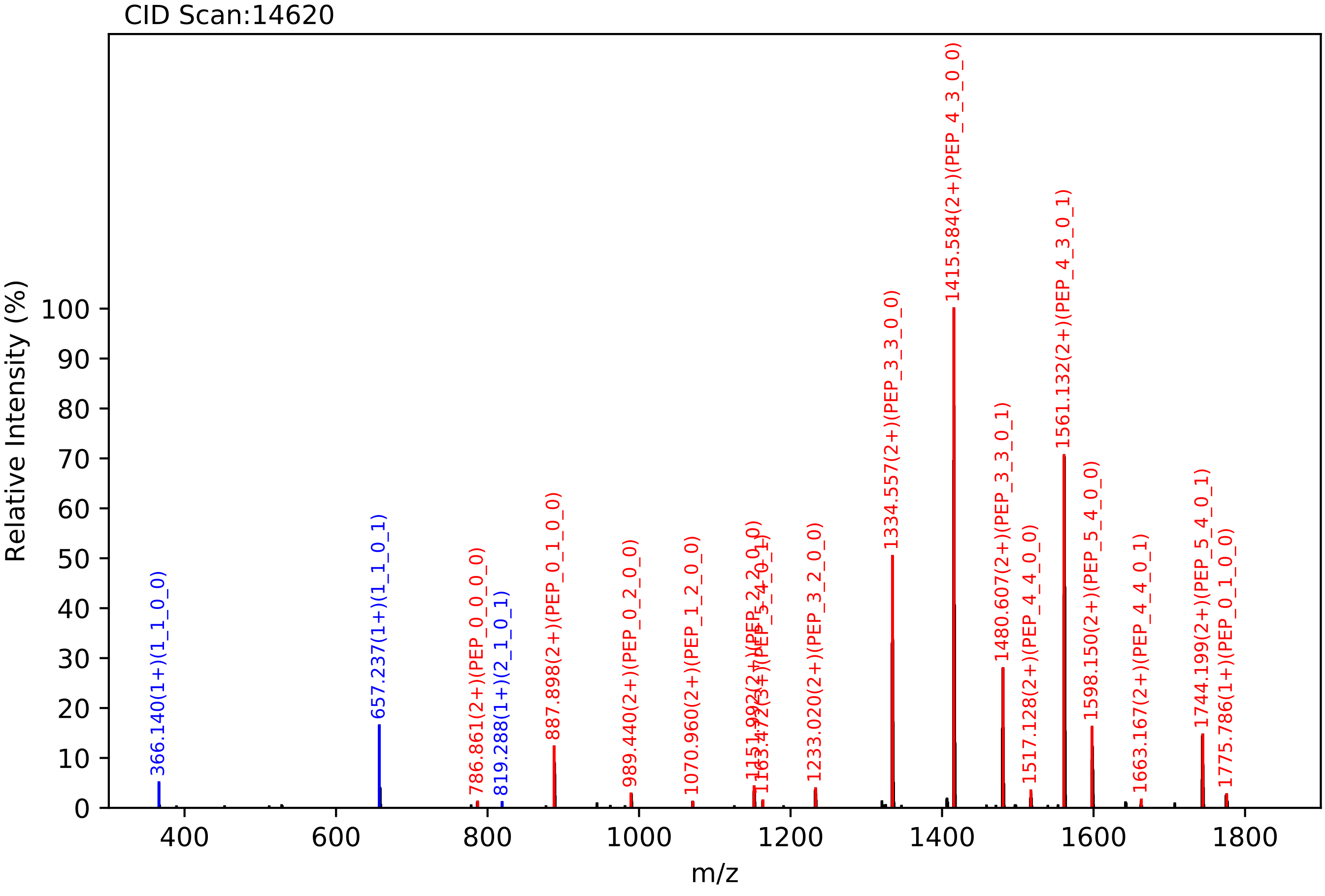

Unknown set no. 211, Experiment: Human Plasma exp_3

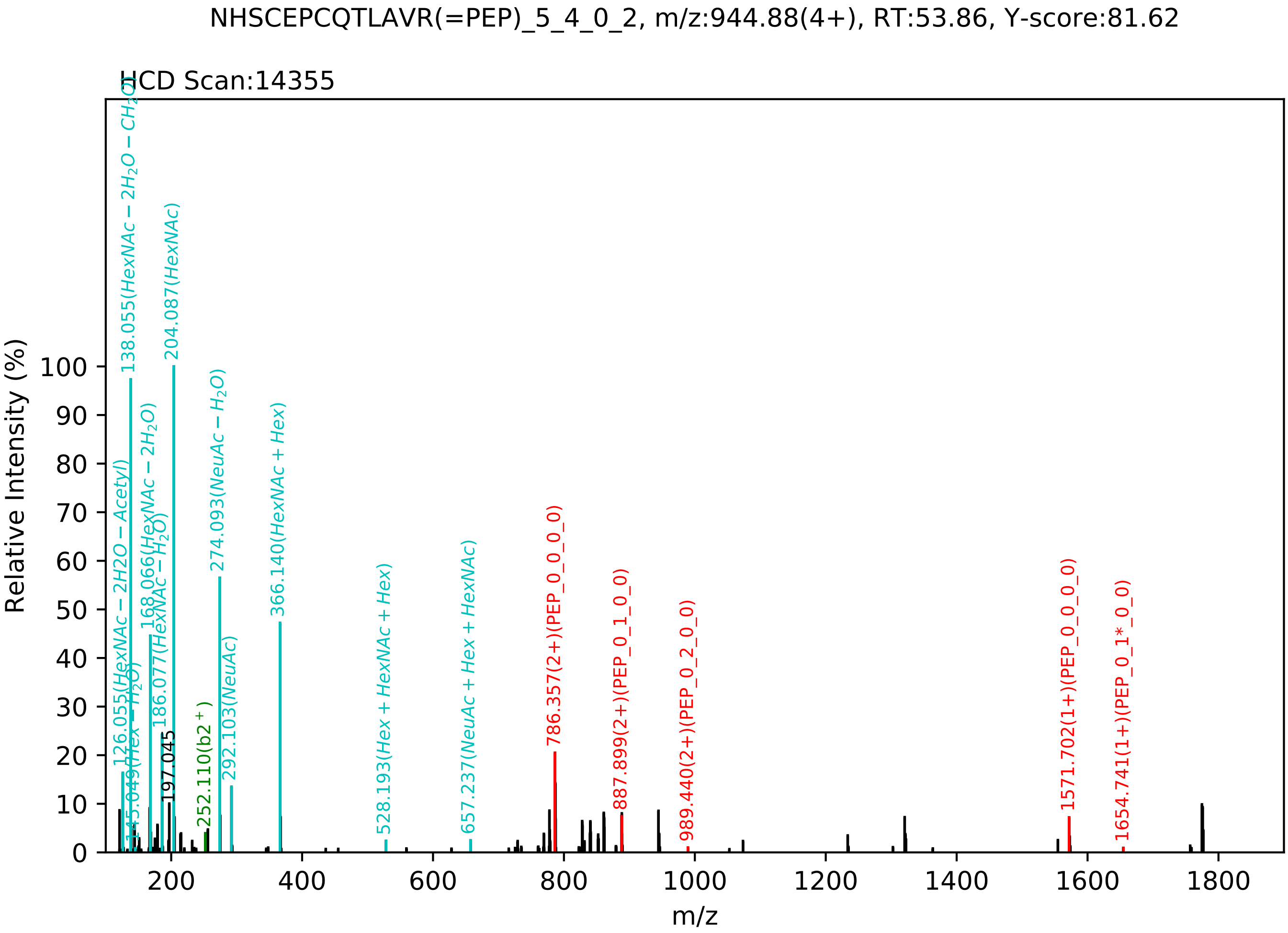

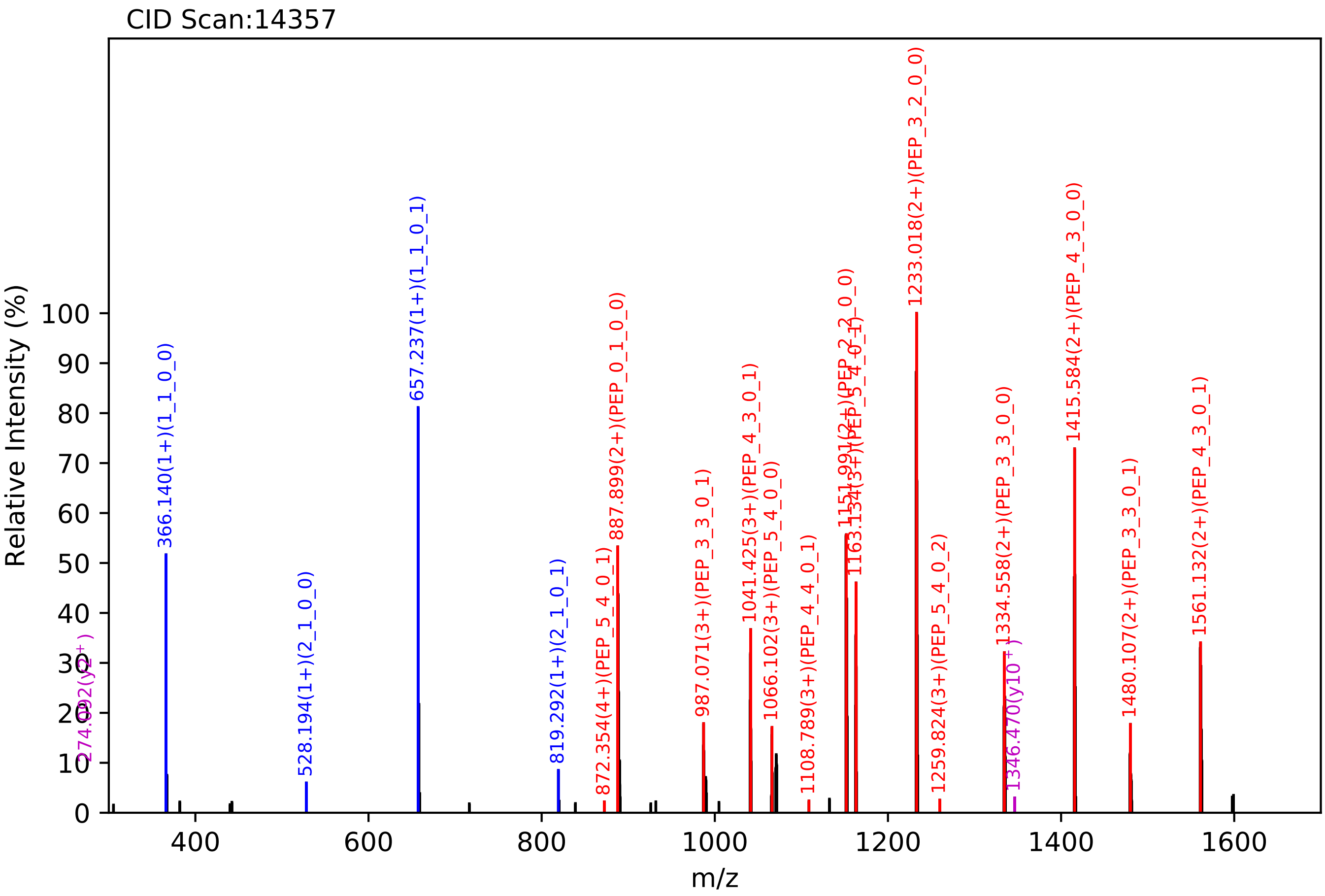

Unknown set no. 212, Experiment: Human Plasma exp_3

NHSCEPCQTLAVR(=PEP)_5_4_0_2, m/z:1259.50(3+), RT:53.77, Y-score:81.92

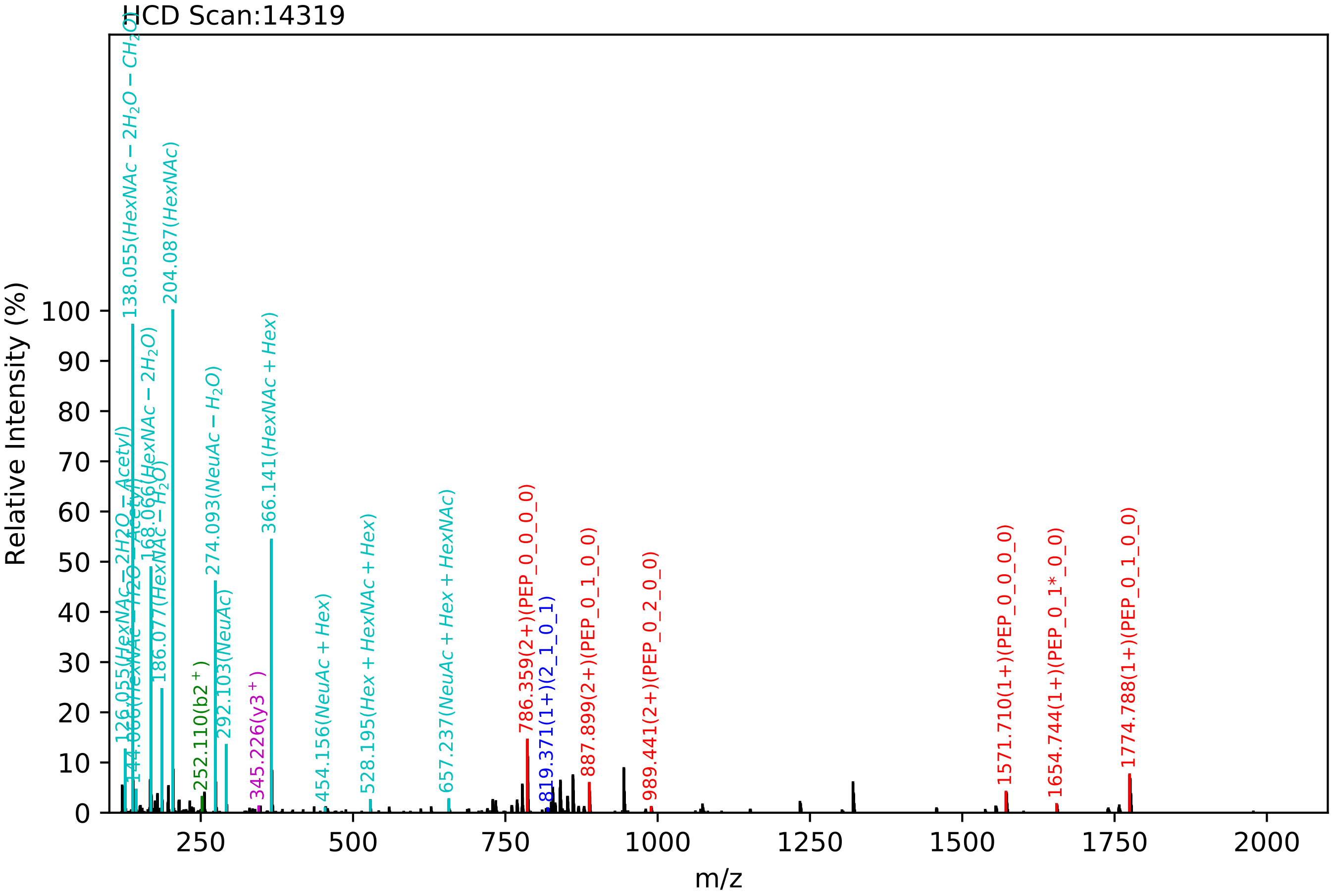

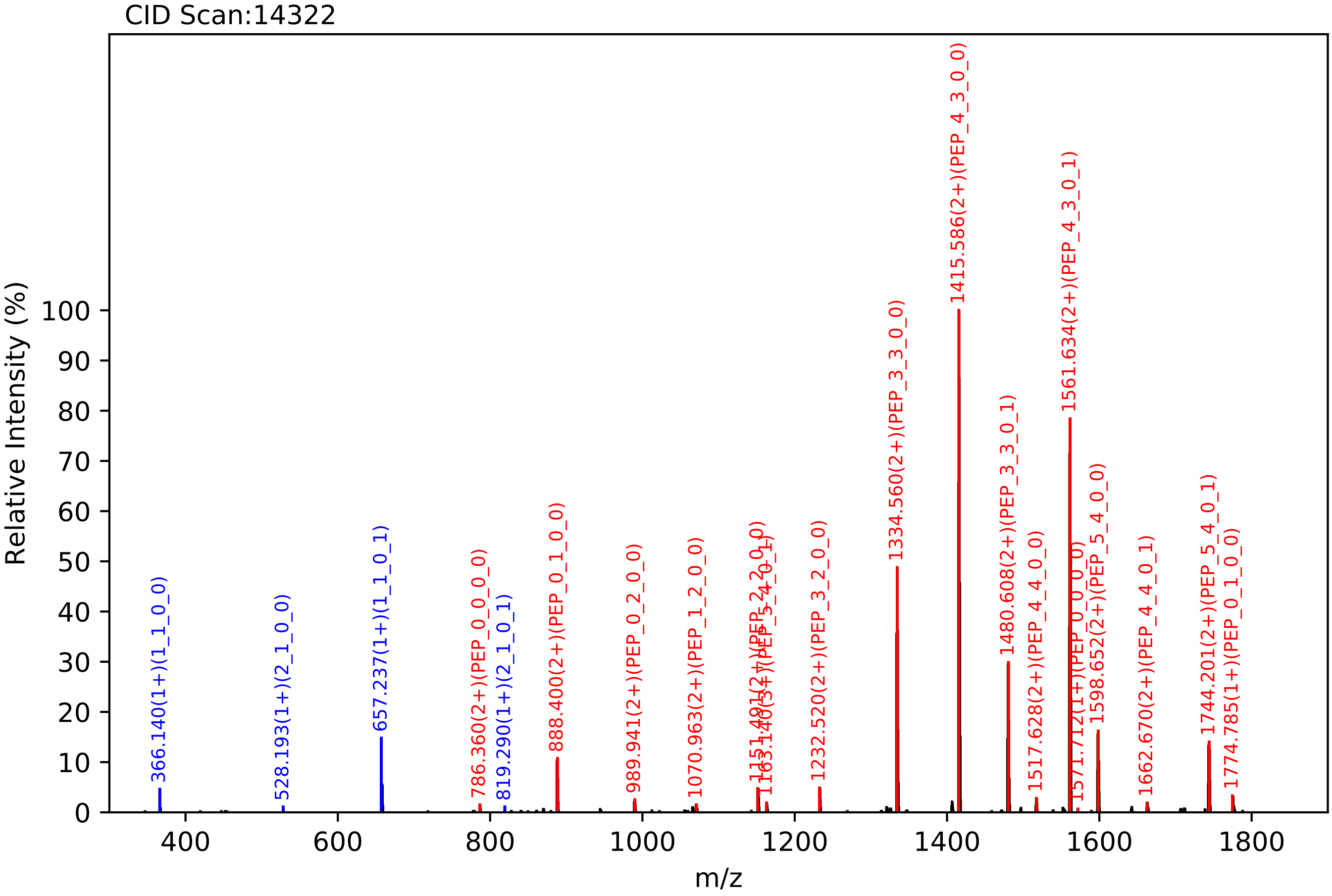

Unknown set no. 213, Experiment: Human Plasma exp_4

NHSCEPCQTLAVR(=PEP)_5_4_0_2, m/z:944.88(4+), RT:54.15, Y-score:80.70

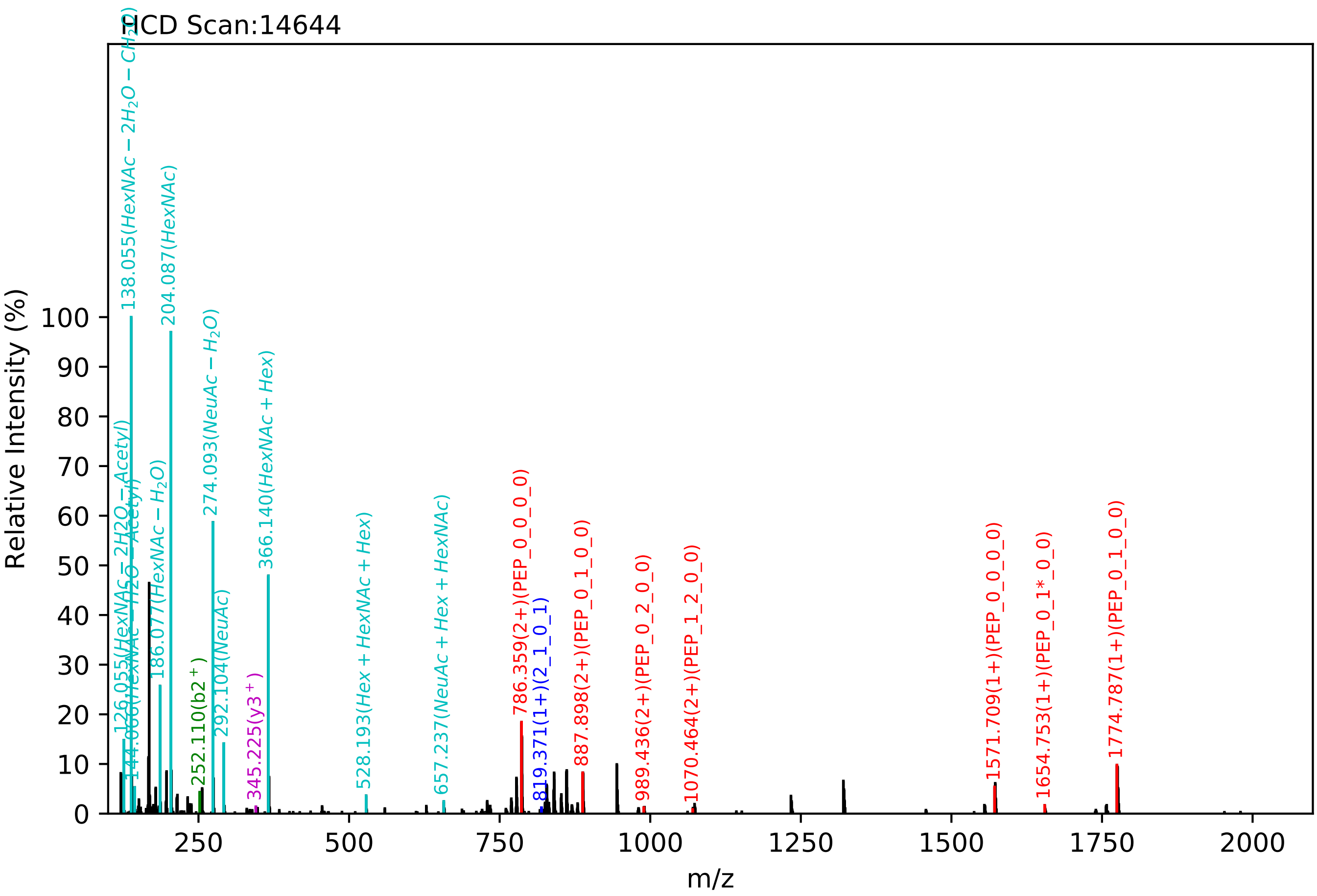

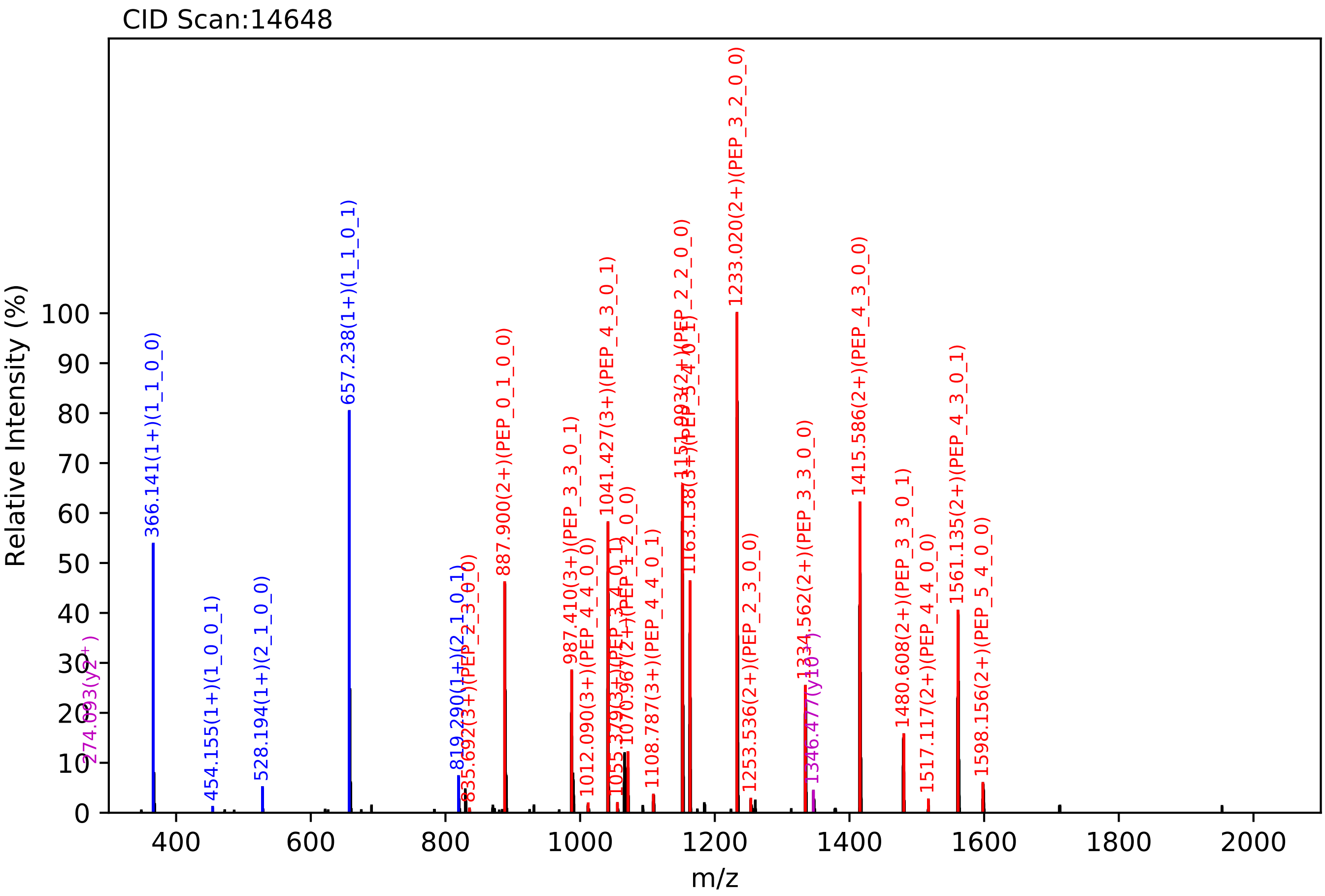

Unknown set no. 214, Experiment: Human Plasma exp_4

NHSCEPCQTLAVR(=PEP)_5_4_0_2, m/z:1259.50(3+), RT:54.14, Y-score:82.64

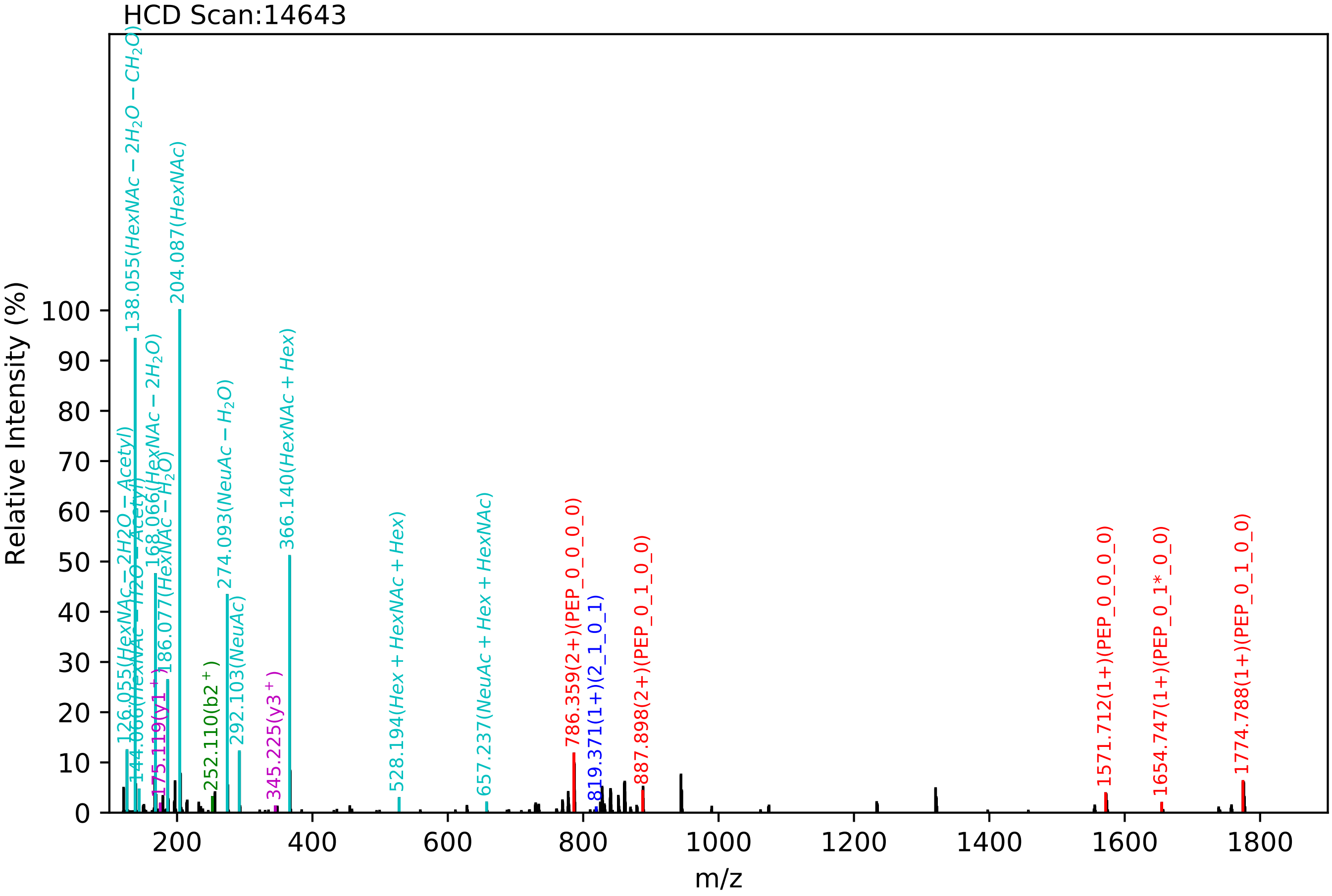

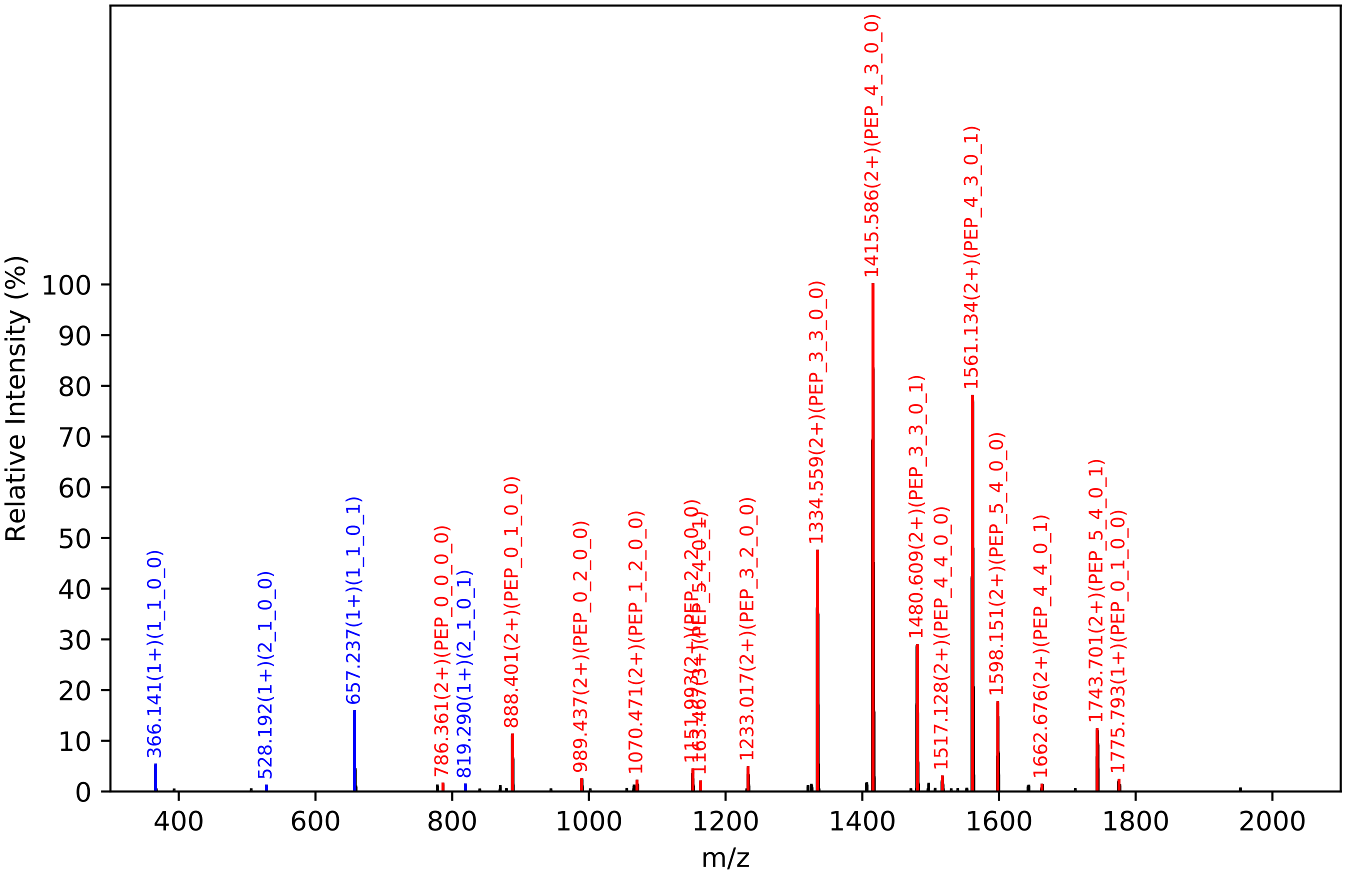

Unknown set no. 215, Experiment: Human Plasma exp_5

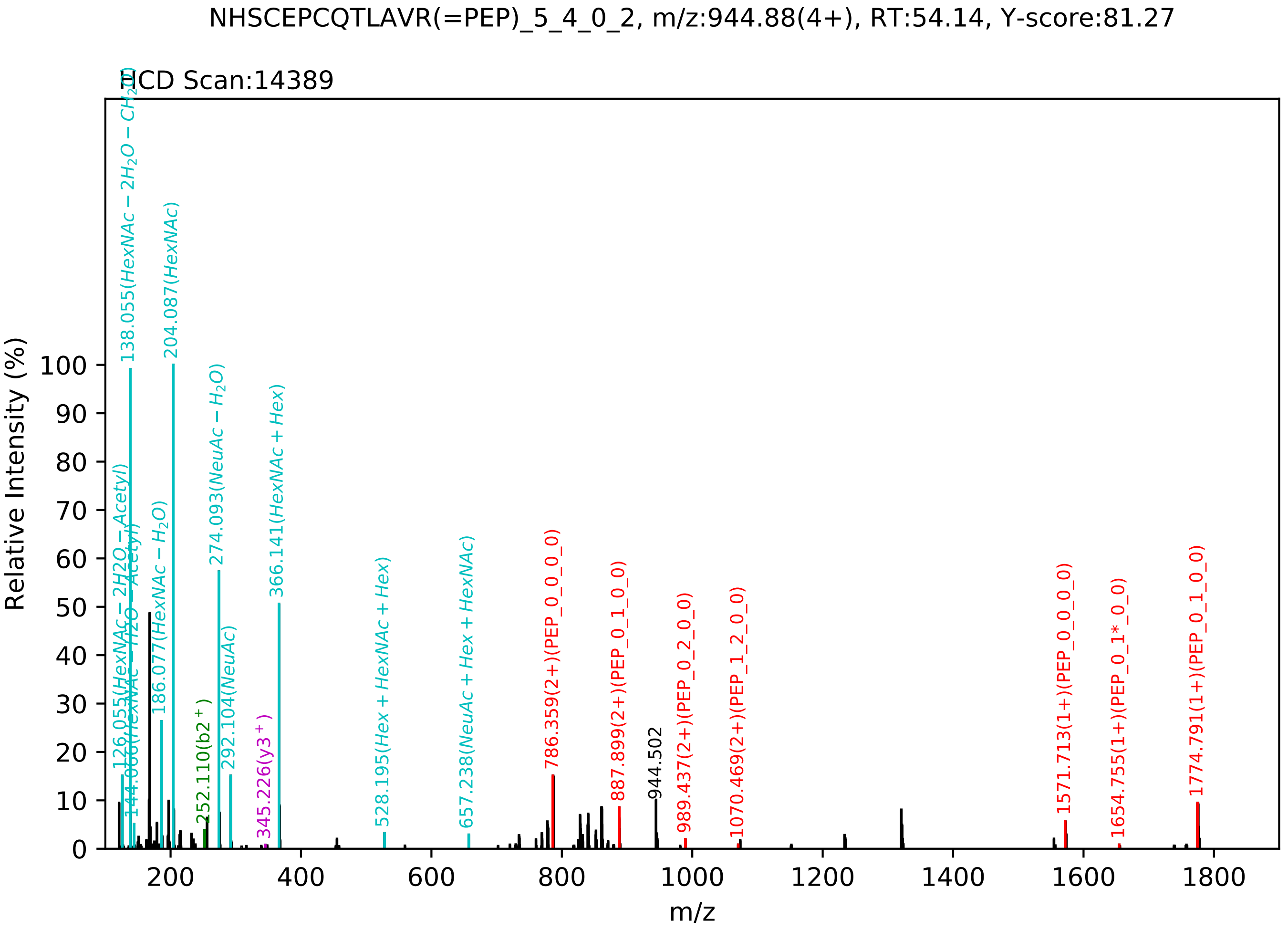

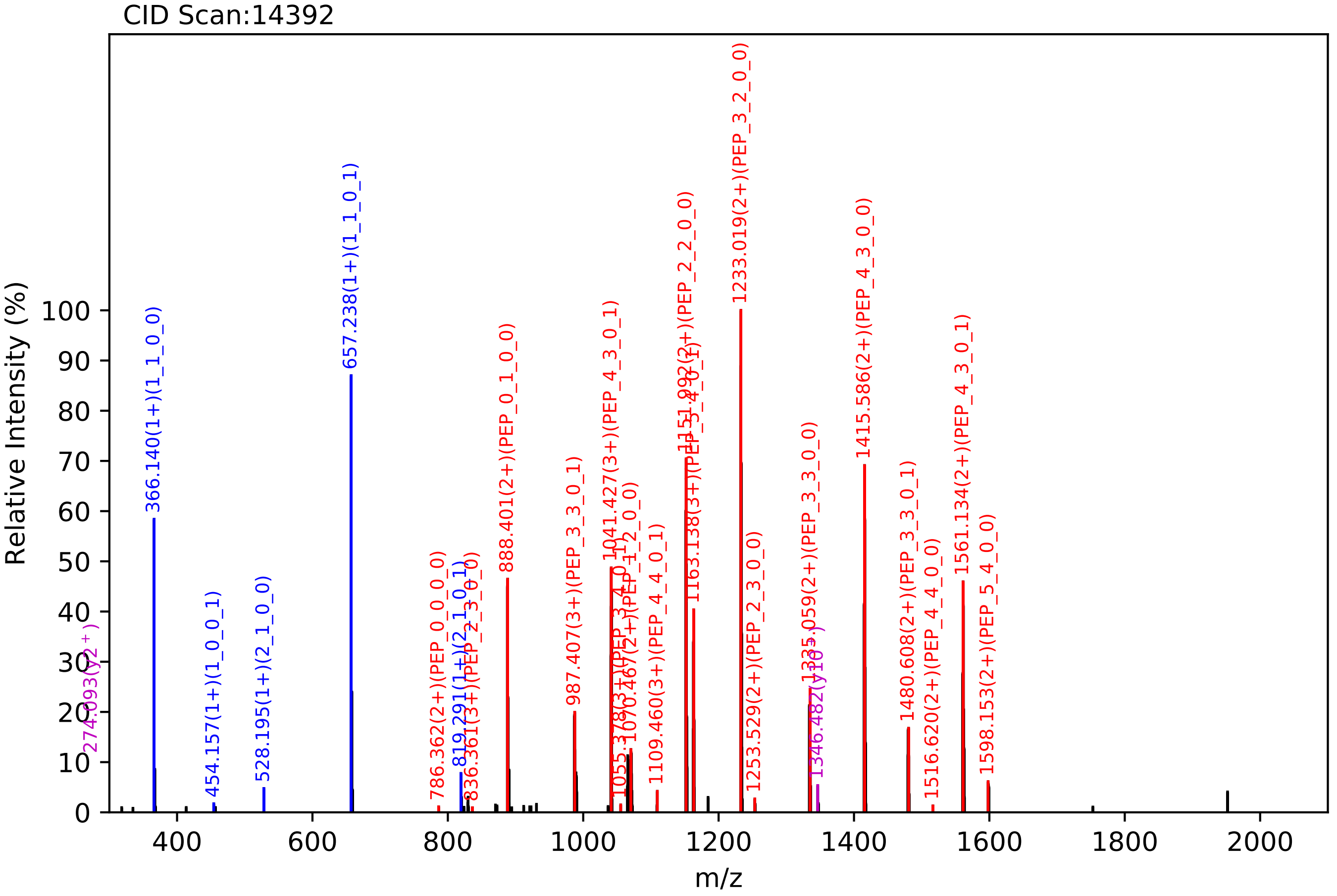

Unknown set no. 216, Experiment: Human Plasma exp_2

NVTAEQAR(=PEP)_5_4_0_1, m/z:934.72(3+), RT:16.73, Y-score:84.82

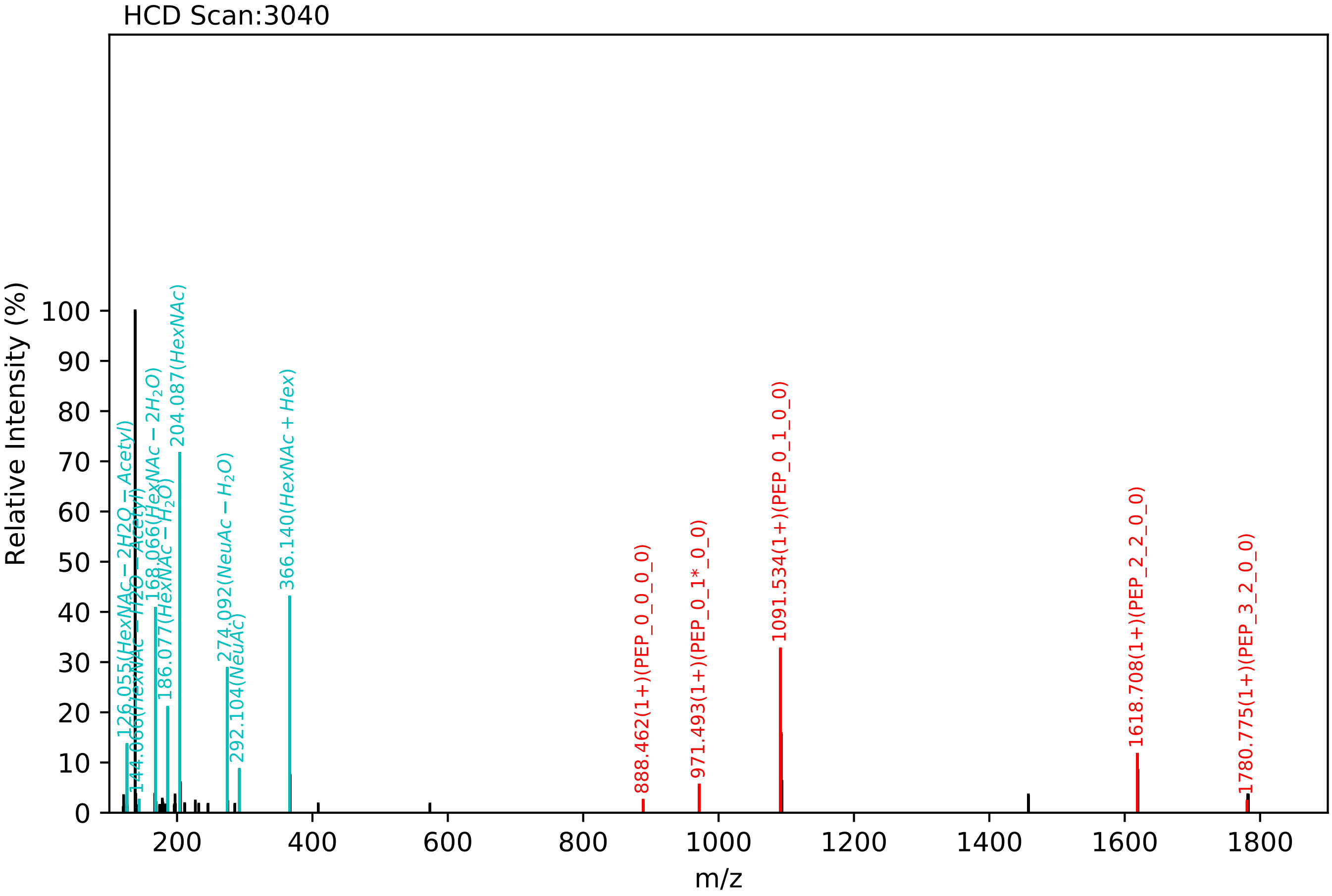

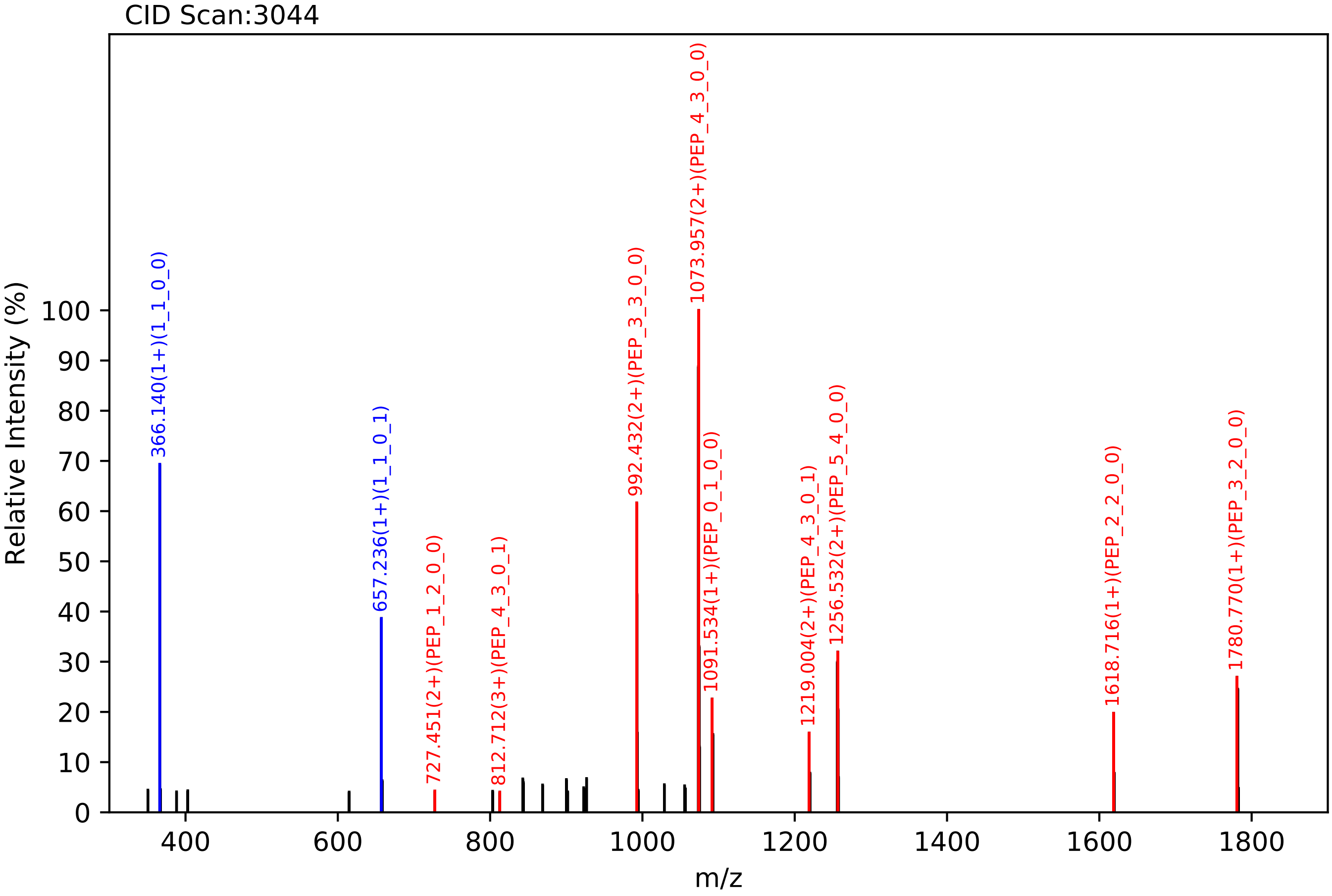

Unknown set no. 217, Experiment: Human Plasma exp_3

NVTAEQAR(=PEP)_5_4_0_1, m/z:934.72(3+), RT:16.80, Y-score:91.50

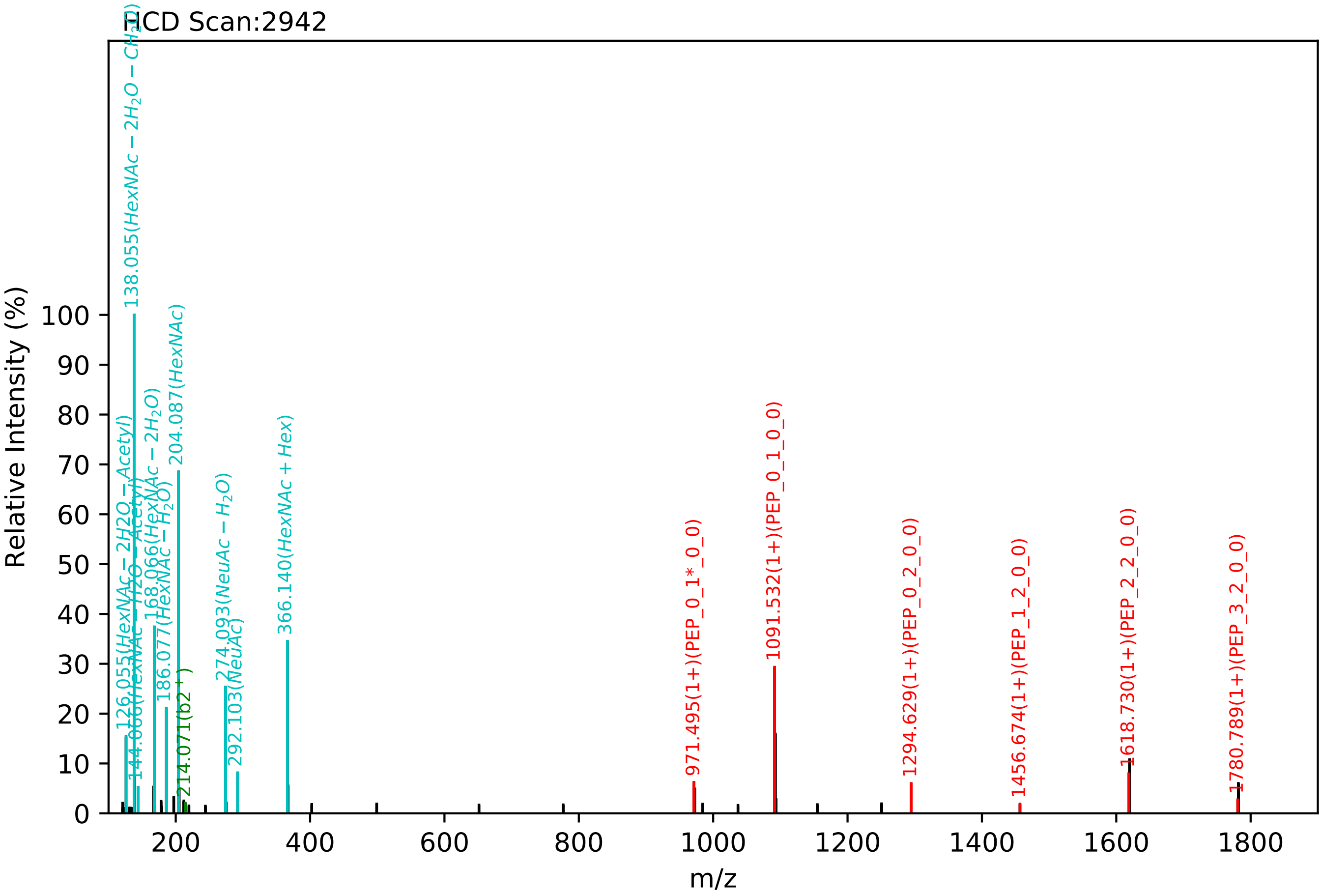

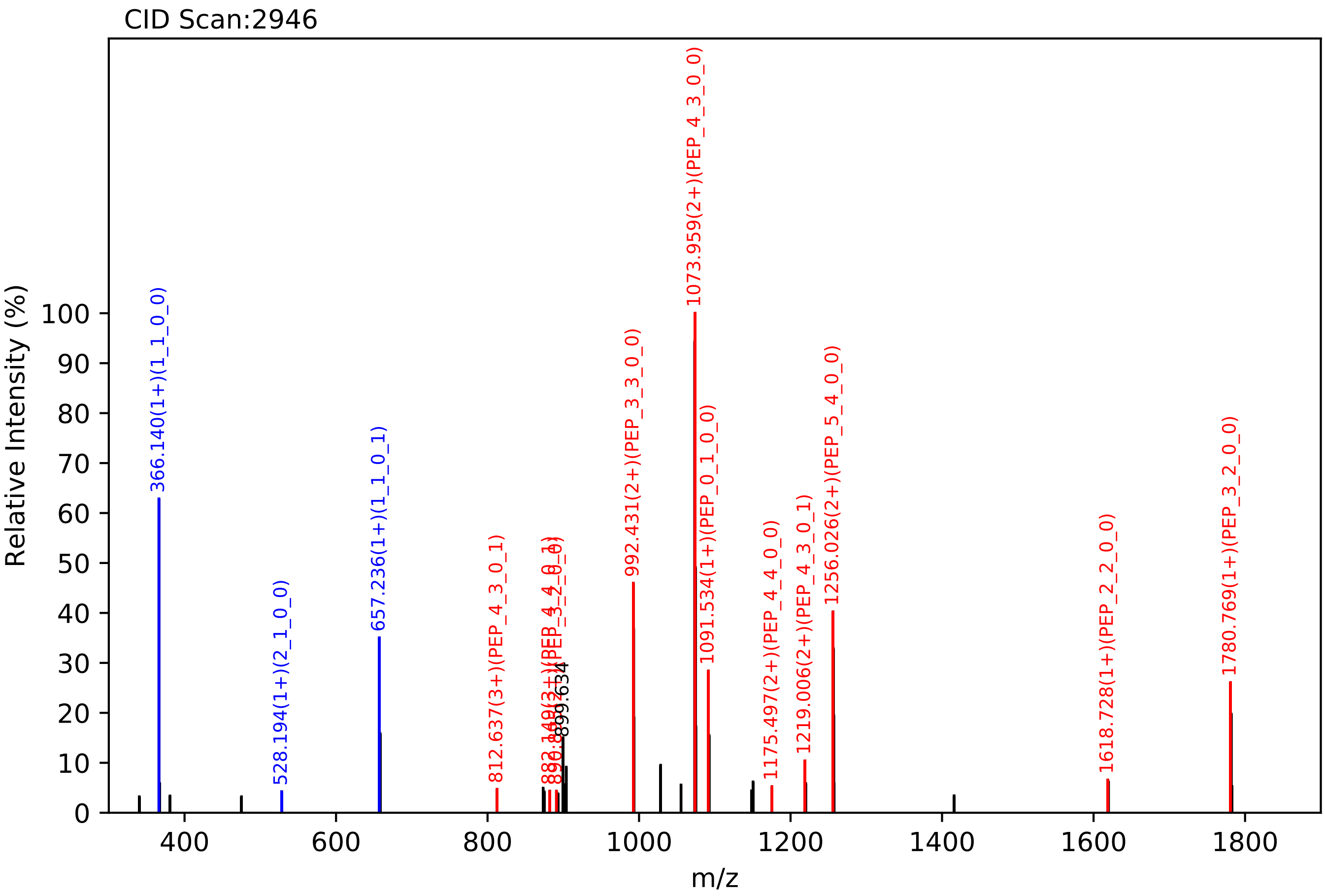

Unknown set no. 218, Experiment: Human Plasma exp_1

NVTAEQAR(=PEP)_5_4_0_2, m/z:1031.75(3+), RT:25.36, Y-score:91.83

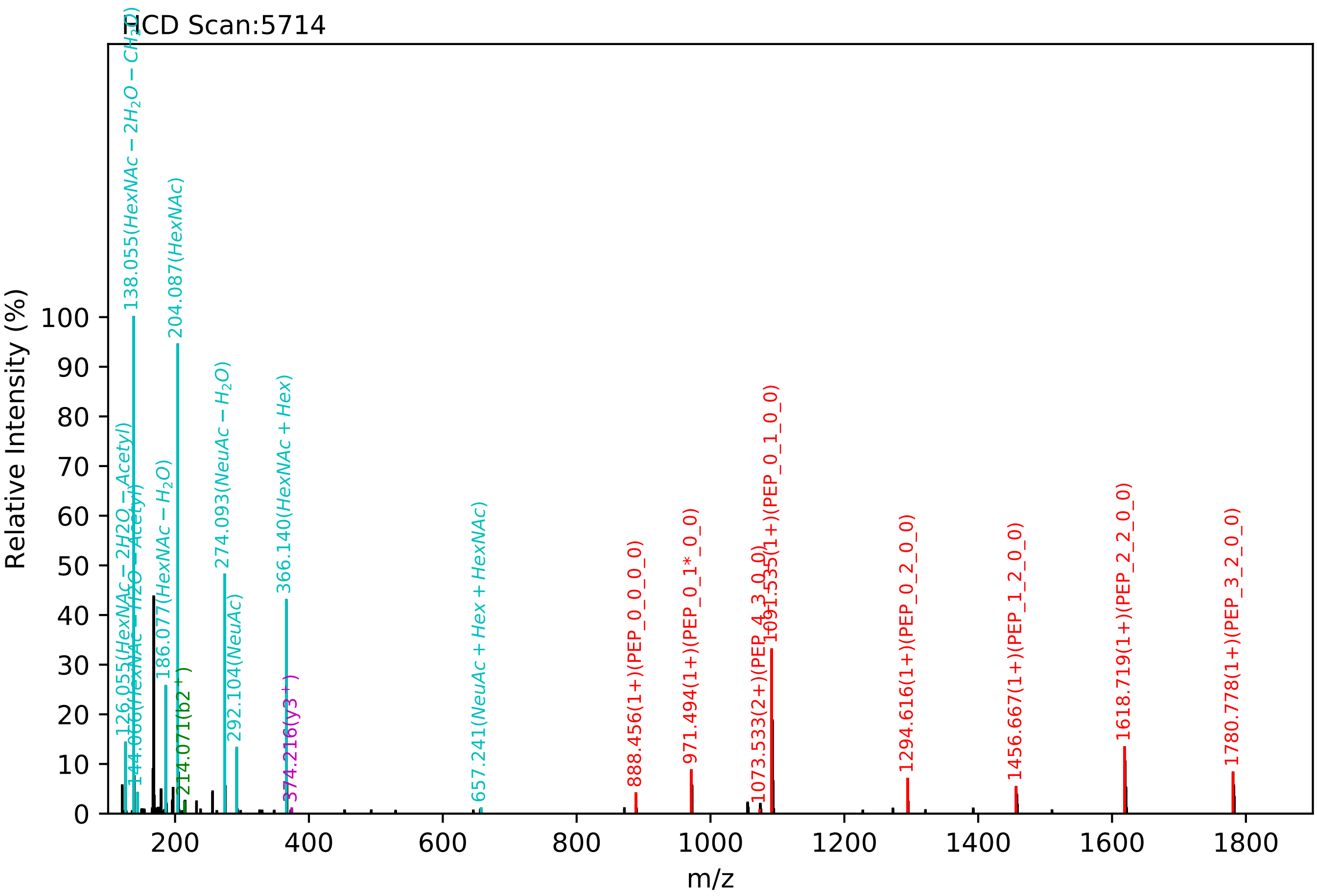

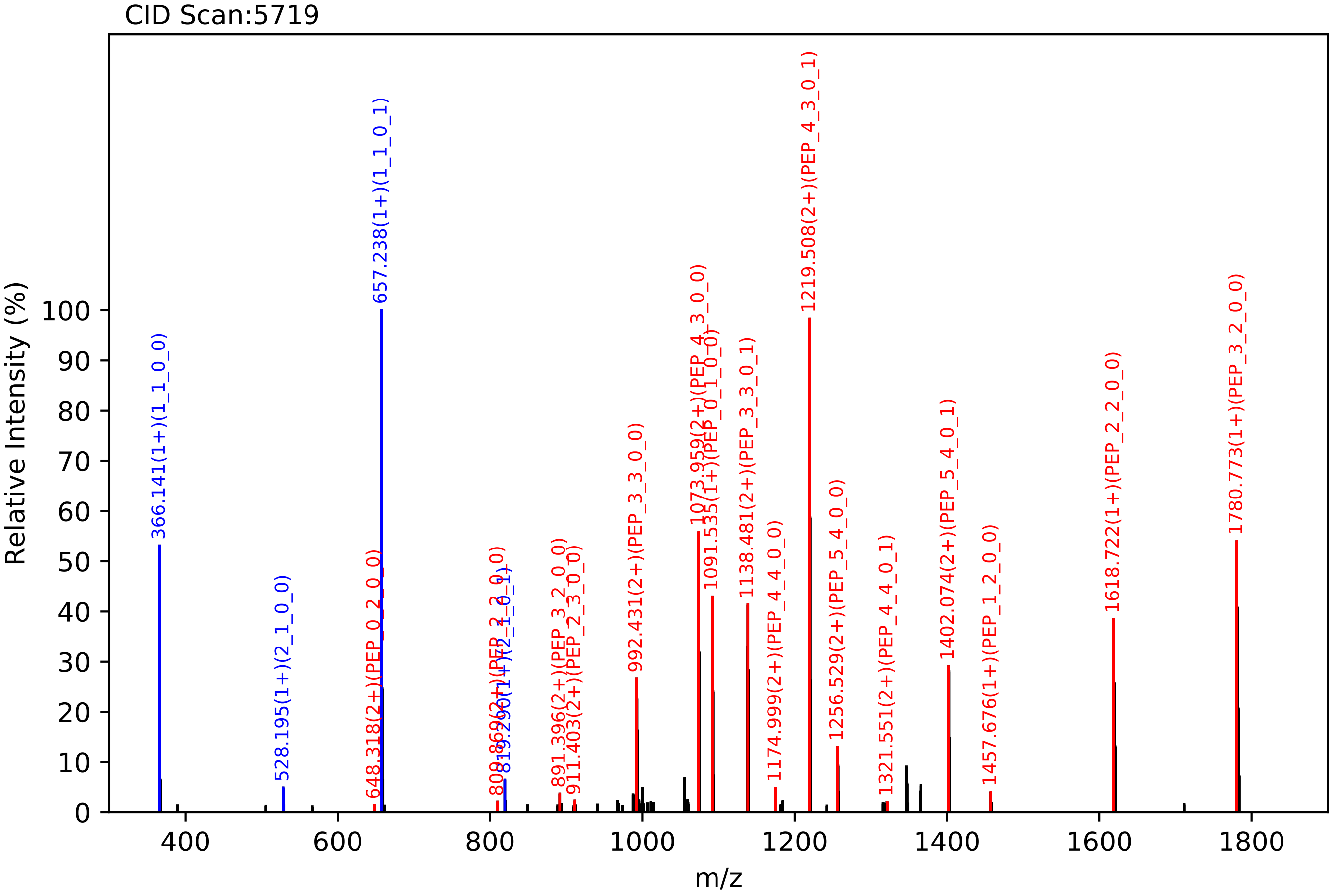

Unknown set no. 219, Experiment: Human Plasma exp_2

NVTAEQAR(=PEP)_5_4_0_2, m/z:1031.75(3+), RT:25.68, Y-score:89.09

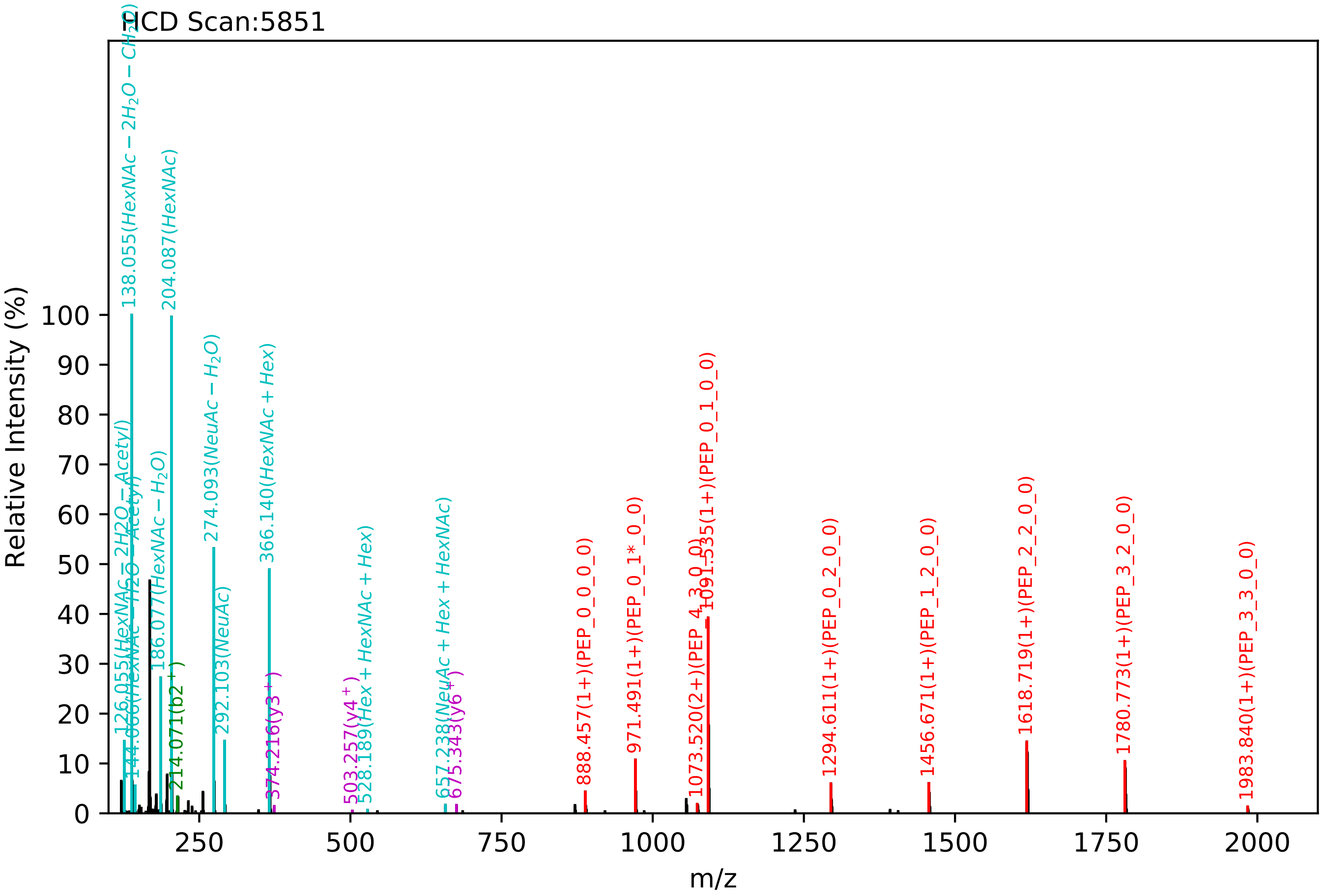

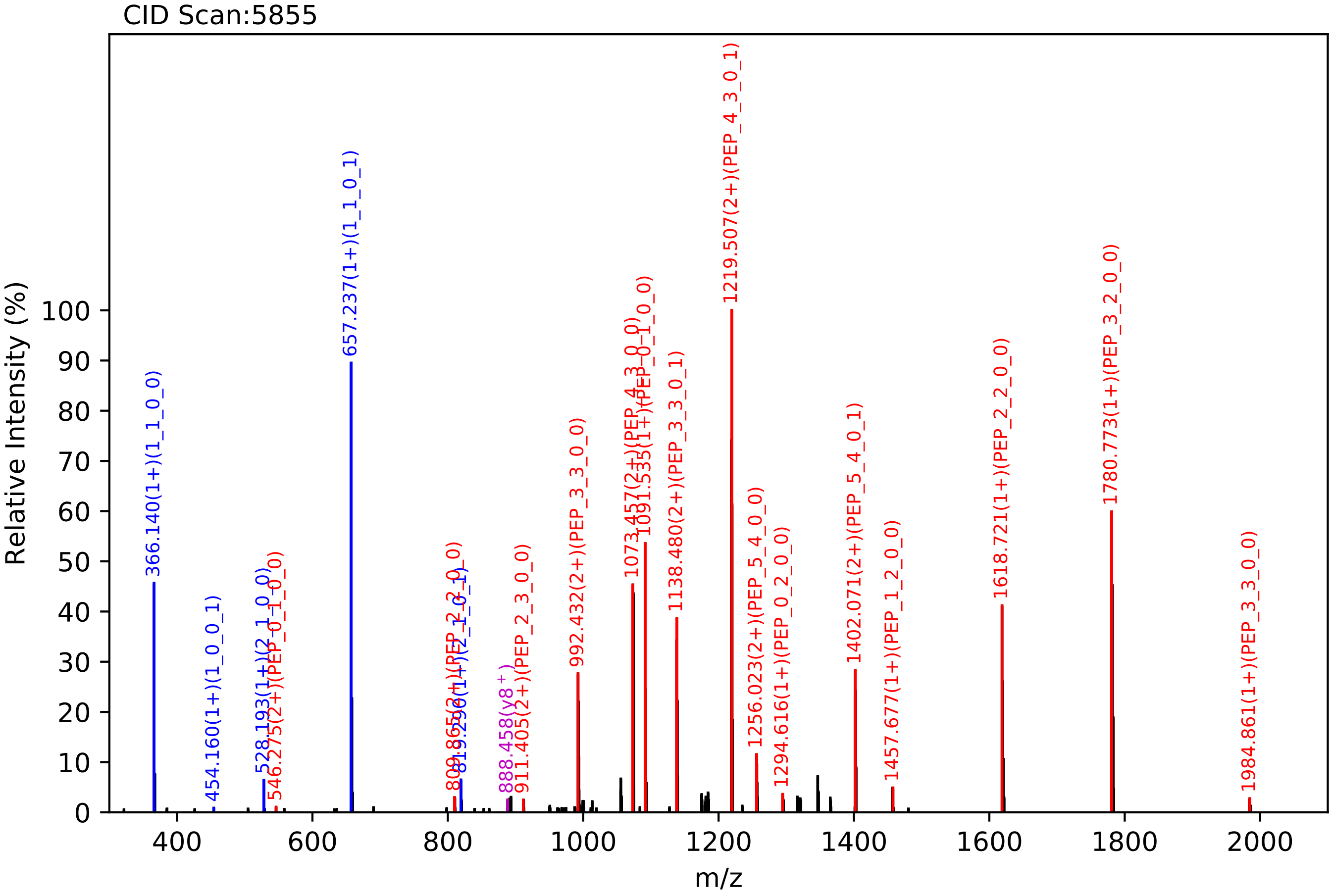

Unknown set no. 220, Experiment: Human Plasma exp_3

NVTAEQAR(=PEP)_5_4_0_2, m/z:1031.75(3+), RT:25.77, Y-score:86.05

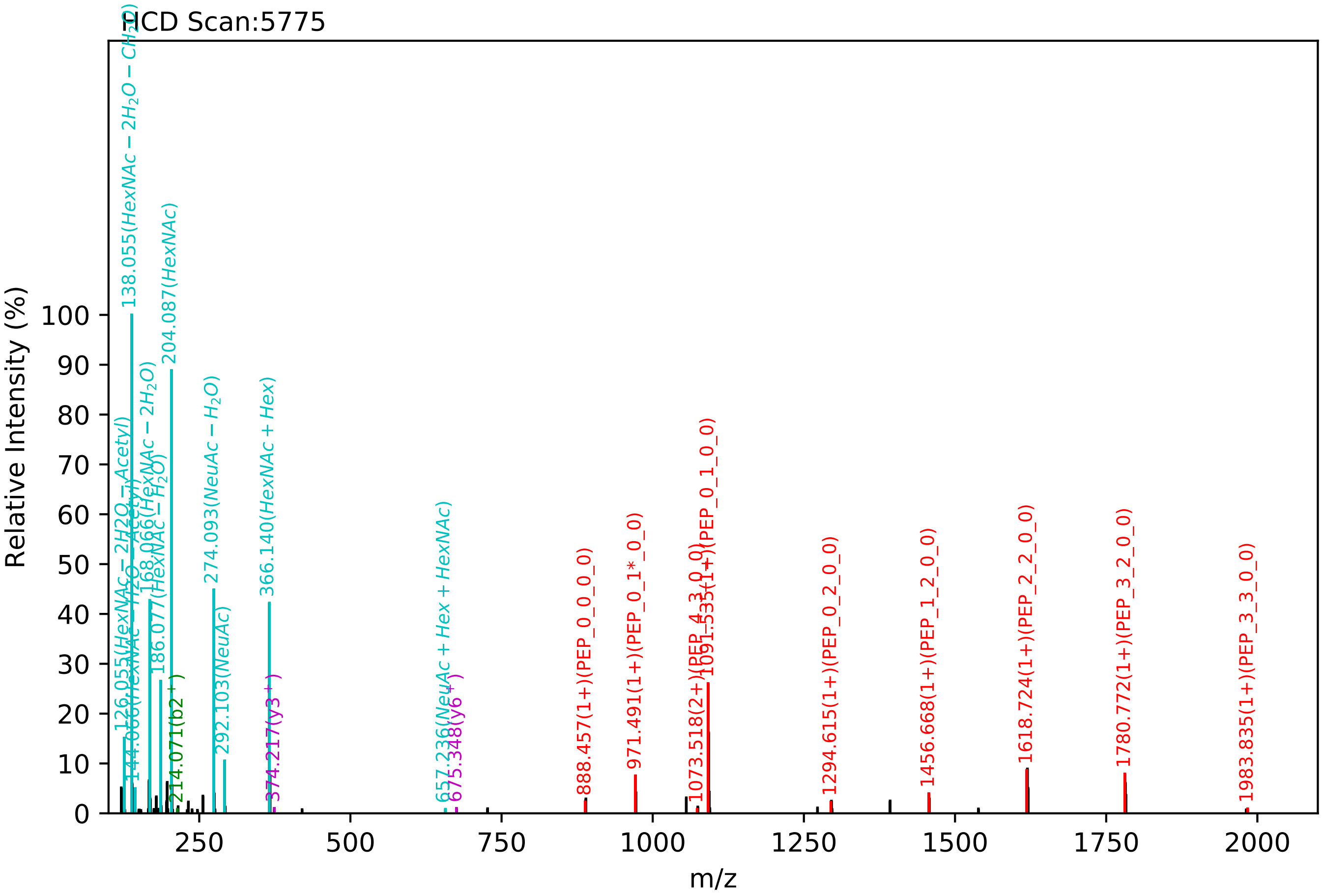

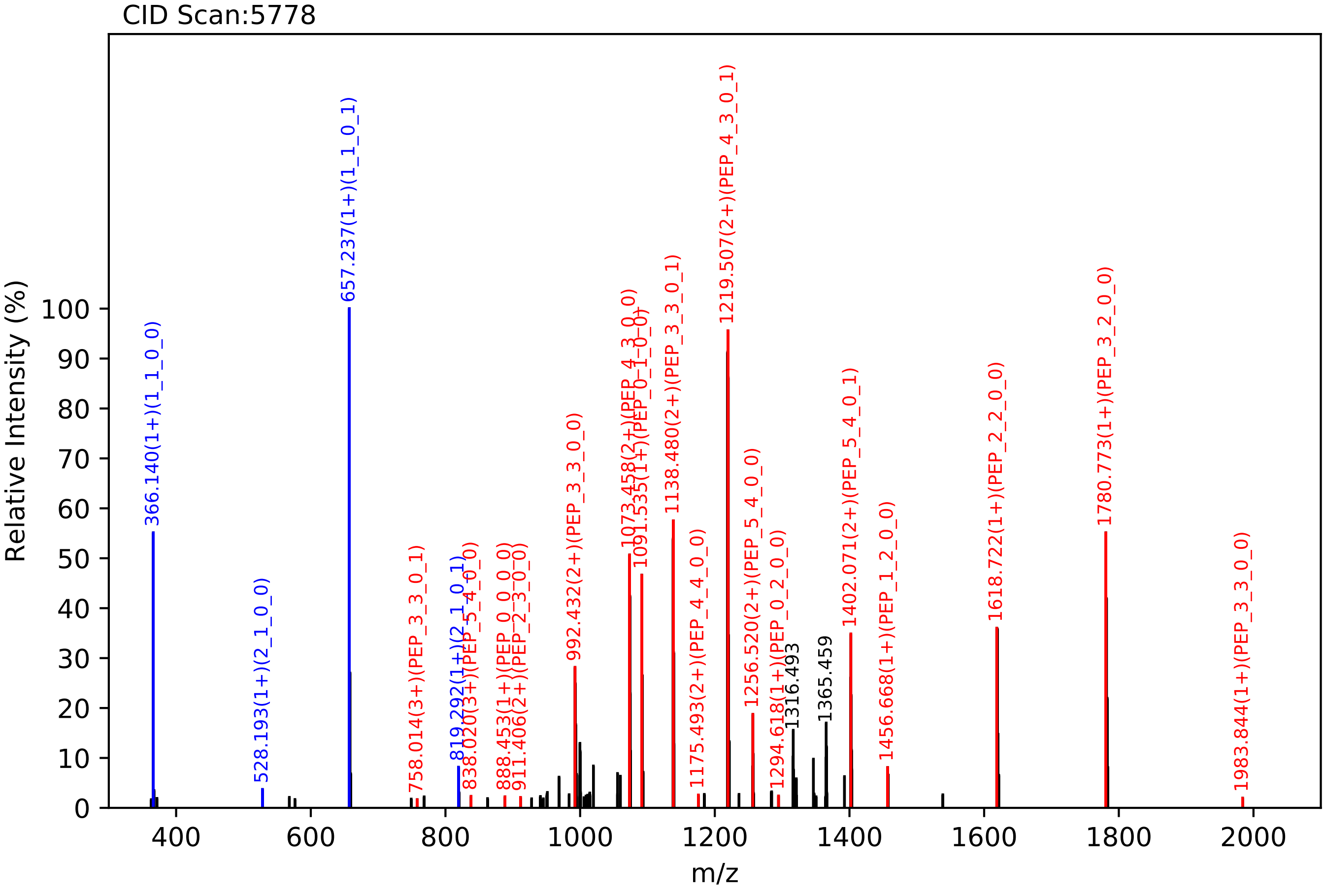

Unknown set no. 221, Experiment: Human Plasma exp_5

NVTAEQAR(=PEP)_5_4_0_2, m/z:1031.75(3+), RT:25.90, Y-score:90.35

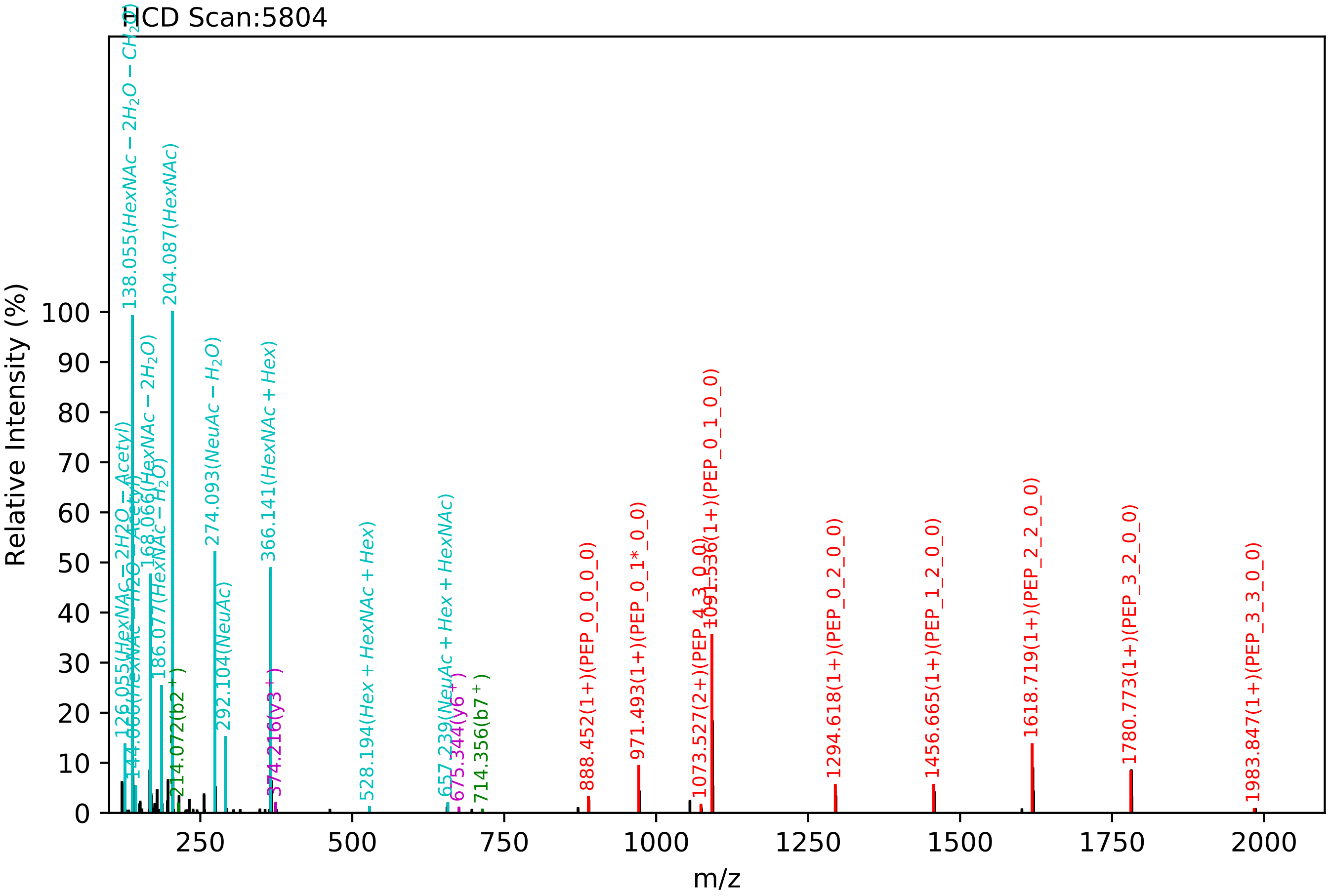

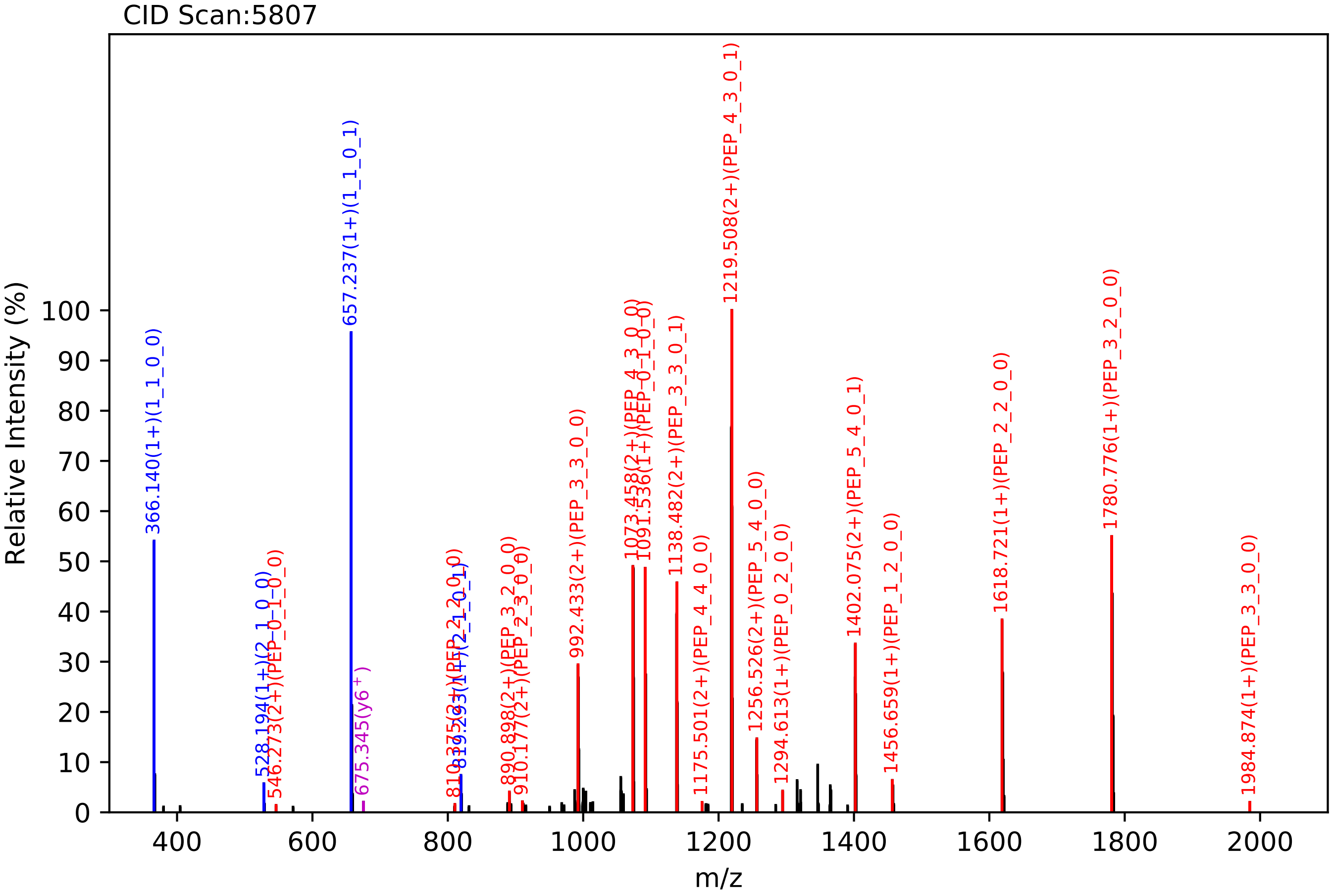

Unknown set no. 222, Experiment: Human Plasma exp_5

NVTAEQAR(=PEP)_6_5_0_3, m/z:1250.49(3+), RT:41.20, Y-score:91.95

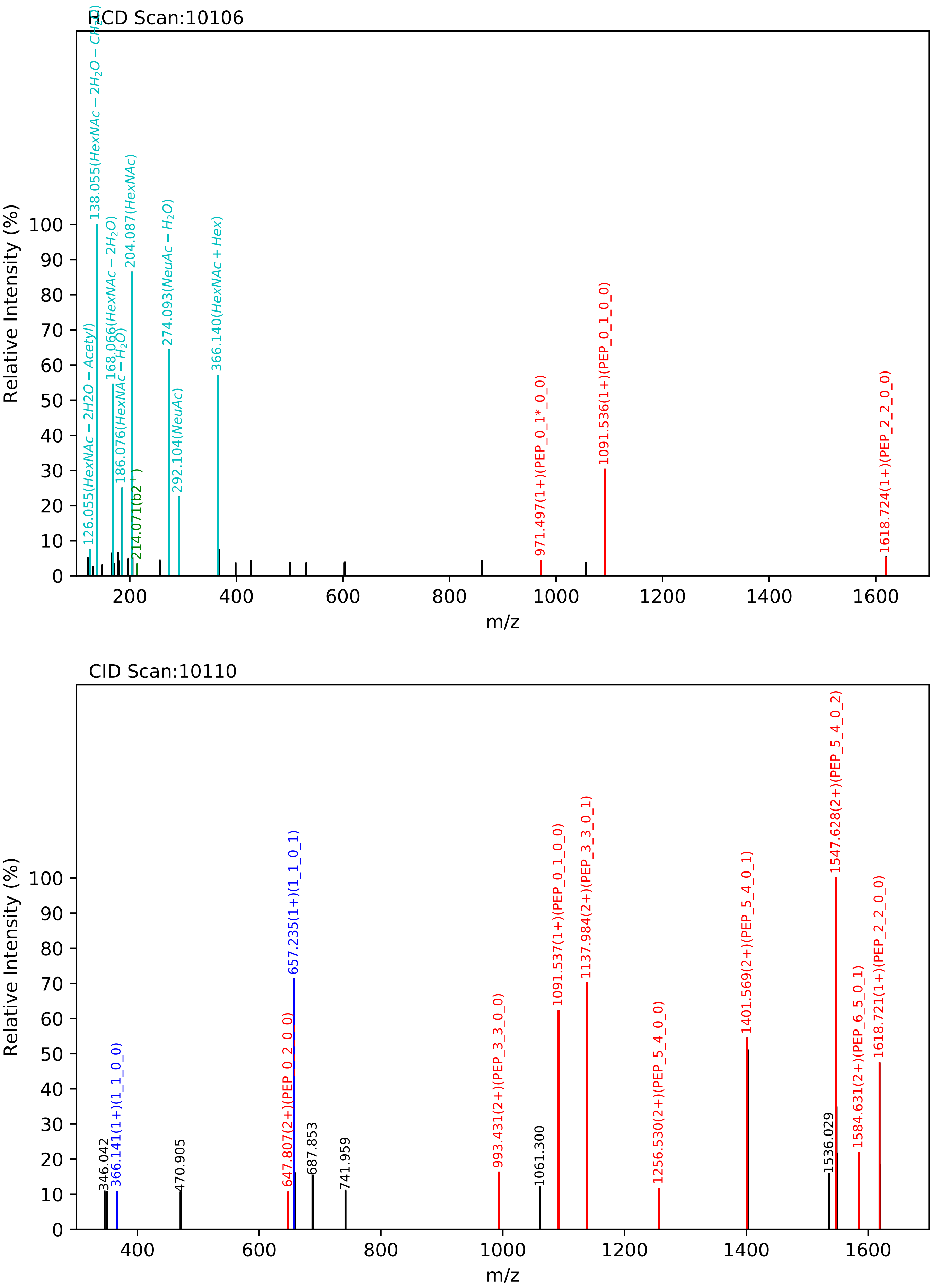

Unknown set no. 223, Experiment: Human Plasma exp_1

NYTLTGR(=PEP)_5_4_1_2, m/z:1059.09(3+), RT:60.01, Y-score:85.58

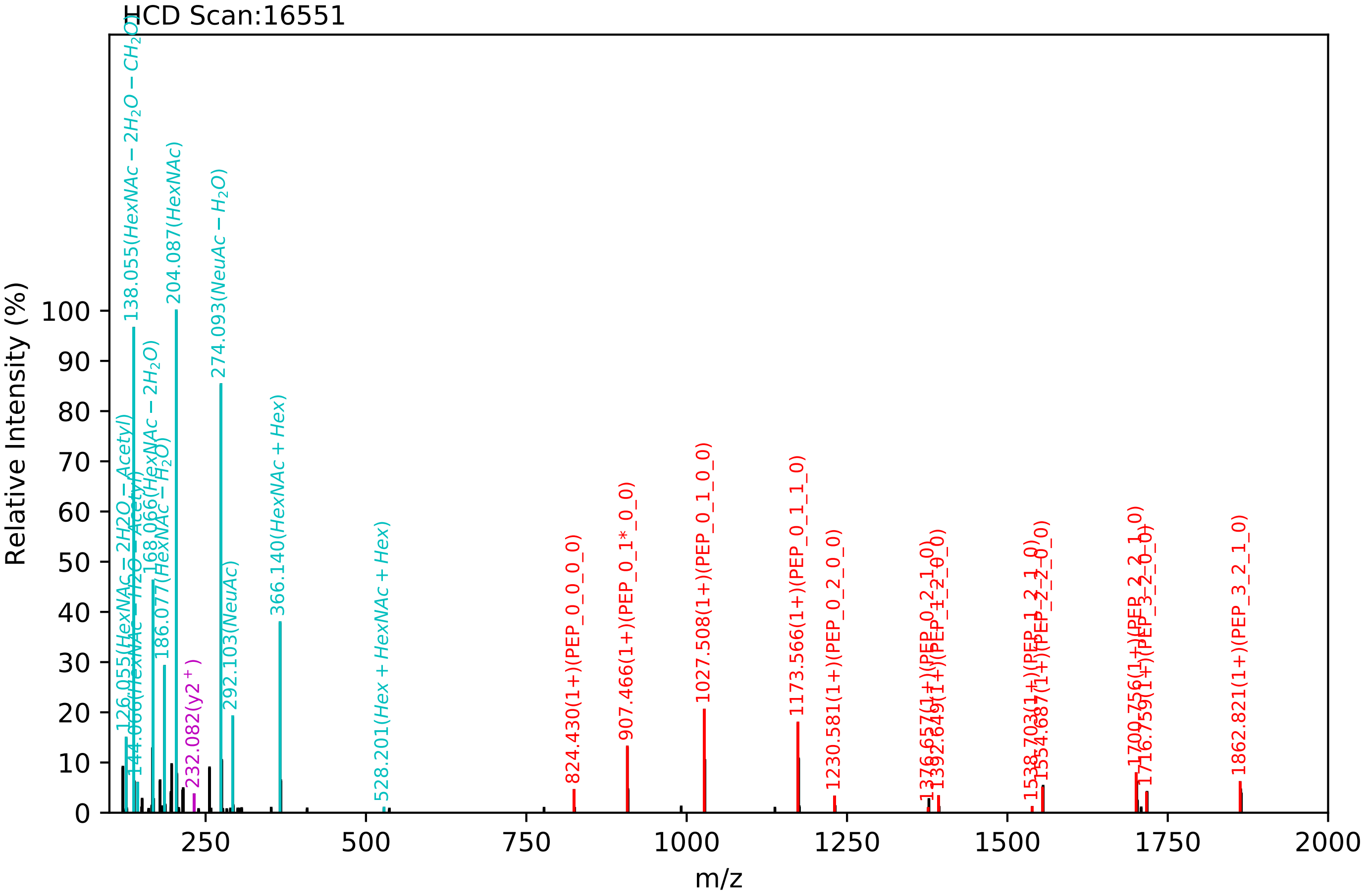

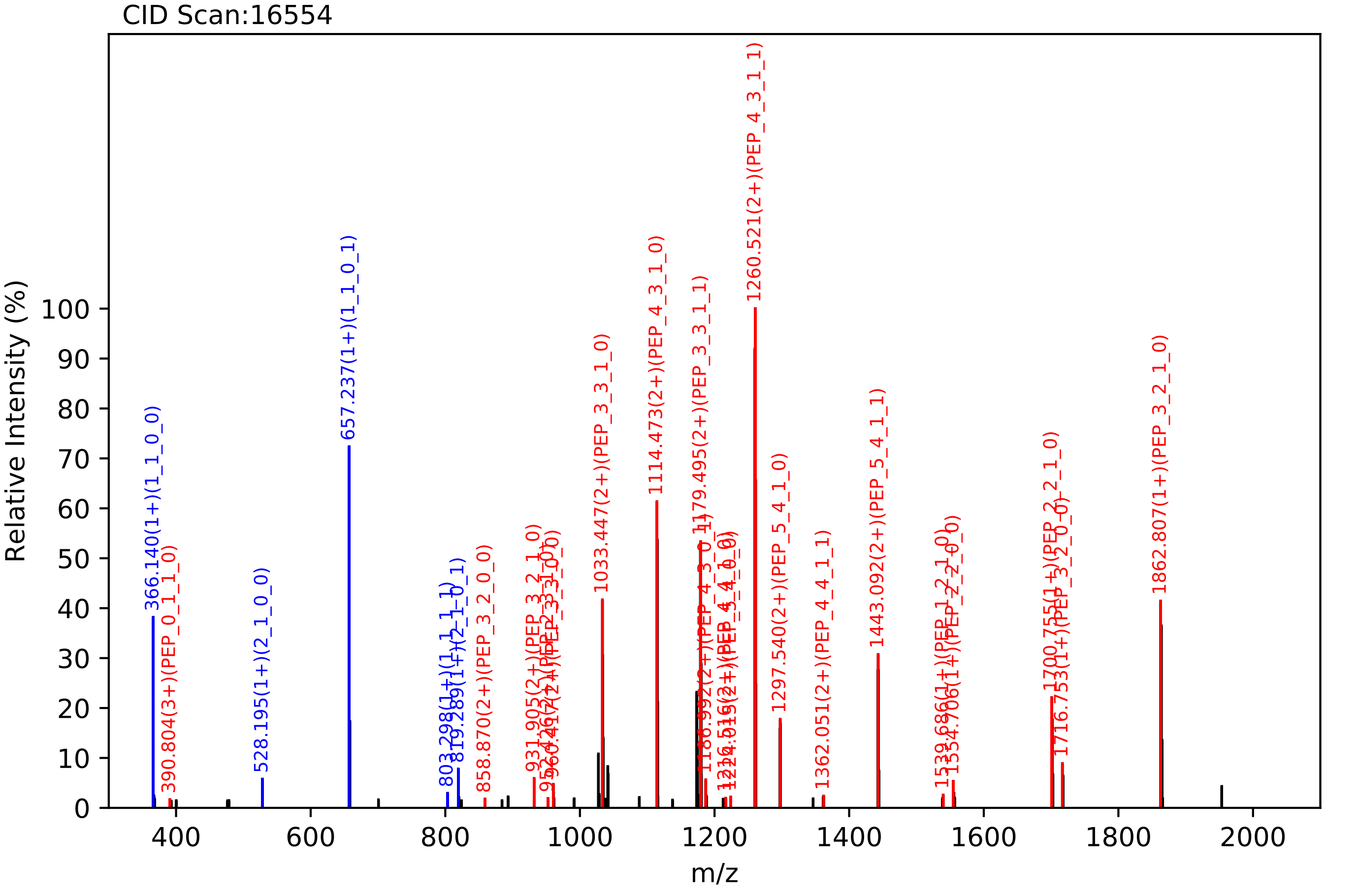

Unknown set no. 224, Experiment: Human Plasma exp_2

NYTLTGR(=PEP)_5_4_1_2, m/z:1059.09(3+), RT:60.04, Y-score:87.72

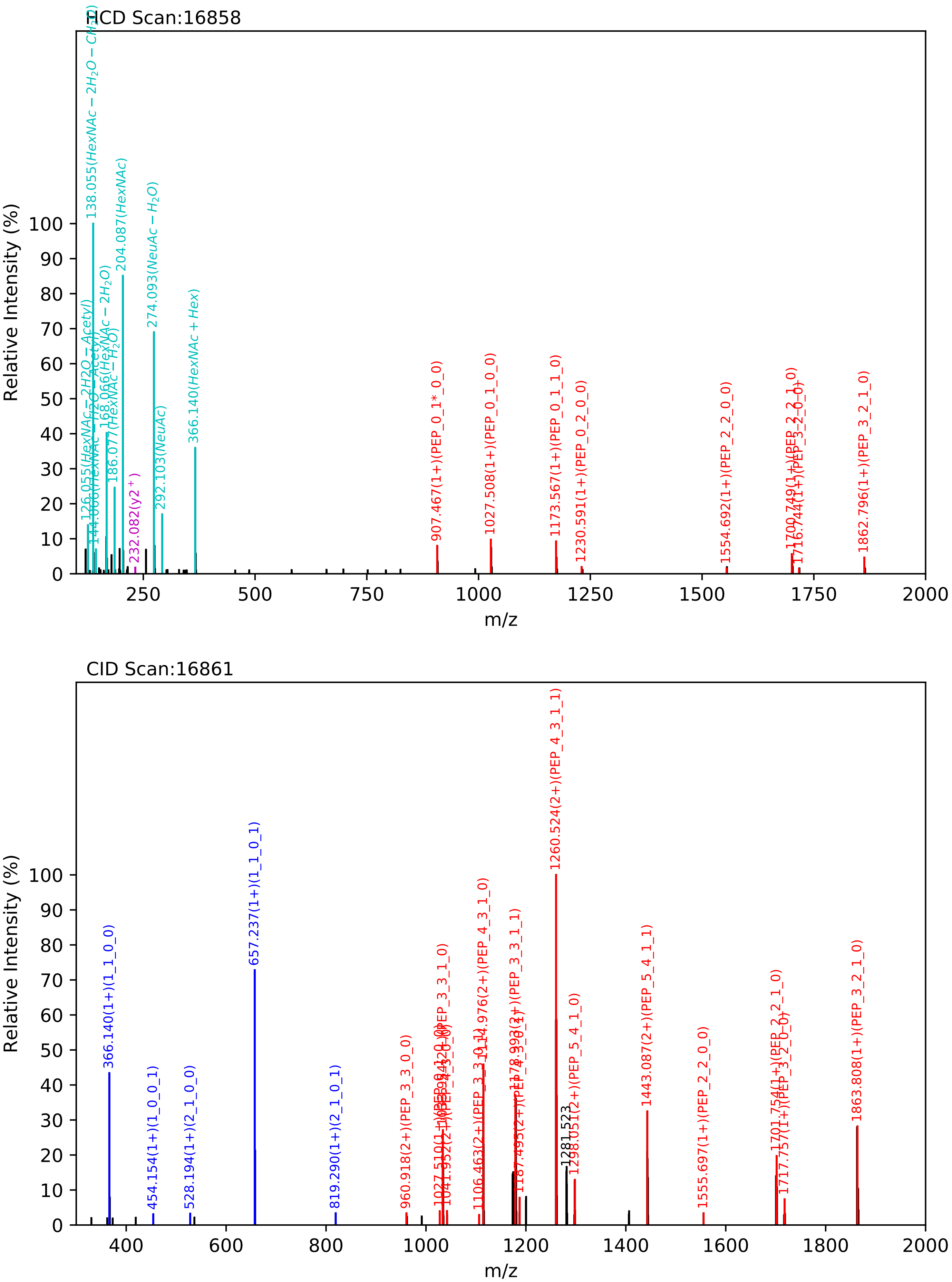

Unknown set no. 225, Experiment: Human Plasma exp_3

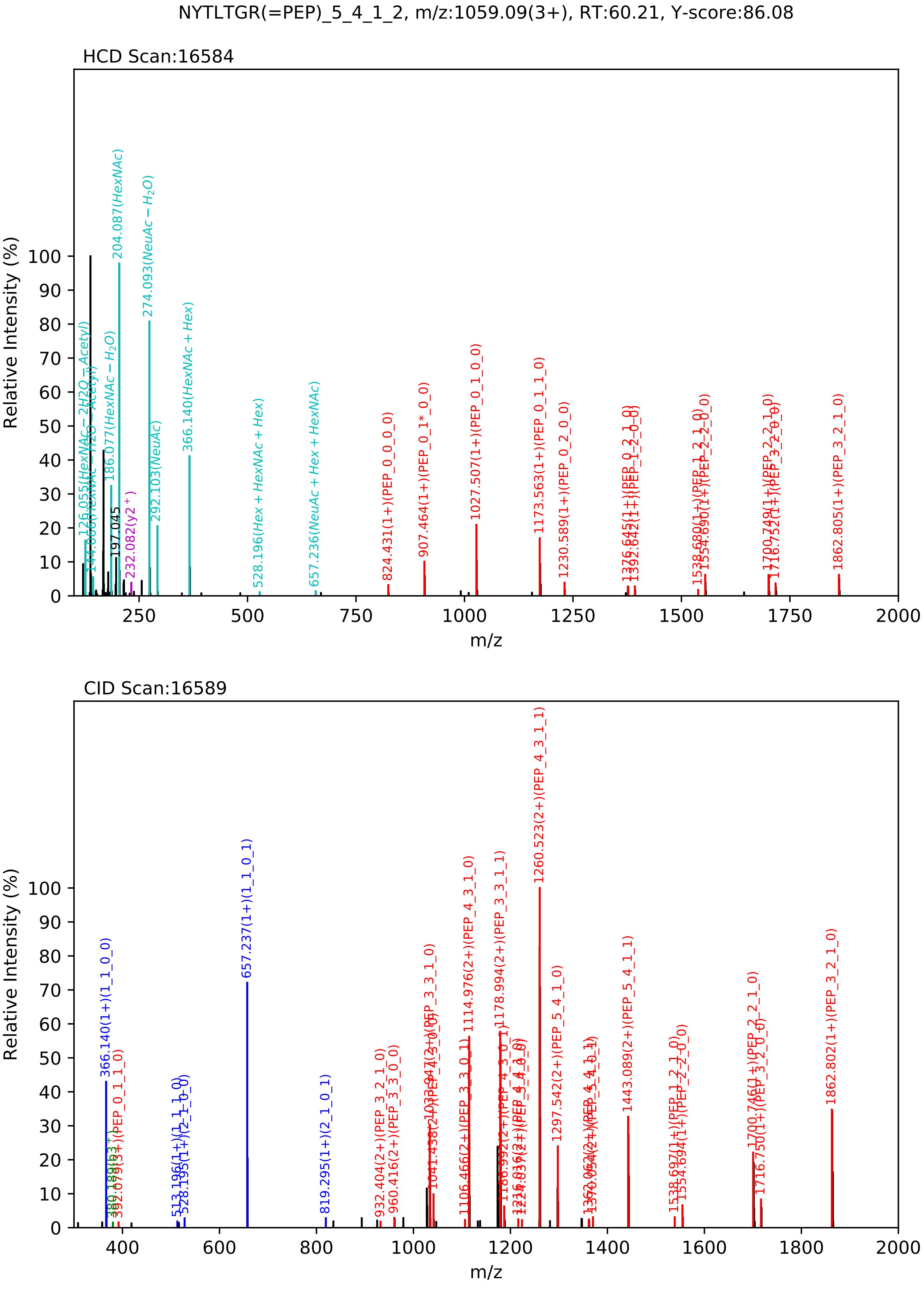

Unknown set no. 226, Experiment: Human Plasma exp_4

NYTLTGR(=PEP)_5_4_1_2, m/z:1059.09(3+), RT:60.32, Y-score:86.01

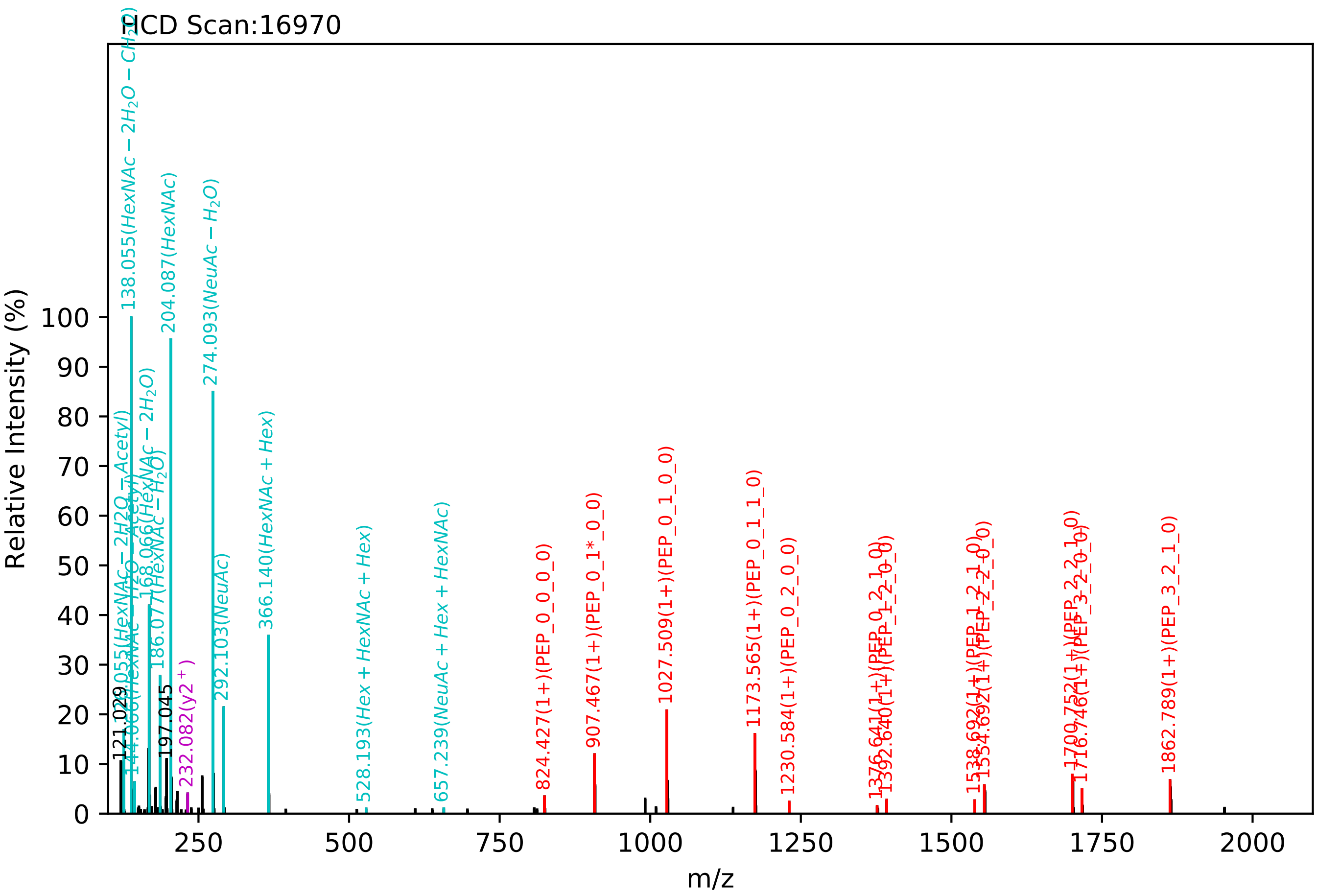

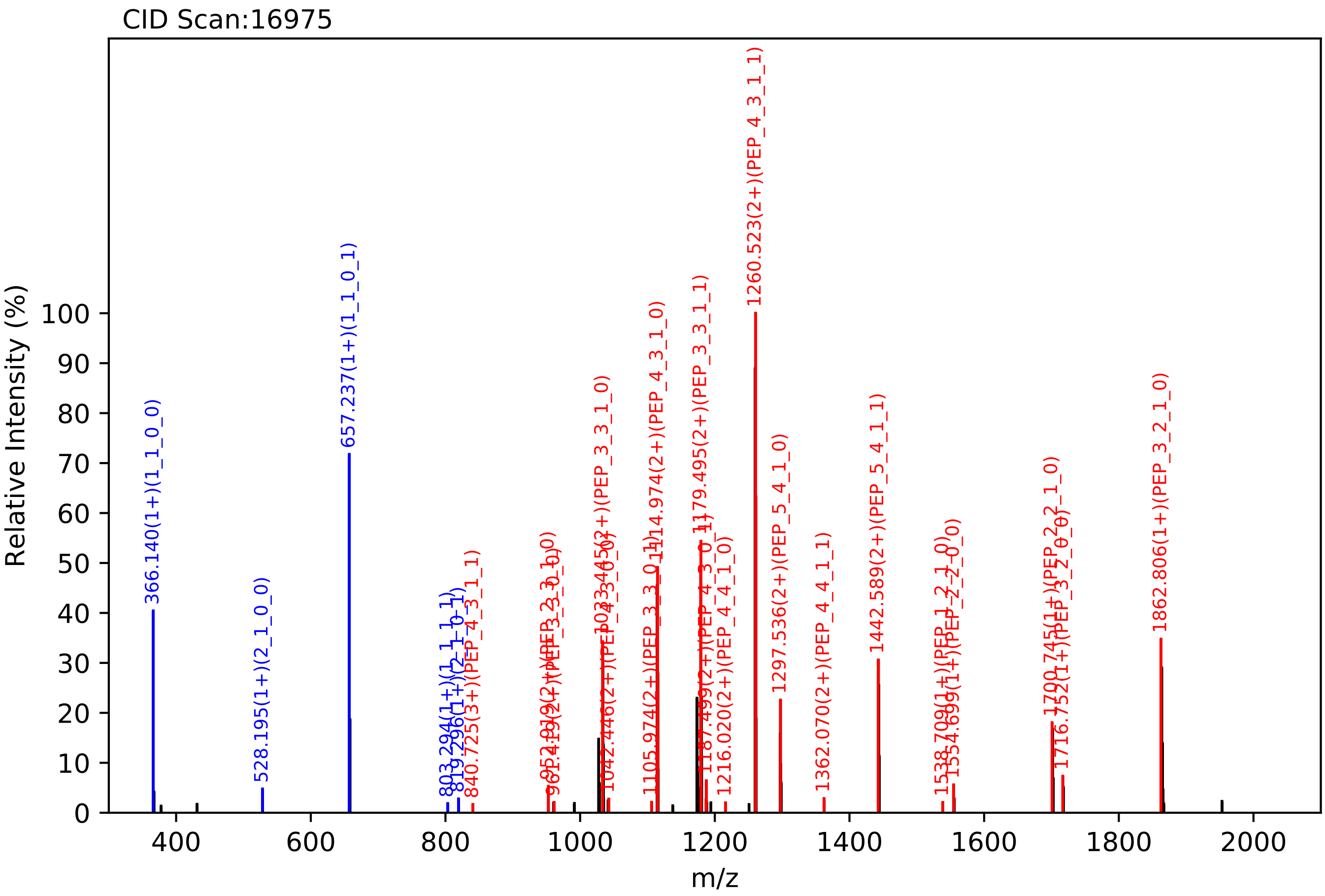

Unknown set no. 227, Experiment: Human Plasma exp_5

NYTLTGR(=PEP)_5_4_1_2, m/z:1059.09(3+), RT:60.28, Y-score:86.19

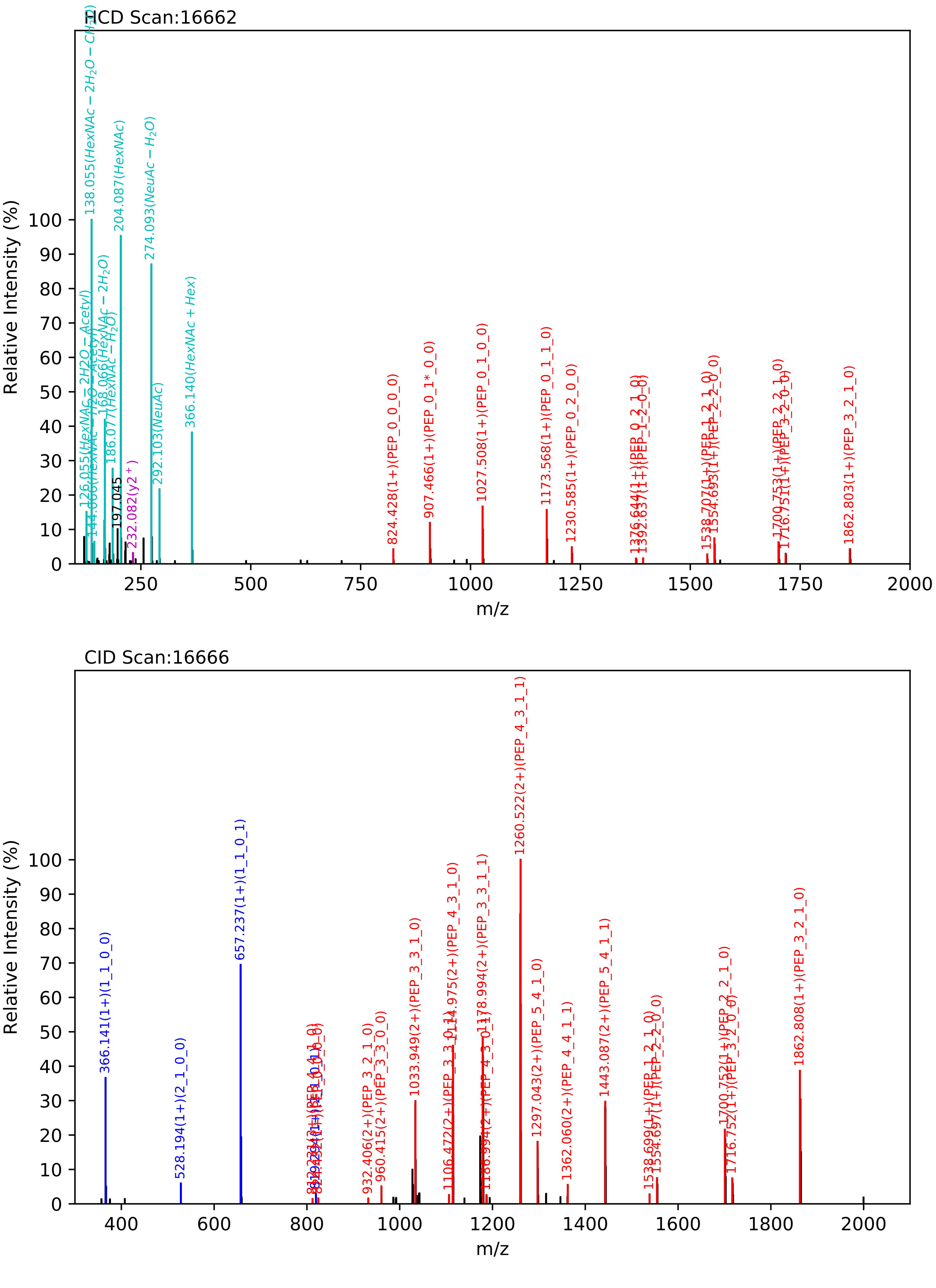

Unknown set no. 228, Experiment: Human Plasma exp_1

GGSSGWSGGLAQNR(=PEP)_5_4_0_2, m/z:885.36(4+), RT:67.31, Y-score:88.23

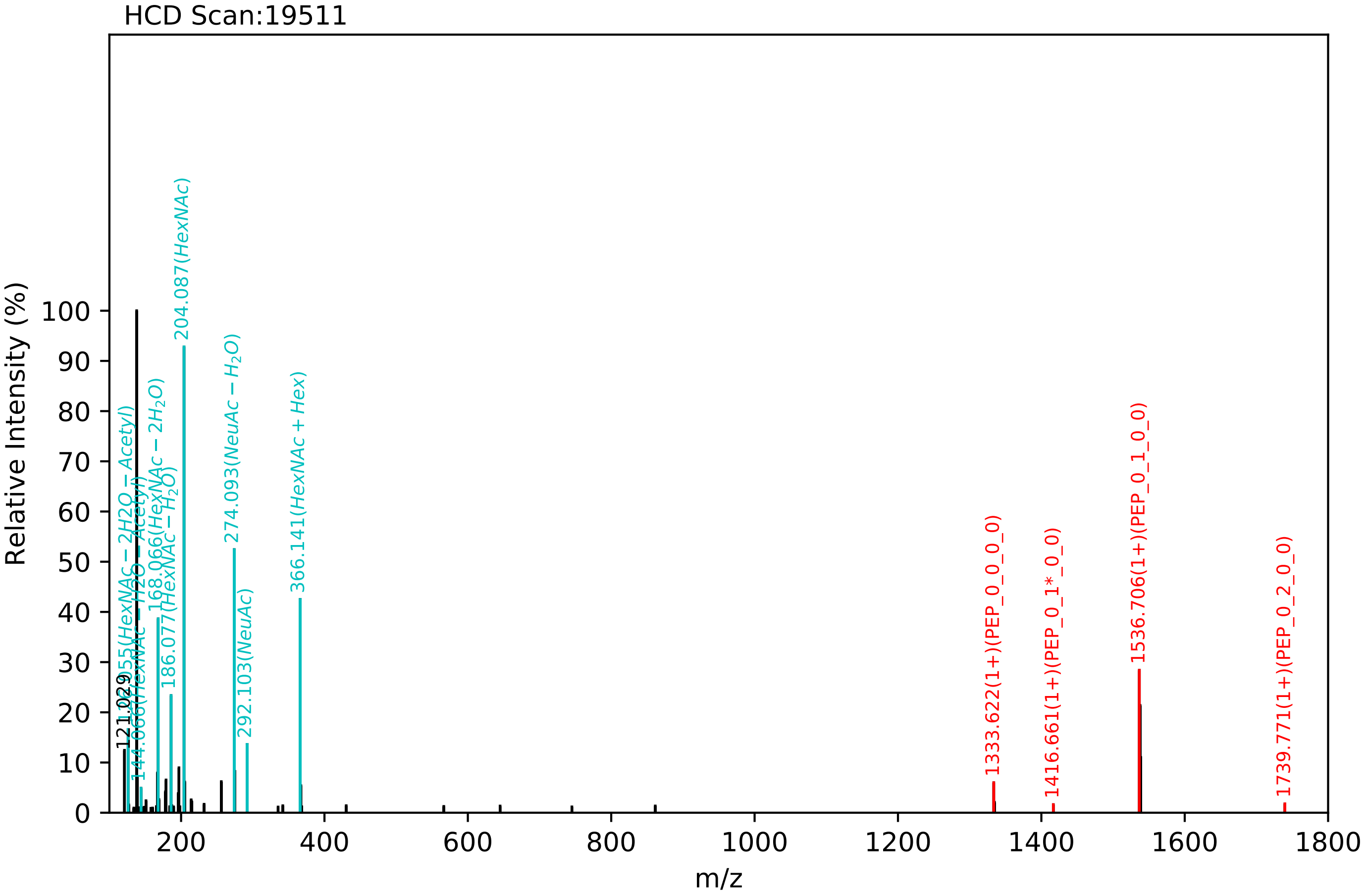

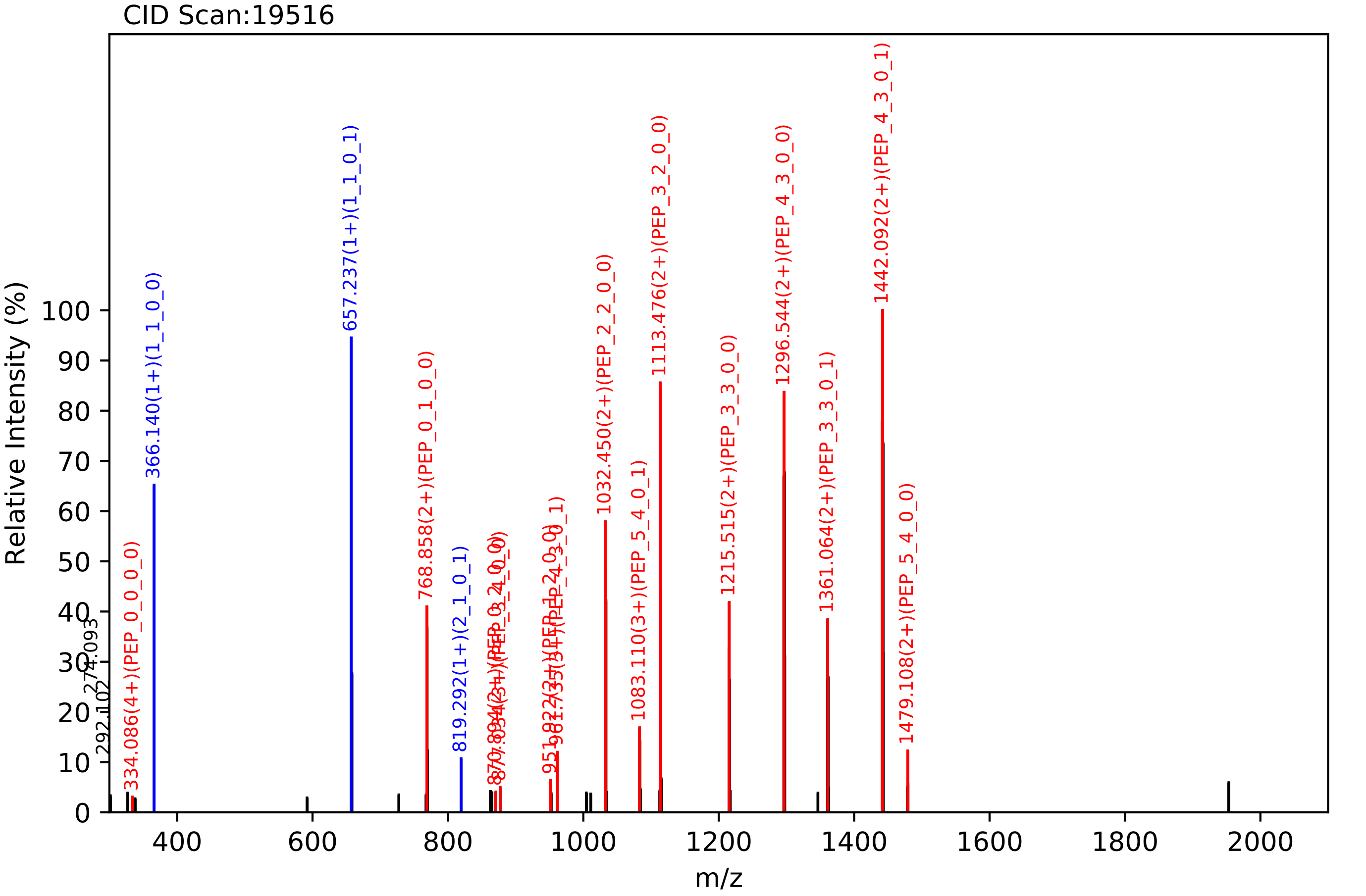

Unknown set no. 229, Experiment: Human Plasma exp_3

GGSSGWSGGLAQNR(=PEP)_5_4_0_2, m/z:885.36(4+), RT:67.29, Y-score:89.94

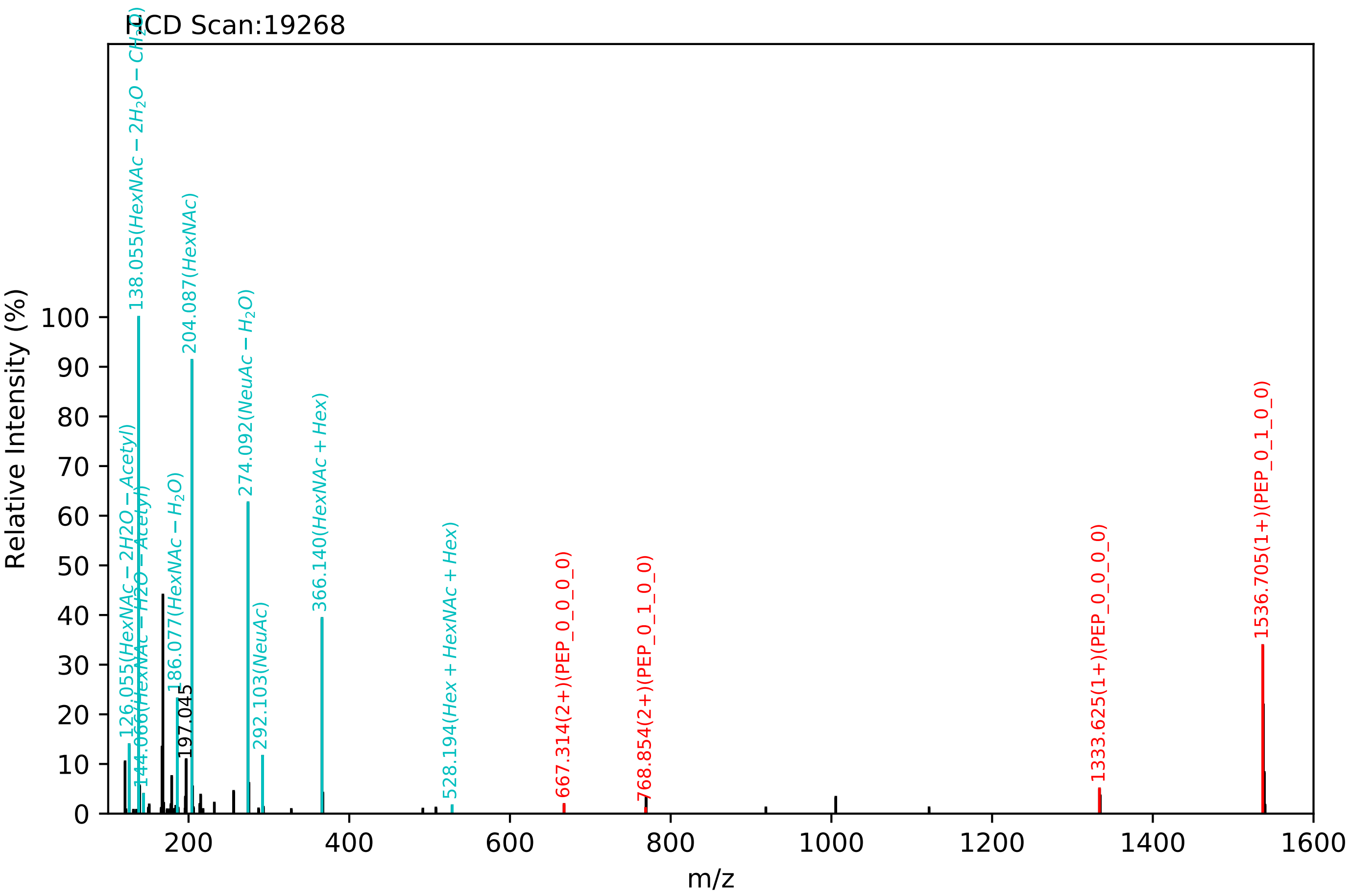

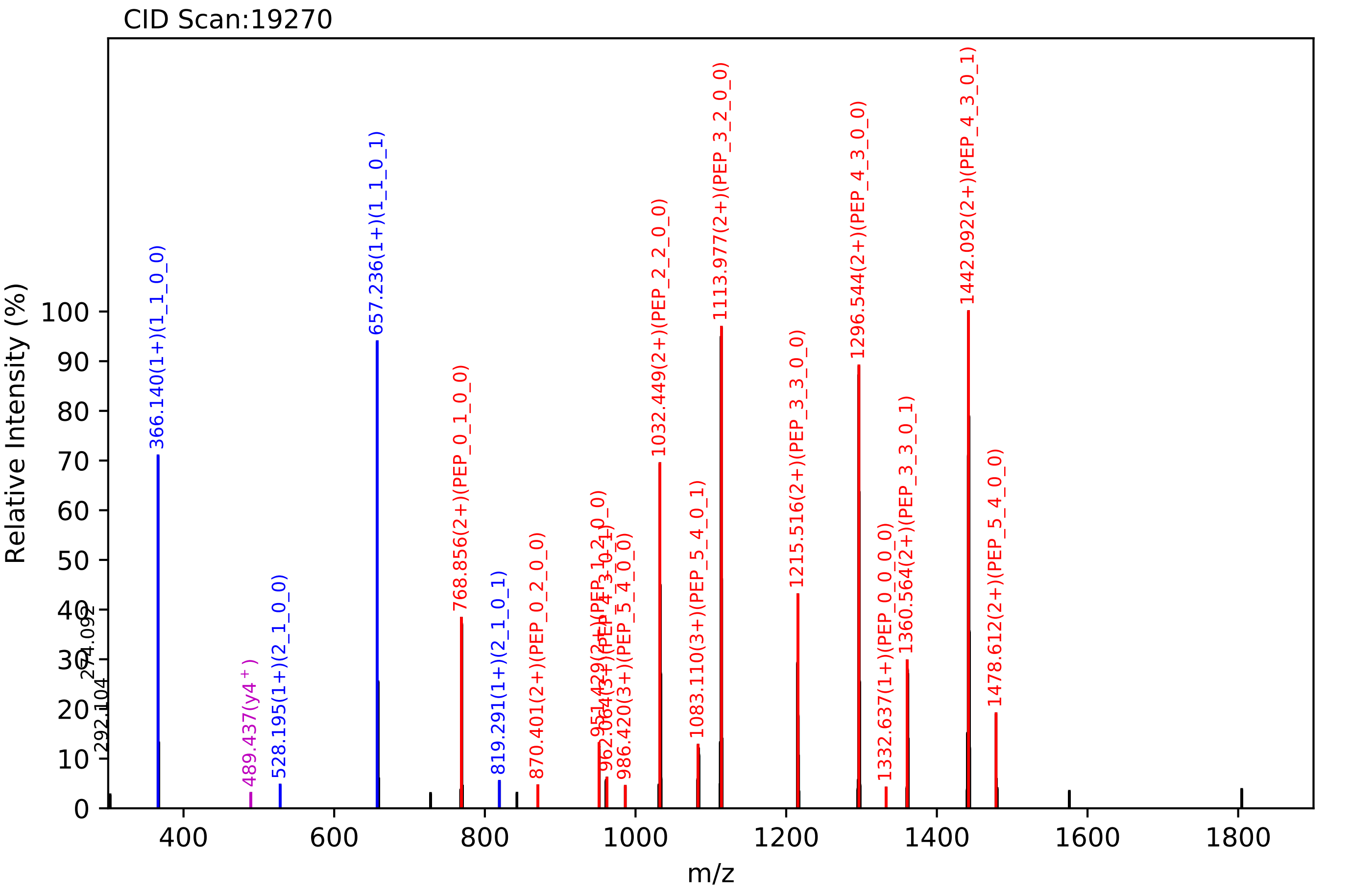

Unknown set no. 230, Experiment: Human Plasma exp_5

GGSSGWSGGLAQNR(=PEP)_5_4_0_2, m/z:885.36(4+), RT:67.16, Y-score:84.93

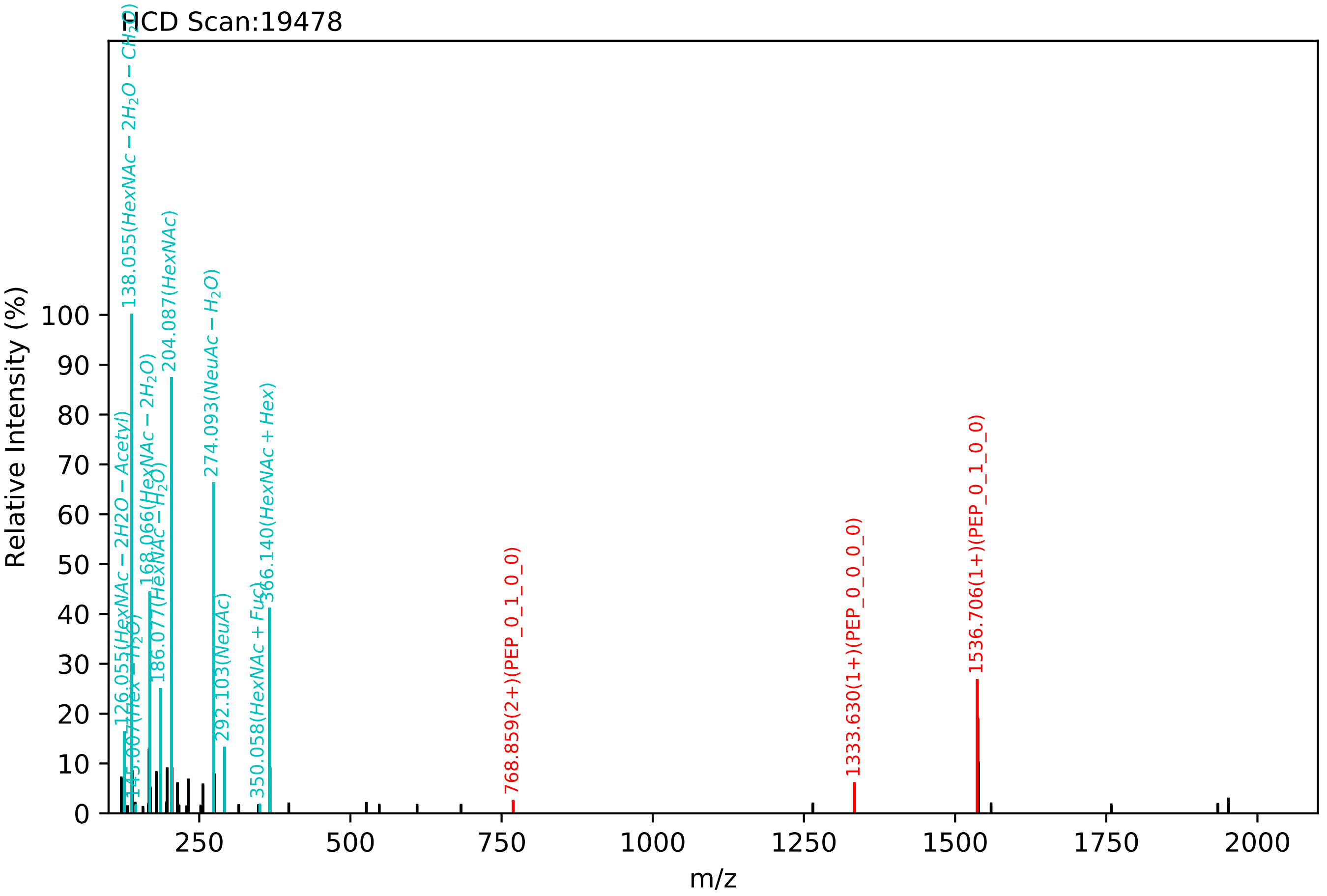

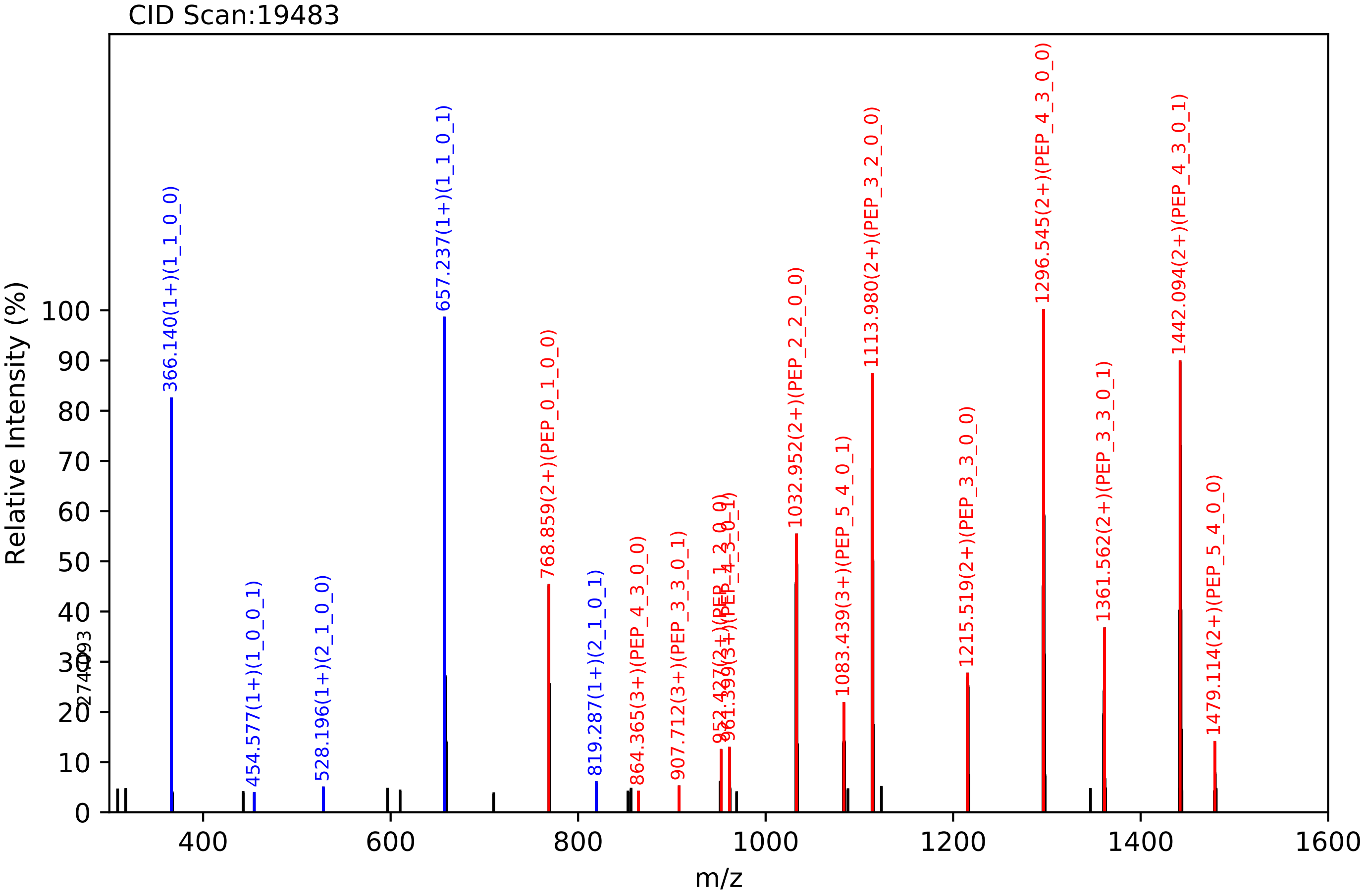

Unknown set no. 231, Experiment: Human Plasma exp_1

NVTEK(=PEP)_4_3_0_1, m/z:1069.93(2+), RT:14.00, Y-score:94.66

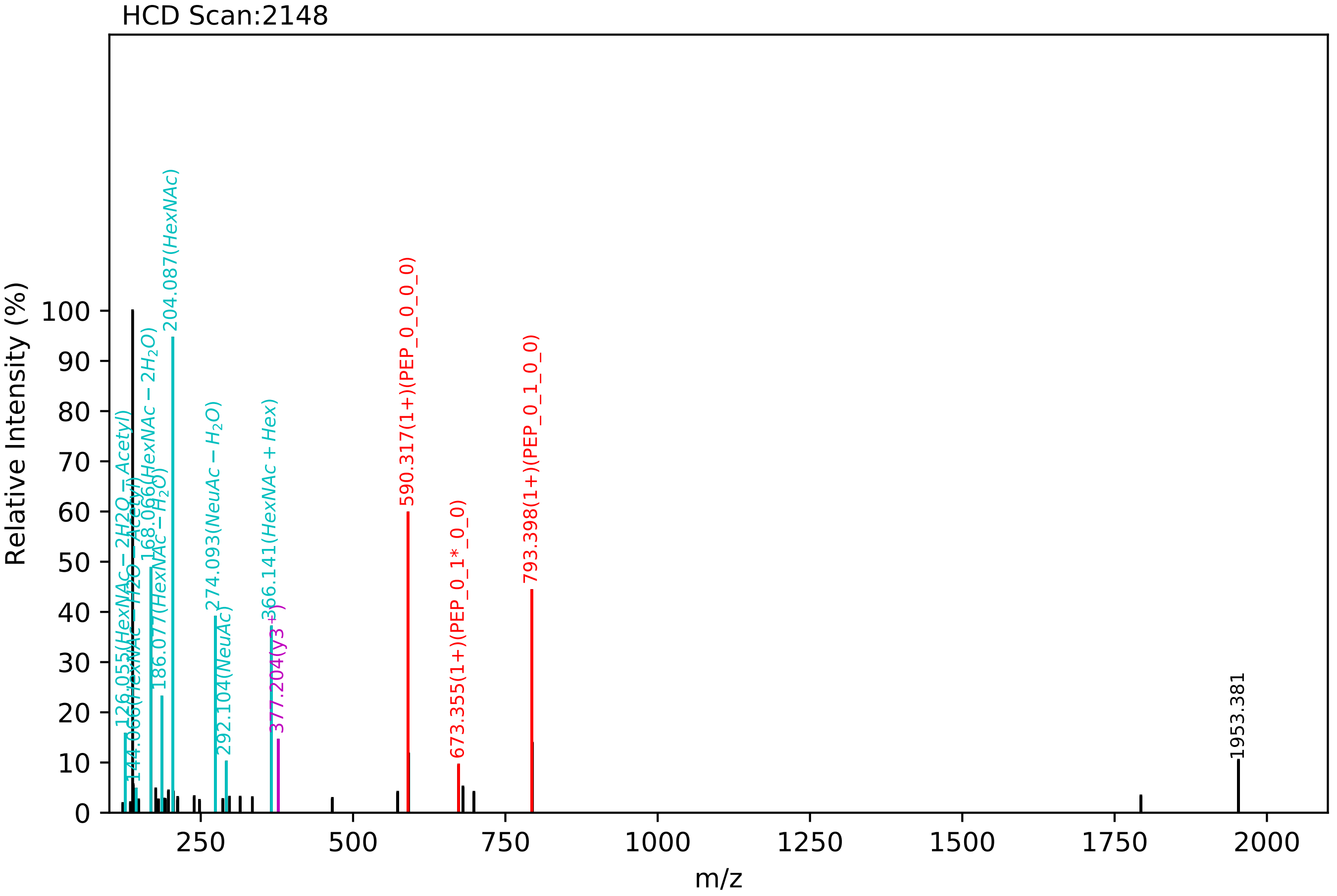

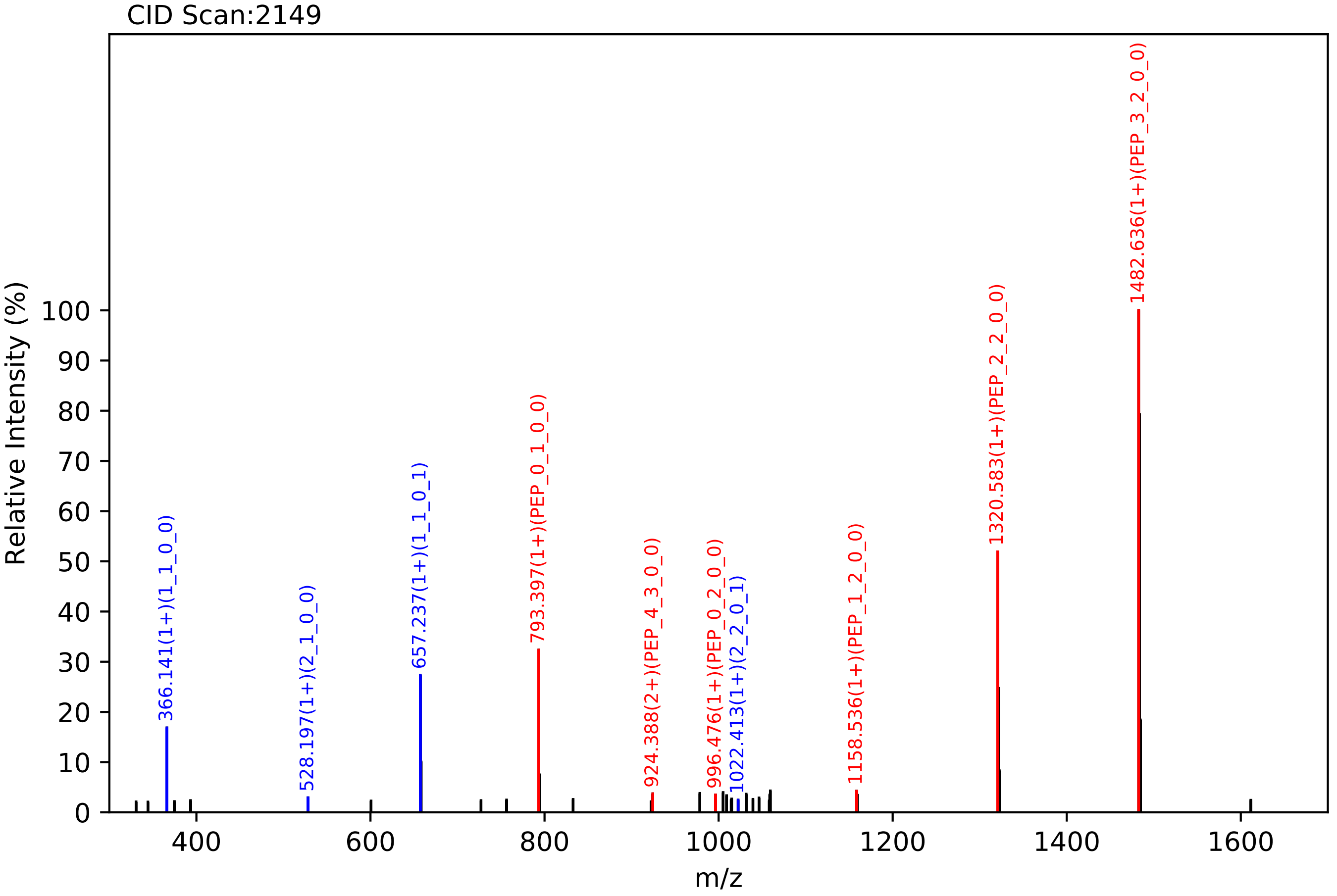

Unknown set no. 232, Experiment: Human Plasma exp_4

NVTEK(=PEP)_4_3_0_1, m/z:1069.93(2+), RT:14.50, Y-score:92.02

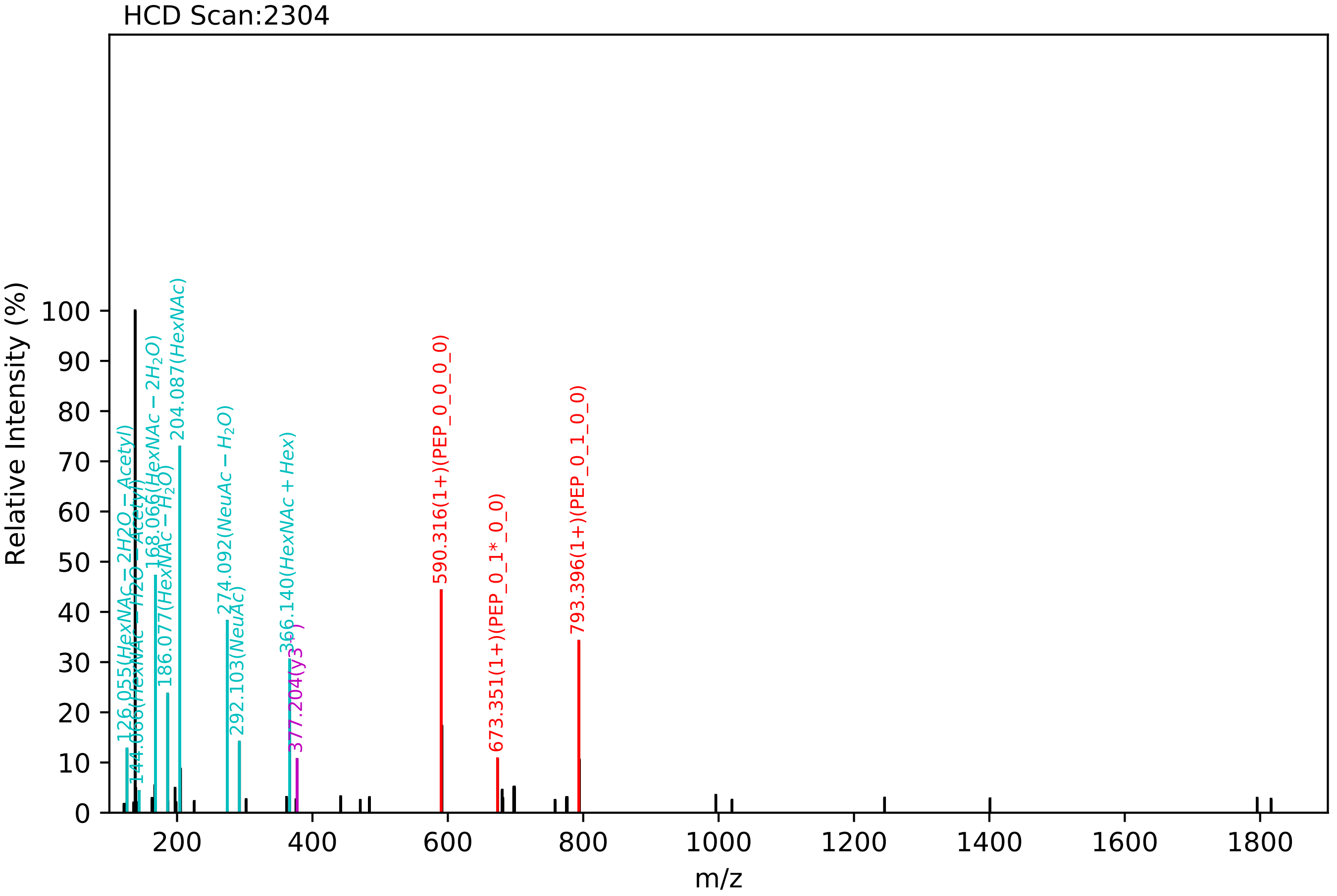

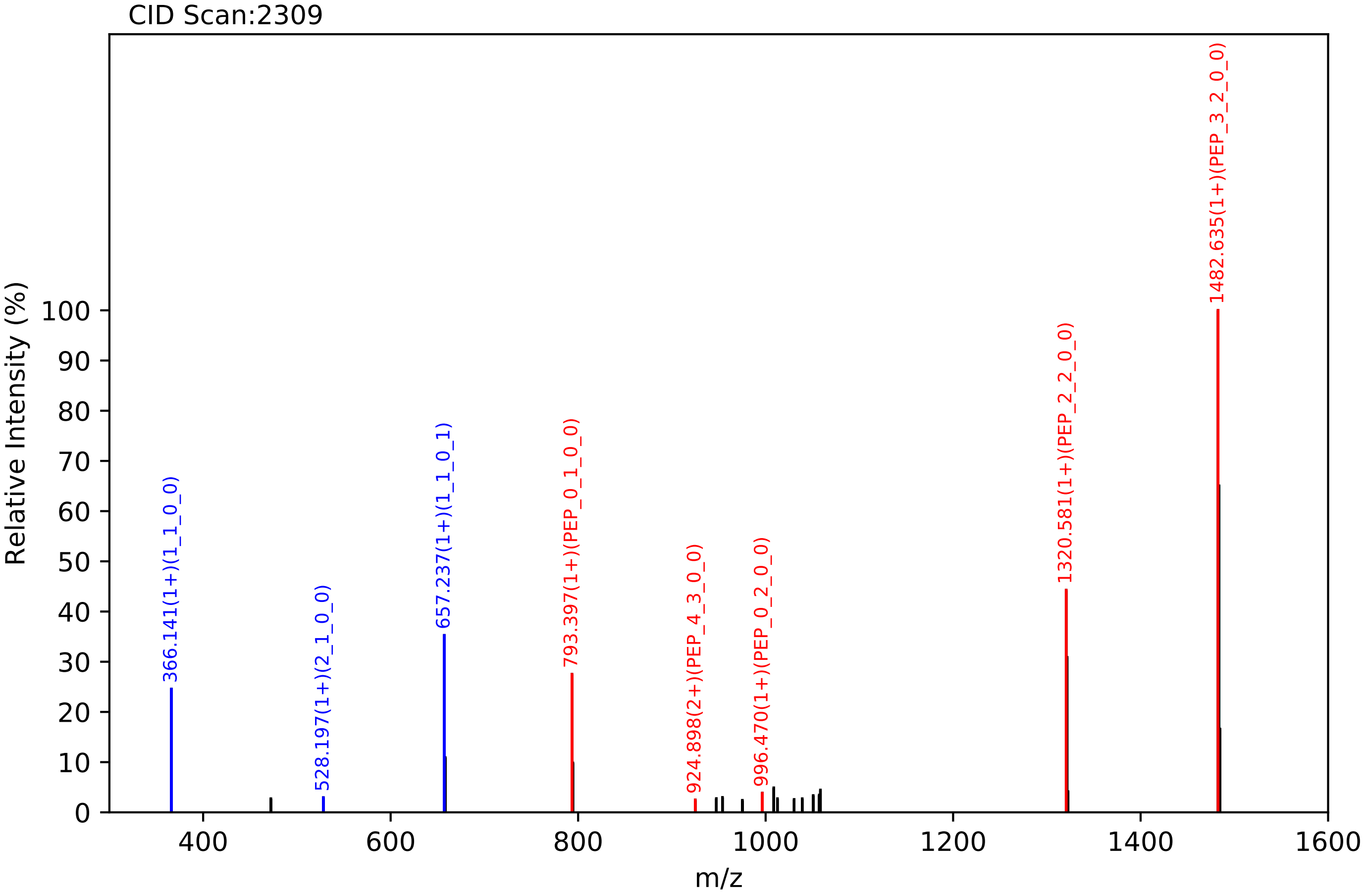

Unknown set no. 233, Experiment: Human Plasma exp_5

NVTEK(=PEP)_4_3_0_1, m/z:1069.93(2+), RT:14.35, Y-score:94.06

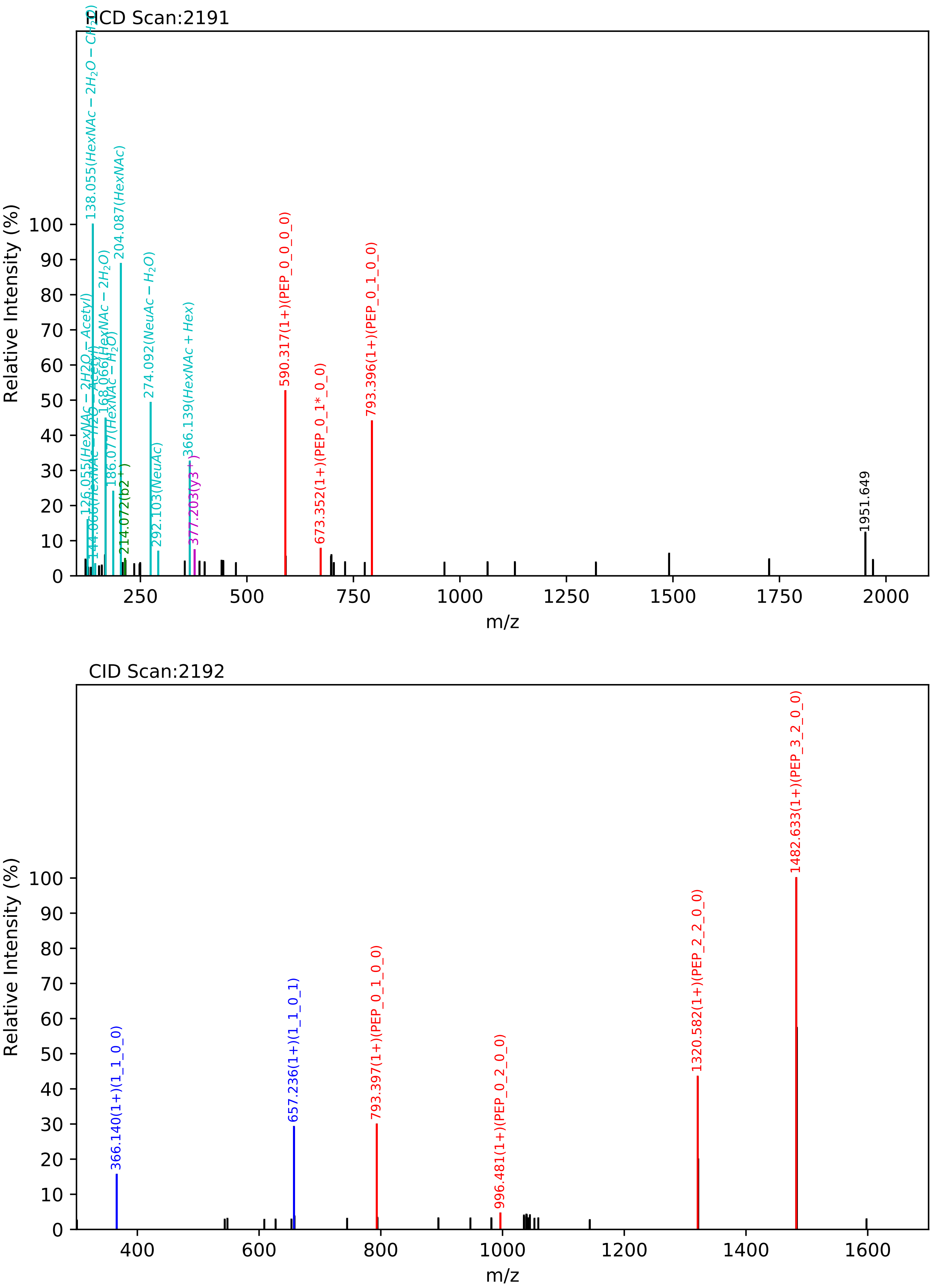

Unknown set no. 234, Experiment: Human Plasma exp_1

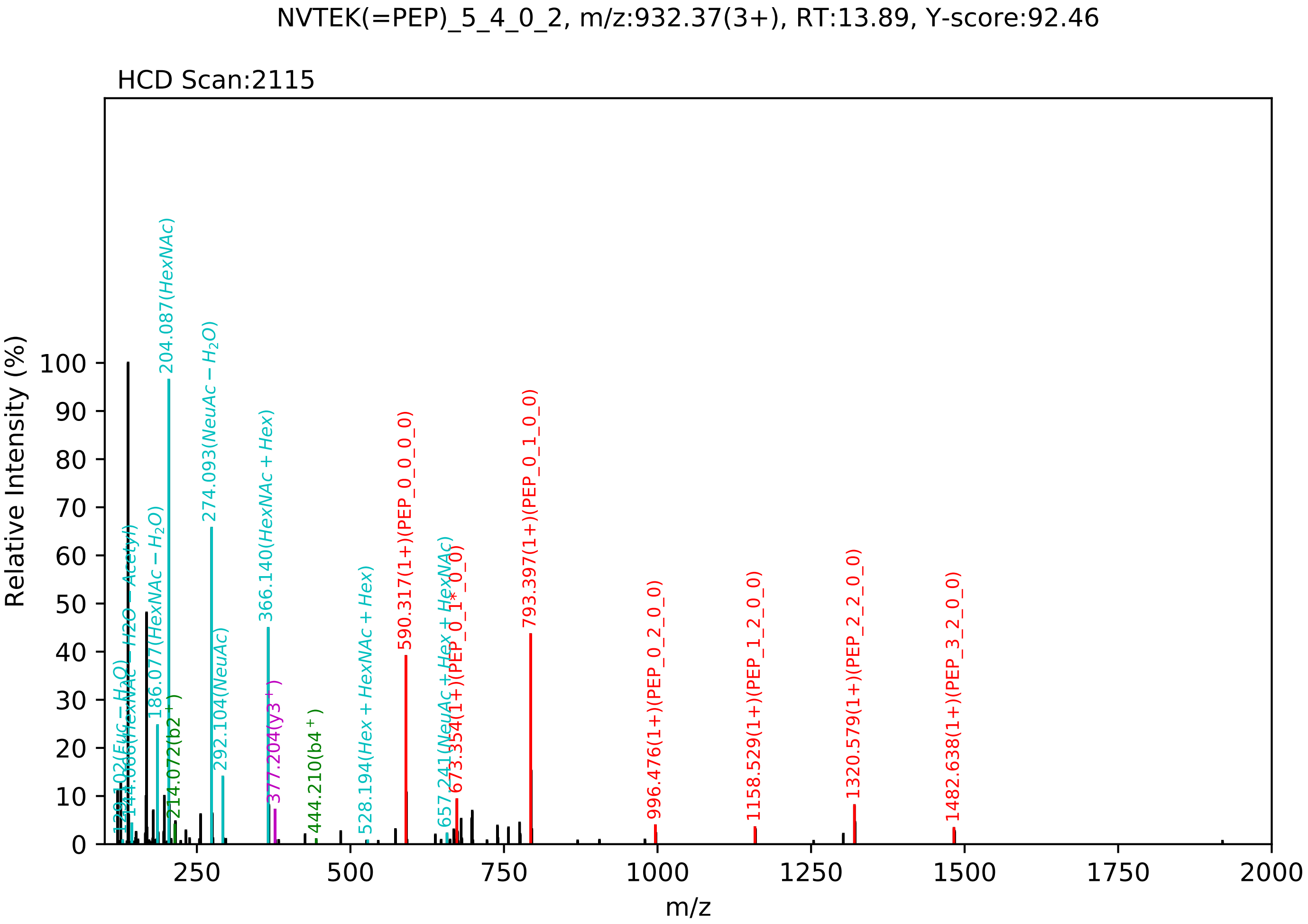

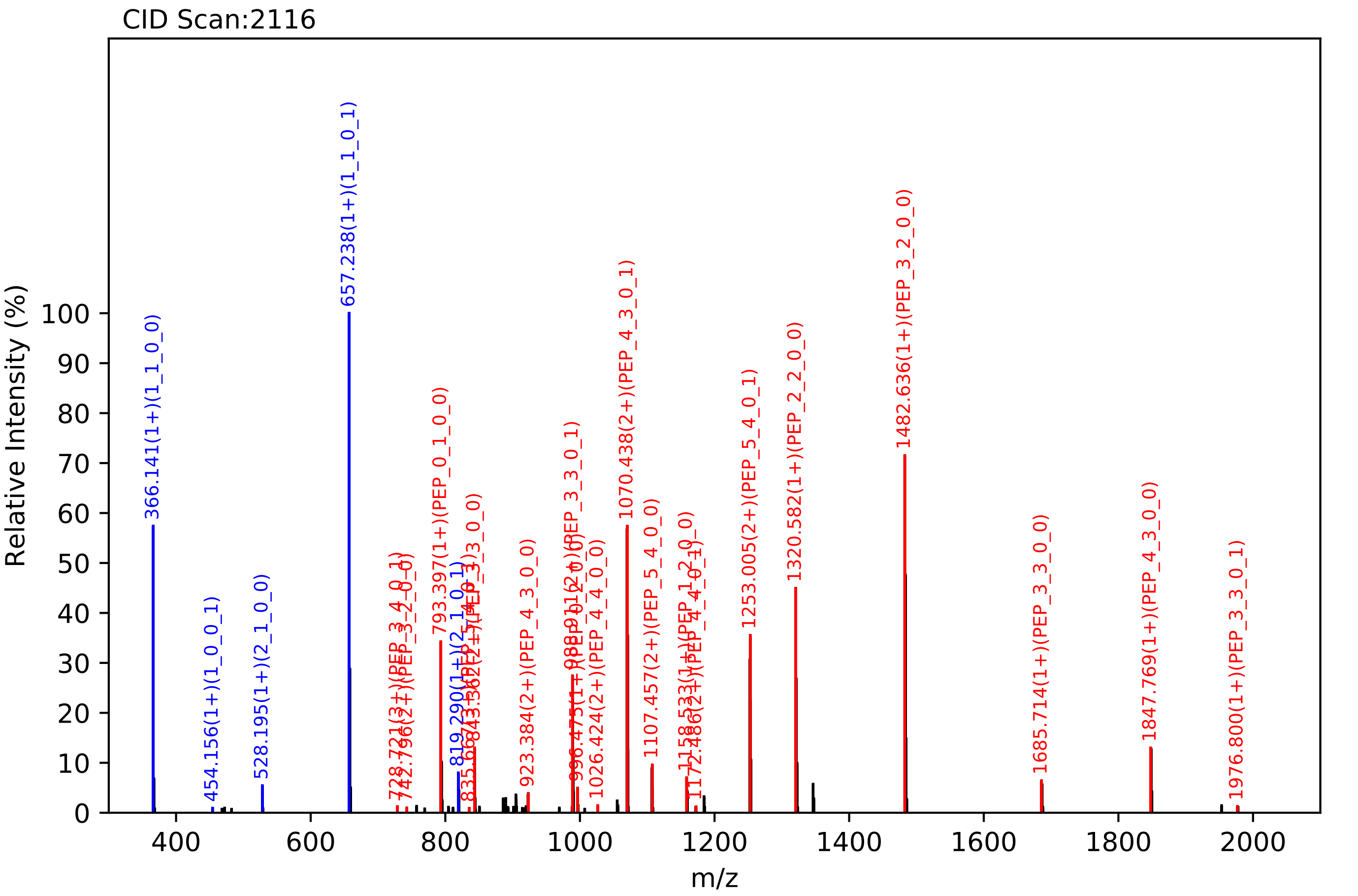

Unknown set no. 235, Experiment: Human Plasma exp_1

NVTEK(=PEP)_5_4_0_2, m/z:1398.05(2+), RT:13.92, Y-score:87.51

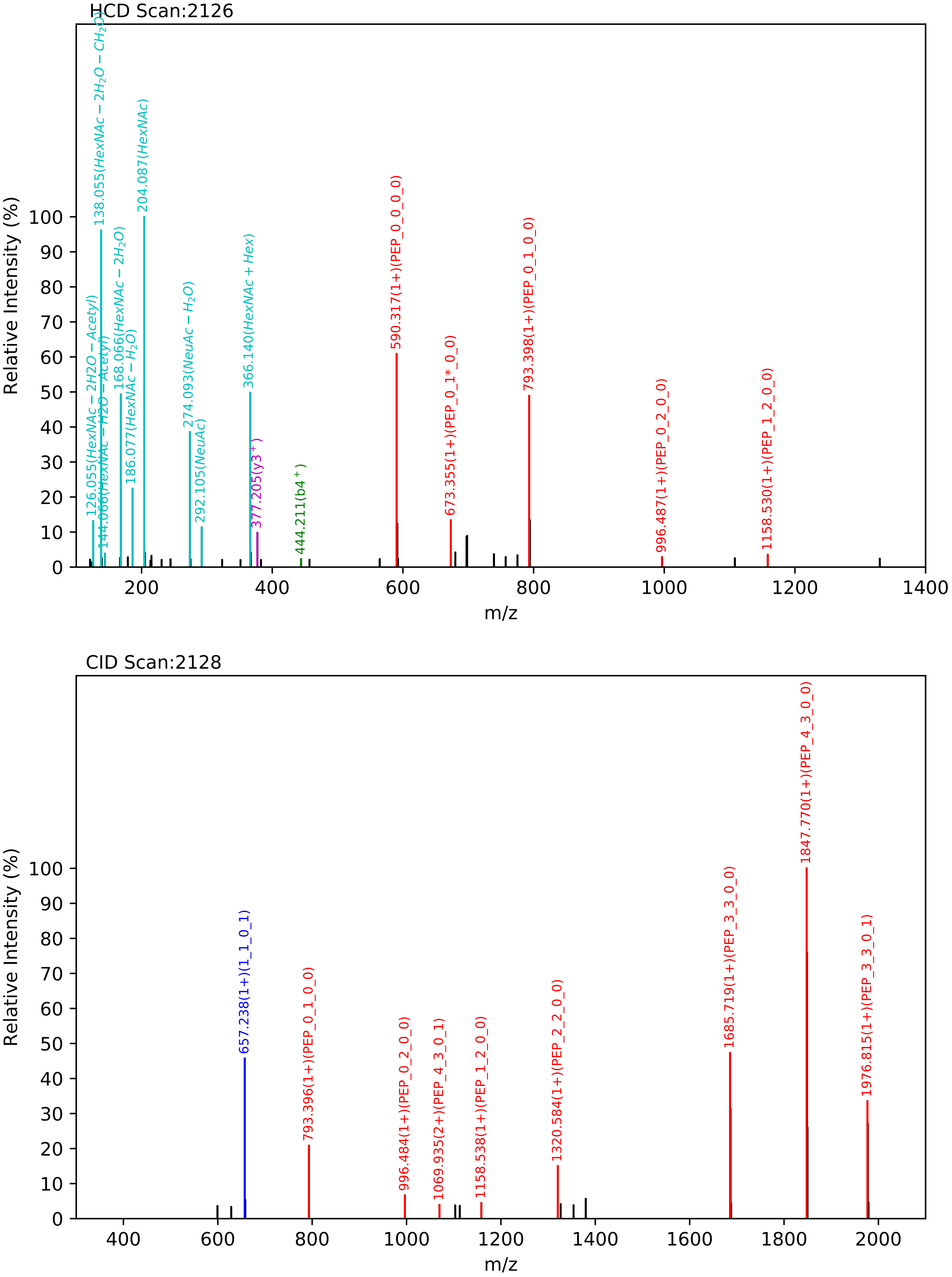

Unknown set no. 236, Experiment: Human Plasma exp_2

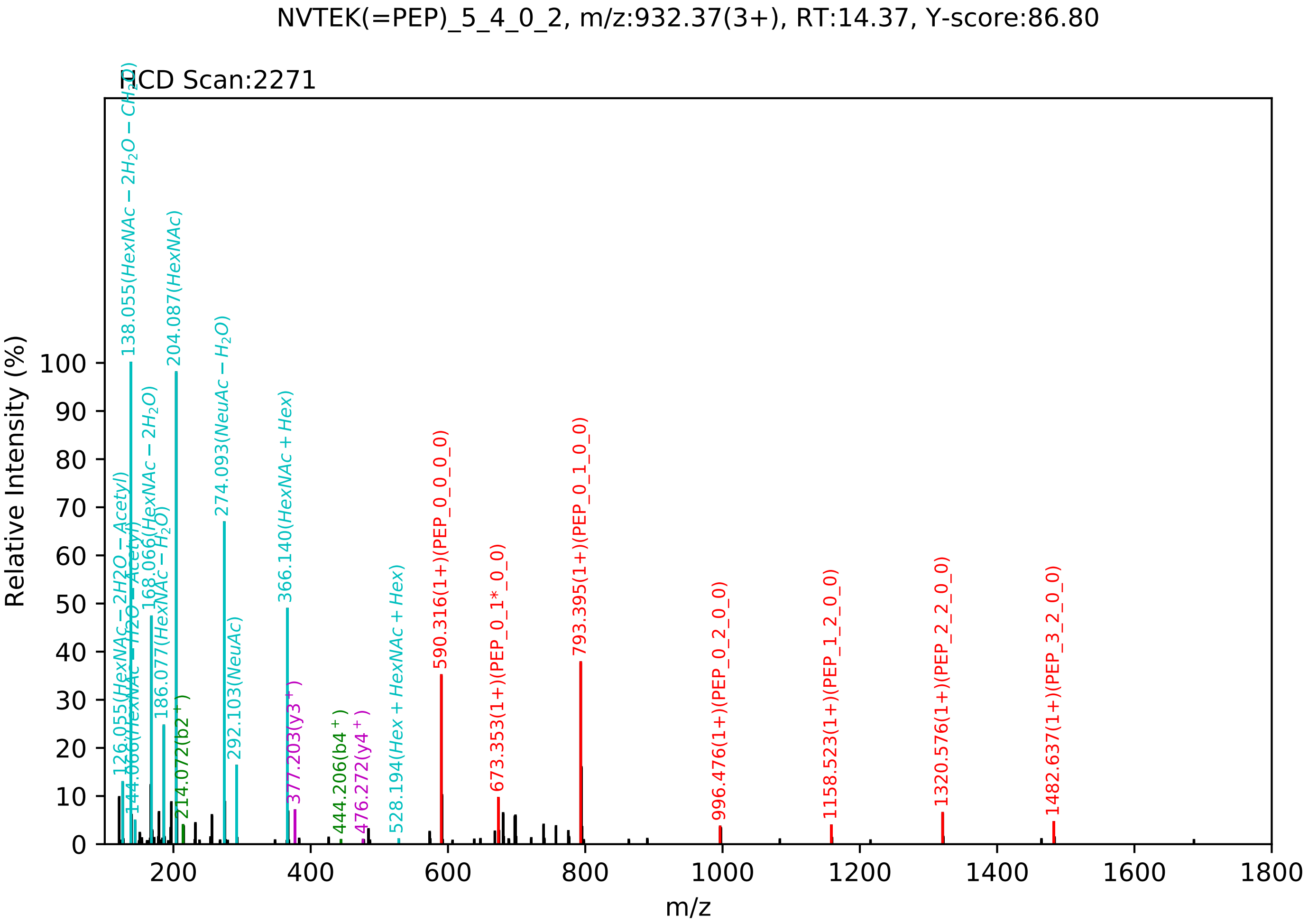

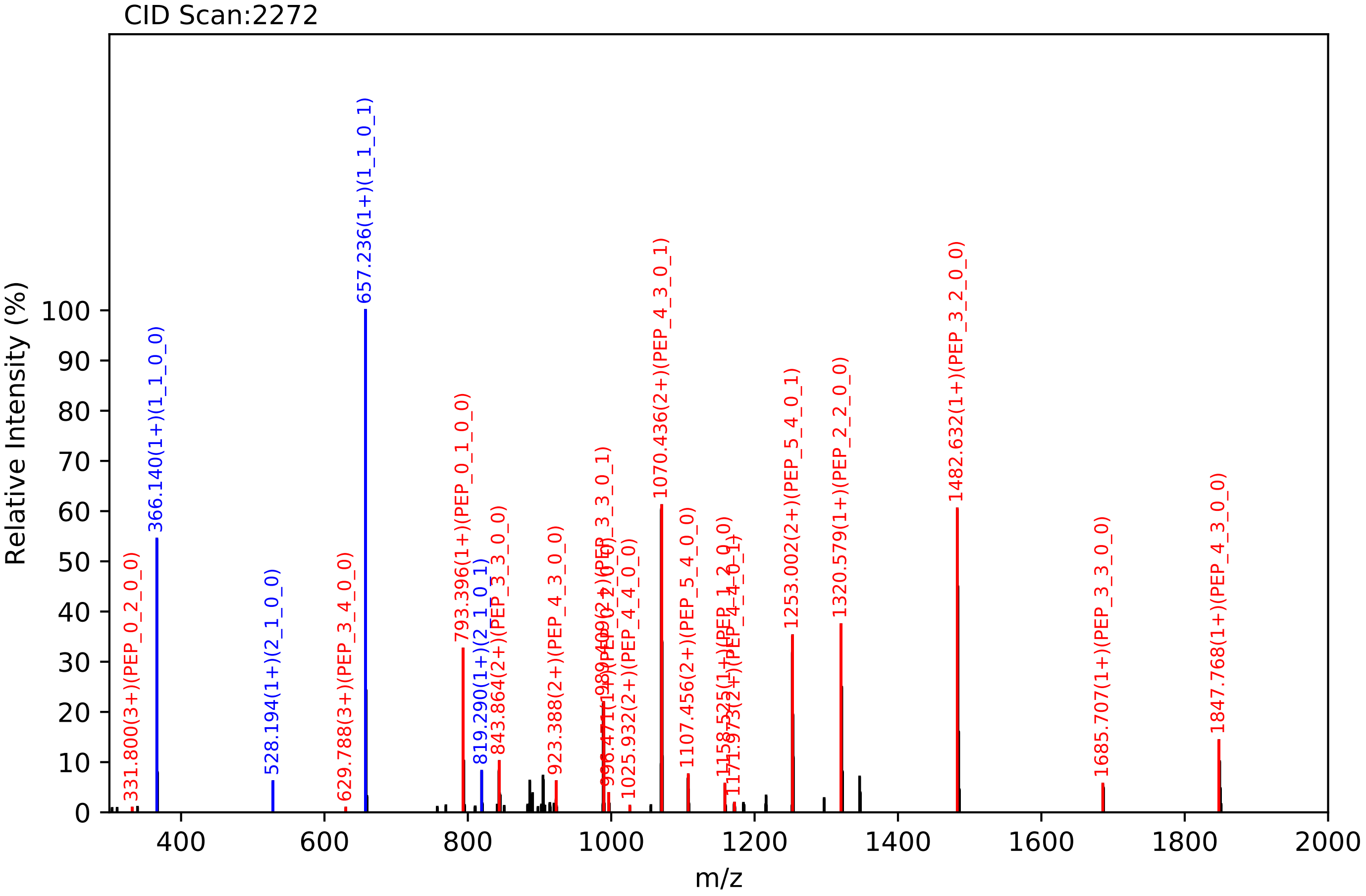

Unknown set no. 237, Experiment: Human Plasma exp_3

NVTEK(=PEP)_5_4_0_2, m/z:932.37(3+), RT:14.12, Y-score:90.86

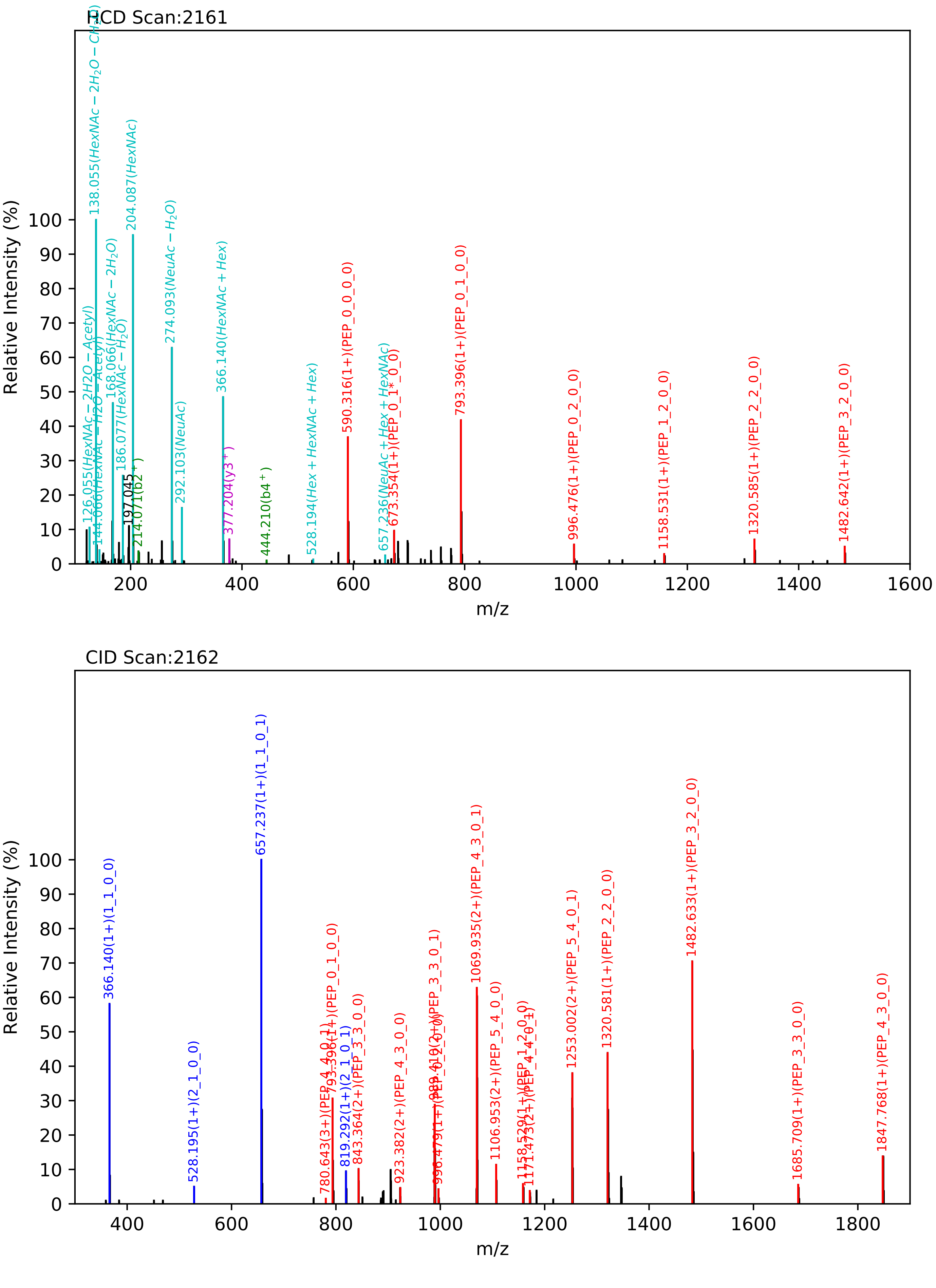

Unknown set no. 238, Experiment: Human Plasma exp_4

NVTEK(=PEP)_5_4_0_2, m/z:932.37(3+), RT:14.37, Y-score:84.23

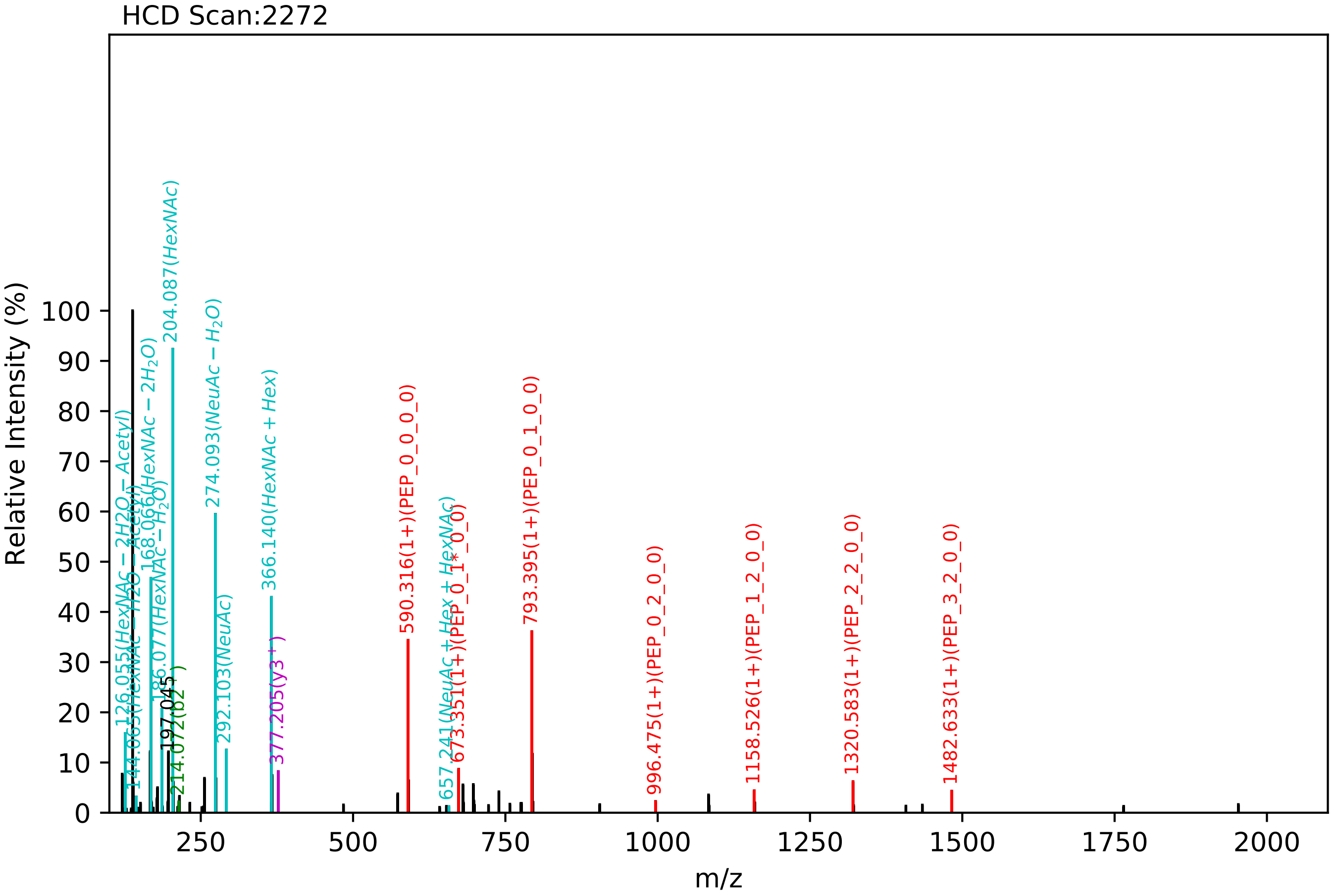

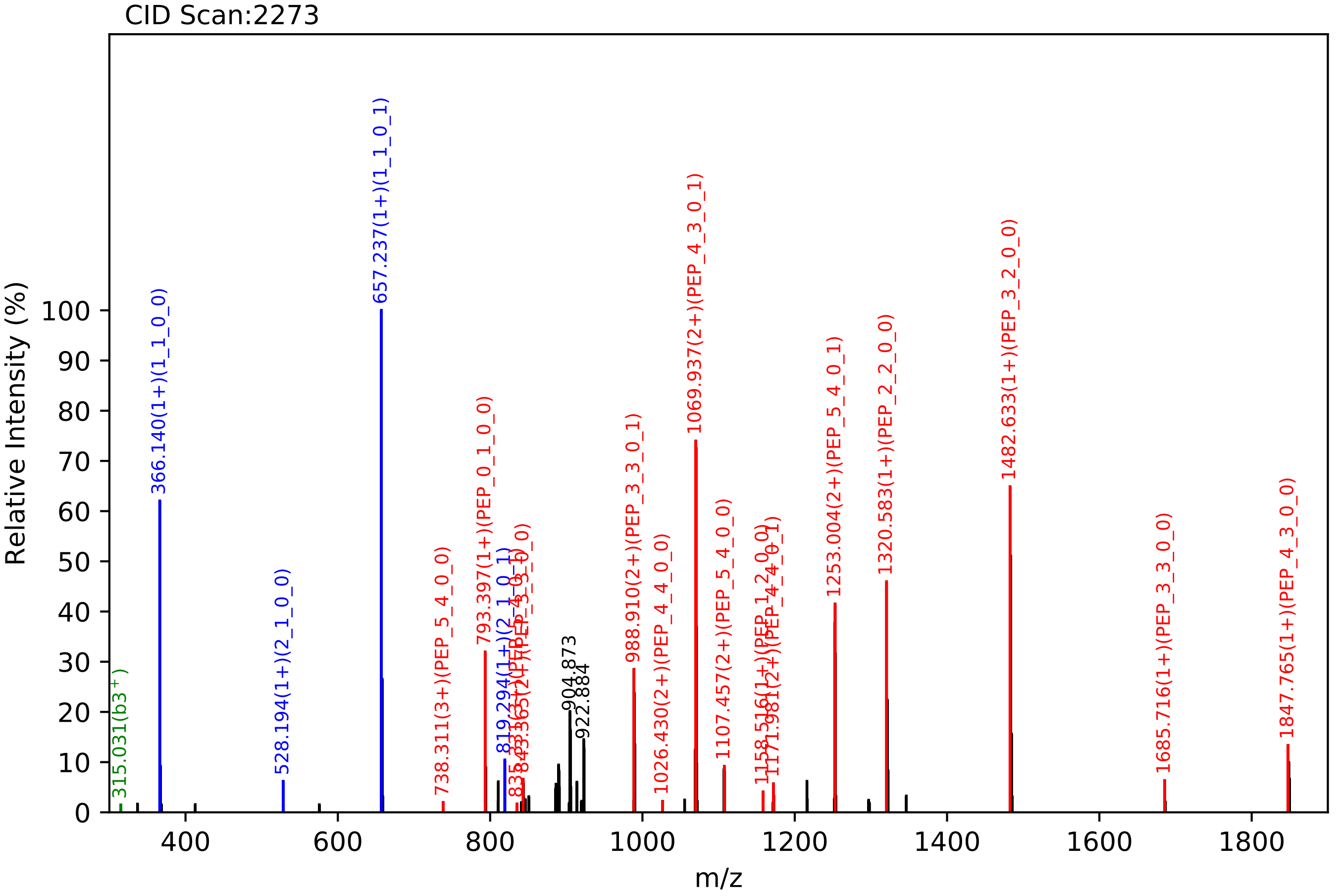

Unknown set no. 239, Experiment: Human Plasma exp_5

NVTEK(=PEP)_5_4_0_2, m/z:932.37(3+), RT:14.70, Y-score:92.73

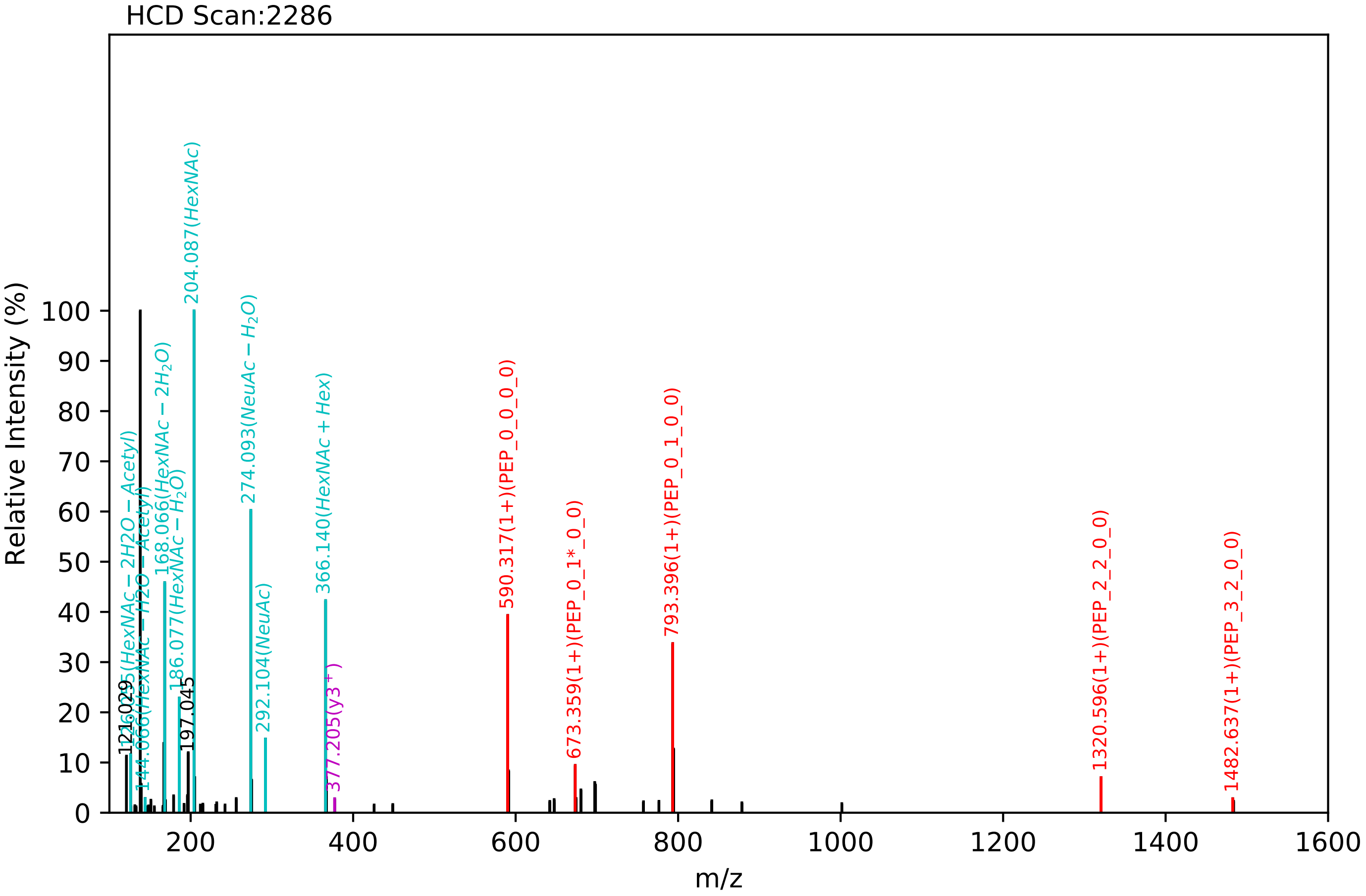

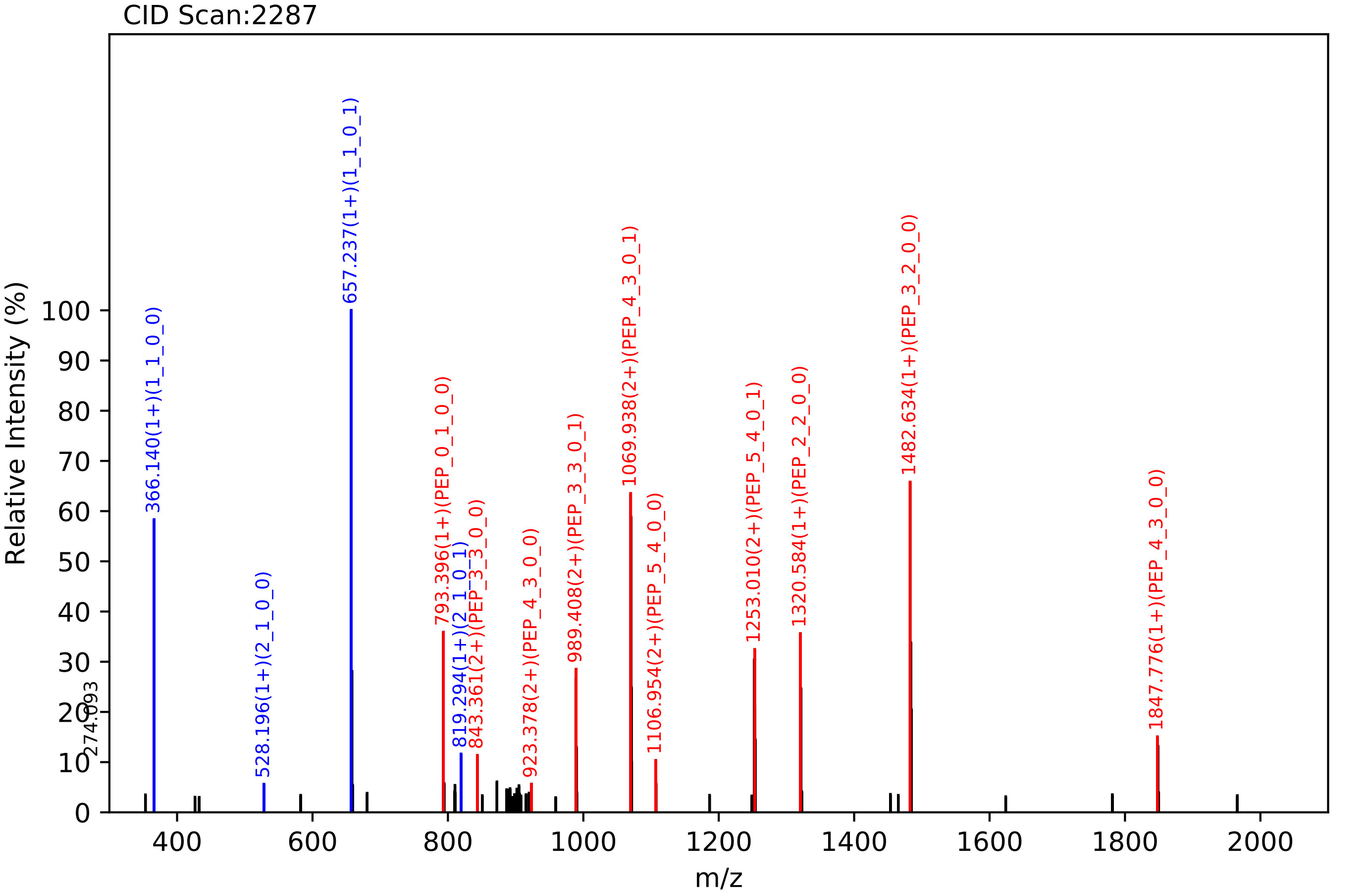

Unknown set no. 240, Experiment: Human Plasma exp_1

FVQGNSTEVACHPGYGLPK(=PEP)_5_4_0_2, m/z:1067.20(4+), RT:73.01, Y-score:78.32

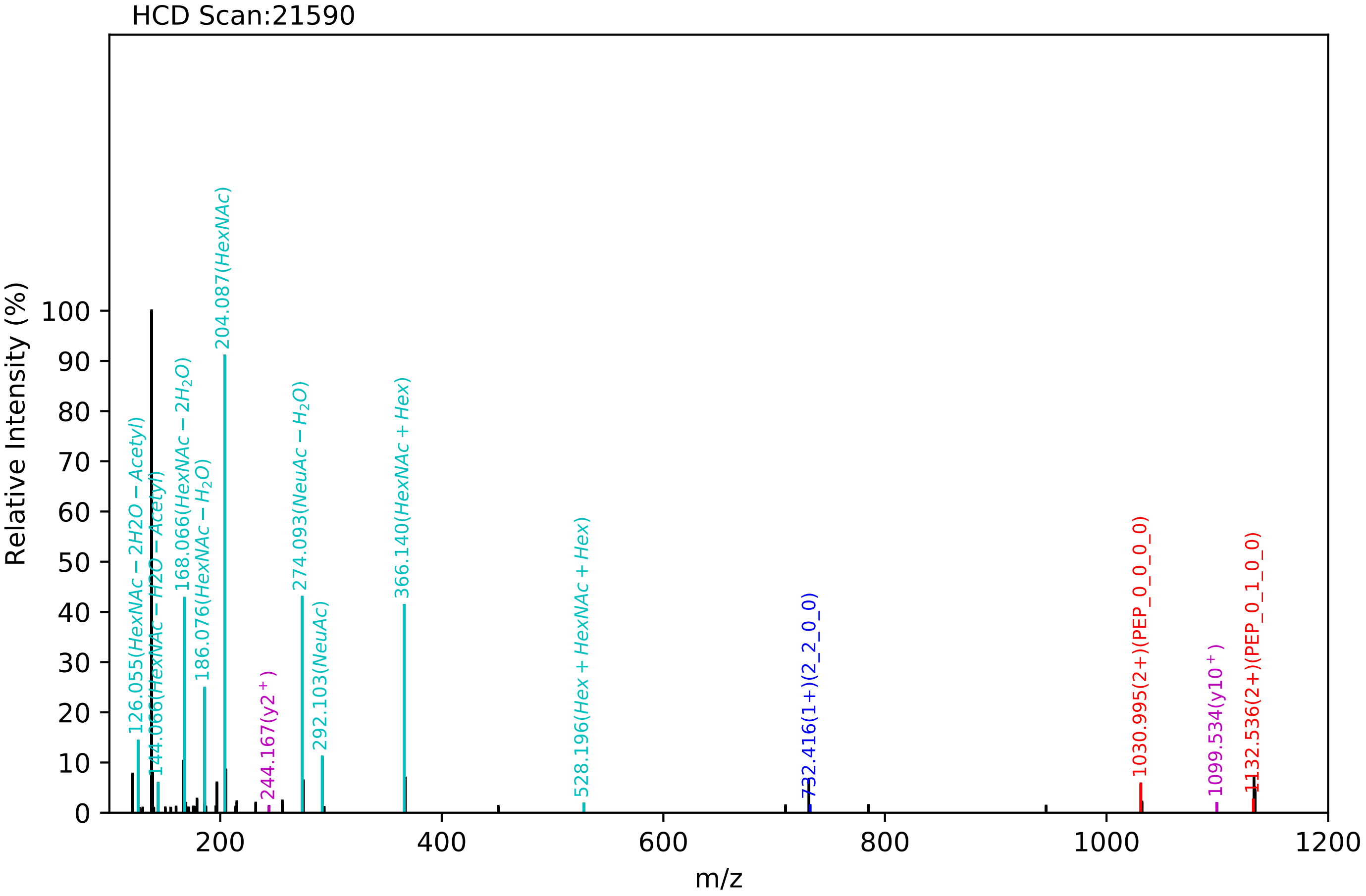

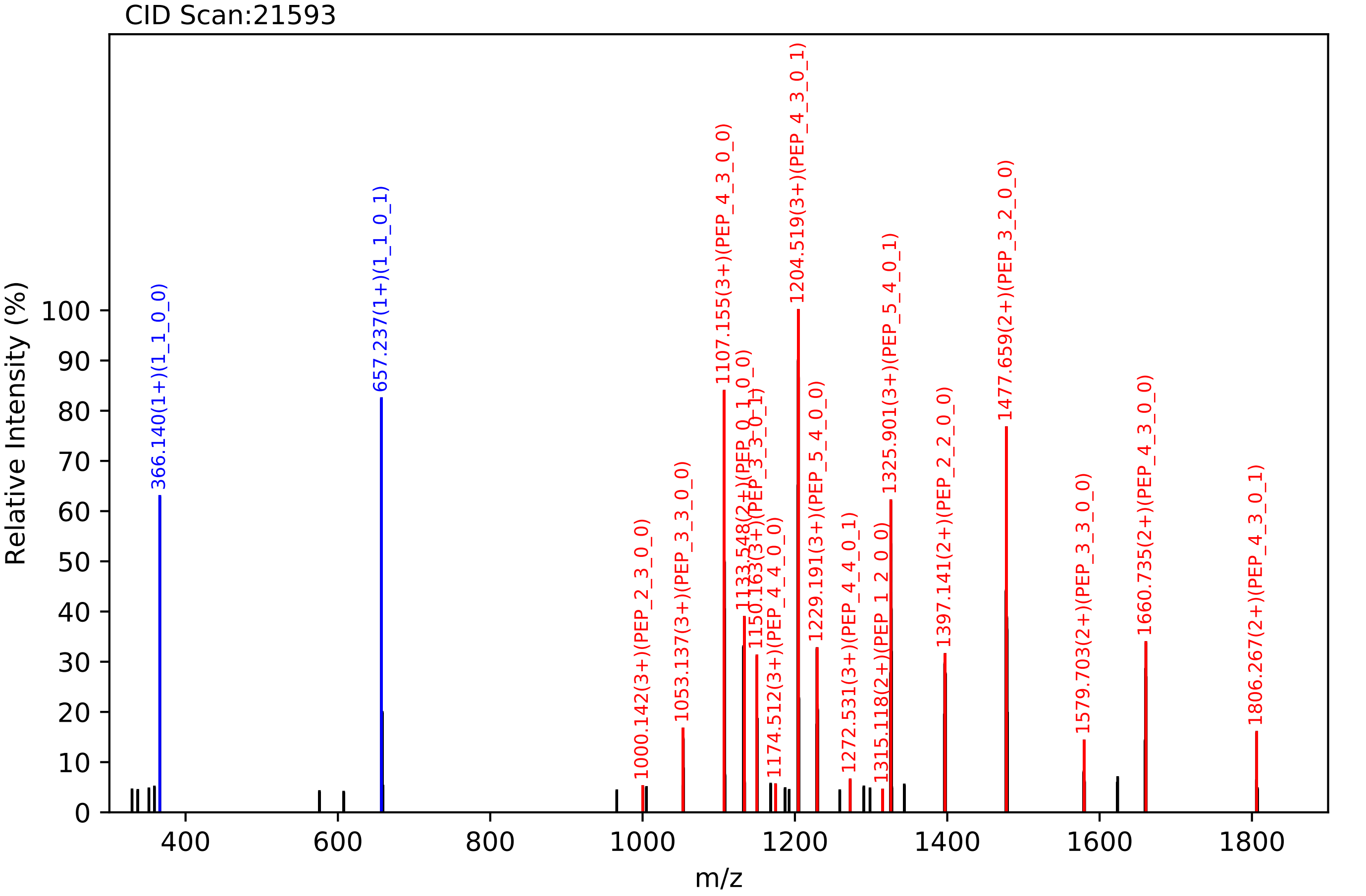

Unknown set no. 241, Experiment: Human Plasma exp_4

FVQGNSTEVACHPGYGLPK(=PEP)_5_4_0_2, m/z:1067.20(4+), RT:73.13, Y-score:80.88

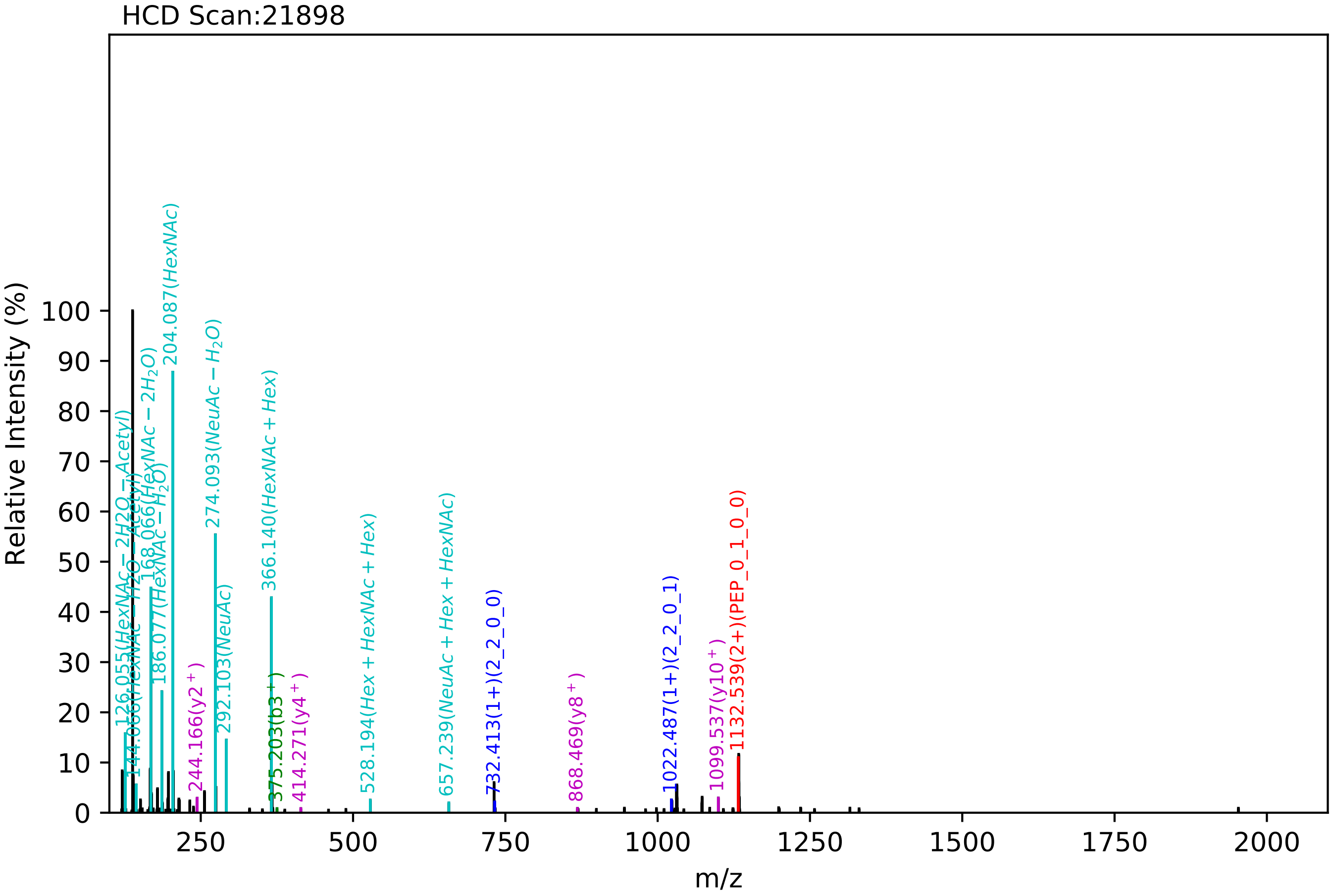

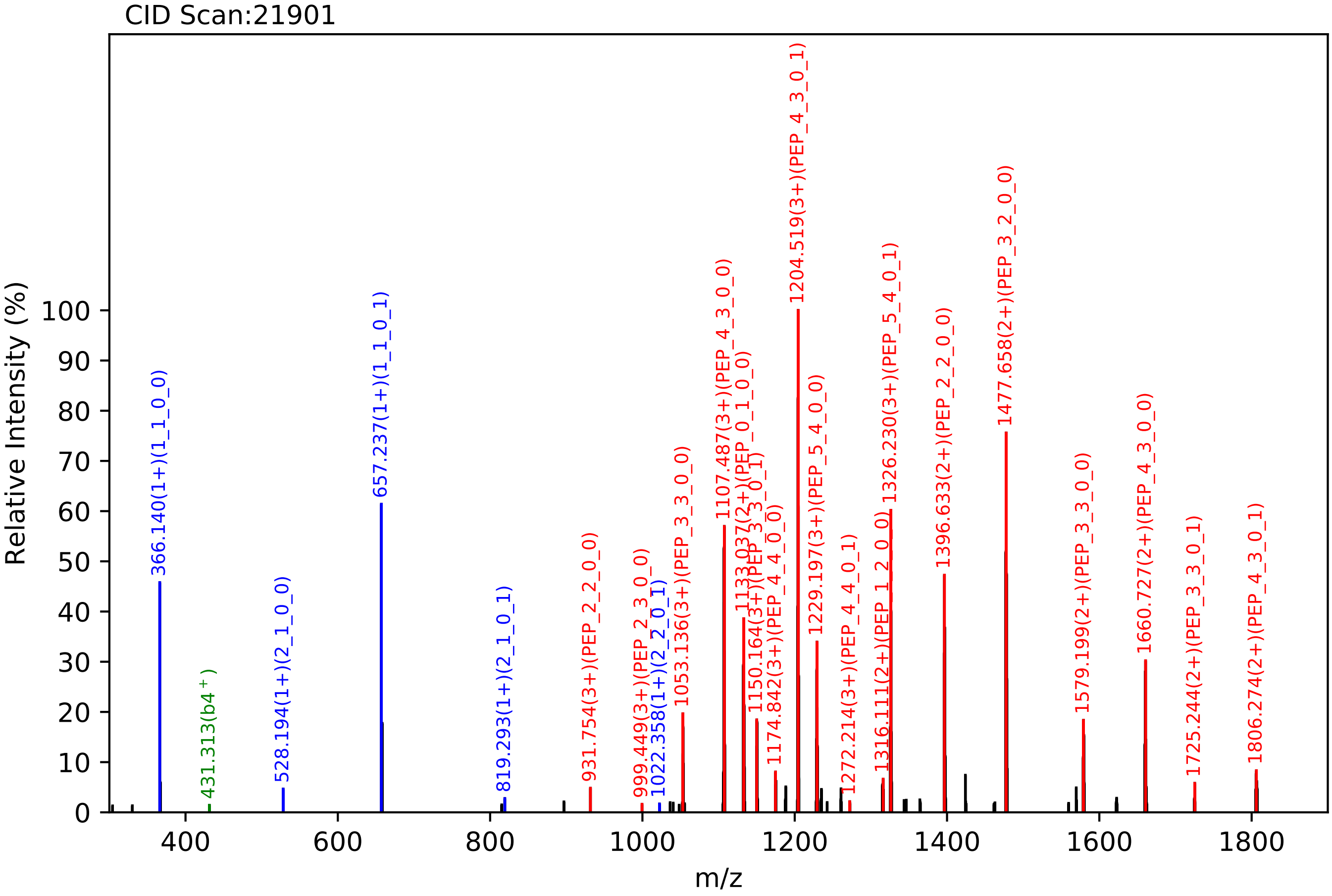

Unknown set no. 242, Experiment: Human Plasma exp_3

FLNNGTCTAEGK(=PEP)_5_4_0_2, m/z:879.85(4+), RT:55.75, Y-score:88.03

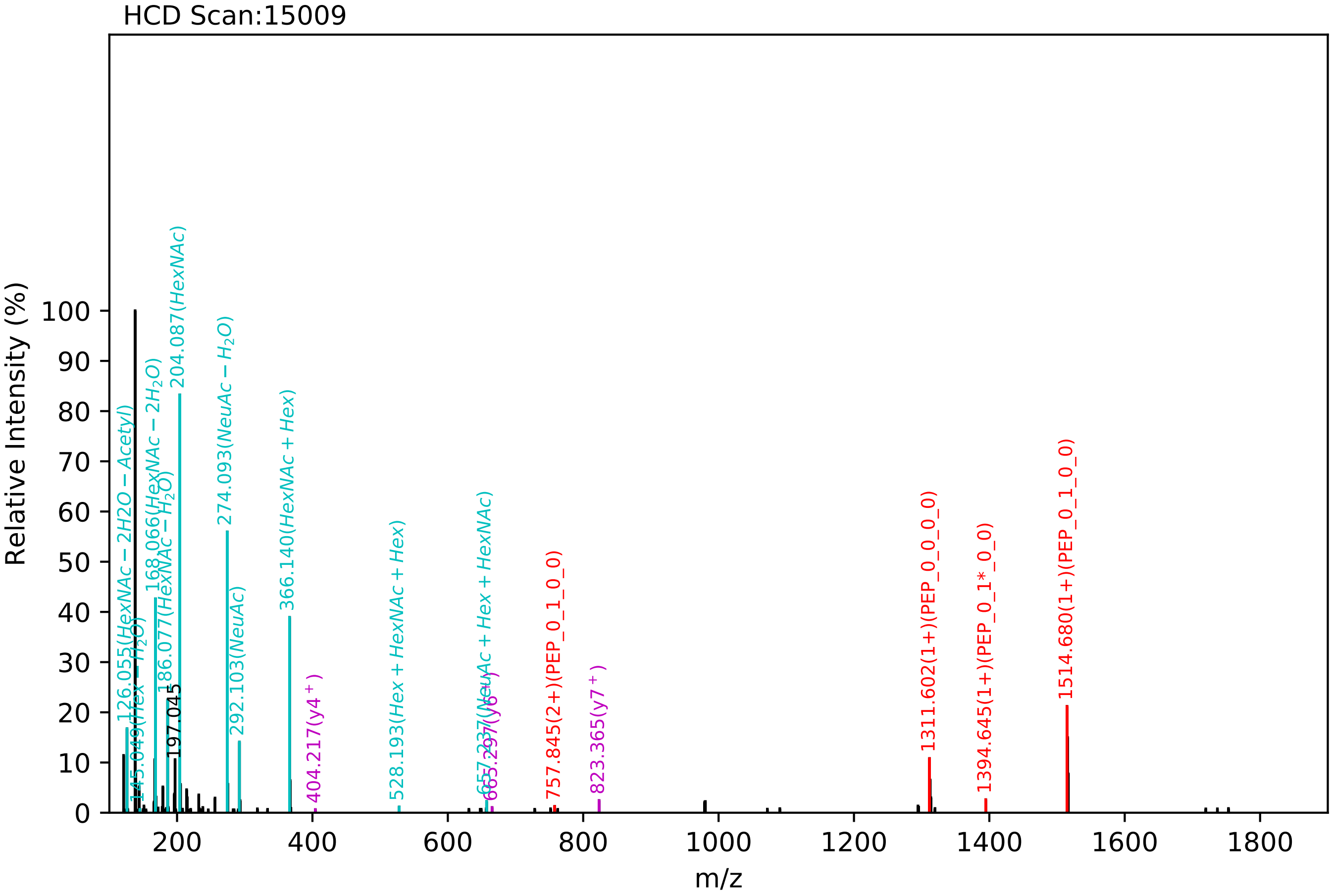

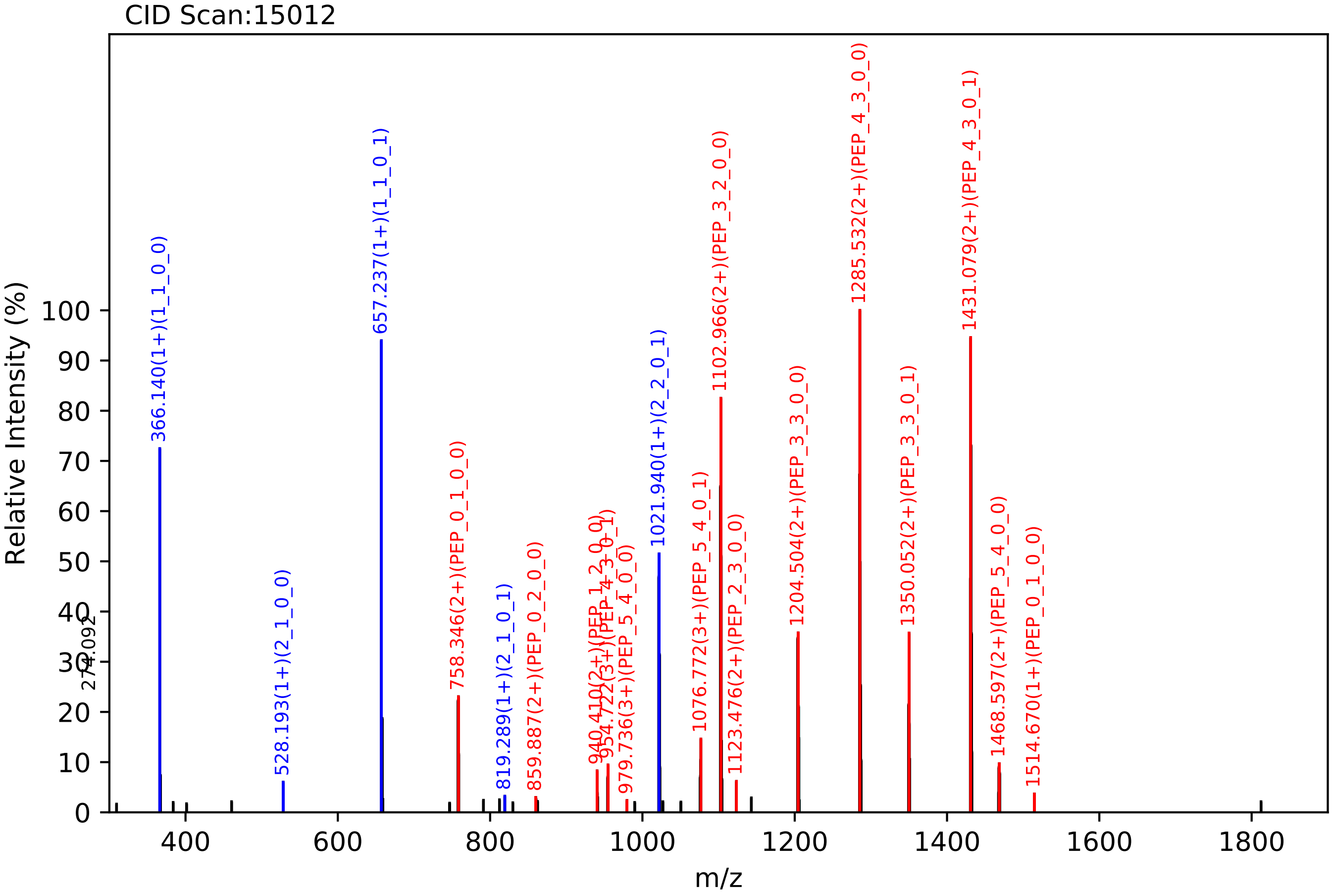

Unknown set no. 243, Experiment: Human Plasma exp_5

FLNNGTCTAEGK(=PEP)_5_4_0_2, m/z:879.85(4+), RT:55.95, Y-score:92.48

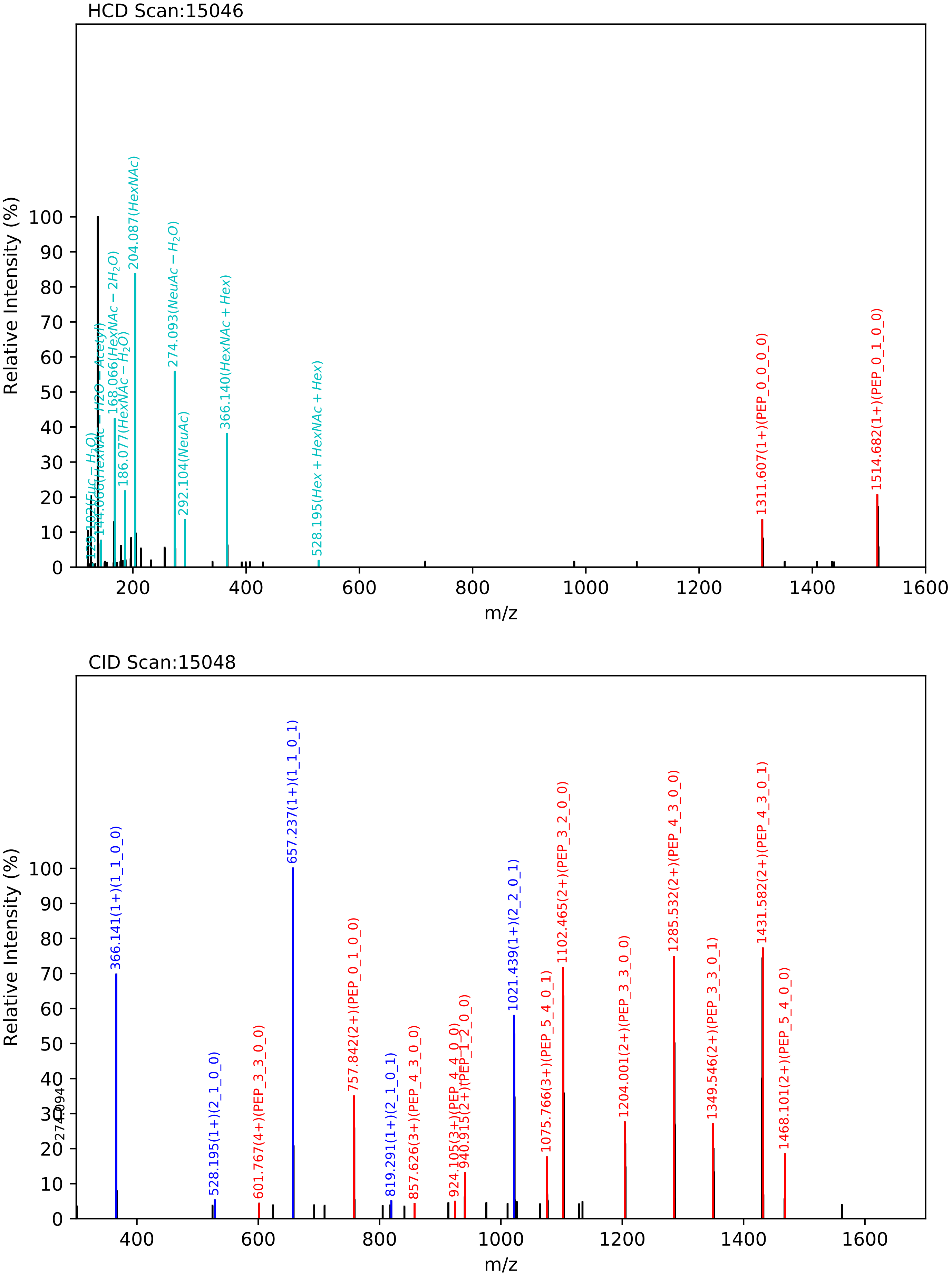

Unknown set no. 244, Experiment: Human Plasma exp_4

NGTAVCATNR(=PEP)_5_4_0_1, m/z:993.07(3+), RT:17.81, Y-score:91.30

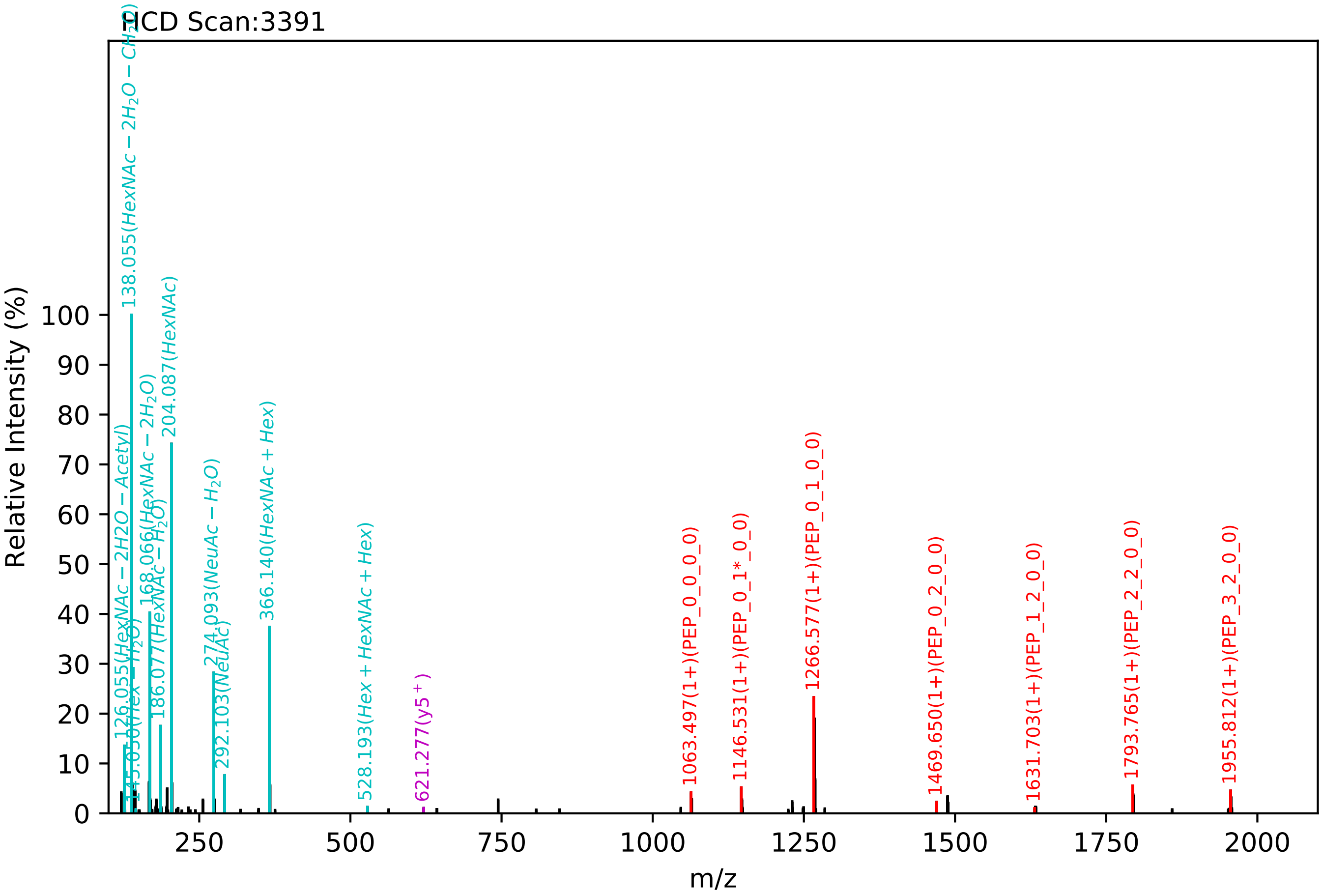

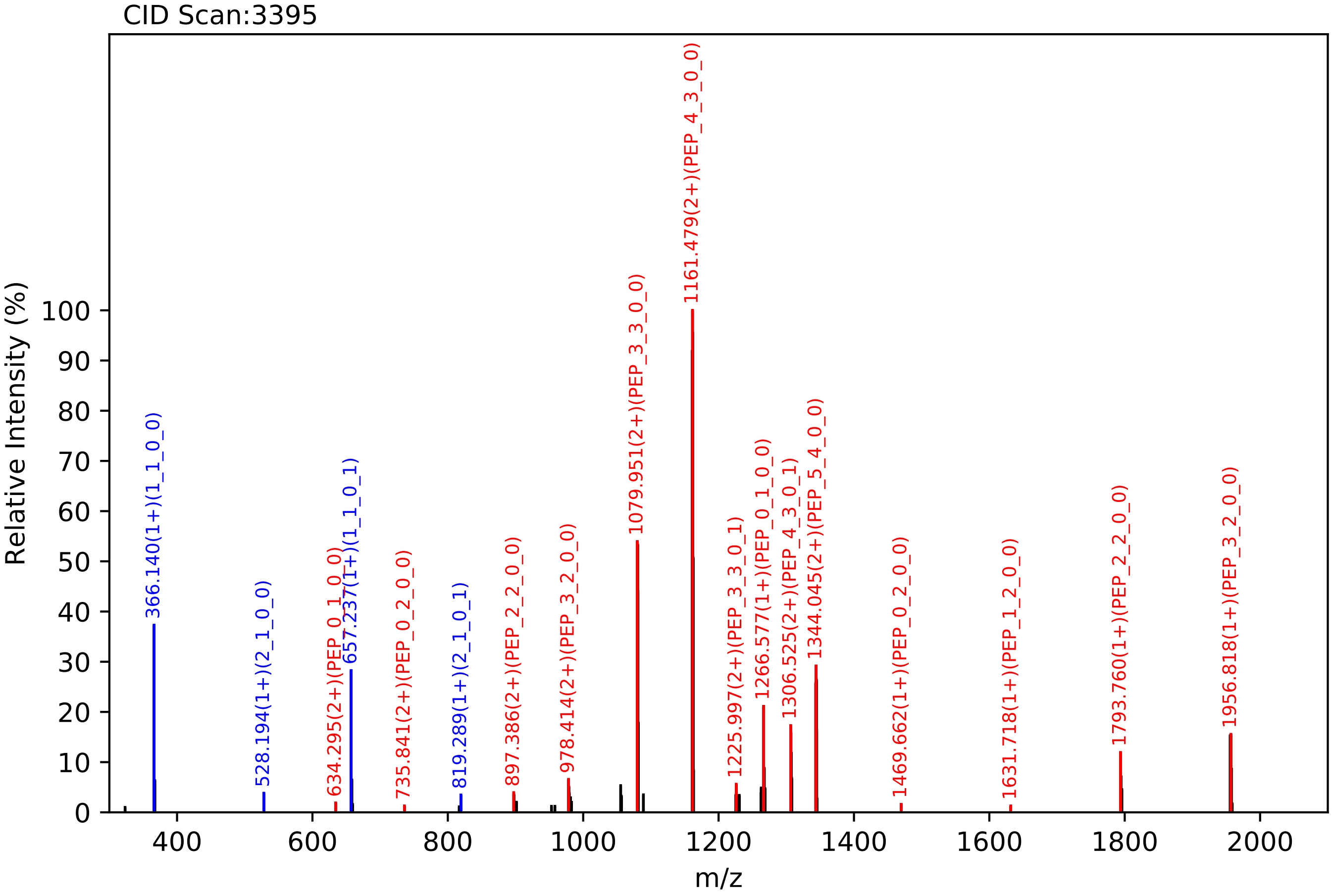

Unknown set no. 245, Experiment: Human Plasma exp_1

NGTAVCATNR(=PEP)_5_4_0_2, m/z:1090.10(3+), RT:28.10, Y-score:93.50

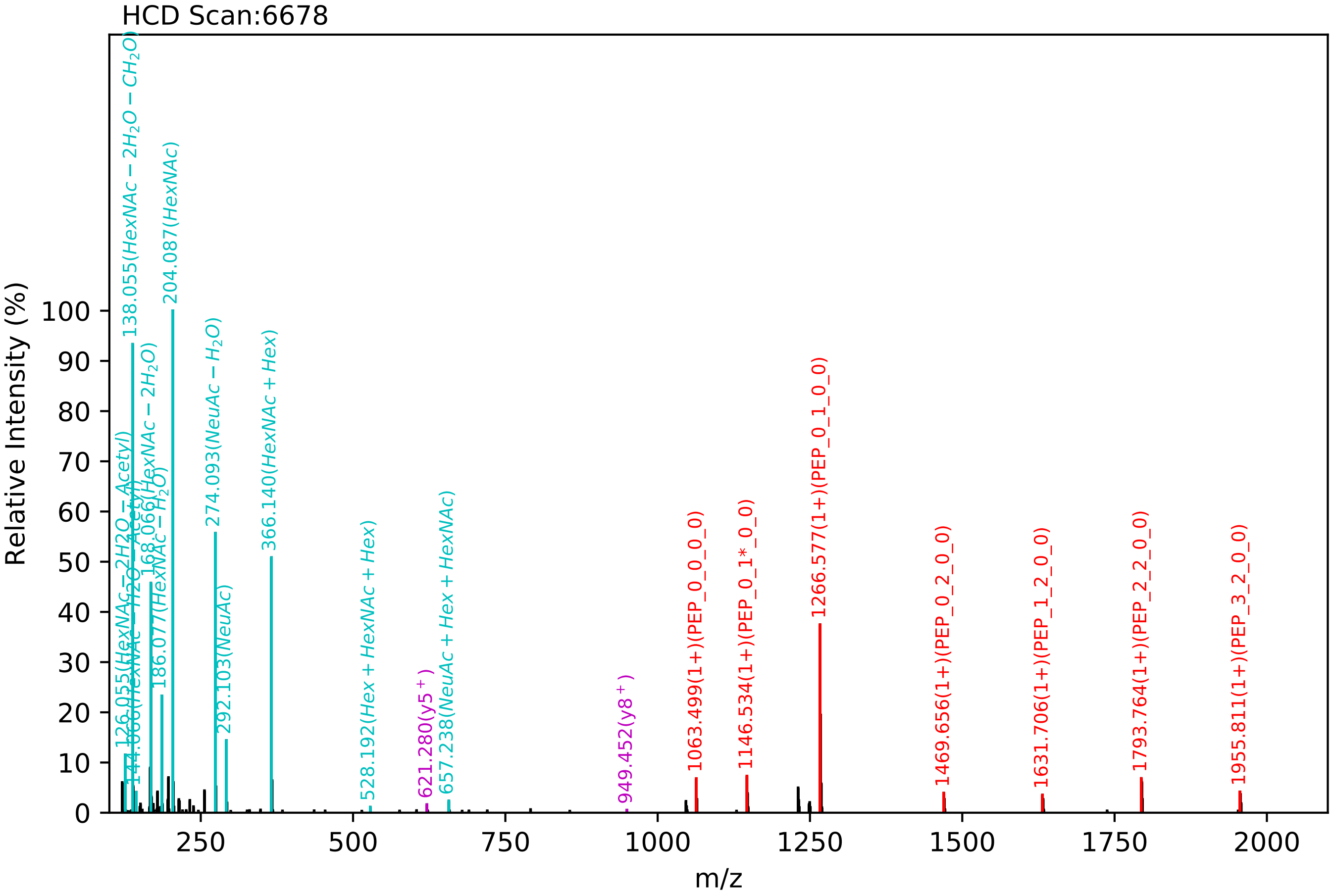

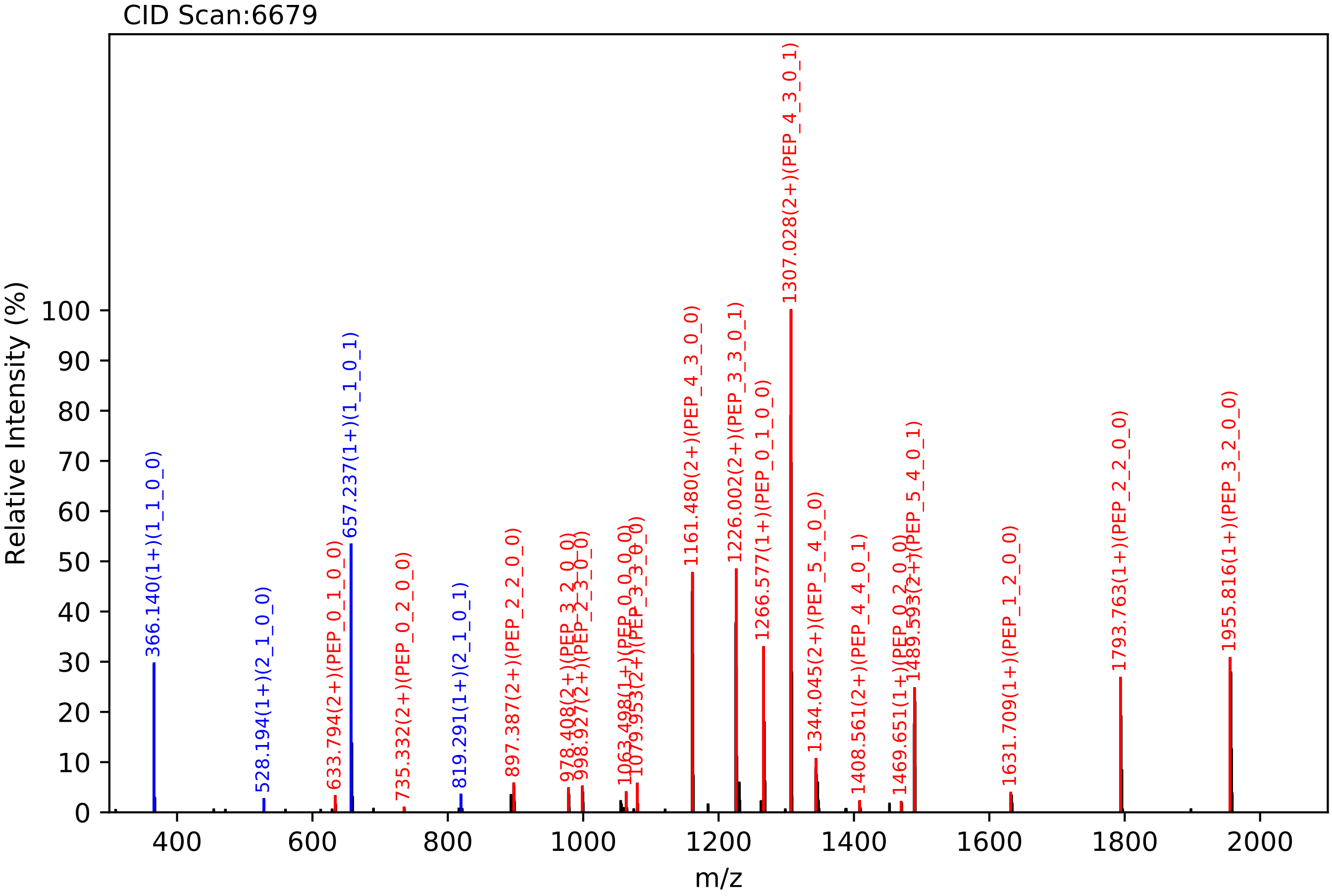

Unknown set no. 246, Experiment: Human Plasma exp_2

NGTAVCATNR(=PEP)_5_4_0_2, m/z:1090.10(3+), RT:27.82, Y-score:92.48

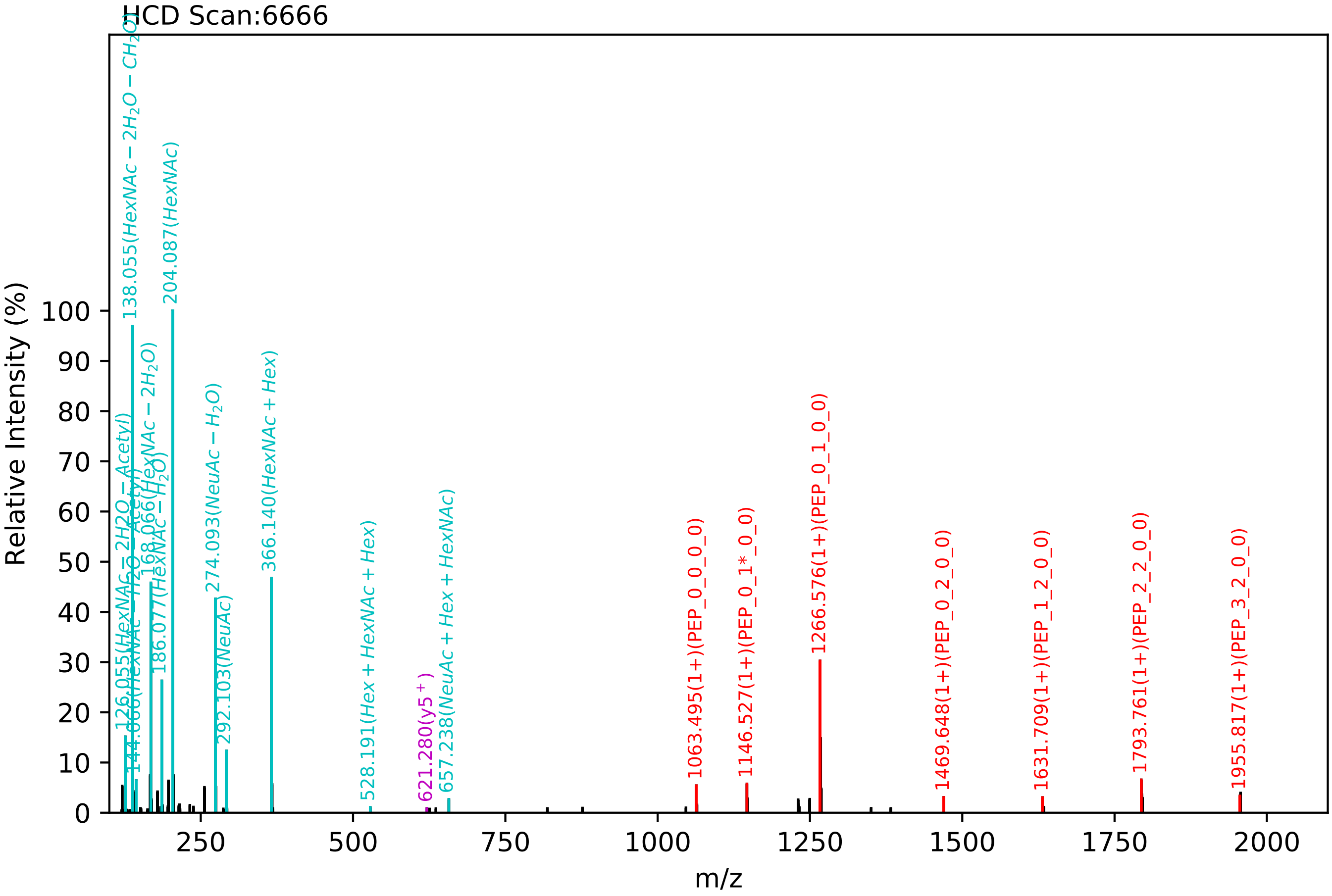

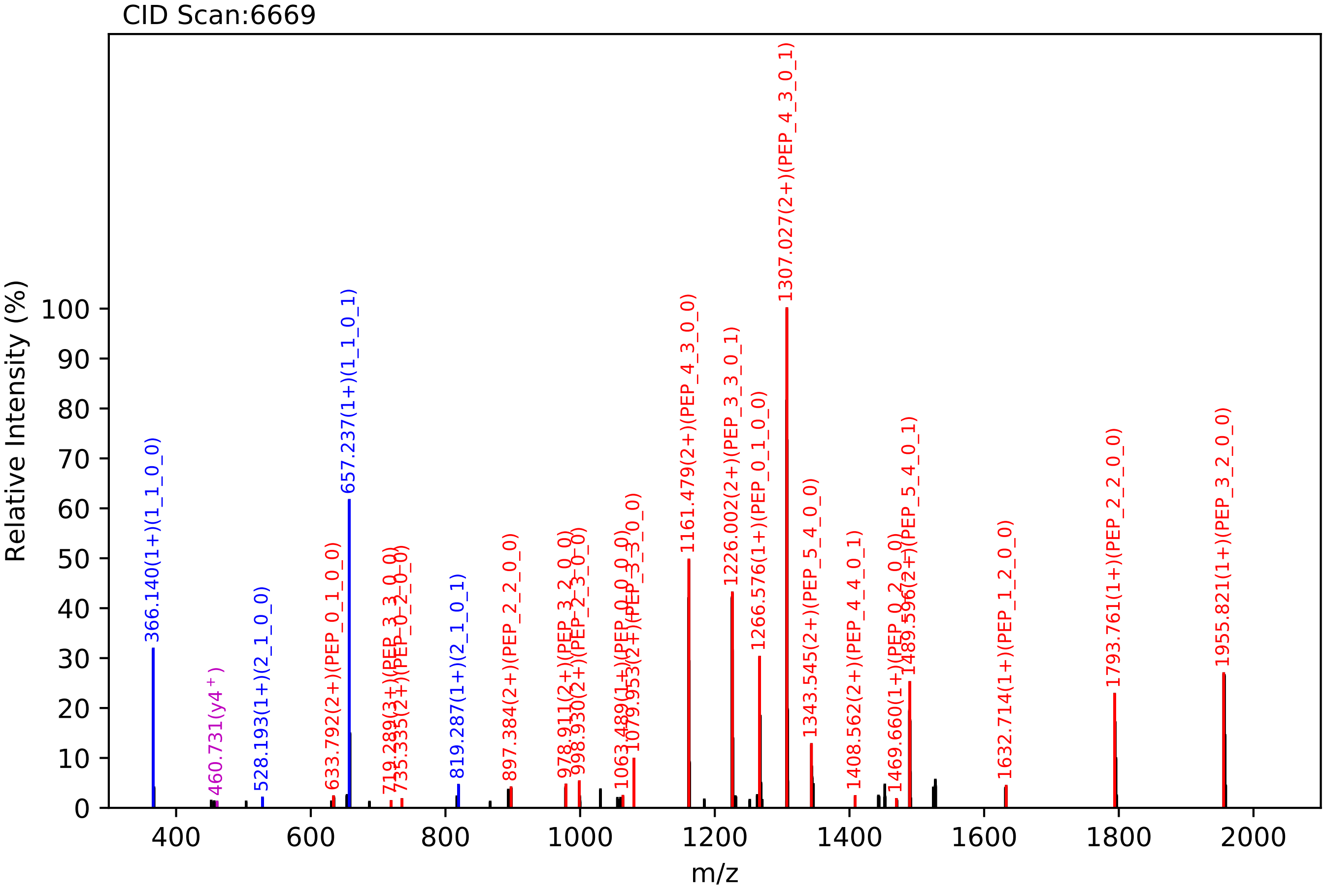

Unknown set no. 247, Experiment: Human Plasma exp_3

NGTAVCATNR(=PEP)_5_4_0_2, m/z:1090.10(3+), RT:28.60, Y-score:93.00

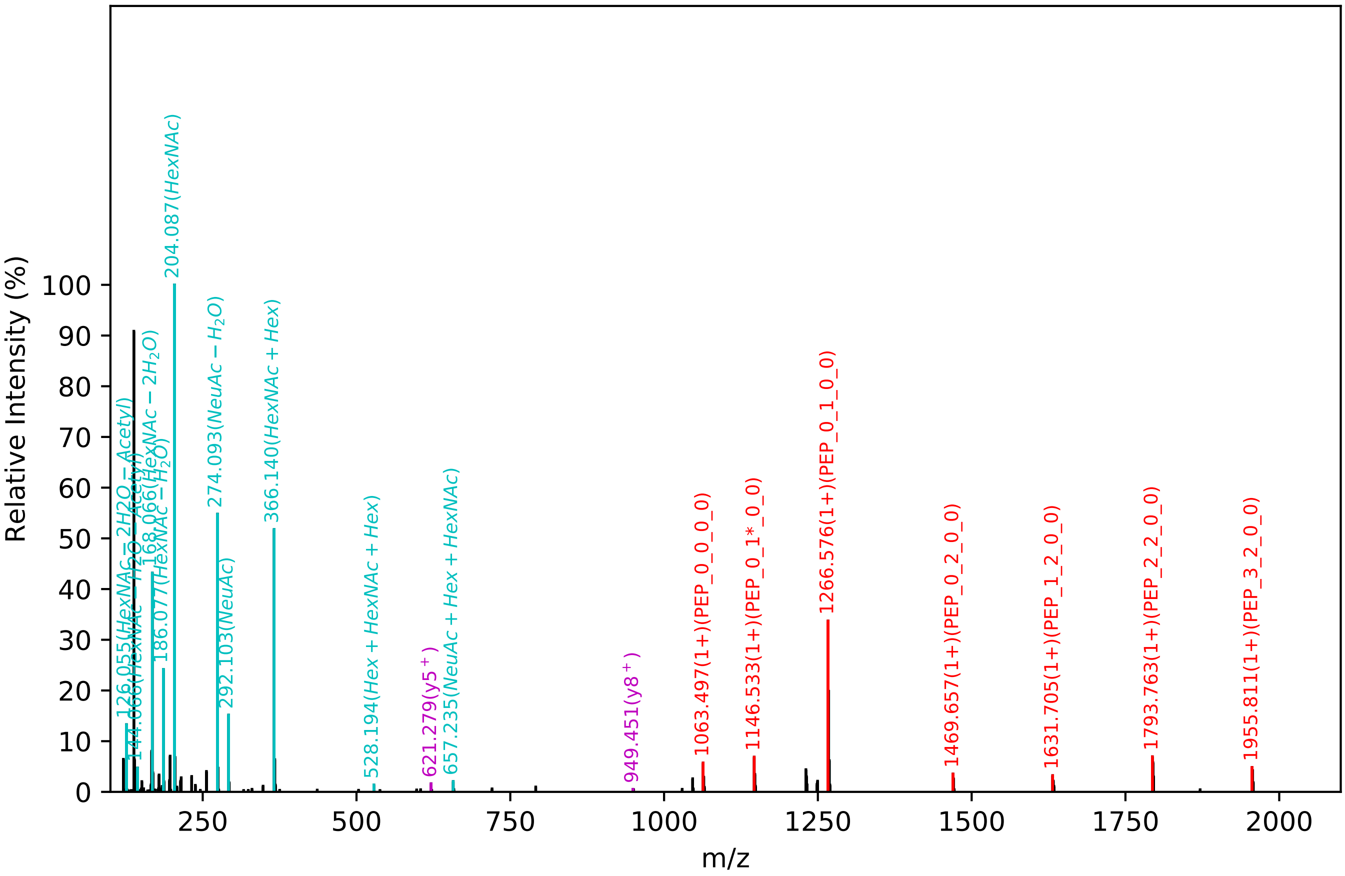

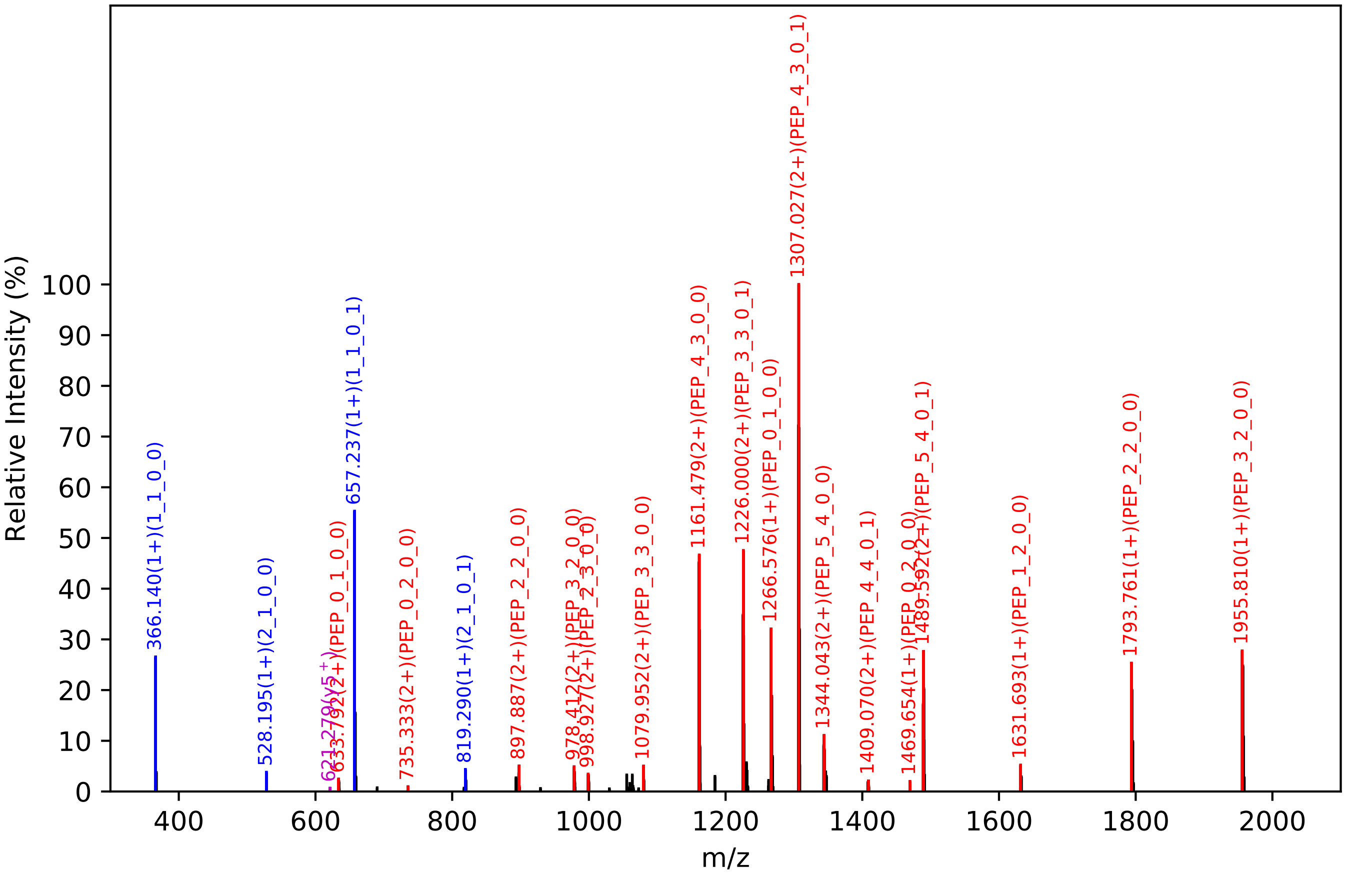

Unknown set no. 248, Experiment: Human Plasma exp_4

NGTAVCATNR(=PEP)_5_4_0_2, m/z:1090.10(3+), RT:28.63, Y-score:92.01

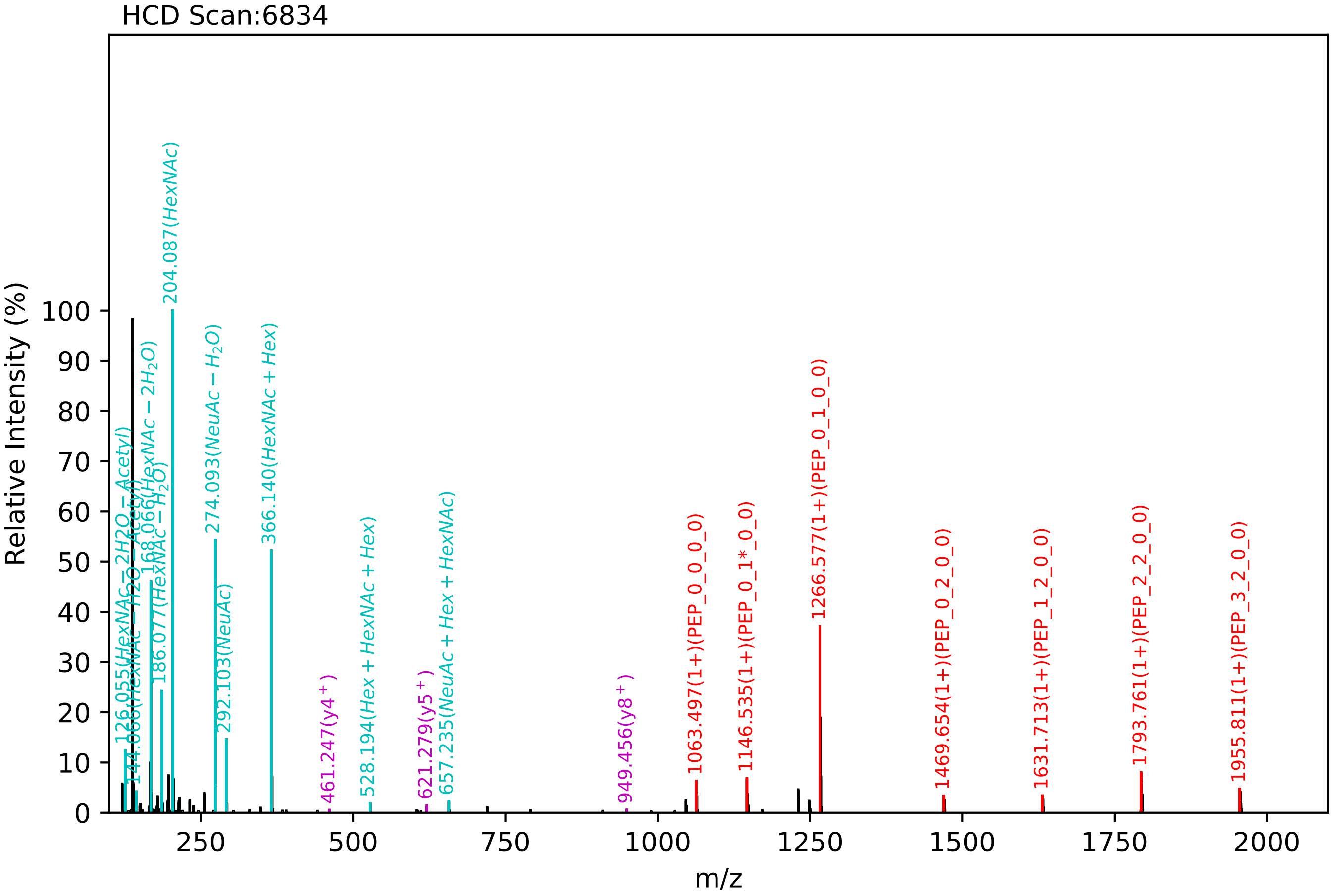

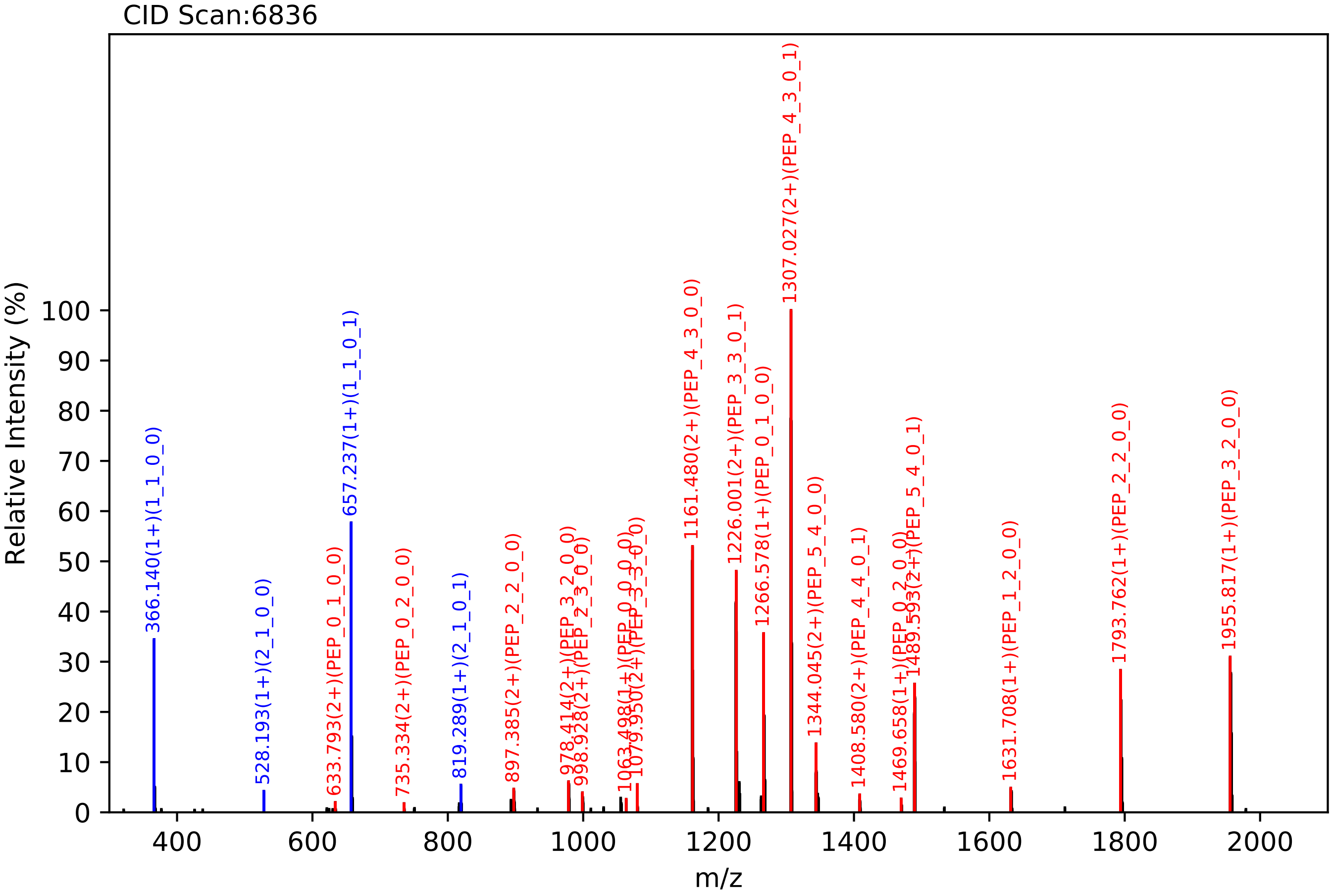

Unknown set no. 249, Experiment: Human Plasma exp_5

NGTAVCATNR(=PEP)_5_4_0_2, m/z:1090.10(3+), RT:28.61, Y-score:93.66

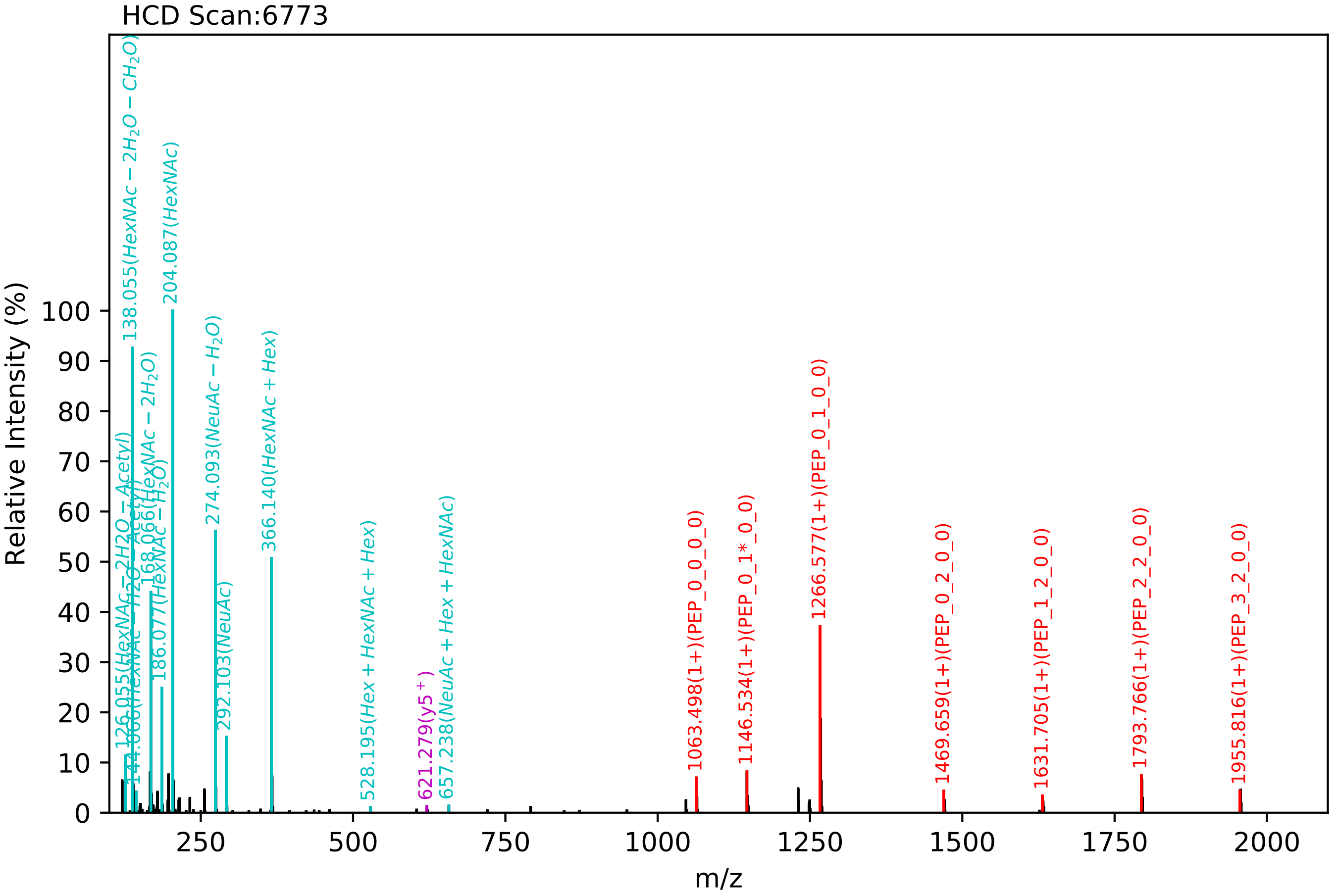

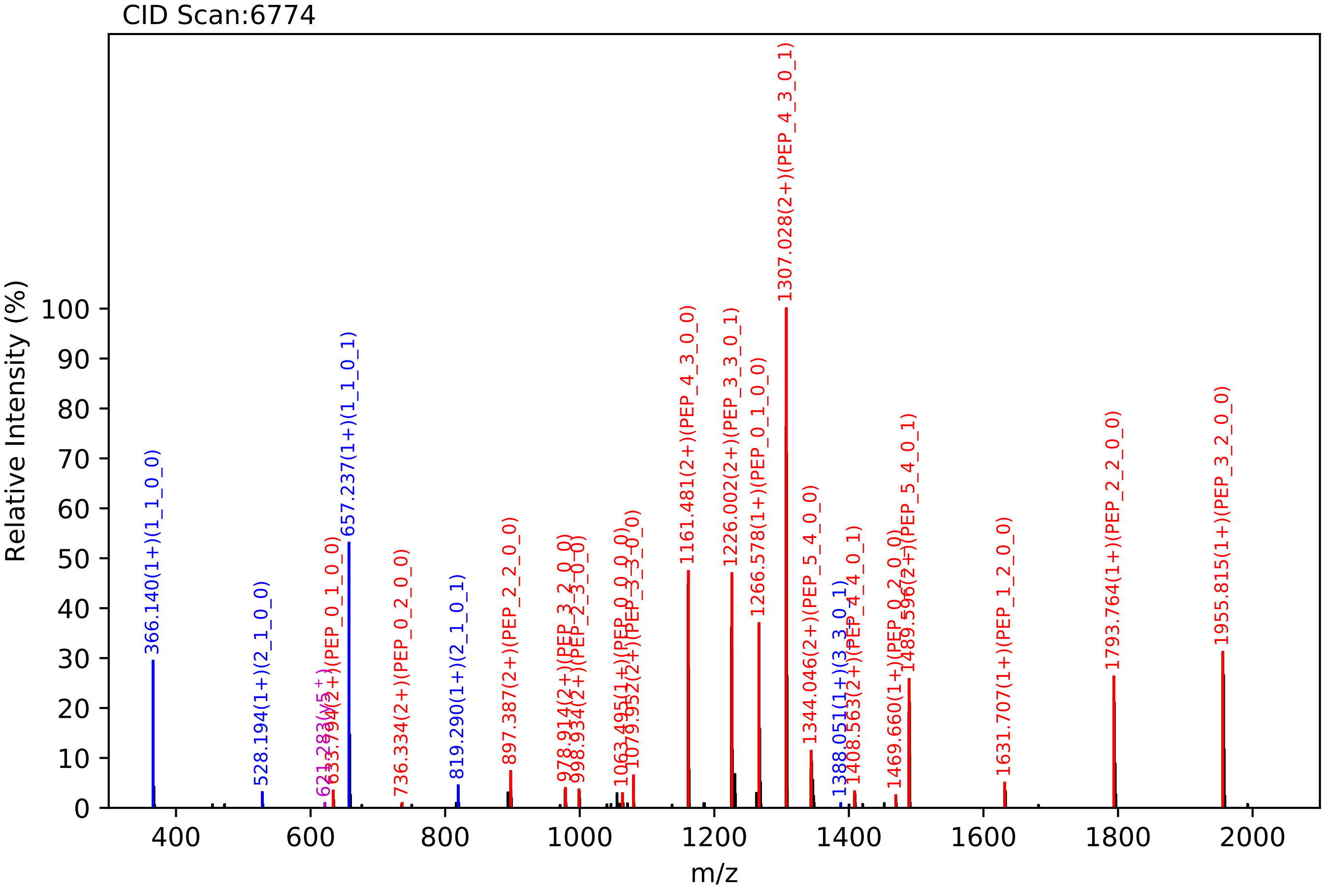

Unknown set no. 250, Experiment: Human Plasma exp_4

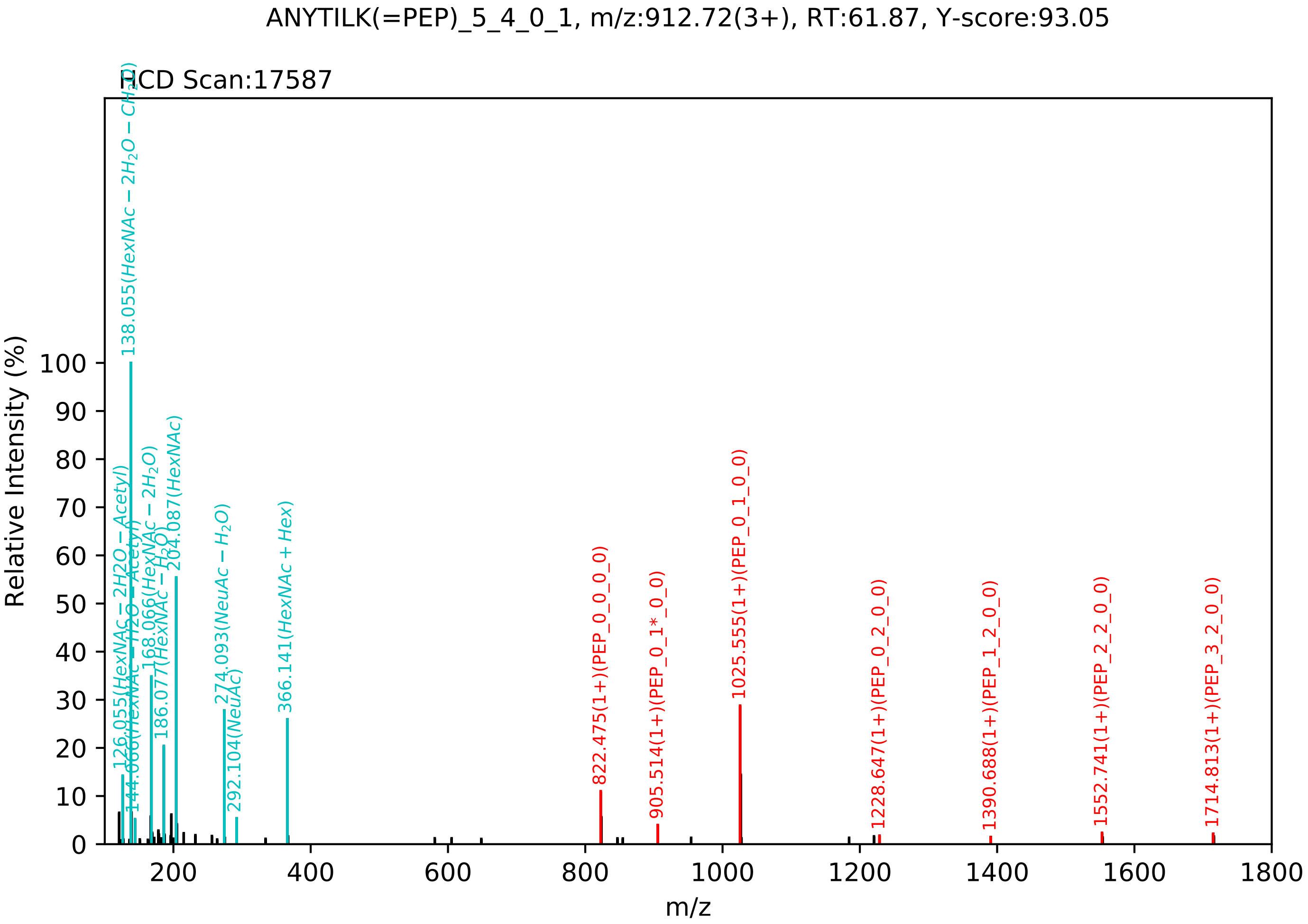

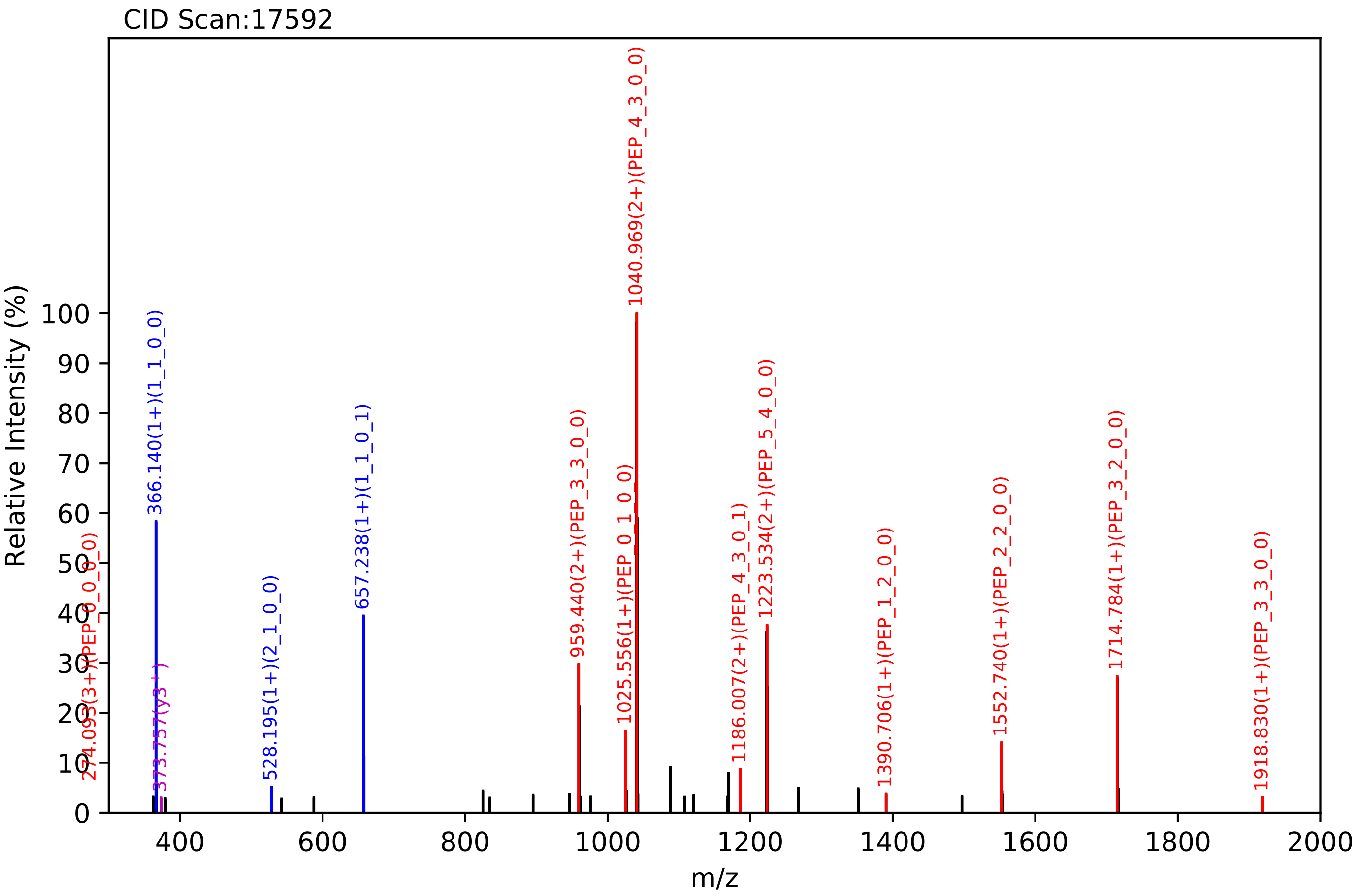

Unknown set no. 251, Experiment: Human Plasma exp_2

ANYTILK(=PEP)_5_4_0_2, m/z:1009.75(3+), RT:74.72, Y-score:94.57

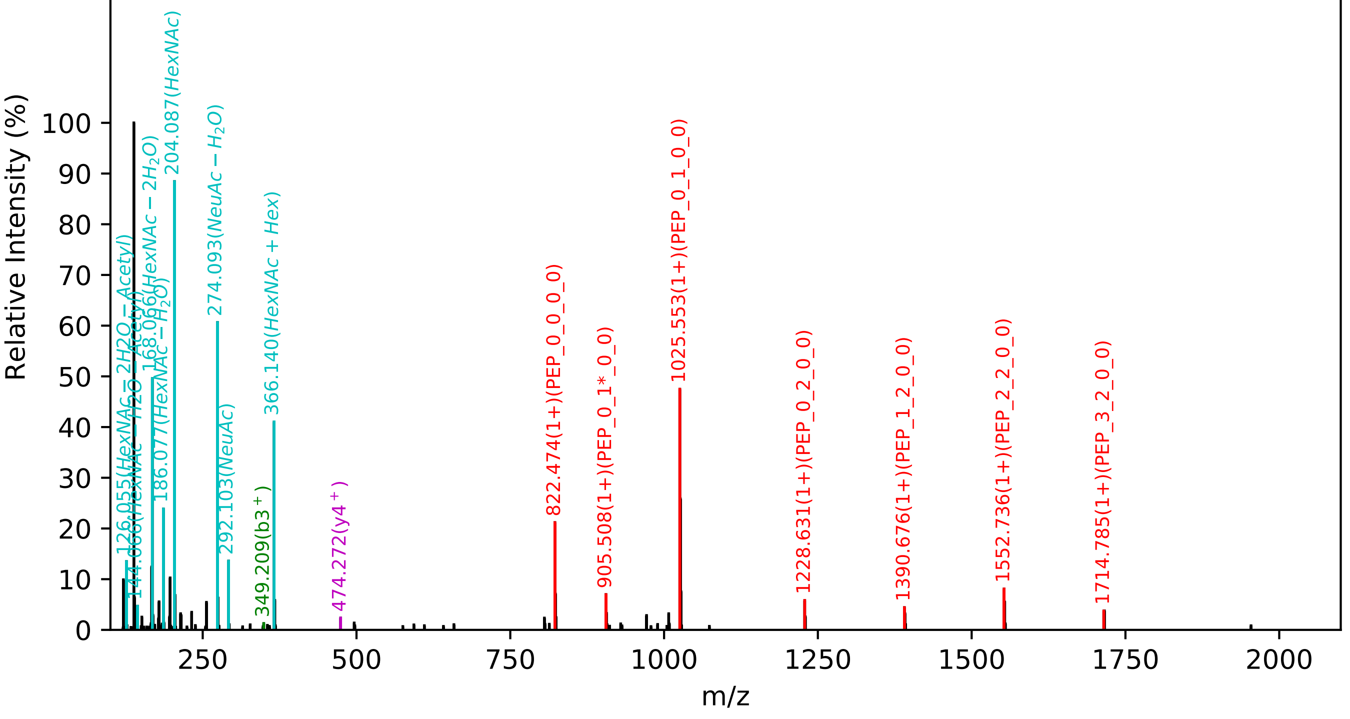

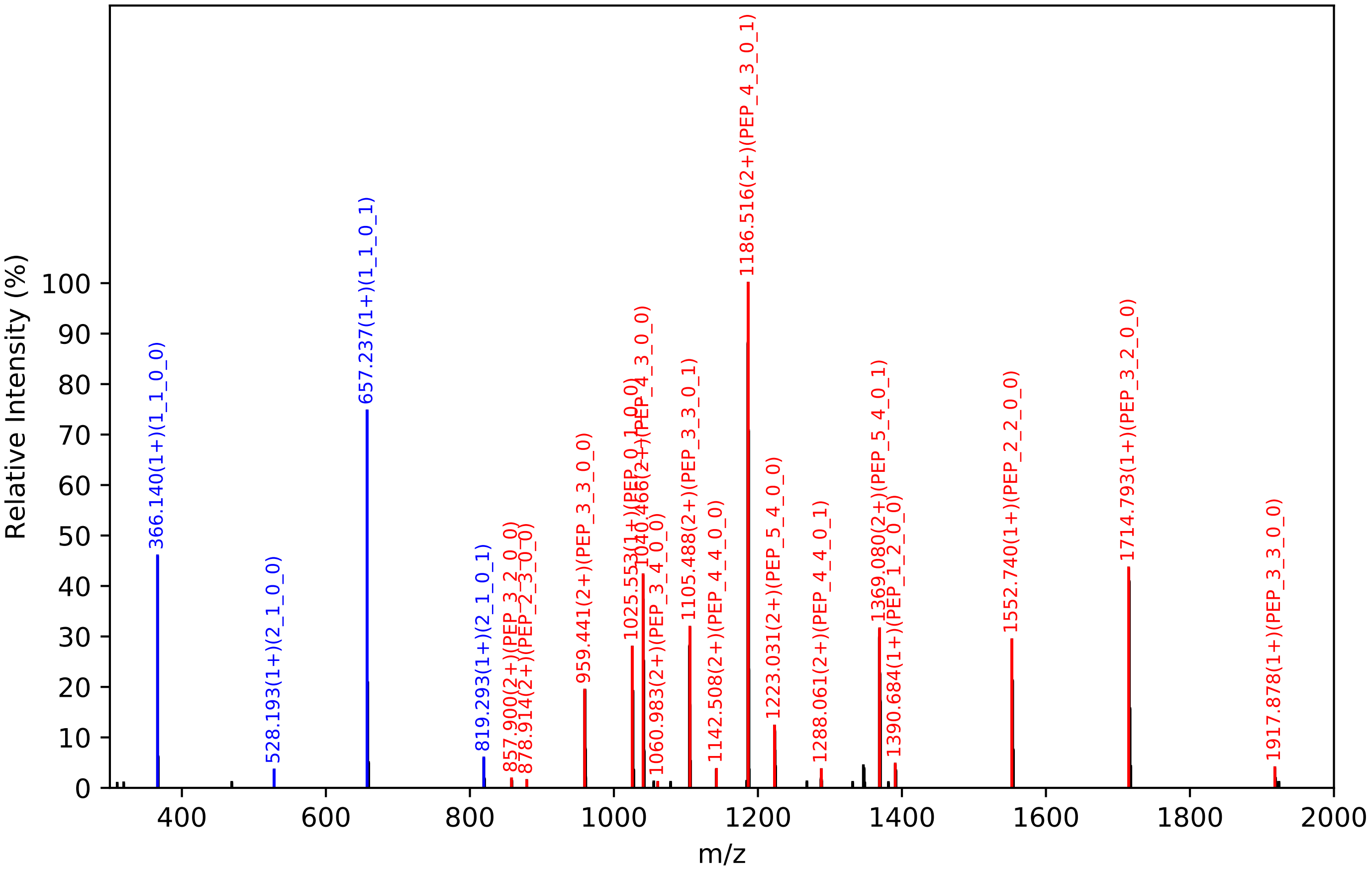

Unknown set no. 252, Experiment: Human Plasma exp_4

ANYTILK(=PEP)_5_4_0_2, m/z:1009.75(3+), RT:74.94, Y-score:92.23

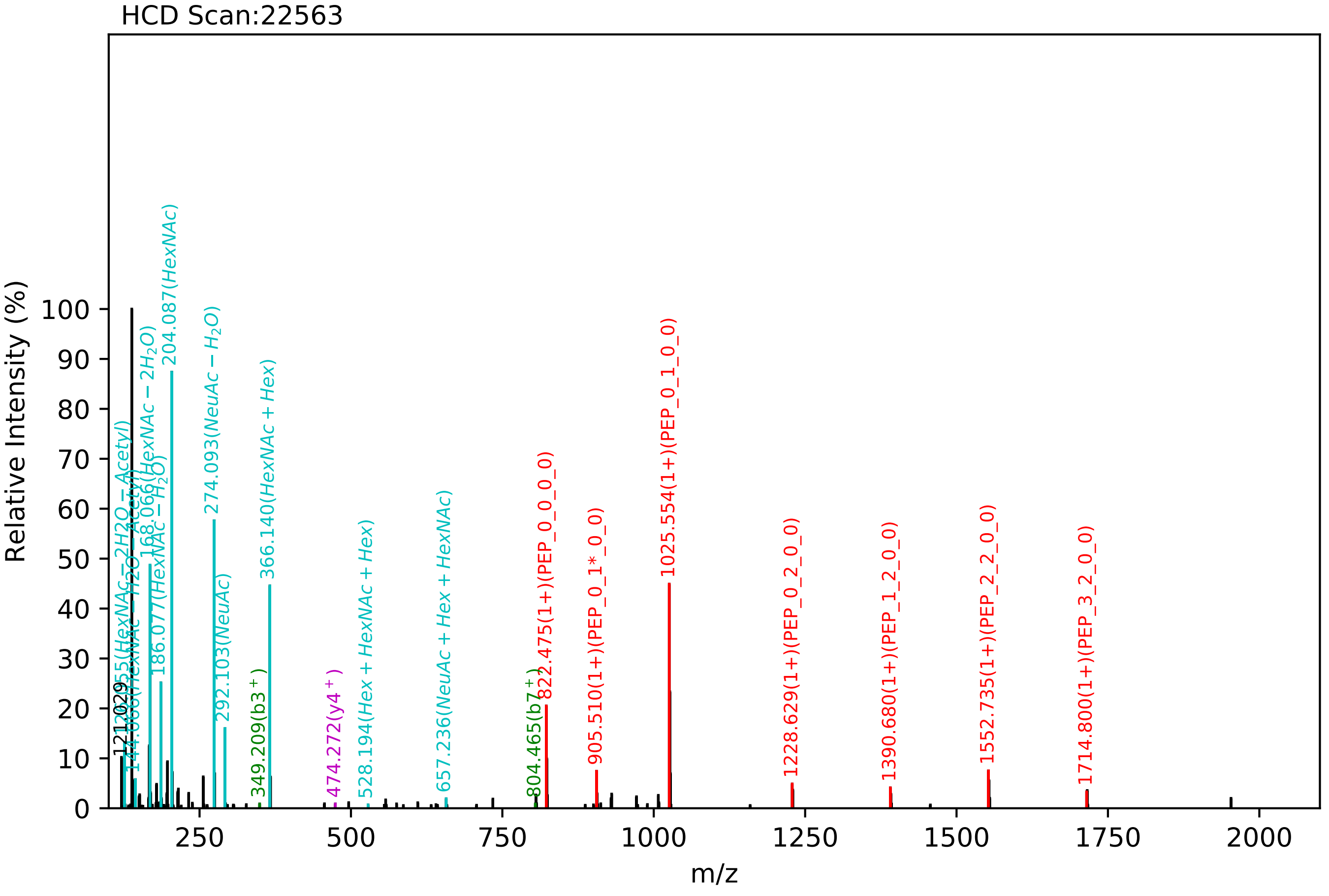

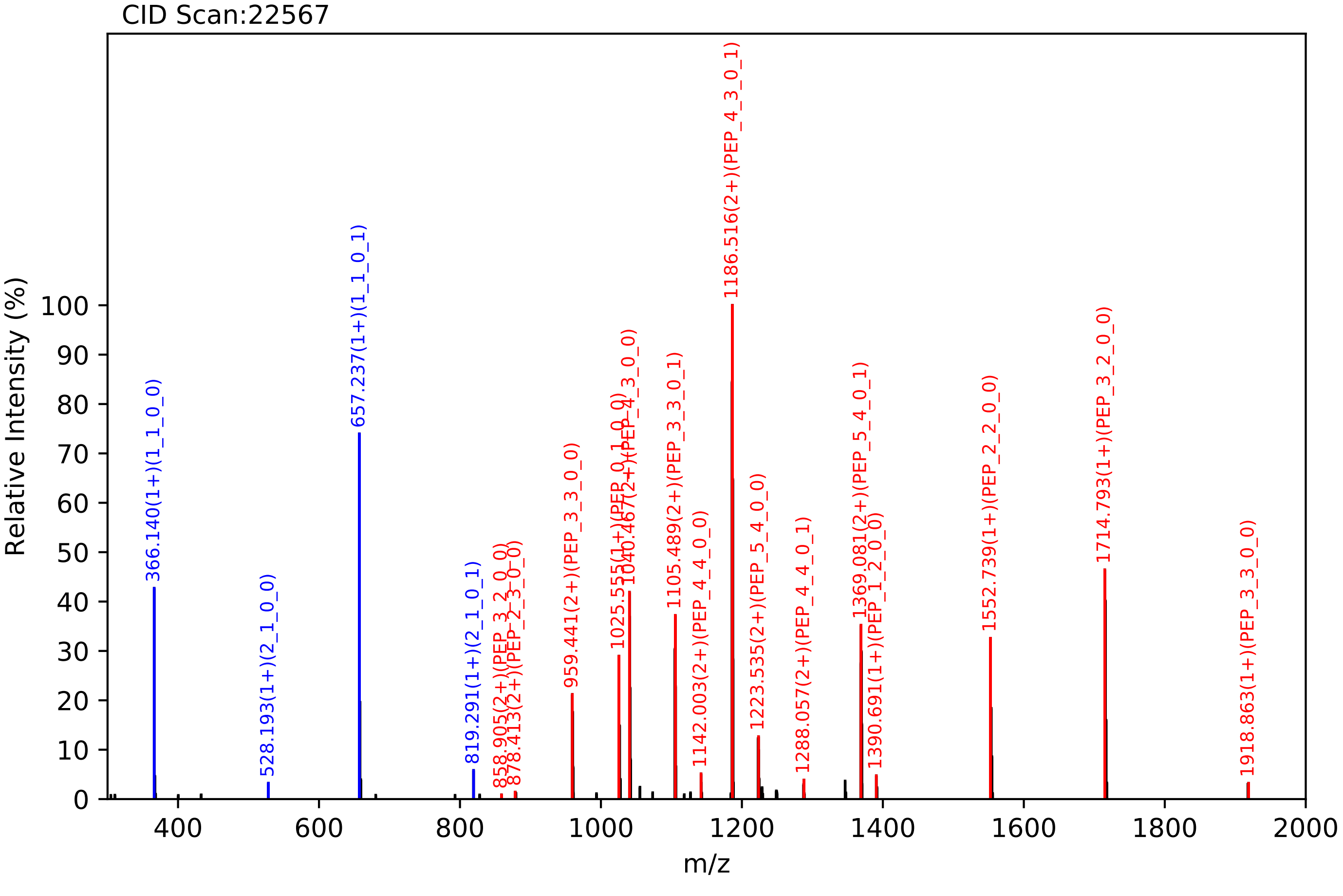

Unknown set no. 253, Experiment: Human Plasma exp_3

DLEITNATLQSEEDSR(=PEP)_4_3_0_1, m/z:1123.81(3+), RT:84.70, Y-score:96.74

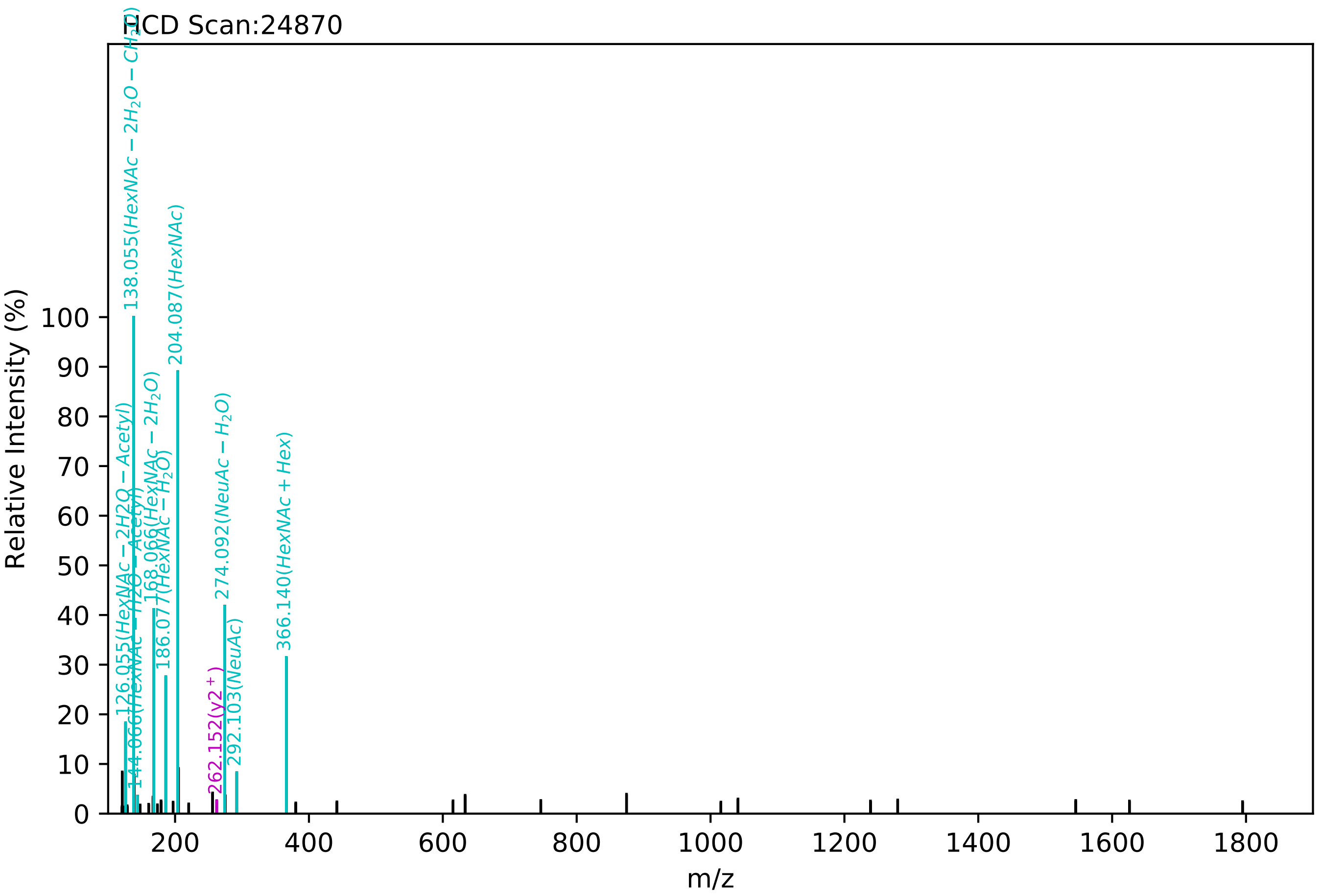

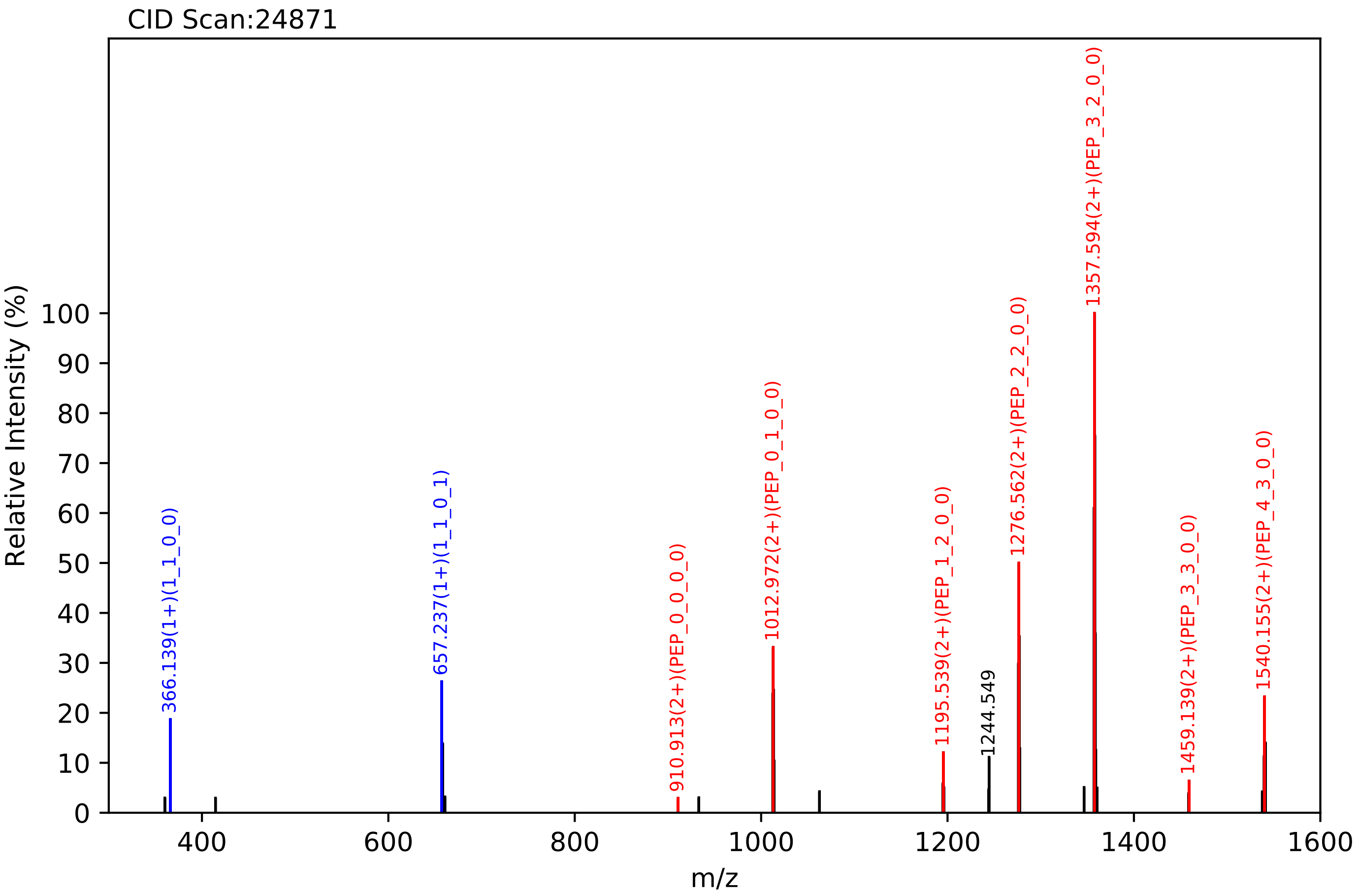

Unknown set no. 254, Experiment: Human Plasma exp_4

DLEITNATLQSEEDSR(=PEP)_5_4_0_2, m/z:1007.16(4+), RT:85.62, Y-score:71.59

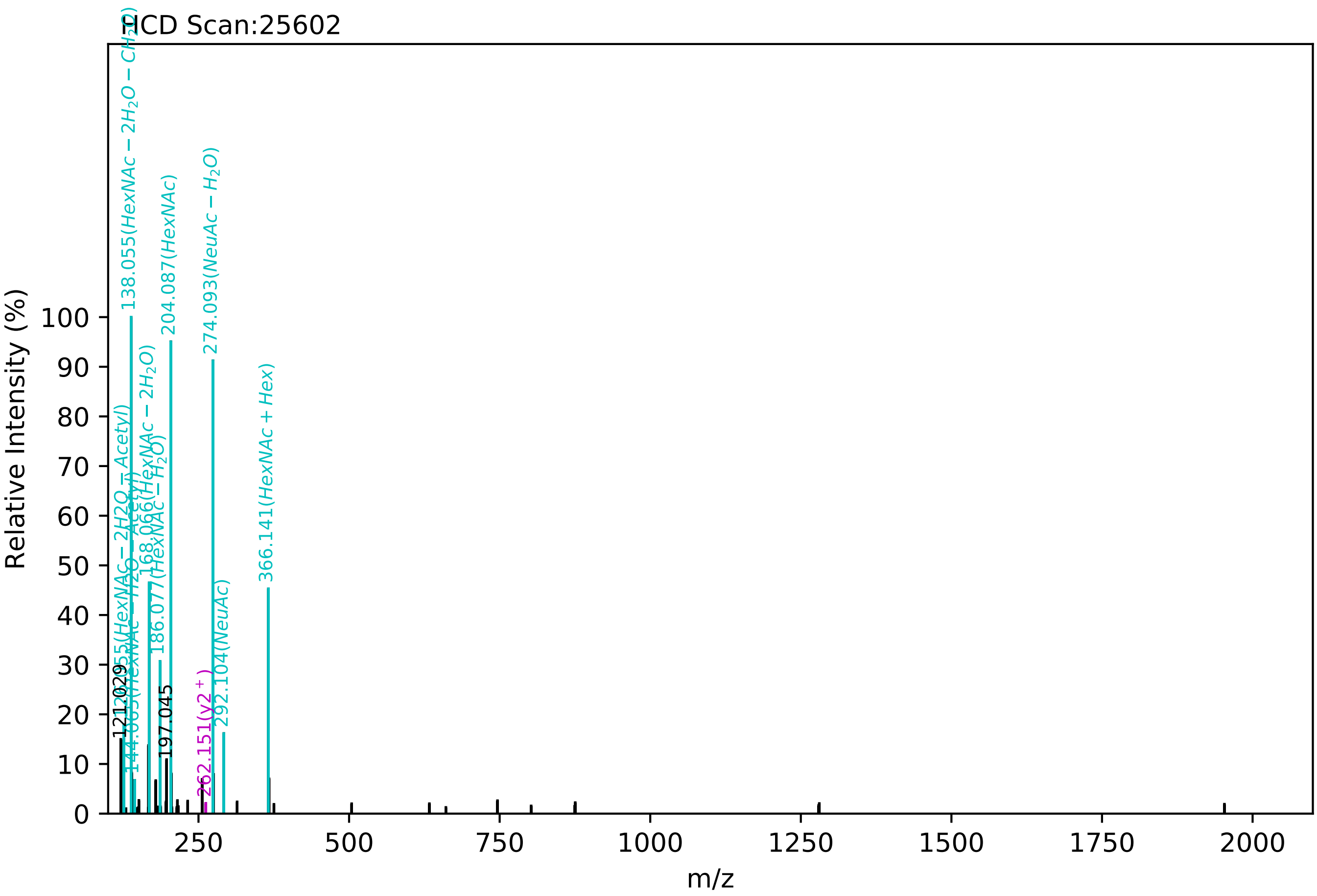

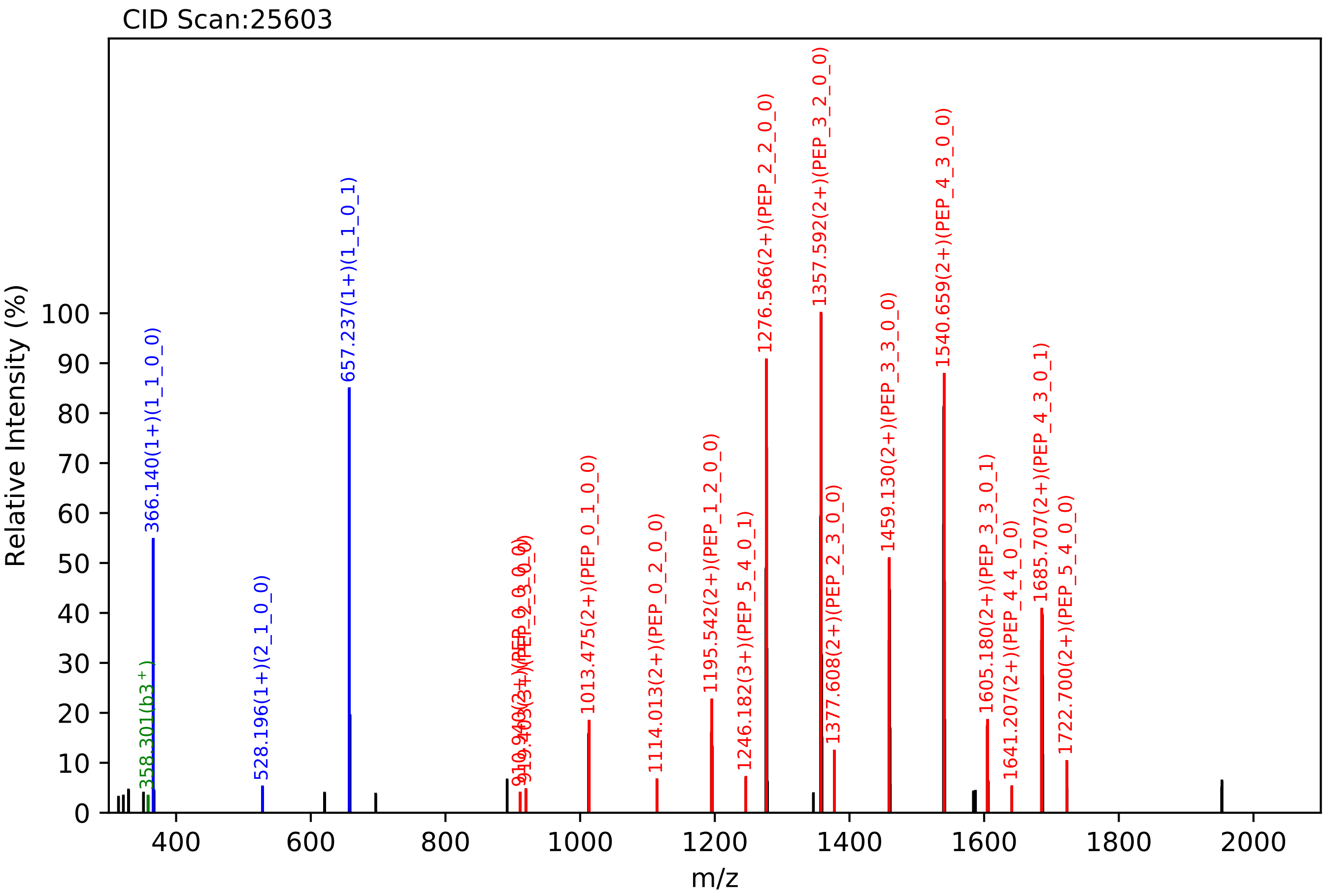

Unknown set no. 255, Experiment: Human Plasma exp_1

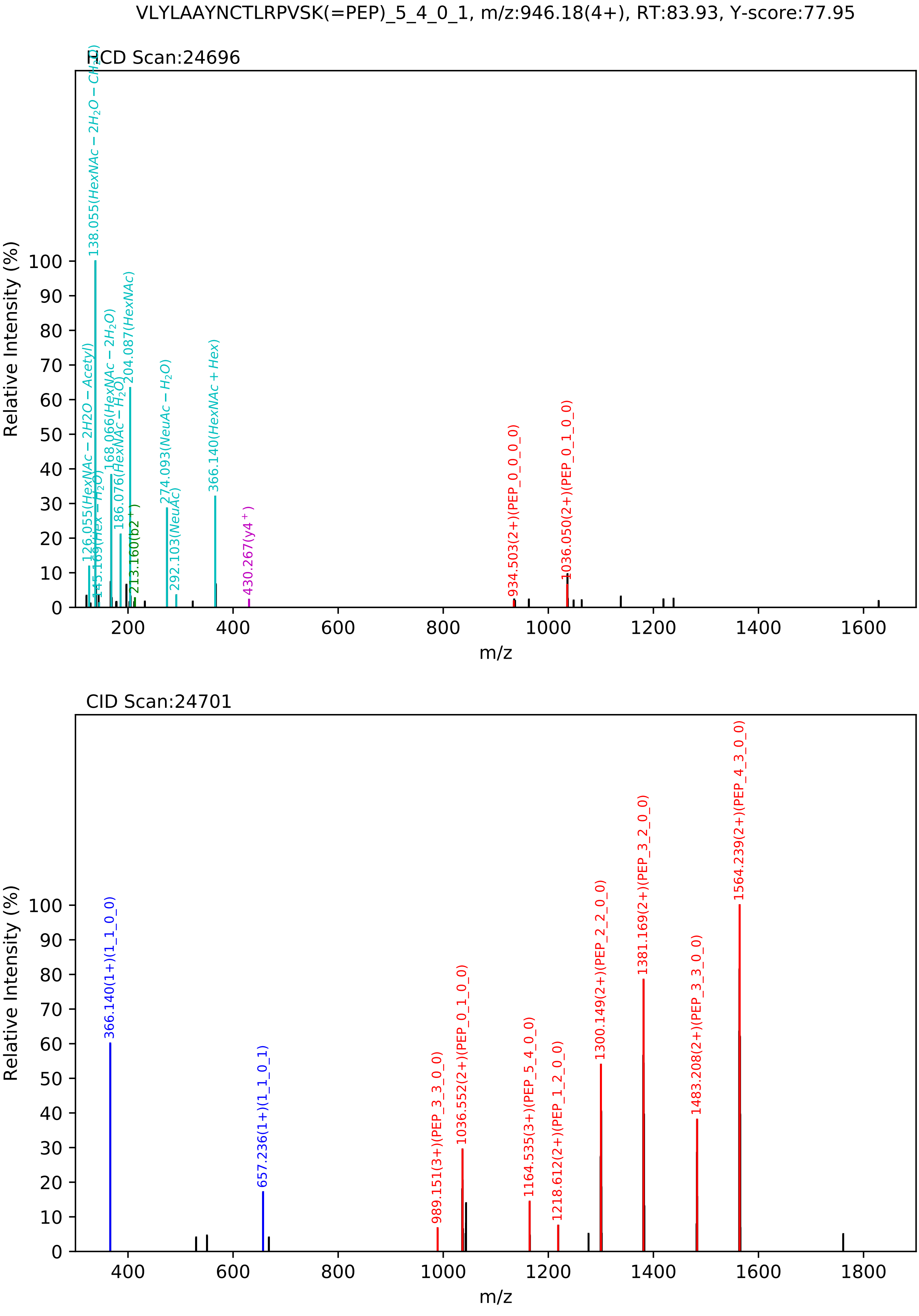

Unknown set no. 256, Experiment: Human Plasma exp_1

VELEDFNGNR(=PEP)_5_4_1_2, m/z:1181.80(3+), RT:76.98, Y-score:95.74

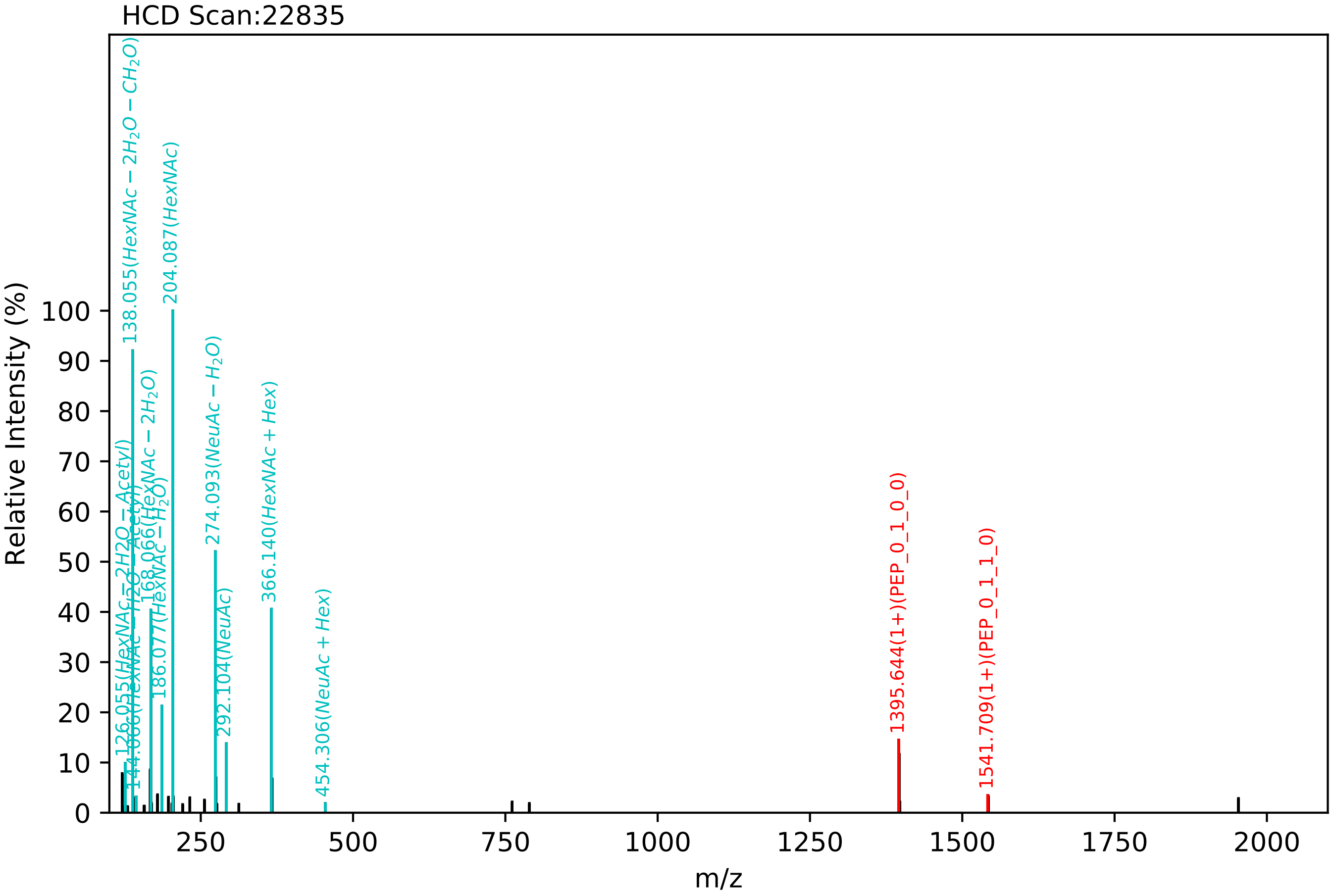

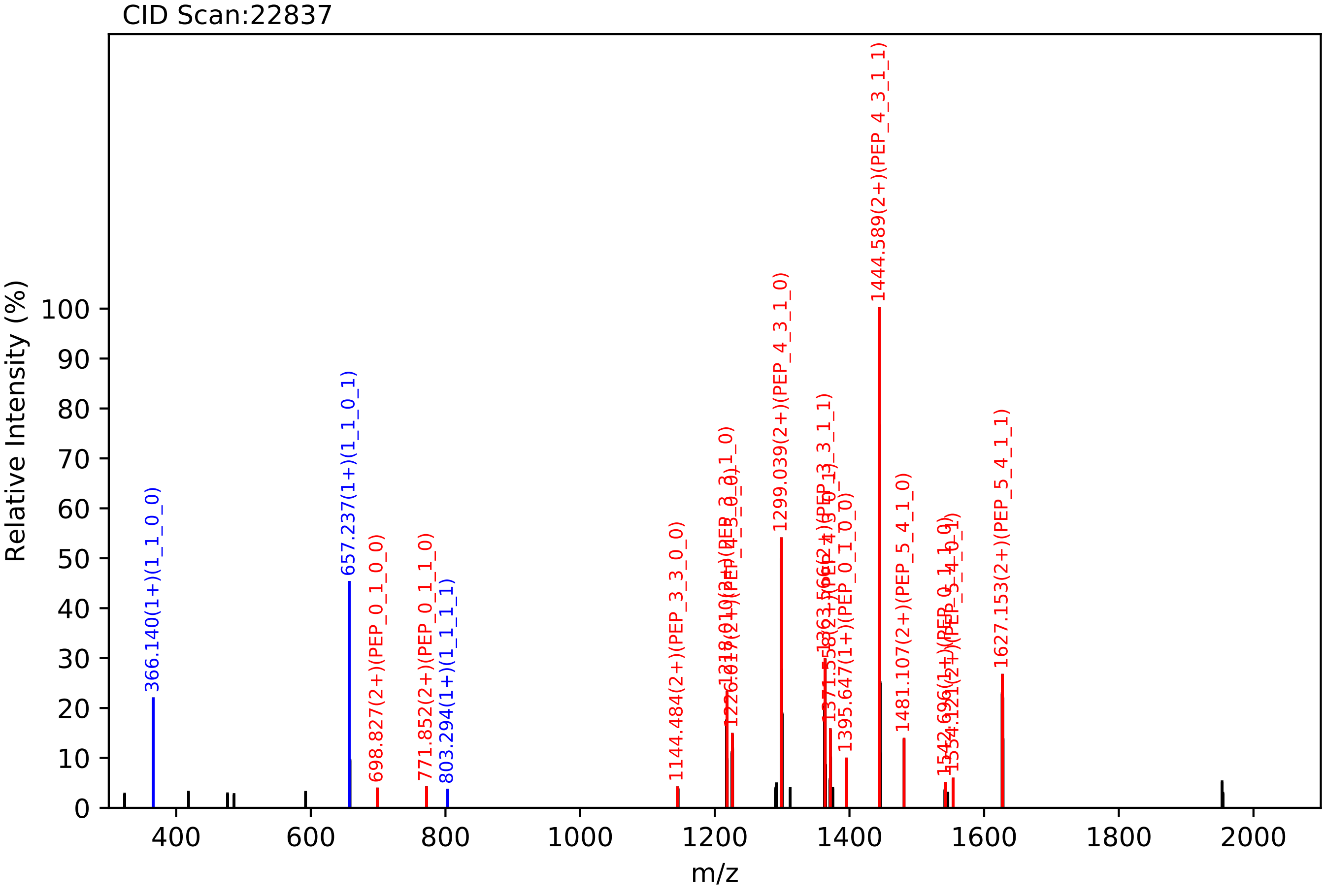

Unknown set no. 257, Experiment: Human Plasma exp_4

VELEDFNGNR(=PEP)_5_4_1_2, m/z:1181.80(3+), RT:77.33, Y-score:87.35

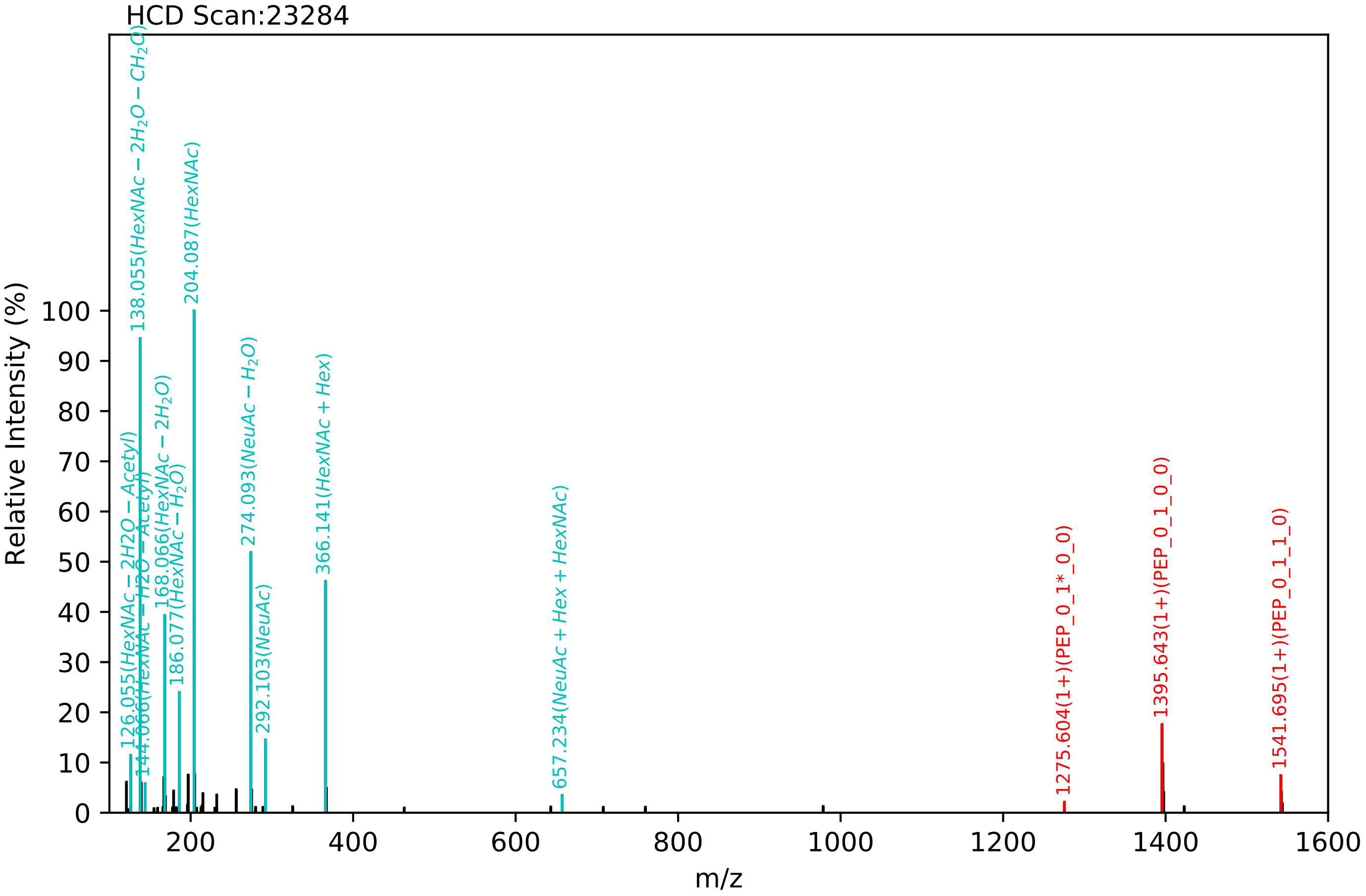

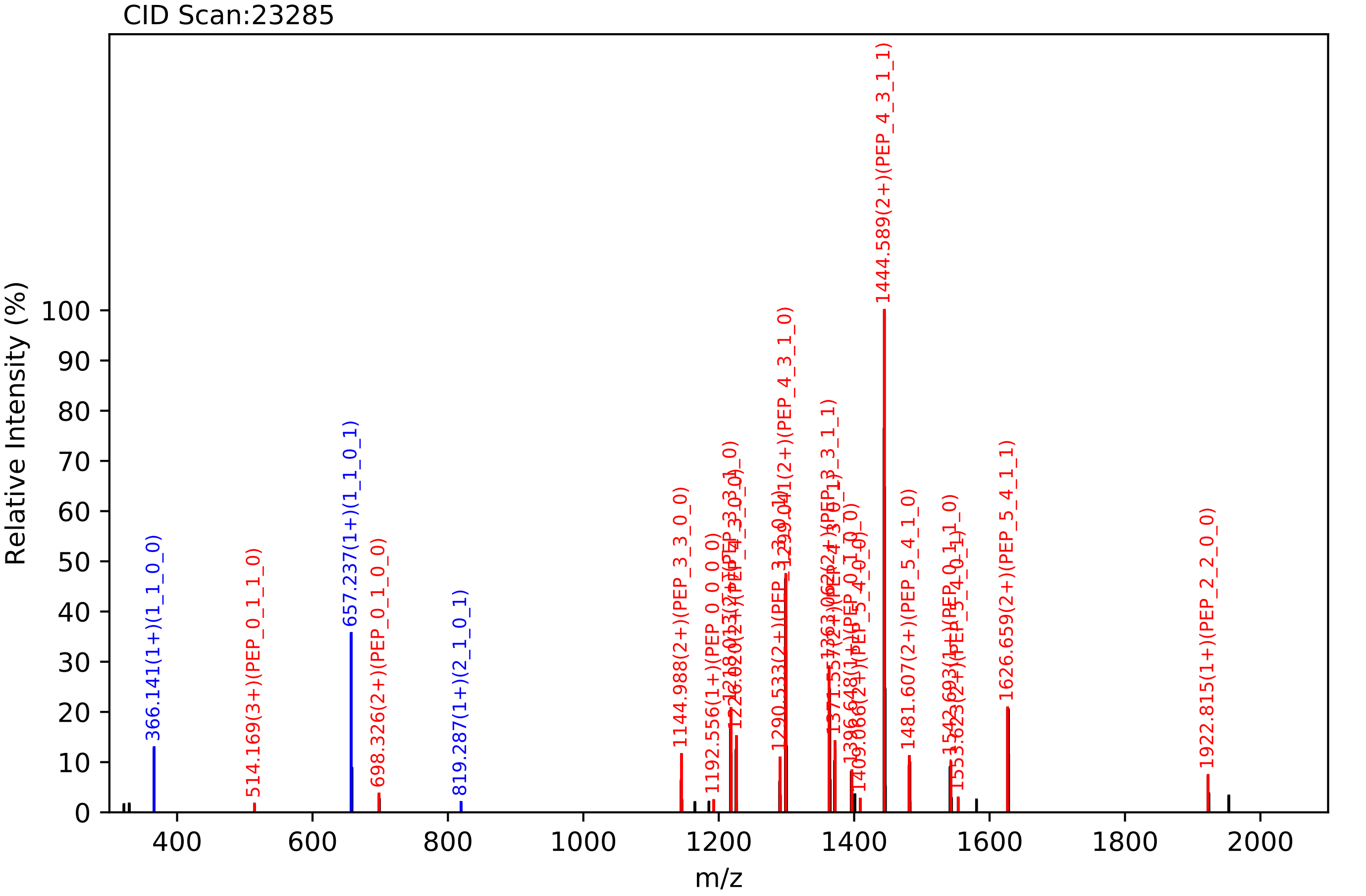

Unknown set no. 258, Experiment: Human Plasma exp_2

MVSHHNLTTGATLINEQWLLTTAK(=PEP)_5_4_0_2, m/z:1221.80(4+), RT:100.10, Y-score:77.93

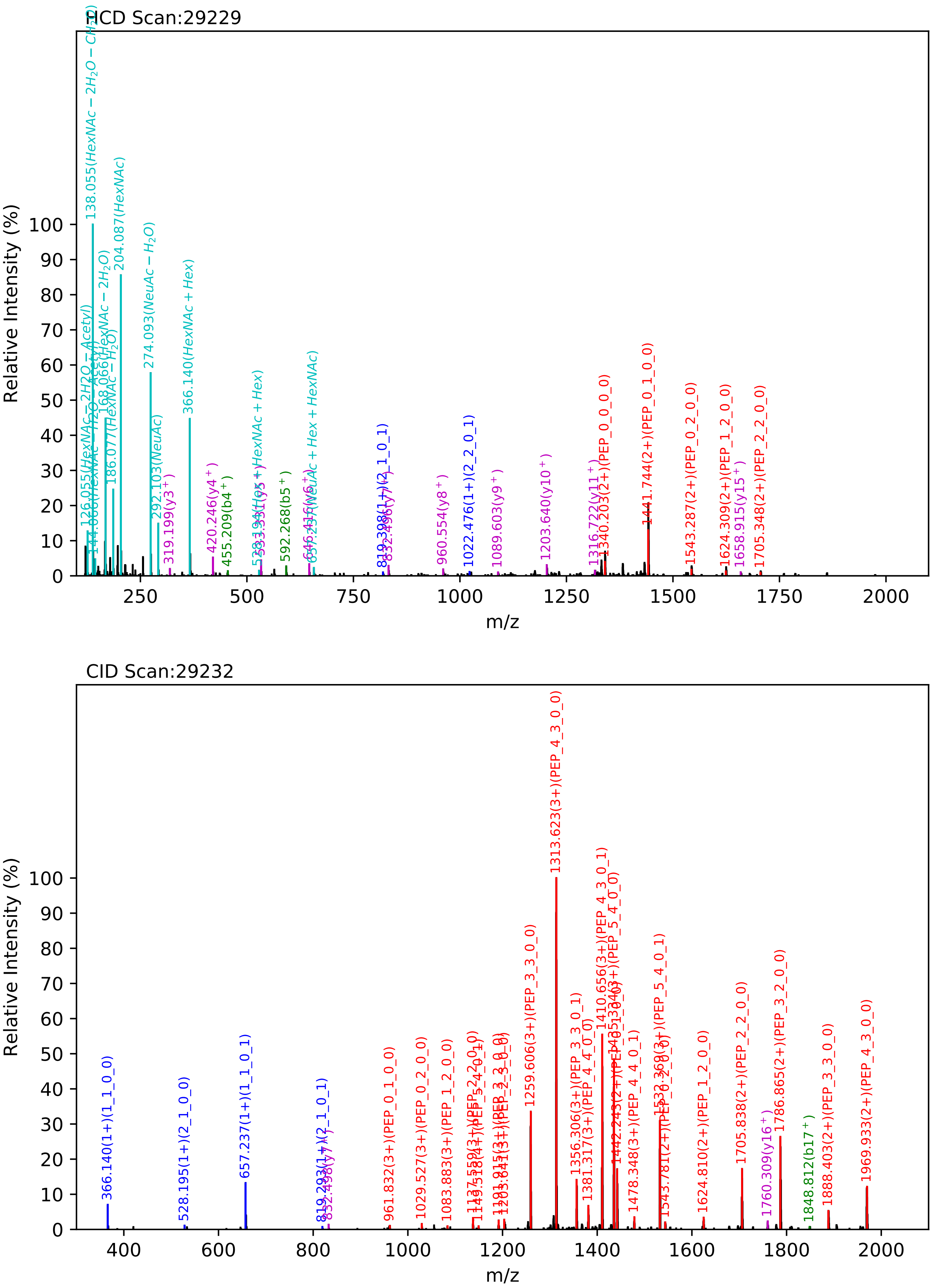

Unknown set no. 259, Experiment: Human Plasma exp_1

VVLHPNYSQVDIGLIK(=PEP)_4_3_0_1, m/z:1115.19(3+), RT:96.98, Y-score:83.29

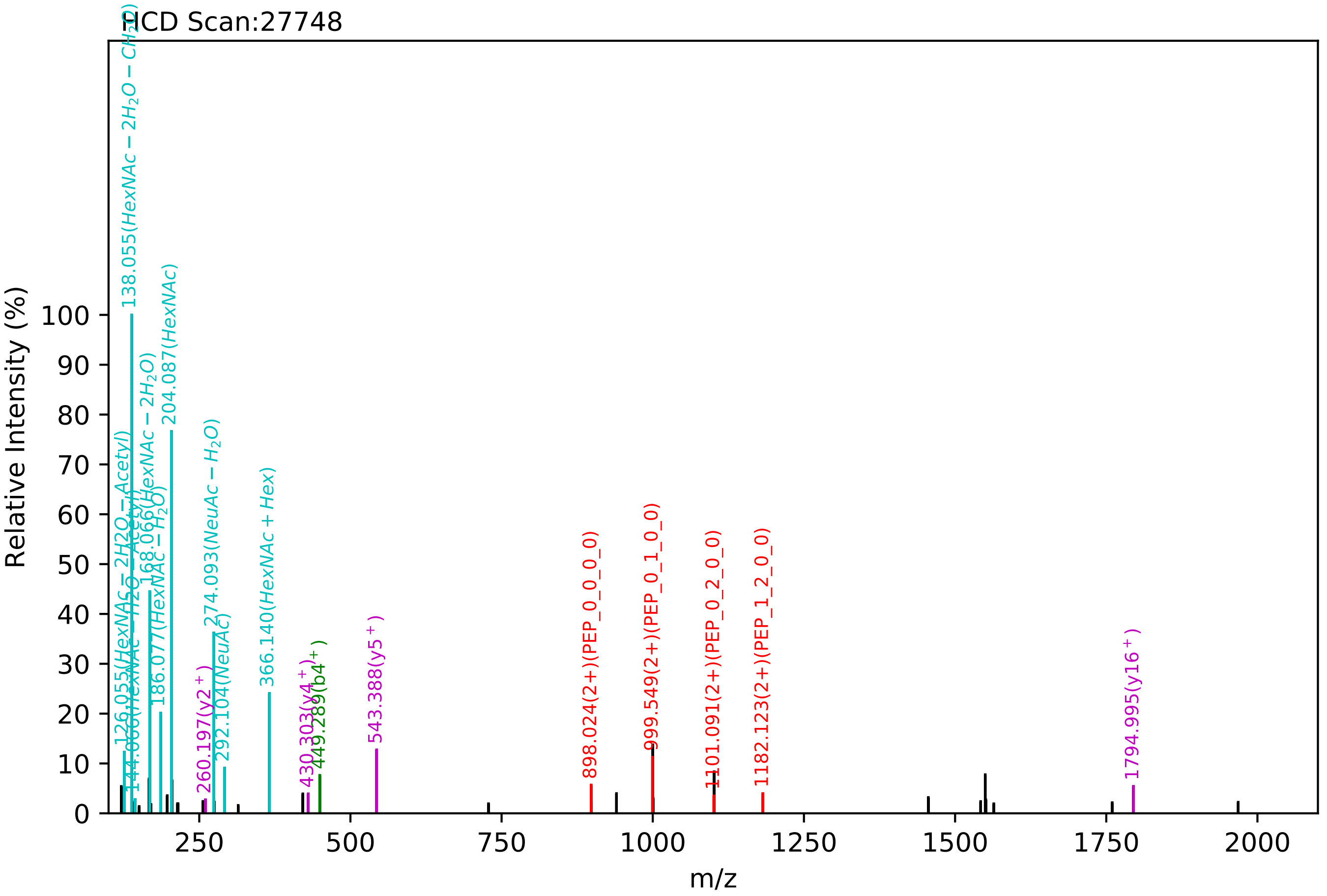

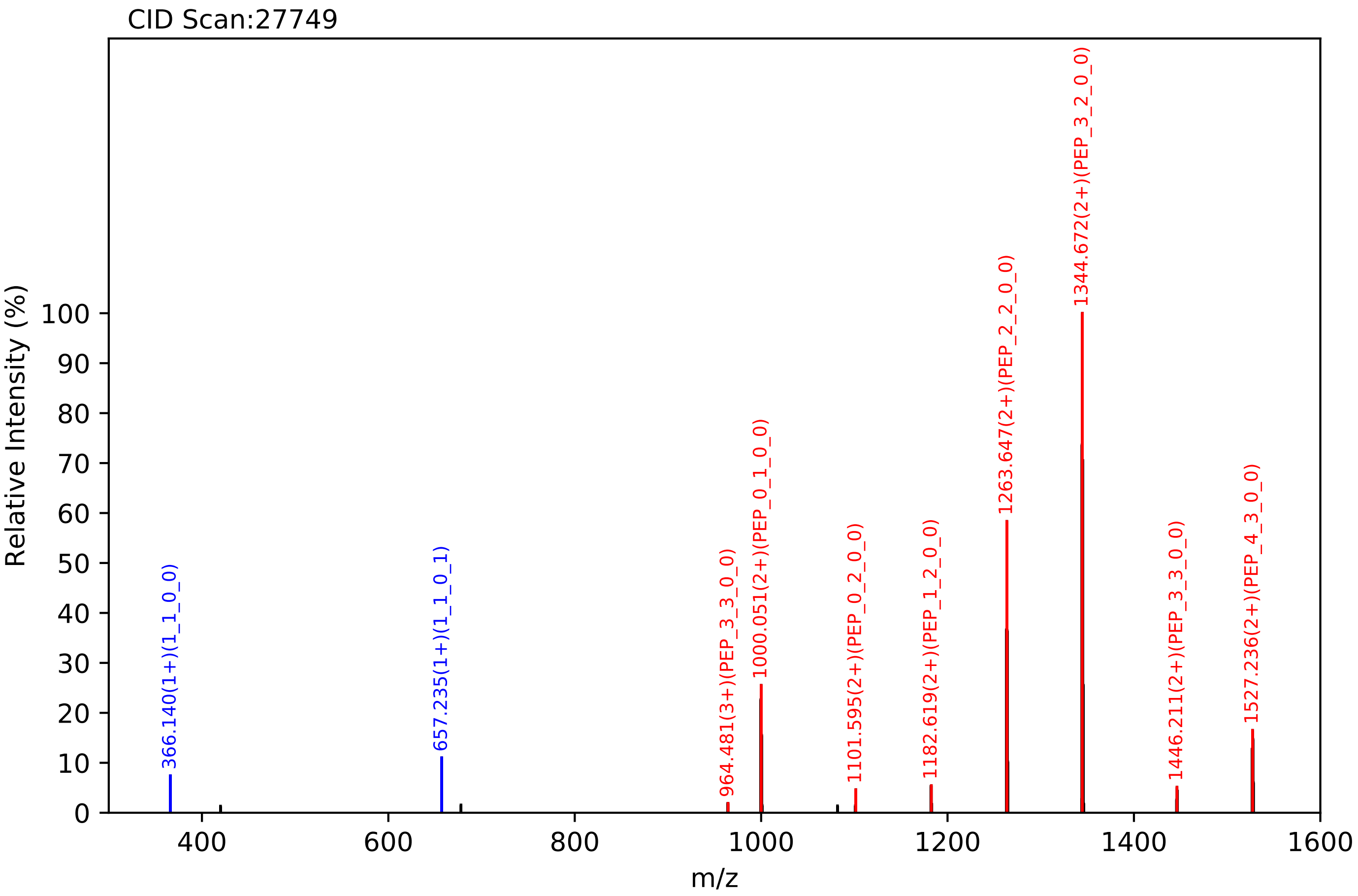

Unknown set no. 260, Experiment: Human Plasma exp_2

VVLHPNYSQVDIGLIK(=PEP)_4_3_0_1, m/z:1115.19(3+), RT:97.11, Y-score:87.97

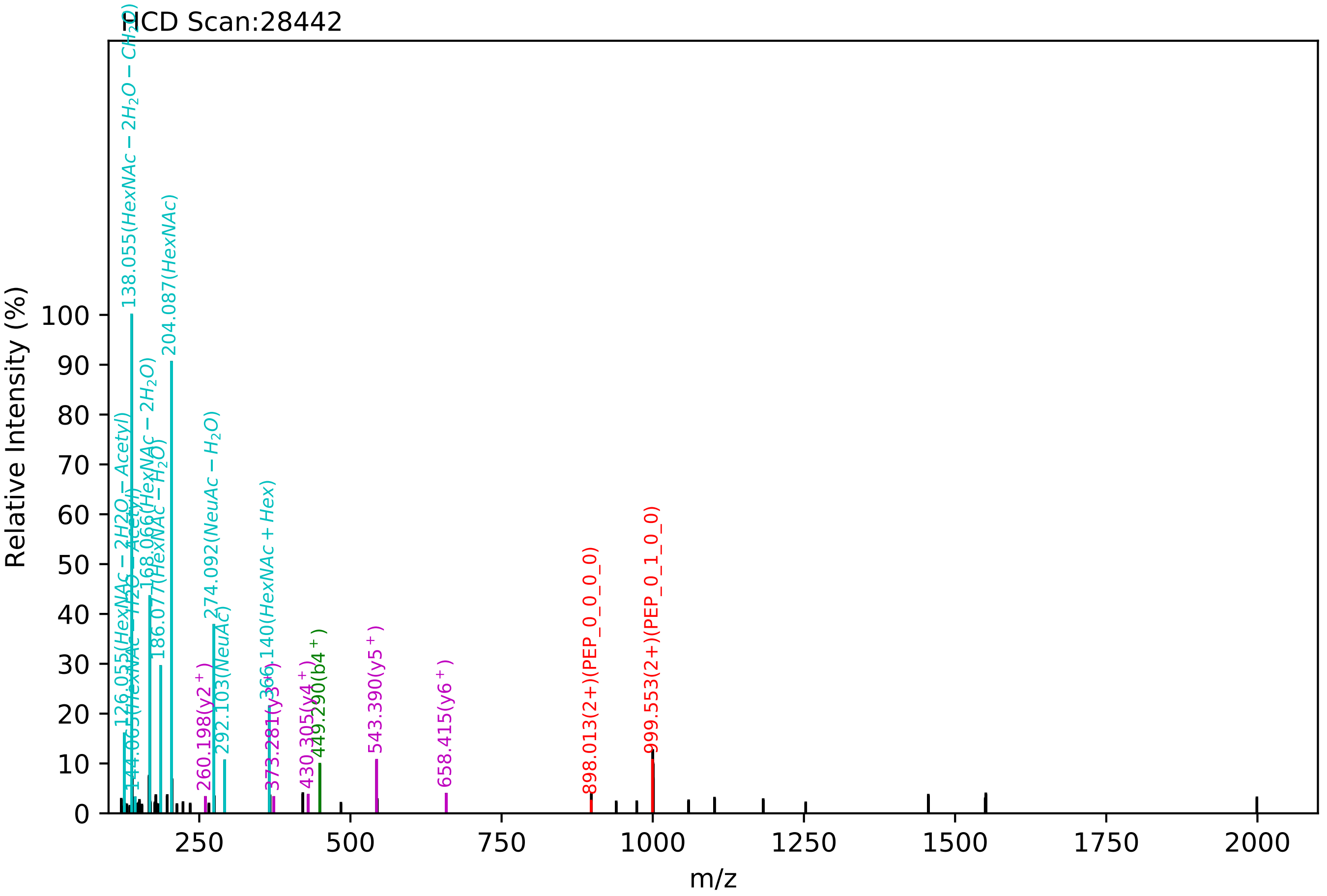

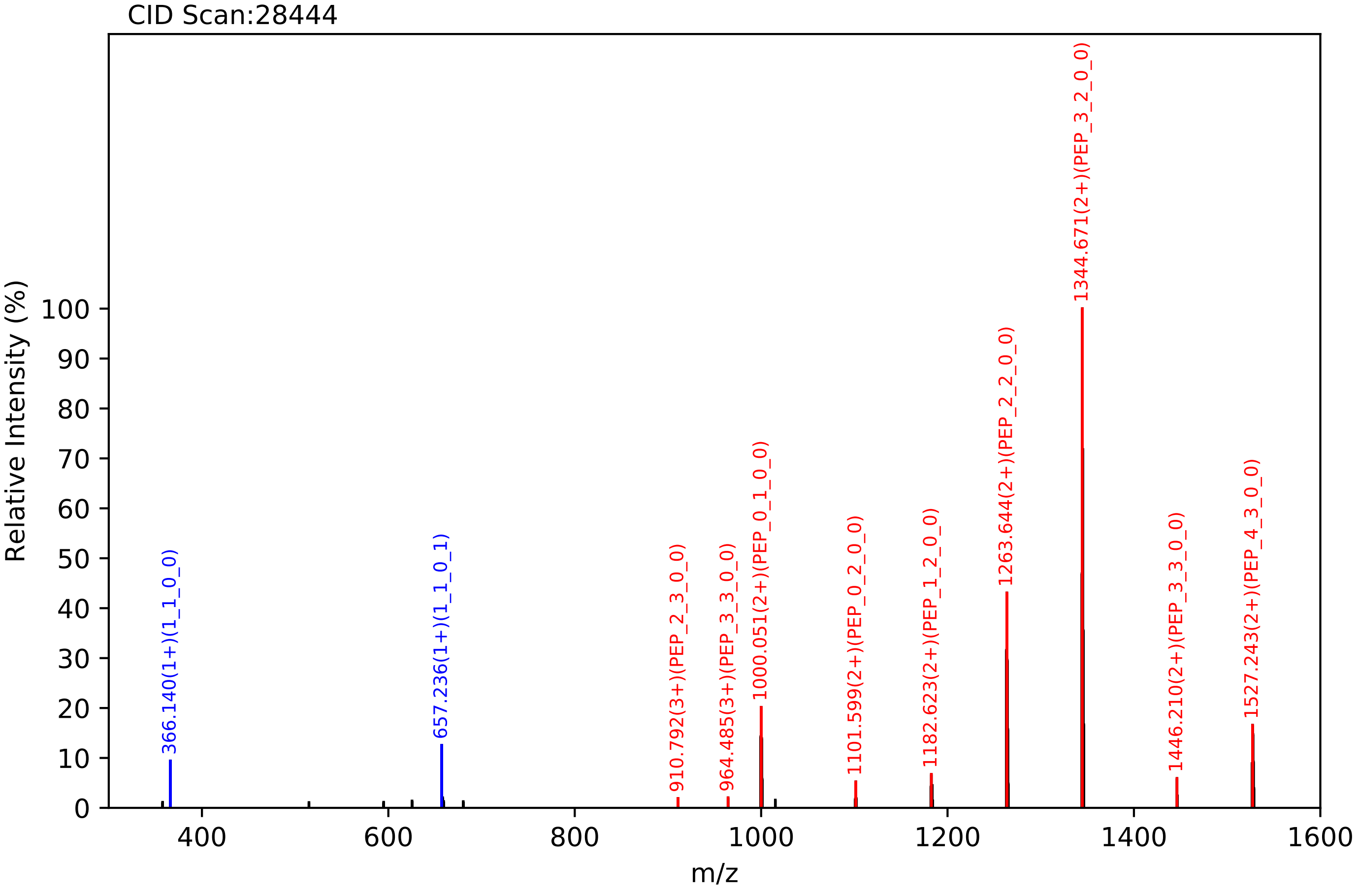

Unknown set no. 261, Experiment: Human Plasma exp_2

VVLHPNYSQVDIGLIK(=PEP)_5_4_0_1, m/z:927.93(4+), RT:88.74, Y-score:78.43

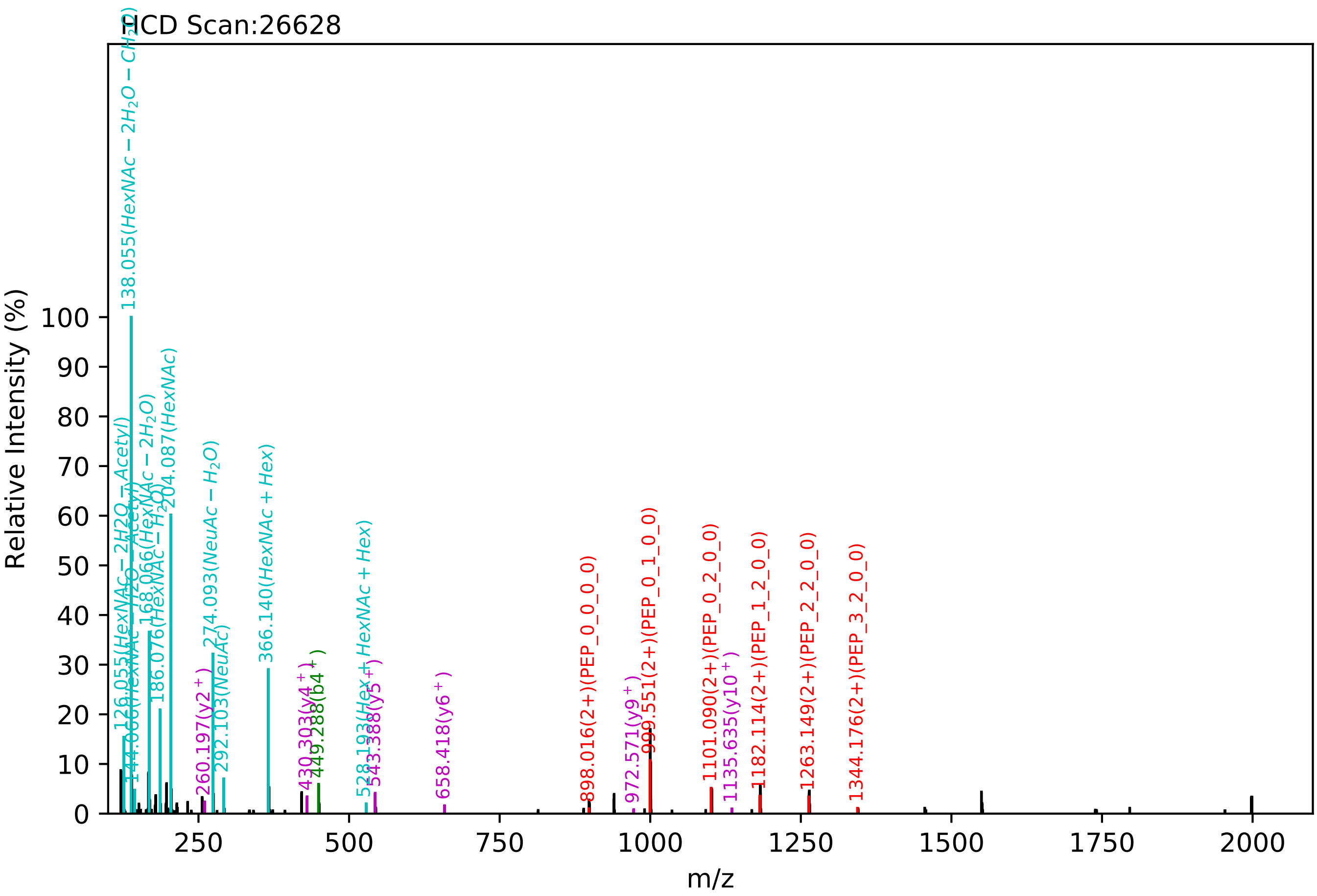

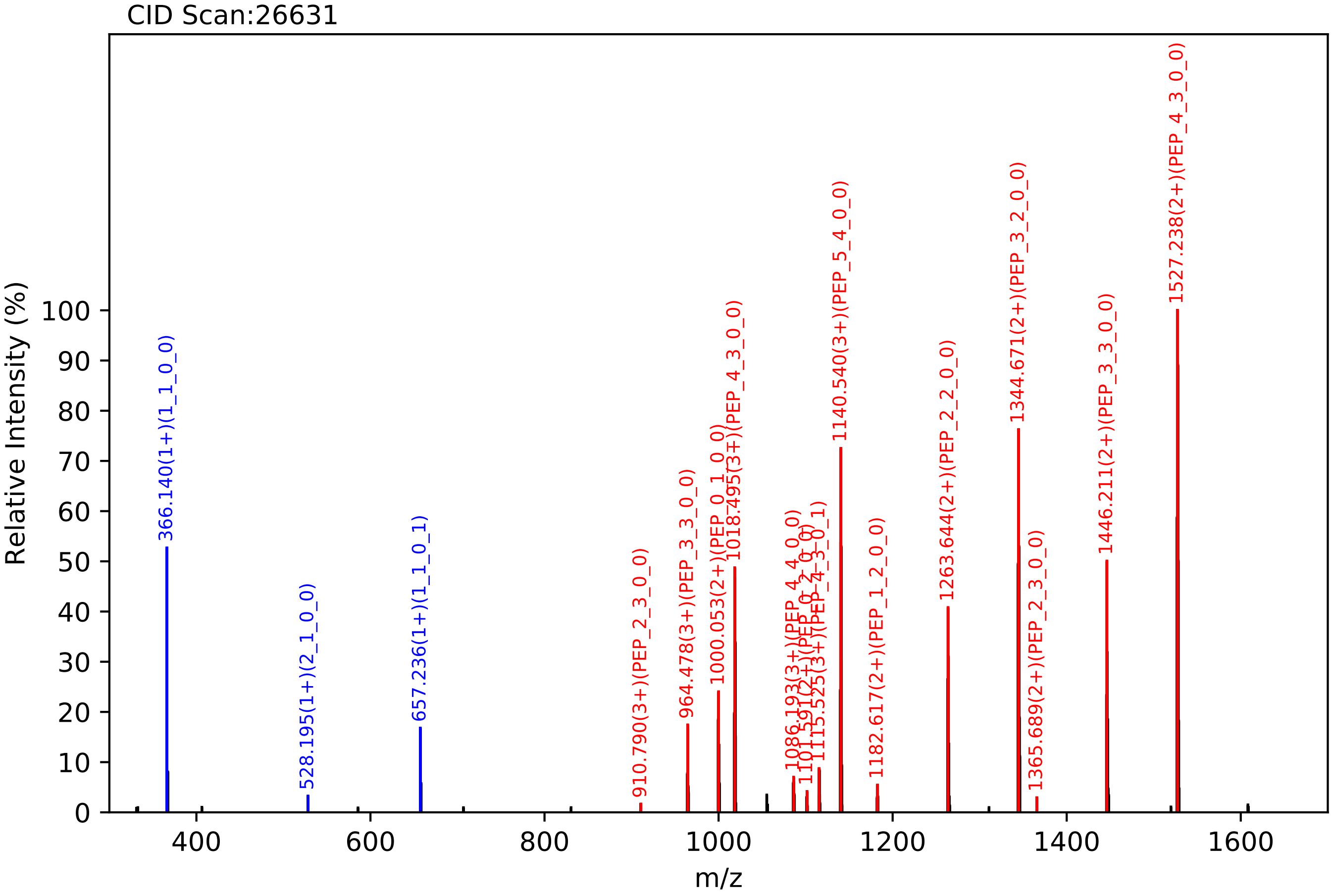

Unknown set no. 262, Experiment: Human Plasma exp_4

VVLHPNYSQVDIGLIK(=PEP)_5_4_0_1, m/z:927.93(4+), RT:88.60, Y-score:80.44

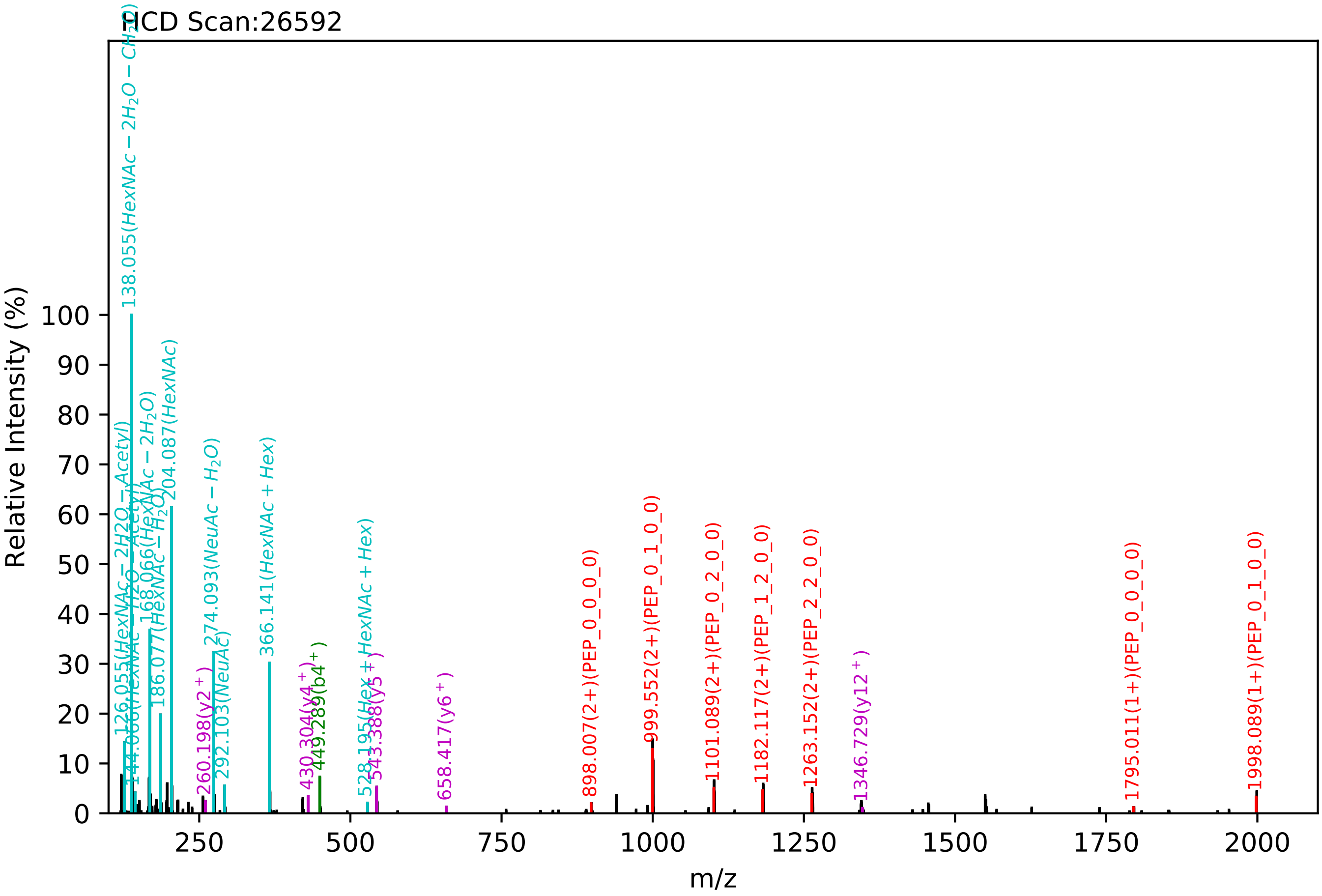

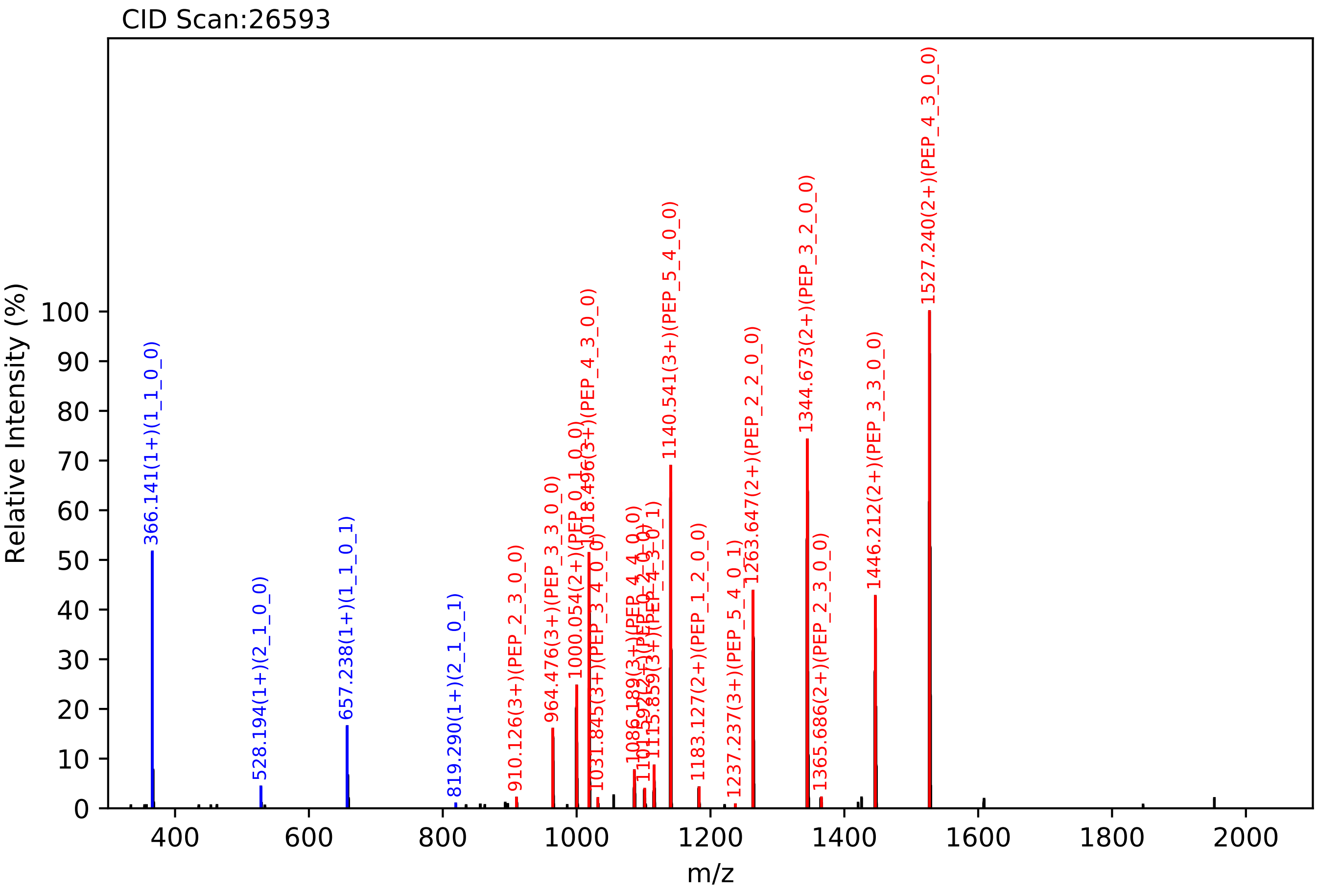

Unknown set no. 263, Experiment: Human Plasma exp_1

VVLHPNYSQVDIGLIK(=PEP)_5_4_0_2, m/z:800.76(5+), RT:97.17, Y-score:80.90

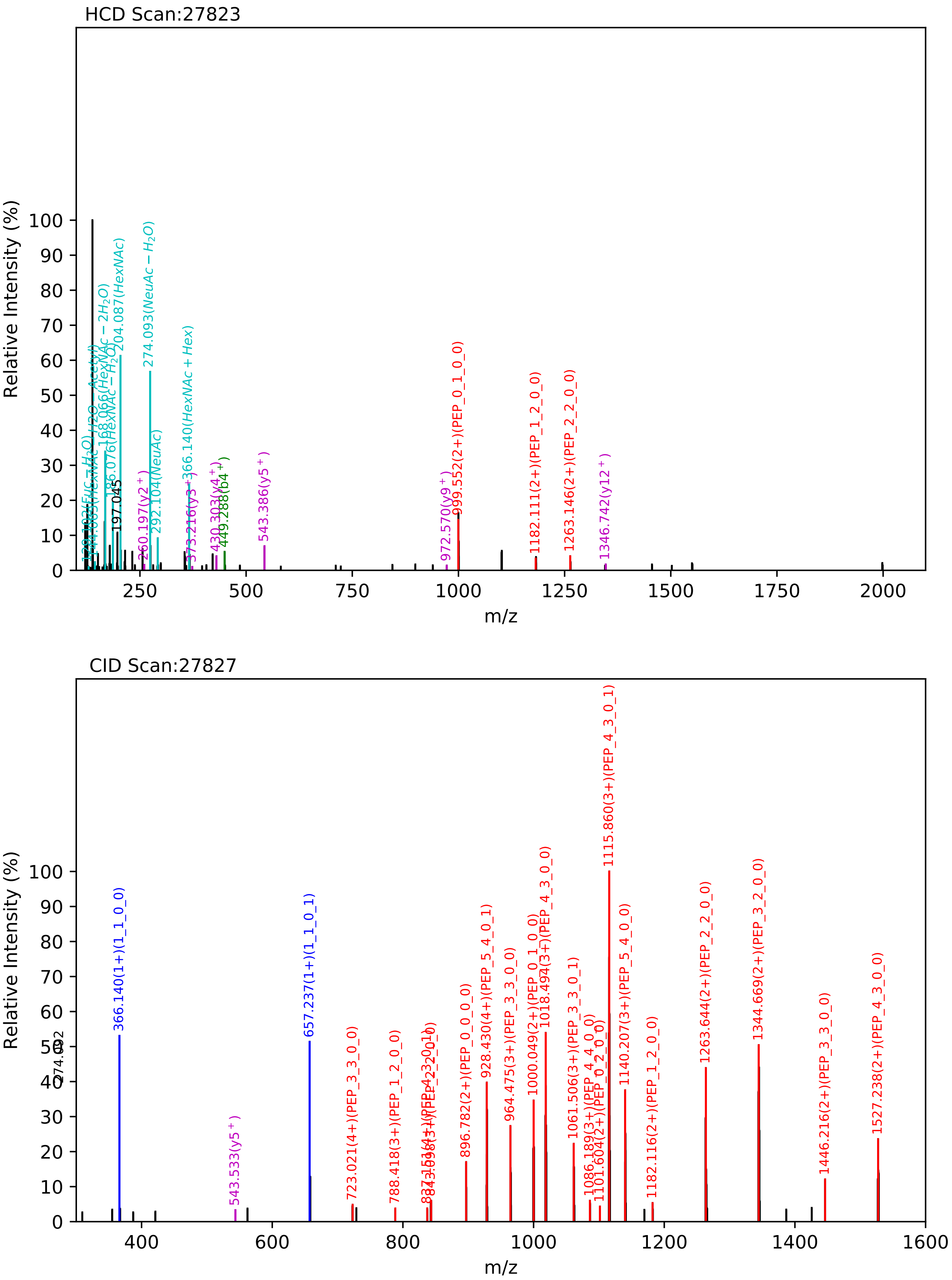

Unknown set no. 264, Experiment: Human Plasma exp_1

VVLHPNYSQVDIGLIK(=PEP)_5_4_0_2, m/z:1000.70(4+), RT:97.53, Y-score:78.97

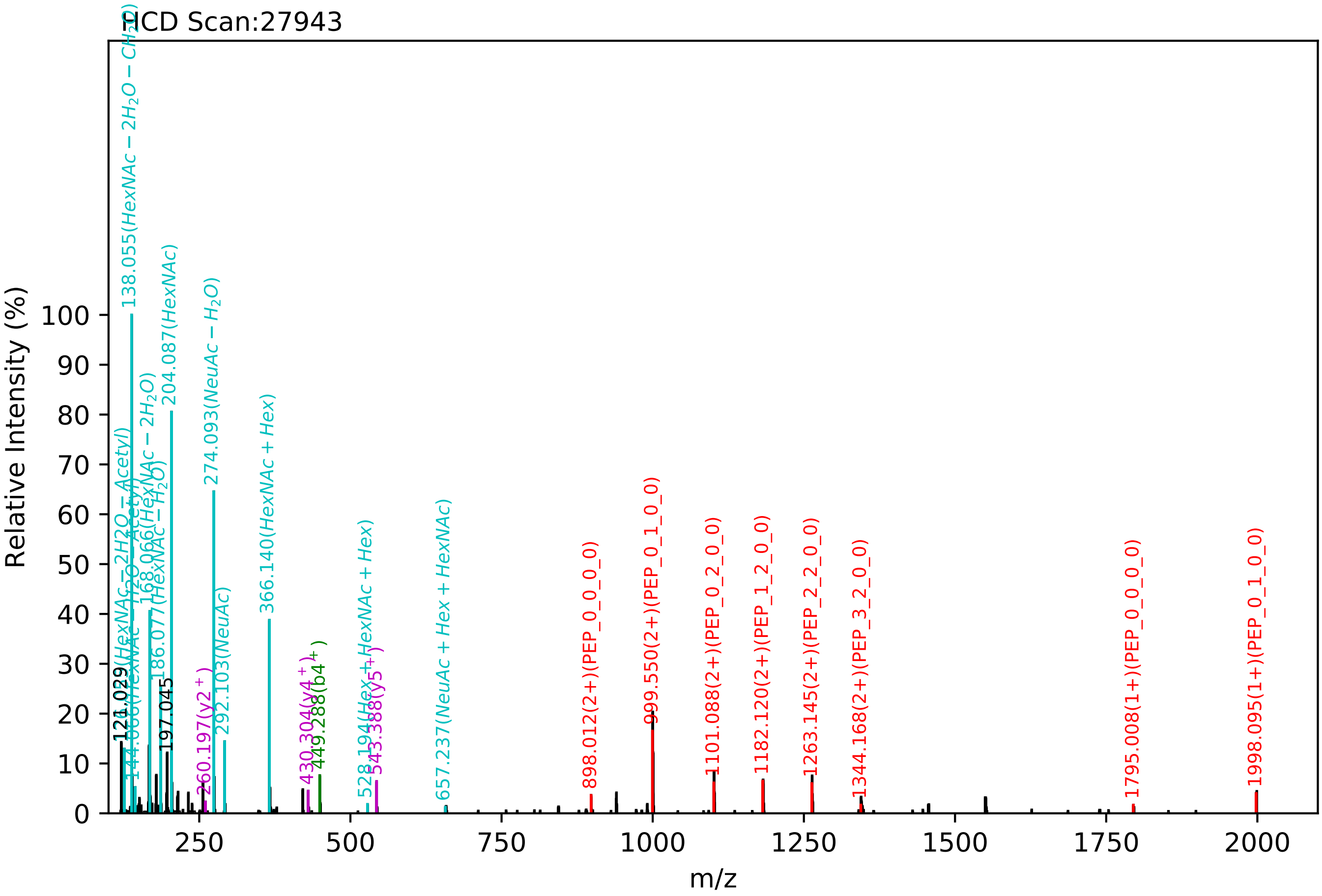

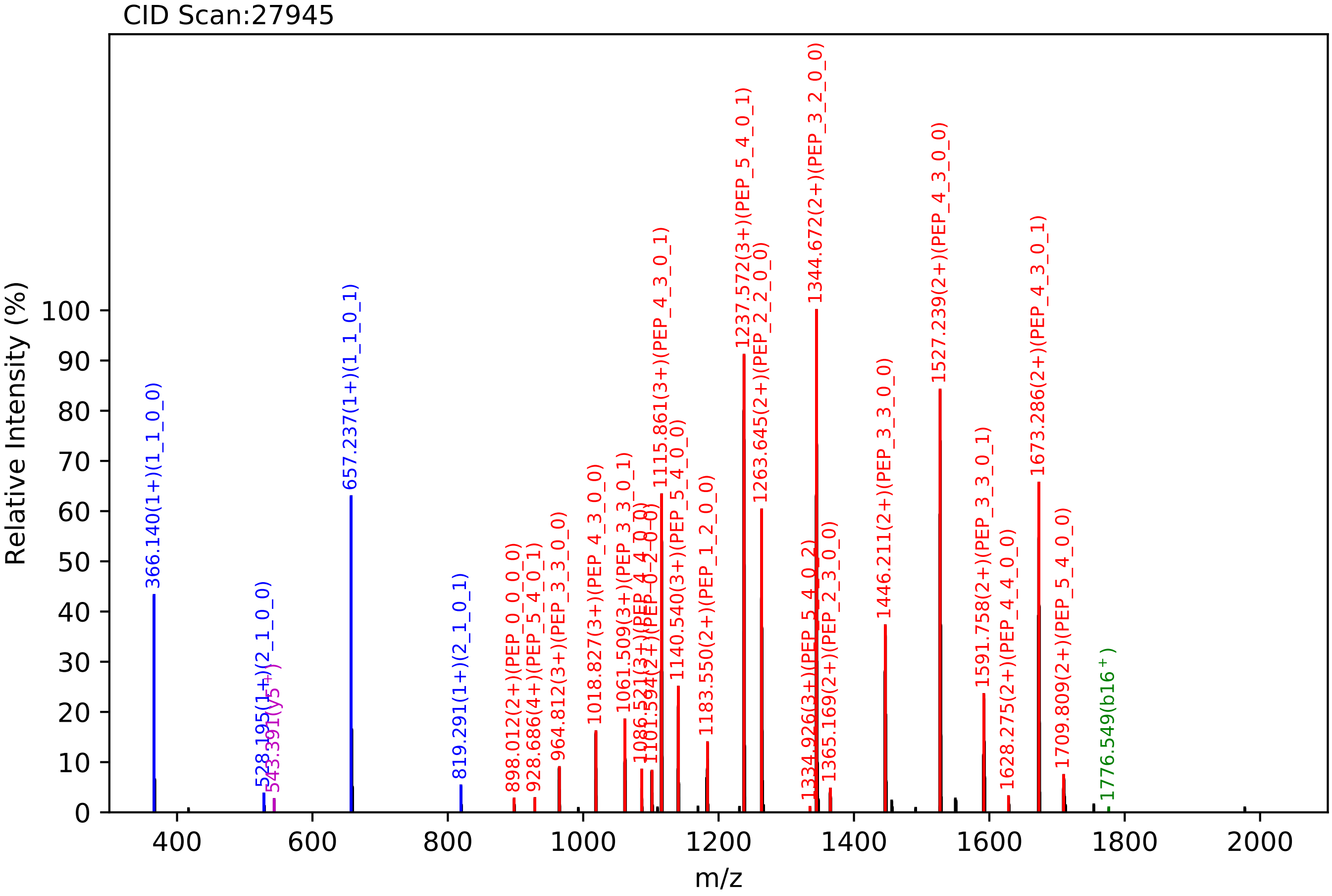

Unknown set no. 265, Experiment: Human Plasma exp_2

VVLHPNYSQVDIGLIK(=PEP)_5_4_0_2, m/z:1000.70(4+), RT:97.82, Y-score:78.56

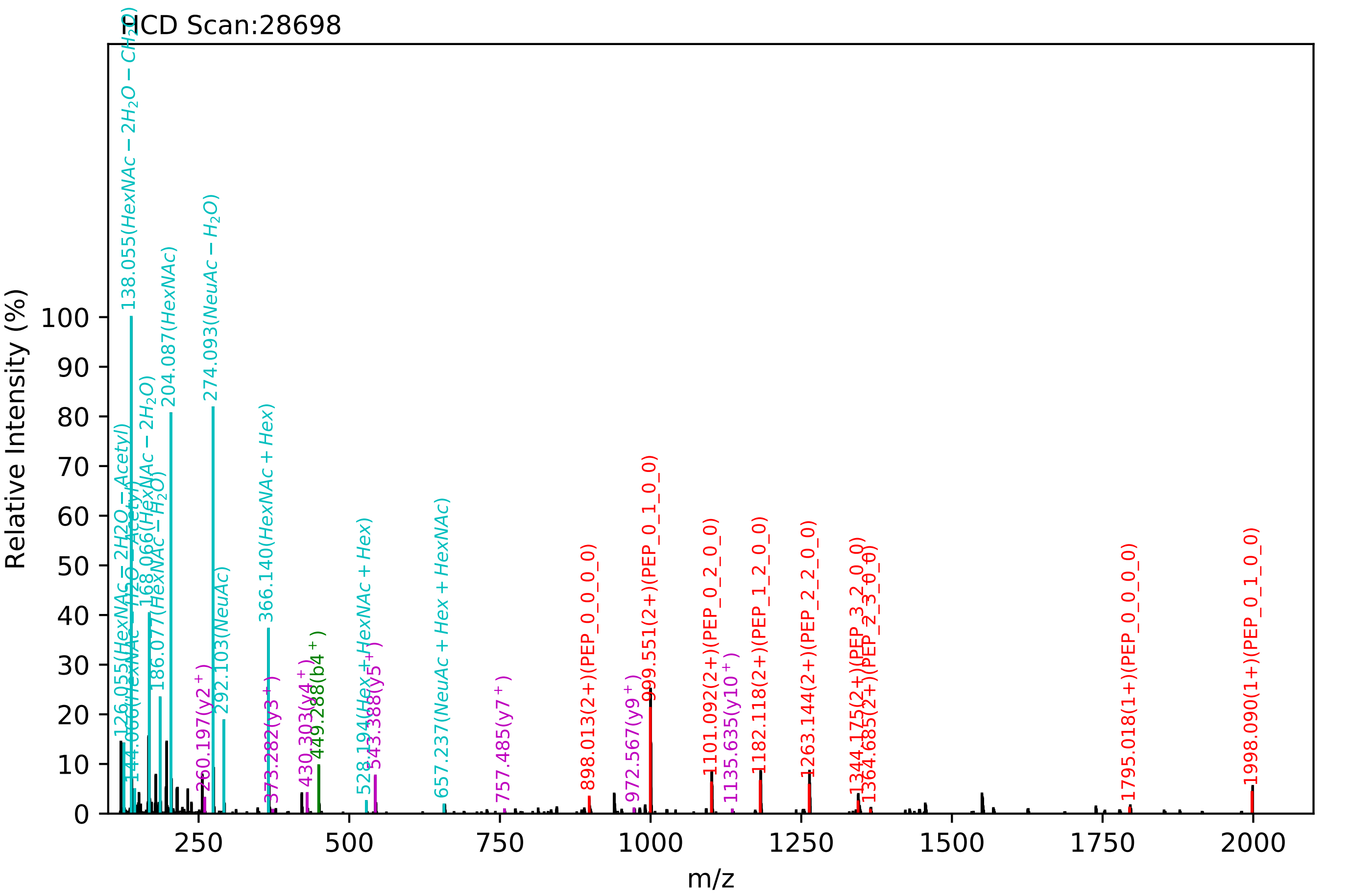

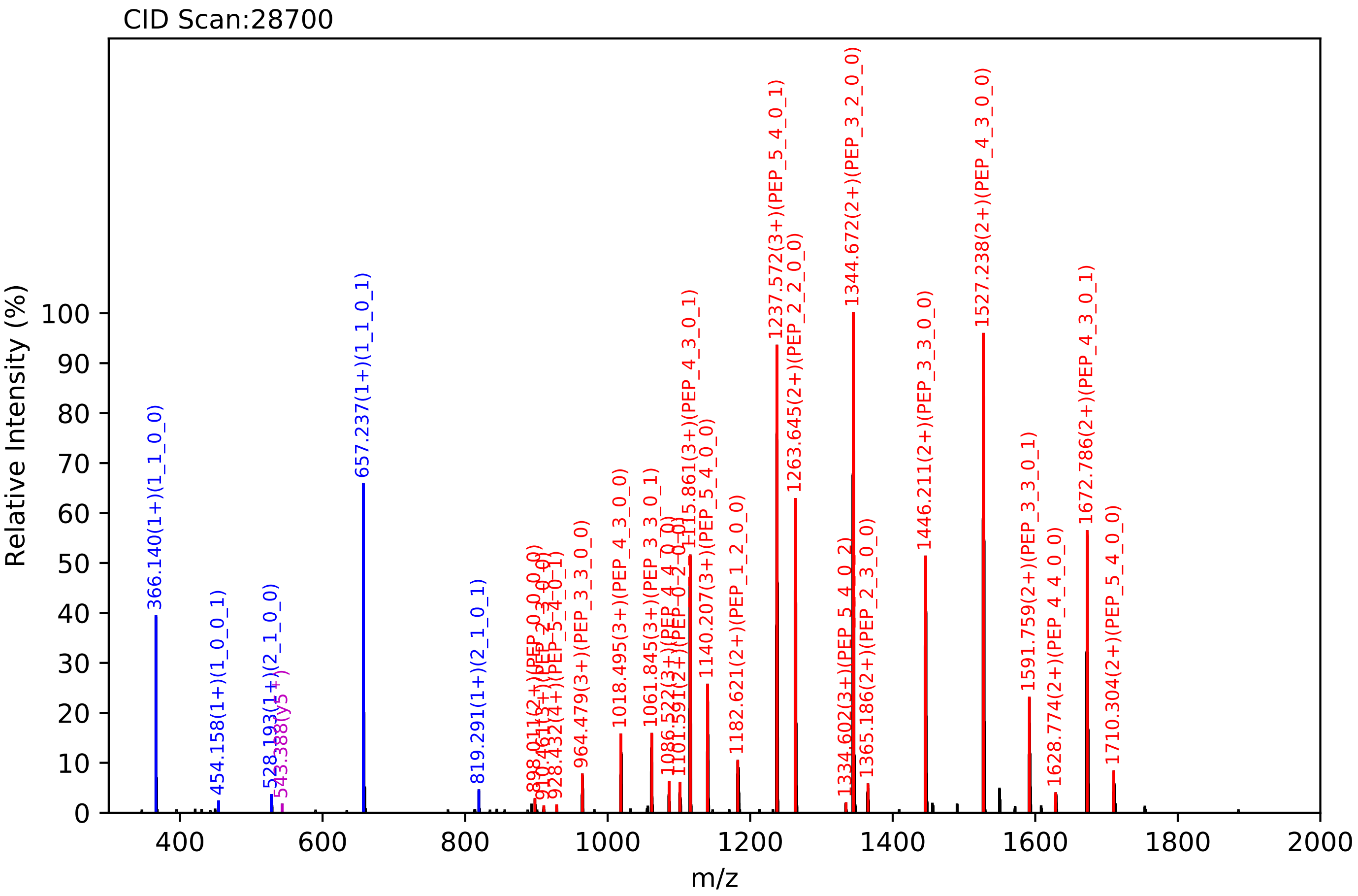

Unknown set no. 266, Experiment: Human Plasma exp_4

VVLHPNYSQVDIGLIK(=PEP)_5_4_0_2, m/z:800.76(5+), RT:97.26, Y-score:71.66

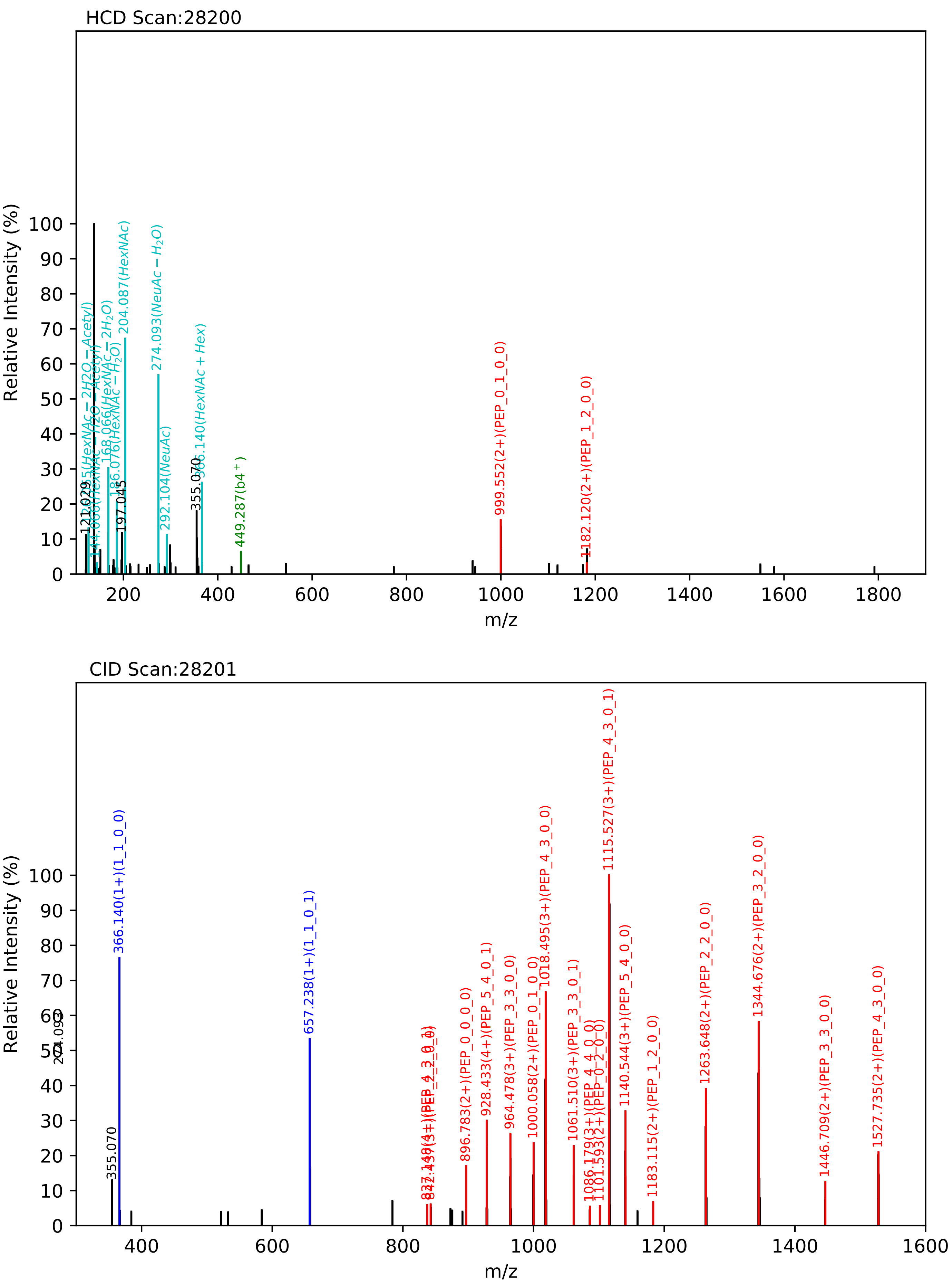

Unknown set no. 267, Experiment: Human Plasma exp_4

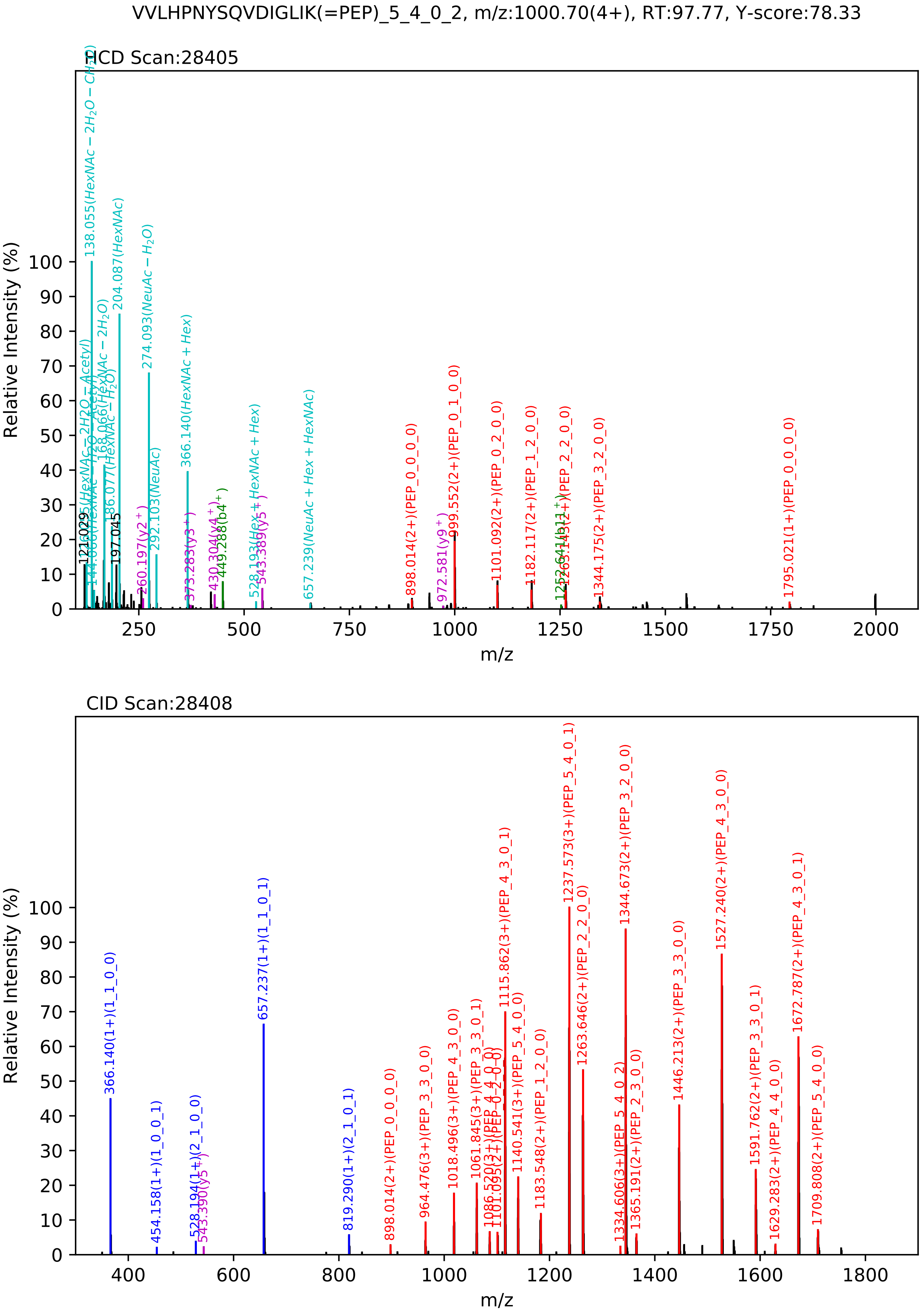

Unknown set no. 268, Experiment: Human Plasma exp_5

VVLHPNYSQVDIGLIK(=PEP)_5_4_0_2, m/z:1000.70(4+), RT:97.41, Y-score:78.54

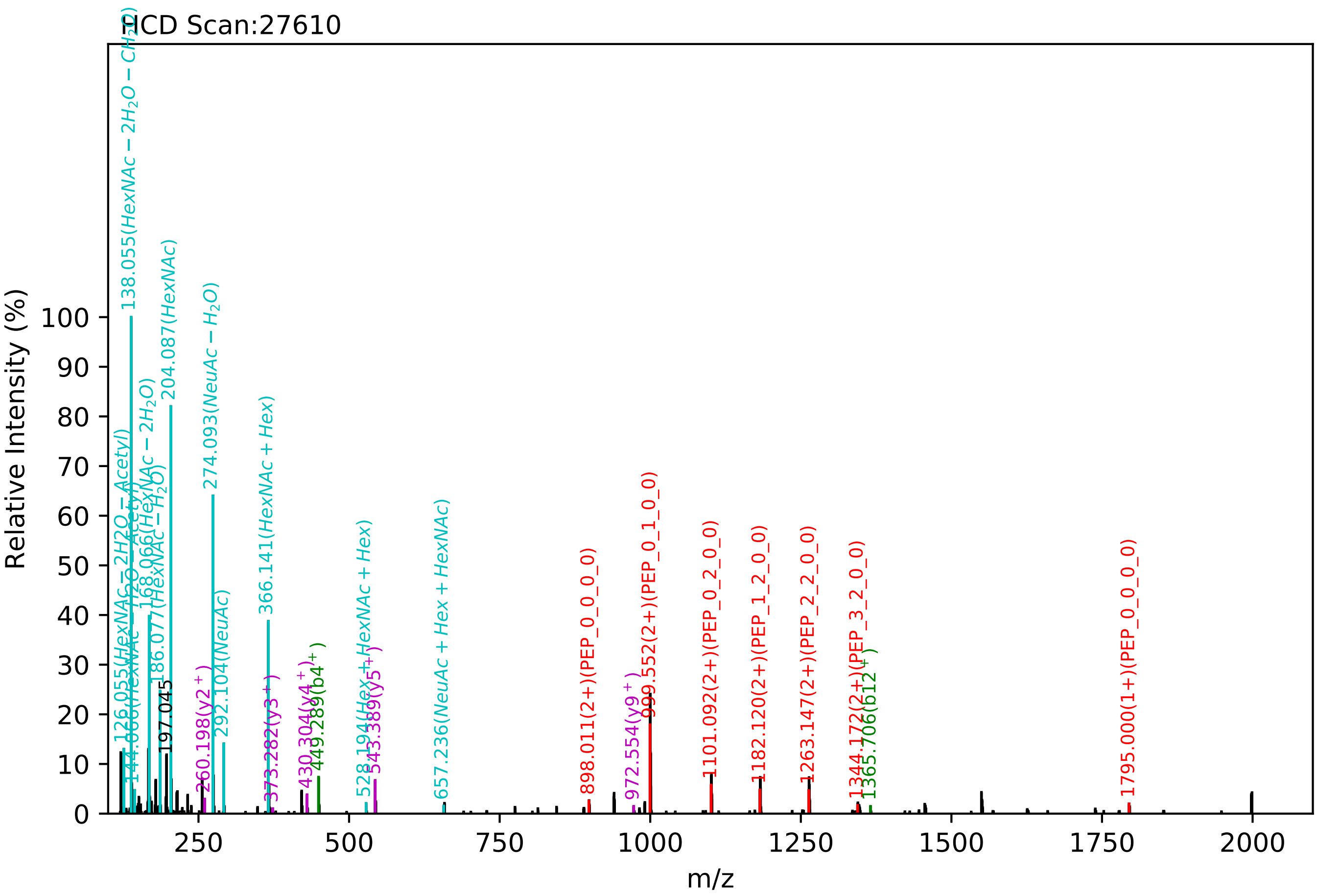

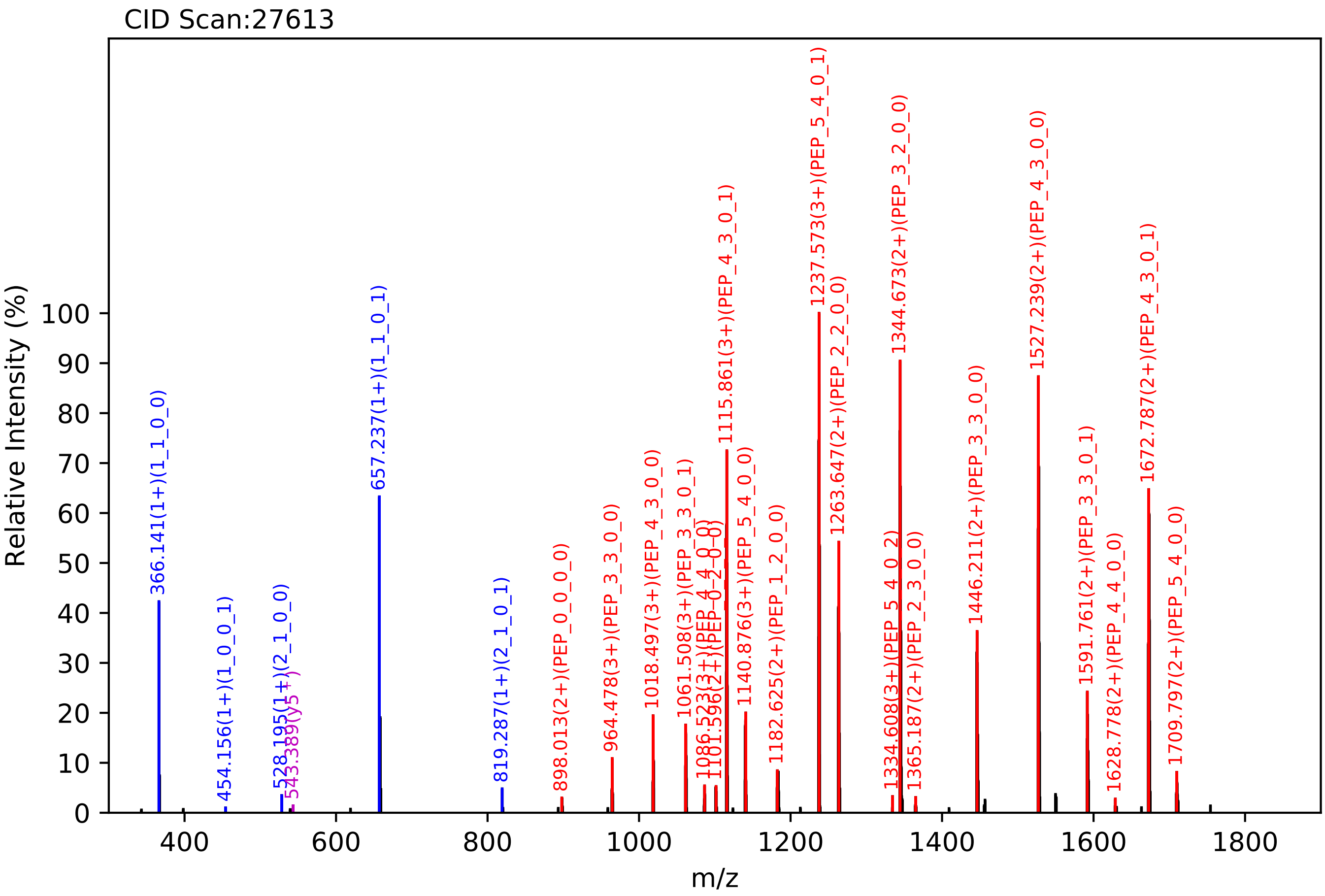

Unknown set no. 269, Experiment: Human Plasma exp_4

VVLHPNYSQVDIGLIK(=PEP)_6_4_0_1, m/z:968.44(4+), RT:88.54, Y-score:77.45

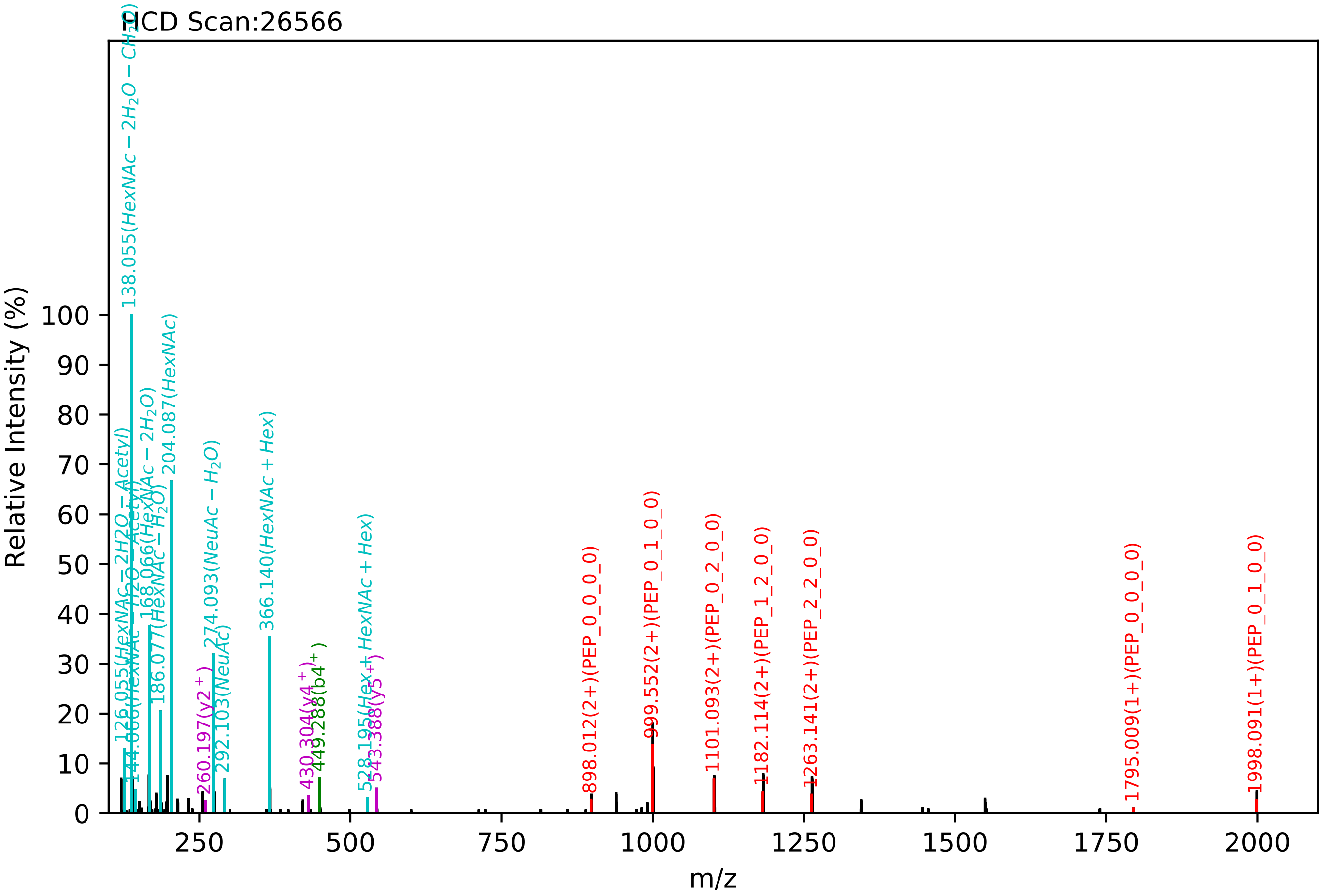

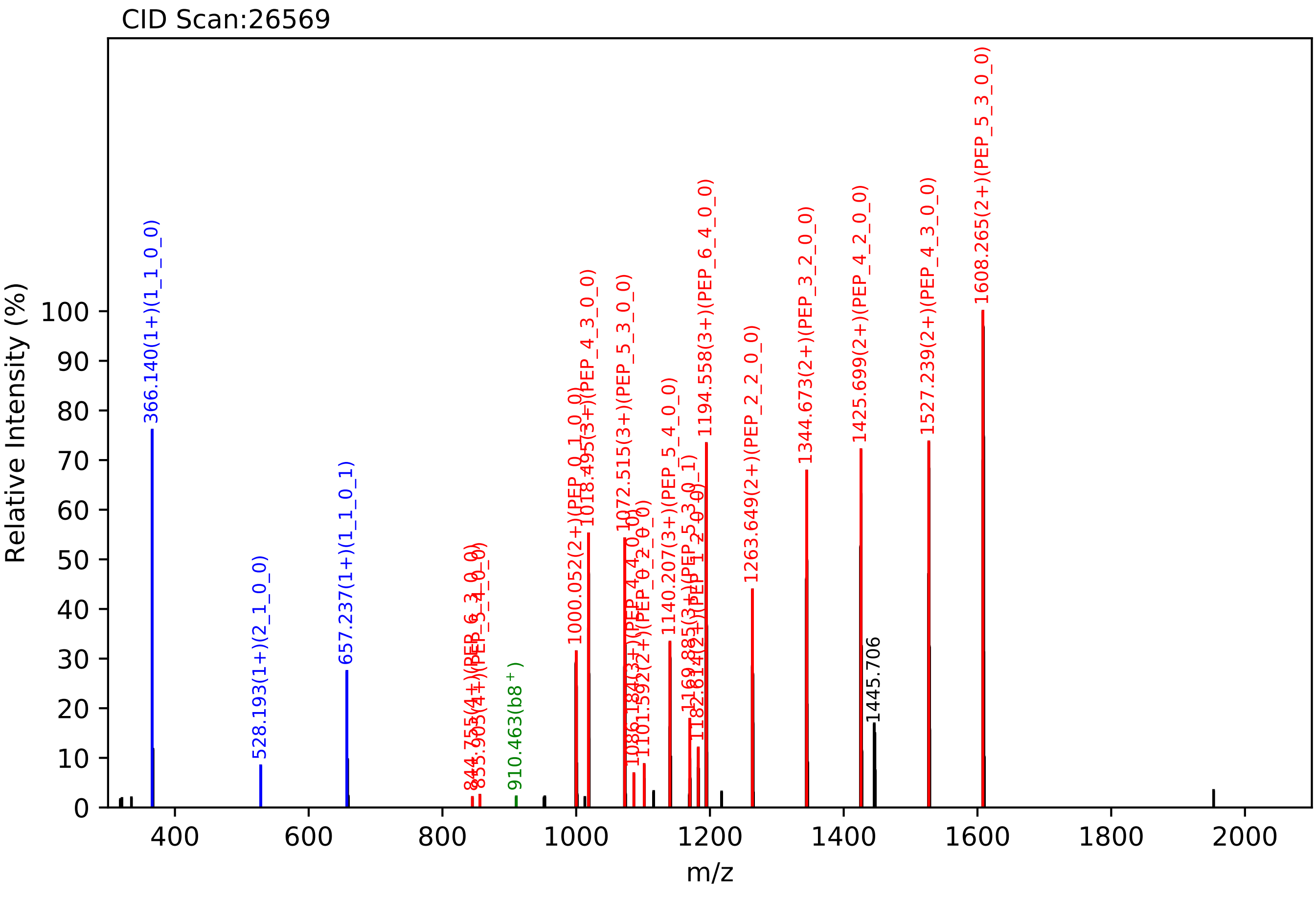

Unknown set no. 270, Experiment: Human Plasma exp_1

VVLHPNYSQVDIGLIK(=PEP)_6_5_0_2, m/z:1091.99(4+), RT:96.55, Y-score:77.70

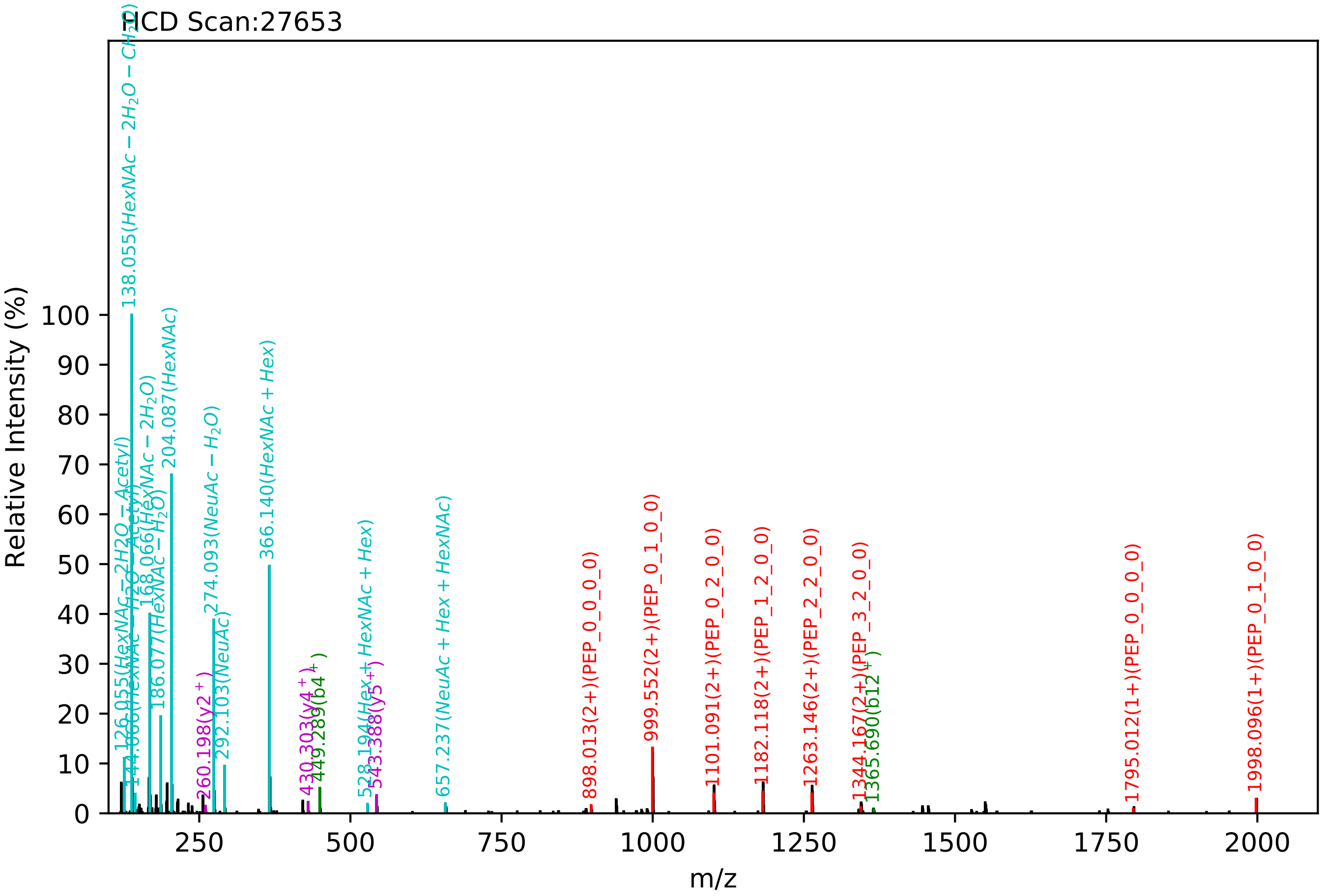

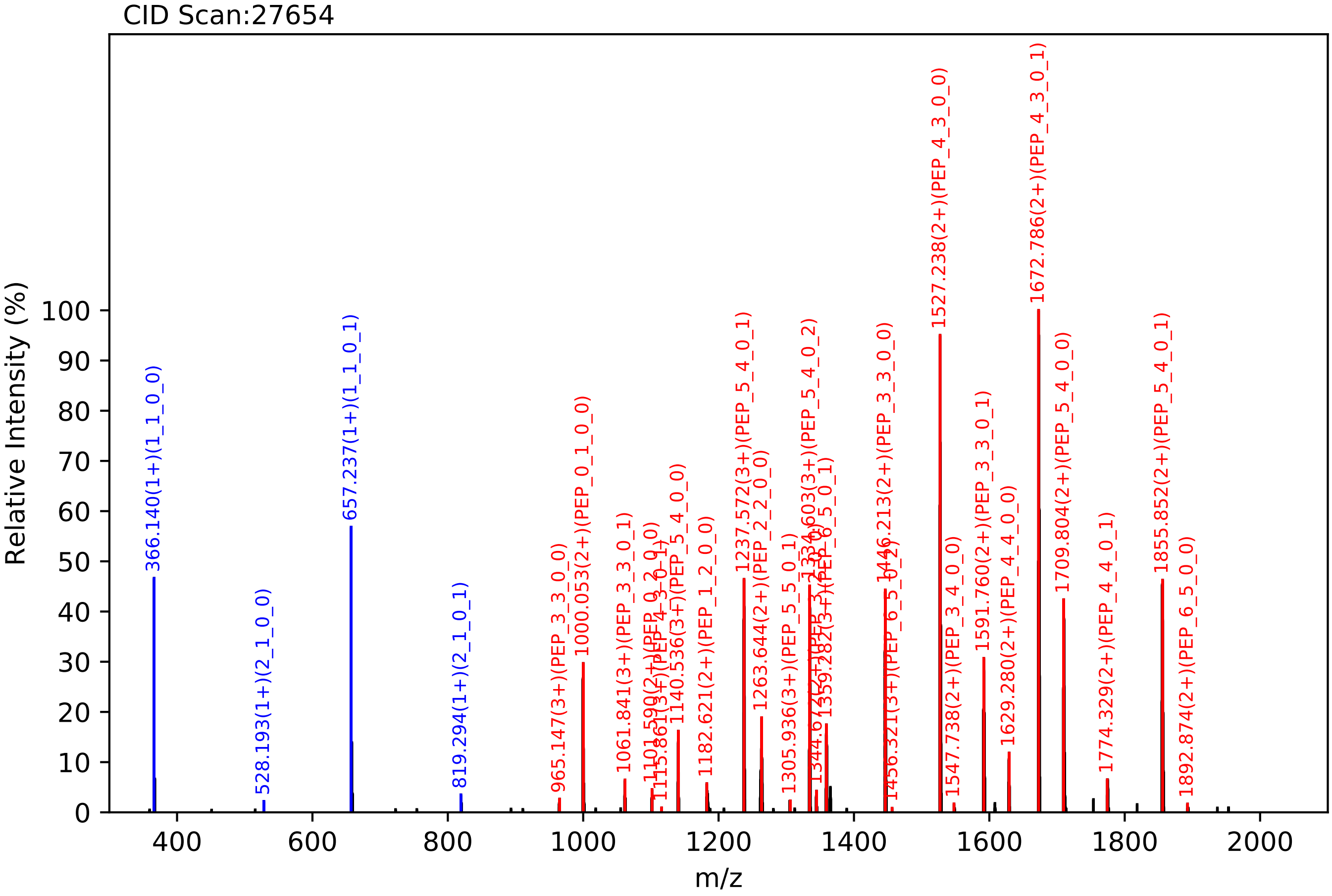

Unknown set no. 271, Experiment: Human Plasma exp_4

VVLHPNYSQVDIGLIK(=PEP)_6_5_0_3, m/z:1164.76(4+), RT:107.12, Y-score:77.51

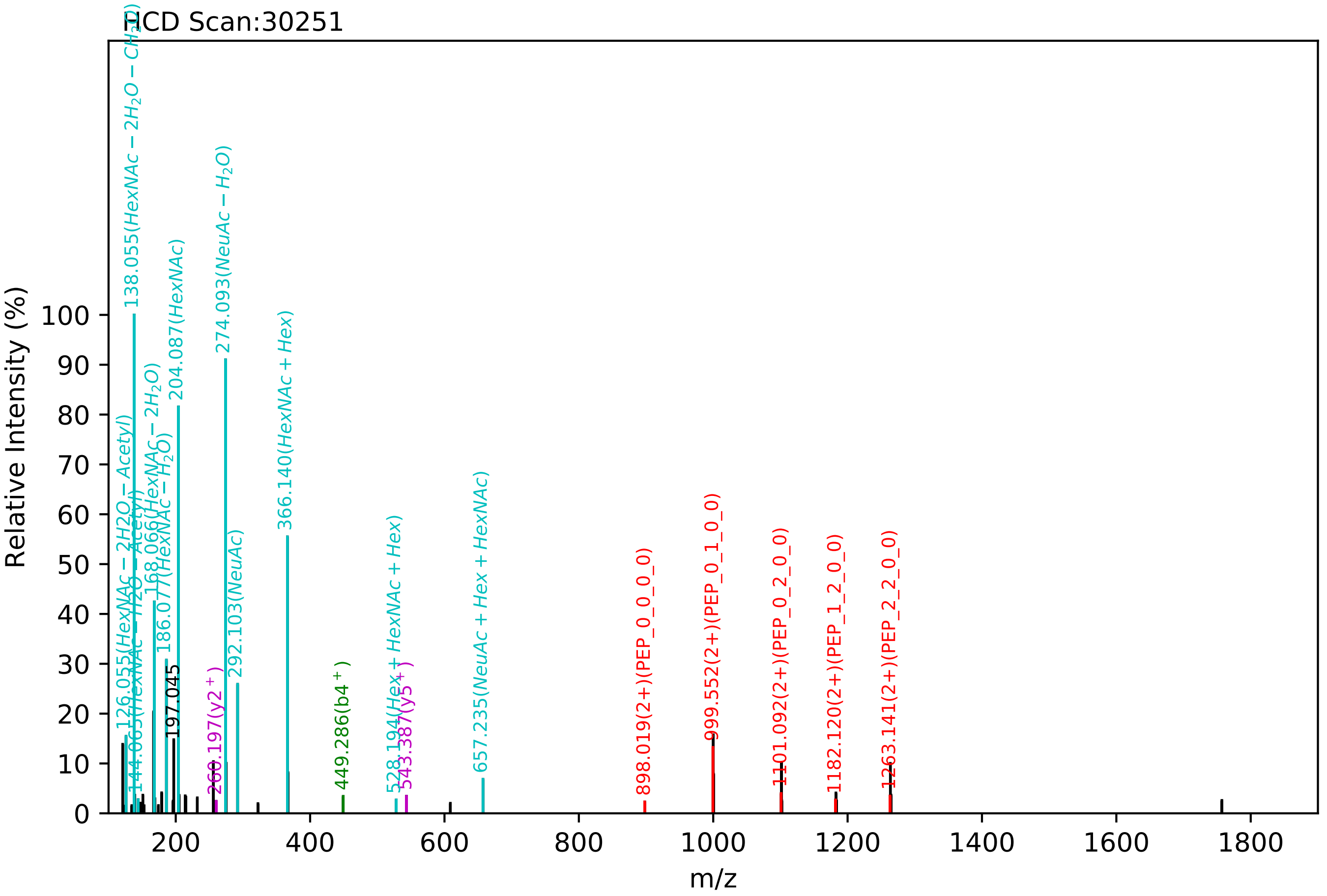

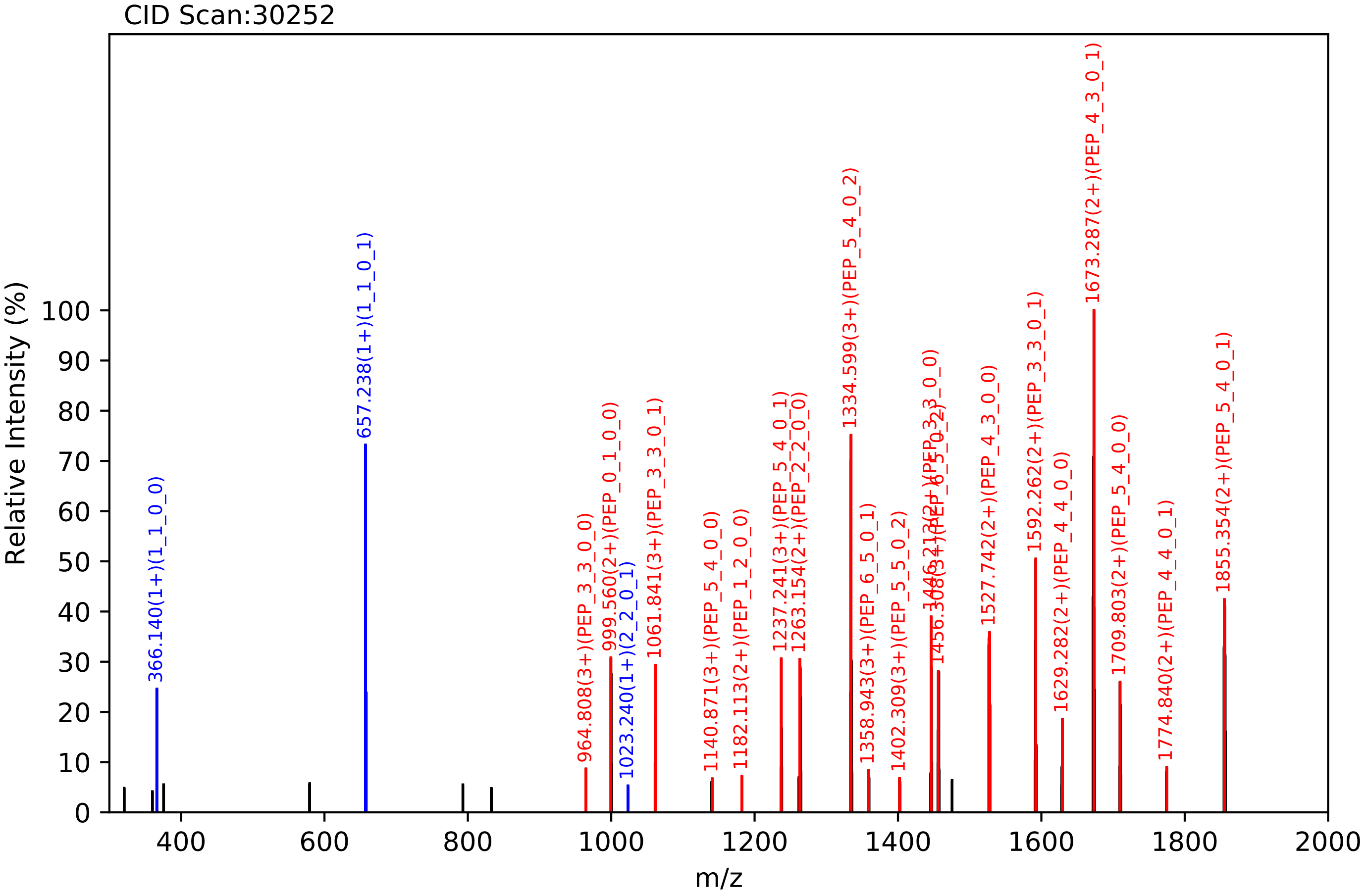

Unknown set no. 272, Experiment: Human Plasma exp_5

VVLHPNYSQVDIGLIK(=PEP)_6_5_0_3, m/z:1164.76(4+), RT:106.05, Y-score:76.84

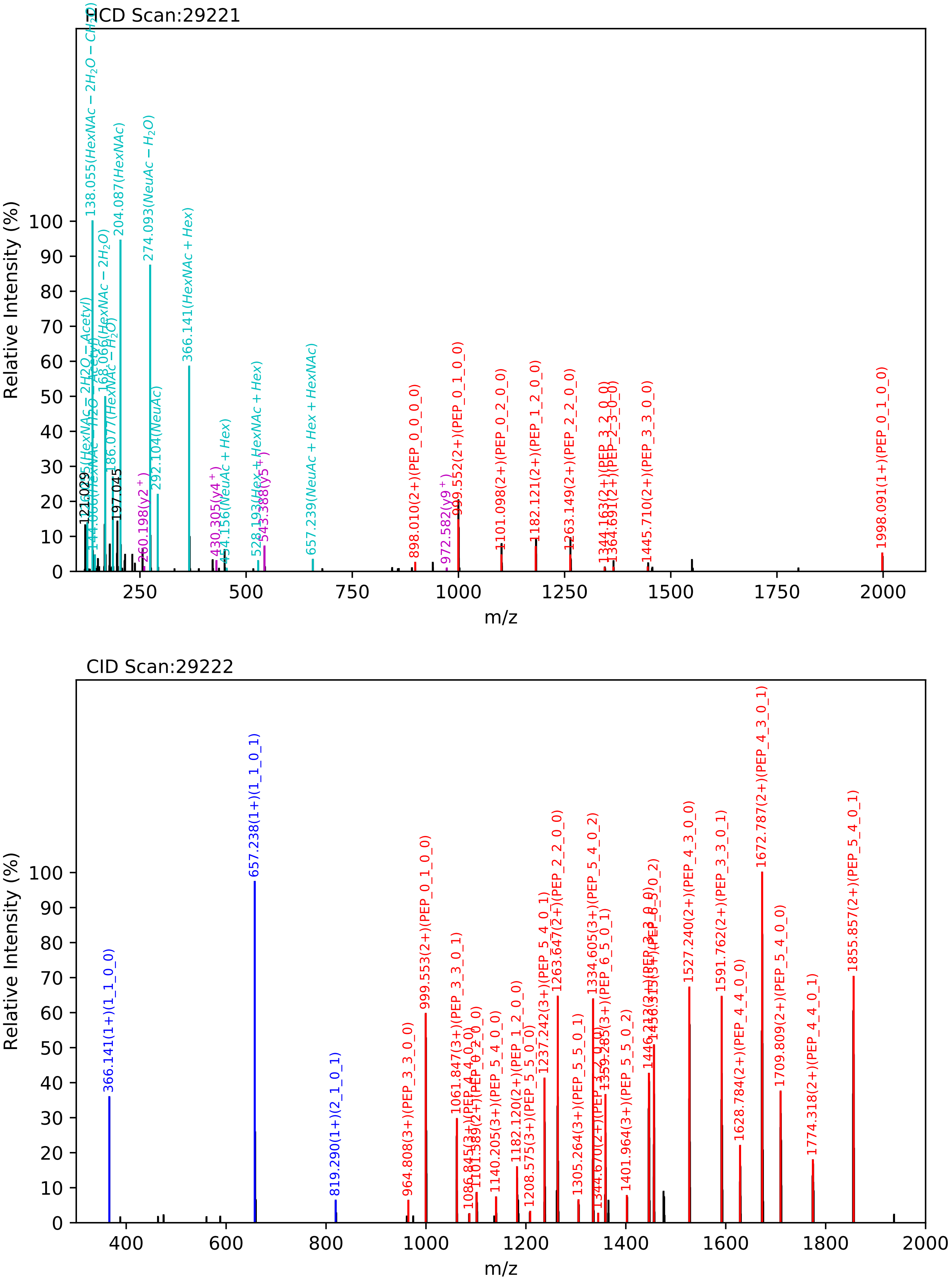

Unknown set no. 273, Experiment: Human Plasma exp_4

VVLHPNYSQVDIGLIK(=PEP)_6_5_1_3, m/z:1201.28(4+), RT:105.99, Y-score:71.80

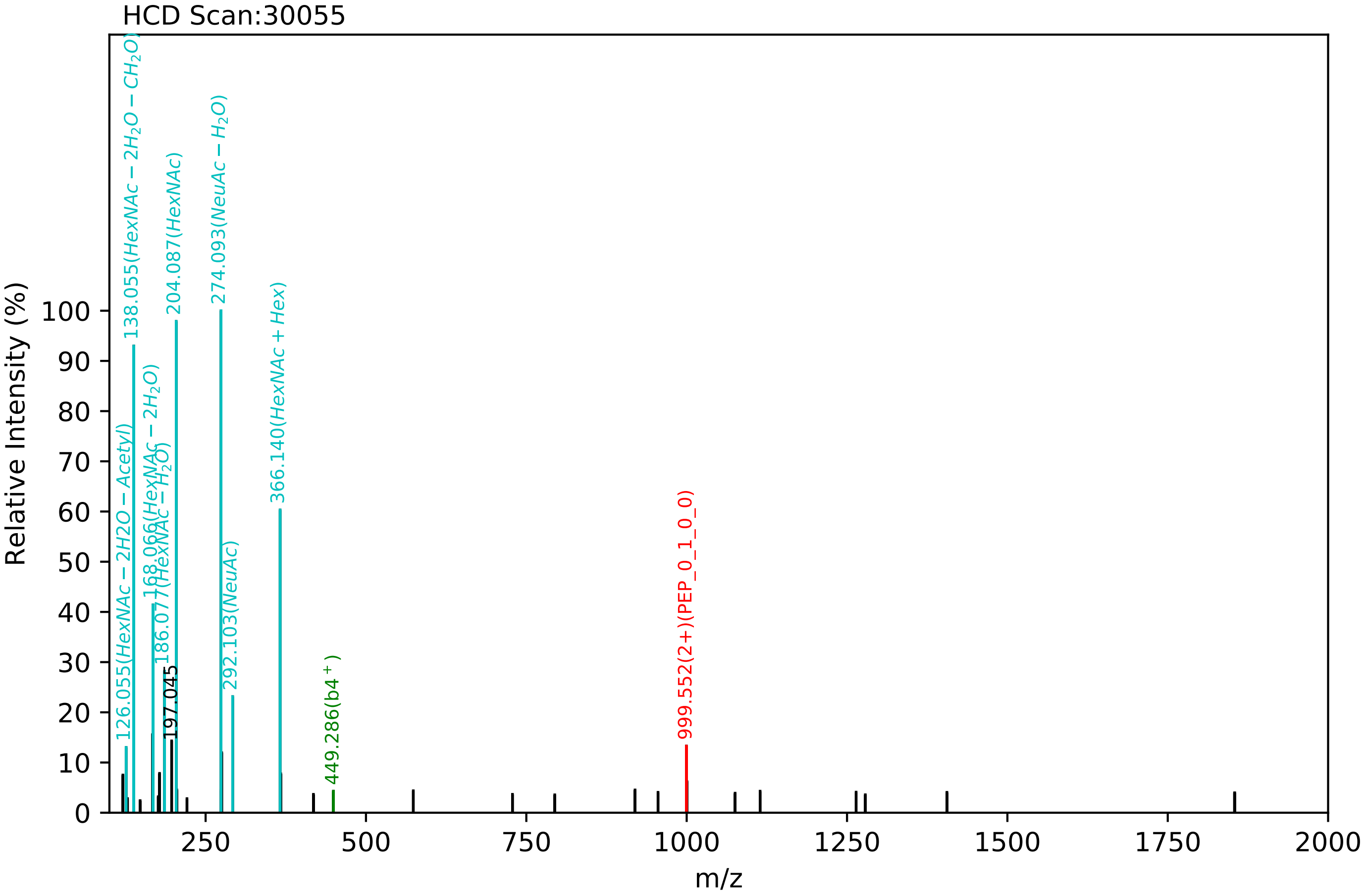

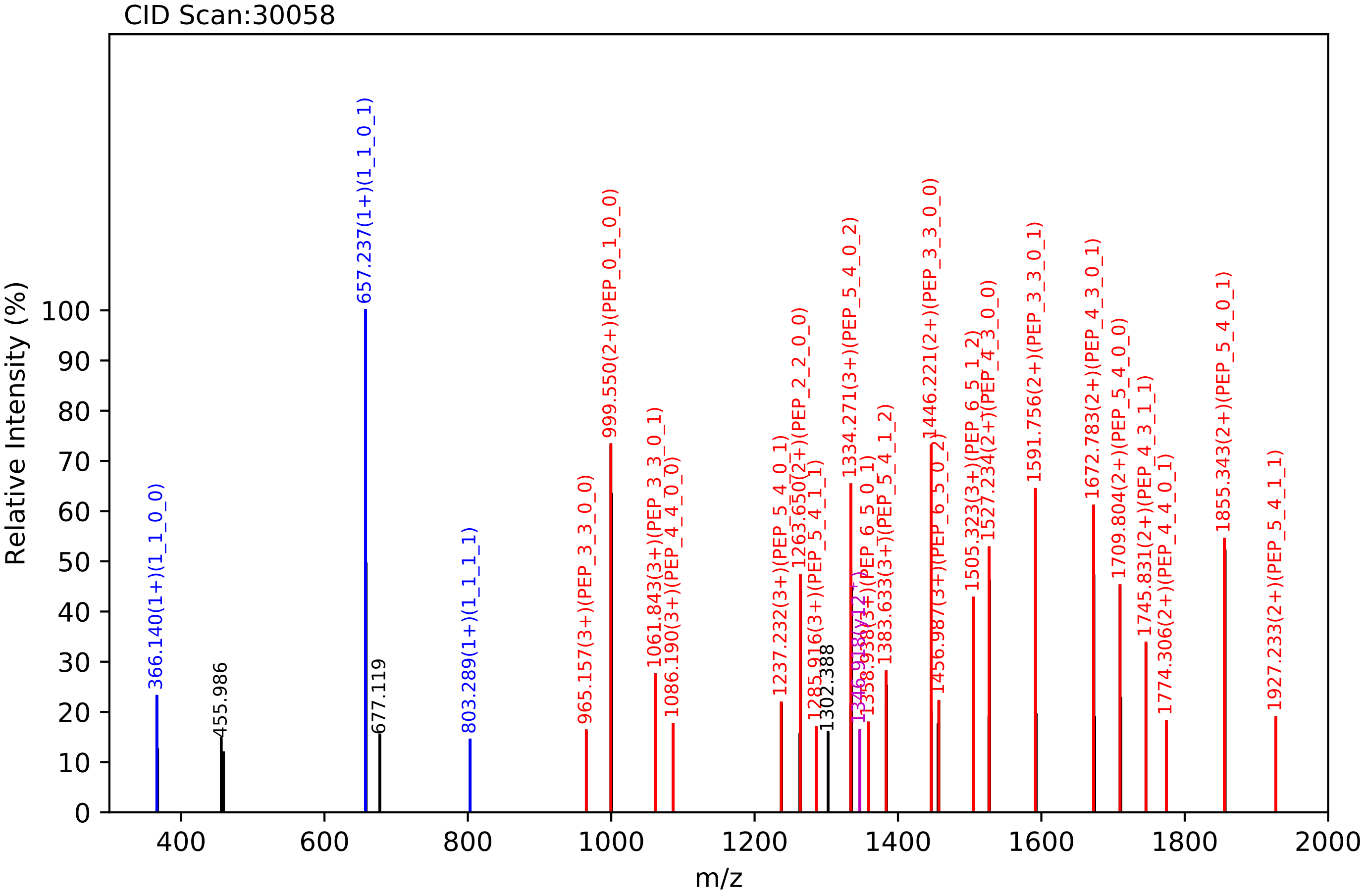

Unknown set no. 274, Experiment: Human Plasma exp_2

ALPQPQNVTSLLGCTH(=PEP)_4_3_0_1, m/z:1095.48(3+), RT:109.07, Y-score:83.11

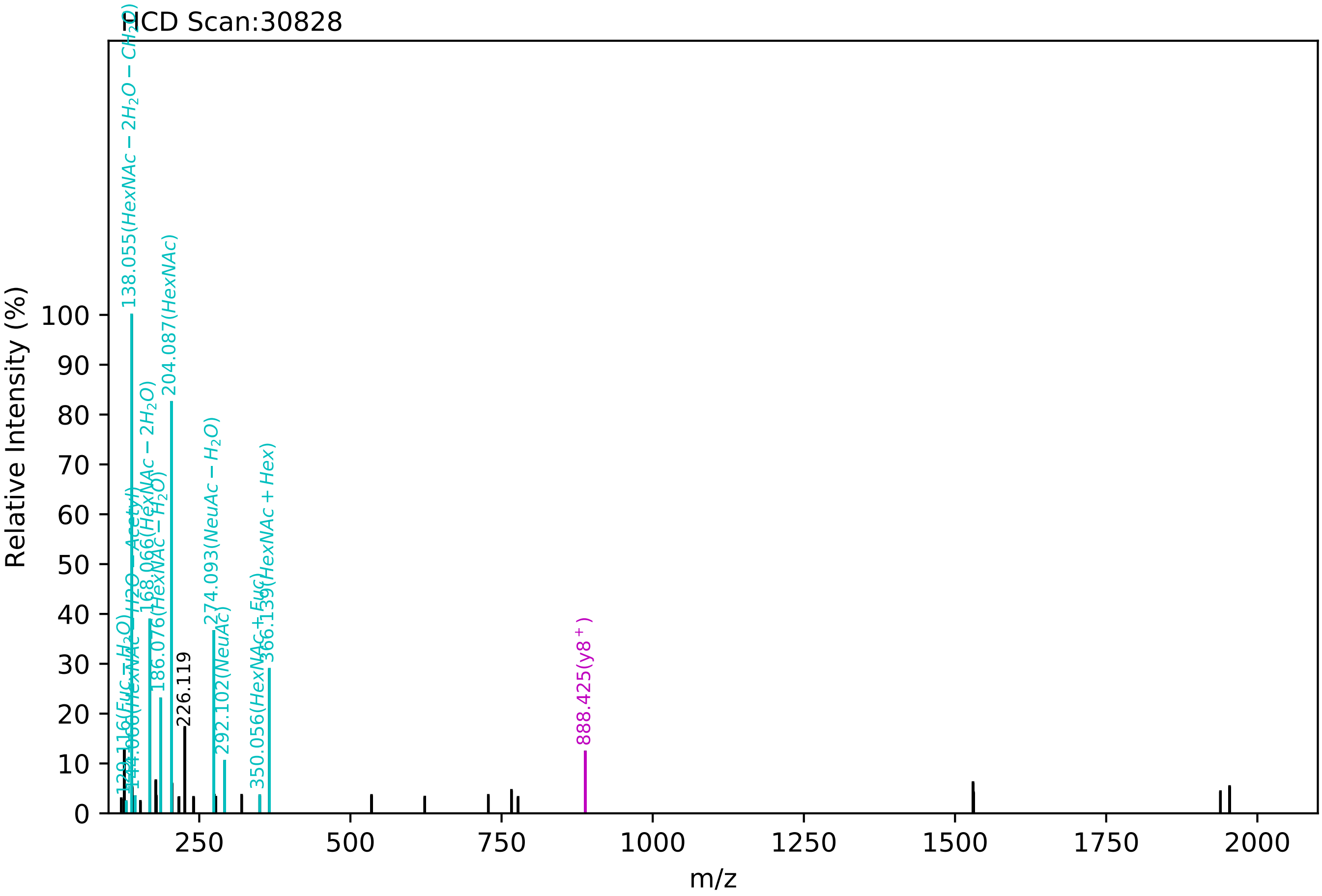

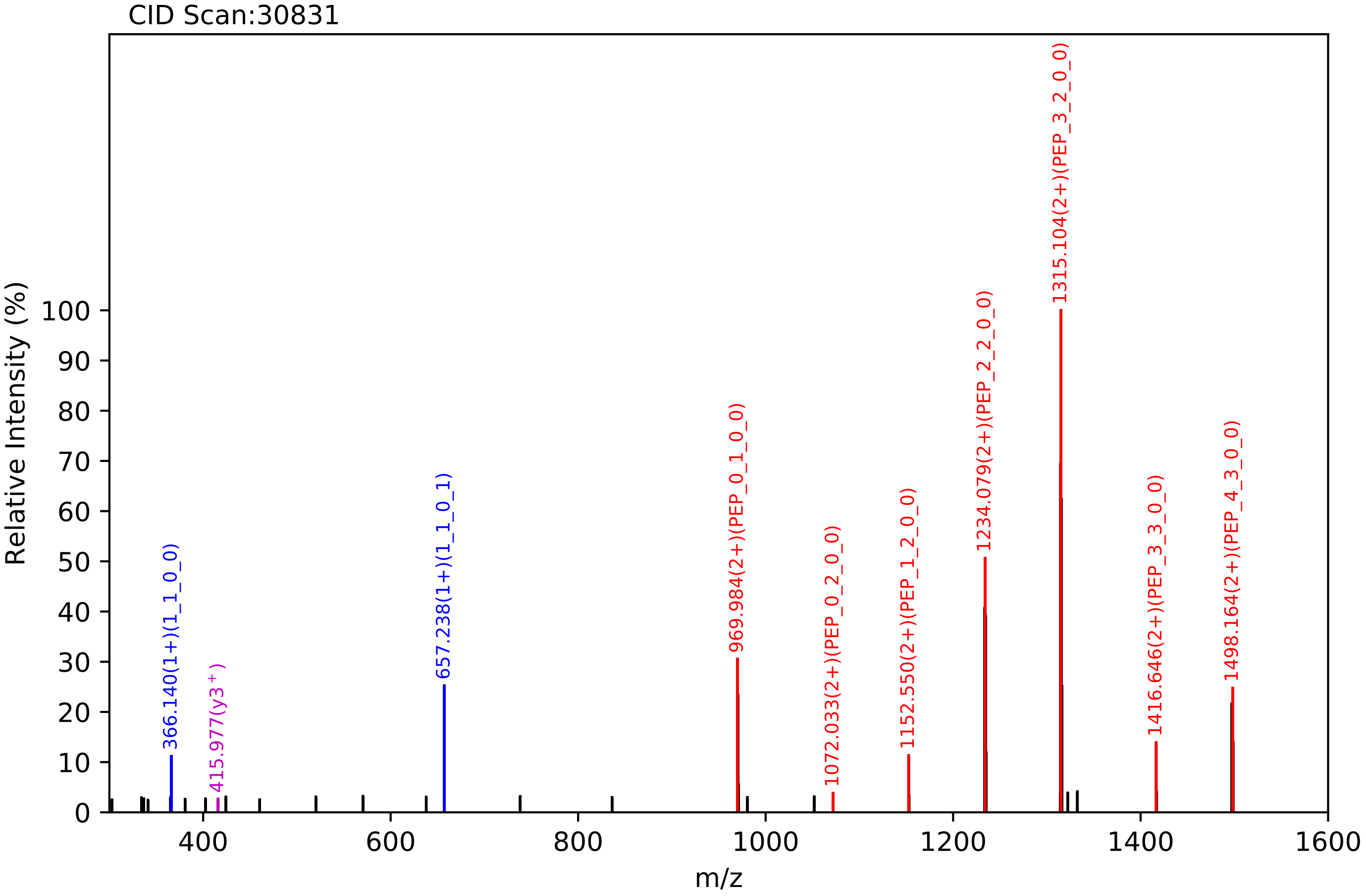

Unknown set no. 275, Experiment: Human Plasma exp_3

ALPQPQNVTSLLGCTH(=PEP)_4_3_0_1, m/z:1095.48(3+), RT:109.16, Y-score:87.28

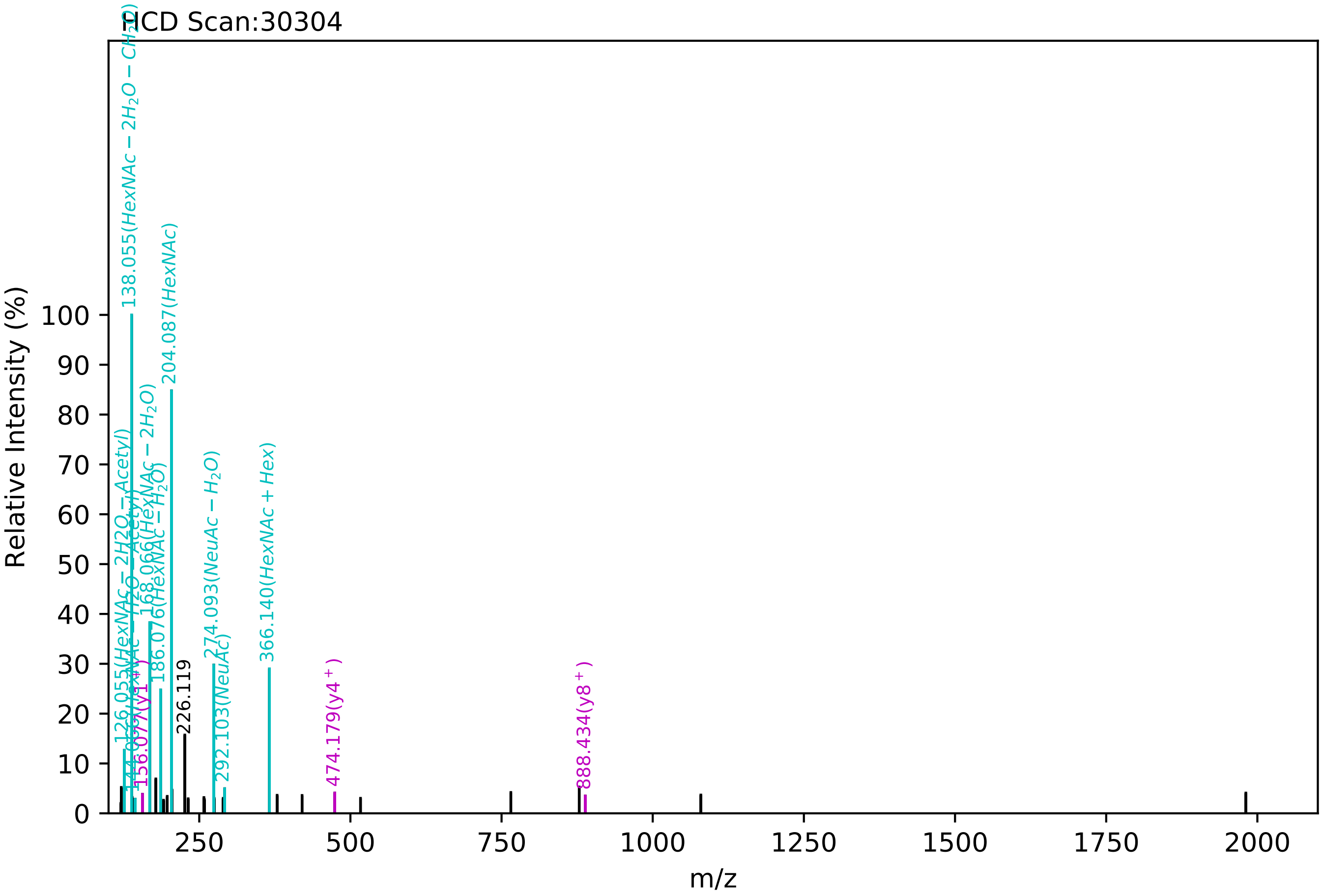

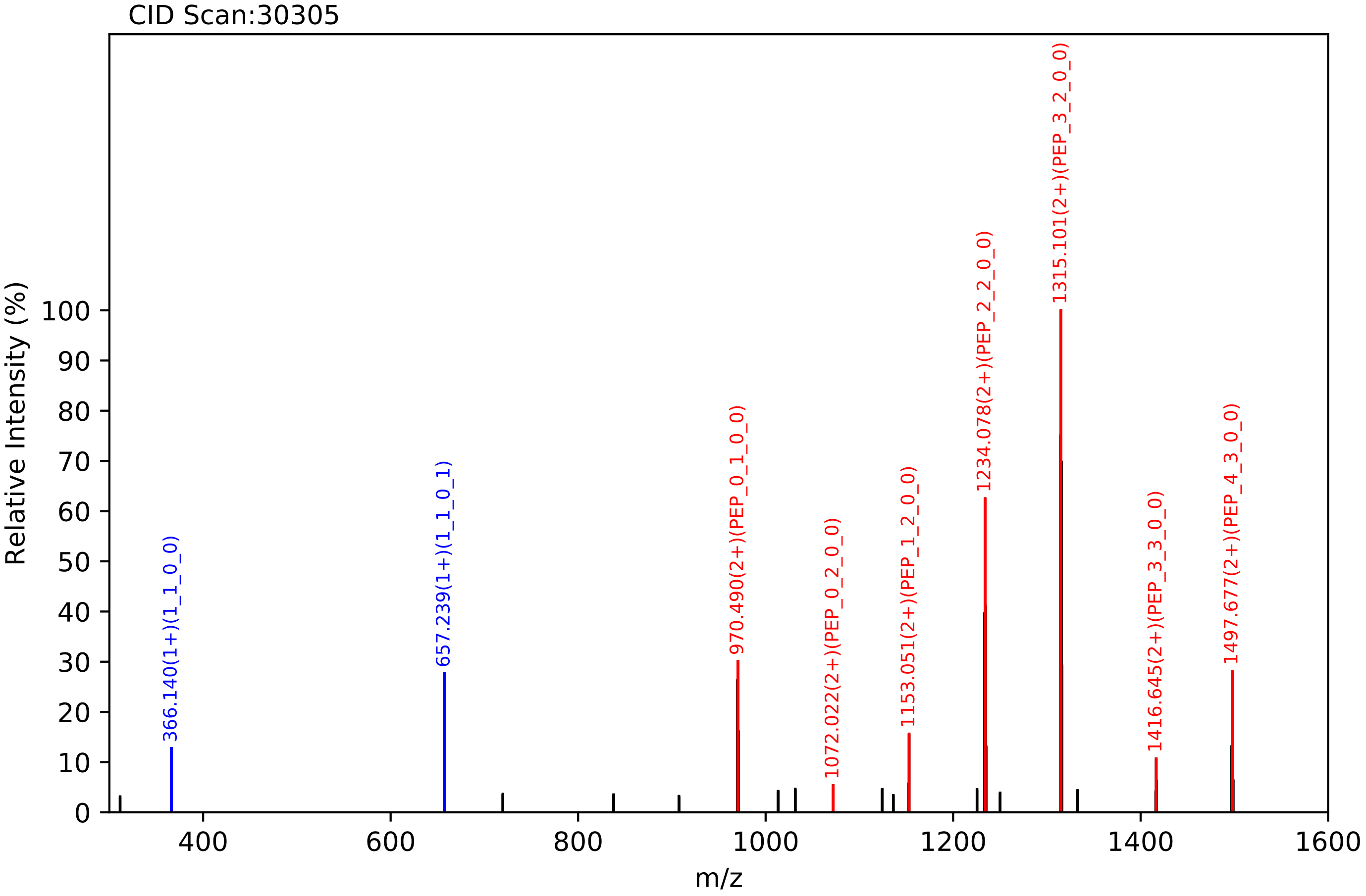

Unknown set no. 276, Experiment: Human Plasma exp_1

ALPQPQNVTSLLGCTH(=PEP)_5_4_0_2, m/z:985.92(4+), RT:109.75, Y-score:84.44

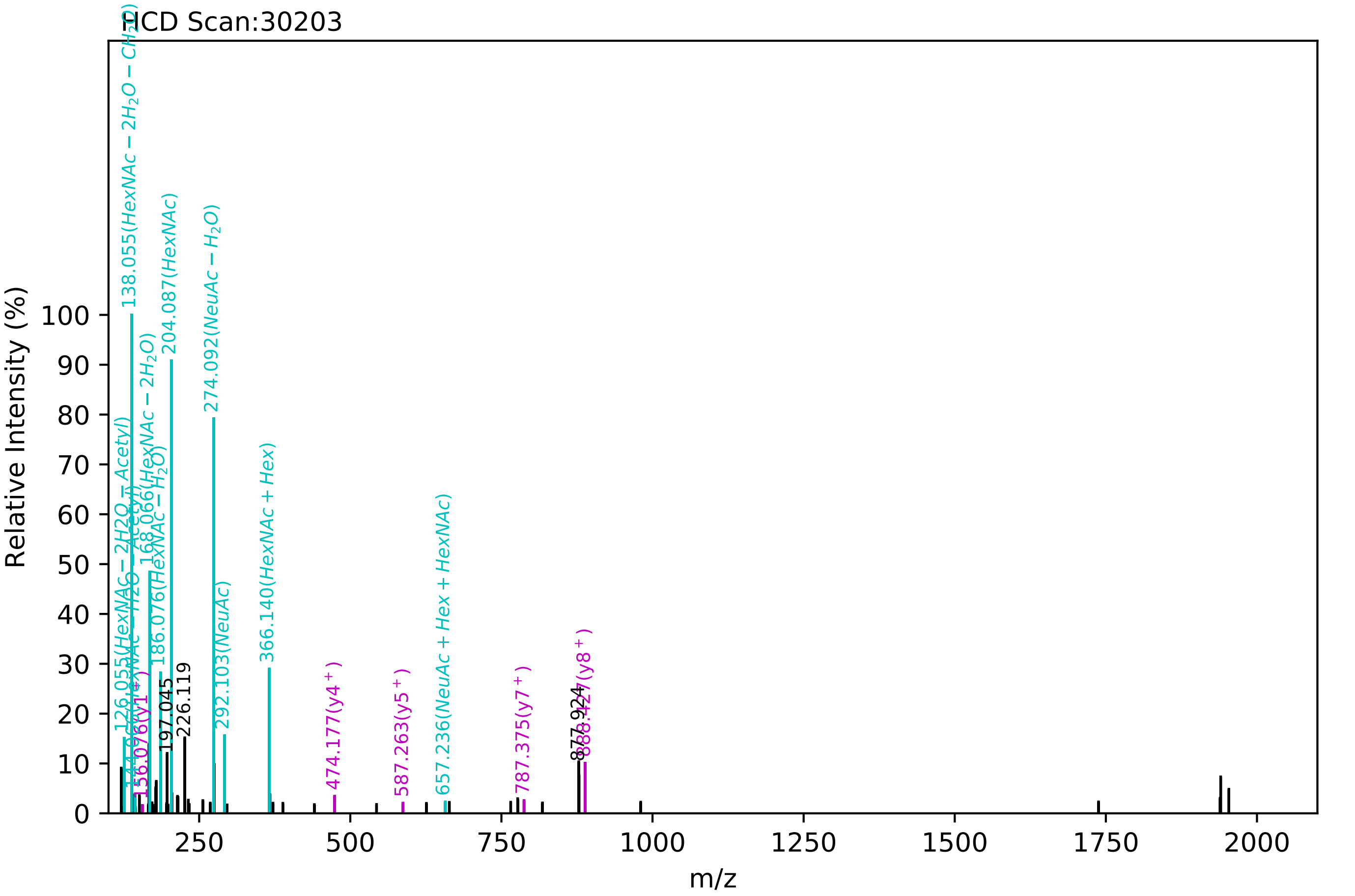

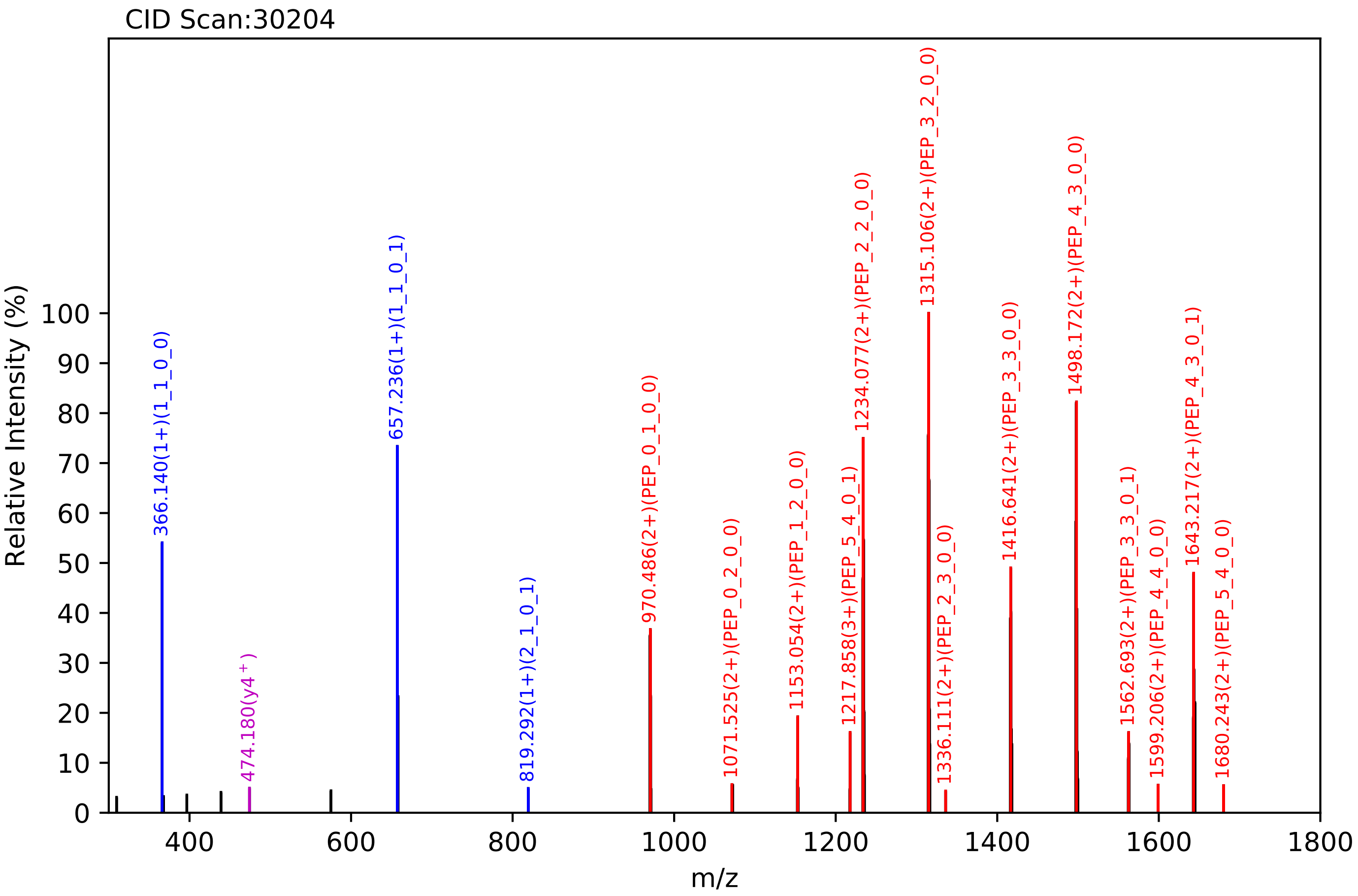

Unknown set no. 277, Experiment: Human Plasma exp_2

ALPQPQNVTSLLGCTH(=PEP)_5_4_0_2, m/z:985.92(4+), RT:109.39, Y-score:85.20

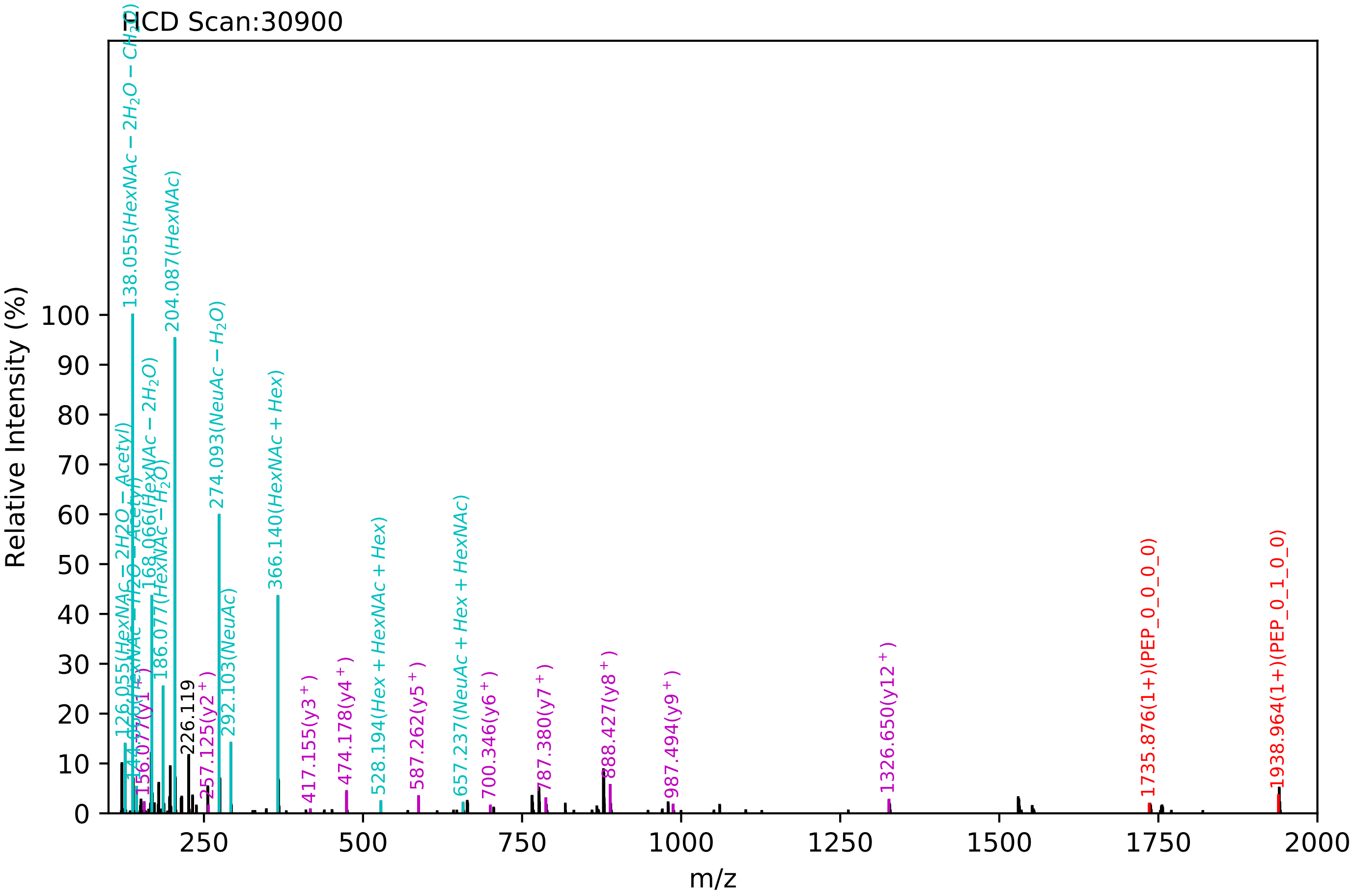

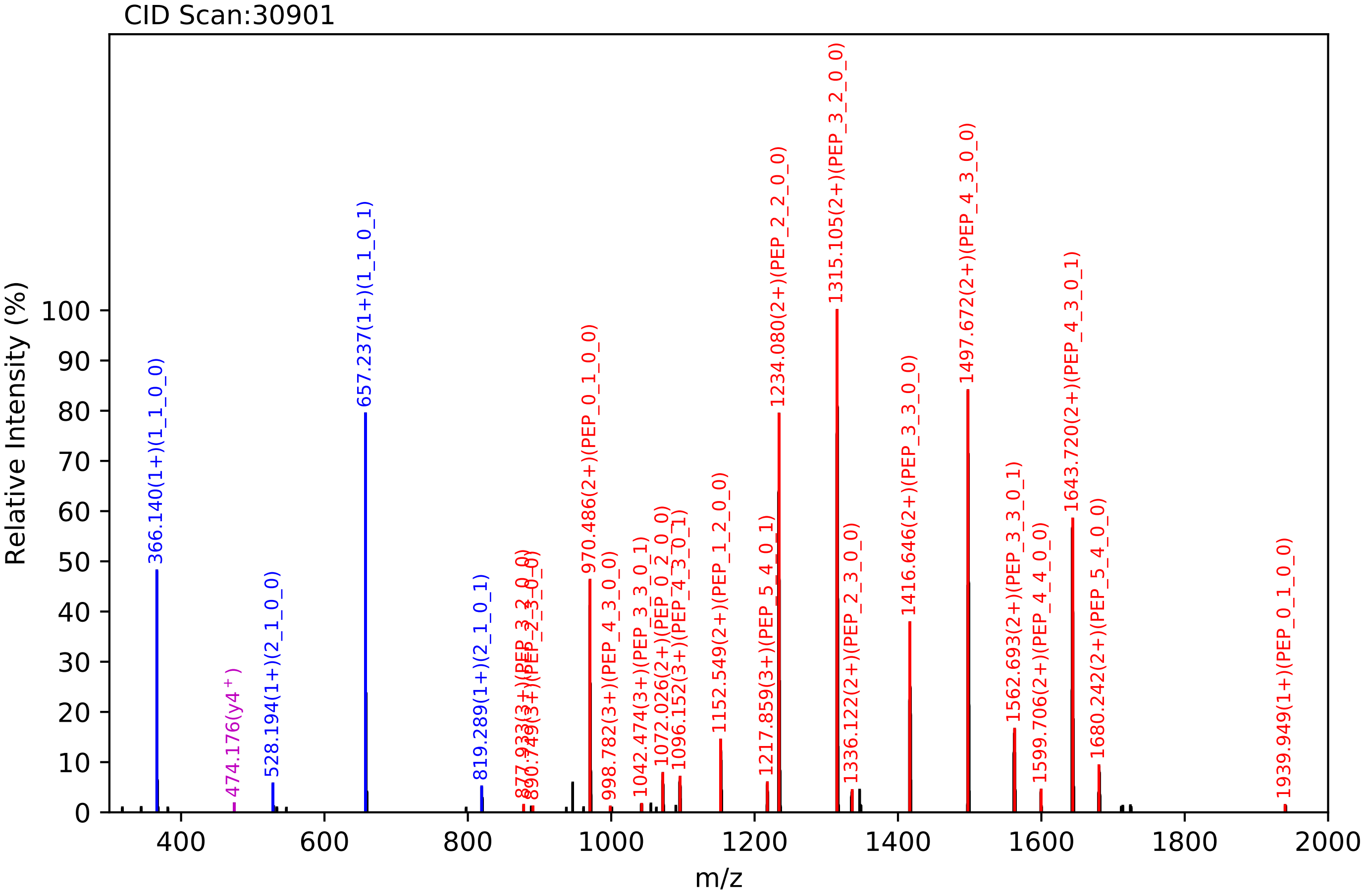

Unknown set no. 278, Experiment: Human Plasma exp_3

ALPQPQNVTSLLGCTH(=PEP)_5_4_0_2, m/z:985.92(4+), RT:110.11, Y-score:85.89

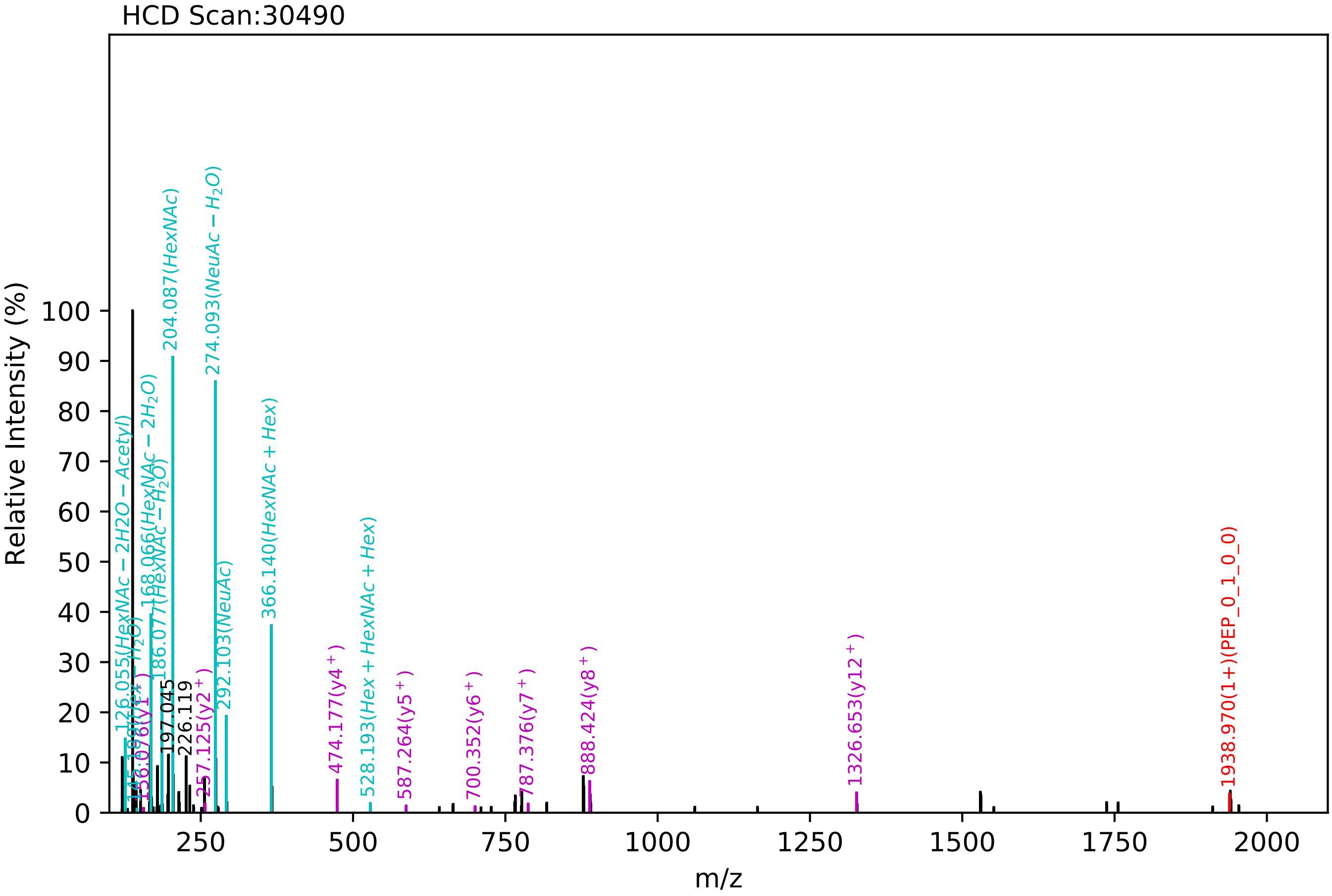

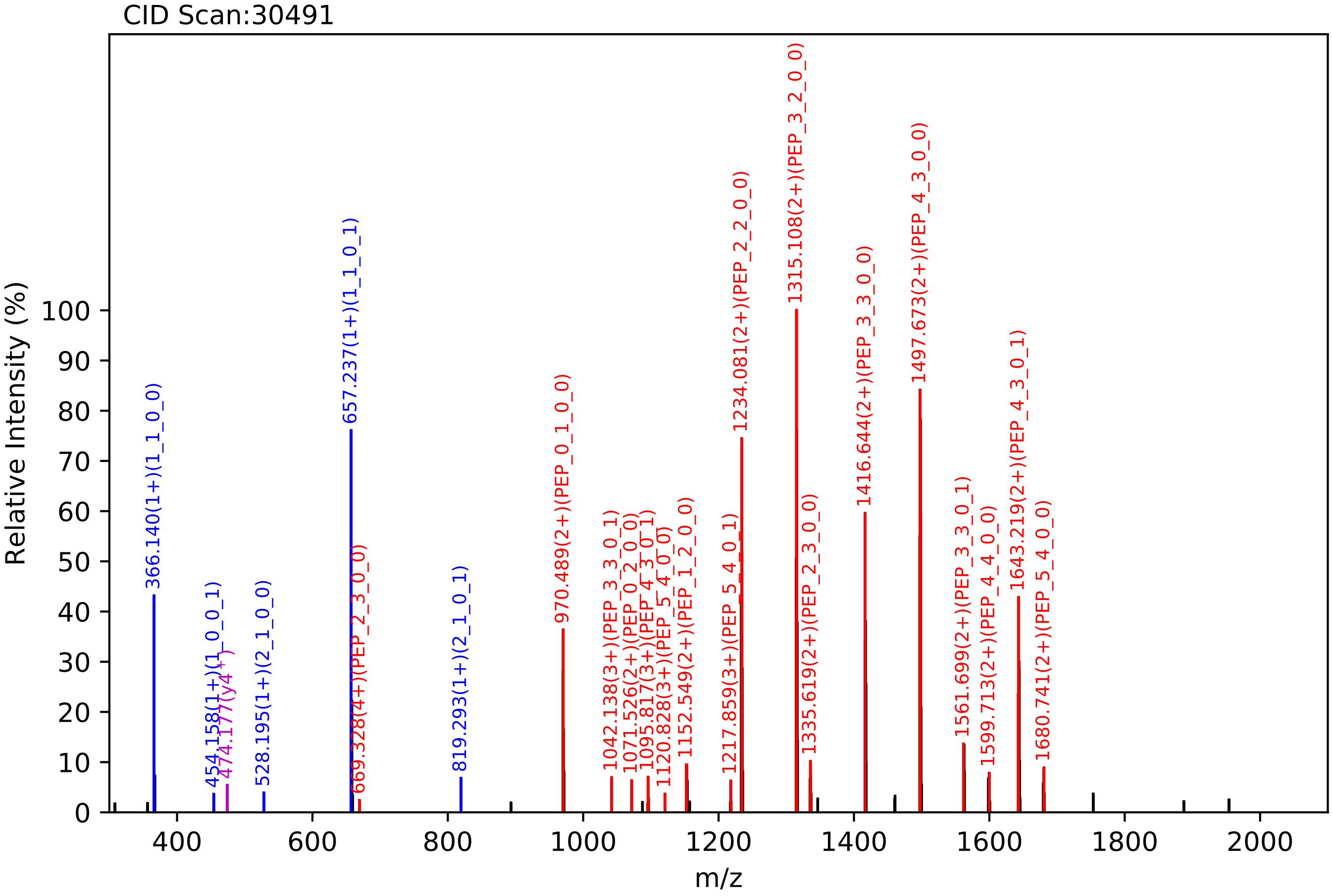

Unknown set no. 279, Experiment: Human Plasma exp_4

ALPQPQNVTSLLGCTH(=PEP)_5_4_0_2, m/z:985.92(4+), RT:108.92, Y-score:82.76

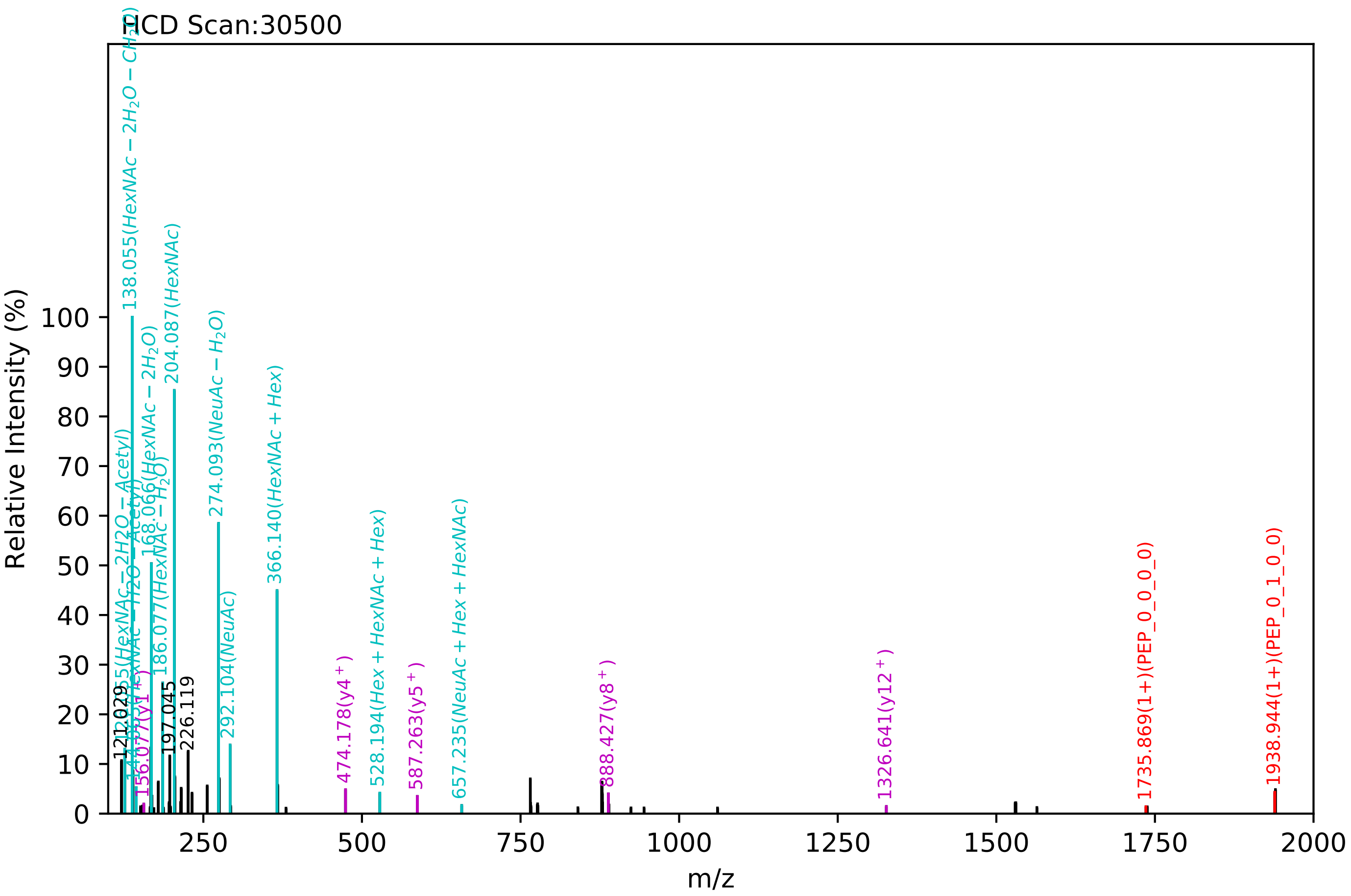

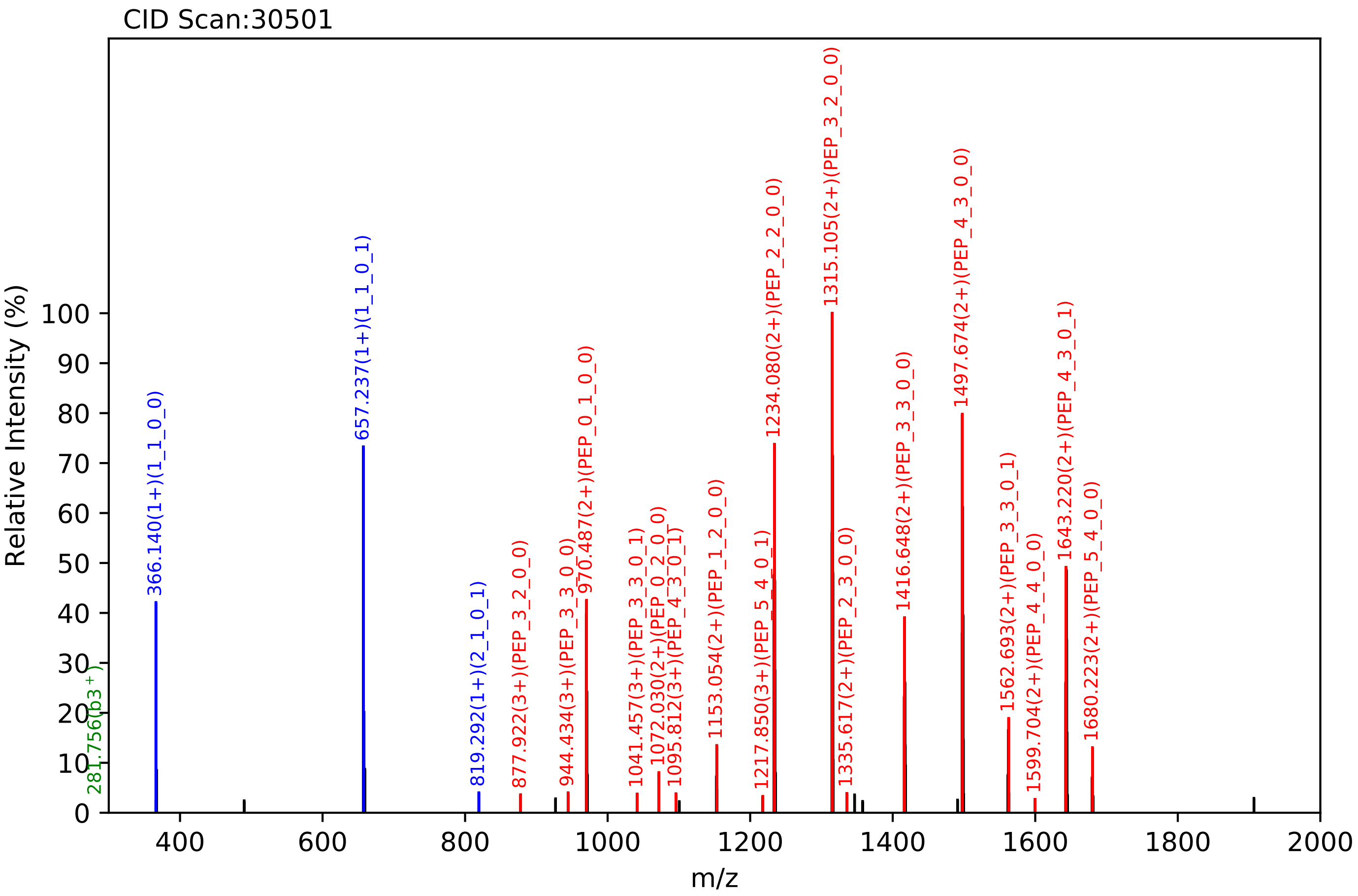

Unknown set no. 280, Experiment: Human Plasma exp_5

ALPQPQNVTSLLGCTH(=PEP)_5_4_0_2, m/z:985.92(4+), RT:109.74, Y-score:87.53

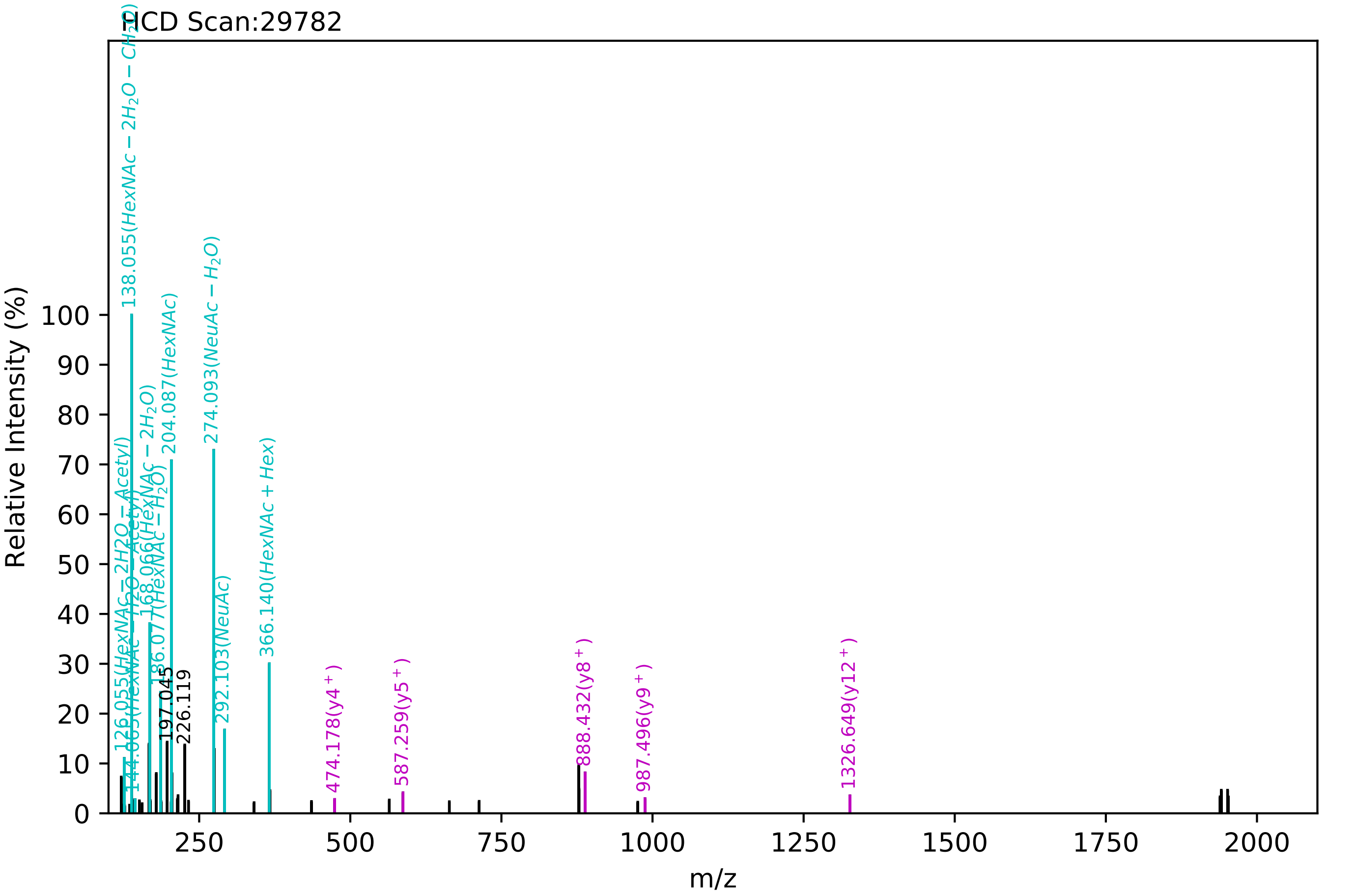

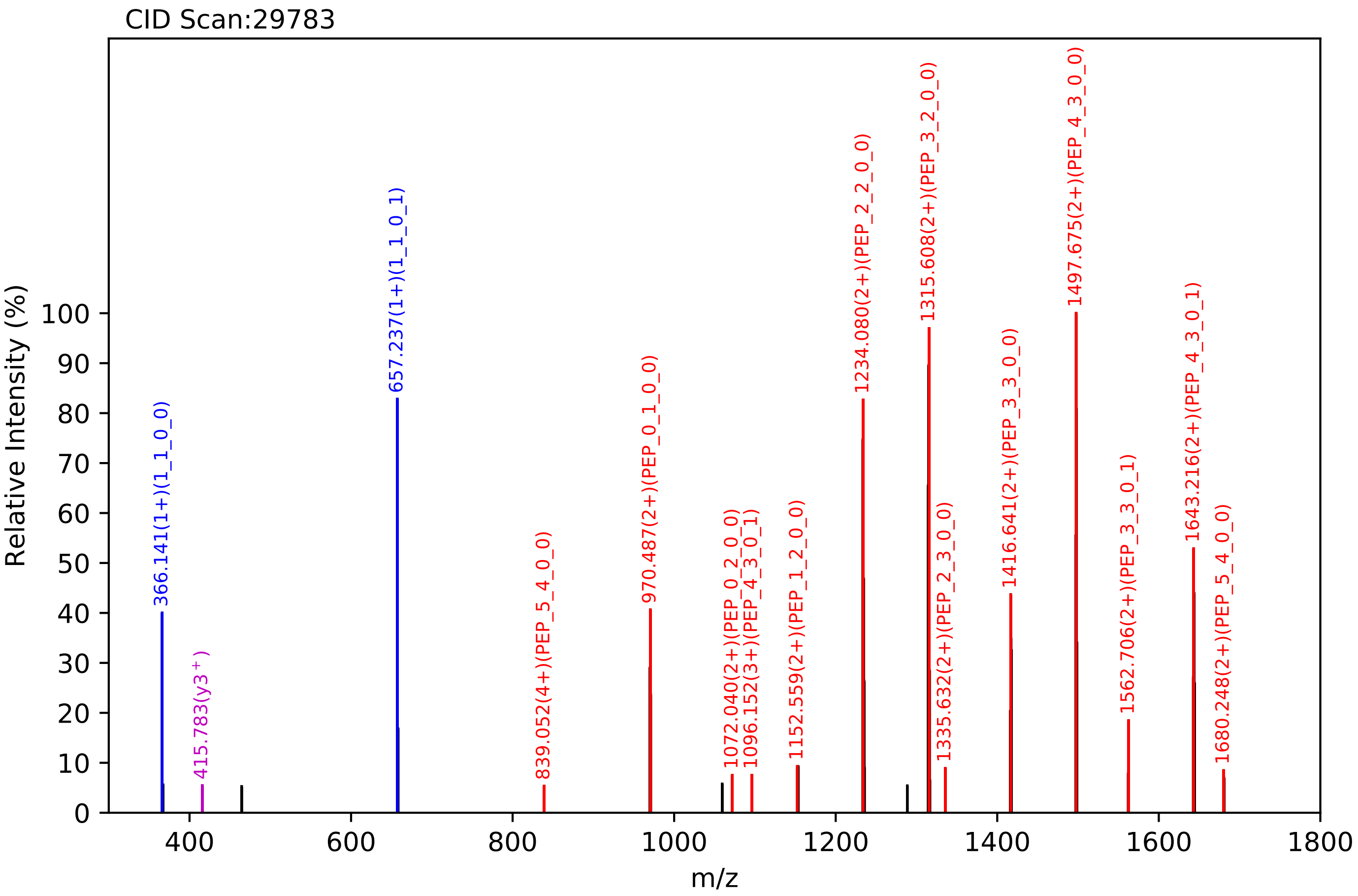

Unknown set no. 281, Experiment: Human Plasma exp_1

SWPAVGNCSSALR(=PEP)_5_4_0_1, m/z:1106.79(3+), RT:75.84, Y-score:89.50

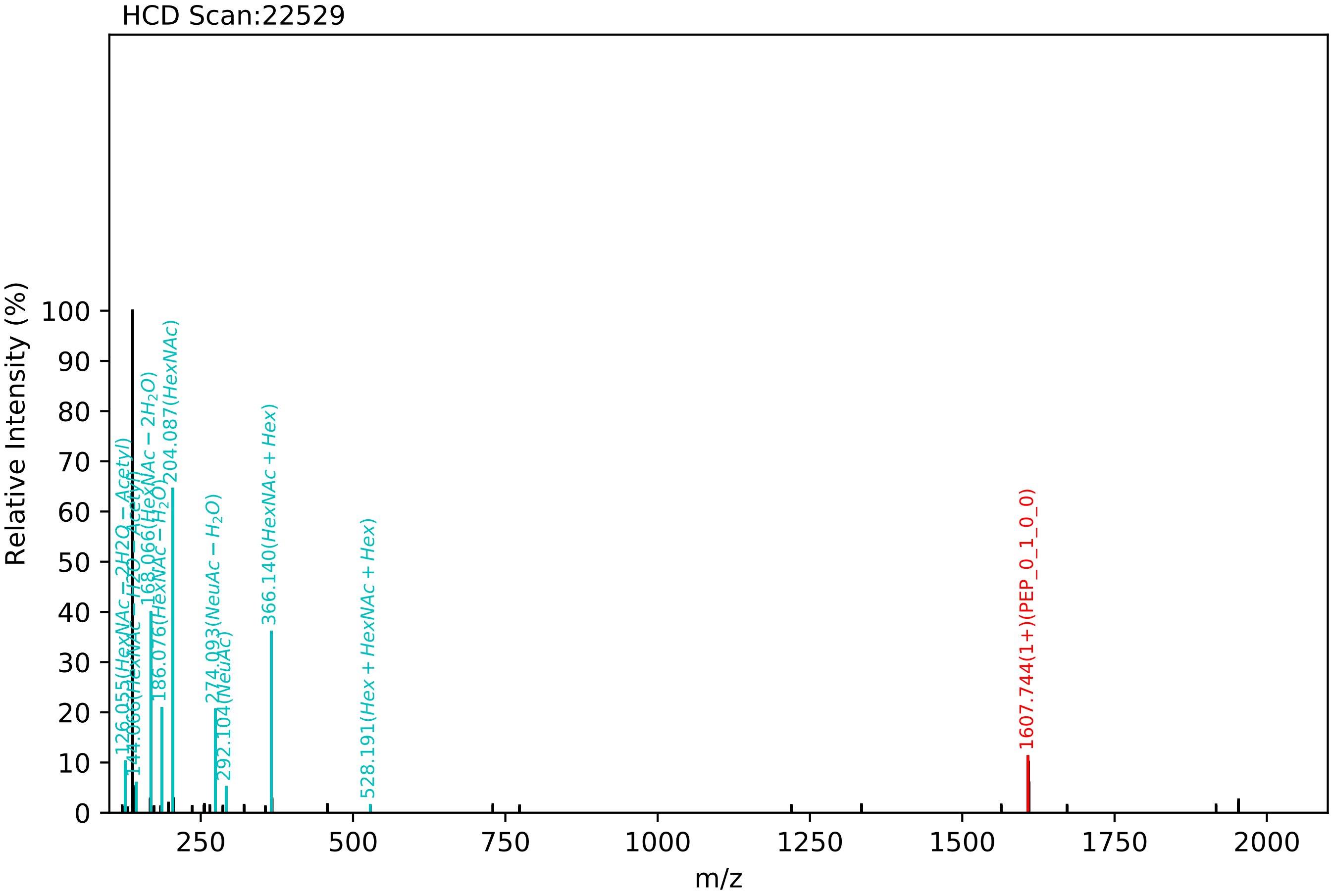

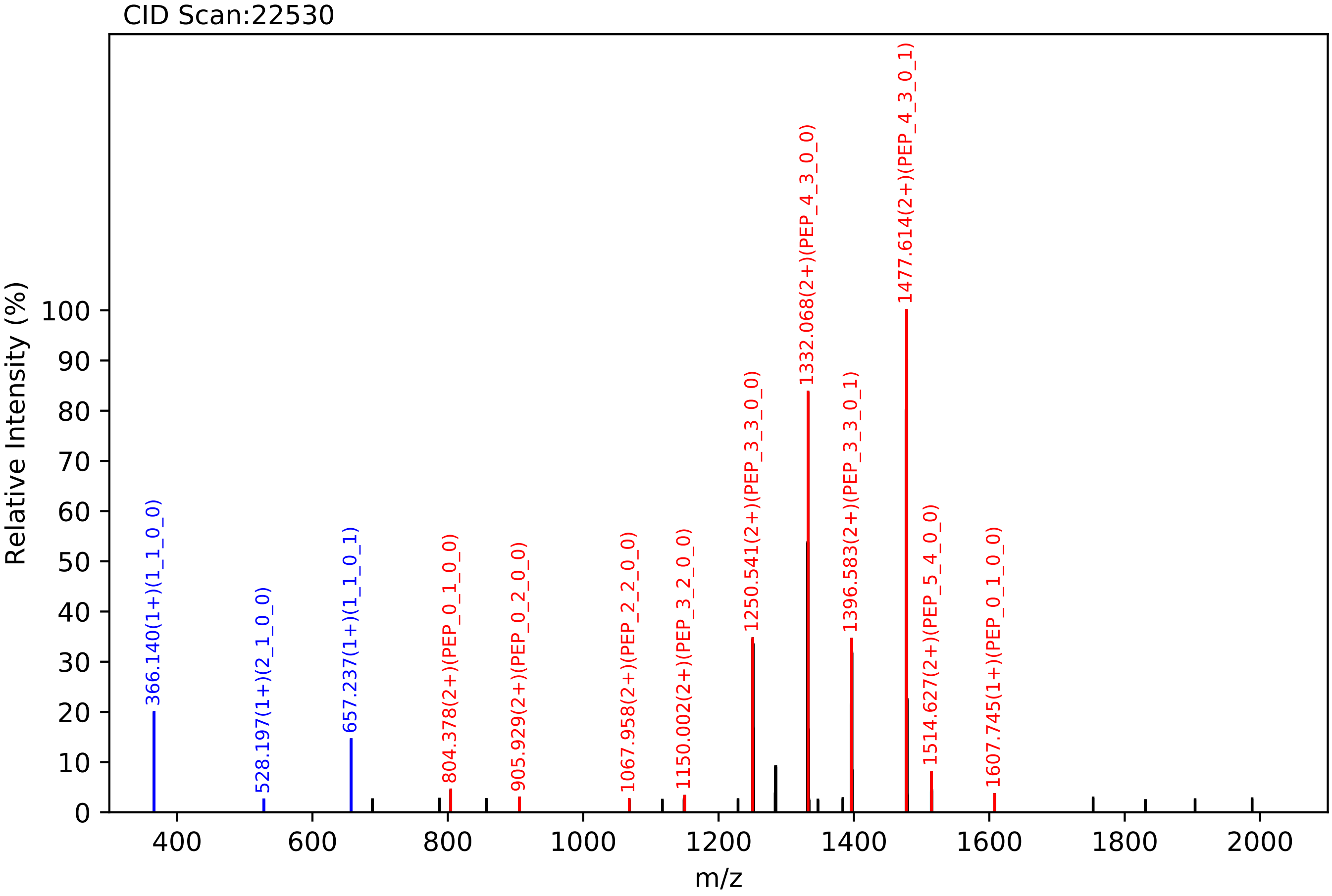

Unknown set no. 282, Experiment: Human Plasma exp_1

SWPAVGNCSSALR(=PEP)_5_4_0_2, m/z:903.12(4+), RT:86.20, Y-score:83.12

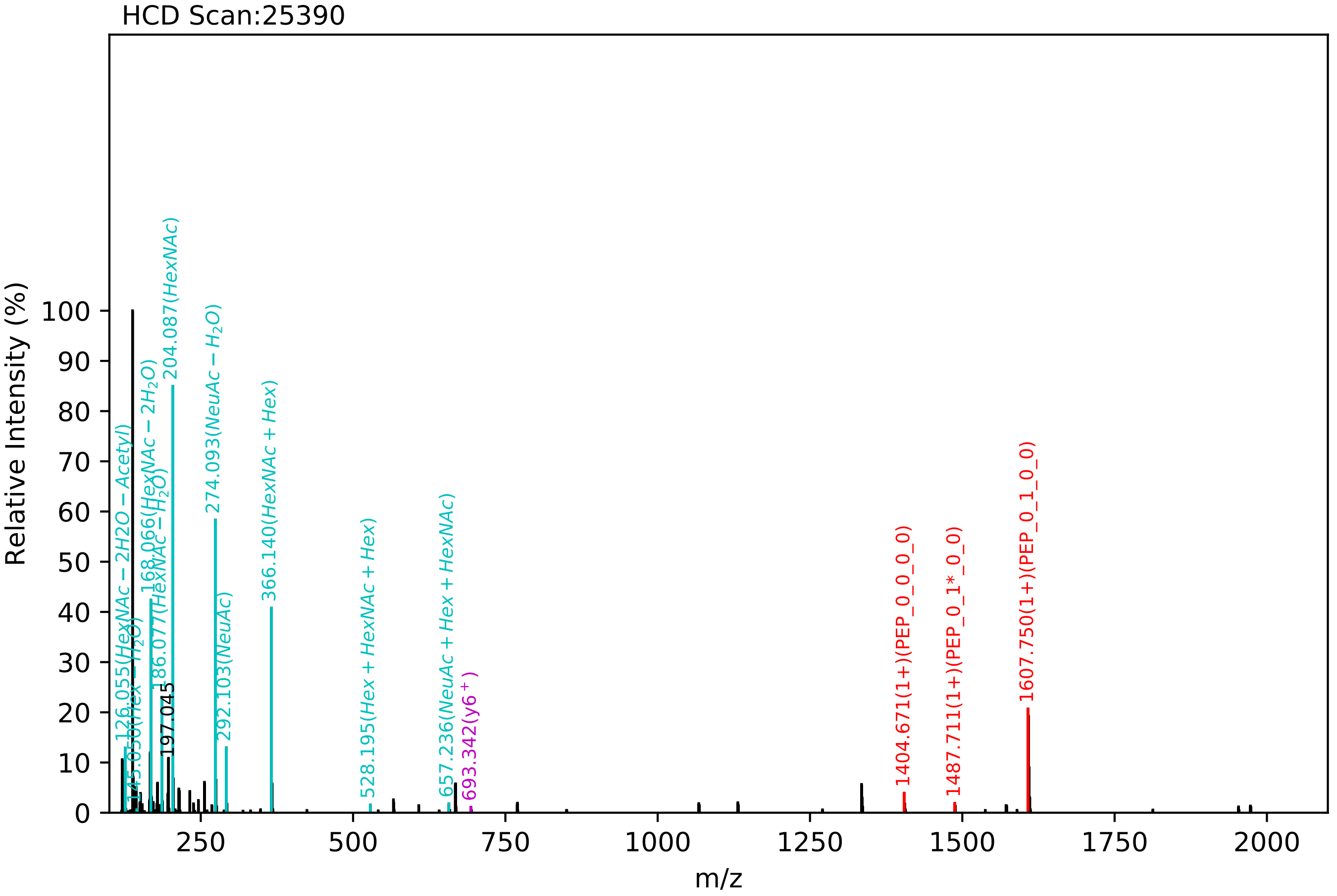

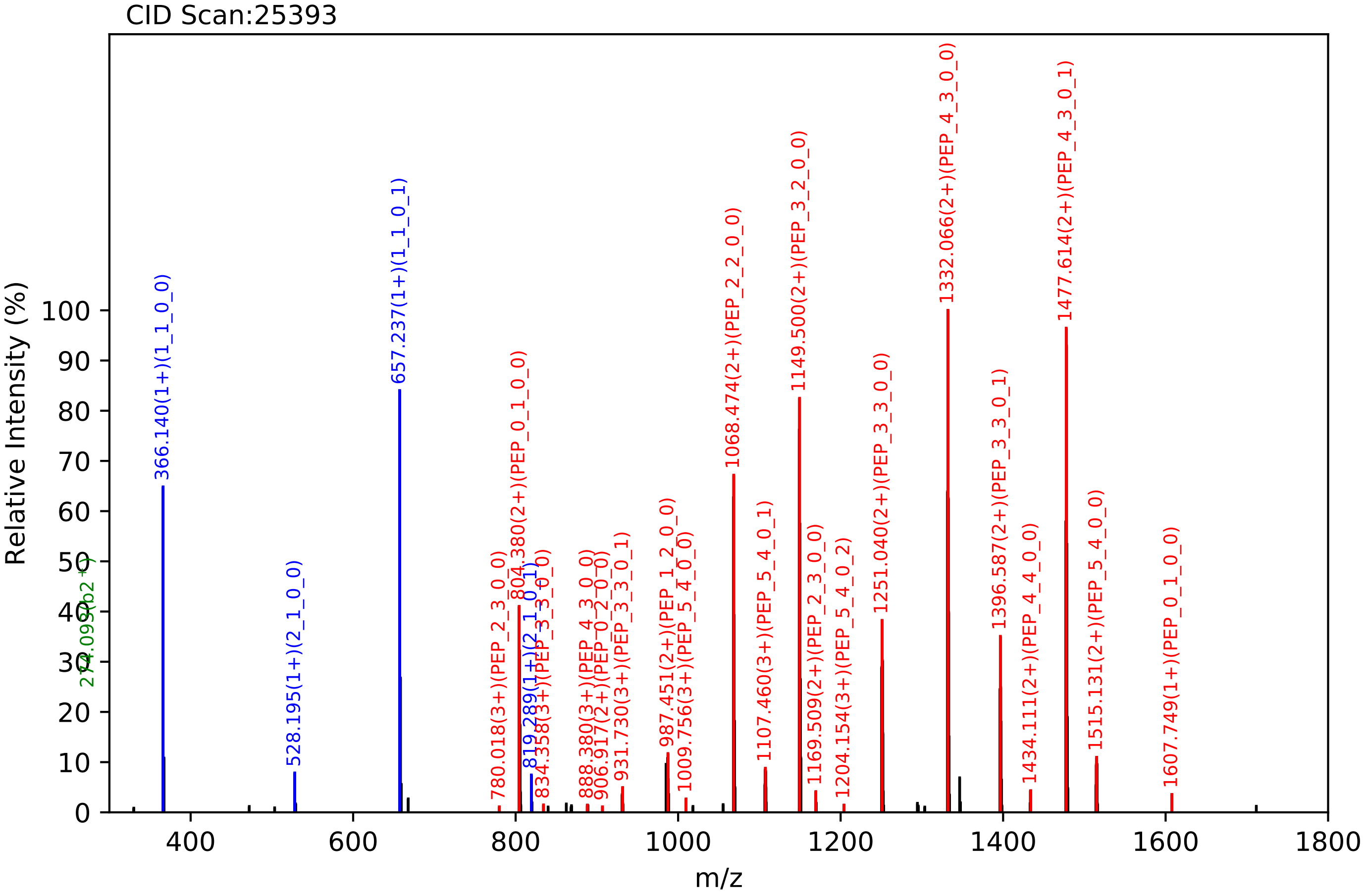

Unknown set no. 283, Experiment: Human Plasma exp_2

SWPAVGNCSSALR(=PEP)_5_4_0_2, m/z:903.12(4+), RT:86.28, Y-score:83.84

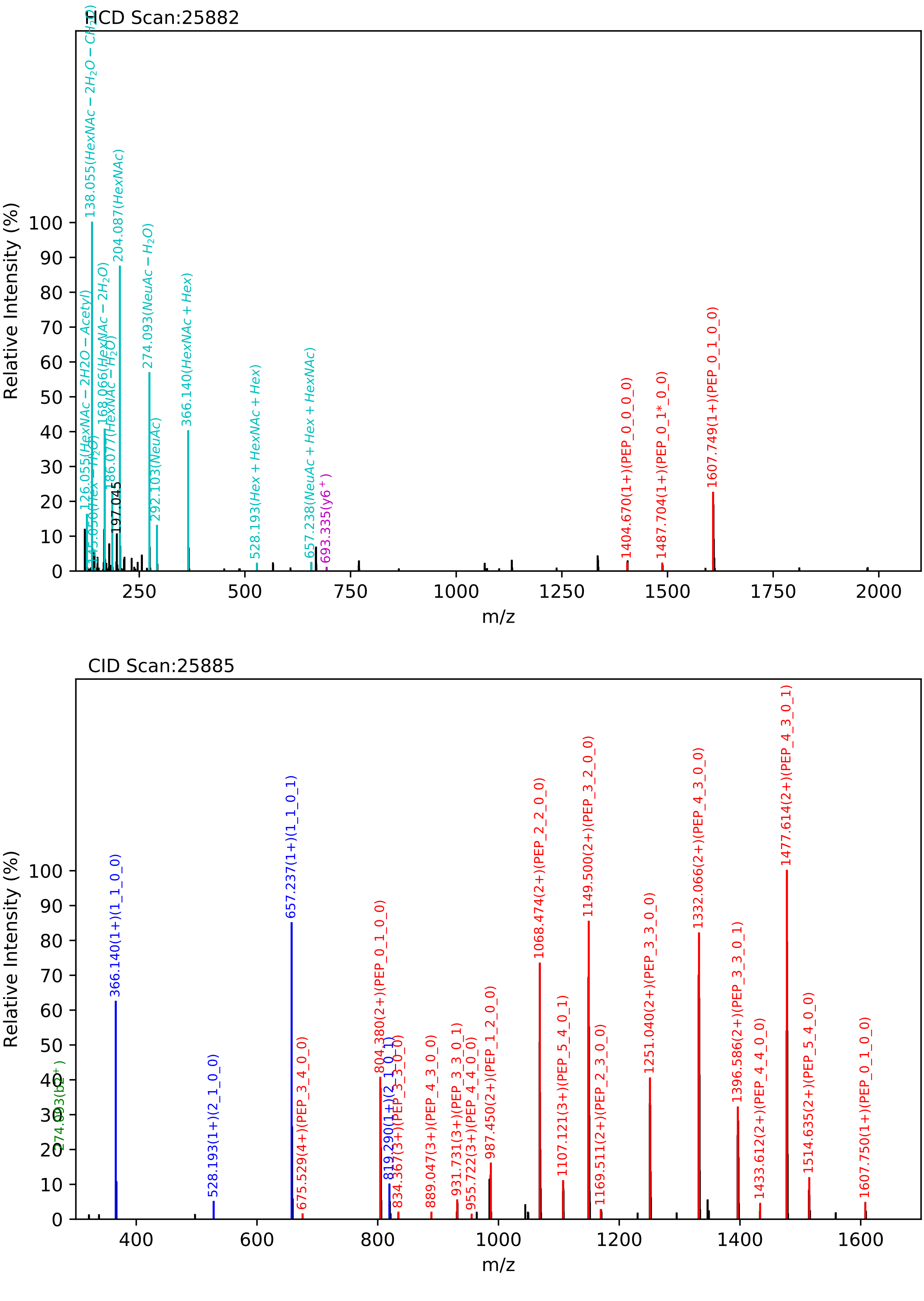

Unknown set no. 284, Experiment: Human Plasma exp_3

SWPAVGNCSSALR(=PEP)_5_4_0_2, m/z:903.12(4+), RT:86.85, Y-score:83.30

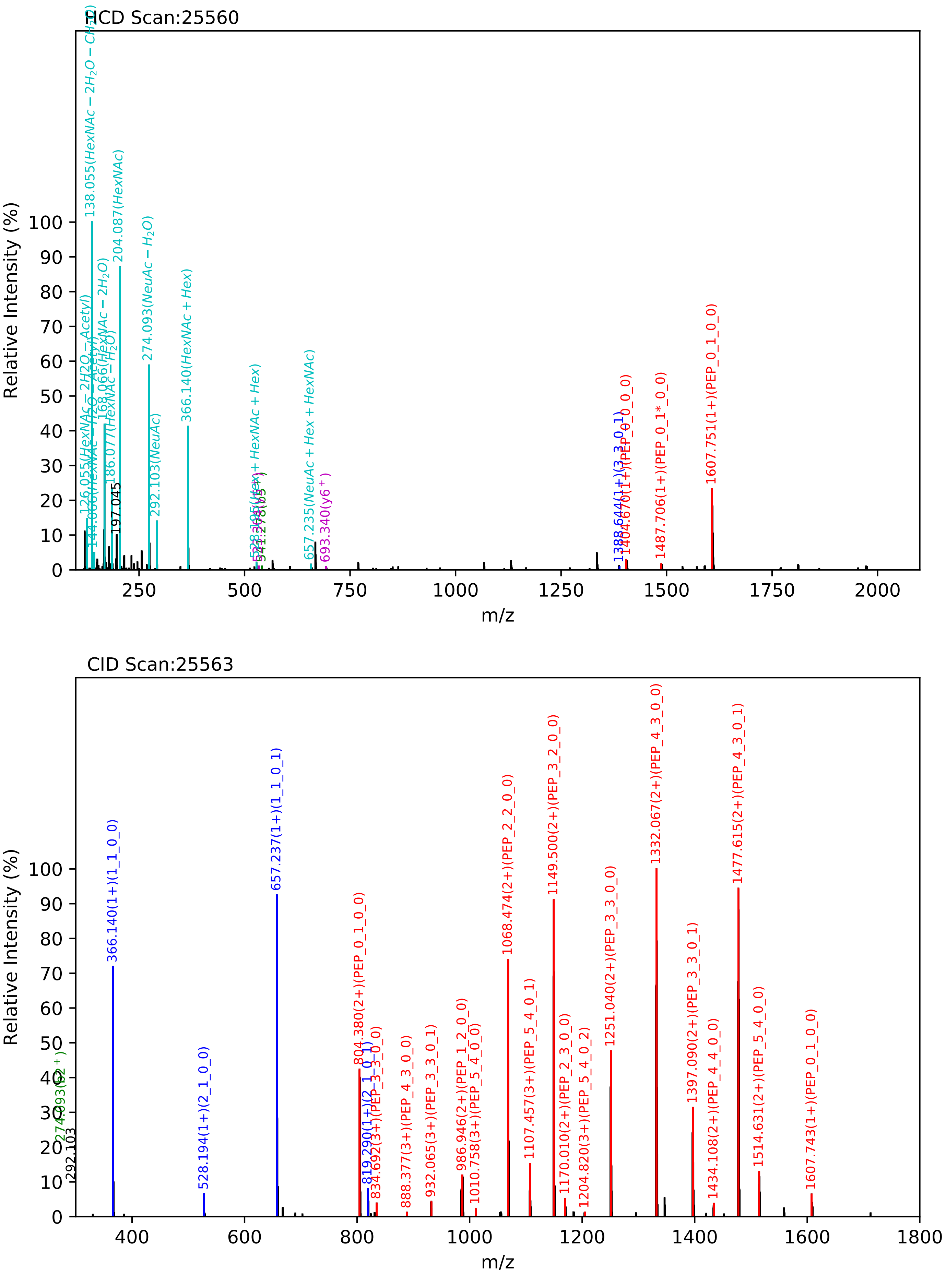

Unknown set no. 285, Experiment: Human Plasma exp_4

SWPAVGNCSSALR(=PEP)_5_4_0_2, m/z:903.12(4+), RT:86.37, Y-score:89.06

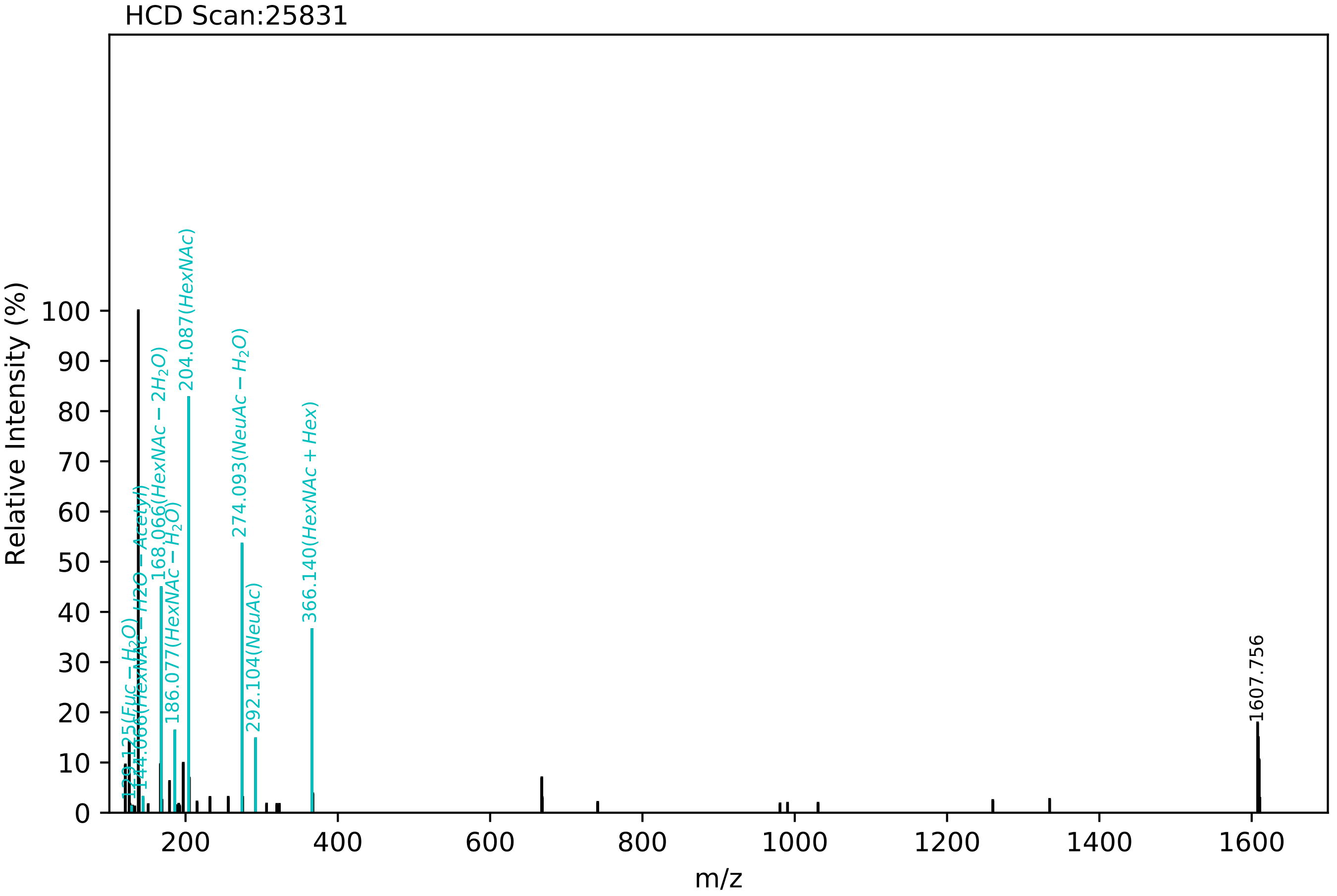

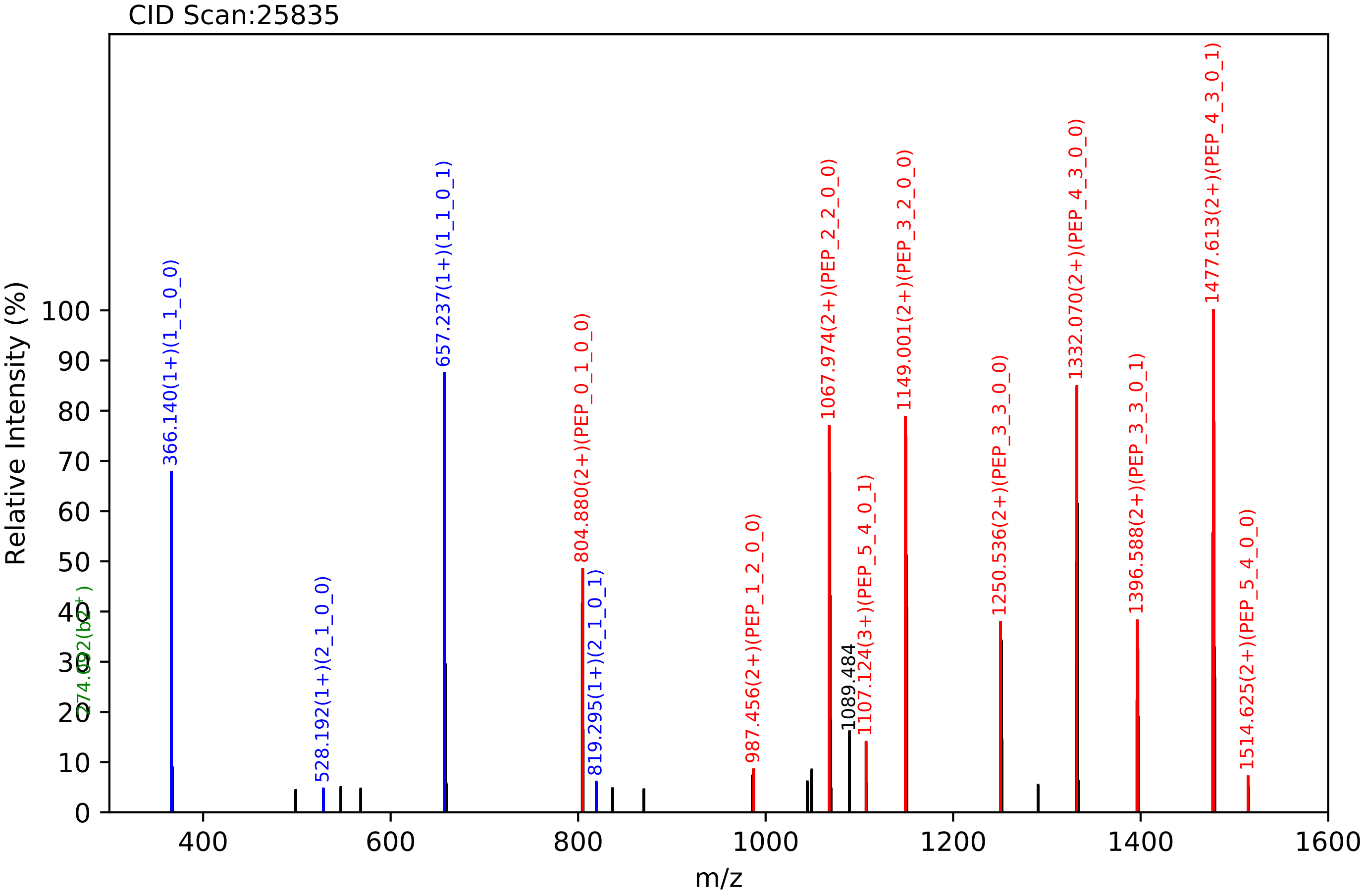

Unknown set no. 286, Experiment: Human Plasma exp_5

SWPAVGNCSSALR(=PEP)_5_4_0_2, m/z:903.12(4+), RT:86.68, Y-score:86.32

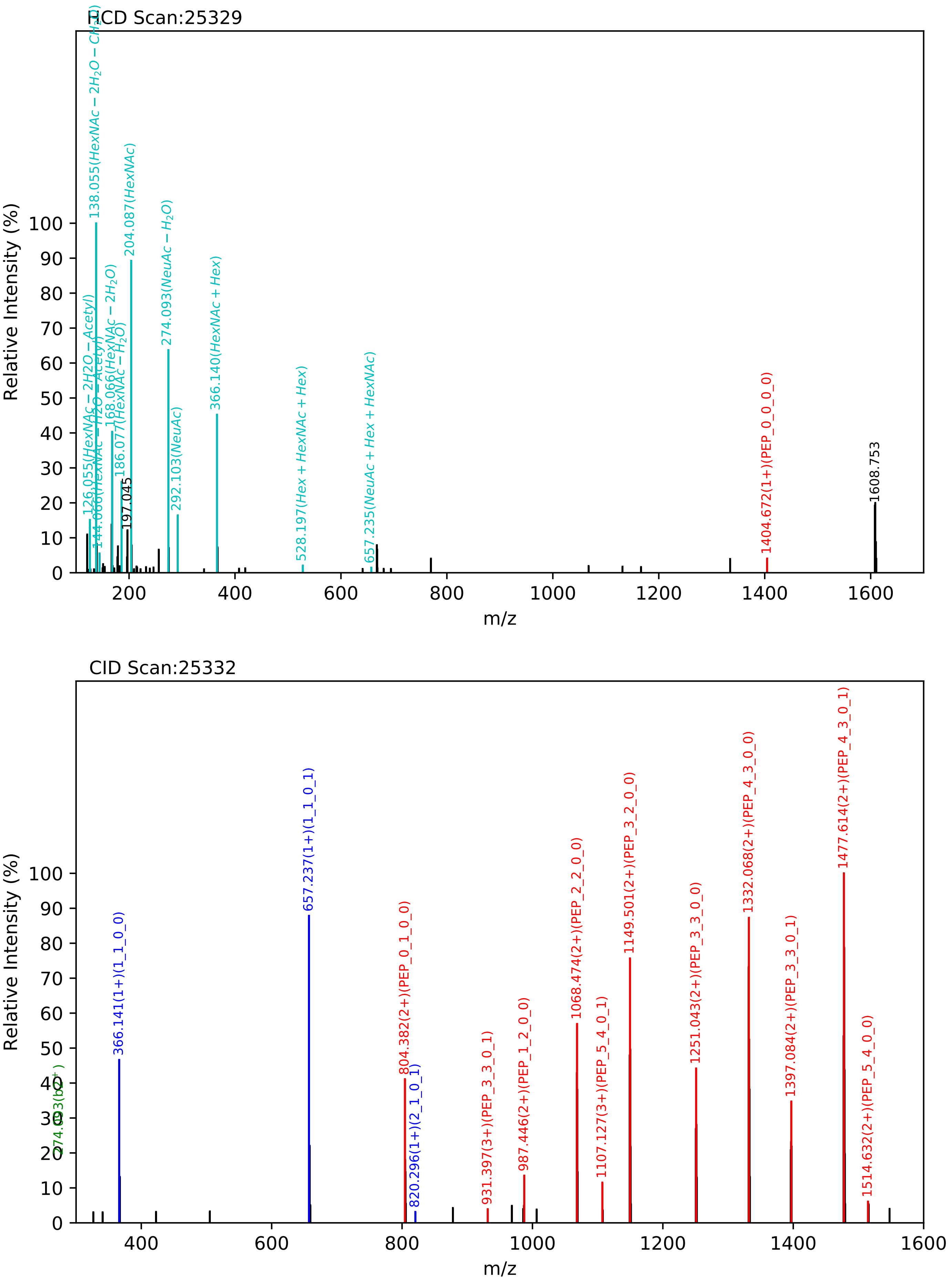

Unknown set no. 287, Experiment: Human Plasma exp_2

SWPAVGNCSSALR(=PEP)_5_4_1_2, m/z:939.63(4+), RT:86.01, Y-score:82.72

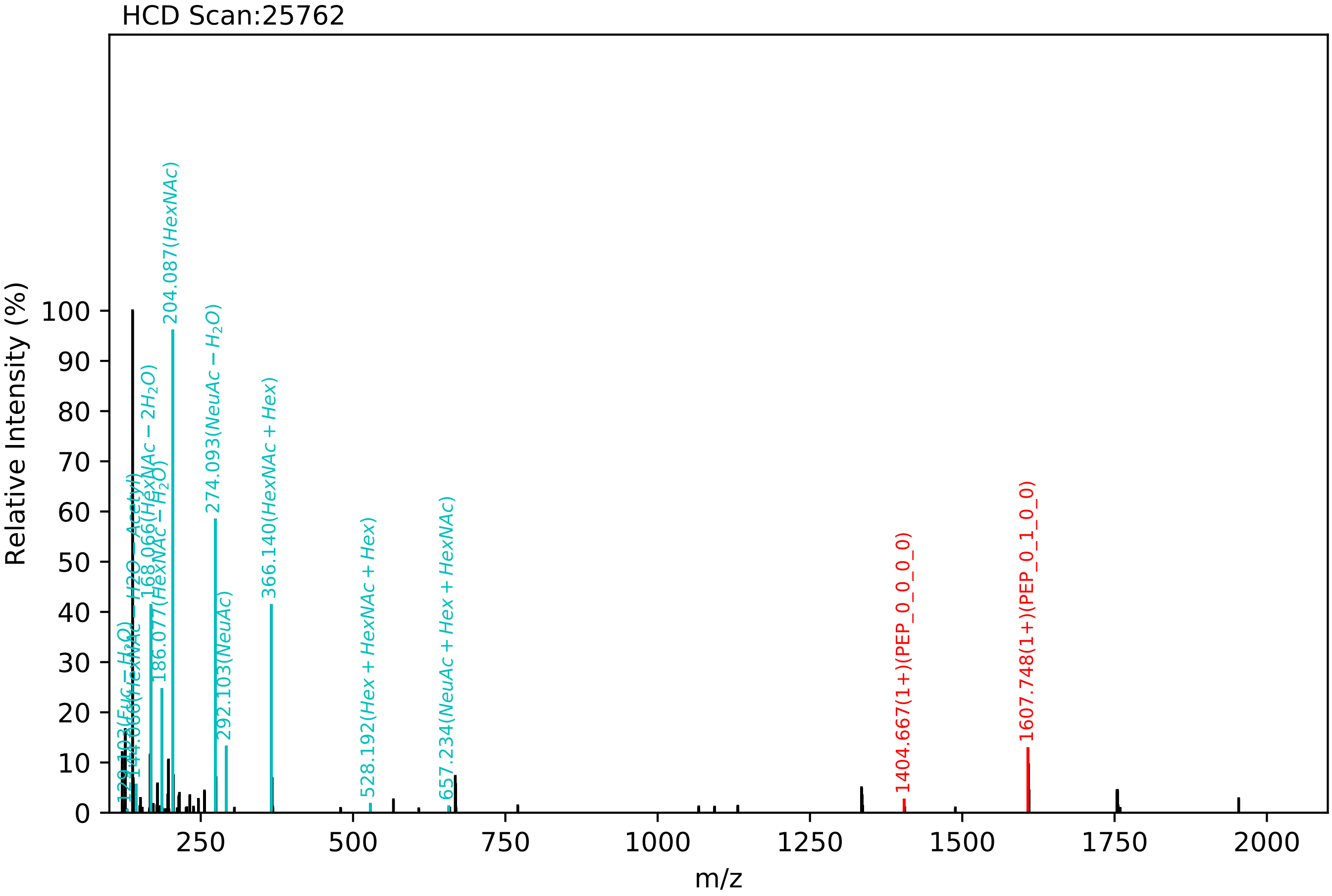

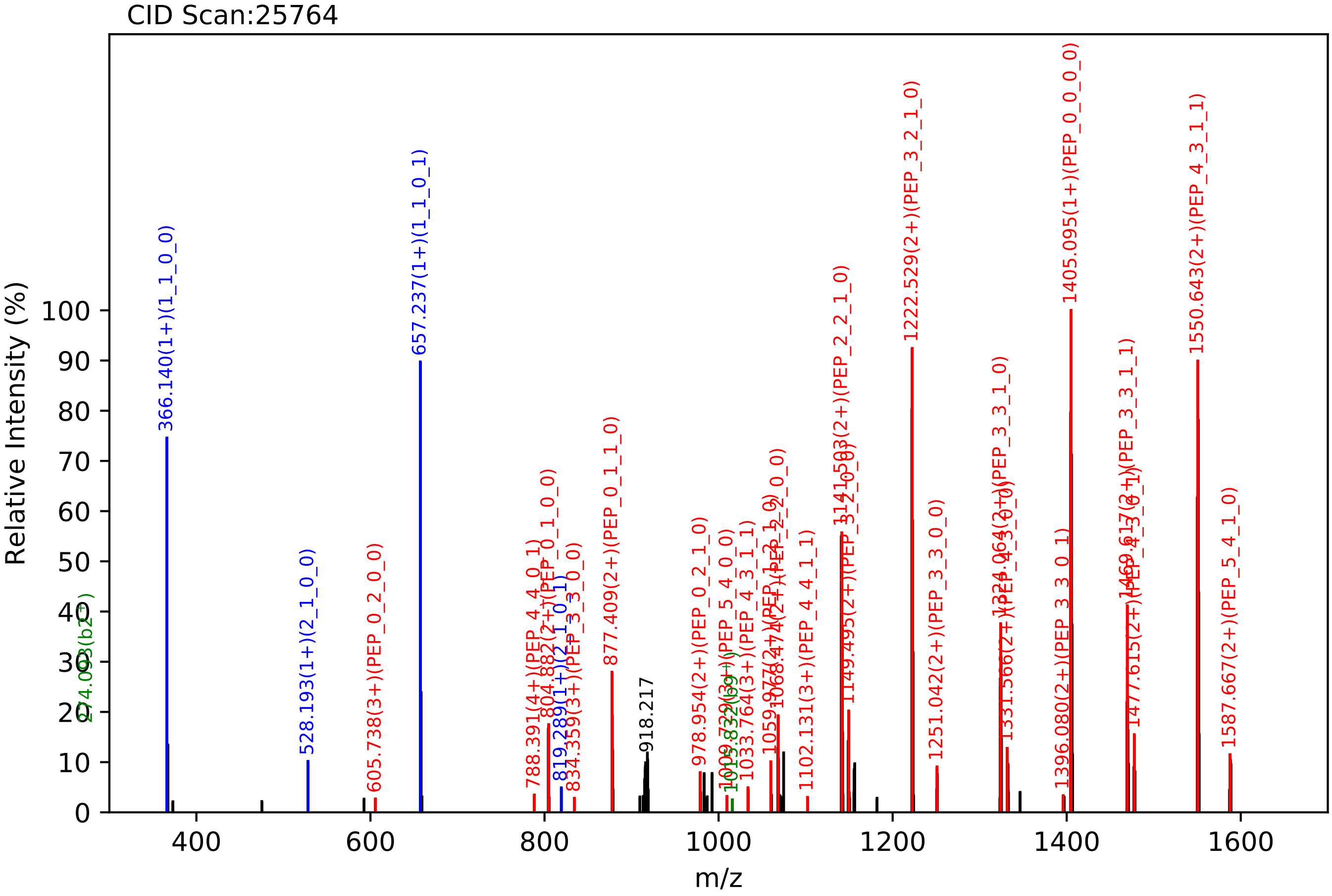

Unknown set no. 288, Experiment: Human Plasma exp_4

SWPAVGNCSSALR(=PEP)_5_4_1_2, m/z:939.63(4+), RT:86.15, Y-score:76.91

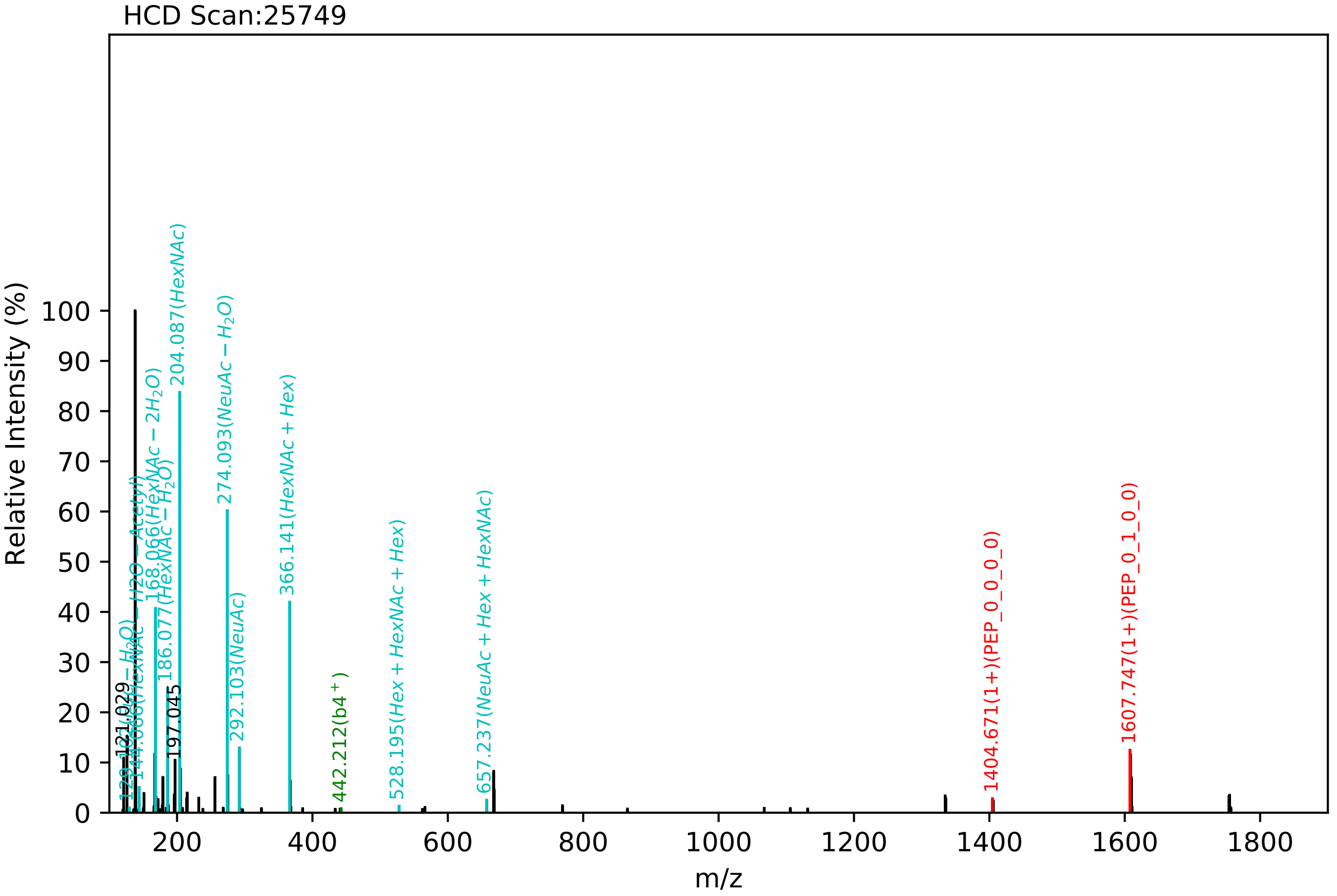

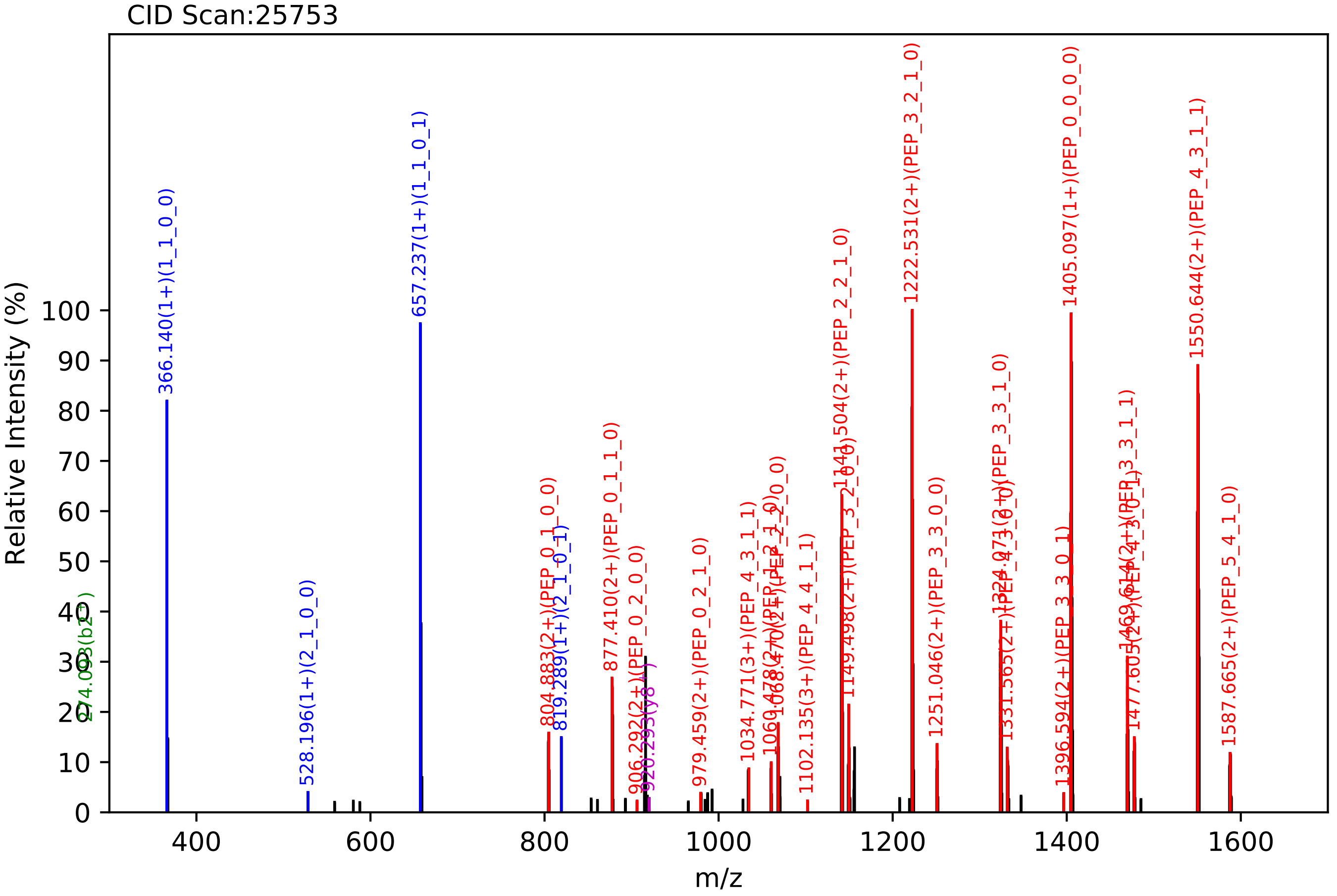

Unknown set no. 289, Experiment: Human Plasma exp_5

SWPAVGNCSSALR(=PEP)_5_4_1_2, m/z:939.63(4+), RT:85.81, Y-score:83.78

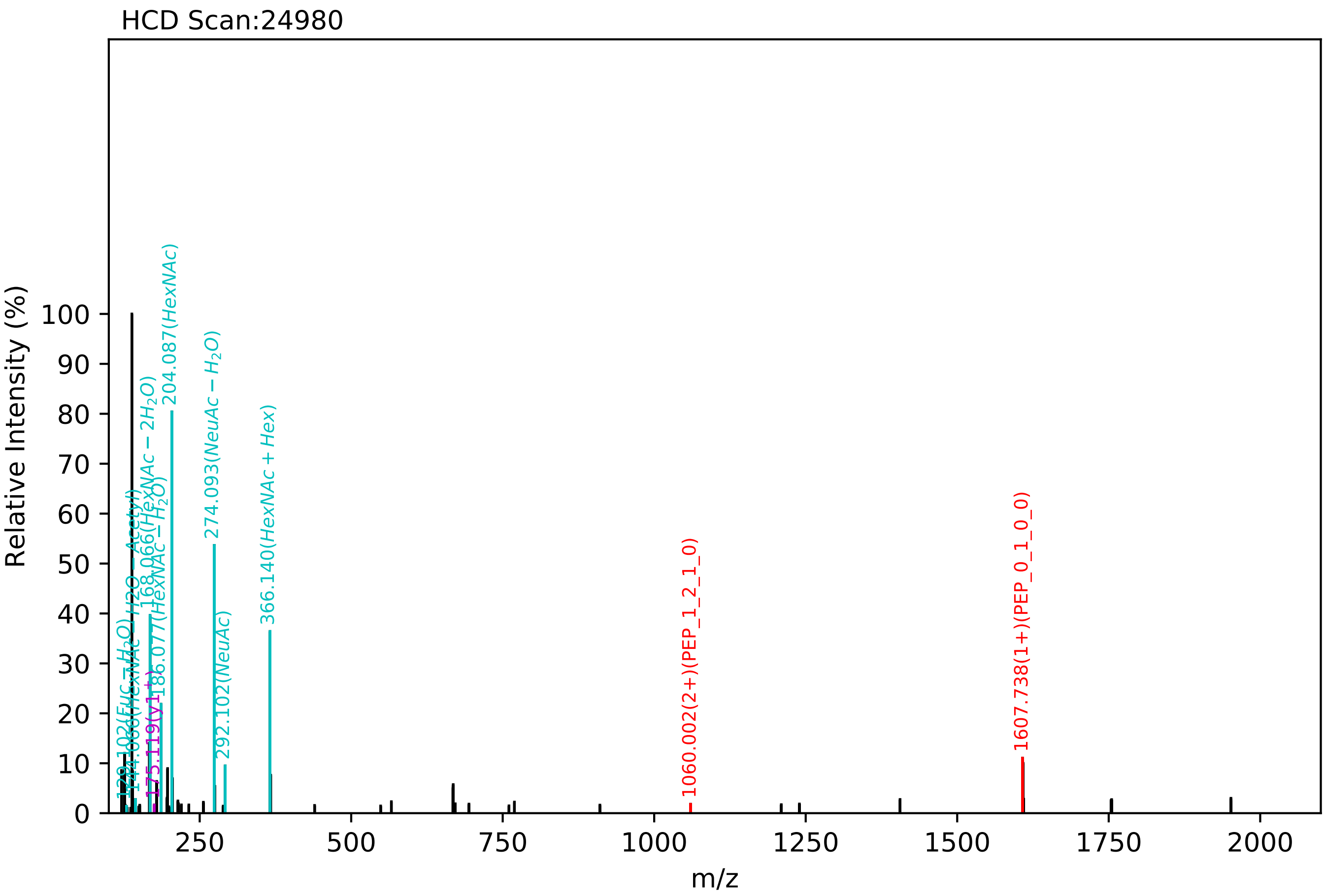

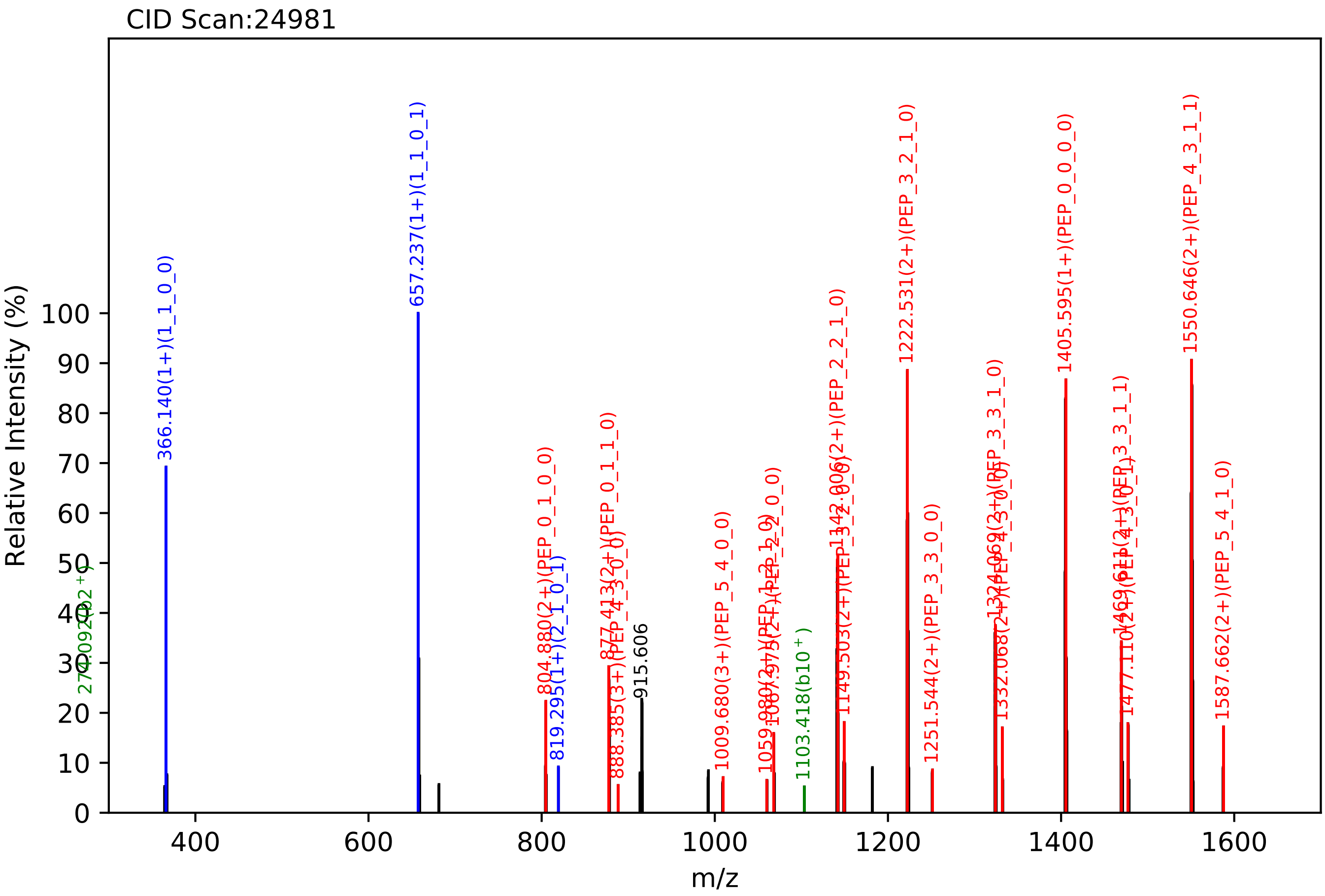

Unknown set no. 290, Experiment: Human Plasma exp_3

SWPAVGNCSSALR(=PEP)_6_5_0_2, m/z:994.40(4+), RT:85.57, Y-score:88.45

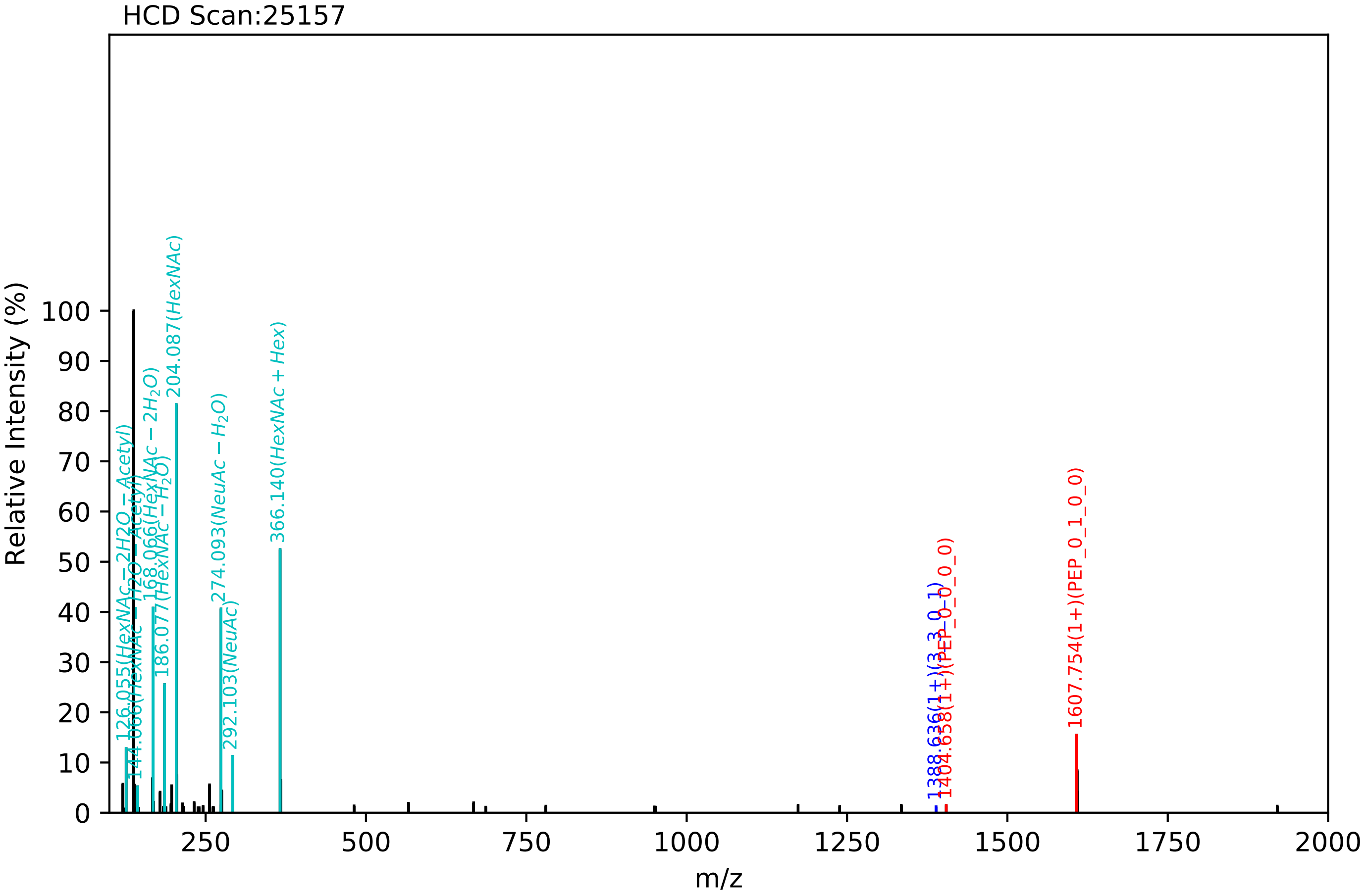

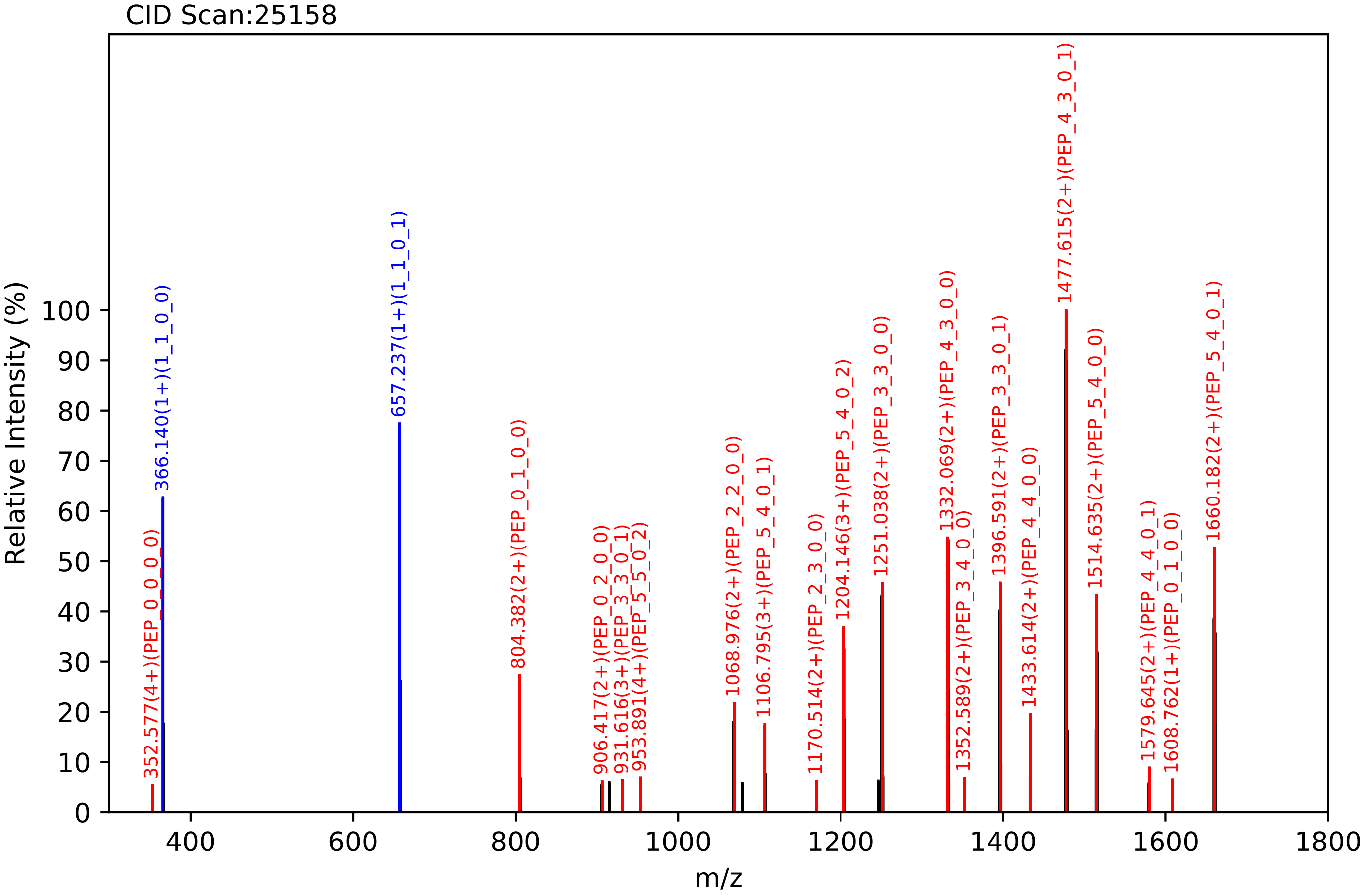

Unknown set no. 291, Experiment: Human Plasma exp_4

SWPAVGNCSSALR(=PEP)_6_5_1_3, m/z:1103.69(4+), RT:97.98, Y-score:78.31

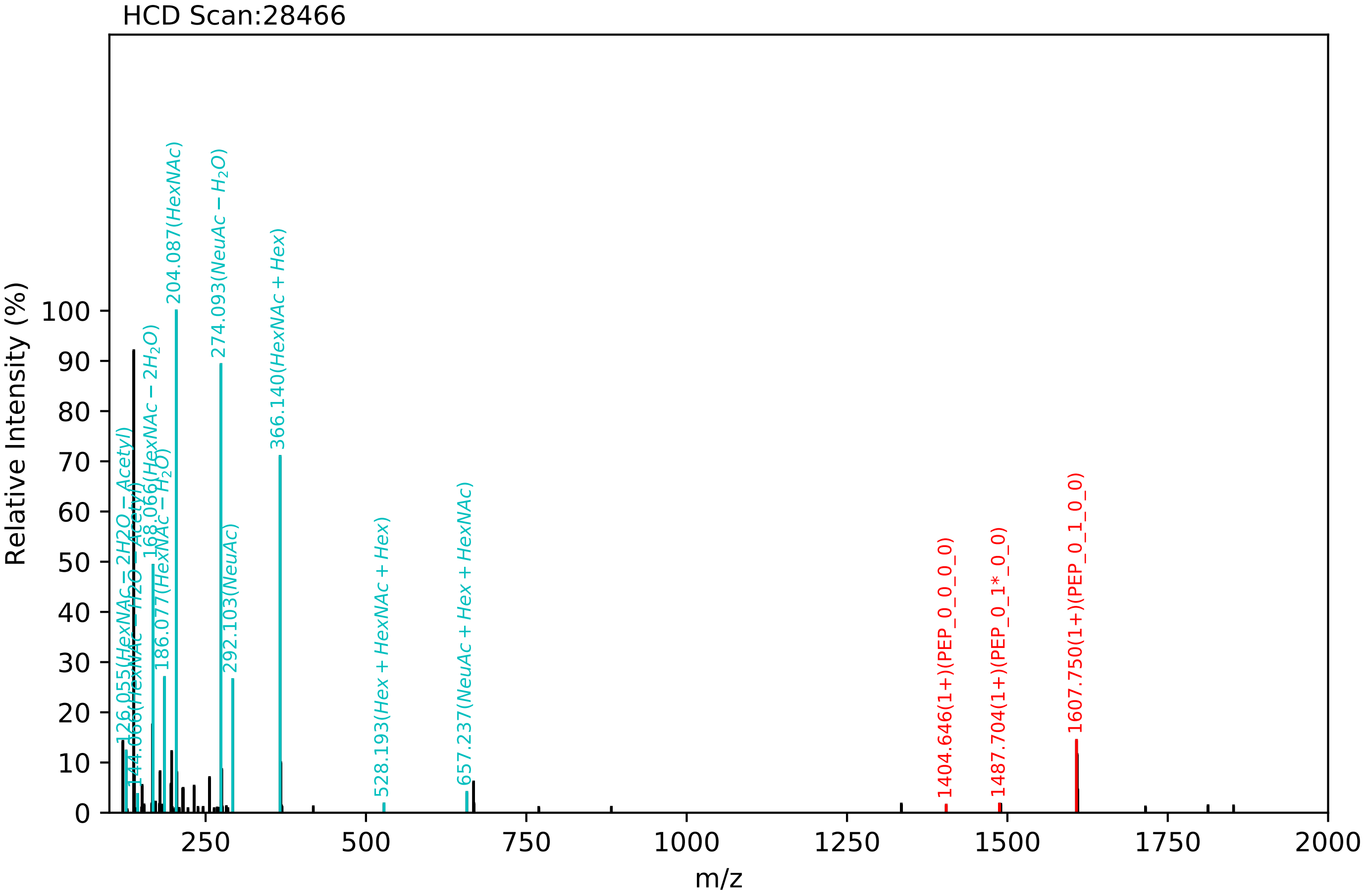

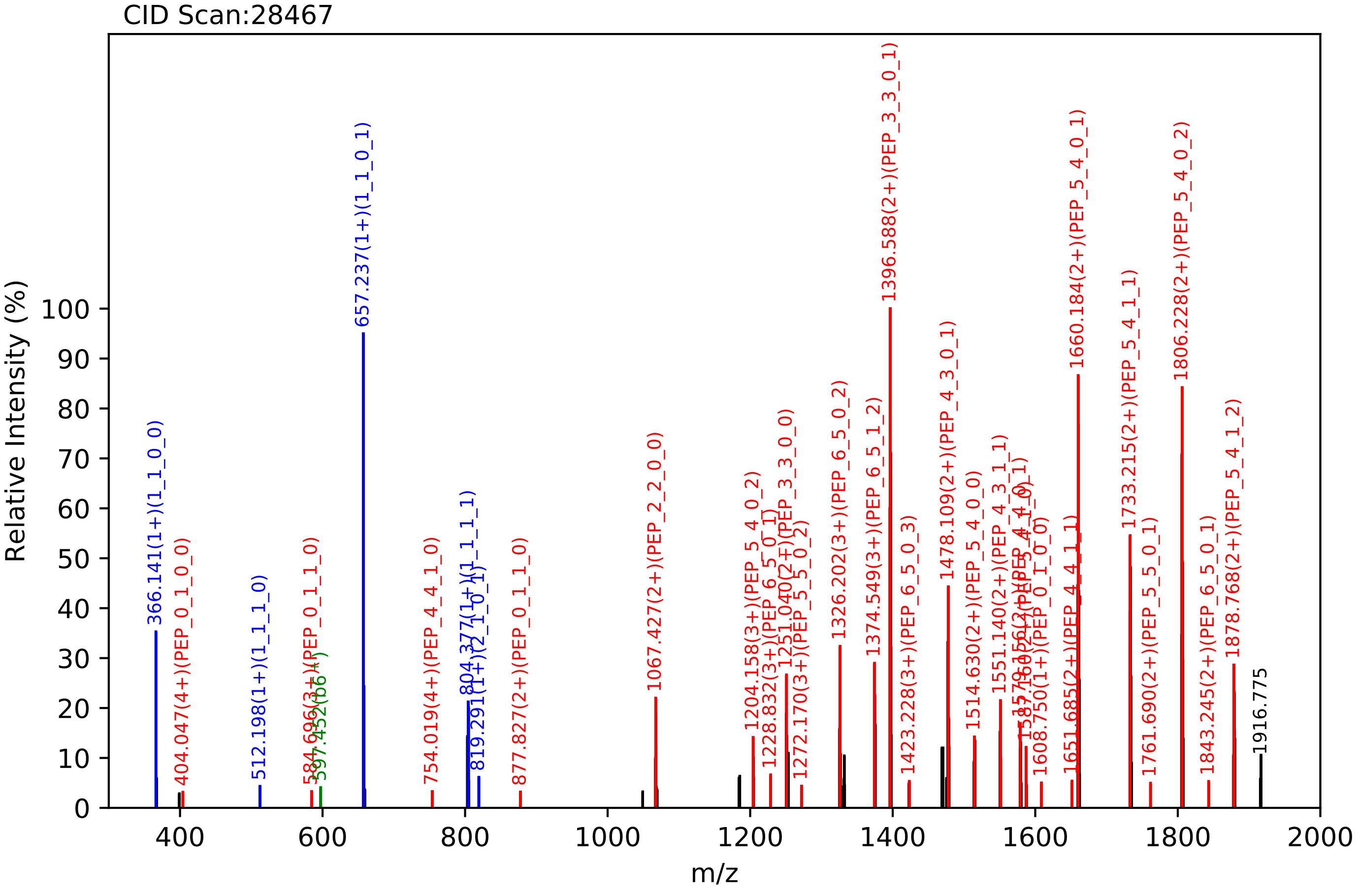

Unknown set no. 292, Experiment: Human Plasma exp_1

DFVNASSK(=PEP)_5_4_0_2, m/z:1024.74(3+), RT:55.83, Y-score:91.29

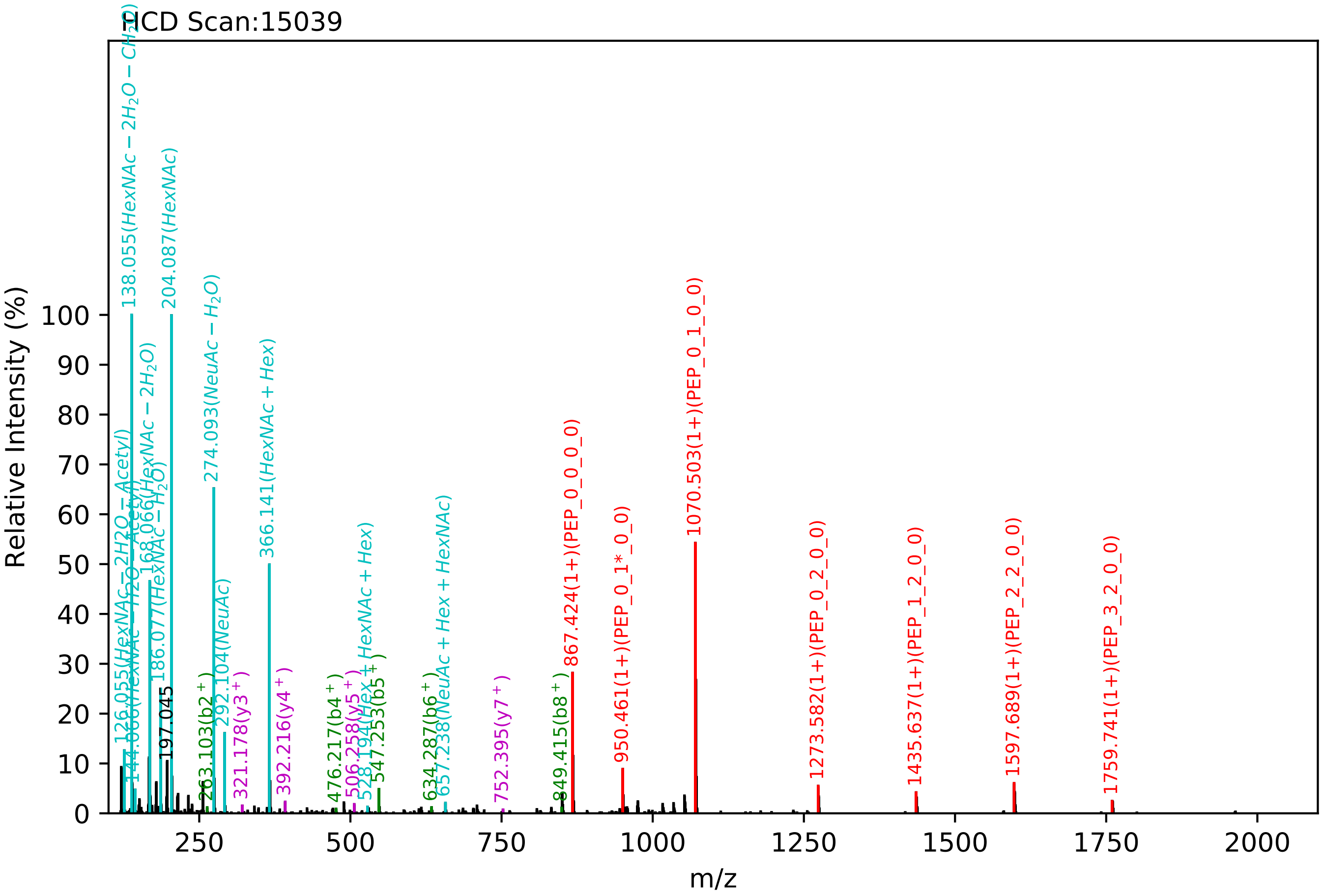

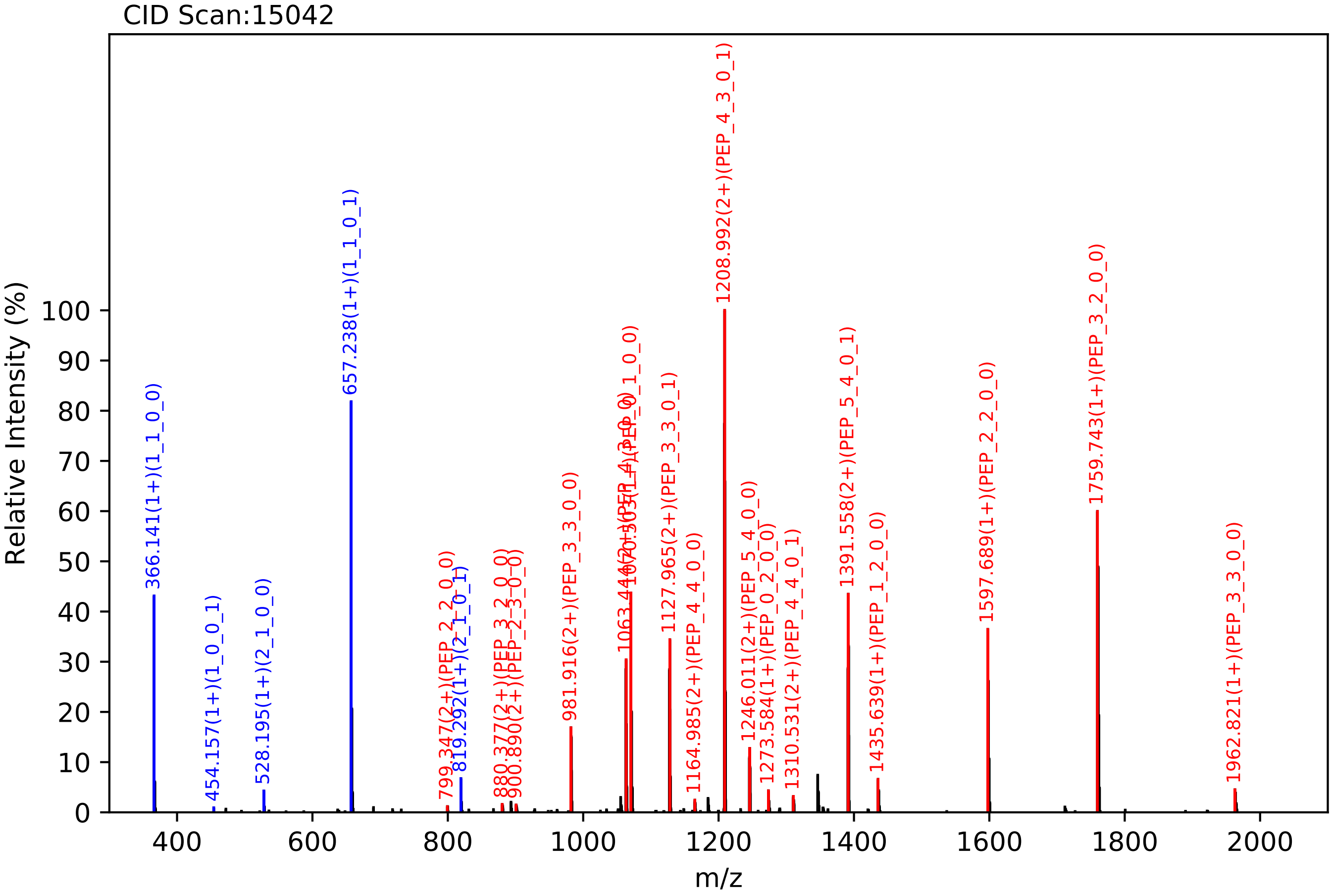

Unknown set no. 293, Experiment: Human Plasma exp_1

DFVNASSK(=PEP)_5_4_0_2, m/z:1536.60(2+), RT:55.74, Y-score:78.74

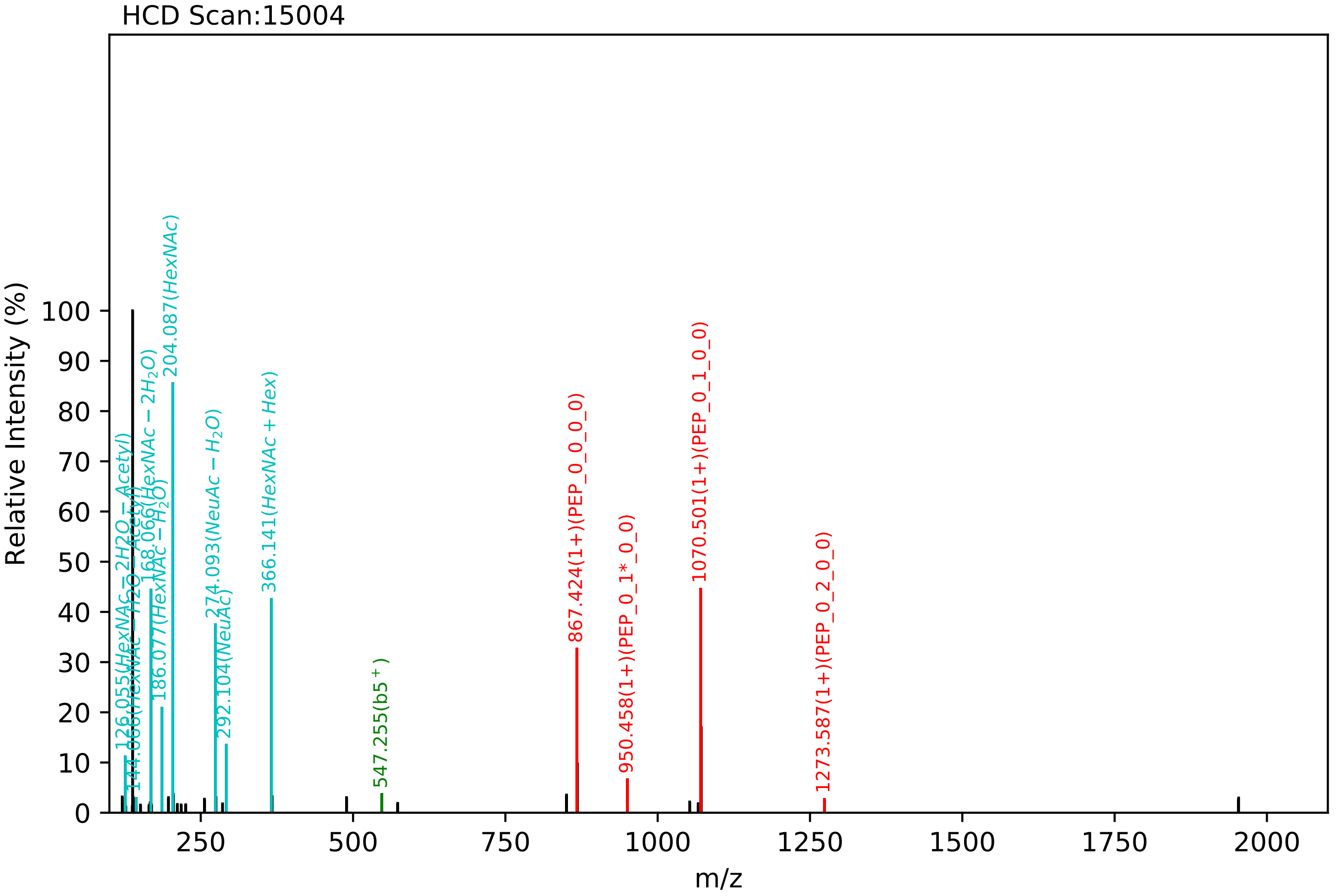

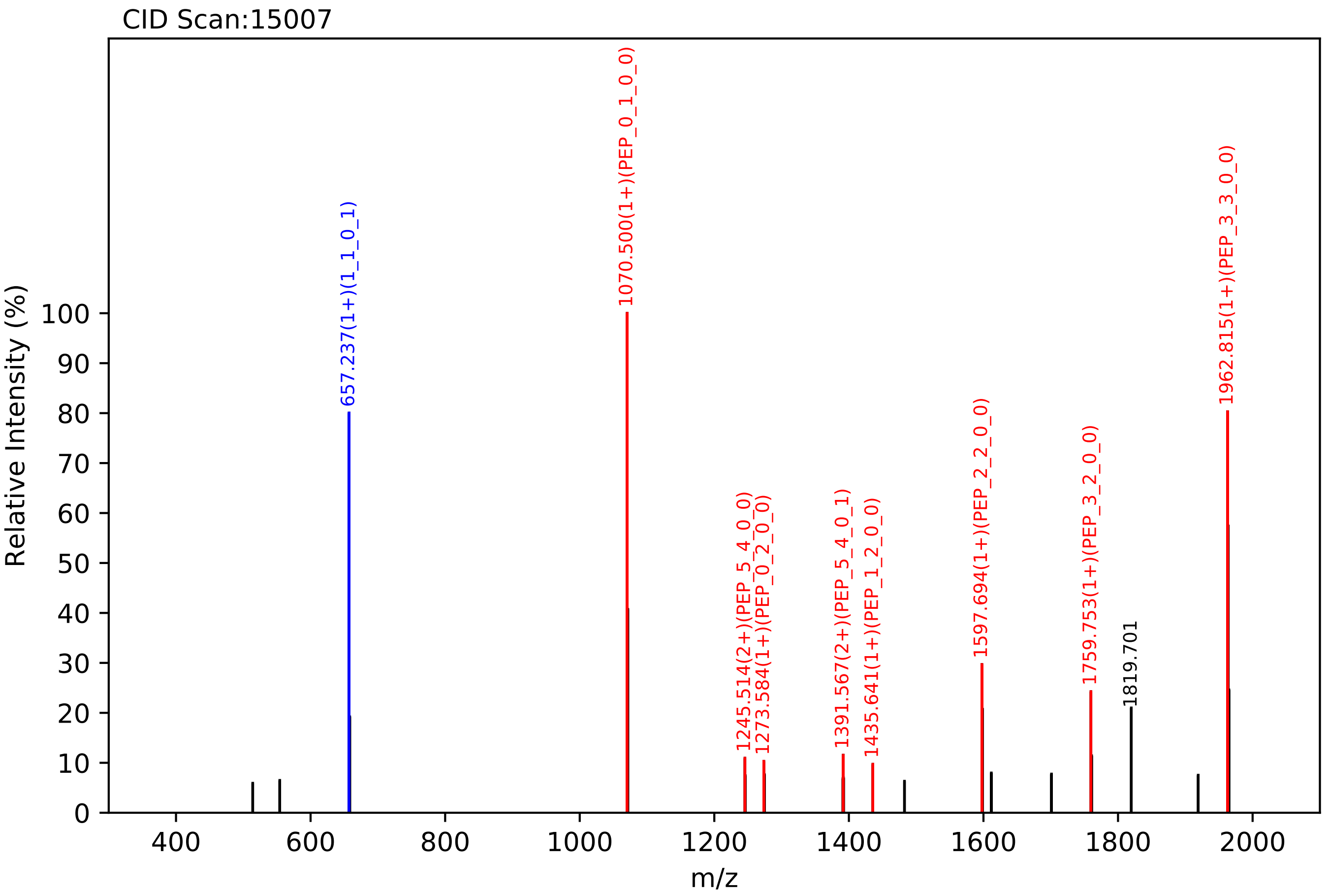

Unknown set no. 294, Experiment: Human Plasma exp_2

DFVNASSK(=PEP)_5_4_0_2, m/z:1024.74(3+), RT:55.94, Y-score:90.77

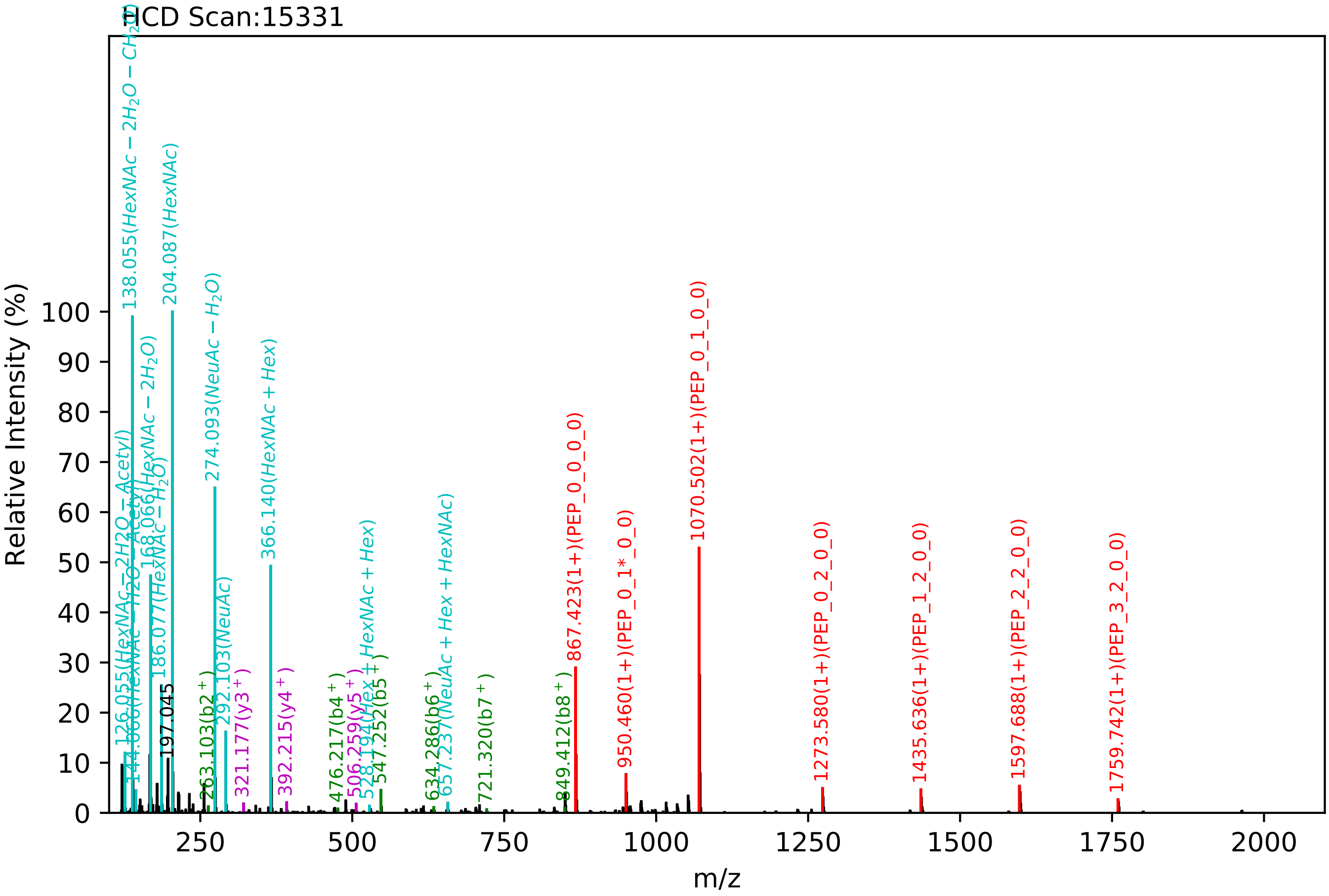

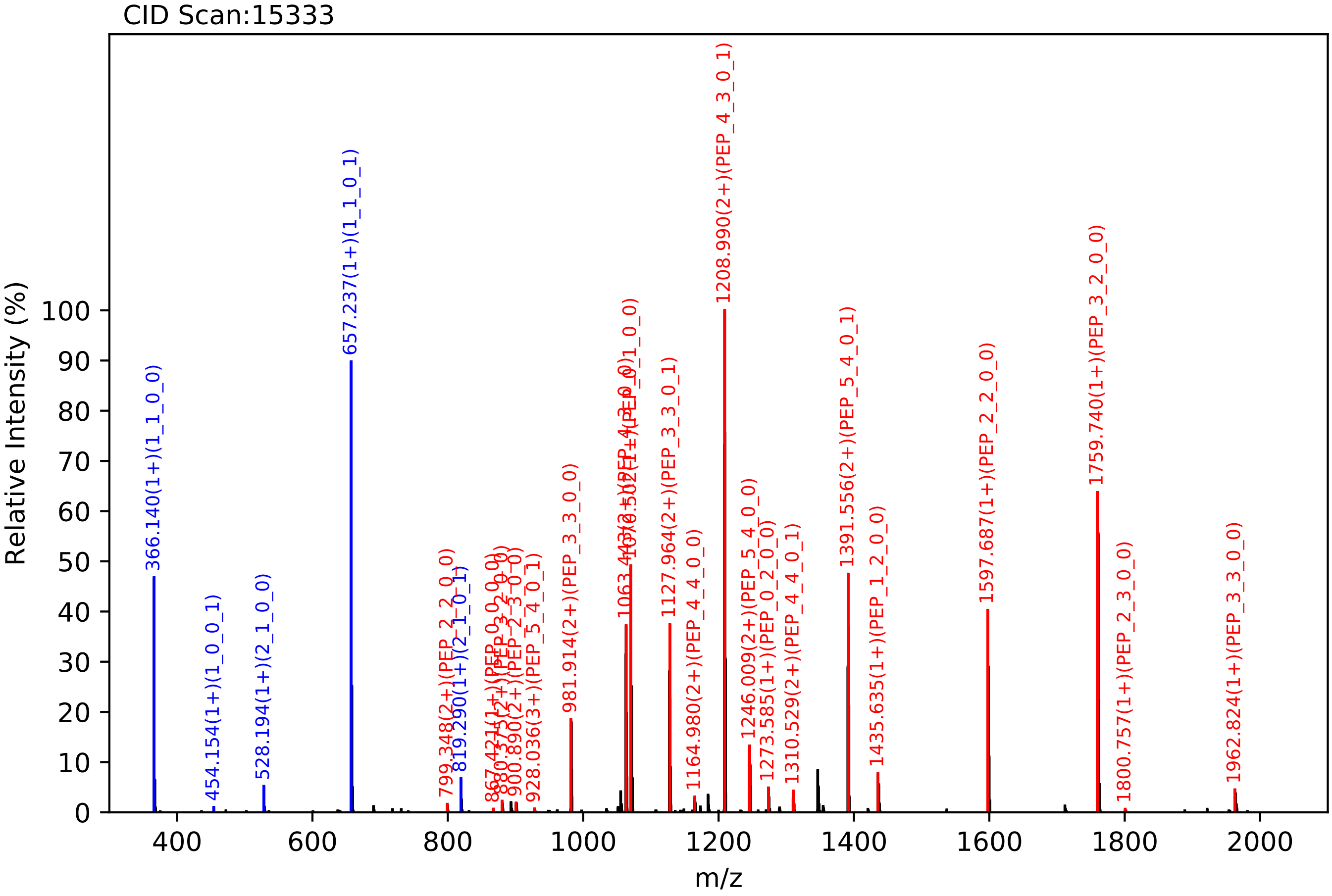

Unknown set no. 295, Experiment: Human Plasma exp_3

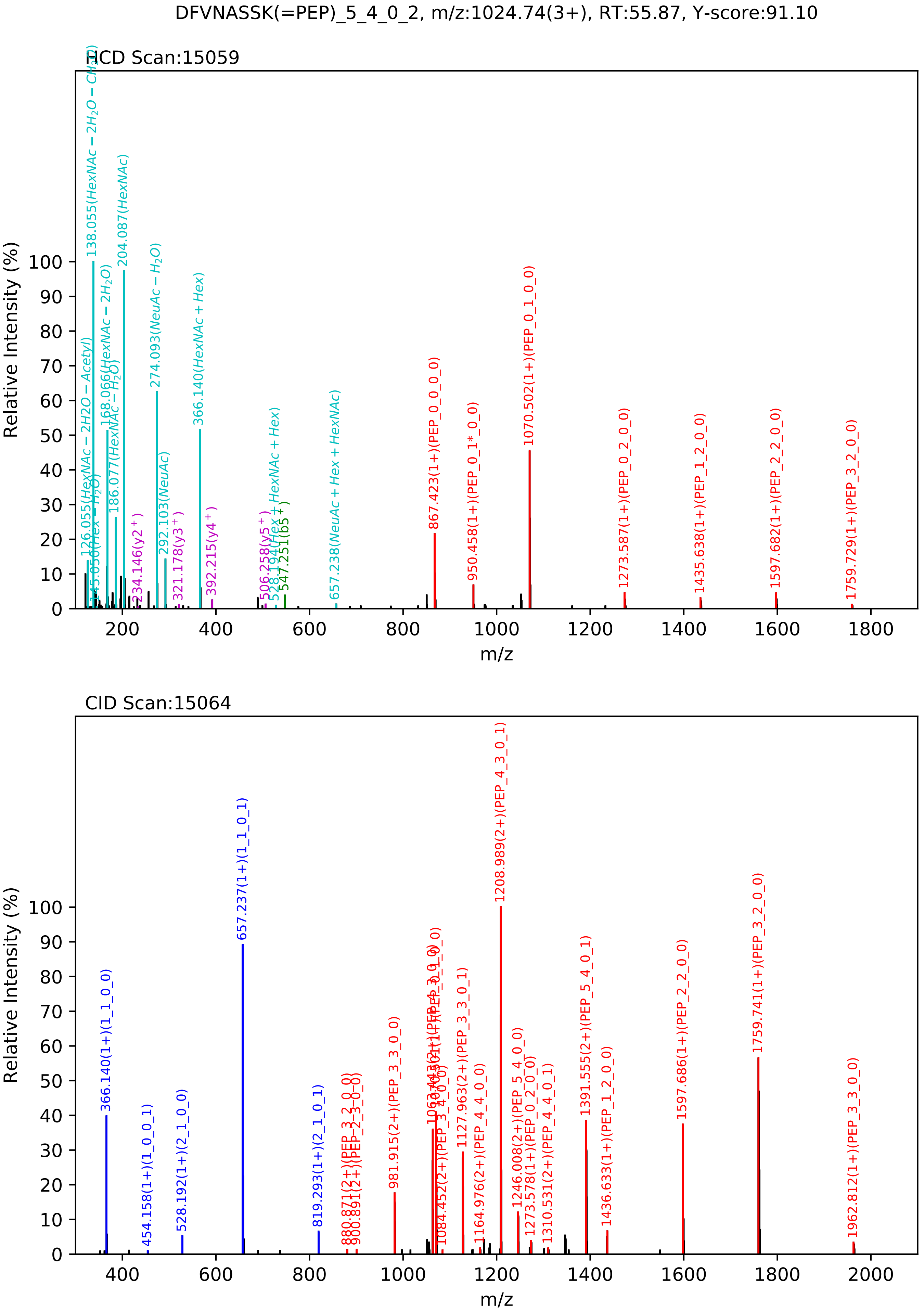

Unknown set no. 296, Experiment: Human Plasma exp_4

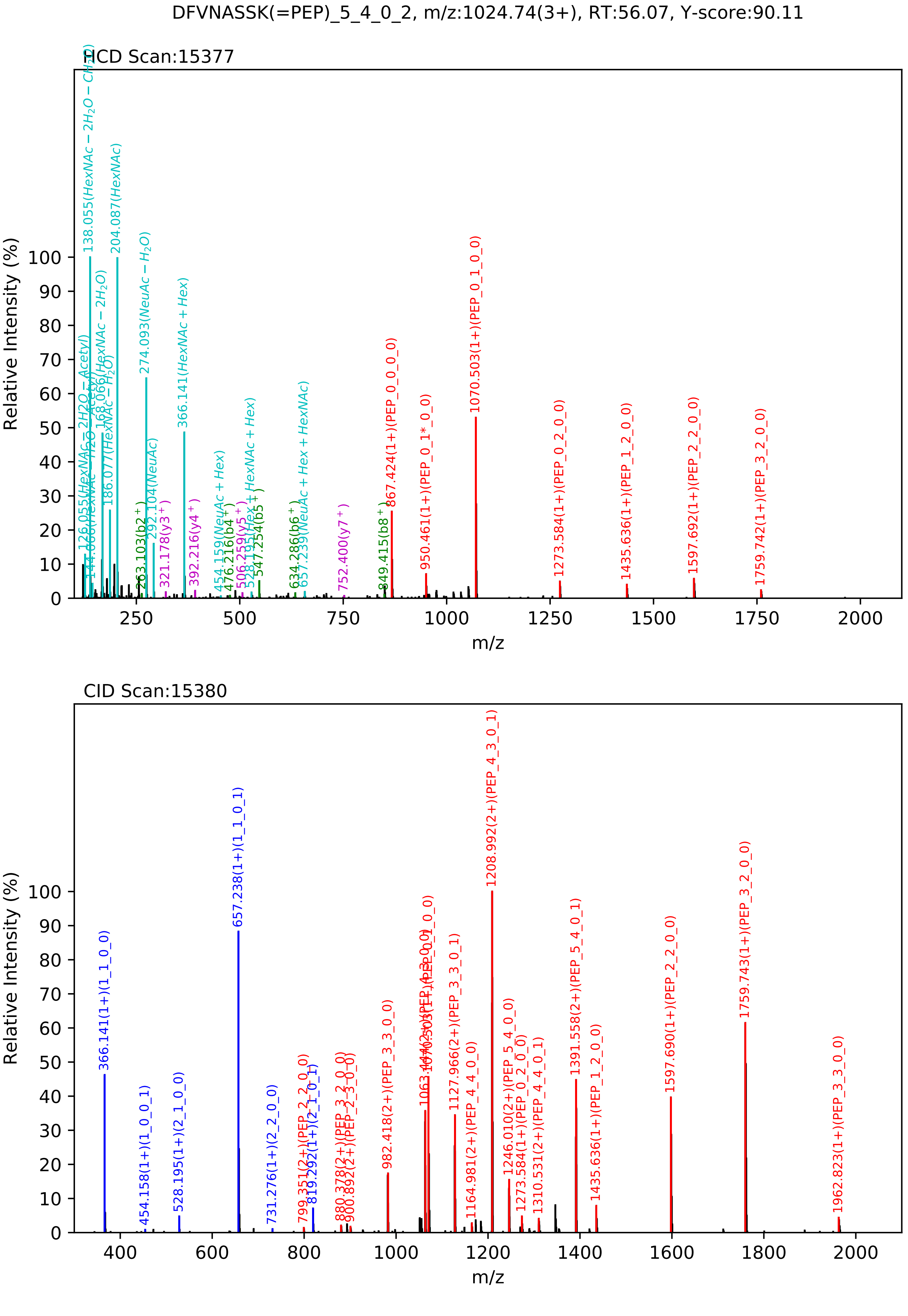

Unknown set no. 297, Experiment: Human Plasma exp_4

DFVNASSK(=PEP)_5_4_0_2, m/z:1536.60(2+), RT:56.08, Y-score:89.85

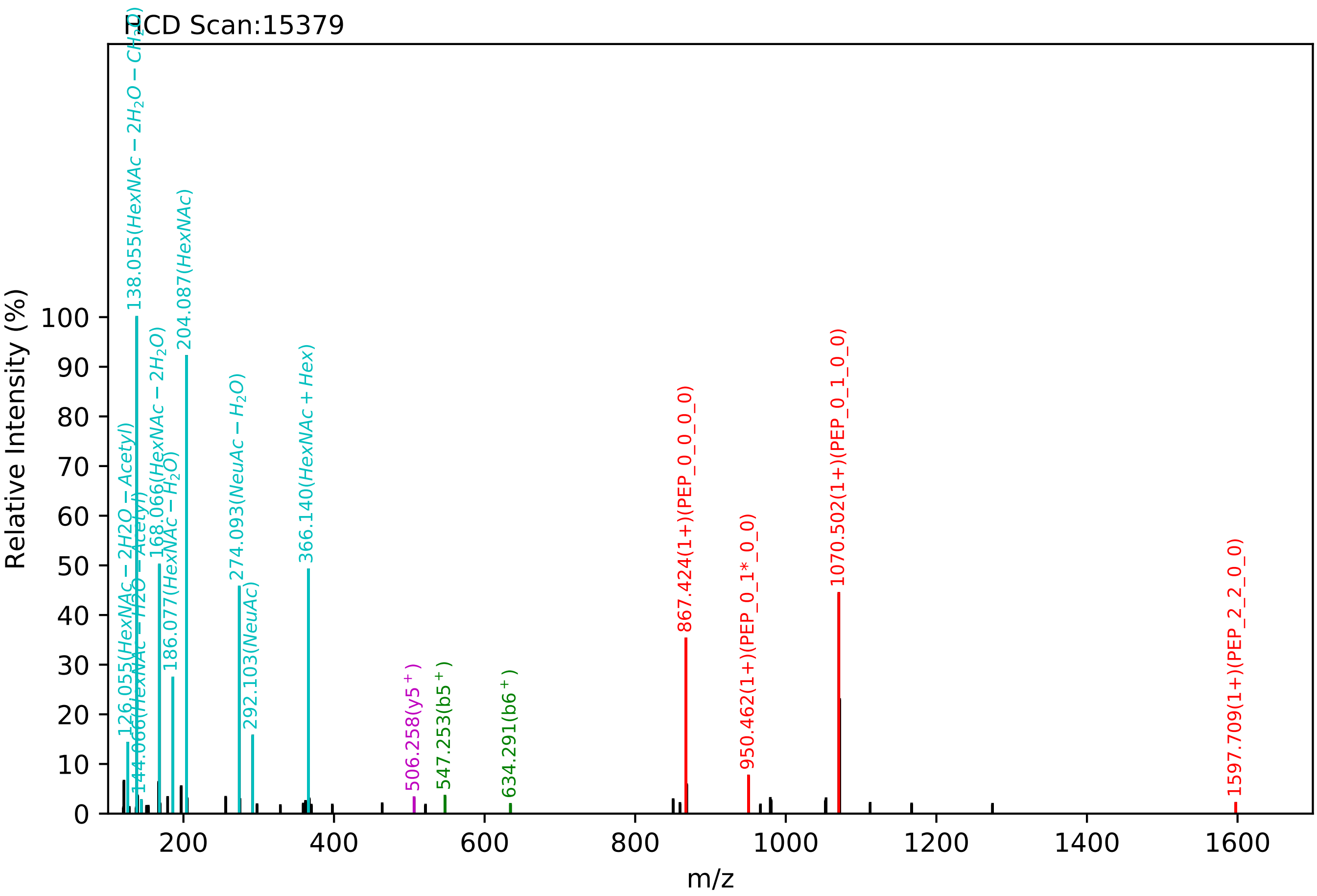

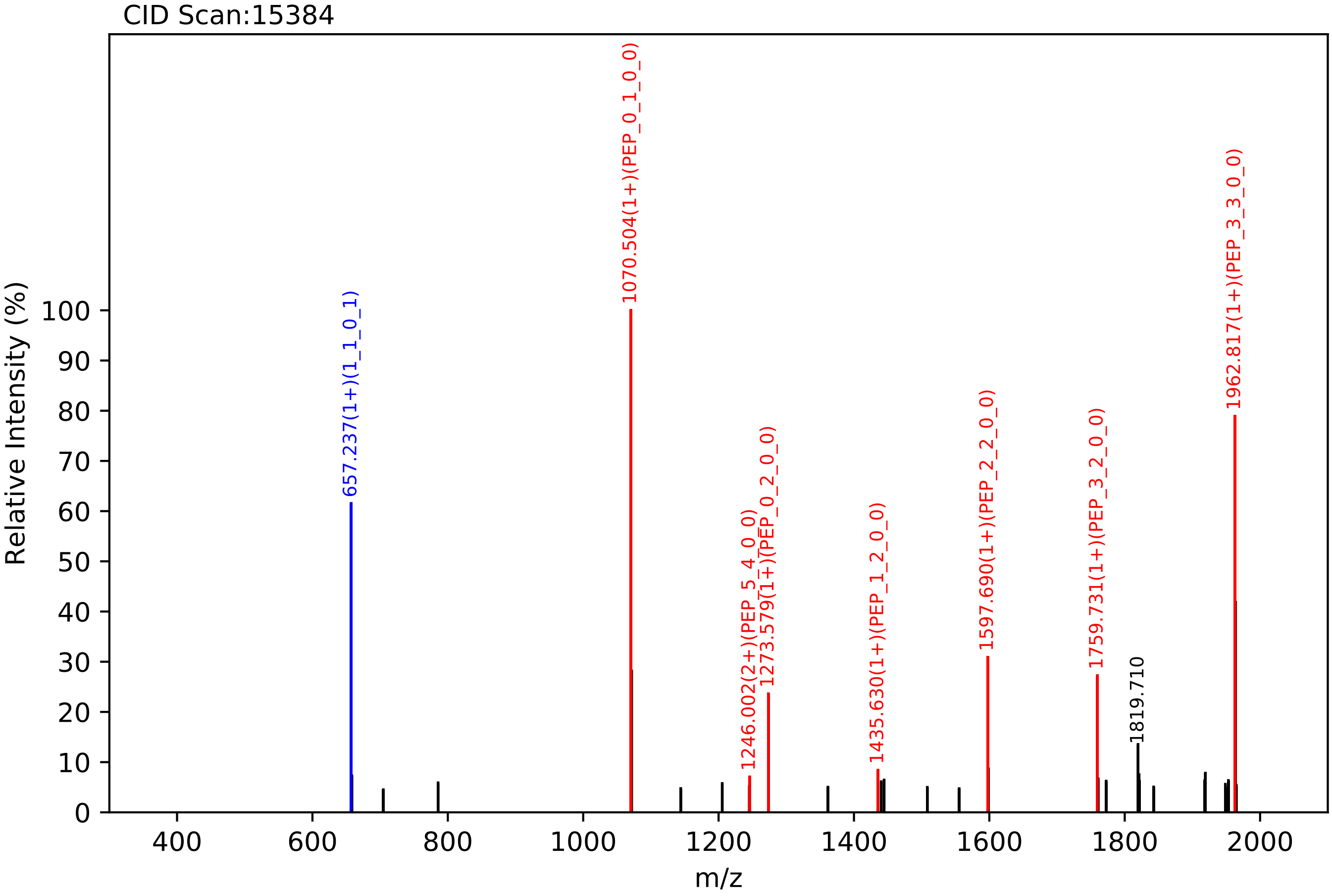

Unknown set no. 298, Experiment: Human Plasma exp_5

DFVNASSK(=PEP)_5_4_0_2, m/z:1536.60(2+), RT:56.20, Y-score:76.55

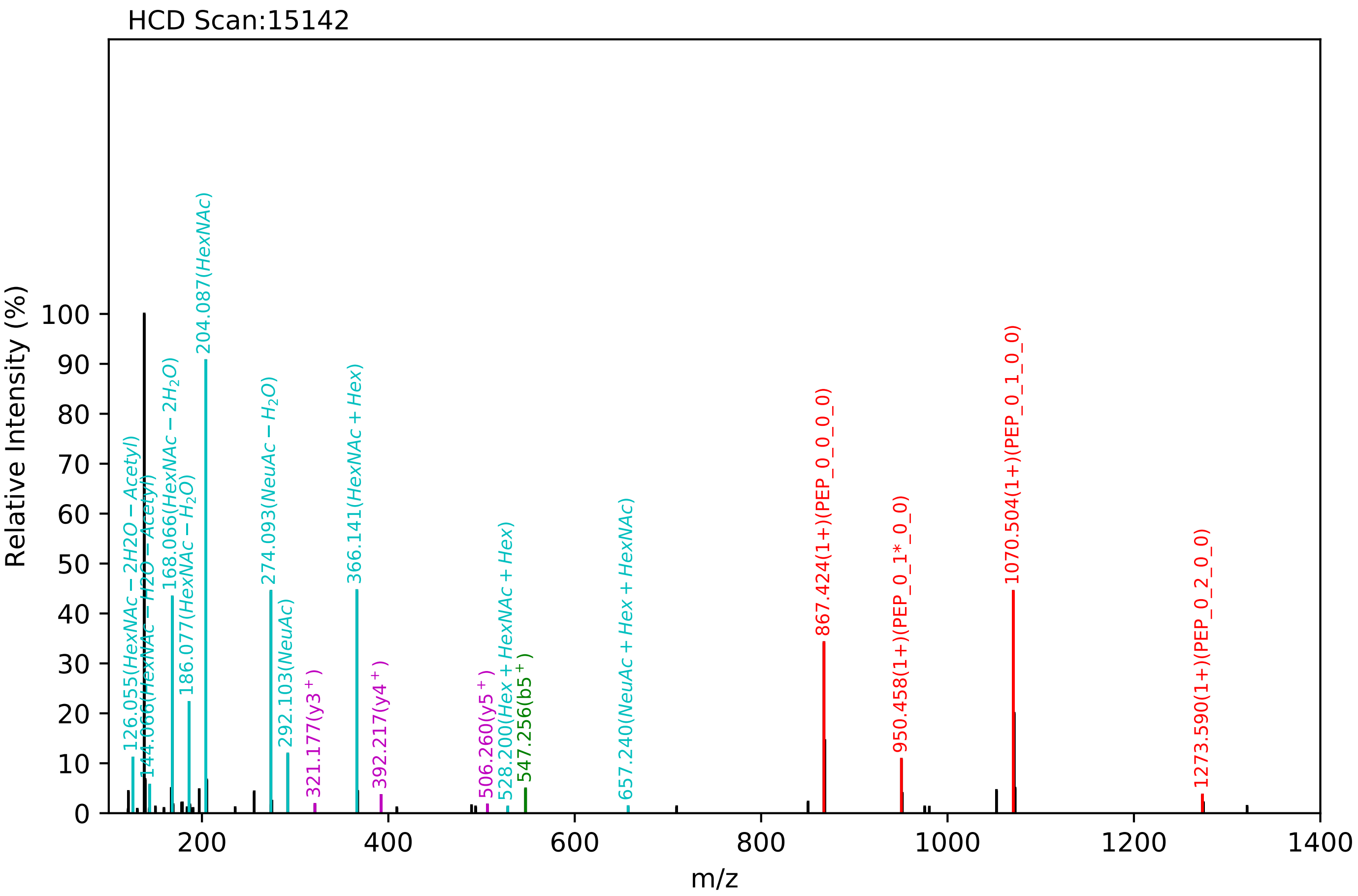

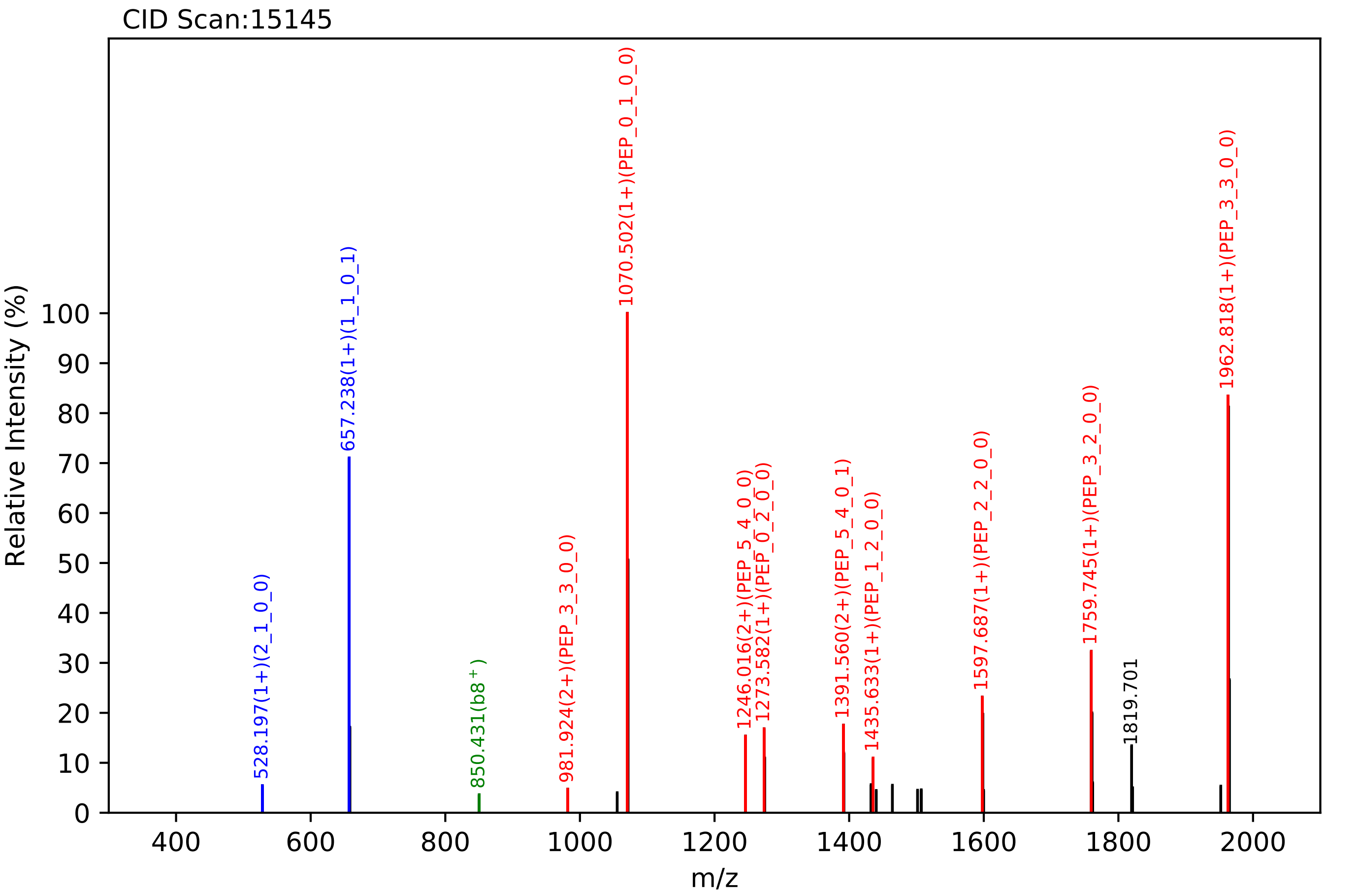

Unknown set no. 299, Experiment: Human Plasma exp_1

DFVNASSK(=PEP)_6_5_0_3, m/z:1243.48(3+), RT:69.14, Y-score:79.08

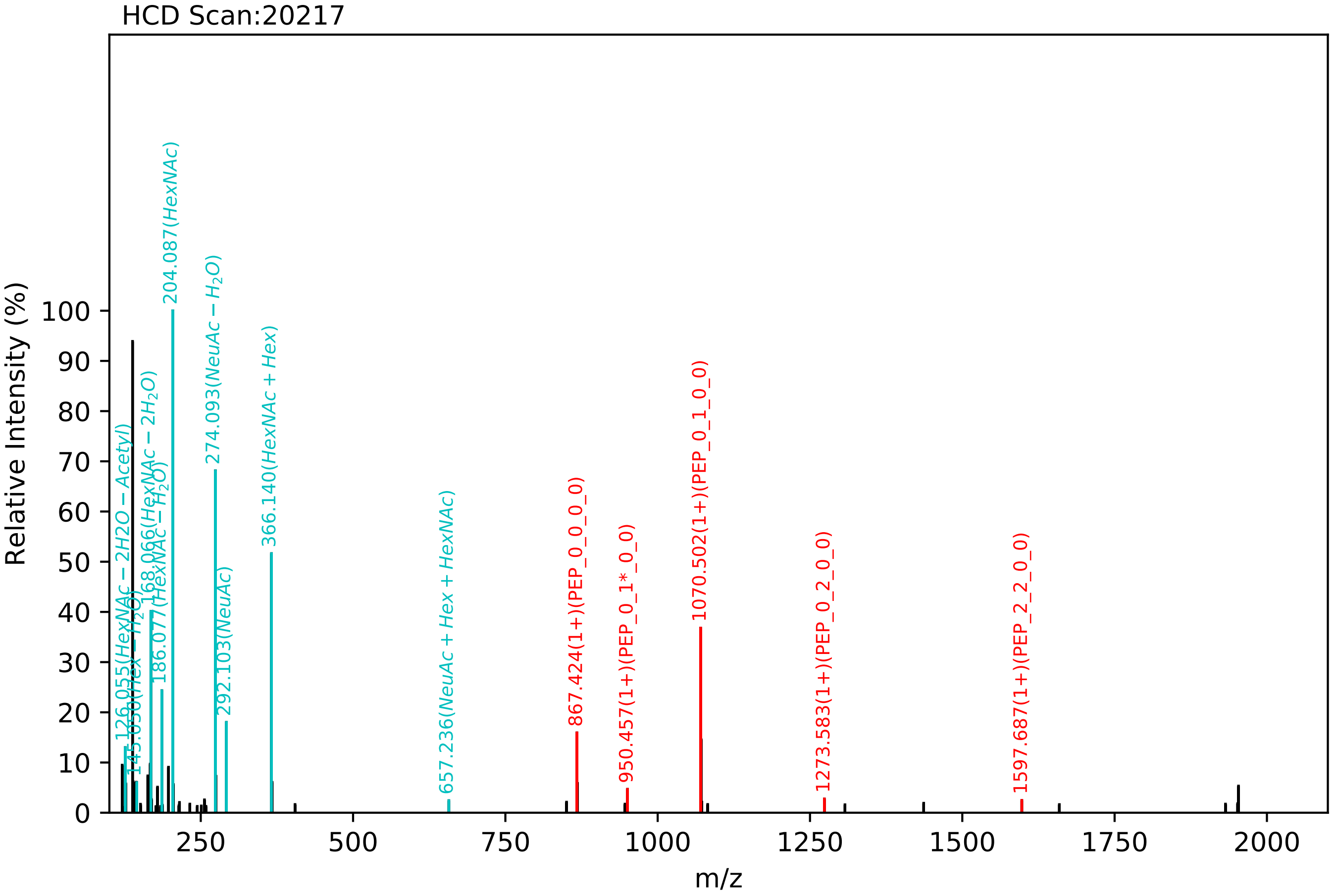

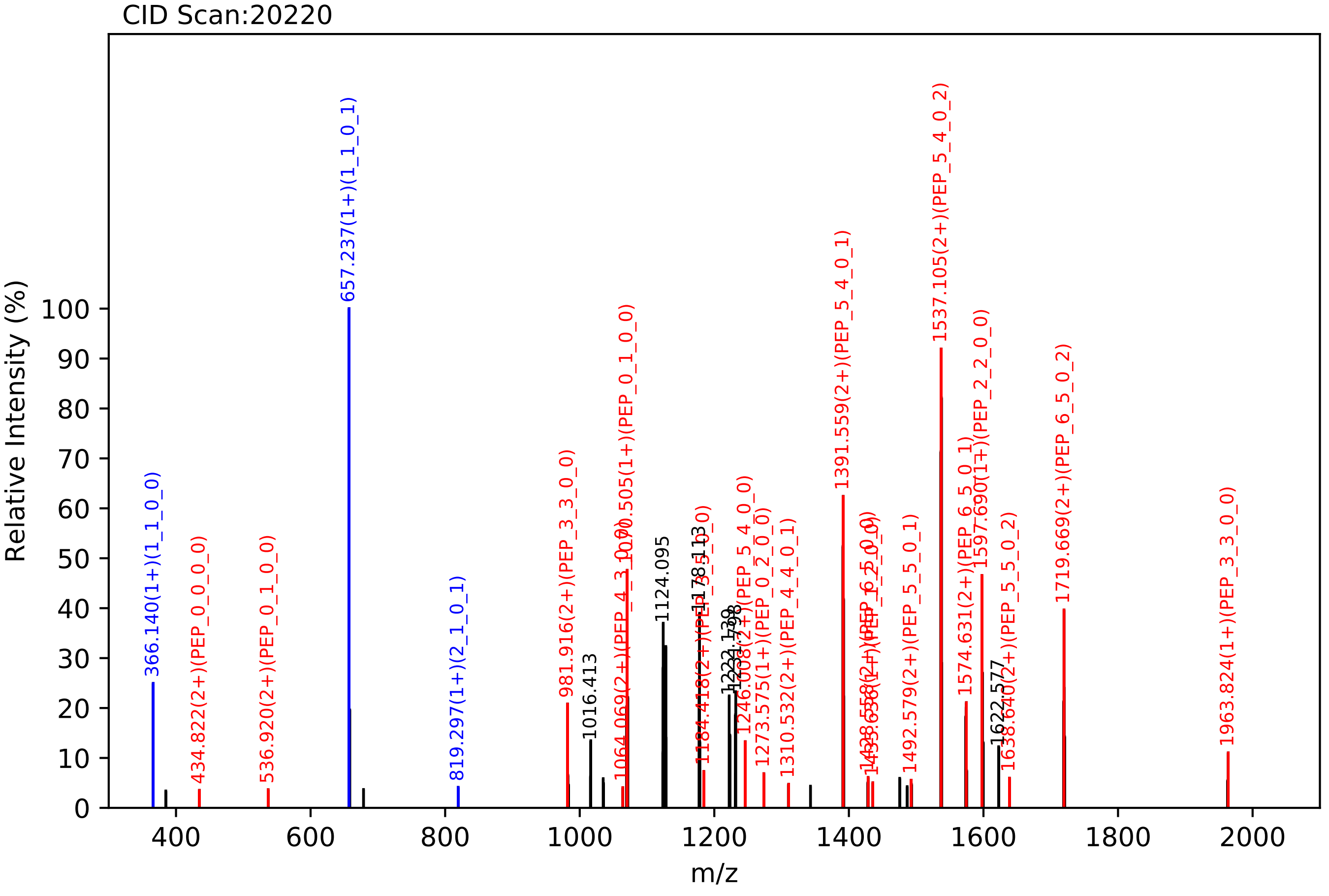

Unknown set no. 300, Experiment: Human Plasma exp_2

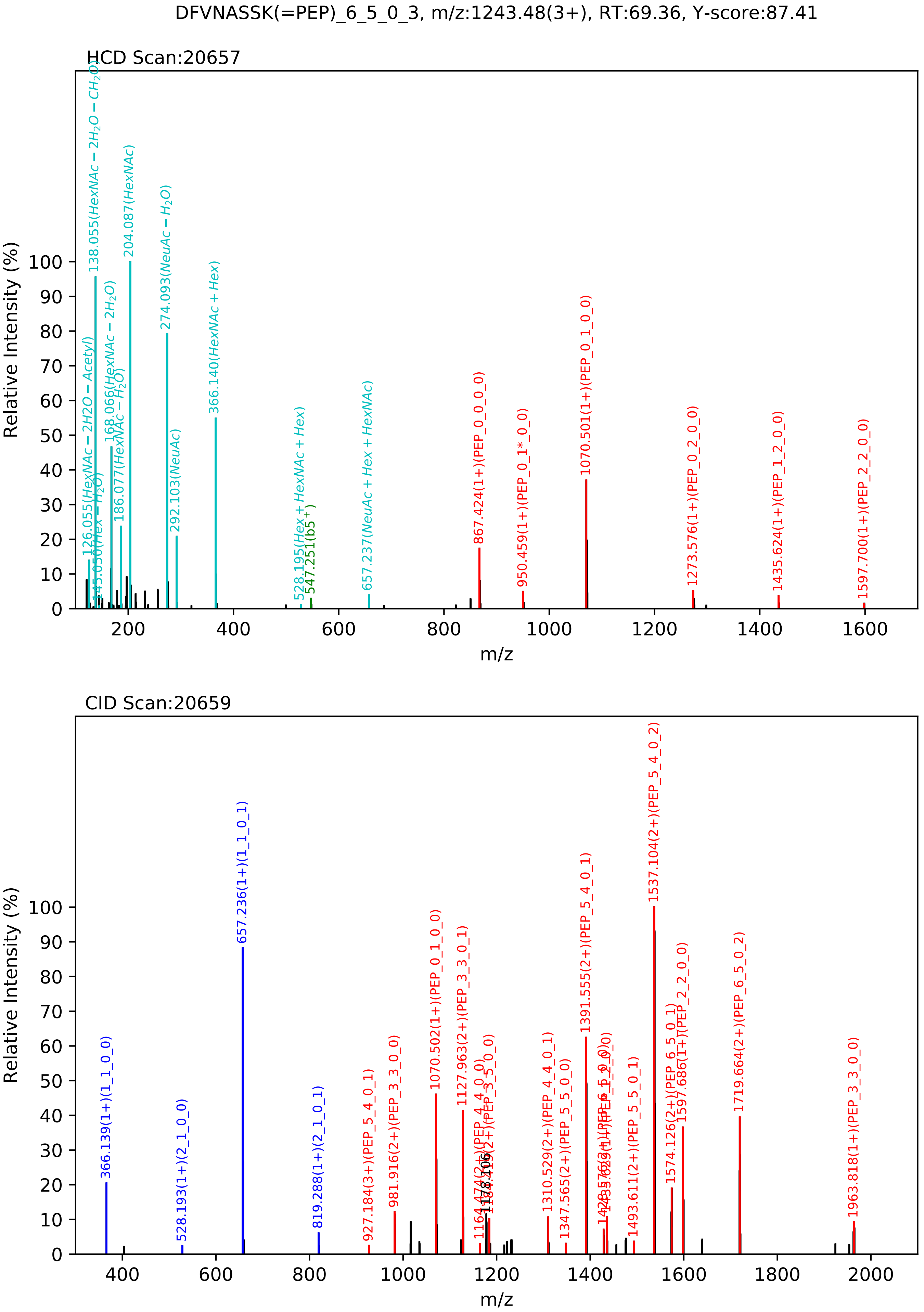

Unknown set no. 301, Experiment: Human Plasma exp_3

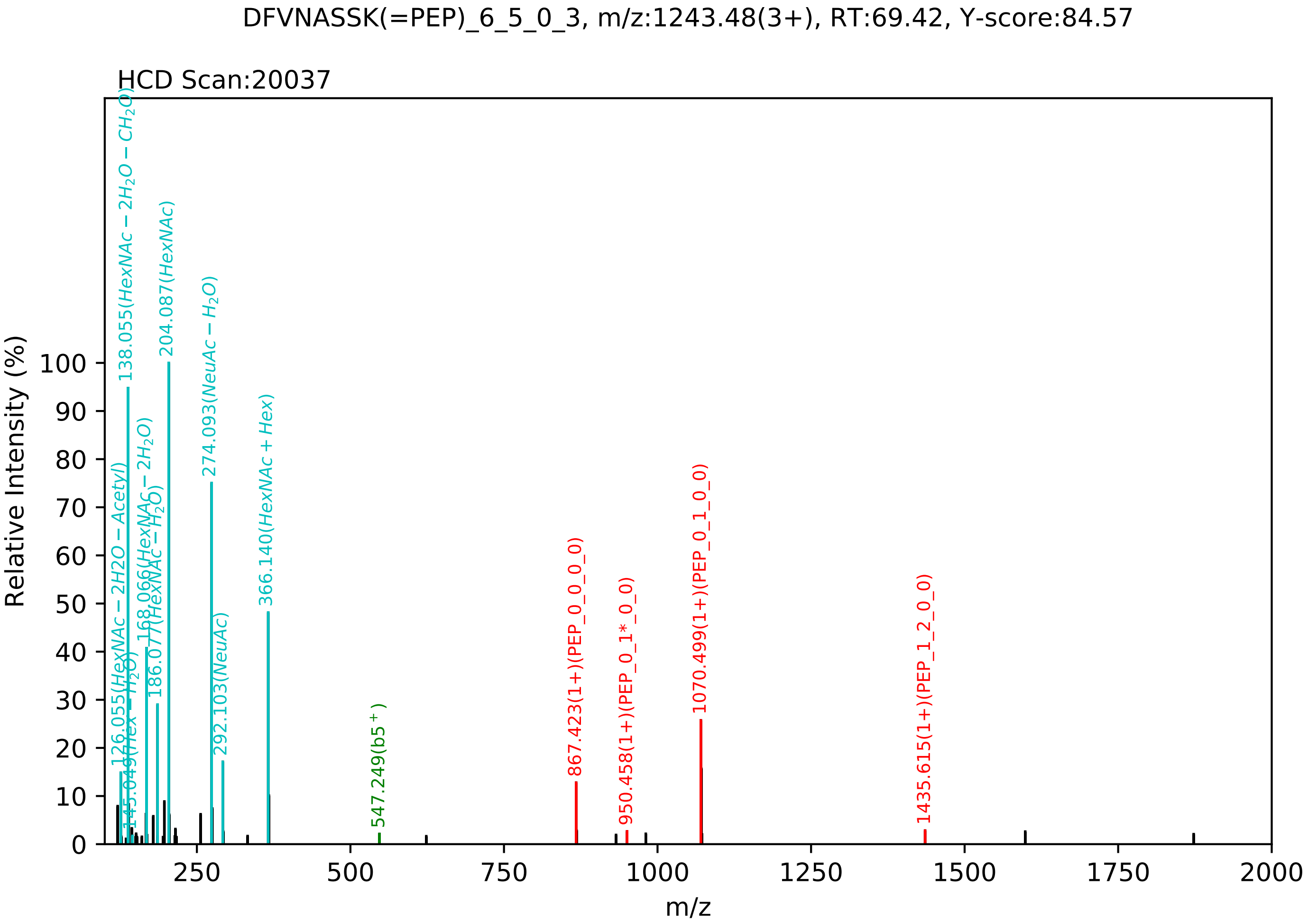

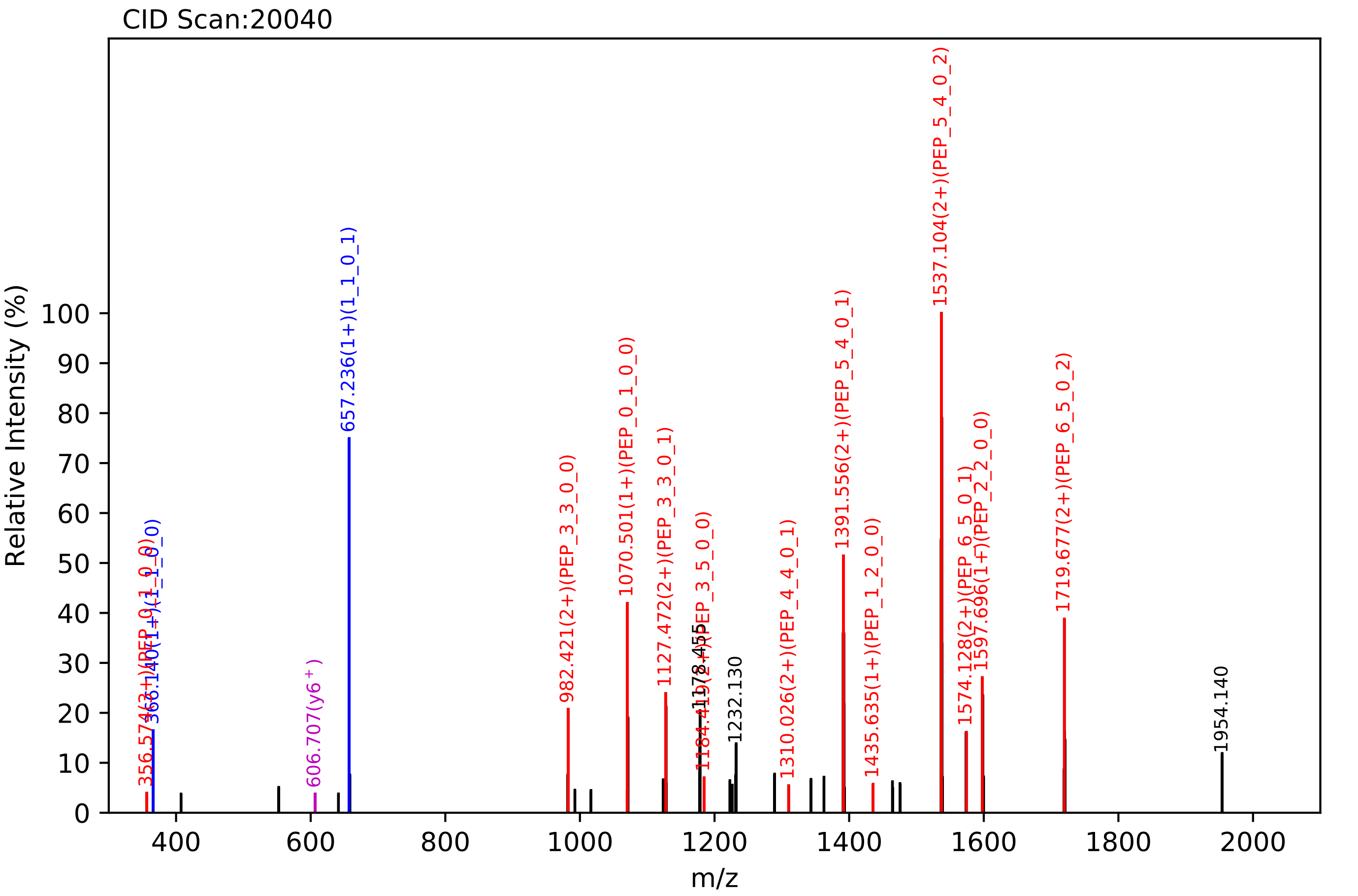

Unknown set no. 302, Experiment: Human Plasma exp_4

DFVNASSK(=PEP)_6_5_0_3, m/z:1243.48(3+), RT:69.43, Y-score:94.60

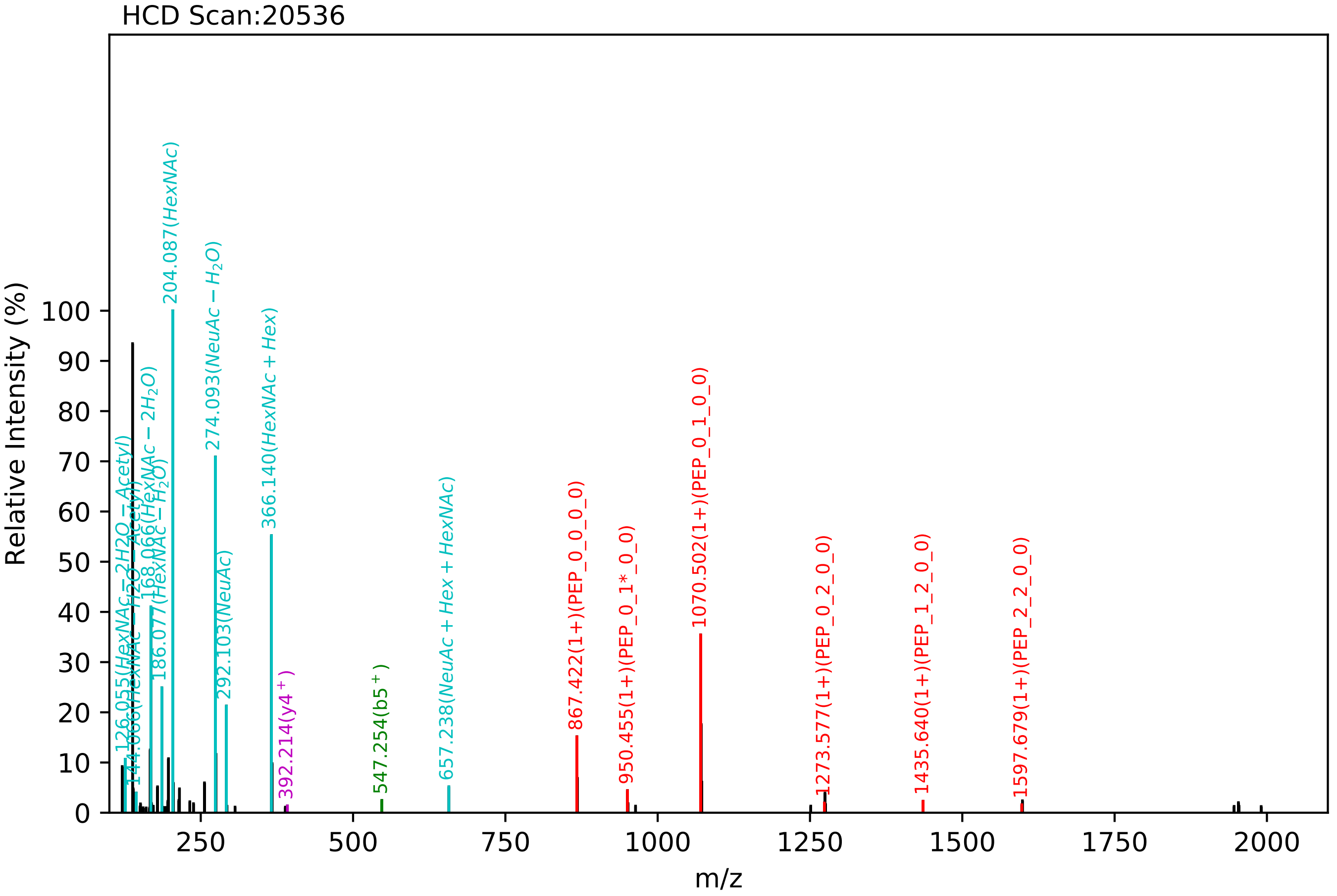

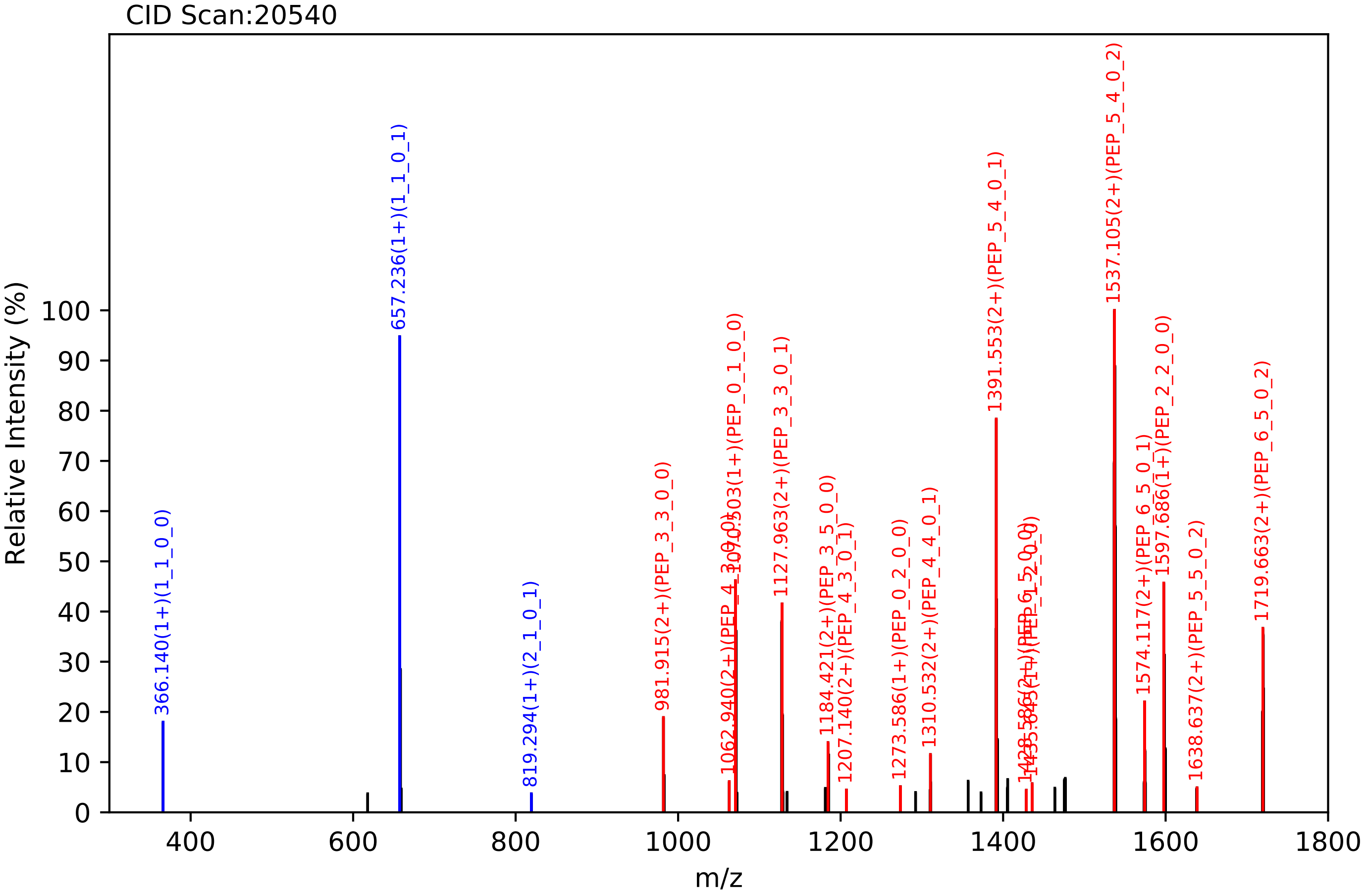

Unknown set no. 303, Experiment: Human Plasma exp_5

DFVNASSK(=PEP)_6_5_0_3, m/z:1243.48(3+), RT:69.26, Y-score:84.72

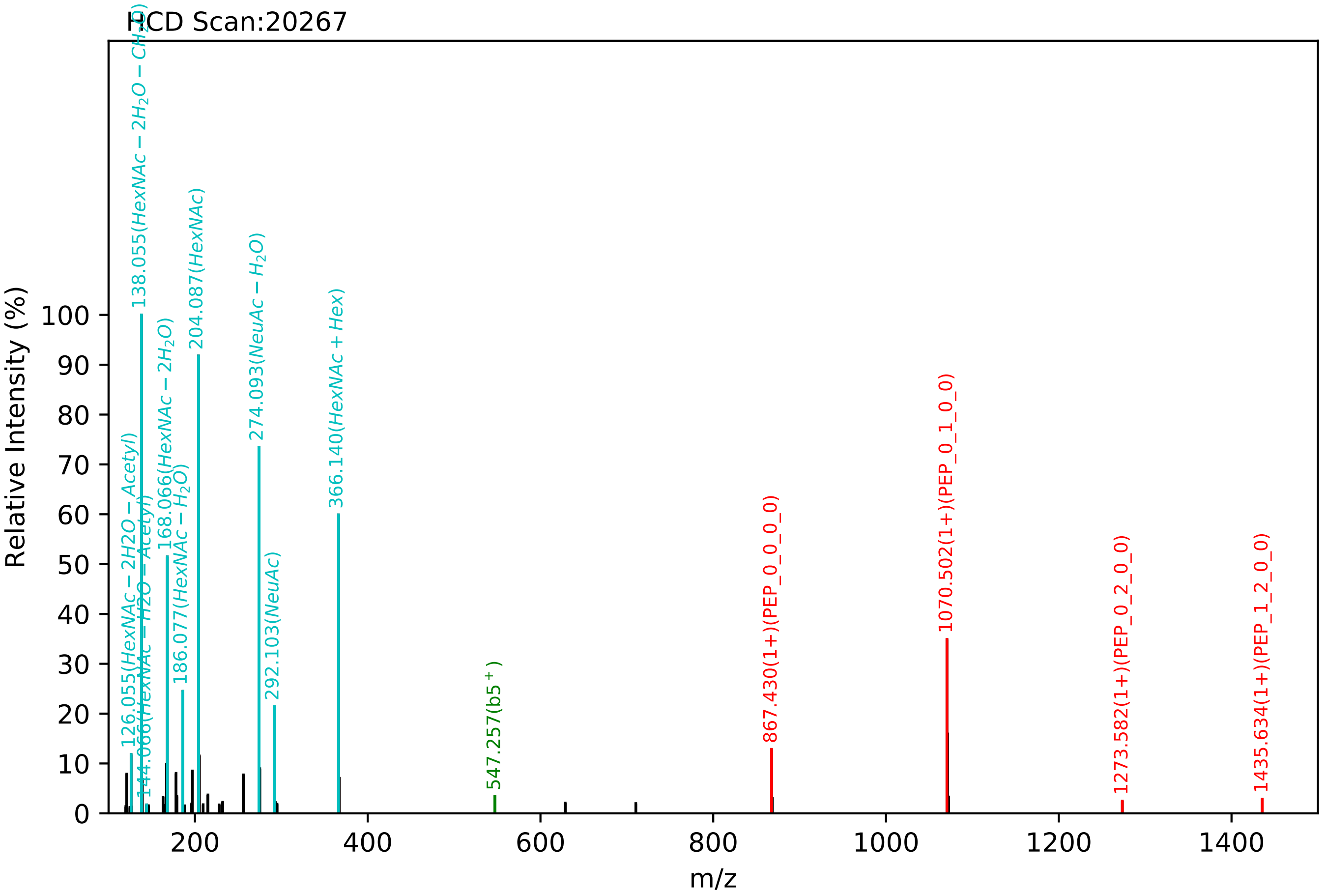

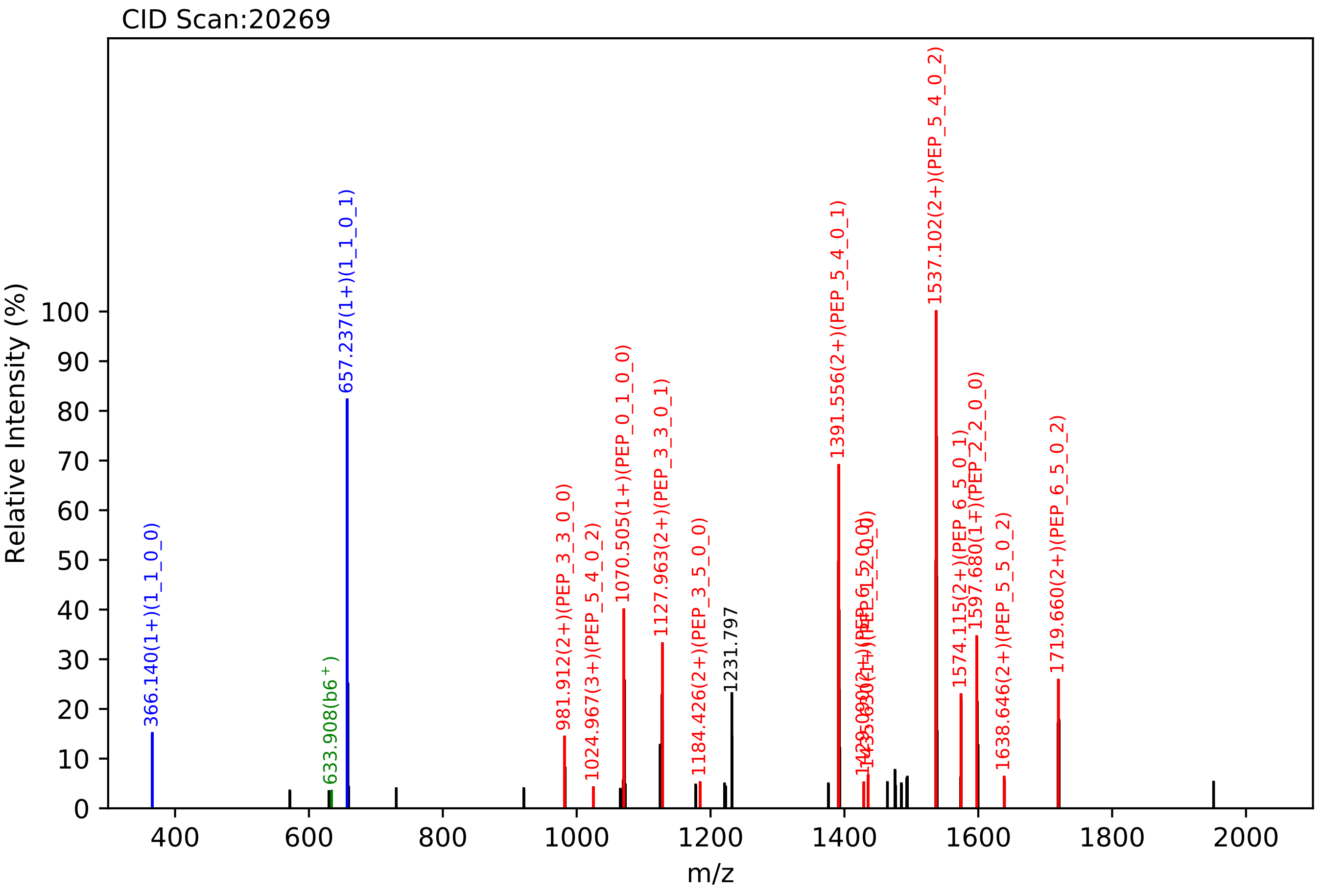

Unknown set no. 304, Experiment: Human Plasma exp_1

NLSMPLLPADFHK(=PEP)_5_4_0_2, m/z:922.64(4+), RT:103.68, Y-score:96.40

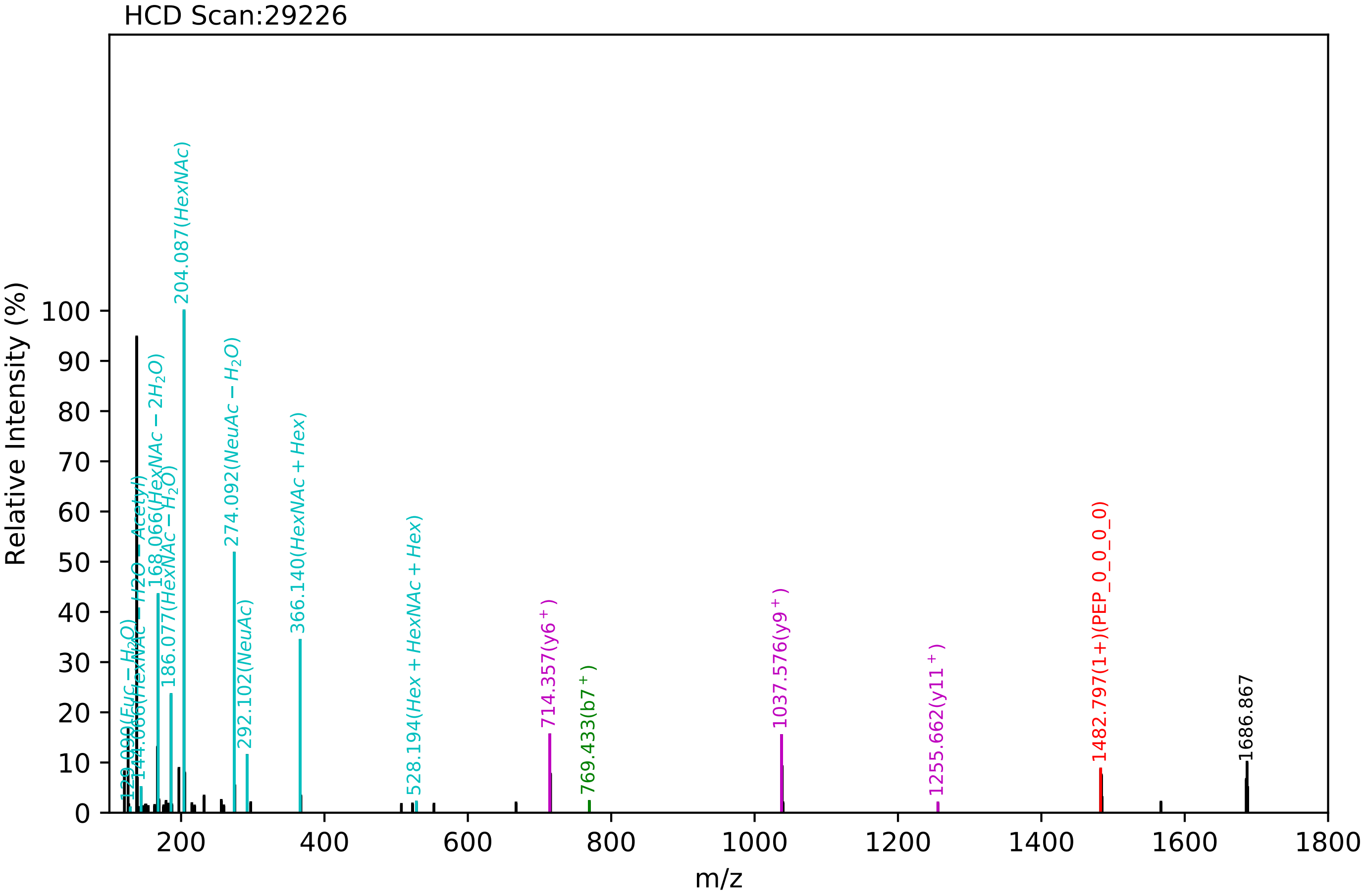

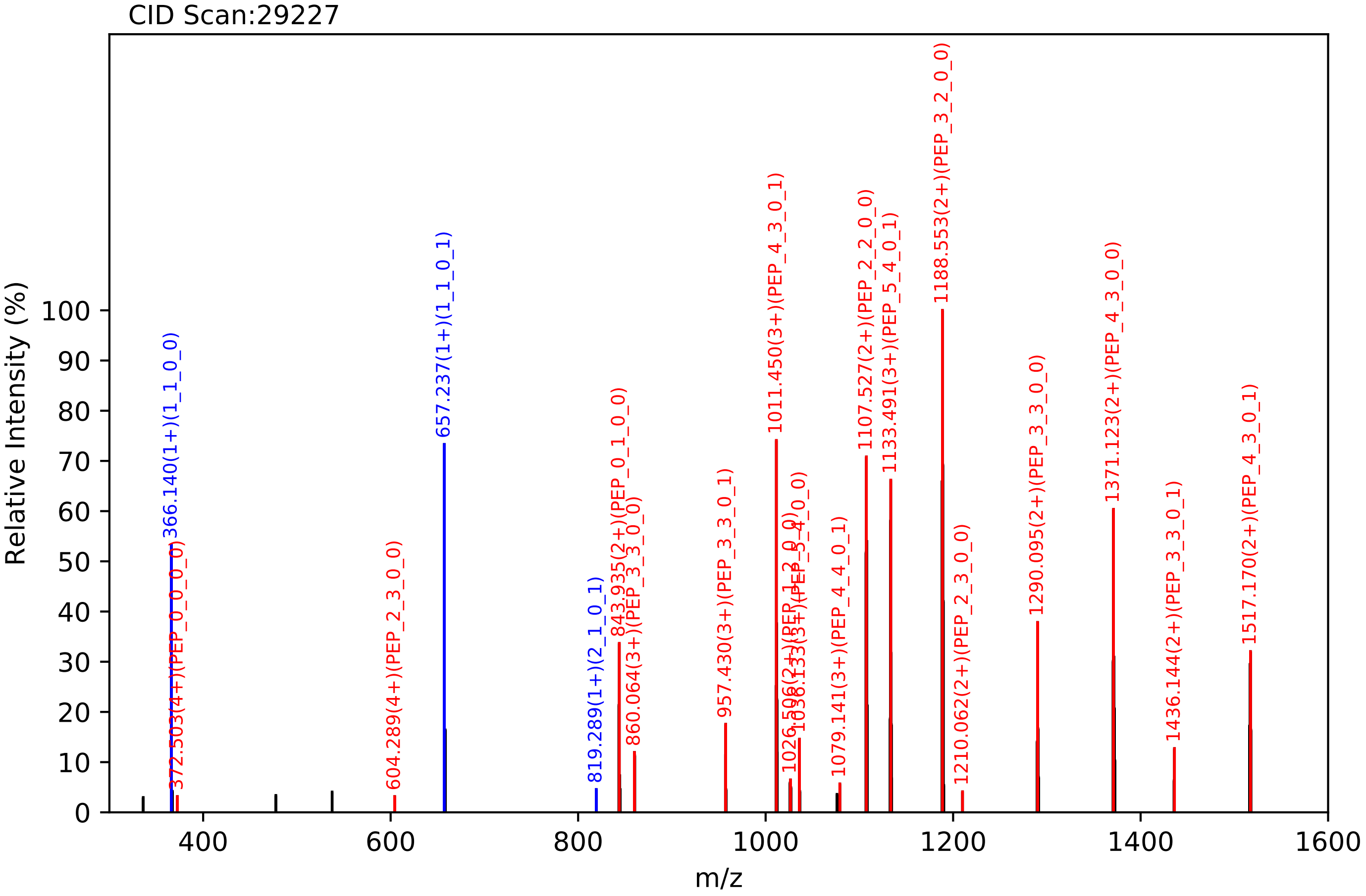

Unknown set no. 305, Experiment: Human Plasma exp_2

NLSMPLLPADFHK(=PEP)_5_4_0_2, m/z:922.65(4+), RT:103.76, Y-score:98.72

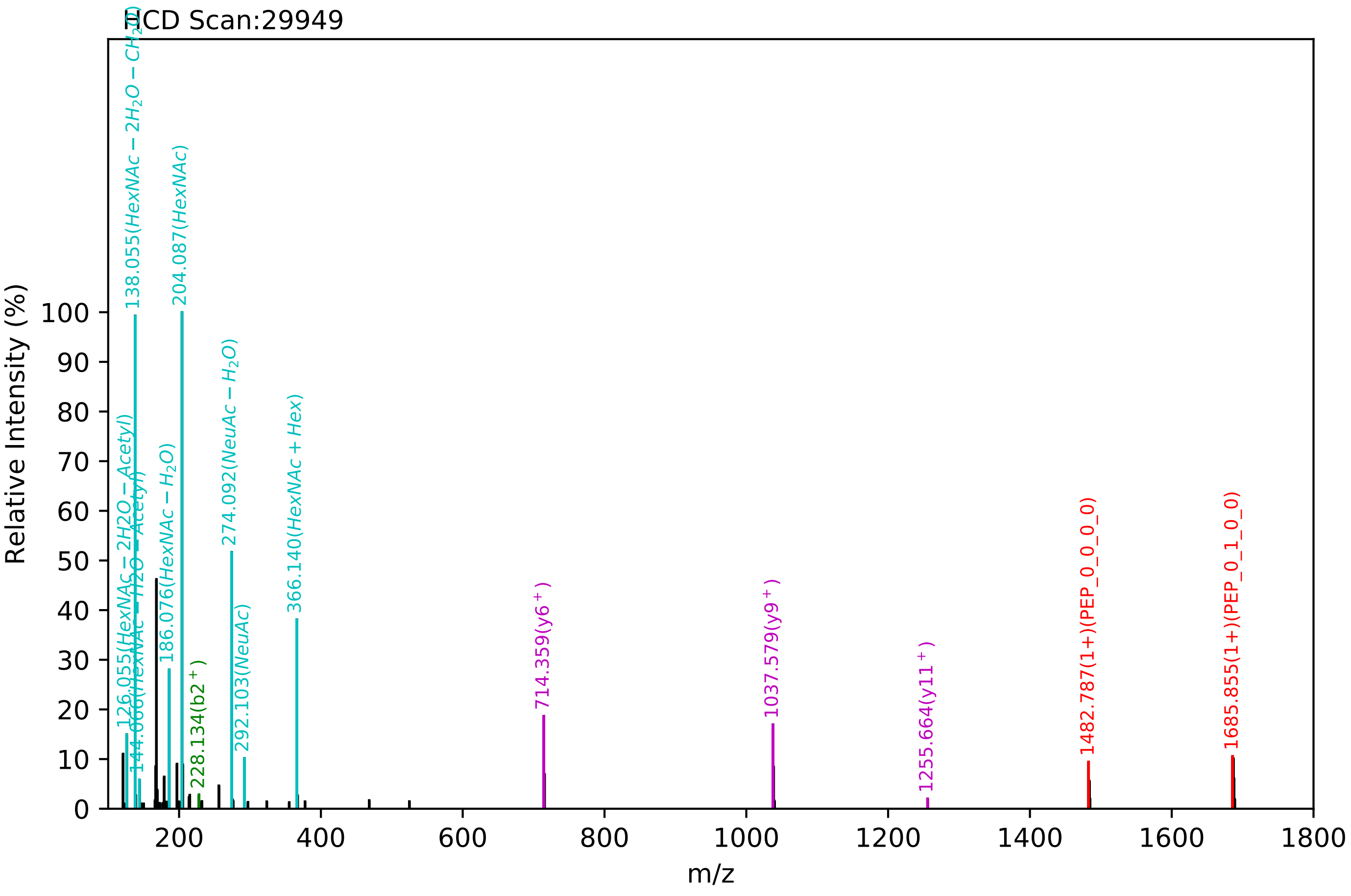

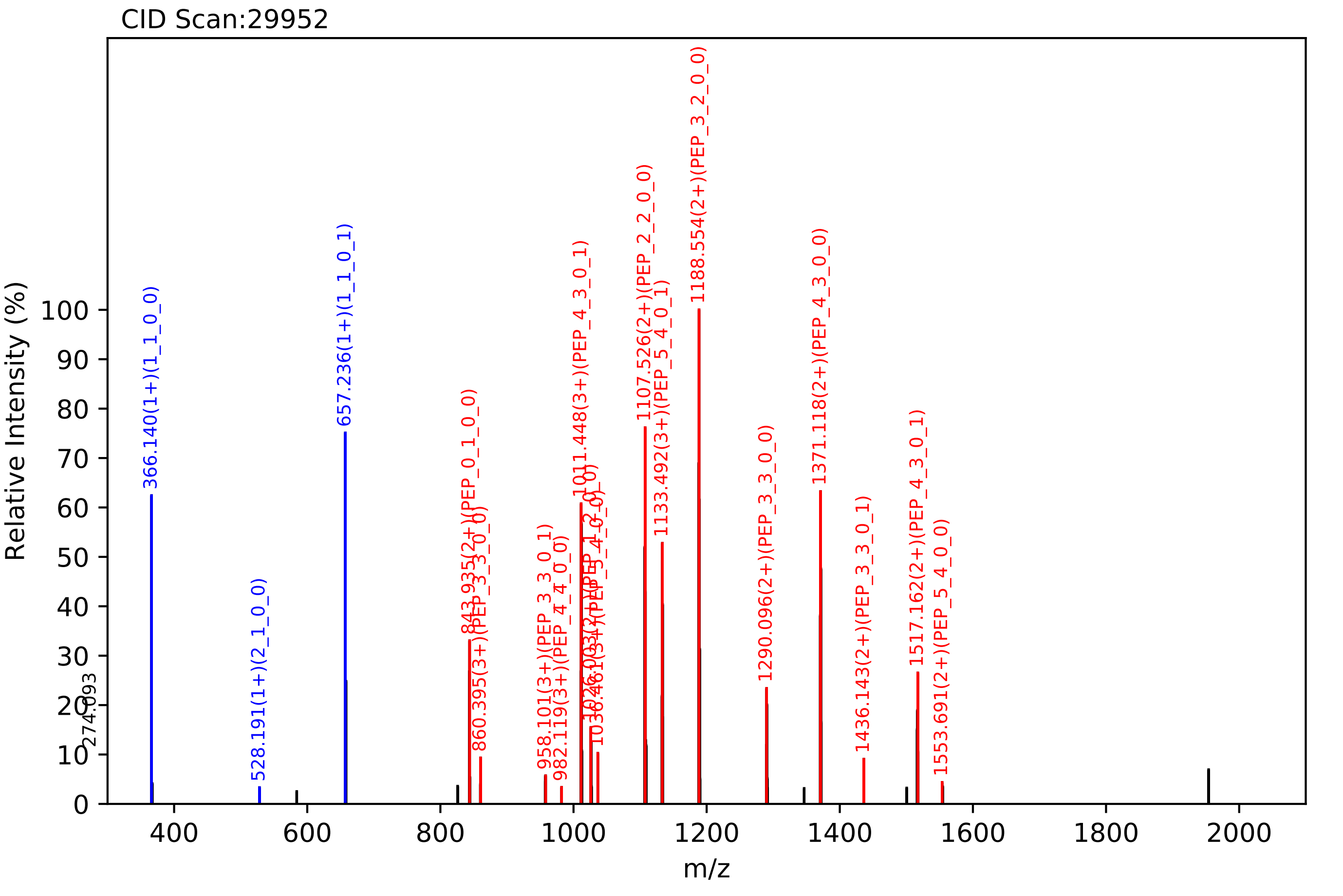

Unknown set no. 306, Experiment: Human Plasma exp_4

NLSMPLLPADFHK(=PEP)_5_4_0_2, m/z:922.64(4+), RT:103.86, Y-score:91.49

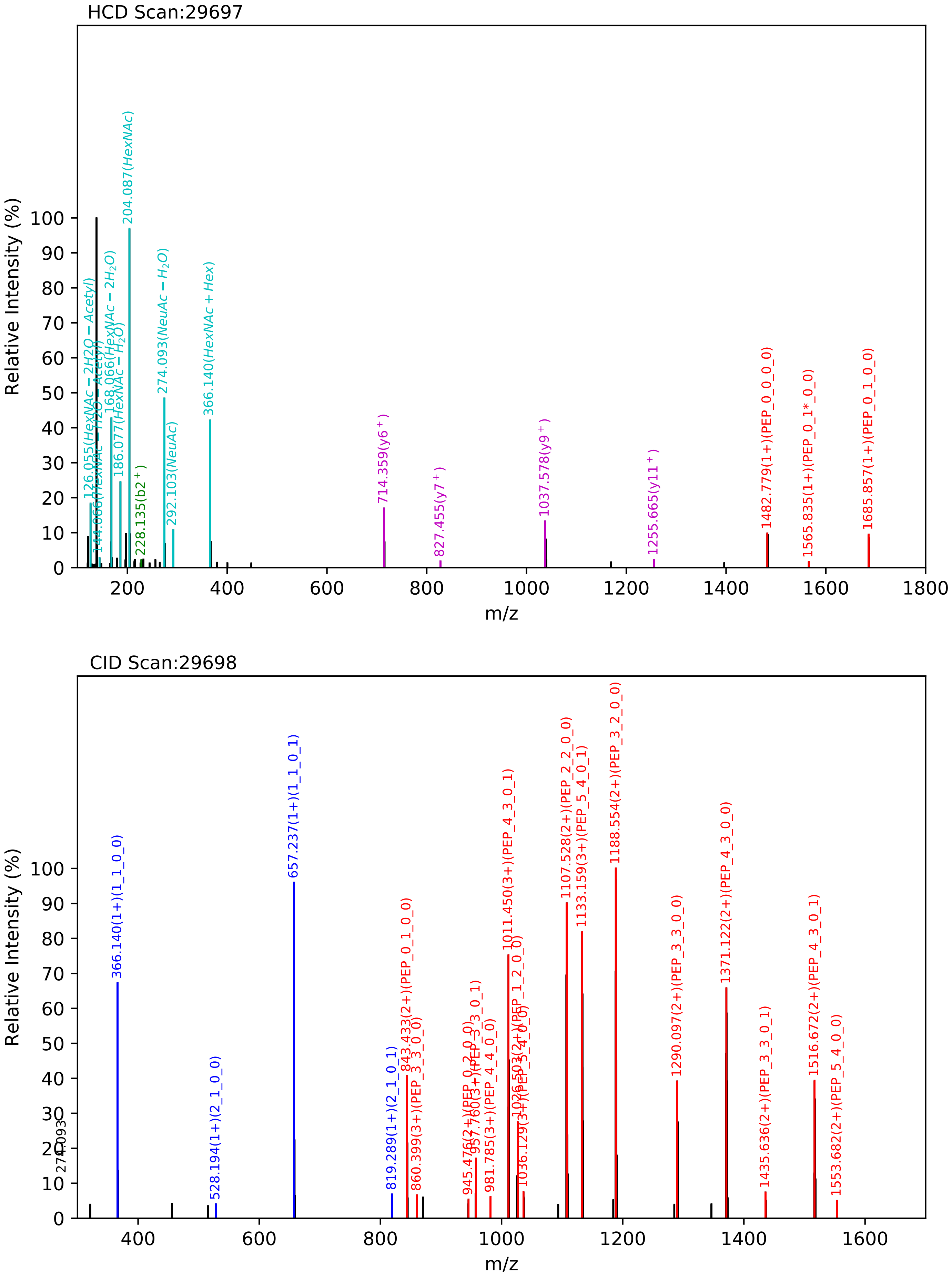

Unknown set no. 307, Experiment: Human Plasma exp_5

NLSMPLLPADFHK(=PEP)_5_4_0_2, m/z:922.64(4+), RT:103.58, Y-score:94.48

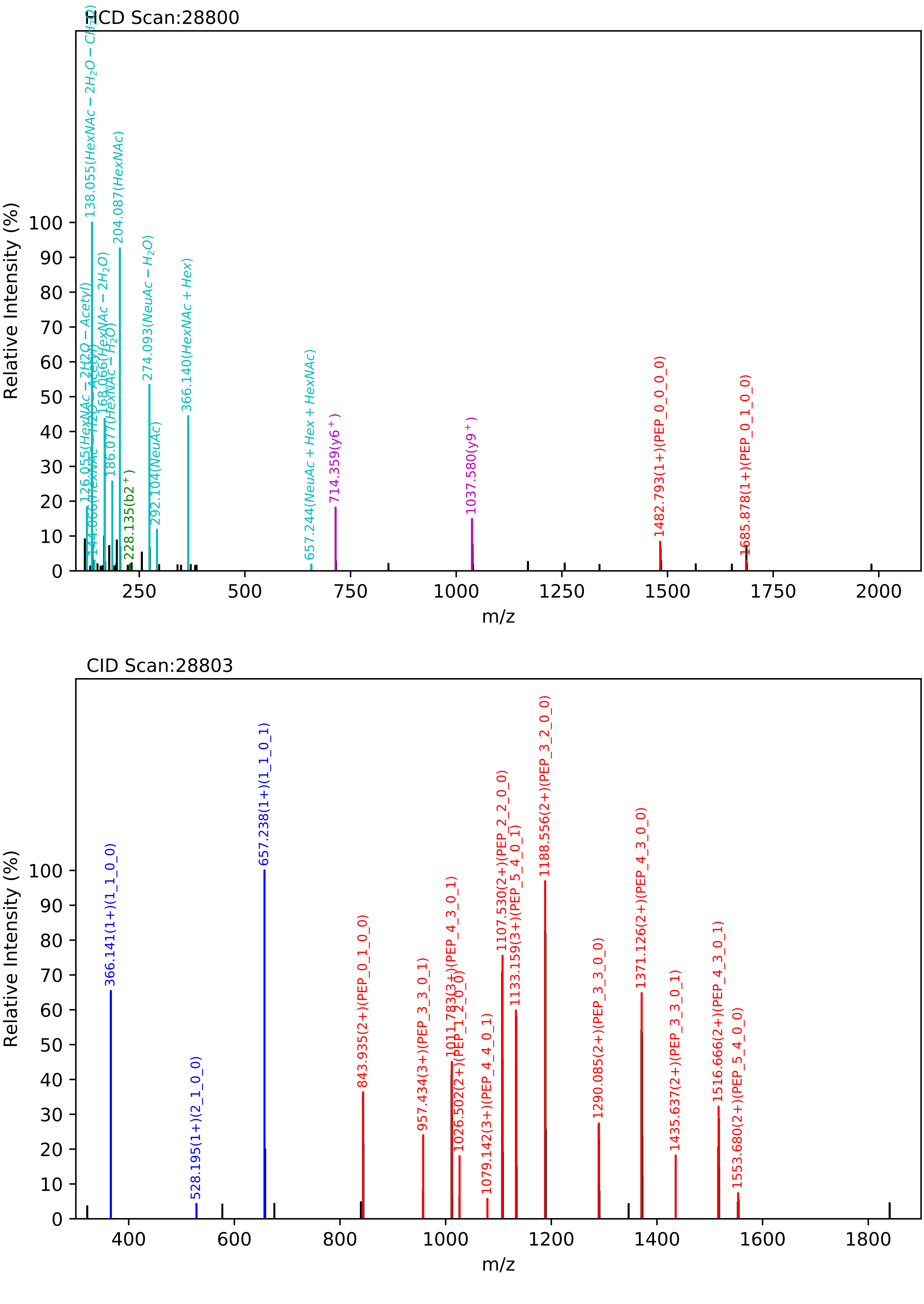

Unknown set no. 308, Experiment: Human Plasma exp_3

SMTNR(=PEP)_4_3_0_1, m/z:1078.92(2+), RT:16.77, Y-score:88.77

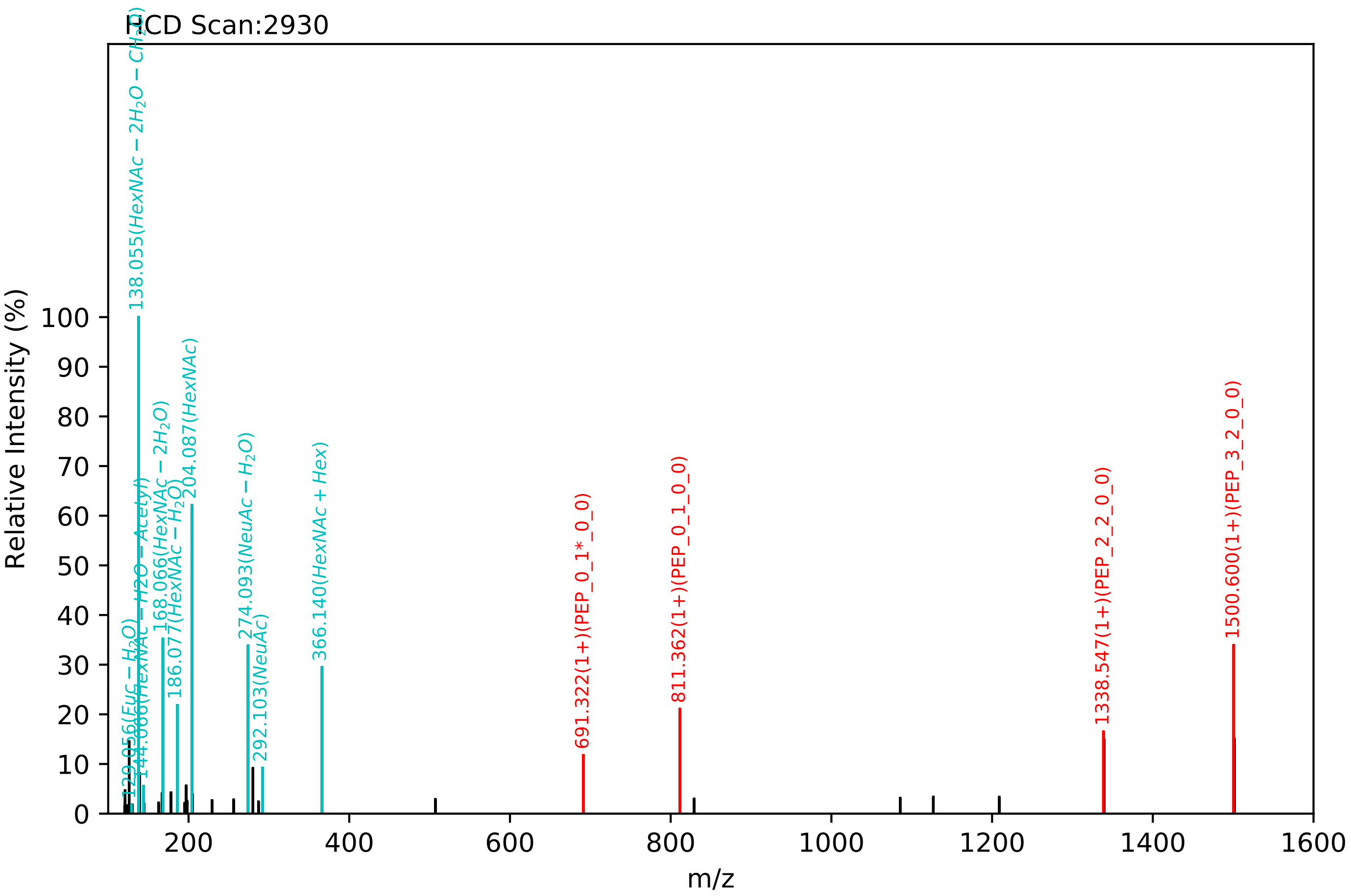

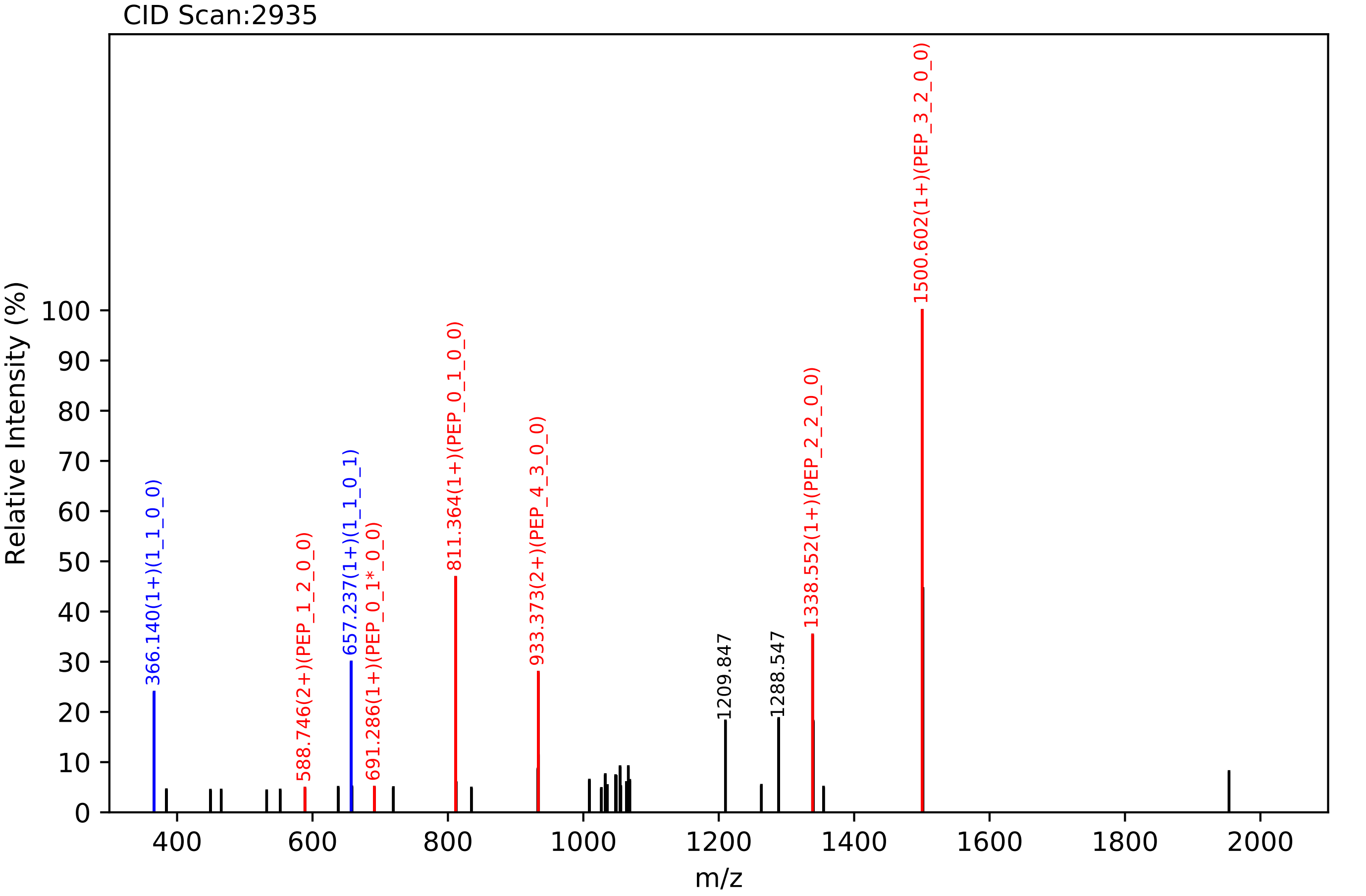

Unknown set no. 309, Experiment: Human Plasma exp_2

SMTNR(=PEP)_5_4_0_2, m/z:938.36(3+), RT:16.78, Y-score:94.31

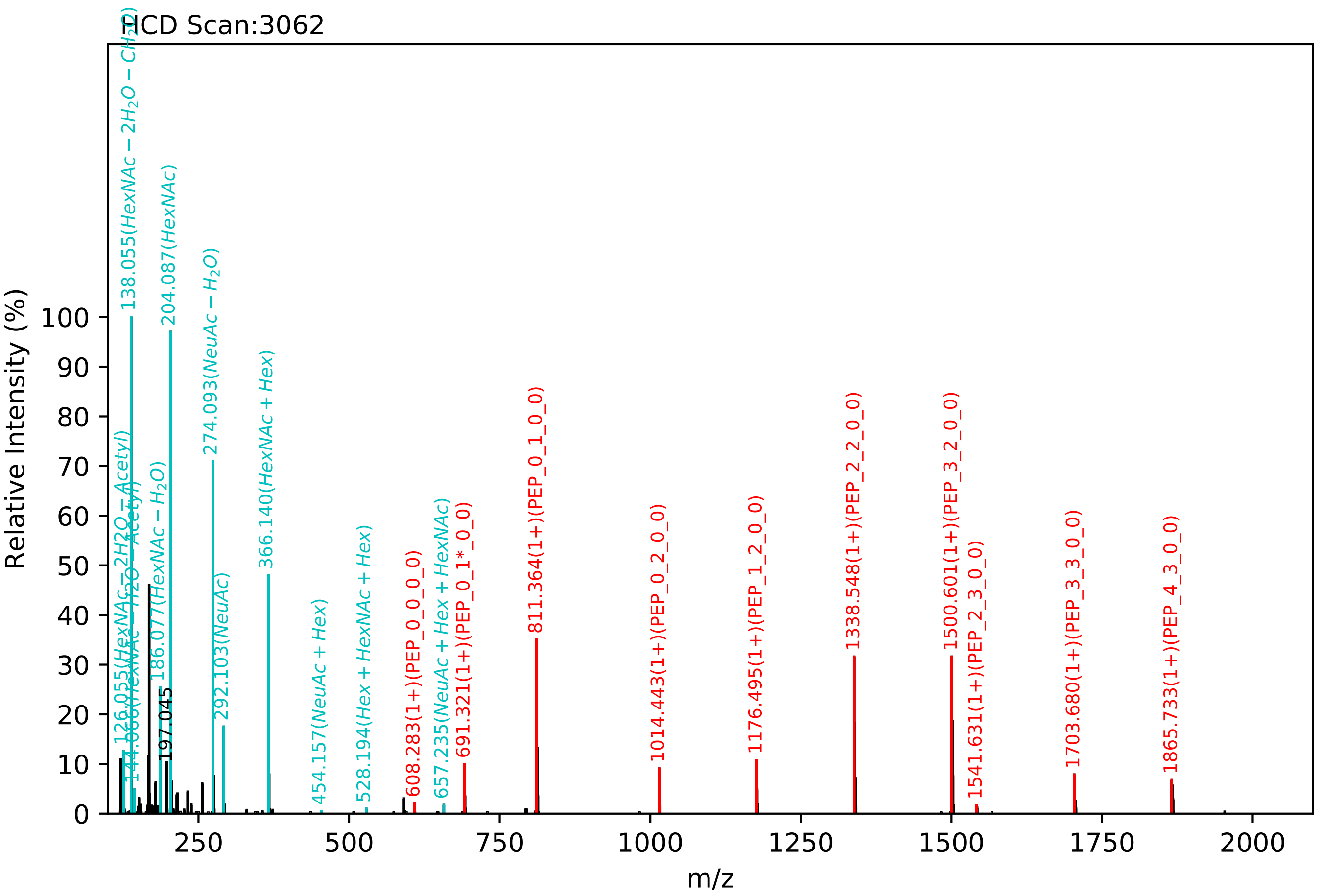

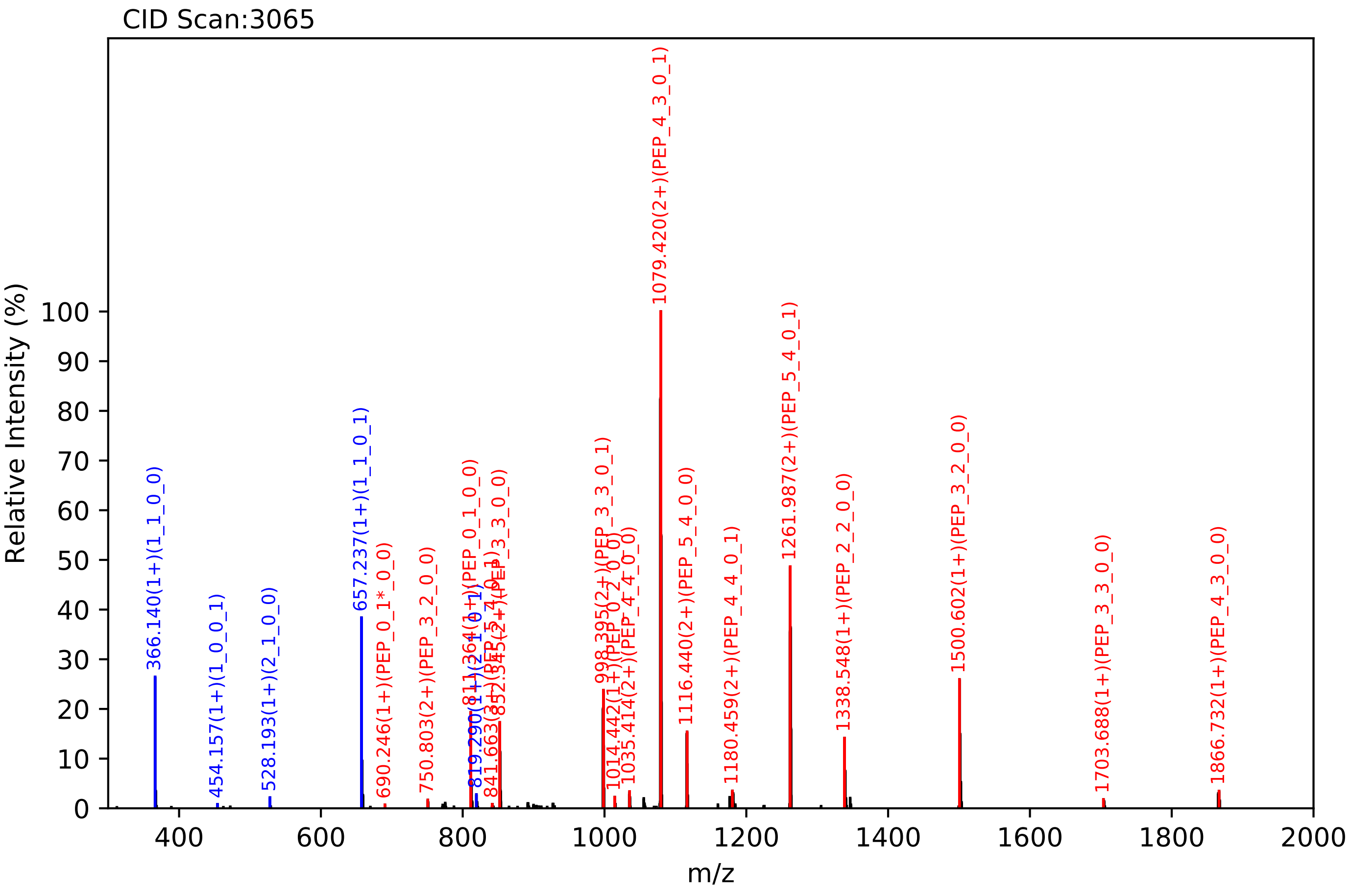

Unknown set no. 310, Experiment: Human Plasma exp_3

SMTNR(=PEP)_5_4_0_2, m/z:938.36(3+), RT:16.93, Y-score:94.59

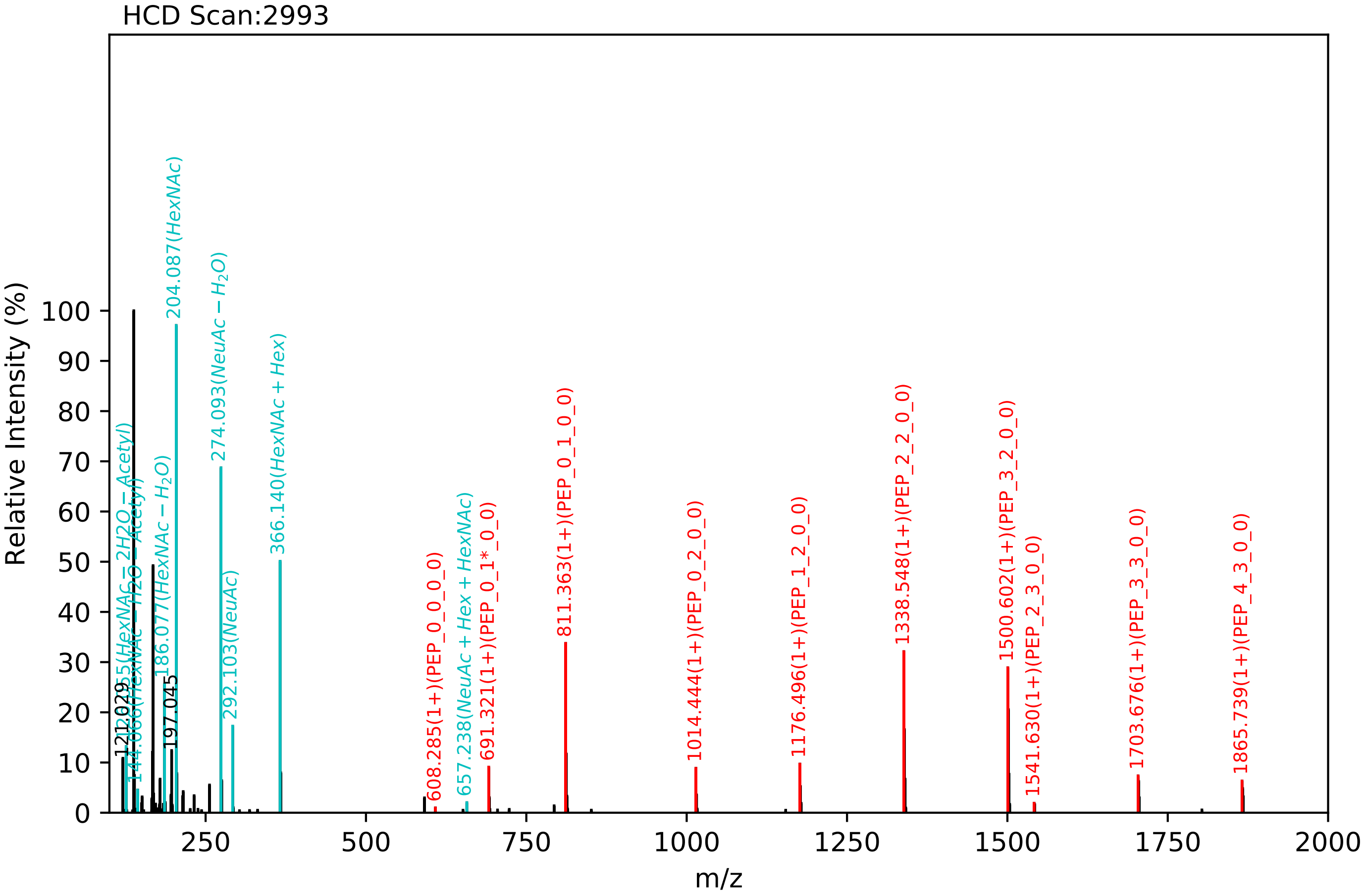

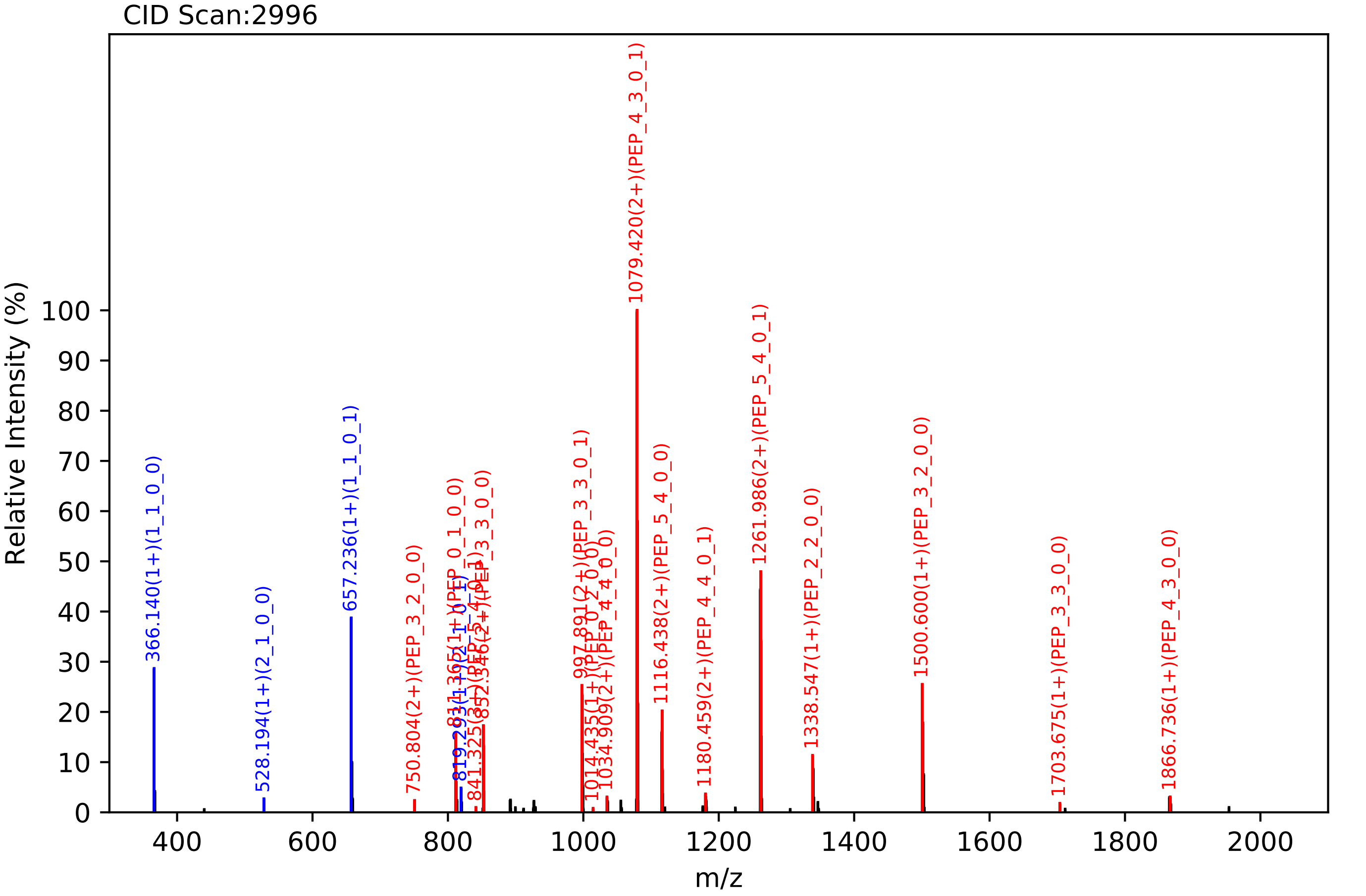

Unknown set no. 311, Experiment: Human Plasma exp_4

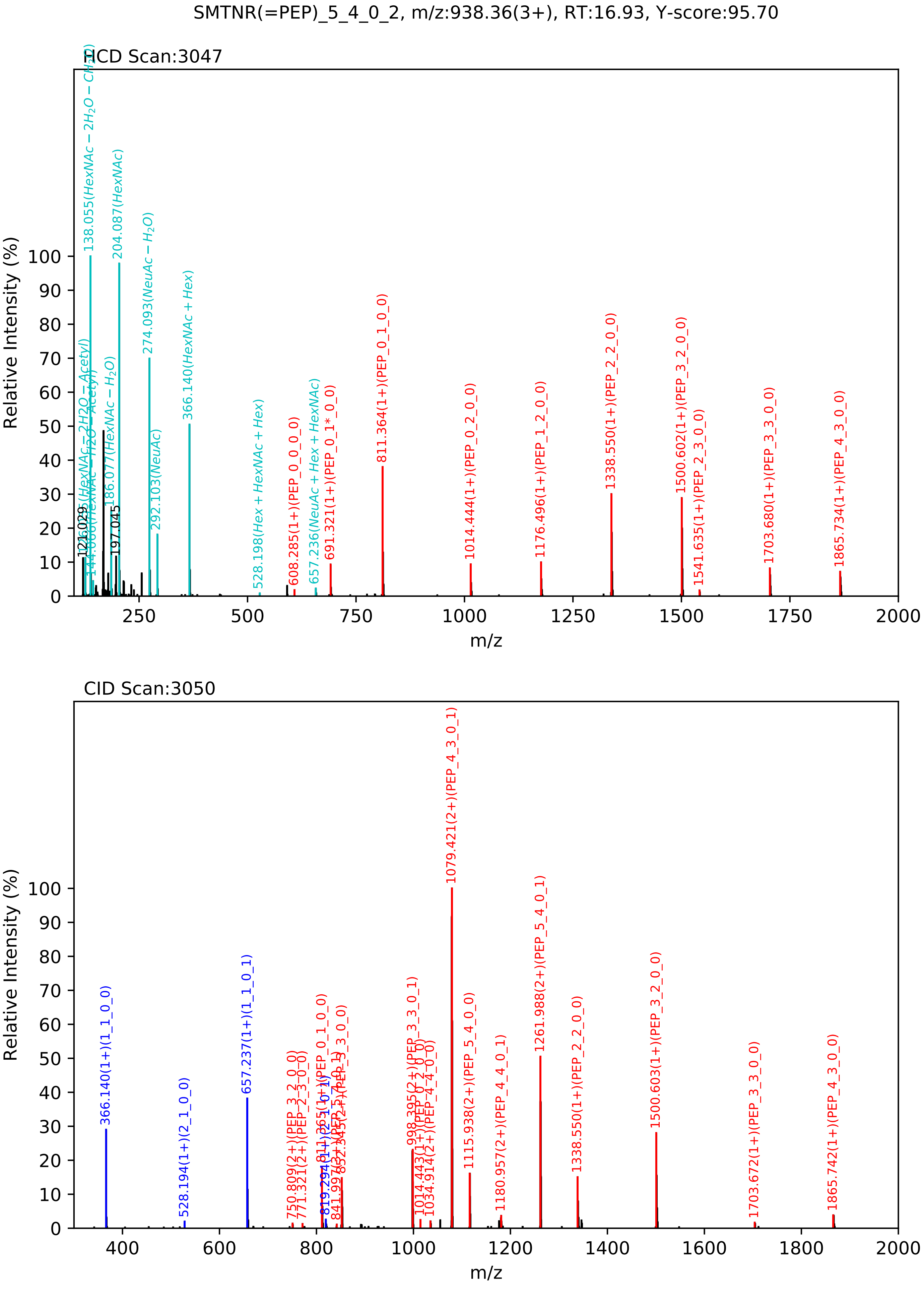

Unknown set no. 312, Experiment: Human Plasma exp_5

SMTNR(=PEP)_5_4_0_2, m/z:938.36(3+), RT:17.00, Y-score:90.47

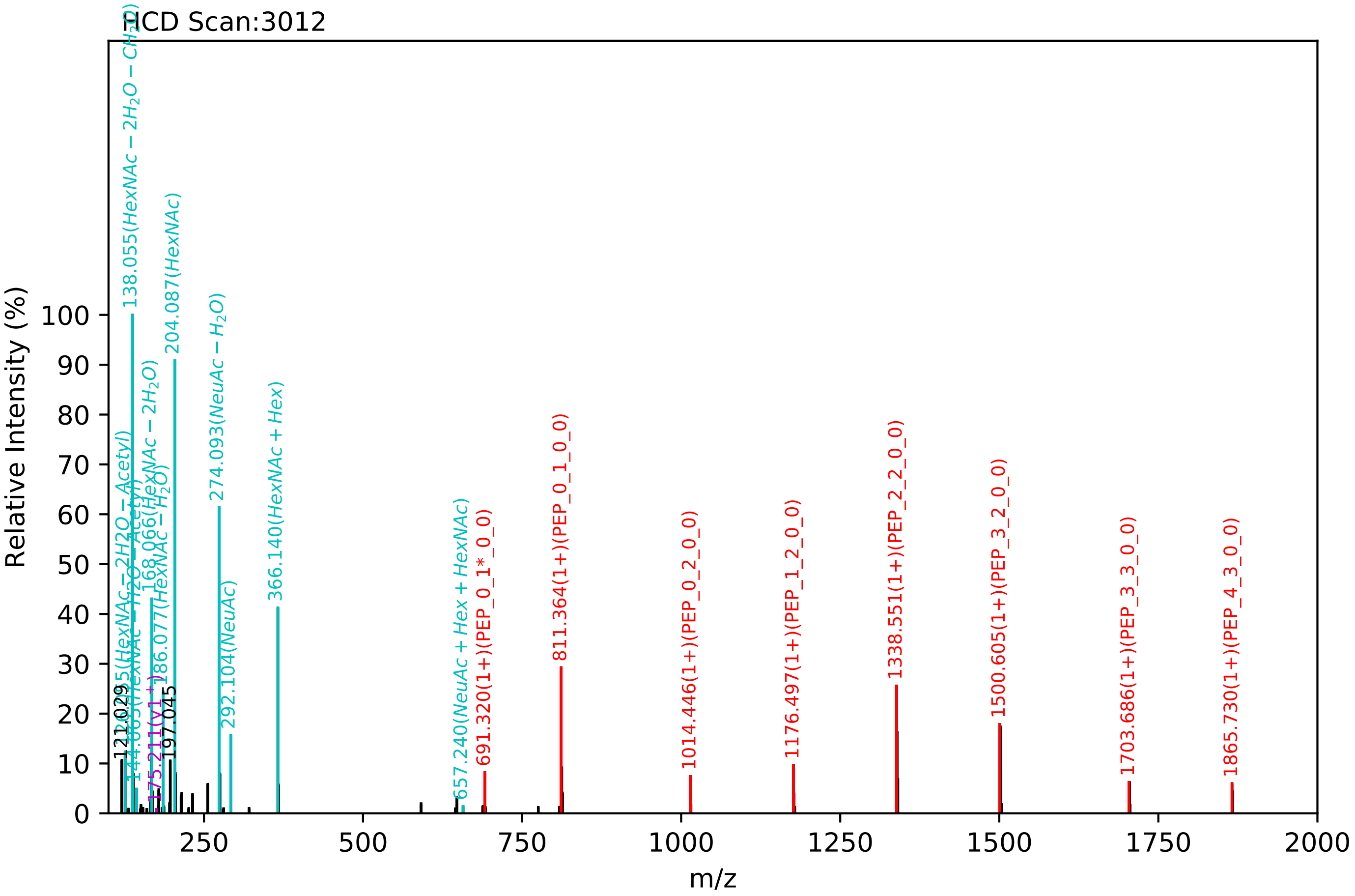

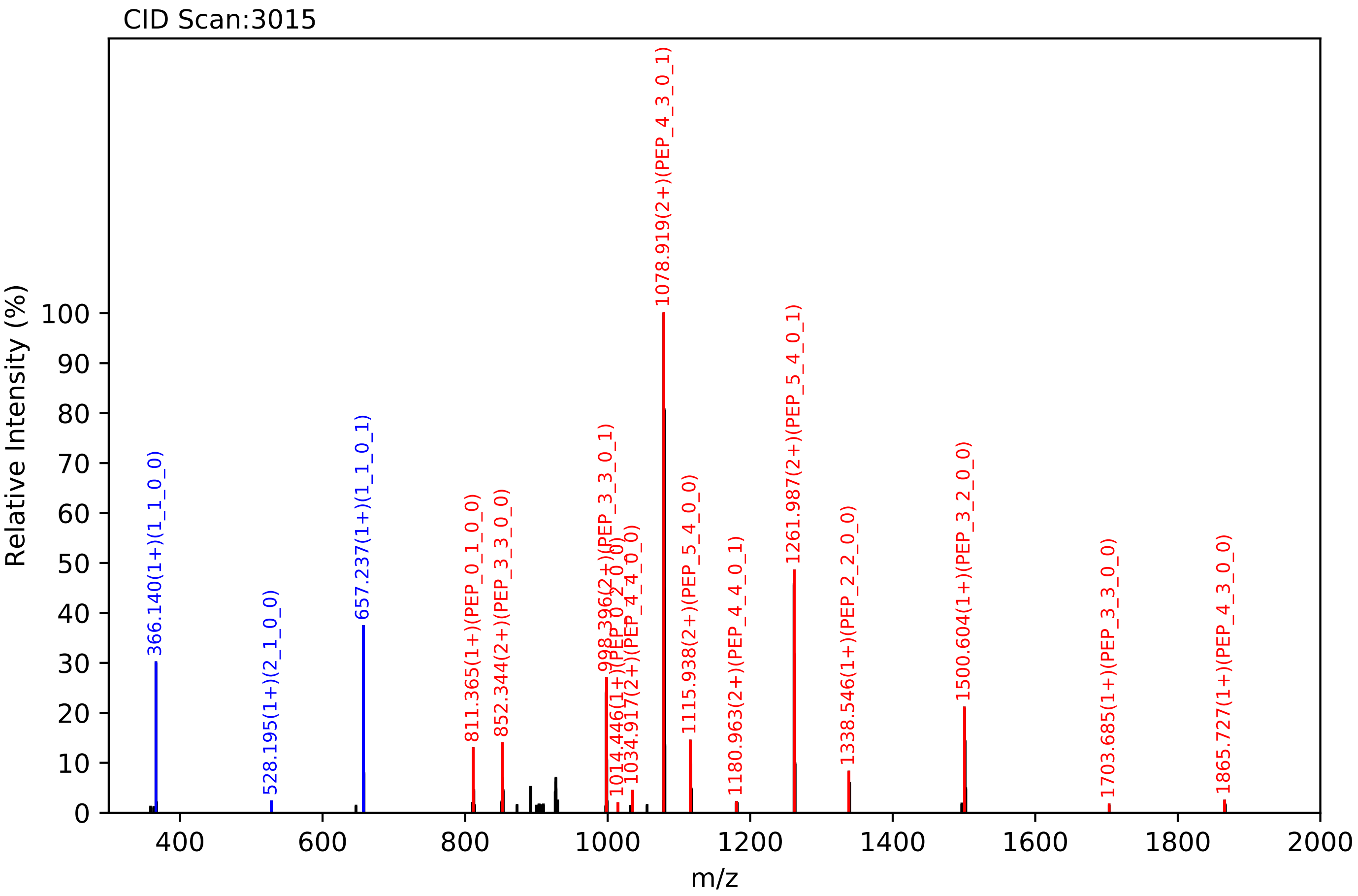

Unknown set no. 313, Experiment: Human Plasma exp_4

DSVSVVLGQHFFNR(=PEP)_5_4_0_2, m/z:953.16(4+), RT:81.95, Y-score:74.30

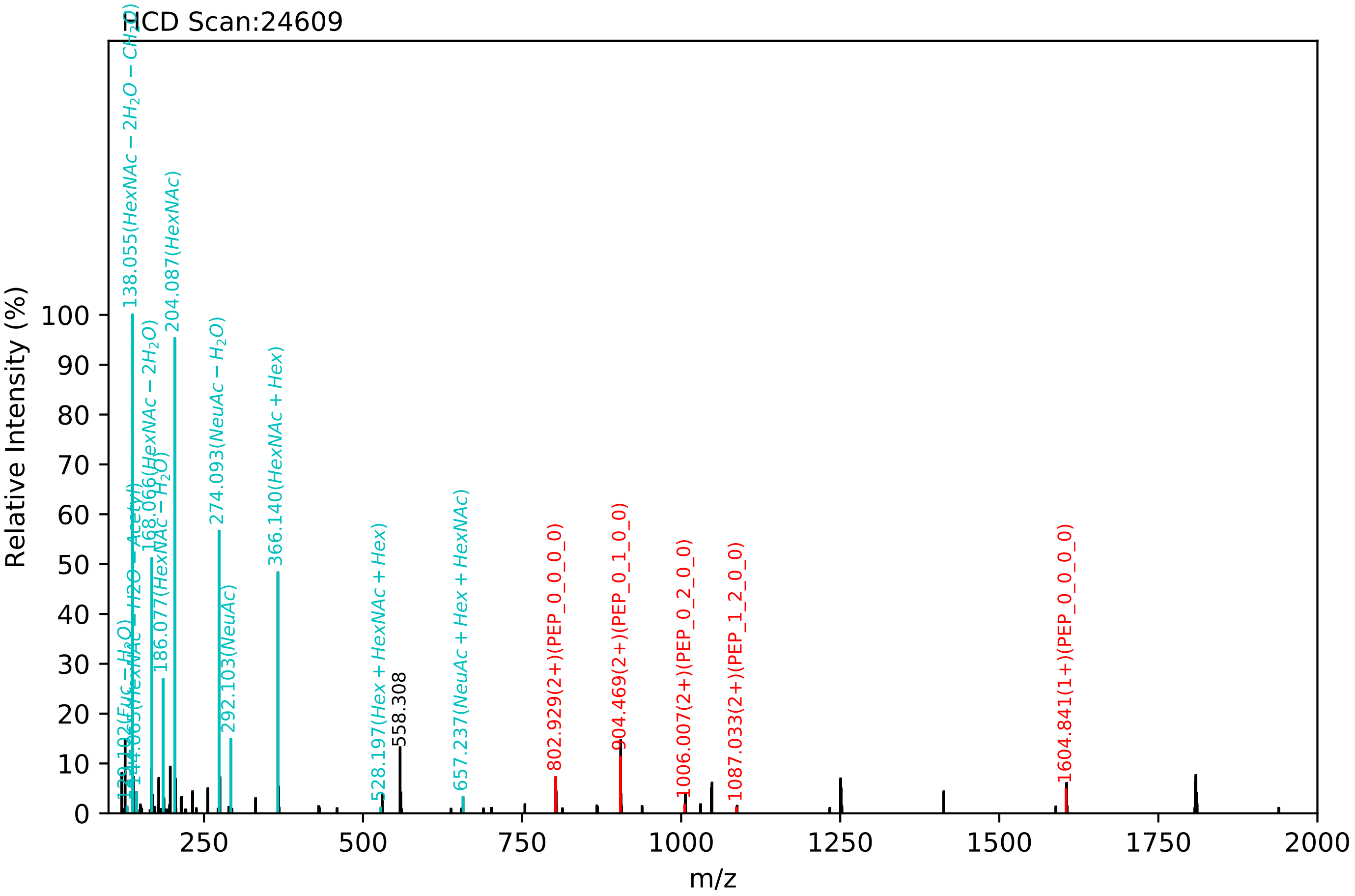

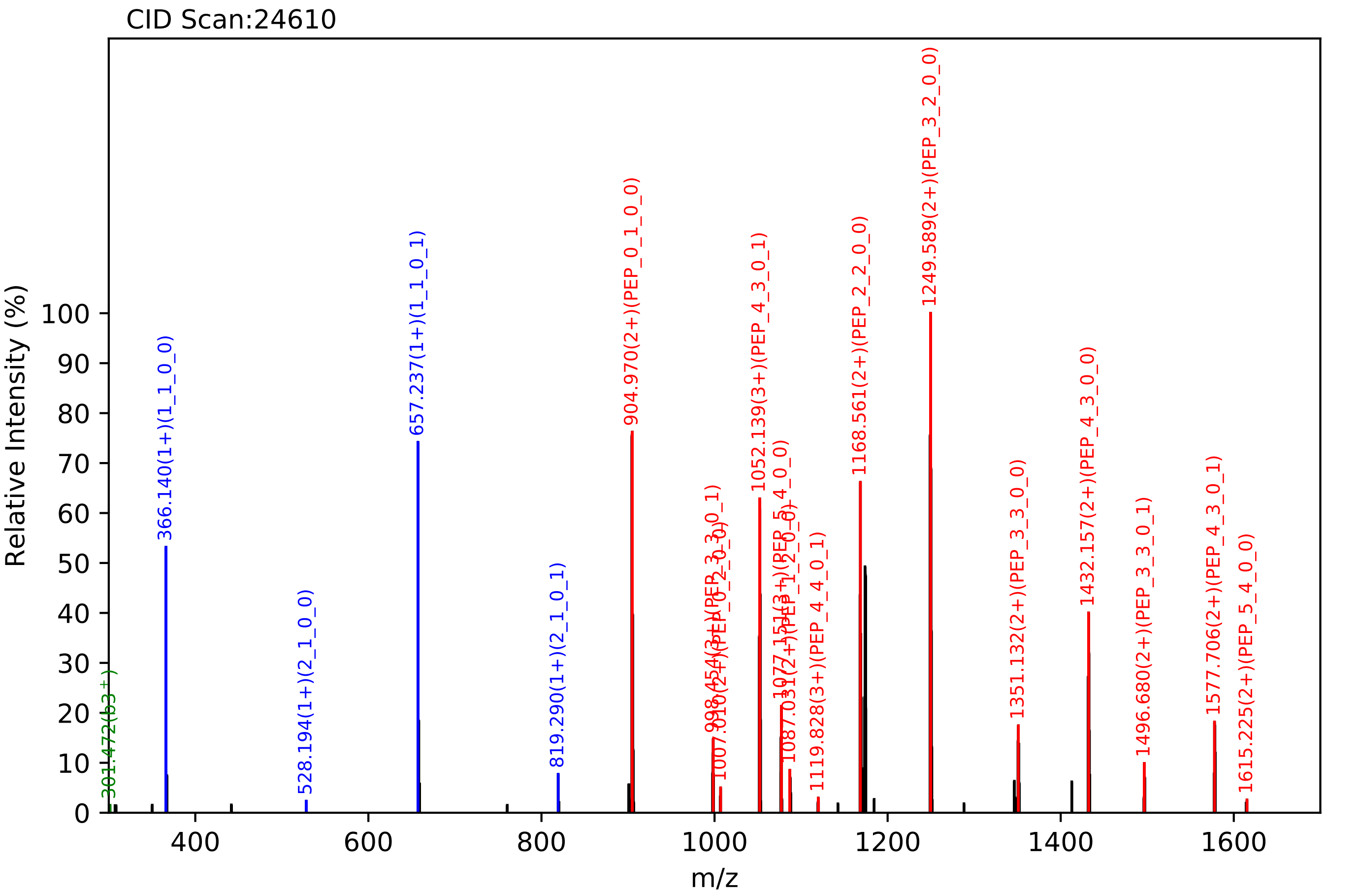

Unknown set no. 314, Experiment: Human Plasma exp_2

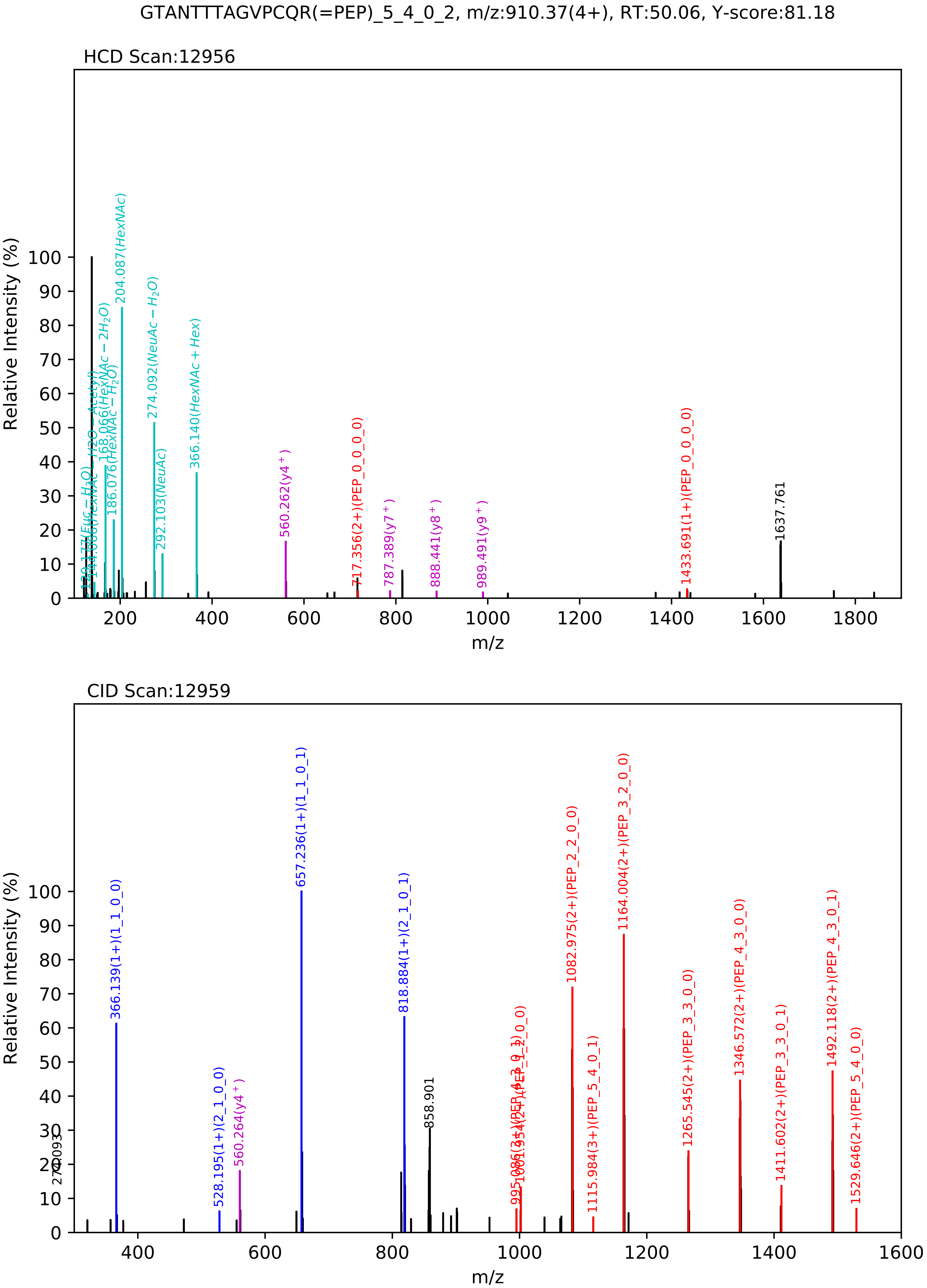

Unknown set no. 315, Experiment: Human Plasma exp_5

GTANTTTAGVPCQR(=PEP)_5_4_0_2, m/z:910.37(4+), RT:50.32, Y-score:88.43

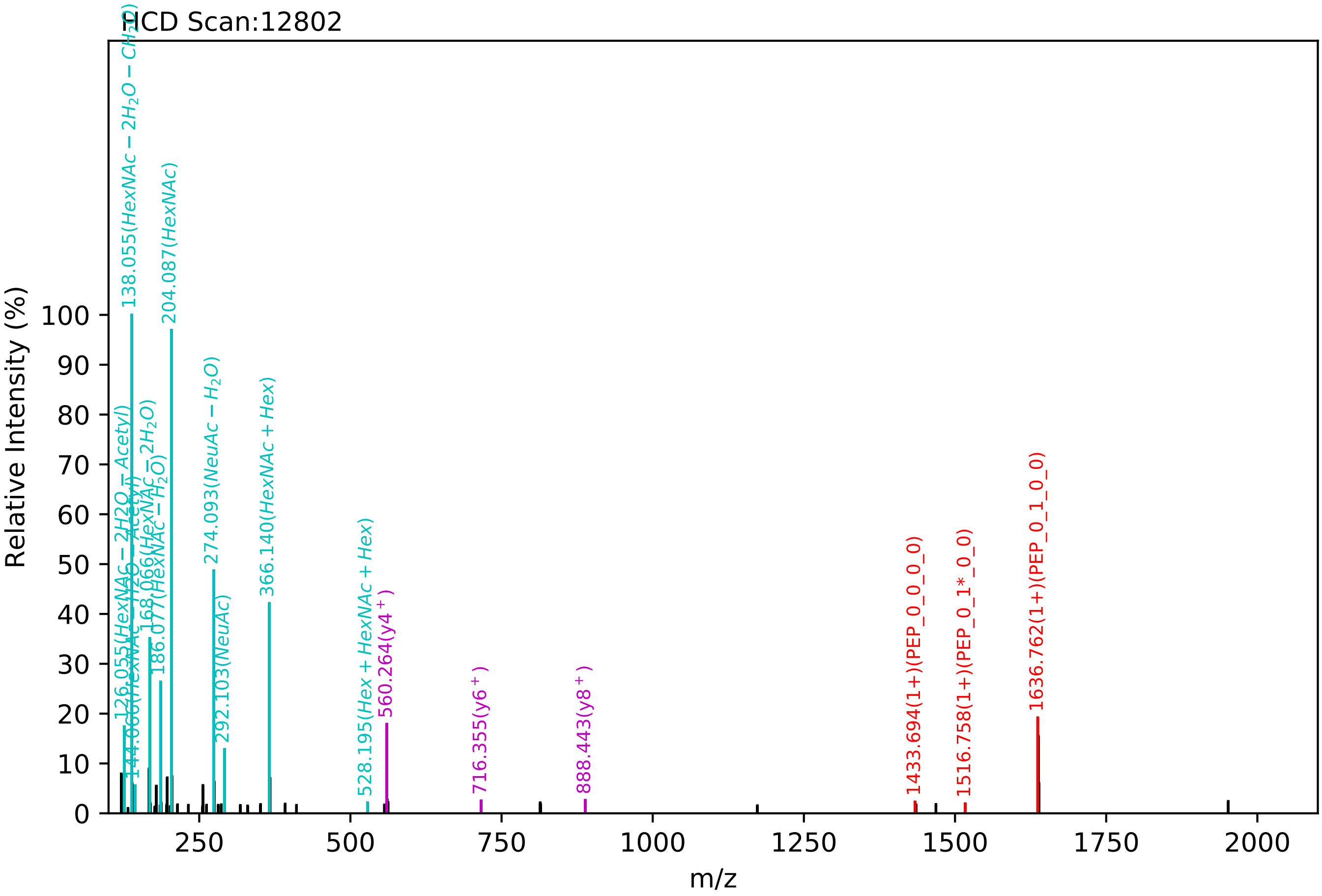

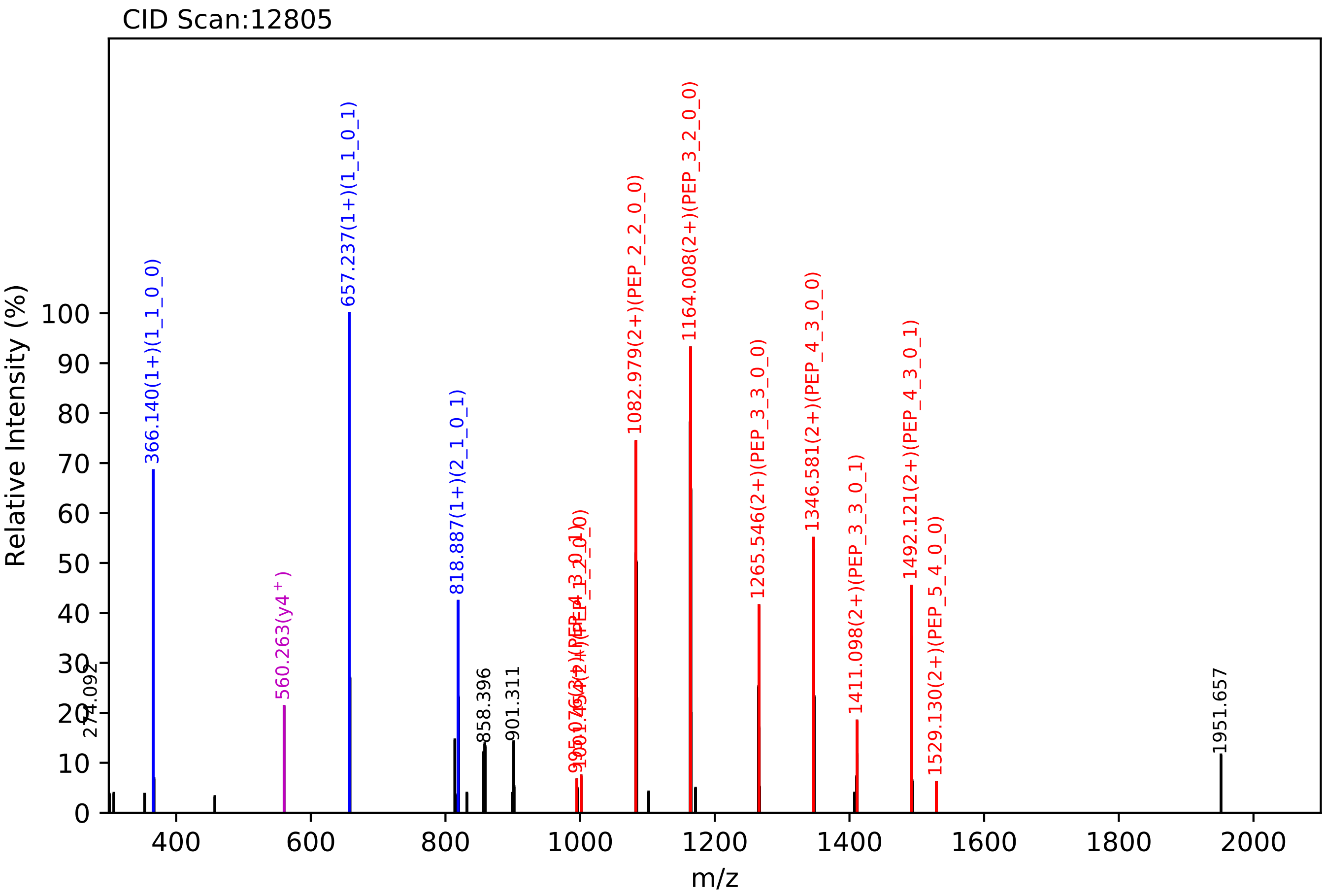

Unknown set no. 316, Experiment: Human Plasma exp_1

VIDFNCTTSSVSSALANTK(=PEP)_5_4_0_1, m/z:982.92(4+), RT:92.35, Y-score:99.39

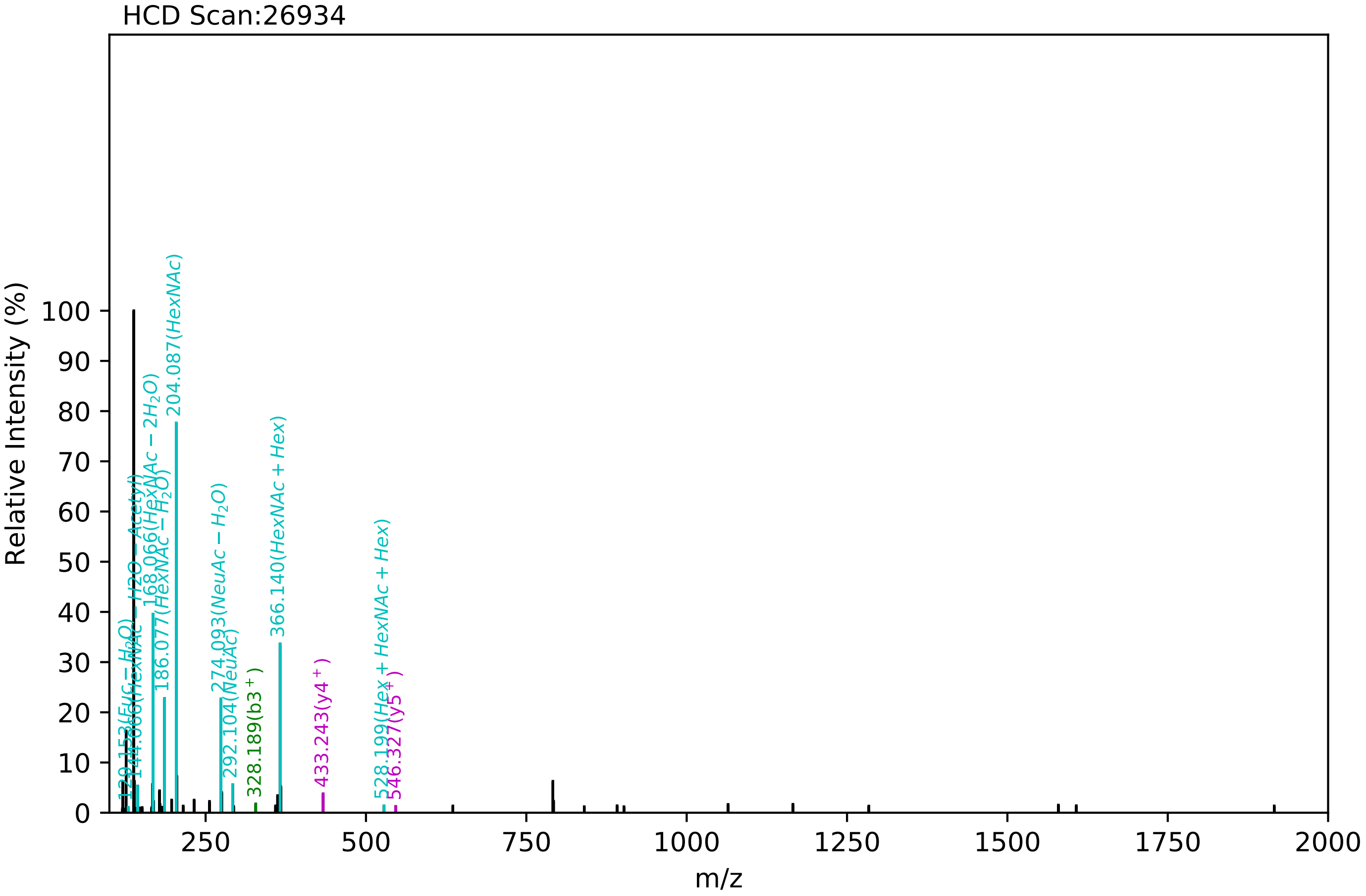

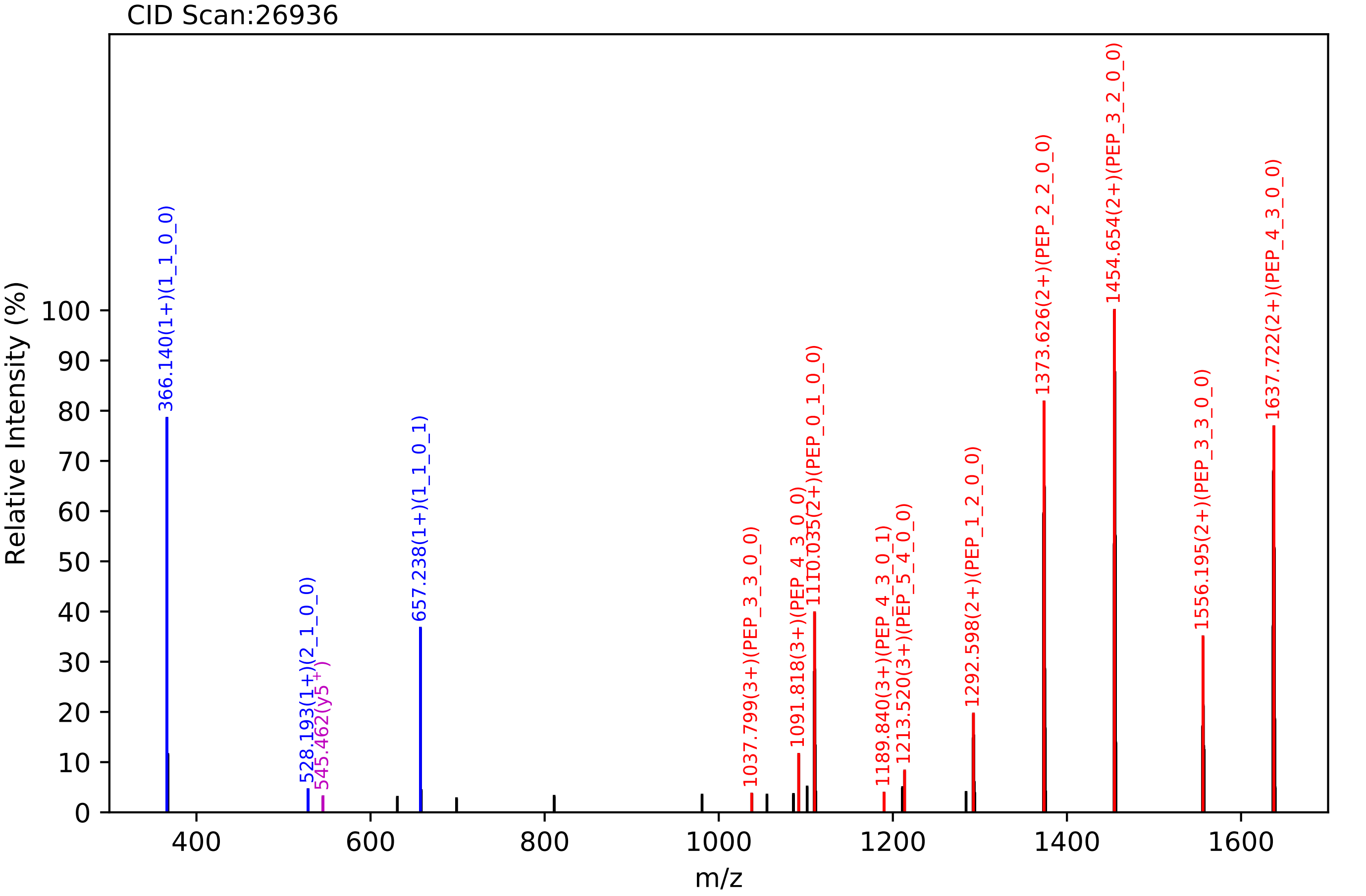

Unknown set no. 317, Experiment: Human Plasma exp_2

VIDFNCTTSSVSSALANTK(=PEP)_5_4_0_1, m/z:982.92(4+), RT:92.39, Y-score:100.00

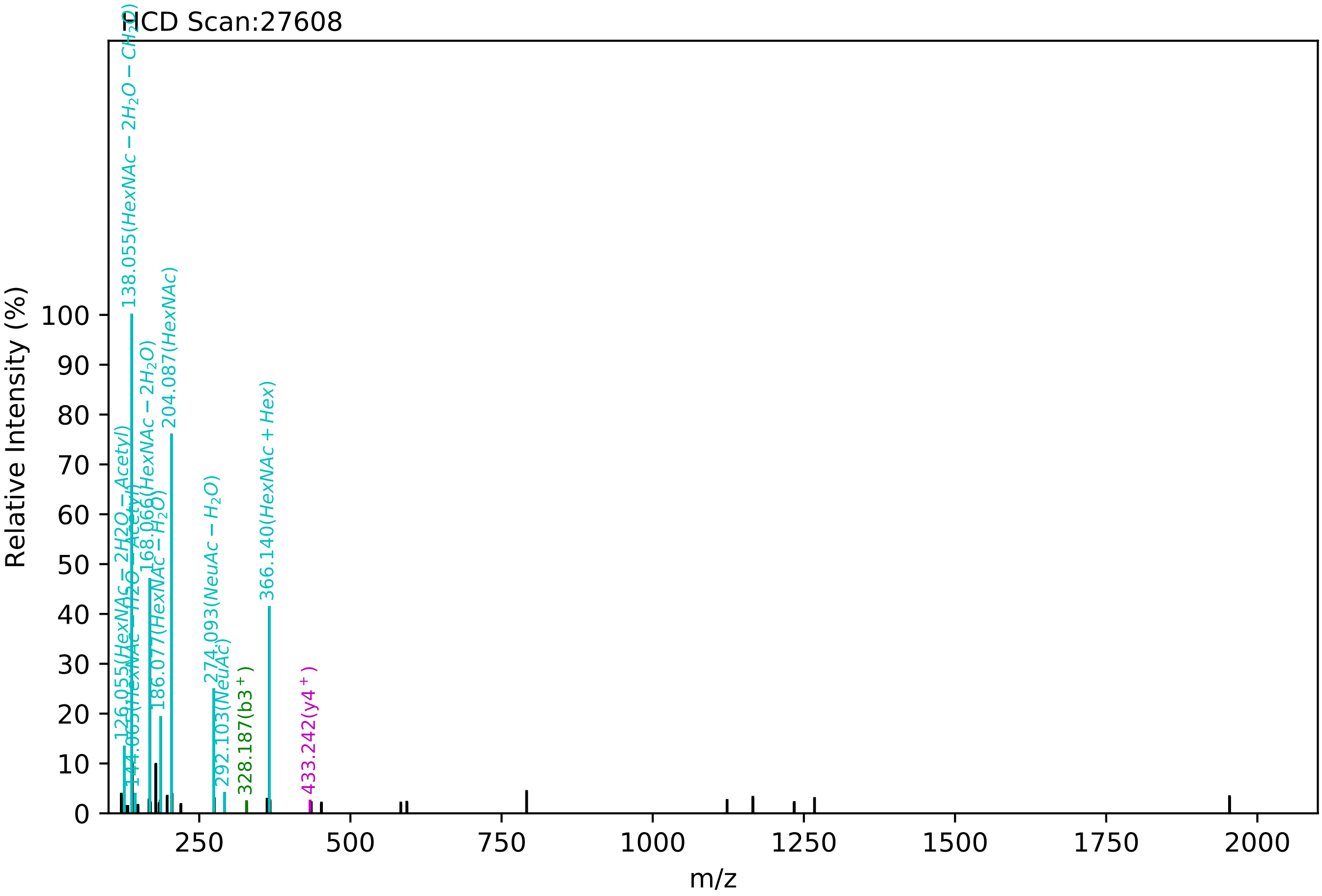

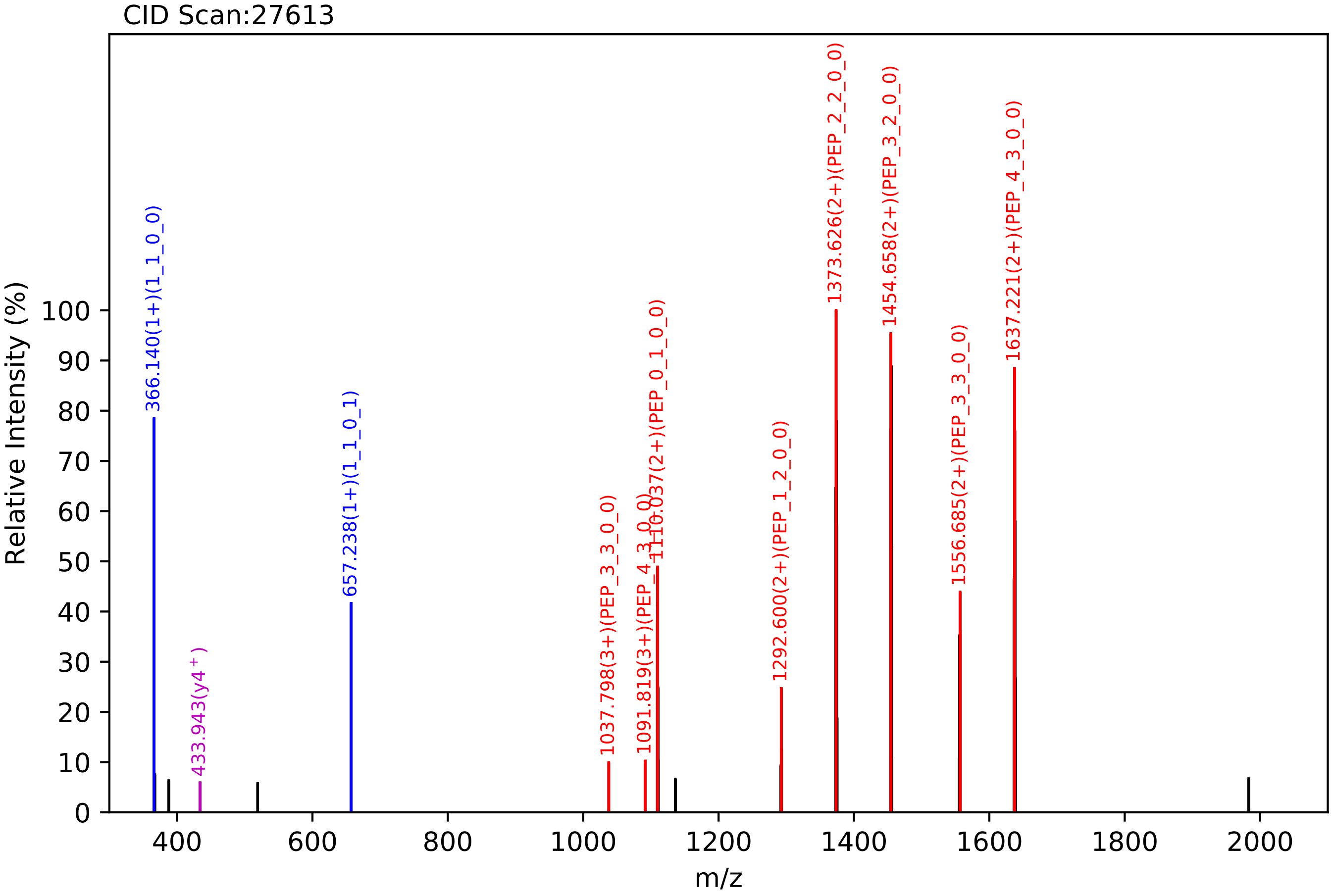

Unknown set no. 318, Experiment: Human Plasma exp_3

VIDFNCTTSSVSSALANTK(=PEP)_5_4_0_1, m/z:982.92(4+), RT:92.50, Y-score:94.95

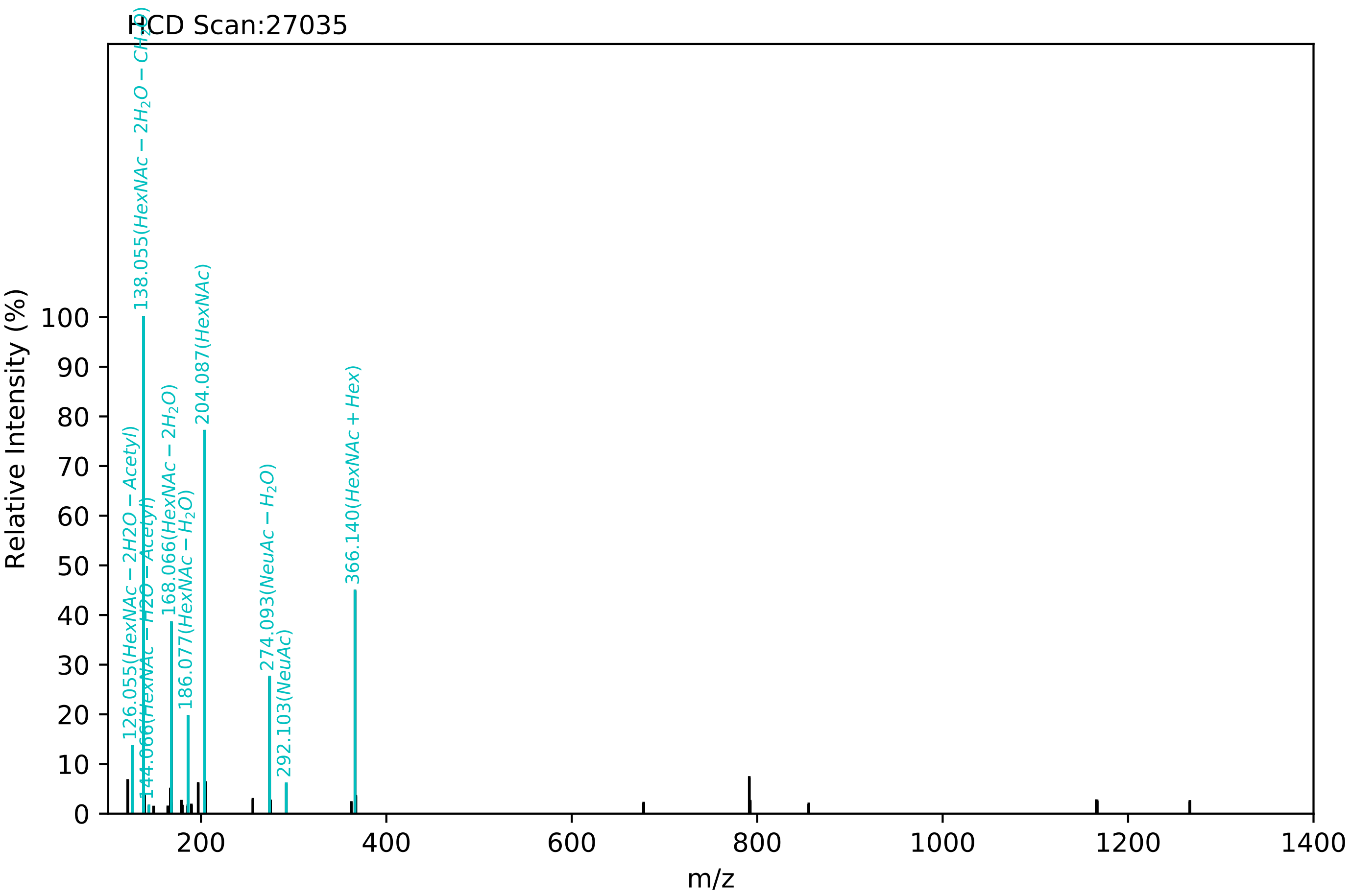

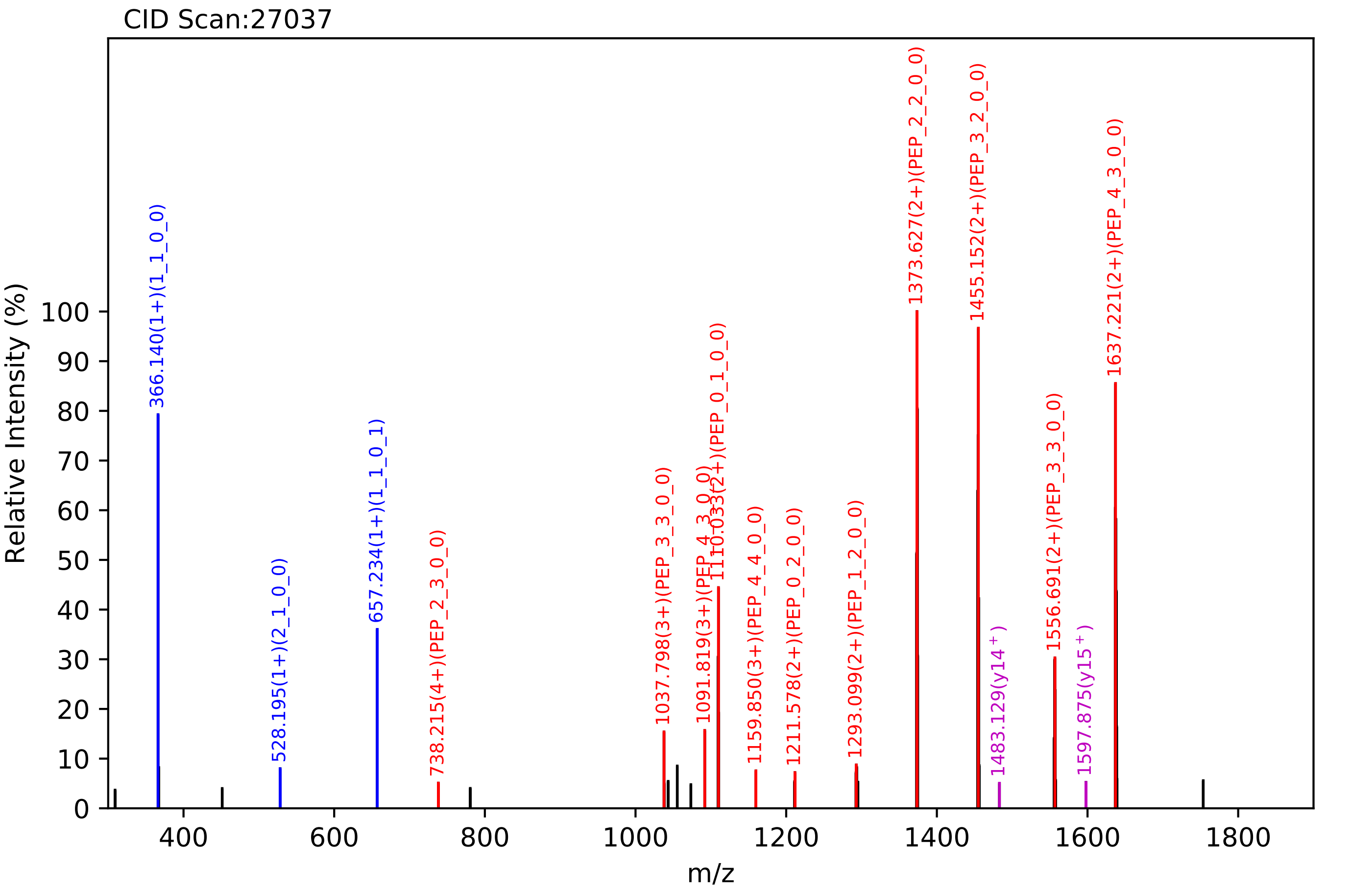

Unknown set no. 319, Experiment: Human Plasma exp_4

VIDFNCTTSSVSSALANTK(=PEP)_5_4_0_1, m/z:982.92(4+), RT:92.35, Y-score:94.40

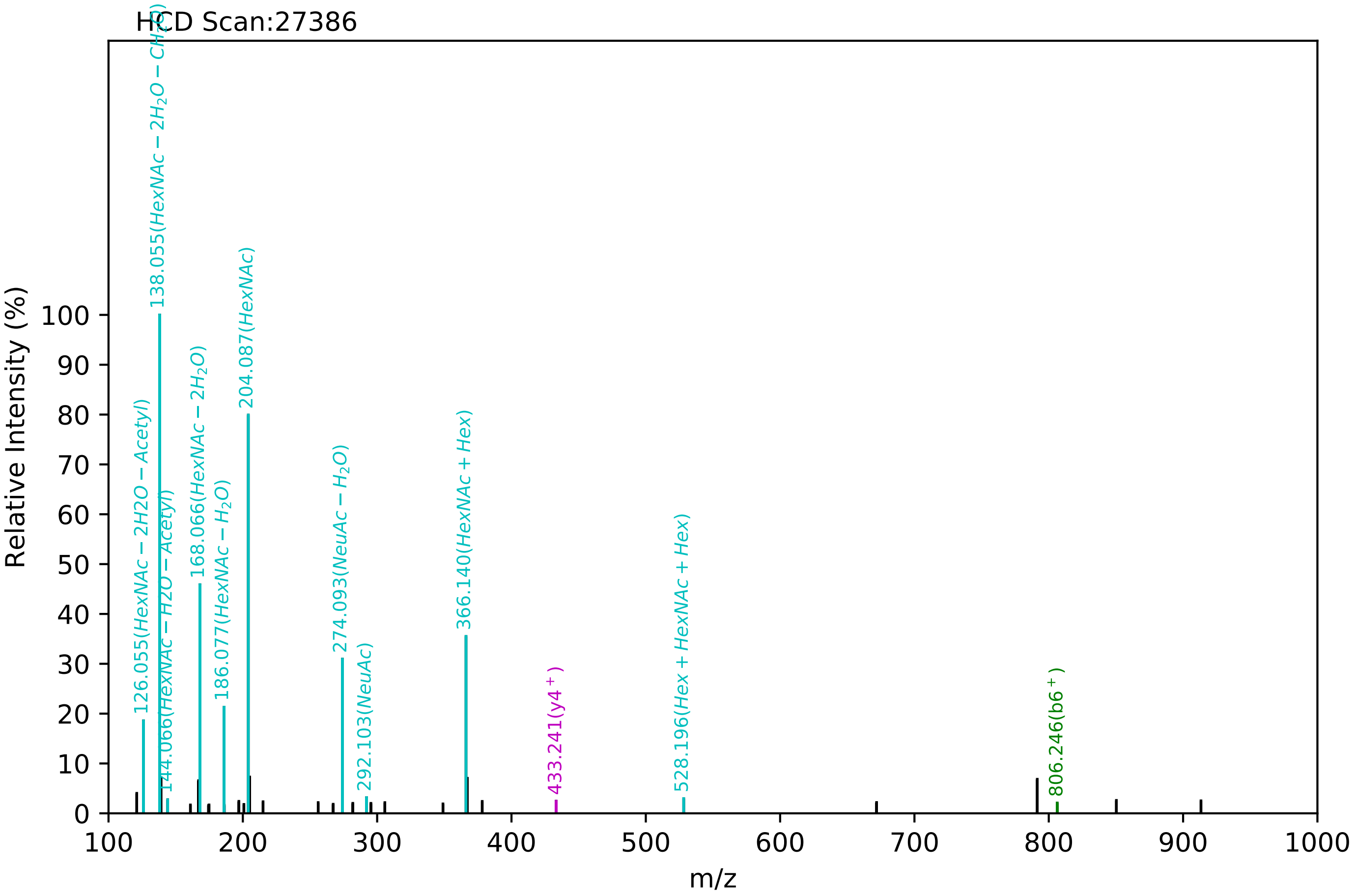

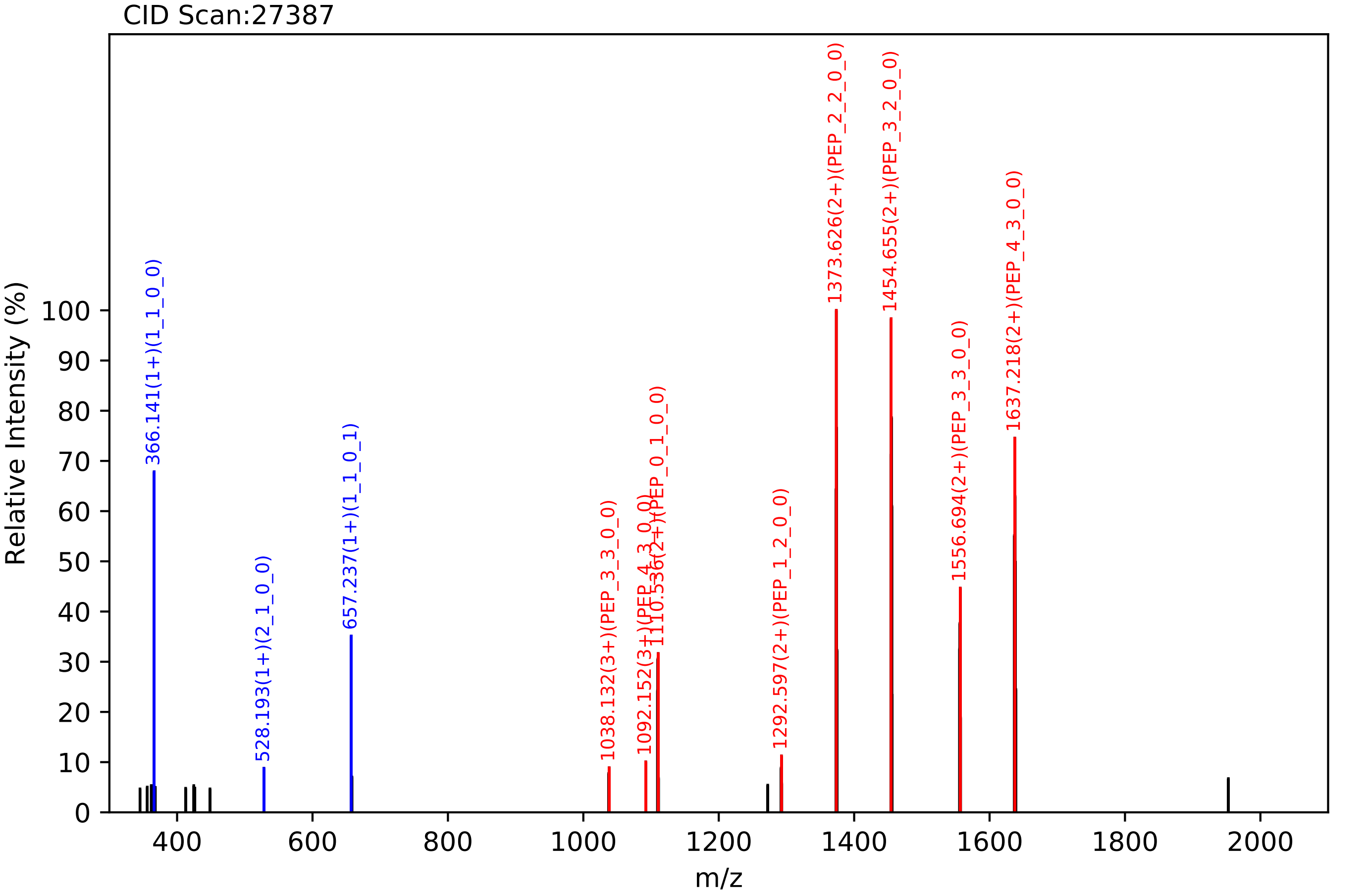

Unknown set no. 320, Experiment: Human Plasma exp_5

VIDFNCTTSSVSSALANTK(=PEP)_5_4_0_1, m/z:982.92(4+), RT:92.13, Y-score:90.74

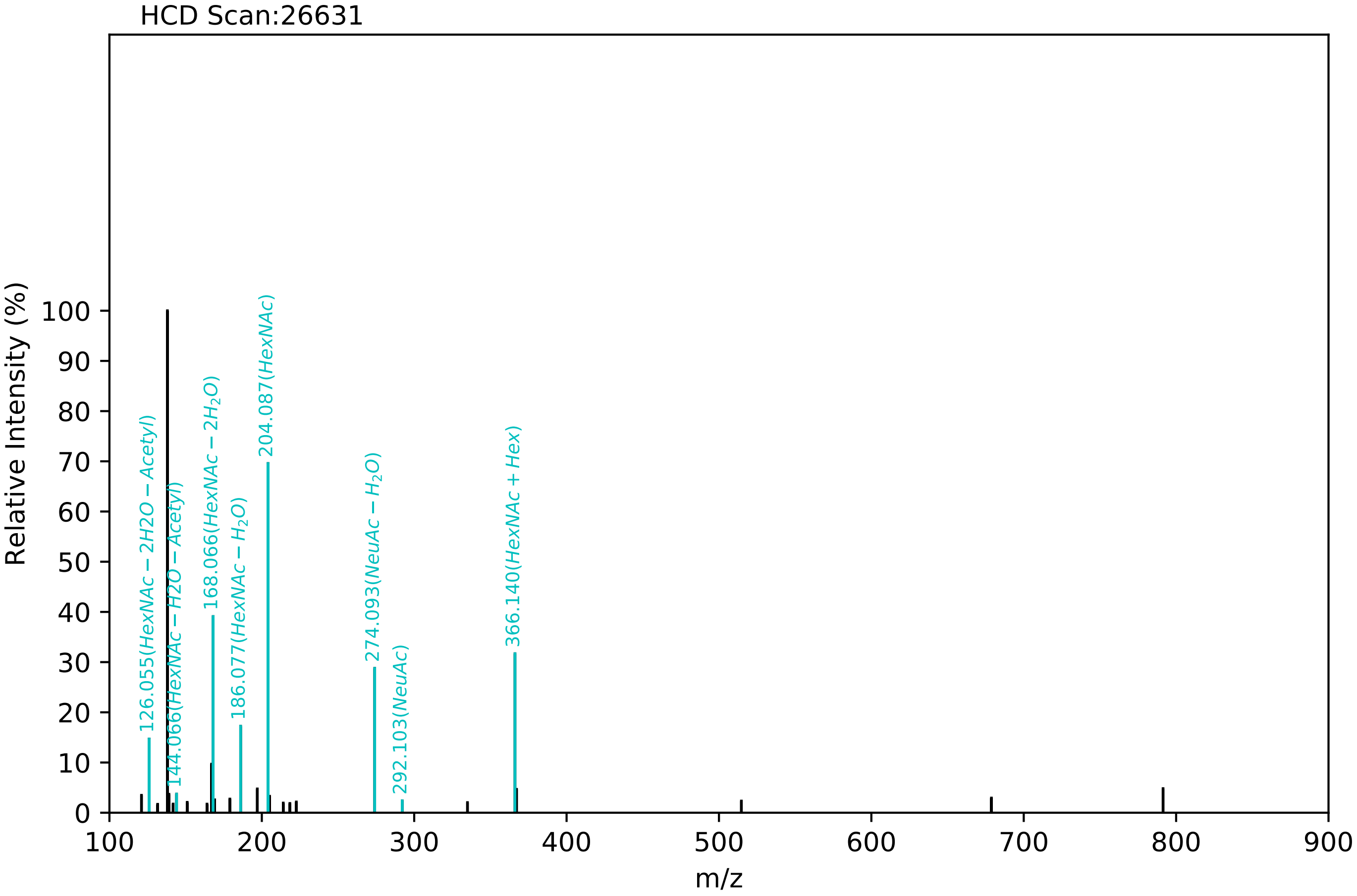

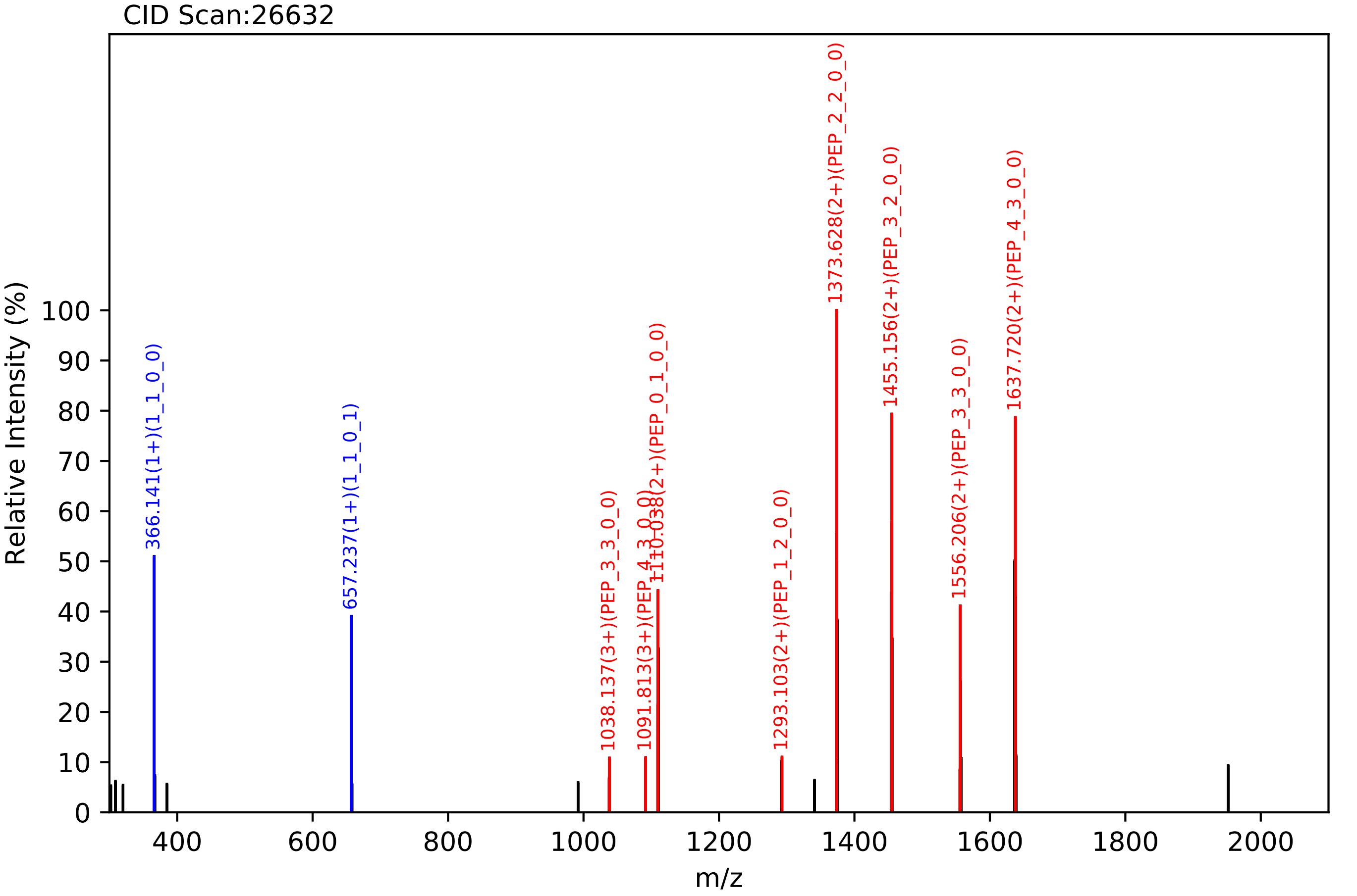

Unknown set no. 321, Experiment: Human Plasma exp_5

VIDFNCTTSSVSSALANTK(=PEP)_5_4_0_1, m/z:1310.22(3+), RT:92.14, Y-score:91.91

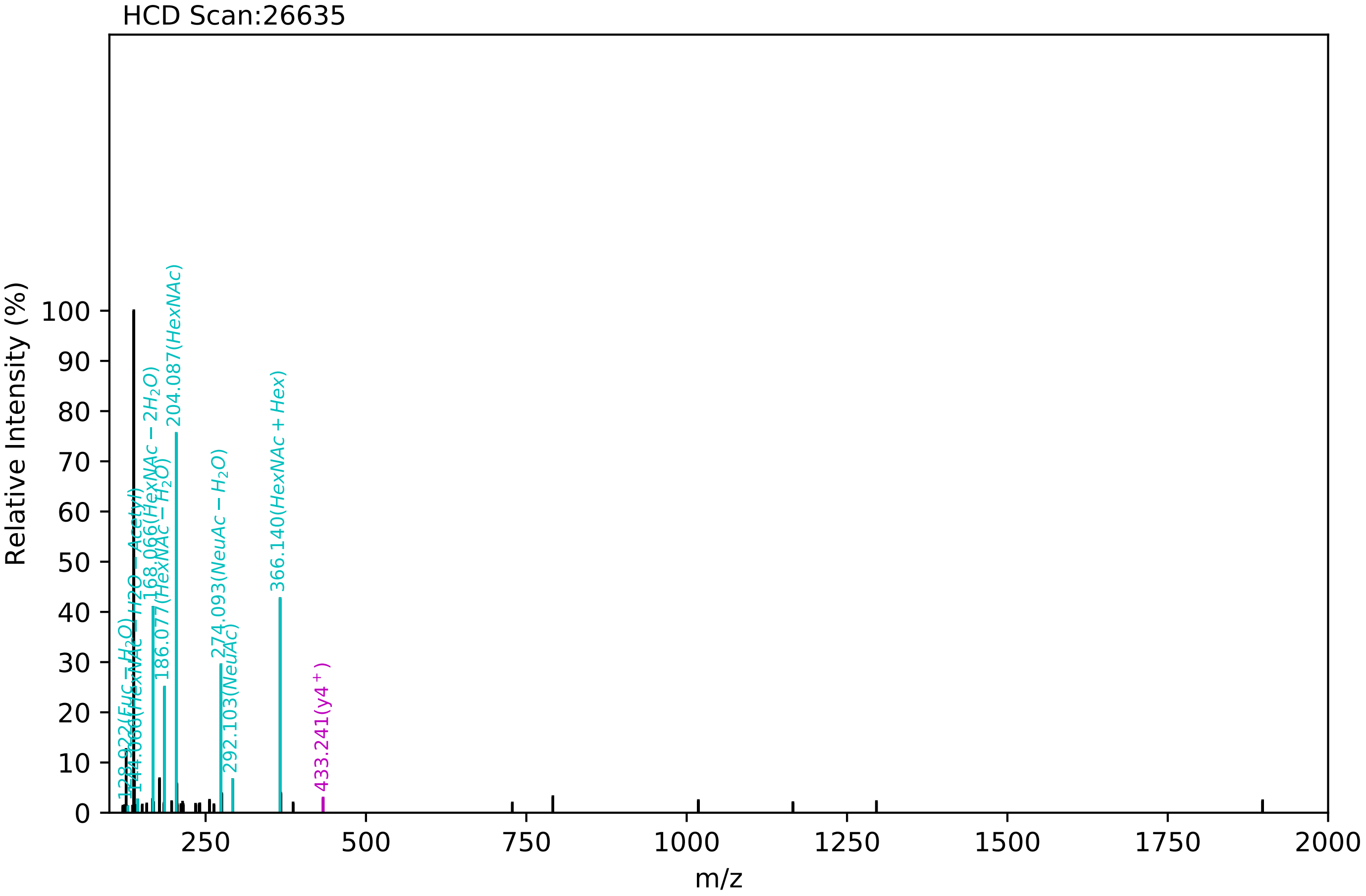

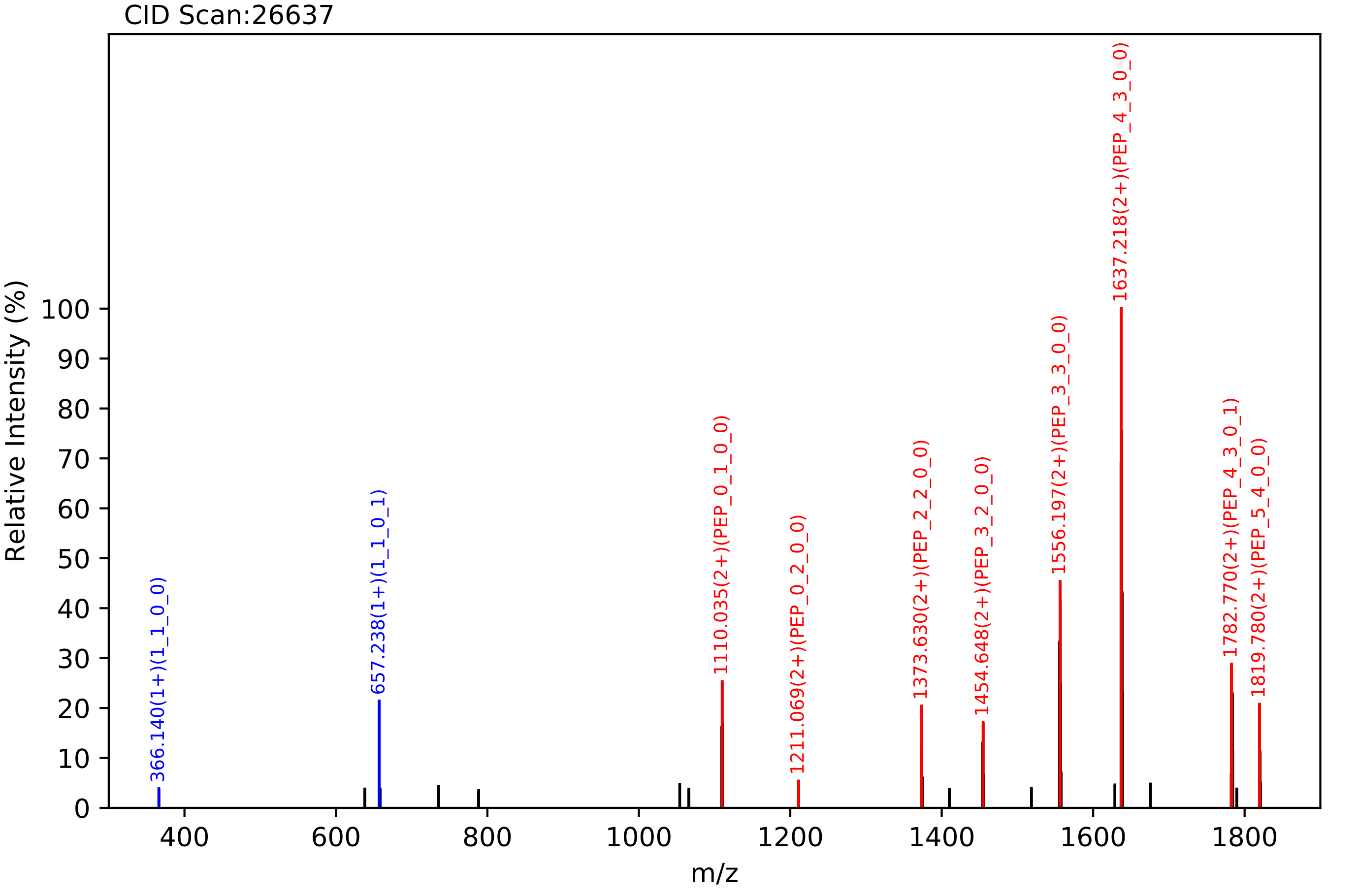

Unknown set no. 322, Experiment: Human Plasma exp_1

VIDFNCTTSSVSSALANTK(=PEP)_5_4_0_2, m/z:1055.69(4+), RT:101.20, Y-score:89.94

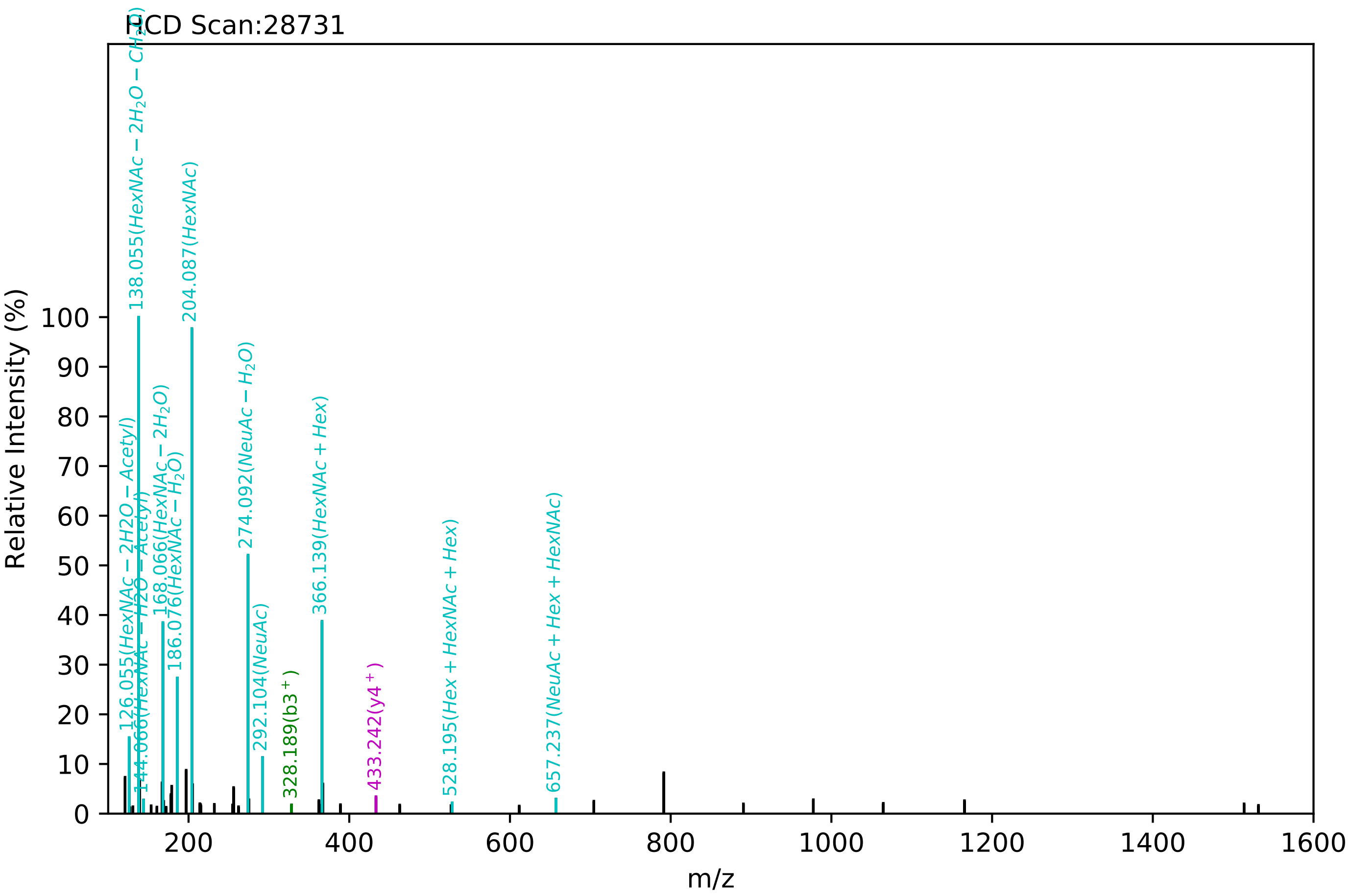

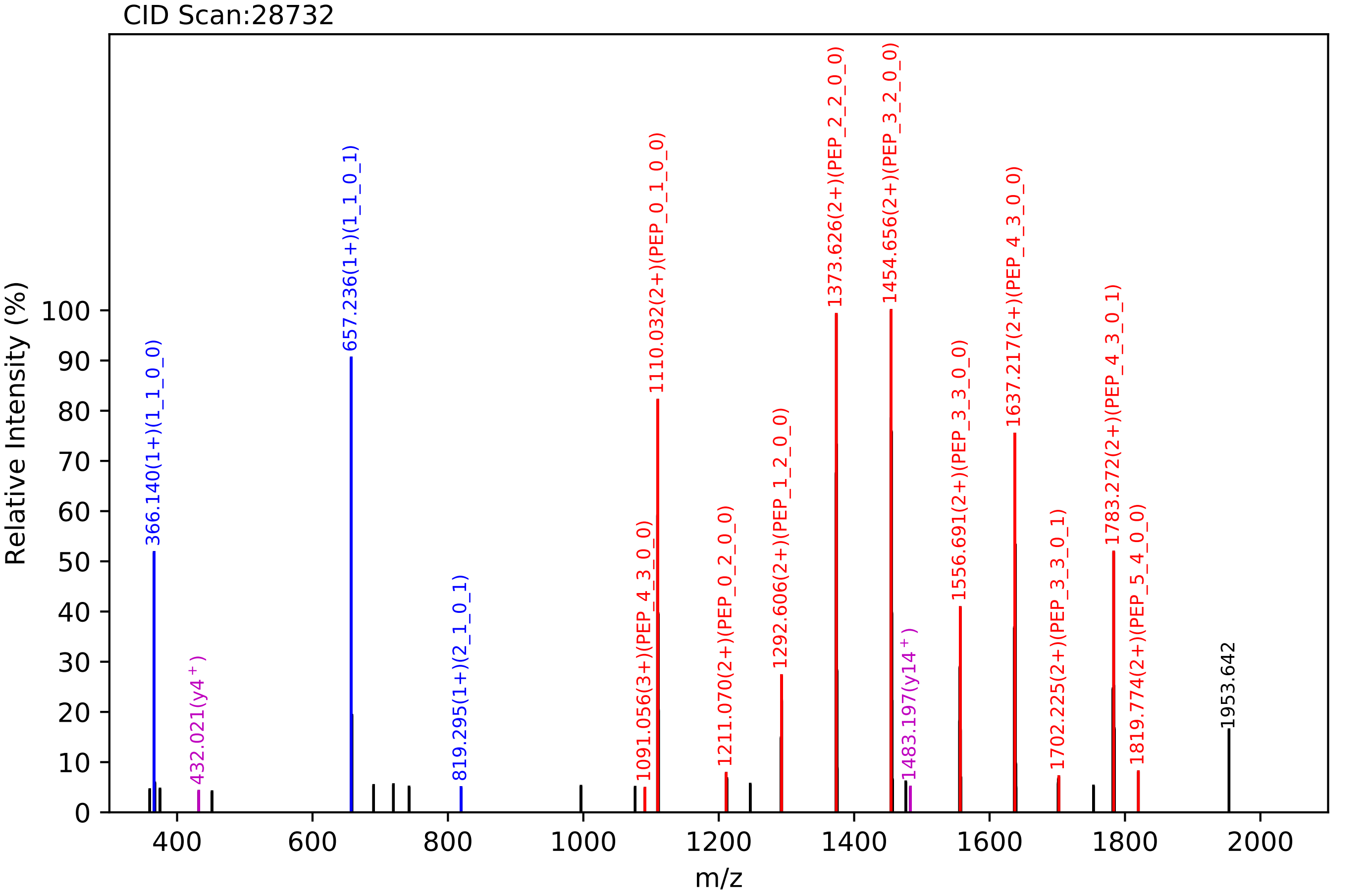

Unknown set no. 323, Experiment: Human Plasma exp_2

VIDFNCTTSSVSSALANTK(=PEP)_5_4_0_2, m/z:1055.69(4+), RT:101.39, Y-score:83.23

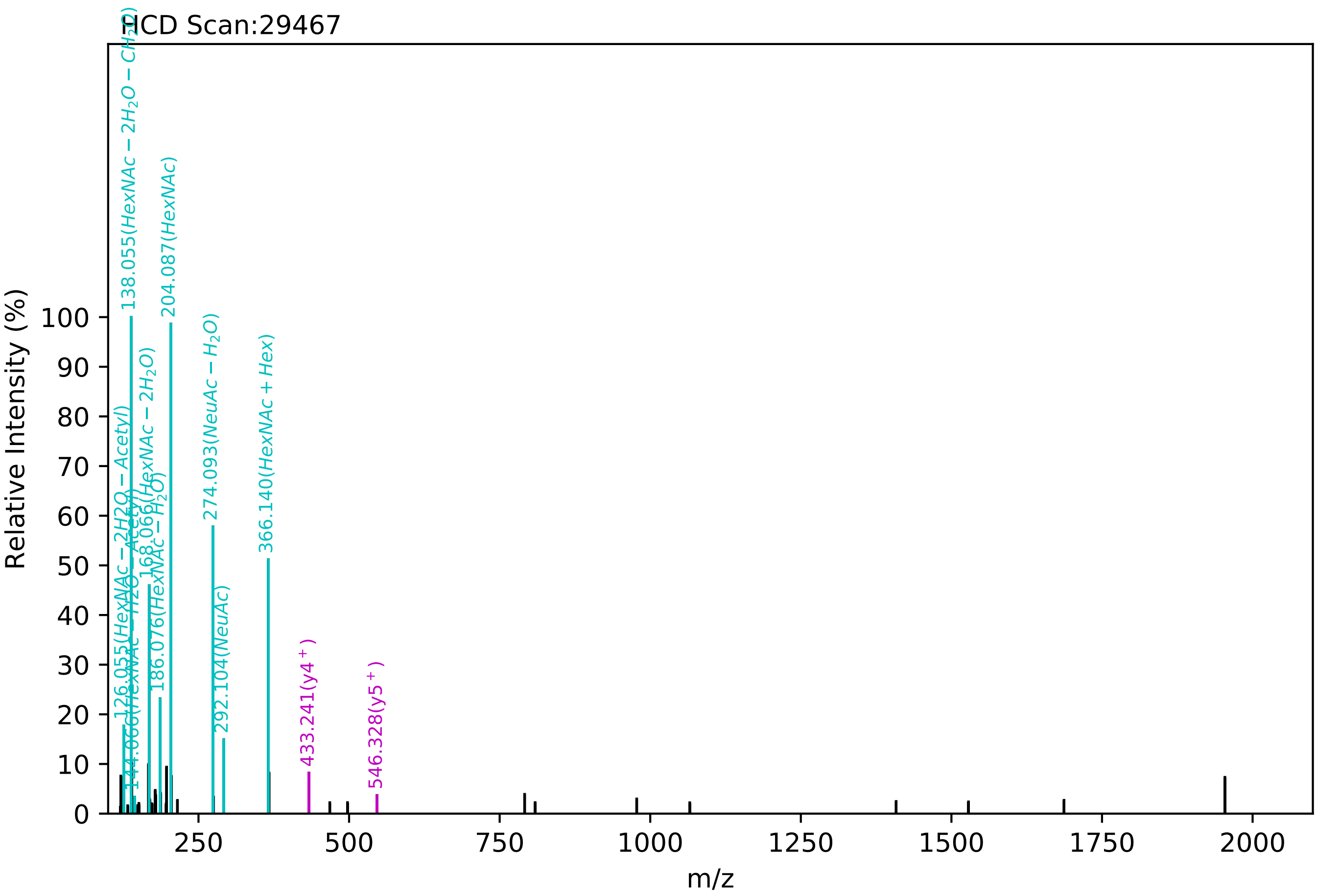

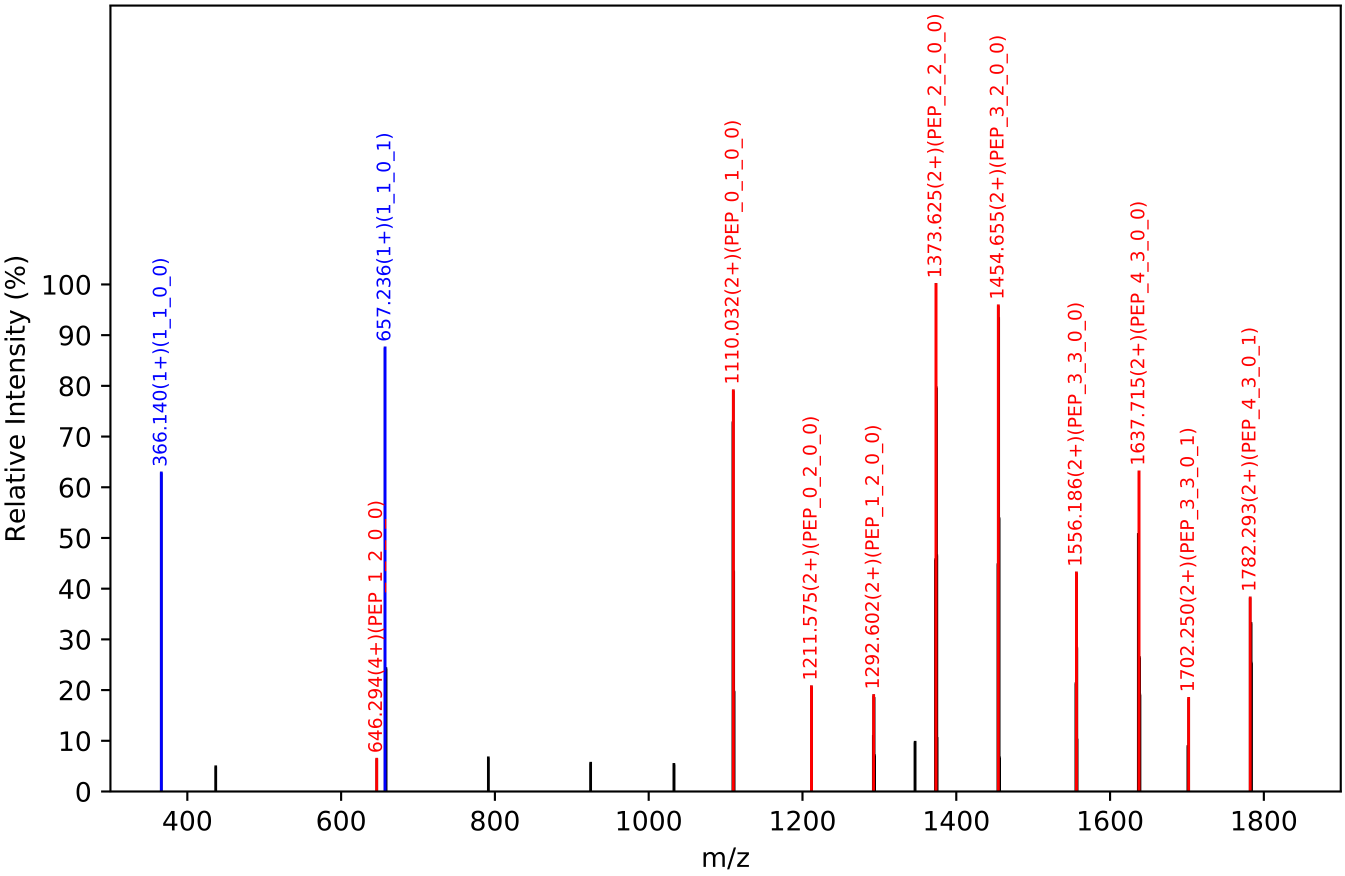

Unknown set no. 324, Experiment: Human Plasma exp_3

VIDFNCTTSSVSSALANTK(=PEP)_5_4_0_2, m/z:1055.69(4+), RT:101.46, Y-score:86.71

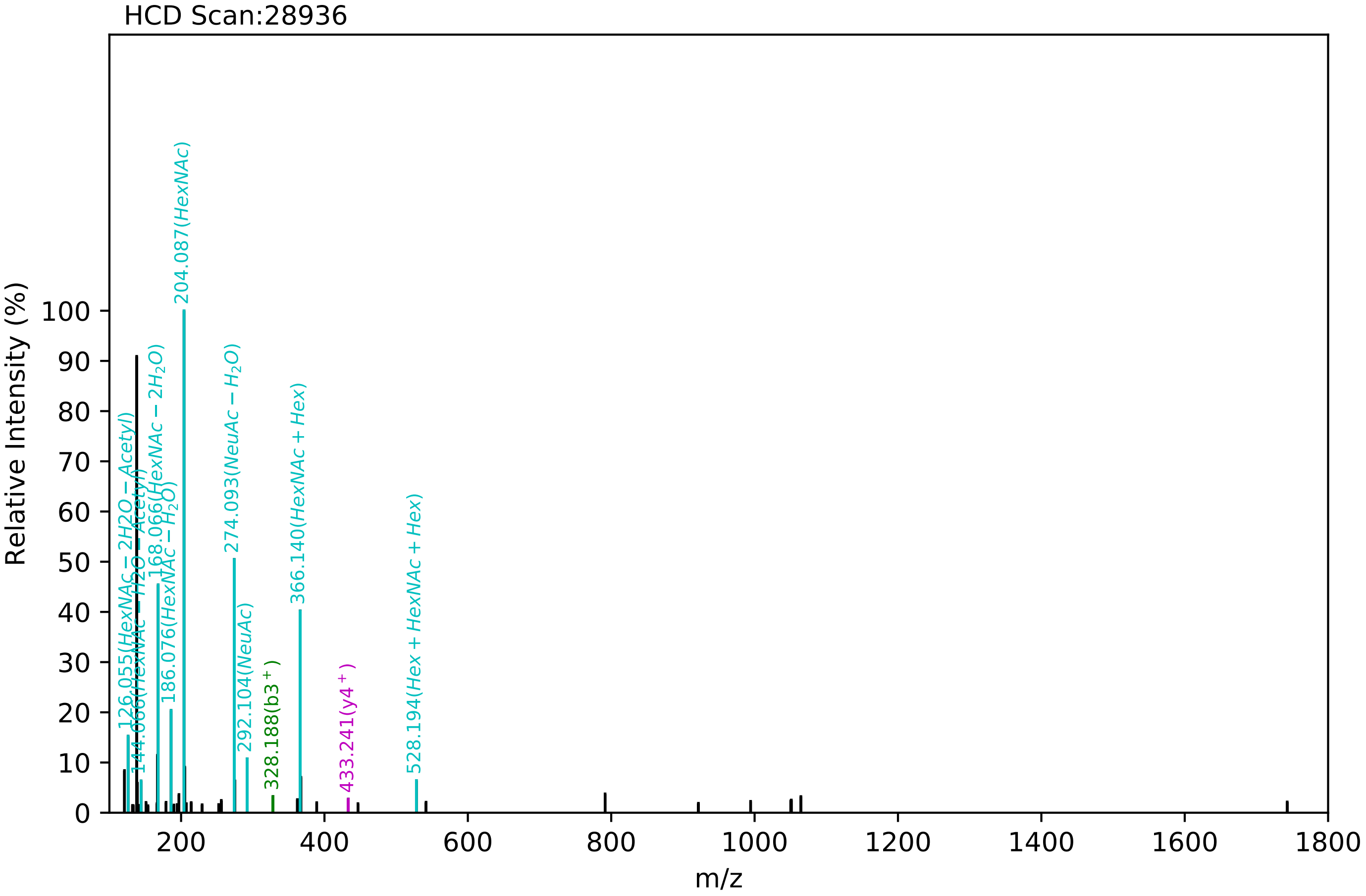

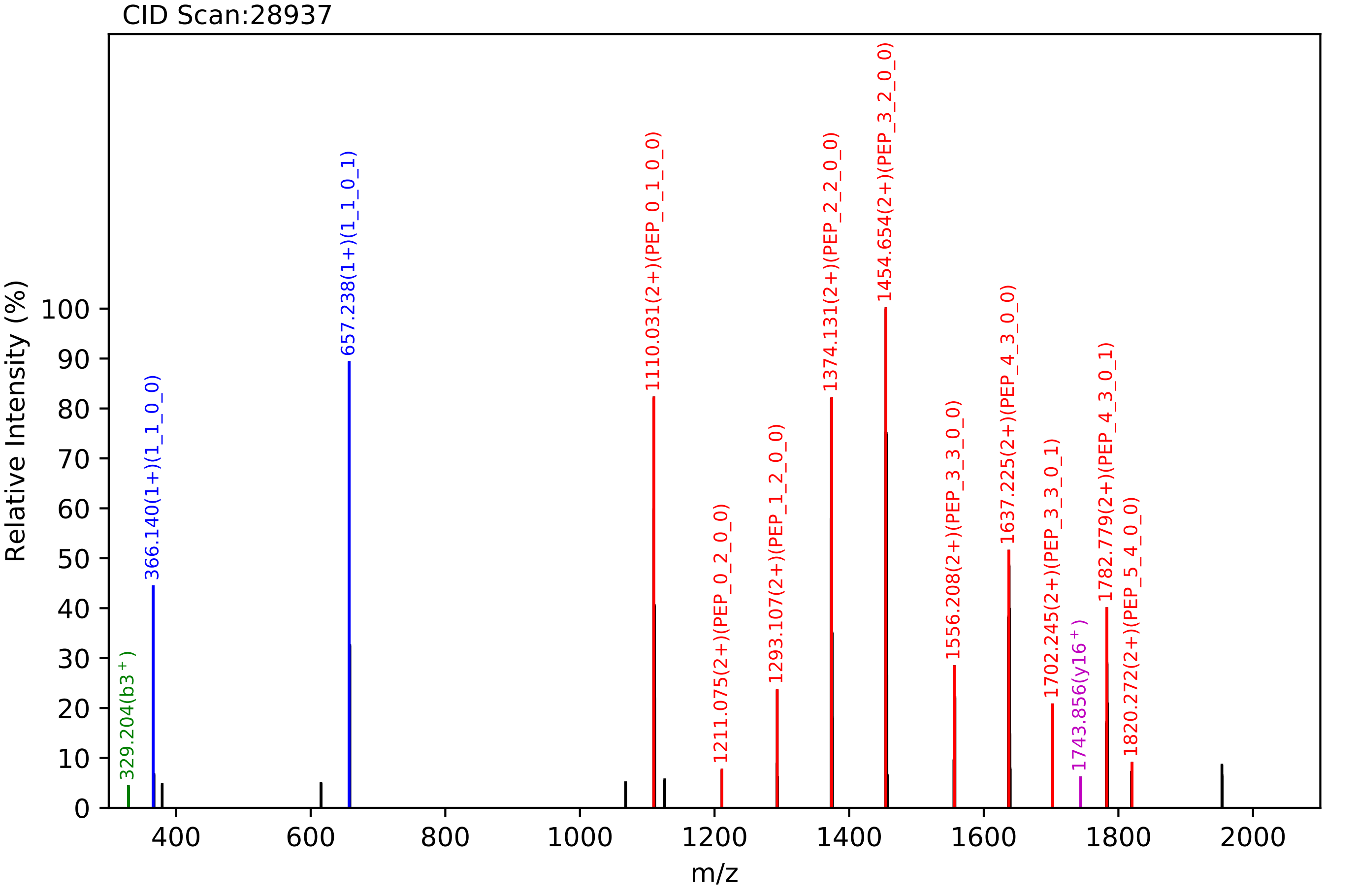

Unknown set no. 325, Experiment: Human Plasma exp_4

VIDFNCTTSSVSSALANTK(=PEP)_5_4_0_2, m/z:1055.69(4+), RT:101.57, Y-score:90.46

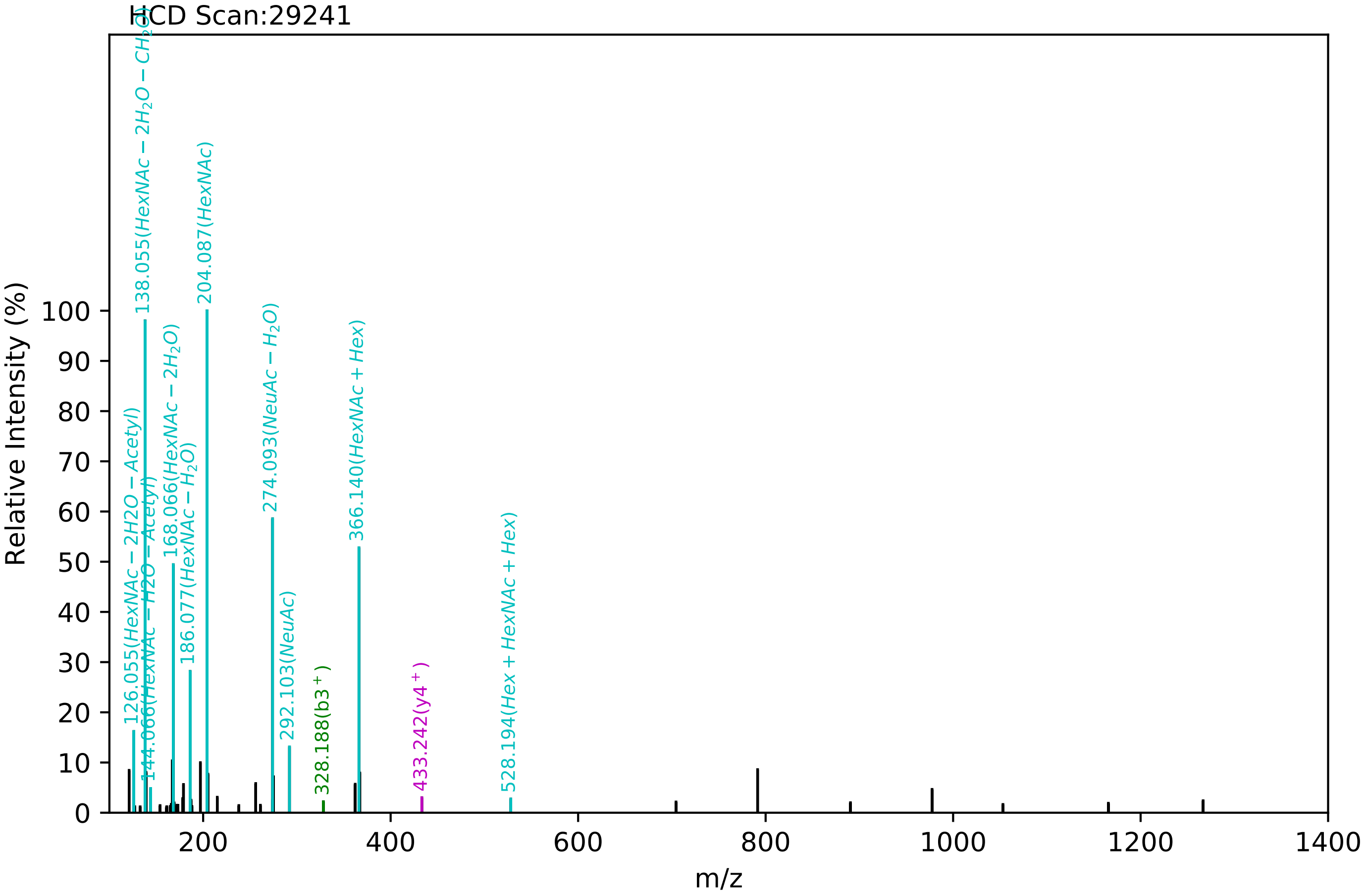

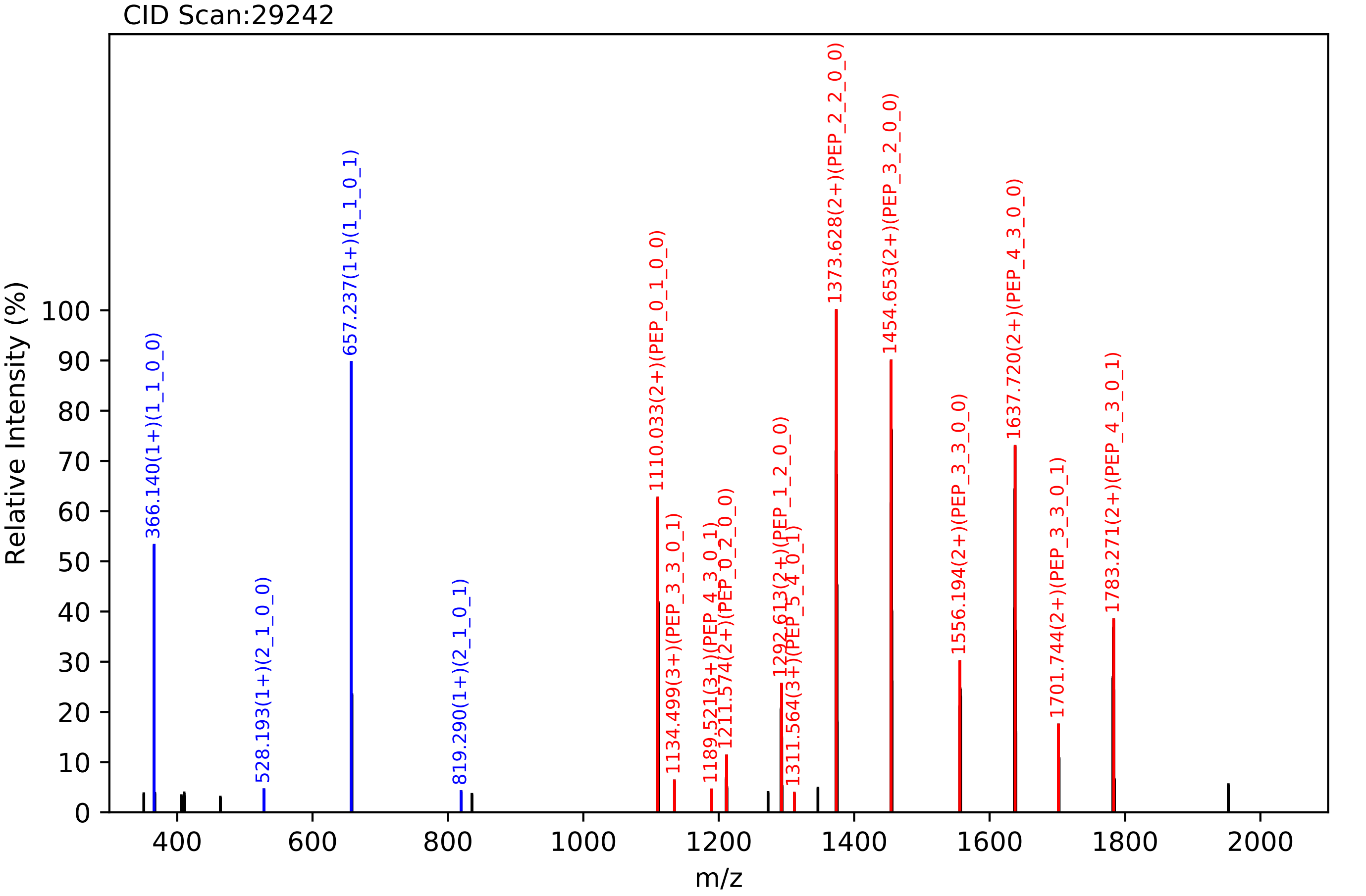

Unknown set no. 326, Experiment: Human Plasma exp_5

VIDFNCTTSSVSSALANTK(=PEP)_5_4_0_2, m/z:1055.69(4+), RT:101.28, Y-score:88.11

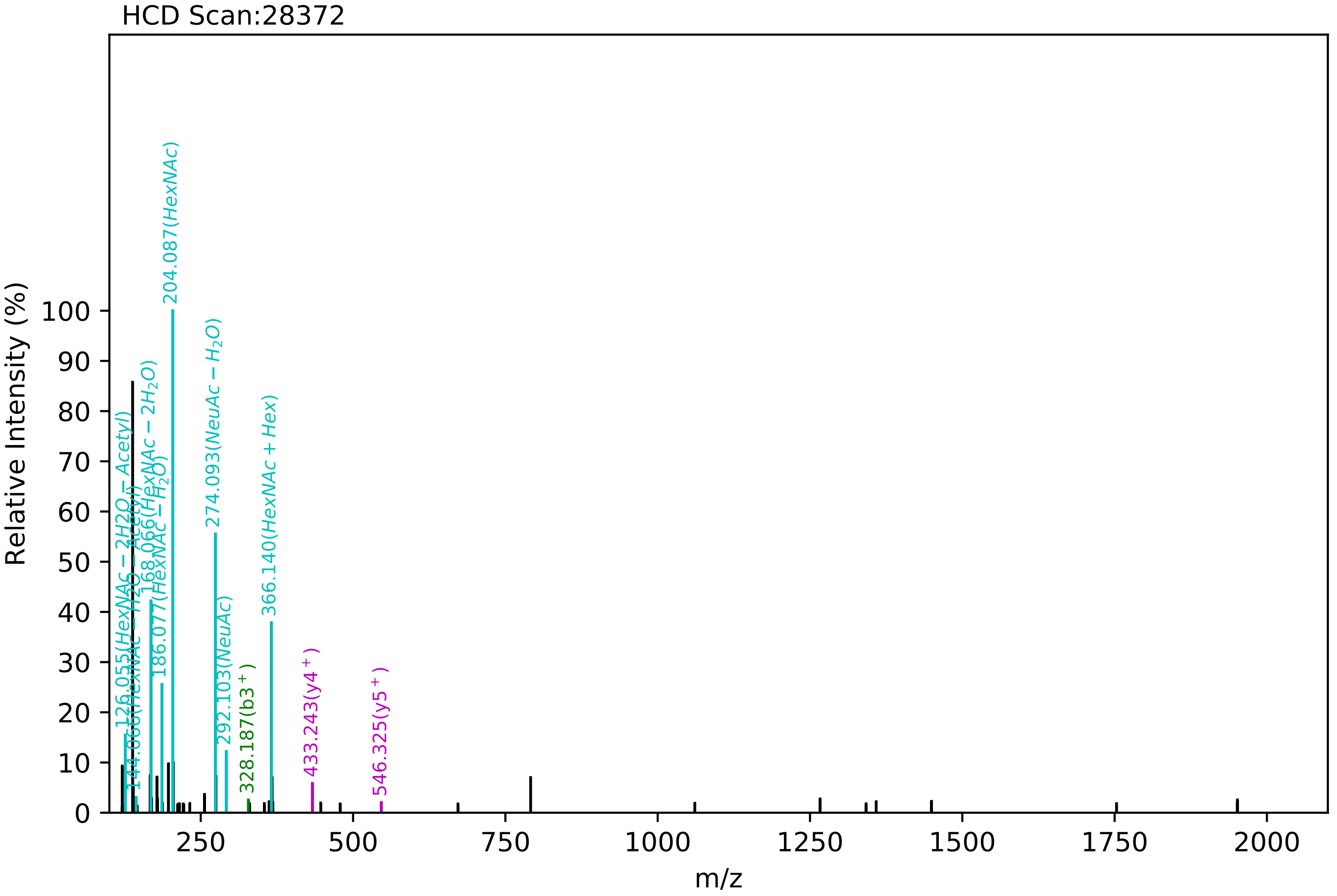

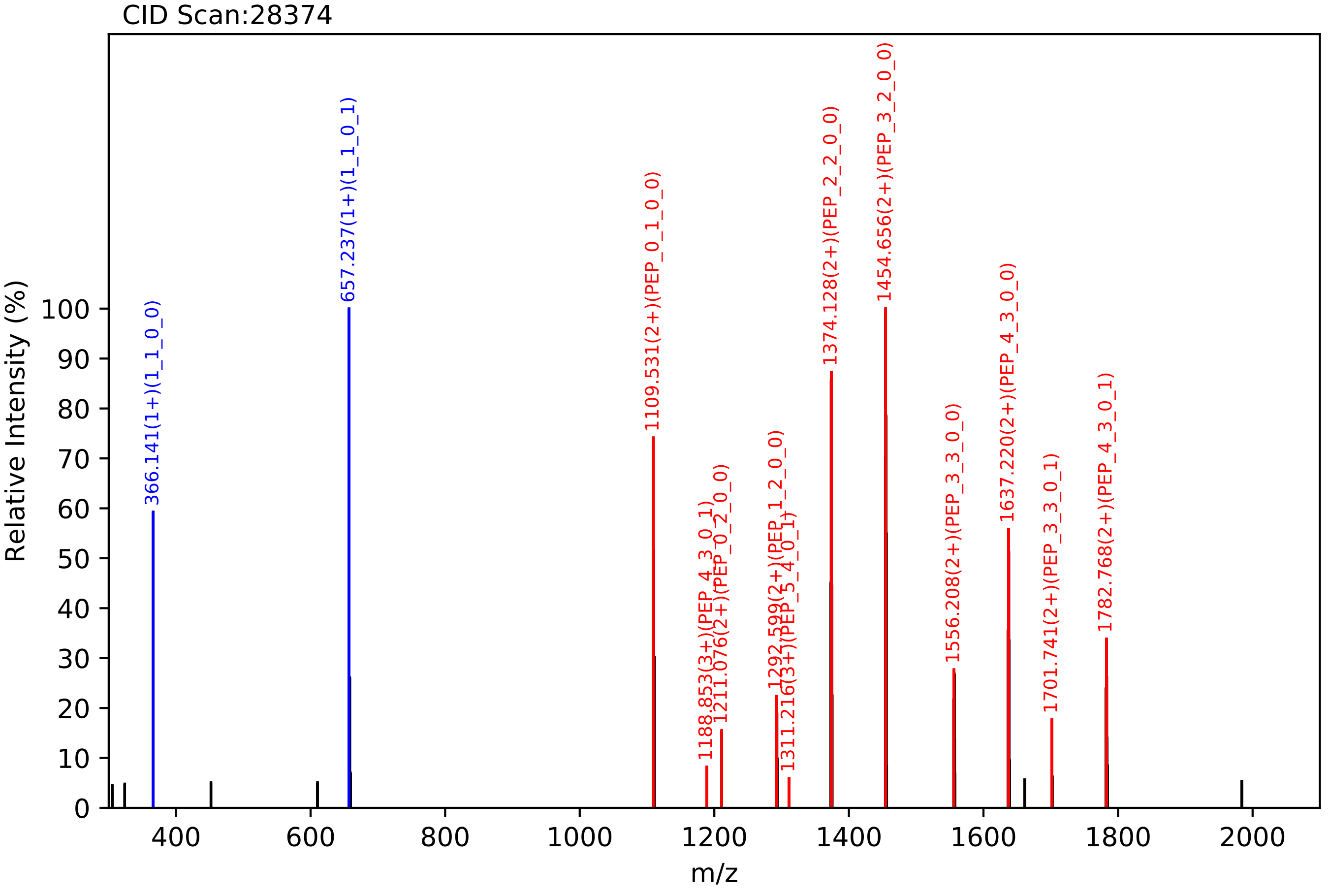

Unknown set no. 327, Experiment: Human Plasma exp_5

LFNVTPQDEQK(=PEP)_5_5_1_1, m/z:896.12(4+), RT:62.03, Y-score:81.96

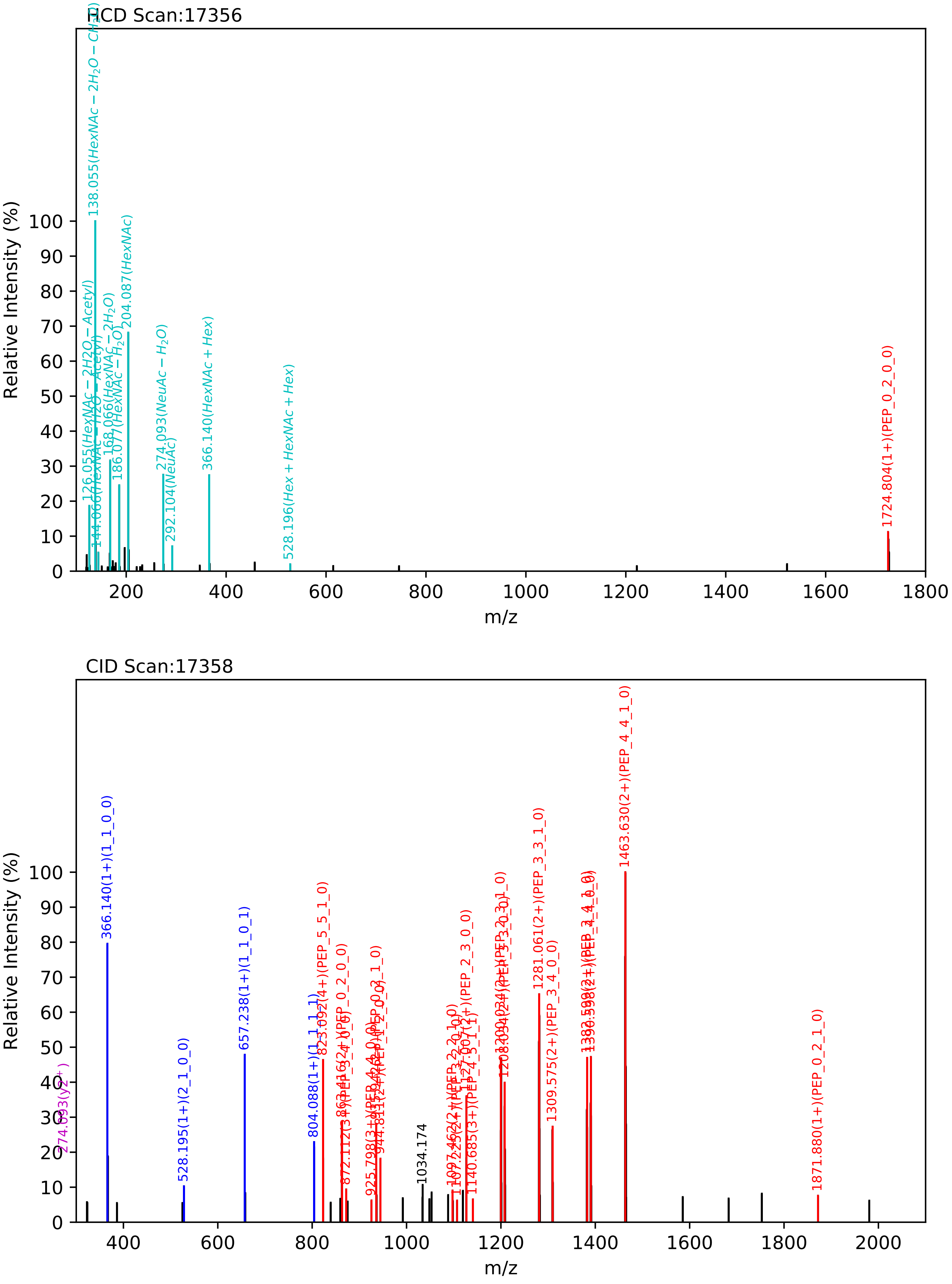

Unknown set no. 328, Experiment: Human Plasma exp_1

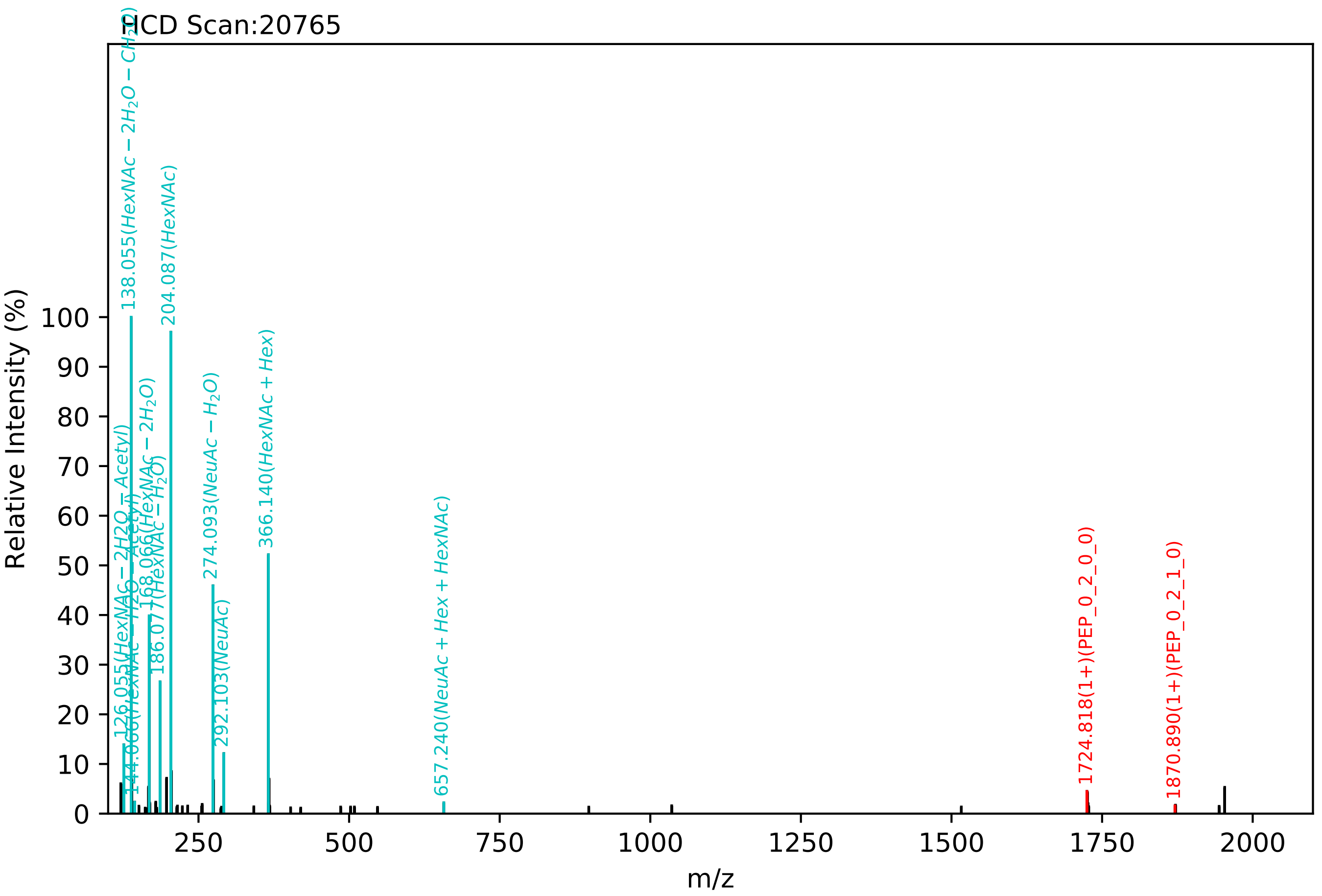

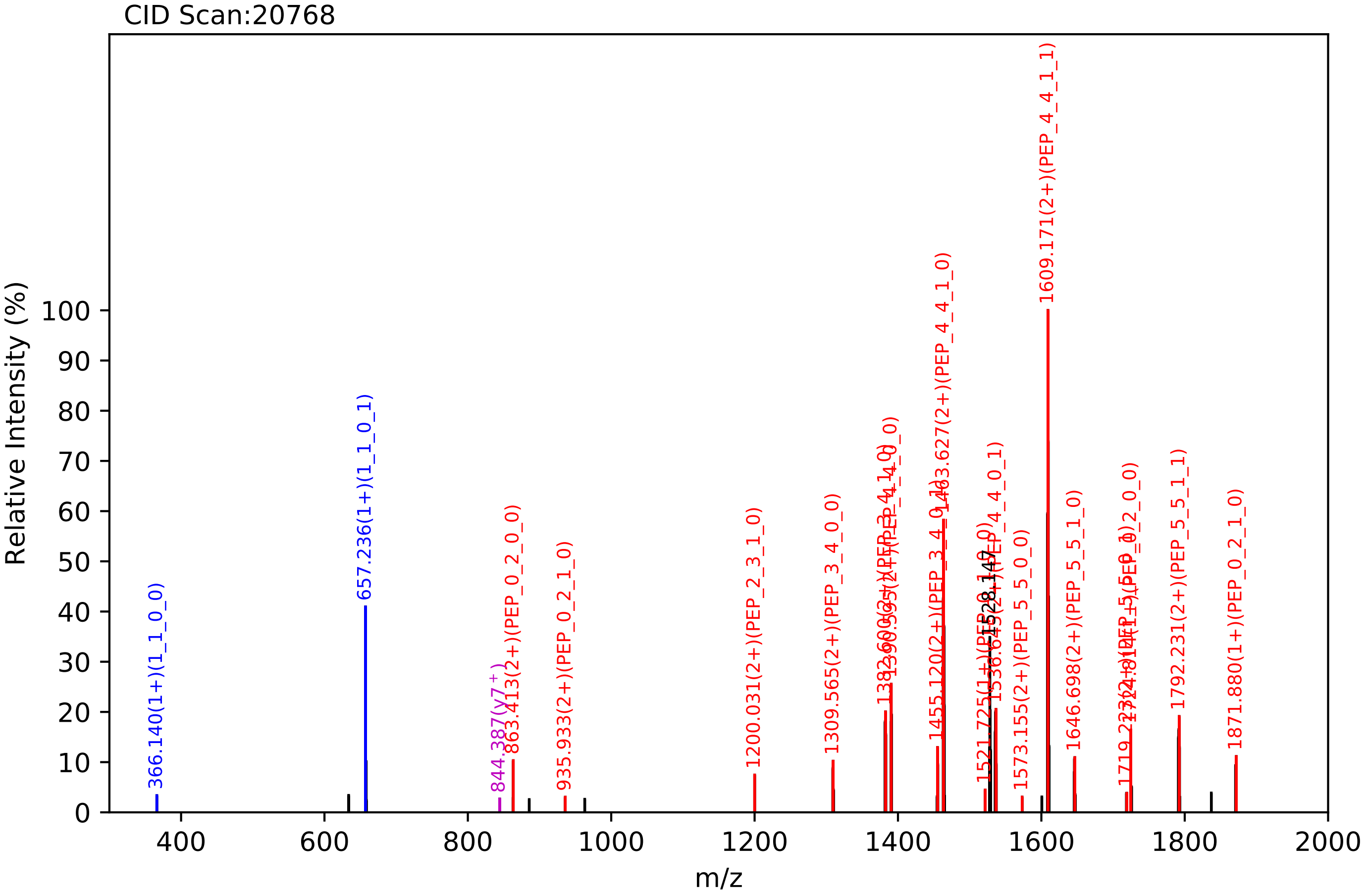

Unknown set no. 329, Experiment: Human Plasma exp_4

LFNVTPQDEQK(=PEP)_5_5_1_2, m/z:968.90(4+), RT:71.16, Y-score:73.10

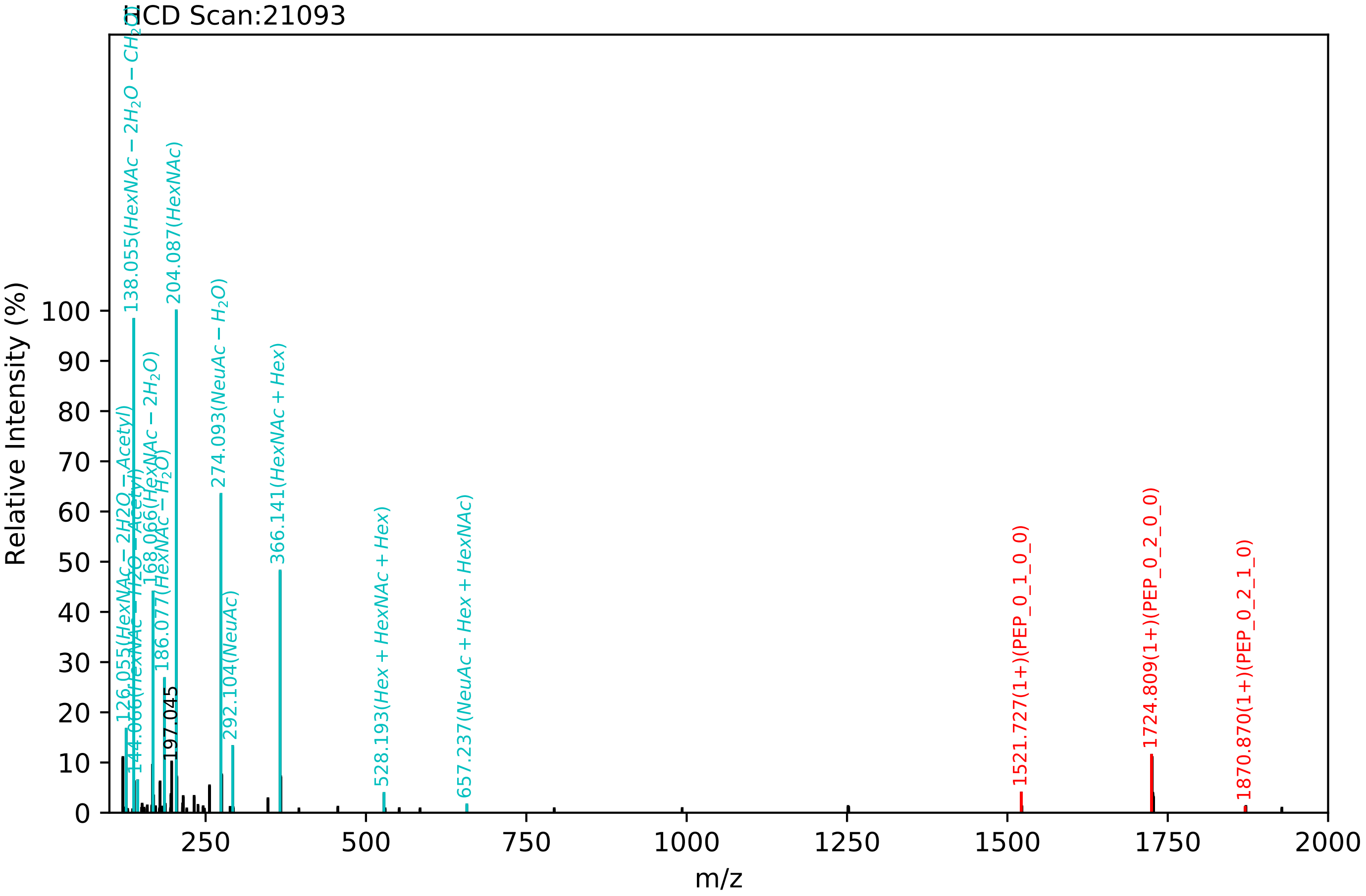

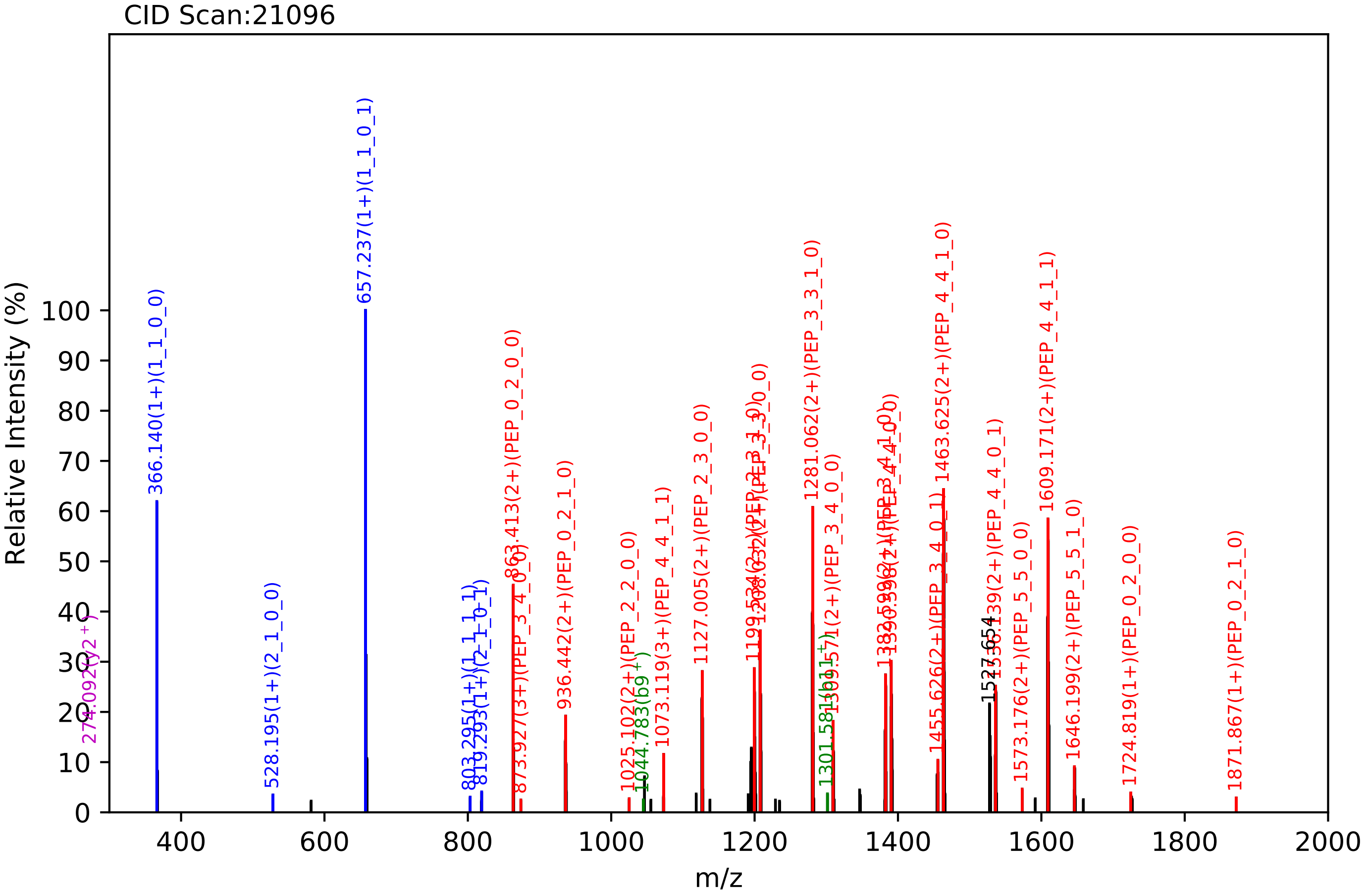

Unknown set no. 330, Experiment: Human Plasma exp_4

LFNVTPQDEQK(=PEP)_5_5_1_2, m/z:1291.53(3+), RT:71.03, Y-score:73.93

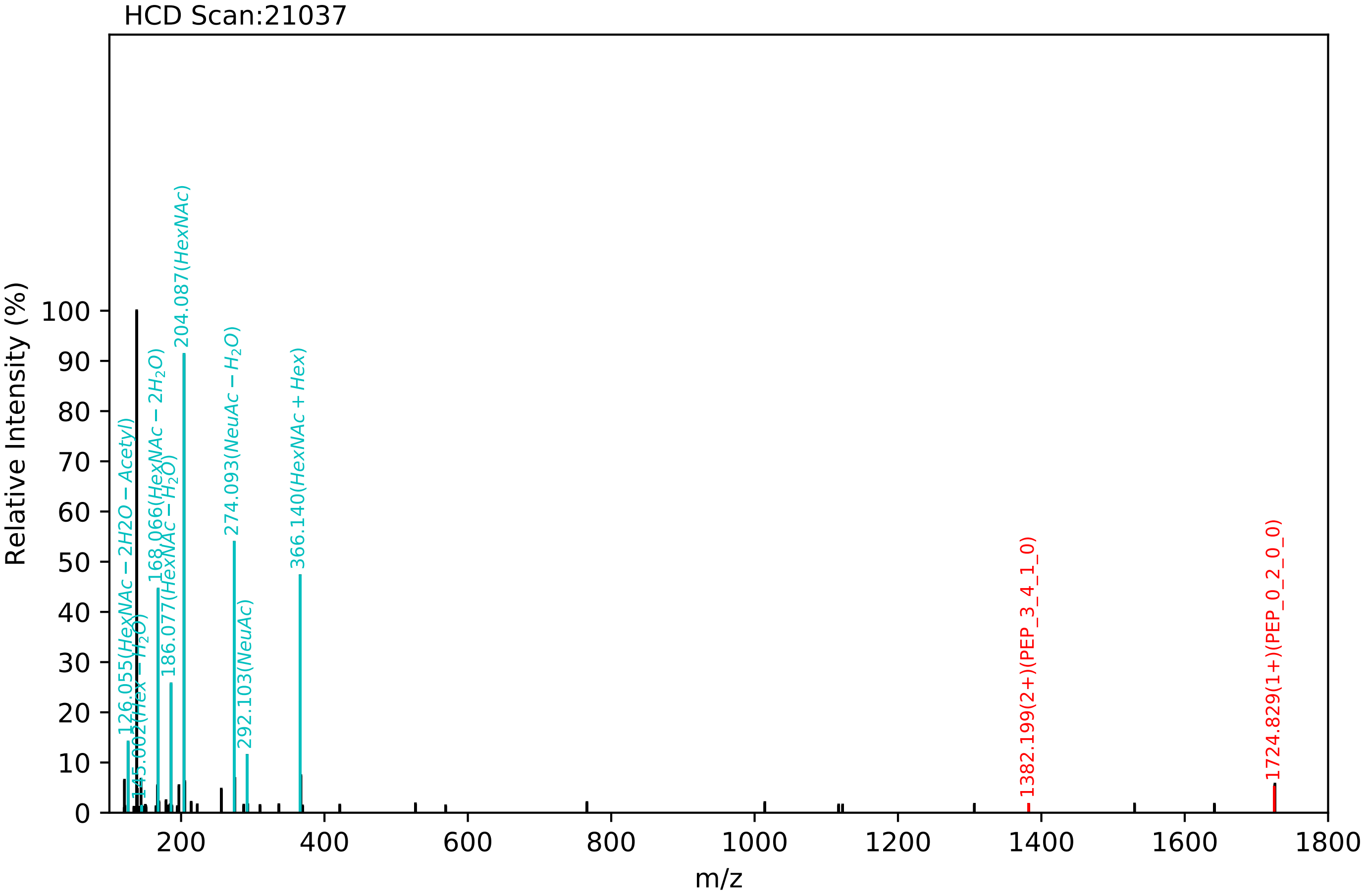

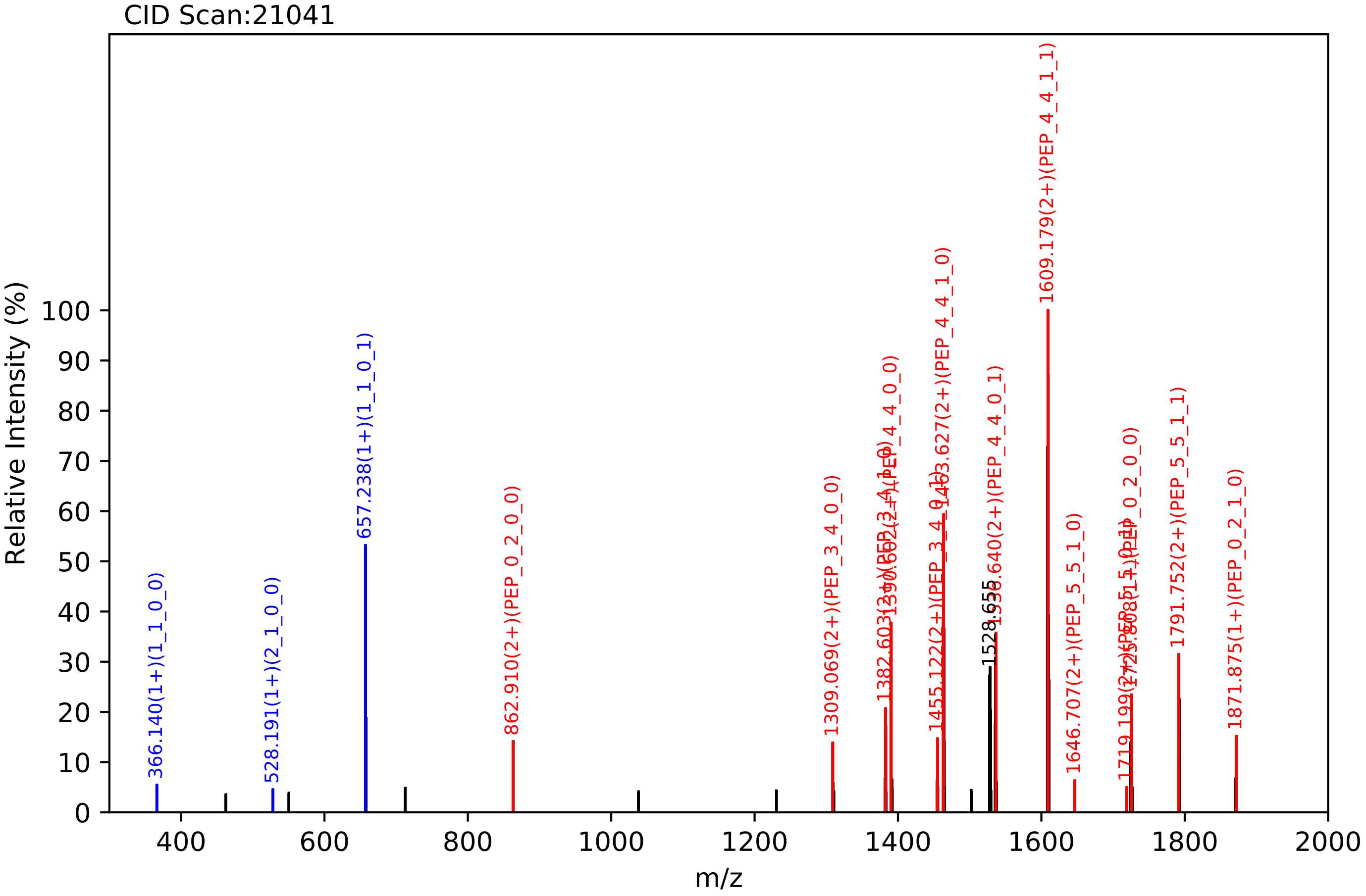

Unknown set no. 331, Experiment: Human Plasma exp_1

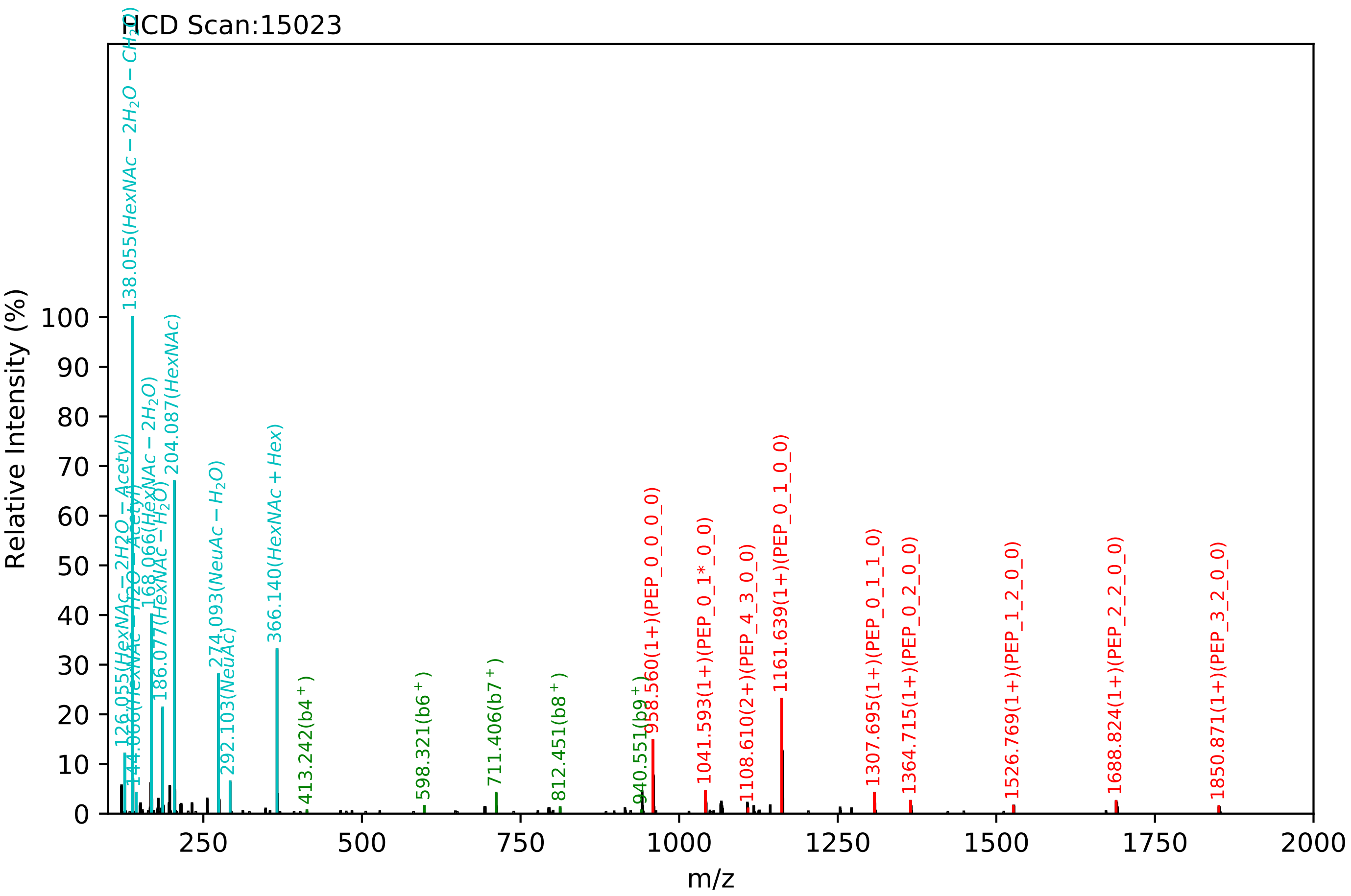

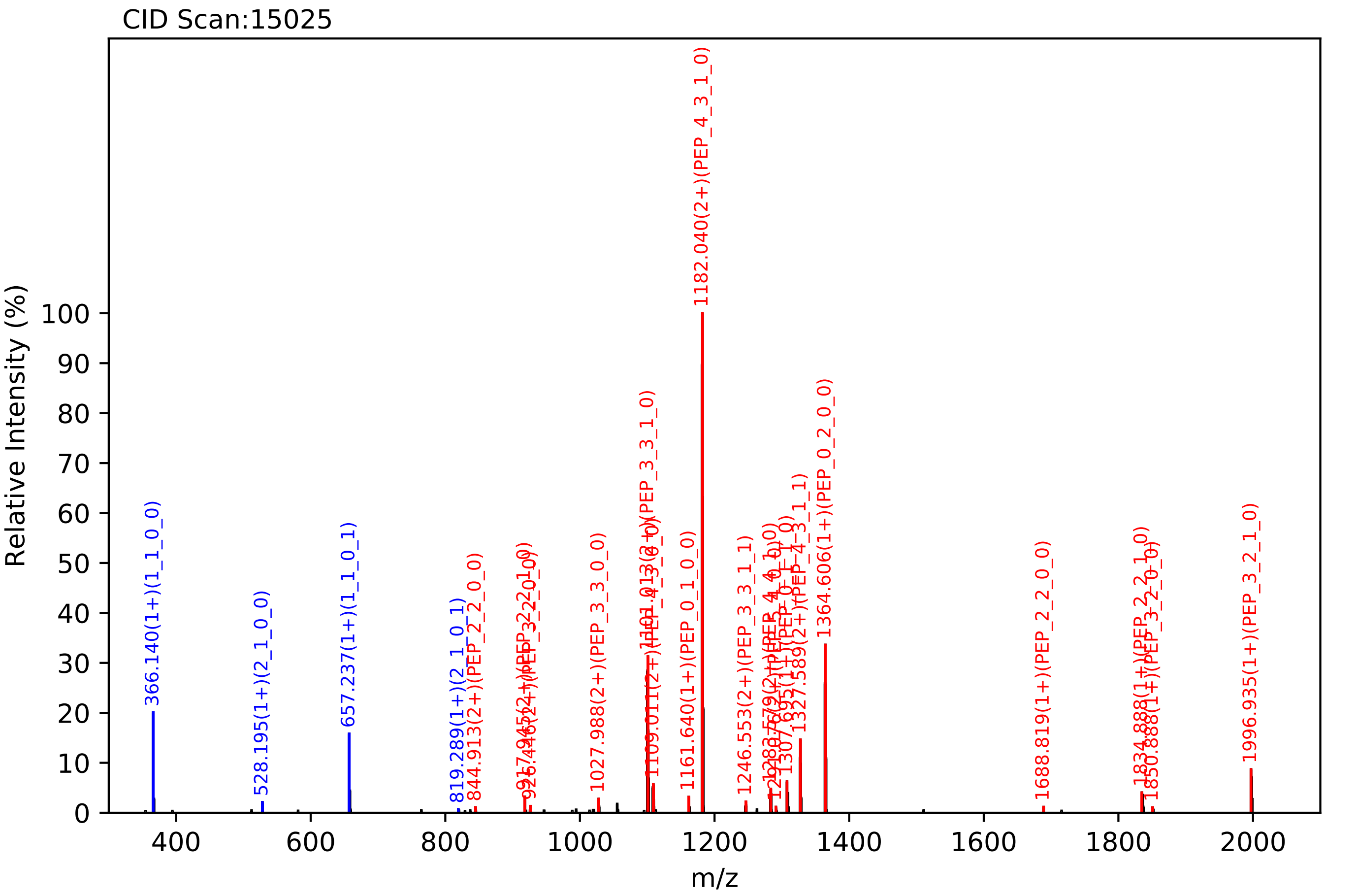

Unknown set no. 332, Experiment: Human Plasma exp_2

TPLTANITK(=PEP)_5_4_1_1, m/z:1006.77(3+), RT:55.94, Y-score:92.85

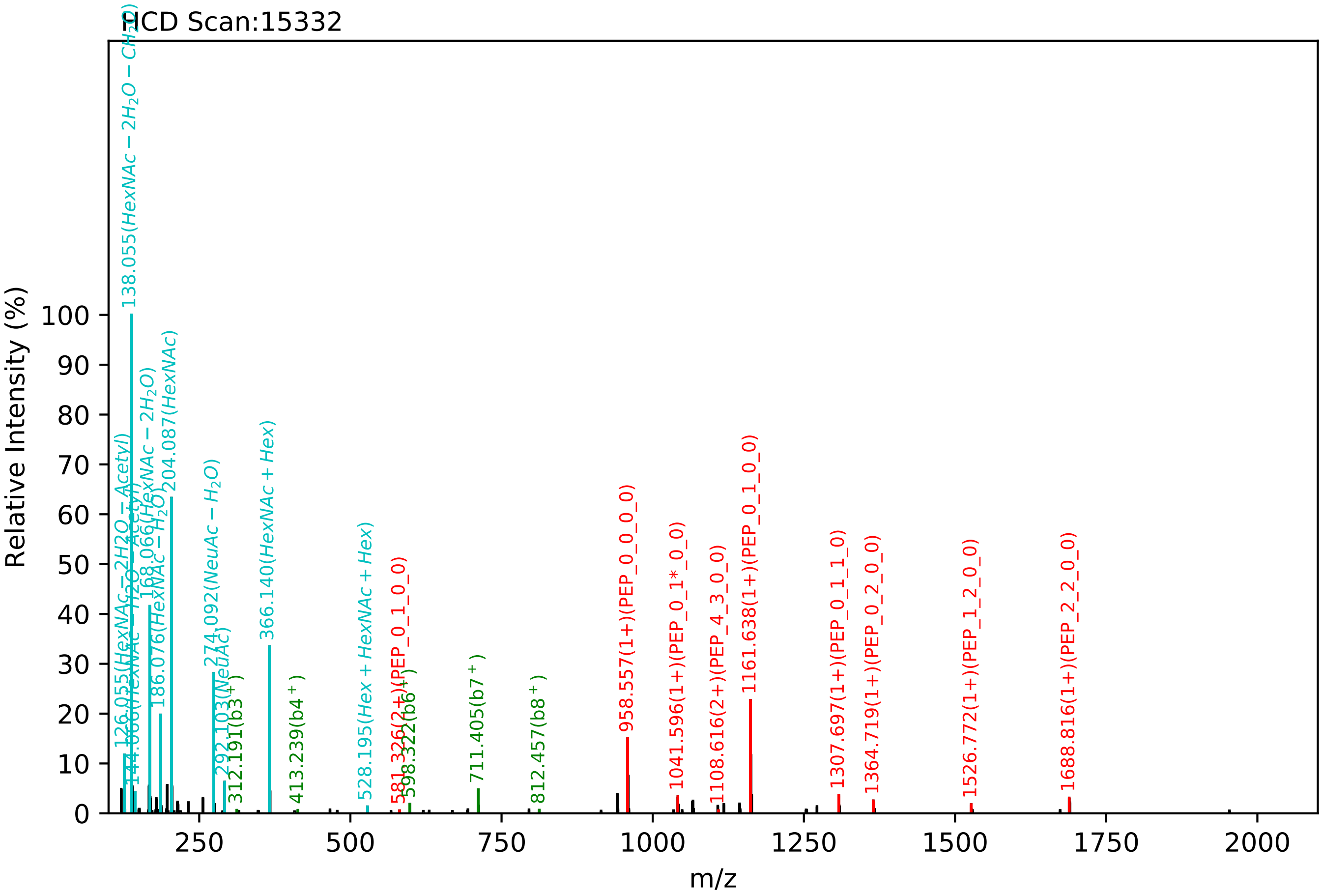

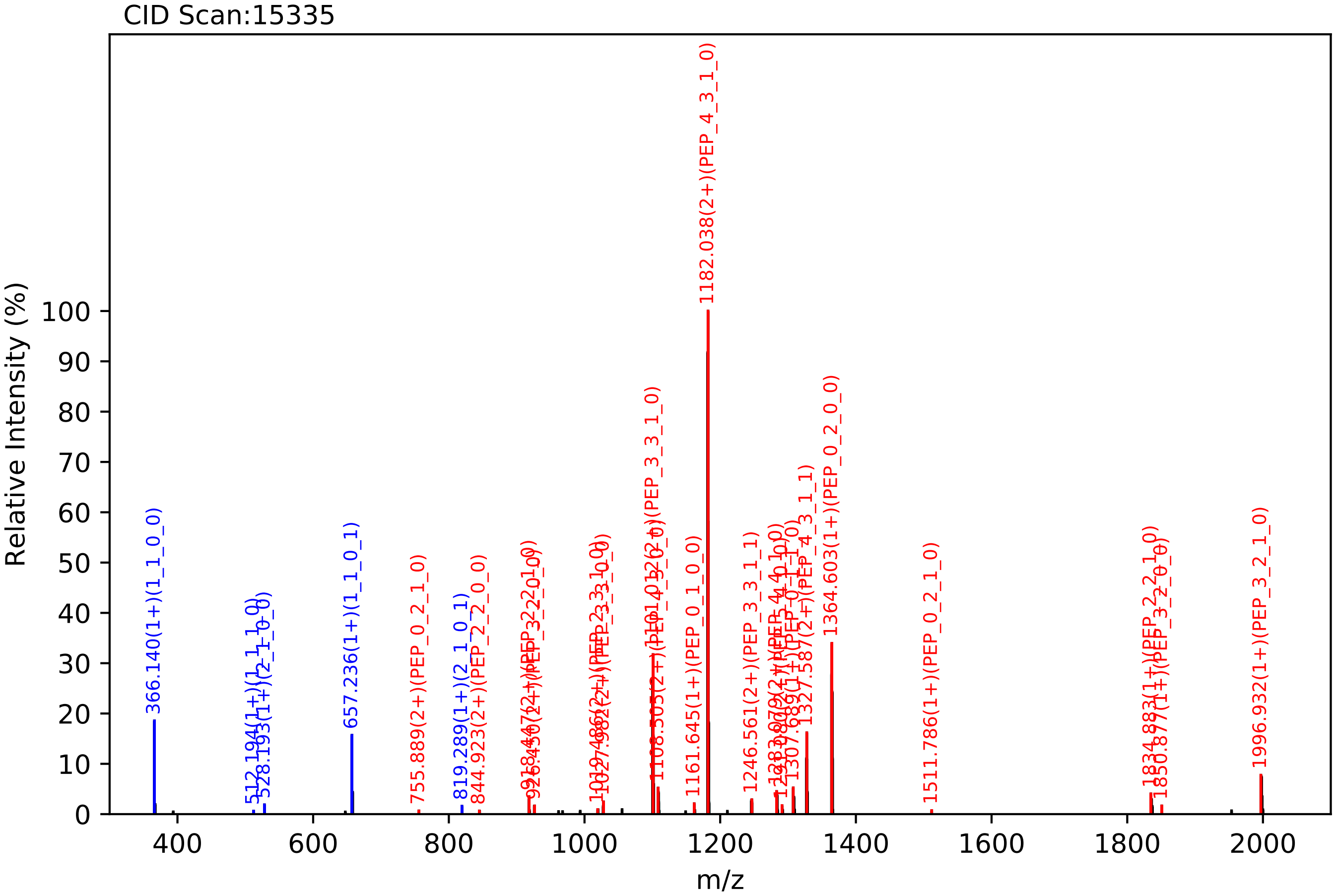

Unknown set no. 333, Experiment: Human Plasma exp_3

TPLTANITK(=PEP)_5_4_1_1, m/z:1509.65(2+), RT:55.42, Y-score:91.90

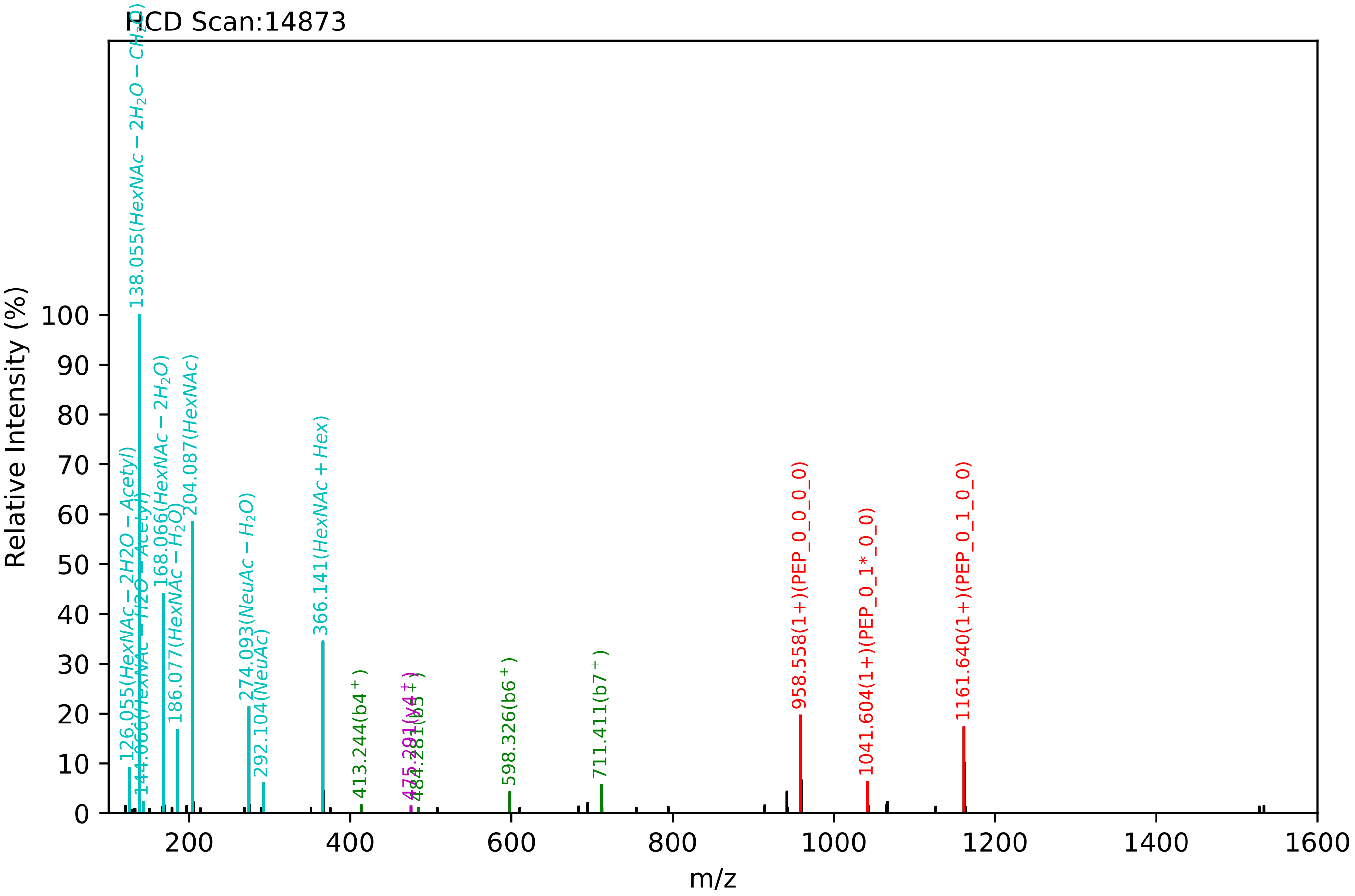

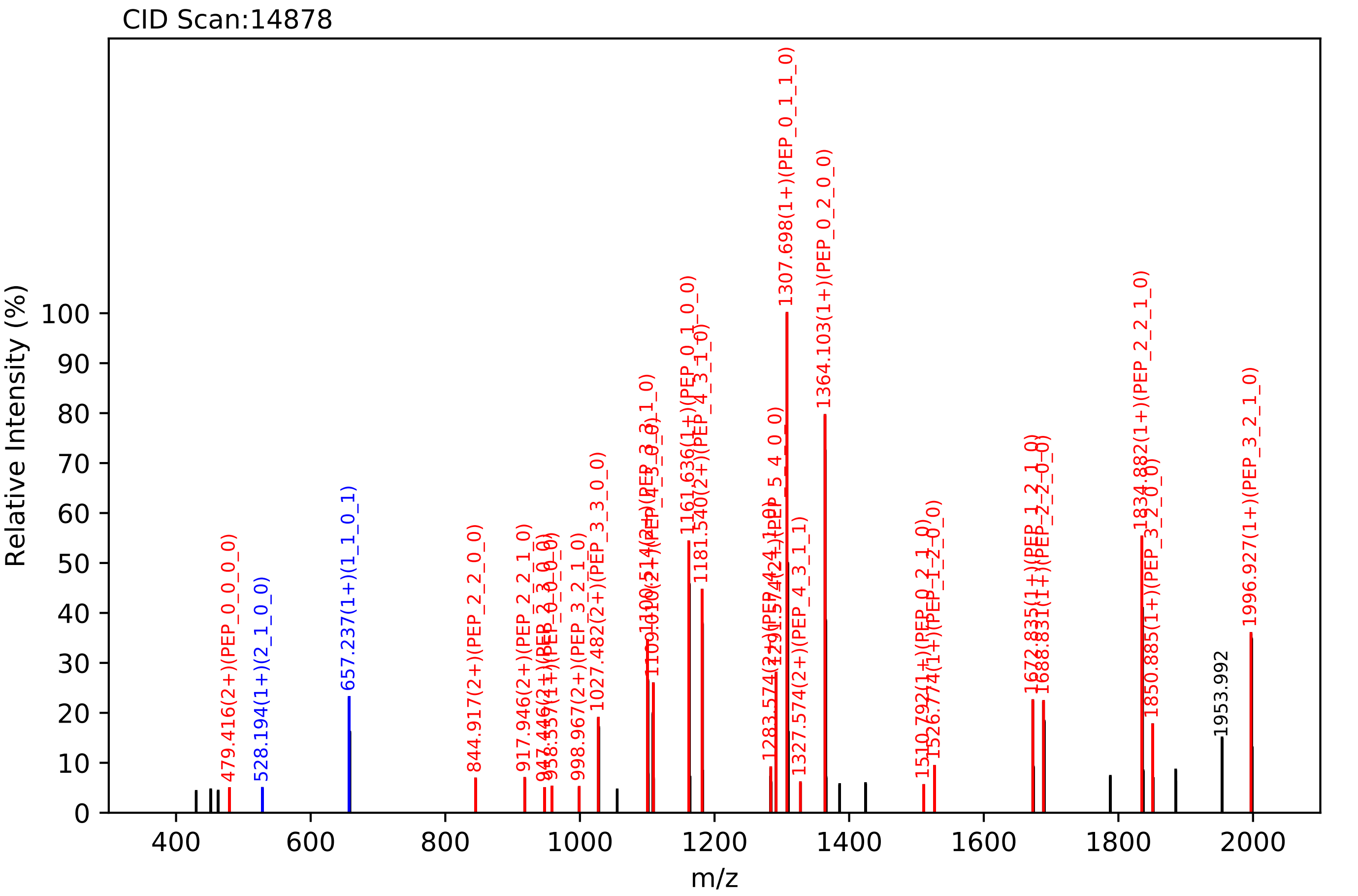

Unknown set no. 334, Experiment: Human Plasma exp_4

TPLTANITK(=PEP)_5_4_1_1, m/z:1509.65(2+), RT:55.57, Y-score:73.04

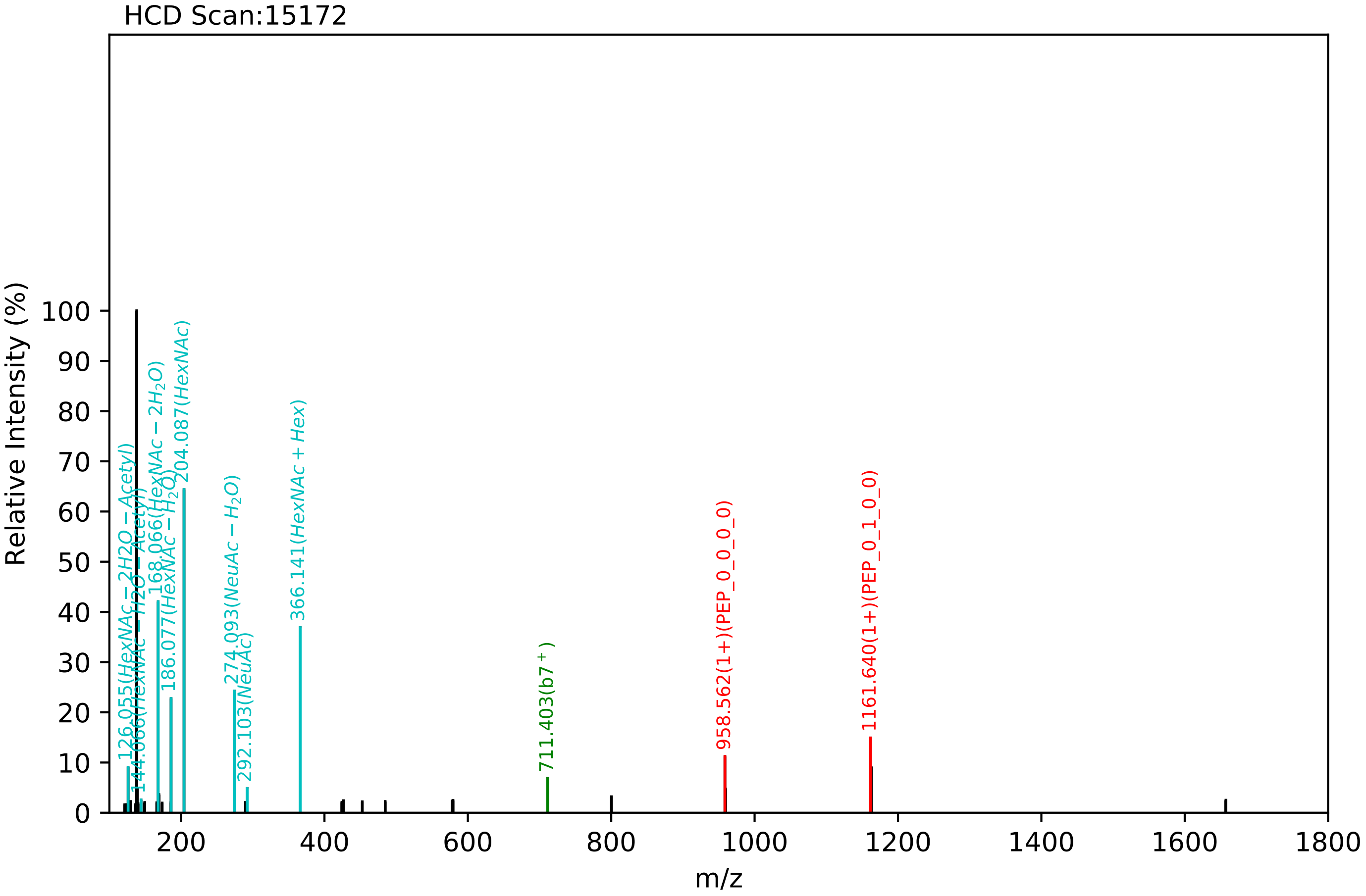

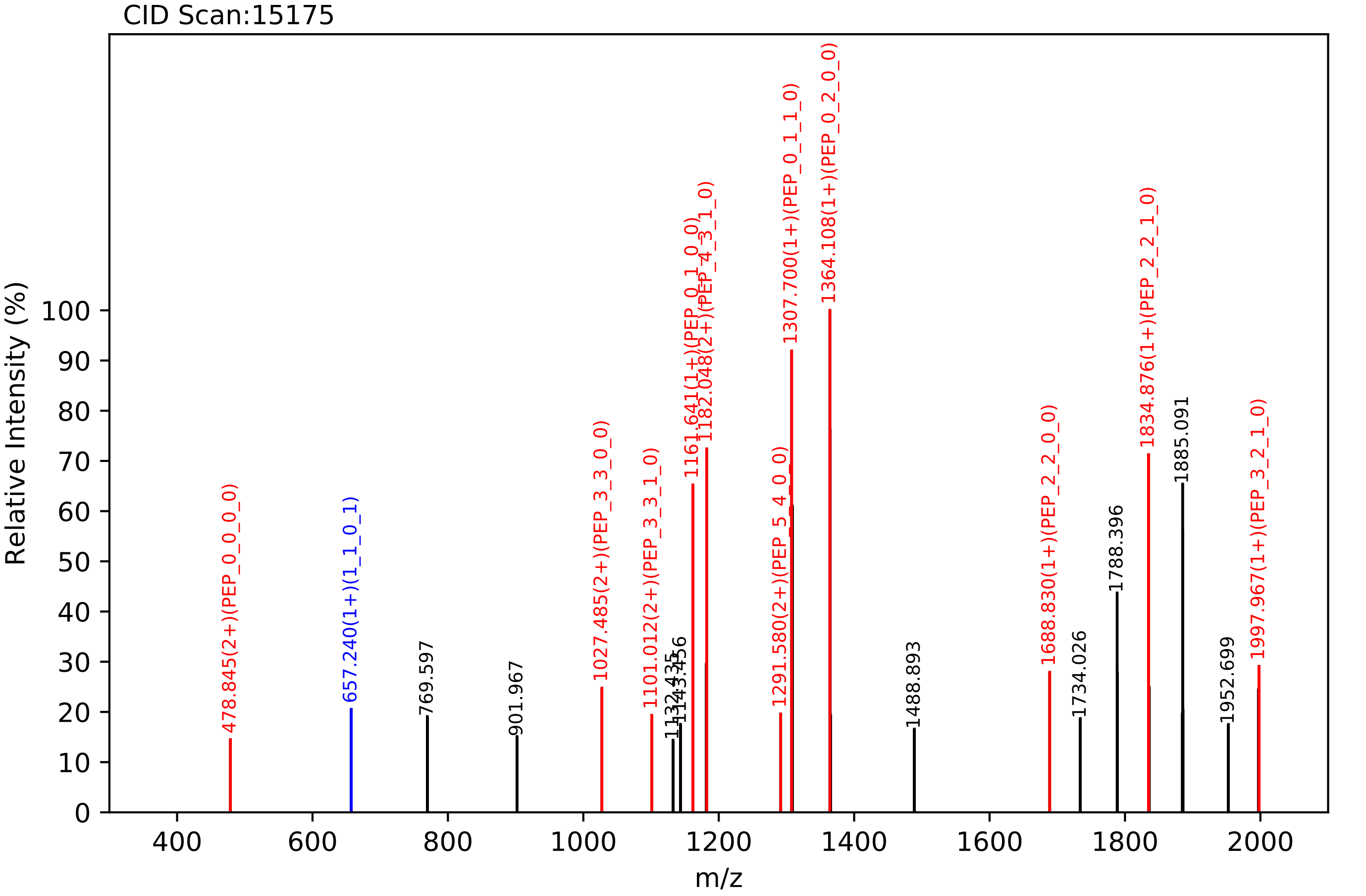

Unknown set no. 335, Experiment: Human Plasma exp_3

TPLTANITK(=PEP)_5_4_1_2, m/z:1655.20(2+), RT:66.19, Y-score:89.16

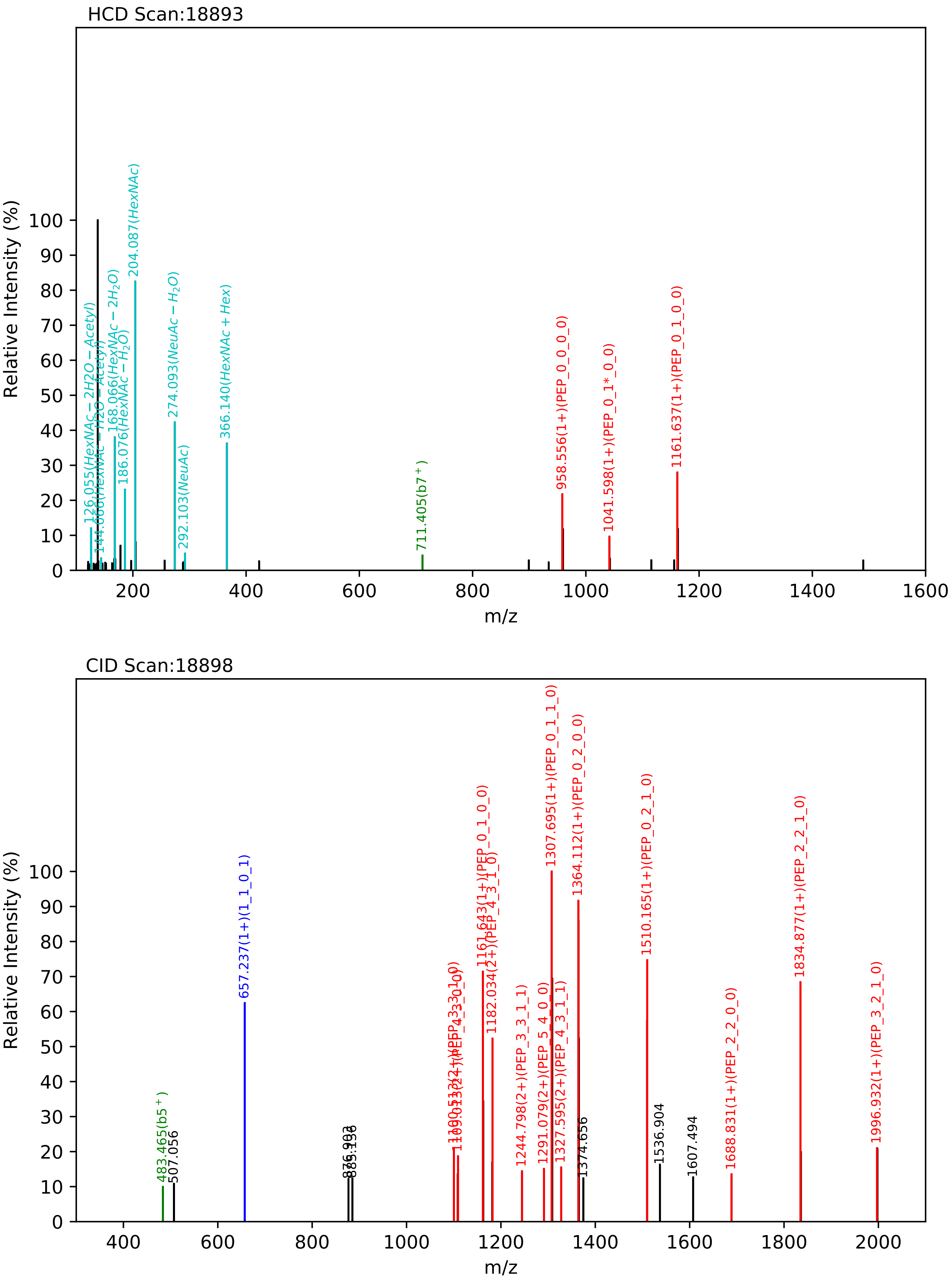

Unknown set no. 336, Experiment: Human Plasma exp_3

TPLTANITK(=PEP)_5_5_1_1, m/z:806.10(4+), RT:55.74, Y-score:92.17

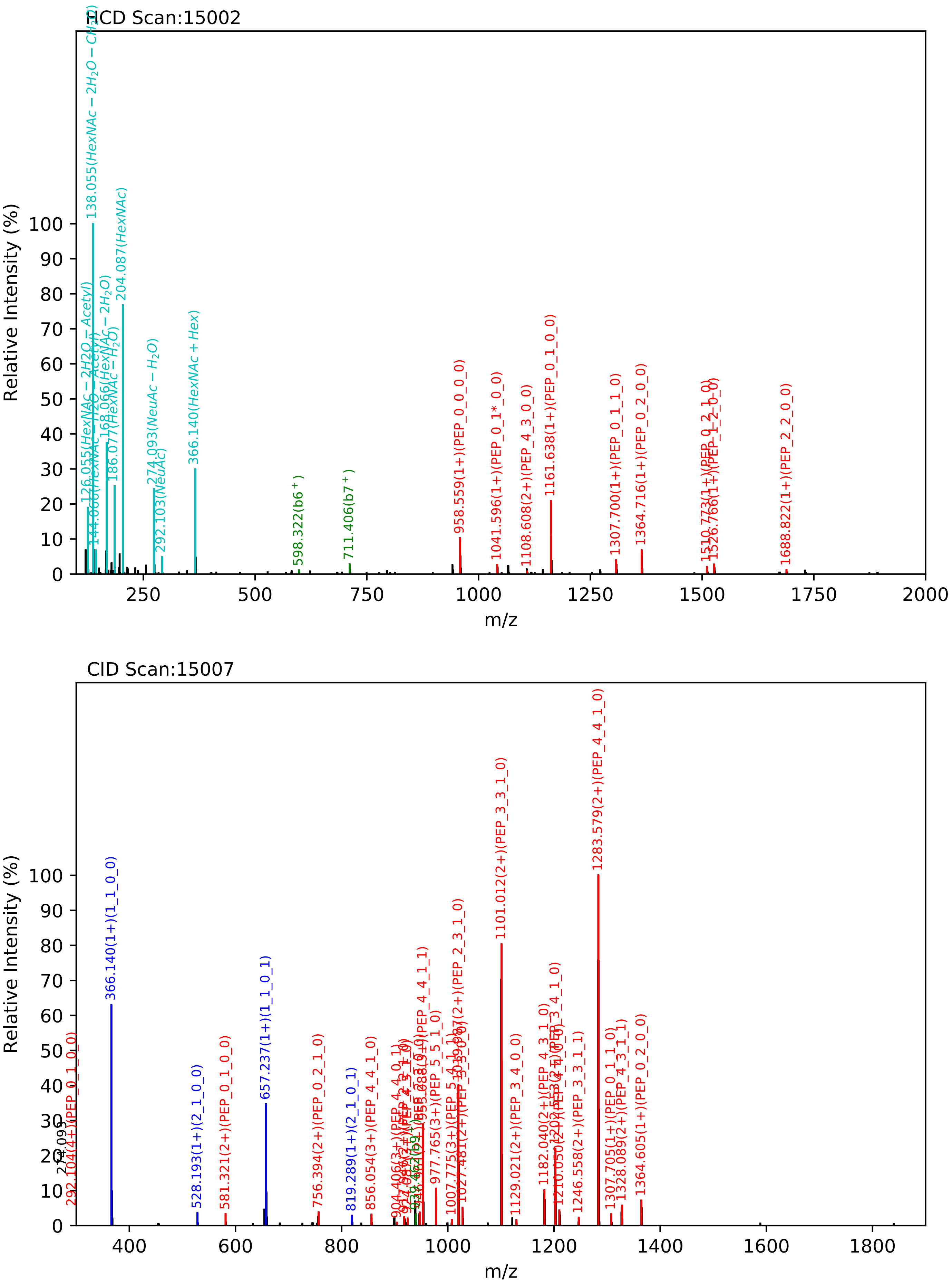

Unknown set no. 337, Experiment: Human Plasma exp_3

TPLTANITK(=PEP)_5_5_1_1, m/z:1611.69(2+), RT:55.71, Y-score:97.31

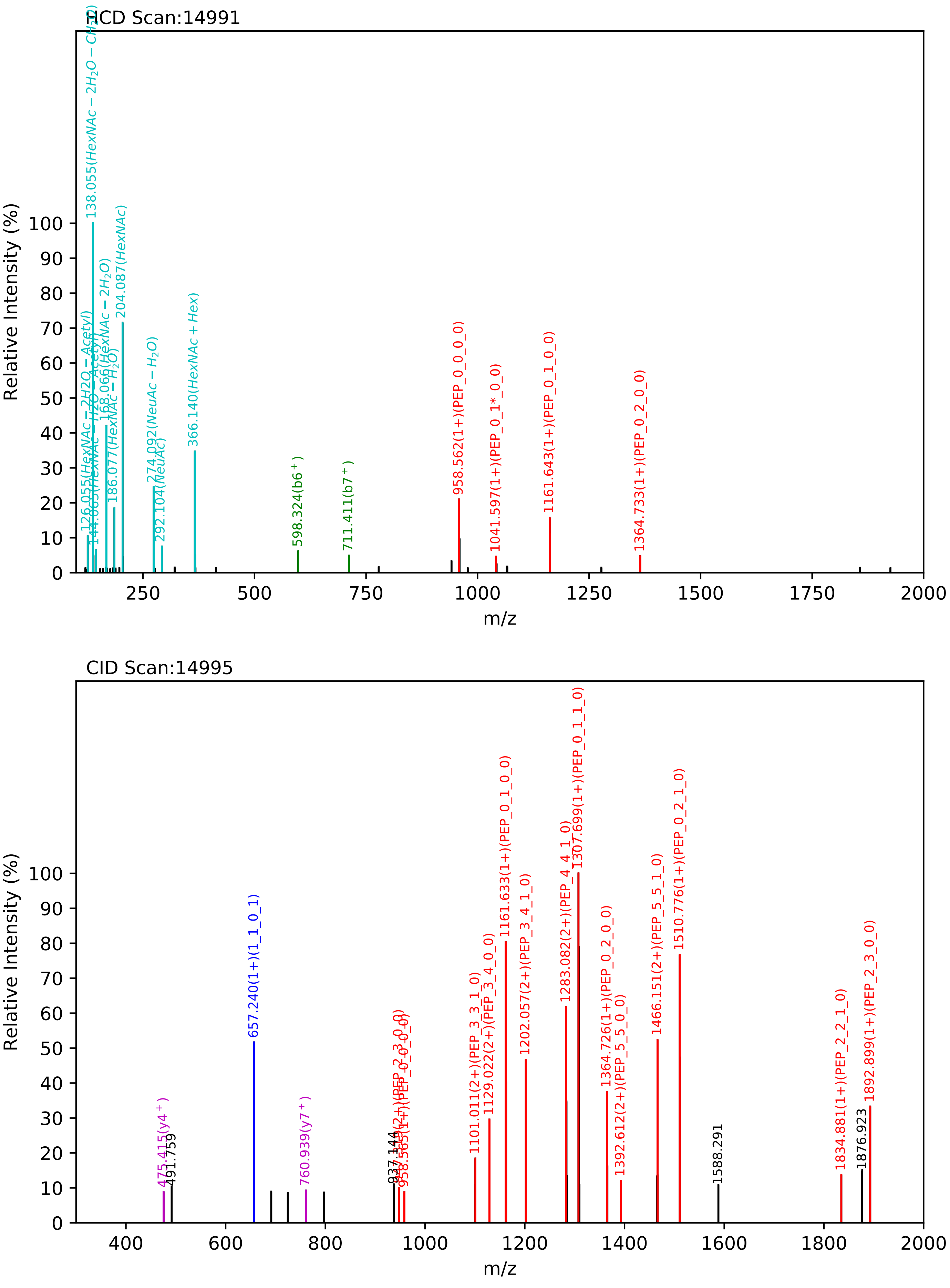

Unknown set no. 338, Experiment: Human Plasma exp_4

TPLTANITK(=PEP)_5_5_1_1, m/z:806.10(4+), RT:55.94, Y-score:91.10

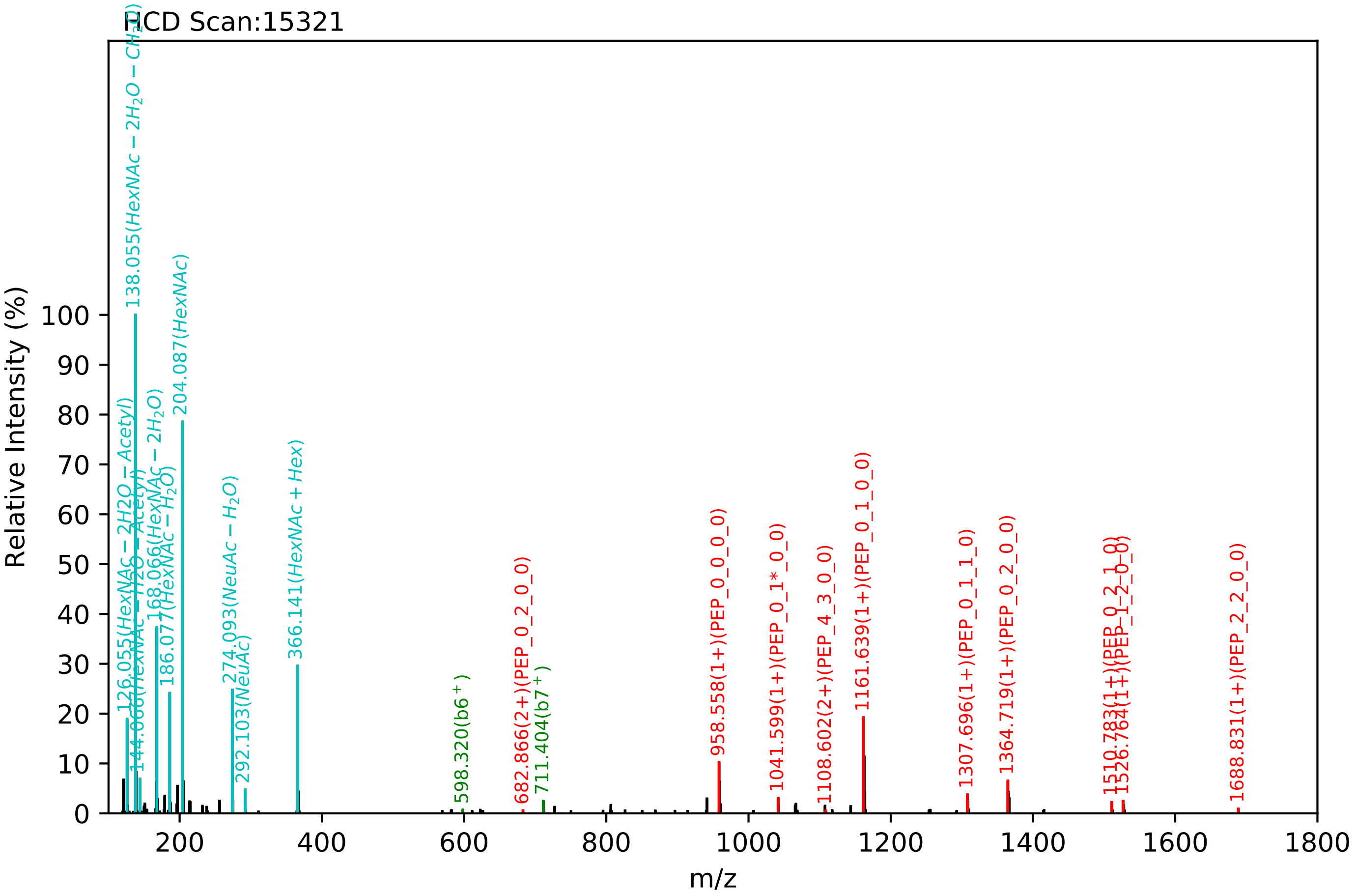

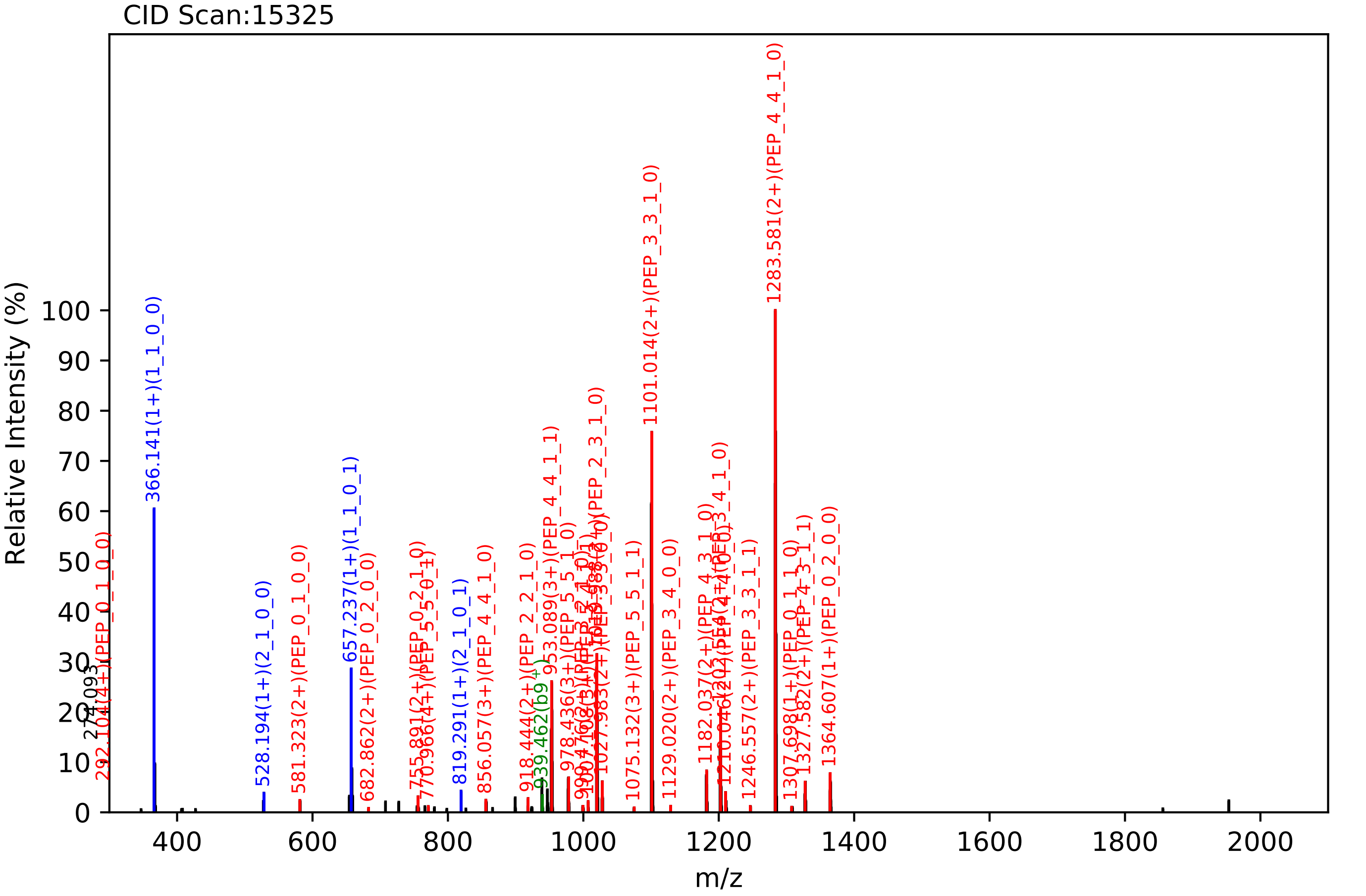

Unknown set no. 339, Experiment: Human Plasma exp_4

TPLTANITK(=PEP)_5_5_1_1, m/z:1611.19(2+), RT:55.92, Y-score:100.00

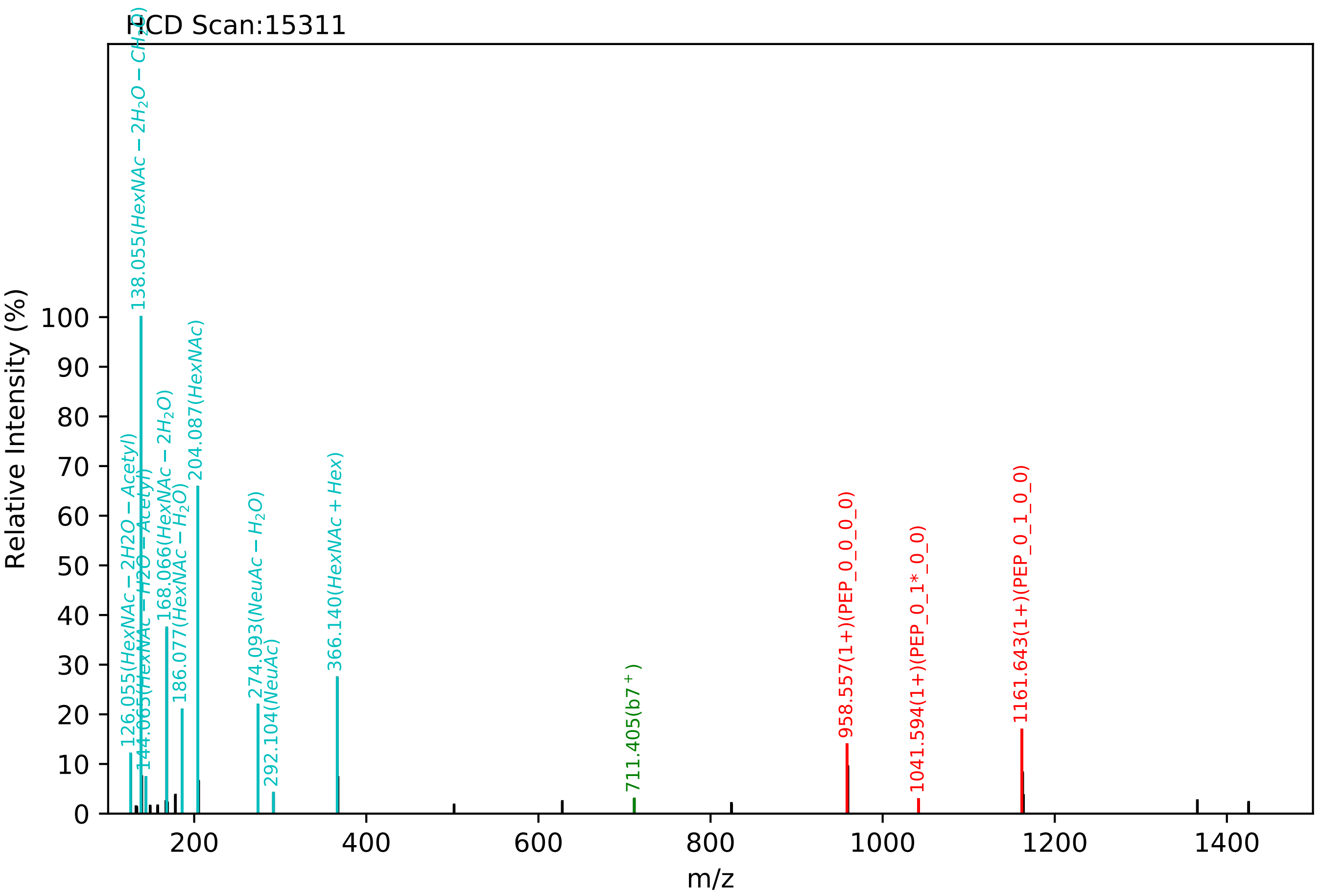

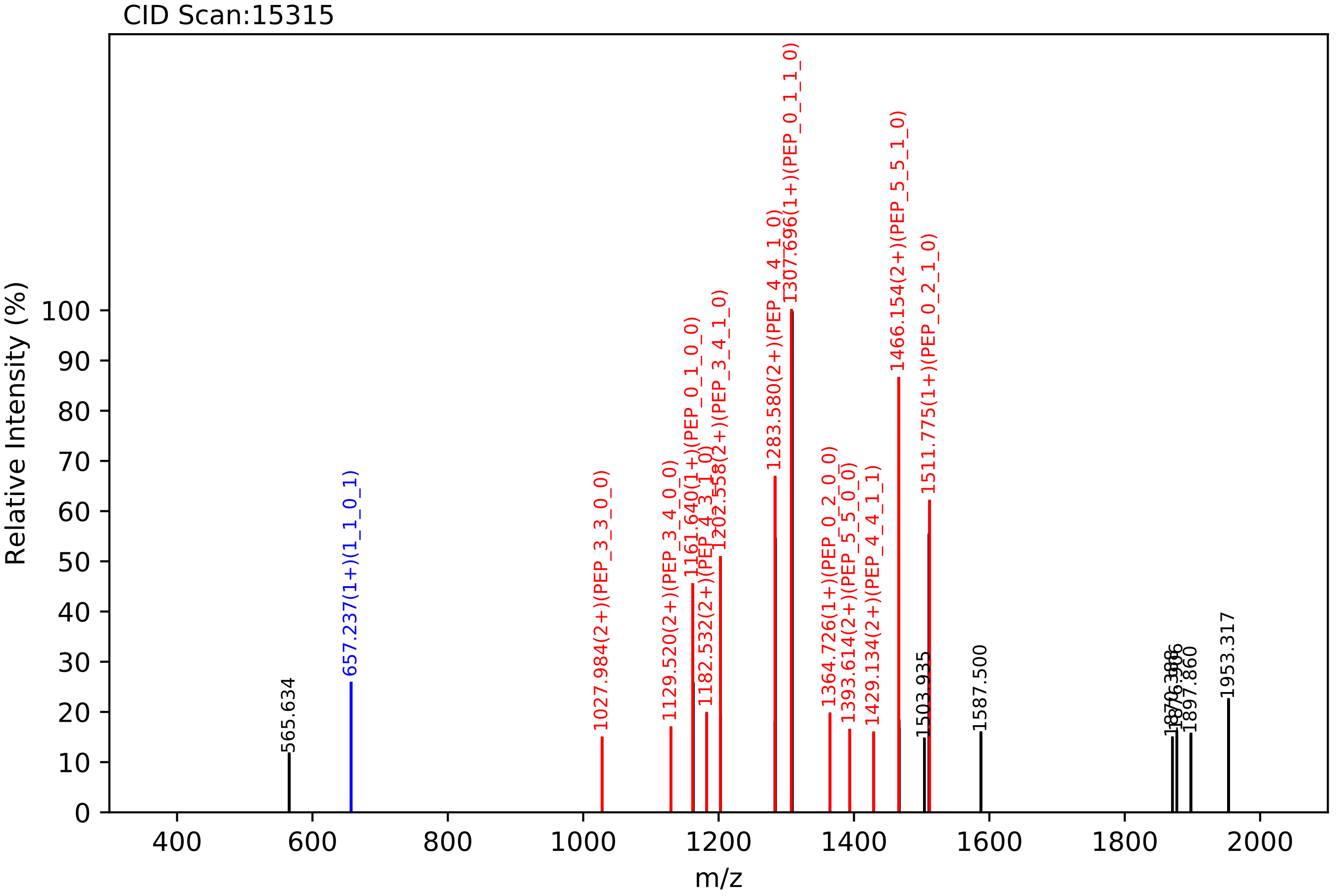

Unknown set no. 340, Experiment: Human Plasma exp_3

TPLTANITK(=PEP)_5_5_1_2, m/z:878.87(4+), RT:66.41, Y-score:86.13

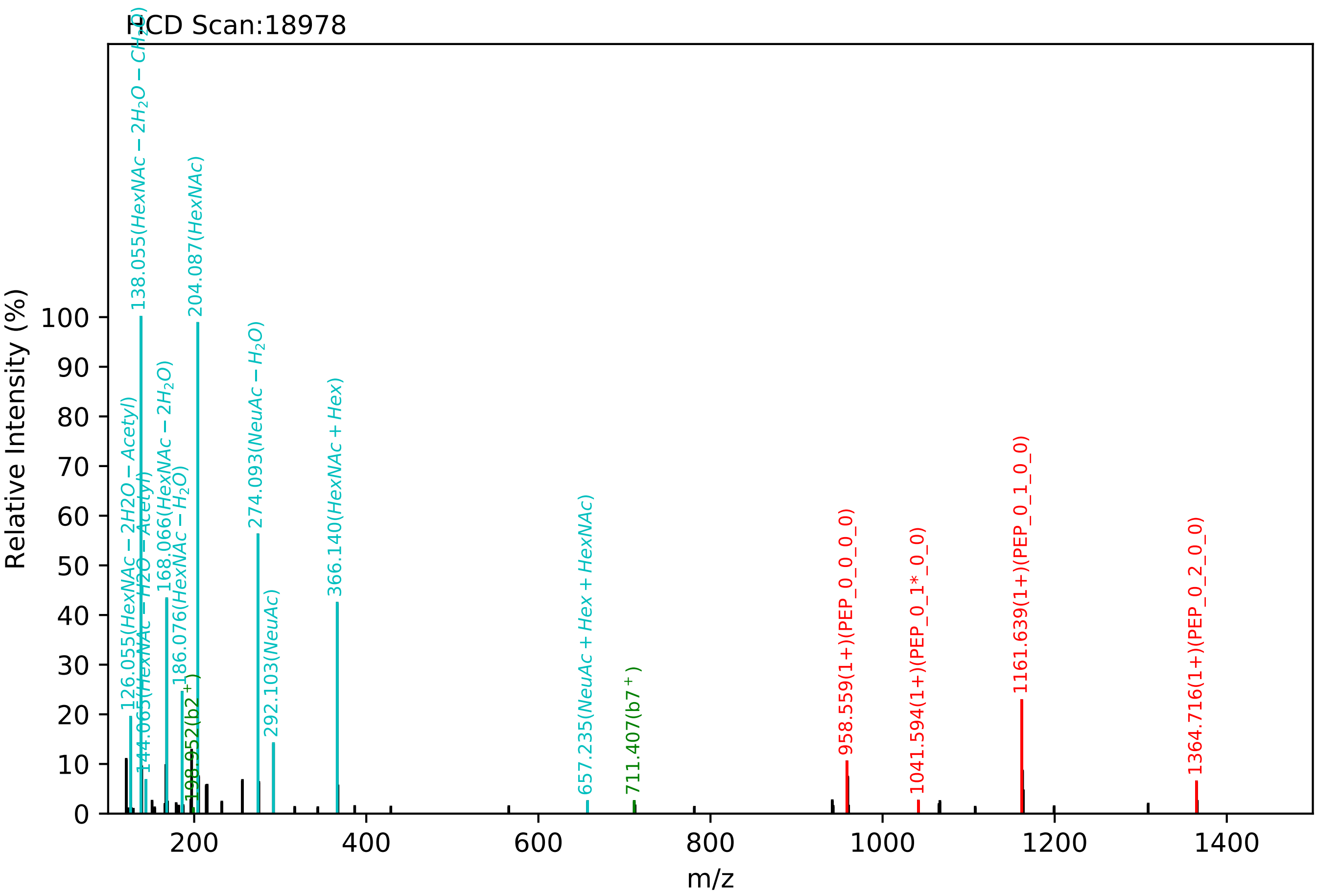

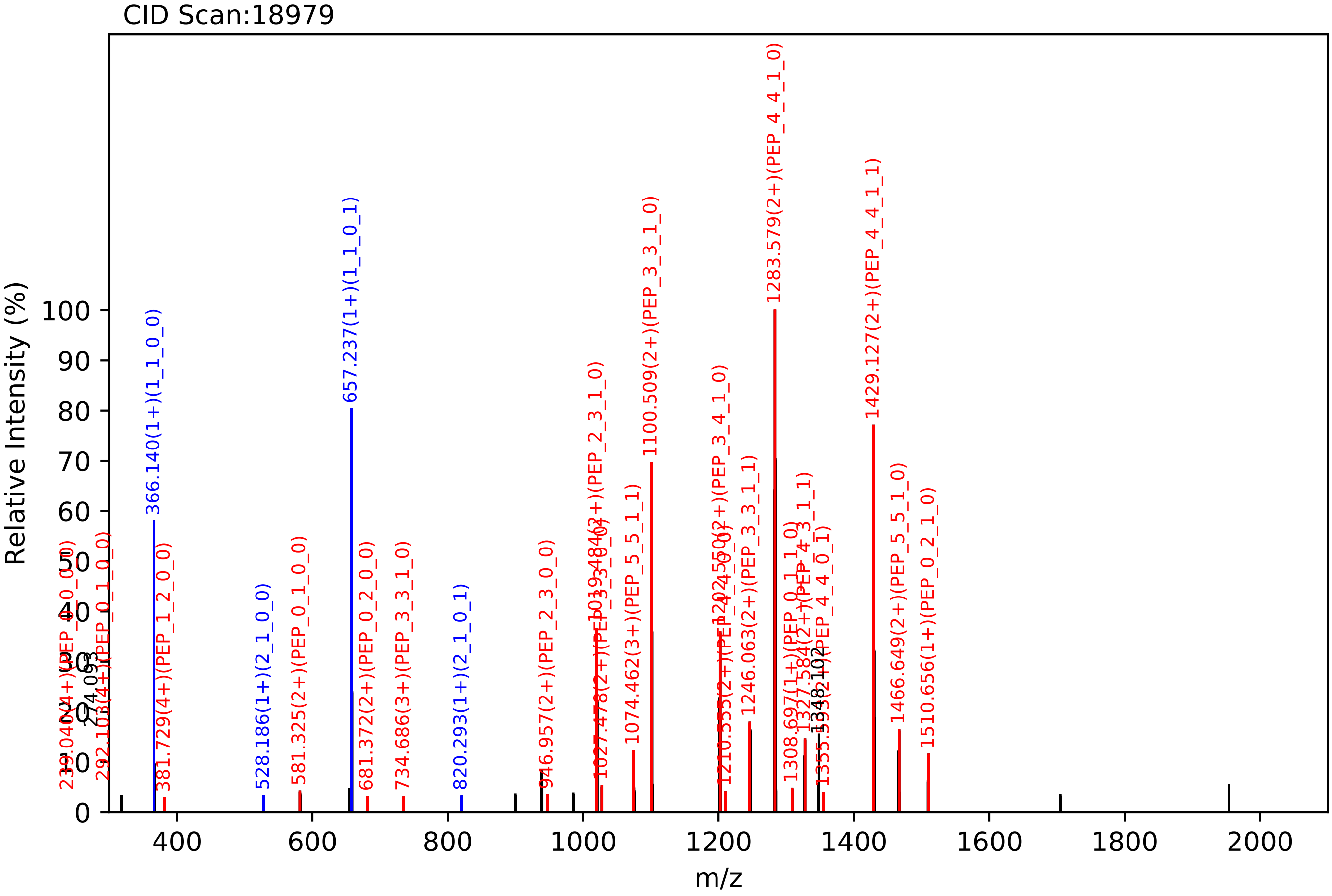

Unknown set no. 341, Experiment: Human Plasma exp_1

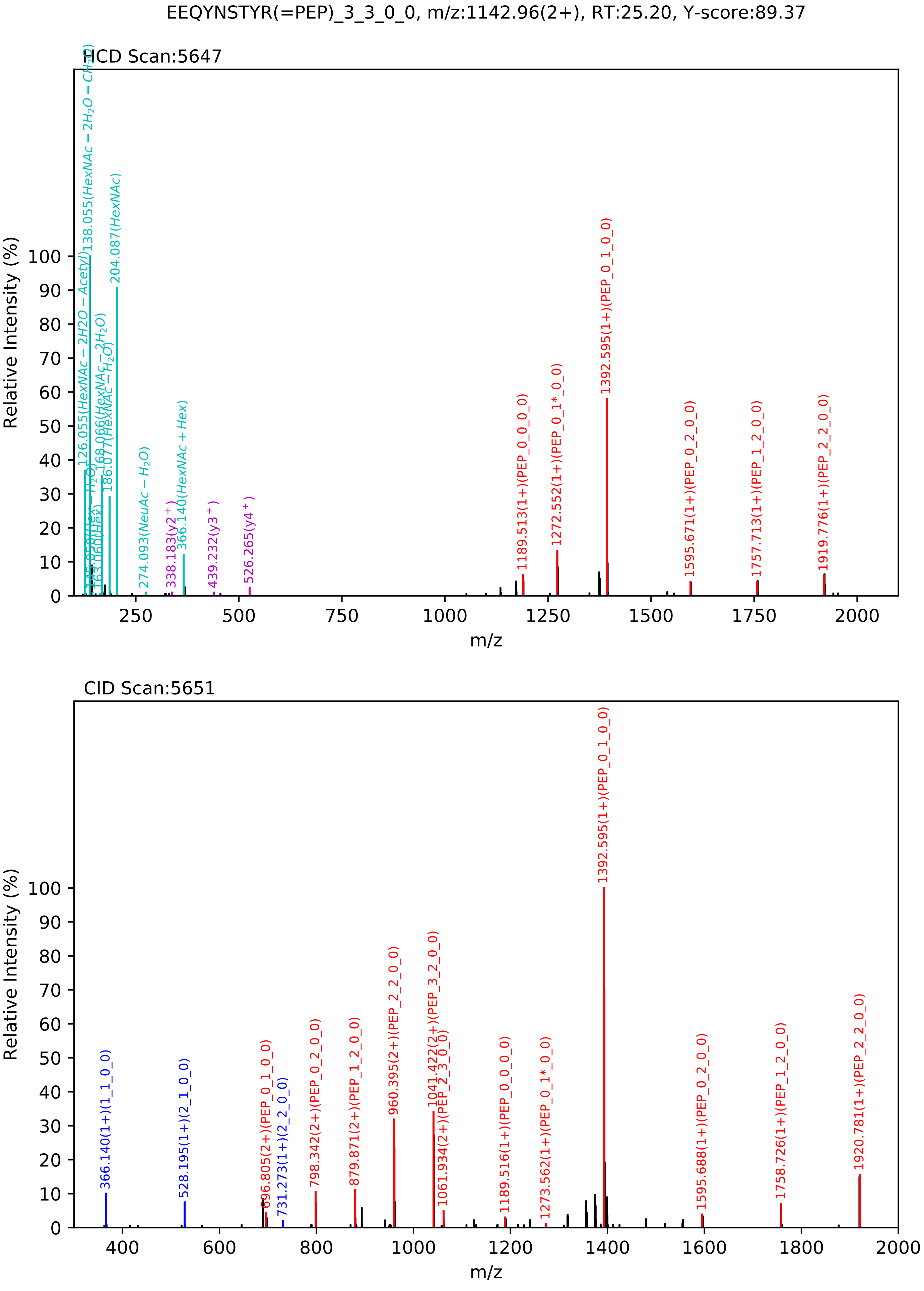

Unknown set no. 342, Experiment: Human Plasma exp_2

EEQYNSTYR(=PEP)_3_3_0_0, m/z:1142.96(2+), RT:25.99, Y-score:94.30

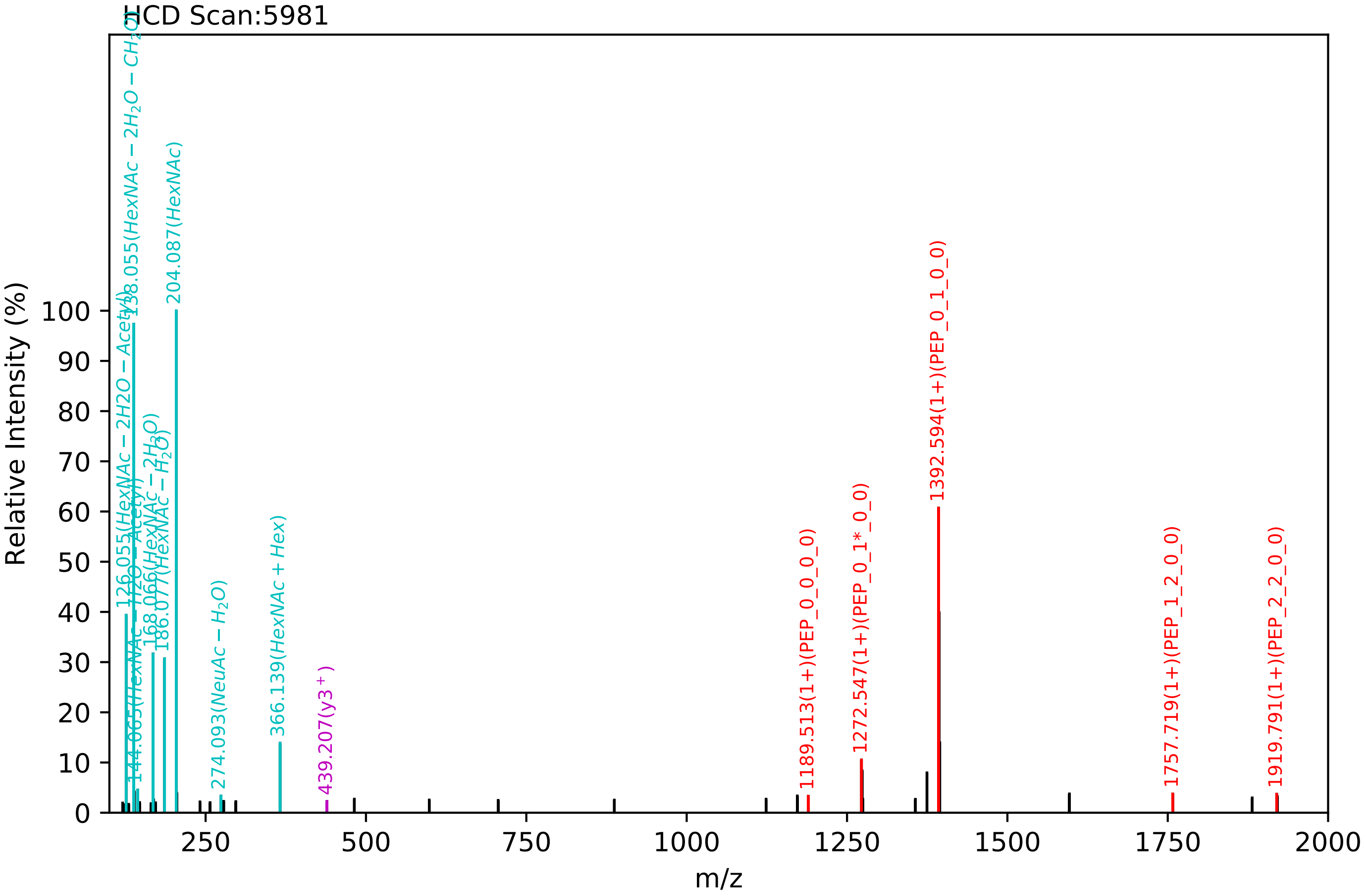

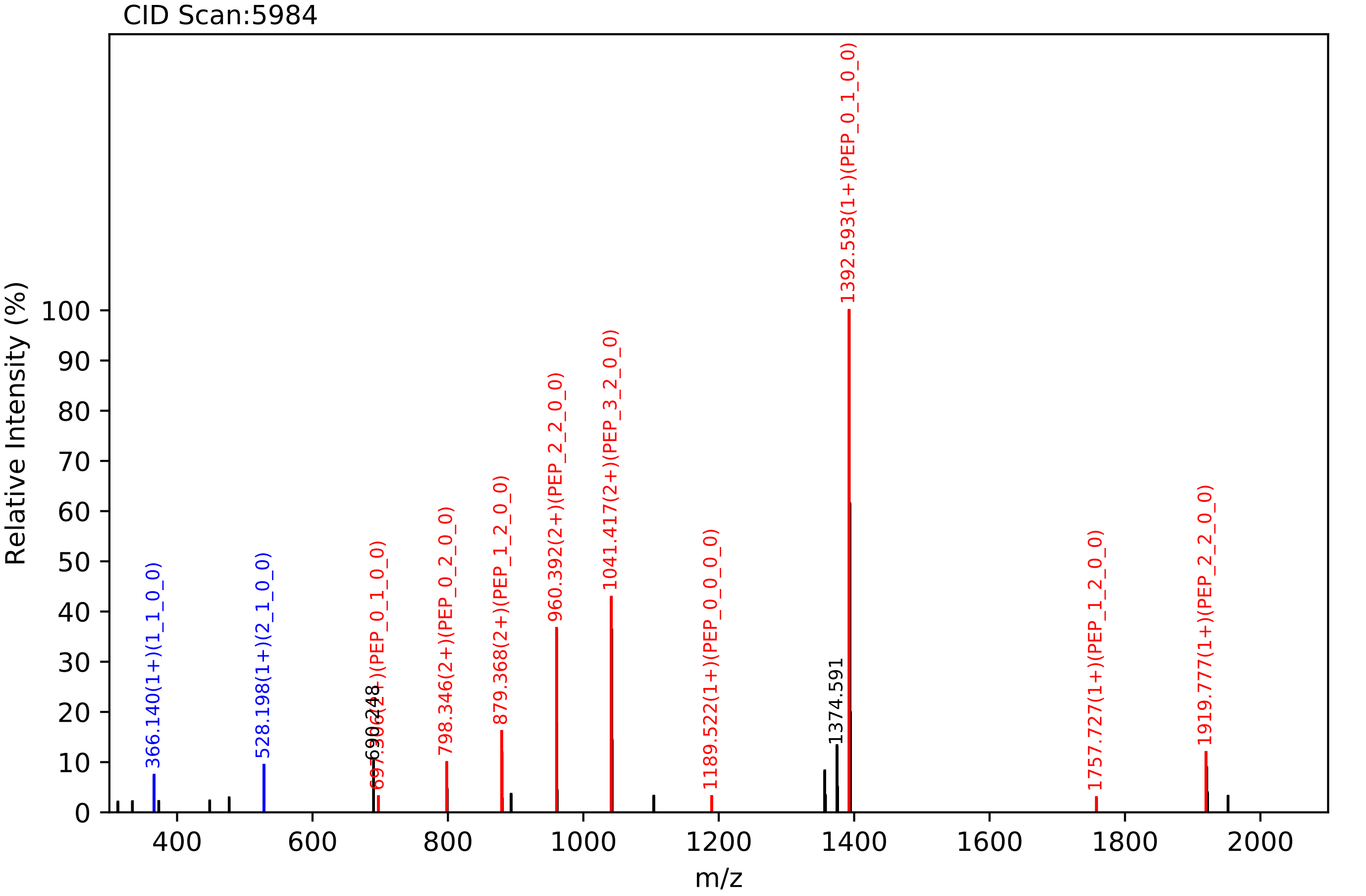

Unknown set no. 343, Experiment: Human Plasma exp_3

EEQYNSTYR(=PEP)_3_3_0_0, m/z:1142.96(2+), RT:26.17, Y-score:91.28

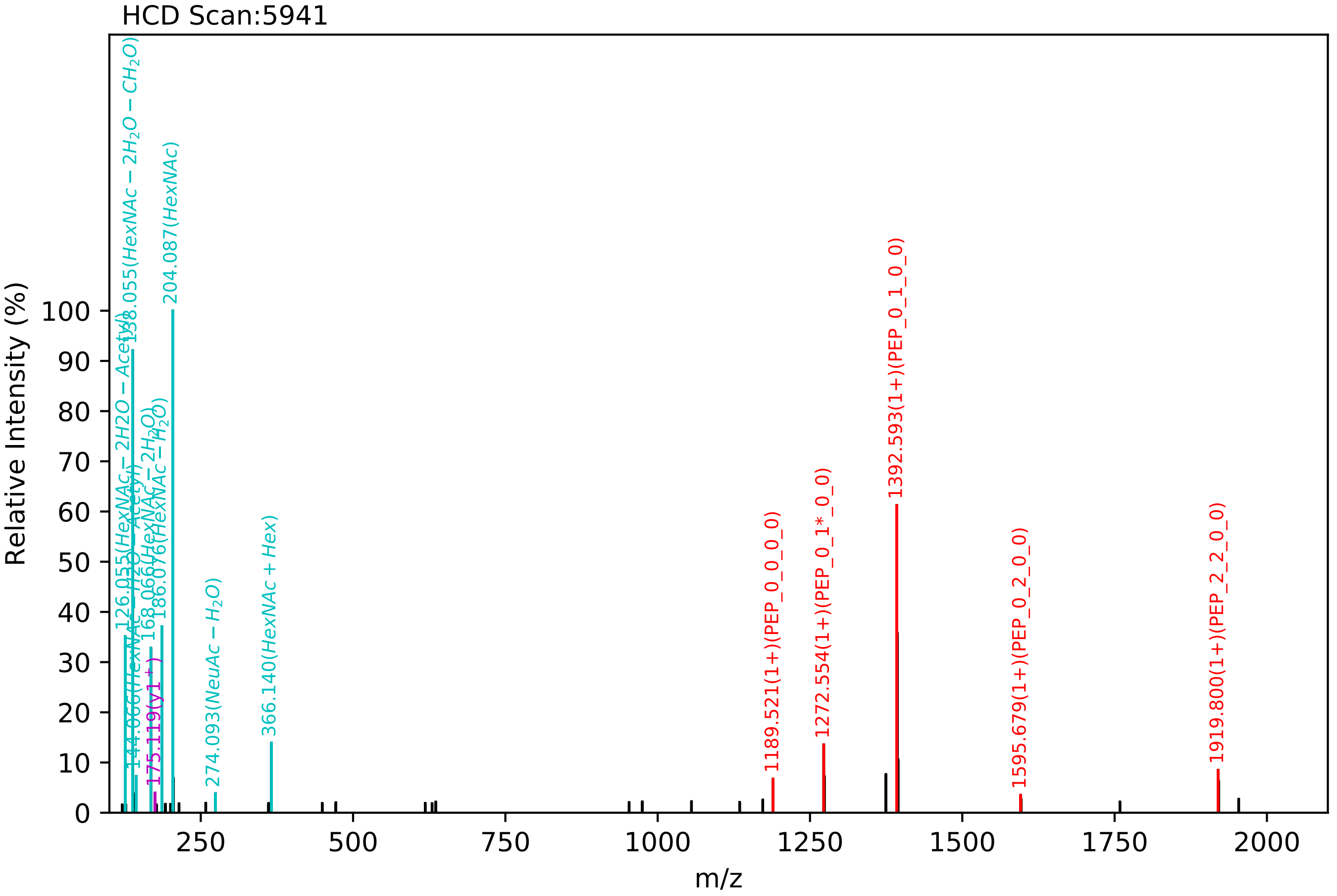

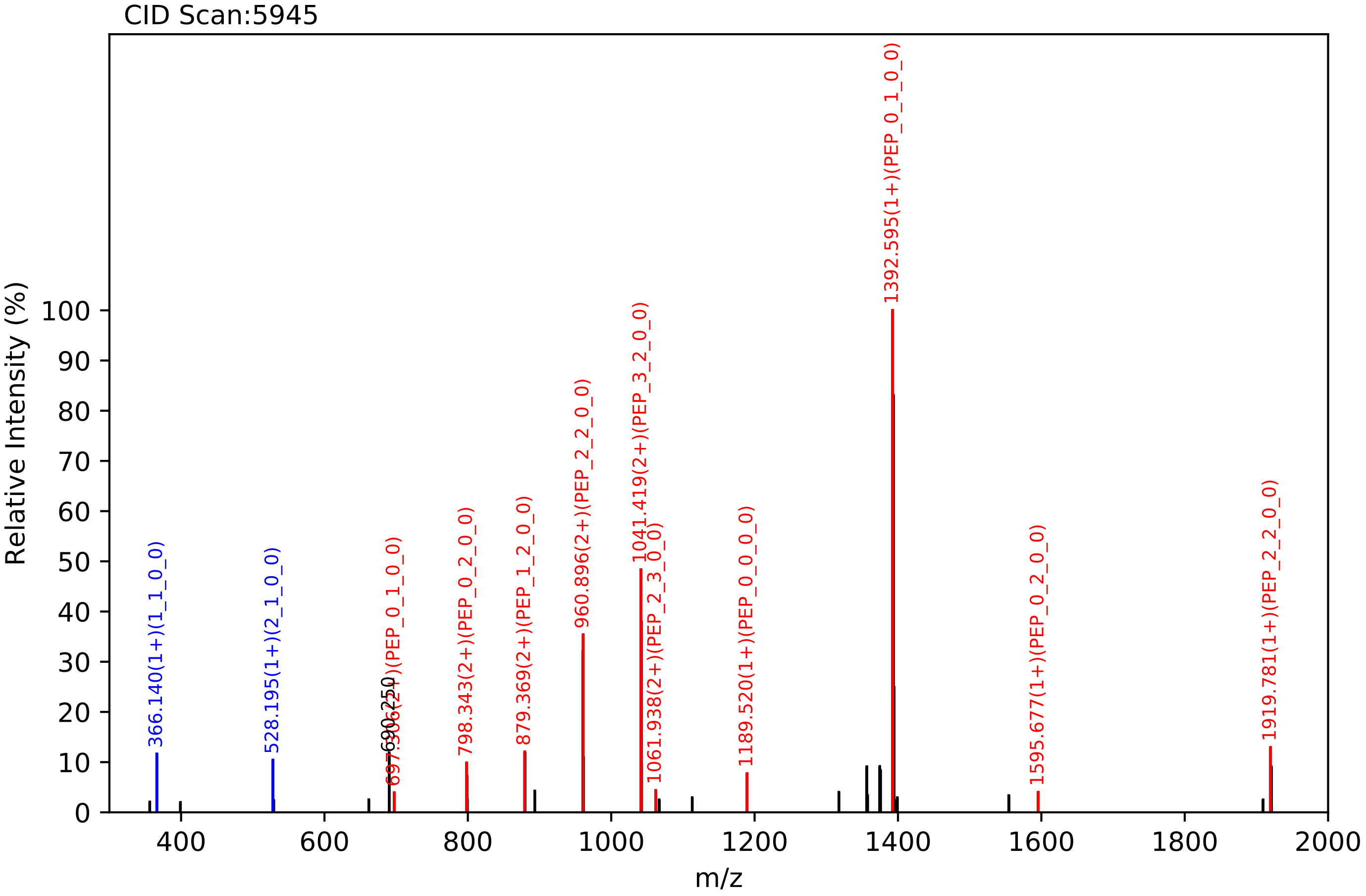

Unknown set no. 344, Experiment: Human Plasma exp_4

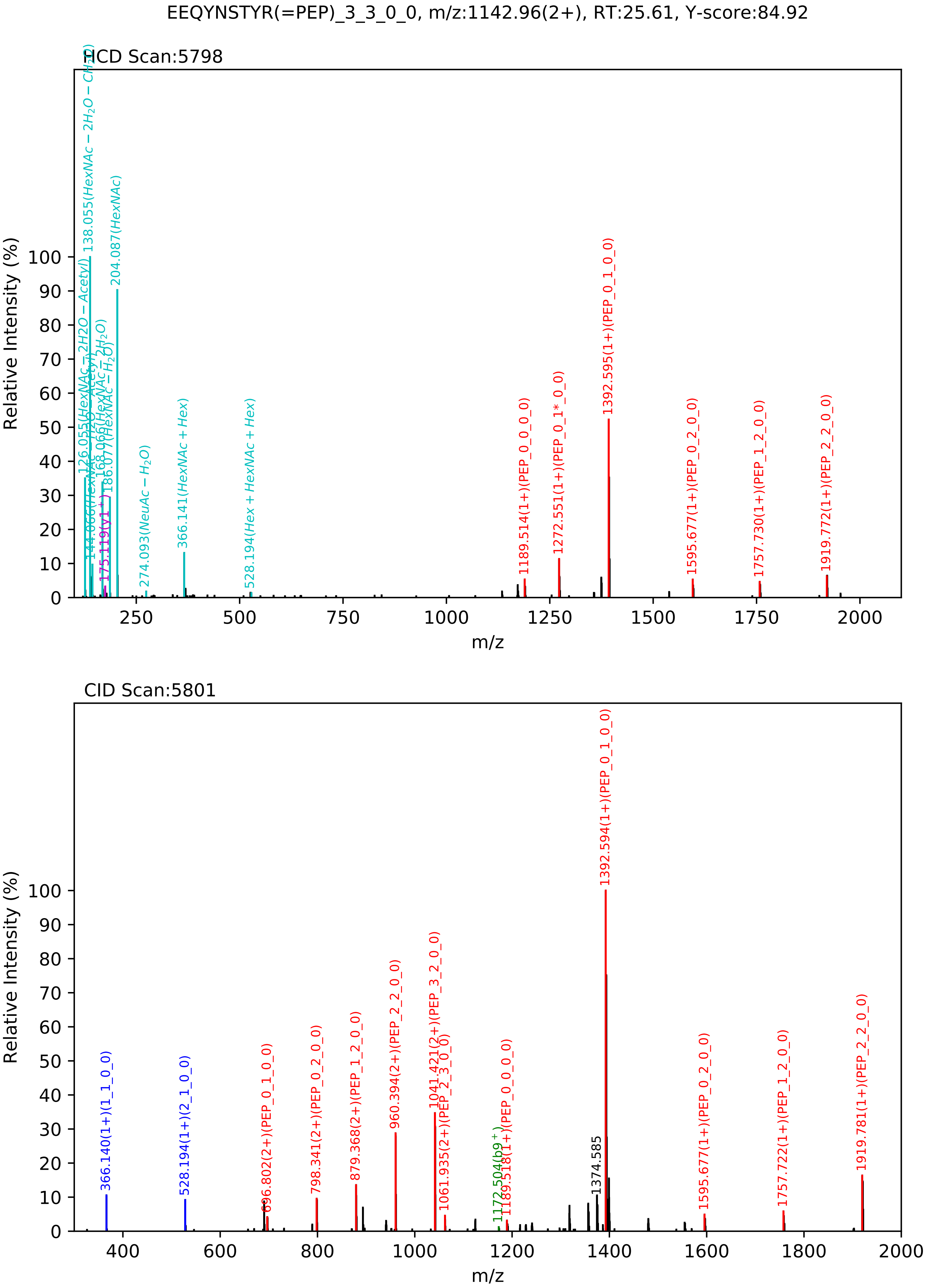

Unknown set no. 345, Experiment: Human Plasma exp_5

EEQYNSTYR(=PEP)_3_3_0_0, m/z:1142.96(2+), RT:26.15, Y-score:89.38

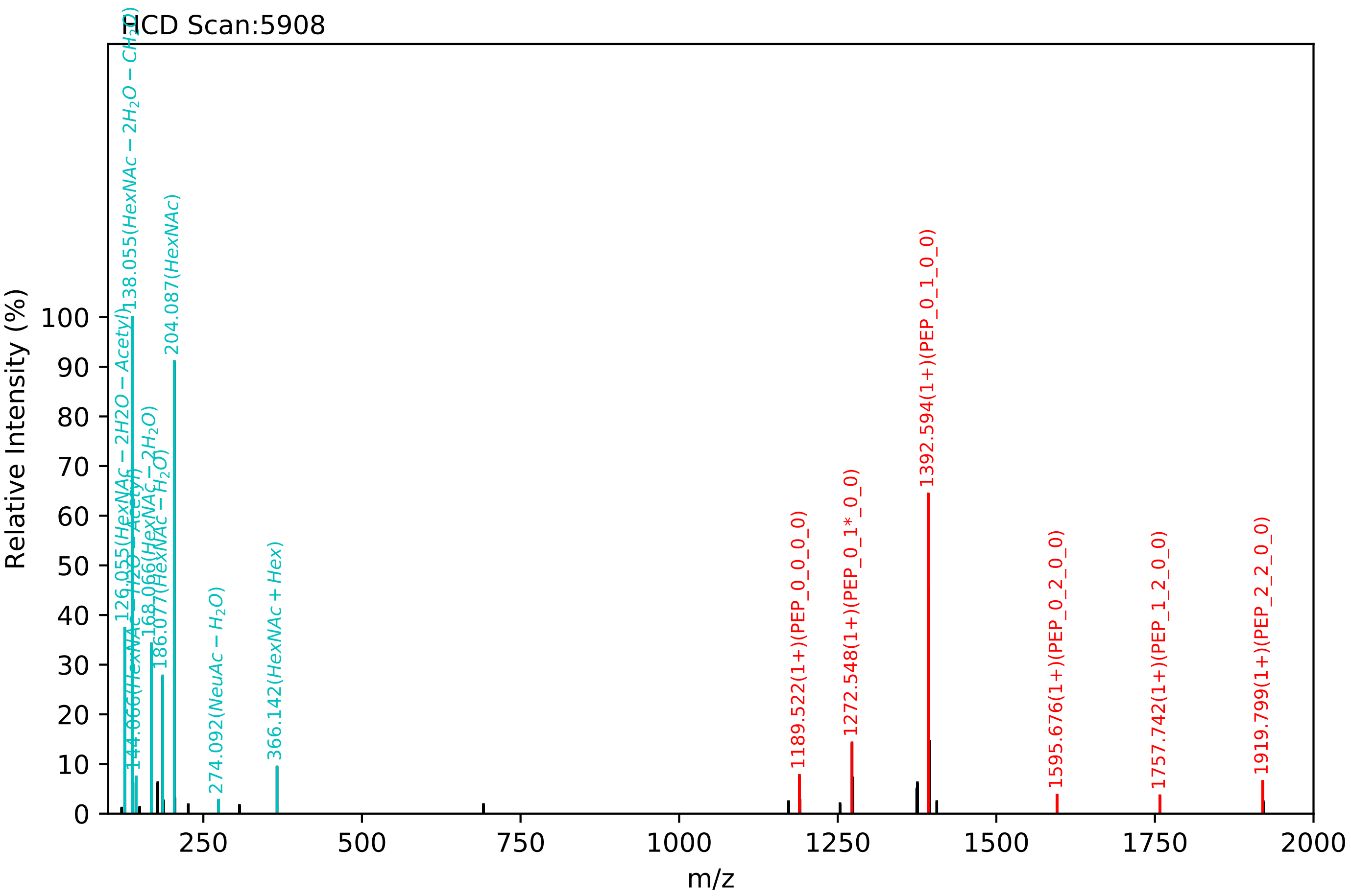

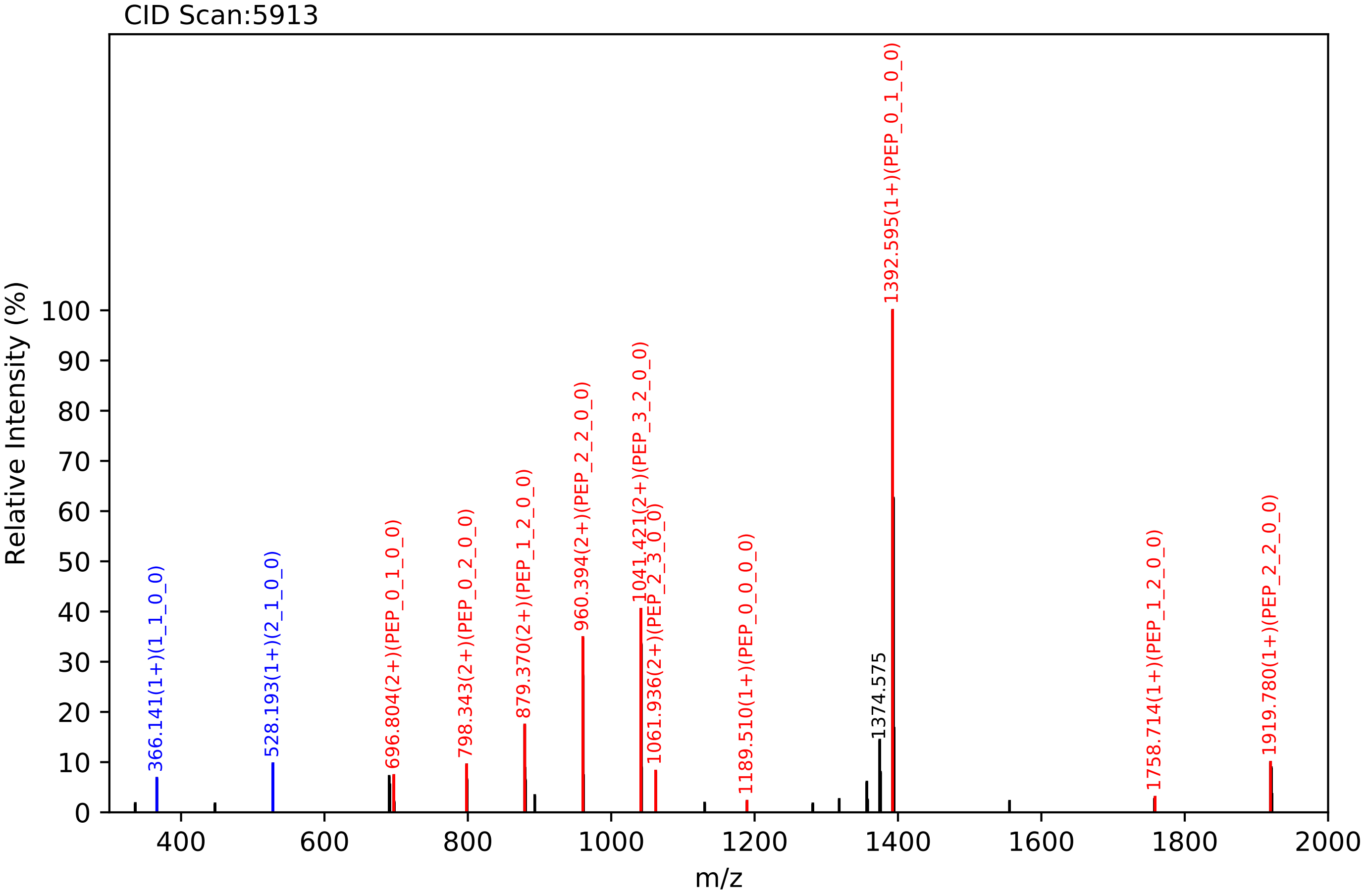

Unknown set no. 346, Experiment: Human Plasma exp_1

EEQYNSTYR(=PEP)_3_3_1_0, m/z:1215.99(2+), RT:23.08, Y-score:100.00

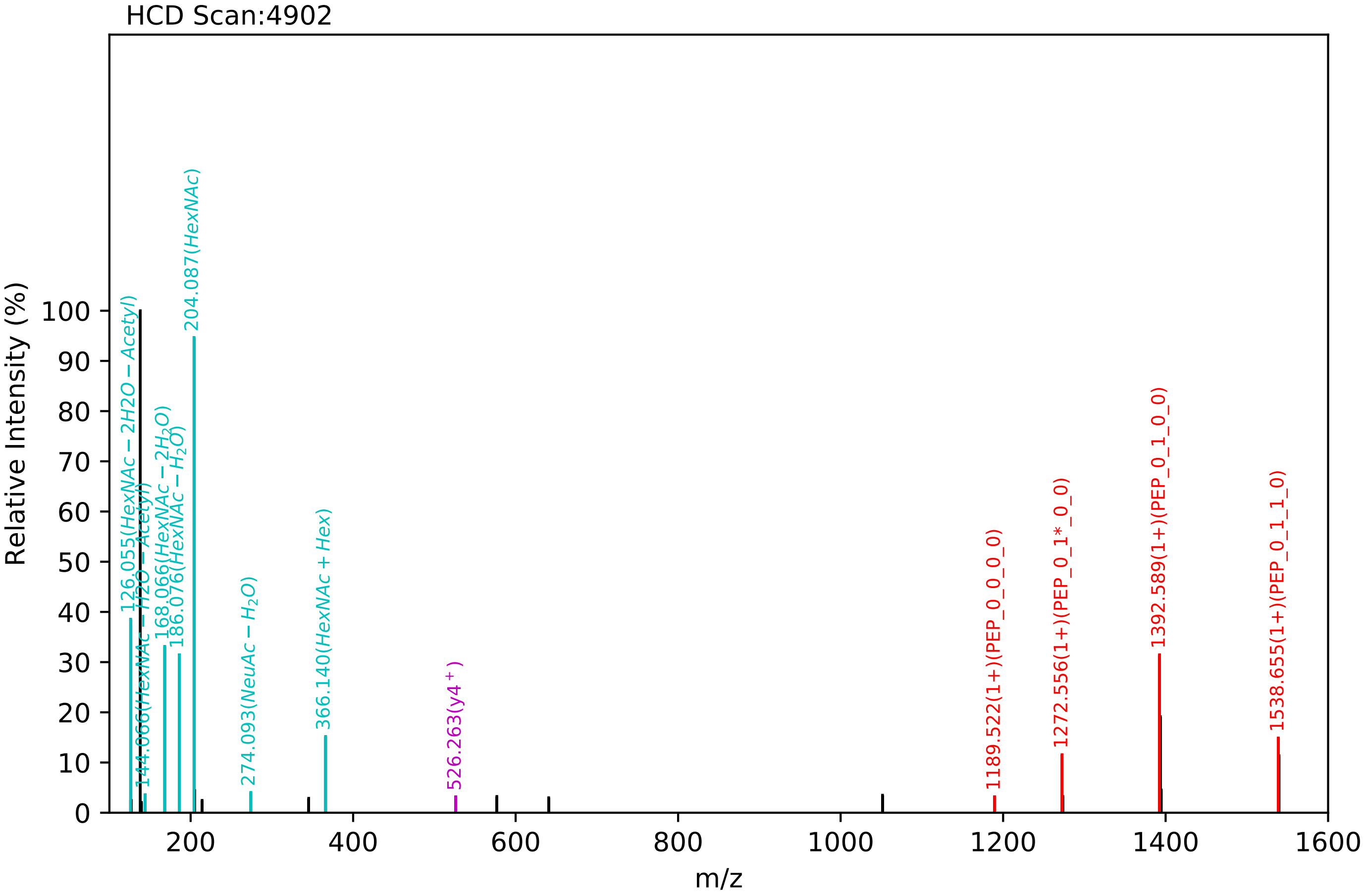

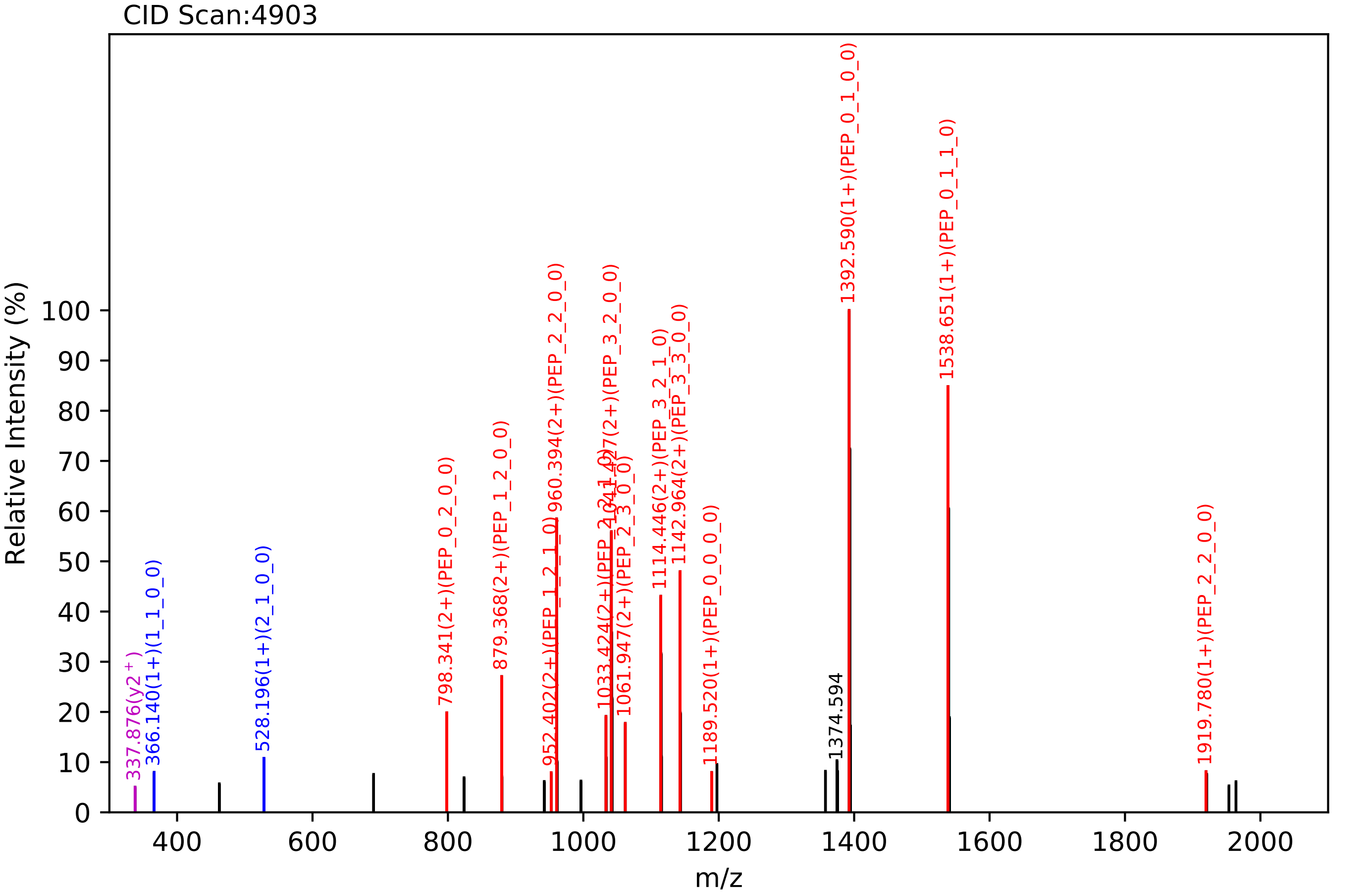

Unknown set no. 347, Experiment: Human Plasma exp_2

EEQYNSTYR(=PEP)_3_3_1_0, m/z:1215.99(2+), RT:24.60, Y-score:98.90

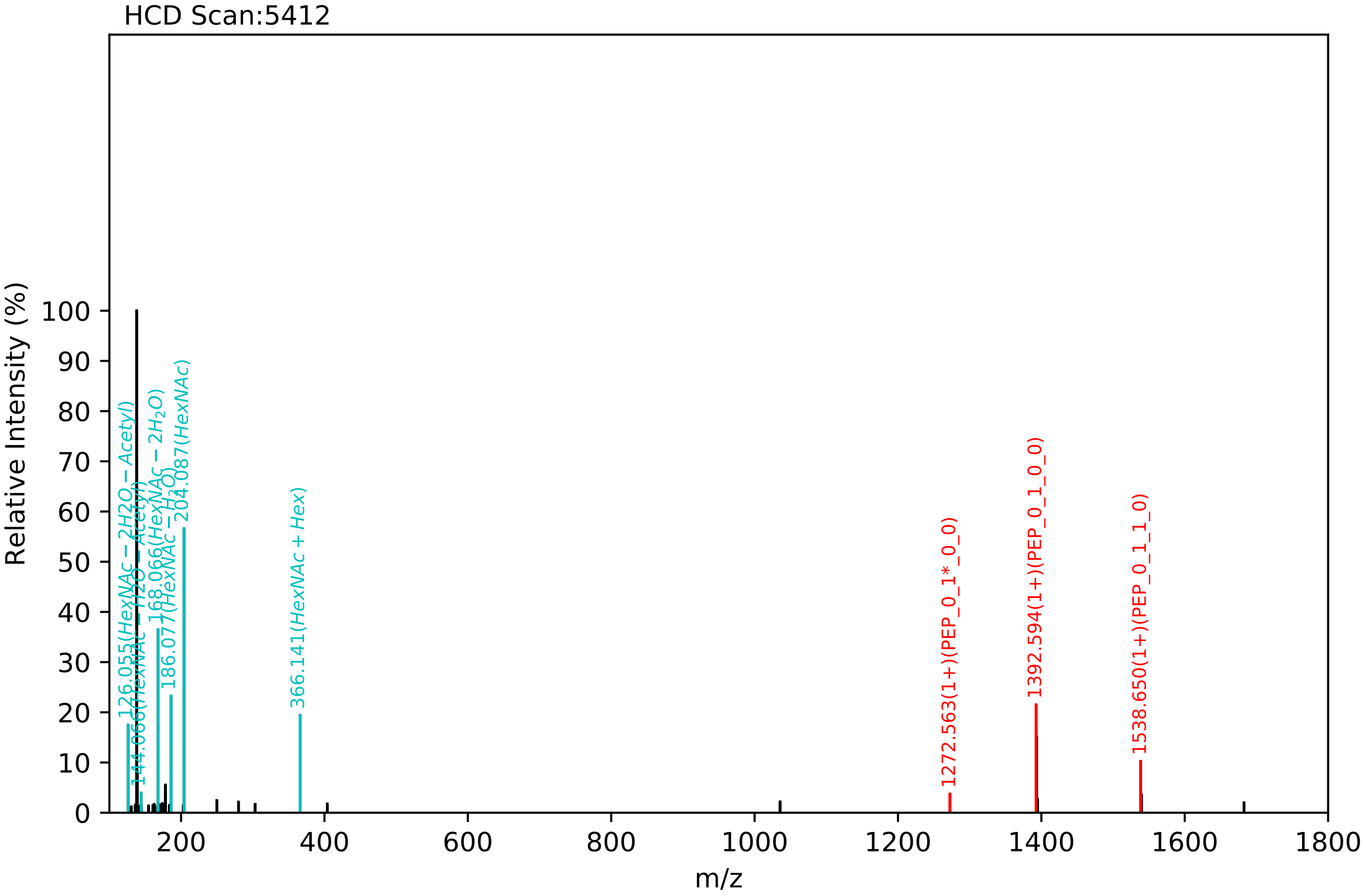

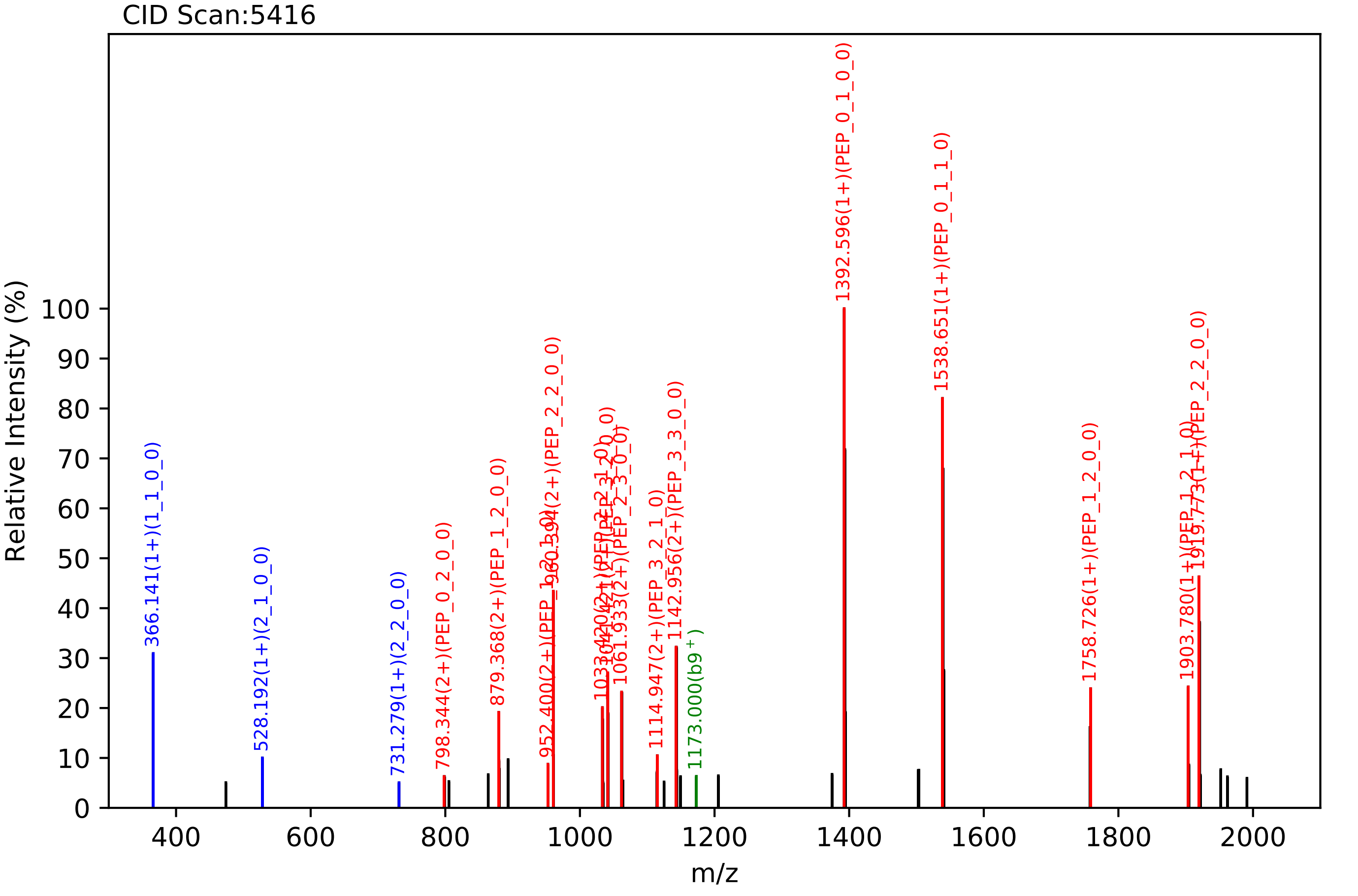

Unknown set no. 348, Experiment: Human Plasma exp_3

EEQYNSTYR(=PEP)_3_3_1_0, m/z:1215.99(2+), RT:24.47, Y-score:97.50

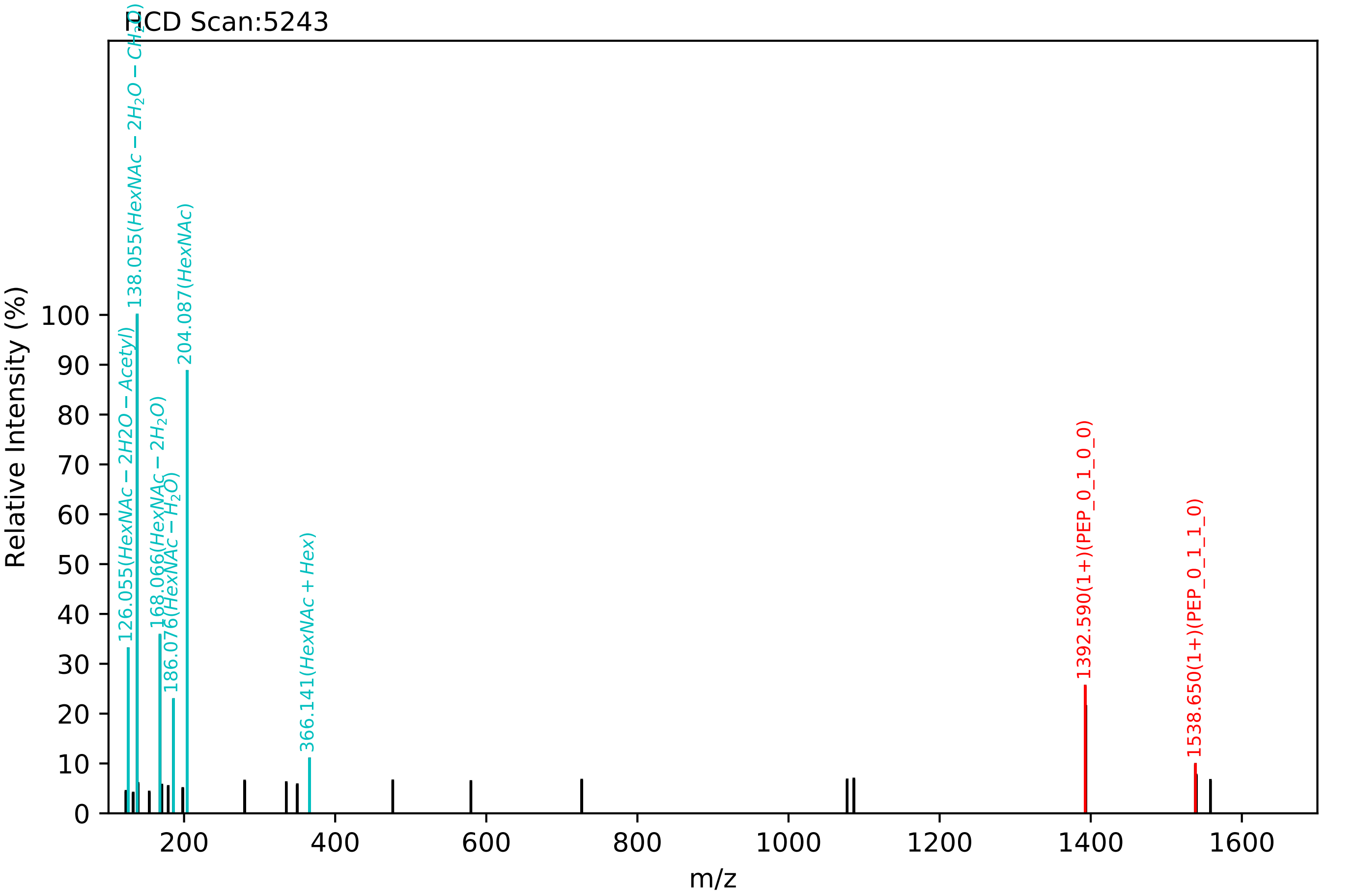

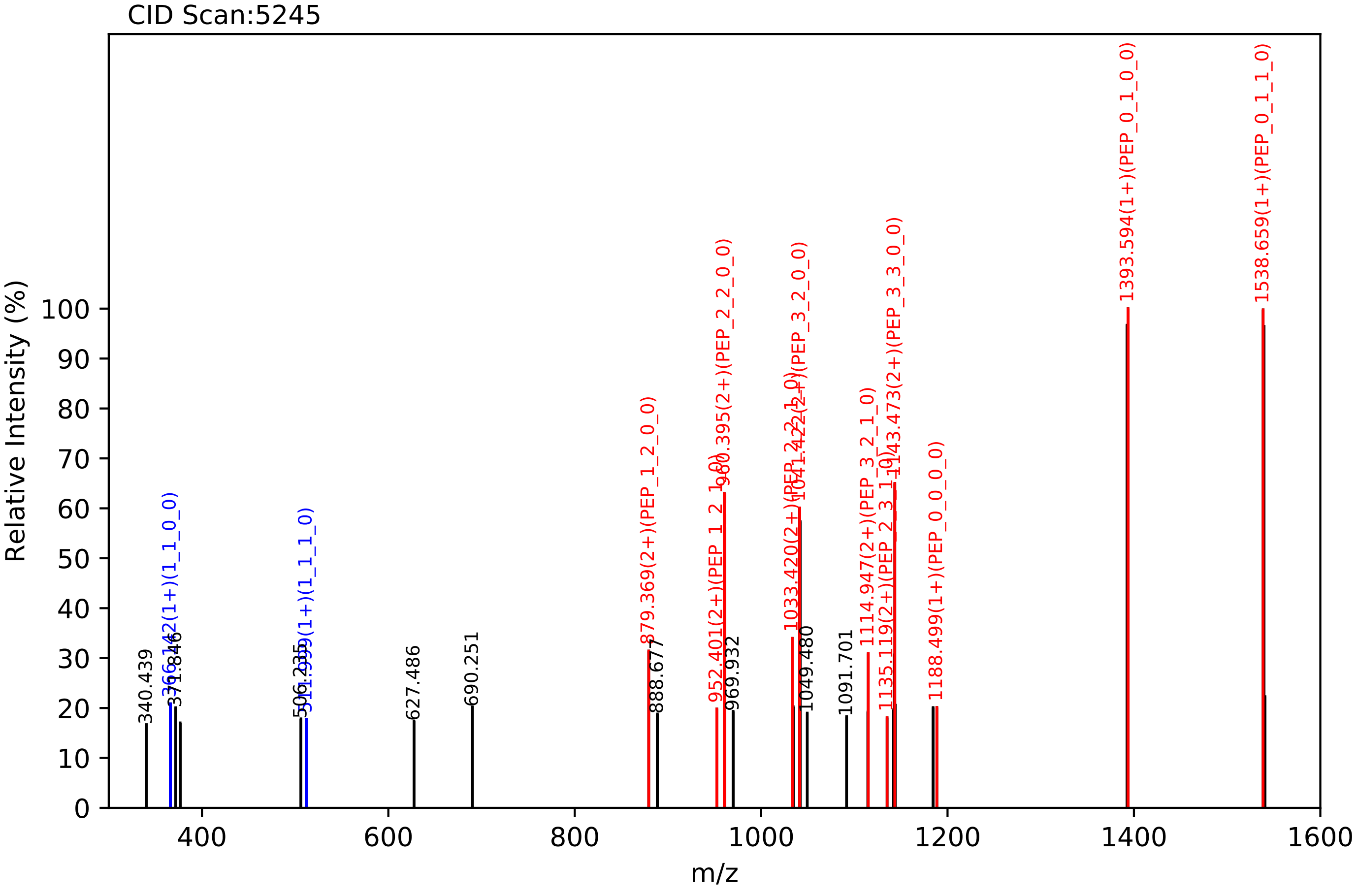

Unknown set no. 349, Experiment: Human Plasma exp_4

EEQYNSTYR(=PEP)_3_3_1_0, m/z:1215.99(2+), RT:25.91, Y-score:91.05

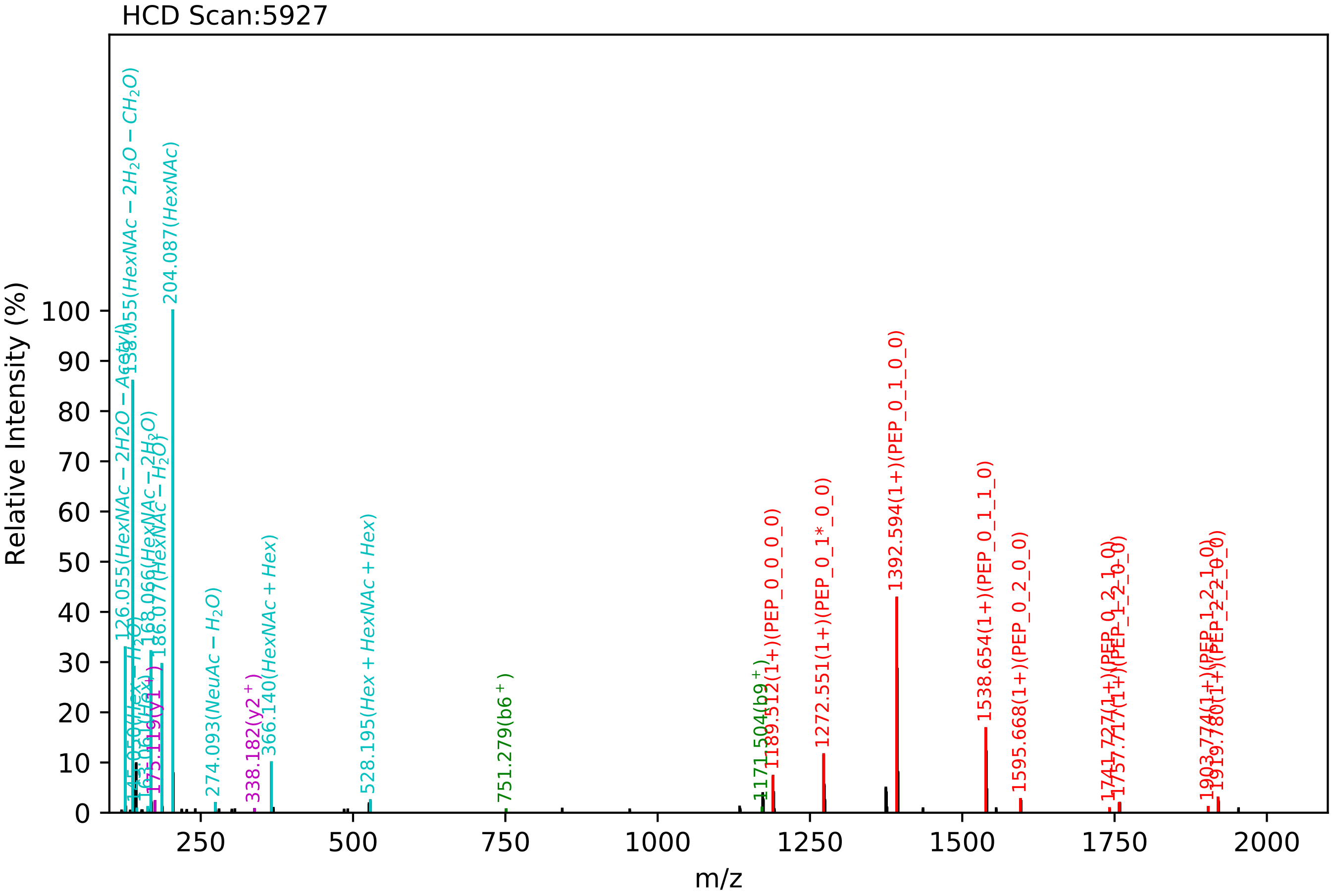

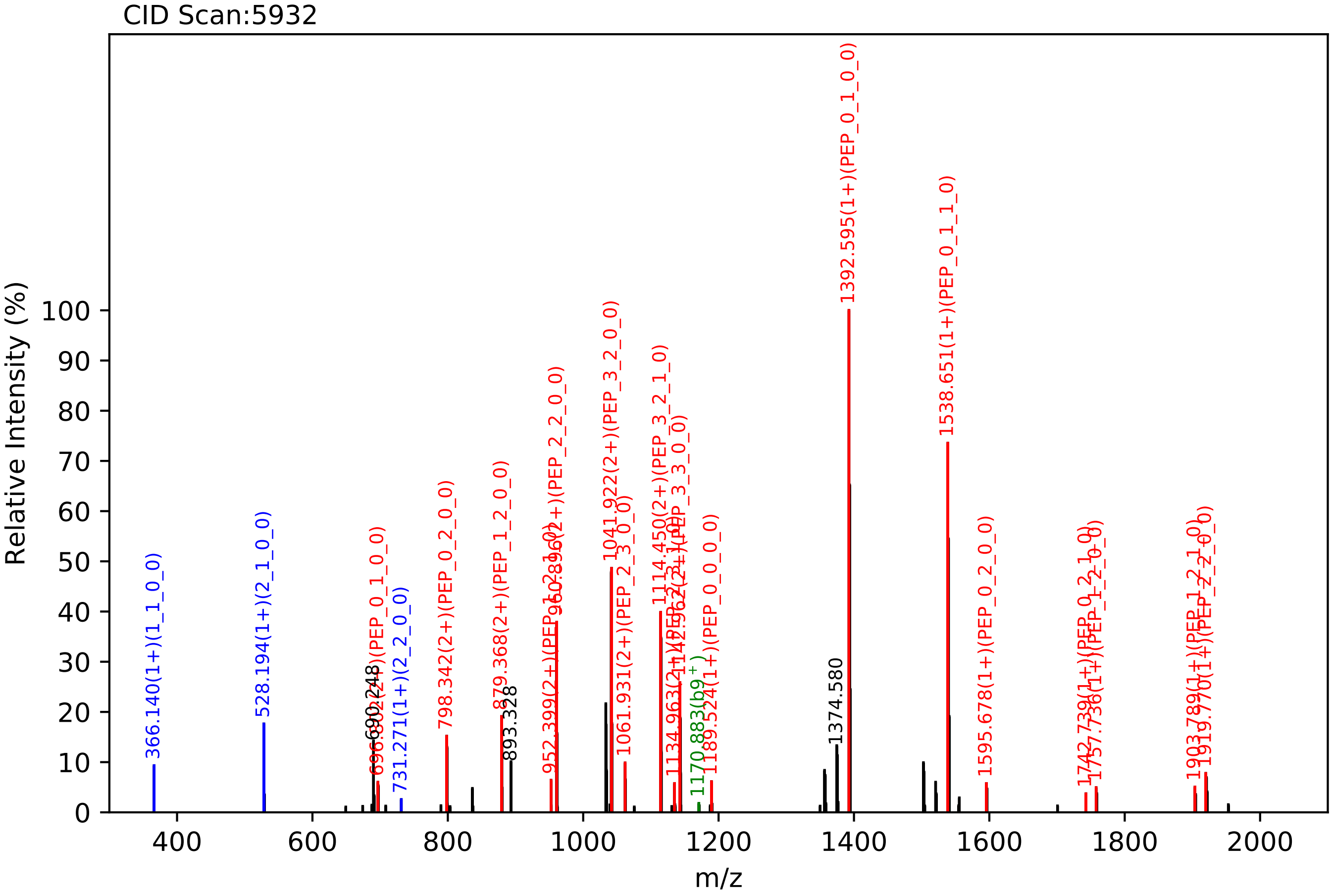

Unknown set no. 350, Experiment: Human Plasma exp_1

EEQYNSTYR(=PEP)_3_4_0_0, m/z:1244.50(2+), RT:25.57, Y-score:93.53

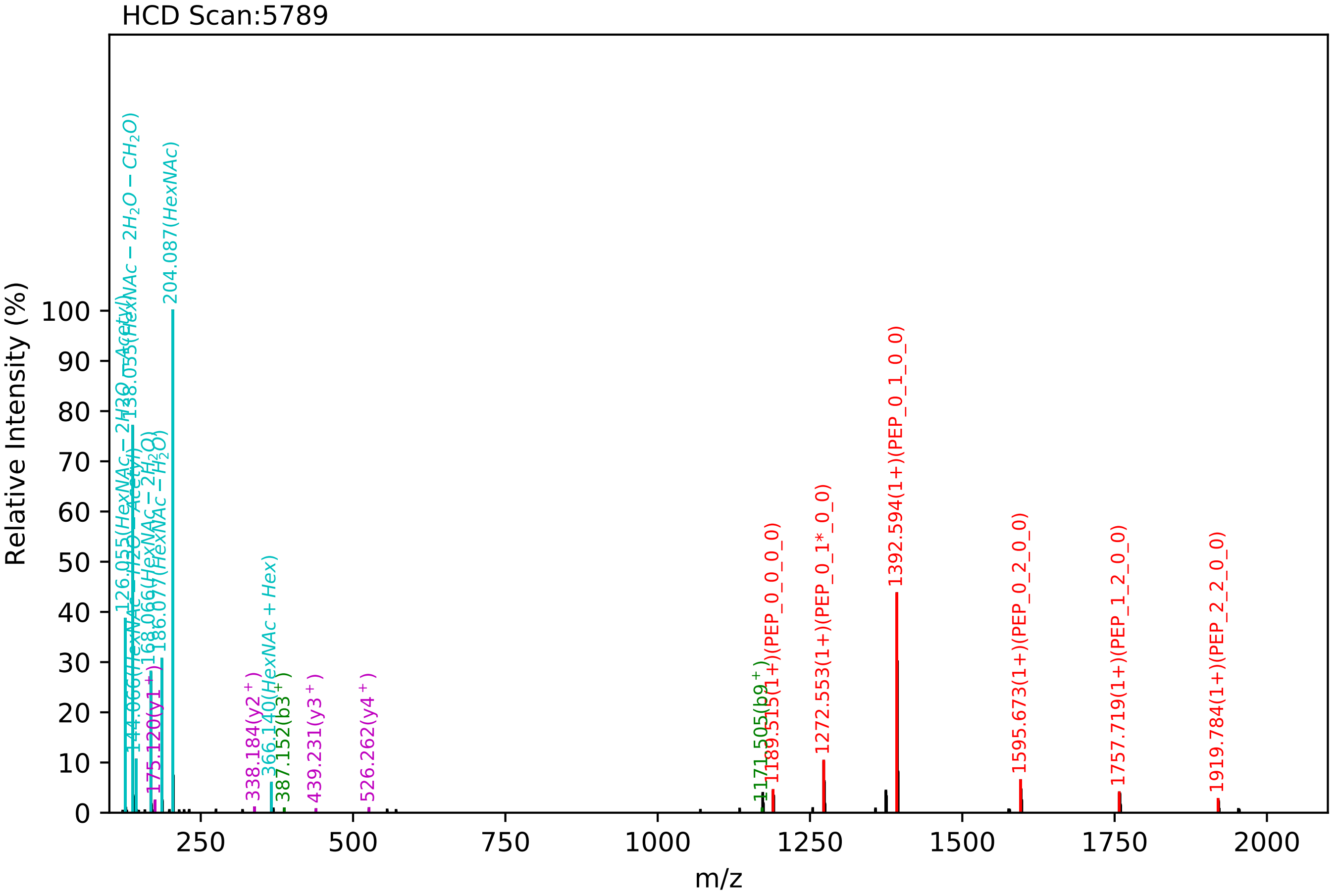

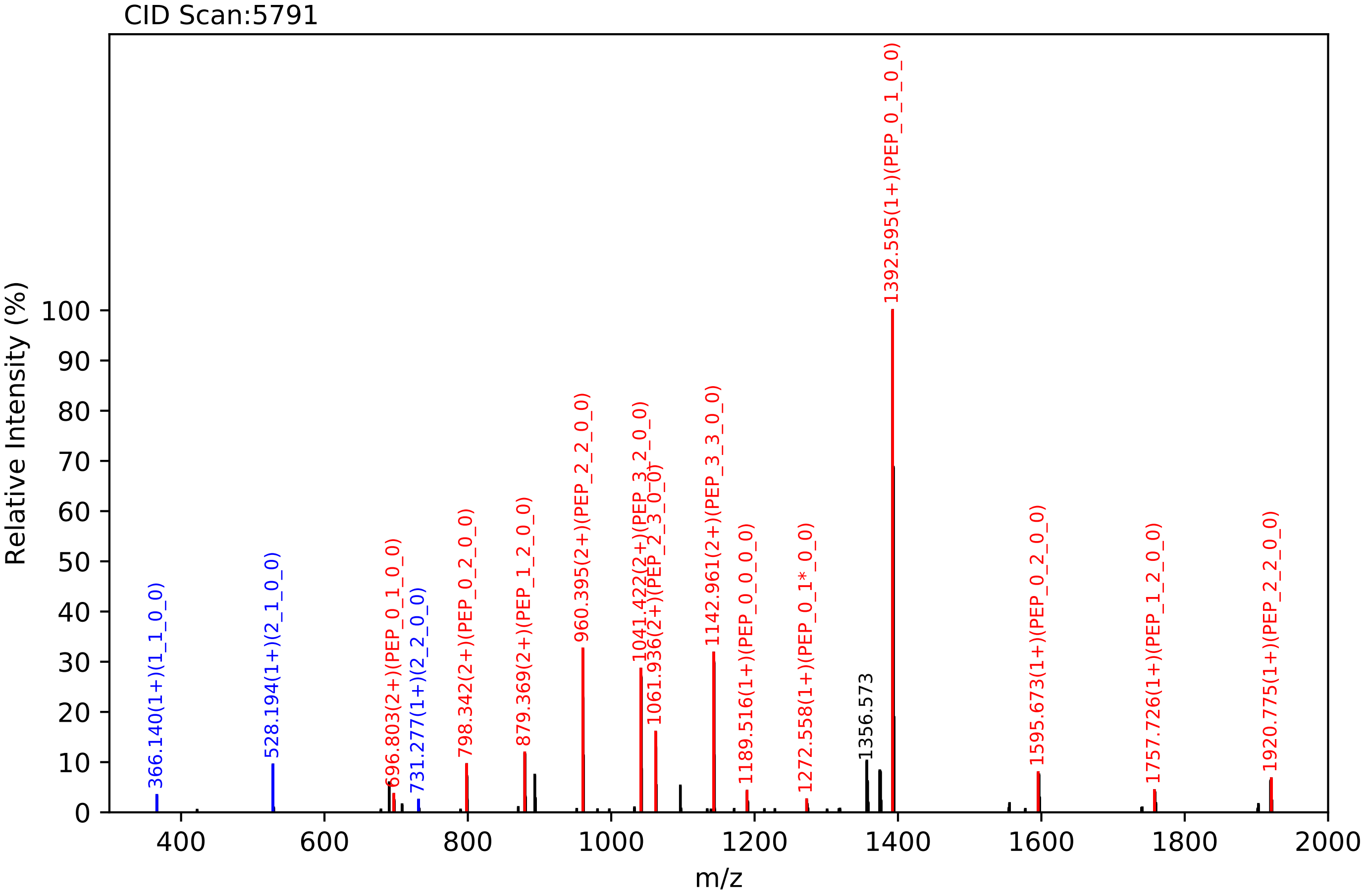

Unknown set no. 351, Experiment: Human Plasma exp_2

EEQYNSTYR(=PEP)_3_4_0_0, m/z:1244.50(2+), RT:25.95, Y-score:90.62

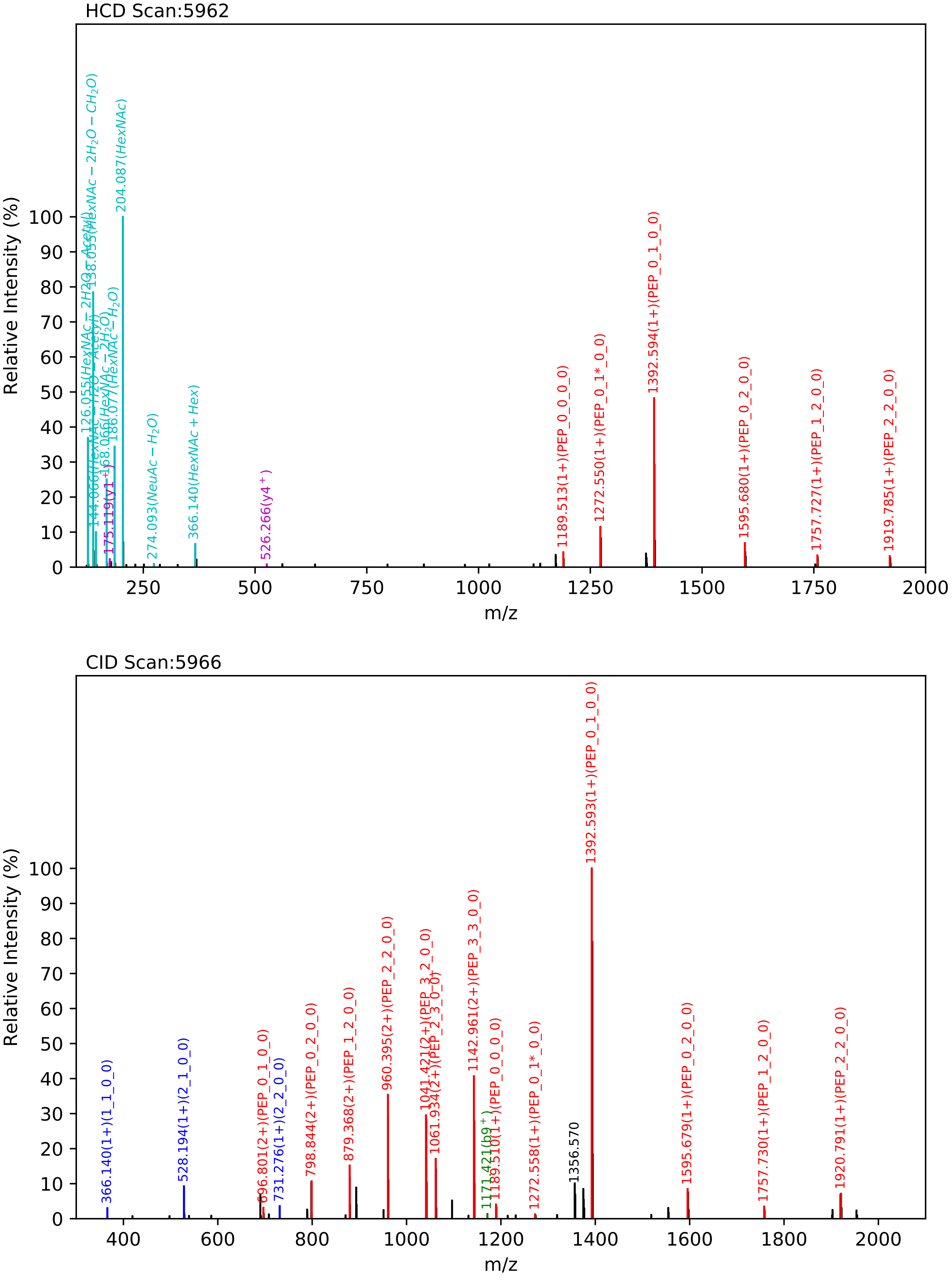

Unknown set no. 352, Experiment: Human Plasma exp_4

EEQYNSTYR(=PEP)_3_4_0_0, m/z:1244.50(2+), RT:25.52, Y-score:86.77

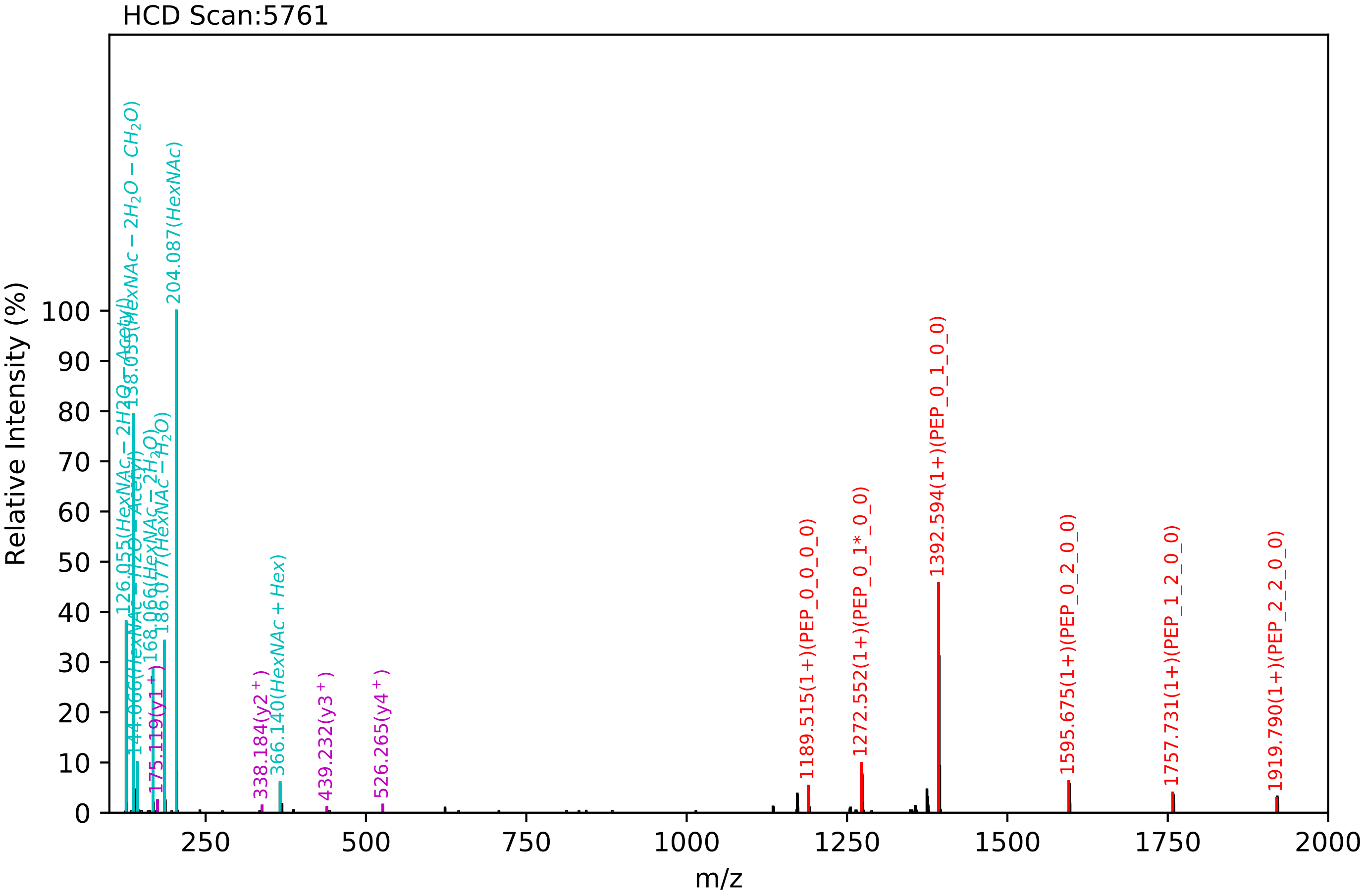

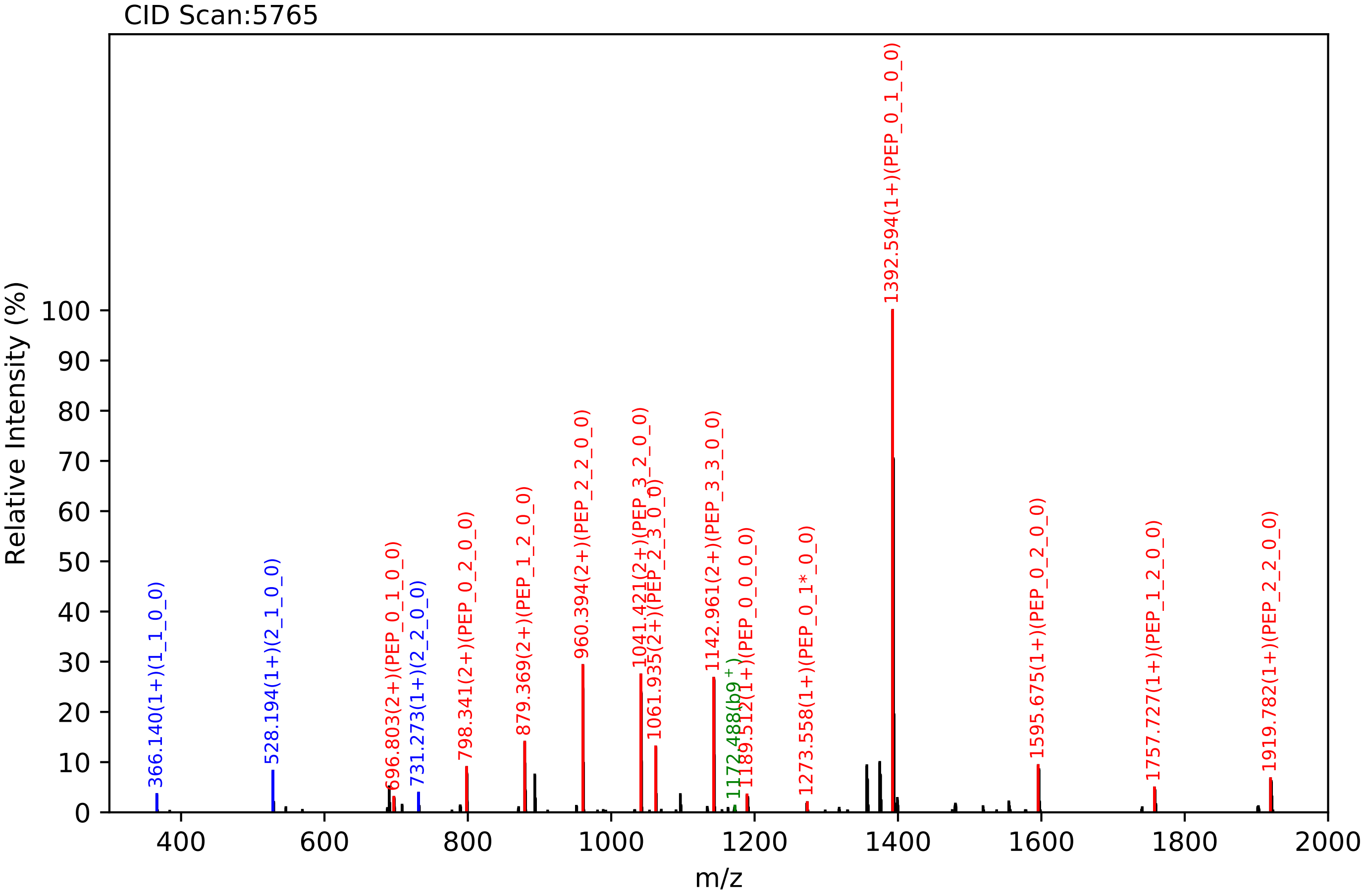

Unknown set no. 353, Experiment: Human Plasma exp_1

EEQYNSTYR(=PEP)_3_4_1_0, m/z:1317.53(2+), RT:25.33, Y-score:89.31

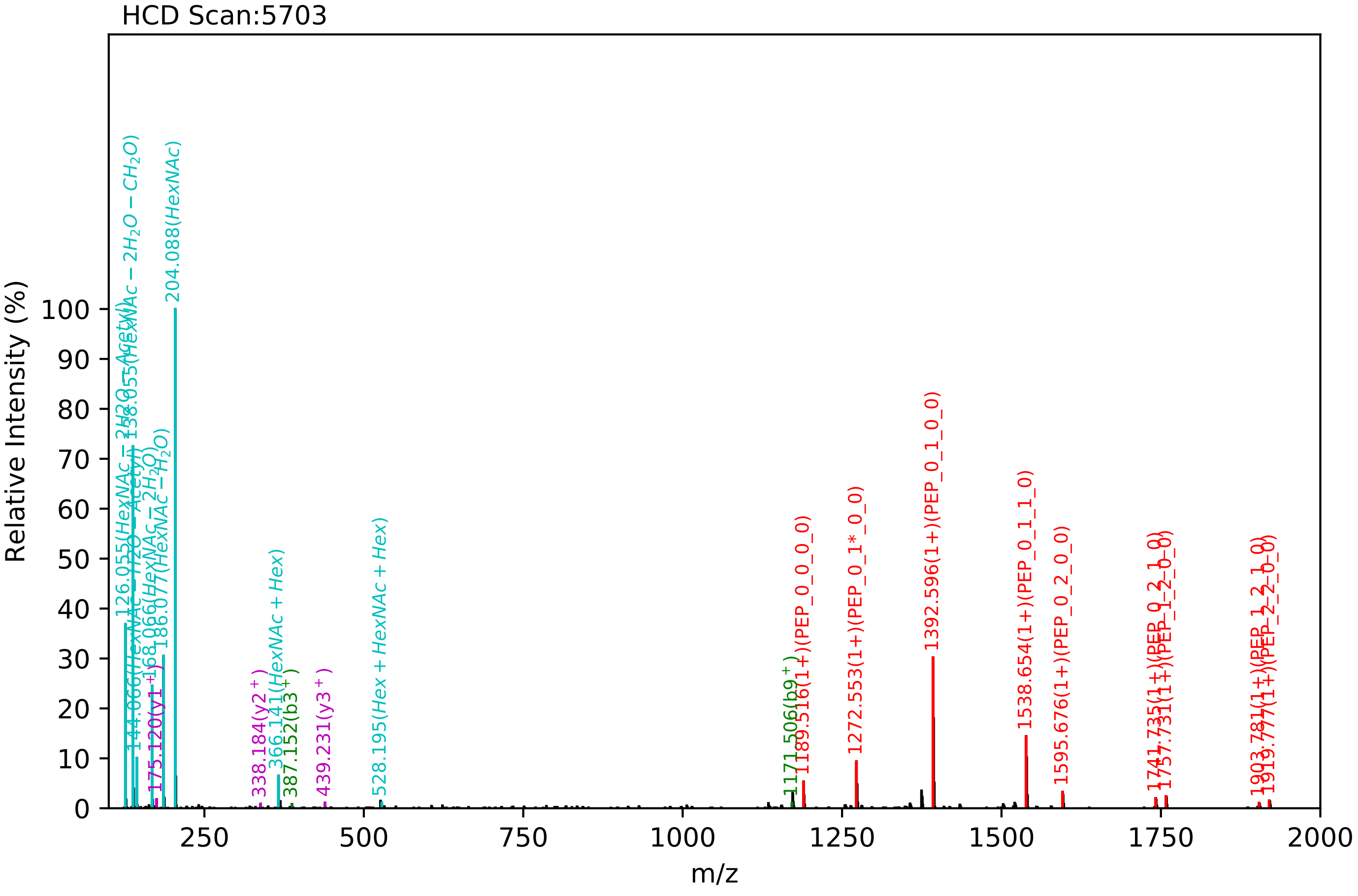

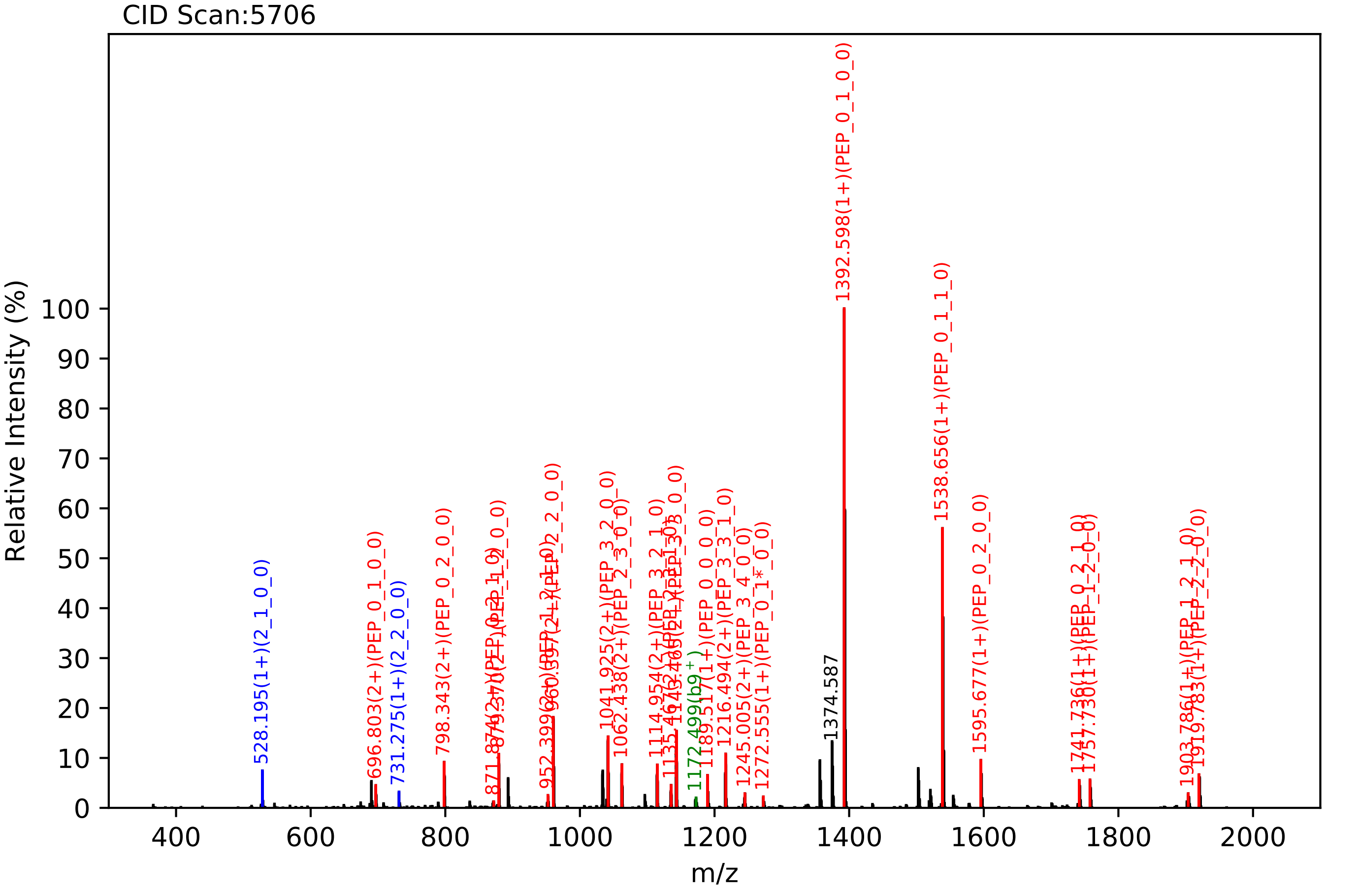

Unknown set no. 354, Experiment: Human Plasma exp_2

EEQYNSTYR(=PEP)_3_4_1_0, m/z:1317.53(2+), RT:25.02, Y-score:89.46

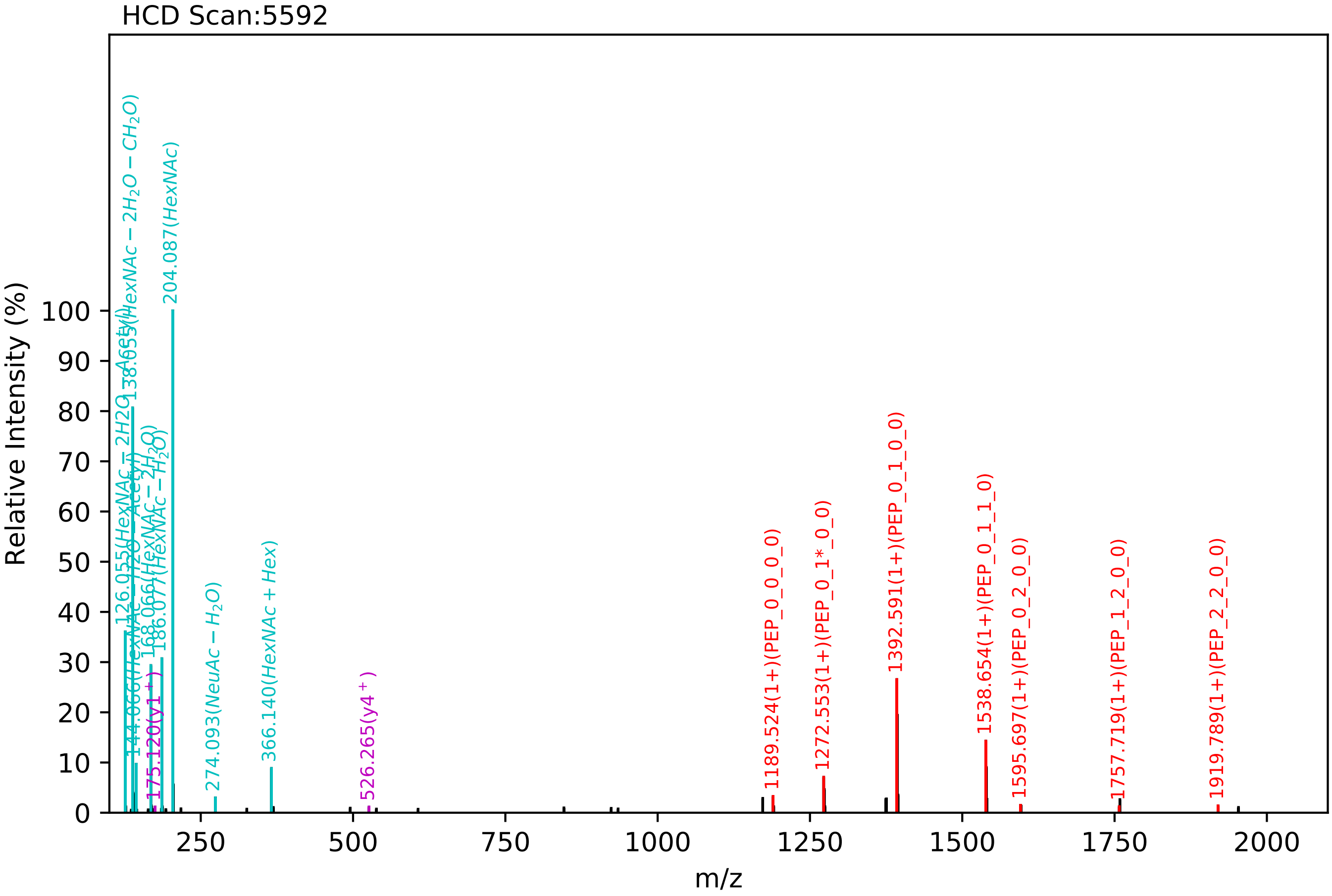

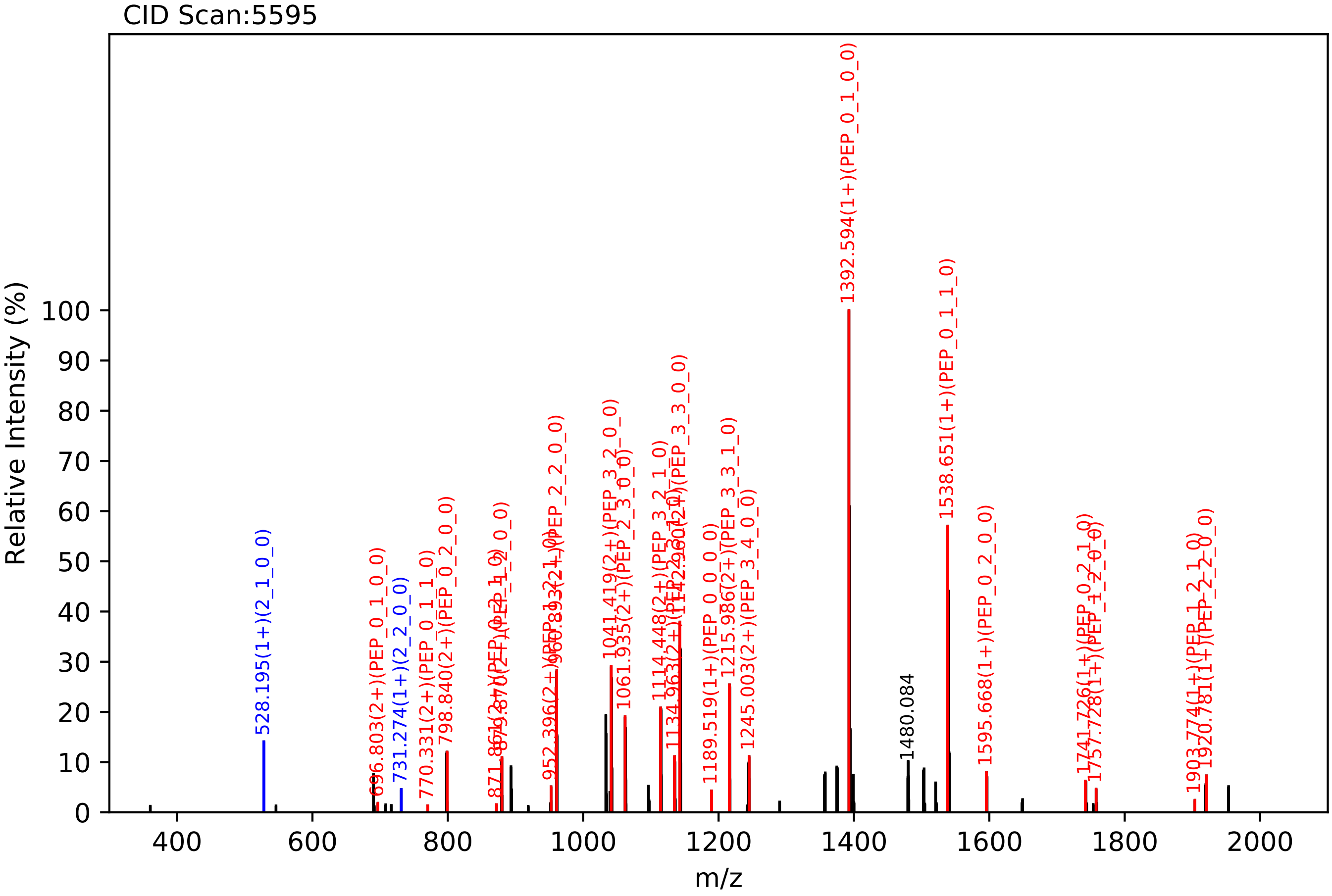

Unknown set no. 355, Experiment: Human Plasma exp_3

EEQYNSTYR(=PEP)_3_4_1_0, m/z:1317.53(2+), RT:26.01, Y-score:89.39

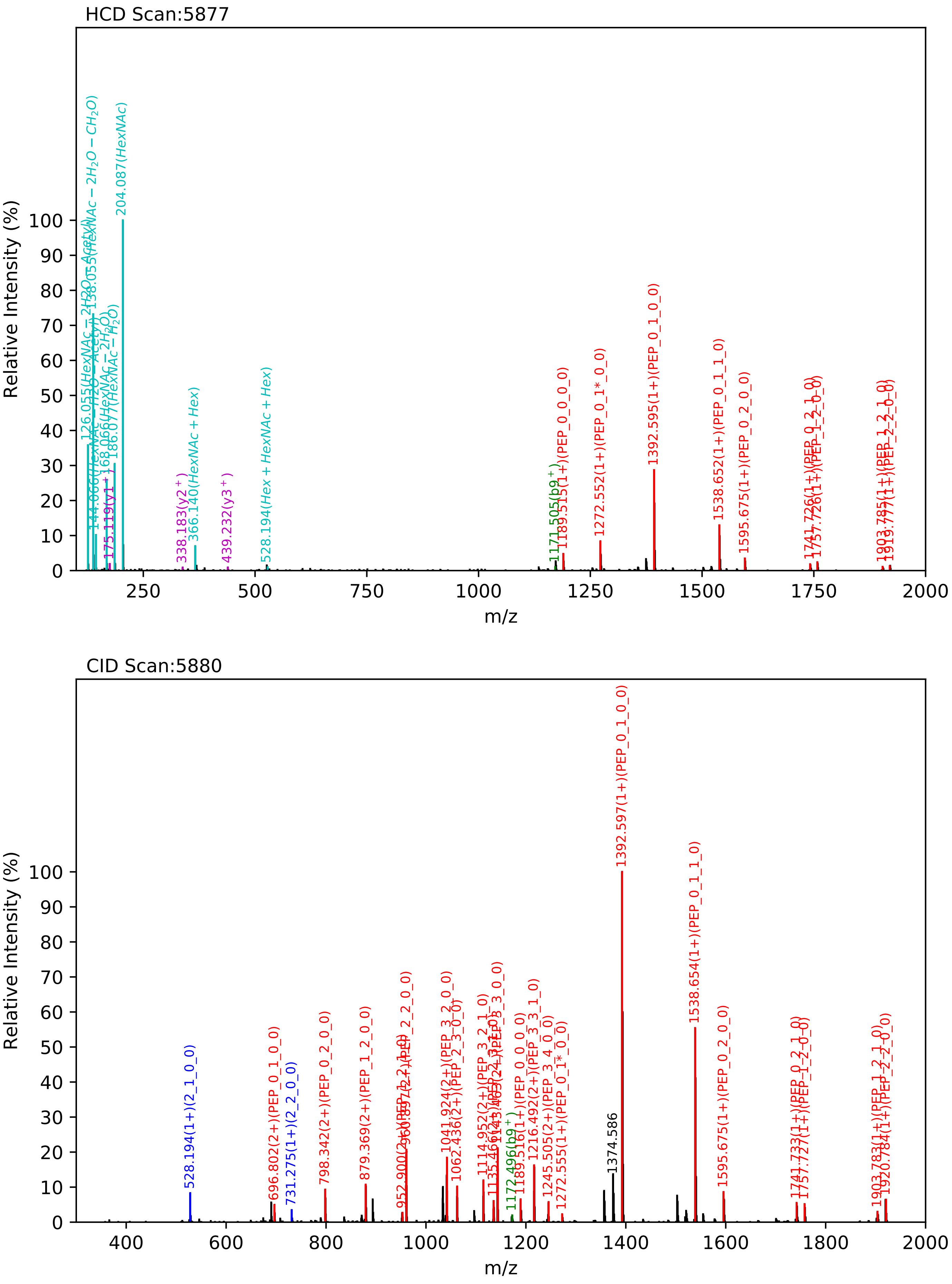

Unknown set no. 356, Experiment: Human Plasma exp_4

EEQYNSTYR(=PEP)_3_4_1_0, m/z:1317.53(2+), RT:26.89, Y-score:95.95

HCD Scan:6190

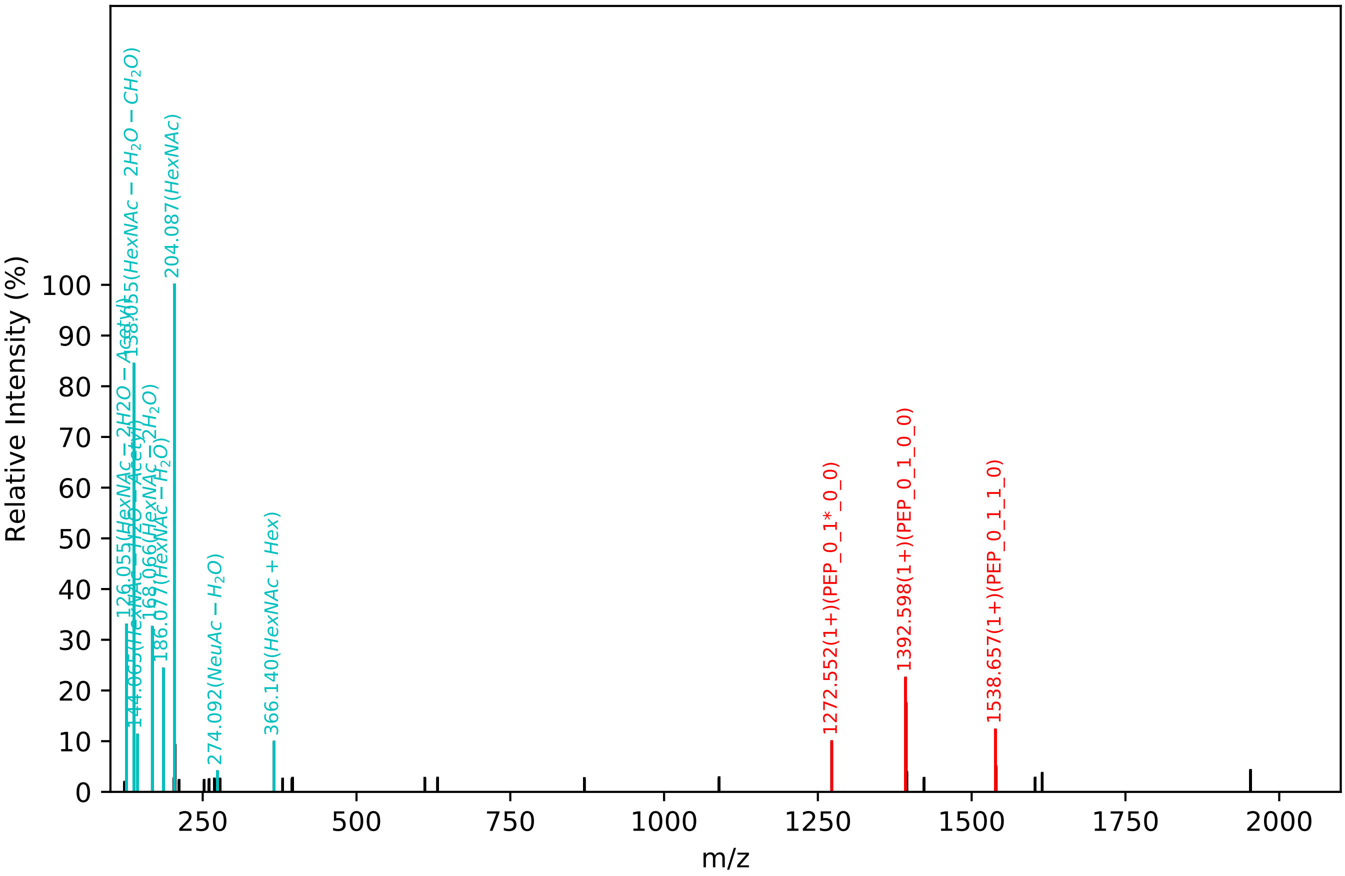

CID Scan:6195

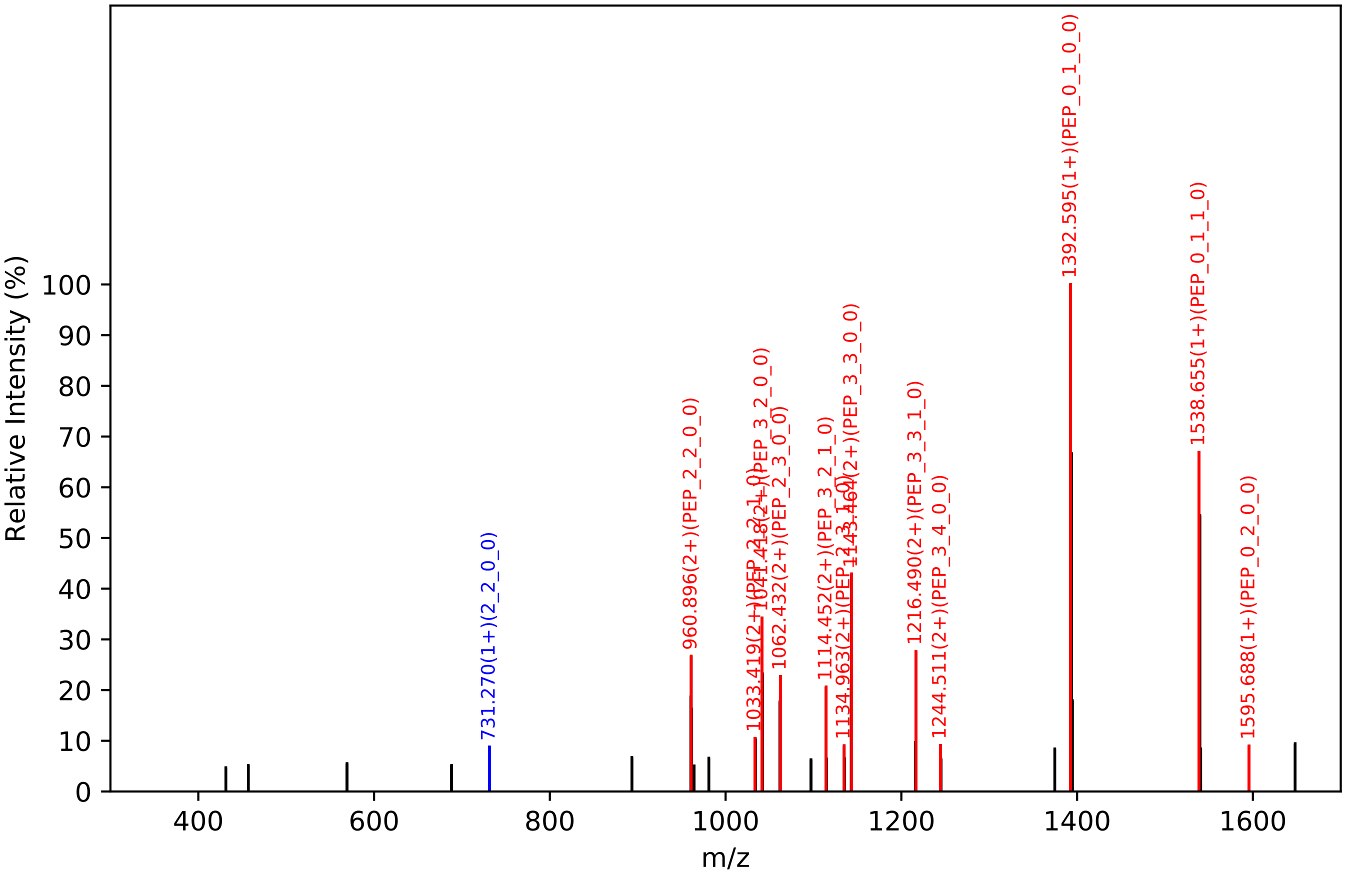

Unknown set no. 357, Experiment: Human Plasma exp_5

EEQYNSTYR(=PEP)_3_4_1_0, m/z:1317.53(2+), RT:25.84, Y-score:89.51

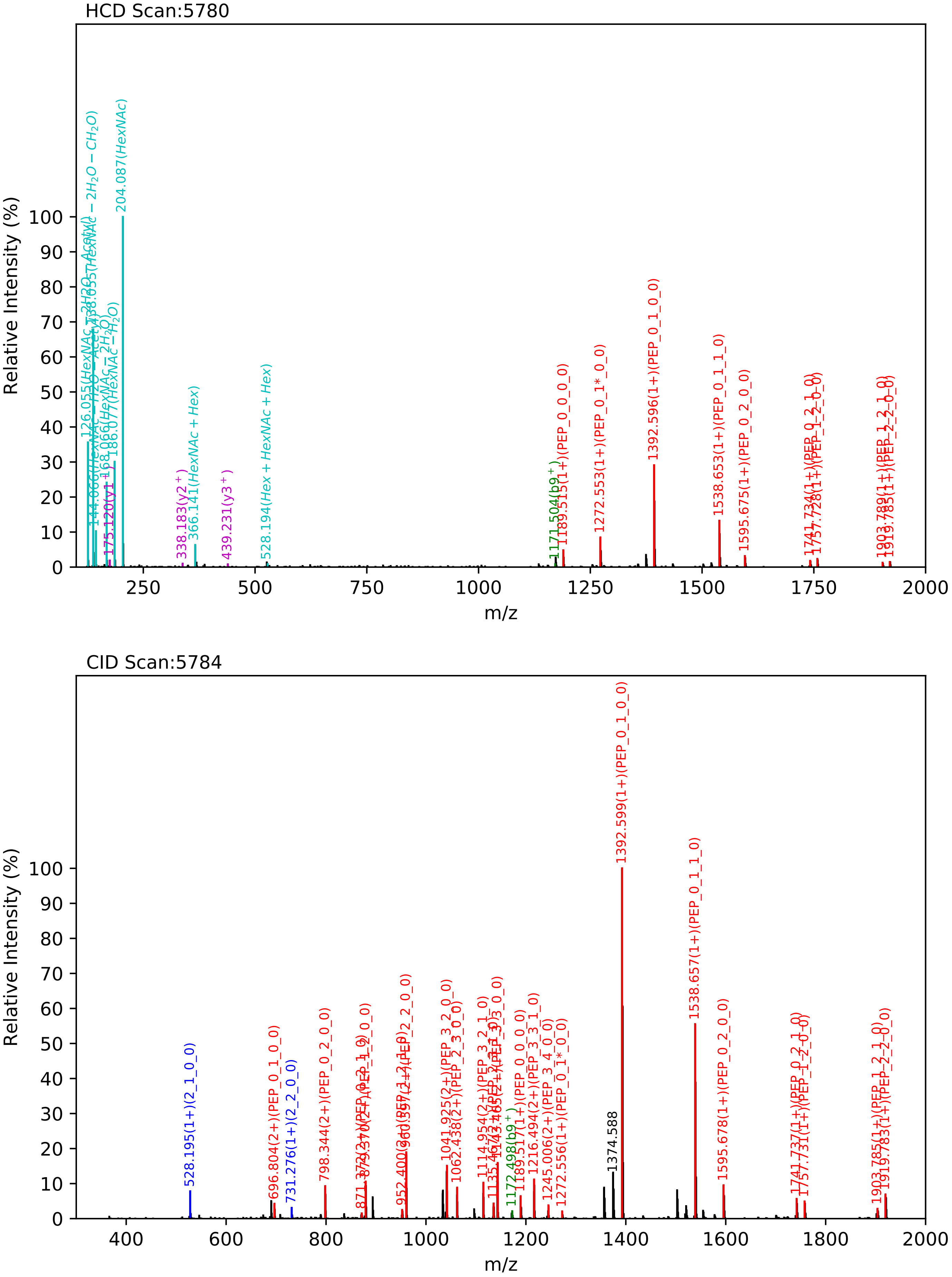

Unknown set no. 358, Experiment: Human Plasma exp_1

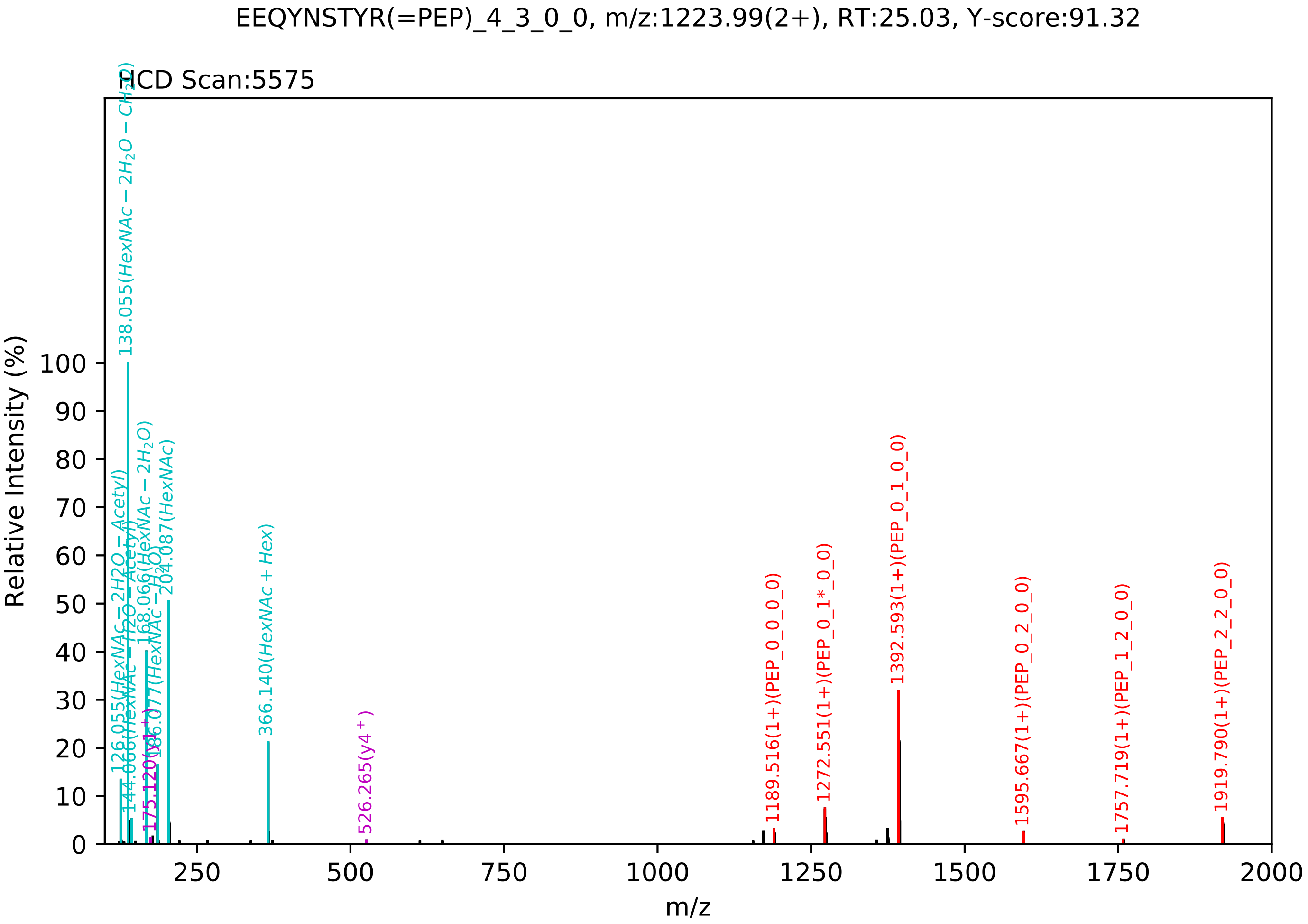

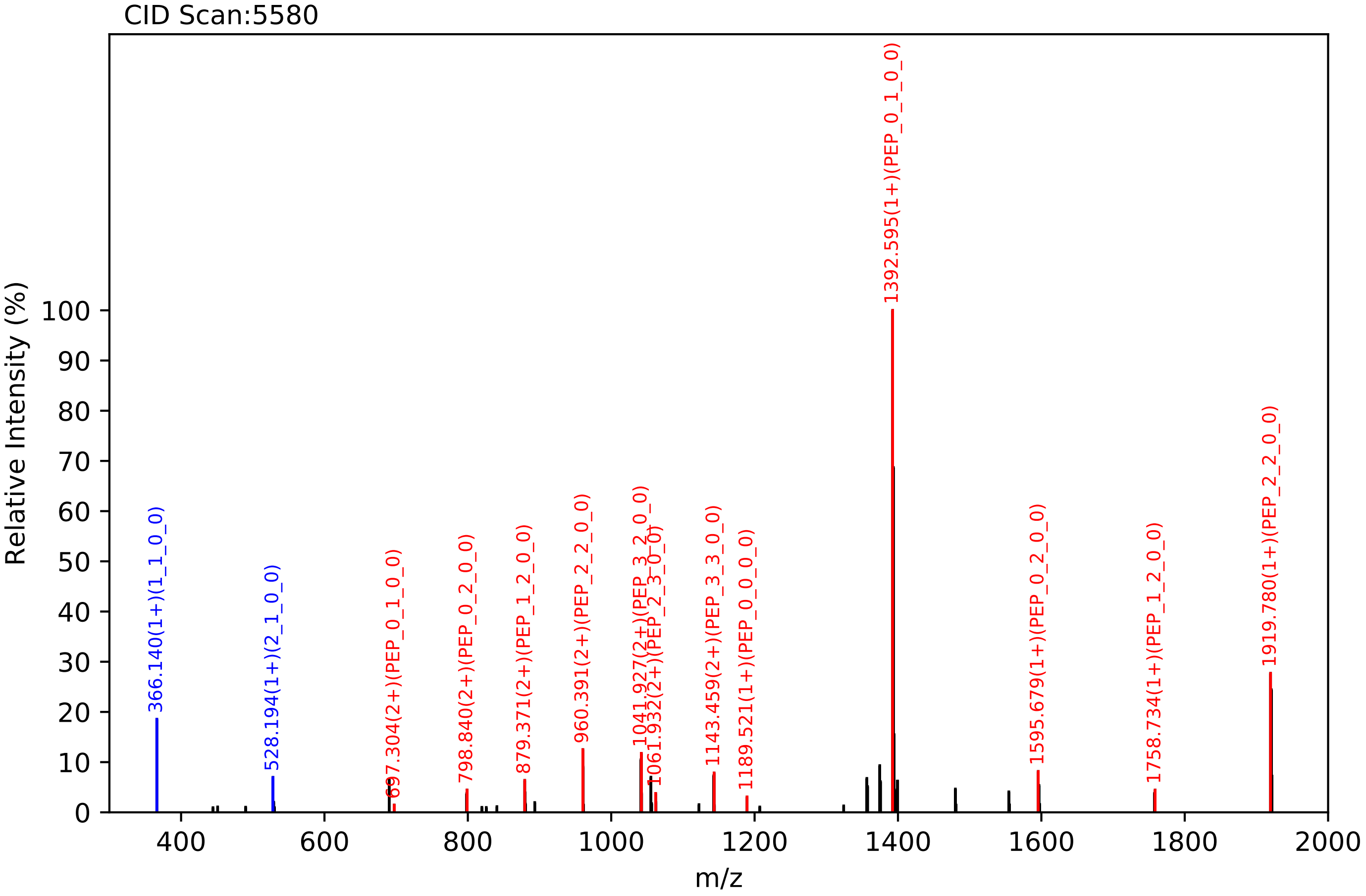

Unknown set no. 359, Experiment: Human Plasma exp_2

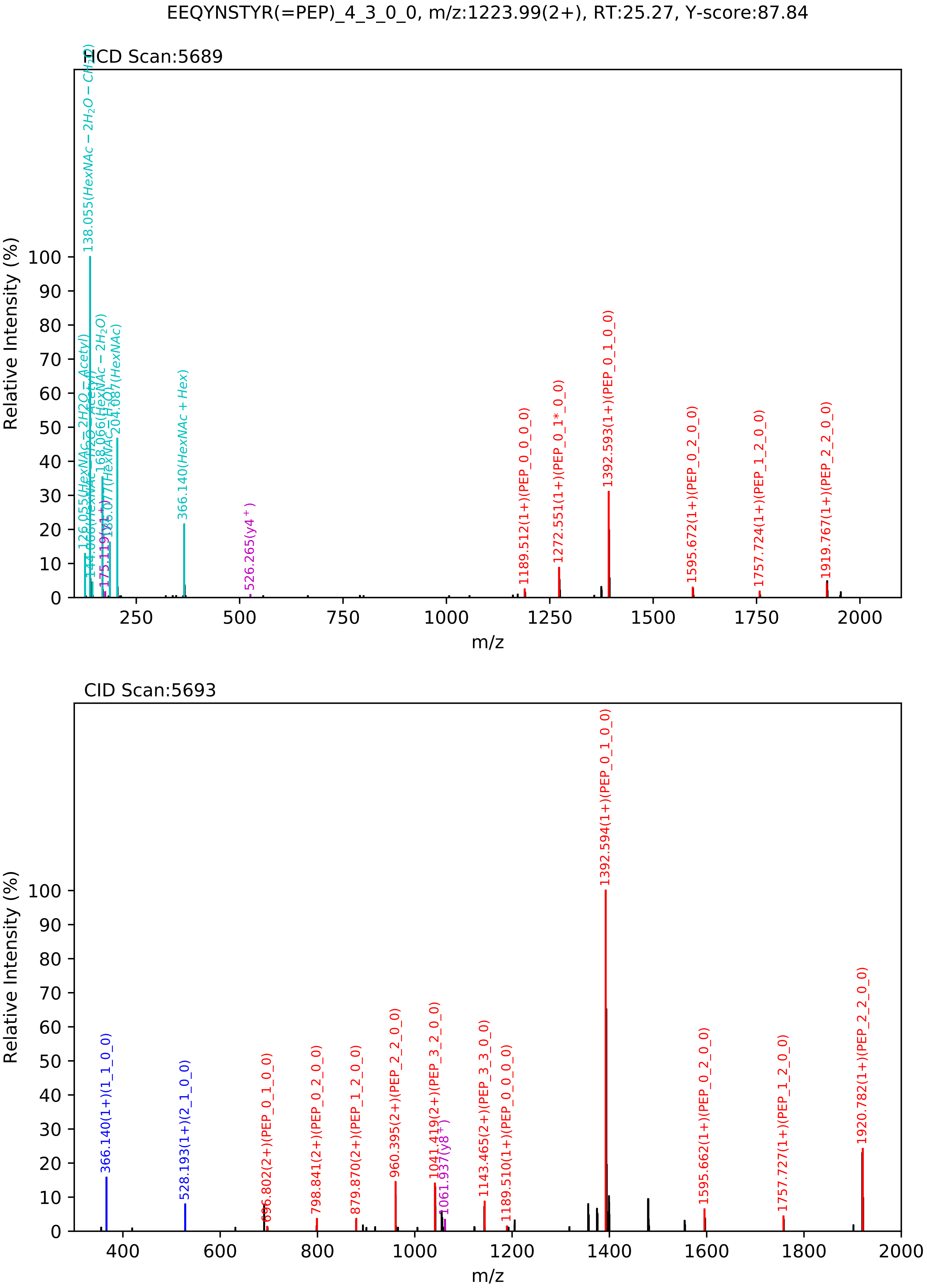

Unknown set no. 360, Experiment: Human Plasma exp_3

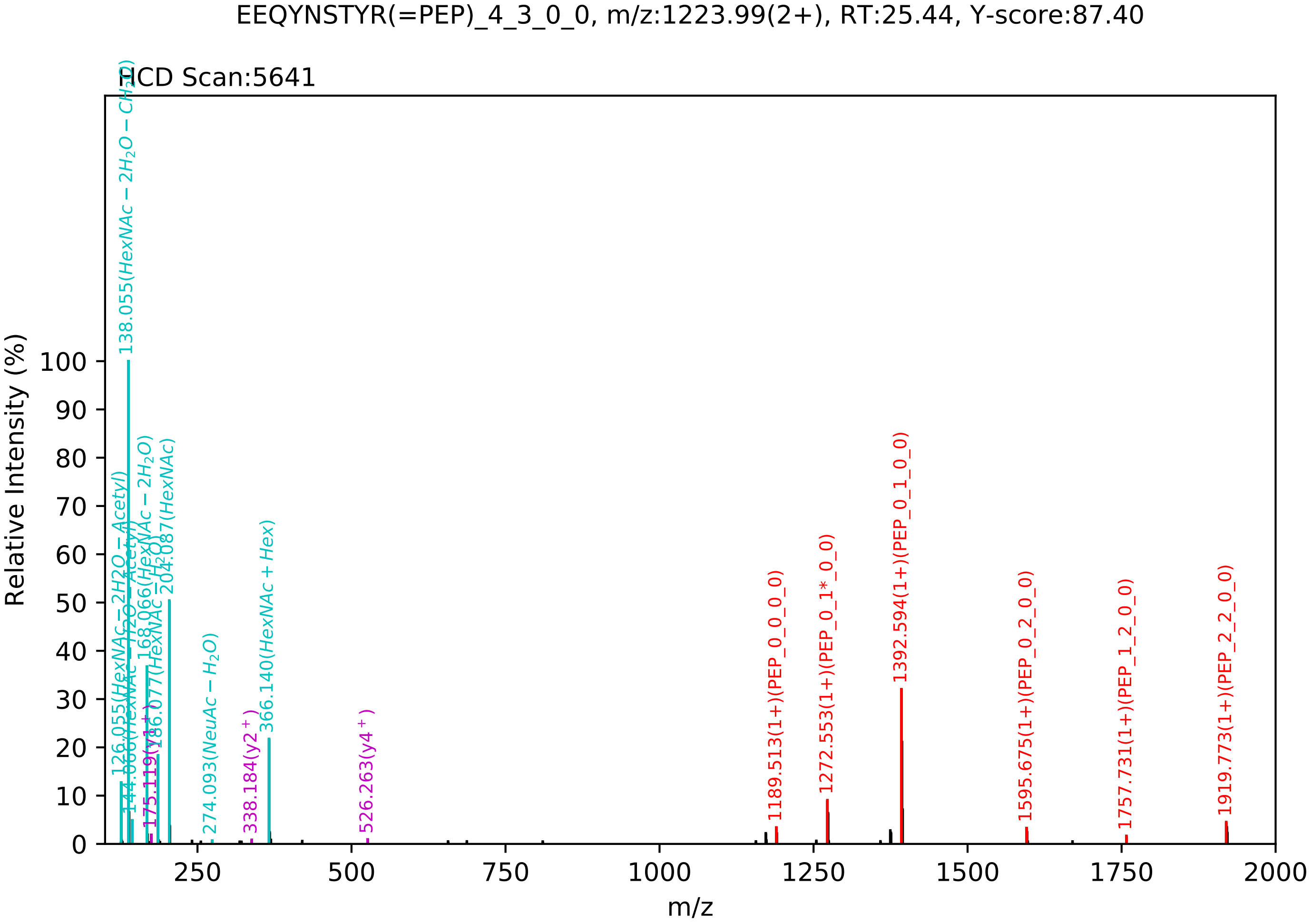

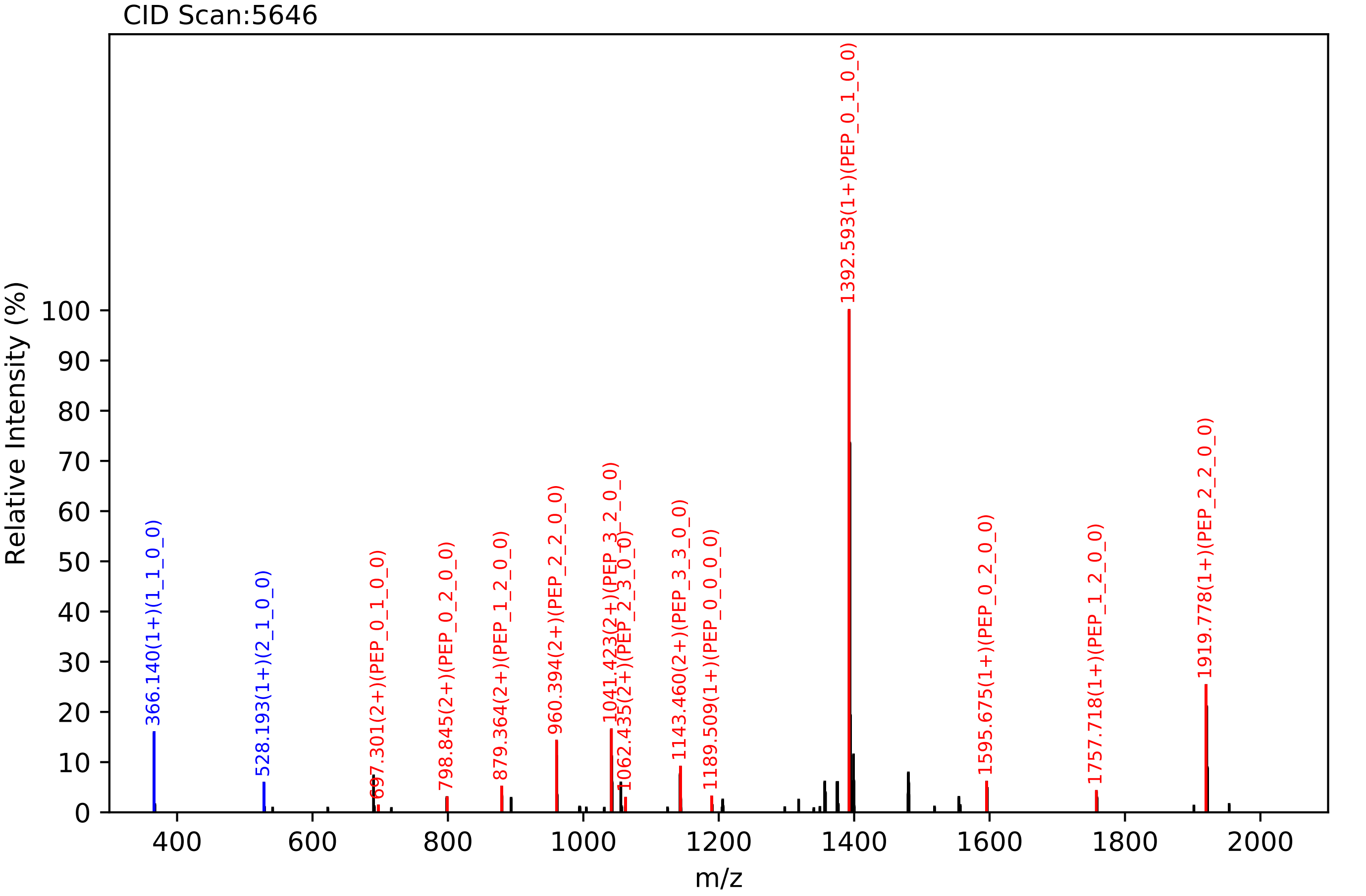

Unknown set no. 361, Experiment: Human Plasma exp_4

EEQYNSTYR(=PEP)_4_3_0_0, m/z:1223.99(2+), RT:25.54, Y-score:90.11

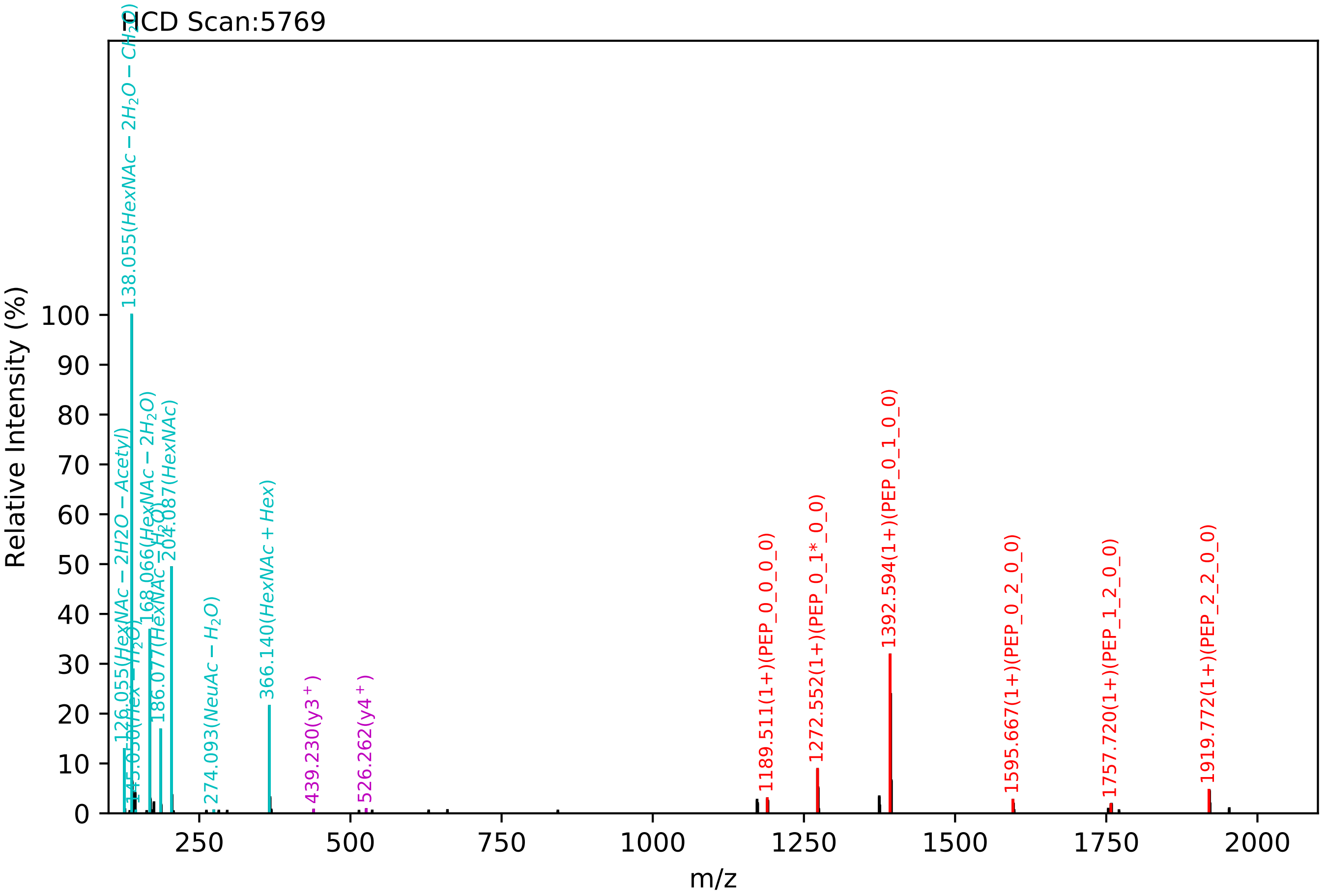

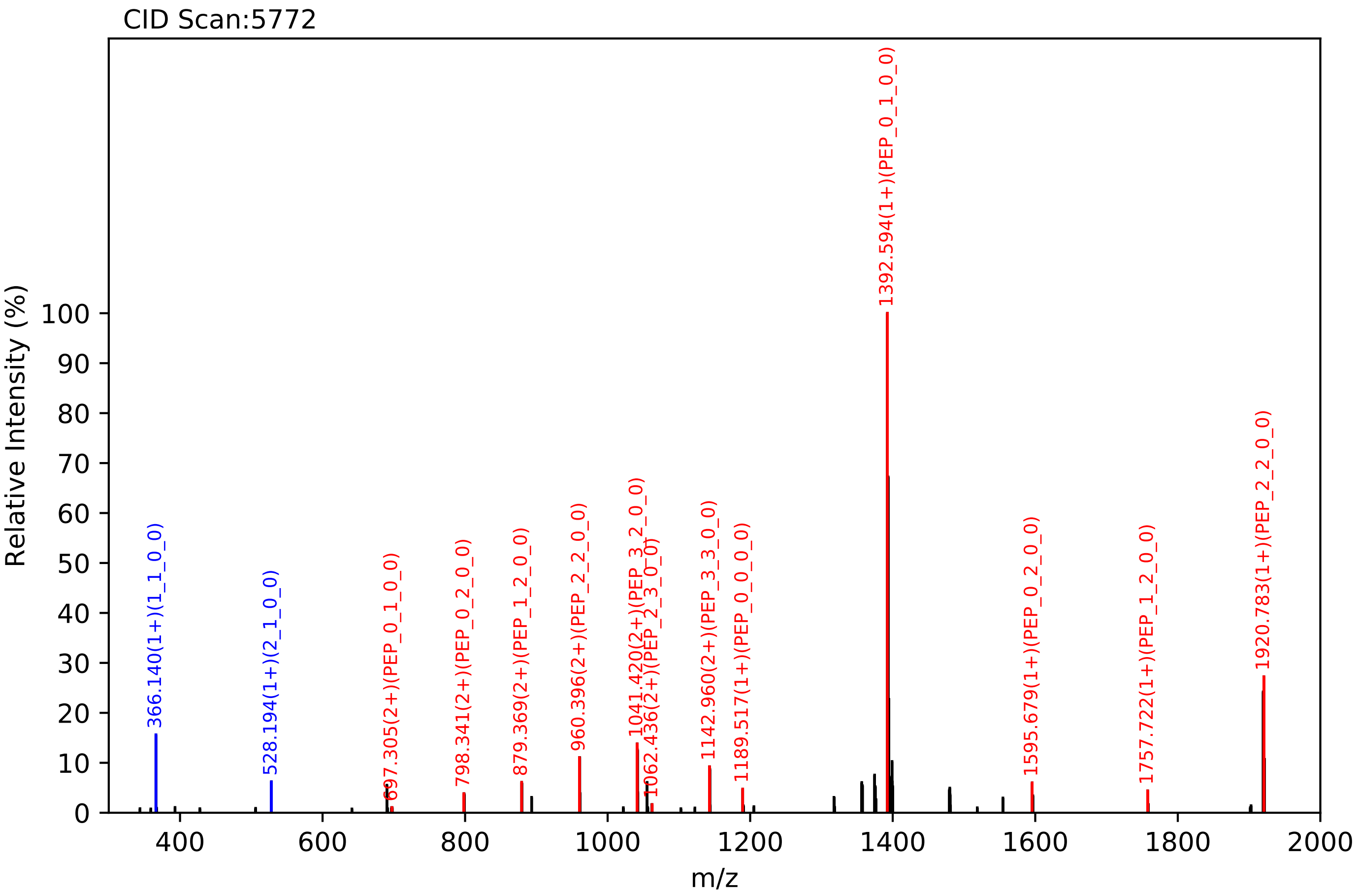

Unknown set no. 362, Experiment: Human Plasma exp_5

EEQYNSTYR(=PEP)_4_3_0_0, m/z:1223.99(2+), RT:25.42, Y-score:91.81

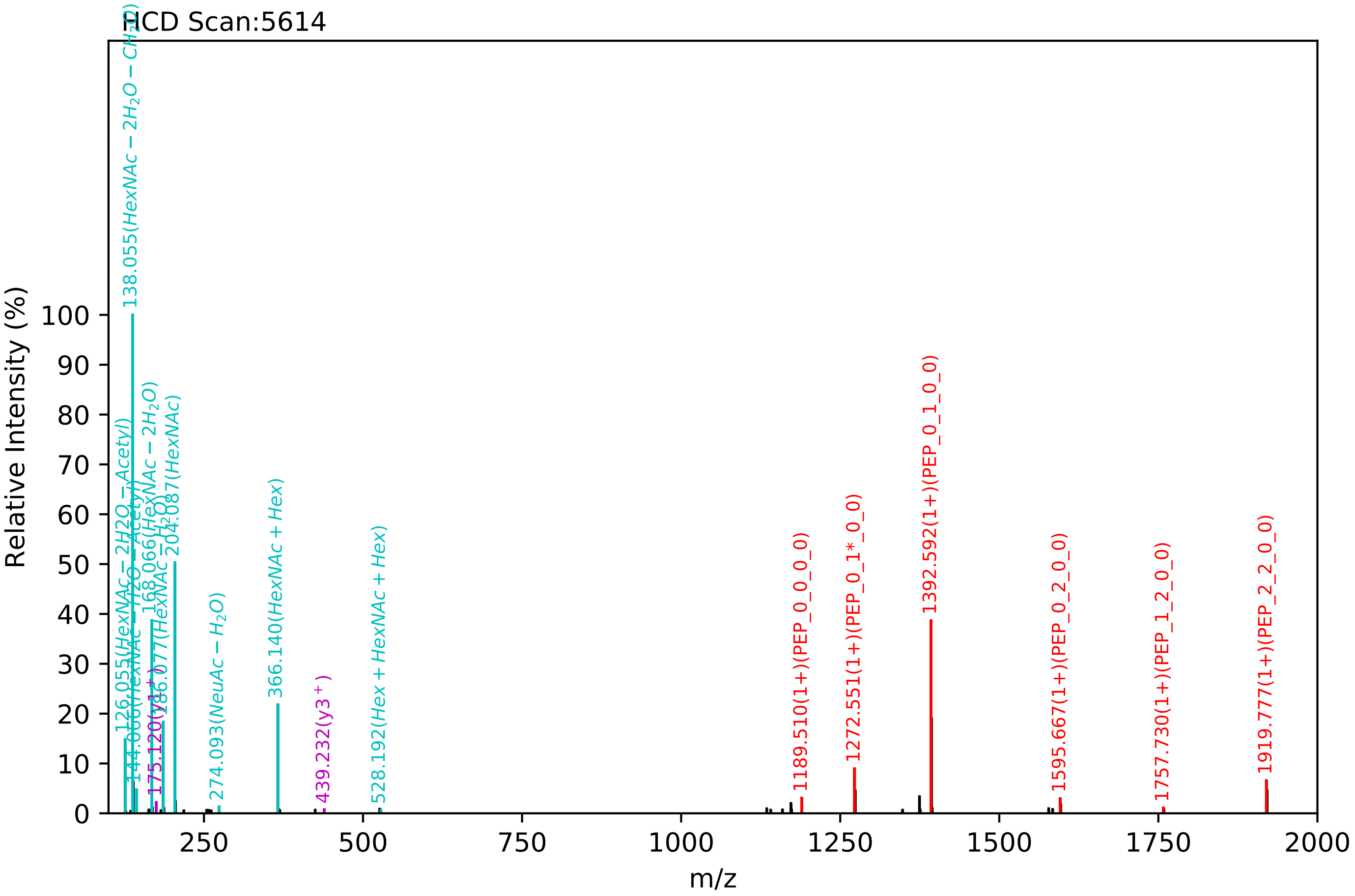

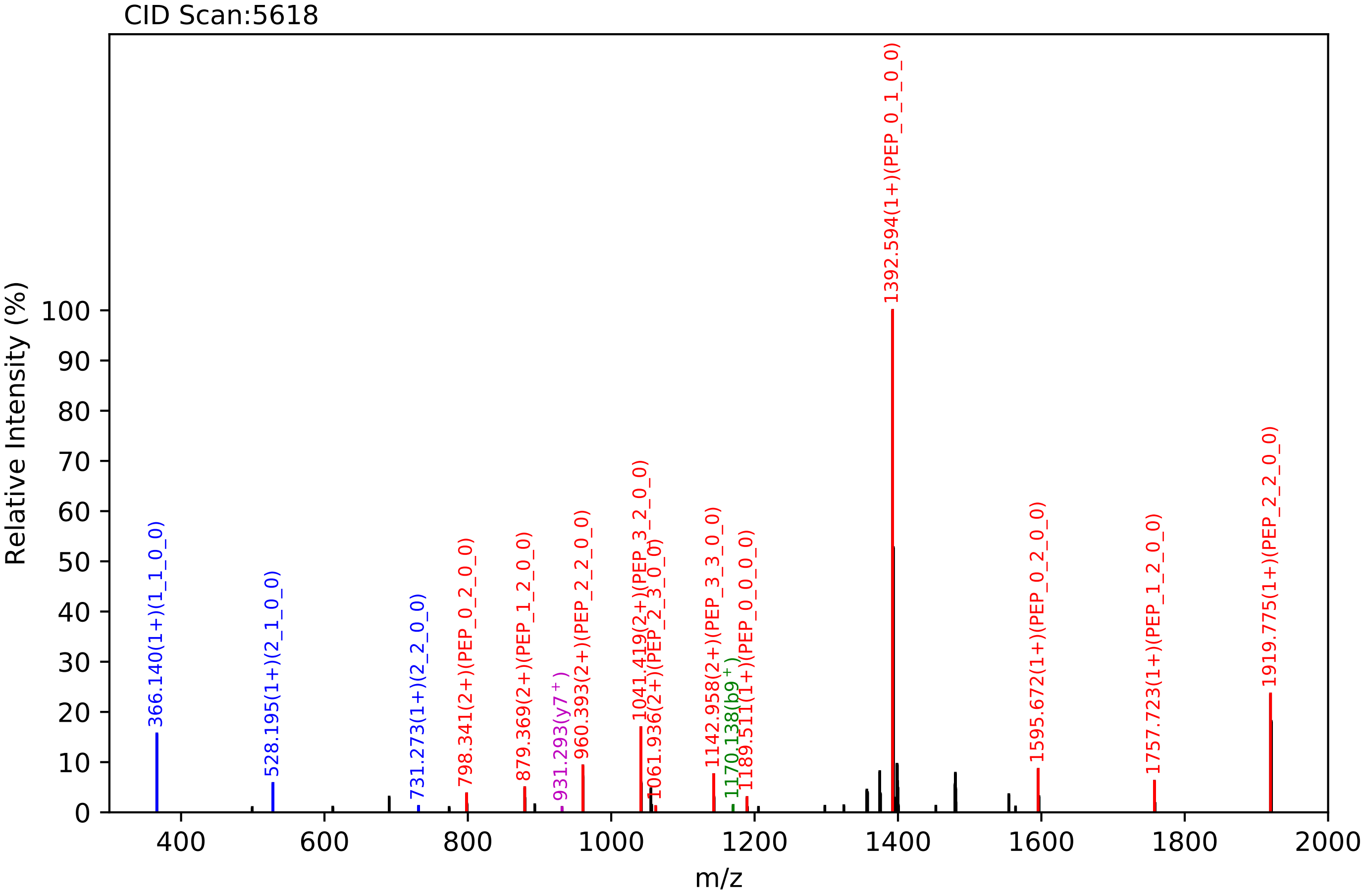

Unknown set no. 363, Experiment: Human Plasma exp_1

EEQYNSTYR(=PEP)_4_3_1_0, m/z:1297.02(2+), RT:25.29, Y-score:90.10

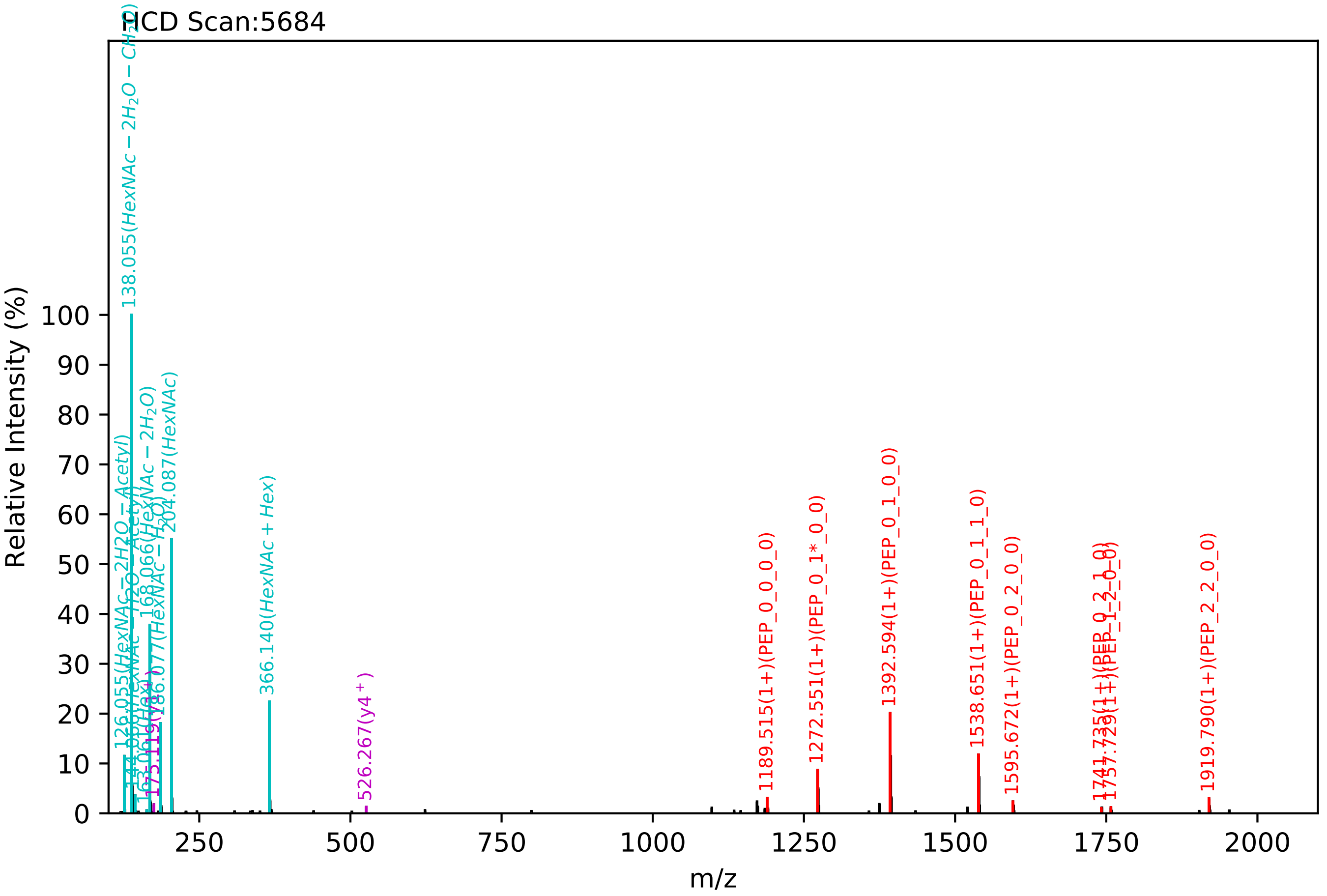

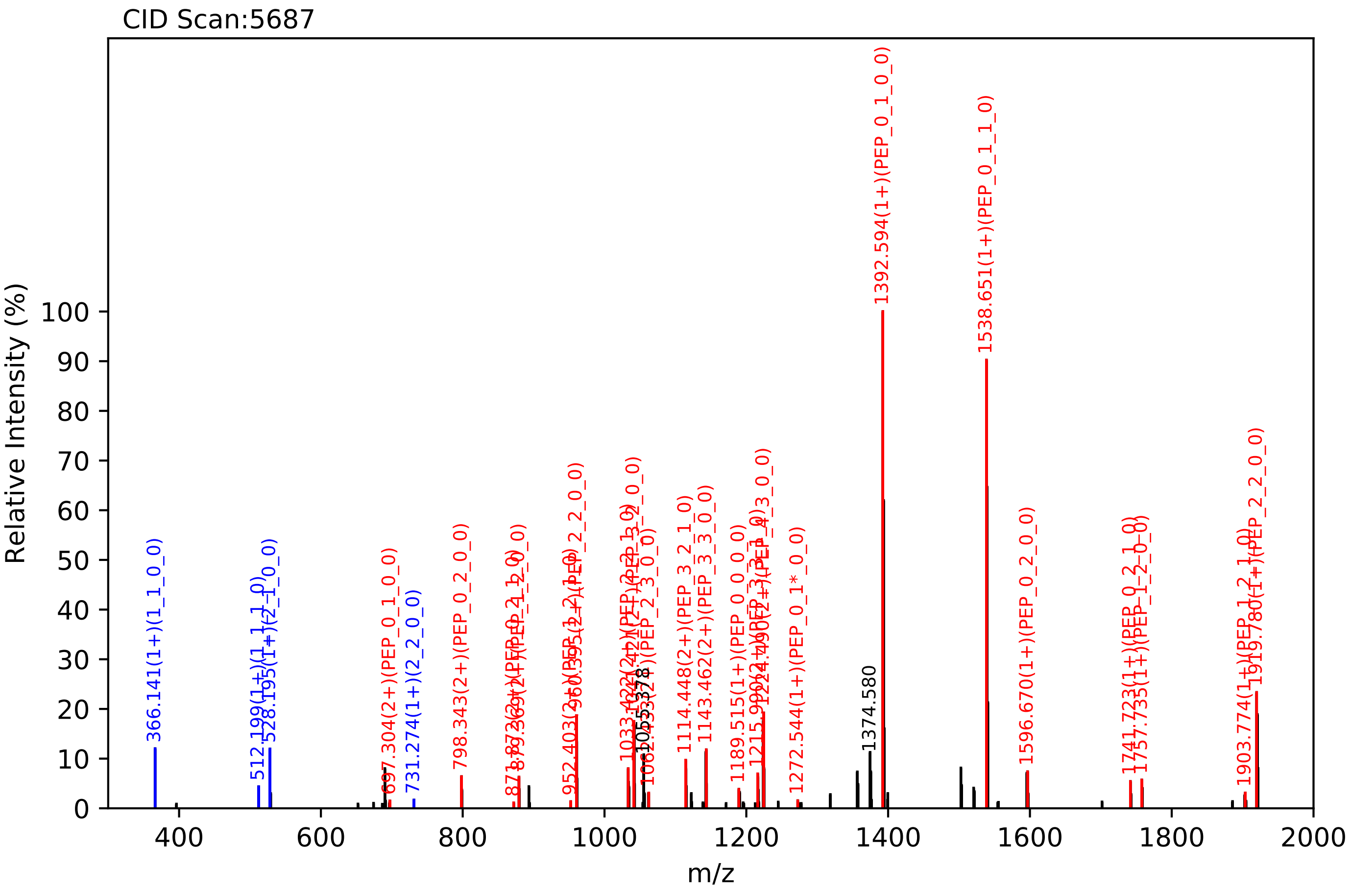

Unknown set no. 364, Experiment: Human Plasma exp_2

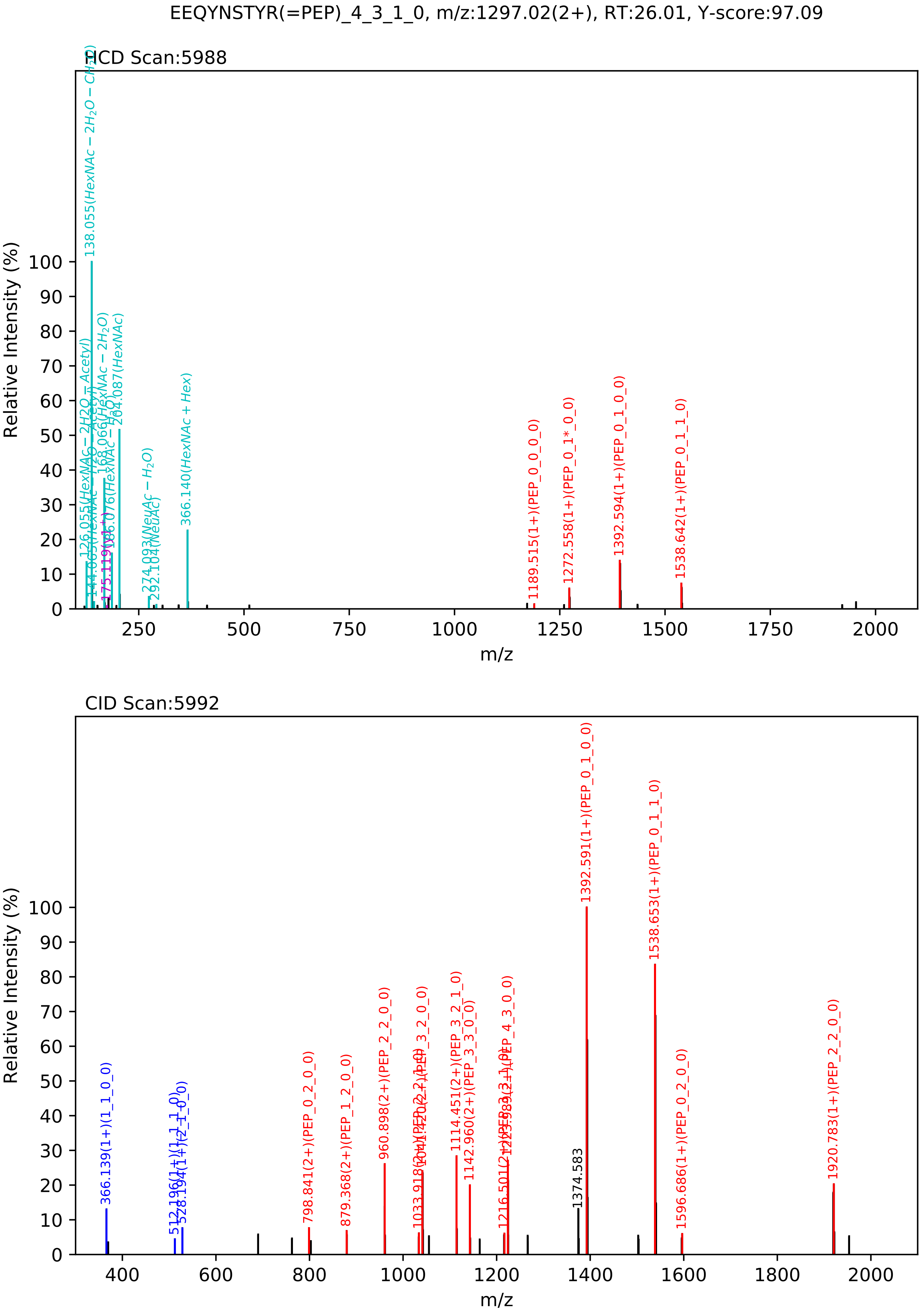

Unknown set no. 365, Experiment: Human Plasma exp_3

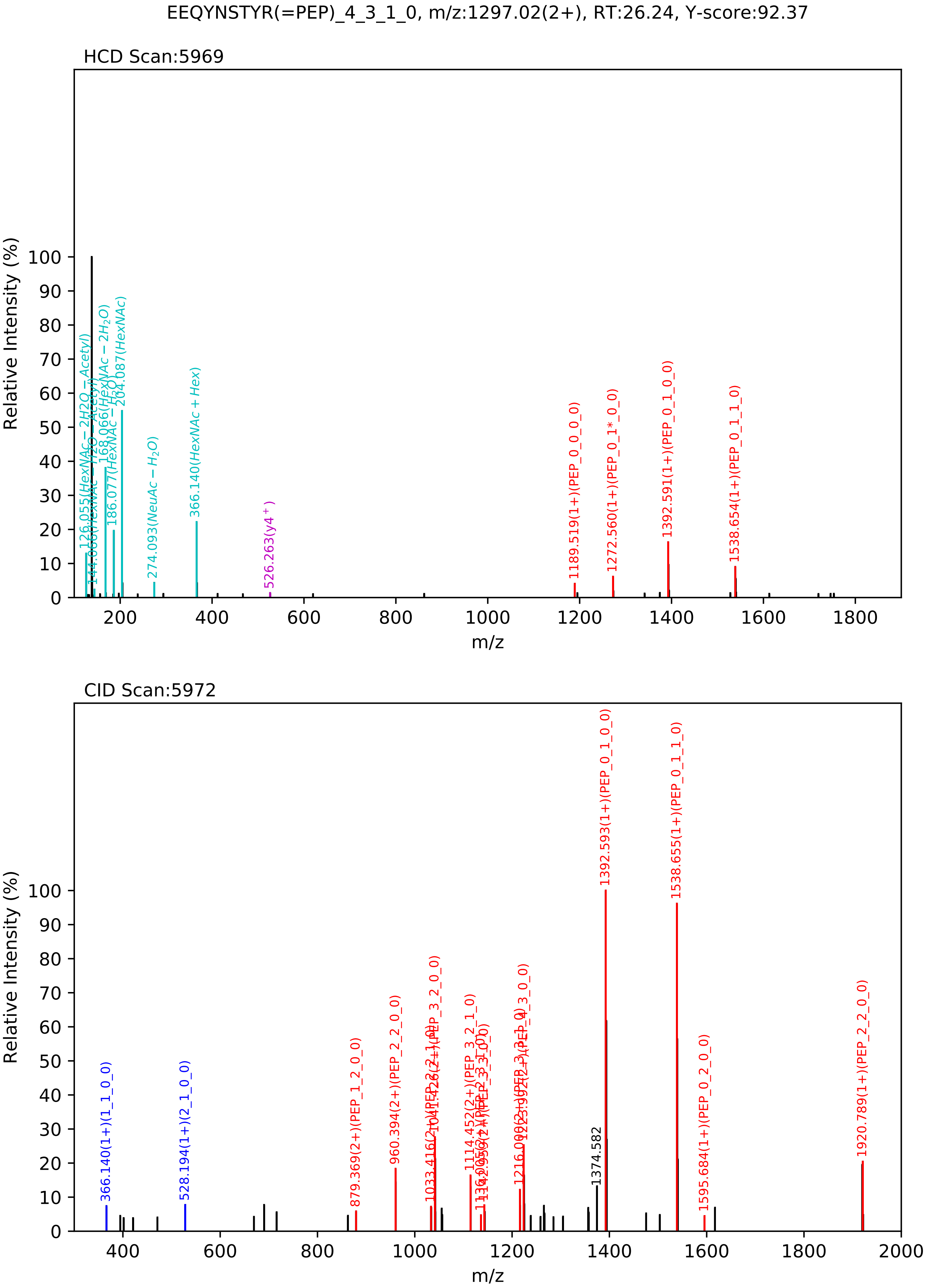

Unknown set no. 366, Experiment: Human Plasma exp_5

EEQYNSTYR(=PEP)_4_3_1_0, m/z:1297.02(2+), RT:25.75, Y-score:90.67

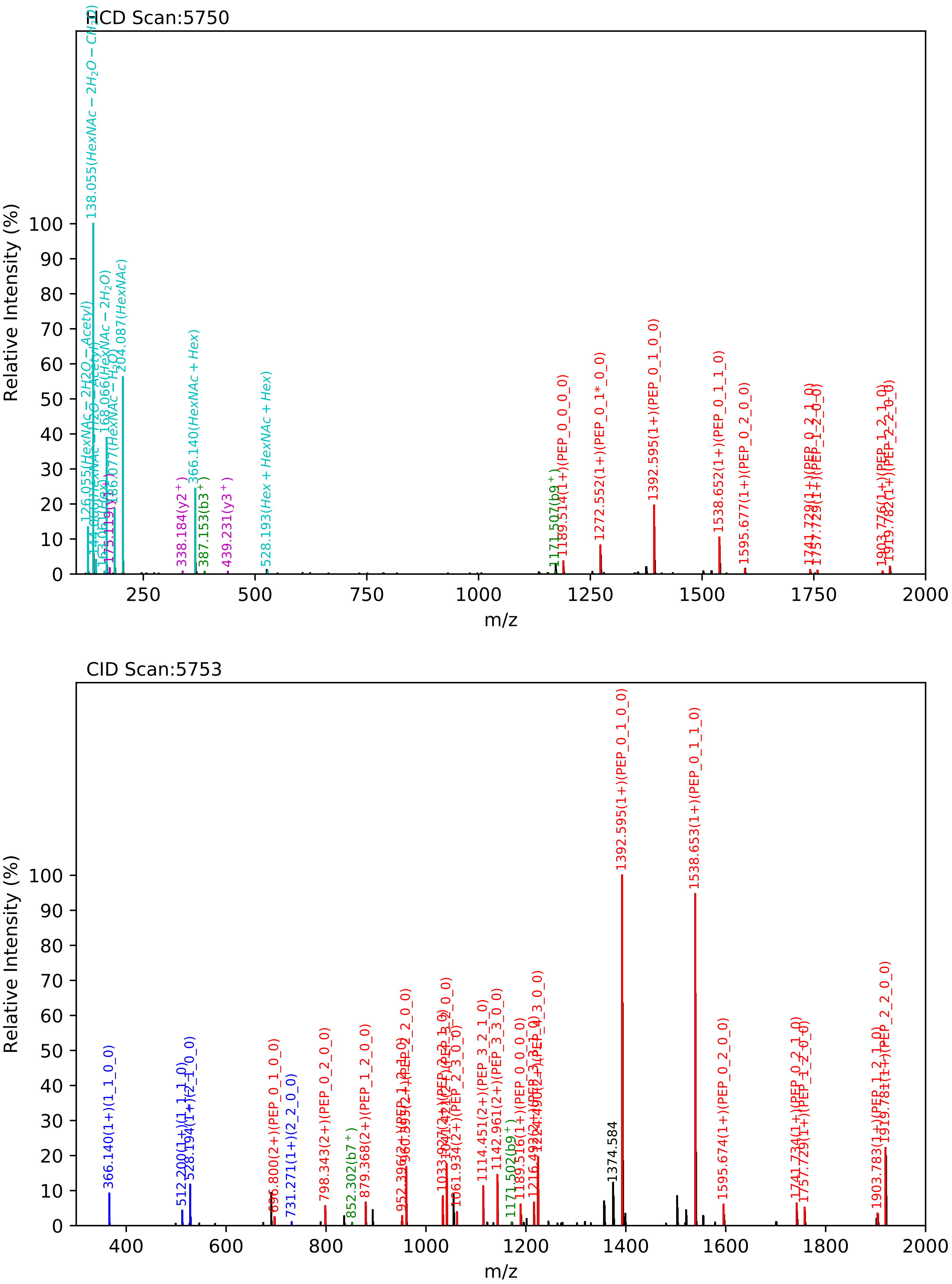

Unknown set no. 367, Experiment: Human Plasma exp_1

EEQYNSTYR(=PEP)_4_3_1_1, m/z:1442.56(2+), RT:37.83, Y-score:96.24

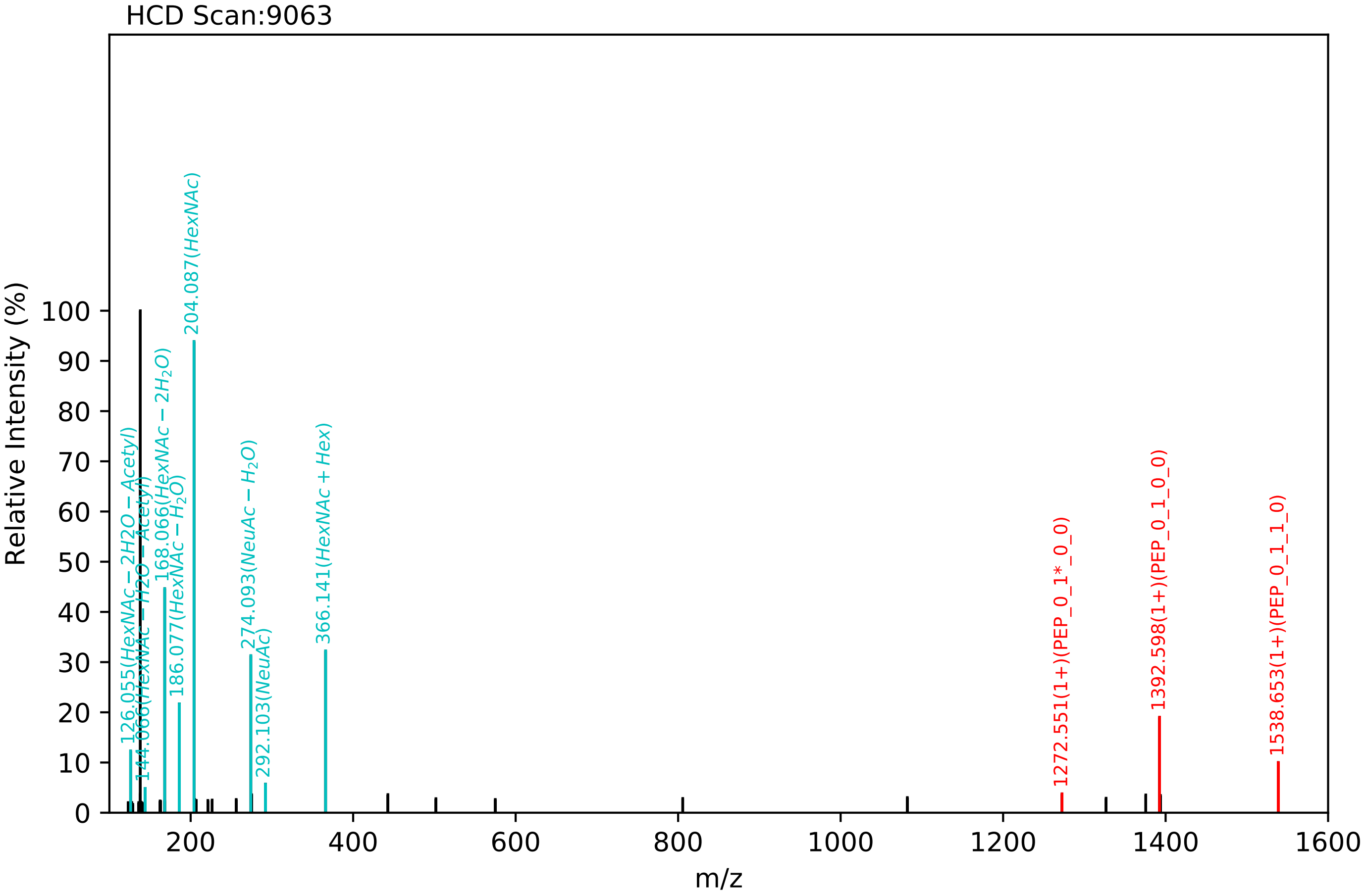

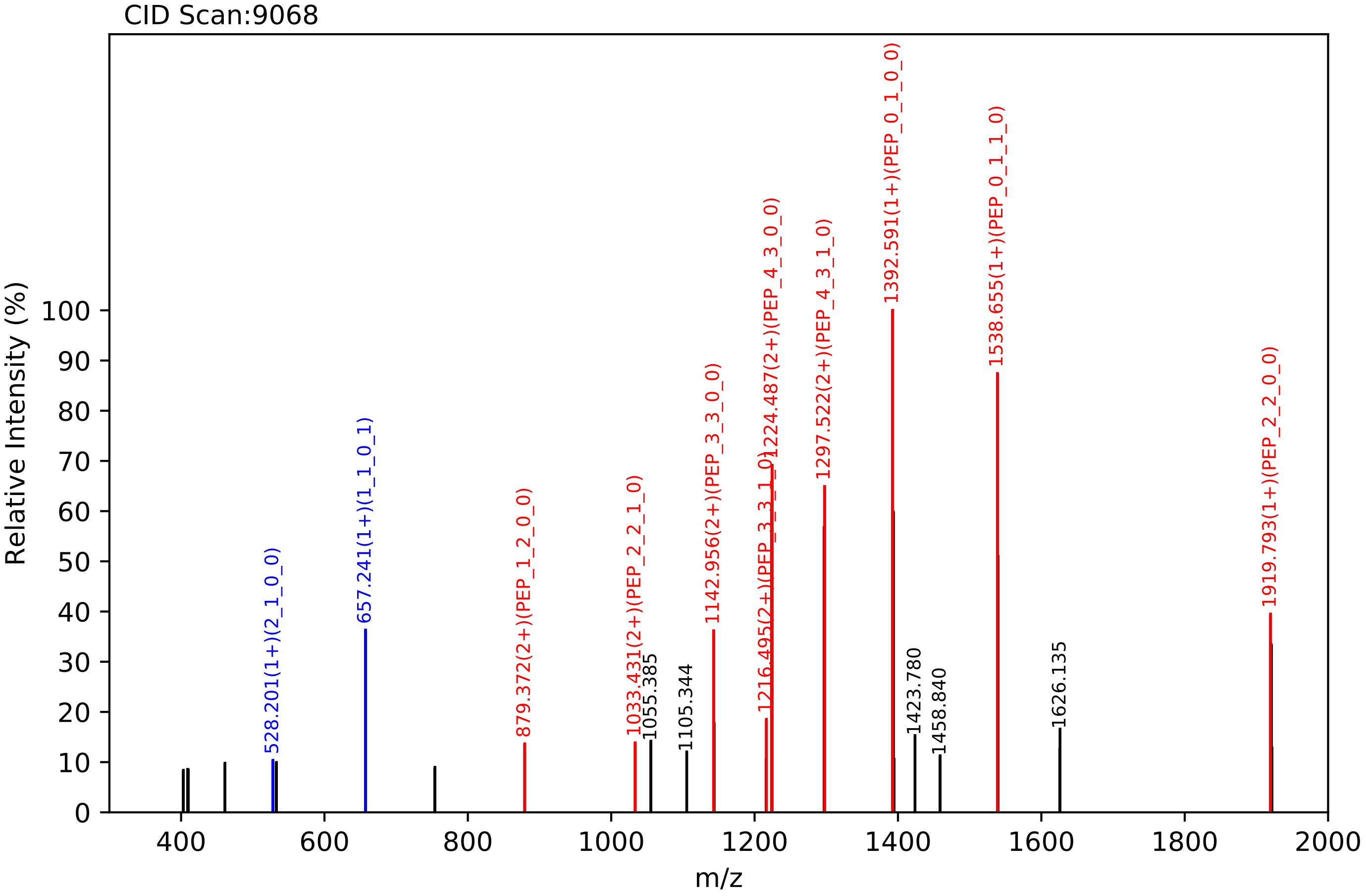

Unknown set no. 368, Experiment: Human Plasma exp_2

EEQYNSTYR(=PEP)_4_3_1_1, m/z:1442.56(2+), RT:38.01, Y-score:93.92

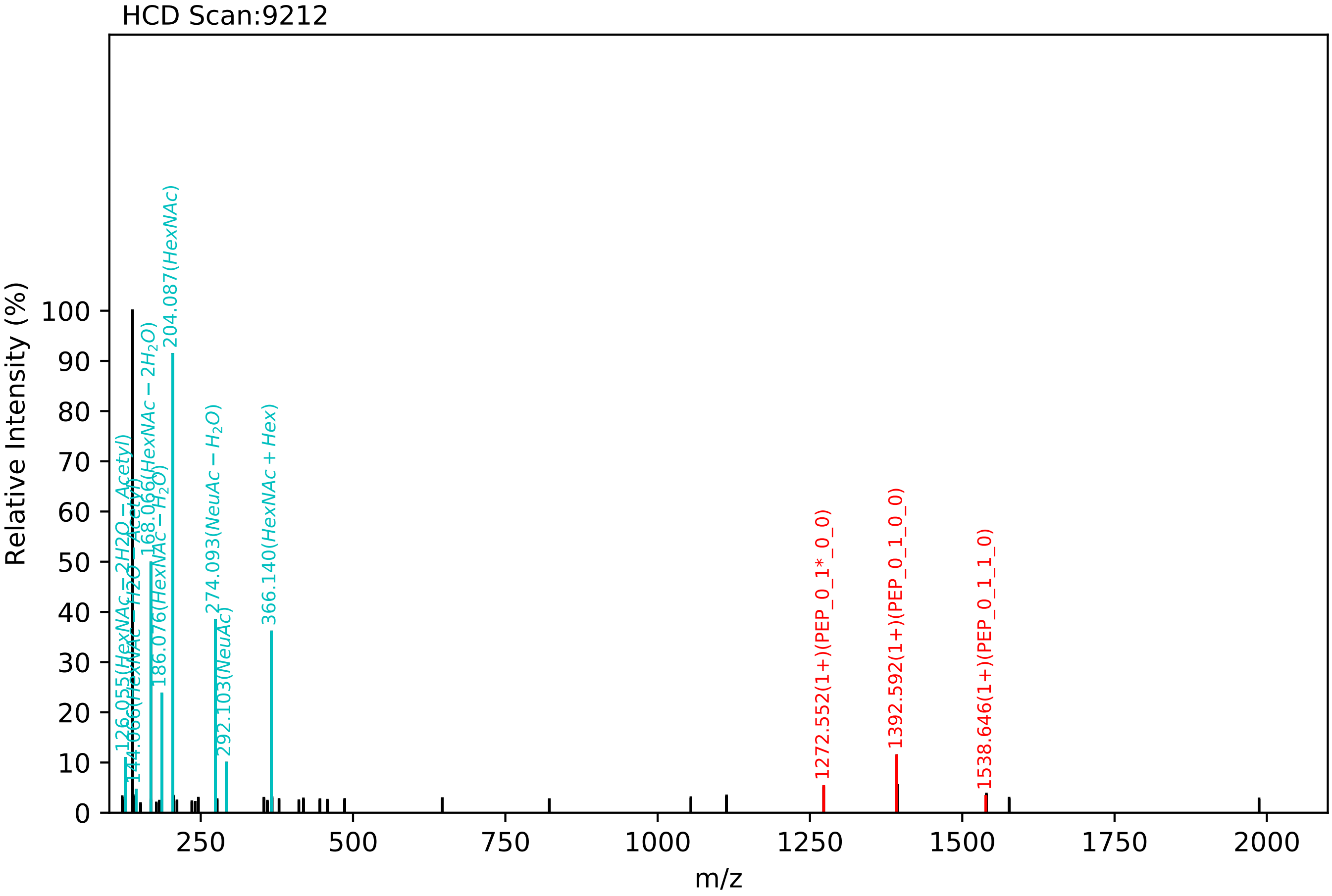

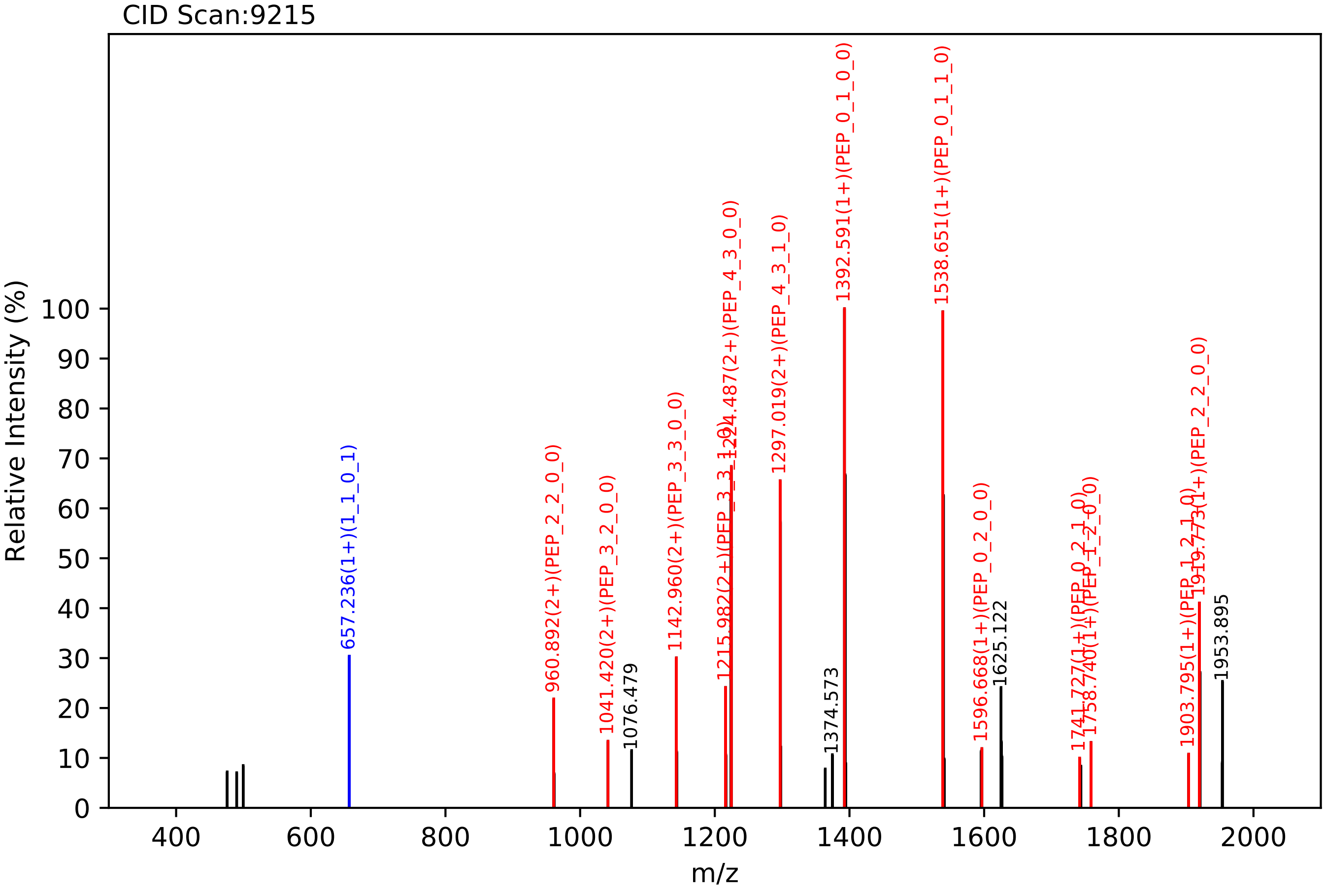

Unknown set no. 369, Experiment: Human Plasma exp_3

EEQYNSTYR(=PEP)_4_3_1_1, m/z:962.04(3+), RT:38.39, Y-score:86.30

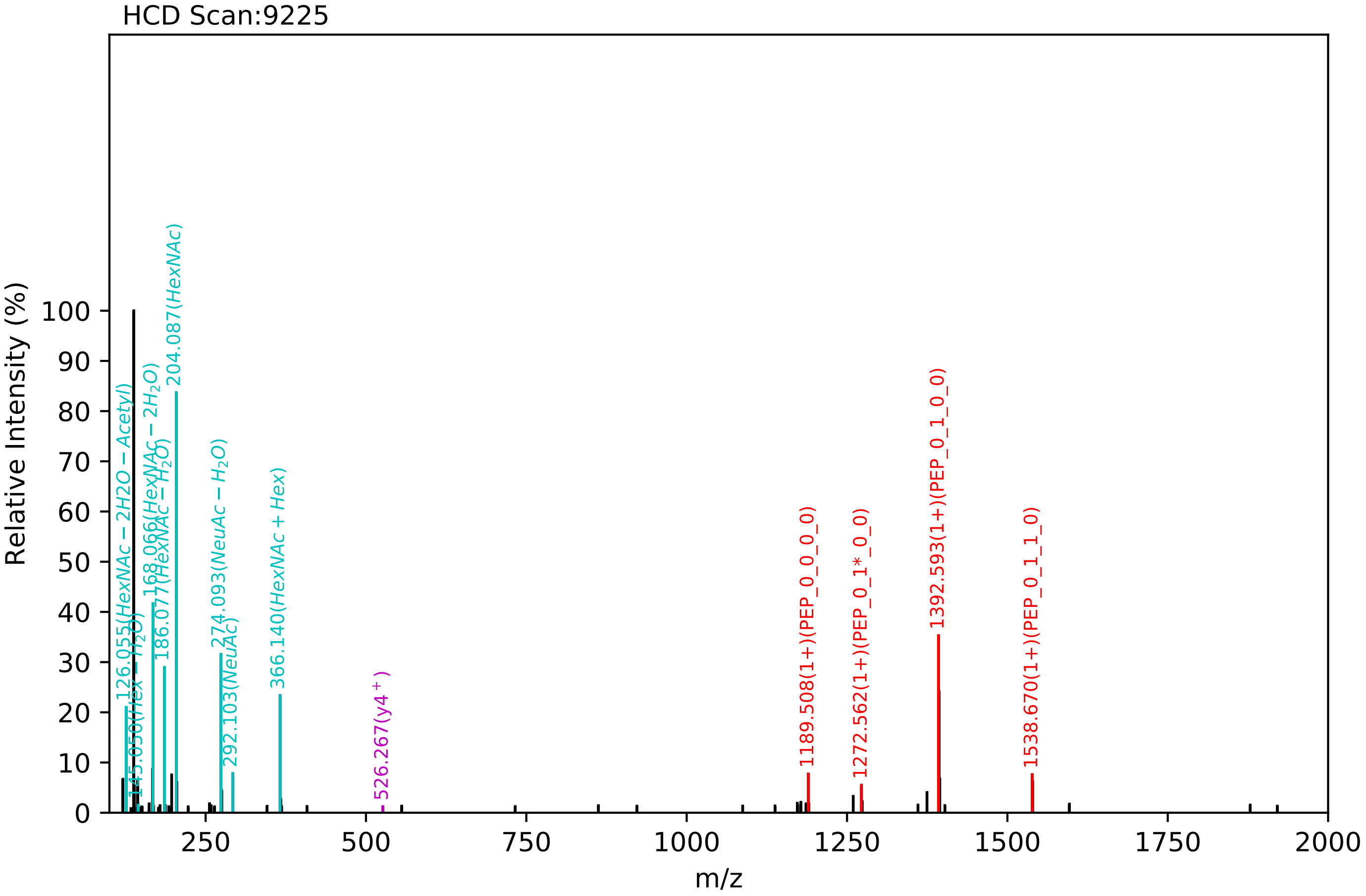

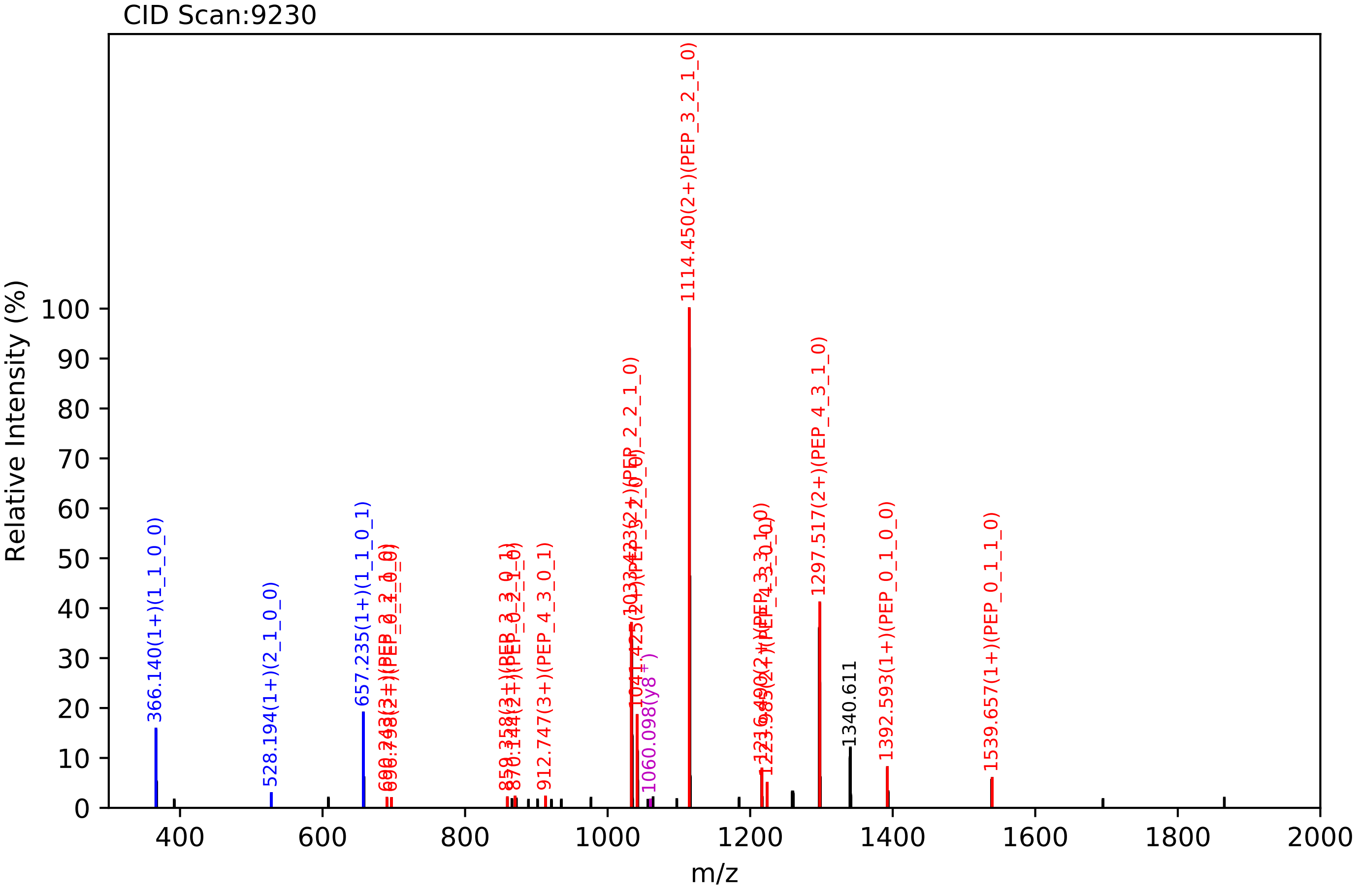

Unknown set no. 370, Experiment: Human Plasma exp_3

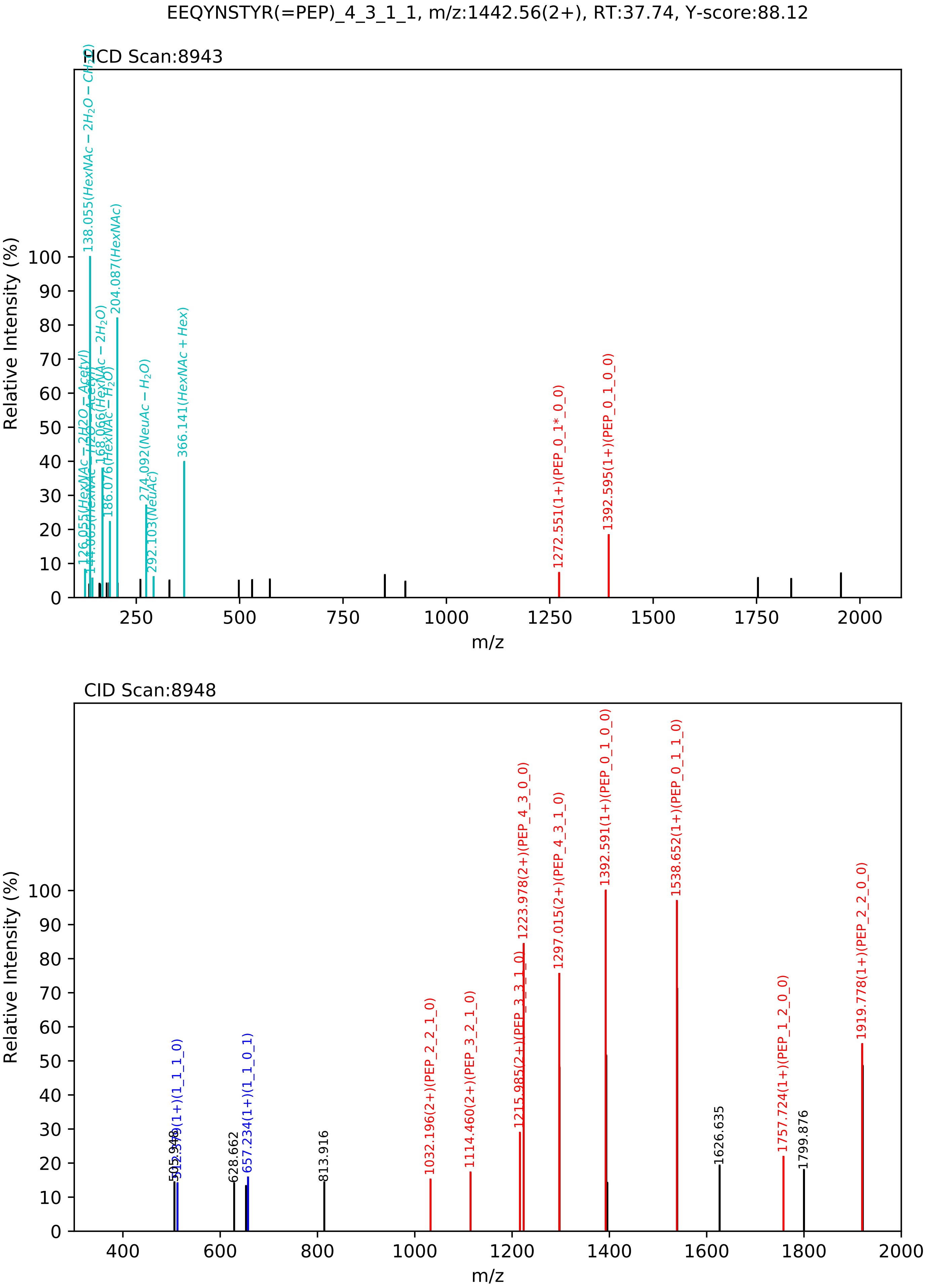

Unknown set no. 371, Experiment: Human Plasma exp_4

EEQYNSTYR(=PEP)_4_3_1_1, m/z:1442.56(2+), RT:38.35, Y-score:89.75

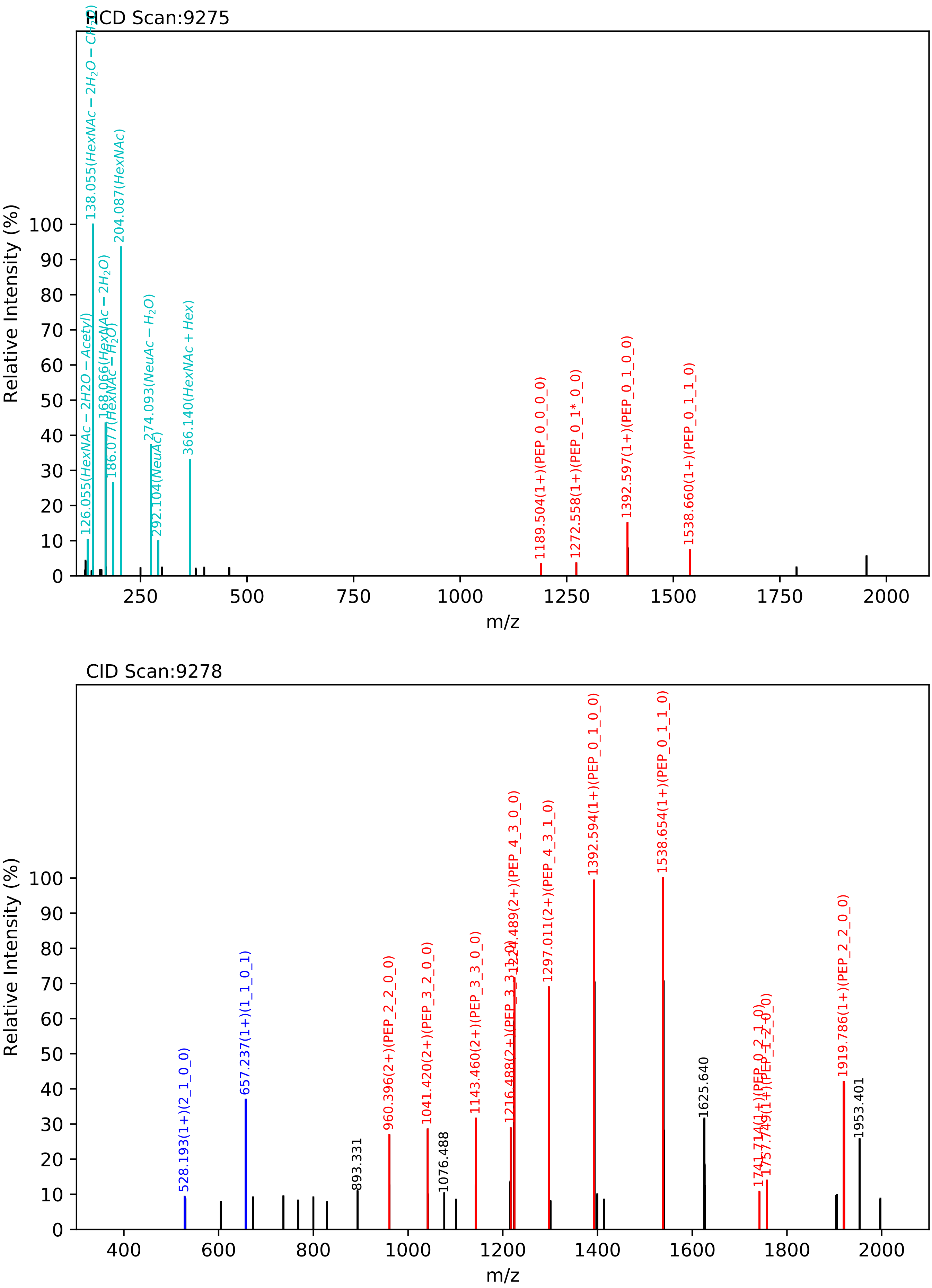

Unknown set no. 372, Experiment: Human Plasma exp_5

EEQYNSTYR(=PEP)_4_3_1_1, m/z:962.04(3+), RT:38.54, Y-score:95.87

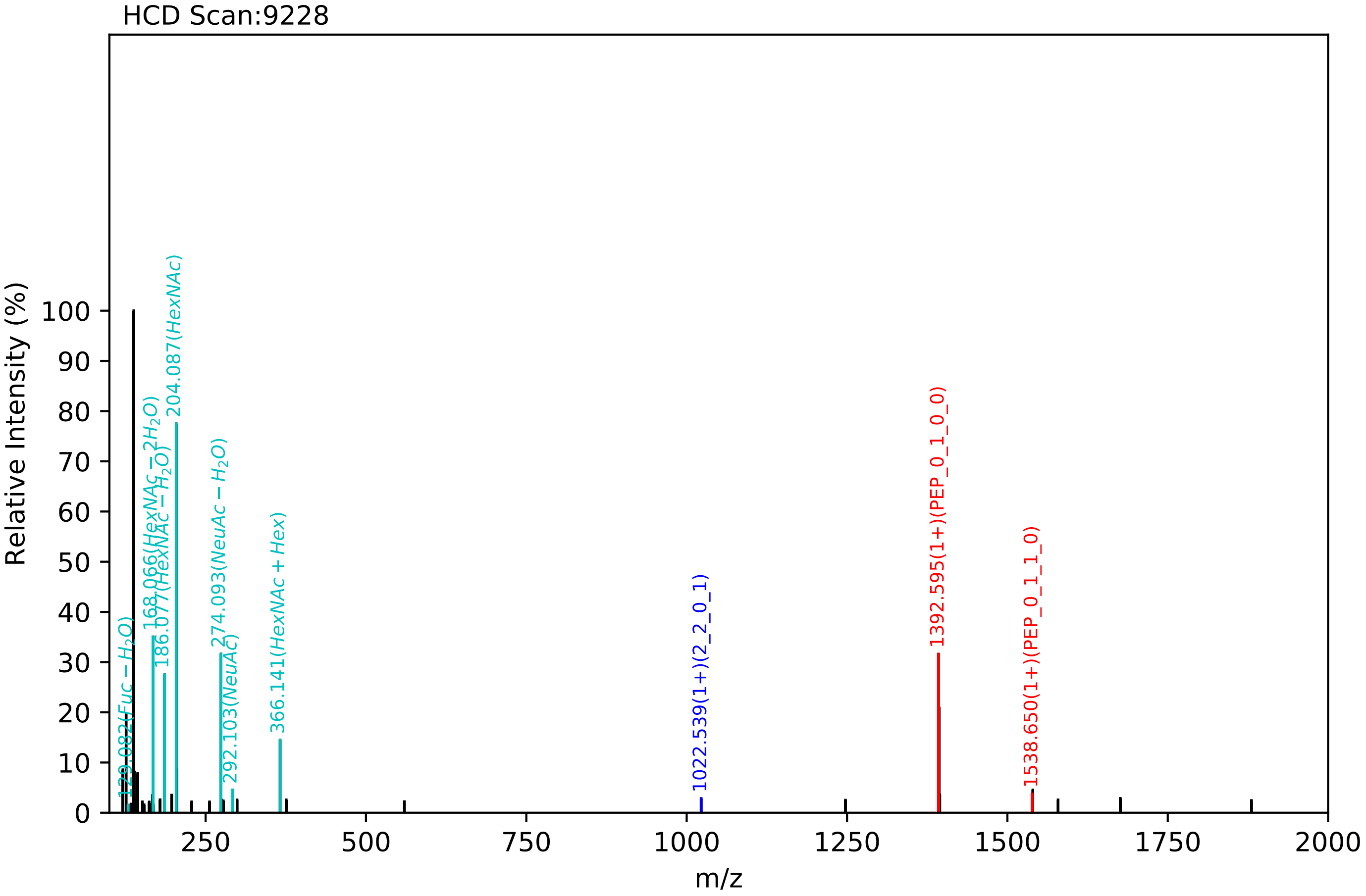

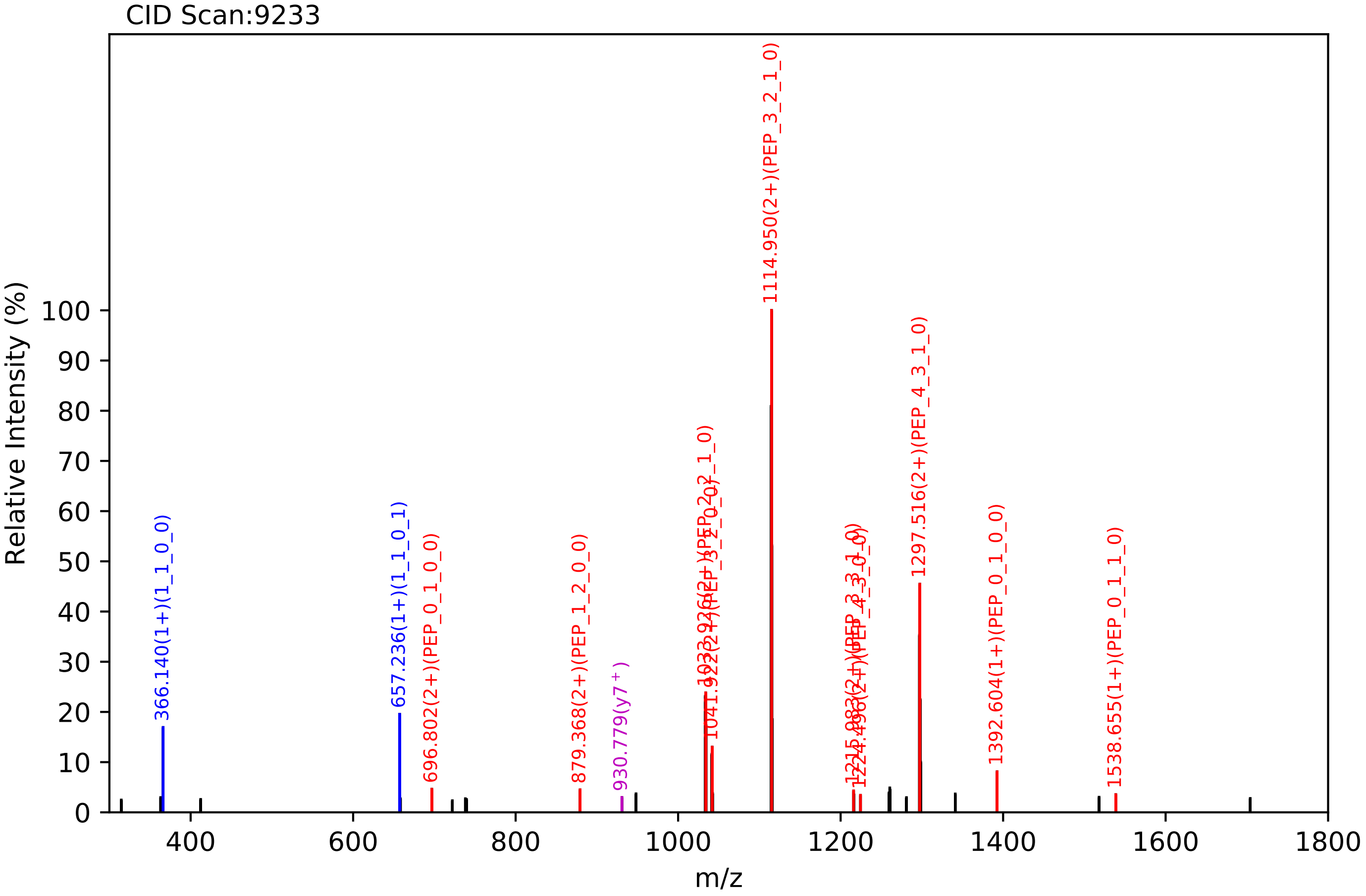

Unknown set no. 373, Experiment: Human Plasma exp_5

EEQYNSTYR(=PEP)_4_3_1_1, m/z:1443.06(2+), RT:38.41, Y-score:90.65

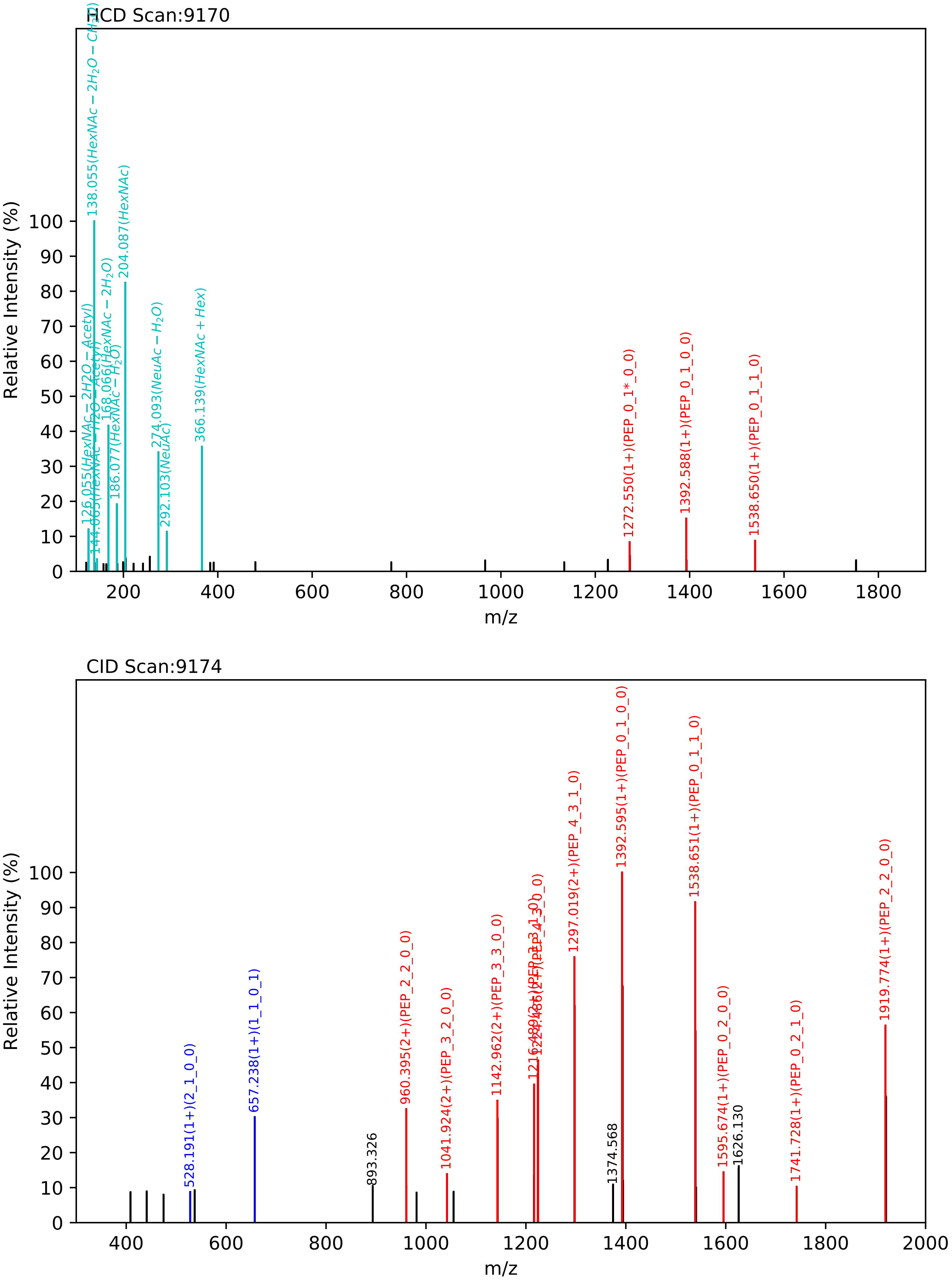

Unknown set no. 374, Experiment: Human Plasma exp_3

EEQYNSTYR(=PEP)_4_4_0_0, m/z:1325.53(2+), RT:24.97, Y-score:88.64

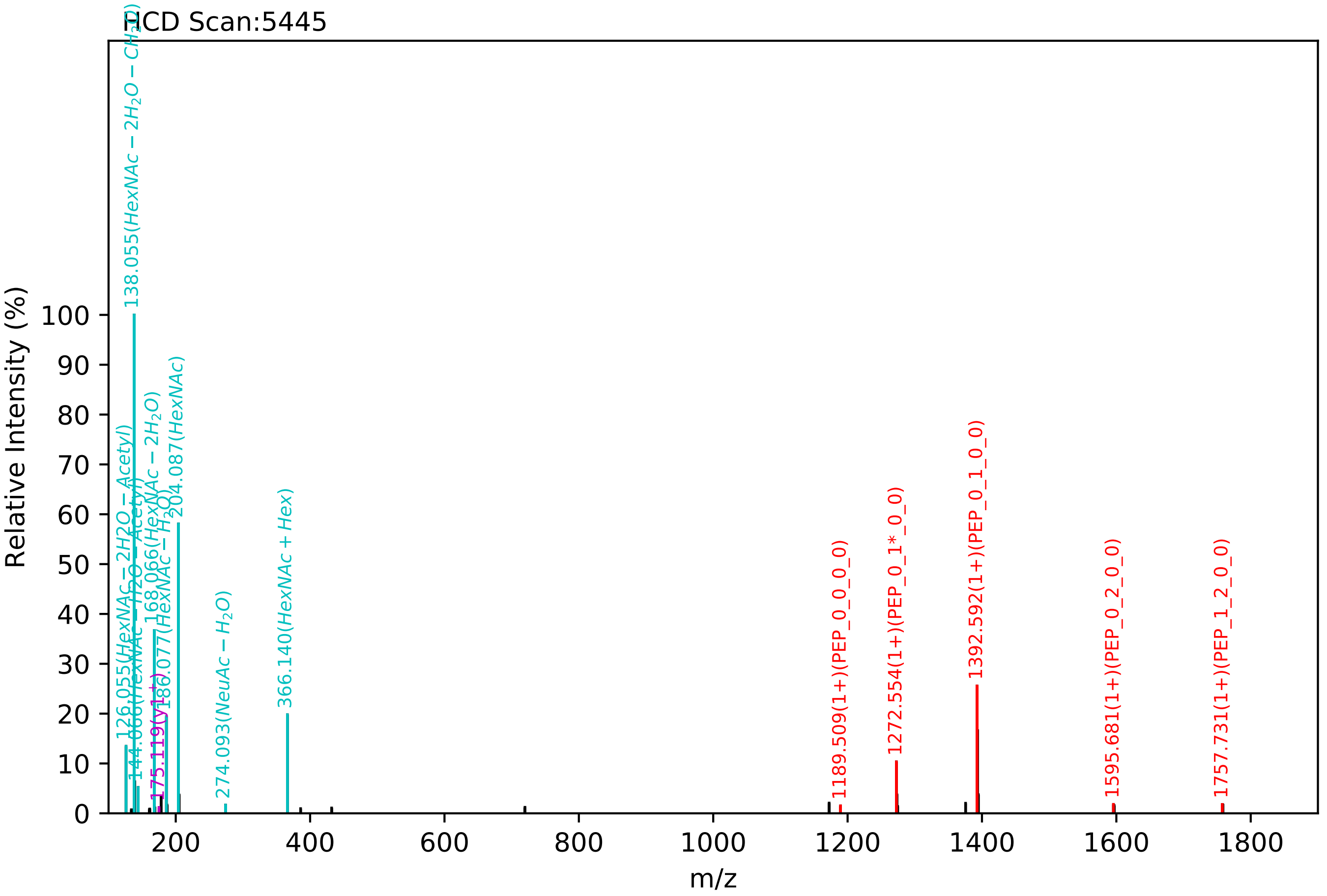

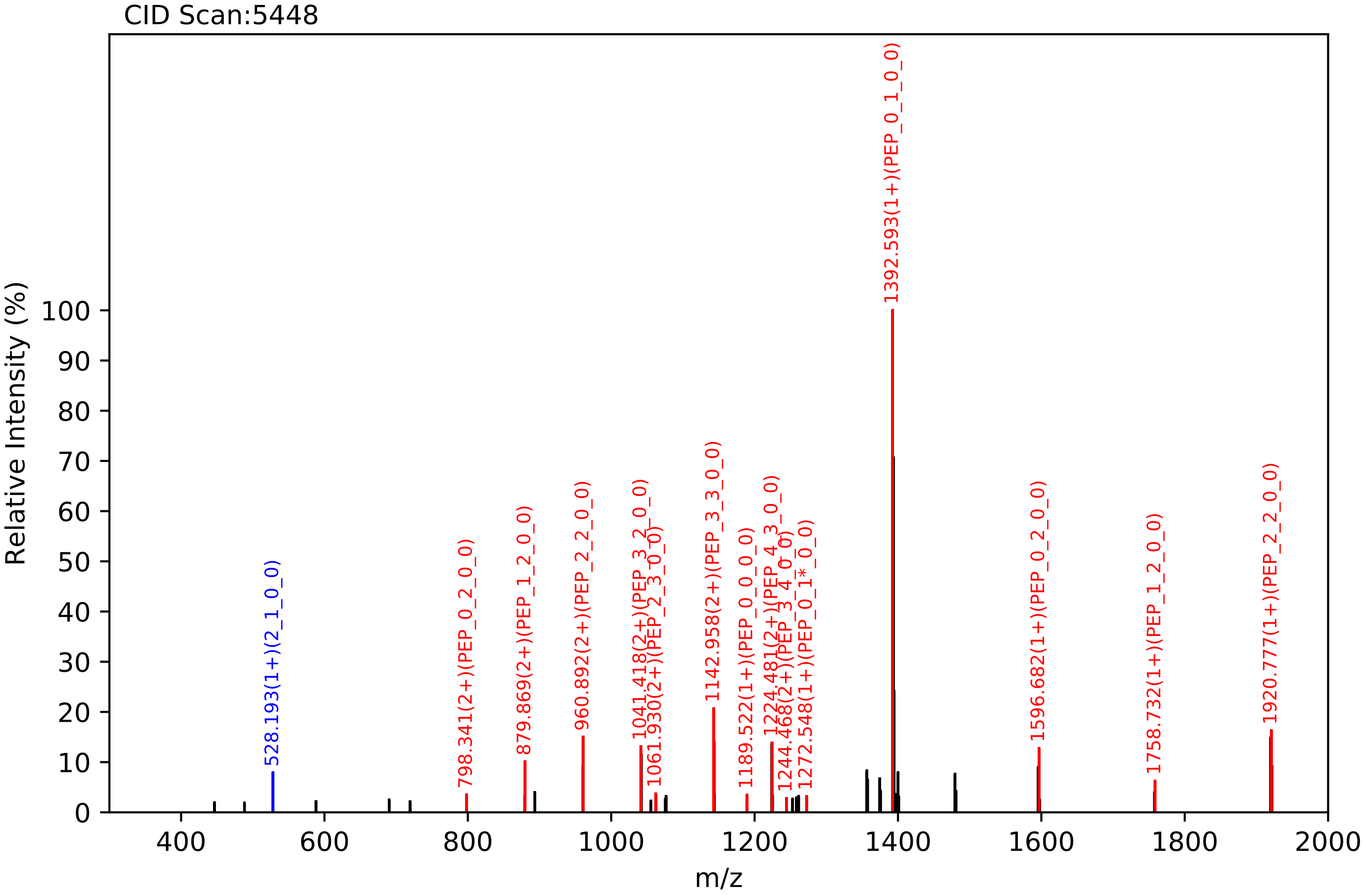

Unknown set no. 375, Experiment: Human Plasma exp_5

EEQYNSTYR(=PEP)_4_4_0_0, m/z:1325.52(2+), RT:24.96, Y-score:86.43

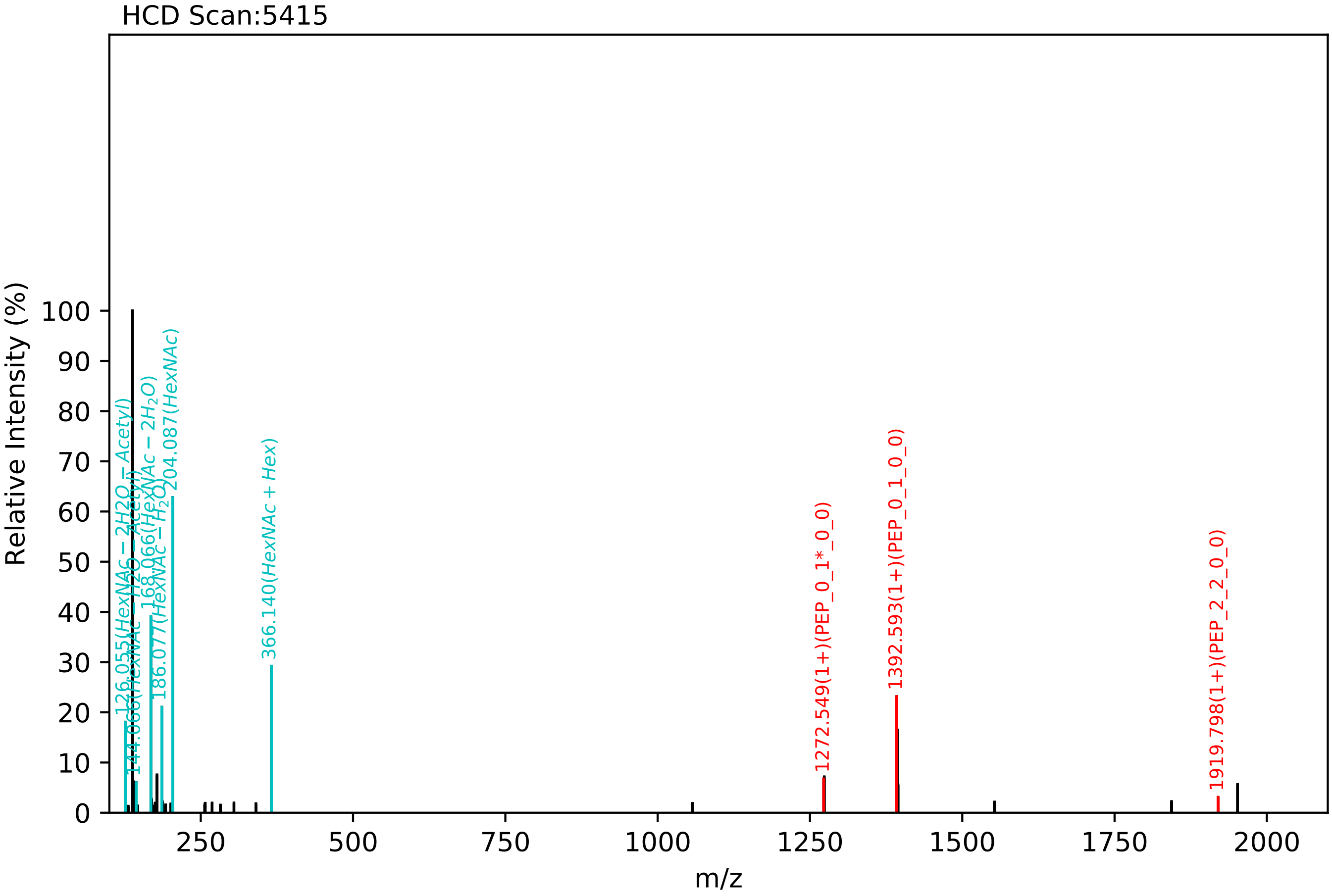

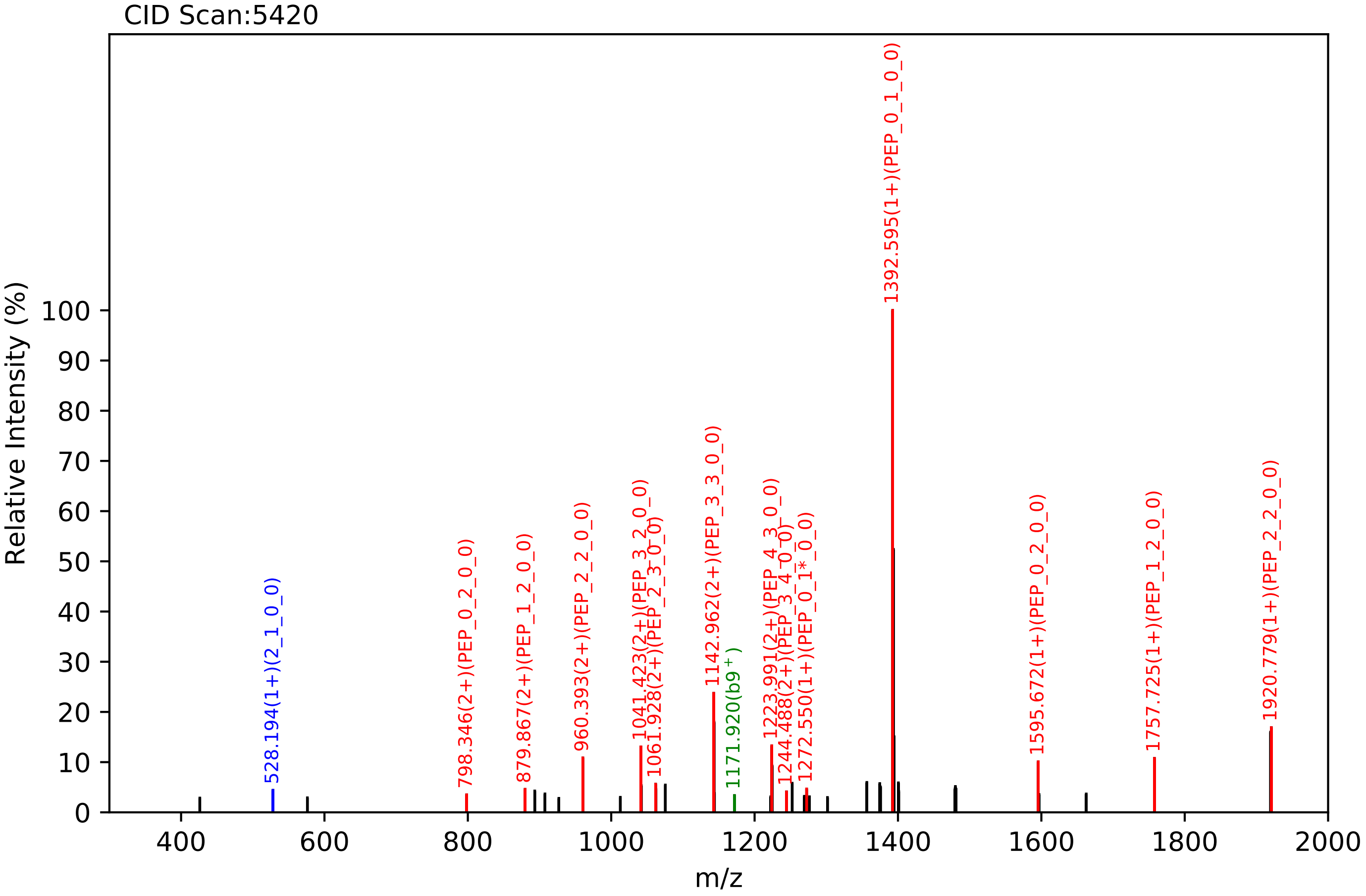

Unknown set no. 376, Experiment: Human Plasma exp_1

EEQYNSTYR(=PEP)_4_4_1_0, m/z:932.71(3+), RT:25.10, Y-score:98.69

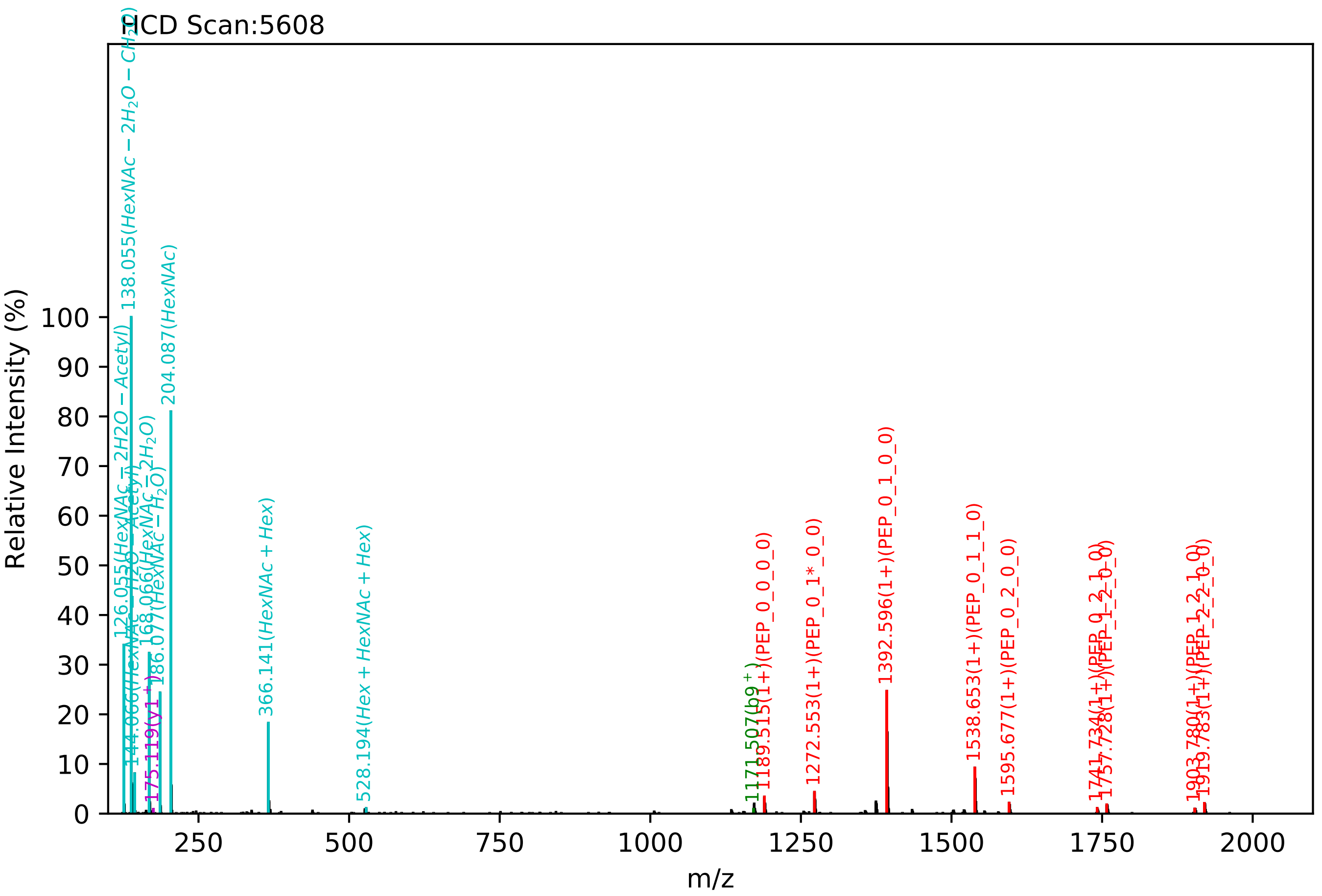

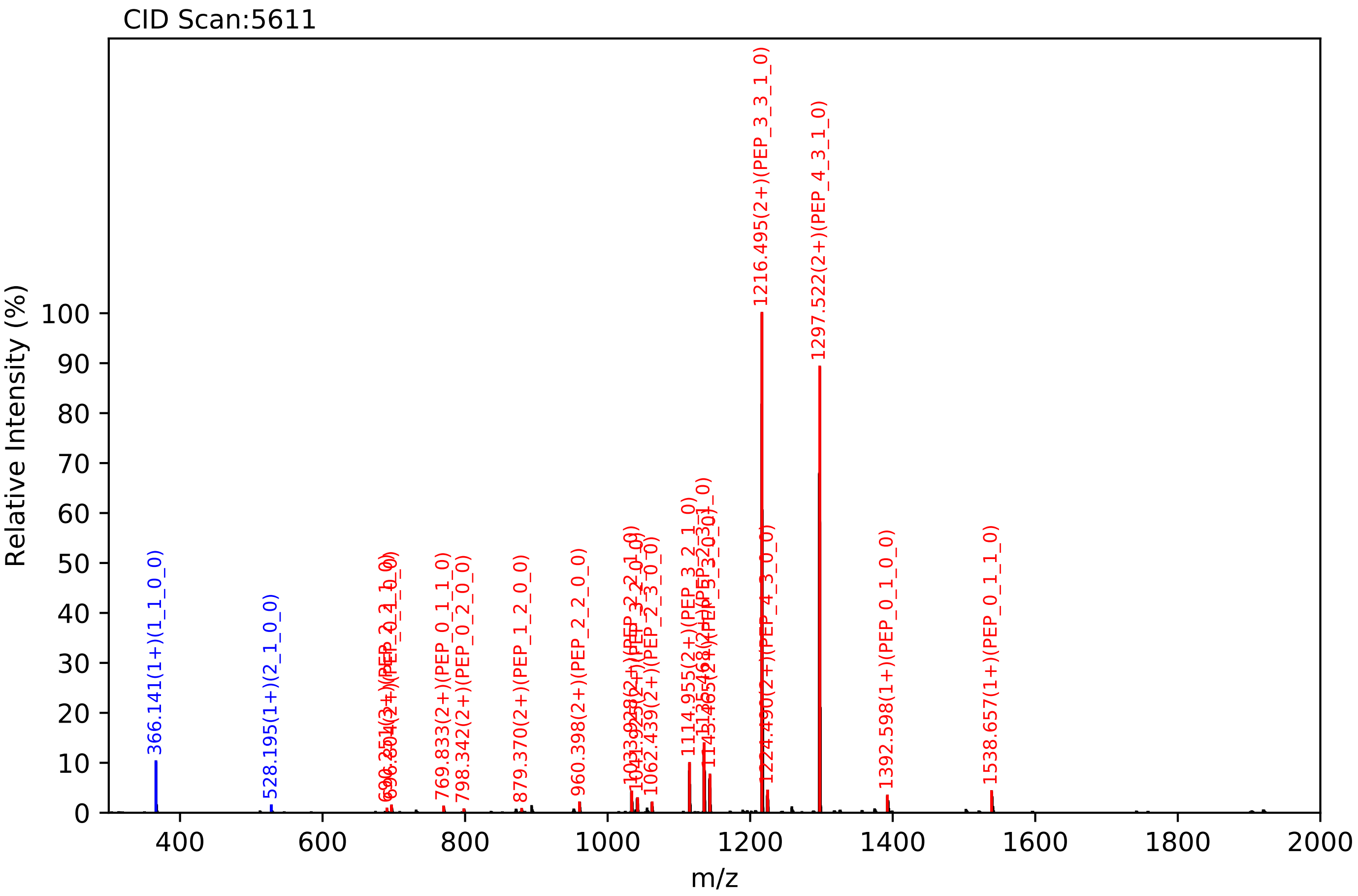

Unknown set no. 377, Experiment: Human Plasma exp_1

EEQYNSTYR(=PEP)_4_4_1_0, m/z:1398.56(2+), RT:22.92, Y-score:97.94

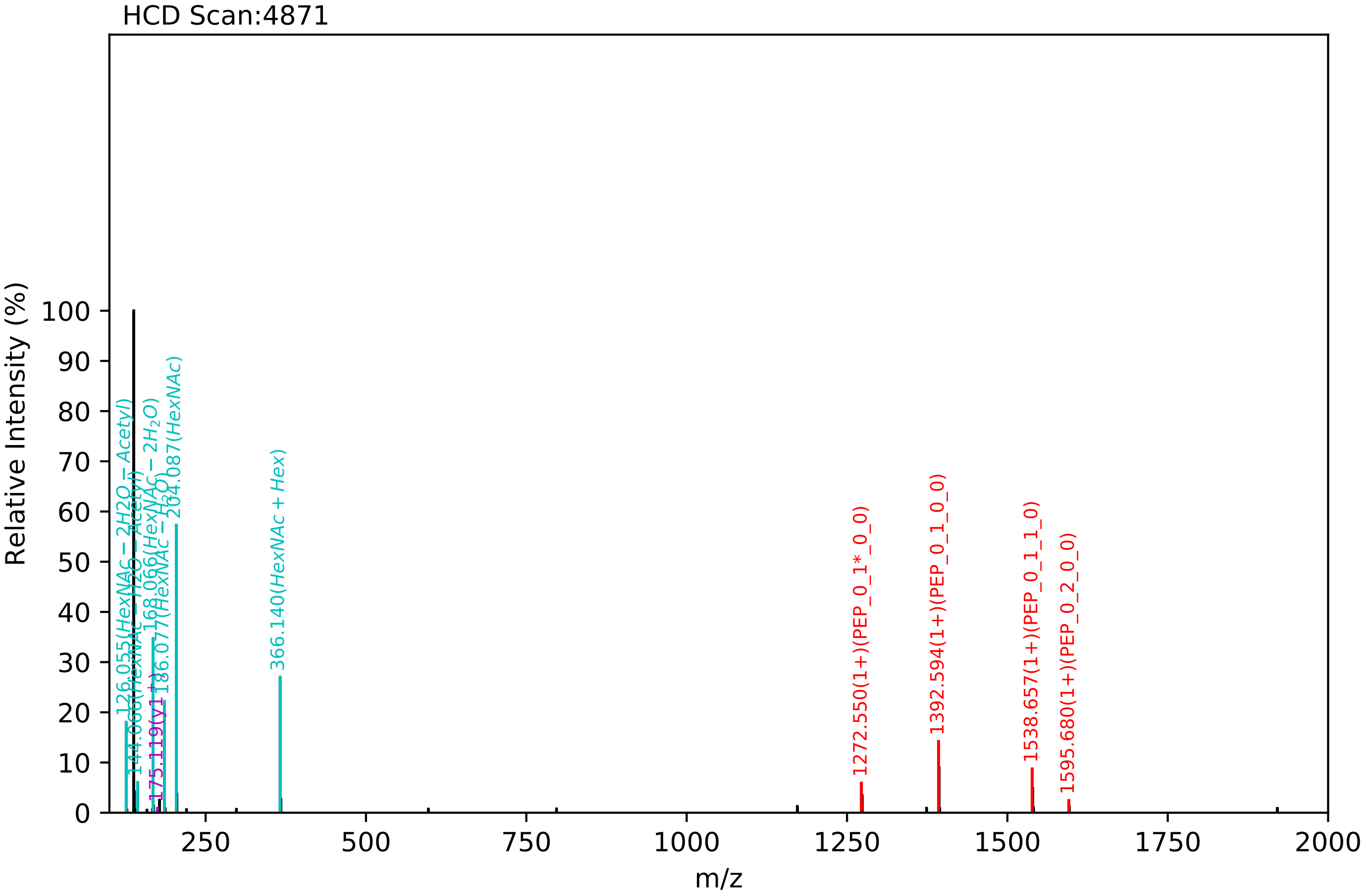

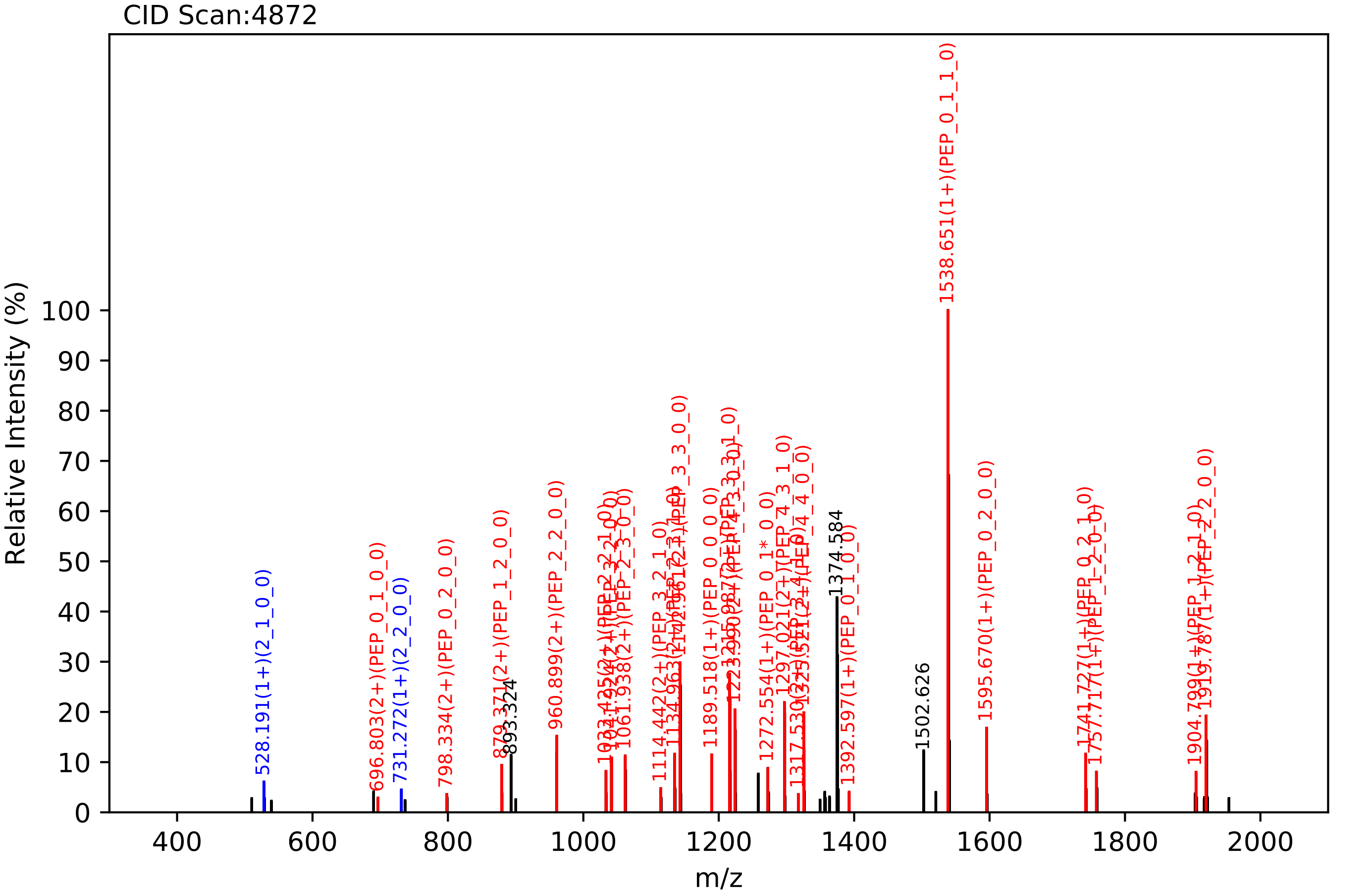

Unknown set no. 378, Experiment: Human Plasma exp_2

EEQYNSTYR(=PEP)_4_4_1_0, m/z:1398.56(2+), RT:26.37, Y-score:95.35

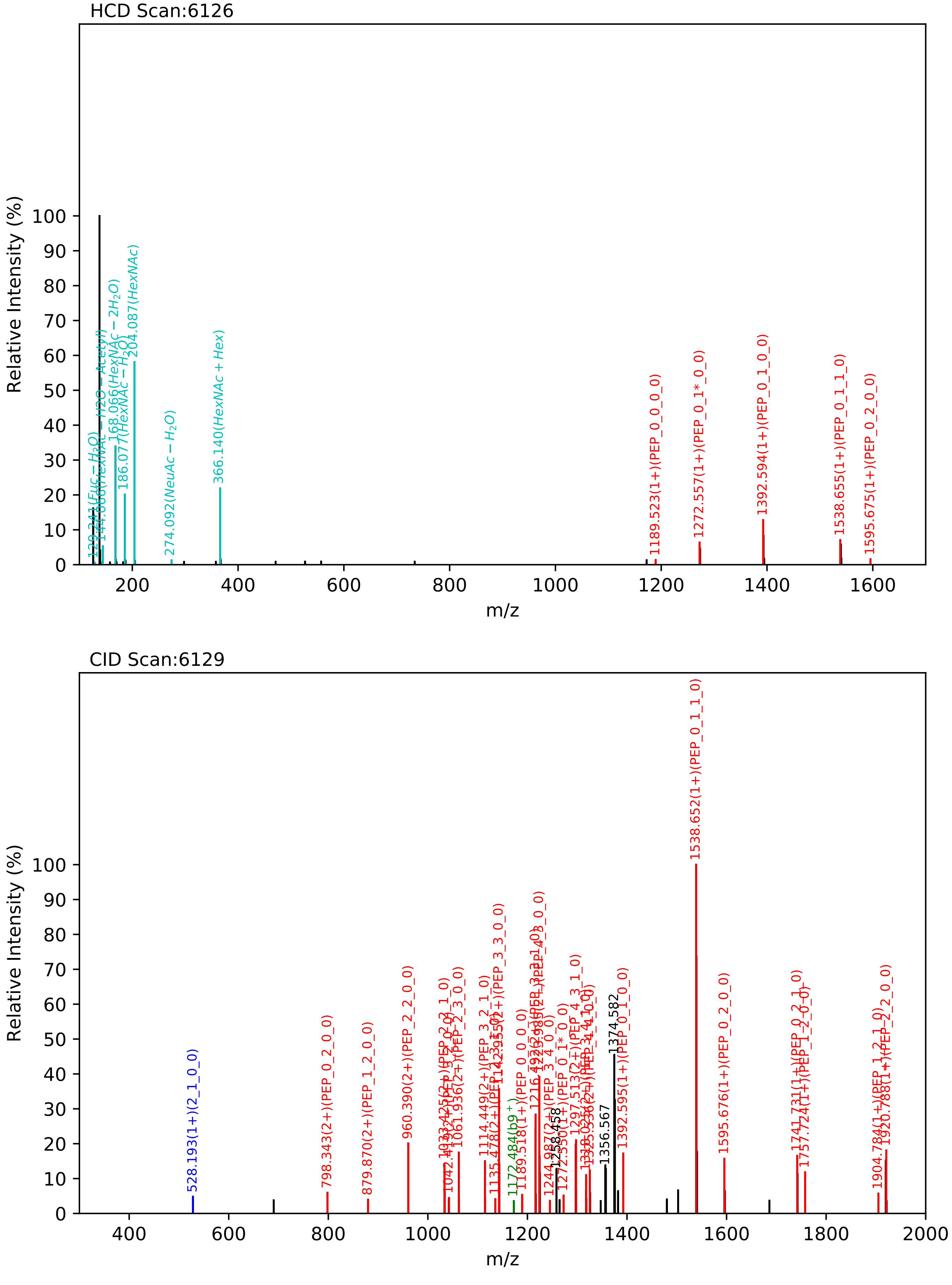

Unknown set no. 379, Experiment: Human Plasma exp_3

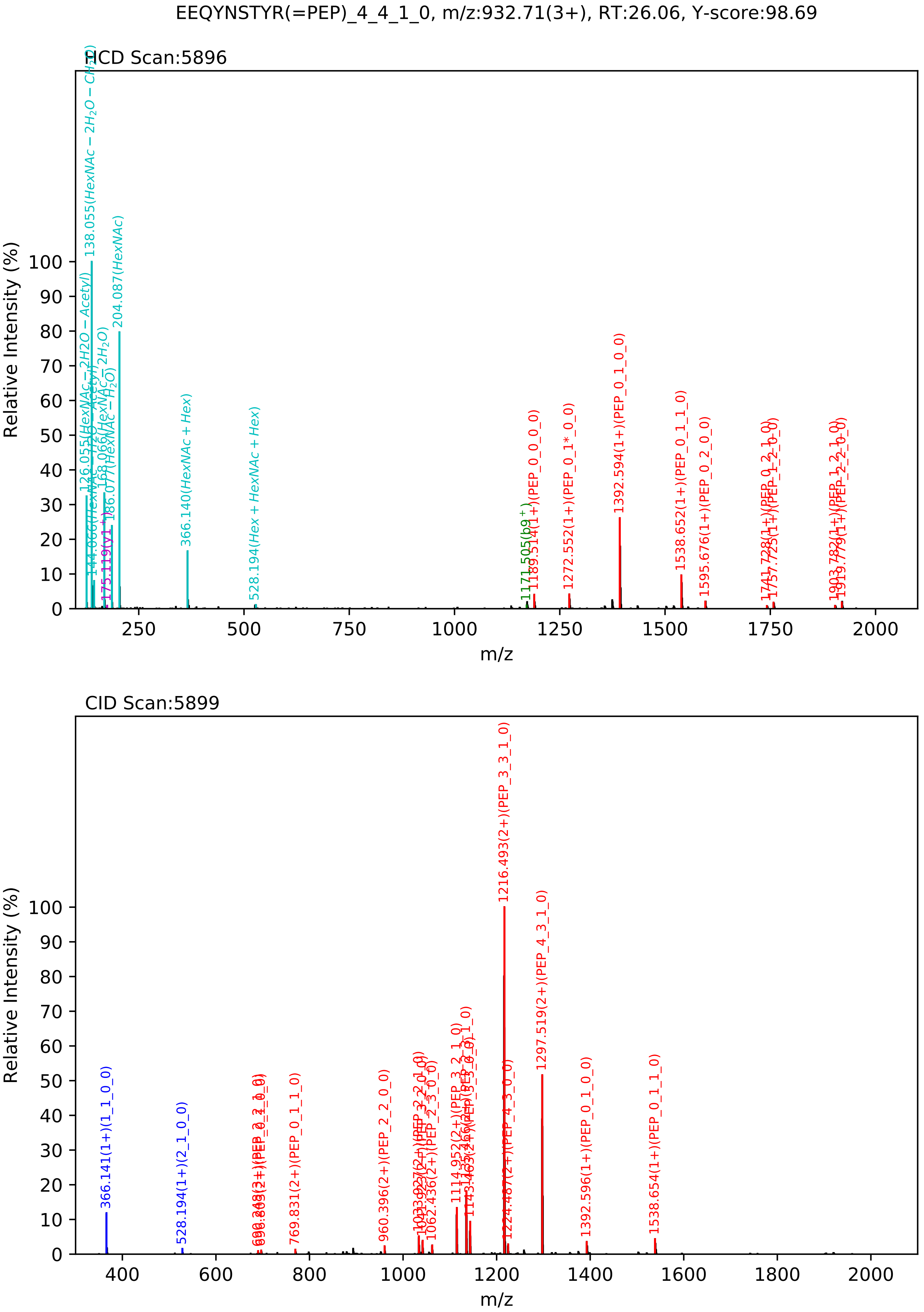

Unknown set no. 380, Experiment: Human Plasma exp_3

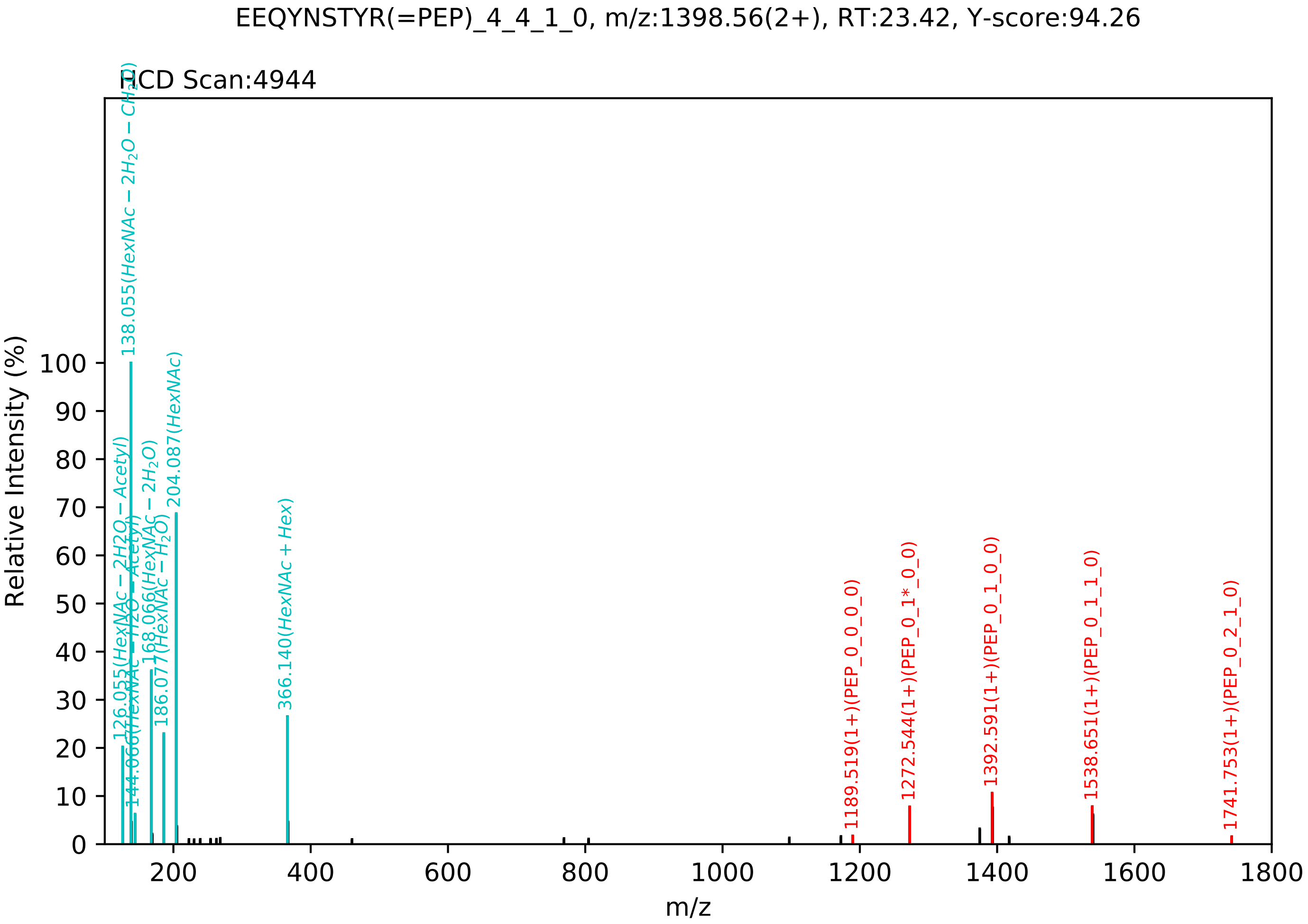

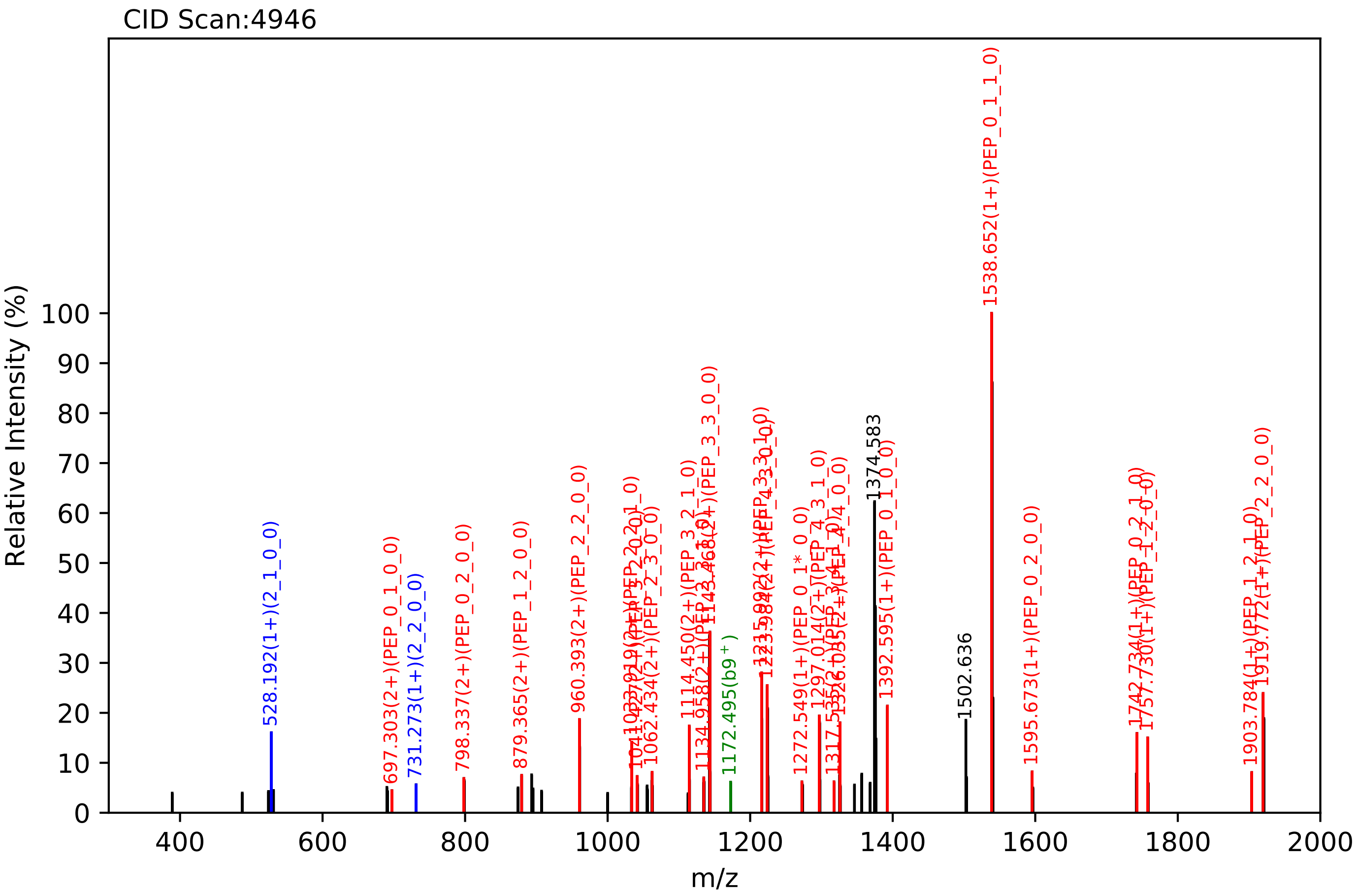

Unknown set no. 381, Experiment: Human Plasma exp_4

EEQYNSTYR(=PEP)_4_4_1_0, m/z:1398.56(2+), RT:26.93, Y-score:100.00

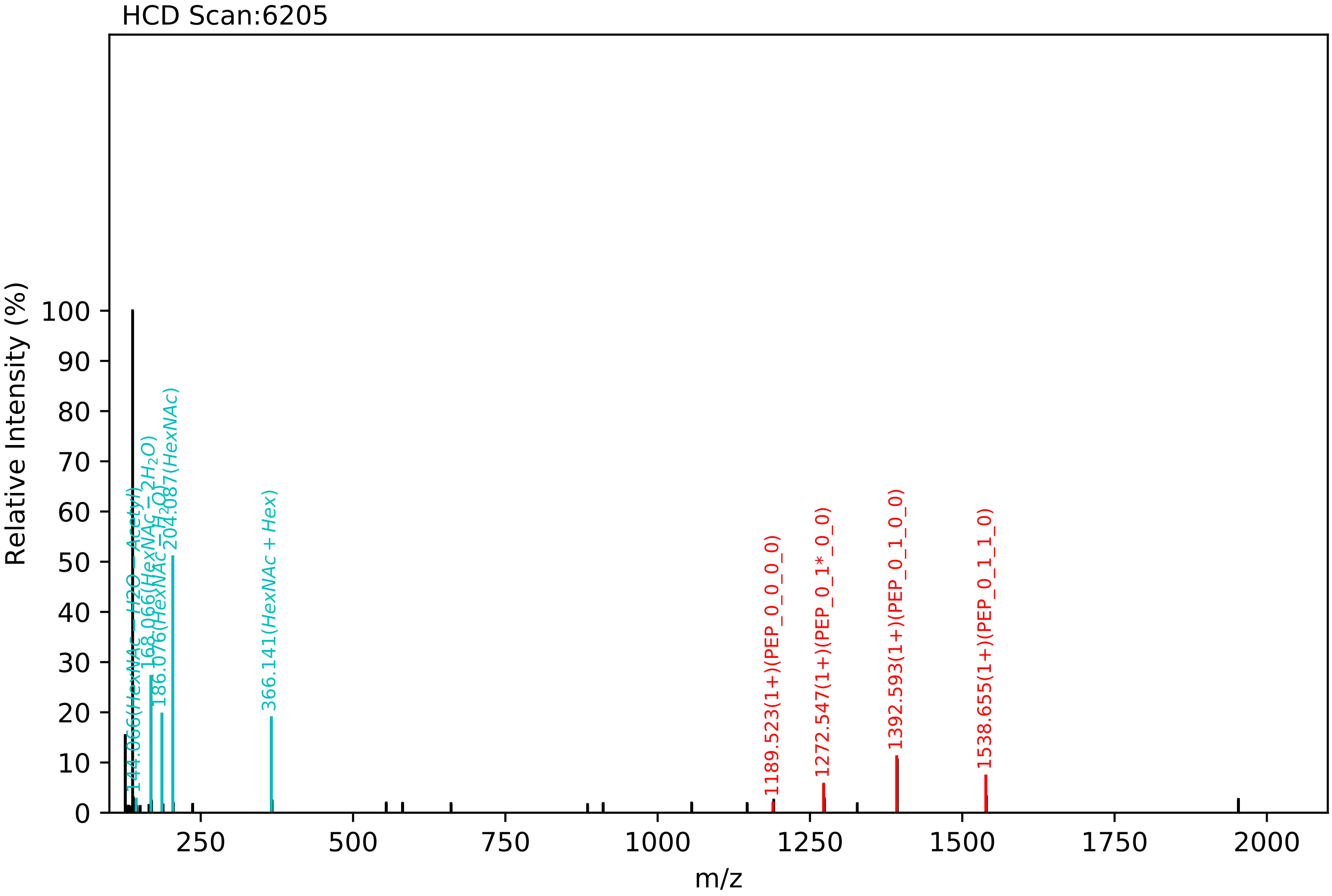

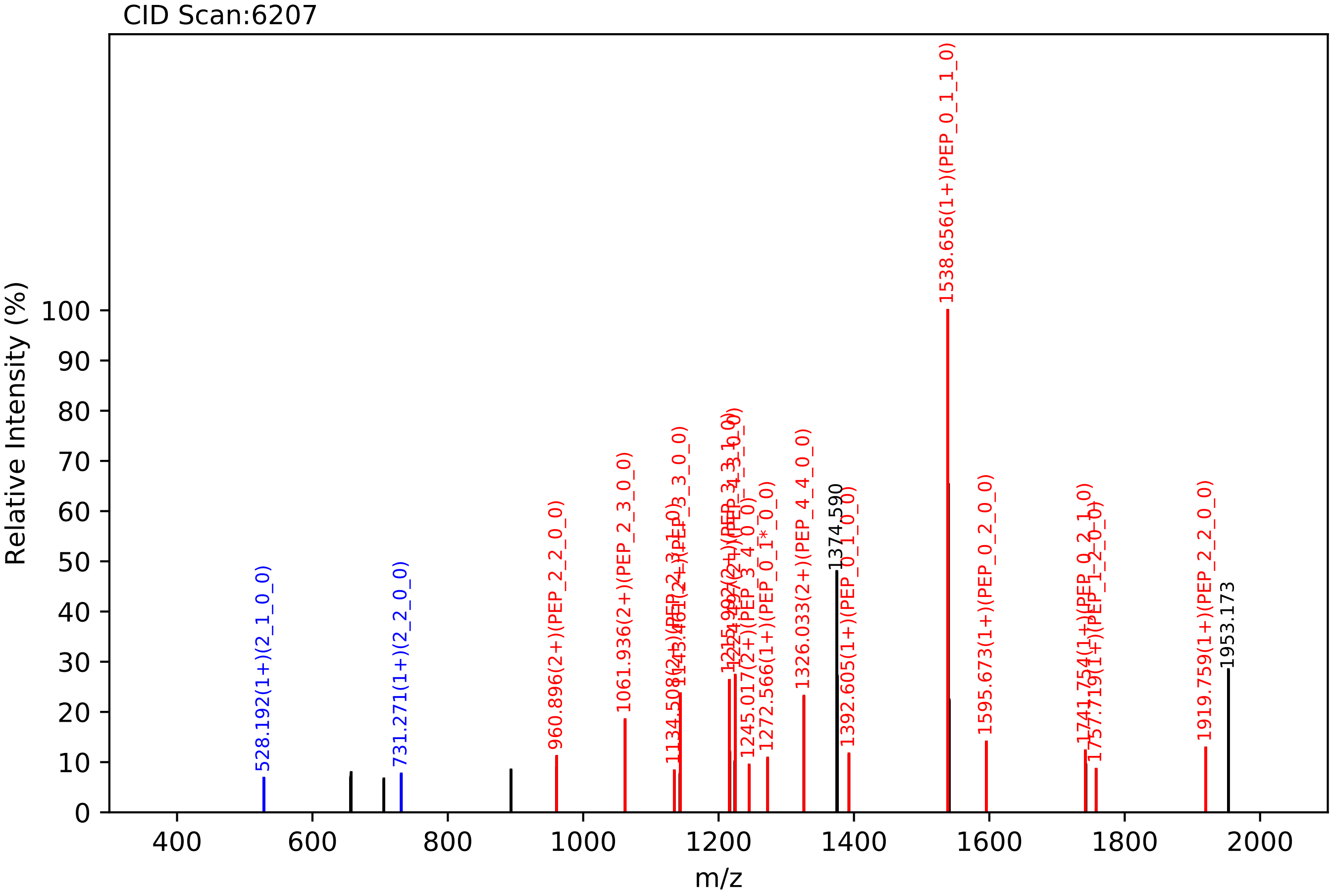

Unknown set no. 382, Experiment: Human Plasma exp_5

EEQYNSTYR(=PEP)_4_4_1_0, m/z:932.71(3+), RT:25.66, Y-score:98.66

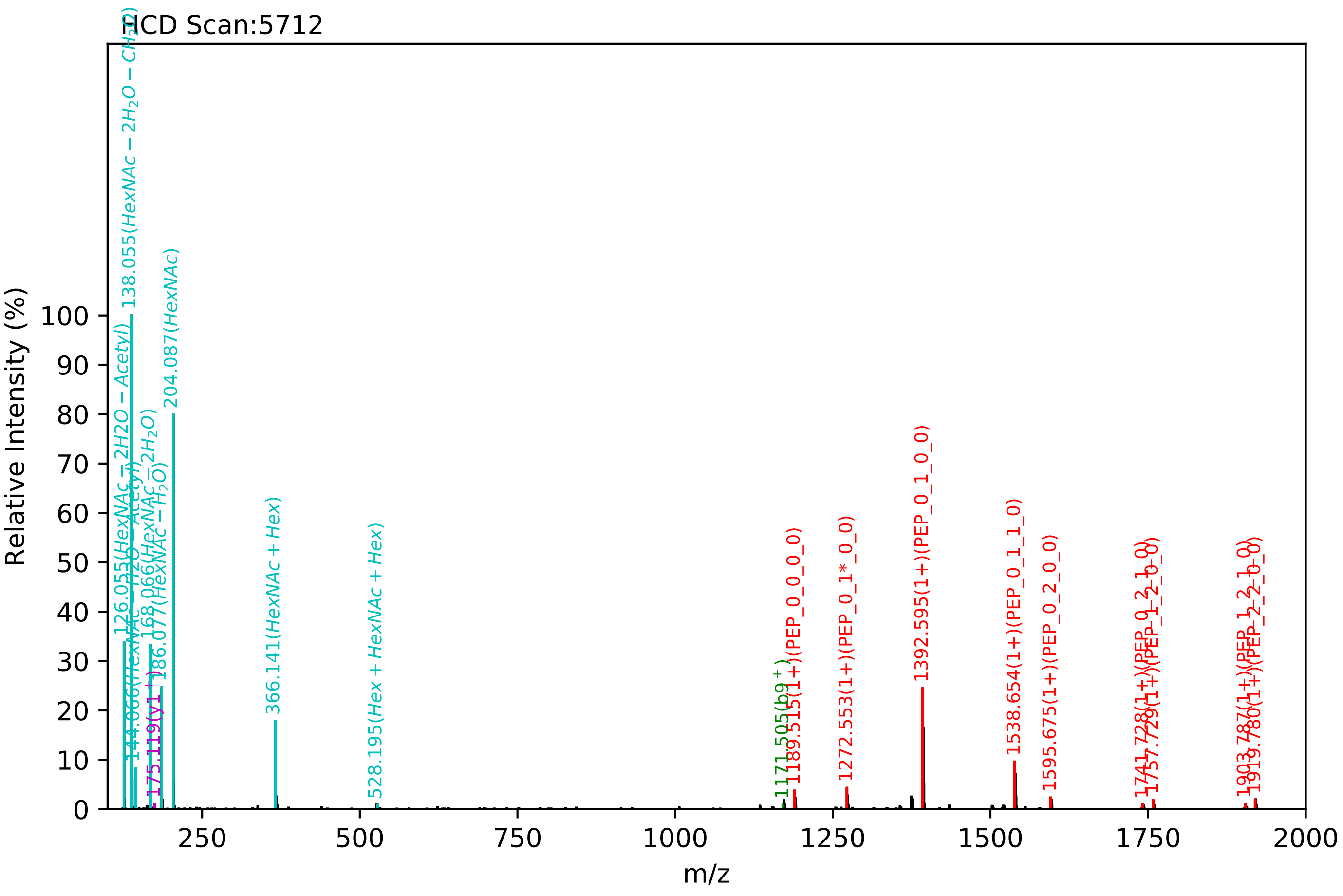

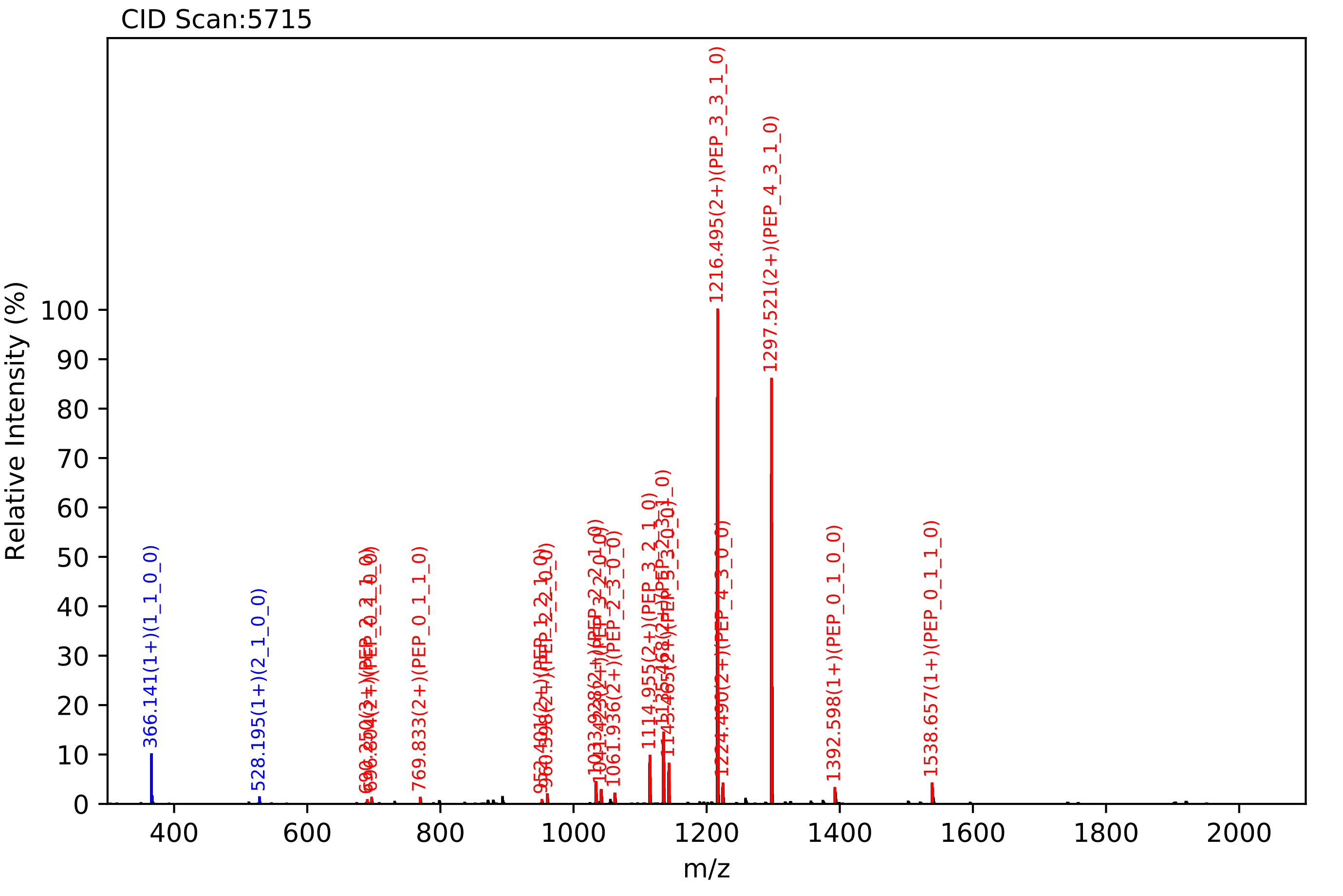

Unknown set no. 383, Experiment: Human Plasma exp_1

EEQYNSTYR(=PEP)_4_4_1_1, m/z:1029.74(3+), RT:38.10, Y-score:93.88

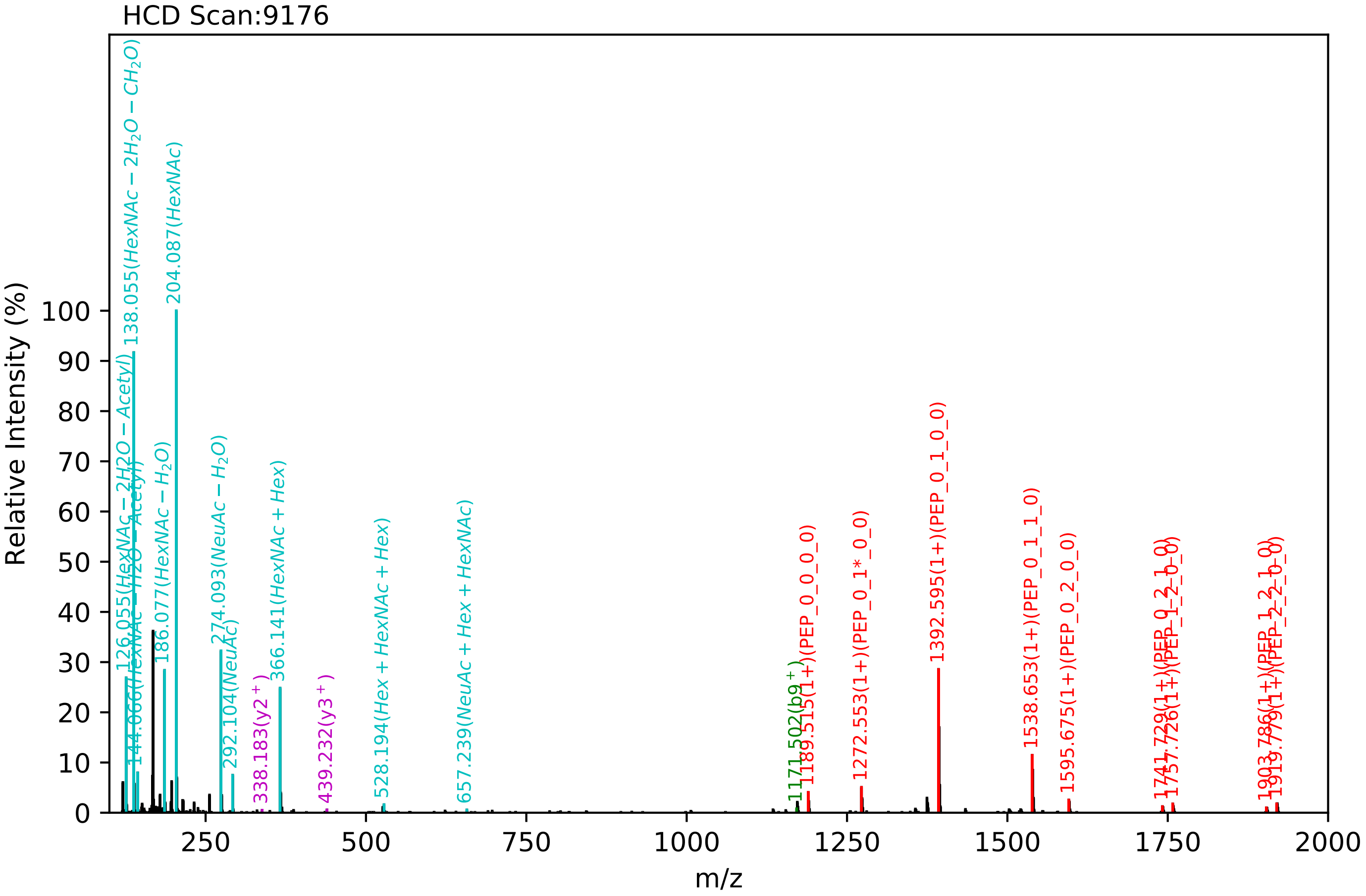

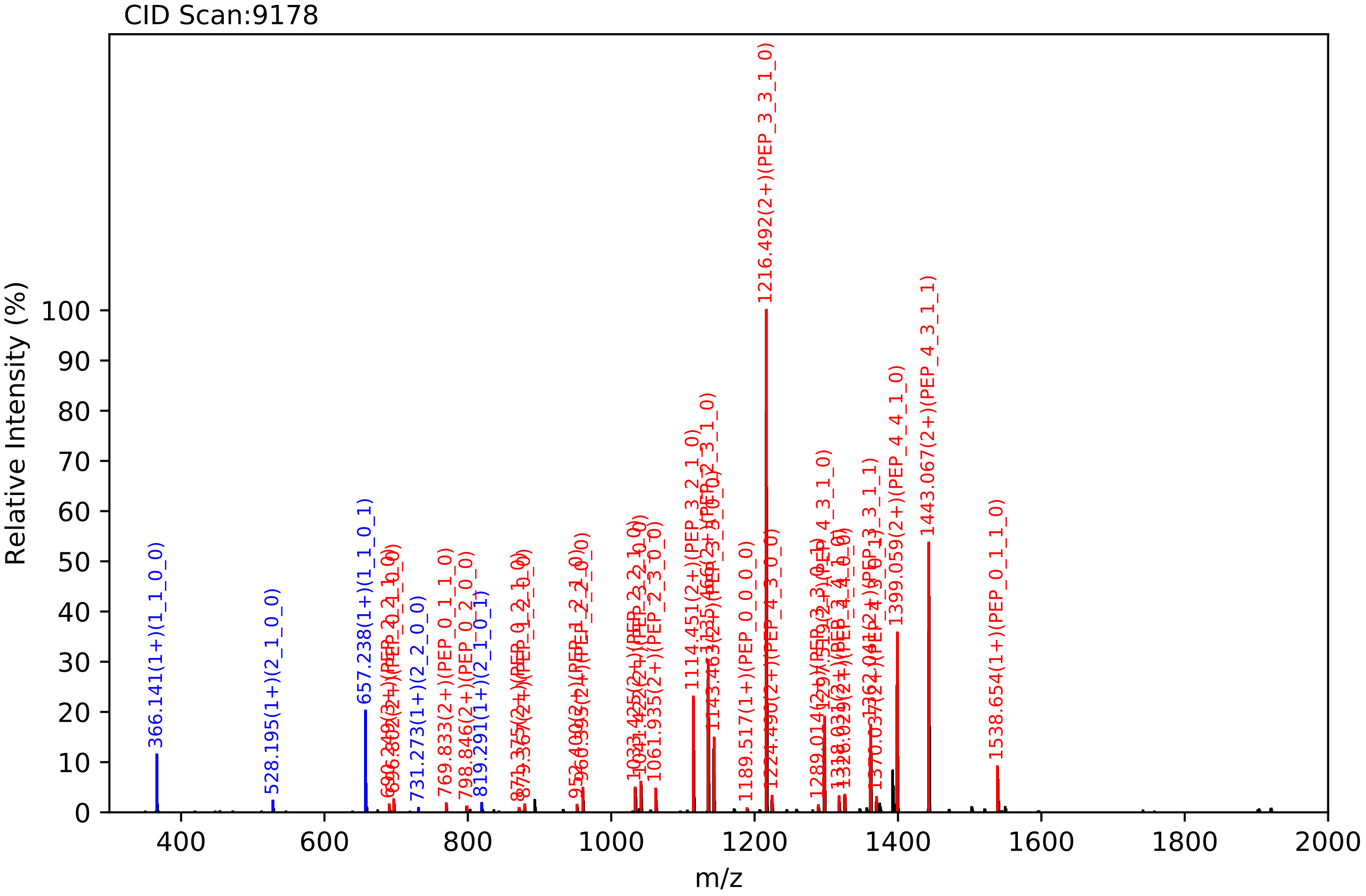

Unknown set no. 384, Experiment: Human Plasma exp_1

EEQYNSTYR(=PEP)_4_4_1_1, m/z:1544.10(2+), RT:38.30, Y-score:88.91

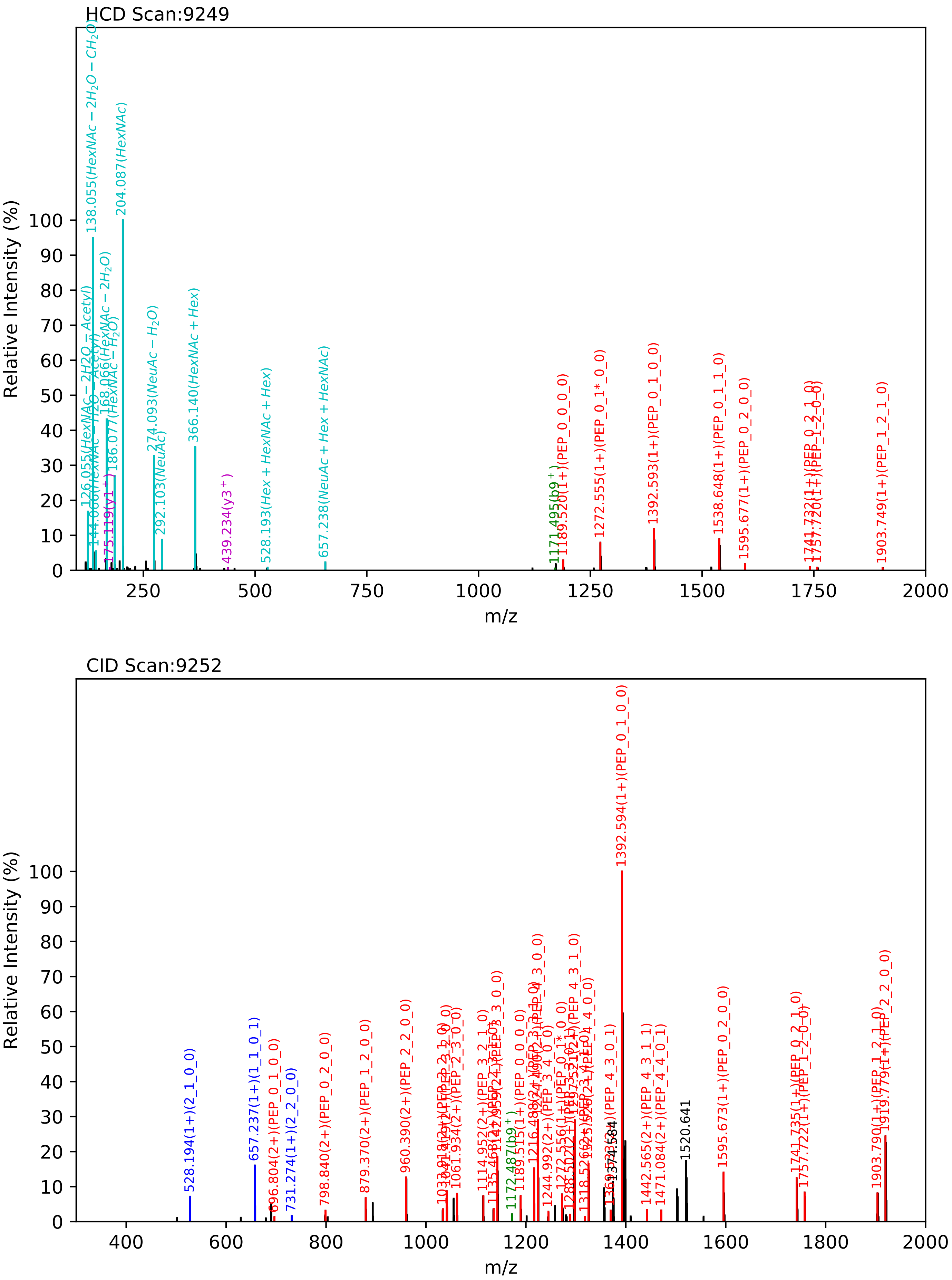

Unknown set no. 385, Experiment: Human Plasma exp_2

EEQYNSTYR(=PEP)_4_4_1_1, m/z:1029.74(3+), RT:38.37, Y-score:93.98

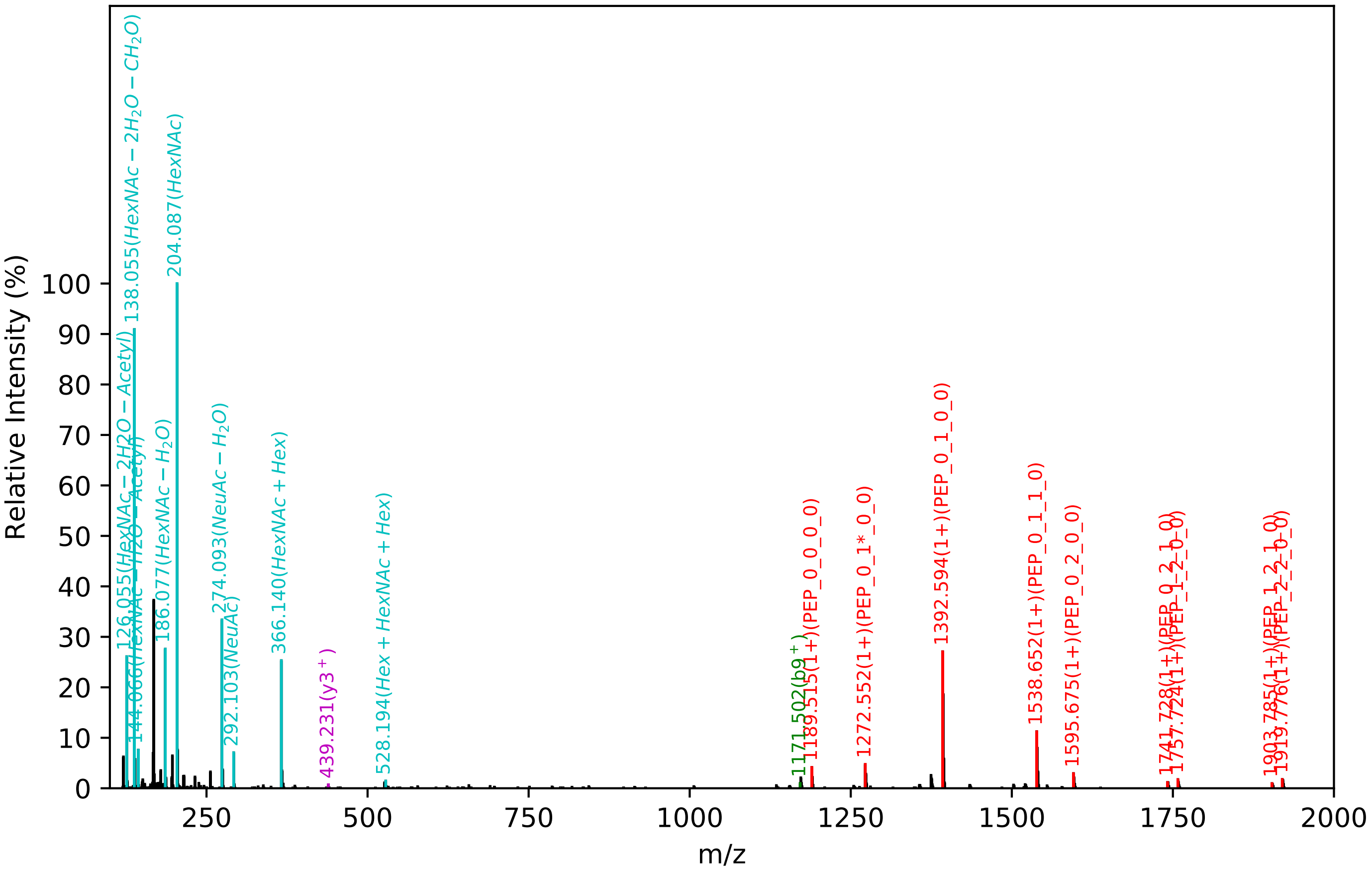

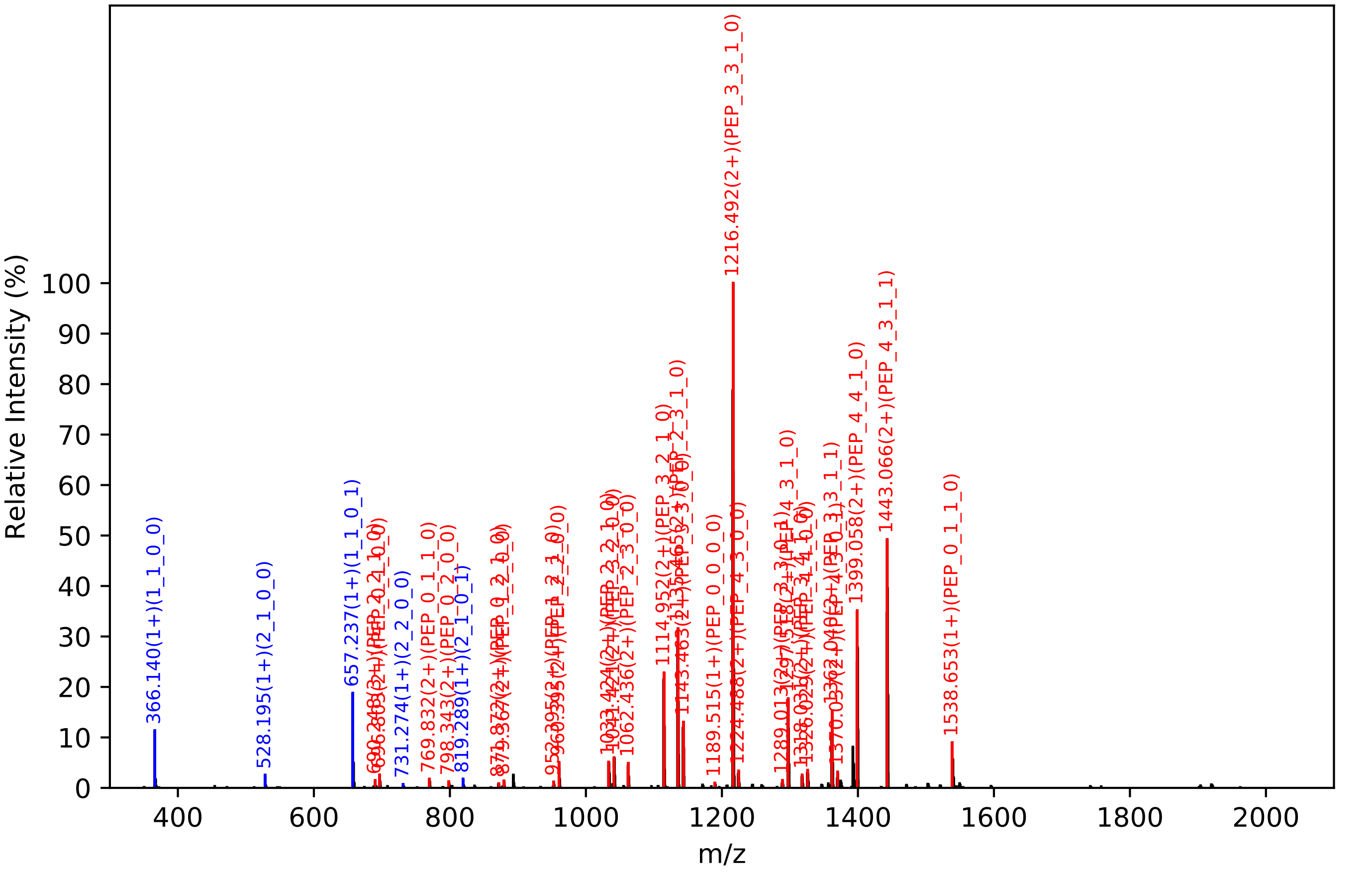

Unknown set no. 386, Experiment: Human Plasma exp_3

EEQYNSTYR(=PEP)_4_4_1_1, m/z:1029.74(3+), RT:38.65, Y-score:93.73

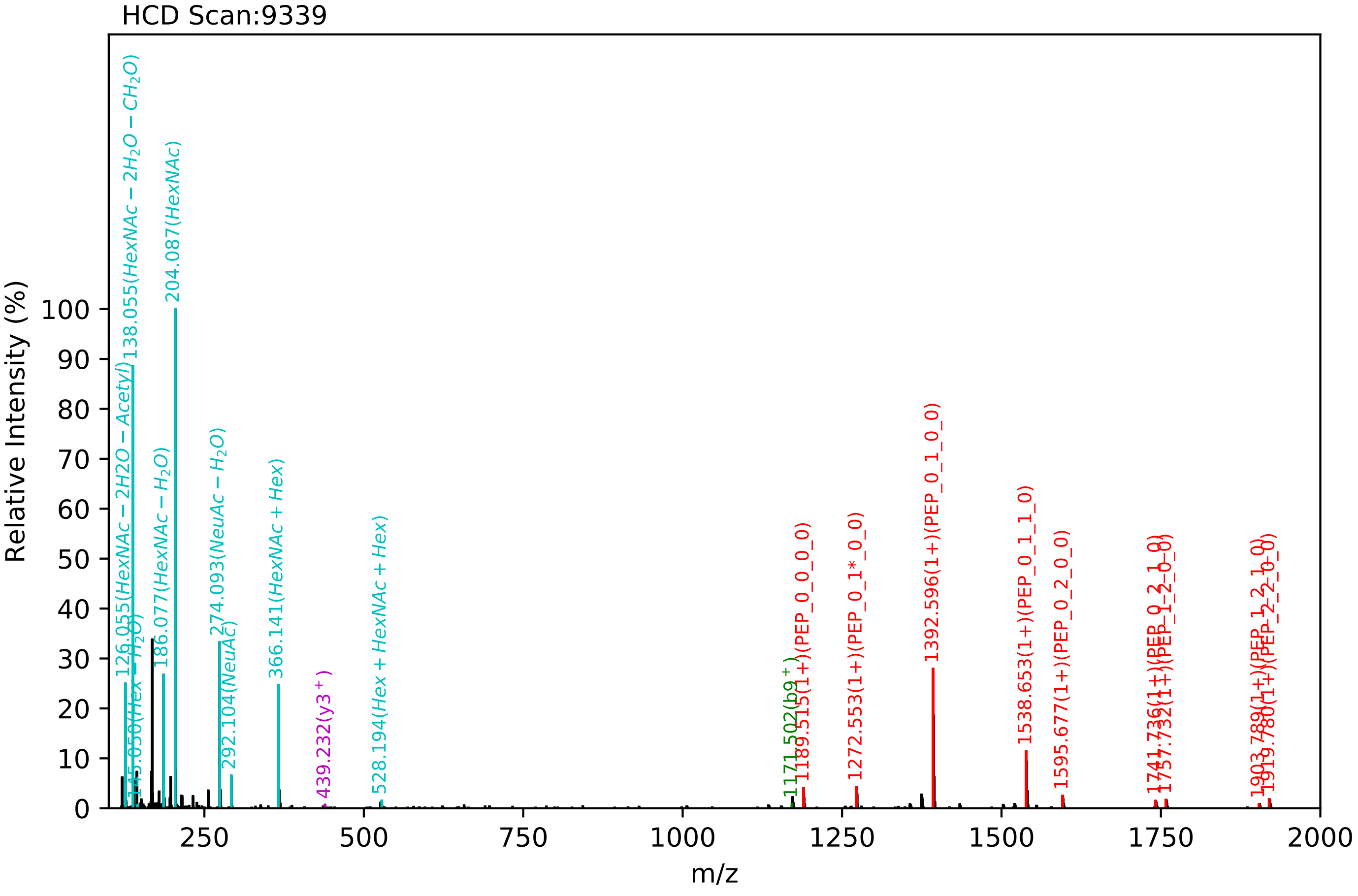

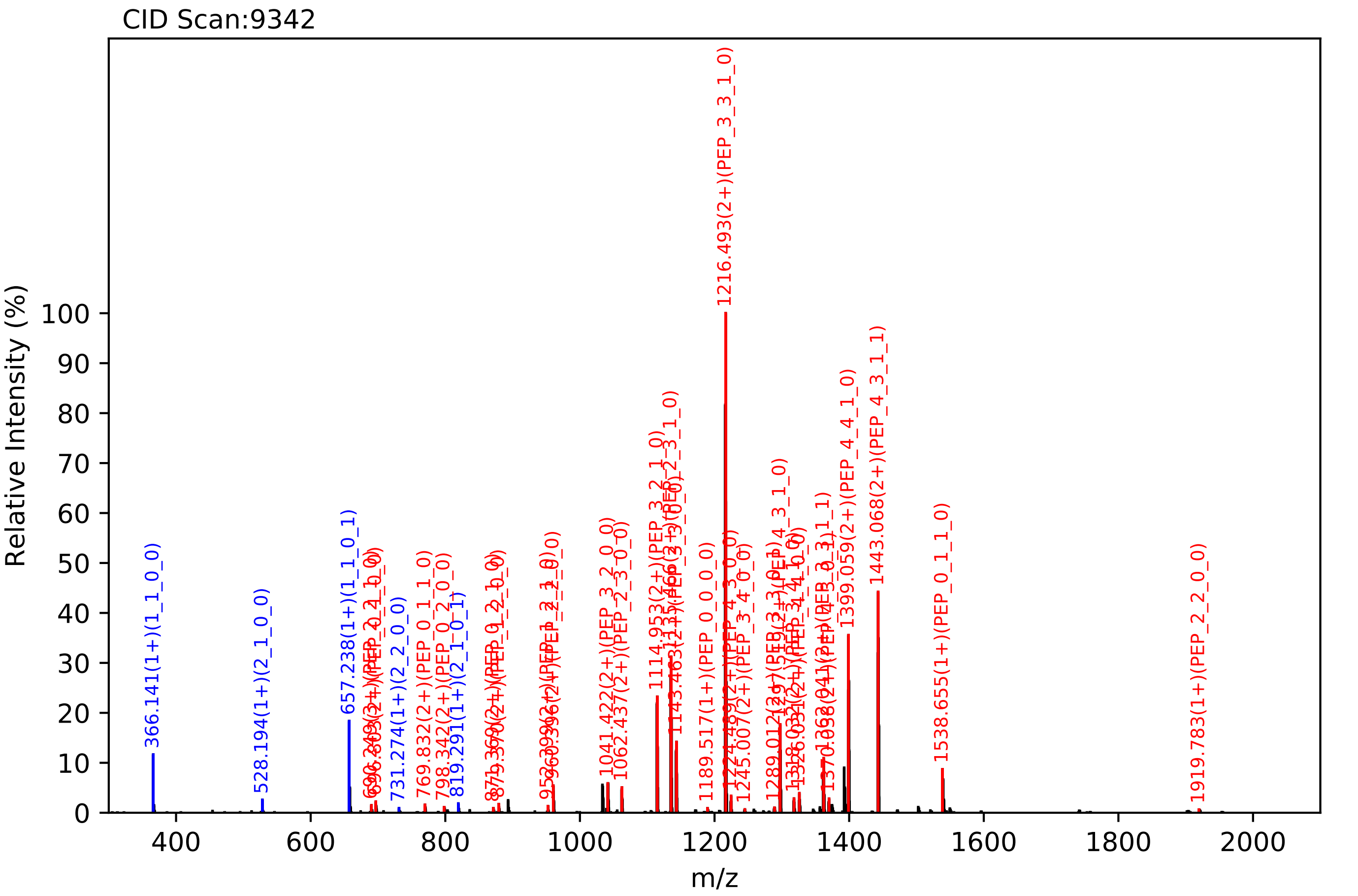

Unknown set no. 387, Experiment: Human Plasma exp_3

EEQYNSTYR(=PEP)_4_4_1_1, m/z:1544.10(2+), RT:38.82, Y-score:90.32

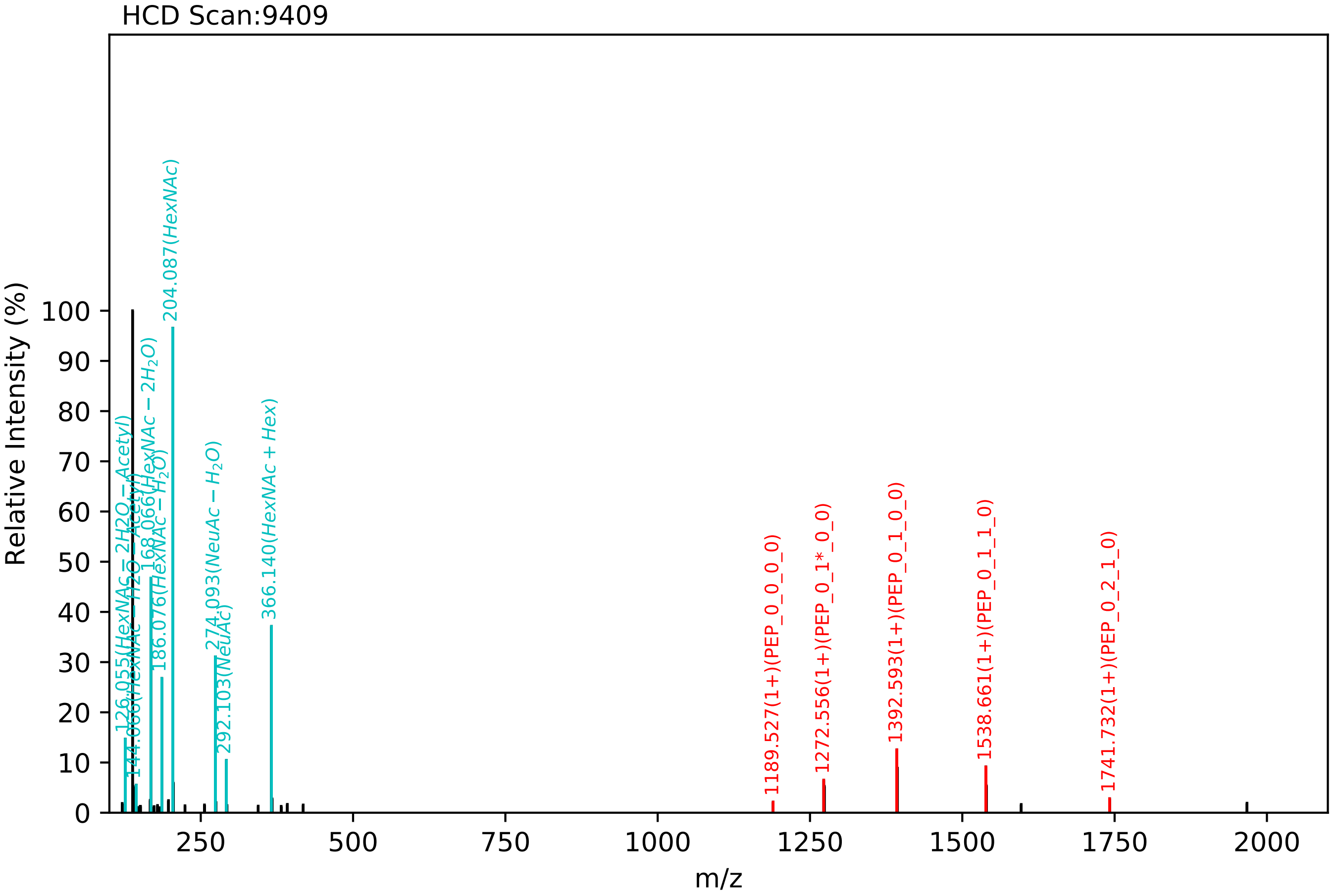

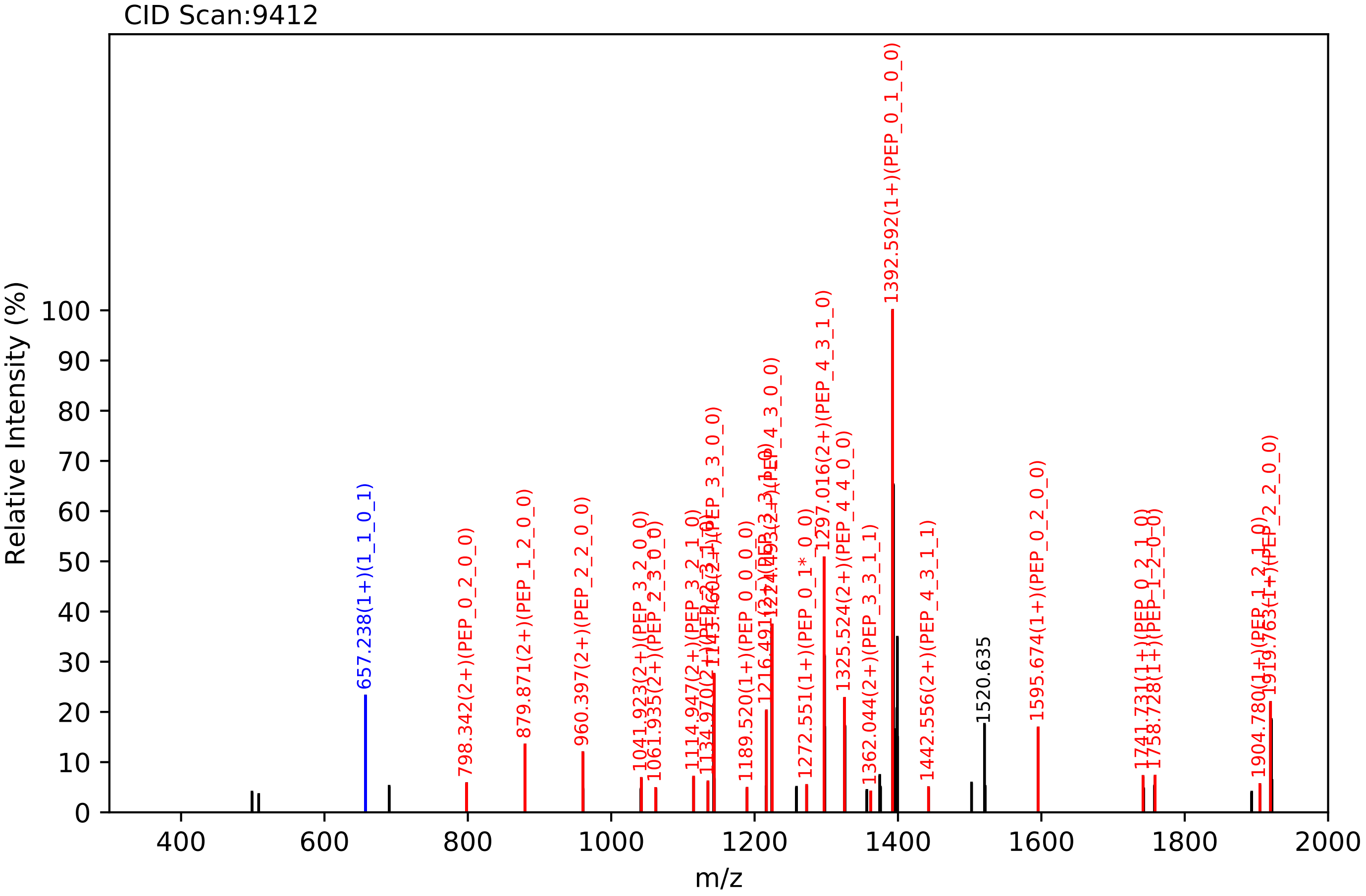

Unknown set no. 388, Experiment: Human Plasma exp_4

EEQYNSTYR(=PEP)_4_4_1_1, m/z:1544.10(2+), RT:38.31, Y-score:87.72

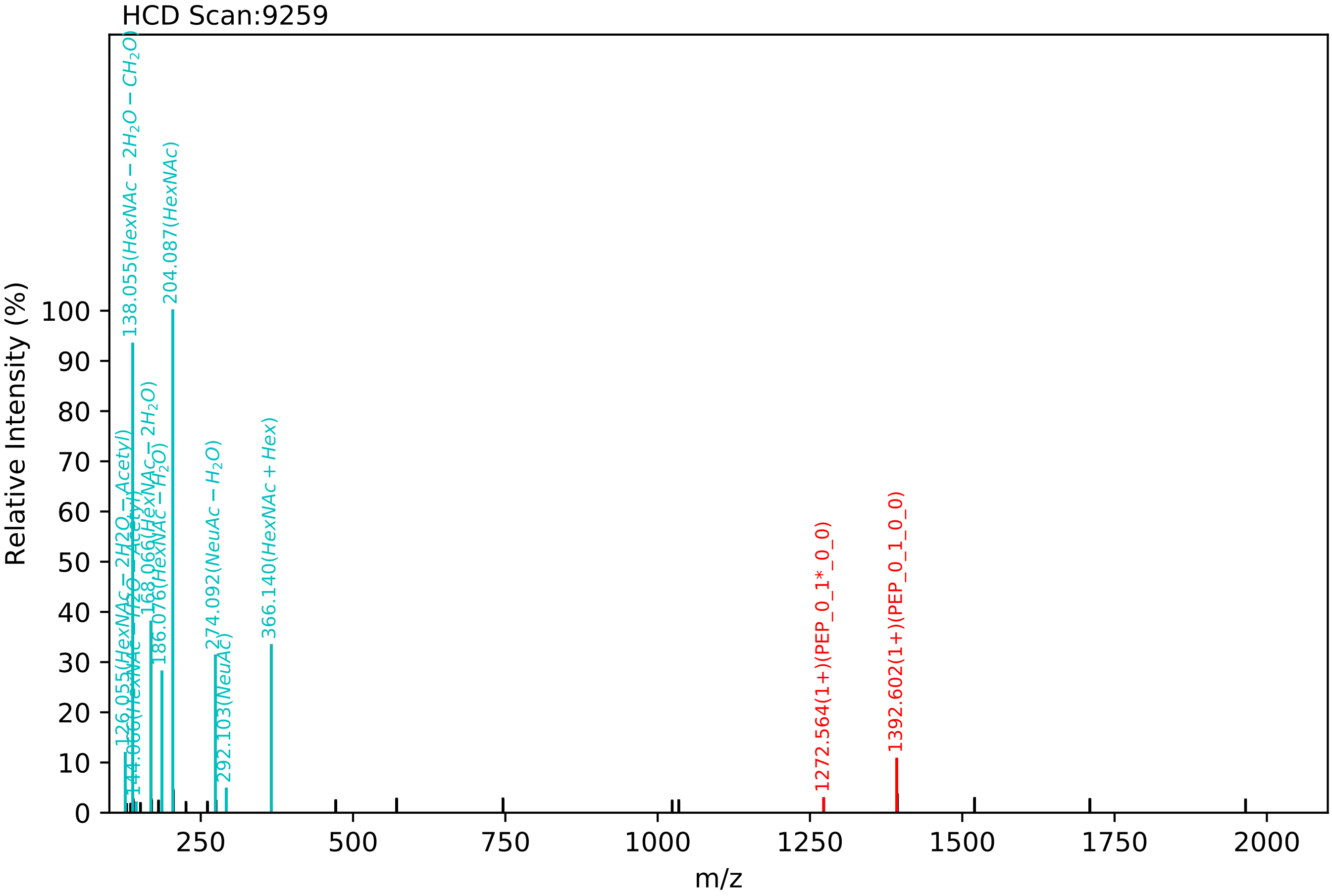

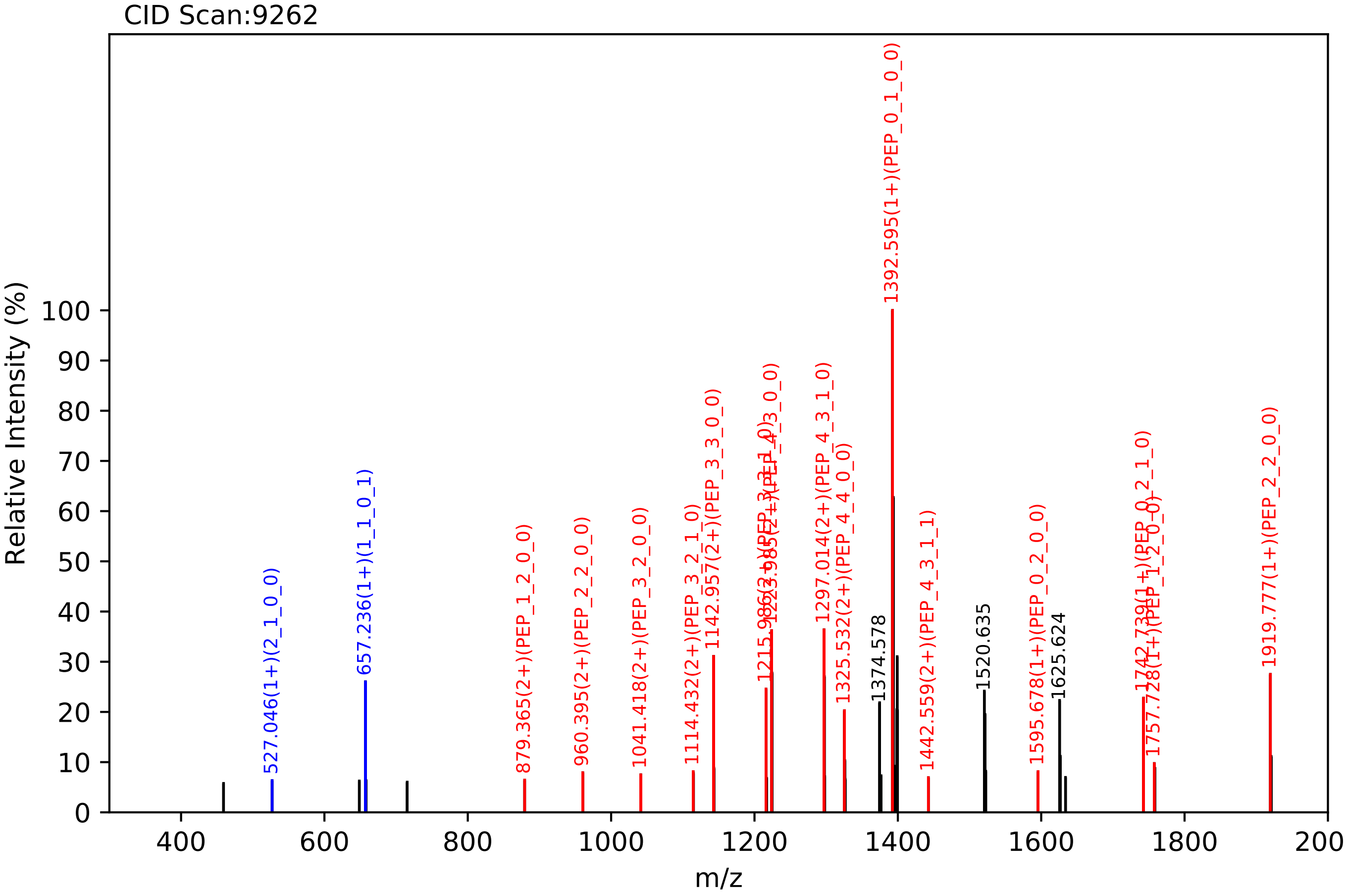

Unknown set no. 389, Experiment: Human Plasma exp_5

EEQYNSTYR(=PEP)_4_4_1_1, m/z:1544.10(2+), RT:38.92, Y-score:79.66

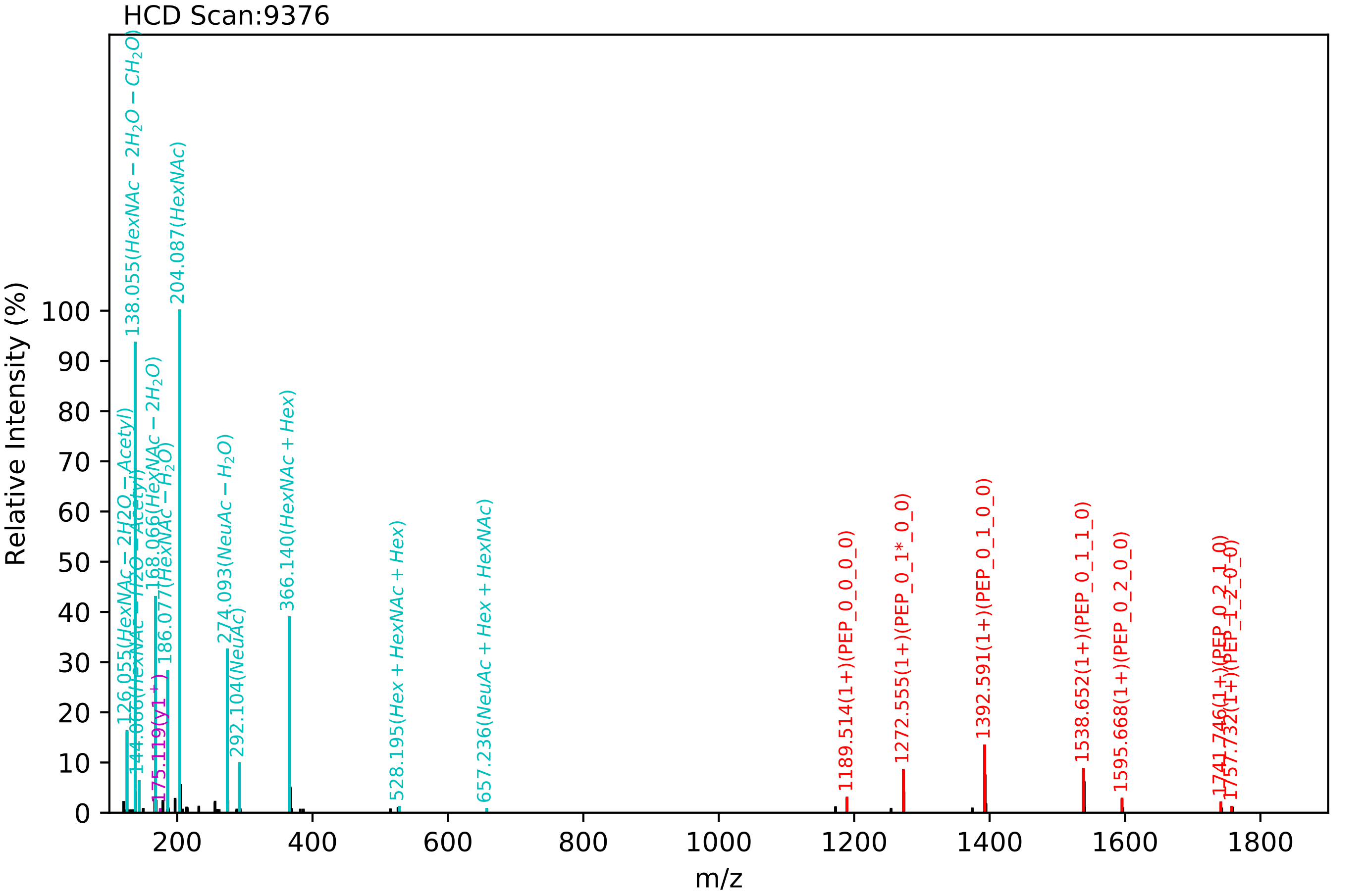

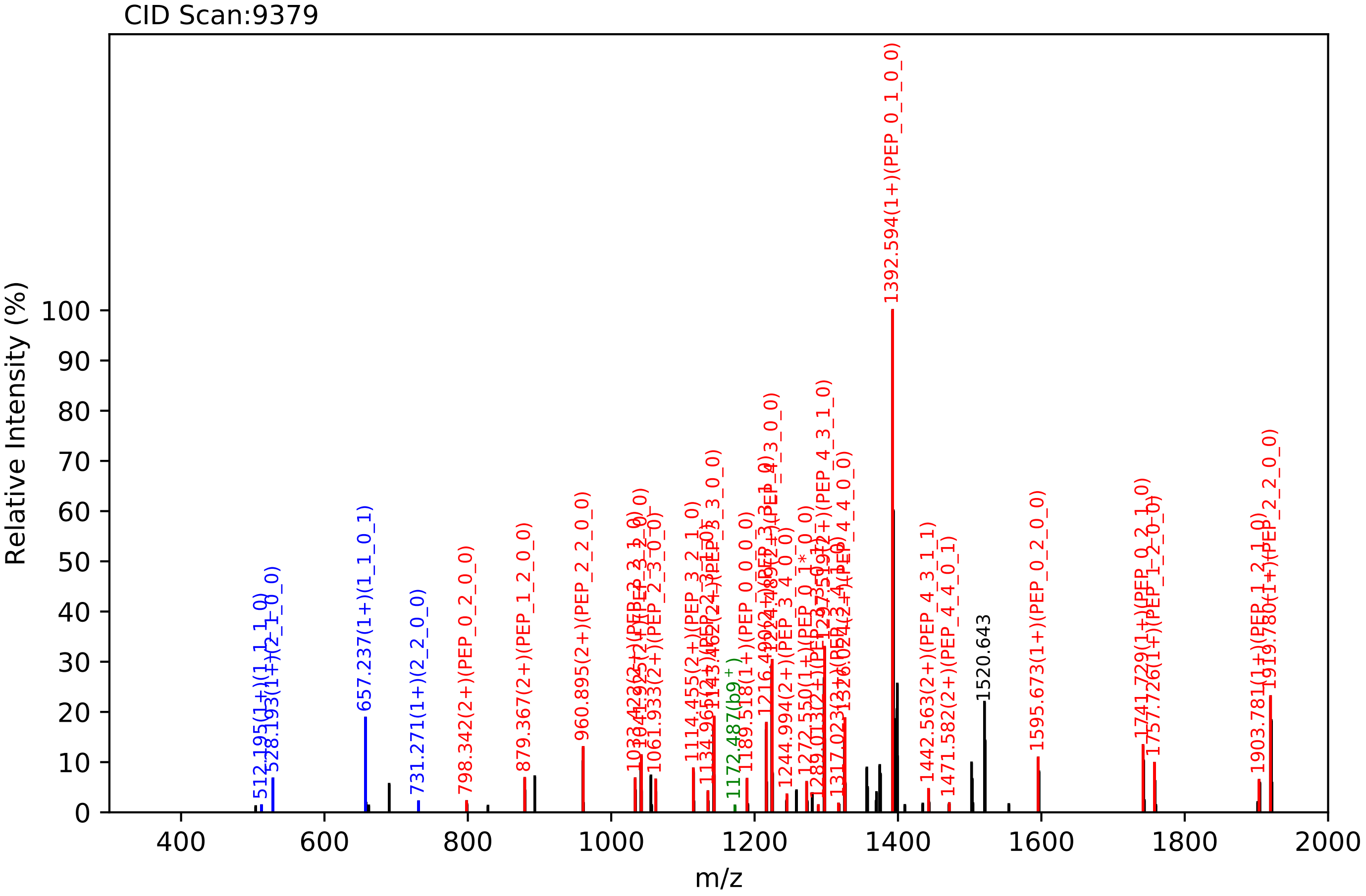

Unknown set no. 390, Experiment: Human Plasma exp_2

EEQYNSTYR(=PEP)_4_5_1_0, m/z:1500.10(2+), RT:26.92, Y-score:96.26

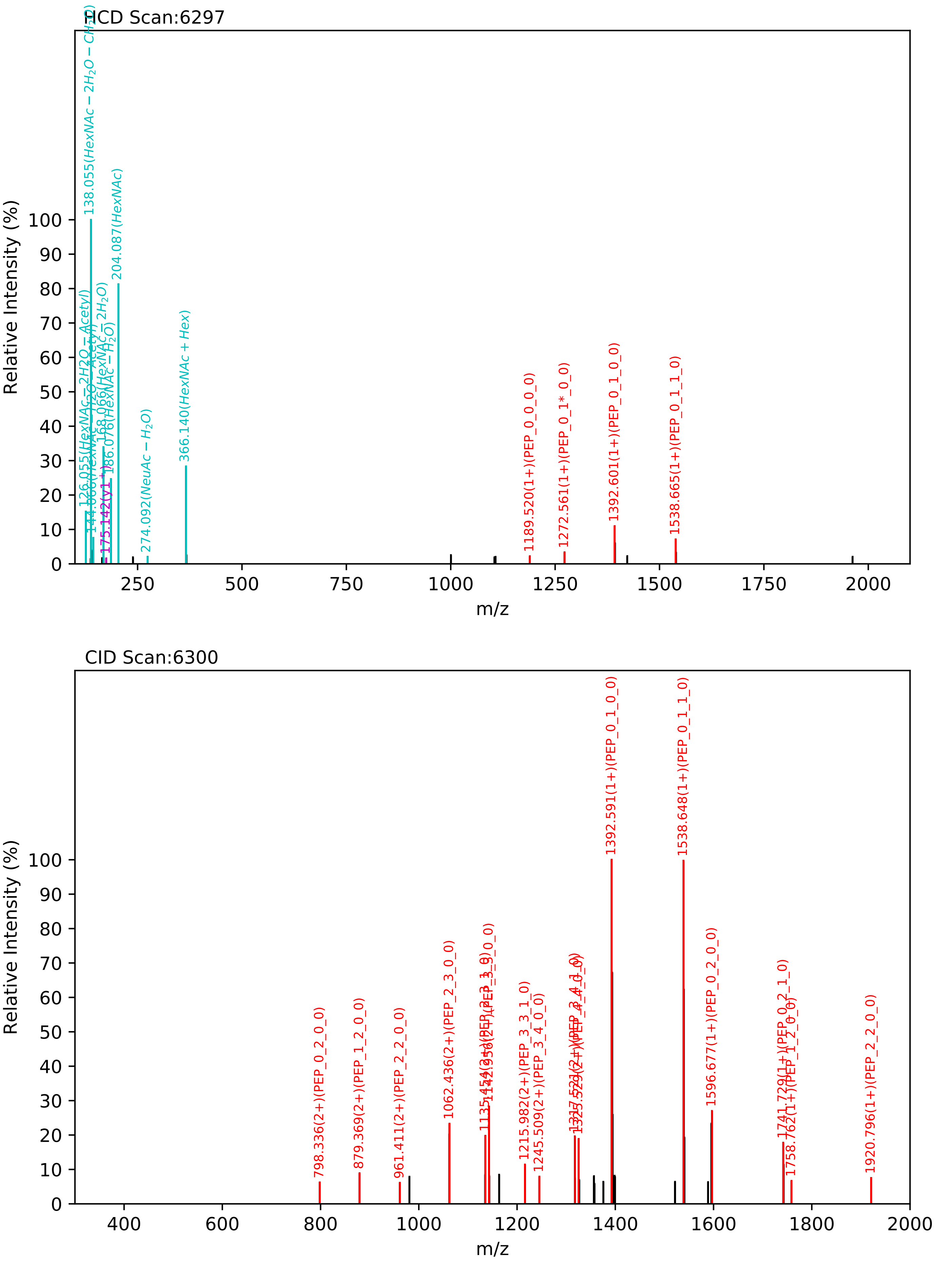

Unknown set no. 391, Experiment: Human Plasma exp_4

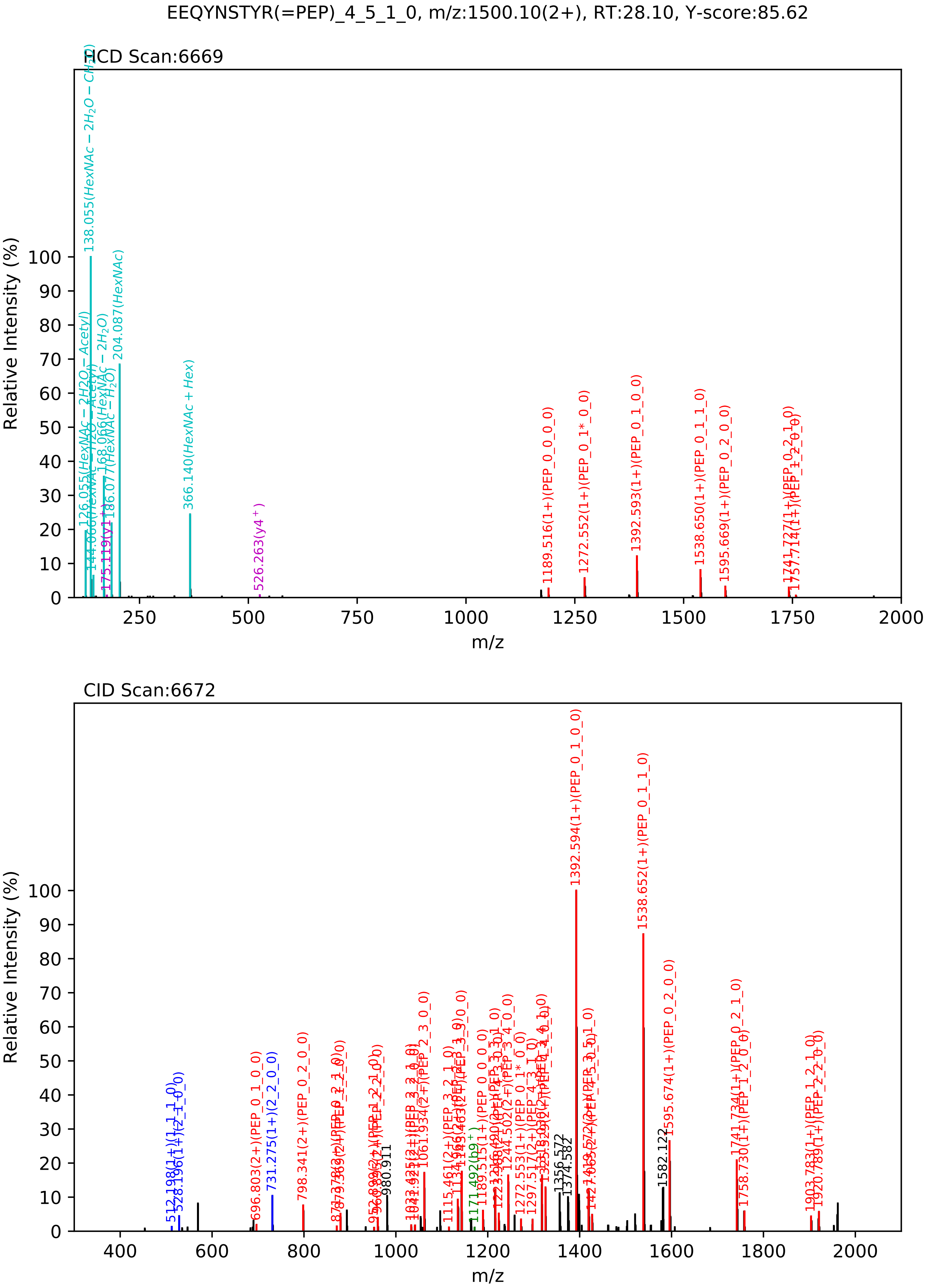

Unknown set no. 392, Experiment: Human Plasma exp_5

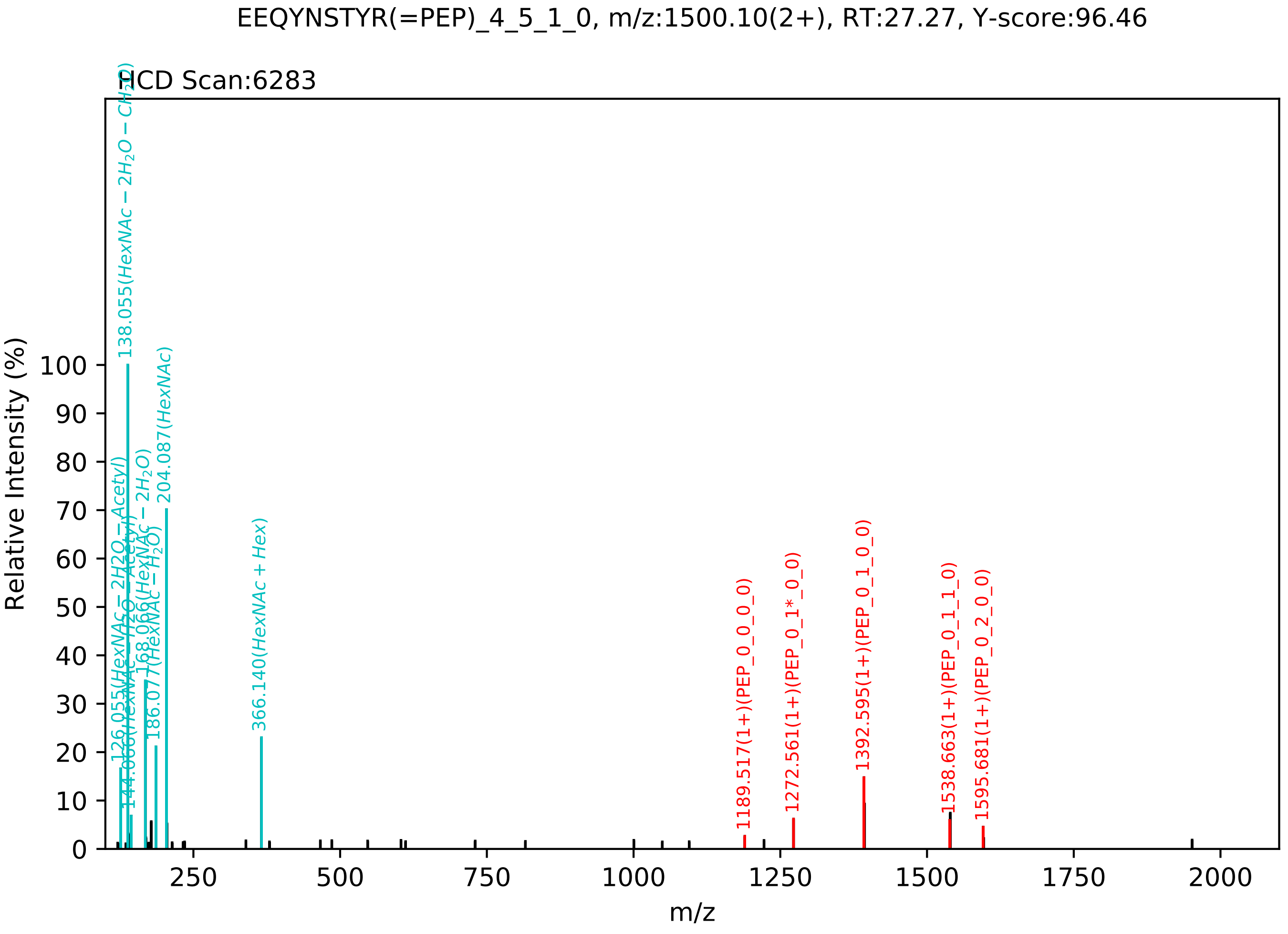

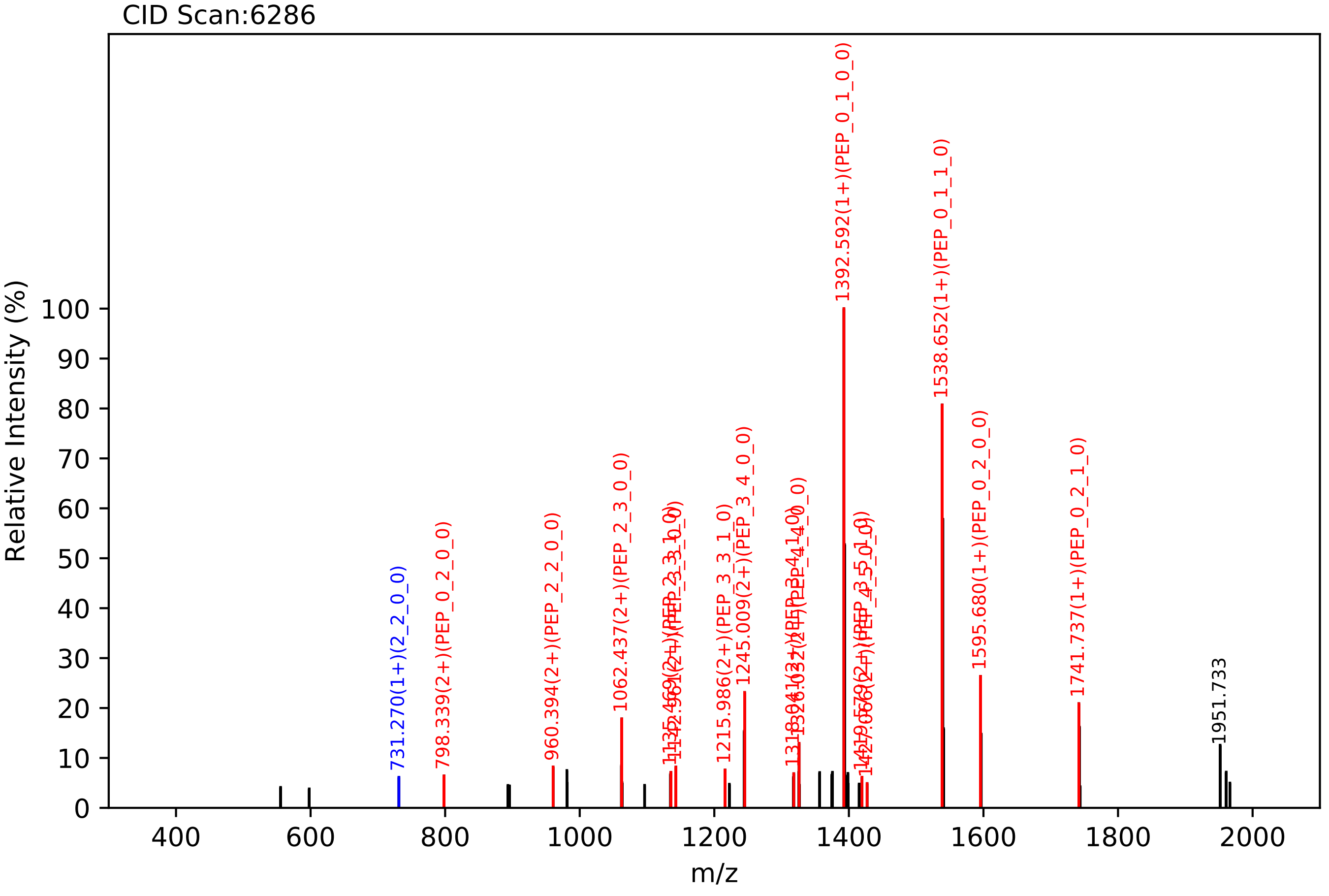

Unknown set no. 393, Experiment: Human Plasma exp_1

EEQYNSTYR(=PEP)_5_3_1_1, m/z:1016.06(3+), RT:37.14, Y-score:89.84

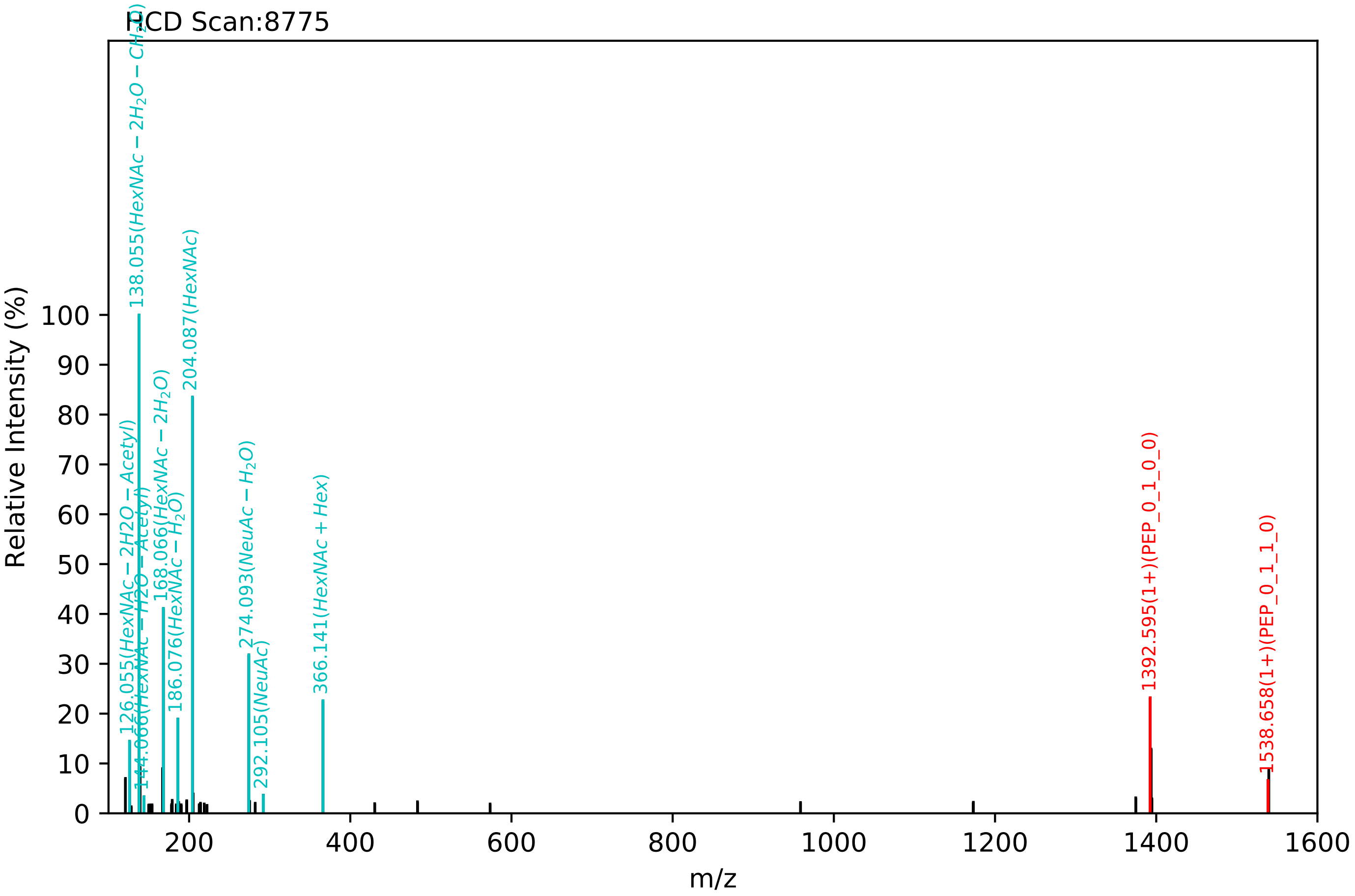

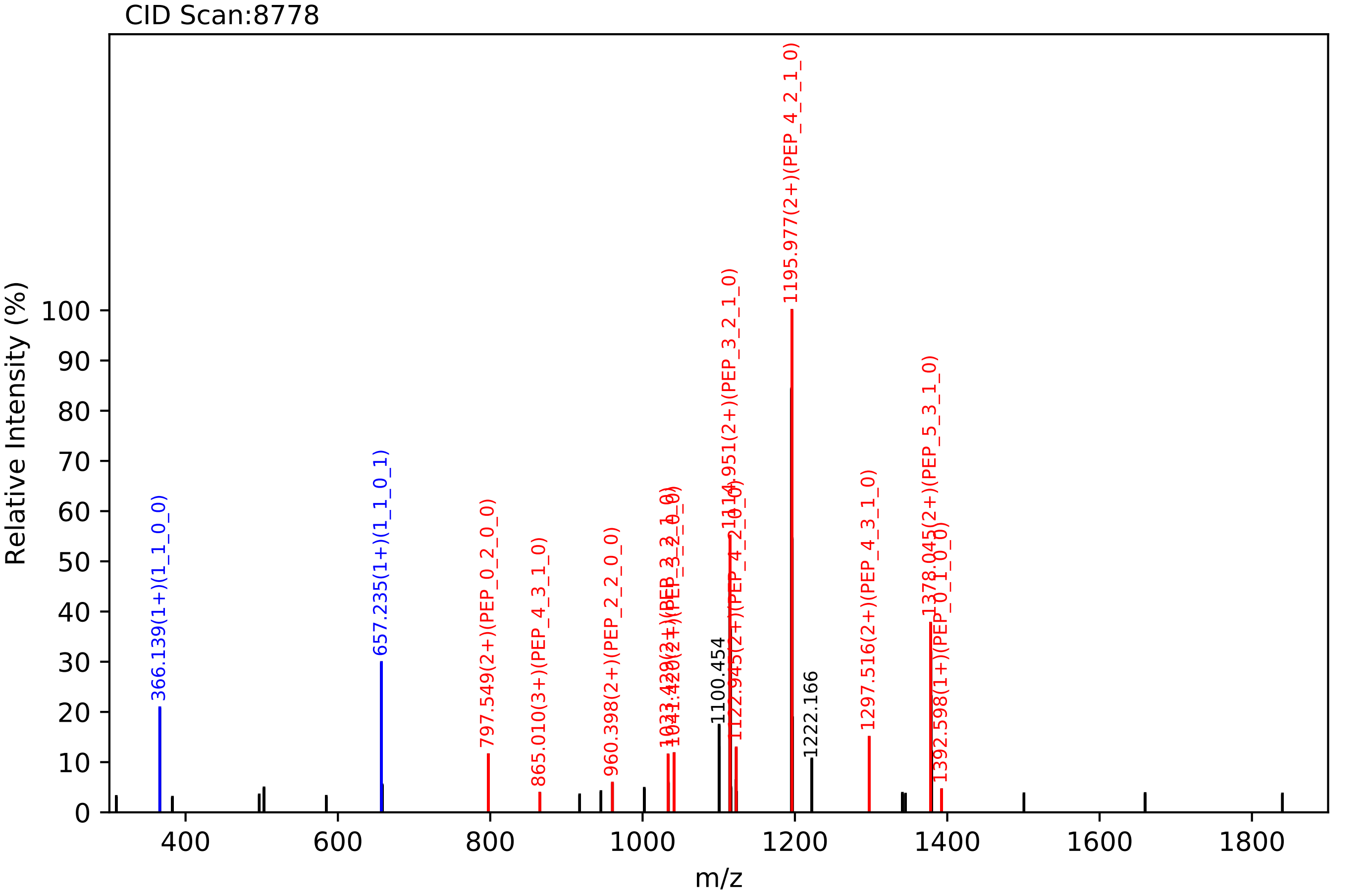

Unknown set no. 394, Experiment: Human Plasma exp_2

EEQYNSTYR(=PEP)_5_3_1_1, m/z:1016.06(3+), RT:37.35, Y-score:79.70

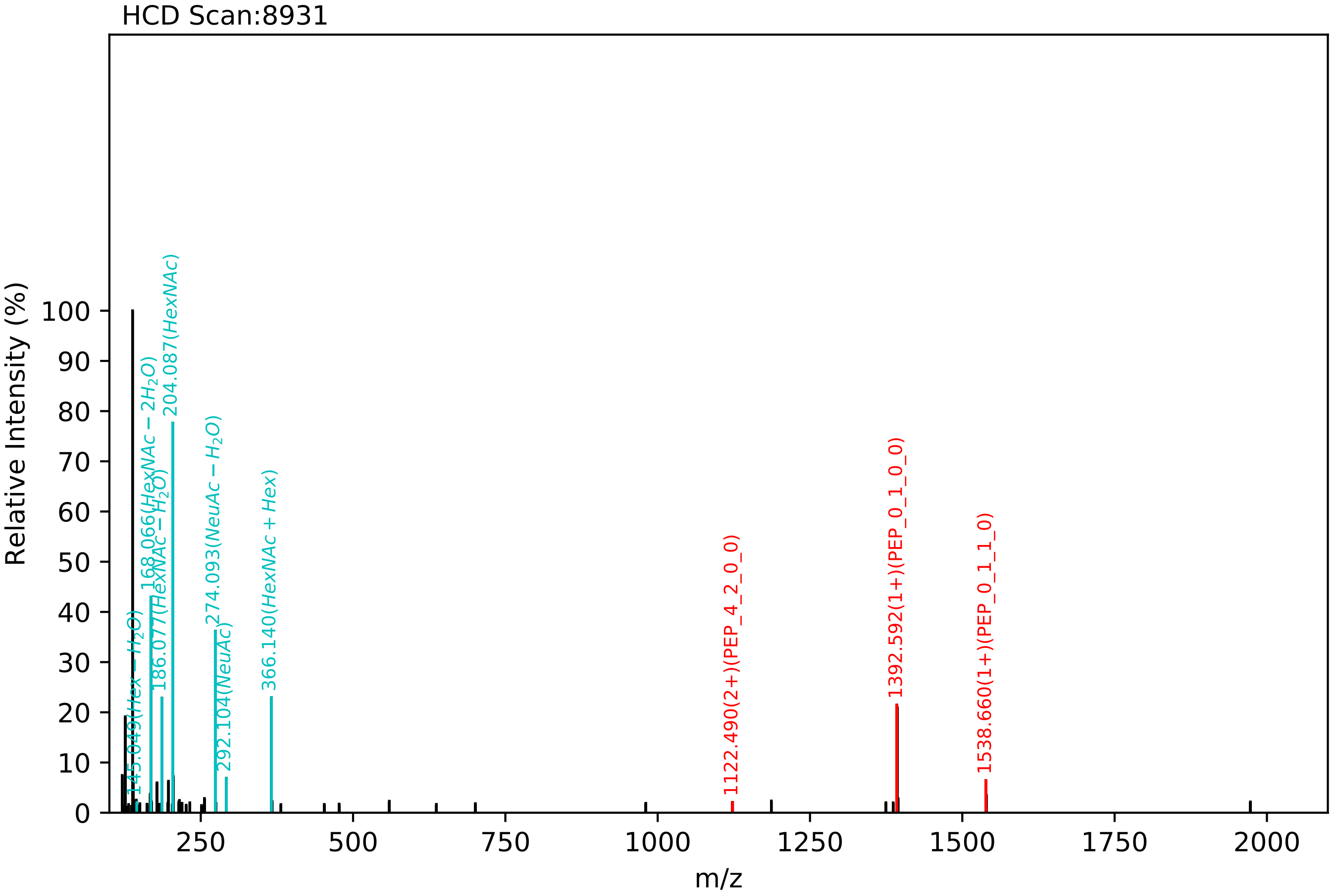

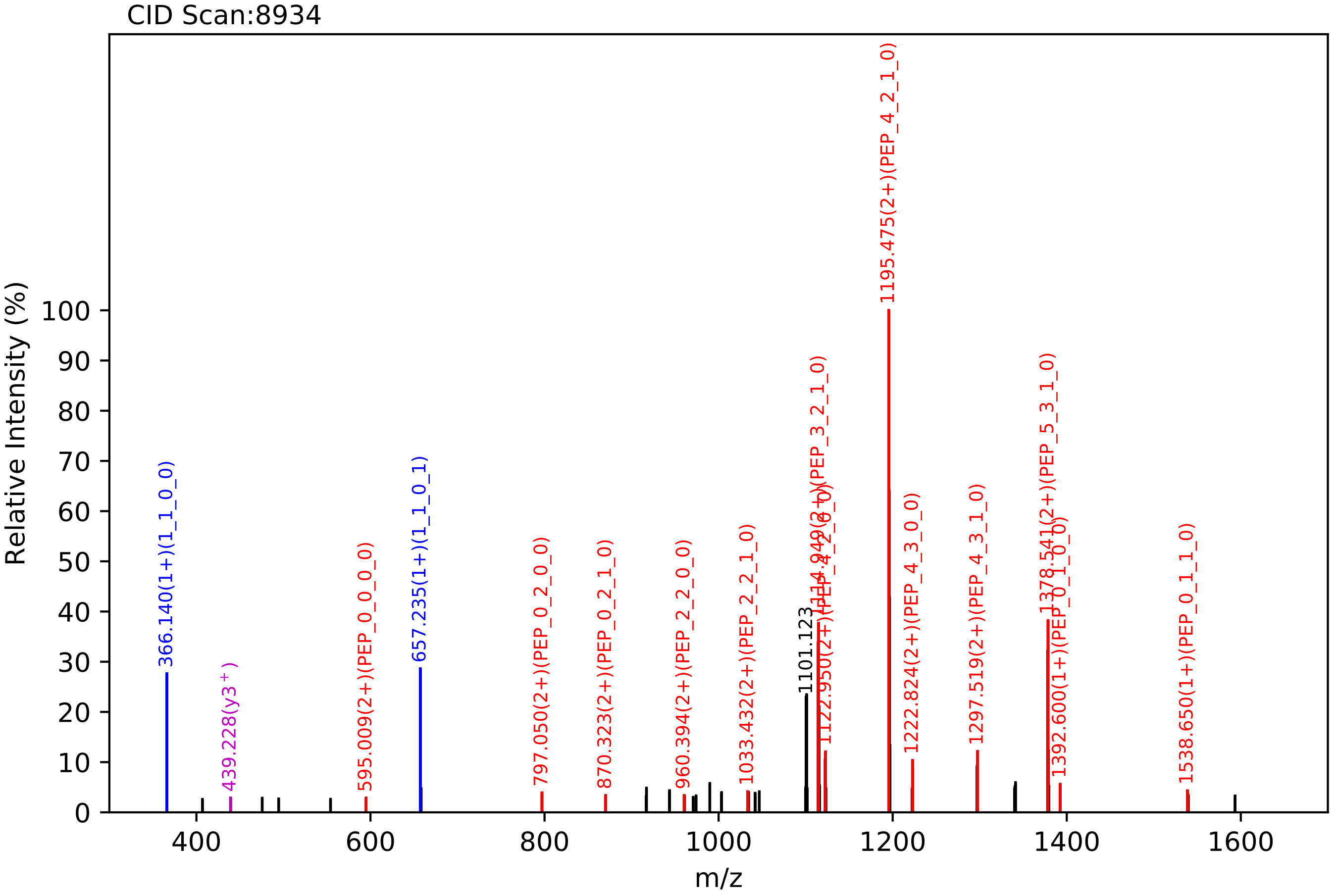

Unknown set no. 395, Experiment: Human Plasma exp_3

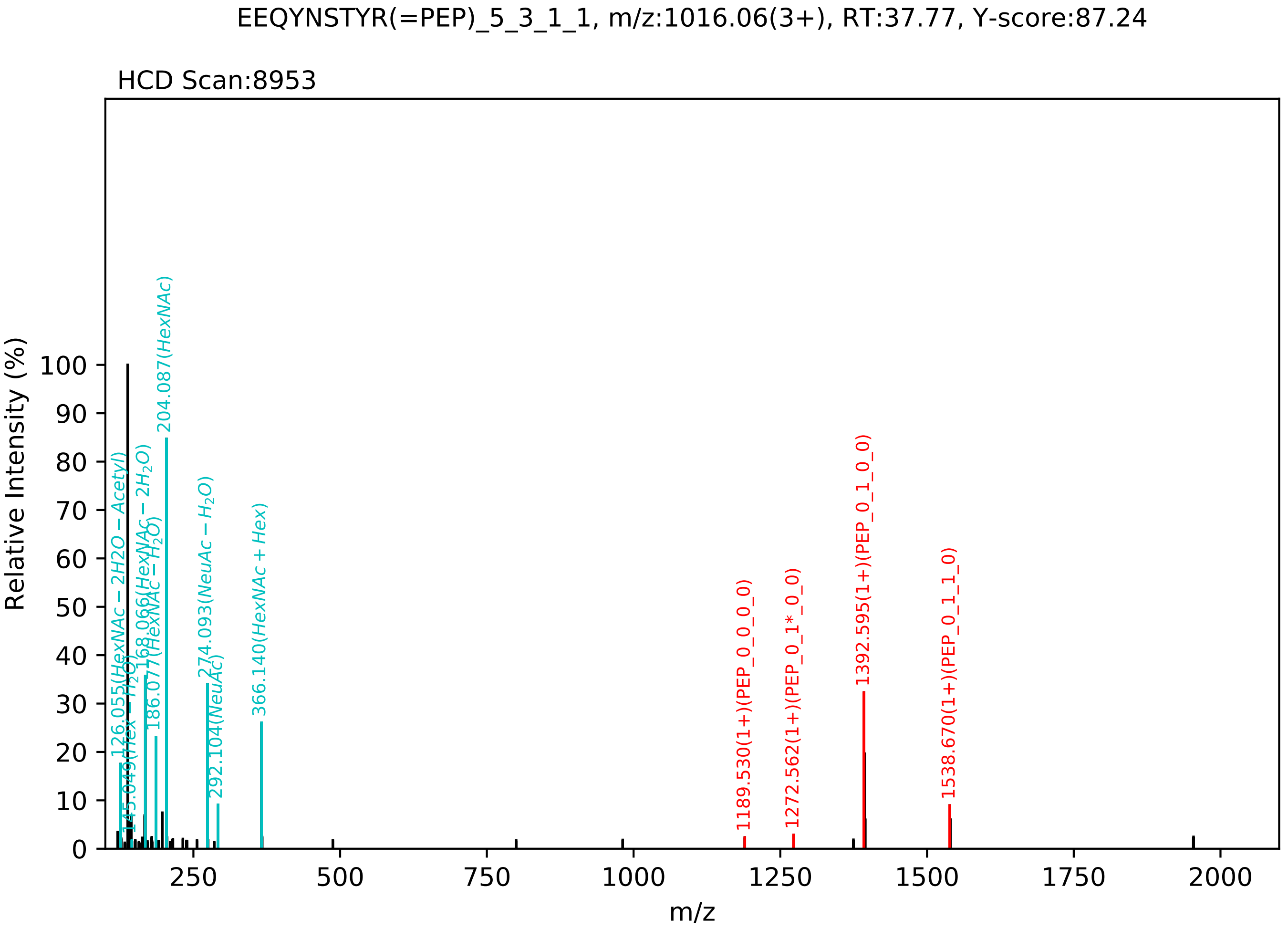

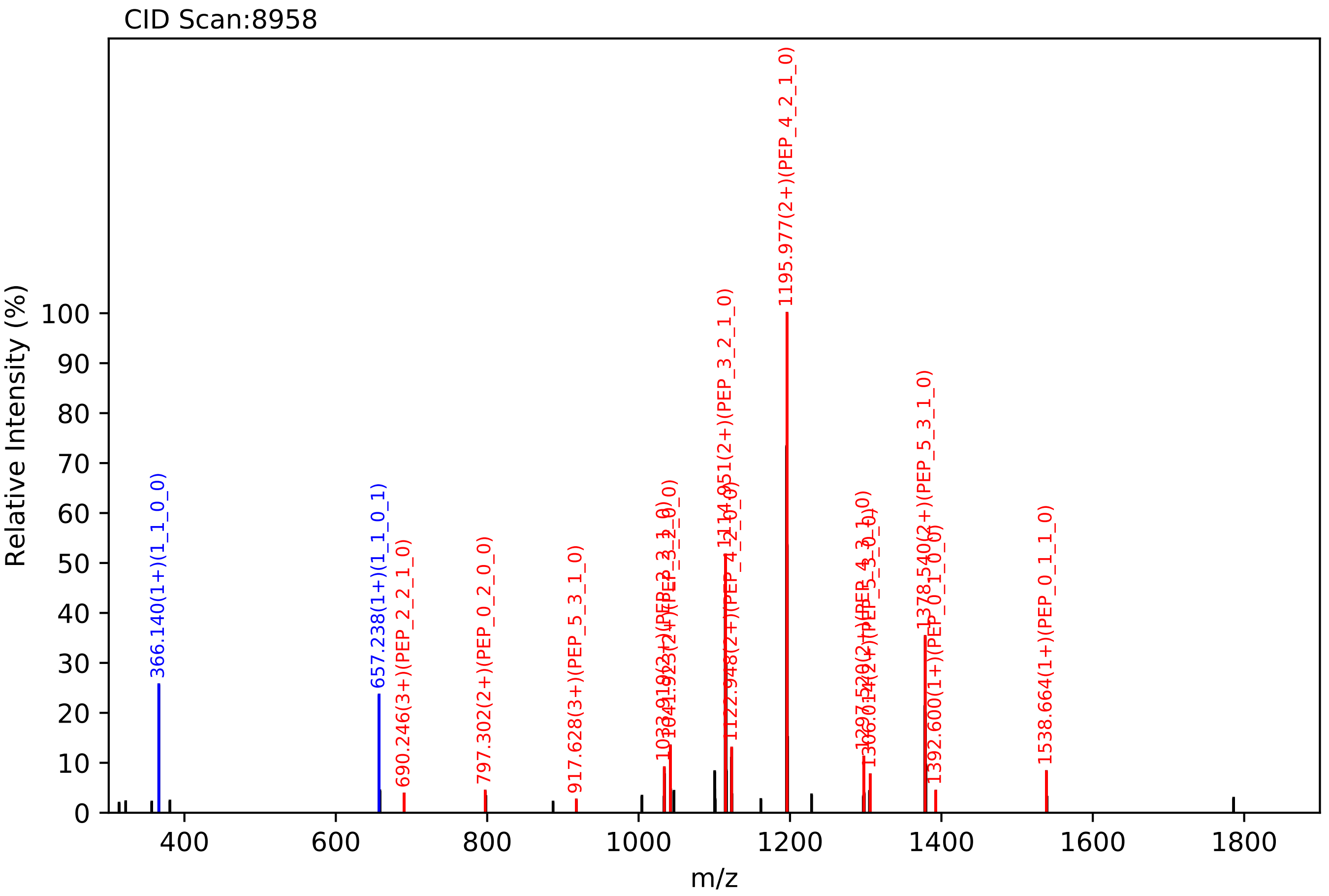

Unknown set no. 396, Experiment: Human Plasma exp_2

EEQYNSTYR(=PEP)_5_4_0_0, m/z:1406.55(2+), RT:24.69, Y-score:83.01

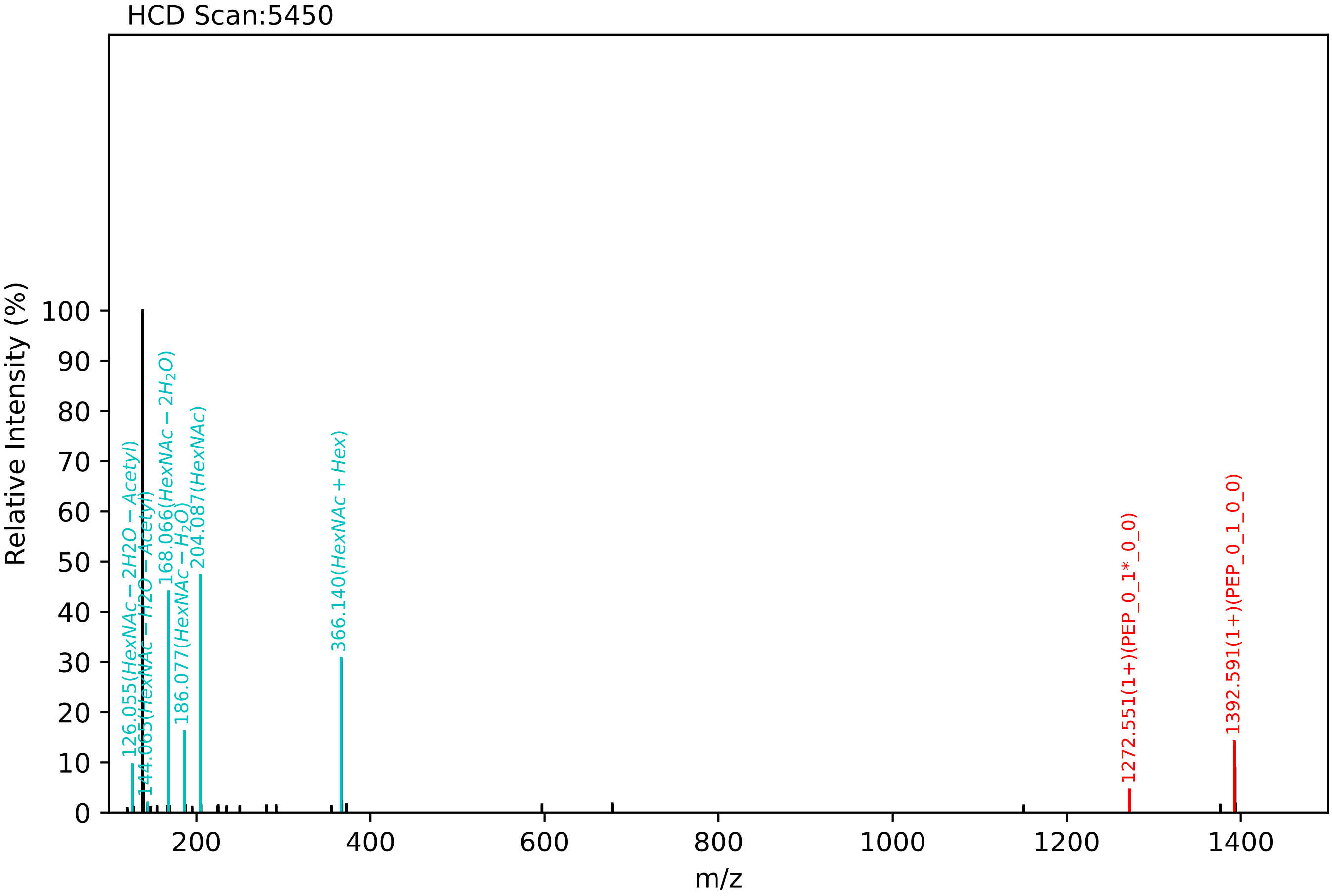

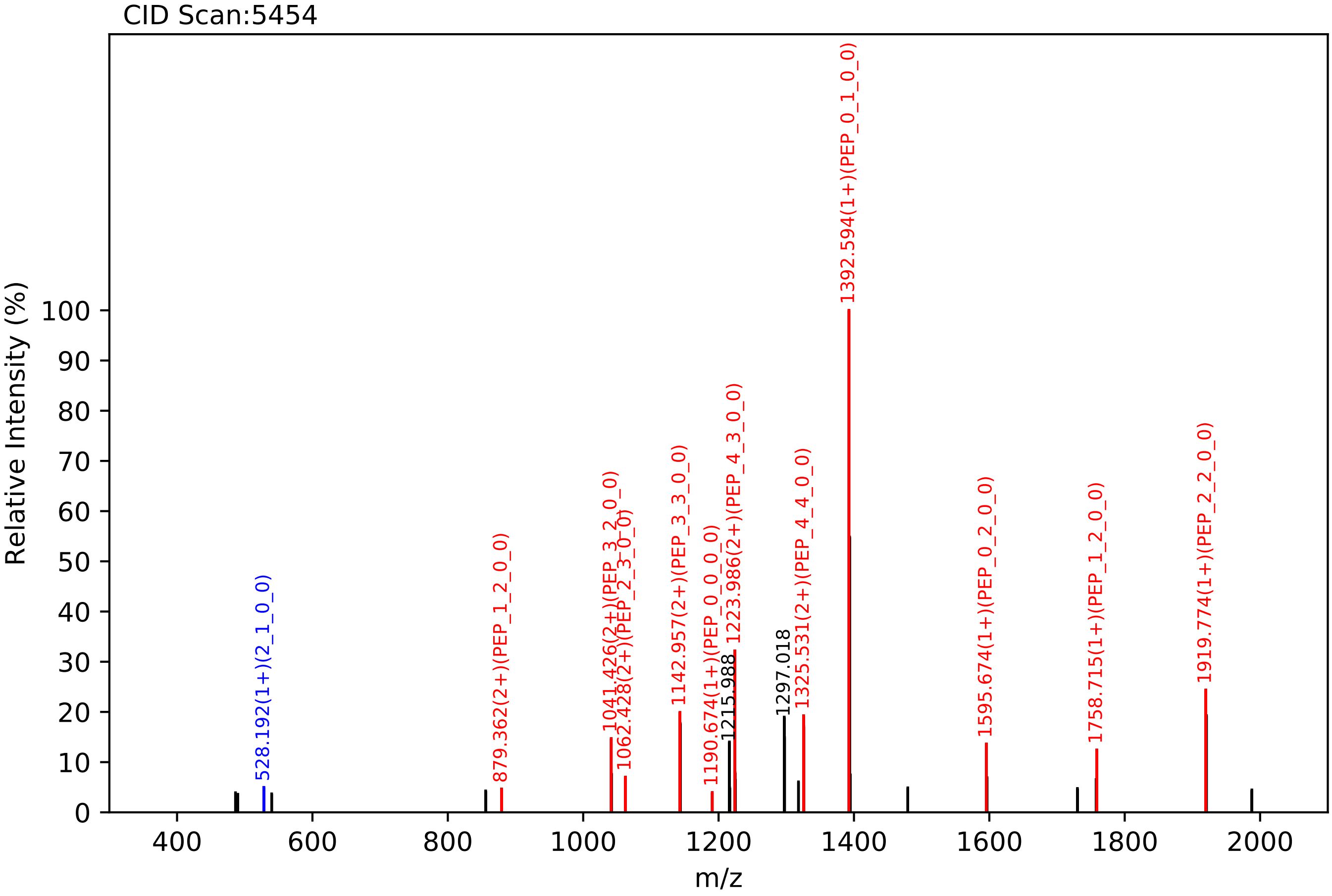

Unknown set no. 397, Experiment: Human Plasma exp_1

EEQYNSTYR(=PEP)_5_4_0_1, m/z:1552.10(2+), RT:38.28, Y-score:96.06

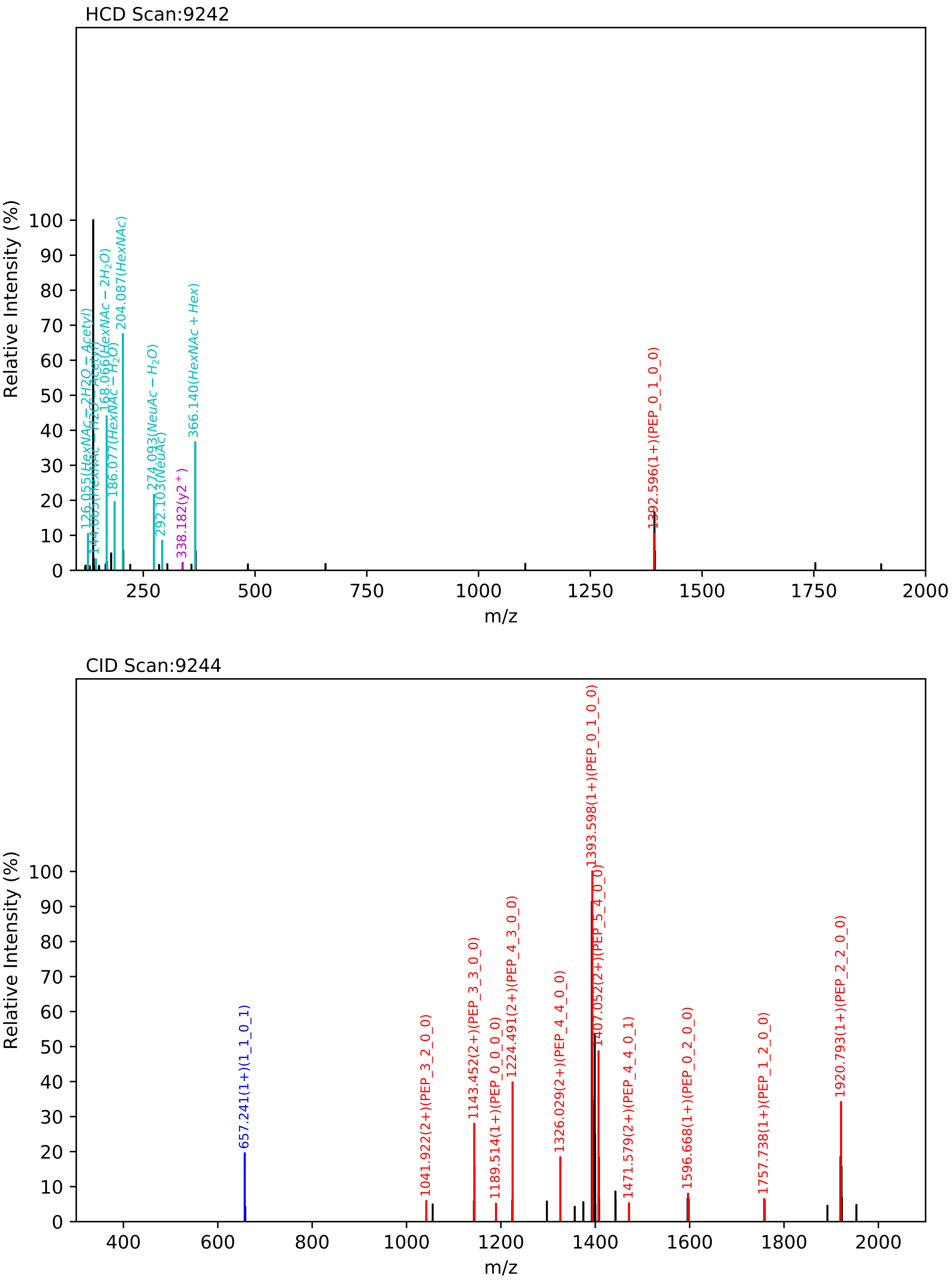

Unknown set no. 398, Experiment: Human Plasma exp_3

EEQYNSTYR(=PEP)_5_4_0_1, m/z:1552.10(2+), RT:38.32, Y-score:82.19

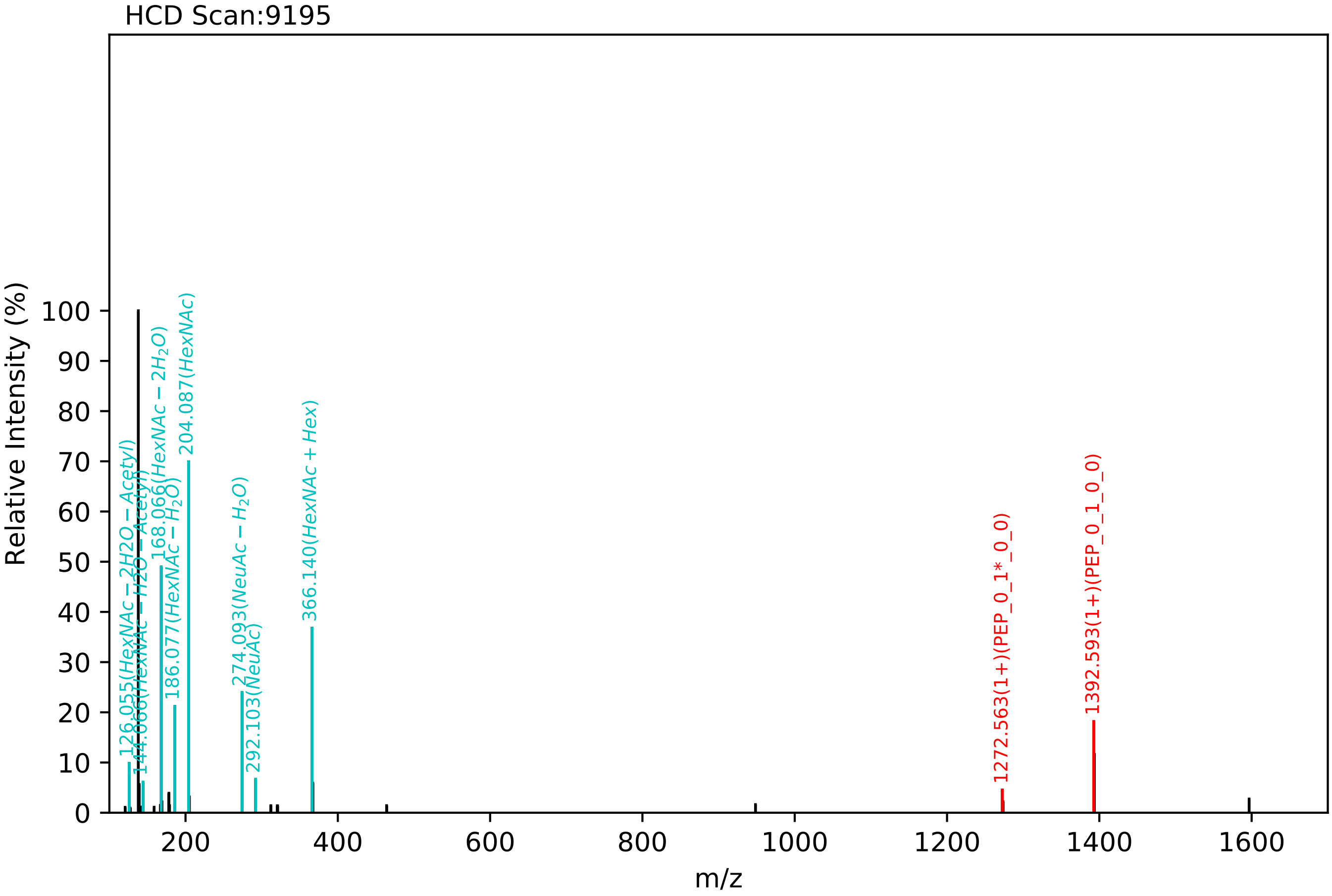

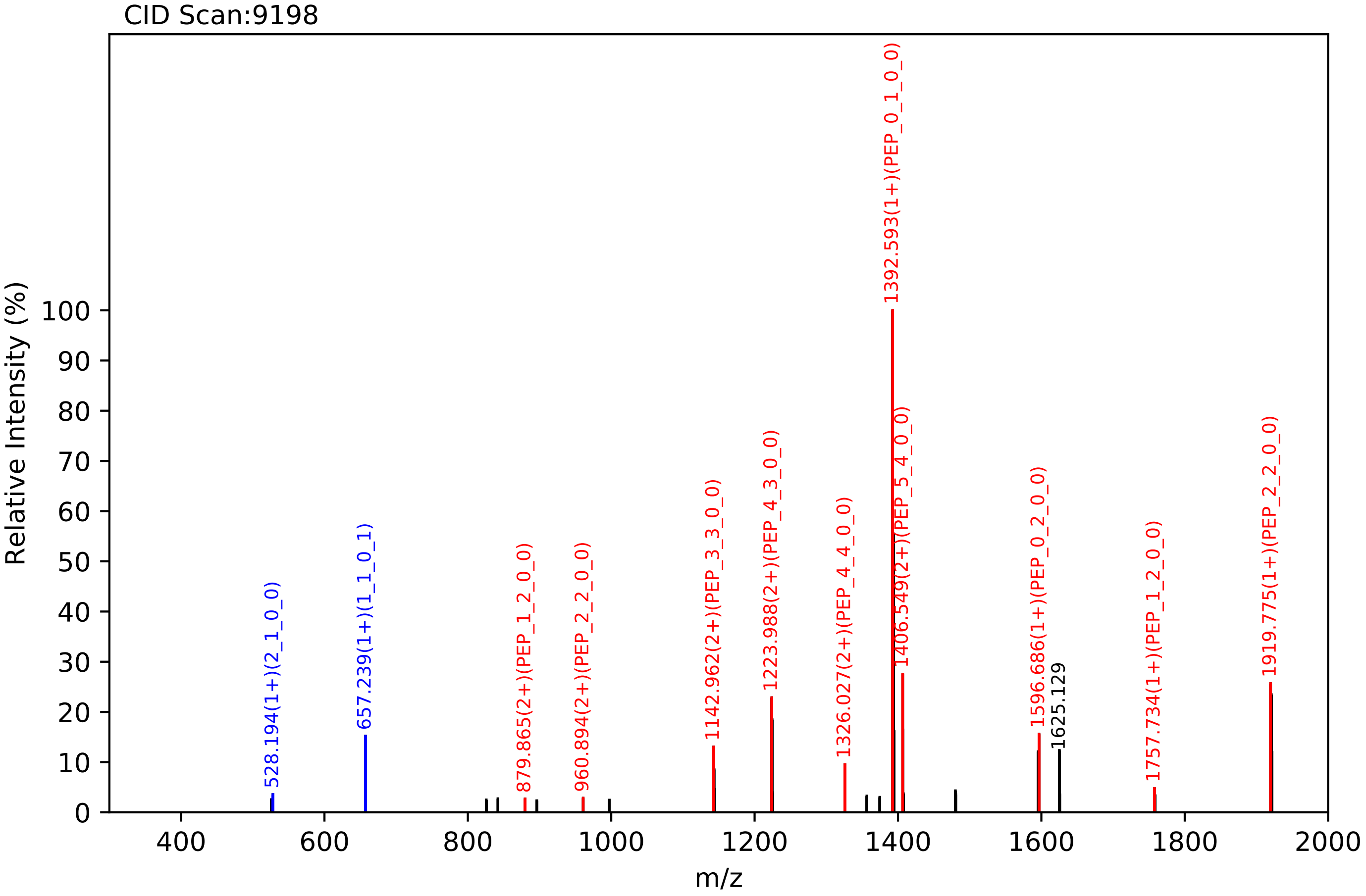

Unknown set no. 399, Experiment: Human Plasma exp_1

EEQYNSTYR(=PEP)_5_4_1_0, m/z:1479.58(2+), RT:25.61, Y-score:100.00

HCD Scan:5806

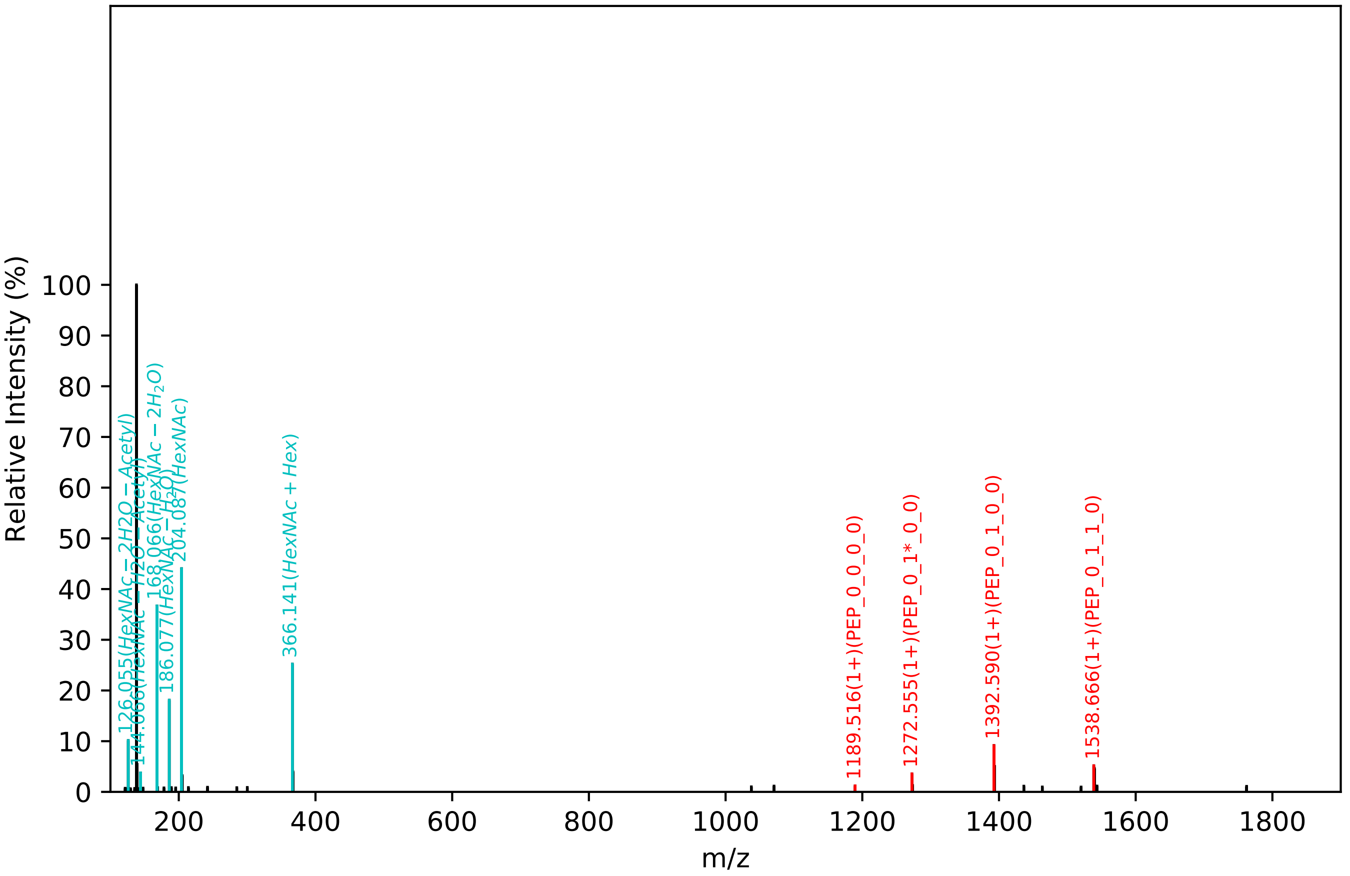

CID Scan:5809

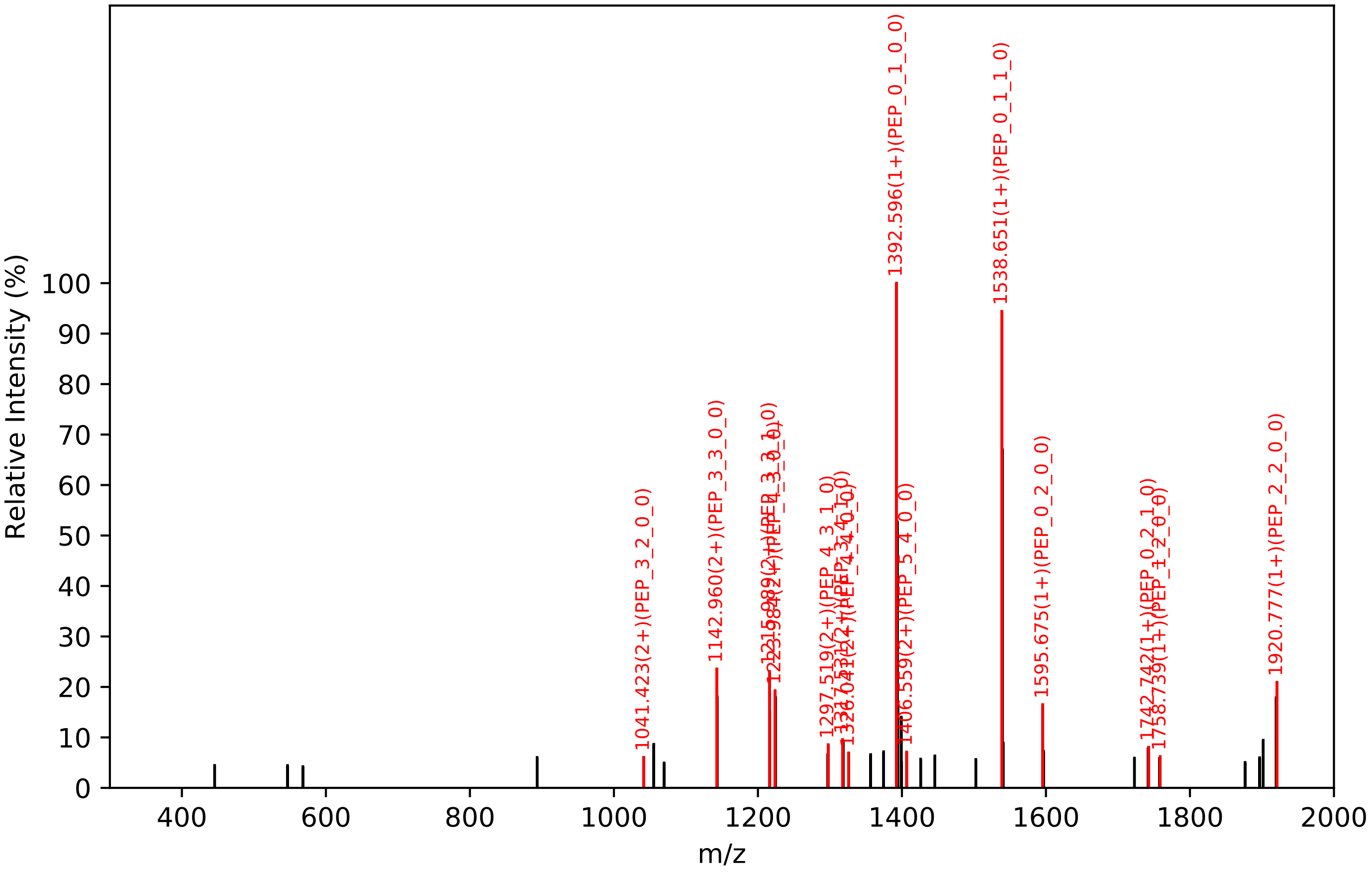

Unknown set no. 400, Experiment: Human Plasma exp_2

EEQYNSTYR(=PEP)_5_4_1_0, m/z:1479.58(2+), RT:25.41, Y-score:84.75

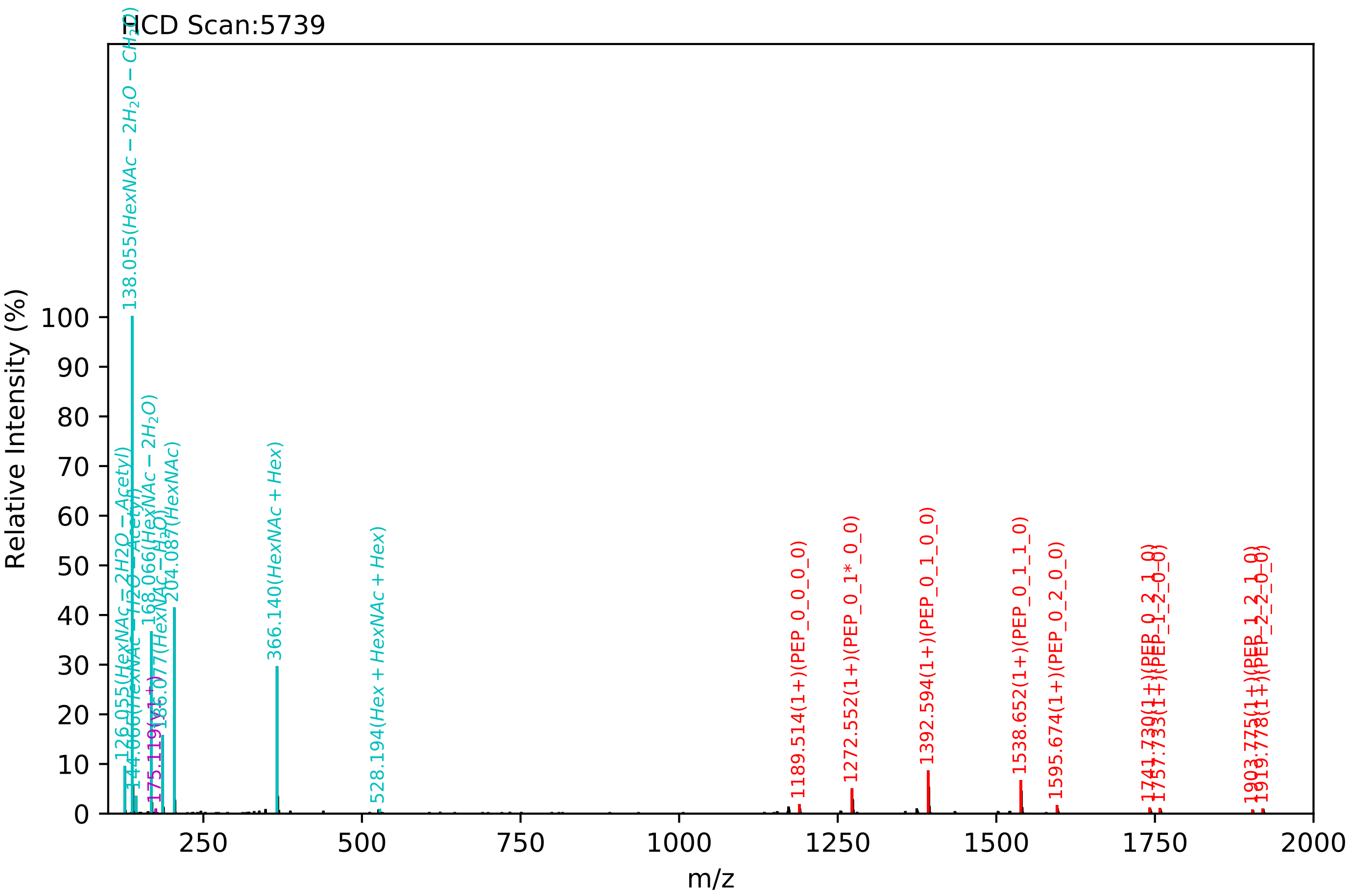

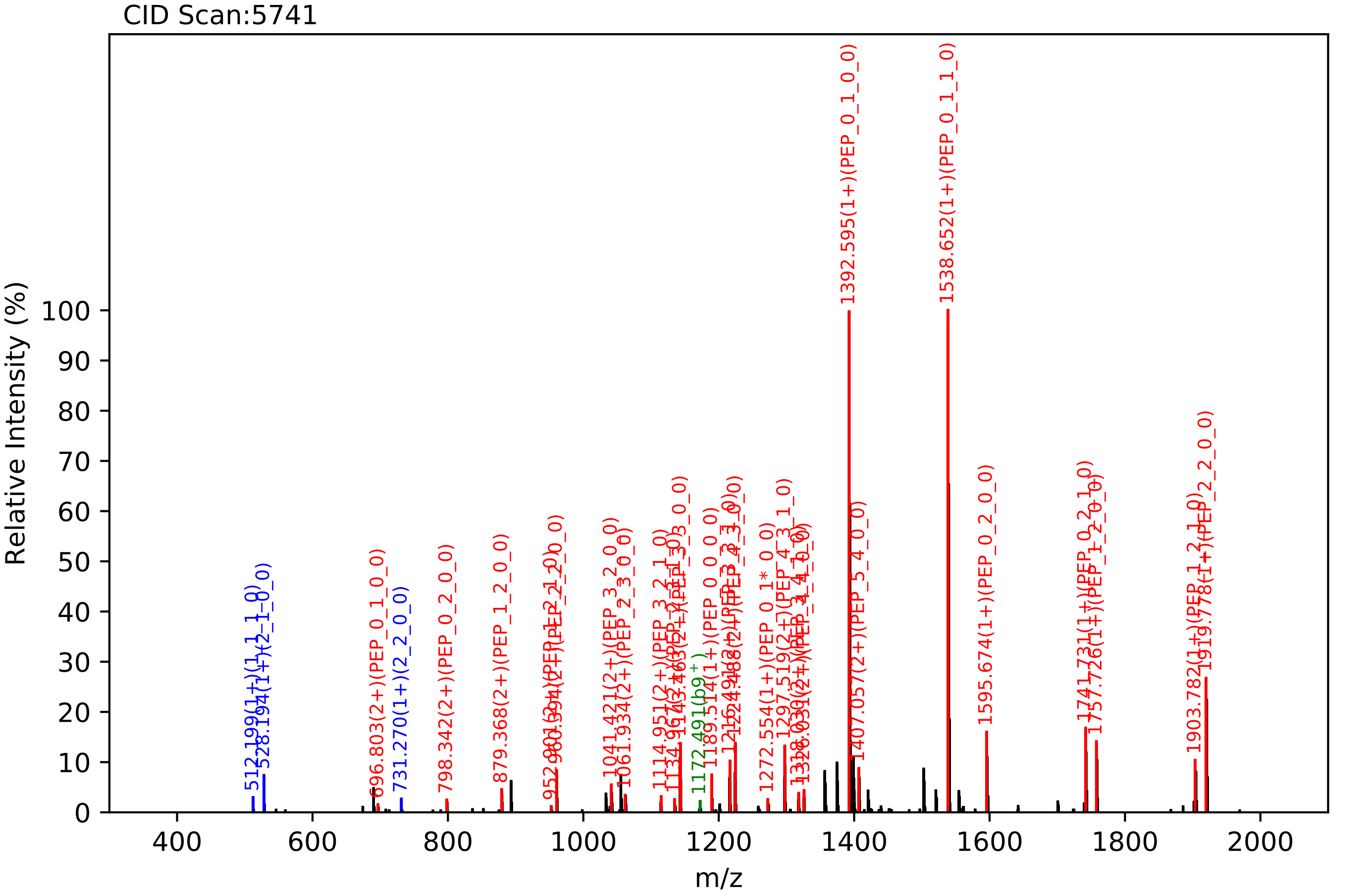

Unknown set no. 401, Experiment: Human Plasma exp_4

EEQYNSTYR(=PEP)_5_4_1_0, m/z:1479.58(2+), RT:25.56, Y-score:90.39

HCD Scan:5779

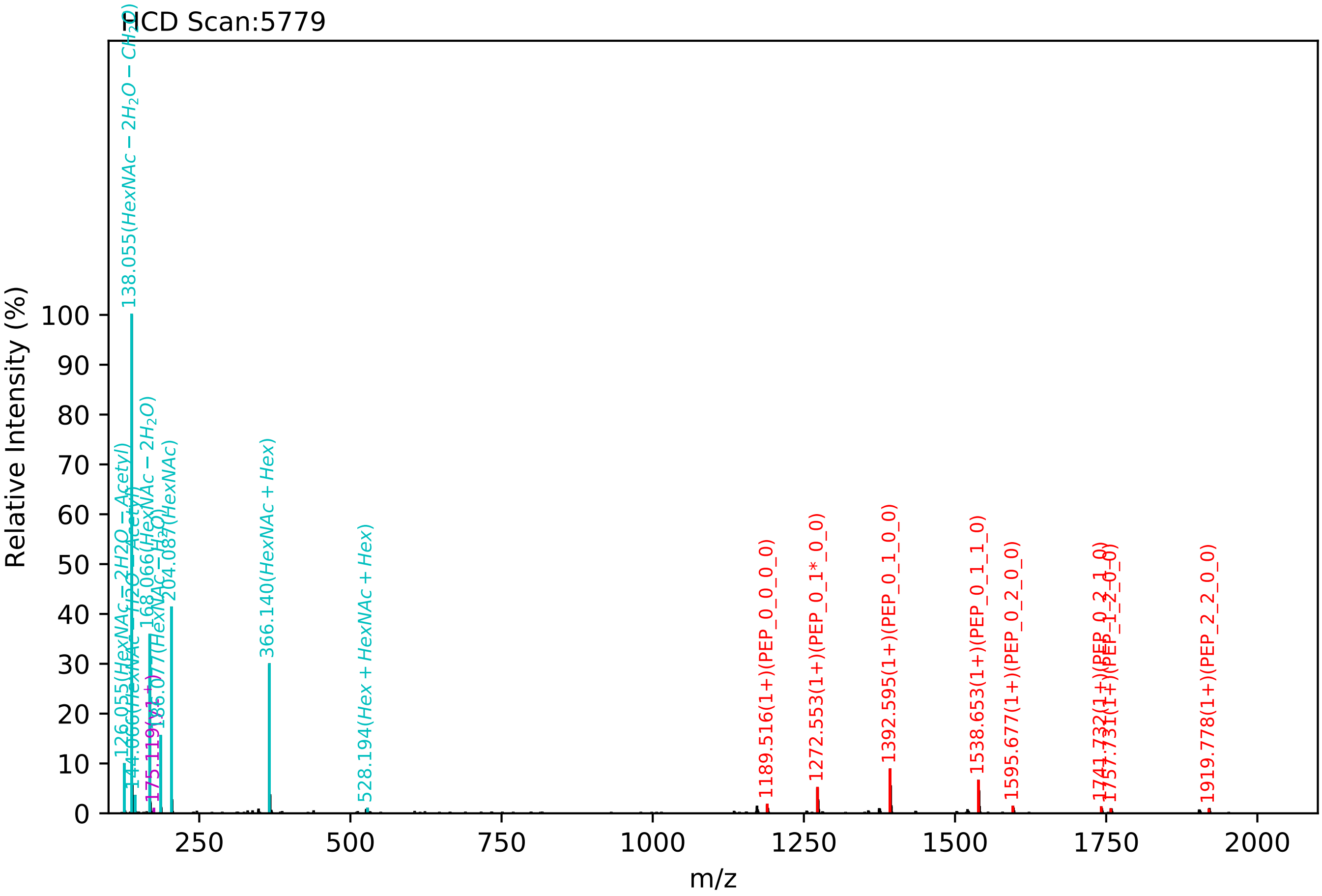

CID Scan:5782

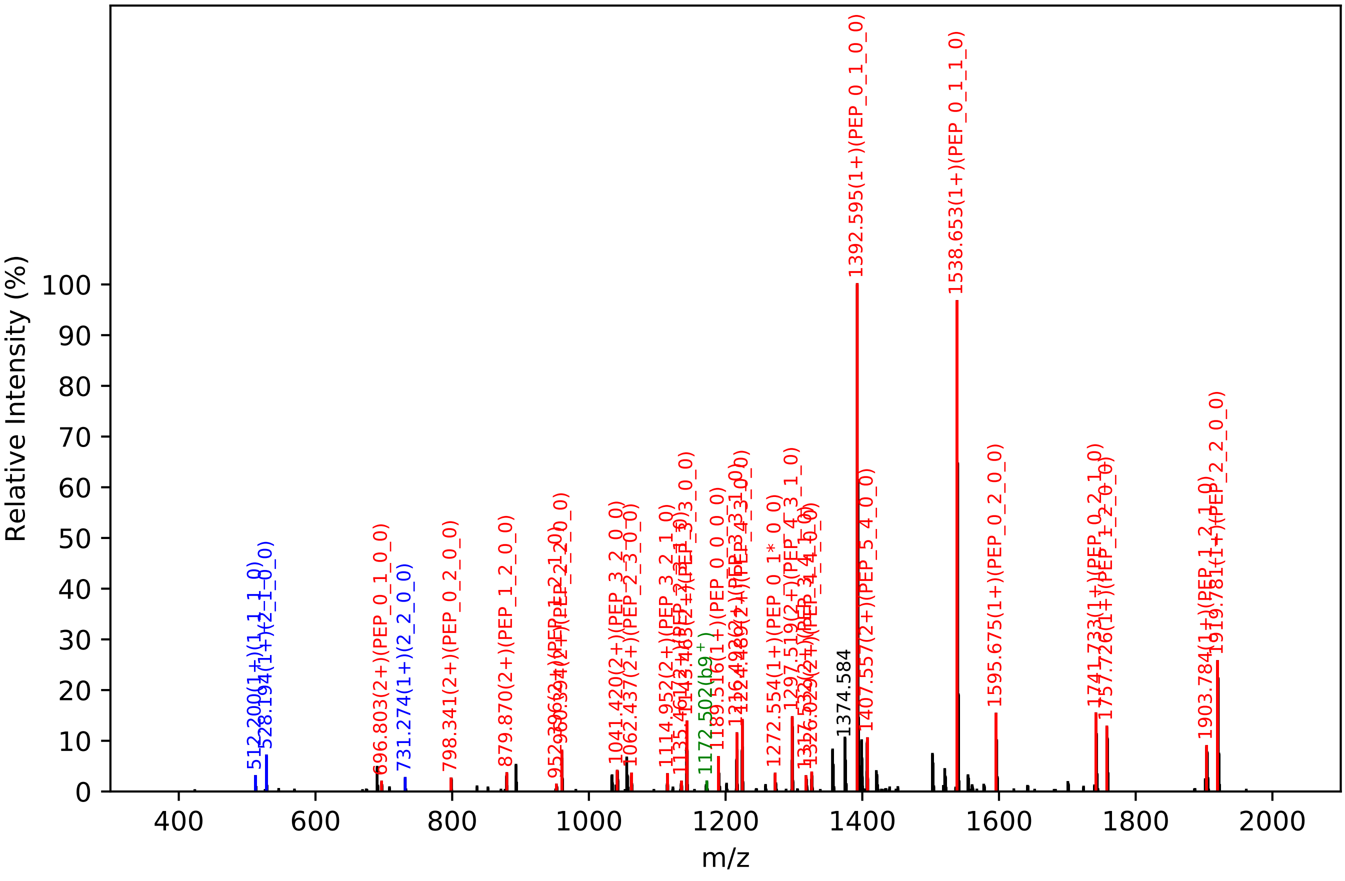

Unknown set no. 402, Experiment: Human Plasma exp_1

EEQYNSTYR(=PEP)_5_4_1_1, m/z:1625.63(2+), RT:37.19, Y-score:95.02

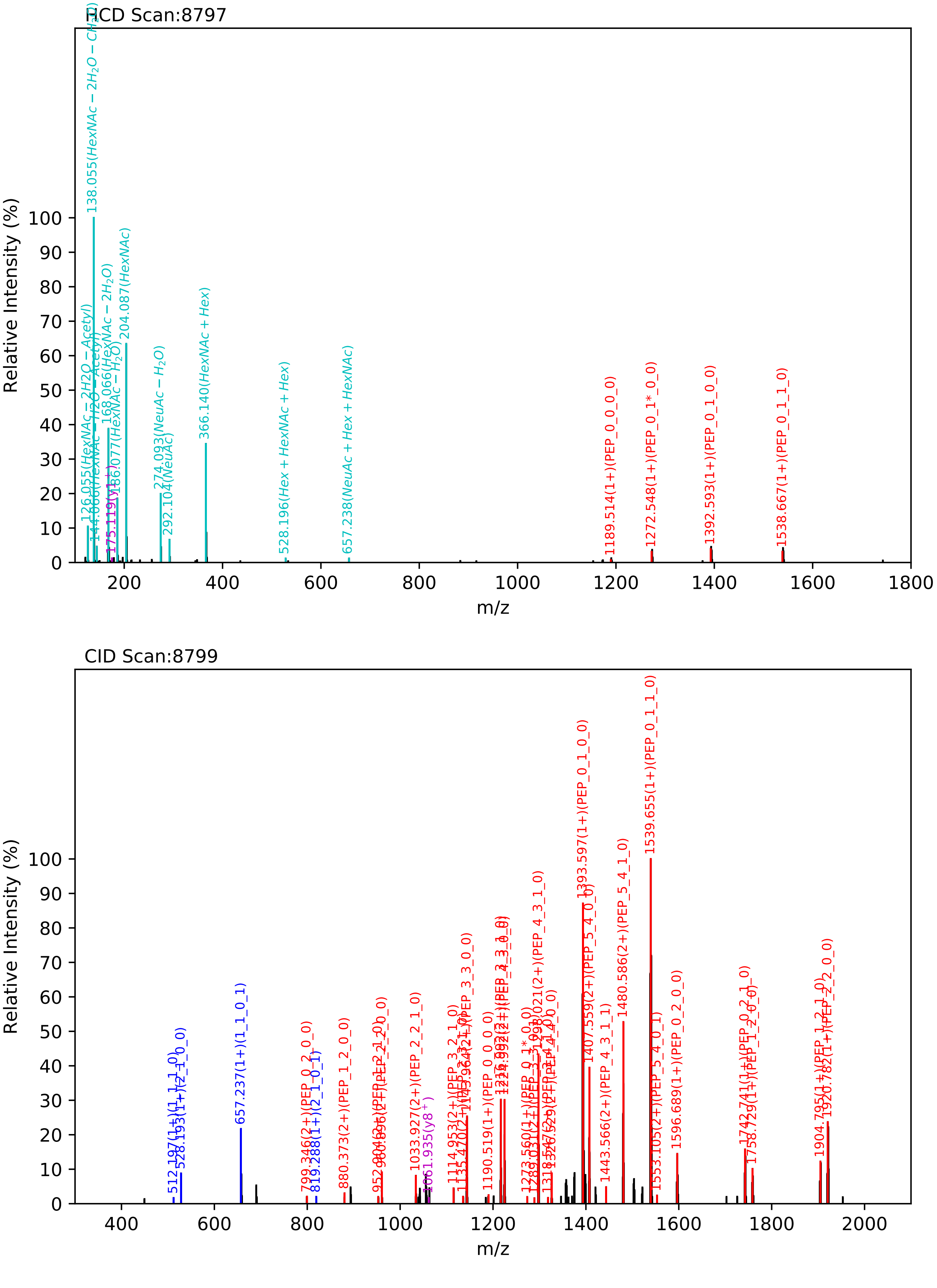

Unknown set no. 403, Experiment: Human Plasma exp_2

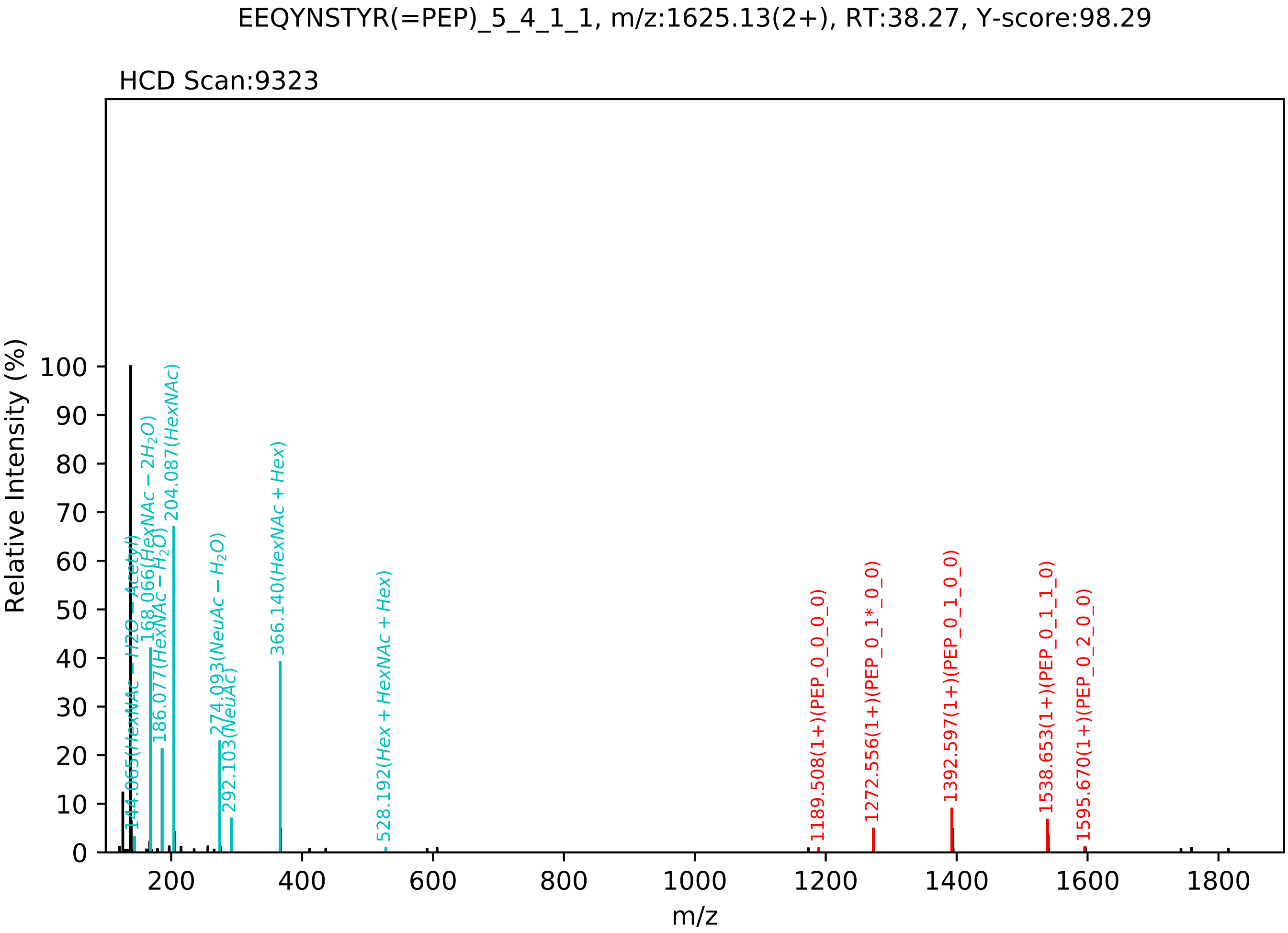

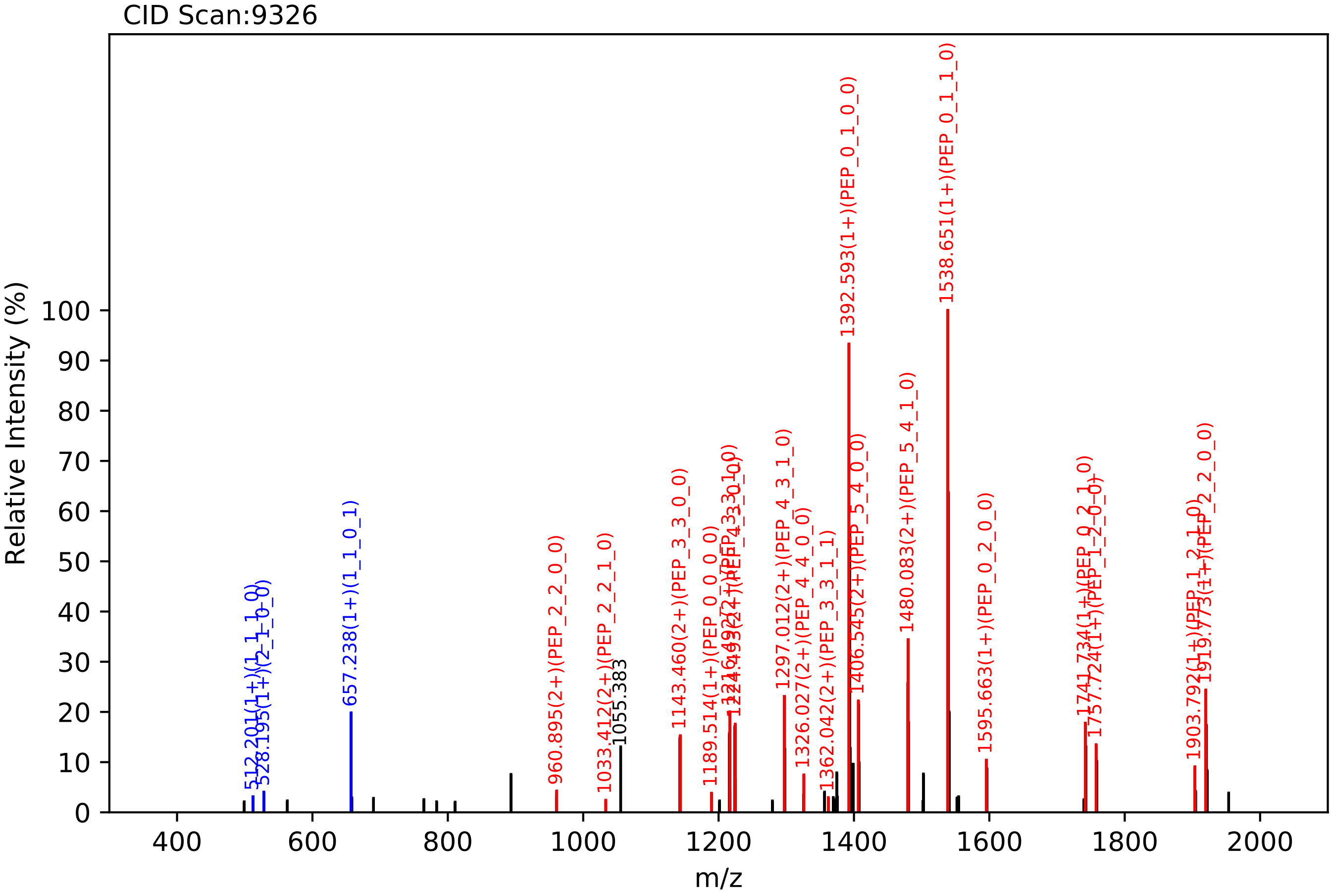

Unknown set no. 404, Experiment: Human Plasma exp_3

EEQYNSTYR(=PEP)_5_4_1_1, m/z:813.07(4+), RT:38.23, Y-score:89.06

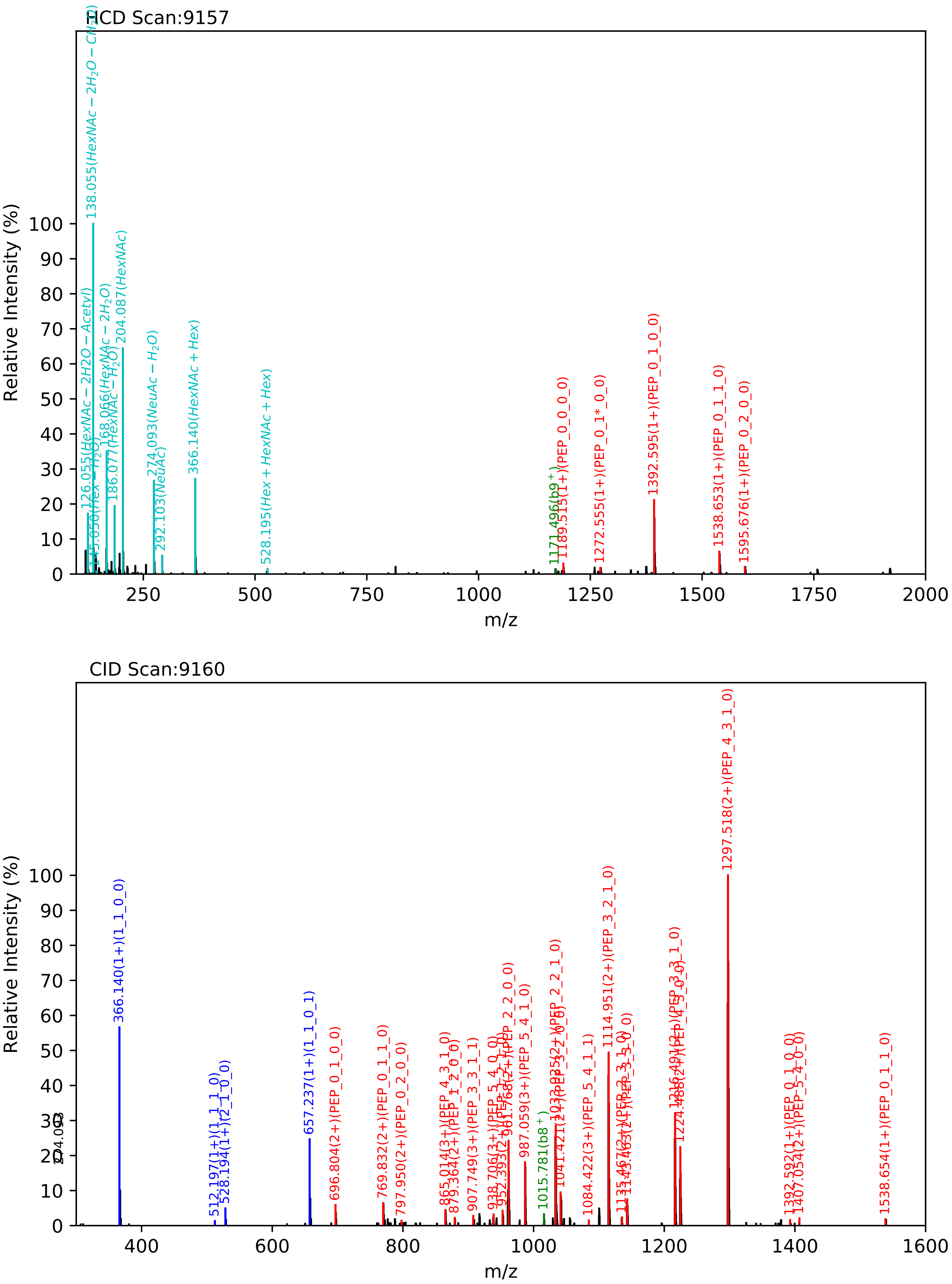

Unknown set no. 405, Experiment: Human Plasma exp_3

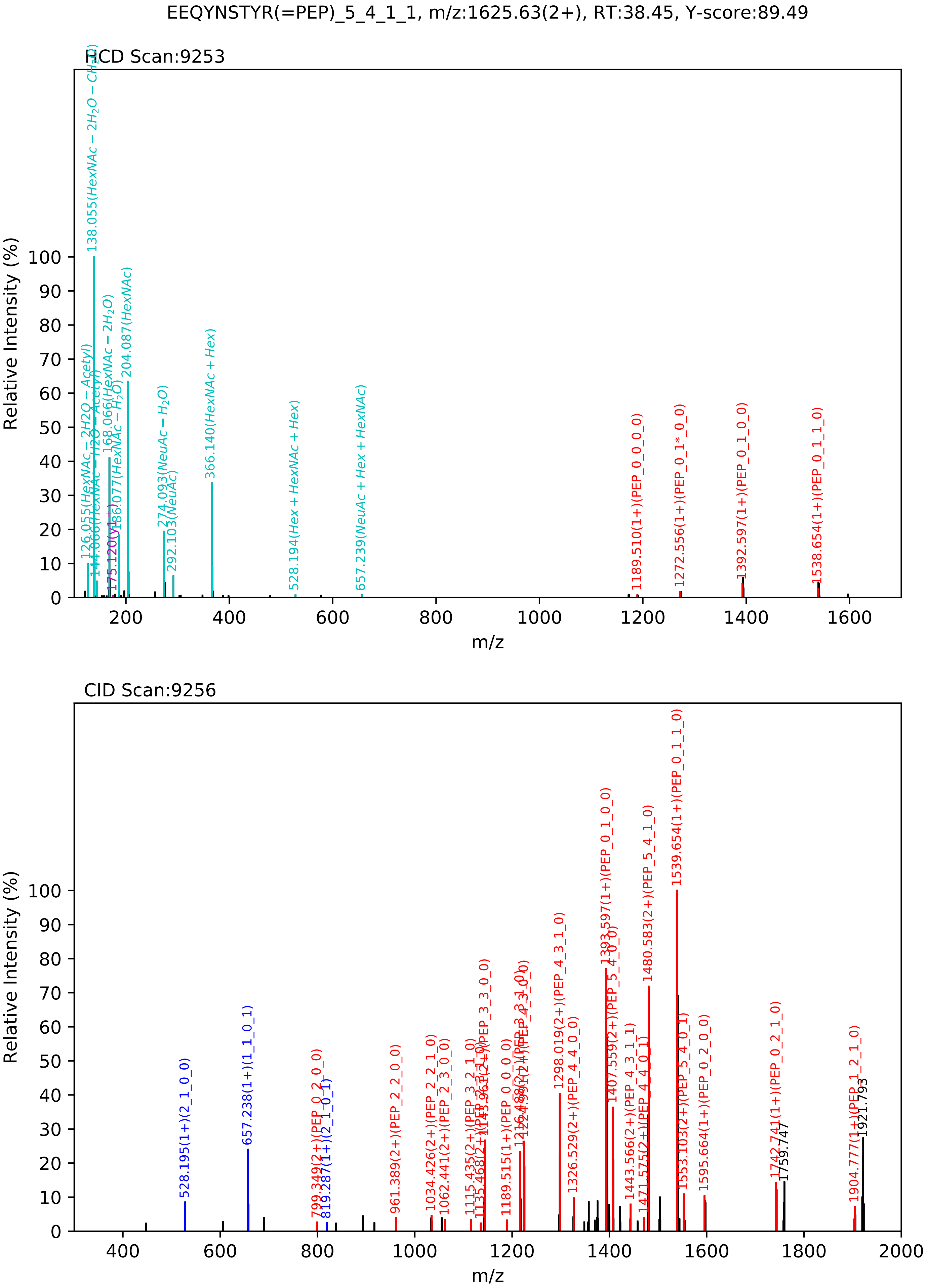

Unknown set no. 406, Experiment: Human Plasma exp_4

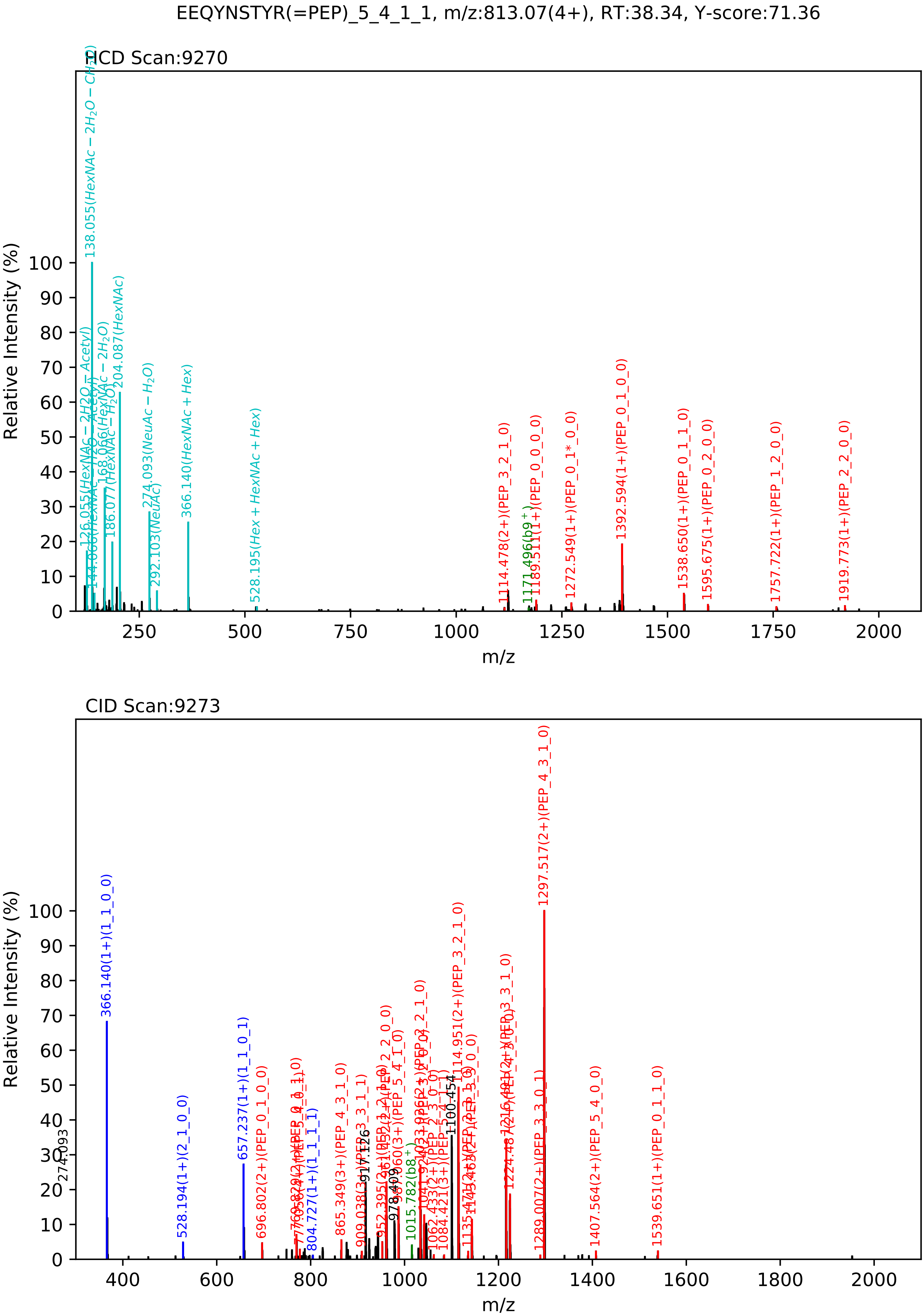

Unknown set no. 407, Experiment: Human Plasma exp_4

EEQYNSTYR(=PEP)_5_4_1_1, m/z:1083.76(3+), RT:37.28, Y-score:94.56

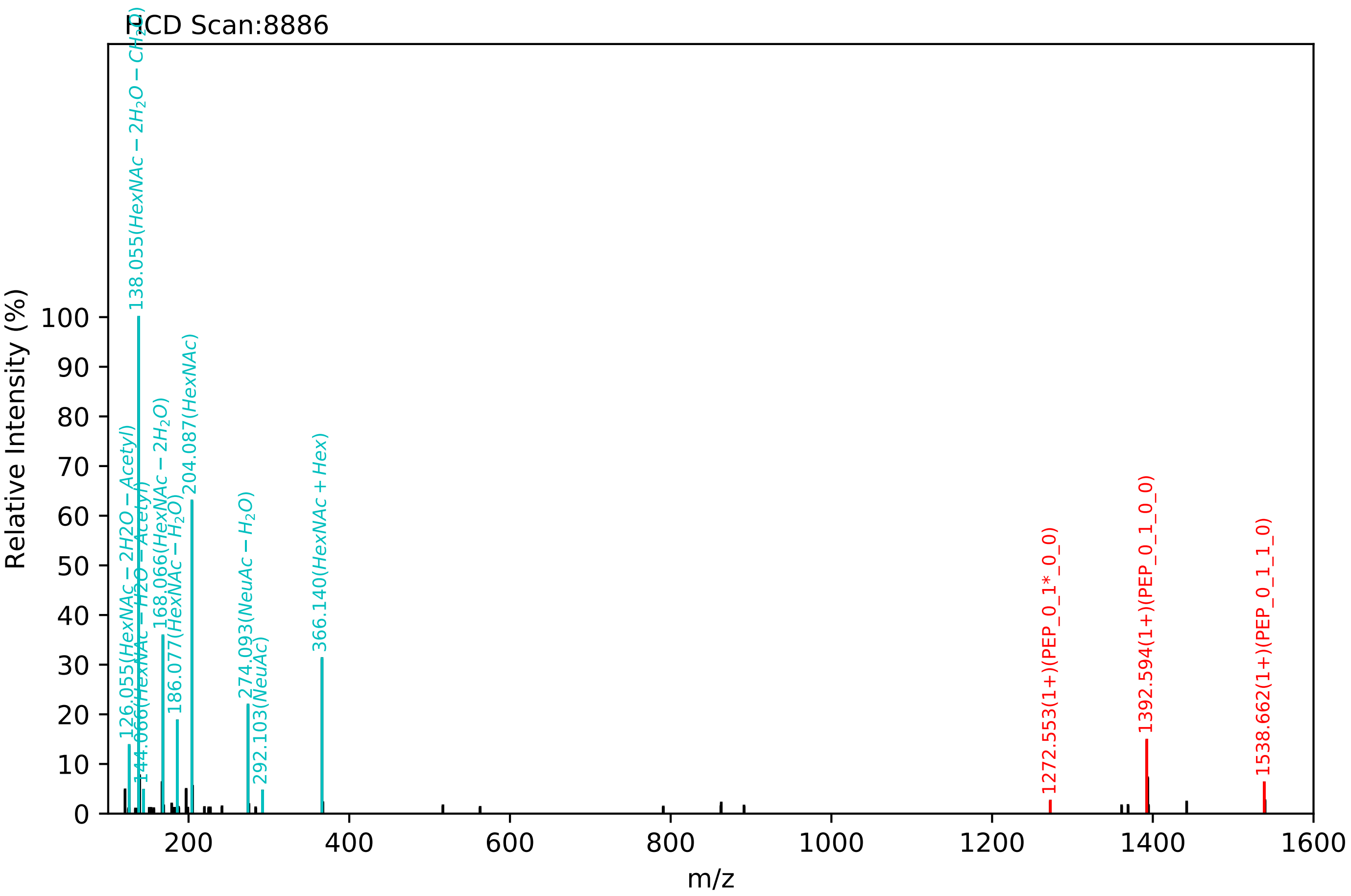

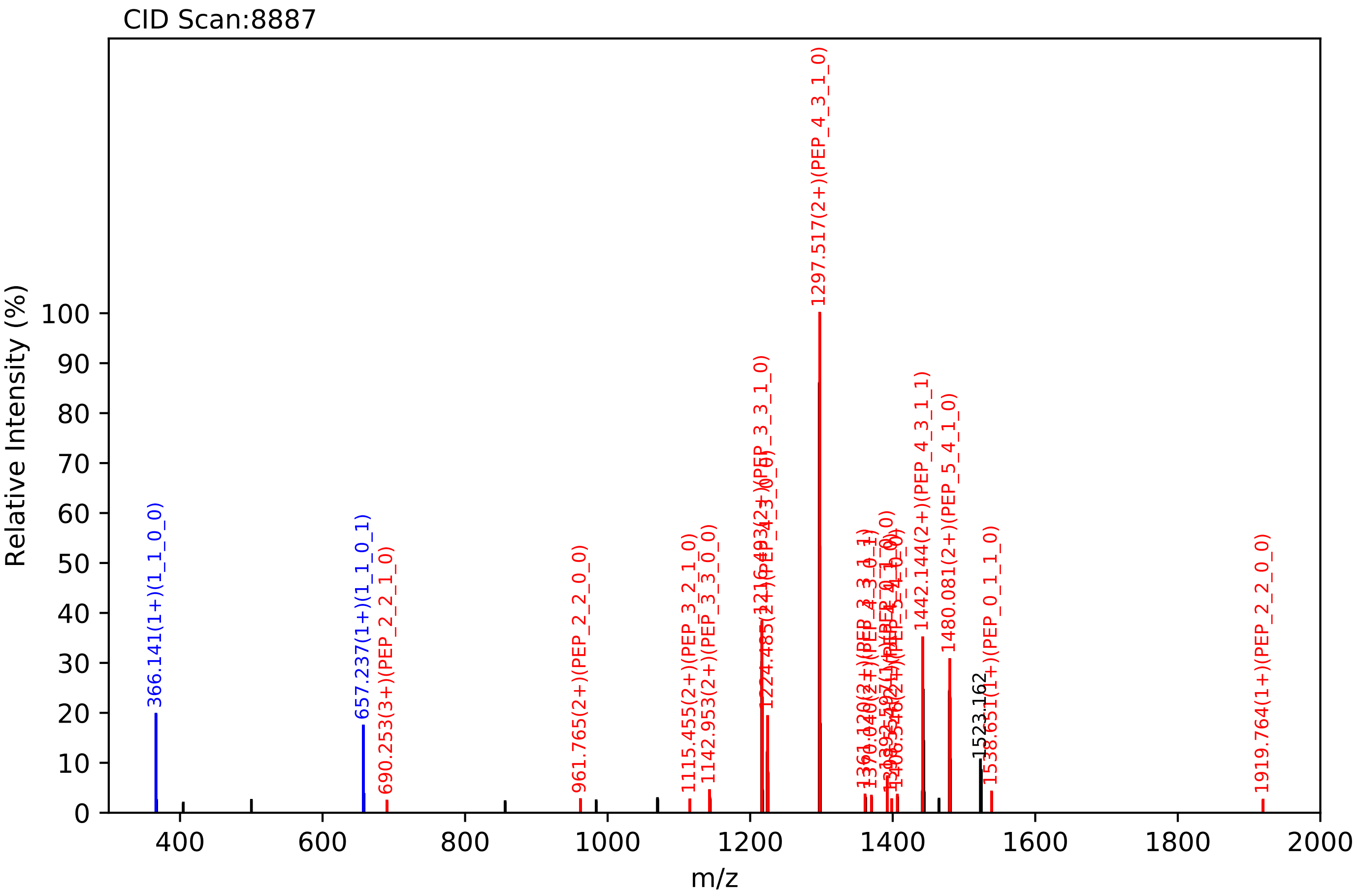

Unknown set no. 408, Experiment: Human Plasma exp_4

EEQYNSTYR(=PEP)_5_4_1_1, m/z:1625.13(2+), RT:38.62, Y-score:95.81

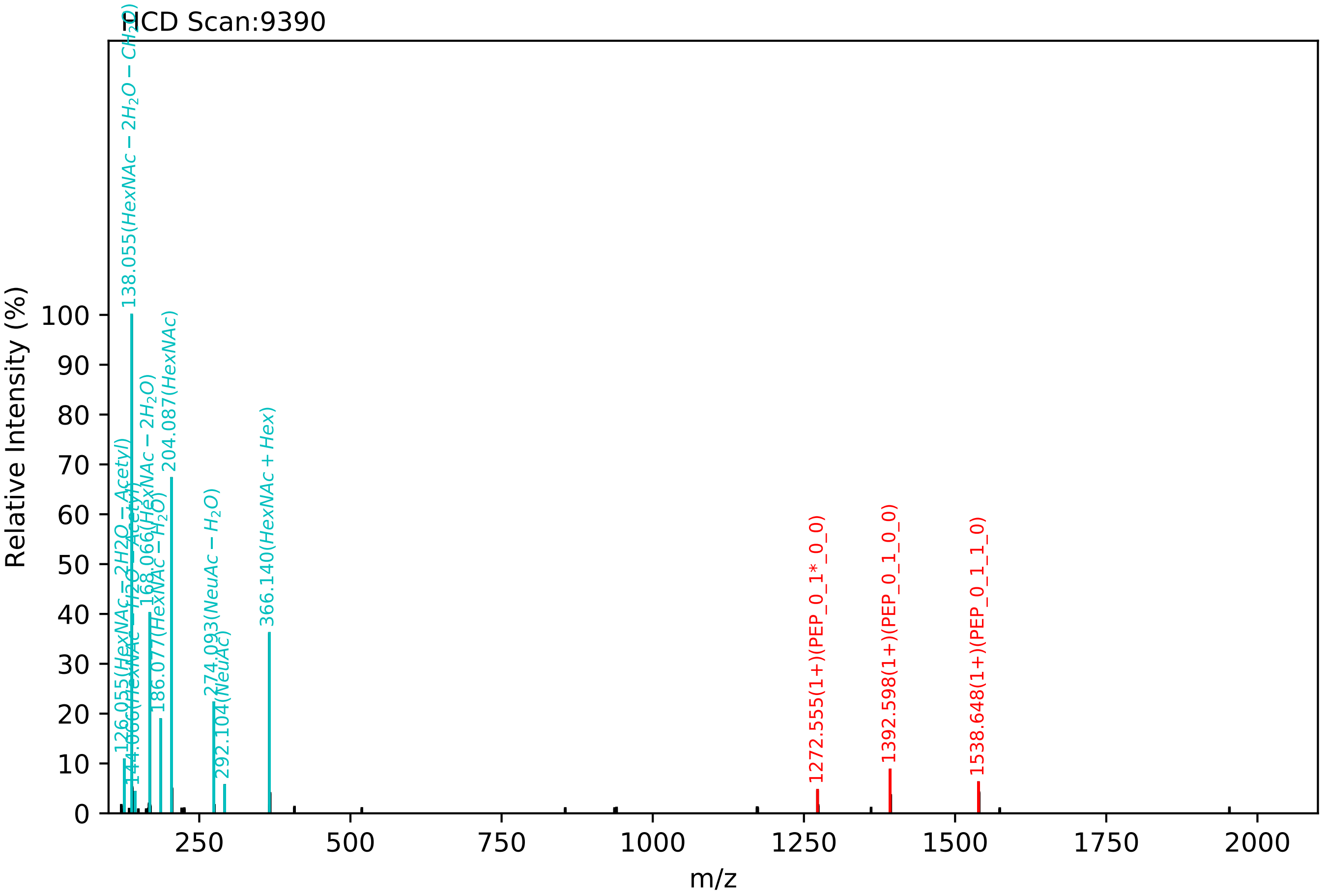

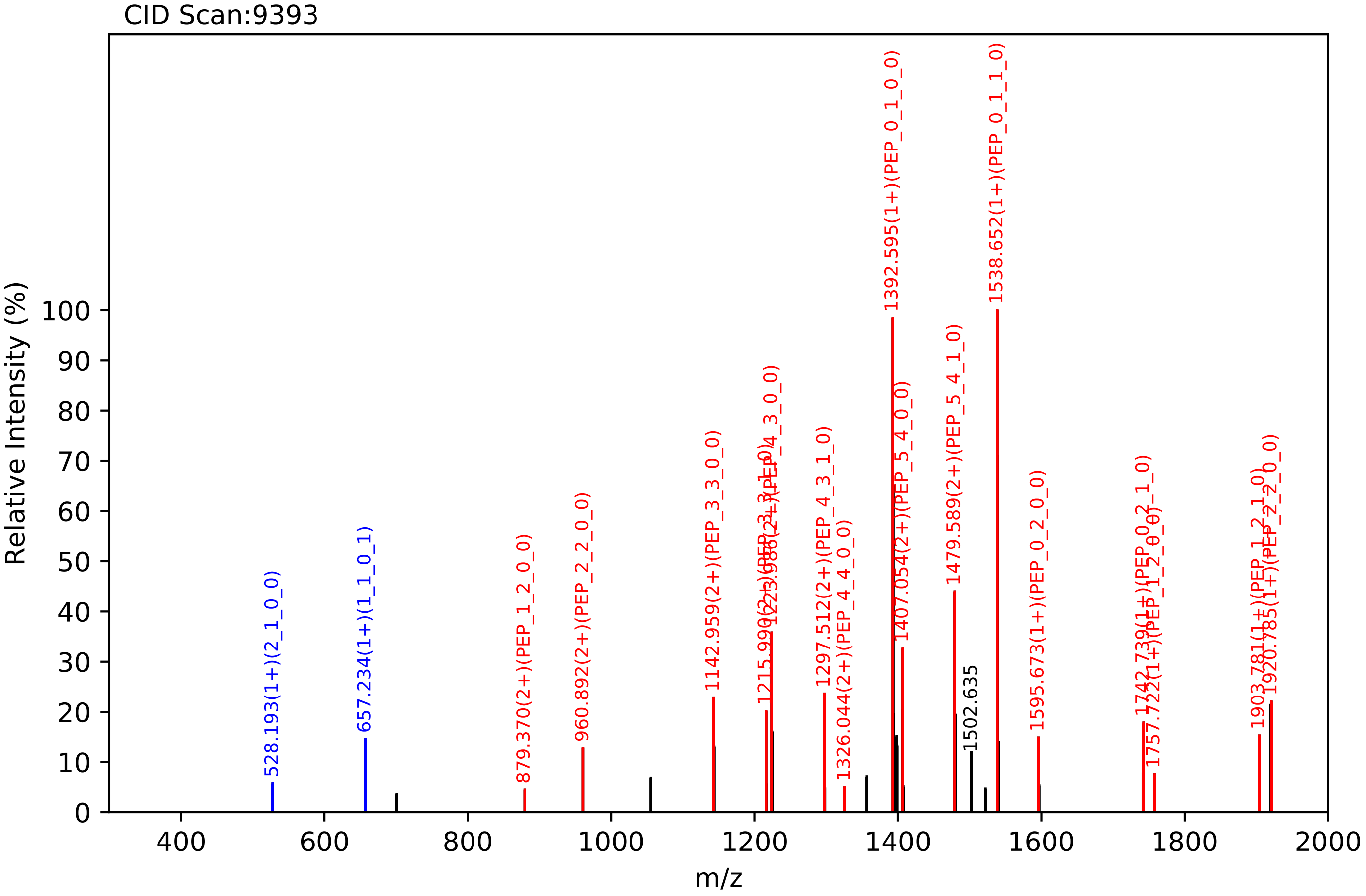

Unknown set no. 409, Experiment: Human Plasma exp_5

EEQYNSTYR(=PEP)_5_4_1_1, m/z:1625.13(2+), RT:38.69, Y-score:92.43

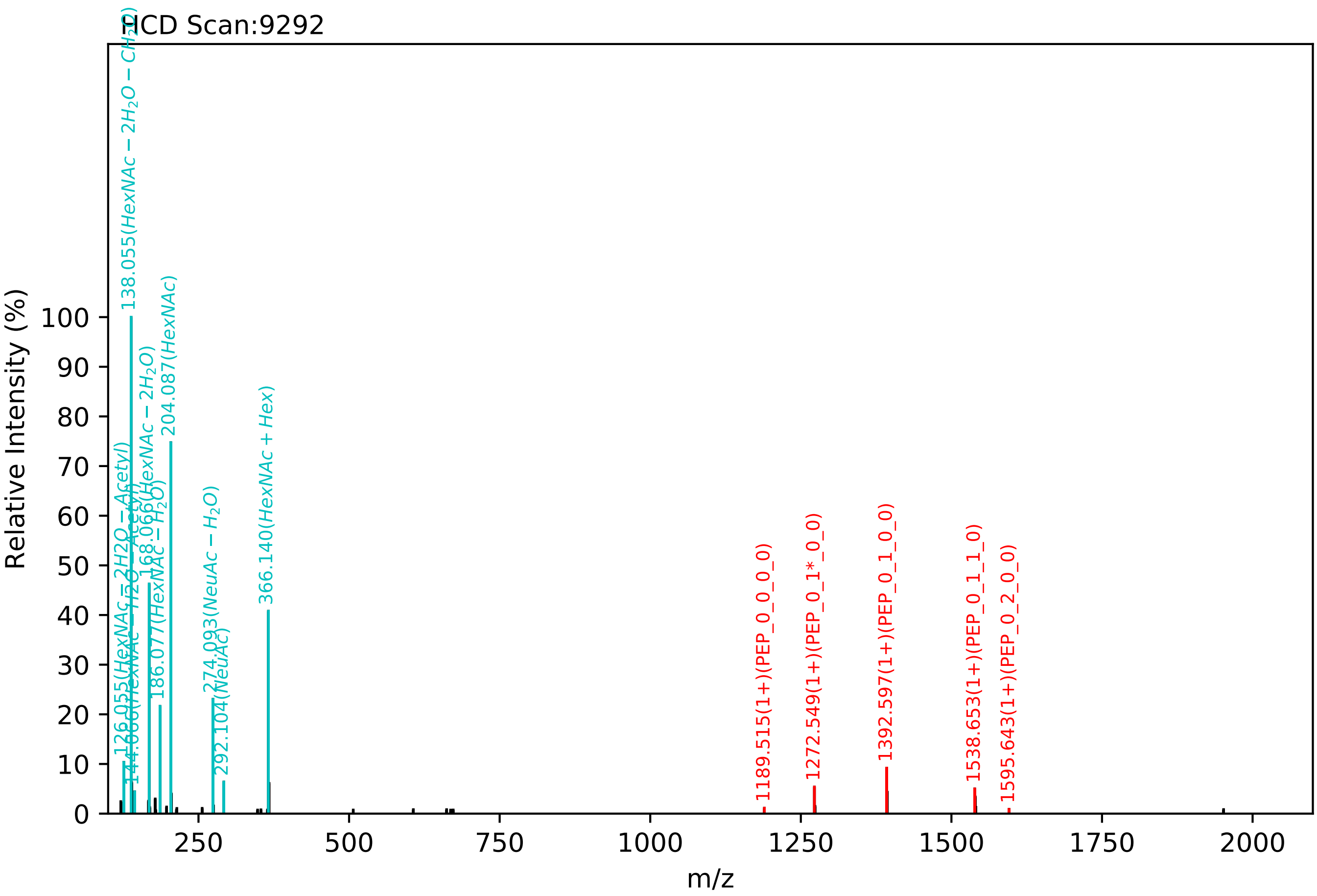

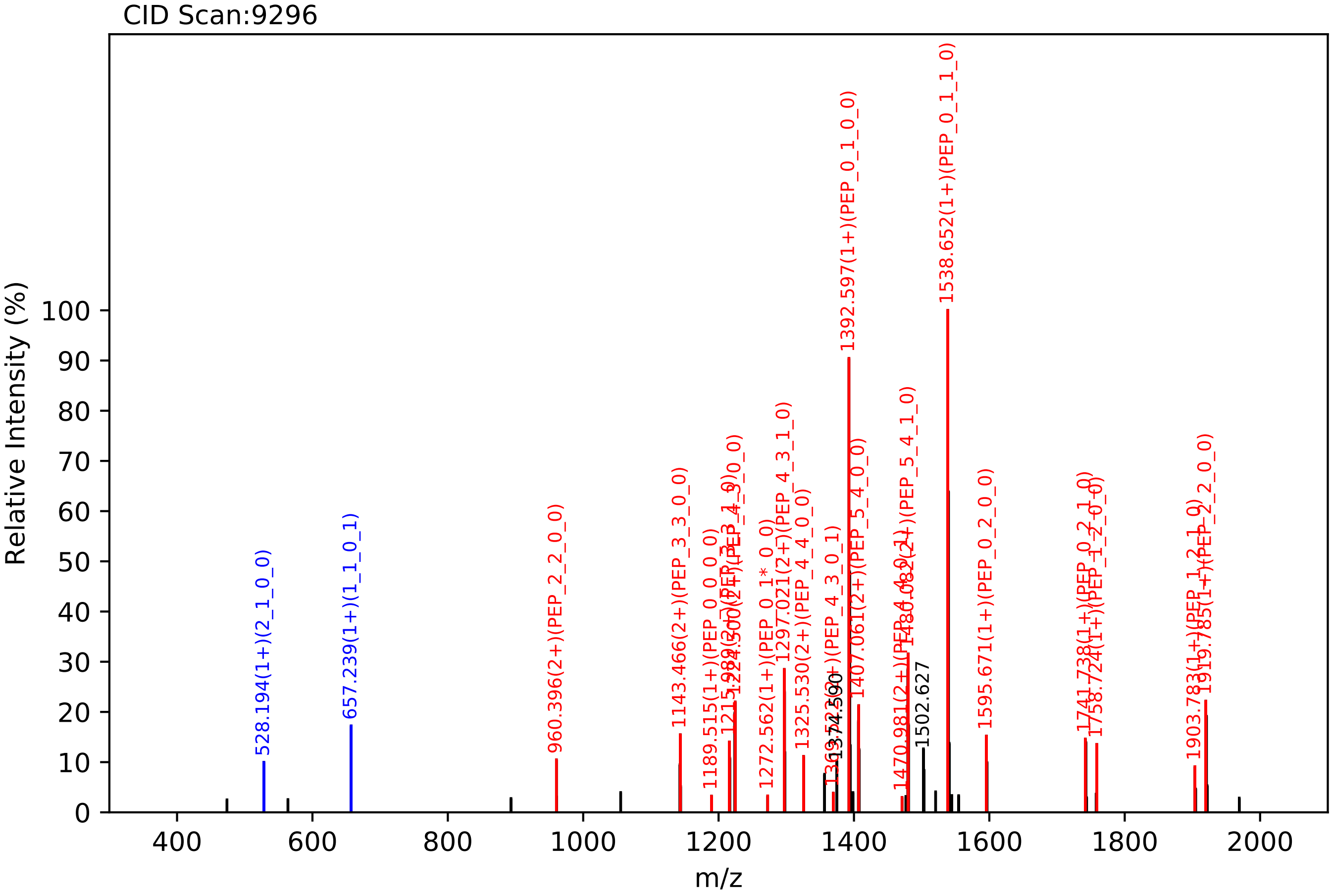

Unknown set no. 410, Experiment: Human Plasma exp_1

EEQYNSTYR(=PEP)_5_4_1_2, m/z:1180.78(3+), RT:49.52, Y-score:86.11

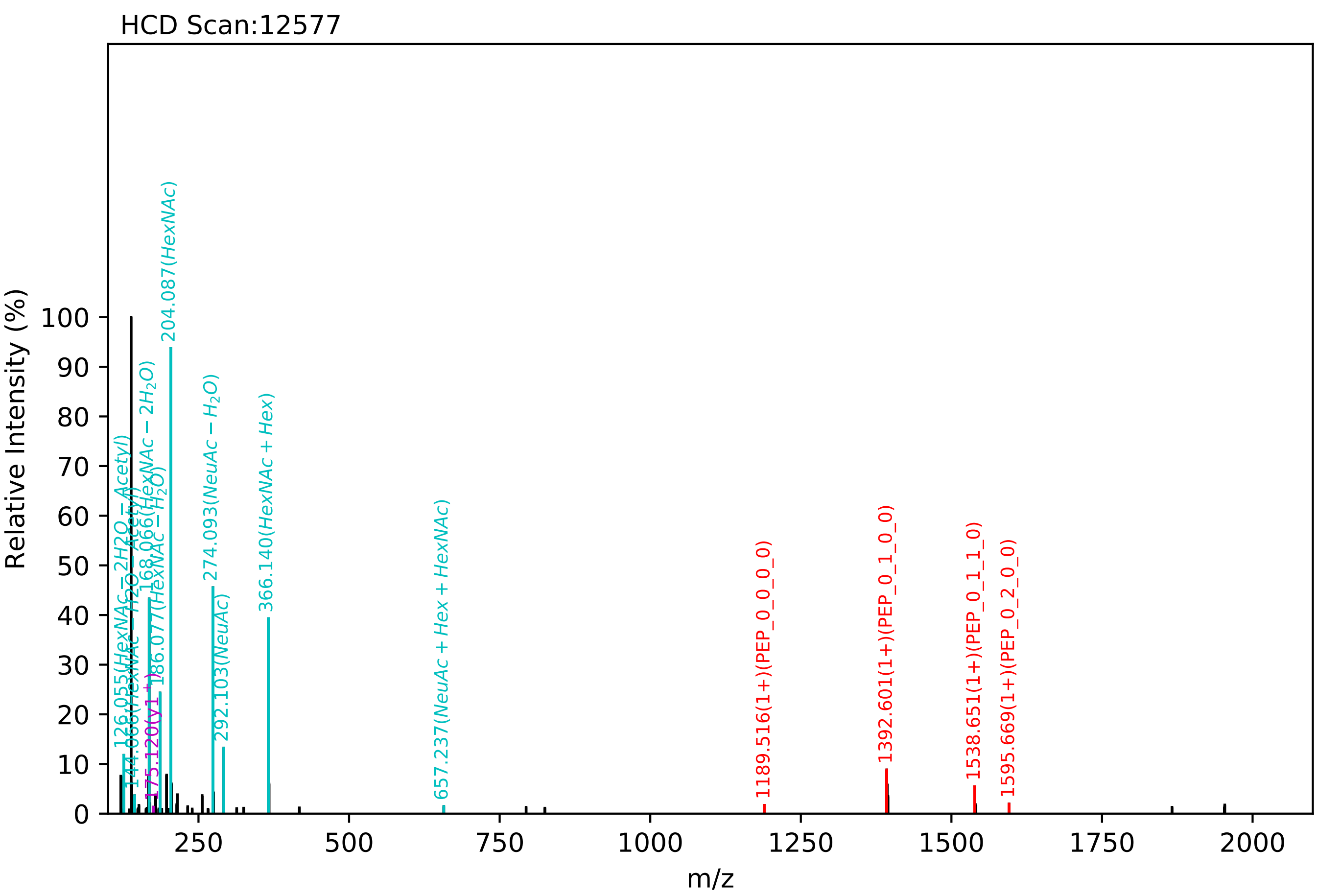

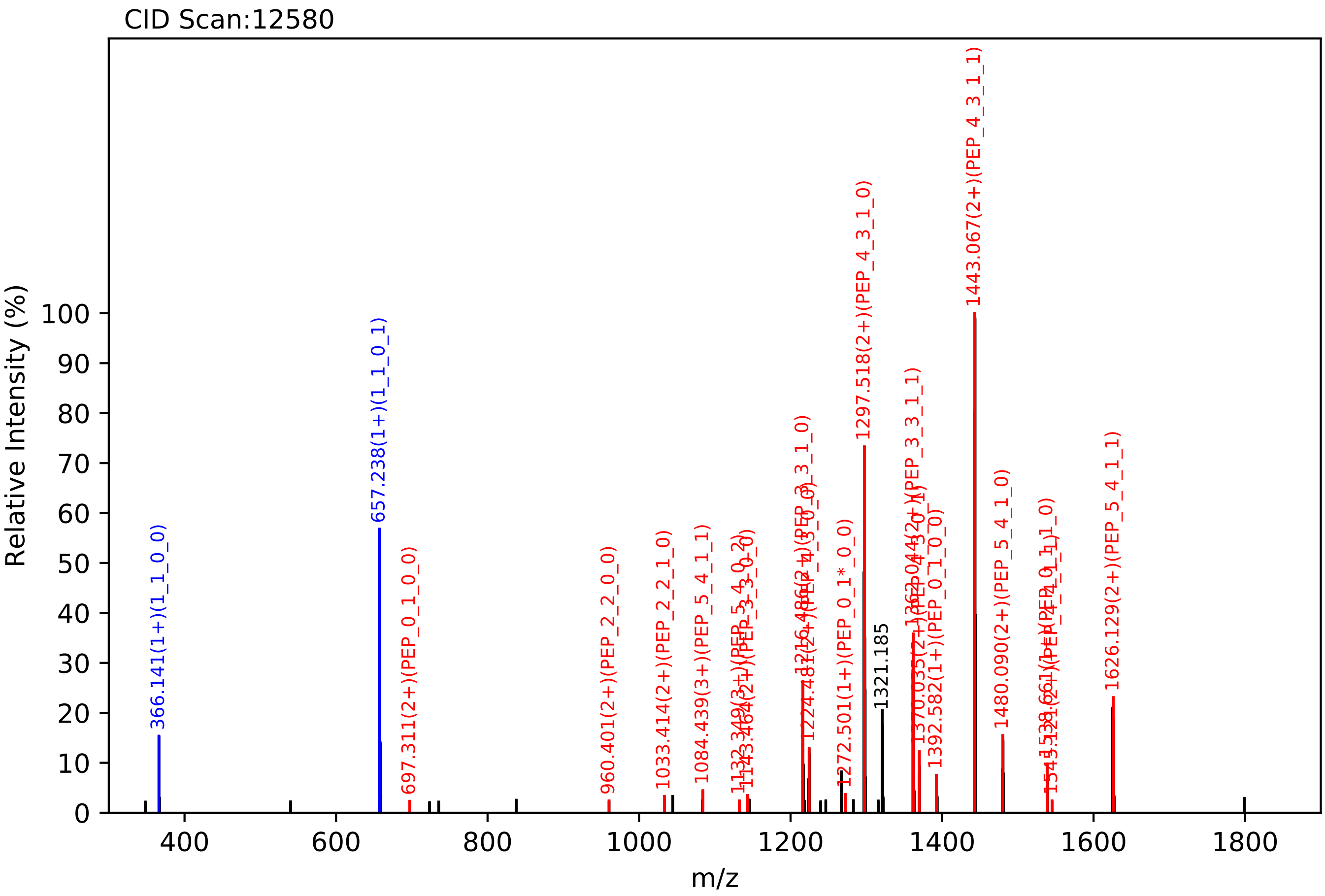

Unknown set no. 411, Experiment: Human Plasma exp_2

EEQYNSTYR(=PEP)_5_4_1_2, m/z:1180.79(3+), RT:50.93, Y-score:85.72

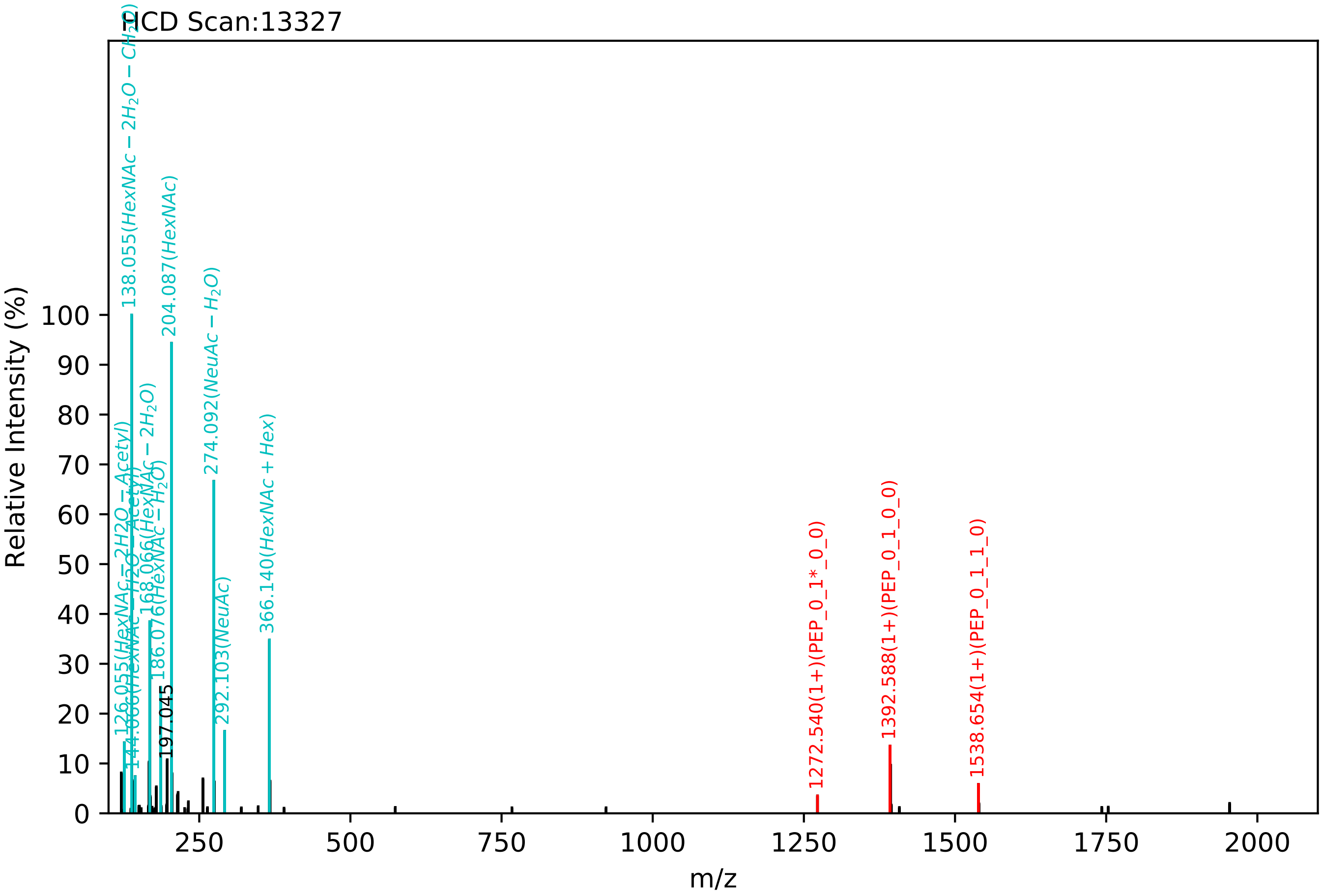

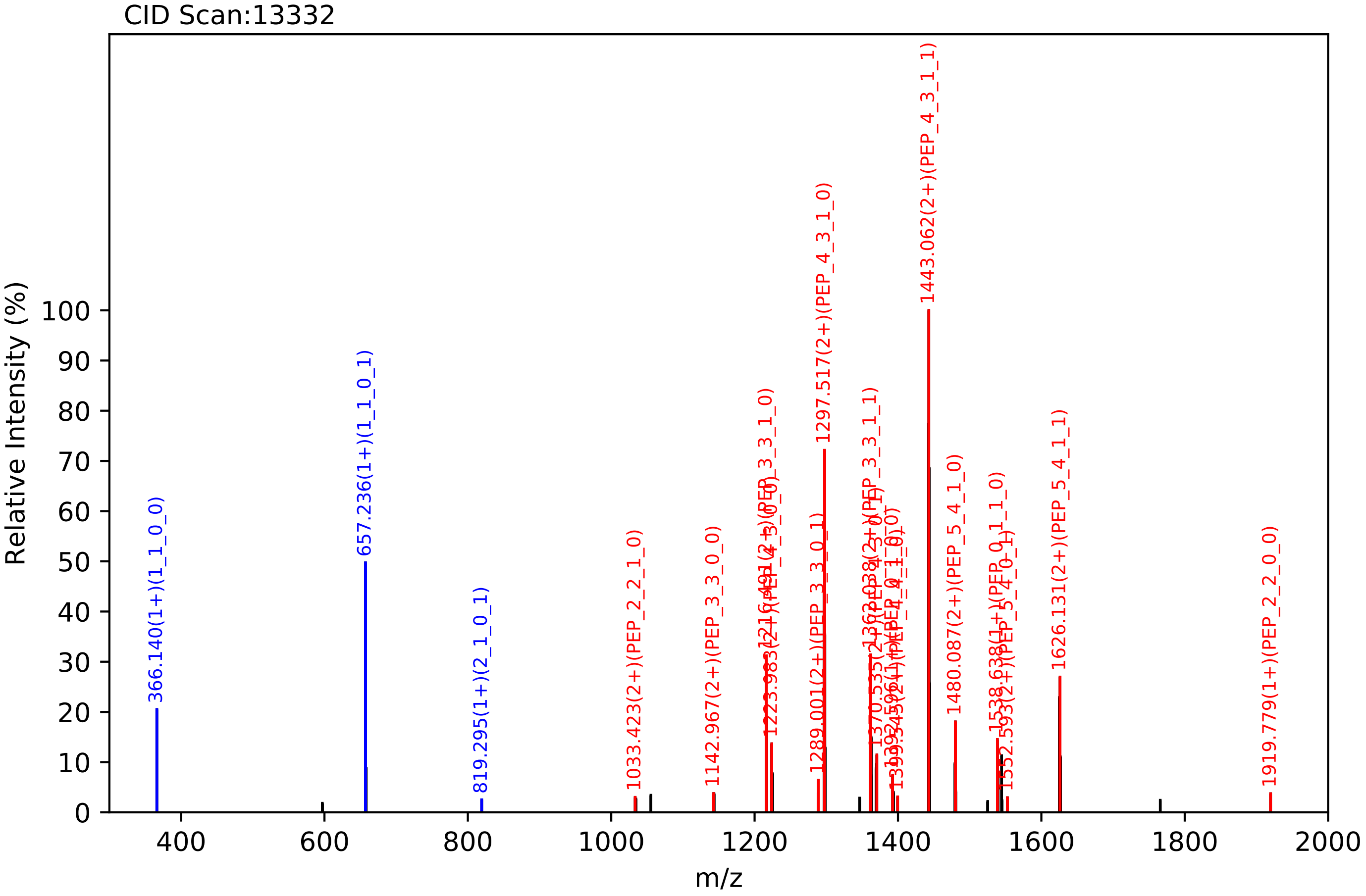

Unknown set no. 412, Experiment: Human Plasma exp_1

EEQYNSTYR(=PEP)_5_4_2_0, m/z:1035.41(3+), RT:23.81, Y-score:92.52

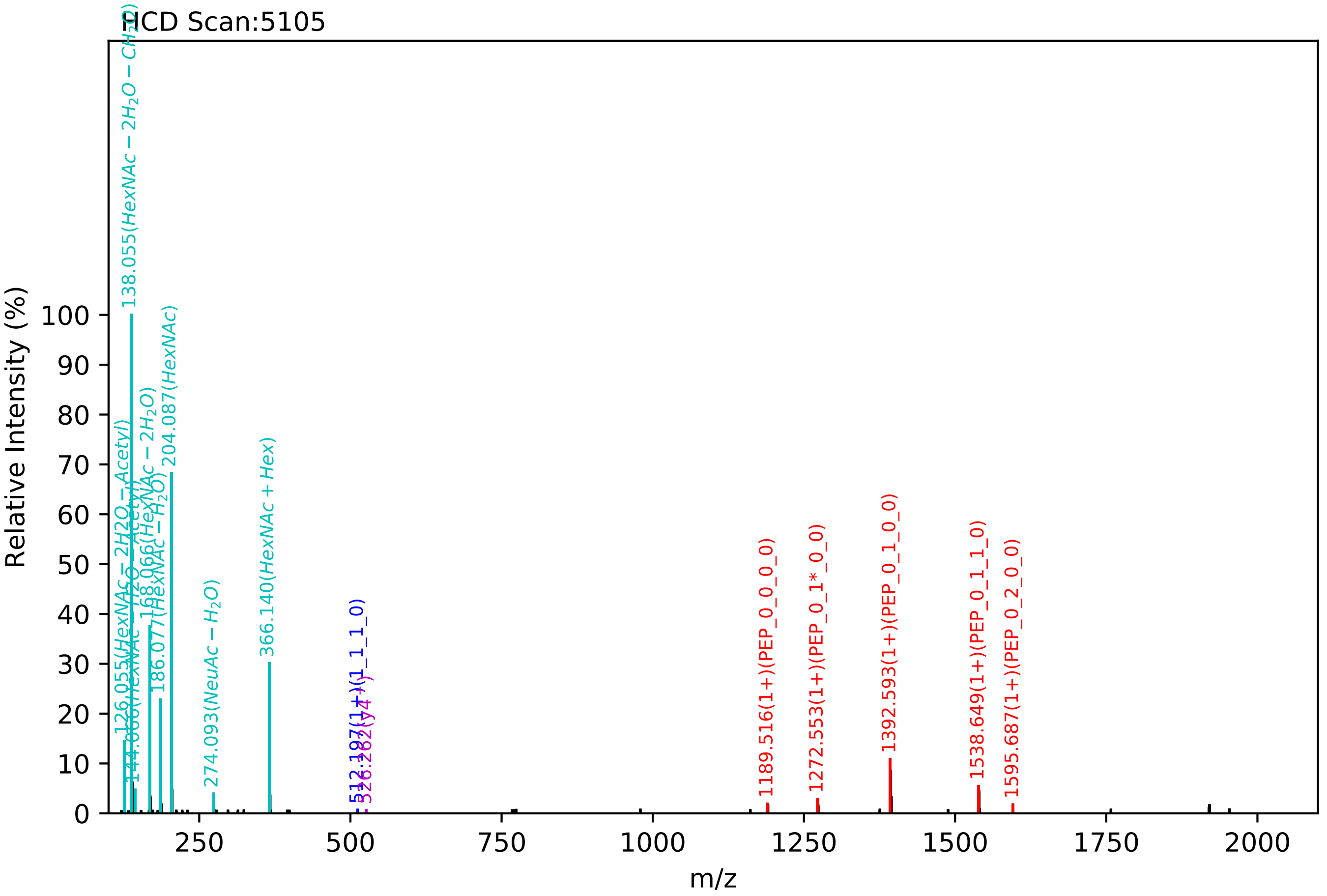

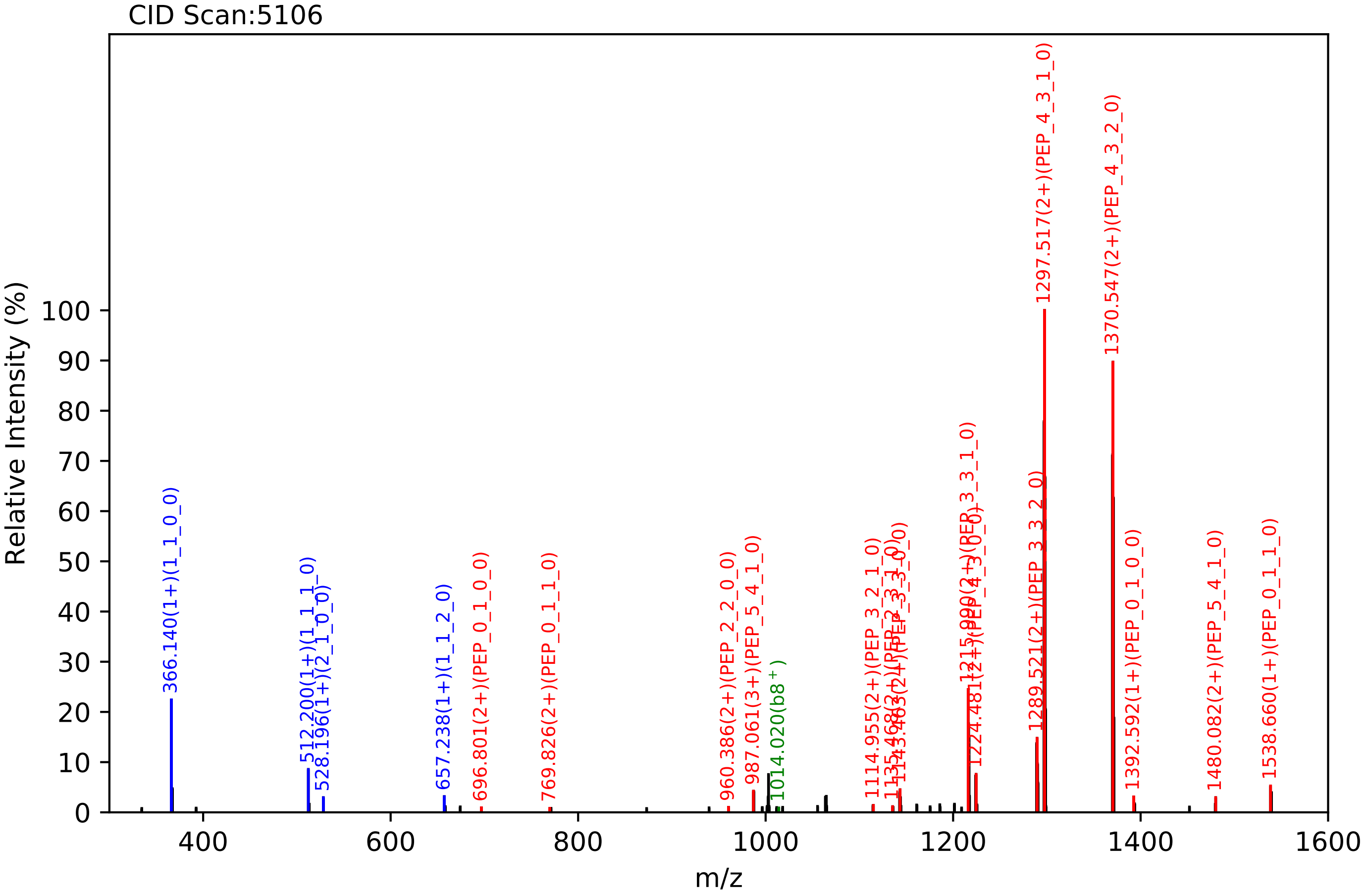

Unknown set no. 413, Experiment: Human Plasma exp_5

EEQYNSTYR(=PEP)_5_4_2_0, m/z:1035.41(3+), RT:24.28, Y-score:96.23

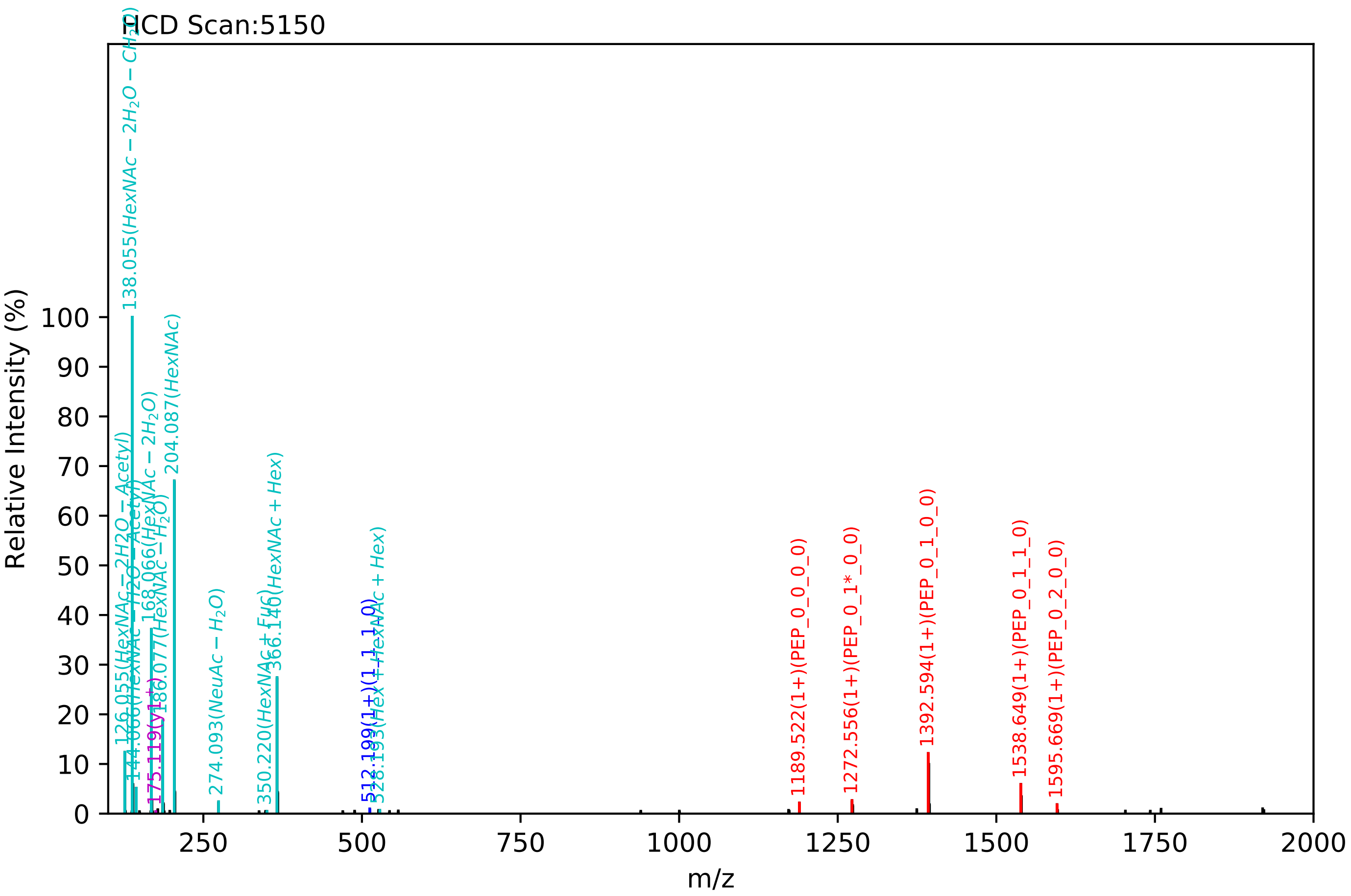

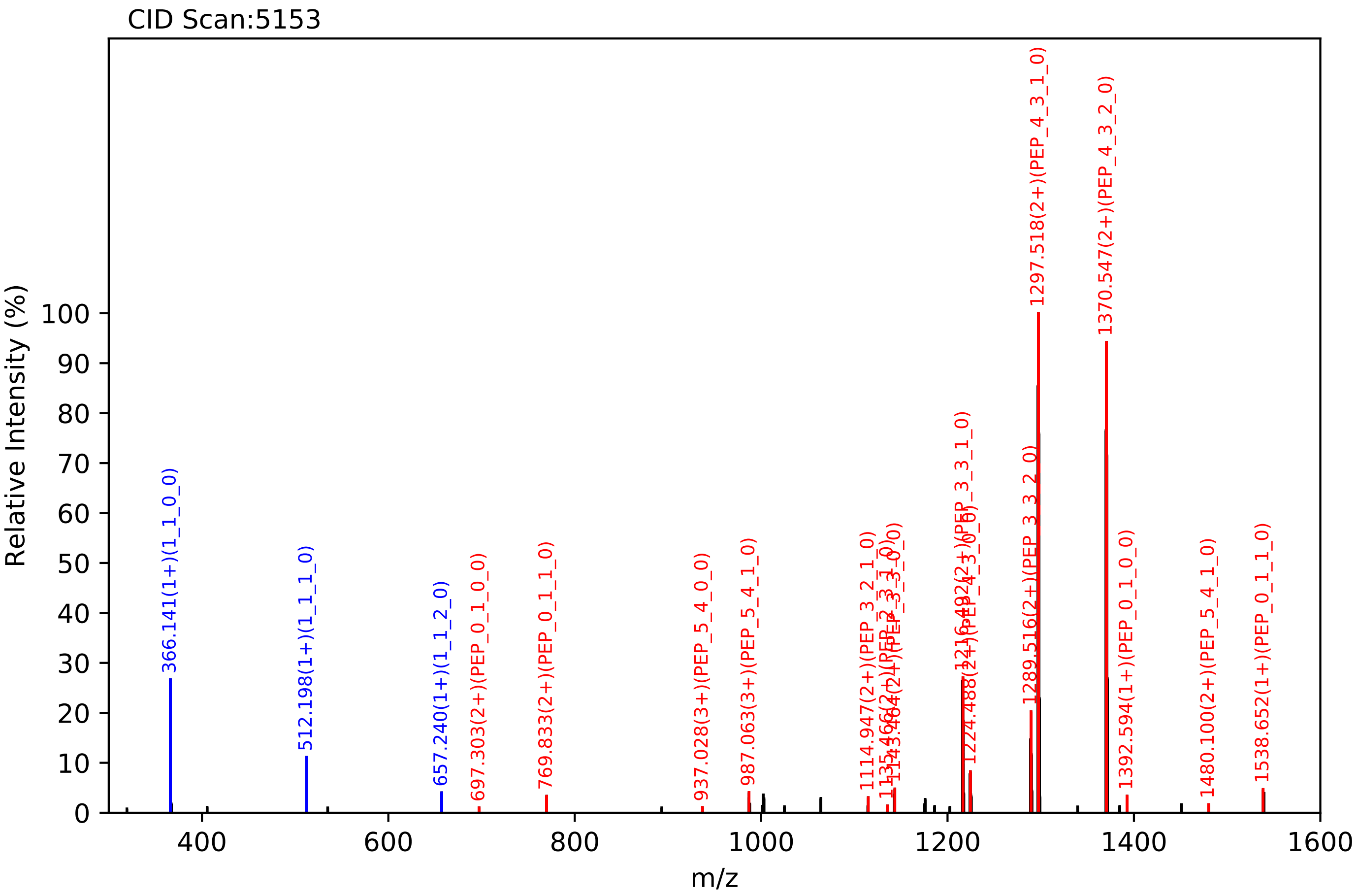

Unknown set no. 414, Experiment: Human Plasma exp_2

EEQYNSTYR(=PEP)_5_4_2_1, m/z:1132.44(3+), RT:36.40, Y-score:87.61

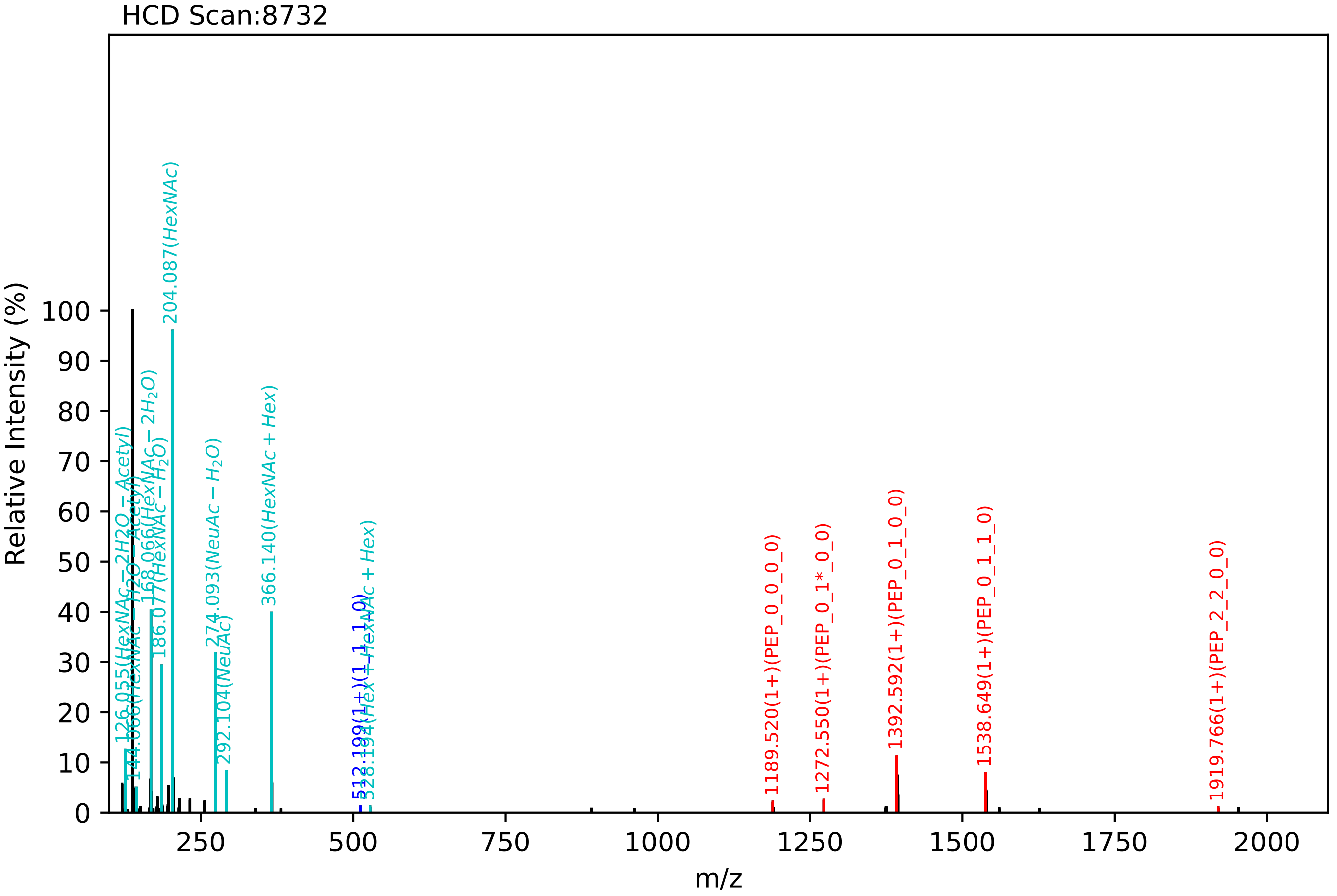

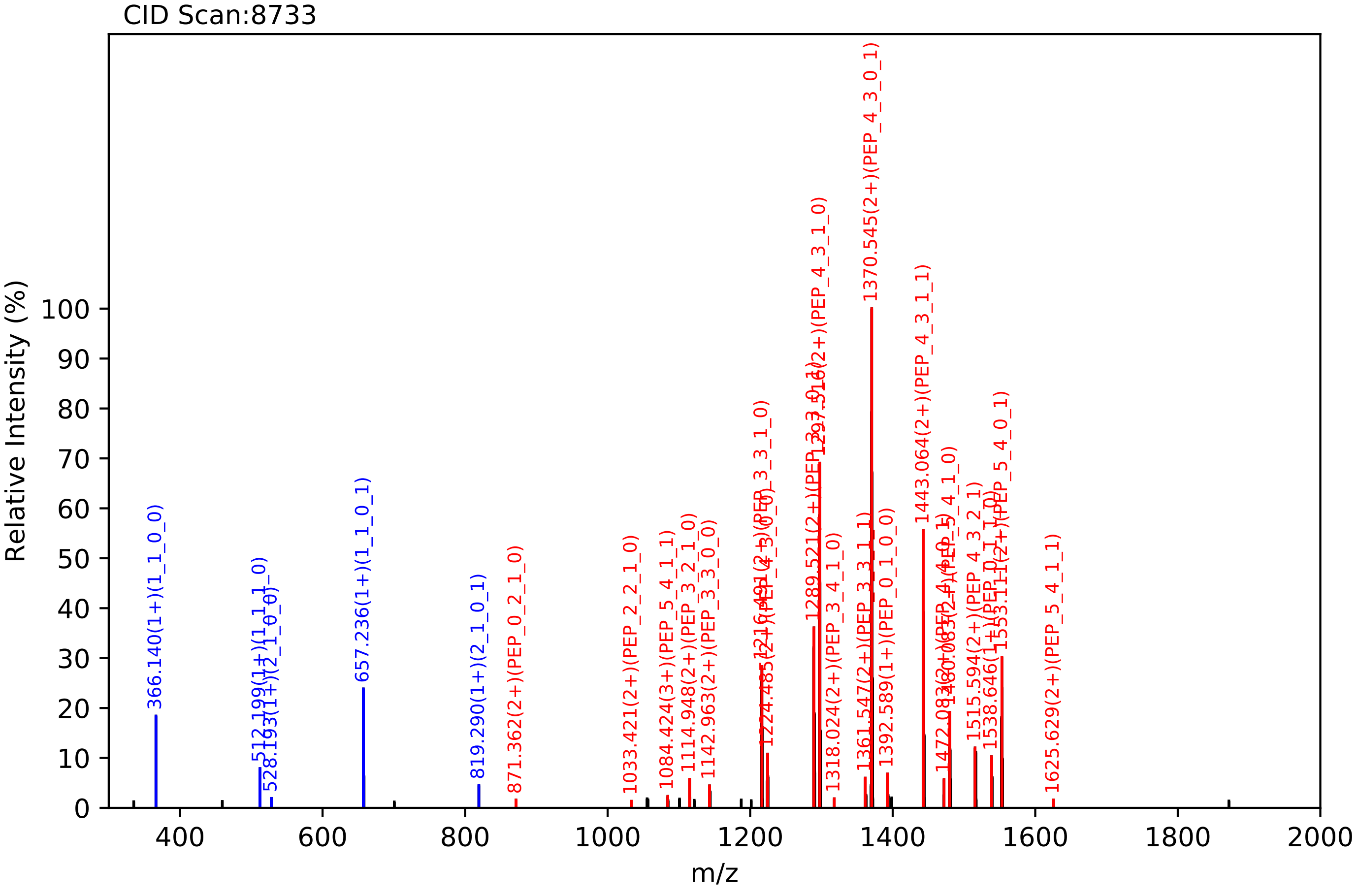

Unknown set no. 415, Experiment: Human Plasma exp_3

EEQYNSTYR(=PEP)_5_4_2_1, m/z:1132.44(3+), RT:36.79, Y-score:88.48

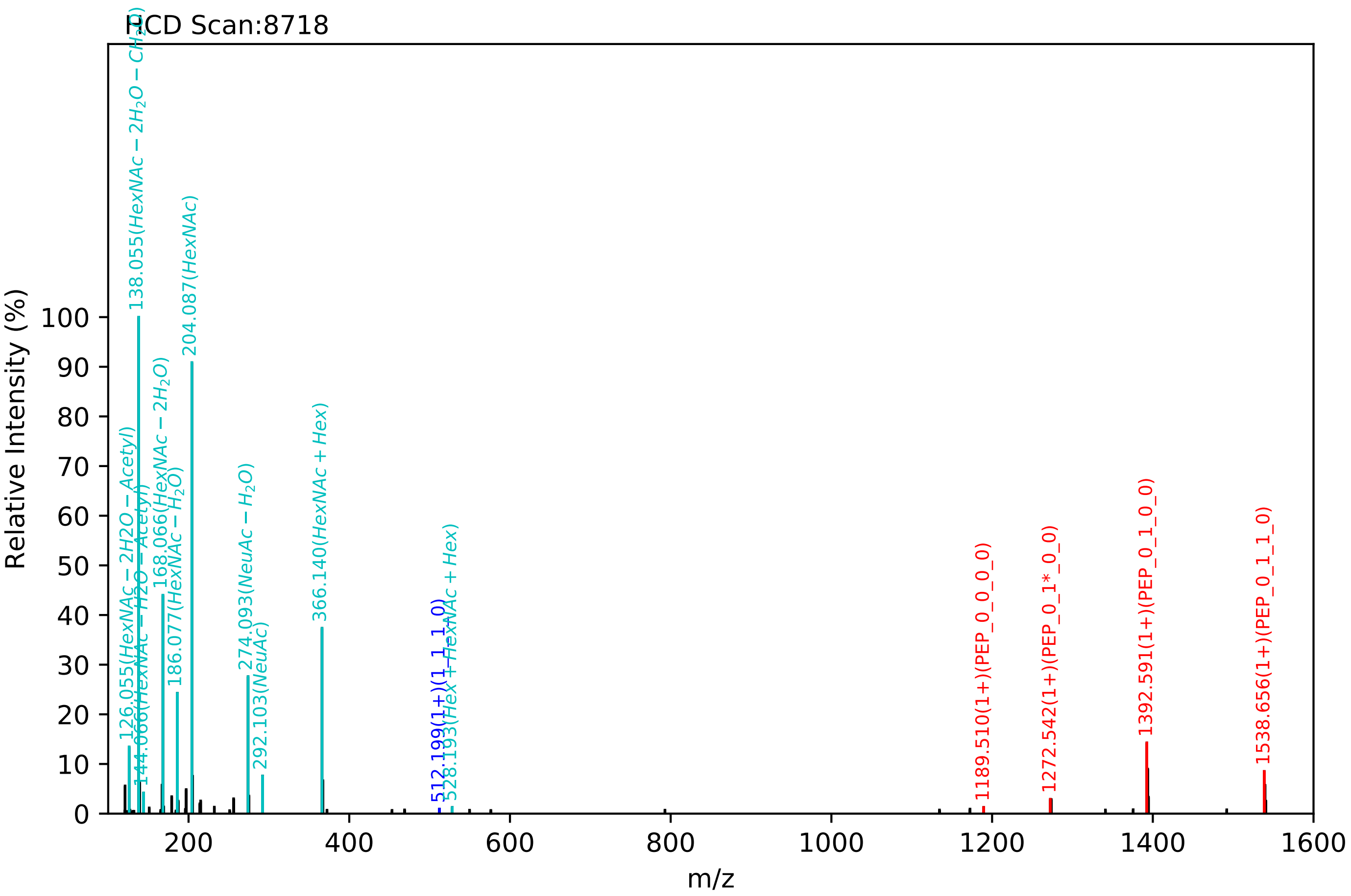

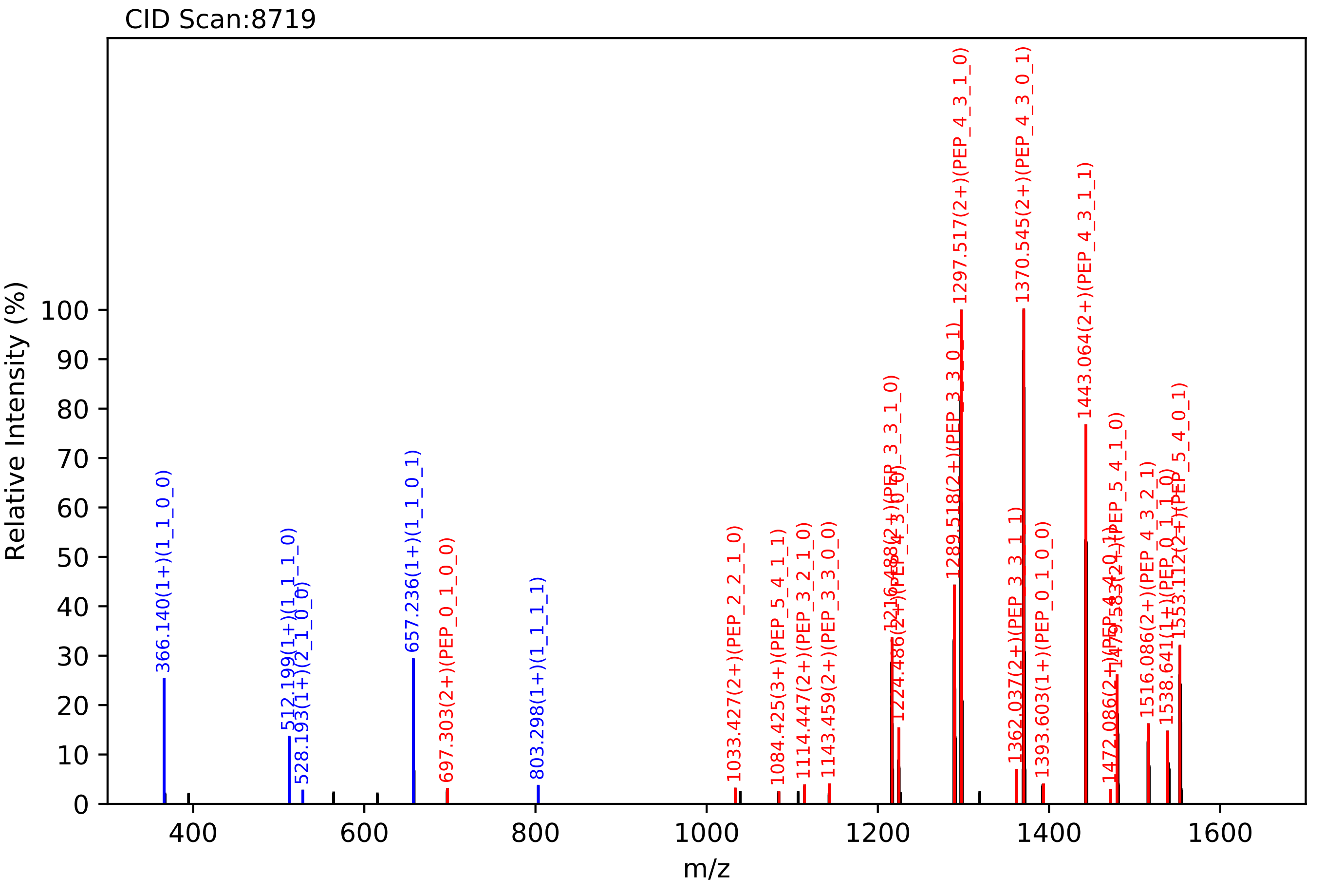

Unknown set no. 416, Experiment: Human Plasma exp_5

EEQYNSTYR(=PEP)_5_4_2_1, m/z:1132.44(3+), RT:36.80, Y-score:93.08

HCD Scan:8690

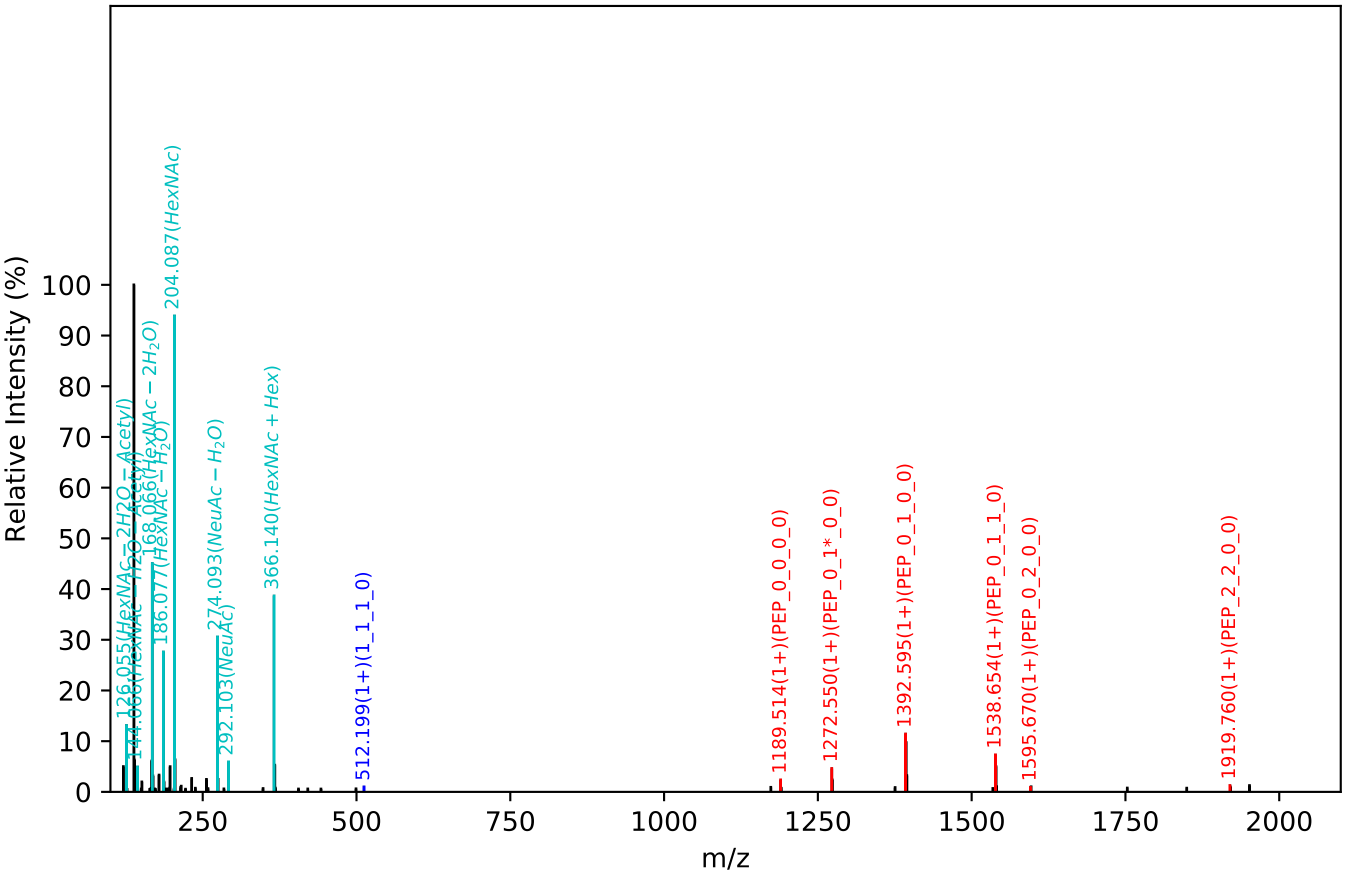

CID Scan:8692

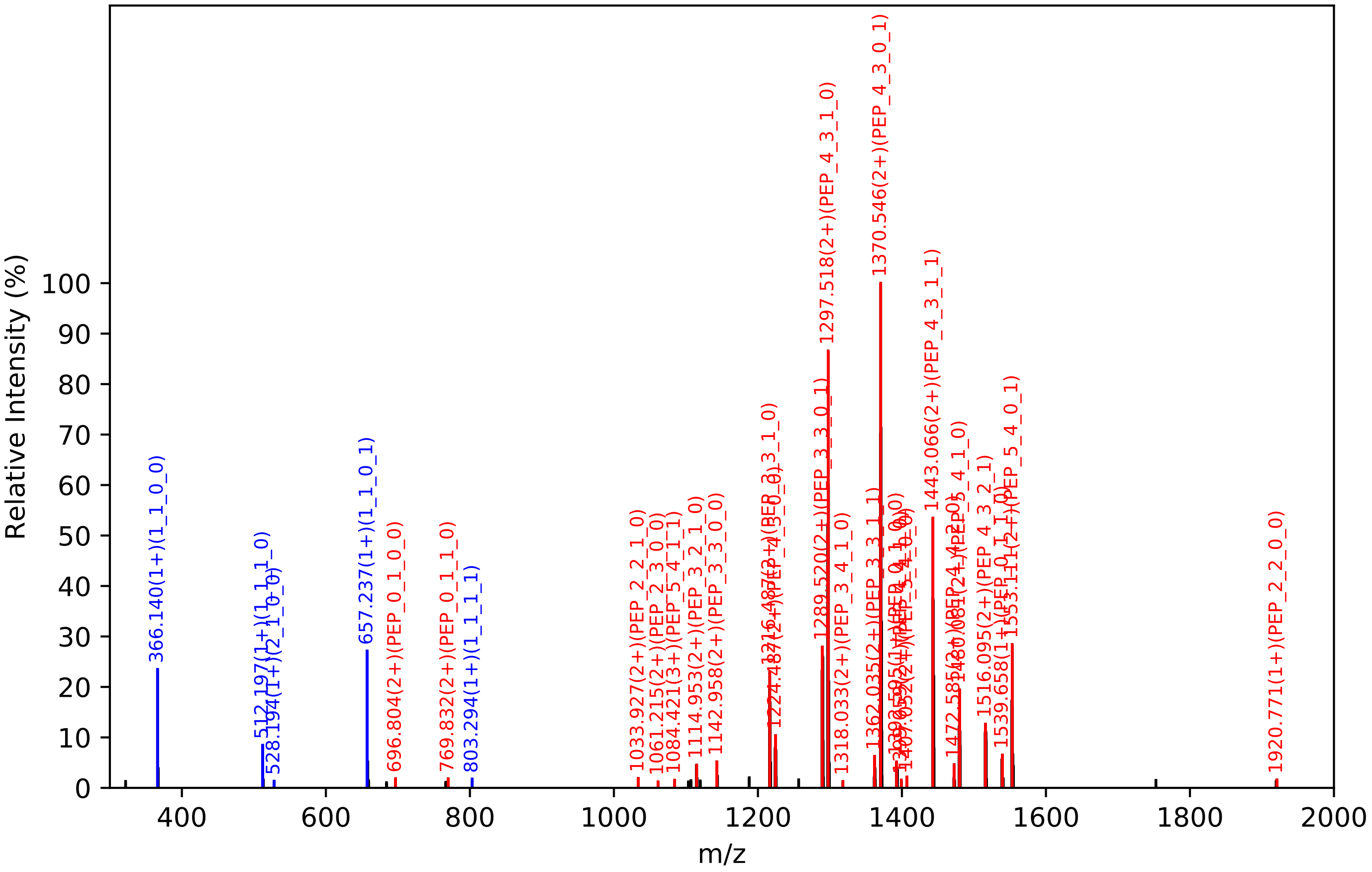

Unknown set no. 417, Experiment: Human Plasma exp_4

EEQYNSTYR(=PEP)_5_5_1_0, m/z:1581.13(2+), RT:27.63, Y-score:82.53

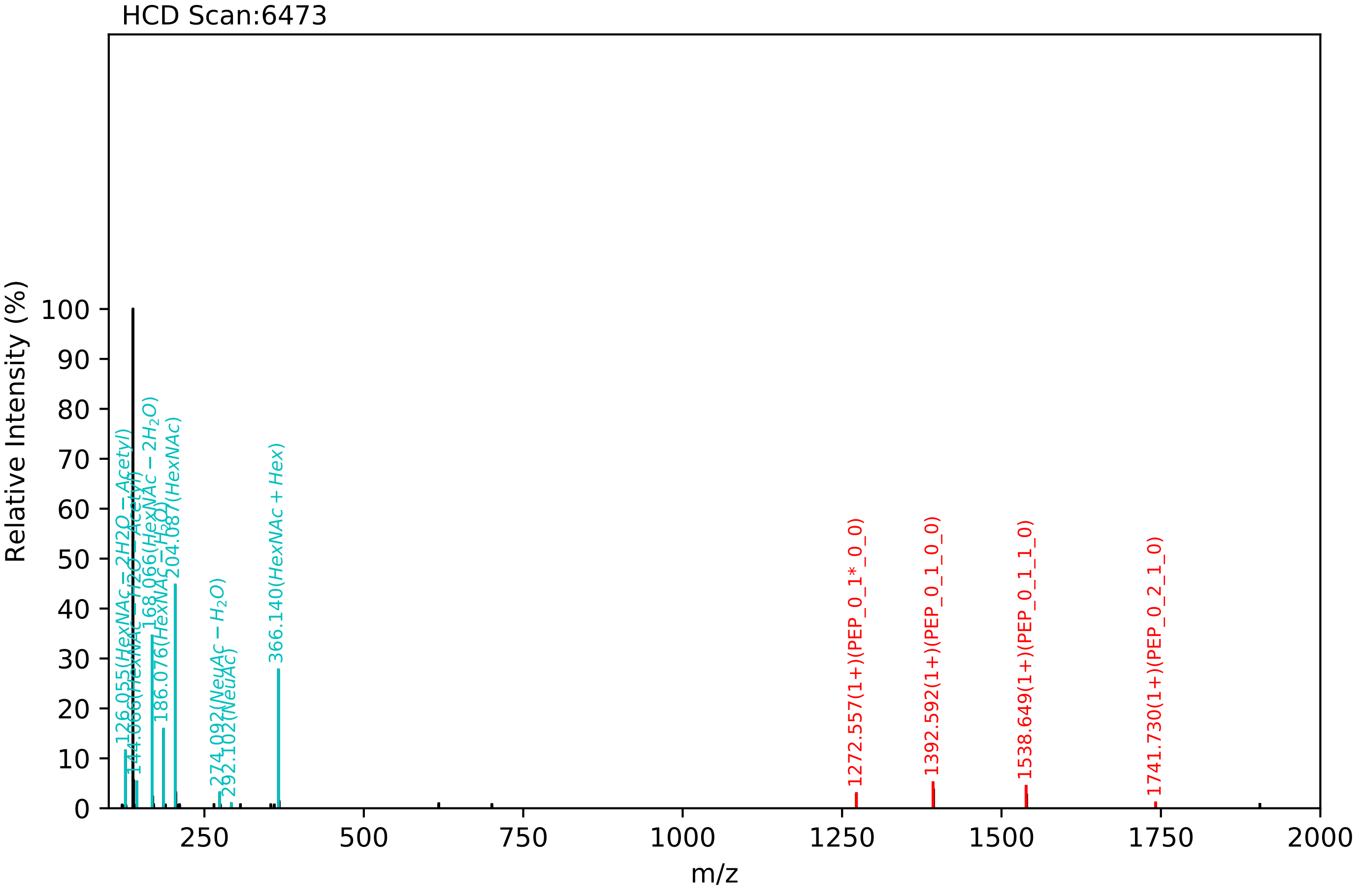

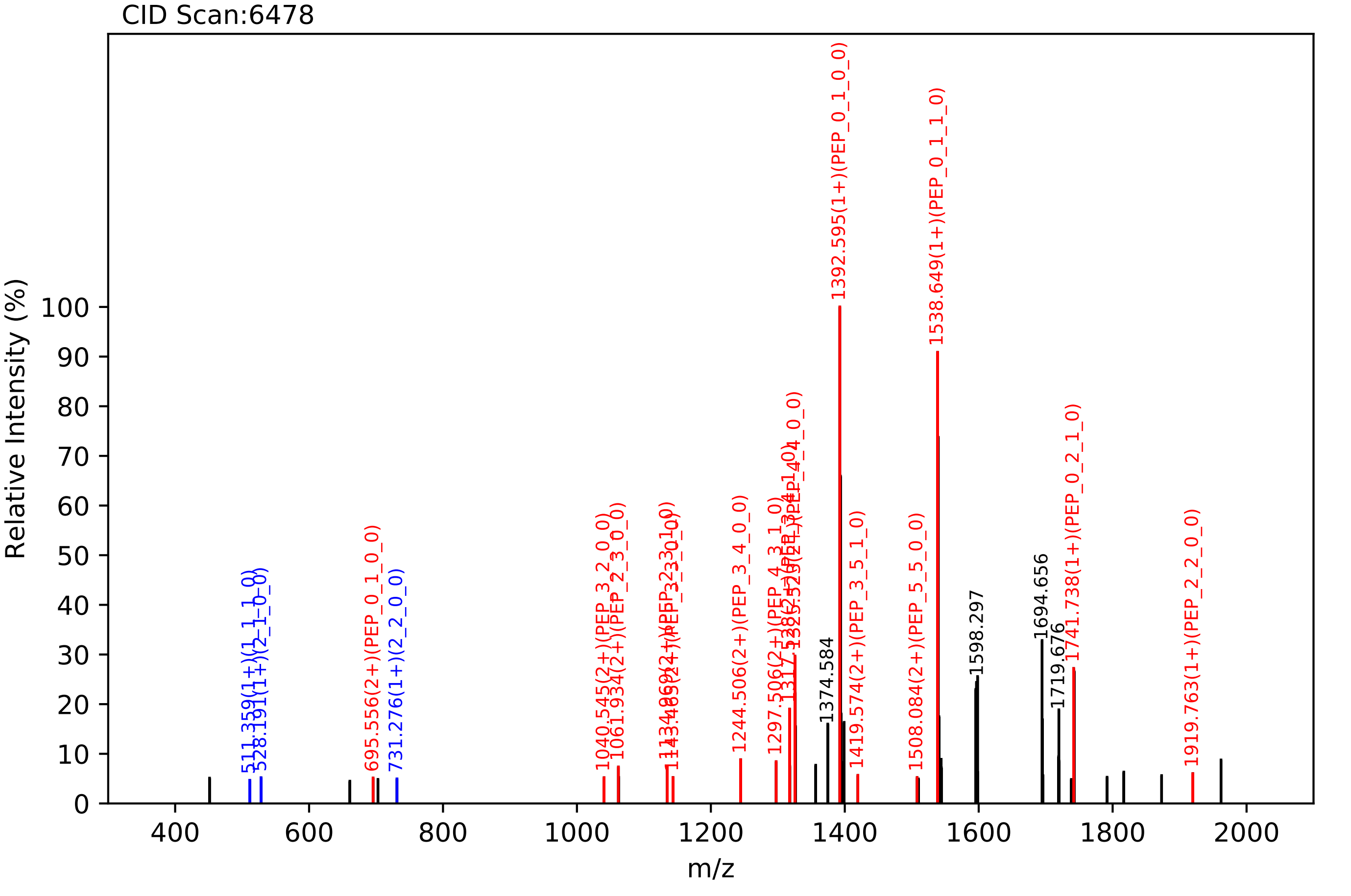

Unknown set no. 418, Experiment: Human Plasma exp_4

EEQYNSTYR(=PEP)_5_5_1_2, m/z:1248.48(3+), RT:50.64, Y-score:75.76

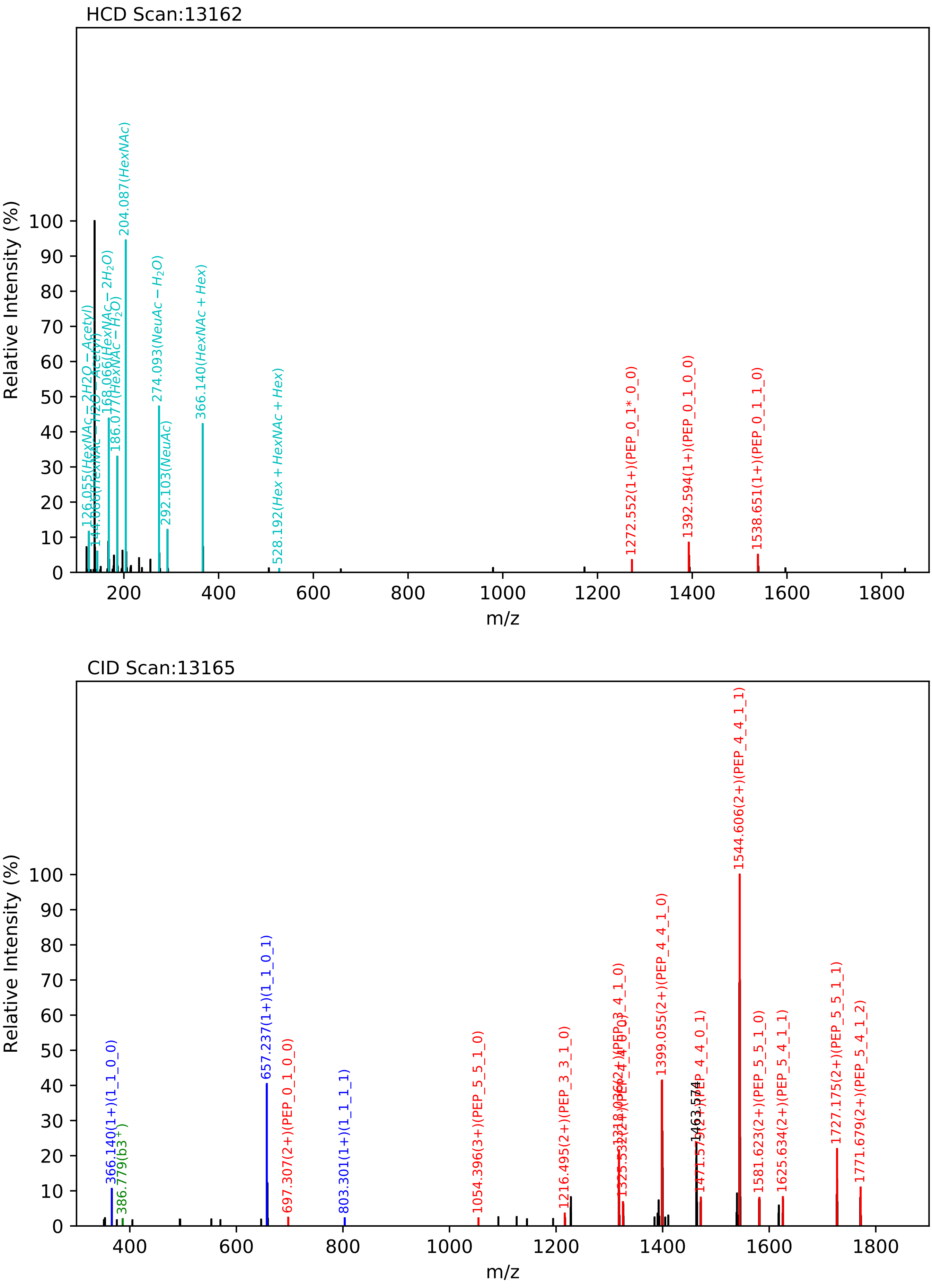

Unknown set no. 419, Experiment: Human Plasma exp_1

EEQFNSTFR(=PEP)_3_3_0_0, m/z:1126.97(2+), RT:52.00, Y-score:89.62

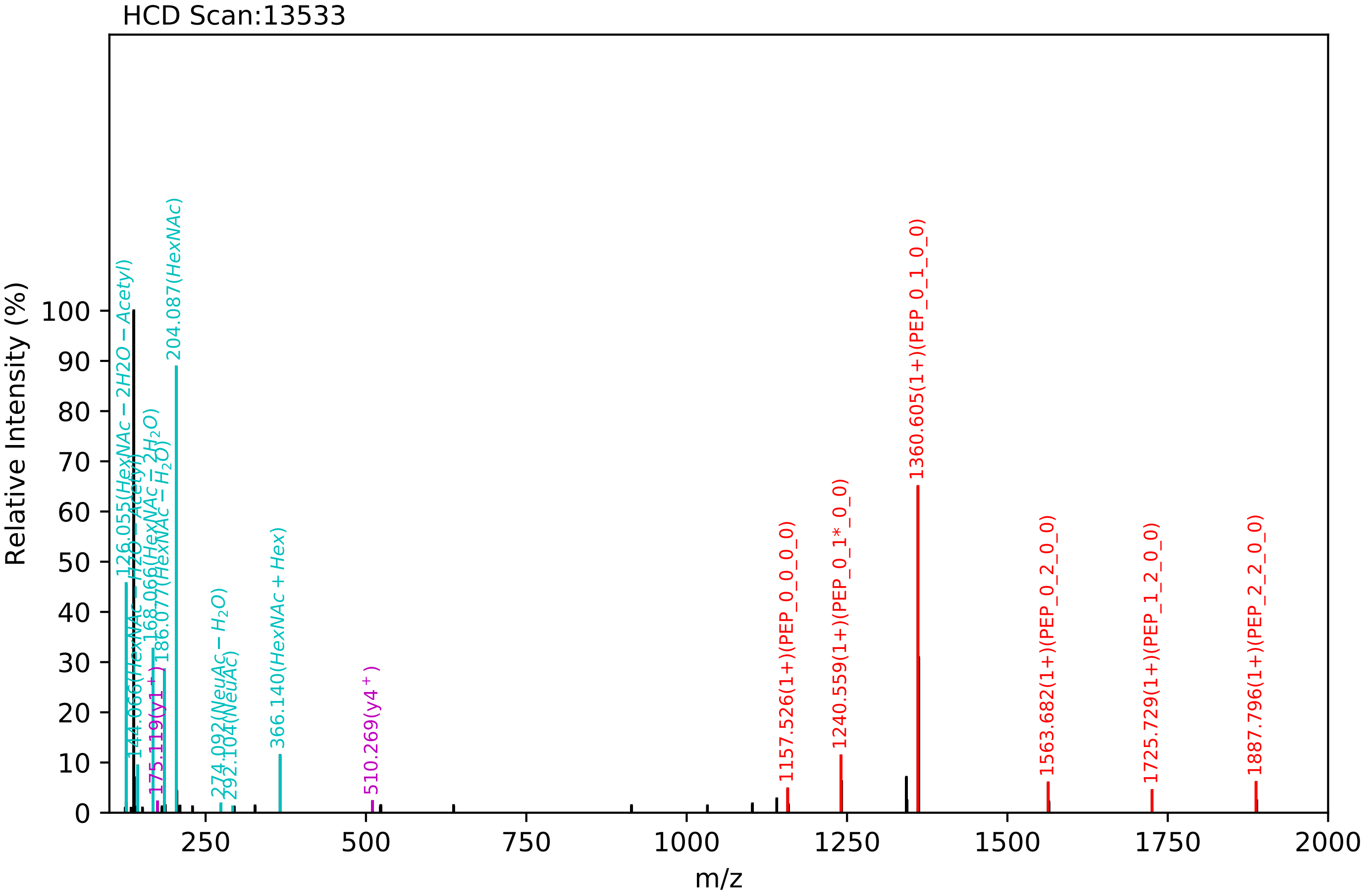

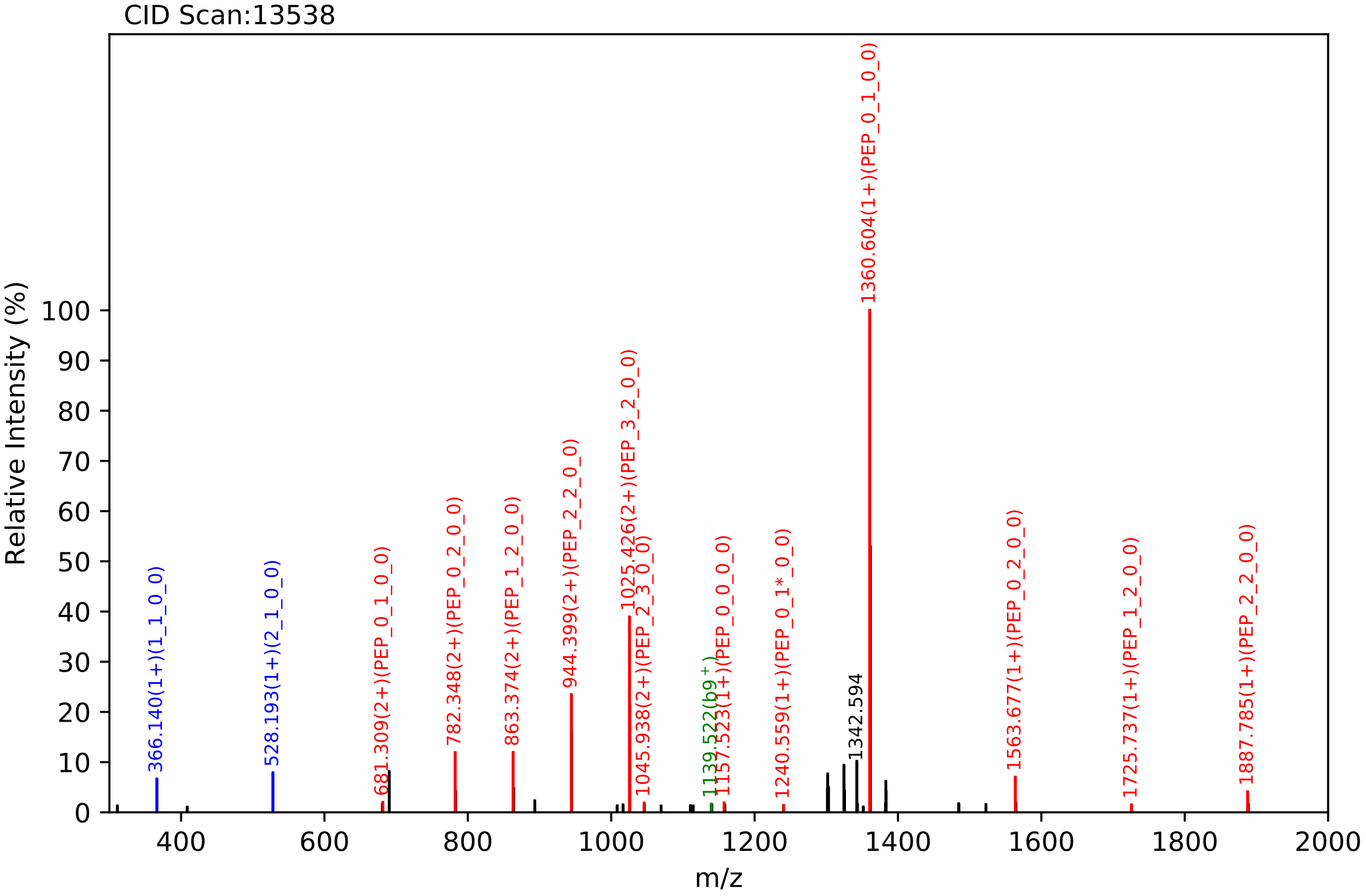

Unknown set no. 420, Experiment: Human Plasma exp_2

EEQFNSTFR(=PEP)_3_3_0_0, m/z:1126.97(2+), RT:52.04, Y-score:88.37

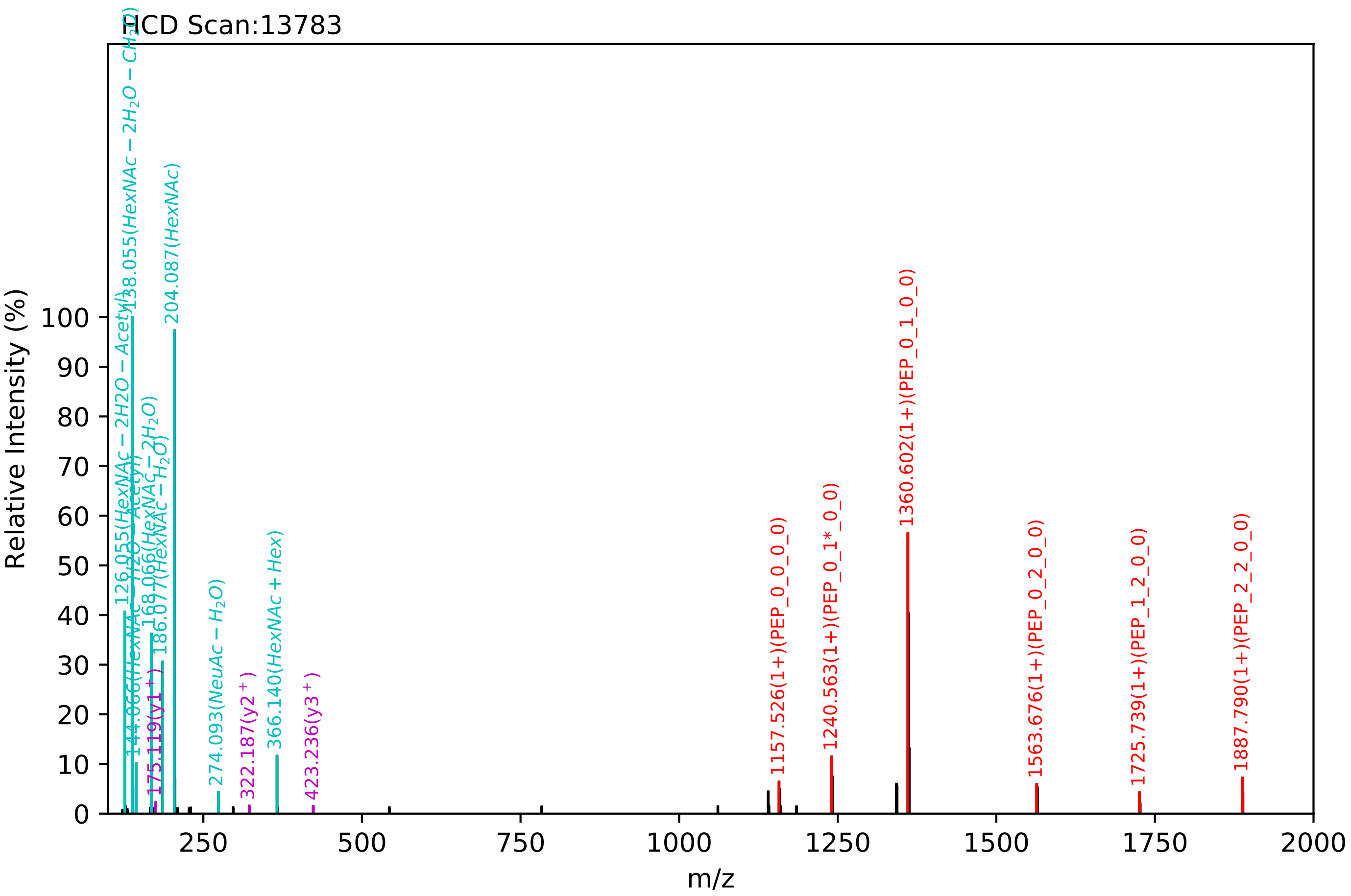

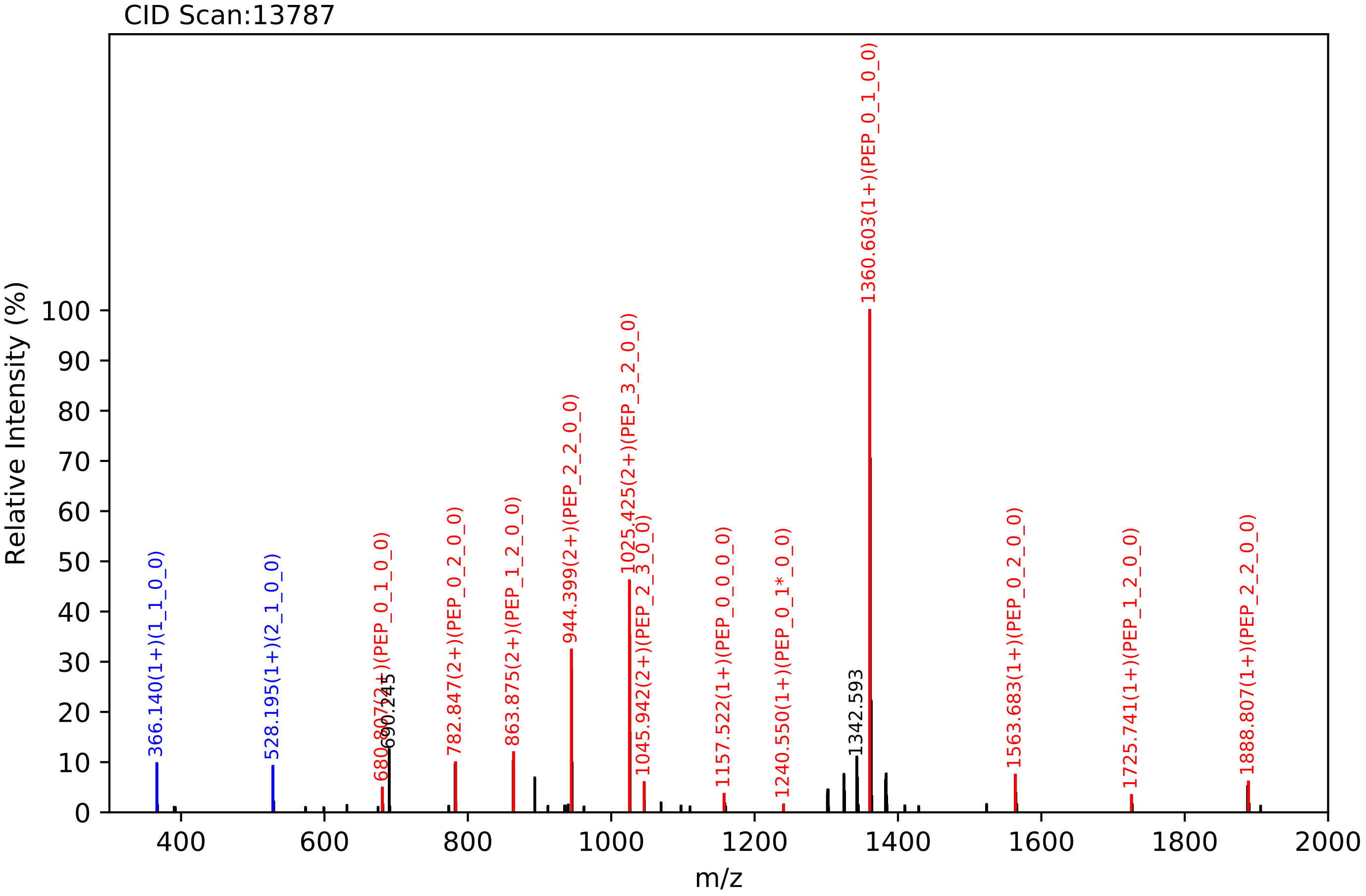

Unknown set no. 421, Experiment: Human Plasma exp_5

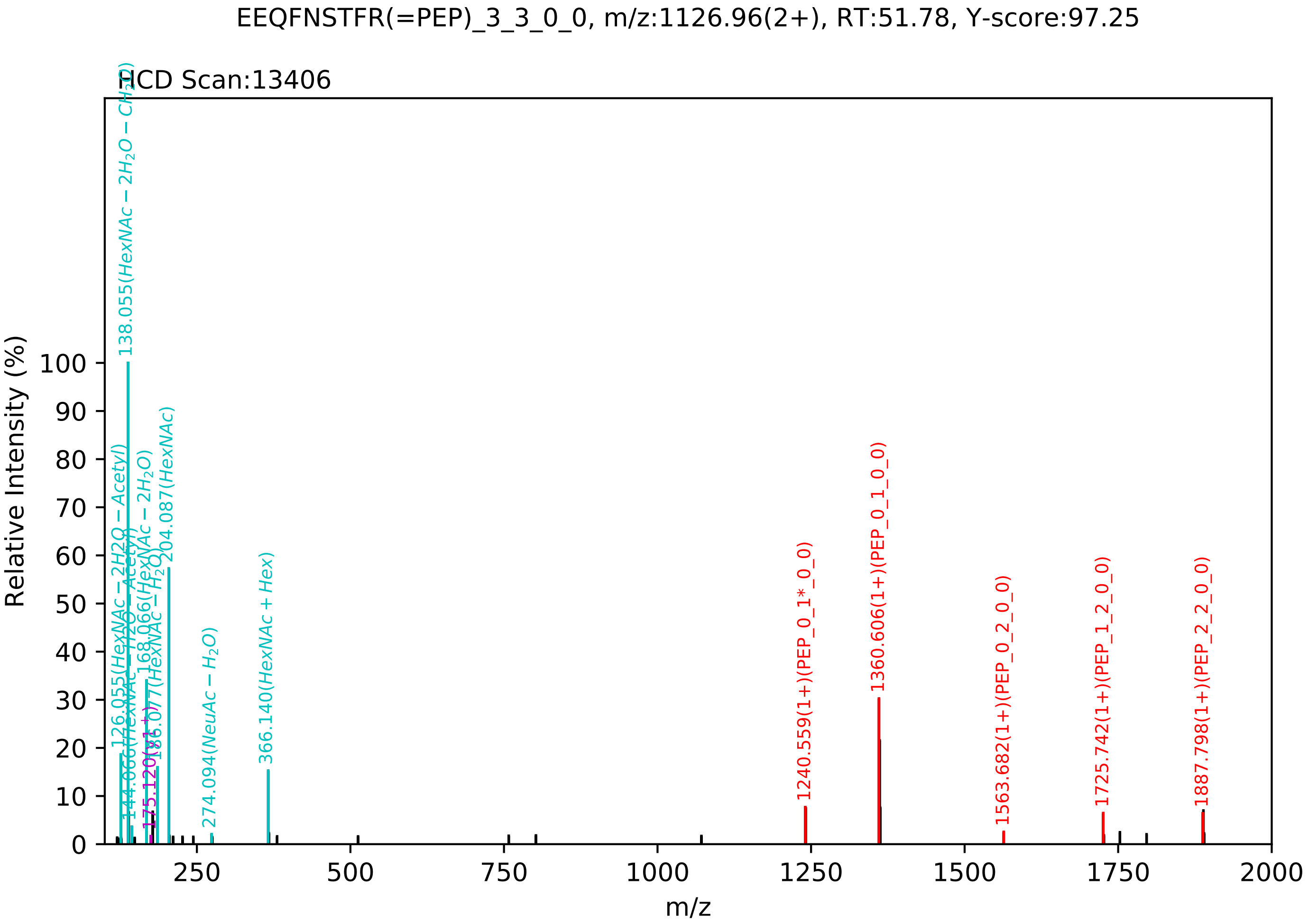

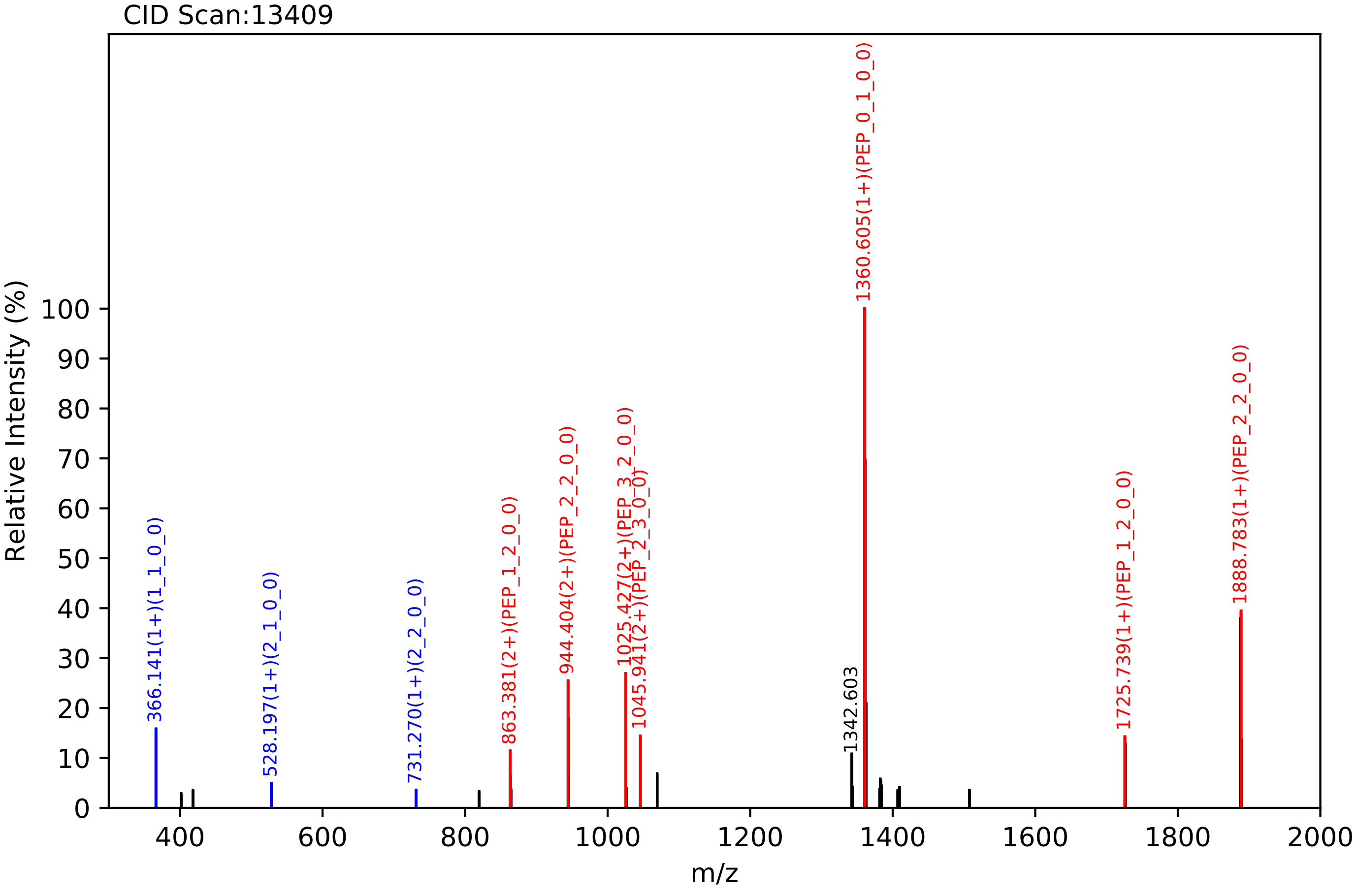

Unknown set no. 422, Experiment: Human Plasma exp_1

EEQFNSTFR(=PEP)_3_3_1_0, m/z:1199.99(2+), RT:51.22, Y-score:90.31

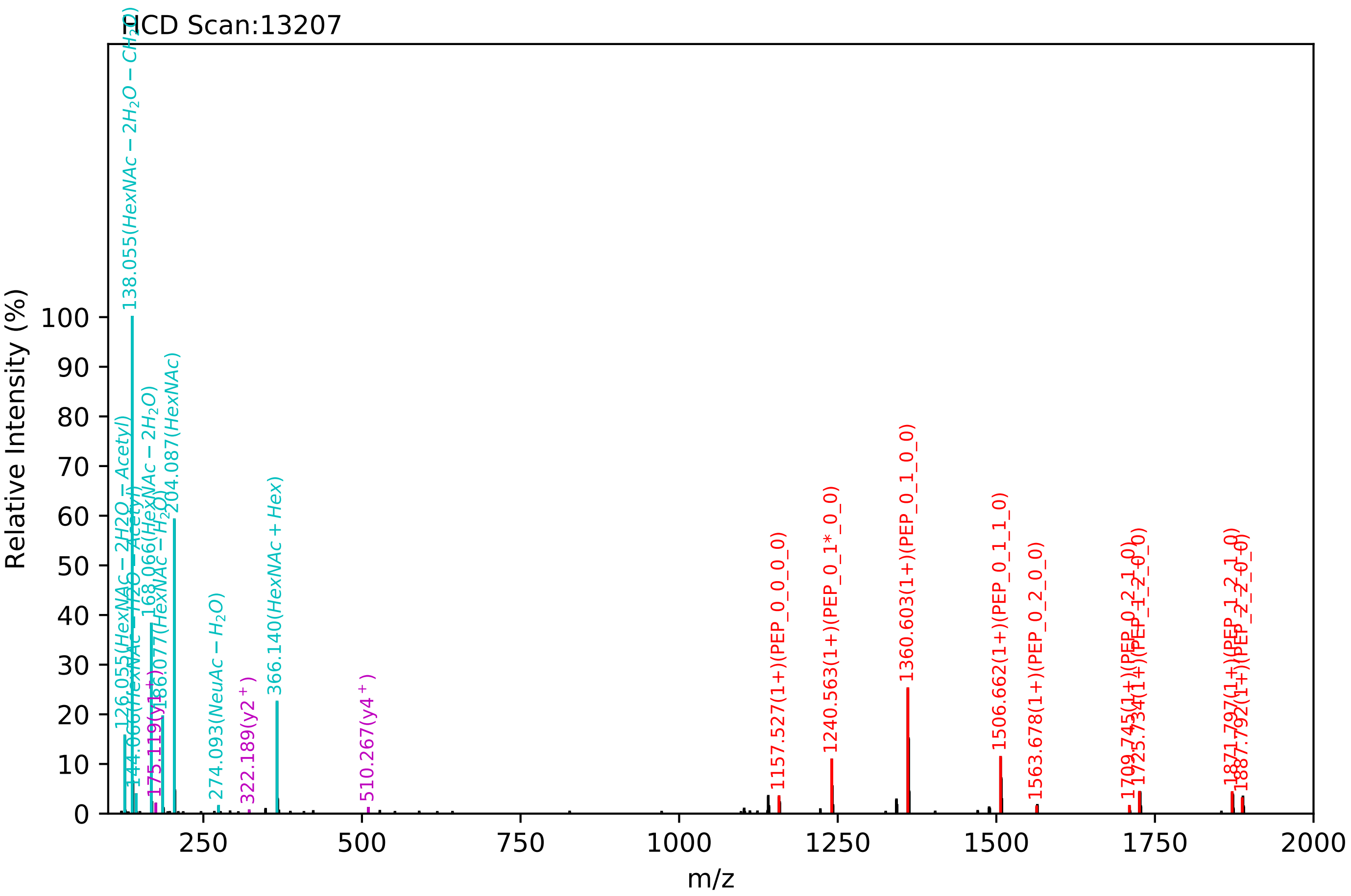

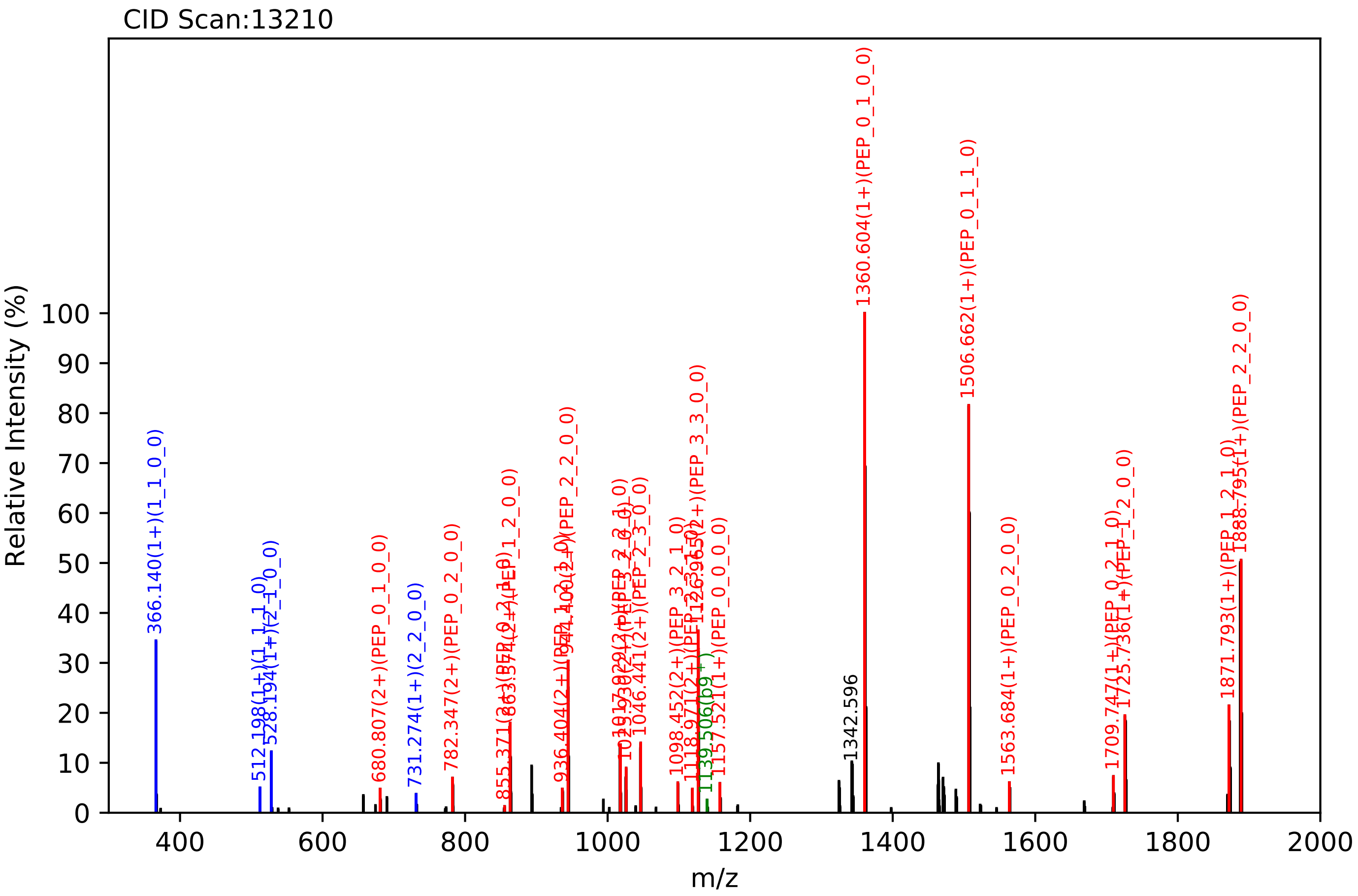

Unknown set no. 423, Experiment: Human Plasma exp_2

EEQFNSTFR(=PEP)_3_3_1_0, m/z:1199.99(2+), RT:51.24, Y-score:90.51

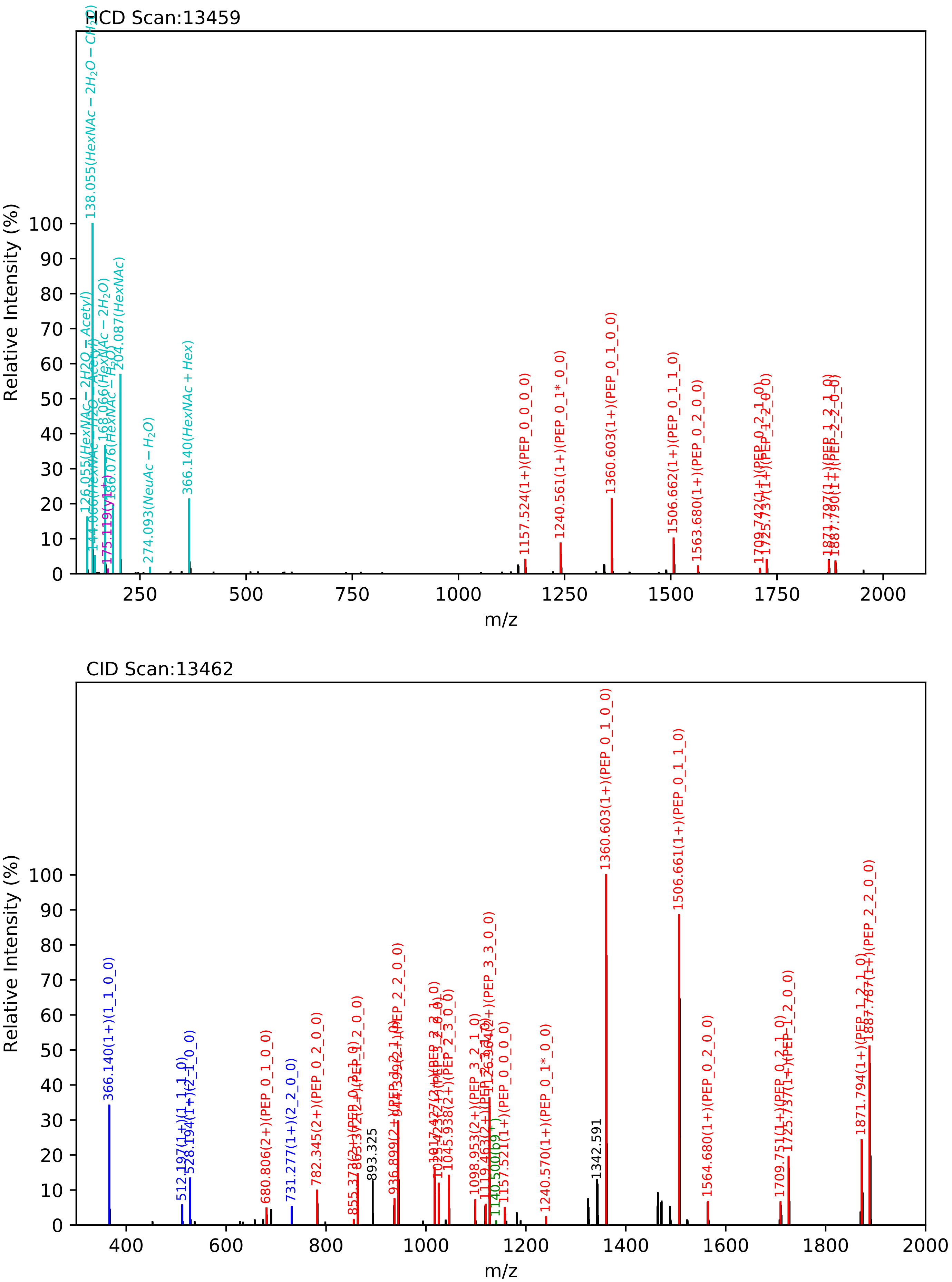

Unknown set no. 424, Experiment: Human Plasma exp_3

EEQFNSTFR(=PEP)_3_3_1_0, m/z:1199.99(2+), RT:51.47, Y-score:93.11

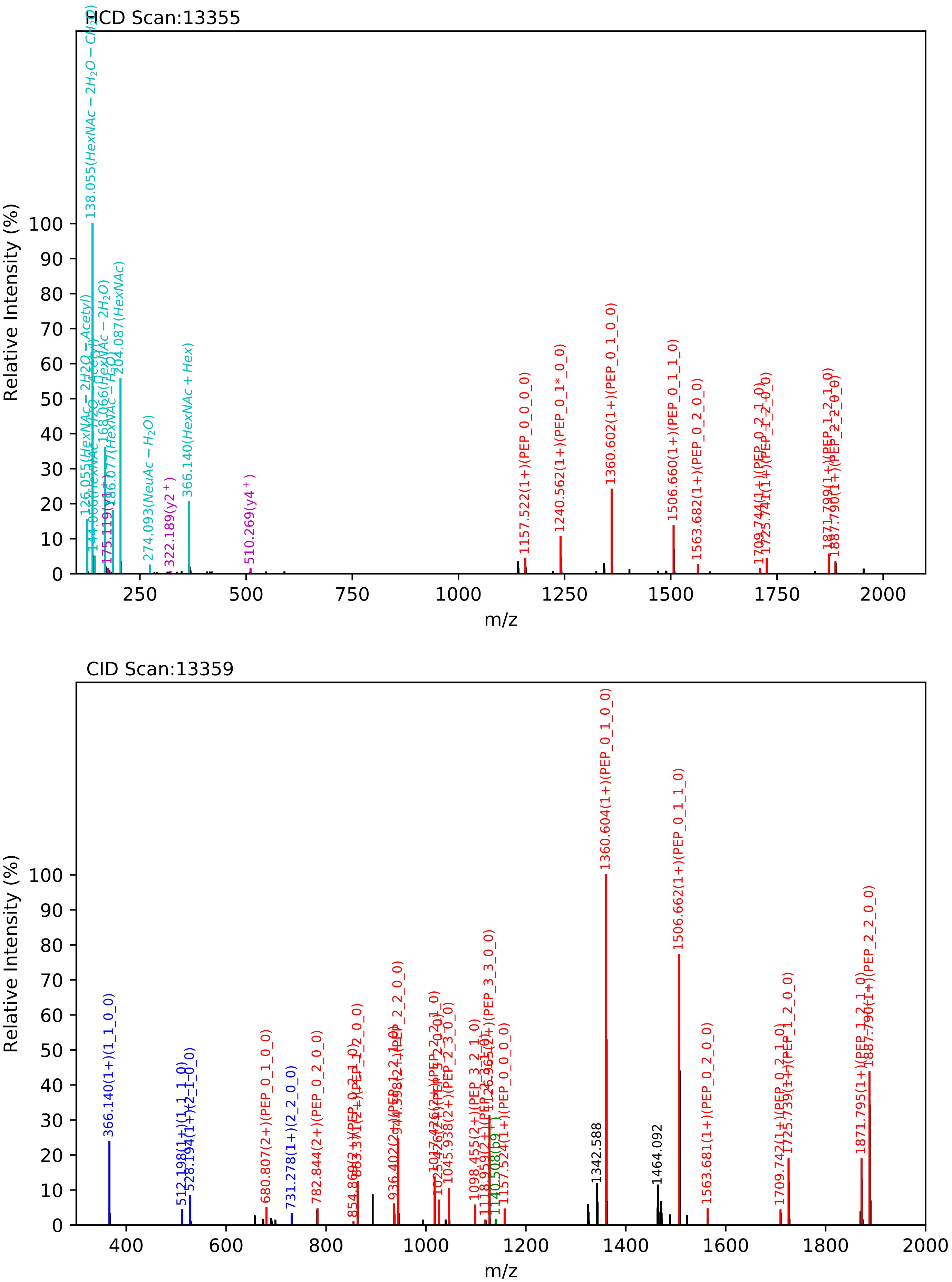

Unknown set no. 425, Experiment: Human Plasma exp_4

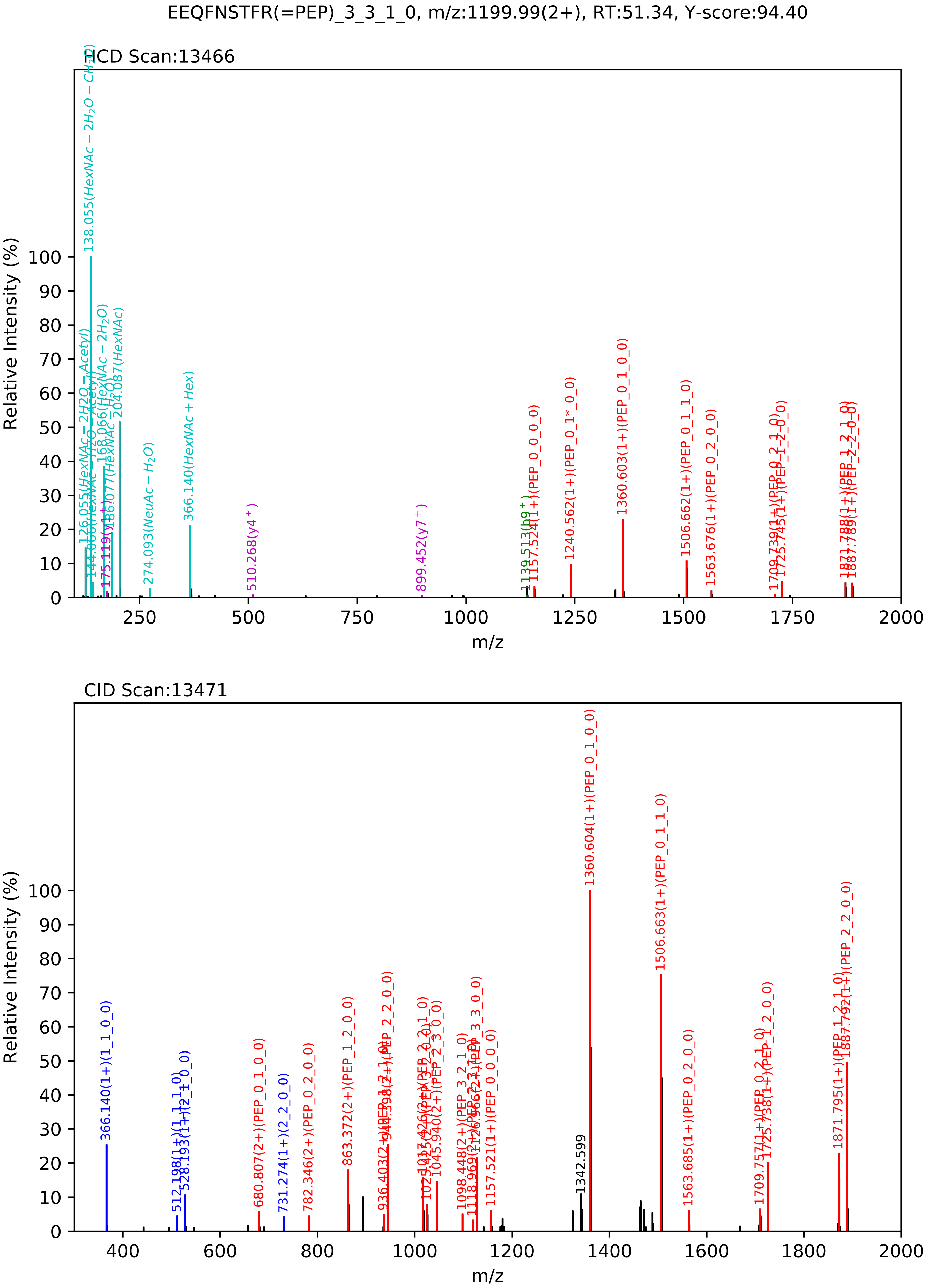

Unknown set no. 426, Experiment: Human Plasma exp_5

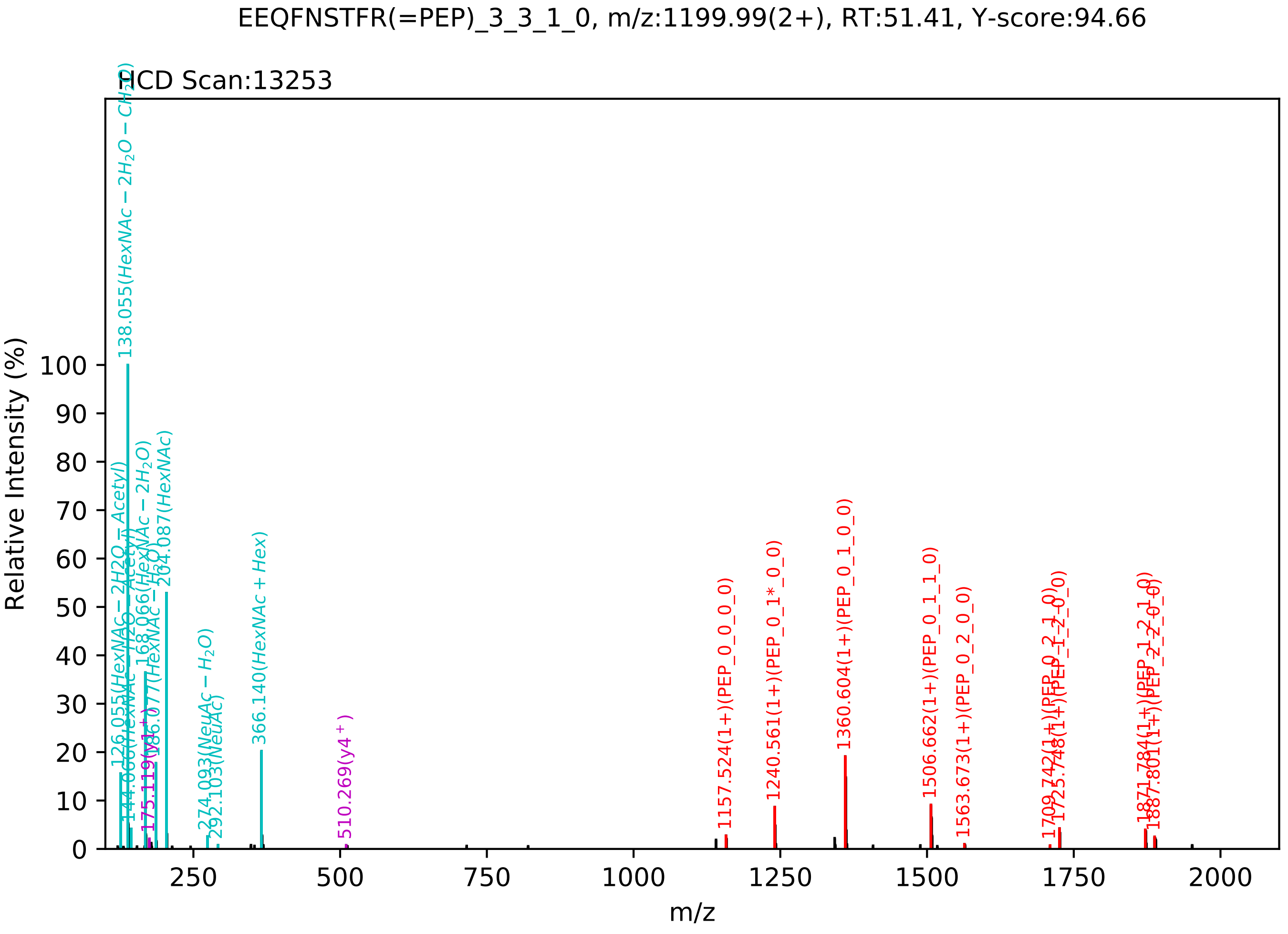

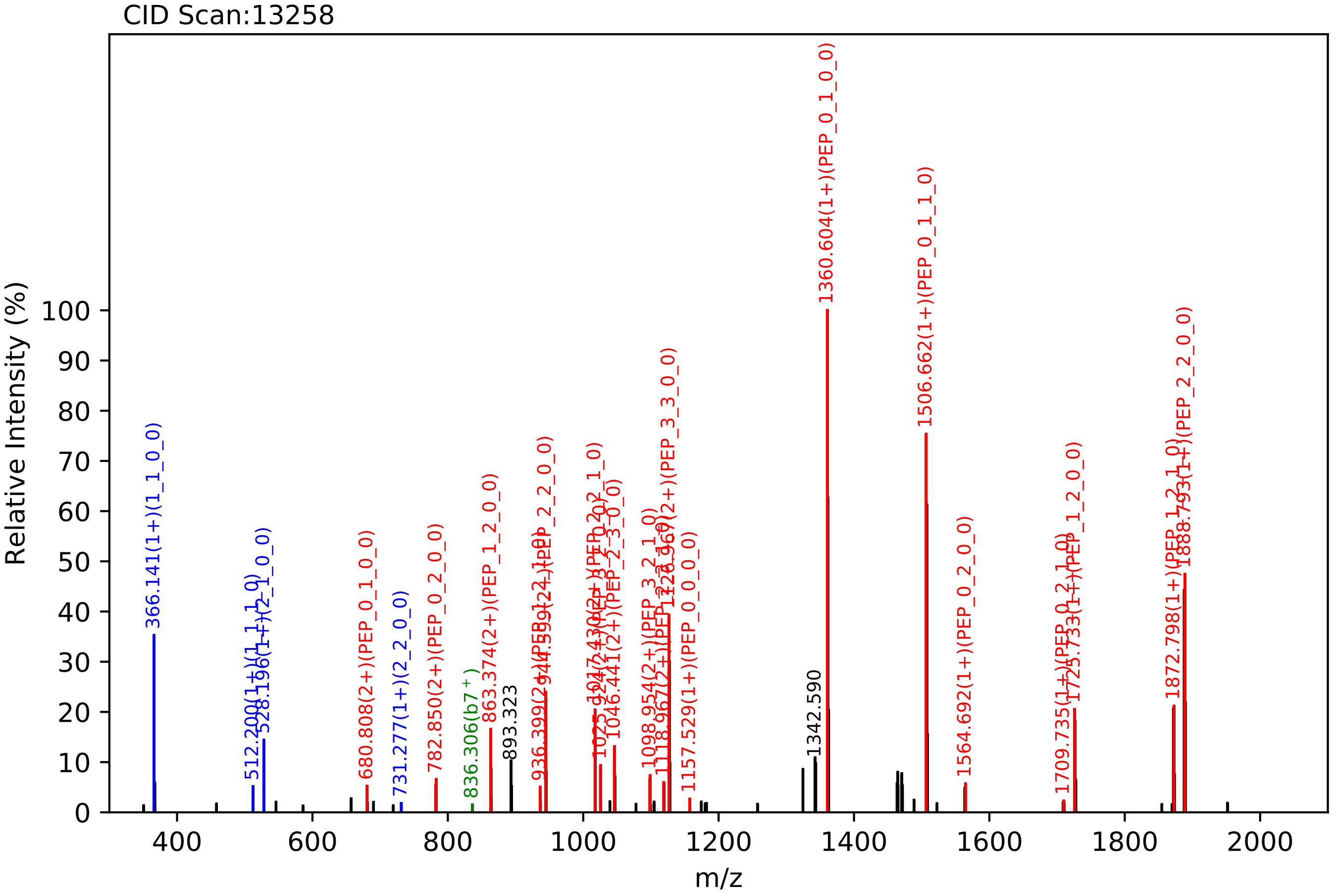

Unknown set no. 427, Experiment: Human Plasma exp_1

EEQFNSTFR(=PEP)_3_4_0_0, m/z:1228.51(2+), RT:52.13, Y-score:89.97

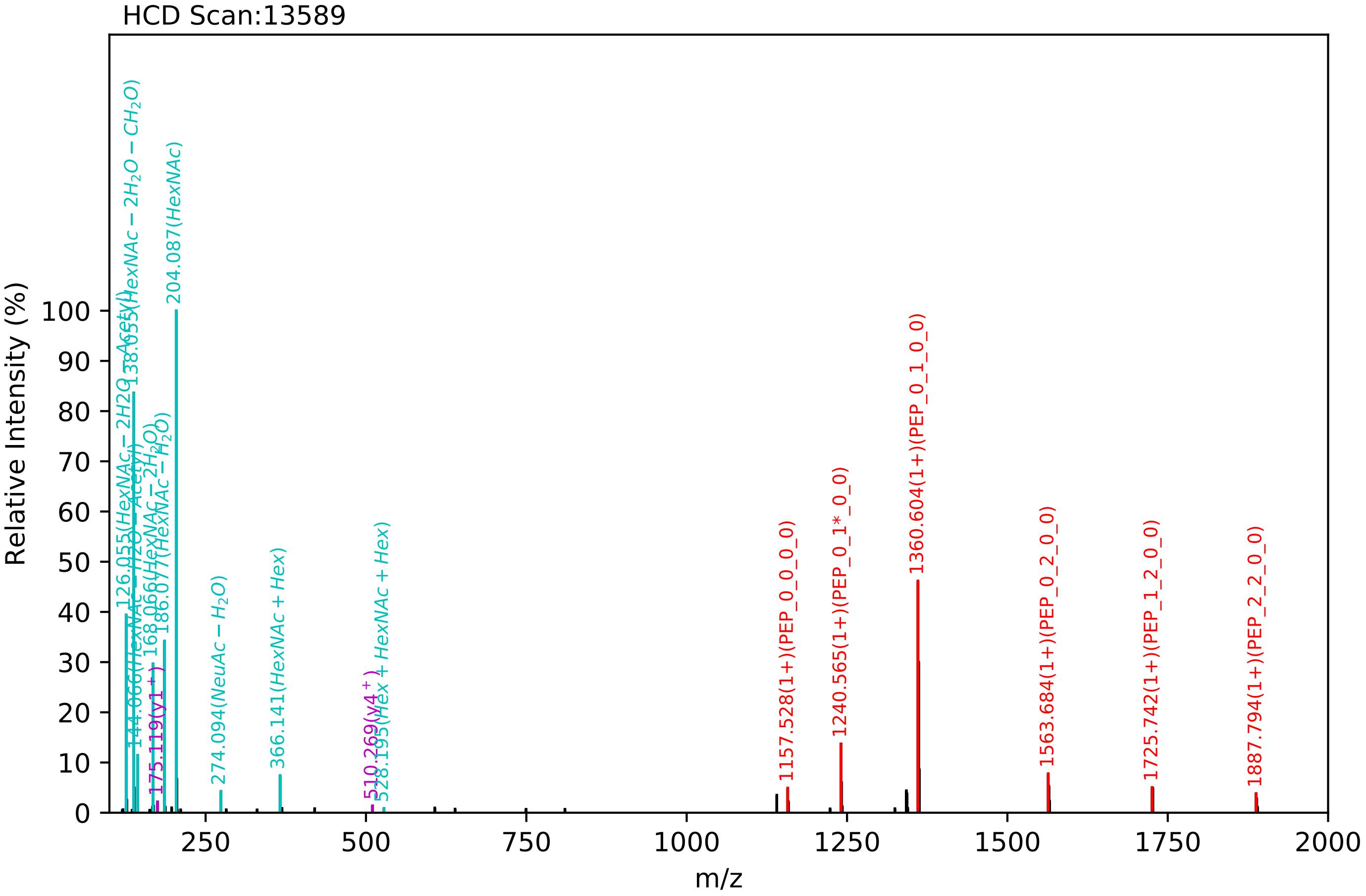

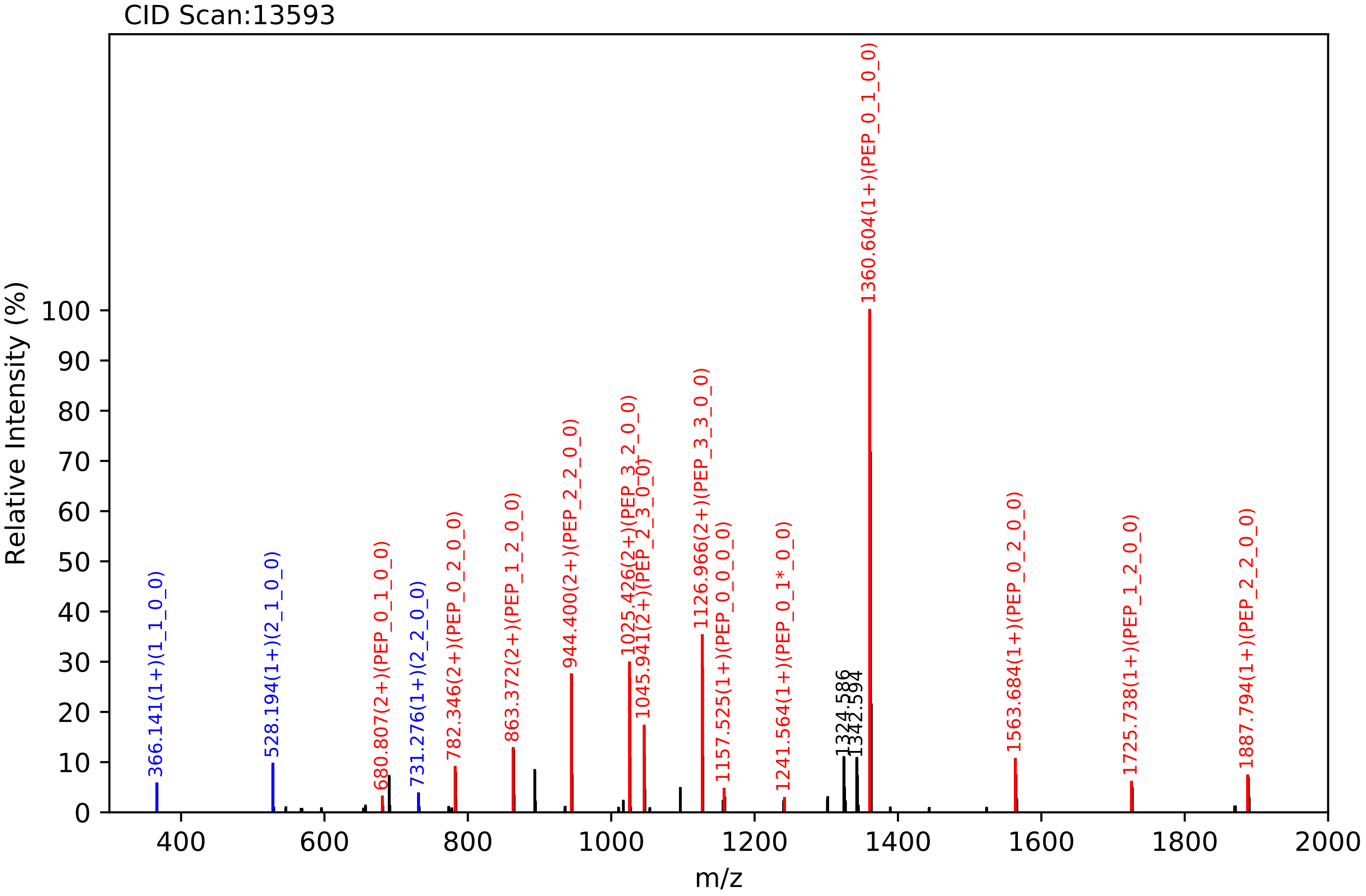

Unknown set no. 428, Experiment: Human Plasma exp_4

EEQFNSTFR(=PEP)_3_4_0_0, m/z:1228.51(2+), RT:52.28, Y-score:85.24

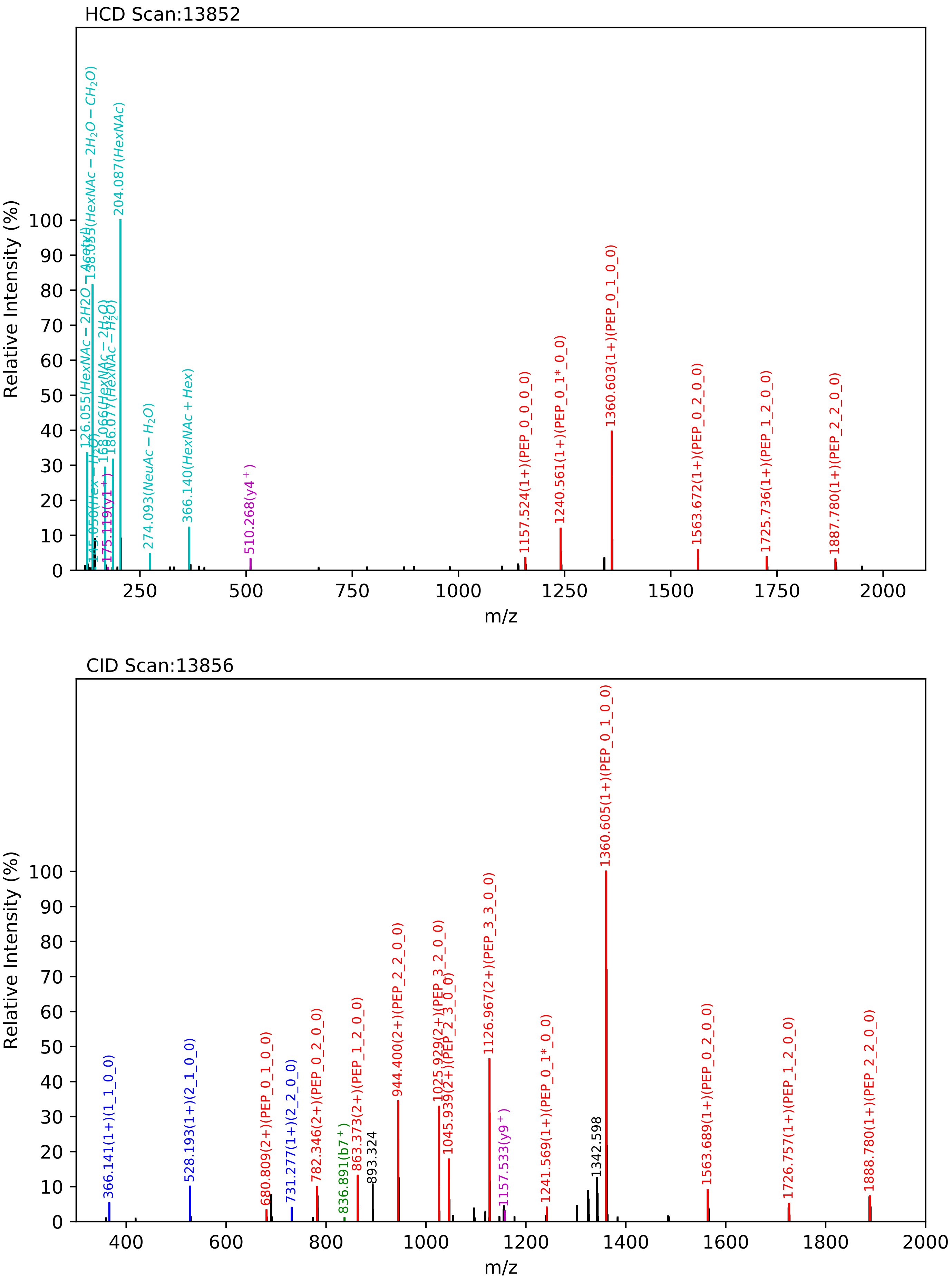

Unknown set no. 429, Experiment: Human Plasma exp_1

EEQFNSTFR(=PEP)_3_4_1_0, m/z:1301.53(2+), RT:52.34, Y-score:89.42

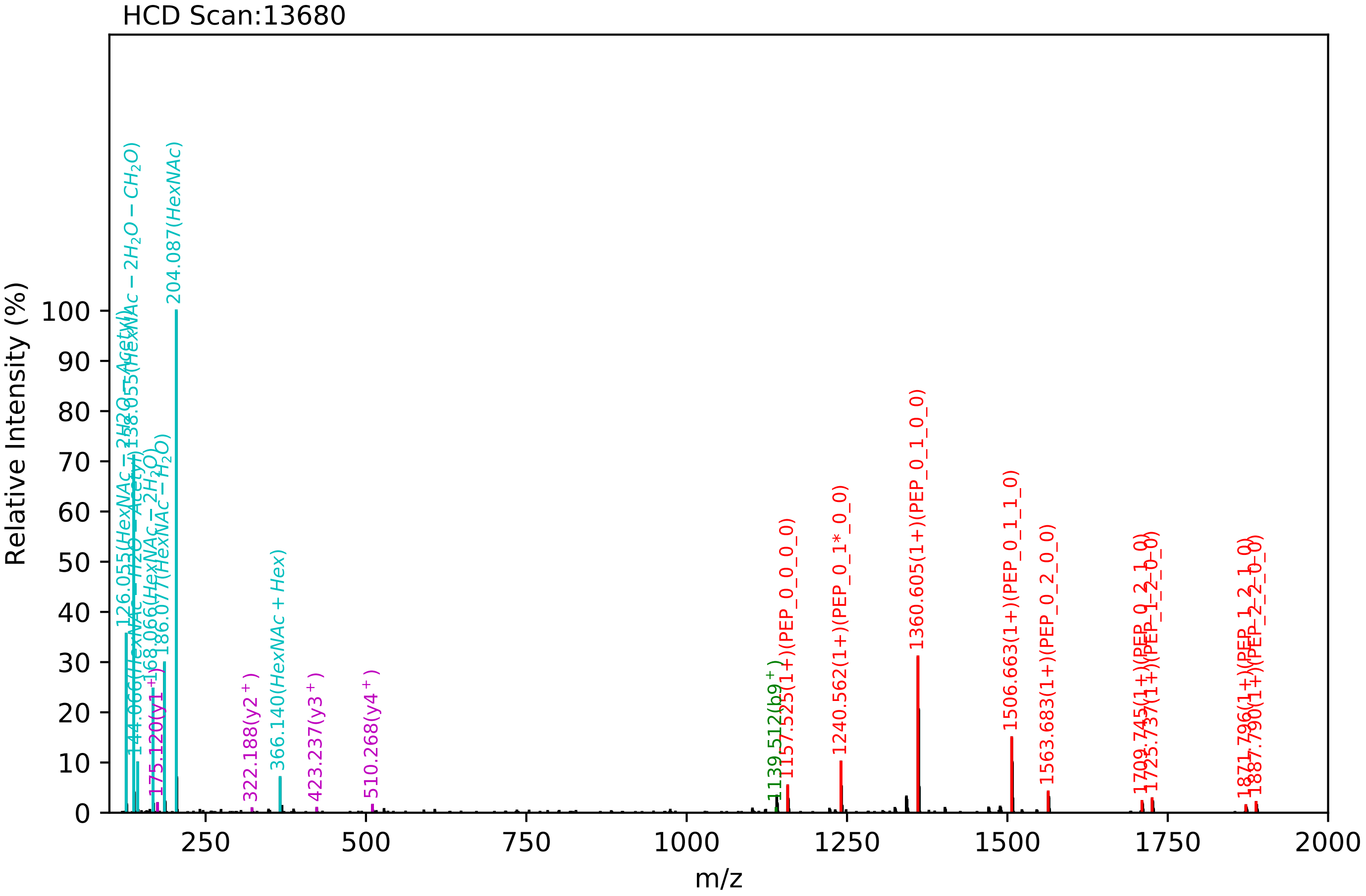

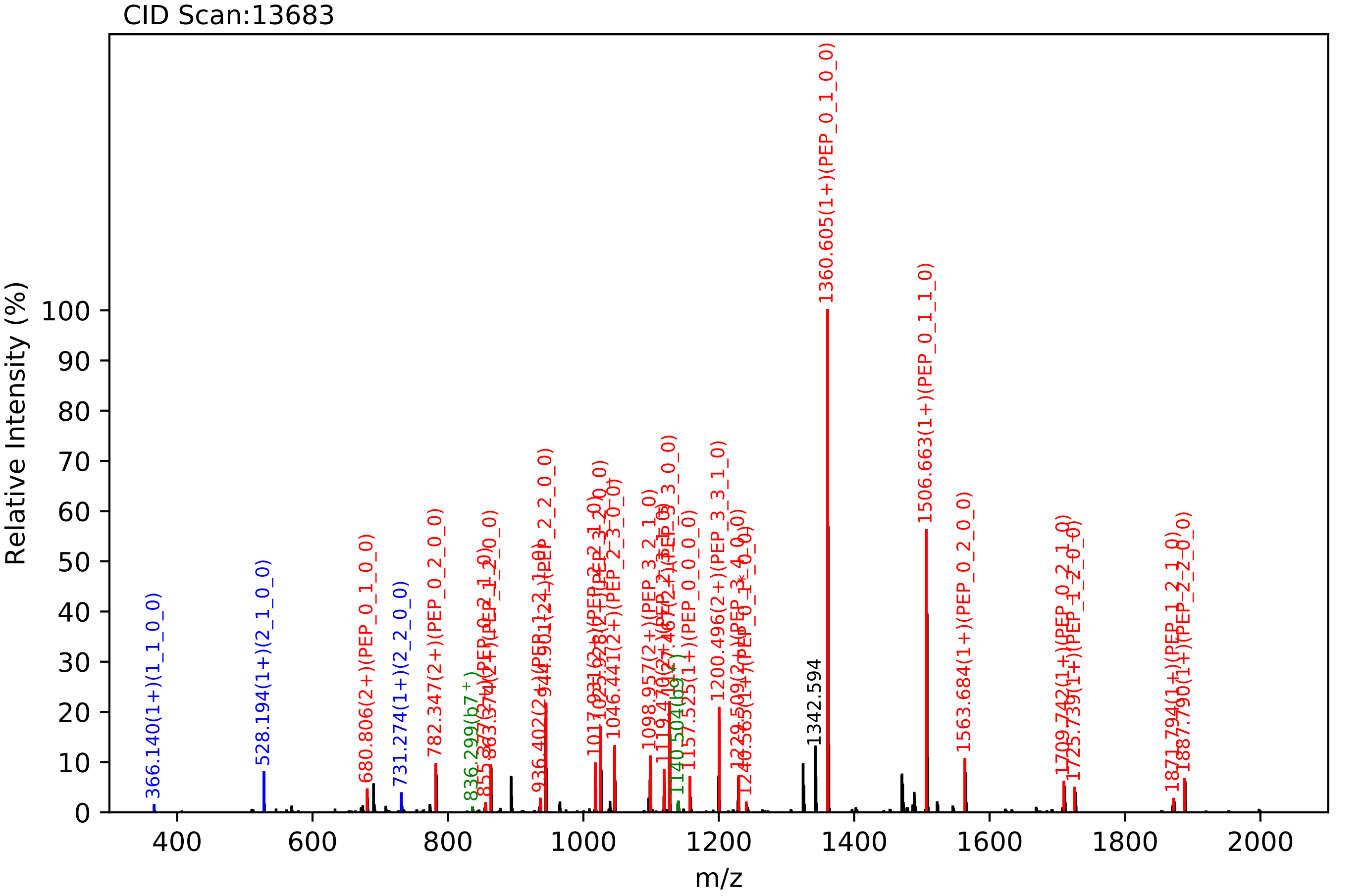

Unknown set no. 430, Experiment: Human Plasma exp_2

EEQFNSTFR(=PEP)_3_4_1_0, m/z:1301.53(2+), RT:52.32, Y-score:89.78

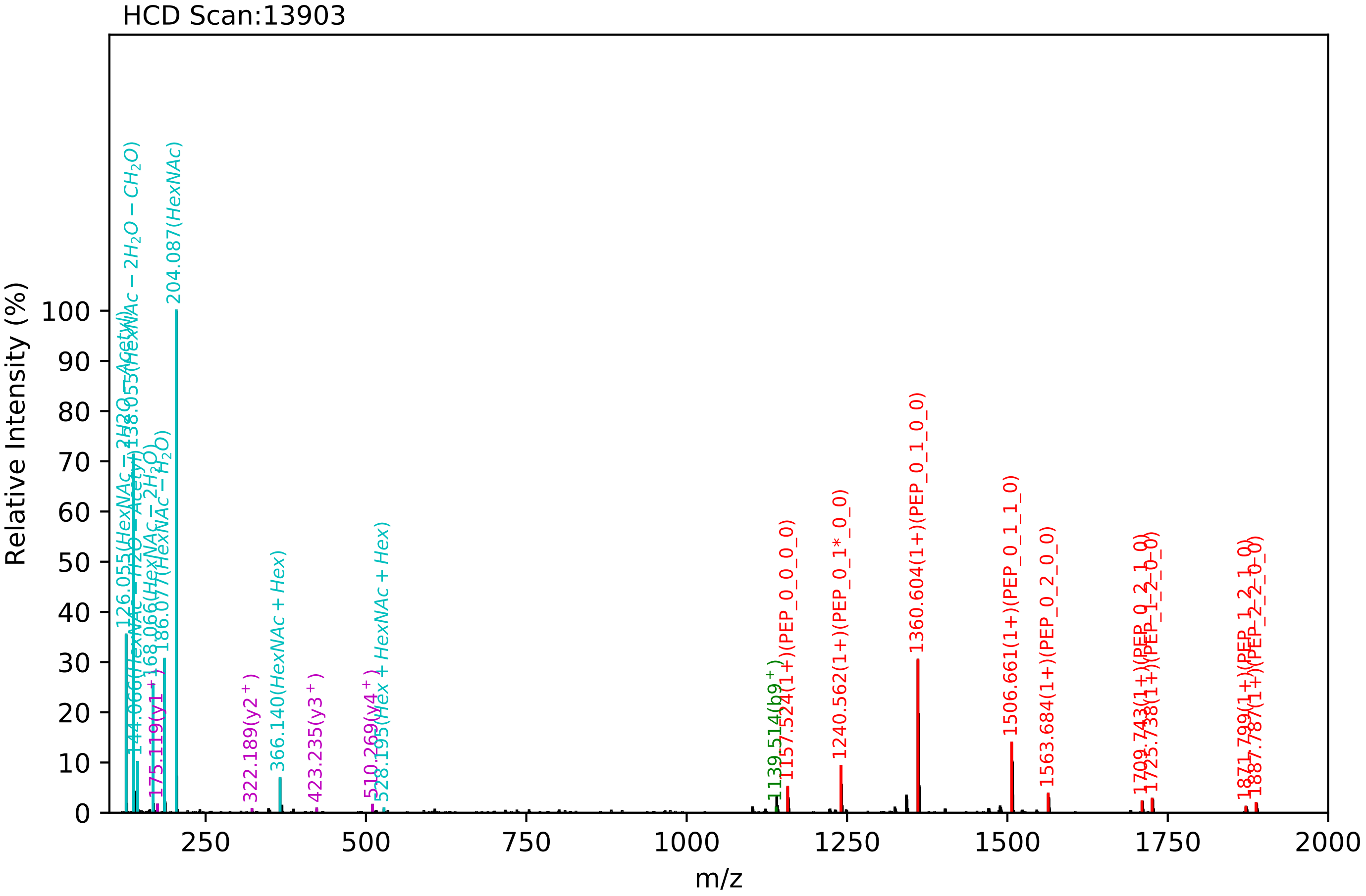

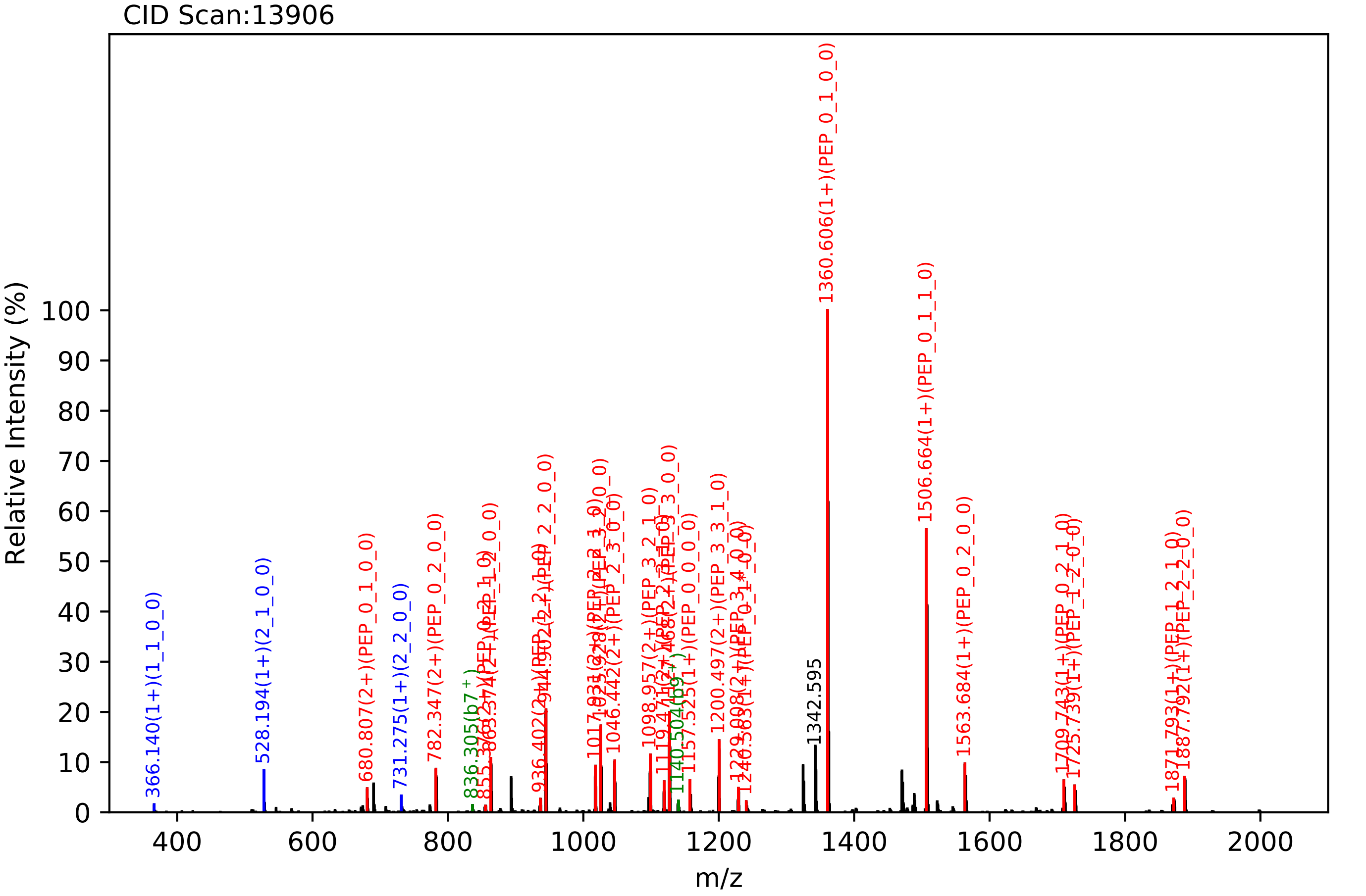

Unknown set no. 431, Experiment: Human Plasma exp_3

EEQFNSTFR(=PEP)_3_4_1_0, m/z:1301.53(2+), RT:52.62, Y-score:89.47

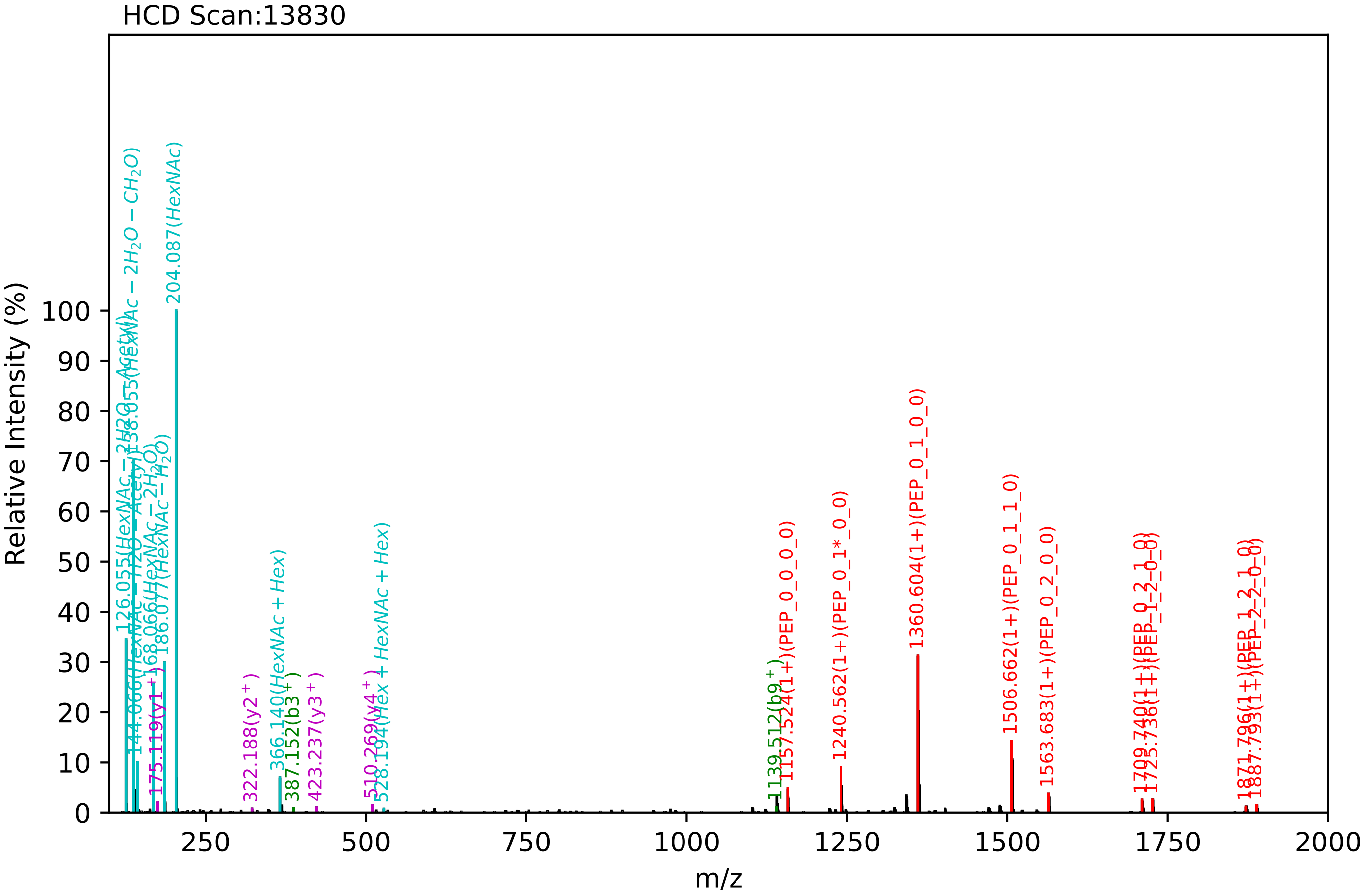

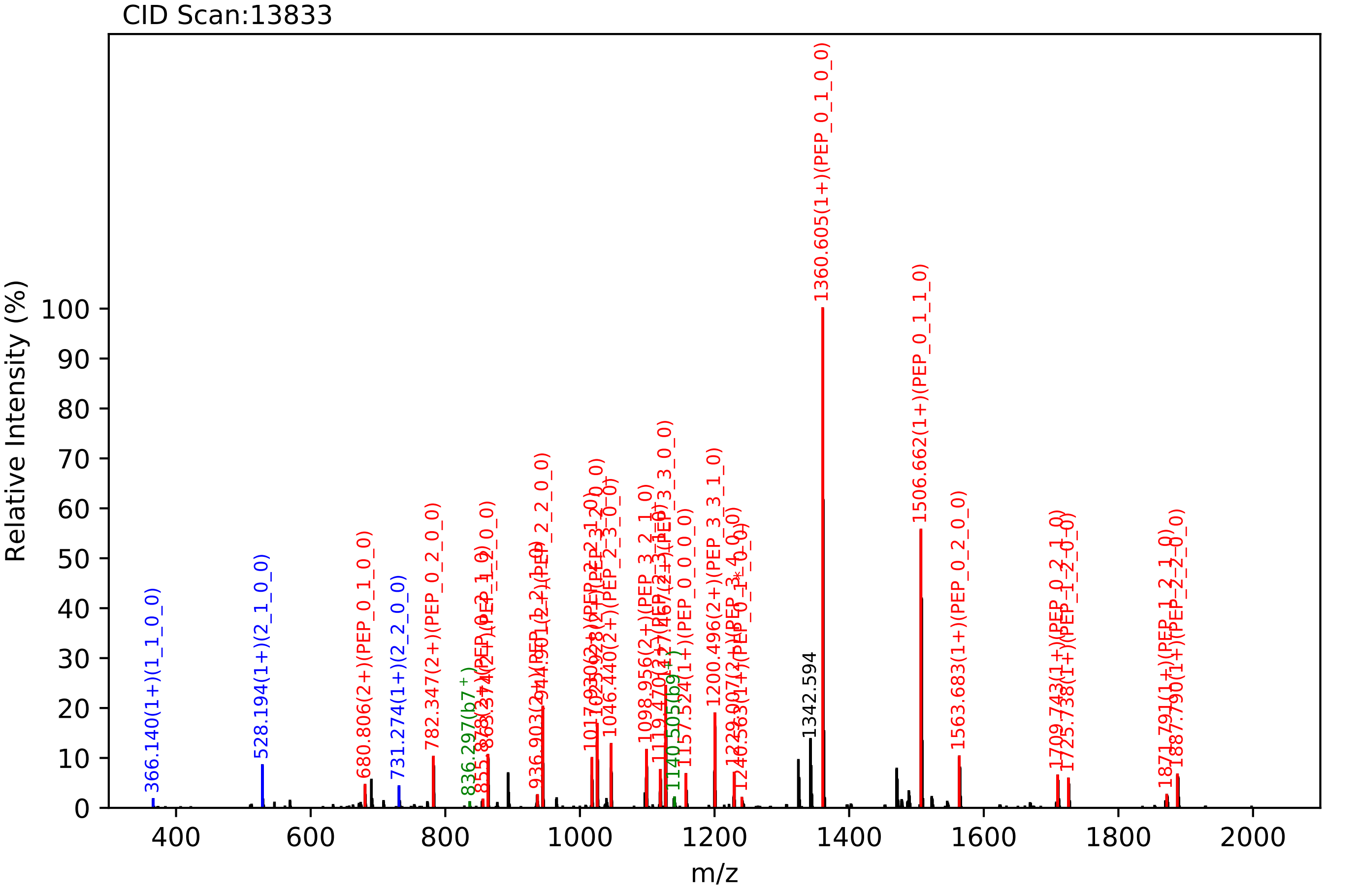

Unknown set no. 432, Experiment: Human Plasma exp_4

EEQFNSTFR(=PEP)_3_4_1_0, m/z:1301.53(2+), RT:52.52, Y-score:89.40

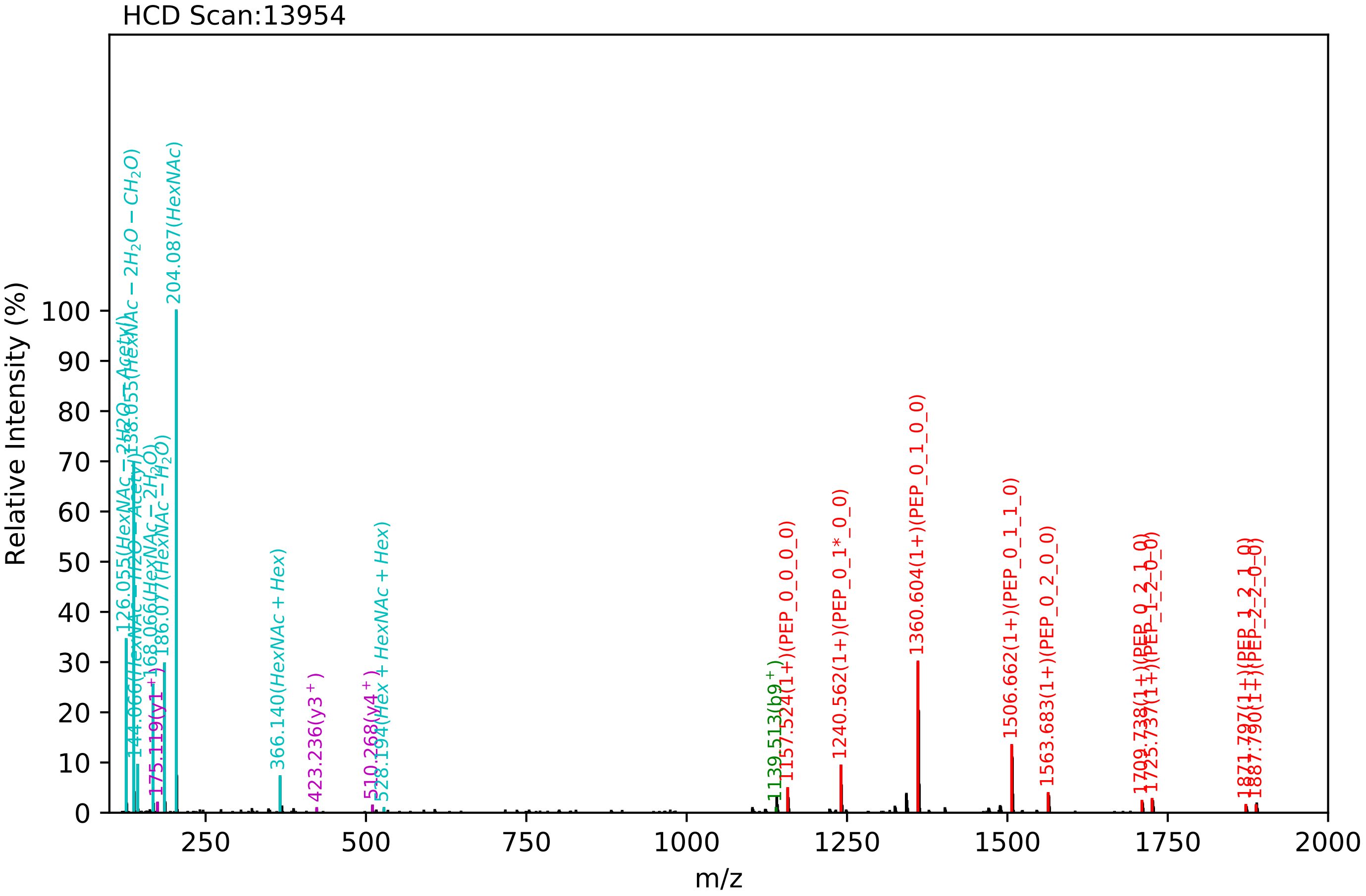

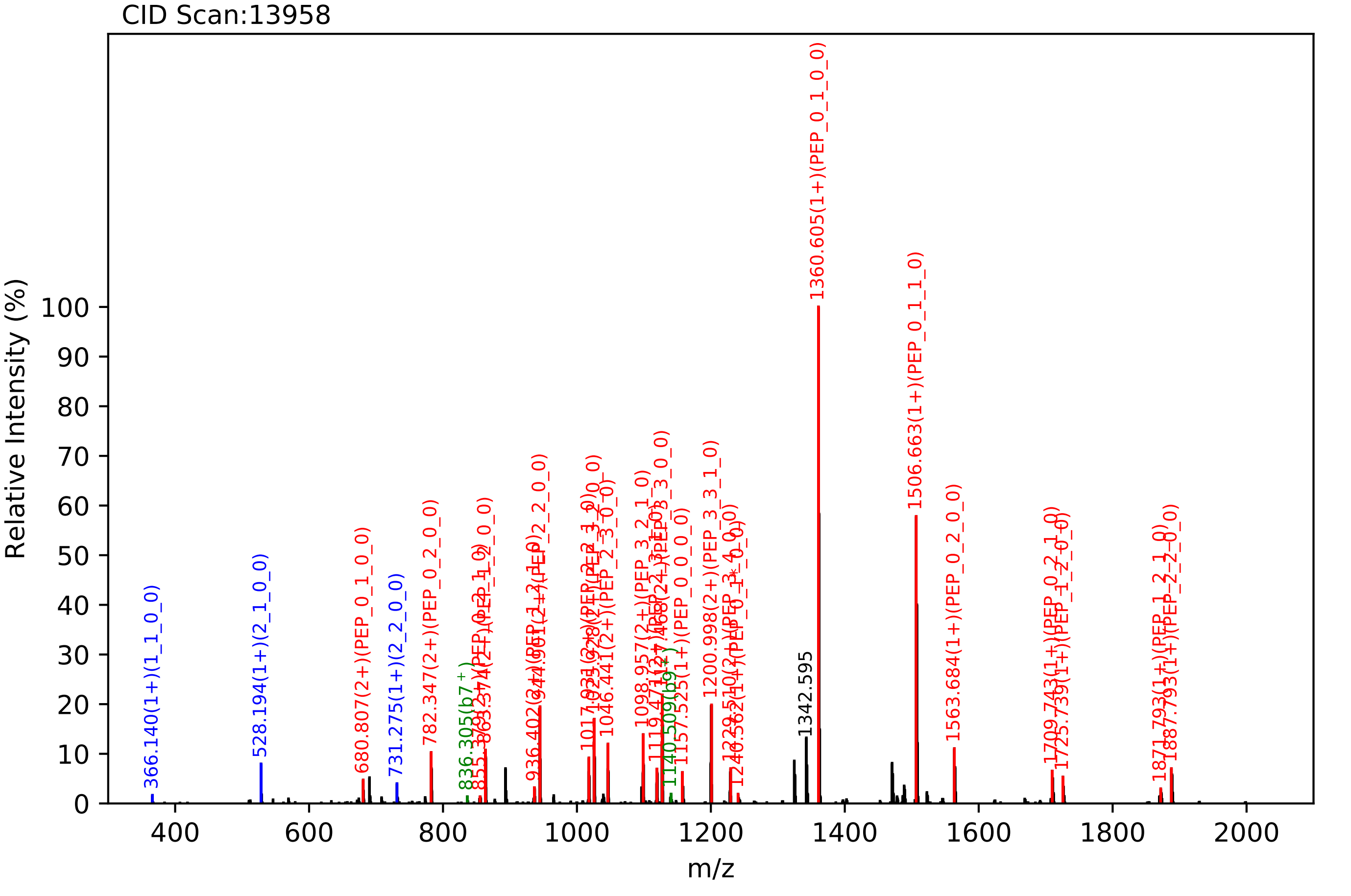

Unknown set no. 433, Experiment: Human Plasma exp_5

EEQFNSTFR(=PEP)_3_4_1_0, m/z:1301.53(2+), RT:52.59, Y-score:89.82

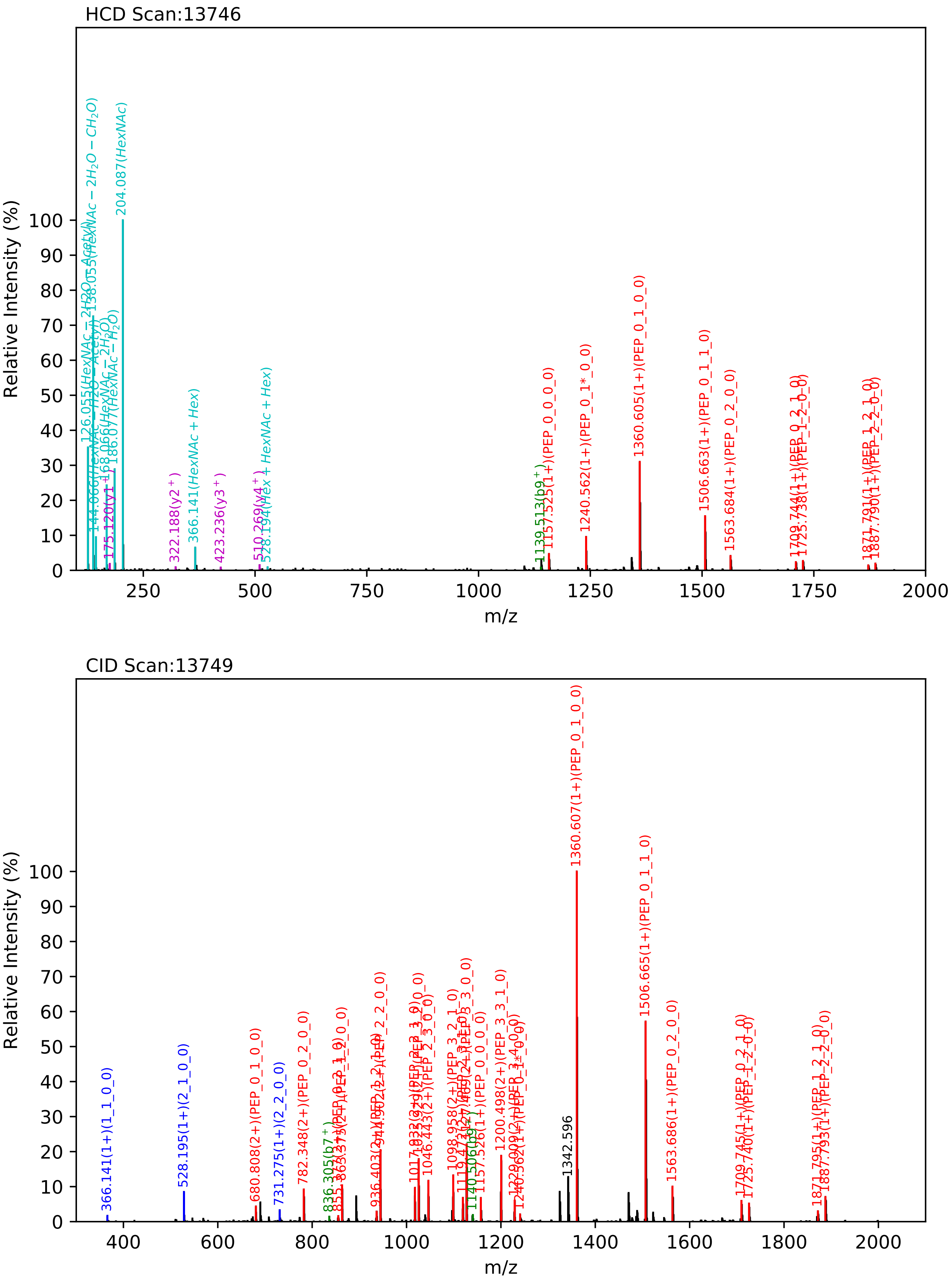

Unknown set no. 434, Experiment: Human Plasma exp_1

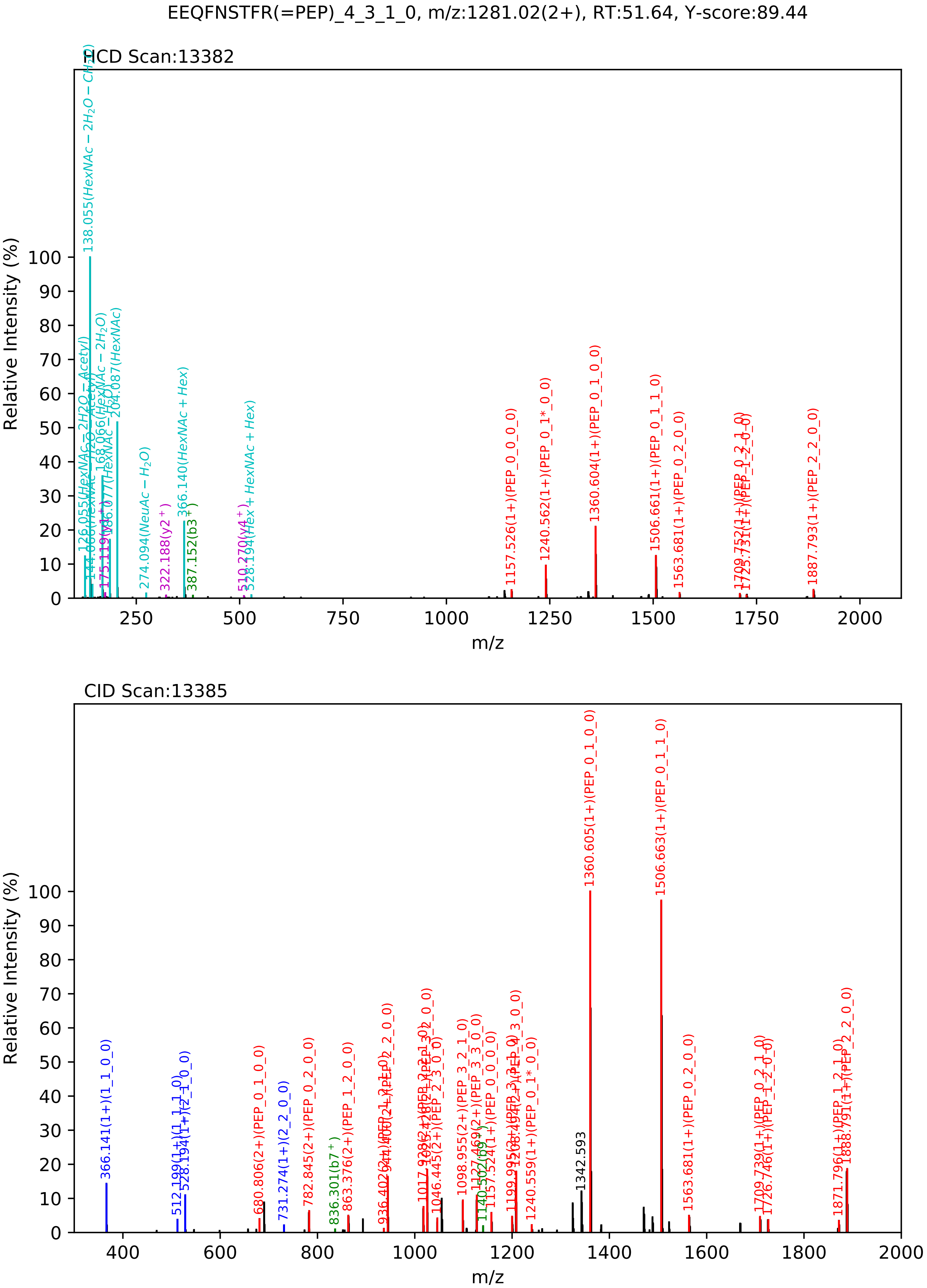

Unknown set no. 435, Experiment: Human Plasma exp_2

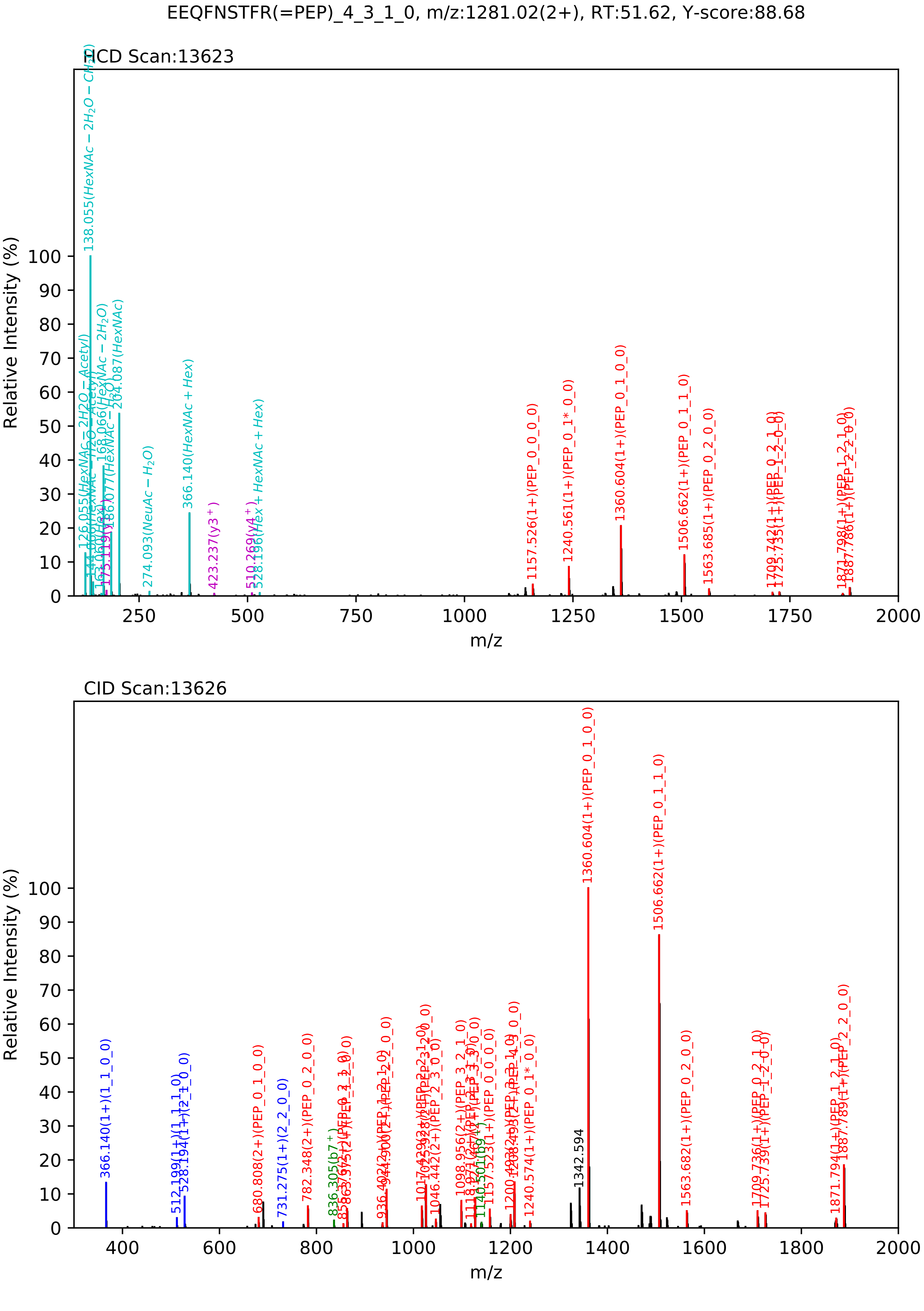

Unknown set no. 436, Experiment: Human Plasma exp_3

EEQFNSTFR(=PEP)_4_3_1_0, m/z:1281.02(2+), RT:51.90, Y-score:89.19

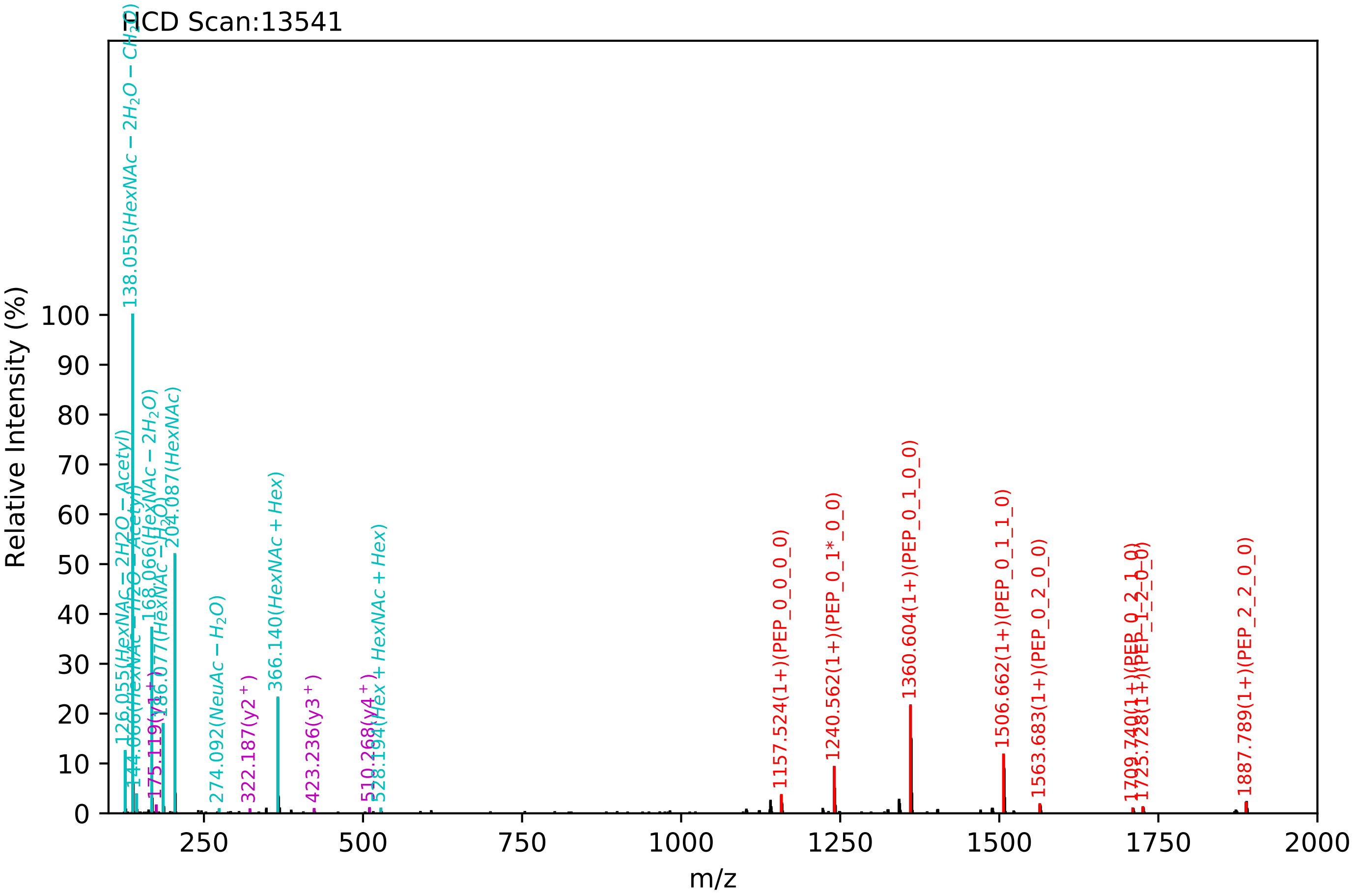

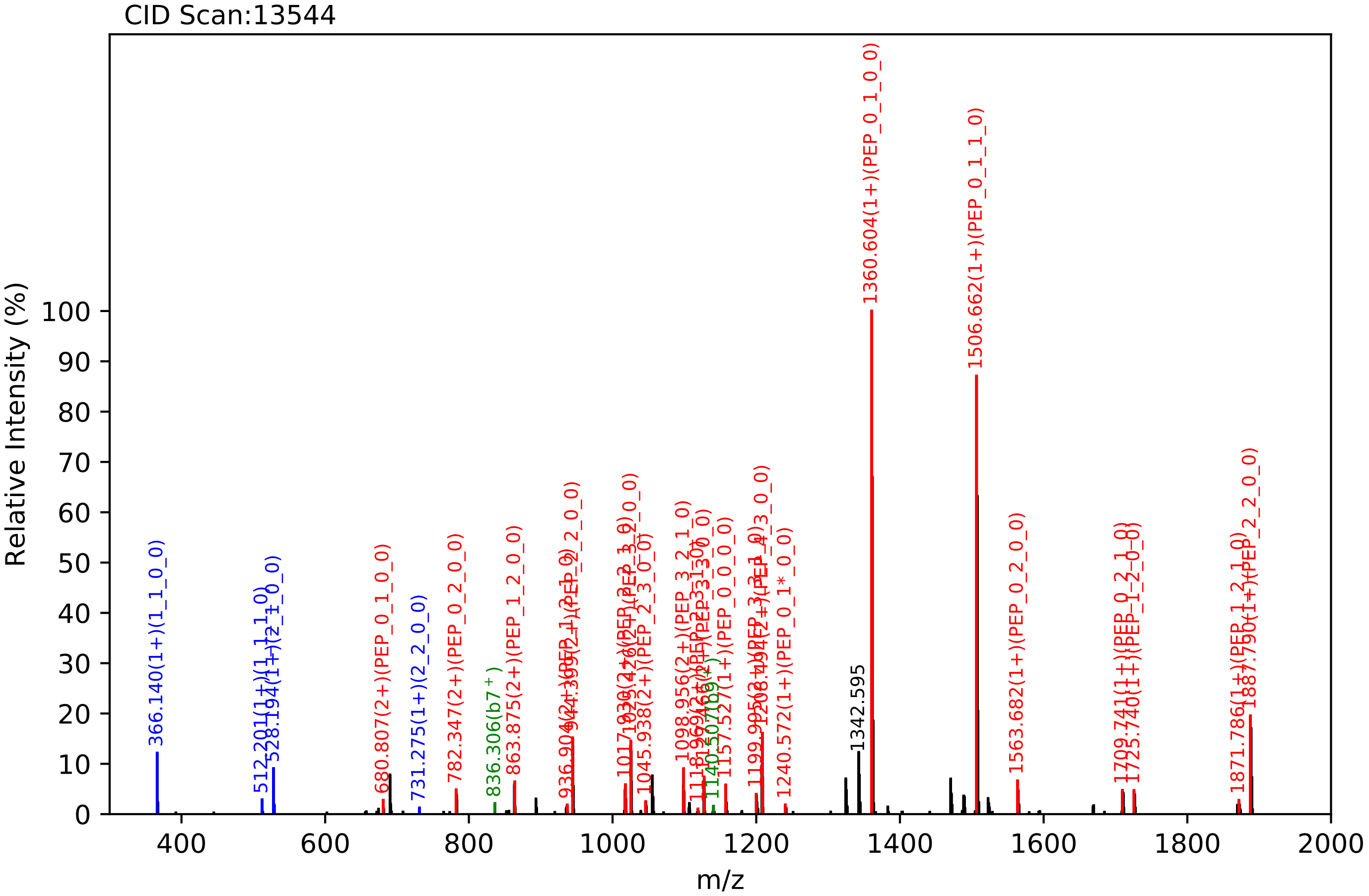

Unknown set no. 437, Experiment: Human Plasma exp_4

EEQFNSTFR(=PEP)_4_3_1_0, m/z:1281.02(2+), RT:51.34, Y-score:89.29

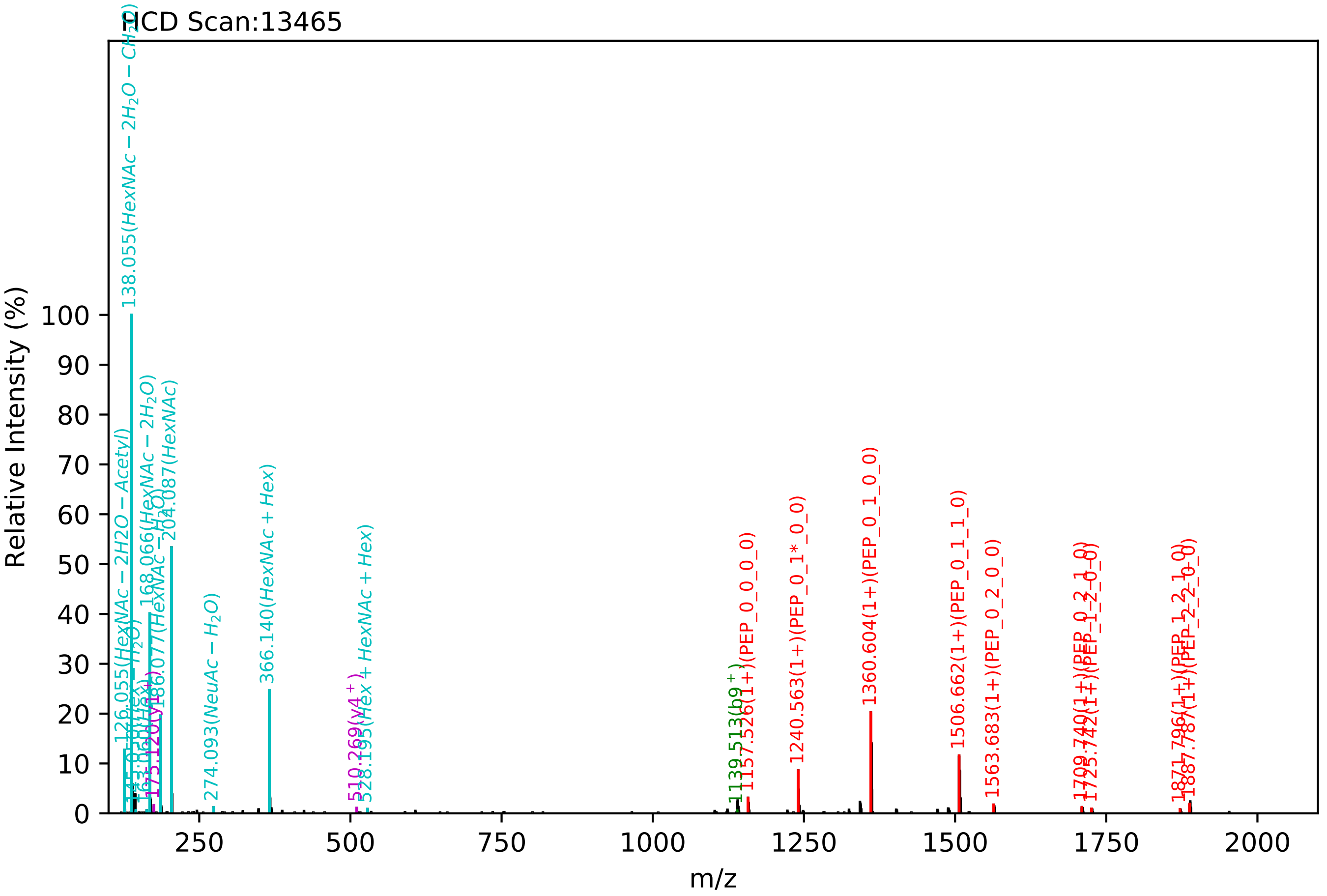

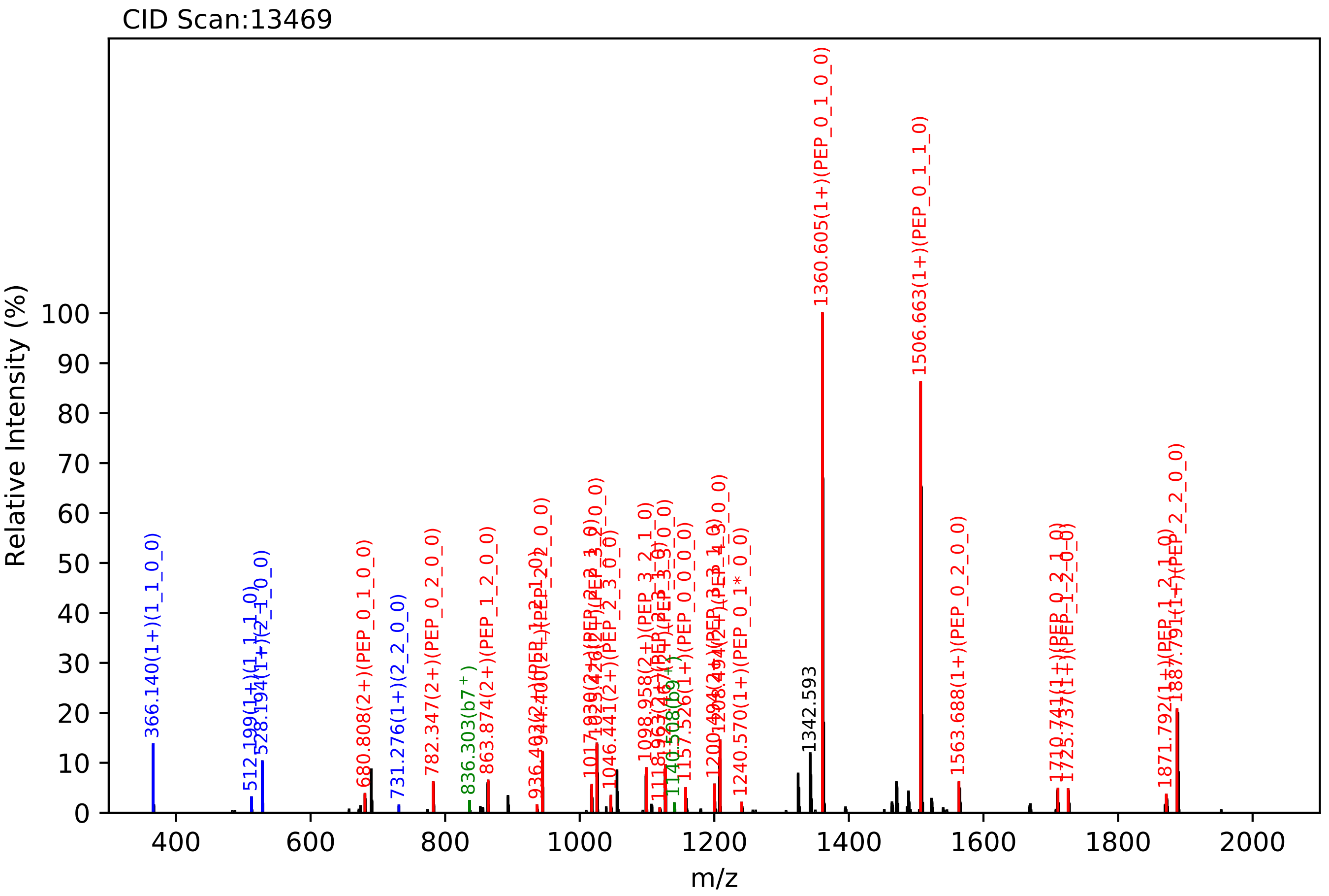

Unknown set no. 438, Experiment: Human Plasma exp_5

EEQFNSTFR(=PEP)_4_3_1_0, m/z:854.35(3+), RT:52.32, Y-score:92.26

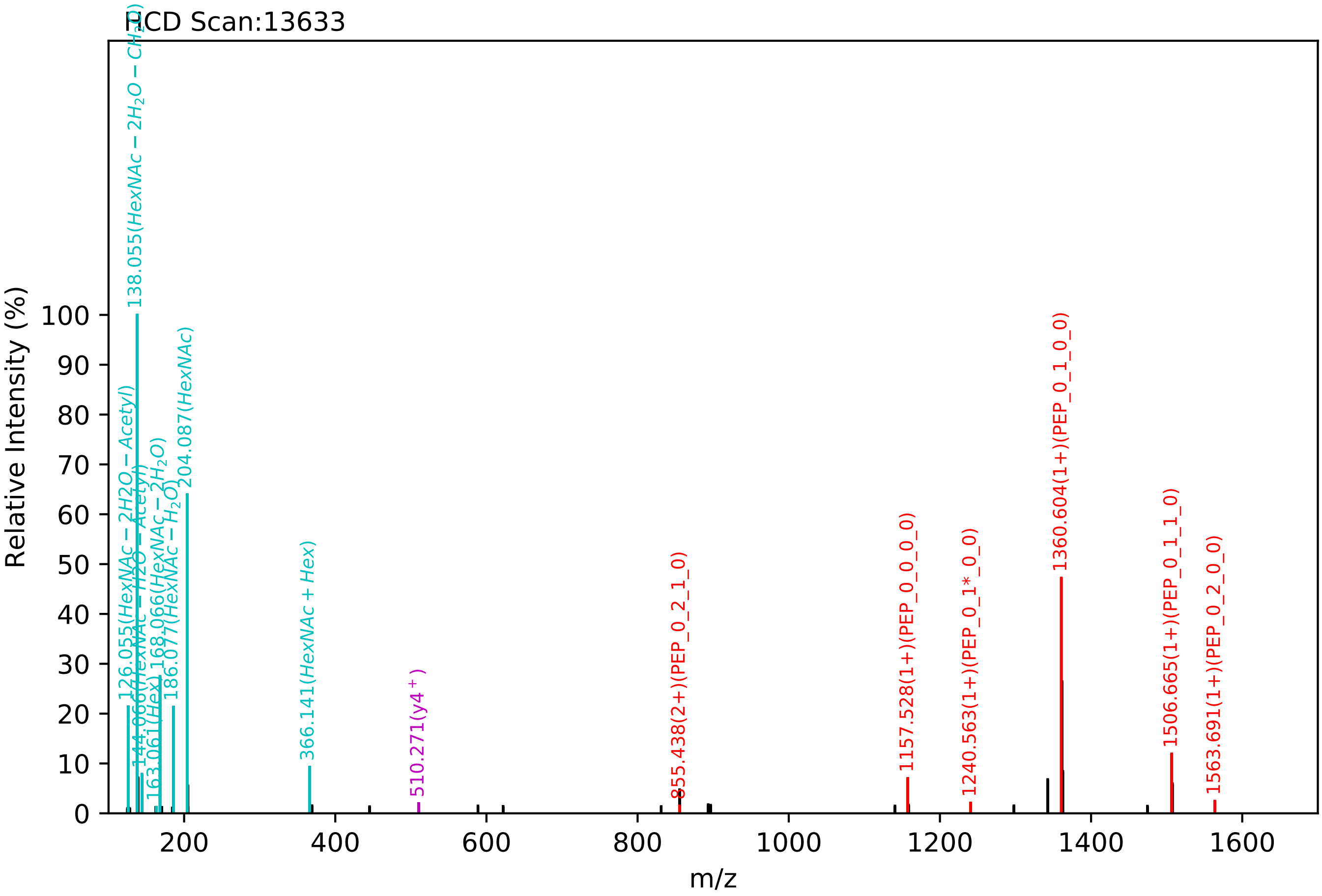

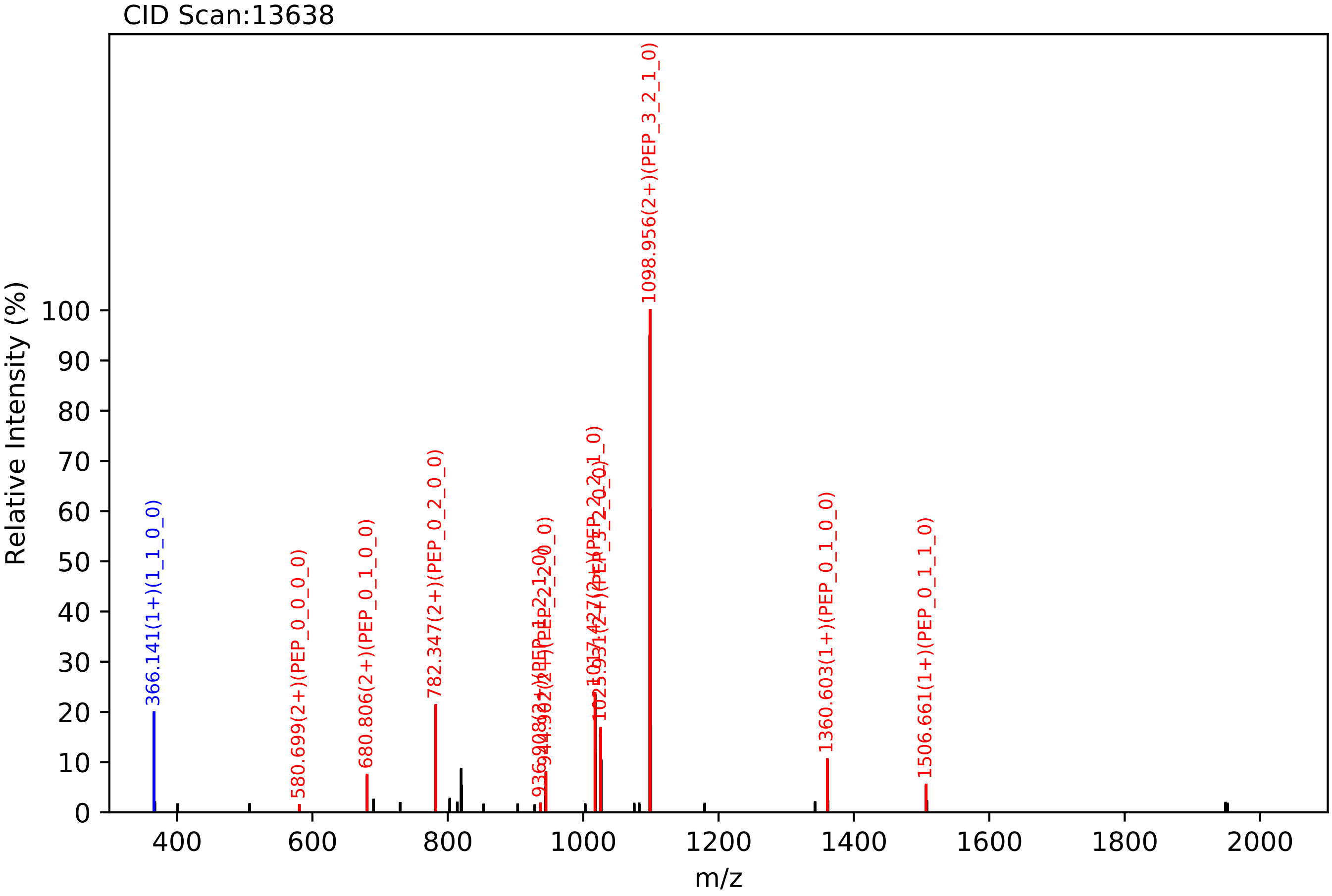

Unknown set no. 439, Experiment: Human Plasma exp_5

EEQFNSTFR(=PEP)_4_3_1_0, m/z:1281.02(2+), RT:51.89, Y-score:89.72

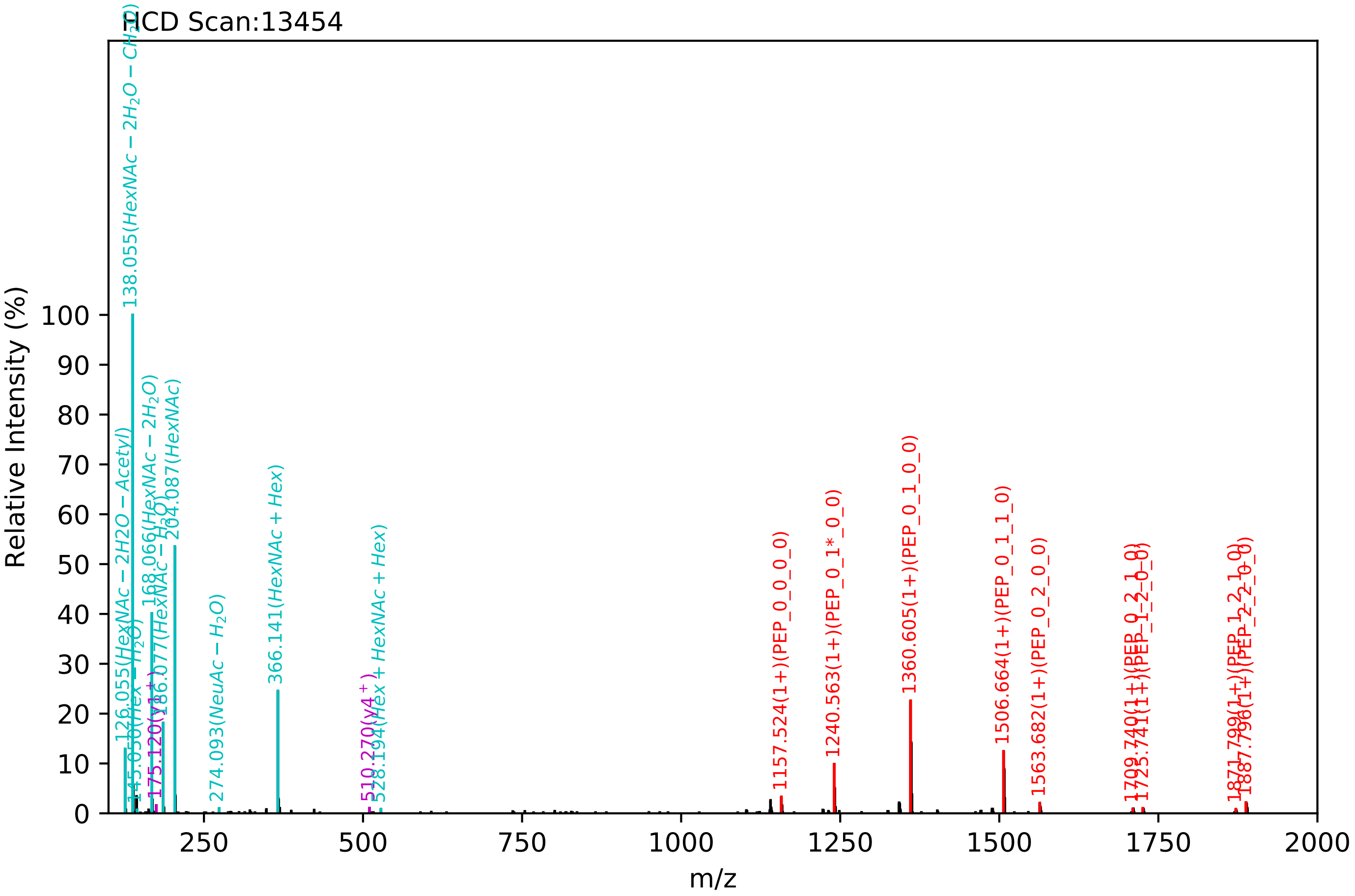

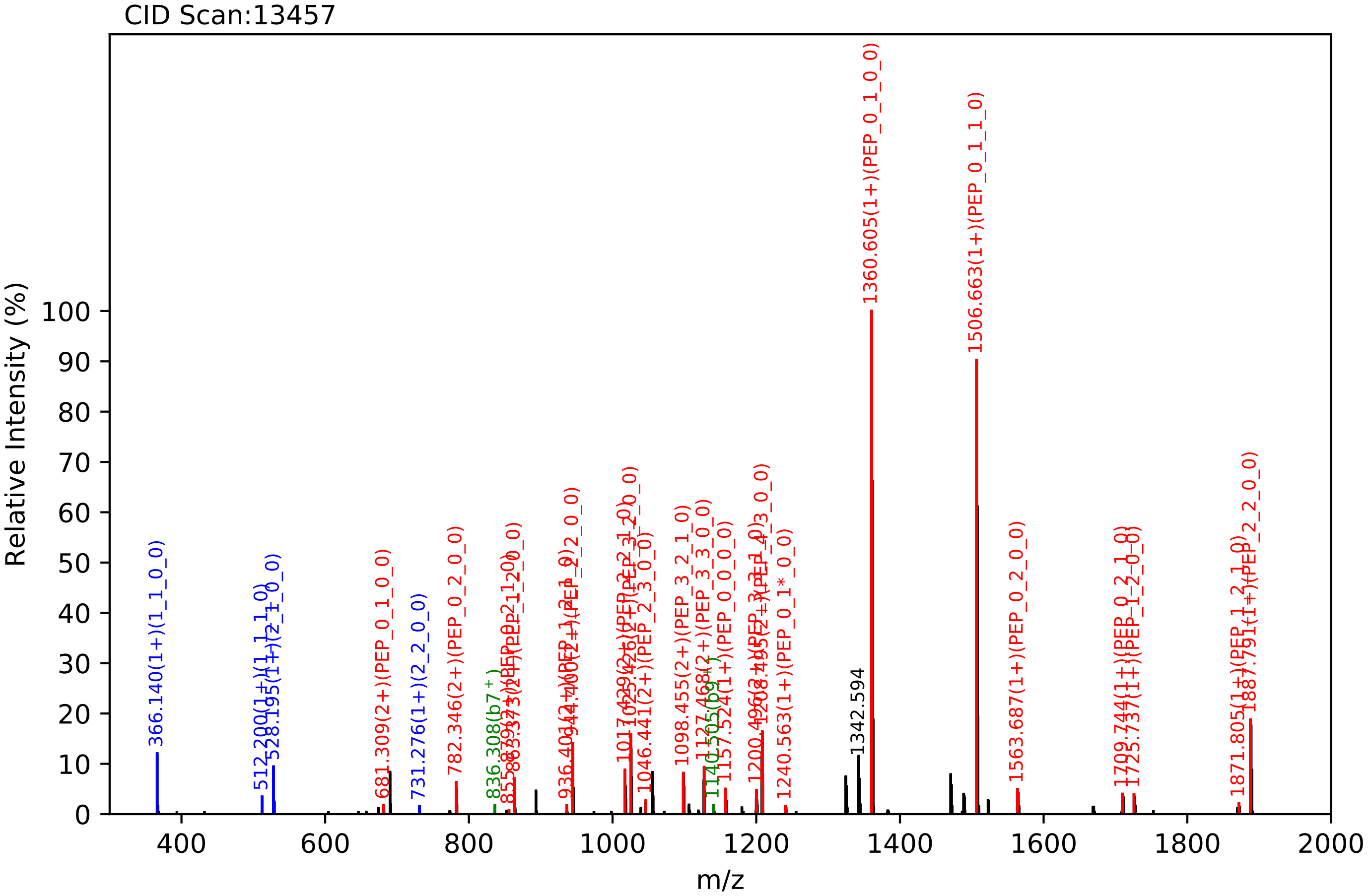

Unknown set no. 440, Experiment: Human Plasma exp_1

EEQFNSTFR(=PEP)_4_3_1_1, m/z:951.38(3+), RT:61.54, Y-score:92.24

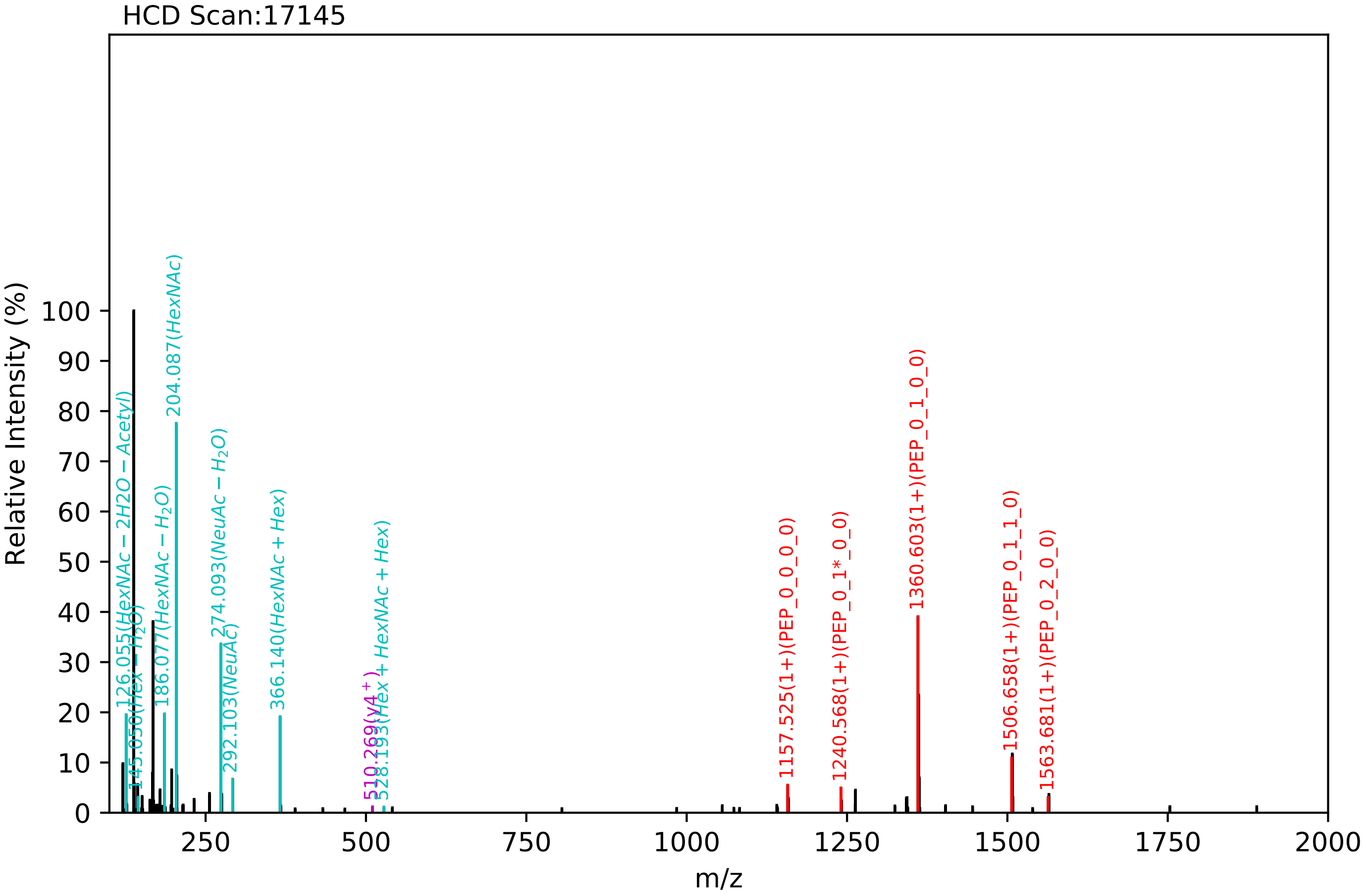

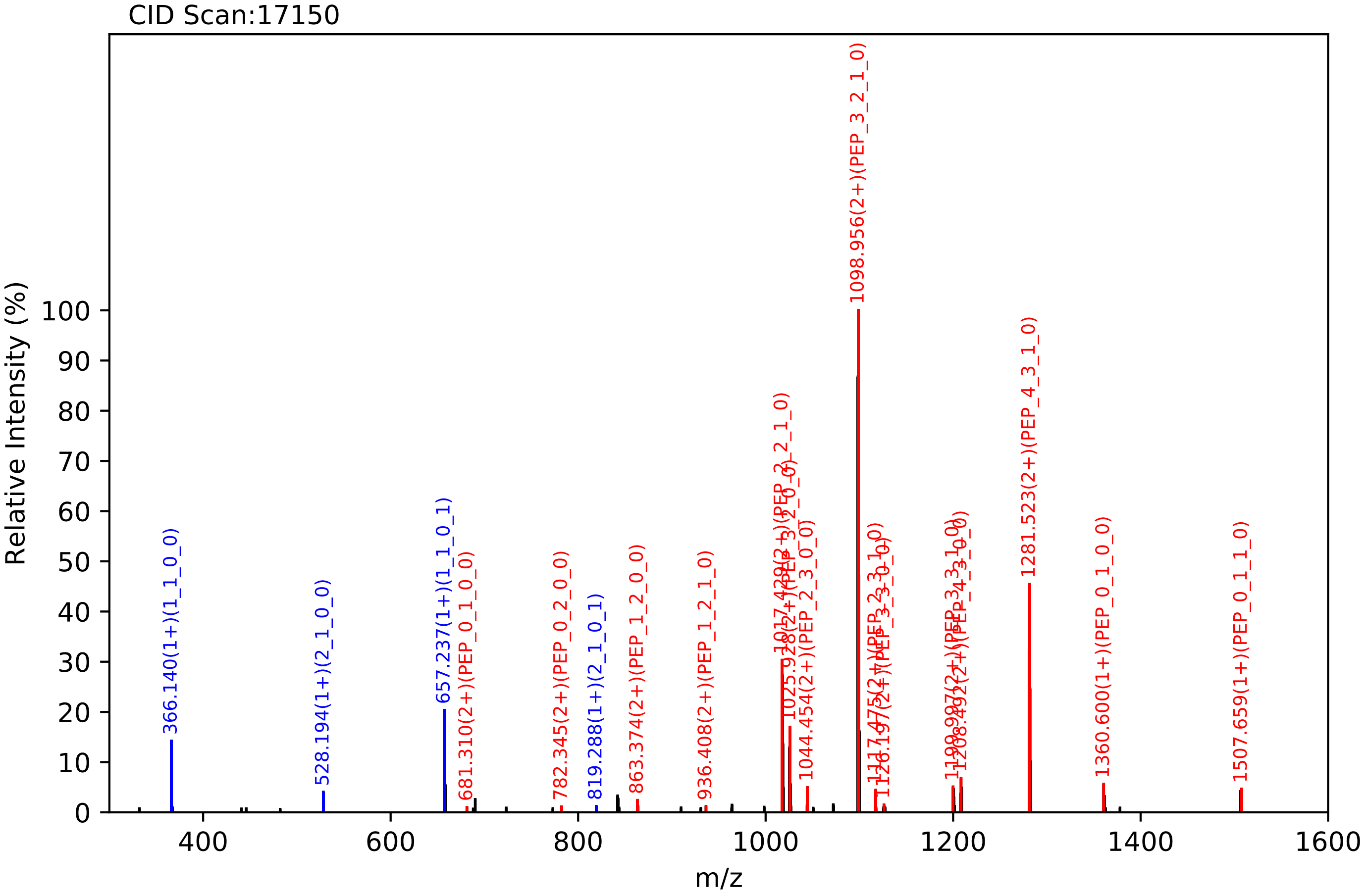

Unknown set no. 441, Experiment: Human Plasma exp_1

EEQFNSTFR(=PEP)_4_3_1_1, m/z:1426.56(2+), RT:60.65, Y-score:92.01

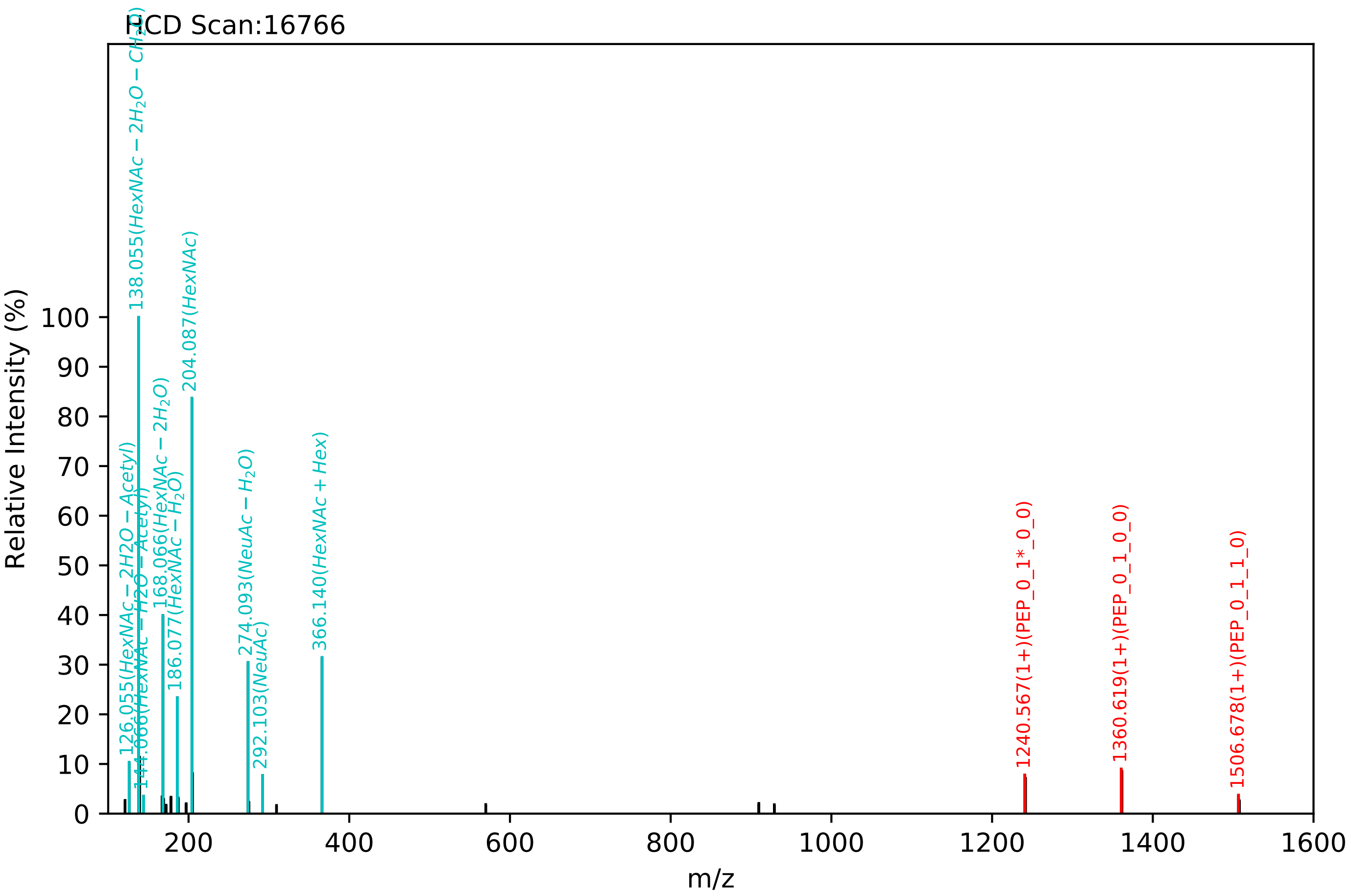

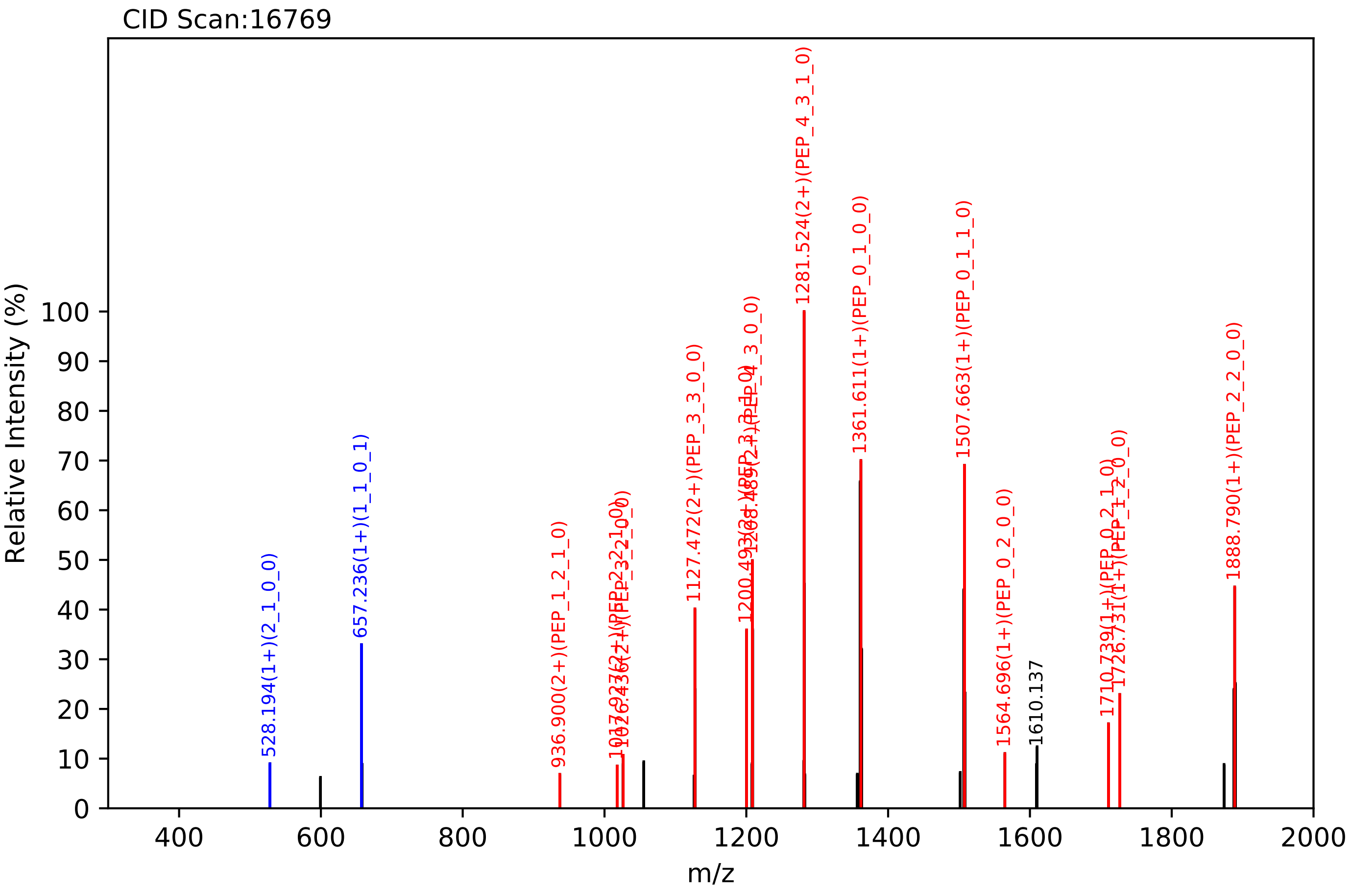

Unknown set no. 442, Experiment: Human Plasma exp_2

EEQFNSTFR(=PEP)_4_3_1_1, m/z:951.38(3+), RT:61.68, Y-score:95.50

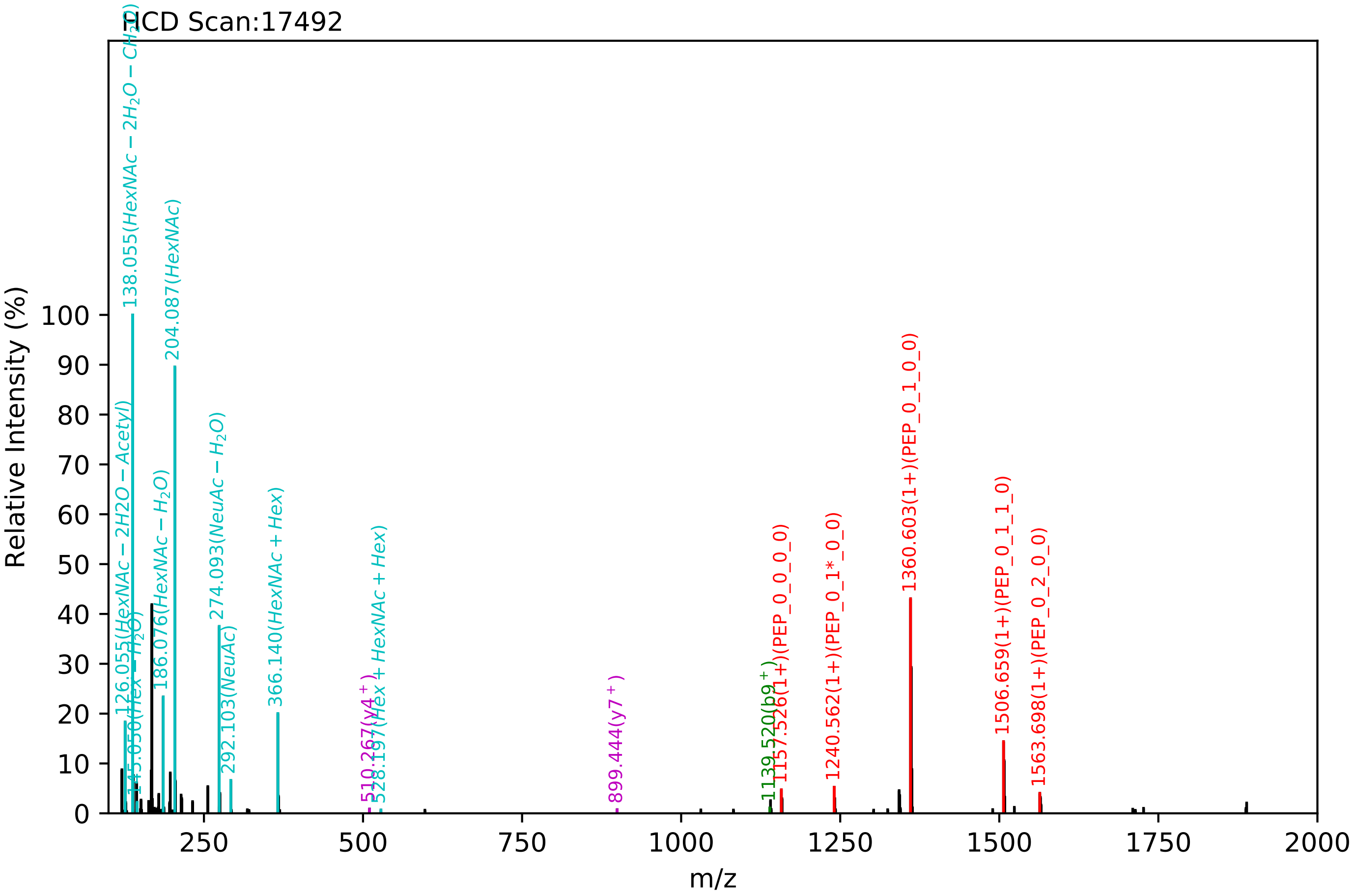

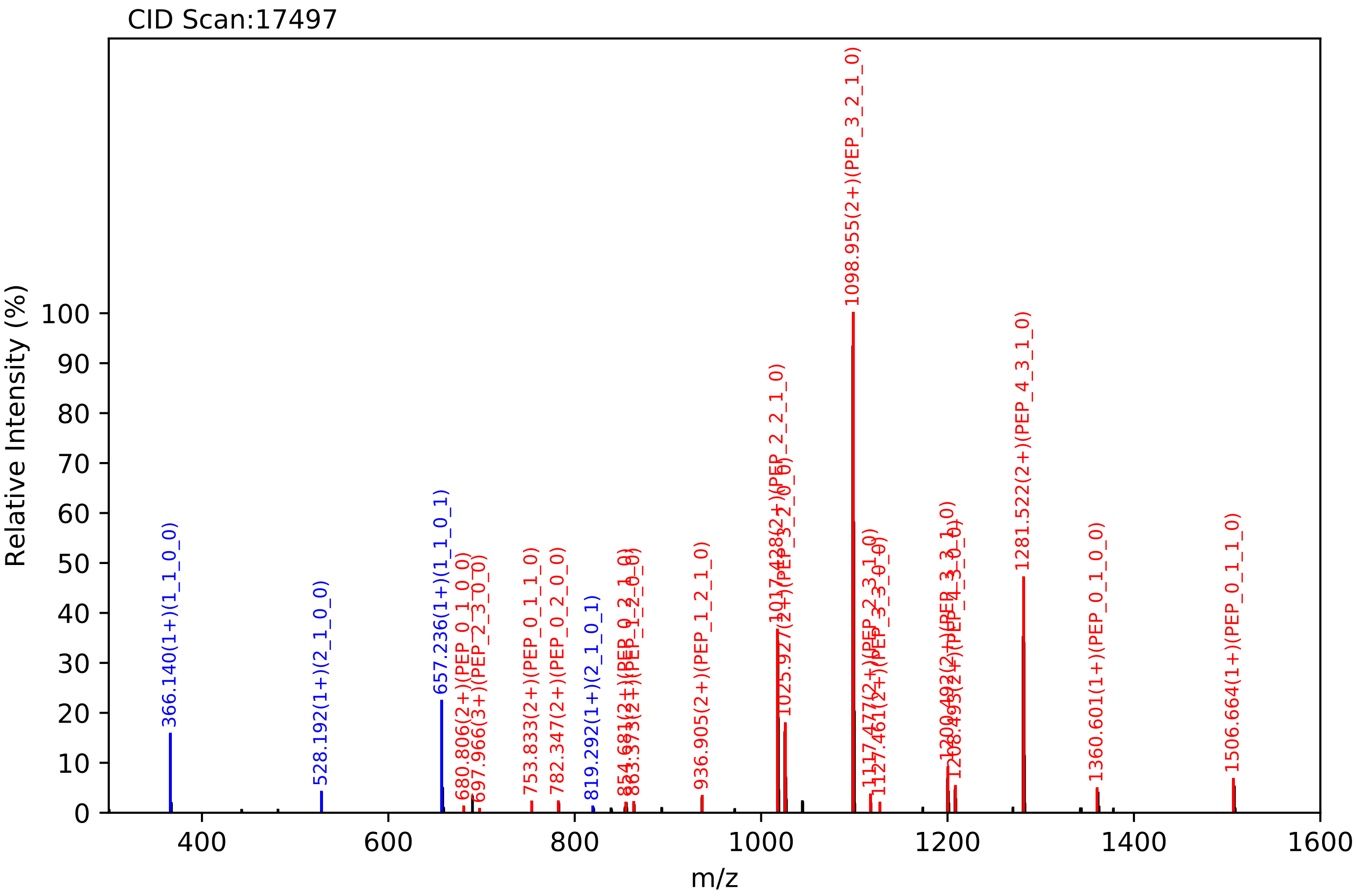

Unknown set no. 443, Experiment: Human Plasma exp_3

EEQFNSTFR(=PEP)_4_3_1_1, m/z:951.38(3+), RT:61.64, Y-score:89.35

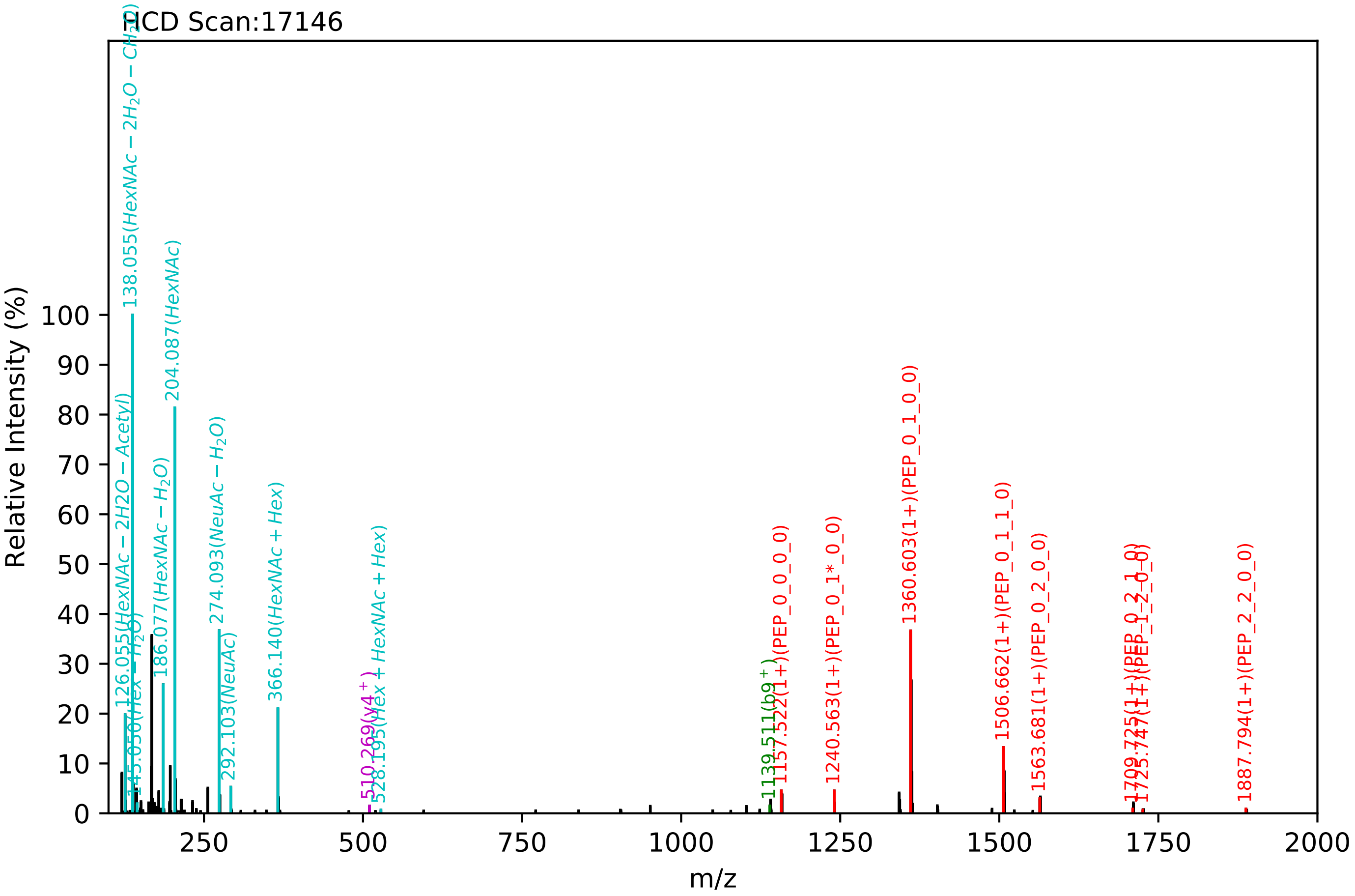

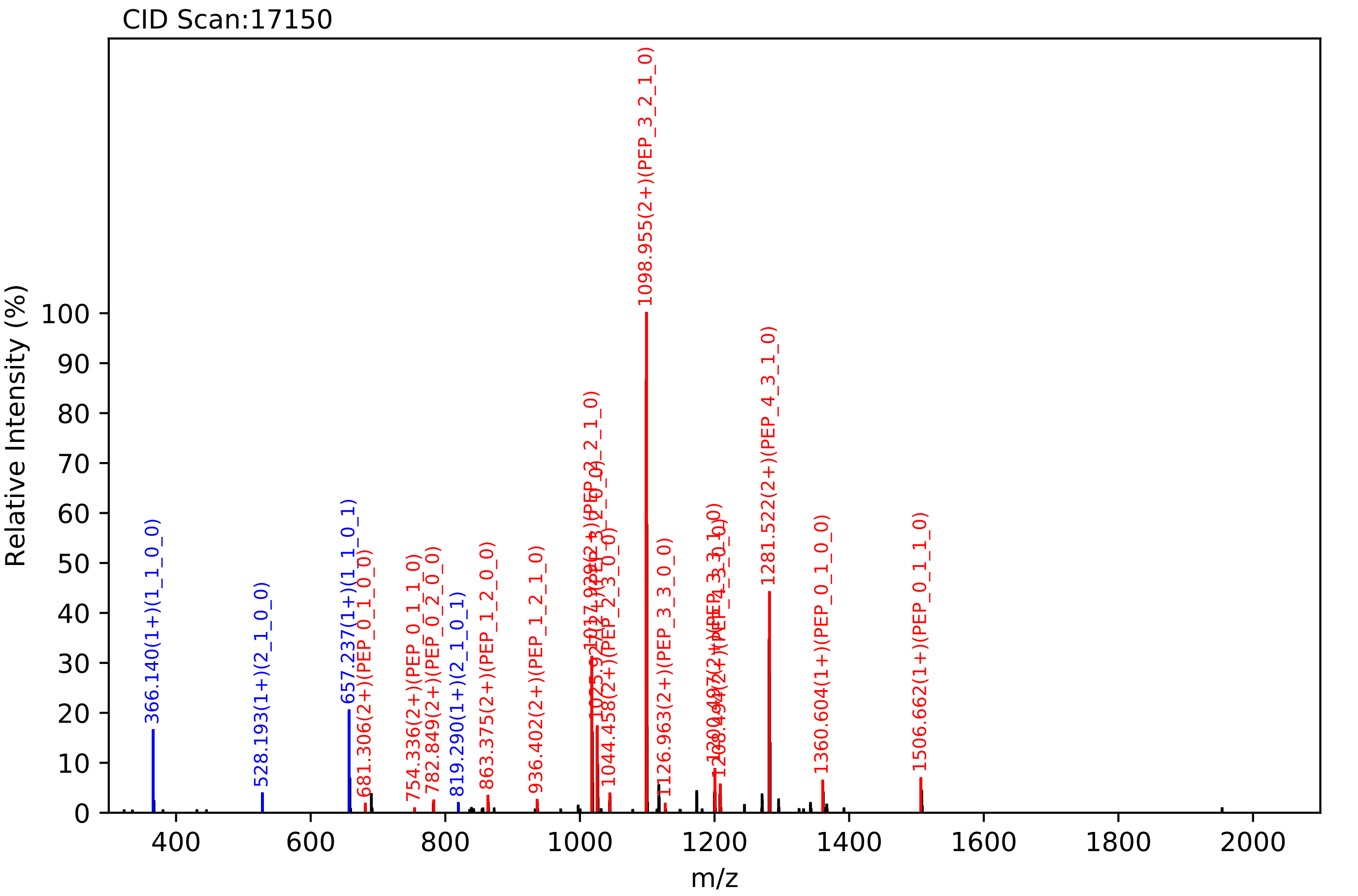

Unknown set no. 444, Experiment: Human Plasma exp_3

EEQFNSTFR(=PEP)_4_3_1_1, m/z:1426.57(2+), RT:61.49, Y-score:89.76

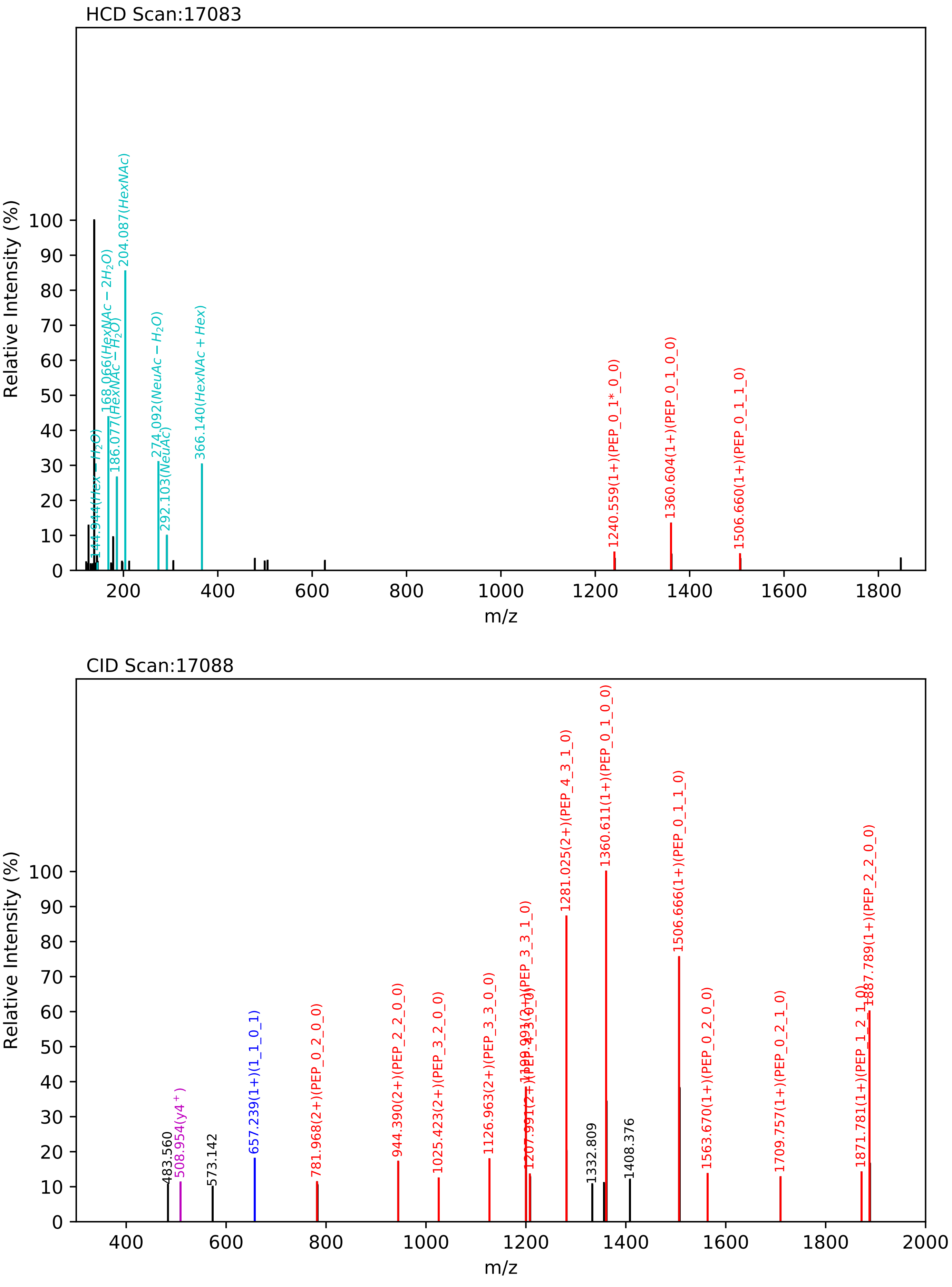

Unknown set no. 445, Experiment: Human Plasma exp_4

EEQFNSTFR(=PEP)_4_3_1_1, m/z:951.38(3+), RT:61.79, Y-score:93.93

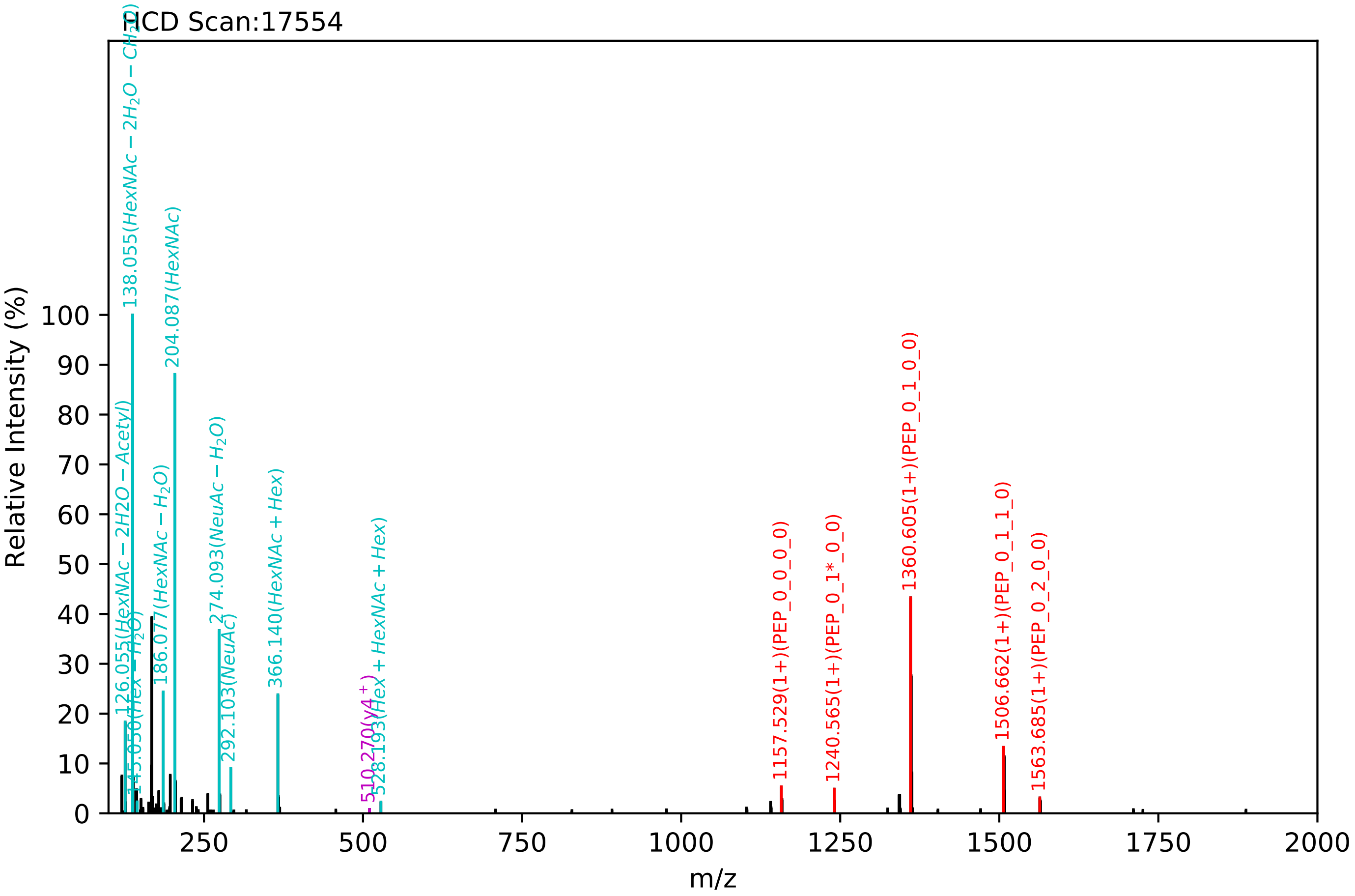

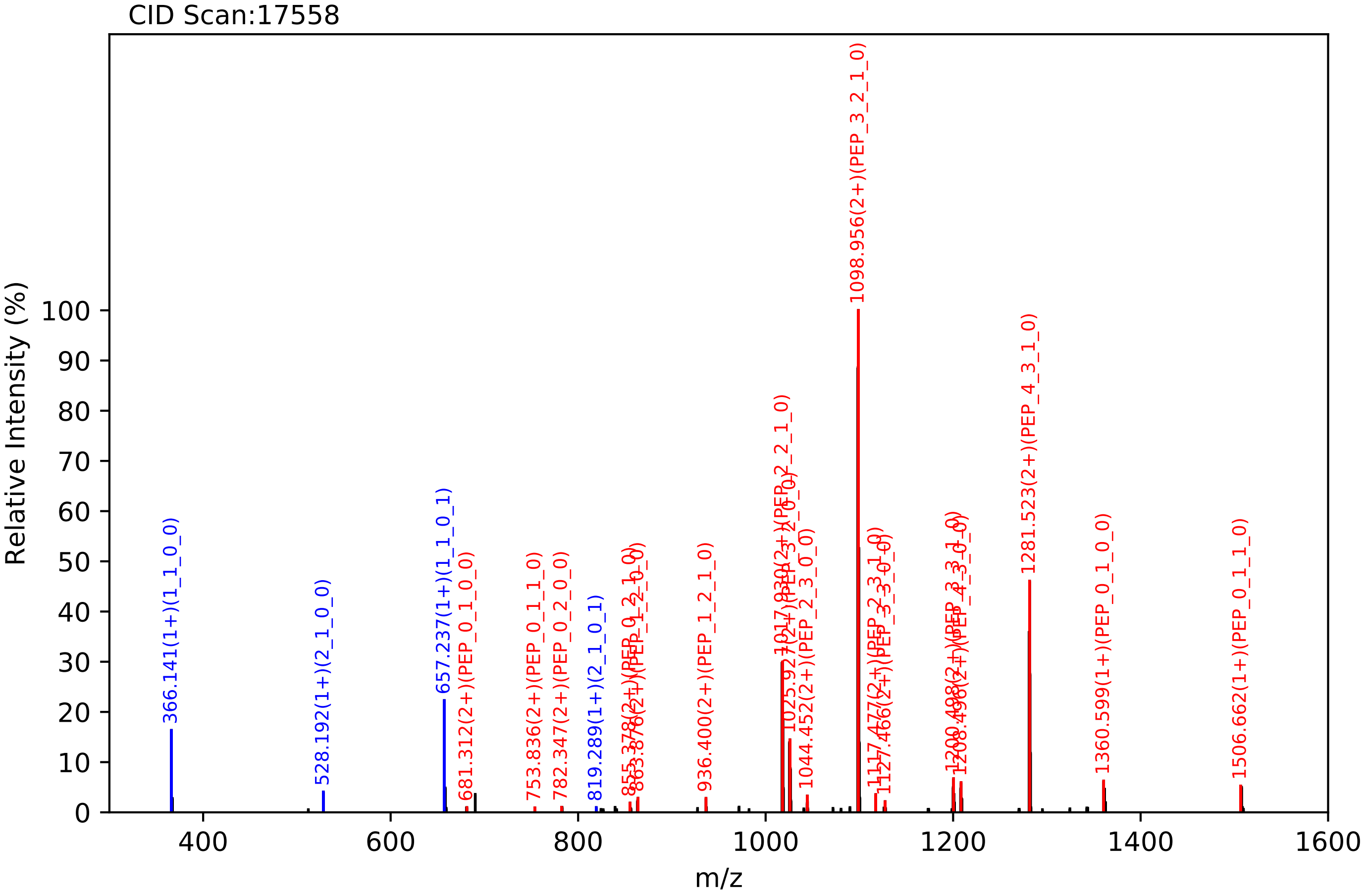

Unknown set no. 446, Experiment: Human Plasma exp_5

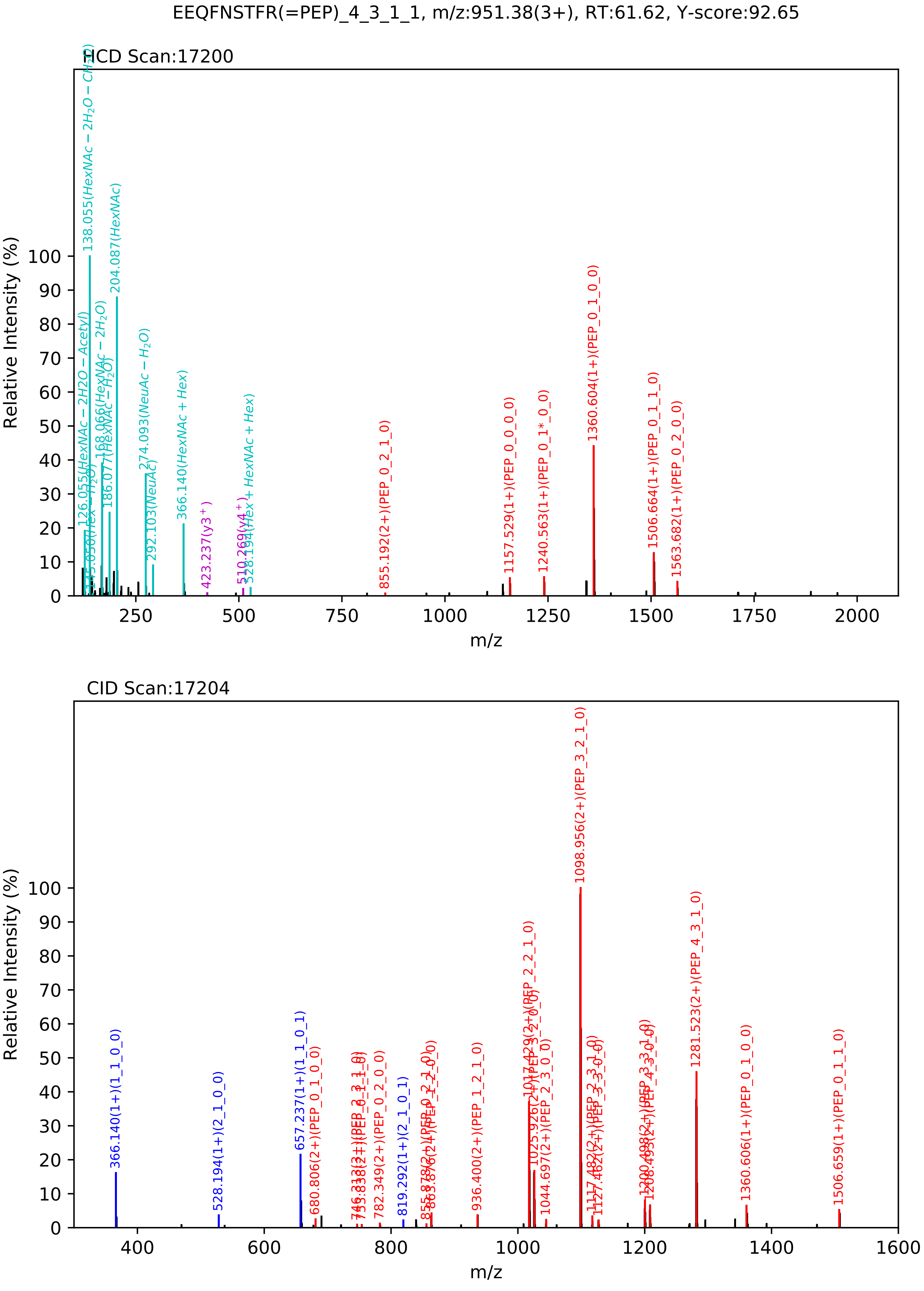

Unknown set no. 447, Experiment: Human Plasma exp_5

EEQFNSTFR(=PEP)_4_3_1_1, m/z:1426.57(2+), RT:60.73, Y-score:84.06

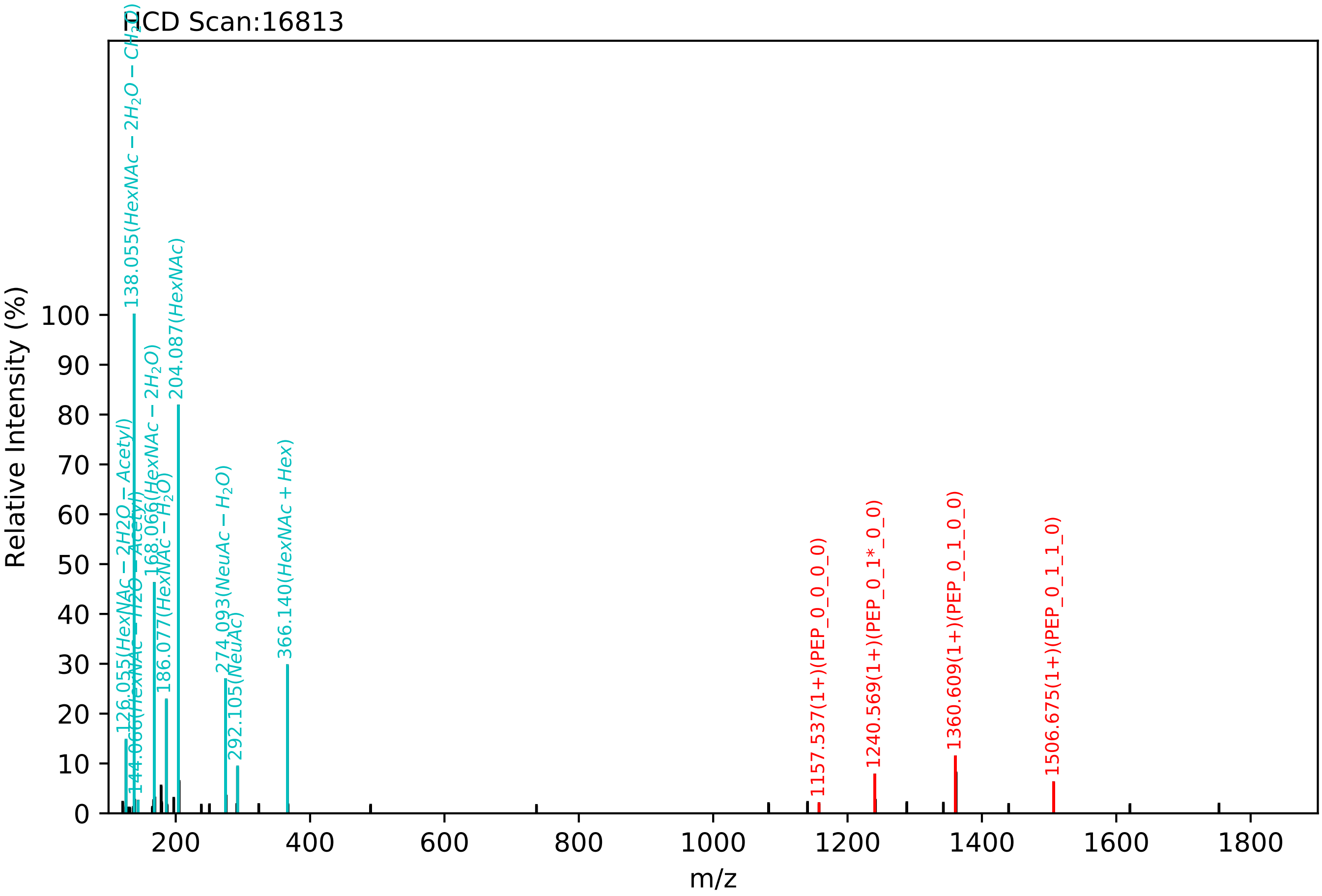

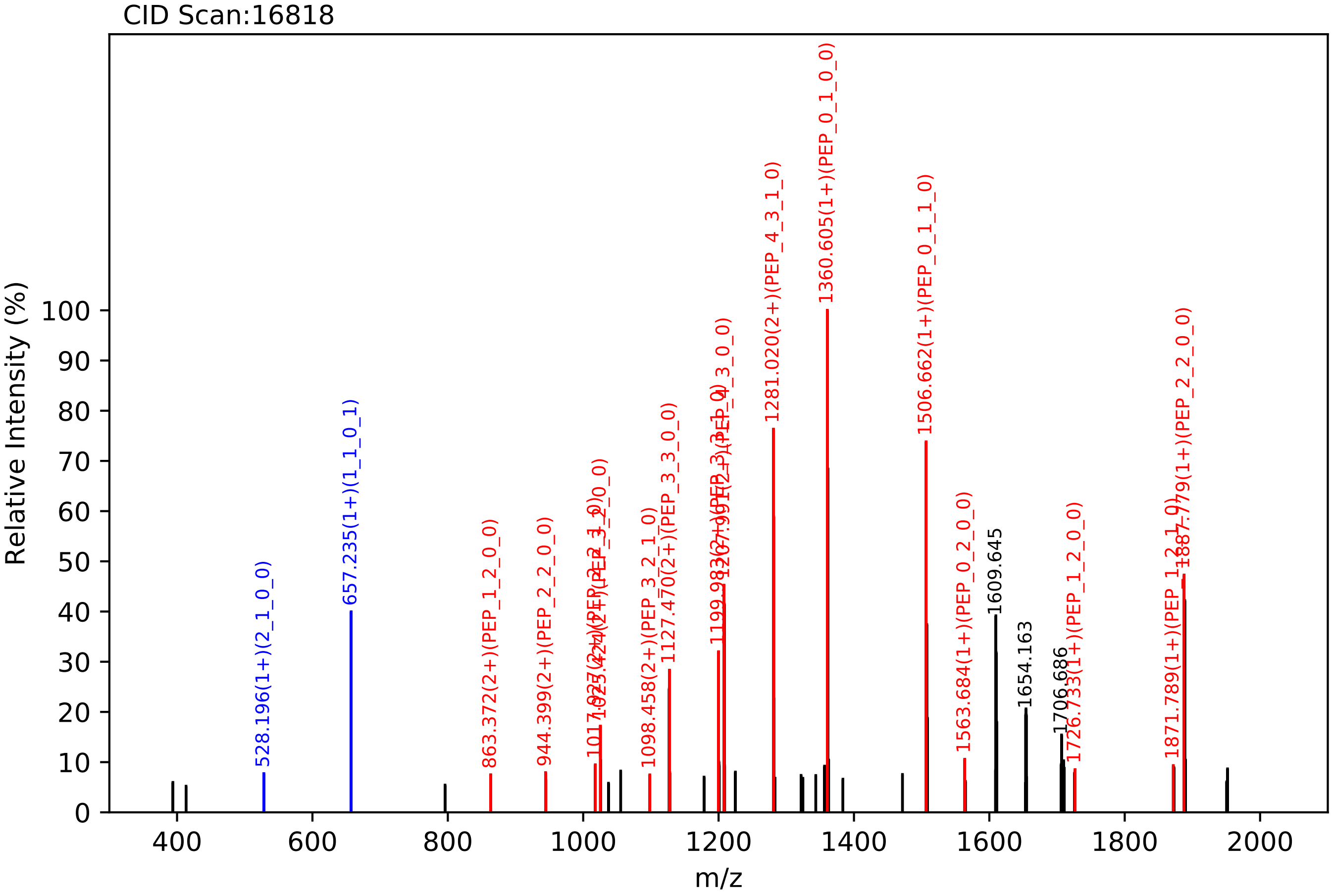

Unknown set no. 448, Experiment: Human Plasma exp_1

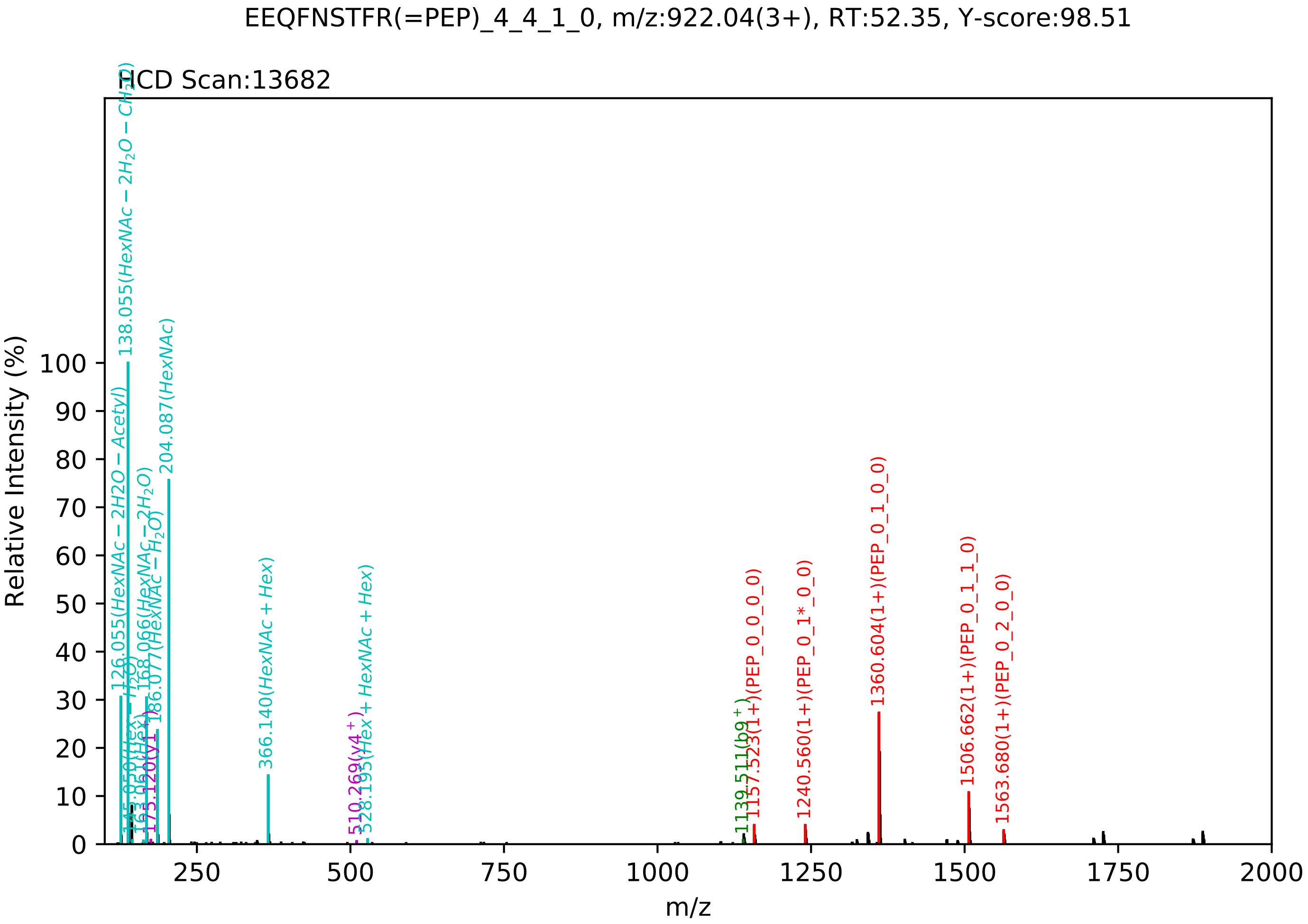

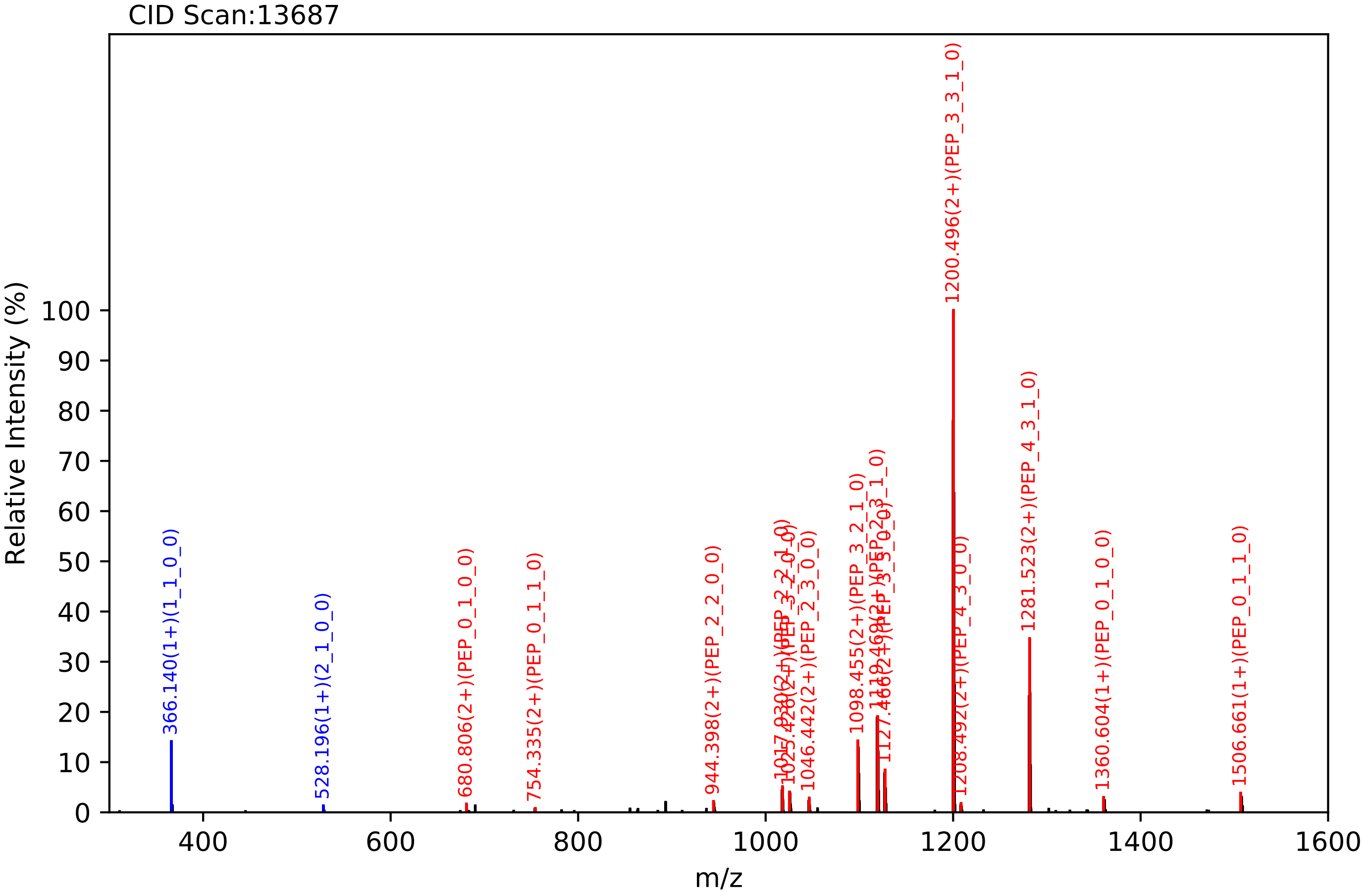

Unknown set no. 449, Experiment: Human Plasma exp_1

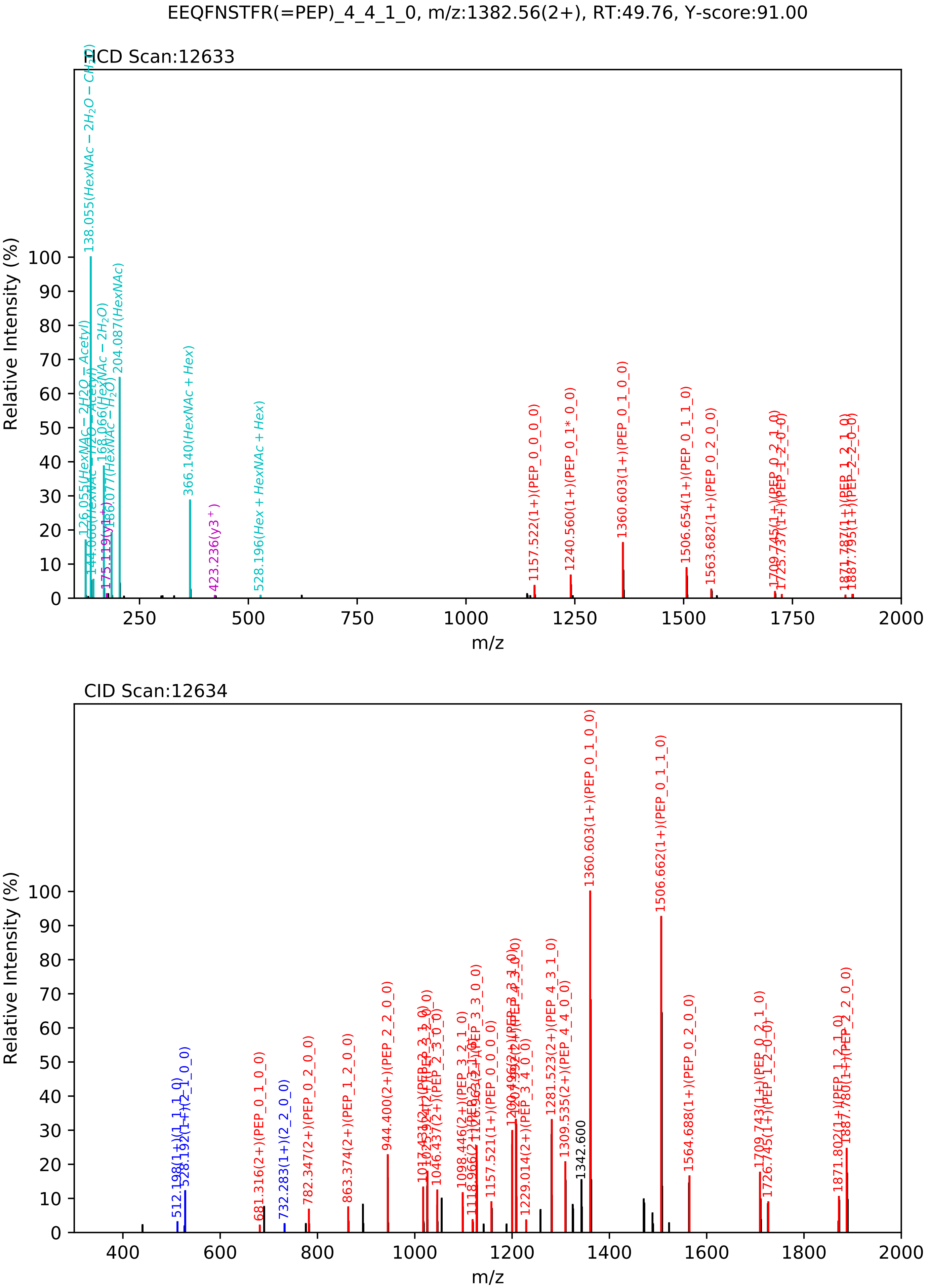

Unknown set no. 450, Experiment: Human Plasma exp_2

EEQFNSTFR(=PEP)_4_4_1_0, m/z:922.04(3+), RT:52.33, Y-score:98.15

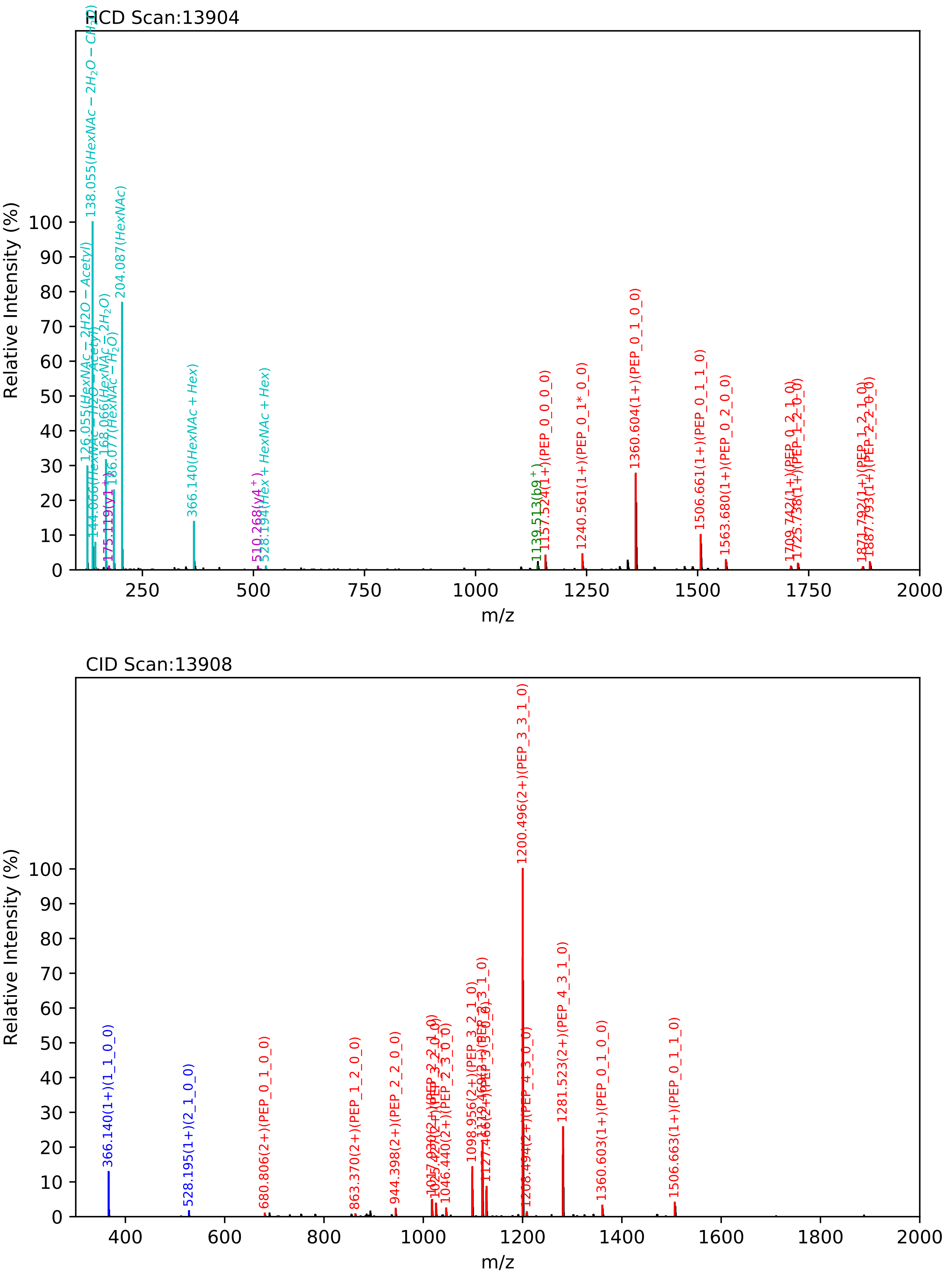

Unknown set no. 451, Experiment: Human Plasma exp_2

EEQFNSTFR(=PEP)_4_4_1_0, m/z:1382.56(2+), RT:49.82, Y-score:96.30

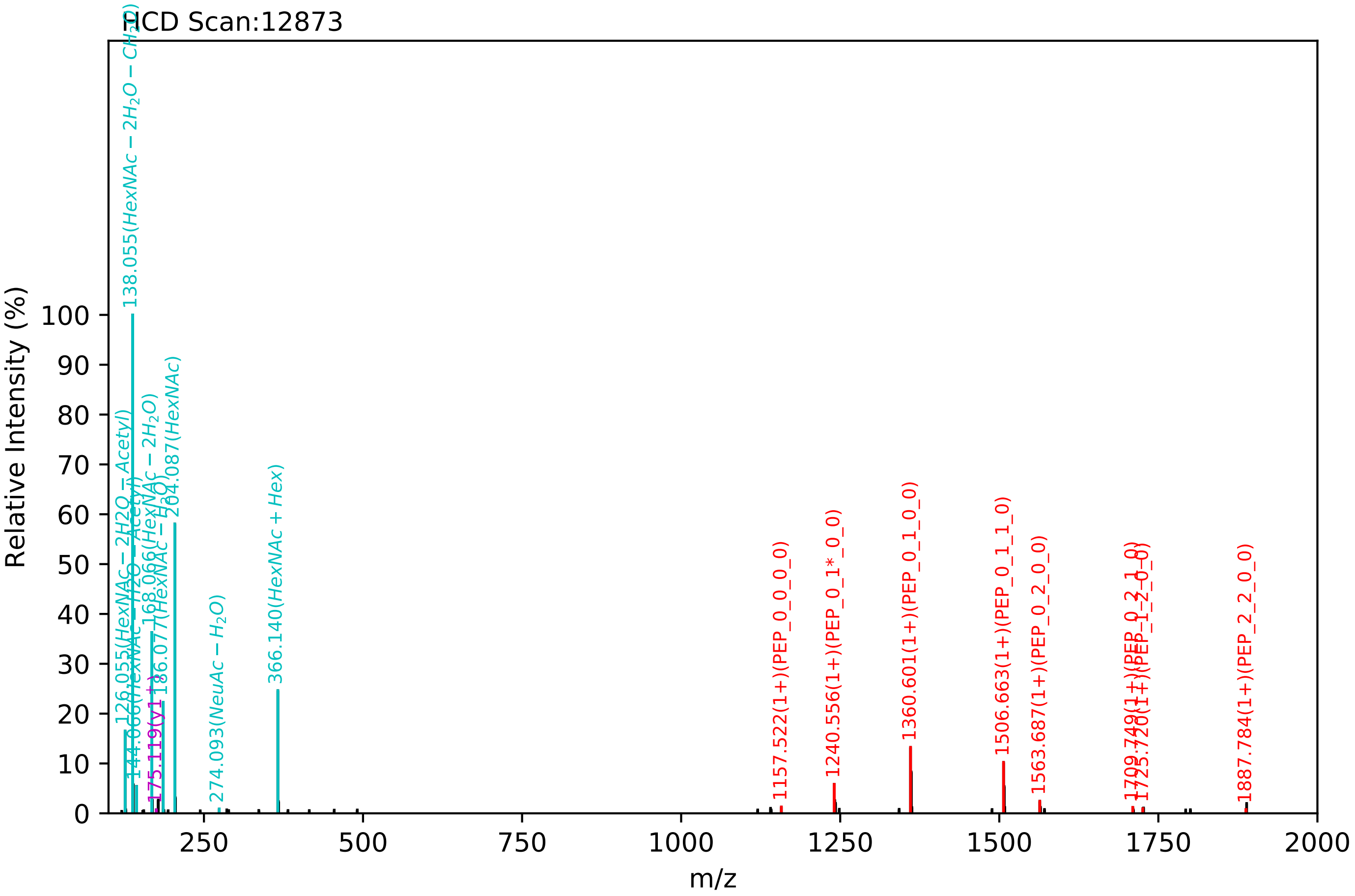

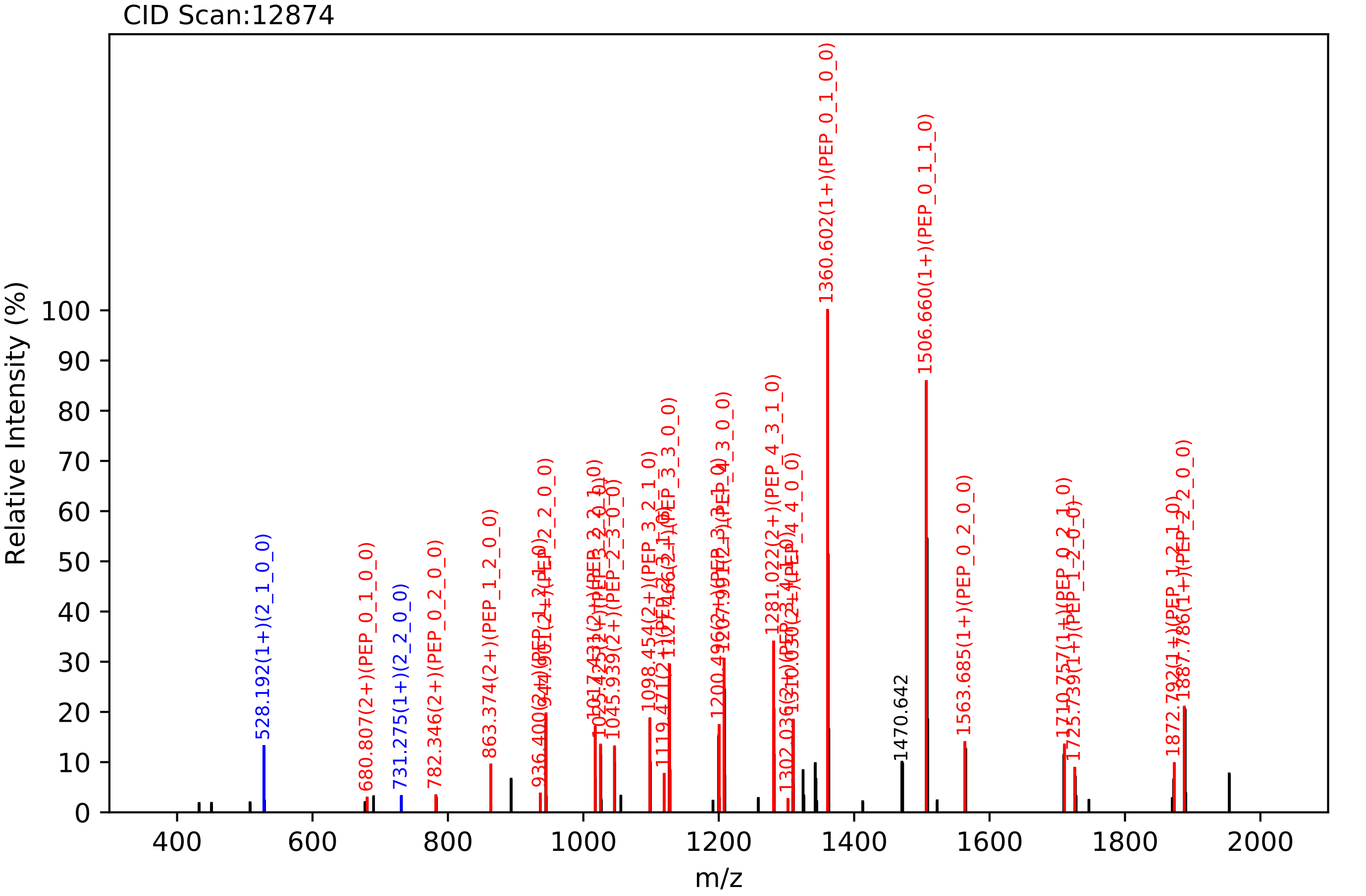

Unknown set no. 452, Experiment: Human Plasma exp_3

EEQFNSTFR(=PEP)_4_4_1_0, m/z:1382.56(2+), RT:52.32, Y-score:89.19

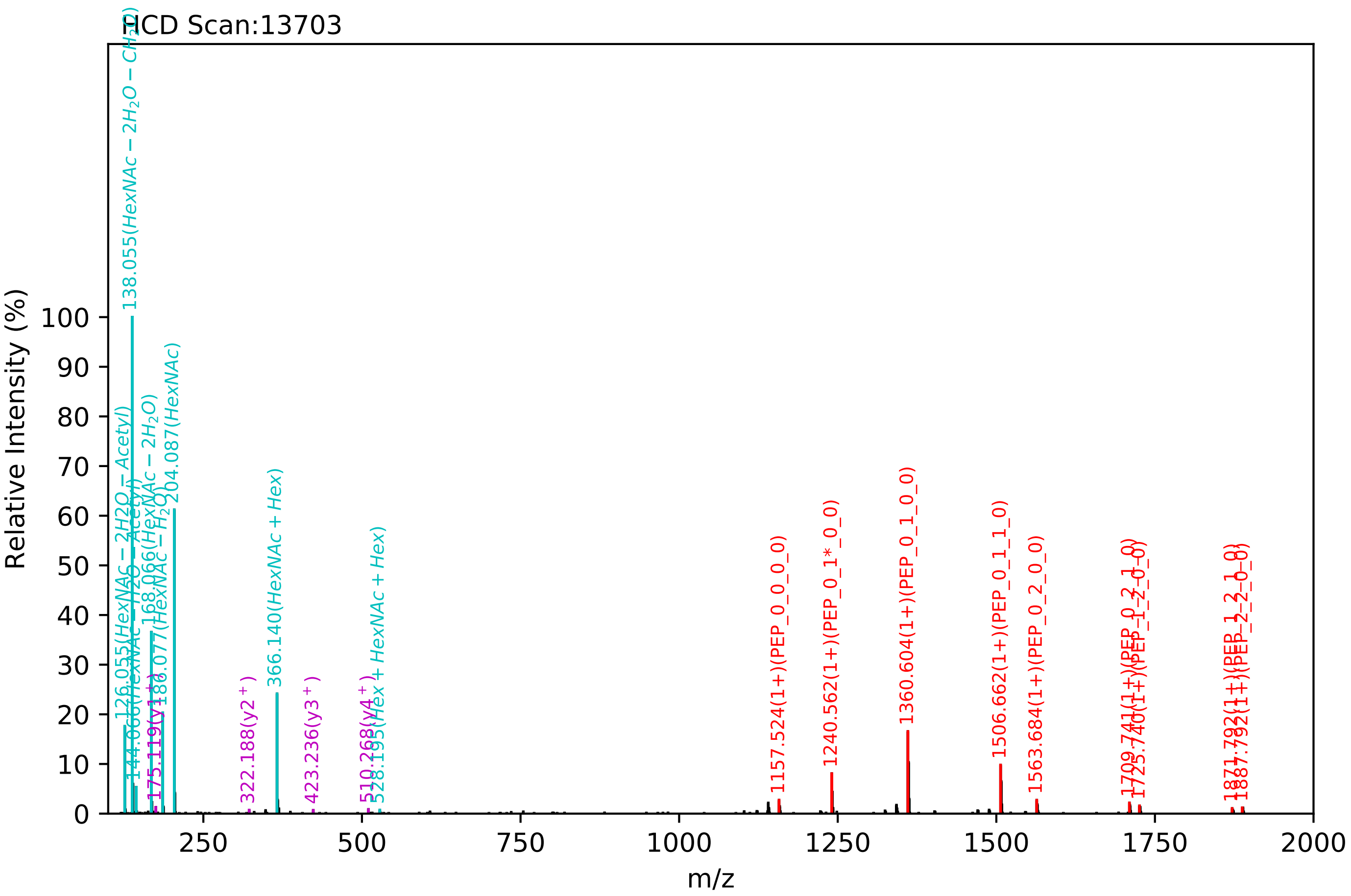

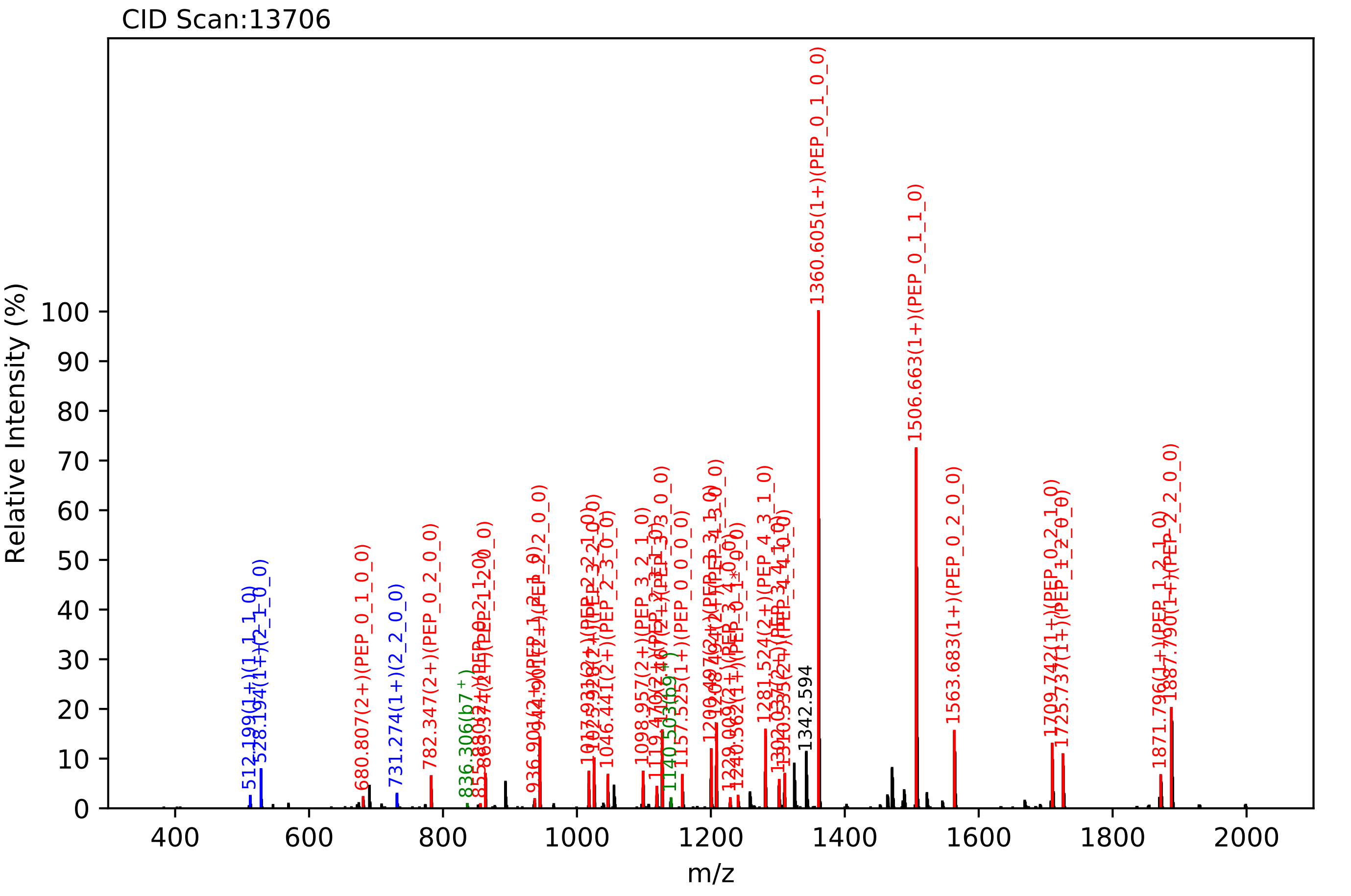

Unknown set no. 453, Experiment: Human Plasma exp_4

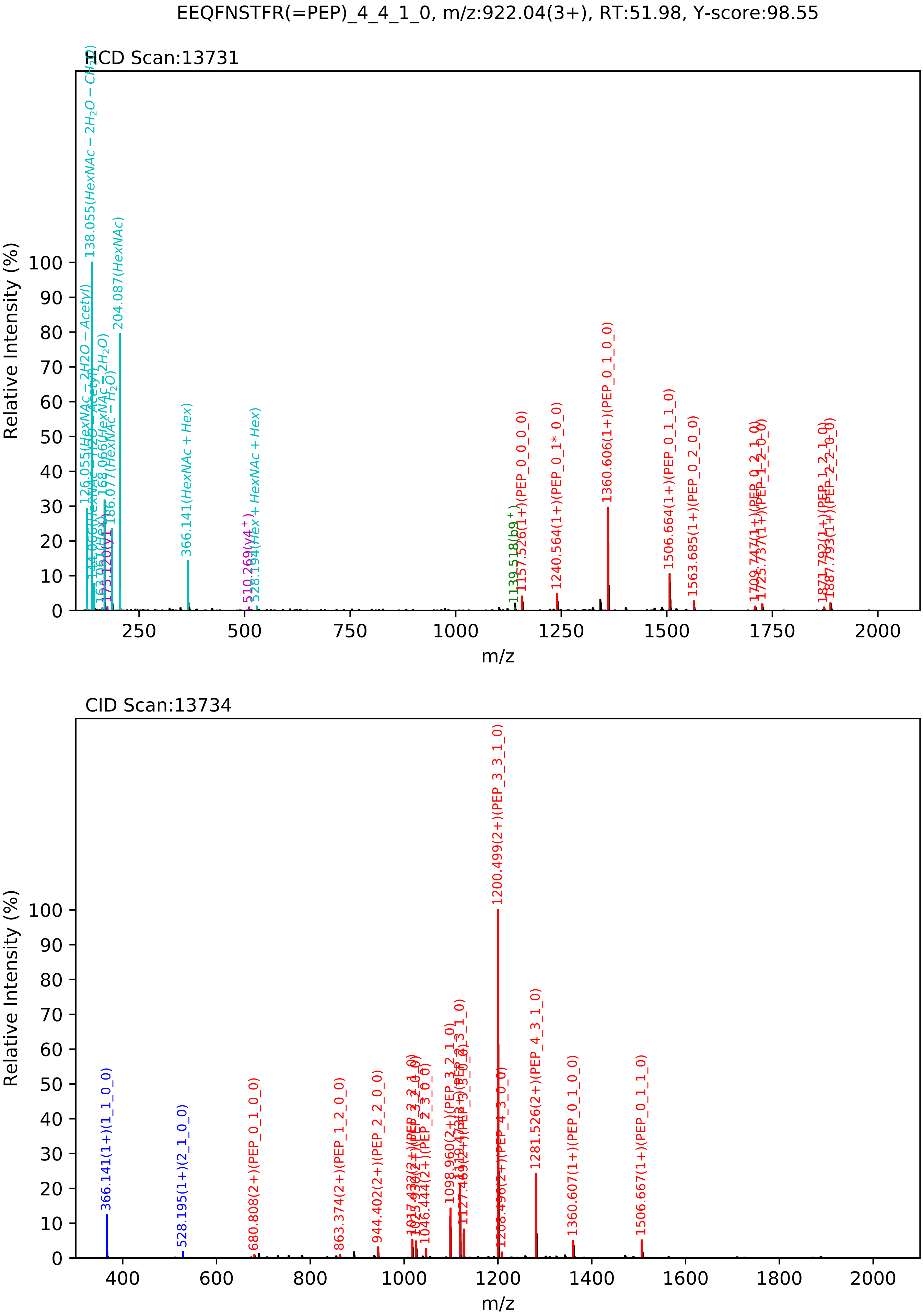

Unknown set no. 454, Experiment: Human Plasma exp_4

EEQFNSTFR(=PEP)_4_4_1_0, m/z:1382.56(2+), RT:51.42, Y-score:91.44

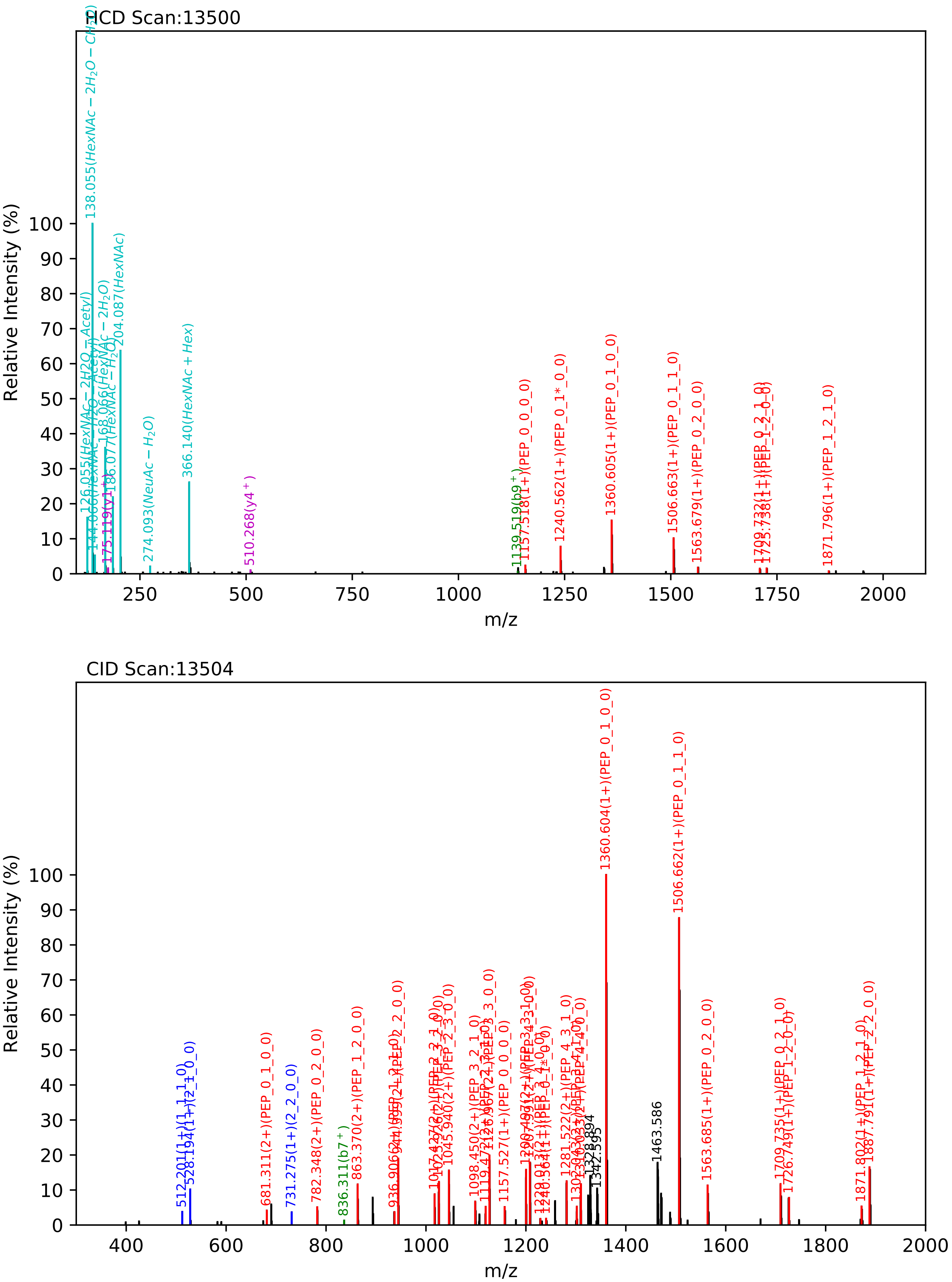

Unknown set no. 455, Experiment: Human Plasma exp_5

EEQFNSTFR(=PEP)_4_4_1_0, m/z:922.04(3+), RT:52.07, Y-score:98.63

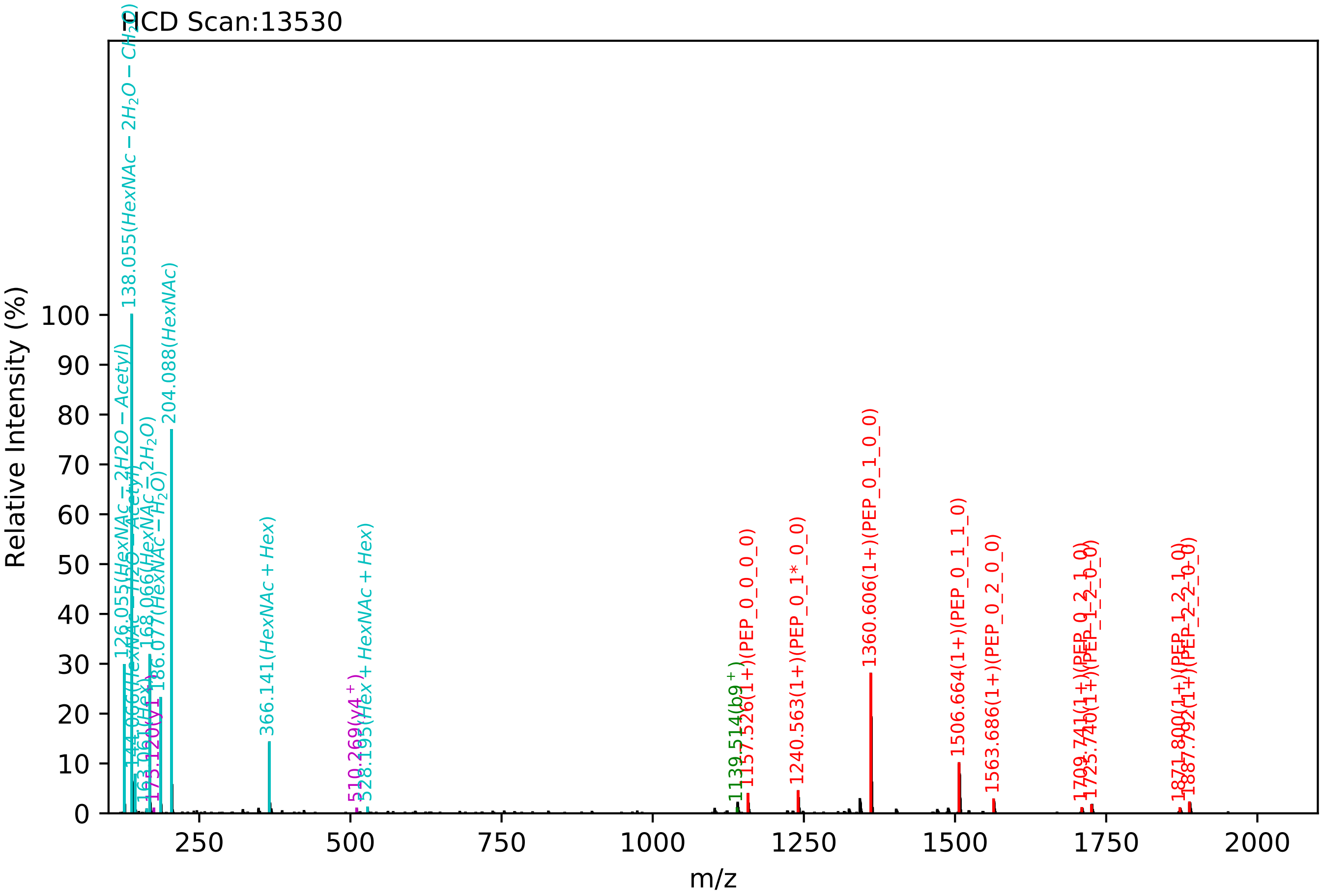

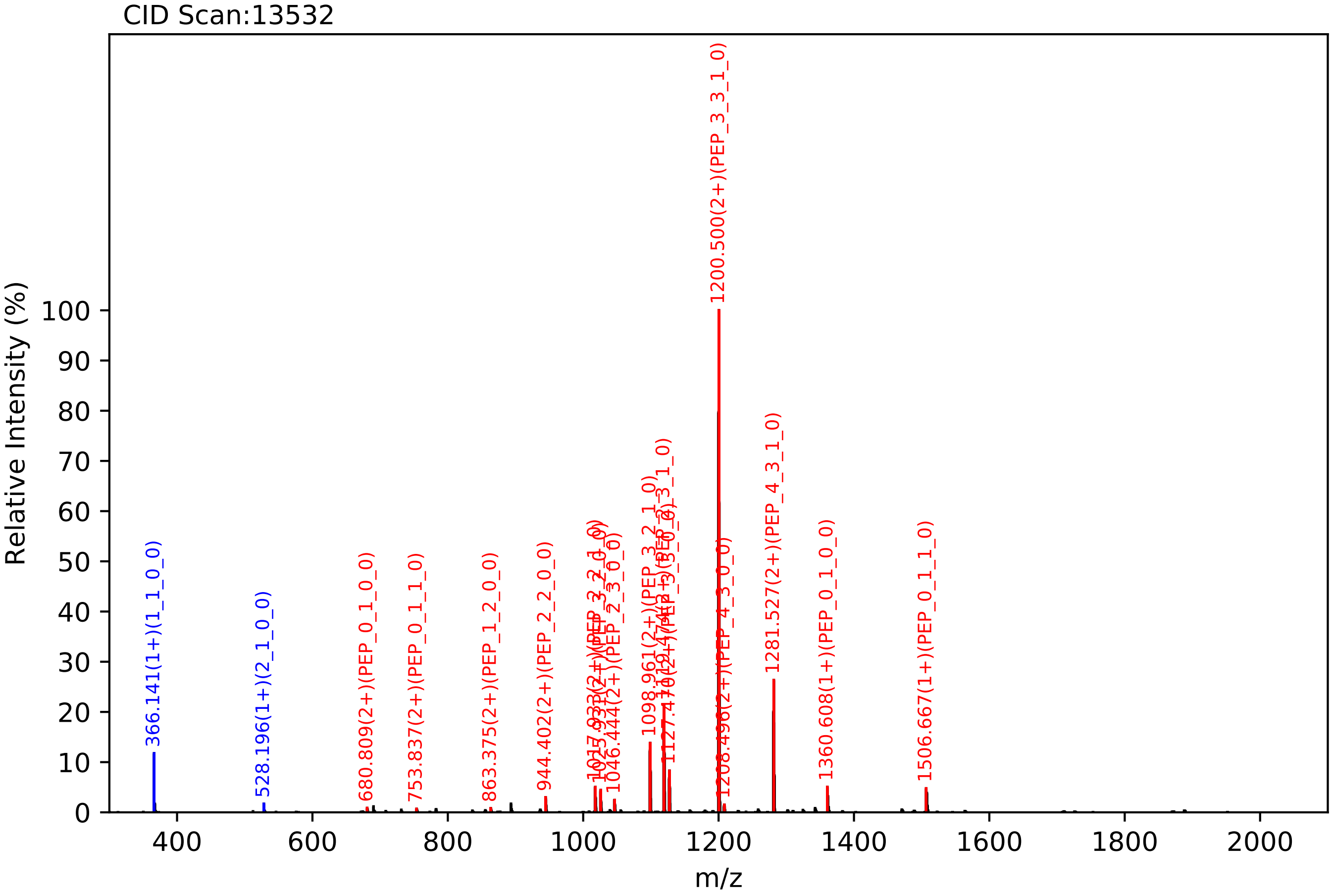

Unknown set no. 456, Experiment: Human Plasma exp_5

EEQFNSTFR(=PEP)_4_4_1_0, m/z:1382.56(2+), RT:50.05, Y-score:97.00

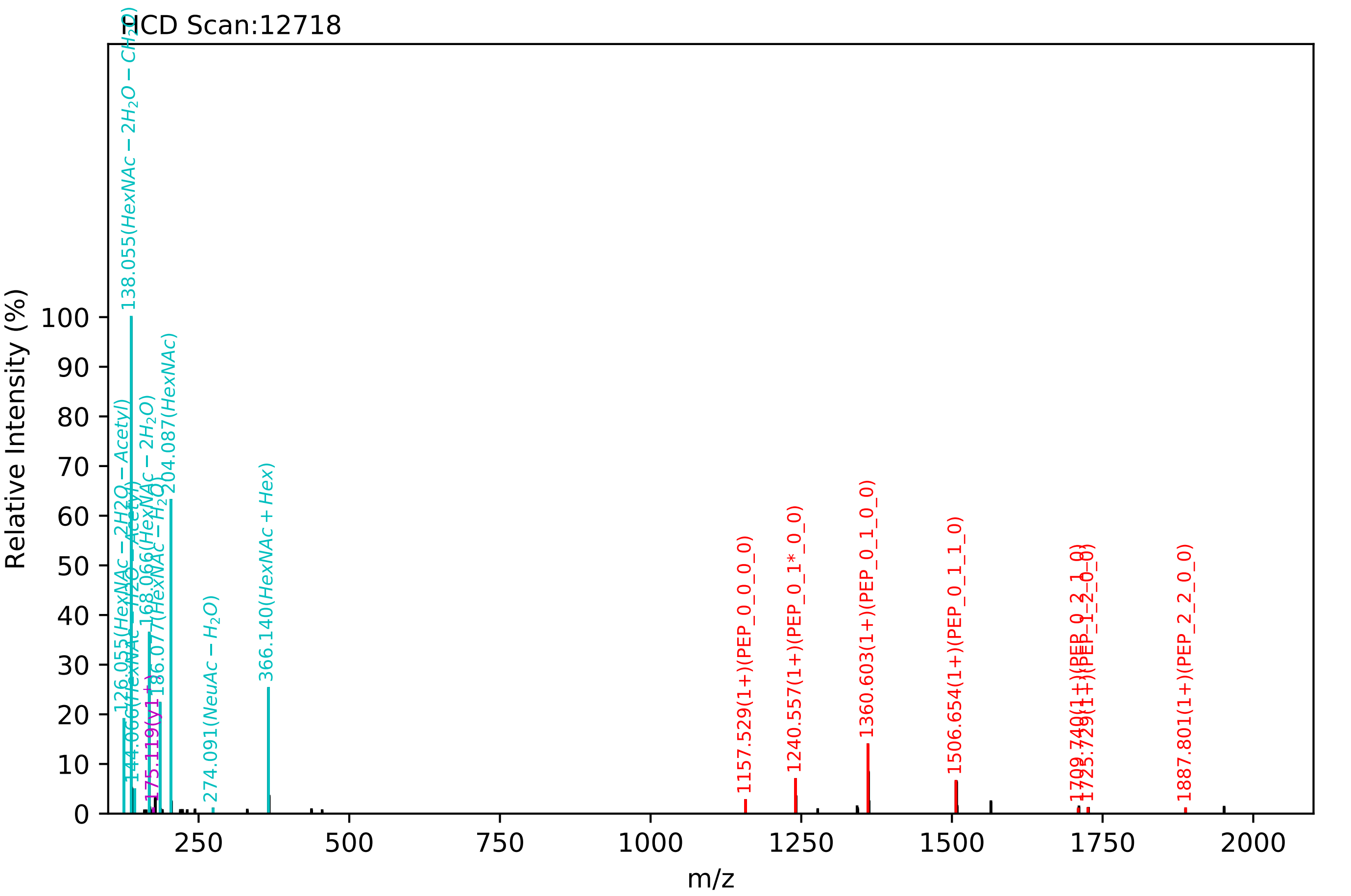

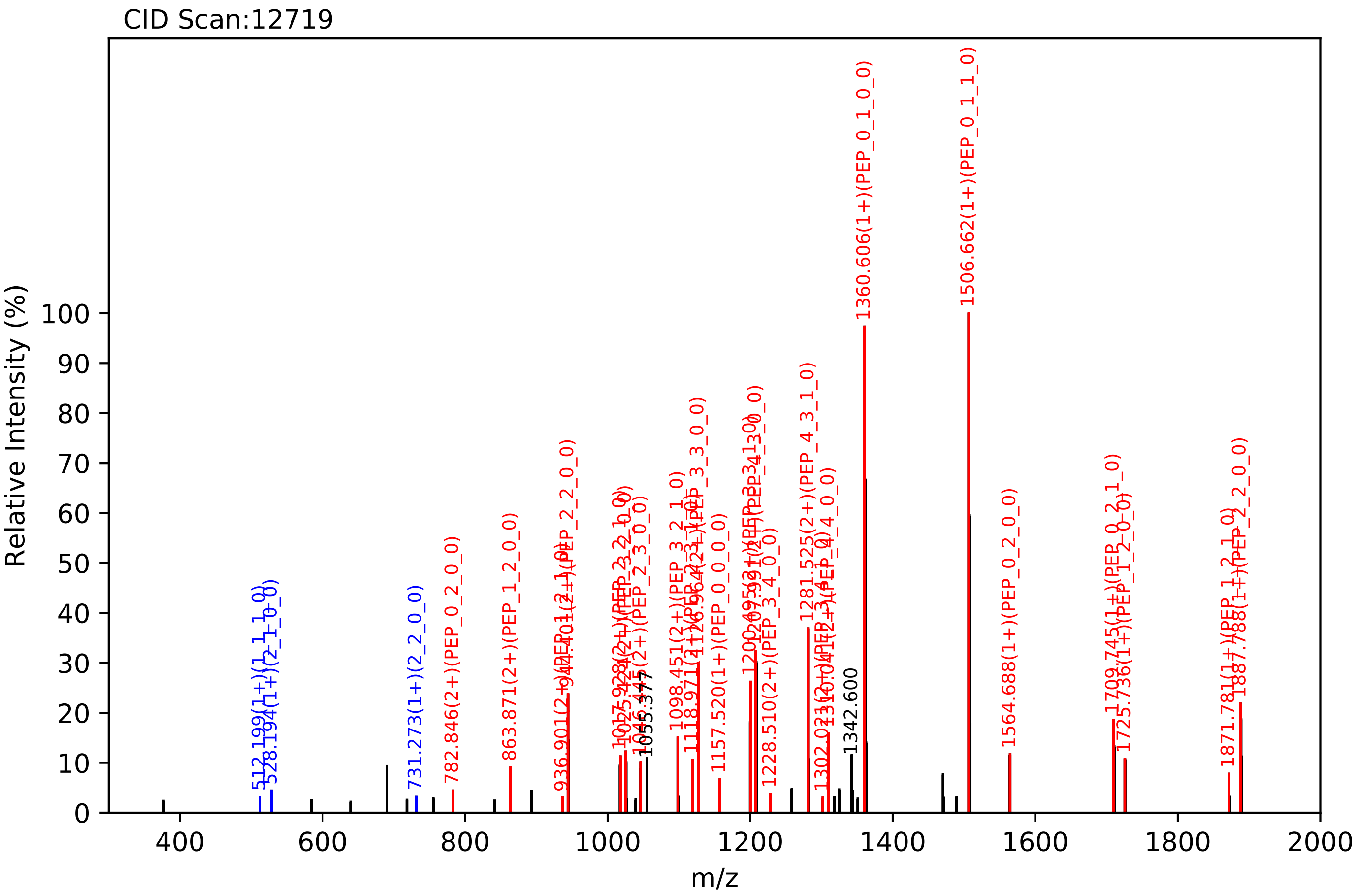

Unknown set no. 457, Experiment: Human Plasma exp_1

EEQFNSTFR(=PEP)_4_4_1_1, m/z:1528.11(2+), RT:61.10, Y-score:97.81

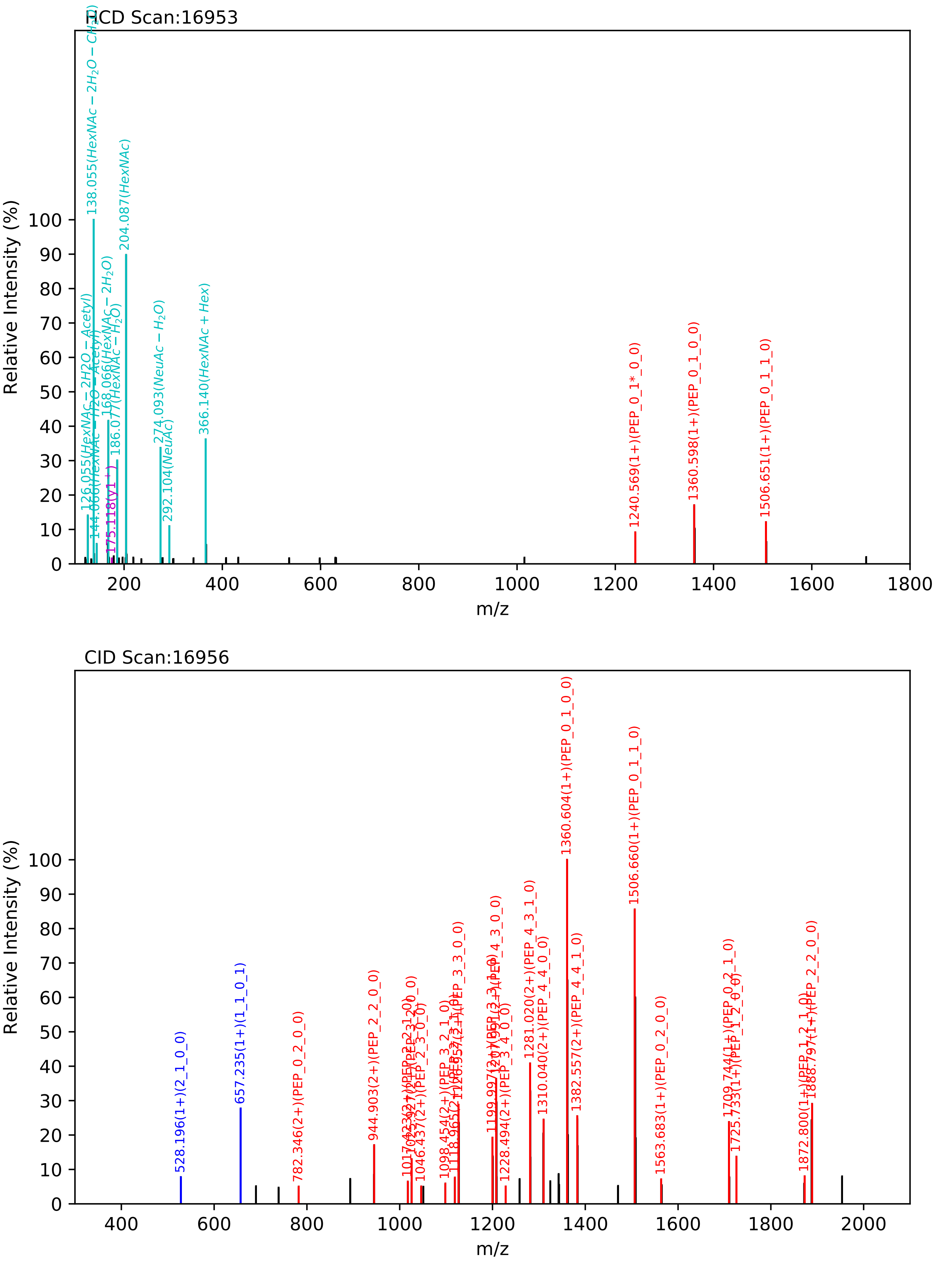

Unknown set no. 458, Experiment: Human Plasma exp_2

EEQFNSTFR(=PEP)_4_4_1_1, m/z:1019.08(3+), RT:61.80, Y-score:93.63

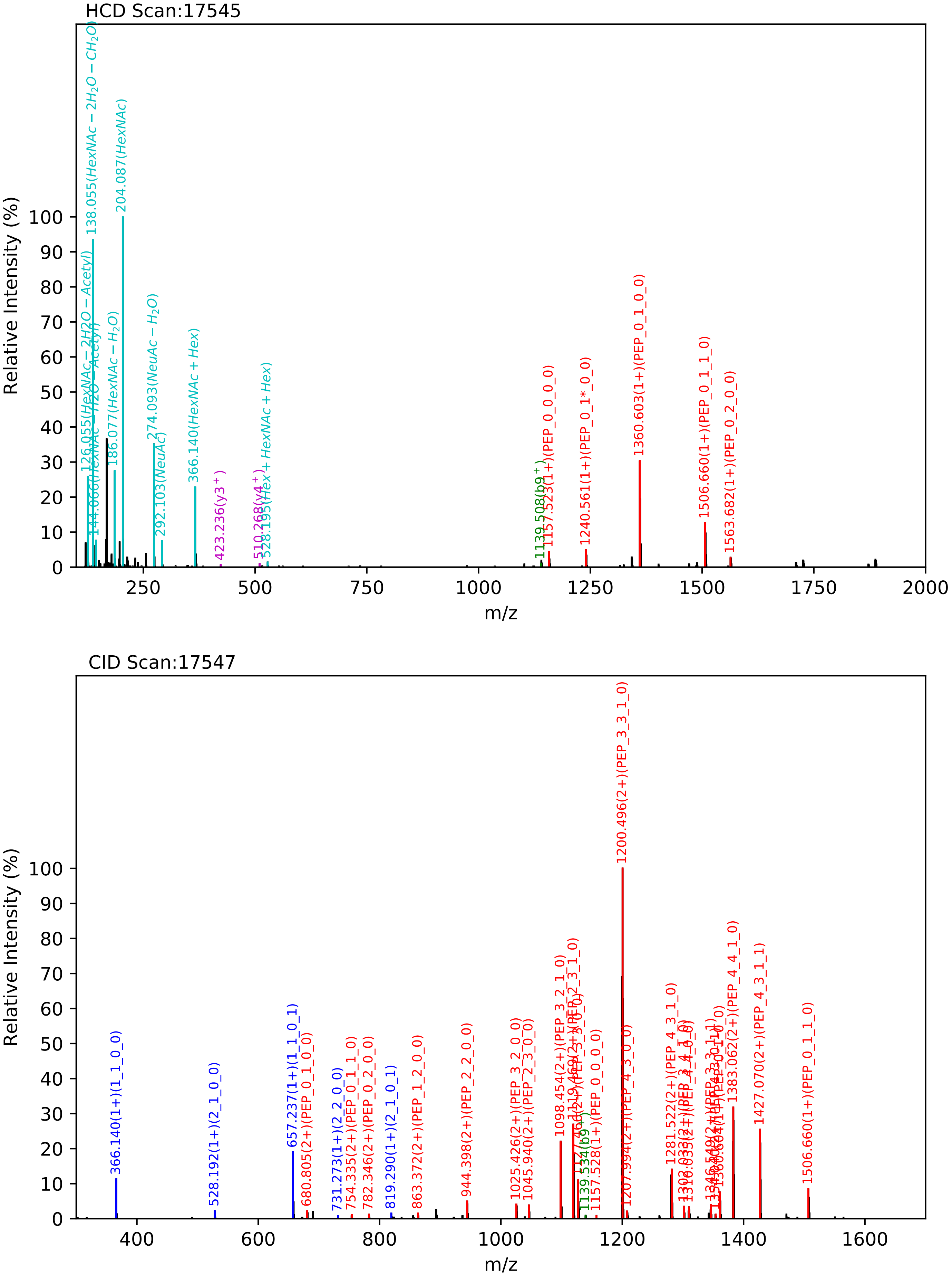

Unknown set no. 459, Experiment: Human Plasma exp_2

EEQFNSTFR(=PEP)_4_4_1_1, m/z:1528.11(2+), RT:61.24, Y-score:94.49

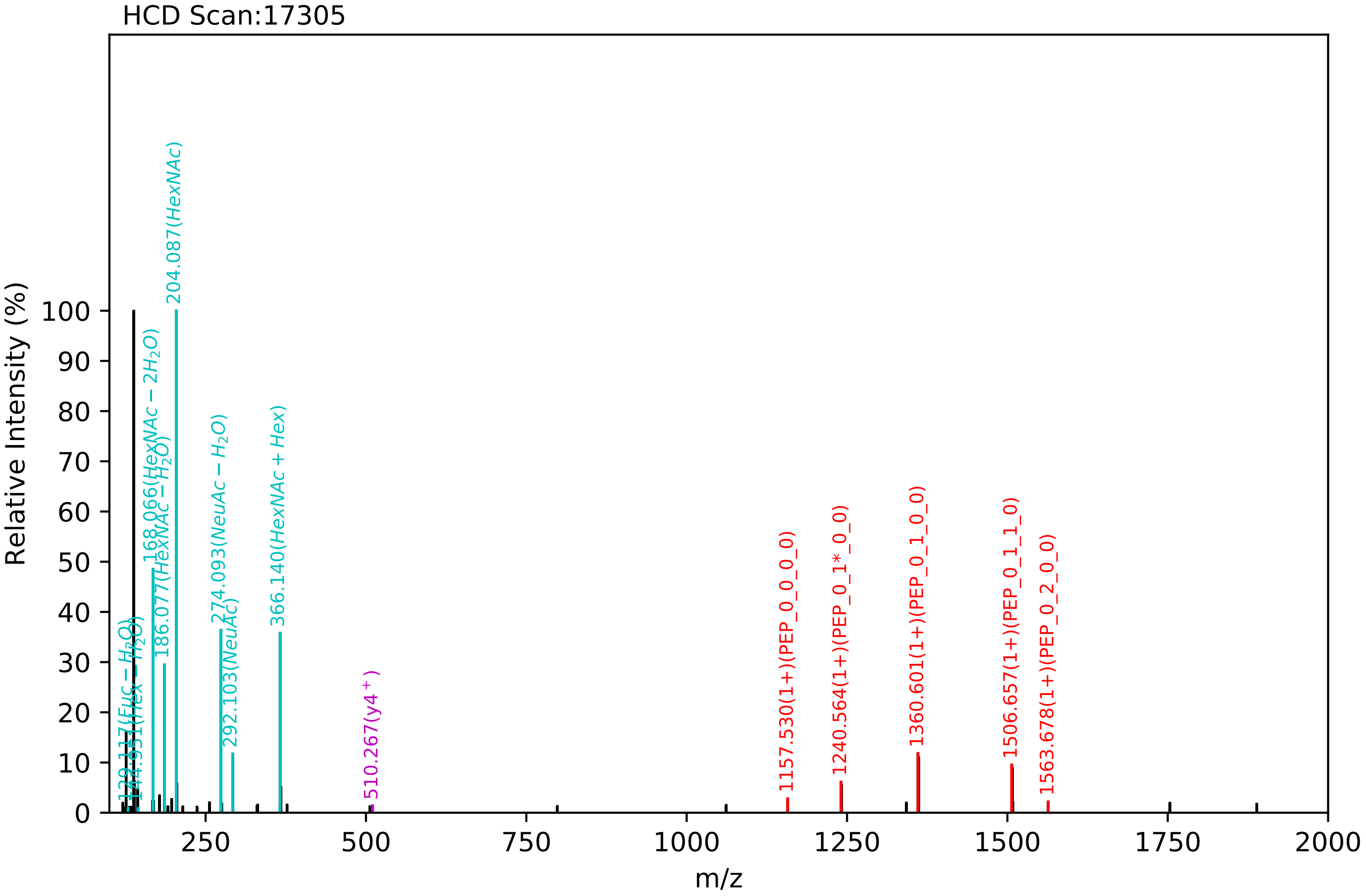

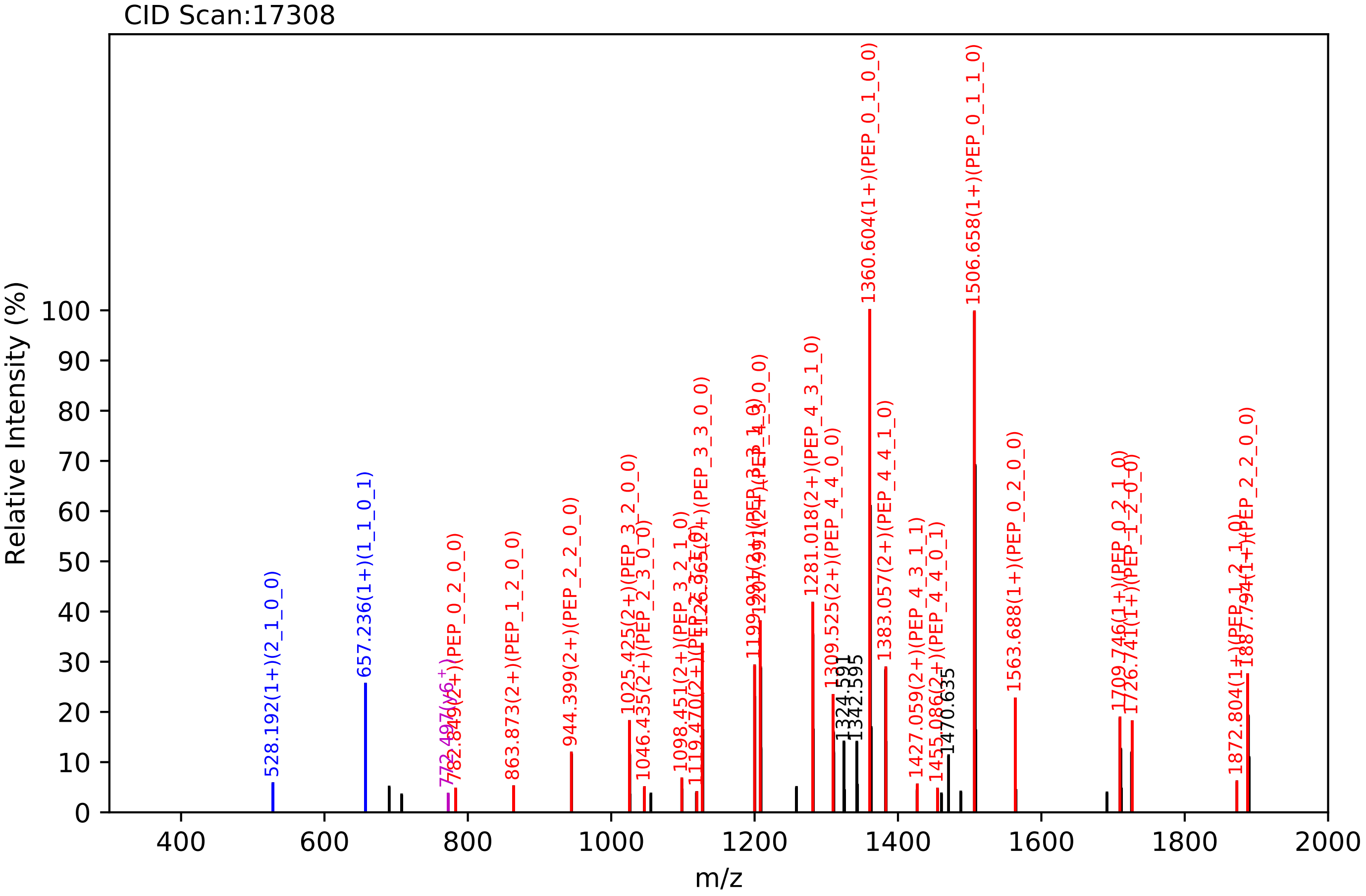

Unknown set no. 460, Experiment: Human Plasma exp_3

EEQFNSTFR(=PEP)_4_4_1_1, m/z:1019.08(3+), RT:61.22, Y-score:93.38

HCD Scan:16964

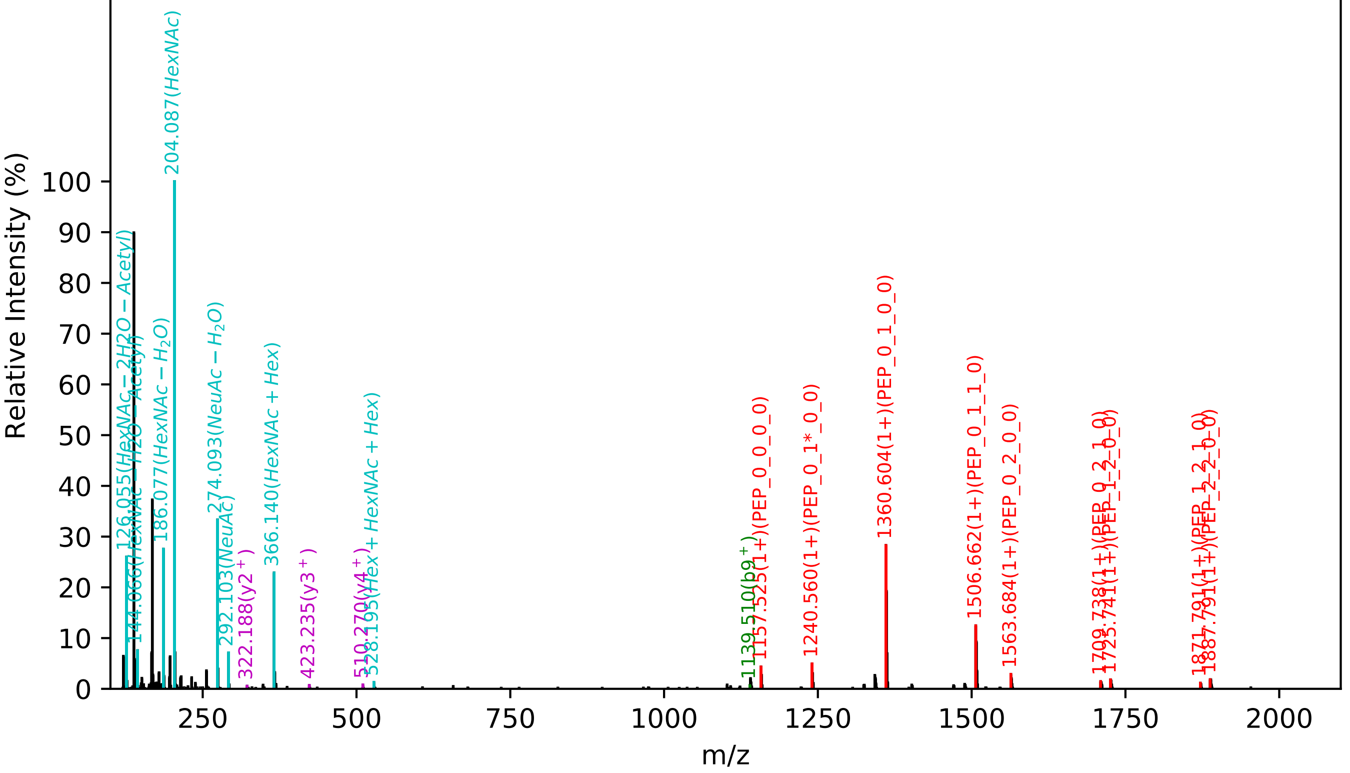

CID Scan:16967

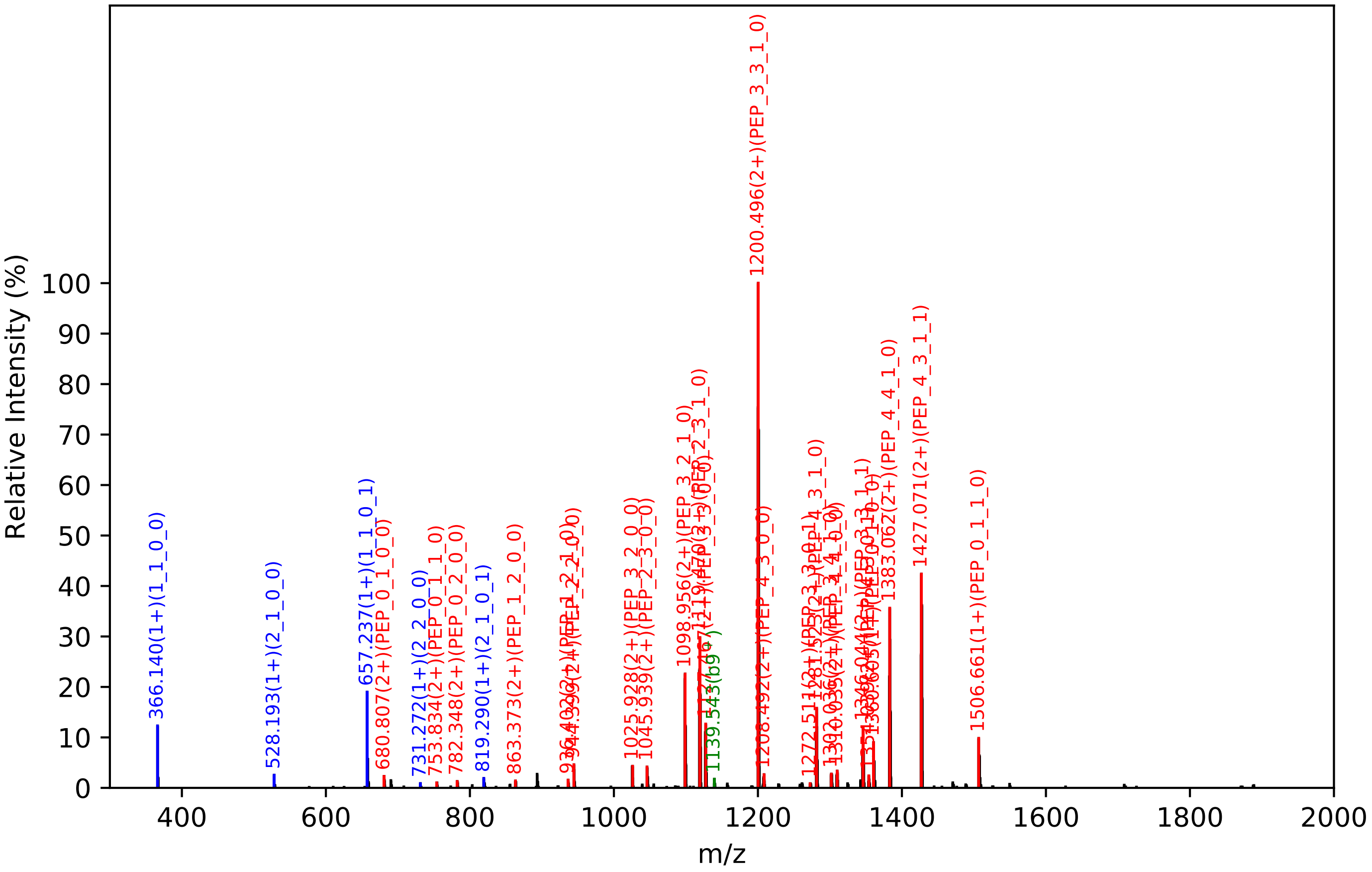

Unknown set no. 461, Experiment: Human Plasma exp_3

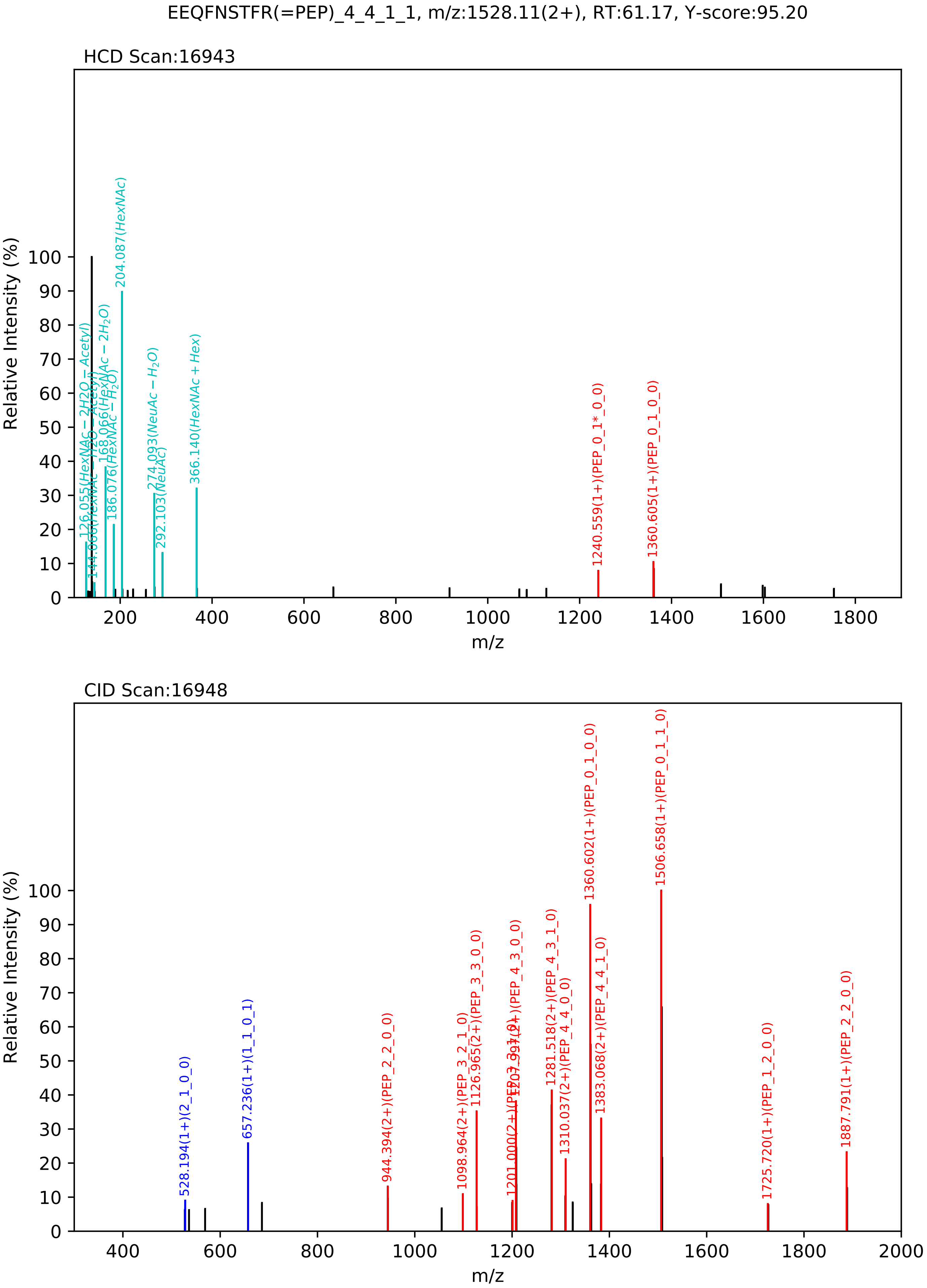

Unknown set no. 462, Experiment: Human Plasma exp_4

EEQFNSTFR(=PEP)_4_4_1_1, m/z:1019.08(3+), RT:61.90, Y-score:93.94

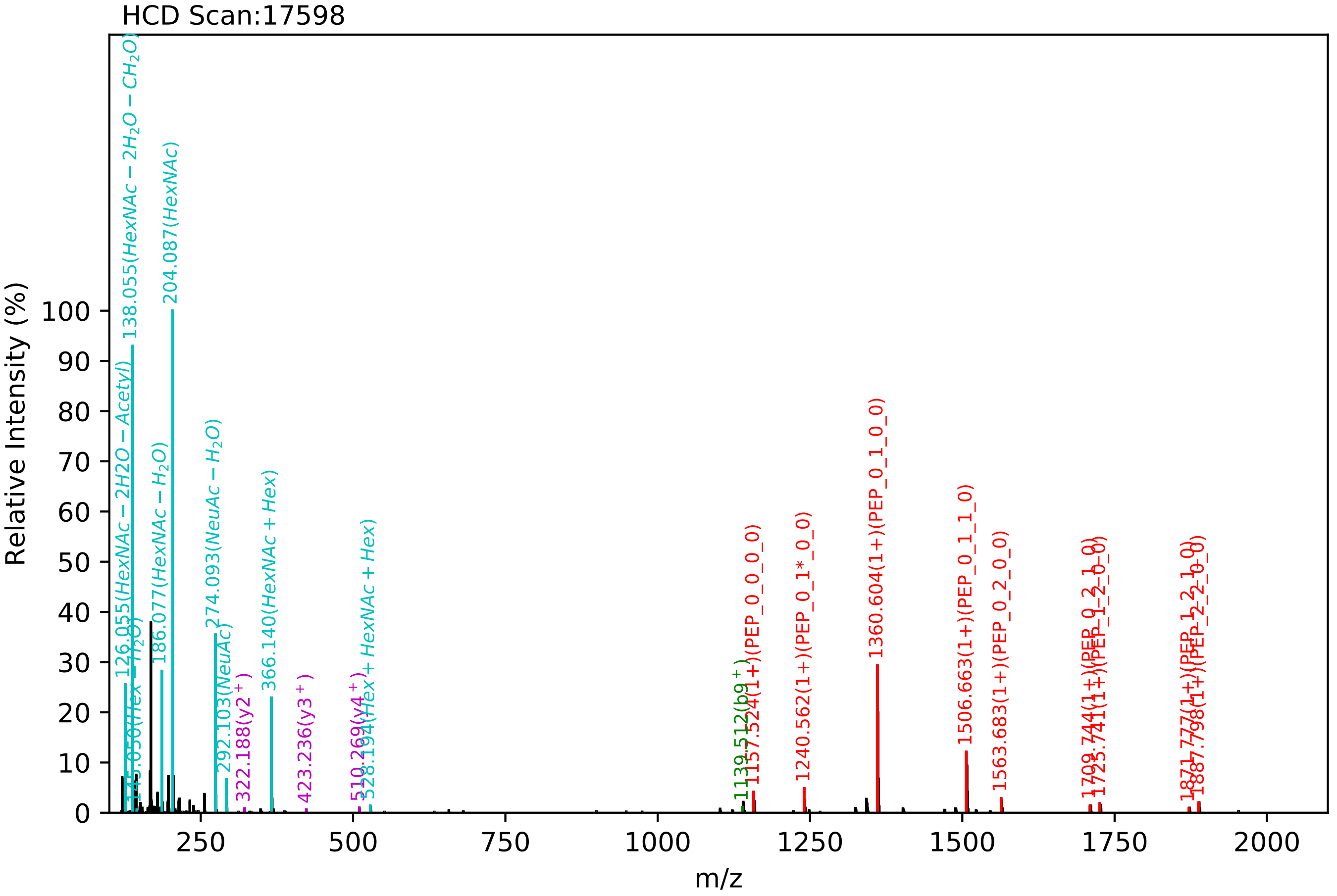

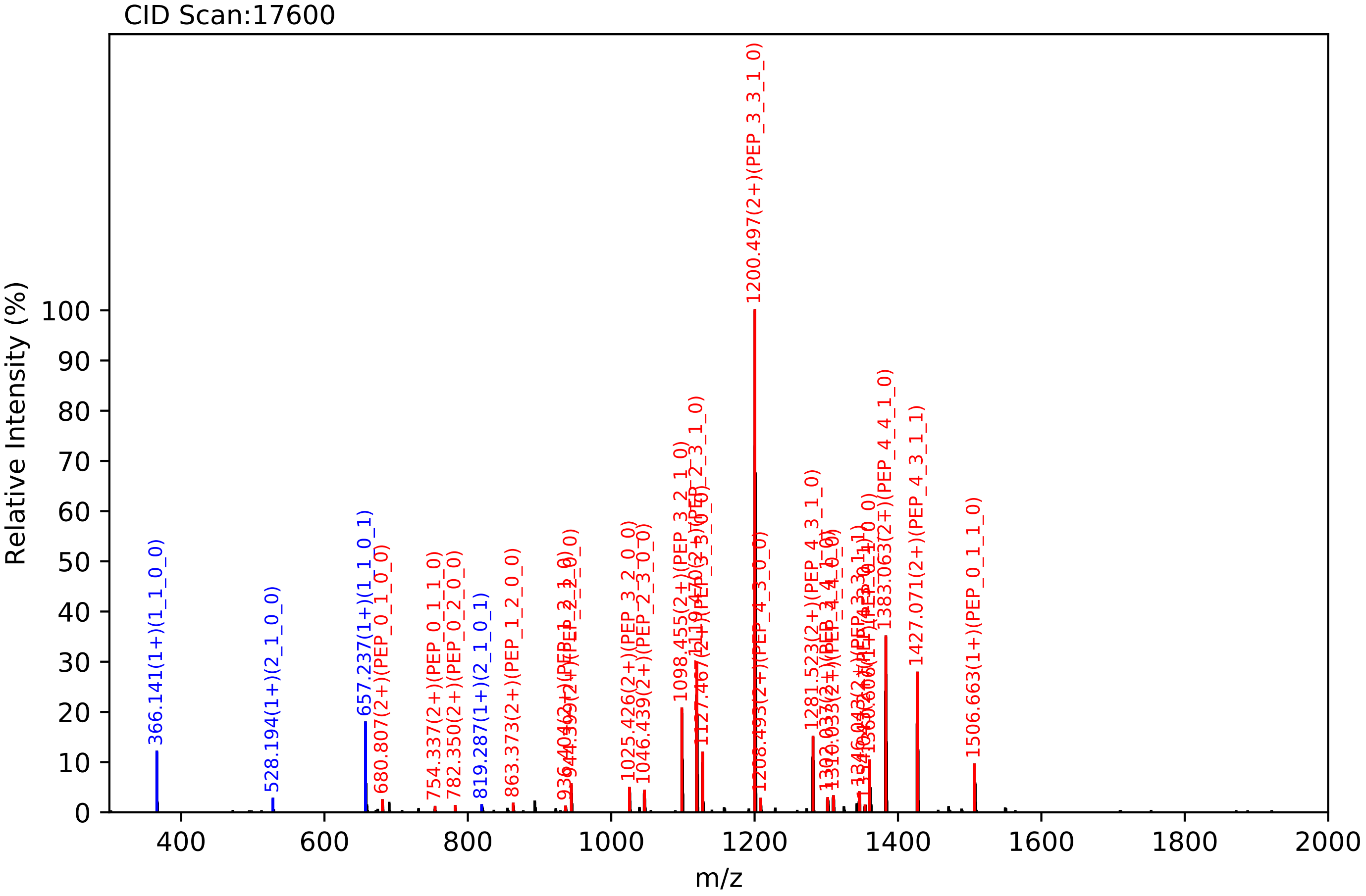

Unknown set no. 463, Experiment: Human Plasma exp_4

EEQFNSTFR(=PEP)_4_4_1_1, m/z:1528.11(2+), RT:61.34, Y-score:95.96

HCD Scan:17364

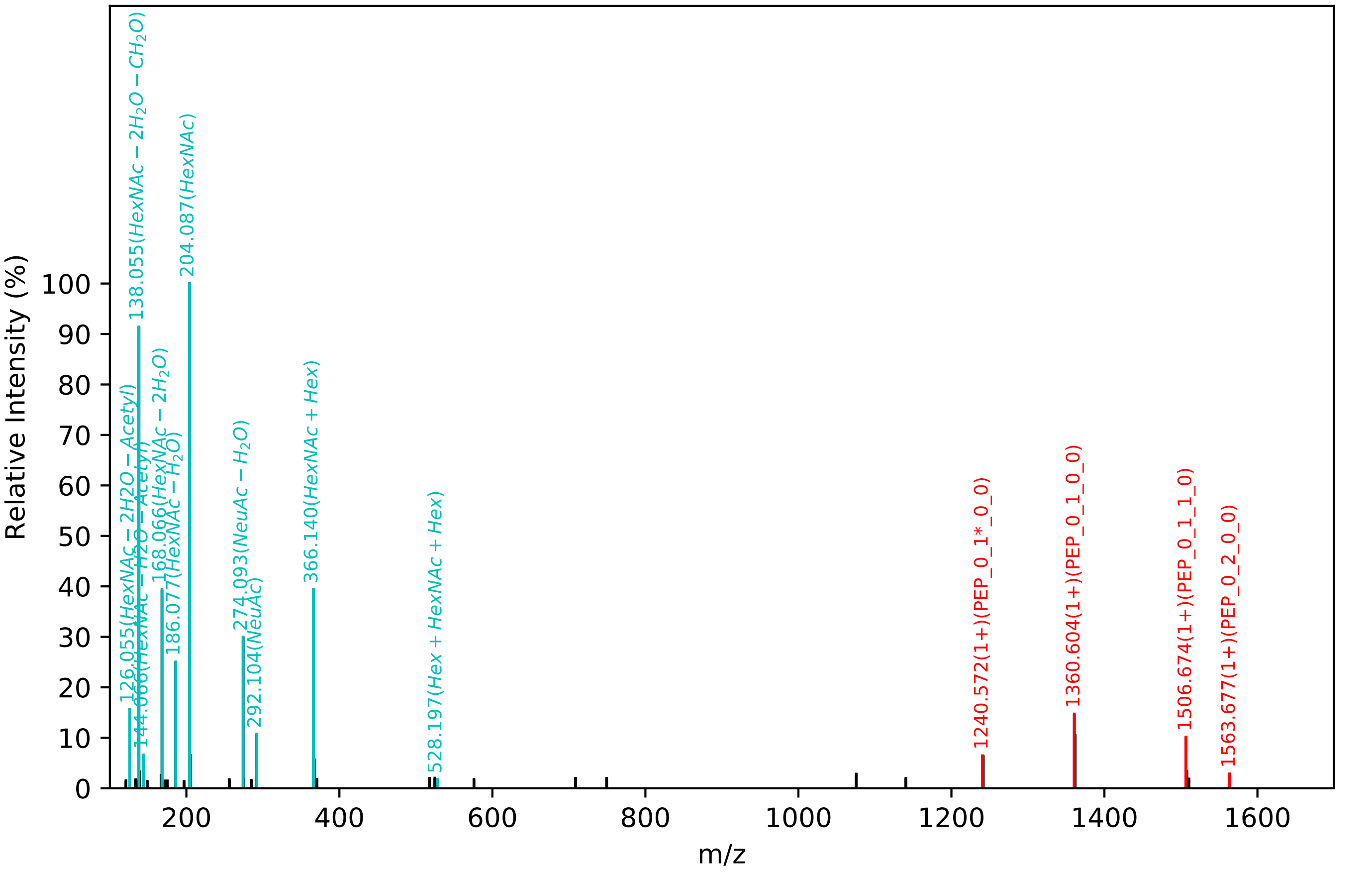

CID Scan:17369

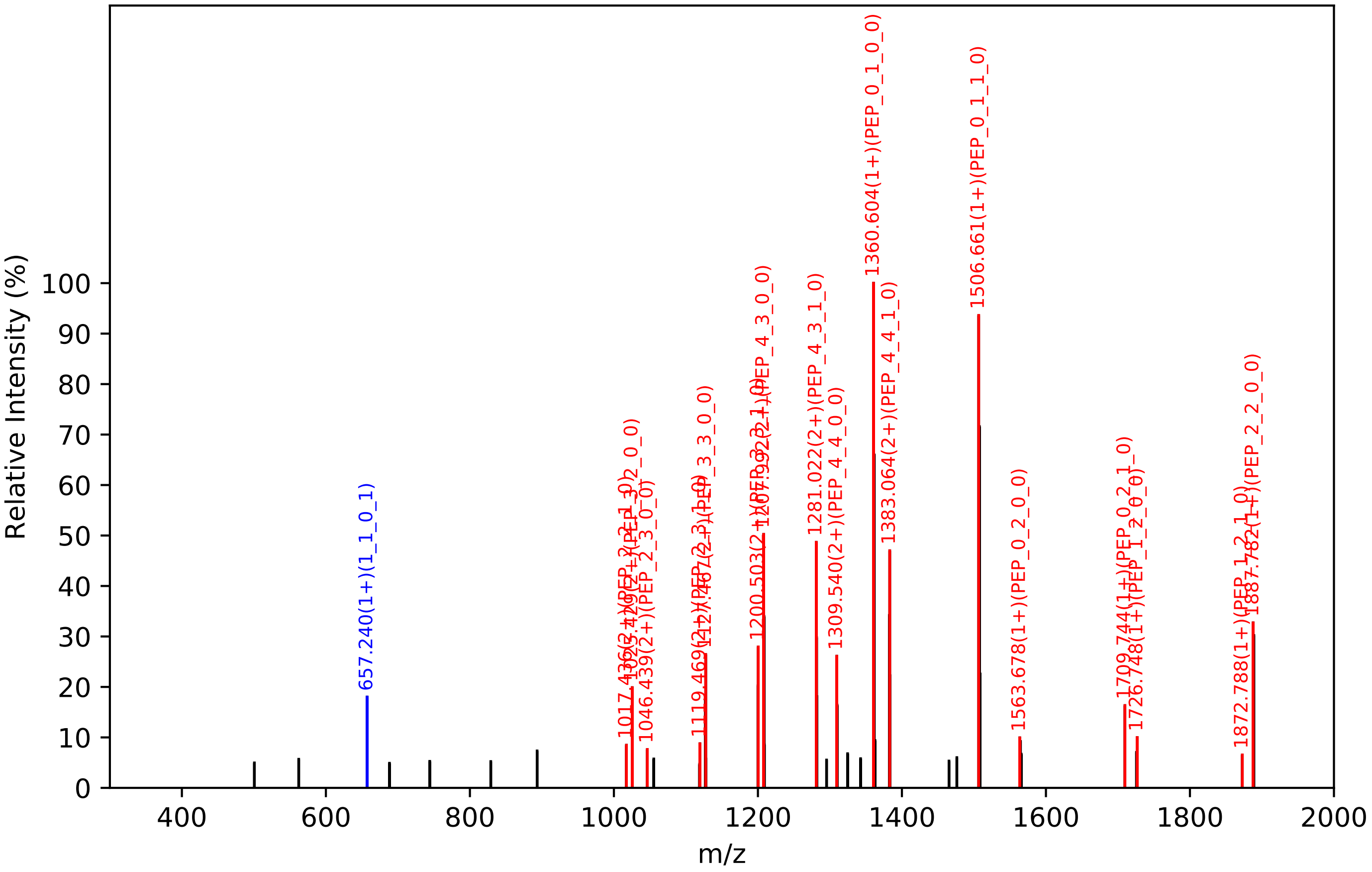

Unknown set no. 464, Experiment: Human Plasma exp_5

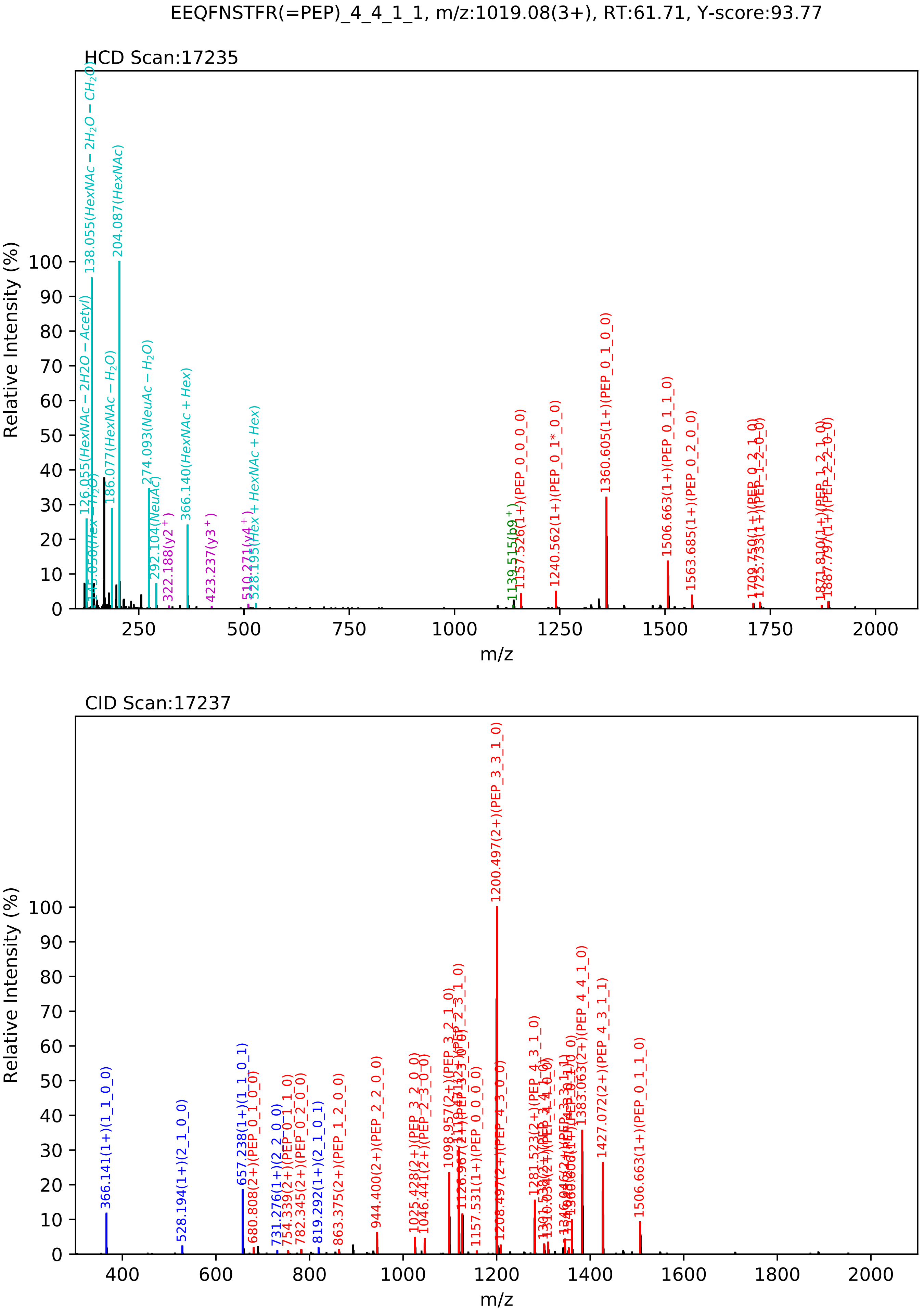

Unknown set no. 465, Experiment: Human Plasma exp_5

EEQFNSTFR(=PEP)_4_4_1_1, m/z:1528.11(2+), RT:61.16, Y-score:92.83

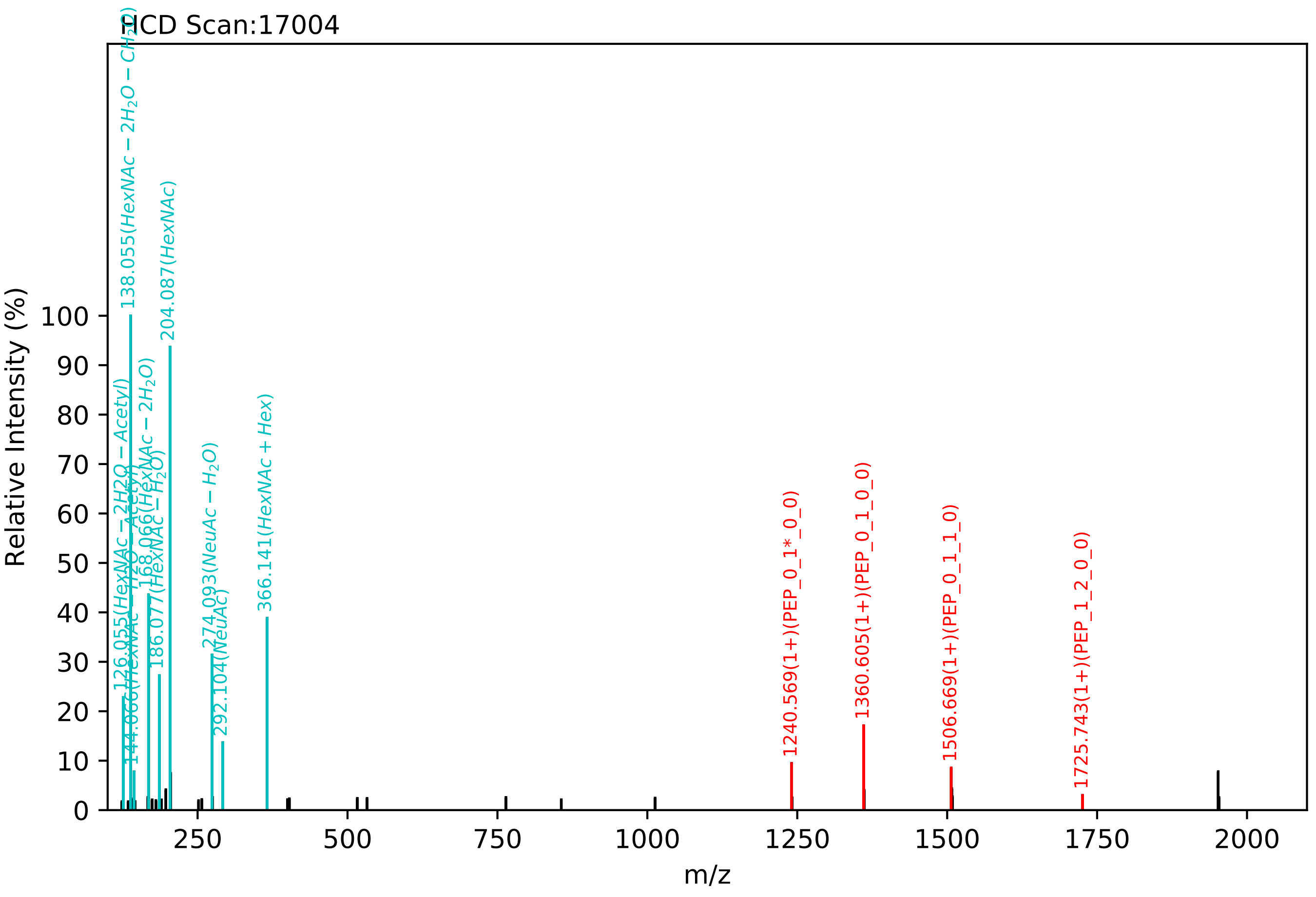

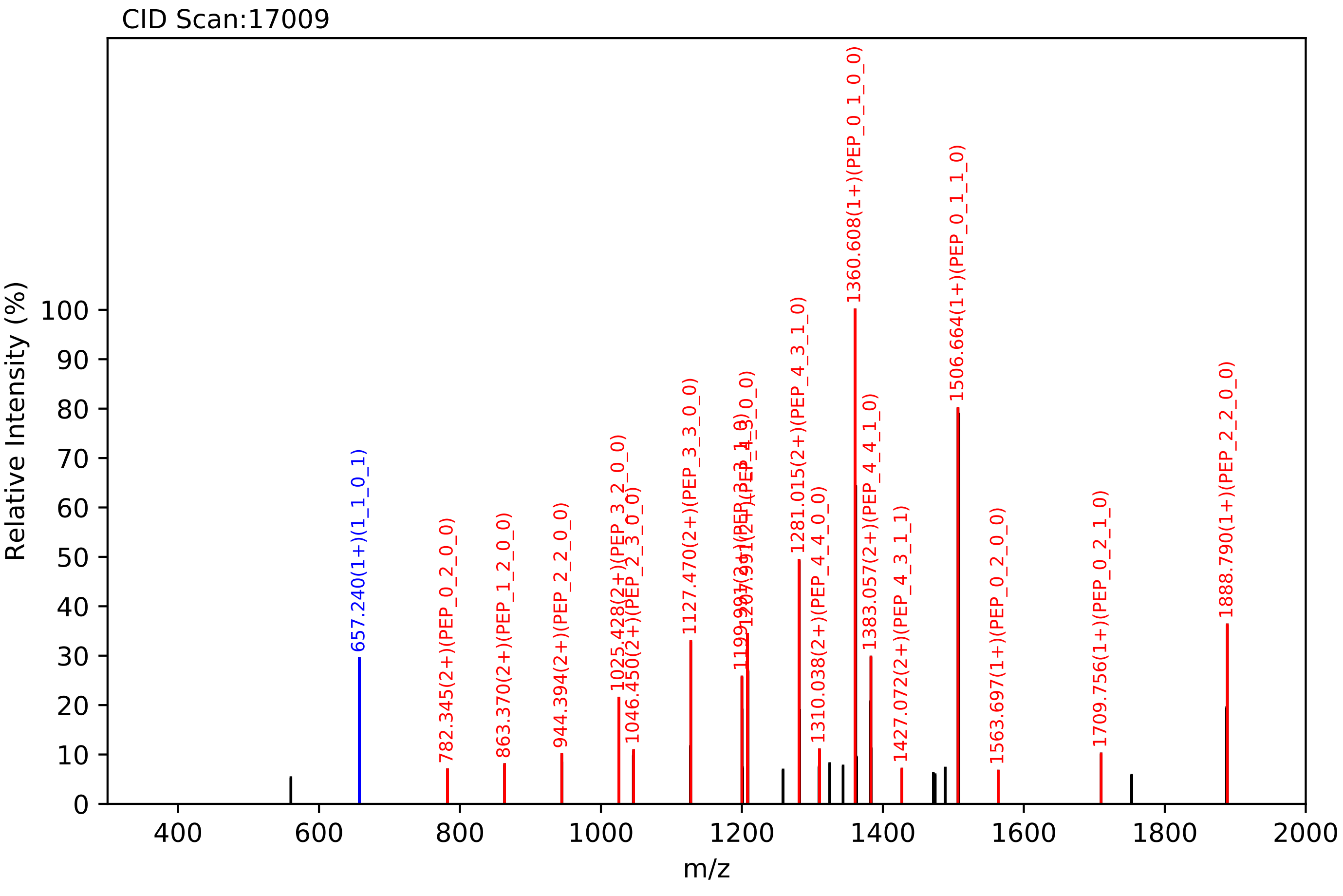

Unknown set no. 466, Experiment: Human Plasma exp_1

EEQFNSTFR(=PEP)_4_5_1_0, m/z:1484.10(2+), RT:51.99, Y-score:90.26

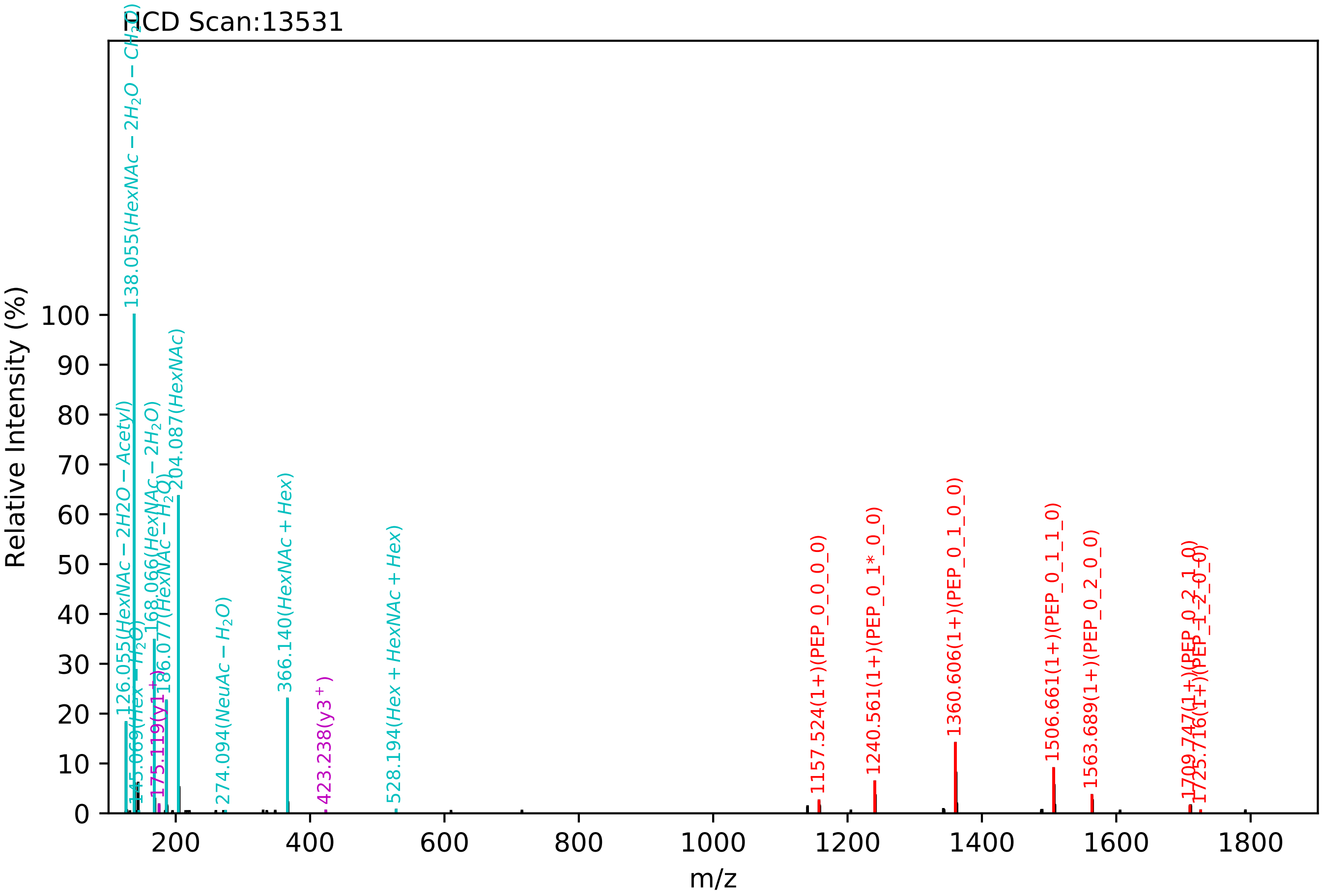

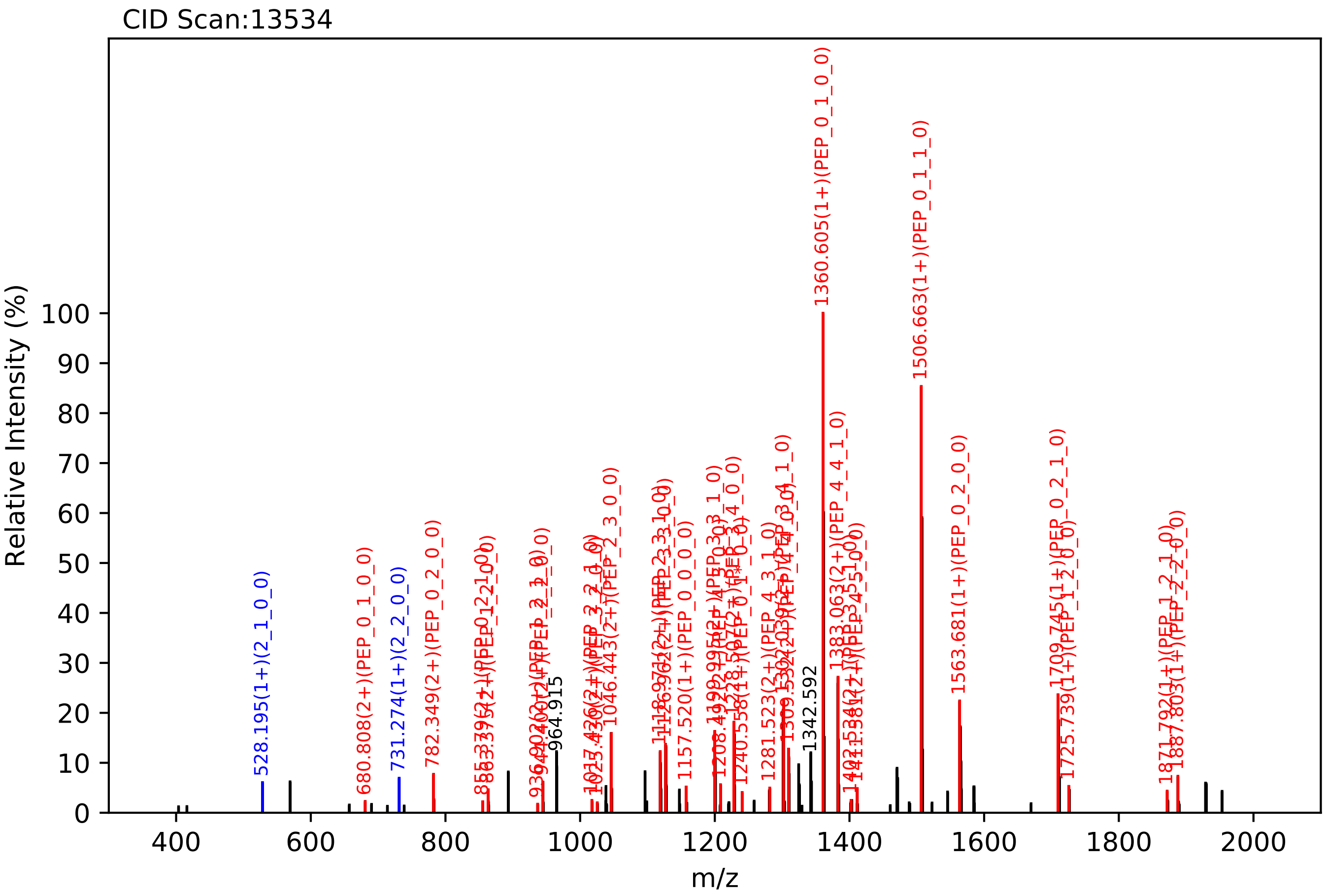

Unknown set no. 467, Experiment: Human Plasma exp_2

EEQFNSTFR(=PEP)_4_5_1_0, m/z:1484.10(2+), RT:52.04, Y-score:91.95

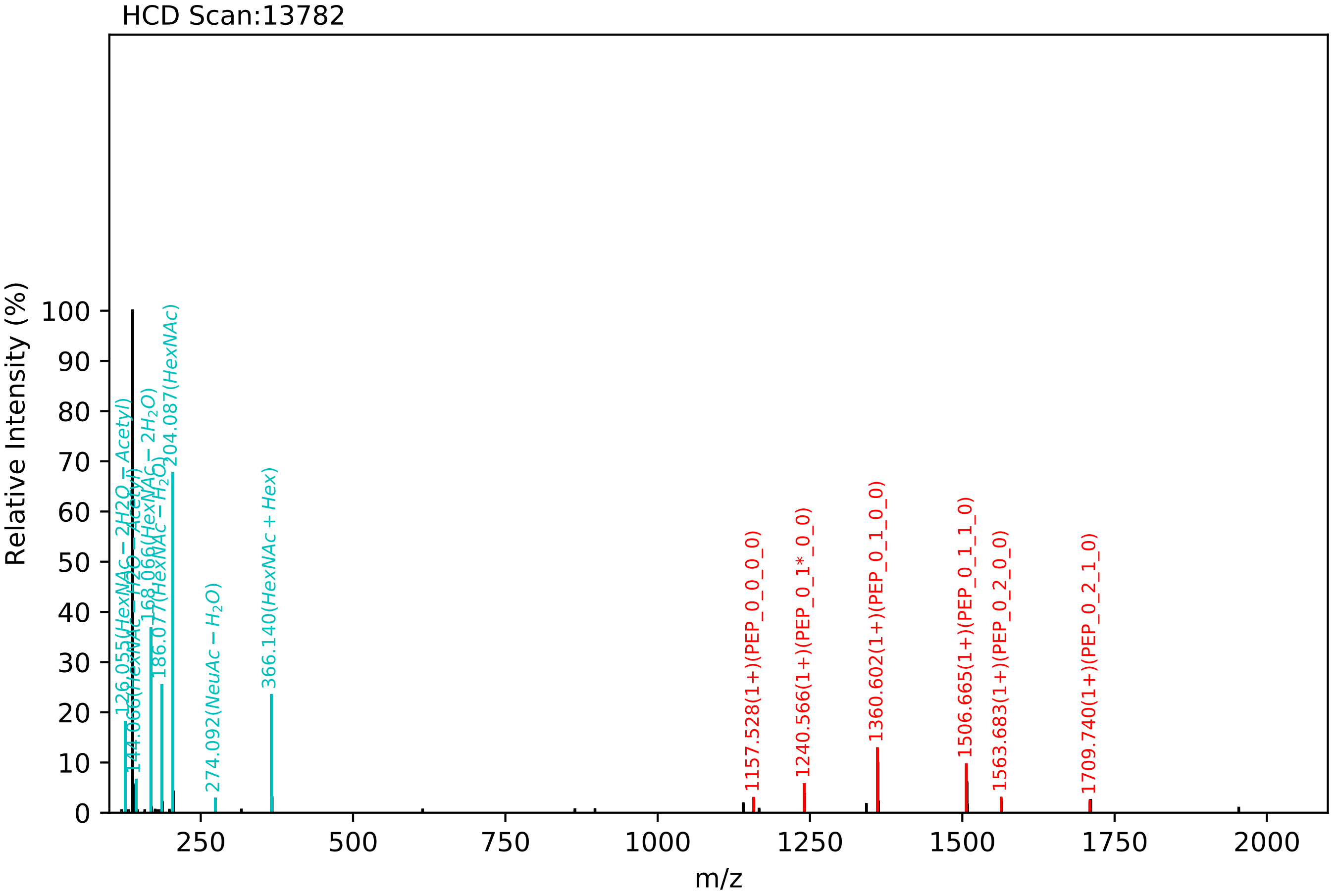

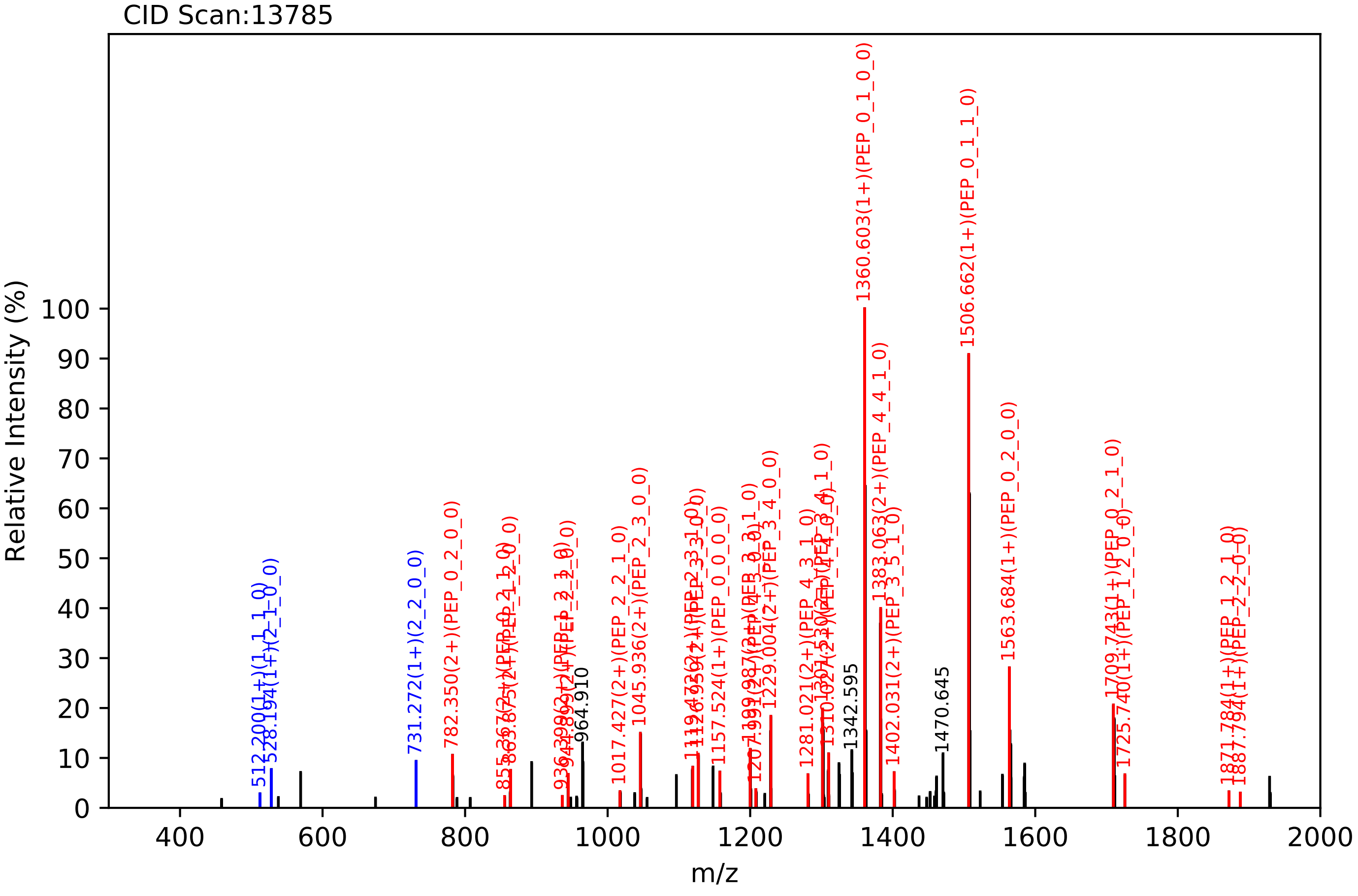

Unknown set no. 468, Experiment: Human Plasma exp_3

EEQFNSTFR(=PEP)_4_5_1_0, m/z:1484.10(2+), RT:52.84, Y-score:97.51

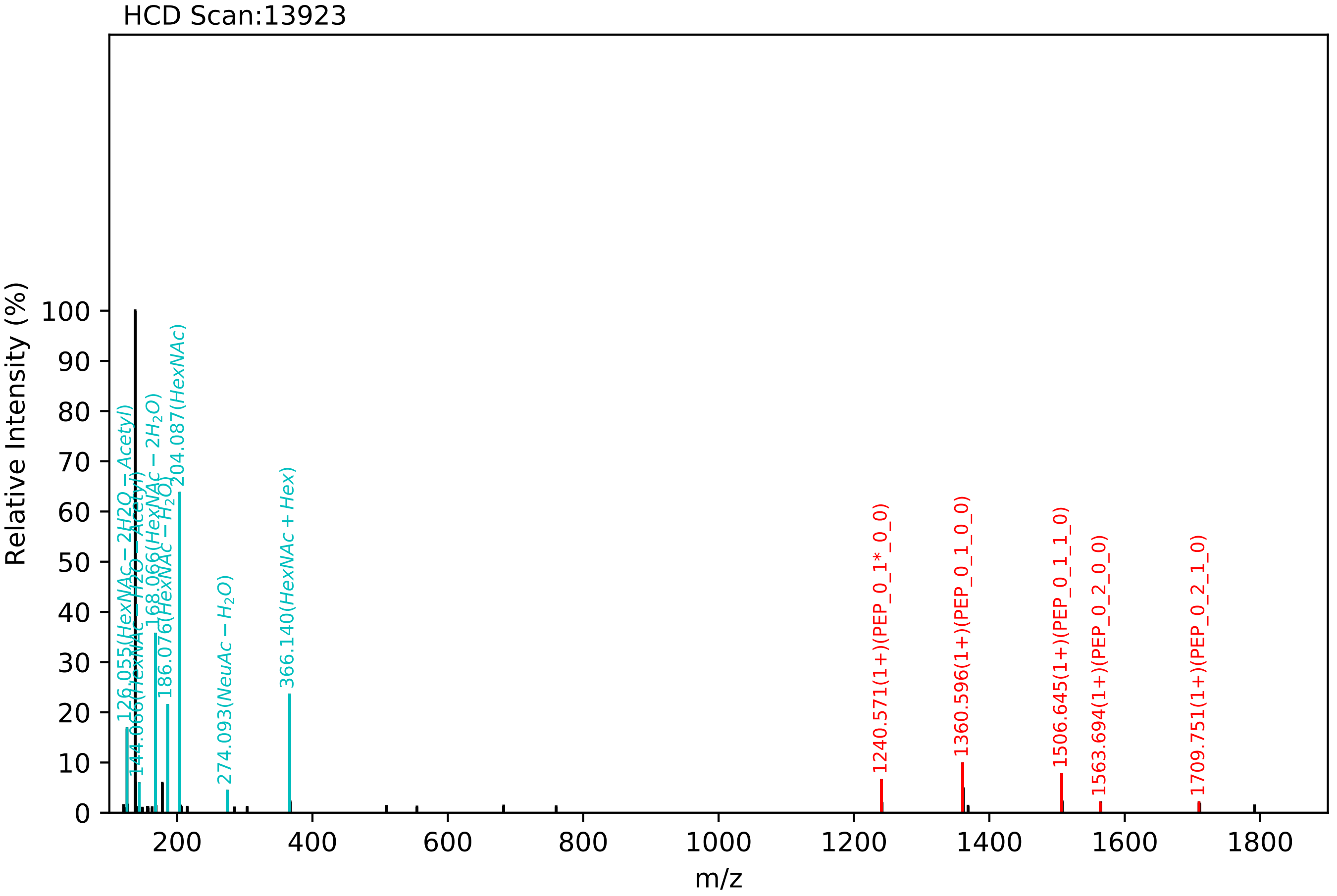

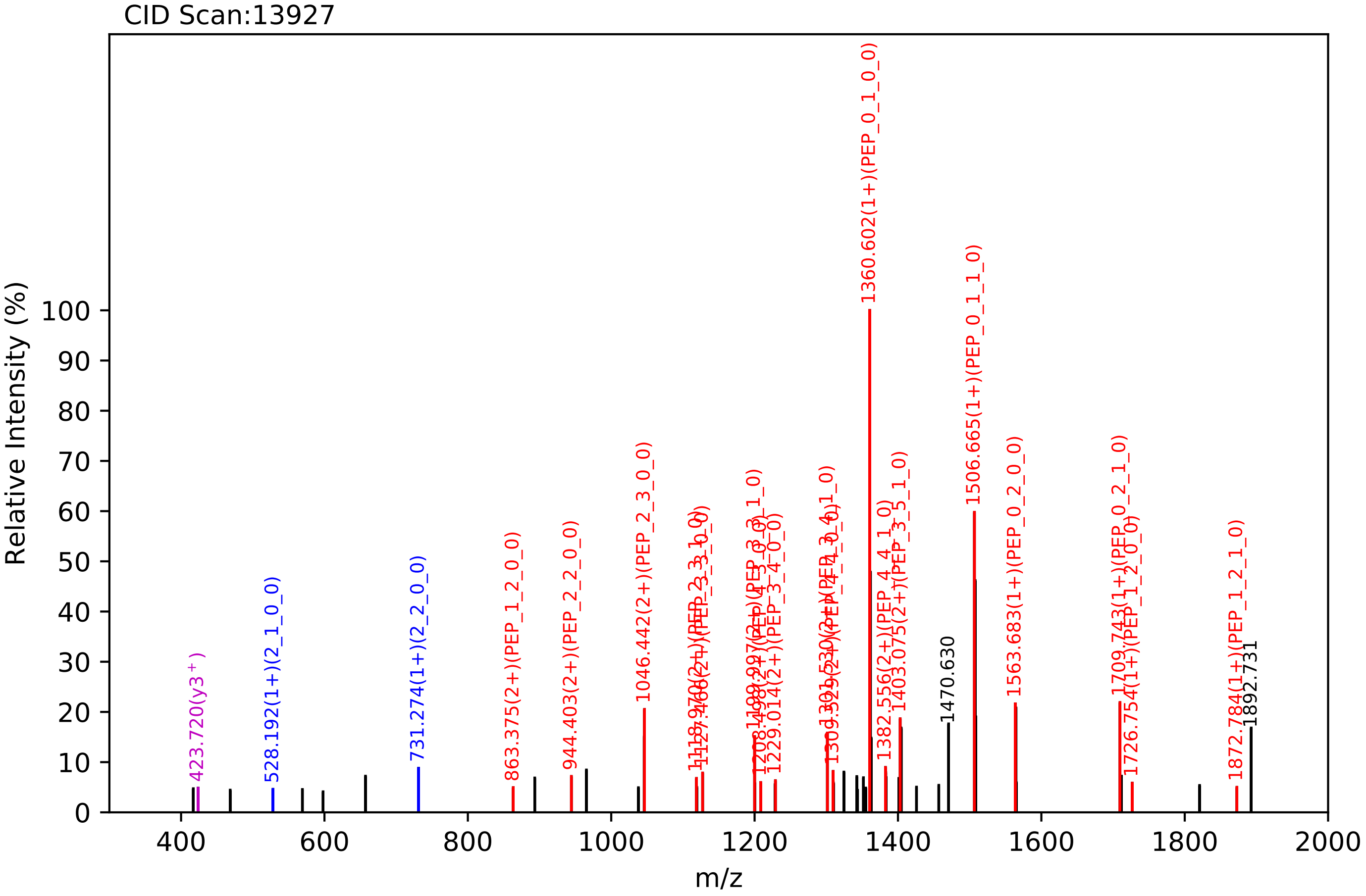

Unknown set no. 469, Experiment: Human Plasma exp_4

EEQFNSTFR(=PEP)_4_5_1_0, m/z:1484.10(2+), RT:52.19, Y-score:88.03

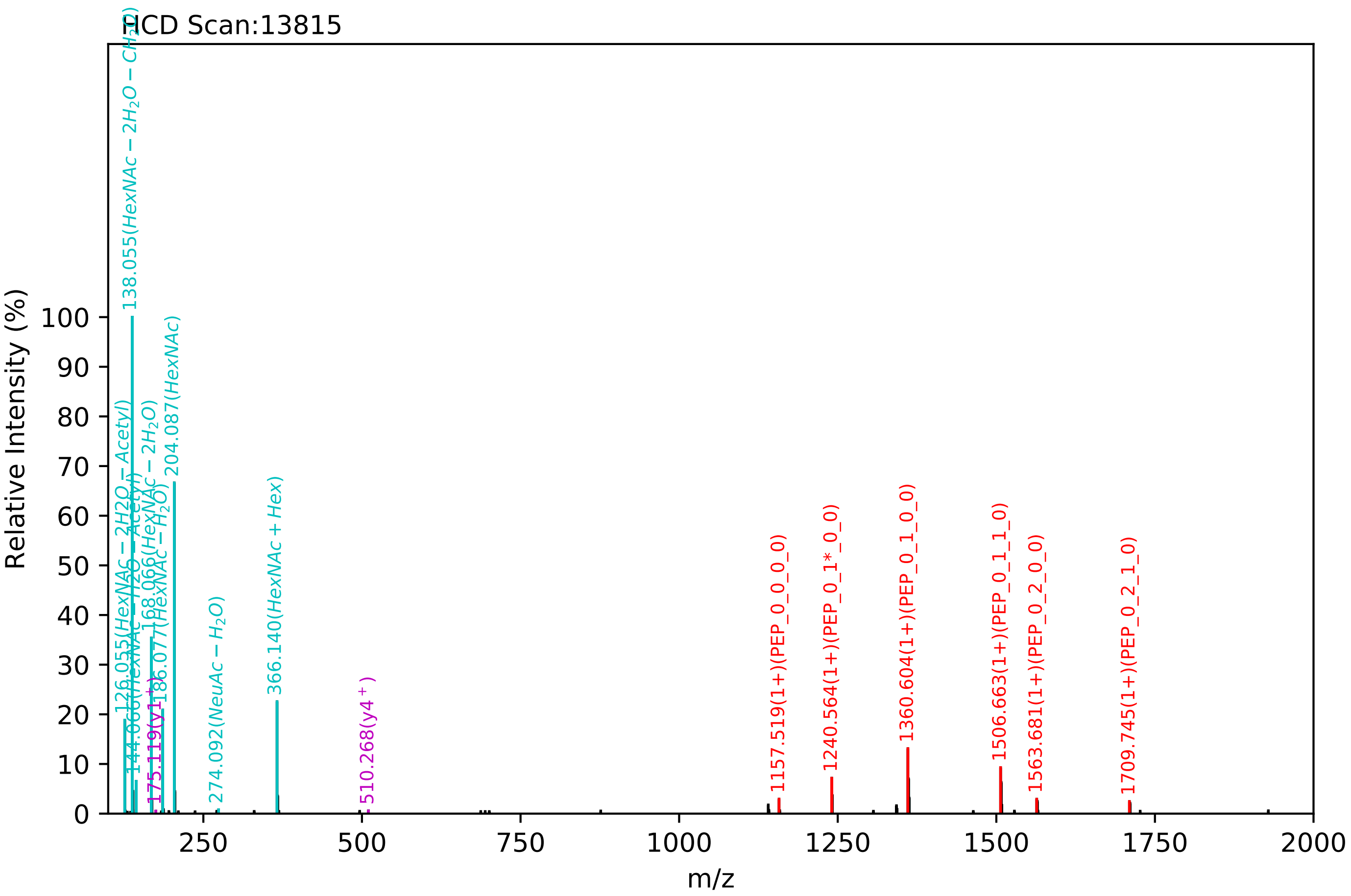

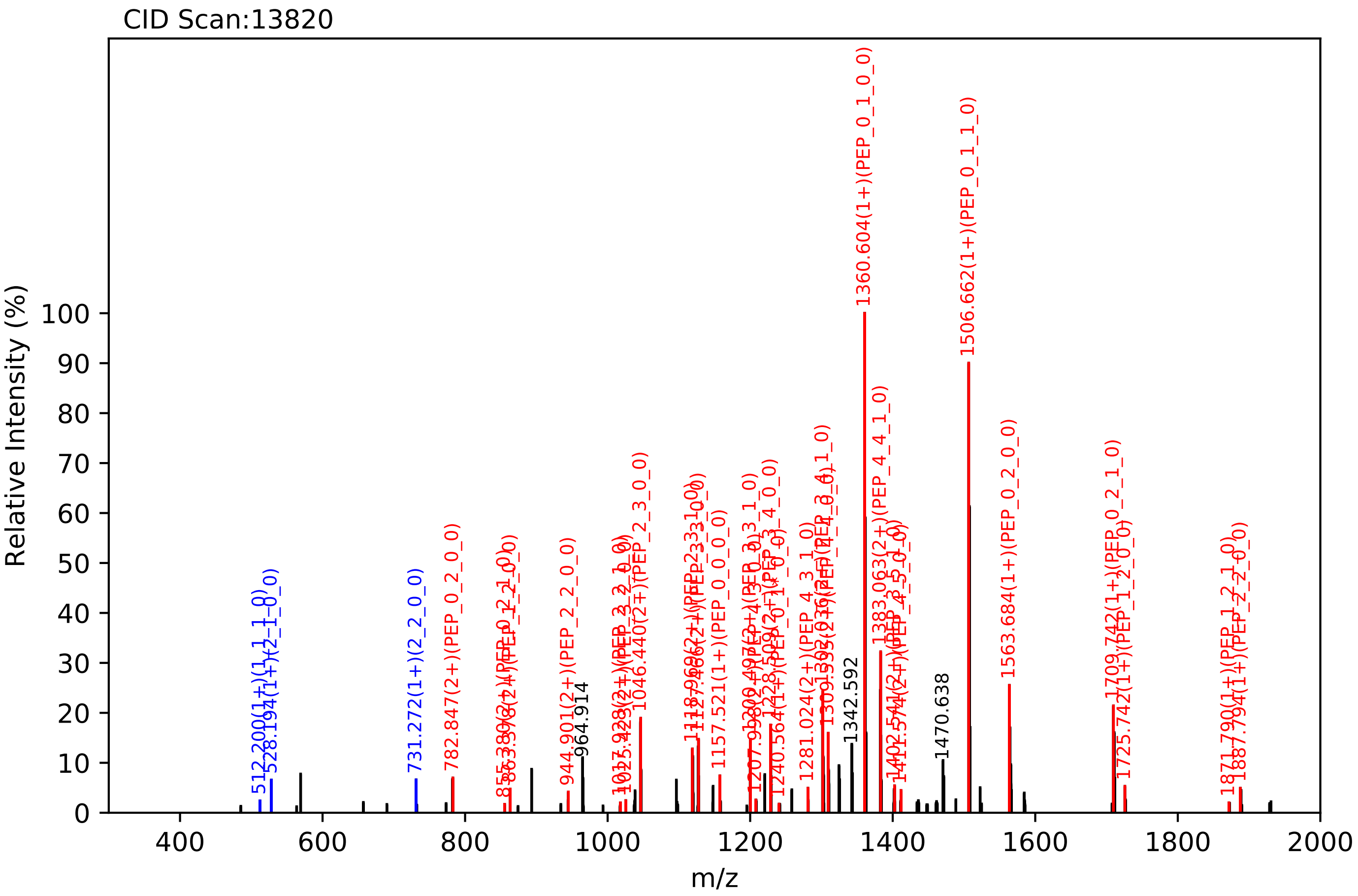

Unknown set no. 470, Experiment: Human Plasma exp_2

EEQFNSTFR(=PEP)_5_3_1_1, m/z:1005.40(3+), RT:61.12, Y-score:93.63

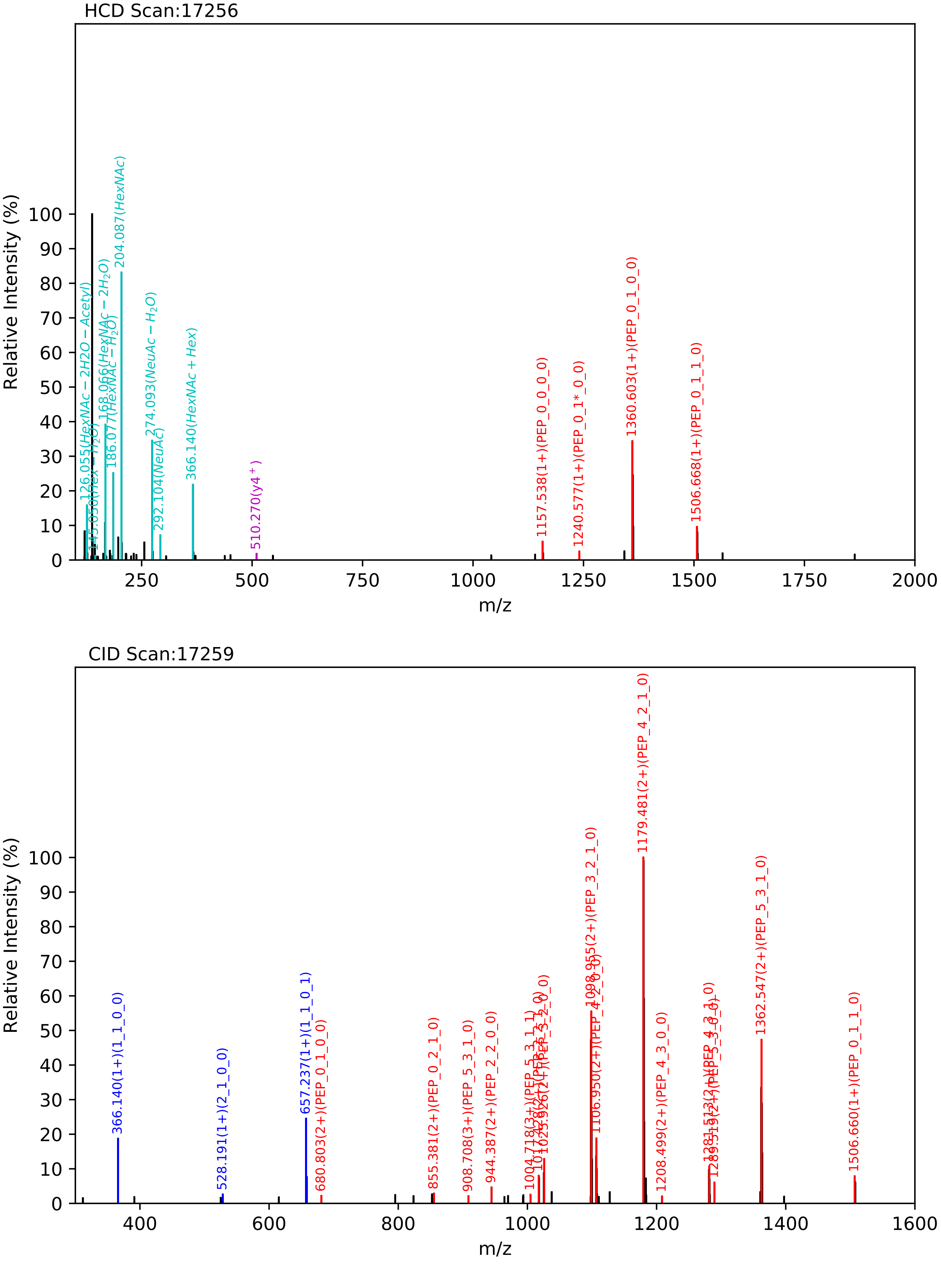

Unknown set no. 471, Experiment: Human Plasma exp_1

EEQFNSTFR(=PEP)_5_4_1_0, m/z:1463.59(2+), RT:51.50, Y-score:86.66

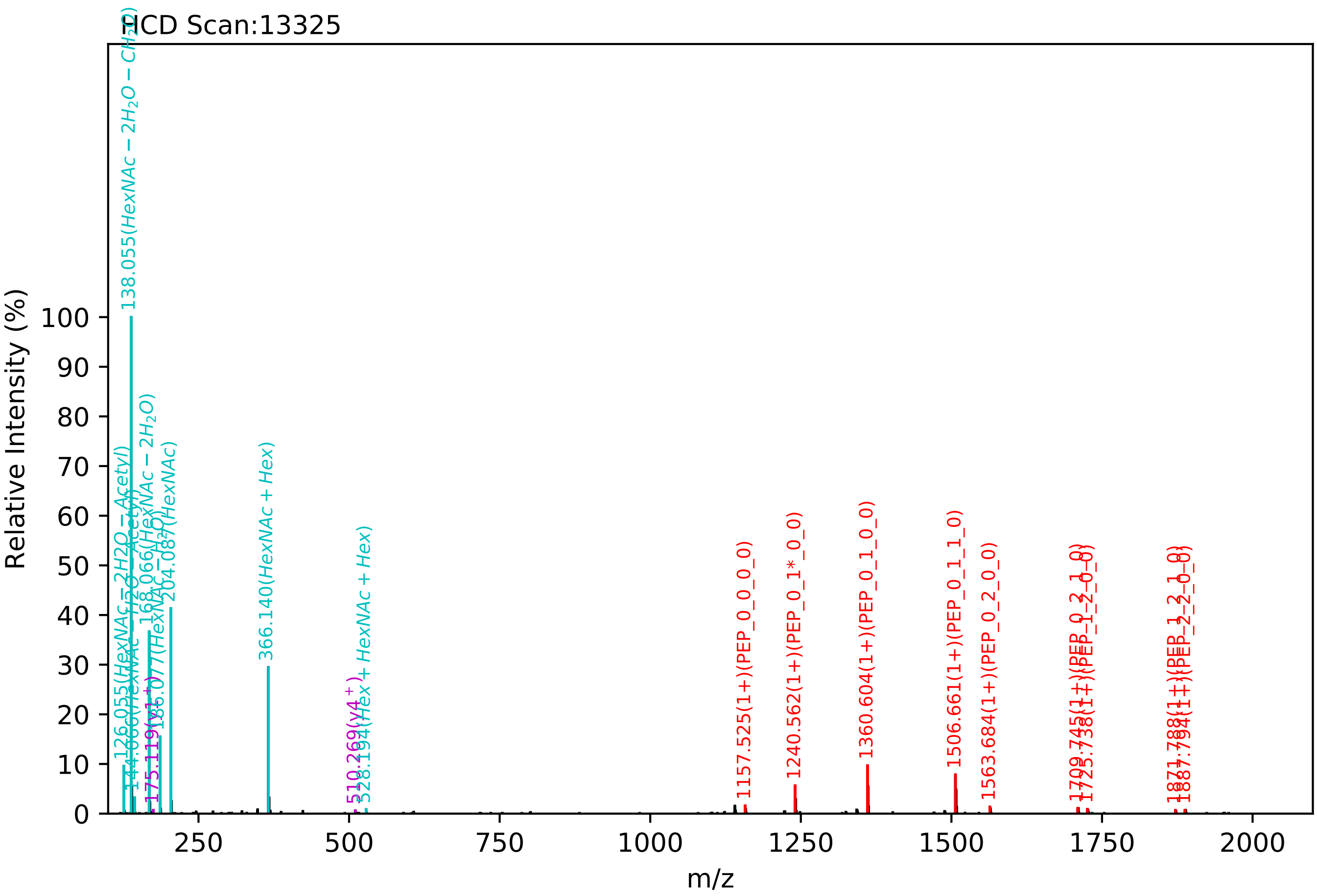

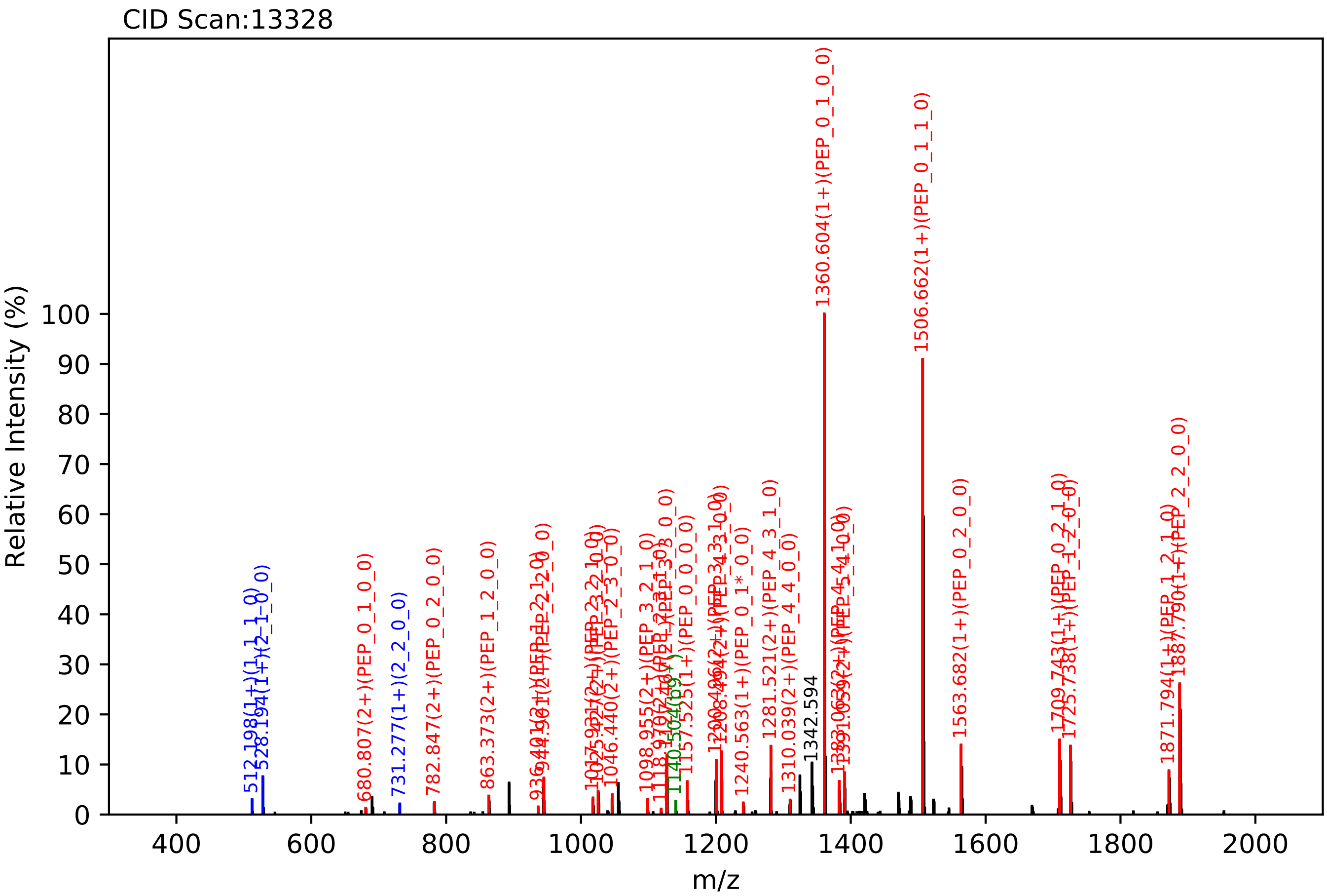

Unknown set no. 472, Experiment: Human Plasma exp_3

EEQFNSTFR(=PEP)_5_4_1_0, m/z:1463.59(2+), RT:60.72, Y-score:96.33

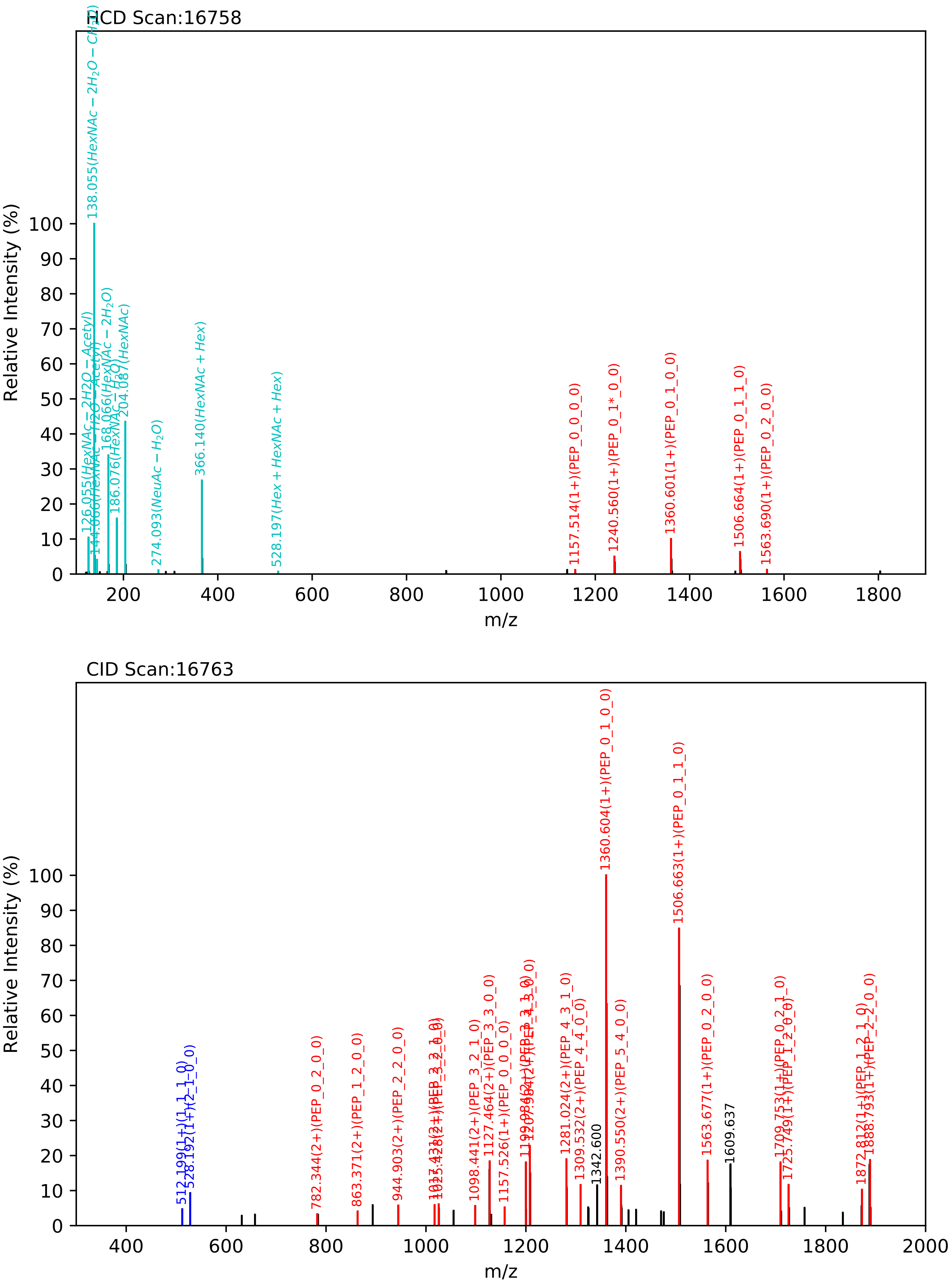

Unknown set no. 473, Experiment: Human Plasma exp_4

EEQFNSTFR(=PEP)_5_4_1_0, m/z:1463.59(2+), RT:51.71, Y-score:89.95

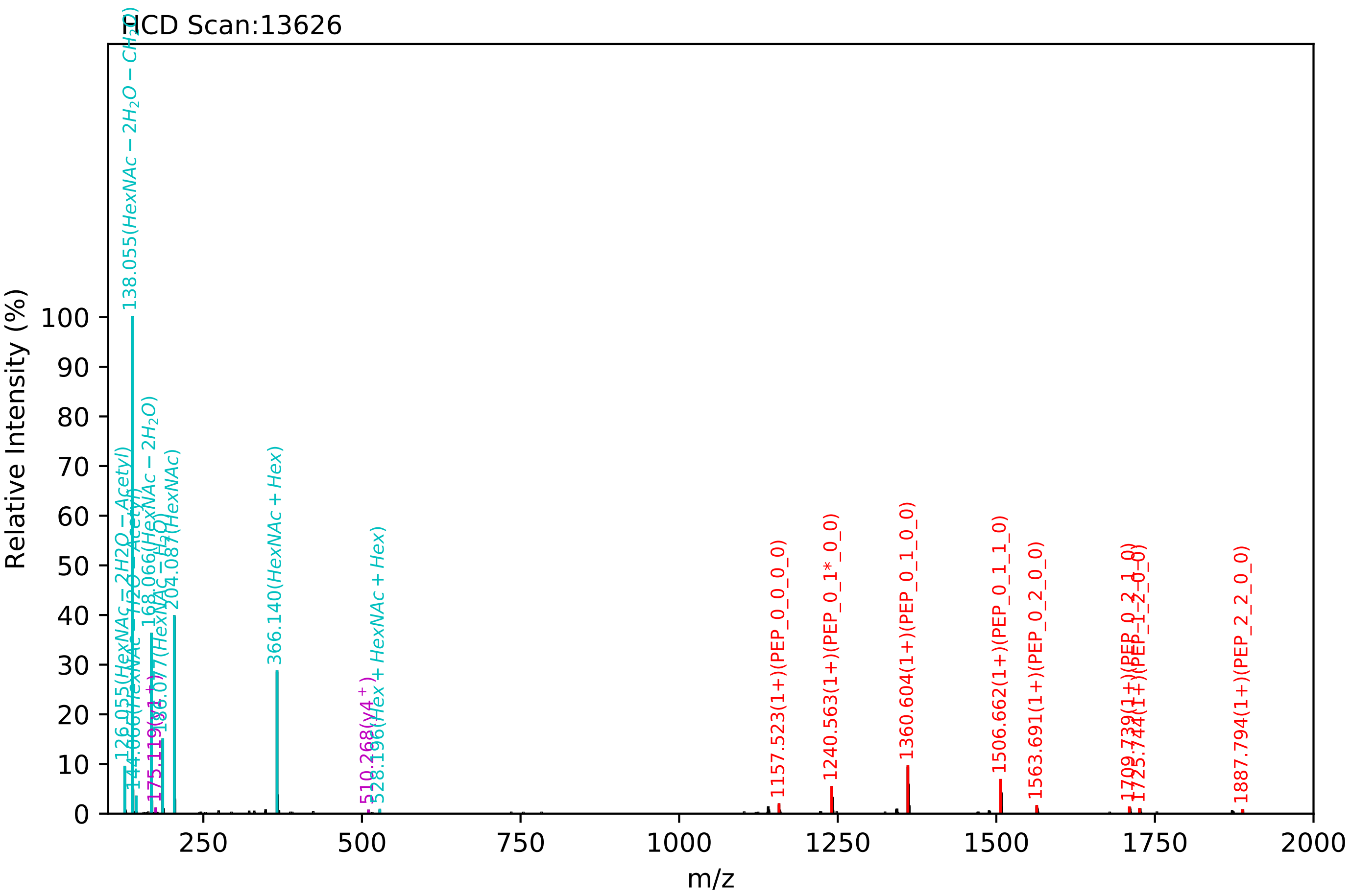

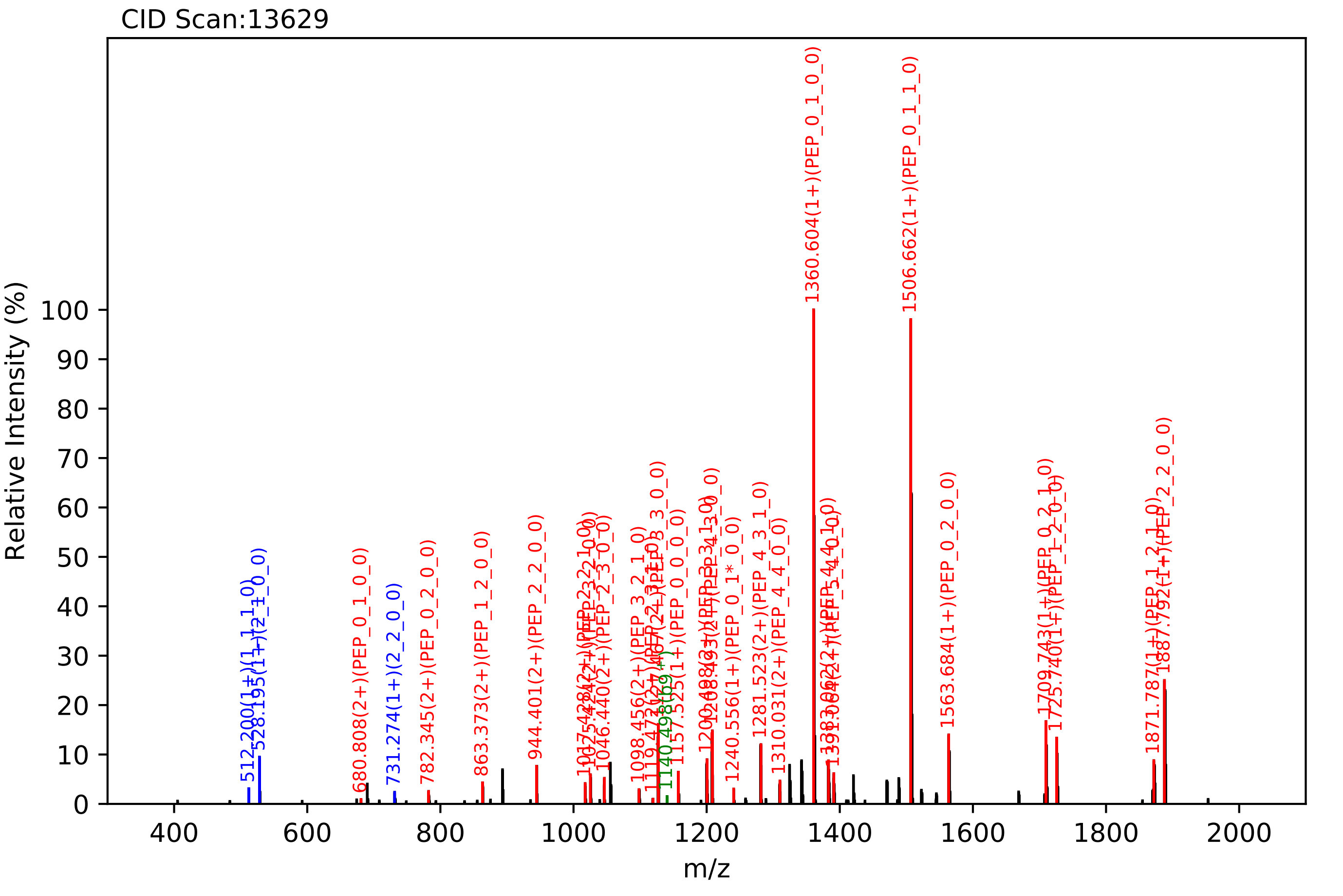

Unknown set no. 474, Experiment: Human Plasma exp_1

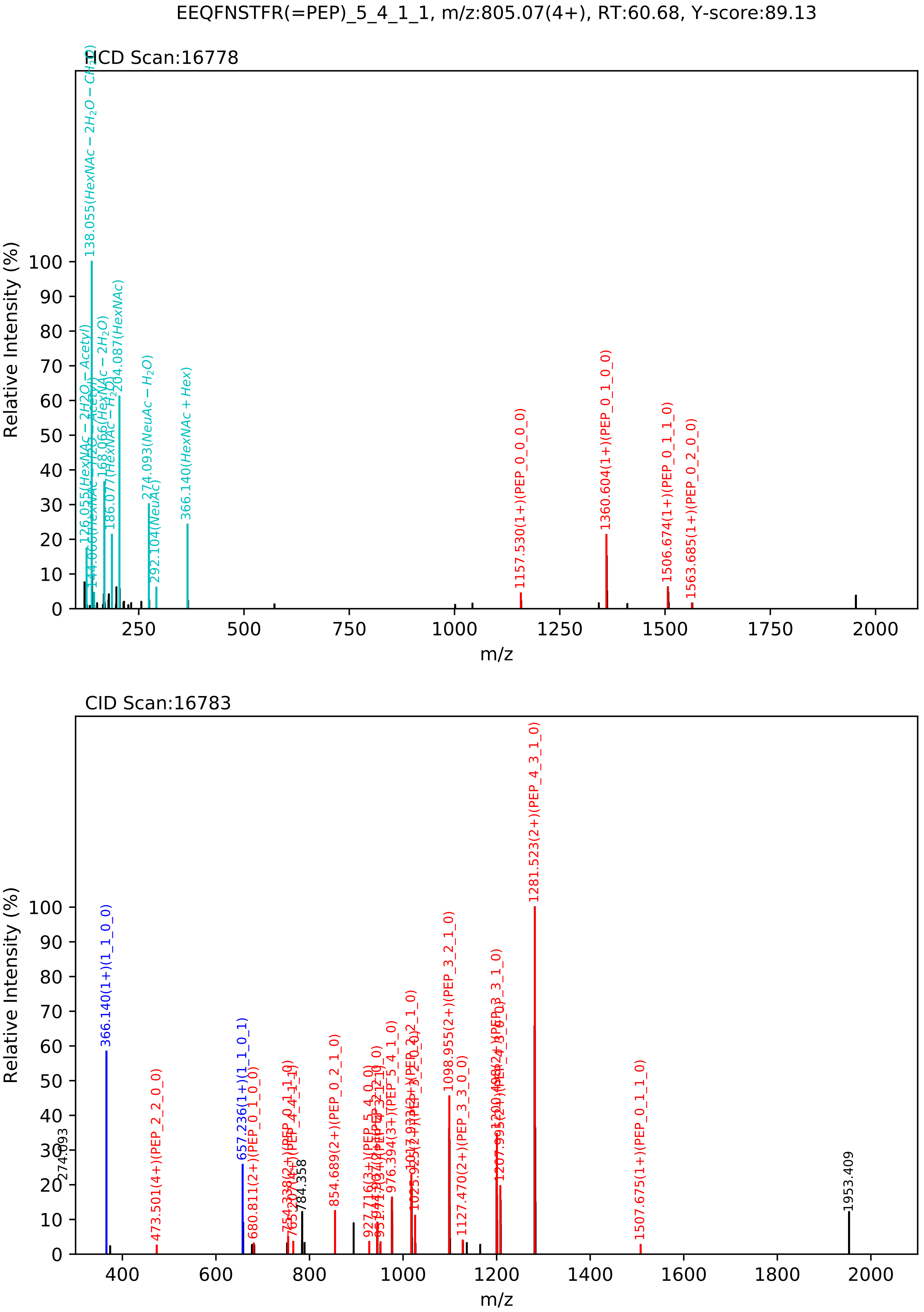

Unknown set no. 475, Experiment: Human Plasma exp_1

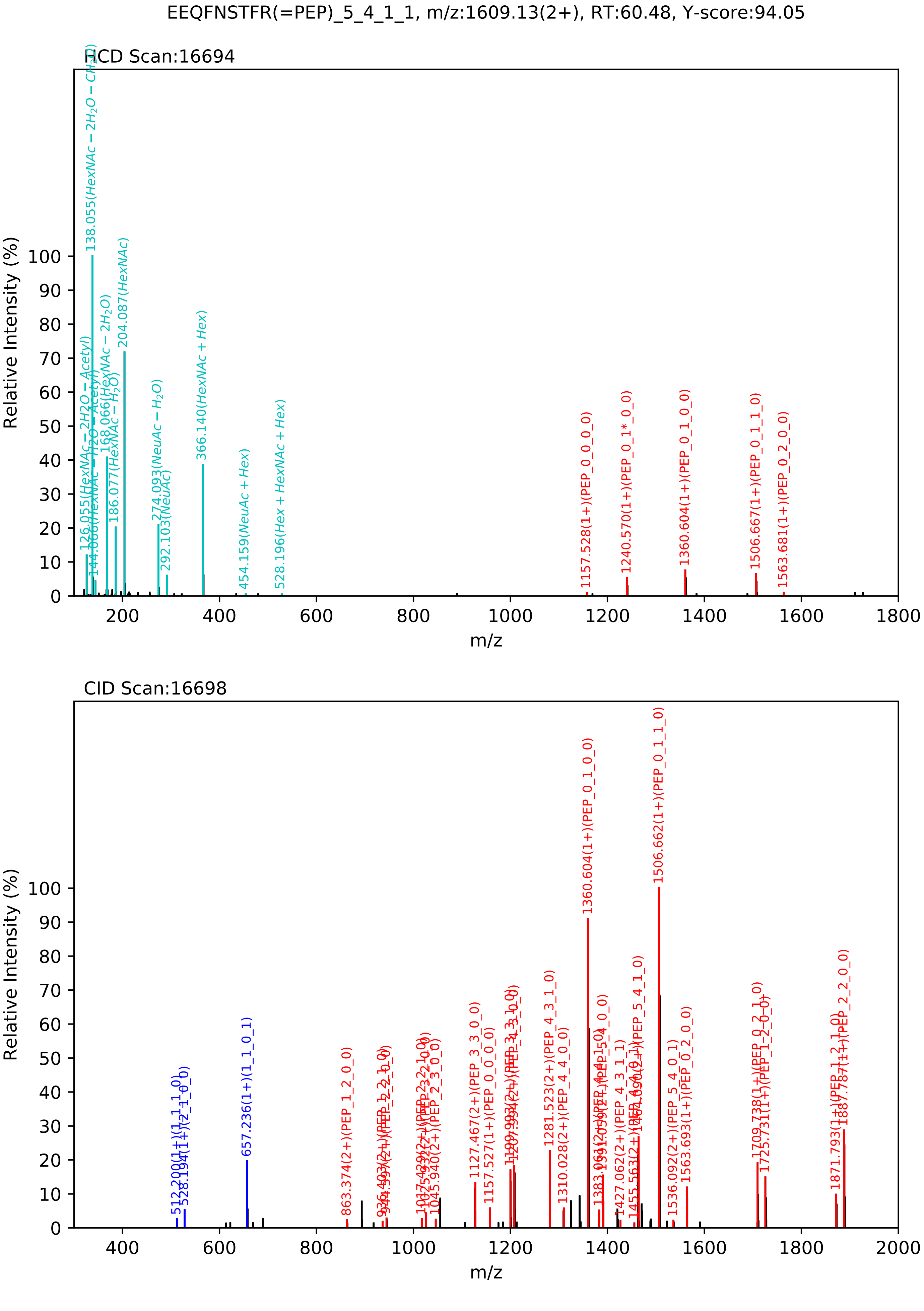

Unknown set no. 476, Experiment: Human Plasma exp_2

EEQFNSTFR(=PEP)_5_4_1_1, m/z:805.07(4+), RT:60.72, Y-score:89.40

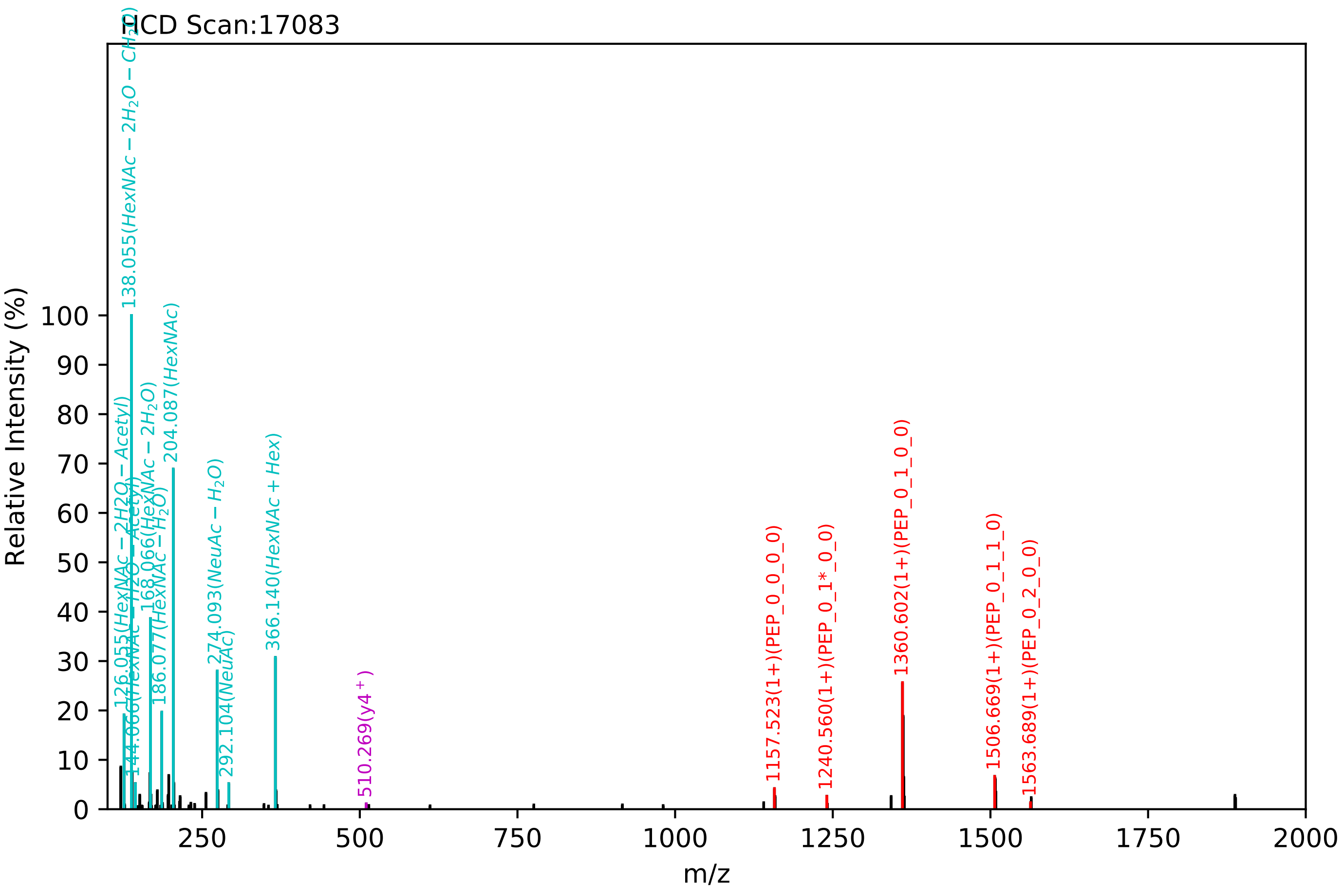

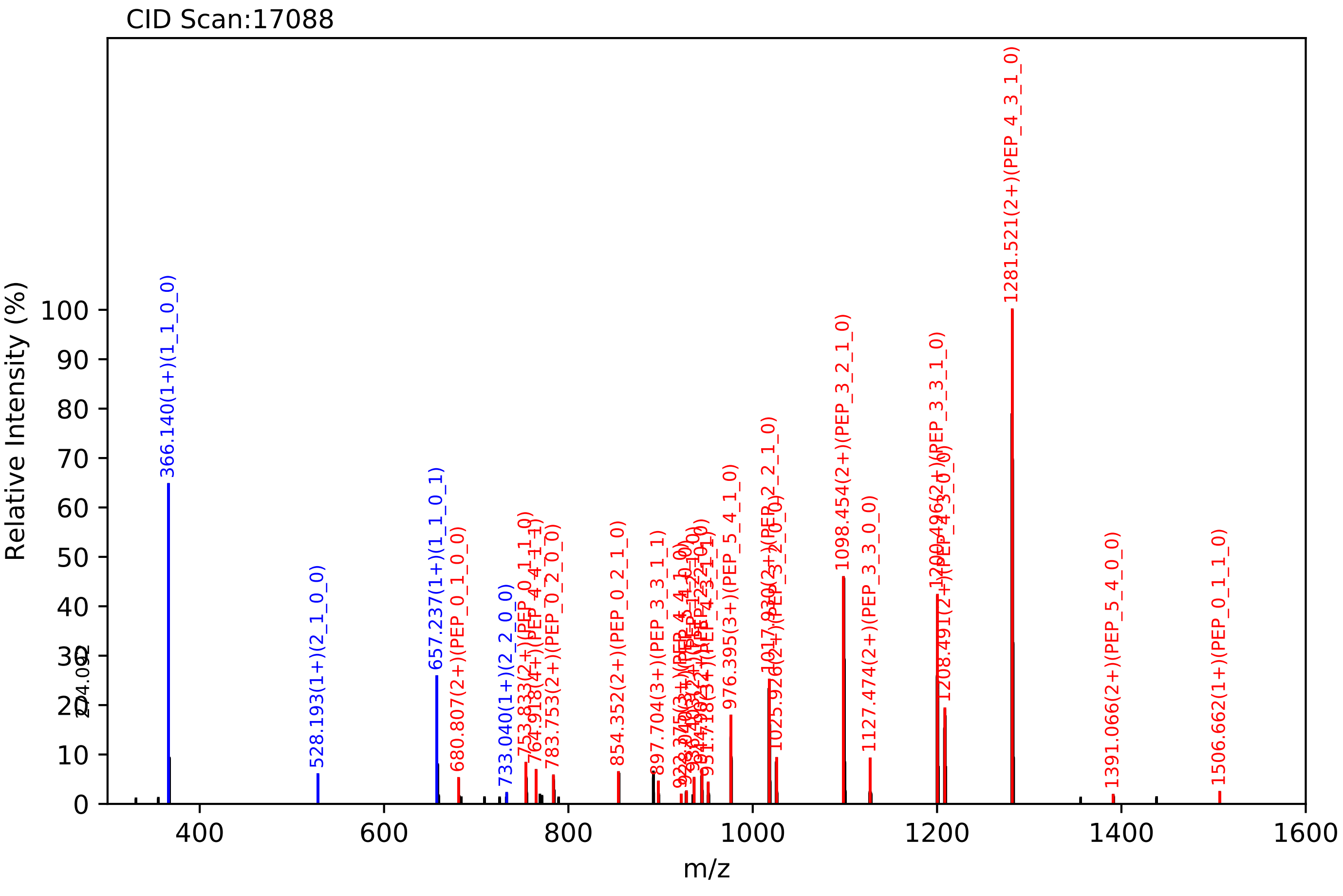

Unknown set no. 477, Experiment: Human Plasma exp_2

EEQFNSTFR(=PEP)_5_4_1_1, m/z:1609.13(2+), RT:61.11, Y-score:98.33

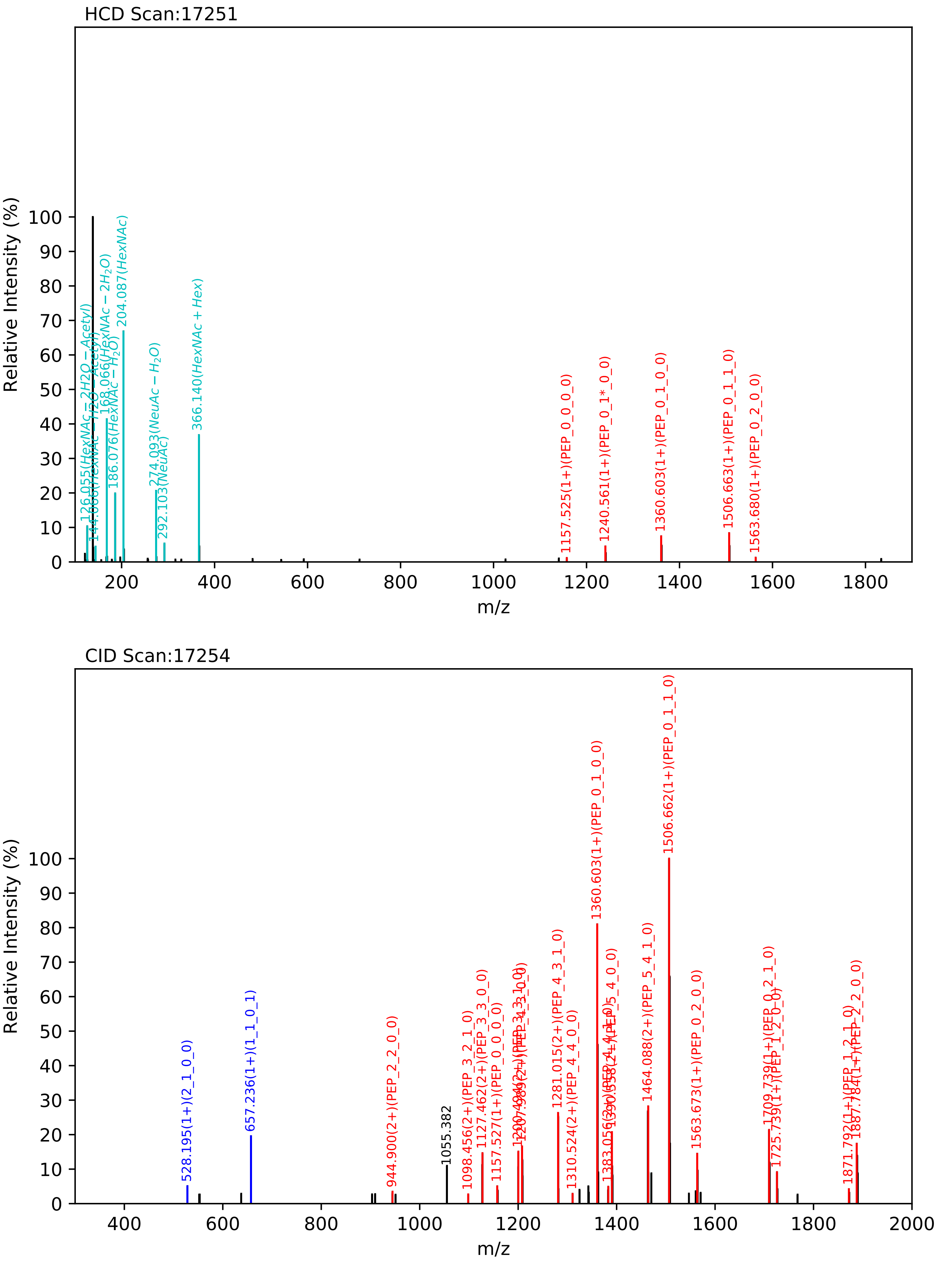

Unknown set no. 478, Experiment: Human Plasma exp_3

EEQFNSTFR(=PEP)_5_4_1_1, m/z:805.07(4+), RT:60.66, Y-score:93.09

HCD Scan:16732

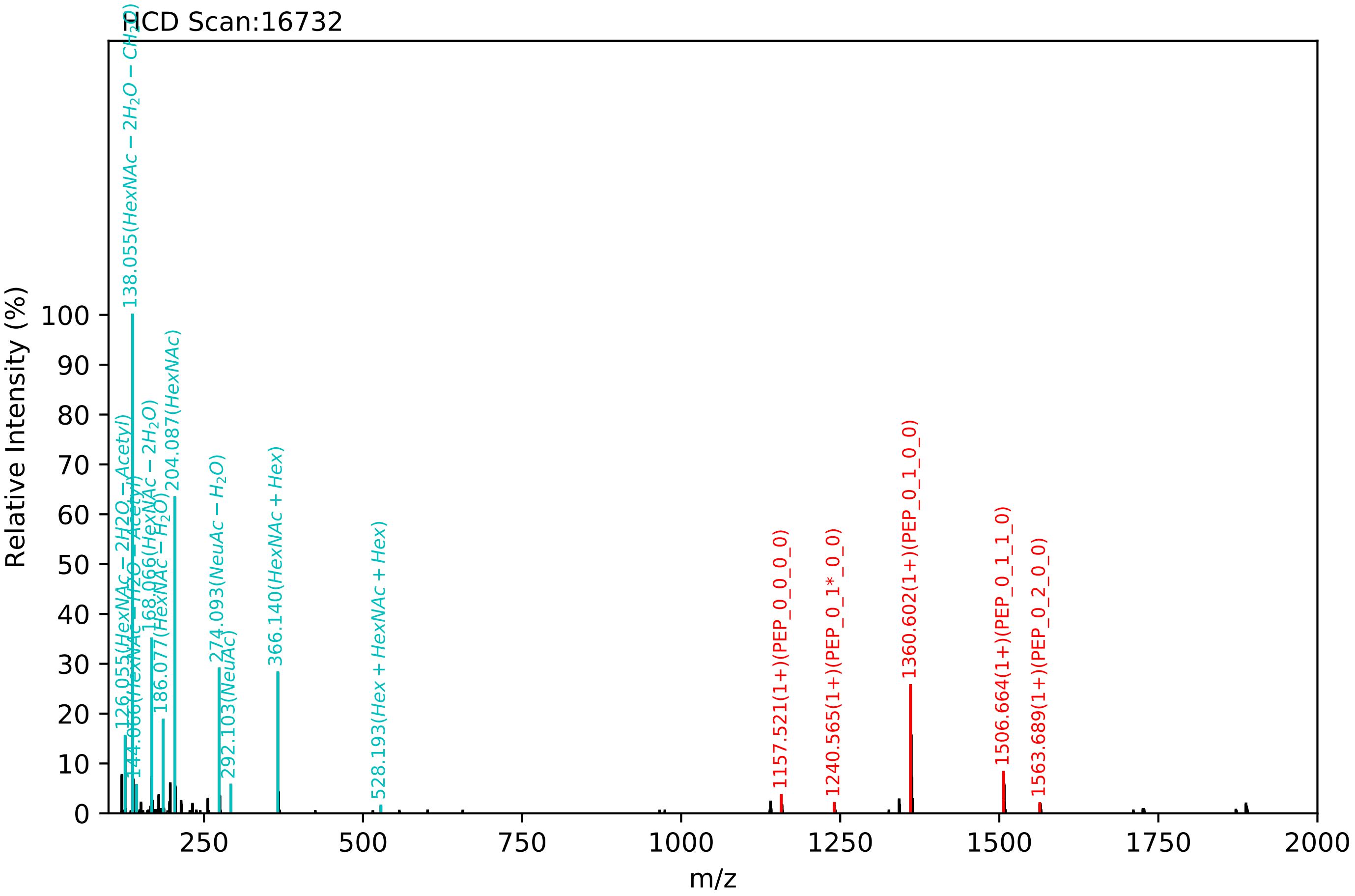

CID Scan:16737

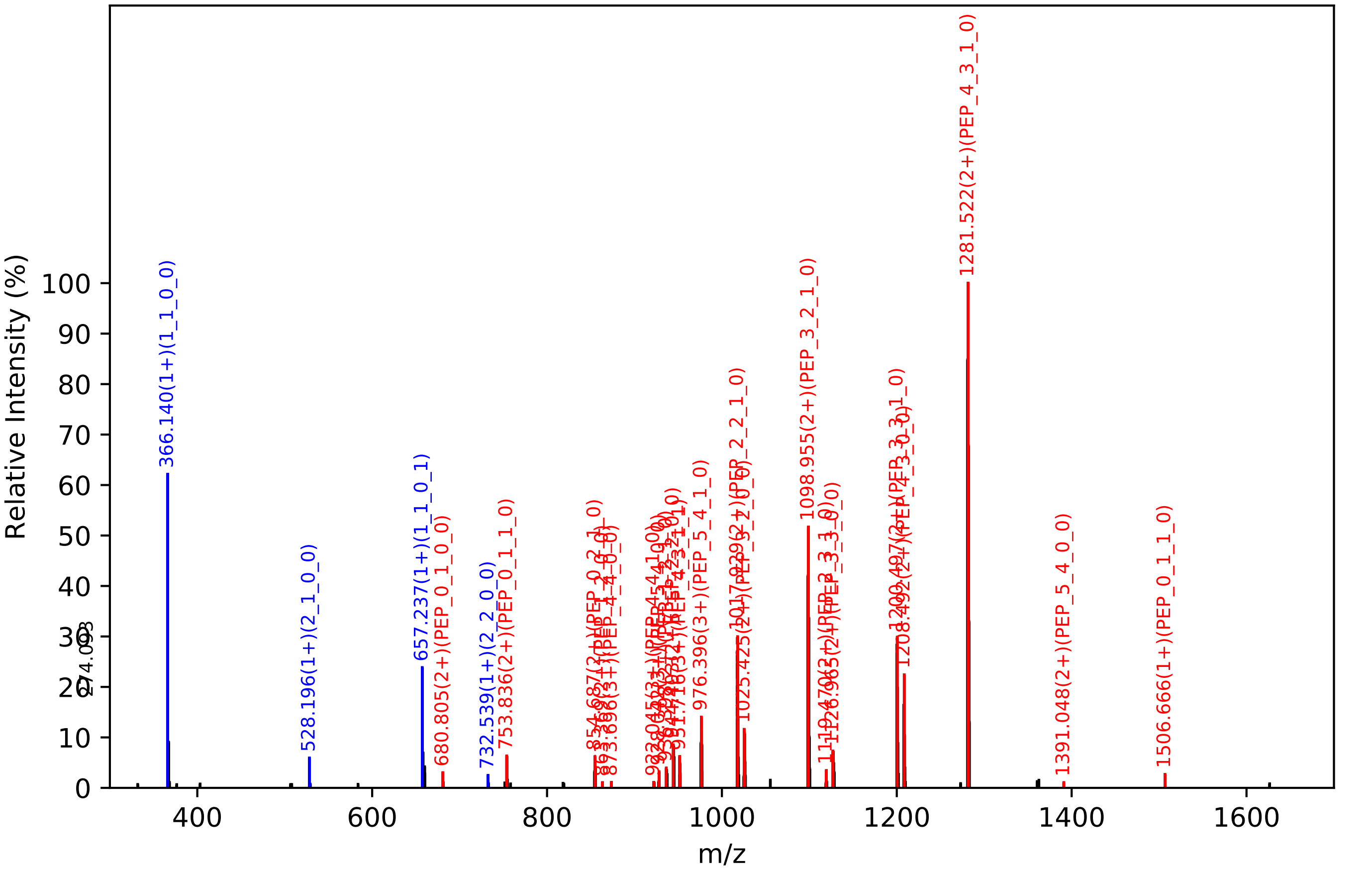

Unknown set no. 479, Experiment: Human Plasma exp_3

EEQFNSTFR(=PEP)_5_4_1_1, m/z:1609.13(2+), RT:60.54, Y-score:94.30

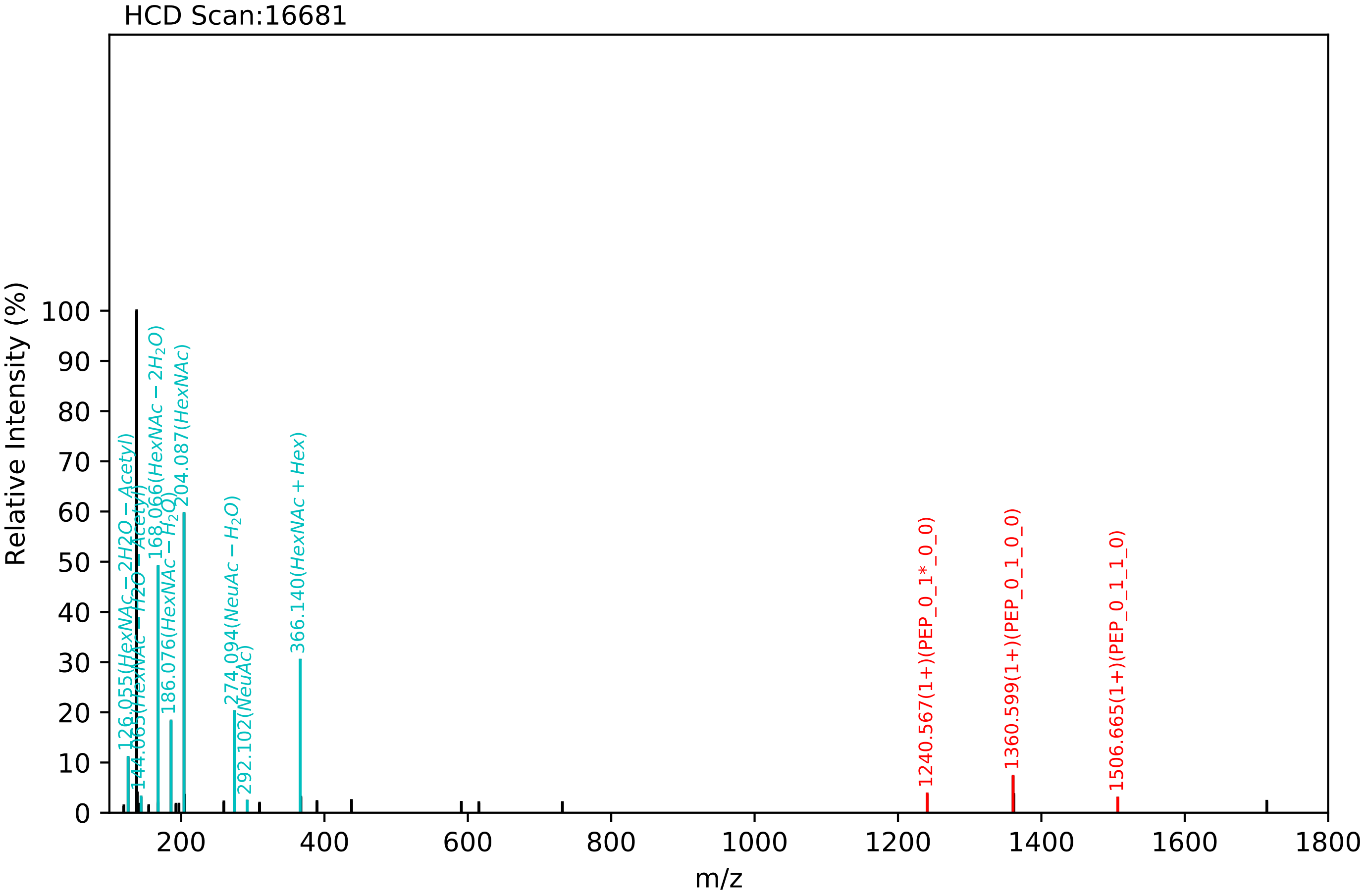

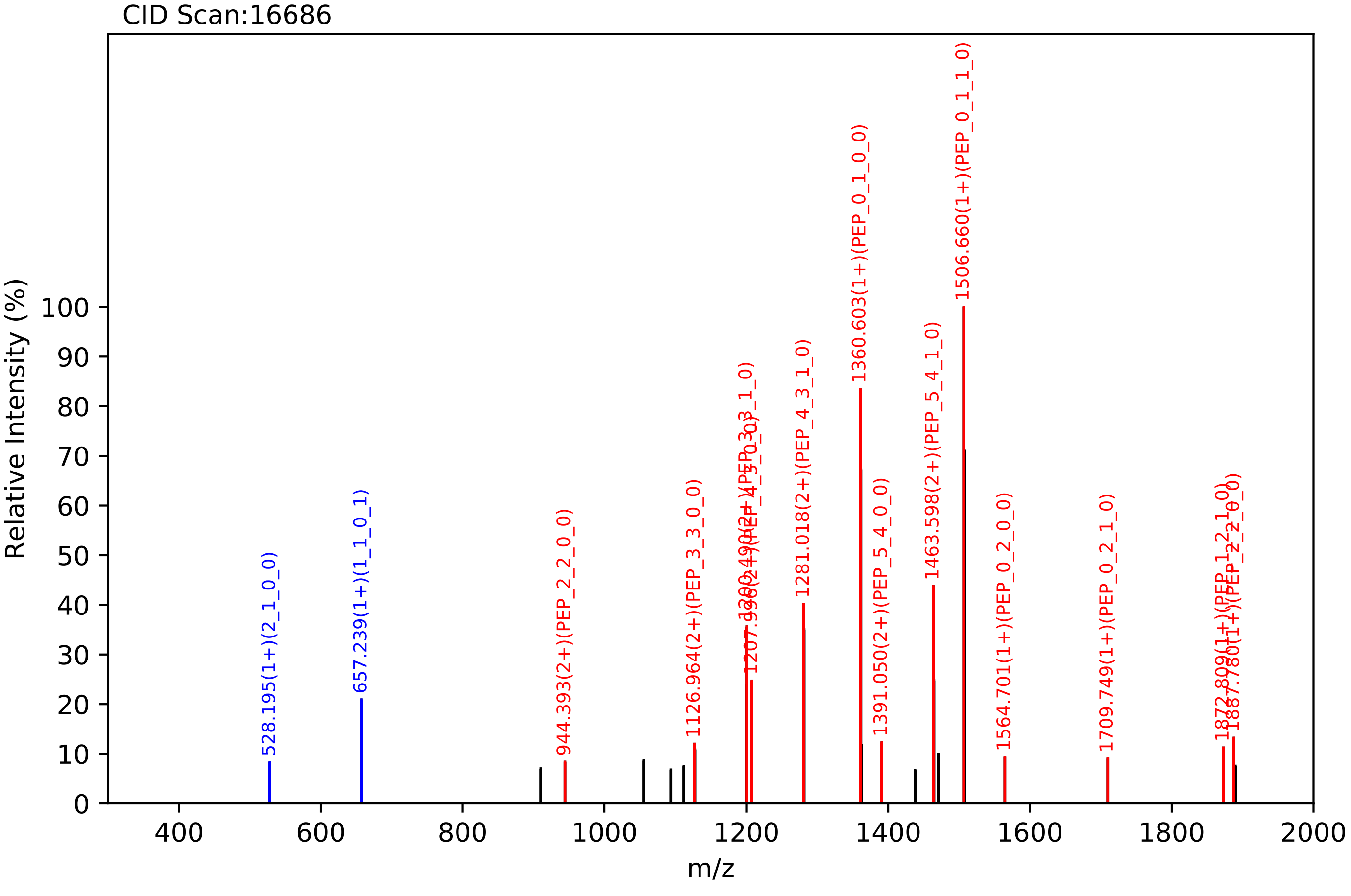

Unknown set no. 480, Experiment: Human Plasma exp_4

EEQFNSTFR(=PEP)_5_4_1_1, m/z:1609.13(2+), RT:61.21, Y-score:99.12

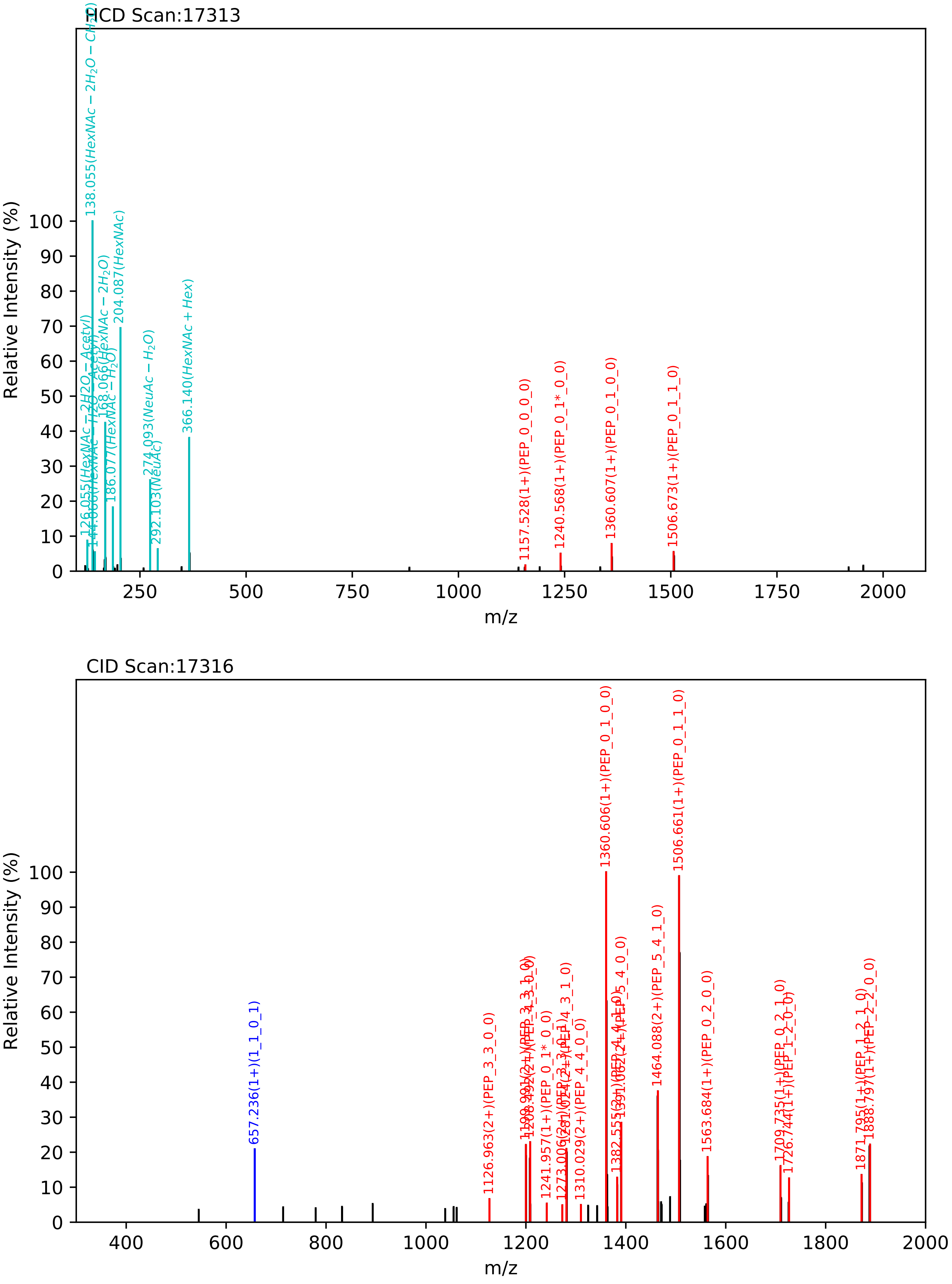

Unknown set no. 481, Experiment: Human Plasma exp_5

EEQFNSTFR(=PEP)_5_4_1_1, m/z:805.07(4+), RT:60.61, Y-score:90.44

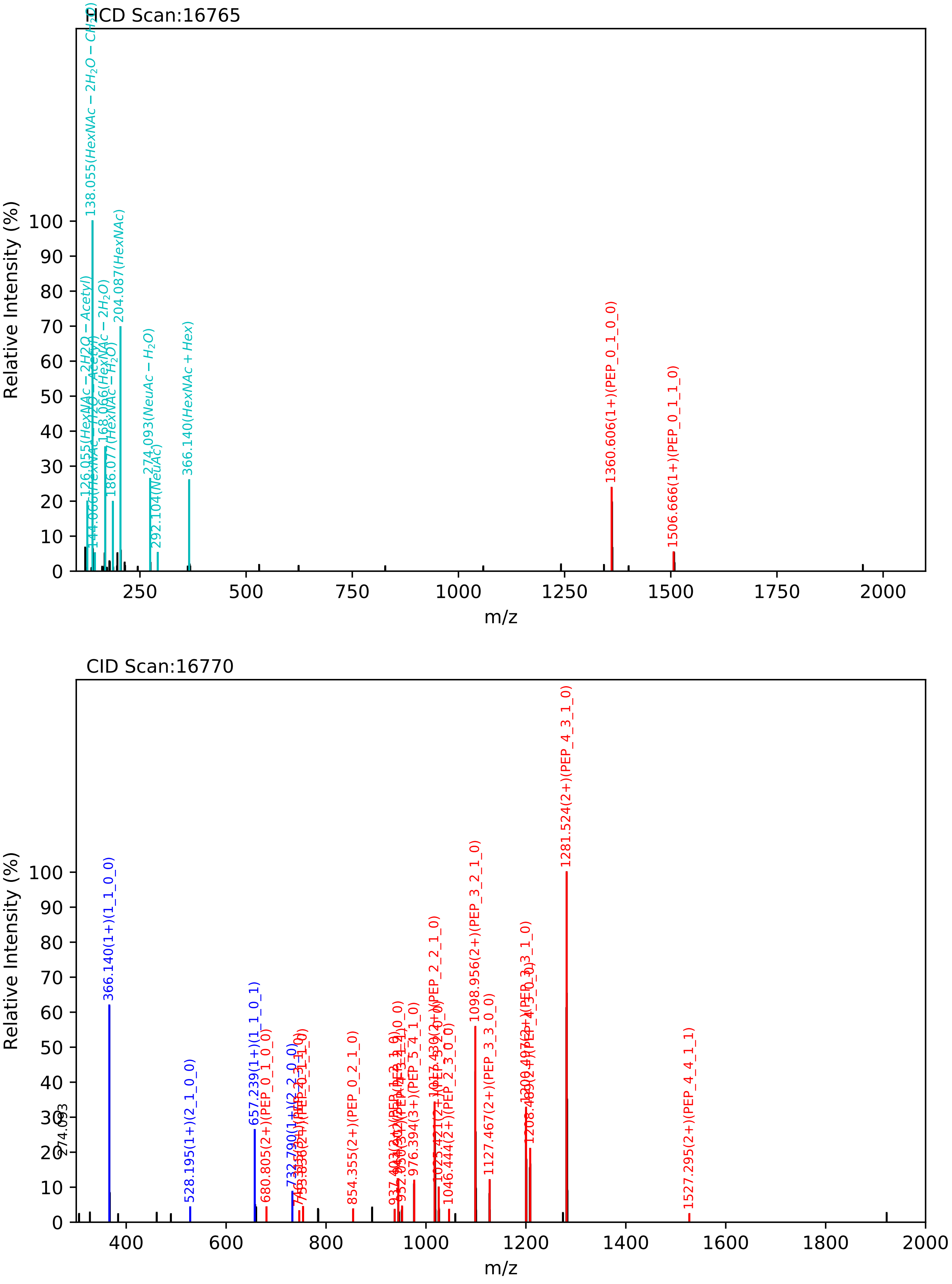

Unknown set no. 482, Experiment: Human Plasma exp_5

EEQFNSTFR(=PEP)_5_4_1_1, m/z:1609.13(2+), RT:60.52, Y-score:96.54

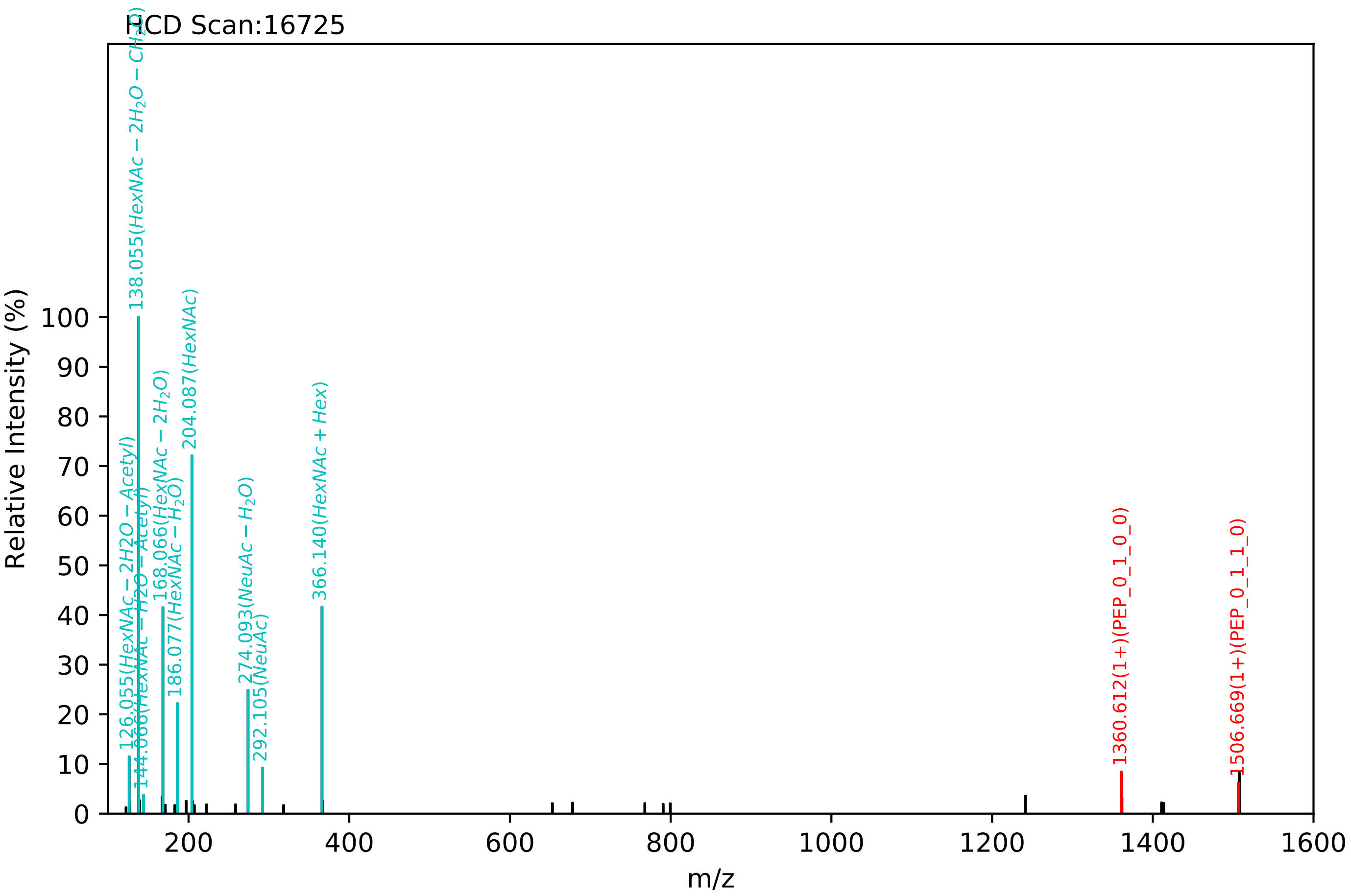

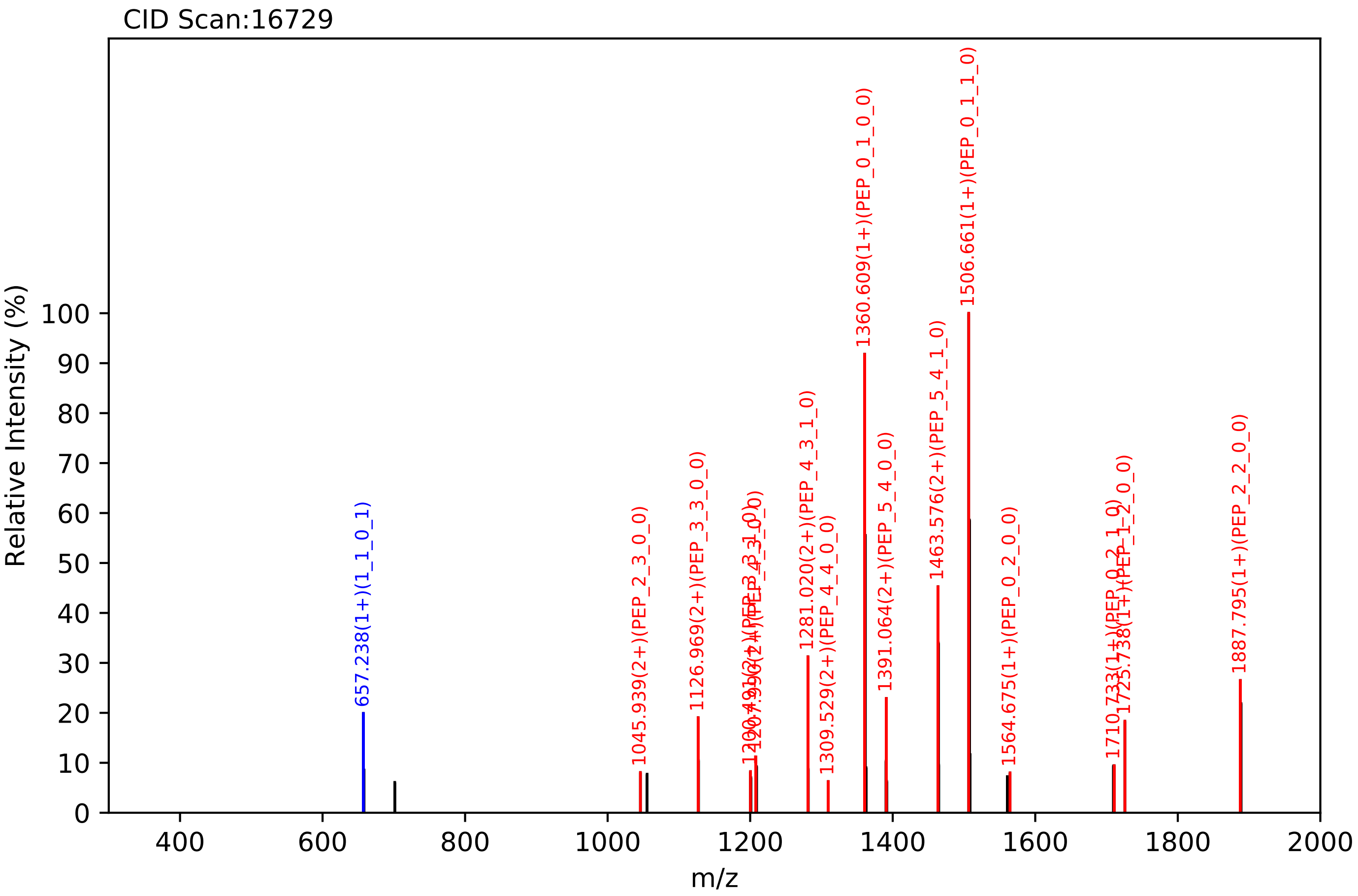

Unknown set no. 483, Experiment: Human Plasma exp_5

EEQFNSTFR(=PEP)_5_4_1_2, m/z:1170.12(3+), RT:71.24, Y-score:88.40

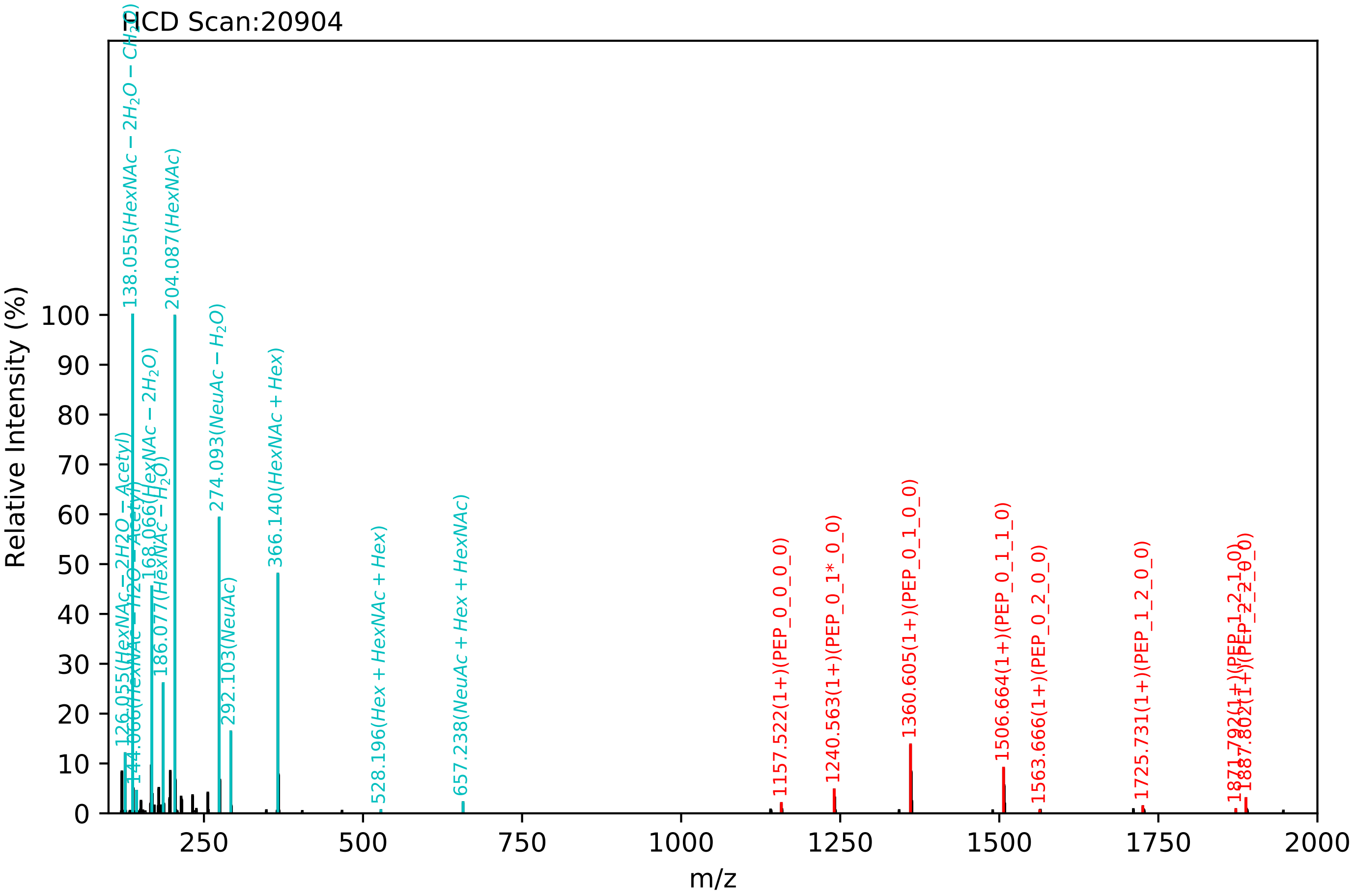

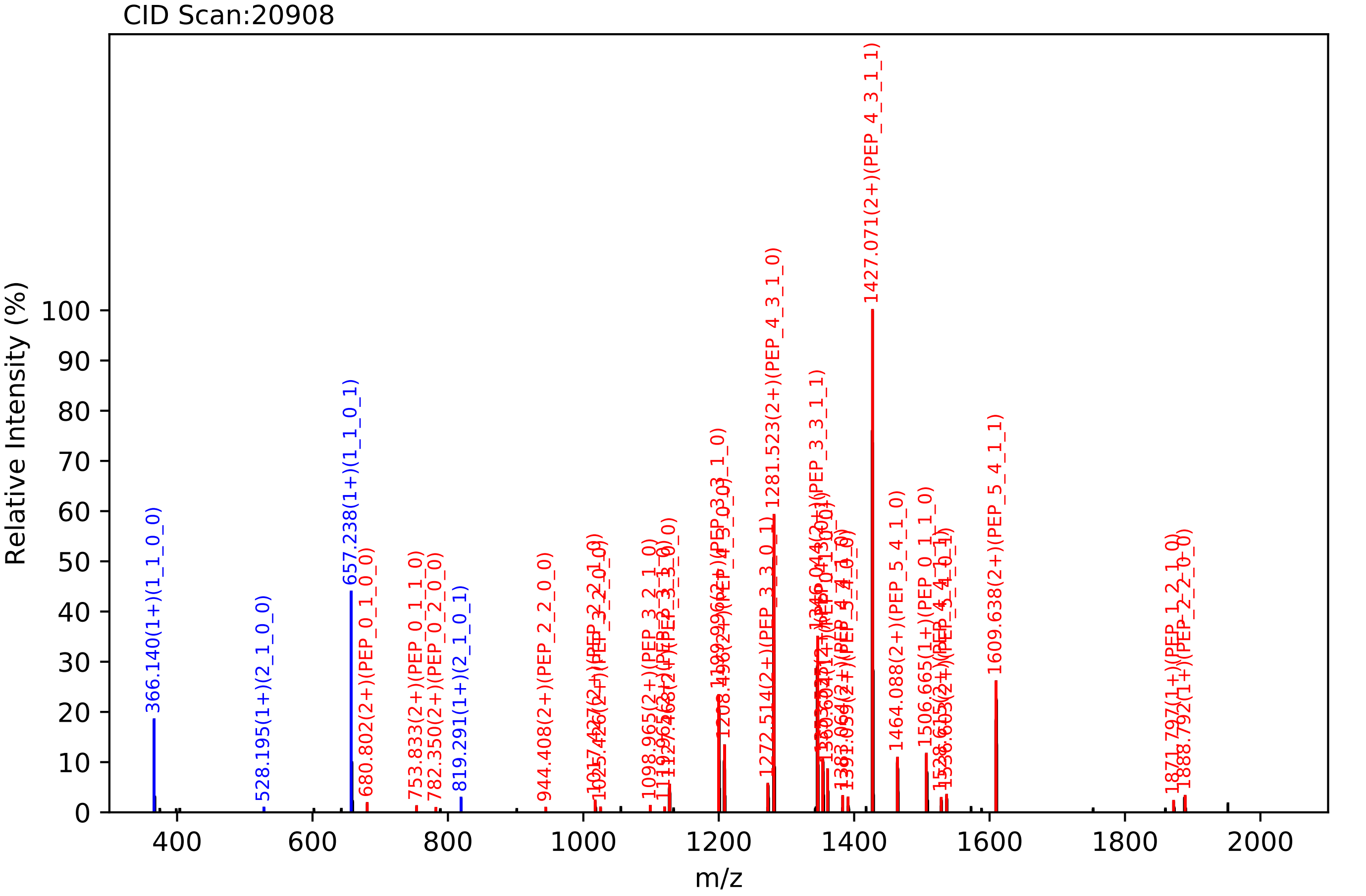

Unknown set no. 484, Experiment: Human Plasma exp_4

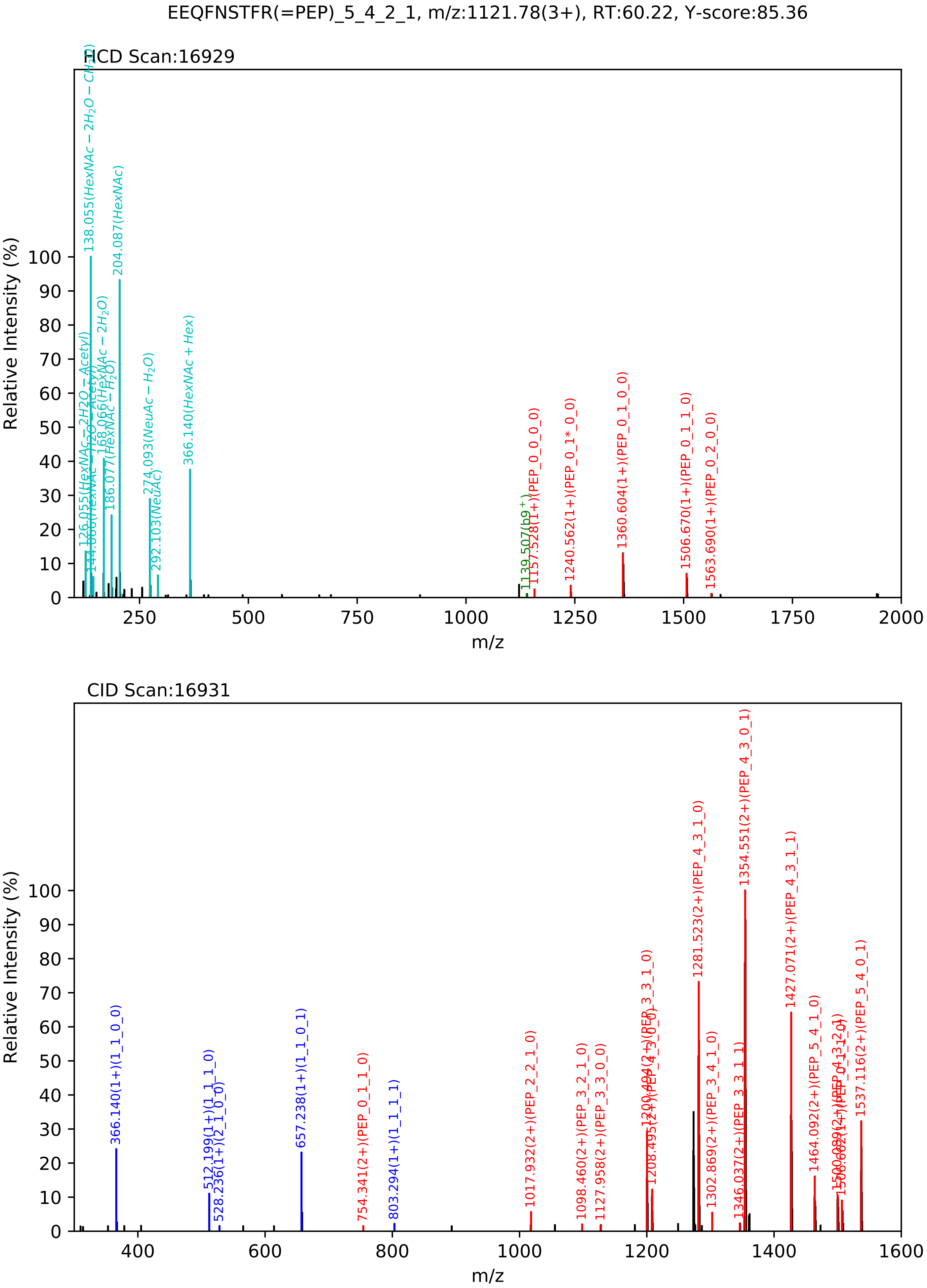

Unknown set no. 485, Experiment: Human Plasma exp_5

EEQFNSTFR(=PEP)_5_4_2_1, m/z:1121.78(3+), RT:60.10, Y-score:86.75

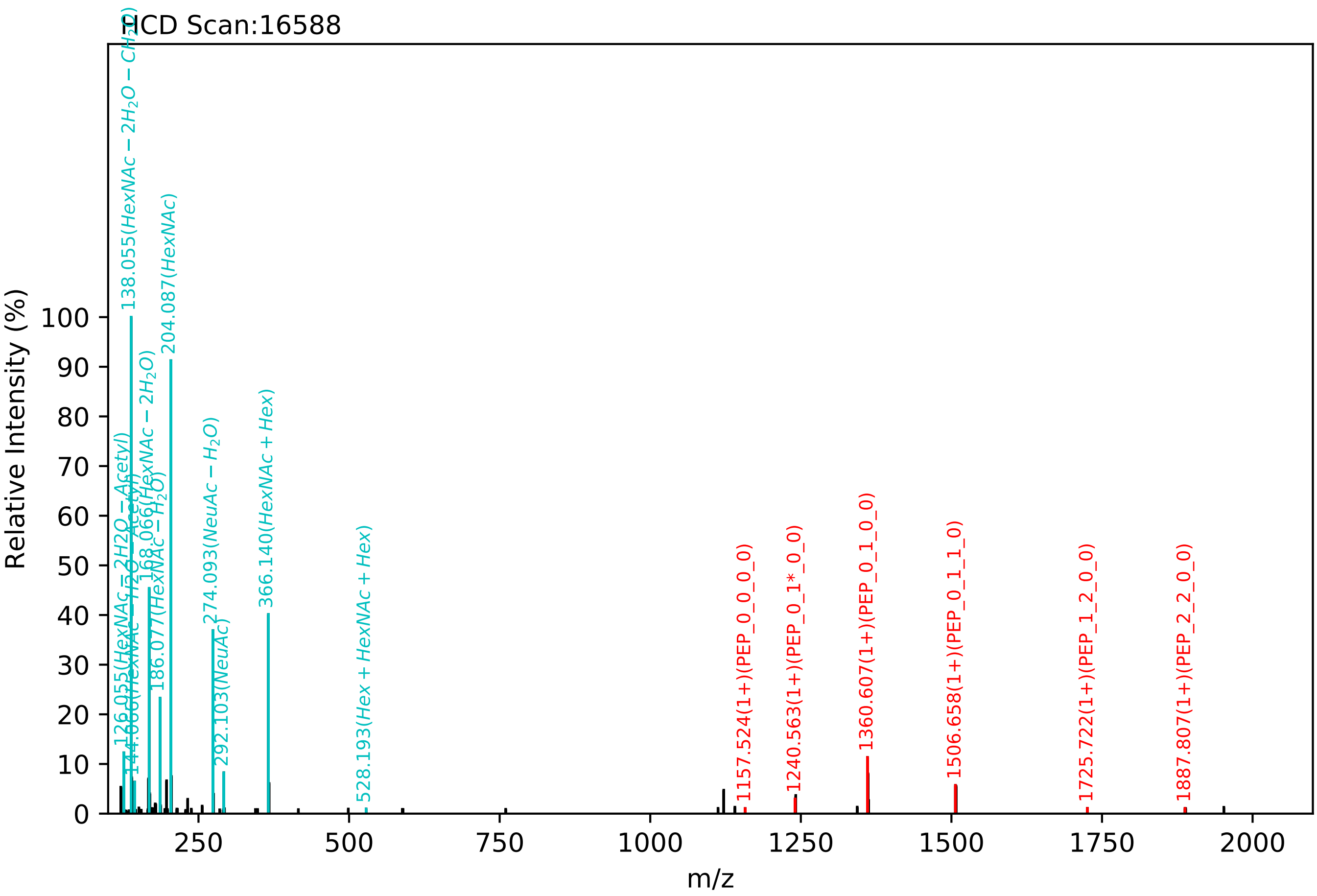

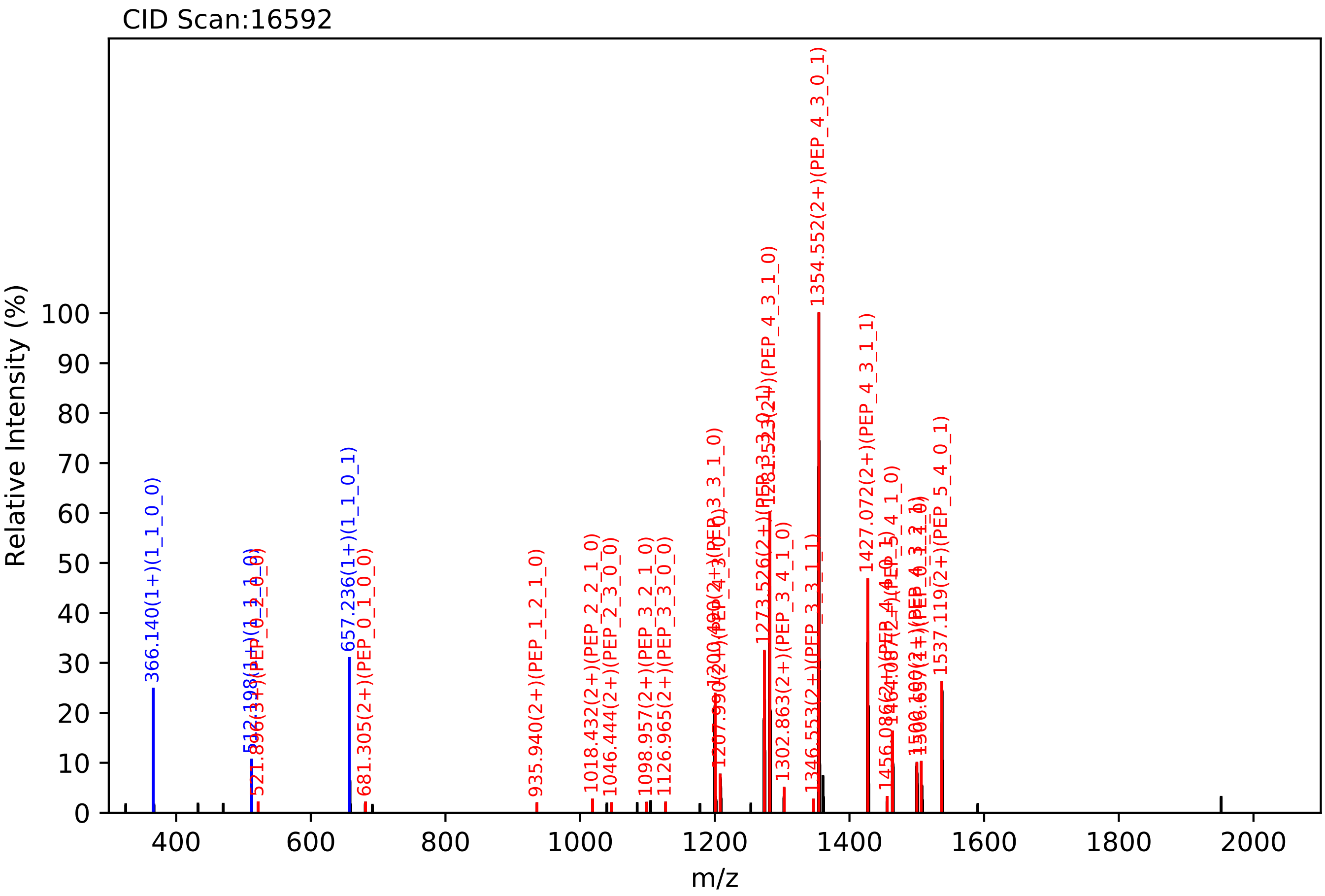

Unknown set no. 486, Experiment: Human Plasma exp_3

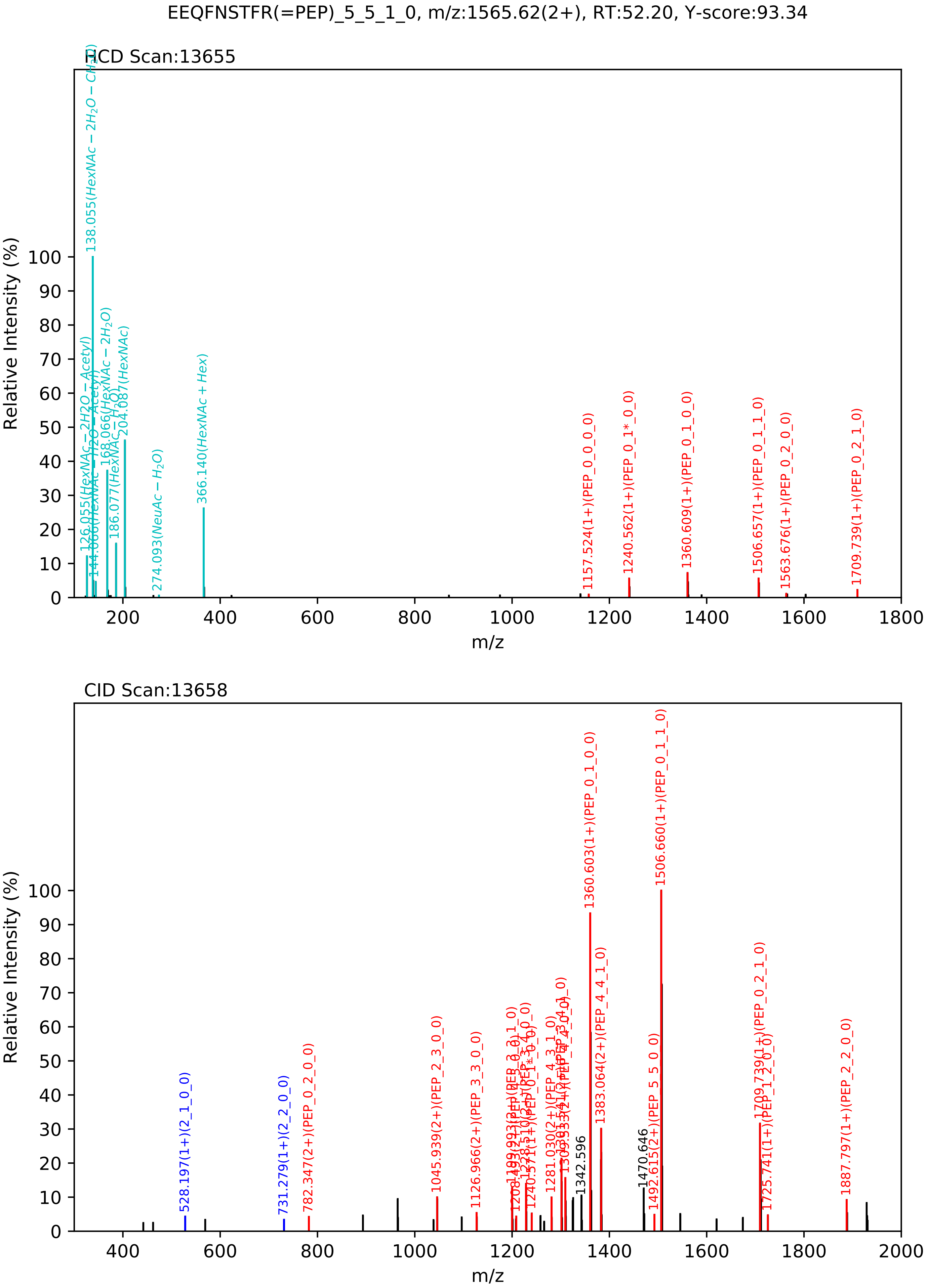

Unknown set no. 487, Experiment: Human Plasma exp_1

EEQFNSTFR(=PEP)_6_3_1_1, m/z:1059.42(3+), RT:60.74, Y-score:88.95

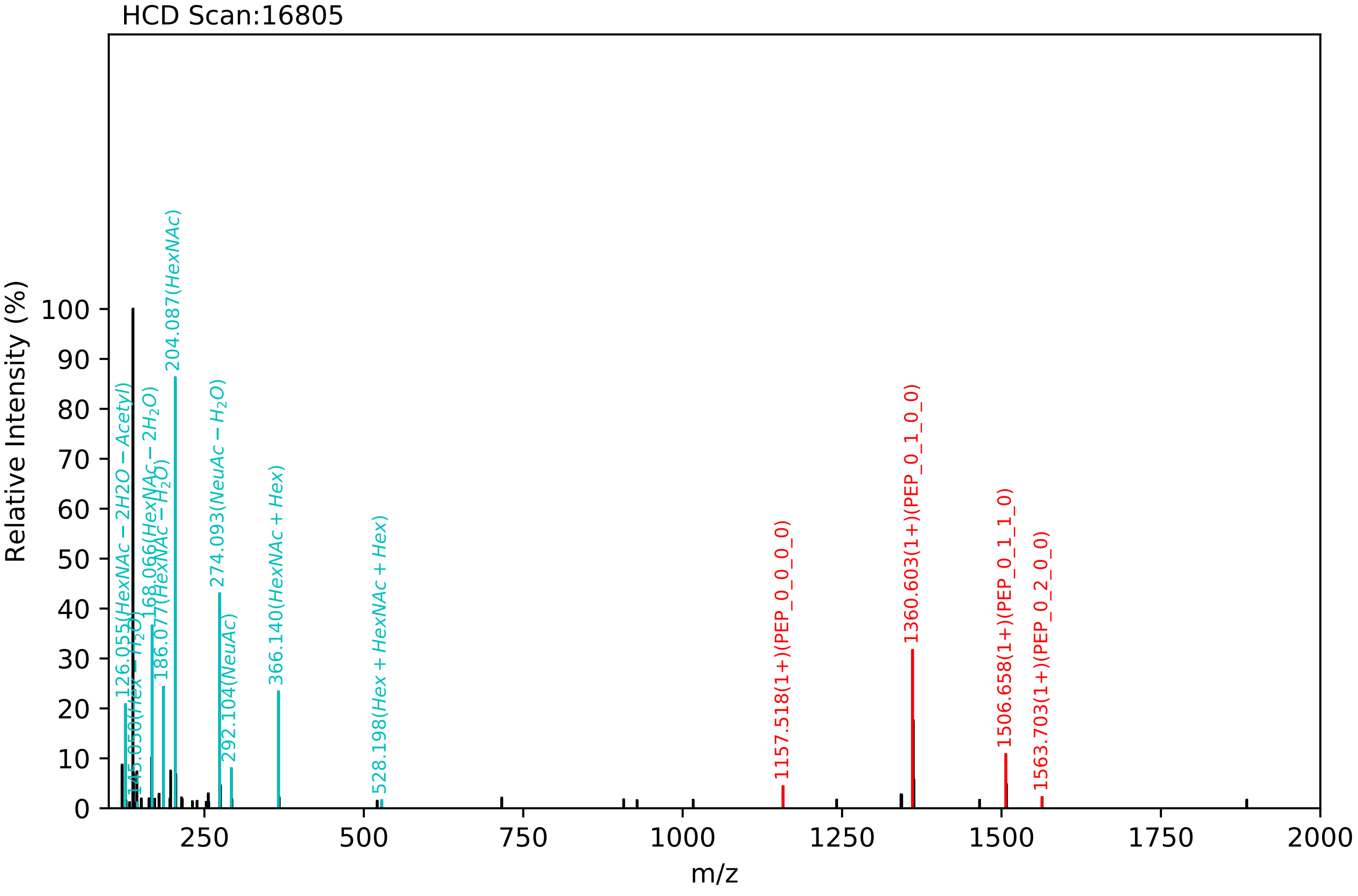

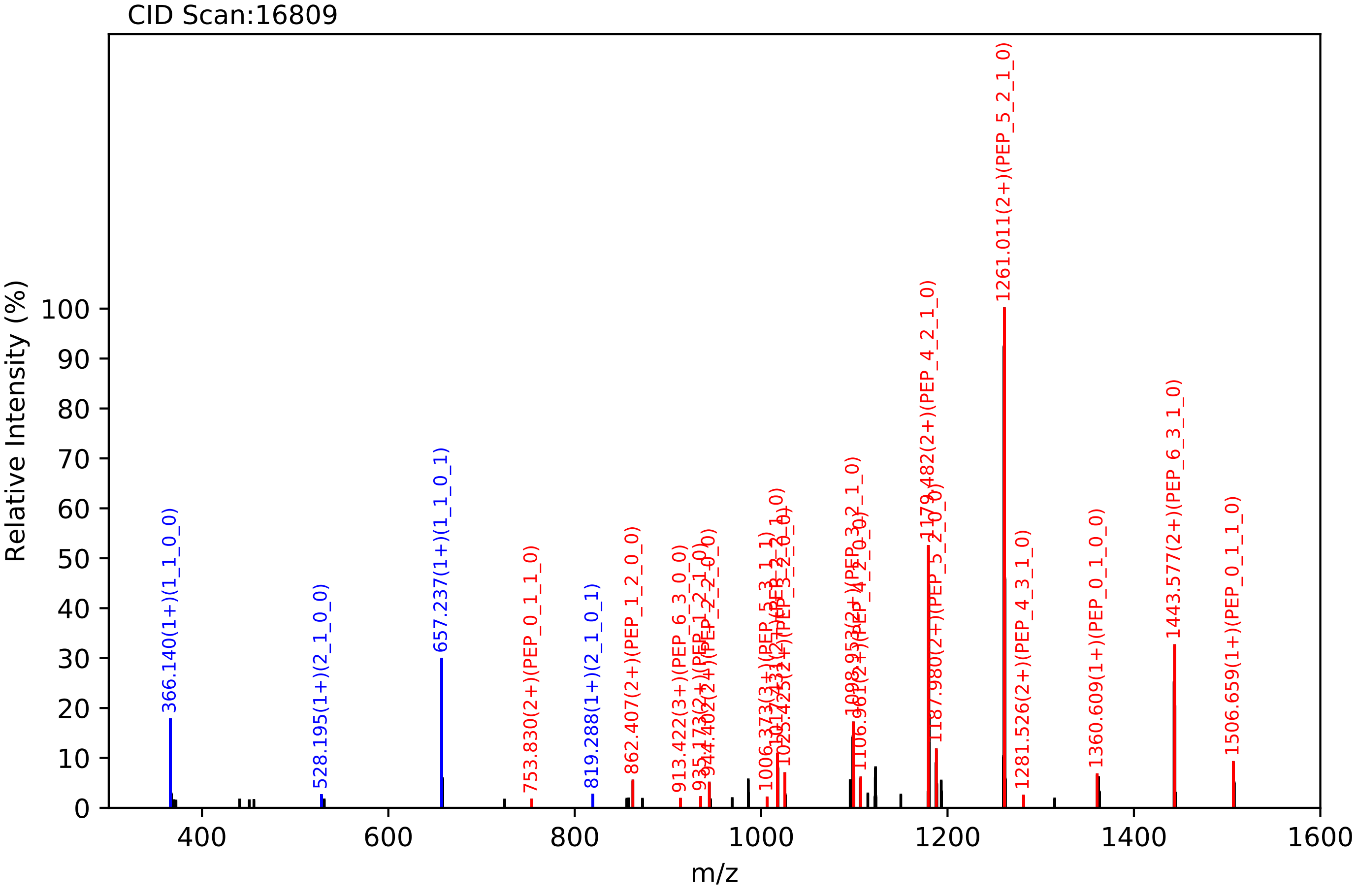

Unknown set no. 488, Experiment: Human Plasma exp_4

EEQFNSTFR(=PEP)_6_3_1_1, m/z:1059.42(3+), RT:60.94, Y-score:82.98

HCD Scan:17192

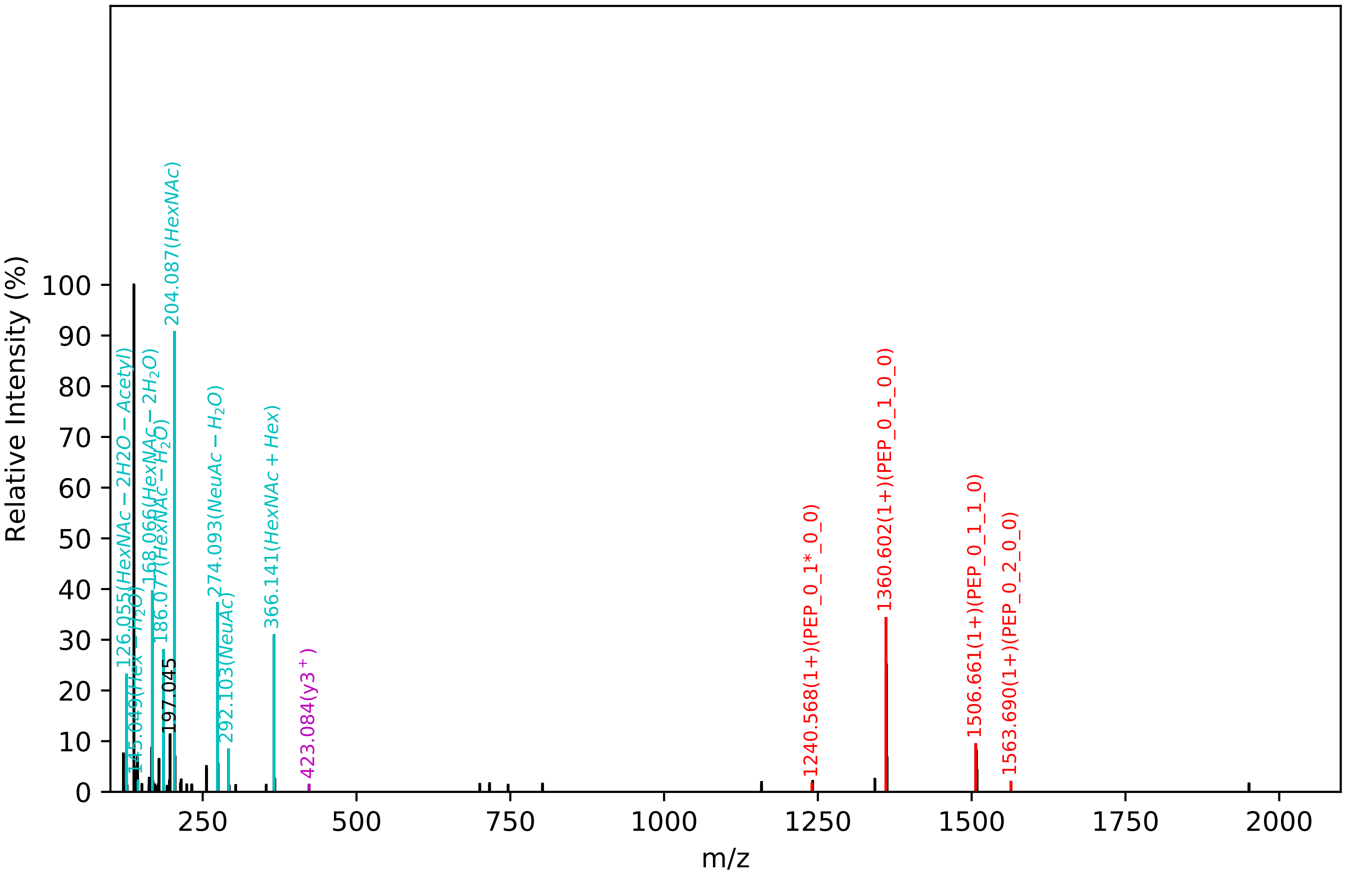

CID Scan:17195

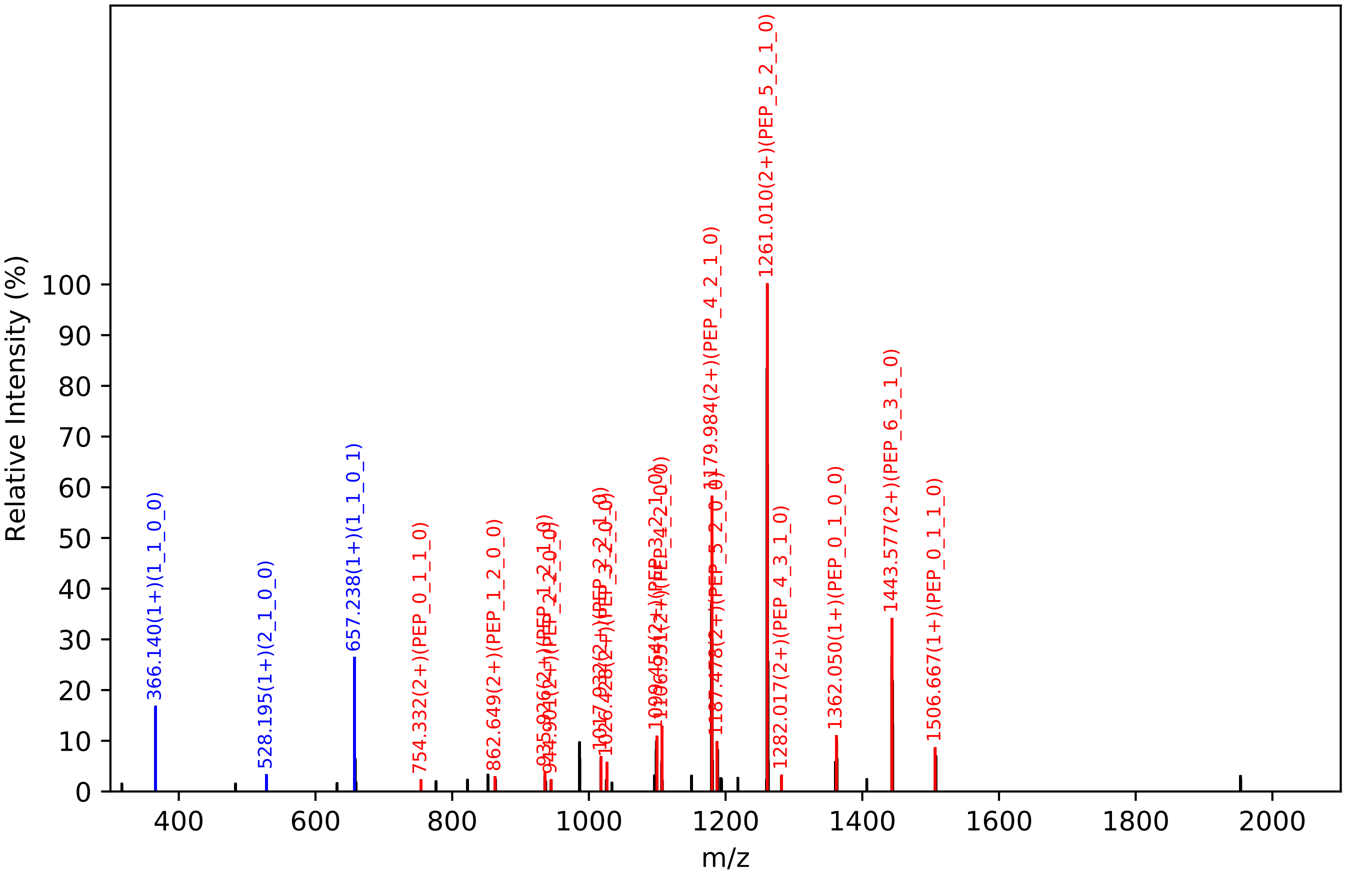

Unknown set no. 489, Experiment: Human Plasma exp_5

EEQFNSTFR(=PEP)_6_3_1_1, m/z:1059.42(3+), RT:60.90, Y-score:91.06

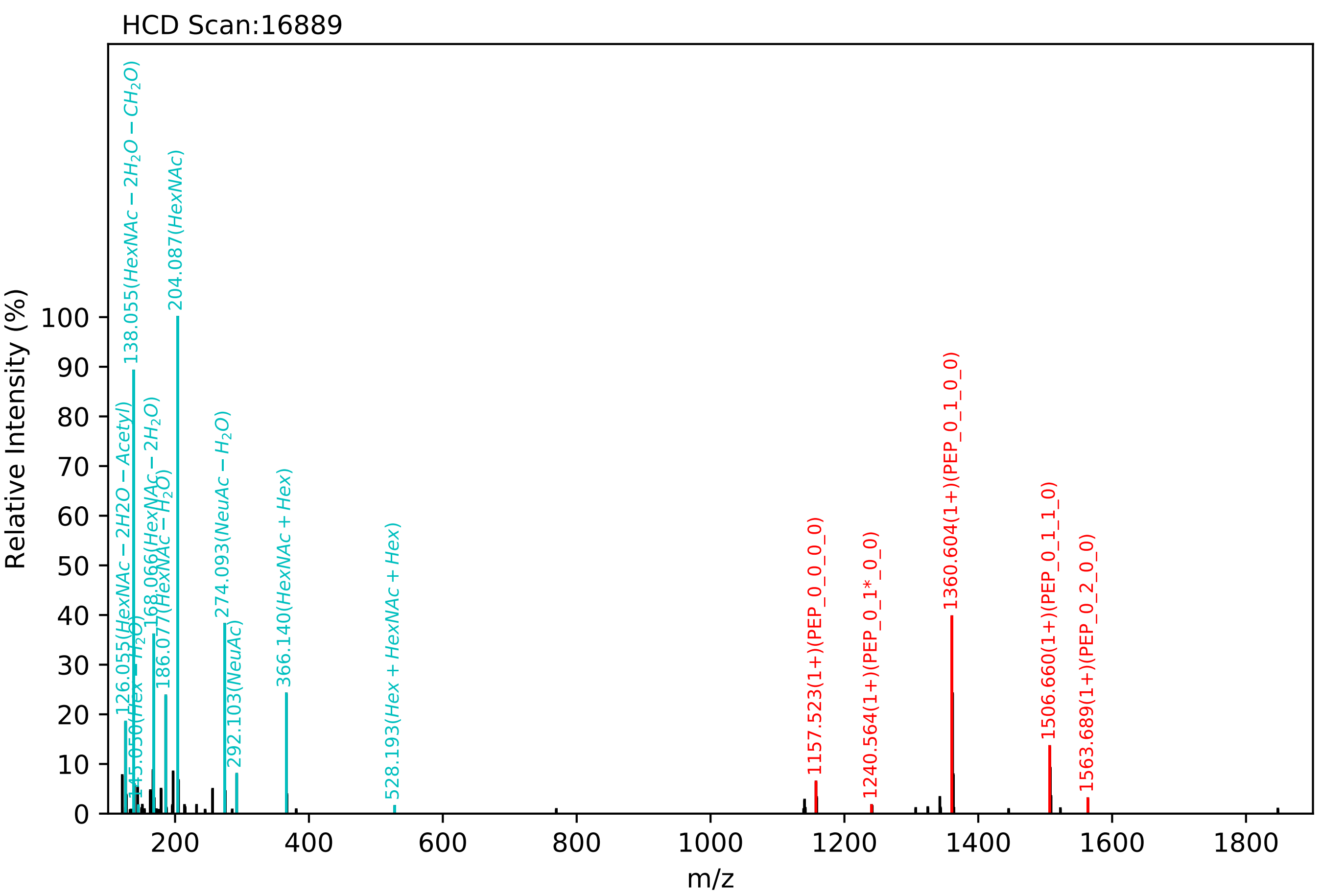

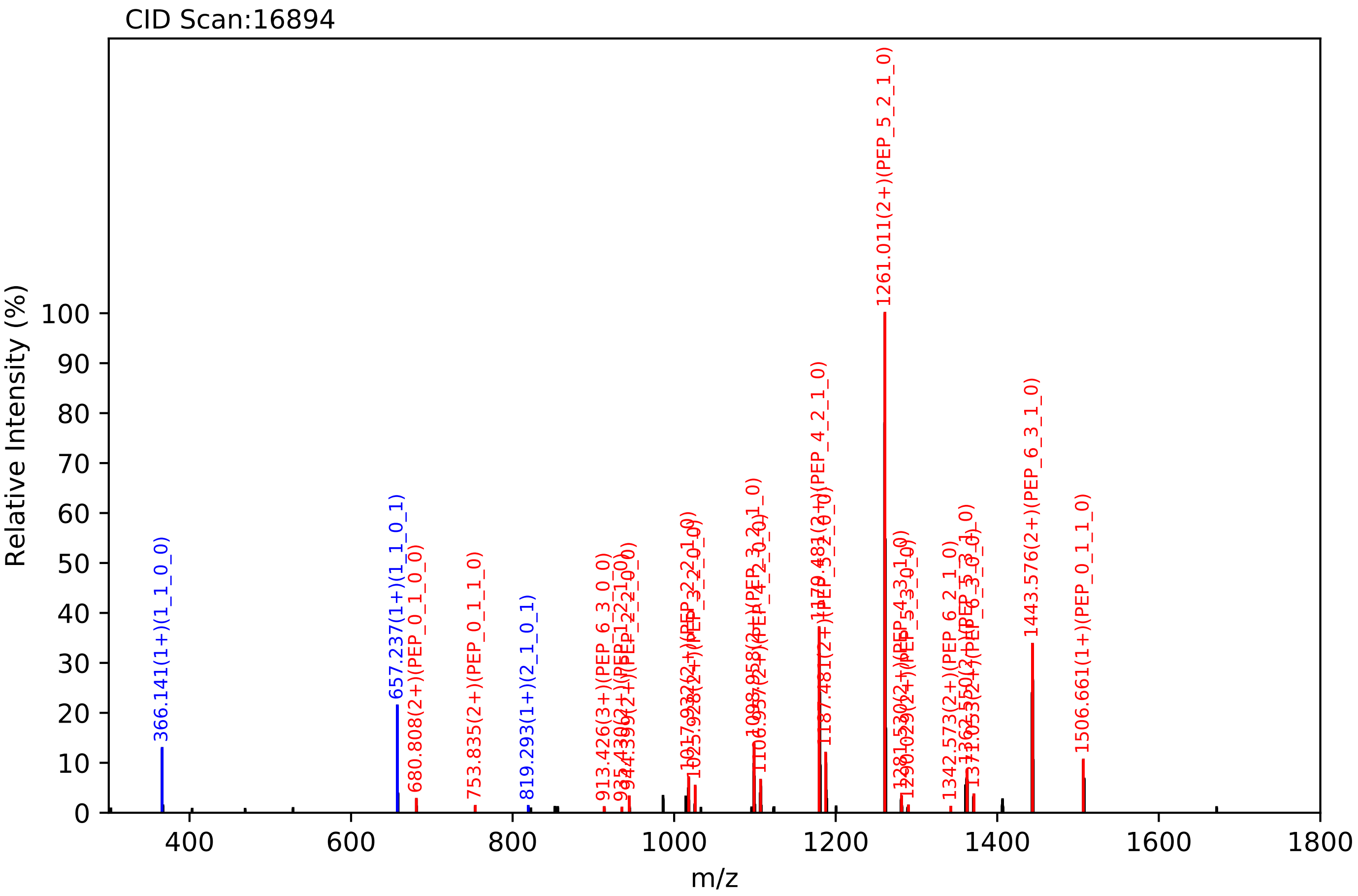

Unknown set no. 490, Experiment: Human Plasma exp_1

EEQYNSTFR(=PEP)_3_4_1_0, m/z:1309.53(2+), RT:41.08, Y-score:87.51

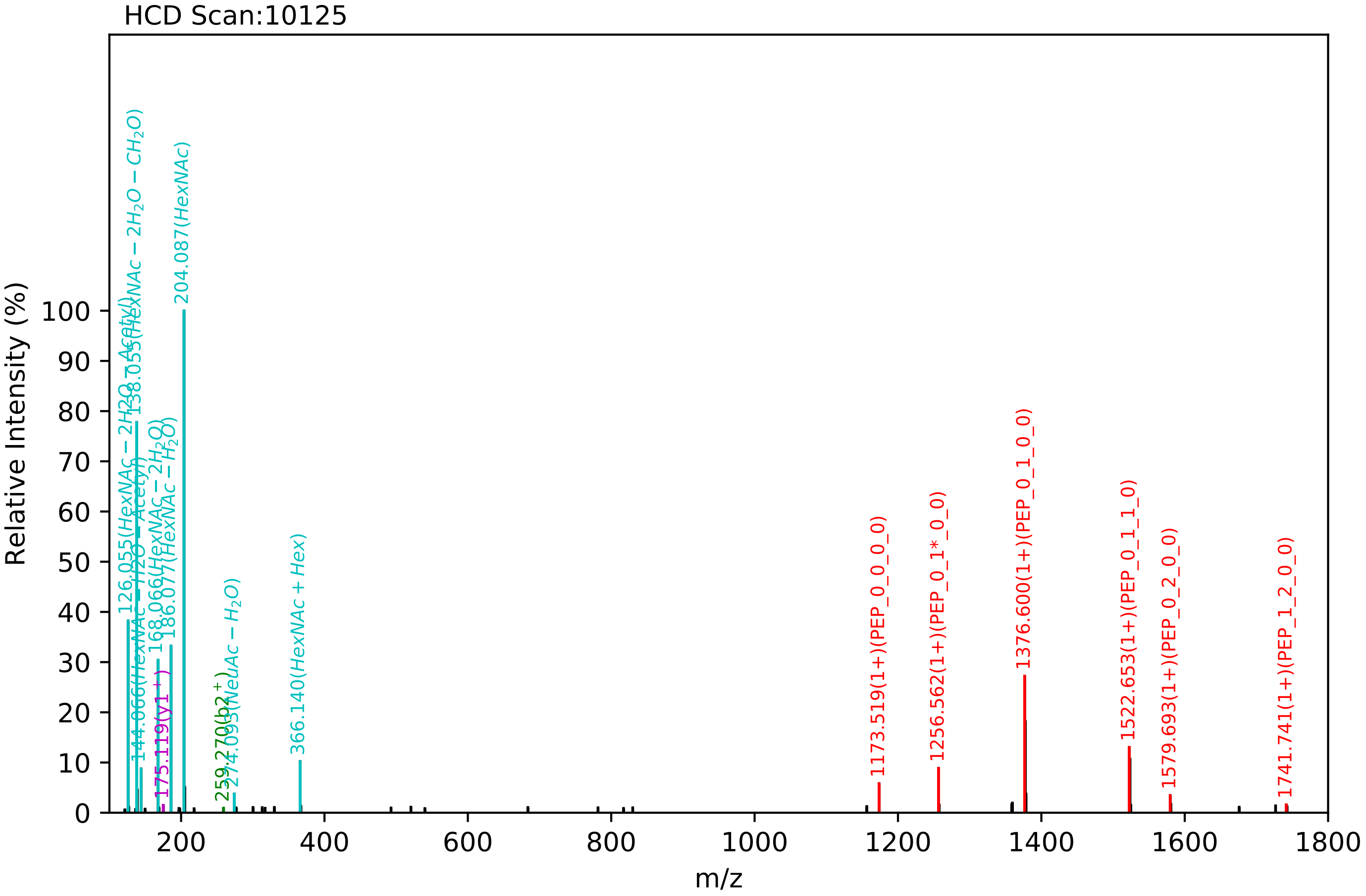

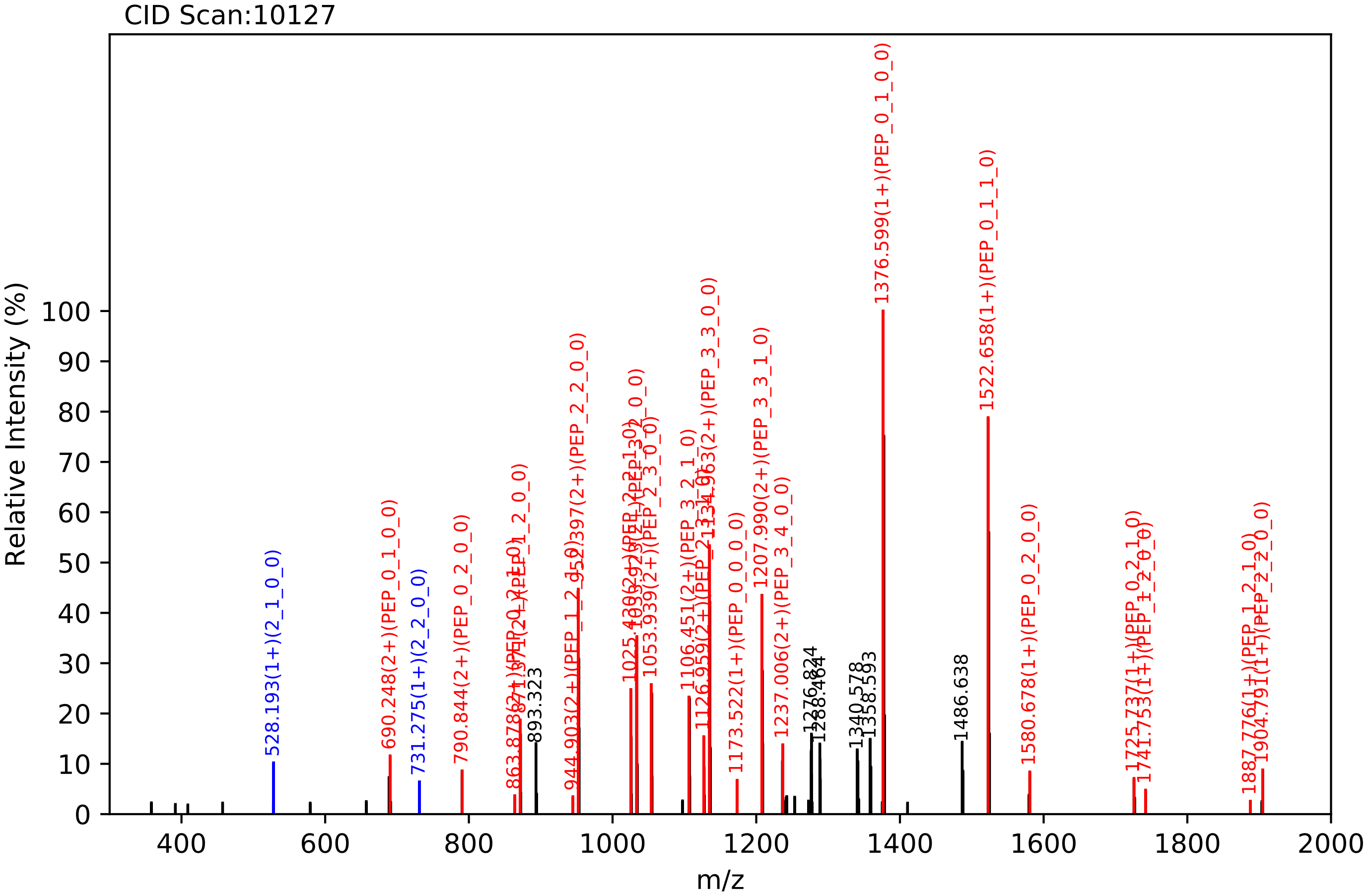

Unknown set no. 491, Experiment: Human Plasma exp_3

EEQYNSTFR(=PEP)_3_4_1_0, m/z:1309.53(2+), RT:40.48, Y-score:92.72

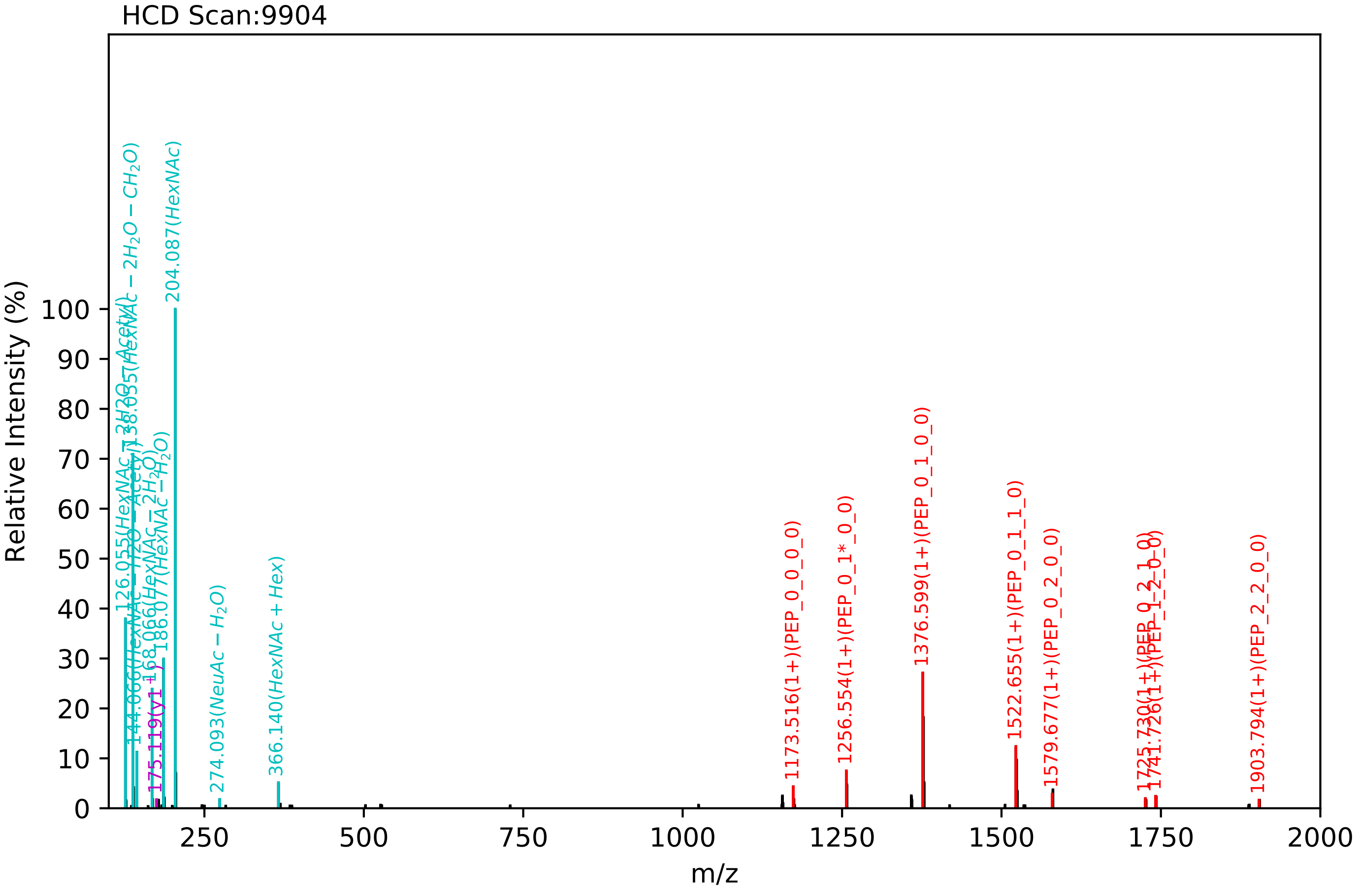

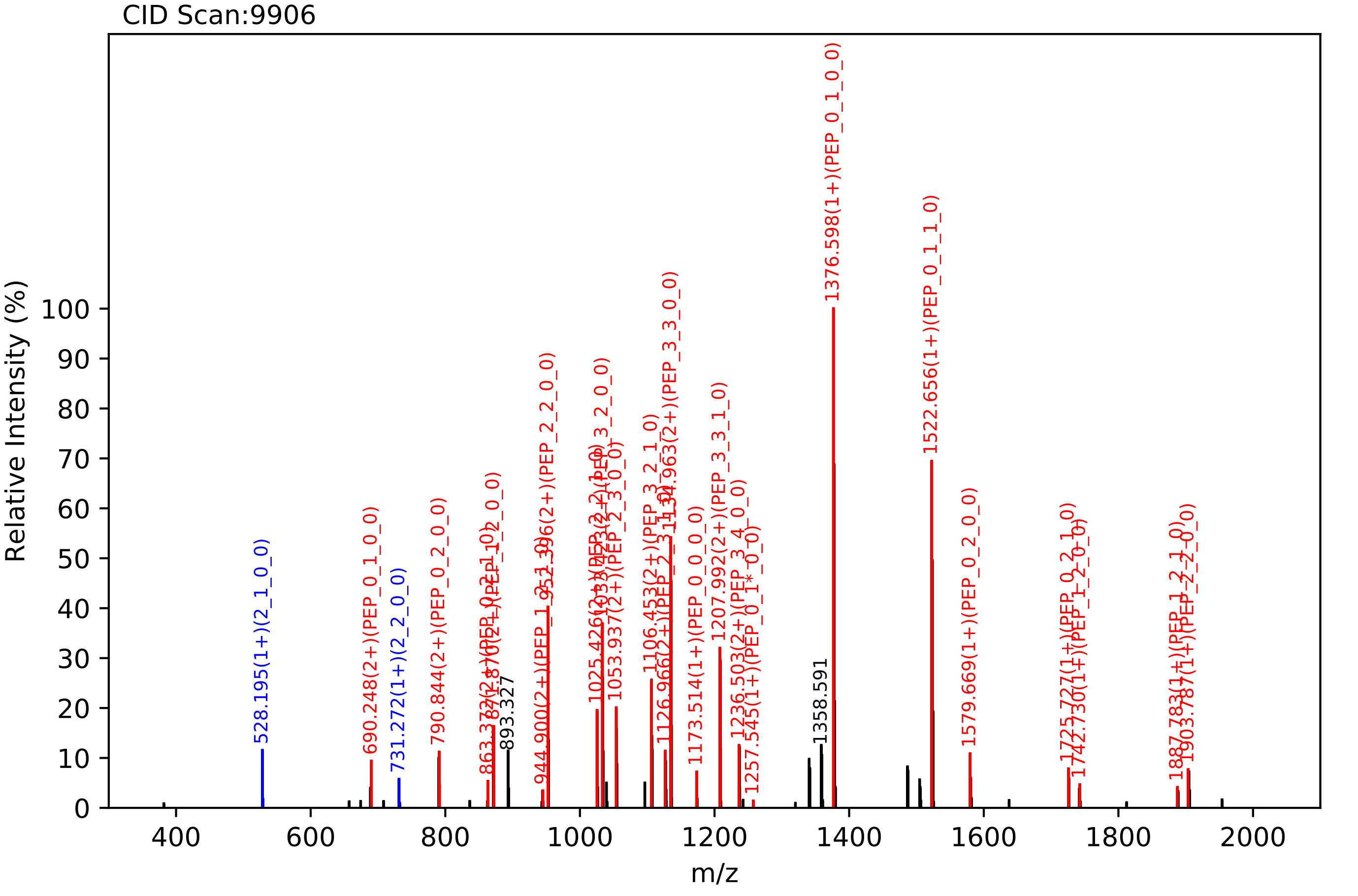

Unknown set no. 492, Experiment: Human Plasma exp_4

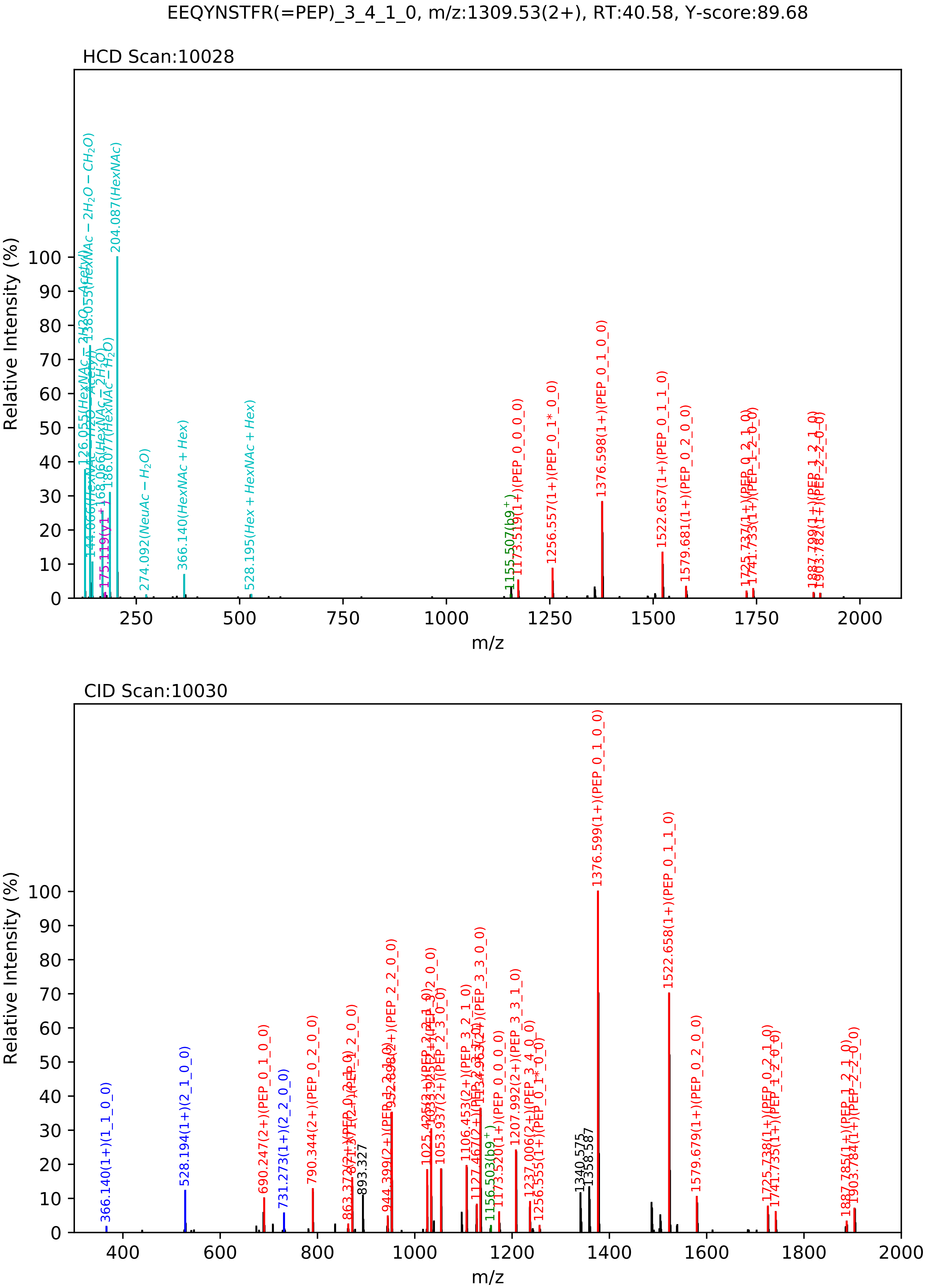

Unknown set no. 493, Experiment: Human Plasma exp_5

EEQYNSTFR(=PEP)_3_4_1_0, m/z:1309.53(2+), RT:41.72, Y-score:87.71

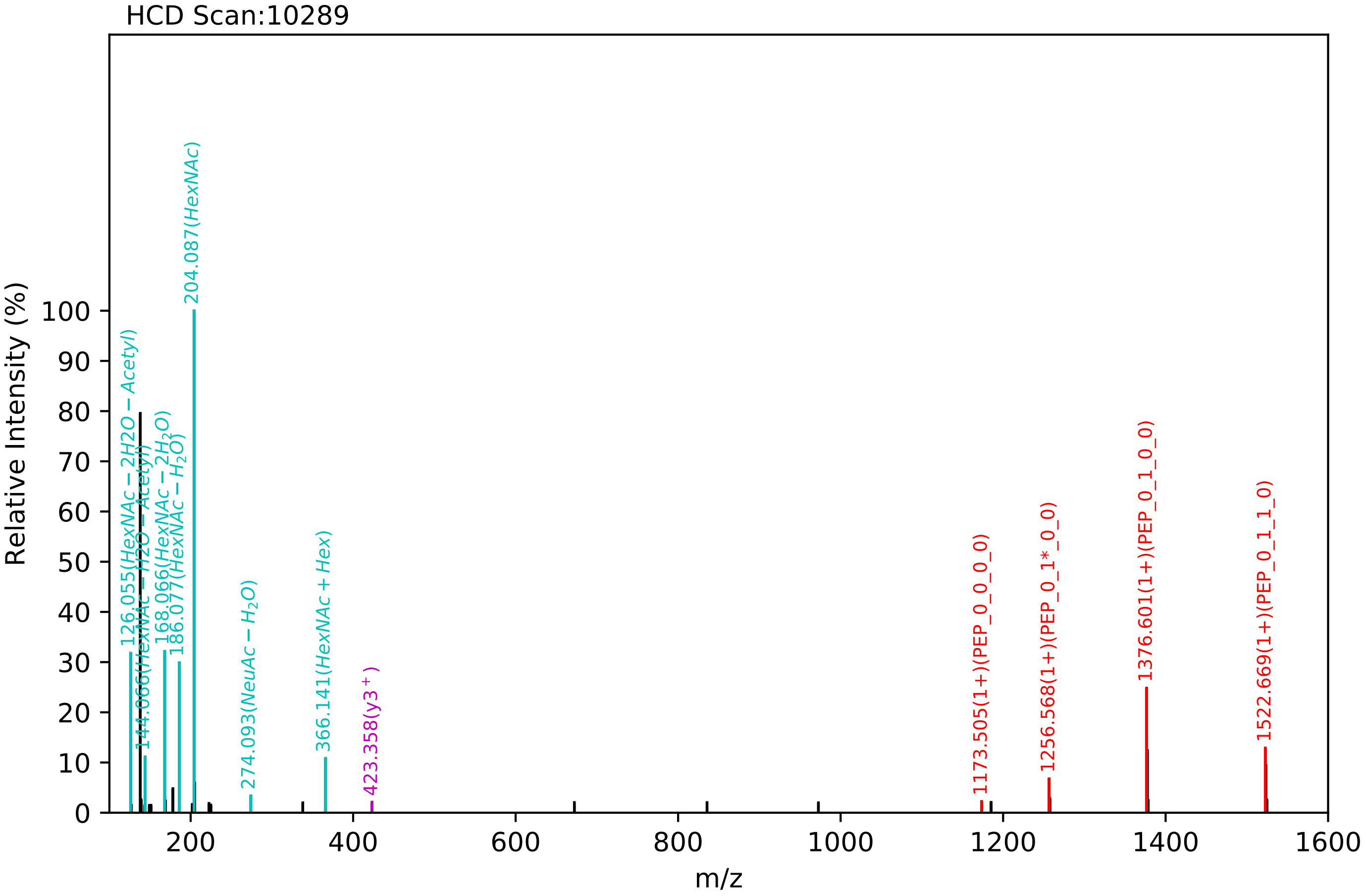

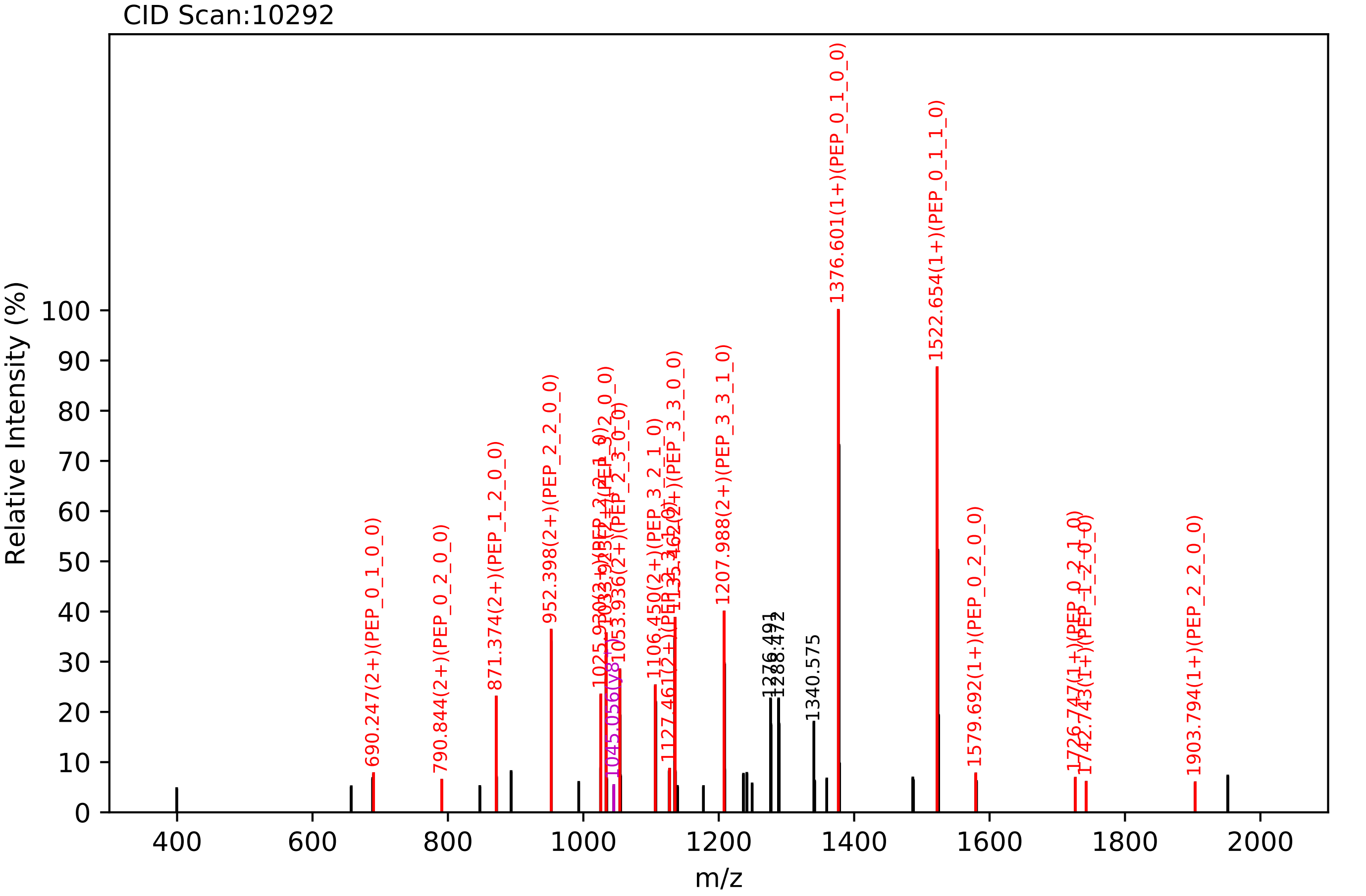

Unknown set no. 494, Experiment: Human Plasma exp_1

EEQYNSTFR(=PEP)_4_3_1_0, m/z:1289.02(2+), RT:39.25, Y-score:95.83

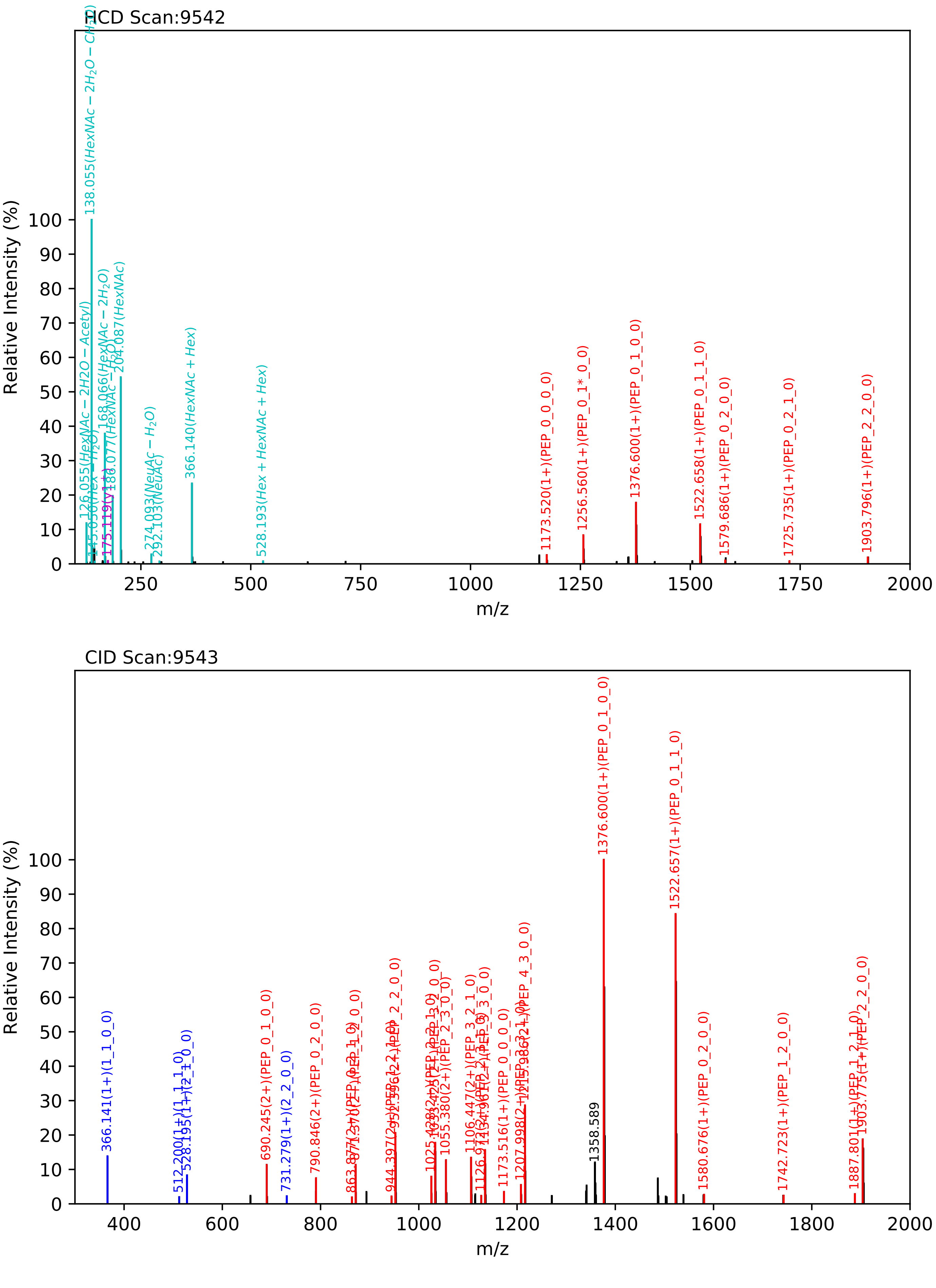

Unknown set no. 495, Experiment: Human Plasma exp_3

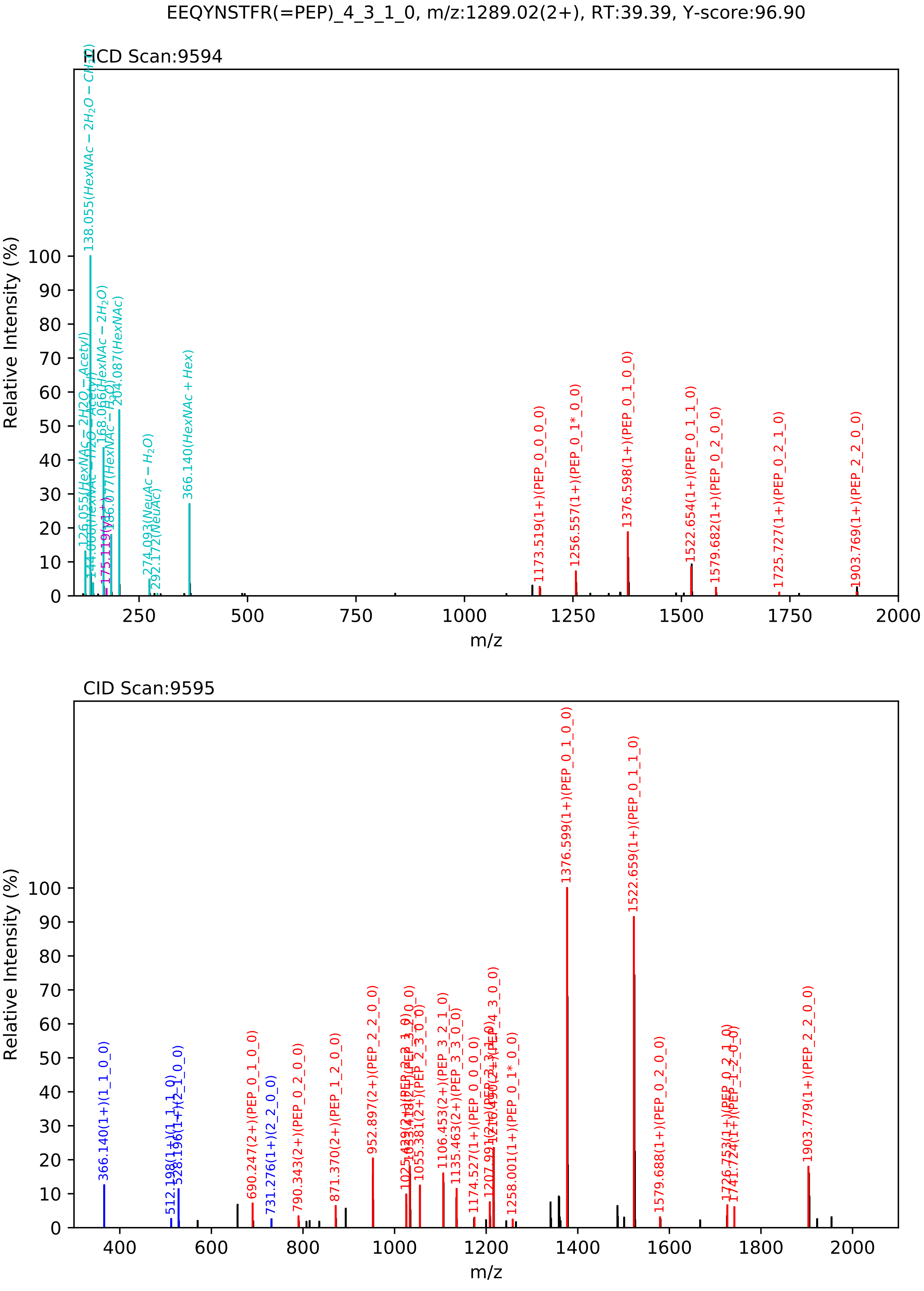

Unknown set no. 496, Experiment: Human Plasma exp_5

EEQYNSTFR(=PEP)_4_3_1_0, m/z:1289.02(2+), RT:39.82, Y-score:94.47

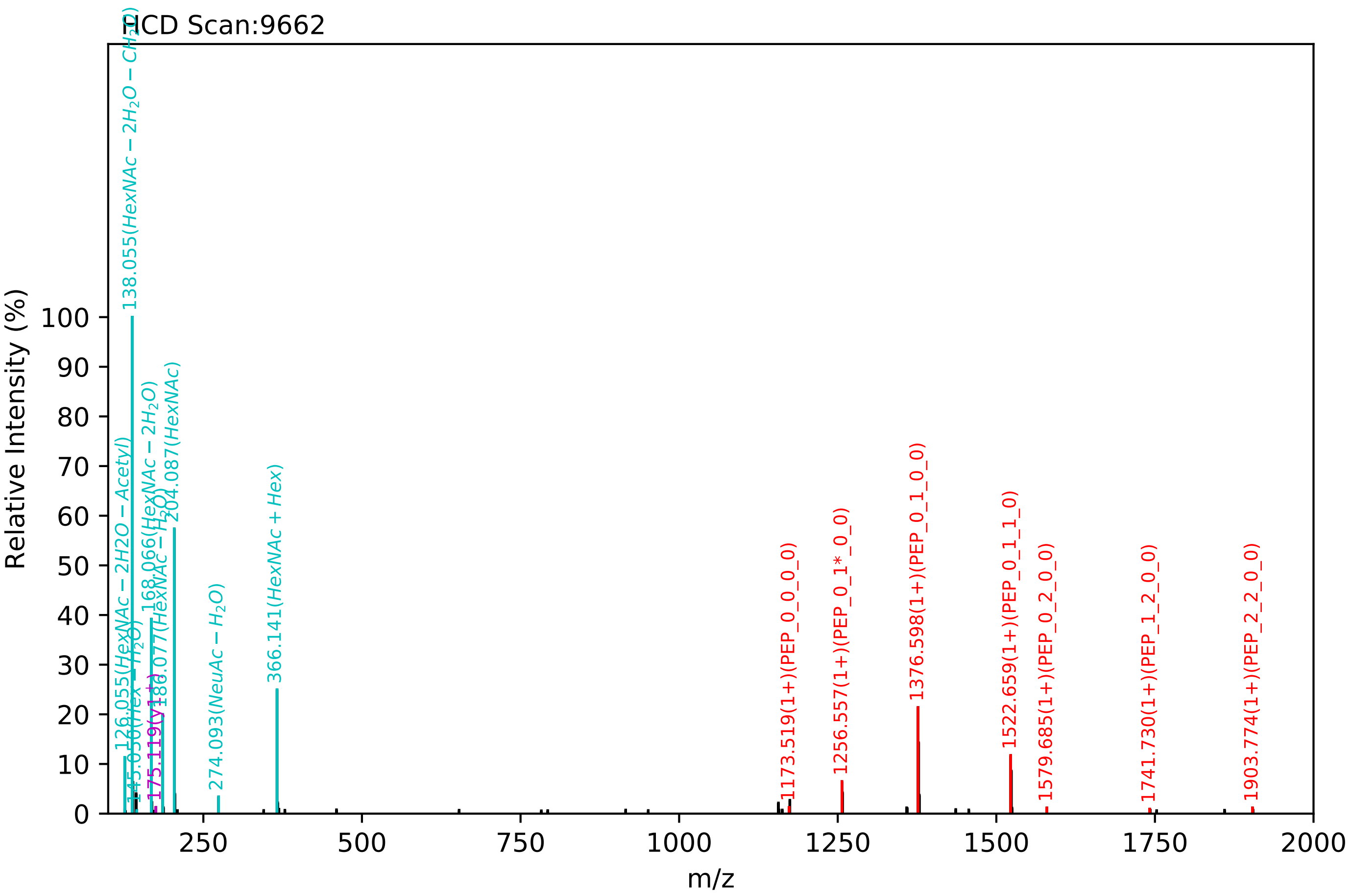

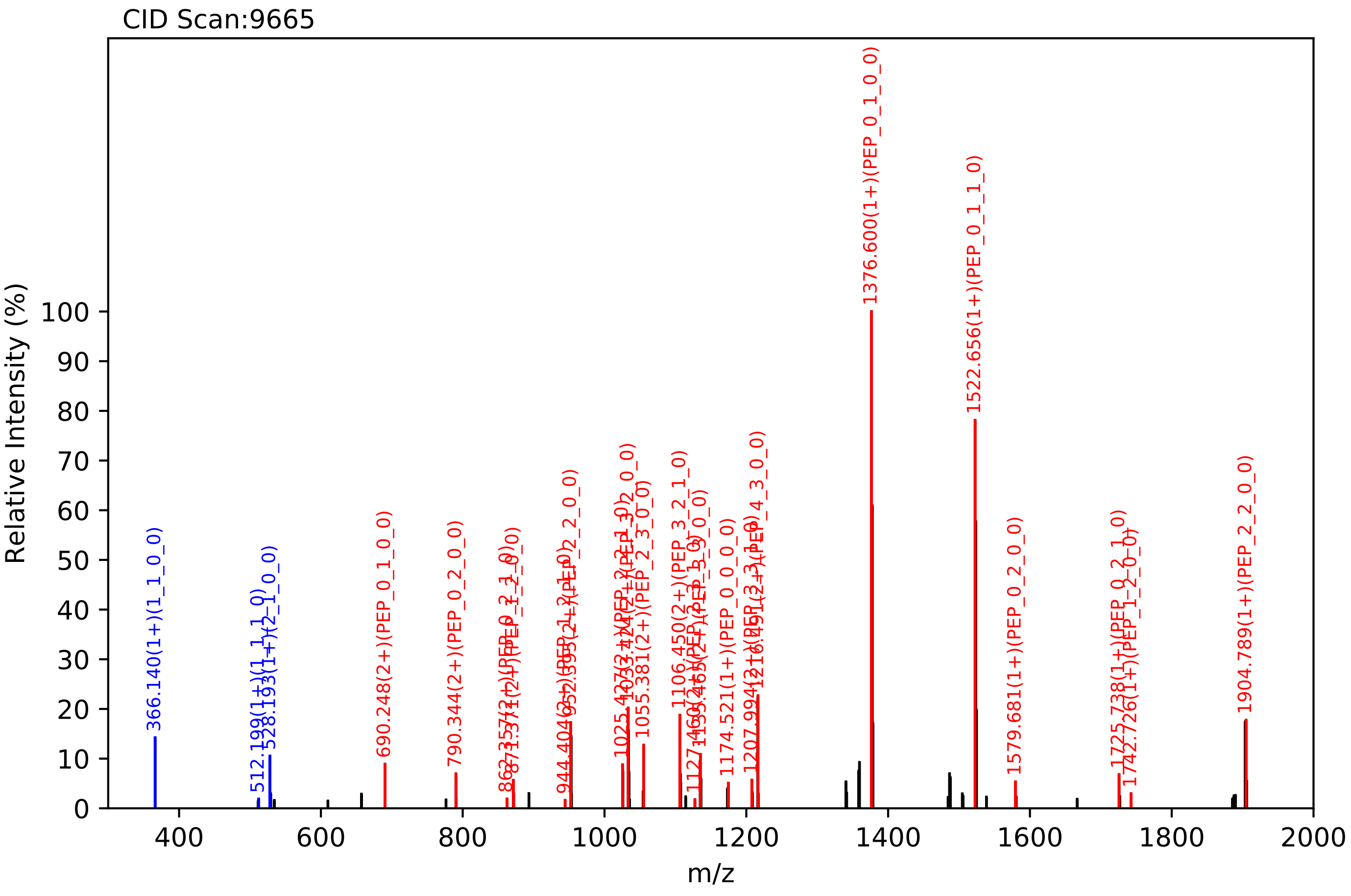

Unknown set no. 497, Experiment: Human Plasma exp_1

EEQYNSTFR(=PEP)_5_4_1_0, m/z:1472.08(2+), RT:39.11, Y-score:96.10

HCD Scan:9504

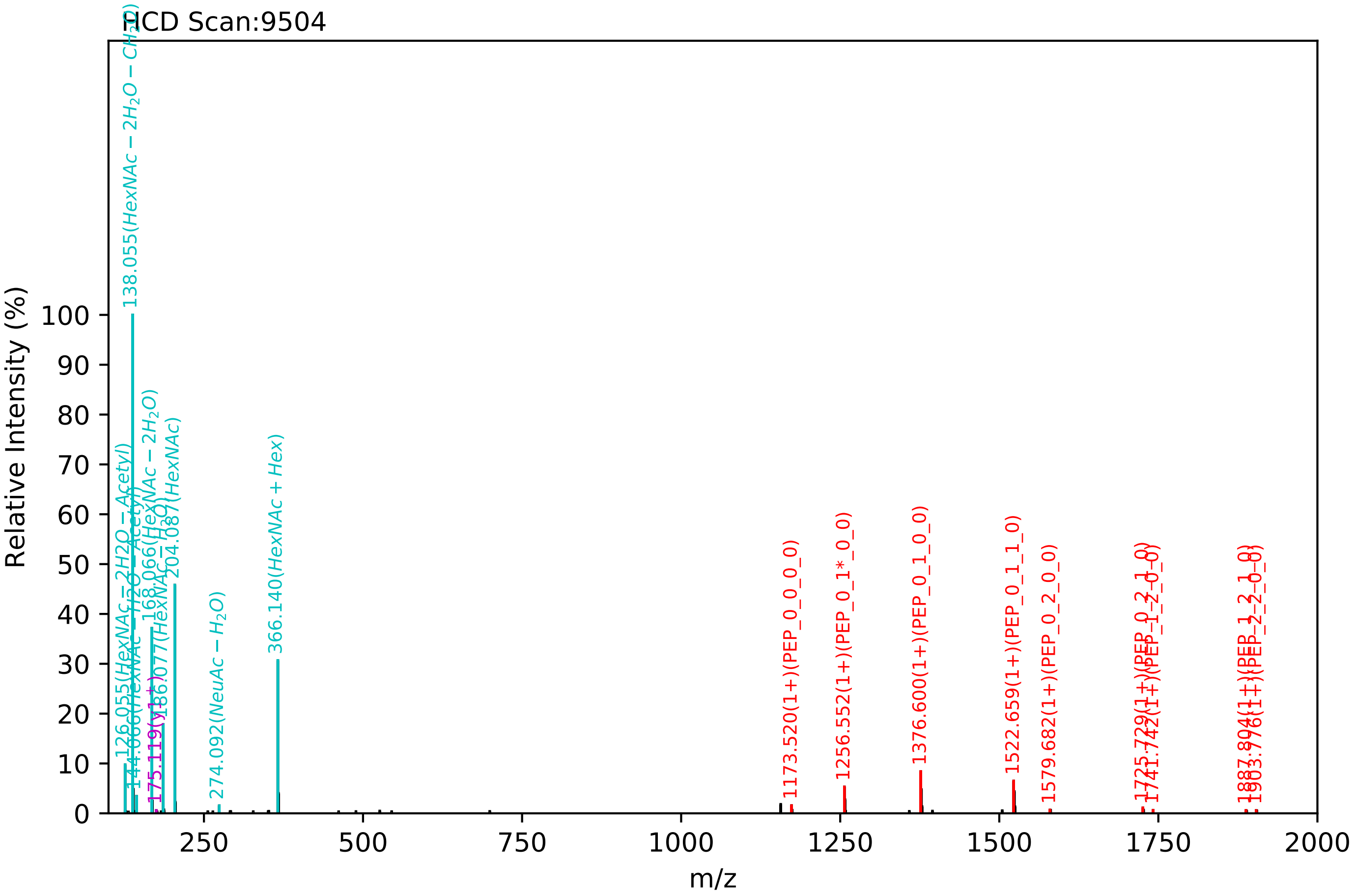

CID Scan:9505

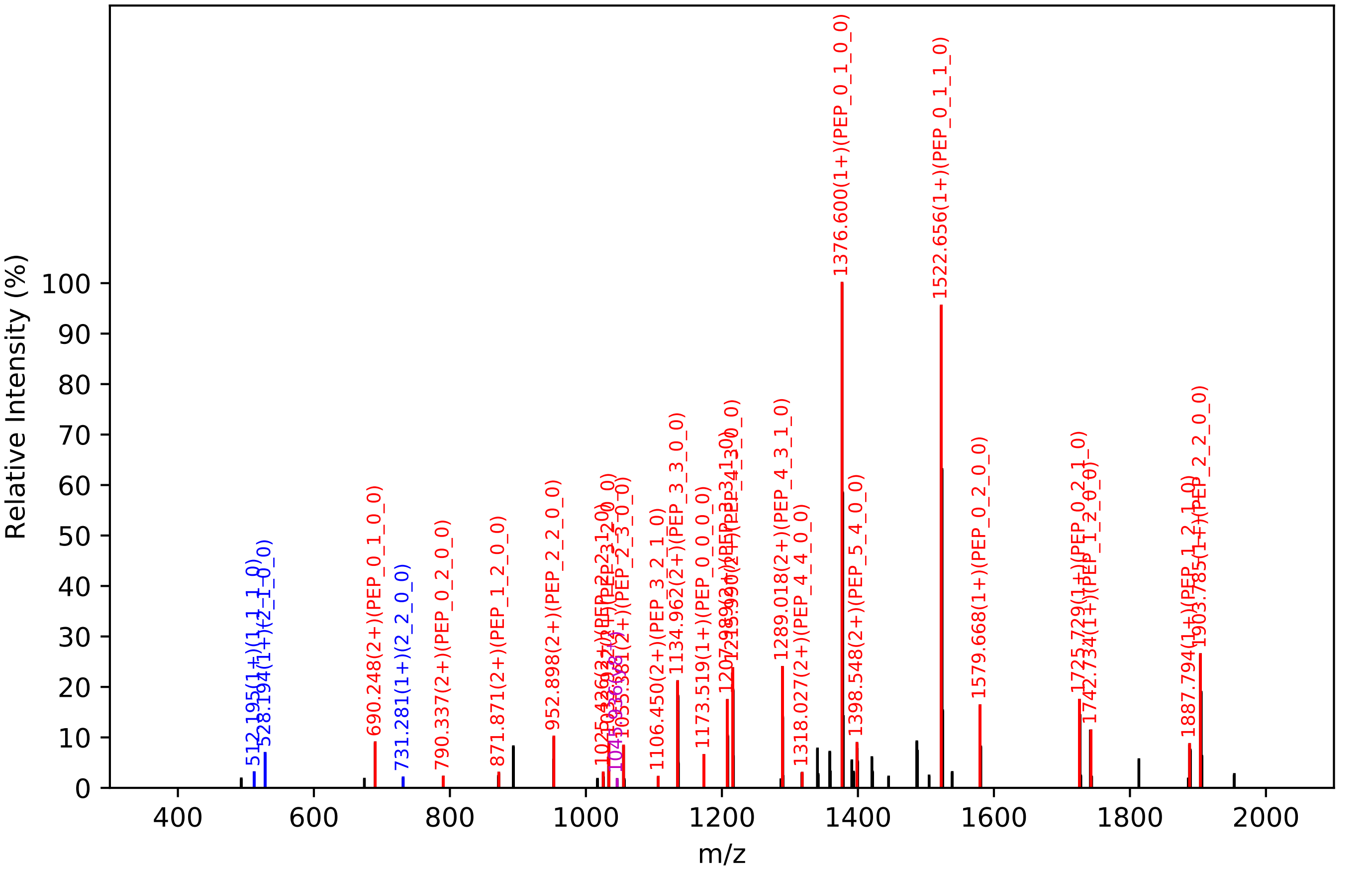

Unknown set no. 498, Experiment: Human Plasma exp_2

EEQYNSTFR(=PEP)_5_4_1_0, m/z:1471.58(2+), RT:39.78, Y-score:96.79

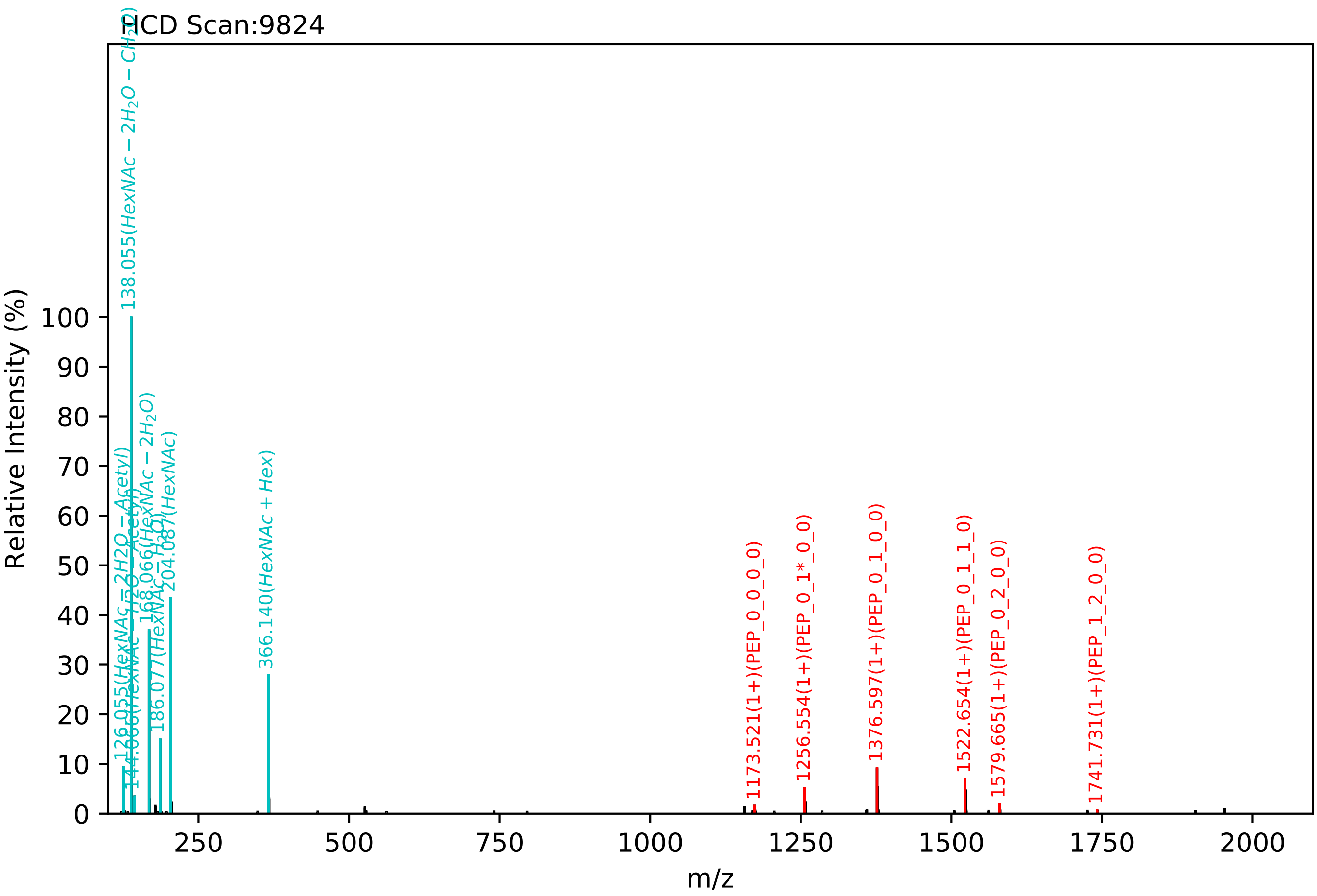

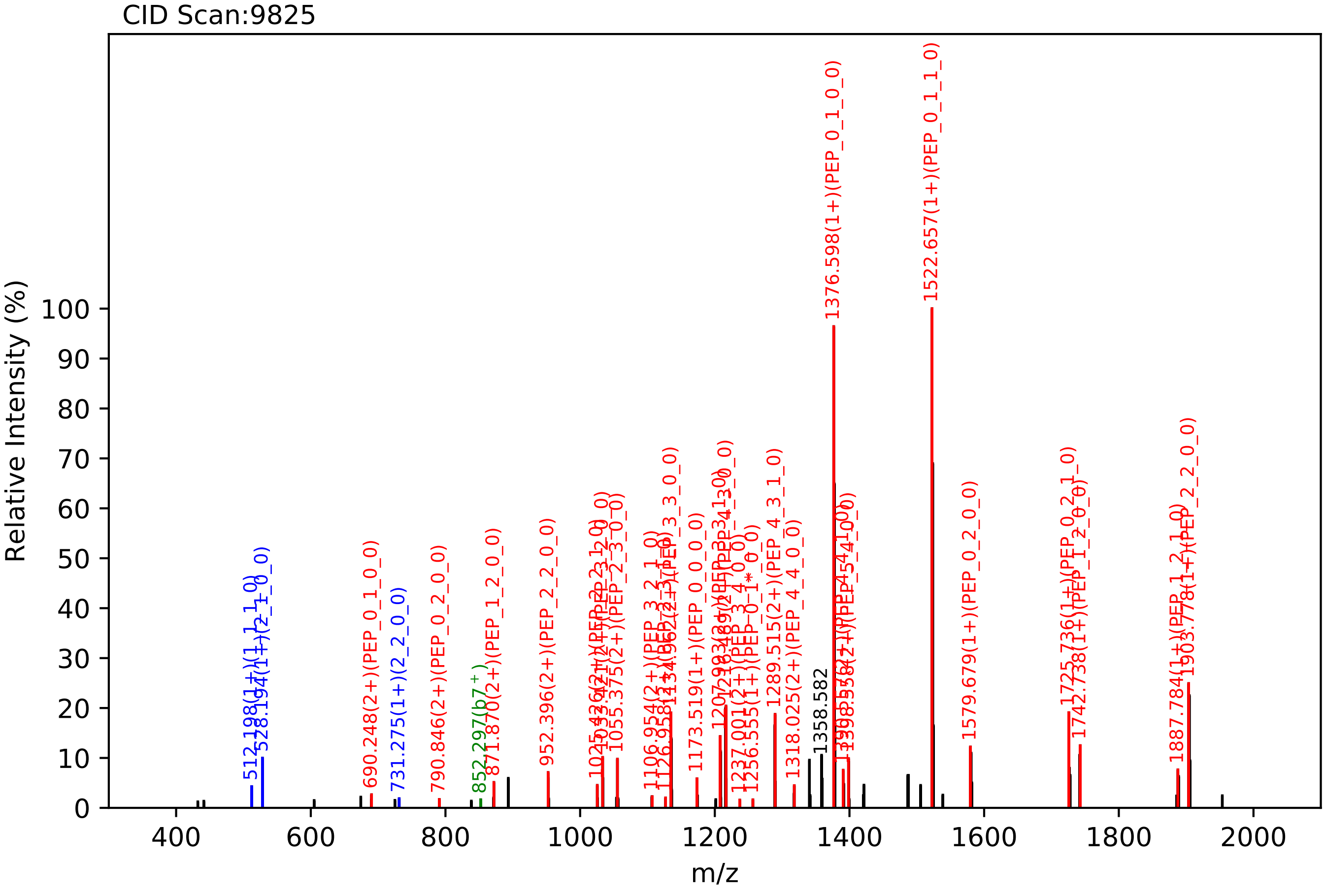

Unknown set no. 499, Experiment: Human Plasma exp_3

EEQYNSTFR(=PEP)_5_4_1_0, m/z:1471.58(2+), RT:39.02, Y-score:81.42

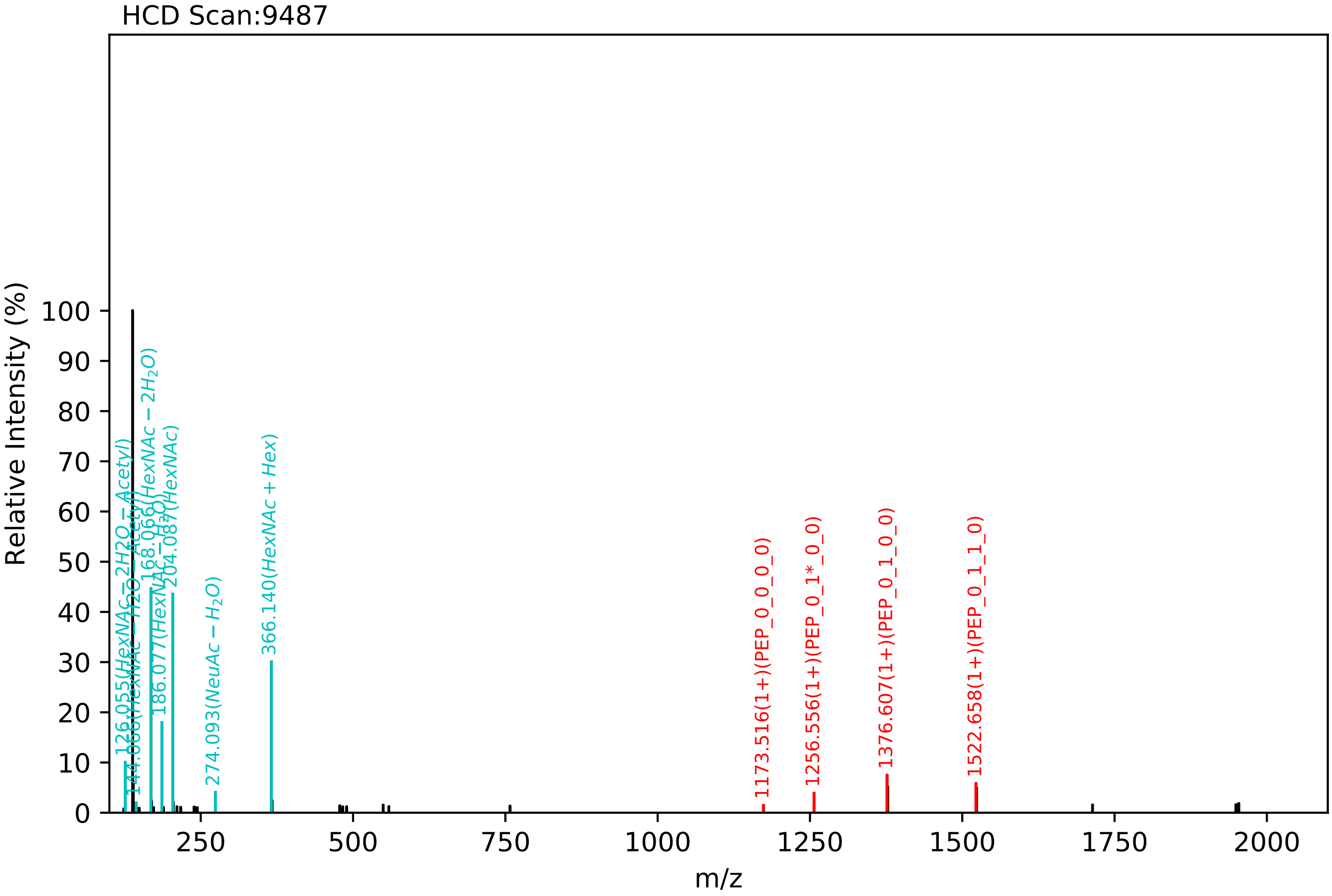

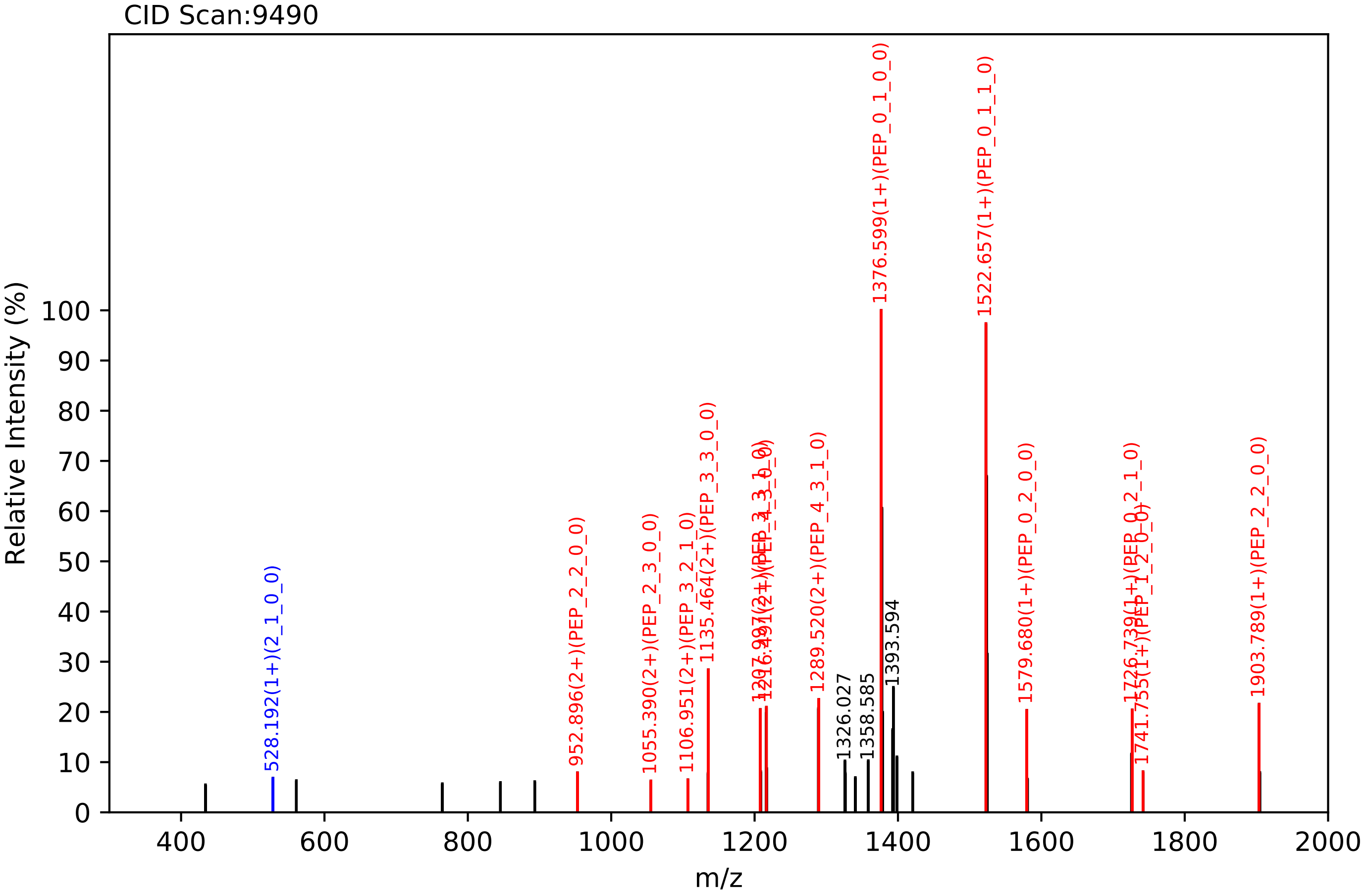

Unknown set no. 500, Experiment: Human Plasma exp_5

EEQYNSTFR(=PEP)_5_4_1_0, m/z:1471.58(2+), RT:39.62, Y-score:86.35

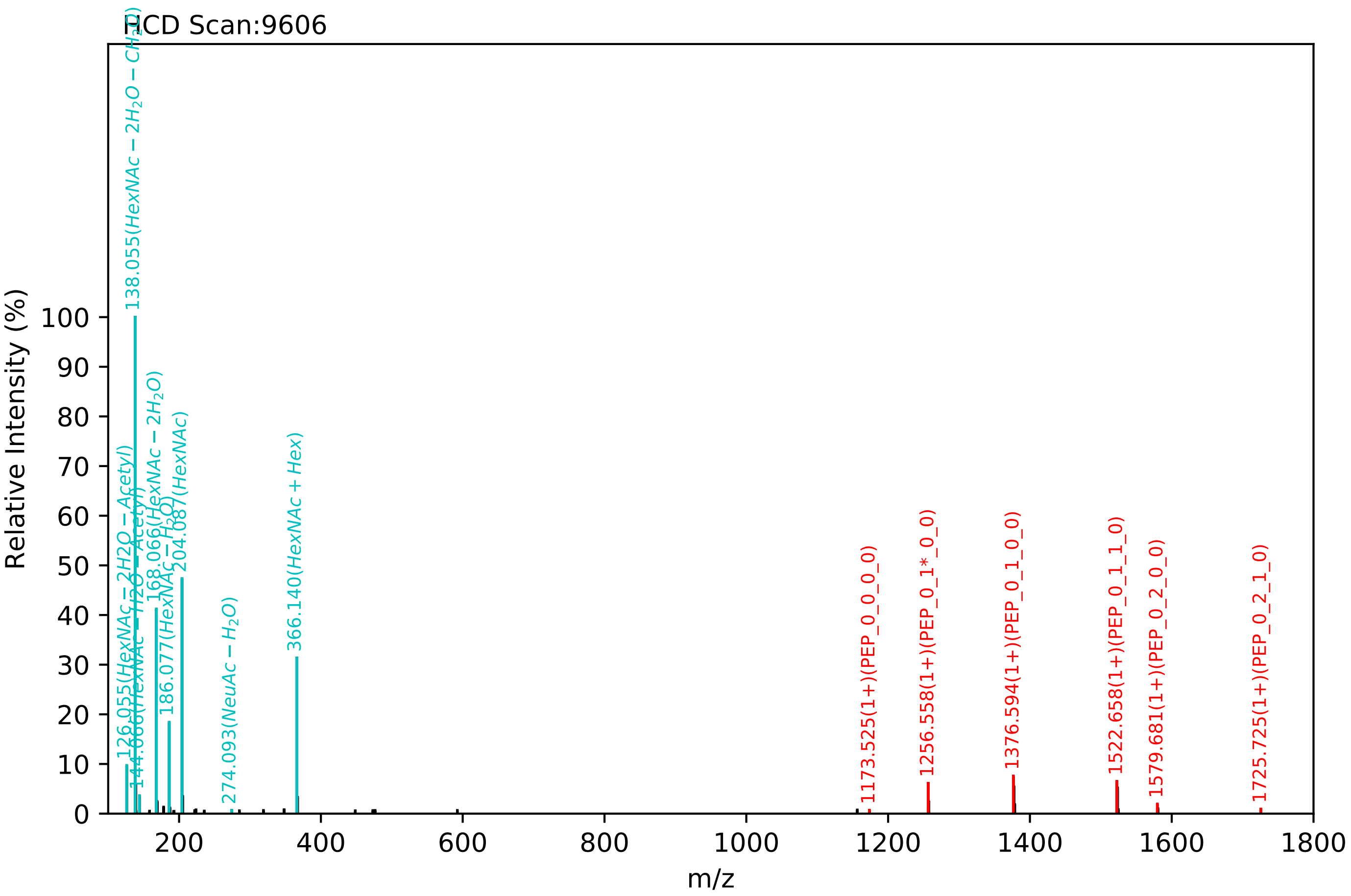

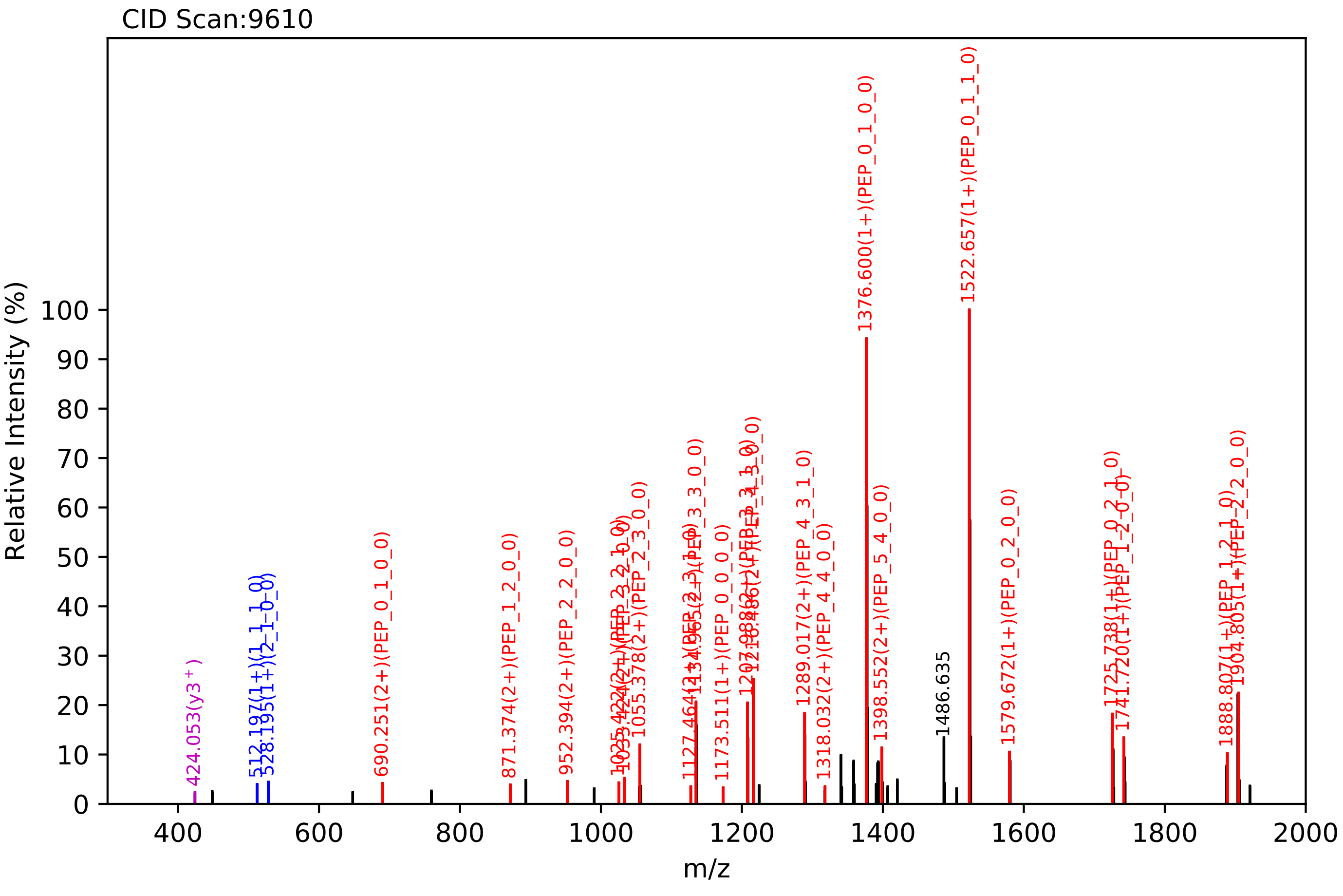

Unknown set no. 501, Experiment: Human Plasma exp_1

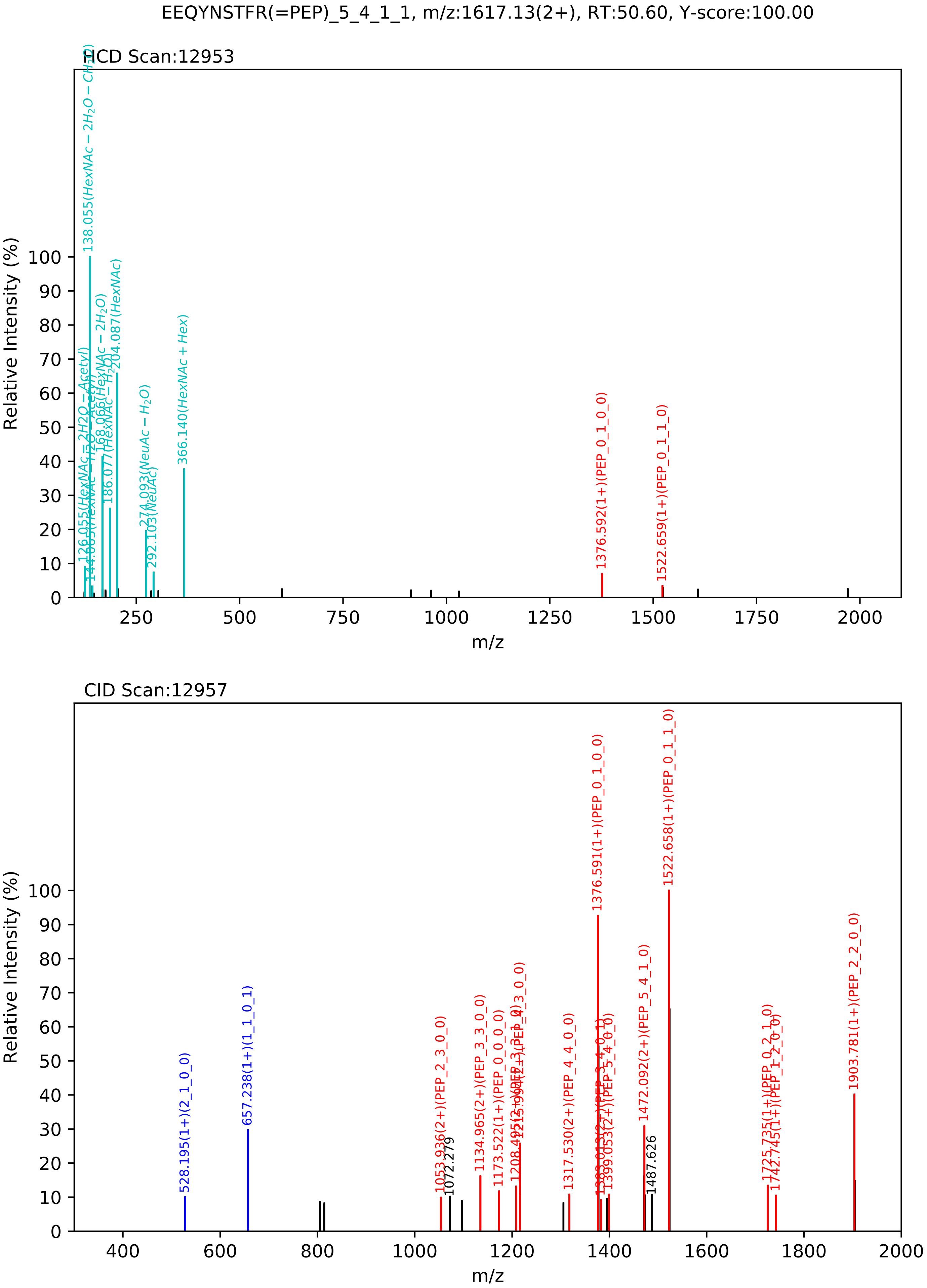

Unknown set no. 502, Experiment: Human Plasma exp_2

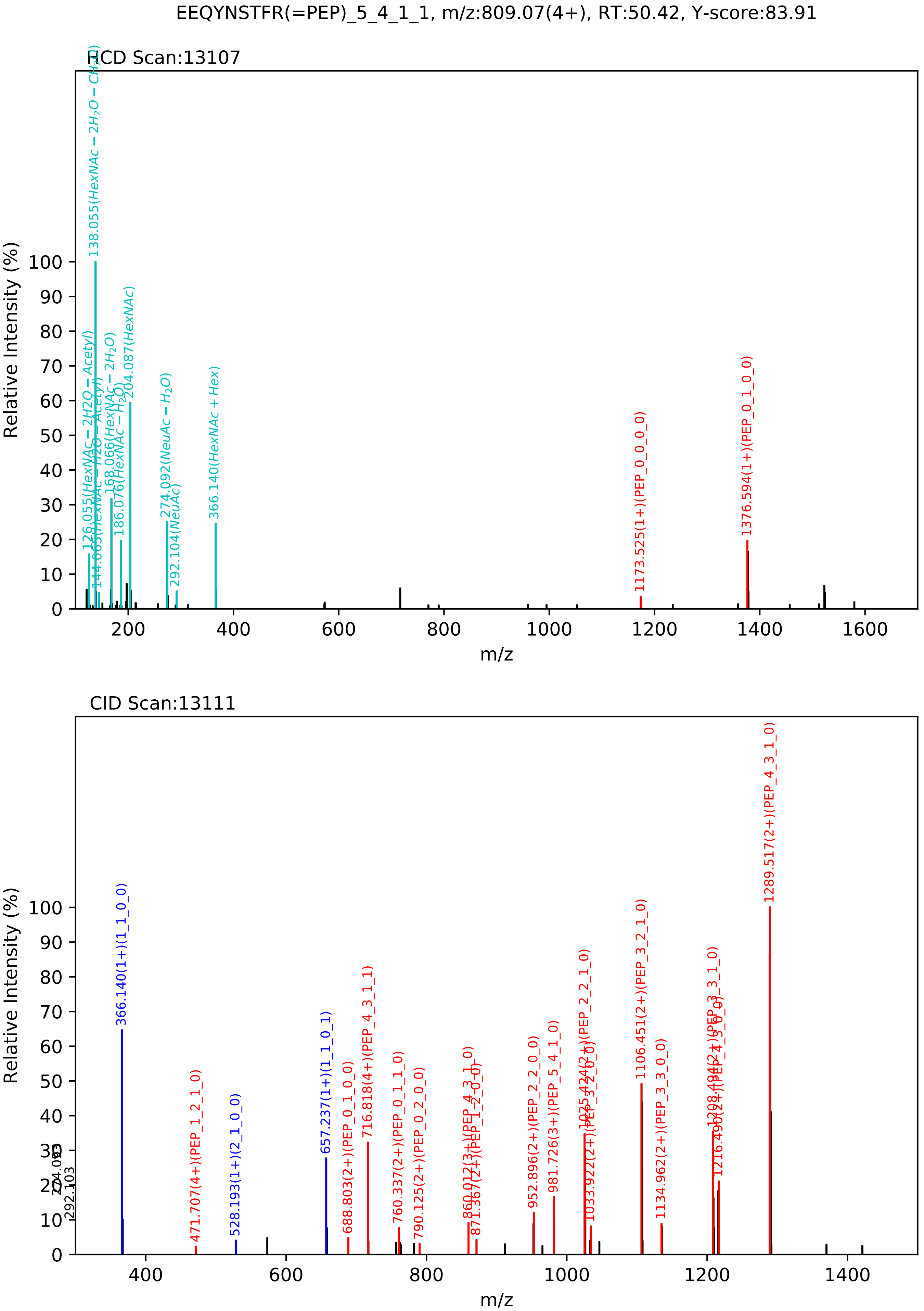

Unknown set no. 503, Experiment: Human Plasma exp_2

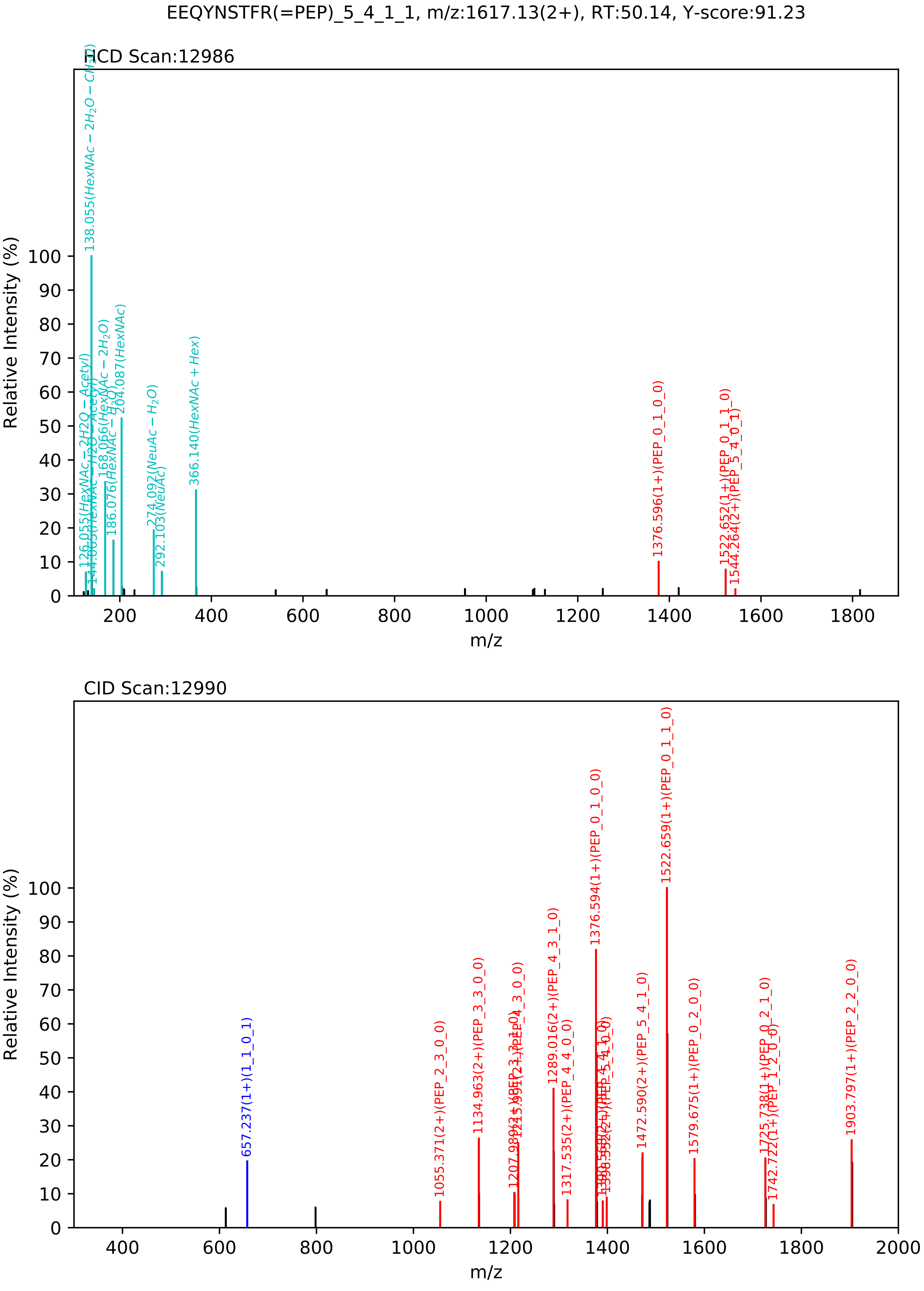

Unknown set no. 504, Experiment: Human Plasma exp_3

EEQYNSTFR(=PEP)_5_4_1_1, m/z:809.07(4+), RT:50.57, Y-score:83.59

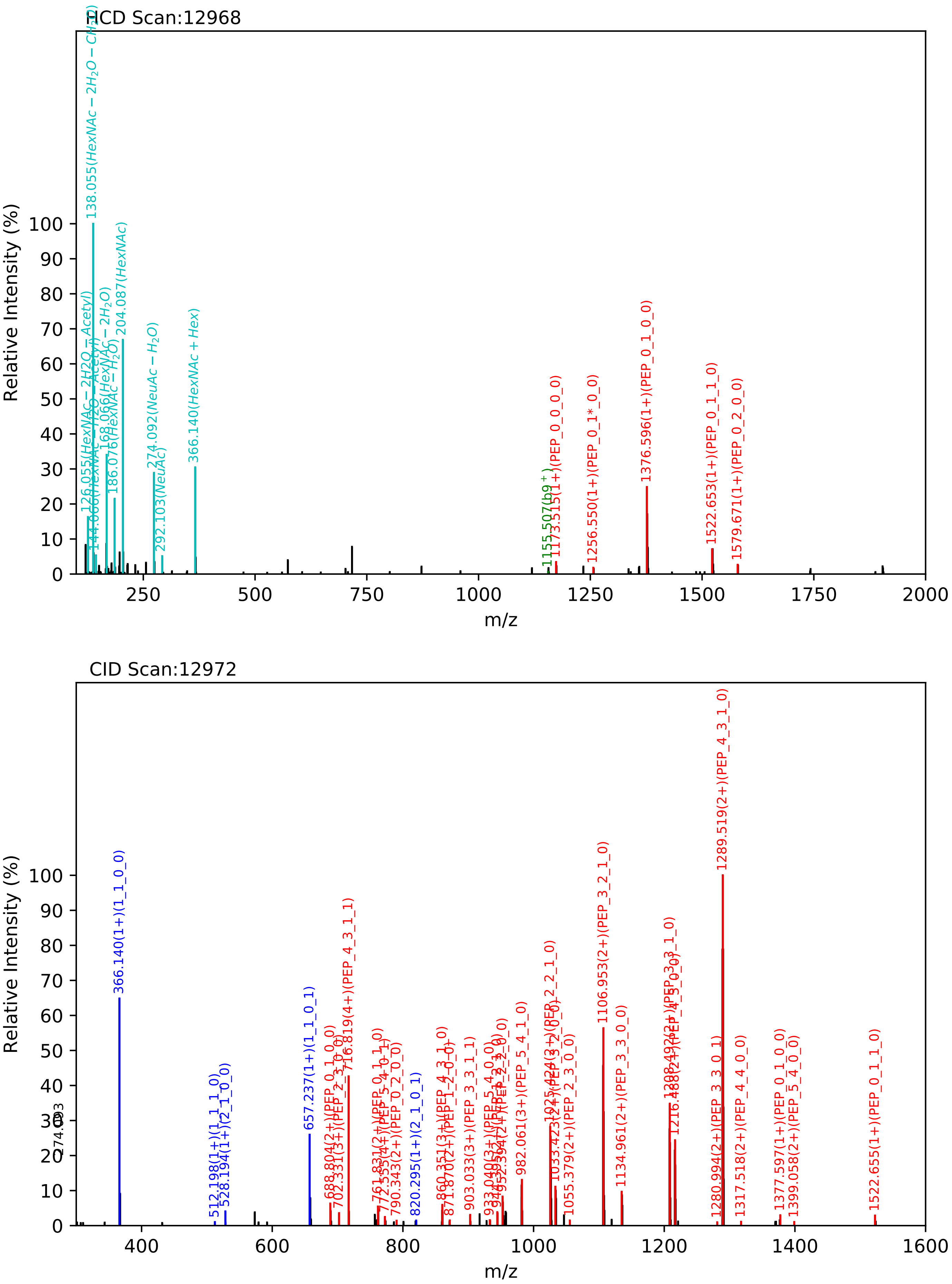

Unknown set no. 505, Experiment: Human Plasma exp_4

EEQYNSTFR(=PEP)_5_4_1_1, m/z:809.07(4+), RT:50.57, Y-score:90.03

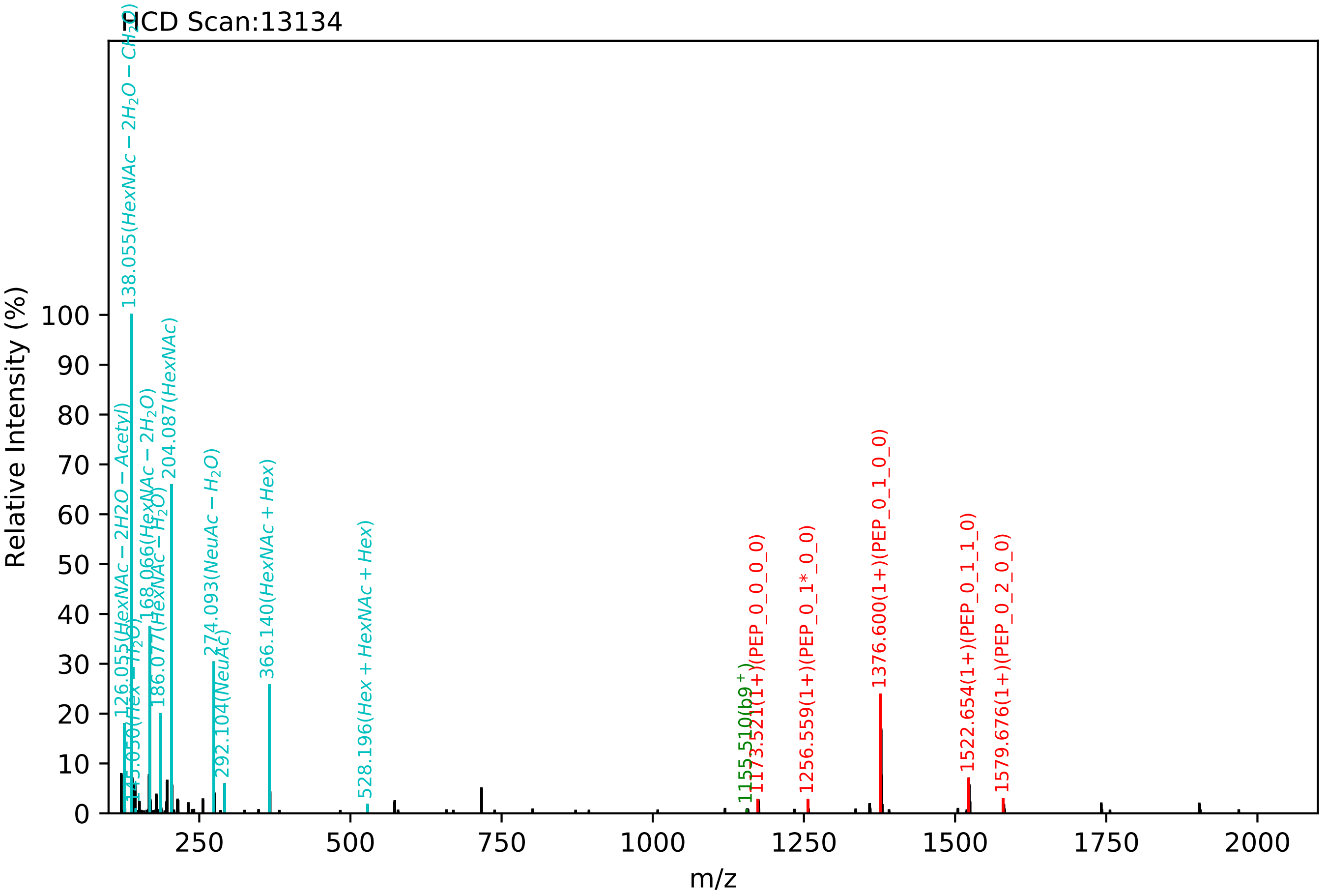

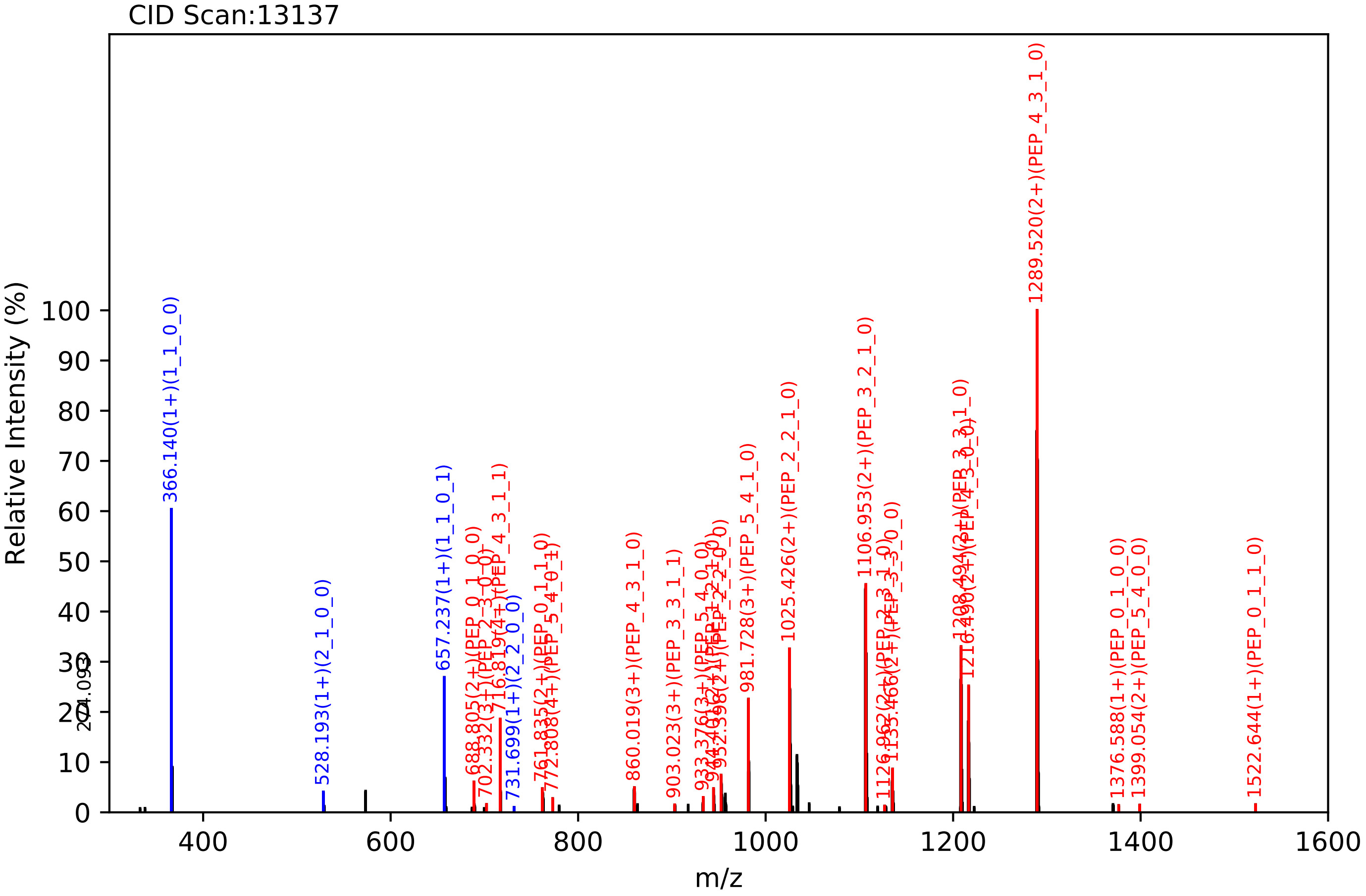

Unknown set no. 506, Experiment: Human Plasma exp_5

EEQYNSTFR(=PEP)_5_4_1_1, m/z:809.07(4+), RT:50.68, Y-score:90.68

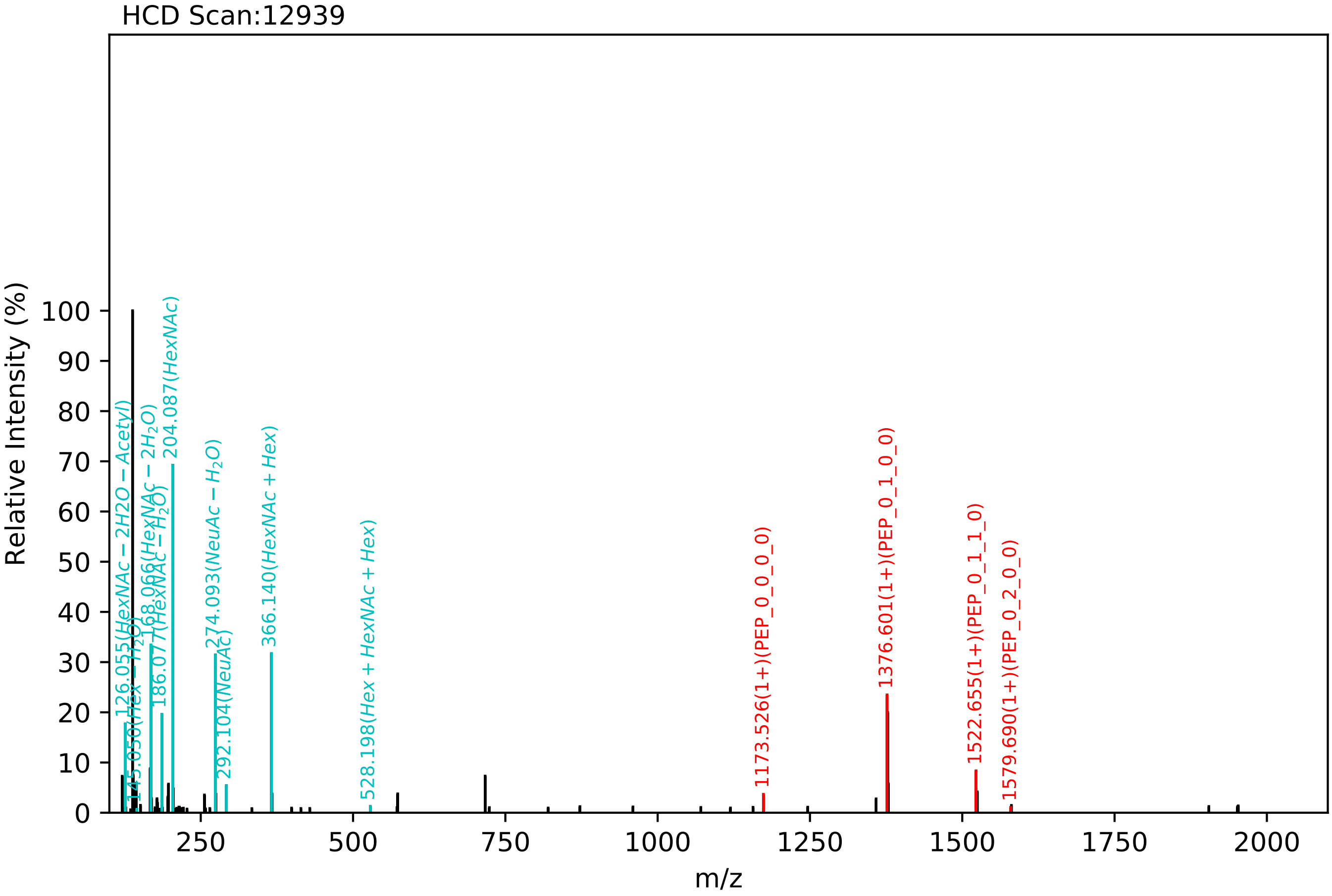

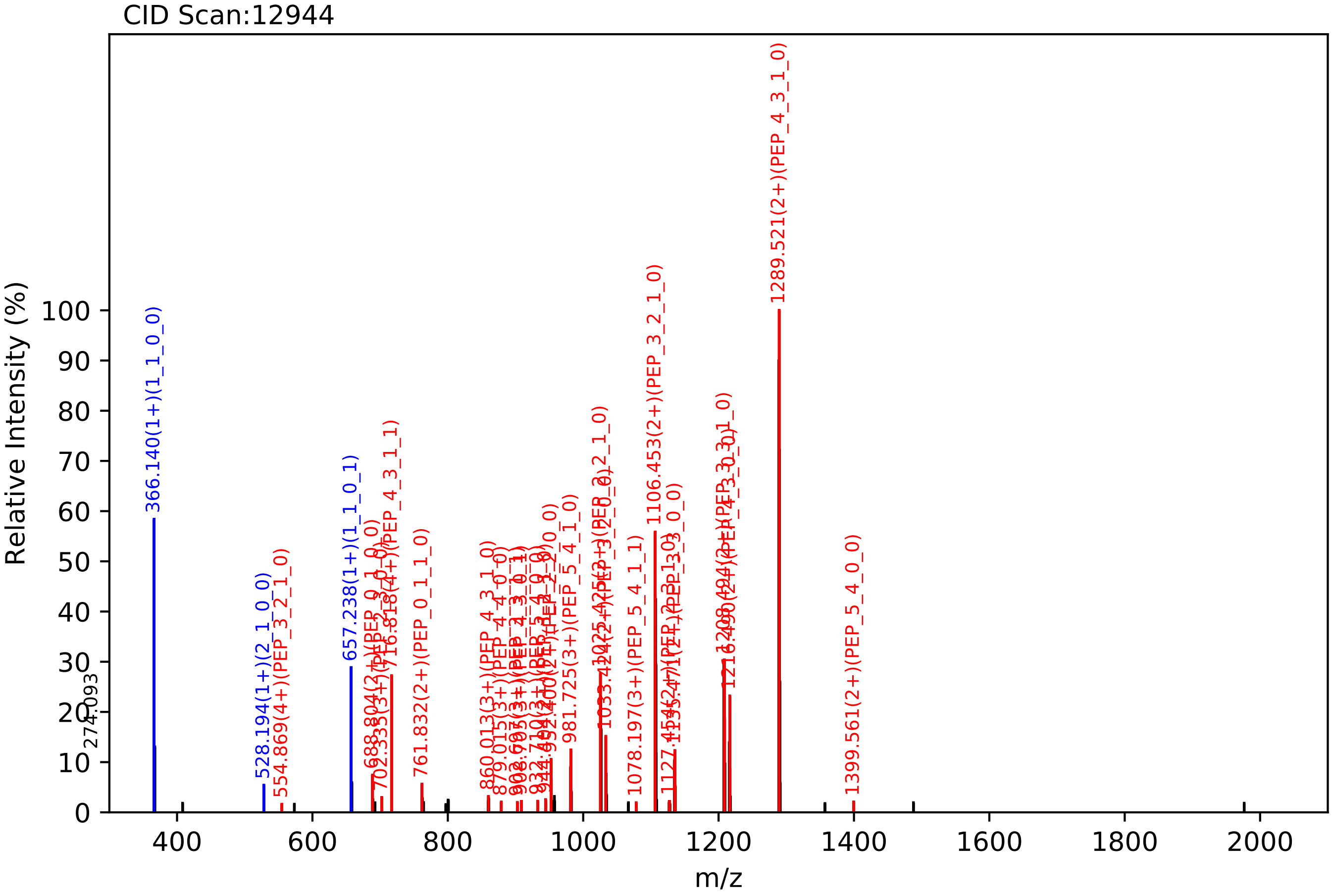

Unknown set no. 507, Experiment: Human Plasma exp_4

EEQYNSTFR(=PEP)_5_4_1_2, m/z:1175.46(3+), RT:60.24, Y-score:85.08

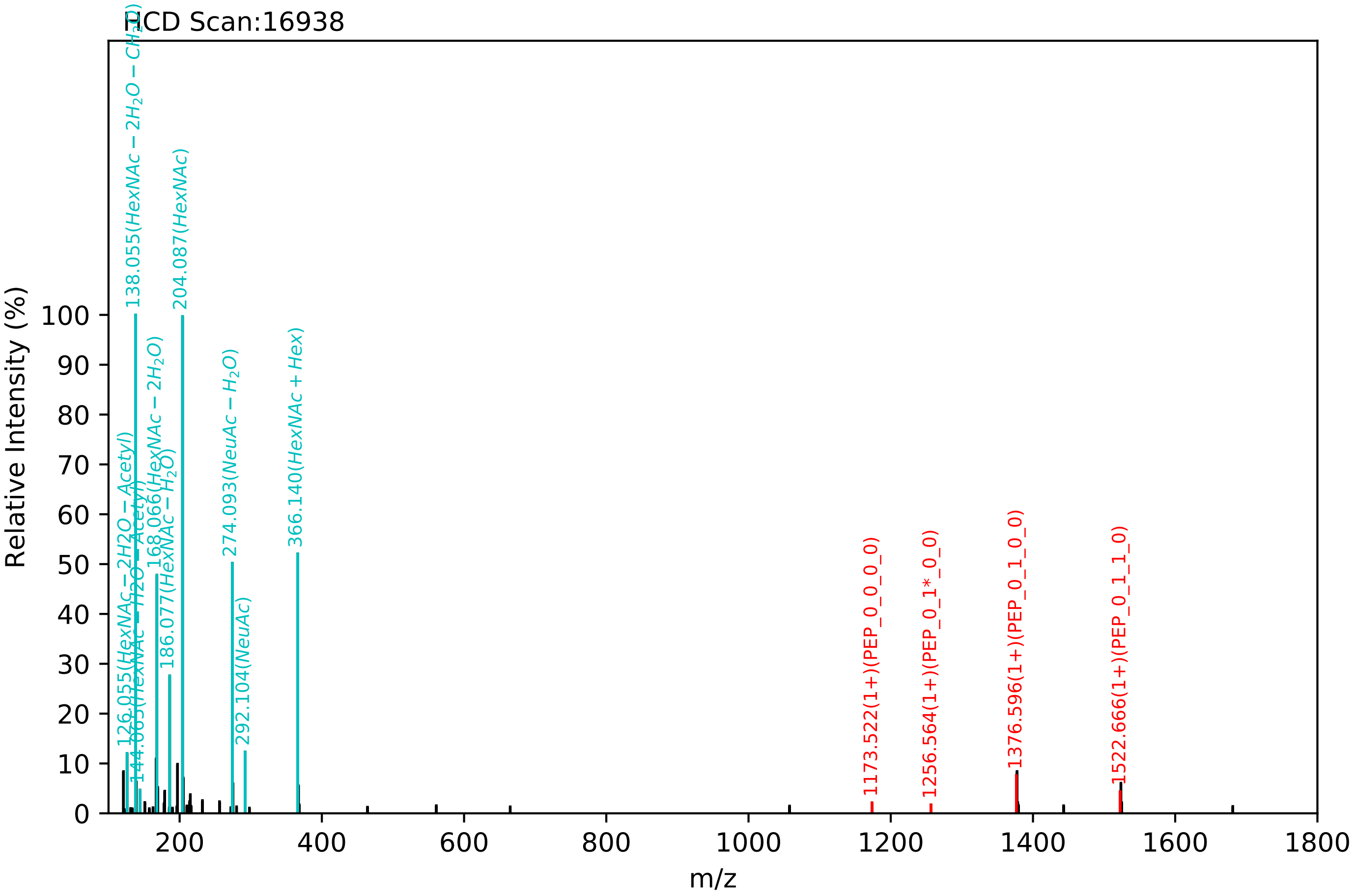

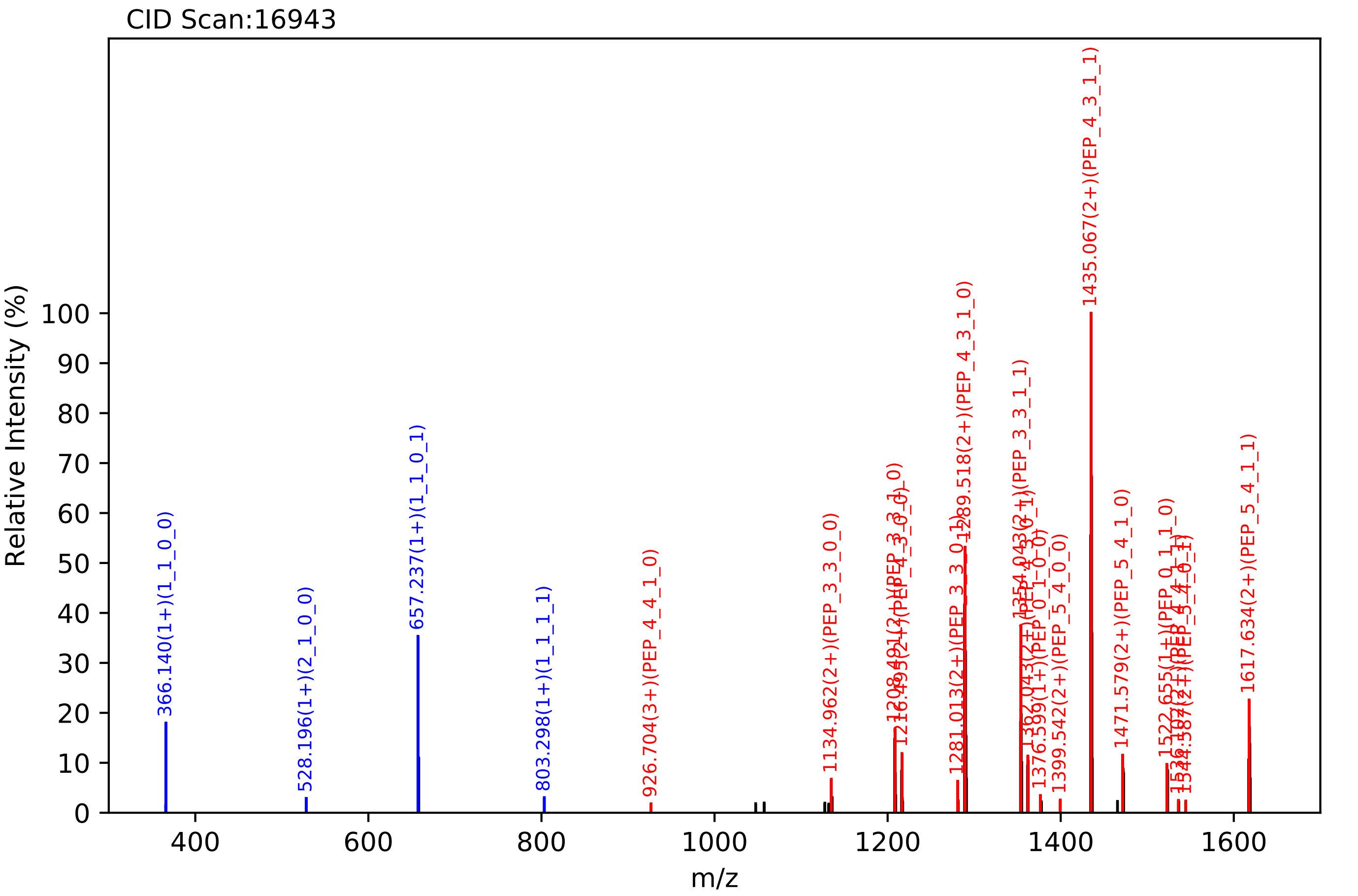

Unknown set no. 508, Experiment: Human Plasma exp_5

EEQYNSTFR(=PEP)_5_4_1_2, m/z:1175.46(3+), RT:60.20, Y-score:84.62

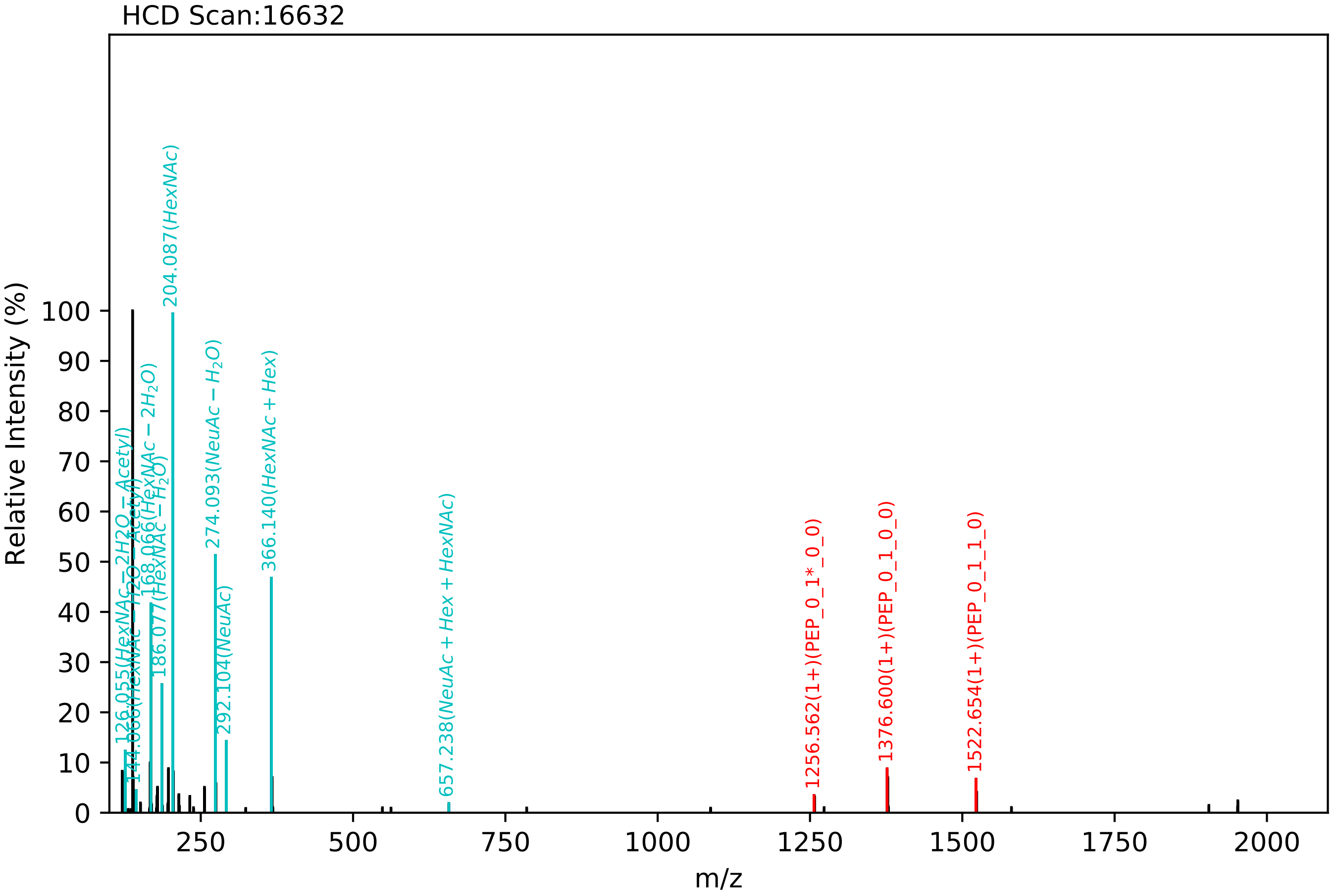

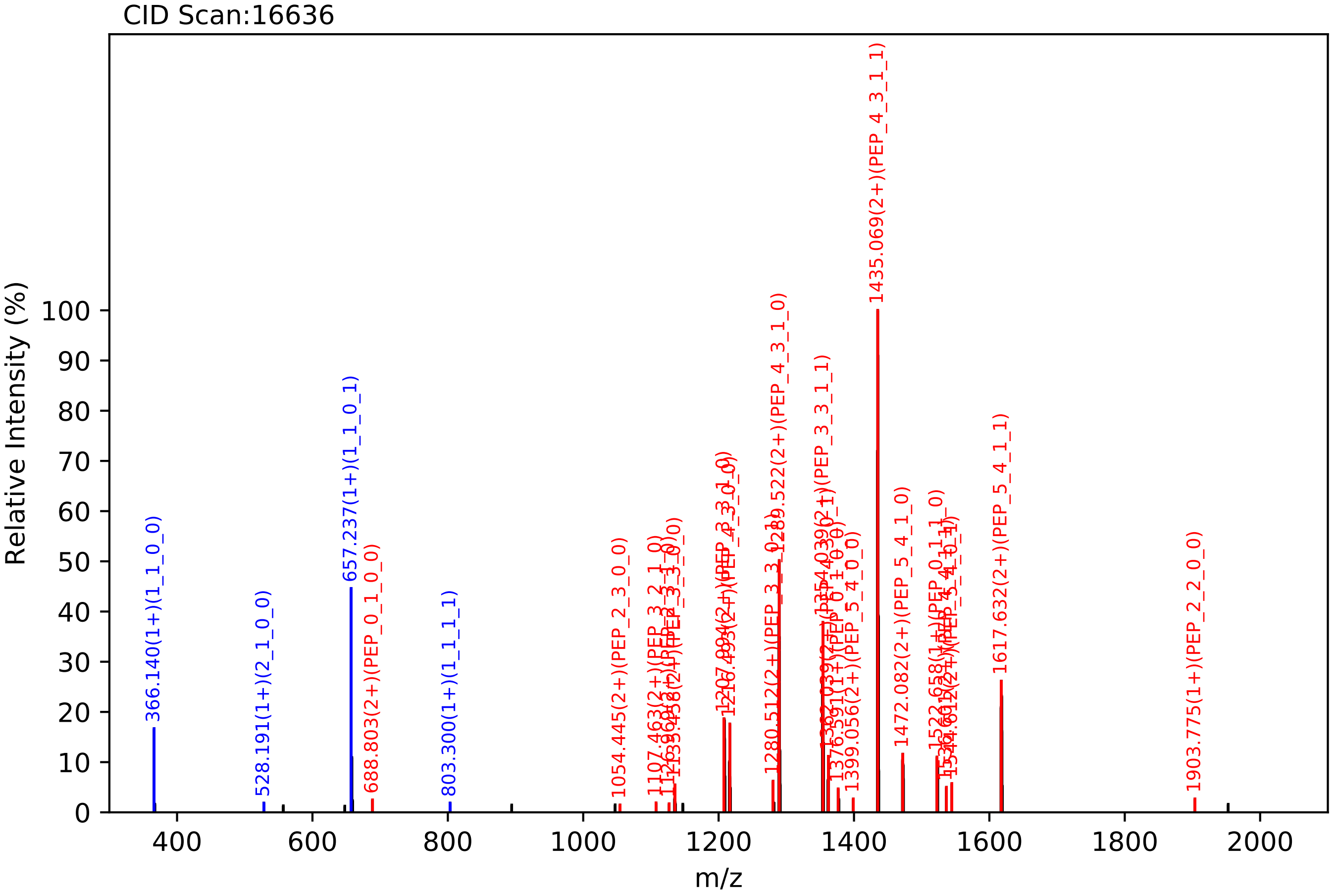

Unknown set no. 509, Experiment: Human Plasma exp_1

EEQFNSTYR(=PEP)_3_3_1_0, m/z:1207.99(2+), RT:39.94, Y-score:93.86

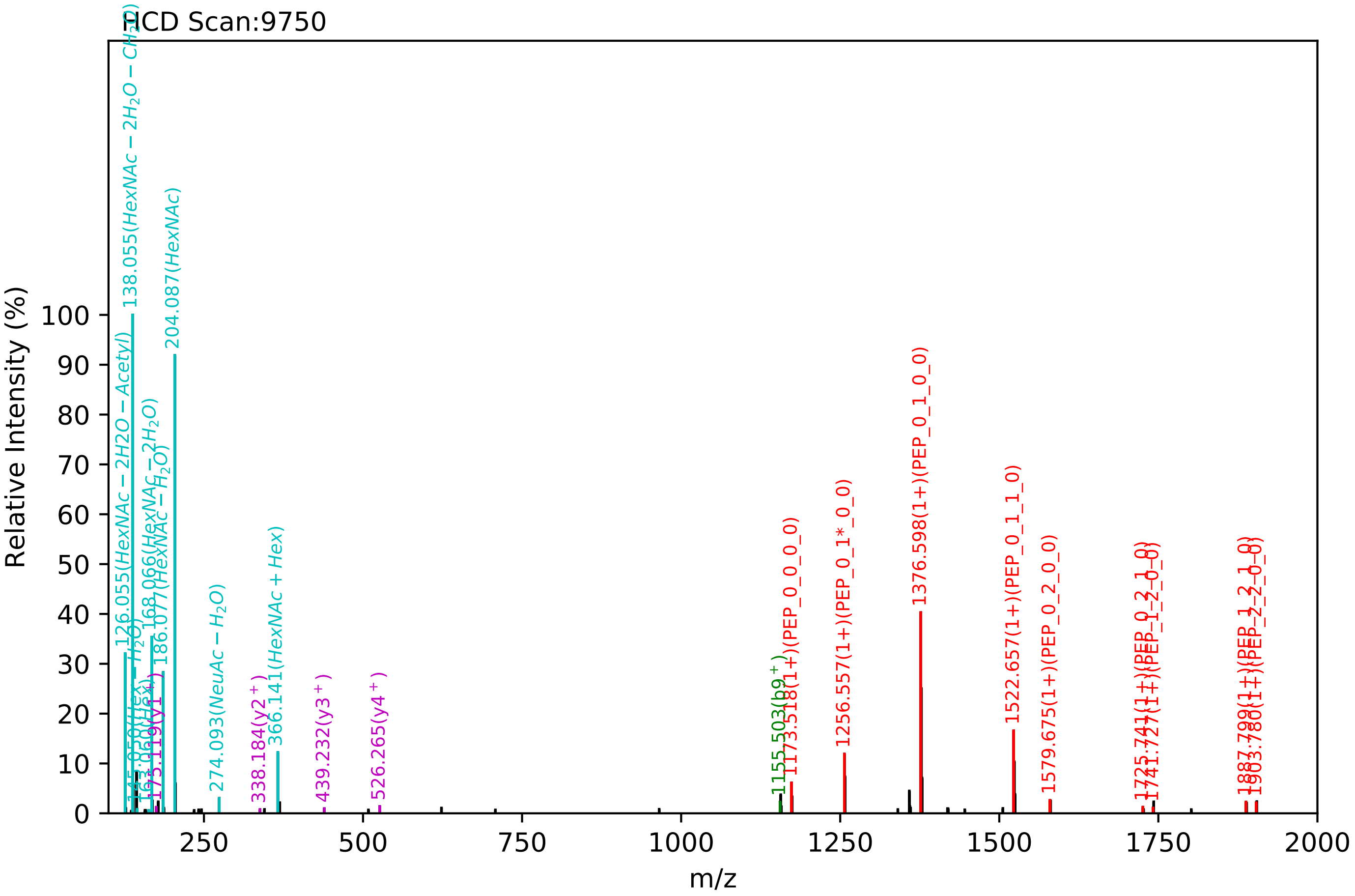

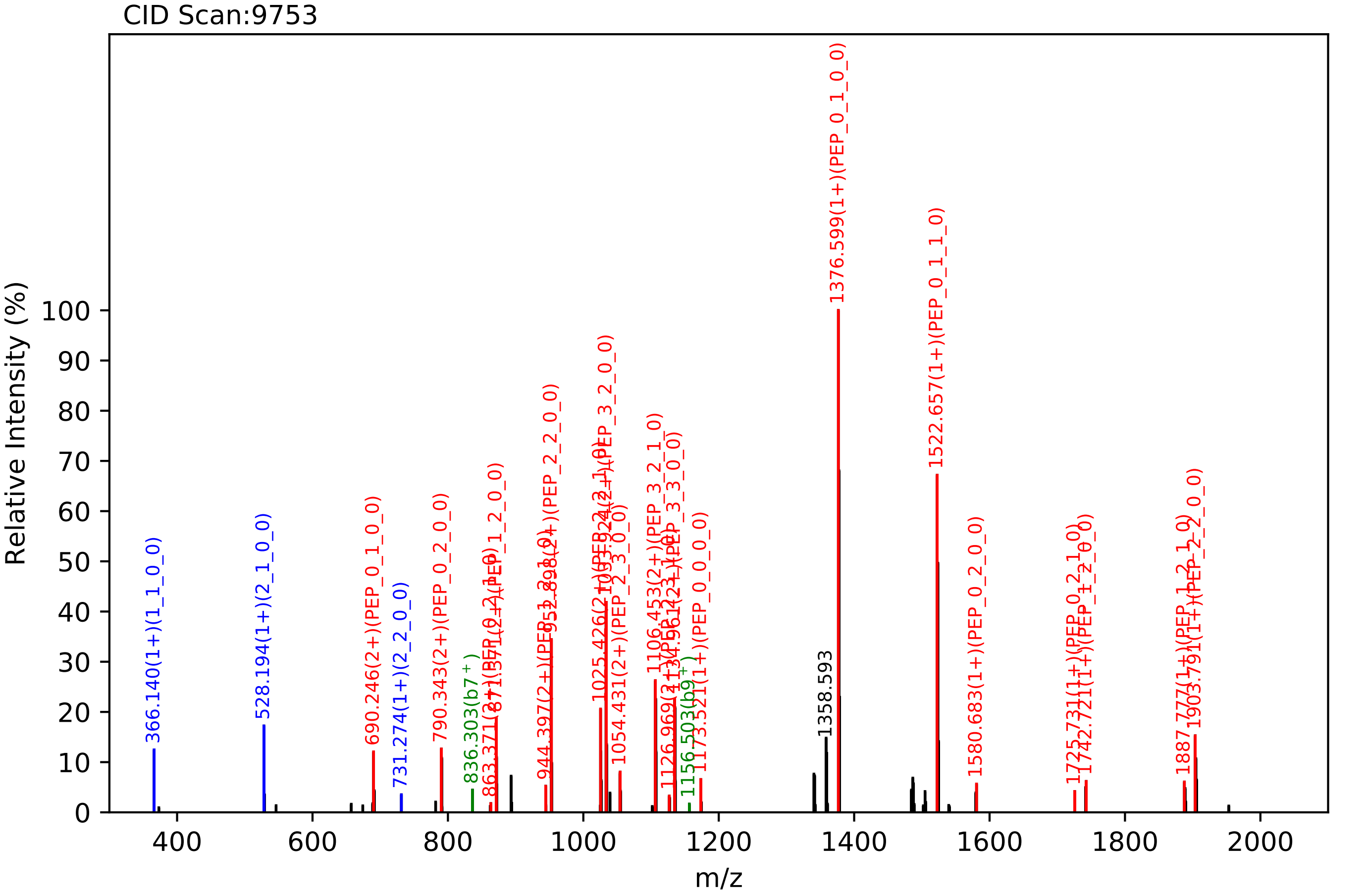

Unknown set no. 510, Experiment: Human Plasma exp_2

EEQFNSTYR(=PEP)_3_3_1_0, m/z:1207.99(2+), RT:40.13, Y-score:91.86

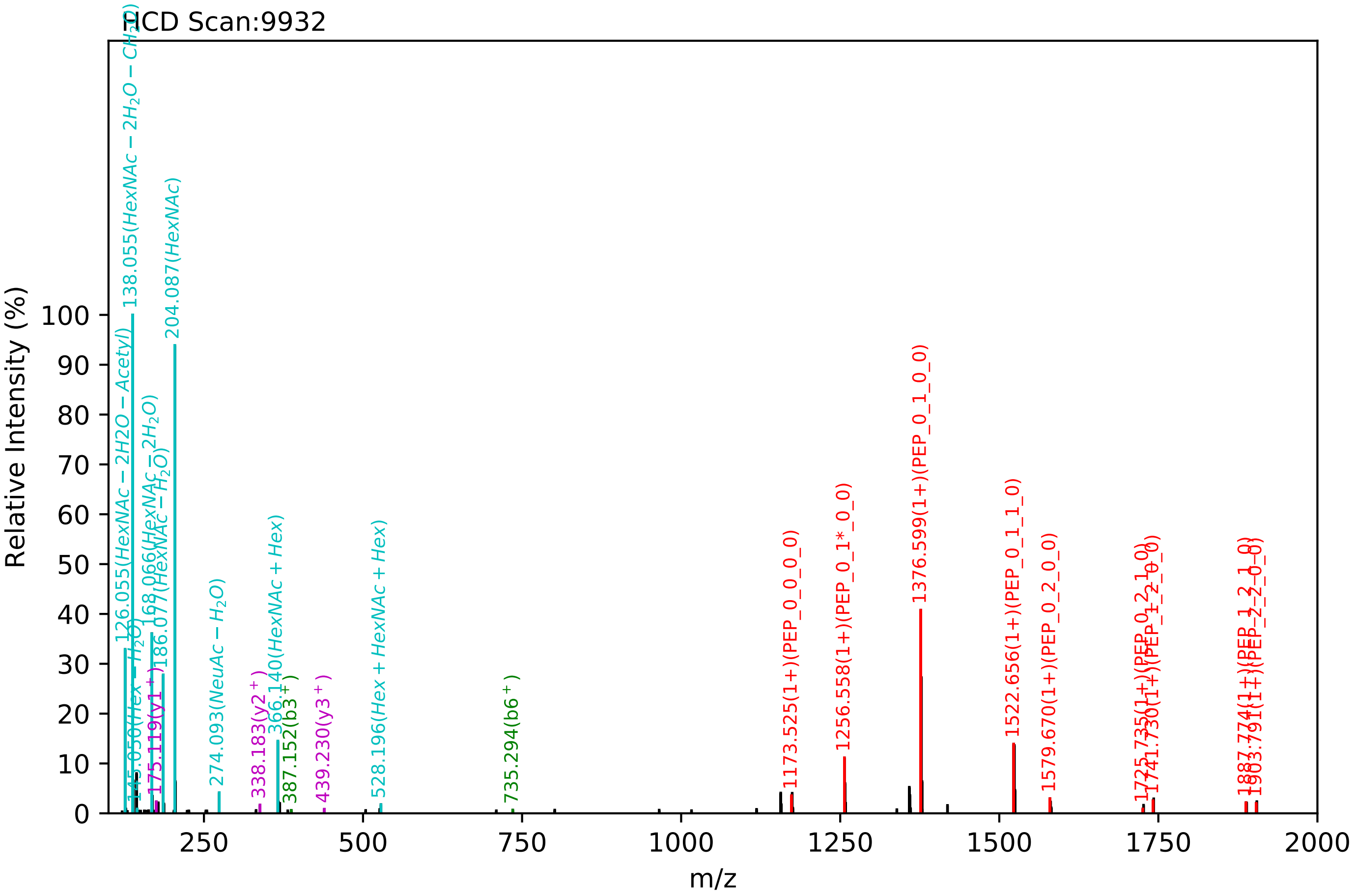

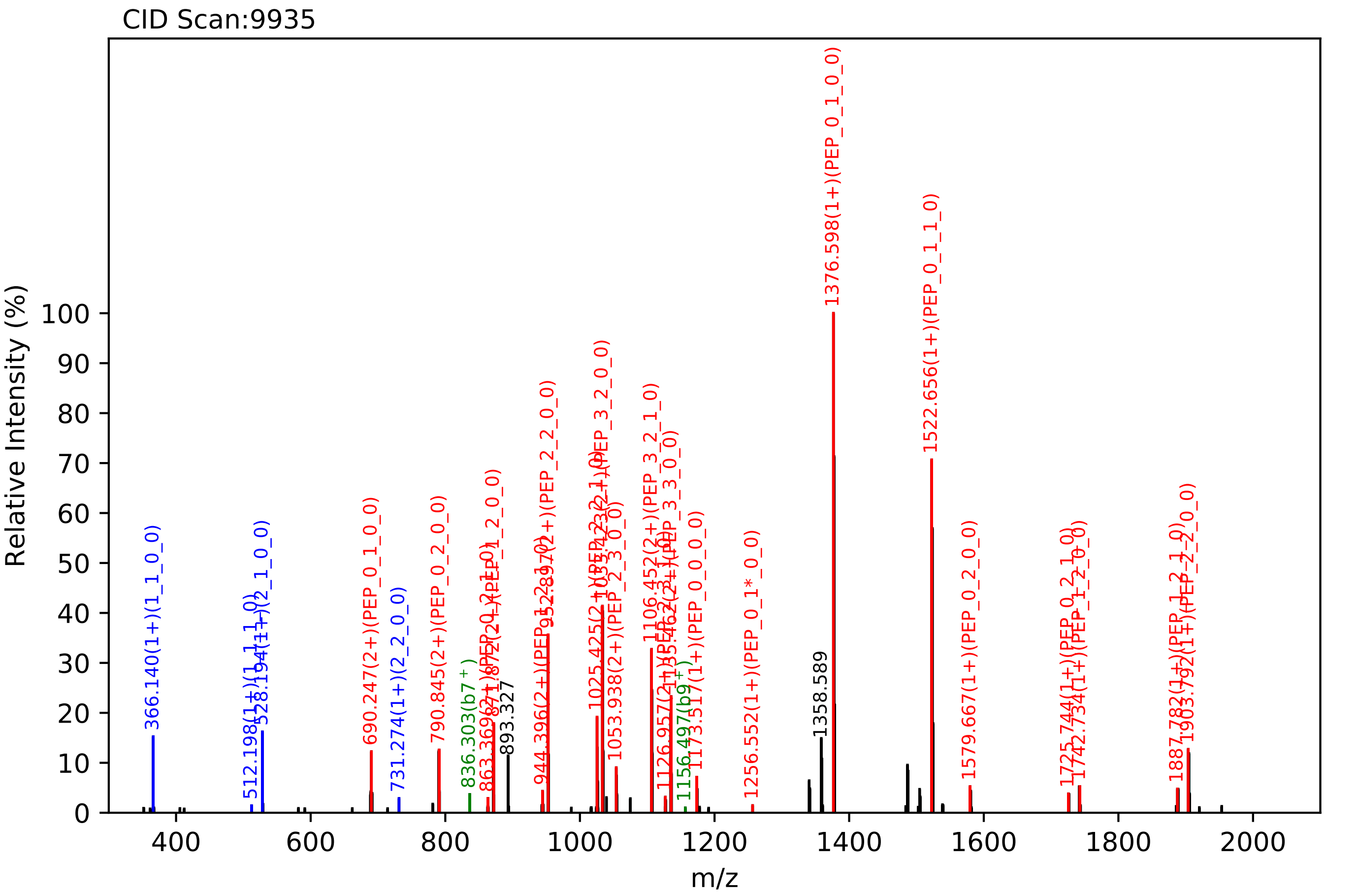

Unknown set no. 511, Experiment: Human Plasma exp_3

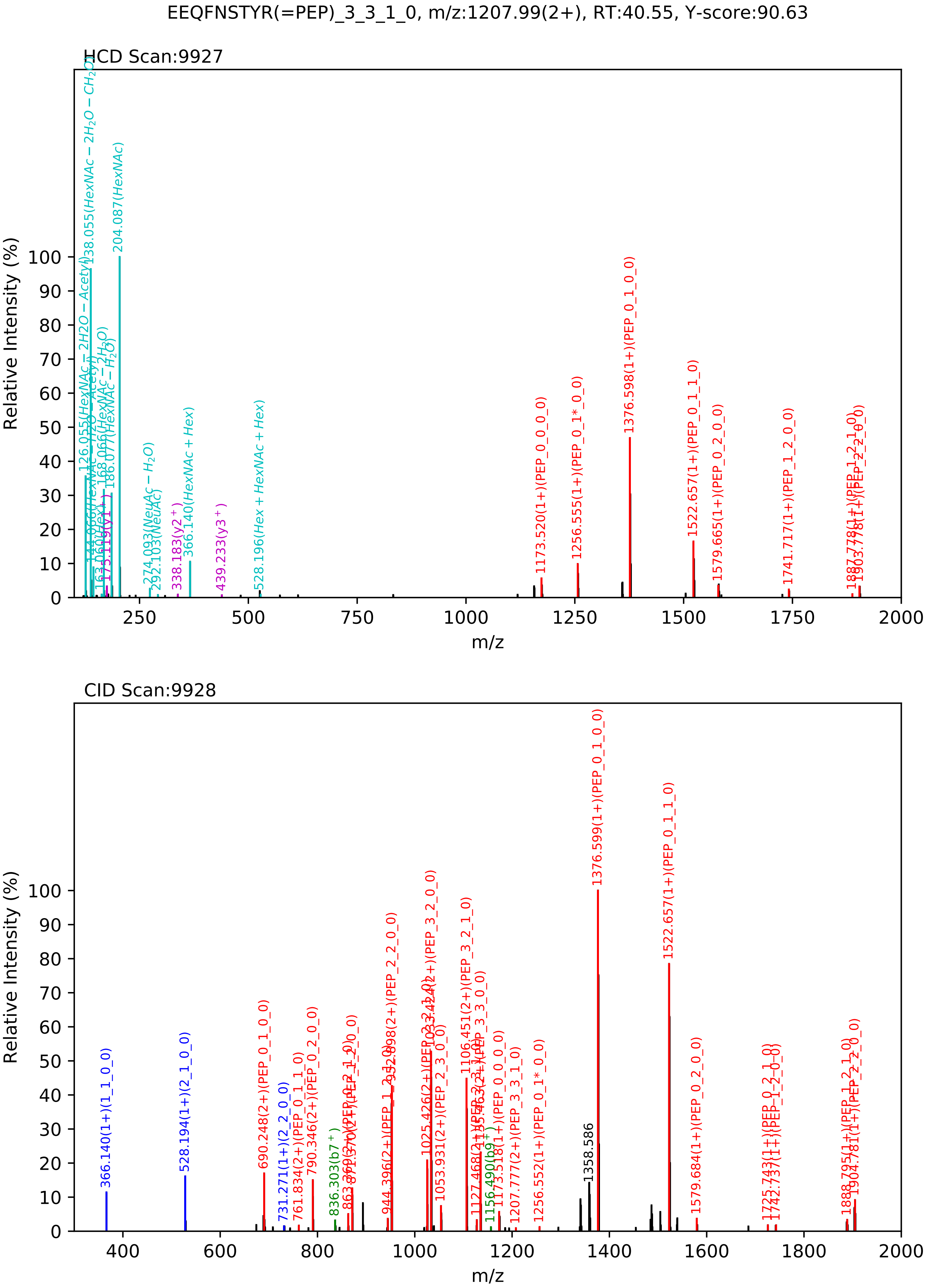

Unknown set no. 512, Experiment: Human Plasma exp_4

EEQFNSTYR(=PEP)_3_3_1_0, m/z:1207.99(2+), RT:40.41, Y-score:93.80

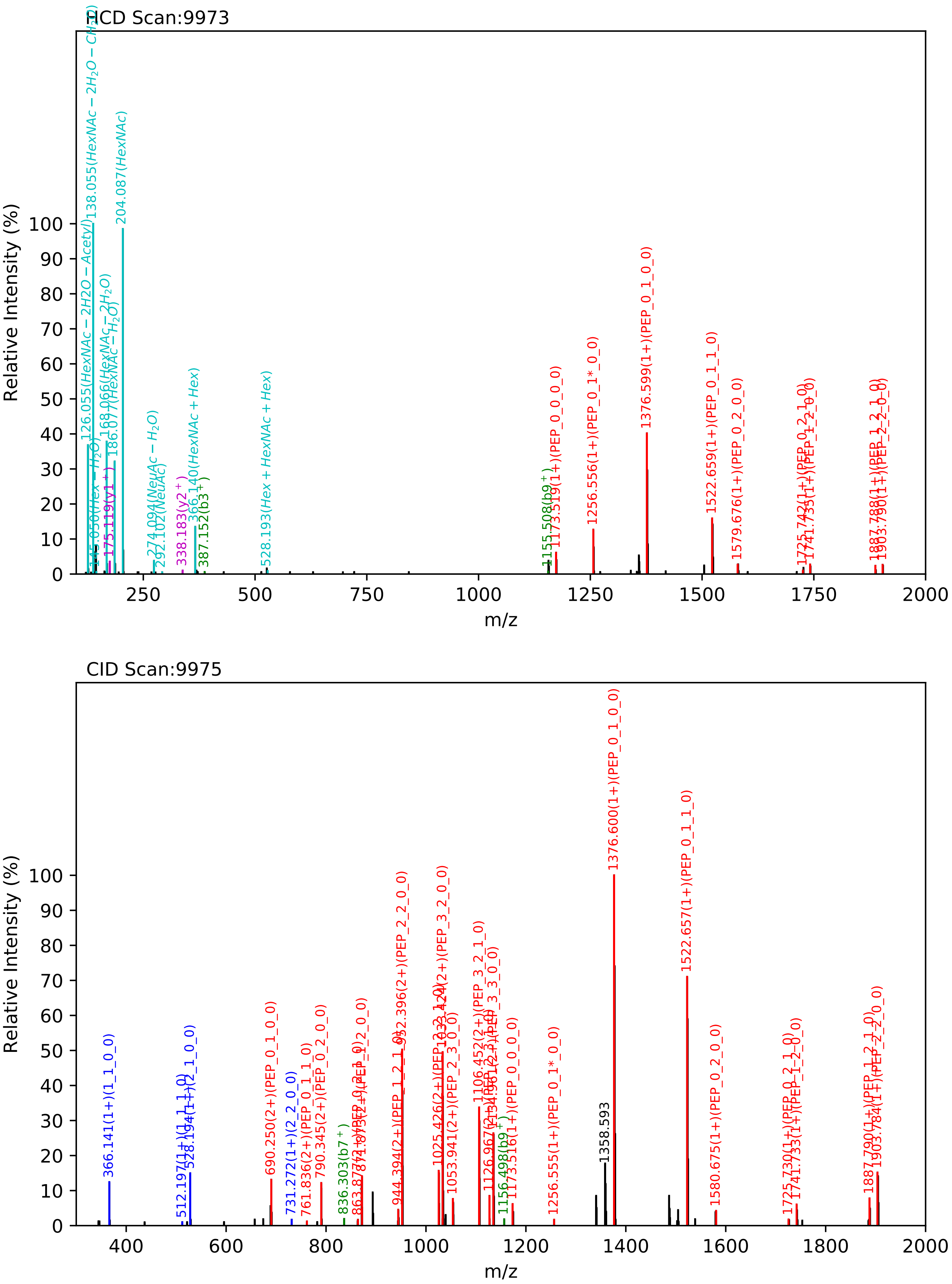

Unknown set no. 513, Experiment: Human Plasma exp_5

EEQFNSTYR(=PEP)_3_3_1_0, m/z:1207.99(2+), RT:40.53, Y-score:92.86

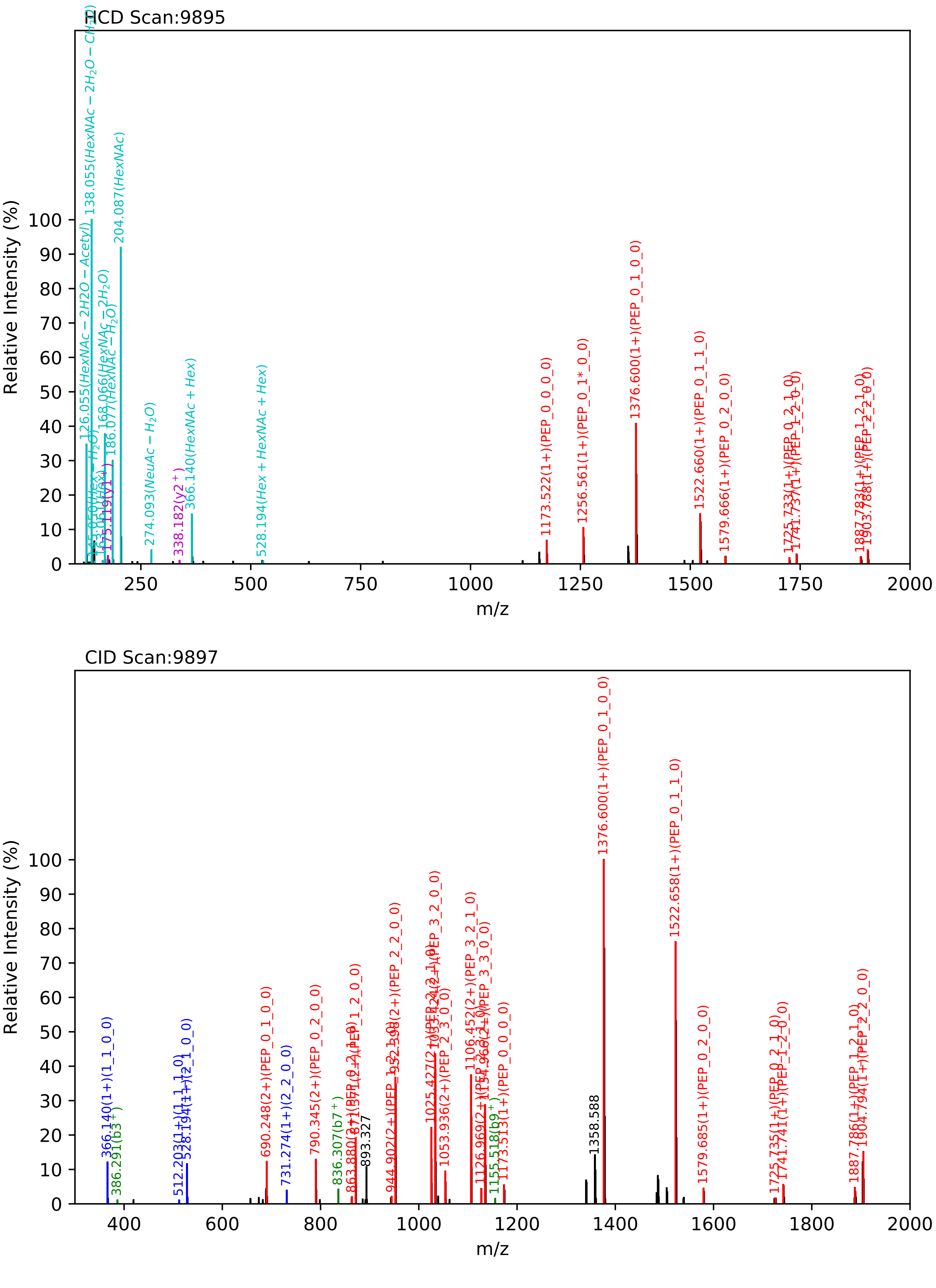

Unknown set no. 514, Experiment: Human Plasma exp_1

EEQFNSTYR(=PEP)_3_4_1_0, m/z:1309.53(2+), RT:40.05, Y-score:91.14

HCD Scan:9786

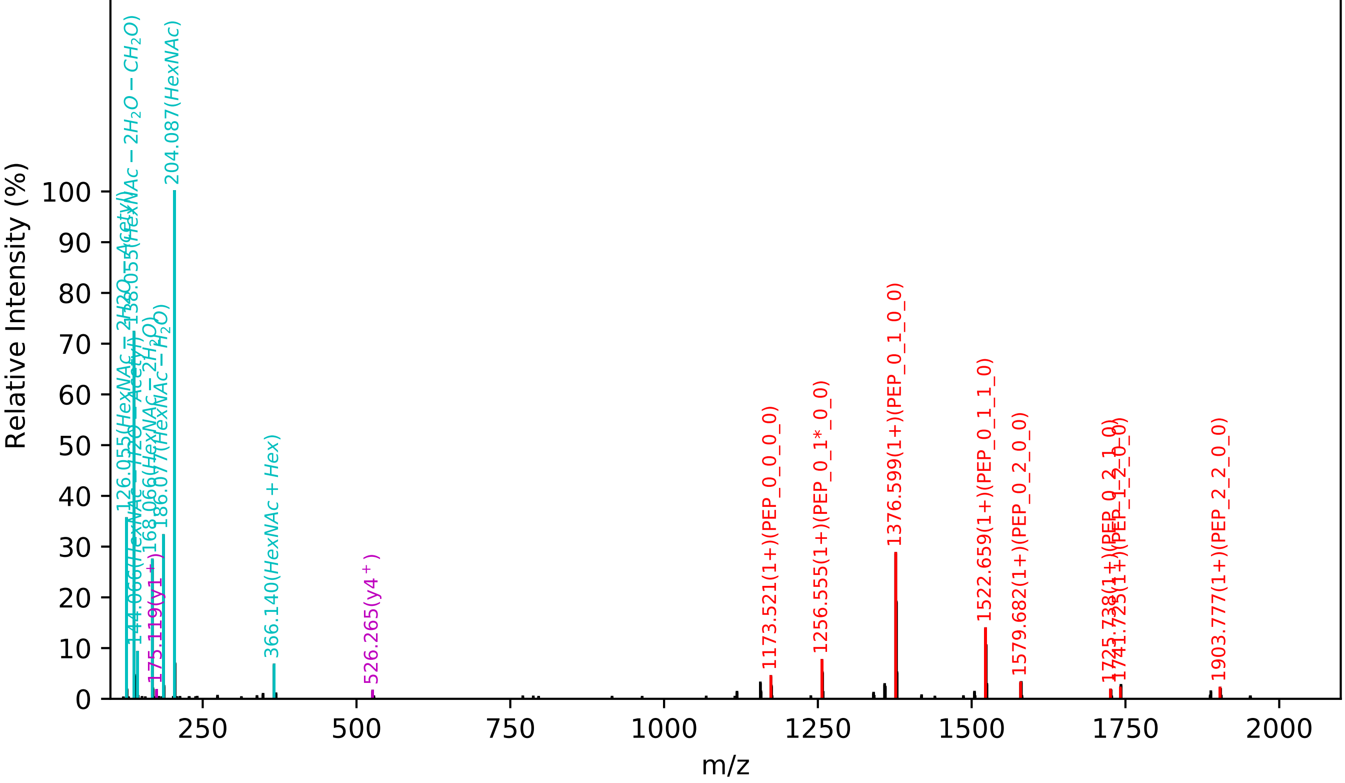

CID Scan:9787

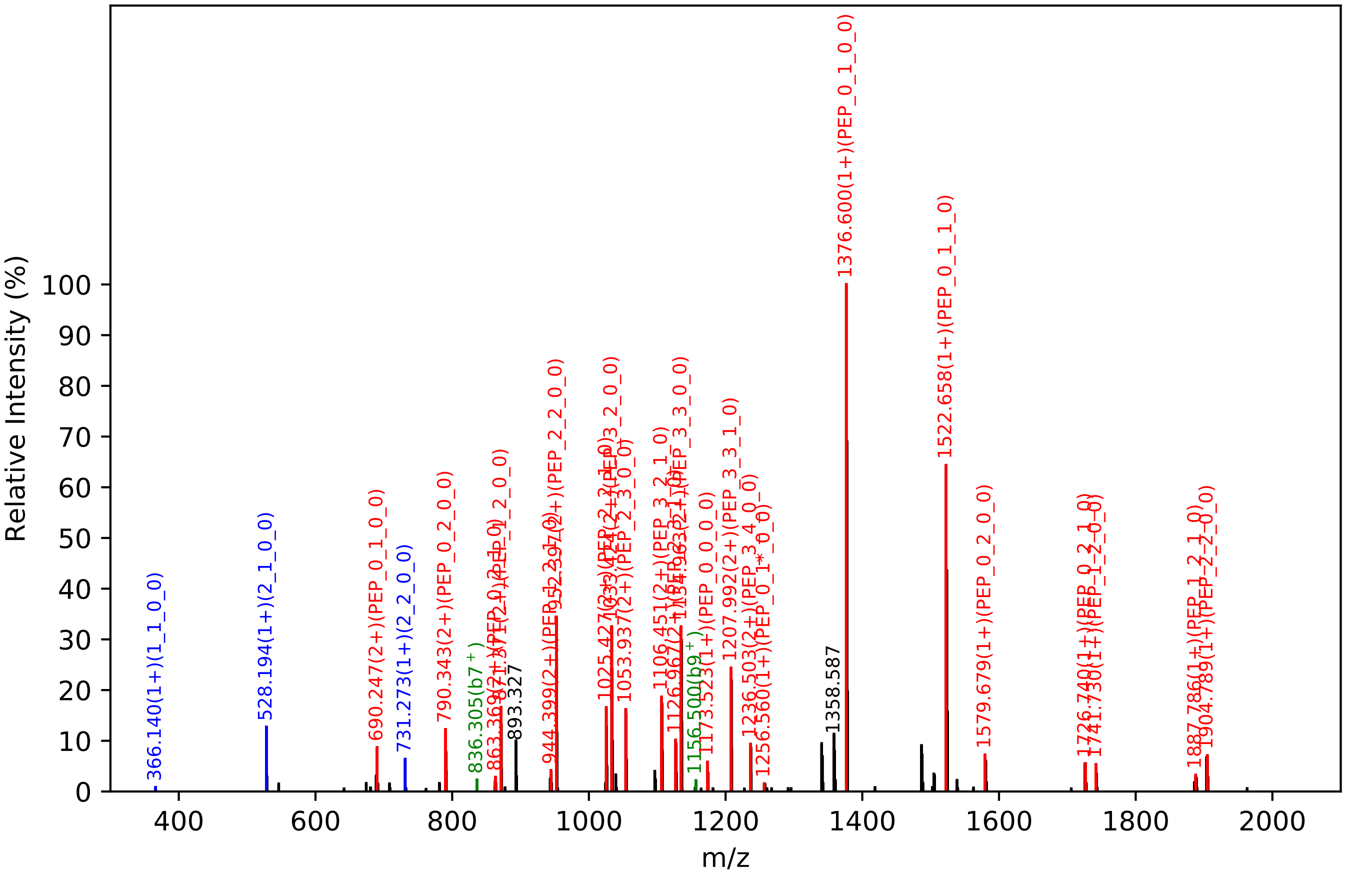

Unknown set no. 515, Experiment: Human Plasma exp_2

EEQFNSTYR(=PEP)_3_4_1_0, m/z:1309.53(2+), RT:40.26, Y-score:91.00

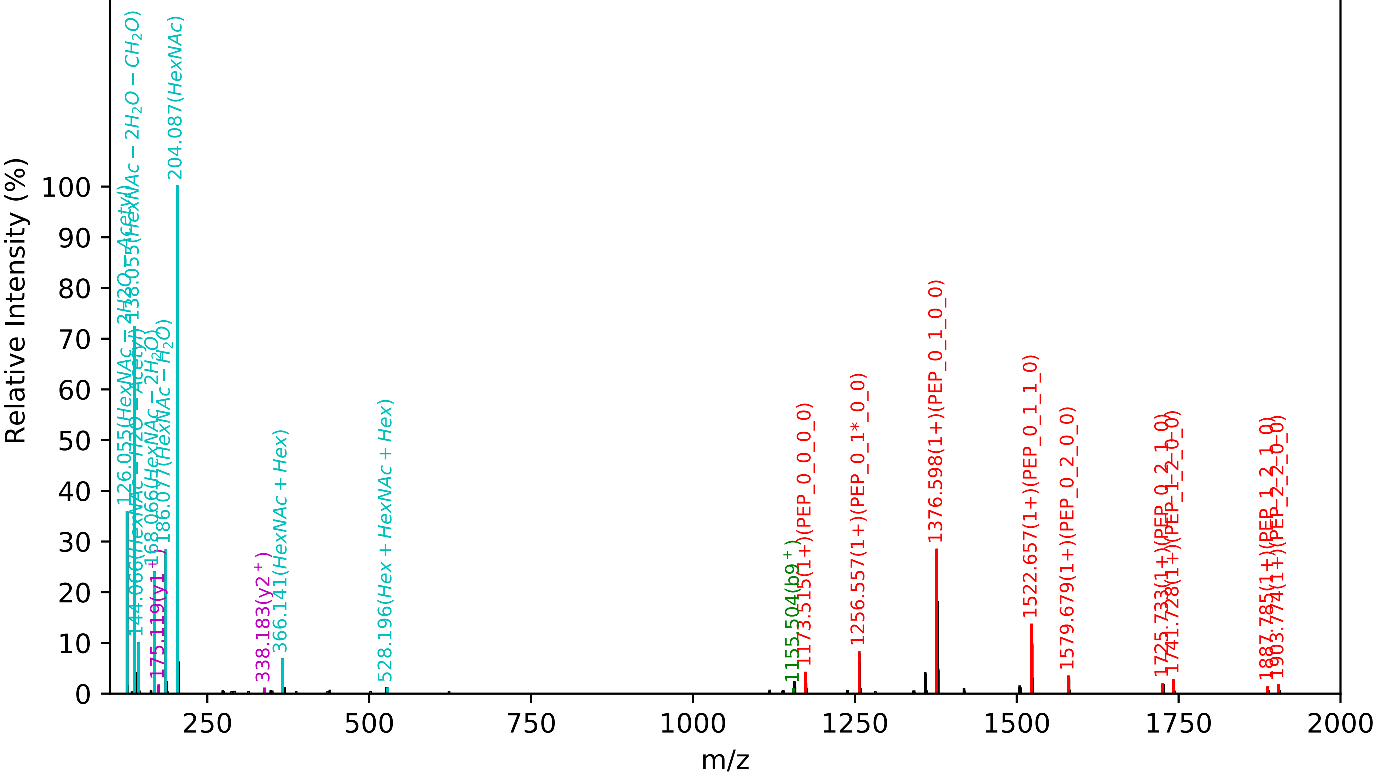

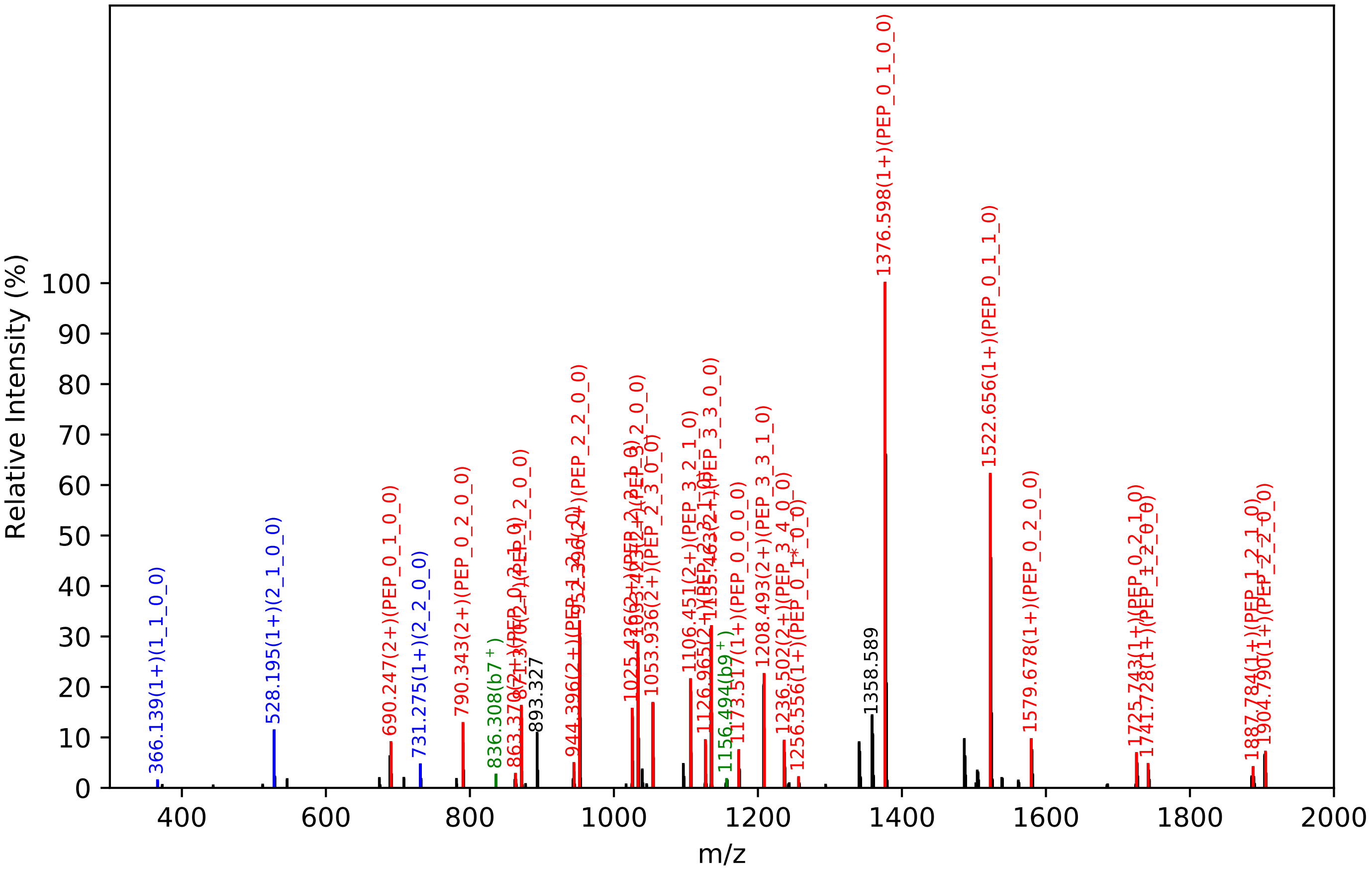

Unknown set no. 516, Experiment: Human Plasma exp_3

EEQFNSTYR(=PEP)_3_4_1_0, m/z:1309.53(2+), RT:41.00, Y-score:91.26

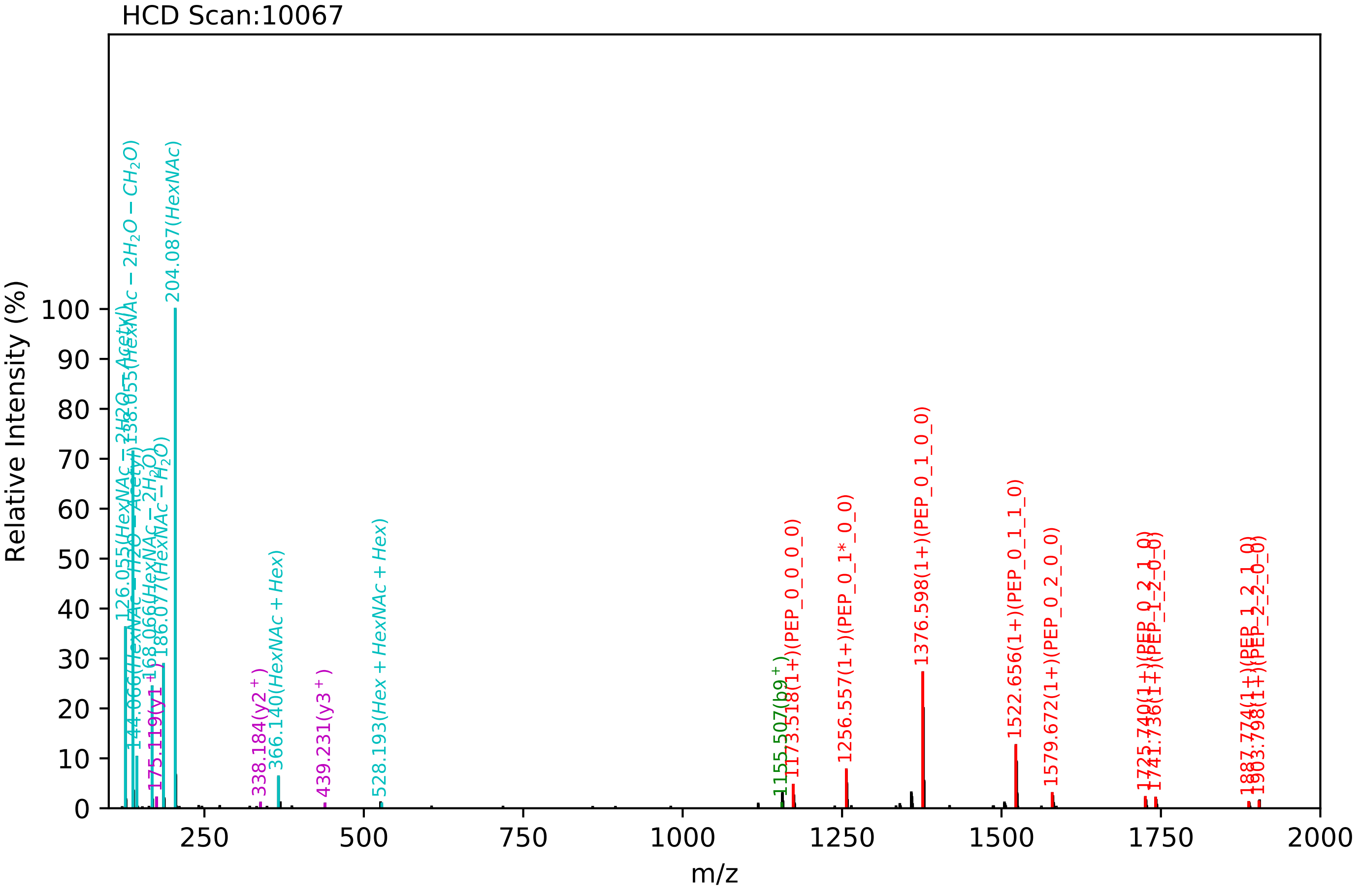

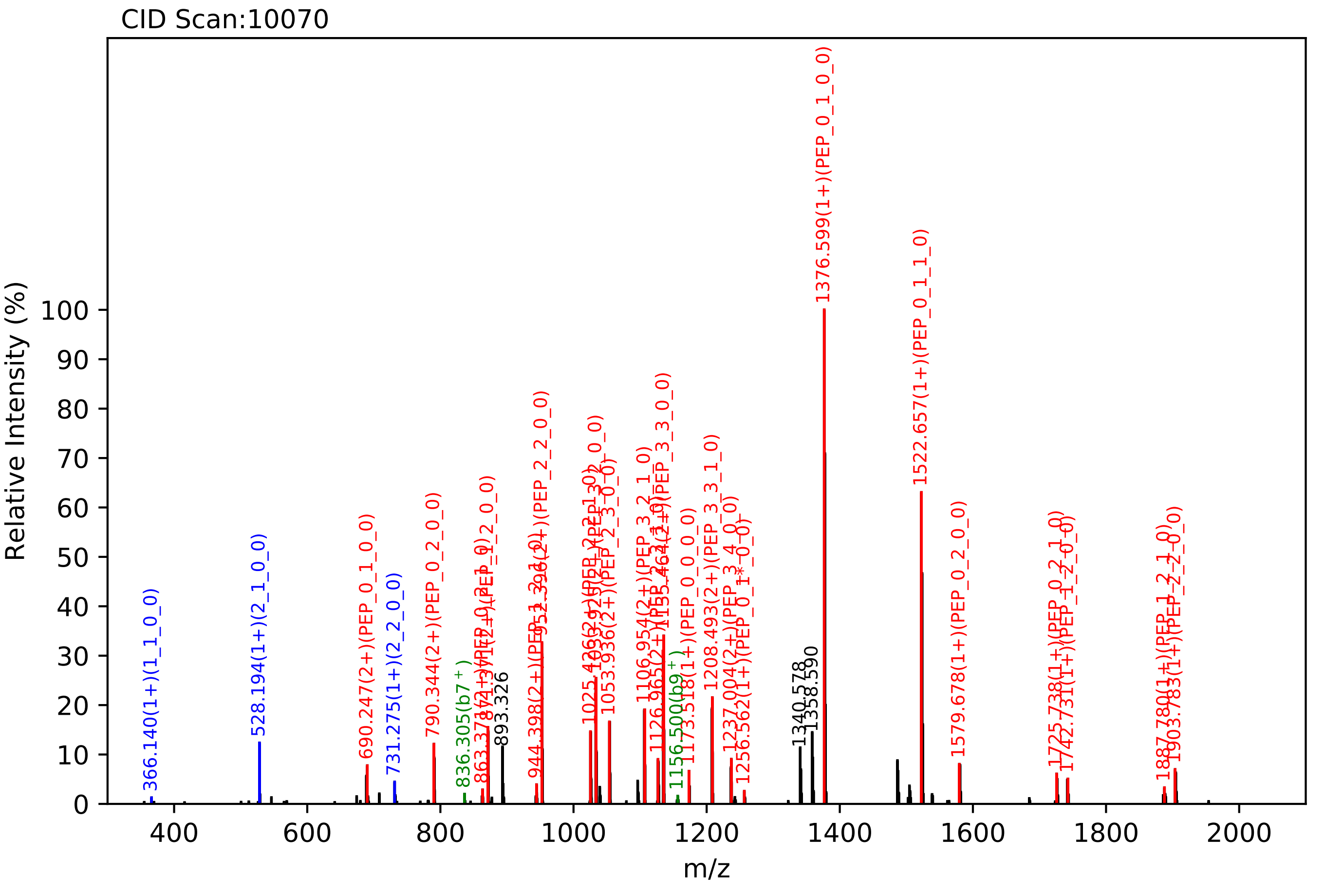

Unknown set no. 517, Experiment: Human Plasma exp_4

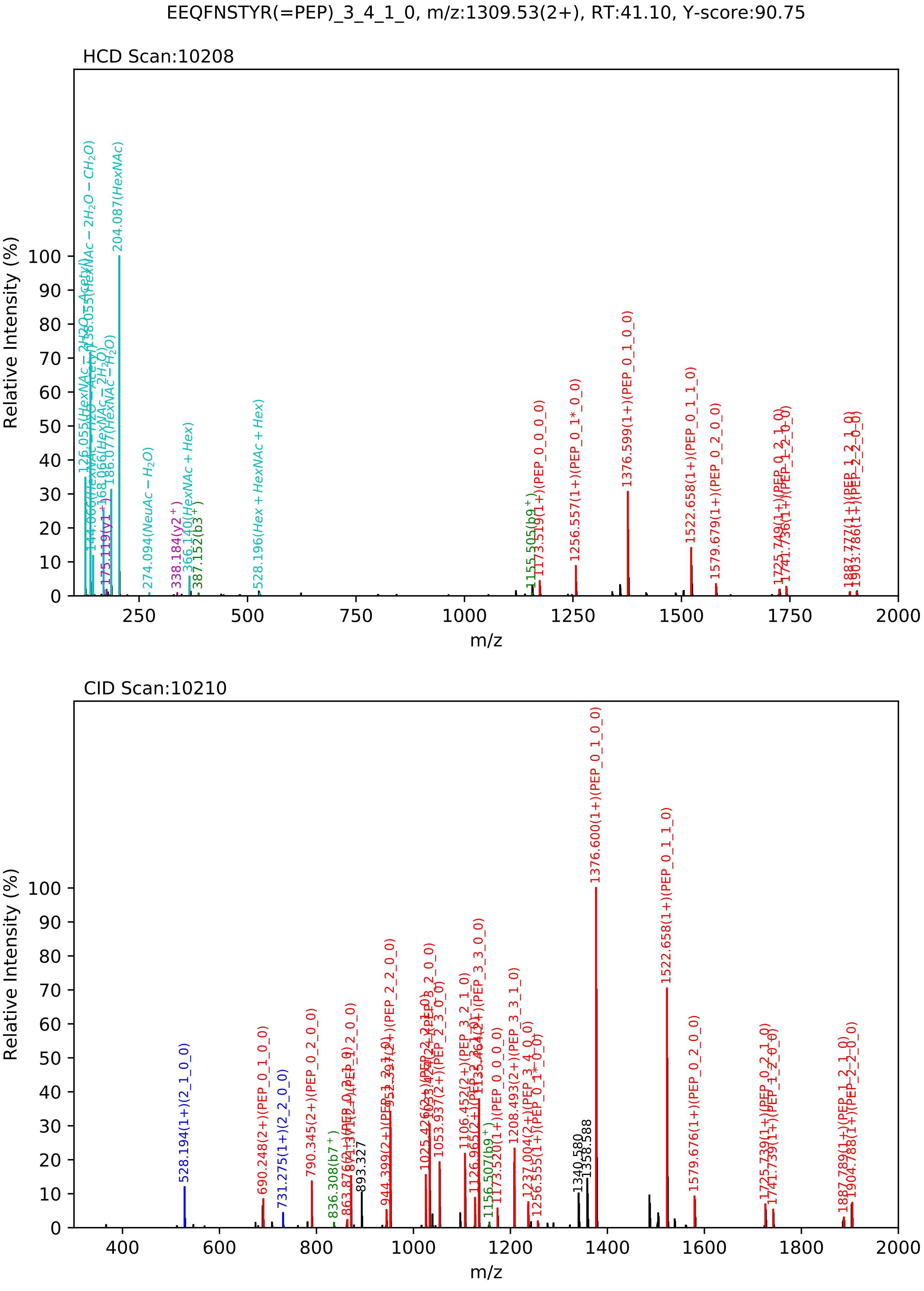

Unknown set no. 518, Experiment: Human Plasma exp_5

EEQFNSTYR(=PEP)_3_4_1_0, m/z:1309.53(2+), RT:41.19, Y-score:91.40

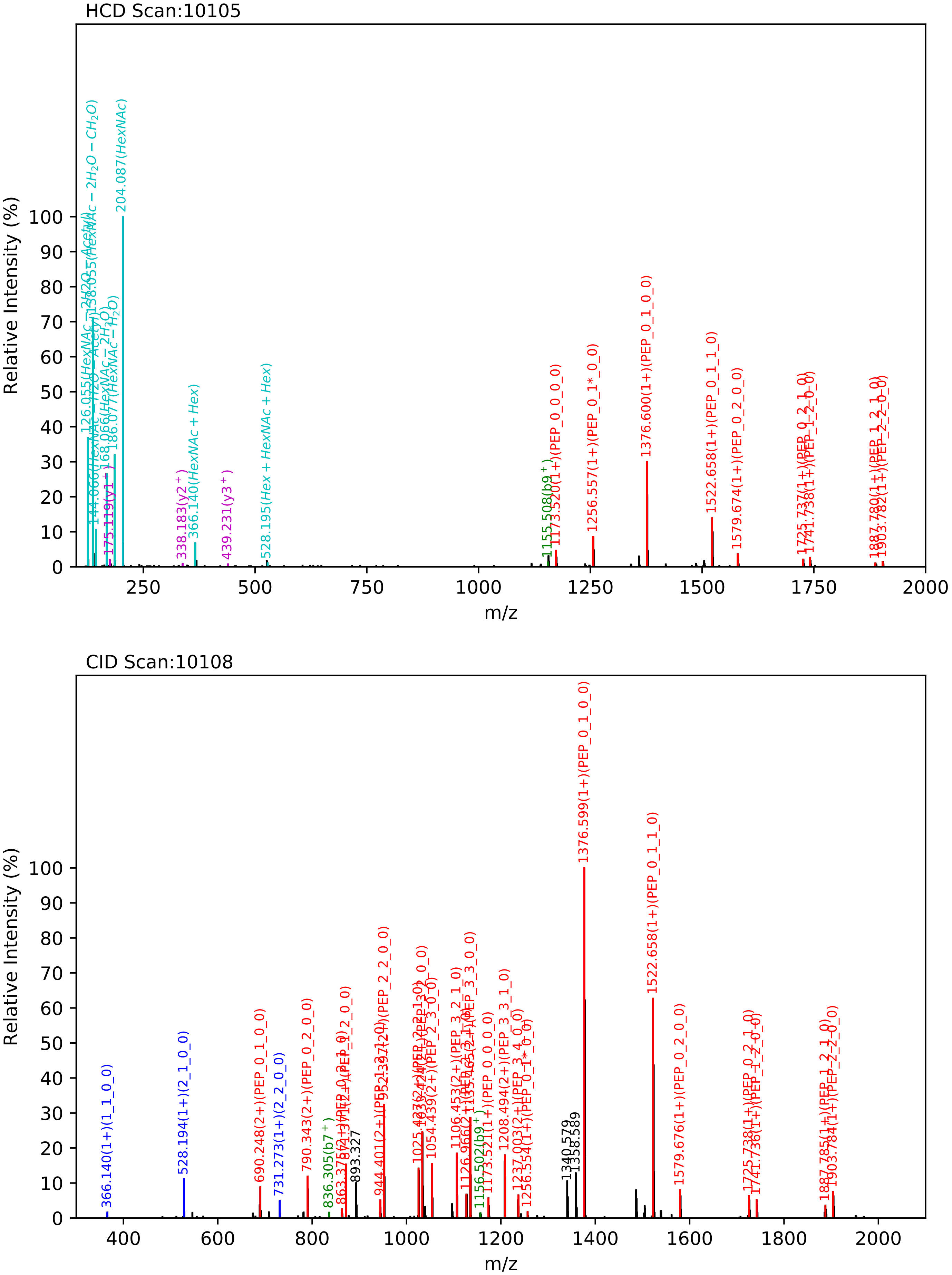

Unknown set no. 519, Experiment: Human Plasma exp_1

EEQFNSTYR(=PEP)_3_5_1_0, m/z:1411.07(2+), RT:42.18, Y-score:90.37

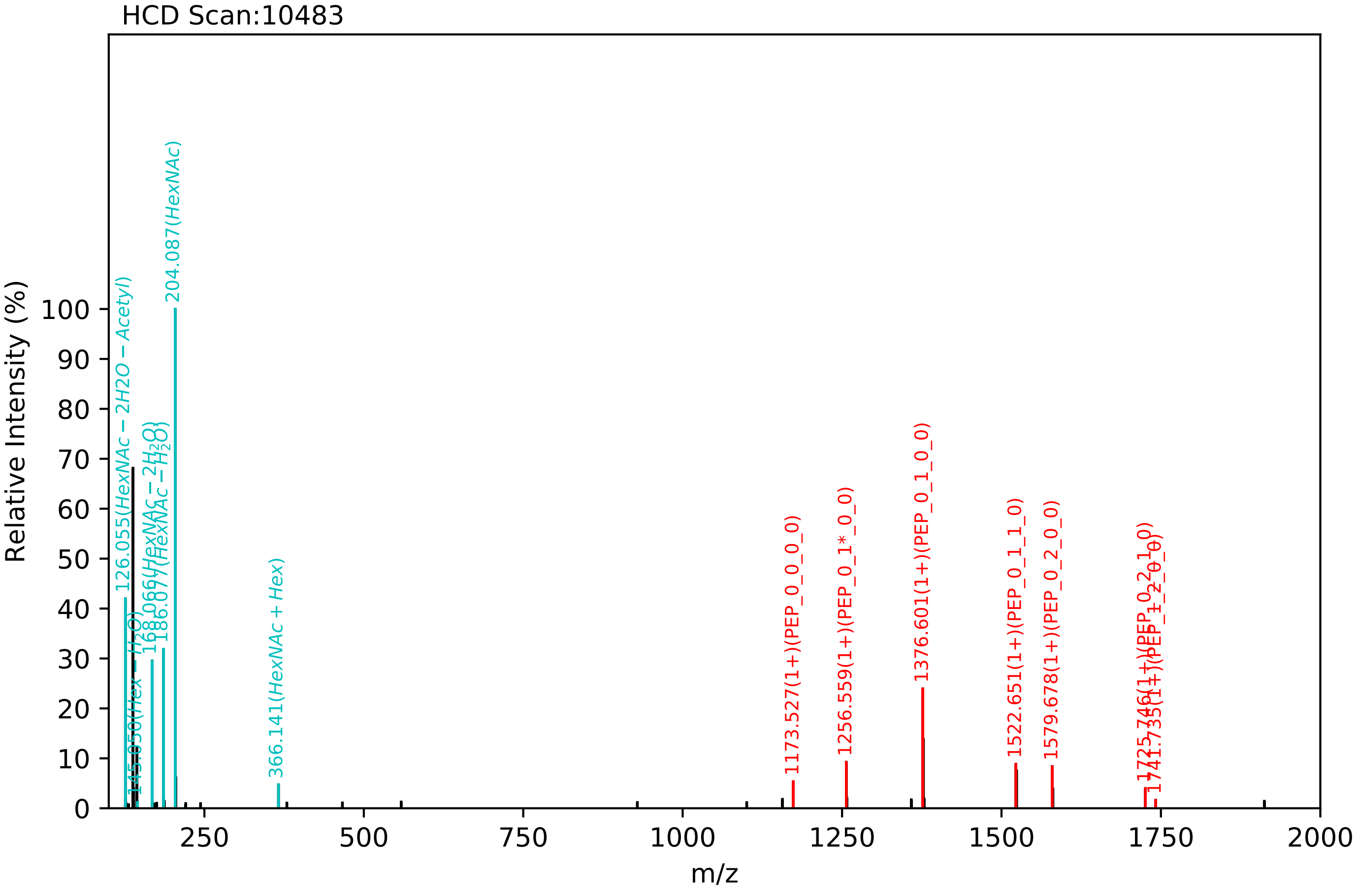

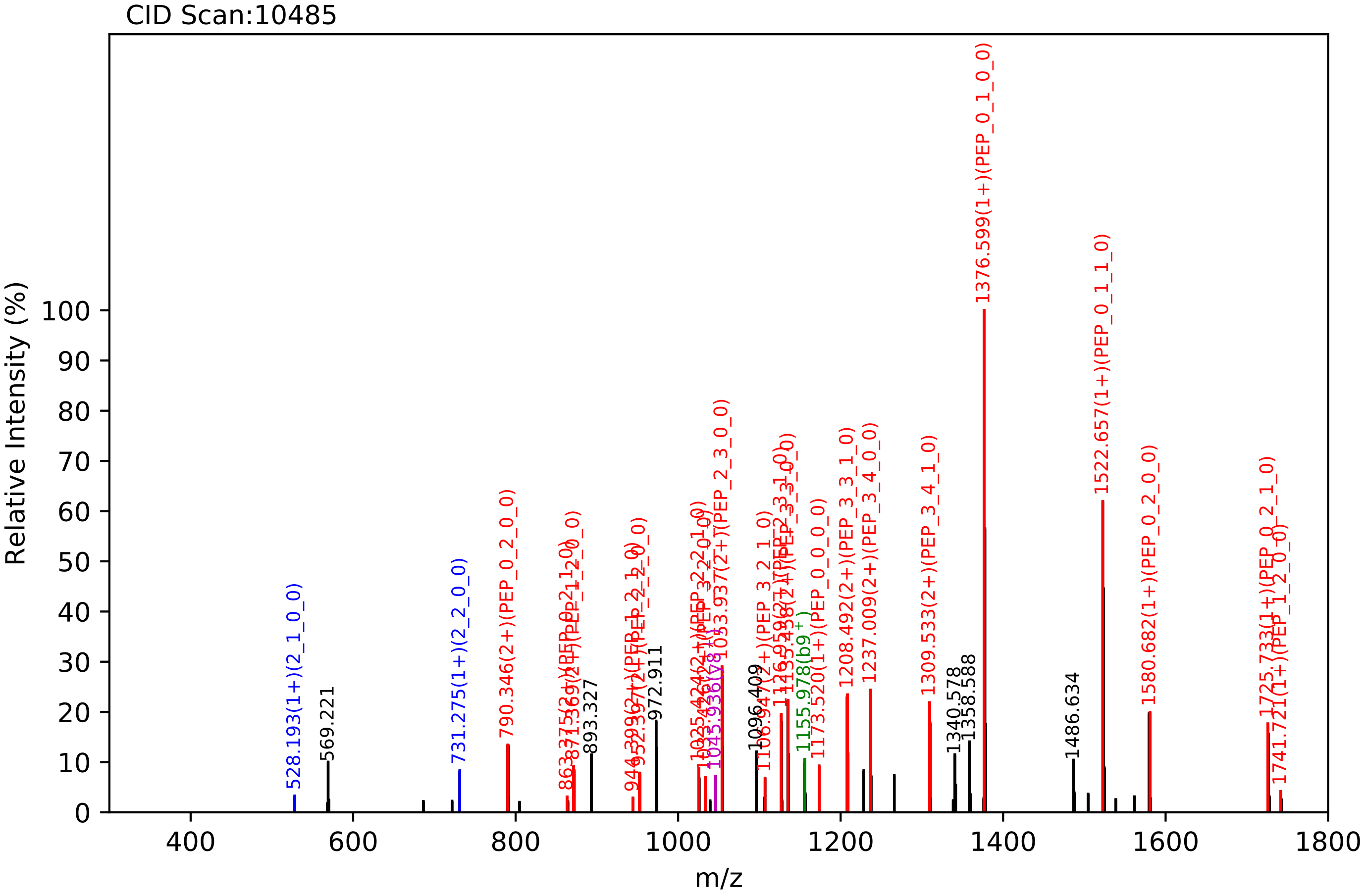

Unknown set no. 520, Experiment: Human Plasma exp_2

EEQFNSTYR(=PEP)_3_5_1_0, m/z:1411.07(2+), RT:42.22, Y-score:89.84

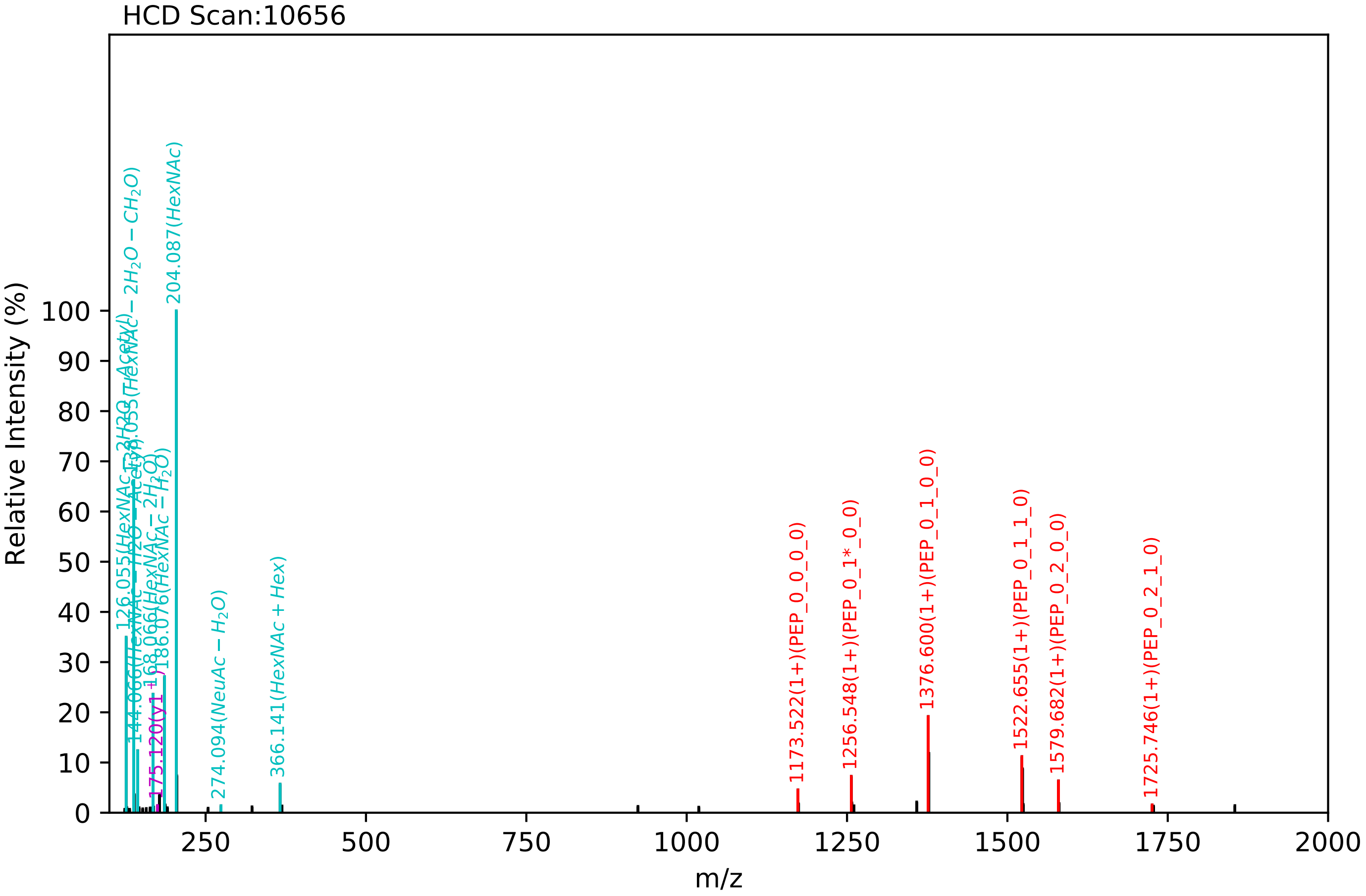

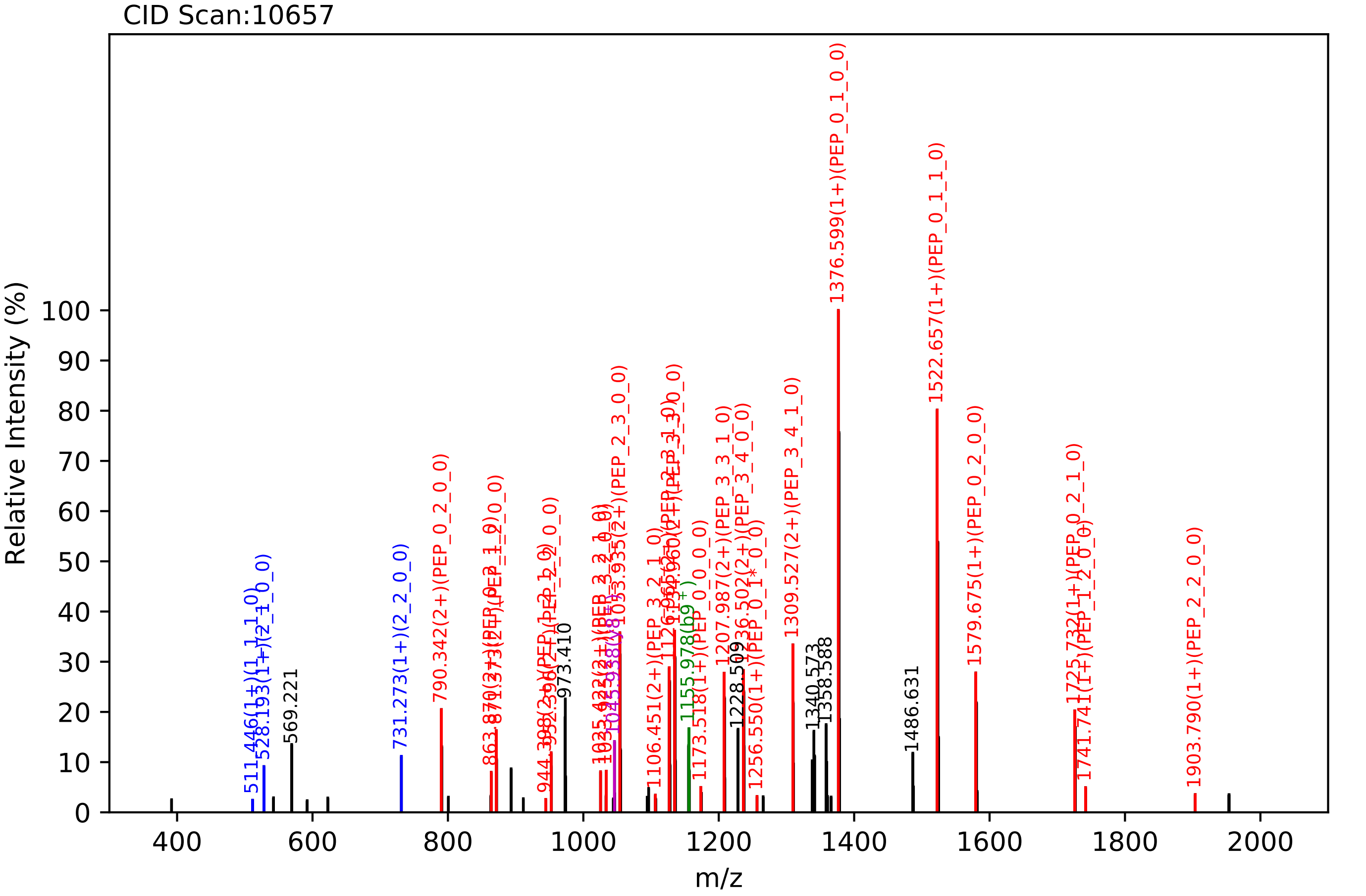

Unknown set no. 521, Experiment: Human Plasma exp_3

EEQFNSTYR(=PEP)_3_5_1_0, m/z:1411.07(2+), RT:42.12, Y-score:89.23

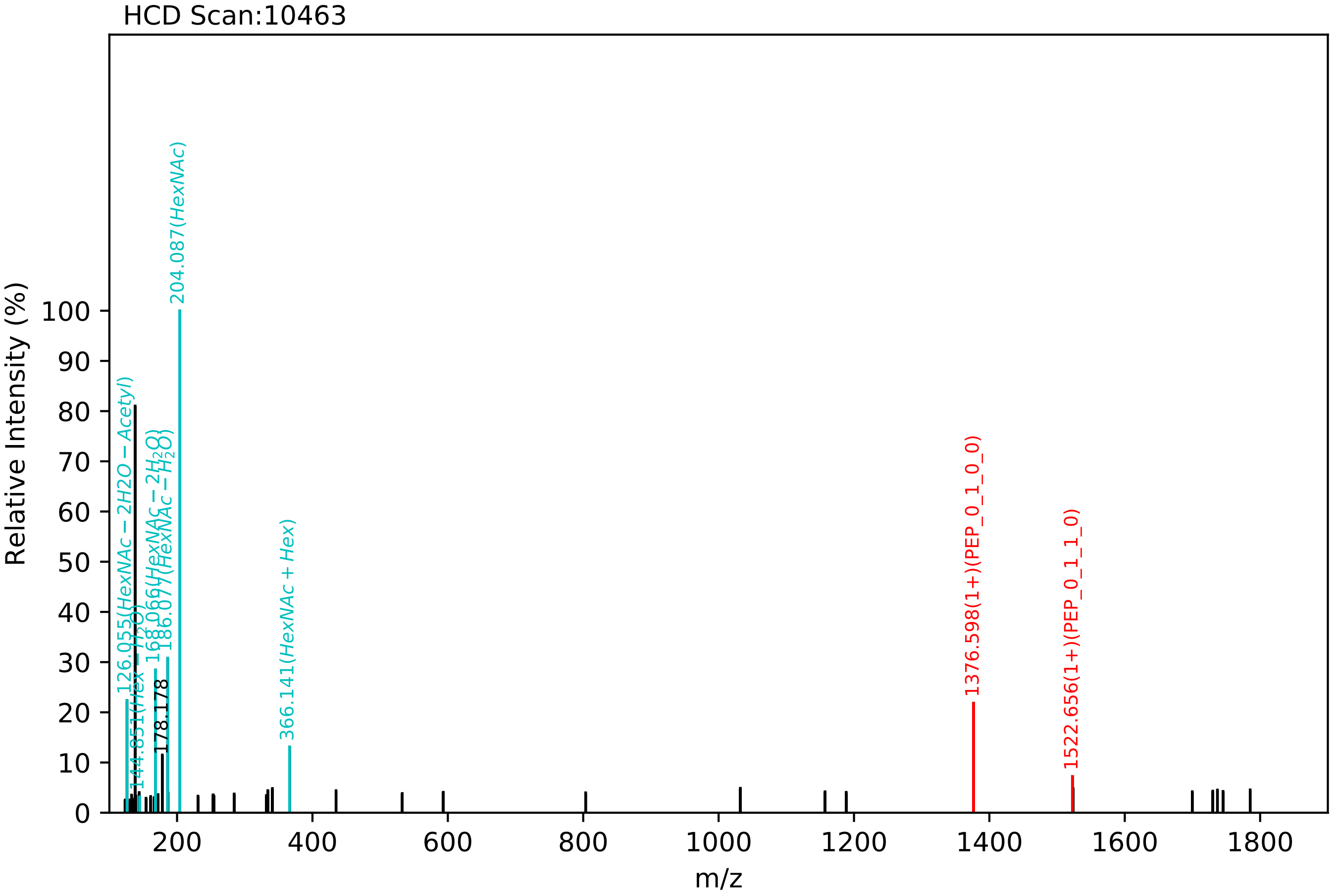

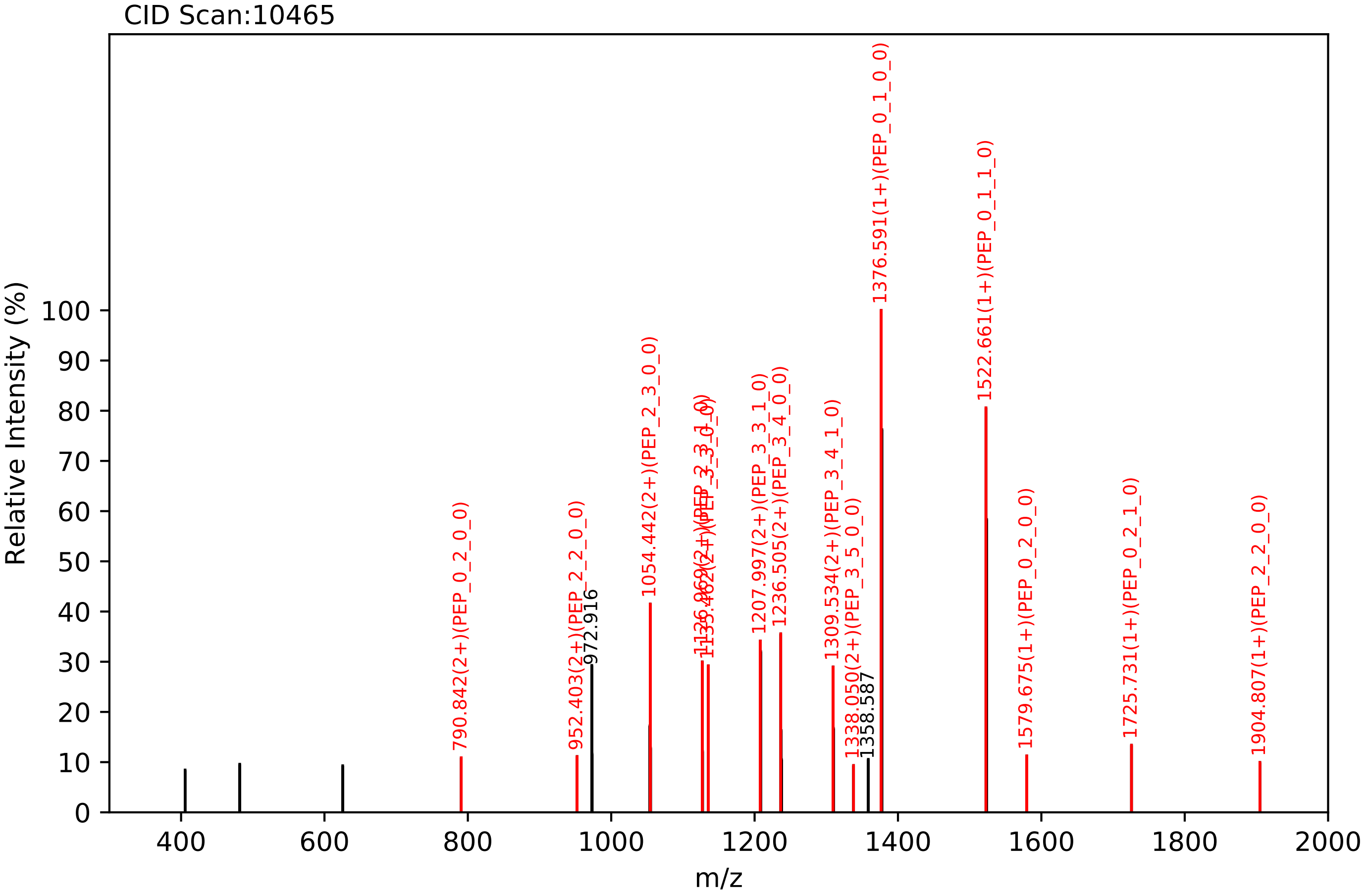

Unknown set no. 522, Experiment: Human Plasma exp_4

EEQFNSTYR(=PEP)_3_5_1_0, m/z:1411.07(2+), RT:42.50, Y-score:82.79

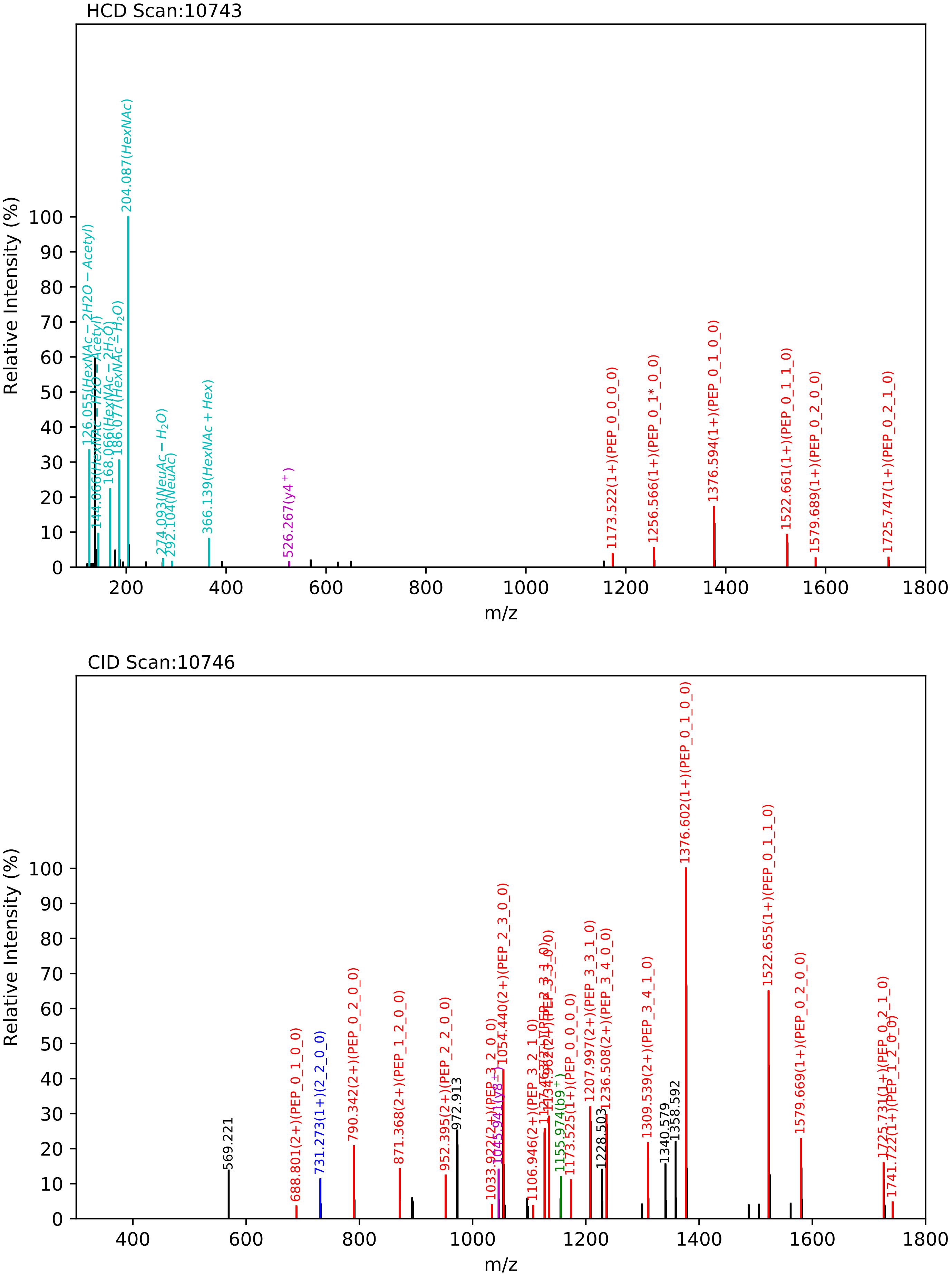

Unknown set no. 523, Experiment: Human Plasma exp_5

EEQFNSTYR(=PEP)_3_5_1_0, m/z:1411.07(2+), RT:42.63, Y-score:80.68

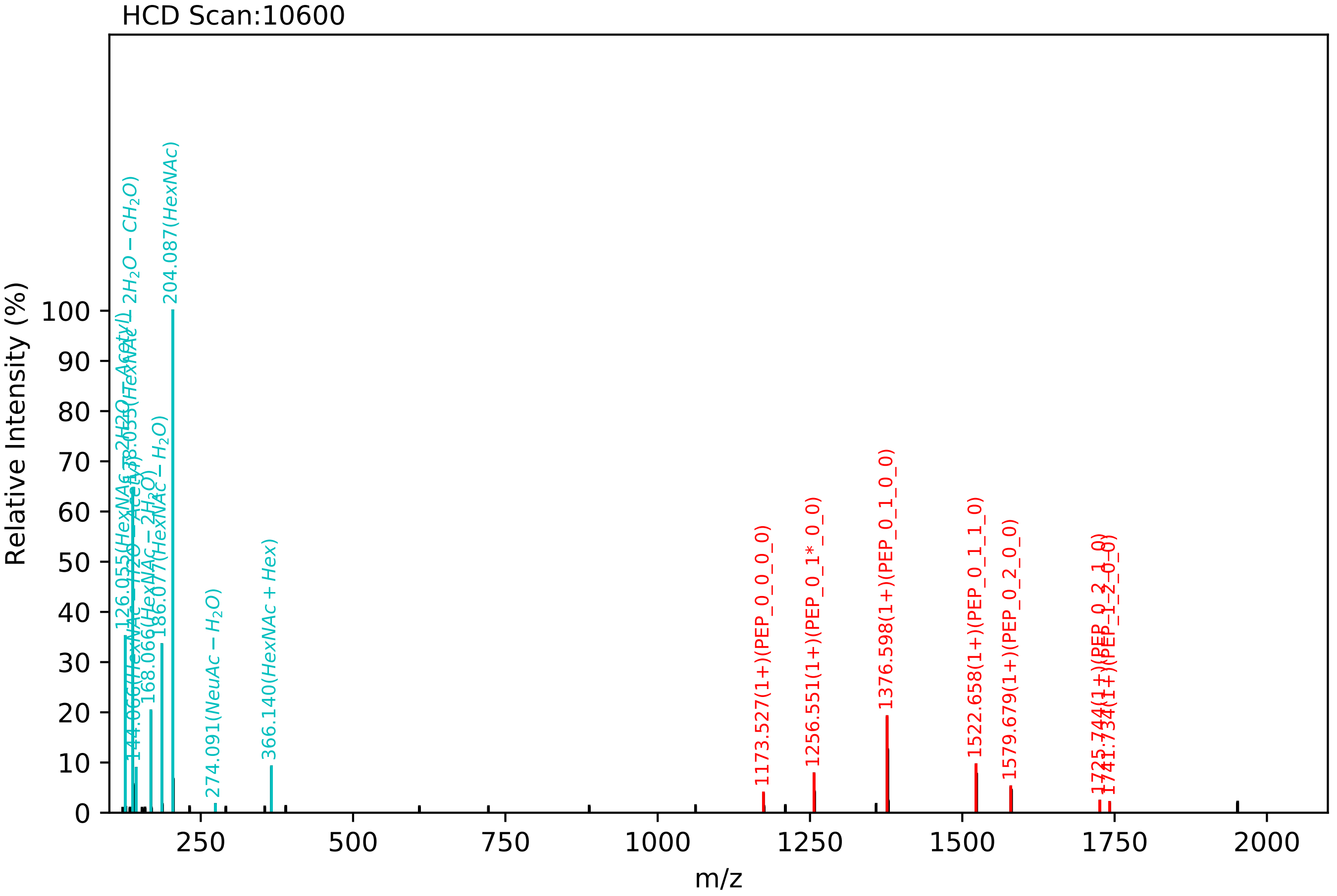

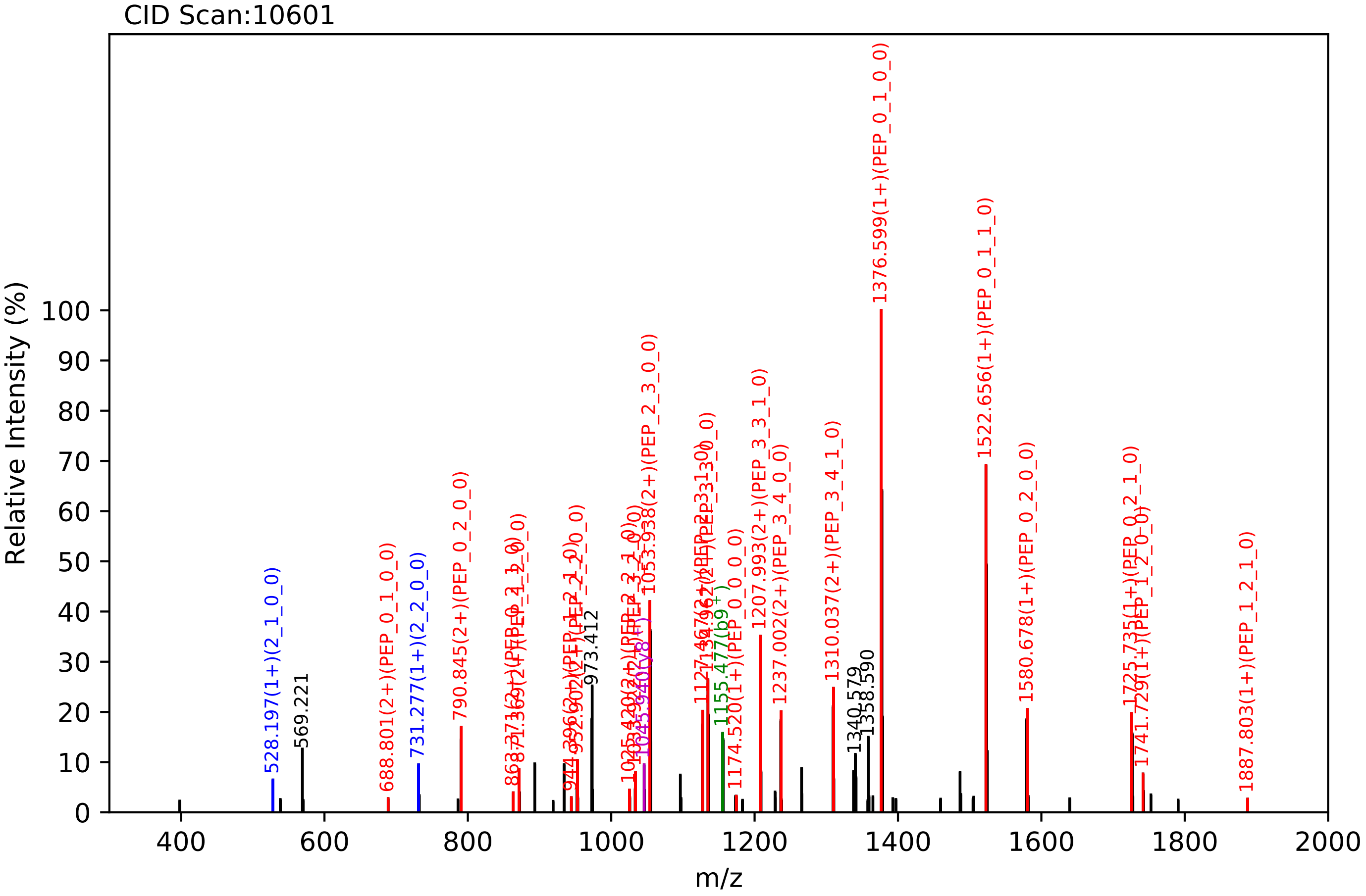

Unknown set no. 524, Experiment: Human Plasma exp_2

EEQFNSTYR(=PEP)_4_3_1_0, m/z:1289.02(2+), RT:39.43, Y-score:94.75

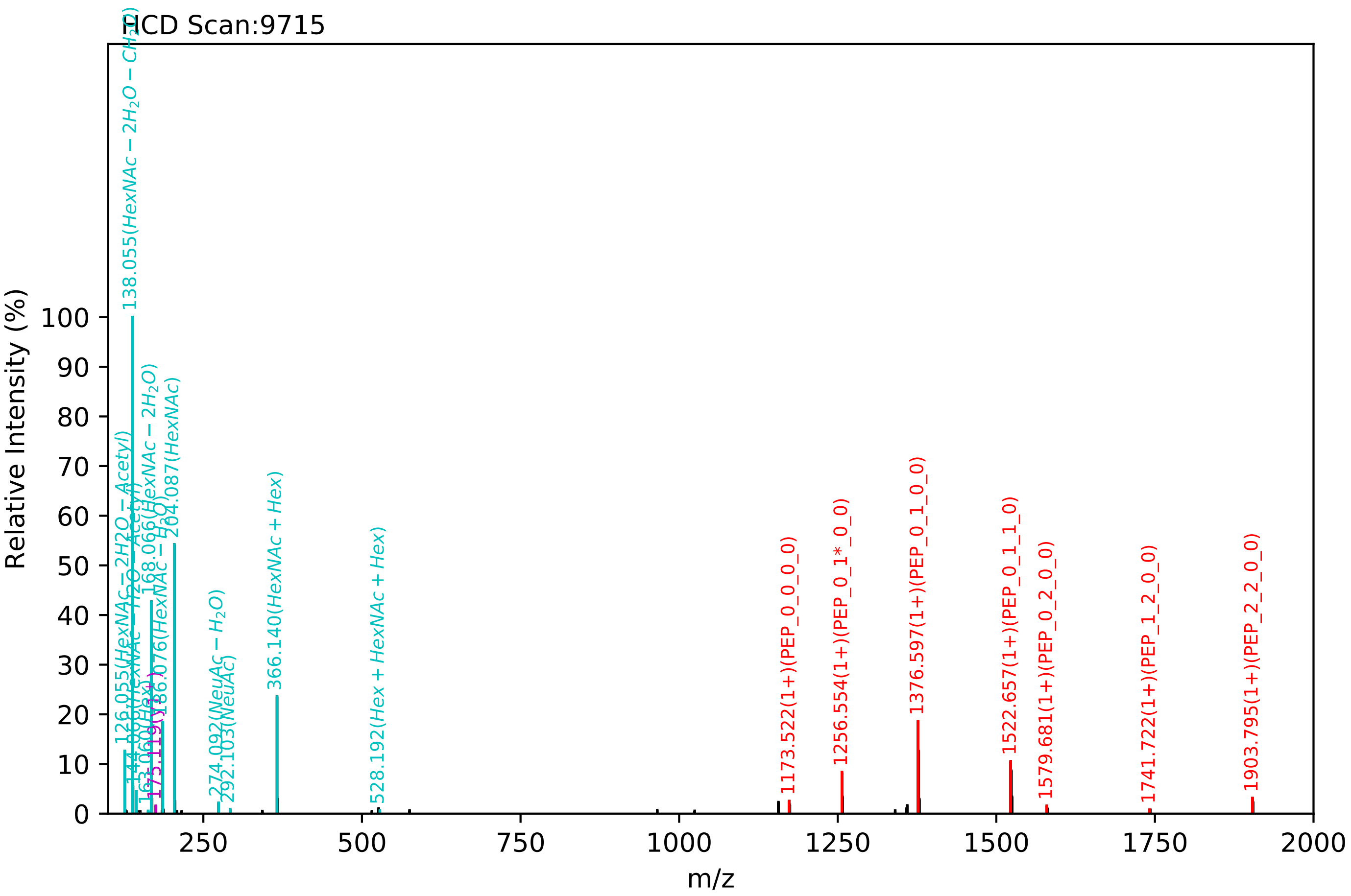

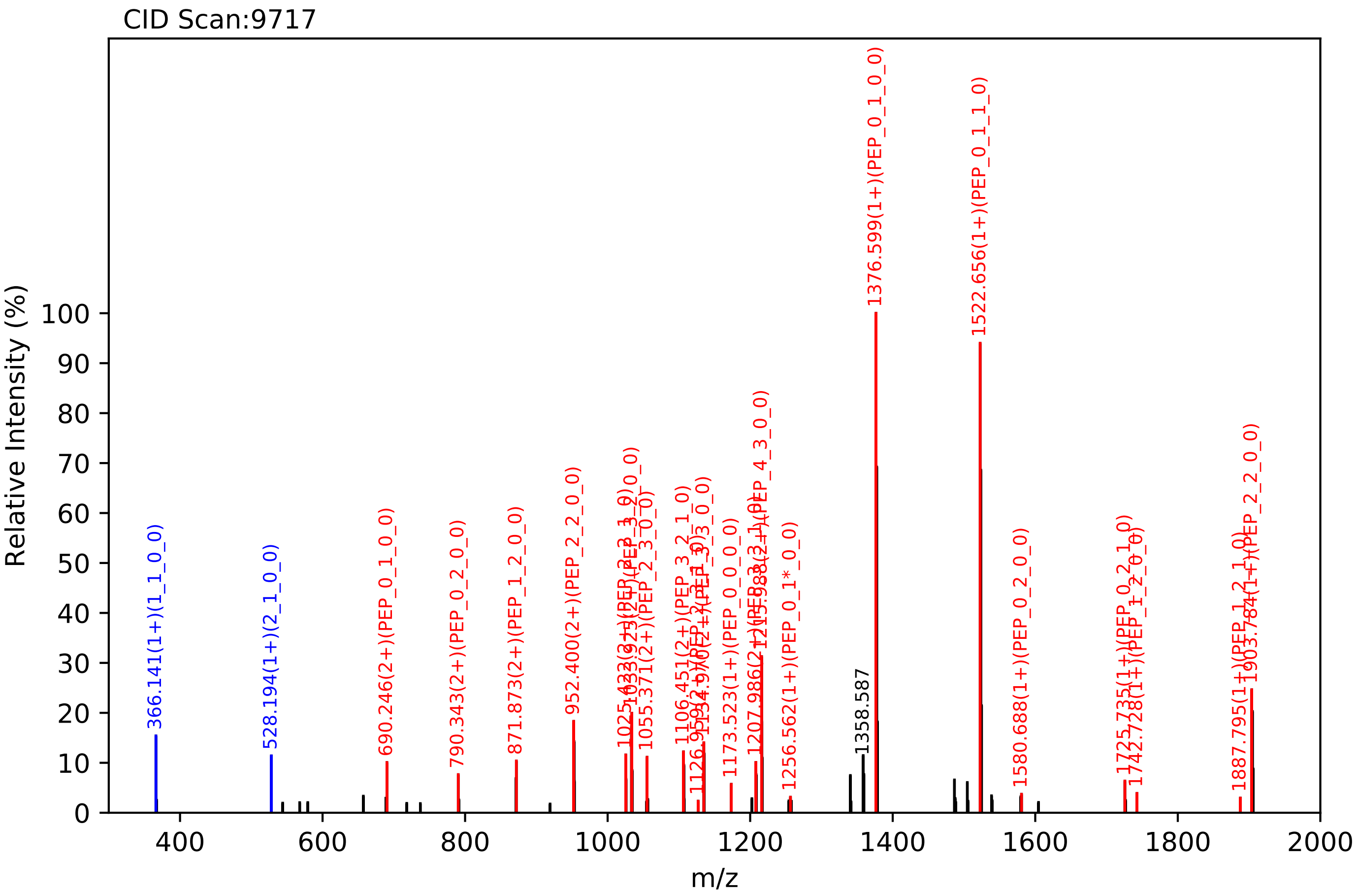

Unknown set no. 525, Experiment: Human Plasma exp_3

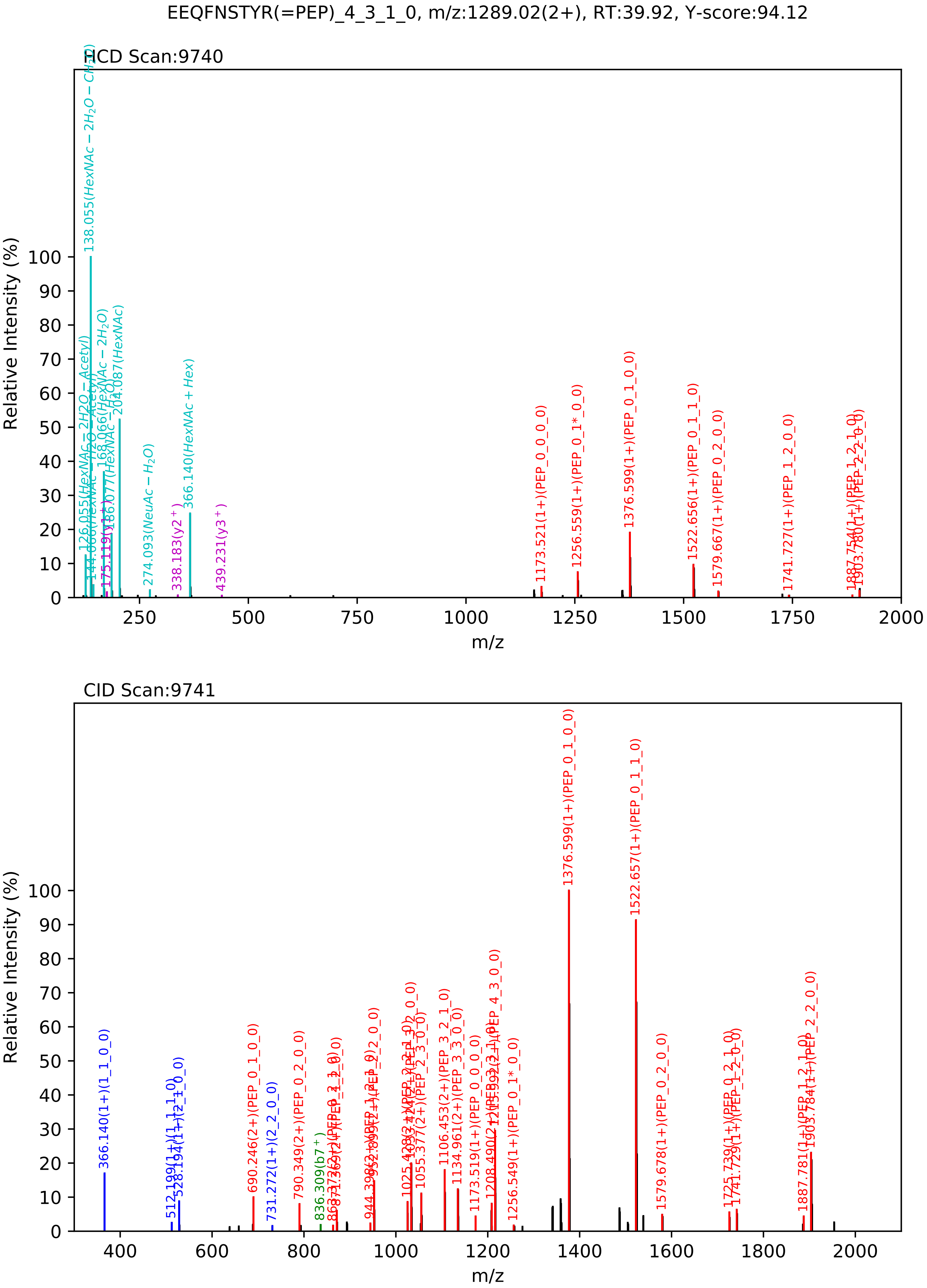

Unknown set no. 526, Experiment: Human Plasma exp_4

EEQFNSTYR(=PEP)_4_3_1_0, m/z:1289.02(2+), RT:40.25, Y-score:89.59

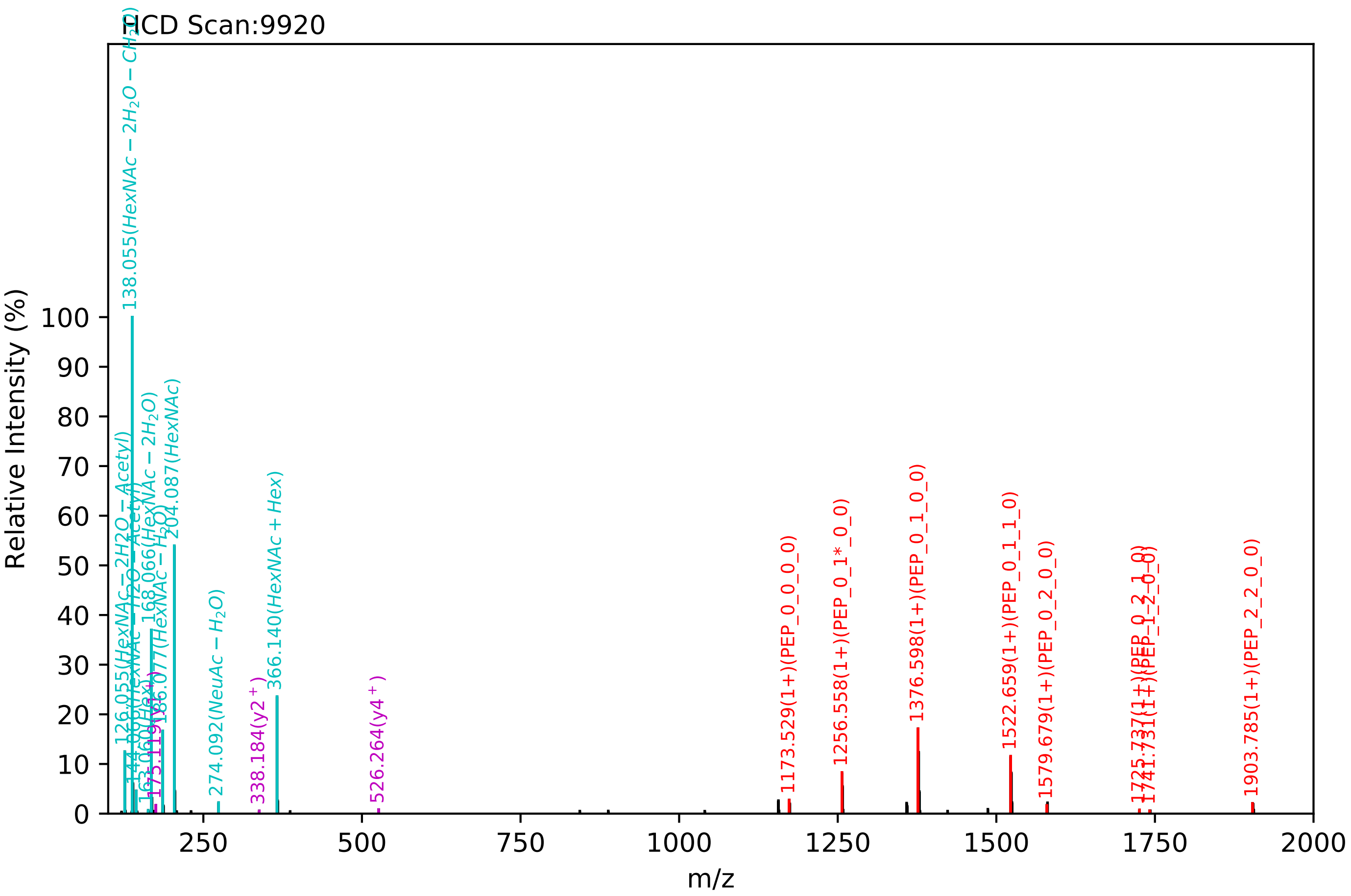

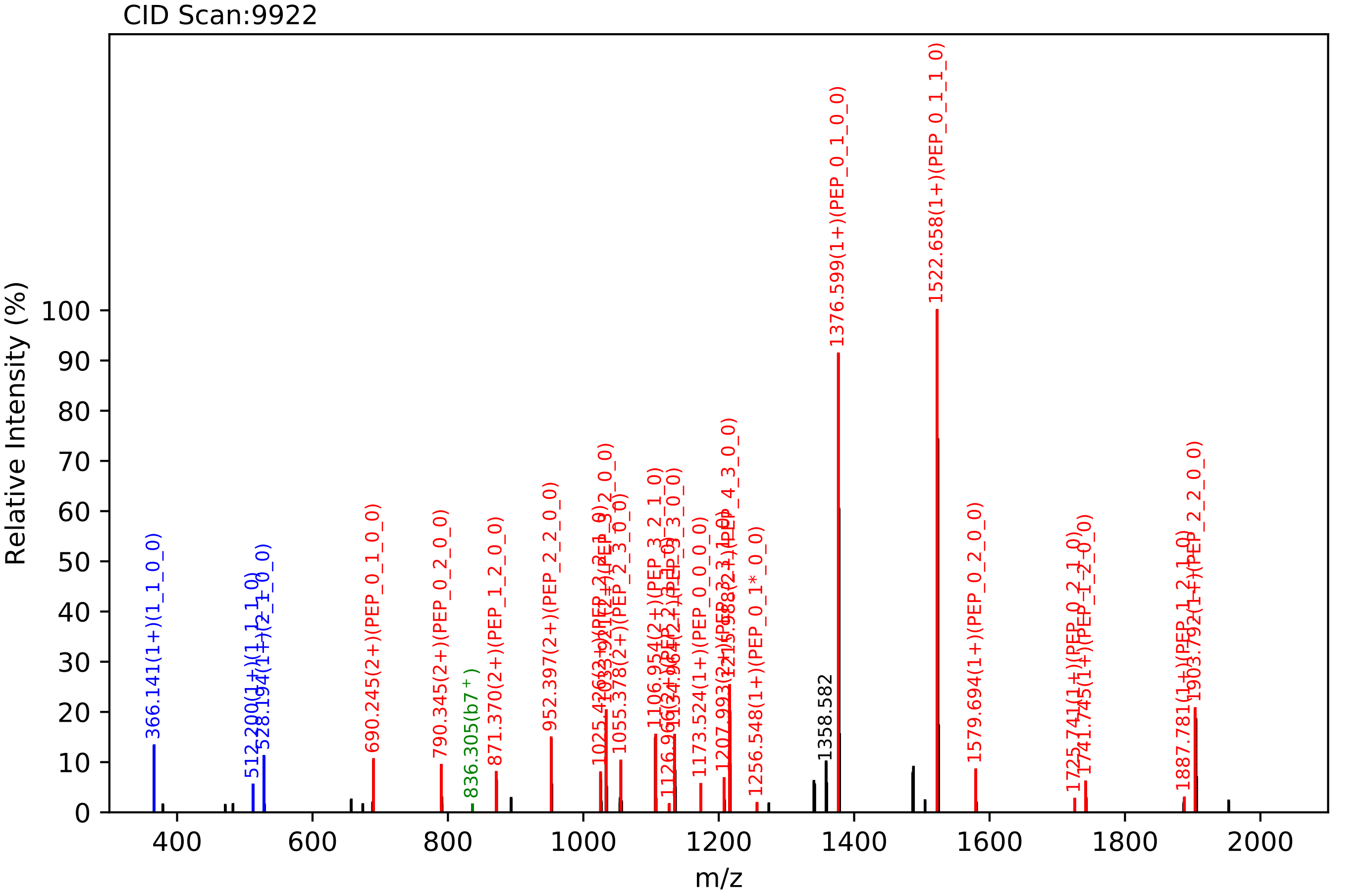

Unknown set no. 527, Experiment: Human Plasma exp_1

EEQFNSTYR(=PEP)_4_4_1_0, m/z:927.38(3+), RT:40.22, Y-score:97.77

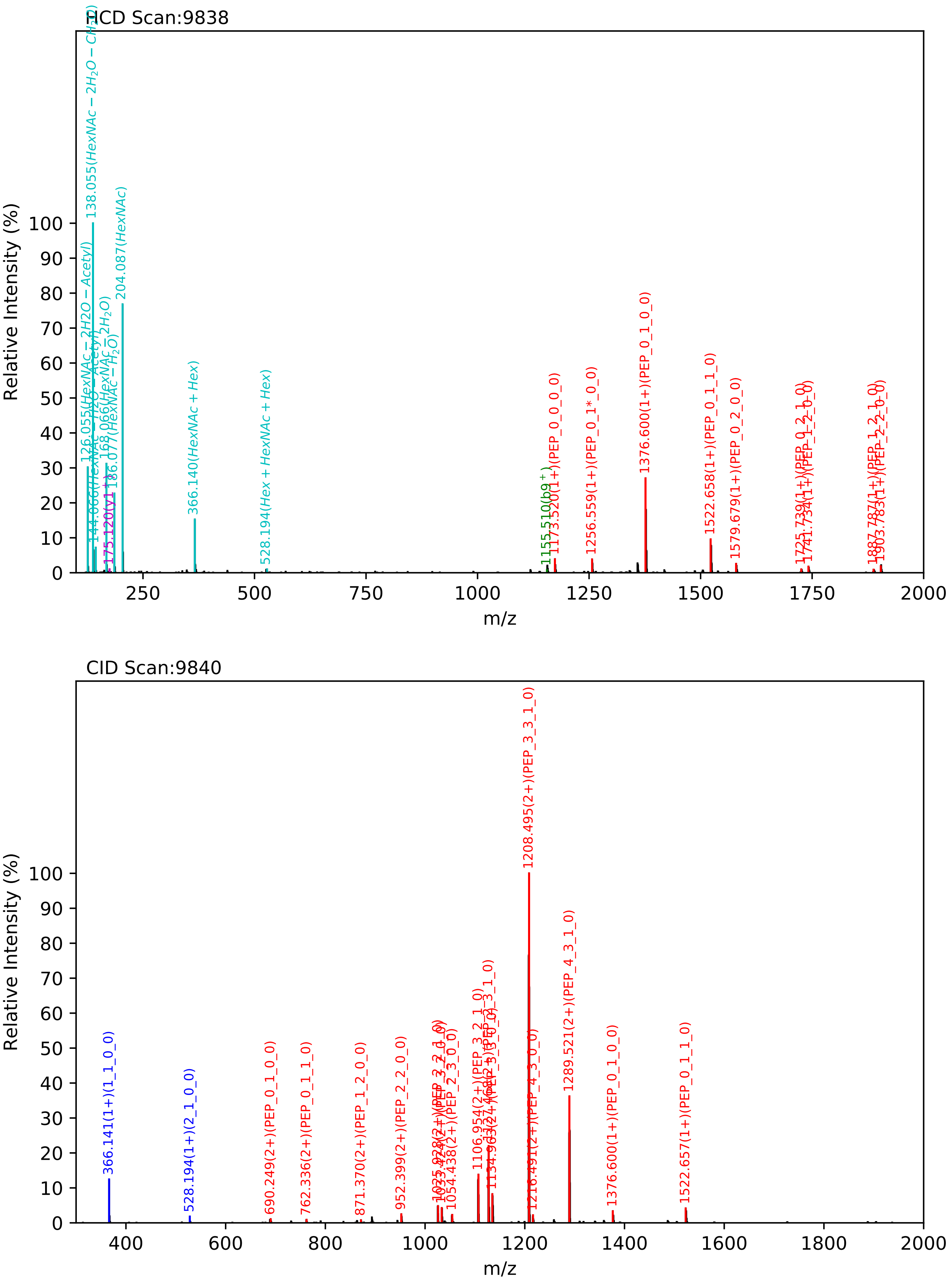

Unknown set no. 528, Experiment: Human Plasma exp_2

EEQFNSTYR(=PEP)_4_4_1_0, m/z:927.38(3+), RT:40.41, Y-score:98.54

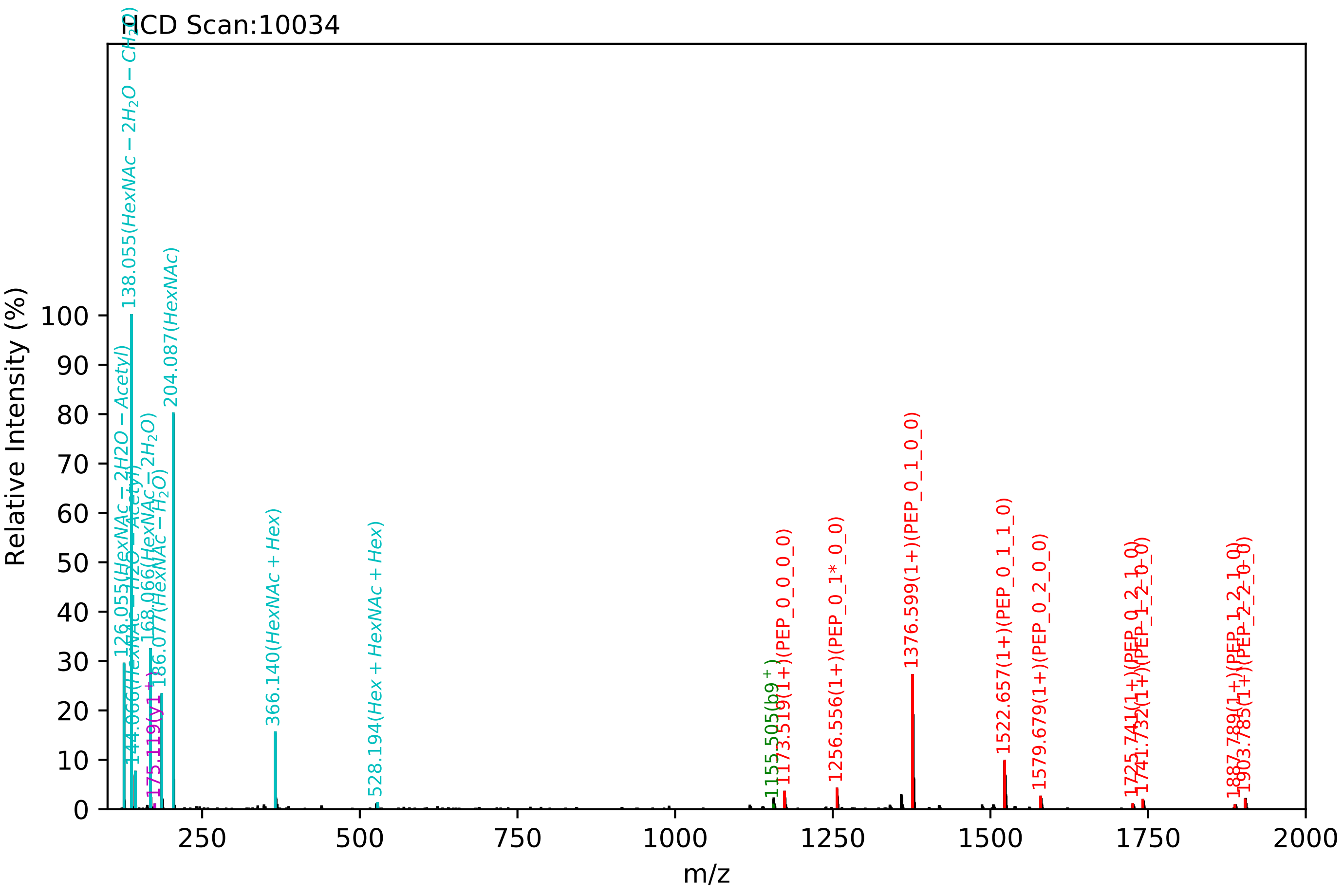

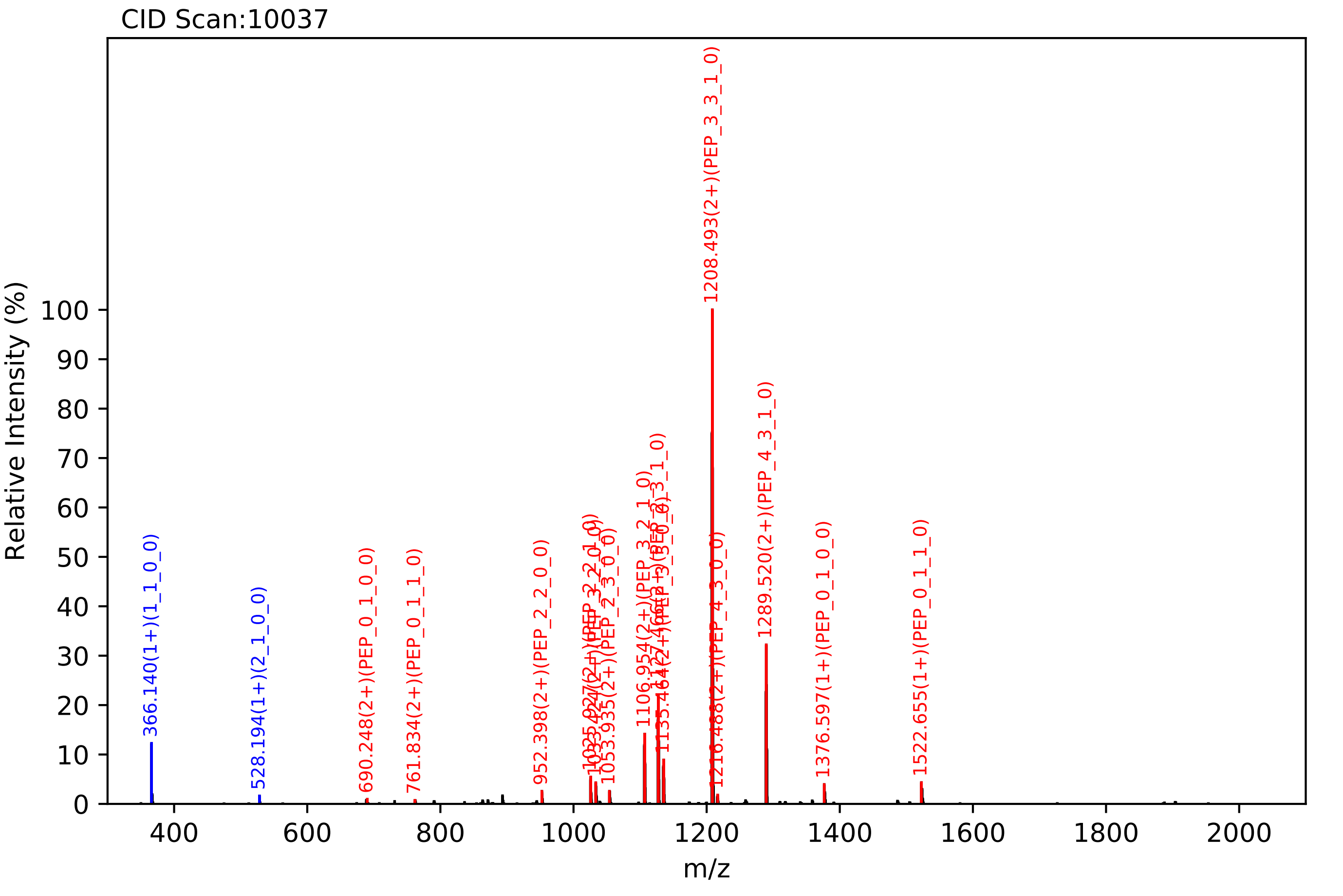

Unknown set no. 529, Experiment: Human Plasma exp_2

EEQFNSTYR(=PEP)_4_4_1_0, m/z:1390.56(2+), RT:41.01, Y-score:97.63

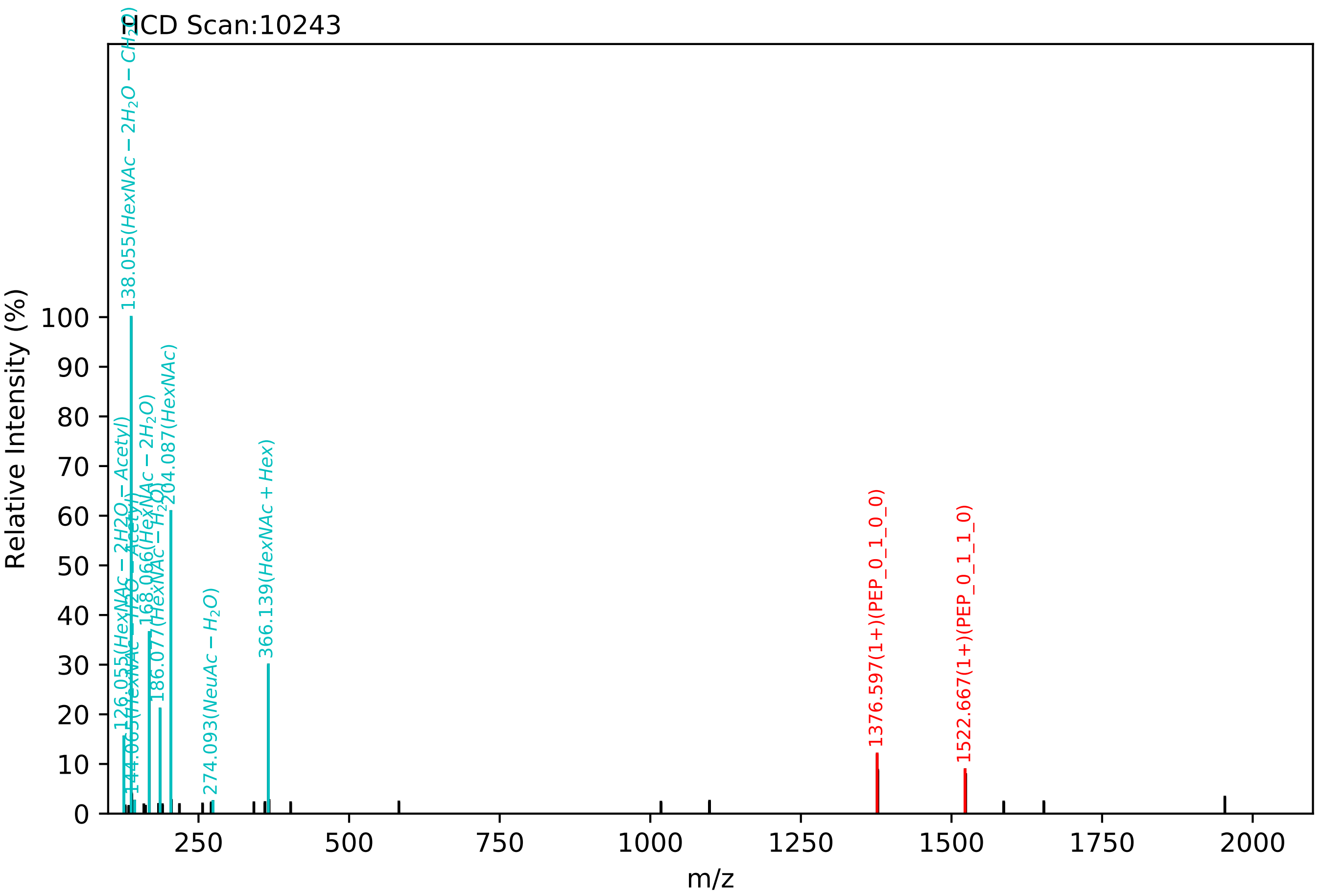

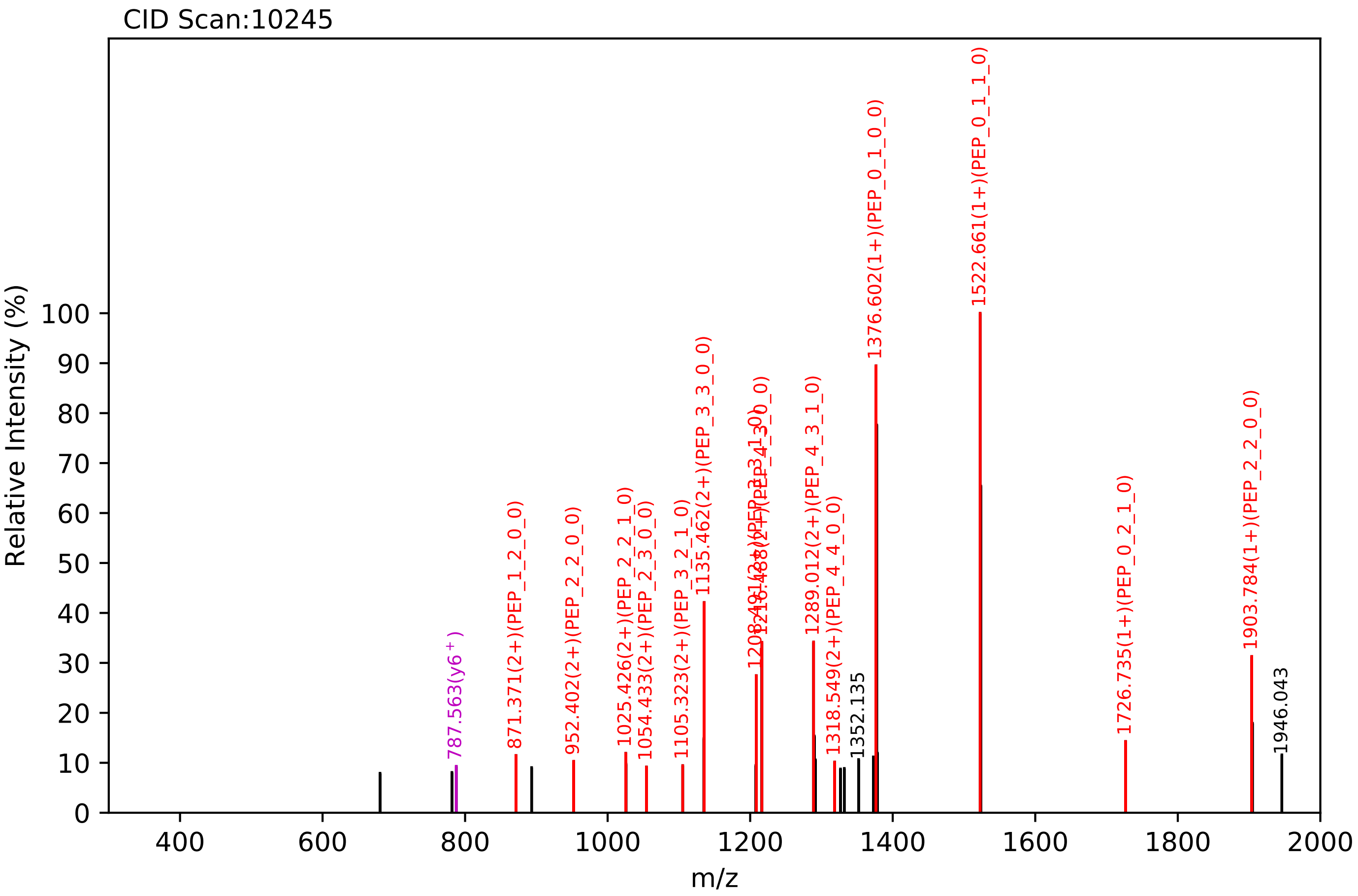

Unknown set no. 530, Experiment: Human Plasma exp_5

EEQFNSTYR(=PEP)_4_4_1_0, m/z:927.38(3+), RT:40.83, Y-score:98.59

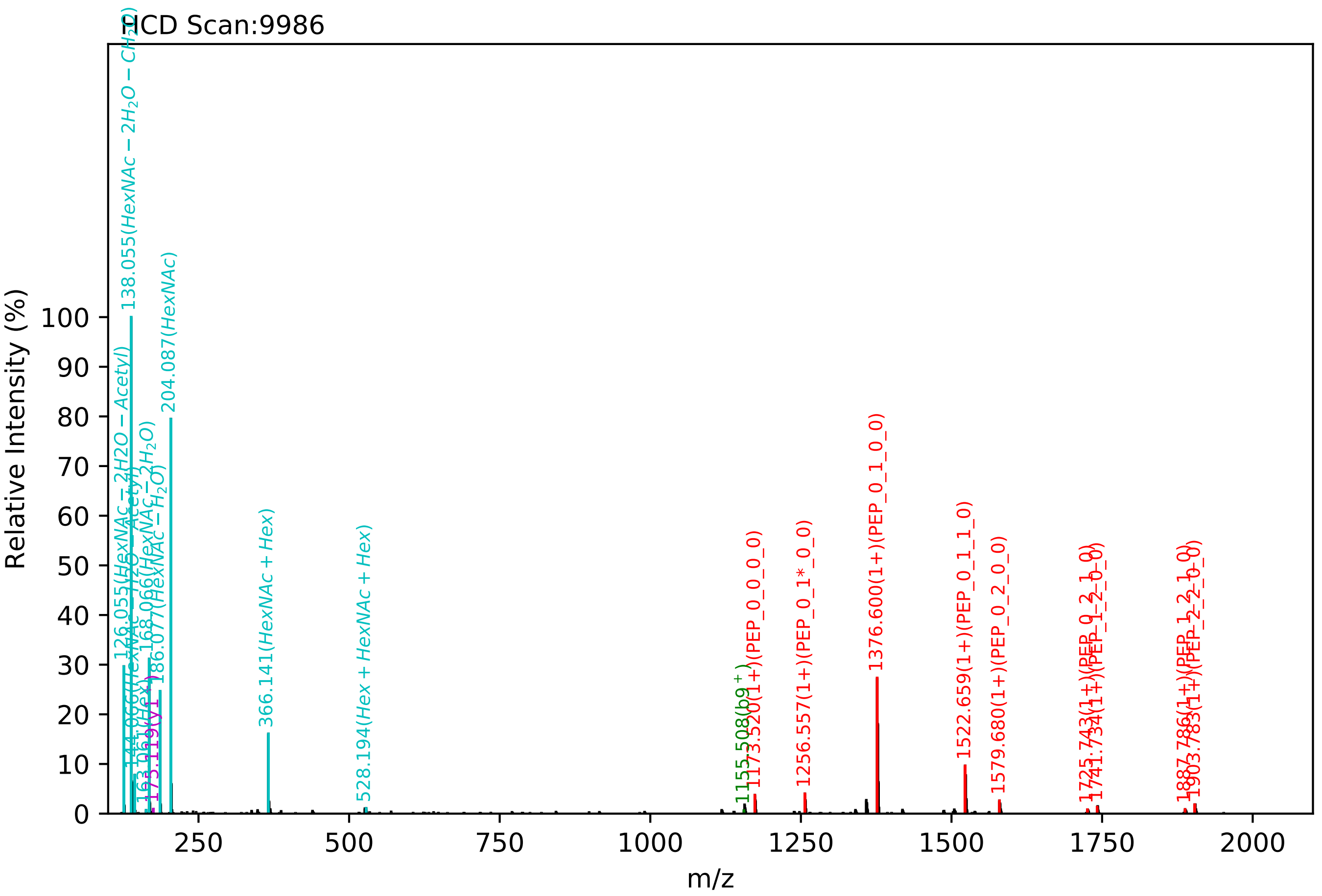

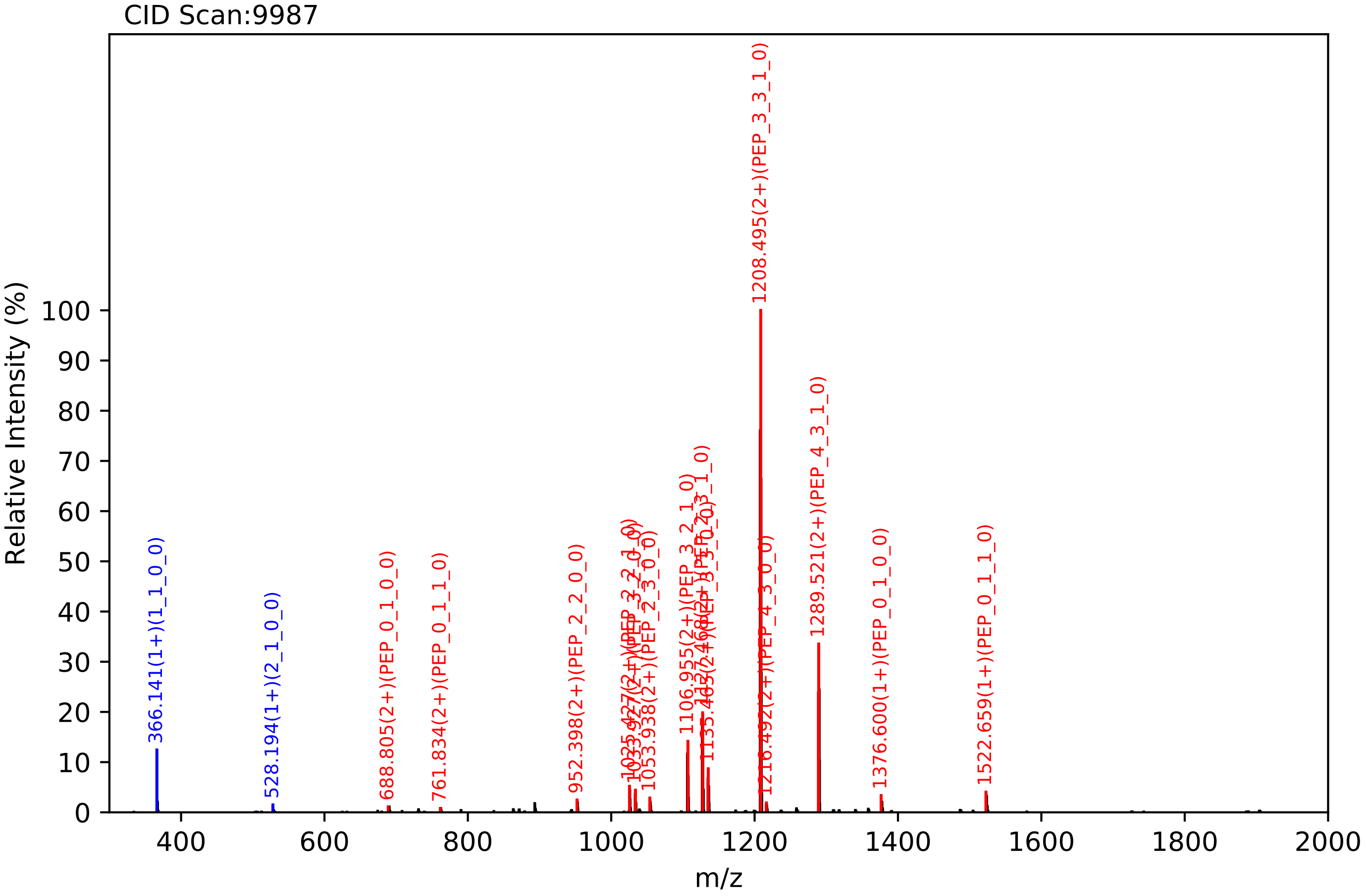

Unknown set no. 531, Experiment: Human Plasma exp_5

EEQFNSTYR(=PEP)_4_4_1_0, m/z:1390.56(2+), RT:40.89, Y-score:94.76

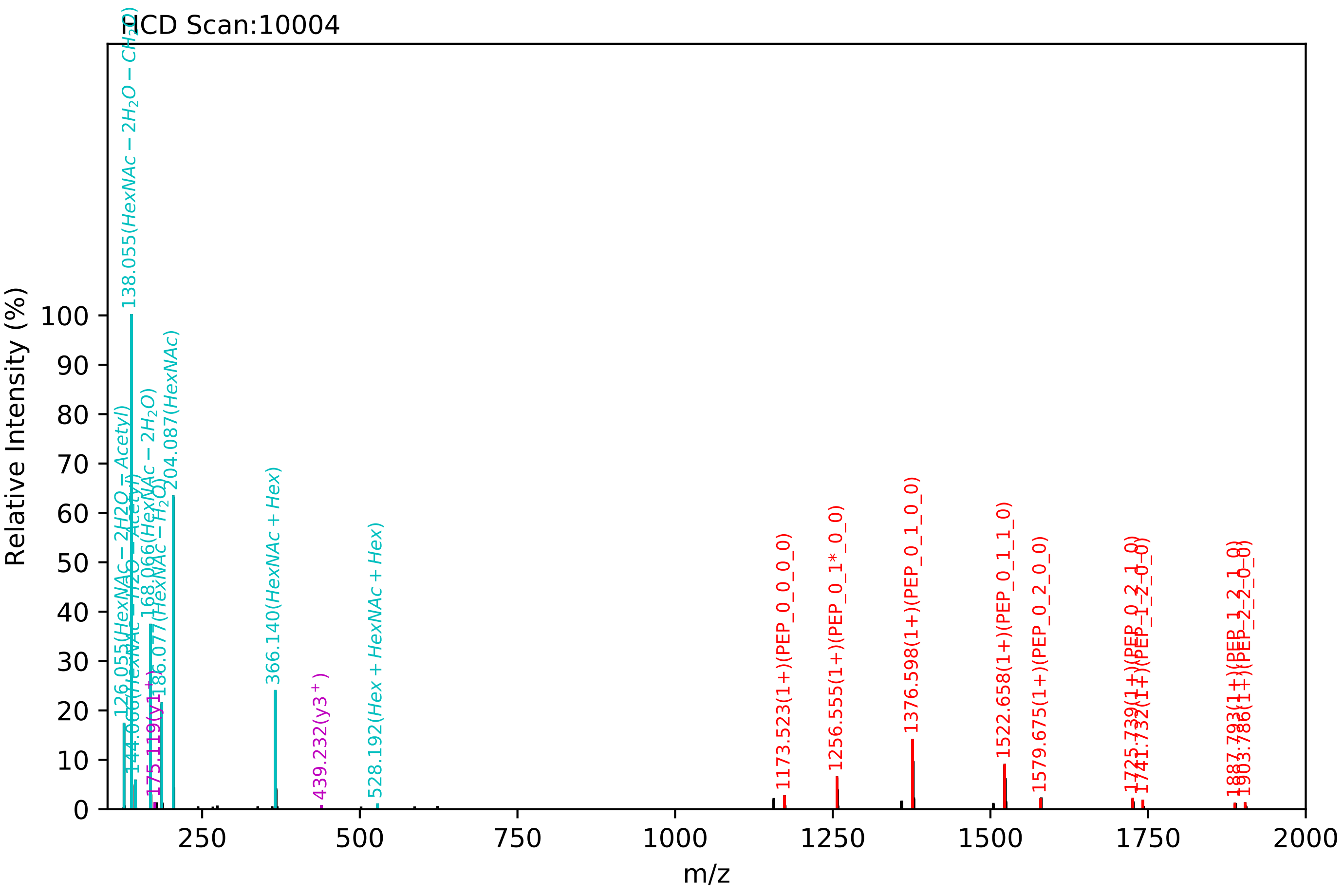

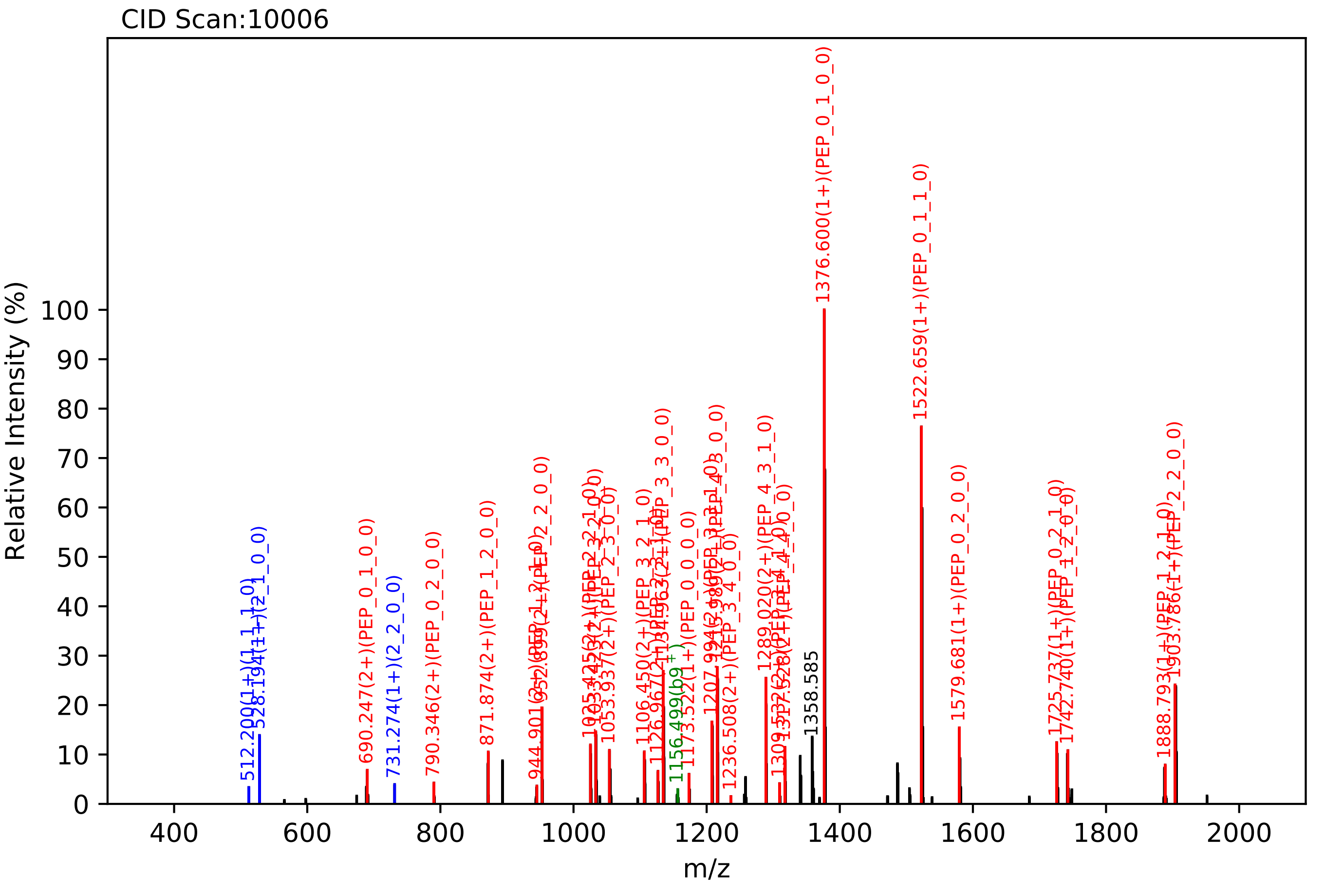

Unknown set no. 532, Experiment: Human Plasma exp_1

EEQFNSTYR(=PEP)_4_4_1_1, m/z:1024.41(3+), RT:50.65, Y-score:82.43

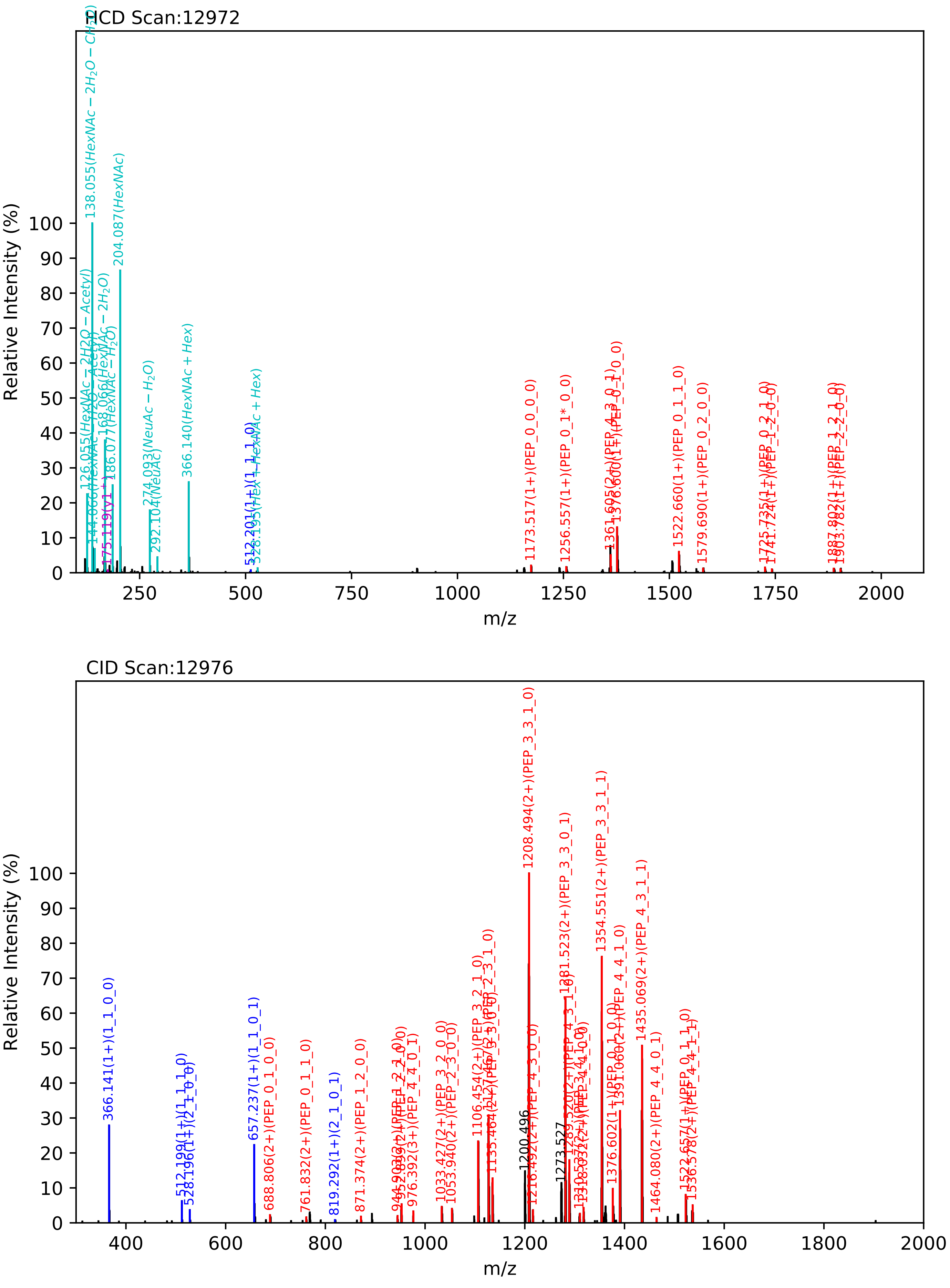

Unknown set no. 533, Experiment: Human Plasma exp_1

EEQFNSTYR(=PEP)_4_4_1_1, m/z:1536.11(2+), RT:50.77, Y-score:81.23

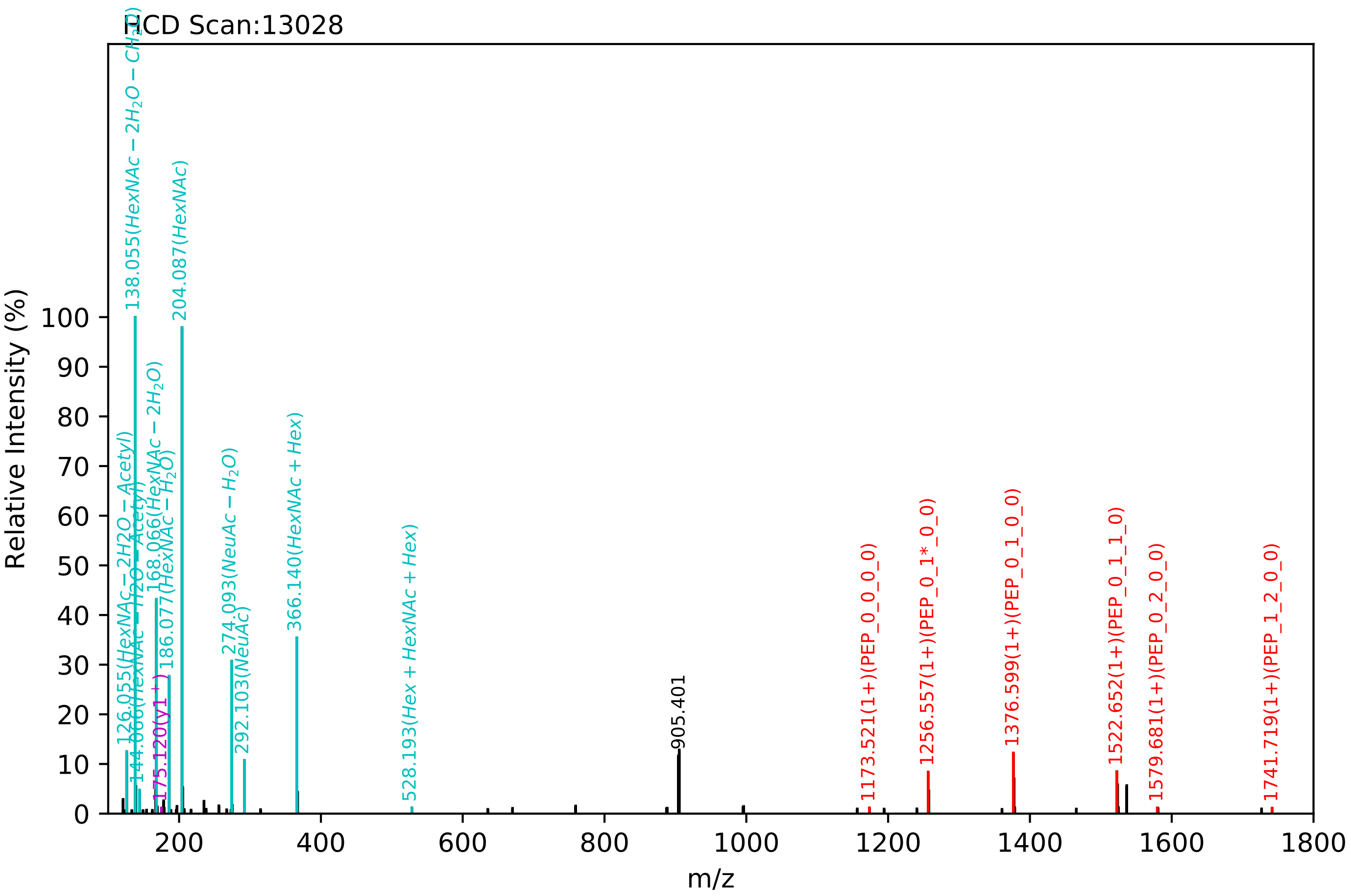

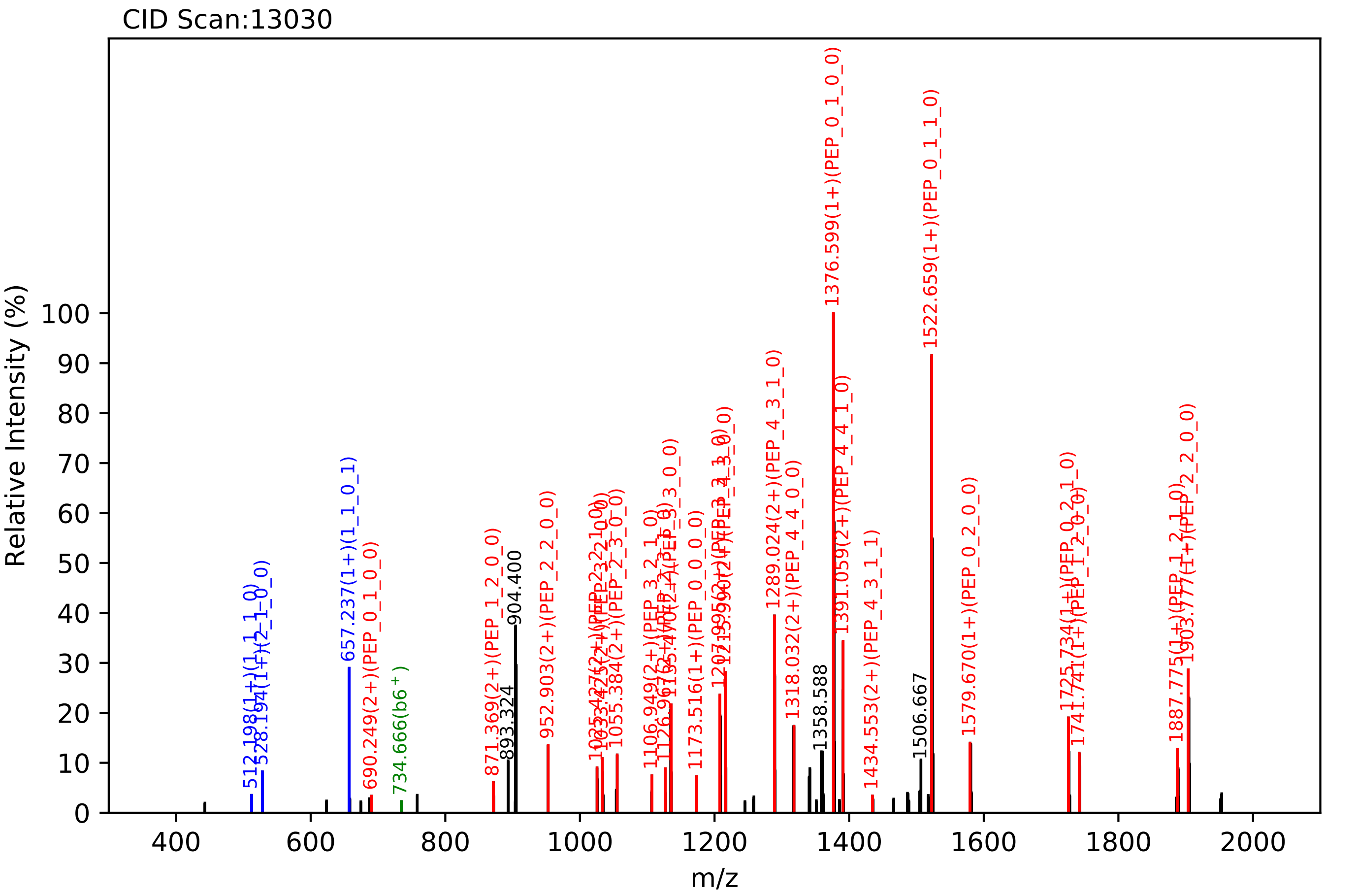

Unknown set no. 534, Experiment: Human Plasma exp_2

EEQFNSTYR(=PEP)_4_4_1_1, m/z:1024.41(3+), RT:50.70, Y-score:81.60

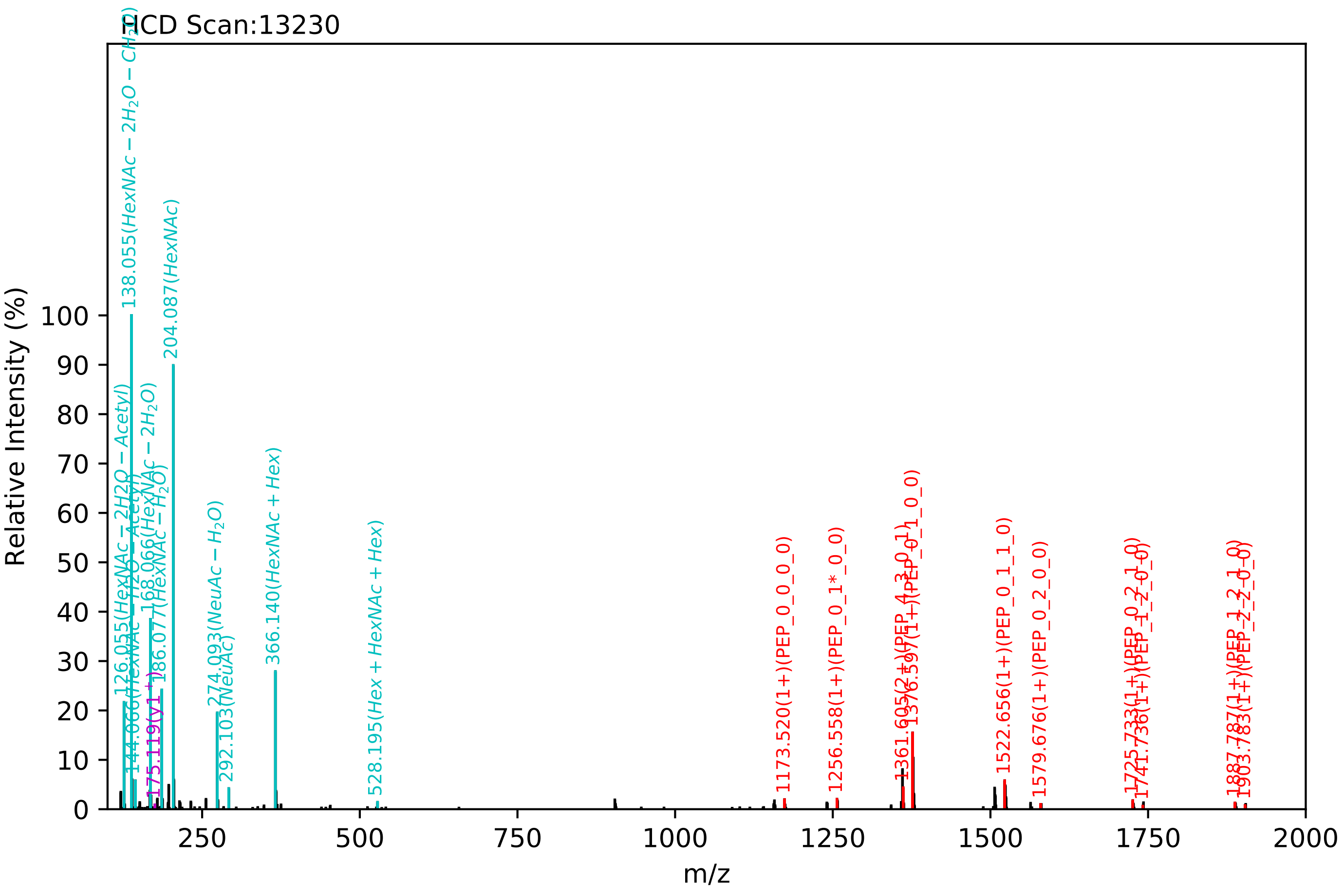

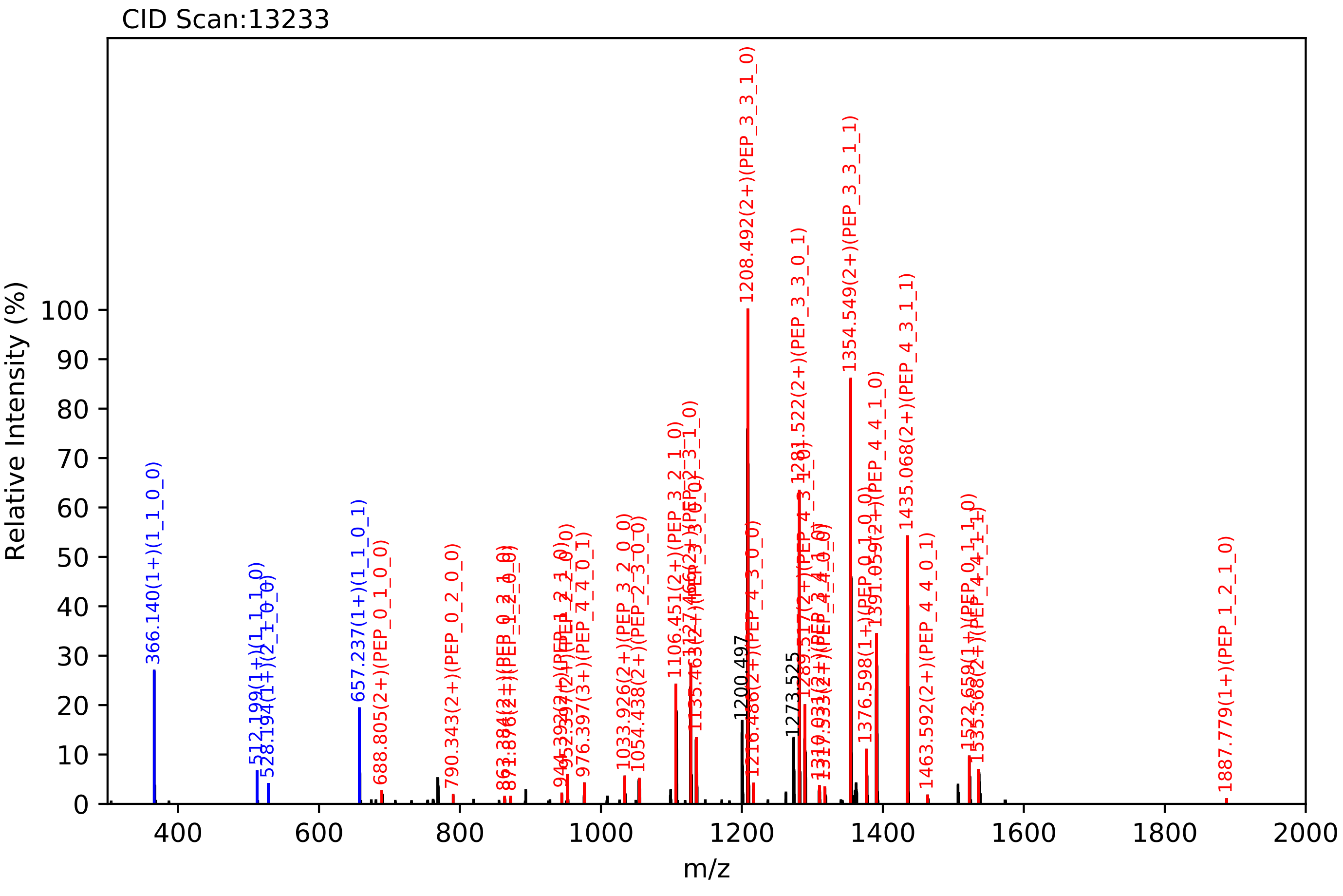

Unknown set no. 535, Experiment: Human Plasma exp_3

EEQFNSTYR(=PEP)_4_4_1_1, m/z:1024.41(3+), RT:50.95, Y-score:83.07

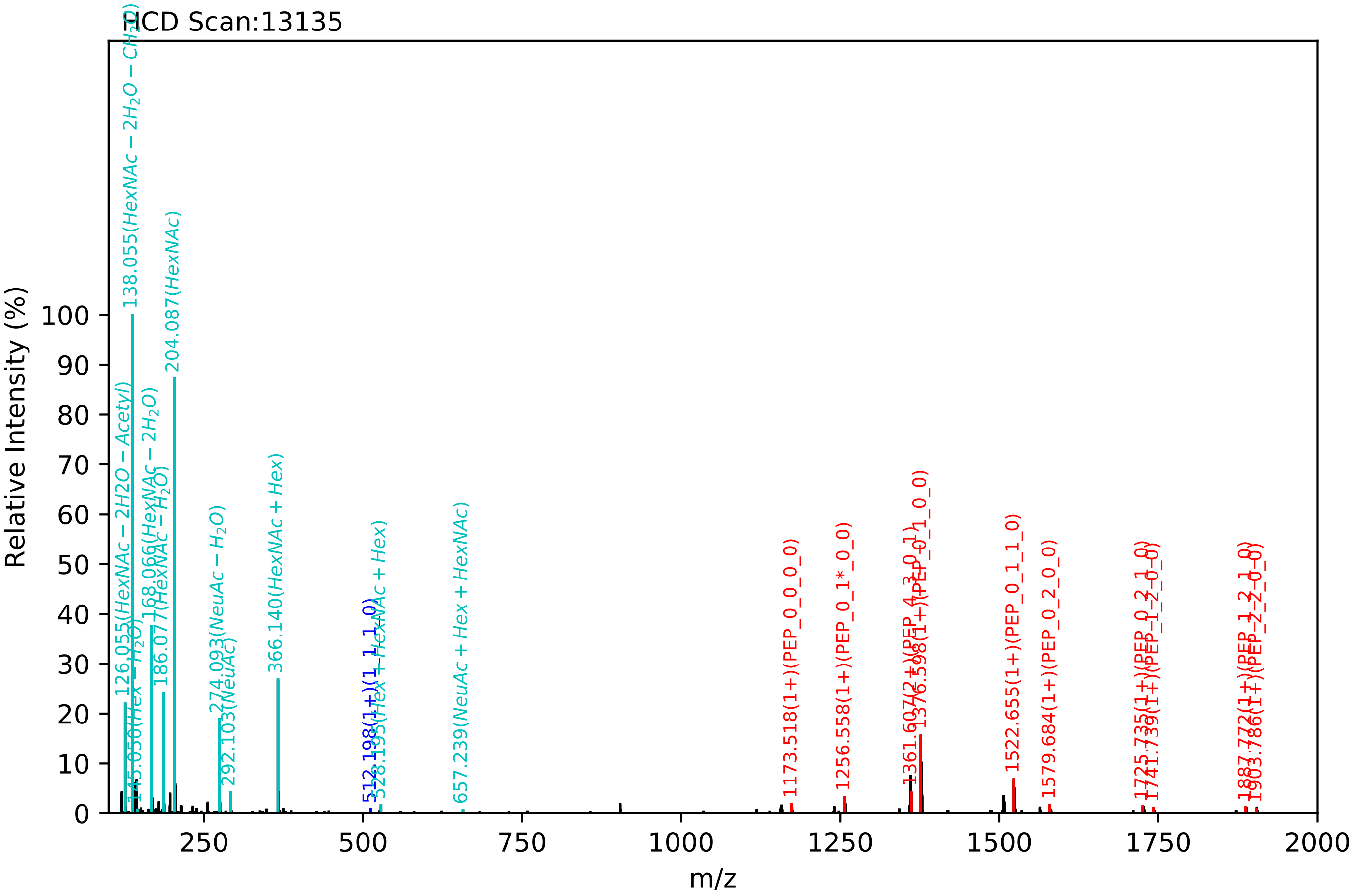

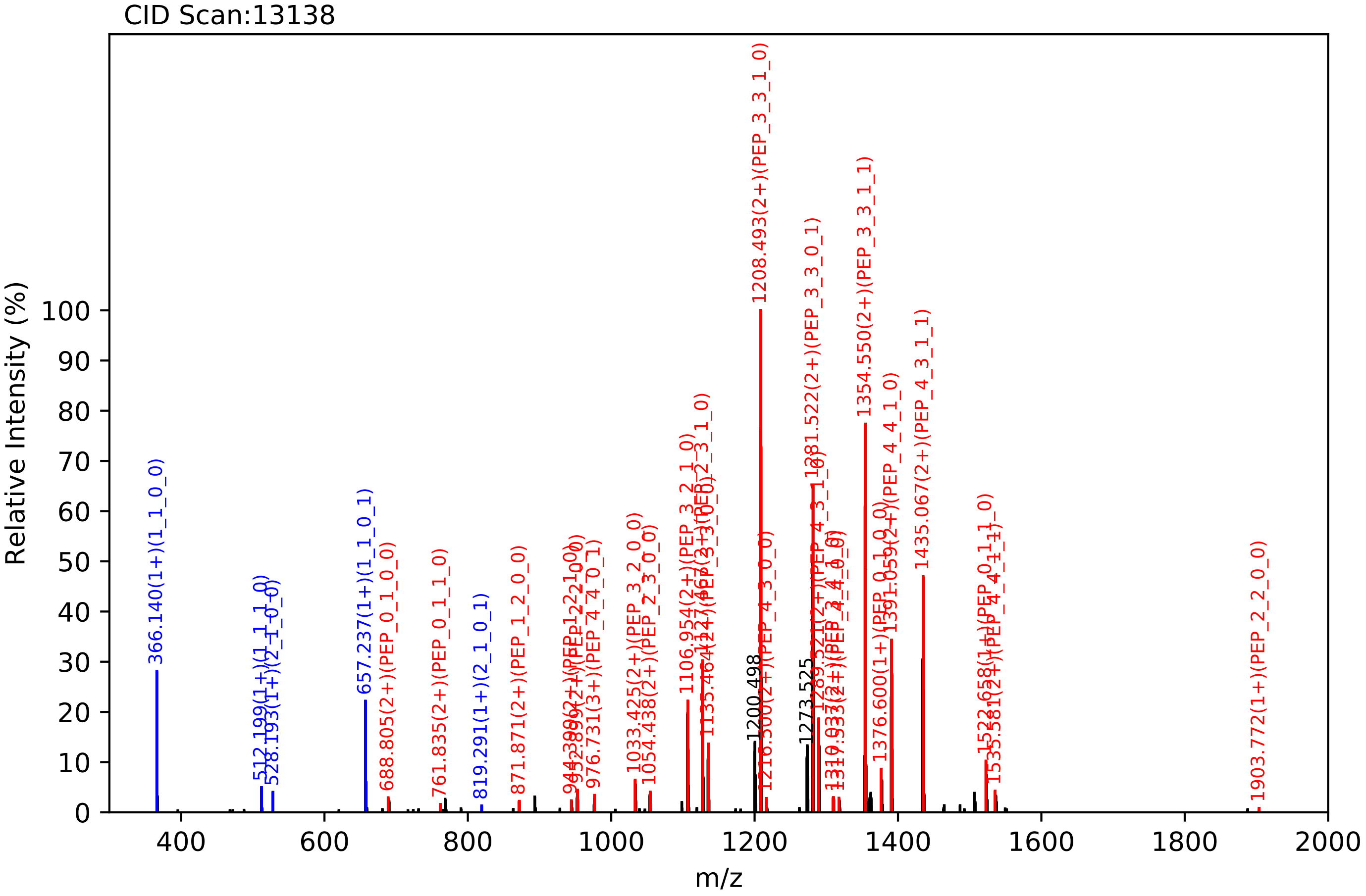

Unknown set no. 536, Experiment: Human Plasma exp_4

EEQFNSTYR(=PEP)_4_4_1_1, m/z:1024.41(3+), RT:50.91, Y-score:84.79

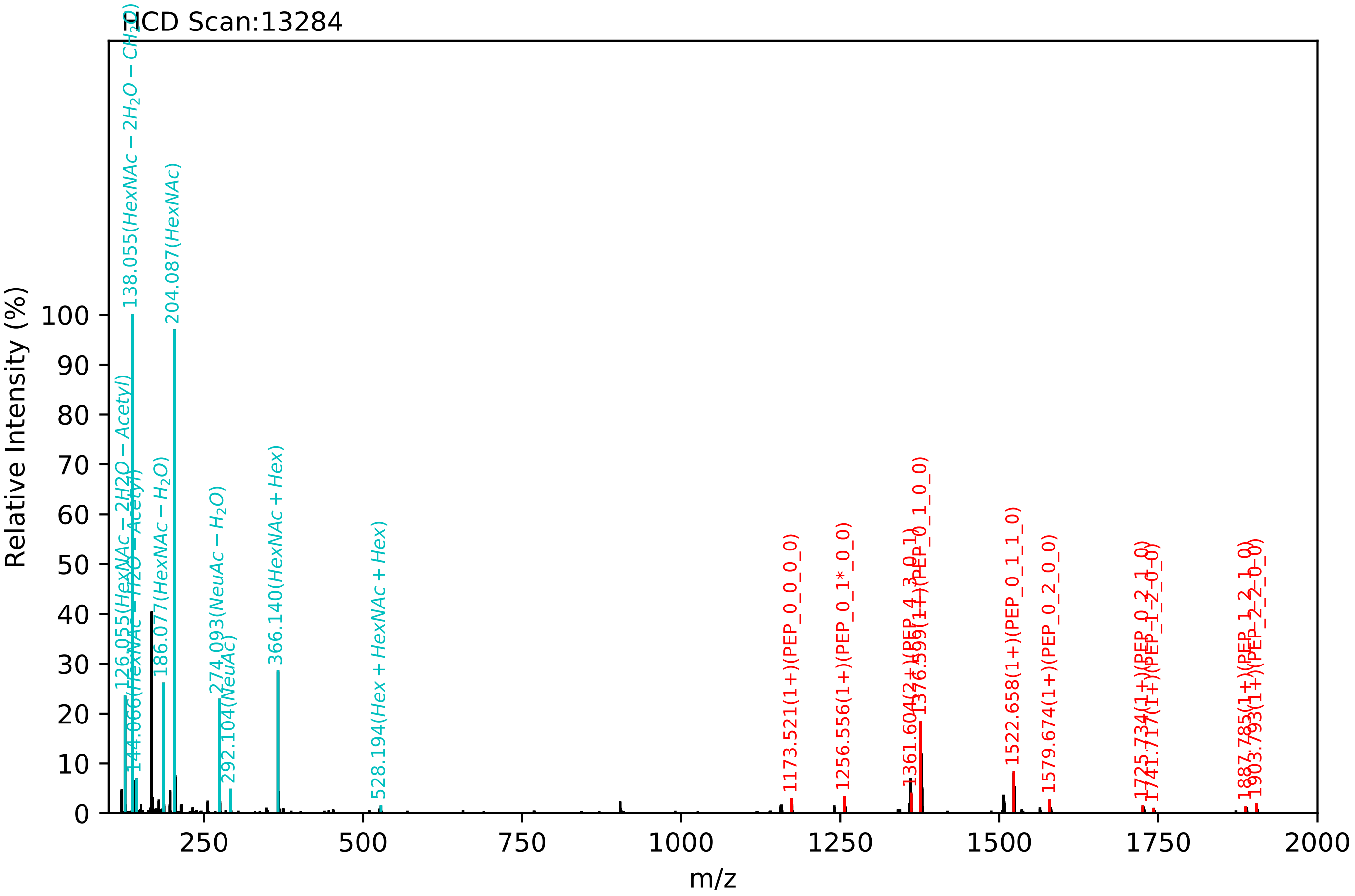

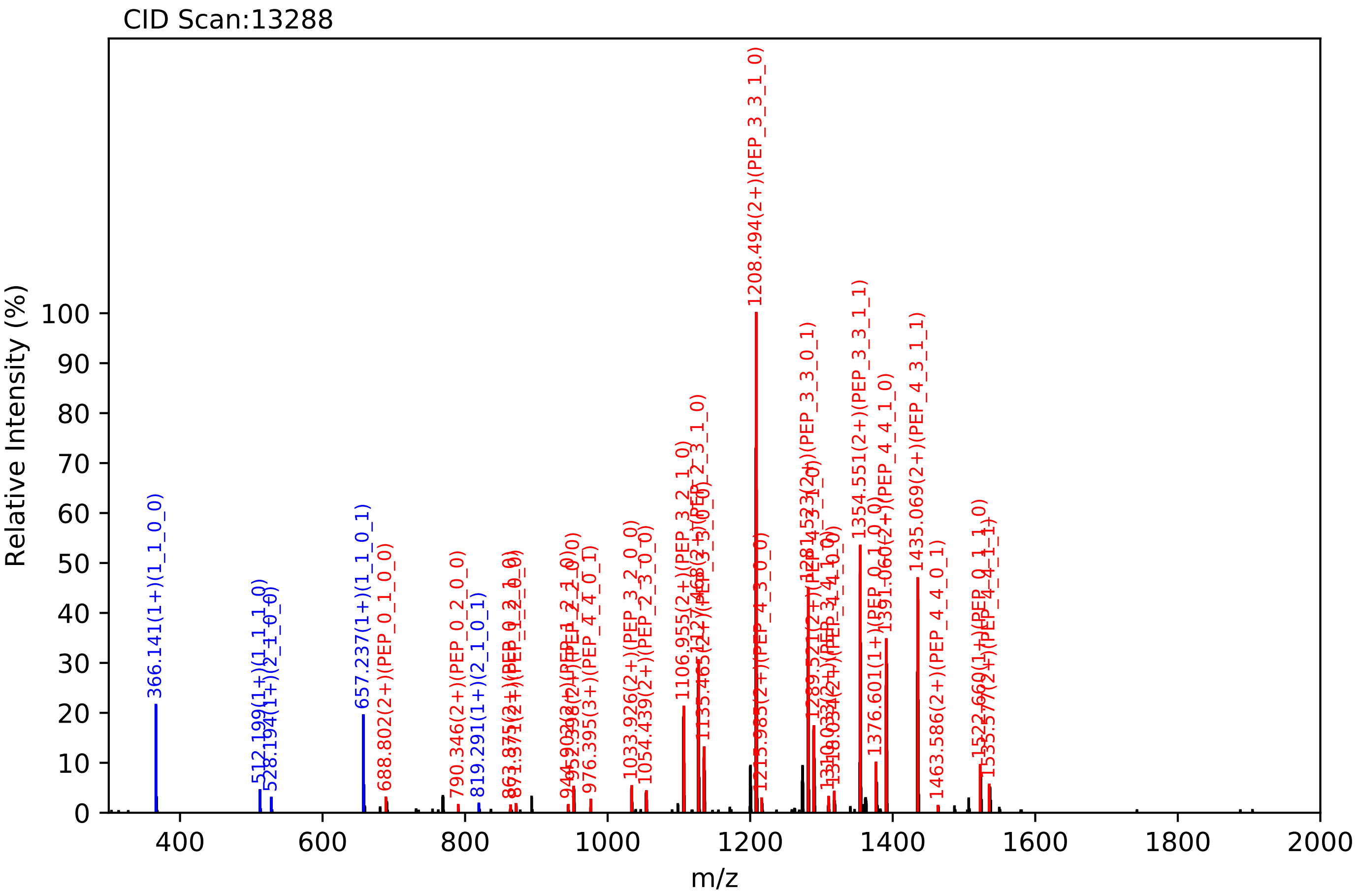

Unknown set no. 537, Experiment: Human Plasma exp_5

EEQFNSTYR(=PEP)_4_4_1_1, m/z:1024.41(3+), RT:51.02, Y-score:85.05

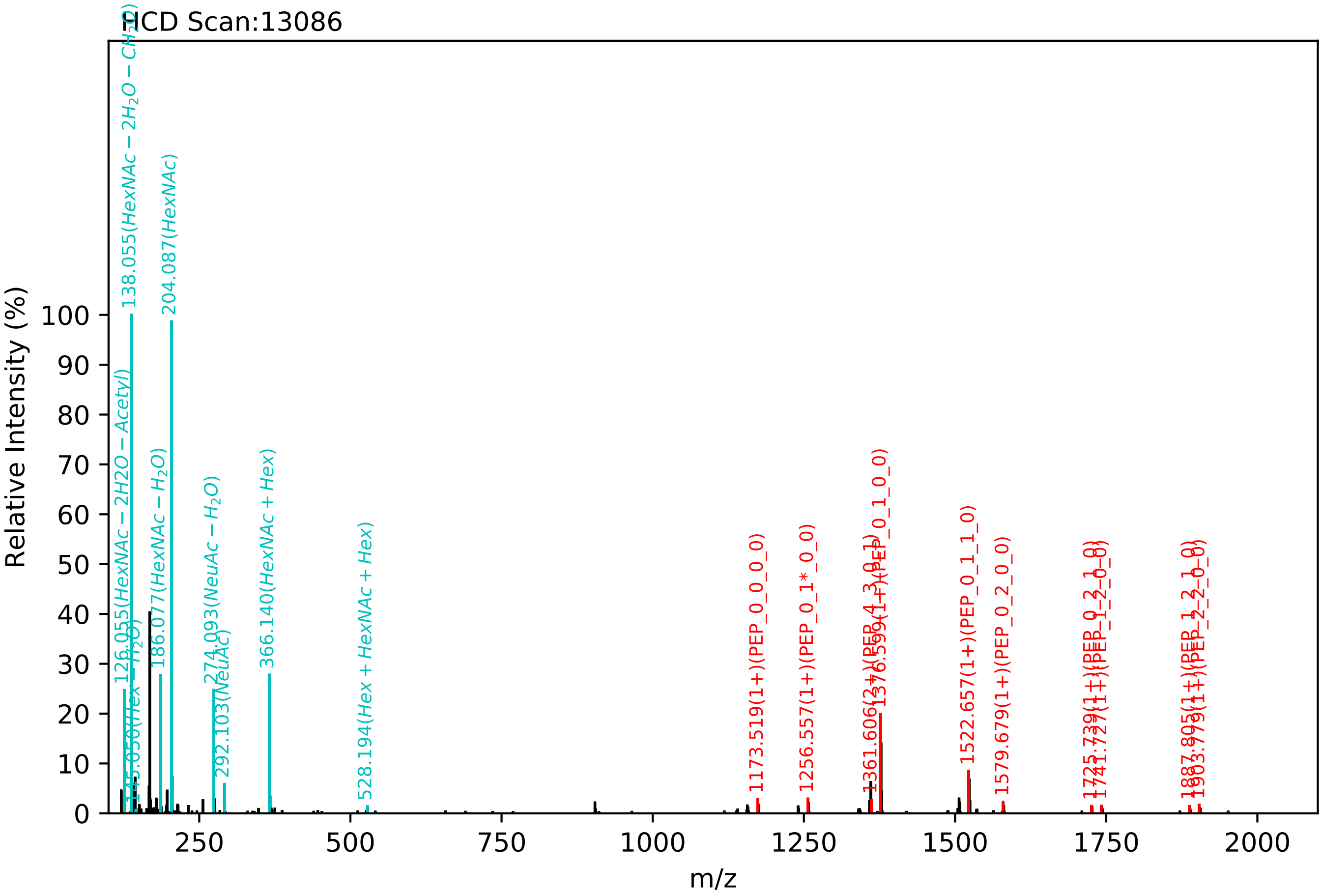

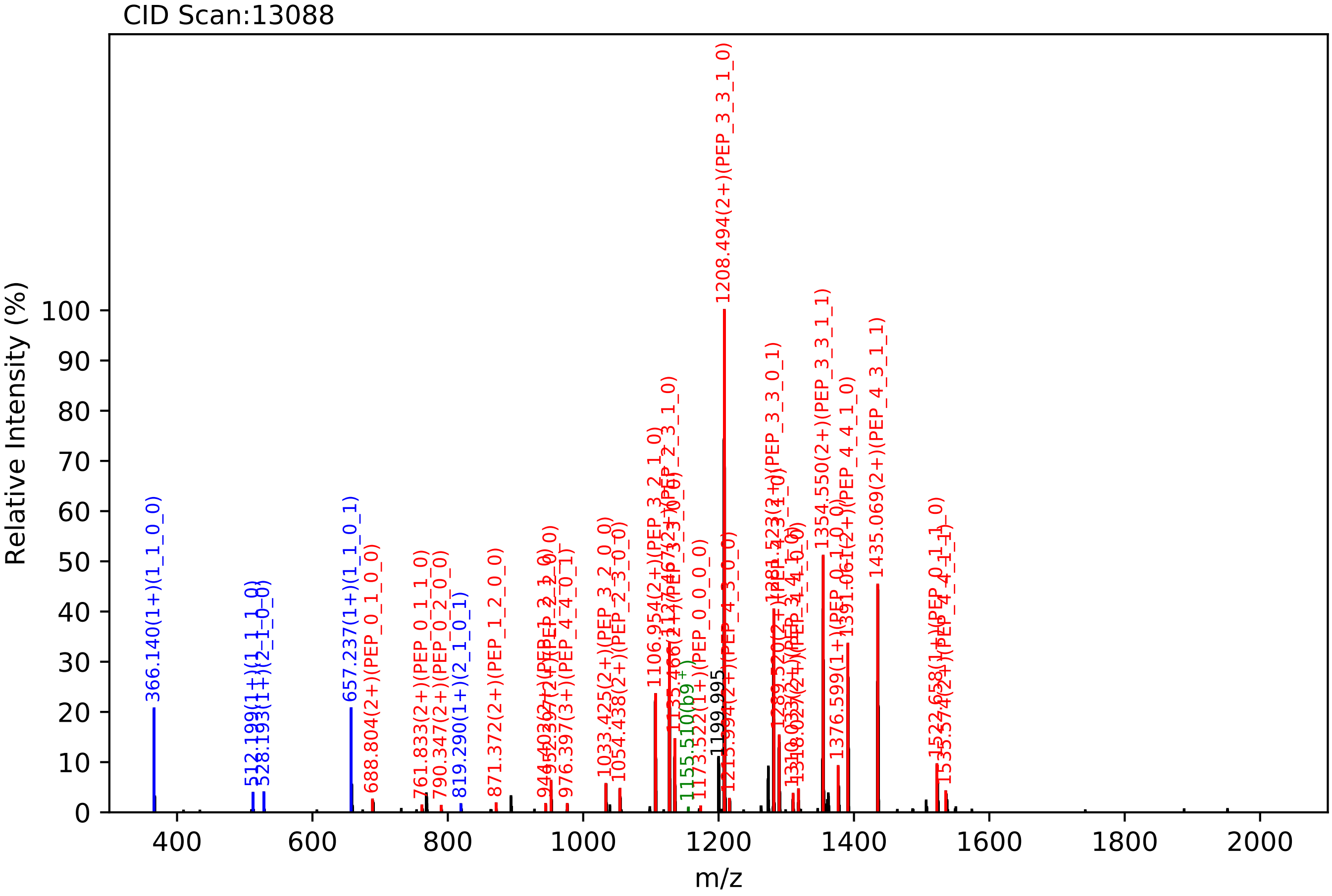

Unknown set no. 538, Experiment: Human Plasma exp_2

EEQFNSTYR(=PEP)_4_5_1_0, m/z:1492.10(2+), RT:41.90, Y-score:100.00

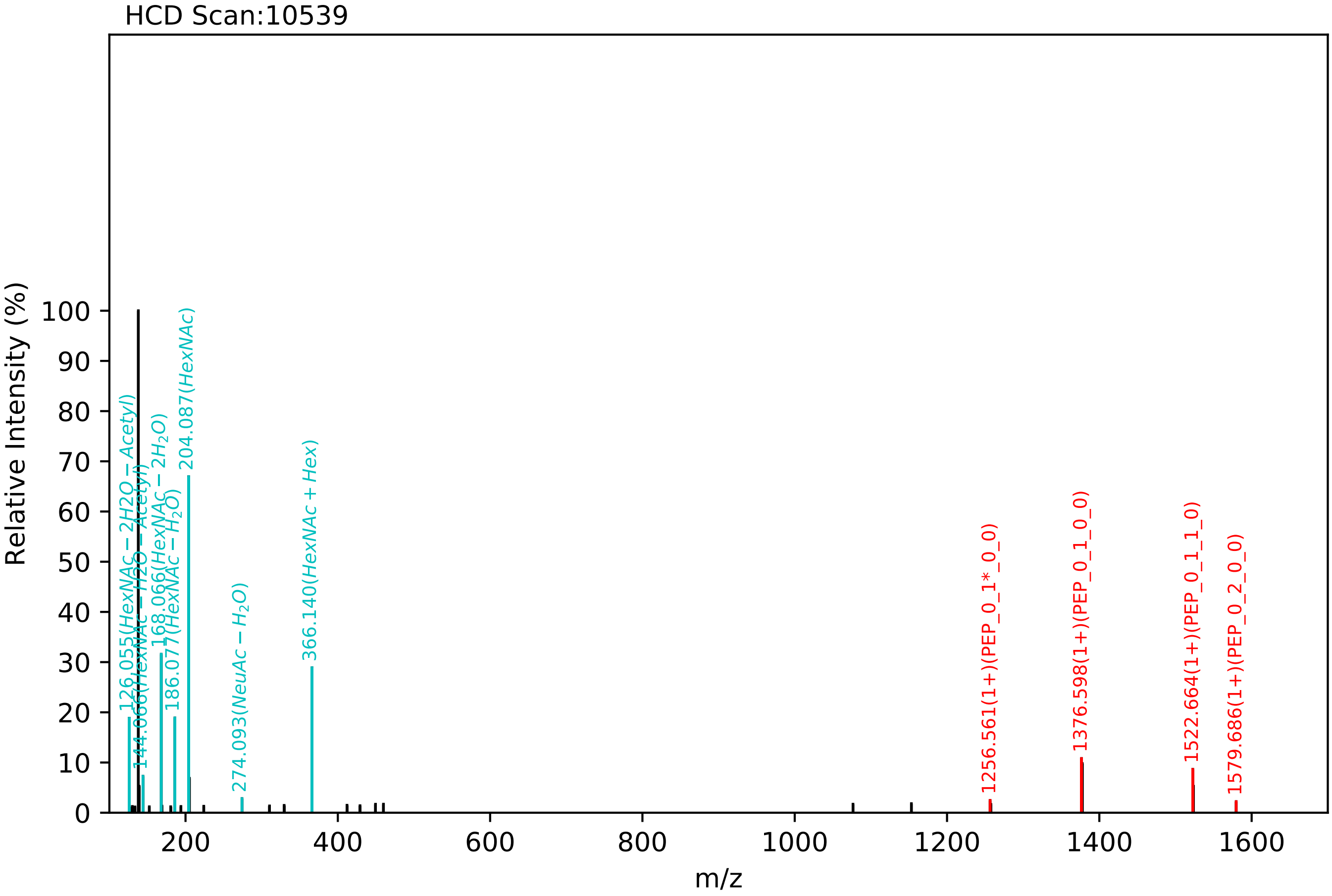

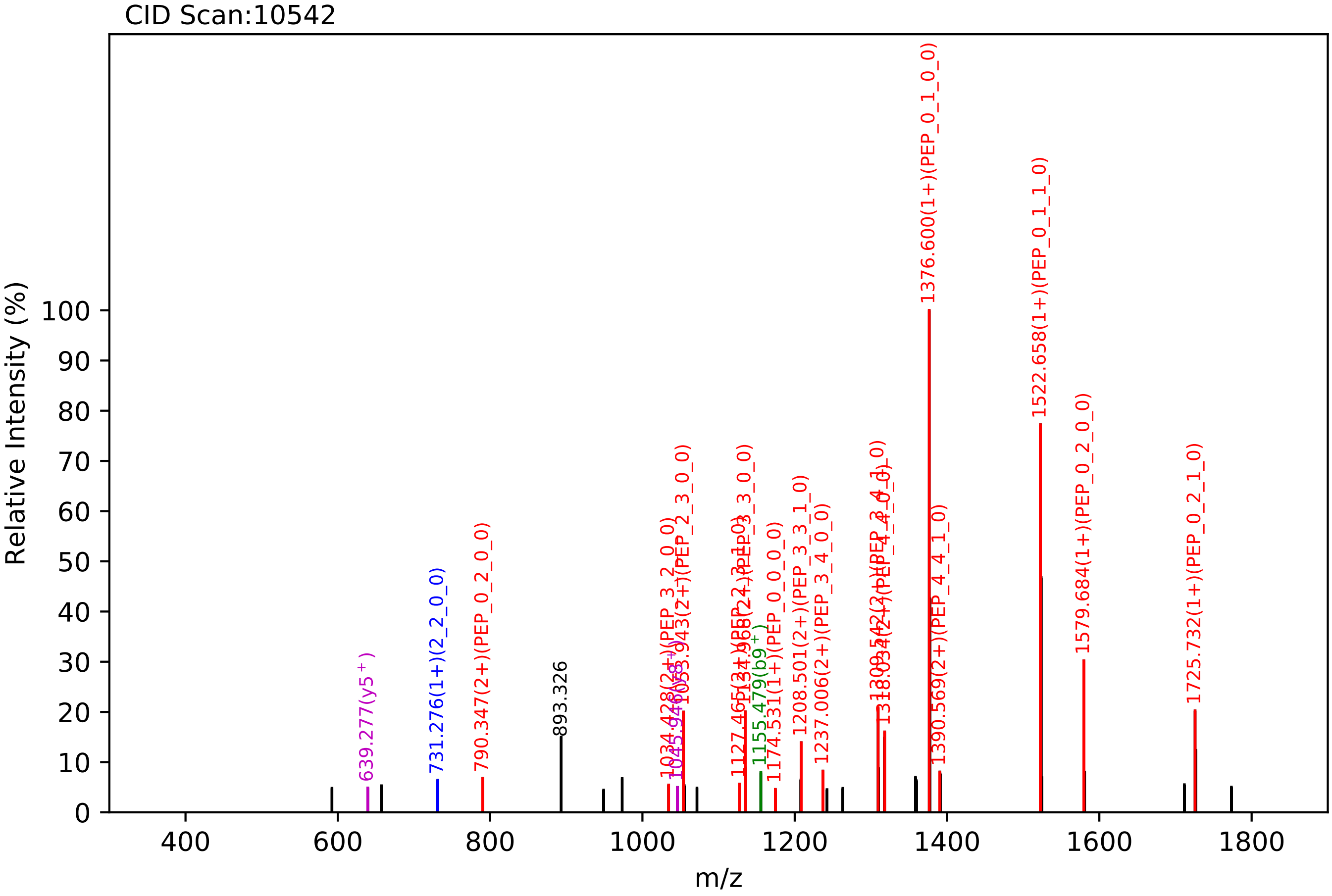

Unknown set no. 539, Experiment: Human Plasma exp_3

EEQFNSTYR(=PEP)_5_4_1_0, m/z:1472.08(2+), RT:39.54, Y-score:91.22

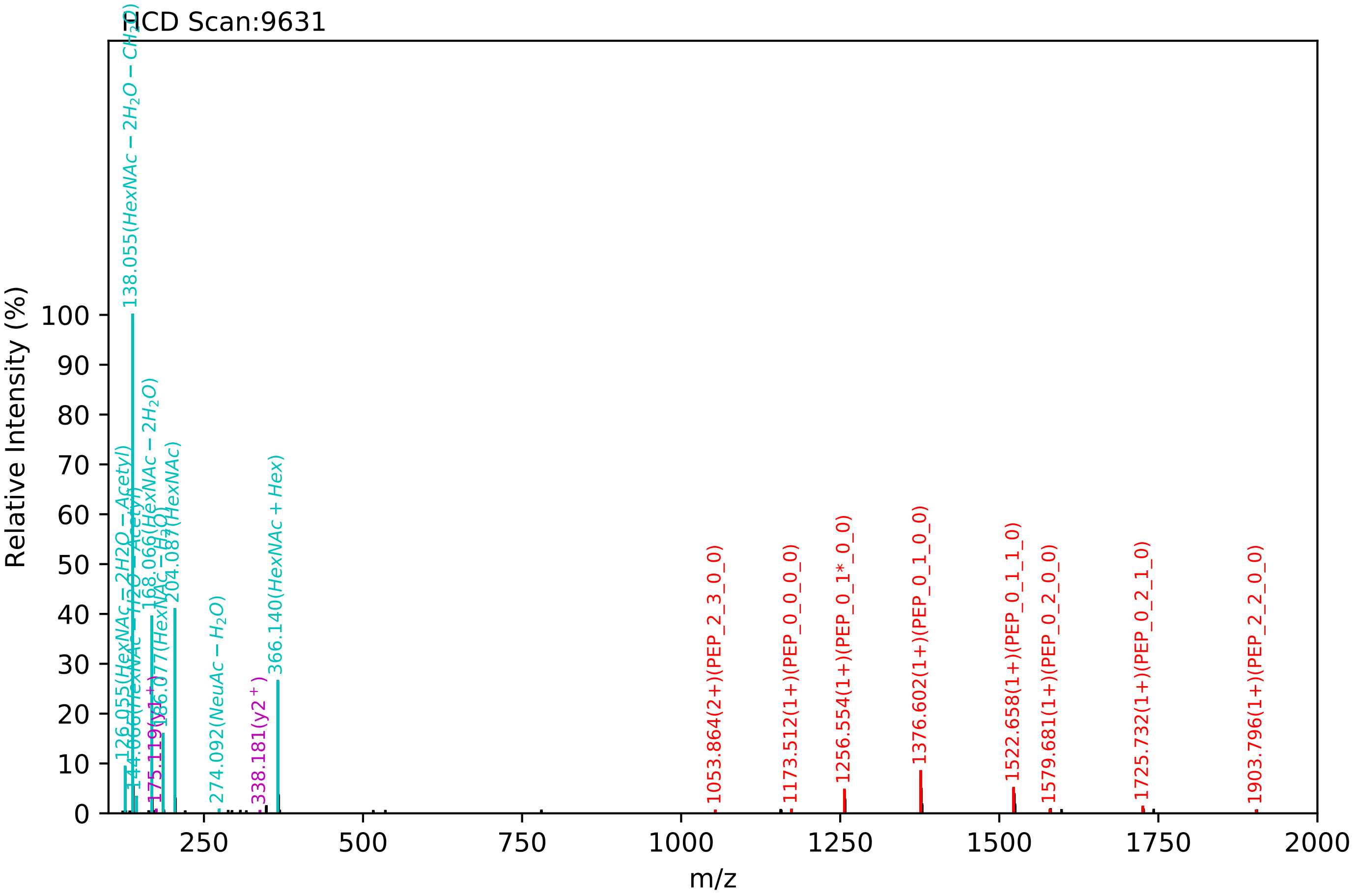

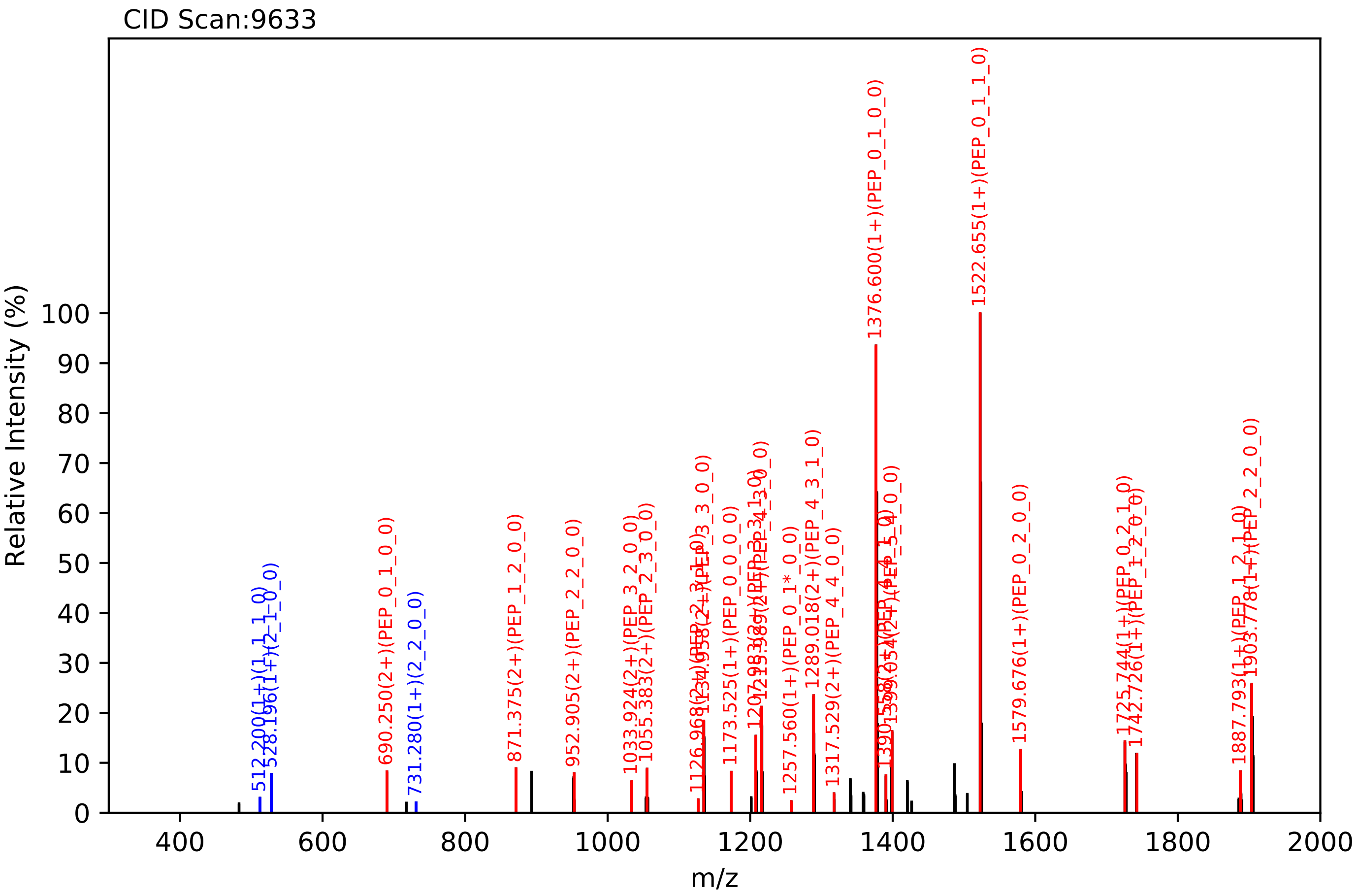

Unknown set no. 540, Experiment: Human Plasma exp_2

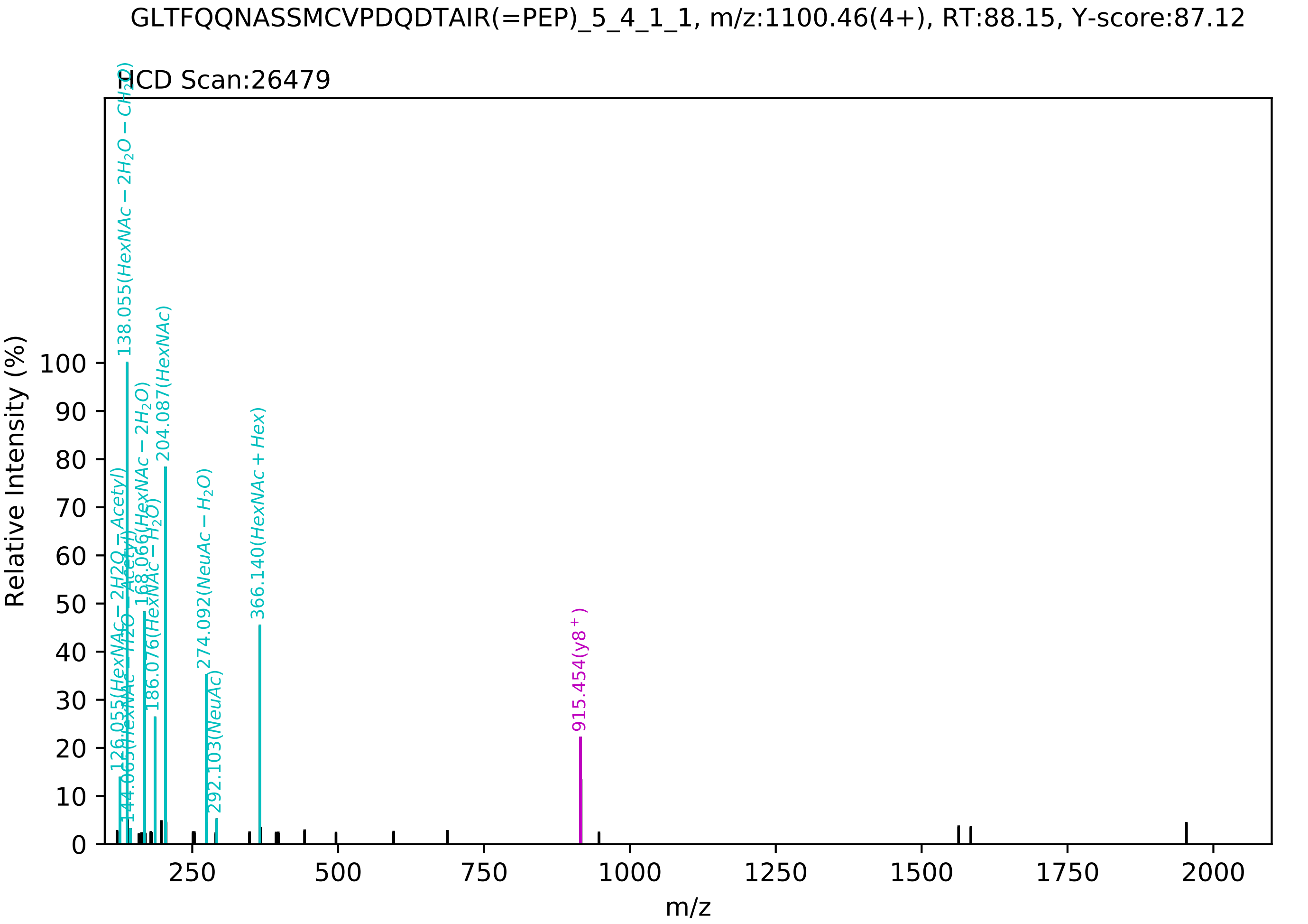

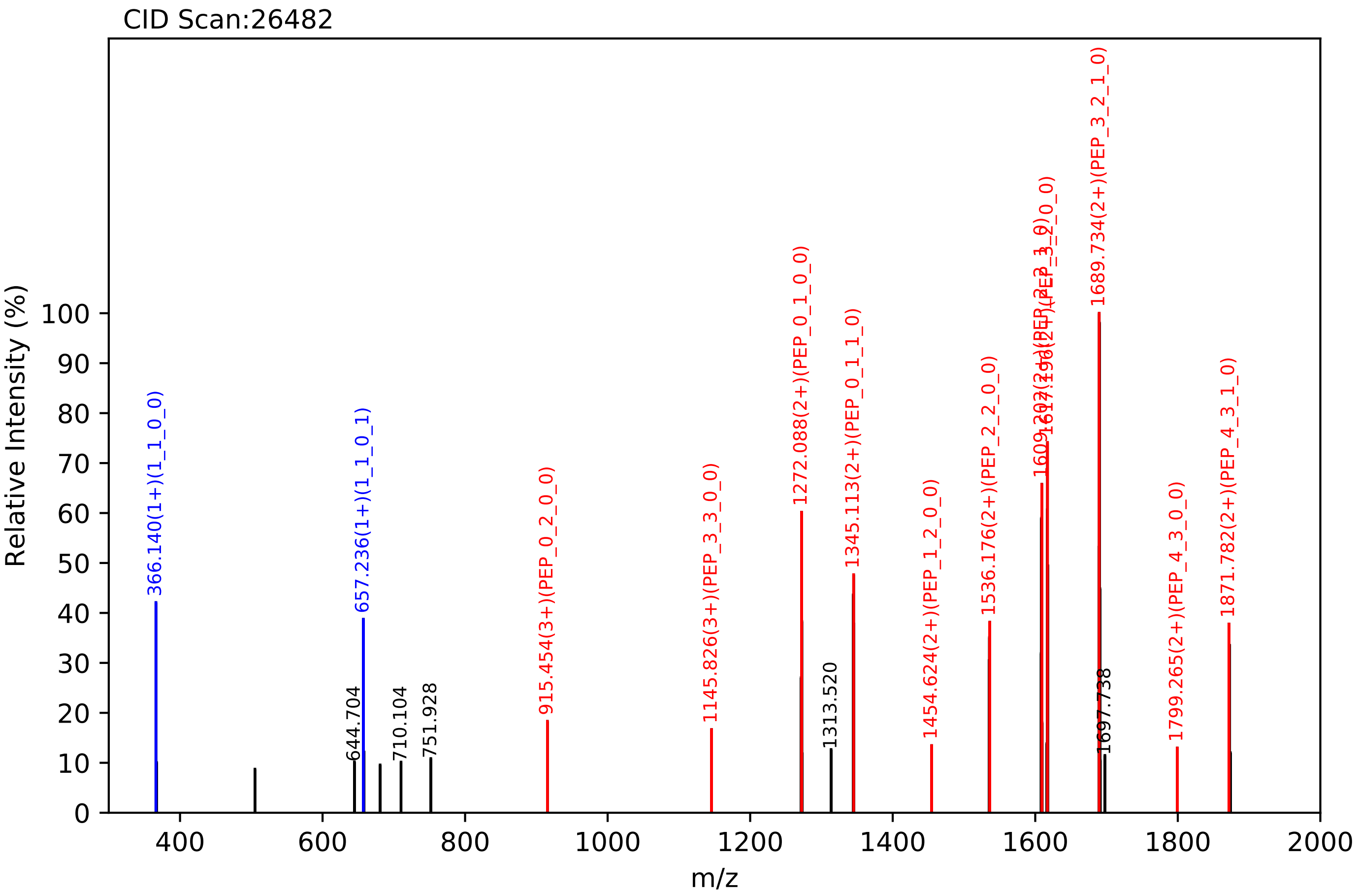

Unknown set no. 541, Experiment: Human Plasma exp_1

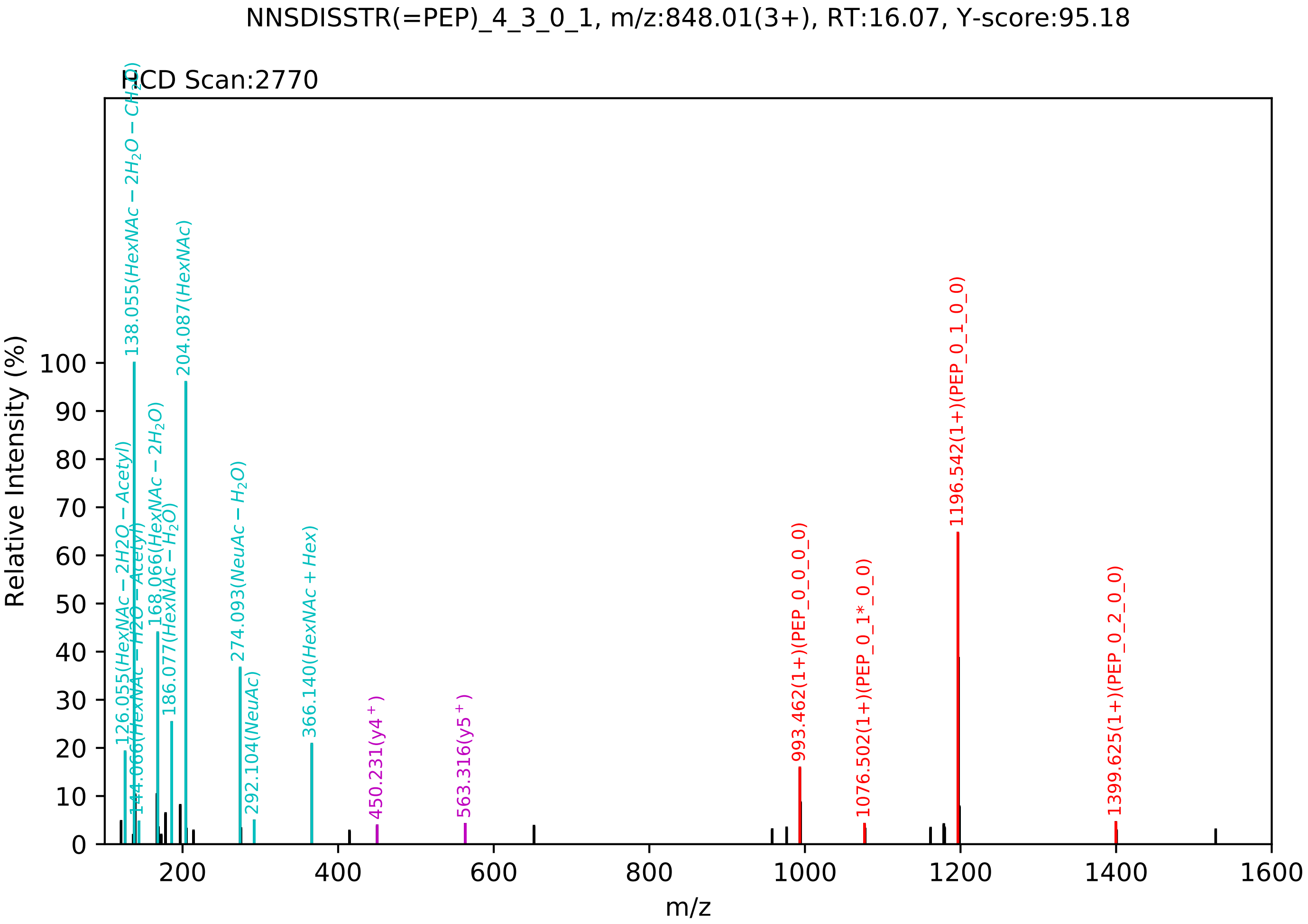

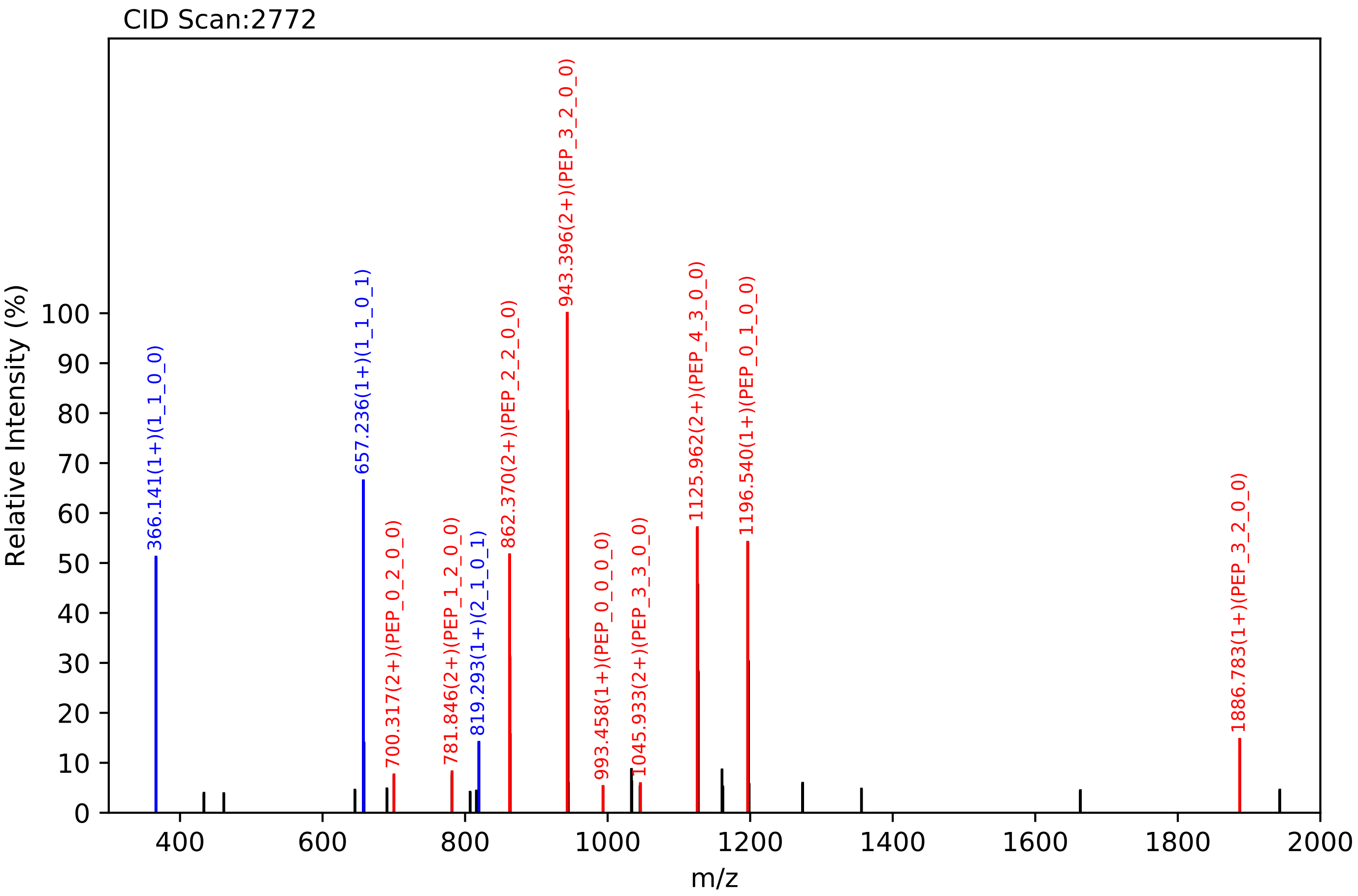

Unknown set no. 542, Experiment: Human Plasma exp_2

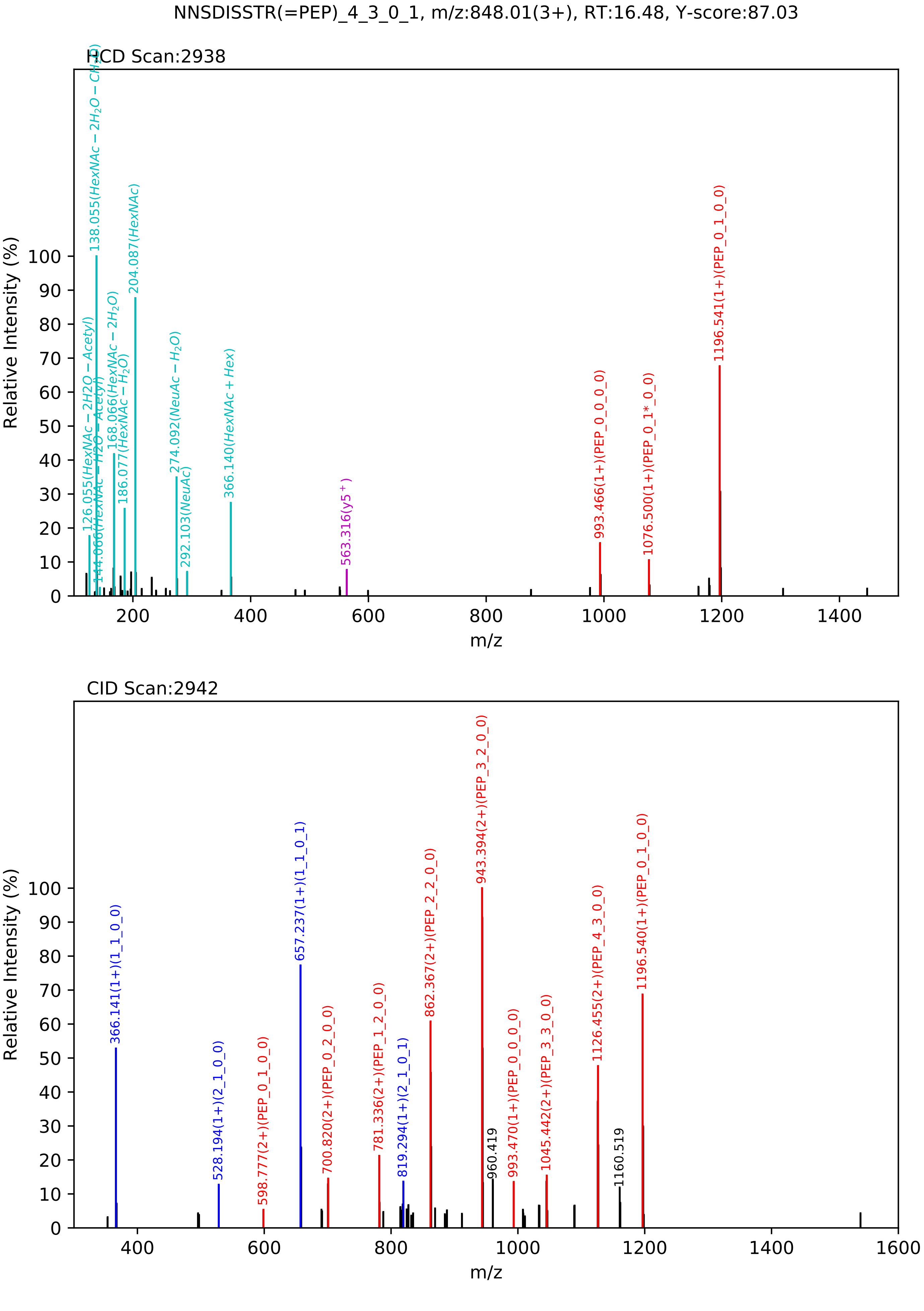

Unknown set no. 543, Experiment: Human Plasma exp_3

NNSDISSTR(=PEP)_4_3_0_1, m/z:848.01(3+), RT:16.54, Y-score:83.00

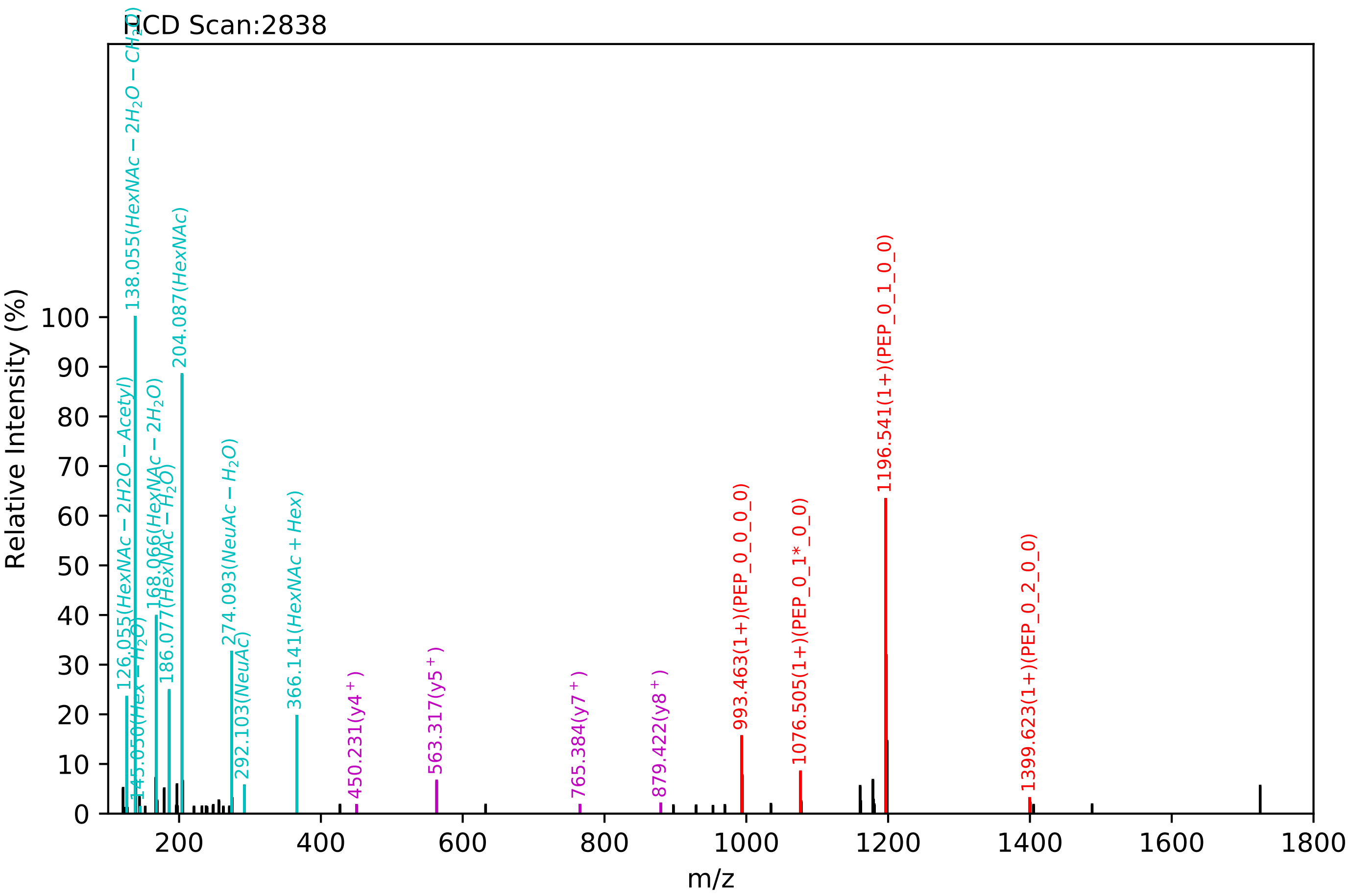

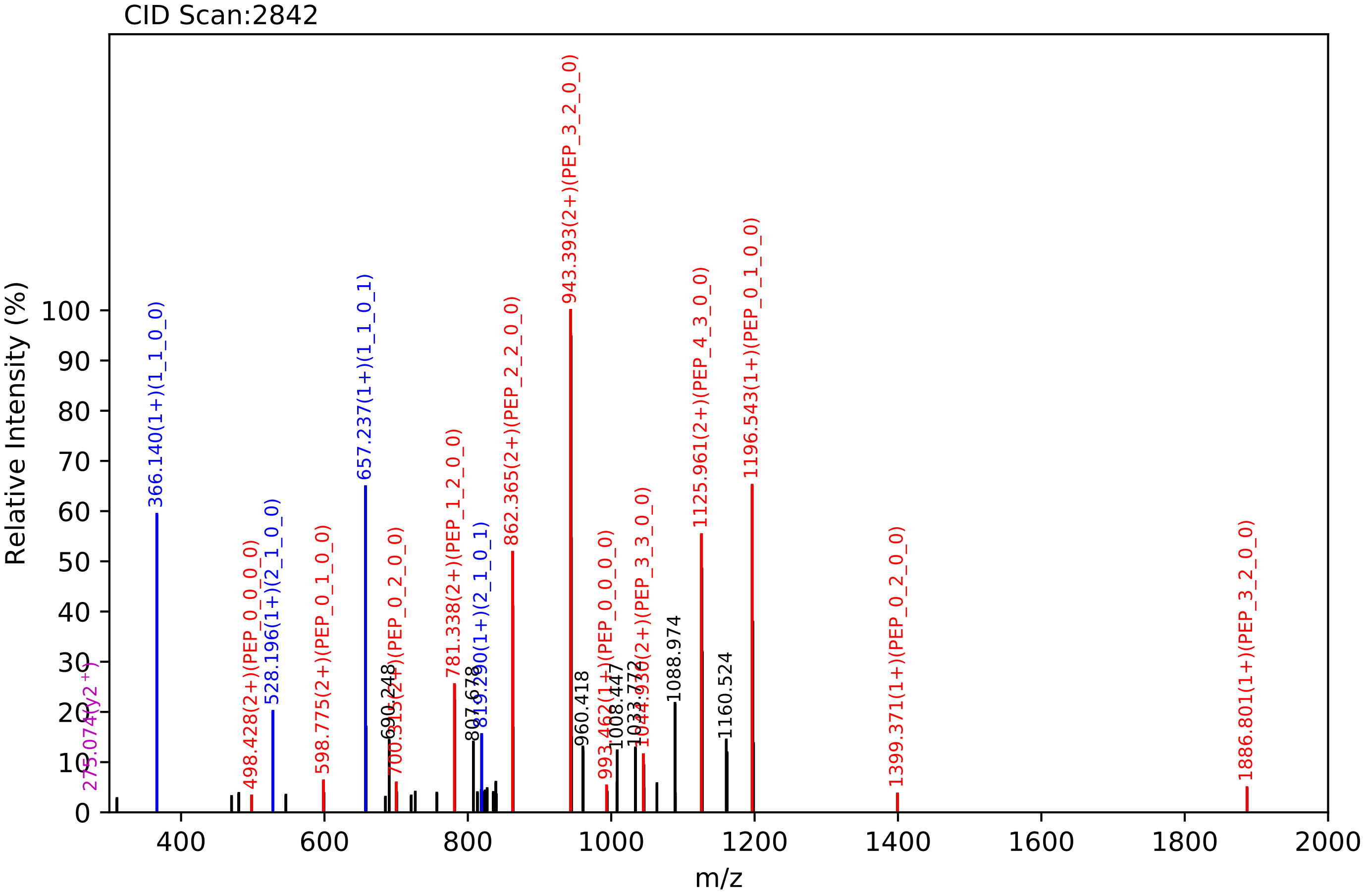

Unknown set no. 544, Experiment: Human Plasma exp_4

NNSDISSTR(=PEP)_4_3_0_1, m/z:848.01(3+), RT:16.57, Y-score:85.63

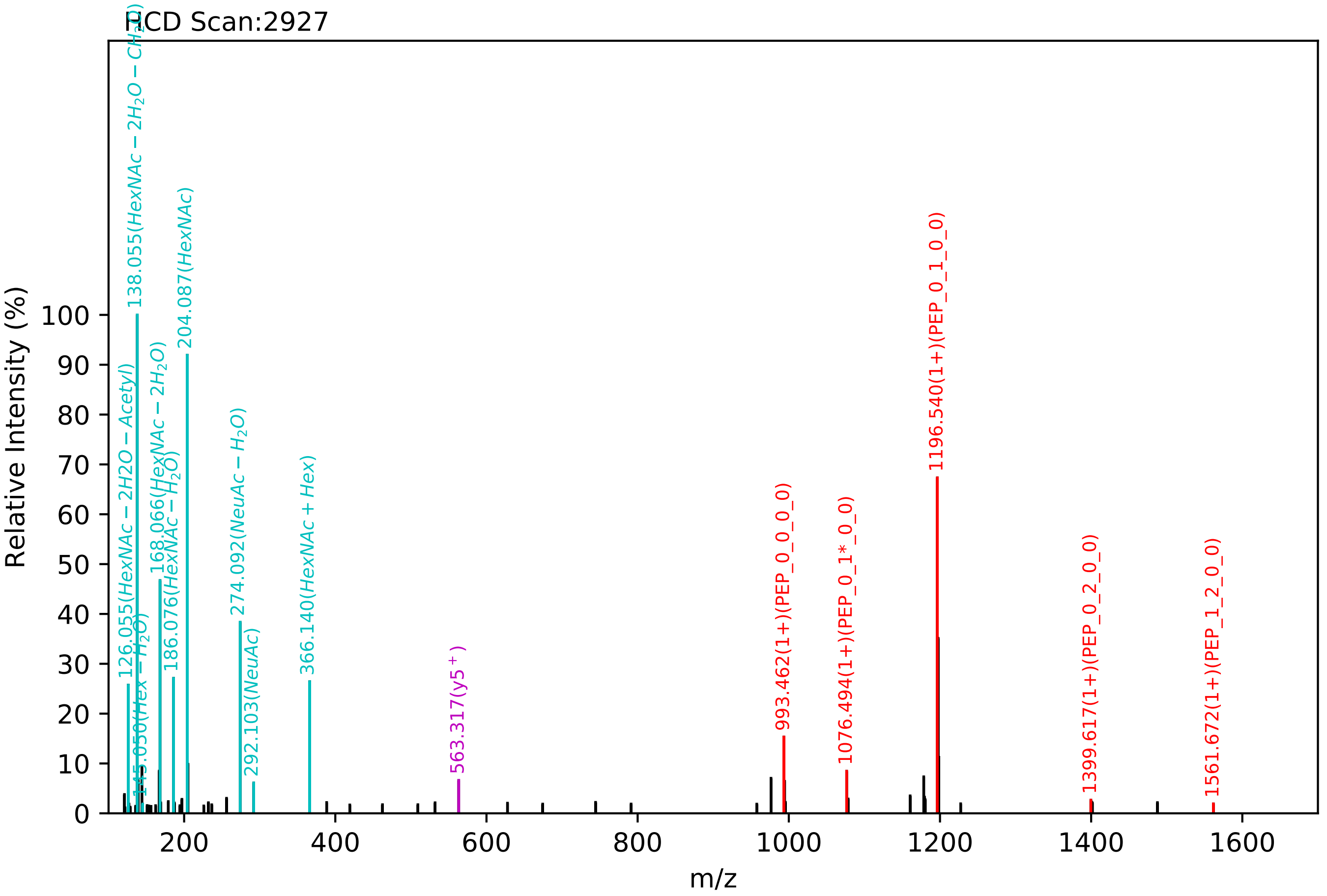

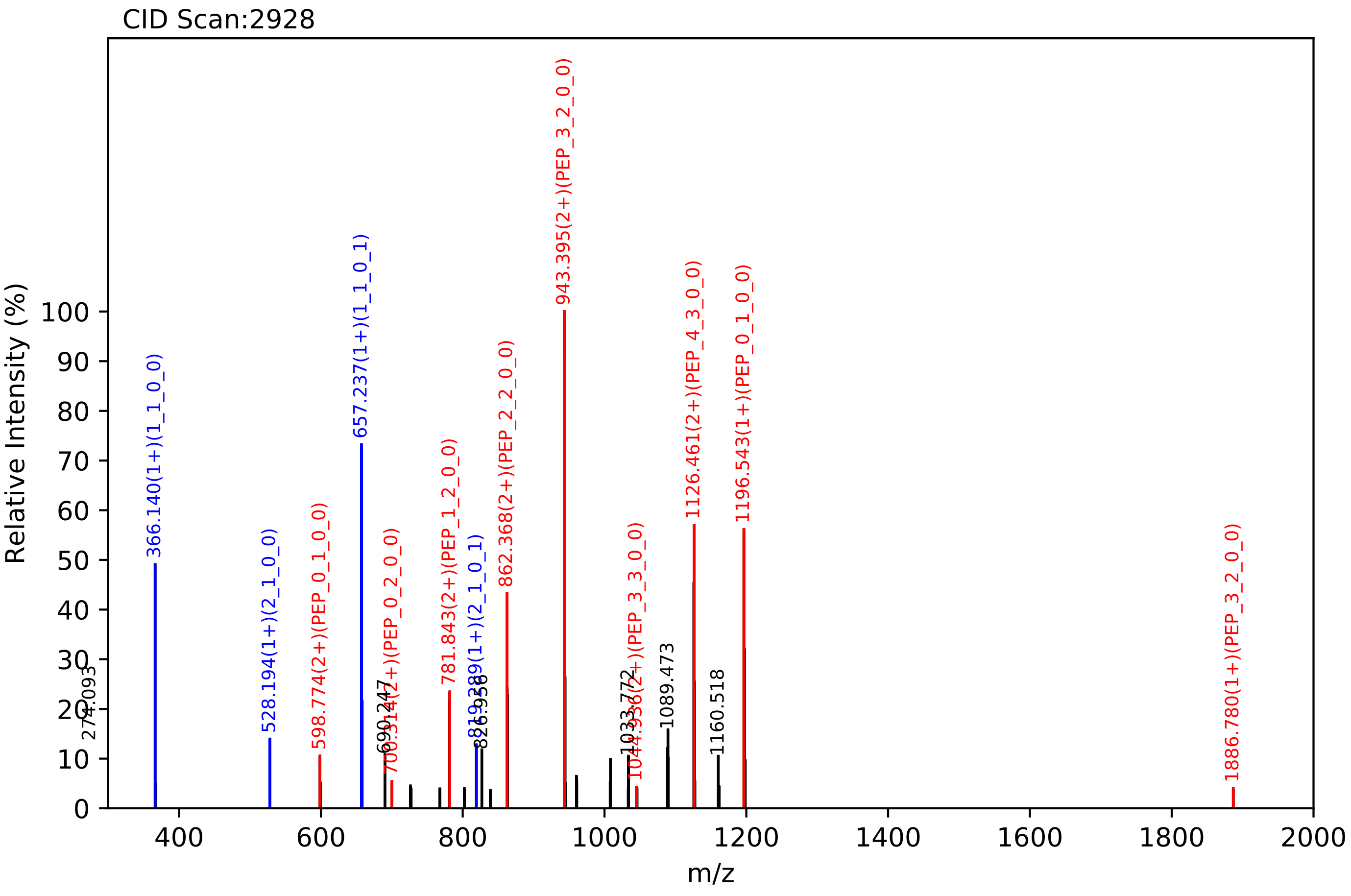

Unknown set no. 545, Experiment: Human Plasma exp_5

NNSDISSTR(=PEP)_4_3_0_1, m/z:848.01(3+), RT:16.48, Y-score:82.68

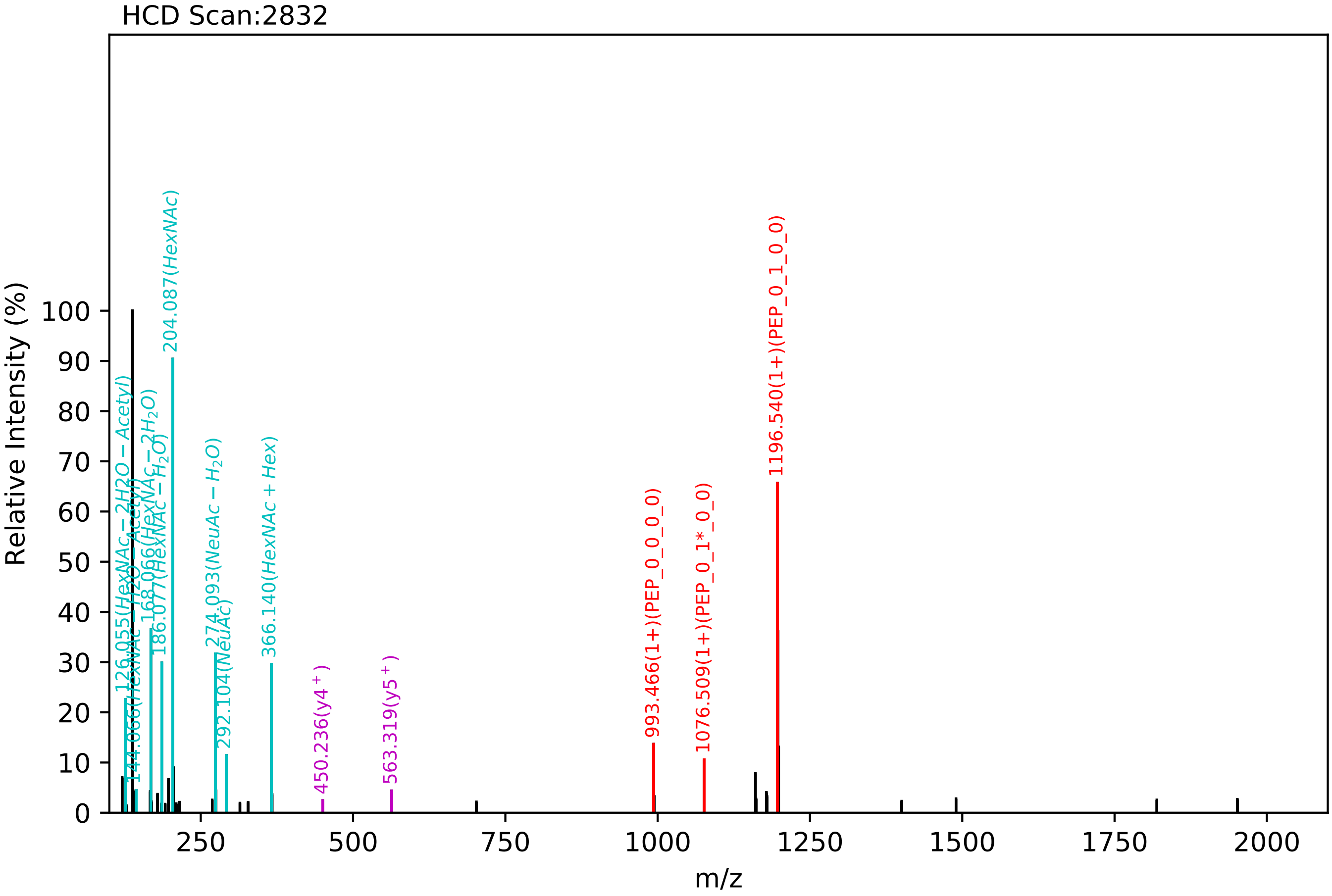

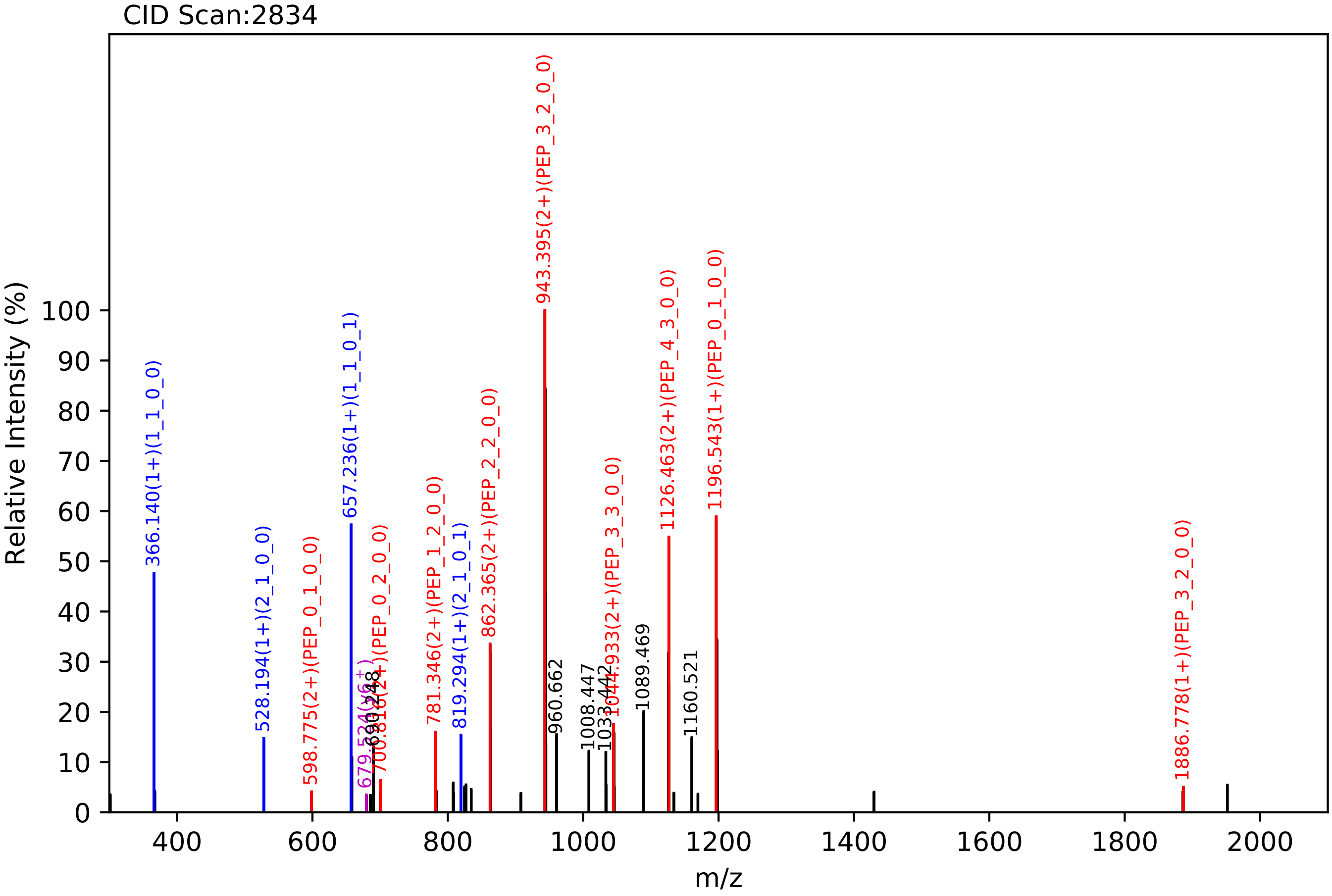

Unknown set no. 546, Experiment: Human Plasma exp_1

NNSDISSTR(=PEP)_4_3_1_1, m/z:896.69(3+), RT:16.72, Y-score:77.63

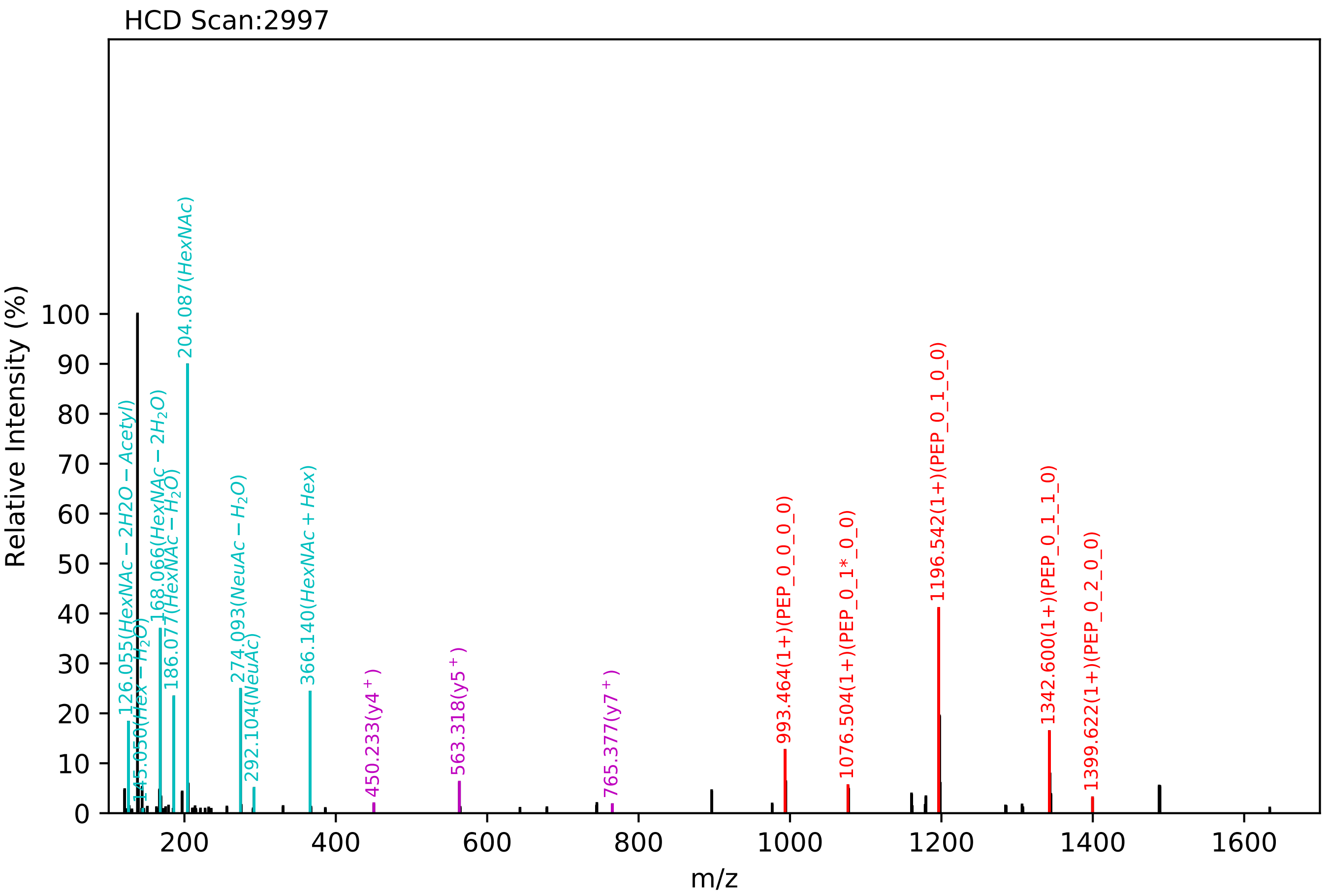

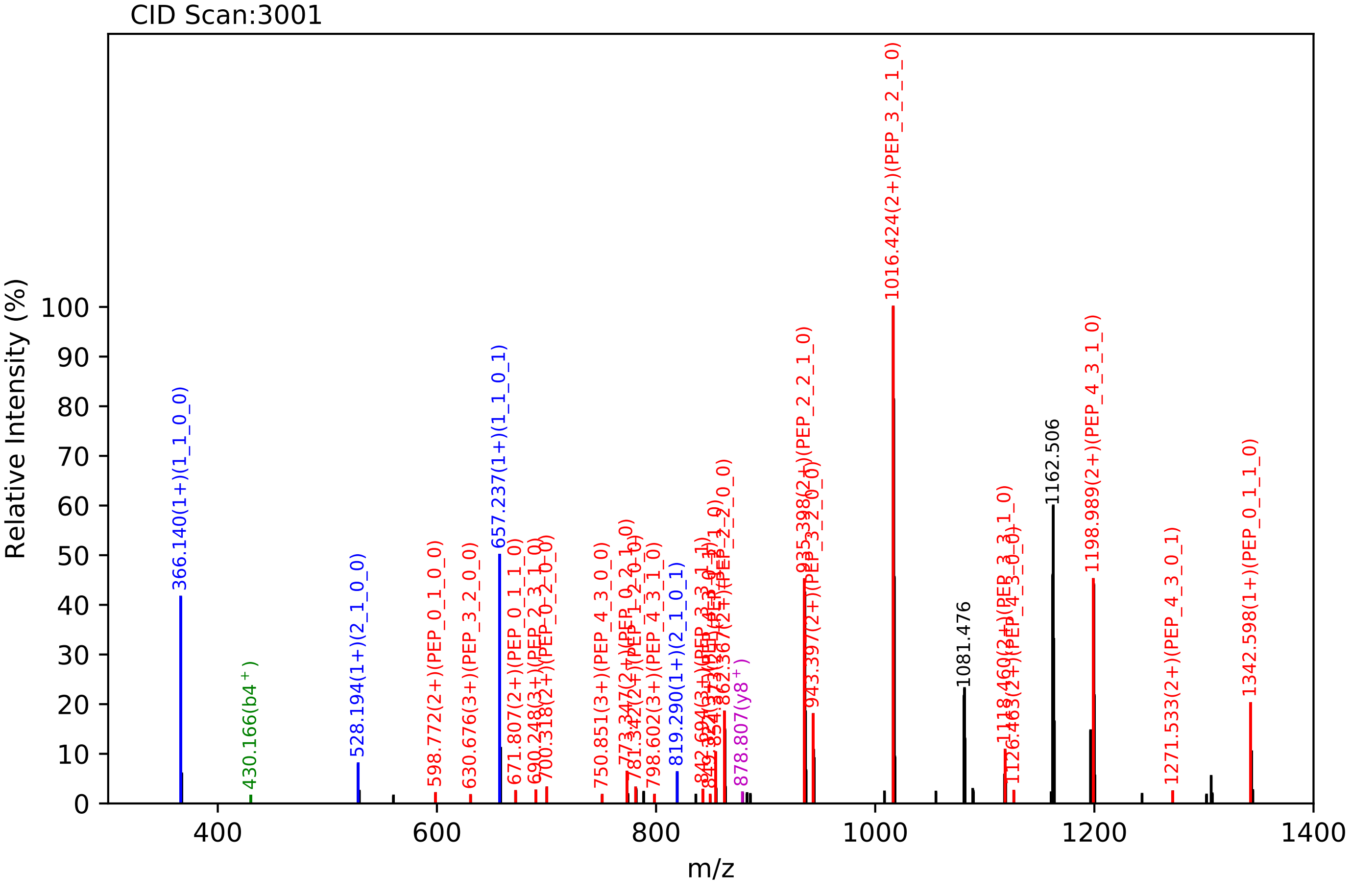

Unknown set no. 547, Experiment: Human Plasma exp_1

NNSDISSTR(=PEP)_5_3_0_1, m/z:902.03(3+), RT:15.81, Y-score:90.09

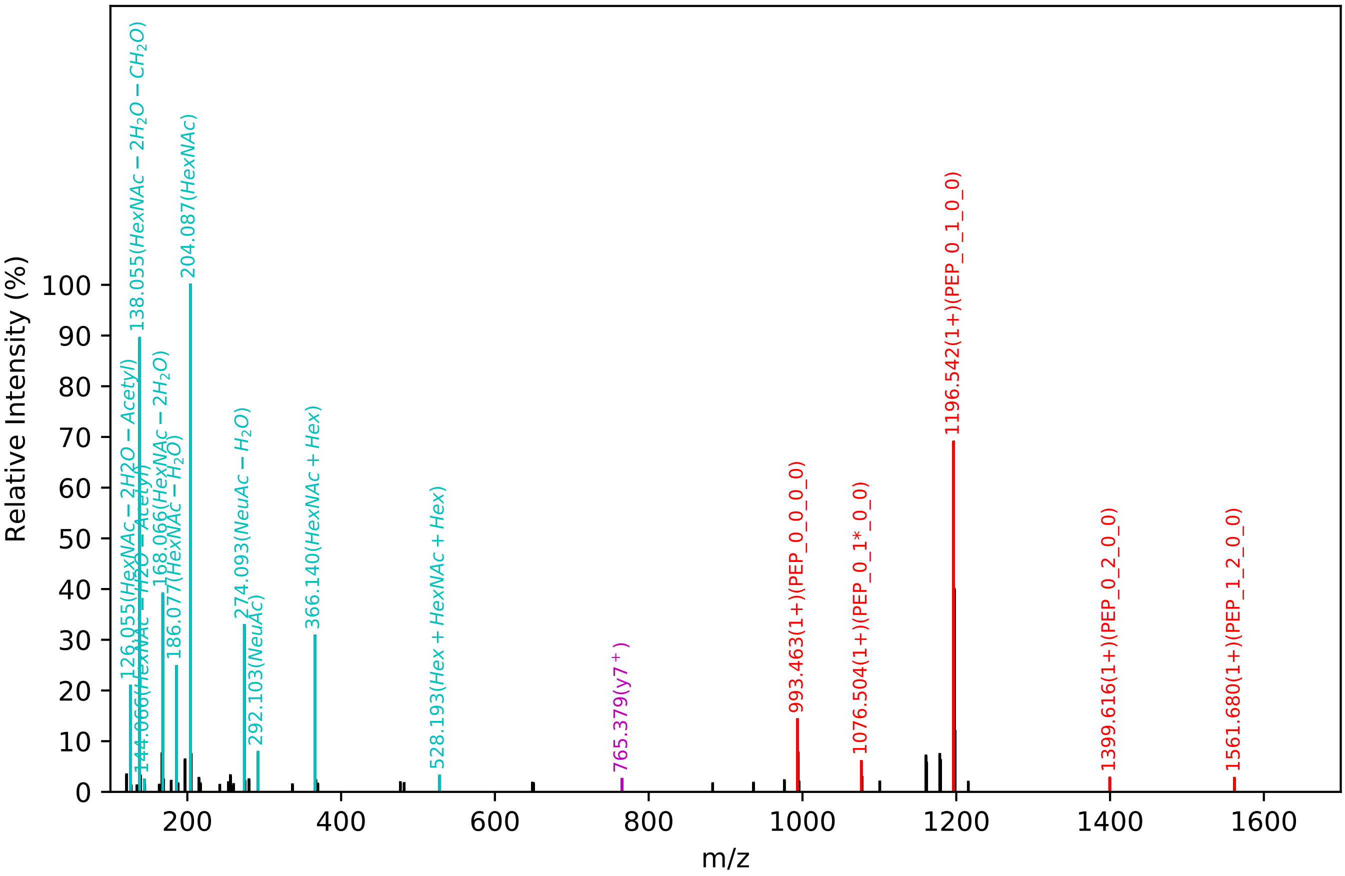

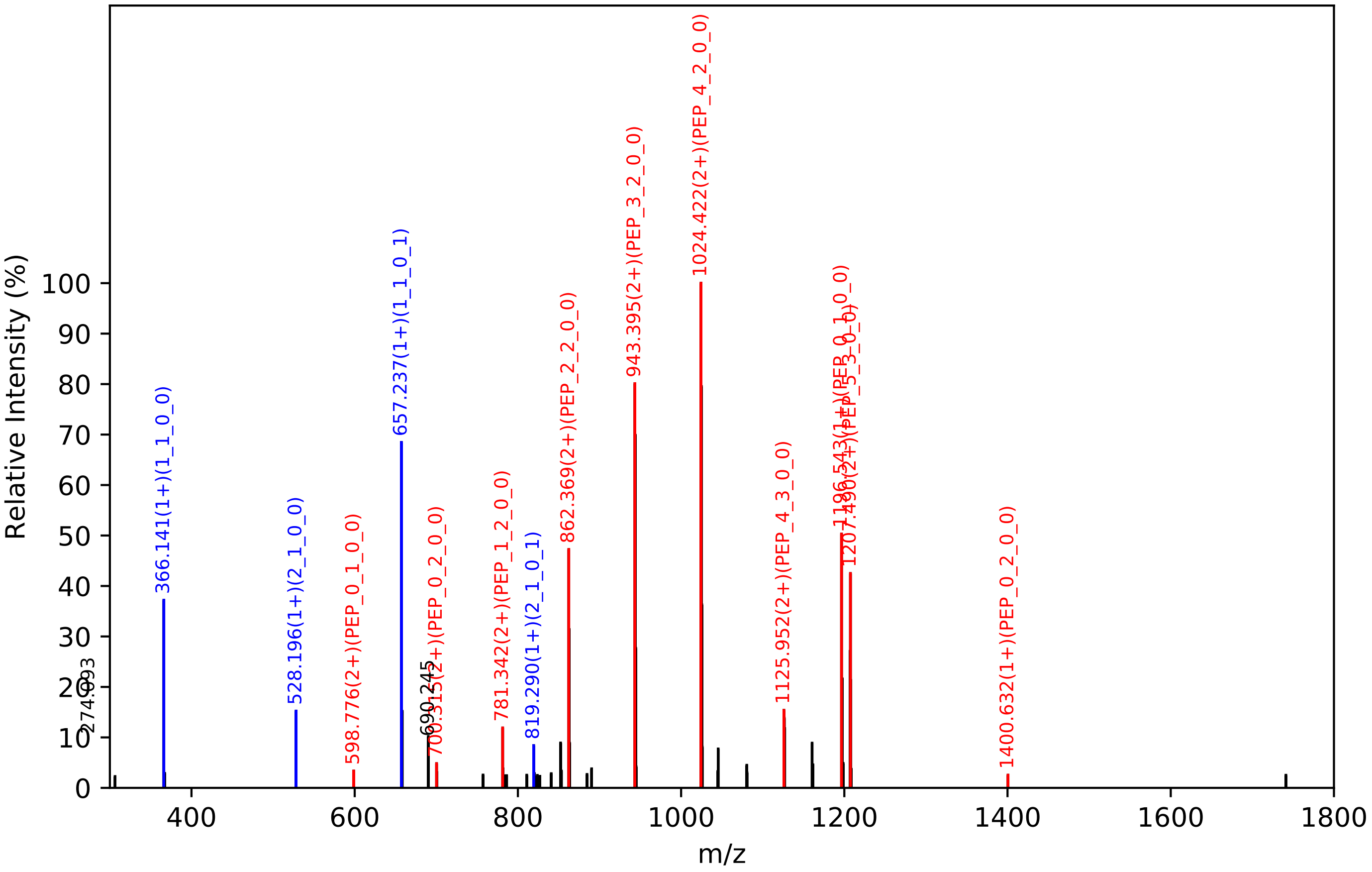

Unknown set no. 548, Experiment: Human Plasma exp_2

NNSDISSTR(=PEP)_5_3_0_1, m/z:902.03(3+), RT:16.22, Y-score:87.43

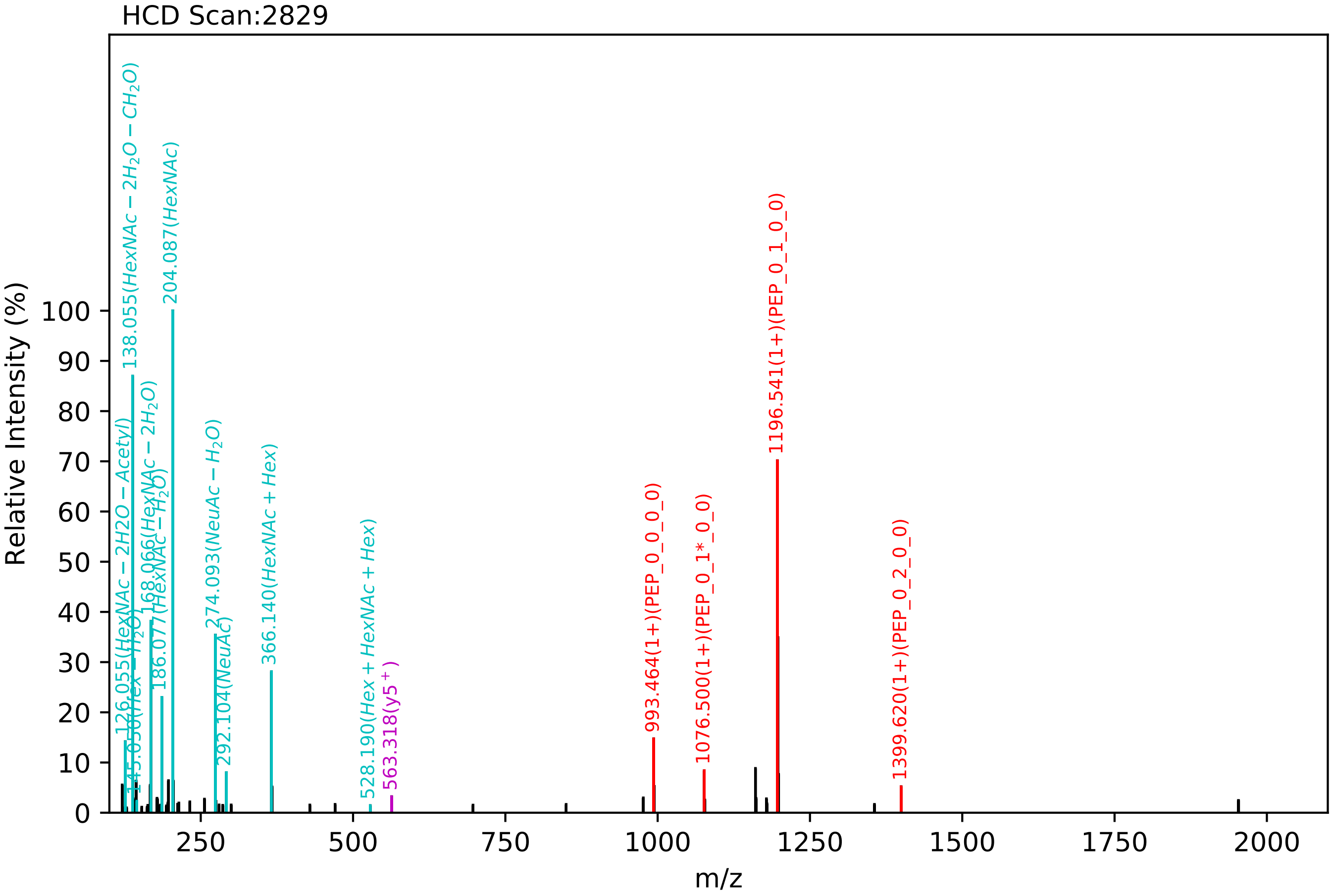

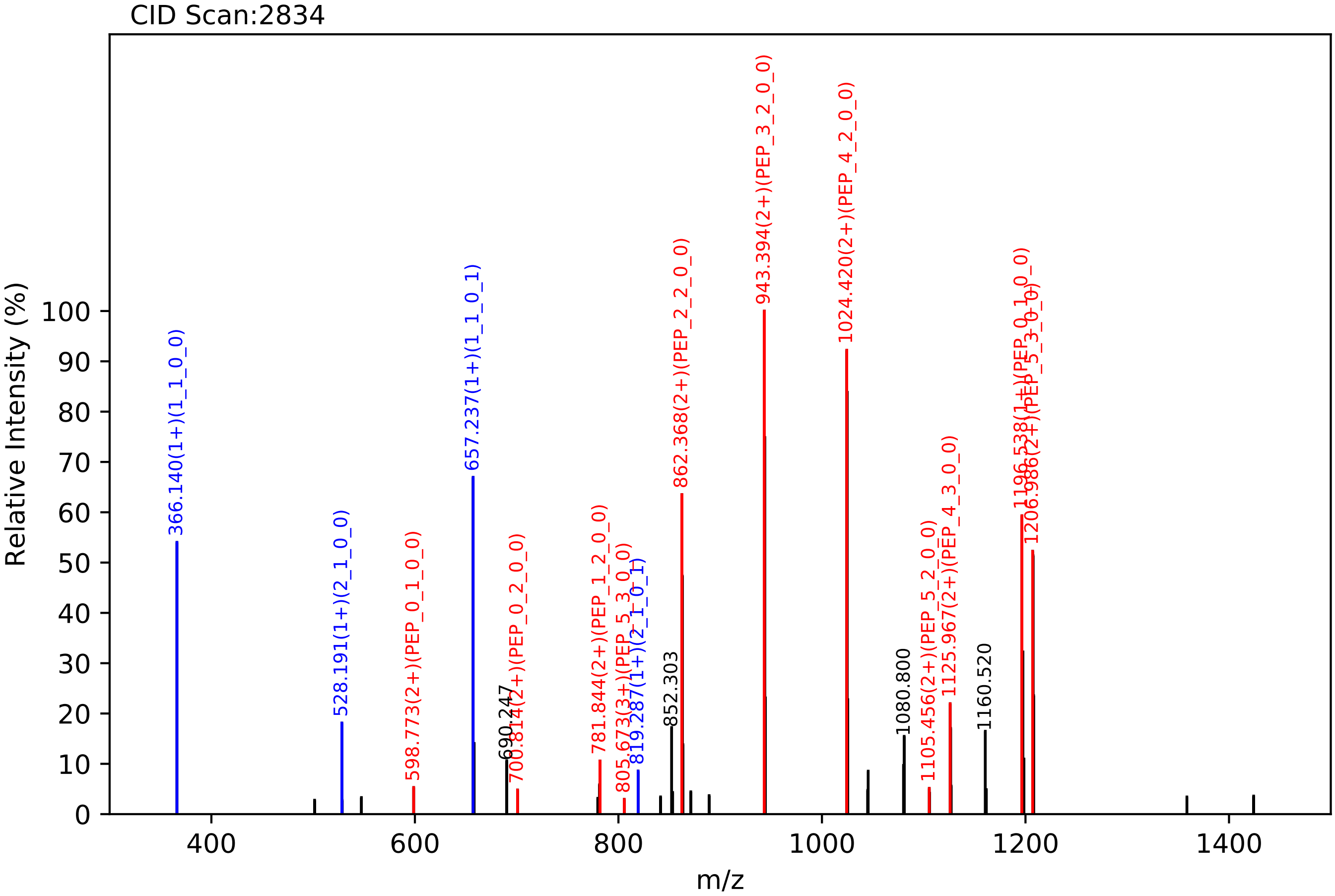

Unknown set no. 549, Experiment: Human Plasma exp_3

NNSDISSTR(=PEP)_5_3_0_1, m/z:902.03(3+), RT:16.30, Y-score:89.77

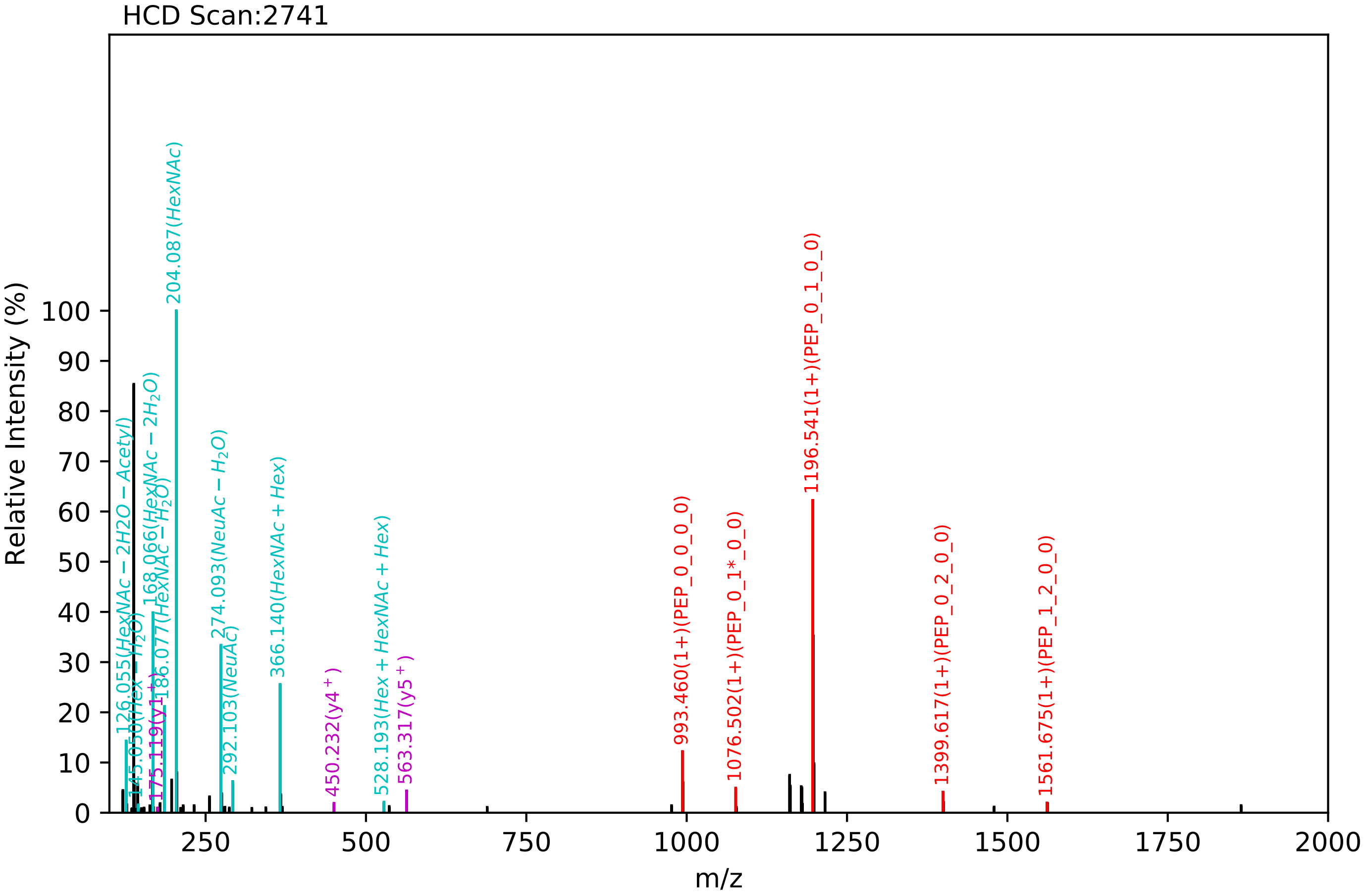

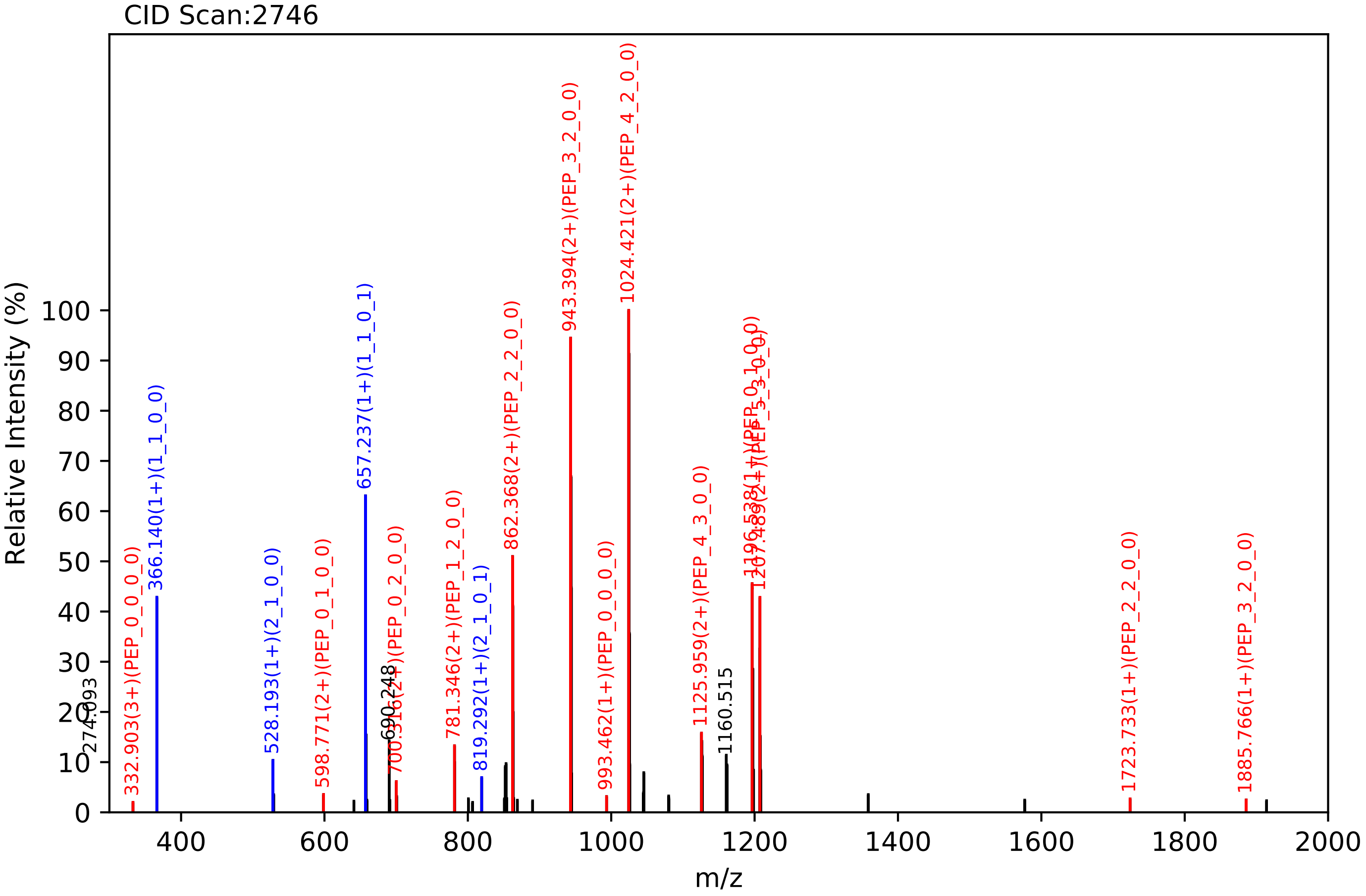

Unknown set no. 550, Experiment: Human Plasma exp_4

NNSDISSTR(=PEP)_5_3_0_1, m/z:902.03(3+), RT:16.33, Y-score:87.19

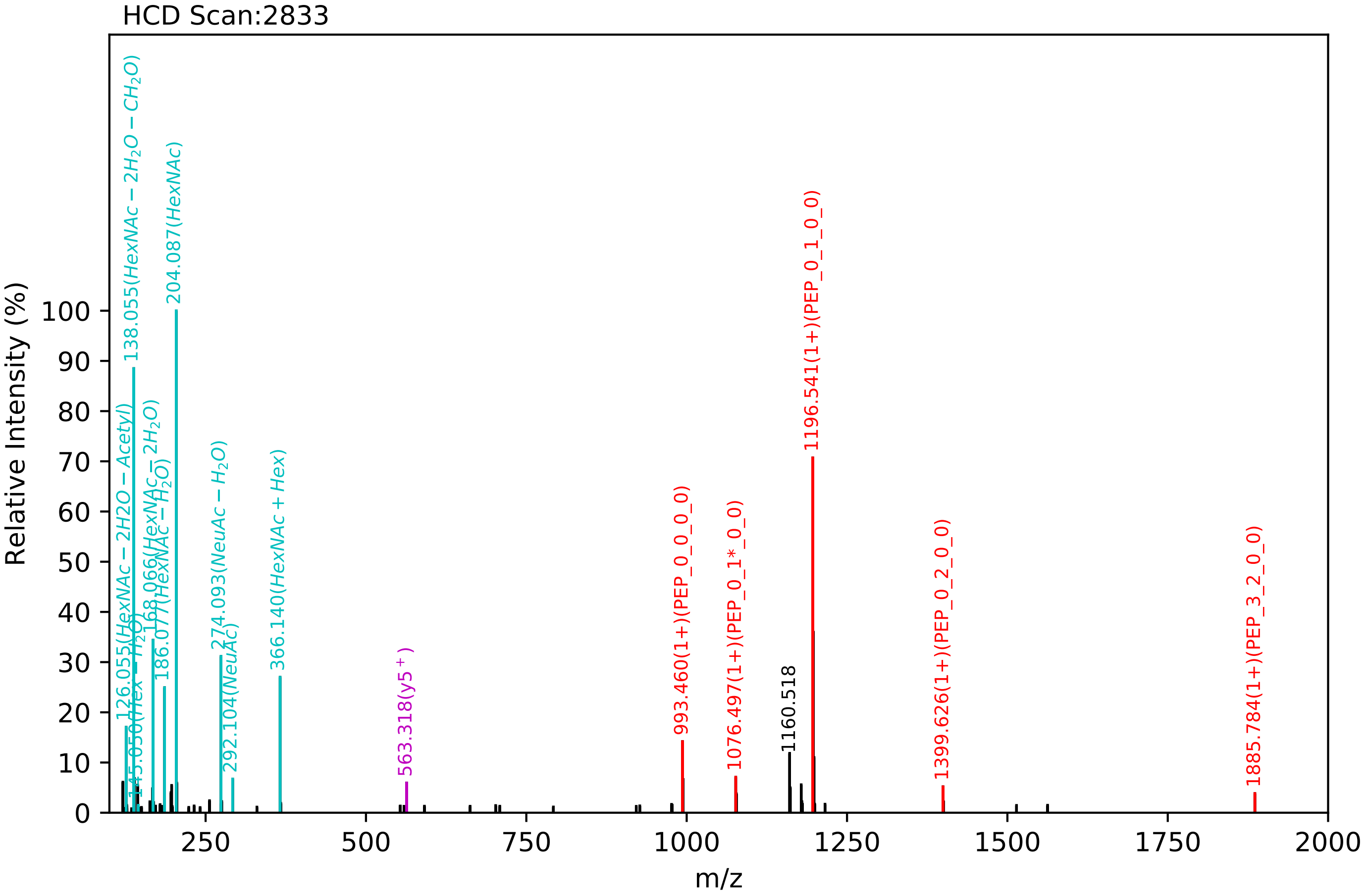

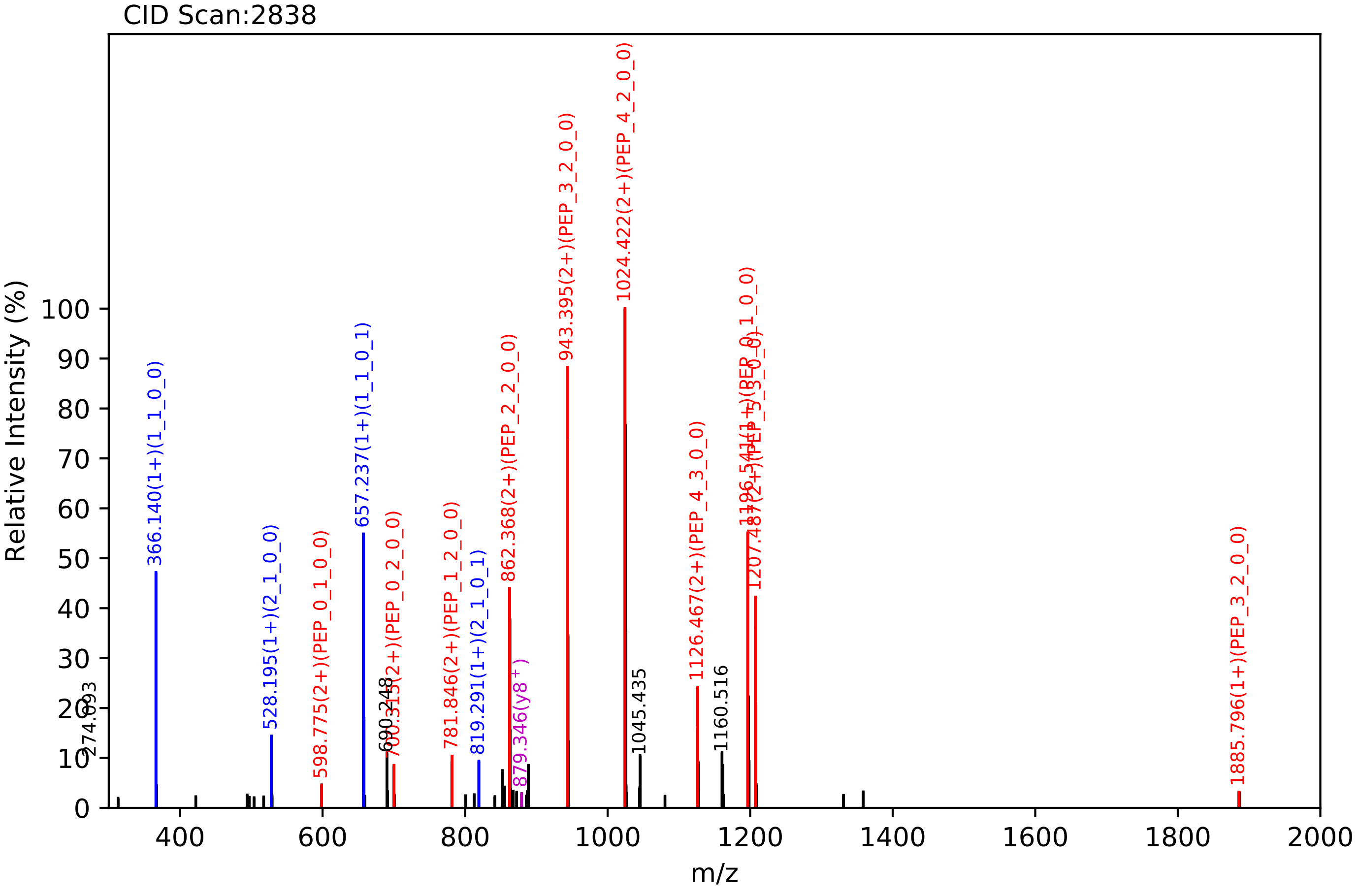

Unknown set no. 551, Experiment: Human Plasma exp_5

NNSDISSTR(=PEP)_5_3_0_1, m/z:902.03(3+), RT:16.26, Y-score:87.98

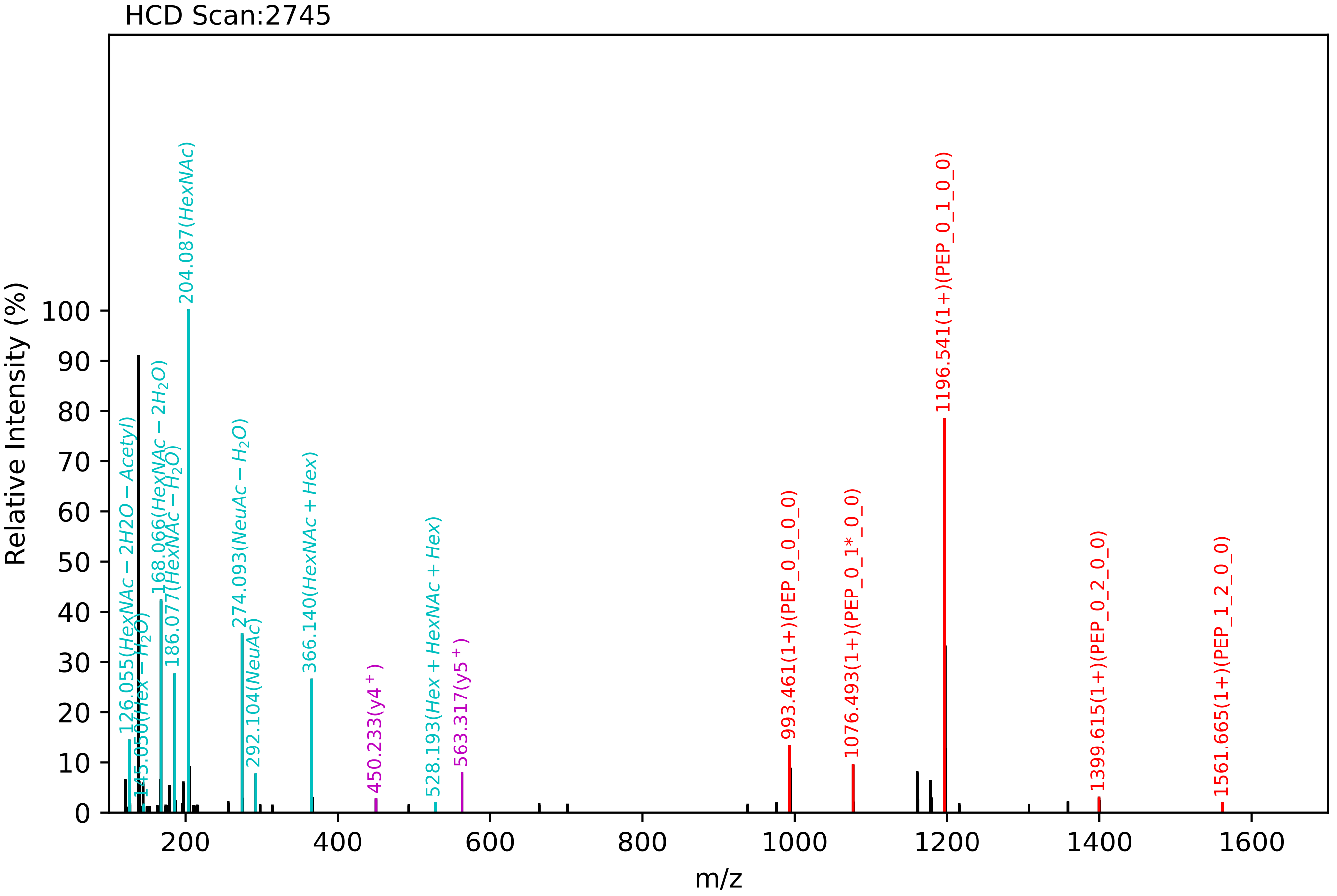

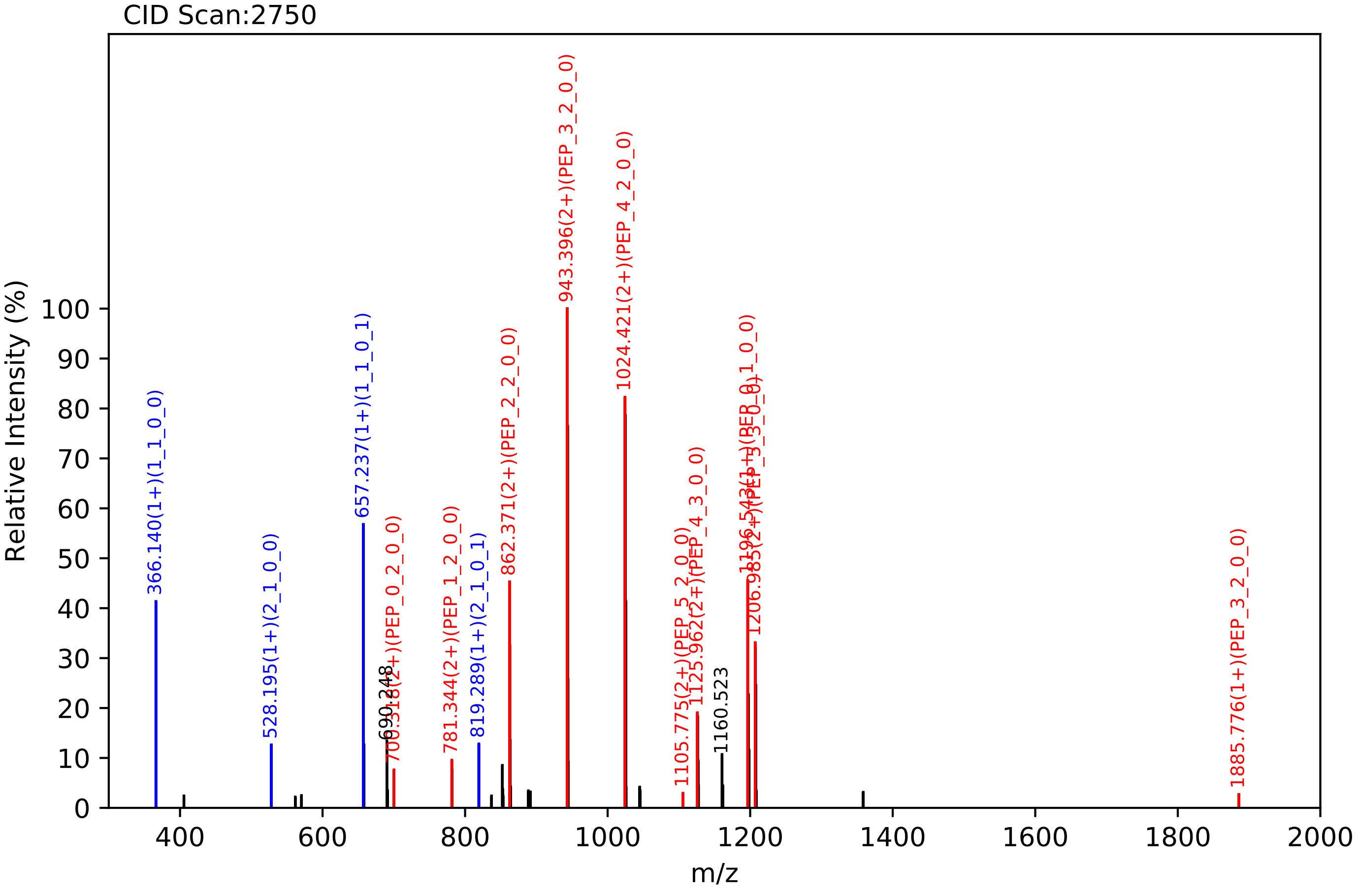

Unknown set no. 552, Experiment: Human Plasma exp_4

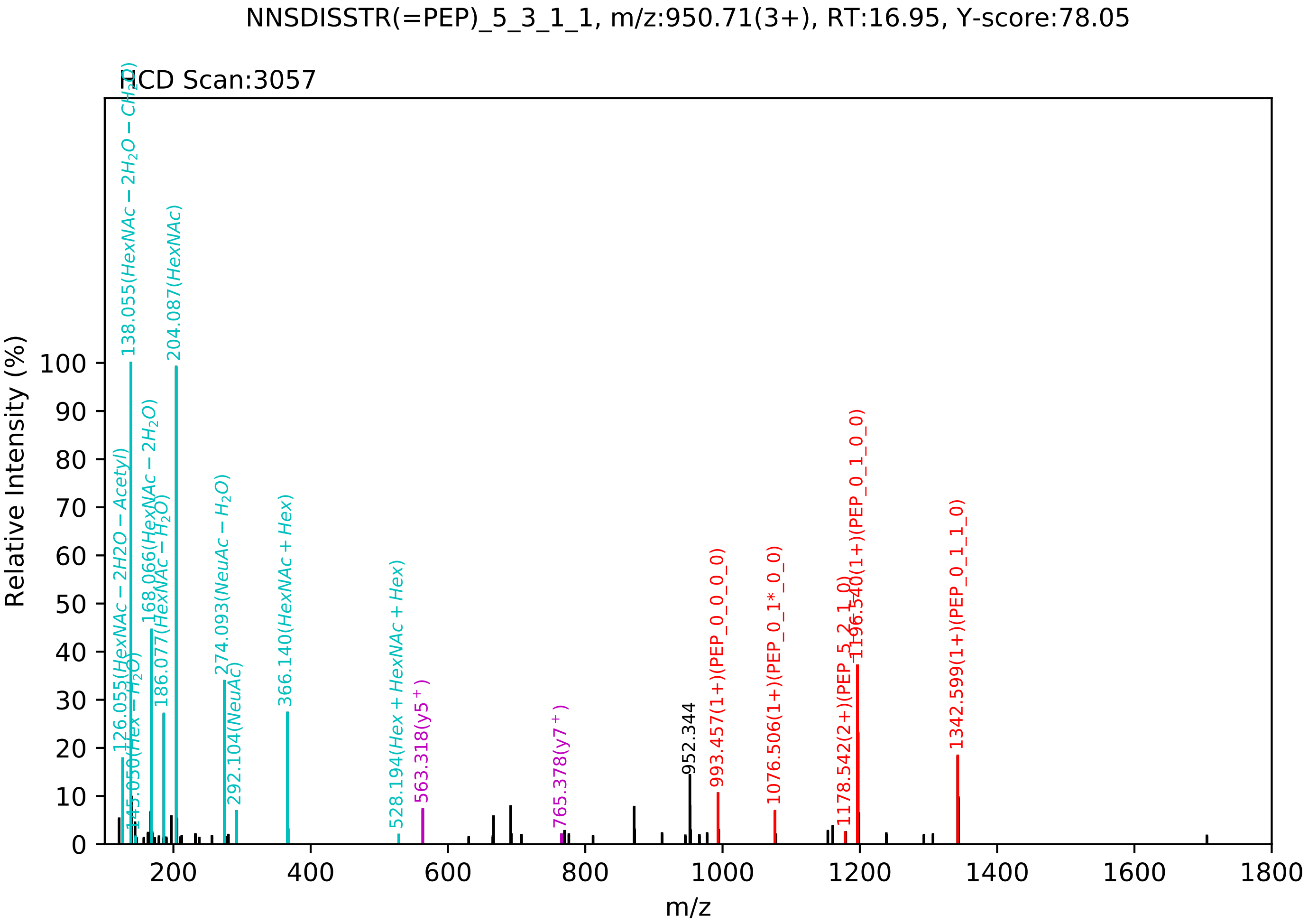

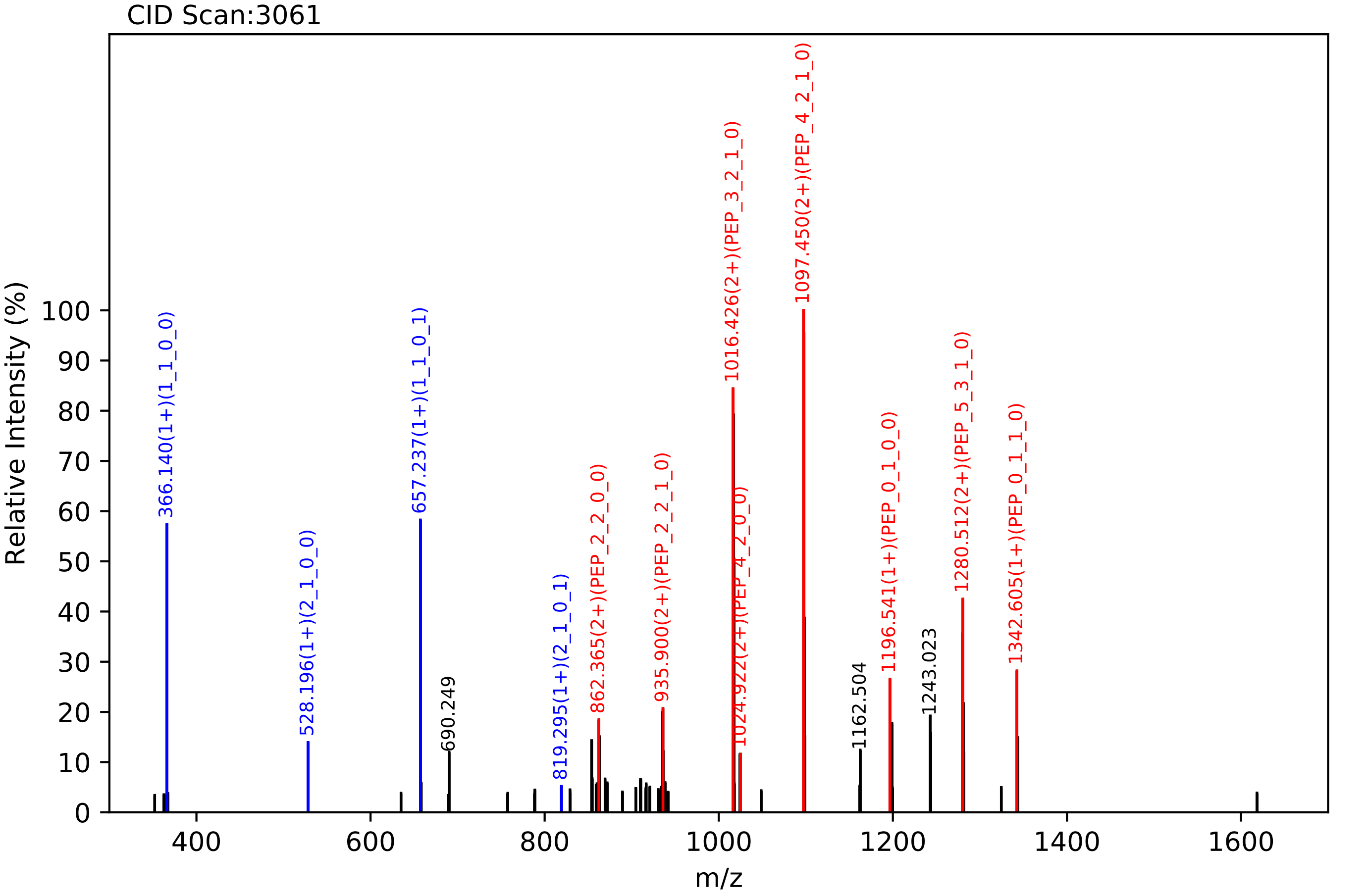

Unknown set no. 553, Experiment: Human Plasma exp_2

NNSDISSTR(=PEP)_5_4_0_1, m/z:969.72(3+), RT:16.64, Y-score:92.07

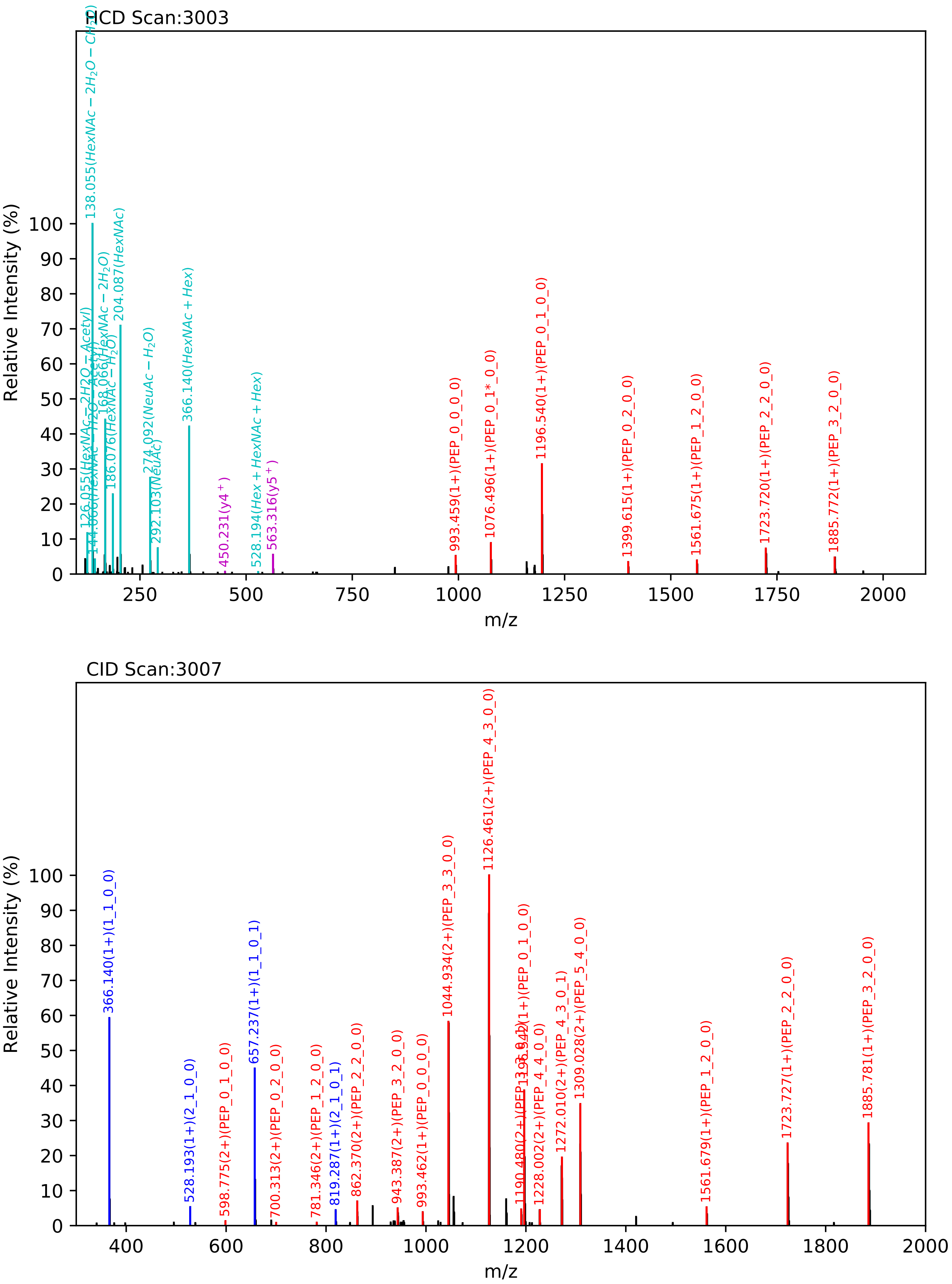

Unknown set no. 554, Experiment: Human Plasma exp_2

NNSDISSTR(=PEP)_5_4_1_1, m/z:1018.40(3+), RT:17.08, Y-score:89.47

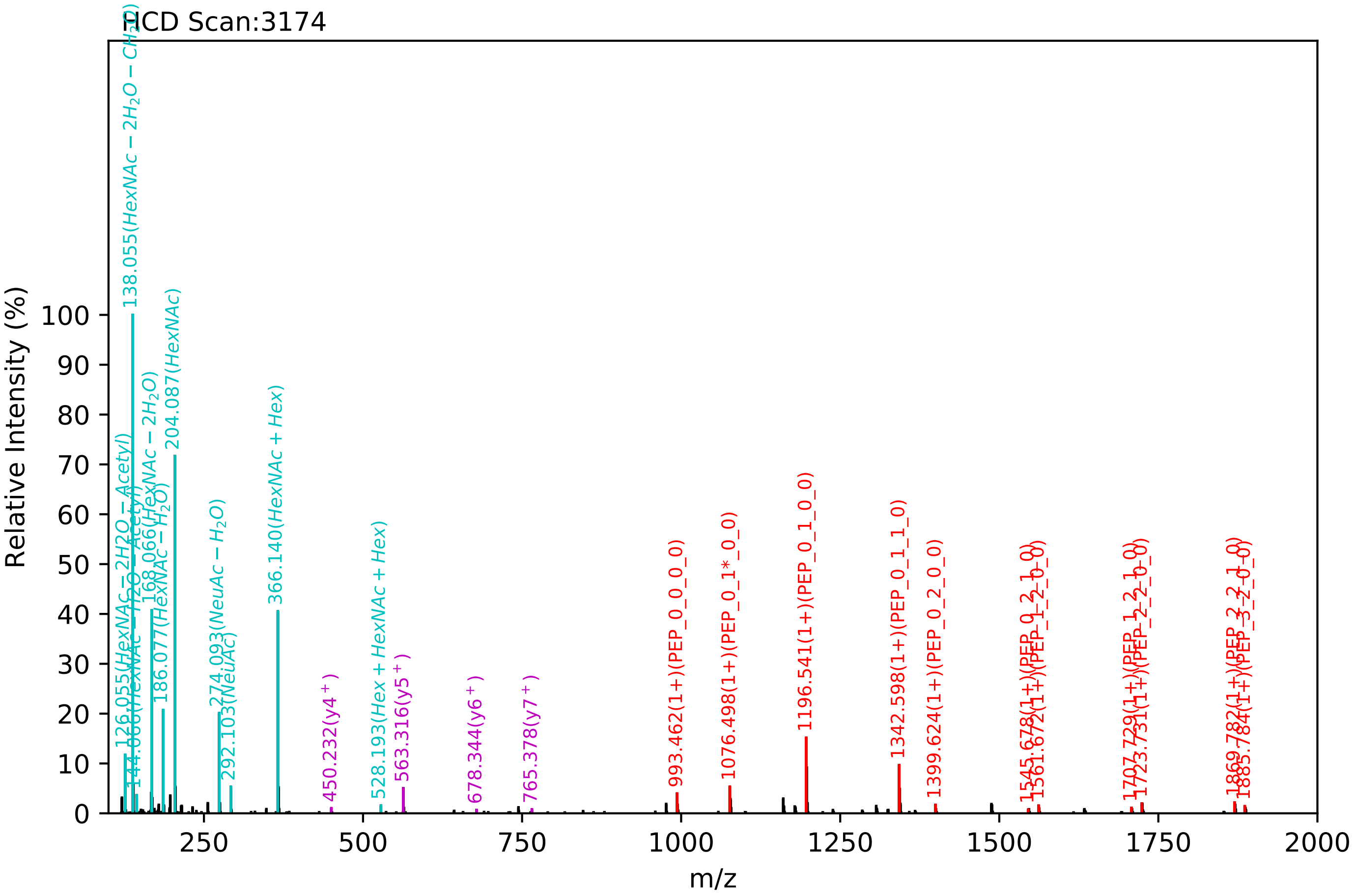

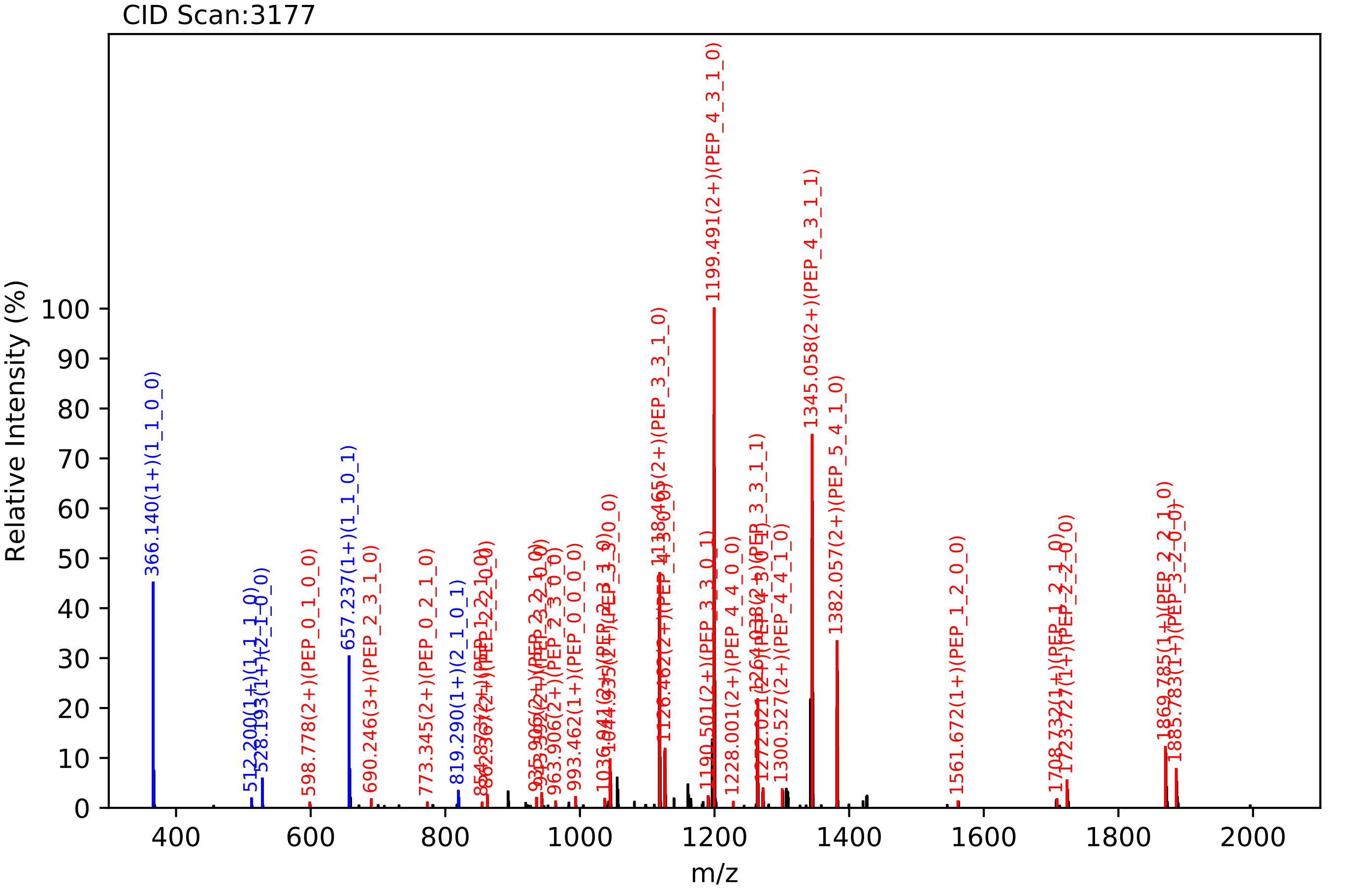

Unknown set no. 555, Experiment: Human Plasma exp_2

NNSDISSTR(=PEP)_5_4_1_1, m/z:1527.10(2+), RT:17.15, Y-score:84.12

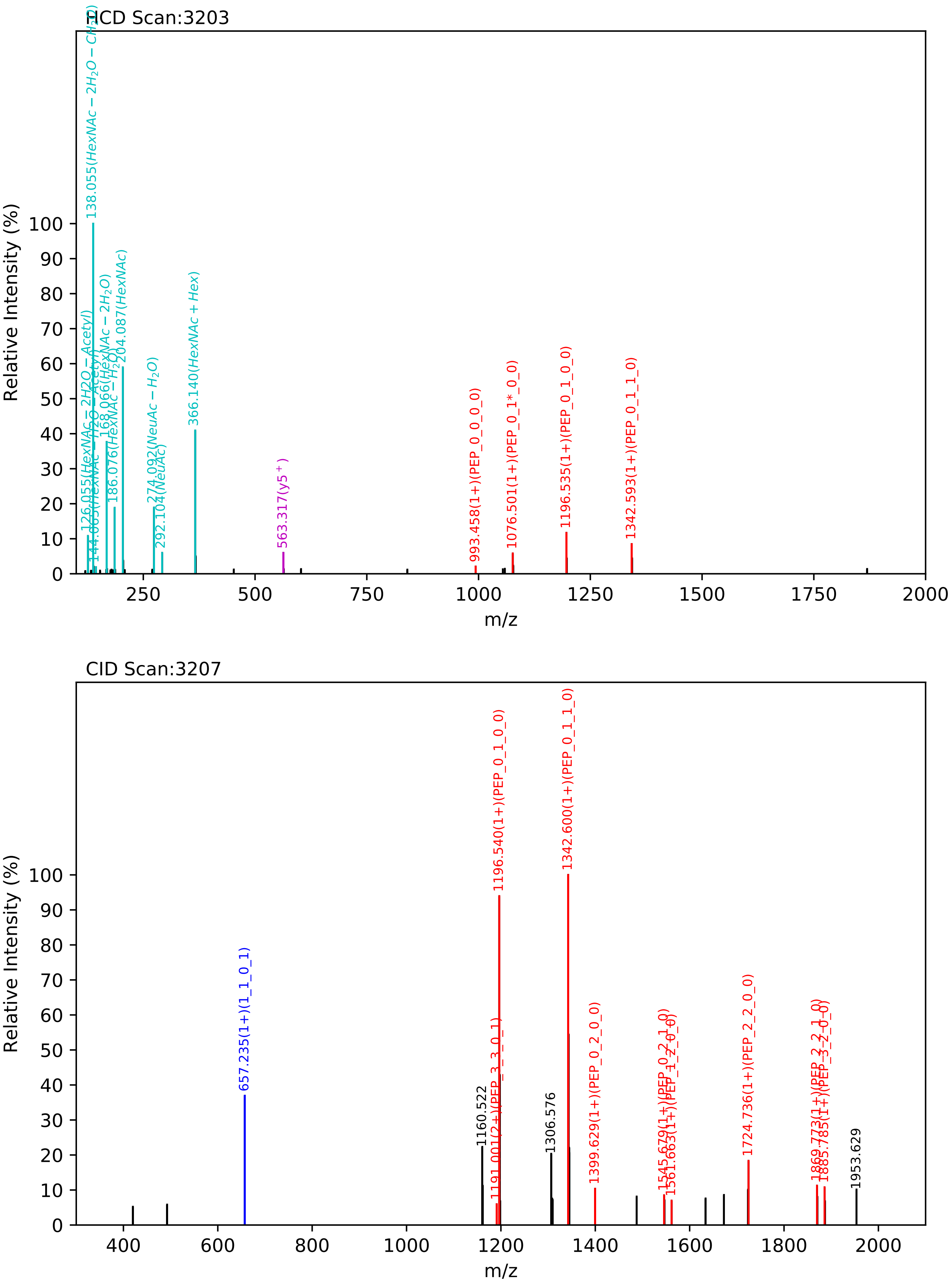

Unknown set no. 556, Experiment: Human Plasma exp_5

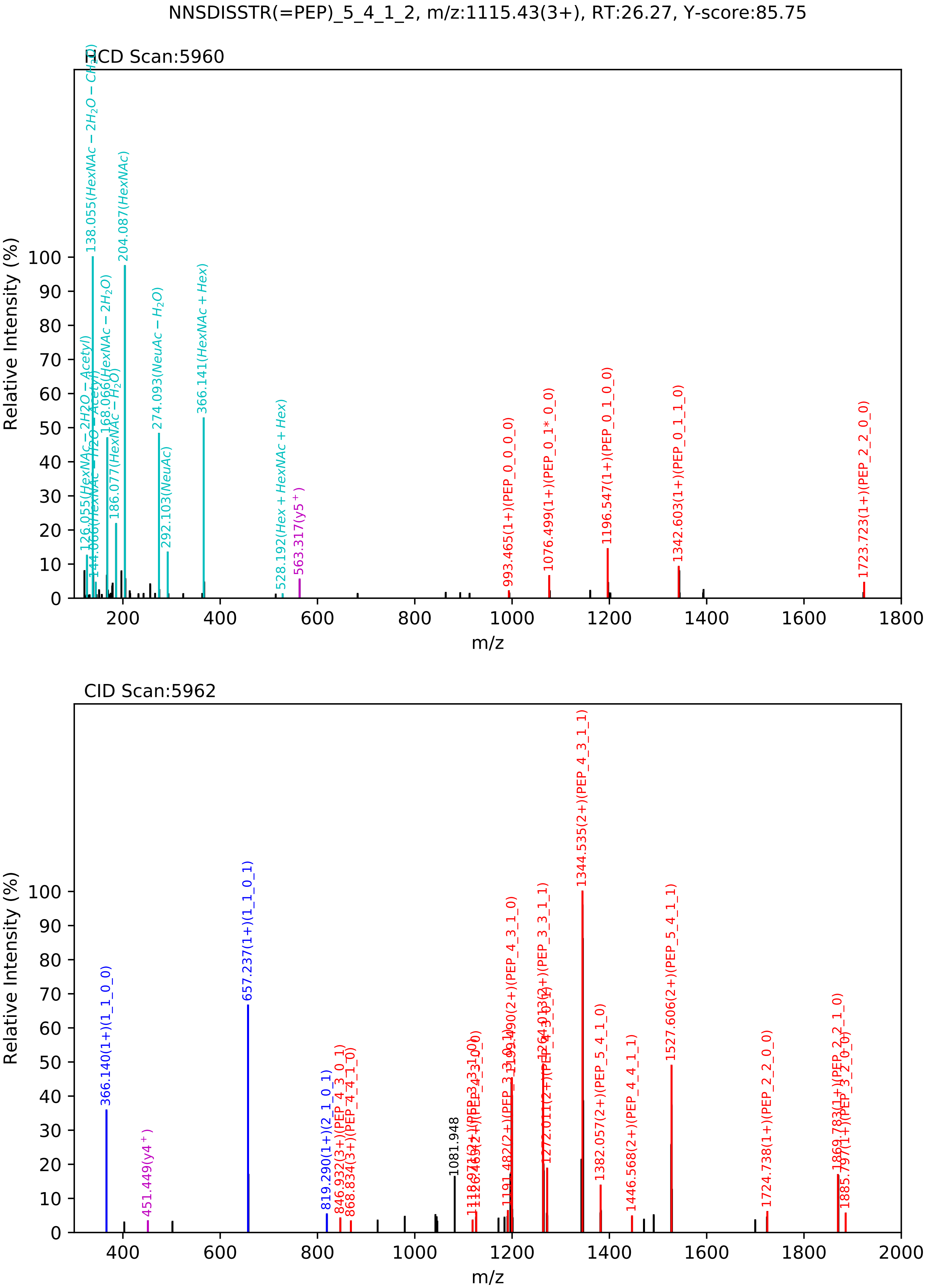

Unknown set no. 557, Experiment: Human Plasma exp_4

NNSDISSTR(=PEP)_5_5_0_1, m/z:1037.41(3+), RT:17.72, Y-score:86.38

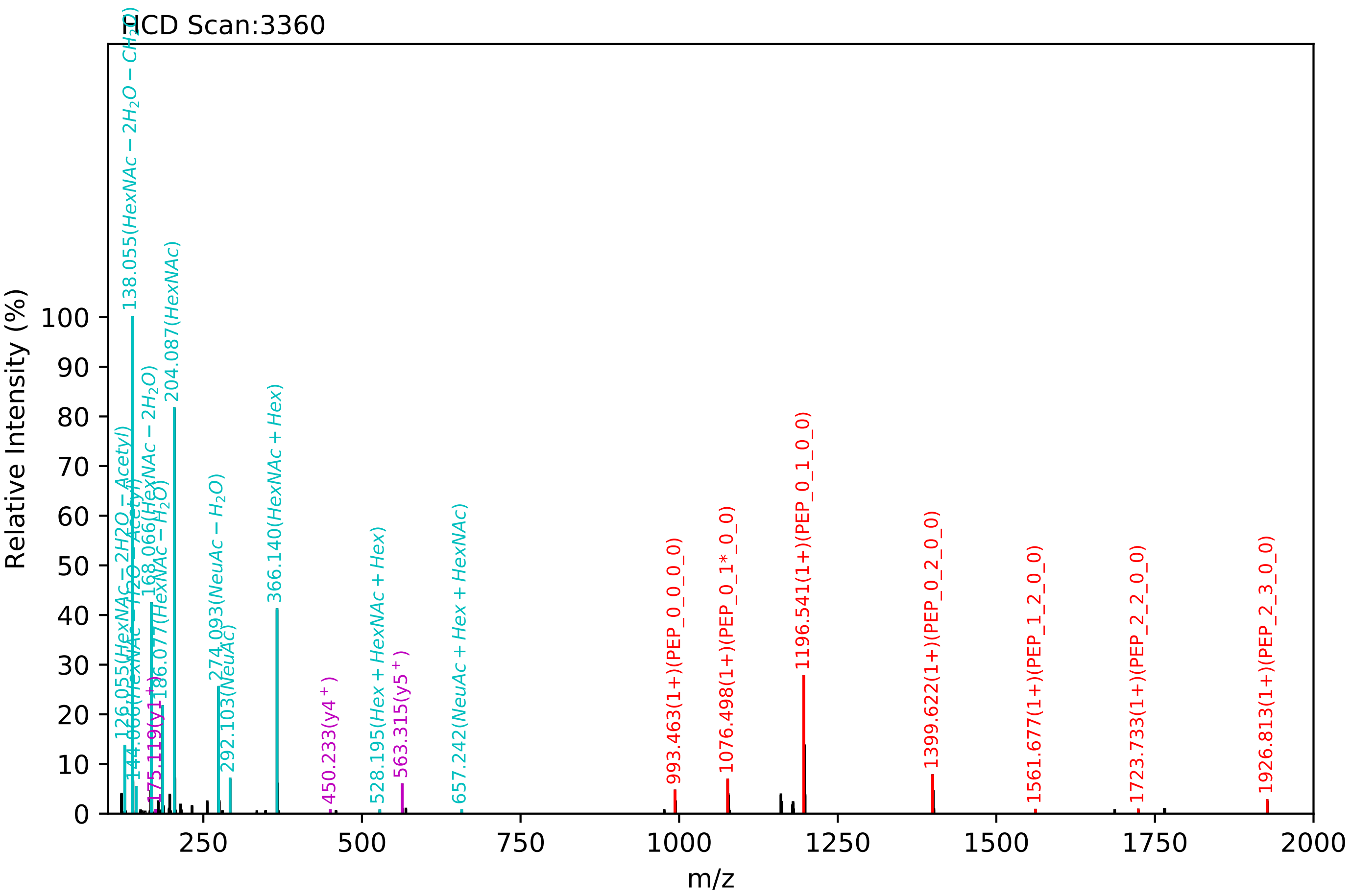

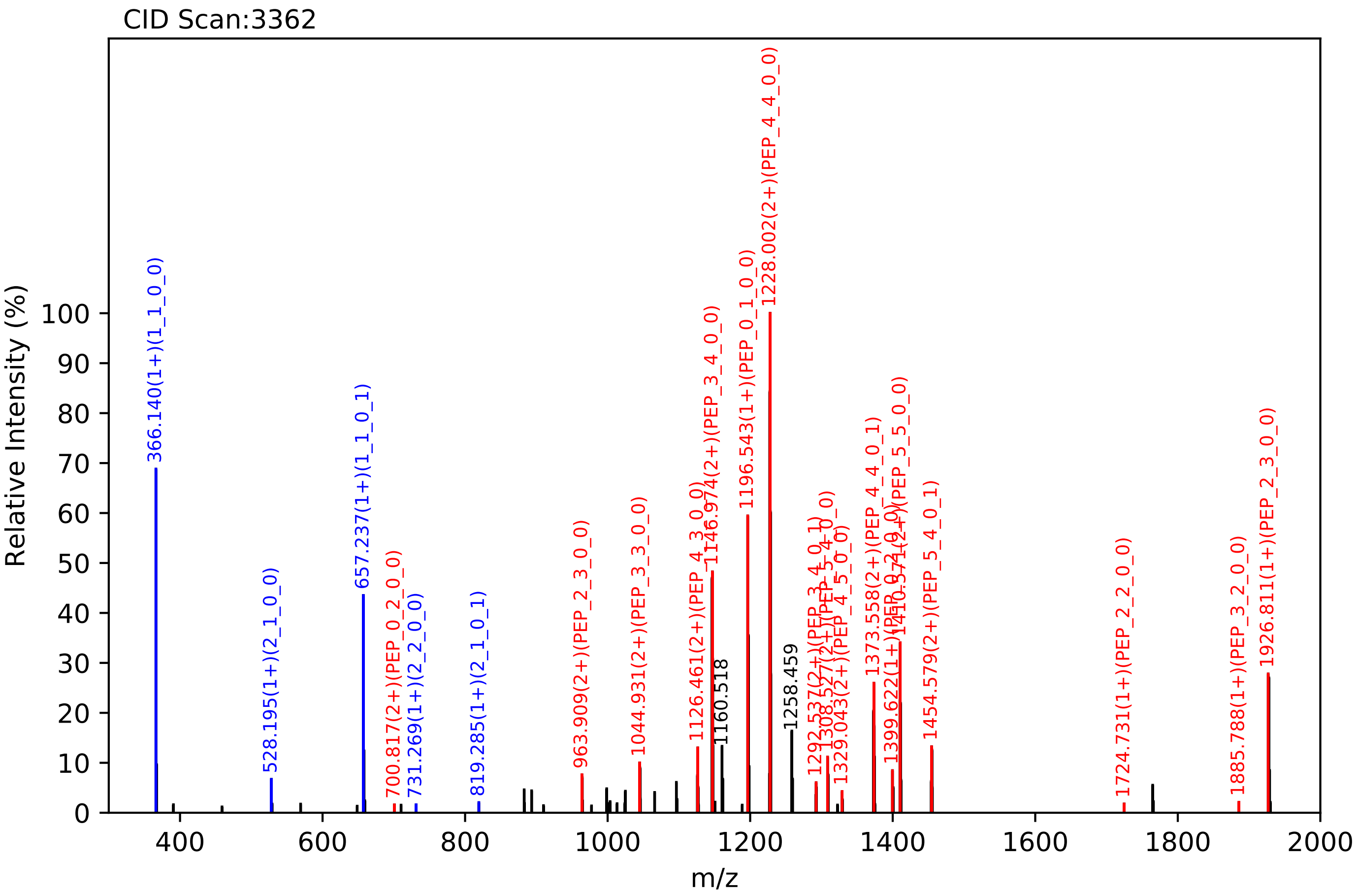

Unknown set no. 558, Experiment: Human Plasma exp_5

NNSDISSTR(=PEP)_5_5_1_1, m/z:1086.10(3+), RT:18.54, Y-score:90.75

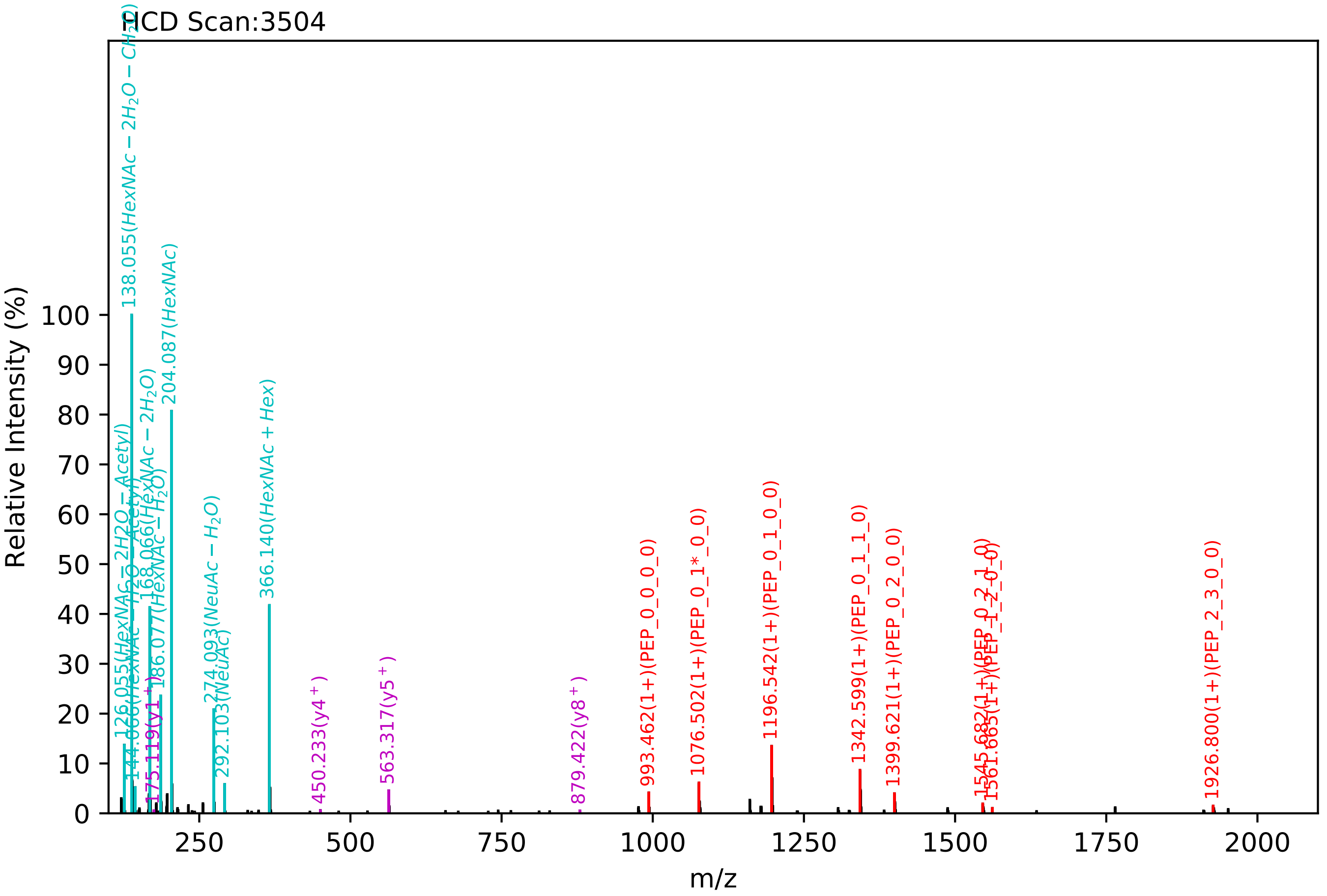

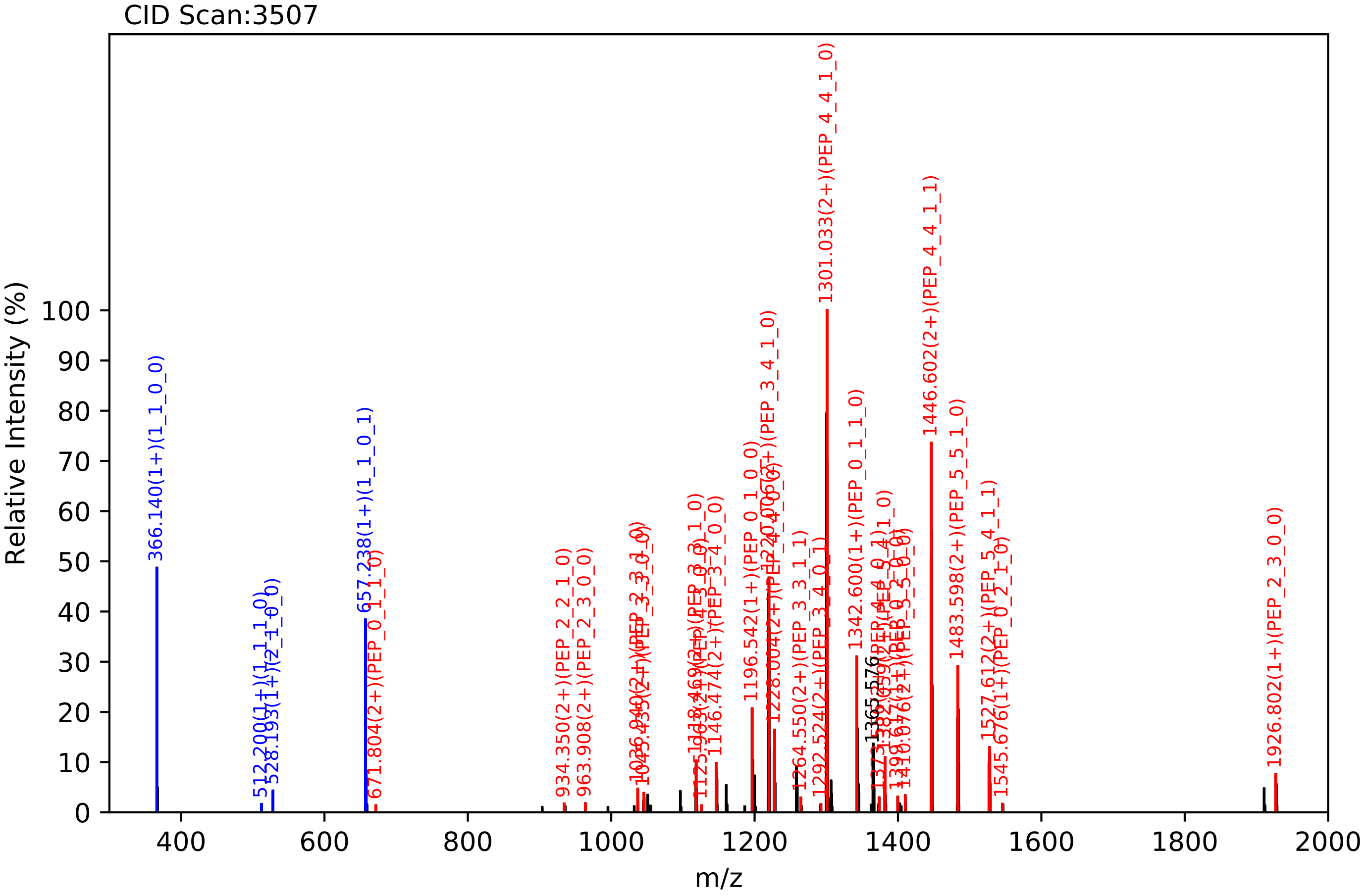

Unknown set no. 559, Experiment: Human Plasma exp_1

NNSDISSTR(=PEP)_6_3_0_1, m/z:956.04(3+), RT:15.57, Y-score:85.79

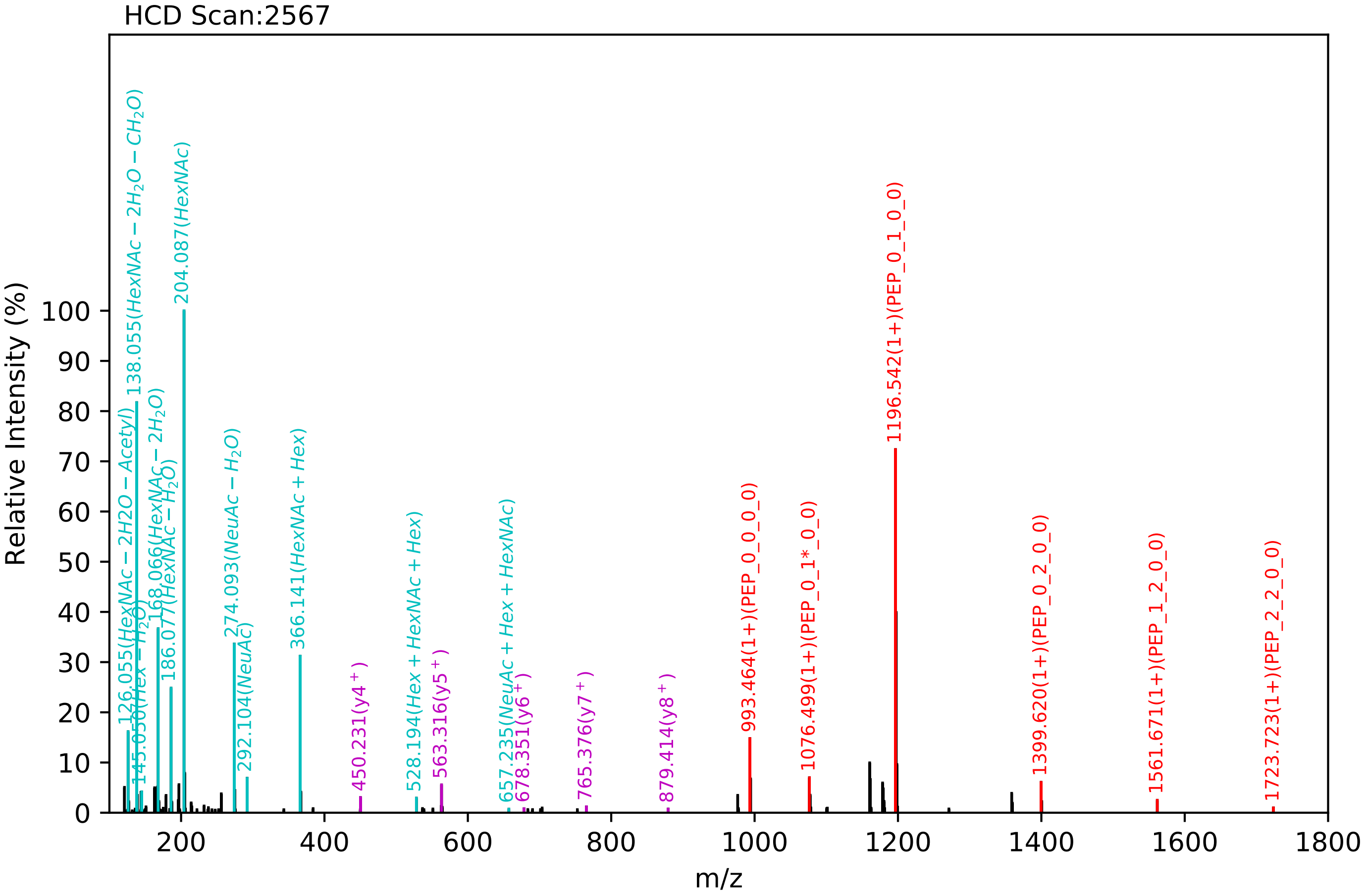

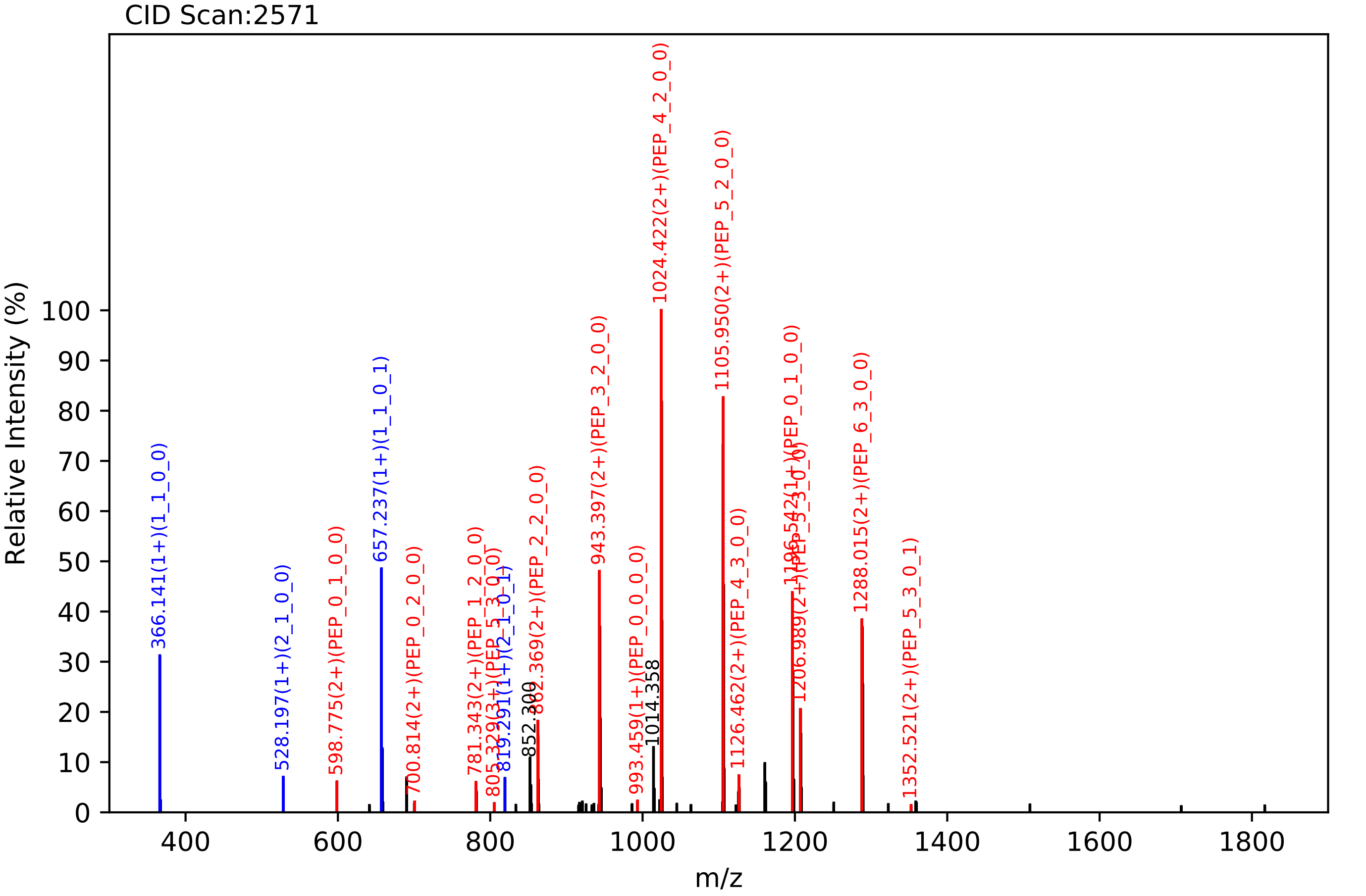

Unknown set no. 560, Experiment: Human Plasma exp_2

NNSDISSTR(=PEP)_6_3_0_1, m/z:956.04(3+), RT:16.10, Y-score:85.45

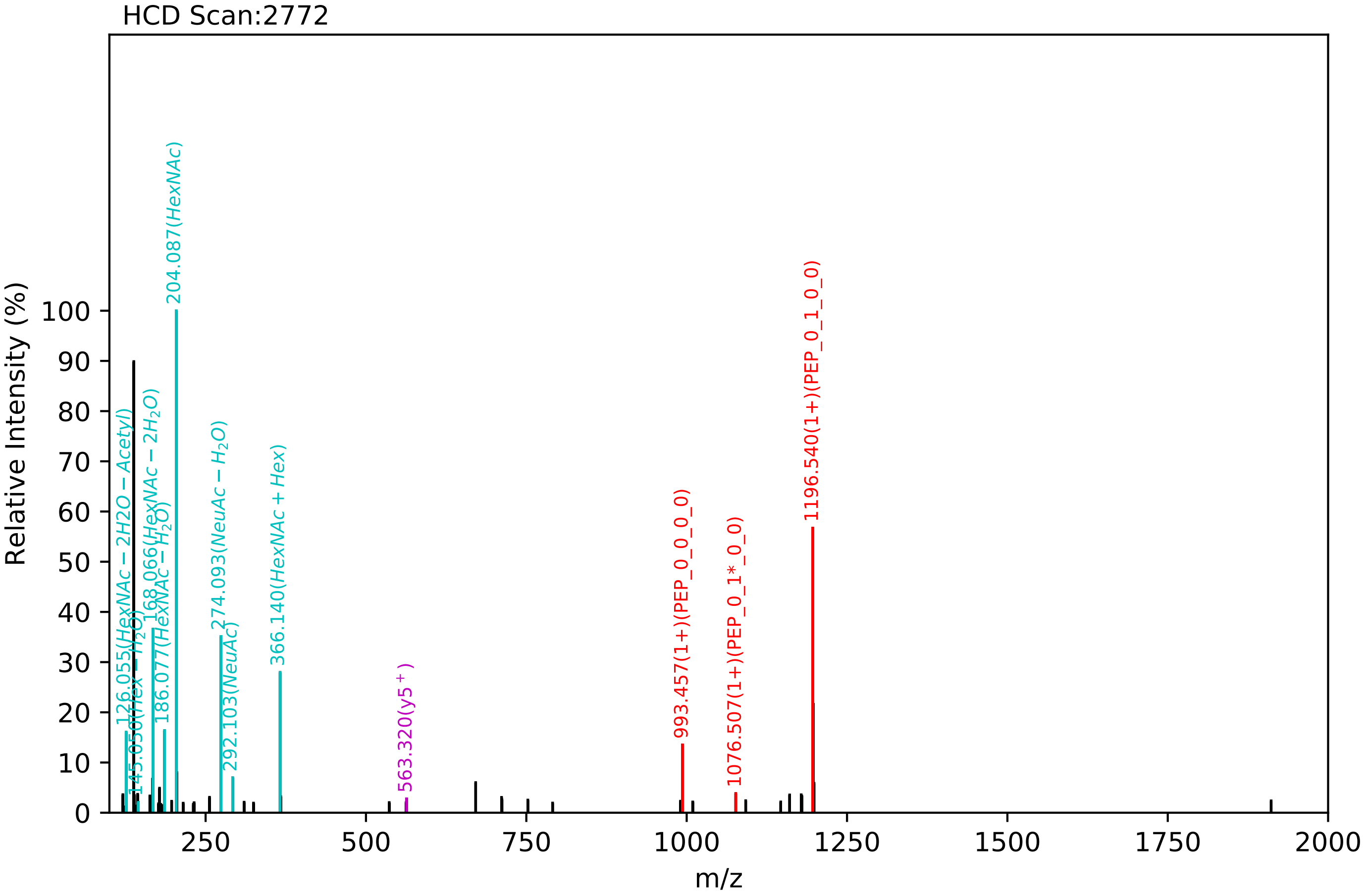

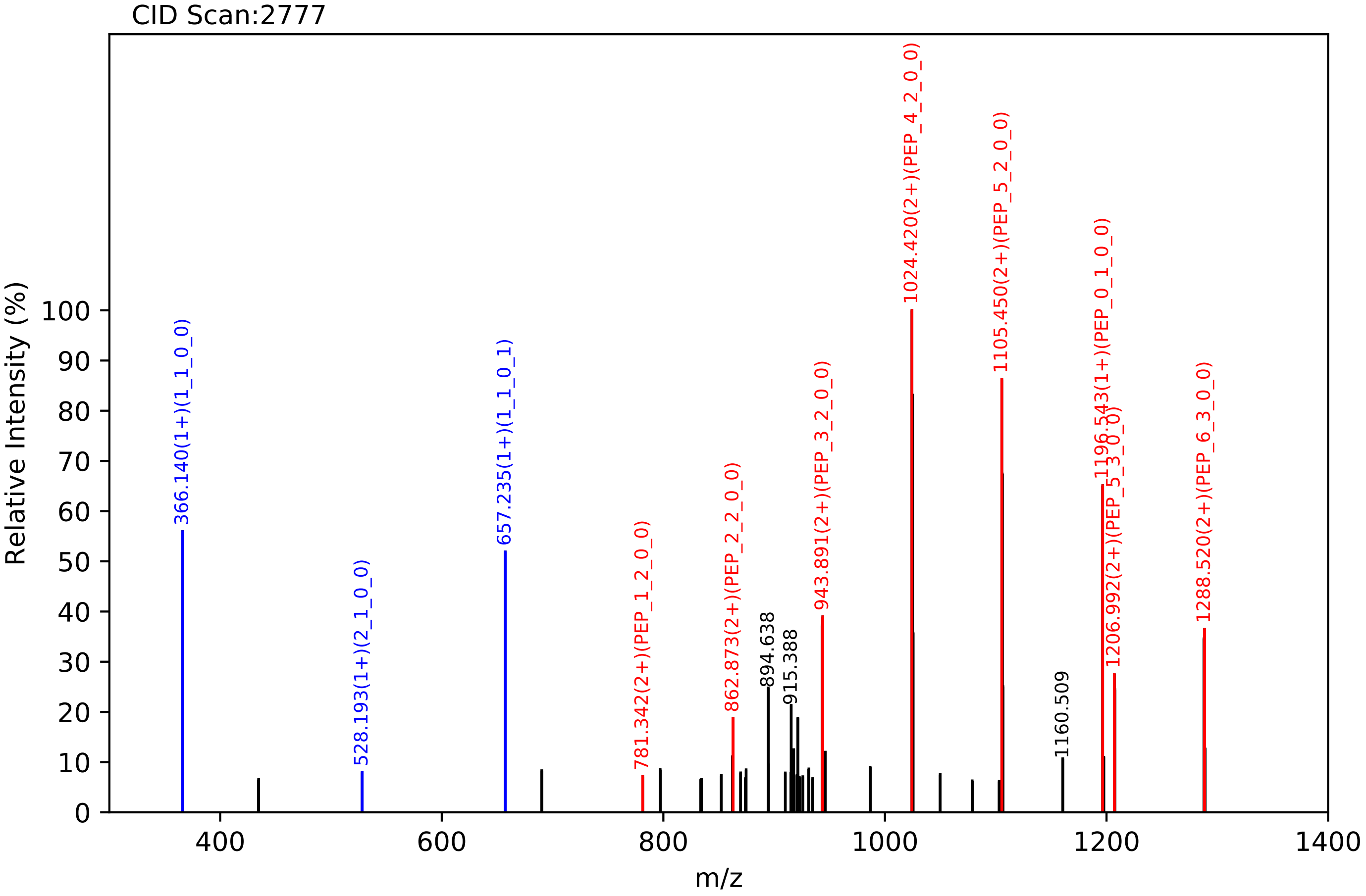

Unknown set no. 561, Experiment: Human Plasma exp_3

NNSDISSTR(=PEP)_6_3_1_1, m/z:1004.73(3+), RT:17.01, Y-score:86.24

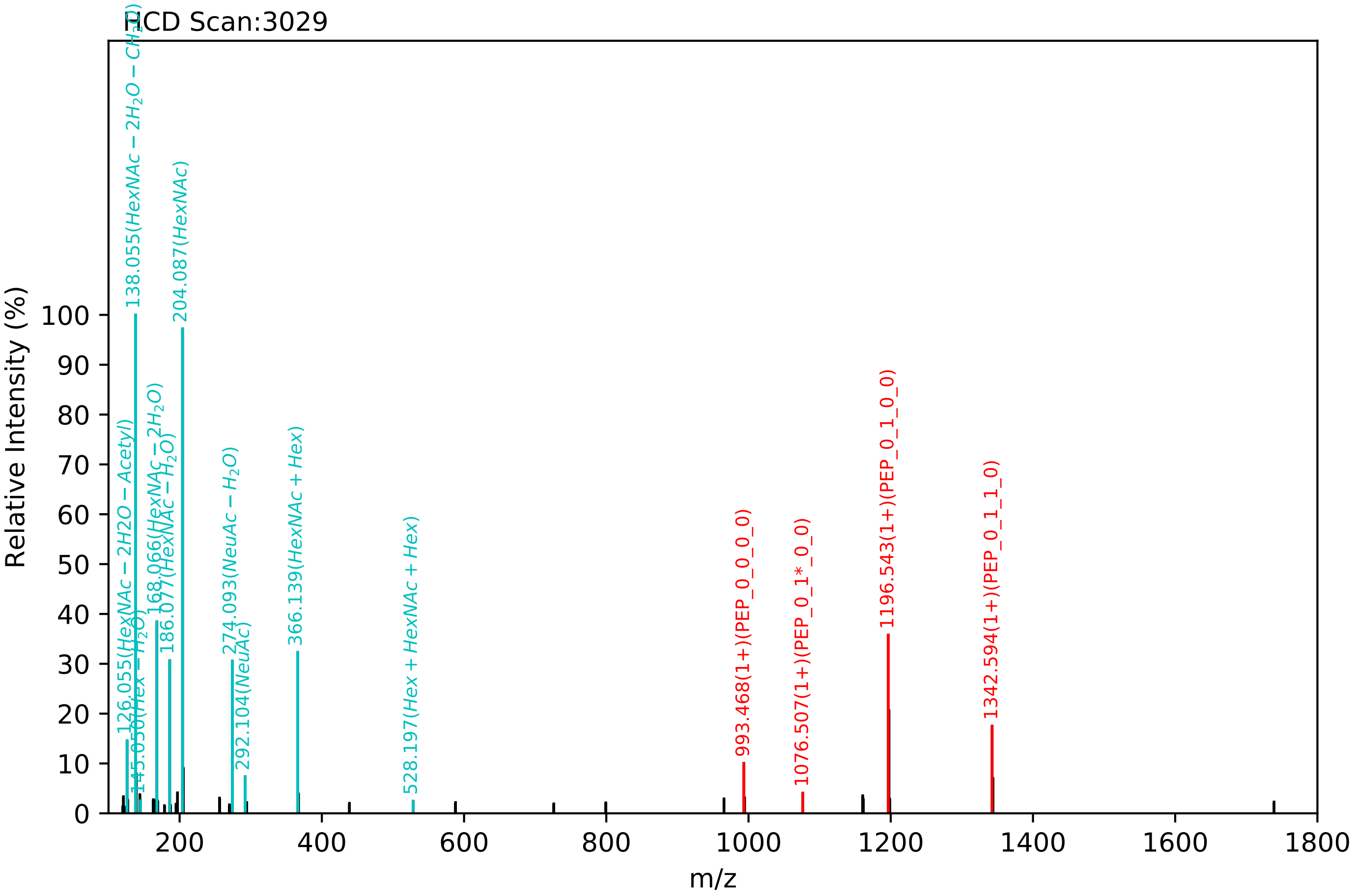

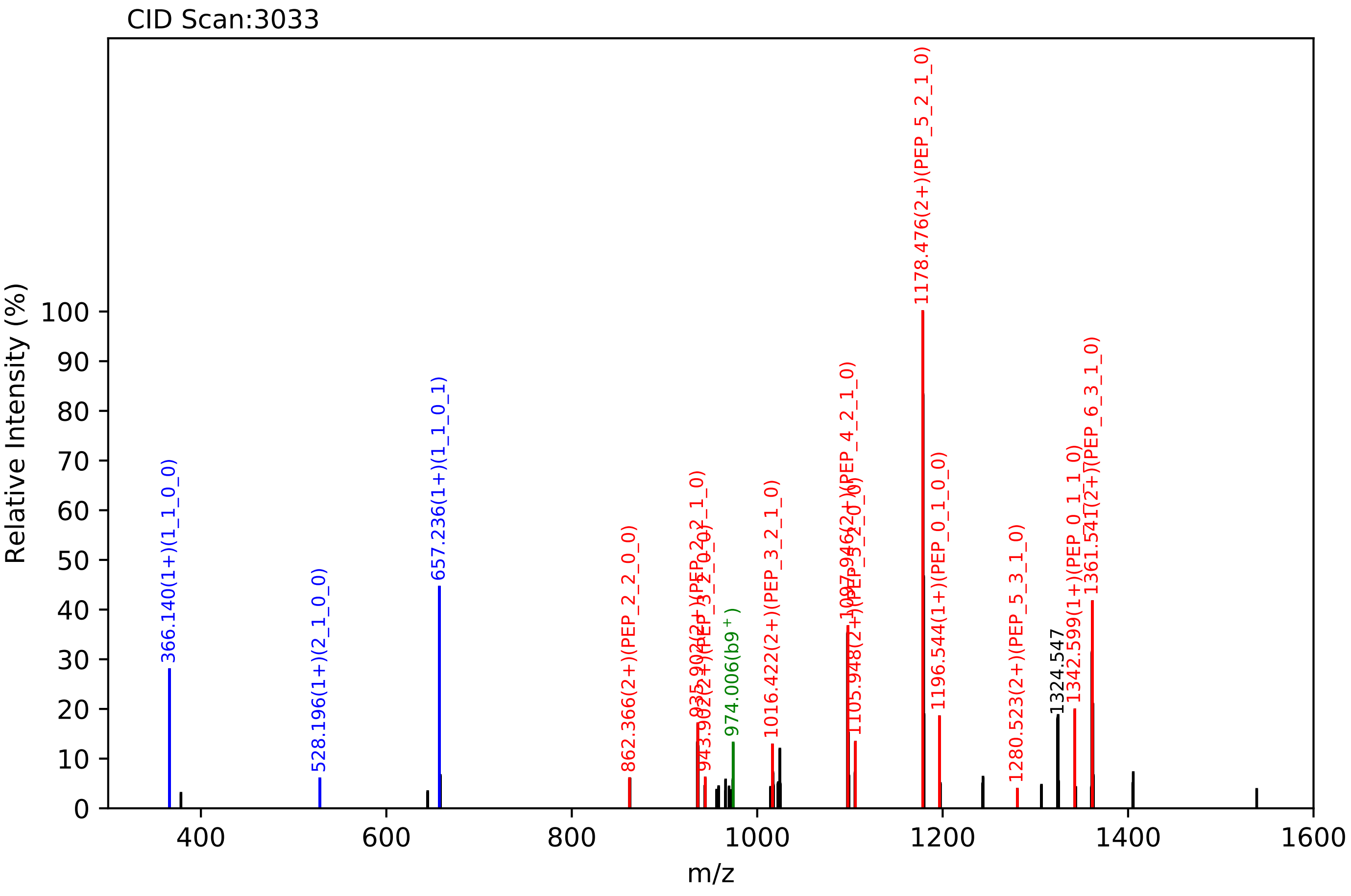

Unknown set no. 562, Experiment: Human Plasma exp_4

NNSDISSTR(=PEP)_6_3_1_1, m/z:1004.73(3+), RT:17.09, Y-score:90.87

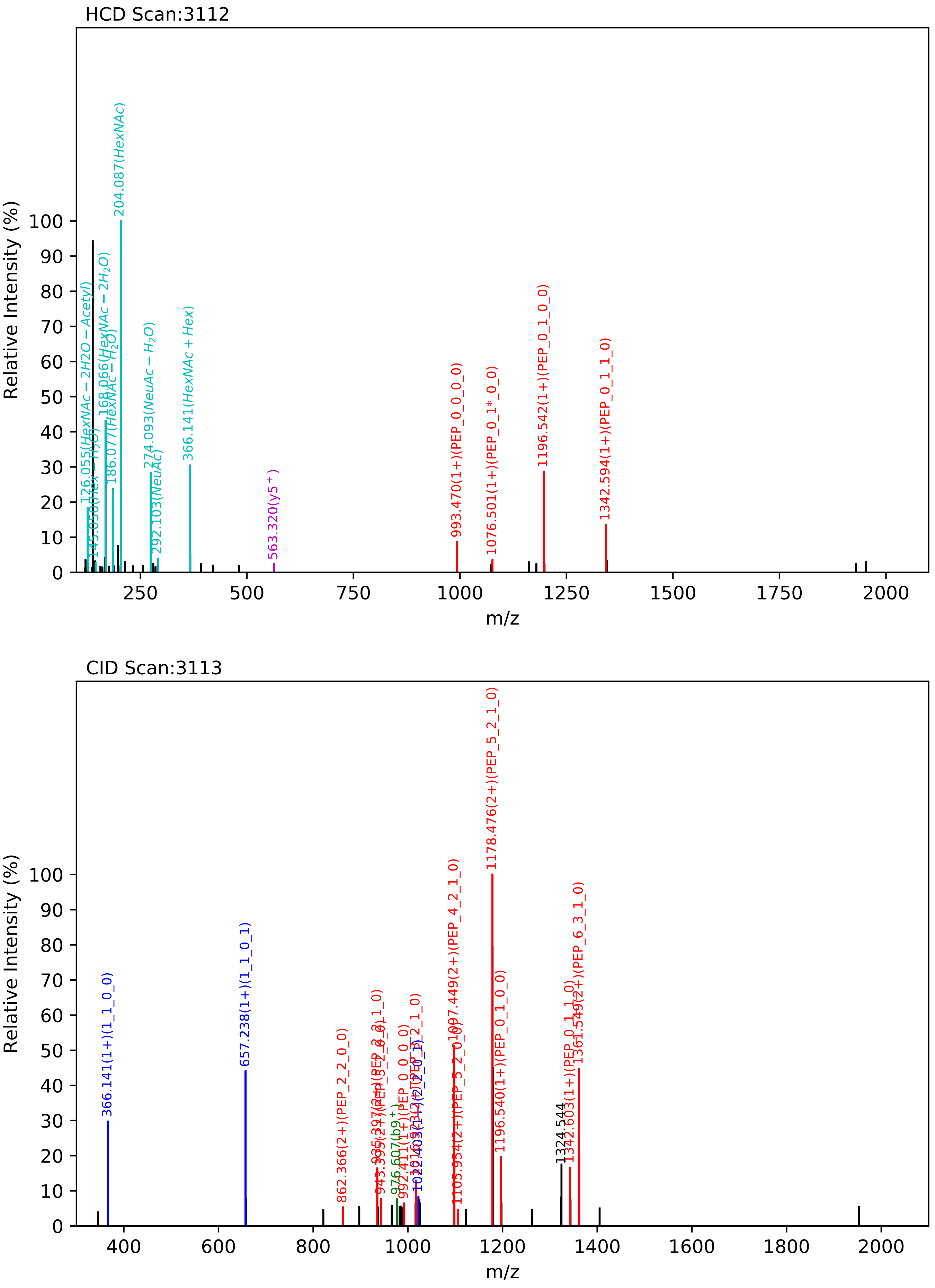

Unknown set no. 563, Experiment: Human Plasma exp_1

ENISDPTSPLR(=PEP)_5_3_1_1, m/z:1029.10(3+), RT:66.38, Y-score:95.83

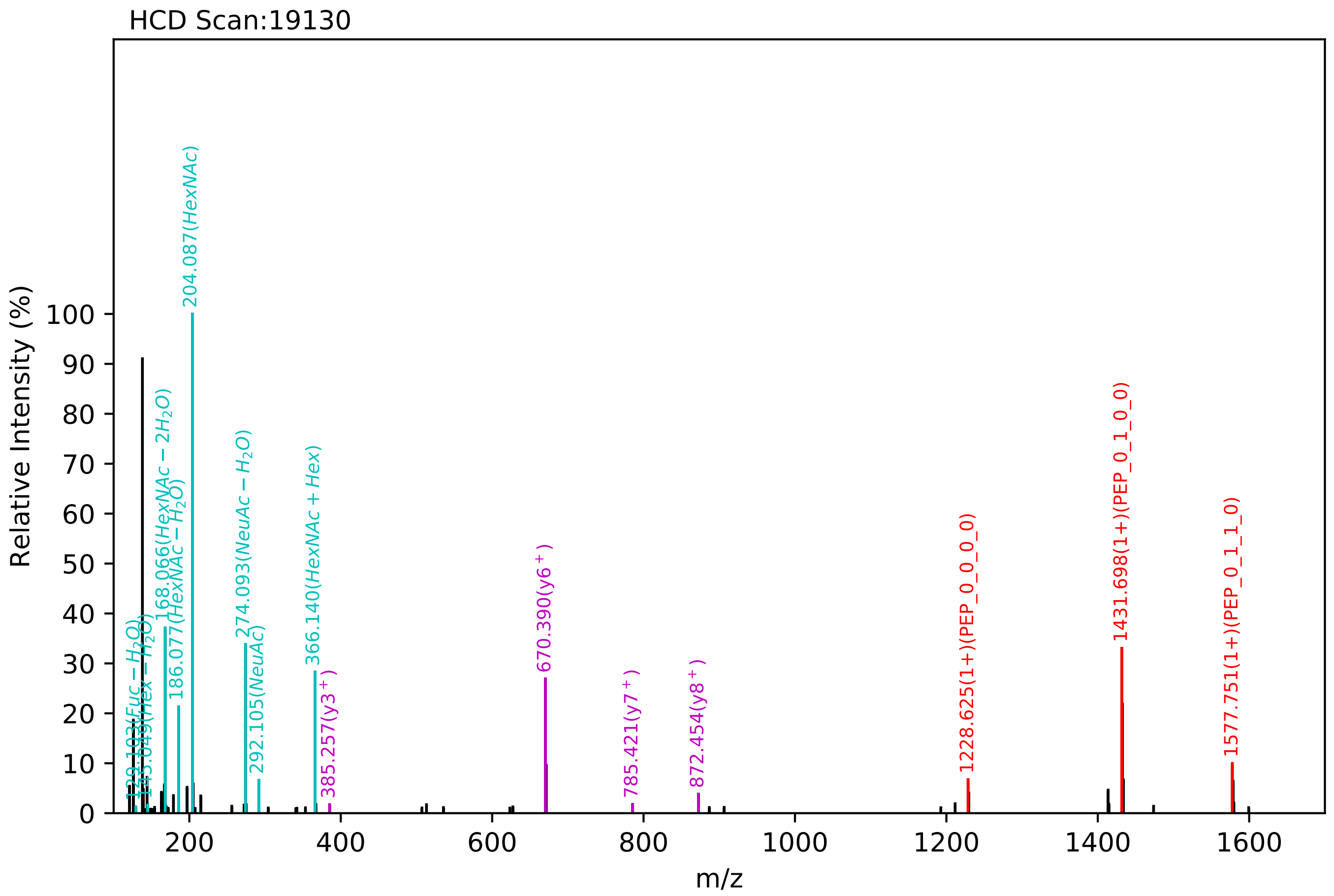

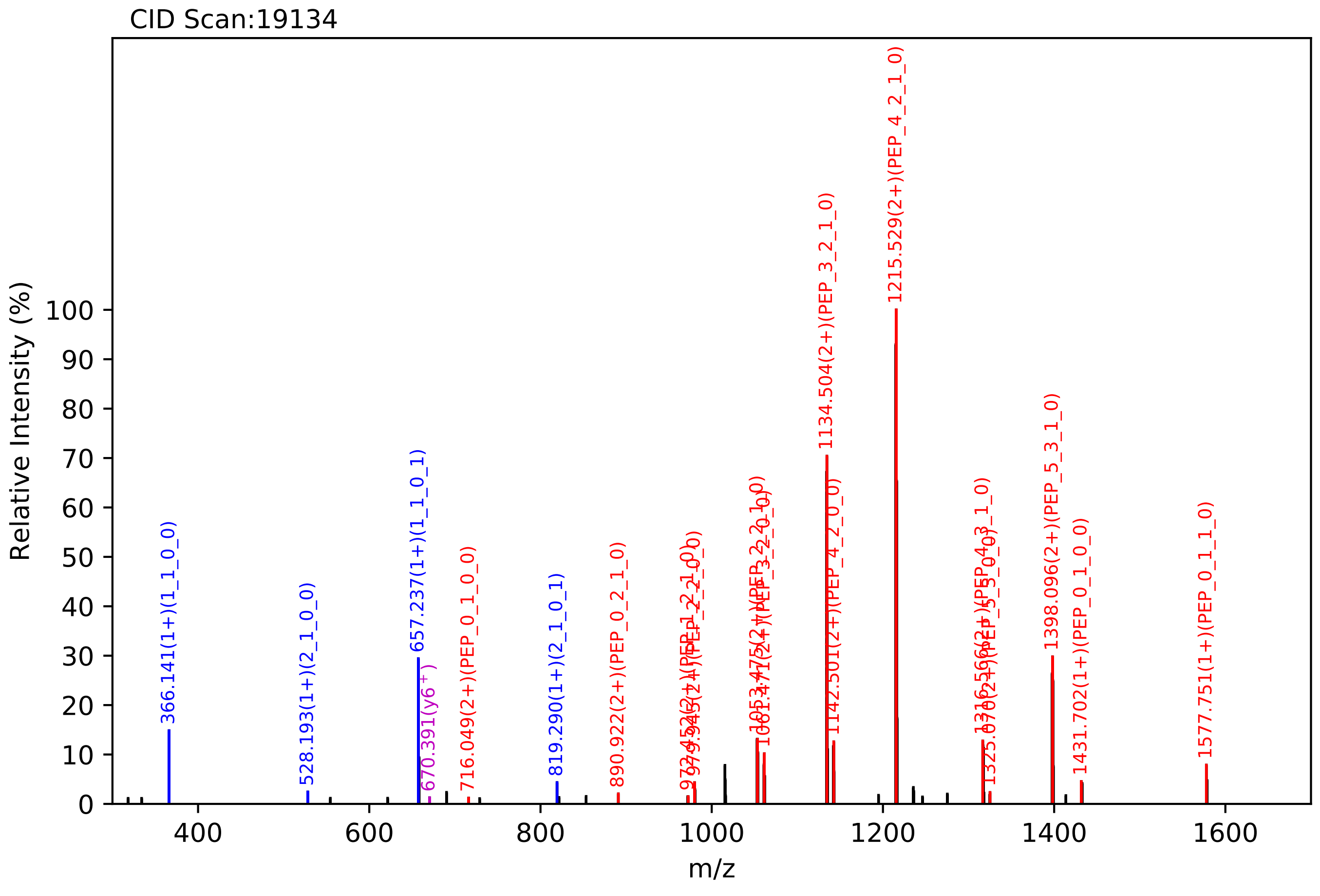

Unknown set no. 564, Experiment: Human Plasma exp_3

ENISDPTSPLR(=PEP)_5_3_1_1, m/z:1029.10(3+), RT:66.22, Y-score:95.11

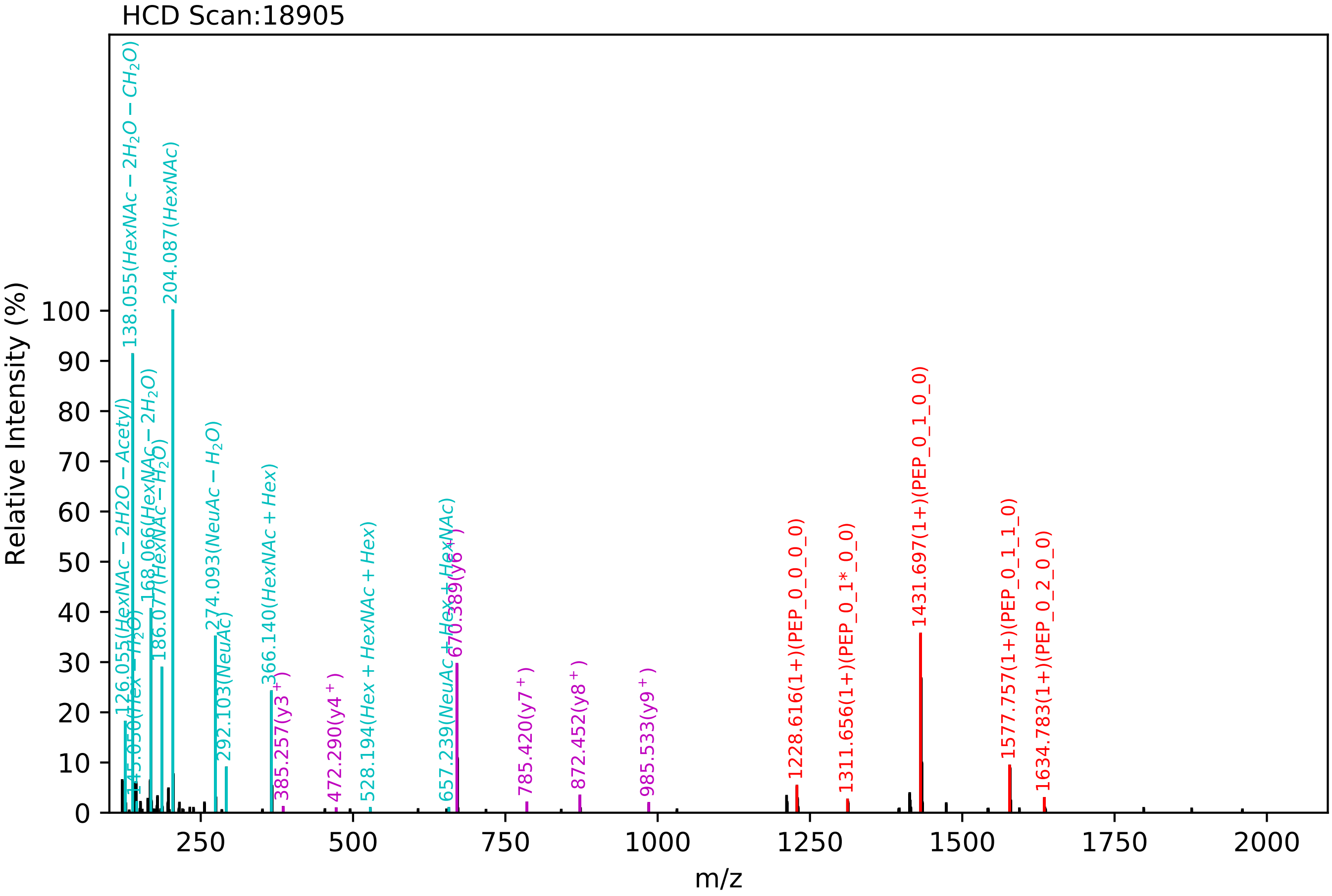

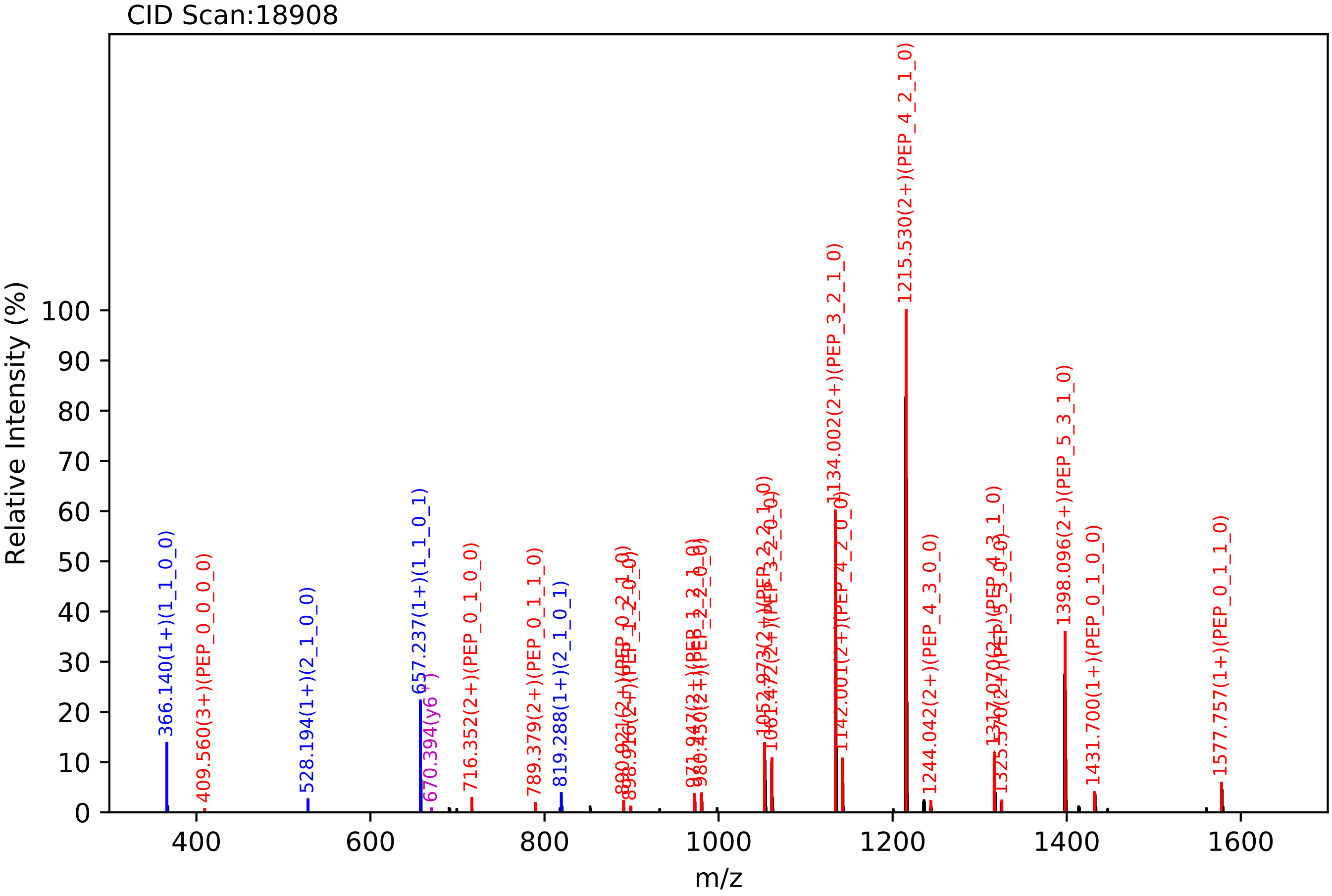

Unknown set no. 565, Experiment: Human Plasma exp_4

ENISDPTSPLR(=PEP)_5_3_1_1, m/z:1029.10(3+), RT:66.61, Y-score:93.46

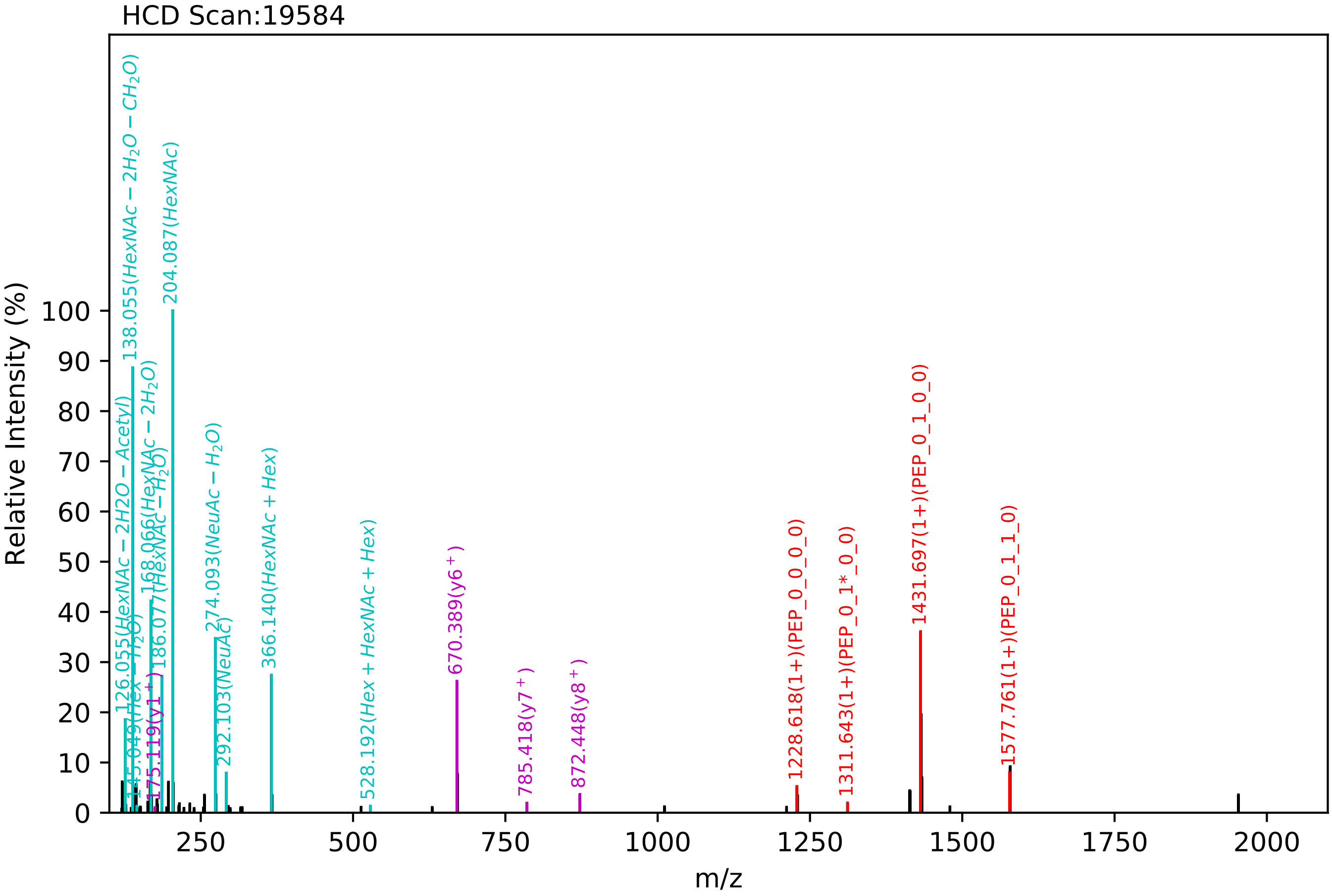

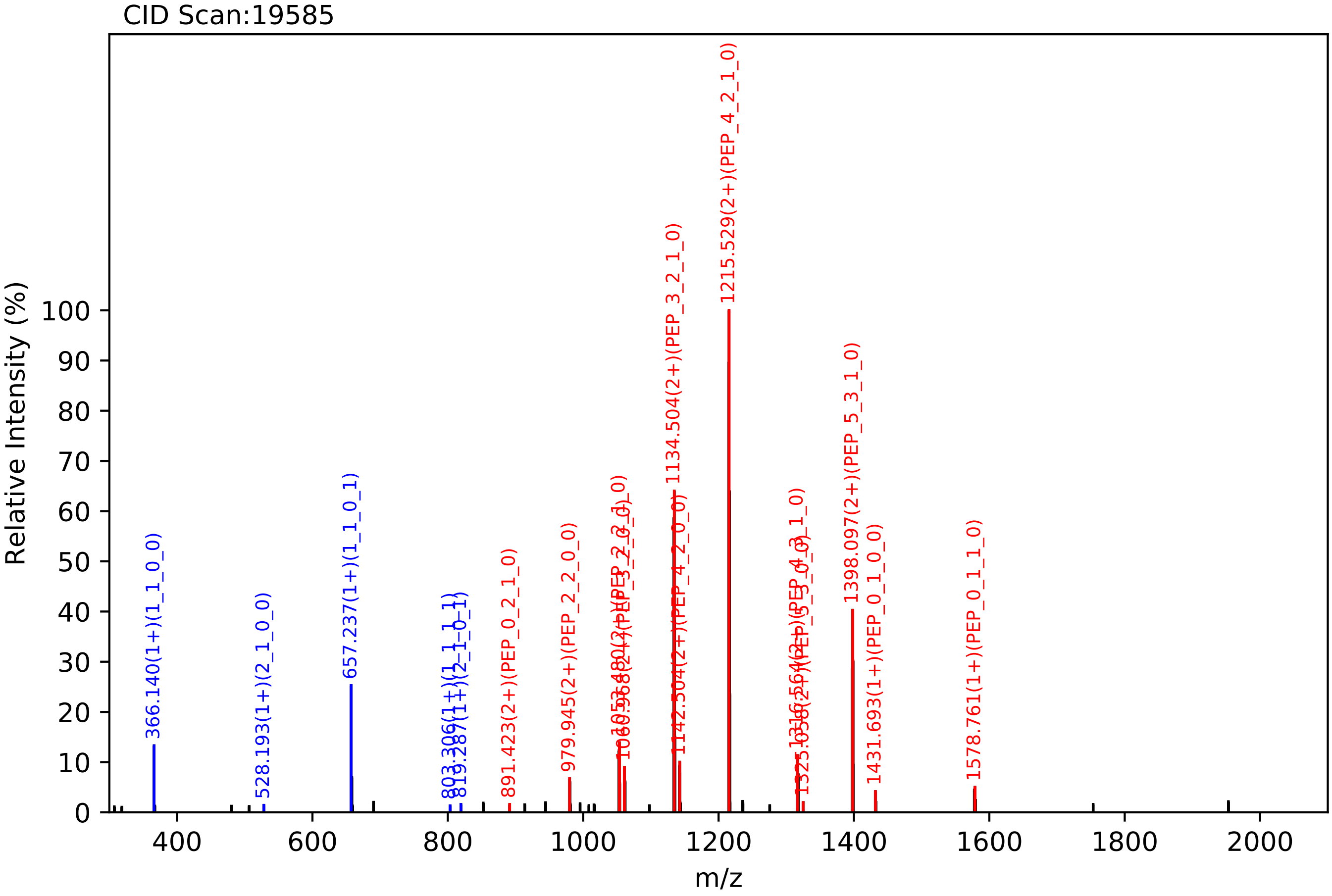

Unknown set no. 566, Experiment: Human Plasma exp_5

ENISDPTSPLR(=PEP)_5_3_1_1, m/z:1029.10(3+), RT:66.30, Y-score:94.83

HCD Scan:19133

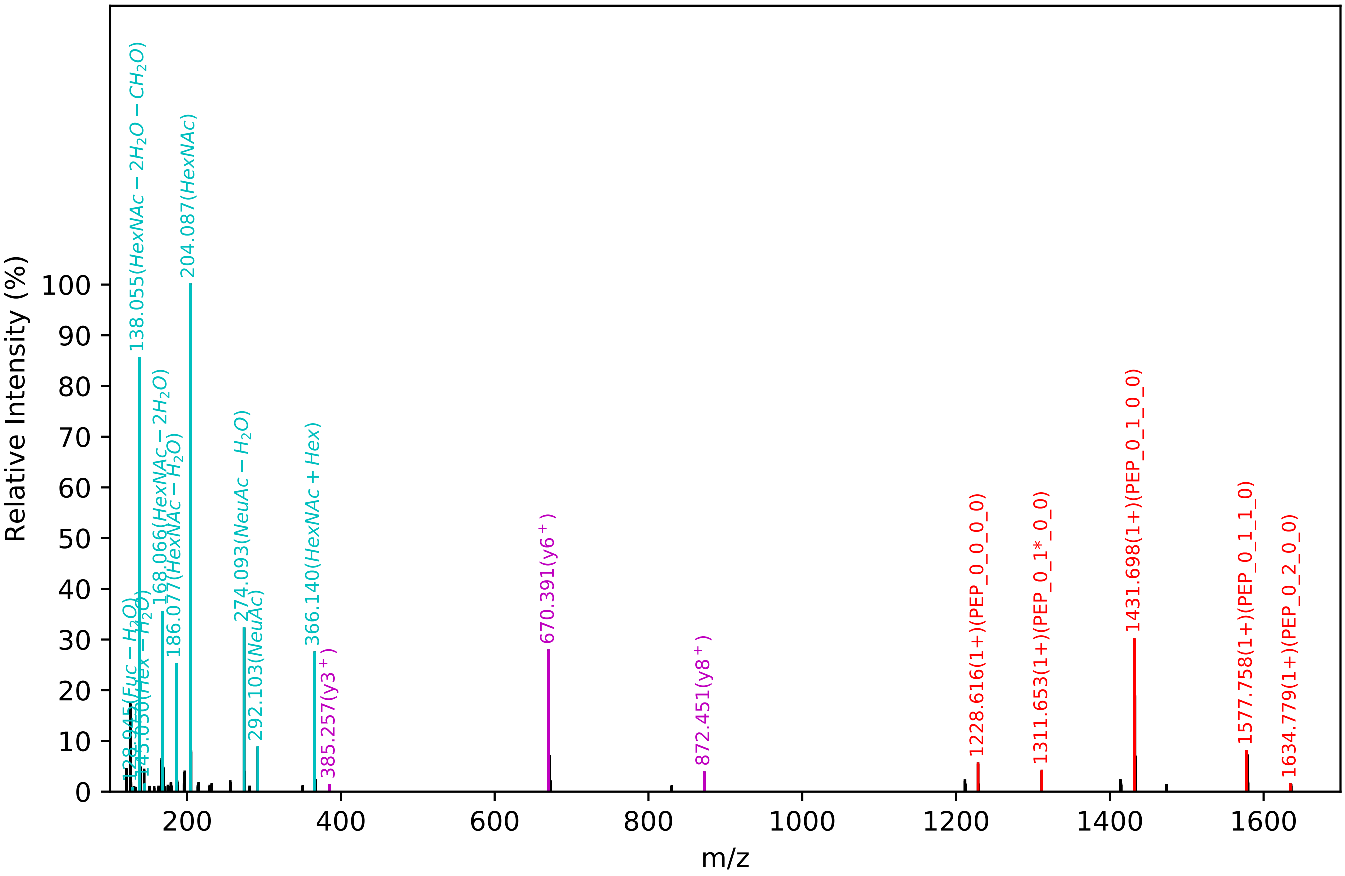

CID Scan:19137

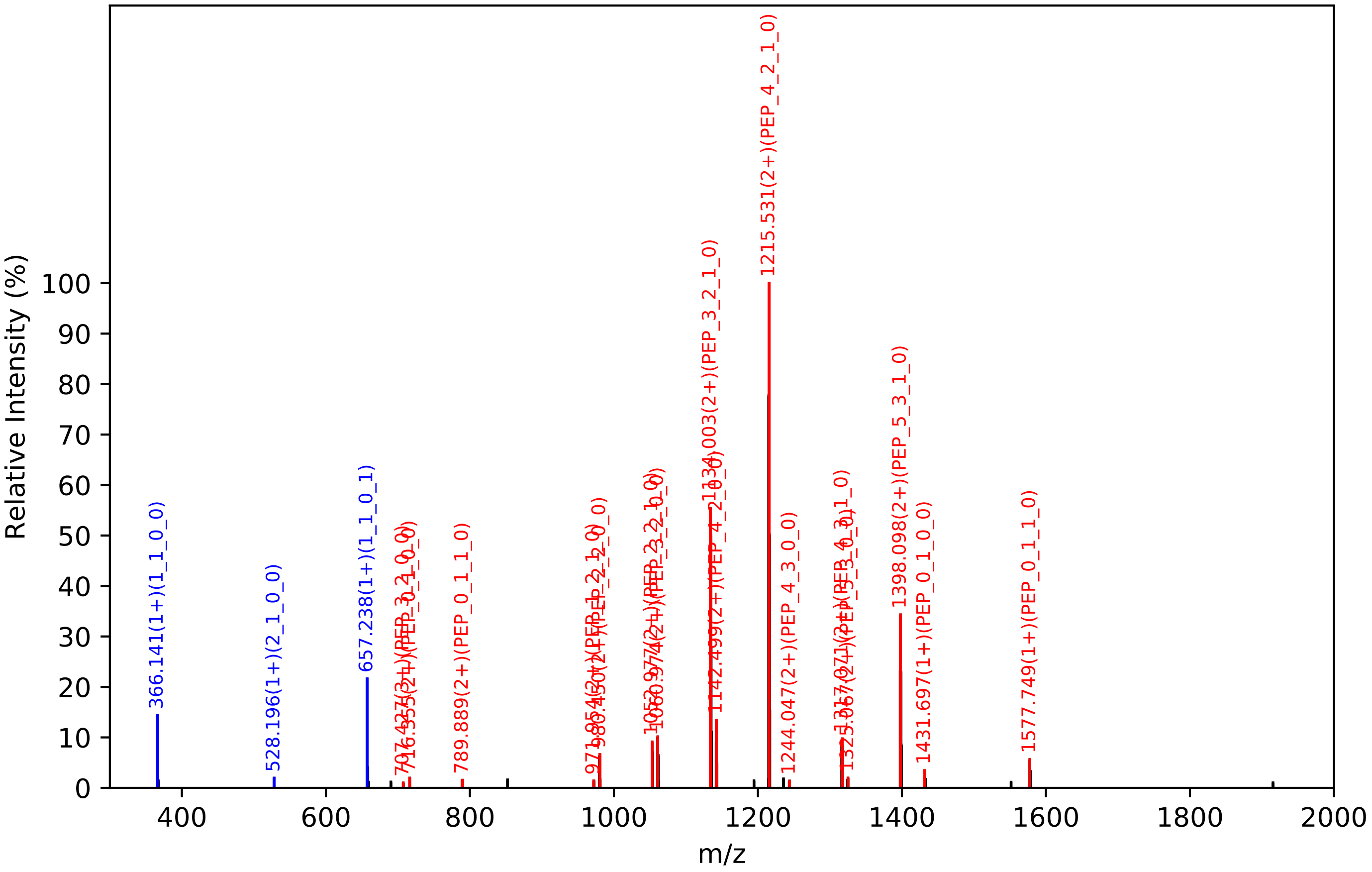

Unknown set no. 567, Experiment: Human Plasma exp_2

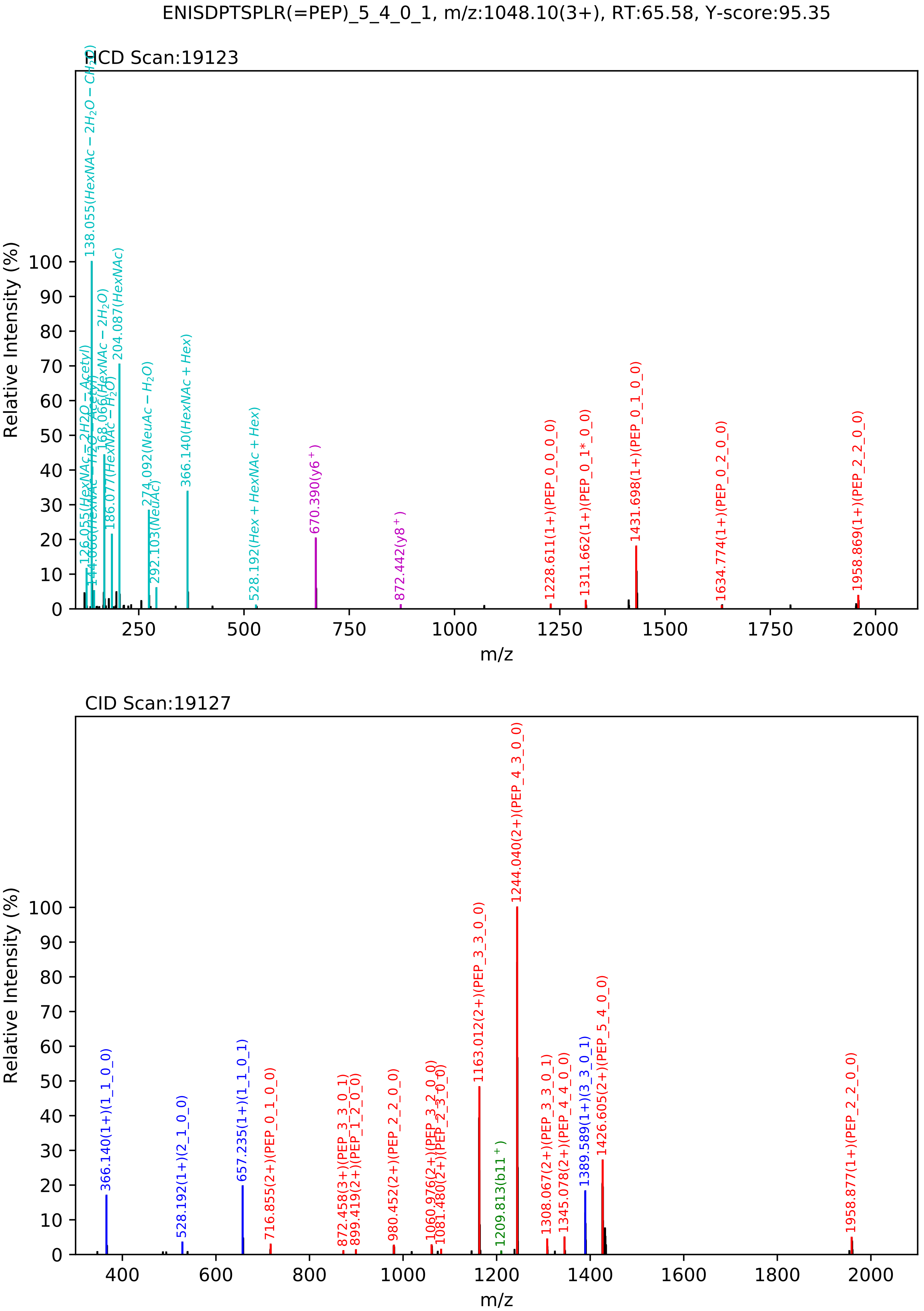

Unknown set no. 568, Experiment: Human Plasma exp_1

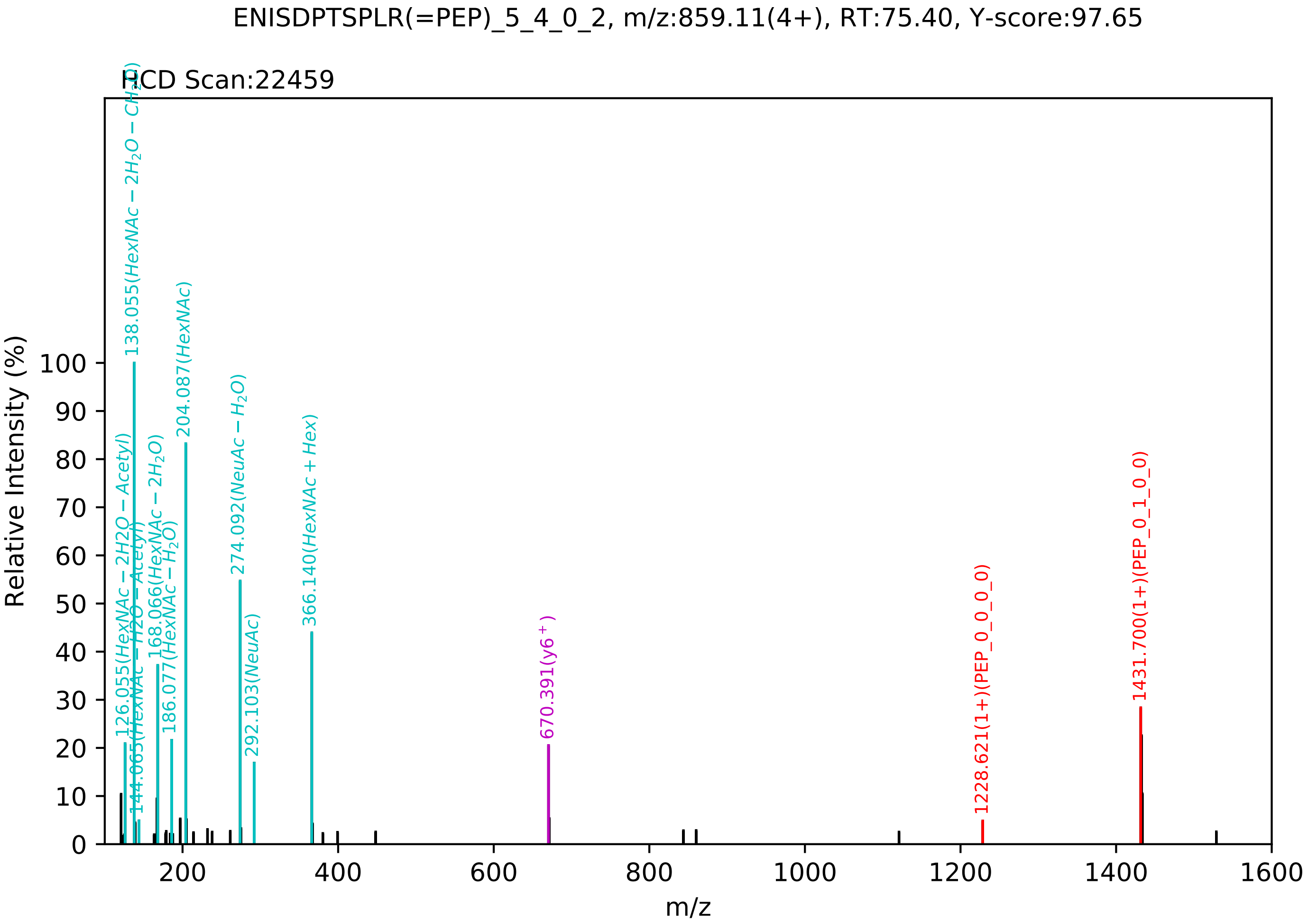

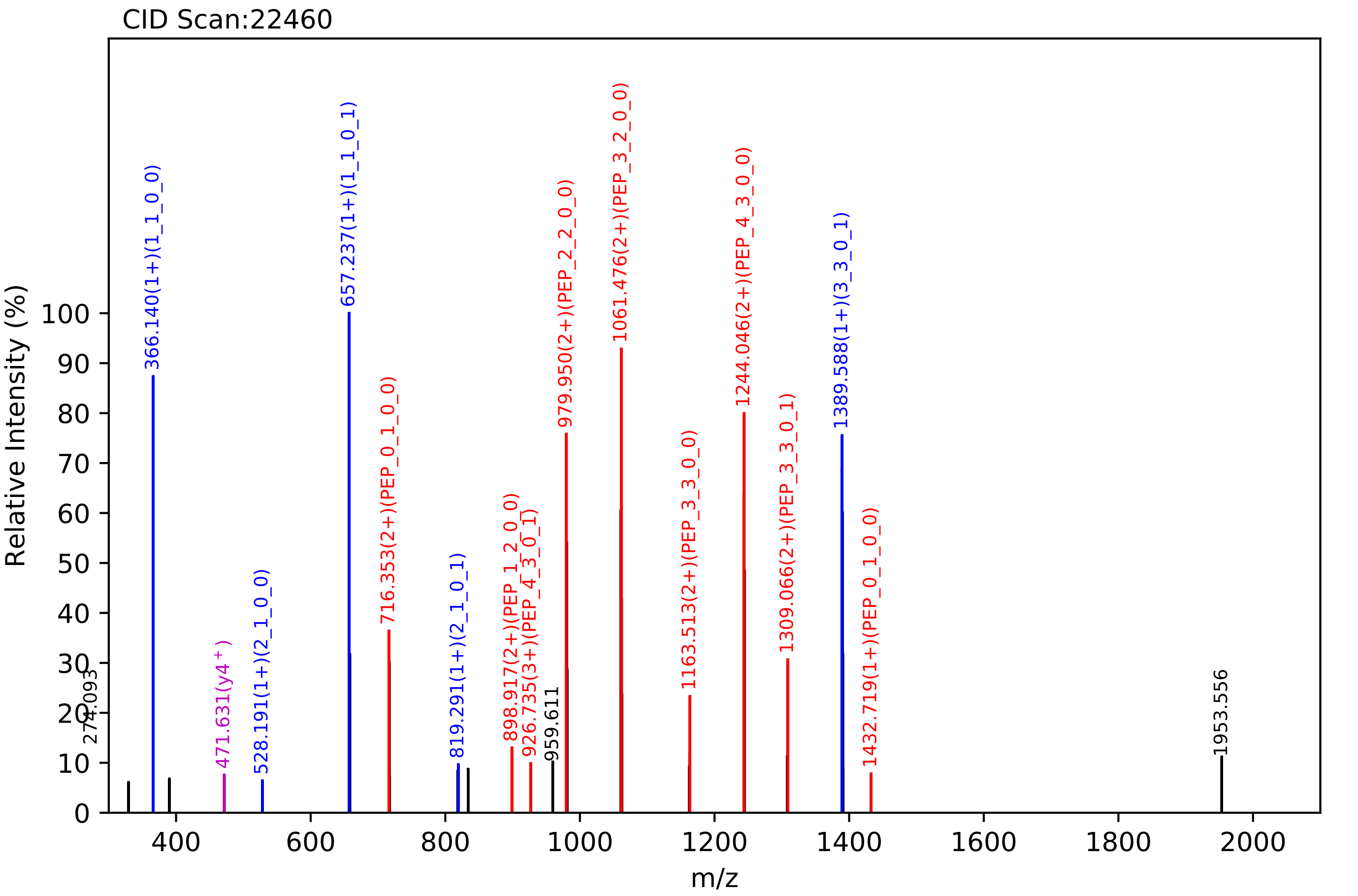

Unknown set no. 569, Experiment: Human Plasma exp_1

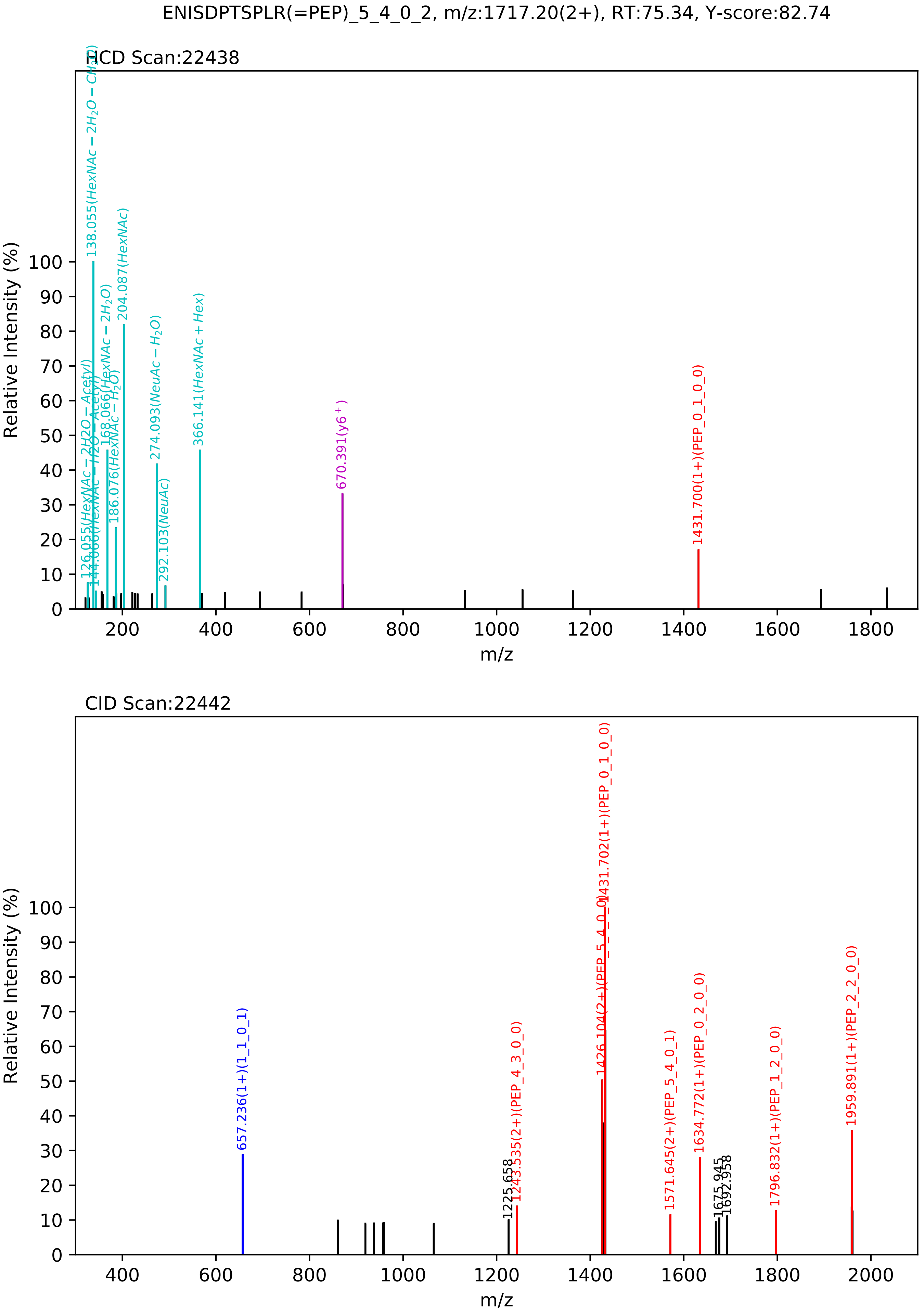

Unknown set no. 570, Experiment: Human Plasma exp_2

ENISDPTSPLR(=PEP)_5_4_0_2, m/z:859.11(4+), RT:75.75, Y-score:86.76

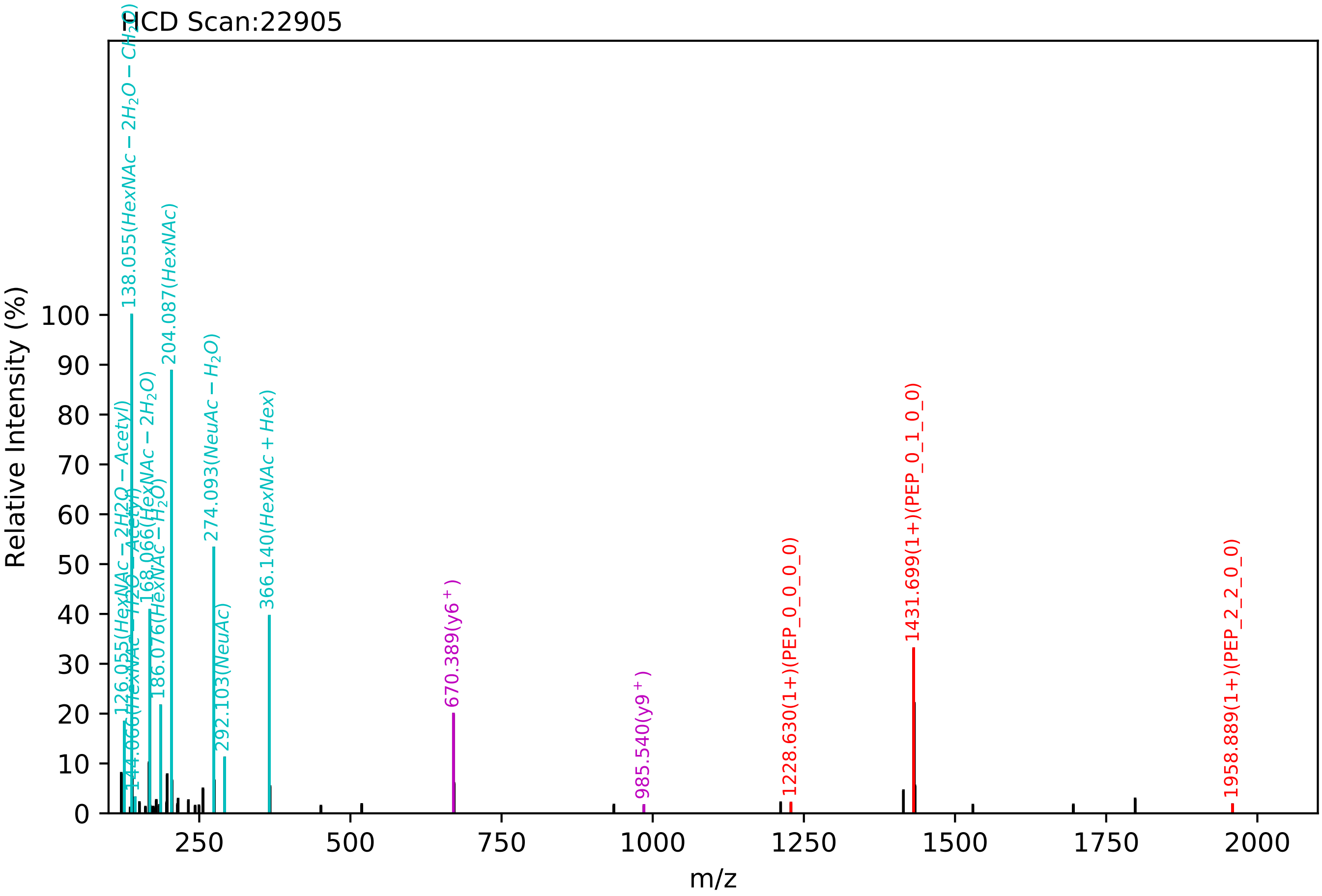

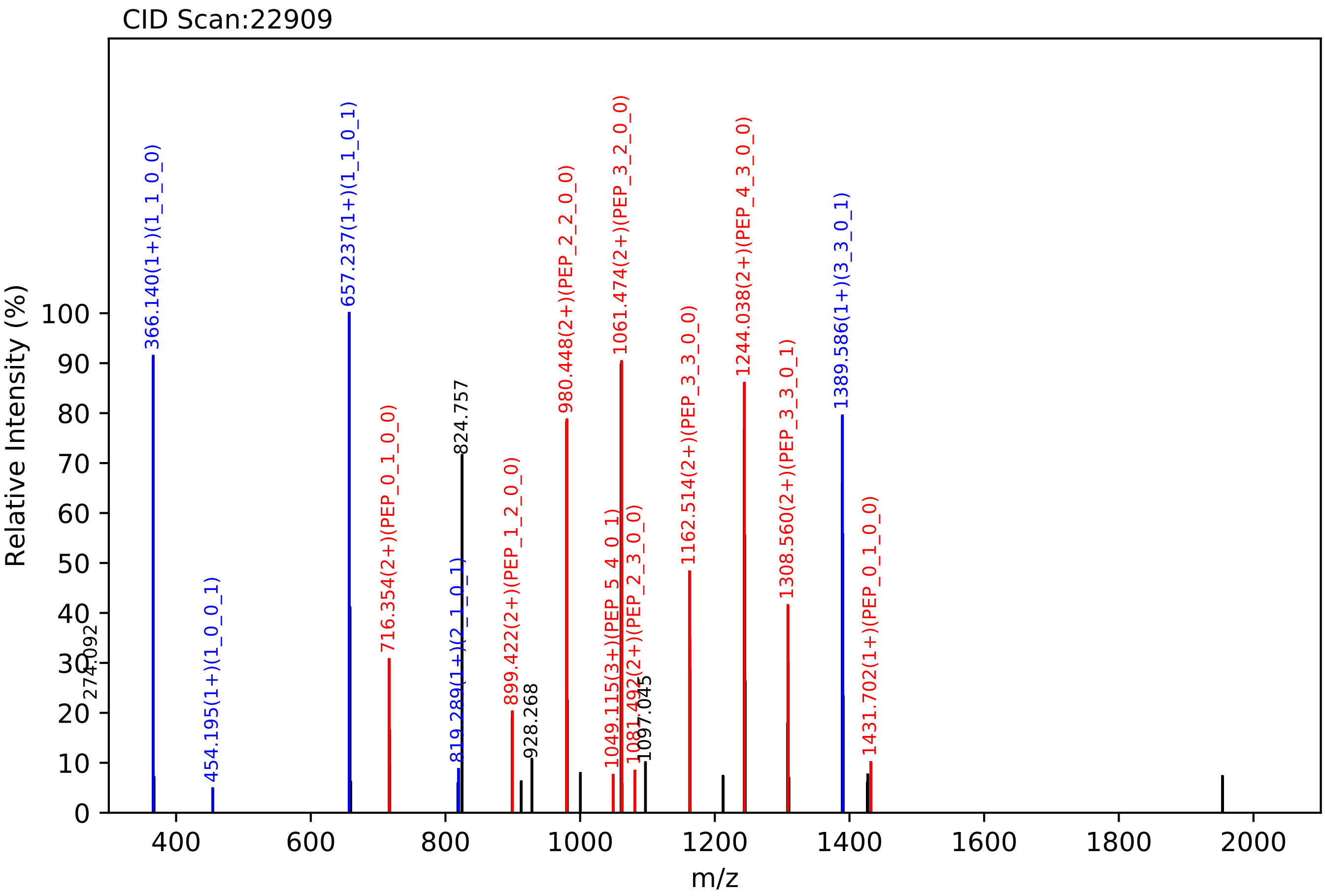

Unknown set no. 571, Experiment: Human Plasma exp_2

ENISDPTSPLR(=PEP)_5_4_0_2, m/z:1145.14(3+), RT:75.35, Y-score:97.56

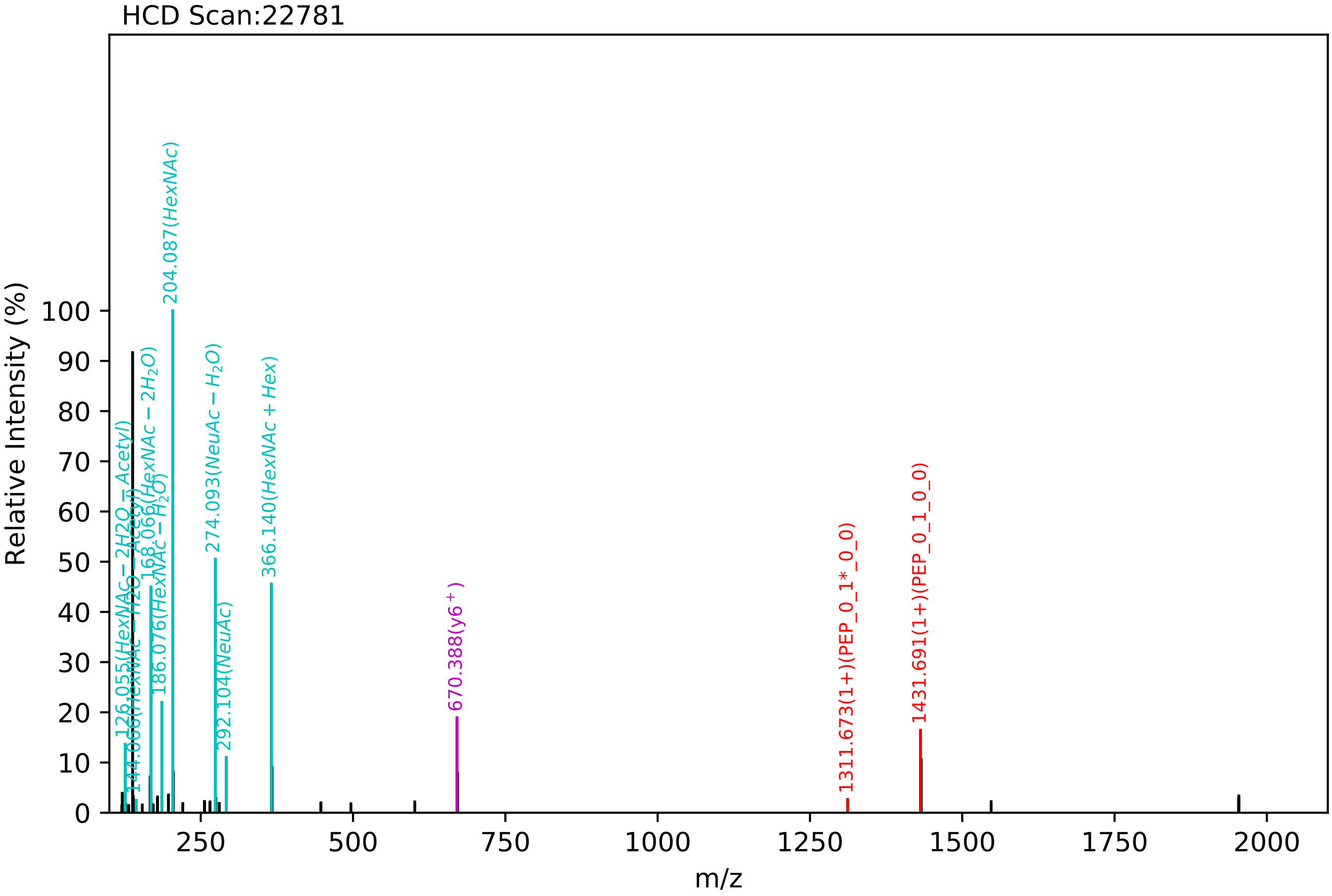

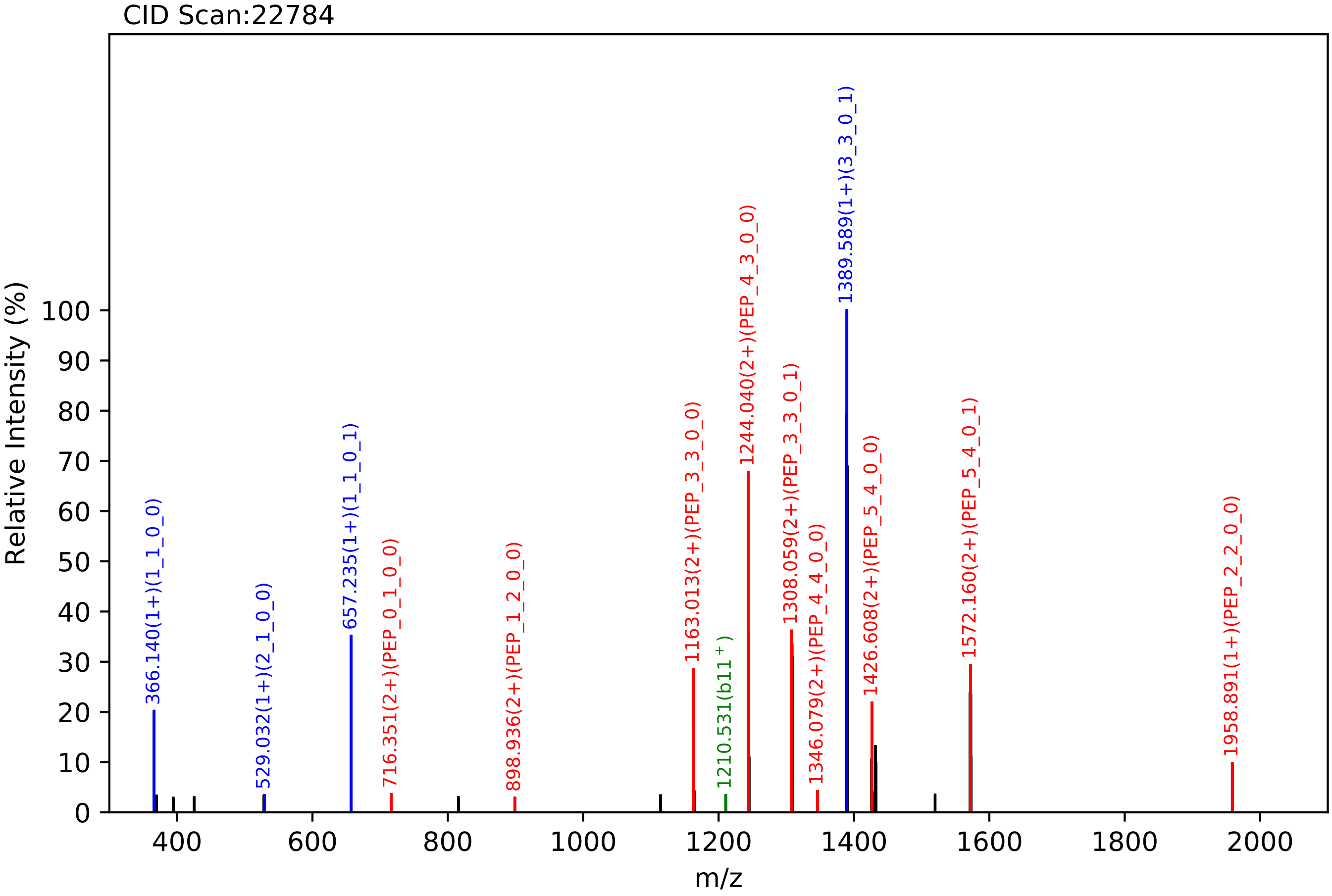

Unknown set no. 572, Experiment: Human Plasma exp_3

ENISDPTSPLR(=PEP)_5_4_0_2, m/z:859.11(4+), RT:75.62, Y-score:90.45

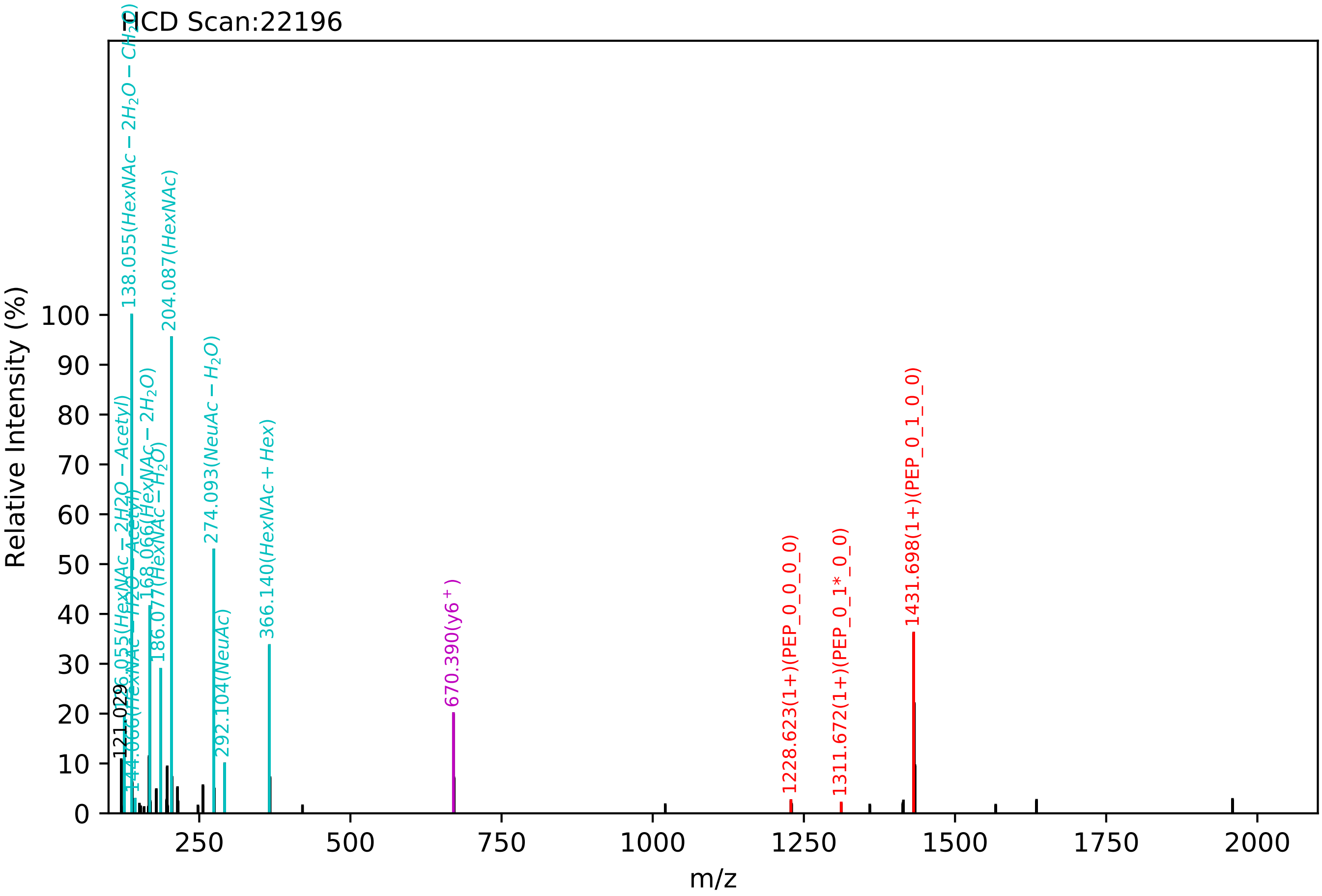

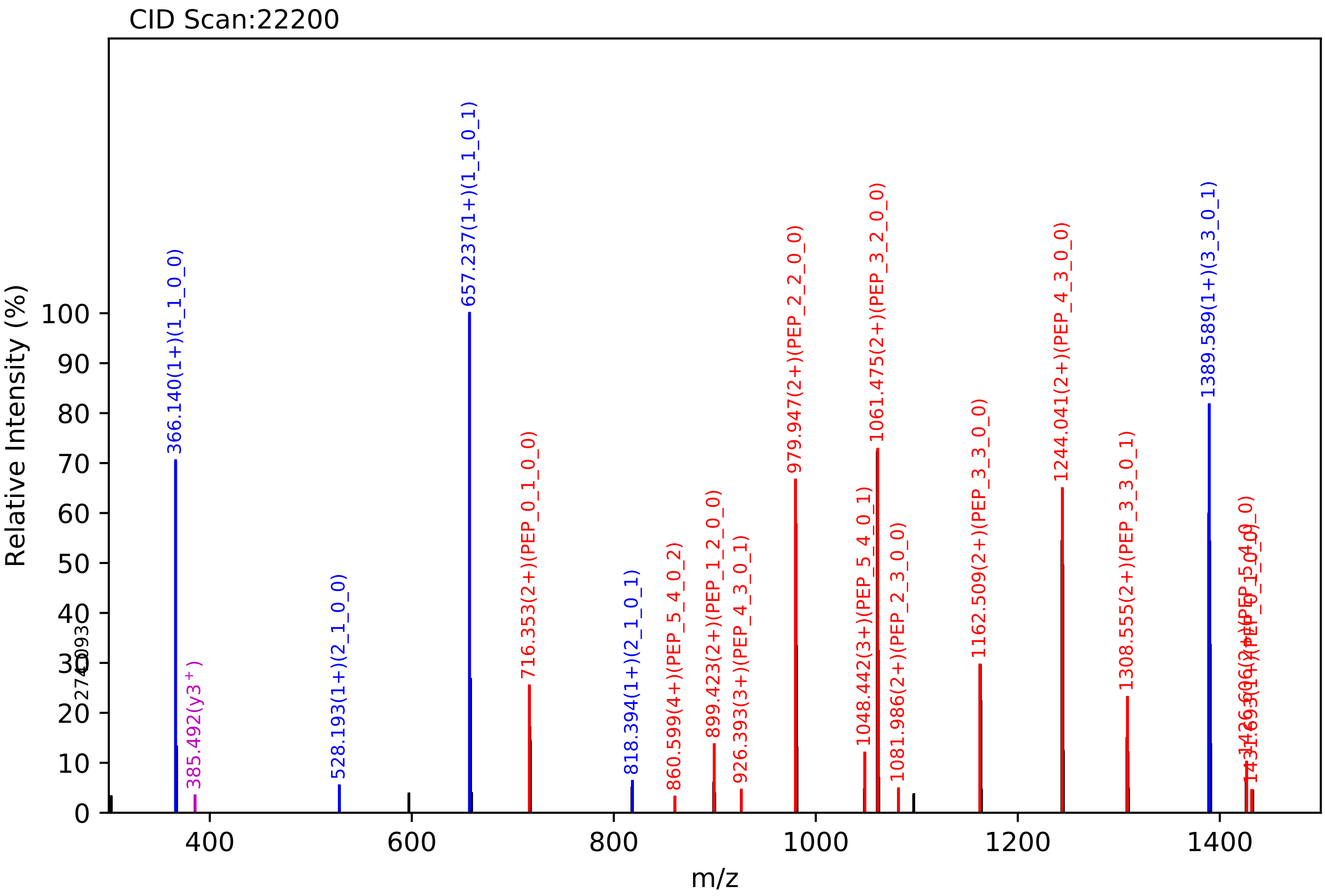

Unknown set no. 573, Experiment: Human Plasma exp_4

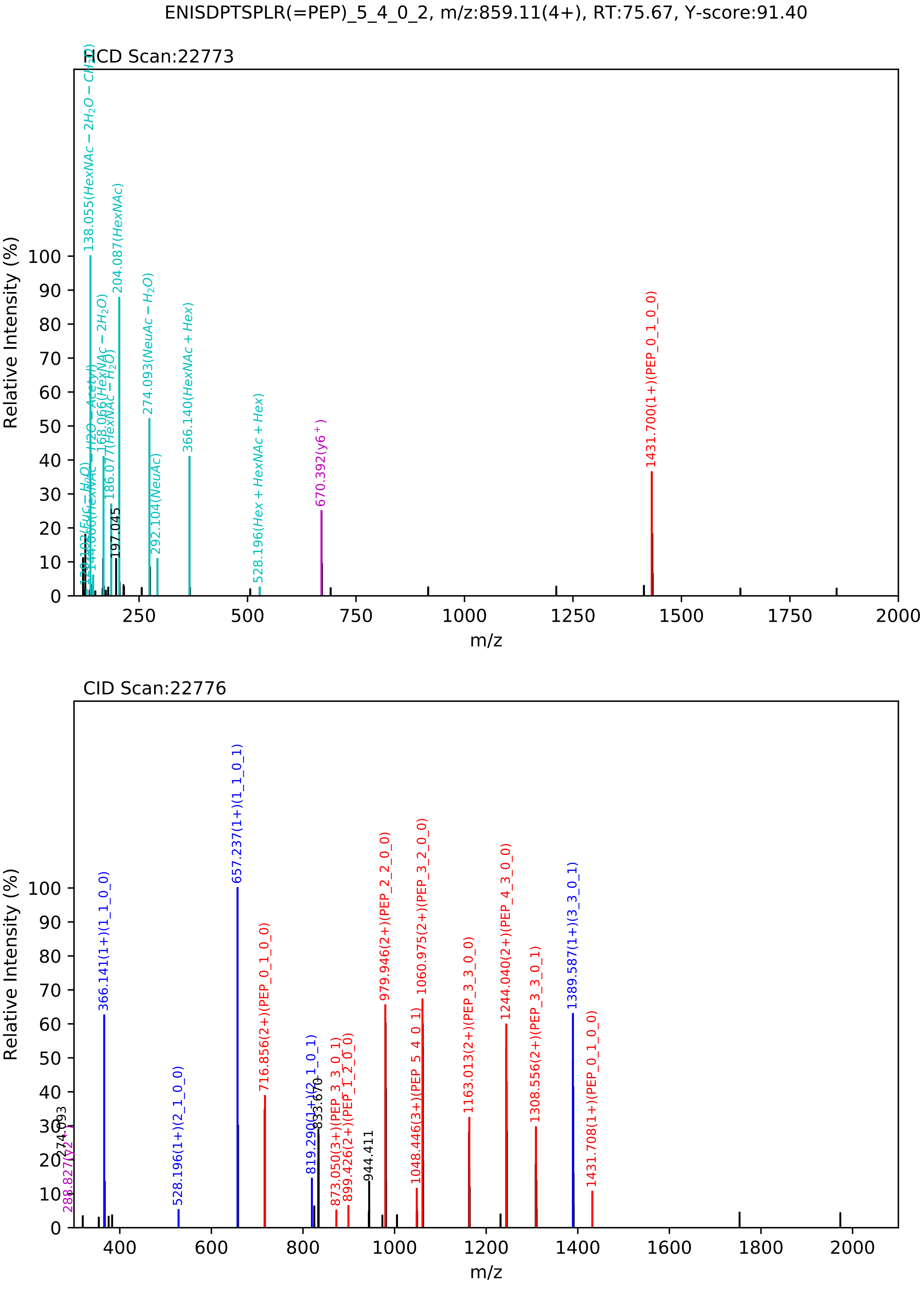

Unknown set no. 574, Experiment: Human Plasma exp_2

GLCVNASAVSR(=PEP)_5_4_0_2, m/z:1113.45(3+), RT:57.05, Y-score:89.31

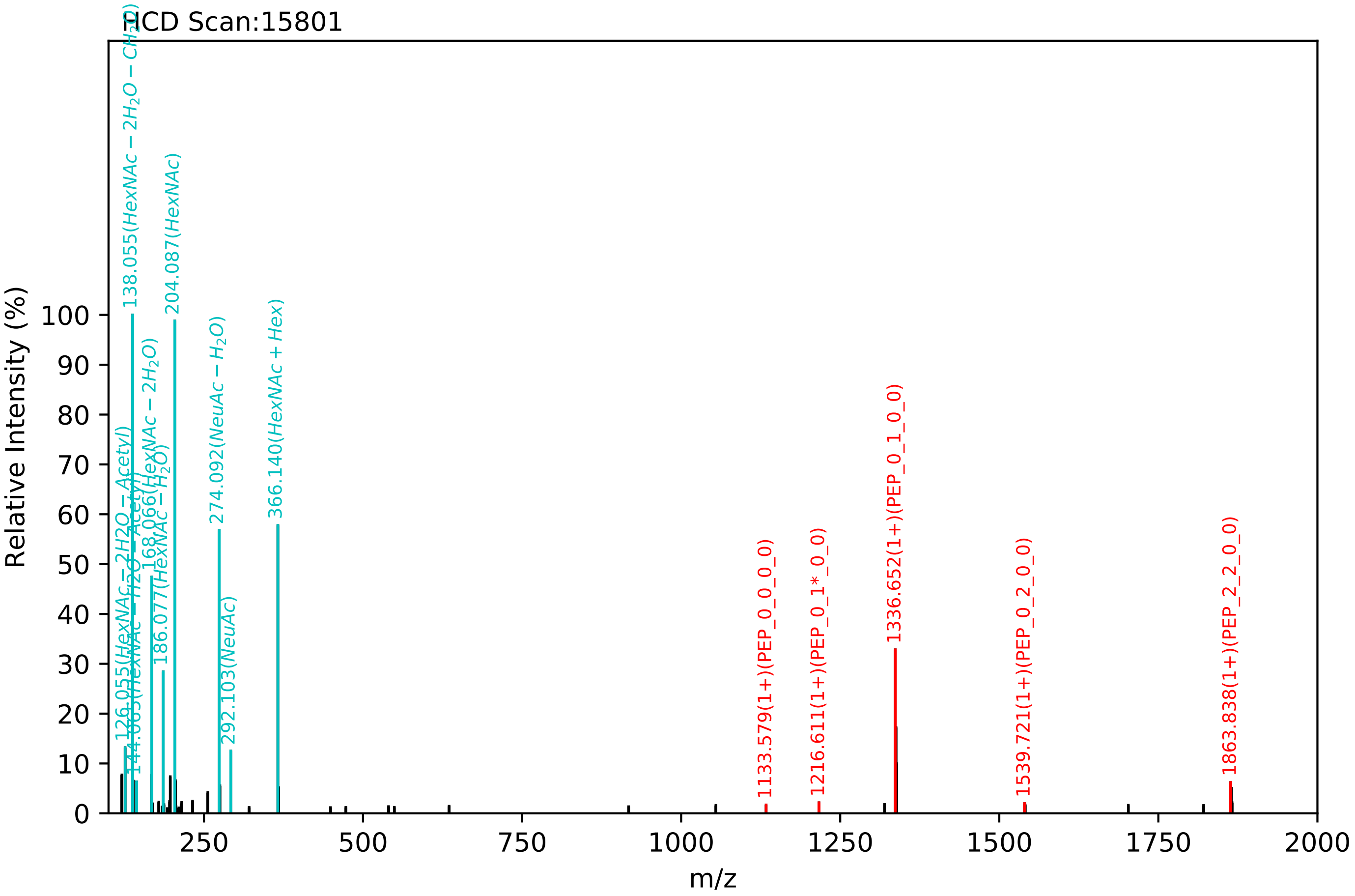

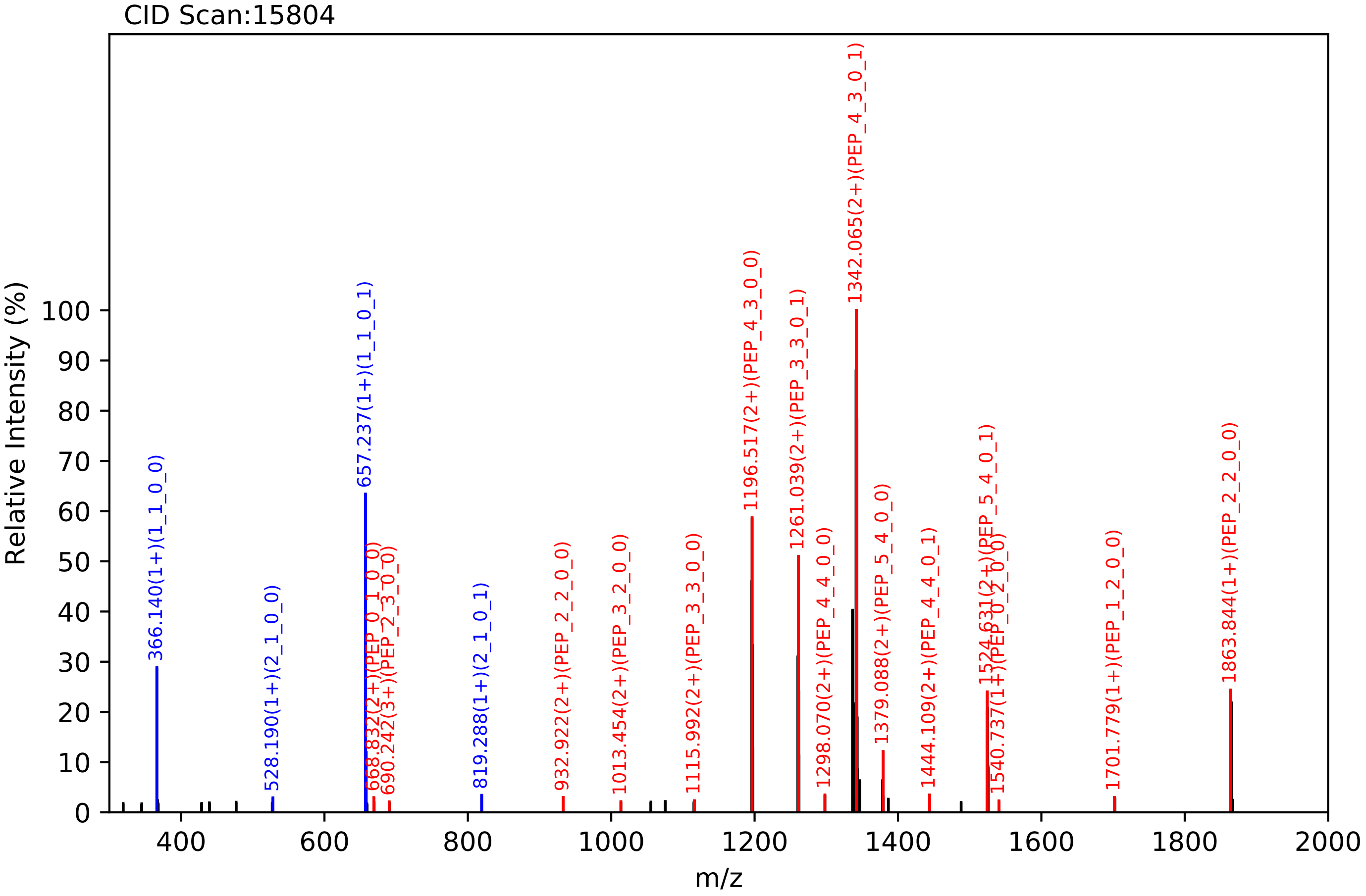

Unknown set no. 575, Experiment: Human Plasma exp_3

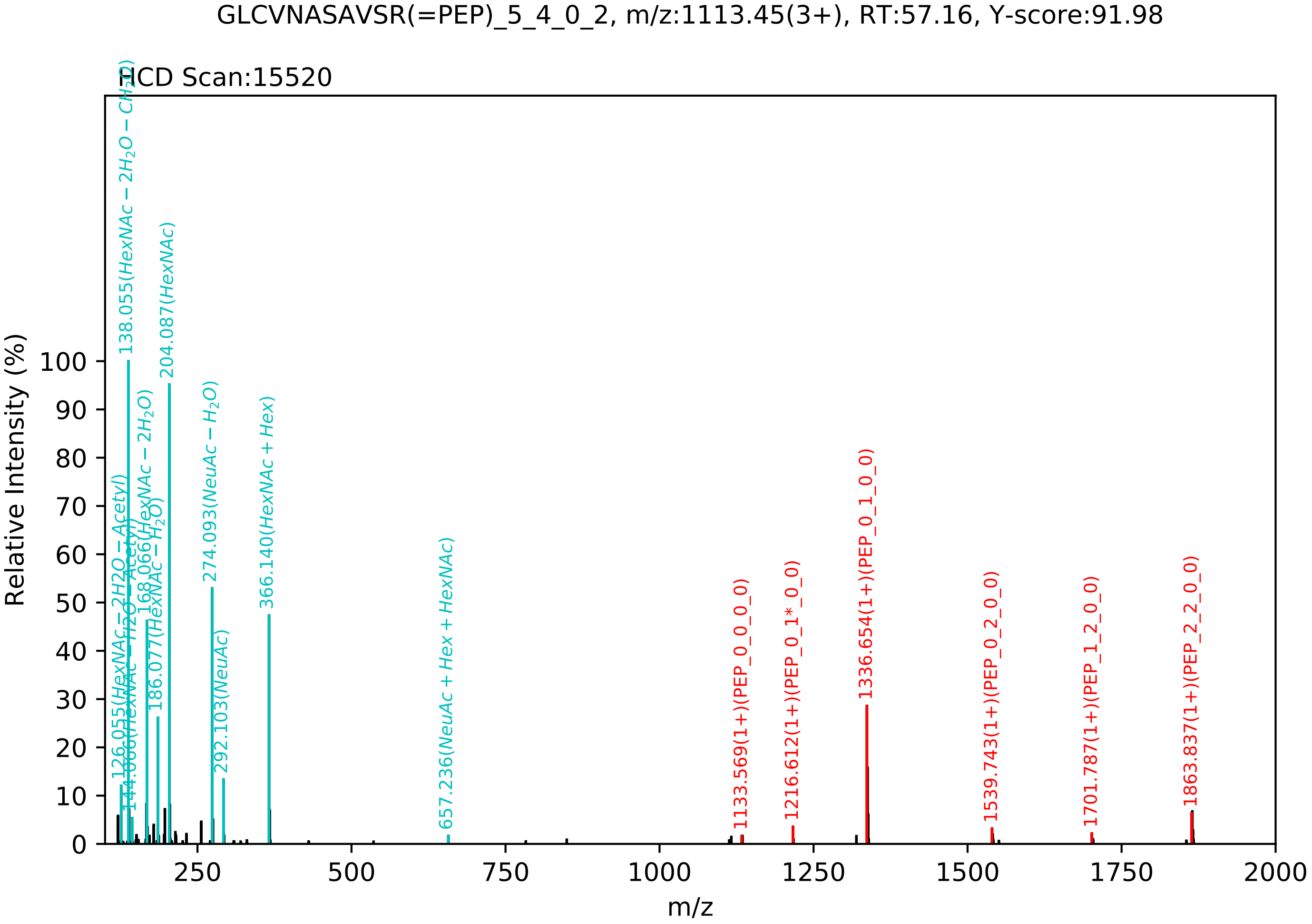

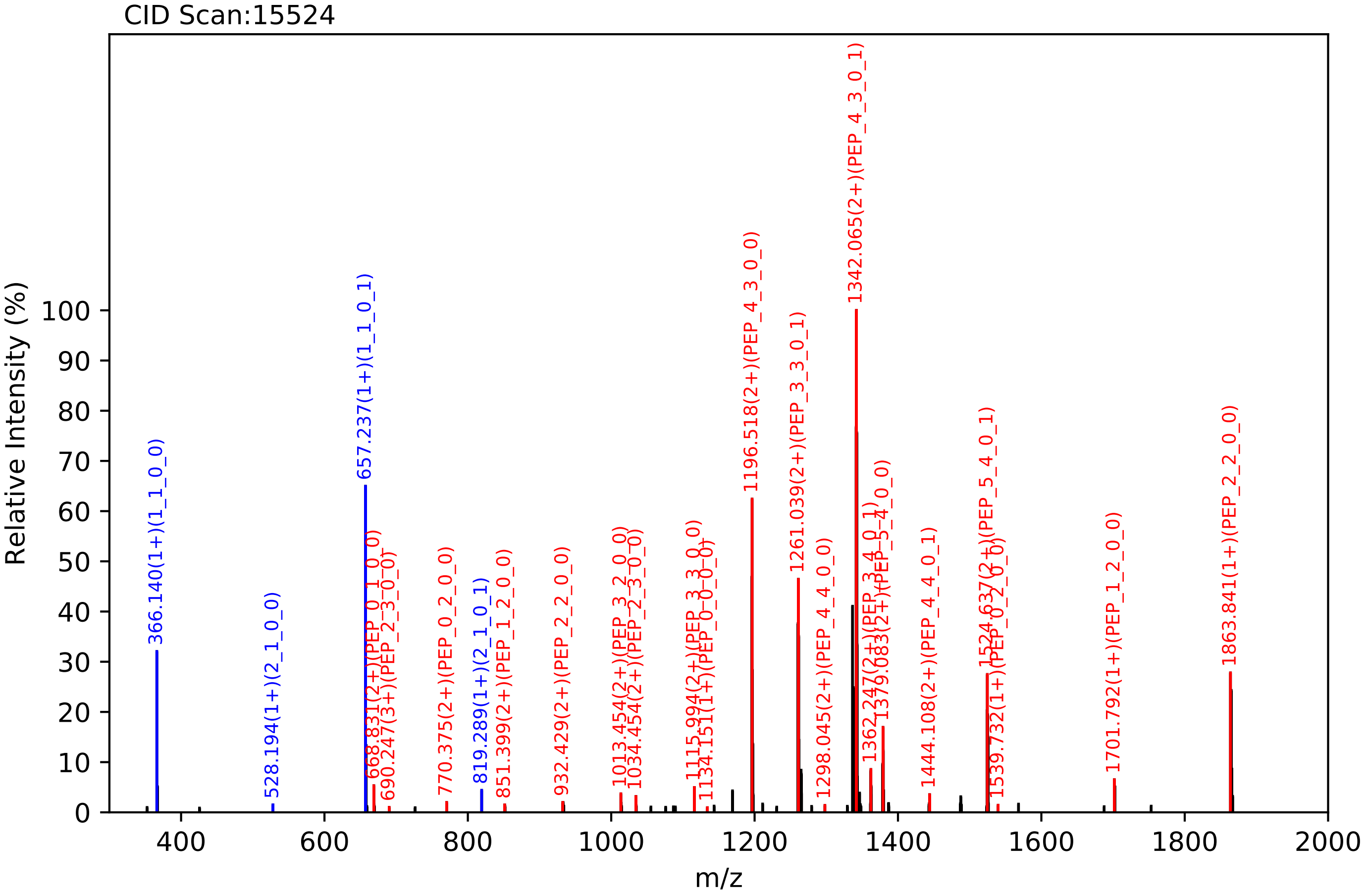

Unknown set no. 576, Experiment: Human Plasma exp_2

NNSLR(=PEP)_4_3_0_1, m/z:1076.44(2+), RT:18.09, Y-score:93.19

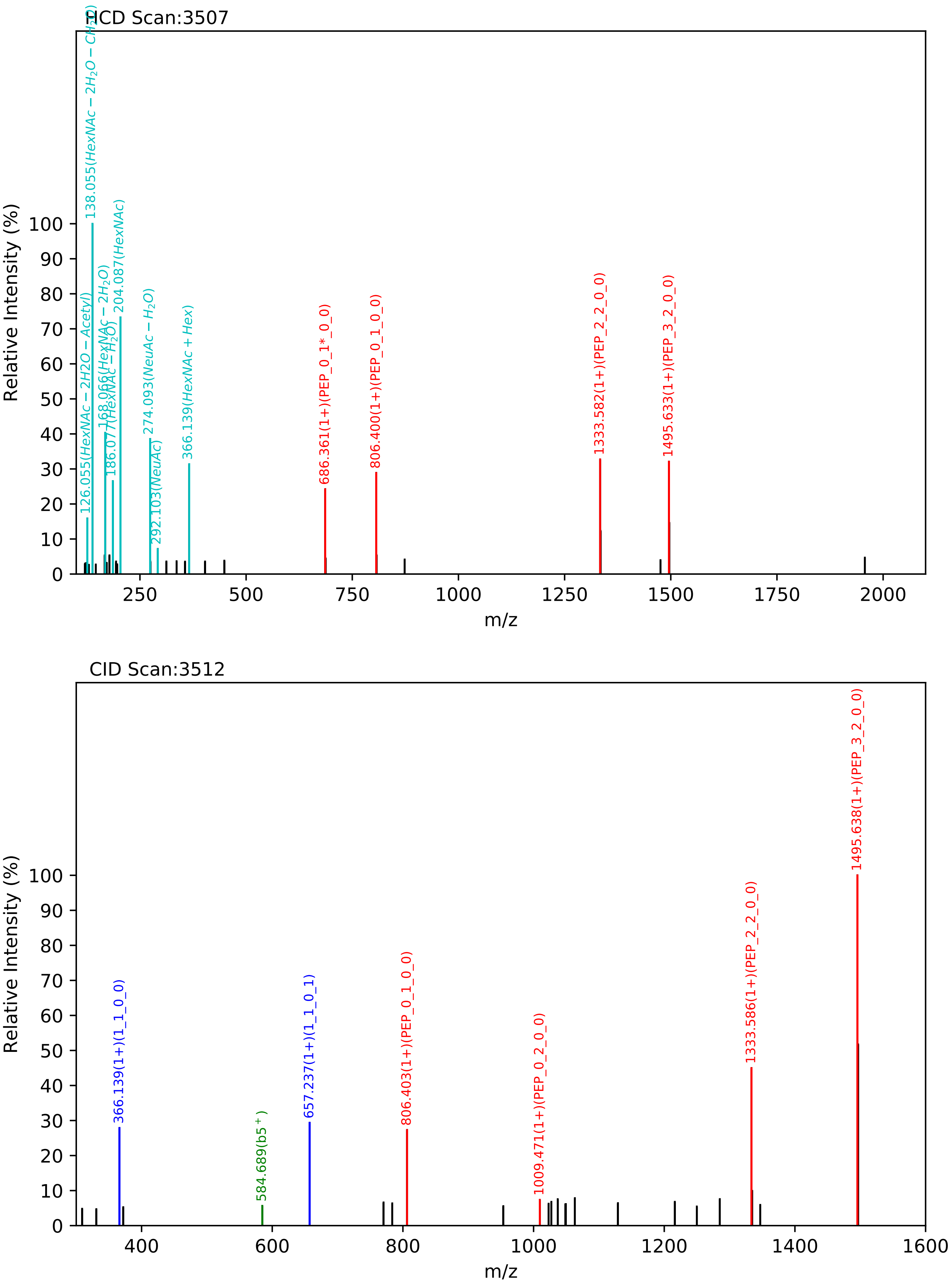

Unknown set no. 577, Experiment: Human Plasma exp_1

NNSLR(=PEP)_5_4_0_2, m/z:936.71(3+), RT:17.77, Y-score:91.70

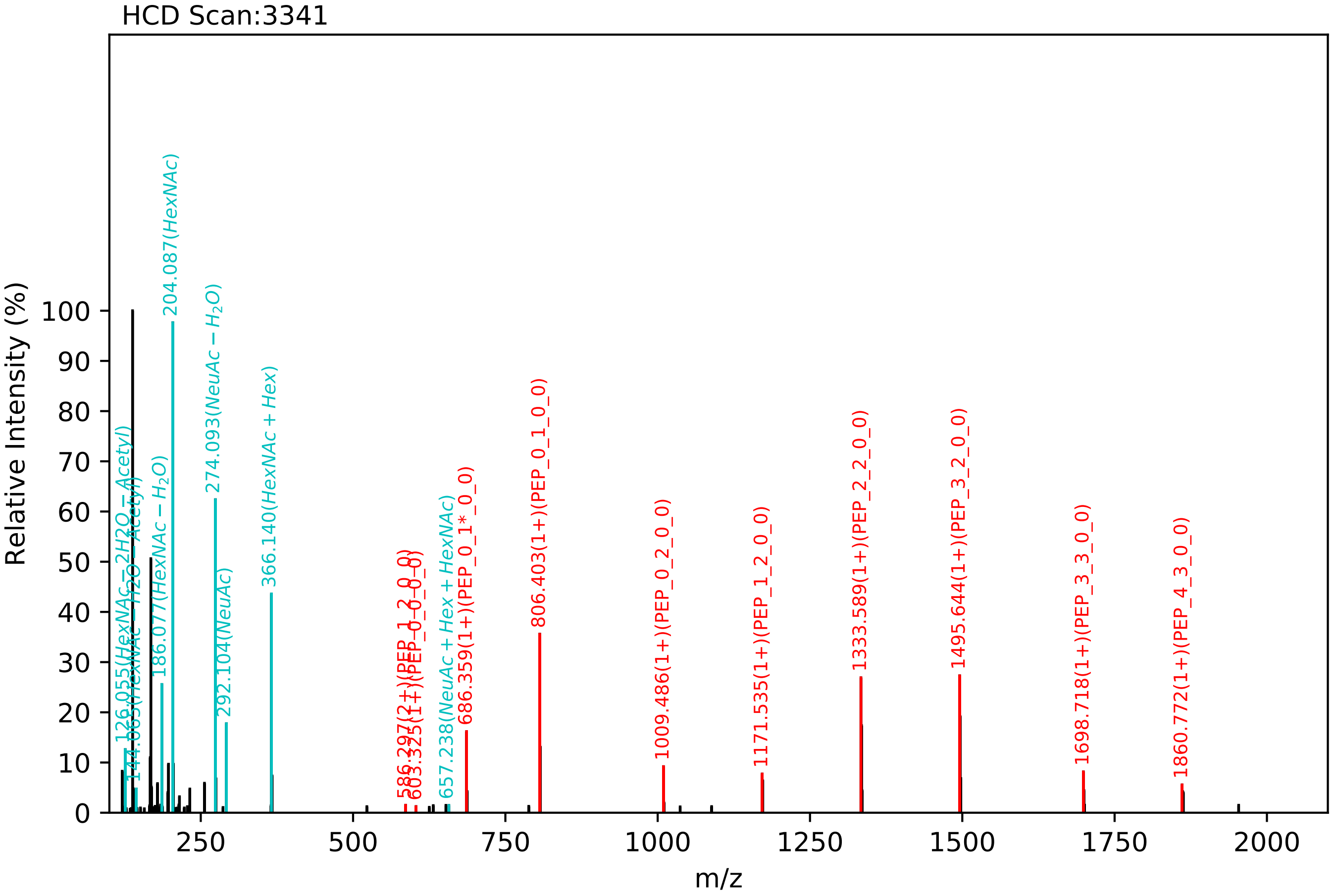

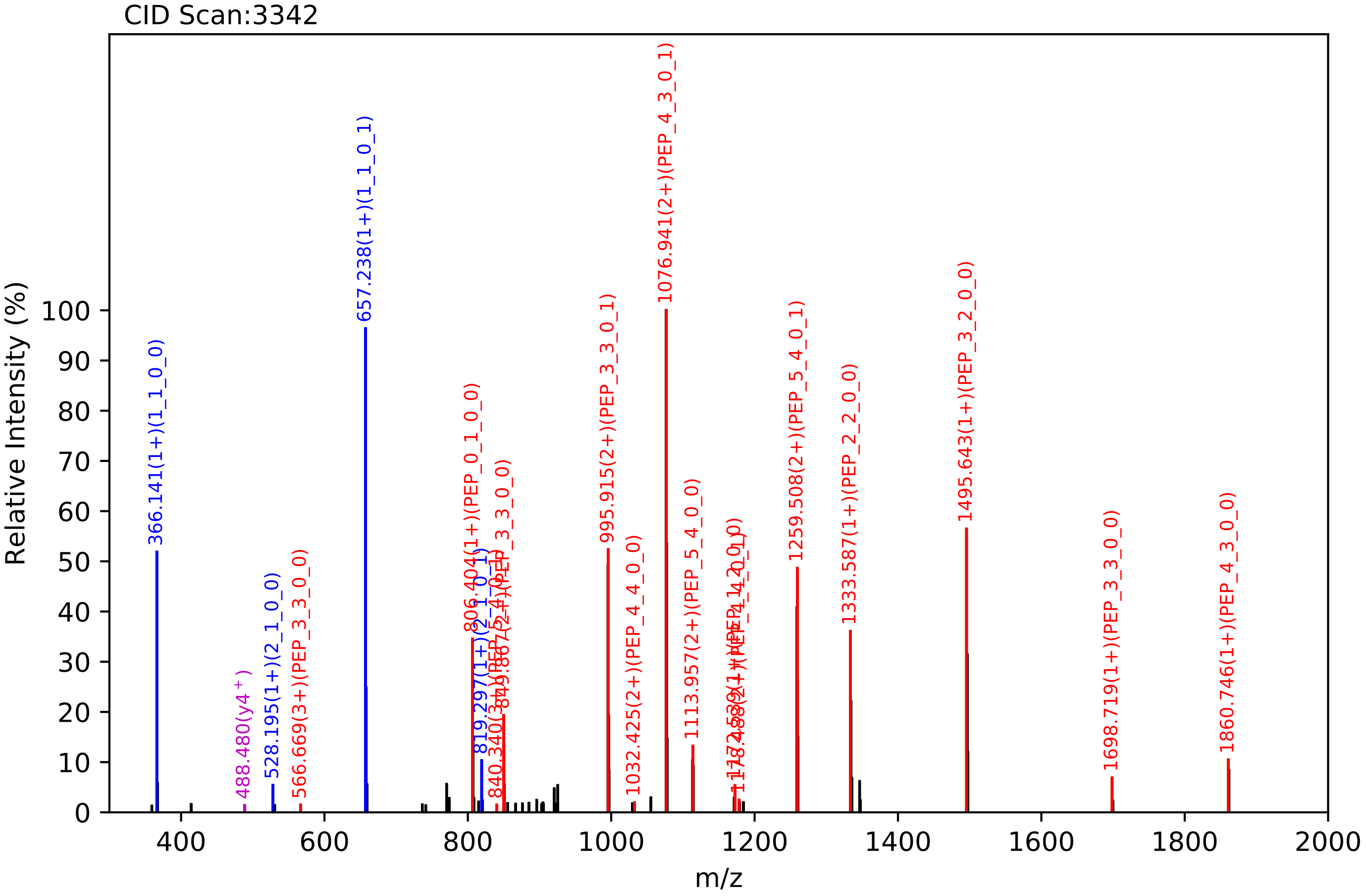

Unknown set no. 578, Experiment: Human Plasma exp_2

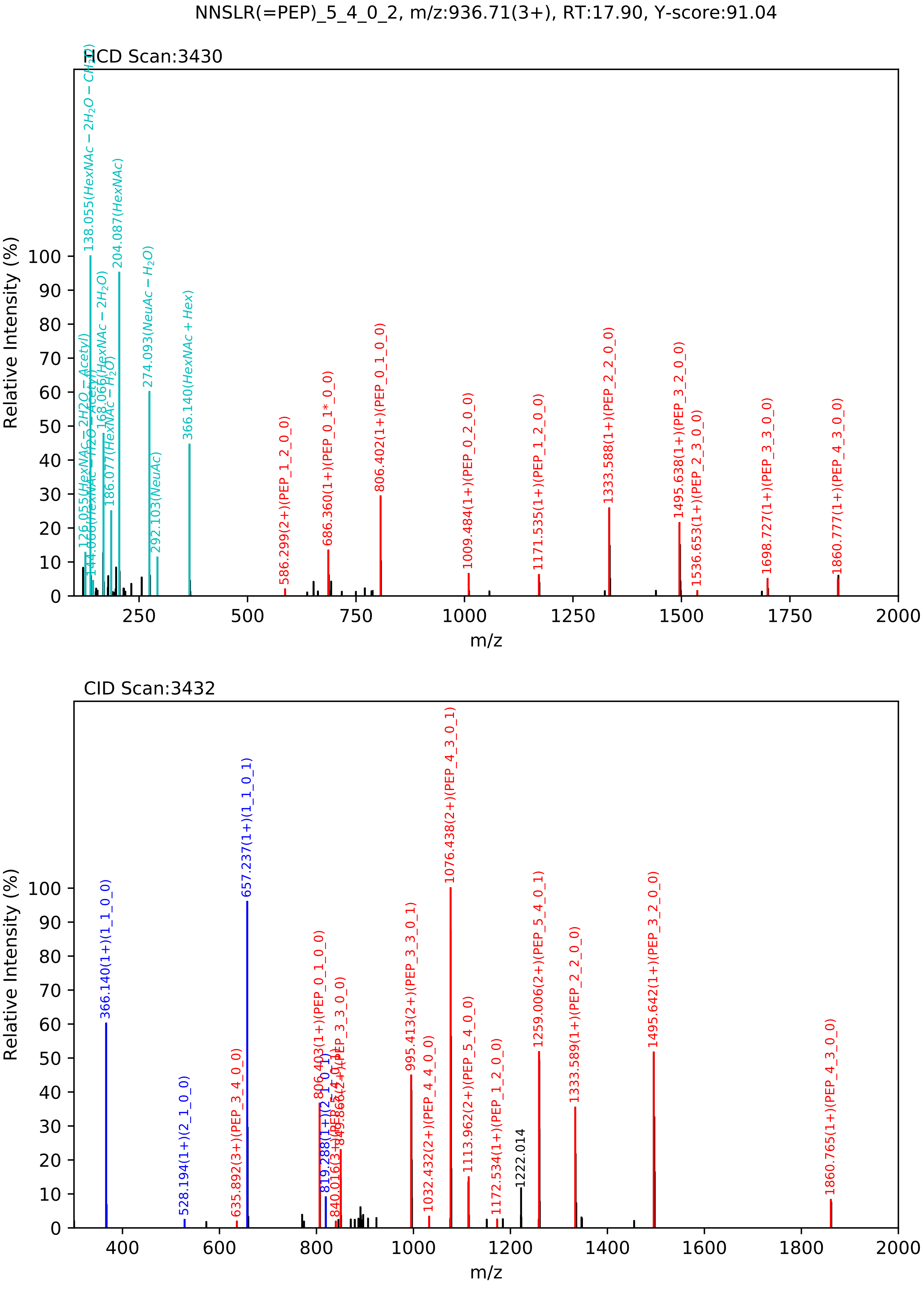

Unknown set no. 579, Experiment: Human Plasma exp_3

NNSLR(=PEP)_5_4_0_2, m/z:936.71(3+), RT:18.03, Y-score:94.17

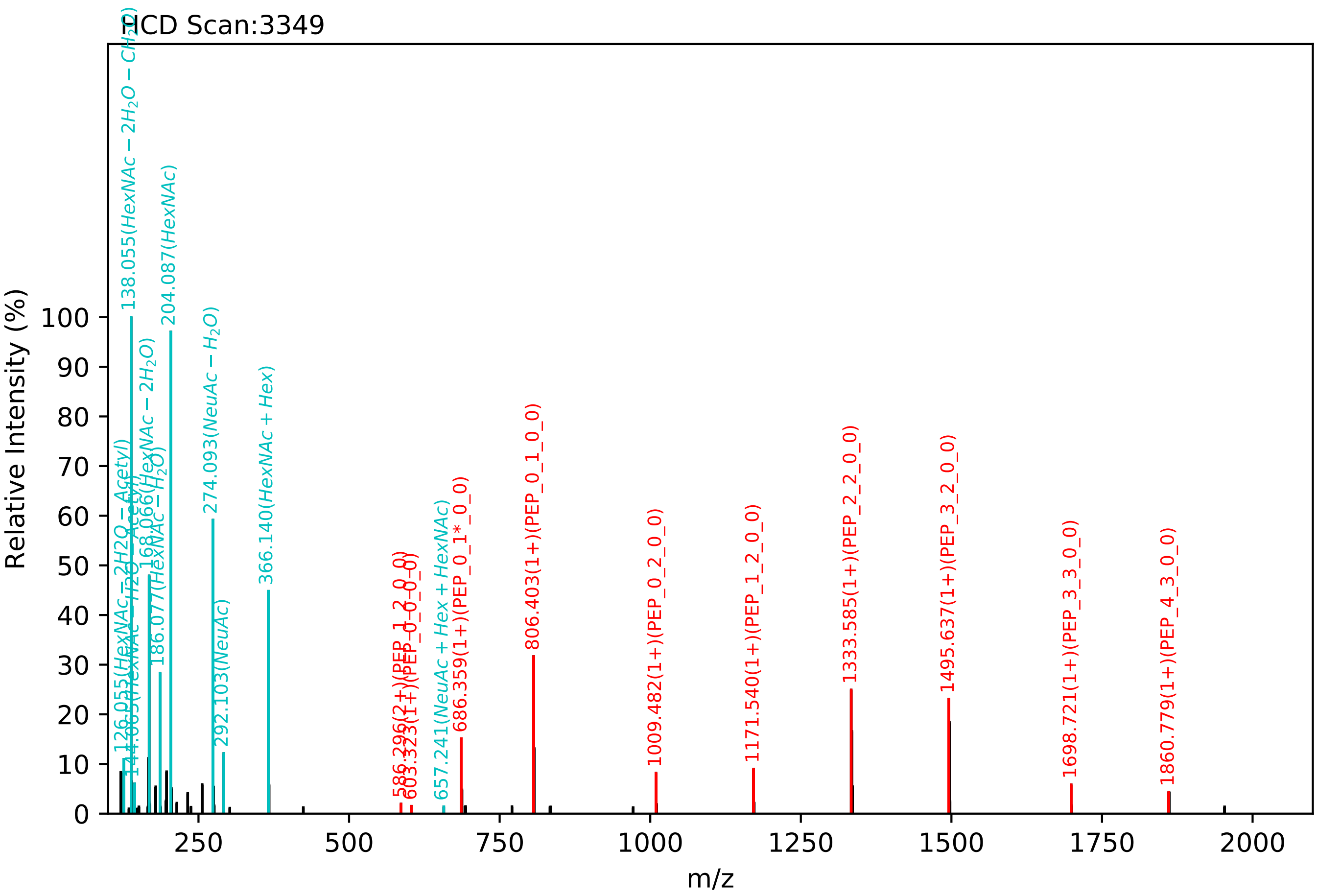

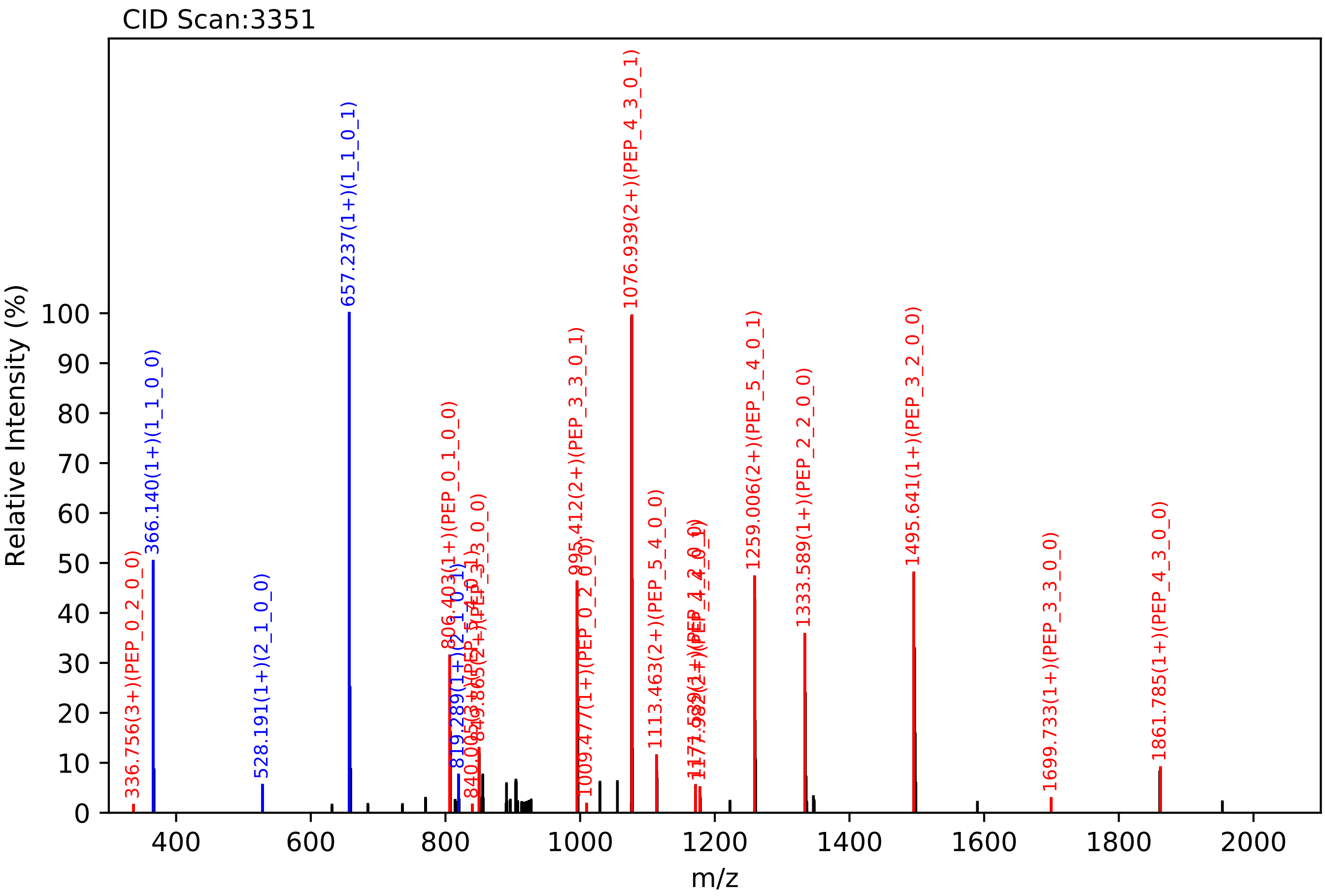

Unknown set no. 580, Experiment: Human Plasma exp_4

NNSLR(=PEP)_5_4_0_2, m/z:936.71(3+), RT:18.63, Y-score:91.19

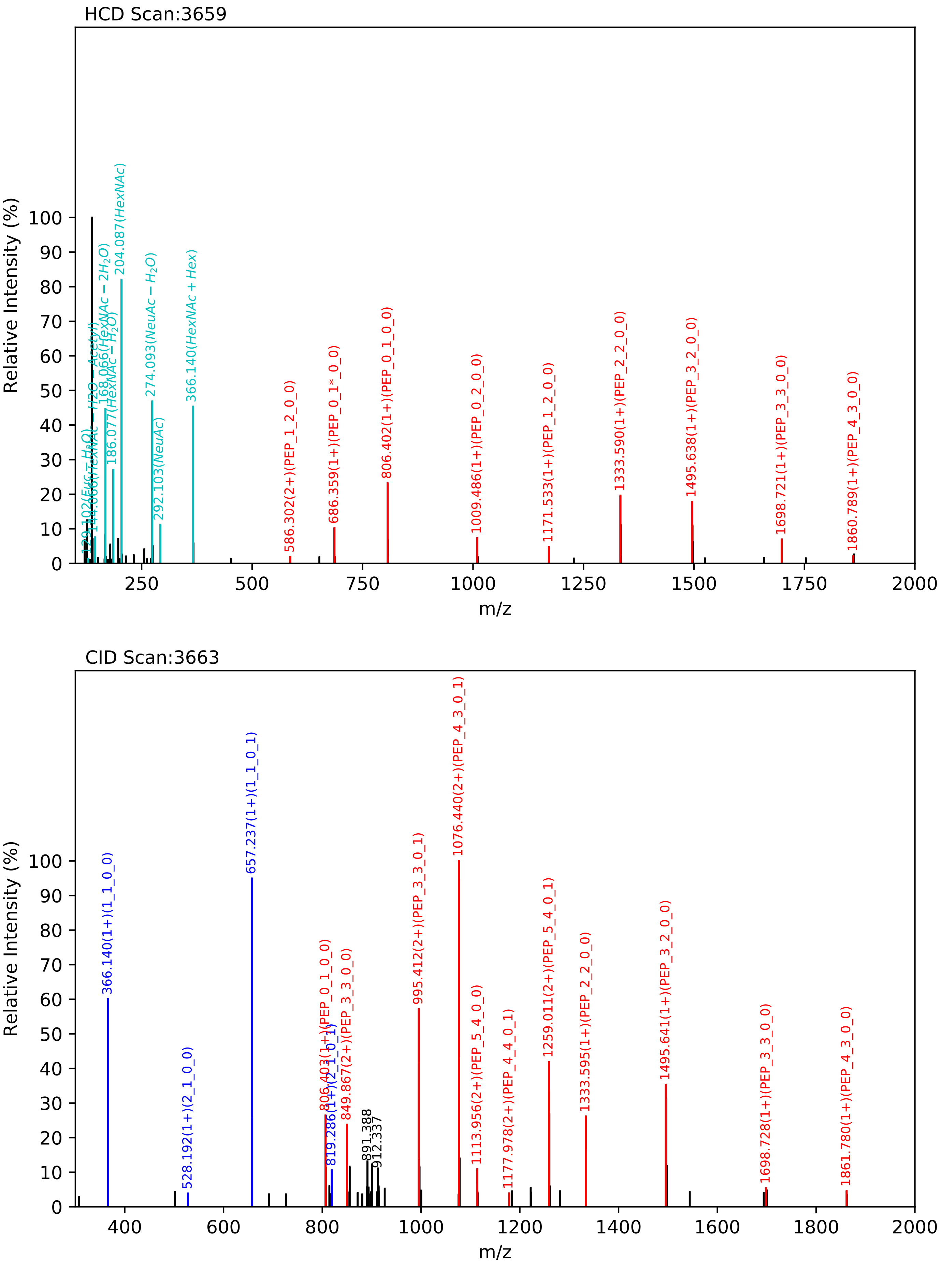

Unknown set no. 581, Experiment: Human Plasma exp_5

NNSLR(=PEP)_5_4_0_2, m/z:936.71(3+), RT:18.09, Y-score:91.21

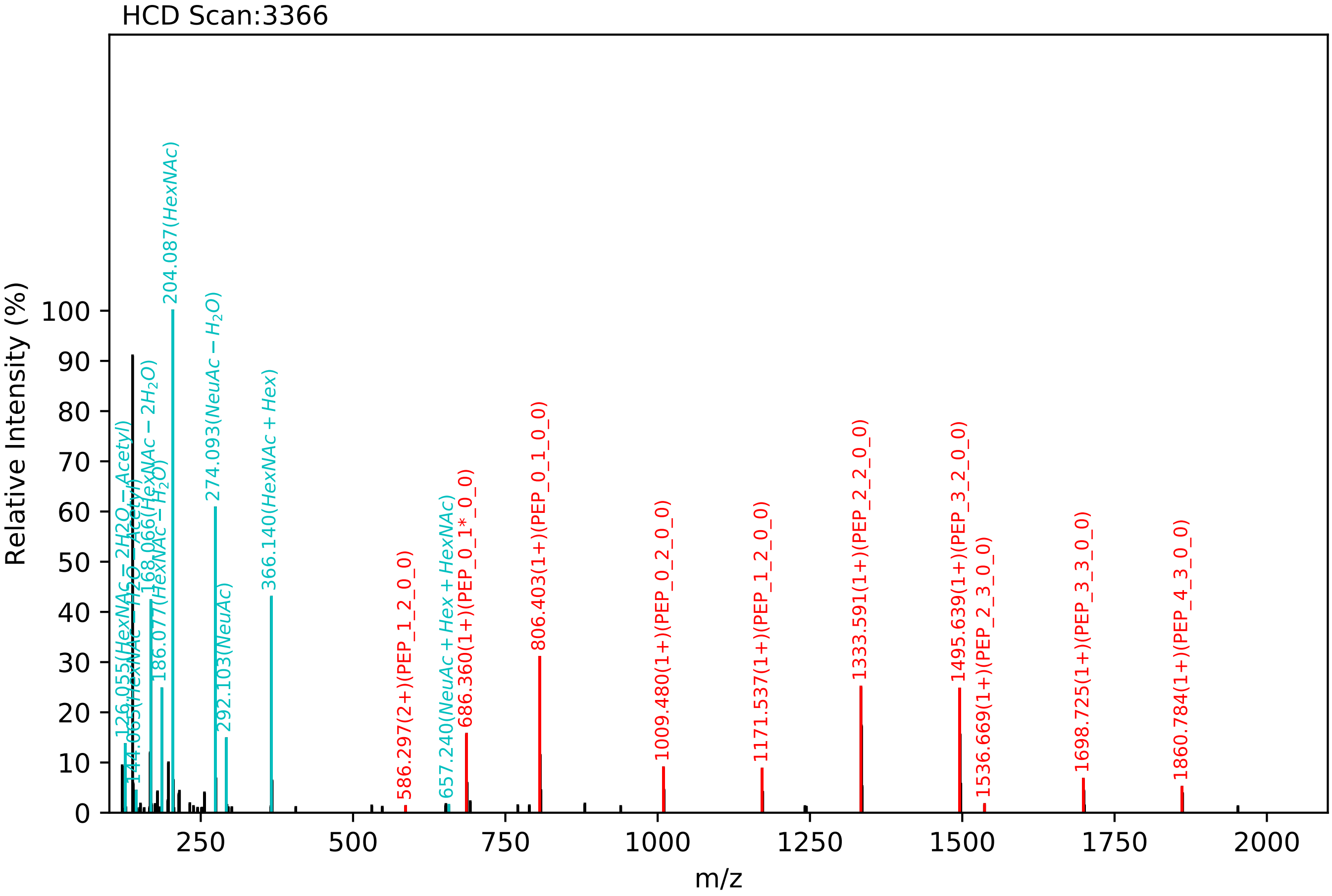

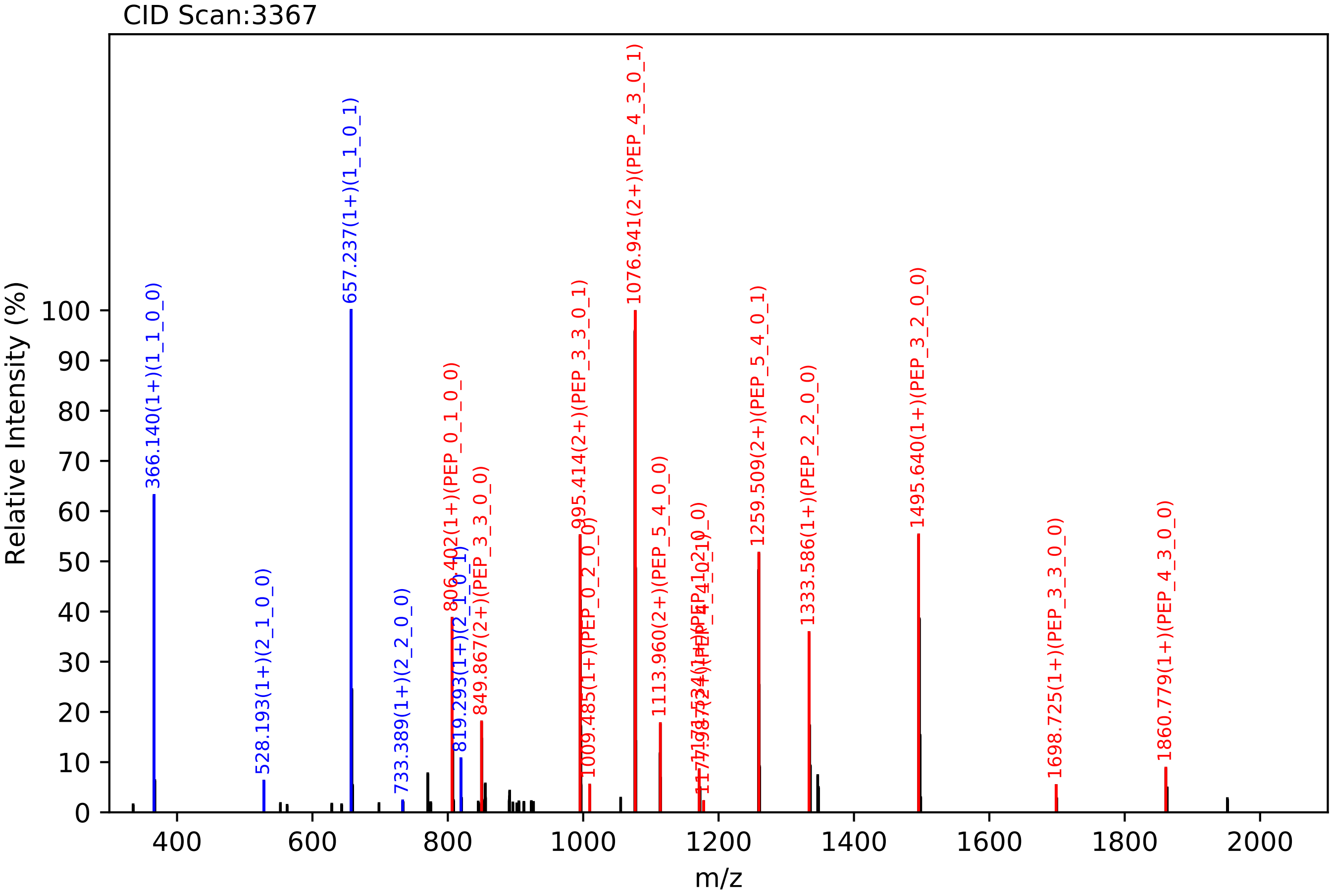

Unknown set no. 582, Experiment: Human Plasma exp_2

ENLTAR(=PEP)_5_4_0_2, m/z:970.05(3+), RT:36.68, Y-score:95.03

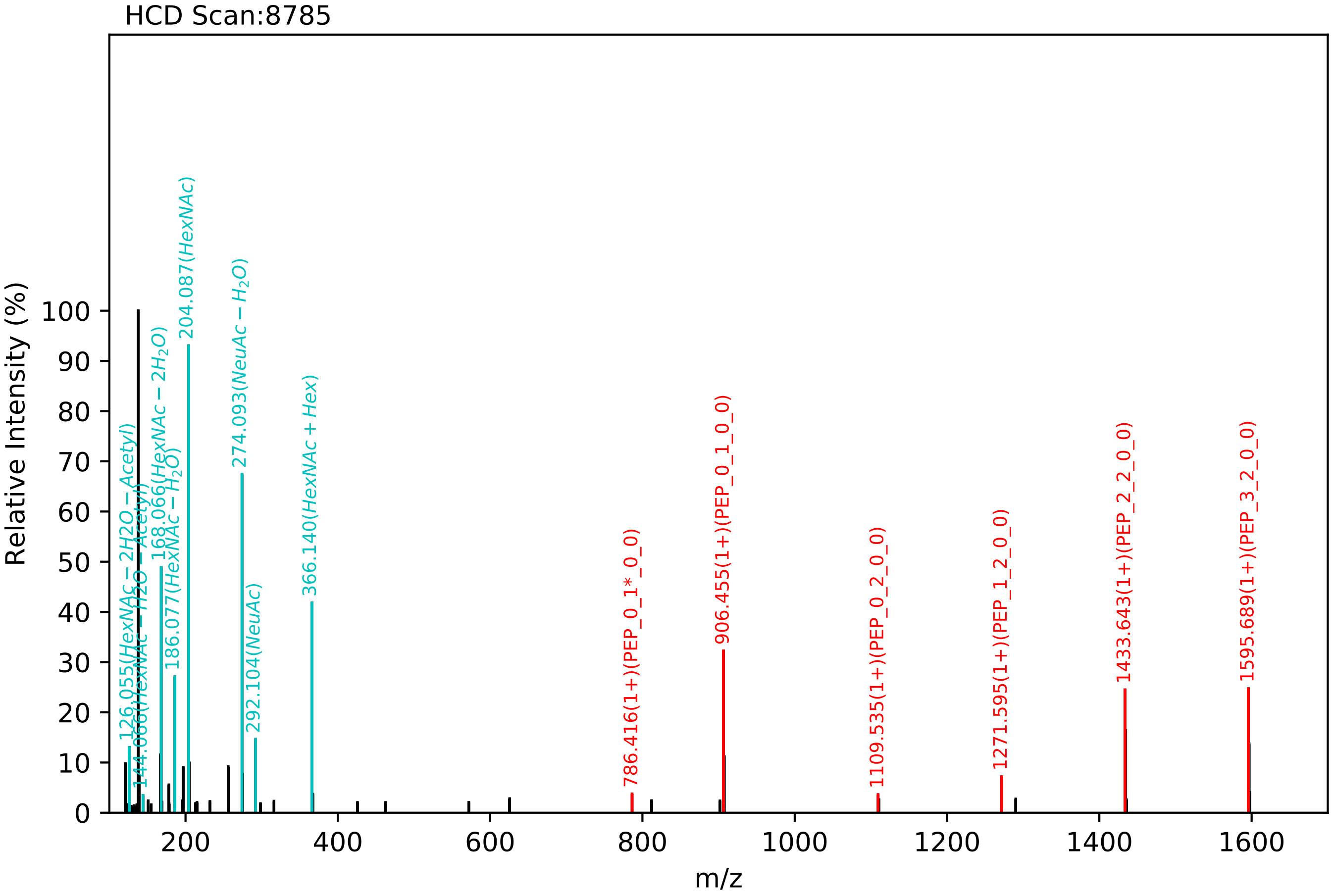

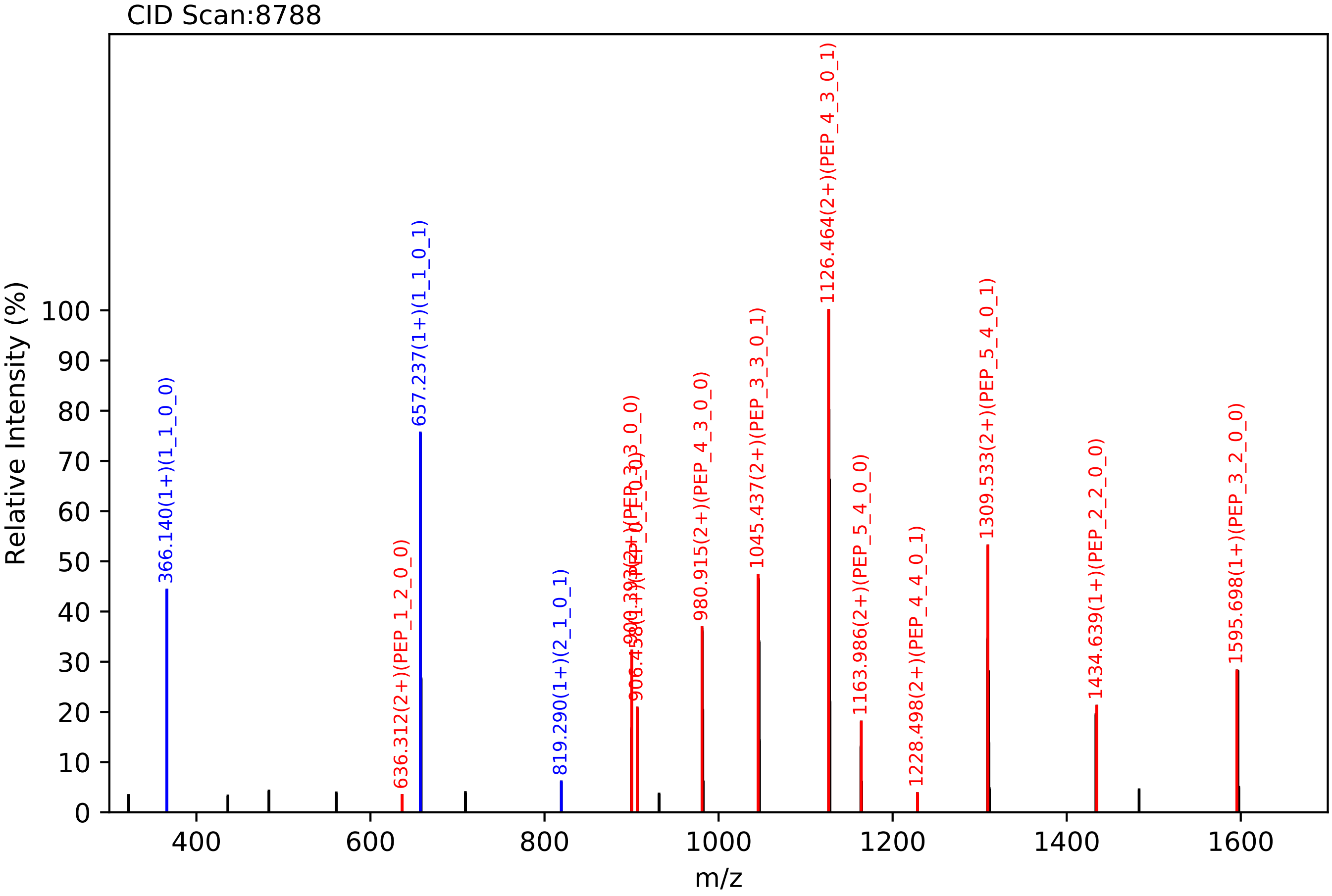

Unknown set no. 583, Experiment: Human Plasma exp_1

ENLTAR(=PEP)_6_5_0_3, m/z:1188.80(3+), RT:52.10, Y-score:90.32

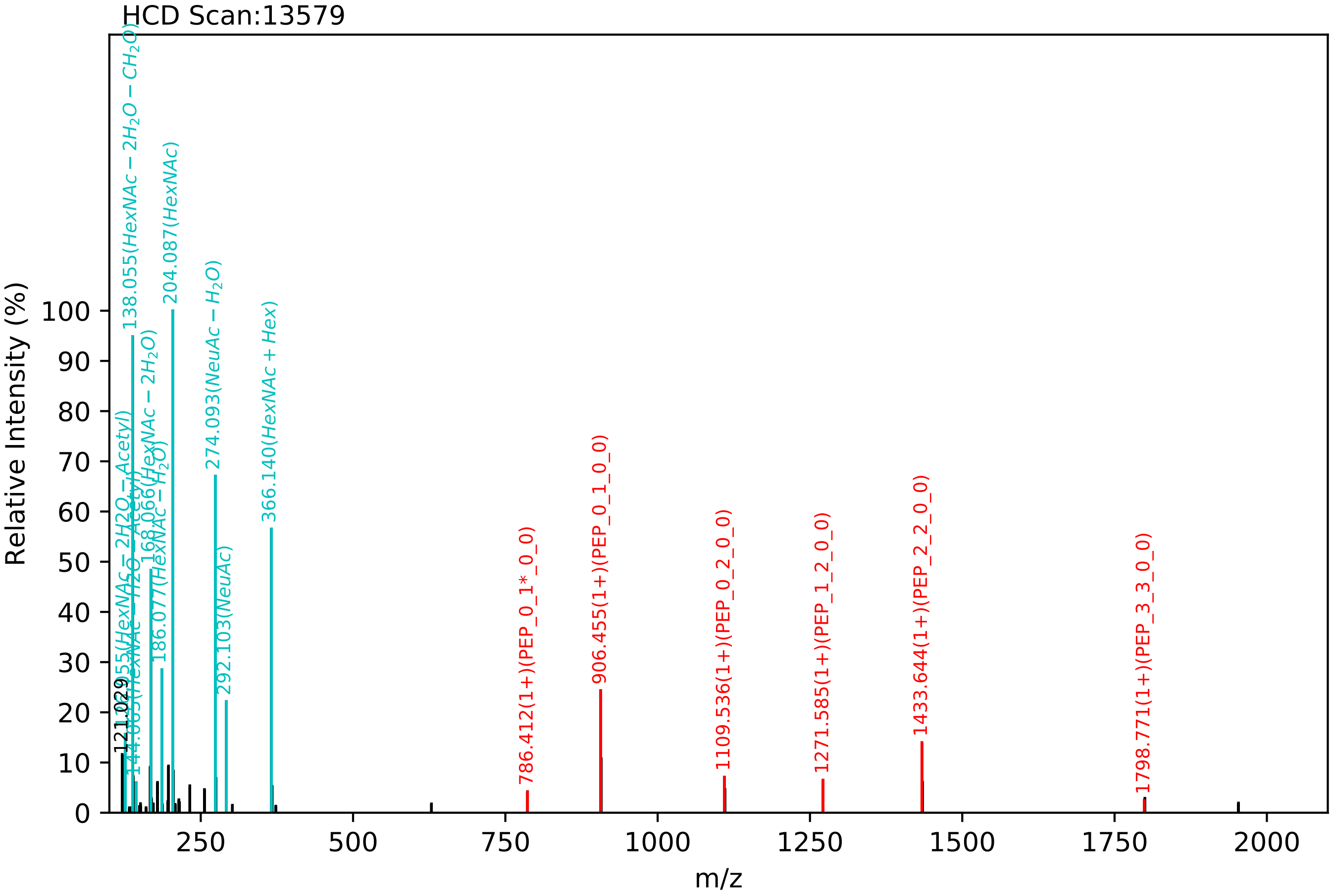

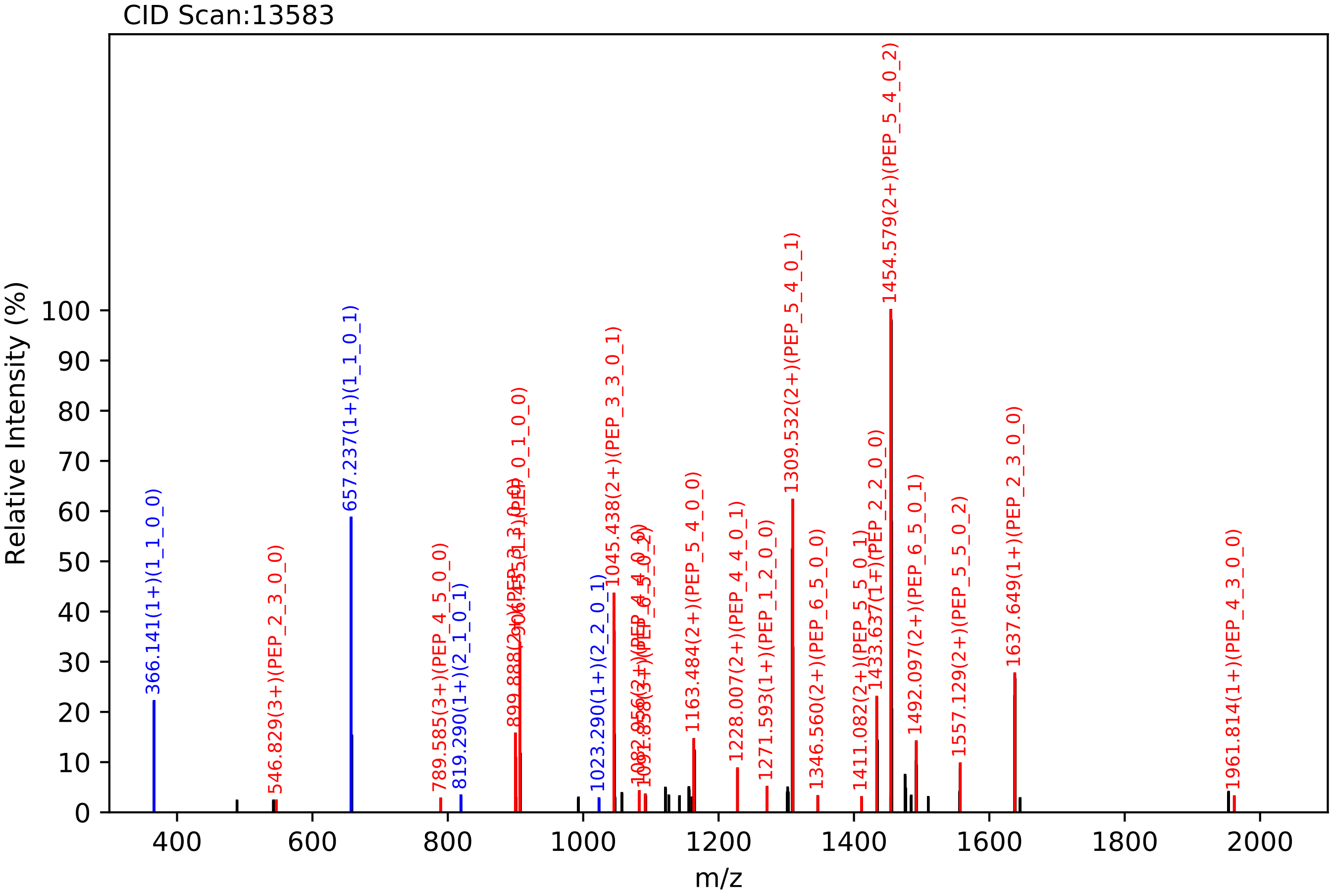

Unknown set no. 584, Experiment: Human Plasma exp_3

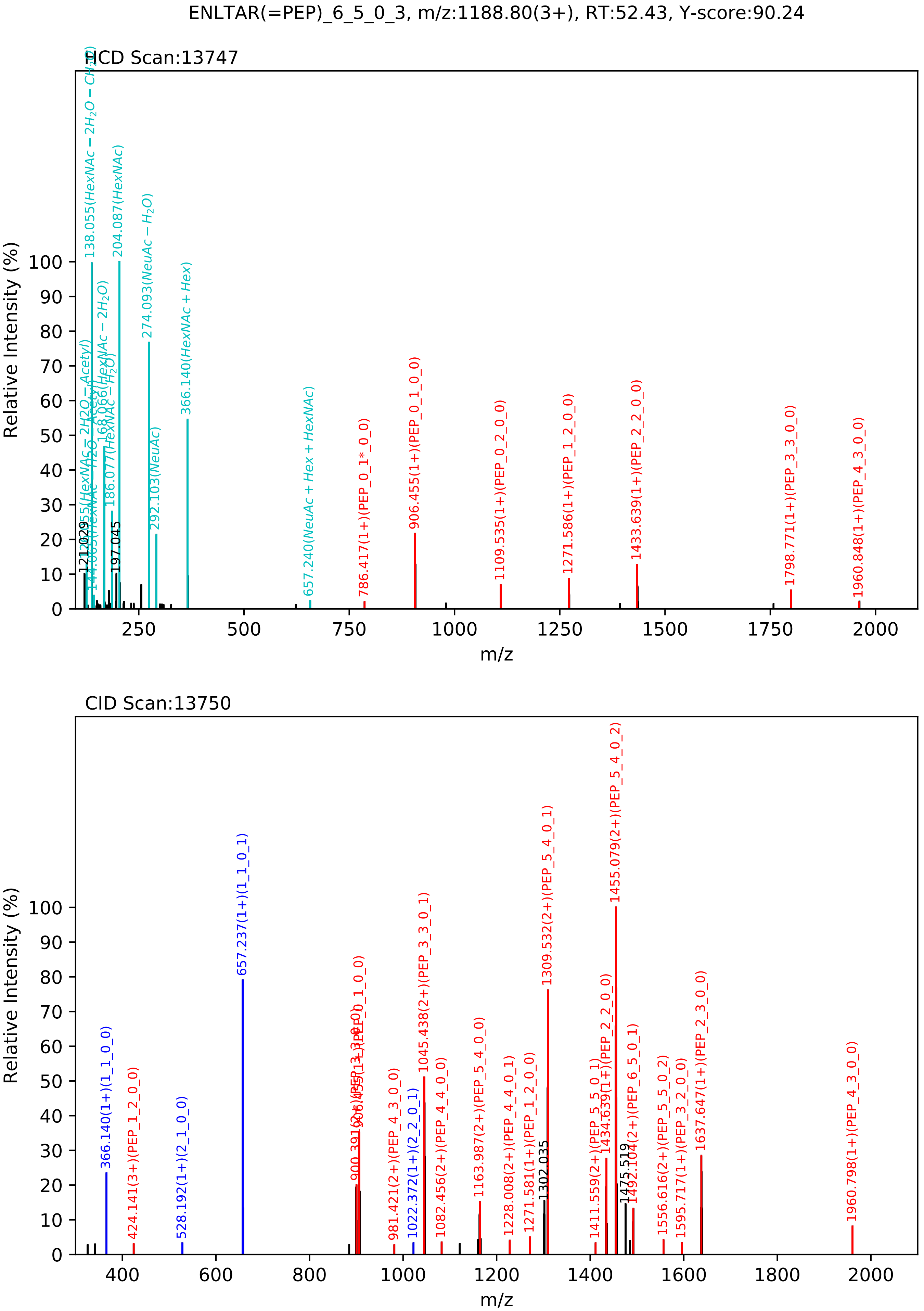

Unknown set no. 585, Experiment: Human Plasma exp_4

ENLTAR(=PEP)_6_5_0_3, m/z:1188.80(3+), RT:52.50, Y-score:92.53

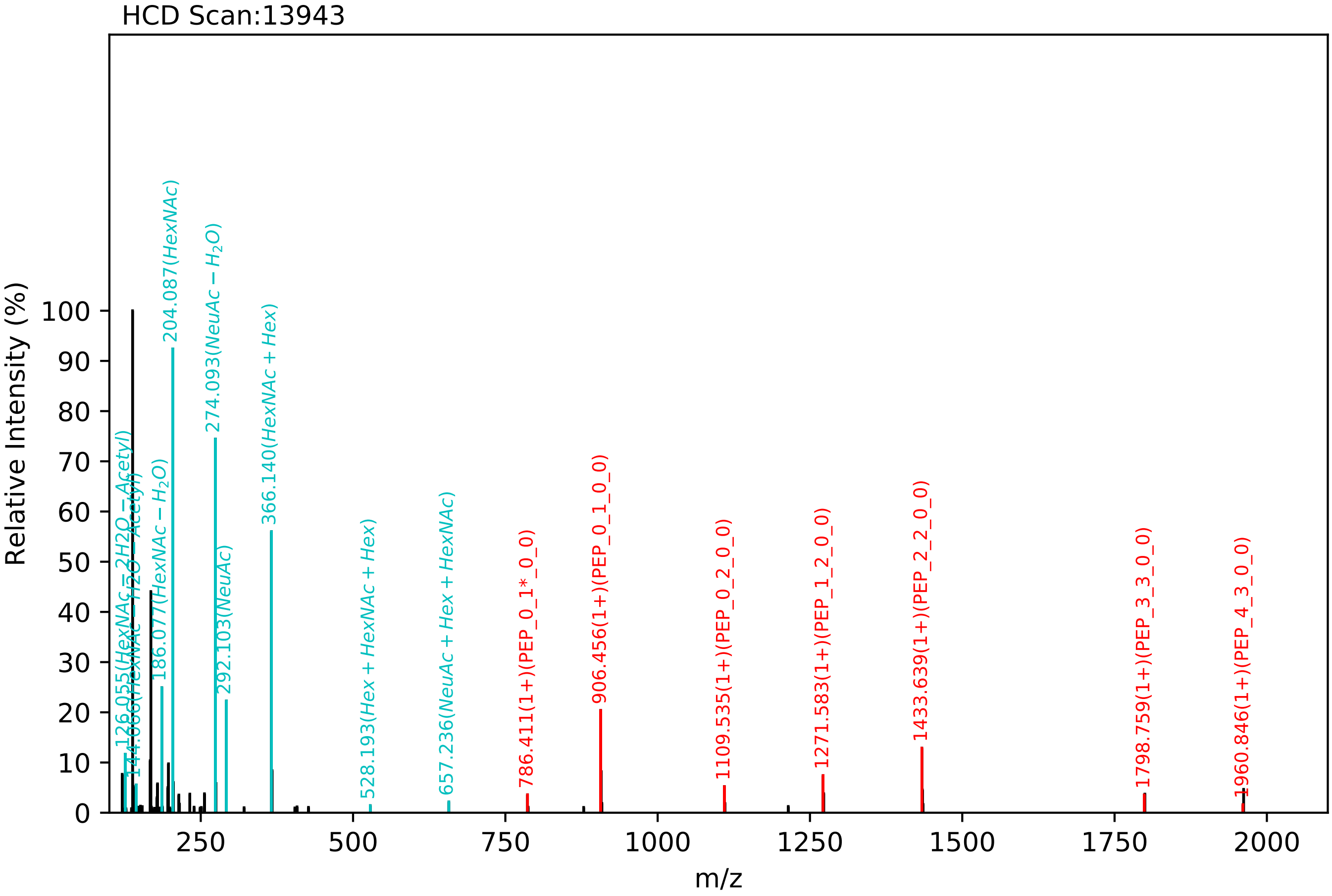

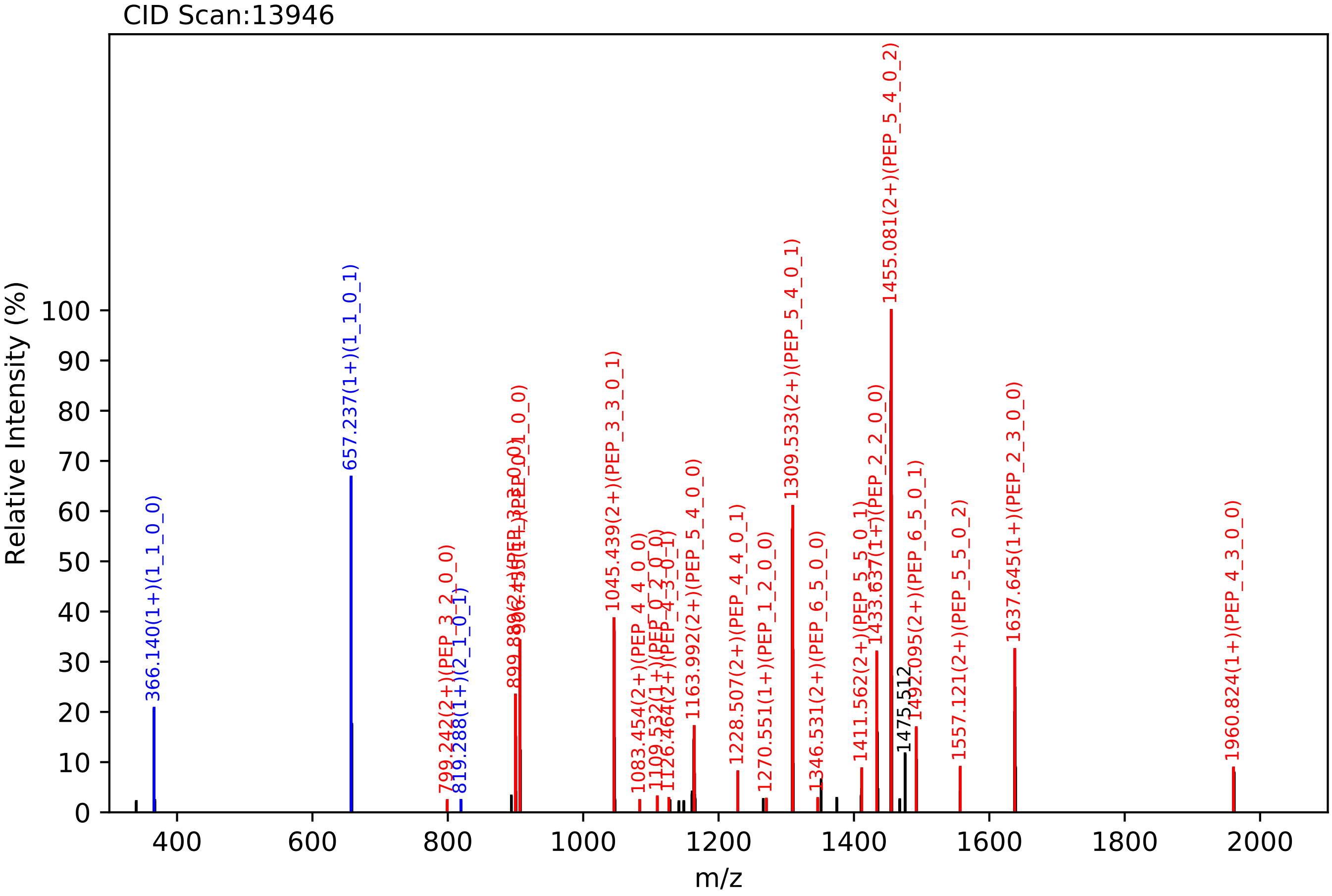

Unknown set no. 586, Experiment: Human Plasma exp_1

ITYSIVQTNCSK(=PEP)_5_4_0_2, m/z:905.38(4+), RT:75.97, Y-score:88.06

HCD Scan:22573

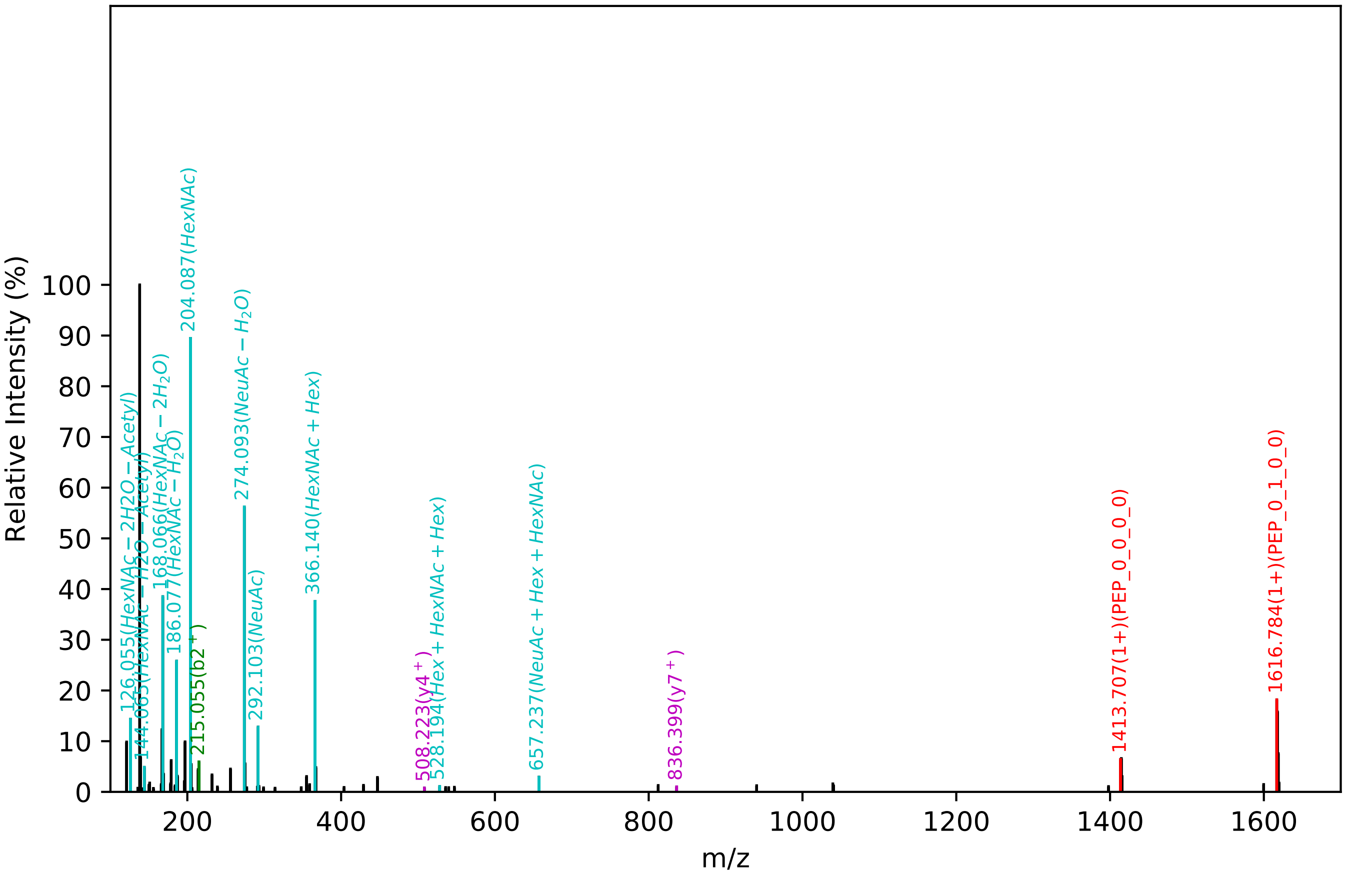

CID Scan:22574

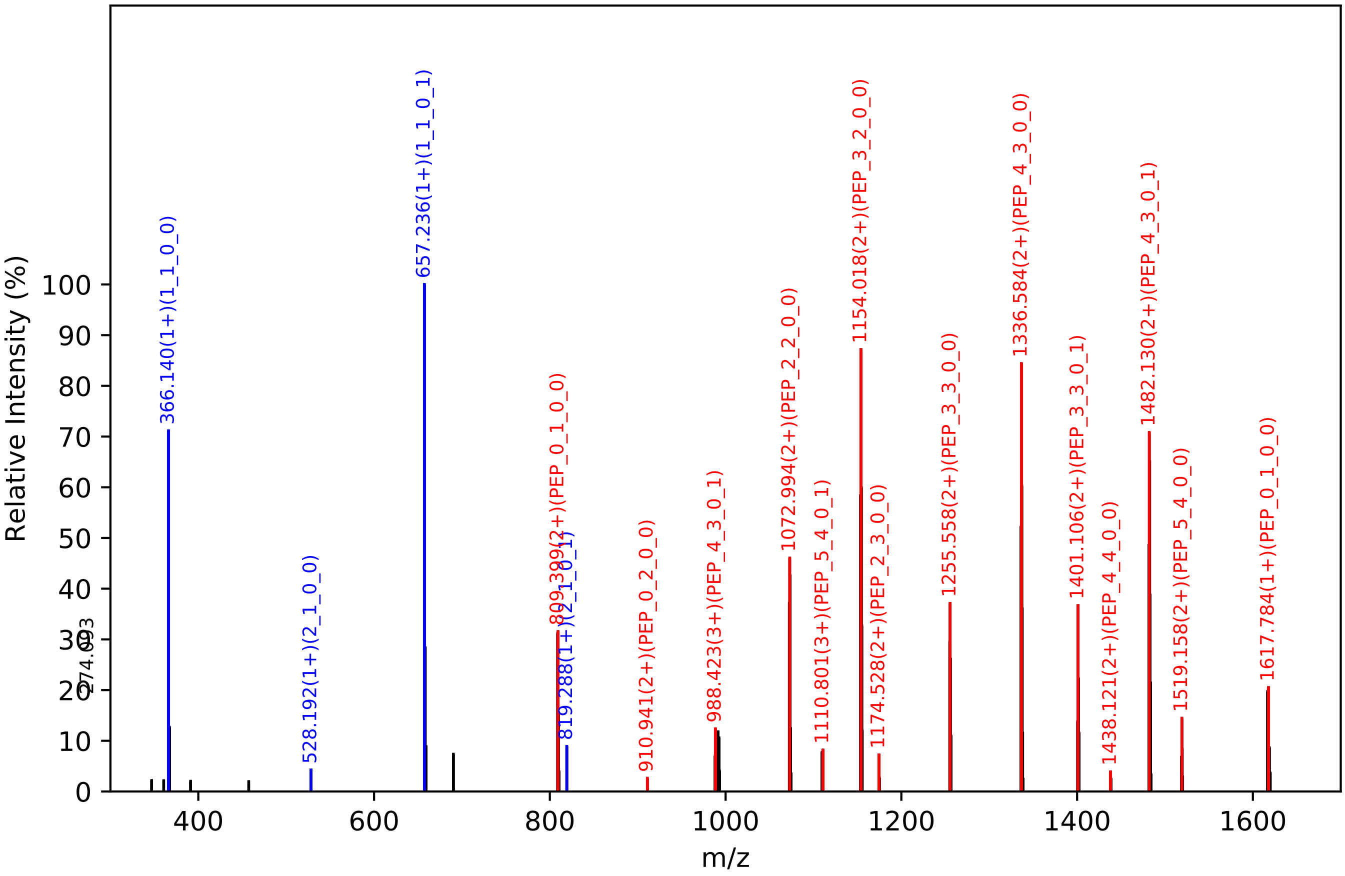

Unknown set no. 587, Experiment: Human Plasma exp_2

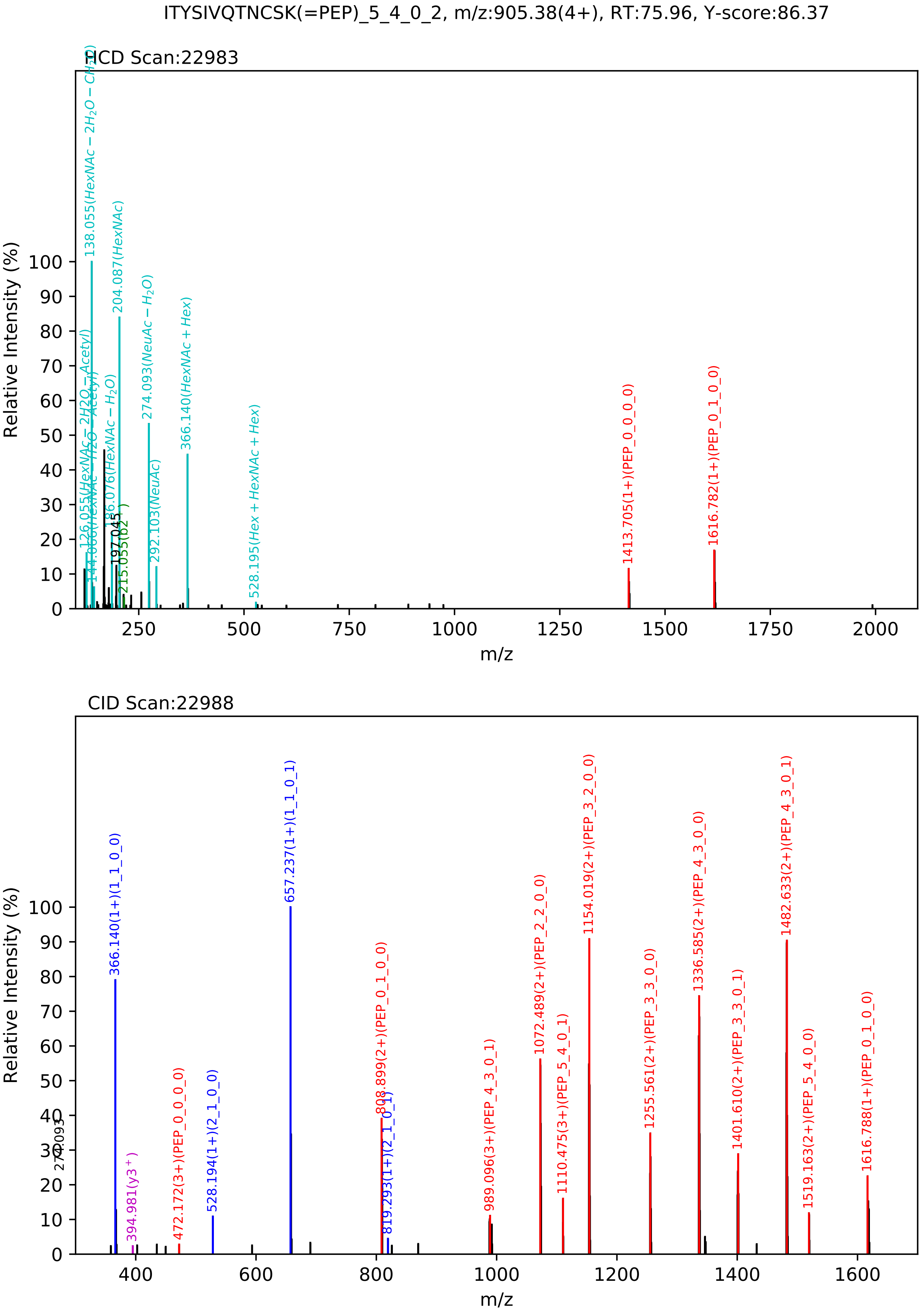

Unknown set no. 588, Experiment: Human Plasma exp_3

ITYSIVQTNCSK(=PEP)_5_4_0_2, m/z:905.38(4+), RT:76.13, Y-score:94.49

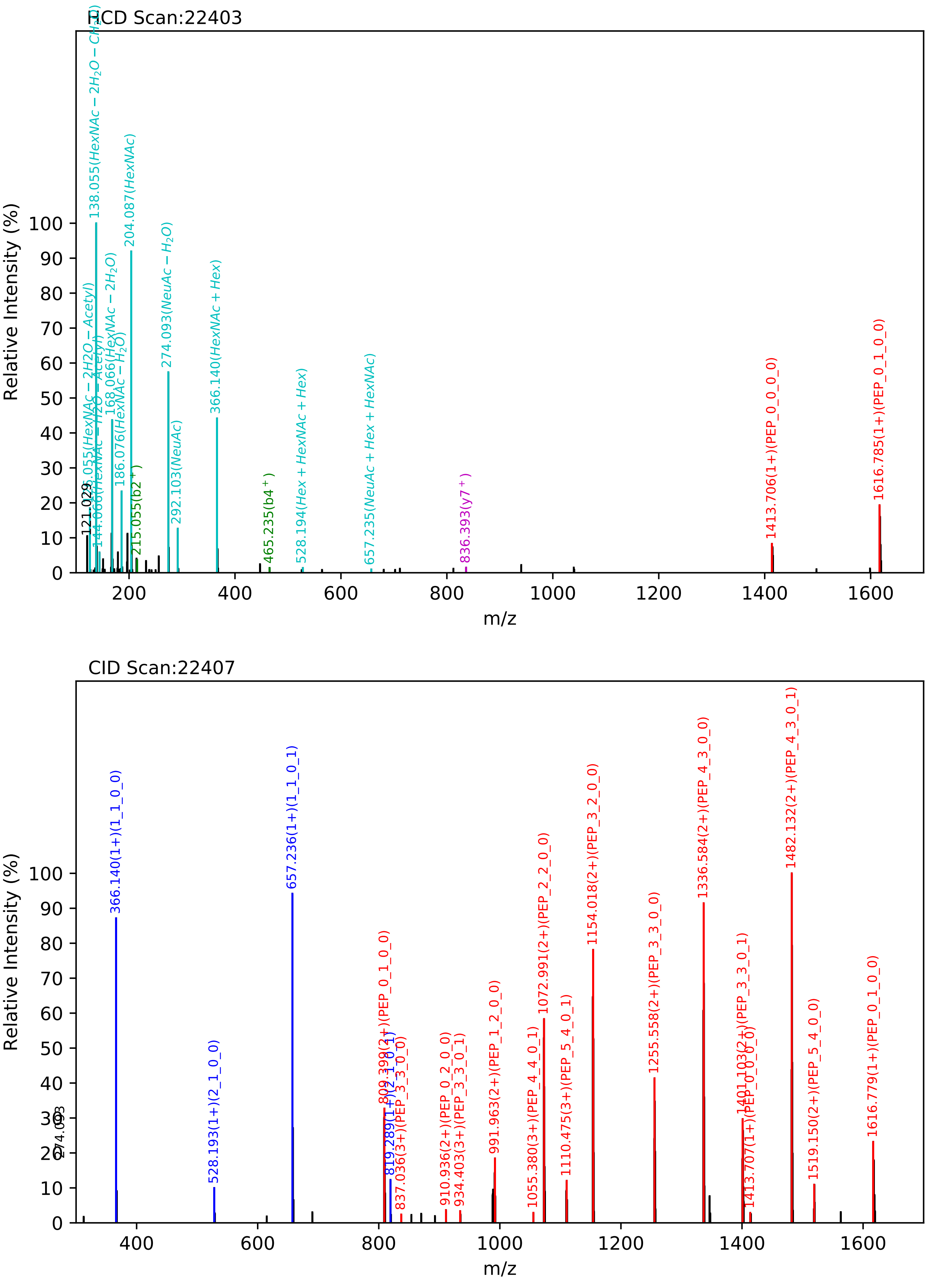

Unknown set no. 589, Experiment: Human Plasma exp_4

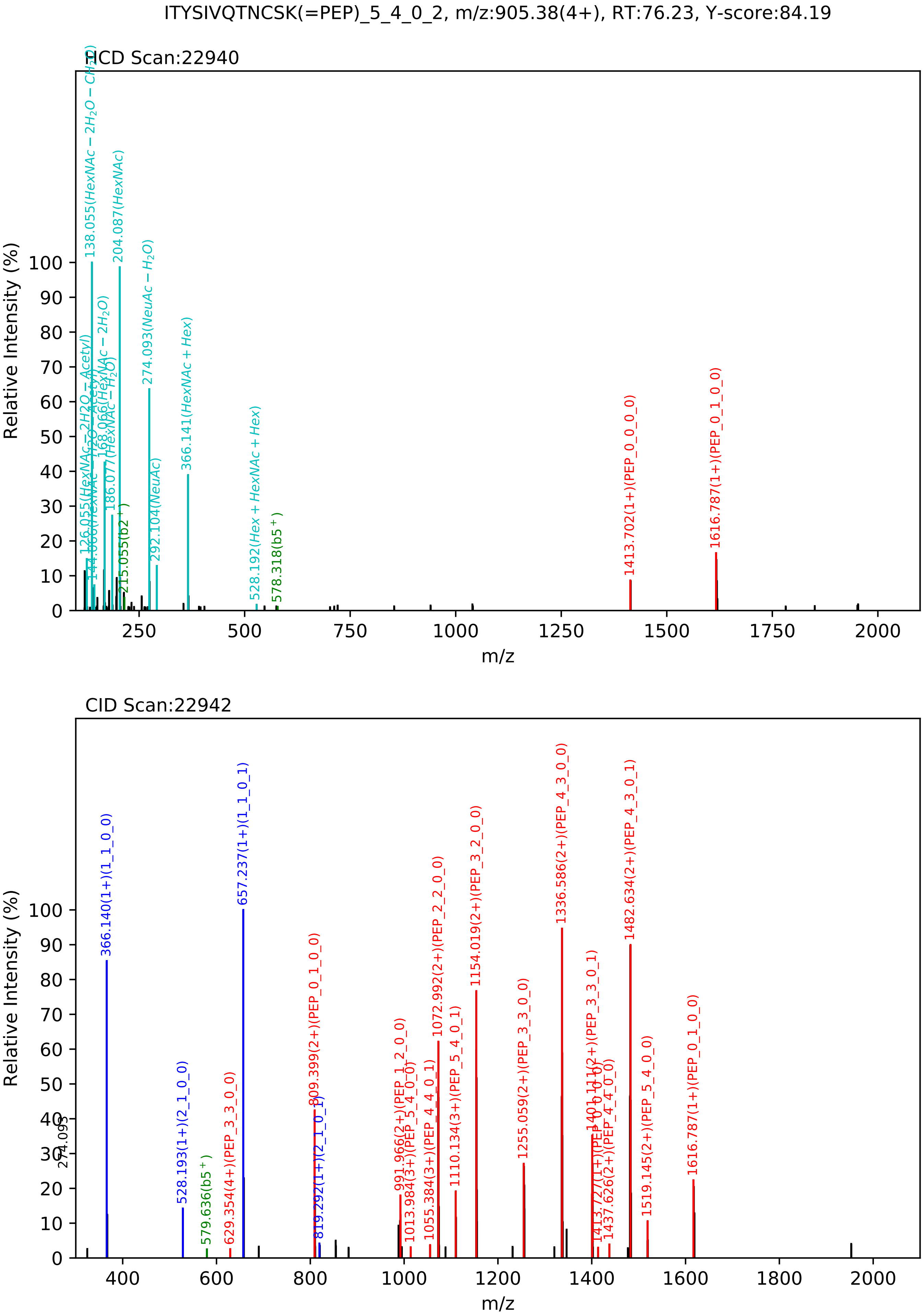

Unknown set no. 590, Experiment: Human Plasma exp_5

ITYSIVQTNCSK(=PEP)_5_4_0_2, m/z:905.38(4+), RT:75.91, Y-score:83.82

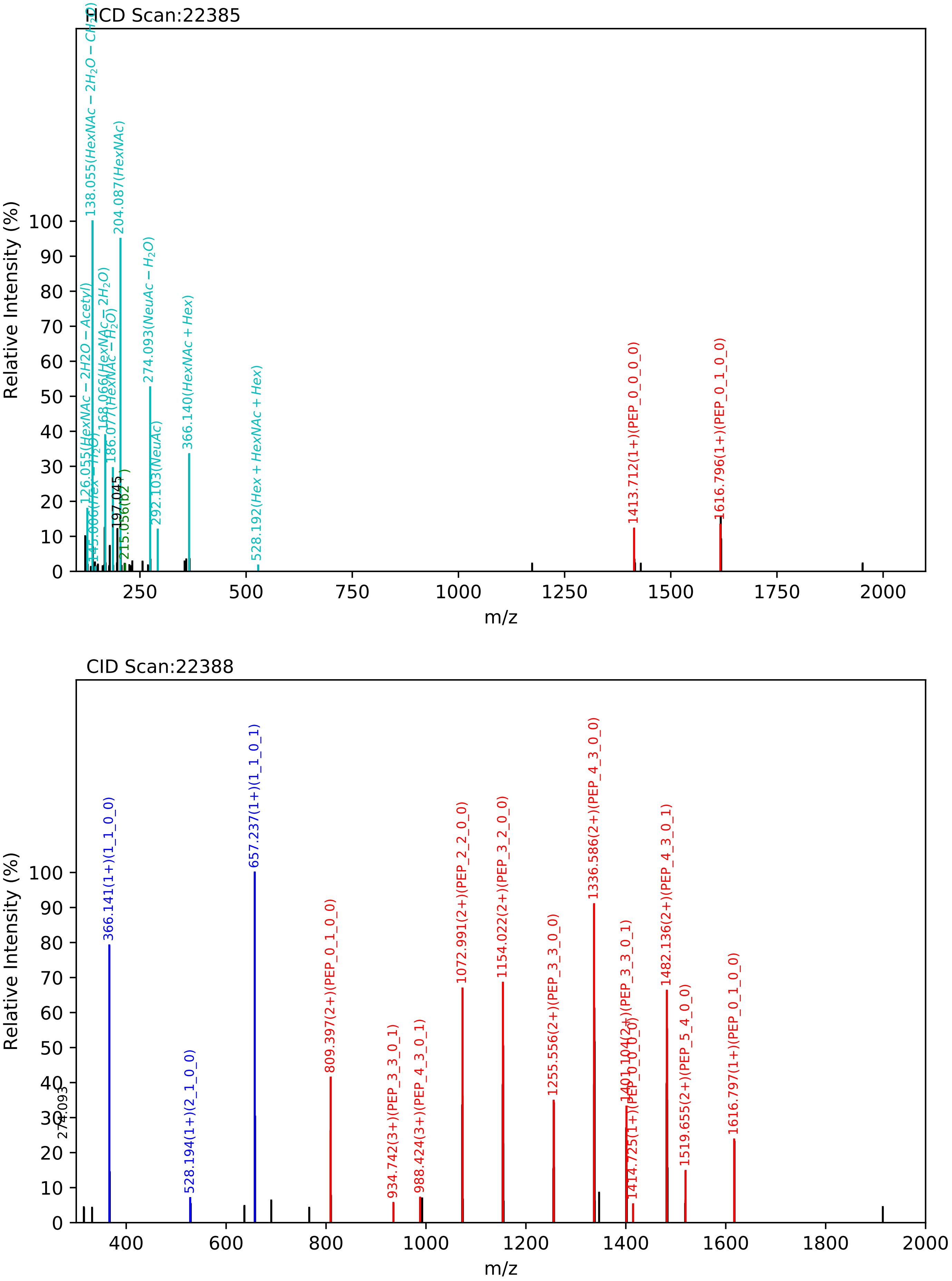

Unknown set no. 591, Experiment: Human Plasma exp_1

ITYSIVQTNCSK(=PEP)_5_4_1_2, m/z:1255.52(3+), RT:75.43, Y-score:87.92

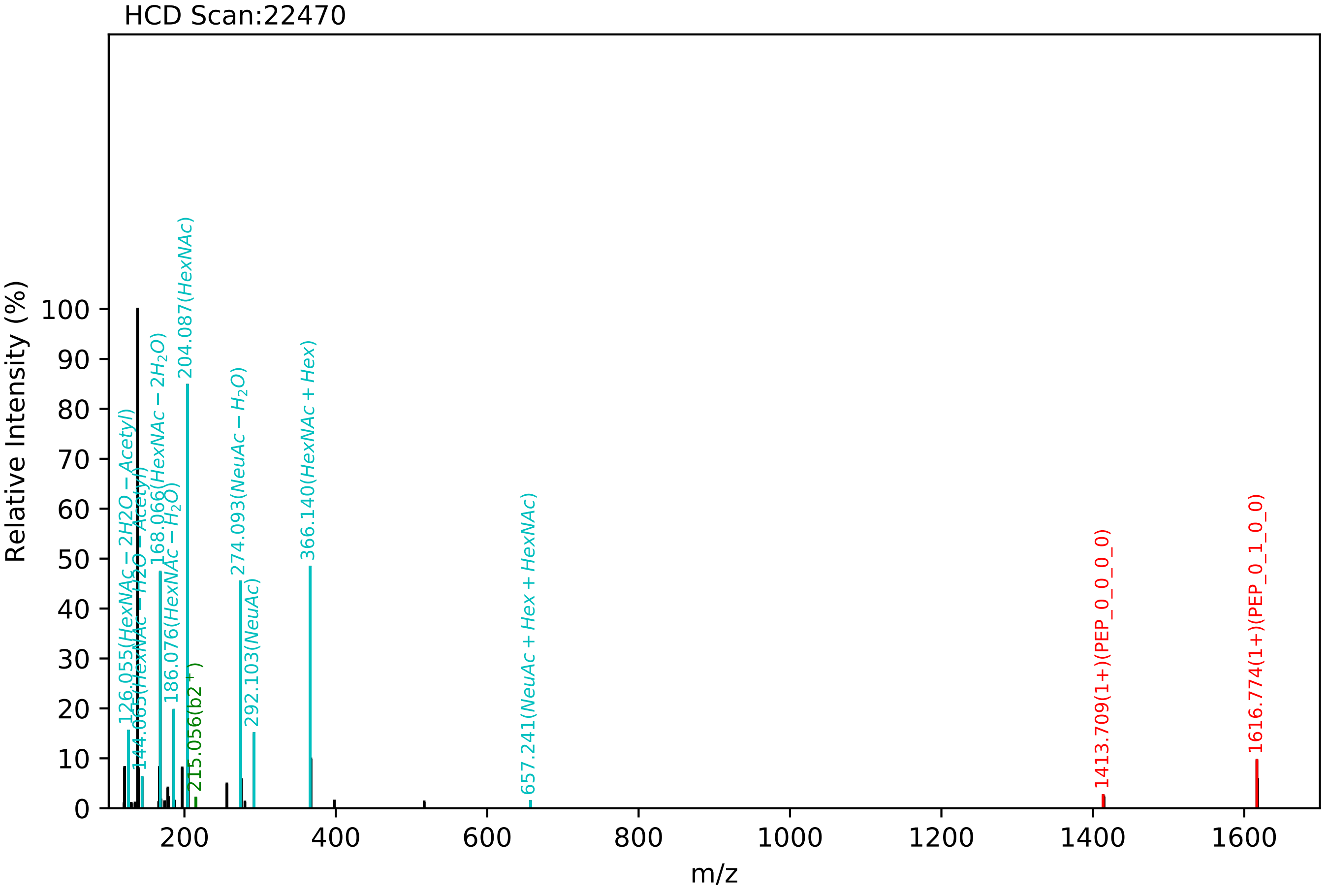

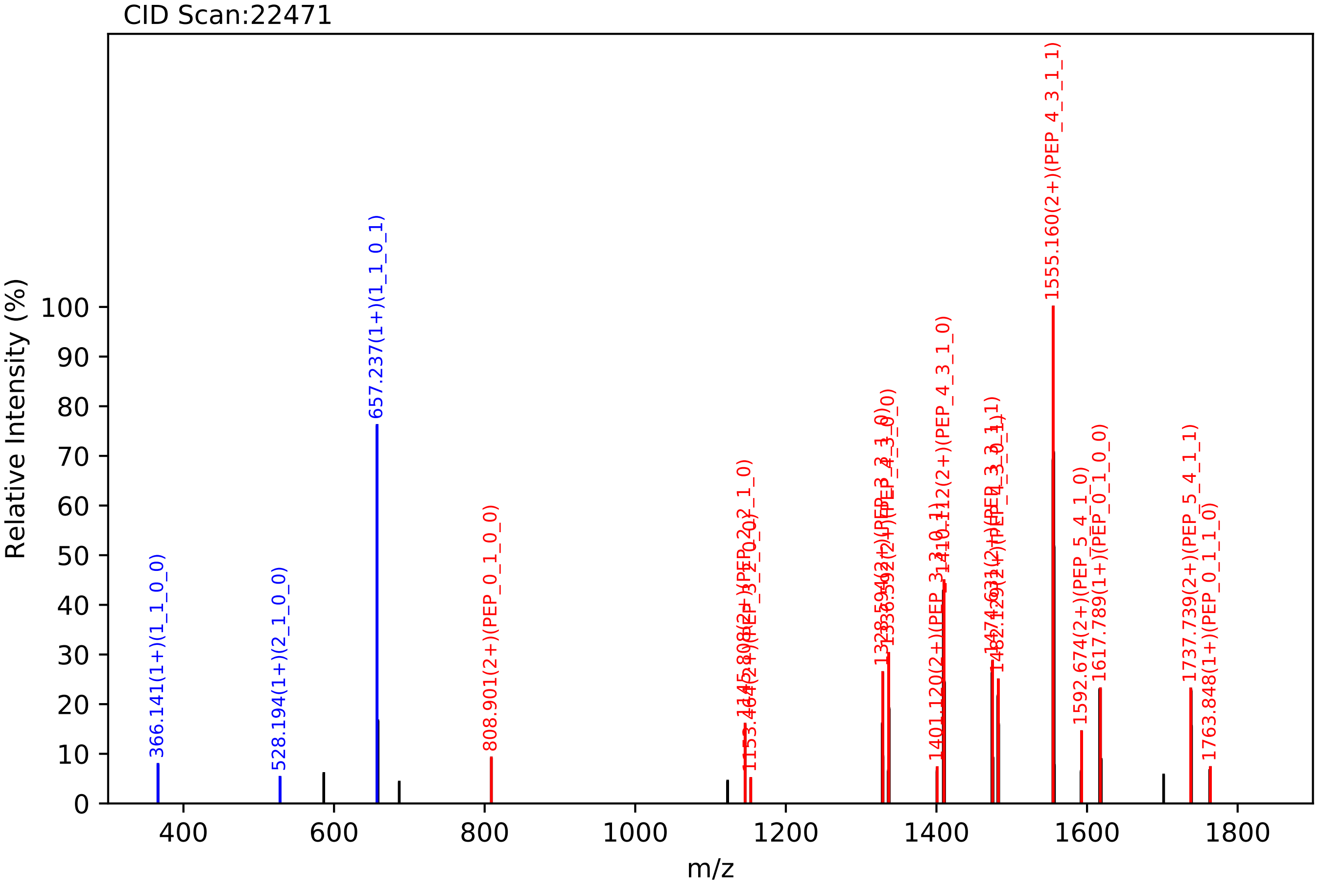

Unknown set no. 592, Experiment: Human Plasma exp_2

ITYSIVQTNCSK(=PEP)_6_5_1_3, m/z:1105.95(4+), RT:87.19, Y-score:79.18

HCD Scan:26234

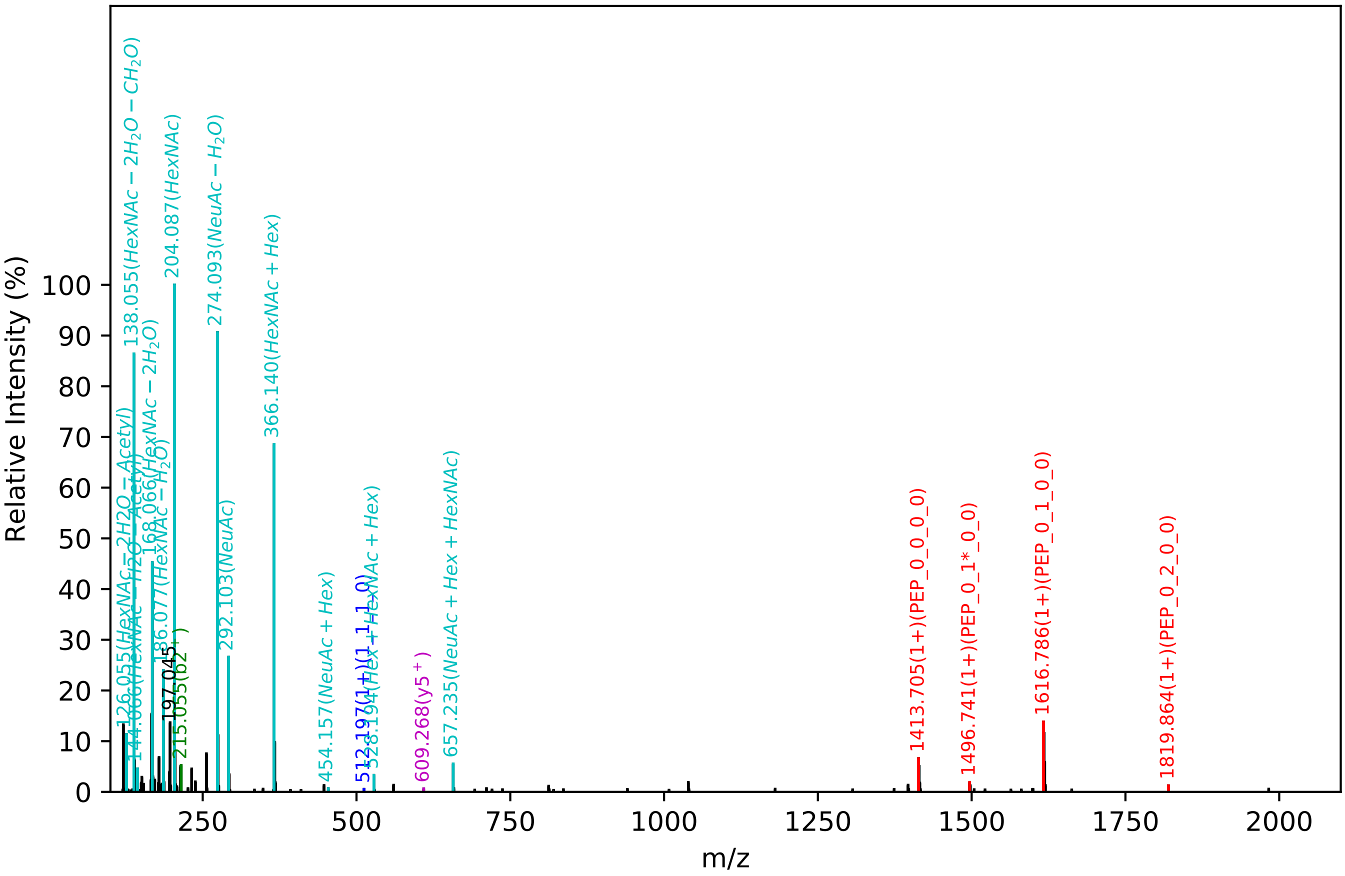

CID Scan:26235

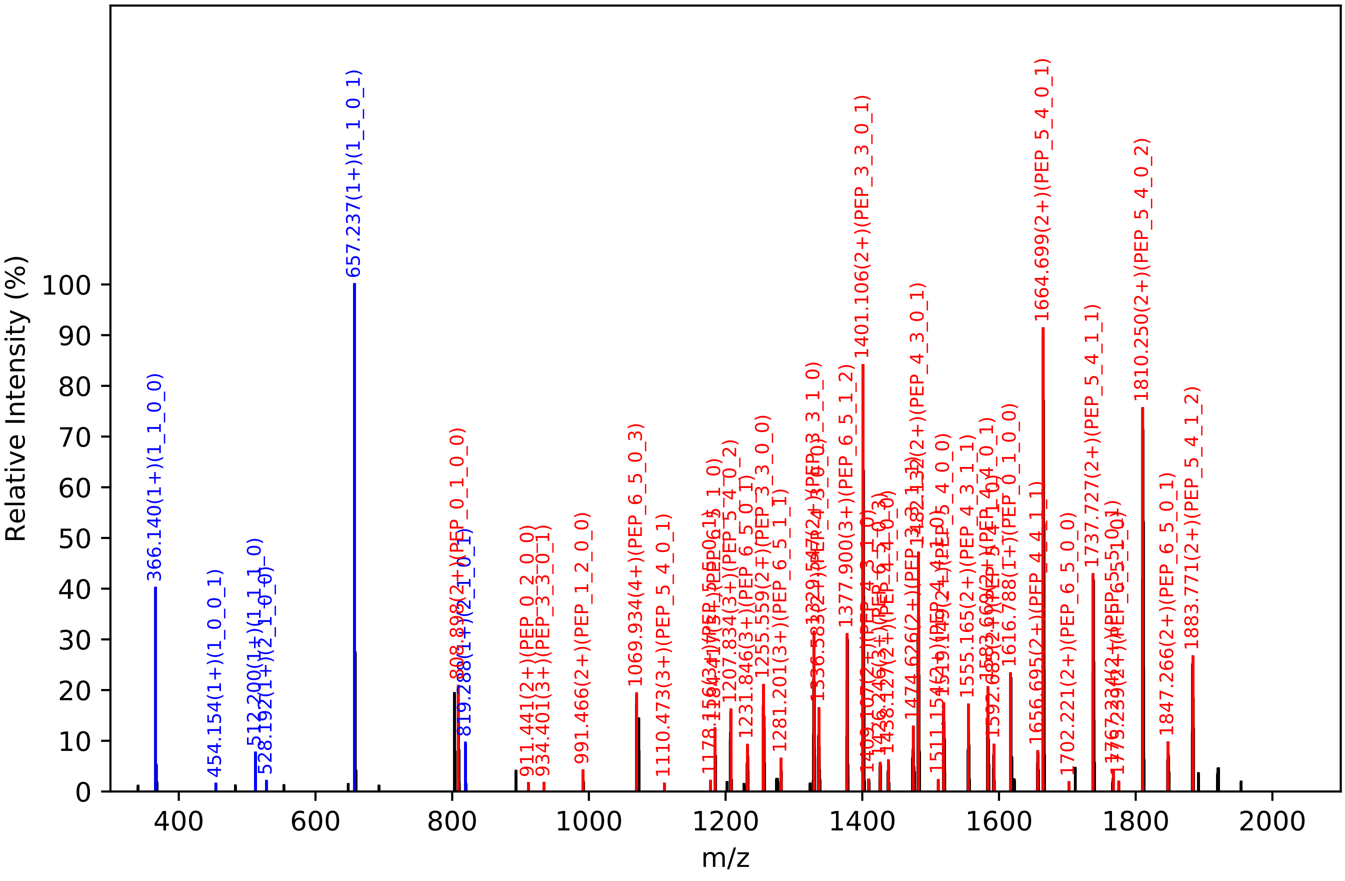

Unknown set no. 593, Experiment: Human Plasma exp_4

ITYSIVQTNCSK(=PEP)_6_5_1_3, m/z:1105.95(4+), RT:87.09, Y-score:78.42

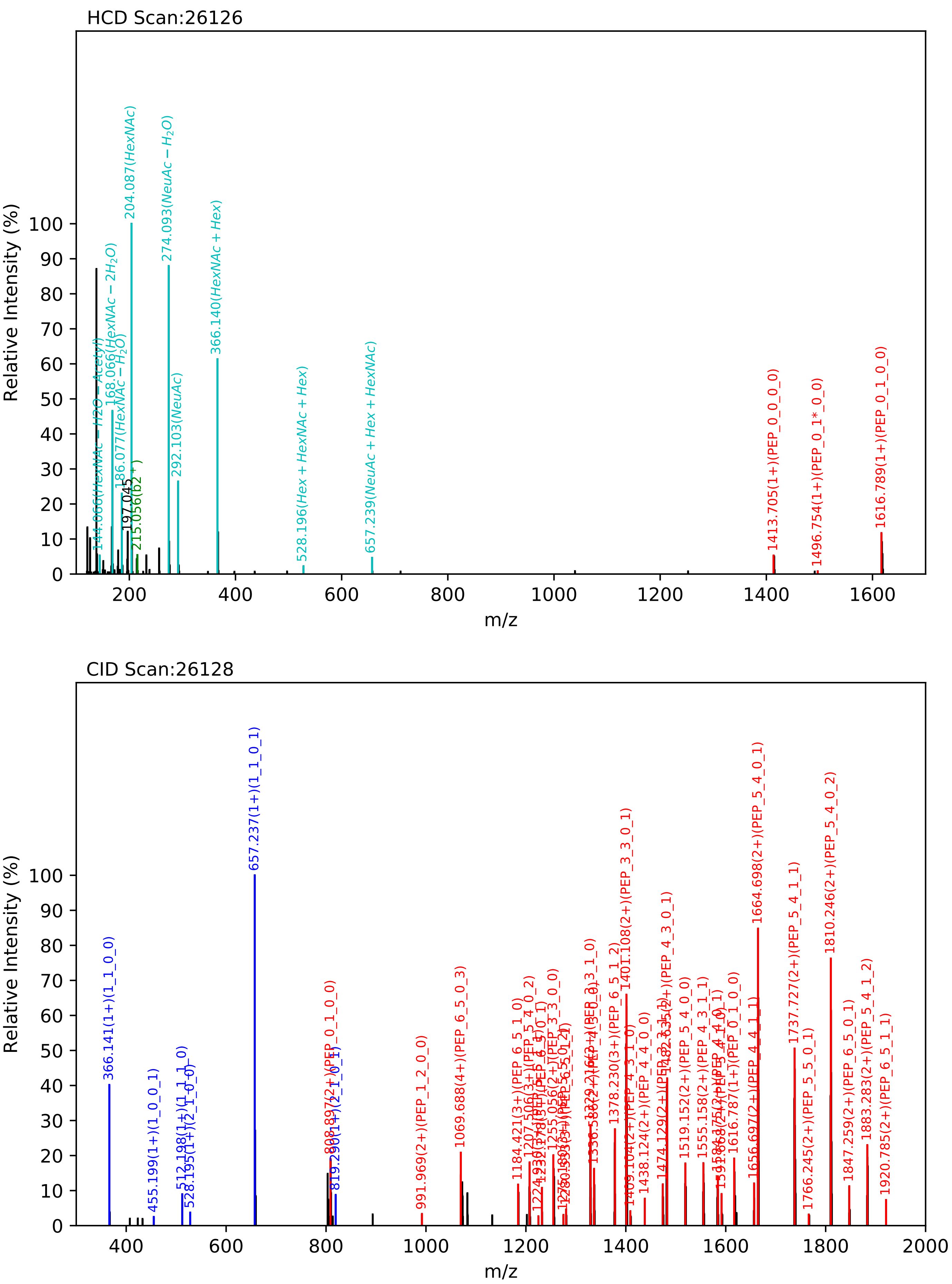

Unknown set no. 594, Experiment: Human Plasma exp_5

ITYSIVQTNCSK(=PEP)_6_5_1_3, m/z:1105.95(4+), RT:86.74, Y-score:79.85

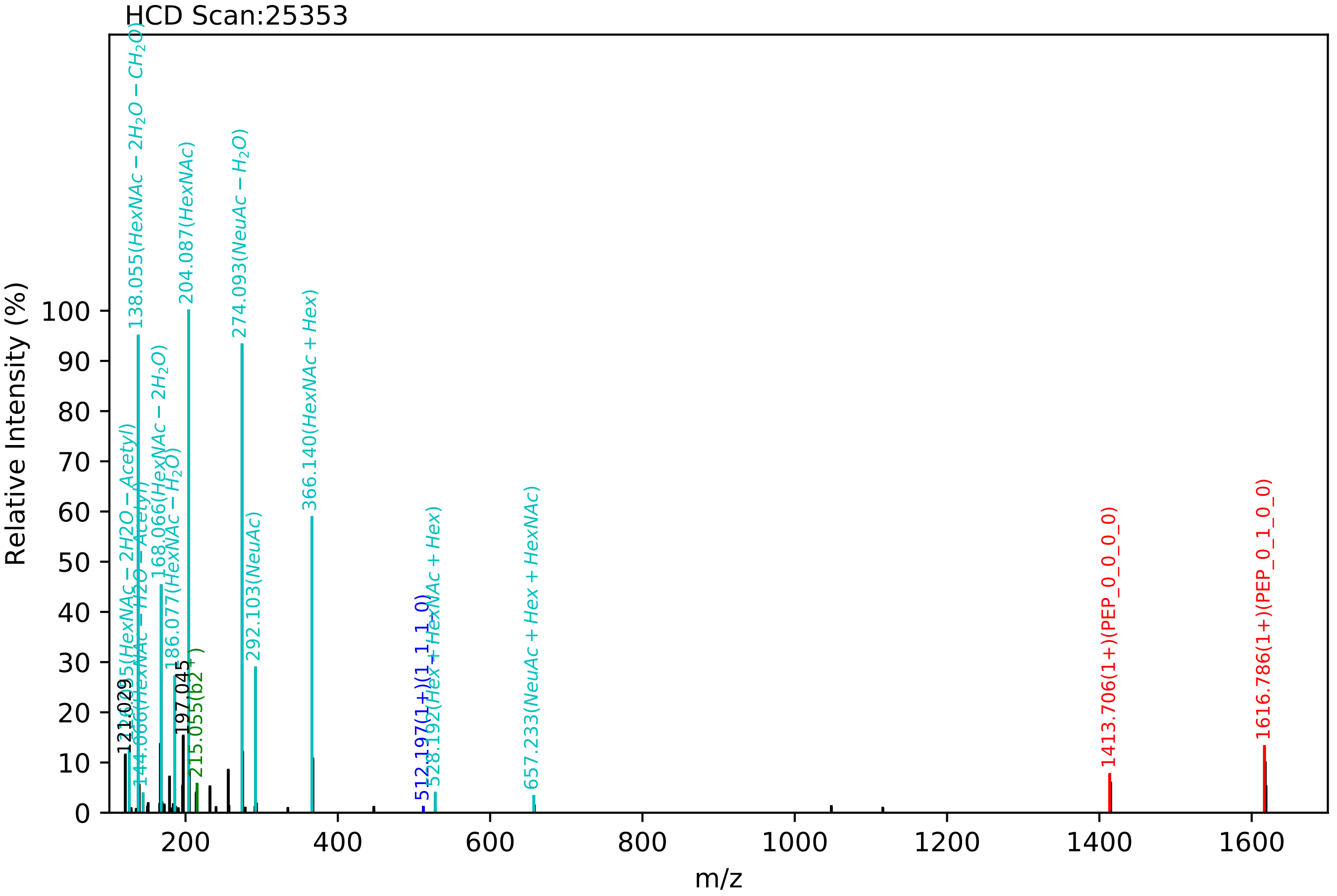

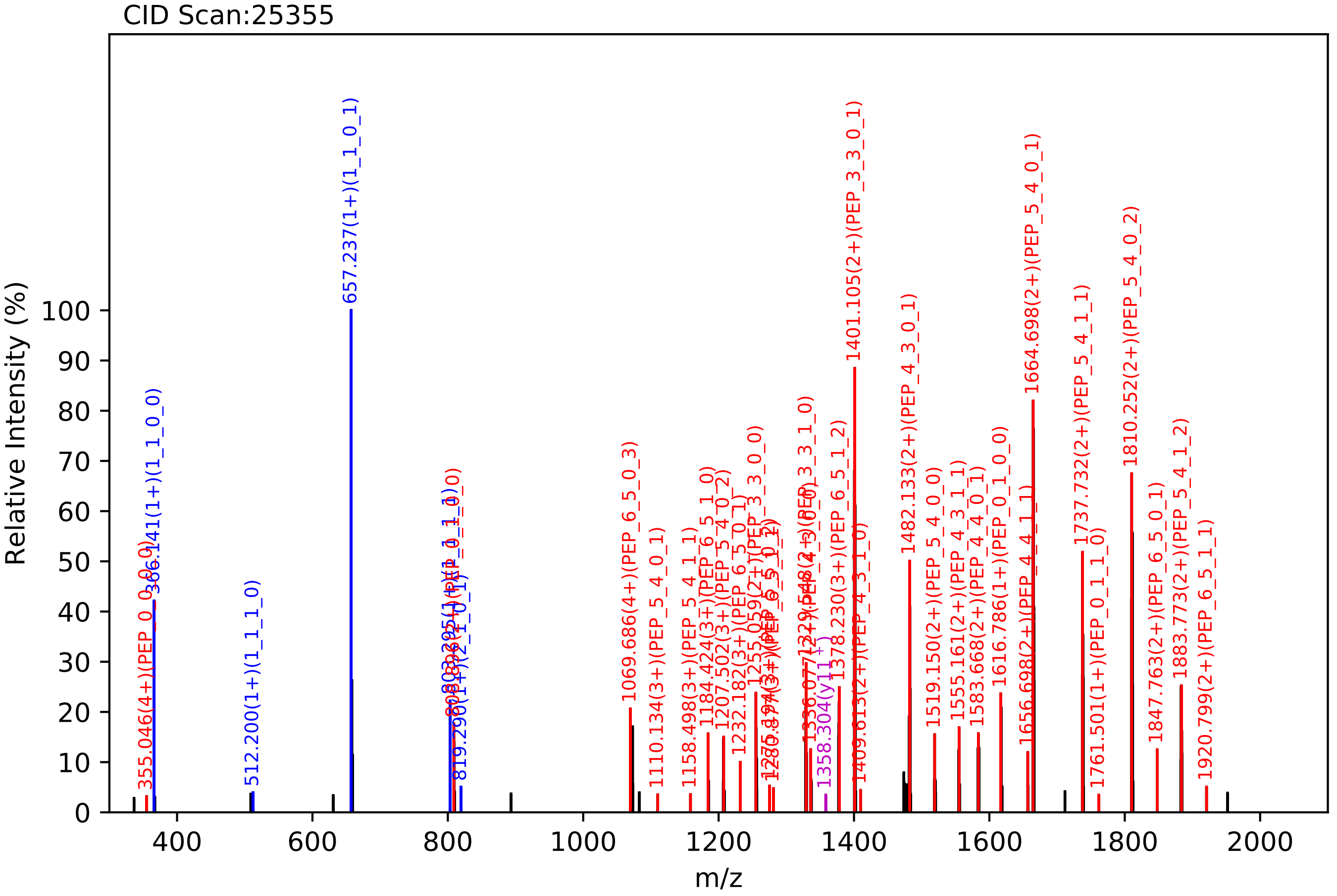

Unknown set no. 595, Experiment: Human Plasma exp_3

LNAENNATFYFK(=PEP)_5_4_0_2, m/z:909.87(4+), RT:91.27, Y-score:82.70

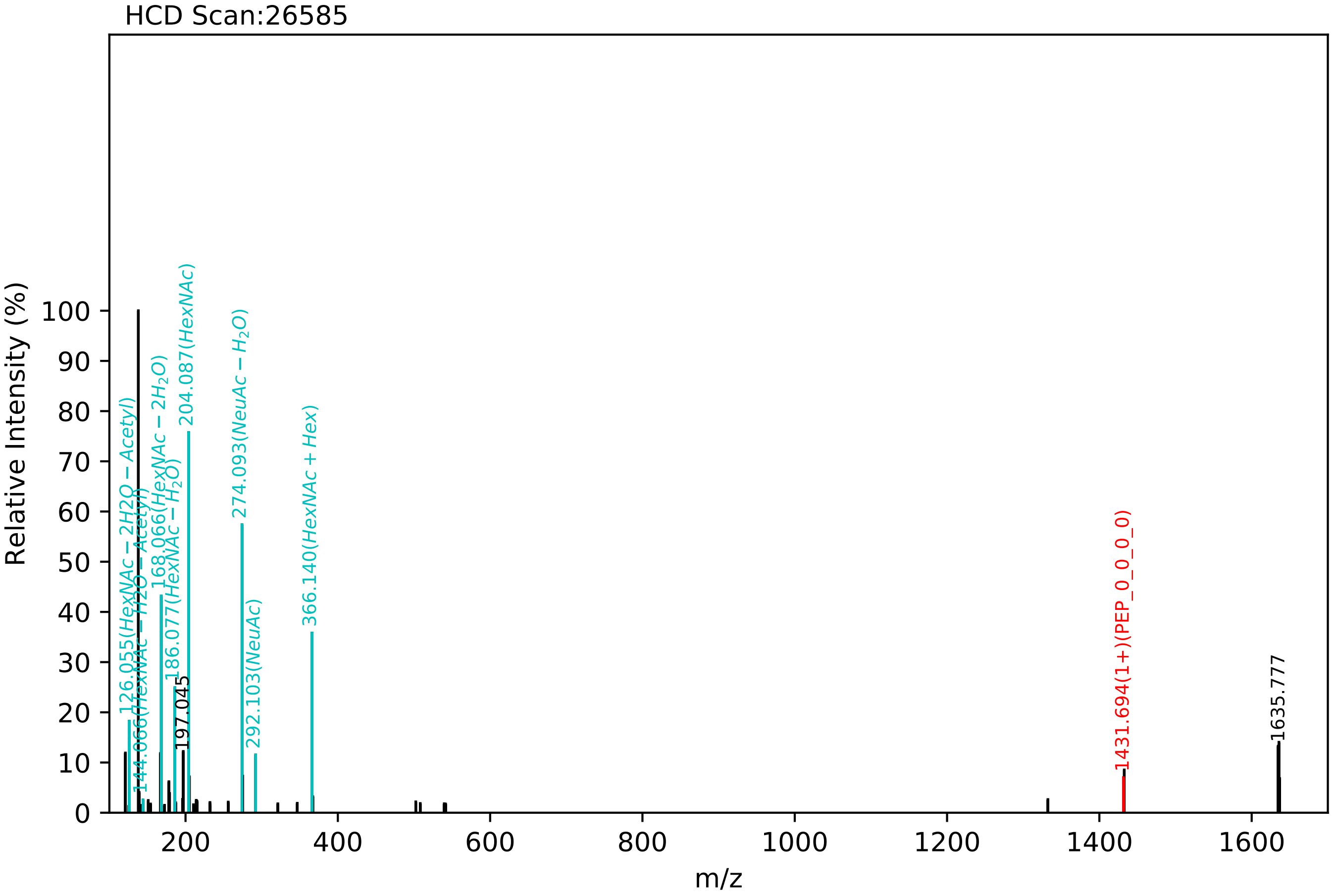

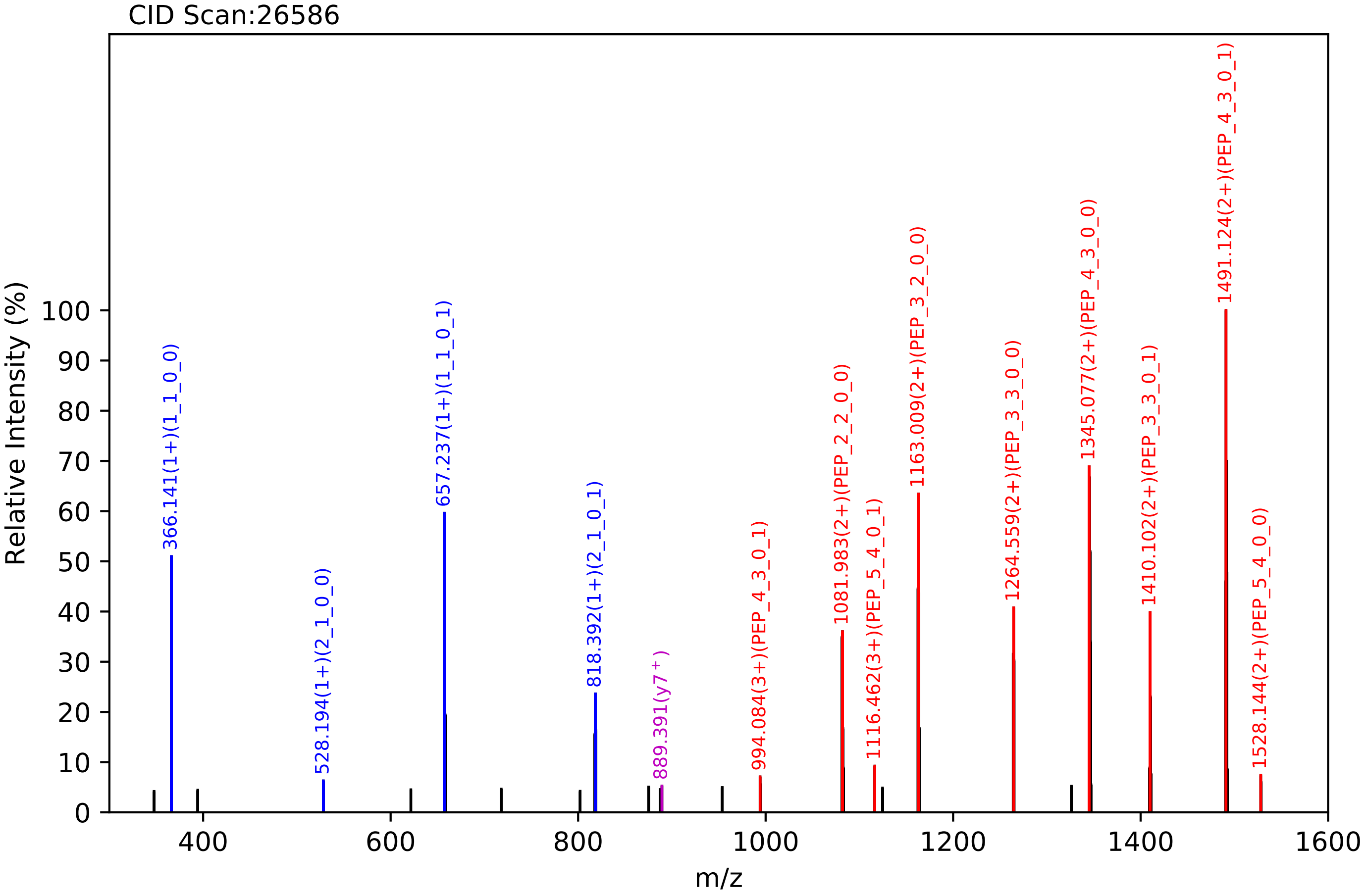

Unknown set no. 596, Experiment: Human Plasma exp_3

LNAENNATFYFK(=PEP)_6_5_1_3, m/z:1110.44(4+), RT:103.47, Y-score:81.39

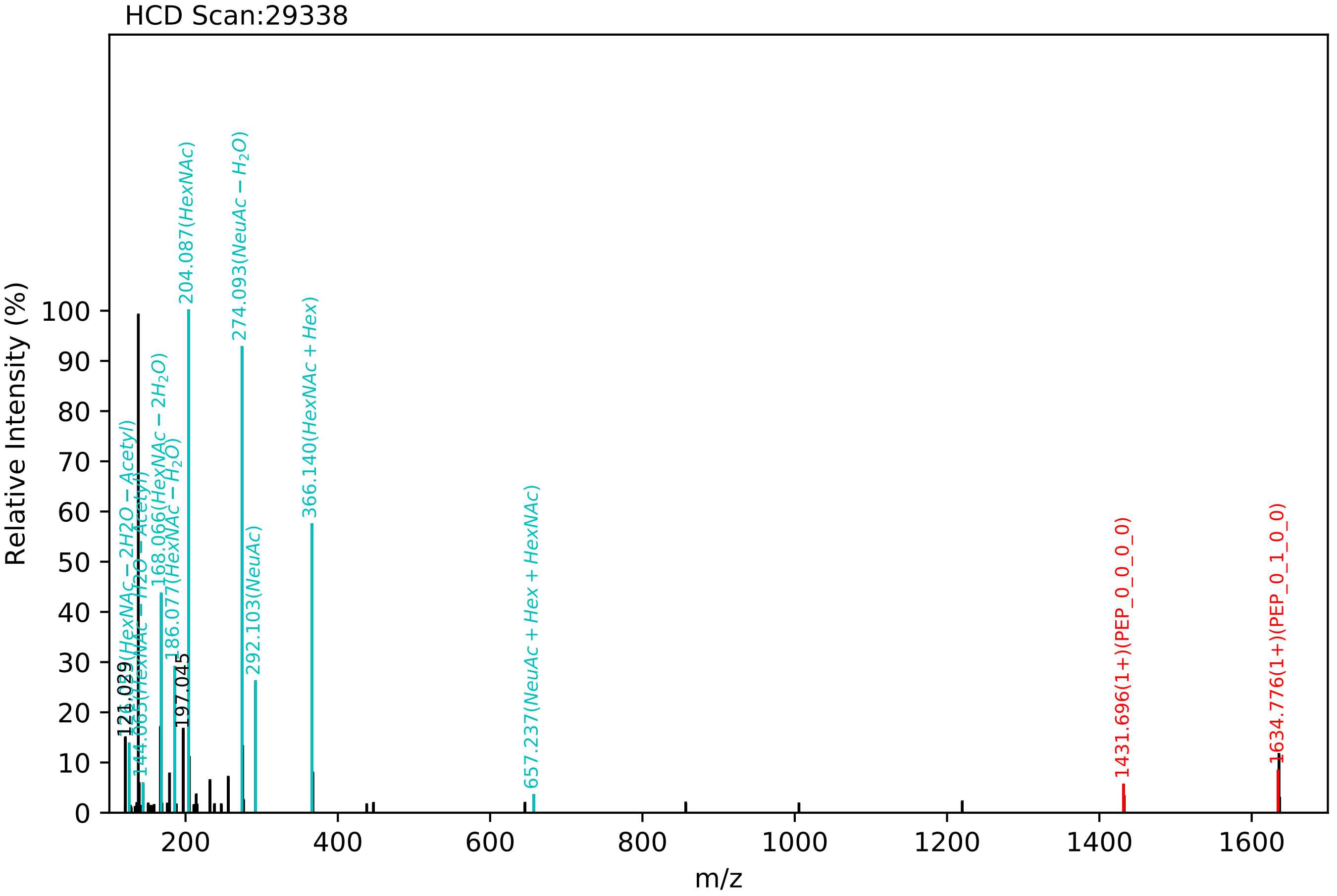

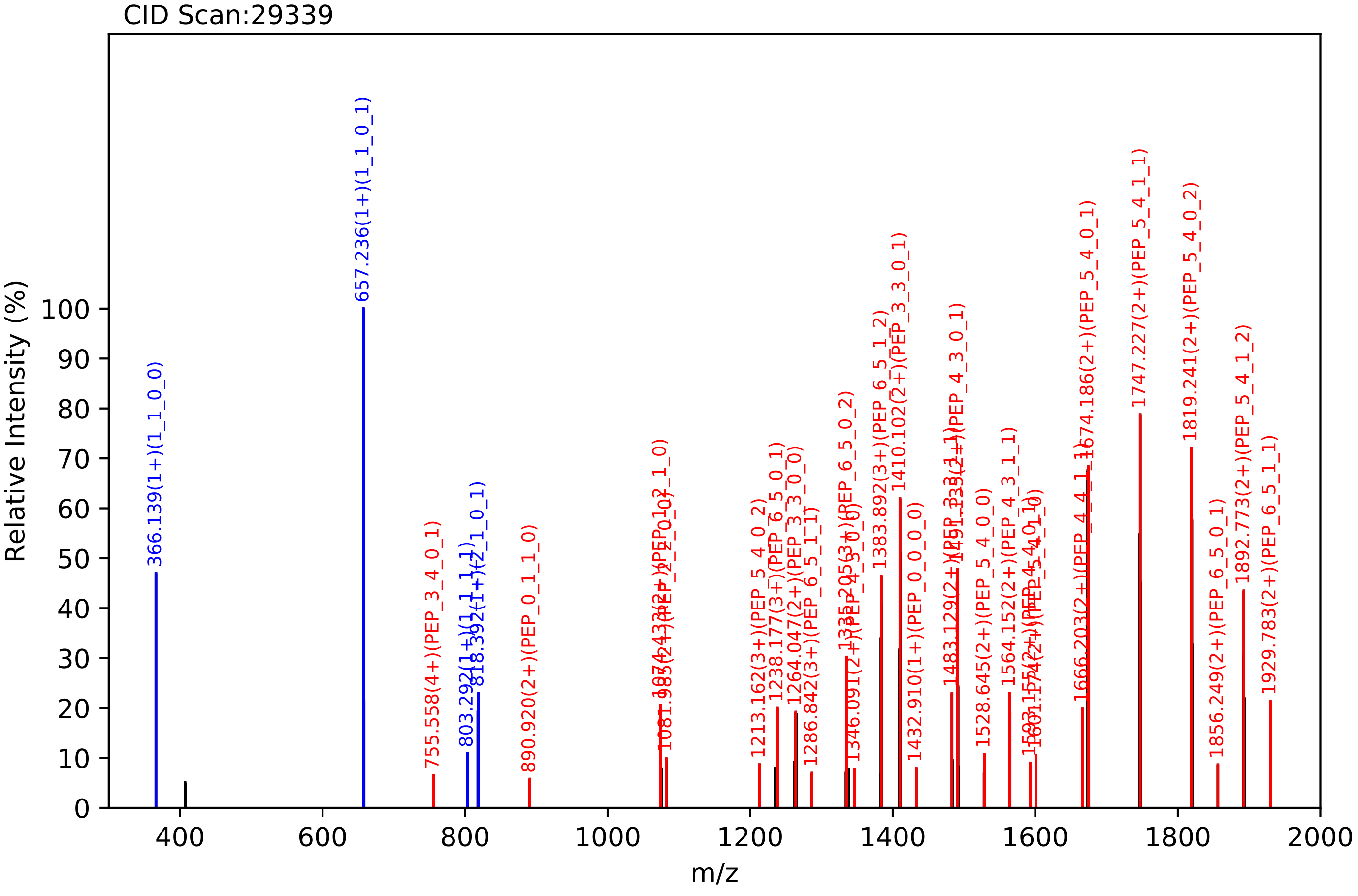

Unknown set no. 597, Experiment: Human Plasma exp_2

YNSQNQSNNQFVLYR(=PEP)_5_4_0_2, m/z:1020.67(4+), RT:86.26, Y-score:89.58

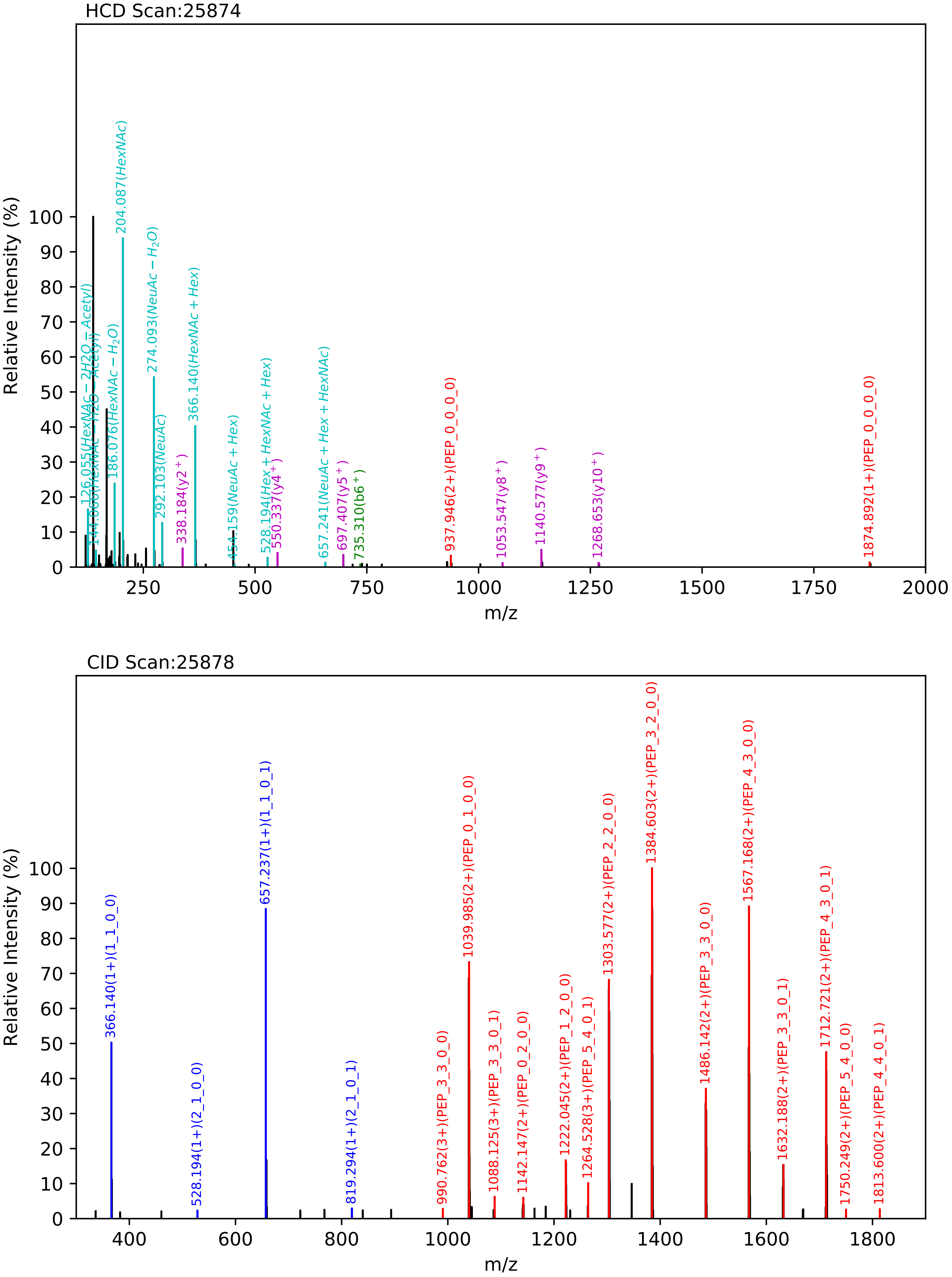

Unknown set no. 598, Experiment: Human Plasma exp_4

YNSQNQSNNQFVLYR(=PEP)_5_4_0_2, m/z:1020.67(4+), RT:86.42, Y-score:84.75

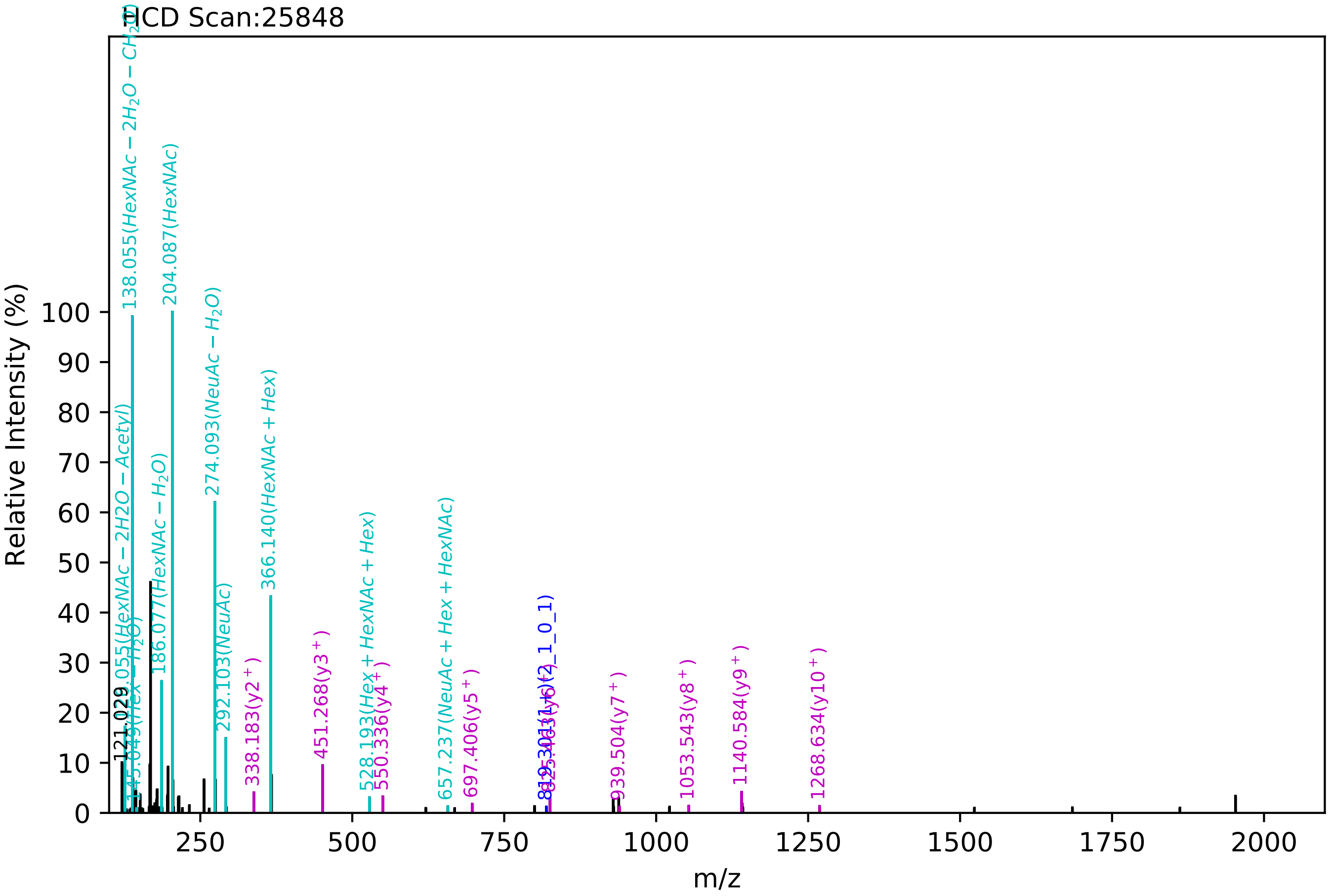

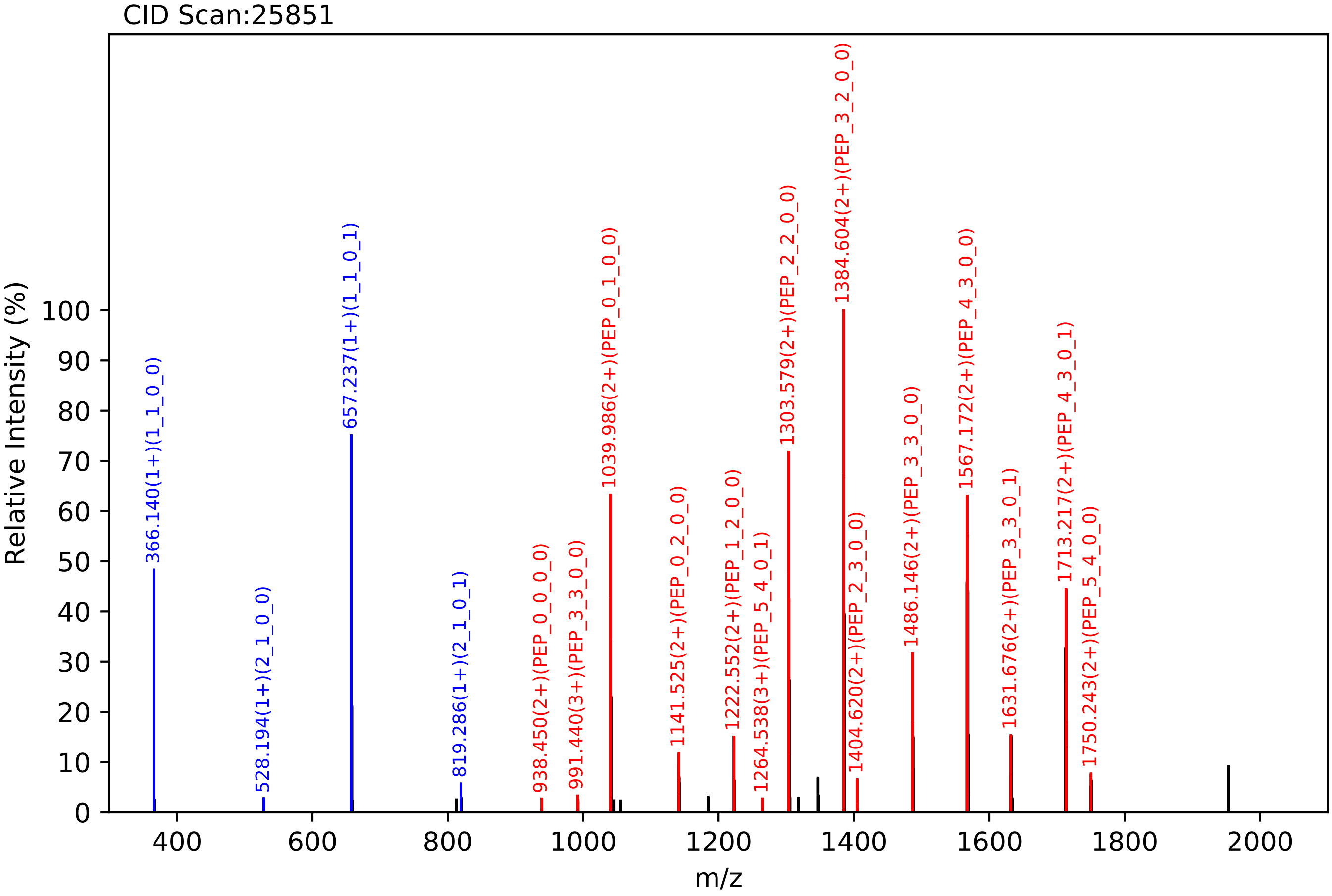

Unknown set no. 599, Experiment: Human Plasma exp_5

YNSQNQSNNQFVLYR(=PEP)_5_4_0_2, m/z:1020.67(4+), RT:86.27, Y-score:87.28

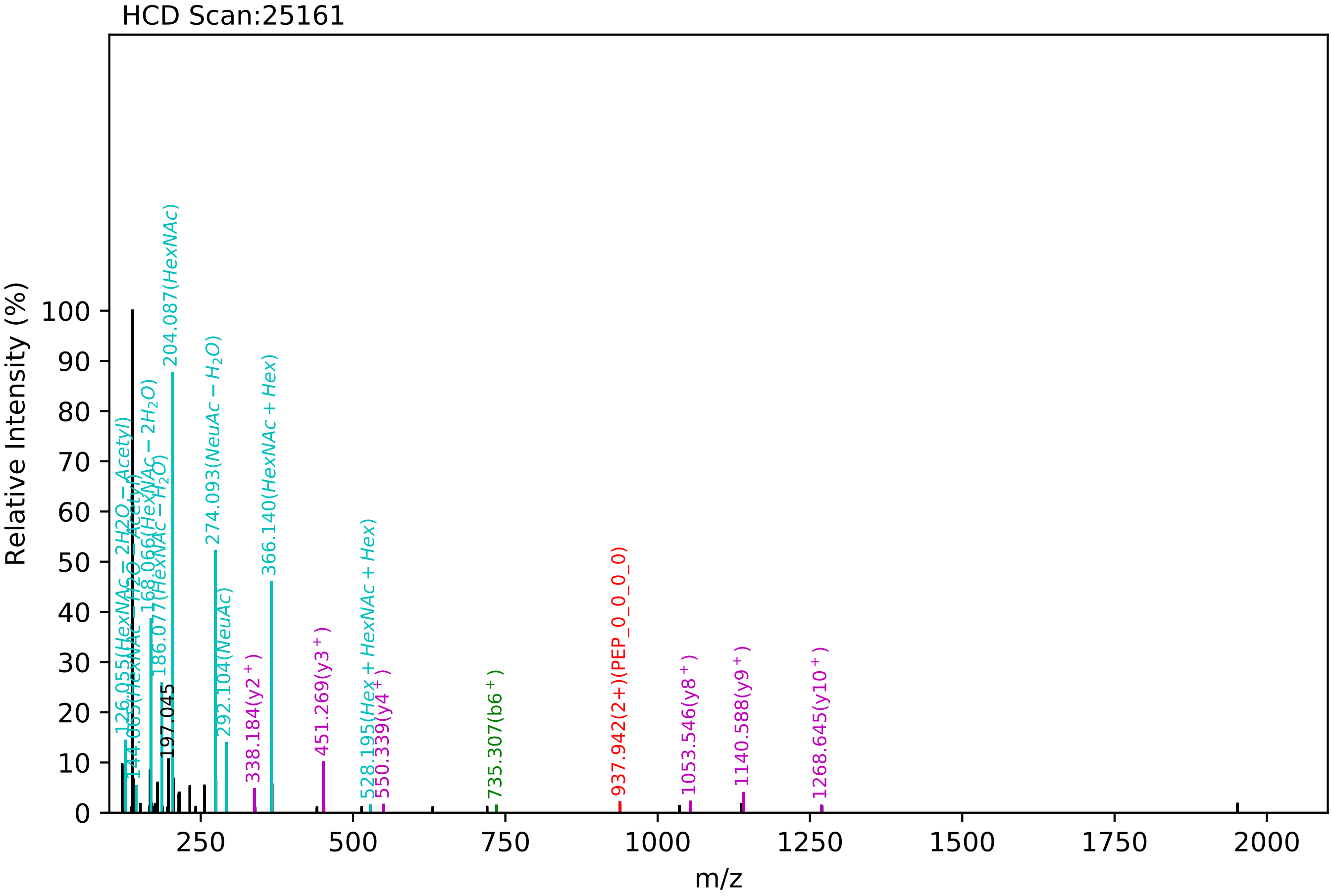

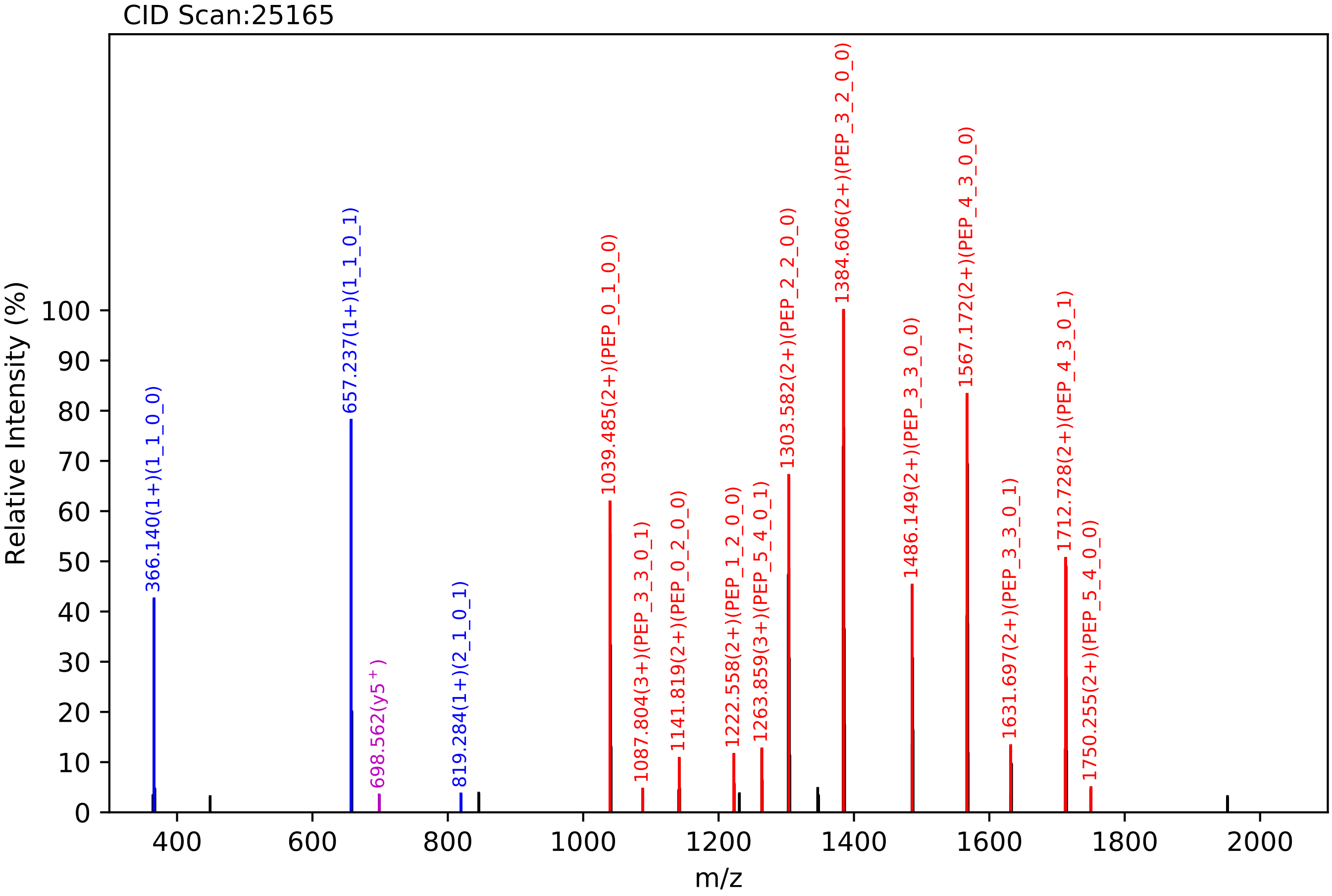

Unknown set no. 600, Experiment: Human Plasma exp_4

DNYTDLVAIQNK(=PEP)_5_4_0_2, m/z:1200.15(3+), RT:86.38, Y-score:92.36

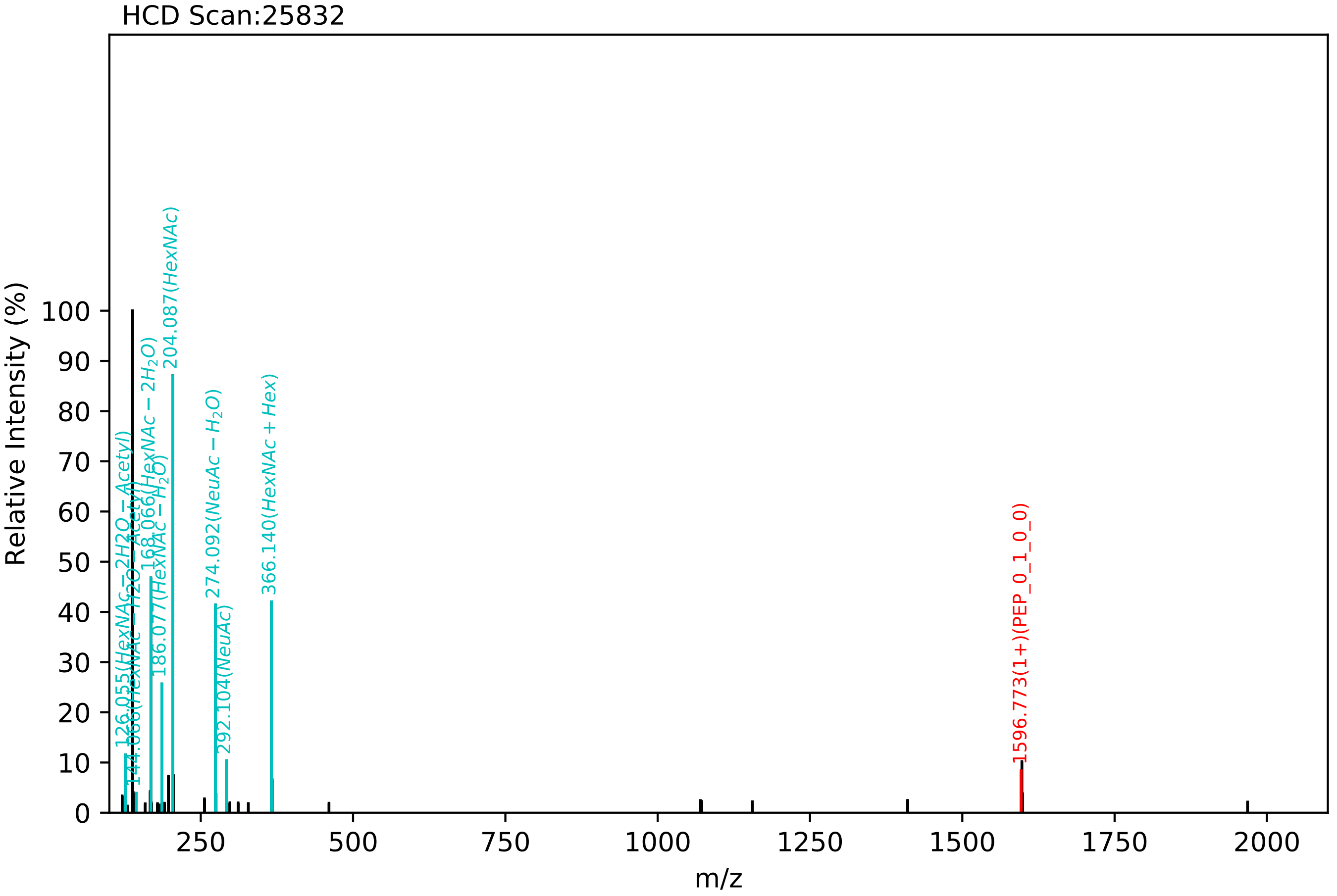

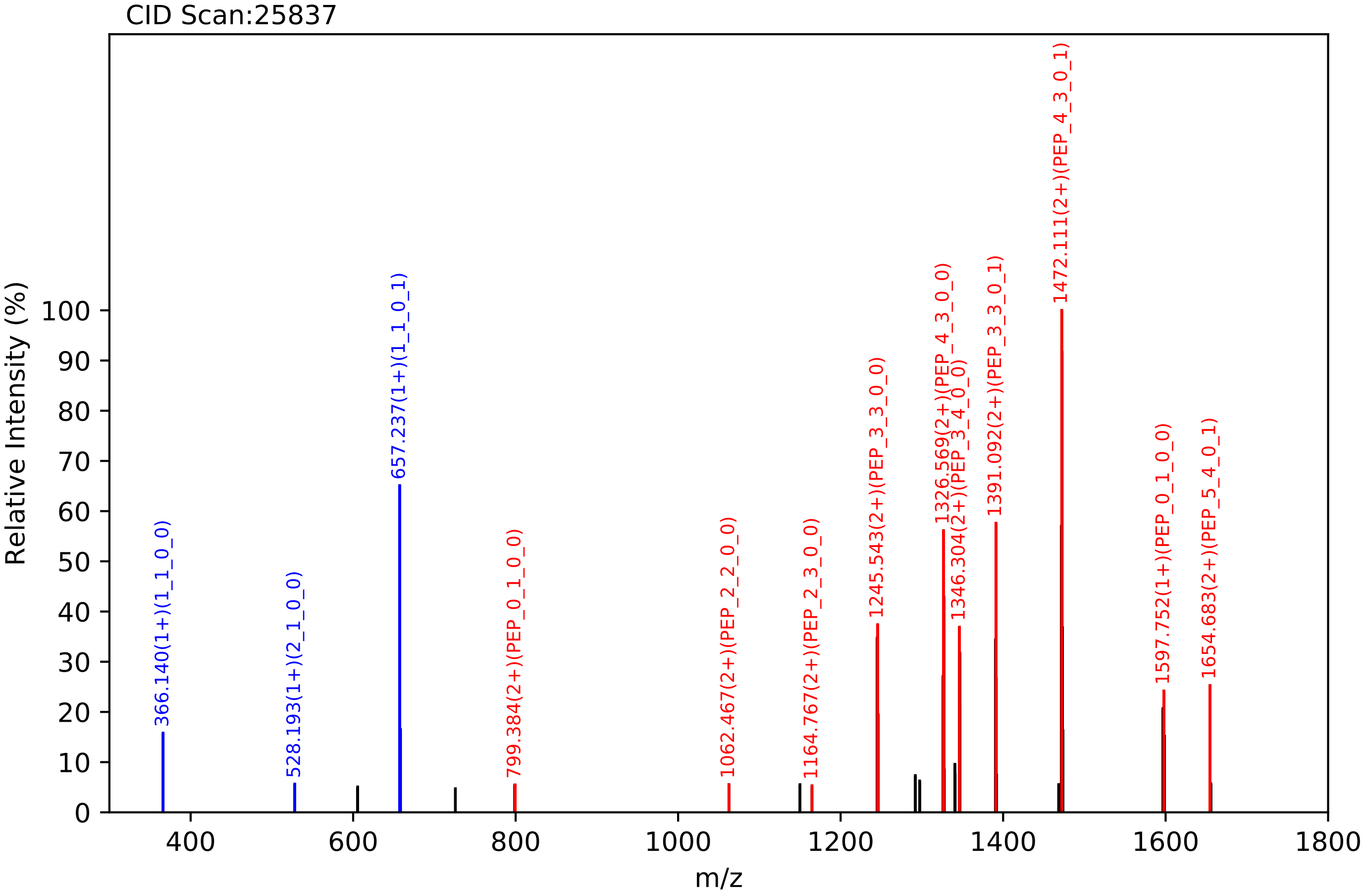

Unknown set no. 601, Experiment: Human Plasma exp_1

VVYNR(=PEP)_6_5_0_3, m/z:1171.13(3+), RT:49.93, Y-score:82.32

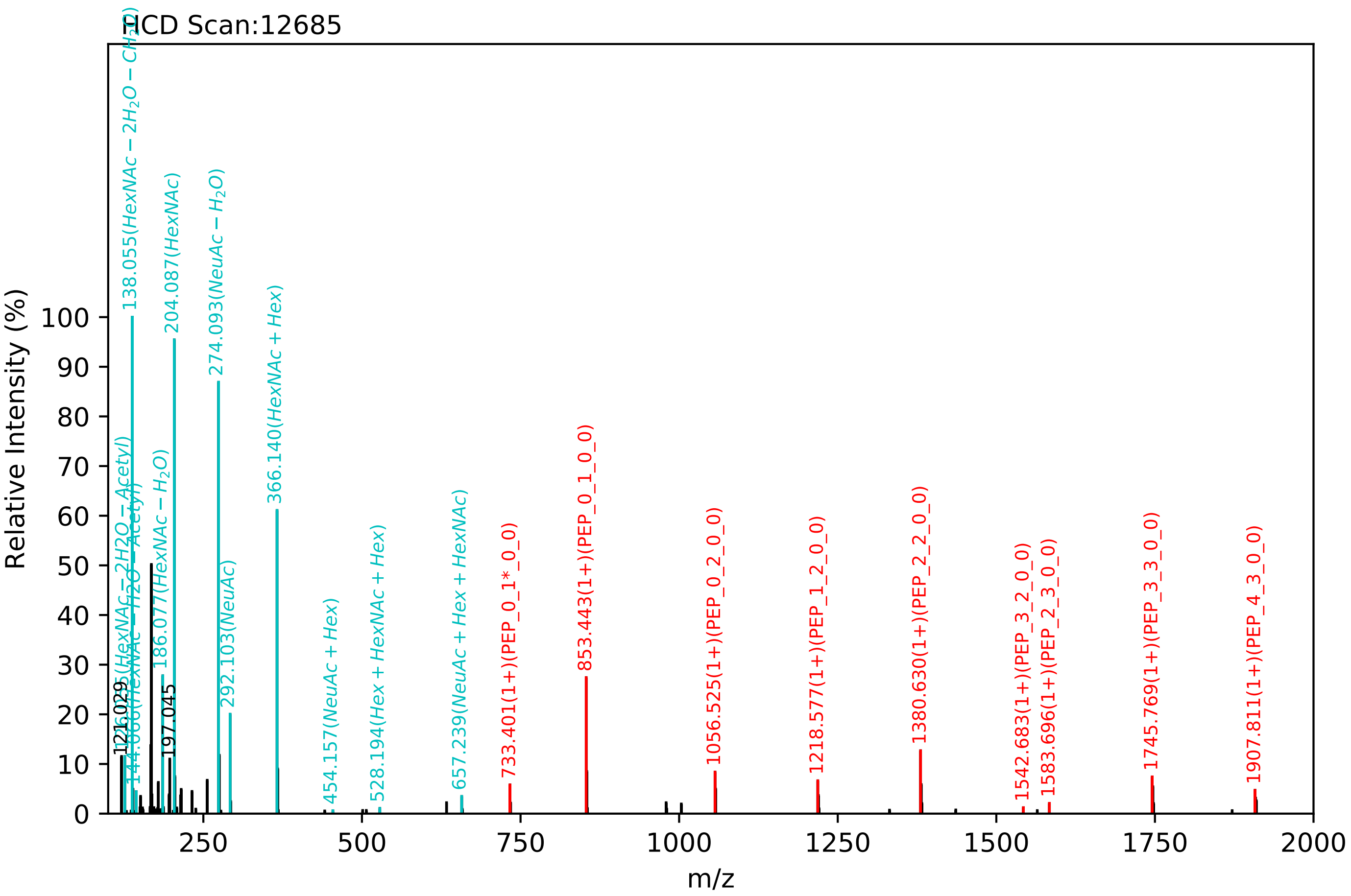

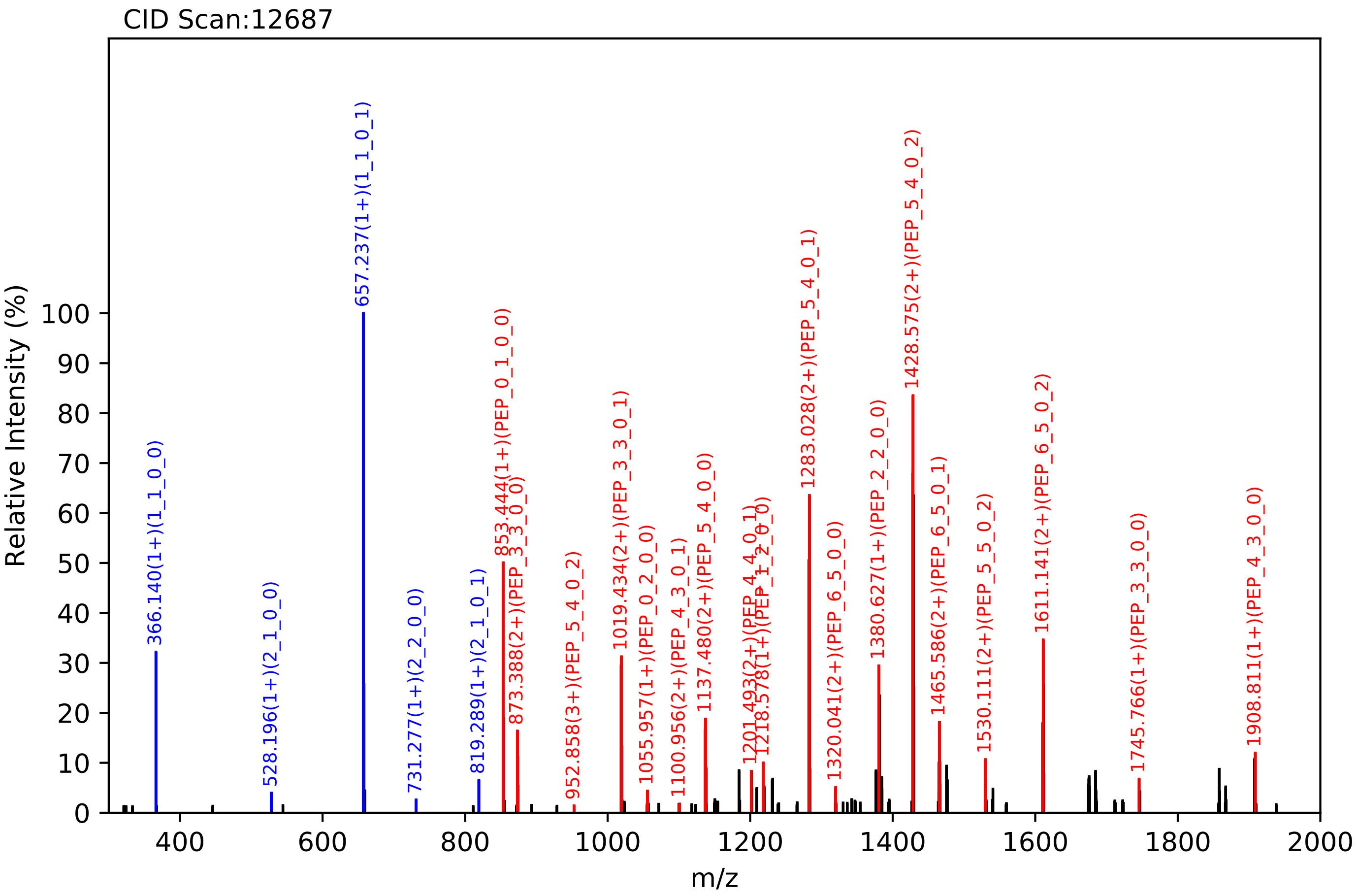

Unknown set no. 602, Experiment: Human Plasma exp_2

VVYNR(=PEP)_6_5_0_3, m/z:1171.13(3+), RT:49.92, Y-score:81.24

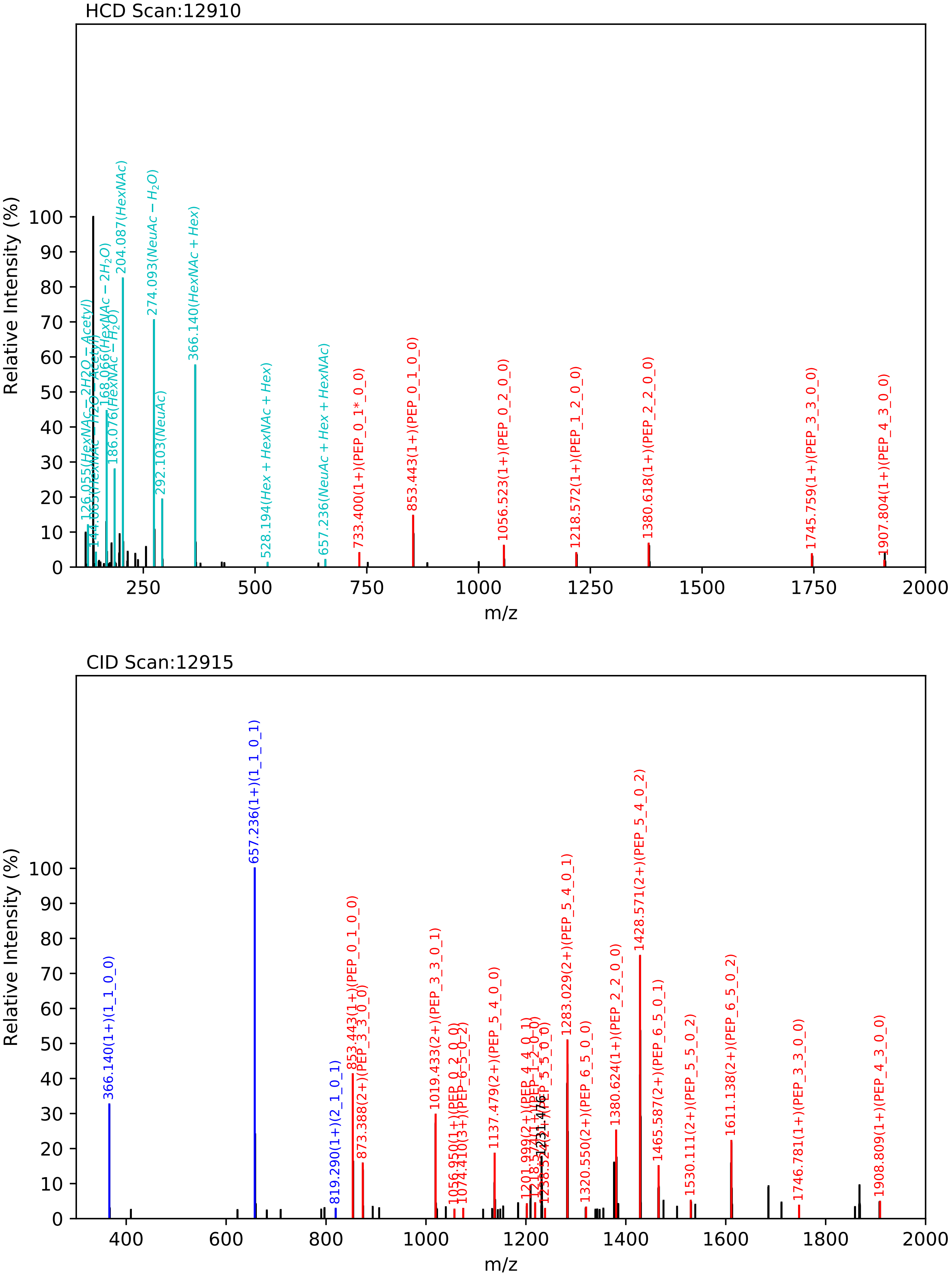

Unknown set no. 603, Experiment: Human Plasma exp_4

VVYNR(=PEP)_6_5_0_3, m/z:1171.13(3+), RT:50.15, Y-score:77.31

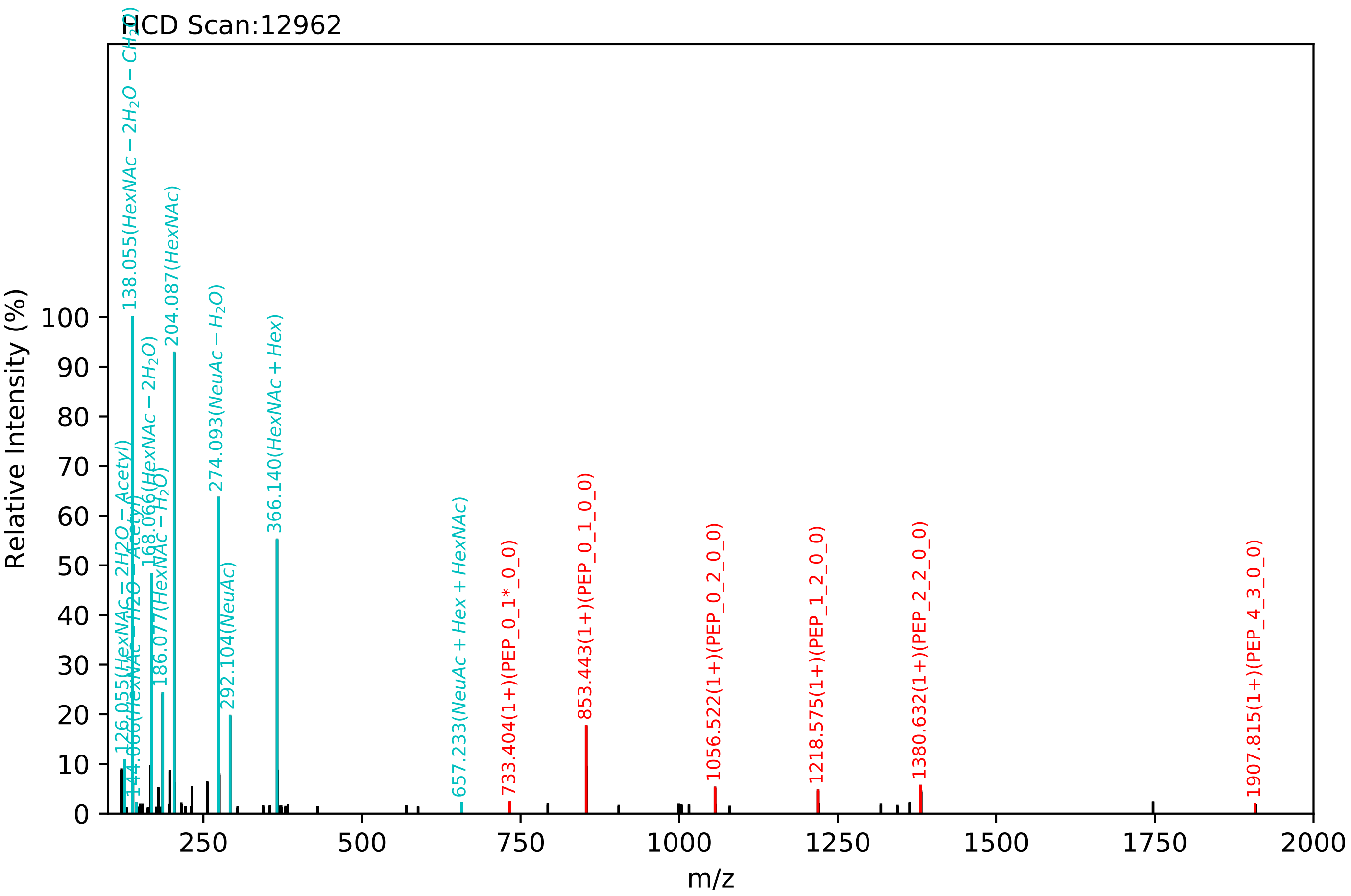

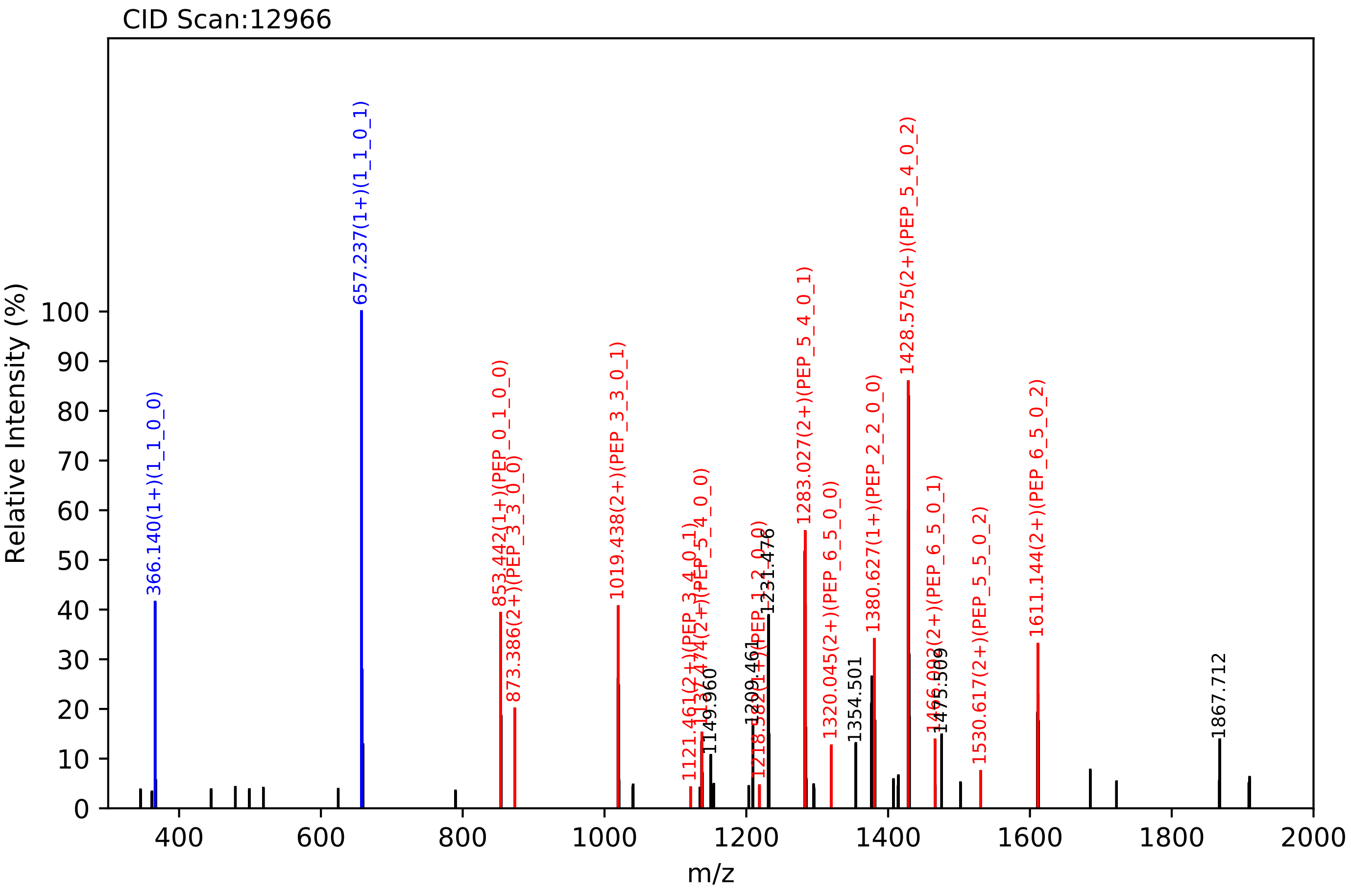

Unknown set no. 604, Experiment: Human Plasma exp_5

VVYNR(=PEP)_6_5_0_3, m/z:1171.13(3+), RT:50.27, Y-score:79.75

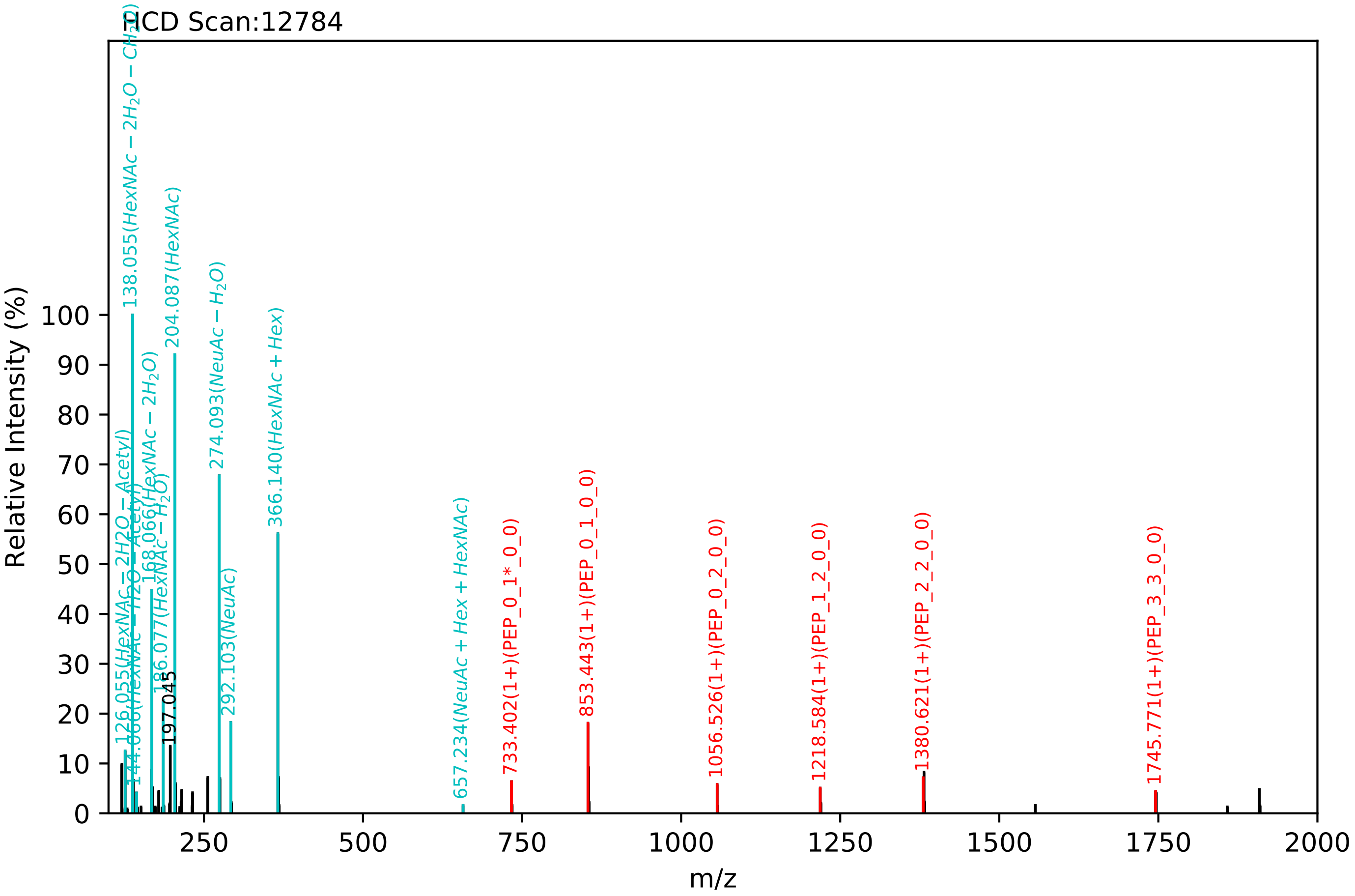

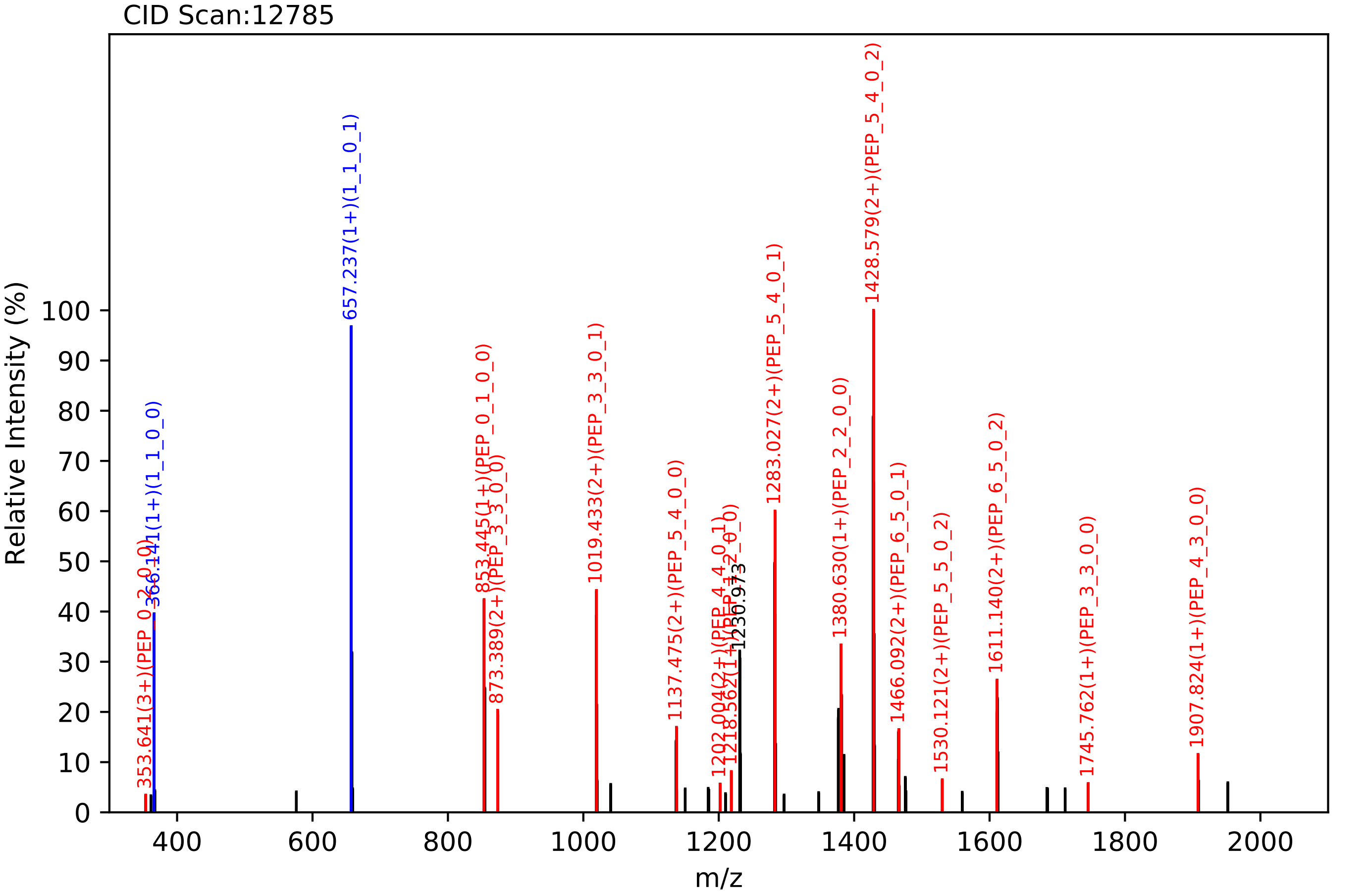

Unknown set no. 605, Experiment: Human Plasma exp_1

NWSLPNR(=PEP)_5_4_1_2, m/z:1079.77(3+), RT:79.06, Y-score:82.06

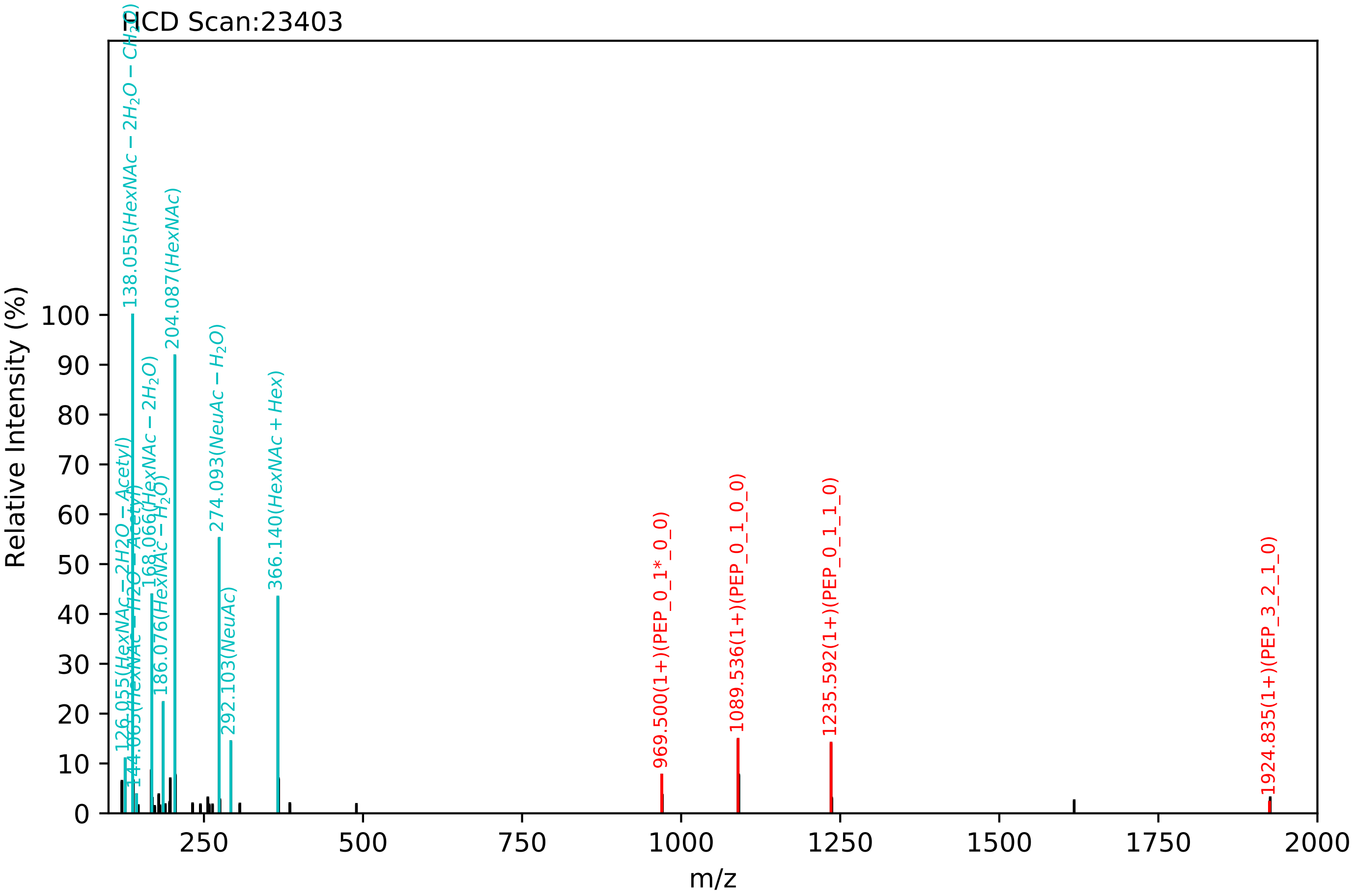

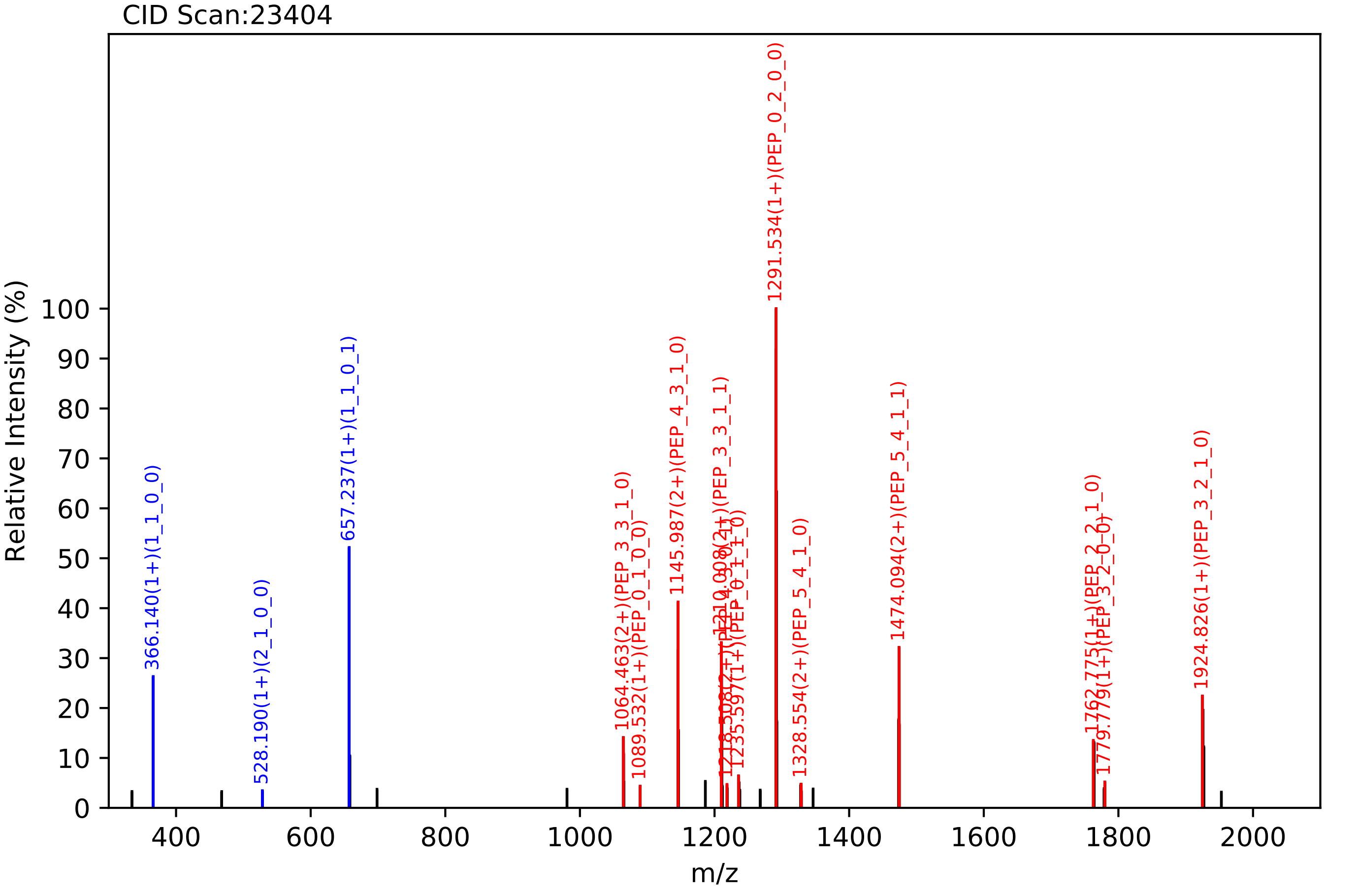

Unknown set no. 606, Experiment: Human Plasma exp_5

NWSLPNR(=PEP)_5_4_1_2, m/z:1079.77(3+), RT:79.02, Y-score:88.57

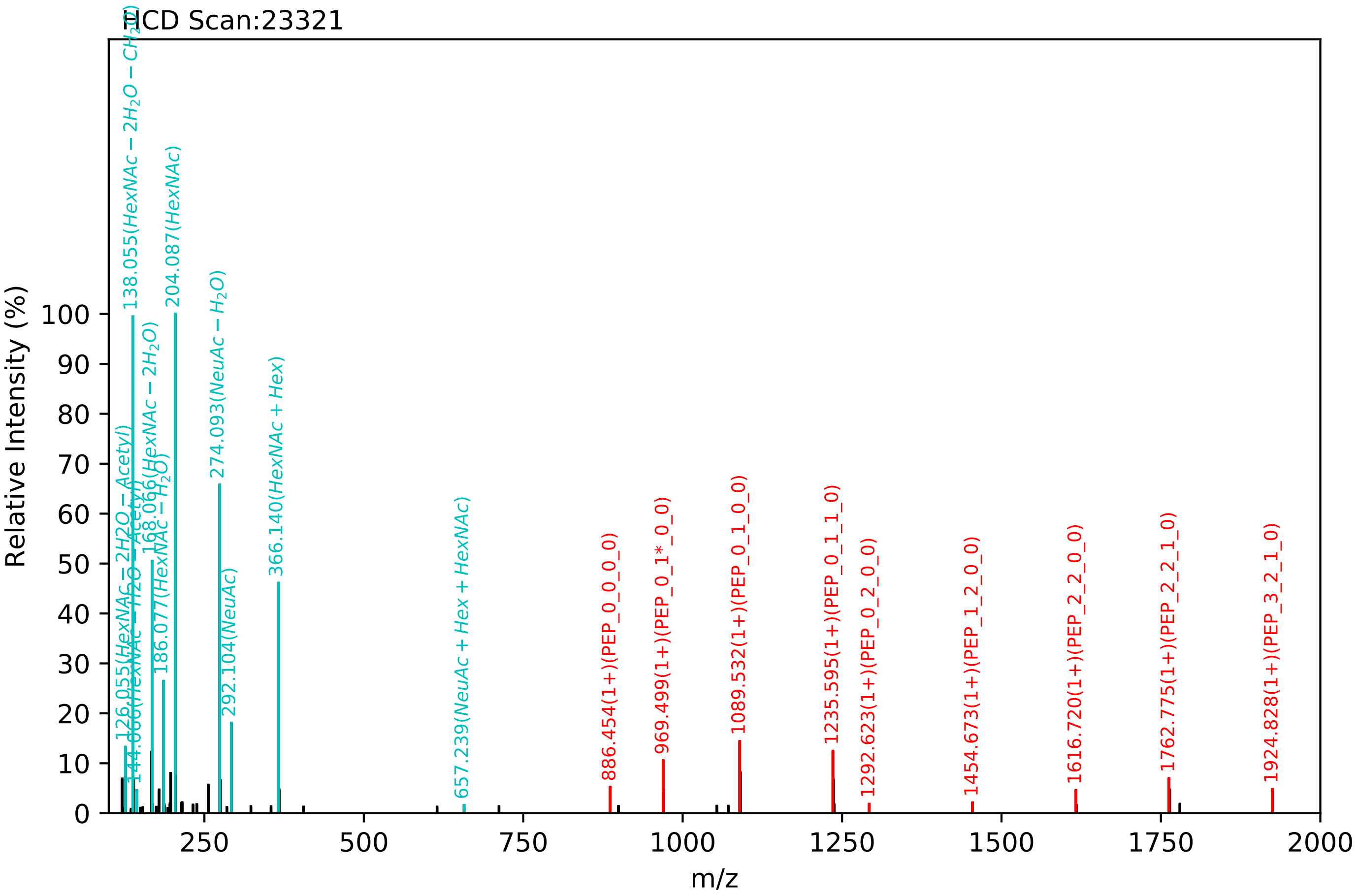

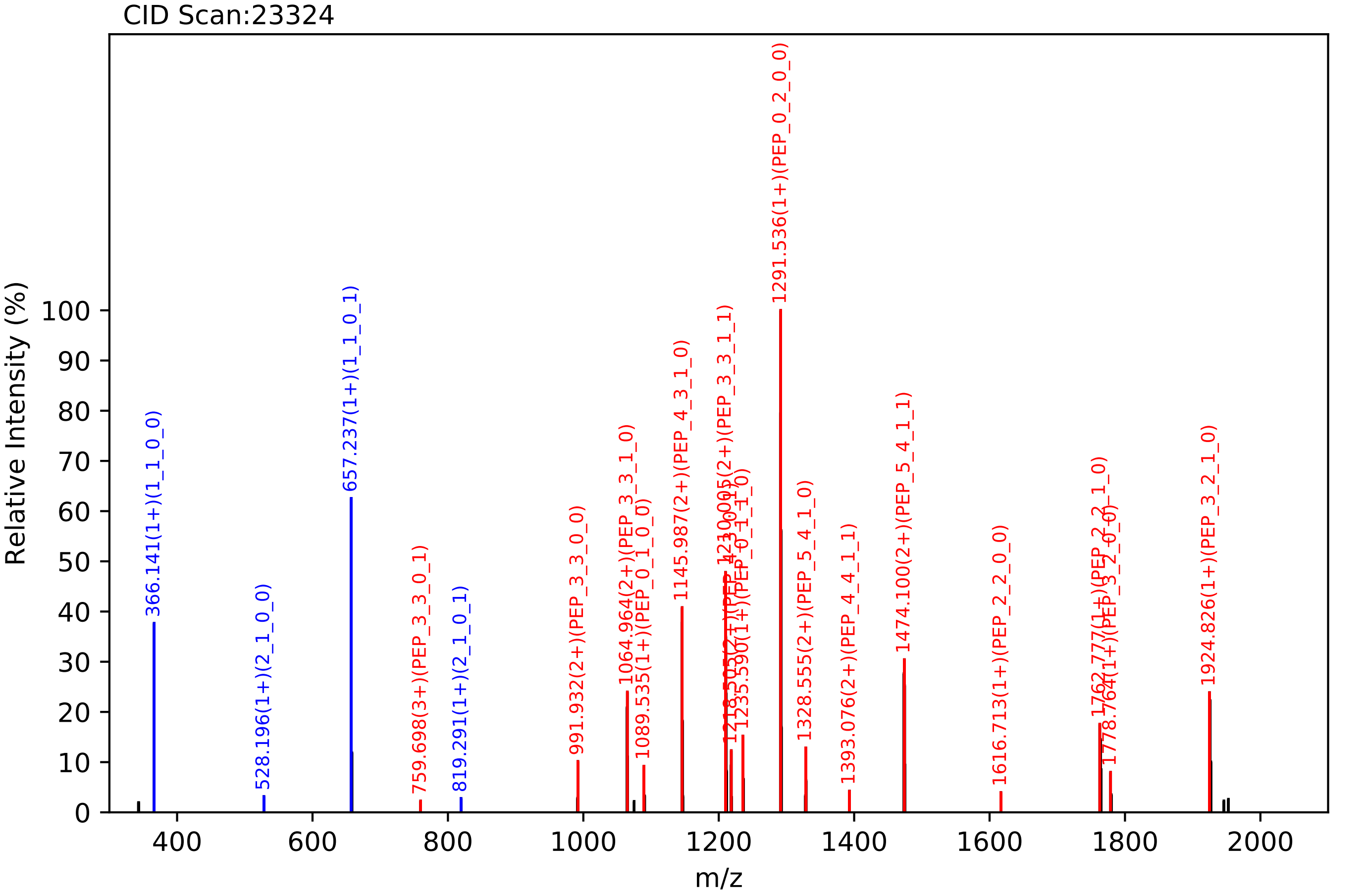

Unknown set no. 607, Experiment: Human Plasma exp_2

GVNFNVSK(=PEP)_5_4_0_2, m/z:1535.12(2+), RT:68.09, Y-score:83.71

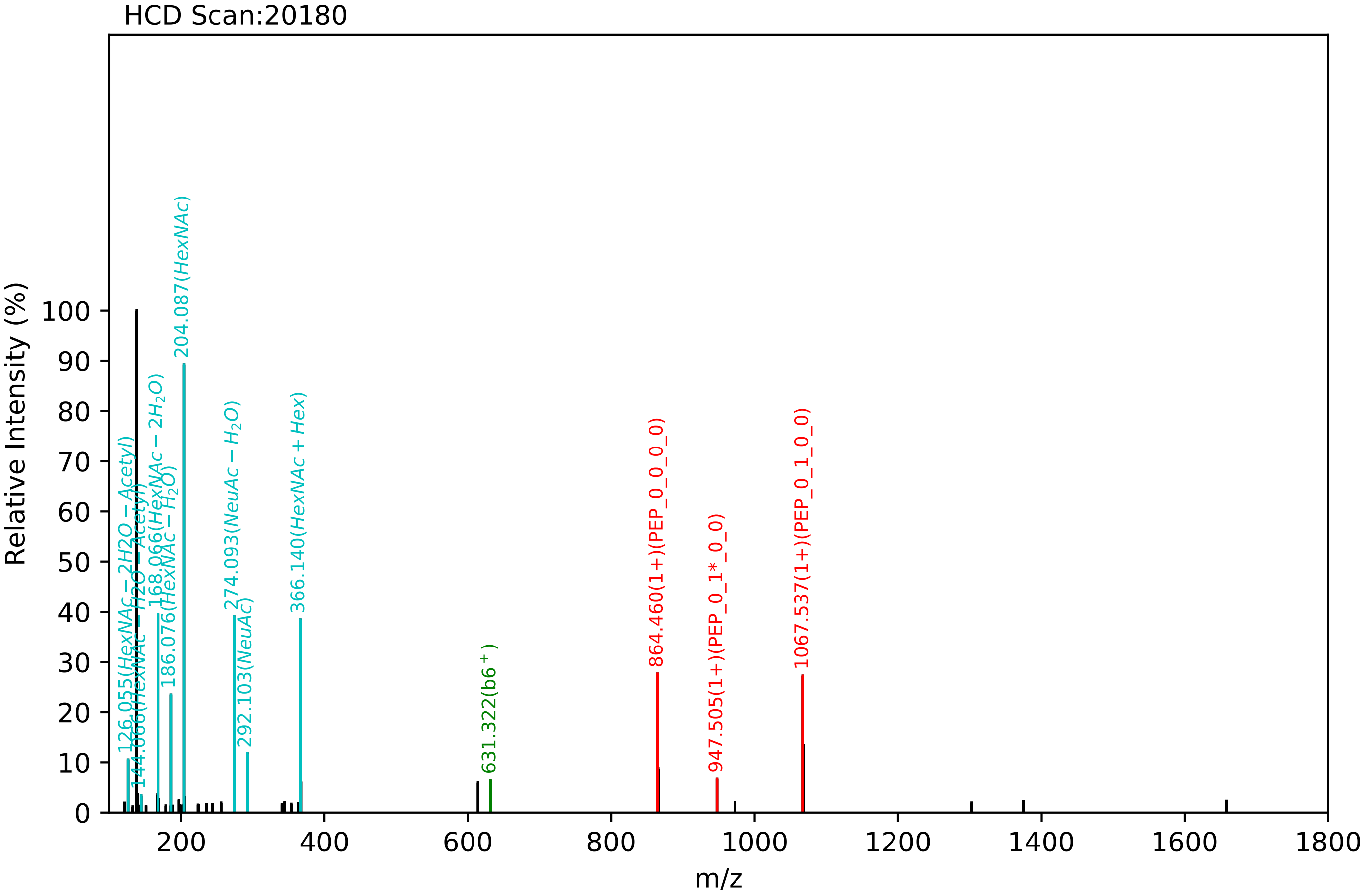

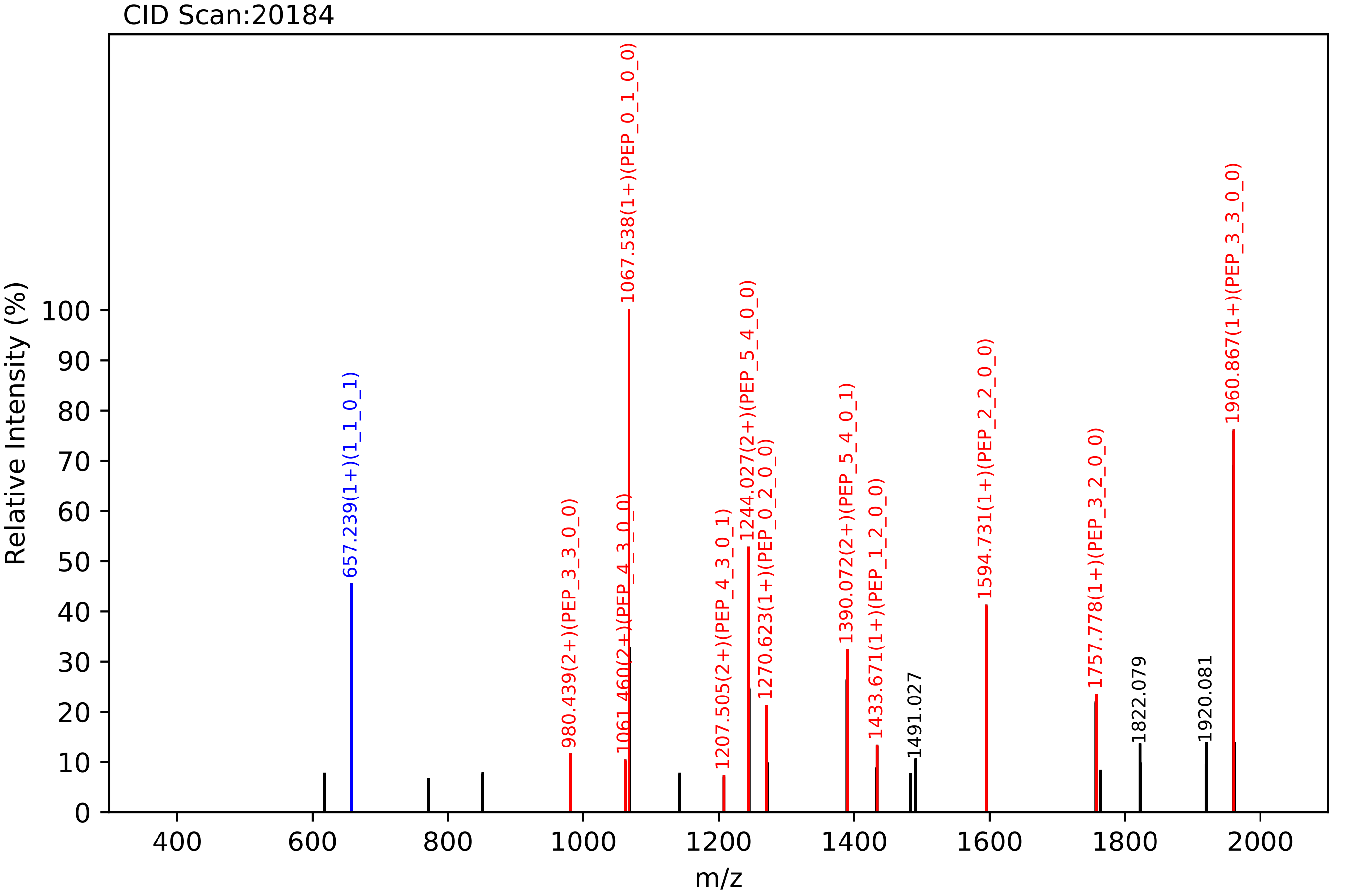

Unknown set no. 608, Experiment: Human Plasma exp_5

GVNFNVSK(=PEP)_5_4_0_2, m/z:1023.75(3+), RT:67.94, Y-score:83.46

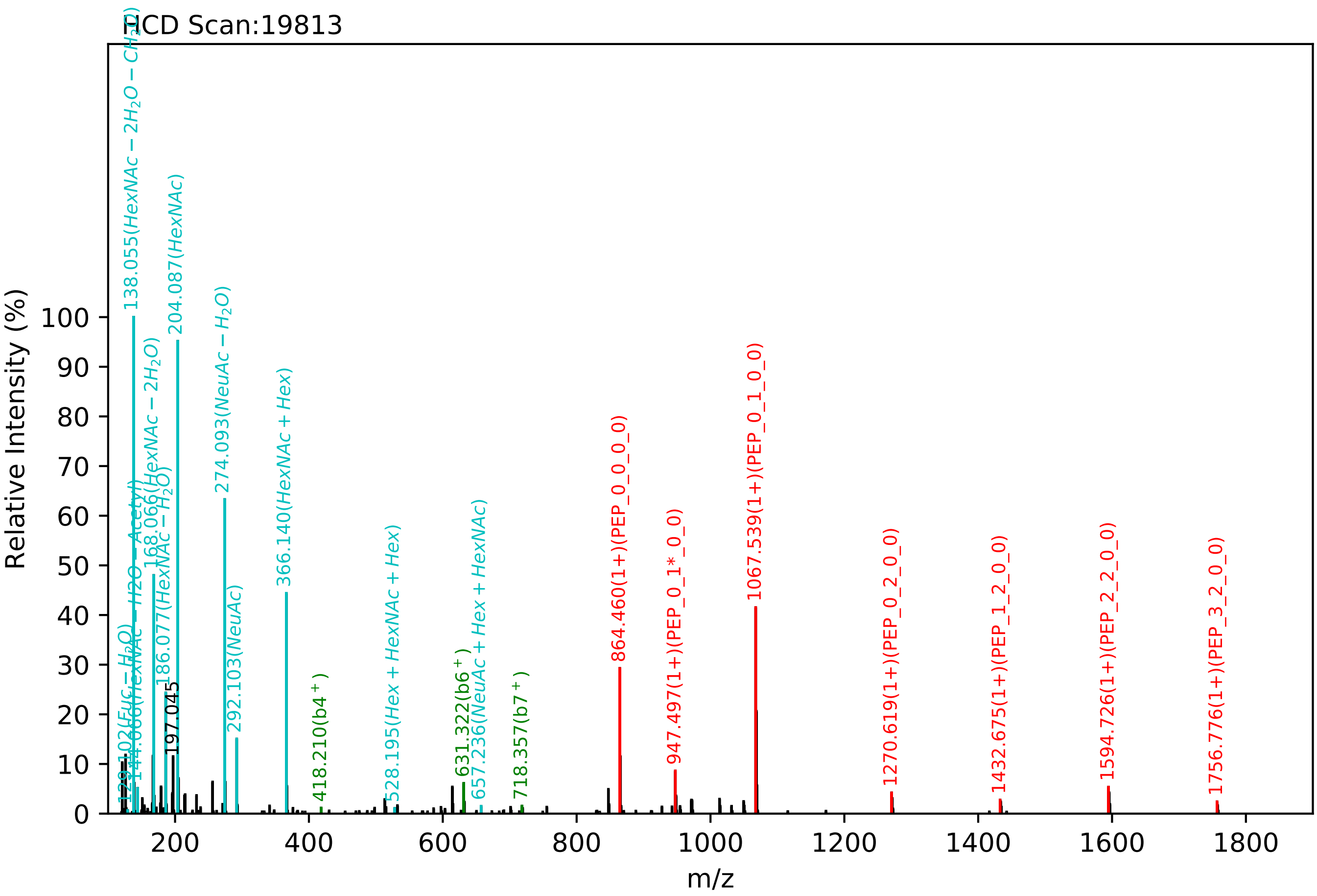

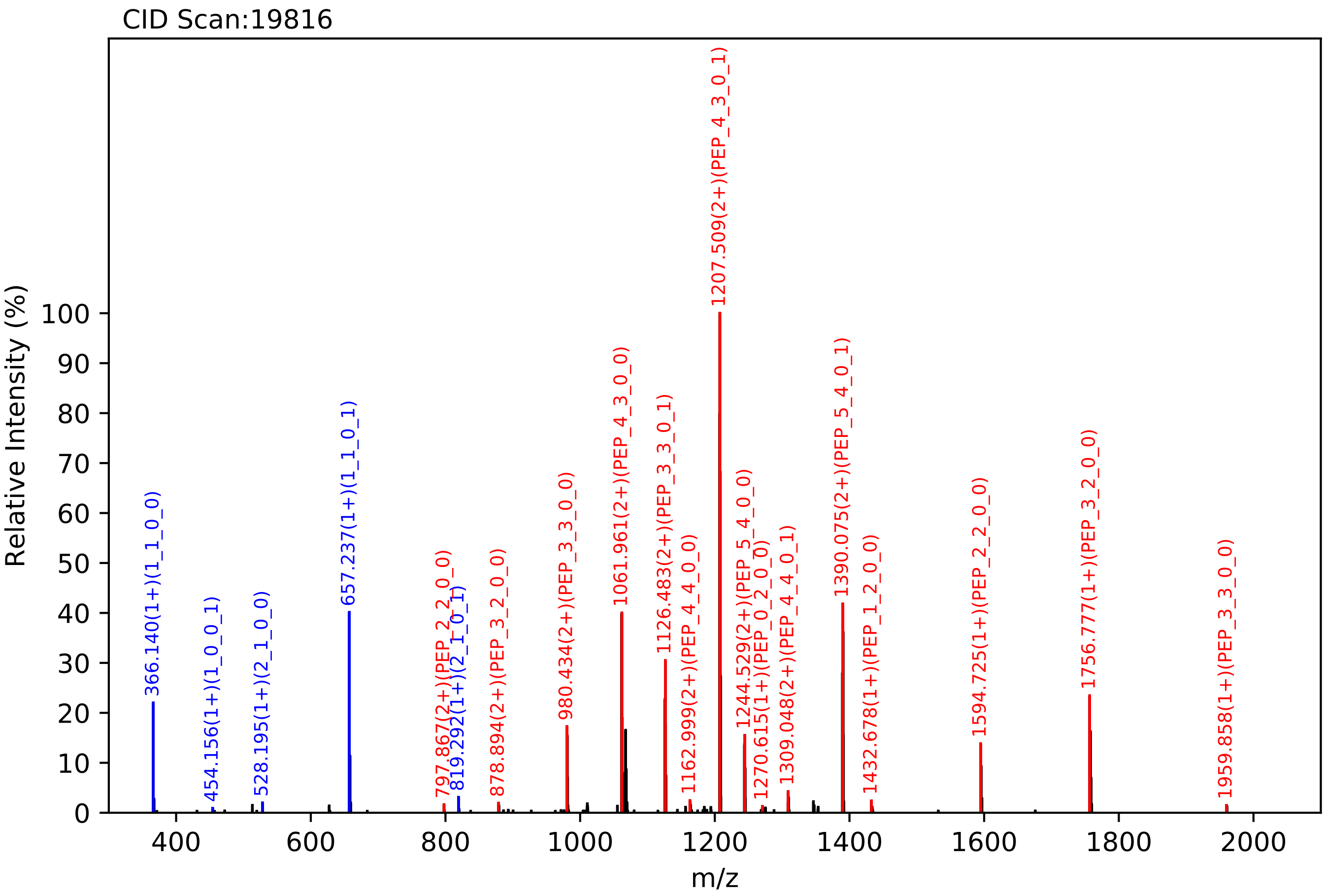

Unknown set no. 609, Experiment: Human Plasma exp_1

NLSCR(=PEP)_5_4_0_2, m/z:952.03(3+), RT:22.84, Y-score:82.37

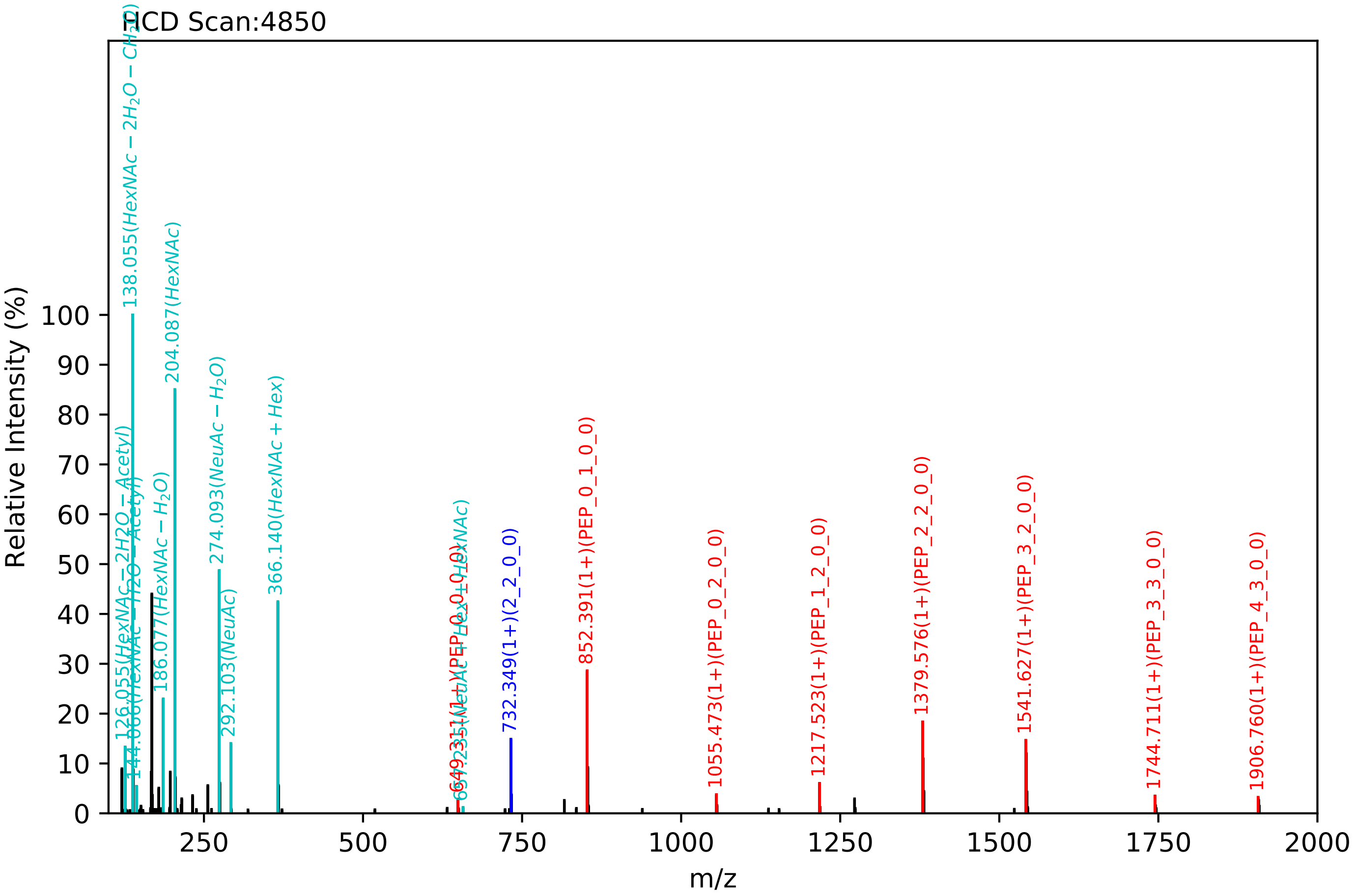

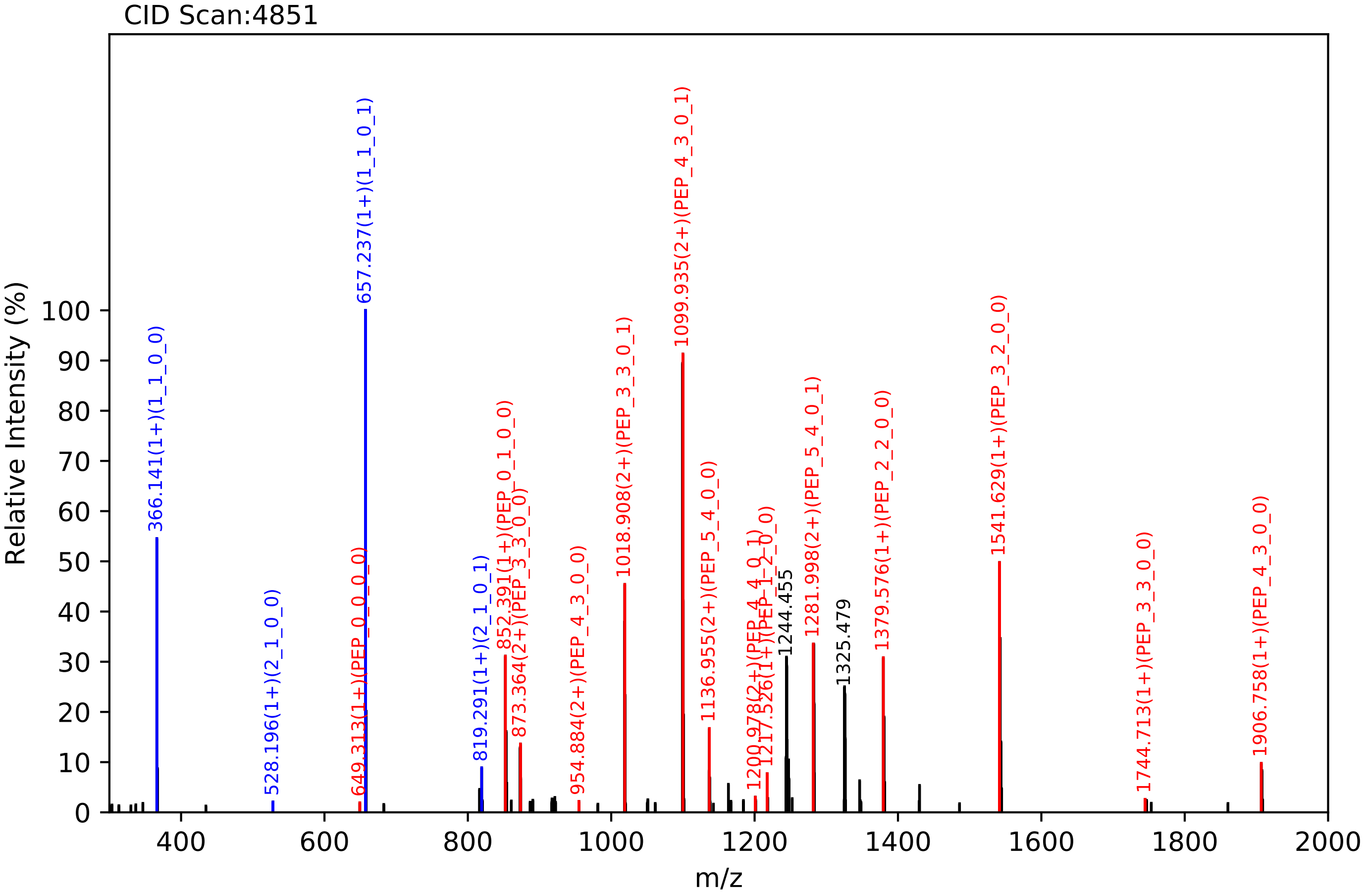

Unknown set no. 610, Experiment: Human Plasma exp_2

NLSCR(=PEP)_5_4_0_2, m/z:952.04(3+), RT:23.00, Y-score:79.39

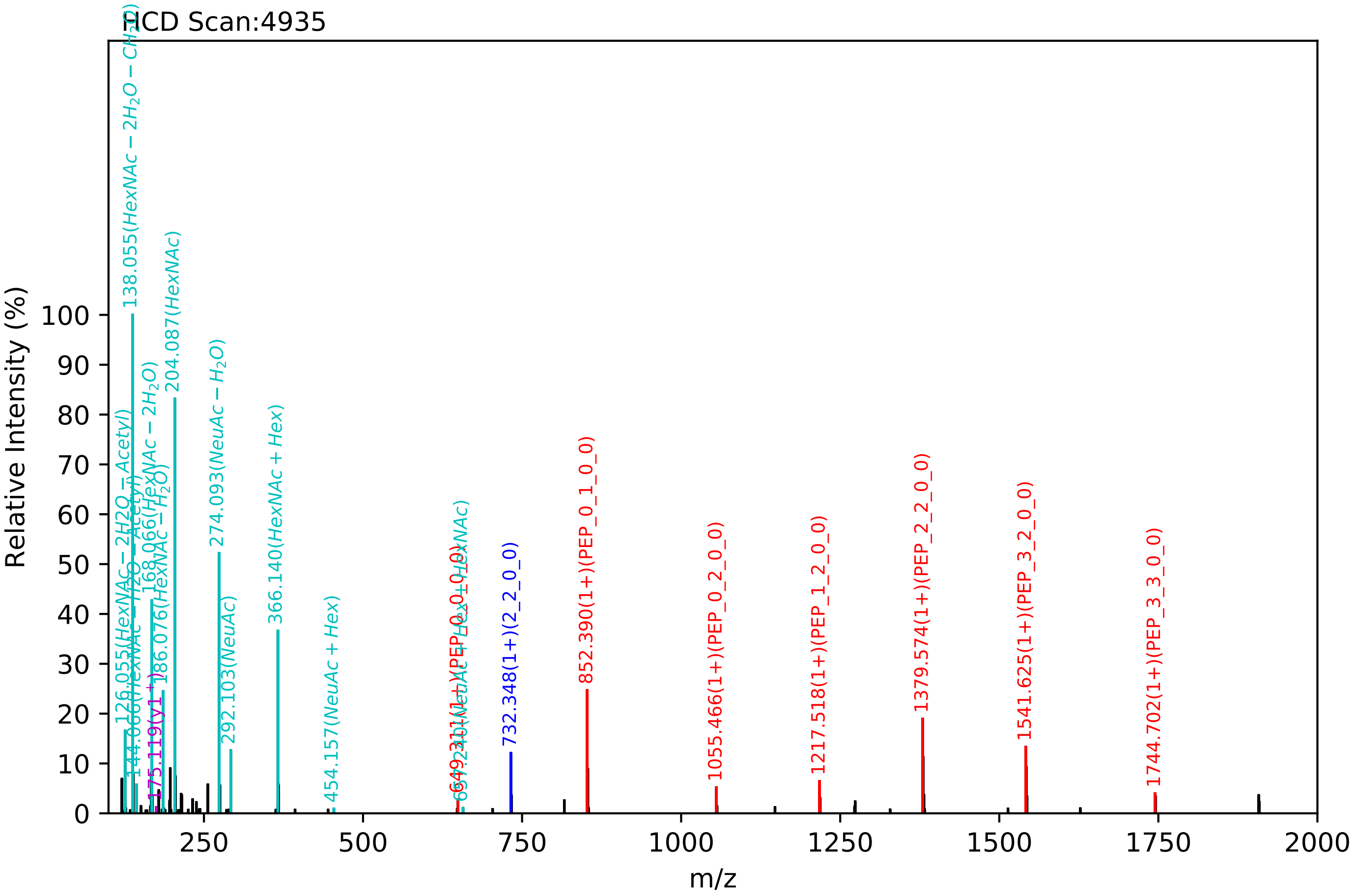

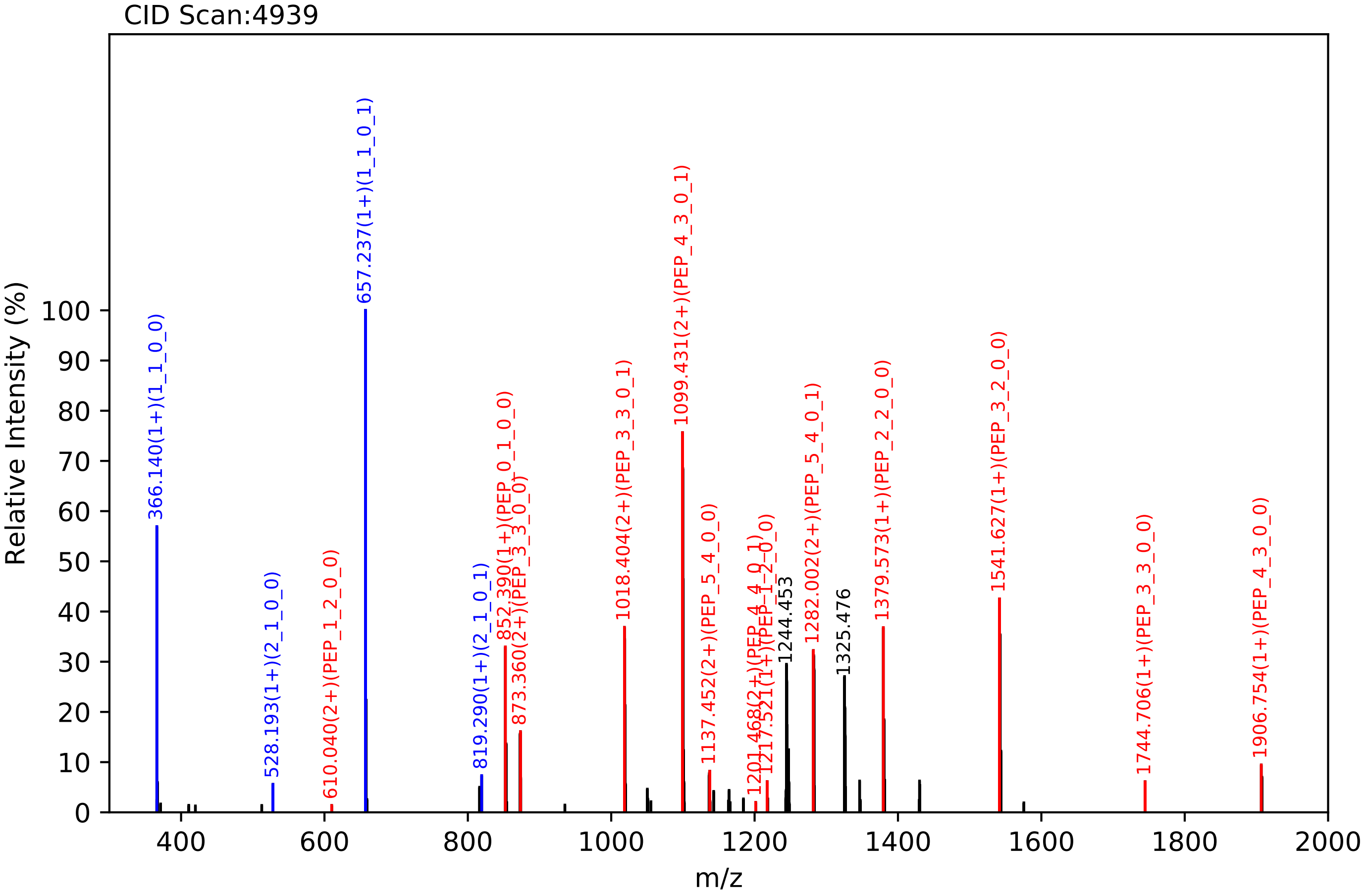

Unknown set no. 611, Experiment: Human Plasma exp_4

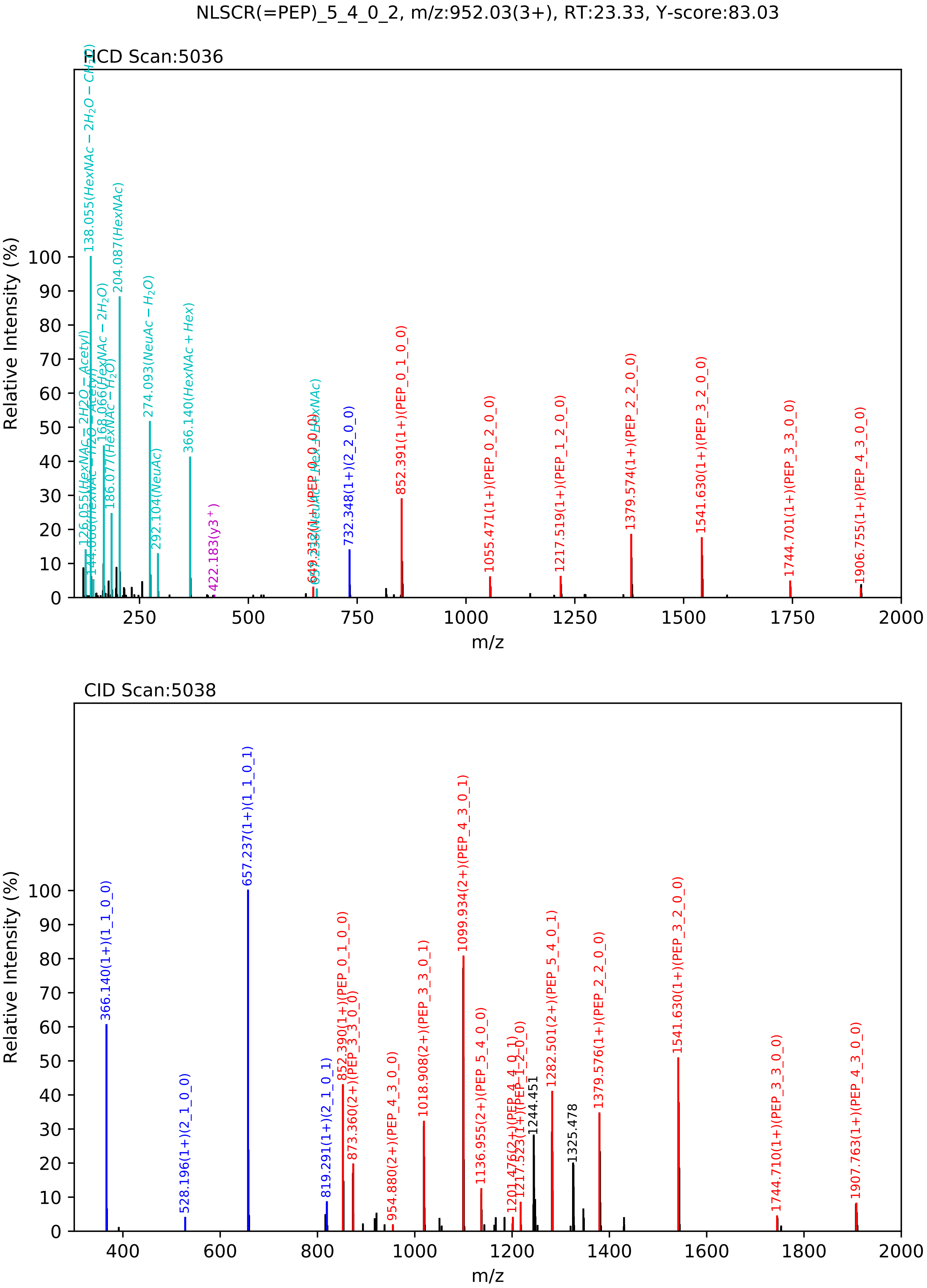

Unknown set no. 612, Experiment: Human Plasma exp_5

NLSCR(=PEP)_5_4_0_2, m/z:952.03(3+), RT:23.23, Y-score:81.71

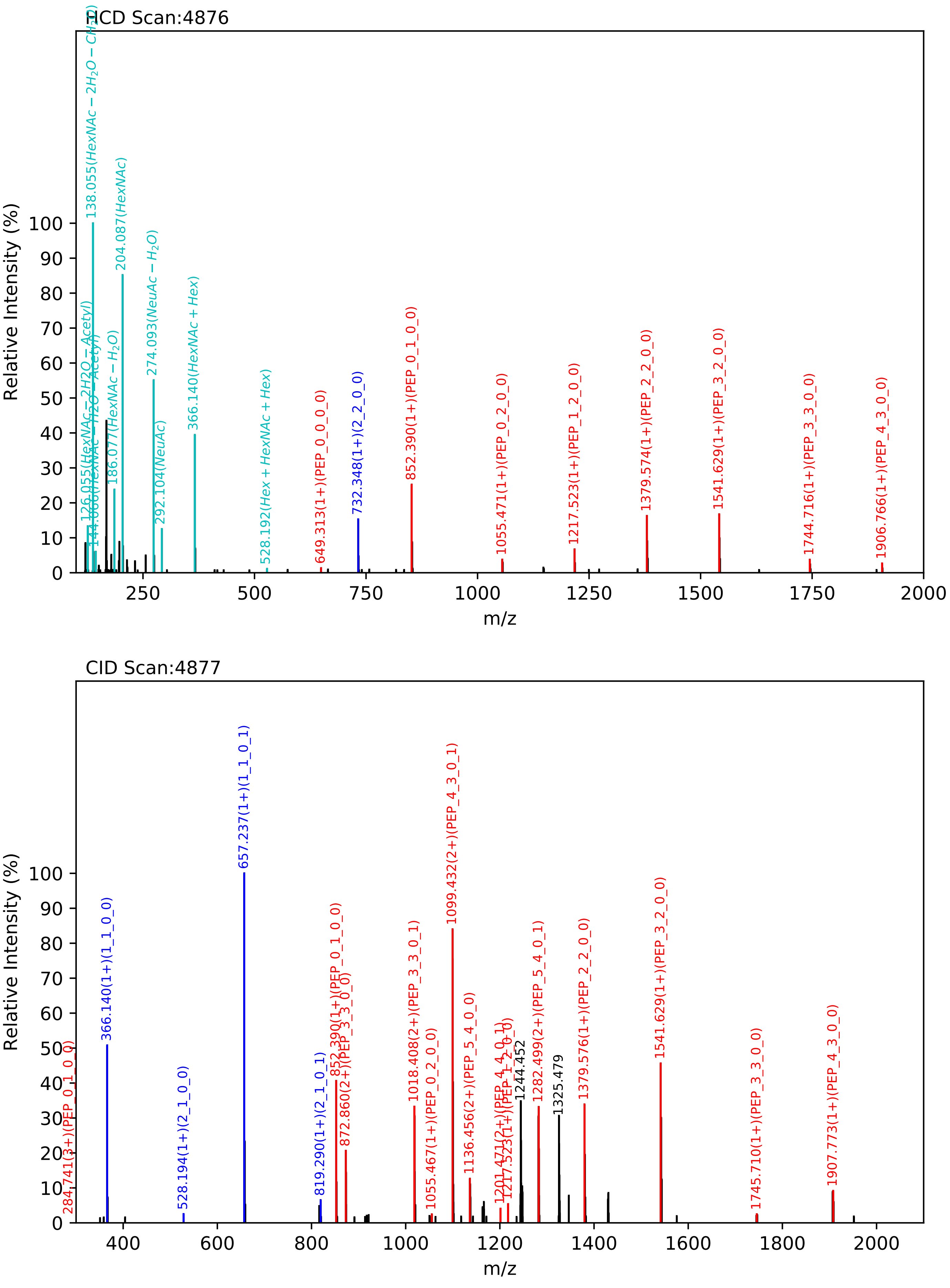

Unknown set no. 613, Experiment: Human Plasma exp_3

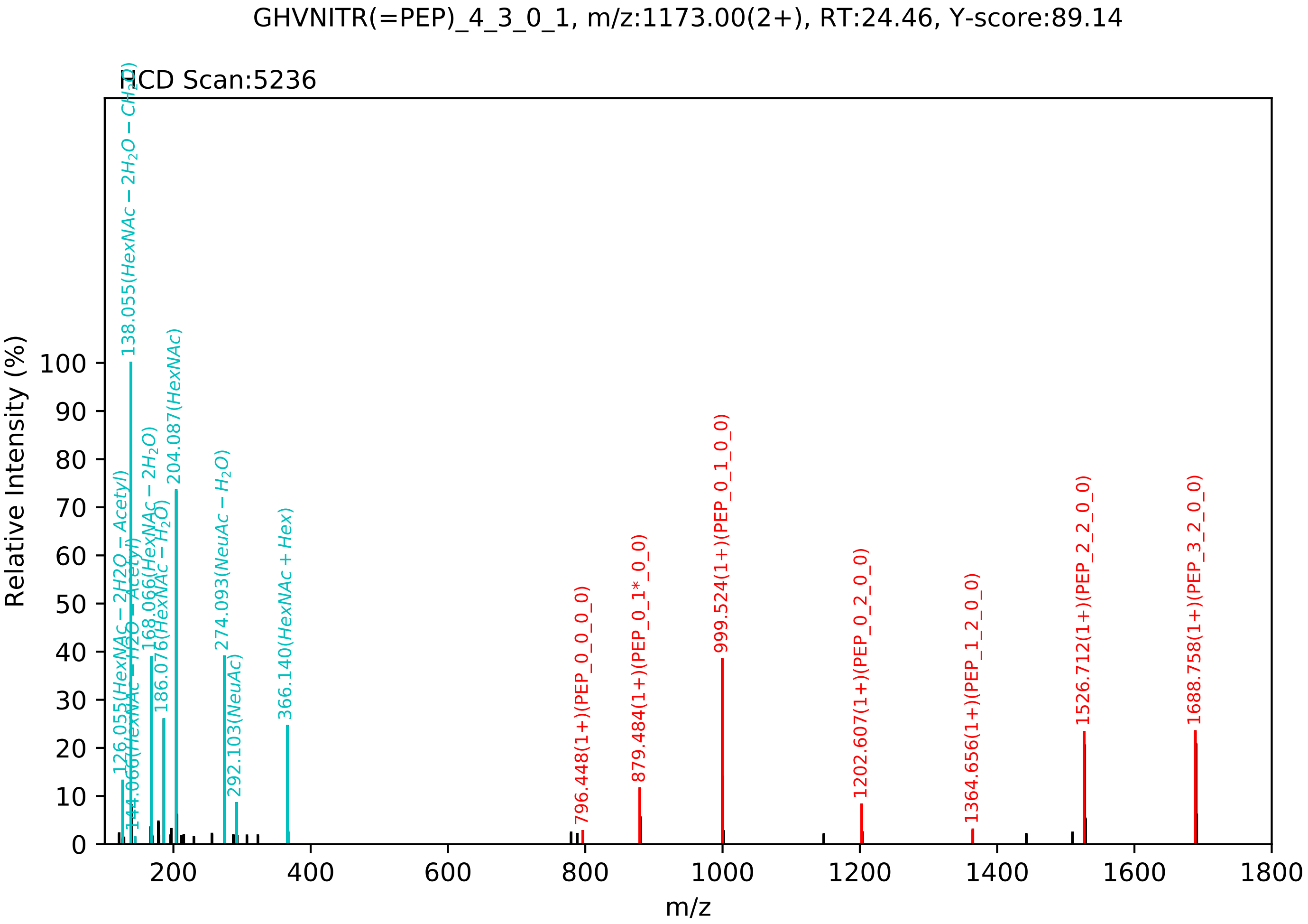

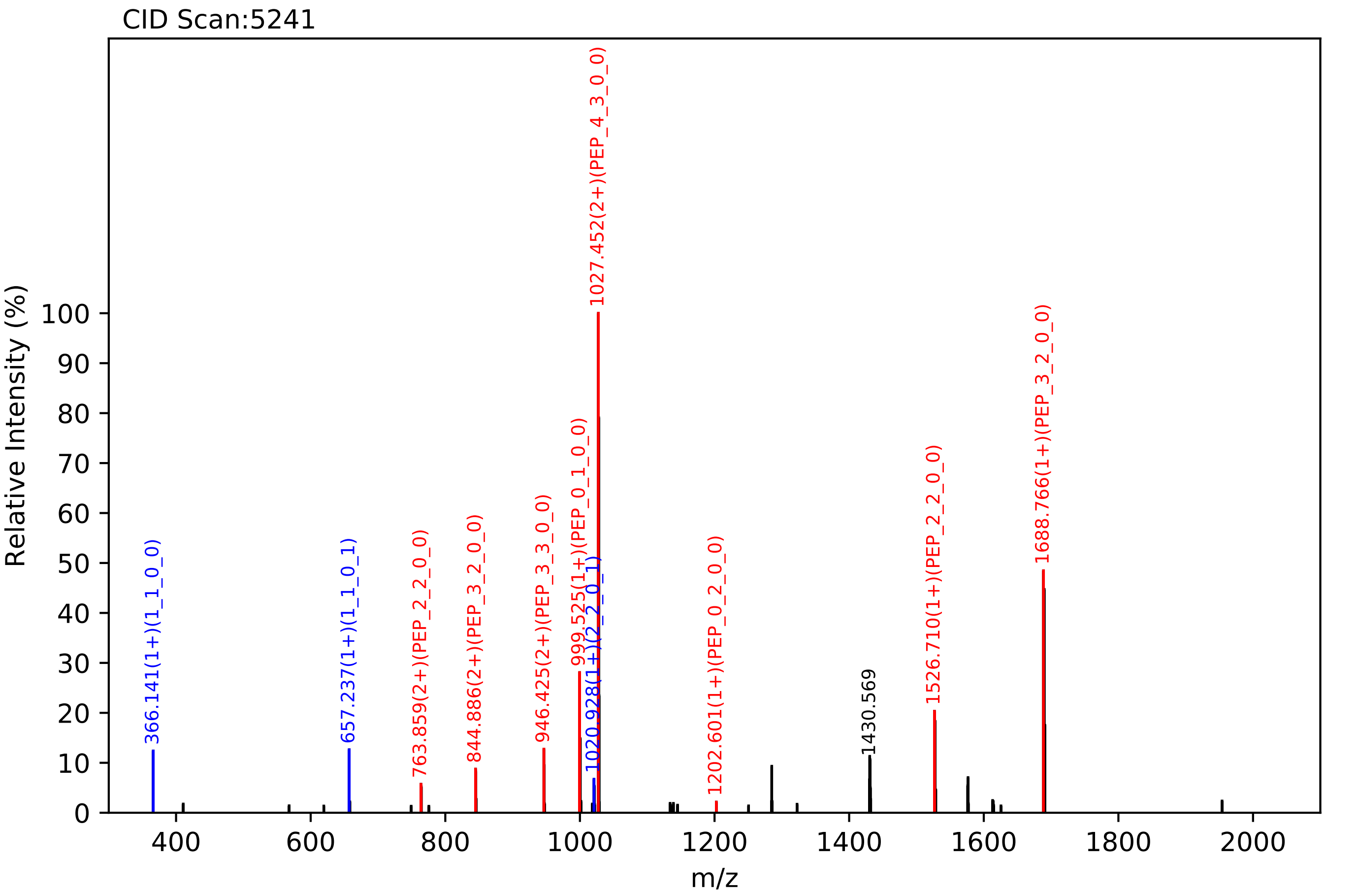

Unknown set no. 614, Experiment: Human Plasma exp_4

GHVNITR(=PEP)_4_3_0_1, m/z:1173.00(2+), RT:24.64, Y-score:91.67

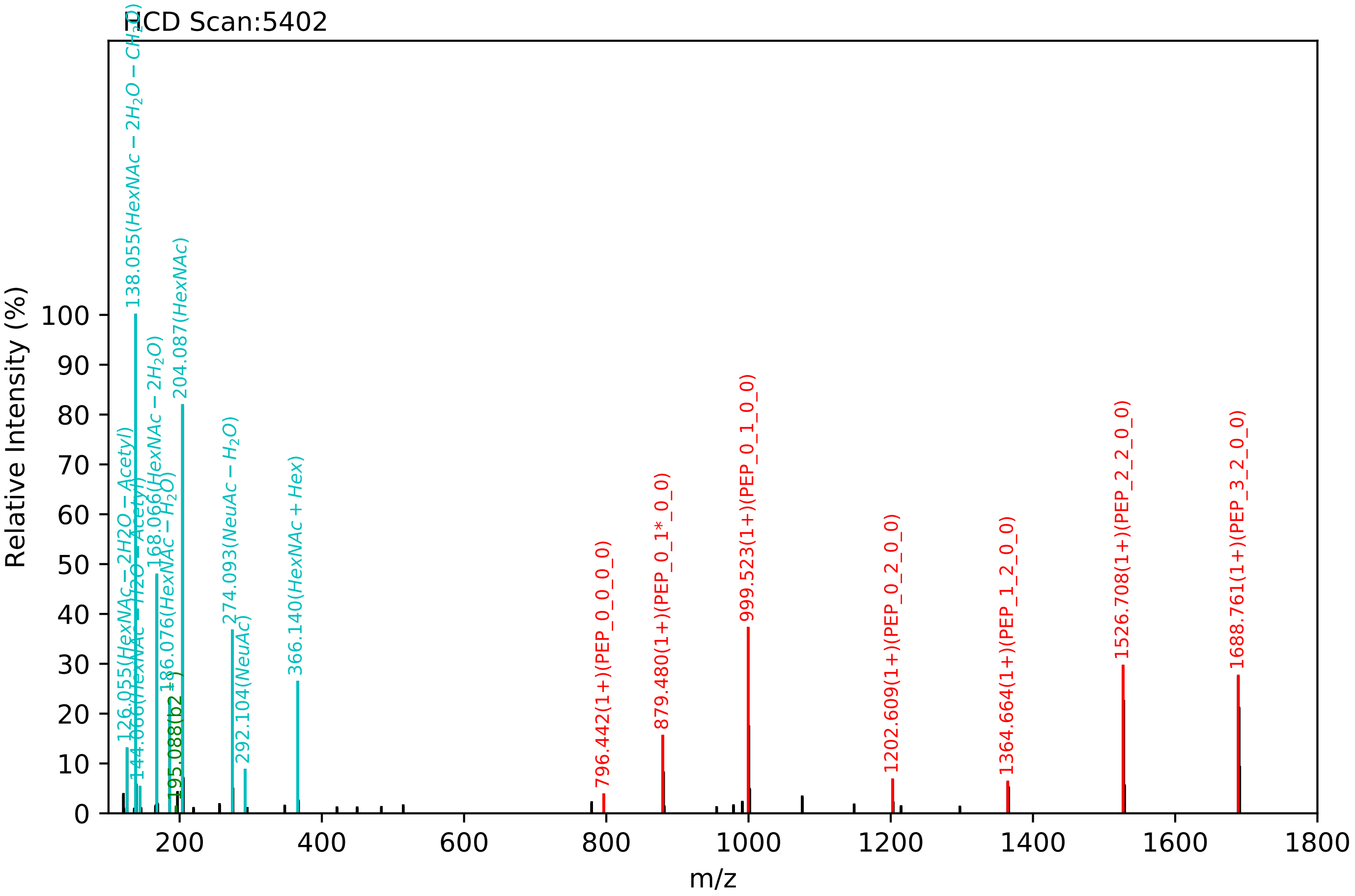

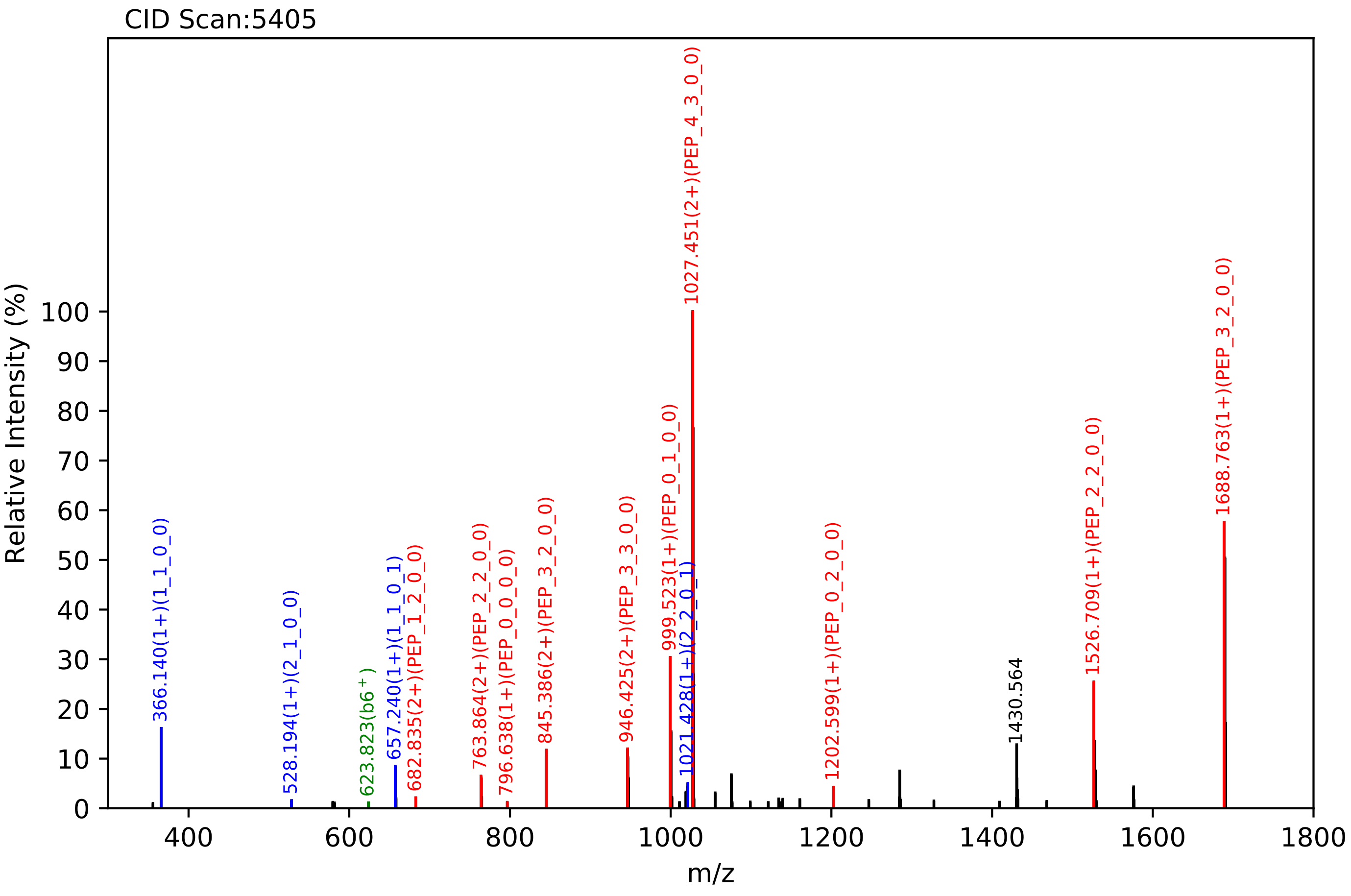

Unknown set no. 615, Experiment: Human Plasma exp_5

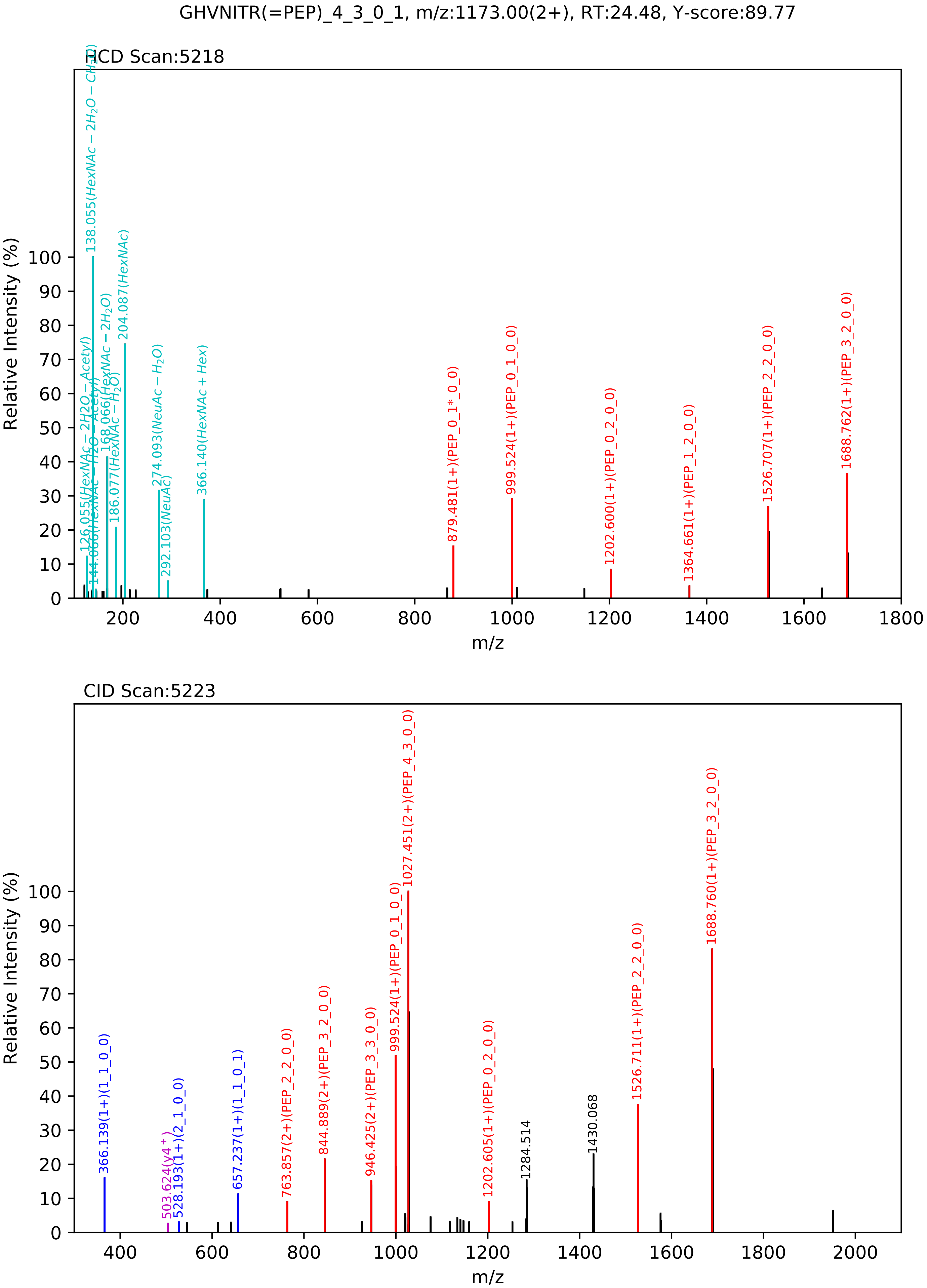

Unknown set no. 616, Experiment: Human Plasma exp_1

GHVNITR(=PEP)_5_4_1_2, m/z:1049.76(3+), RT:24.43, Y-score:86.94

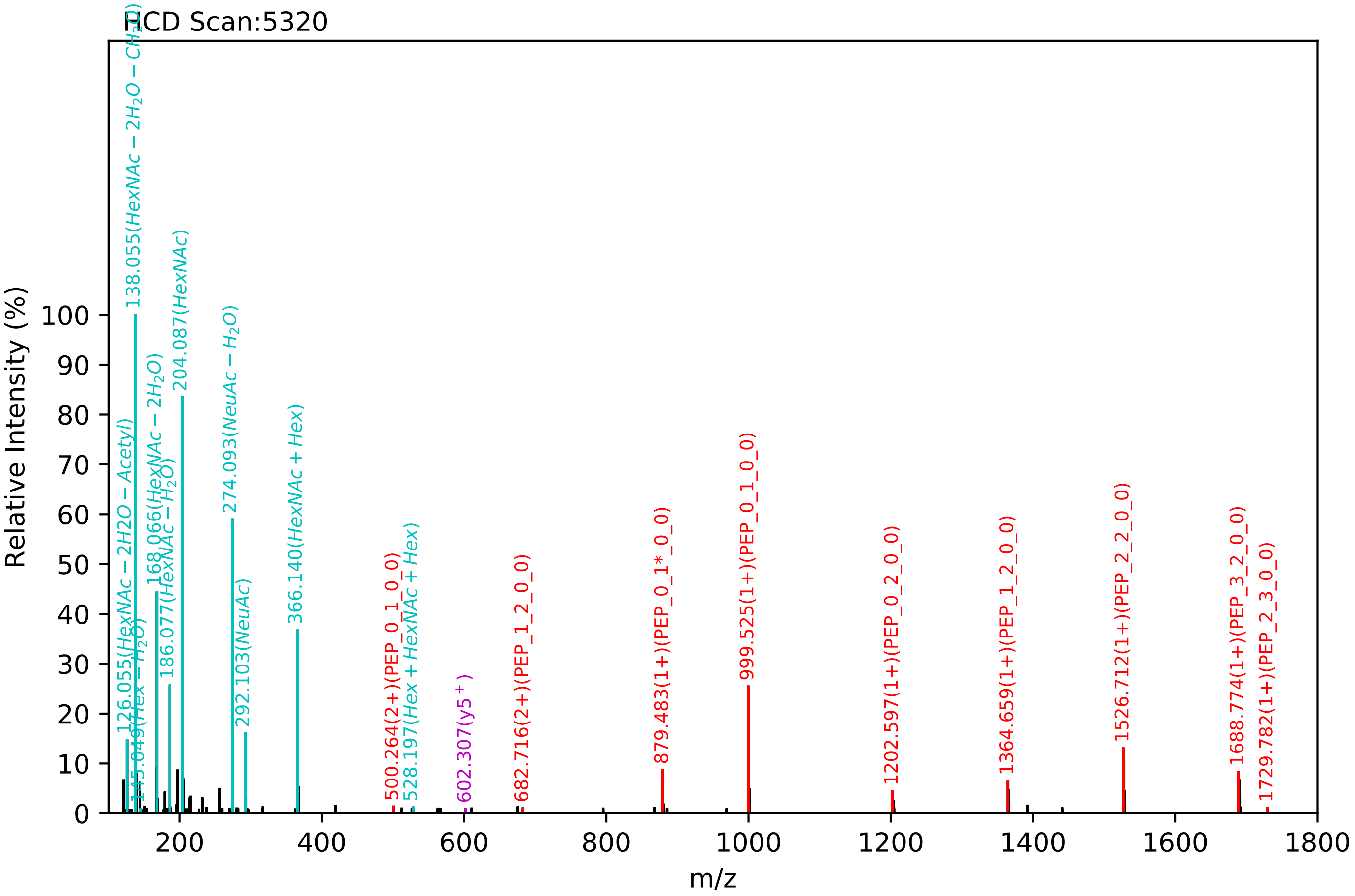

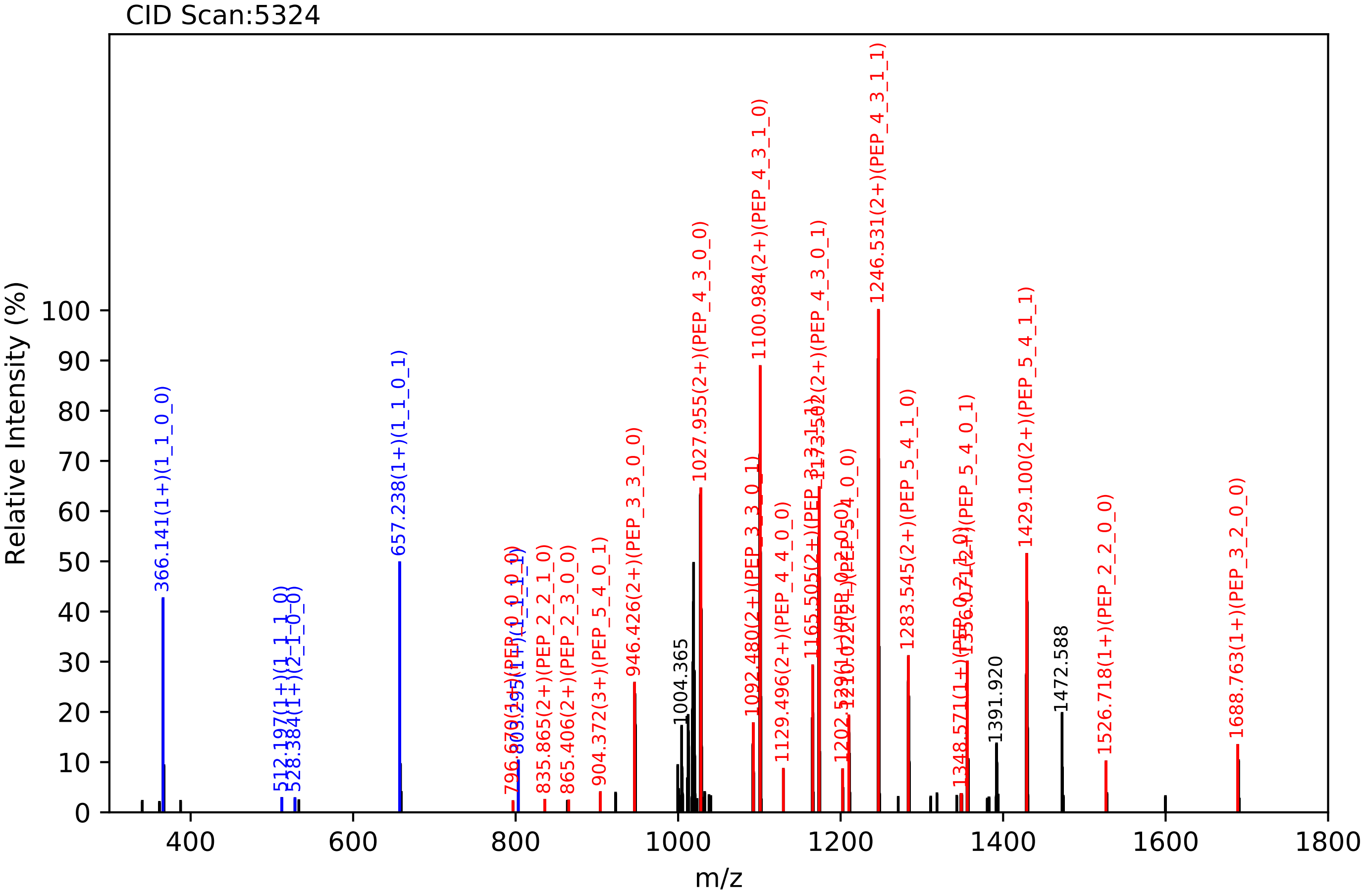

Unknown set no. 617, Experiment: Human Plasma exp_4

GHVNITR(=PEP)_5_4_1_2, m/z:1049.76(3+), RT:24.82, Y-score:92.24

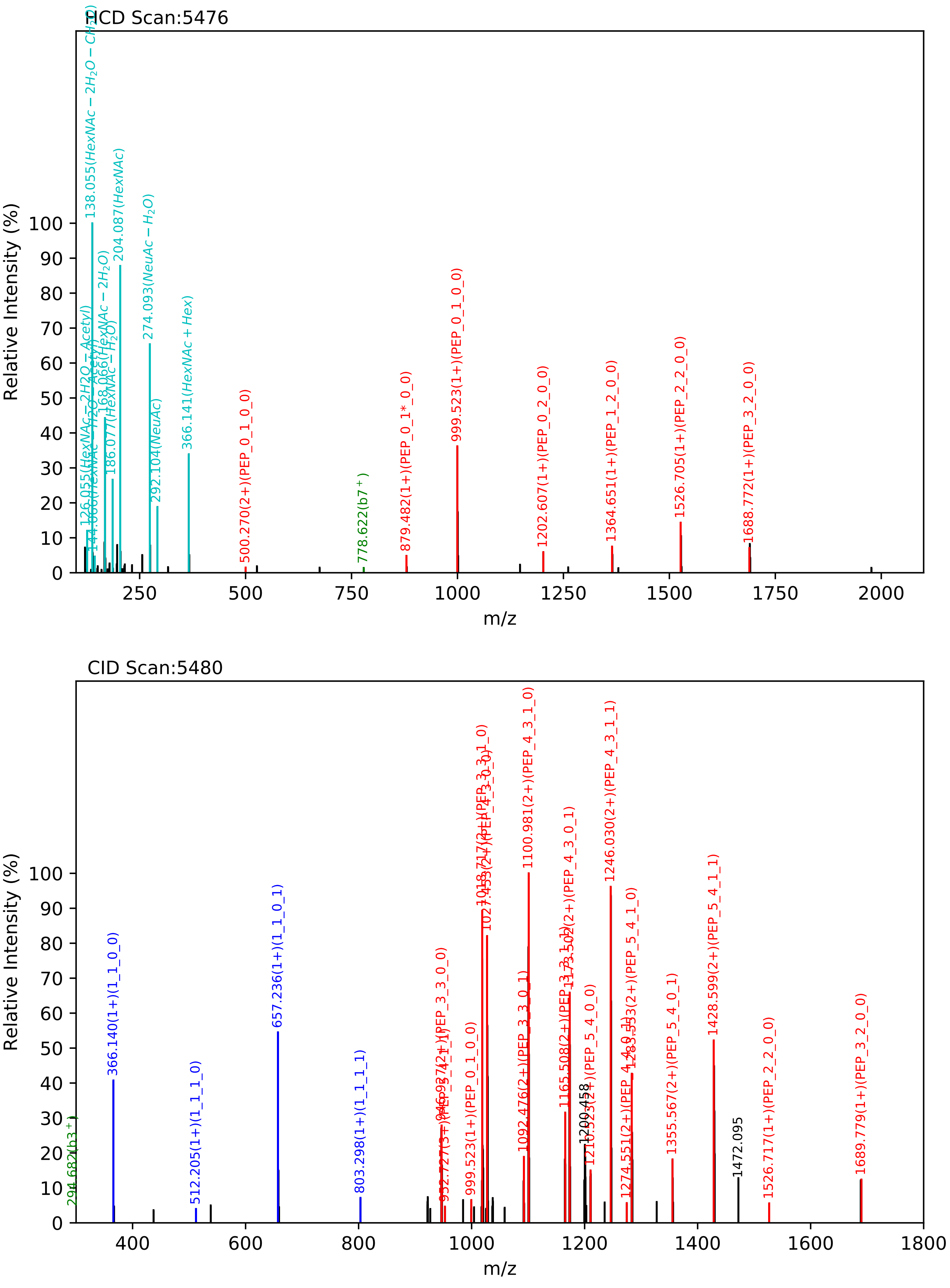

Unknown set no. 618, Experiment: Human Plasma exp_2

NFTENDLLVR(=PEP)_5_4_0_2, m/z:857.11(4+), RT:93.51, Y-score:90.24

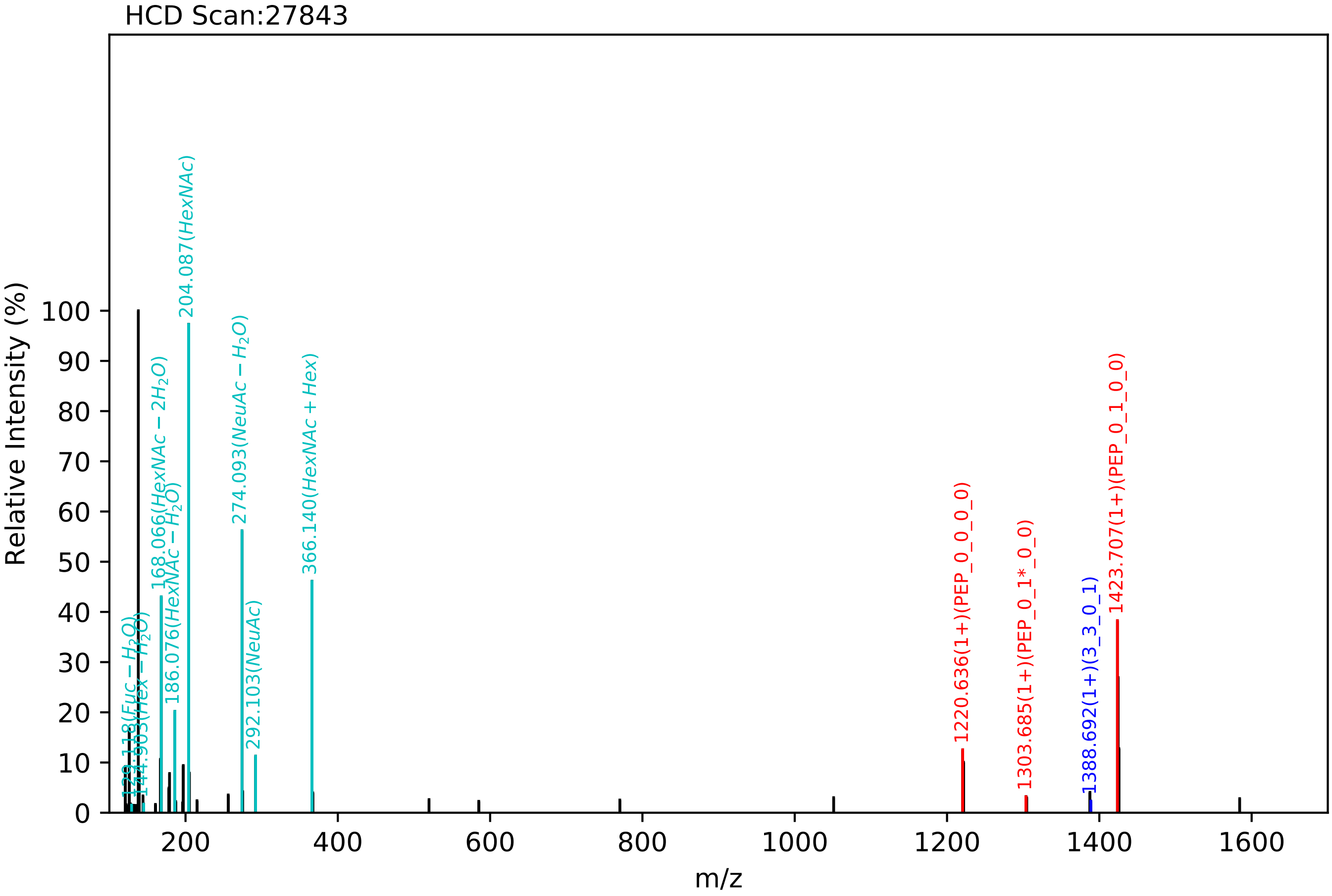

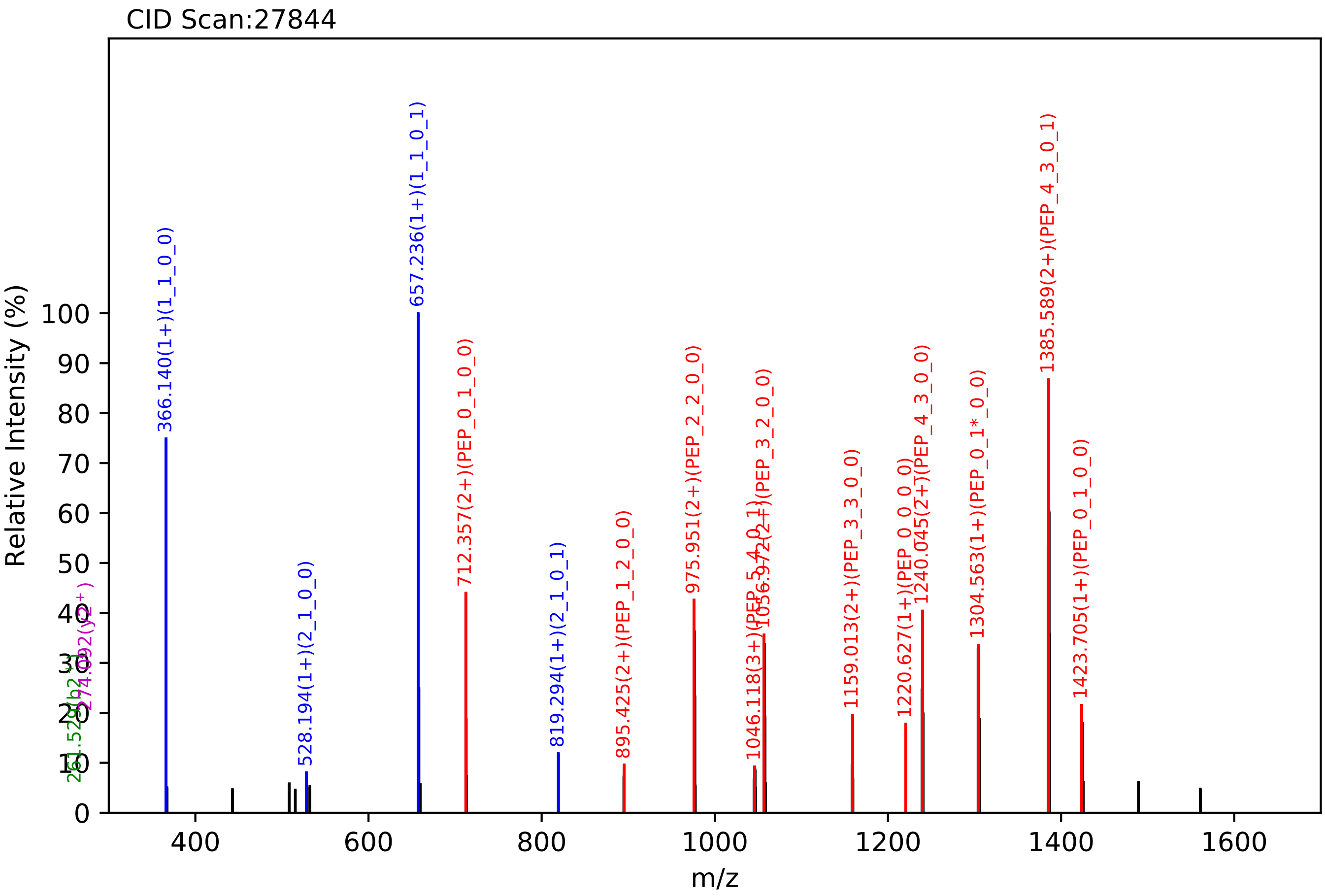

Unknown set no. 619, Experiment: Human Plasma exp_2

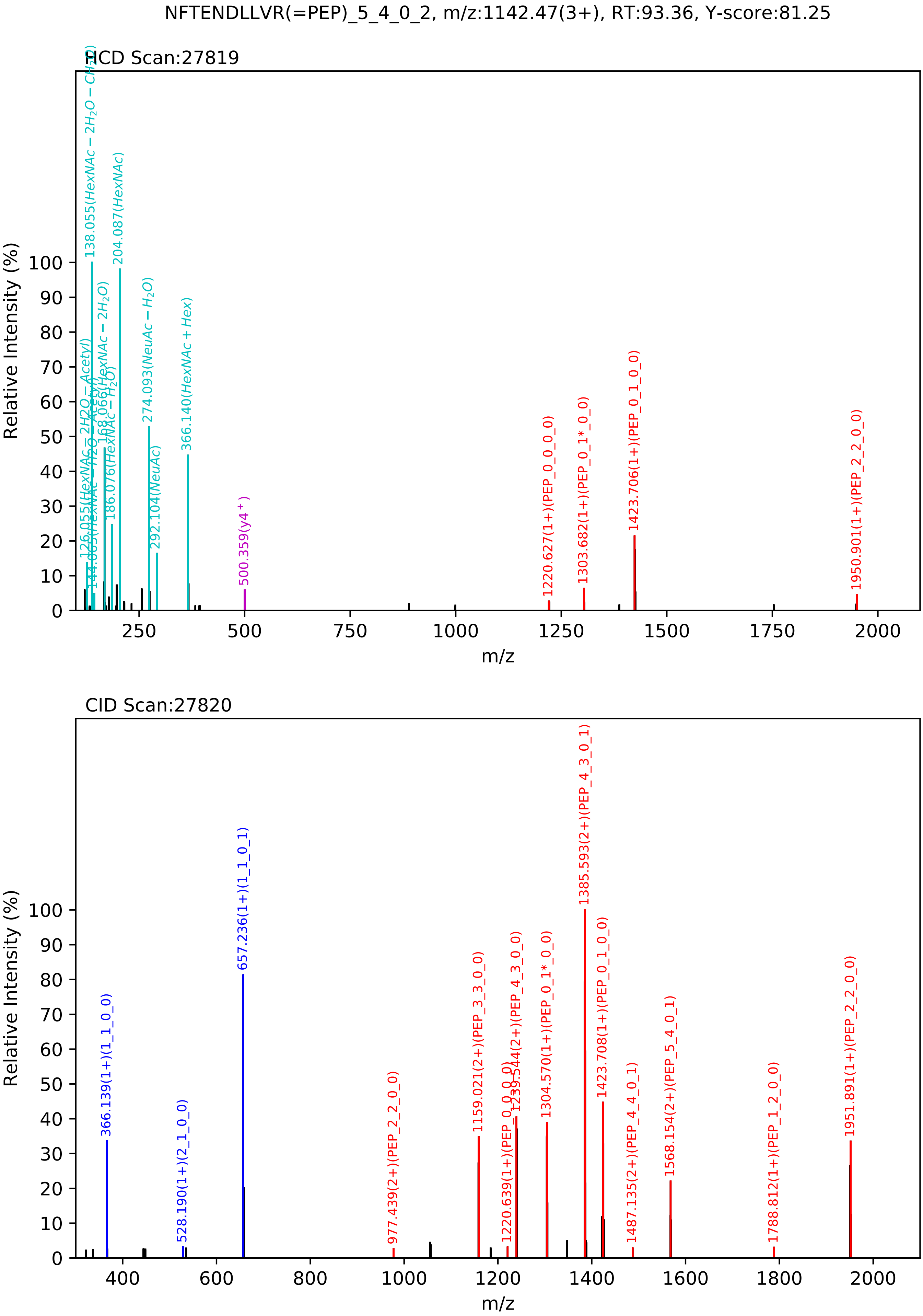

Unknown set no. 620, Experiment: Human Plasma exp_3

NFTENDLLVR(=PEP)_5_4_0_2, m/z:857.11(4+), RT:93.58, Y-score:98.69

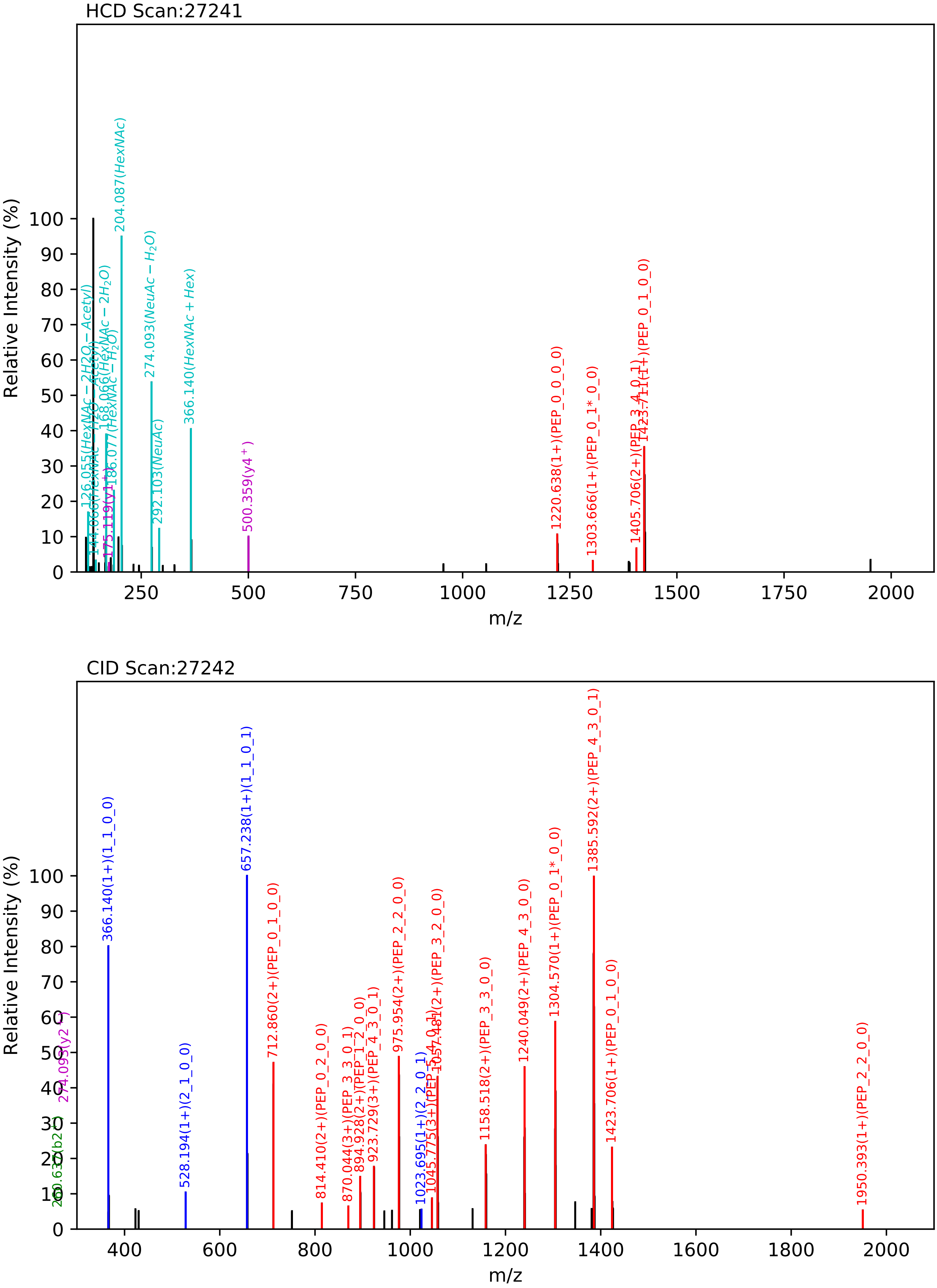

Unknown set no. 621, Experiment: Human Plasma exp_3

NFTENDLLVR(=PEP)_5_4_0_2, m/z:1142.47(3+), RT:93.48, Y-score:92.21

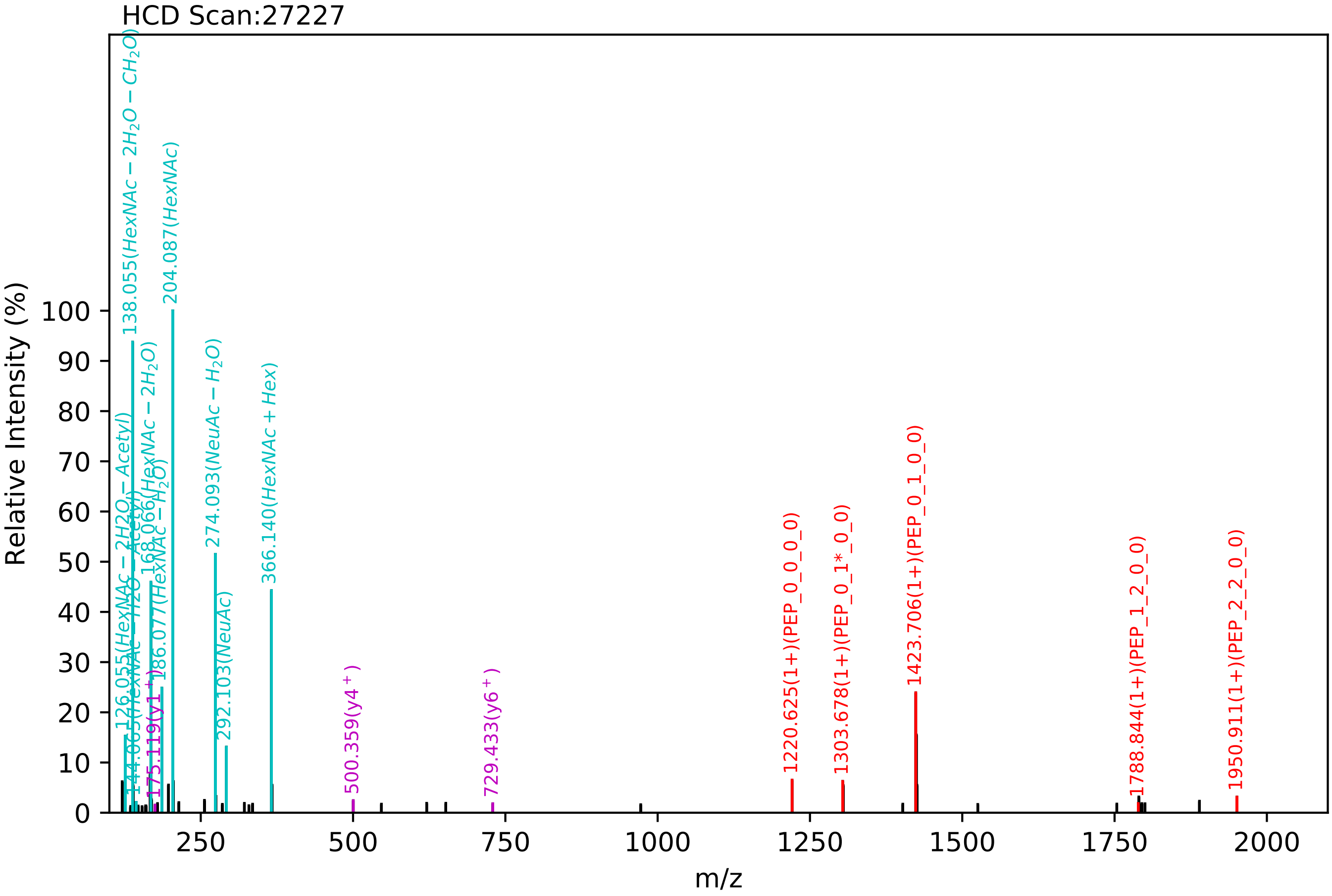

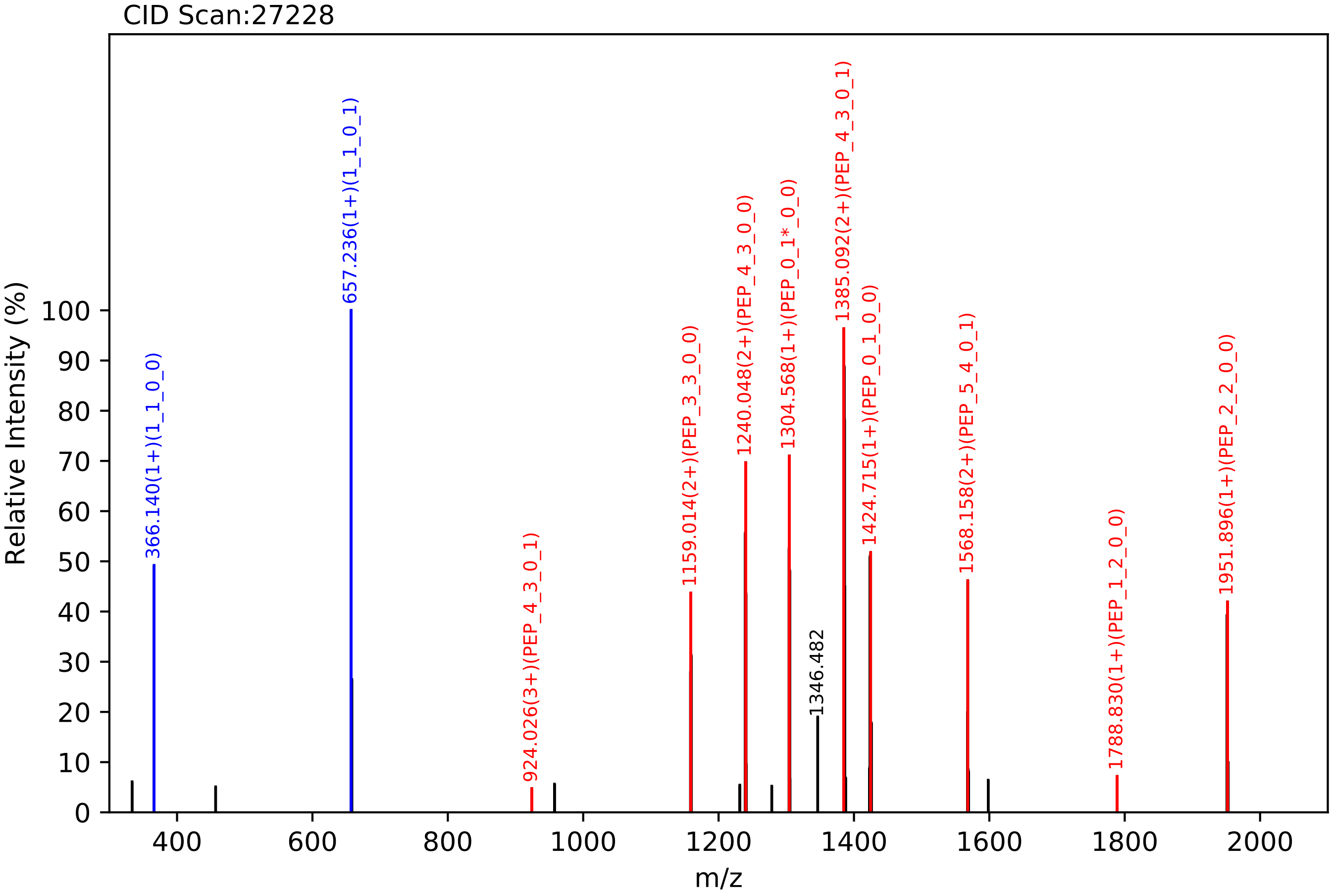

Unknown set no. 622, Experiment: Human Plasma exp_4

NFTENDLLVR(=PEP)_5_4_0_2, m/z:857.11(4+), RT:93.73, Y-score:90.92

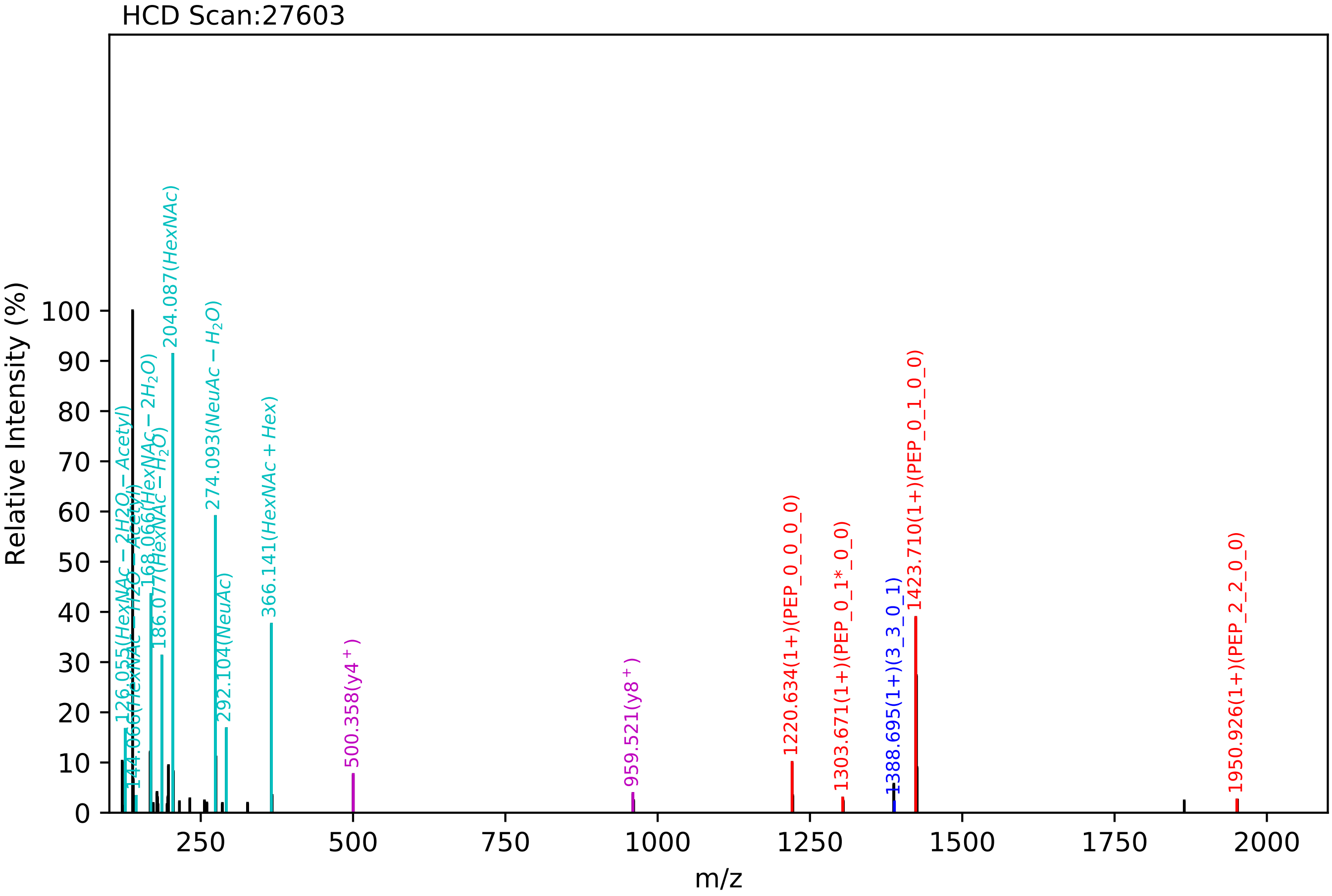

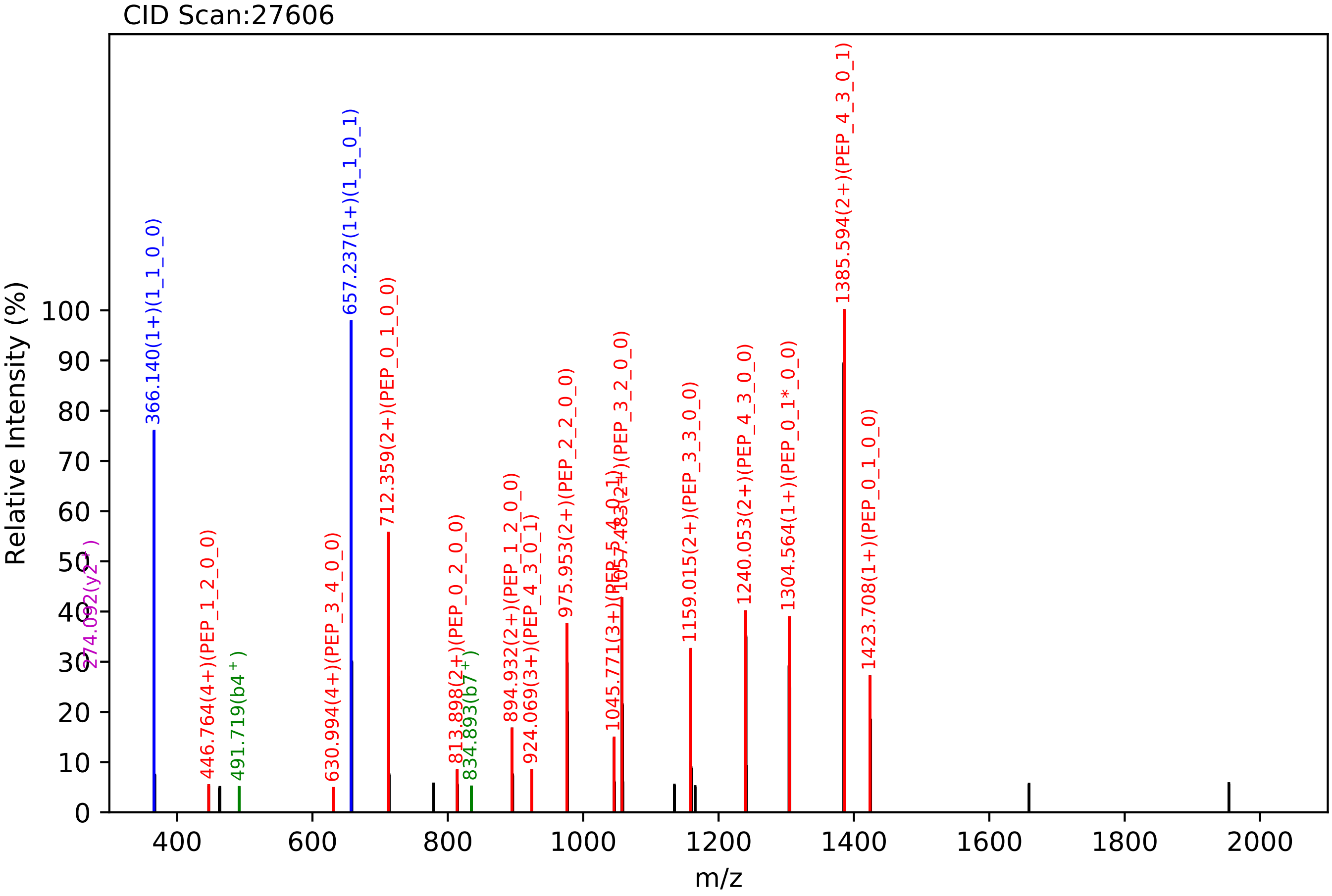

Unknown set no. 623, Experiment: Human Plasma exp_1

CGLVPVLAENYNK(=PEP)_4_3_0_1, m/z:1009.10(3+), RT:99.63, Y-score:92.93

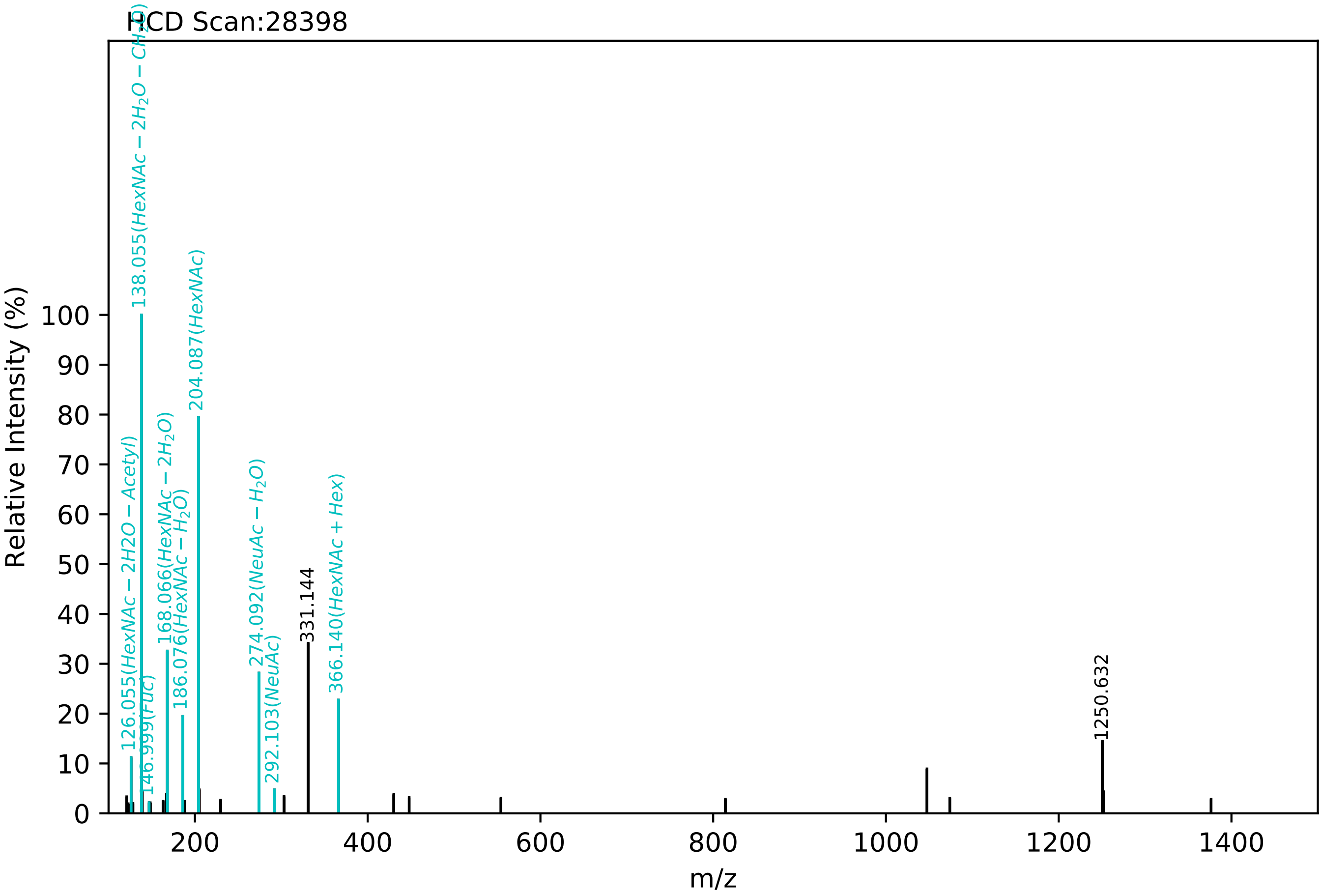

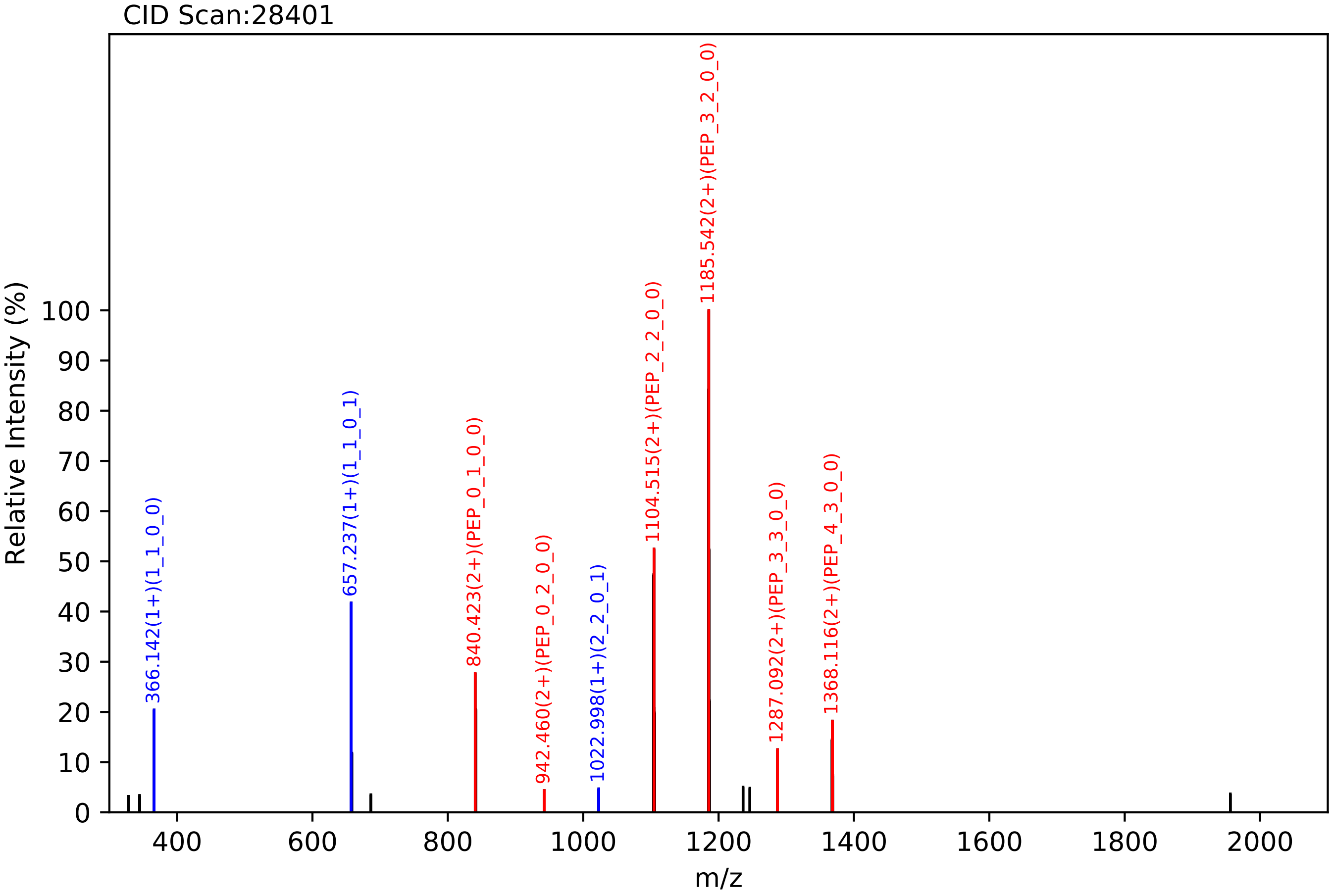

Unknown set no. 624, Experiment: Human Plasma exp_2

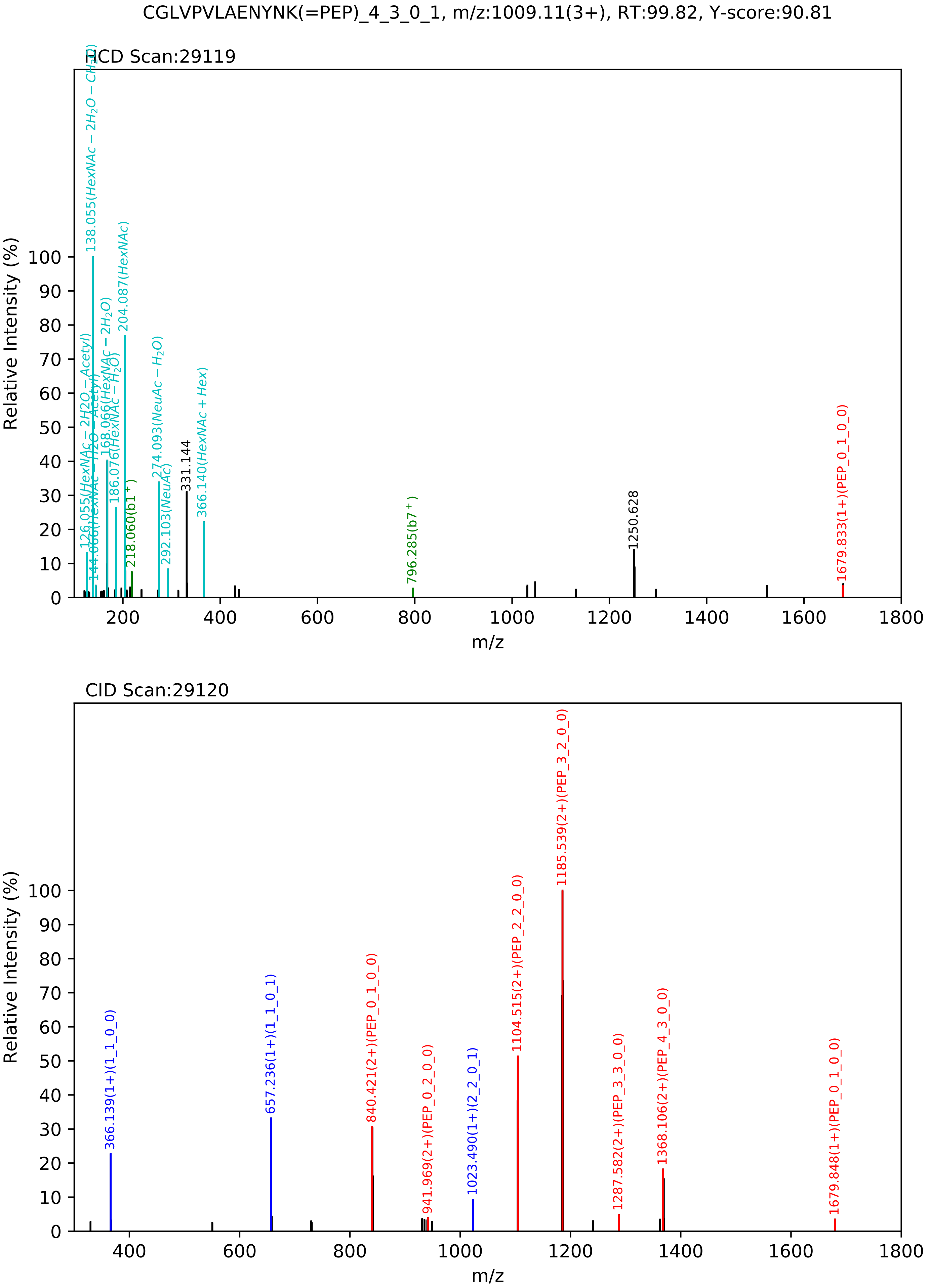

Unknown set no. 625, Experiment: Human Plasma exp_3

CGLVPVLAENYNK(=PEP)_4_3_0_1, m/z:1009.11(3+), RT:99.87, Y-score:88.39

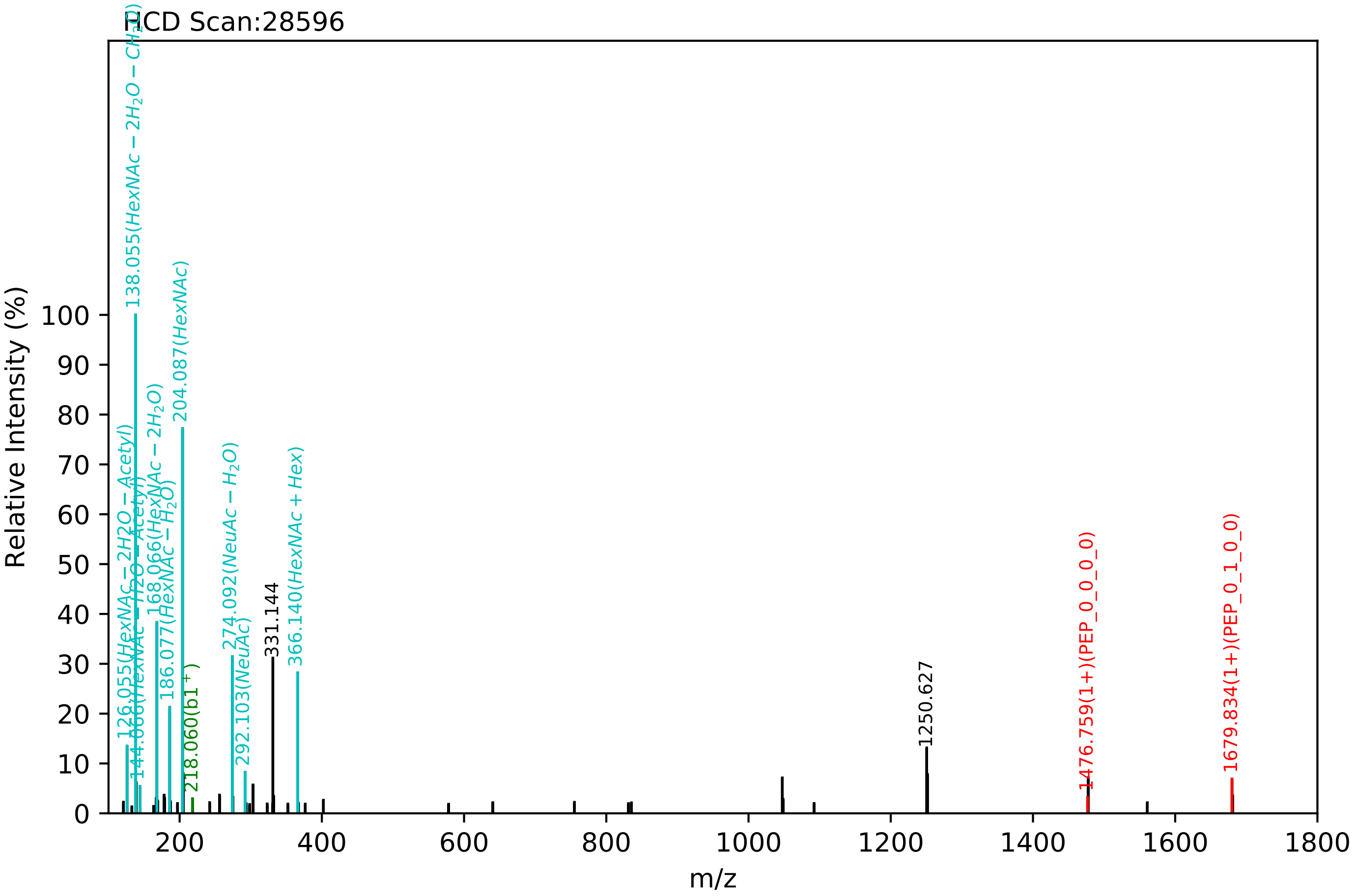

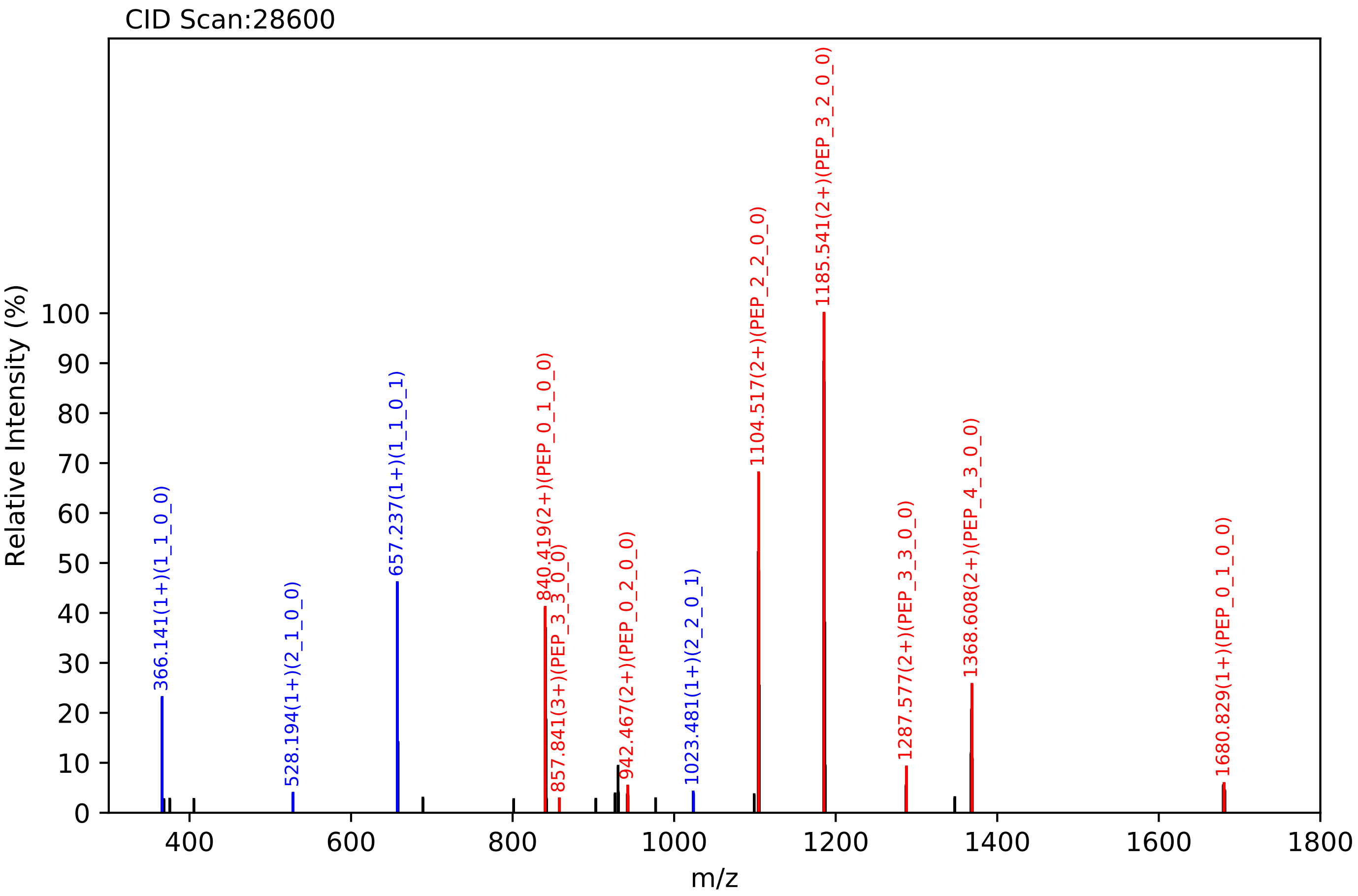

Unknown set no. 626, Experiment: Human Plasma exp_4

CGLVPVLAENYNK(=PEP)_4_3_0_1, m/z:1009.11(3+), RT:99.95, Y-score:88.64

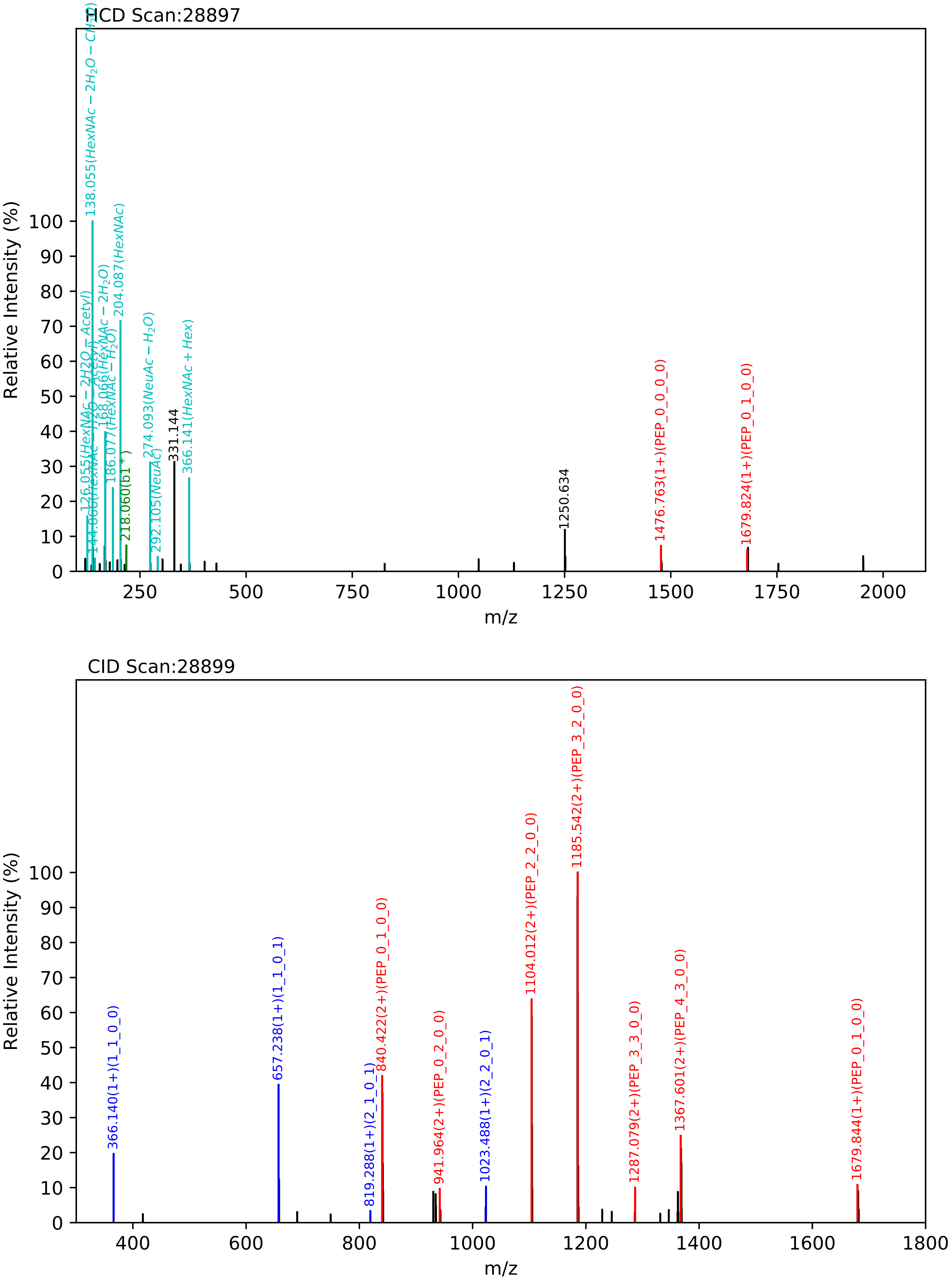

Unknown set no. 627, Experiment: Human Plasma exp_5

CGLVPVLAENYNK(=PEP)_4_3_0_1, m/z:1009.11(3+), RT:99.67, Y-score:89.31

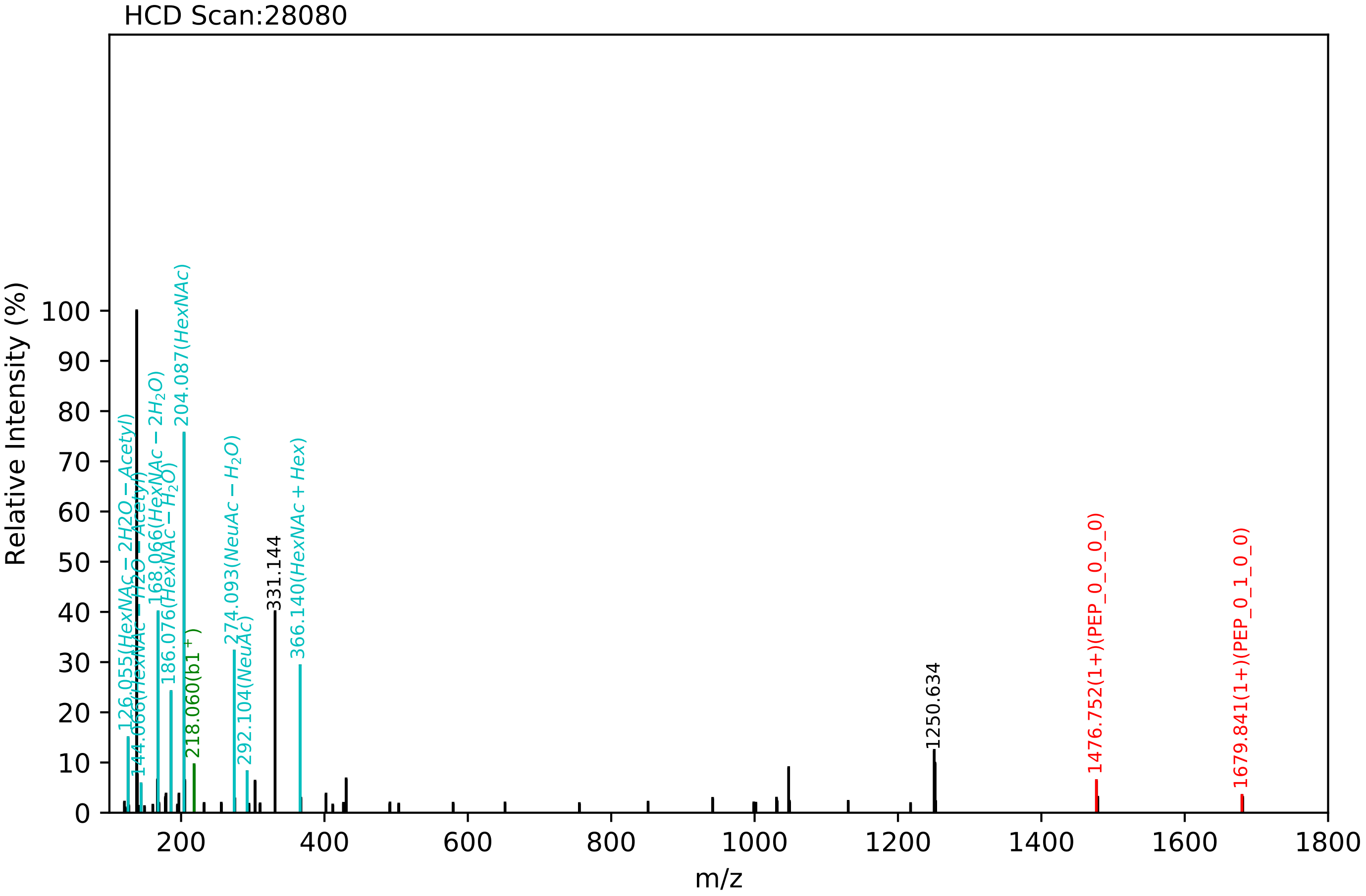

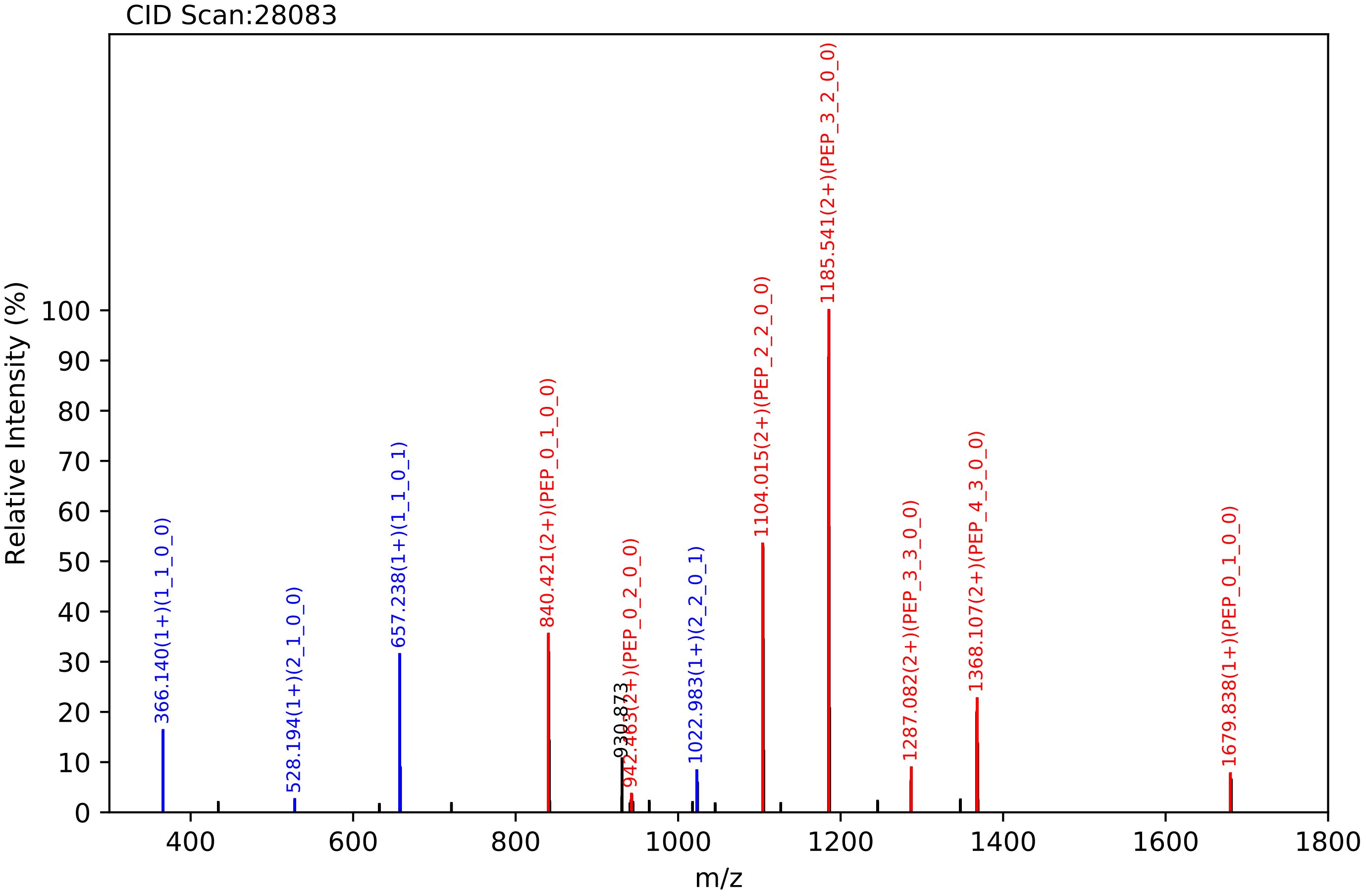

Unknown set no. 628, Experiment: Human Plasma exp_4

CGLVPVLAENYNK(=PEP)_5_4_0_1, m/z:848.37(4+), RT:88.17, Y-score:92.27

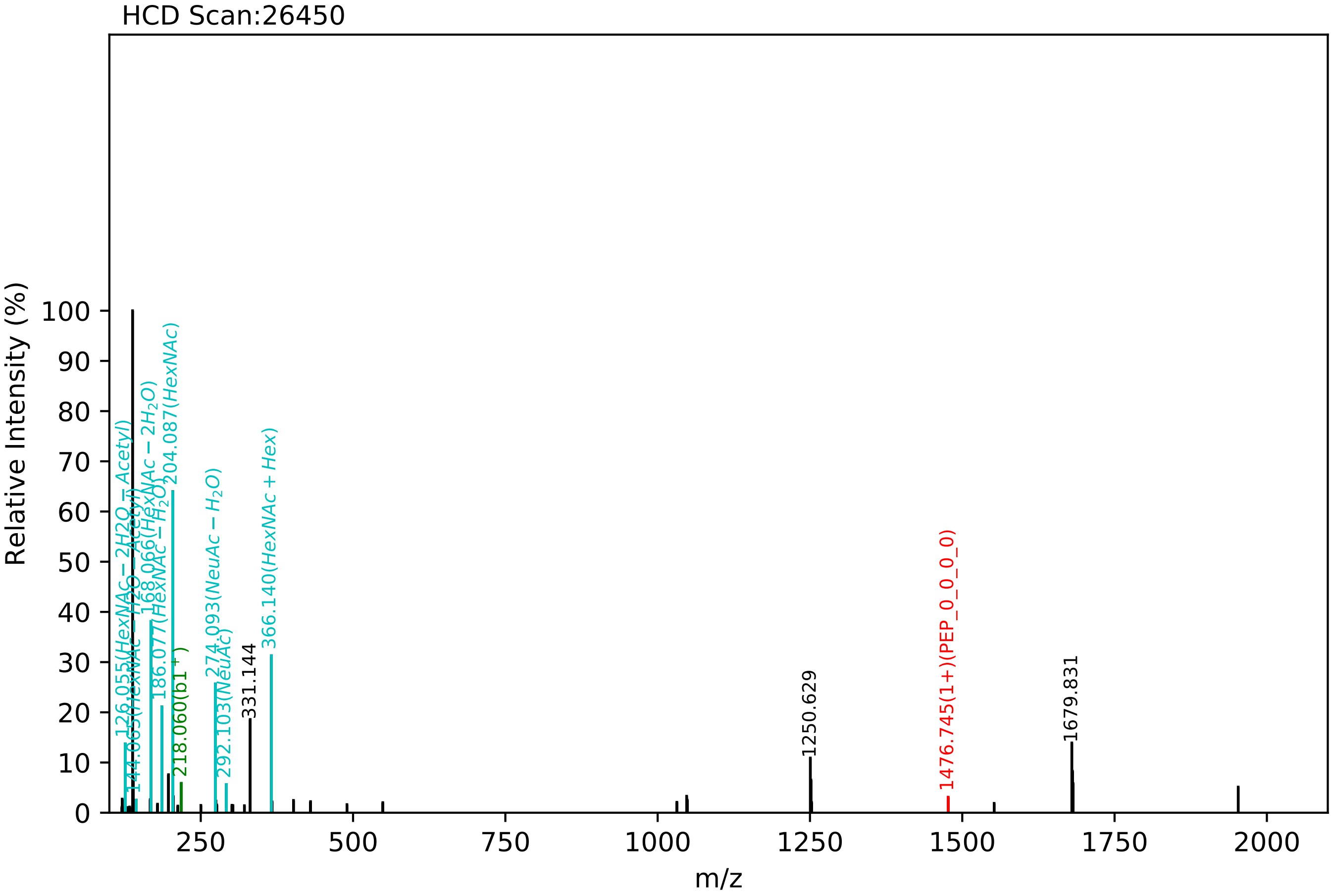

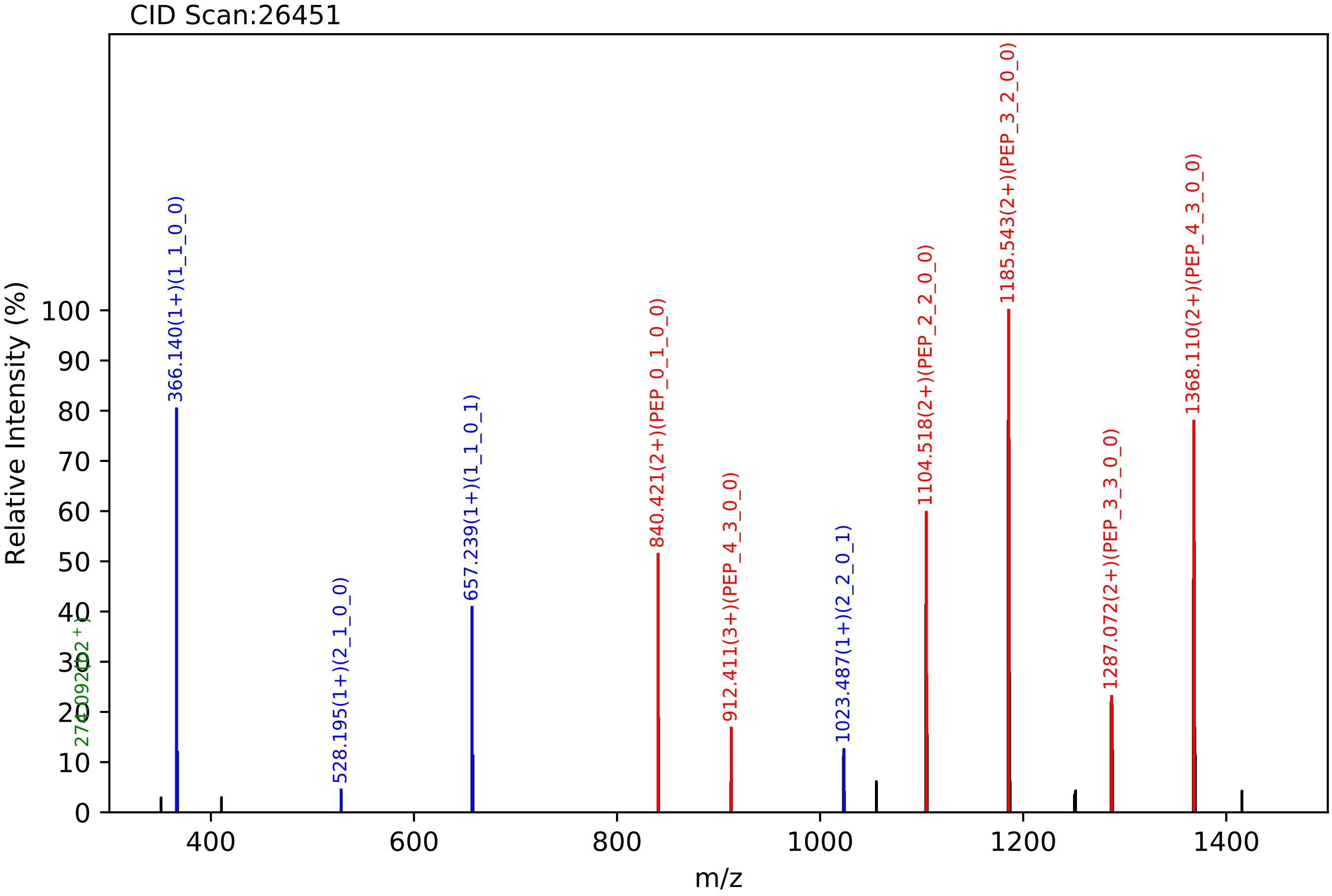

Unknown set no. 629, Experiment: Human Plasma exp_1

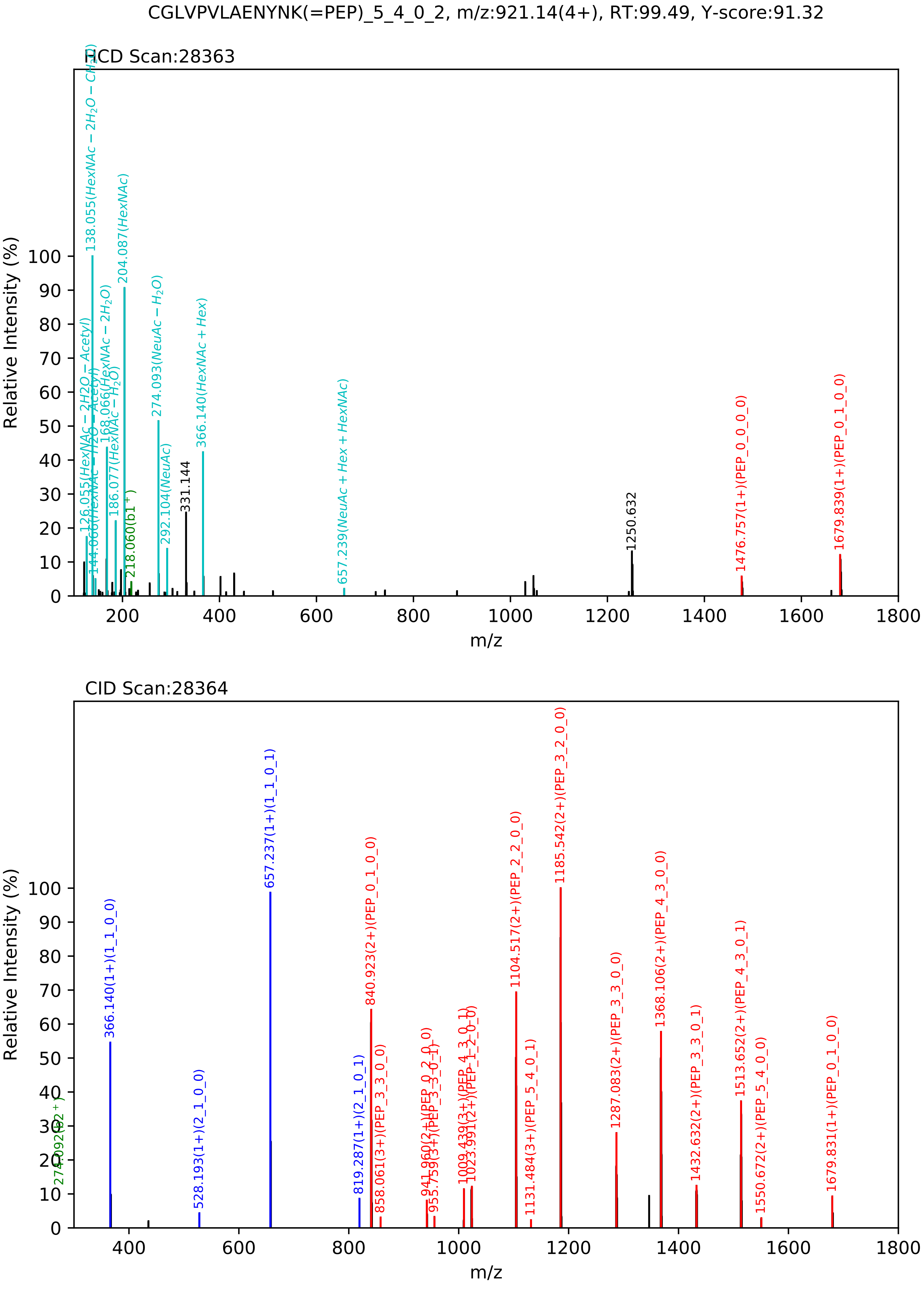

Unknown set no. 630, Experiment: Human Plasma exp_2

CGLVPVLAENYNK(=PEP)_5_4_0_2, m/z:921.14(4+), RT:100.75, Y-score:92.10

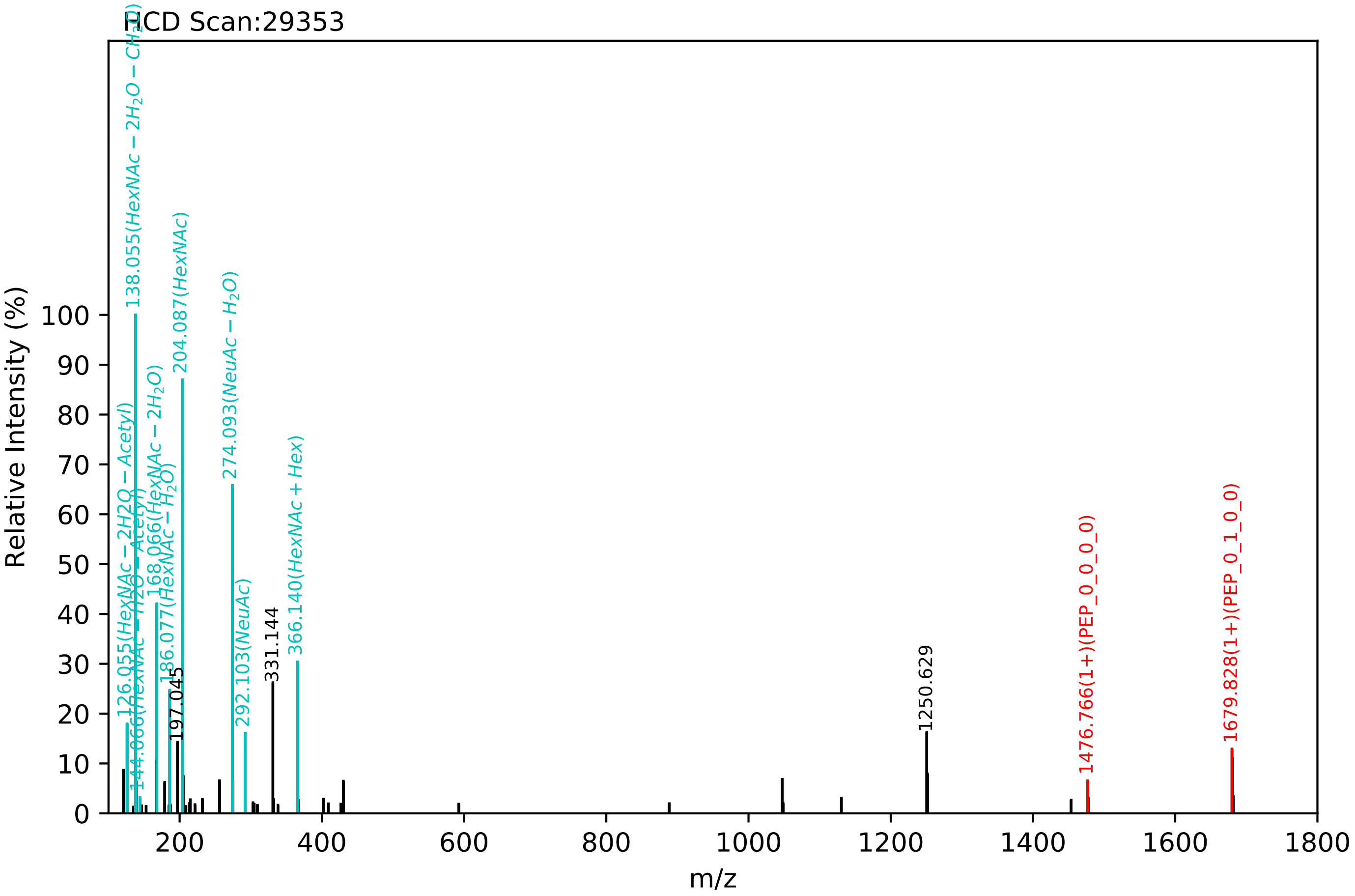

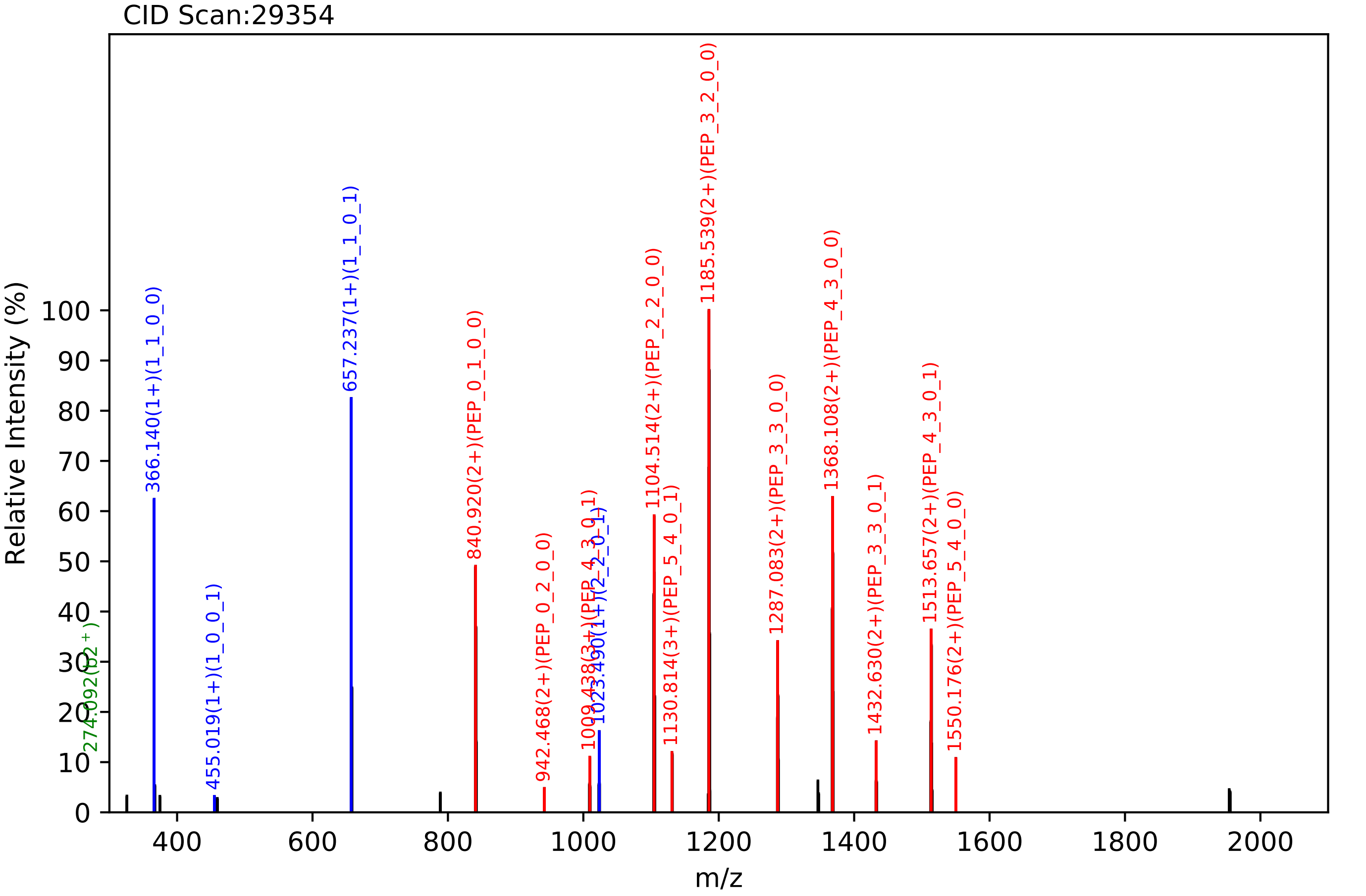

Unknown set no. 631, Experiment: Human Plasma exp_3

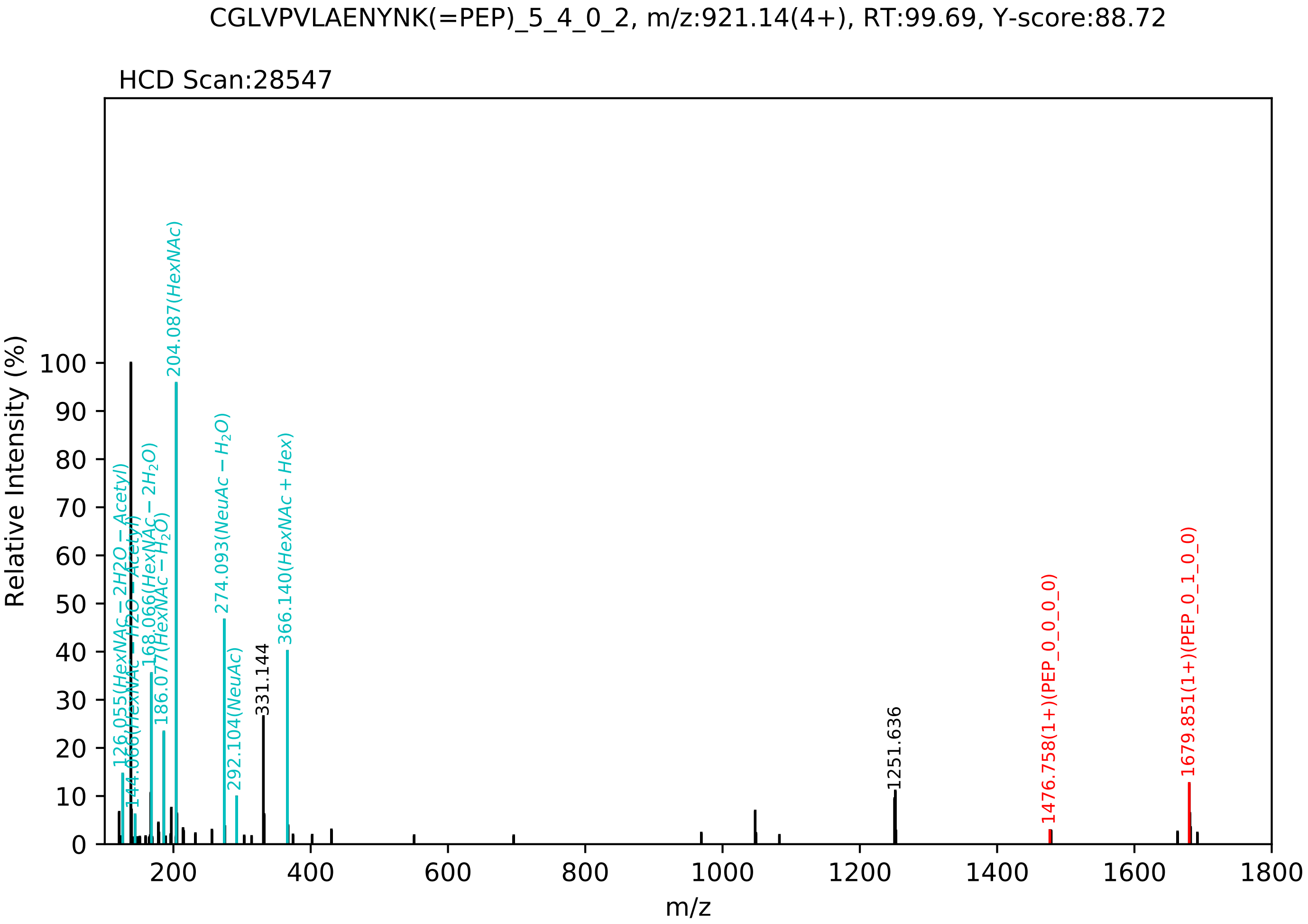

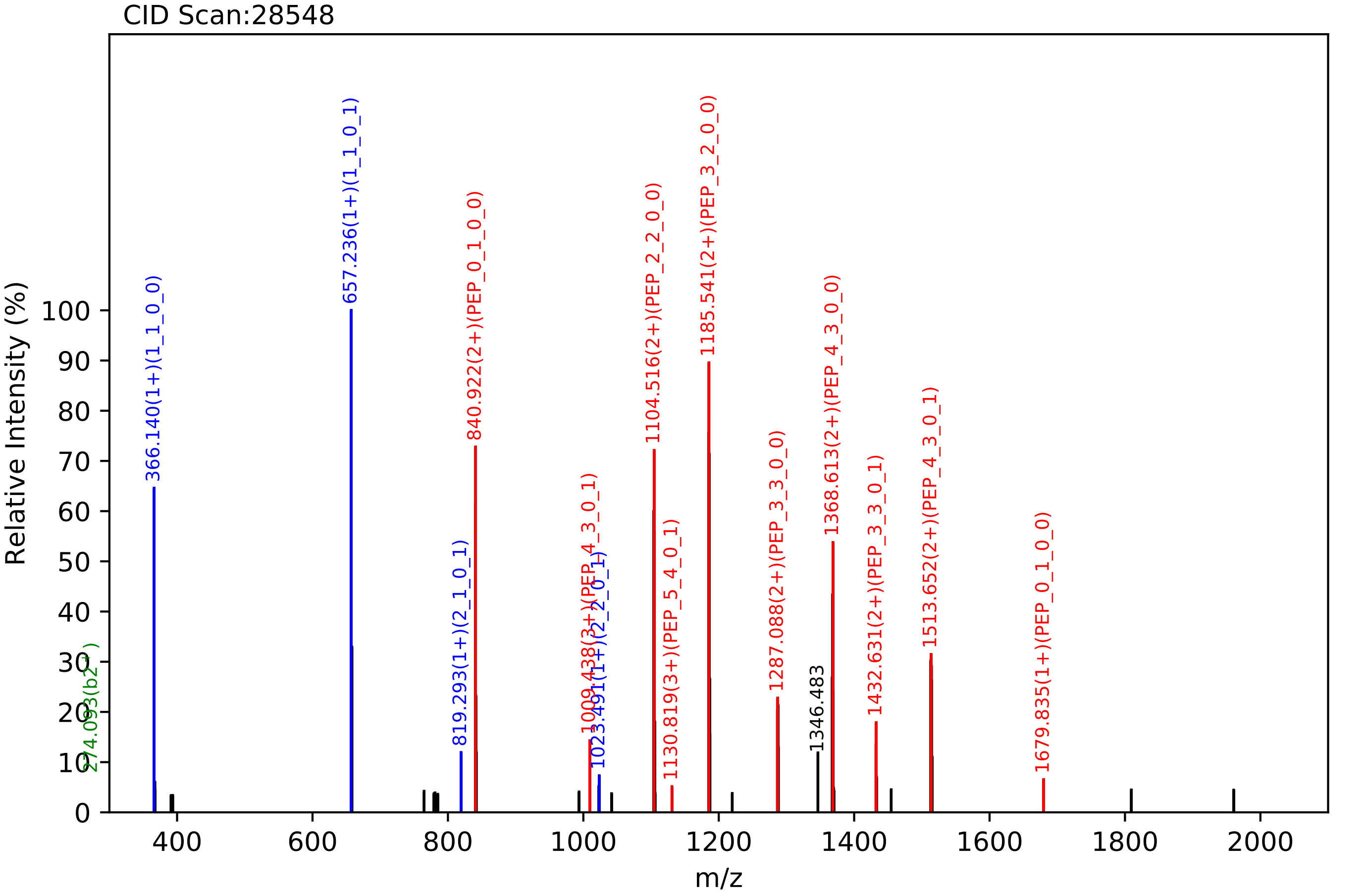

Unknown set no. 632, Experiment: Human Plasma exp_4

CGLVPVLAENYNK(=PEP)_5_4_0_2, m/z:921.14(4+), RT:100.90, Y-score:91.57

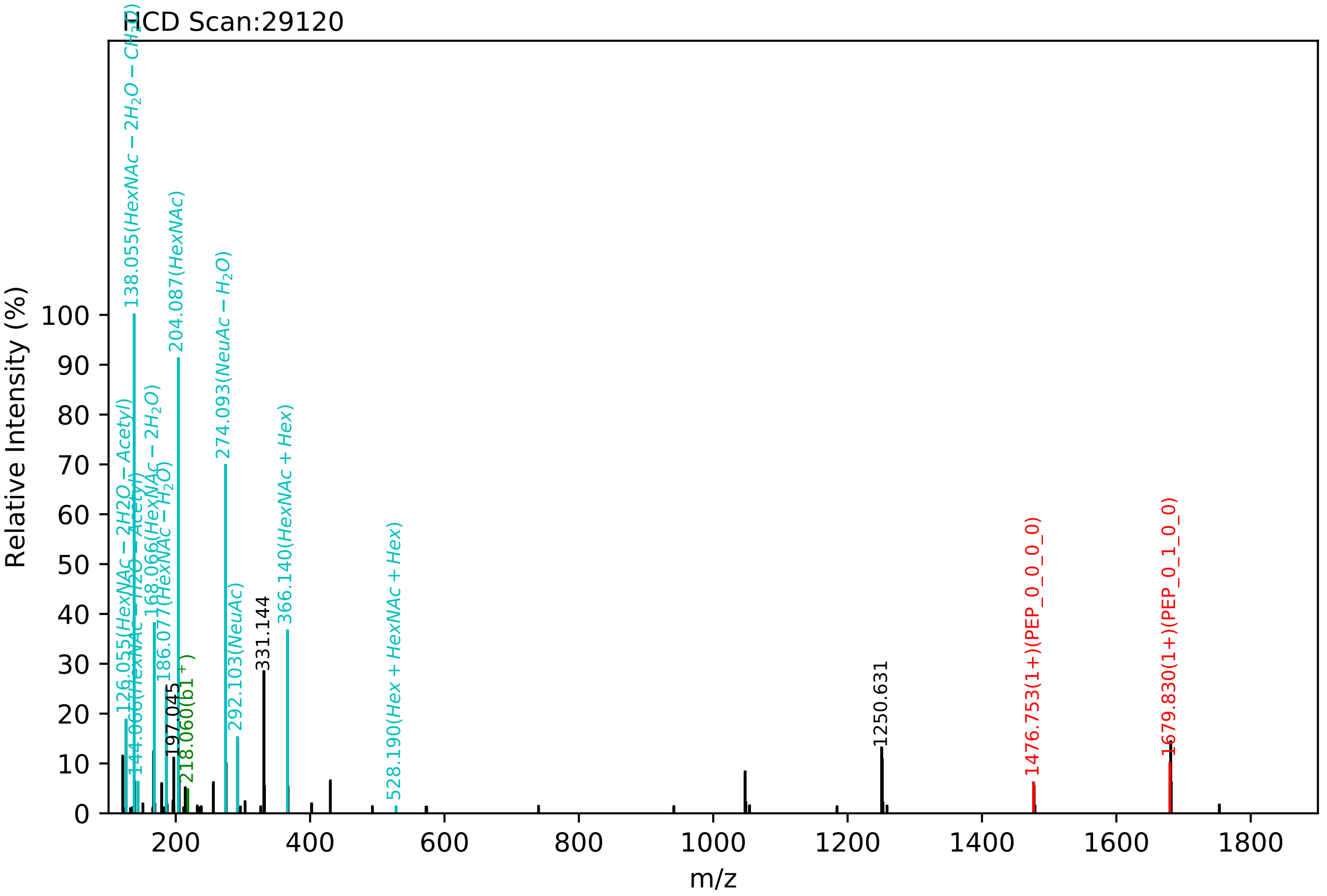

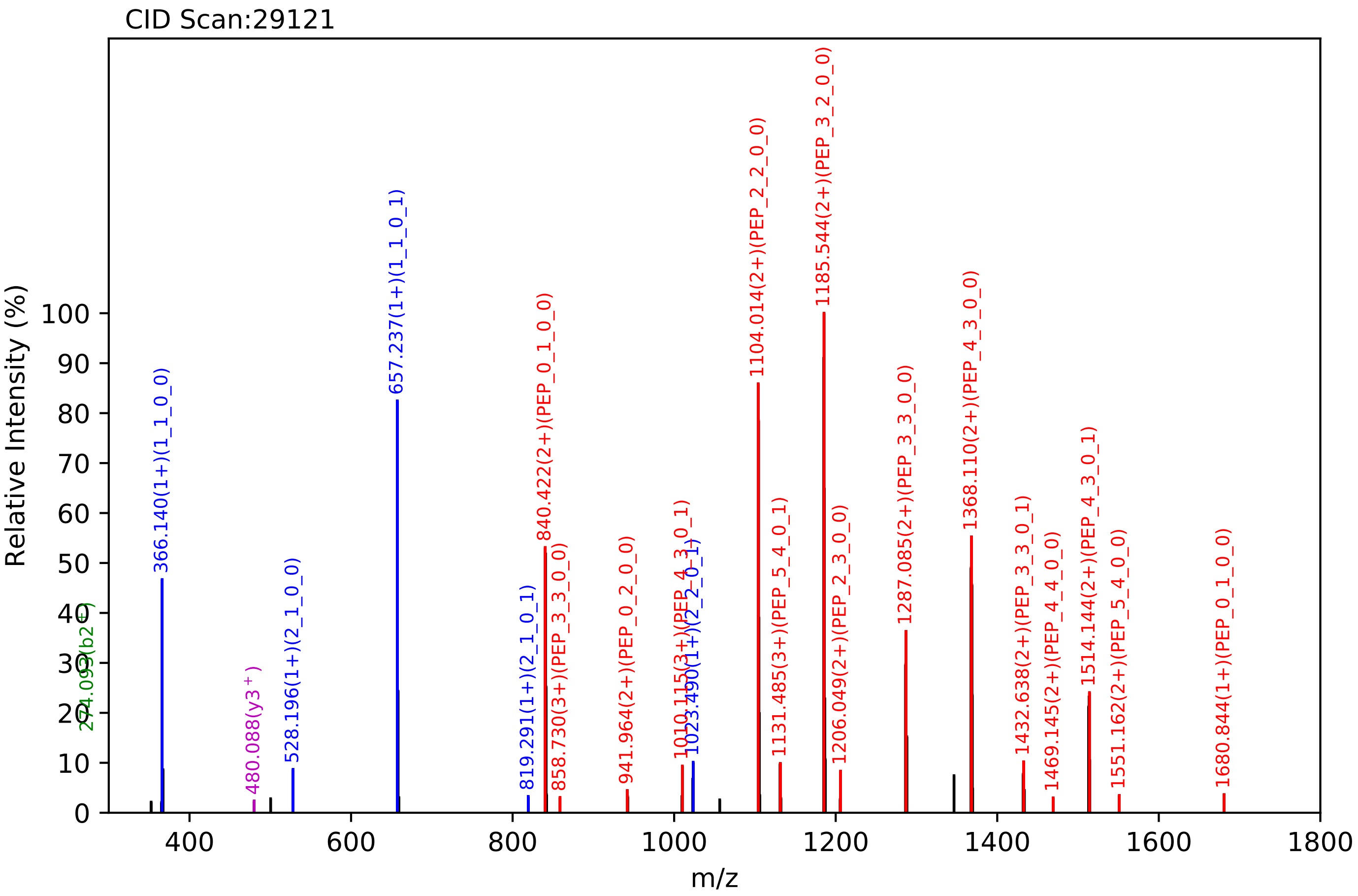

Unknown set no. 633, Experiment: Human Plasma exp_5

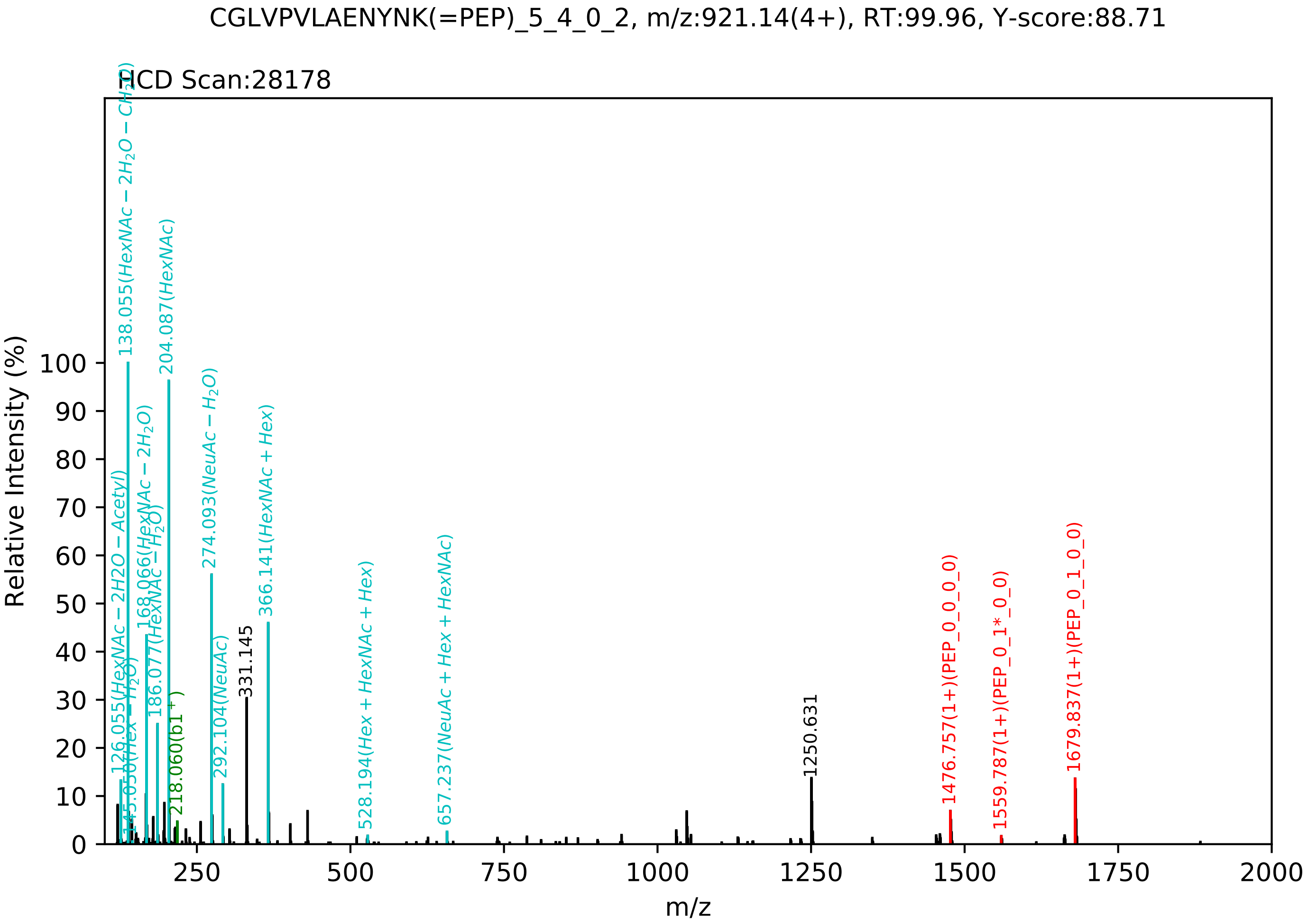

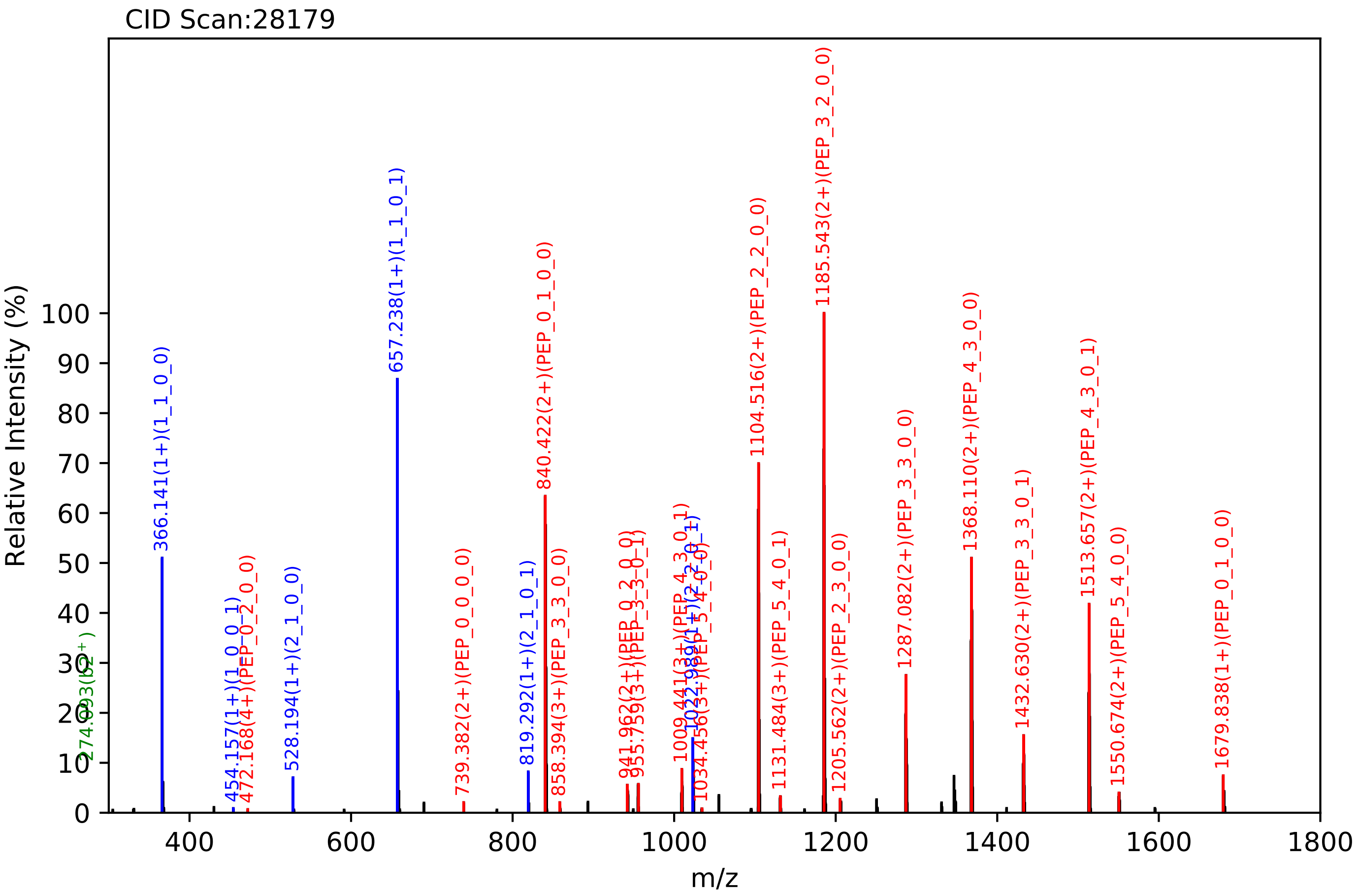

Unknown set no. 634, Experiment: Human Plasma exp_3

LDVDQALNR(=PEP)_5_4_0_2, m/z:1083.45(3+), RT:78.91, Y-score:83.38

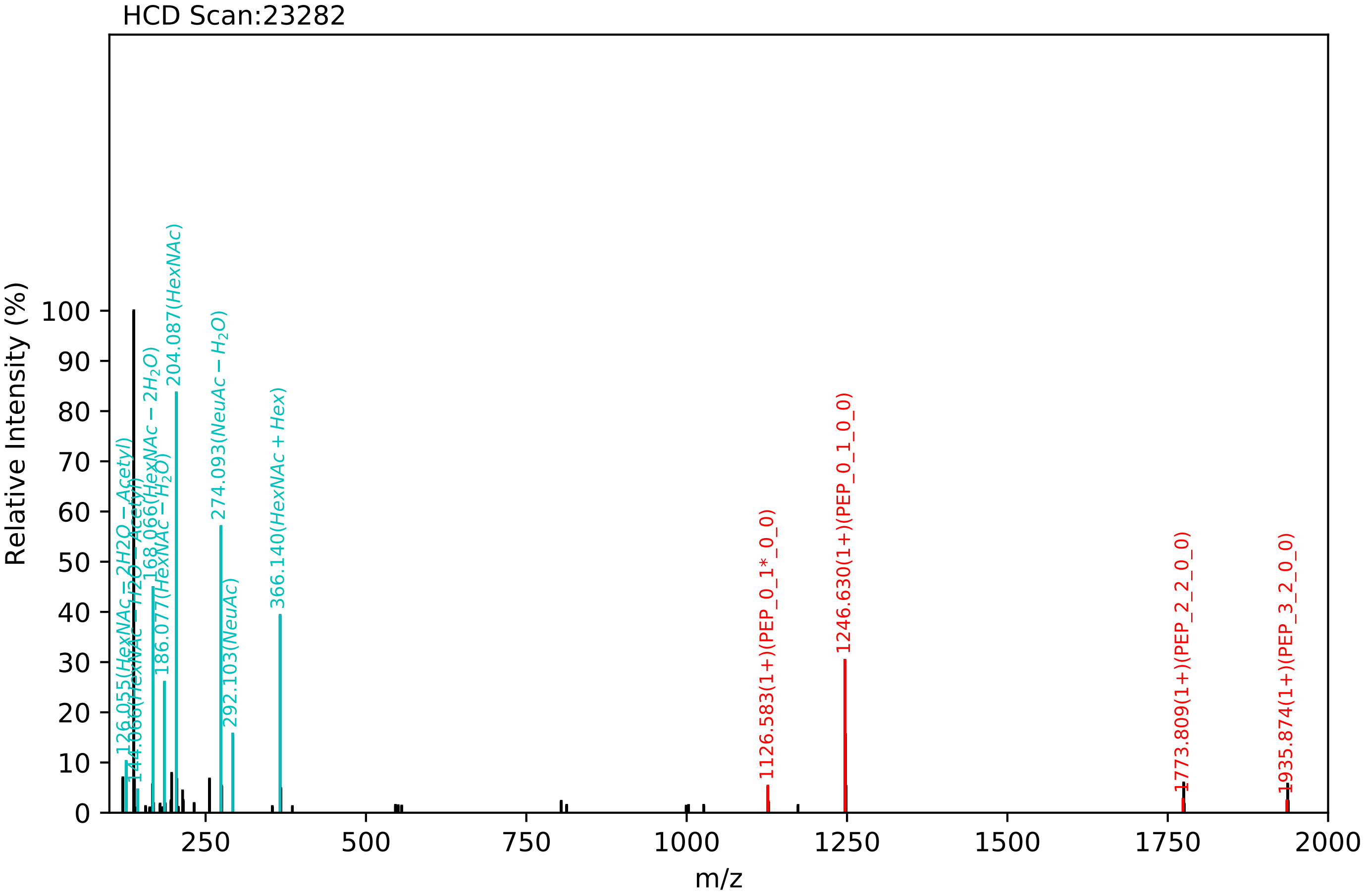

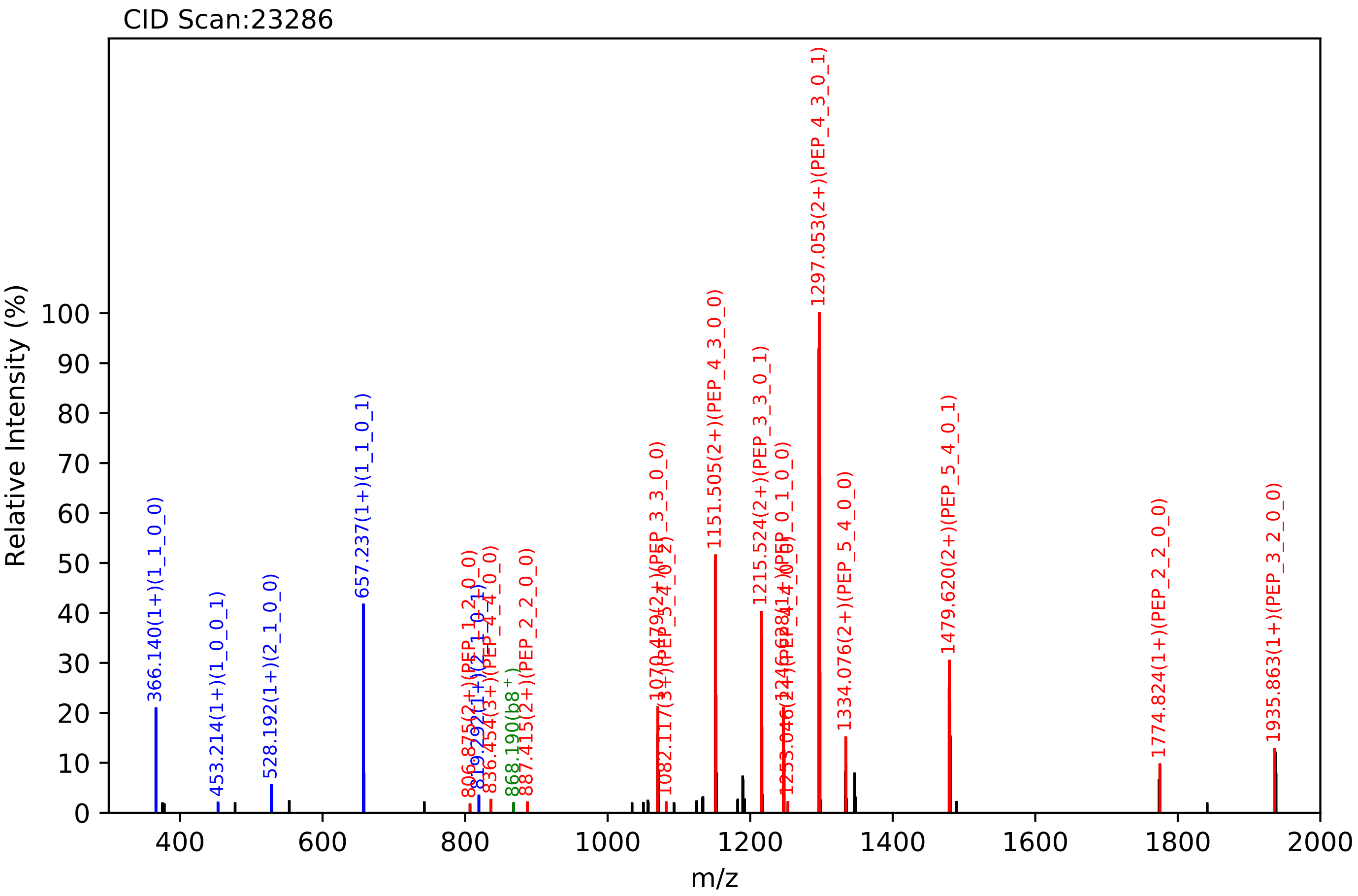

Unknown set no. 635, Experiment: Human Plasma exp_4

VTACHSSQPNATLYK(=PEP)_5_4_0_2, m/z:1294.54(3+), RT:51.44, Y-score:76.39

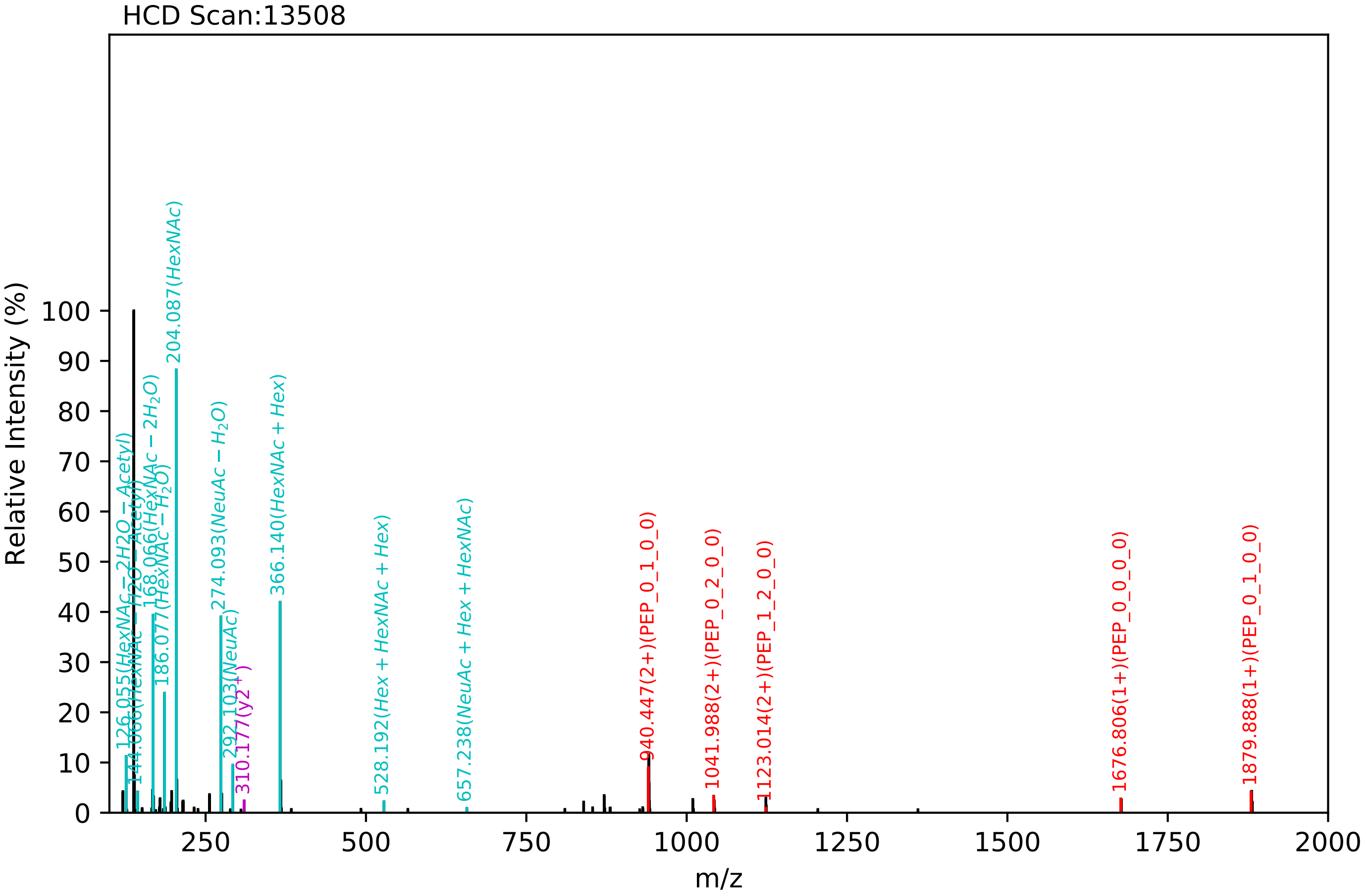

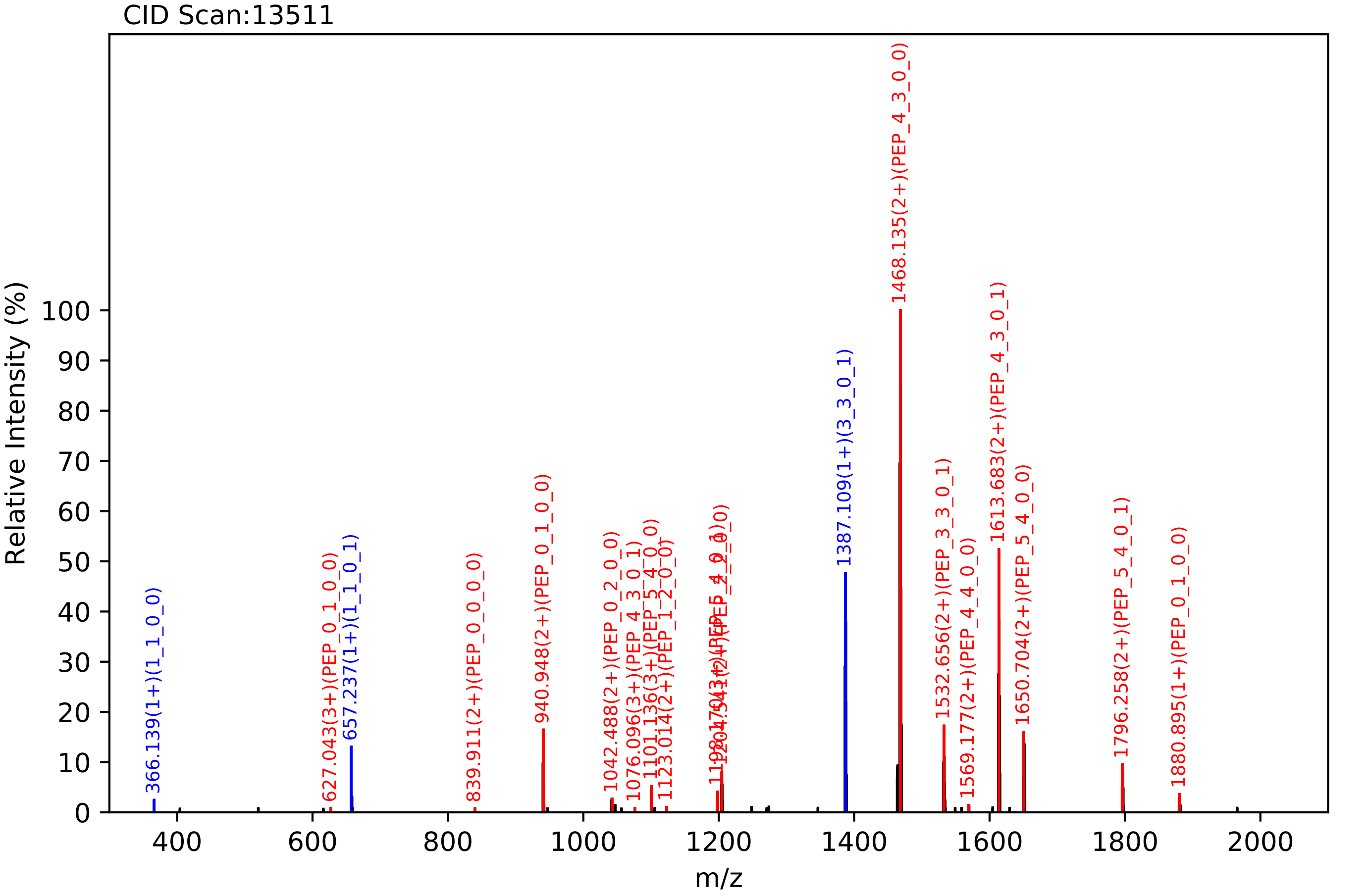

Unknown set no. 636, Experiment: Human Plasma exp_1

EVFVHPNYSK(=PEP)_5_4_0_2, m/z:856.86(4+), RT:61.43, Y-score:83.48

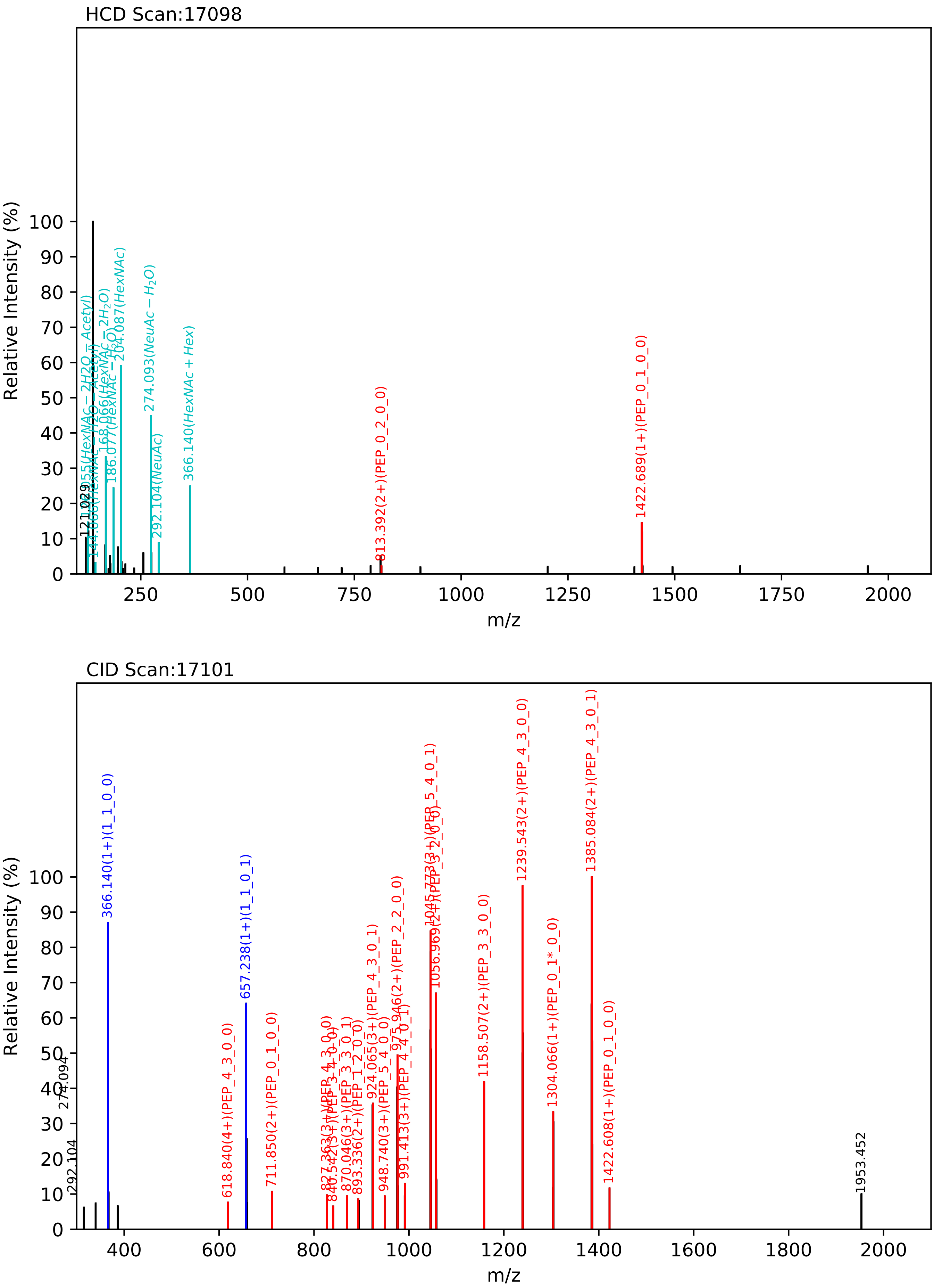

Unknown set no. 637, Experiment: Human Plasma exp_2

EVFVHPNYSK(=PEP)_5_4_0_2, m/z:856.85(4+), RT:61.57, Y-score:84.91

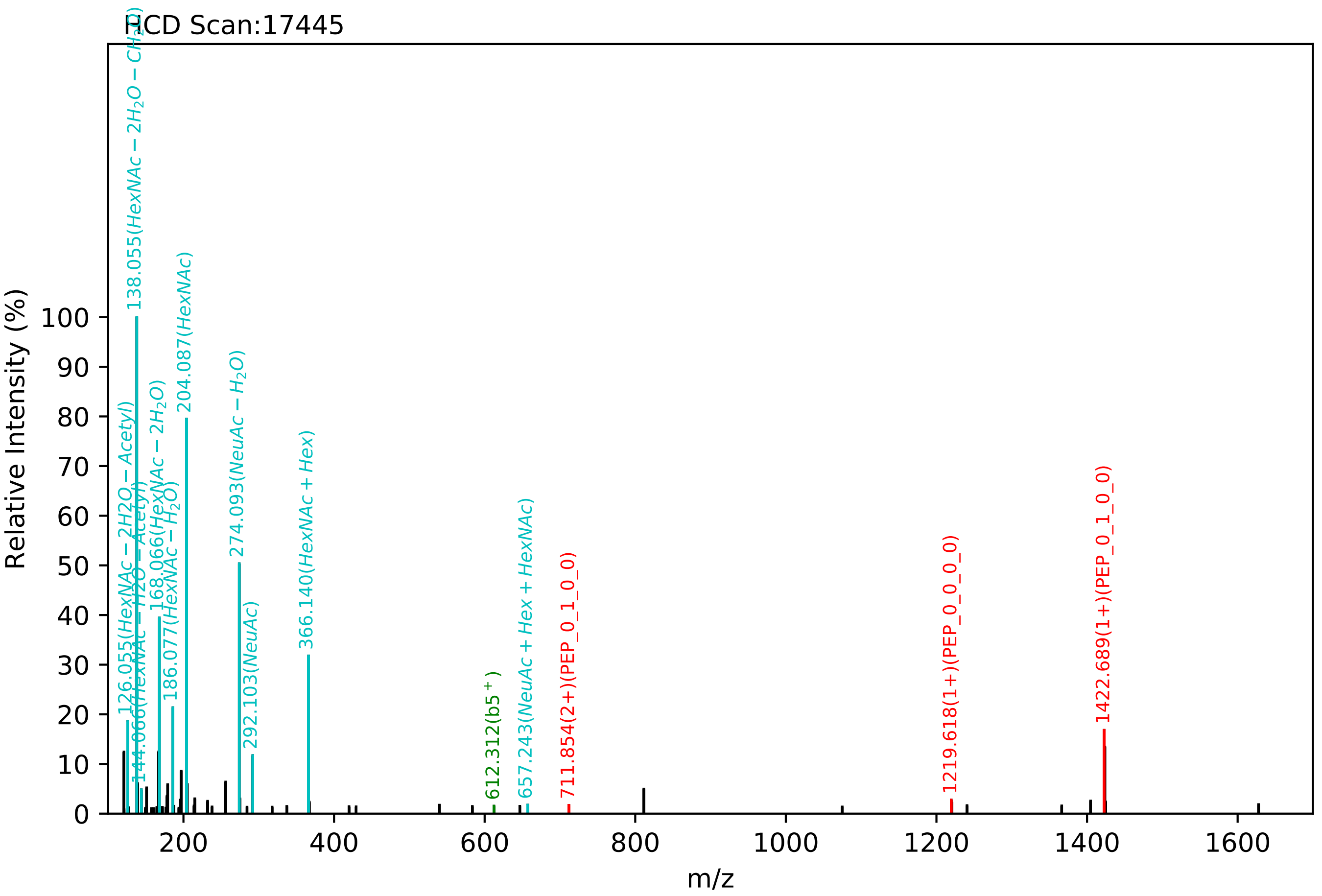

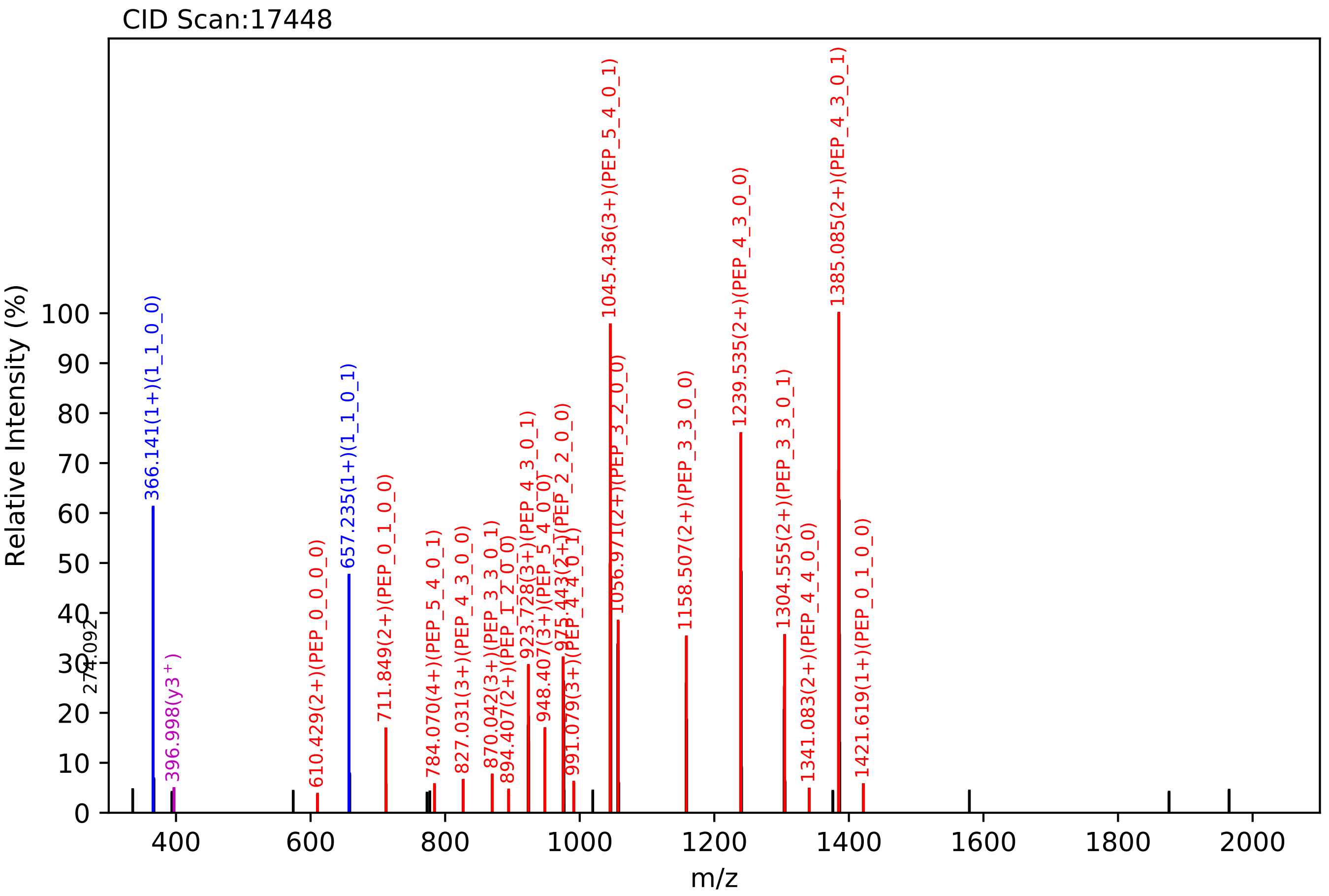

Unknown set no. 638, Experiment: Human Plasma exp_1

NGSLFAFR(=PEP)_4_3_0_1, m/z:820.68(3+), RT:84.48, Y-score:94.31

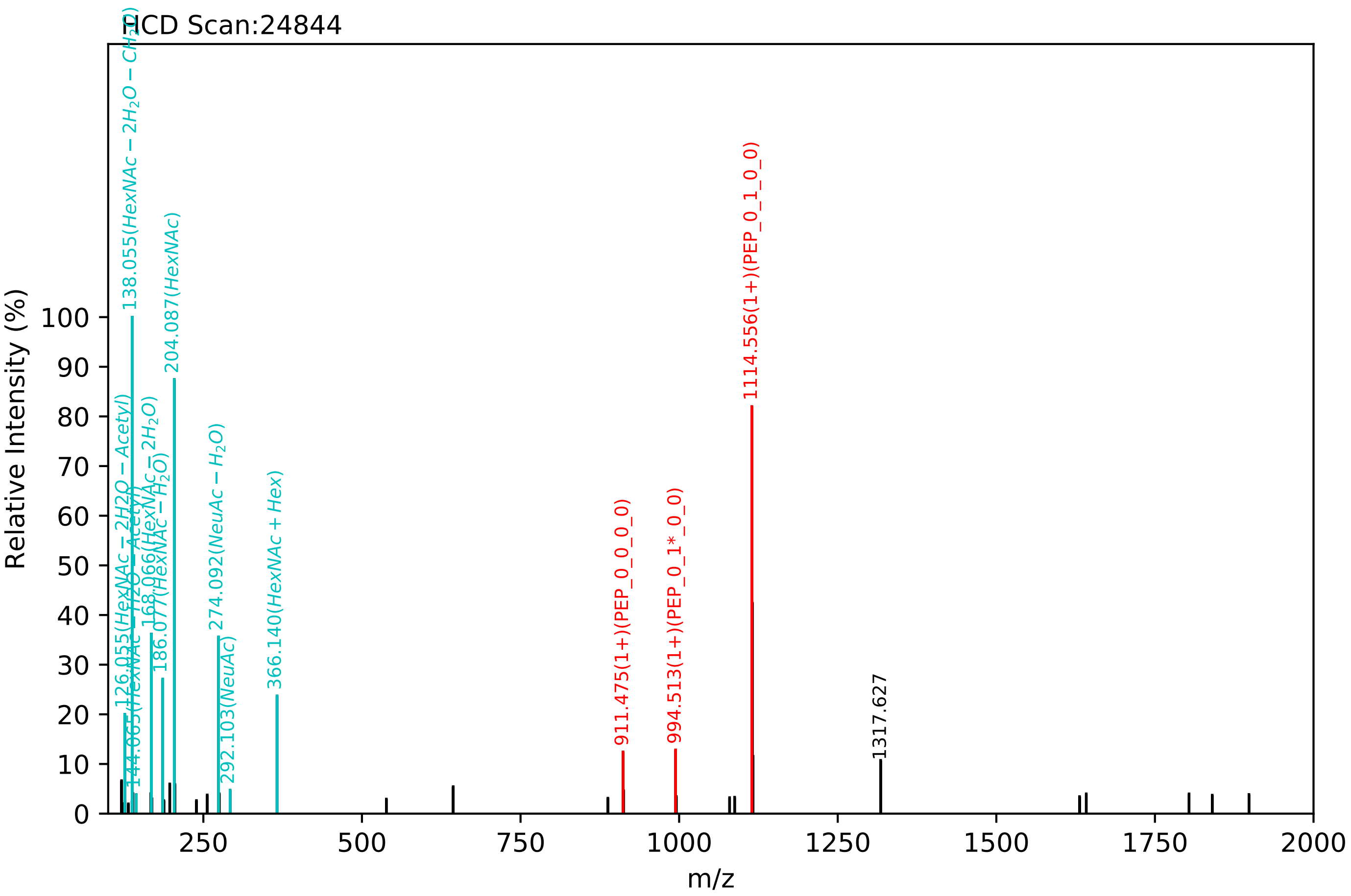

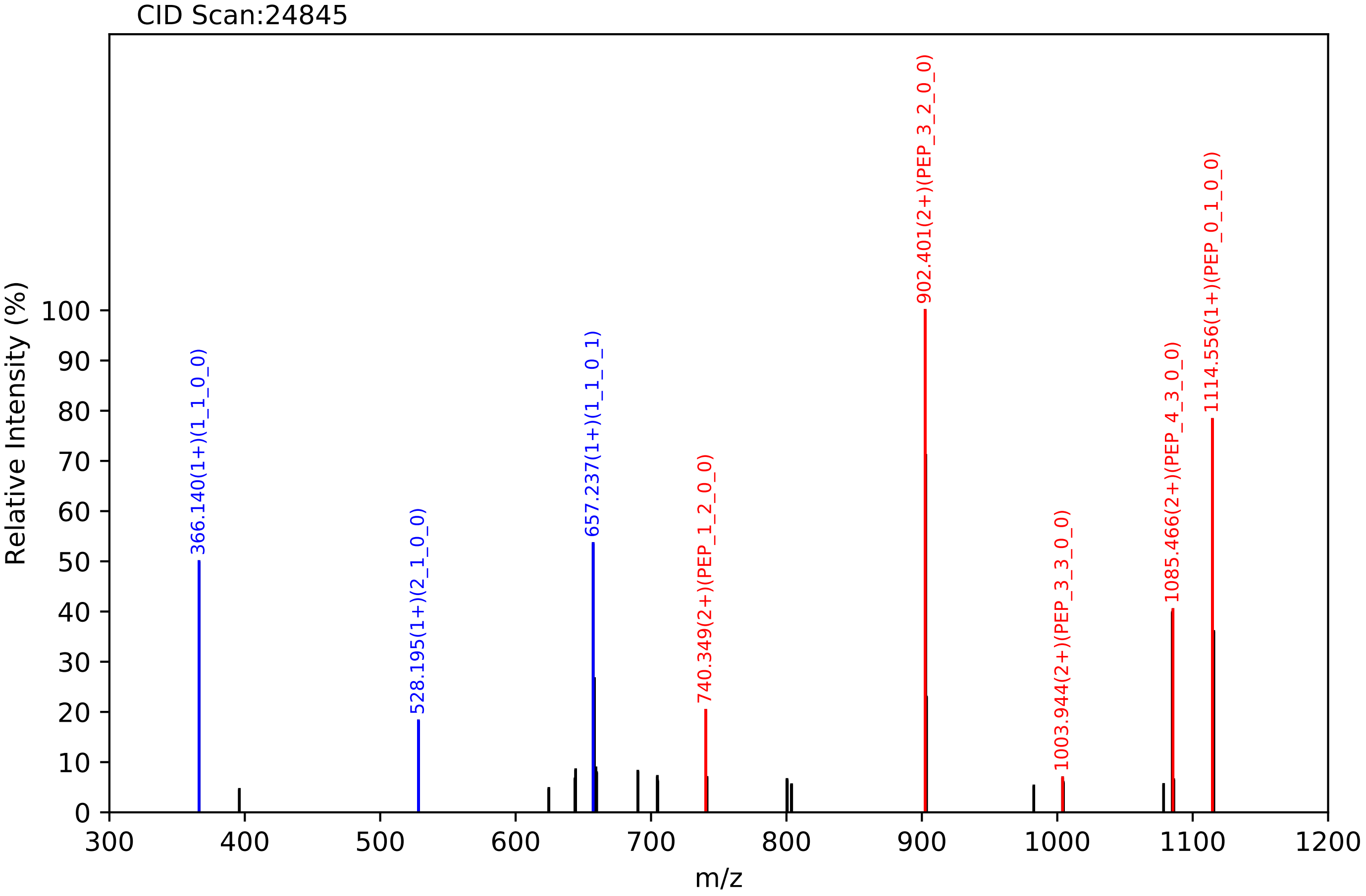

Unknown set no. 639, Experiment: Human Plasma exp_2

NGSLFAFR(=PEP)_4_3_0_1, m/z:820.68(3+), RT:84.45, Y-score:89.95

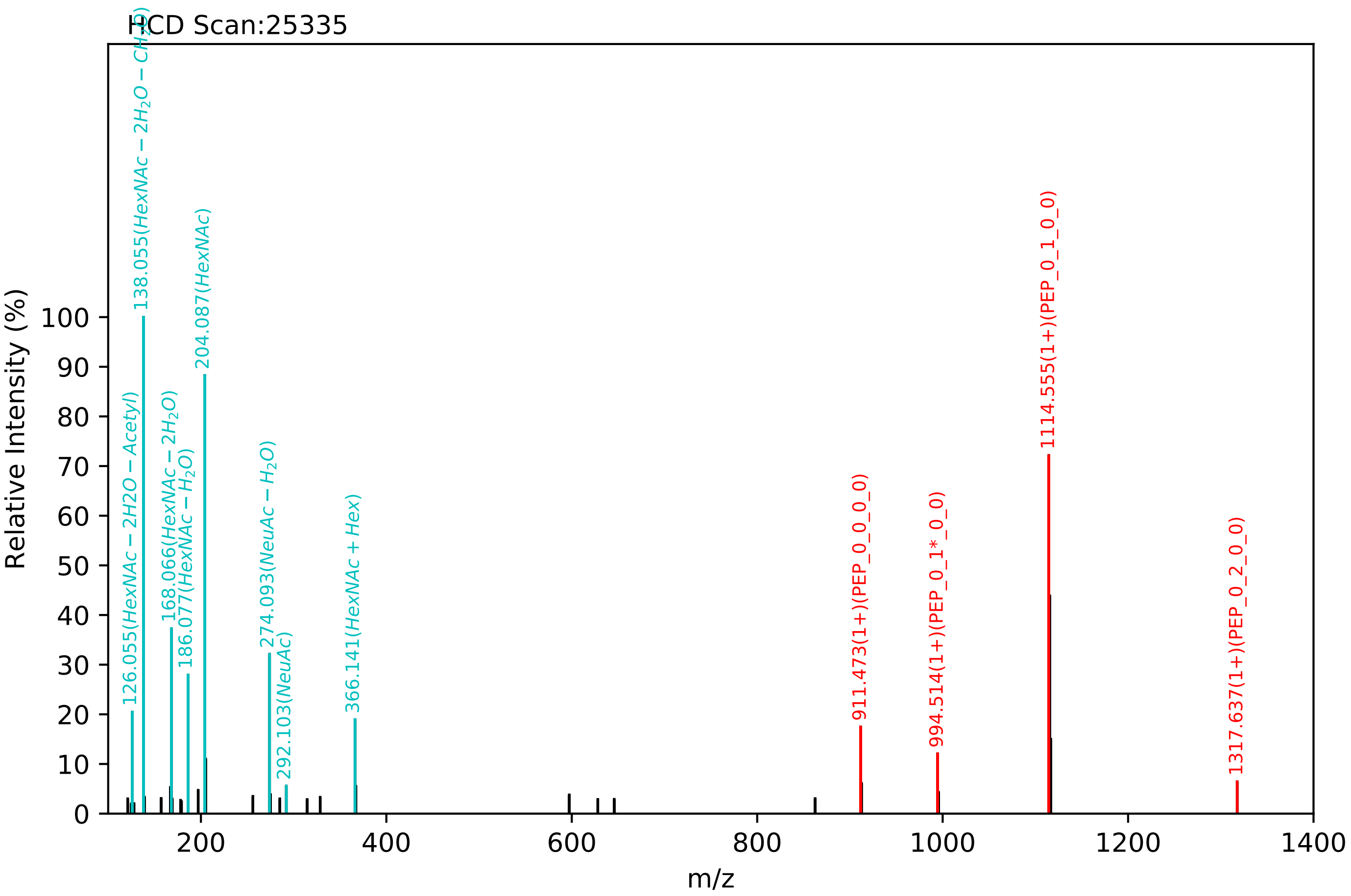

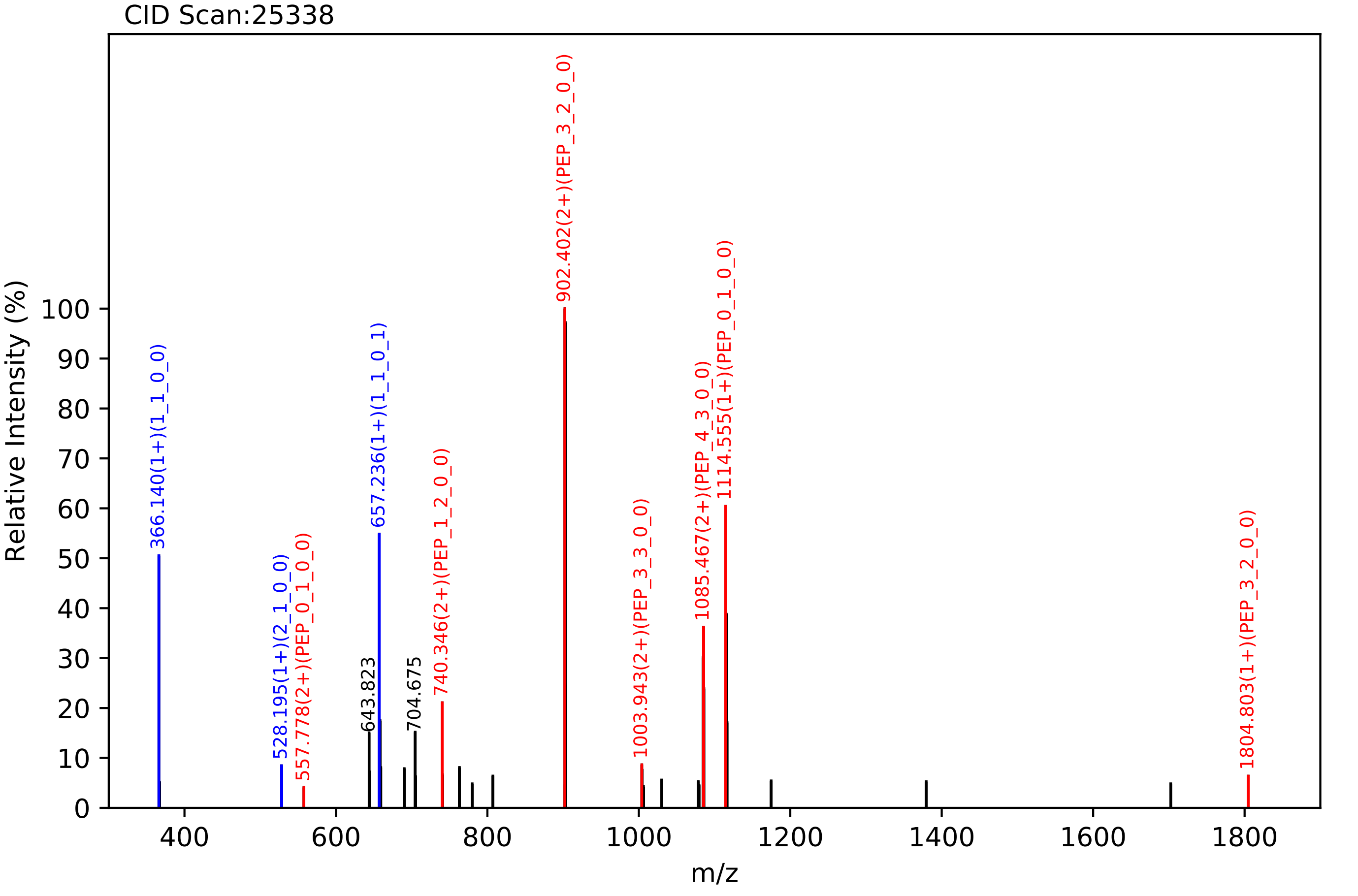

Unknown set no. 640, Experiment: Human Plasma exp_3

NGSLFAFR(=PEP)_4_3_0_1, m/z:820.68(3+), RT:84.55, Y-score:89.37

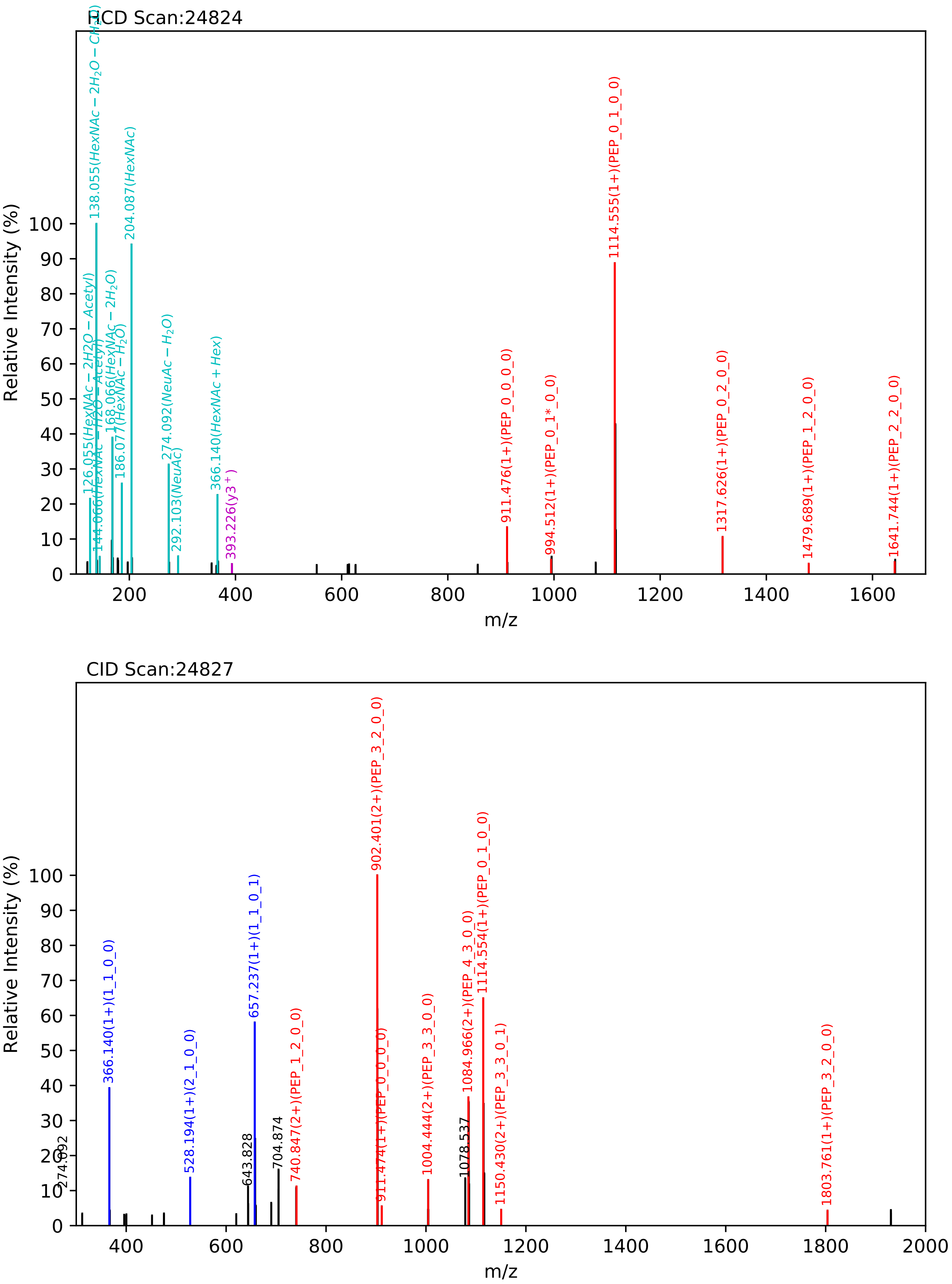

Unknown set no. 641, Experiment: Human Plasma exp_4

NGSLFAFR(=PEP)_4_3_0_1, m/z:820.68(3+), RT:84.64, Y-score:94.90

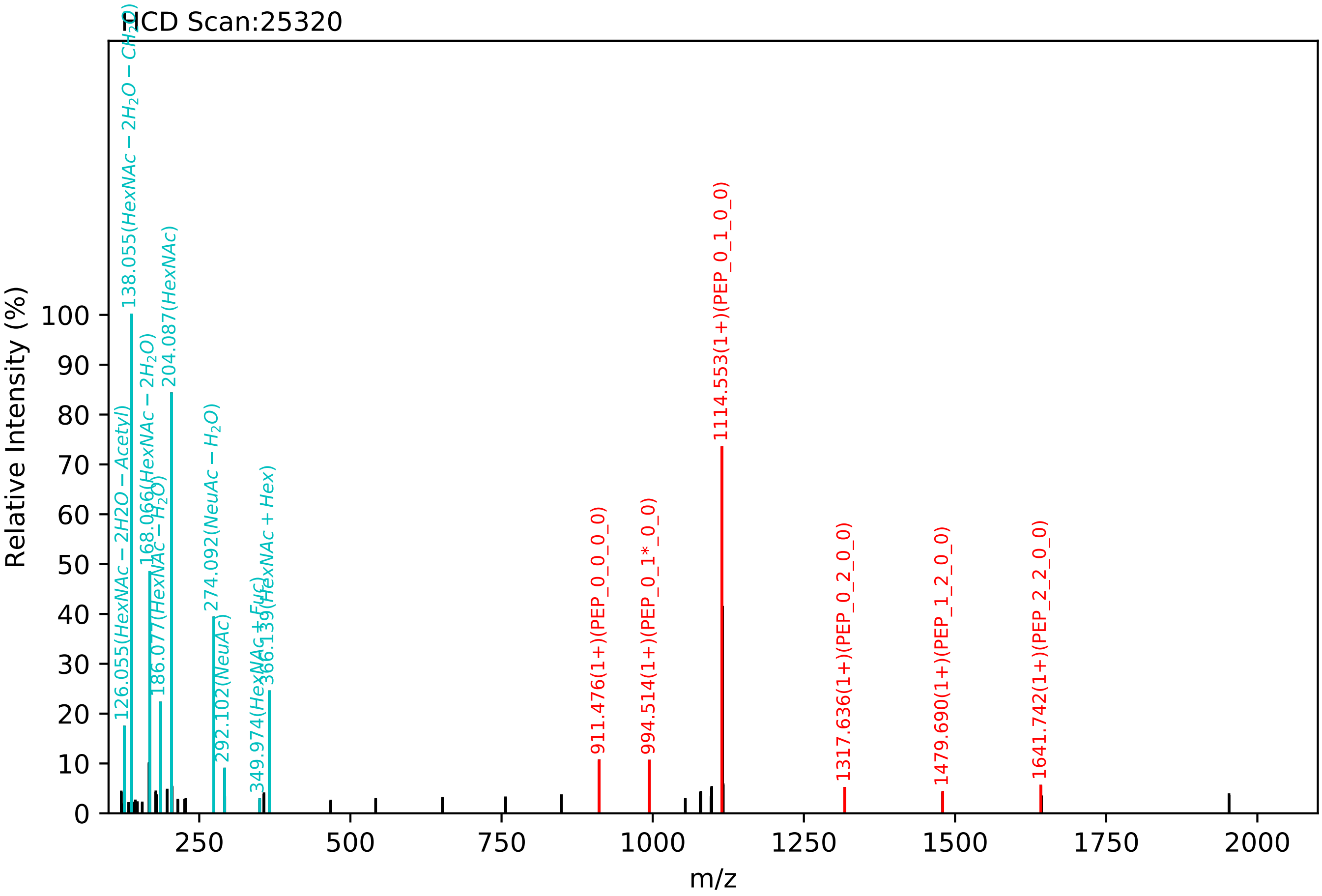

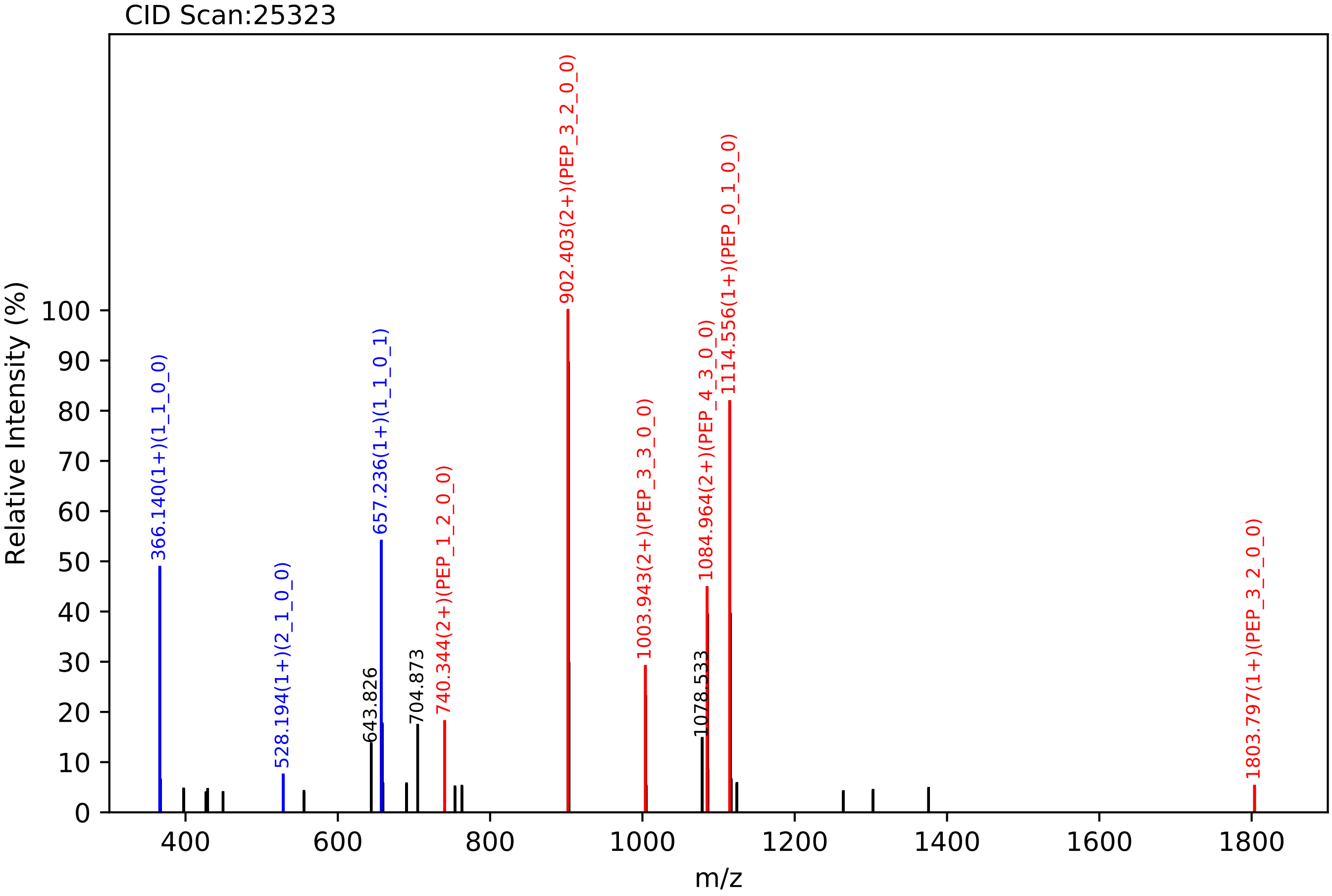

Unknown set no. 642, Experiment: Human Plasma exp_5

NGSLFAFR(=PEP)_4_3_0_1, m/z:820.68(3+), RT:84.27, Y-score:97.96

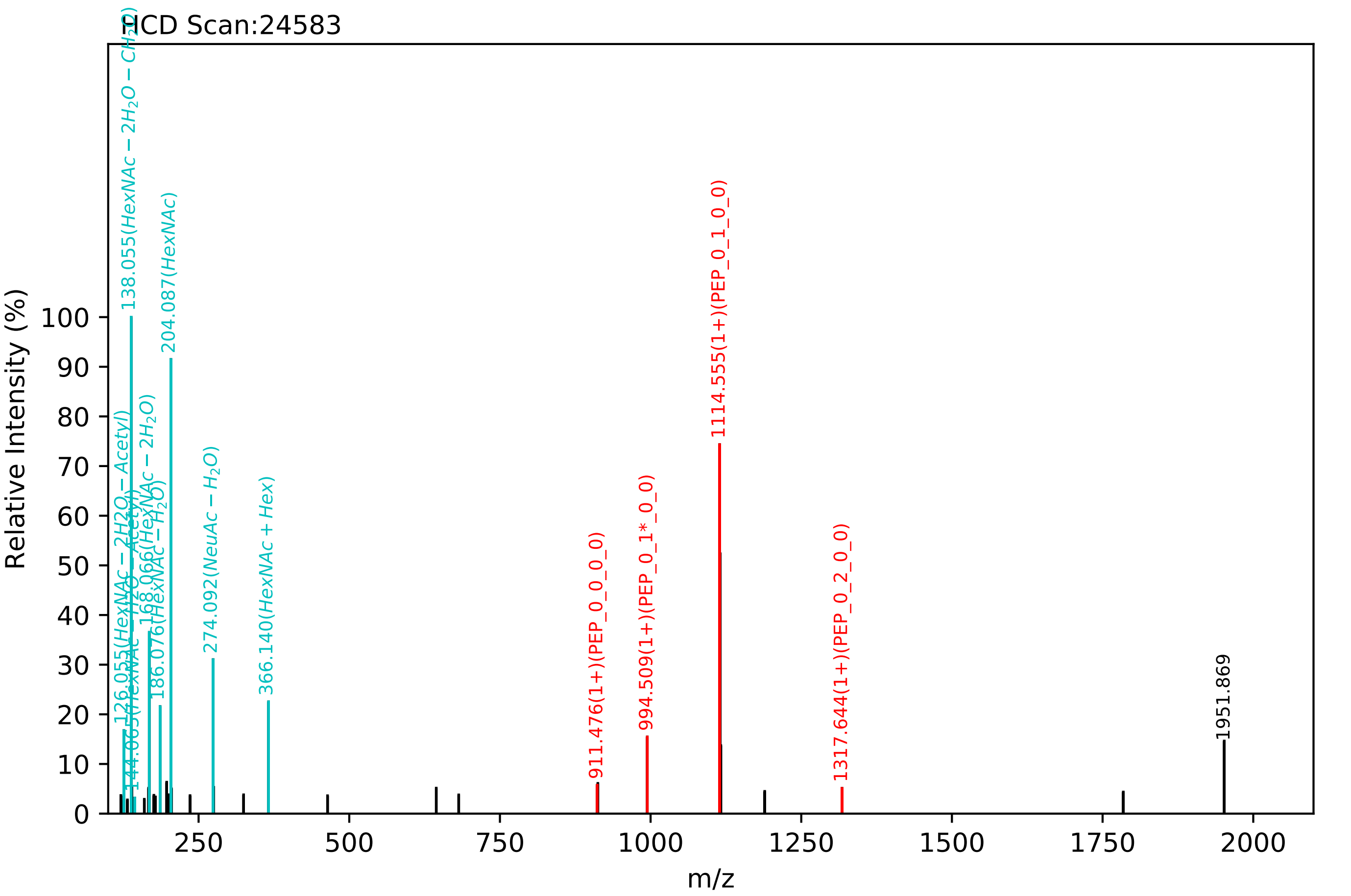

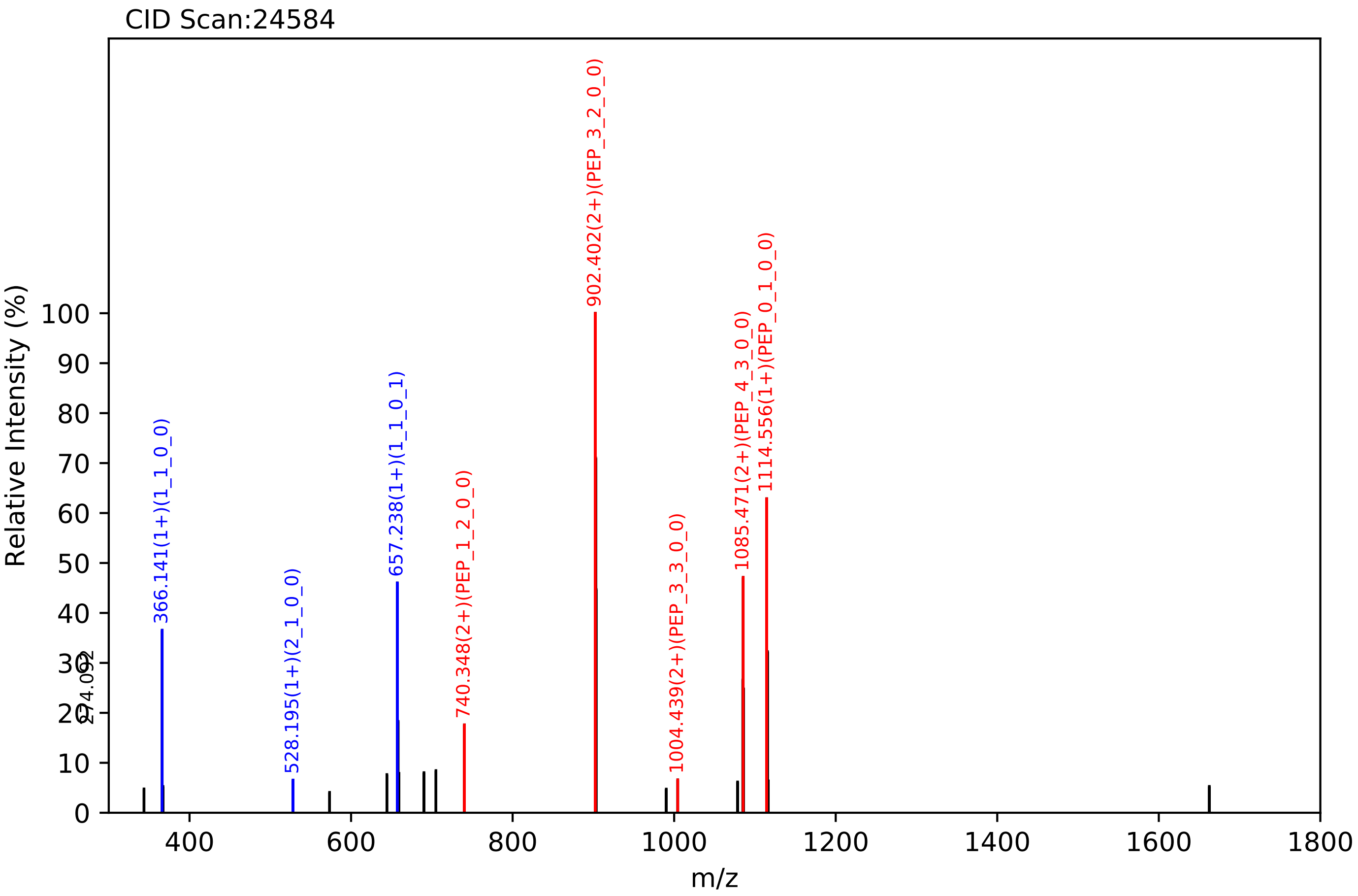

Unknown set no. 643, Experiment: Human Plasma exp_1

NGSLFAFR(=PEP)_5_3_0_1, m/z:874.70(3+), RT:83.68, Y-score:92.11

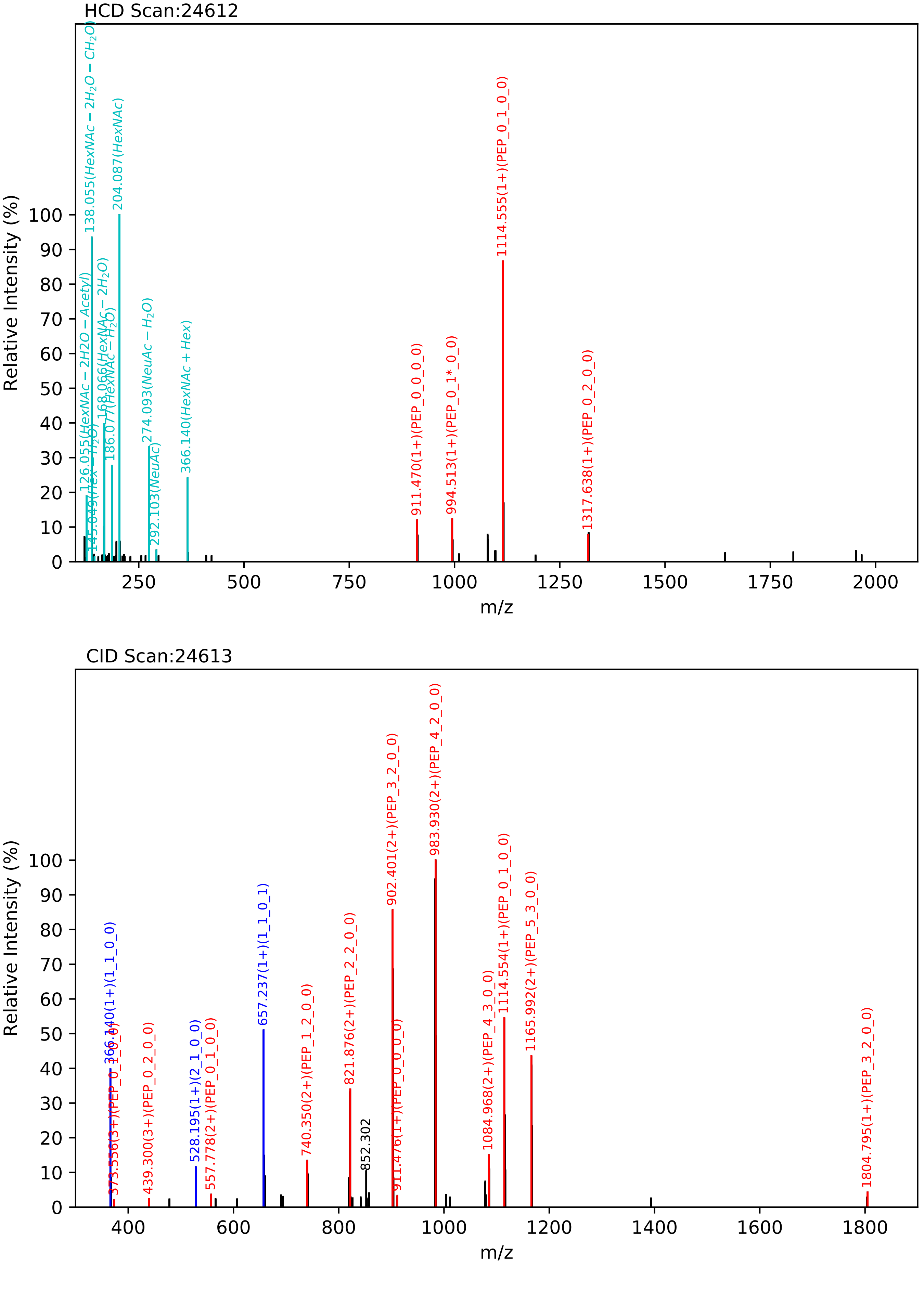

Unknown set no. 644, Experiment: Human Plasma exp_2

NGSLFAFR(=PEP)_5_3_0_1, m/z:874.70(3+), RT:83.65, Y-score:94.72

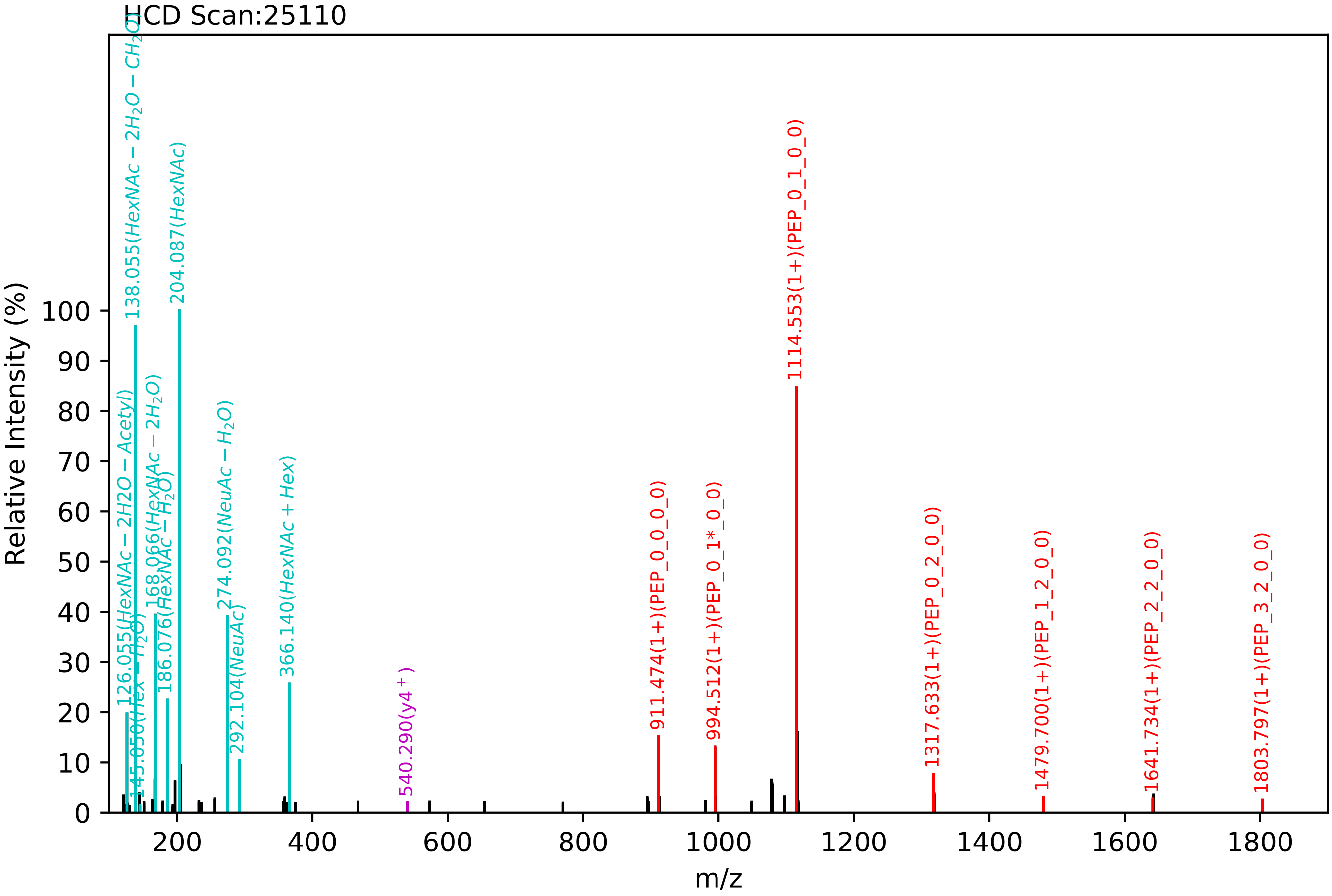

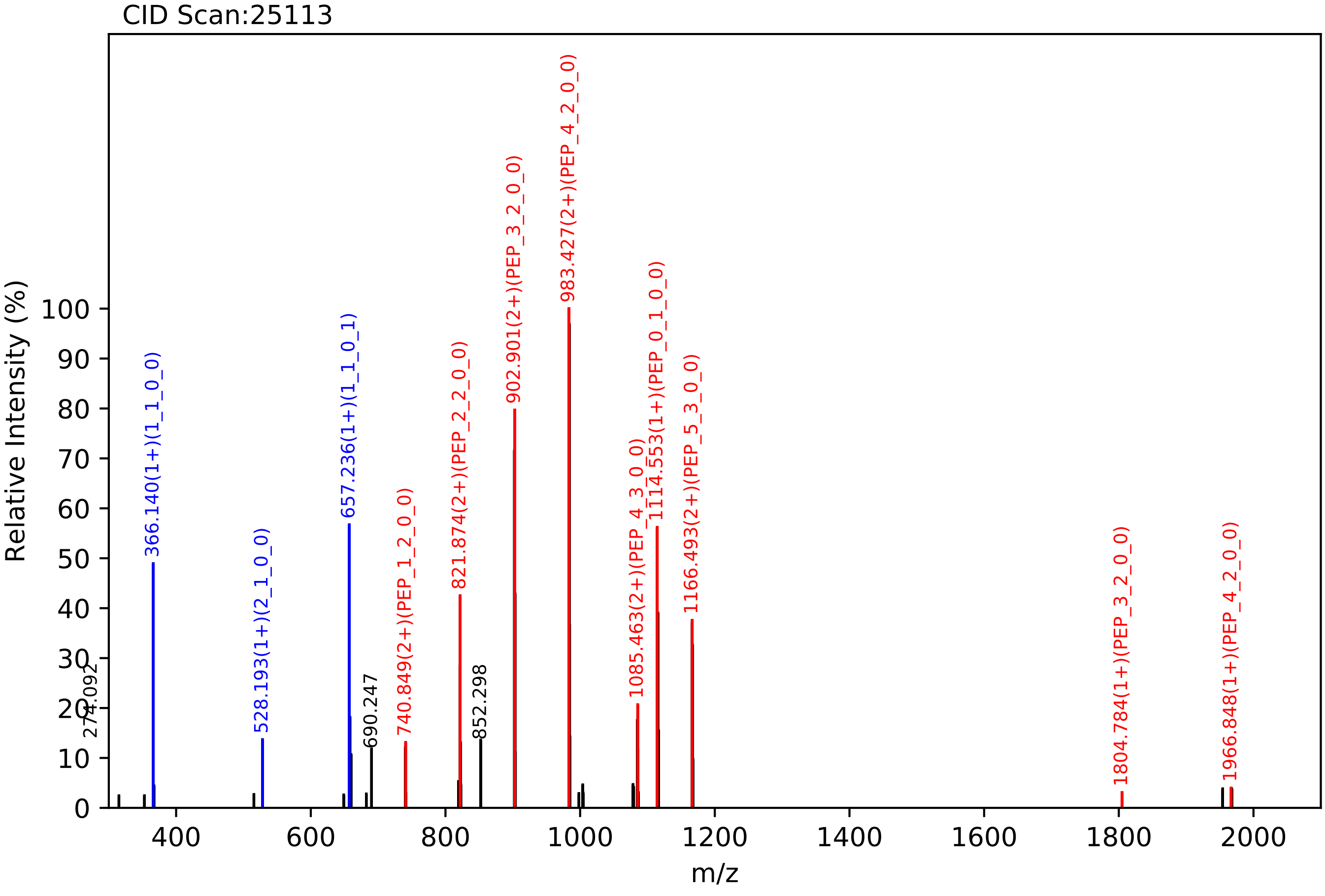

Unknown set no. 645, Experiment: Human Plasma exp_3

NGSLFAFR(=PEP)_5_3_0_1, m/z:874.70(3+), RT:83.79, Y-score:96.13

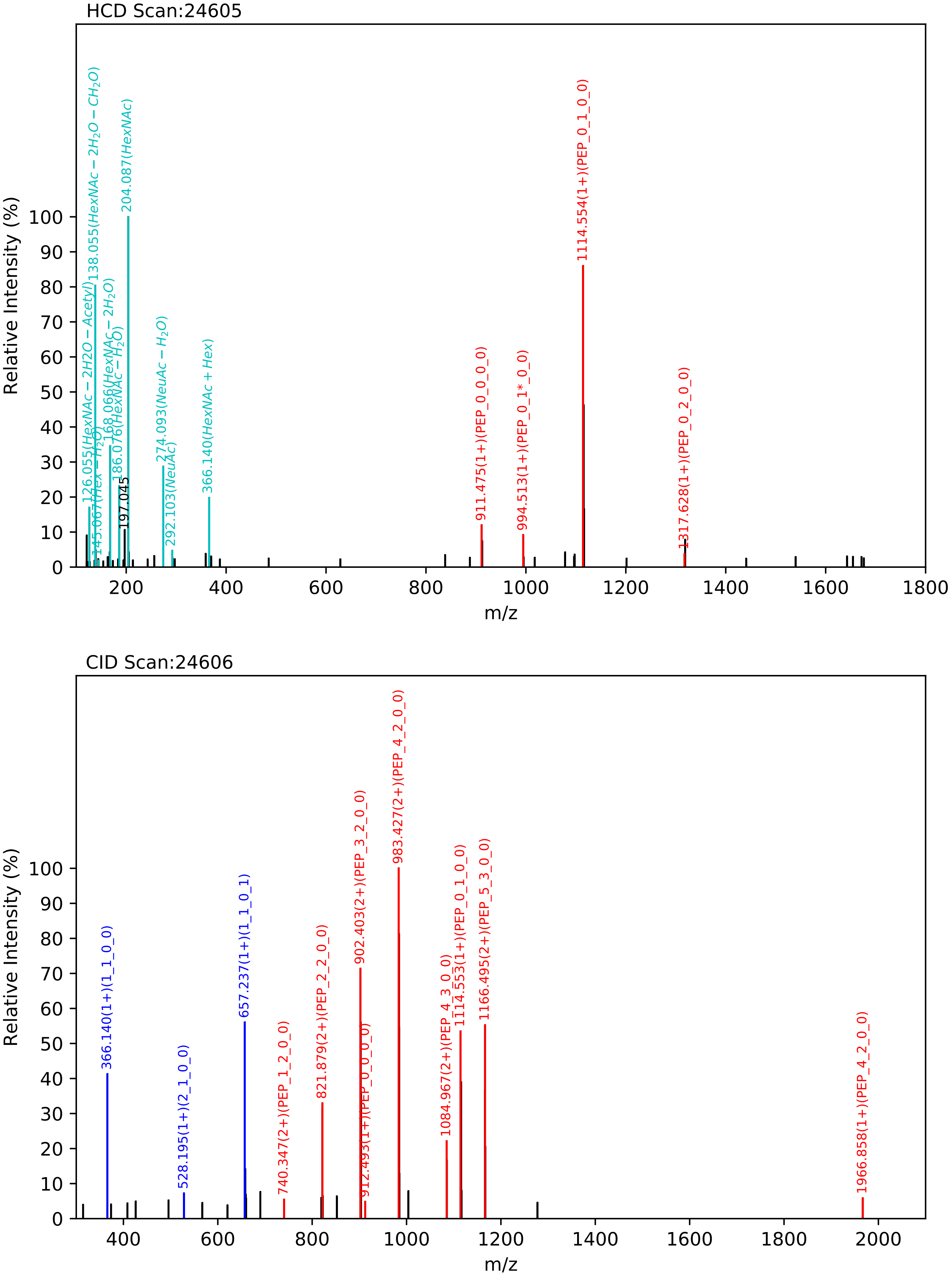

Unknown set no. 646, Experiment: Human Plasma exp_4

NGSLFAFR(=PEP)_5_3_0_1, m/z:874.70(3+), RT:83.89, Y-score:96.23

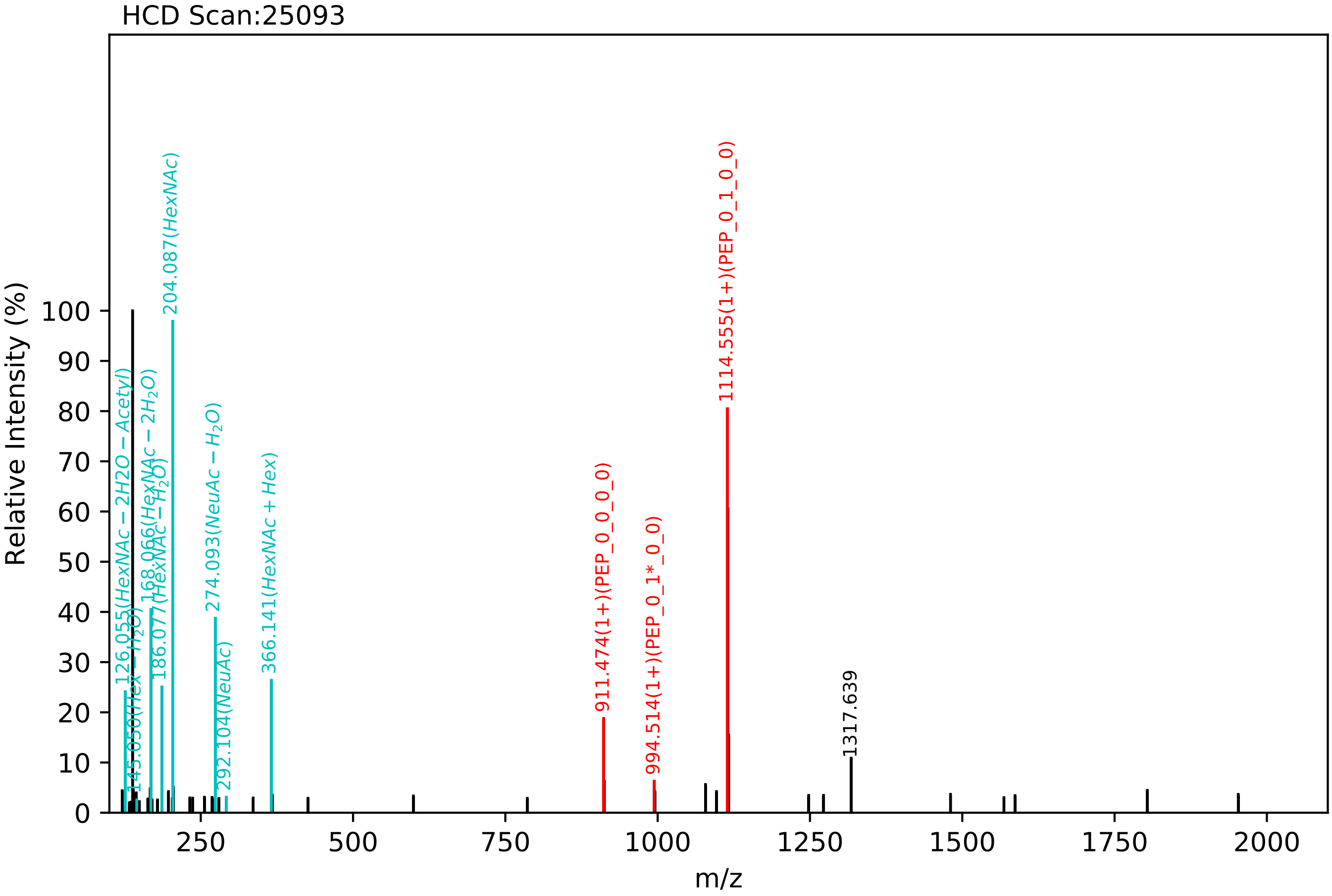

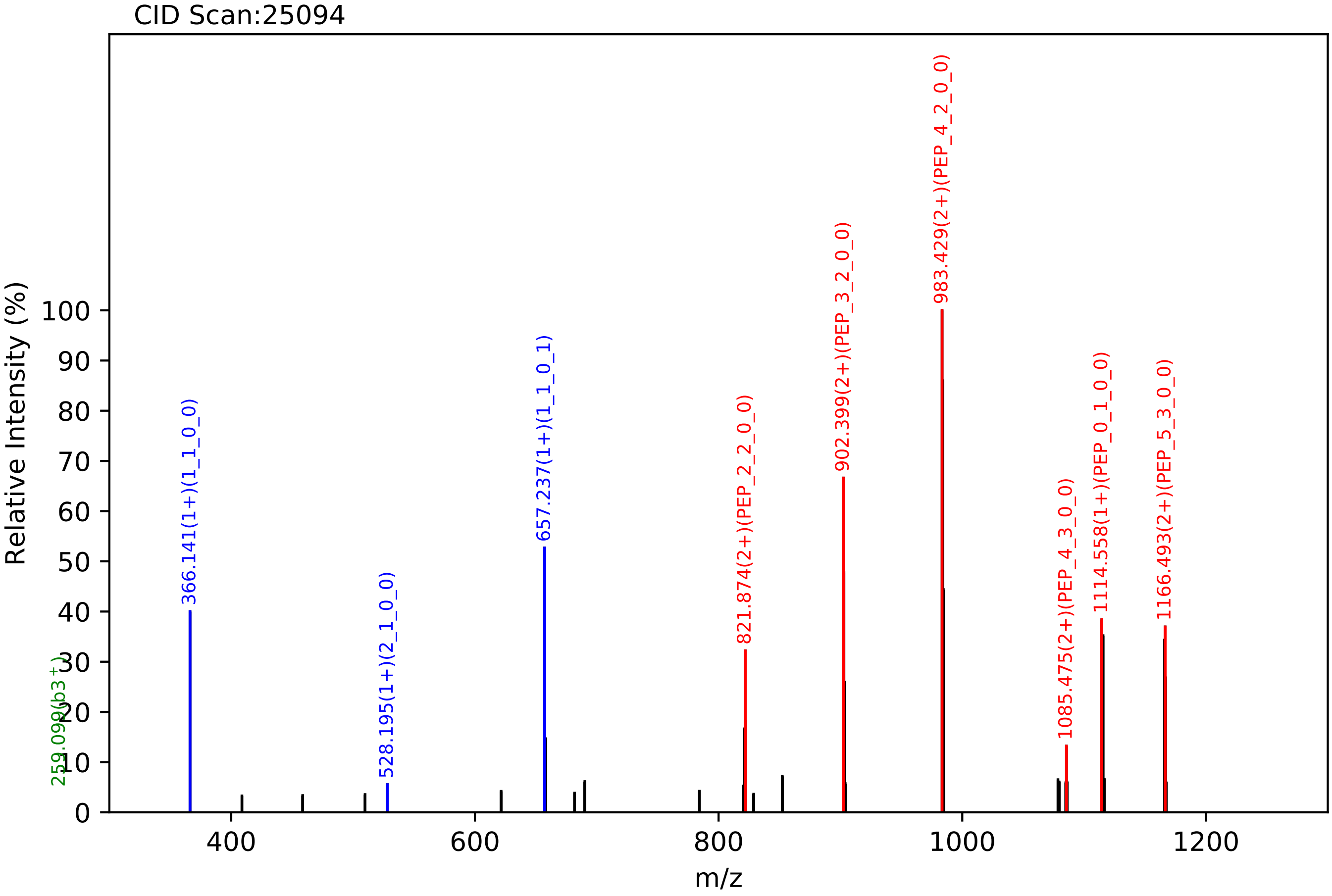

Unknown set no. 647, Experiment: Human Plasma exp_5

NGSLFAFR(=PEP)_5_3_0_1, m/z:874.70(3+), RT:83.62, Y-score:91.22

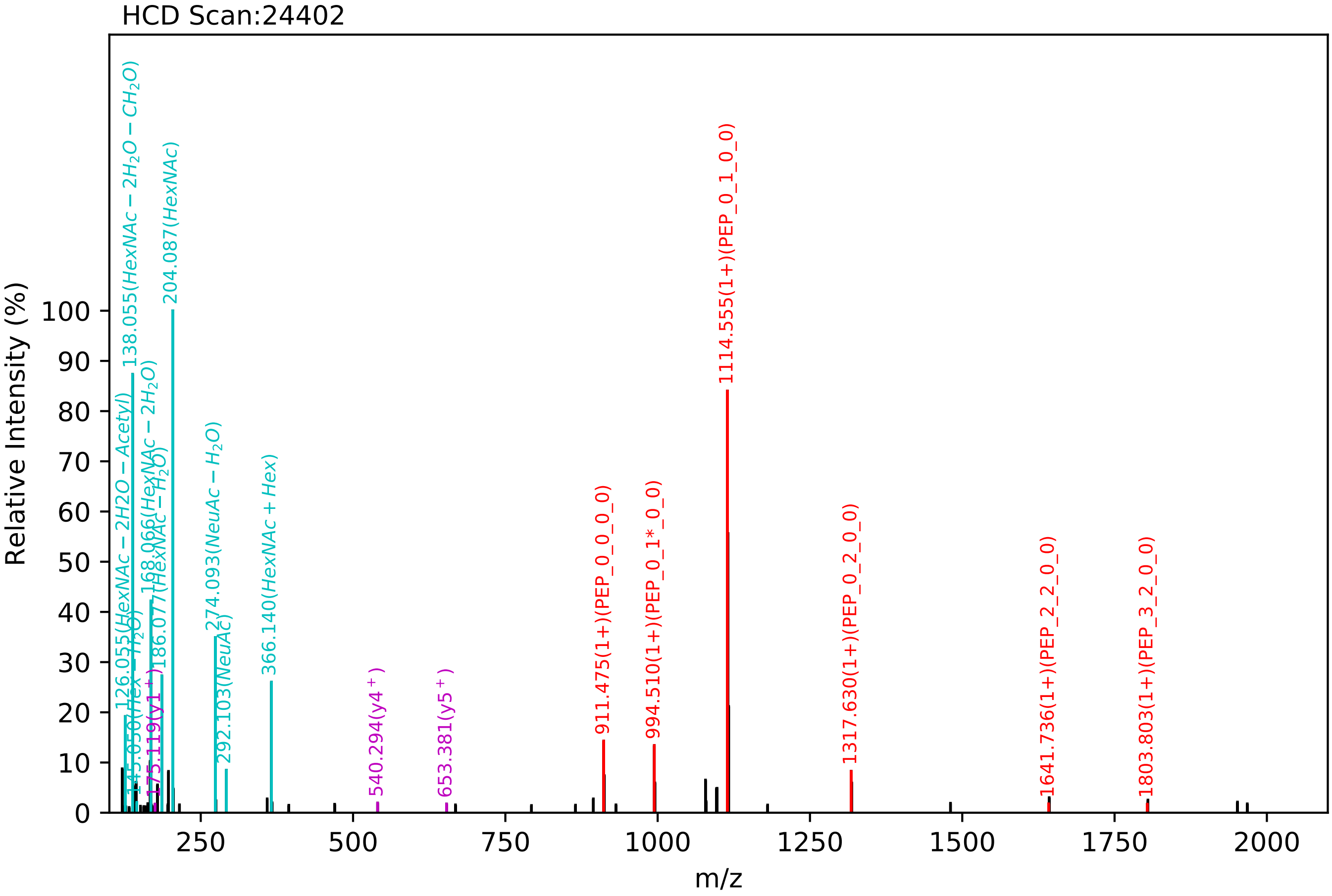

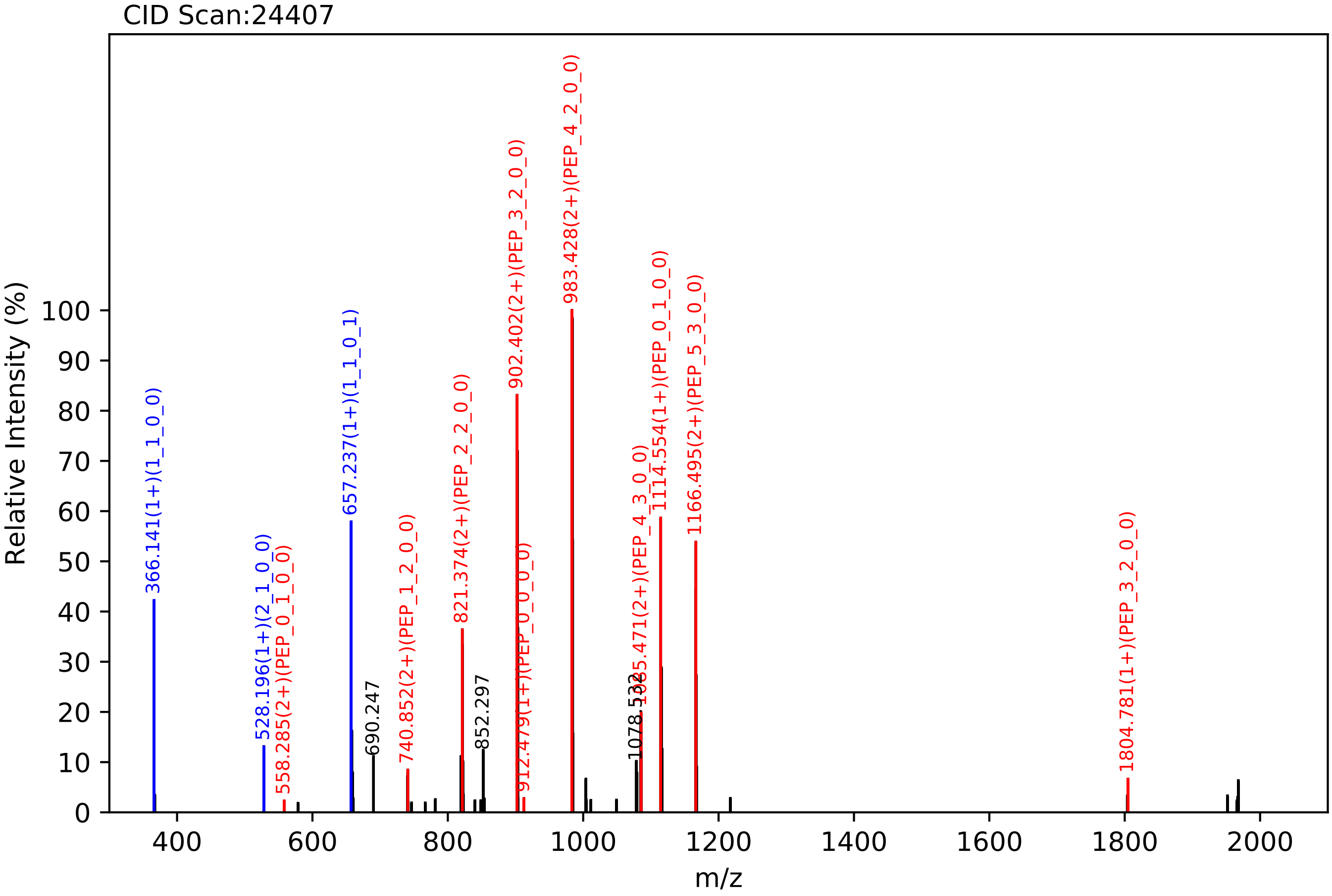

Unknown set no. 648, Experiment: Human Plasma exp_1

NGSLFAFR(=PEP)_5_4_0_1, m/z:942.39(3+), RT:82.93, Y-score:94.57

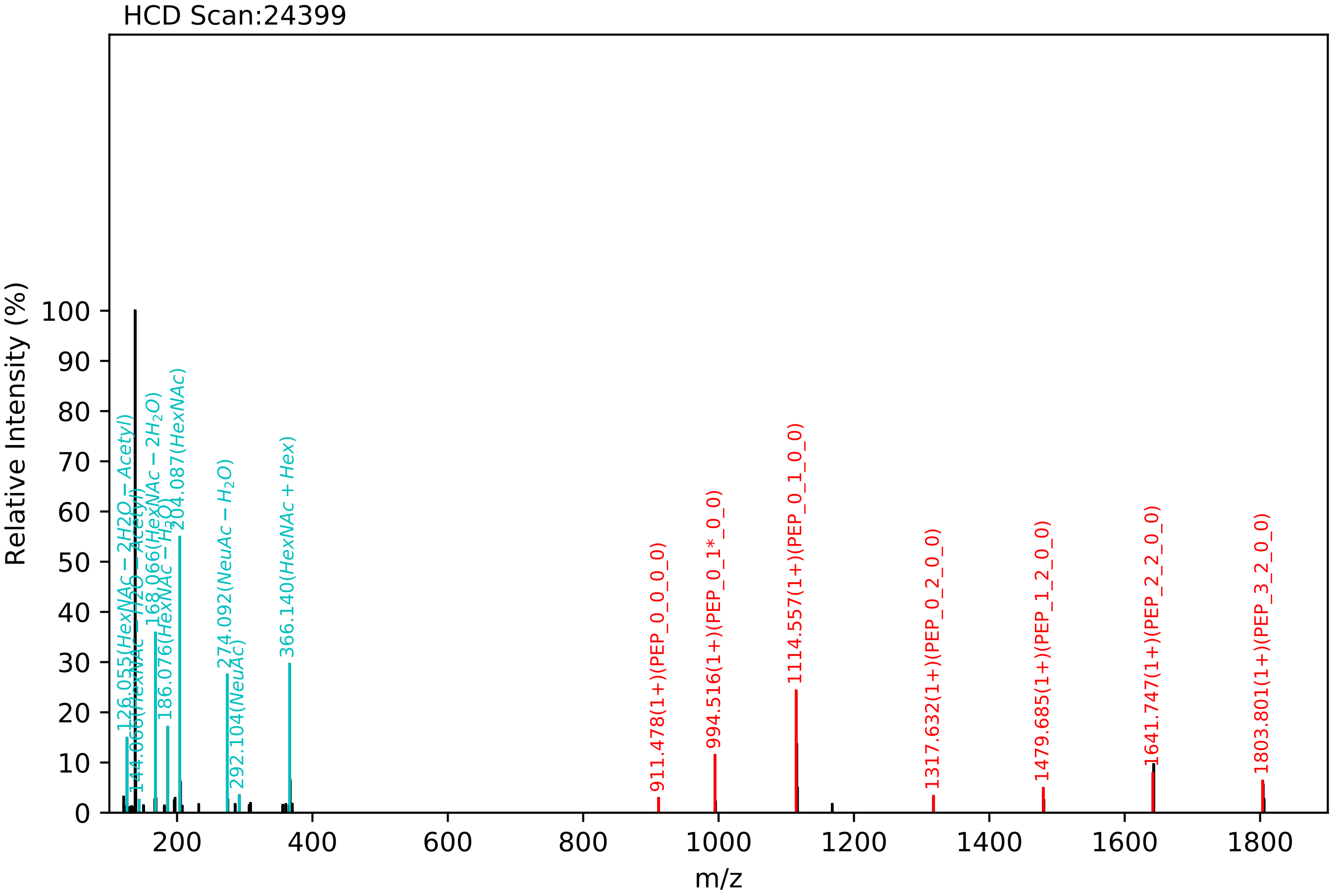

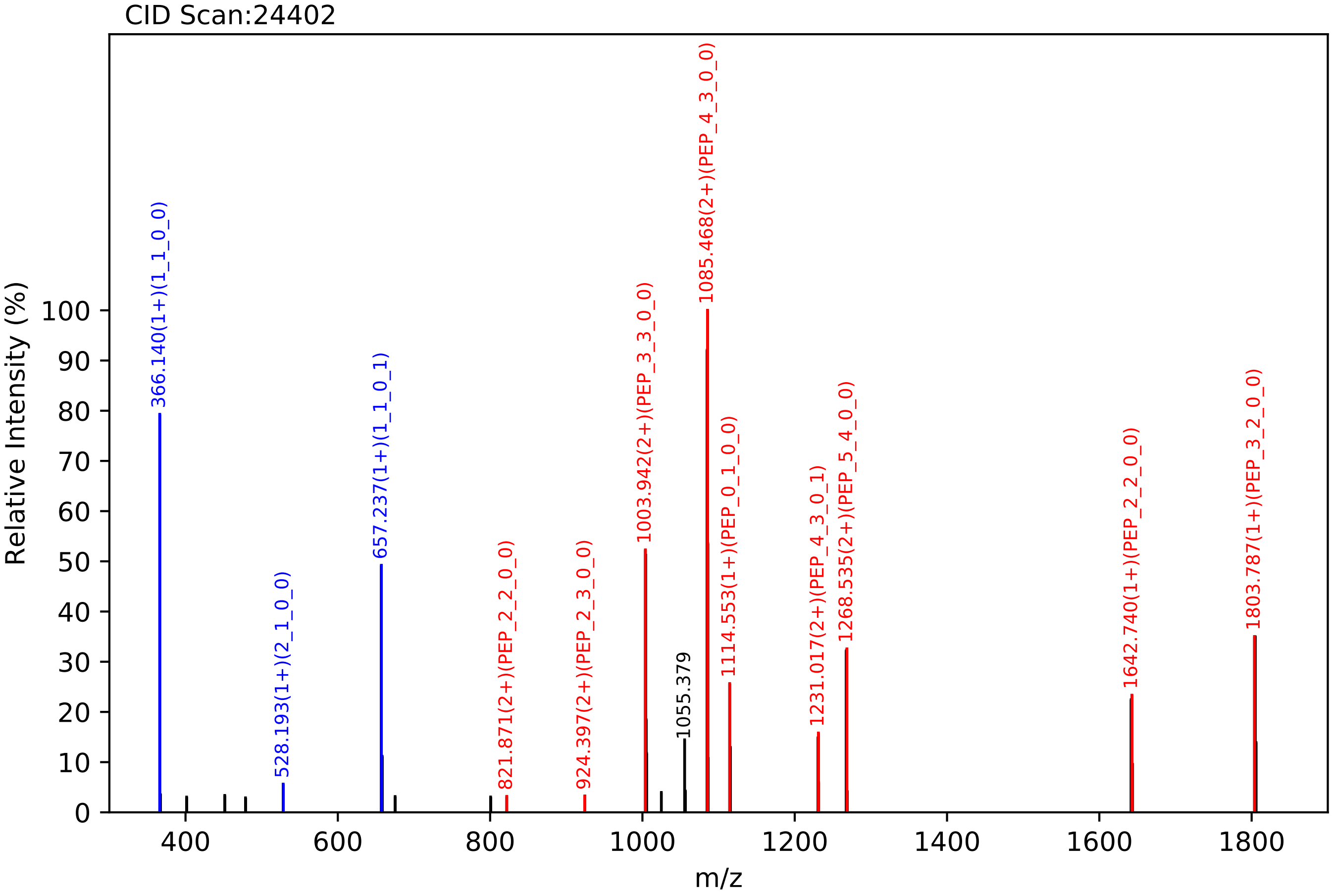

Unknown set no. 649, Experiment: Human Plasma exp_3

NGSLFAFR(=PEP)_5_4_0_1, m/z:942.39(3+), RT:83.20, Y-score:93.21

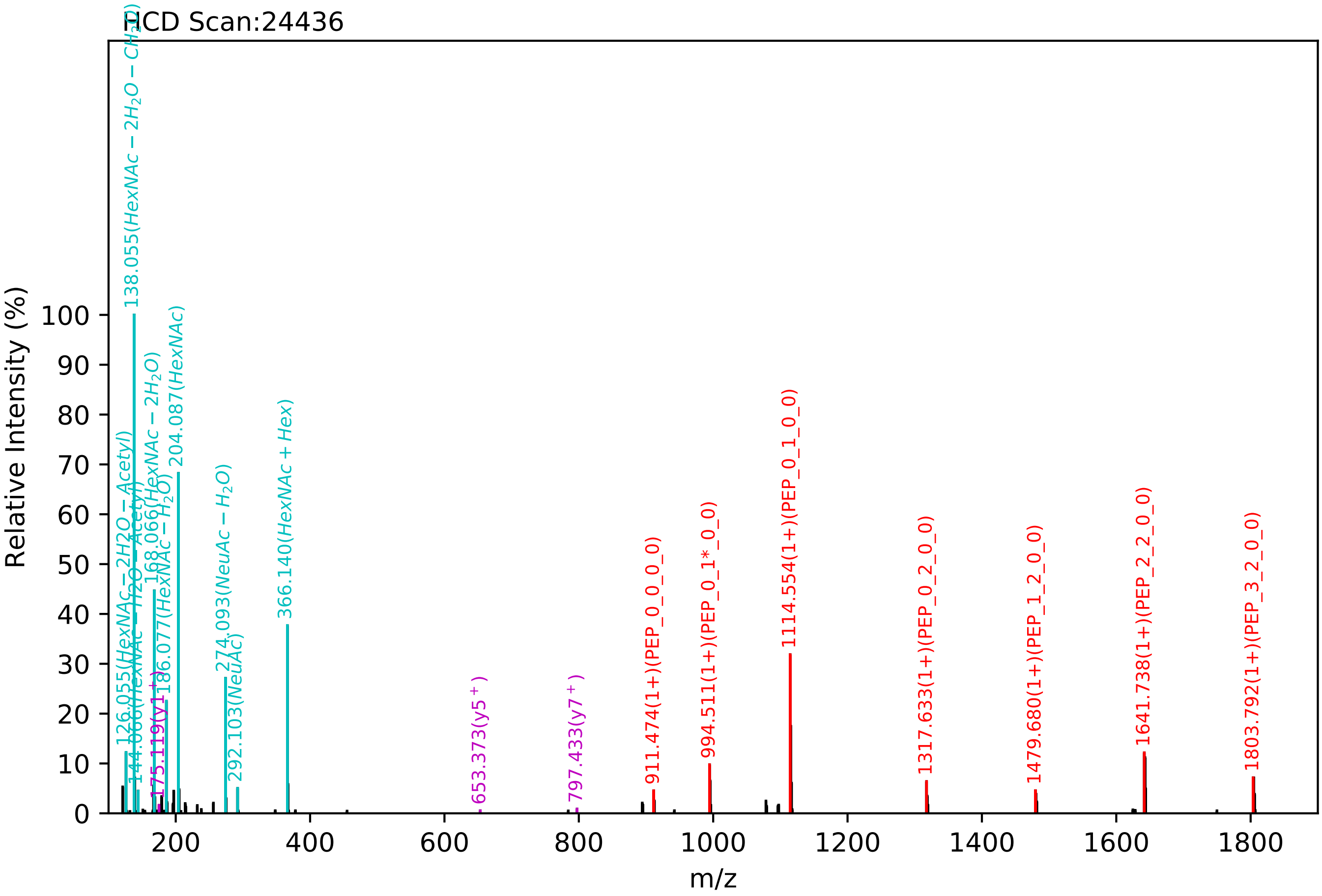

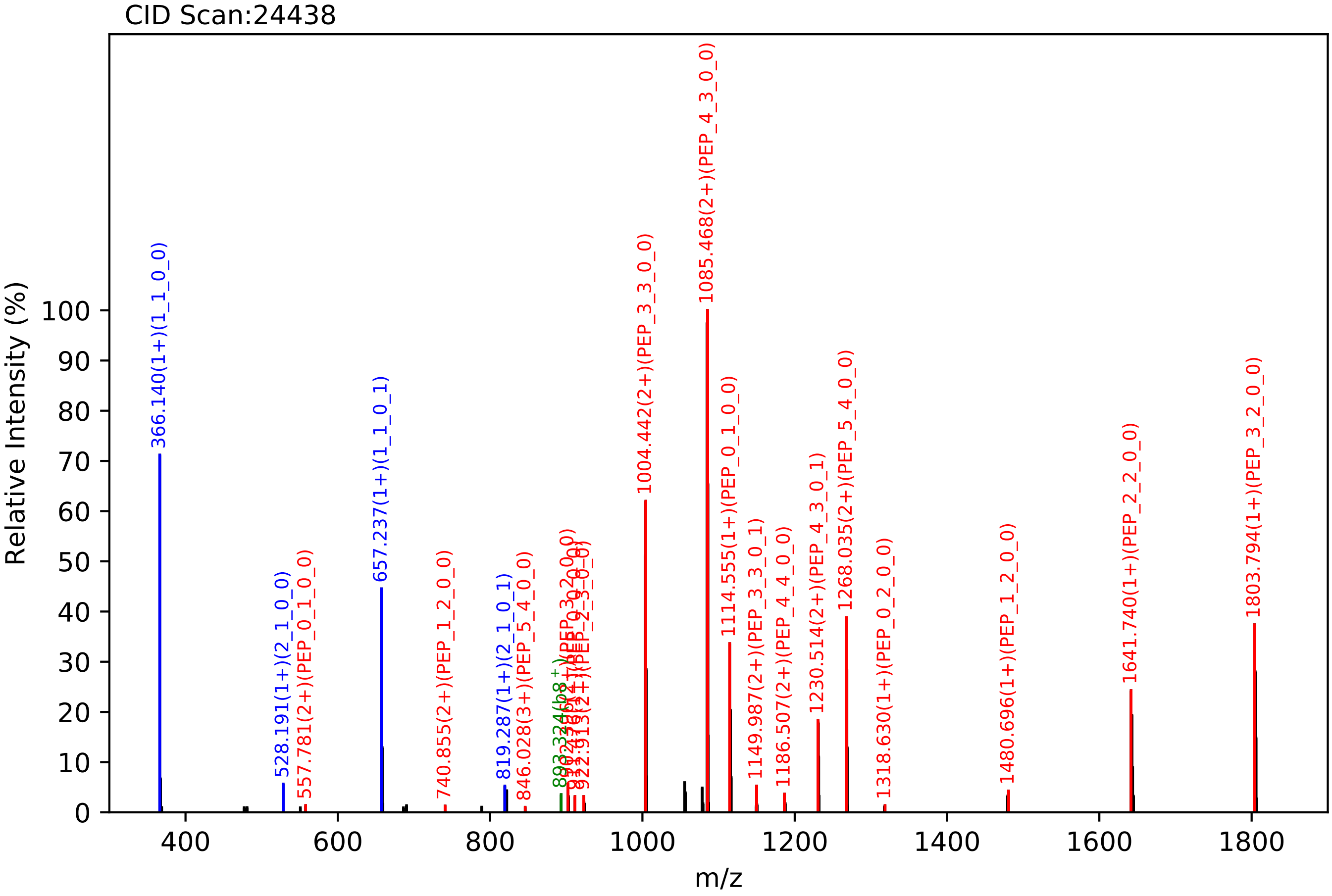

Unknown set no. 650, Experiment: Human Plasma exp_4

NGSLFAFR(=PEP)_5_4_0_1, m/z:942.39(3+), RT:83.71, Y-score:96.07

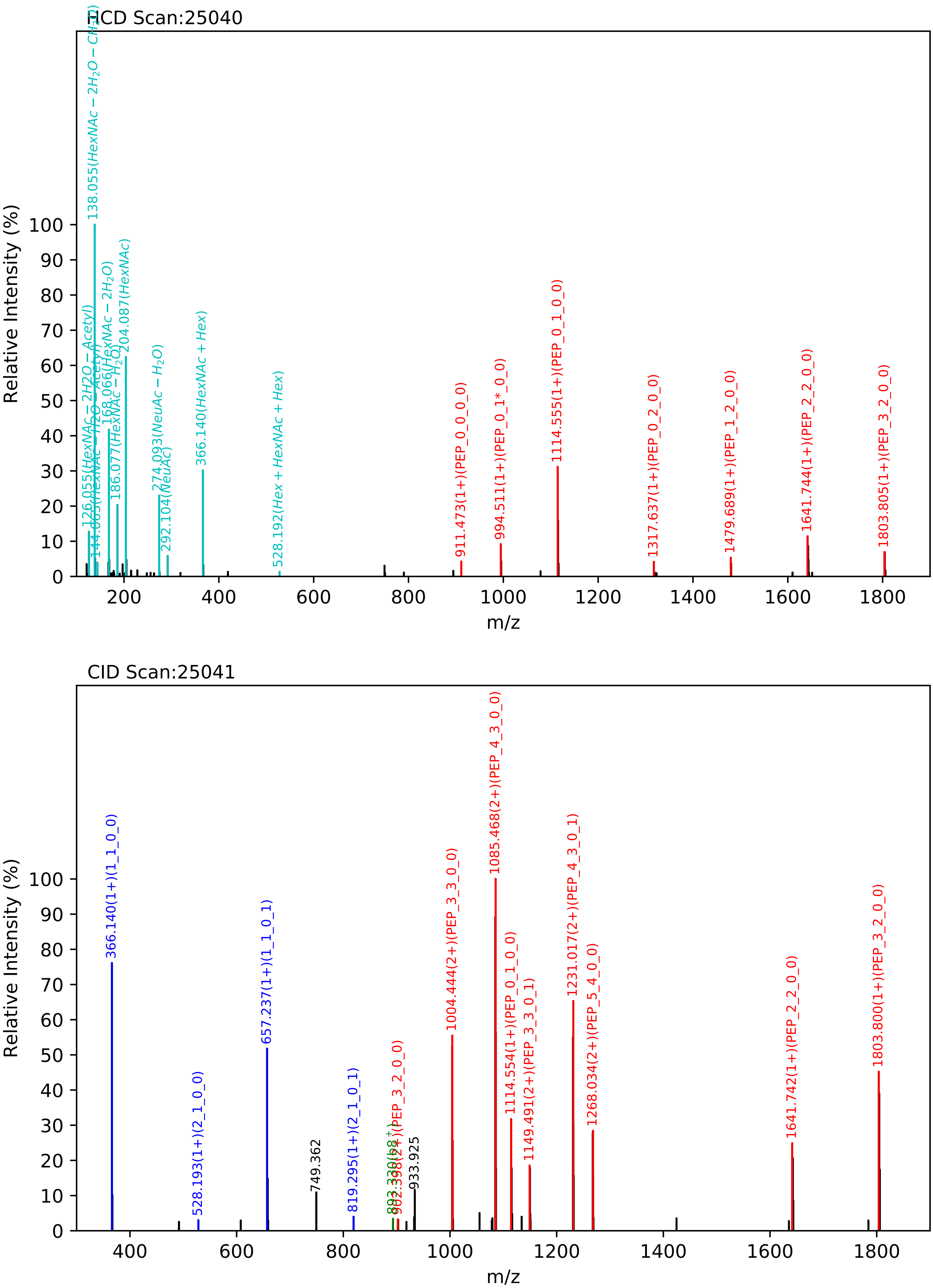

Unknown set no. 651, Experiment: Human Plasma exp_5

NGSLFAFR(=PEP)_5_4_0_1, m/z:942.39(3+), RT:82.79, Y-score:98.96

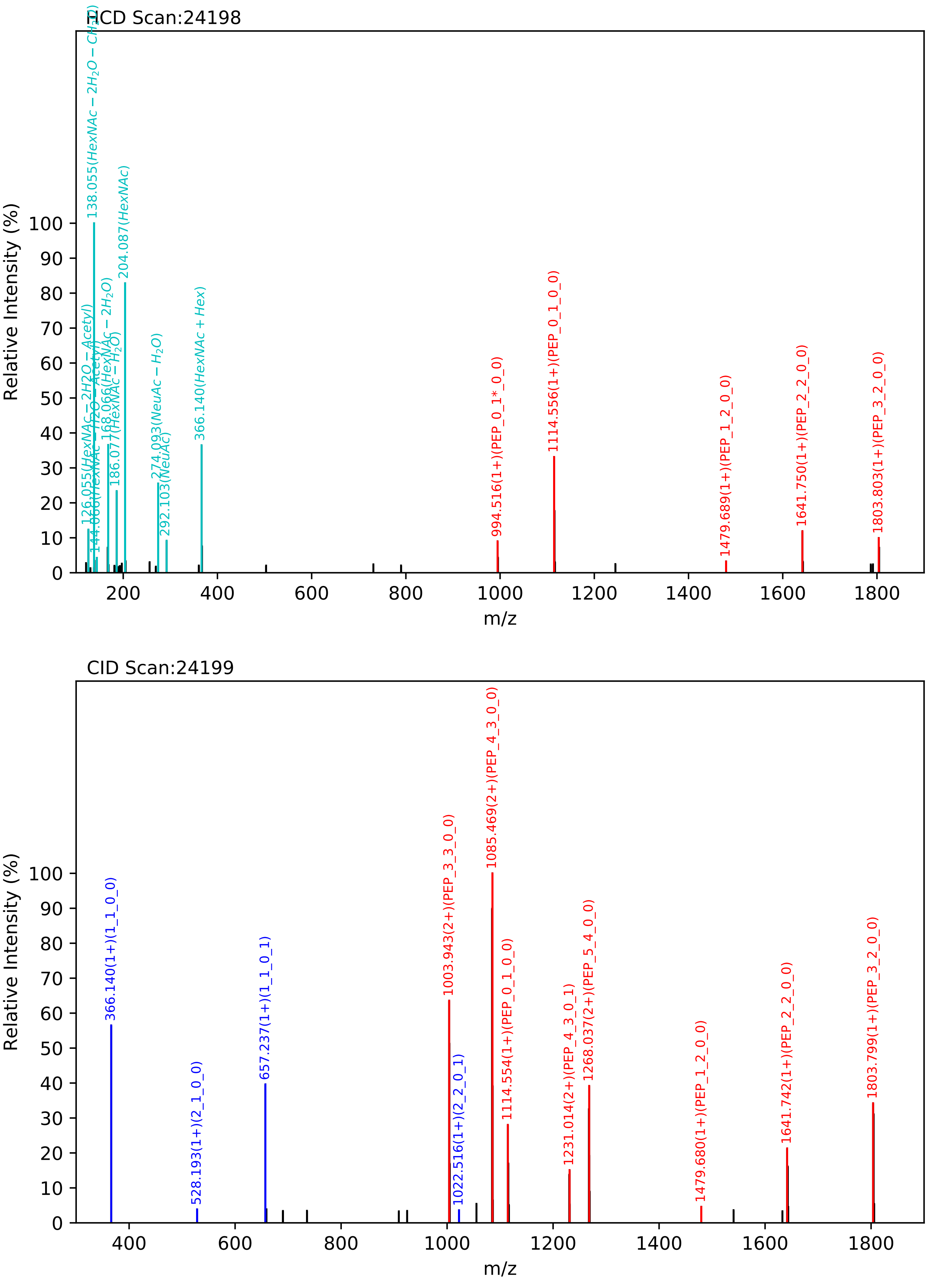

Unknown set no. 652, Experiment: Human Plasma exp_1

NGSLFAFR(=PEP)_5_4_0_2, m/z:1039.42(3+), RT:97.39, Y-score:92.43

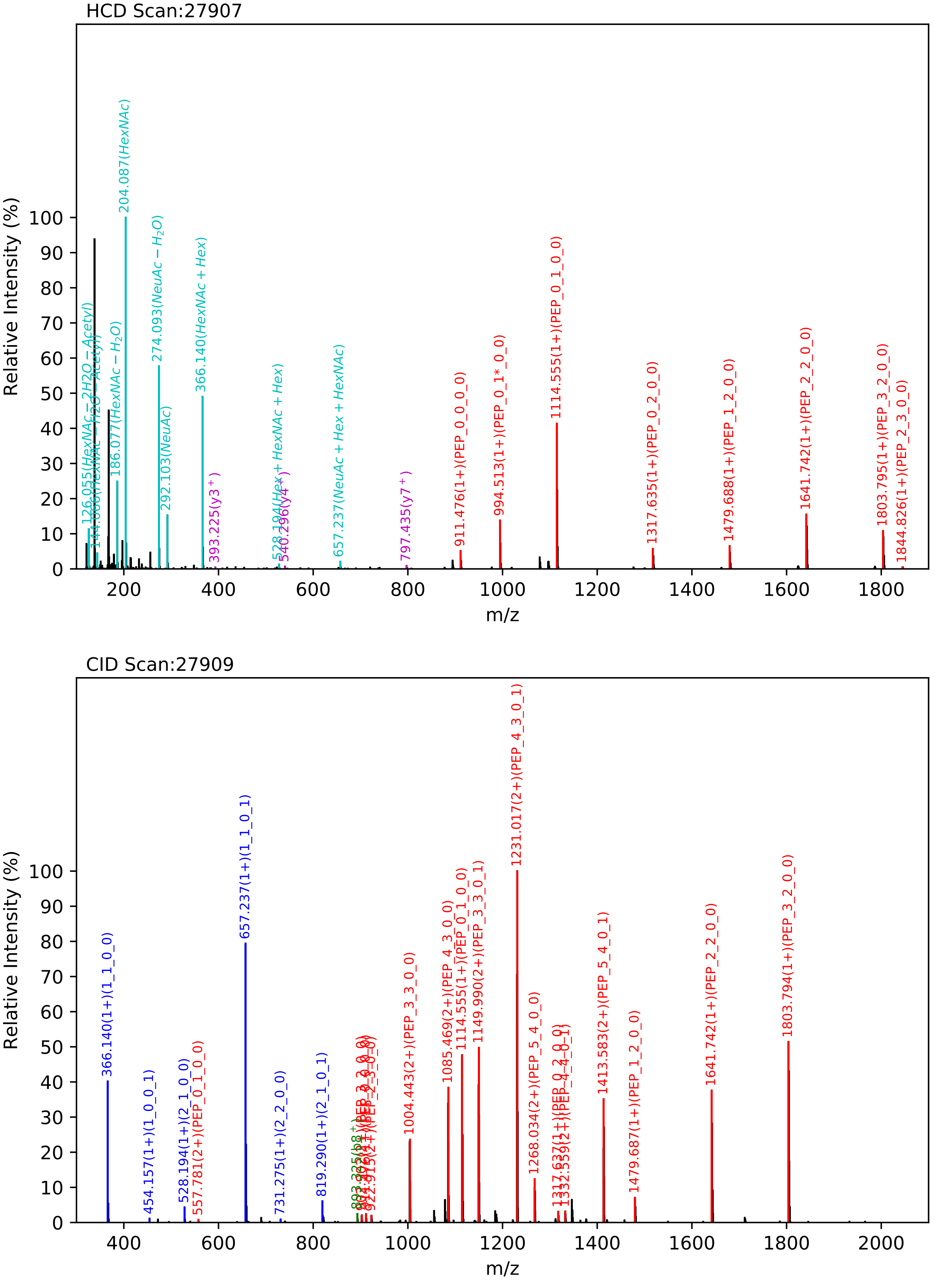

Unknown set no. 653, Experiment: Human Plasma exp_1

NGSLFAFR(=PEP)_5_4_0_2, m/z:1558.63(2+), RT:97.47, Y-score:91.95

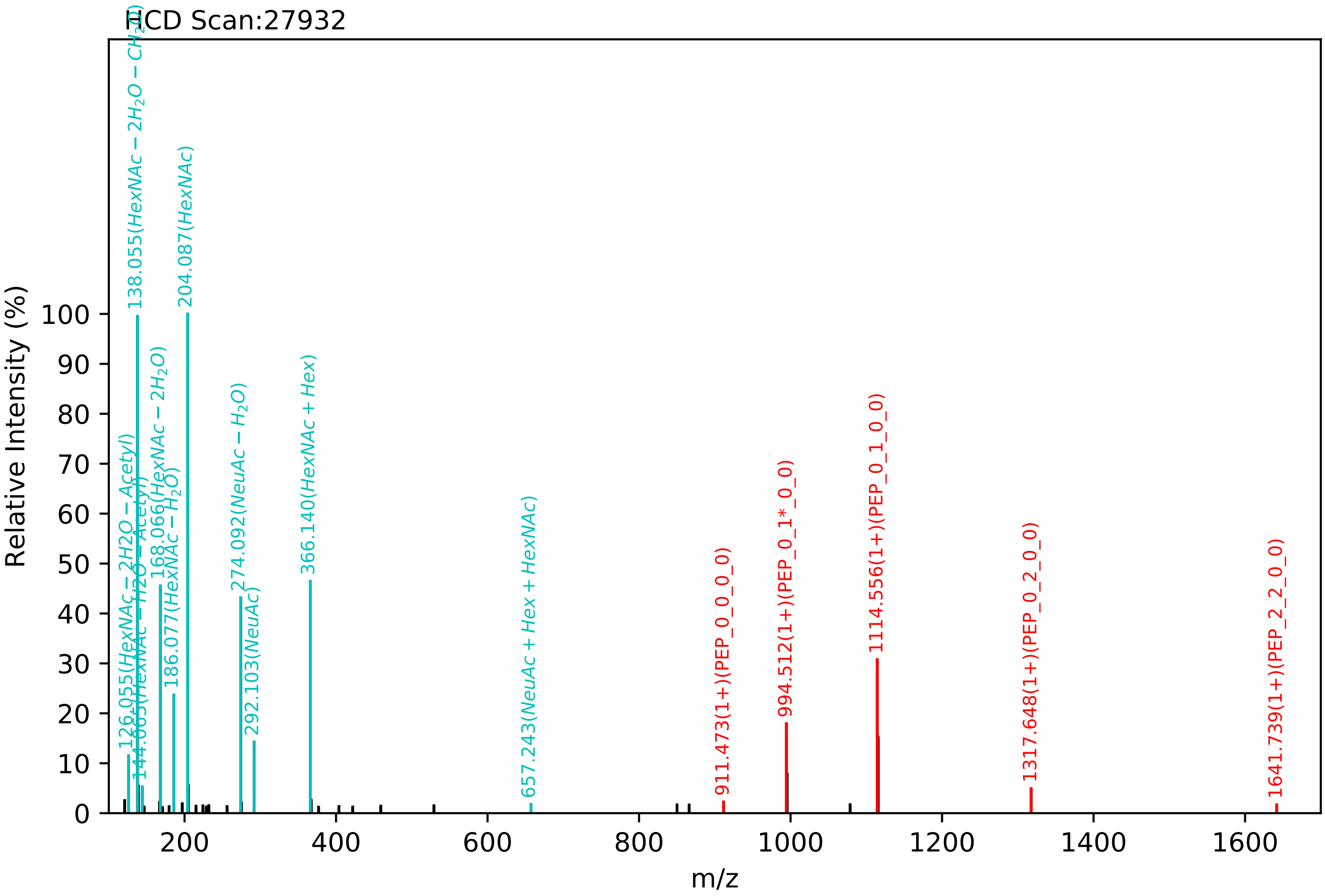

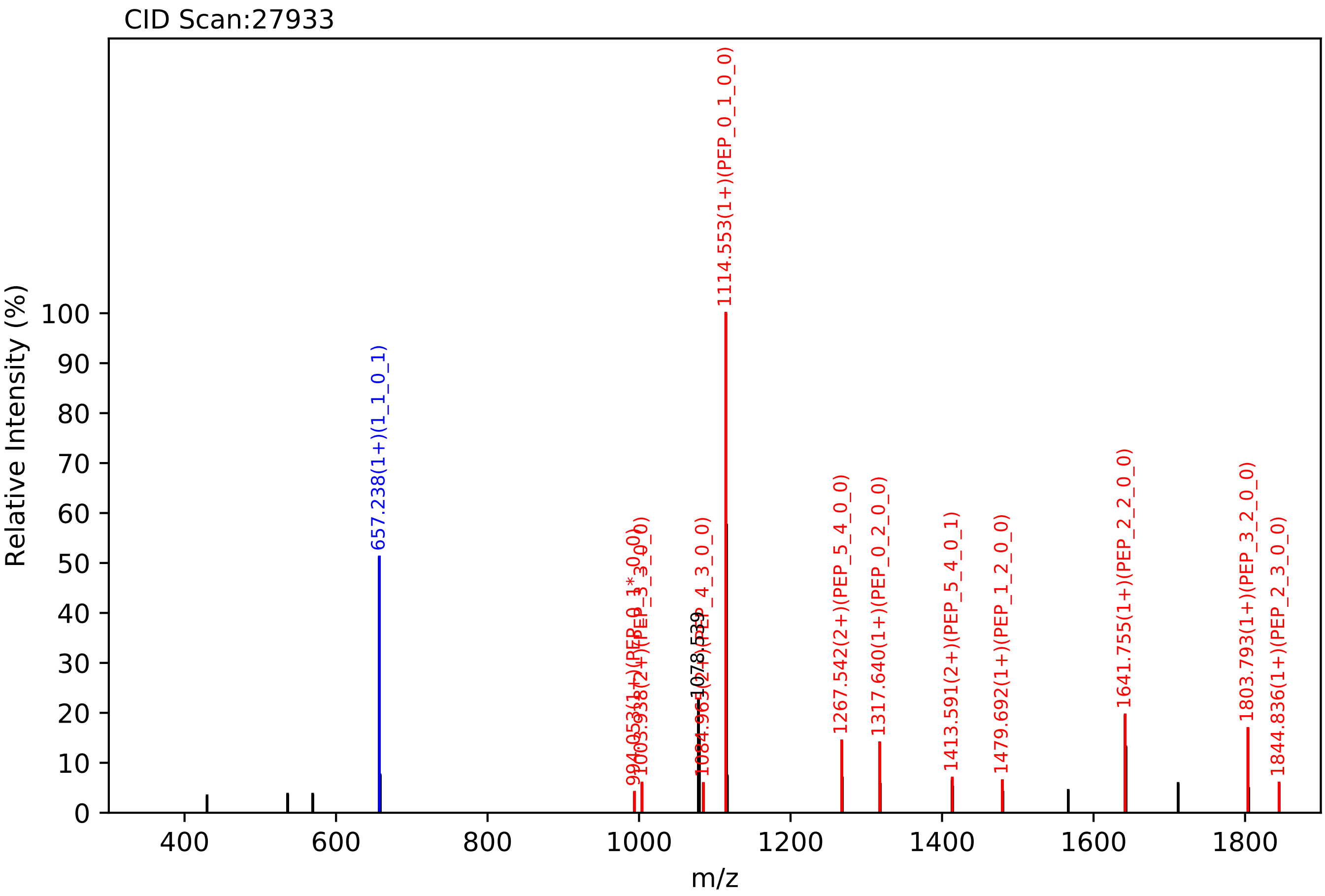

Unknown set no. 654, Experiment: Human Plasma exp_2

NGSLFAFR(=PEP)_5_4_0_2, m/z:1039.42(3+), RT:97.52, Y-score:92.37

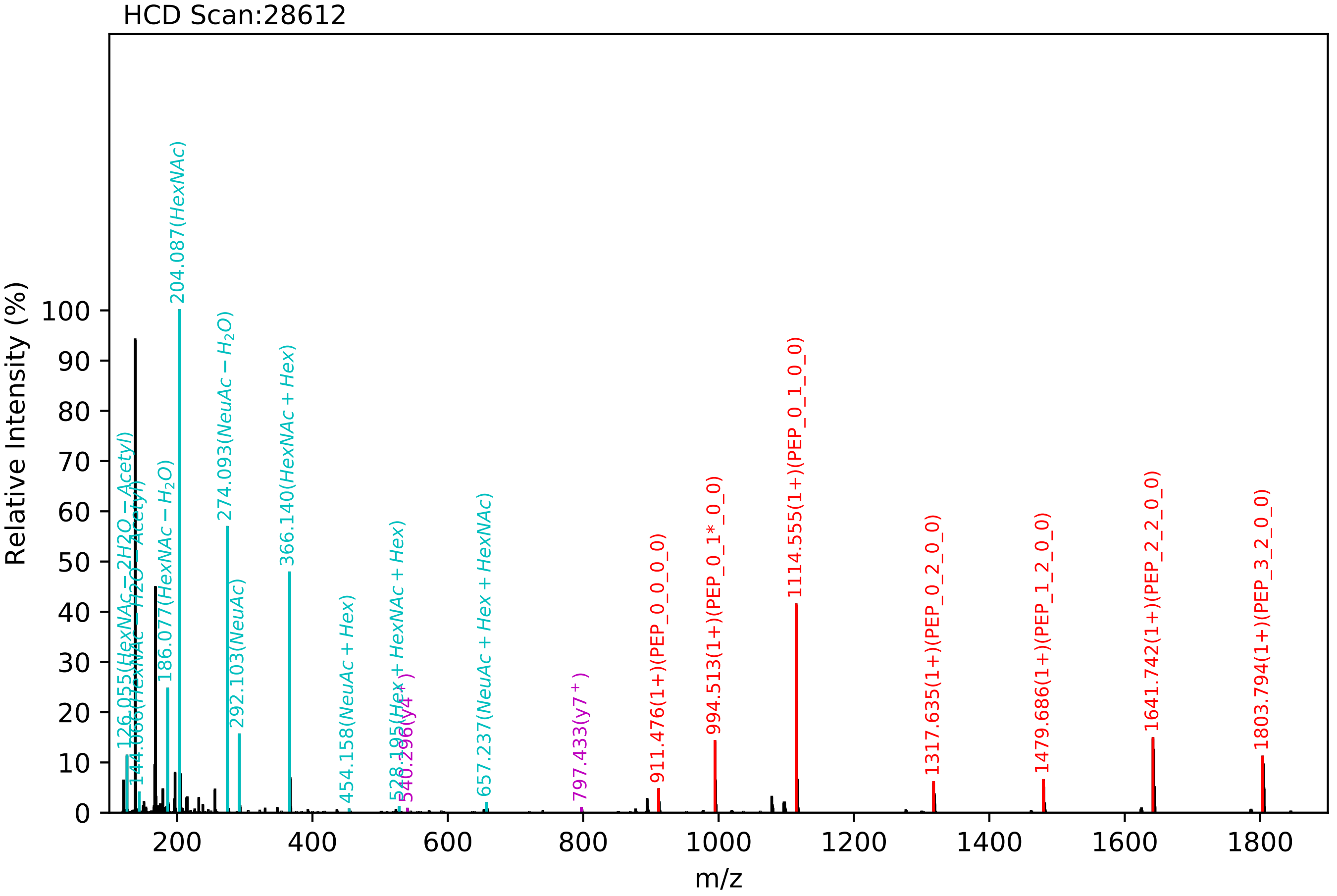

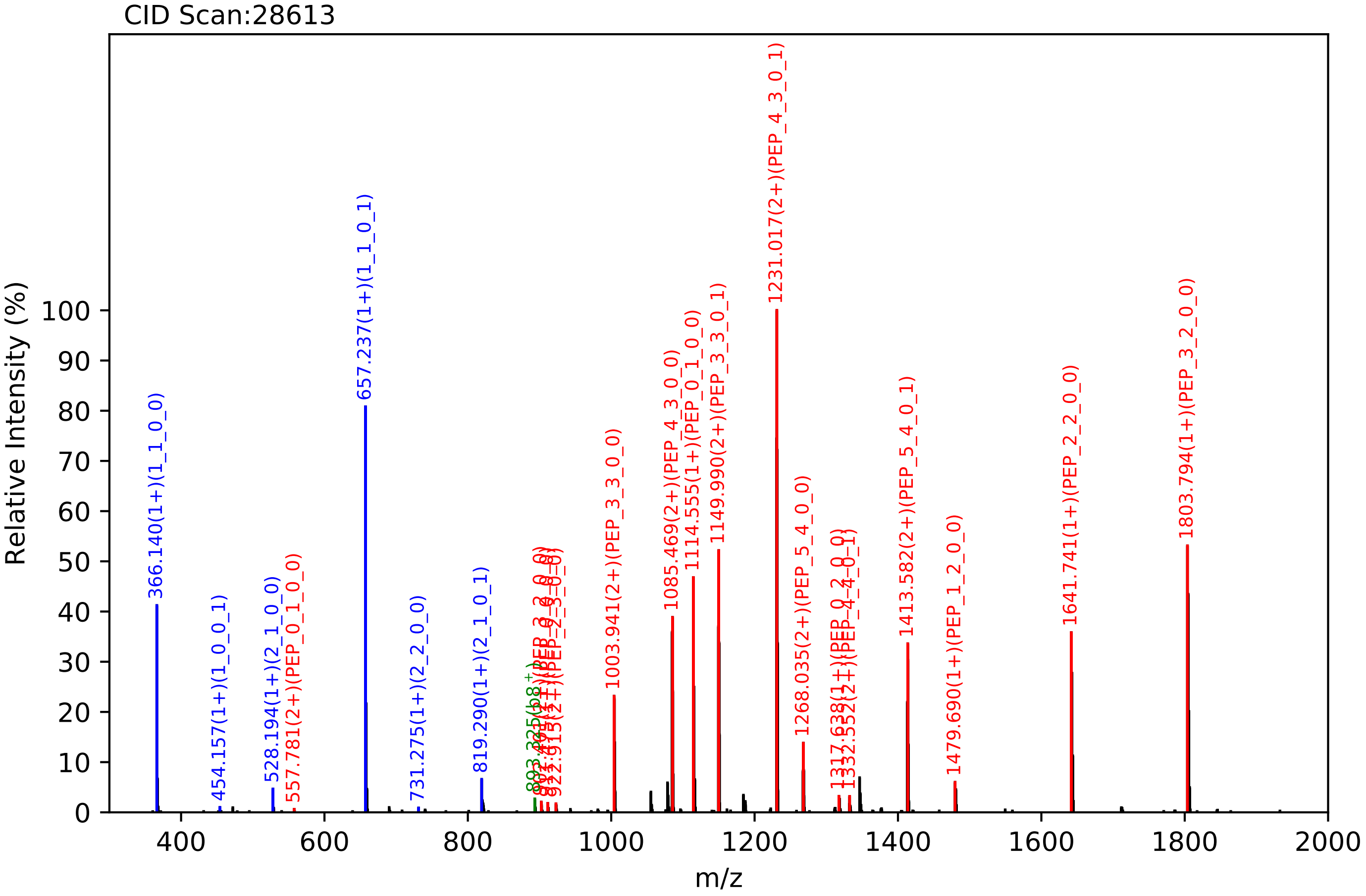

Unknown set no. 655, Experiment: Human Plasma exp_5

NGSLFAFR(=PEP)_5_4_0_2, m/z:1039.42(3+), RT:97.33, Y-score:91.44

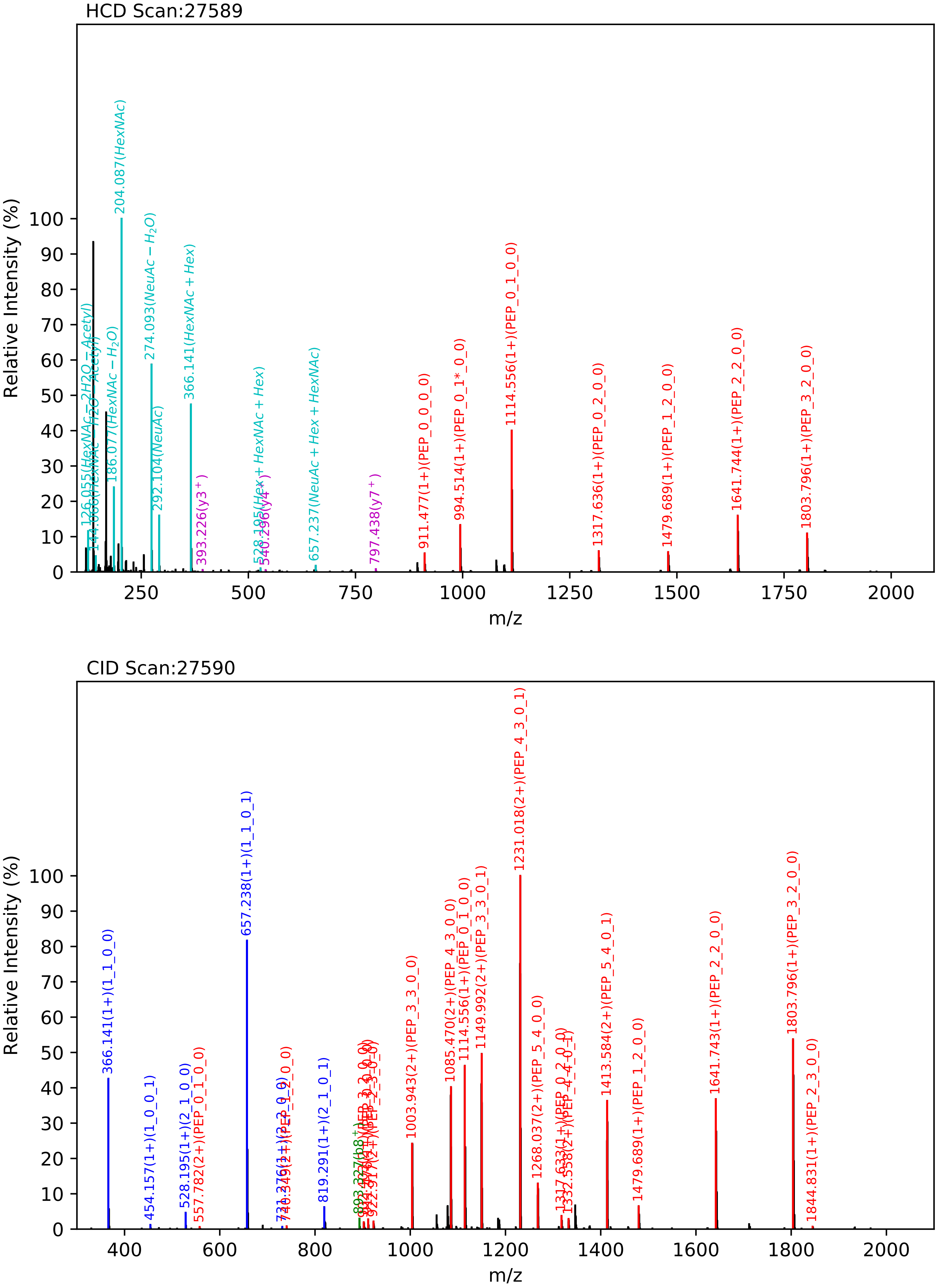

Unknown set no. 656, Experiment: Human Plasma exp_4

NGSLFAFR(=PEP)_5_4_1_2, m/z:1088.11(3+), RT:97.96, Y-score:87.27

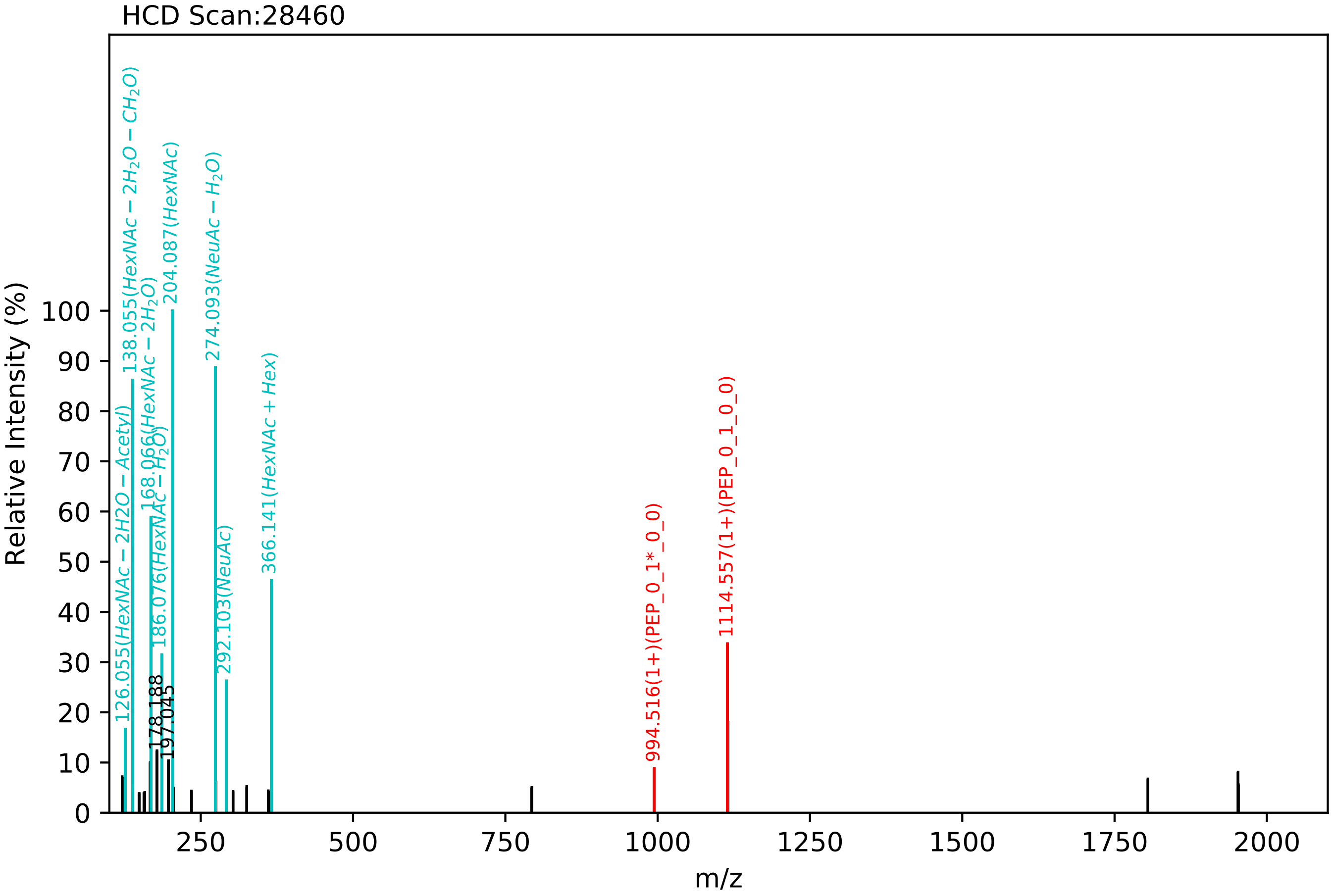

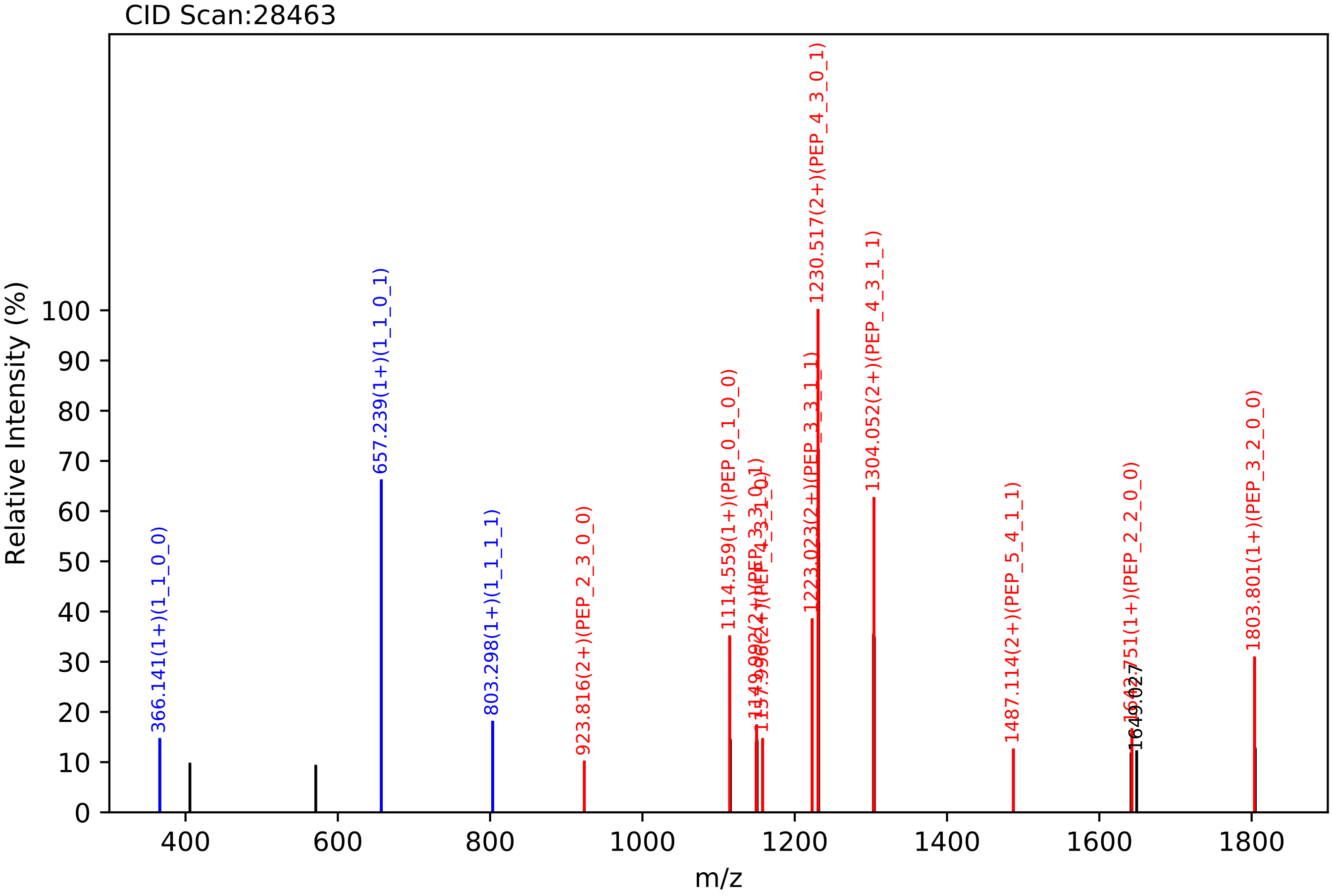

Unknown set no. 657, Experiment: Human Plasma exp_1

NGSLFAFR(=PEP)_6_3_0_1, m/z:928.72(3+), RT:83.09, Y-score:95.53

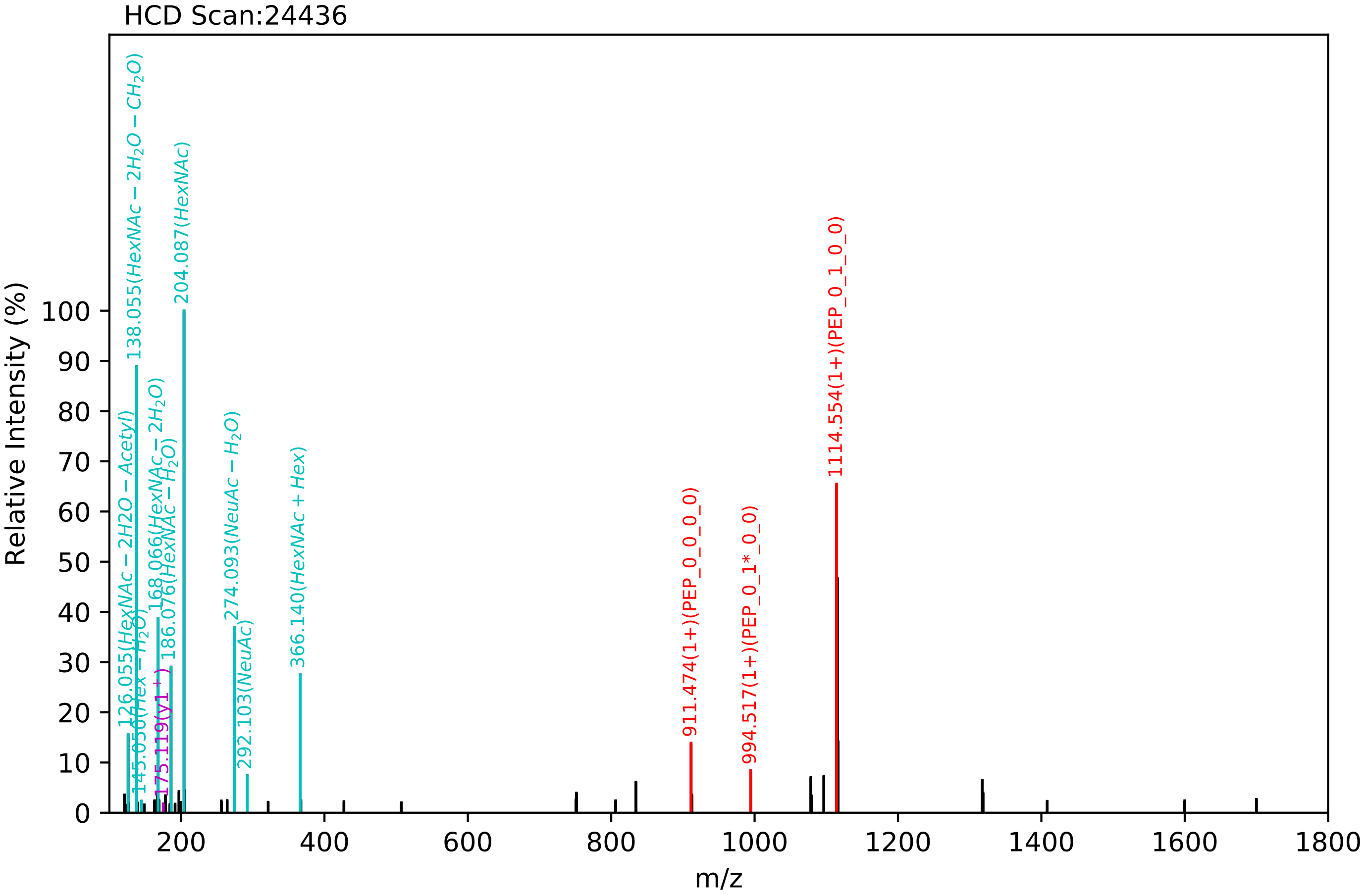

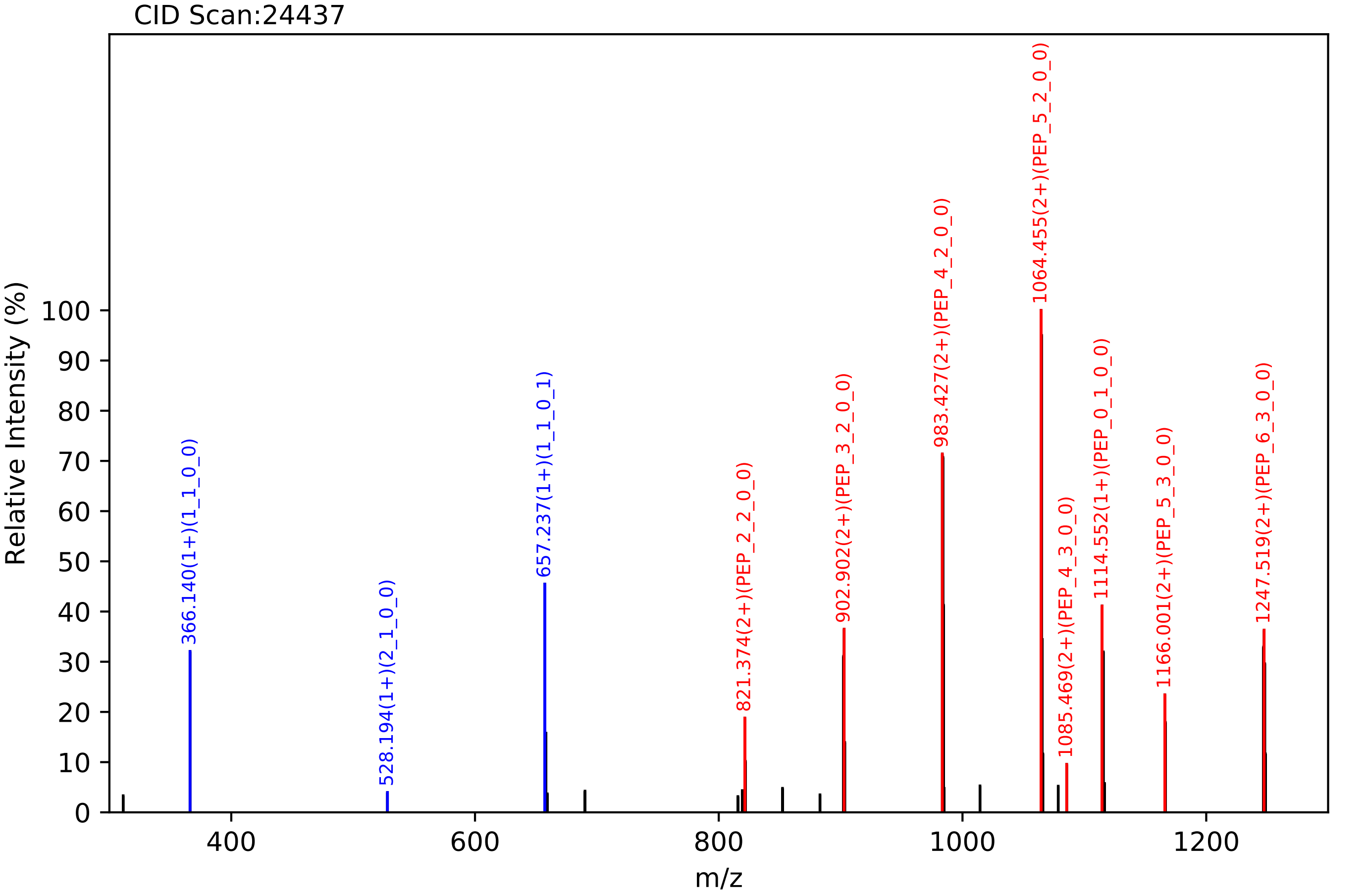

Unknown set no. 658, Experiment: Human Plasma exp_2

NGSLFAFR(=PEP)_6_3_0_1, m/z:928.72(3+), RT:83.15, Y-score:98.03

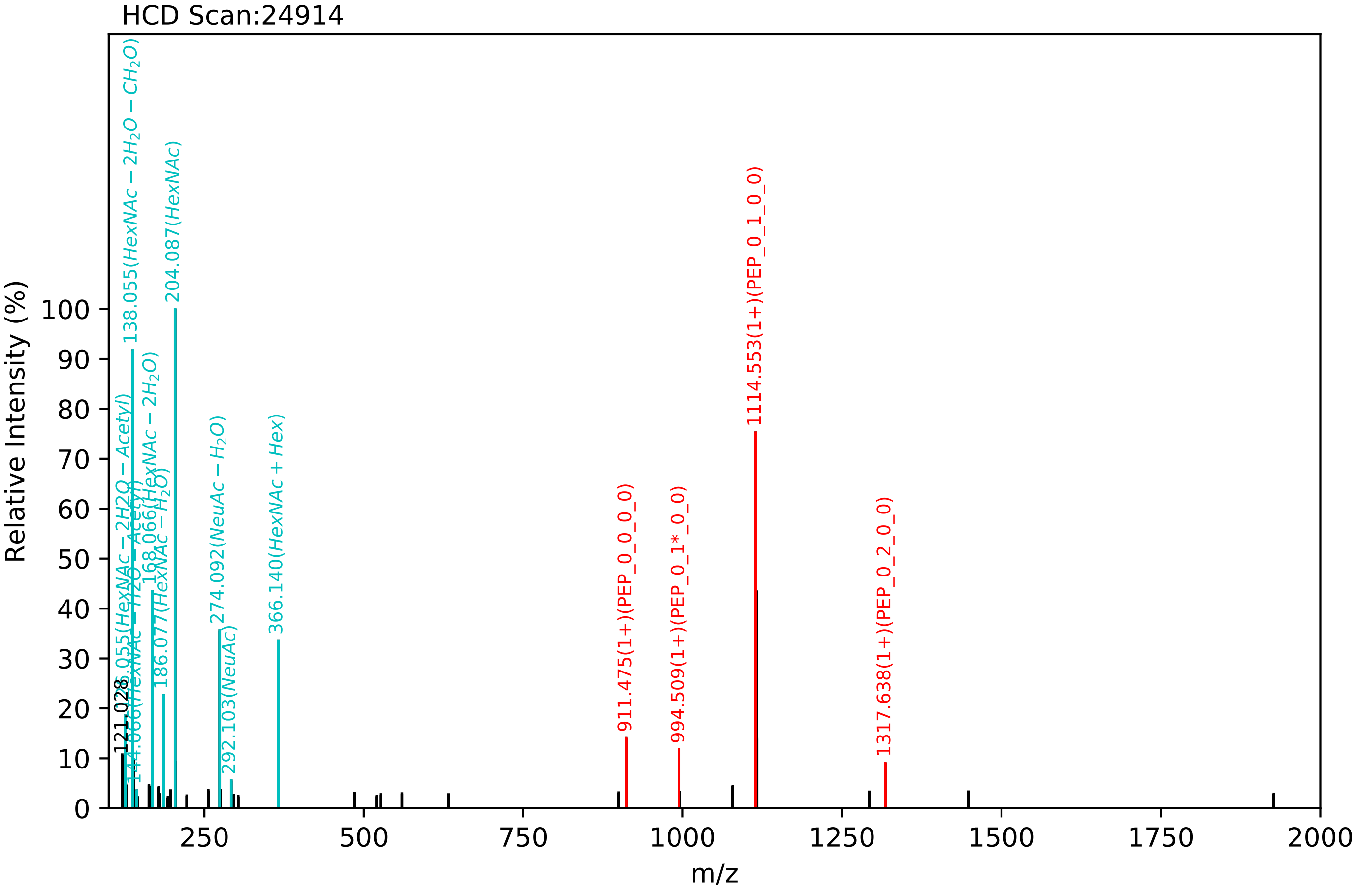

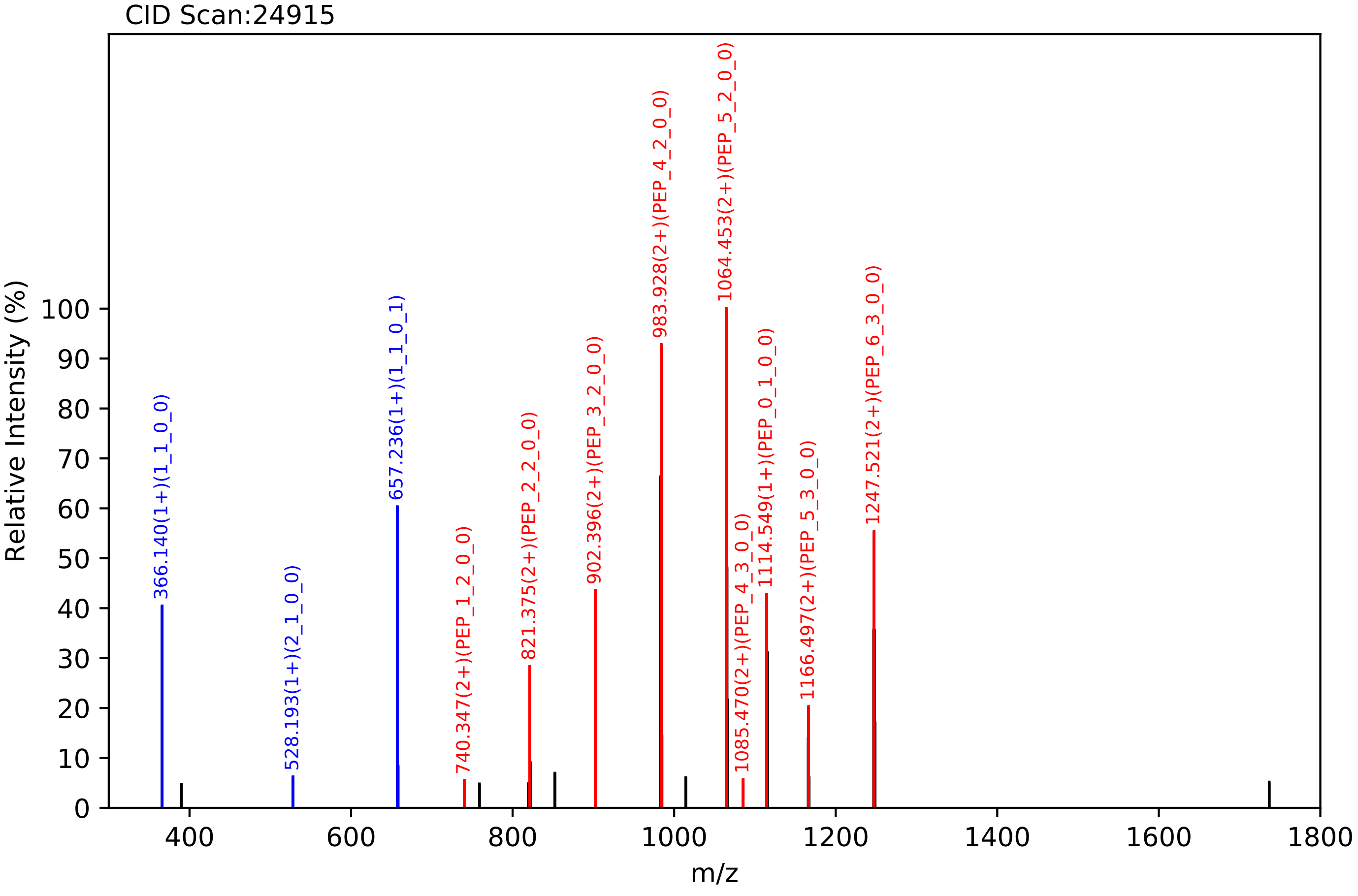

Unknown set no. 659, Experiment: Human Plasma exp_3

NGSLFAFR(=PEP)_6_3_0_1, m/z:928.72(3+), RT:83.24, Y-score:93.90

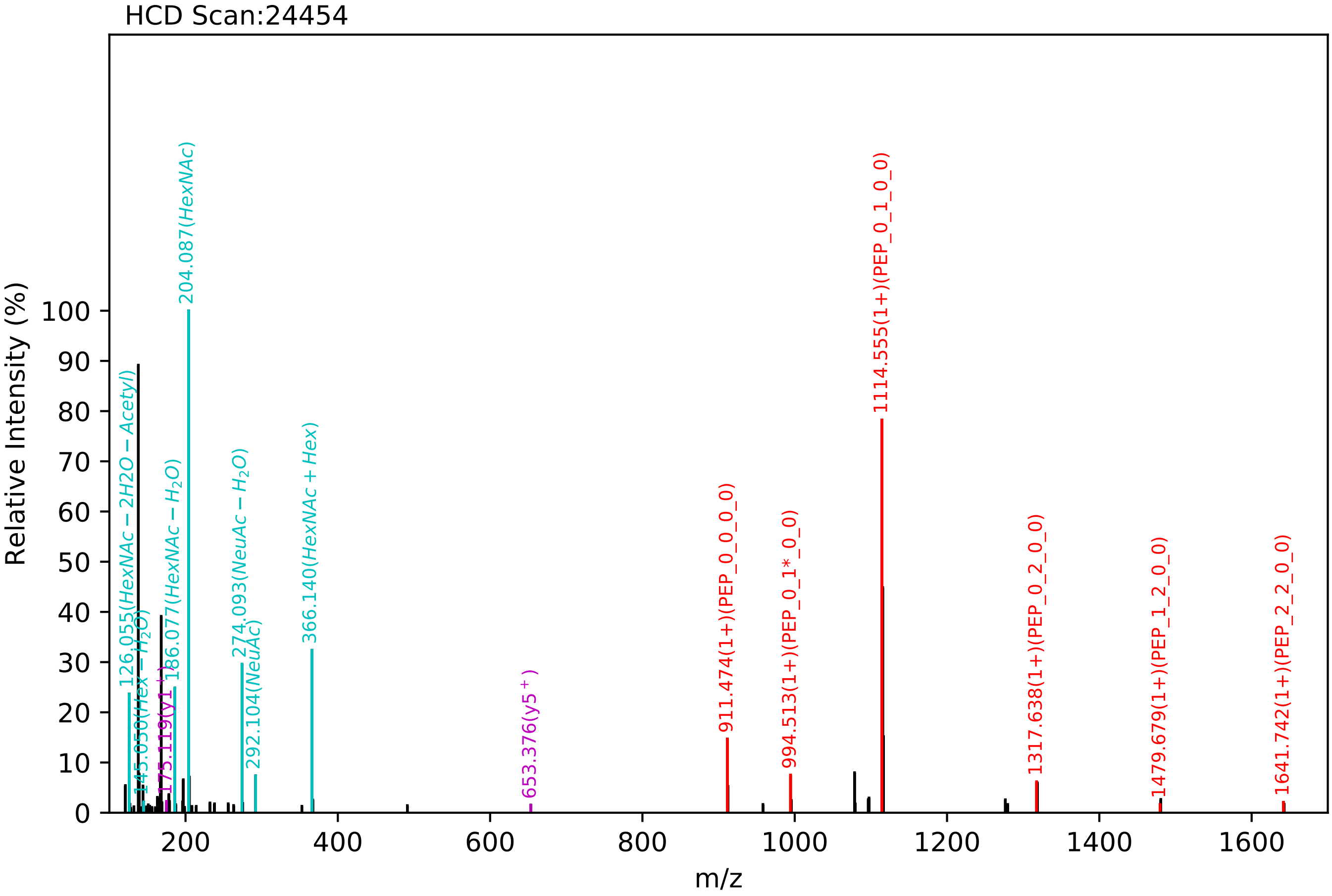

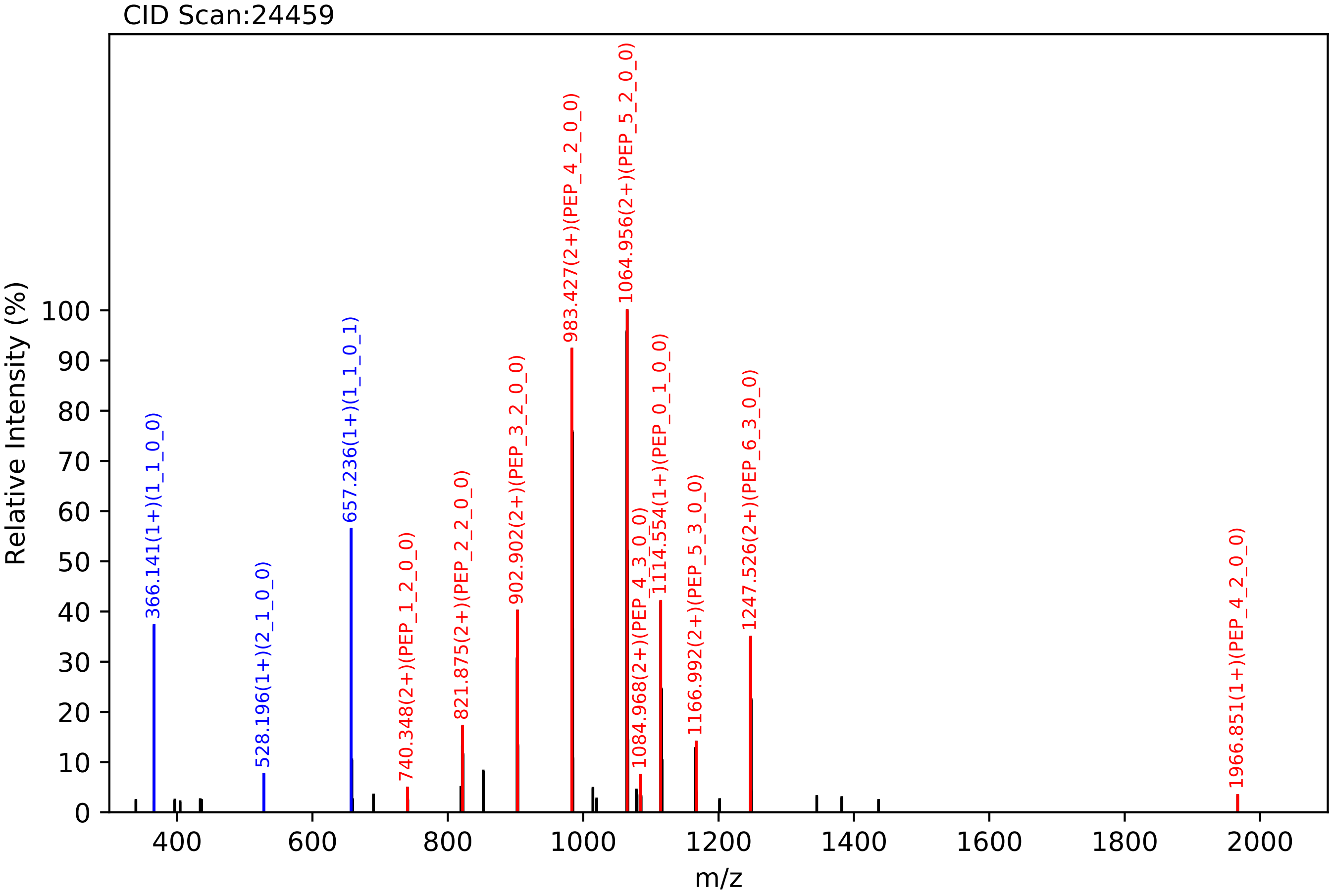

Unknown set no. 660, Experiment: Human Plasma exp_4

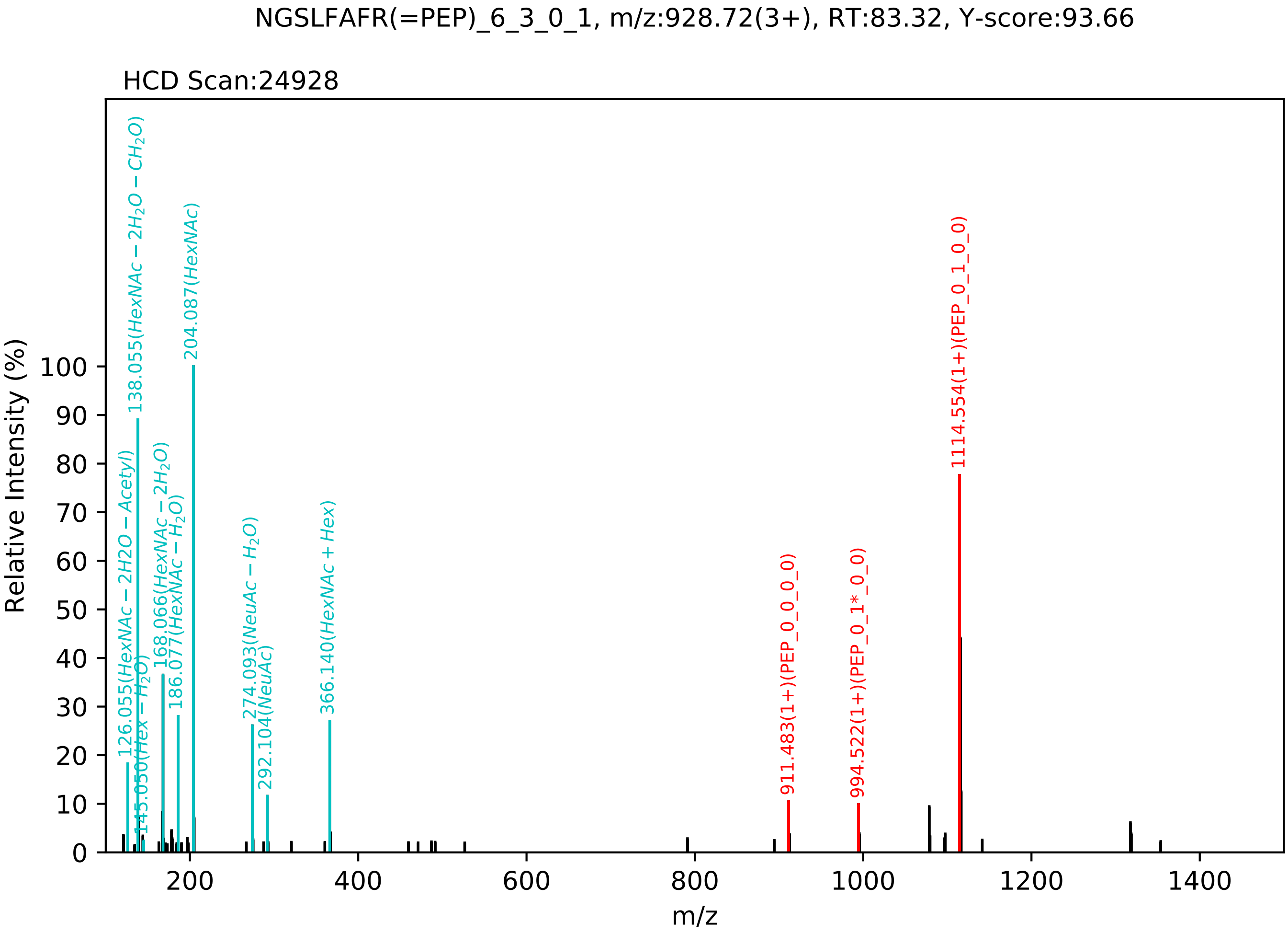

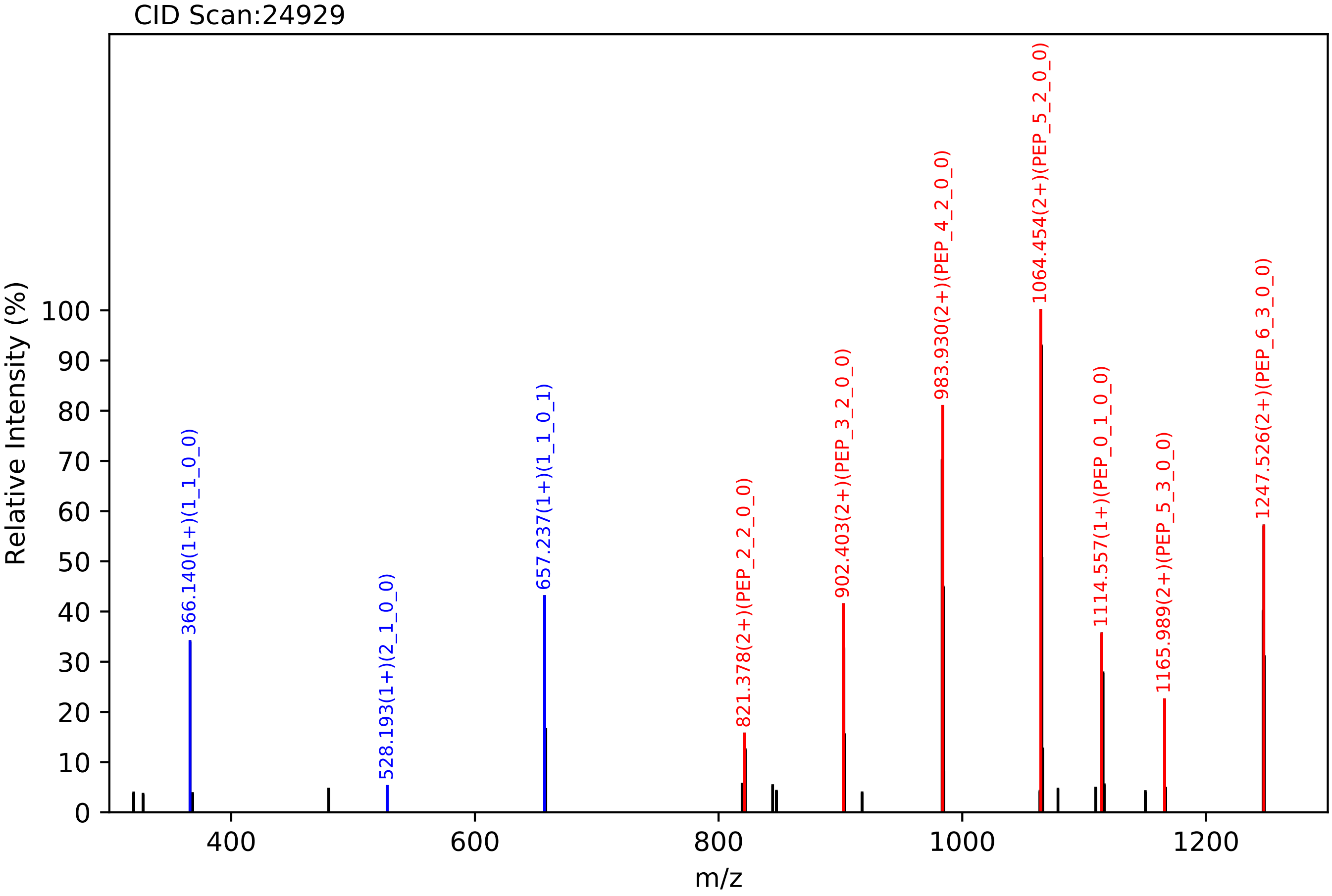

Unknown set no. 661, Experiment: Human Plasma exp_5

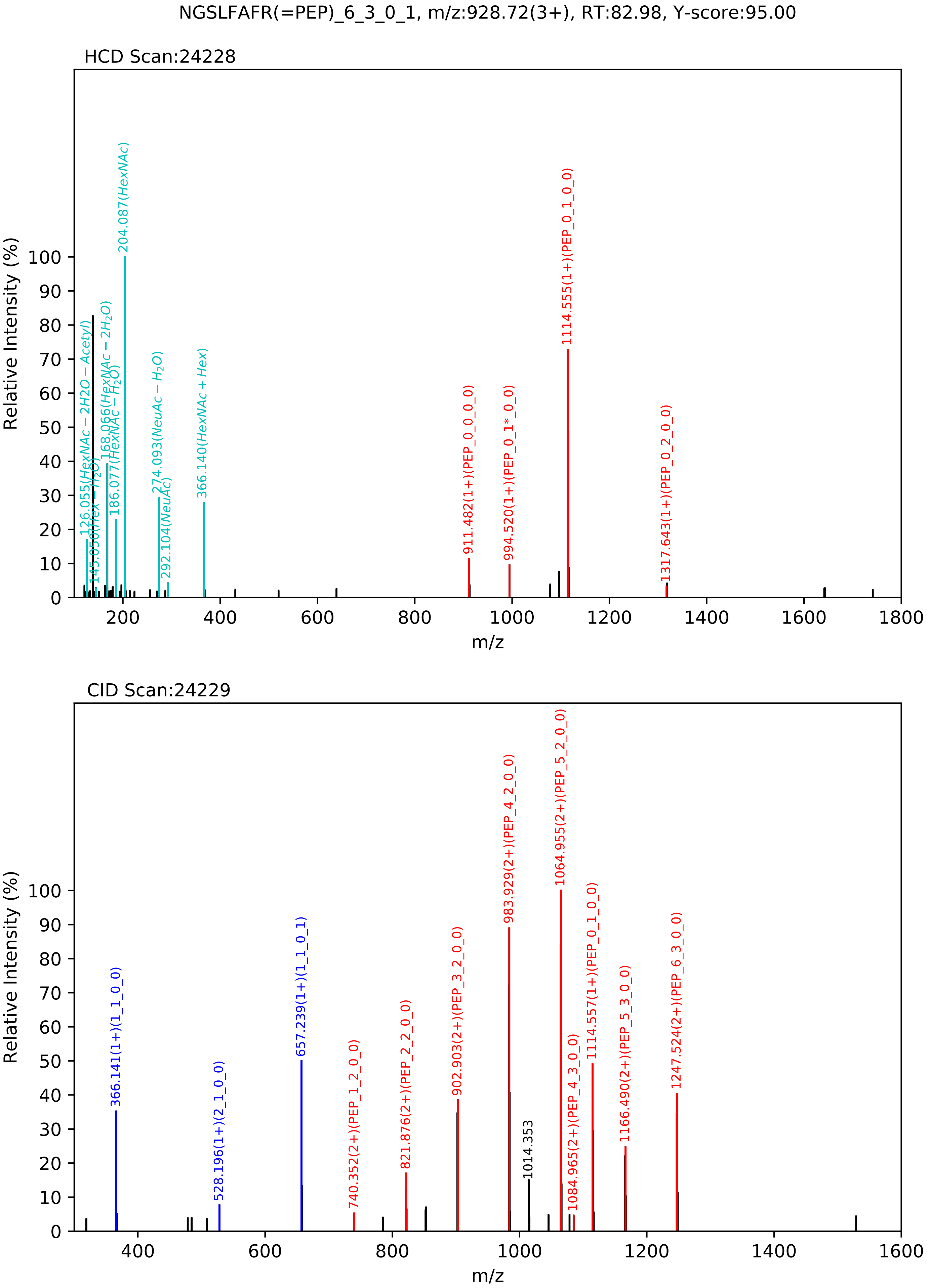

Unknown set no. 662, Experiment: Human Plasma exp_2

NGSLFAFR(=PEP)_6_5_1_3, m/z:1306.85(3+), RT:111.89, Y-score:95.95

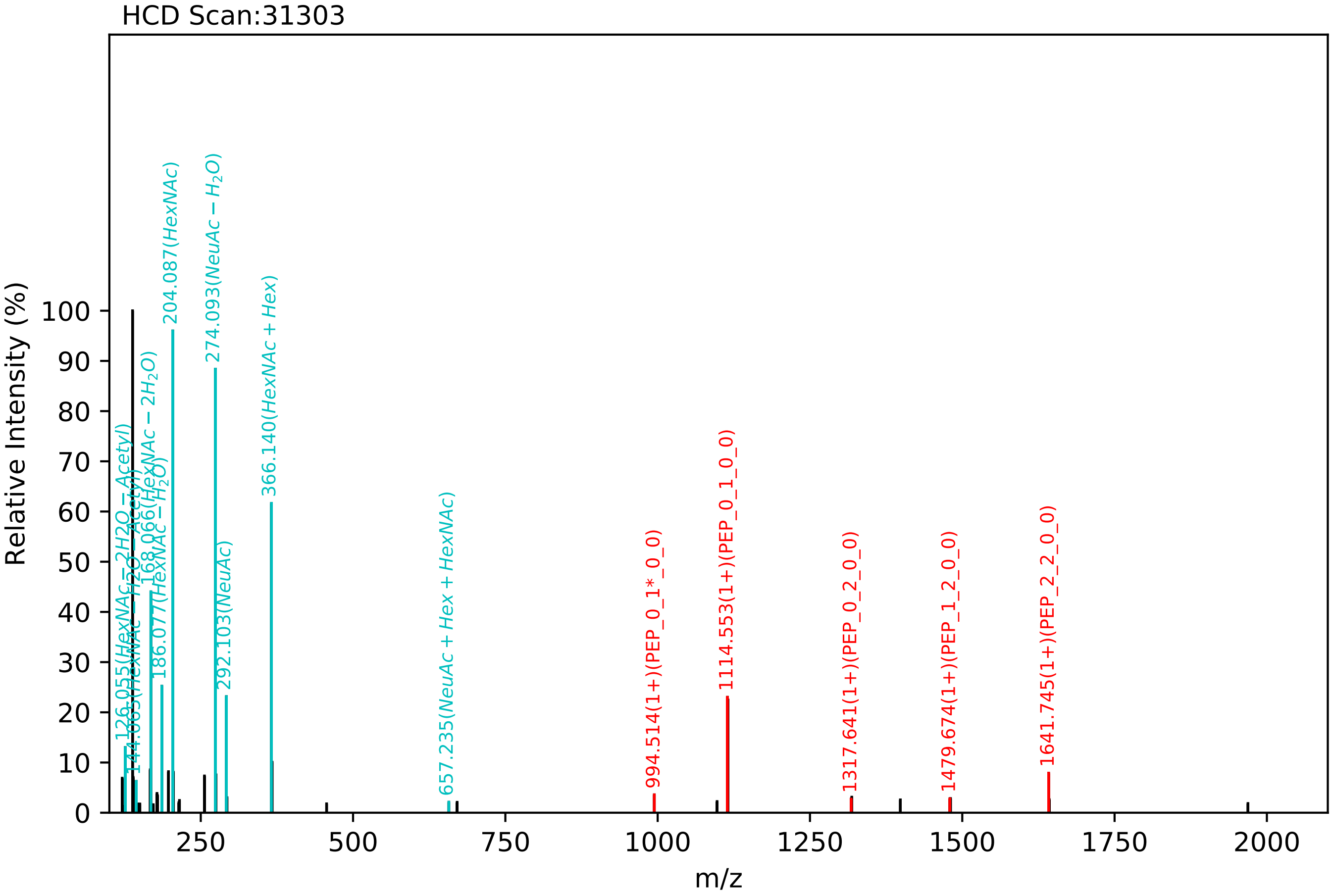

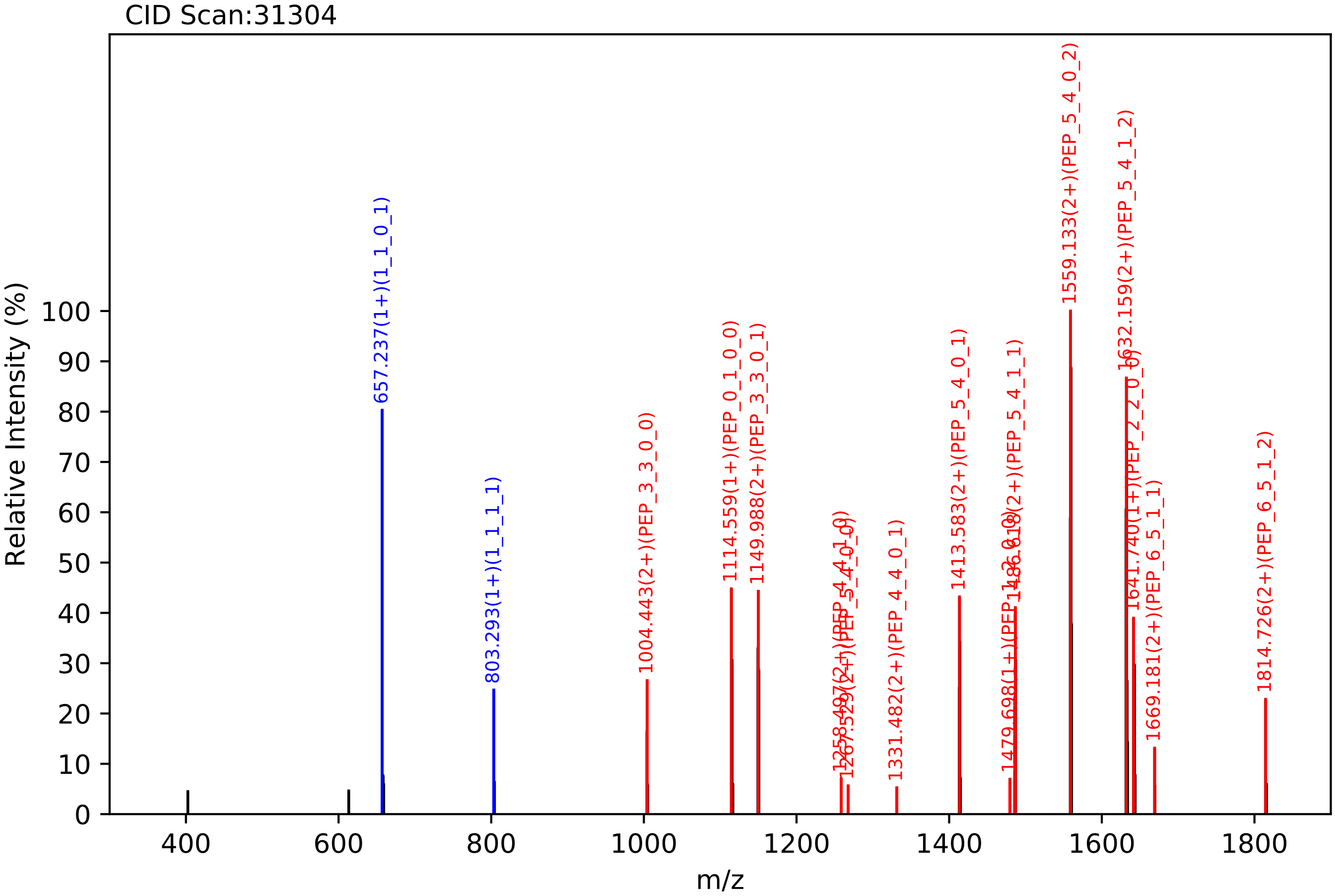

Unknown set no. 663, Experiment: Human Plasma exp_3

NGSLFAFR(=PEP)_6_5_1_3, m/z:1306.85(3+), RT:112.16, Y-score:86.73

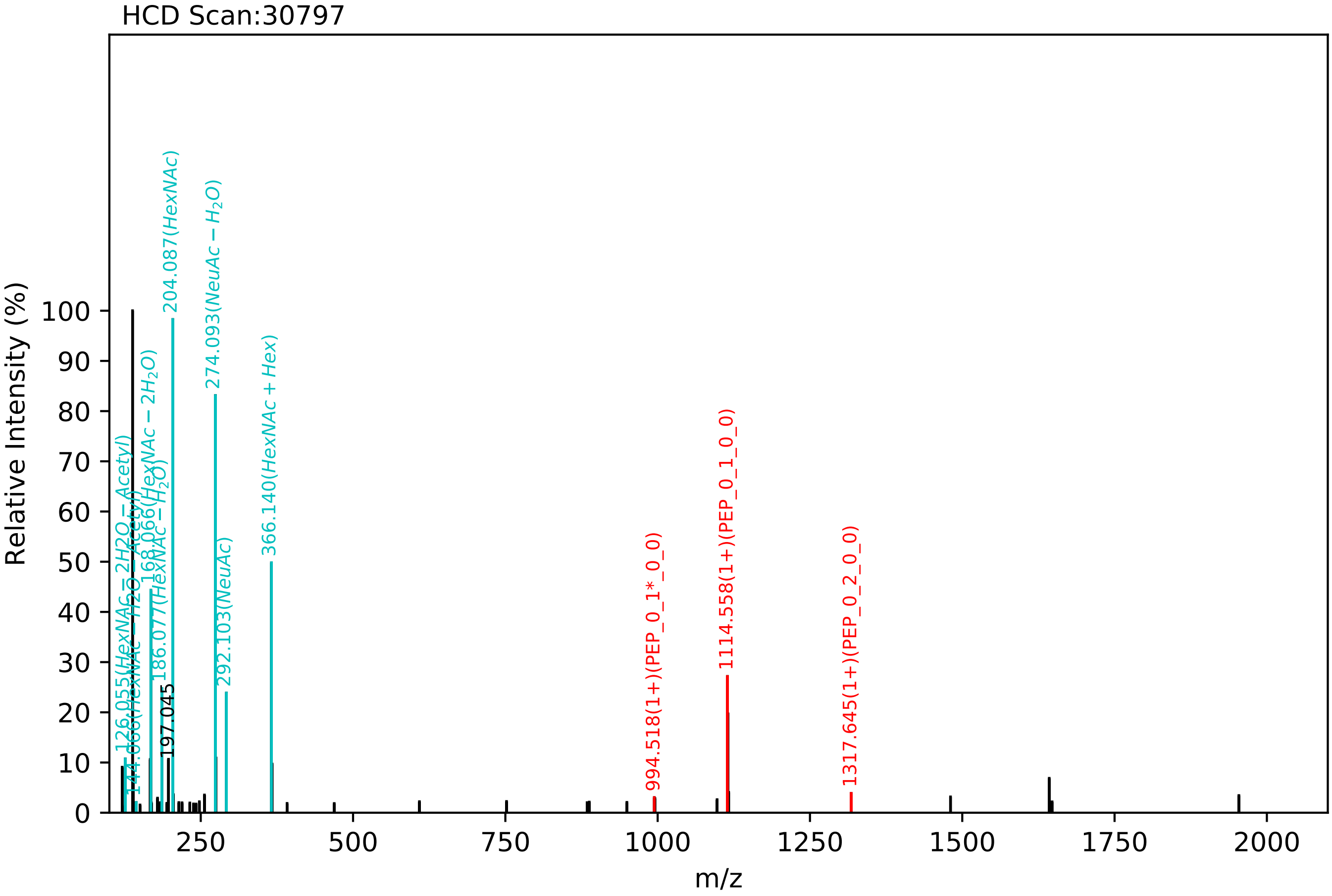

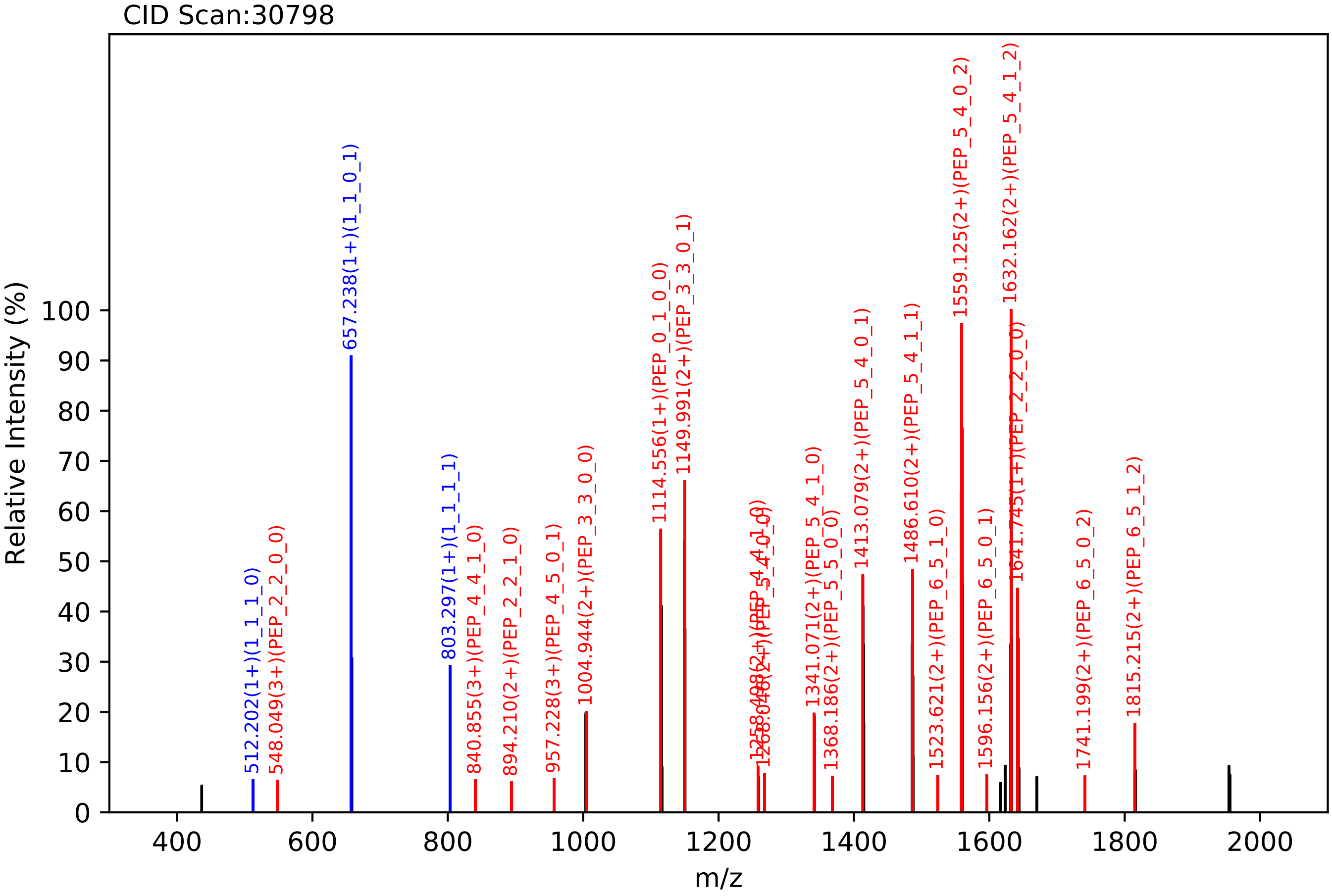

Unknown set no. 664, Experiment: Human Plasma exp_4

NGSLFAFR(=PEP)_6_5_1_3, m/z:1306.85(3+), RT:112.11, Y-score:92.35

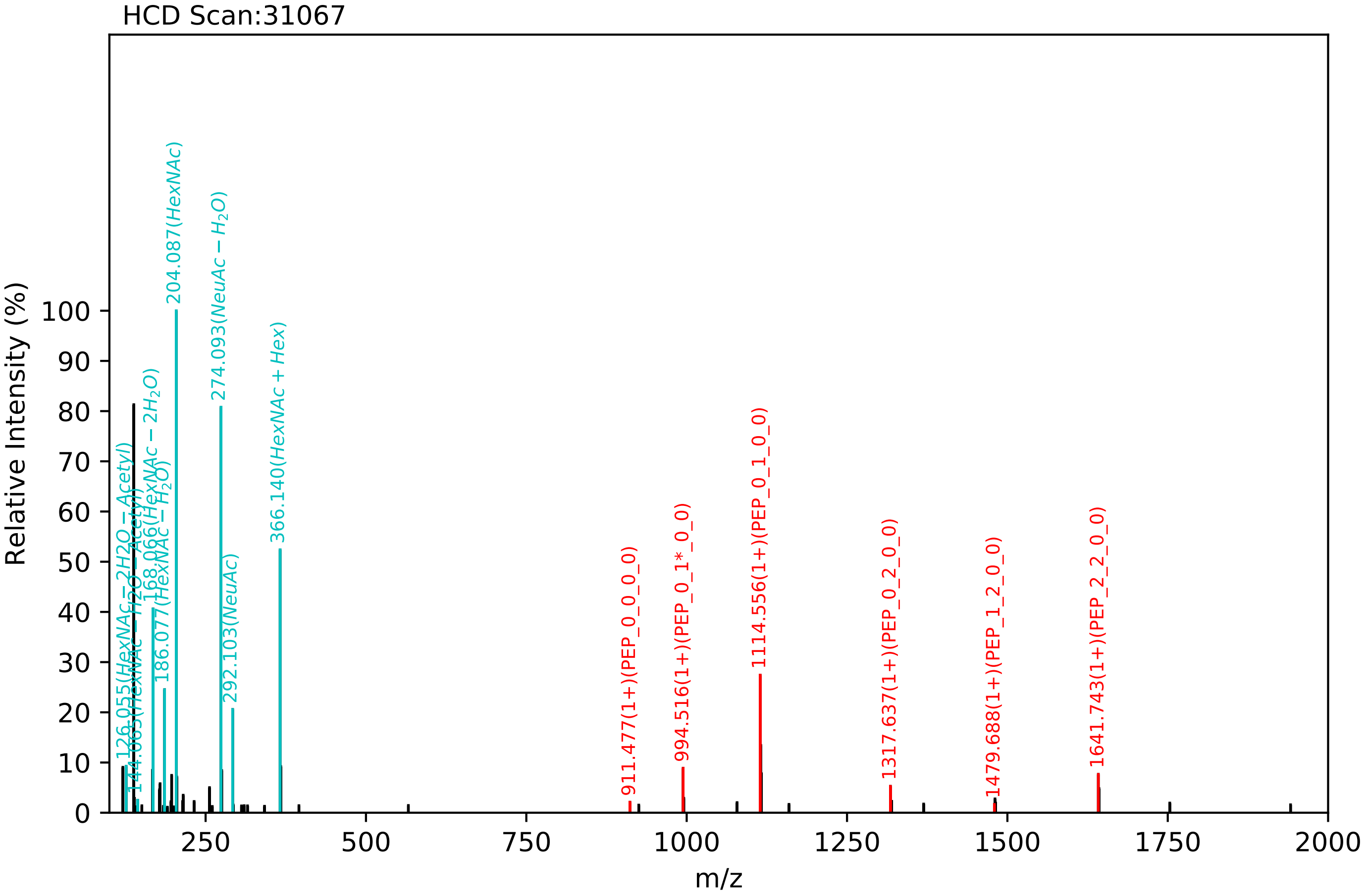

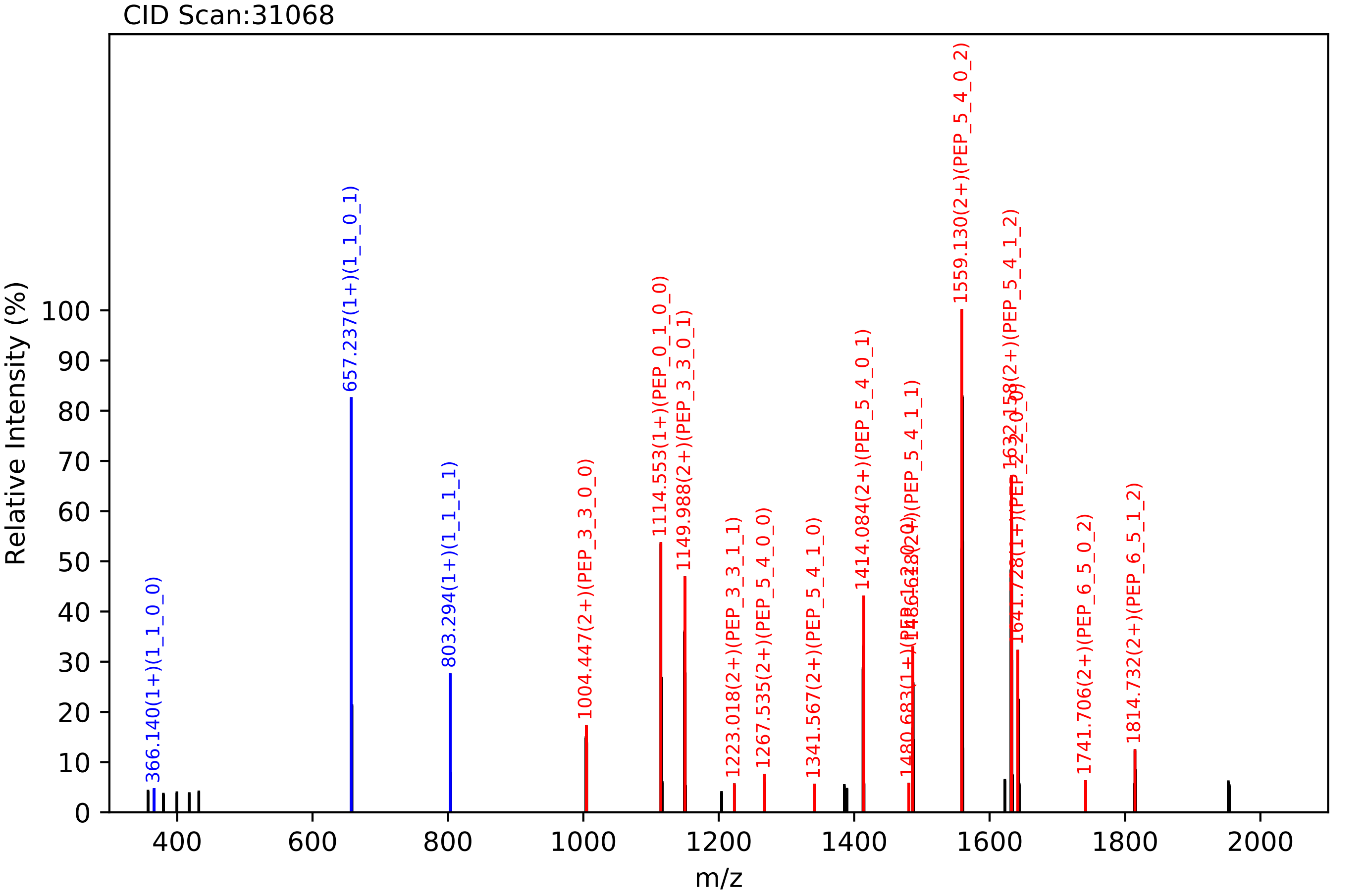

Unknown set no. 665, Experiment: Human Plasma exp_3

NNATVHEQVGGPSLTSDLQAQSK(=PEP)_5_4_0_2, m/z:918.00(5+), RT:72.48, Y-score:81.69

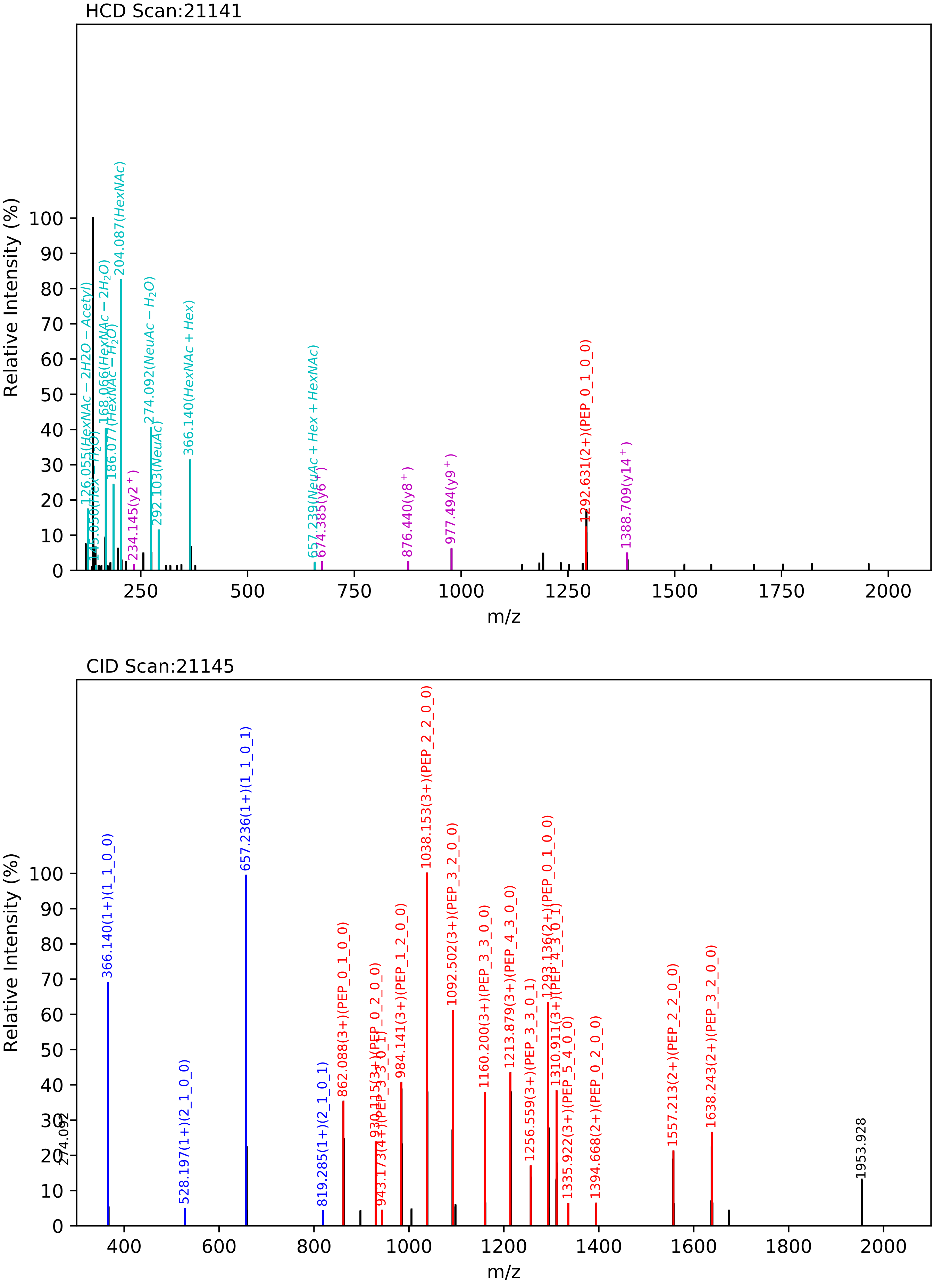

Unknown set no. 666, Experiment: Human Plasma exp_4

NNATVHEQVGGPSLTSDLQAQSK(=PEP)_5_4_0_2, m/z:918.00(5+), RT:72.76, Y-score:80.70

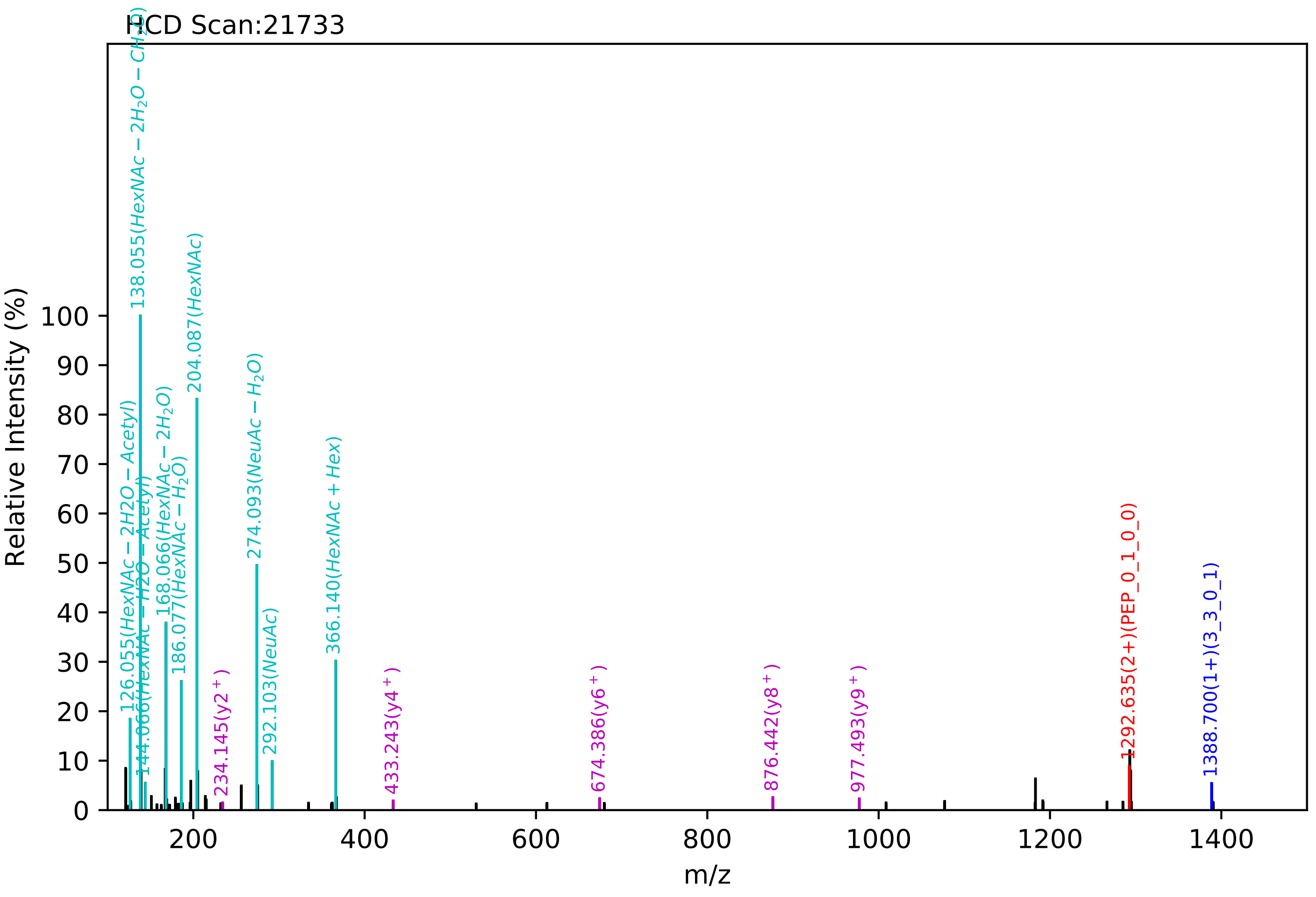

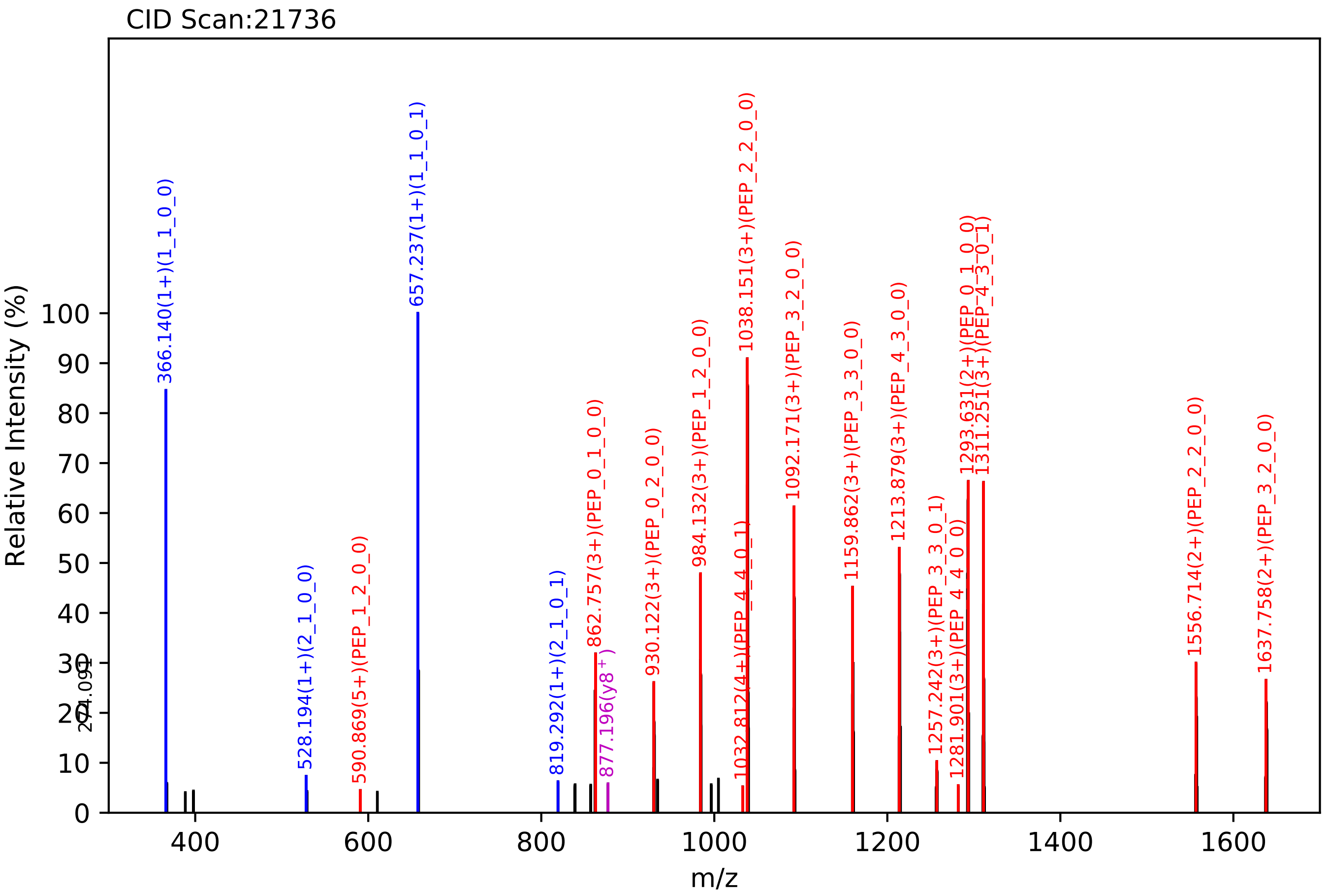

Unknown set no. 667, Experiment: Human Plasma exp_1

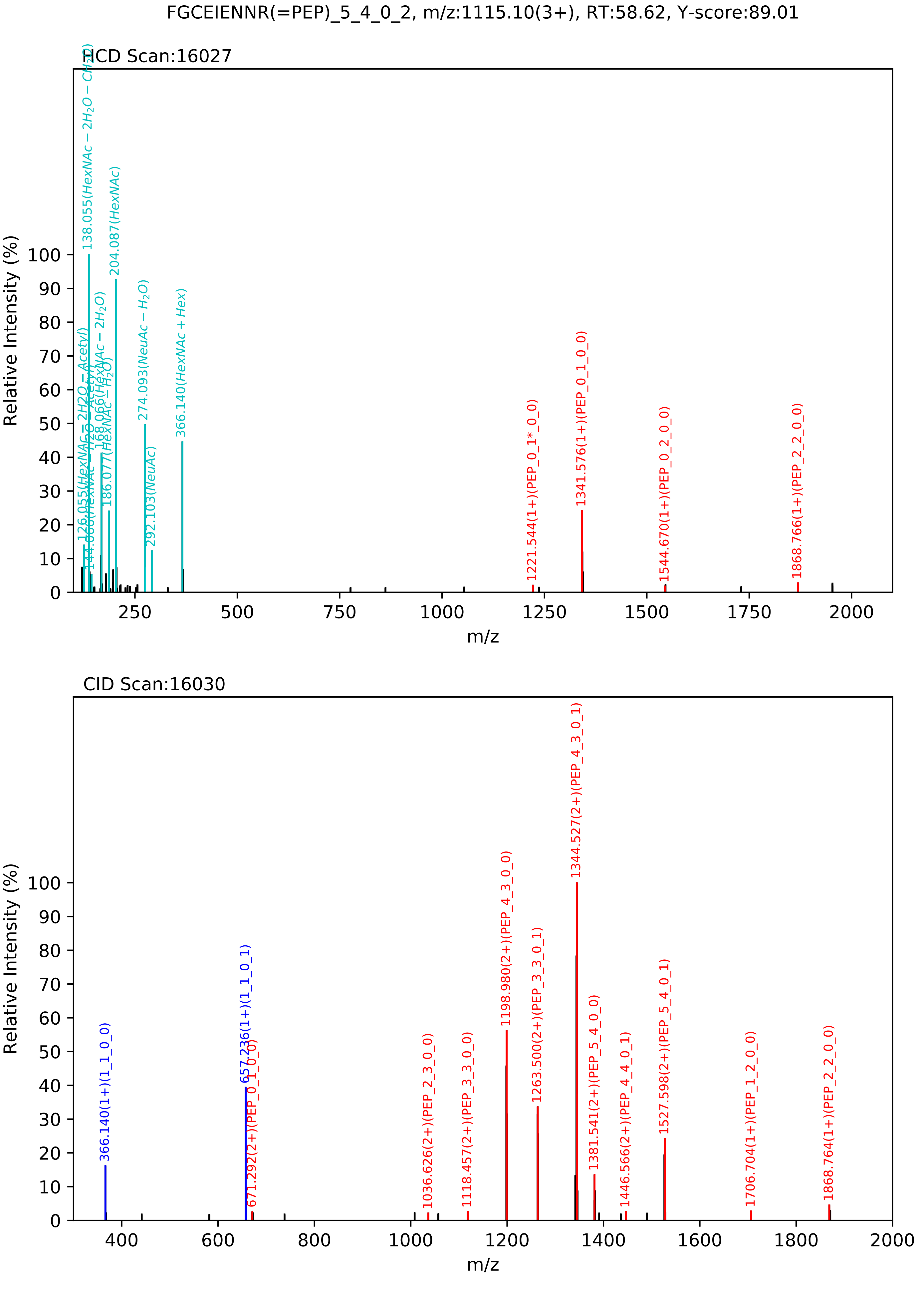

Unknown set no. 668, Experiment: Human Plasma exp_2

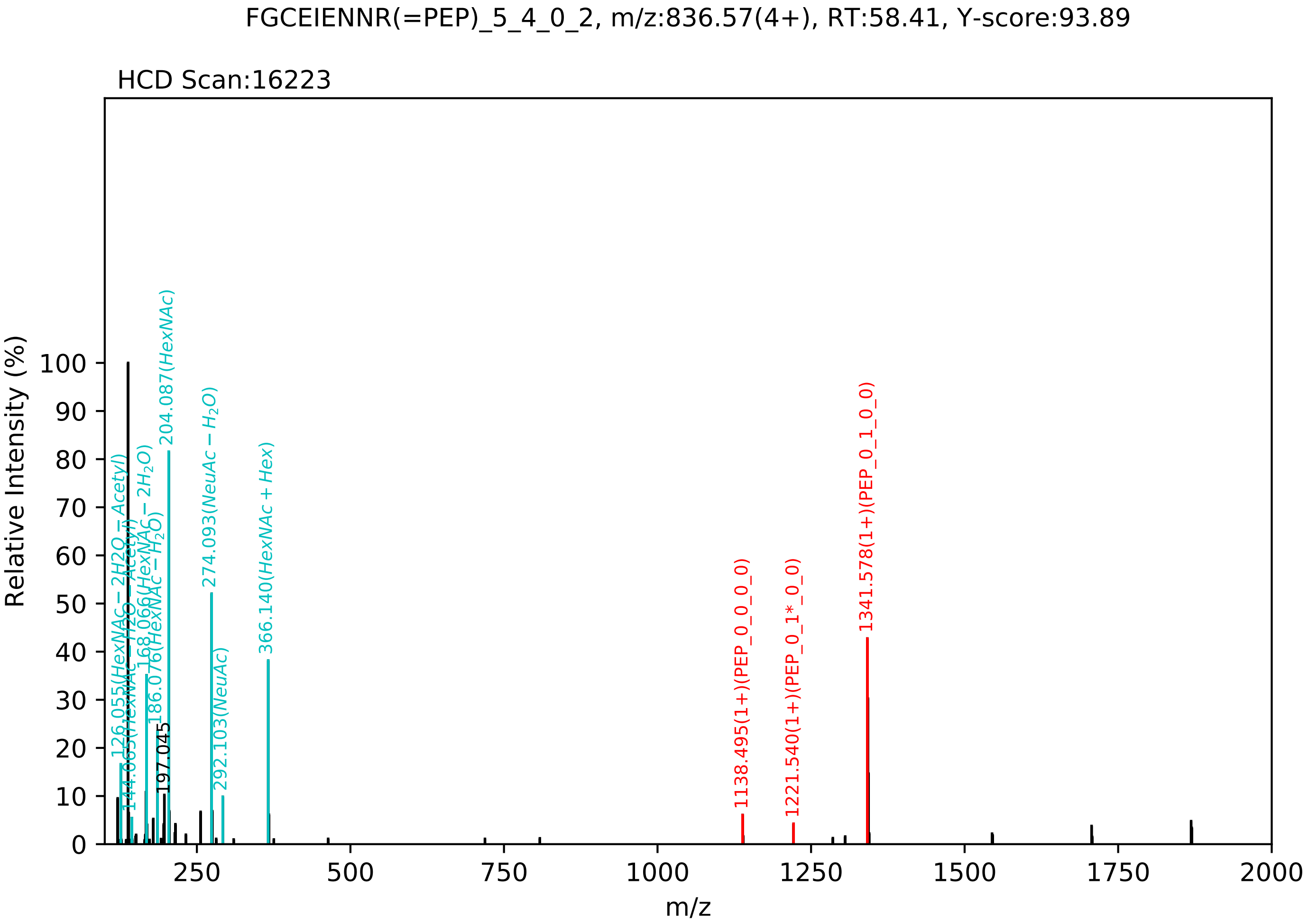

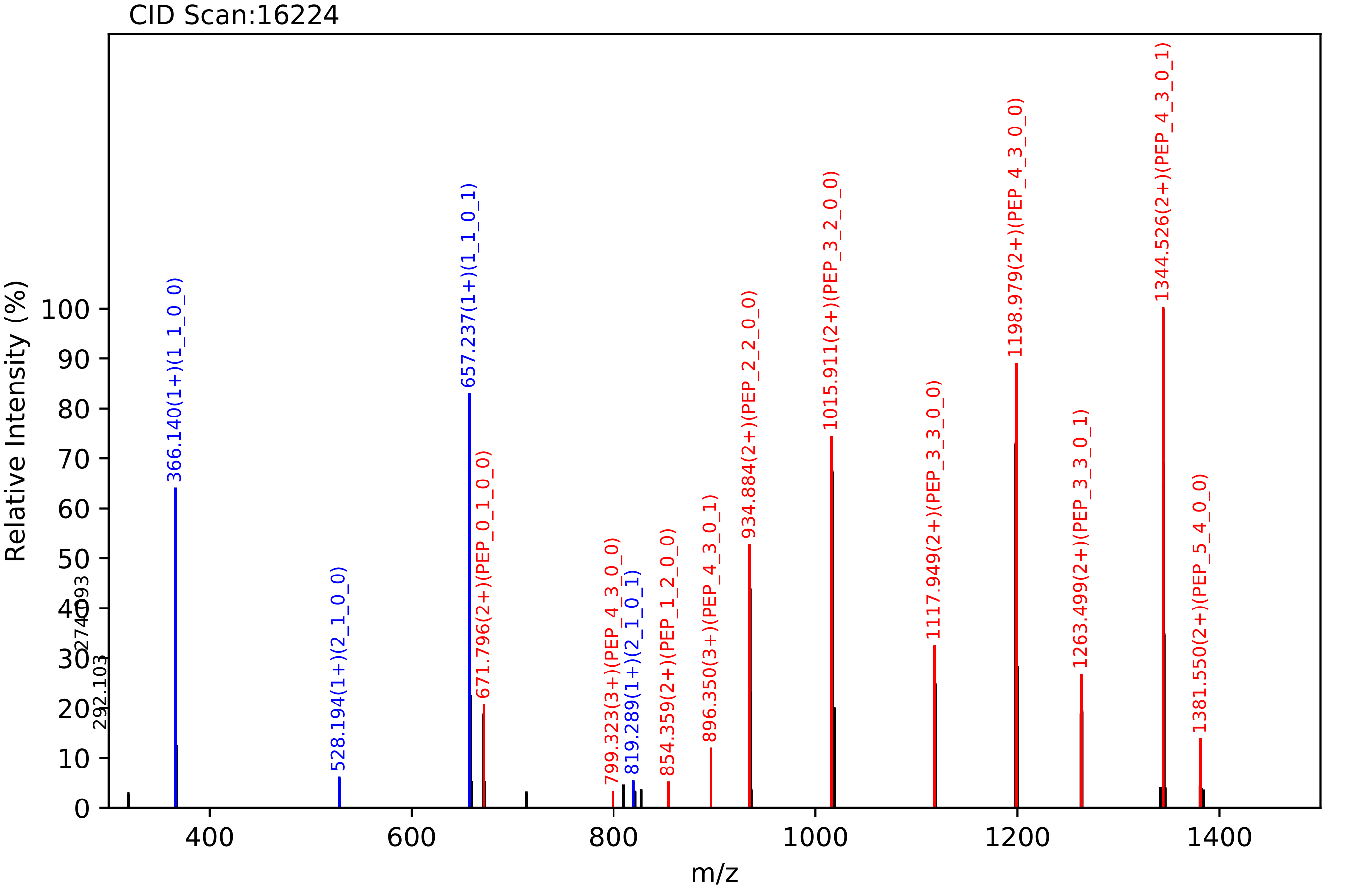

Unknown set no. 669, Experiment: Human Plasma exp_3

FGCEIENNR(=PEP)_5_4_0_2, m/z:836.57(4+), RT:58.43, Y-score:92.53

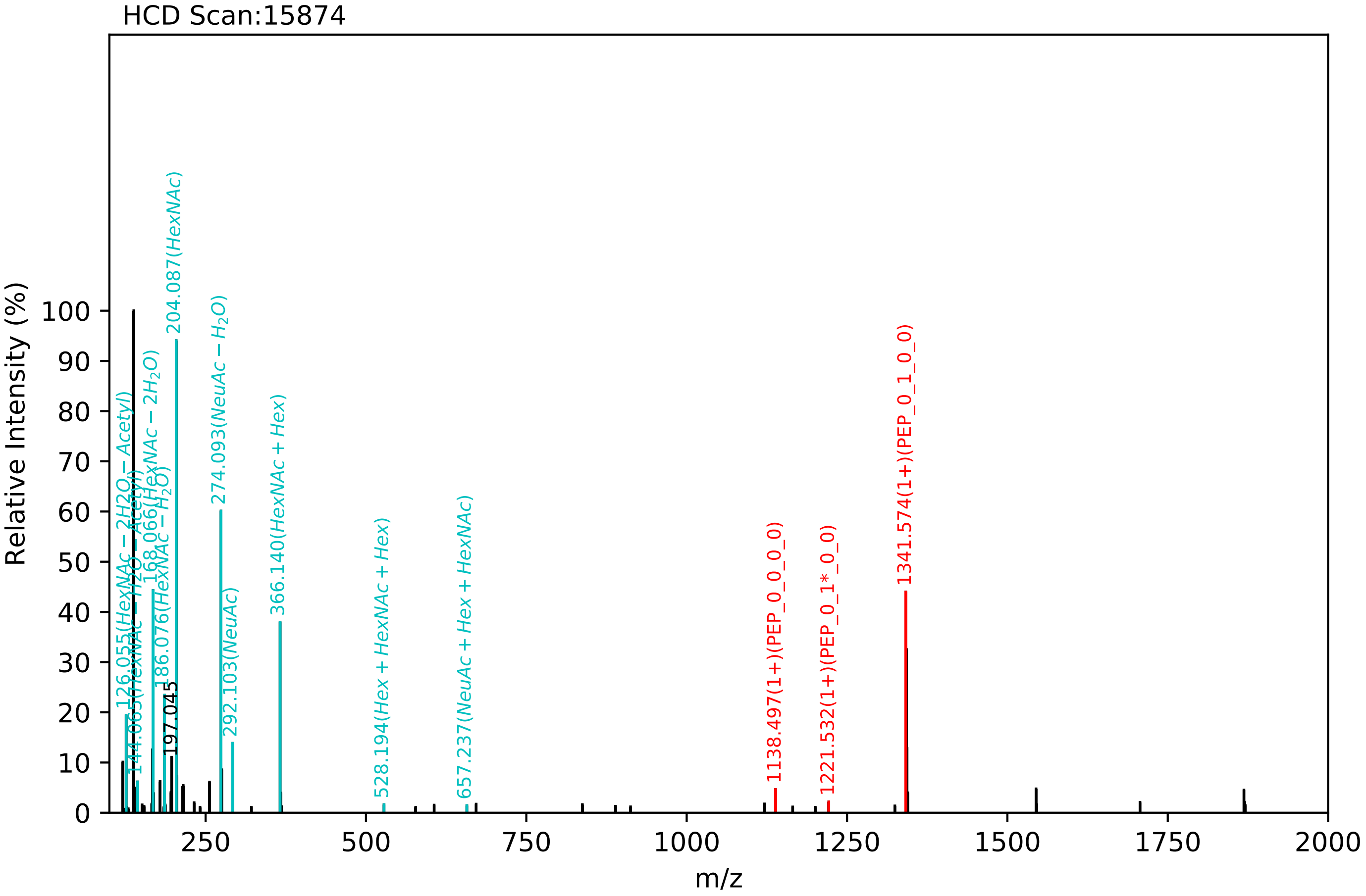

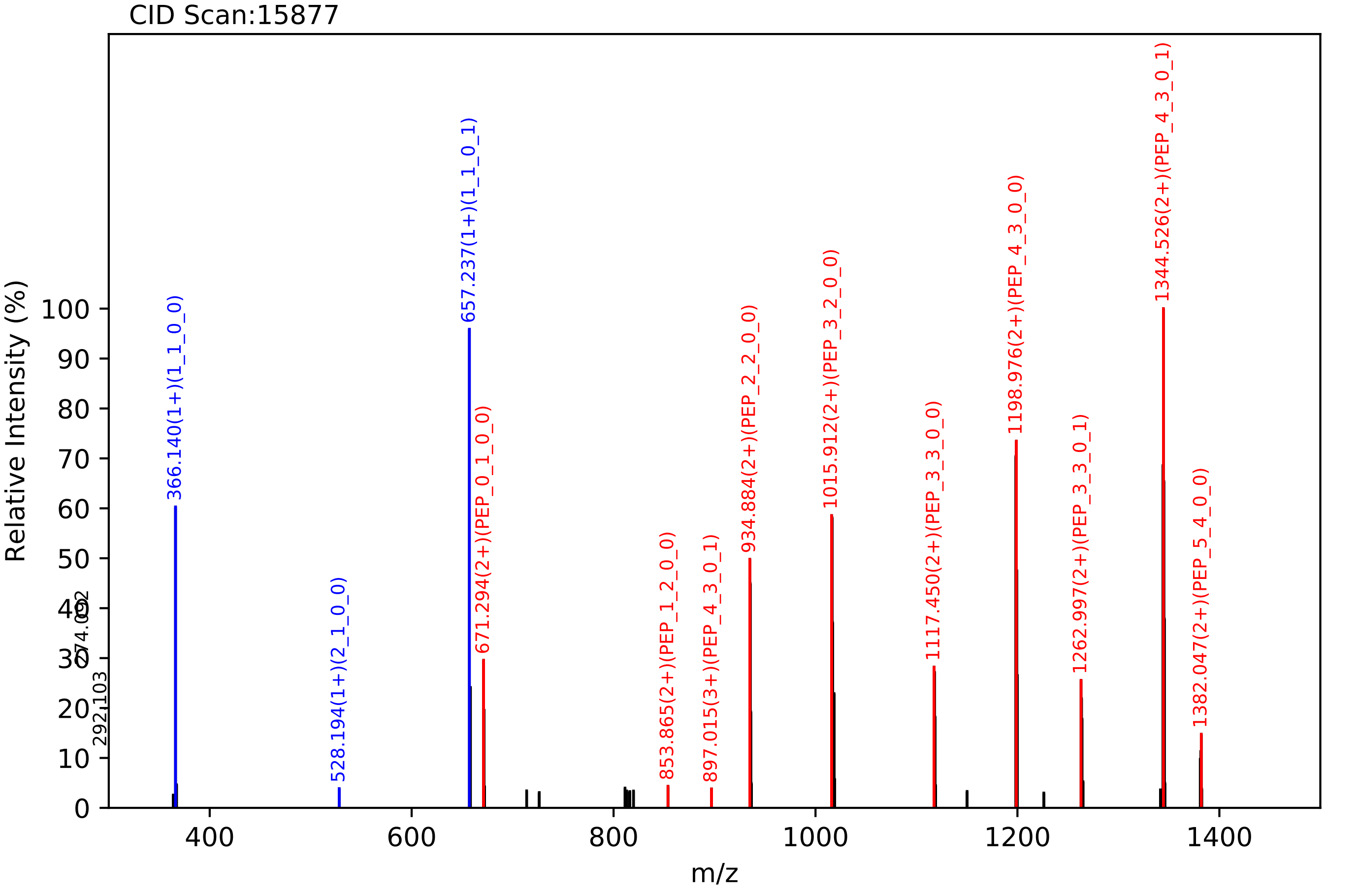

Unknown set no. 670, Experiment: Human Plasma exp_4

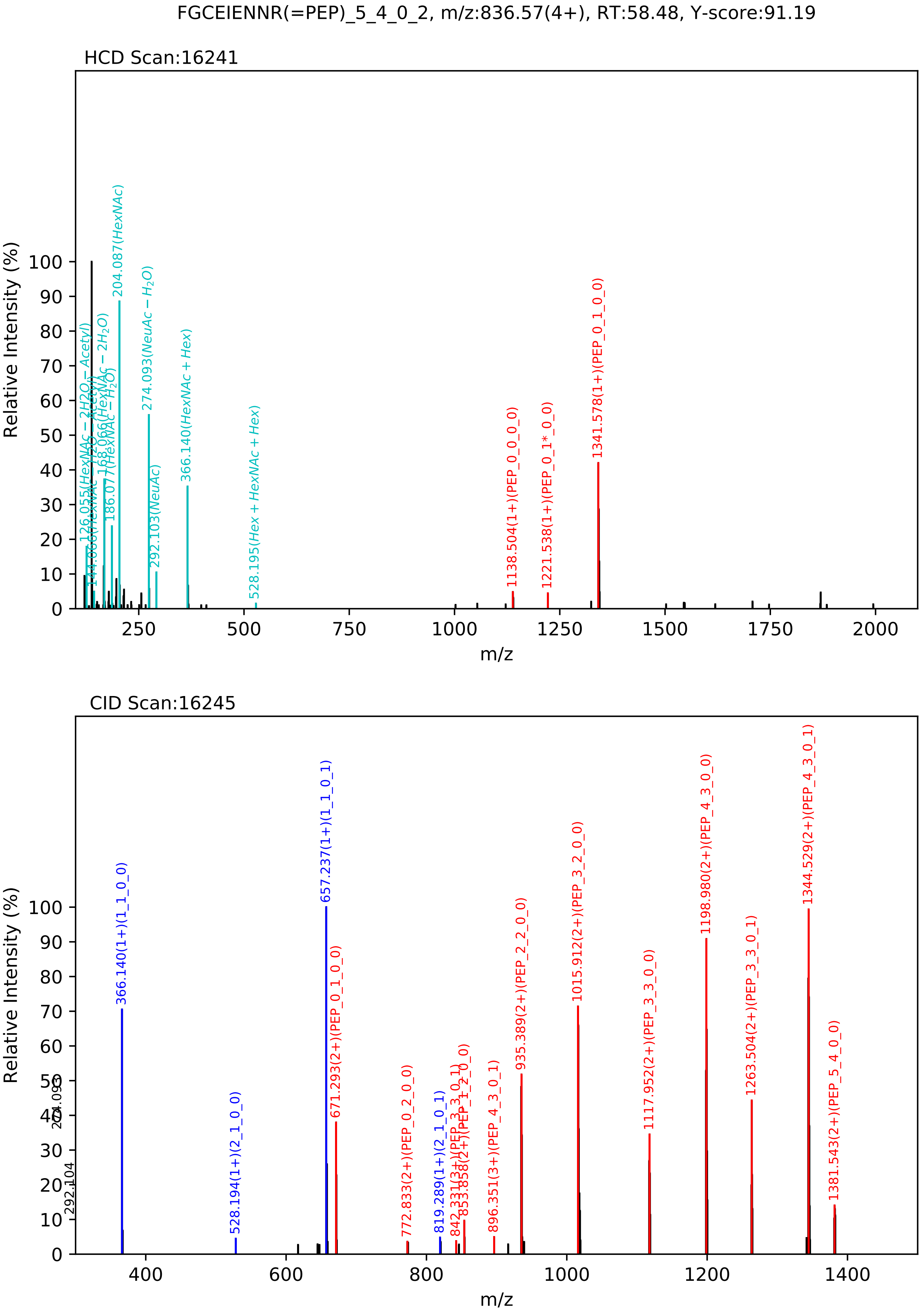

Unknown set no. 671, Experiment: Human Plasma exp_5

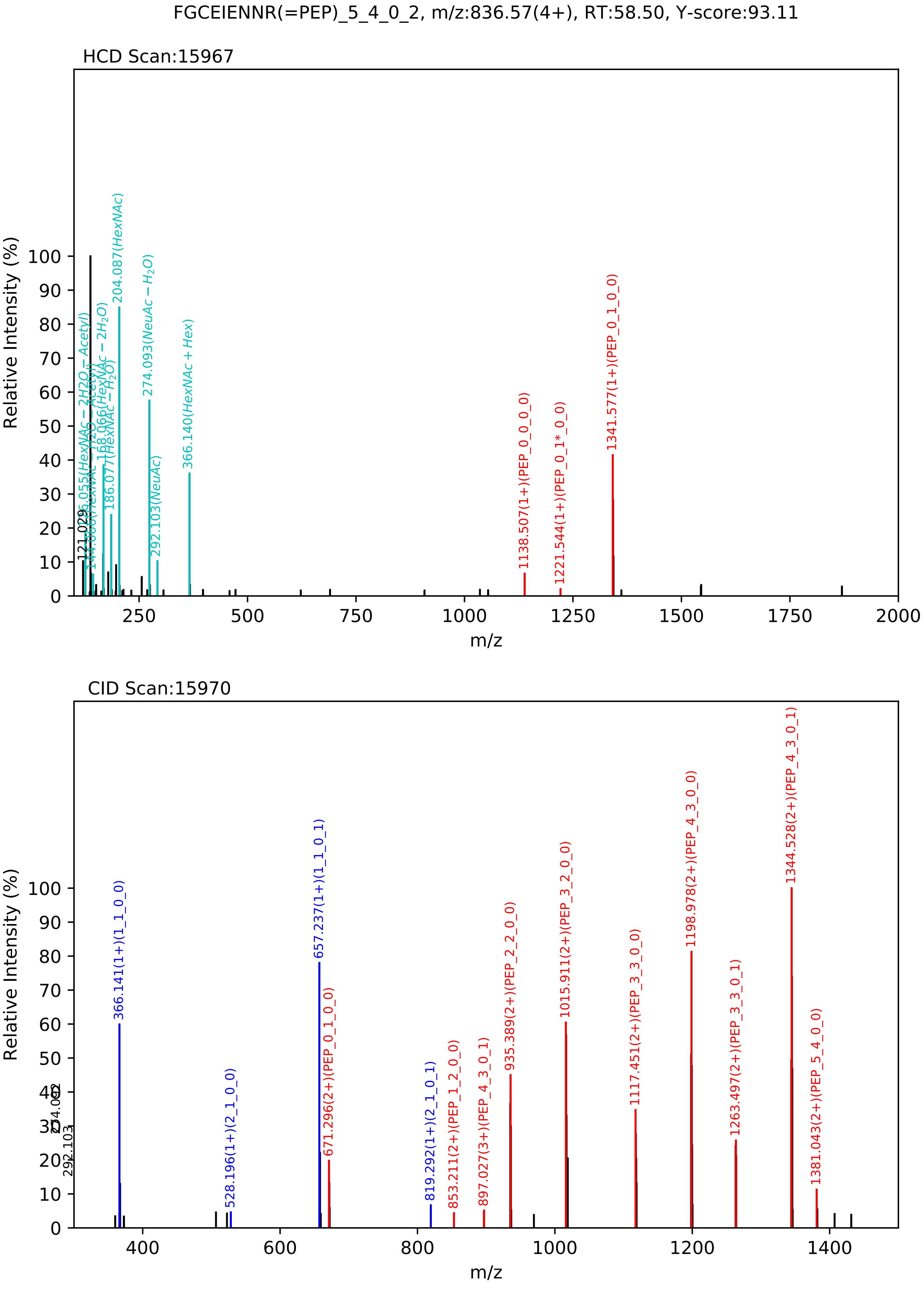

Supplementary Figure 5. Model selection of (a) deep neural network and (b) support vector machine.

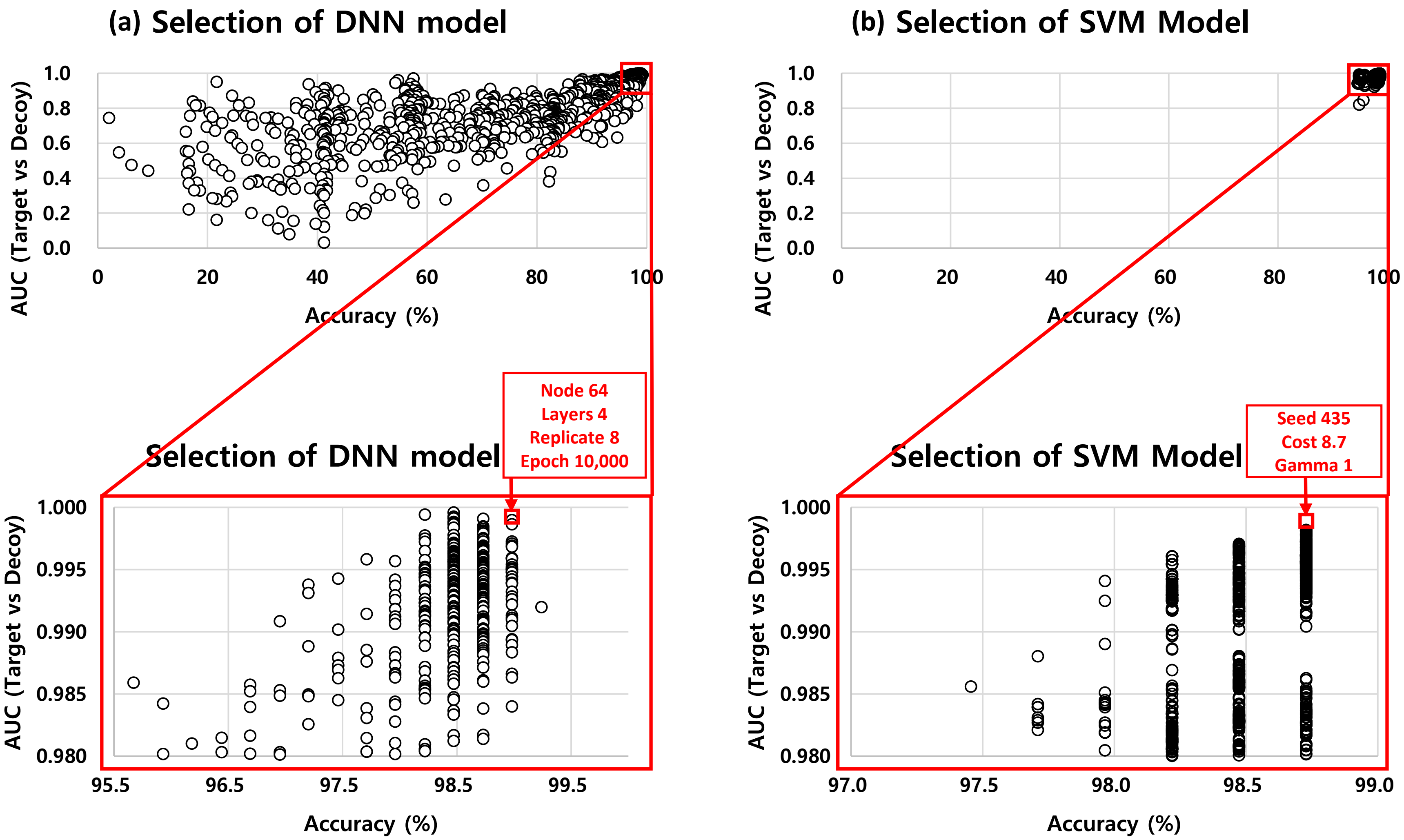

Supplement: Supplementary file 1 — Supplementary figures and tables. [file 41598_2019_57274_MOESM1_ESM.pdf]
